# Genomics, molecular and evolutionary perspective of NAC transcription factors

**DOI:** 10.1371/journal.pone.0231425

**Published:** 2020-04-10

**Authors:** Tapan Kumar Mohanta, Dhananjay Yadav, Adil Khan, Abeer Hashem, Baby Tabassum, Abdul Latif Khan, Elsayed Fathi Abd_Allah, Ahmed Al-Harrasi

**Affiliations:** 1 Natural and Medicinal Plant Sciences Research Center, University of Nizwa, Nizwa, Oman; 2 Dept. of Medical Biotechnology, Yeungnam University, Gyeongsan, Republic of Korea; 3 Botany and Microbiology Department, College of Science, King Saud University, Riyadh, Saudi Arabia; 4 Mycology and Plant Disease Survey Department, Plant Pathology Research Institute, ARC, Giza, Egypt; 5 Department of Zoology, Toxicology laboratory, Raza P.G. College, Rampur, Uttar Pradesh, India; 6 Plant Production Department, College of Food and Agricultural Sciences, King Saud University, Riyadh, Saudi Arabia; University of Naples Federico II, ITALY

## Abstract

NAC (NAM, ATAF1,2, and CUC2) transcription factors are one of the largest transcription factor families found in the plants and are involved in diverse developmental and signalling events. Despite the availability of comprehensive genomic information from diverse plant species, the basic genomic, biochemical, and evolutionary details of NAC TFs have not been established. Therefore, NAC TFs family proteins from 160 plant species were analyzed in the current study. Study revealed, *Brassica napus* (410) encodes highest number and *Klebsormidium flaccidum* (3) encodes the lowest number of TFs. The study further revealed the presence of NAC TF in the Charophyte algae *K*. *flaccidum*. On average, the monocot plants encode higher number (141.20) of NAC TFs compared to the eudicots (125.04), gymnosperm (75), and bryophytes (22.66). Furthermore, our analysis revealed that several NAC TFs are membrane bound and contain monopartite, bipartite, and multipartite nuclear localization signals. NAC TFs were also found to encode several novel chimeric proteins and regulate a complex interactome network. In addition to the presence of NAC domain, several NAC proteins were found to encode other functional signature motifs as well. Relative expression analysis of *NAC TFs* in *A*. *thaliana* revealed root tissue treated with urea and ammonia showed higher level of expression and leaf tissues treated with urea showed lower level of expression. The synonymous codon usage is absent in the NAC TFs and it appears that they have evolved from orthologous ancestors and undergone vivid duplications to give rise to paralogous NAC TFs. The presence of novel chimeric NAC TFs are of particular interest and the presence of chimeric NAC domain with other functional signature motifs in the NAC TF might encode novel functional properties in the plants.

## Introduction

Next-generation sequencing (NGS) has fostered the sequencing of many plant genomes. The availability of so many genomes has allowed researchers to readily identify genes, examine genetic diversity within a species, and gain insight into the evolution of genes and gene families. Gene expression is regulated in part by different families of proteins known as transcription factors (TFs) [1–4]. The TFs are involved in inducing the transcription of DNA into RNA [5–8]. They include numerous and diverse proteins, all of which contain one or more DNA-binding motifs [8–10]. The DNA-binding domain enables them to bind to the promoter or repressor sequence of DNA that is present either at the upstream, downstream, or within an intron region of a coding gene [11,12]. Some TFs bind to a DNA promoter region located near the transcription start site of a gene and help to form the transcription initiation complex [13–16]. Other TFs bind to regulatory enhancer sequences and stimulate or repress transcription of the related genes [17–19]. Regulating transcription is of paramount importance to controlling gene expression and TFs enable the expression of an individual gene in a unique manner, such as during different stages of development or in response to biotic or abiotic stress [20–22]. TFs act as a molecular switch for temporal and spatial gene regulation [23,24]. A considerable portion of a genome consists of genes encoding transcription factors. For example, there are at least 52 different TF families in the *Arabidopsis thaliana*, and the NAC (no apical meristem (NAM) TF family is one of them.

NAC TFs are characterised by the presence of a conserved N-terminal NAC domain comprising approximately 150 amino acids and a diversified C-terminal end. The DNA binding NAC domain is divided into five sub-domains designated A-E. Sub-domain A is apparently involved in the formation of functional dimers, while sub-domains B and E appear to be responsible for the functional divergence of NAC genes [25–28]. The dimeric architecture of NAC proteins can remain stable even at a concentration of 5M NaCl [28]. The dimerization is established by Leu14-Thr23, and Glu26-Tyr31 amino acid residues. The dimeric form is responsible for the functional unit of stress-responsive SNAC1 and can modulate DNA-binding specificity [28–30]. Sub-domains C and D contain positively charged amino acids that bind to DNA [28]. The crystal structure of the SNAC1 TF revealed the presence of a central semi-β-barrel formed from seven twisted anti-parallel β-strands with three α-helices [28]. The NAC domain is most responsible for DNA binding activity that lies between amino acids Val119-Ser183, Lys123-Lys126, with Lys79, Arg85, and Arg88 reside within different strands of β-sheets [26,31,32]. The remaining portion of the NAC domain contains a loop region composed of the amino acids, Gly144-Gly149 and Lys180-Asn183, which are very flexible in nature [28]. The loop region of SNAC1 is quite long and different from the loop region of ANAC, an abscisic-acid-responsive NAC, and could underlie the basis for different biological functions. NAC TFs possesses mono or bipartite nuclear localization signals which contain a Lys residue in sub-domain D [25,32–34]. In addition, NAC proteins, as part of a mechanism of self-regulation, also modulate the expression of several other proteins [32,35]. The D subunit of a few NAC TFs contain a hydrophobic negative regulatory domain (NRD), comprised of L-V-F-Y amino acids, which is involved in suppressing transcriptional activity [36]. For example, the NRD domain can suppress the transcriptional activity of Dof, WRKY, and APETALA 2/dehydration responsive elements (AP2/DRE) TFs [36].

Studies indicate that the diverse C-terminal domain contains a transcription regulatory region (TRR) which has several group-specific motifs that can activate or repress transcription activity [37–40]. The C-terminal region imparts differences in the function of individual NAC proteins by regulating the interaction of NAC TFs with various target proteins. Although the C-terminal region of NAC TFs is varied greatly, it also contains group-specific conserved motifs [41]. Although various aspects of NAC TFs have been studied [42,43], most studies were limited within a few plant species. For example, Zhu et al., (2012) has studied with only 16 species where in few cases they used expressed sequence tag (EST) as well [42] and Pereira-Santana et al., (2015) used 24 land plant species [43] where they were included the genome sequences of unicellular organisms including algae and bacteria. However, Pereira-Santana et al., (2015) did not find any NAC TFs in the algae and bacteria [43]. Therefore, a detailed comparative study of the genomic, molecular biology, and evolution of NAC TFs has across the lineage level of plant kingdom has not been conducted so far. Therefore, a comprehensive analysis of NAC TFs is presented in the current study. We analysed nucleotide and protein data of the NAC TFs to find out the genomic diversity, biochemical, evolutionary, and expression analysis of NAC TFs from 160 plant species.

## Materials and methods

### Identification of NAC TFs

NAC genes from 160 plant species (9 algae, 3 bryophytes, 1 pteridophyte, 5 gymnosperms, and 142 higher plants) were obtained from searches in the National Centre for Biotechnology Information (https://www.ncbi.nlm.nih.gov/), Phytozome, and Plant Genome databases [44,45]. BLASTP (E-value cut-off was 1E-5) and hidden Markov model were used to identify the NAC TFs in different species using AtNAC1 and AtNAC2 as the query sequences [46]. BLASTP analysis was conducted against the respected proteome of the individual species to find the best hit to minimize the error rate [44]. Protein and CDS sequences of each species were collected and further analysed. Protein sequences of the NAC TFs were subjected to BLASTP analysis against the reference databases NCBI, Phytozome, and Plant Genome Database [44,45] to reconfirm them as a NAC TF of the respective identified species. All of the NAC TF protein sequences in the examined species were also subjected to ScanProsite and InterProScan to confirm the presence of a NAC domain [47,48]. Sequences that were found to contain a NAC domain were considered as NAC TFs. The presence of multiple NAC domains, along with the presence of chimeric NAC domains, were determined through ScanProsite and InterProScans [47,48]. The presence of multiple functional sites in NAC TFs were also analysed using ScanProsite software [48].

### Analysis of membrane attachment and nuclear localization signal sequences

The presence of transmembrane domains in NAC TFs of all of the examined species were identified using TMHMM server v. 2.0 [49]. Nuclear localization signal sequences in NAC TFs were identified using NLStradamus software, which uses a hidden Markov model for the prediction of nuclear localization signals [50]. The NAC TF protein sequences were uploaded in FASTA format to run the program. The parameters used to run the NLS analysis were; HMM state emission and transition frequencies, 2 state HMM static; prediction type Viterbi and posterior, prediction cut-off 0.4; prediction display, and image and graphic [50].

### Interactome analysis of NAC TFs

*A*. *thaliana* NAC TFs were used to examine the complex interactome network of NAC TFs. The individual interaction network of each NAC TF in *A*. *thaliana* was searched in a string database that contains 9.6 million proteins from 2031 organisms [51,52]. The interactome network of each of NAC TF were noted and the results were later used to construct the interactome network of *A*. *thaliana* NAC TFs. The presented interactome network was based on an experimentally validated network, co-expressed network, and a mined network [52]. These outputs were used to construct the interactome network. The NAC TFs used to construct the interactome network were subjected to GO (gene ontology) and cellular process analyses [52].

### Gene expression analysis

Differential gene expression of NAC TFs was analysed to elucidate their role in growth, development, and nitrogen assimilation. *A*. *thaliana* NAC TFs were used to examine differential gene expression. The transcriptome data from *A*. *thaliana* treated with ammonia, nitrate, and urea were utilized from the PhytoMine database in Phytozome [44]. The experimental conditions were as follows; the *A*. *thaliana* seeds were cold stratified in water for 3 days and sown in pots. The pots were placed in the growth chamber (22^o^ C day/20^o^ C night, 14 hrs light with flux density of 350 μmol m^-2^s^-1^) and later thinned one plant per pot. When rosette was achieved 7–8 leaves, treatment was conducted. The plants were watered with nutrient solution containing 5mM urea, 10 mM KNO_3_ (potassium nitrate), and 10 mM (NH_4_)_3_PO_4_ (ammonium phosphate) for each of individual experiment. The nutrient solutions were supplied at three days interval for four weeks. After four weeks, the leaf, stem, and root tissues were harvested for expression analysis. The expression pattern of NAC TFs for leaf and root tissues in the treated *A*. *thaliana* plants were analysed separately. The expression was measured in fragments per kilobase of exon per million fragments mapped (FPKM). Transcripts with a zero value were discarded from the study.

### Construction of a phylogenetic tree

Two approaches were used to construct the phylogenetic trees. In the first approach, a phylogenetic tree was constructed using the NAC TFs of individual species. In the second approach, the NAC TFs of all of the examined species were combined to construct a phylogenetic tree. The phylogenetic tree for individual species was constructed to determine the deletion and duplication events in NAC TFs within individual species. We excluded the short sequences from the study those resulted in error during the alignment. Prior to the construction of the phylogenetic trees, a model selection was carried out in MEGA6 software [53]. The following parameters were used in the model, analysis, model selection; tree to use, automatic (neighbour joining), statistical method, maximum likelihood; substitution type, nucleotides; gaps/missing data treatment, partial deletion; site coverage cut-off (%), 95; codons included, 1^st^+2^nd^+3^rd^+non-coding. Based on the lowest BIC values of model selection, phylogenetic trees of NAC TFs were carried out using the neighbour joining method, a GTR statistical model, and 1000 bootstrap replicates.

### Analysis of transition and transversion rates

Transition and transversion rates in NAC TFs within individual species were analysed using MEGA6 software [53]. The converted MEGA file format of individual species was used to determine the rate of transition and transversion. The following statistical parameters were used to study the transition/transversion rate: estimate transition/transversion bias; maximum composite likelihood estimates of the pattern of nucleotide substitution; substitution type, nucleotides; model/method, Tamura-Nei; gaps/missing data treatment, pairwise deletion; codon position, 1^st^, 2^nd^, 3^rd^, and non-coding sites.

### Analysis of gene deletion and duplication

Prior to the analysis of deletion and duplication events in NAC TFs, a species tree was constructed in the NCBI taxonomy browser (https://www.ncbi.nlm.nih.gov/Taxonomy/CommonTree/wwwcmt.cgi). All of the studied species were used to construct the species tree. The resulting phylogenetic trees of individual species in a nwk file format were uploaded in Notung 2.9 software [54] as a gene tree and reconciled as a gene tree with the species tree to obtain duplicated and deleted genes. Deletion and duplication events were analysed in all of the studied species individually.

## Results and discussion

### NAC transcription factors exhibit diverse genomic and biochemical features

Advancements in genome sequencing technology have enabled the discovery of the genomic details of large number of plant species. The availability the genome sequence data allowed us to study the genomic details of NAC TFs in diverse plant species. The presence of NAC TFs in 160 species (18774 NAC sequences) was identified and served as the basis of the conducted analyses. Comparisons of NAC sequences revealed that *Brassica napus* has the highest number (410) of NAC TFs, while the pteridophyte plant, *Marchantia polymorpha*, was found to contain the lowest number (9) (Table 1). On average, monocot plants contain a higher (141.20) number of NAC TFs relative to dicot plants (125.56). Except for *Hordeum vulgare* (76), *Saccharum officinarum* (44), and *Zostera marina* (62) all other monocot species possess more than one hundred NAC TFs each (Table 1). Lower eukaryotic plants, bryophytes and pteridophytes also possess NAC TFs. In addition, the algal species, *Klebsormidium flaccidum*, also contains NAC TFs and this finding represents the first report of NAC TFs in algae (Table 1). A NAC TF in *Trifolium pratense* (Tp57577_TGAC_v2_mRNA14116) was found to be the largest NAC TF, comprising 3101 amino acids, while a NAC TF in *Fragaria x ananassa* (FANhyb_icon00034378_a.1.g00001.1) was found to be the smallest NAC TF, comprising only 25 amino acids. Although it only contains a 25 amino acid sequence, it still encodes a NAC domain. Typically, NAC TFs contain a single NAC domain located near the N-terminal region of the protein. The current analysis, however, also identified NAC TFs with two NAC domains. At least 77 of the 160 studied species were found to contain two NAC domains (Table 1).

**Table 1:** Genomic details of NAC TFs of plants. NAC TFs have not undergone conditional duplication and none of a NAC TF gene has lost. In addition, transfer of NAC TFs was not observed from one species to another.

Sl. No	Name of the species	No. of double domain NAC TF	No. of Novel chimeric NAC TFs	Total No. of NAC TFs	No. of duplicated genes	No. of paralogous genes
**Monocots**						
**1**	*Aegilops tauschii*		4	117	114	114
**2**	*Brachypodium distachyon*	2	1	137	135	135
**3**	*Brachypodium stacei*	1	1	128	127	127
**4**	*Hordeum vulgare*			76	76	76
**5**	*Leersia perrieri*	5	2	163	162	162
**6**	*Oropetium thomaeum*	1		118	103	103
**7**	*Oryza barthii*		4	134	138	138
**8**	*Oryza brachyantha*	1	1	118	110	110
**9**	*Oryza glaberrima*	1		116	110	110
**10**	*Oryza glumipatula*	2		140	139	139
**11**	*Oryza longistaminata*	1	6	125	98	98
**12**	*Oryza meridionalis*	2	2	127	123	123
**13**	*Oryza nivara*	4	1	146	130	130
**14**	*Oryza punctata*	6	1	135	133	133
**15**	*Oryza rufipogon*	4	3	136	129	129
**16**	*Oryza sativa subsp. indica*	1	3	157	156	156
**17**	*Oryza sativa subsp. japonica*	1		139	138	138
**18**	*Panicum hallii*	3	6	139	126	126
**19**	*Panicum virgatum*	9	6	310	309	309
**20**	*Phoenix dactylifera*	3	1	124	123	123
**21**	*Phyllostachys edulis*			125	124	124
**22**	*Phyllostachys heterocycla*	2	2	125	124	124
**23**	*Saccharum officinarum*			44	33	33
**24**	*Setaria italica*	4		139	134	134
**25**	*Setaria viridis*	1		135	118	118
**26**	*Sorghum bicolor*	1		141	134	134
**27**	*Spirodela polyrhiza*			55	48	48
**28**	*Triticum aestivum*	2	2	263	209	209
**29**	*Triticum urartu*		1	103	74	74
**30**	*Zea mays*	1	1	130	119	119
**31**	*Zostera marina*	1		62	55	55
**32**	*Zoysia japonica*		4	176	160	160
**33**	*Zoysia matrella*	1	3	313	230	230
**34**	*Zoysia pacifica*	1	2	205	183	183
**Dicots**						
**35**	*Actinidia chinensis*	1	5	167	166	166
**36**	*Aethionema arabicum*	3		85	84	84
**37**	*Amaranthus hypochondriacus*	1		44	37	37
**38**	*Amborella trichopoda*			46	45	45
**39**	*Ananas comosus*		1	73	72	72
**40**	*Aquilegia coerulea*			80	79	79
**41**	*Arabidopsis halleri*	2		94	93	93
**42**	*Arabidopsis lyrata*	4	1	122	121	121
**43**	*Arabidopsis thaliana*	5		113	112	112
**44**	*Arabis alpina*	1		82	81	81
**45**	*Arachis duranensis*			82	81	81
**46**	*Arachis hypogaea*			162	161	161
**47**	*Arachis ipaensis*			83	81	81
**48**	*Artemisia annua*			28	27	27
**49**	*Azadirachta indica*			183	182	182
**50**	*Beta vulgaris*			53	52	52
**51**	*Boechera stricta*	2		123	122	122
**52**	*Brassica napus*	10	7	410	409	409
**53**	*Brassica oleracea*	4	3	271	270	270
**54**	*Brassica rapa*	4	2	256	255	255
**55**	*Cajanus cajan*			96	95	95
**56**	*Camelina sativa*	17	3	341	330	330
**57**	*Cannabis sativa*			58	57	57
**58**	*Capsella grandiflora*	2		95	94	94
**59**	*Capsella rubella*	5		119	118	118
**60**	*Capsicum annum*			96	95	95
**61**	*Carica papaya*			82	81	81
**62**	*Castanea mollissima*	4		91	78	78
**63**	*Catharanthus roseus*		2	121	120	120
**64**	*Chenopodium quinoa*		1	96	95	95
**65**	*Cicer arietinum*			96	95	95
**66**	*Citrullus lanatus*			80	79	79
**67**	*Citrus clementina*			129	128	128
**68**	*Citrus sinensis*	2		145	143	143
**69**	*Coffea canephora*			63	62	62
**70**	*Cucumis melo*			92	91	91
**71**	*Cuccumis sativus*			83	80	80
**72**	*Daucus carota*		2	96	95	95
**73**	*Dianthus caryophyllus*			79	77	77
**74**	*Dichanthelium oligosanthes*	8	2	131	100	100
**75**	*Dorcoceras hygrometricum*		2	83	76	76
**76**	*Elaeis guineensis*	2	1	170	167	167
**77**	*Eragrostis tef*	8	3	172	165	165
**78**	*Eucalyptus camaldulensis*			200	124	124
**79**	*Eucalyptus grandis*			164	150	150
**80**	*Eutrema salsugineum*	2		122	104	104
**81**	*Fragaria vesca*	3	6	127	123	123
**82**	*Fragaria x ananassa*	2	1	98	97	97
**83**	*Genlisea aurea*		1	45	42	42
**84**	*Glycine max*			180	175	175
**85**	*Glycine soja*		1	173	166	166
**86**	*Gossypium arboreum*			150	146	146
**87**	*Gossypium hirsutum*	1	2	306	296	296
**88**	*Gossypium raimondii*			153	145	145
**89**	*Helianthus annuus*			21	20	20
**90**	*Humulus lupulus*			74	68	68
**91**	*Ipomoea trifida*	1	2	131	123	123
**92**	*Jatropha curcas*		1	97	93	93
**93**	*Juglans regia*	3		92	81	81
**94**	*Kalanchoe laxiflora*			166	165	165
**95**	*Kalanchoe marnieriana*			179	178	178
**96**	*Lactuca sativa*			54	52	52
**97**	*Linum usitatissimum*	1	1	191	187	187
**98**	*Lotus japonicus*	2		98	92	92
**99**	*Malus domestica*	2	9	253	232	232
**100**	*Manihot esculenta*			130	128	128
**101**	*Medicago truncatula*	1		97	90	90
**102**	*Mimulus guttatus*			114	113	113
**103**	*Morus notabilis*		2	78	77	77
**104**	*Musa acuminata*	1	1	170	164	164
**105**	*Nelumbo nucifera*			88	79	79
**106**	*Nicotiana benthamiana*	2	2	227	185	185
**107**	*Nicotiana sylvestris*			156	149	149
**108**	*Nicotiana tabacum*			280	279	279
**109**	*Nicotiana tomentosiformis*			172	162	162
**110**	*Ocimum tenuiflorum*	2	1	110	82	82
**111**	*Petunia axillaris*	3		131	108	108
**112**	*Petunia inflata*			157	147	147
**113**	*Phaseolus vulgaris*			85	84	84
**114**	*Populus euphratica*	2	3	155	149	149
**115**	*Populus trichocarpa*		1	169	149	149
**116**	*Prunus mume*	1		129	128	128
**117**	*Prunus persica*	1	1	115	114	114
**118**	*Pyrus bretschneideri*	1	5	185	183	183
**119**	*Raphanus raphanistrum*	4	3	207	206	206
**120**	*Raphanus sativus*	5	1	217	197	197
**121**	*Ricinus communis*			95	87	87
**122**	*Salix purpurea*			175	152	152
**123**	*Salvia miltiorrhiza*	1	2	87	81	81
**124**	*Sesamum indicum*			105	104	104
**125**	*Sisymbrium irio*	2	2	121	118	118
**126**	*Solanum lycopersicum*			101	94	94
**127**	*Solanum melongena*	1	3	95	85	85
**128**	*Solanum pennellii*		2	102	98	98
**129**	*Solanum pimpinellifolium*			97	90	90
**130**	*Solanum tuberosum*	1		129	115	115
**131**	*Spinacia oleracea*			45	43	43
**132**	*Tarenaya hassleriana*	1		178	177	177
**133**	*Thellungiella halophila*	2		122	121	121
**134**	*Thellungiella parvula*	1		92	91	91
**135**	*Theobroma cacao*			132	131	131
**136**	*Trifolium pratense*	2	2	97	76	76
**137**	*Utricularia gibba*		1	74	73	73
**138**	*Vigna angularis*			98	97	97
**139**	*Vigna radiata*	2		82	81	81
**140**	*Vigna unguiculata*			20	19	19
**141**	*Ziziphus jujuba*			101	100	100
**142**	*Vitis vinifera*	1		70	79	79
**Gymnosperms**						
**143**	*Picea abies*	1		100	73	73
**144**	*Picea glauca*			32	31	31
**145**	*Picea sitchensis*			16	15	15
**146**	*Pinus taeda*			31	27	27
**147**	*Pseudotsuga menziesii*	5	3	196	195	195
**Pteridophyte**						
**148**	*Selaginella moellendorffii*			22	21	21
**Bryophytes**						
**149**	*Marchantia polymorpha*			9		
**150**	*Physcomitrella patens*			33	32	32
**151**	*Sphagnum fallax*			26	25	25
**Algae**						
**152**	*Bathycoccus prasinos*			0	0	0
**153**	*Chlamydomonas reinhardtii*			0	0	0
**154**	*Chlorella sp*. NC64A			0	0	0
**155**	*Coccomyxa sp*.			0	0	0
**156**	*Dunaliella salina*			0	0	0
**157**	*Klebsormidium flaccidum*			3	0	0
**158**	*Micromonas pusilla*			0	0	0
**159**	*Ostreococcus lucimarinus*			0	0	0
**160**	*Volvox carteri*			0	0	0

Multiple sequence alignment revealed the presence of a conserved consensus sequence at the N-terminus. The major conserved consensus sequences are P-G-F-R-F-H-P-T-D-D/E-L-I/V, Y-L-x_2_-K, D-L-x-K-x_2_-P-W-x-L-P, E-W-Y-F-F, G-Y-W-K-A/T-T-G-x-D-x _1-2_-I/V, G-x-K-K-x-L-V-F-Y, and T-x-W-x-M-H-E-Y. Among these consensus sequences, D-D/E-L-I/V, E-W-Y-F-F, G-Y-W-K, and M-H-E-Y are the conserved motifs most observed. The D-D/E-L motif is a characteristic feature of the calcium-binding motifs present in the EF-hand of calcium-dependent protein kinases and the presence of this motif in NAC TFs indicates that they have the potential to regulate Ca^2+^ signalling events in cells [55]. The D-D-E/E motif is located in the β’ sheet whereas the Y-L-x_2_-K motif is in the α1a/b chain. Except for G-F-R-F-H-P-T-D-D/E-L-I/V, the conserved consensus sequences contain the positively charged amino acids Lys (L) and Arg (K) that can bind to negatively charged DNA. Welner et al. (2012) published the crystal structure of ANAC019 and reported that Y^94^-W-K-A-T-G-T-D in β3, I^11^-K-K-A-L-V-F-Y of β4, K^123^-A-P-K-G-T-K-T-N-W in the loop between β4 and β5, and I^133^-M-H-E-Y-R of β5 and Y^160^-K-K-Q at the C-terminal end are located close to the bound DNA and are associated with DNA binding activity [56]. They reported that Y^94^-W-K-A-T-G-T-D is responsible for the specific recognition of DNA and binds at the major groove within DNA, whereas I^11^-K-K-A-L-V-F-Y, K^123^-A-P-K-G-T-K-T-N-W, I^133^-M-H-E-Y-R, and Y^160^-K-K-Q bind to the backbone of the DNA molecule and provide affinity for DNA binding activity [56]. In the present analysis of 160 plant species, the identification of the conserved consensus sequences G-Y-W-K-A/T-T-G-x-D-x_1-2_-I/V, G-x-K-K-x-L-V-F-Y, and T-x-W-x-M-H-E-Y is in agreement with Welner et al (2012); suggesting that NAC TFs contain conserved consensus sequences for specific DNA recognition and increasing the affinity for DNA binding.

Hao et al., (2010) reported that the D subunit of NAC TFs contain a hydrophobic L-V-F-Y amino acid motif that partially suppresses the WRKY, Dof, and APETALA2 transcriptional regulators [36]. This suggests that NAC TFs function as a negative regulator of transcription for WRKY, Dof, and APETALA 2/ dehydration responsive element. The sequence alignment, however, revealed the presence L-V-F-Y transcriptional repressor motif in NAC TF family proteins in diverse plant species. If all the NAC TF with L-V-F-Y motif will supress the transcriptional activity of WRKY, Dof, and APETALA 2, it will be challenging for the plants to sustain its cellular and biological activities.

**Fig 1:**
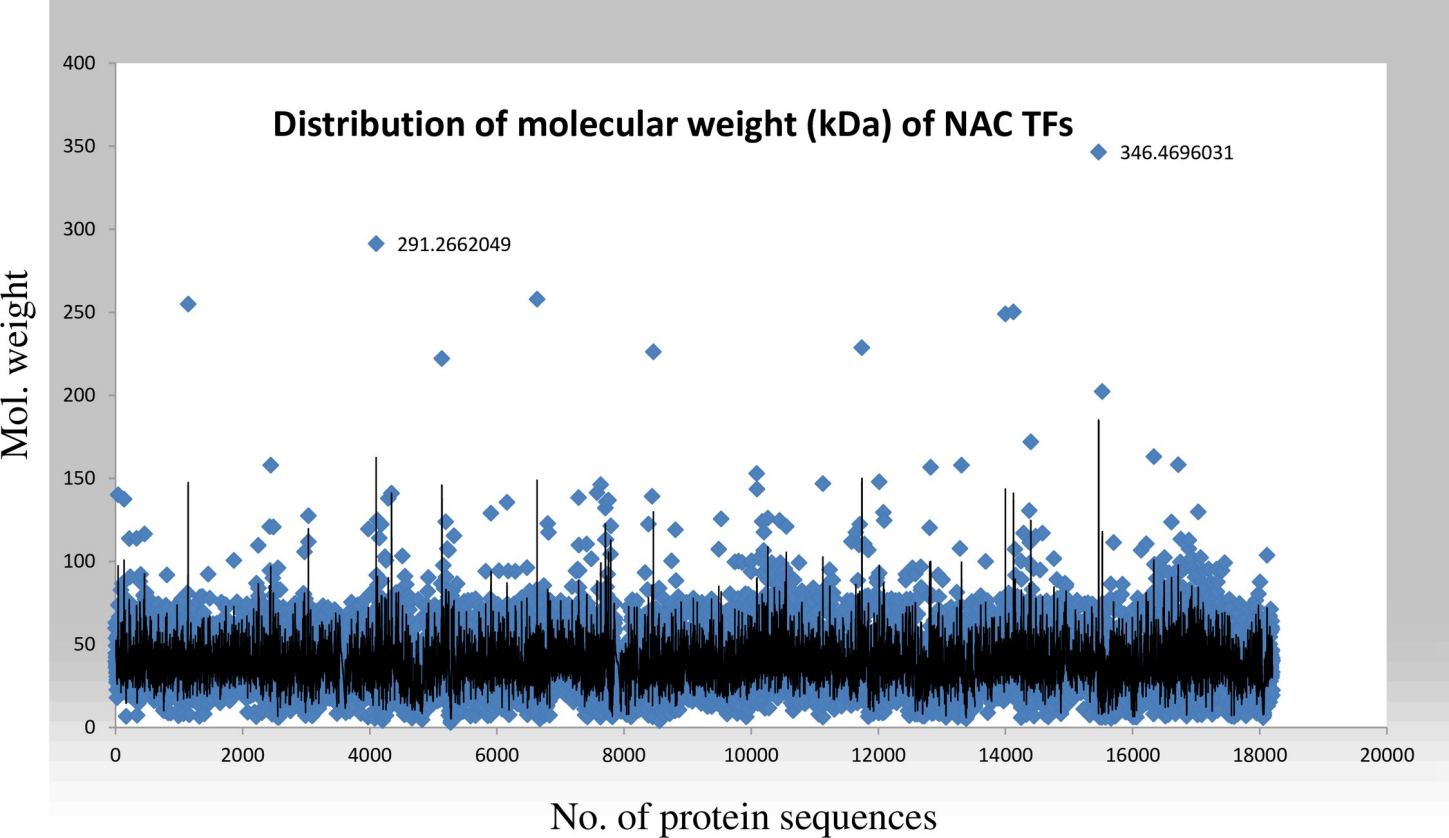
The distribution of the molecular weight of NAC TFs. The molecular weight of NAC TFs ranged from 2.94 kDa (*Fragaria* x *ananassa*, FANhyb_icon00034378_a.1.g00001.1) to 346.46 kDa (*Trifolium pratense*, Tp57577_TGAC_v2_mRNA14116). The average molecular weight of NAC TFs was 38.72 kDa. In total, 17158 NAC TFs were utilized in the analysis of molecular weight. The analysis was conducted using a protein isoelectric point calculator (http://isoelectric.org/).

The molecular weight of NAC TFs ranged from 2.94 kDa (*Fragaria* x *ananassa*_FANhyb_icon00034378_a.1.g00001.1) to 346.46 kilodaltons (kDa) (*Trifolium pratense*_Tp57577_TGAC_v2) (Fig 1). Among the studied NAC TFs, only 10 NAC proteins have a molecular weight (MW) more than 200 kDa and 99 are between 100 to 200 kDa. The MW of the majority of the NAC proteins range between 40 to 55 kDa (Fig 1). The average molecular weight of the plant proteins falls in the same range (average 48.256 kDa) as found in the case of *A*. *thaliana* proteome) [57].

**Fig 2:**
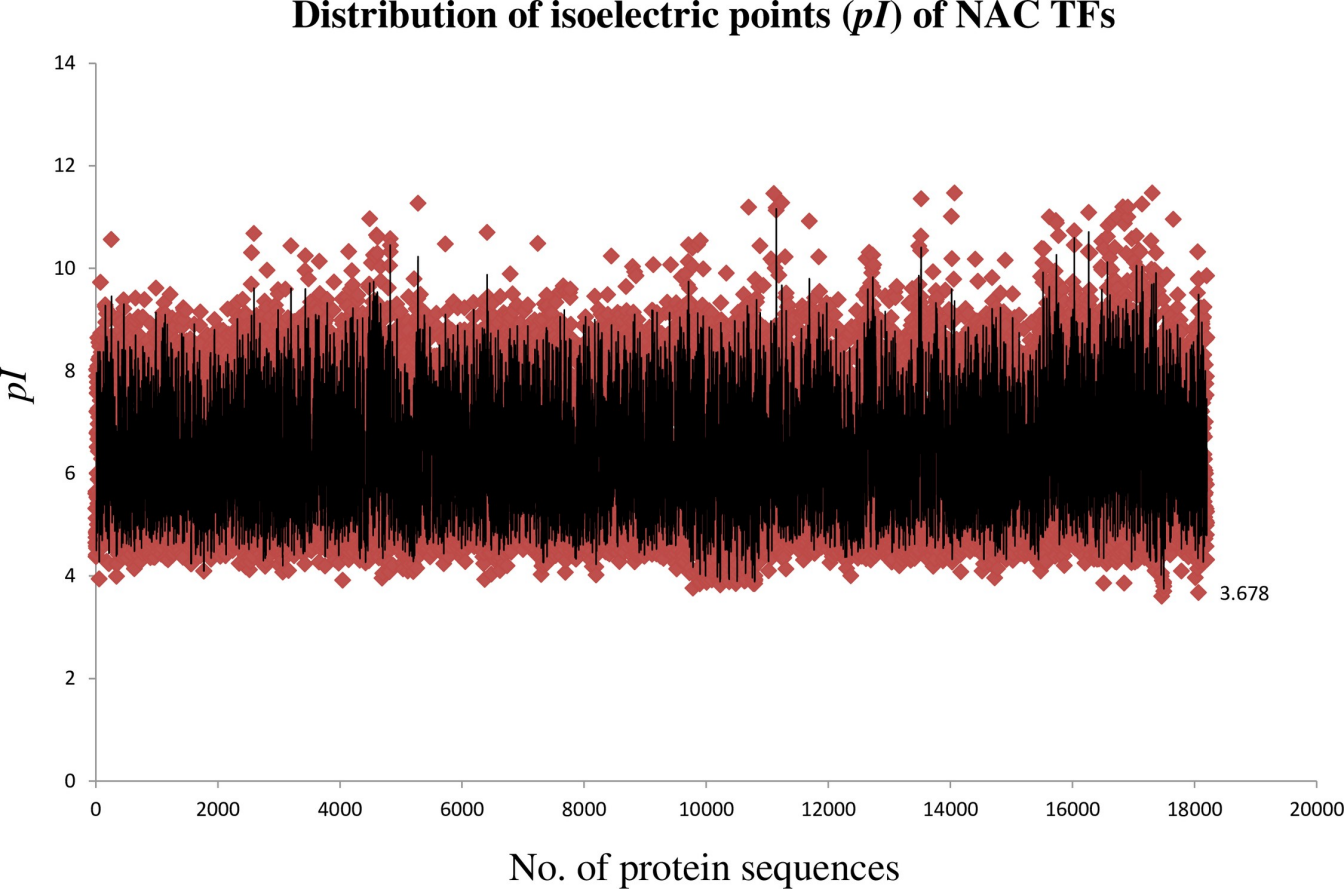
The distribution of the isoelectric point of NAC TFs. The isoelectric point of NAC TFs ranged from pI 3.78 (OB07G17140.1, *Oryza brachyantha*) to pI 11.47 (Sevir.3G242500, *Setaria viridis*). The average isoelectric point of NAC TFs was 6.38. A total of 17158 NAC TFs were utilized in the analysis of the pI of NAC TFs. The analysis of pI was conducted using a protein isoelectric point calculator (http://isoelectric.org/).

The Isoelectric point (pI) of the NAC proteins ranged from 11.47 (Brast01G304500.1.p, (*Brachypodium stacei*) to 3.60 (ObartAA03S_FGP19036, *Oryza barthii*). The majority of the NAC TFs fell within a *pI* rage of 5–8 (Fig 2). Among the 18774 analysed NAC TFs, the *pI* of 99 proteins were ≥ 10. Approximately 69.28% of the NAC TFs had a *pI* that was in an acidic range, whereas the remaining 30.72% had a *pI* within in a basic range. A protein with a pH below the *pI* carries a net positive charge, whereas a protein with a pH above the *pI* carries a net negative charge. The *pI* of a protein determines its transport, solubility, and sub-cellular localization [57–60]. Biomembranes, such as those surrounding the nucleus, are negatively charged; as a result, positively charged (acidic pI) NAC TFs are readily attracted to the nuclear membrane and subsequently transported into the nucleus to function in transcriptional regulation. There are, however, approximately 30.72% NAC TFs that possess a basic *pI*; suggesting that they are localized in the cytosol or plasma membrane of the cell. The major role of the TFs is to bind to specific DNA sequences to regulate transcription. The majority of the proteins have either an acidic or basic *pI* and those with a neutral *pI* close to 7.4 are few because proteins tend to be insoluble, unreactive, and unstable at a pH close to its *pI*. This is the main reason why among the 18774 NAC TFs analysed, only two (XP_010925972.1, *Elaeis guineensis*; Lus10008200, *Linum usitatissimum)* had a *pI* 7.4. The existence of NAC proteins with a *pI* above 10 led us to speculate whether these TFs function while attached to a transmembrane domain. Therefore, additional analyses were conducted to determine if NAC TFs also have the potential to bind to the transmembrane domain or if the NAC TFs with a basic *pI* remain within the cytosol.

### NAC TF proteins are membrane bound

Transcription factors regulate diverse cellular events at transcriptional, translational, and posttranslational levels. They are also involved in nuclear transport and posttranslational modifications. In several cases, TFs are synthesized but remain inactive in the cytoplasm and are only induced into activity through non-covalent interactions [61,62]. TFs are able to remain inactive through their physical association with intracellular membranes and are released by proteolytic cleavage. NAC TFs are a family of proteins whose numbers are in the hundreds in the majority of plant species. The fact that NAC TFs are such a large protein family, it is not surprising that NAC TFs have evolved diverse functional roles. Therefore, it is plausible that NAC TFs may be associated with sub-cellular organelle other than the nucleus to fulfil their diverse functional roles. It is essential, however, to confirm if NAC TFs contain signalling sequences for transmembrane localization. Therefore, we analysed the NAC gene sequences to determine if the signalling sequences present in NAC TFs possess a transmembrane domain.

Results indicated that at least 2190 (8.57%) NAC TFs possess a transmembrane domain (S1 Fig, S1 File). Transmembrane domains were found at both the N- and C-terminal ends of NAC proteins. In the majority of the cases, however, the transmembrane domain was located towards the C-terminal end. Seo et al., (2008) indicated the presence of a transmembrane domain in TFs and suggested that transmembrane domain functions through two proteolytic mechanisms, commonly known as regulated ubiquitin/proteasome-dependent (RUP) and regulated intramenbrane proteolysis (RIP) [63,64]. The bZIP plant TF is present as an integral membrane protein associated with stress response in the endoplasmic reticulum (ER) [65–68]. Studies suggest that the majority of membrane bound TFs are associated with the ER and a membrane bound TF was also found to be involved in cell division [69,70]. At least 10% of the TFs in *Arabidopsis thaliana* have been reported to be transmembrane bound [70]. The collective evidence clearly indicates that membrane-mediated transcriptional regulation is a common stress response and that NAC TFs play a vital role in stress resistance in the ER. Therefore, these membrane-bound NAC TFs can be of great importance for the manipulation of stress resistance using biotechnology.

### NAC TF contain monopartite, bipartite, non-canonical, and nuclear export signal sequences

The import of NAC TFs into the nucleus is mediated by nuclear membrane-bound importins and exportins that form a ternary complex consisting of importin α, importin β1, and a cargo molecule. Importin α serve as an adaptor molecule of importin β1 and recognises the nuclear localization signal (NLS) of the cargo protein needing to be imported. Importin β1 and β2, however, also recognize the NLS directly and bind to the cargo protein. Although the NLS of TFs have been widely studied in the animal kingdom, their study in plants has been more restricted. Therefore, the NLS of NAC TFs was examined in the current study. Results indicate that NAC TFs contain diverse NLS. The NLS were found in the N- and C-terminal regions of NAC TF proteins. Some NAC TFs were found to contain only one NLS whereas other contain multiple NLS. At least 3579 of the total NAC TFs analysed were found to contain either one or multiple NLS (S2 Fig, S2 File). More specifically, 2604 NAC TFs were found to possess only one NLS at the N-terminal end of the NAC protein, whereas 975 were found to possess two NLS, 254 possess three NLS, and 48 were possess four NLS. The NLS were located towards the N-terminal end in the majority of NAC proteins.

NLS motifs are rich in positively charged amino acids and bind to importin α to be imported into the nucleus. The NLS motifs are classified as monopartite or bipartite. A monopartite NLS contains a single cluster of positively charged amino acids and are grouped into two subclasses, class-I and class-II. Class-I possesses four consecutives positively charged amino acids and class-II contains three positively charged amino acids, represented by K(K/R)-x-K/R; where x represents any amino acid that is present after two basic amino acids. Bipartite NLS motifs contain two clusters of positively charged amino acids separated by a 10–12 amino acid linker sequence. Bipartite NLS motifs are characterised by the consensus sequence K-R-P-A-A-T-K-K-A-G-Q-A-K-K-K-K. In addition to monopartite and bipartite NLS motifs, importin α also recognises non-canonical NLS motifs. Non-canonical NLS motifs are longer and considerably variable relative to monopartite and bipartite NLS motifs and are classified as class-III and class-IV NLS. Non-canonical NLS motifs are usually present in the C-terminal end and bind with importin β2. Class-III and class-IV NLS motifs contain K-R-x(W/F/Y)-x_2_-A-F and (P/R)-x_2_-K-R-(K/R) consensus sequences, respectively. We identified at least 1702 unique NLS consensus sequences in the N-terminal region of NAC TFs. The monopartite class I NLS motifs were found to contain more than four consecutive basic amino acids with the number of their consecutive basic amino acids ranging from four to fourteen (K-K-K-K-K-K-K-K-K-K-K-K-K-K-K). The bipartite NLS motifs contain two clusters of consecutive basic amino acids separated by up to twenty-four linker amino acids (K-K-K-x_3_-R- x_2_-R- x_4_-K- x_3_-K- x_3_-K-x-K- x_2_-R-K-K).

The non-canonical NLS motifs contain at least six centrally-located, positively charged amino acids (K-x-R-R-R-P-R-R-x_2_-R-K) flanked by positively charged amino acids on both sides. Our analysis of the N-terminal NLS of NAC TFs, however, did not identify any NAC TFs containing this consensus sequence. Instead, several new variants of this consensus sequence were identified with multiple clusters of positively charged amino acids. These NLS were designated as multipartite NLS motifs (Table 2, S2 Fig, S2 File). Much of the diversity of NLS motifs is associated with the sequence of the variable linker amino acids. In our analysis, we removed the linker amino acid sequences, represented as x, to obtain a more concise picture of NLS diversity. Removing the linker amino acids present in monopartite, bipartite, and multipartite NLS motifs resulted in the identification of 97 different NLS consensus sequences in the N-terminal region of NAC TFs (S2 Fig, S2 File). The R-K-R-R-K consensus sequence was found to be present 347 times, K-K-K 297 times, K-R-K 185 times, K-K-R 165 times, K-R-R 153 times, R-R-R 96 times, R-K-K 95 times, R-K-R 83 times, K-K-K-K 75 times, R-R-K 74 times, R-R-R-R 58 times, K-K-R-K 49 times, K-K-R-K-R 49 times, and K-R-K-R 40 times. At least 27 NLS amino acid consensus sequences were only found once among the 160 studied species (S2 File).

**Table 2:** Putative multipartite nuclear localization signal sequences of NAC transcription factor proteins. The underlined amino acids are designated as NLS and letter x denoted as any amino acid.

C-terminal multipartite NLS	N-terminal multipartite NLS
R-K-R-x-R-x-R-K-K-x_4_-K-x-K-K-K-R-x_3_-K-x_3_-K-K-x_3_-R-R-K-x_2_-K	K-K-K-K-x_7_-K-K-K-K-x_7_-K-K-K-K
R-R-R-x_4_-K-K-x_6_-R-x_2_-R-x_2_-R-R-x_4_-R-R-R-x_6_-R-x_2_-R-R-x_9_-R-R-R-R-R-R-R-x_2_-R-R	K-K-K-K-x-K-x_5_-K-x-K-K-x_7_-K-K-K-K-x_2_-K-K-K
K-K-K-x_4_-K-K-x-K-x_5_-K-x_4_-K-K-K-R-x-K-R-K-x-K-x_4_-K-K-K-R-K-K	K-K-K-x_2_-K-K-x-K-x_5_-K-x_4_-K-K-K-R-x-K-R-K-x-K-x_4_-K-K-K-R-K-K
K-K-R-x_4_-K-x_2_-K-x-K-x_2_-K-K-R-x-R-K-x_4_-K-x_2_-K-x-K-K-R-x-R-K-x_4_-K-x_2_-K-x-K-x-R	K-K-R-x-R-K-x_2_-K-x-K-x_2_-K-K-K-x-RK-x_2_-K-R-R-x_2_-K-K-K-x-R
K-K-R-x-R-K-x_2_-K-x-K-x_2_-K-K-K-x-R-K-x_2_-K-R-R-x_2_-K-K-K-x-R	K-K-R-x-R-K-x_2_-K-x-K-x_2_-K-K-R-x-R-K-x_2_-K-x-K-x_2_-K-K-R-x-R-K-x_2_-K-x_2_-K-x-K-x-R
K-K-R-x-R-K-x_2_-K-x-K-x_2_-K-K-R-x-R-K-x_2_-K-x-K-x_2_-K-K-R	K-x_2_-K-K-K-x_3_-K-K-K-K-K-x-K-x_8_-K-x_9_-K-x_2_-K-K-R-x_2_-K-K-K-K-x-K
R-K-R-x-R-x_3_-K-K-R-R-x_2_-K-x_9_-K-x_4_-R-x-K-x_2_-R-x-R-R-x_5_-K-K-R	K-x_2_-K-K-K-x_3_-K-x-K-K-K-x-K-K-K-x_2_-K-K-K-x-K
R-K-R-x-R-x-R-x_5_-K-x-K-K-K-R-x_3_-K-x_4_-K-R-x_2_-R-R-K	R-K-R-x-R-x-R-K-K-x_2_-K-x-K-K-K-R-x_2_-K-x_2_-KK-x_2_-R-R-K-x_2_-K
R-R-x-R-R-R-x-R-R-x_8_-R-x_6_-R-R-x_5_-R-R-R-x-R-x_5_-R-x_8_-R-R-R-R	R-K-R-x-R-x-R-x_2_-K-x-K-K-K-R-x_2_-K-x4-K-R-x_2_-R-R-K-x-K-x_2_-R
R-R-x-R-R-x-R-x-R-R-R-x_9_-R-x_2_-R-R-K-R-K-x-R-x_4_-R-R-R-R-R-R-x_4_-R-K	
R-x-R-R-R-R-x_6_-R-x_11_-R-x_8_-R-R-x_3_-R-R-R-x_2_-R-R-x-R-x-R-x_6_-R-R-R-R-R-x_4_-R-R-x_2_-R	
R-x-R-R-x_3_-K-R-R-R-x_2_-R-x-R-R-x-R-x-R-x_7_-R-x_3_-R-R-R-x_7_-R-x_2_-R-R-R-R	
R-x-R-x-R-R-R-x_3_-R-R-R-x_3_-R-x-R-x_2_-R-x_4_-R-R-R-x_5_-R-K-x-R-x_3_-R-R- x_13_-R-R-x-K-x_5_-R-R-x_6_-K-R-R	

The C-terminal end of NAC TF proteins also contain monopartite, bipartite, and multipartite NLS motifs (Table 2, S2 Fig, S2 File). Removal of the linker amino acids present in between the consecutive basic amino acids, resulted in the identification of 94 unique consensus sequences. Some of the important NLS found in the C-terminal end were K-K-K (144), K-K-R (83), R-R-R (65), K-R-K (60), K-K-R-K-R (58) and others (Table 2, S2 Fig, S2 File). A comparison of the 97 NLS consensus sequence present in N-terminal region with the 94 NLS sequences present in the C-terminal region indicated that 84 NLS consensus sequences were shared between the N-terminal and C-terminal regions. This indicates that there is a close relationship between the NLS sequences in these two regions. An analysis of the unique NLS consensus sequence in the N-and C-terminal regions indicated that 13 NLS consensus sequences were unique to the N-terminal region whereas nine NLS consensus sequences were unique to the C-terminal region (Table 2, S2 Fig, S2 File). Up to six classes of NLS have been reported to be associated with importin α subunit [71]. To the best of our knowledge, this is the first report describing such a high level of diversity and dynamism in the NLS consensus sequences of NAC TFs and plant transcription factors in general. This is also the first report of the presence of unique NLSs in the N-and C-terminal regions of NAC TFs.

Several nuclear-associated proteins contain NLS, as well as nuclear export signals (NESs). Proteins that perform their function within the nucleus need to be exported out of the nucleus and into the cytoplasm to undergo proteosomal degradation. Therefore, a NES is required in addition to an NLS. A Ran-GTP complex binds directly to an NES and mediates the nuclear export process of the cargo molecules [72]. NES sequences contain a hydrophobic, conserved L-V-F-Y (substitute L-V/I-F-M) motif separated by variable linker amino acids at both ends [73]. The presence of an L-V-F-Y motif in all NAC proteins, suggests that all NAC proteins have the potential to be exported out of the nucleus. Hao et al. (2010), however, reported that the hydrophobic L-V-F-Y motif functions as a transcriptional repressor of WRKY, Dof, and APETALA TFs. If the L-V-F-Y motif (S3 Fig) acts as a transcriptional repressor, then the transcriptional activity of these TFs would be affected; resulting supressed transcriptional activities. Therefore, we feel that the L-V-F-Y motifs might not function as a transcriptional repressor for WRKY, Dof, and APETALA 2 transcription factor. Instead it act as a nuclear export signal sequence as reported by Kosugi et al. (2008) [73].

### NAC TFs possess a complex interactome network

**Fig 3:**
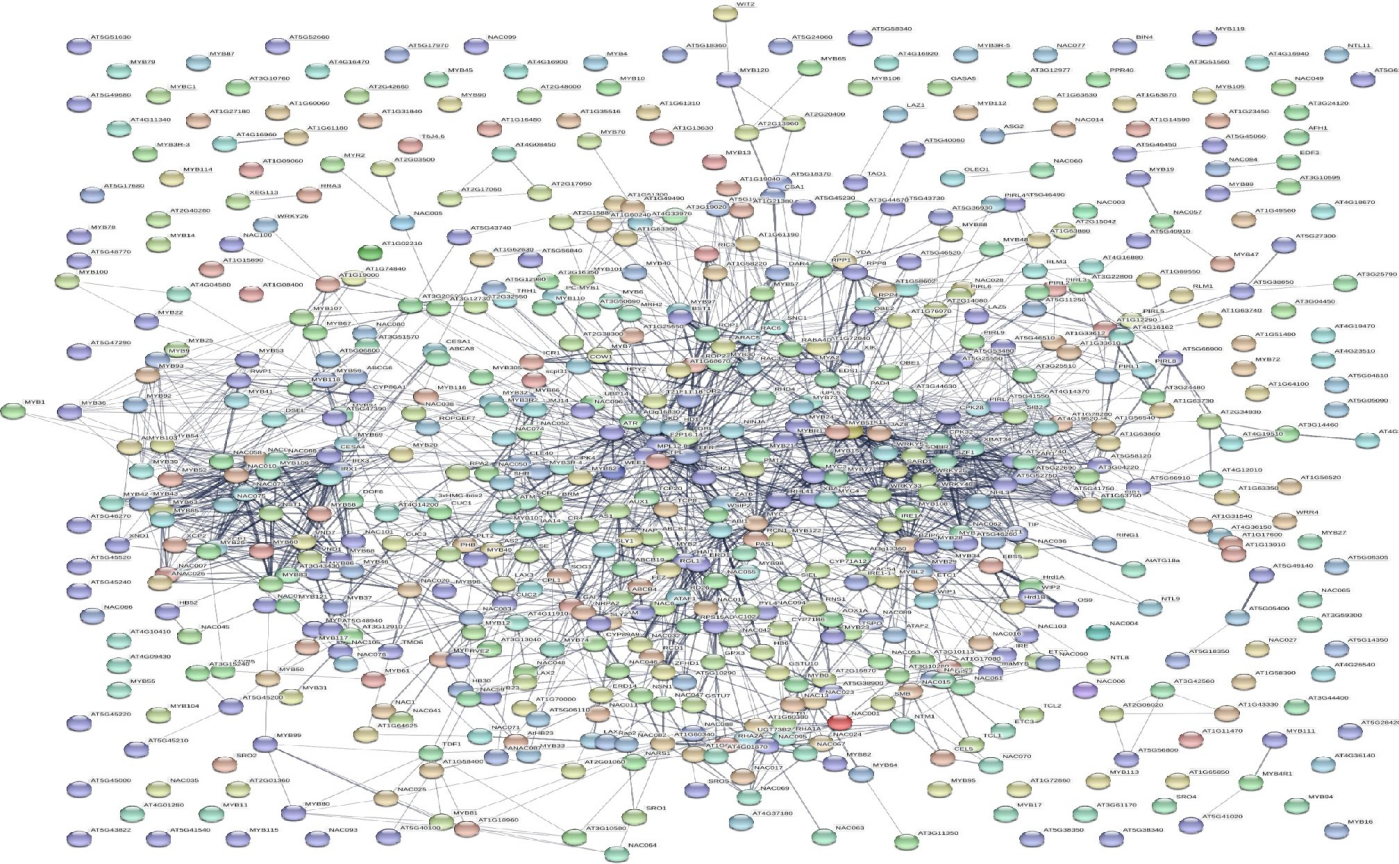
Interactome network of NAC TFs. The interactome network of NAC TF reflects a diverse complex of interacting proteins. The NAC TFs of *A*. *thaliana* were utilized in the interactome network analysis. The interactome map of *A*. *thaliana* was determined using the string database (https://string-db.org).

The interacting partner of a protein can provide significant information about its potential function and an entire protein-protein interactome network can greatly assist in unravelling the signalling cascade of the proteins. Different cascades are interlinked in signalling systems and form intricate constellations that provide information about cell response and function. Thus, the interactome network of NAC TFs in *A*. *thaliana* were explored. The presence of a dynamic network was revealed, and a diverse set of interacting protein partners of NAC TFs were identified (Fig 3, Table 3). The NAC TFs frequently interact with *ABI* (*ABSCISIC ACID INSENSITIVE*), *VND7* (*VASCULAR RELATED NAC DOMAIN*), *MYB* (*MYELOBLASTOSIS*), *DREB2A* (*DEHYDRATION RESPONSIVE ELEMENT BINDING*), *DREB2G*, *WRKY*, *JMJ* (*JUMONJI*), *LEA* (*LATE EMBRYOGENESIS ABUNDANT*), *KNAT* (*KNOX TAIL*), *CUC* (*CUP SHAPED COTYLEDON*), *MC5* (*METACASPASES 5*) and other important genes involved in plant growth, development, and stress responses (Table 3). In addition, NAC TFs was also found to interact with other NAC TFs as well (Table 3).

**Table 3:** Interacting partners of NAC TFs in plants. *A*. *thaliana* NAC TFs was used to construct the interactome network. Asterisk indicates no interaction.

NAC TFs	Experimental Interactions	Co-expression	Text mining Interactions
NAC1	RNS1, AT3G10260, AT1G17080		NAC024, NAC095, ARV1, AT2G01410, AT1G60380, AT1G60340
NAC2	ERD14	NAC32, NAC102, DREB2A	NAC32, NAC102
NAC3	***	****	NTL
NAC4	***	****	NTL, PLP transferase
NAC5	****	****	CYP96A2, MYB
NAC7	VND7	XCP1, XCP2	VND7, MYB46
NAC8	***	ATM, ATR	ATM, ATR
NAC10	***	MYB83, MYB63	MYB83, MYB85, MYB46, MY63, MYB58, MYB52, MYB69, KNAT
NAC11	****	****	NAC95
NAC12	*	IRX1	MYB46, MYB83, MYB58, MYB63, IRX9, APL, KNAT7
NAC13	RCD1	AOX1A, RCD1	AOX1A, RCD1, NAC88
NAC14		ASG2	HB4, LZF1, NTL, BZIP61, MYB30, RSW3
NAC16			NYE, NYC1, EEL, ABF2, PAP20, UTR1, TAG1
NAC17			TAG1, UTR1, UTR3, WRKY15, RGF6, FRU, AOX1A, NTL
NAC18	GAI		NAM, NAC
NAC19	ZFHD1, TCP20, CPL1, TCP8, NAC32, RHA1A, RHA2A	NAC32, ERD1	ZFHD1, TCP20, CPL1, TCP8, NAC32, RHA1A, RHA2A, ERD1
NAC20	AT3G43430, SHR, PHB, PLT2, MYB59, HB23, HB30	TMO6, DOF6, SHR, PLT2	TMO6, DOF6, SHR, PLT2, AT1G64620, AT3G43430
NAC23	****	*****	NAC95, AT3G01030, AT5G27880, AT5G01860, MYB64
NAC24	****	*****	NAC95, NAC47
NAC25	****	At1g75910, GRP20, CYP86C4	At1g75910, GRP20, CYP86C4
NAC26	VND7	VND7, MYB83, XCP1, AT4G08160	VND7, MYB46, MYB85, MYB83, XCP1
NAC028	*****	*******	TOM2A, TOM2B, TOM3, ARLA1C, ARLA1D, DBP1, PDLP2, OBE2
NAC29	NAC6, GRL, IAA14,	NAC6, HAI1	NAC6, HAI1, SAG12, PI
NAC32	HAI1, NAC019, ABI1, NAM, RVE2, PYL4	ATAF1, HAI1, NAC019, GSTU7,	NAC102, NAM, NAC19ATAF1
NAC36	*****	AT5G52760, XBAT34, AT5G52750, SOBIR1, RING1, WRKY53, WRKY46, SARD1,	AT5G42050
NAC38	BRM	MYB69, CIPK4, ABCA8	AT4G29770, AIP2, SDE3
NAC40			NTL, MEE59, NPX, SCP2, SCO1, PUB18, PUB19, LB20
NAC41	NAC83	NAC83, AT1G12810	NAC83, GSTF3, AT1G12810
NAC42	****	CYP71A12, GSTU10, AT5G38900, CYP71B6	CYP71A12, GSTU10, AT5G38900
NAC44	****	****	AT1G54890, NAC90
NAC45	HB52, NAC97	NAC97	CYP71B34, WAK5, NAC97
NAC46	RCD1, BRM	CYP89A9, AT4G11910	RCD1, AT1G78040, bHLH11,
NAC47	***	HAI1, Rap2.6L, NAC6	NAC5, NAC24, HAI1, AT1G60380
NAC48	****	*****	CYP89A9, STAY-GREEN2
NAC49	****	*****	ERF115, WOX5, LBD19
NAC50	JMJ14, NAC052, GAI, TPL	NAC52, JMJ14	JMJ14, PPR, NAC52, AT5G41650, CYP71A25
NAC52	JMJ14, NAC50	JMJ14, PPR, UBP14	JMJ14, NAC50, PPR, CRCK2, PPD6, MFDX1, CYP71A25
NAC53	****	BZIP60, UGT73B, DREB2A, MYB27	NTL, PUM4, MYB103,
NAC55	ZFHD1, HAI1, F2P16.14	ERD1, AT2G31945, MYB2	ZFHD1, ERD1, HAI1, ABF2, bZIP, MYC2
NAC57	*****	*****	MYB19, AT3G58090, AT1G07730, AT4G13580, AT3G13650
NAC58	*****	RWP1, ABCG6, CYP86A1	PPR, RWP1, ABCG6, MYB86, MYB26
NAC60	****	ABI4, DREB2G, WOX12	NACA5, NTL, SCP2, SCO1, ZFP3, GRF7
NAC61	****	NAC90, ACS4,	NAC44, LEA, NAC85, NAC95, NAC90,
NAC62	****	BZIP60, CZF, WRKY33, TIP, SZF1, CPK32, CPK28, TET8,	BZIP60, WRKY33, TIP
NAC63	*****	******	LRR, NAC95, ATPMEPCRD,
NAC64	*****	*****	AT3G59880, AT5G50540, AT2G44010, sks16, SKS6
NAC66	*****	*****	MYB26, MYB46, MYB83, MYB85, MYB63, MYB58, KNAT7, WRKY12
NAC67	*****	****	NAM, AT1G78040, NAC95
NAC68	*****	BZIp60, NAC62	NTL, LPP gamma, LINC2, DEG9, S1P, ENODL17, RPL23AB
NAC69	****	NAC95	NTL, IAA30, RIN3, SPT16, RLP18
NAC71	****	WNK, TM6, AT1G64625	Rap2.6L, AT2G41870, RAP2.4
NAC73	****	MYB46, MYB83, IRX1, IRX3, CESA4	MYB46, MYB83, IRX1, IRX3, MYB63, CESA4
NAC74	F2P16.14, TOPLESS, BRM	DSEL, scpl31, HXXXD type	SCRL20, F-ox/LLR, sks11
NAC75	*****	RING/U-box	GATA5, LBD15, GATA12, JLO, scpl48, RNS3, EIF3E, SHM7
NAC76	VND7, NAC83	****	VND7, NAC83, UBQ, MYB46
NAC77	******	******	DOT5, NAC23, LBD10, NF-YB7, MYB84, GRF5, GRF7, RR8
NAC78	******	PIP-3	NTL, MAYB27, MYB103, PUM4, KNAT2, KNAT6, SUF4, GH9B8
NAC80	BRM	*****	PPR, TT7, 4CL3, BRM
NAC82	SRO1, RCD1	*****	UBX, WW
NAC83	VND7, NAC41, CUC2, VND1, NAC105, NAC76, NAC101, NAC1	*****	VND7, NAC41, CUC2, VND1, NAC105, NAC76, MYB83, MYB46
NAC84	****	EDF3	ZFP10, Delta9, EDF3, SPT16, GS1
NAC85	****	****	LEA, PUP4, NAC90, NAC61, XERO1
NAC87	****	****	SWAP, WRKY36, TIR-NBS, NBS-LRR, BHLH11
NAC88	****	****	UBC18, NAC17, NAC13, NAC53
NAC89	VAP27-1, TSPO, TI1,	*****	BZIP28, BZIP60, MC5
NAC90	*****	AT3G57460, MPK11	DTA4, CHI, NAC44, NAC85, LEA
NAC94	*****	*****	MC5, D111, RML, BAG6, LCAT3, AATP1, BZIP28
NAC95	*****	NAC24, NAM	NAC23, NAM, NAC24, MAY64, NAC69
NAC96	T21F11.18	*****	ABF2, Dna-J, TOPLESS,
NAC97	NAC45, LRR, BRM	*****	******
NAC100	*****	*****	AT4G27850, AT1G26410, GRP20, TT7, 4CL3,
NAC101	RPA2, VND7, VR-NAC, NAC83	*****	NVD7, NAC83, XCP1, UBQ, RNS3
NAC102	****	ATAF1, tolB, NAC32, RHL41, ZAT6, UGT73B2	ATAF1, NAC32
NAC103	****	*****	BZIP60, BZIP28, D111, CLPTM1, NAC44
NAC105	VND7, NAC83,	*****	VND7, GH, NAC83, UBQ, LAC1, MYB46, RIC4

The expression of several of *NAC* genes are either up- or down-regulated by auxin, ethylene, or ABA, suggesting that NAC TFs play a role in plant hormonal signalling [74–76]. One of the most challenging aspects of a protein-protein interactome network is that the interaction can vary depending upon the cell and its environment [77]. Therefore, it is necessary to investigate the dynamic interactions of proteins in different cells and environmental conditions to completely understand their interacting partner and the cellular function of the TF. NAC TFs regulate *ERD* and *NCED* (ABA biosynthesis) genes through a direct interaction with their promoters [78,79]. NAC TFs (ANAC019, ANAC055, and ANAC072) interact with *ERD1* which encodes a Clp protease regulatory subunit [80]. The overexpression of one of these three NAC TFs, however, did not induce the up-regulation of *ERD1* because the induction of *ERD1* depends on the co-expression of a zinc finger homeodomain TF, ZFHD1 [80]. ANAC019 and ANAC055 interact with ABI (abscisic acid insensitive), and at least five MYB TFs can bind to the NAC TF promoter region [81,82]. In this case, the NAC DNA binding domain mediates the interaction with RHA2A and ZFHD1 [82].

### NAC TFs encodes chimeric proteins and contain multiple binding sites

**Fig 4:**
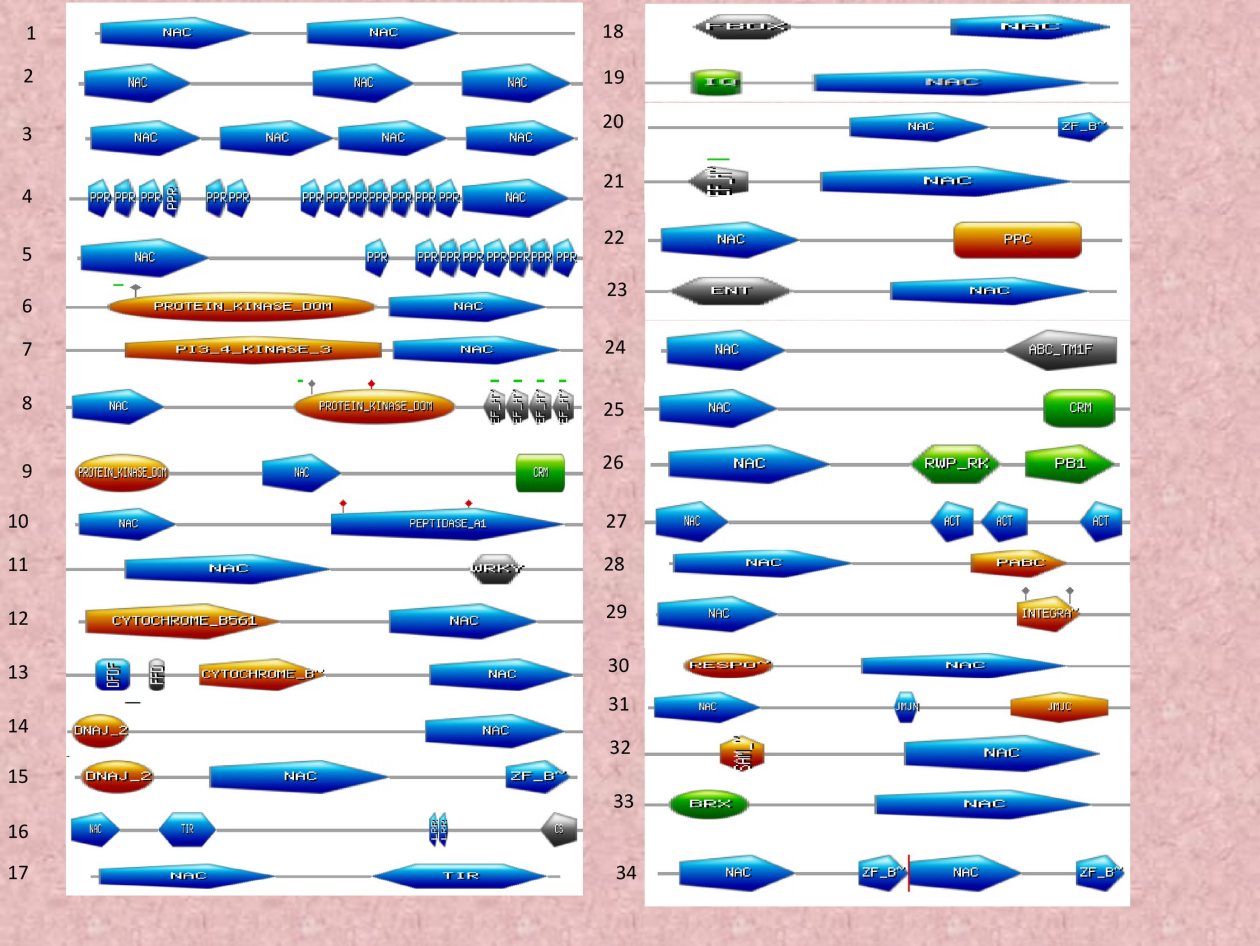
Chimeric NAC domains. NAC TFs possess chimeric NAC domains with at least 34 diverse chimeric NAC domains identified in the studied species. (1) two NAC domain (2) three NAC domain (3) four NAC domain (4) 13 PPR repeats followed by a NAC (5) NAC domain followed by eight PPR repeats (6) protein kinase domain followed by NAC (7) PI3_kinase_3 domain followed by NAC (8) NAC domain followed by kinase and EF-hand domain (9) protein kinase domain followed by NAC and CRM domain (10) NAC domain followed by peptidase A1 domain (11) NAC domain followed by WRKY domain (12) cytochrome B561 domain followed by NAC (13) two DFDF domain followed by cytochrome B and NAC (14) DNA_J2 domain followed by NAC (15) DNA_J2 domain followed by NAC and ZF_B domain (16) NAC domain followed by a TIR, two LRR and a CS domain (17) NAC followed by TIR domain (18) F-box domain followed by NAC (19) IQ domain followed by NAC (20) NAC domain followed by ZF_B domain (21) EF-hand domain followed by NAC (22) NAC domain followed by PPC domain (23) ENT domain followed by NAC (24) NAC domain followed by ABC_TM1F domain (25) NAC domain followed by CRM domain (26) NAC domain followed by RWP_RK and PB1 domain (27) NAC domain followed by three ACT domain (28) NAC domain followed by PABC domain (29) NAC domain followed by INTEGRA domain (30) RESPO domain followed by NAC (31) NAC domain followed by JMJN and JMJC domain (32) SAM domain followed by NAC (33) BRX domain followed by NAC and (34) repeat of NAC and ZF_domain. The identification of chimeric NAC domain sequences was determined using the ScanProsite and InterProScan server. The details regarding the presence of chimeric NAC TF in different taxa can be found in S1 Table.

**Fig 5:**
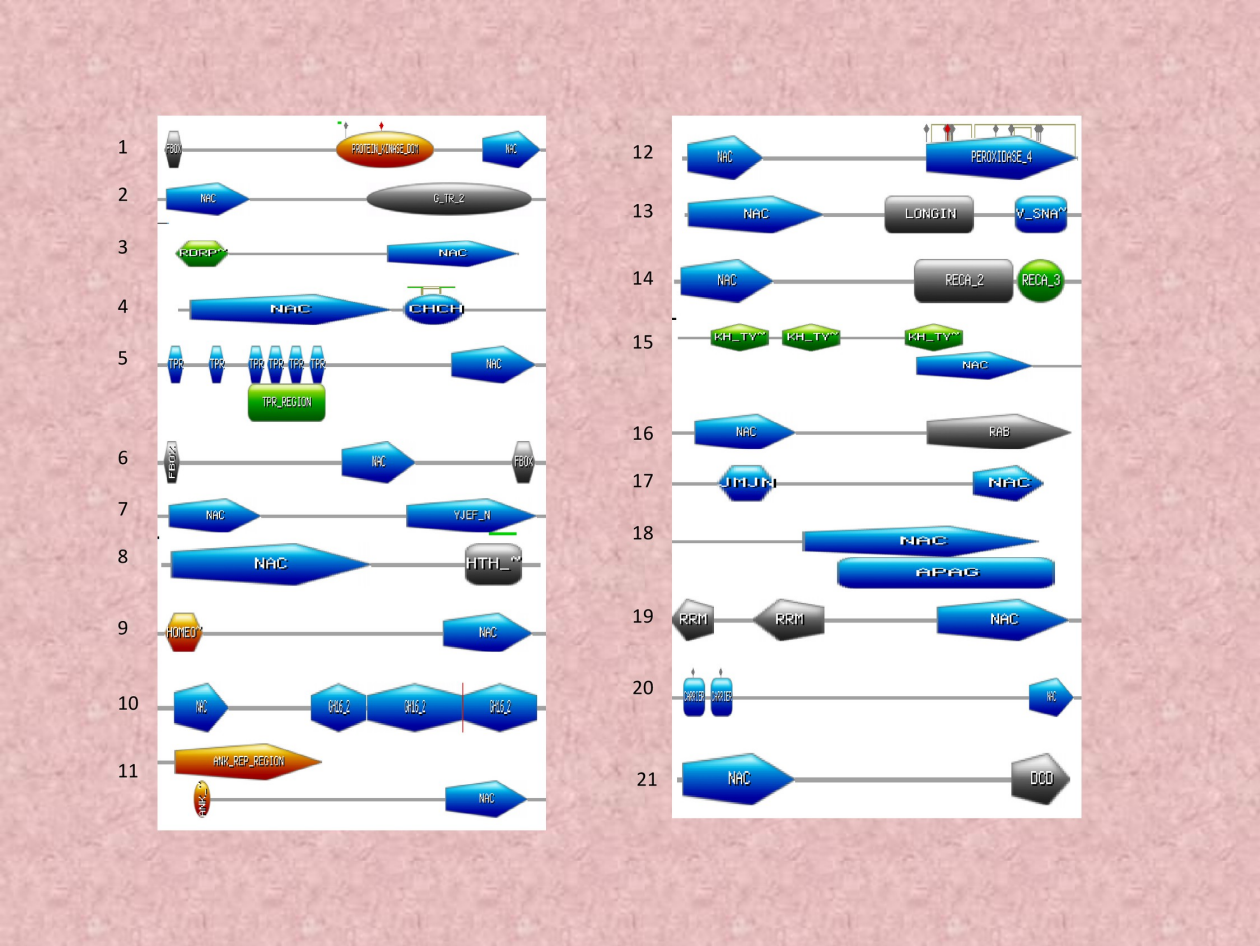
Chimeric NAC domains NAC TFs possess chimeric NAC domains with at least 21 diverse chimeric NAC domains identified in the studied species. (1) F-box domain followed by protein kinase and NAC domain (2) NAC domain followed by G_TR_2 domain (3) RDRP domain followed by NAC (4) NAC domain followed by CHCH domain (5) TPR repeats followed by NAC domain (6) F-box domain followed by NAC and F-box domain (7) NAC domain followed by YJEF_N domain (8) NAC domain followed by HTH domain (9) Homeobox domain followed by NAC domain (10) NAC domain followed by three GH6.2 domain (11) ANK repeat domain followed by NAC domain (12) NAC domain followed by peroxidase domain (13) NAC domain followed by LONGIN and V_SNA domain (14) NAC domain followed by RECA_2 and RECA_3 domain (15) KH_TY repeats followed by NAC domain (16) NAC domain followed by RAB domain (17) JMJN domain followed by NAC domain (18) NAC domain followed by APAG domain (19) two RRM domain followed by NAC domain (20) carrier domain followed by NAC domain and (21) NAC domain followed by DCO domain. The identification of chimeric NAC domain sequences was determined using the ScanProsite and InterProScan server. The details regarding the presence of chimeric NAC TF in different taxa can be found in S1 Table.

NAC TFs are characterised by the presence of a DNA binding domain. Several NAC TFs, however, contain more than one NAC domain. Chimeric NAC TFs have also been identified. At least 45 variants of chimeric NAC TFs were identified in our analysis (Fig 4). Several of the NAC TFs were also found to possess as many as three or four NAC DNA binding domains. Furthermore, the NAC domains were found to be associated with PPR (pentatricopeptide), protein kinase, PI3_4_kinase_3, EF-hands (elongation factor), CRM, peptidase A1, WRKY, cytochrome B561, OFOF, FFO, Dna_J2, ZF_B, TIR, LRR, CS, F-box, IQ, PPC, ENT, ABC_TM1F, RWP_RK, PB1, PABC, ACT, INTEGRA, RESPO, JMJC, SAM, BRX, G_TR_2, RORP, CHCH, TPR, YJEF_N, HTH, HOMEO, GH16, ANK_REP_REGION, Peroxidase, LONGIN, V_SNA, RECA_2, KH_TY, APAG, RRM, carrier, and a DCO domain. At least four NAC TFs from *A*. *thaliana*, ten from *B*. *napus*, four from *B*. *rapa*, two from *M*. *domestica*, four from *P*. *virgatum*, 17 from *C*. *sativa*, eight from *D*. *oligosanthes*, eight from *E*. *tef*, and five from *L*. *perrieri* were found to possess 2 NAC domains (S1 Table). NAC TFs in several other species were also found to contain two NAC domains (S1 Table). When two NAC domains were present, both domains were located towards the N-terminal end. NAC TFs of at least three species, *O*. *rufipogon*, *B*. *stacei*, and *Camelina sativa* were found to possess three NAC domains whereas the NAC TFs in *A*. *lyrata* (gene id: 338342), *C*. *sativa* (Csa16g052260.1), and *E*. *tef* (462951506) were found to possess four NAC domains (Fig 4). Other chimeric domains were also identified in different regions of the NAC protein (Fig 4). The F-box and protein kinase domain was followed by a NAC domain and the NAC domain was followed by a G_TR_2 domain (Fig 5).

The presence of chimeric domains within NAC TFs is of particular interest, especially for understanding why they are there and how they impact the function of a specific NAC TF. The most common domains, such as PPR, TIR, WRKY, protein kinase, ZF_B, EF-hands, cytochrome B, DNAJ, F-box, peroxidase, and GH16 are involved in diverse cellular processes, including transcriptional regulation of plant development and stress response [83–91]. The association of a TIR domain with an NBS-LRR domain is an example of the association of TF domains with other domains to form chimeric proteins [92]. The presence of different domains with the NAC domain could potentially enable the NAC domain to assist in the function of the associated domains and vice versa. For example, NAC TFs could have the potential to regulate peroxidase by possessing a peroxidase domain within the NAC TF, instead of regulating it separately with another TF. The presence of multiple domains can enable the co-regulation of diverse functional sites within the NAC TFs. The presence of chimeric TFs has been recently reported in WRKY TFs as well [93,94]. Therefore, the presence of chimeric domains in NAC TFs can impart a significant dynamic aspect to the ability of NAC TFs to regulate gene expression.

In addition to the presence of multiple chimeric domains, NAC TFs were also found to contain diverse active/binding motifs for several other proteins. It is possible that NAC TFs may play a dual role as a transcription factor and as an enzyme. At least 404 NAC TFs were found to possess other functional motifs comprising 101 unique functional sequences (S2 Table). Some of the highly abundant functional motifs of NAC TFs were 7,8-dihydro-6-hydroxymethylpterin-pyrophosphokinase signature, aldehyde dehydrogenase glutamic acid active site, lipocalin signature, phosphopantetheine attachment site, cysteine protease inhibitor, ATP synthase alpha and beta subunit signature, aminotransferase class II-pyridoxal-phosphate attachment site and others (S2 Table). This is the first study to report the presence of such a diverse number of functional sites and signature motifs in NAC TFs. Although majority of the functional domains are associated with a specific function in plants, the presence of a histocompatibility complex and a translationally controlled tumour protein (TCTP) sequence are of very interesting. These proteins are specifically found in animal systems and the histocompatibility complex is the major contributing factor regulating the binding of antigens. More specifically, TCTP is a highly conserved protein that is involved in microtubule stabilization, calcium binding, and apoptosis and is associated with the early growth phase of tumours [95]. The presence of MHC and TCTP in association with NAC domains suggests that this combination may be playing a crucial role in the plant immune system and in uncontrolled cell growth. The presence of diverse functional sites in NAC TFs indicates that NAC TFs are involved in diverse cellular functions and metabolic pathways. This statement is supported by the large number of NAC TFs that are present in plant genomes.

### NAC TFs are involved in diverse cellular processes

NAC TFs are known to possess diverse chimeric domains, as a result, it is more than likely that NAC TFs are also involved in the regulation of diverse cellular pathways and cellular processes. To help substantiate this premise, the interactome associated with NAC TFs in *A*. *thaliana* was analysed. Results indicated that NAC TFs are potentially involved in a least 289 different cellular processes and pathways (S3 Table). The majority are related to cell, tissue, and organ (root, stem, meristem) development, as well as signalling processes. Several NAC TFs also appear to be associated with phytohormone signalling, including auxin, gibberellin, jasmonic acid, and salicylic acid signalling pathways. NAC TFs were also found to be associated with pathways involved in the response to bacterial, fungal, UV, heat and other biotic and abiotic stresses (S3 Table). At least 202 genes in the NAC TF interactome network were found to be associated with pathways related to the nucleus, 239 were associated with intracellular membranes, and 241 were associated with intracellular organelles, 20 with the endoplasmic reticulum, and 3 with the nuclear matrix. If the association is designated based on the description of a pathway, 127 genes were found to be associated with transcription factor activity and sequence-specific DNA binding, 143 with DNA binding, 146 with nucleic acid binding, 220 with organic cyclic compound binding, 220 with heterocyclic compound binding, 65 with ATP binding, 49 with macromolecular complex binding, 48 with chromatin binding, 35 with ADP binding, 25 with sequence-specific DNA binding, 18 with transcription regulatory region binding, 8 with structural constituents of the cell wall, 11 with auxin transport activity, 2 with LRR binding, and 2 with bHLH transcription factor binding. These data clearly indicate that NAC TFs are involved in diverse cellular processes. The identification of LRR protein in the pathway description of NAC TFs agrees with the presence of an LRR domain in a chimeric NAC domain of NAC TFs.

### NAC TFs are expressed in a spatiotemporal manner

**Fig 6:**
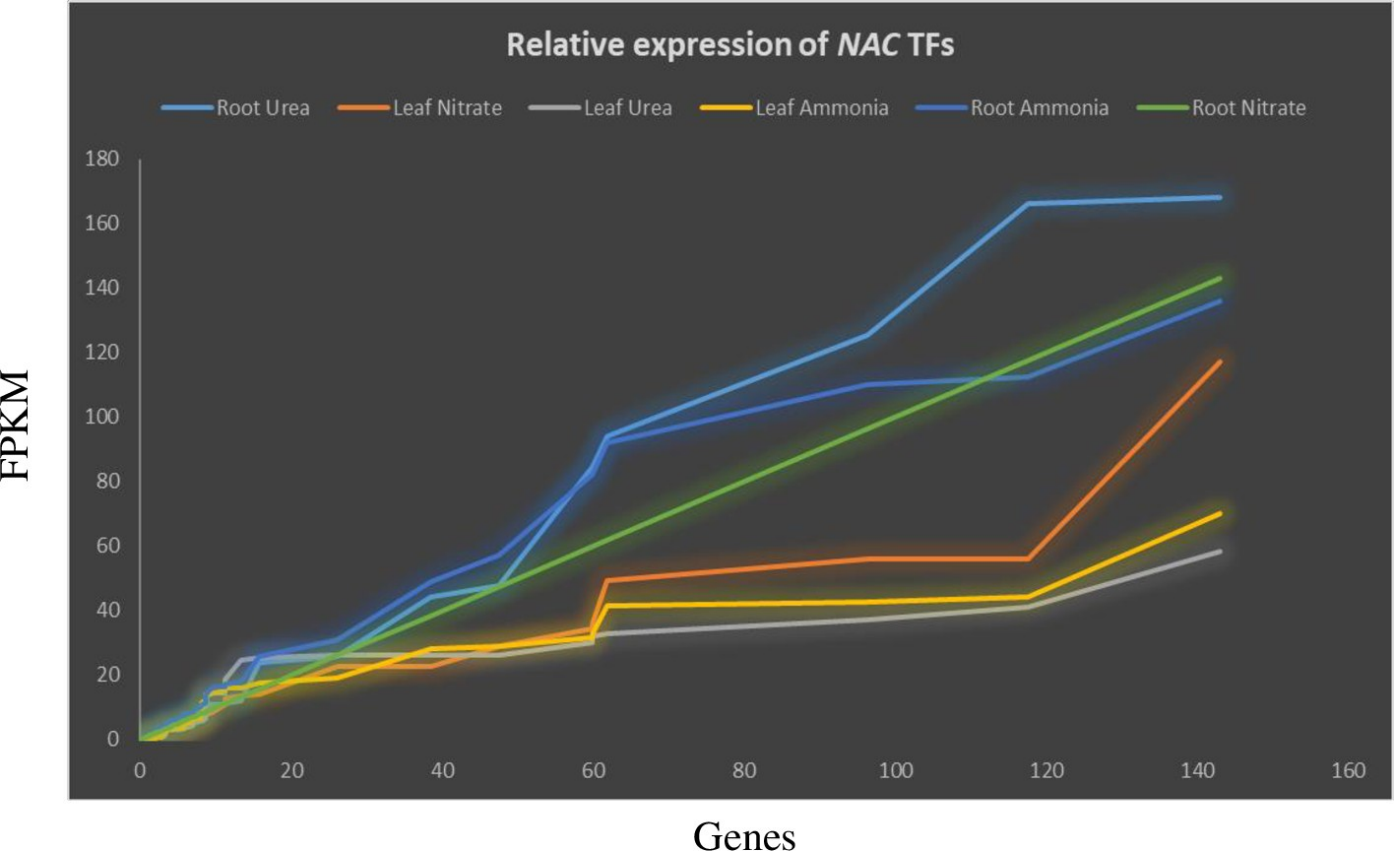
Differential expression of NAC TFs in leaves and roots of *A*. *thaliana* plants treated with ammonia, nitrate, and urea. The expression of *A*. *thaliana NAC* TFs was analysed to determine their response to different sources of nitrogen. Urea and ammonia in root tissue show higher expression level whereas urea treated leaf tissue showed low level of NAC expression. The expression data were obtained from the PhytoMine database in Phytozome and presented as FPKM (Fragments per Kilobase of transcripts per million mapped reads). The X-axis represents the *NAC* TF genes and Y-axis represent the Fragments per Kilobase of transcripts per million mapped reads.

Plant uses ammonia, nitrate, and urea as the source of nitrogen for its growth and development. Nitrogen is also associated with an increased rate of photosynthesis. Therefore, the role of ammonia source in the growth and development of the plants is very important. Nitrate is readily available as nitrogen source for plants and the uptake of nitrate is high in the acidic pH whereas the uptake of ammonia is high in the neutral pH. Studying the expression pattern of *NAC TFs* in nitrate and ammonia treated plant can explains how different nitrogen source modulate the expression of *NAC TFs* and give the glimpse of their role in plants growing in the acidic and neutral pH soil. Urea is applied as an artificial nitrogen sources for the plants when there is a lack of nitrate or ammonia in the soil. Therefore, patterns of *NAC TF* gene expression were analysed in leaf and root tissues of *A*. *thaliana* treated with ammonia, nitrate, or urea (Fig 6). Among a total of 120 *NAC* TFs, 95, 97, and 98 were differentially expressed in leaf tissue treated with ammonia, nitrate, or urea, respectively. Leaf tissues treated with ammonia, nitrate and urea exhibited 70.14, 117.11, and 58.35 FPKM expression values for *AtNAC1* (AT1G01010.1), *AtNAC4* (AT1G02230.1), and *AtNAC1* (AT1G01010.1), respectively. At least 46 genes in leaves exhibited expression of more than one FPKM in response to ammonia, 54 in response to nitrate, and 44 in response to urea. *AtNAC1* was highly expressed in ammonia and urea treated leaves. At least 24, 26, and 25 *NAC* TFs did not exhibit any expression in leaf tissues treated with ammonia, nitrate, or urea. The *AtNAC1* is involved in auxin signaling and modulates lateral root formation [74,96,97]. The higher expression of *AtNAC1* with response to treatment of nitrogenous compound reflects it role in plant development. *AtNAC4* is reported to be involved in nitrate transport and its higher expression in nitrate treated plant directly indicate its active role nitrogen transport and assimilation [98].

Relative to leaf tissues, the expression of *NAC* TFs in root tissues was more dynamic. Root tissue treated with urea exhibited the highest expression of NAC TFs relative to leaves treated with ammonia or nitrate (Fig 6). The number of *AtNAC* TFs whose expression was one or more FPKM in response to ammonia, nitrate, or urea were 75, 71, and 70, respectively. *AtNAC8* (AT5G08790.1) was highly expressed in ammonia-treated roots, whereas, *AtNAC91* (AT5G24590.2) was highly expressed in nitrate- and urea-treated roots. Urea, ammonia and nitrate (UAN) commonly serve as a source of nitrogen (N) for plants. Analysis of the levels of gene expression indicate that ammonia and nitrate modulate the expression of *NAC* TFs more than urea. A study utilizing *Pinus taeda* revealed that fertilization with ammonium, nitrate, or urea produces different effects on growth and drought tolerance [99]. Results of the current analysis indicate that *AtNAC8* and *AtNAC91* are the major *NAC* TFs involved in nitrogen assimilation during plant growth. The TaNAC8 was reported to be associated with strip rust and abiotic stress responses [100,101].

### Codon usage in NAC TF is dynamic

Codon usage bias in NAC TFs of the examined species were studied. separately. Among 61 sense codons, only 14 were found in the all species. These included AAG (K), ACU (R), AGA (R), AGG (R), UCU (S), AUC (I), AUG (M), CAA (Q), CCU (P), GAA (E), GCU (A), GGA (G), UGG (0), and UUC (F) (Table 4). The most abundant codon was UCU (S), which was found 30 times in in *Humulus lupulus* NAC TFs (Table 4). The codons CGA (R), CGC (R), CGG (R), CGU (R) were absent in 127 of the 160 examined species. ACG (T), UCG (S), CAG (Q), CAC (H), CCA (P), CCC (P), CCG (P), and GCG (A) were absent in 126 of the examined species (S4 File). The highest relative synonymous codon usage bias (RSCU) was found to be 1.35, 1.23, 1.29 for the codon AAA (K) in *Ocimum tenufolium*, *Picea sitchensis*, and *Ipomea trifida*. Synonymous codon-usage was not observed in NAC TFs. Relative codon usage is determined by dividing the ratio of observed frequency of codons by the expected frequency, provided that all of the synonymous codons for the same amino acids are used equally. Relative Synonymous Codon Usage (RSCU), however, is not related to the usage of amino acids. An RSCU > 1 indicates the occurrence of codons more frequently than expected, while an RSCU < 1 indicates that the codon occurs less frequently than expected [102,103]. Non-synonymous substitution in organisms is subject to natural selection [104,105]. Genes with lower non-synonymous selection leads to functional diversity of a gene. The presence of a low level of nonsynonymous codon usage in NAC TFs indicates that they are functional and have evolved from paralogous ancestors.

**Table 4:** Codon usage of NAC TFs in plants.

Codons	Codon present in No. of species	Codon absent in No. of species	Average abundance of codons	Highest no. of codons	Name of the species with highest no. of codons
AAA (K)	126	20	4.77	9.9	*Glycine soja*
AAG (K)	146	0	10.75	24.2	*Sphagnum fallax*
AAC (N)	144	2	3.66	14.2	*Beta vulgaris*
AAU (N)	127	19	9.25	20.5	*Spinacia oleracea*
ACA (T)	139	7	2.33	15.2	*Citrus sinensis*
ACC (T)	137	9	2.4	17	*Amborella trichopoda*
ACG (T)	20	126	5.91	13	*Dorcoceras hygrometricum*
ACU (T)	146	0	7.42	16.6	*Sesamum indicum*
AGA (R)	146	0	10.92	24.3	*Klebsormidium flaccidum*
AGG (R)	146	0	4.12	18.8	*Amborella trichopoda*
CGA (R)	19	127	5.22	13.9	*Linum usitatissimum*
CGC (R)	19	127	2.47	6	*Linum usitatissimum*
CGG (R)	19	127	3.93	8.6	*Citrullus lanatus*
CGU (R)	19	127	2.06	4.7	*Linum usitatissimum*
AGC (S)	143	3	3.54	24.2	*Beta vulgaris*
AGU (S)	144	2	1.83	5.2	*Dorcoceras hygrometricum*
UCC (S)	141	5	4.51	12.3	*Aegilops tauschii*
UCG (S)	20	126	2.64	6.4	*Dorcoceras hygrometricum*
UCU (S)	146	0	4.65	30.5	*Humulus lupulus*
UCA (S)	139	7	5.09	15.1	*Morus notabilis*
AUA (I)	124	22	4.80	15.3	*Sphagnum fallax*
AUC (I)	146	0	5.10	16.7	*Sphagnum fallax*
AUU (I)	126	20	8.71	15.9	*Spinacia oleracea*
AUG (M)	146	0	7.81	22.8	*Sphagnum fallax*
CAA (Q)	146	0	5.31	15.4	*Fragaria vesca*
CAG (Q)	20	126	13.3	22.6	*Linum usitatissimum*
CAC (H)	20	126	6.64	10.9	*Beta vulgaris*
CAU (H)	144	2	4.45	9.7	*Setaria viridis*
CCA (P)	20	126	11.09	16.3	*Dorcoceras hygrometricum*
CCC (P)	20	126	14.18	19.2	*Amborella trichopoda*
CCG (P)	20	126	5.10	11.1	*Dorcoceras hygrometricum*
CCU (P)	146	0	8.00	24.7	*Klebsormidium flaccidum*
CUA (L)	143	3	5.83	28.3	*Sphagnum fallax*
CUC (L)	123	23	5.74	23.6	*Sphagnum fallax*
CUG (L)	142	4	5.87	43.9	*Sphagnum fallax*
CUU (L)	145	1	5.94	32.6	*Sphagnum fallax*
UUG (L)	125	21	5.94	24.4	*Sphagnum fallax*
UAA (L)	124	22	5.37	17.2	*Sphagnum fallax*
GAA (E)	146	0	4.62	27	*Klebsormidium flaccidum*
GAG (E)	145	1	5.54	18.1	*Sphagnum fallax*
GAC (D)	145	1	5.05	14.9	*Beta vulgaris*
GAU (D)	144	2	5.86	21.7	*Spinacia oleracea*
GCA (A)	135	11	5.49	18.5	*Citrus sinensis*
GCC (A)	130	16	5.05	15	*Amborella trichopoda*
GCG (A)	20	126	4.64	11.2	*Dorcoceras hygrometricum*
GCU (A)	146	0	4.65	31.1	*Setaria viridis*
GGA (G)	146	0	4.63	27.5	*Setaria viridis*
GGC (G)	141	5	5.41	17.1	*Amborella trichopoda*
GGG (G)	145	1	2.7	6.7	*Elaeis guineensis*
GGU (G)	145	1	2.40	5.9	*Elaeis guineensis*
GUA (V)	140	6	1.46	3.5	*Sphagnum fallax*
GUC (V)	123	23	0.93	2	*Morus notabilis*
GUG (V)	142	4	4.35	11.8	*Beta vulgaris*
GUU (V)	143	3	5.38	16.6	*Klebsormidium flaccidum*
UAC (Y)	138	8	3.84	10.1	*Morus notabilis*
UAU (Y)	126	20	6.23	14.8	*Solanum melongena*
UGG (W)	147	0	3.89	14.5	*Vitis vinifera*
UGC (C)	143	3	5.14	15.6	*Oropetium thomaeum*
UGU (C)	145	1	3.9	9.6	*Zoysia matrella*
UUC (F)	146	0	4.60	25.4	*Picea glauca*
UUU (F)	126	20	10.67	19.2	*Sphagnum fallax*

### Rate of transition of NAC TFs is higher than the rate of transversion

Nucleotide mutation is an integral part of the evolution of a genome and leads to the acquisition of required traits and the elimination of detrimental traits from the genome. It is a regular process and hundreds of thousands of nucleotides have undergone addition or deletion events in the evolution of a genome. The alteration or conversion of a nucleotide occurs either through a transition or a transversion. A transition event involves the interchange of two-ring purines (A and G) or of one-ring pyrimidines (C and T). Transversion events the exchange of a purine for a pyrimidine or vice versa. The rate at which these two events occur is important to understanding of the evolution of a gene. Therefore, the rate of nucleotide substitution in NAC TFs was analysed. Results indicated that the rate of transition in NAC TFs is higher than the rate of transversion. The substitution of adenine with guanine was found to be highest in *Linum usitatissimum* (15.82), while the substitution of guanine to adenine was found to be the highest in *Lotus japonicas* (19.07). The lowest rate of substitution from adenine to guanine and vice versa was found in *Trifolium pratense* (9.73) and *Amborella trichopoda* (10.8), respectively (S4 Table). The highest rate of substitution from thiamine to cytosine and vice versa was found in *Klebsormidium flaccidum* (7.19) and *Pseudotsuga menziesii* (11.59), respectively. The lowest rate of substitutions from thiamine to cytosine and vice versa was found in *Capsella grandiflora* (2.41) and *Cicer arietinum* (1.62), respectively (S4 Table). These data make it evident that the rates of transition of purine (adenine and guanine) nucleotides are higher than the rates of pyrimidines. The highest rate of transversion from adenine to thiamine and vice versa was found in *Capsella grandiflora* (12.34 for adenine to thiamine and 9.91 for thiamine to adenine) (S4 Table). The rate of substitution by transversion is slower relative to the rate of substitution by transition.

*Capsella grandiflora* is a close relative of *Arabidopsis thaliana* and is predicted to be the progenitor of *Capsella bursa-pastoris*. *Capsella grandiflora* is a self-pollinating plant and is used as a model organism in evolutionary studies and the change from self-incompatibility into self-compatibility. The genomic consequences of the evolution of selfing, however, is poorly understood. *Capsella rubella*, a close relative of *Capsella grandiflora*, that evolved self-compatibility 200,000 years ago [106] also exhibits a high rate of transversion from adenine to thiamine (11.19). Thus, the higher rate of transversion from adenine to thiamine in *Capsella grandiflora* and *Capsella rubella* may be a possible factor in the evolution of self-pollination. Higher rates of transversion were also found in *Solanum pimpinellifolium* (11.4) and *Castanea mollissima* ((11.31) Chinese chestnut). *Solanum pimpinellifolium* is self-pollinating and exhibits high levels of stress tolerance [107]. *Castanea mollissima* has evolved over a period of time in coexistence with chestnut blight and is resistant to the pathogen. This indicates that higher rates of transversion from adenine to thiamine and vice versa are associated with self-pollination and stress tolerance in plants. The highest rate of substitution from guanine to cytosine and vice versa was found in *Arachis hypogaea* (11.07), and *Camelina sativa* (11.46), respectively (S4 Table). The lowest rate of substitution from adenine to thiamine and vice versa was found in *Linum usitatissimum* (3.72) and *Klebsormidium flaccidum* (6.67), respectively. Notably, the highest rate of substitution from thiamine to cytosine was found in *Klebsormidium flaccidum* and the highest rate of substitution from adenine to guanine was found in *Linum usitatissimum*. This indicates that organisms which exhibit the highest rate of transition possess the lowest rate of transversion.

### NAC TFs evolved from orthologous ancestors

**Fig 7:**
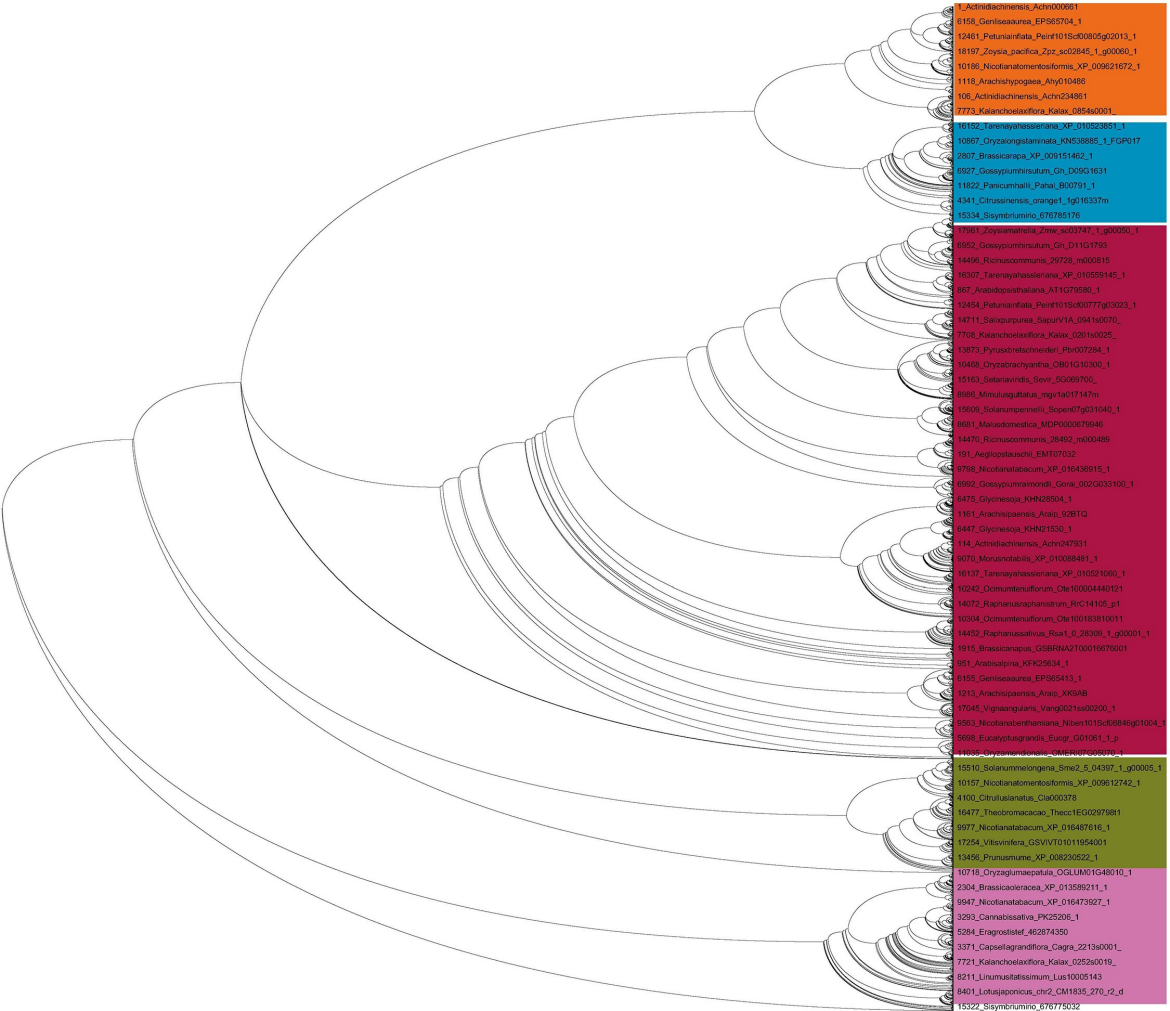
Phylogenetic tree of NAC TFs. A phylogenetic tree of NAC TF reveals the presence of seven clustered orthologous groups (COGs). Each group also possesses two or more sub-groups. The phylogenetic tree shows lineage (monocot/dicot) specific grouping of NAC TFs. The phylogenetic tree was constructed using the neighbour-joining method with 1000 bootstrap replicates.

A phylogenetic tree of NAC TFs was constructed to understand their evolutionary relationships. A model selection was conducted before constructing the phylogenetic tree using the maximum likelihood statistical method. The phylogenetic tree revealed the presence of at least seven phylogenetic clustered orthologous groups (COGs) originating from a common, orthologous ancestor (Fig 7). Each phylogenetic cluster was further divided into two or more sub-groups. A phylogenetic tree of each individual species was subsequently constructed to examine the duplication and loss events in NAC TFs. The phylogenetic tree of each species was independently reconciled with the collective species tree. This analysis indicated that NAC TFs in all of the species were duplicated and none of a NAC TFs was found to be lost. This suggest that NAC TFs evolved from common ancestors (orthology) and underwent numerous duplication events during the divergence and speciation (paralogy) events, which gave rise to diverse gene functions in plant development and growth. The NAC TFs of *K*. *flaccidum* might be the most possible common ancestors of some plant species and the NAC TFs of other algal species could have contributed towards the evolution of other NAC TFs in plants. If the duplication would have disrupted the normal functioning of the cell, the organism might have reduced its reproductive fitness and would have been died. However, the duplication of NAC TFs possesses beneficial character thus providing the fitness advantage. Gene duplication contribute to the evolution that provides new genetic content for mutation, selection, and drift to act and to create new evolutionary opportunities [108]. Genome duplication is a common event in plants and multiple event of genome duplication have occurred during the diversification of angiosperms [109]. Genome duplication sometimes followed by the increased rate of evolution of some important genes [109]. The duplicated genes is responsible for the functional divergence and may play role in escaping the extinction [109,110]. In addition, duplication can lead to decreased probabilities of extinction, increase genetic variation, mutational robustness, and tolerance to changing environmental conditions [109]. The genetic variation incurred by duplication contribute to selection pressure and provide the opportunities for survival diverse environmental stress. Being, NAC TFs are highly duplicated, they might be providing such genetic variability in the plant kingdom to evade diverse environmental responses.

We also checked for the presence of potential foreign or homologous sequences (xenologs) in NAC TFs. No primary xenologs, sibling donor xenologs, sibling recipient xenologs, incompatible xenologs, autoxenologs, or paraxenologs were identified in NAC TFs. Although the phylogenetic tree indicates the evolution NAC TFs from common ancestors, none of the NAC genes in the examined species were found to have been transferred from one species to another. Previous studies of NAC TFs in six plant species also reported a high level of duplication and divergent evolution [111]. The expansion of TF families was associated with an increase in the structural complexity of the organism [112]. Previous studies reported the lineage-specific grouping of transcription factors [93,111]. The phylogenetic tree of NAC TFs also revealed the presence of lineage-specific clustering as well. In a few cases, however, order-specific clustering of NAC TFs was also observed. For example, NAC TFs in dicot species of the Brassica lineage, including *A*. *thaliana*, *A*. *halleri*, *B*. *napus*, *B*. *rapa*, *R*. *sativus*, *R*. *raphanistrum*, *C*. *rubella*, *A*. *alpine*, and others, grouped together. Similarly, NAC TFs in monocot plant species, including *O*. *sativa*, *O*. *nivara*, *B*. *distachyon*, and others, also grouped together.

## Conclusion

NAC TFs are present in higher plants, as well as in a few species of algae. The number of NAC TFs per genome and their structural and functional properties increased with the complexity of the organism. The algae *Klebsormidium flaccidum*, a charophyte, was also found to possess NAC TFs; suggesting that the evolution of NAC TFs was associated with the adaptation of plant life from an aquatic to a terrestrial form. The paralogous evolution of NAC TFs underlies their diverse functional role in plant growth and development. Duplication events in NAC TFs were greater than deletion events and the absence of any loss of NAC TFs in different plant species indicates their evolution in recent times. As NAC TFs play a pivotal role within the nucleus regulating gene expression, the presence of bipartite and multipartite nuclear localization signals is of particular interest and provides the basis for further investigation of their functional roles.

## Supporting information

S1 Table Supplementary table showing different chimeric domains of NAC TFs. (DOCX)

S2 Table NAC TFs showing the presence of novel functional domain along with NAC domains. (PDF)

S3 Table NAC TFs showing their involvement in different pathways and biological process. (PDF)

S4 Table Substitution rate of NAC TFs of plants. (DOCX)

S1 File Accession number of transmembrane domains containing NAC TF proteins. (XLSX)

S2 File Nuclear localization signal sequences of NAC TFs. Sheet 1 of the file show all the raw N-terminal NLS consensus sequences, unique NLS with linker amino acids, and unique NLS post removal of linker amino acids. Sheet 2 represents the number of occurrences of N-terminal NLS and sheet 3 represents C-terminal NLS, number of occurrences, C-terminal unique NLS, and N-and C-terminal unique NLS.
(XLSX)

S3 File Accession number and species details of NAC TF proteins containing multi-functional binding sites. (XLSX)

S4 File Details of codon usage of NAC TFs in plants. (XLSX)

S1 Fig Transmembrane bound NAC TF proteins. (XZ)

S2 Fig Graphical presentation of nuclear localization signal sequences of NAC TF proteins. (XZ)

S3 Fig The presence of L-V-F-Y/H conserved motif in NAC TFs of plants (*A*. *thaliana*). (RAR)
