# Supplementary material for: Genomics, molecular and evolutionary perspective of NAC transcription factors
Source: PLoS One. 2020 Apr 10;15(4):e0231425. doi: 10.1371/journal.pone.0231425 (PMC7147800; doi:10.1371/journal.pone.0231425)
Supplement: S2 Table — (PDF) [file pone.0231425.s002.pdf]

Supplementary Table 2

NAC TFs showing the presence of novel functional domain along with NAC domains.

NAC TFs	Species	Active site
Pavir.Ha00781	*Panicum virgatum*	*Aldehyde dehydrogenases cysteine active site*
Carubv10005139m	*Capsella rubella*	*Aldehyde dehydrogenases glutamic acid active site*
491912	*Arabidopsis lyrata*	*Aldehyde dehydrogenases glutamic acid active site*
GSBRNA2T00058100001	*Brassica napus*	*Aldehyde dehydrogenases glutamic acid active site*
GSBRNA2T00078811001	*Brassica napus*	*Aldehyde dehydrogenases glutamic acid active site*
XP_013638893.1	*Brassica oleracea*	*Antenna complexes beta subunits signature*
Prupe.2G196600.1.p	*Prunus persica*	*ATP synthase alpha and beta subunits signature*
Pavir.J01198	*Panicum virgatum*	*Cysteine proteases inhibitors signature*
GSMUA_Achr9P20400_001	*Musa acuminata*	*Endopeptidase Clp serine active site*
ACHN289231	*Actinidia chinensis*	*Heavy-metal-associated domain*
LOC_Os01g47670	*Oryza sativa*	*Inorganic pyrophosphatase signature*
Thhalv10009374m	*Eutrema salsugineum*	*Inorganic pyrophosphatase signature*
Niben101Scf07168g02012.1	*Nicotiana benthamiana*	*Lipocalin signature*
XP_011088938.1	*Sesamum indicum*	*Pancreatic trypsin inhibitor (Kunitz) family signature*
KHN08560.1	*Glycine soja*	*Putative AMP-binding domain signature*
MLOC_75795.1	*Hordeum vulgare*	*Zinc carboxypeptidases, zinc-binding region 2 signature*
Vradi01g03390.1	*Vigna radiata*	*2Fe-2S ferredoxin-type iron-sulfur binding region signature*
FANhyb_rscf00000665.1.g00005.1	*Fragaria ananassa*	*2-oxo acid dehydrogenases acyltransferase component lipoyl binding site*
XP_010916182.1	*Elaeis guineensi*	*4Fe-4S ferredoxin-type iron-sulfur binding domain profile*
XP_010916183.1	*Elaeis guineensi*	*4Fe-4S ferredoxin-type iron-sulfur binding domain profile*
GSMUA_Achr11P03780_001	*Musa acuminata*	*7,8-dihydro-6-hydroxymethylpterin-pyrophosphokinase signature*
GSMUA_Achr11P17510_001	*Musa acuminata*	*7,8-dihydro-6-hydroxymethylpterin-pyrophosphokinase signature*
GSMUA_Achr6P36840_001	*Musa acuminata*	*7,8-dihydro-6-hydroxymethylpterin-pyrophosphokinase signature*
GSMUA_Achr7P06640_001	*Musa acuminata*	*7,8-dihydro-6-hydroxymethylpterin-pyrophosphokinase signature*

GSMUA_Achr8P11590_001	*Musa acuminata*	*7,8-dihydro-6-hydroxymethylpterin-pyrophosphokinase signature*
NNU_008894-RA	*Nelumbo nucifera*	*7,8-dihydro-6-hydroxymethylpterin-pyrophosphokinase signature*
NNU_012069-RA	*Nelumbo nucifera*	*7,8-dihydro-6-hydroxymethylpterin-pyrophosphokinase signature*
Phvul.011G005700	*Phaseolus vulgaris*	*7,8-dihydro-6-hydroxymethylpterin-pyrophosphokinase signature*
PDK_30s1016001g009	*Phoenix dactylifera*	*7,8-dihydro-6-hydroxymethylpterin-pyrophosphokinase signature*
Spipo1G0086600	*Spirodela polyrhiza*	*7,8-dihydro-6-hydroxymethylpterin-pyrophosphokinase signature*
Zosma265g00020	*Zostera marina*	*7,8-dihydro-6-hydroxymethylpterin-pyrophosphokinase signature*
ACHN002691	*Actinidia chinensis*	*7,8-dihydro-6-hydroxymethylpterin-pyrophosphokinase signature*
ACHN093311	*Actinidia chinensis*	*7,8-dihydro-6-hydroxymethylpterin-pyrophosphokinase signature*
ACHN157011	*Actinidia chinensis*	*7,8-dihydro-6-hydroxymethylpterin-pyrophosphokinase signature*
ACHN317511	*Actinidia chinensis*	*7,8-dihydro-6-hydroxymethylpterin-pyrophosphokinase signature*
ACO007609.1	*Ananus comosus*	*7,8-dihydro-6-hydroxymethylpterin-pyrophosphokinase signature*
Aqcoe7G078200.1.p	*Aquilegiacoerulea*	*7,8-dihydro-6-hydroxymethylpterin-pyrophosphokinase signature*
Bv5_097990_fpfn.t1	*Beta vulgaris*	*7,8-dihydro-6-hydroxymethylpterin-pyrophosphokinase signature*
Brast07G026300.1.p	*Brachypodium stacei*	*7,8-dihydro-6-hydroxymethylpterin-pyrophosphokinase signature*
XP_004514529.1	*Cicer arietinum*	*7,8-dihydro-6-hydroxymethylpterin-pyrophosphokinase signature*
XP_010933718.1	*Elaeis guineensi*	*7,8-dihydro-6-hydroxymethylpterin-pyrophosphokinase signature*
Glyma-09G231700.1.p	*Glycine max*	*7,8-dihydro-6-hydroxymethylpterin-pyrophosphokinase signature*
KHN11660.1	*Glycine soja*	*7,8-dihydro-6-hydroxymethylpterin-pyrophosphokinase signature*
Medtr4g036030.1	*Medicago truncatula*	*7,8-dihydro-6-hydroxymethylpterin-pyrophosphokinase signature*
XP_010108028.1	*Morus notabilis*	*7,8-dihydro-6-hydroxymethylpterin-pyrophosphokinase signature*
PDK_30s1016001g009	*Phoenix dactylifera*	*7,8-dihydro-6-hydroxymethylpterin-pyrophosphokinase signature*
Tp57577_TGAC_v2_mRNA3761	*Trifolium pratense*	*7,8-dihydro-6-hydroxymethylpterin-pyrophosphokinase signature*
Vang05g07280	*Vigna angularis*	*7,8-dihydro-6-hydroxymethylpterin-pyrophosphokinase signature*
C.cajan_20892	*Cajanus cajan*	*7,8-dihydro-6-hydroxymethylpterin-pyrophosphokinase signature*
GSVIVT01001264001	*Vitis vinifera*	*7,8-dihydro-6-hydroxymethylpterin-pyrophosphokinase signature*
Aradu.Y1DM8	*Arachis duranensis*	*ABC transporters family signature*
Araip.HYM8C	*Arachis ipaensis*	*ABC transporters family signature*

mrna03833-1-v1-0-hybrid	*Fragaria vesca*	*Adenosine and AMP deaminase signature*
PSME_00042313-RA	*Pseudotsuga menziesii*	*Adenosine and AMP deaminase signature*
Zosma69g00740	*Zostera marina*	*Adipokinetic hormone family signature*
Pavir.8NG107600.1.p	*Panicum virgatum*	*Aldehyde dehydrogenases cysteine active site*
Pavir.8NG113600.1.p	*Panicum virgatum*	*Aldehyde dehydrogenases cysteine active site*
RrC2674_p3	*Raphanus raphanistrum*	*Aldehyde dehydrogenases cysteine active site*
Rsa1.0_00285.1_g00018.1	*Raphanus sativus*	*Aldehyde dehydrogenases cysteine active site*
Araha-3365s0007	*Arabidopsis halleri*	*Aldehyde dehydrogenases glutamic acid active site*
AT4G28530	*Arabidopsis thaliana*	*Aldehyde dehydrogenases glutamic acid active site*
Bra010362	*Brassica rapa*	*Aldehyde dehydrogenases glutamic acid active site*
Bra011037	*Brassica rapa*	*Aldehyde dehydrogenases glutamic acid active site*
Cagra-1225s0038	*Capsella grandiflora*	*Aldehyde dehydrogenases glutamic acid active site*
evm.model.supercontig_80.94	*Carica papaya*	*Aldehyde dehydrogenases glutamic acid active site*
Kalax.0013s0167	*Kalanchoe laxiflora*	*Aldehyde dehydrogenases glutamic acid active site*
Kalax.0154s0005	*Kalanchoe laxiflora*	*Aldehyde dehydrogenases glutamic acid active site*
Thhalv10025606m	*Thellungiella halophila*	*Aldehyde dehydrogenases glutamic acid active site*
Araha.3365s0007.1.p	*Arabidopsis halleri*	*Aldehyde dehydrogenases glutamic acid active site*
KFK29397.1	*Arabis alpina*	*Aldehyde dehydrogenases glutamic acid active site*
XP_013603495.1	*Brassica oleracea*	*Aldehyde dehydrogenases glutamic acid active site*
XP_009109087.1	*Brassica rapa*	*Aldehyde dehydrogenases glutamic acid active site*
XP_009129548.1	*Brassica rapa*	*Aldehyde dehydrogenases glutamic acid active site*
Csa10g014090.1	*Camelina sativa*	*Aldehyde dehydrogenases glutamic acid active site*
Csa11g015230.1	*Camelina sativa*	*Aldehyde dehydrogenases glutamic acid active site*
Csa12g021980.1	*Camelina sativa*	*Aldehyde dehydrogenases glutamic acid active site*
Carubv10005139m	*Capsella rubella*	*Aldehyde dehydrogenases glutamic acid active site*
Thhalv10025606m	*Eutrema salsugineum*	*Aldehyde dehydrogenases glutamic acid active site*
Kalax-0013s0167.1.p	*Kalanchoe marnieriana*	*Aldehyde dehydrogenases glutamic acid active site*
Kalax-0154s0005.1.p	*Kalanchoe marnieriana*	*Aldehyde dehydrogenases glutamic acid active site*

RrC20539_p1	*Raphanus raphanistrum*	*Aldehyde dehydrogenases glutamic acid active site*
Rsa1.0_13977.1_g00001.1	*Raphanus sativus*	*Aldehyde dehydrogenases glutamic acid active site*
GSBRNA2T00104607001	*Brassica napus*	*Aldehyde dehydrogenases glutamic acid active site*
Bv3_050780_usim.t1	*Beta vulgaris*	*Aldo/keto reductase family putative active site signature*
FANhyb_rscf00000207.1.g00009.1	*Fragaria ananassa*	*Aldo/keto reductase family putative active site signature*
Zmw_sc01138.1.g00130.1	*Zoysia matrella*	*Aldo/keto reductase family putative active site signature*
Zpz_sc00095.1.g00090.1.am.mk	*Zoysia pacifica*	*Aldo/keto reductase family putative active site signature*
LOC_Os12g22940	*Oryza sativa*	*Alkaline phosphatase active site*
OMERI12G11070.1	*Oryza meridionalis*	*Alkaline phosphatase active site*
ORUFI12G10910.1	*Oryza rufipogon*	*Alkaline phosphatase active site*
BGIOSGA037293.1	*Oryza sativa Indica*	*Alkaline phosphatase active site*
LOC_Os12g22940.1	*Oryza sativa japonica*	*Alkaline phosphatase active site*
Carubv10012206m	*Capsella rubella*	*Aminoacyl-transfer RNA synthetases class-I signature*
Oropetium_20150105_22096	*Oropetium thomaeum*	*Aminotransferases class-II pyridoxal-phosphate attachment site*
ObartAA03S_FGP10288	*Oryza barthii*	*Aminotransferases class-II pyridoxal-phosphate attachment site*
ORGLA12G0169600.1	*Oryza glaberrima*	*Aminotransferases class-II pyridoxal-phosphate attachment site*
OpuncBB_FGP10000	*Oryza punctata*	*Aminotransferases class-II pyridoxal-phosphate attachment site*
LOC_Os12g43530	*Oryza sativa*	*Aminotransferases class-II pyridoxal-phosphate attachment site*
Oropetium_20150105_22096A	*Oropetium thomaeum*	*Aminotransferases class-II pyridoxal-phosphate attachment site*
ORGLA12G0169600.1	*Oryza glaberrima*	*Aminotransferases class-II pyridoxal-phosphate attachment site*
OGLUM12G21360.1	*Oryza glumaepatula*	*Aminotransferases class-II pyridoxal-phosphate attachment site*
KN539001.1	*Oryza longistaminata*	*Aminotransferases class-II pyridoxal-phosphate attachment site*
OMERI12G14510	*Oryza meridionalis*	*Aminotransferases class-II pyridoxal-phosphate attachment site*
ONIVA12G18630.1	*Oryza nivara*	*Aminotransferases class-II pyridoxal-phosphate attachment site*
OPUNC12G17870.1	*Oryza punctata*	*Aminotransferases class-II pyridoxal-phosphate attachment site*
ORUFI12G21970.1	*Oryza rufipogon*	*Aminotransferases class-II pyridoxal-phosphate attachment site*
BGIOSGA035784.1	*Oryza sativa Indica*	*Aminotransferases class-II pyridoxal-phosphate attachment site*
LOC_Os12g43530.1	*Oryza sativa japonica*	*Aminotransferases class-II pyridoxal-phosphate attachment site*

XP_009146239.1	*Brassica rapa*	*Antenna complexes beta subunits signature*
Zmw_sc05821.1.g00010.1	*Zoysia matrella*	*ArgE / dapE / ACY1 / CPG2 / yscS family signature 1*
Zpz_sc00055.1.g00240.1.am.mkhc	*Zoysia pacifica*	*ArgE / dapE / ACY1 / CPG2 / yscS family signature 1*
Medtr4g134460.1	*Medicago truncatula*	*Aspartate and glutamate racemases signature 1*
XP_010545324.1	*Tarenaya hassleriana*	*Aspartate and glutamate racemases signature 1*
XP_010545325.1	*Tarenaya hassleriana*	*Aspartate and glutamate racemases signature 1*
Brast02G382000.1.p	*Brachypodium stacei*	*Aspartokinase signature*
Brast02G383700.1.p	*Brachypodium stacei*	*Aspartokinase signature*
EMT33859	*Aegilops tauschii*	ATP Binding site and proton acceptor
Gorai.008G130300.1	*Gossypium raimondii*	*ATP synthase alpha and beta subunits signature*
PH01000820G0540	*Phyllostachys heterocycla*	*ATP synthase alpha and beta subunits signature*
Pta007882	*Pinus taeda*	*ATP synthase alpha and beta subunits signature*
ppa020746m	*Prunus persica*	*ATP synthase alpha and beta subunits signature*
Solyc08g008660.2.1	*Solanum lycopersicum*	*ATP synthase alpha and beta subunits signature*
PGSC0003DMP400010296	*Solanum tuberosum*	*ATP synthase alpha and beta subunits signature*
PGSC0003DMP400010297	*Solanum tuberosum*	*ATP synthase alpha and beta subunits signature*
CA11g08290	*Capsicum annuum*	*ATP synthase alpha and beta subunits signature*
Cotton_A_26426_BGI-A2_v1.0	*Gossypium arboreum*	*ATP synthase alpha and beta subunits signature*
XP_010100656.1	*Morus notabilis*	*ATP synthase alpha and beta subunits signature*
PH01000820G0540	*Phyllostachys heterocycla*	*ATP synthase alpha and beta subunits signature*
XP_008233055.1	*Prunus mume*	*ATP synthase alpha and beta subunits signature*
Sme2.5_02517.1_g00007.1	*Solanum melongena*	*ATP synthase alpha and beta subunits signature*
Sopen08g004490.1	*Solanum pennellii*	*ATP synthase alpha and beta subunits signature*
Zmw_sc04326.1.g00020.1	*Zoysia matrella*	*ATP synthase alpha and beta subunits signature*
Ciclev10019282m	*Citrus clementina*	*ATP synthase alpha and beta subunits signature*
Gh_A12G1049	*Gossypium hirsutum*	*ATP synthase alpha and beta subunits signature*
Sopim08g008660.0.1	*Solanum pimpinellifolium*	*ATP synthase alpha and beta subunits signature*
Pbr032225.1	*Pyrus bretschneideri*	*ATP-dependent DNA ligase AMP-binding site*

Pbr032225.1	*Pyrus bretschneideri*	*ATP-dependent DNA ligase AMP-binding site*
Spipo1G0084400	*Spirodela polyrhiza*	*Bacterial regulatory proteins, araC family signature*
MLOC_19933.1	*Hordeum vulgare*	*Beta-ketoacyl synthases active site*
MLOC_19933.1	*Hordeum vulgare*	*Beta-ketoacyl synthases active site*
Thecc1EG018508t	*Theobroma cacao*	*C-5 cytosine-specific DNA methylases active site*
Aqcoe1G058100.1.p	*Aquilegiacoerulea*	*Cadherin domain signature*
KFK30695.1	*Arabis alpina*	*Carbamoyl-phosphate synthase subdomain signature 2*
ObartAA03S_FGP25558	*Oryza barthii*	*Cysteine proteases inhibitors signature*
ORGLA06G0236900.1	*Oryza glaberrima*	*Cysteine proteases inhibitors signature*
OpuncBB_FGP25111	*Oryza punctata*	*Cysteine proteases inhibitors signature*
LOC_Os06g51070	*Oryza sativa*	*Cysteine proteases inhibitors signature*
462845378	*Eragrostis tef*	*Cysteine proteases inhibitors signature*
ORGLA06G0236900.1	*Oryza glaberrima*	*Cysteine proteases inhibitors signature*
OGLUM06G29360.1	*Oryza glumaepatula*	*Cysteine proteases inhibitors signature*
OMERI06G27920.1	*Oryza meridionalis*	*Cysteine proteases inhibitors signature*
ONIVA06G30950.1	*Oryza nivara*	*Cysteine proteases inhibitors signature*
OPUNC06G25510.1	*Oryza punctata*	*Cysteine proteases inhibitors signature*
ORUFI06G29930.1	*Oryza rufipogon*	*Cysteine proteases inhibitors signature*
BGIOSGA020507.1	*Oryza sativa Indica*	*Cysteine proteases inhibitors signature*
LOC_Os06g51070.1	*Oryza sativa japonica*	*Cysteine proteases inhibitors signature*
Pavir.4NG342400.1.p	*Panicum virgatum*	*Cysteine proteases inhibitors signature*
Pavir.7KG085500.1.p	*Panicum virgatum*	*Cysteine proteases inhibitors signature*
Zmw_sc02823.1.g00180.1	*Zoysia matrella*	*Cysteine proteases inhibitors signature*
Zpz_sc01253.1.g00030.1.sm.mk	*Zoysia pacifica*	*Cysteine proteases inhibitors signature*
Zjn_sc00068.1.g03510.1.sm.mk	*Zoysia japonica*	*Cysteine proteases inhibitors signature*
OGLUM01G06950.1	*Oryza glumaepatula*	*Cytochrome P450 cysteine heme-iron ligand signature*
KN539290.1	*Oryza longistaminata*	*Cytochrome P450 cysteine heme-iron ligand signature*
KN539290.1	*Oryza longistaminata*	*Cytochrome P450 cysteine heme-iron ligand signature*

OMERI07G10400	*Oryza meridionalis*	*Cytochrome P450 cysteine heme-iron ligand signature*
Zmw_sc00276.1.g00230.1	*Zoysia matrella*	*Cytochrome P450 cysteine heme-iron ligand signature*
Zmw_sc02343.1.g00060.1	*Zoysia matrella*	*Cytochrome P450 cysteine heme-iron ligand signature*
Zpz_sc03671.1.g00030.1.am.mk	*Zoysia pacifica*	*Cytochrome P450 cysteine heme-iron ligand signature*
Sobic.003G105800.1.p	*Sorghum bicolor*	*Endopeptidase Clp serine active site*
GRMZM2G123246_P01	*Vzea mays*	*Endopeptidase Clp serine active site*
EMT14807	*Aegilops tauschii*	*Eukaryotic and viral aspartyl proteases active site*
Tae025010	*Triticum aestivum*	*Eukaryotic and viral aspartyl proteases active site*
Tae030617	*Triticum aestivum*	*Eukaryotic and viral aspartyl proteases active site*
Tae045052	*Triticum aestivum*	*Eukaryotic and viral aspartyl proteases active site*
EMS63374	*Triticum urartu*	*Eukaryotic and viral aspartyl proteases active site*
Itr_sc004419.1_g00003.1	*Ipomea trifida*	*Eukaryotic and viral aspartyl proteases active site*
Traes_7DL_BDD45DB24.2	*Triticum aestivum*	*Eukaryotic and viral aspartyl proteases active site*
Zjn_sc00092.1.g00060.1.am.mk	*Zoysia japonica*	*Eukaryotic and viral aspartyl proteases active site*
Zmw_sc03455.1.g00010.1	*Zoysia matrella*	*Eukaryotic and viral aspartyl proteases active site*
Zpz_sc03692.1.g00060.1.sm.mk	*Zoysia pacifica*	*Eukaryotic and viral aspartyl proteases active site*
Niben101Scf07508g02004.1	*Nicotiana benthamiana*	*FGGY family of carbohydrate kinases signature 2*
MA_18939g0010	*Picea abies*	*Fumarate lyases signature*
Rsa1.0_01603.1_g00007.1	*Raphanus sativus*	*Fumarate lyases signature*
GSMUA_Achr10P10790_001	*Musa acuminata*	*GHMP kinases putative ATP-binding domain*
Peinf101Scf00337g03028.1	*Petunia inflata*	*Glucoamylase active site region signature*
Cucsa.083540.1	*Cucumis sativus*	*Glyceraldehyde 3-phosphate dehydrogenase active*
ONIVA05G06750.1	*Oryza nivara*	*Glycoprotease family signature*
Cc08_g16900	*Coffea canephora*	*Glycosyl hydrolases family 5 signature*
30147.m014211	*Ricinus communis*	*Glycosyl hydrolases family 9 active sites signature 2*
Kalax-0686s0005.1.p	*Kalanchoe marnieriana*	*Hemopexin domain signature*
Kalax-0609s0006.1.p	*Kalanchoe marnieriana*	*Hemopexin domain signature*
Scf00060.g6196.t1	*Utricularia gibba*	*Histone H4 signature*

XP_016648489.1	*Prunus mume*	*Histone H4 signature*
Prupe.4G138500.1.p	*Prunus persica*	*Histone H4 signature*
Zmw_sc06792.1.g00040.1	*Zoysia matrella*	*Histone H4 signature*
ObartAA03S_FGP17273	*Oryza barthii*	*HMG-I and HMG-Y DNA-binding domain (A+T-hook)*
LOC_Os03g59730	*Oryza sativa*	*HMG-I and HMG-Y DNA-binding domain (A+T-hook)*
Bradi1g04229.1.p	*Brachypodium distachyon*	*HMG-I and HMG-Y DNA-binding domain (A+T-hook)*
Brast02G355100.1.p	*Brachypodium stacei*	*HMG-I and HMG-Y DNA-binding domain (A+T-hook)*
OGLUM03G38320.1	*Oryza glumaepatula*	*HMG-I and HMG-Y DNA-binding domain (A+T-hook)*
OMERI03G35410.1	*Oryza meridionalis*	*HMG-I and HMG-Y DNA-binding domain (A+T-hook)*
ORUFI03G40160.1	*Oryza rufipogon*	*HMG-I and HMG-Y DNA-binding domain (A+T-hook)*
BGIOSGA009581.1	*Oryza sativa Indica*	*HMG-I and HMG-Y DNA-binding domain (A+T-hook)*
LOC_Os03g59730.1	*Oryza sativa japonica*	*HMG-I and HMG-Y DNA-binding domain (A+T-hook)*
Pbr020079.1	*Pyrus bretschneideri*	*Immunoglobulins and major histocompatibility complex proteins signature*
EcC046909.20	*Eucalyptus camaldulensis*	*Immunoglobulins and major histocompatibility complex proteins signature*
EcS504758.10	*Eucalyptus camaldulensis*	*Immunoglobulins and major histocompatibility complex proteins signature*
Pbr020079.1	*Pyrus bretschneideri*	*Immunoglobulins and major histocompatibility complex proteins signature*
ORGLA01G0211000.1	*Oryza glaberrima*	*Inorganic pyrophosphatase signature*
Pbr026815.1	*Pyrus bretschneideri*	*Inorganic pyrophosphatase signature*
Thhalv10009374m	*Thellungiella halophila*	*Inorganic pyrophosphatase signature*
XP_010108843.1	*Morus notabilis*	*Inorganic pyrophosphatase signature*
ORGLA01G0211000.1	*Oryza glaberrima*	*Inorganic pyrophosphatase signature*
OGLUM01G29620.1	*Oryza glumaepatula*	*Inorganic pyrophosphatase signature*
OMERI01G23440.1	*Oryza meridionalis*	*Inorganic pyrophosphatase signature*
ONIVA01G29020.1	*Oryza nivara*	*Inorganic pyrophosphatase signature*
ORUFI01G28690.1	*Oryza rufipogon*	*Inorganic pyrophosphatase signature*

LOC_Os01g47670.1	*Oryza sativa japonica*	*Inorganic pyrophosphatase signature*
Pbr026815.1	*Pyrus bretschneideri*	*Inorganic pyrophosphatase signature*
GSVIVT01026495001	*Vitis vinifera*	*Iron-containing alcohol dehydrogenases signature 1*
OB07G24770.1	*Oryza brachyantha*	*Legume lectins beta-chain signature*
ORGLA03G0364100.1	*Oryza glaberrima*	*Legume lectins beta-chain signature*
OGLUM03G39450.1	*Oryza glumaepatula*	*Legume lectins beta-chain signature*
OGLUM03G39510.1	*Oryza glumaepatula*	*Legume lectins beta-chain signature*
KN538833.1	*Oryza longistaminata*	*Legume lectins beta-chain signature*
OMERI03G36390.1	*Oryza meridionalis*	*Legume lectins beta-chain signature*
OMERI03G36430.1	*Oryza meridionalis*	*Legume lectins beta-chain signature*
ONIVA03G41430.1	*Oryza nivara*	*Legume lectins beta-chain signature*
ONIVA03G41480.1	*Oryza nivara*	*Legume lectins beta-chain signature*
OPUNC03G36710.1	*Oryza punctata*	*Legume lectins beta-chain signature*
OPUNC03G36760.1	*Oryza punctata*	*Legume lectins beta-chain signature*
BGIOSGA013858.1	*Oryza sativa Indica*	*Legume lectins beta-chain signature*
BGIOSGA013866.1	*Oryza sativa Indica*	*Legume lectins beta-chain signature*
LOC_Os03g61249.1	*Oryza sativa japonica*	*Legume lectins beta-chain signature*
LOC_Os03g61319.1	*Oryza sativa japonica*	*Legume lectins beta-chain signature*
Nta011914	*Nicotiana tabacum*	*Lipocalin signature*
Niben101Ctg05879g00002.1	*Nicotiana benthamiana*	*Lipocalin signature*
Niben101Scf01337g00001.1	*Nicotiana benthamiana*	*Lipocalin signature*
Niben101Scf08665g00007.1	*Nicotiana benthamiana*	*Lipocalin signature*
Niben101Scf13438g00001.1	*Nicotiana benthamiana*	*Lipocalin signature*
Niben101Scf15279g00002.1	*Nicotiana benthamiana*	*Lipocalin signature*
XP_009762991.1	*Nicotiana sylvestris*	*Lipocalin signature*
XP_009763090.1	*Nicotiana sylvestris*	*Lipocalin signature*
XP_009782335.1	*Nicotiana sylvestris*	*Lipocalin signature*
XP_009792222.1	*Nicotiana sylvestris*	*Lipocalin signature*

XP_009794230.1	*Nicotiana sylvestris*	*Lipocalin signature*
XP_016449500.1	*Nicotiana tabacum*	*Lipocalin signature*
XP_016469037.1	*Nicotiana tabacum*	*Lipocalin signature*
XP_016474545.1	*Nicotiana tabacum*	*Lipocalin signature*
XP_016477162.1	*Nicotiana tabacum*	*Lipocalin signature*
XP_016490755.1	*Nicotiana tabacum*	*Lipocalin signature*
XP_016504577.1	*Nicotiana tabacum*	*Lipocalin signature*
XP_016510726.1	*Nicotiana tabacum*	*Lipocalin signature*
XP_009598748.1	*Nicotiana tomentosiformis*	*Lipocalin signature*
XP_009601217.1	*Nicotiana tomentosiformis*	*Lipocalin signature*
XP_009602879.1	*Nicotiana tomentosiformis*	*Lipocalin signature*
XP_009628837.1	*Nicotiana tomentosiformis*	*Lipocalin signature*
KFK42489.1	*Arabis alpina*	*Mannitol dehydrogenases signature*
OB09G21580.1	*Oryza brachyanta*	*N-6 Adenine-specific DNA methylases signature*
Sobic.003G110900.1.p	*Sorghum bicolor*	*N-6 Adenine-specific DNA methylases signature*
Zosma53g00110	*Zostera marina*	*N-6 Adenine-specific DNA methylases signature*
Aqcoe2G375500.1.p	*Aquilegiacoerulea*	*N-6 Adenine-specific DNA methylases signature*
scaffold07459-snap-gene-0.9	*Castanea mollissima*	*N-6 Adenine-specific DNA methylases signature*
OB04G27030.1	*Oryza brachyantha*	*N-6 Adenine-specific DNA methylases signature*
OB09G21580.1	*Oryza brachyantha*	*N-6 Adenine-specific DNA methylases signature*
Csa16g023250.1	*Camelina sativa*	*Neutral zinc metallopeptidases, zinc-binding region signature*
Ote100091280041	*Ocimum tenuiflorum*	*Neutral zinc metallopeptidases, zinc-binding region signature*
Neem_8133_f_4	*Azadirachta indica*	*Nt-dnaJ domain signature*
FANhyb_rscf00000018.1.g00013.1	*Fragaria ananassa*	*Nt-dnaJ domain signature*
EPS67213.1	*Genlisea aurea*	*Peroxidases active site signature*
Ciclev10008619m	*Citrus clementina*	*pfkB family of carbohydrate kinases signature 1*
Pavir.J21162	*Panicum virgatum*	*pfkB family of carbohydrate kinases signature 2*
Thhalv10006408m	*Thellungiella halophila*	*pfkB family of carbohydrate kinases signature 2*

Thhalv10006408m	*Eutrema salsugineum*	*pfkB family of carbohydrate kinases signature 2*
Zpz_sc00266.1.g00320.1.am.mk	*Zoysia pacifica*	*Phospholipase A2 histidine active site*
GSMUA_Achr11P08970_001	*Musa acuminata*	*Phosphopantetheine attachment site*
ObartAA03S_FGP27339	*Oryza barthii*	*Phosphopantetheine attachment site*
ORGLA07G0243600.1	*Oryza glaberrima*	*Phosphopantetheine attachment site*
OpuncBB_FGP26901	*Oryza punctata*	*Phosphopantetheine attachment site*
LOC_Os07g37920	*Oryza sativa*	*Phosphopantetheine attachment site*
Sobic.002G342100.1.p	*Sorghum bicolor*	*Phosphopantetheine attachment site*
Sobic.002G342100.1.p	*Sorghum bicolor*	*Phosphopantetheine attachment site*
GRMZM2G179885_P01	*Zea mays*	*Phosphopantetheine attachment site*
scaffold07067-augustus-gene-0.6	*Castanea mollissima*	*Phosphopantetheine attachment site*
Glyma-13G174700.1.p	*Glycine max*	*Phosphopantetheine attachment site*
KHN09961.1	*Glycine soja*	*Phosphopantetheine attachment site*
LPERR07G16320.1	*Leersia perrieri*	*Phosphopantetheine attachment site*
ORGLA07G0243600.1	*Oryza glaberrima*	*Phosphopantetheine attachment site*
OGLUM07G18780.1	*Oryza glumaepatula*	*Phosphopantetheine attachment site*
KN538693.1	*Oryza longistaminata*	*Phosphopantetheine attachment site*
OMERI07G15720.1	*Oryza meridionalis*	*Phosphopantetheine attachment site*
ONIVA07G17340.1	*Oryza nivara*	*Phosphopantetheine attachment site*
OPUNC07G17890.1	*Oryza punctata*	*Phosphopantetheine attachment site*
ORUFI07G19770.1	*Oryza rufipogon*	*Phosphopantetheine attachment site*
BGIOSGA024071.1	*Oryza sativa Indica*	*Phosphopantetheine attachment site*
LOC_Os07g37920.1	*Oryza sativa japonica*	*Phosphopantetheine attachment site*
KFK41460.1	*Arabis alpina*	*Polygalacturonase active site*
Rsa1.0_00061.1_g00015.1	*Raphanus sativus*	*Polygalacturonase active site*
Sobic.005G041000.1.p	*Sorghum bicolor*	*Polyprenyl synthases signature 1*
.XP_010536267.1	*Tarenaya hassleriana*	*PPM-type phosphatase domain signature*
Solyc04g079940.2.1	*Solanum lycopersicum*	*Prokaryotic membrane lipoprotein lipid attachment site profile*

Scf00187.g11893.t1	*Utricularia gibba*	*Prokaryotic membrane lipoprotein lipid attachment site profile*
Niben101Scf01153g00009.1	*Nicotiana benthamiana*	*Prokaryotic membrane lipoprotein lipid attachment site profile*
Niben101Scf01153g00009.1	*Nicotiana benthamiana*	*Prokaryotic membrane lipoprotein lipid attachment site profile*
XP_009789698.1	*Nicotiana sylvestris*	*Prokaryotic membrane lipoprotein lipid attachment site profile*
XP_009789699.1	*Nicotiana sylvestris*	*Prokaryotic membrane lipoprotein lipid attachment site profile*
XP_016453035.1	*Nicotiana tabacum*	*Prokaryotic membrane lipoprotein lipid attachment site profile*
XP_016471015.1	*Nicotiana tabacum*	*Prokaryotic membrane lipoprotein lipid attachment site profile*
XP_016487616.1	*Nicotiana tabacum*	*Prokaryotic membrane lipoprotein lipid attachment site profile*
XP_016487617.1	*Nicotiana tabacum*	*Prokaryotic membrane lipoprotein lipid attachment site profile*
XP_009603640.1	*Nicotiana tomentosiformis*	*Prokaryotic membrane lipoprotein lipid attachment site profile*
Sopim04g079940.0.1	*Solanum pimpinellifolium*	*Prokaryotic membrane lipoprotein lipid attachment site profile*
Glyma-13G243200.1.p	*Glycine max*	*Putative AMP-binding domain signature*
Si039061m	*Setaria italica*	*Regulator of chromosome condensation (RCC1) signature 2*
Seita.9G169600.1.p	*Setaria italica*	*Regulator of chromosome condensation (RCC1) signature 2*
GRMZM2G156977_P01	*Zea mays*	*Ribosomal protein L24e signature*
Cotton_A_00183_BGI-A2_v1.0	*Gossypium arboreum*	*Ribosomal protein L31 signature*
Gh_A01G0250	*Gossypium hirsutum*	*Ribosomal protein L31 signature*
KN539025.1	*Oryza longistaminata*	*Ribosomal protein L36 signature*
RrC831_p3	*Raphanus raphanistrum*	*Ribosomal protein S10 signature*
Cotton_A_09649_BGI-A2_v1.0	*Gossypium arboreum*	*Ribosomal protein S19e signature*
Zjn_sc00034.1.g02530.1.am.mk	*Zoysia japonica*	*Ribosomal protein S8 signature*
Spipo0G0093000	*Spirodela polyrhiza*	*Ribosome-binding factor A signature*
PH01000491G0390	*Phyllostachys heterocycla*	*Rubredoxin signature*
PH01000491G0390	*Phyllostachys heterocycla*	*Rubredoxin signature*
Bradi3g12470.1	*Brachypodium distachyon*	*Serine proteases, subtilase family, aspartic acid active site*
Pahal.F00337	*Panicum hallii*	*Serine proteases, subtilase family, aspartic acid active site*
Pavir.Fa00048	*Panicum virgatum*	*Serine proteases, subtilase family, aspartic acid active site*
Si013361m	*Setaria italica*	*Serine proteases, subtilase family, aspartic acid active site*

Si013378m	*Setaria italica*	*Serine proteases, subtilase family, aspartic acid active site*
Si013524m	*Setaria italica*	*Serine proteases, subtilase family, aspartic acid active site*
Sevir.6G256500	*Setaria viridis*	*Serine proteases, subtilase family, aspartic acid active site*
GRMZM2G125777_P01	*Zea mays*	*Serine proteases, subtilase family, aspartic acid active site*
Bradi3g12470.1.p	*Brachypodium distachyon*	*Serine proteases, subtilase family, aspartic acid active site*
Brast03G309200.1.p	*Brachypodium stacei*	*Serine proteases, subtilase family, aspartic acid active site*
Do003945.1	*Dichanthelium oligosanthes*	*Serine proteases, subtilase family, aspartic acid active site*
Itr_sc000194.1_g00003.1	*Ipomea trifida*	*Serine proteases, subtilase family, aspartic acid active site*
Pahal.F00337.1	*Panicum hallii*	*Serine proteases, subtilase family, aspartic acid active site*
Pavir.6KG413300.1.p	*Panicum virgatum*	*Serine proteases, subtilase family, aspartic acid active site*
Pavir.6NG362800.1.p	*Panicum virgatum*	*Serine proteases, subtilase family, aspartic acid active site*
Seita.6G251900.1.p	*Setaria italica*	*Serine proteases, subtilase family, aspartic acid active site*
Sme2.5_05012.1_g00010.1	*Solanum melongena*	*Serine proteases, subtilase family, aspartic acid active site*
XP_010543143.1	*Tarenaya hassleriana*	*Serine proteases, subtilase family, aspartic acid active site*
XP_010543151.1	*Tarenaya hassleriana*	*Serine proteases, subtilase family, aspartic acid active site*
XP_010543159.1	*Tarenaya hassleriana*	*Serine proteases, subtilase family, aspartic acid active site*
Traes_5BL_3BA7EA9A3	*Triticum aestivum*	*Serine proteases, subtilase family, aspartic acid active site*
cassava4-1_025821m	*Manihot esculenta*	*Serine proteases, trypsin family, serine active site*
KZV46422.1	*Dorcoceras hygrometricum*	*Serine/Threonine protein kinases active-site signature*
Gh_D11G0701	*Gossypium hirsutum*	*Serine/Threonine protein kinases active-site signature*
XP_010551540.1	*Tarenaya hassleriana*	*Serine/threonine specific protein phosphatases signature*
OMERI04G25580.2	*Oryza meridionalis*	*Sigma-54 interaction domain ATP-binding region A signature*
RrC3821_p1	*Raphanus raphanistrum*	*Sigma-54 interaction domain ATP-binding region A signature*
AUR62000898	*Chenopodium quinoa*	*Signal peptidases I serine active site*
Cucsa.284890.1	*Cucumis sativus*	*Signal peptidases I serine active site*
scaffold02306-augustus-gene-0.21	*Castanea mollissima*	*Signal peptidases I serine active site*
OB07G32110.1	*Oryza brachyanta*	*Signal peptidases I signature 3*
GSBRNA2T00106219001	*Brassica napus*	*Signal peptidases I signature 3*

OB07G32110.1	*Oryza brachyantha*	*Signal peptidases I signature 3*
XP_013583708.1	*Brassica oleracea*	*Signal peptidases I signature 3*
Tp57577_TGAC_v2_mRNA33521	*Trifolium pratense*	*Soybean trypsin inhibitor (Kunitz) protease inhibitors family signature*
Aqcoe3G142100.1.p	*Aquilegiacoerulea*	*SRP54-type proteins GTP-binding domain signature*
EcC047434.20	*Eucalyptus camaldulensis*	*Sugar transport proteins signature 2*
Eucgr-I01940.1.p	*Eucalyptus grandis*	*Sugar transport proteins signature 2*
KHN21163.1	*Glycine soja*	*Synaptobrevin signature*
TRAES3BF073600100CFD_t1	*Triticum aestivum*	*Syntaxin / epimorphin family signature*
PH01002656G0020	*Phyllostachys heterocycla*	*TonB-dependent receptor proteins signature 1*
AA11G00017	*Aethionema arabicum*	*TonB-dependent receptor proteins signature 1*
AA32G01100	*Aethionema arabicum*	*TonB-dependent receptor proteins signature 1*
PH01002656G0020	*Phyllostachys heterocycla*	*TonB-dependent receptor proteins signature 1*
XP_010520901.1	*Tarenaya hassleriana*	*TonB-dependent receptor proteins signature 1*
XP_010539252.1	*Tarenaya hassleriana*	*TonB-dependent receptor proteins signature 1*
C.cajan_41044	*Cajanus cajan*	*TonB-dependent receptor proteins signature 1*
Zosma1g02650	*Zostera marina*	*Translationally controlled tumor protein (TCTP) domain signature 2*
Spipo0G0093000	*Spirodela polyrhiza*	*Trp-Asp (WD) repeats signature*
Tp4g25130	*Thellungiella parvula*	*Trp-Asp (WD) repeats signature*
483400	*Arabidopsis lyrata*	*Trp-Asp (WD) repeats signature*
Bostr.25993s0479.1.p	*Boechera stricta*	*Trp-Asp (WD) repeats signature*
GSBRNA2T00045062001	*Brassica napus*	*Trp-Asp (WD) repeats signature*
GSBRNA2T00084755001	*Brassica napus*	*Trp-Asp (WD) repeats signature*
XP_013634618.1	*Brassica oleracea*	*Trp-Asp (WD) repeats signature*
XP_013634619.1	*Brassica oleracea*	*Trp-Asp (WD) repeats signature*
.XP_013634620.1	*Brassica oleracea*	*Trp-Asp (WD) repeats signature*
XP_009142151.1	*Brassica rapa*	*Trp-Asp (WD) repeats signature*
XP_008221120.1	*Prunus mume*	*Trp-Asp (WD) repeats signature*
KFK37142.1	*Arabis alpina*	*Trp-Asp (WD) repeats signature*

Kalax-0139s0008.1.p	*Kalanchoe marnieriana*	*Tubulin subunits alpha, beta, and gamma signature*
Kalax-0355s0005.1.p	*Kalanchoe marnieriana*	*Tubulin subunits alpha, beta, and gamma signature*
XP_016502907.1	*Nicotiana tabacum*	*Tubulin-beta mRNA autoregulation signal*
Zmw_sc03169.1.g00010.1	*Zoysia matrella*	*Tubulin-beta mRNA autoregulation signal*
GSMUA_Achr7P09510_001	*Musa acuminata*	*Zinc carboxypeptidases, zinc-binding region 2 signature*
Pavir.Ab01115	*Panicum virgatum*	*Zinc carboxypeptidases, zinc-binding region 2 signature*
Pavir.J12496	*Panicum virgatum*	*Zinc carboxypeptidases, zinc-binding region 2 signature*
PDK_30s745071g005	*Phoenix dactylifera*	*Zinc carboxypeptidases, zinc-binding region 2 signature*
MLOC_75795.1	*Hordeum vulgare*	*Zinc carboxypeptidases, zinc-binding region 2 signature*
Pavir-1KG158700.1.p	*Panicum virgatum*	*Zinc carboxypeptidases, zinc-binding region 2 signature*
Pavir-1KG168100.1.p	*Panicum virgatum*	*Zinc carboxypeptidases, zinc-binding region 2 signature*
PDK_30s745071g005	*Phoenix dactylifera*	*Zinc carboxypeptidases, zinc-binding region 2 signature*
Traes_3AS_74E386CE7.1	*Triticum aestivum*	*Zinc carboxypeptidases, zinc-binding region 2 signature*
TRAES3BF096900070CFD_t1	*Triticum aestivum*	*Zinc carboxypeptidases, zinc-binding region 2 signature*
Do016529.1	*Dichanthelium oligosanthes*	*Zinc finger BED-type profile*
Araip.9MG9F	*Arachis ipaensis*	*Zinc finger C2H2 type domain signature*
PDK_30s663981g010	*Phoenix dactylifera*	*Zinc-containing alcohol dehydrogenases signature*
PDK_30s663981g010	*Phoenix dactylifera*	*Zinc-containing alcohol dehydrogenases signature*
Araha.61839s0001.1.p	*Arabidopsis halleri*	
OB11G21460.1	*Oryza brachyantha*	
