# Supplementary material for: Genomics, molecular and evolutionary perspective of NAC transcription factors
Source: PLoS One. 2020 Apr 10;15(4):e0231425. doi: 10.1371/journal.pone.0231425 (PMC7147800; doi:10.1371/journal.pone.0231425)
Supplement: S3 Table — (PDF) [file pone.0231425.s003.pdf]

Supplementary Table 3

NAC TFs showing their involvement in different pathways and biological process.

Pathways of NAC TFs	Gene Ontology identifier	False discovery rate
Abscisic acid-activated signaling pathway	GO.0009738	0.042
Aerenchyma formation	GO.0010618	0.00901
Aging	GO.0007568	8.89E-06
Anatomical structure development	GO.0048856	7.77E-21
Anatomical structure formation involved in morphogenesis	GO.0048646	0.00169
Anatomical structure morphogenesis	GO.0009653	1.23E-16
Androecium development	GO.0048466	0.0436
Anthocyanin accumulation in tissues in response to UV light	GO.0043481	0.0239
Anthocyanin-containing compound biosynthetic process	GO.0009718	0.00442
Aromatic compound biosynthetic process	GO.0019438	1.44E-35
Asymmetric cell division	GO.0008356	0.0265
Auxin influx	GO.0060919	1.46E-06
Auxin polar transport	GO.0009926	0.0357
Auxin transport	GO.0060918	0.0128
Auxin-activated signaling pathway	GO.0009734	0.00338
Biological regulation	GO.0065007	7.85E-33
Biological_process	GO.0008150	0.000629
Biosynthetic process	GO.0009058	1.44E-16
Cell communication	GO.0007154	1.07E-11
Cell development	GO.0048468	2.09E-09
Cell differentiation	GO.0030154	1.97E-12
Cell fate commitment	GO.0045165	0.015
Cell growth	GO.0016049	0.000551
Cell morphogenesis	GO.0000902	0.000244
Cell morphogenesis involved in differentiation	GO.0000904	0.000137
Cell tip growth	GO.0009932	0.0014
Cell wall organization	GO.0071555	0.00345
Cell wall organization or biogenesis	GO.0071554	8.29E-06
Cellular aromatic compound metabolic process	GO.0006725	1.46E-22
Cellular biosynthetic process	GO.0044249	2.58E-18
Cellular component organization	GO.0016043	0.021
Cellular component organization or biogenesis	GO.0071840	0.000535
Cellular developmental process	GO.0048869	1.19E-10
Cellular heat acclimation	GO.0070370	0.00717
Cellular macromolecule biosynthetic process	GO.0034645	9.31E-34
Cellular macromolecule metabolic process	GO.0044260	4.50E-16
Cellular metabolic process	GO.0044237	5.22E-06
Cellular nitrogen compound biosynthetic process	GO.0044271	7.48E-28
Cellular nitrogen compound metabolic process	GO.0034641	5.23E-18
Cellular process	GO.0009987	1.86E-07
Cellular response to acid chemical	GO.0071229	2.33E-07
Cellular response to auxin stimulus	GO.0071365	0.00188
Cellular response to chemical stimulus	GO.0070887	9.87E-07
Cellular response to gibberellin stimulus	GO.0071370	3.53E-06
Cellular response to hormone stimulus	GO.0032870	2.11E-06
Cellular response to jasmonic acid stimulus	GO.0071395	0.000857

Cellular response to lipid	GO.0071396	2.97E-05
Cellular response to organic substance	GO.0071310	2.15E-07
Cellular response to oxygen-containing compound	GO.1901701	2.66E-06
Cellular response to stimulus	GO.0051716	3.41E-14
Cellular response to stress	GO.0033554	3.01E-07
Cellular response to sulfur starvation	GO.0010438	0.00231
Defense response	GO.0006952	1.78E-13
Defense response to bacterium	GO.0042742	0.00012
Defense response to bacterium, incompatible interaction	GO.0009816	0.00141
Defense response to fungus, incompatible interaction	GO.0009817	0.0192
Defense response to other organism	GO.0098542	0.000304
Defense response, incompatible interaction	GO.0009814	3.74E-06
Detection of bacterium	GO.0016045	0.0239
Developmental cell growth	GO.0048588	0.00412
Developmental growth	GO.0048589	0.00171
Developmental growth involved in morphogenesis	GO.0060560	0.00161
Developmental maturation	GO.0021700	6.83E-06
Developmental process	GO.0032502	5.95E-23
Developmental process involved in reproduction	GO.0003006	5.00E-05
Embryo development ending in seed dormancy	GO.0009793	0.00221
Embryonic meristem development	GO.0048508	2.69E-08
Embryonic meristem initiation	GO.0090421	3.54E-10
ERAD pathway	GO.0036503	0.0138
ER-associated ubiquitin-dependent protein catabolic process	GO.0030433	0.0138
Floral organ development	GO.0048437	0.00856
Floral whorl development	GO.0048438	0.00115
Flower development	GO.0009908	0.0245
Formation of organ boundary	GO.0010160	0.000179
Fruit development	GO.0010154	0.000277
Gene expression	GO.0010467	1.59E-28
Gibberellic acid mediated signaling pathway	GO.0009740	2.10E-06
Gravitropism	GO.0009630	0.0182
Heat acclimation	GO.0010286	0.015
Heterocycle metabolic process	GO.0046483	1.46E-22
Hormone-mediated signaling pathway	GO.0009755	1.34E-06
Immune system process	GO.0002376	7.48E-08
Indole glucosinolate biosynthetic process	GO.0009759	0.0239
Innate immune response	GO.0045087	5.60E-08
Integument development	GO.0080060	0.00717
Intercellular transport	GO.0010496	0.0427
Jasmonic acid mediated signaling pathway	GO.0009867	0.000531
Lateral root development	GO.0048527	6.03E-06
Lateral root morphogenesis	GO.0010102	0.00115
Leaf development	GO.0048366	4.16E-05
Leaf senescence	GO.0010150	4.15E-06
Macromolecule metabolic process	GO.0043170	3.11E-13
Meristem development	GO.0048507	5.08E-06
Meristem initiation	GO.0010014	7.99E-10
Meristem maintenance	GO.0010073	0.0474
Meristem structural organization	GO.0009933	9.68E-08
Metabolic process	GO.0008152	0.00882

Multicellular organismal development	GO.0007275	3.66E-20
Multicellular organismal process	GO.0032501	2.78E-21
Multi-organism process	GO.0051704	2.29E-07
Negative regulation of biological process	GO.0048519	2.74E-08
Negative regulation of cell communication	GO.0010648	2.40E-05
Negative regulation of cell differentiation	GO.0045596	0.00254
Negative regulation of cellular biosynthetic process	GO.0031327	8.59E-05
Negative regulation of cellular macromolecule biosynthetic process	GO.2000113	0.00017
Negative regulation of cellular metabolic process	GO.0031324	0.000298
Negative regulation of cellular process	GO.0048523	9.51E-10
Negative regulation of developmental process	GO.0051093	0.000857
Negative regulation of gene expression	GO.0010629	0.00156
Negative regulation of gibberellic acid mediated signaling pathway	GO.0009938	0.0107
Negative regulation of macromolecule metabolic process	GO.0010605	0.00221
Negative regulation of metabolic process	GO.0009892	0.00221
Negative regulation of multicellular organismal process	GO.0051241	0.00882
Negative regulation of nitrogen compound metabolic process	GO.0051172	2.00E-05
Negative regulation of nucleobase-containing compound metabolic process	GO.0045934	0.000157
Negative regulation of response to stimulus	GO.0048585	0.00345
Negative regulation of signal transduction	GO.0009968	0.00373
Negative regulation of signaling	GO.0023057	2.40E-05
Negative regulation of transcription, DNA-templated	GO.0045892	0.000231
Negative regulation of trichome patterning	GO.1900033	0.000278
Nitrogen compound metabolic process	GO.0006807	3.32E-15
Nucleic acid metabolic process	GO.0090304	4.93E-31
Nucleobase-containing compound metabolic process	GO.0006139	1.92E-25
Organ development	GO.0048513	1.76E-23
Organ formation	GO.0048645	0.000144
Organ morphogenesis	GO.0009887	0.00116
Organic cyclic compound biosynthetic process	GO.1901362	1.31E-33
Organic cyclic compound metabolic process	GO.1901360	1.51E-21
Organic substance biosynthetic process	GO.1901576	8.42E-18
Organic substance metabolic process	GO.0071704	0.00021
Pattern specification process	GO.0007389	4.24E-08
Phyllome development	GO.0048827	1.12E-08
Plant epidermis development	GO.0090558	1.76E-07
Plant epidermis morphogenesis	GO.0090626	0.0177
Plant-type cell wall biogenesis	GO.0009832	1.71E-06
Plant-type hypersensitive response	GO.0009626	0.00901
Plant-type secondary cell wall biogenesis	GO.0009834	2.75E-09
Positive gravitropism	GO.0009958	0.0312
Positive regulation of biological process	GO.0048518	2.40E-17
Positive regulation of cell death	GO.0010942	2.86E-05
Positive regulation of cellular biosynthetic process	GO.0031328	2.32E-17
Positive regulation of cellular component biogenesis	GO.0044089	0.000106
Positive regulation of cellular metabolic process	GO.0031325	2.38E-17
Positive regulation of cellular process	GO.0048522	8.92E-23
Positive regulation of defense response	GO.0031349	0.0257

Positive regulation of defense response to bacterium	GO.1900426	0.0151
Positive regulation of leaf senescence	GO.1900057	0.00439
Positive regulation of macromolecule metabolic process	GO.0010604	4.58E-16
Positive regulation of metabolic process	GO.0009893	6.63E-13
Positive regulation of nitrogen compound metabolic process	GO.0051173	3.21E-18
Positive regulation of nucleobase-containing compound metabolic process	GO.0045935	2.35E-18
Positive regulation of programmed cell death	GO.0043068	0.0265
Positive regulation of response to stimulus	GO.0048584	0.00169
Positive regulation of secondary cell wall biogenesis	GO.1901348	7.35E-09
Positive regulation of sequence-specific DNA binding transcription factor activity	GO.0051091	8.89E-06
Positive regulation of signal transduction	GO.0009967	0.042
Positive regulation of transcription, DNA-templated	GO.0045893	1.23E-18
Post-embryonic development	GO.0009791	0.000358
Post-embryonic morphogenesis	GO.0009886	0.00169
Post-embryonic organ development	GO.0048569	6.79E-07
Primary metabolic process	GO.0044238	2.86E-05
Primary shoot apical meristem specification	GO.0010072	1.02E-08
Programmed cell death	GO.0012501	3.38E-06
Regionalization	GO.0003002	1.11E-07
Regulation of abscisic acid-activated signaling pathway	GO.0009787	0.00901
Regulation of actin filament depolymerization	GO.0030834	0.0427
Regulation of biological process	GO.0050789	2.14E-40
Regulation of cell communication	GO.0010646	9.30E-09
Regulation of cell death	GO.0010941	0.0069
Regulation of cell differentiation	GO.0045595	1.12E-06
Regulation of cell fate commitment	GO.0010453	0.000108
Regulation of cell wall organization or biogenesis	GO.1903338	1.87E-10
Regulation of cellular biosynthetic process	GO.0031326	1.89E-44
Regulation of cellular component biogenesis	GO.0044087	8.91E-06
Regulation of cellular macromolecule biosynthetic process	GO.2000112	8.08E-45
Regulation of cellular metabolic process	GO.0031323	1.84E-44
Regulation of cellular process	GO.0050794	1.00E-44
Regulation of defense response	GO.0031347	0.000101
Regulation of defense response to insect	GO.2000068	0.0239
Regulation of developmental process	GO.0050793	3.37E-06
Regulation of embryonic development	GO.0045995	0.0265
Regulation of epidermal cell differentiation	GO.0045604	0.0432
Regulation of flavonoid biosynthetic process	GO.0009962	0.00442
Regulation of gene expression	GO.0010468	1.45E-44
Regulation of gibberellic acid mediated signaling pathway	GO.0009937	0.00579
Regulation of glucosinolate biosynthetic process	GO.0010439	0.00102
Regulation of leaf development	GO.2000024	0.000146
Regulation of leaf senescence	GO.1900055	0.00442
Regulation of macromolecule biosynthetic process	GO.0010556	4.27E-44
Regulation of macromolecule metabolic process	GO.0060255	5.52E-43
Regulation of metabolic process	GO.0019222	5.74E-41
Regulation of multicellular organismal development	GO.2000026	6.67E-06
Regulation of multicellular organismal process	GO.0051239	6.89E-06
Regulation of multi-organism process	GO.0043900	0.0177

Regulation of nitrogen compound metabolic process	GO.0051171	7.17E-46
Regulation of nucleic acid-templated transcription	GO.1903506	3.26E-46
Regulation of nucleobase-containing compound metabolic process	GO.0019219	3.26E-46
Regulation of organ morphogenesis	GO.2000027	0.00717
Regulation of primary metabolic process	GO.0080090	9.66E-43
Regulation of reactive oxygen species metabolic process	GO.2000377	0.0312
Regulation of response to biotic stimulus	GO.0002831	0.00626
Regulation of response to external stimulus	GO.0032101	0.0109
Regulation of response to stimulus	GO.0048583	1.43E-09
Regulation of response to stress	GO.0080134	9.34E-05
Regulation of salicylic acid biosynthetic process	GO.0080142	0.0432
Regulation of salicylic acid metabolic process	GO.0010337	0.0265
Regulation of secondary cell wall biogenesis	GO.2000652	6.82E-10
Regulation of secondary metabolite biosynthetic process	GO.1900376	0.000119
Regulation of sequence-specific DNA binding transcription factor activity	GO.0051090	4.22E-06
Regulation of shoot system development	GO.0048831	0.00282
Regulation of signal transduction	GO.0009966	6.42E-07
Regulation of signaling	GO.0023051	6.28E-09
Regulation of sulfur metabolic process	GO.0042762	0.000179
Regulation of transcription, DNA-templated	GO.0006355	9.79E-47
Regulation of trichome morphogenesis	GO.2000039	0.0239
Reproductive process	GO.0022414	4.46E-05
Reproductive shoot system development	GO.0090567	0.0139
Reproductive structure development	GO.0048608	0.000238
Reproductive system development	GO.0061458	0.000238
Response to abiotic stimulus	GO.0009628	1.56E-10
Response to abscisic acid	GO.0009737	1.03E-05
Response to acid chemical	GO.0001101	1.10E-22
Response to auxin	GO.0009733	1.38E-07
Response to bacterium	GO.0009617	0.000157
Response to chemical	GO.0042221	3.74E-17
Response to chitin	GO.0010200	9.07E-05
Response to endogenous stimulus	GO.0009719	1.23E-16
Response to endoplasmic reticulum stress	GO.0034976	0.000173
Response to ethylene	GO.0009723	0.00538
Response to external stimulus	GO.0009605	7.93E-07
Response to fungus	GO.0009620	0.0254
Response to gibberellin	GO.0009739	1.56E-15
Response to hormone	GO.0009725	7.57E-14
Response to hydrogen peroxide	GO.0042542	0.022
Response to inorganic substance	GO.0010035	0.00168
Response to insect	GO.0009625	0.000146
Response to jasmonic acid	GO.0009753	1.16E-17
Response to lipid	GO.0033993	2.73E-13
Response to nitrogen compound	GO.1901698	0.0184
Response to organic cyclic compound	GO.0014070	4.11E-12
Response to organic substance	GO.0010033	1.22E-20
Response to osmotic stress	GO.0006970	1.12E-06
Response to other organism	GO.0051707	1.46E-06

Response to oxygen-containing compound	GO.1901700	1.92E-22
Response to radiation	GO.0009314	0.0455
Response to salicylic acid	GO.0009751	3.96E-16
Response to salt stress	GO.0009651	1.39E-06
Response to stimulus	GO.0050896	1.23E-16
Response to stress	GO.0006950	1.16E-17
Response to unfolded protein	GO.0006986	0.0151
Response to UV	GO.0009411	0.0334
Response to water deprivation	GO.0009414	0.000207
Response to wounding	GO.0009611	0.000108
RNA metabolic process	GO.0016070	4.00E-34
Root cap development	GO.0048829	1.88E-09
Root development	GO.0048364	1.88E-15
Root epidermal cell differentiation	GO.0010053	1.15E-05
Root hair cell development	GO.0080147	0.000551
Root hair cell differentiation	GO.0048765	1.20E-05
Root hair cell tip growth	GO.0048768	0.00741
Root hair elongation	GO.0048767	0.0048
Root morphogenesis	GO.0010015	1.05E-09
Root system development	GO.0022622	2.08E-15
Seed development	GO.0048316	0.000723
Shoot system development	GO.0048367	1.02E-08
Shoot system morphogenesis	GO.0010016	0.000919
Sieve element differentiation	GO.0090603	0.00901
Sieve element enucleation	GO.0090602	0.00901
Signal transduction	GO.0007165	1.61E-10
Single organism reproductive process	GO.0044702	7.43E-06
Single organism signaling	GO.0044700	3.53E-11
Single-organism cellular process	GO.0044763	0.0279
Single-organism developmental process	GO.0044767	4.17E-23
Single-organism process	GO.0044699	0.000756
Specification of symmetry	GO.0009799	0.0432
Stamen development	GO.0048443	0.0436
Stamen filament development	GO.0080086	0.0107
Stem cell differentiation	GO.0048863	0.0432
System development	GO.0048731	4.40E-16
Tissue development	GO.0009888	2.58E-18
Transcription, DNA-templated	GO.0006351	8.08E-45
Transport of virus in host, cell to cell	GO.0046740	0.00901
Trichoblast differentiation	GO.0010054	1.85E-05
Trichome differentiation	GO.0010026	5.88E-05
Trichome morphogenesis	GO.0010090	0.0109
Trichome patterning	GO.0048629	0.000278
Unidimensional cell growth	GO.0009826	0.00531
Xylem development	GO.0010089	4.16E-06
Xylem vessel member cell differentiation	GO.0048759	1.43E-09
