# Supplementary material for: Genomics, molecular and evolutionary perspective of NAC transcription factors
Source: PLoS One. 2020 Apr 10;15(4):e0231425. doi: 10.1371/journal.pone.0231425 (PMC7147800; doi:10.1371/journal.pone.0231425)
Supplement: S4 Table — (DOCX) [file pone.0231425.s004.docx]

***Supplementary Table 4:*** Substitution rate of NAC TFs of plants.

|  | | A | | T | | | C | G |  | A | | | T | | C | G | |  | A | T | | C | G |  | A | | T | | C | G |
| --- | --- | --- | --- | --- | --- | --- | --- | --- | --- | --- | --- | --- | --- | --- | --- | --- | --- | --- | --- | --- | --- | --- | --- | --- | --- | --- | --- | --- | --- | --- |
| *Actinidia chinensis* | | | | | | | | |  | *Aegilops tauschi* | | | | | | | |  | [*Aethionema arabicum*](http://planttfdb.cbi.pku.edu.cn/index.php?sp=Aar) | | | | |  | *Amaranthus hypochondriacus* | | | | | |
| A | - | | | 10.62 | | 4.26 | | **10.83** |  | - | | 10.36 | | | 4.71 | | **12.32** |  | - | | 11.43 | 4.3 | **10.15** |  | - | 11.69 | | 4.83 | | **10.45** |
| T | 8.34 | | | - | | **4.34** | | 5.38 |  | 7.78 | | - | | | **4.37** | | 5.96 |  | 9.01 | | - | **3.54** | 5.7 |  | 8.54 | - | | **3.45** | | 5.16 |
| C | 8.34 | | | **10.83** | | - | | 5.38 |  | 7.78 | | **9.62** | | | - | | 5.96 |  | 9.01 | | **9.39** | - | 5.7 |  | 8.54 | **8.35** | | - | | 5.16 |
| G | **16.79** | | | 10.62 | | 4.26 | | - |  | **16.09** | | 10.36 | | | 4.71 | | - |  | **16.04** | | 11.43 | 4.3 | - |  | **17.32** | 11.69 | | 4.83 | | - |
|  | *Amborella trichopoda* | | | | | | | |  | *Ananas comosus* | | | | | | | |  | *Aquilegia coerulea* | | | | |  | *Arabidopsis halleri* | | | | | |
| A | - | | 3.99 | | 10.98 | | | **14.14** |  | - | 3.73 | | | 10.95 | | | **15.36** |  | - | | 11 | 4.33 | **11.11** |  | - | 10.93 | | 4.15 | | **11.07** |
| T | 8.17 | | - | | **5.41** | | | 10.7 |  | 7.66 | - | | | **5.91** | | | 10.32 |  | 8.67 | | - | **3.44** | 5.32 |  | 8.98 | - | | **3.3** | | 5.73 |
| C | 8.17 | | **1.97** | | - | | | 10.7 |  | 7.66 | **2.01** | | | - | | | 10.32 |  | 8.67 | | **8.73** | - | 5.32 |  | 8.98 | **8.71** | | - | | 5.73 |
| G | **10.8** | | 3.99 | | 10.98 | | | - |  | **11.39** | 3.73 | | | 10.95 | | | - |  | **18.09** | | 11 | 4.33 | - |  | **17.34** | 1.93 | | 4.15 | | - |
|  | *Arabidopsis lyrata* | | | | | | | |  | *Arabidopsis thaliana* | | | | | | | |  | *Arabis alpina* | | | | |  | *Arachis duranensis* | | | | | |
| A | - | | 4.03 | | 10.6 | | | **14.35** |  | - | 4.06 | | | 10.4 | | | **14.86** |  | - | | 11.04 | 4.25 | **10.62** |  | - | 11.17 | | 4.43 | | **10.3** |
| T | 8.63 | | - | | **4.51** | | | 10.64 |  | 8.41 | - | | | **4.65** | | | 10.52 |  | 8.88 | | - | **3.63** | 5.67 |  | 8.76 | - | | **3.82** | | 5.37 |
| C | 8.63 | | **1.71** | | - | | | 10.64 |  | 8.41 | **1.82** | | | - | | | 10.52 |  | 8.88 | | **9.42** | - | 5.67 |  | 8.76 | **9.62** | | - | | 5.37 |
| G | **11.63** | | 4.03 | | 10.6 | | | - |  | **11.89** | 4.06 | | | 10.4 | | | - |  | **16.63** | | 11.04 | 4.25 | - |  | **16.79** | 11.17 | | 4.43 | | - |
|  | *Arachis hypogaea* | | | | | | | |  | *Arachis ipaensis* | | | | | | | |  | *Artemisia annua* | | | | |  | *Azadirachta indica* | | | | | |
| A | - | | 4 | | 11.07 | | | **14.21** |  | - | 3.93 | | | 10.85 | | | **14.63** |  | - | | 10.85 | 4.08 | **10.93** |  | - | 10.98 | | 4.22 | | **10.34** |
| T | 8.16 | | - | | **5.53** | | | 10.24 |  | 8.39 | - | | | **4.95** | | | 10 |  | 8.24 | | - | **4.22** | 5.62 |  | 8.91 | - | | **3.82** | | 5.6 |
| C | 8.16 | | **2** | | - | | | 10.24 |  | 8.39 | **1.79** | | | - | | | 10 |  | 8.24 | | **11.24** | - | 5.62 |  | 8.91 | **9.95** | | - | | 5.6 |
| G | **11.32** | | 4 | | 11.07 | | | - |  | **12.28** | 3.93 | | | 10.85 | | | - |  | **16.01** | | 10.85 | 4.08 | - |  | **16.44** | 10.98 | | 4.22 | | - |
|  | *Beta vulgaris* | | | | | | | |  | *Boechera stricta* | | | | | | | |  | *Brachypodium distachyon* | | | | |  | *Brachypodium stacei* | | | | | |
| A | - | | 3.85 | | 10.95 | | | **14.38** |  | - | 11.02 | | | 4.23 | | | **10.86** |  | - | | 10.6 | 4.93 | **12.57** |  | - | 10.52 | | 4.96 | | **12.65** |
| T | 8.62 | | - | | **4.72** | | | 10 |  | 8.77 | - | | | **3.57** | | | 5.58 |  | 7.75 | | - | **4.05** | 6.19 |  | 7.68 | - | | **4.16** | | 6.21 |
| C | 8.62 | | **1.66** | | - | | | 10 |  | 8.77 | **9.29** | | | - | | | 5.58 |  | 7.75 | | **8.71** | - | 6.19 |  | 7.68 | **8.83** | | - | | 6.21 |
| G | **12.4** | | 3.85 | | 10.95 | | | - |  | **17.09** | 11.02 | | | 4.23 | | | - |  | **15.74** | | 10.6 | 4.93 | - |  | **15.63** | 10.52 | | 4.96 | | - |
|  | *Brassica napus* | | | | | | | |  | *Brassica oleracea* | | | | | | | |  | *Brassica rapa* | | | | |  | *Cajanus cajan* | | | | | |
| A | - | | 10.83 | | 4.26 | | | **10.71** |  | - | 10.83 | | | 4.29 | | | **10.72** |  | - | | 3.98 | 10.73 | **14.47** |  | - | 11.31 | | 4.53 | | **10.3** |
| T | 8.61 | | - | | **4.01** | | | 5.57 |  | 8.7 | - | | | **3.91** | | | 5.57 |  | 8.48 | | - | **4.63** | 10.6 |  | 8.63 | - | | **3.9** | | 5.53 |
| C | 8.61 | | **10.18** | | - | | | 5.57 |  | 8.7 | **9.87** | | | - | | | 5.57 |  | 8.48 | | **1.72** | - | 10.6 |  | 8.63 | **9.73** | | - | | 5.53 |
| G | **16.57** | | 10.83 | | 4.26 | | | - |  | **16.73** | 10.83 | | | 4.29 | | | - |  | **11.58** | | 3.98 | 10.73 | - |  | **16.07** | 11.31 | | 4.53 | | - |
|  | *Camelina sativa* | | | | | | | |  | *Cannabis sativa* | | | | | | | |  | *Capsella grandiflora* | | | | |  | *Capsella rubella* | | | | | |
| A | - | | 3.88 | | 10.44 | | | **14.9** |  | - | 11.17 | | | 4.35 | | | **10.49** |  | - | | 12.34 | 4.92 | **9.49** |  | - | 11.19 | | 4.35 | | **10.48** |
| T | 8.41 | | - | | **4.9** | | | 10.46 |  | 9.25 | - | | | **3.24** | | | 5.72 |  | 9.91 | | - | **2.41** | 6.34 |  | 8.81 | - | | **3.69** | | 5.65 |
| C | 8.41 | | **1.82** | | - | | | 10.46 |  | 9.25 | **8.33** | | | - | | | 5.72 |  | 9.91 | | **6.2** | - | 6.34 |  | 8.81 | **9.48** | | - | | 5.65 |
| G | **11.99** | | 3.88 | | 10.44 | | | - |  | **16.97** | 11.17 | | | 4.35 | | | - |  | **14.83** | | 12.34 | 4.92 | - |  | **16.33** | 11.19 | | 4.35 | | - |

|  | *Capsicum annuum* | | | |  | *Carica papaya* | | | |  | *Castanea mollissima* | | | |  | *Catharanthus roseus* | | | |
| --- | --- | --- | --- | --- | --- | --- | --- | --- | --- | --- | --- | --- | --- | --- | --- | --- | --- | --- | --- |
| A | - | 10.95 | 3.95 | **10.8** |  | - | 10.43 | 4.05 | **11.49** |  | - | 11.31 | 4.53 | **10.03** |  | - | 10.54 | 4.13 | **11.35** |
| T | 8.91 | - | **3.47** | 5.49 |  | 8.39 | - | **3.98** | 5.47 |  | 8.98 | - | **3.75** | 5.73 |  | 8.6 | - | **3.82** | 5.69 |
| C | 8.91 | **9.62** | - | 5.49 |  | 8.39 | **10.24** | - | 5.47 |  | 8.98 | **9.37** | - | 5.73 |  | 8.6 | **9.76** | - | 5.69 |
| G | **17.51** | 10.95 | 3.95 | - |  | **17.63** | 10.43 | 4.05 | - |  | **15.72** | 11.31 | 4.53 | - |  | **17.14** | 10.54 | 4.13 | - |
|  | *Chenopodium quinoa* | | | |  | *Cicer arietinum* | | | |  | *Citrullus lanatus* | | | |  | *Citrus clementina* | | | |
| A | - | 11.2 | 4.48 | **10.46** |  | - | 4.05 | 10.91 | **13.8** |  | - | 3.95 | 10.82 | **14.62** |  | - | 3.9 | 10.56 | **14.72** |
| T | 8.77 | - | **3.76** | 5.67 |  | 8.93 | - | **4.36** | 10.17 |  | 8.25 | - | **5.19** | 10.24 |  | 8.33 | - | **5.26** | 10.29 |
| C | 8.77 | **9.4** | - | 5.67 |  | 8.93 | **1.62** | - | 10.17 |  | 8.25 | **1.89** | - | 10.24 |  | 8.33 | **1.94** | - | 10.29 |
| G | **16.16** | 11.2 | 4.48 | - |  | **12.11** | 4.05 | 10.91 | - |  | **11.77** | 3.95 | 10.82 | - |  | **11.92** | 3.9 | 10.56 | - |
|  | *Citrus sinensis* | | | |  | *Coffea canephora* | | | |  | *Cucumis melo* | | | |  | *Cucumis sativus* | | | |
| A | - | 3.93 | 10.49 | **14.37** |  | - | 11.22 | 4.64 | **10.3** |  | - | 11.31 | 4.53 | **10.21** |  | - | 4.18 | 10.97 | **14.12** |
| T | 8.39 | - | **5.72** | 10.12 |  | 8.63 | - | **4.03** | 5.76 |  | 8.5 | - | **4.07** | 5.39 |  | 8.45 | - | **4.75** | 10.2 |
| C | 8.39 | **2.14** | - | 10.12 |  | 8.63 | **9.75** | - | 5.76 |  | 8.5 | **10.17** | - | 5.39 |  | 8.45 | **1.81** | - | 10.2 |
| G | **11.91** | 3.93 | 10.49 | - |  | **15.42** | 11.22 | 4.64 | - |  | **16.09** | 11.31 | 4.53 | - |  | **11.69** | 4.18 | 10.97 | - |
|  | *Daucus carota* | | | |  | *Dianthus caryophyllus* | | | |  | *Dichanthelium oligosanthes* | | | |  | *Dorcoceras hygrometricum* | | | |
| A | - | 4.04 | 10.99 | **13.8** |  | - | 3.93 | 10.74 | **14.26** |  | - | 10.55 | 4.97 | **12.66** |  | - | 4.04 | 10.85 | **14.14** |
| T | 8.52 | - | **5.16** | 10.34 |  | 8.57 | - | **5.02** | 10.25 |  | 7.49 | - | **4.32** | 5.97 |  | 8.47 | - | **5.04** | 10.3 |
| C | 8.52 | **1.89** | - | 10.34 |  | 8.57 | **1.84** | - | 10.25 |  | 7.49 | **9.16** | - | 5.97 |  | 8.47 | **1.88** | - | 10.3 |
| G | **11.37** | 4.04 | 10.99 | - |  | **11.91** | 3.93 | 10.74 | - |  | **15.88** | 10.55 | 4.97 | - |  | **11.62** | 4.04 | 10.85 | - |
|  | *Elaeis guineensis* | | | |  | *Eragrostis tef* | | | |  | *Eucalyptus camaldulensis* | | | |  | *Eucalyptus grandis* | | | |
| A | - | 3.85 | 10.79 | **15.28** |  | - | 10.72 | 4.7 | **11.48** |  | - | 10.84 | 4.28 | **10.81** |  | - | 3.95 | 10.68 | **14.94** |
| T | 7.88 | - | **5.23** | 10.63 |  | 8.34 | - | **3.99** | 6.09 |  | 8.47 | - | **4.13** | 5.7 |  | 7.87 | - | **5.87** | 10.31 |
| C | 7.88 | **1.86** | - | 10.63 |  | 8.34 | **9.12** | - | 6.09 |  | 8.47 | **10.44** | - | 5.7 |  | 7.87 | **2.17** | - | 10.31 |
| G | **11.33** | 3.85 | 10.79 | - |  | **15.72** | 10.72 | 4.7 | - |  | **16.05** | 10.84 | 4.28 | - |  | **11.4** | 3.95 | 10.68 | - |
|  | *Eutrema salsugineum* | | | |  | *Fragaria vesca* | | | |  | *Fragaria x ananassa* | | | |  | *Genlisea aurea* | | | |
| A | - | 11.04 | 4.27 | **10.67** |  | - | 10.85 | 4.49 | **10.67** |  | - | 10.22 | 4.07 | **11.19** |  | - | 10.5 | 3.98 | **11.58** |
| T | 8.8 | - | **3.67** | 5.62 |  | 8.64 | - | **3.96** | 5.4 |  | 8.26 | - | **4.48** | 5.34 |  | 9.08 | - | **3.34** | 6.52 |
| C | 8.8 | **9.49** | - | 5.62 |  | 8.64 | **9.56** | - | 5.4 |  | 8.26 | **11.24** | - | 5.34 |  | 9.08 | **8.79** | - | 6.52 |
| G | **16.7** | 11.04 | 4.27 | - |  | **17.06** | 10.85 | 4.49 | - |  | **17.32** | 10.22 | 4.07 | - |  | **16.12** | 10.5 | 3.98 | - |
|  | *Glycine max* | | | |  | *Glycine soja* | | | |  | *Gossypium arboreum* | | | |  | *Gossypium hirsutum* | | | |
| A | - | 11.43 | 4.62 | **10.18** |  | - | 11.53 | 4.72 | **10.07** |  | - | 11.05 | 4.39 | **10.56** |  | - | 11.06 | 4.4 | **10.51** |
| T | 8.78 | - | **3.73** | 5.59 |  | 8.98 | - | **3.55** | 5.68 |  | 8.64 | - | **3.89** | 5.5 |  | 8.6 | - | **3.98** | 5.55 |
| C | 8.78 | **9.24** | - | 5.59 |  | 8.98 | **8.67** | - | 5.68 |  | 8.64 | **9.8** | - | 5.5 |  | 8.6 | **10.02** | - | 5.55 |
| G | **16.01** | 11.43 | 4.62 | - |  | **15.92** | 11.53 | 4.72 | - |  | **16.58** | 11.05 | 4.39 | - |  | **16.27** | 11.06 | 4.4 | - |

|  | *Gossypium raimondii* | | | |  | *Helianthus annuus* | | | |  | *Hordeum vulgare* | | | |  | *Humulus lupulus* | | | |
| --- | --- | --- | --- | --- | --- | --- | --- | --- | --- | --- | --- | --- | --- | --- | --- | --- | --- | --- | --- |
| A | - | 11.28 | 4.52 | **10.22** |  | - | 10.39 | 4.31 | **10.79** |  | - | 10.74 | 4.81 | **11.64** |  | - | 11.21 | 4.63 | **10.52** |
| T | 8.85 | - | **3.77** | 5.62 |  | 8.23 | - | **4.74** | 5.46 |  | 8.41 | - | **3.82** | 6.48 |  | 8.7 | - | **3.68** | 5.37 |
| C | 8.85 | **9.39** | - | 5.62 |  | 8.23 | **11.42** | - | 5.46 |  | 8.41 | **8.53** | - | 6.48 |  | 8.7 | **8.92** | - | 5.37 |
| G | **16.09** | 11.28 | 4.52 | - |  | **16.28** | 10.39 | 4.31 | - |  | **15.12** | 10.74 | 4.81 | - |  | **17.06** | 11.21 | 4.63 | - |
|  | *Ipomoea trifida* | | | |  | *Jatropha curcas* | | | |  | *Juglans regia* | | | |  | *Kalanchoe laxiflora* | | | |
| A | - | 10.79 | 4.47 | **10.94** |  | - | 11.19 | 4.34 | **10.66** |  | - | 11.09 | 4.45 | **10.82** |  | - | 11.52 | 4.93 | **9.82** |
| T | 8.33 | - | **4.22** | 5.6 |  | 8.68 | - | **3.66** | 5.5 |  | 8.45 | - | **3.93** | 5.64 |  | 8.72 | - | **4.03** | 5.74 |
| C | 8.33 | **10.19** | - | 5.6 |  | 8.68 | **9.44** | - | 5.5 |  | 8.45 | **9.79** | - | 5.64 |  | 8.72 | **9.4** | - | 5.74 |
| G | **16.27** | 10.79 | 4.47 | - |  | **16.83** | 11.19 | 4.34 | - |  | **16.22** | 11.09 | 4.45 | - |  | **14.92** | 11.52 | 4.93 | - |
|  | *Kalanchoe marnieriana* | | | |  | *Klebsormidium flaccidum* | | | |  | *Lactuca sativa* | | | |  | *Leersia perrieri* | | | |
| A | - | 12.08 | 5.24 | **9.86** |  | - | 7.73 | 4.03 | **13.08** |  | - | 11.39 | 4.57 | **9.9** |  | - | 10.31 | 4.57 | **13.27** |
| T | 9.37 | - | **2.86** | 6.19 |  | 6.67 | - | **7.19** | 5.04 |  | 9.13 | - | **3.67** | 5.91 |  | 7.33 | - | **4.28** | 5.82 |
| C | 9.37 | **6.59** | - | 6.19 |  | 6.67 | **13.78** | - | 5.04 |  | 9.13 | **9.16** | - | 5.91 |  | 7.33 | **9.66** | - | 5.82 |
| G | **14.93** | 12.08 | 5.24 | - |  | **18.28** | 7.73 | 4.03 | - |  | **15.28** | 11.39 | 4.57 | - |  | **16.73** | 10.31 | 4.57 | - |
|  | *Linum usitatissimum* | | | |  | *Lotus japonicus* | | | |  | *Malus domestica* | | | |  | *Manihot esculenta* | | | |
| A | - | 3.72 | 10.39 | **15.82** |  | - | 10.31 | 3.72 | **12.64** |  | - | 10.65 | 4.23 | **11.21** |  | - | 11.22 | 4.54 | **10.48** |
| T | 7.89 | - | **5.6** | 10.09 |  | 8.2 | - | **3.43** | 5.44 |  | 8.18 | - | **4.17** | 5.24 |  | 8.65 | - | **3.82** | 5.57 |
| C | 7.89 | **2.01** | - | 10.09 |  | 8.2 | **9.51** | - | 5.44 |  | 8.18 | **10.5** | - | 5.24 |  | 8.65 | **9.45** | - | 5.57 |
| G | **12.37** | 3.72 | 10.39 | - |  | **19.07** | 10.31 | 3.72 | - |  | **17.5** | 10.65 | 4.23 | - |  | **16.29** | 11.22 | 4.54 | - |
|  | *Medicago truncatula* | | | |  | *Mimulus guttatus* | | | |  | *Morus notabilis* | | | |  | *Musa acuminata* | | | |
| A | - | 11.24 | 4.3 | **10.19** |  | - | 10.72 | 4.02 | **10.51** |  | - | 11.16 | 4.54 | **10.64** |  | - | 10.93 | 4.62 | **10.89** |
| T | 8.9 | - | **3.68** | 5.4 |  | 8.91 | - | **3.91** | 5.67 |  | 8.54 | - | **3.8** | 5.32 |  | 8.14 | - | **4.42** | 5.9 |
| C | 8.9 | **9.62** | - | 5.4 |  | 8.91 | **10.42** | - | 5.67 |  | 8.54 | **9.34** | - | 5.32 |  | 8.14 | **10.47** | - | 5.9 |
| G | **16.79** | 11.24 | 4.3 | - |  | **16.51** | 10.72 | 4.02 | - |  | **17.1** | 11.16 | 4.54 | - |  | **15.05** | 10.93 | 4.62 | - |
|  | *Nelumbo nucifera* | | | |  | *Nicotiana benthamiana* | | | |  | *Nicotiana sylvestris* | | | |  | *Nicotiana tabacum* | | | |
| A | - | 10.88 | 4.4 | **10.9** |  | - | 10.88 | 4.11 | **10.39** |  | - | 10.88 | 4.26 | **10.4** |  | - | 11.2 | 4.31 | **10.21** |
| T | 8.43 | - | **4.04** | 5.56 |  | 8.82 | - | **3.89** | 5.38 |  | 8.87 | - | **3.86** | 5.44 |  | 9.07 | - | **3.57** | 5.55 |
| C | 8.43 | **9.99** | - | 5.56 |  | 8.82 | **10.29** | - | 5.38 |  | 8.87 | **9.87** | - | 5.44 |  | 9.07 | **9.28** | - | 5.55 |
| G | **16.55** | 10.88 | 4.4 | - |  | **17.03** | 10.88 | 4.11 | - |  | **16.98** | 10.88 | 4.26 | - |  | **16.69** | 11.2 | 4.31 | - |
|  | *Nicotiana tomentosiformis* | | | |  | *Ocimum tenuiflorum* | | | |  | *Oropetium thomaeum* | | | |  | *Oryza brachyantha* | | | |
| A | - | 11.05 | 4.3 | **10.43** |  | - | 11.02 | 4.32 | **10.59** |  | - | 10.52 | 4.82 | **12.41** |  | - | 10.51 | 4.85 | **12.98** |
| T | 8.93 | - | **3.64** | 5.43 |  | 8.44 | - | **4.15** | 5.68 |  | 7.46 | - | **4.56** | 5.93 |  | 7.3 | - | **4.37** | 5.92 |
| C | 8.93 | **9.36** | - | 5.43 |  | 8.44 | **10.58** | - | 5.68 |  | 7.46 | **9.97** | - | 5.93 |  | 7.3 | **9.47** | - | 5.92 |
| G | **17.16** | 11.05 | 4.3 | - |  | **15.74** | 11.02 | 4.32 | - |  | **15.61** | 10.52 | 4.82 | - |  | **16.01** | 10.51 | 4.85 | - |

|  | *Oryza glaberrima* | | | |  | *Oryza glumaepatula* | | | |  | *Oryza longistaminata* | | | |  | *Oryza meridionalis* | | | |
| --- | --- | --- | --- | --- | --- | --- | --- | --- | --- | --- | --- | --- | --- | --- | --- | --- | --- | --- | --- |
| A | - | 10.65 | 5.01 | **12.86** |  | - | 10.71 | 5 | **12.47** |  | - | 10.69 | 4.74 | **12.11** |  | - | 10.42 | 4.87 | **13.3** |
| T | 7.52 | - | **4.08** | 6.14 |  | 7.61 | - | **4.18** | 6.16 |  | 7.96 | - | **4.03** | 6.09 |  | 7.43 | - | **4.1** | 6.12 |
| C | 7.52 | **8.69** | - | 6.14 |  | 7.61 | **8.96** | - | 6.16 |  | 7.96 | **9.09** | - | 6.09 |  | 7.43 | **8.79** | - | 6.12 |
| G | **15.73** | 10.65 | 5.01 | - |  | **15.42** | 10.71 | 5 | - |  | **15.82** | 10.69 | 4.74 | - |  | **16.14** | 10.42 | 4.87 | - |
|  | *Oryza nivara* | | | |  | *Oryza punctata* | | | |  | *Oryza rufipogon* | | | |  | *Oryza sativa* | | | |
| A | - | 10.64 | 5.01 | **12.49** |  | - | 10.71 | 4.94 | **12.37** |  | - | 10.67 | 5.04 | **12.84** |  | - | 10.86 | 5.12 | **12.22** |
| T | 7.6 | - | **4.25** | 6.17 |  | 7.66 | - | **4.2** | 6.23 |  | 7.47 | - | **4.14** | 6.18 |  | 7.64 | - | **4.21** | 6.33 |
| C | 7.6 | **9.01** | - | 6.17 |  | 7.66 | **9.12** | - | 6.23 |  | 7.47 | **8.76** | - | 6.18 |  | 7.64 | **8.94** | - | 6.33 |
| G | **15.39** | 10.6 | 5.01 | - |  | **15.22** | 10.71 | 4.94 | - |  | **15.53** | 10.67 | 5.04 | - |  | **17.73** | 10.86 | 5.12 | - |
|  | *Panicum hallii* | | | |  | *Panicum virgatum* | | | |  | *Petunia axillaris* | | | |  | *Petunia inflata* | | | |
| A | - | 10.66 | 5 | **12.63** |  | - | 10.4 | 5.02 | **12.72** |  | - | 10.03 | 4.18 | **10.07** |  | - | 11.13 | 4.28 | **10.28** |
| T | 7.75 | - | **3.97** | 6.32 |  | 7.53 | - | **4.39** | 6.14 |  | 8.77 | - | **4.05** | 5.35 |  | 8.83 | - | **3.79** | 5.41 |
| C | 7.75 | **8.47** | - | 6.32 |  | 7.53 | **9.12** | - | 6.14 |  | 8.77 | **10.68** | - | 5.35 |  | 8.83 | **9.86** | - | 5.41 |
| G | **15.48** | 10.66 | 5 | - |  | **15.6** | 10.4 | 5.02 | - |  | **16.52** | 10.03 | 4.18 | - |  | **16.77** | 11.13 | 4.28 | - |
|  | *Phaselous vulgaris* | | | |  | *Phoenix dactylifera* | | | |  | *Phyllostachys heterocycla* | | | |  | *Physcomitrella patens* | | | |
| A | - | 11.4 | 4.62 | **10.24** |  | - | 10.71 | 4.44 | **11.16** |  | - | 11.03 | 4.97 | **11.6** |  | - | 10.61 | 4.64 | **10.77** |
| T | 8.75 | - | **3.78** | 5.69 |  | 8.2 | - | **4.31** | 5.71 |  | 8.1 | - | **3.94** | 6.27 |  | 8.1 | - | **4.77** | 5.53 |
| C | 8.75 | **9.32** | - | 5.69 |  | 8.2 | **10.39** | - | 5.71 |  | 8.1 | **8.73** | - | 6.27 |  | 8.1 | **10.92** | - | 5.53 |
| G | **15.75** | 11.4 | 4.62 | - |  | **16.0** | 10.71 | 4.44 | - |  | **14.99** | 11.03 | 4.97 | - |  | **15.77** | 10.61 | 4.64 | - |
|  | *Picea abies* | | | |  | *Picea glauca* | | | |  | *Picea sitchensis* | | | |  | *Pinus taeda* | | | |
| A | - | 10.82 | 4.54 | **10.29** |  | - | 10.85 | 4.61 | **10.35** |  | - | 10.35 | 4.45 | **10.58** |  | - | 10.08 | 4.37 | **11.67** |
| T | 8.53 | - | **4.42** | 5.48 |  | 8.42 | - | **4.47** | 5.42 |  | 8.02 | - | **5.11** | 5.12 |  | 8.18 | - | **4.39** | 5.35 |
| C | 8.53 | **10.53** | - | 5.48 |  | 8.42 | **10.52** | - | 5.42 |  | 8.02 | **11.87** | - | 5.12 |  | 8.18 | **10.11** | - | 5.35 |
| G | **16** | 10.82 | 4.54 | - |  | **16.08** | 10.85 | 4.61 | - |  | **16.56** | 10.35 | 4.45 | - |  | **17.86** | 10.08 | 4.37 | - |
|  | *Populus euphratica* | | | |  | *Populus trichocarpa* | | | |  | *Prunus mume* | | | |  | *Prunus persica* | | | |
| A | - | 10.86 | 4.53 | **10.85** |  | - | 10.98 | 4.52 | **10.68** |  | - | 11.12 | 4.43 | **10.65** |  | - | 11.38 | 4.53 | **10.23** |
| T | 8.33 | - | **4.15** | 5.31 |  | 8.47 | - | **4.01** | 5.35 |  | 8.91 | - | **3.49** | 5.55 |  | 8.95 | - | **3.55** | 5.6 |
| C | 8.33 | **9.95** | - | 5.31 |  | 8.47 | **9.74** | - | 5.35 |  | 8.91 | **8.76** | - | 5.55 |  | 8.95 | **8.92** | - | 5.6 |
| G | **17** | 10.86 | 4.53 | - |  | **16.93** | 10.98 | 4.52 | - |  | **17.07** | 11.12 | 4.43 | - |  | **16.36** | 11.38 | 4.53 | - |
|  | *Pseudotsuga menziesii* | | | |  | *Pyrus bretschneideri* | | | |  | *Raphanus raphanistrum* | | | |  | *Raphanus sativus* | | | |
| A | - | 10.74 | 4.44 | **9.98** |  | - | 11.1 | 4.51 | **10.78** |  | - | 11.07 | 4.4 | **10.51** |  | - | 10.96 | 4.35 | **10.71** |
| T | 8.49 | - | **4.79** | 5.68 |  | 8.57 | - | **3.77** | 5.47 |  | 8.72 | - | **3.85** | 5.6 |  | 8.47 | - | **4.06** | 5.52 |
| C | 8.49 | **11.59** | - | 5.68 |  | 8.57 | **9.26** | - | 5.47 |  | 8.72 | **9.7** | - | 5.6 |  | 8.47 | **10.23** | - | 5.52 |
| G | **14.93** | 10.74 | 4.44 | - |  | **16.89** | 11.1 | 4.51 | - |  | **16.36** | 11.07 | 4.4 | - |  | **16.44** | 10.96 | 4.35 | - |

|  | *Ricinus communis* | | | |  | *Saccharum officinarum* | | | |  | *Salix purpurea* | | | |  | *Salvia miltiorrhiza* | | | |
| --- | --- | --- | --- | --- | --- | --- | --- | --- | --- | --- | --- | --- | --- | --- | --- | --- | --- | --- | --- |
| A | - | 10.87 | 4.28 | **11.05** |  | - | 9.95 | 4.48 | **12.78** |  | - | 10.85 | 4.5 | **10.98** |  | - | 11.27 | 4.64 | **10.63** |
| T | 8.56 | - | **3.76** | 5.48 |  | 8.25 | - | **3.94** | 6.61 |  | 8.24 | - | **4.16** | 5.32 |  | 8.49 | - | **3.89** | 5.7 |
| C | 8.56 | **3.76** | - | 5.48 |  | 8.25 | **8.76** | - | 6.61 |  | 8.24 | **10.04** | - | 5.32 |  | 8.49 | **9.44** | - | 5.7 |
| G | **17.27** | 10.87 | 4.28 | - |  | **15.95** | 9.95 | 4.48 | - |  | **17.01** | 10.85 | 4.5 | - |  | **15.82** | 11.27 | 4.64 | - |
|  | *Selaginella moellendorffii* | | | |  | *Sesamum indicum* | | | |  | *Setaria italica* | | | |  | *Setaria viridis* | | | |
| A | - | 10.76 | 4.13 | **11.65** |  | - | 11.3 | 4.82 | **10.17** |  | - | 10.82 | 5.13 | **12.29** |  | - | 10.6 | 5.05 | **12.45** |
| T | 8.65 | - | **3.52** | 6.3 |  | 8.63 | - | **4.03** | 5.65 |  | 7.69 | - | **4.12** | 6.22 |  | 7.67 | - | **4.22** | 6.1 |
| C | 8.65 | **9.15** | - | 6.3 |  | 8.63 | **9.45** | - | 5.65 |  | 7.69 | **8.67** | - | 6.22 |  | 7.67 | **8.87** | - | 6.1 |
| G | **16.01** | 10.76 | 4.13 | - |  | **15.54** | 11.3 | 4.82 | - |  | **15.19** | 10.82 | 5.13 | - |  | **15.65** | 10.6 | 5.05 | - |
|  | *Sisymbrium irio* | | | |  | *Solanum lycopersicum* | | | |  | *Solanum melongena* | | | |  | *Solanum pennellii* | | | |
| A | - | 10.74 | 4.29 | **11.05** |  | - | 11.17 | 4.12 | **10.13** |  | - | 10.72 | 4.04 | **10.67** |  | - | 11.07 | 4.11 | **10.25** |
| T | 8.45 | - | **3.96** | 5.37 |  | 9.1 | - | **3.58** | 5.45 |  | 8.67 | - | **3.95** | 5.3 |  | 8.96 | - | **3.7** | 5.39 |
| C | 8.45 | **9.91** | - | 5.37 |  | 9.1 | **9.7** | - | 5.45 |  | 8.67 | **10.48** | - | 5.3 |  | 8.96 | **9.95** | - | 5.39 |
| G | **17.37** | 10.74 | 4.29 | - |  | **16.9** | 11.17 | 4.12 | - |  | **17.46** | 10.72 | 4.04 | - |  | **17.03** | 11.07 | 4.11 | - |
|  | *Solanum pimpinellifolium* | | | |  | *Solanum tuberosum* | | | |  | *Sorghum bicolor* | | | |  | *Sphagnum fallax* | | | |
| A | - | 11.4 | 4.23 | **9.8** |  | - | 10.96 | 4.13 | **10.34** |  | - | 10.64 | 5.07 | **12.24** |  | - | 10.95 | 4.92 | **10.65** |
| T | 9.3 | - | **3.48** | 5.58 |  | 8.83 | - | **3.86** | 5.38 |  | 7.76 | - | **4.23** | 6.21 |  | 8.03 | - | **4.69** | 5.7 |
| C | 9.3 | **9.38** | - | 5.58 |  | 8.83 | **10.24** | - | 5.38 |  | 7.76 | **8.88** | - | 6.21 |  | 8.03 | **10.44** | - | 5.7 |
| G | **16.33** | 11.4 | 4.23 | - |  | **16.98** | 10.96 | 4.13 | - |  | **15.31** | 10.64 | 5.07 | - |  | **15** | 10.95 | 4.92 | - |
|  | *Spinacia oleracea* | | | |  | *Spirodela polyrhiza* | | | |  | *Tarenaya hassleriana* | | | |  | *Thellungiella parvula* | | | |
| A | - | 11.37 | 4.57 | **10.2** |  | - | 11.01 | 4.84 | **10.69** |  | - | 11.05 | 4.52 | **10.66** |  | - | 11.34 | 4.46 | **10.21** |
| T | 8.74 | - | **3.76** | 5.4 |  | 8.45 | - | **4.21** | 6.38 |  | 8.73 | - | **3.81** | 5.81 |  | 8.69 | - | **3.9** | 5.62 |
| C | 8.74 | **9.35** | - | 5.4 |  | 8.45 | **9.59** | - | 6.38 |  | 8.73 | **9.31** | - | 5.81 |  | **8.69** | **9.9** | - | 5.62 |
| G | **16.51** | 11.37 | 4.57 | - |  | **14.16** | 11.01 | 4.84 | - |  | **16.02** | 11.05 | 4.52 | - |  | **15.78** | 11.34 | 4.46 | - |
|  | *Theobroma cacao* | | | |  | *Trifolium pratense* | | | |  | *Triticum aestivum* | | | |  | *Triticum urartu* | | | |
| A | - | 11.04 | 4.45 | **10.52** |  | - | 11.21 | 4.26 | **9.73** |  | - | 10.65 | 4.85 | **11.2** |  | - | 10.78 | 4.94 | **11.4** |
| T | 8.53 | - | **4.04** | 5.43 |  | 9.05 | - | **3.89** | 5.43 |  | 8.28 | - | **4.34** | 6.16 |  | 8.12 | - | **4.27** | 6.19 |
| C | 8.53 | **10.02** | - | 5.43 |  | 9.05 | **10.24** | - | 5.43 |  | 8.28 | **9.54** | - | 6.16 |  | 8.12 | **9.32** | - | 6.19 |
| G | **16.53** | 11.04 | 4.45 | - |  | **16.24** | 11.21 | 4.26 | - |  | **15.04** | 10.65 | 4.85 | - |  | **14.95** | 10.78 | 4.94 | - |
|  | *Utricularia gibba* | | | |  | *Vigna angularis* | | | |  | *Vigna radiata* | | | |  | *Vigna unguiculata* | | | |
| A | - | 10.97 | 4.56 | **10.67** |  | - | 11.18 | 4.45 | **10.4** |  | - | 10.76 | 4.33 | **10.64** |  | - | 10.21 | 3.95 | **11.11** |
| T | 8.41 | - | **4.26** | 6 |  | 8.87 | - | **3.72** | 5.74 |  | 8.58 | - | **4.16** | 5.59 |  | 8.54 | - | **4.35** | 5.86 |
| C | 8.41 | **10.23** | - | 6 |  | 8.87 | **9.34** | - | 5.74 |  | 8.58 | **10.34** | - | 5.59 |  | 8.54 | **11.24** | - | 5.86 |
| G | **14.96** | 10.97 | 4.56 | - |  | **16.08** | 11.18 | 4.45 | - |  | **16.33** | 10.76 | 4.33 | - |  | **16.19** | 10.21 | 3.95 | - |

|  | *Vitis vinifera* | | | |  | *Zea mays* | | | |  | *Ziziphus jujuba* | | | |  | *Zoysia japonica* | | | |
| --- | --- | --- | --- | --- | --- | --- | --- | --- | --- | --- | --- | --- | --- | --- | --- | --- | --- | --- | --- |
| A | - | 11.27 | 4.64 | **10.21** |  | - | 10.77 | 5.14 | **12.1** |  | - | 11.03 | 5.54 | **10.71** |  | - | 10.83 | 5.02 | **12.08** |
| T | 8.69 | - | **3.98** | 5.74 |  | 7.82 | - | **4.15** | 6.34 |  | 8.6 | - | **3.84** | 5.47 |  | 7.87 | - | **4.04** | 6.18 |
| C | 8.69 | **9.68** | - | 5.74 |  | 7.82 | **8.69** | - | 6.34 |  | 8.6 | **9.33** | - | 5.47 |  | 7.87 | **8.72** | - | 6.18 |
| G | **15.45** | 11.27 | 4.64 | - |  | **14.91** | 10.77 | 5.14 | - |  | **16.84** | 11.03 | 5.54 | - |  | **15.38** | 10.83 | 5.02 | - |
|  | *Zoysia matrella* | | | |  | *Zoysia pacifica* | | | |  |  |  |  |  |  |  |  |  |  |
| A | - | 10.65 | 5 | **12.29** |  | - | 10.41 | 4.88 | **12.67** |  |  |  |  |  |  |  |  |  |  |
| T | 7.73 | - | **4.21** | 6.11 |  | 7.57 | - | **4.35** | 6.04 |  |  |  |  |  |  |  |  |  |  |
| C | 7.73 | **8.98** | - | 6.11 |  | 7.57 | **9.28** | - | 6.04 |  |  |  |  |  |  |  |  |  |  |
| G | **15.54** | 10.65 | 5 | - |  | **18.89** | 10.41 | 4.88 | - |  |  |  |  |  |  |  |  |  |  |
