# Supplementary material for: Genomics, molecular and evolutionary perspective of NAC transcription factors
Source: PLoS One. 2020 Apr 10;15(4):e0231425. doi: 10.1371/journal.pone.0231425 (PMC7147800; doi:10.1371/journal.pone.0231425)
Supplement: S1 Fig — (XZ) [file S1 Fig.pdf › S1 Fig.pdf]

TMHMM result

HELP with output formats

```
# Actinidiachinensis_Achn000661 Length: 537
# Actinidiachinensis_Achn000661 Number of predicted TMHs:  0
# Actinidiachinensis_Achn000661 Exp number of AAs in TMHs: 0
# Actinidiachinensis_Achn000661 Exp number, first 60 AAs:  0
# Actinidiachinensis_Achn000661 Total prob of N-in:        0.00014
Actinidiachinensis_Achn000661   TMHMM2.0        outside      1   537
```

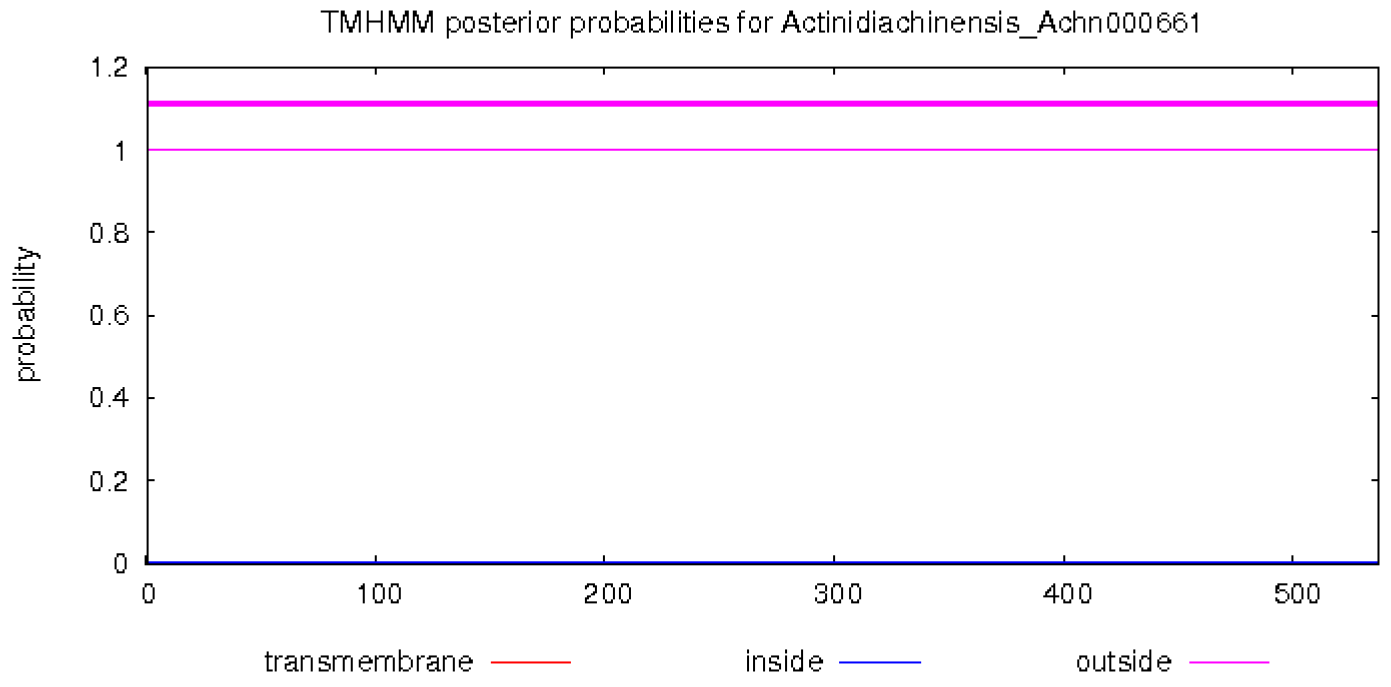

plot in postscript, script for making the plot in gnuplot, data for plot

---

```
# Actinidiachinensis_Achn002691 Length: 342
# Actinidiachinensis_Achn002691 Number of predicted TMHs:  0
# Actinidiachinensis_Achn002691 Exp number of AAs in TMHs: 0
# Actinidiachinensis_Achn002691 Exp number, first 60 AAs:  0
# Actinidiachinensis_Achn002691 Total prob of N-in:        0.00455
Actinidiachinensis_Achn002691   TMHMM2.0        outside      1   342
```

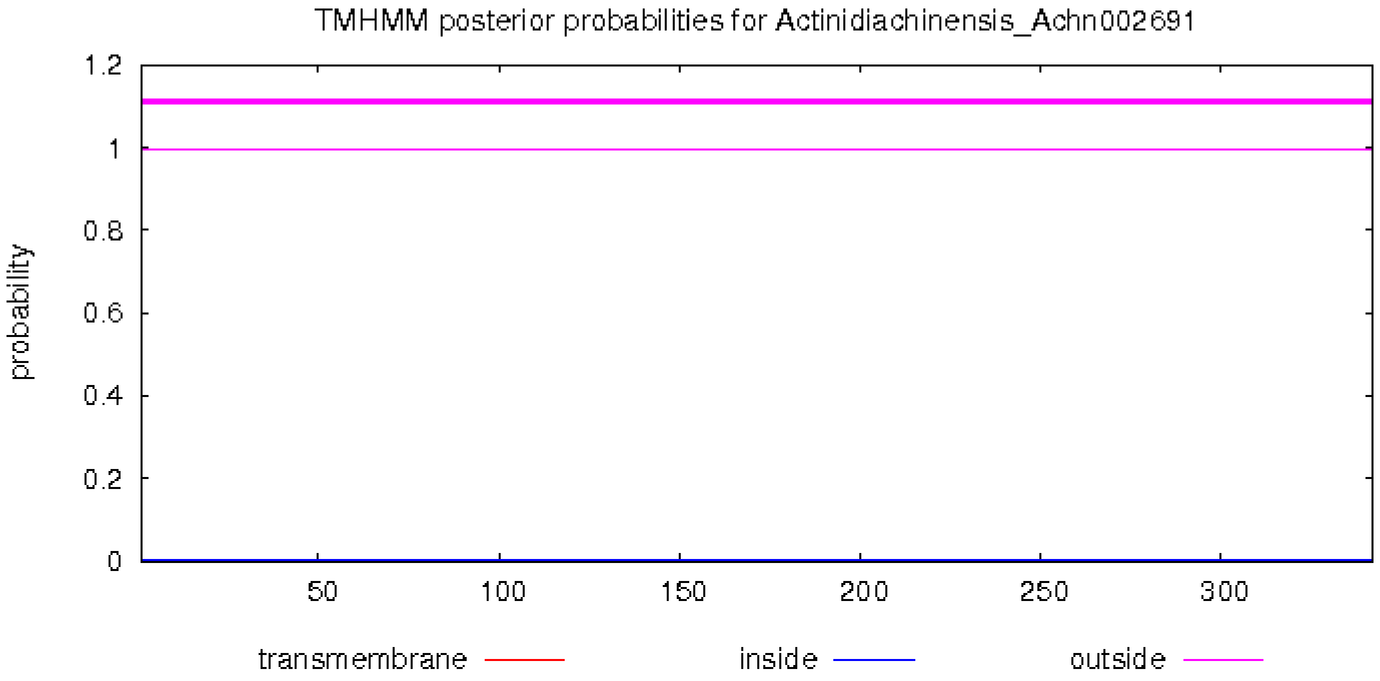

plot in postscript, script for making the plot in gnuplot, data for plot

---

```
# Actinidiachinensis_Achn003771 Length: 398
# Actinidiachinensis_Achn003771 Number of predicted TMHs:  0
# Actinidiachinensis_Achn003771 Exp number of AAs in TMHs: 0.00037
# Actinidiachinensis_Achn003771 Exp number, first 60 AAs:  0.00019
# Actinidiachinensis_Achn003771 Total prob of N-in:        0.00420
Actinidiachinensis_Achn003771	TMHMM2.0	outside	     1   398
```

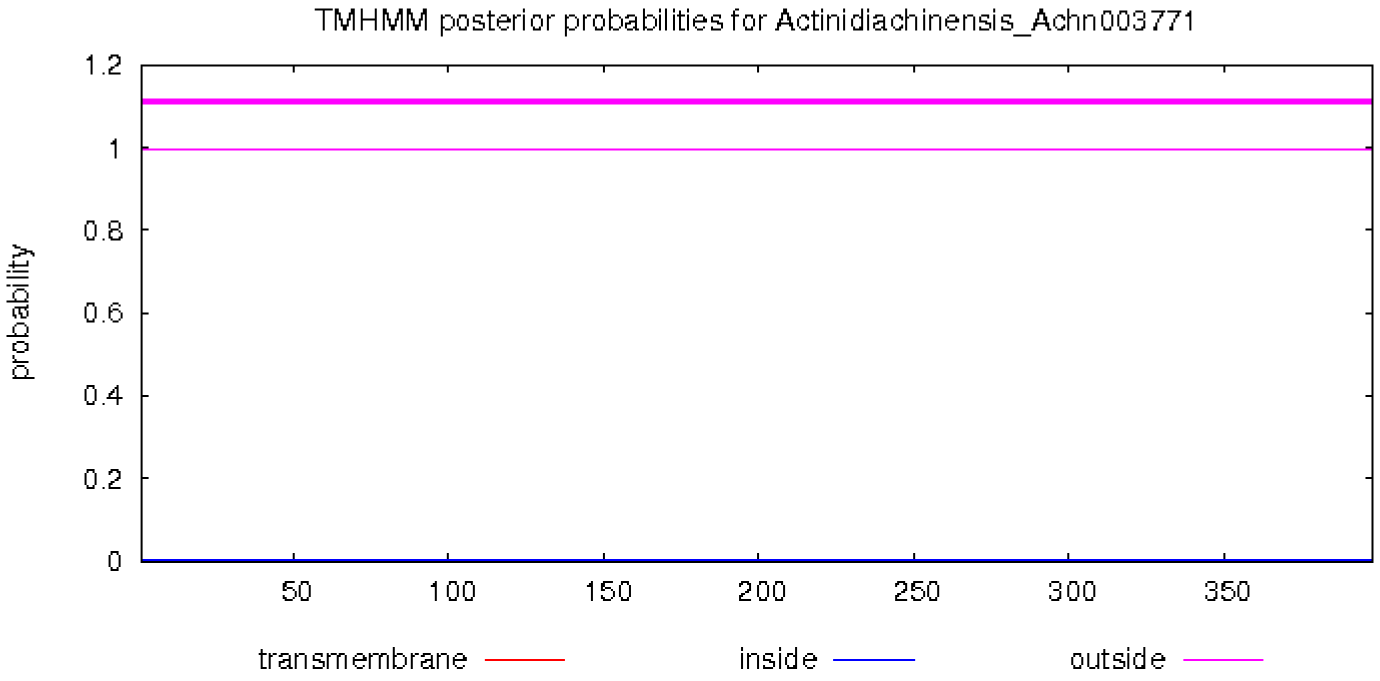

plot in postscript, script for making the plot in gnuplot, data for plot

---

```
# Actinidiachinensis_Achn003781 Length: 537
# Actinidiachinensis_Achn003781 Number of predicted TMHs:  1
# Actinidiachinensis_Achn003781 Exp number of AAs in TMHs: 17.16907
# Actinidiachinensis_Achn003781 Exp number, first 60 AAs:  0.00243
# Actinidiachinensis_Achn003781 Total prob of N-in:        0.08984
Actinidiachinensis_Achn003781	TMHMM2.0	outside	     1   503
```

```
Actinidiachinensis_Achn003781   TMHMM2.0        TMhelix    504   521
Actinidiachinensis_Achn003781   TMHMM2.0        inside     522   537
```

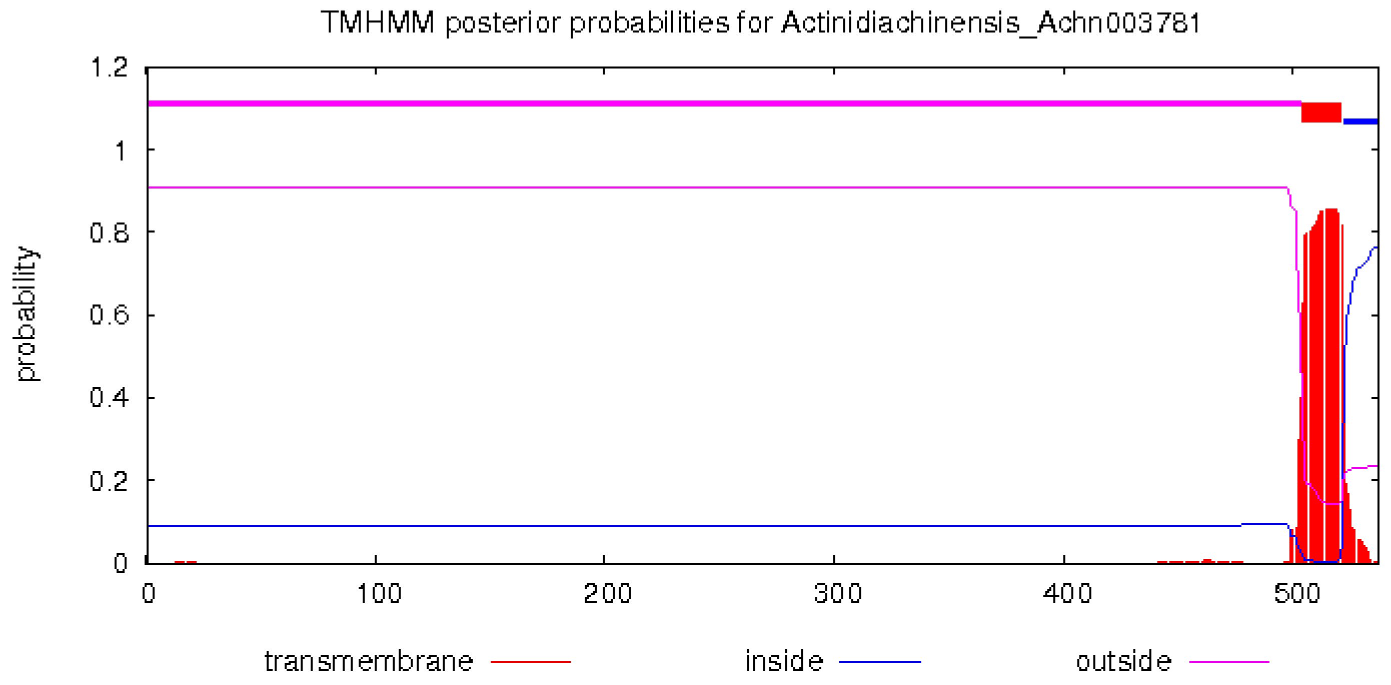

plot in postscript, script for making the plot in gnuplot, data for plot

---

```
# Actinidiachinensis_Achn004191 Length: 360
# Actinidiachinensis_Achn004191 Number of predicted TMHs:  0
# Actinidiachinensis_Achn004191 Exp number of AAs in TMHs: 0.00296
# Actinidiachinensis_Achn004191 Exp number, first 60 AAs:  0
# Actinidiachinensis_Achn004191 Total prob of N-in:        0.00143
Actinidiachinensis_Achn004191   TMHMM2.0        outside      1   360
```

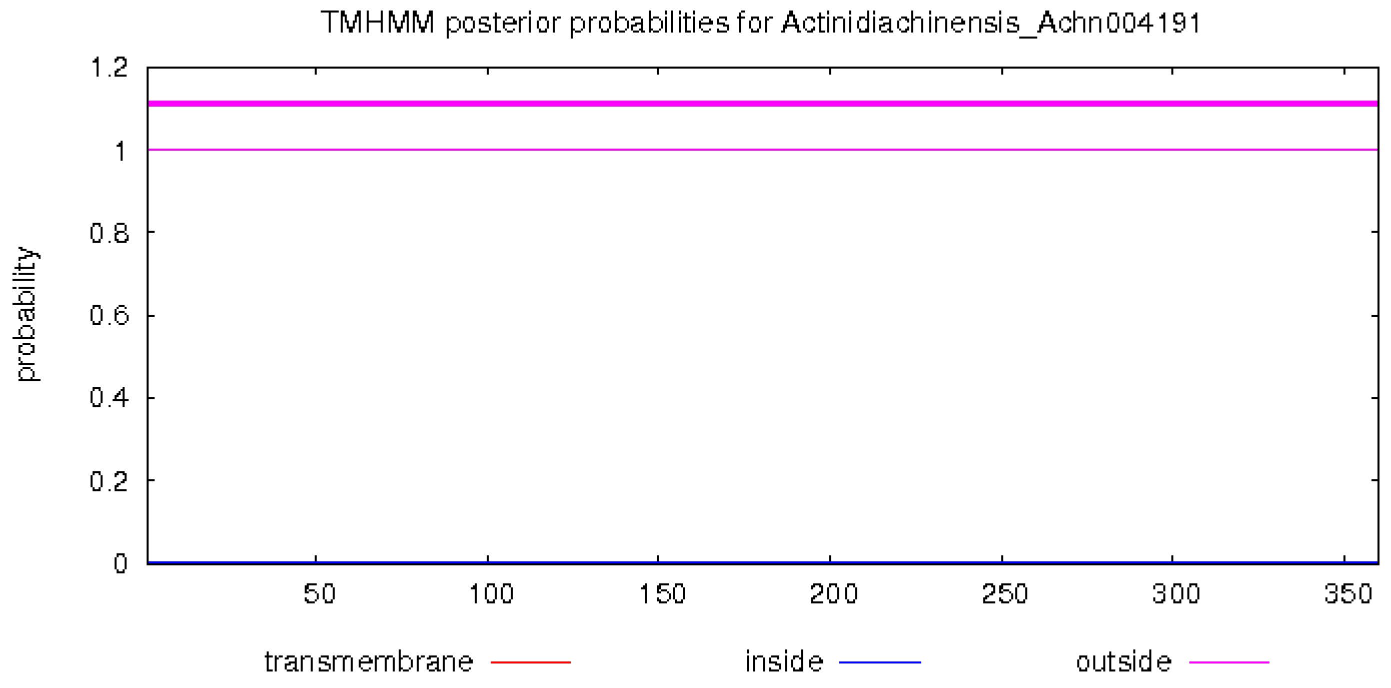

plot in postscript, script for making the plot in gnuplot, data for plot

---

```
# Actinidiachinensis_Achn004841 Length: 558
# Actinidiachinensis_Achn004841 Number of predicted TMHs:  0
# Actinidiachinensis_Achn004841 Exp number of AAs in TMHs: 0.00091
# Actinidiachinensis_Achn004841 Exp number, first 60 AAs:  0.00011
```

```
# Actinidiachinensis_Achn004841 Total prob of N-in:        0.00063
Actinidiachinensis_Achn004841   TMHMM2.0        outside      1   558
```

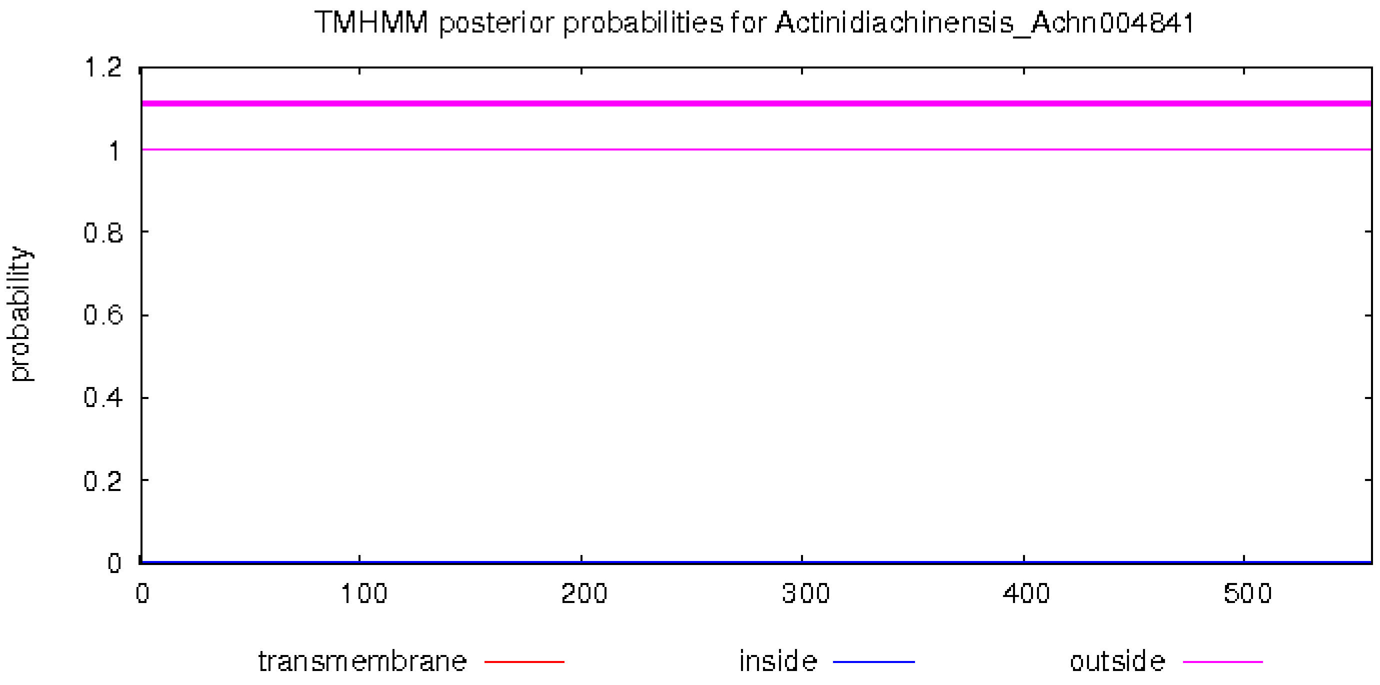

plot in postscript, script for making the plot in gnuplot, data for plot

---

```
# Actinidiachinensis_Achn008531 Length: 321
# Actinidiachinensis_Achn008531 Number of predicted TMHs:  0
# Actinidiachinensis_Achn008531 Exp number of AAs in TMHs: 0.00022
# Actinidiachinensis_Achn008531 Exp number, first 60 AAs:  0
# Actinidiachinensis_Achn008531 Total prob of N-in:        0.00115
Actinidiachinensis_Achn008531   TMHMM2.0        outside      1   321
```

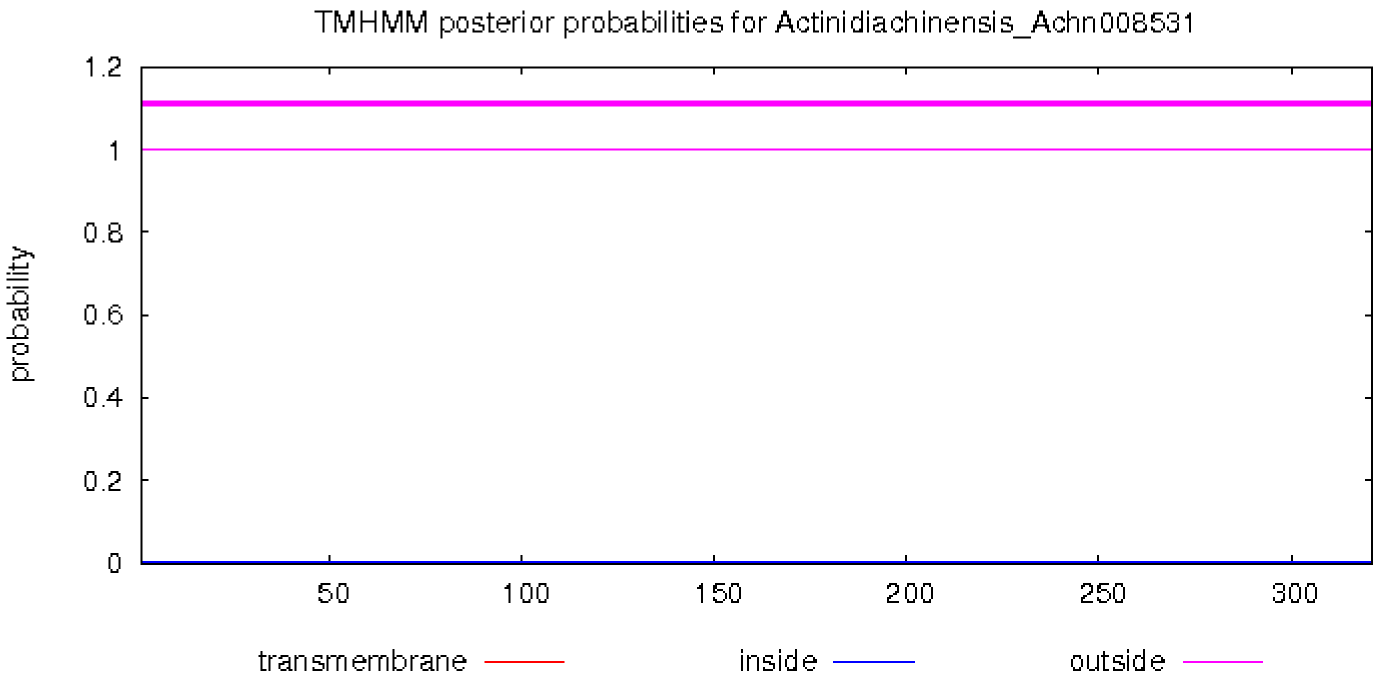

plot in postscript, script for making the plot in gnuplot, data for plot

---

```
# Actinidiachinensis_Achn008541 Length: 481
# Actinidiachinensis_Achn008541 Number of predicted TMHs:  0
# Actinidiachinensis_Achn008541 Exp number of AAs in TMHs: 0.00056
# Actinidiachinensis_Achn008541 Exp number, first 60 AAs:  0
```

```
# Actinidiachinensis_Achn008541 Total prob of N-in:        0.00040
Actinidiachinensis_Achn008541   TMHMM2.0        outside      1   481
```

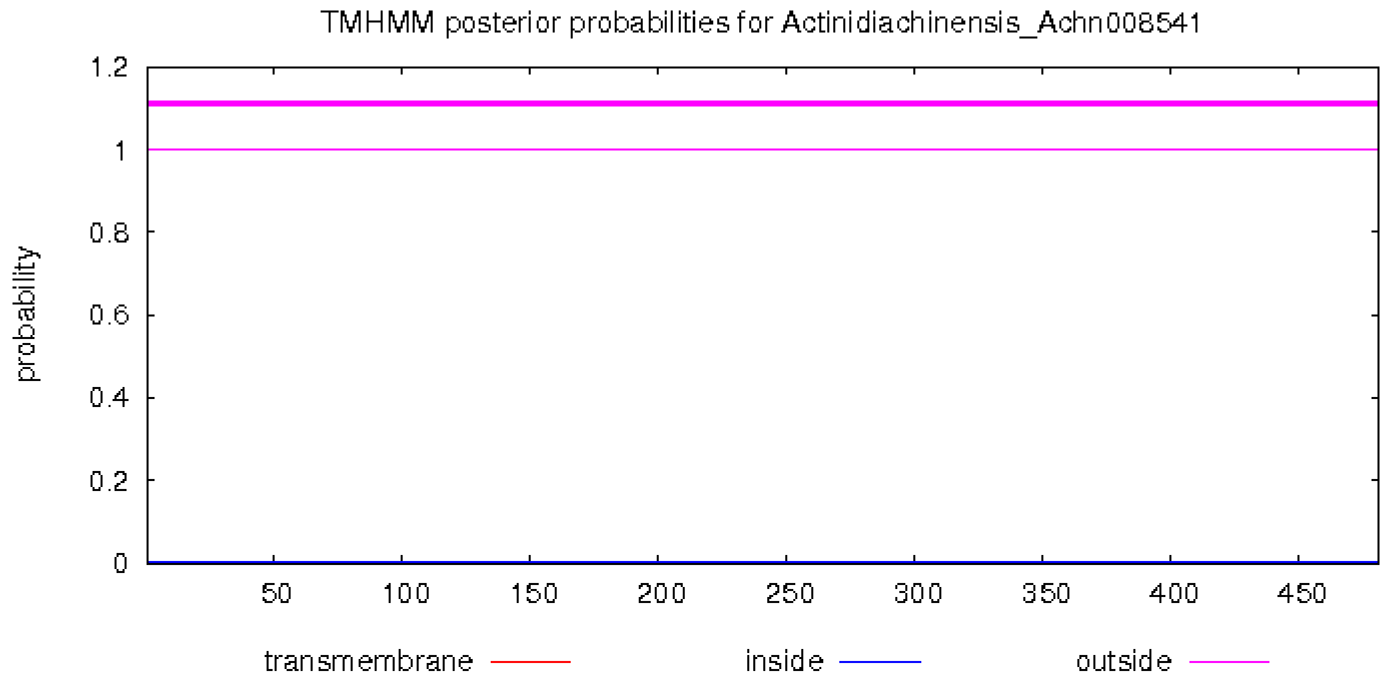

plot in postscript, script for making the plot in gnuplot, data for plot

```
# Actinidiachinensis_Achn008561 Length: 439
# Actinidiachinensis_Achn008561 Number of predicted TMHs:  0
# Actinidiachinensis_Achn008561 Exp number of AAs in TMHs: 0.00738
# Actinidiachinensis_Achn008561 Exp number, first 60 AAs:  0.00684
# Actinidiachinensis_Achn008561 Total prob of N-in:        0.00925
Actinidiachinensis_Achn008561   TMHMM2.0        outside      1   439
```

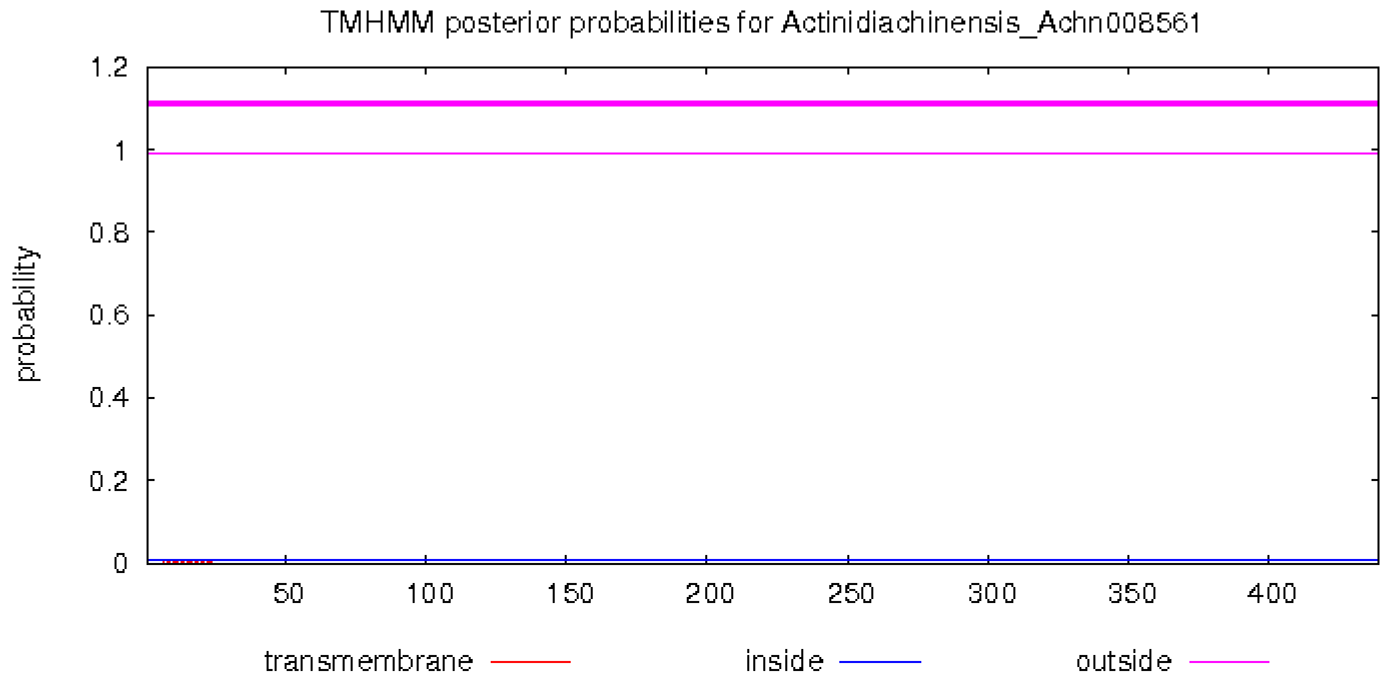

plot in postscript, script for making the plot in gnuplot, data for plot

```
# Actinidiachinensis_Achn008571 Length: 375
# Actinidiachinensis_Achn008571 Number of predicted TMHs:  0
# Actinidiachinensis_Achn008571 Exp number of AAs in TMHs: 0.00225
# Actinidiachinensis_Achn008571 Exp number, first 60 AAs:  0
```

```
# Actinidiachinensis_Achn008571 Total prob of N-in:        0.01789
Actinidiachinensis_Achn008571	TMHMM2.0	outside	     1   375
```

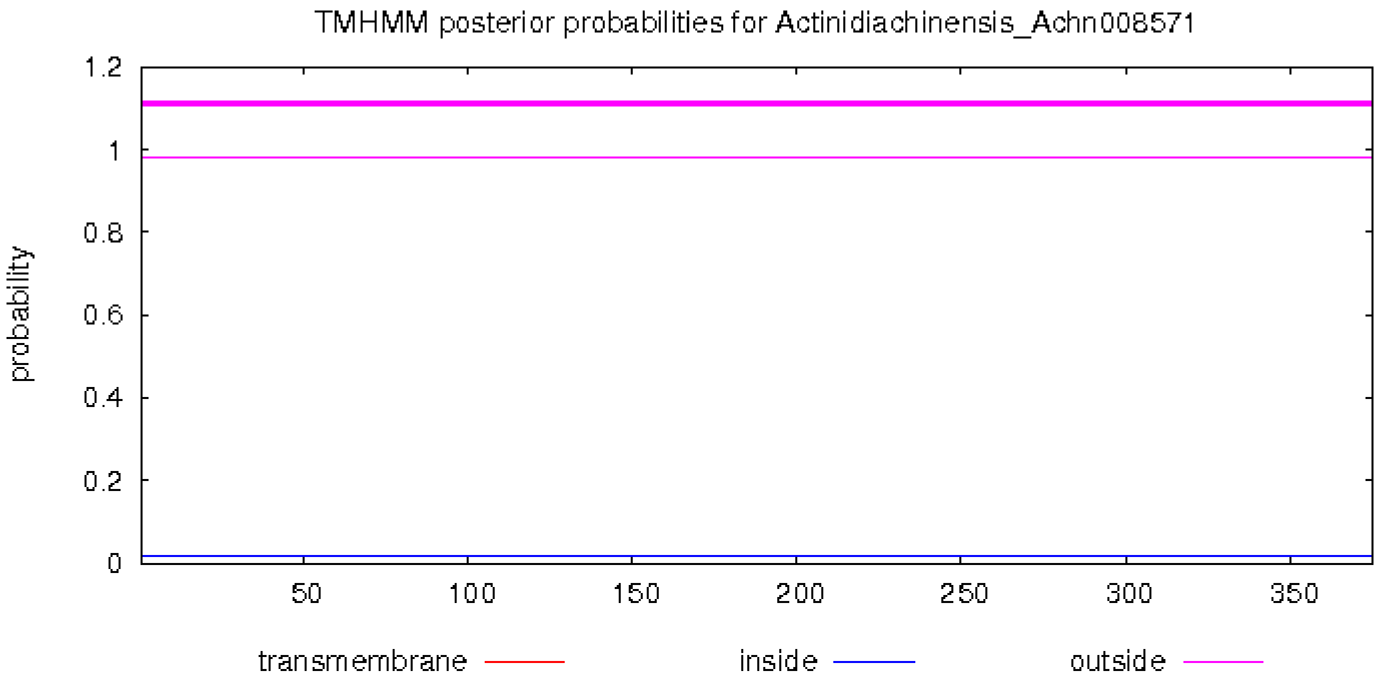

plot in postscript, script for making the plot in gnuplot, data for plot

---

```
# Actinidiachinensis_Achn009451 Length: 380
# Actinidiachinensis_Achn009451 Number of predicted TMHs:  0
# Actinidiachinensis_Achn009451 Exp number of AAs in TMHs: 0.00037
# Actinidiachinensis_Achn009451 Exp number, first 60 AAs:  0
# Actinidiachinensis_Achn009451 Total prob of N-in:        0.00110
Actinidiachinensis_Achn009451	TMHMM2.0	outside	     1   380
```

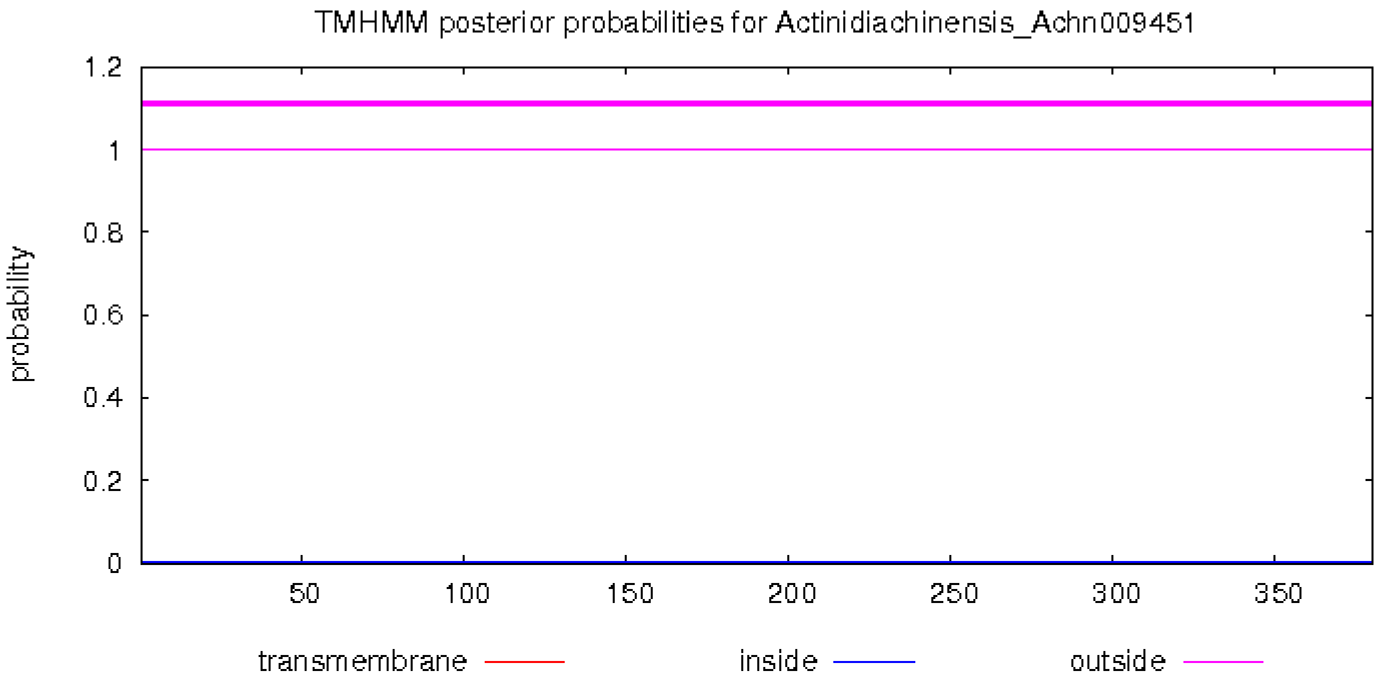

plot in postscript, script for making the plot in gnuplot, data for plot

---

```
# Actinidiachinensis_Achn009461 Length: 344
# Actinidiachinensis_Achn009461 Number of predicted TMHs:  0
# Actinidiachinensis_Achn009461 Exp number of AAs in TMHs: 0.000910000000000000001
# Actinidiachinensis_Achn009461 Exp number, first 60 AAs:  0.0003
```

```
# Actinidiachinensis_Achn009461 Total prob of N-in:        0.02851
Actinidiachinensis_Achn009461   TMHMM2.0        outside      1   344
```

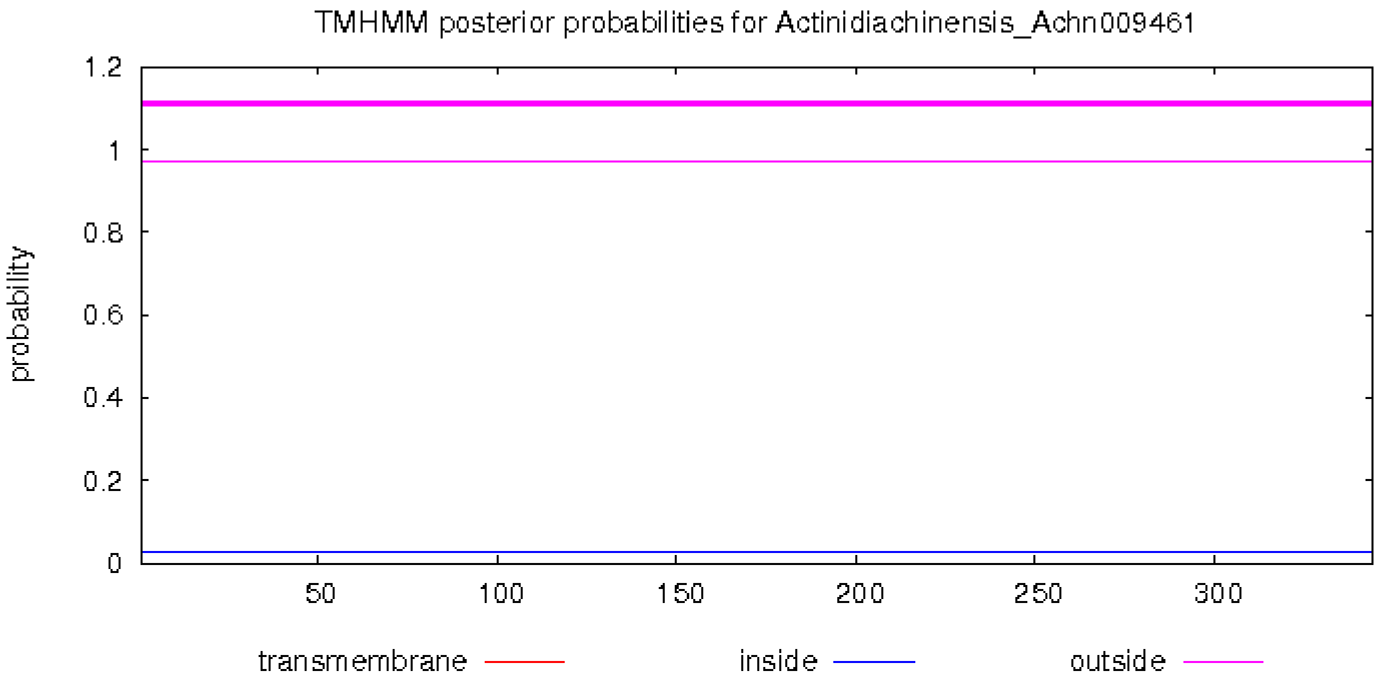

plot in postscript, script for making the plot in gnuplot, data for plot

---

```
# Actinidiachinensis_Achn009471 Length: 380
# Actinidiachinensis_Achn009471 Number of predicted TMHs:  0
# Actinidiachinensis_Achn009471 Exp number of AAs in TMHs: 0.00056
# Actinidiachinensis_Achn009471 Exp number, first 60 AAs:  0
# Actinidiachinensis_Achn009471 Total prob of N-in:        0.00117
Actinidiachinensis_Achn009471   TMHMM2.0        outside      1   380
```

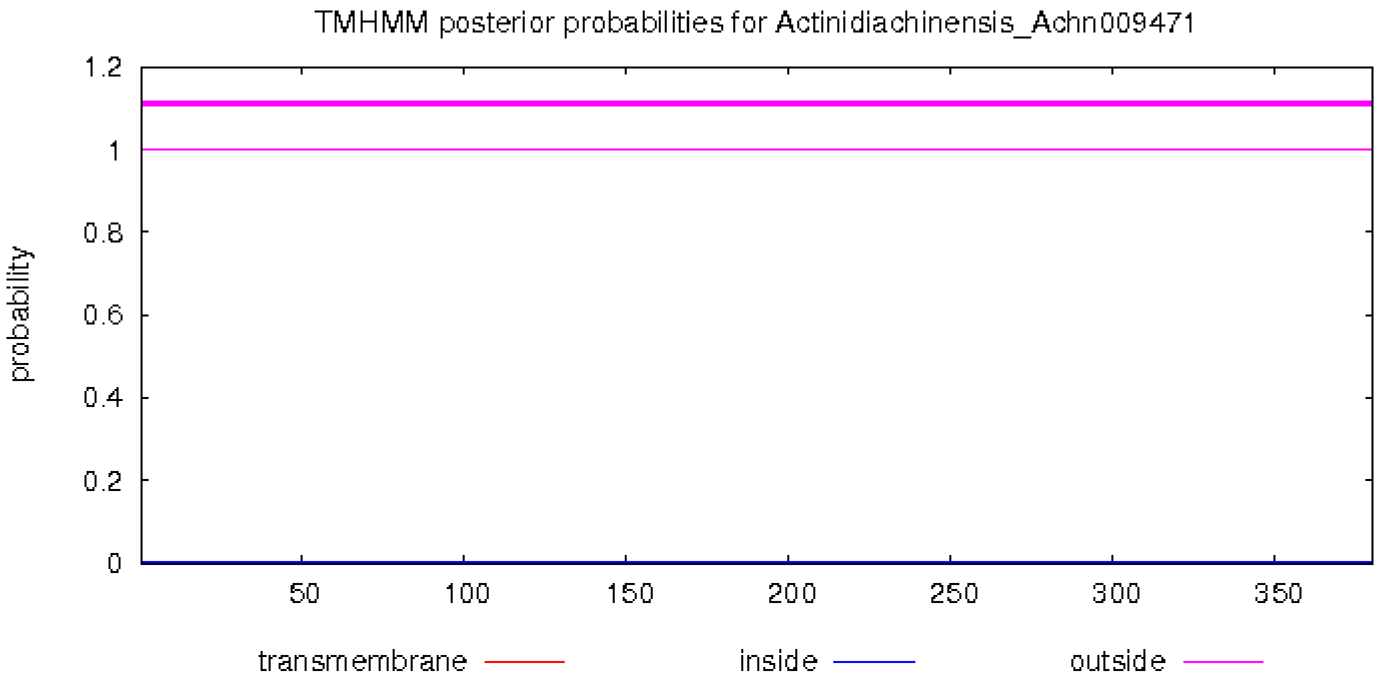

plot in postscript, script for making the plot in gnuplot, data for plot

---

```
# Actinidiachinensis_Achn009481 Length: 334
# Actinidiachinensis_Achn009481 Number of predicted TMHs:  0
# Actinidiachinensis_Achn009481 Exp number of AAs in TMHs: 0.00106
# Actinidiachinensis_Achn009481 Exp number, first 60 AAs:  0.0003
```

```
# Actinidiachinensis_Achn009481 Total prob of N-in:        0.03114
Actinidiachinensis_Achn009481   TMHMM2.0        outside      1   334
```

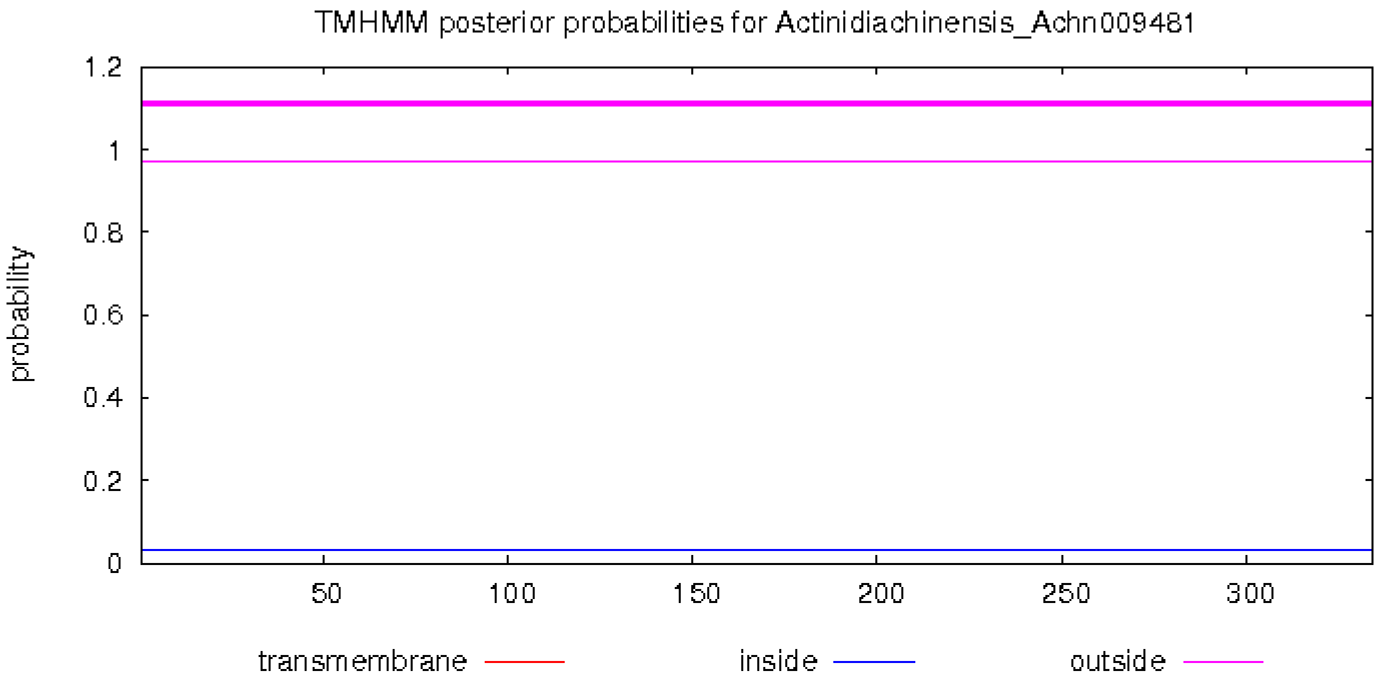

plot in postscript, script for making the plot in gnuplot, data for plot

---

```
# Actinidiachinensis_Achn014031 Length: 358
# Actinidiachinensis_Achn014031 Number of predicted TMHs:  0
# Actinidiachinensis_Achn014031 Exp number of AAs in TMHs: 0.03414
# Actinidiachinensis_Achn014031 Exp number, first 60 AAs:  0
# Actinidiachinensis_Achn014031 Total prob of N-in:        0.01077
Actinidiachinensis_Achn014031   TMHMM2.0        outside      1   358
```

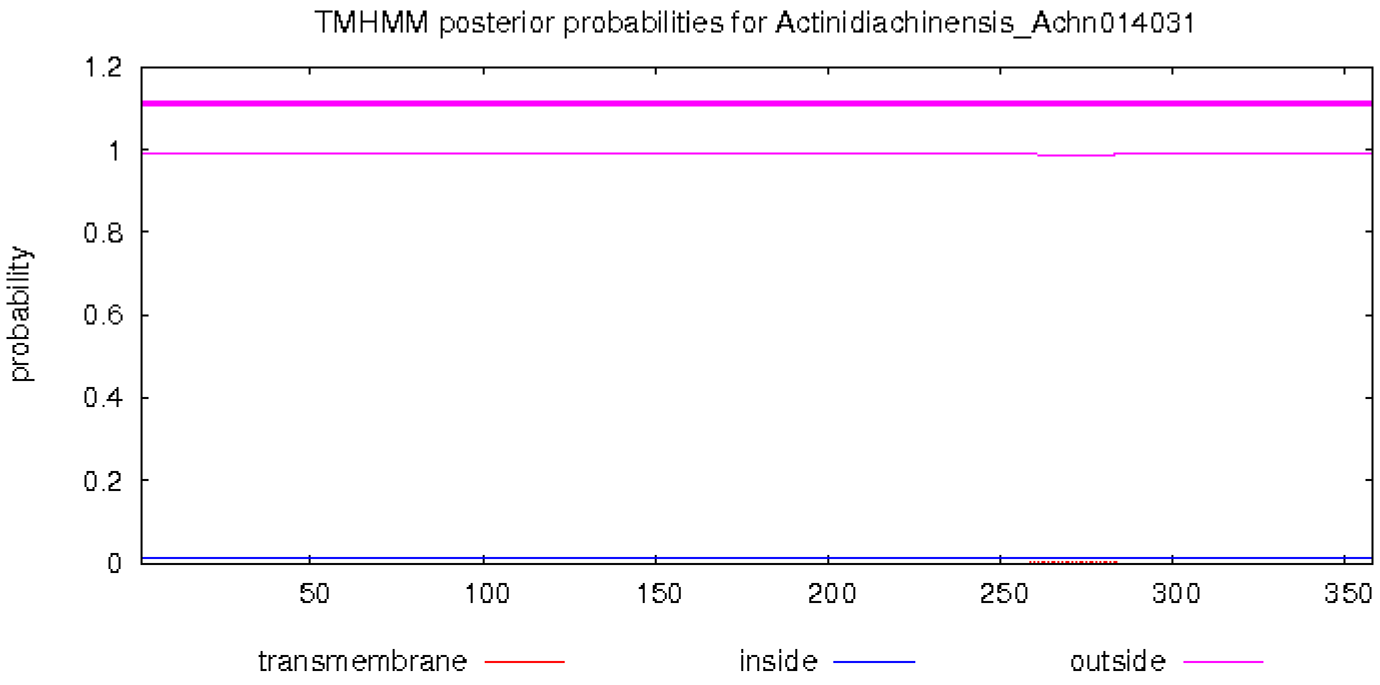

plot in postscript, script for making the plot in gnuplot, data for plot

---

```
# Actinidiachinensis_Achn016981 Length: 239
# Actinidiachinensis_Achn016981 Number of predicted TMHs:  0
# Actinidiachinensis_Achn016981 Exp number of AAs in TMHs: 0.00231
# Actinidiachinensis_Achn016981 Exp number, first 60 AAs:  0.00231
```

```
# Actinidiachinensis_Achn016981 Total prob of N-in:        0.05012
Actinidiachinensis_Achn016981   TMHMM2.0        outside      1   239
```

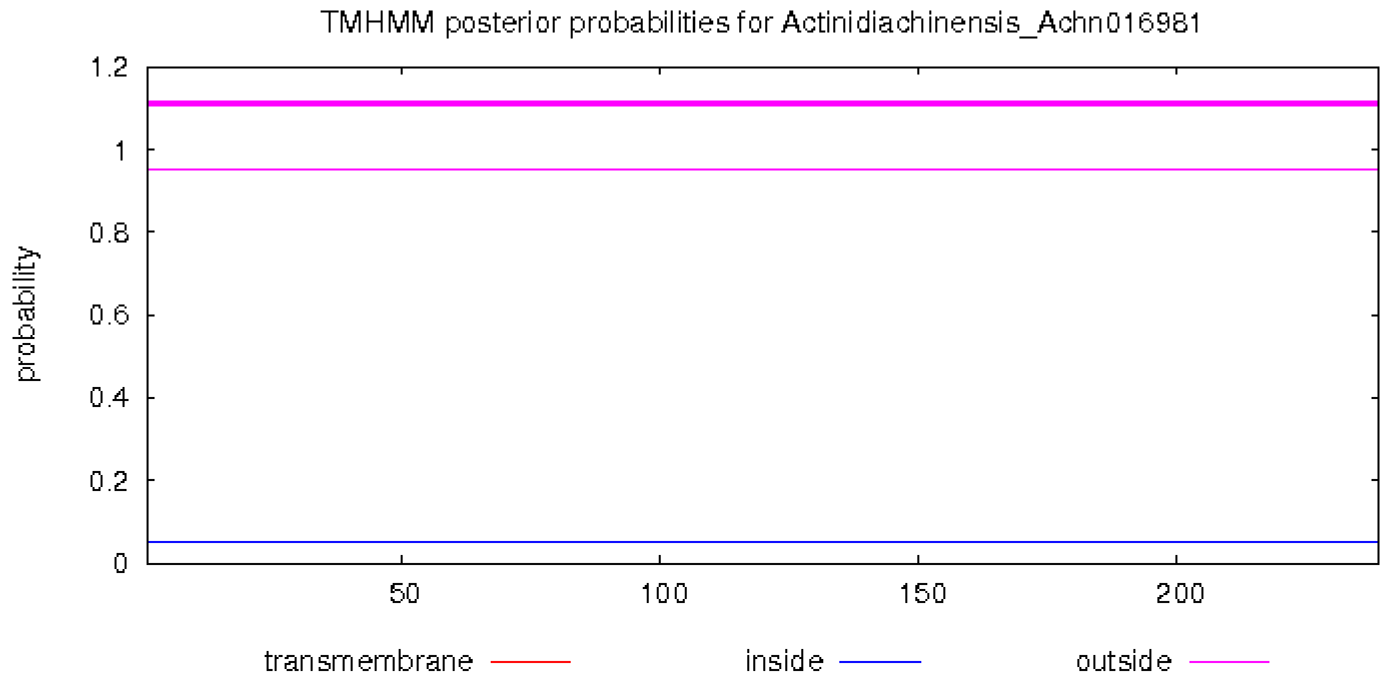

plot in postscript, script for making the plot in gnuplot, data for plot

---

```
# Actinidiachinensis_Achn019581 Length: 286
# Actinidiachinensis_Achn019581 Number of predicted TMHs:  0
# Actinidiachinensis_Achn019581 Exp number of AAs in TMHs: 0.00092
# Actinidiachinensis_Achn019581 Exp number, first 60 AAs:  0.00092
# Actinidiachinensis_Achn019581 Total prob of N-in:        0.02646
Actinidiachinensis_Achn019581   TMHMM2.0        outside      1   286
```

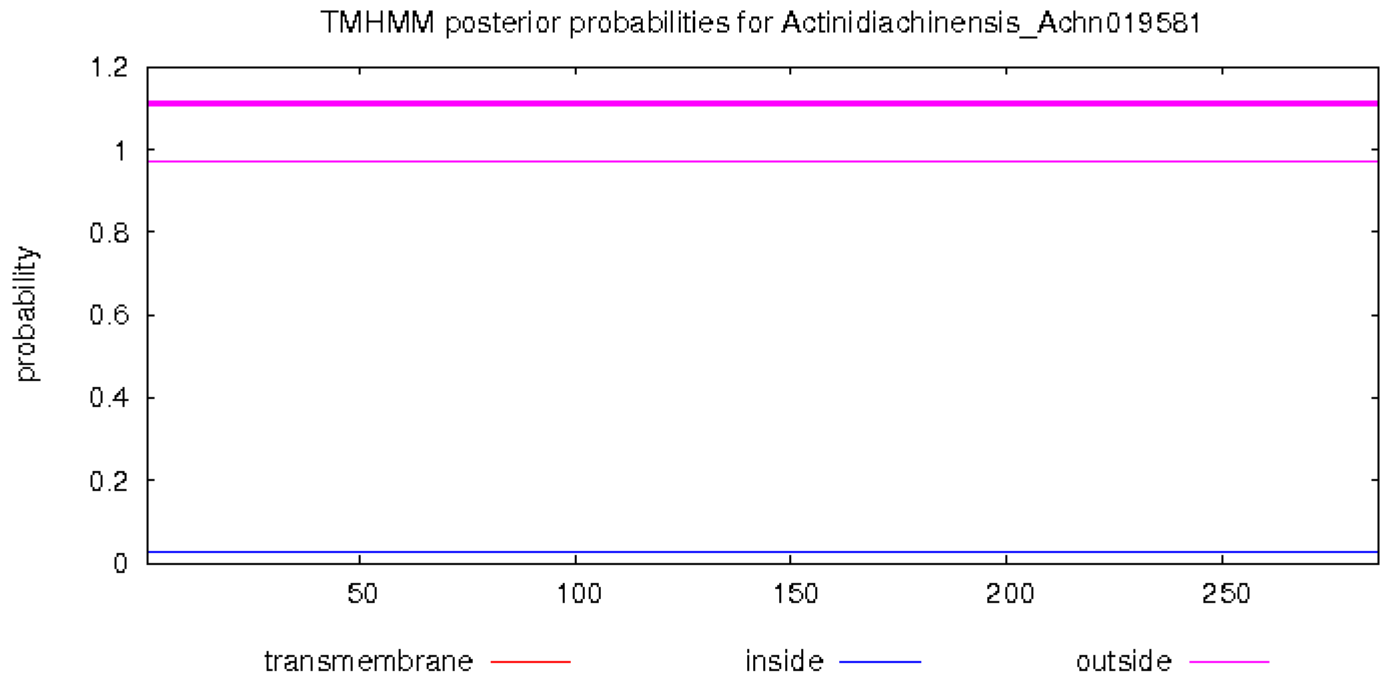

plot in postscript, script for making the plot in gnuplot, data for plot

---

```
# Actinidiachinensis_Achn021021 Length: 302
# Actinidiachinensis_Achn021021 Number of predicted TMHs:  0
# Actinidiachinensis_Achn021021 Exp number of AAs in TMHs: 0.68722
# Actinidiachinensis_Achn021021 Exp number, first 60 AAs:  0.00061
```

```
# Actinidiachinensis_Achn021021 Total prob of N-in:        0.01862
Actinidiachinensis_Achn021021   TMHMM2.0        outside      1   302
```

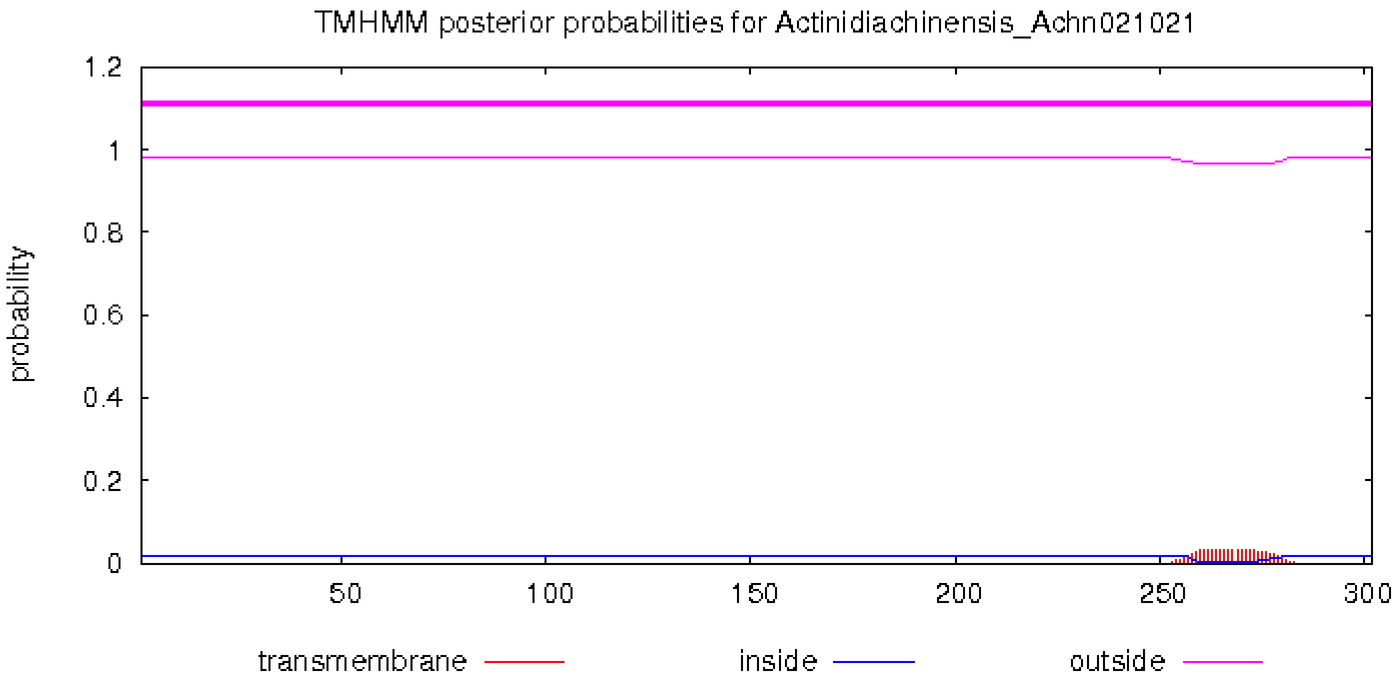

plot in postscript, script for making the plot in gnuplot, data for plot

---

```
# Actinidiachinensis_Achn022601 Length: 203
# Actinidiachinensis_Achn022601 Number of predicted TMHs:  0
# Actinidiachinensis_Achn022601 Exp number of AAs in TMHs: 0.01385
# Actinidiachinensis_Achn022601 Exp number, first 60 AAs:  0
# Actinidiachinensis_Achn022601 Total prob of N-in:        0.00942
Actinidiachinensis_Achn022601   TMHMM2.0        outside      1   203
```

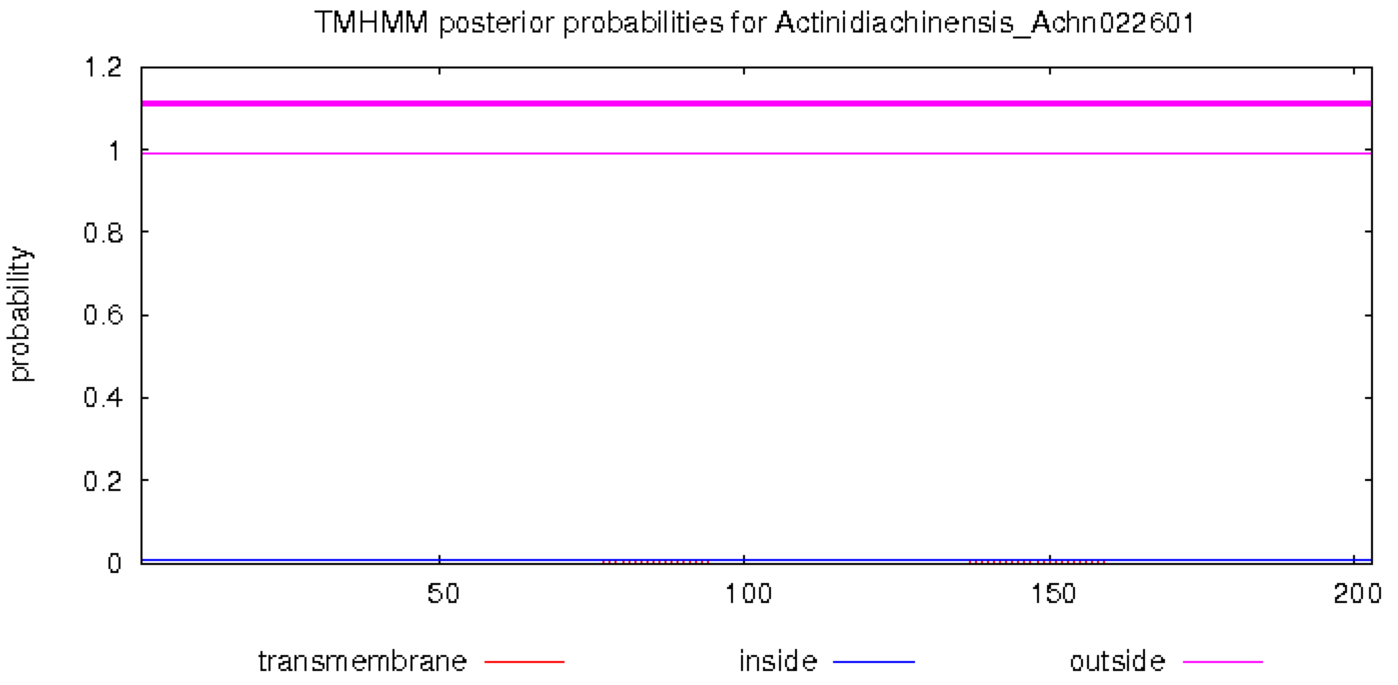

plot in postscript, script for making the plot in gnuplot, data for plot

---

```
# Actinidiachinensis_Achn022611 Length: 372
# Actinidiachinensis_Achn022611 Number of predicted TMHs:  0
# Actinidiachinensis_Achn022611 Exp number of AAs in TMHs: 18.22854
# Actinidiachinensis_Achn022611 Exp number, first 60 AAs:  0
```

```
# Actinidiachinensis_Achn022611 Total prob of N-in:        0.75137
Actinidiachinensis_Achn022611   TMHMM2.0        outside      1   372
```

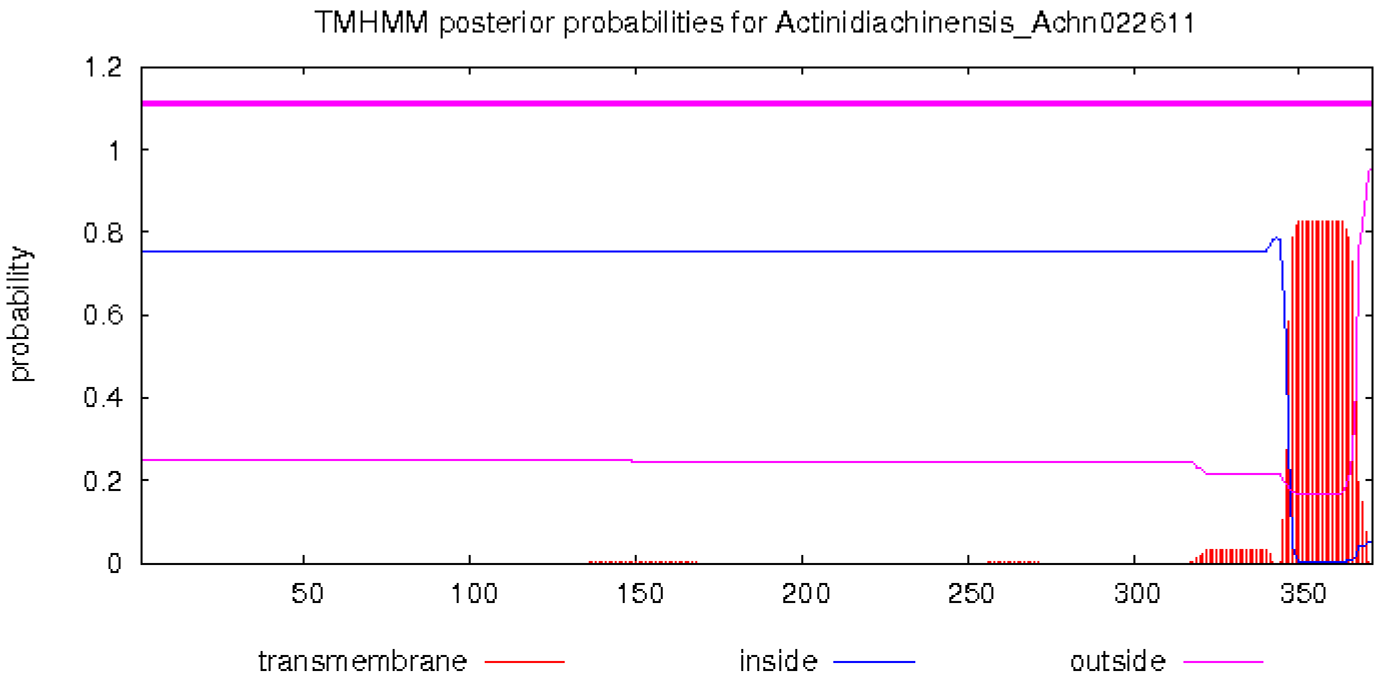

plot in postscript, script for making the plot in gnuplot, data for plot

---

```
# Actinidiachinensis_Achn029231 Length: 269
# Actinidiachinensis_Achn029231 Number of predicted TMHs:  0
# Actinidiachinensis_Achn029231 Exp number of AAs in TMHs: 0.00354
# Actinidiachinensis_Achn029231 Exp number, first 60 AAs:  0.00043
# Actinidiachinensis_Achn029231 Total prob of N-in:        0.18757
Actinidiachinensis_Achn029231   TMHMM2.0        outside      1   269
```

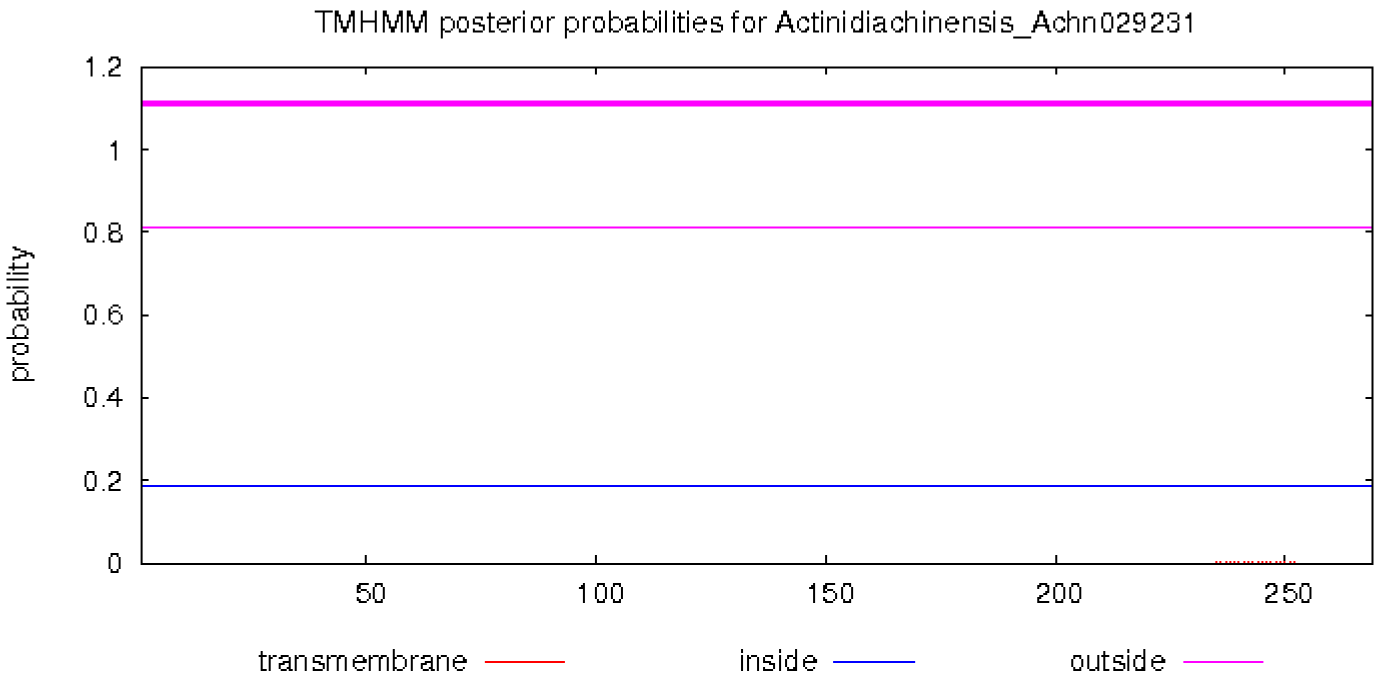

plot in postscript, script for making the plot in gnuplot, data for plot

---

```
# Actinidiachinensis_Achn029521 Length: 250
# Actinidiachinensis_Achn029521 Number of predicted TMHs:  0
# Actinidiachinensis_Achn029521 Exp number of AAs in TMHs: 0.02283
# Actinidiachinensis_Achn029521 Exp number, first 60 AAs:  0.02283
```

```
# Actinidiachinensis_Achn029521 Total prob of N-in:        0.03497
Actinidiachinensis_Achn029521   TMHMM2.0        outside      1   250
```

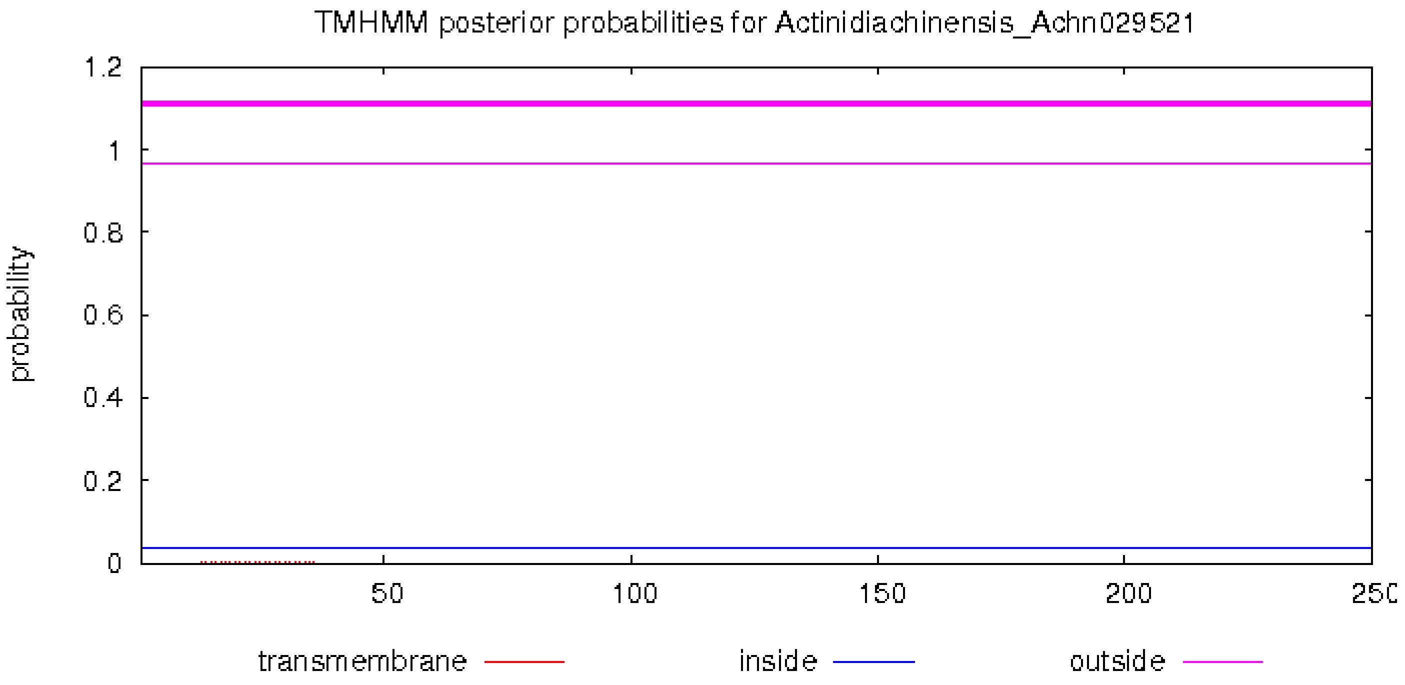

plot in postscript, script for making the plot in gnuplot, data for plot

---

```
# Actinidiachinensis_Achn033651 Length: 310
# Actinidiachinensis_Achn033651 Number of predicted TMHs:  0
# Actinidiachinensis_Achn033651 Exp number of AAs in TMHs: 0.23162
# Actinidiachinensis_Achn033651 Exp number, first 60 AAs:  0
# Actinidiachinensis_Achn033651 Total prob of N-in:        0.00729
Actinidiachinensis_Achn033651   TMHMM2.0        outside      1   310
```

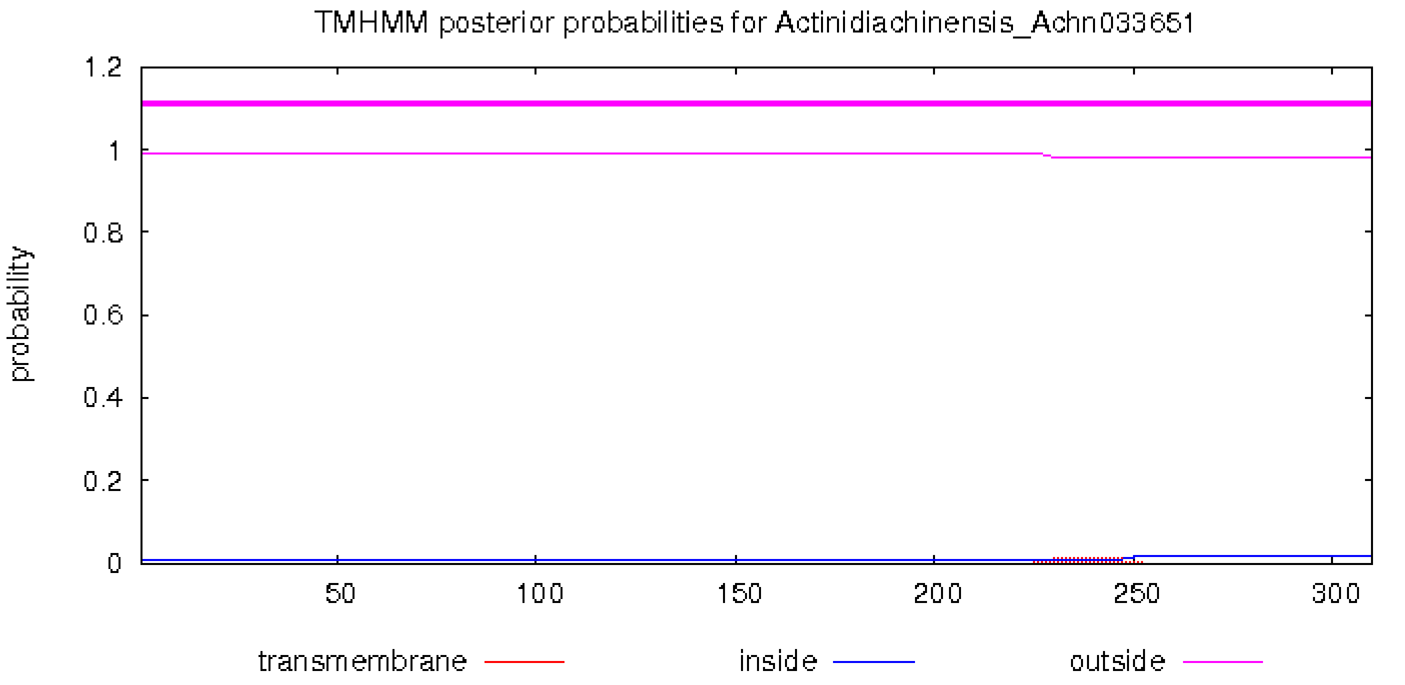

plot in postscript, script for making the plot in gnuplot, data for plot

---

```
# Actinidiachinensis_Achn048661 Length: 270
# Actinidiachinensis_Achn048661 Number of predicted TMHs:  0
# Actinidiachinensis_Achn048661 Exp number of AAs in TMHs: 0.84503
# Actinidiachinensis_Achn048661 Exp number, first 60 AAs:  0.84386
```

```
# Actinidiachinensis_Achn048661 Total prob of N-in:        0.00806
Actinidiachinensis_Achn048661   TMHMM2.0        outside      1   270
```

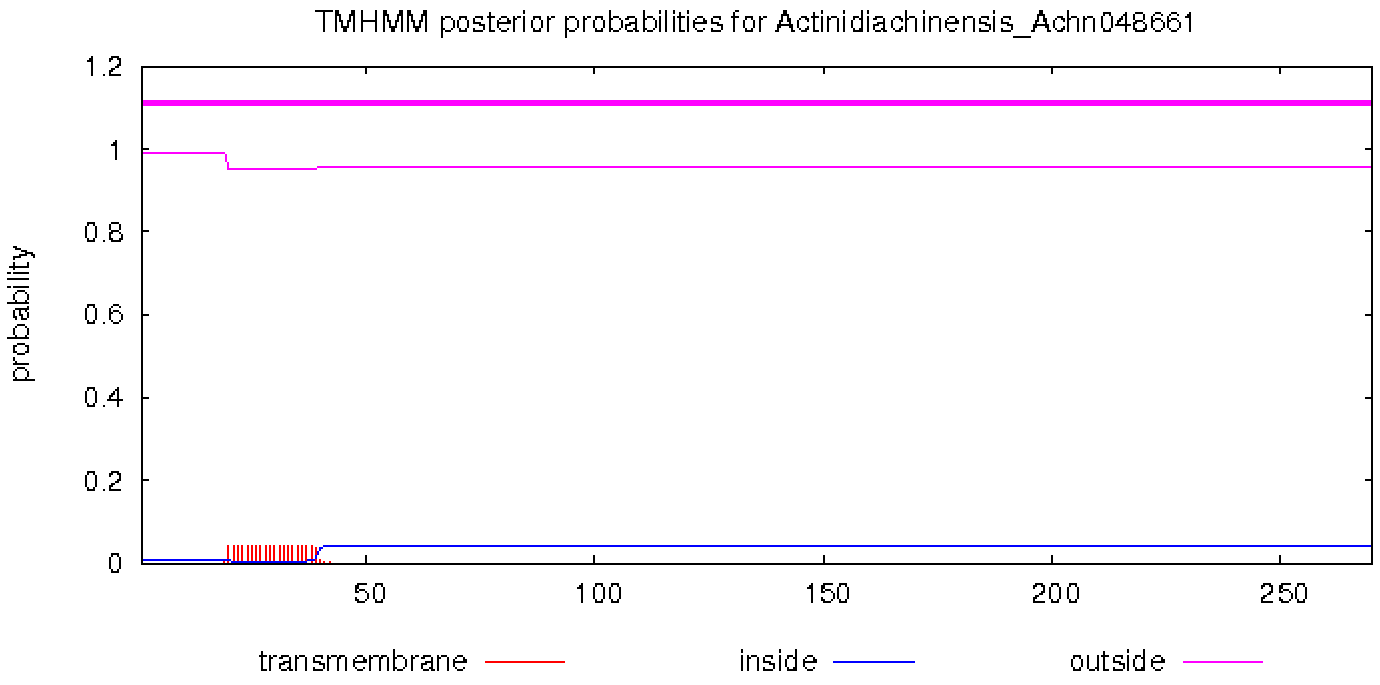

plot in postscript, script for making the plot in gnuplot, data for plot

---

```
# Actinidiachinensis_Achn051151 Length: 297
# Actinidiachinensis_Achn051151 Number of predicted TMHs:  0
# Actinidiachinensis_Achn051151 Exp number of AAs in TMHs: 0.00013
# Actinidiachinensis_Achn051151 Exp number, first 60 AAs:  0
# Actinidiachinensis_Achn051151 Total prob of N-in:        0.03570
Actinidiachinensis_Achn051151   TMHMM2.0        outside      1   297
```

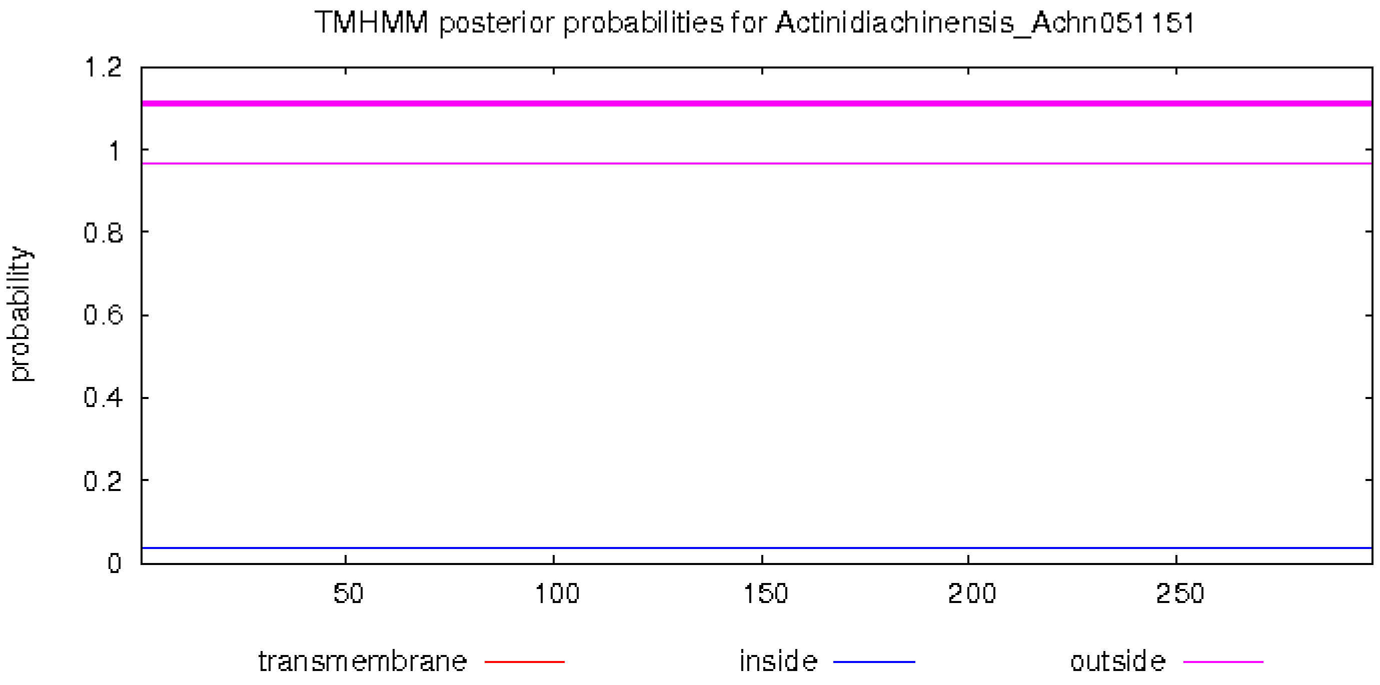

plot in postscript, script for making the plot in gnuplot, data for plot

---

```
# Actinidiachinensis_Achn053241 Length: 153
# Actinidiachinensis_Achn053241 Number of predicted TMHs:  0
# Actinidiachinensis_Achn053241 Exp number of AAs in TMHs: 0
# Actinidiachinensis_Achn053241 Exp number, first 60 AAs:  0
```

```
# Actinidiachinensis_Achn053241 Total prob of N-in:        0.08496
Actinidiachinensis_Achn053241   TMHMM2.0        outside      1   153
```

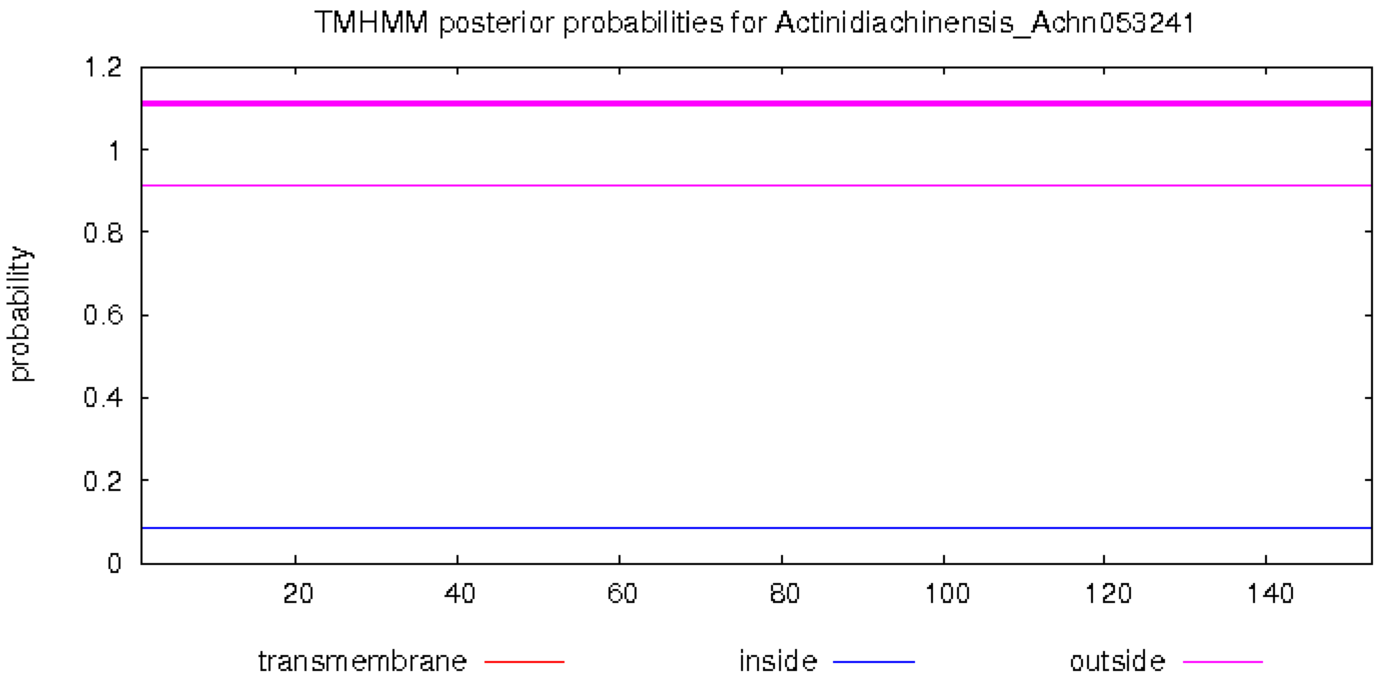

plot in postscript, script for making the plot in gnuplot, data for plot

---

```
# Actinidiachinensis_Achn054031 Length: 403
# Actinidiachinensis_Achn054031 Number of predicted TMHs:  0
# Actinidiachinensis_Achn054031 Exp number of AAs in TMHs: 0.00059
# Actinidiachinensis_Achn054031 Exp number, first 60 AAs:  0.00041
# Actinidiachinensis_Achn054031 Total prob of N-in:        0.00377
Actinidiachinensis_Achn054031   TMHMM2.0        outside      1   403
```

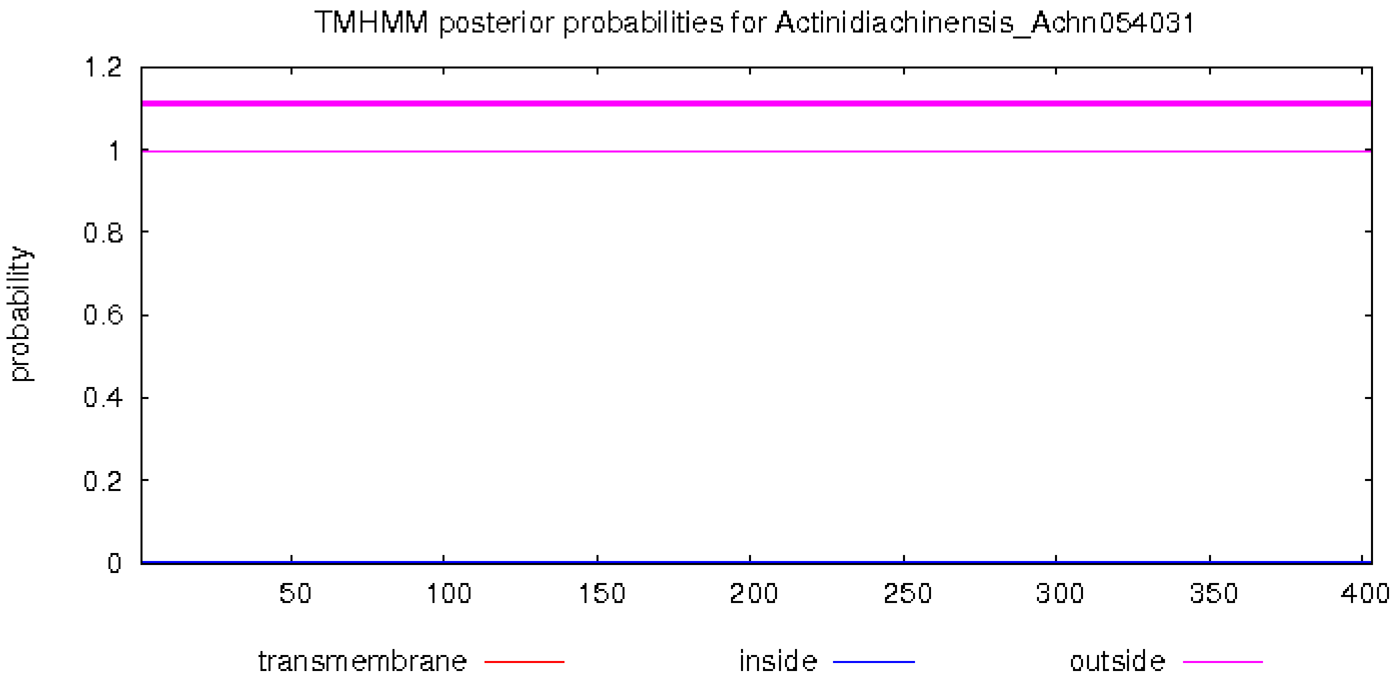

plot in postscript, script for making the plot in gnuplot, data for plot

---

```
# Actinidiachinensis_Achn054041 Length: 531
# Actinidiachinensis_Achn054041 Number of predicted TMHs:  1
# Actinidiachinensis_Achn054041 Exp number of AAs in TMHs: 21.65707
# Actinidiachinensis_Achn054041 Exp number, first 60 AAs:  0
```

```
# Actinidiachinensis_Achn054041 Total prob of N-in:        0.00271
Actinidiachinensis_Achn054041   TMHMM2.0        outside      1   503
Actinidiachinensis_Achn054041   TMHMM2.0        TMhelix    504   526
Actinidiachinensis_Achn054041   TMHMM2.0        inside     527   531
```

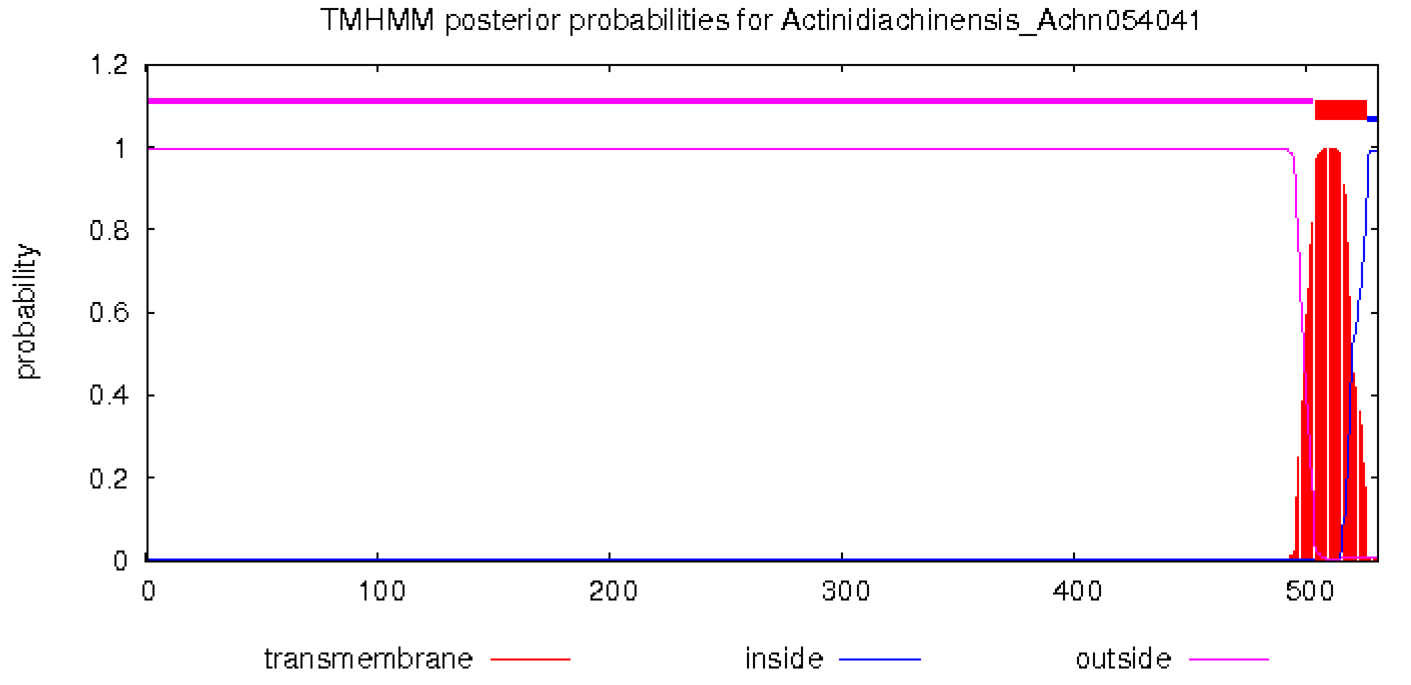

plot in postscript, script for making the plot in gnuplot, data for plot

---

```
# Actinidiachinensis_Achn059641 Length: 403
# Actinidiachinensis_Achn059641 Number of predicted TMHs:  0
# Actinidiachinensis_Achn059641 Exp number of AAs in TMHs: 0.00058
# Actinidiachinensis_Achn059641 Exp number, first 60 AAs:  0.0004
# Actinidiachinensis_Achn059641 Total prob of N-in:        0.00510
Actinidiachinensis_Achn059641   TMHMM2.0        outside      1   403
```

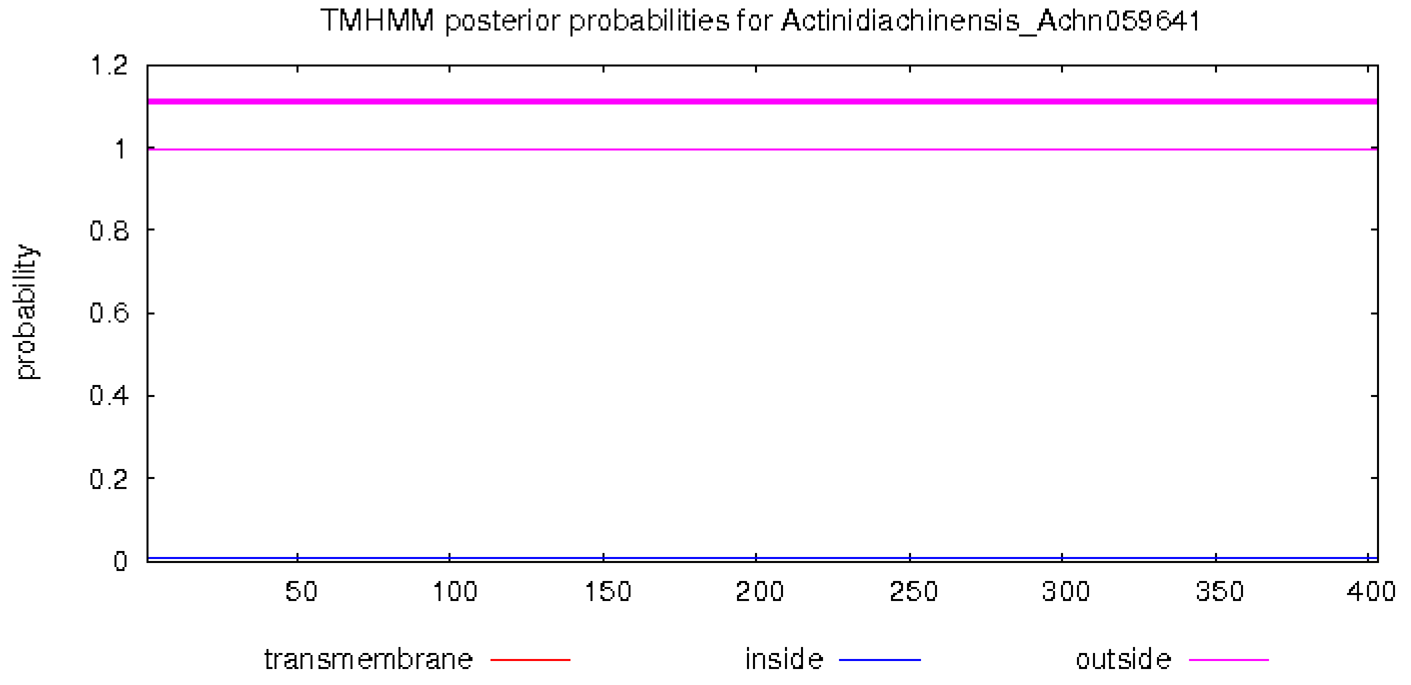

plot in postscript, script for making the plot in gnuplot, data for plot

---

```
# Actinidiachinensis_Achn069281 Length: 354
# Actinidiachinensis_Achn069281 Number of predicted TMHs:  0
```

```
# Actinidiachinensis_Achn069281 Exp number of AAs in TMHs: 0.00018
# Actinidiachinensis_Achn069281 Exp number, first 60 AAs:  0
# Actinidiachinensis_Achn069281 Total prob of N-in:        0.00875
Actinidiachinensis_Achn069281   TMHMM2.0        outside      1   354
```

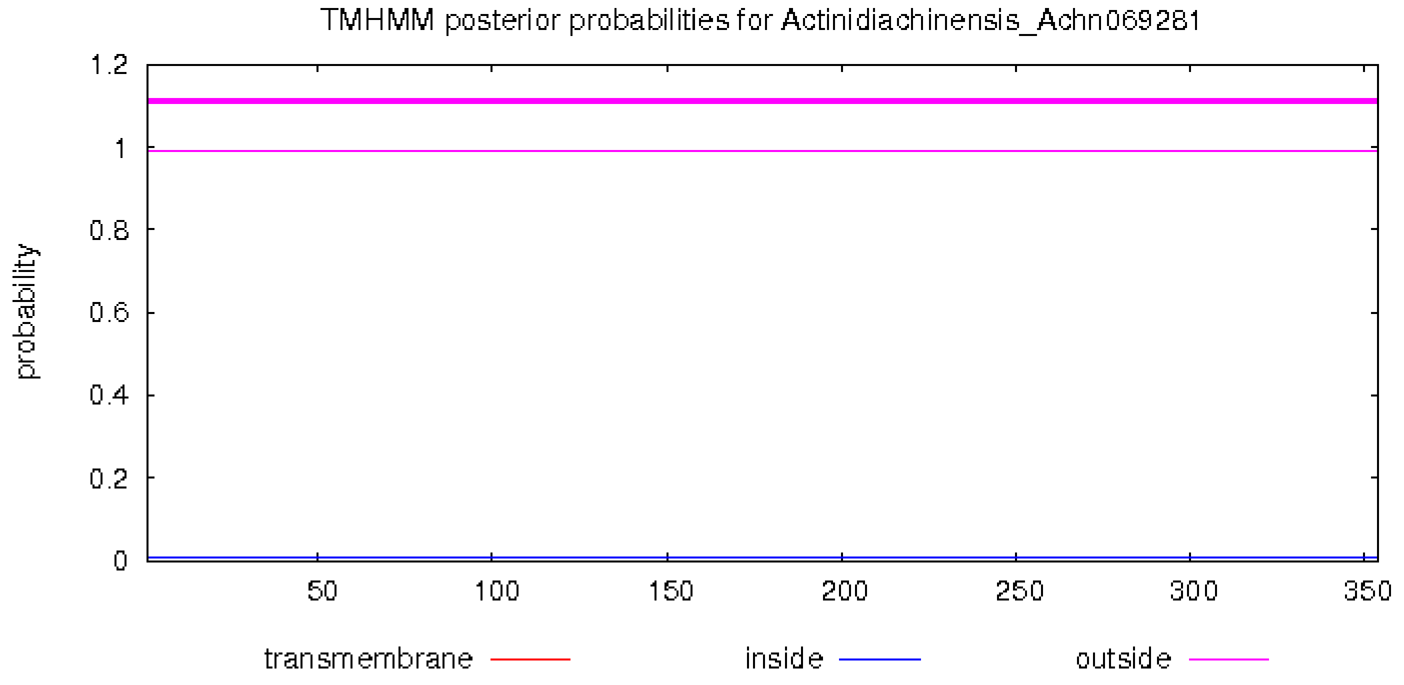

plot in postscript, script for making the plot in gnuplot, data for plot

---

```
# Actinidiachinensis_Achn069791 Length: 472
# Actinidiachinensis_Achn069791 Number of predicted TMHs:  0
# Actinidiachinensis_Achn069791 Exp number of AAs in TMHs: 0.00132
# Actinidiachinensis_Achn069791 Exp number, first 60 AAs:  0.00132
# Actinidiachinensis_Achn069791 Total prob of N-in:        0.00086
Actinidiachinensis_Achn069791   TMHMM2.0        outside      1   472
```

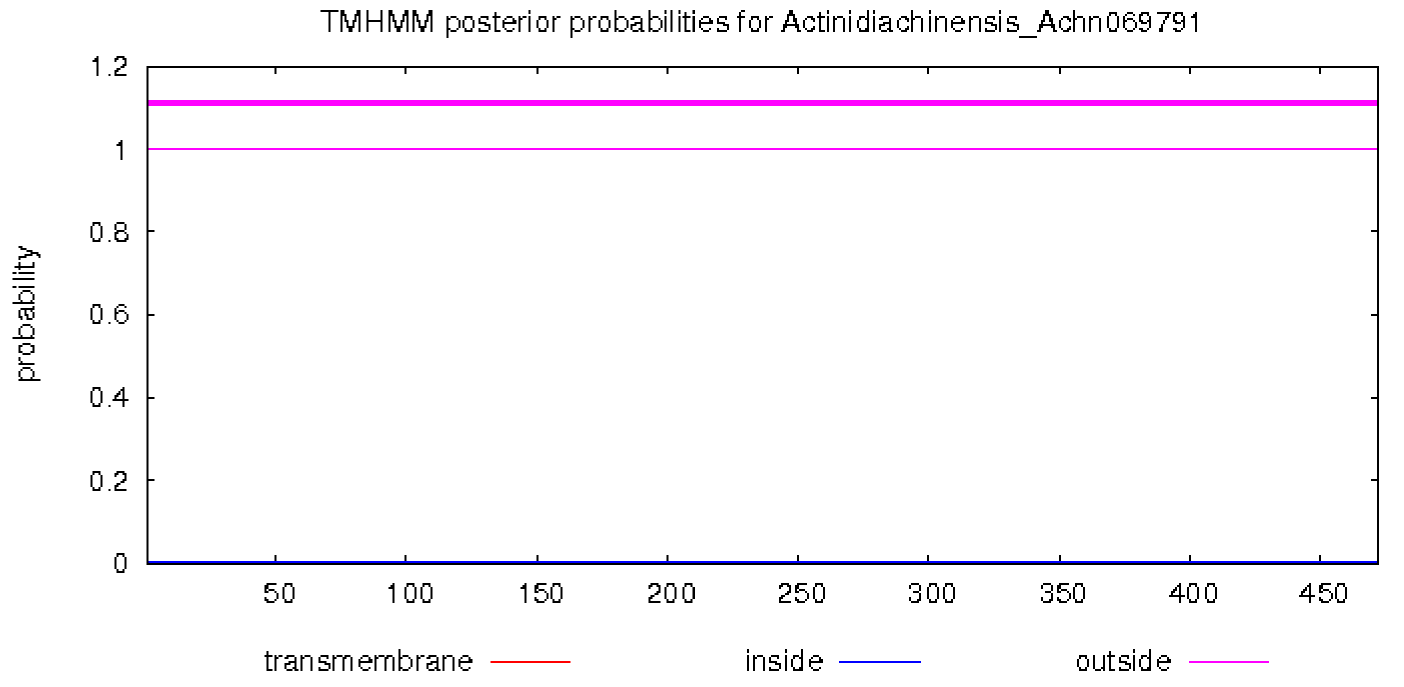

plot in postscript, script for making the plot in gnuplot, data for plot

---

```
# Actinidiachinensis_Achn070531 Length: 501
# Actinidiachinensis_Achn070531 Number of predicted TMHs:  0
```

```
# Actinidiachinensis_Achn070531 Exp number of AAs in TMHs: 0.0015
# Actinidiachinensis_Achn070531 Exp number, first 60 AAs:  0
# Actinidiachinensis_Achn070531 Total prob of N-in:        0.00231
Actinidiachinensis_Achn070531   TMHMM2.0        outside      1   501
```

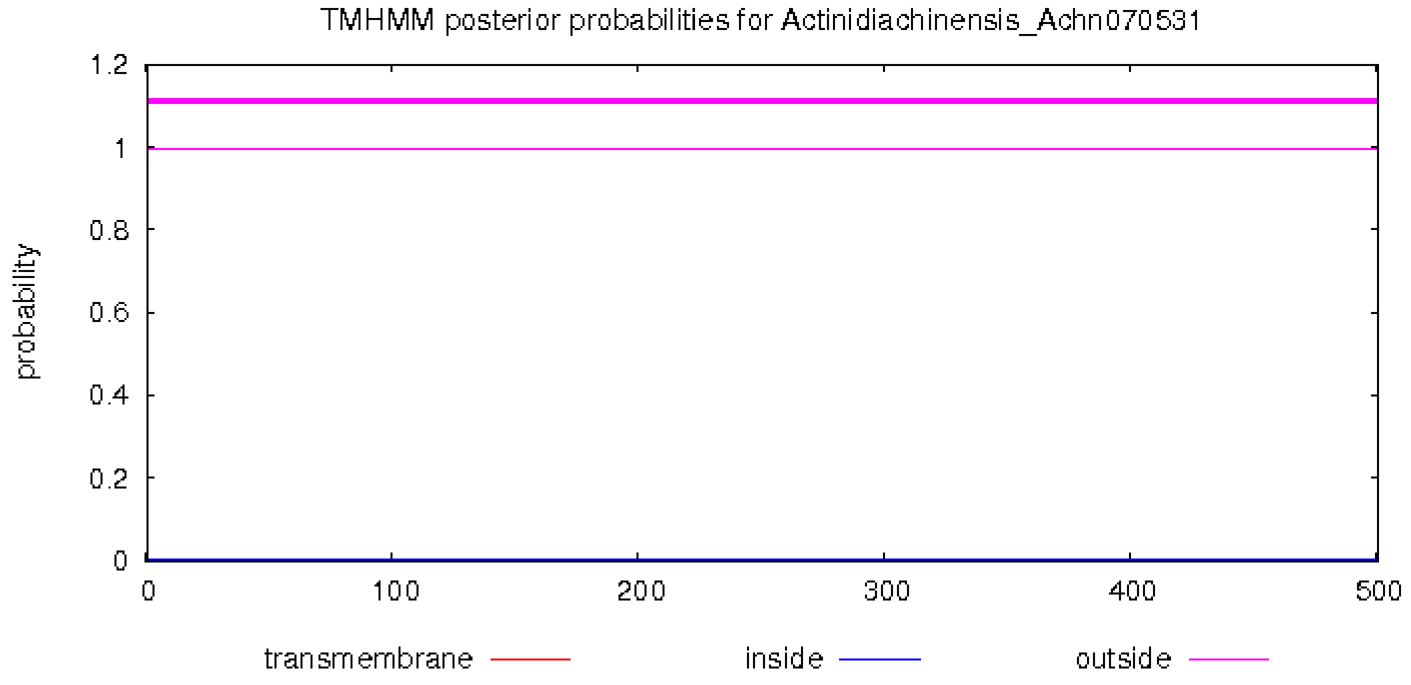

plot in postscript, script for making the plot in gnuplot, data for plot

---

```
# Actinidiachinensis_Achn073421 Length: 257
# Actinidiachinensis_Achn073421 Number of predicted TMHs:  0
# Actinidiachinensis_Achn073421 Exp number of AAs in TMHs: 0.00103
# Actinidiachinensis_Achn073421 Exp number, first 60 AAs:  0
# Actinidiachinensis_Achn073421 Total prob of N-in:        0.01993
Actinidiachinensis_Achn073421   TMHMM2.0        outside      1   257
```

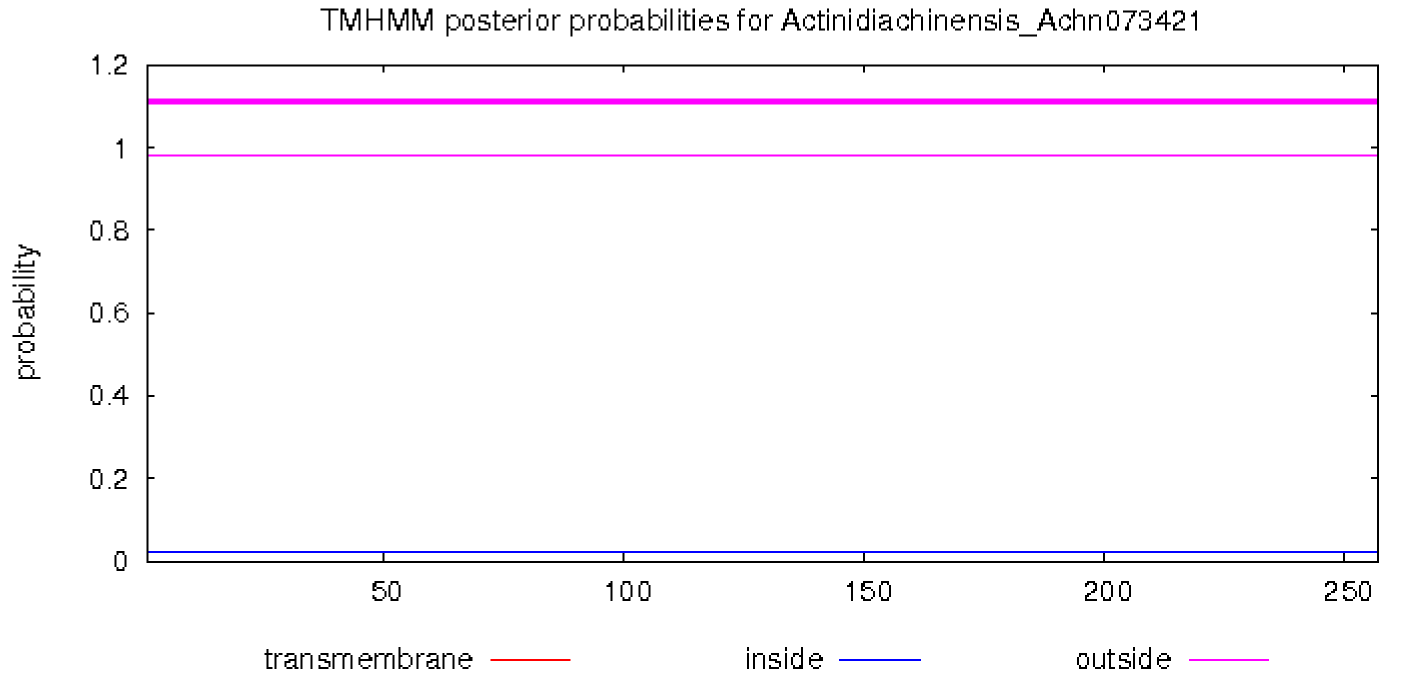

plot in postscript, script for making the plot in gnuplot, data for plot

---

```
# Actinidiachinensis_Achn074521 Length: 397
# Actinidiachinensis_Achn074521 Number of predicted TMHs:  0
```

```
# Actinidiachinensis_Achn074521 Exp number of AAs in TMHs: 0.00333
# Actinidiachinensis_Achn074521 Exp number, first 60 AAs:  0.00059
# Actinidiachinensis_Achn074521 Total prob of N-in:        0.02178
Actinidiachinensis_Achn074521   TMHMM2.0        outside      1   397
```

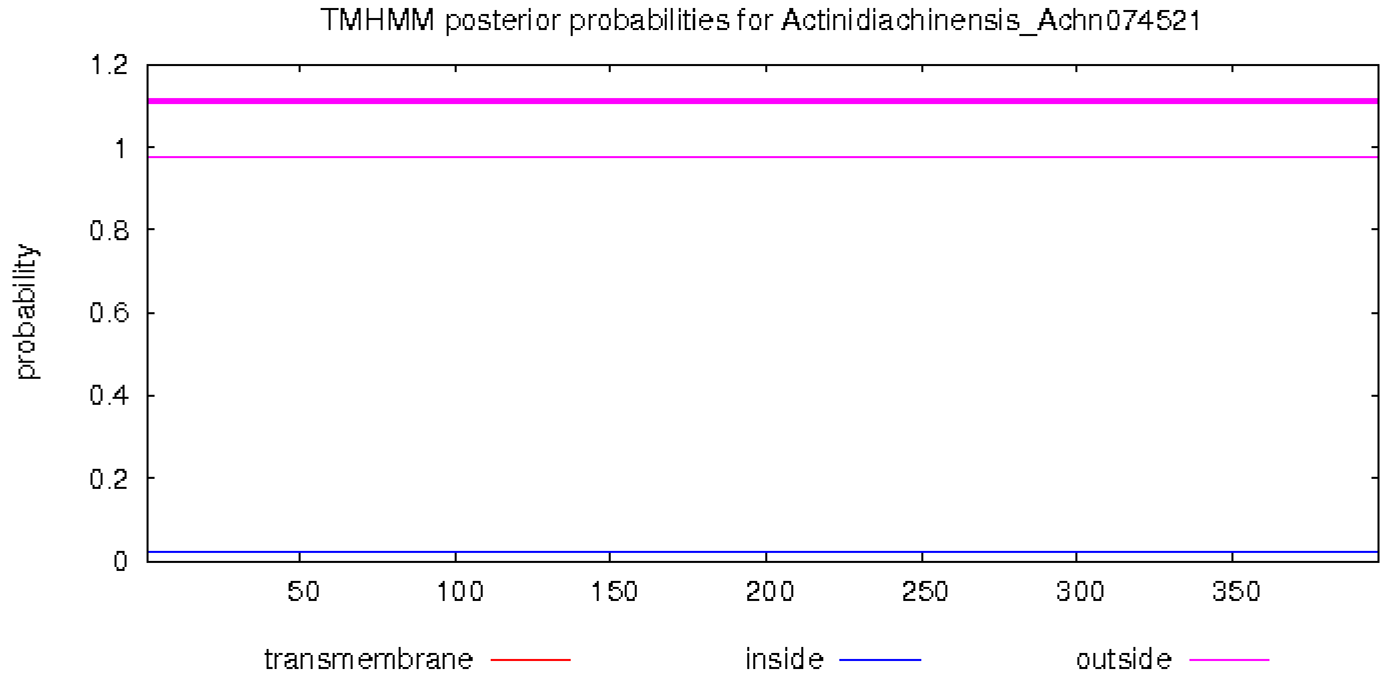

plot in postscript, script for making the plot in gnuplot, data for plot

---

```
# Actinidiachinensis_Achn077531 Length: 294
# Actinidiachinensis_Achn077531 Number of predicted TMHs:  0
# Actinidiachinensis_Achn077531 Exp number of AAs in TMHs: 0.00344
# Actinidiachinensis_Achn077531 Exp number, first 60 AAs:  0
# Actinidiachinensis_Achn077531 Total prob of N-in:        0.01693
Actinidiachinensis_Achn077531   TMHMM2.0        outside      1   294
```

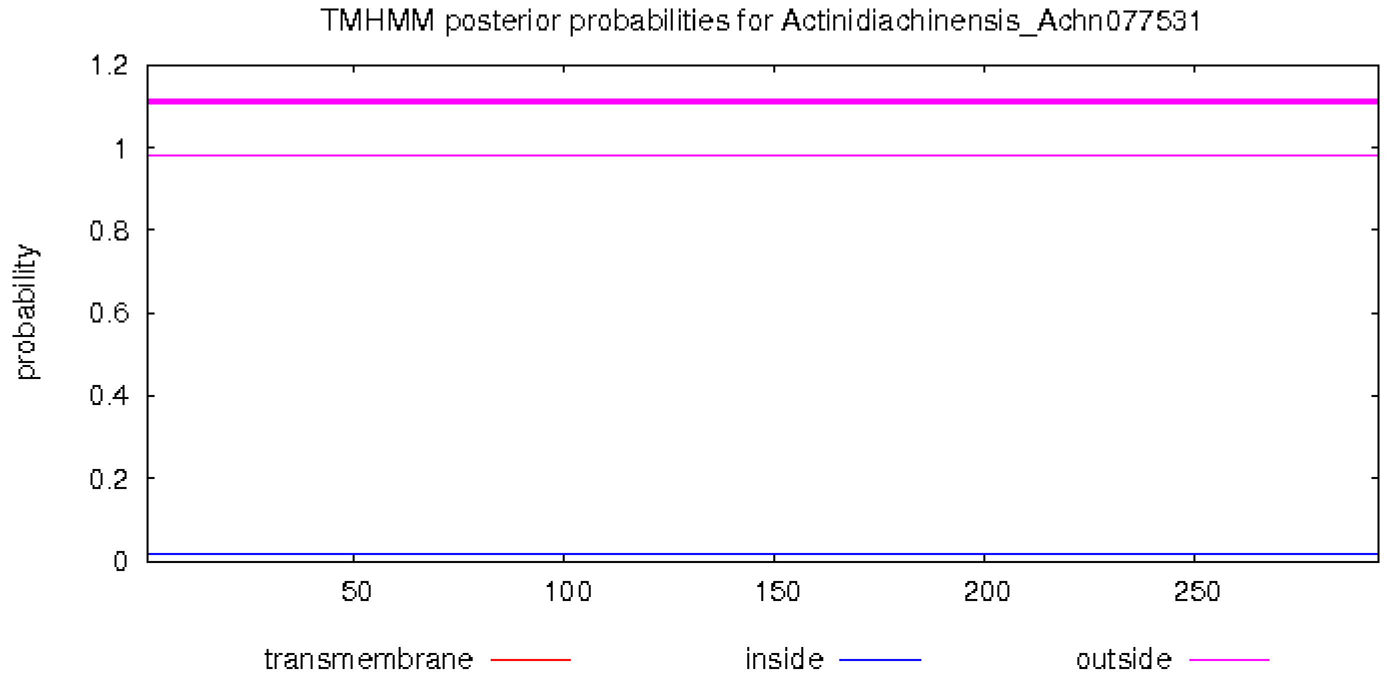

plot in postscript, script for making the plot in gnuplot, data for plot

---

```
# Actinidiachinensis_Achn079241 Length: 332
# Actinidiachinensis_Achn079241 Number of predicted TMHs:  0
```

```
# Actinidiachinensis_Achn079241 Exp number of AAs in TMHs: 0.00015
# Actinidiachinensis_Achn079241 Exp number, first 60 AAs:  0
# Actinidiachinensis_Achn079241 Total prob of N-in:        0.01807
Actinidiachinensis_Achn079241   TMHMM2.0        outside      1   332
```

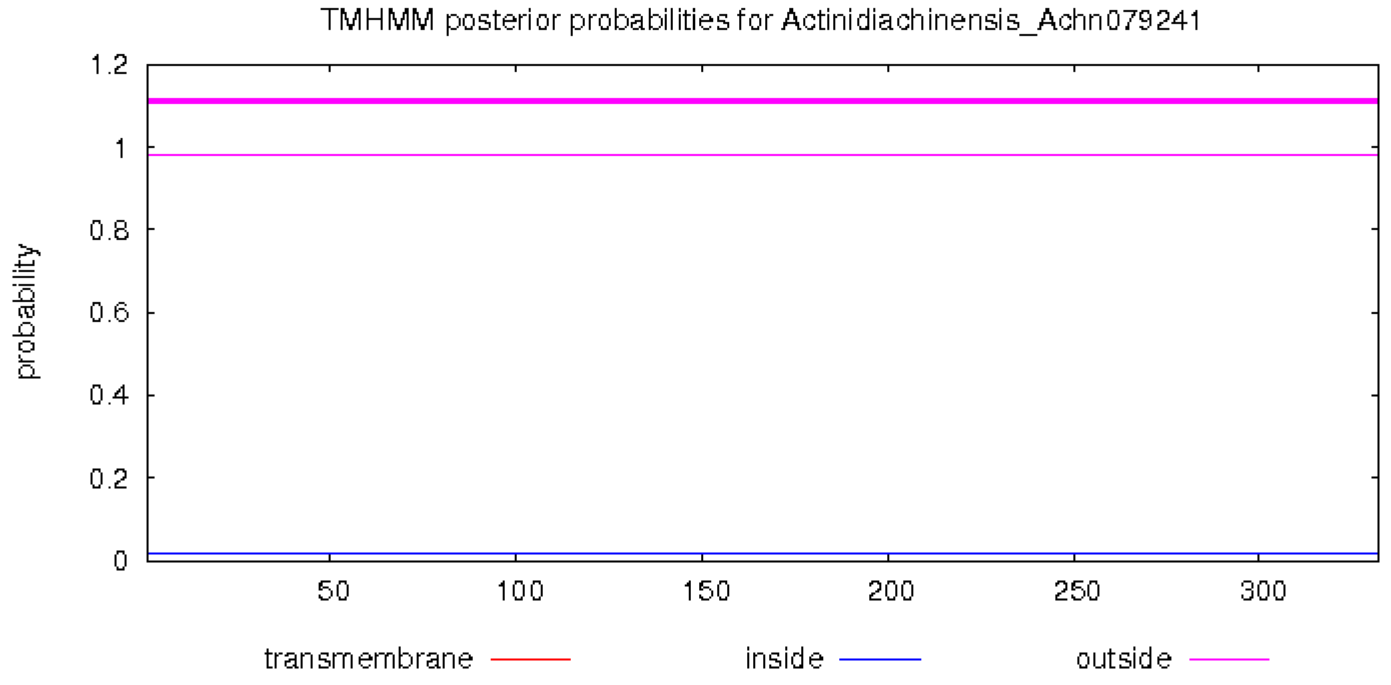

plot in postscript, script for making the plot in gnuplot, data for plot

---

```
# Actinidiachinensis_Achn080521 Length: 469
# Actinidiachinensis_Achn080521 Number of predicted TMHs:  0
# Actinidiachinensis_Achn080521 Exp number of AAs in TMHs: 0.00862
# Actinidiachinensis_Achn080521 Exp number, first 60 AAs:  0.00814
# Actinidiachinensis_Achn080521 Total prob of N-in:        0.00642
Actinidiachinensis_Achn080521   TMHMM2.0        outside      1   469
```

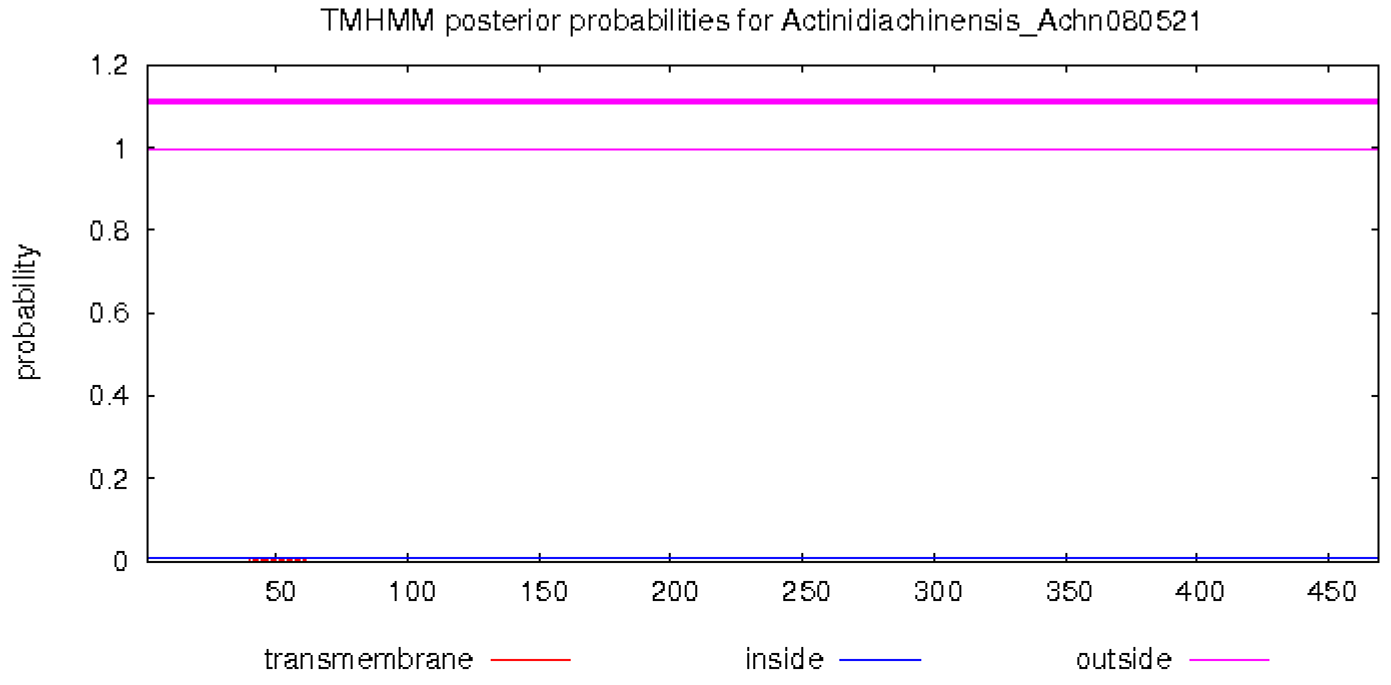

plot in postscript, script for making the plot in gnuplot, data for plot

---

```
# Actinidiachinensis_Achn080531 Length: 320
# Actinidiachinensis_Achn080531 Number of predicted TMHs:  0
```

```
# Actinidiachinensis_Achn080531 Exp number of AAs in TMHs: 0.00793
# Actinidiachinensis_Achn080531 Exp number, first 60 AAs:  0.00512
# Actinidiachinensis_Achn080531 Total prob of N-in:        0.04011
Actinidiachinensis_Achn080531   TMHMM2.0        outside      1   320
```

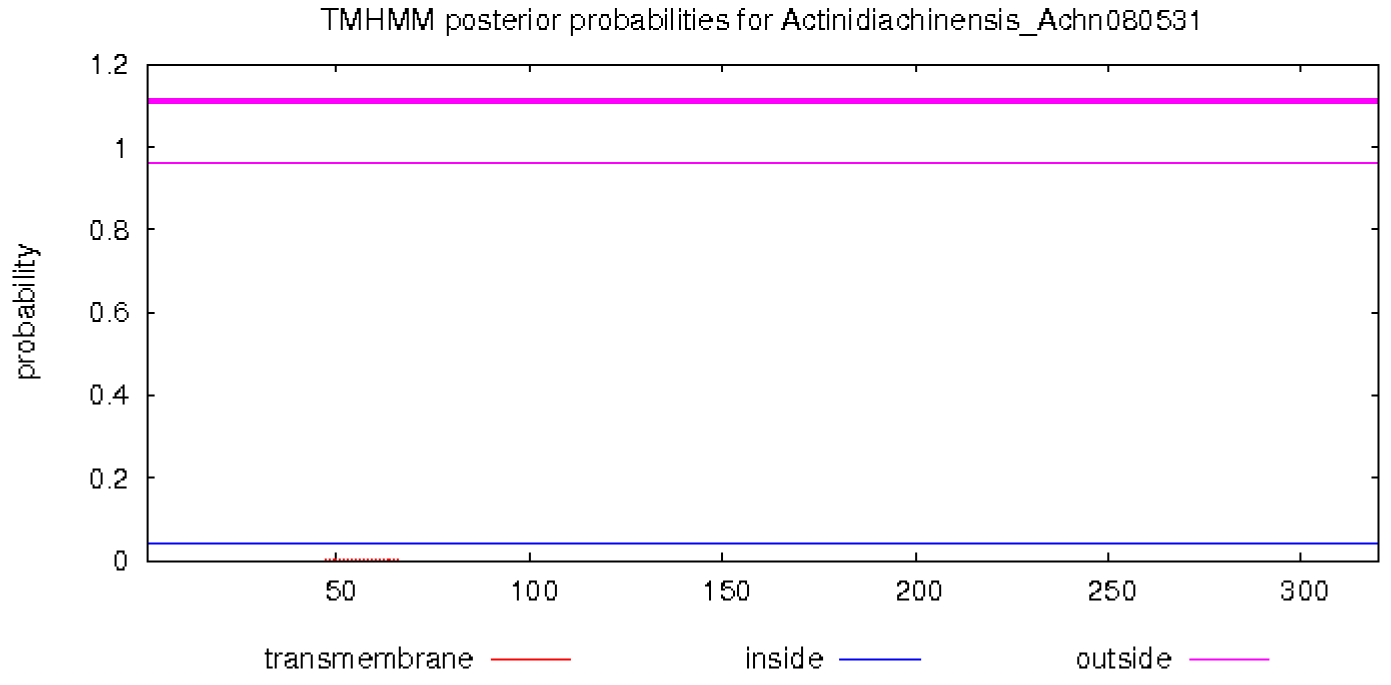

plot in postscript, script for making the plot in gnuplot, data for plot

---

```
# Actinidiachinensis_Achn080541 Length: 373
# Actinidiachinensis_Achn080541 Number of predicted TMHs:  0
# Actinidiachinensis_Achn080541 Exp number of AAs in TMHs: 1.17378
# Actinidiachinensis_Achn080541 Exp number, first 60 AAs:  1.17269
# Actinidiachinensis_Achn080541 Total prob of N-in:        0.06295
Actinidiachinensis_Achn080541   TMHMM2.0        outside      1   373
```

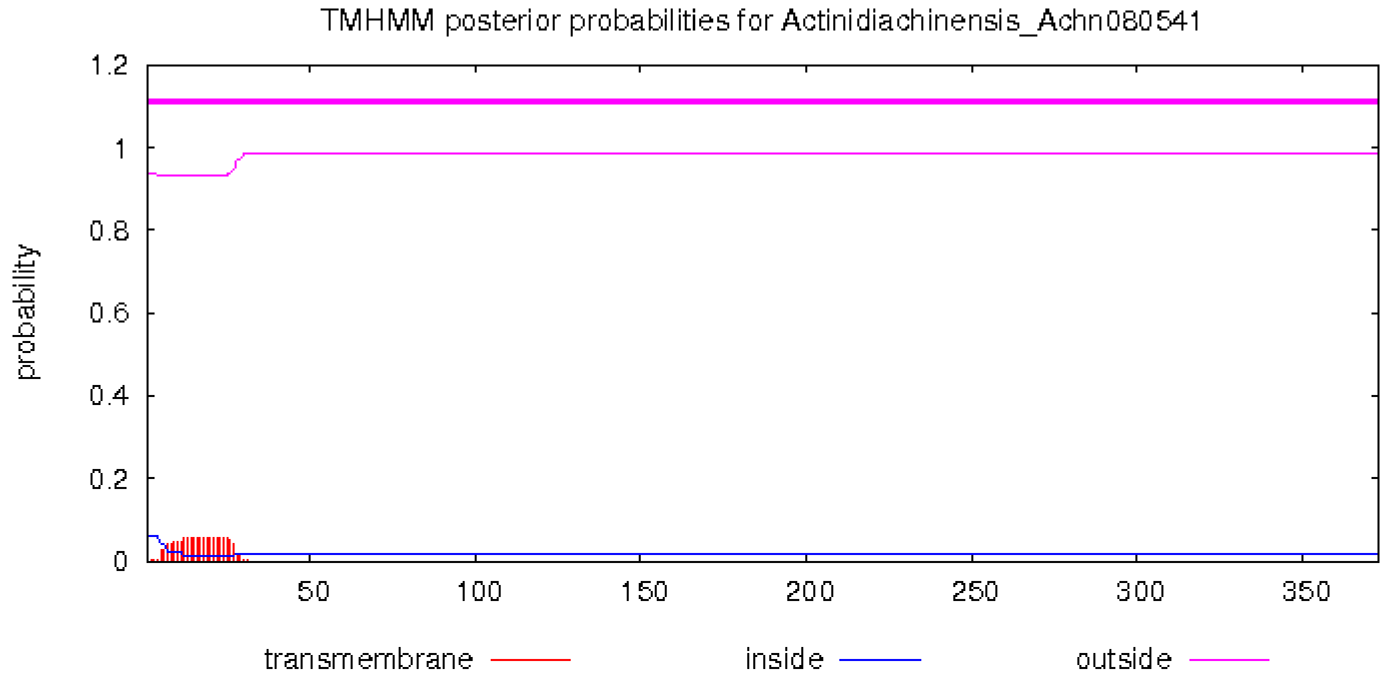

plot in postscript, script for making the plot in gnuplot, data for plot

---

```
# Actinidiachinensis_Achn080851 Length: 469
# Actinidiachinensis_Achn080851 Number of predicted TMHs:  0
```

```
# Actinidiachinensis_Achn080851 Exp number of AAs in TMHs: 0.0004
# Actinidiachinensis_Achn080851 Exp number, first 60 AAs:  0
# Actinidiachinensis_Achn080851 Total prob of N-in:        0.00165
Actinidiachinensis_Achn080851   TMHMM2.0        outside      1   469
```

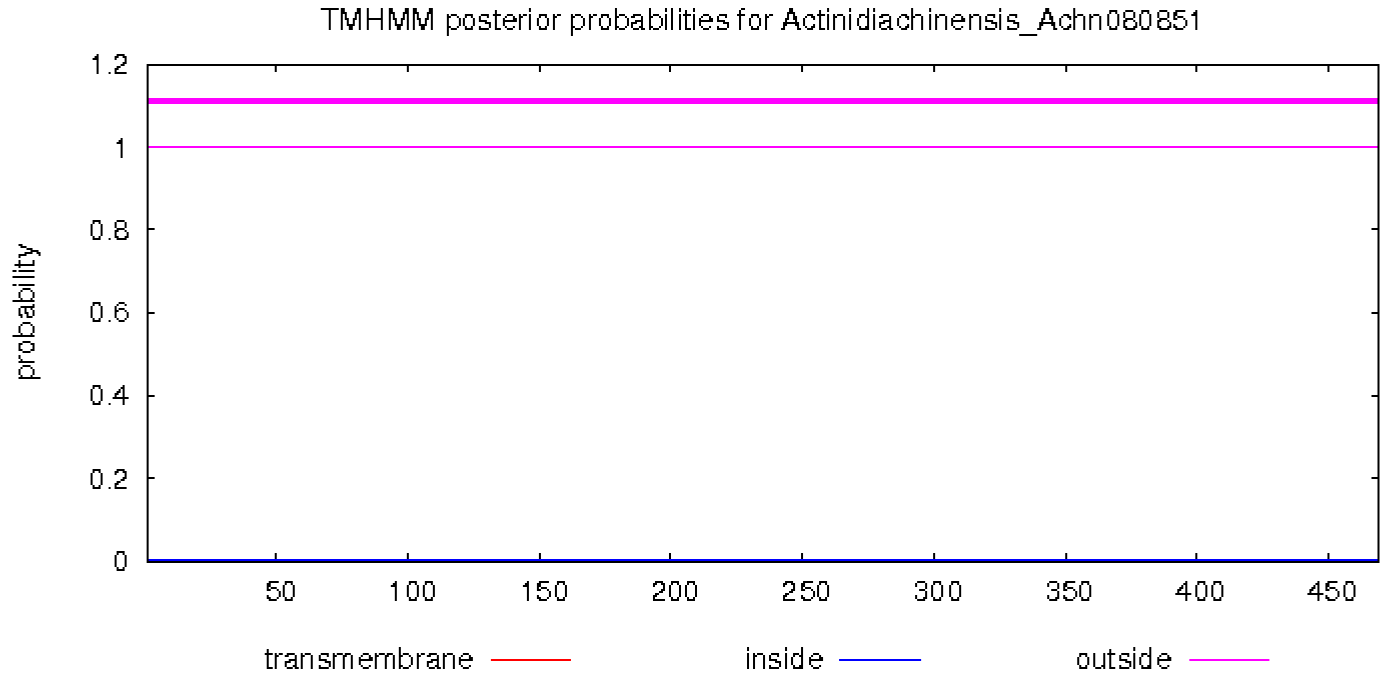

plot in postscript, script for making the plot in gnuplot, data for plot

---

```
# Actinidiachinensis_Achn089081 Length: 1240
# Actinidiachinensis_Achn089081 Number of predicted TMHs:  0
# Actinidiachinensis_Achn089081 Exp number of AAs in TMHs: 0.0841999999999999999
# Actinidiachinensis_Achn089081 Exp number, first 60 AAs:  0.02554
# Actinidiachinensis_Achn089081 Total prob of N-in:        0.00350
Actinidiachinensis_Achn089081   TMHMM2.0        outside      1  1240
```

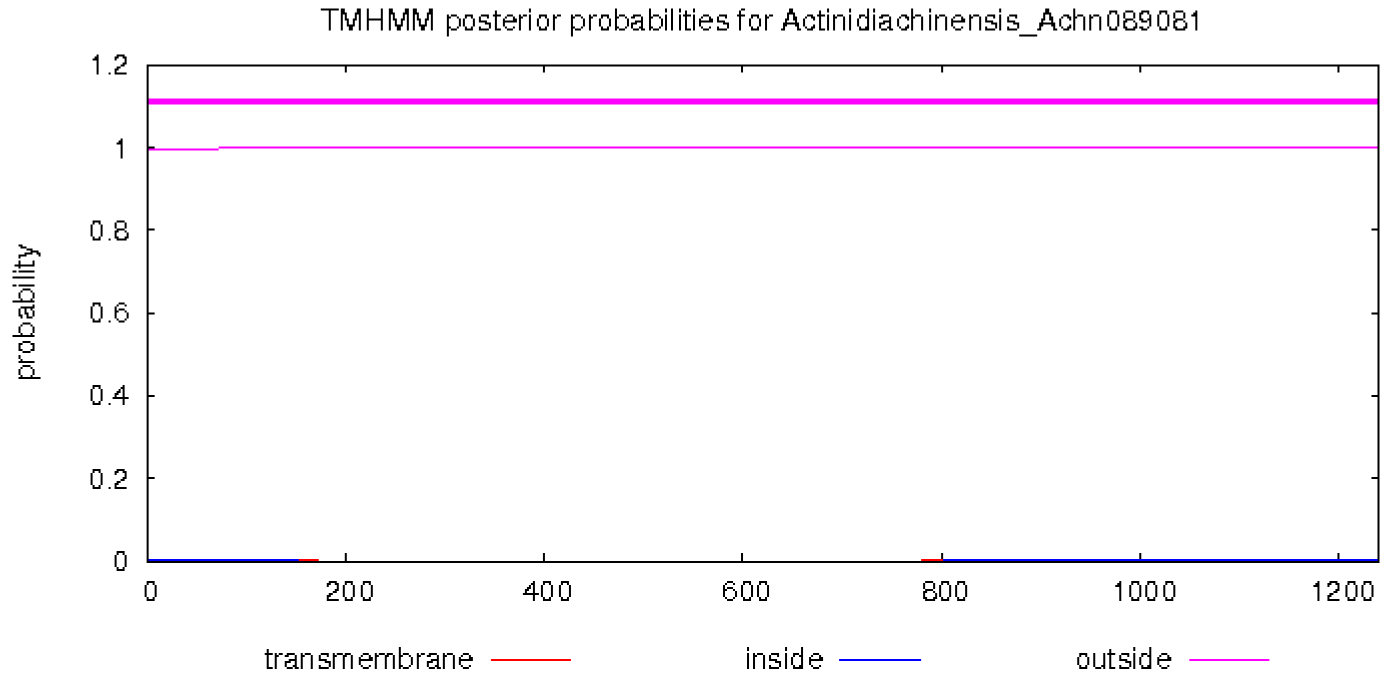

plot in postscript, script for making the plot in gnuplot, data for plot

---

```
# Actinidiachinensis_Achn093311 Length: 303
# Actinidiachinensis_Achn093311 Number of predicted TMHs:  0
```

```
# Actinidiachinensis_Achn093311 Exp number of AAs in TMHs: 0
# Actinidiachinensis_Achn093311 Exp number, first 60 AAs:  0
# Actinidiachinensis_Achn093311 Total prob of N-in:        0.03131
Actinidiachinensis_Achn093311   TMHMM2.0        outside      1   303
```

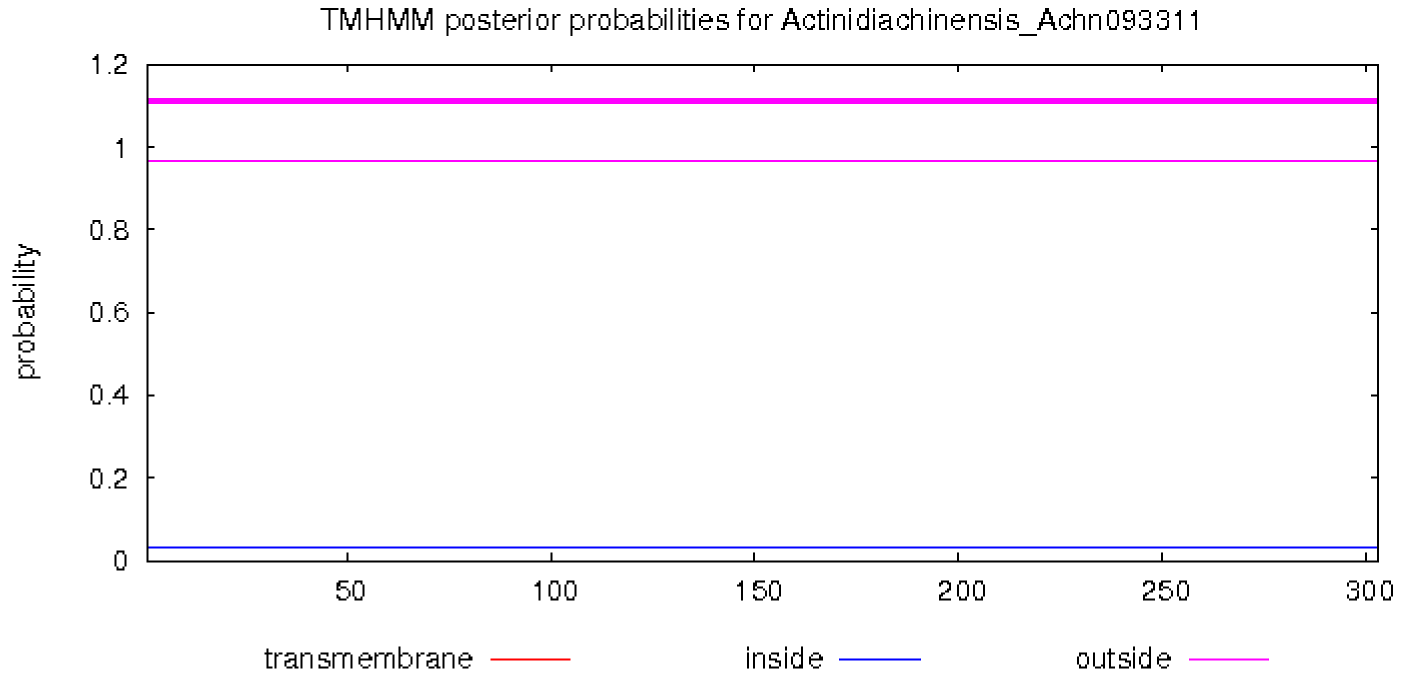

plot in postscript, script for making the plot in gnuplot, data for plot

---

```
# Actinidiachinensis_Achn095321 Length: 405
# Actinidiachinensis_Achn095321 Number of predicted TMHs:  0
# Actinidiachinensis_Achn095321 Exp number of AAs in TMHs: 0.03786
# Actinidiachinensis_Achn095321 Exp number, first 60 AAs:  0.01068
# Actinidiachinensis_Achn095321 Total prob of N-in:        0.00211
Actinidiachinensis_Achn095321   TMHMM2.0        outside      1   405
```

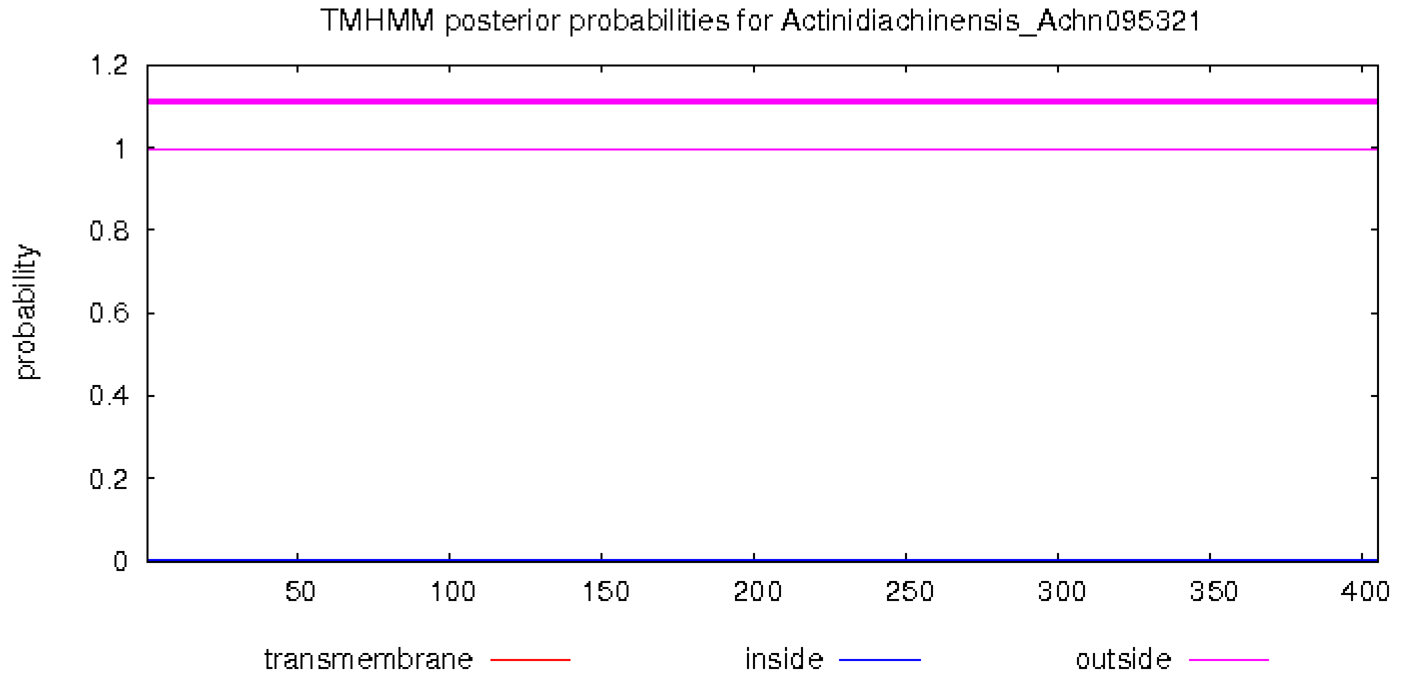

plot in postscript, script for making the plot in gnuplot, data for plot

---

```
# Actinidiachinensis_Achn104221 Length: 444
# Actinidiachinensis_Achn104221 Number of predicted TMHs:  0
```

```
# Actinidiachinensis_Achn104221 Exp number of AAs in TMHs: 0.00042
# Actinidiachinensis_Achn104221 Exp number, first 60 AAs:  0.00042
# Actinidiachinensis_Achn104221 Total prob of N-in:        0.00113
Actinidiachinensis_Achn104221   TMHMM2.0        outside      1   444
```

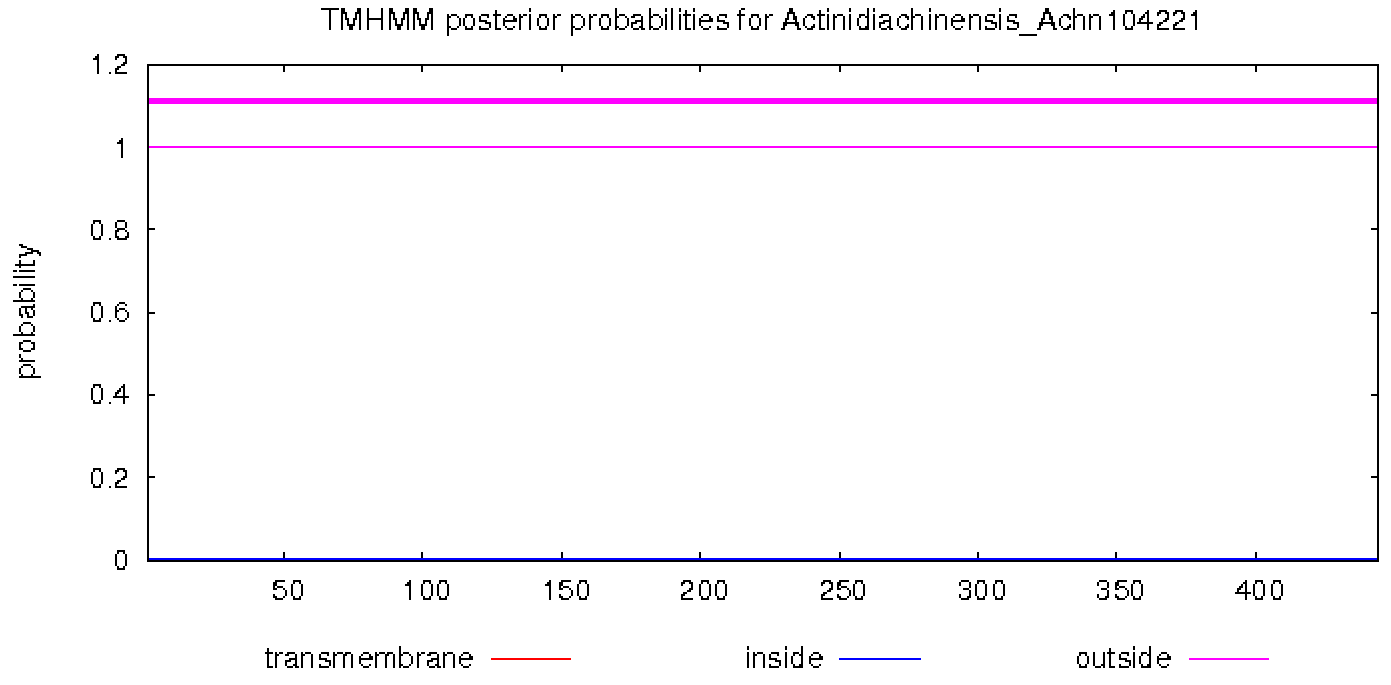

plot in postscript, script for making the plot in gnuplot, data for plot

---

```
# Actinidiachinensis_Achn106991 Length: 653
# Actinidiachinensis_Achn106991 Number of predicted TMHs:  0
# Actinidiachinensis_Achn106991 Exp number of AAs in TMHs: 0.00555
# Actinidiachinensis_Achn106991 Exp number, first 60 AAs:  0
# Actinidiachinensis_Achn106991 Total prob of N-in:        0.00069
Actinidiachinensis_Achn106991   TMHMM2.0        outside      1   653
```

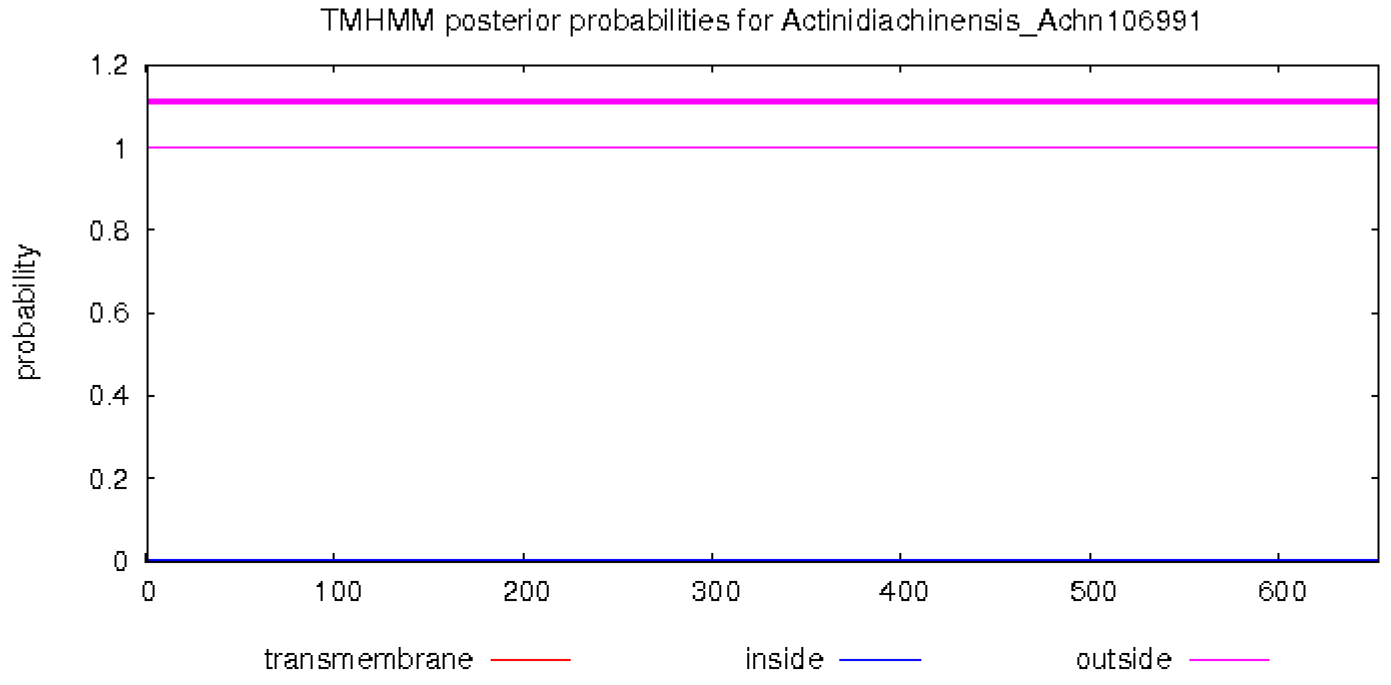

plot in postscript, script for making the plot in gnuplot, data for plot

---

```
# Actinidiachinensis_Achn107641 Length: 387
# Actinidiachinensis_Achn107641 Number of predicted TMHs:  0
```

```
# Actinidiachinensis_Achn107641 Exp number of AAs in TMHs: 0.01406
# Actinidiachinensis_Achn107641 Exp number, first 60 AAs:  0.00083
# Actinidiachinensis_Achn107641 Total prob of N-in:        0.00905
Actinidiachinensis_Achn107641   TMHMM2.0        outside      1   387
```

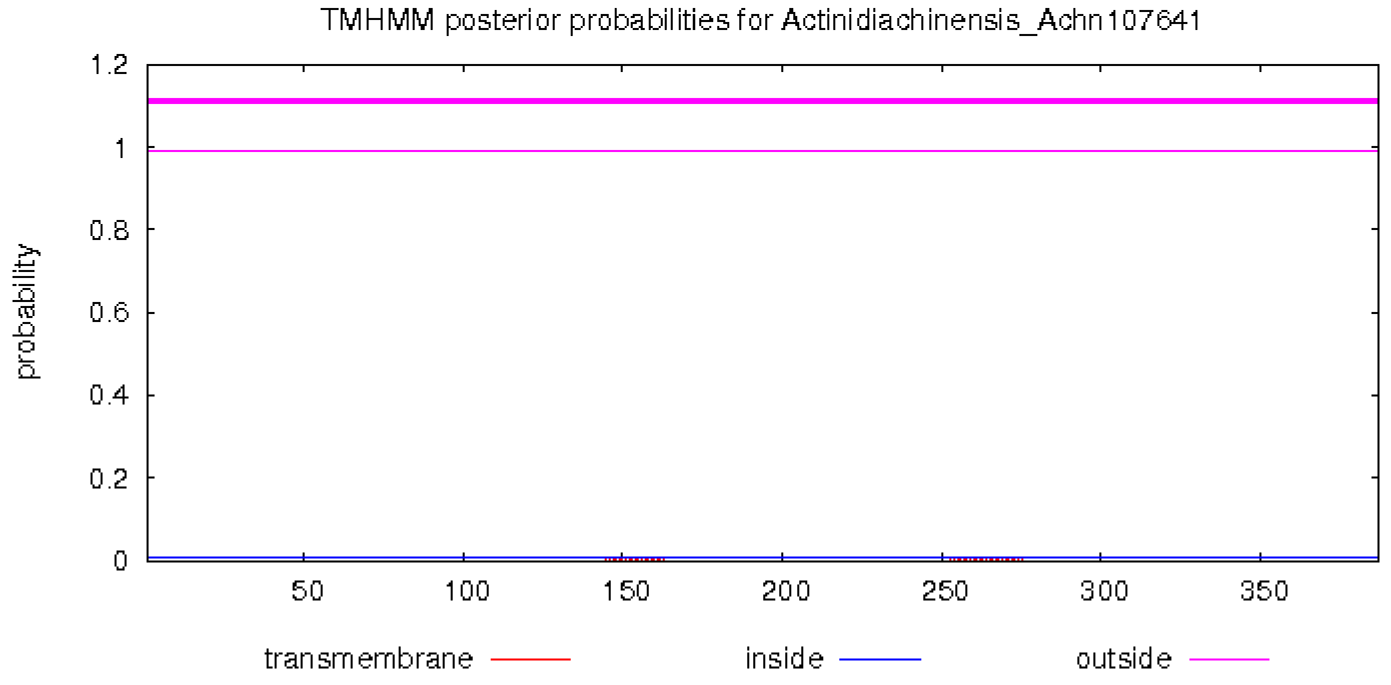

plot in postscript, script for making the plot in gnuplot, data for plot

---

```
# Actinidiachinensis_Achn111051 Length: 322
# Actinidiachinensis_Achn111051 Number of predicted TMHs:  0
# Actinidiachinensis_Achn111051 Exp number of AAs in TMHs: 0.00018
# Actinidiachinensis_Achn111051 Exp number, first 60 AAs:  0.00018
# Actinidiachinensis_Achn111051 Total prob of N-in:        0.00312
Actinidiachinensis_Achn111051   TMHMM2.0        outside      1   322
```

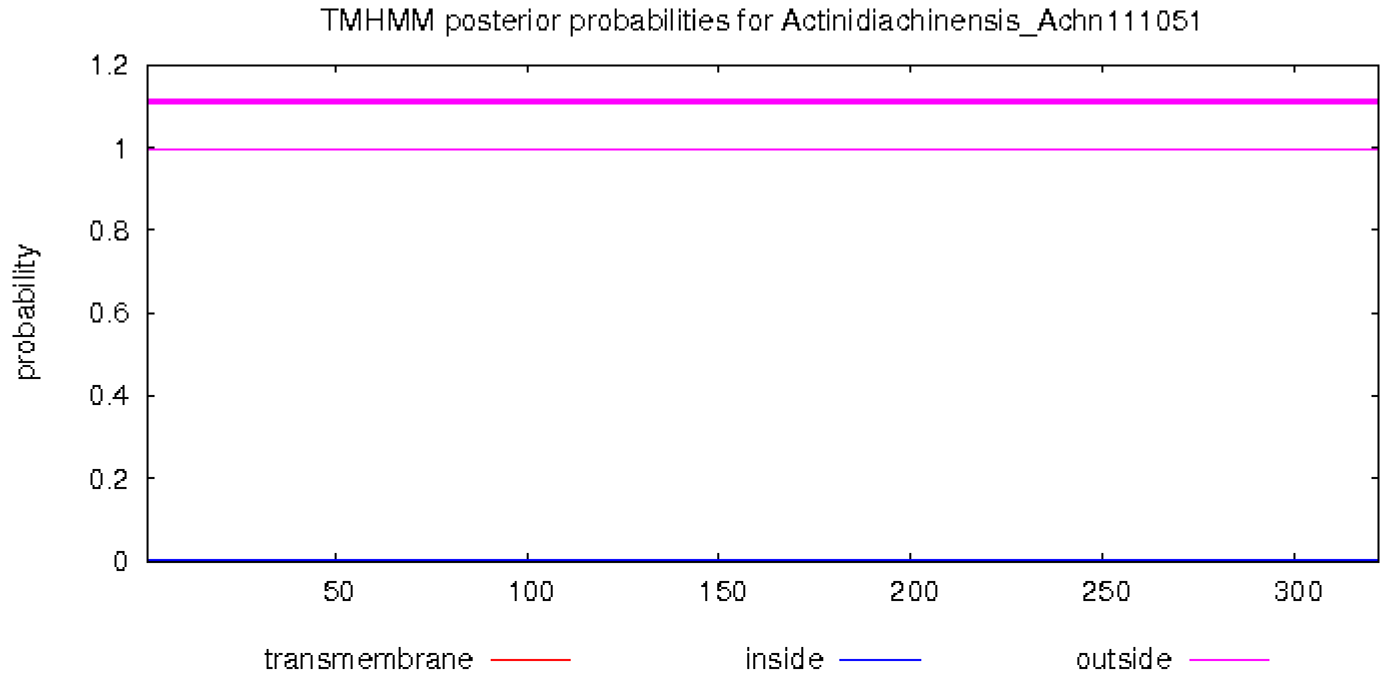

plot in postscript, script for making the plot in gnuplot, data for plot

---

```
# Actinidiachinensis_Achn118631 Length: 410
# Actinidiachinensis_Achn118631 Number of predicted TMHs:  1
```

```
# Actinidiachinensis_Achn118631 Exp number of AAs in TMHs: 17.78239
# Actinidiachinensis_Achn118631 Exp number, first 60 AAs:  0
# Actinidiachinensis_Achn118631 Total prob of N-in:        0.00667
Actinidiachinensis_Achn118631   TMHMM2.0        outside      1   386
Actinidiachinensis_Achn118631   TMHMM2.0        TMhelix    387   409
Actinidiachinensis_Achn118631   TMHMM2.0        inside     410   410
```

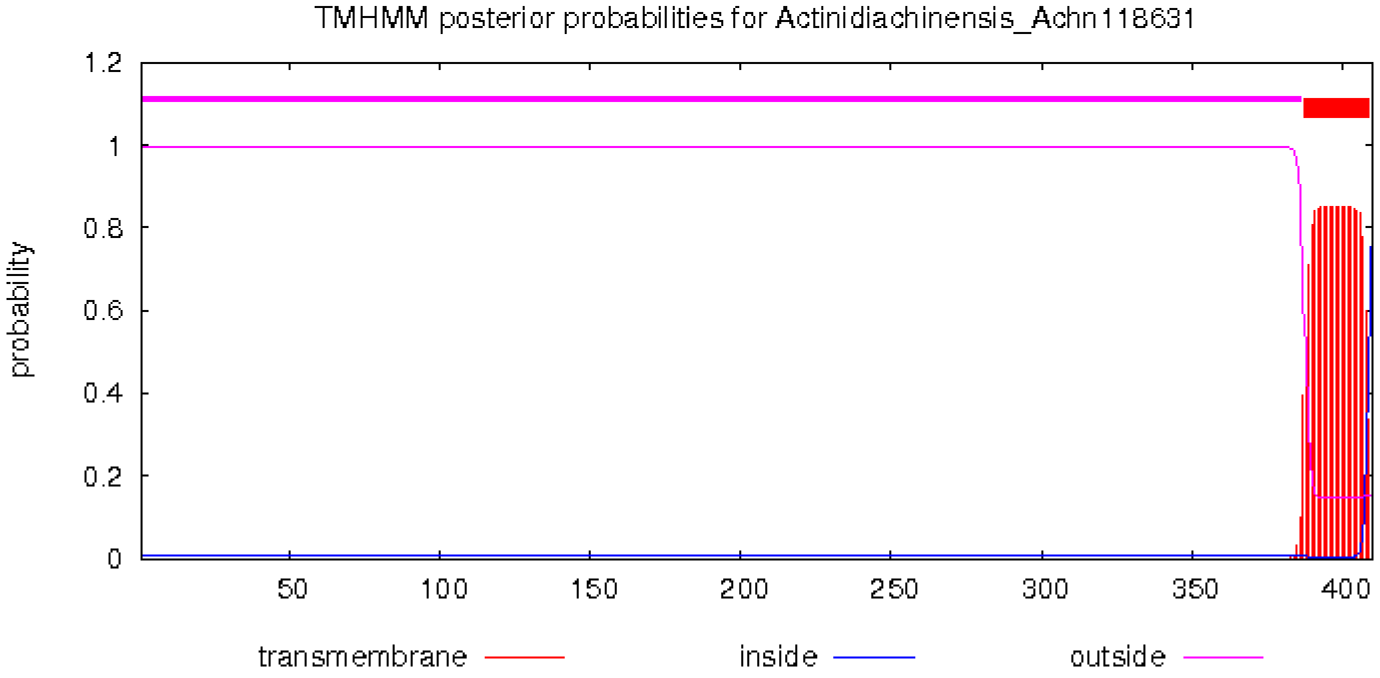

plot in postscript, script for making the plot in gnuplot, data for plot

---

```
# Actinidiachinensis_Achn123251 Length: 290
# Actinidiachinensis_Achn123251 Number of predicted TMHs:  0
# Actinidiachinensis_Achn123251 Exp number of AAs in TMHs: 0.00019
# Actinidiachinensis_Achn123251 Exp number, first 60 AAs:  0
# Actinidiachinensis_Achn123251 Total prob of N-in:        0.00710
Actinidiachinensis_Achn123251   TMHMM2.0        outside      1   290
```

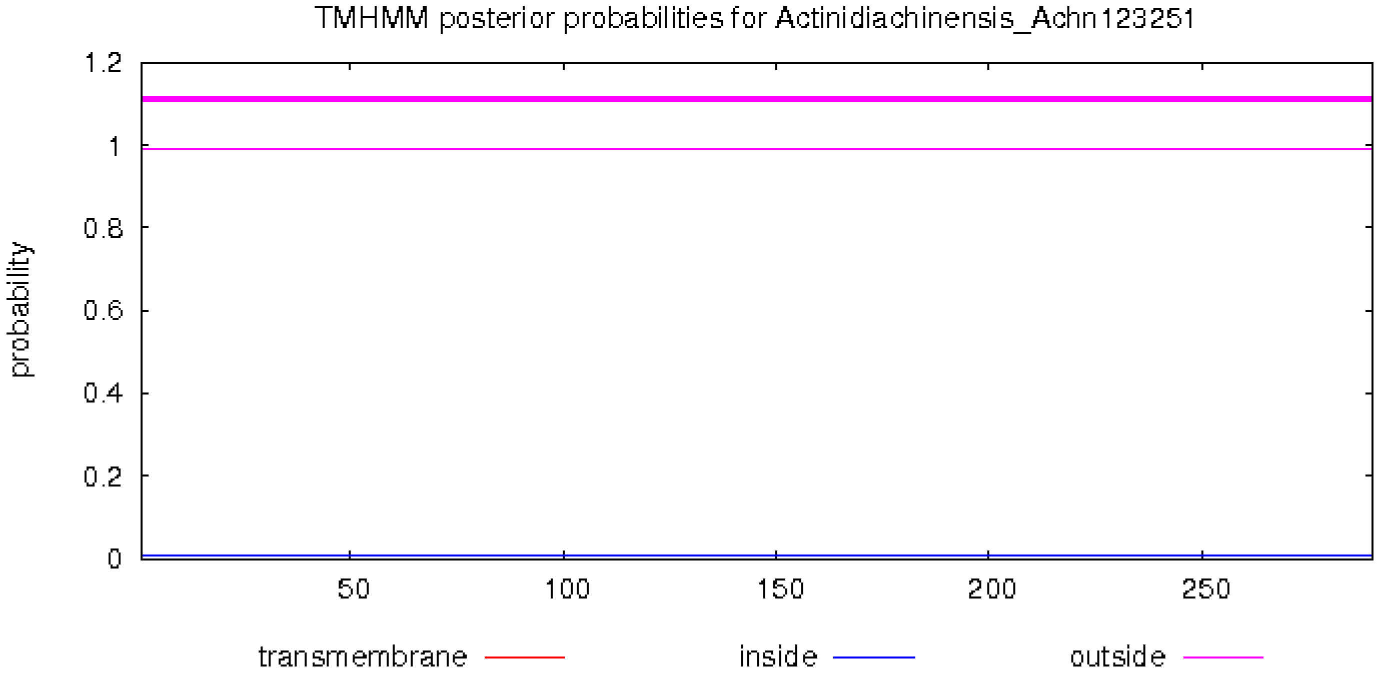

plot in postscript, script for making the plot in gnuplot, data for plot

---

```
# Actinidiachinensis_Achn125861 Length: 335
# Actinidiachinensis_Achn125861 Number of predicted TMHs:  0
# Actinidiachinensis_Achn125861 Exp number of AAs in TMHs: 0.0012
# Actinidiachinensis_Achn125861 Exp number, first 60 AAs:  0
# Actinidiachinensis_Achn125861 Total prob of N-in:        0.01205
Actinidiachinensis_Achn125861   TMHMM2.0        outside      1   335
```

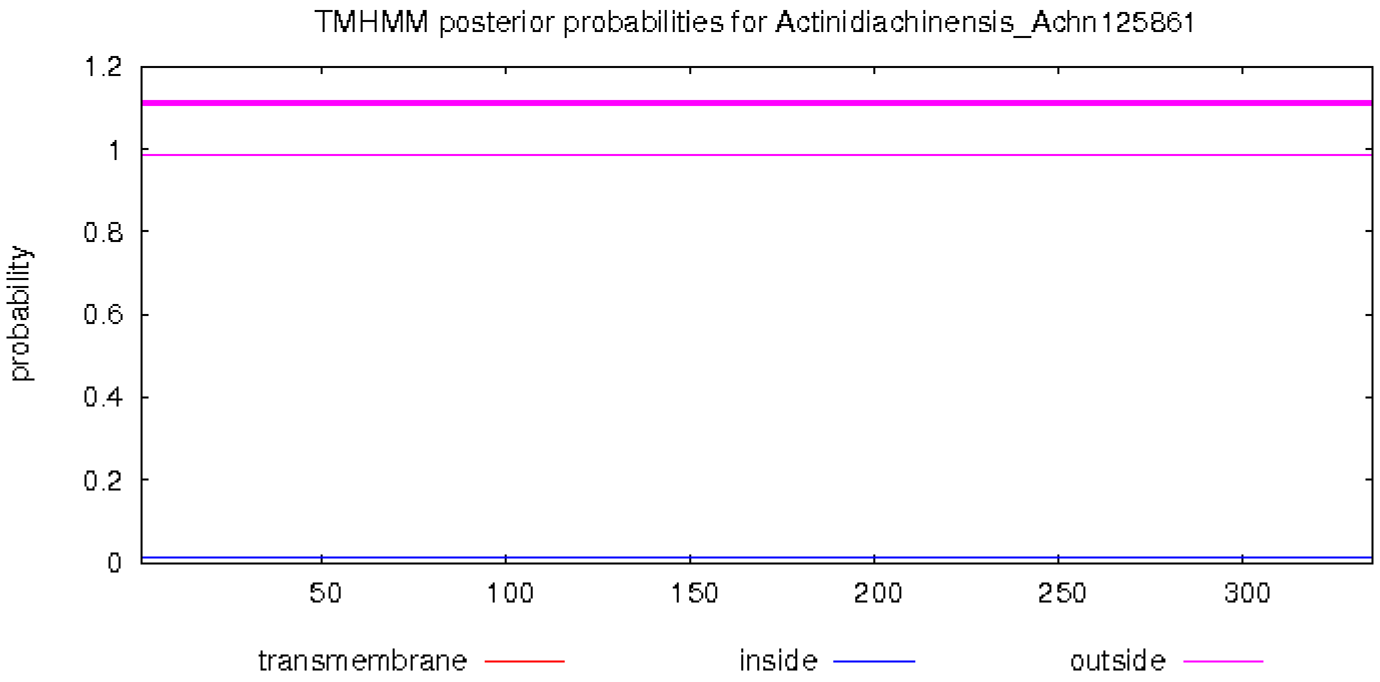

plot in postscript, script for making the plot in gnuplot, data for plot

---

```
# Actinidiachinensis_Achn126301 Length: 221
# Actinidiachinensis_Achn126301 Number of predicted TMHs:  0
# Actinidiachinensis_Achn126301 Exp number of AAs in TMHs: 0.0004
# Actinidiachinensis_Achn126301 Exp number, first 60 AAs:  0.0002
# Actinidiachinensis_Achn126301 Total prob of N-in:        0.07847
Actinidiachinensis_Achn126301   TMHMM2.0        outside      1   221
```

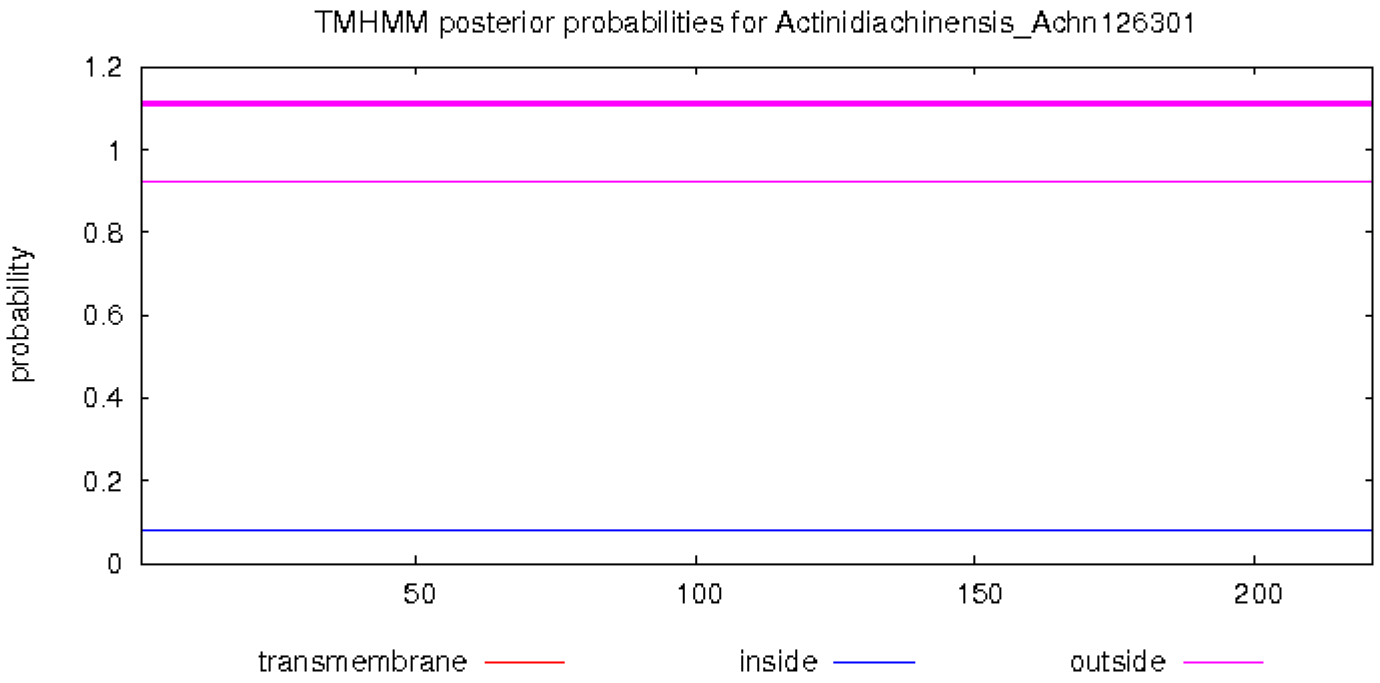

plot in postscript, script for making the plot in gnuplot, data for plot

---

```
# Actinidiachinensis_Achn133971 Length: 315
# Actinidiachinensis_Achn133971 Number of predicted TMHs:  0
# Actinidiachinensis_Achn133971 Exp number of AAs in TMHs: 0.00195
# Actinidiachinensis_Achn133971 Exp number, first 60 AAs:  0
# Actinidiachinensis_Achn133971 Total prob of N-in:        0.02227
Actinidiachinensis_Achn133971   TMHMM2.0        outside      1   315
```

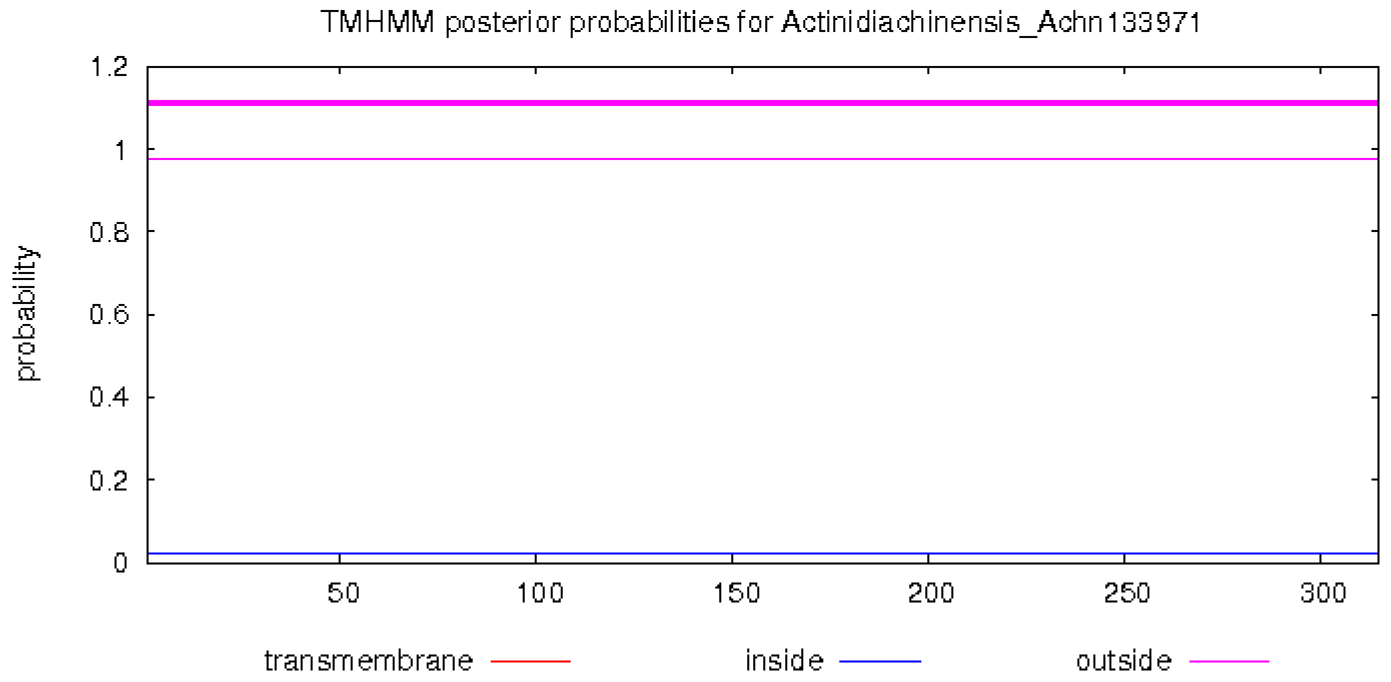

plot in postscript, script for making the plot in gnuplot, data for plot

---

```
# Actinidiachinensis_Achn134171 Length: 319
# Actinidiachinensis_Achn134171 Number of predicted TMHs:  0
# Actinidiachinensis_Achn134171 Exp number of AAs in TMHs: 0.00812
# Actinidiachinensis_Achn134171 Exp number, first 60 AAs:  0.00812
# Actinidiachinensis_Achn134171 Total prob of N-in:        0.01445
Actinidiachinensis_Achn134171   TMHMM2.0        outside      1   319
```

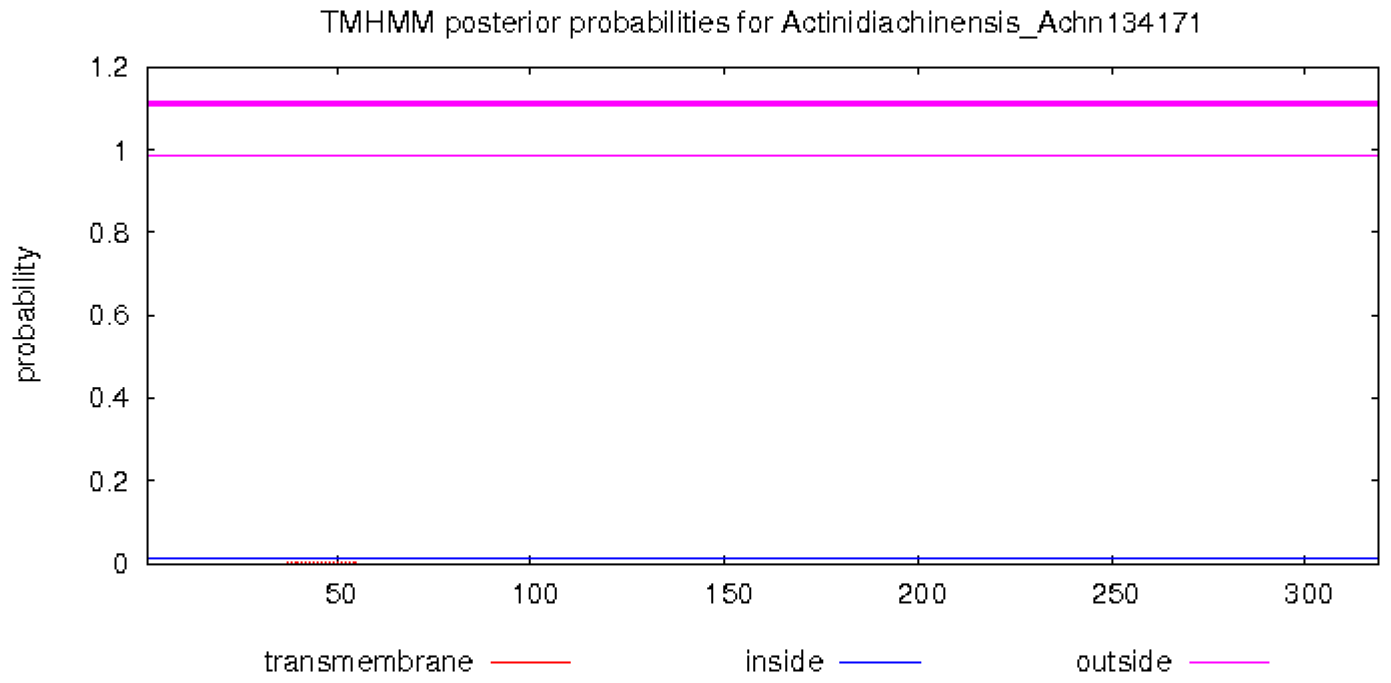

plot in postscript, script for making the plot in gnuplot, data for plot

---

```
# Actinidiachinensis_Achn141961 Length: 288
# Actinidiachinensis_Achn141961 Number of predicted TMHs:  0
# Actinidiachinensis_Achn141961 Exp number of AAs in TMHs: 0.00064
# Actinidiachinensis_Achn141961 Exp number, first 60 AAs:  0
# Actinidiachinensis_Achn141961 Total prob of N-in:        0.00927
Actinidiachinensis_Achn141961	TMHMM2.0	outside	     1   288
```

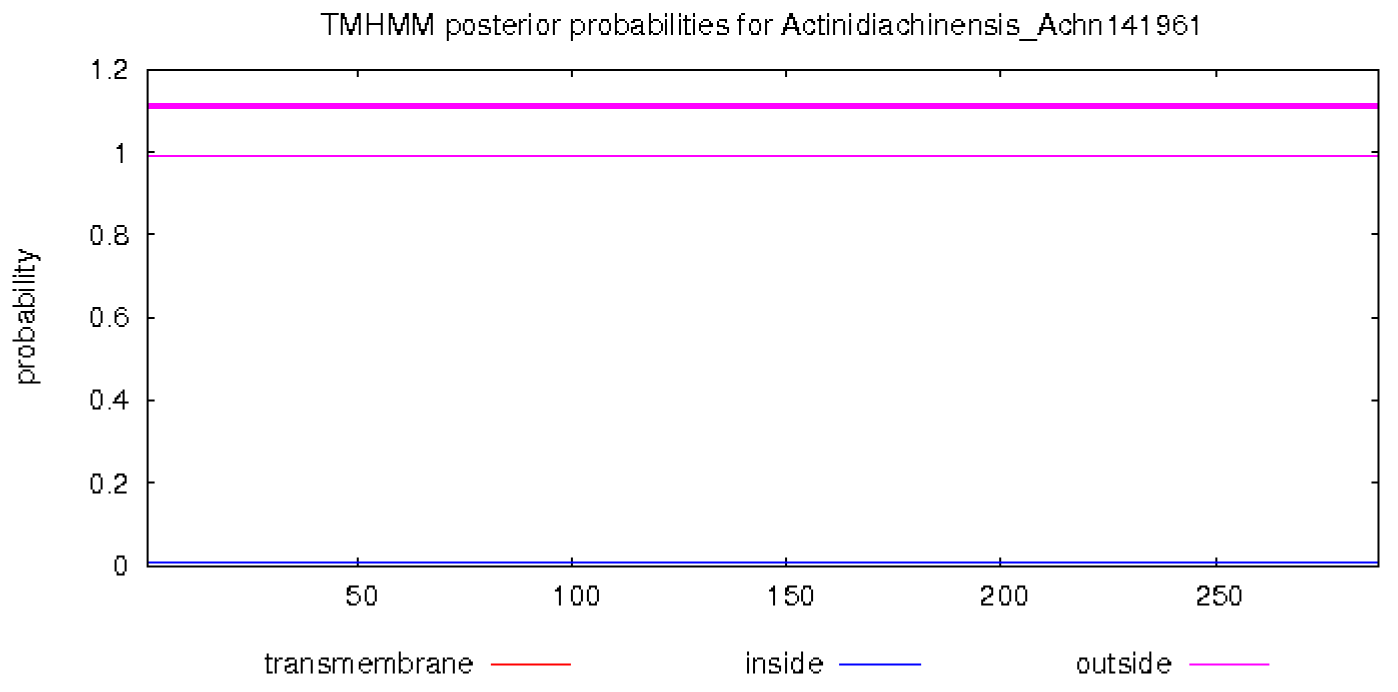

plot in postscript, script for making the plot in gnuplot, data for plot

---

```
# Actinidiachinensis_Achn146511 Length: 327
# Actinidiachinensis_Achn146511 Number of predicted TMHs:  0
# Actinidiachinensis_Achn146511 Exp number of AAs in TMHs: 0
# Actinidiachinensis_Achn146511 Exp number, first 60 AAs:  0
# Actinidiachinensis_Achn146511 Total prob of N-in:        0.01558
Actinidiachinensis_Achn146511	TMHMM2.0	outside	     1   327
```

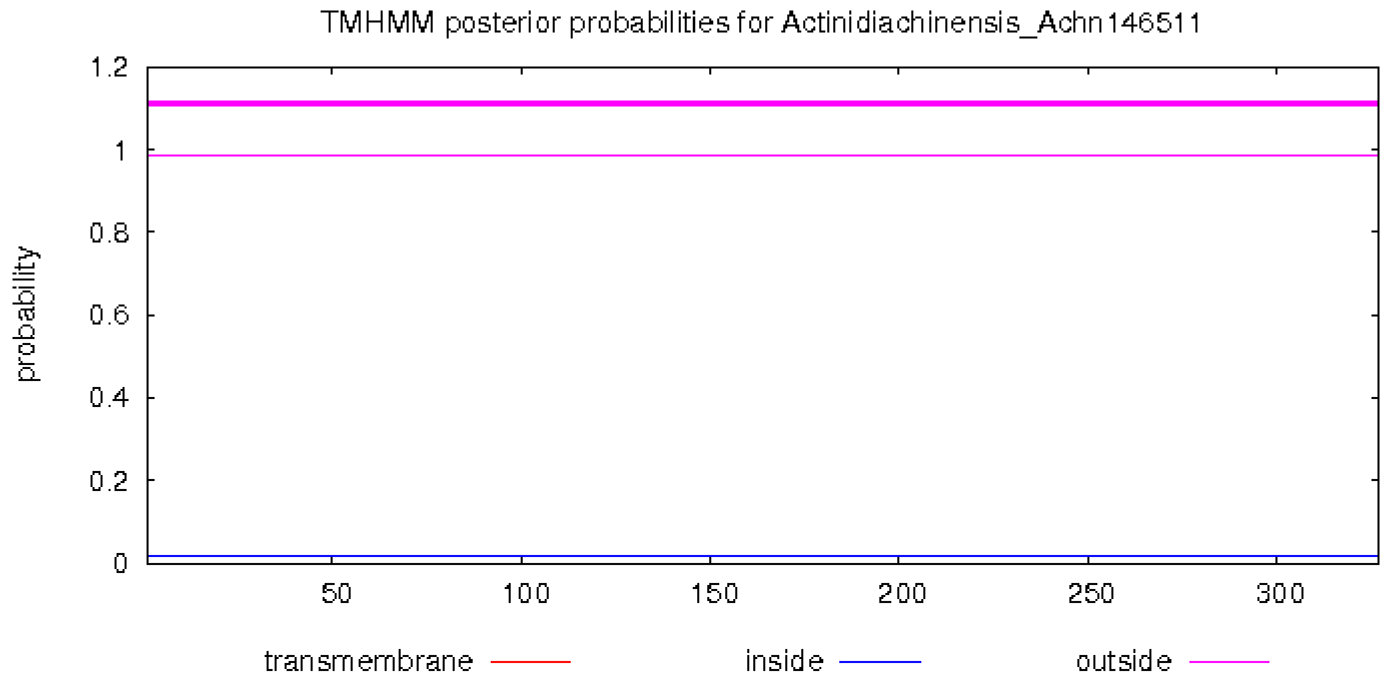

plot in postscript, script for making the plot in gnuplot, data for plot

---

```
# Actinidiachinensis_Achn160251 Length: 347
# Actinidiachinensis_Achn160251 Number of predicted TMHs:  0
# Actinidiachinensis_Achn160251 Exp number of AAs in TMHs: 0.00018
# Actinidiachinensis_Achn160251 Exp number, first 60 AAs:  0.00018
# Actinidiachinensis_Achn160251 Total prob of N-in:        0.02175
Actinidiachinensis_Achn160251	TMHMM2.0	outside	     1   347
```

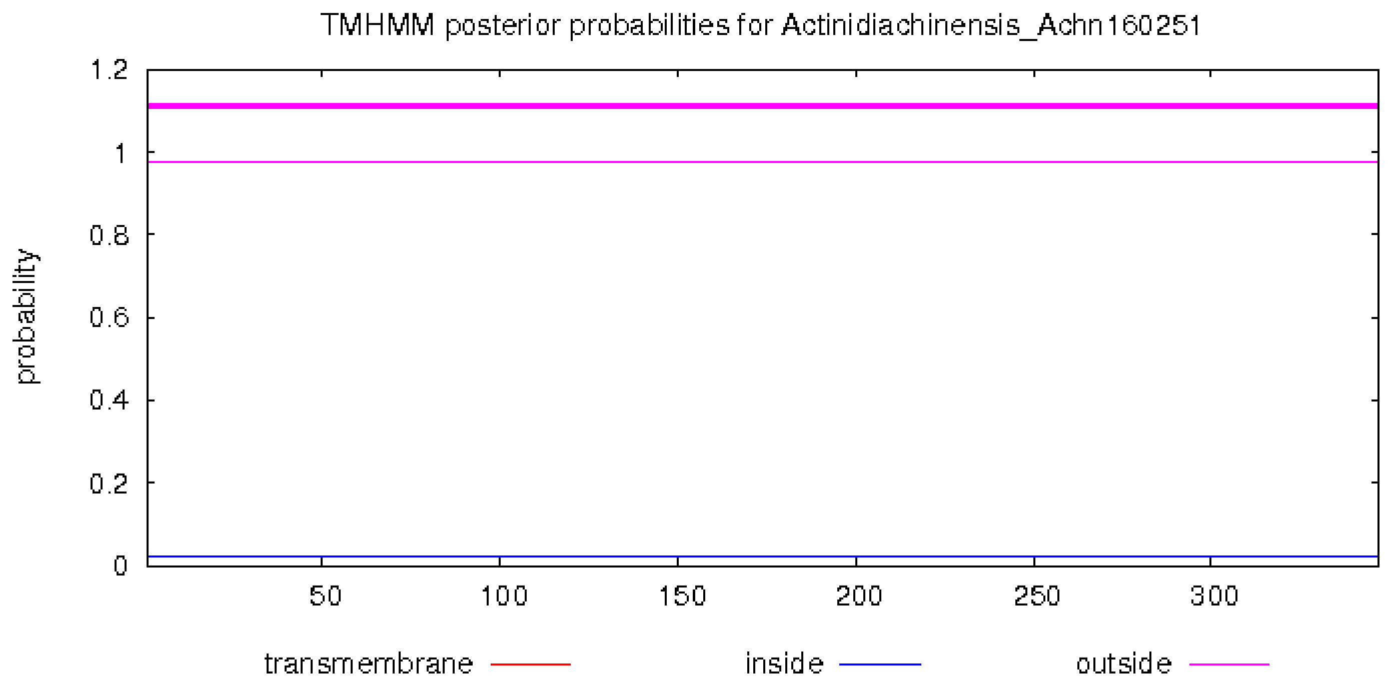

plot in postscript, script for making the plot in gnuplot, data for plot

---

```
# Actinidiachinensis_Achn160261 Length: 190
# Actinidiachinensis_Achn160261 Number of predicted TMHs:  0
# Actinidiachinensis_Achn160261 Exp number of AAs in TMHs: 0.01555
# Actinidiachinensis_Achn160261 Exp number, first 60 AAs:  0.01553
# Actinidiachinensis_Achn160261 Total prob of N-in:        0.12706
Actinidiachinensis_Achn160261	TMHMM2.0	outside	     1   190
```

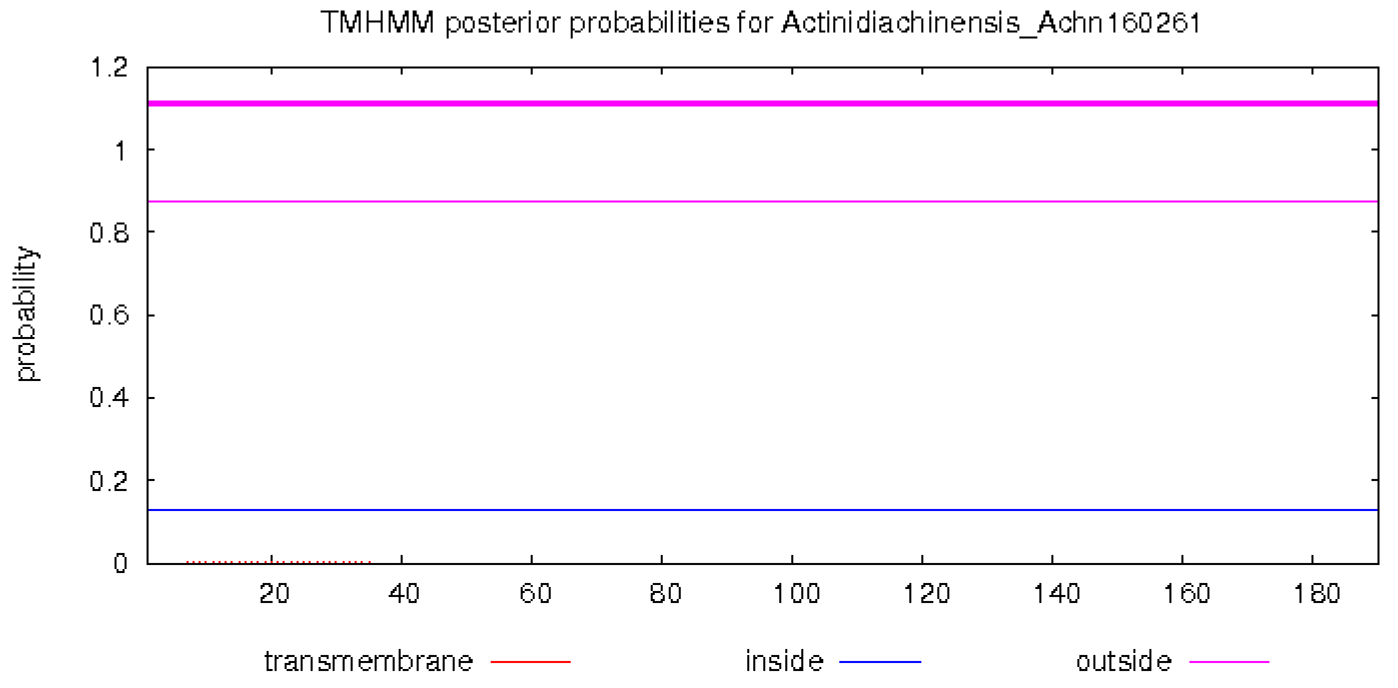

plot in postscript, script for making the plot in gnuplot, data for plot

---

```
# Actinidiachinensis_Achn161331 Length: 597
# Actinidiachinensis_Achn161331 Number of predicted TMHs:  1
# Actinidiachinensis_Achn161331 Exp number of AAs in TMHs: 22.08342
# Actinidiachinensis_Achn161331 Exp number, first 60 AAs:  0.00308
# Actinidiachinensis_Achn161331 Total prob of N-in:        0.01790
Actinidiachinensis_Achn161331   TMHMM2.0        outside      1   565
Actinidiachinensis_Achn161331   TMHMM2.0        TMhelix    566   588
Actinidiachinensis_Achn161331   TMHMM2.0        inside     589   597
```

TMHMM posterior probabilities for Actinidiachinensis_Achn161331

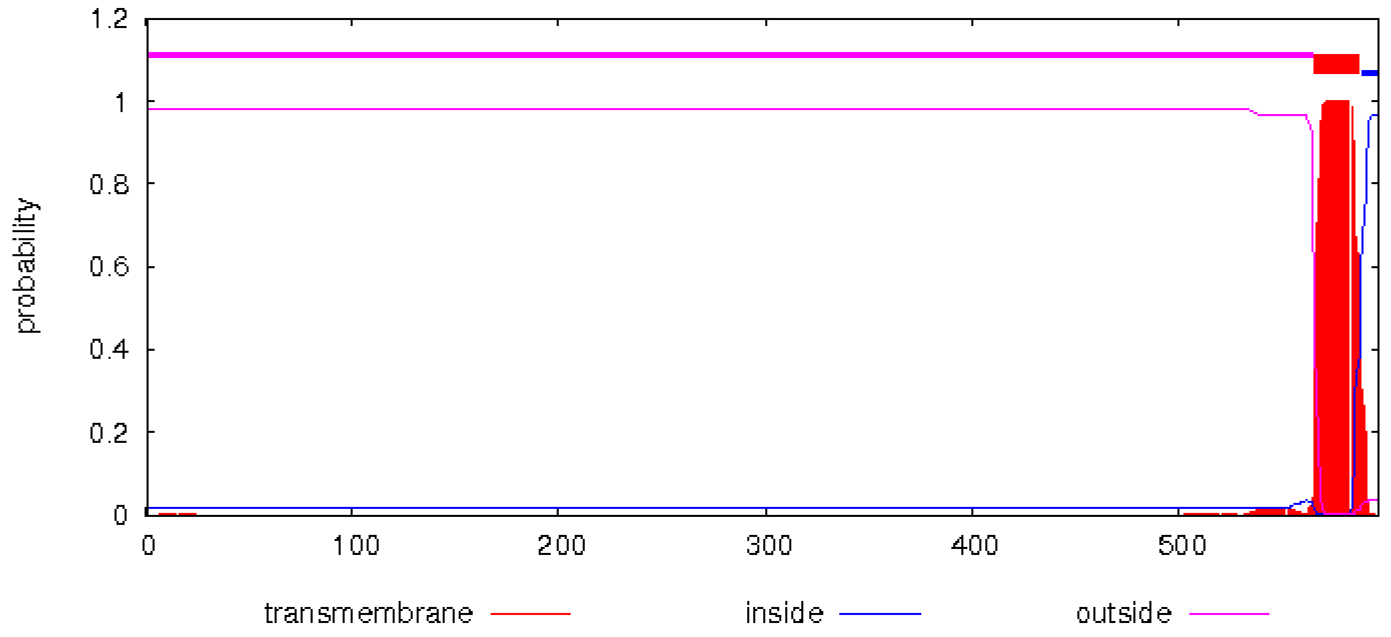

plot in postscript, script for making the plot in gnuplot, data for plot

---

```
# Actinidiachinensis_Achn163571 Length: 268
# Actinidiachinensis_Achn163571 Number of predicted TMHs:  0
# Actinidiachinensis_Achn163571 Exp number of AAs in TMHs: 0.00164
# Actinidiachinensis_Achn163571 Exp number, first 60 AAs:  0.0004
# Actinidiachinensis_Achn163571 Total prob of N-in:        0.01629
Actinidiachinensis_Achn163571   TMHMM2.0        outside      1   268
```

TMHMM posterior probabilities for Actinidiachinensis_Achn163571

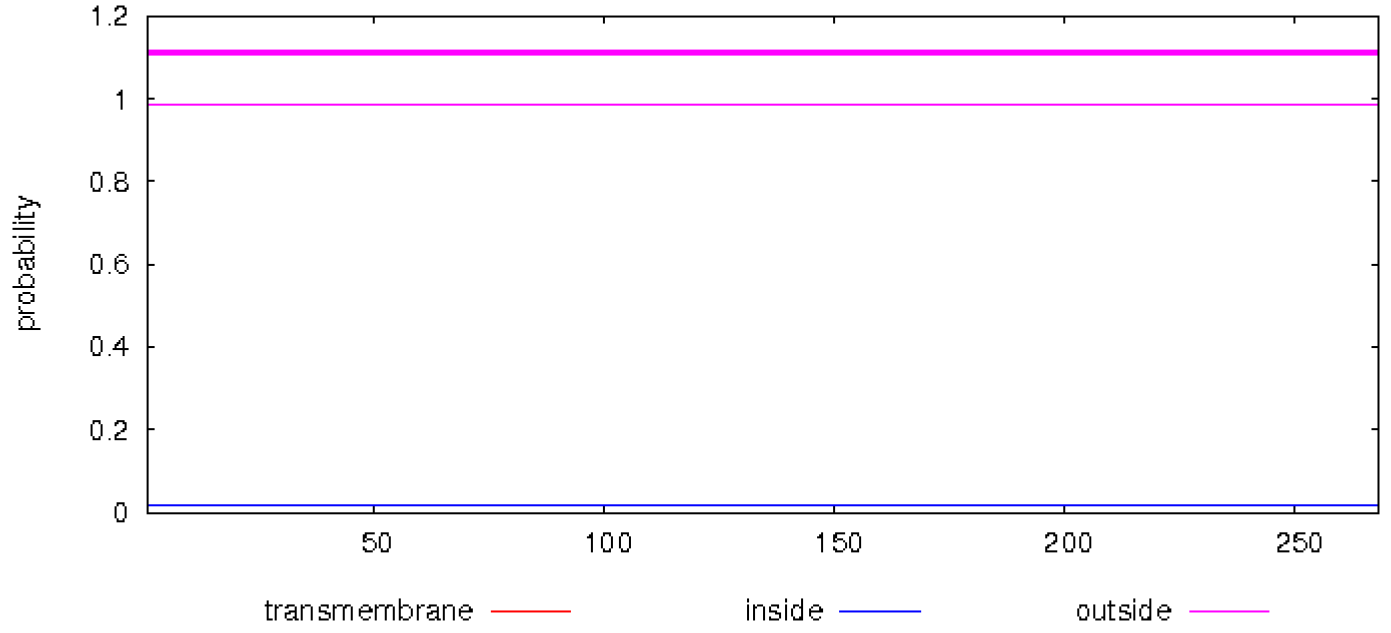

plot in postscript, script for making the plot in gnuplot, data for plot

```
# Actinidiachinensis_Achn163771 Length: 315
# Actinidiachinensis_Achn163771 Number of predicted TMHs:  0
# Actinidiachinensis_Achn163771 Exp number of AAs in TMHs: 0.00487
# Actinidiachinensis_Achn163771 Exp number, first 60 AAs:  0.00042
# Actinidiachinensis_Achn163771 Total prob of N-in:        0.00416
Actinidiachinensis_Achn163771	TMHMM2.0	outside	     1   315
```

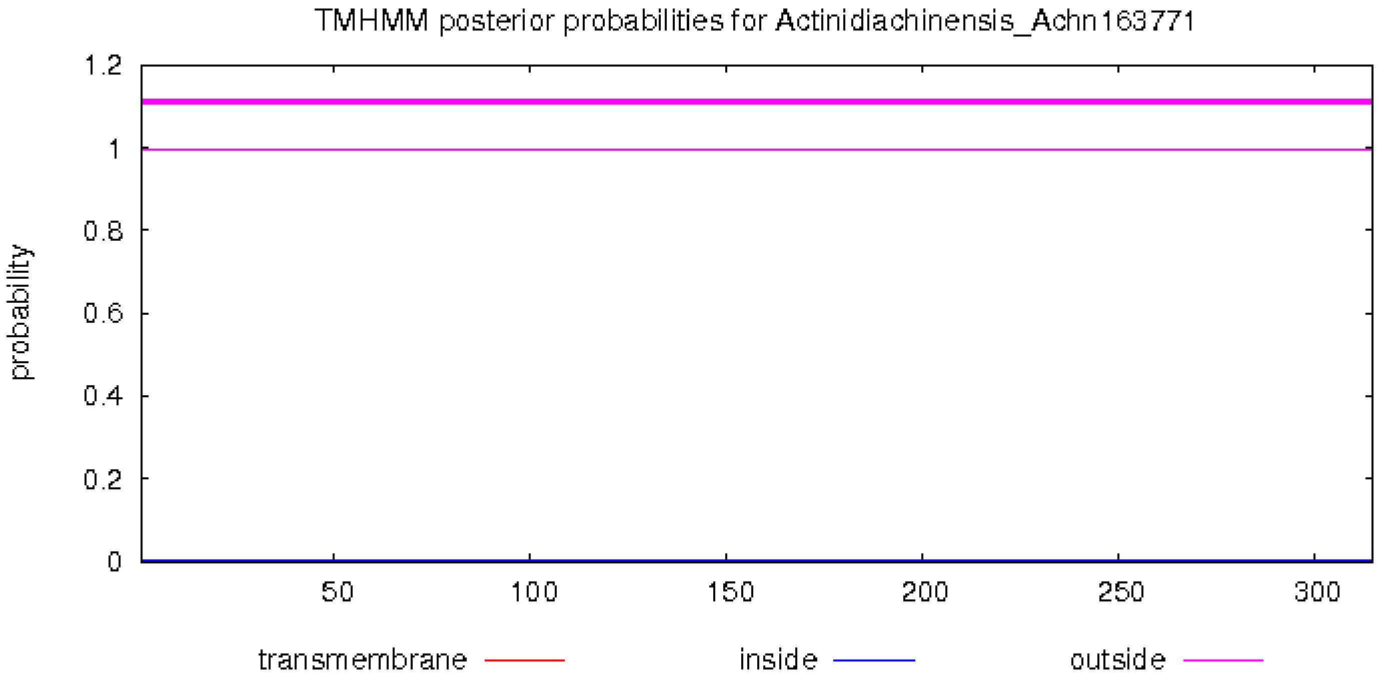

plot in postscript, script for making the plot in gnuplot, data for plot

```
# Actinidiachinensis_Achn166541 Length: 244
# Actinidiachinensis_Achn166541 Number of predicted TMHs:  0
# Actinidiachinensis_Achn166541 Exp number of AAs in TMHs: 0
# Actinidiachinensis_Achn166541 Exp number, first 60 AAs:  0
# Actinidiachinensis_Achn166541 Total prob of N-in:        0.00169
Actinidiachinensis_Achn166541	TMHMM2.0	outside	     1   244
```

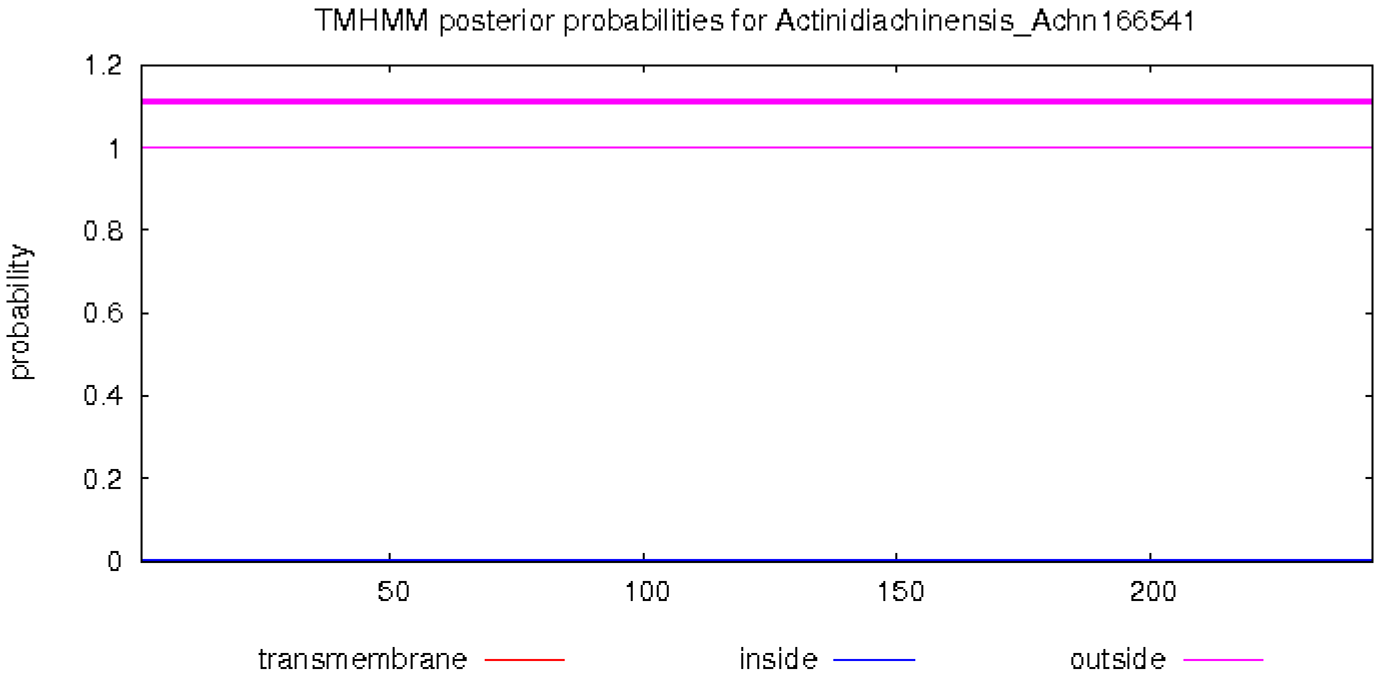

plot in postscript, script for making the plot in gnuplot, data for plot

```
# Actinidiachinensis_Achn166731 Length: 394
# Actinidiachinensis_Achn166731 Number of predicted TMHs:  0
# Actinidiachinensis_Achn166731 Exp number of AAs in TMHs: 0.02493
# Actinidiachinensis_Achn166731 Exp number, first 60 AAs:  0.01887
# Actinidiachinensis_Achn166731 Total prob of N-in:        0.01046
Actinidiachinensis_Achn166731	TMHMM2.0	outside	     1   394
```

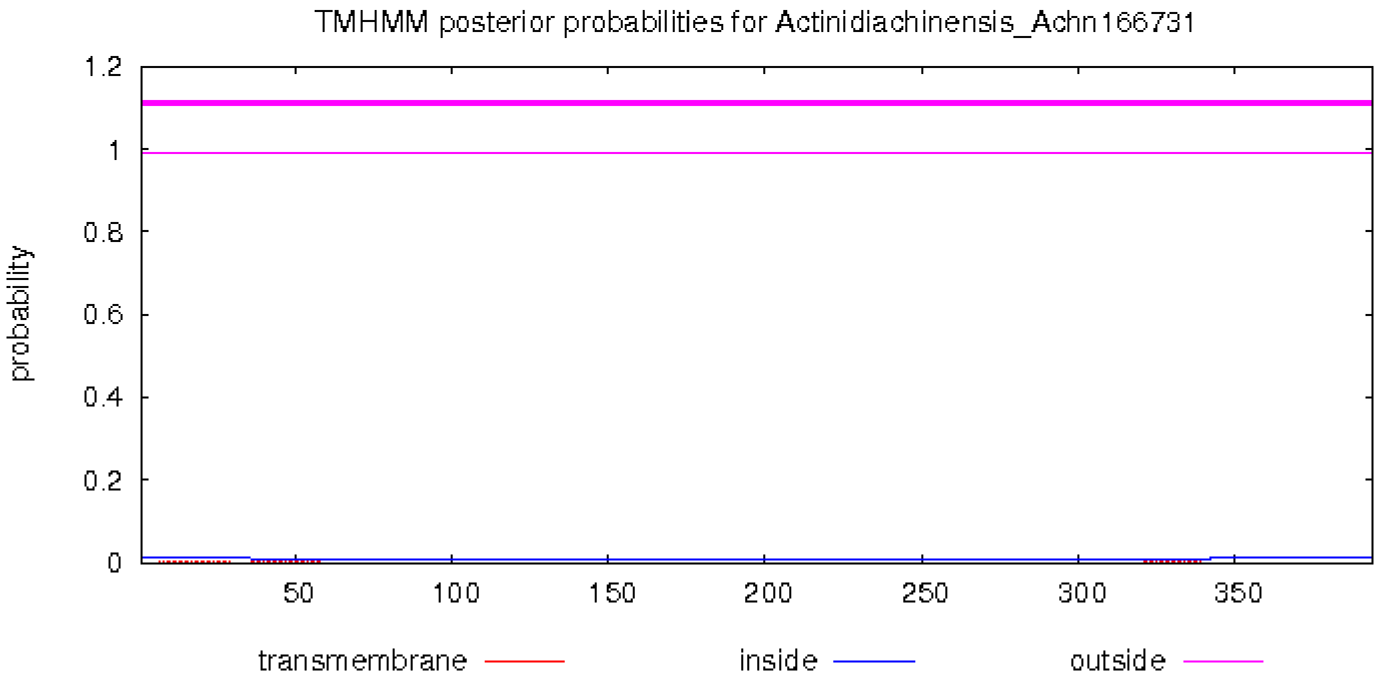

plot in postscript, script for making the plot in gnuplot, data for plot

---

```
# Actinidiachinensis_Achn169421 Length: 282
# Actinidiachinensis_Achn169421 Number of predicted TMHs:  0
# Actinidiachinensis_Achn169421 Exp number of AAs in TMHs: 0.00018
# Actinidiachinensis_Achn169421 Exp number, first 60 AAs:  0
# Actinidiachinensis_Achn169421 Total prob of N-in:        0.02041
Actinidiachinensis_Achn169421	TMHMM2.0	outside	     1   282
```

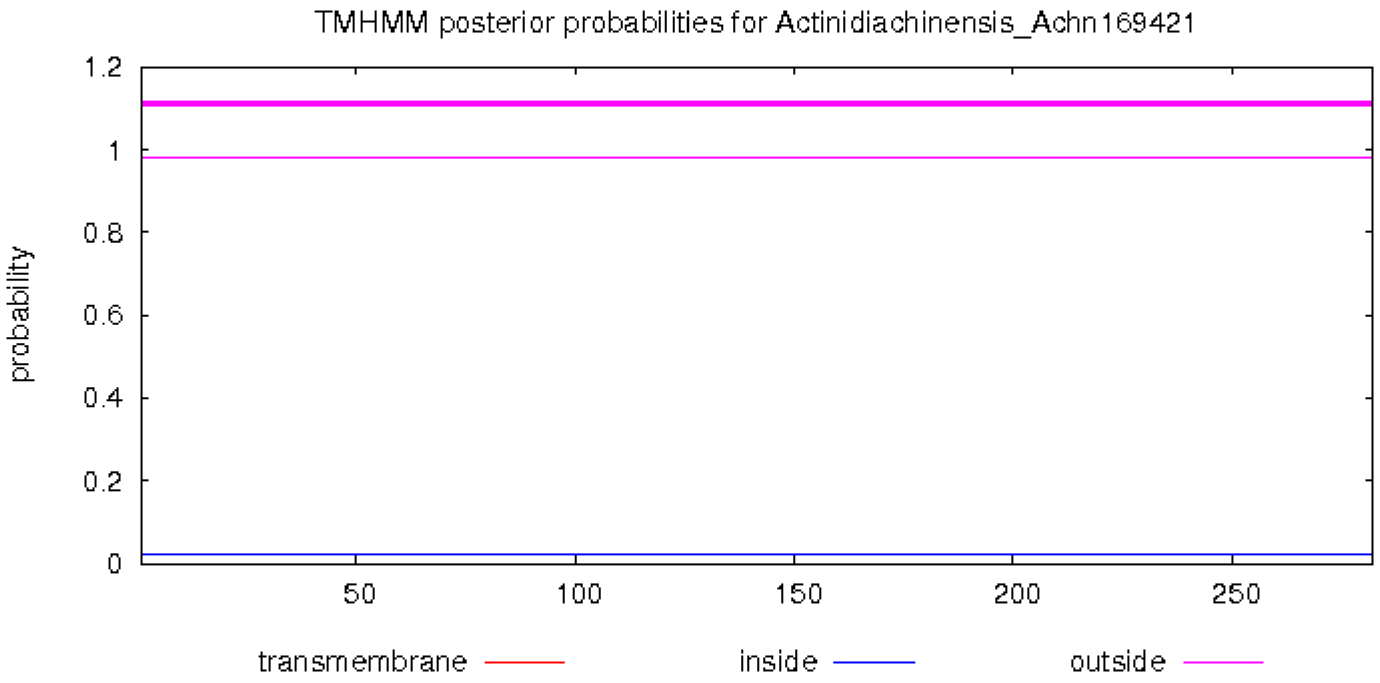

plot in postscript, script for making the plot in gnuplot, data for plot

---

```
# Actinidiachinensis_Achn169681 Length: 245
# Actinidiachinensis_Achn169681 Number of predicted TMHs:  0
# Actinidiachinensis_Achn169681 Exp number of AAs in TMHs: 0.00019
# Actinidiachinensis_Achn169681 Exp number, first 60 AAs:  0
# Actinidiachinensis_Achn169681 Total prob of N-in:        0.06272
Actinidiachinensis_Achn169681   TMHMM2.0        outside      1   245
```

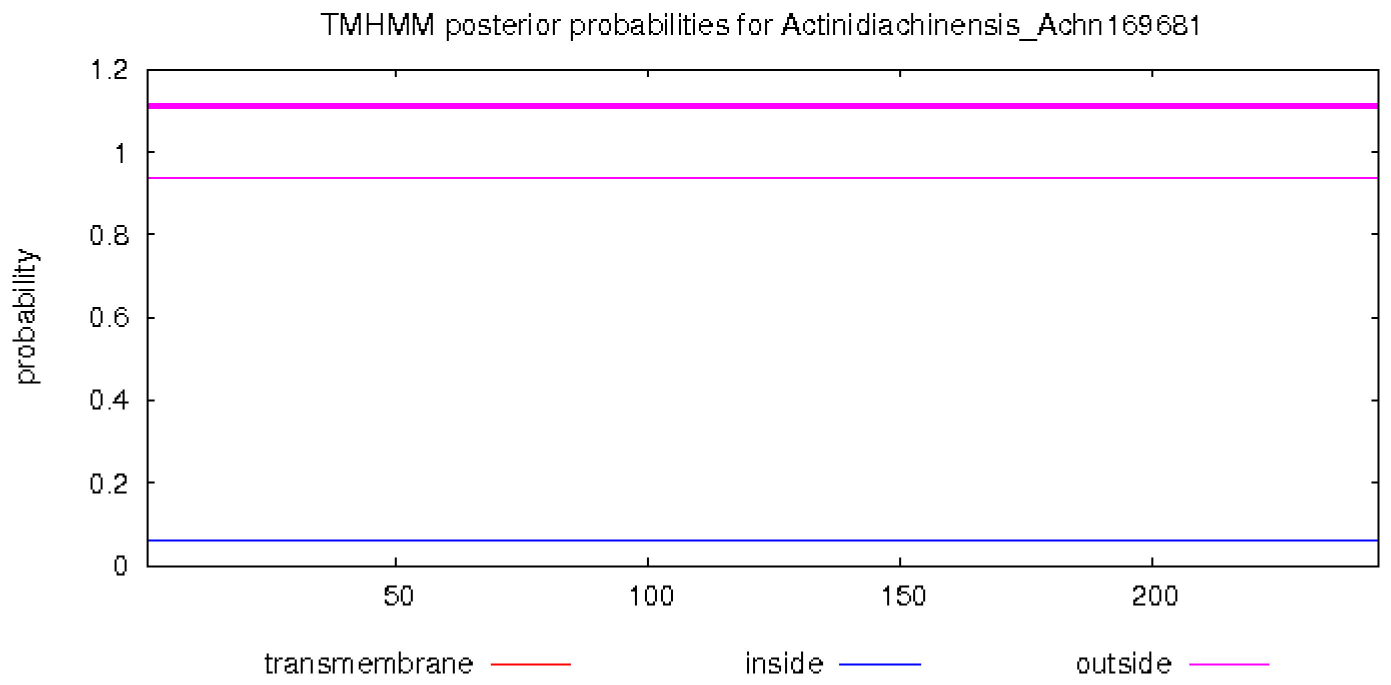

plot in postscript, script for making the plot in gnuplot, data for plot

---

```
# Actinidiachinensis_Achn170411 Length: 382
# Actinidiachinensis_Achn170411 Number of predicted TMHs:  0
# Actinidiachinensis_Achn170411 Exp number of AAs in TMHs: 0.00013
# Actinidiachinensis_Achn170411 Exp number, first 60 AAs:  0
# Actinidiachinensis_Achn170411 Total prob of N-in:        0.00243
Actinidiachinensis_Achn170411   TMHMM2.0        outside      1   382
```

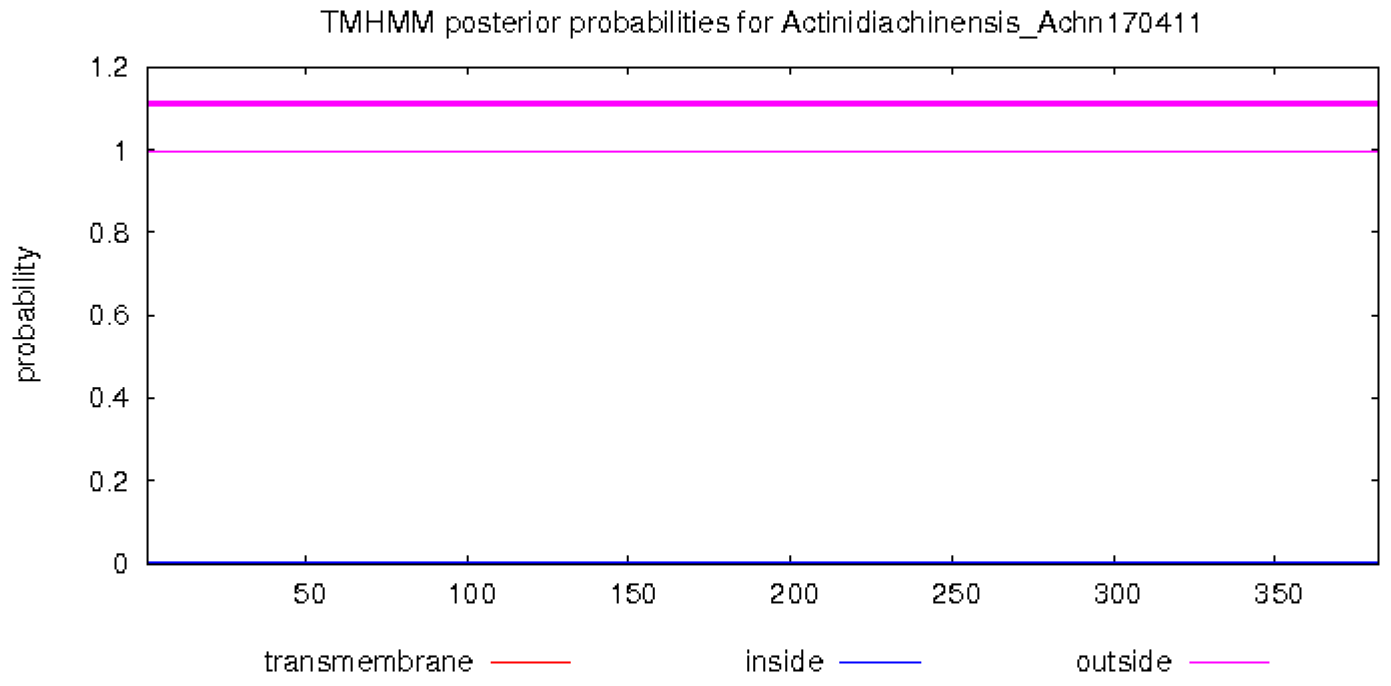

plot in postscript, script for making the plot in gnuplot, data for plot

---

```
# Actinidiachinensis_Achn179541 Length: 245
# Actinidiachinensis_Achn179541 Number of predicted TMHs:  0
# Actinidiachinensis_Achn179541 Exp number of AAs in TMHs: 0.00036
# Actinidiachinensis_Achn179541 Exp number, first 60 AAs:  0
# Actinidiachinensis_Achn179541 Total prob of N-in:        0.10012
Actinidiachinensis_Achn179541   TMHMM2.0        outside      1   245
```

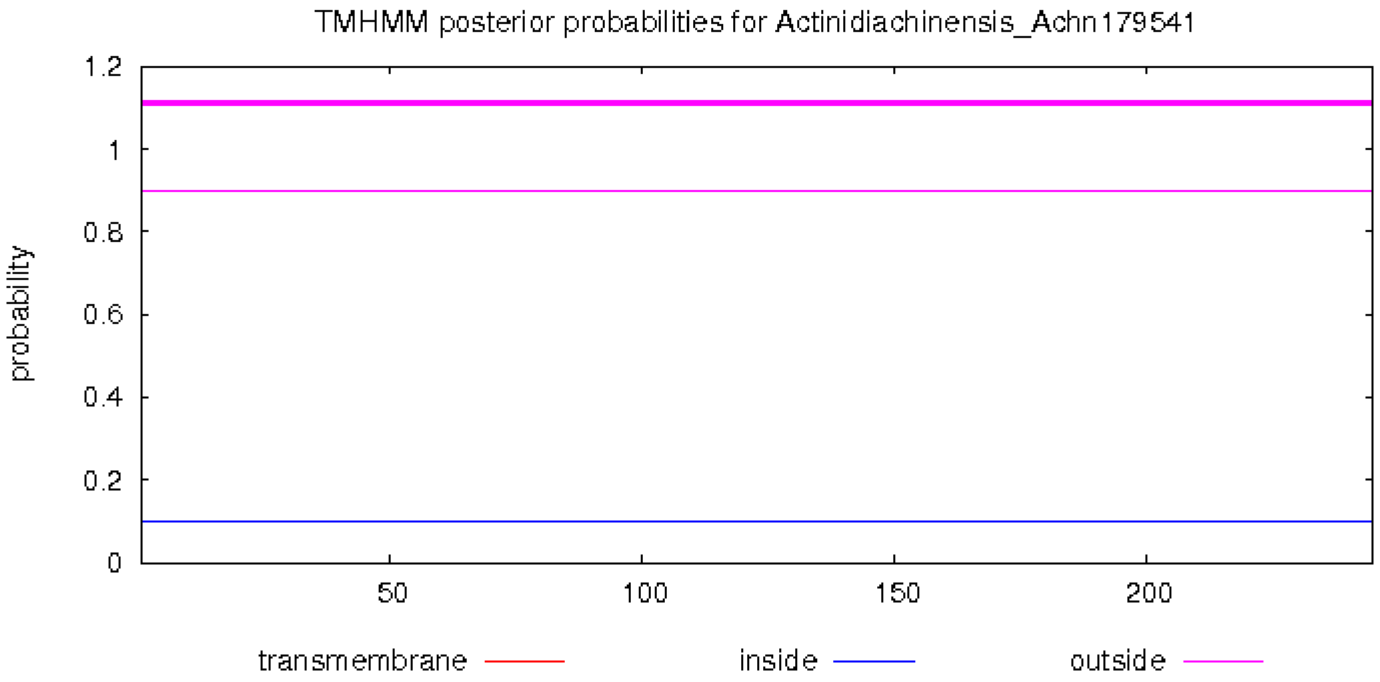

plot in postscript, script for making the plot in gnuplot, data for plot

---

```
# Actinidiachinensis_Achn181121 Length: 492
# Actinidiachinensis_Achn181121 Number of predicted TMHs:  0
# Actinidiachinensis_Achn181121 Exp number of AAs in TMHs: 0.00016
# Actinidiachinensis_Achn181121 Exp number, first 60 AAs:  0.00016
# Actinidiachinensis_Achn181121 Total prob of N-in:        0.00016
Actinidiachinensis_Achn181121   TMHMM2.0        outside      1   492
```

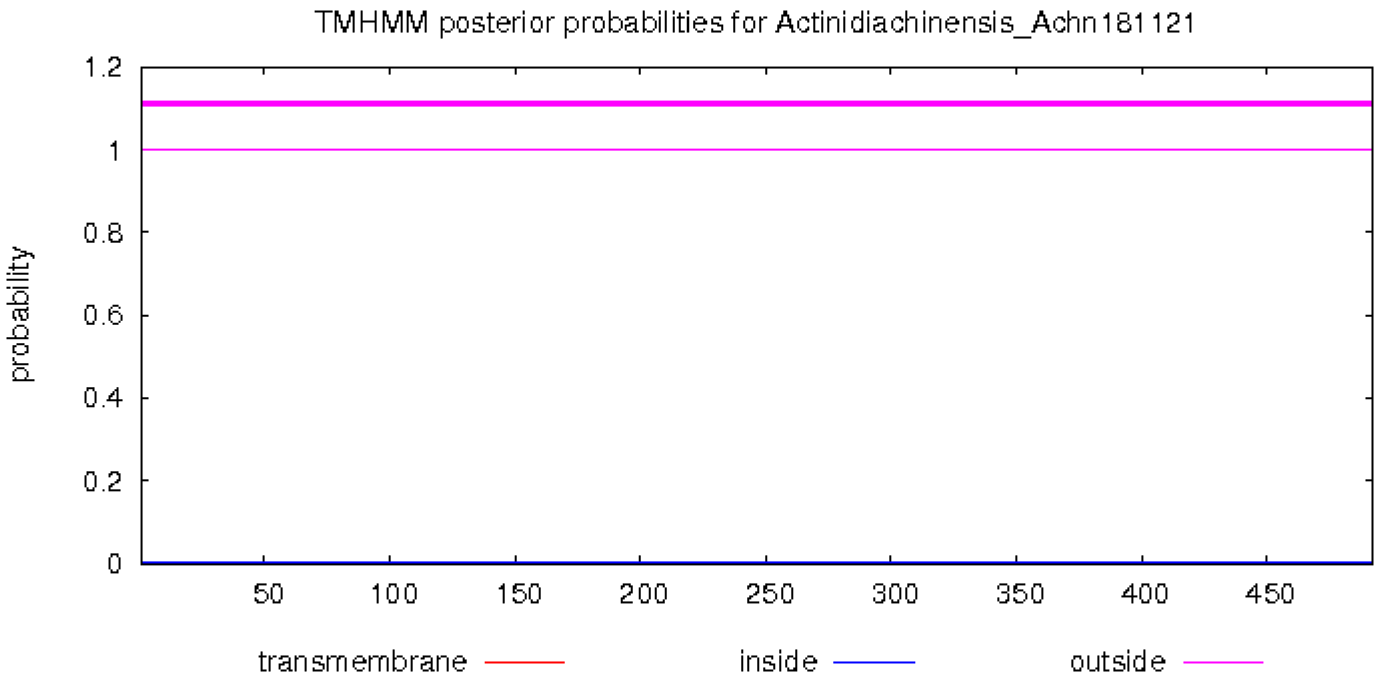

plot in postscript, script for making the plot in gnuplot, data for plot

---

```
# Actinidiachinensis_Achn181381 Length: 324
# Actinidiachinensis_Achn181381 Number of predicted TMHs:  0
# Actinidiachinensis_Achn181381 Exp number of AAs in TMHs: 4.49543
# Actinidiachinensis_Achn181381 Exp number, first 60 AAs:  4.48555
# Actinidiachinensis_Achn181381 Total prob of N-in:        0.24268
Actinidiachinensis_Achn181381   TMHMM2.0        outside      1   324
```

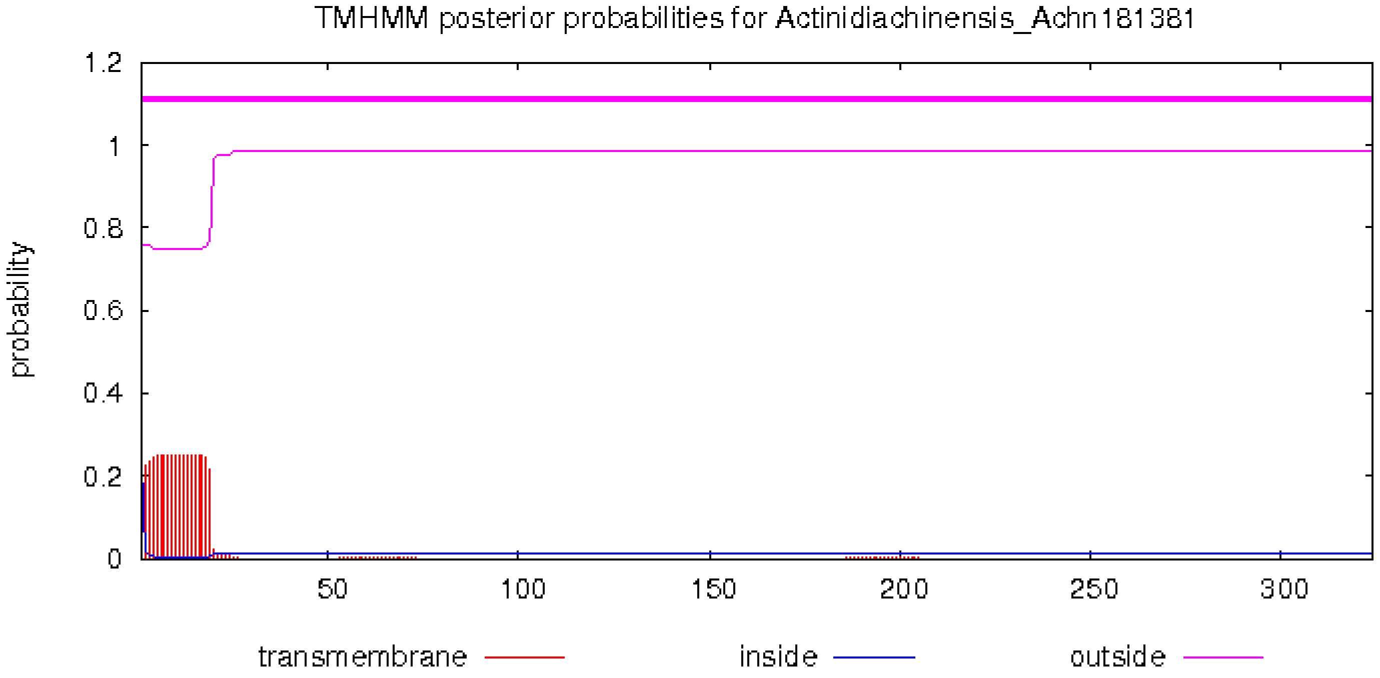

plot in postscript, script for making the plot in gnuplot, data for plot

```
# Actinidiachinensis_Achn182861 Length: 272
# Actinidiachinensis_Achn182861 Number of predicted TMHs:  0
# Actinidiachinensis_Achn182861 Exp number of AAs in TMHs: 0.00089000000000000001
# Actinidiachinensis_Achn182861 Exp number, first 60 AAs:  0.00016
# Actinidiachinensis_Achn182861 Total prob of N-in:        0.03032
Actinidiachinensis_Achn182861   TMHMM2.0        outside      1   272
```

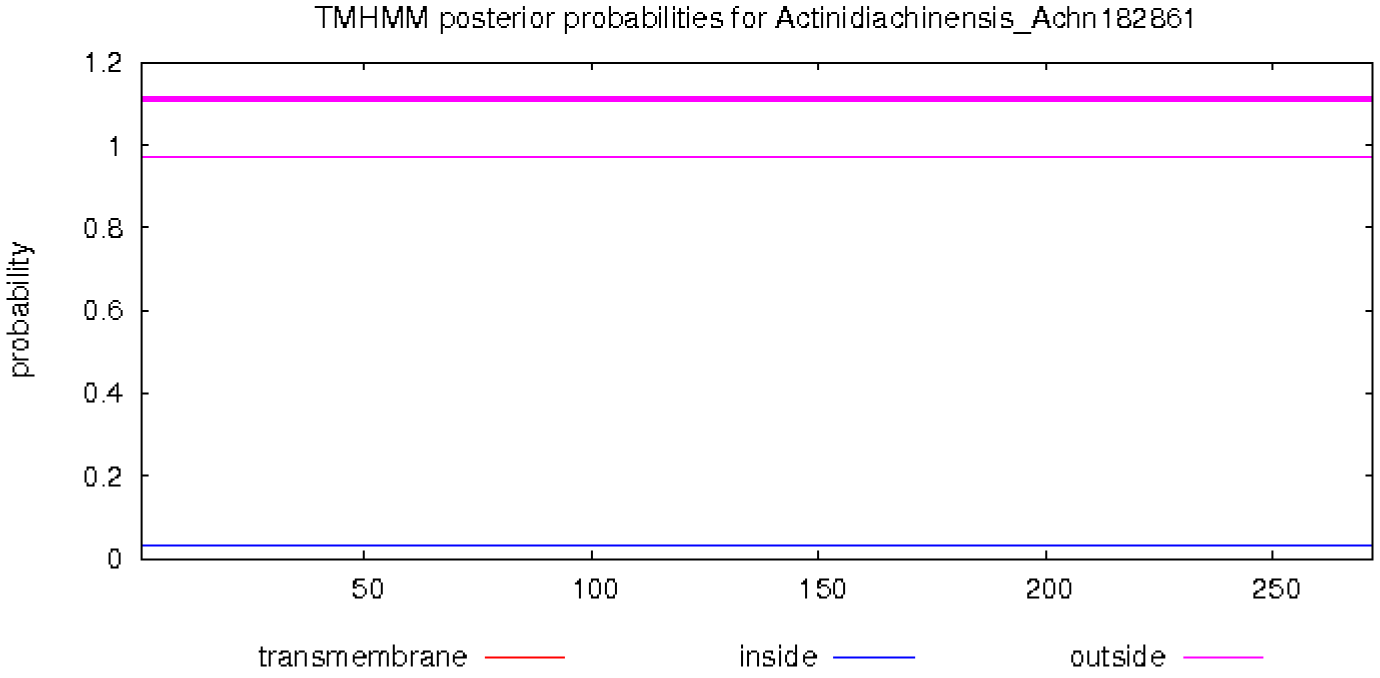

plot in postscript, script for making the plot in gnuplot, data for plot

```
# Actinidiachinensis_Achn183781 Length: 402
# Actinidiachinensis_Achn183781 Number of predicted TMHs:  0
# Actinidiachinensis_Achn183781 Exp number of AAs in TMHs: 0.00297
# Actinidiachinensis_Achn183781 Exp number, first 60 AAs:  0
# Actinidiachinensis_Achn183781 Total prob of N-in:        0.05951
Actinidiachinensis_Achn183781   TMHMM2.0        outside      1   402
```

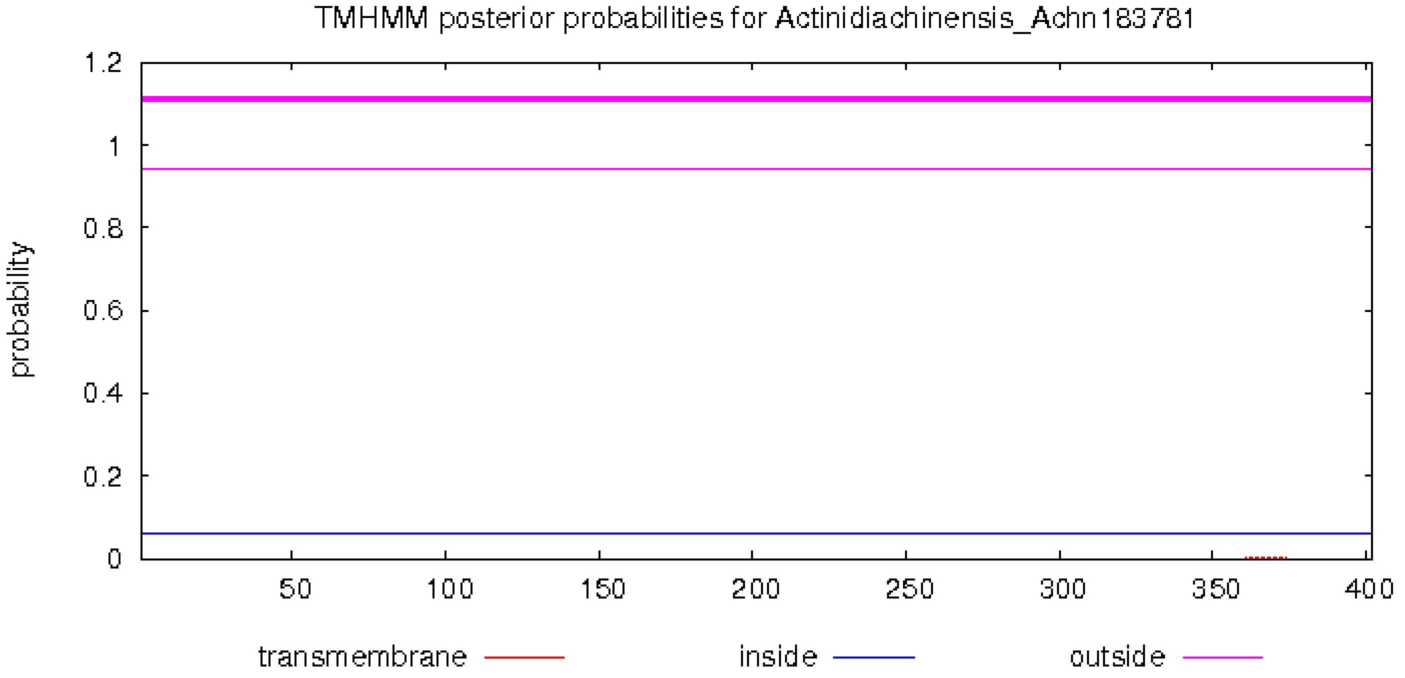

plot in postscript, script for making the plot in gnuplot, data for plot

---

```
# Actinidiachinensis_Achn185001 Length: 353
# Actinidiachinensis_Achn185001 Number of predicted TMHs:  0
# Actinidiachinensis_Achn185001 Exp number of AAs in TMHs: 0.00025
# Actinidiachinensis_Achn185001 Exp number, first 60 AAs:  0
# Actinidiachinensis_Achn185001 Total prob of N-in:        0.01235
Actinidiachinensis_Achn185001   TMHMM2.0        outside      1   353
```

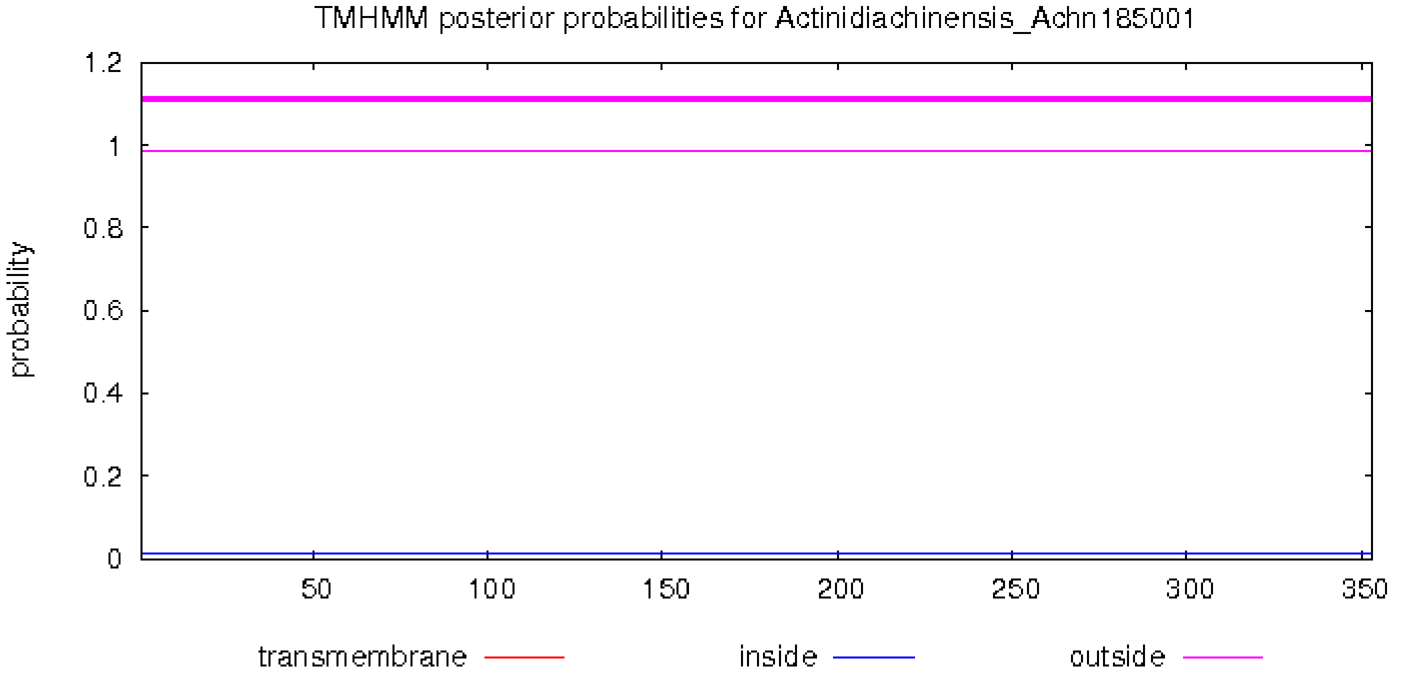

plot in postscript, script for making the plot in gnuplot, data for plot

---

```
# Actinidiachinensis_Achn185081 Length: 393
# Actinidiachinensis_Achn185081 Number of predicted TMHs:  0
# Actinidiachinensis_Achn185081 Exp number of AAs in TMHs: 0.00034
# Actinidiachinensis_Achn185081 Exp number, first 60 AAs:  0.00034
# Actinidiachinensis_Achn185081 Total prob of N-in:        0.00324
Actinidiachinensis_Achn185081	TMHMM2.0	outside	     1   393
```

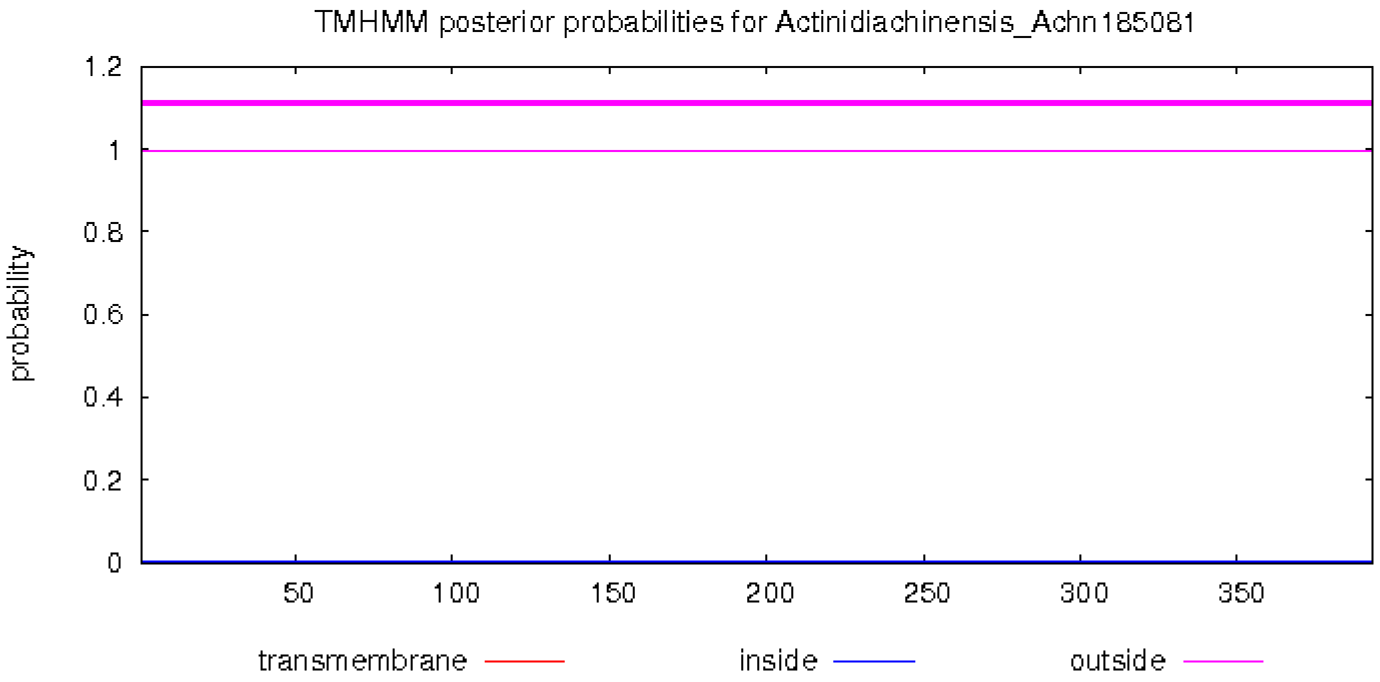

plot in postscript, script for making the plot in gnuplot, data for plot

```
# Actinidiachinensis_Achn190001 Length: 299
# Actinidiachinensis_Achn190001 Number of predicted TMHs:  0
# Actinidiachinensis_Achn190001 Exp number of AAs in TMHs: 0.00079
# Actinidiachinensis_Achn190001 Exp number, first 60 AAs:  4e-05
# Actinidiachinensis_Achn190001 Total prob of N-in:        0.01723
Actinidiachinensis_Achn190001	TMHMM2.0	outside	     1   299
```

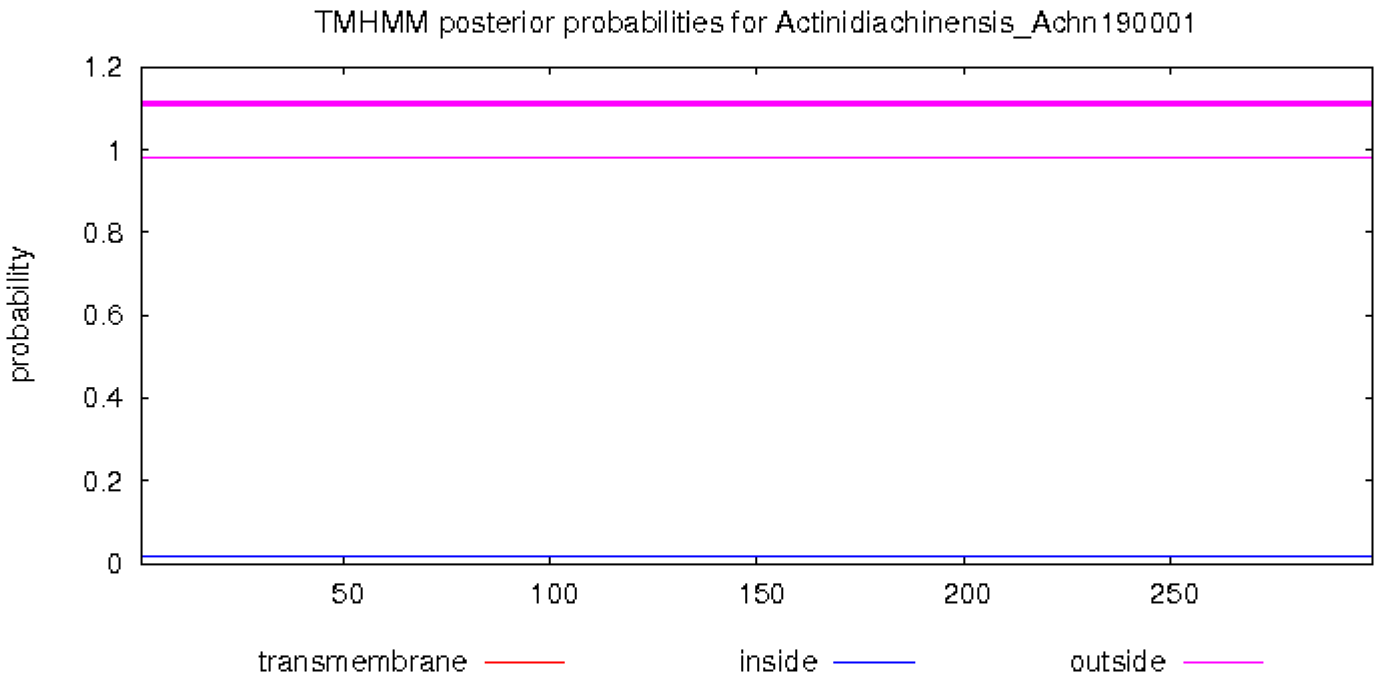

plot in postscript, script for making the plot in gnuplot, data for plot

```
# Actinidiachinensis_Achn193461 Length: 245
# Actinidiachinensis_Achn193461 Number of predicted TMHs:  0
# Actinidiachinensis_Achn193461 Exp number of AAs in TMHs: 0.02307
# Actinidiachinensis_Achn193461 Exp number, first 60 AAs:  0.02307
# Actinidiachinensis_Achn193461 Total prob of N-in:        0.03711
Actinidiachinensis_Achn193461   TMHMM2.0        outside      1   245
```

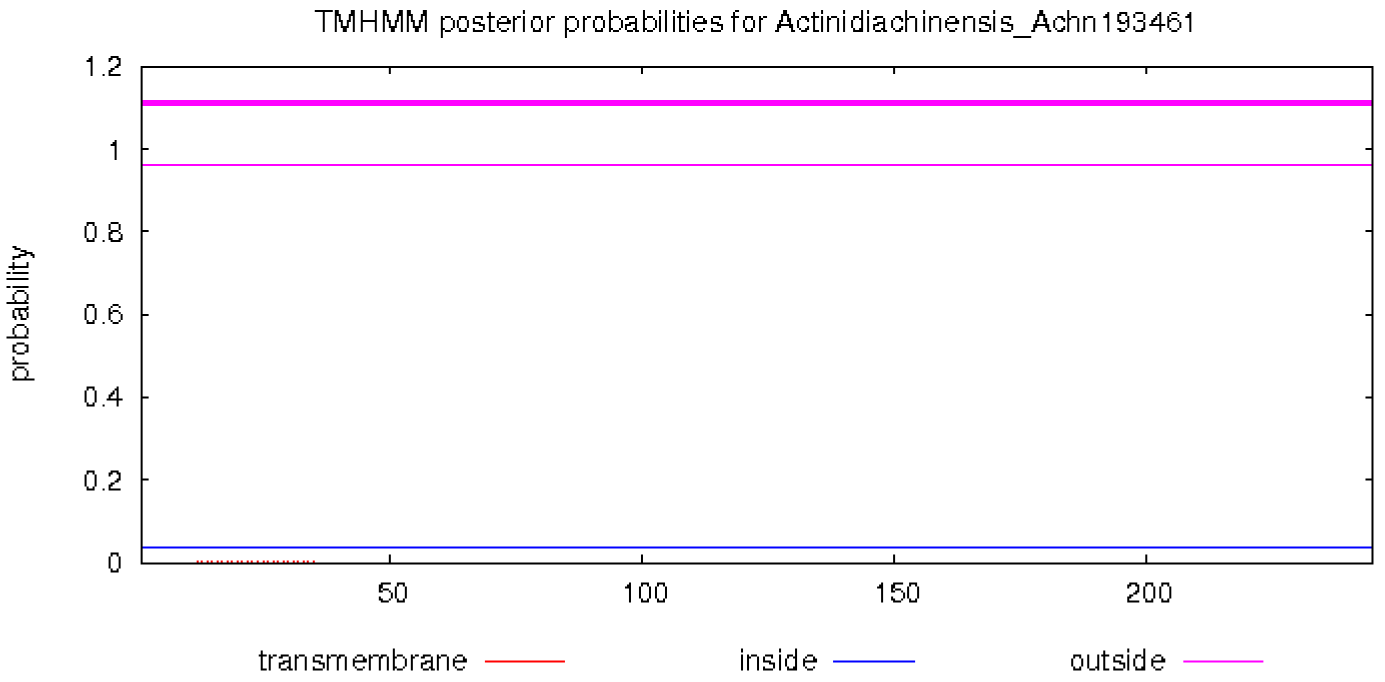

plot in postscript, script for making the plot in gnuplot, data for plot

---

```
# Actinidiachinensis_Achn196961 Length: 310
# Actinidiachinensis_Achn196961 Number of predicted TMHs:  0
# Actinidiachinensis_Achn196961 Exp number of AAs in TMHs: 0.00234
# Actinidiachinensis_Achn196961 Exp number, first 60 AAs:  0.00234
# Actinidiachinensis_Achn196961 Total prob of N-in:        0.05413
Actinidiachinensis_Achn196961   TMHMM2.0        outside      1   310
```

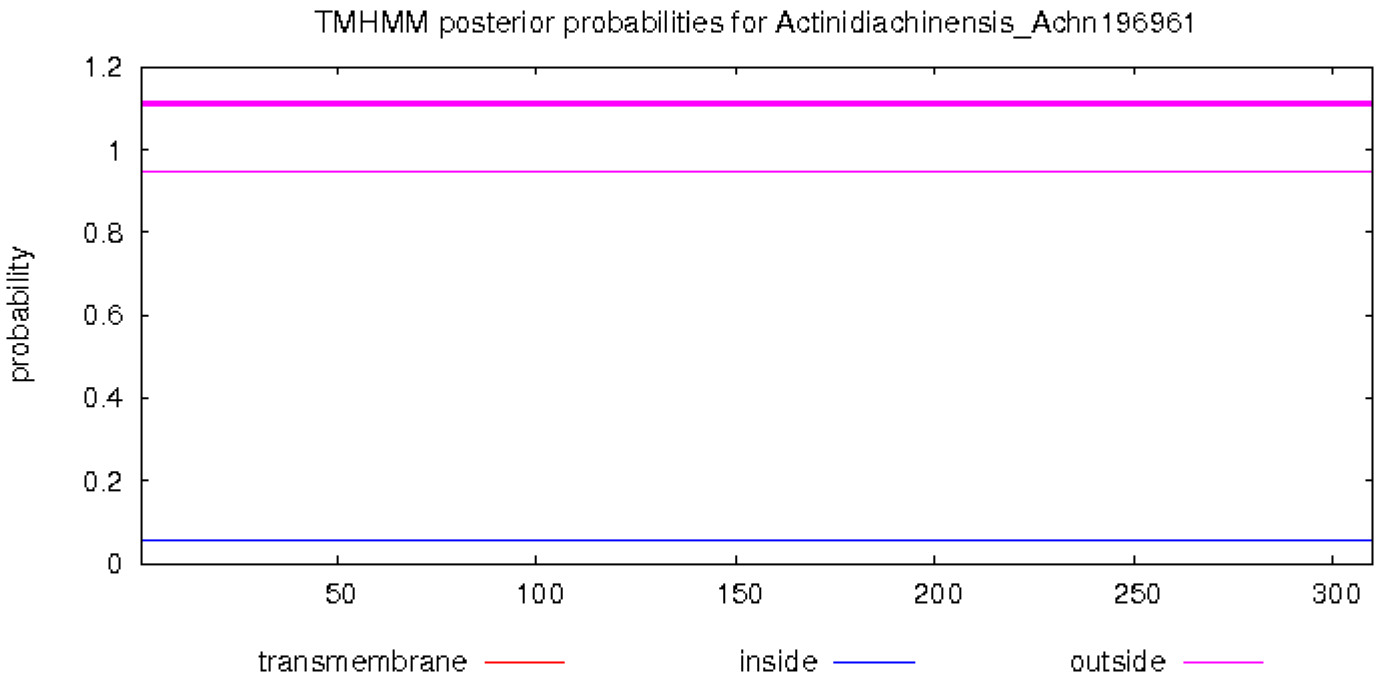

plot in postscript, script for making the plot in gnuplot, data for plot

---

```
# Actinidiachinensis_Achn197411 Length: 442
# Actinidiachinensis_Achn197411 Number of predicted TMHs:  0
# Actinidiachinensis_Achn197411 Exp number of AAs in TMHs: 0.00866
# Actinidiachinensis_Achn197411 Exp number, first 60 AAs:  0.00013
# Actinidiachinensis_Achn197411 Total prob of N-in:        0.00379
Actinidiachinensis_Achn197411   TMHMM2.0        outside      1   442
```

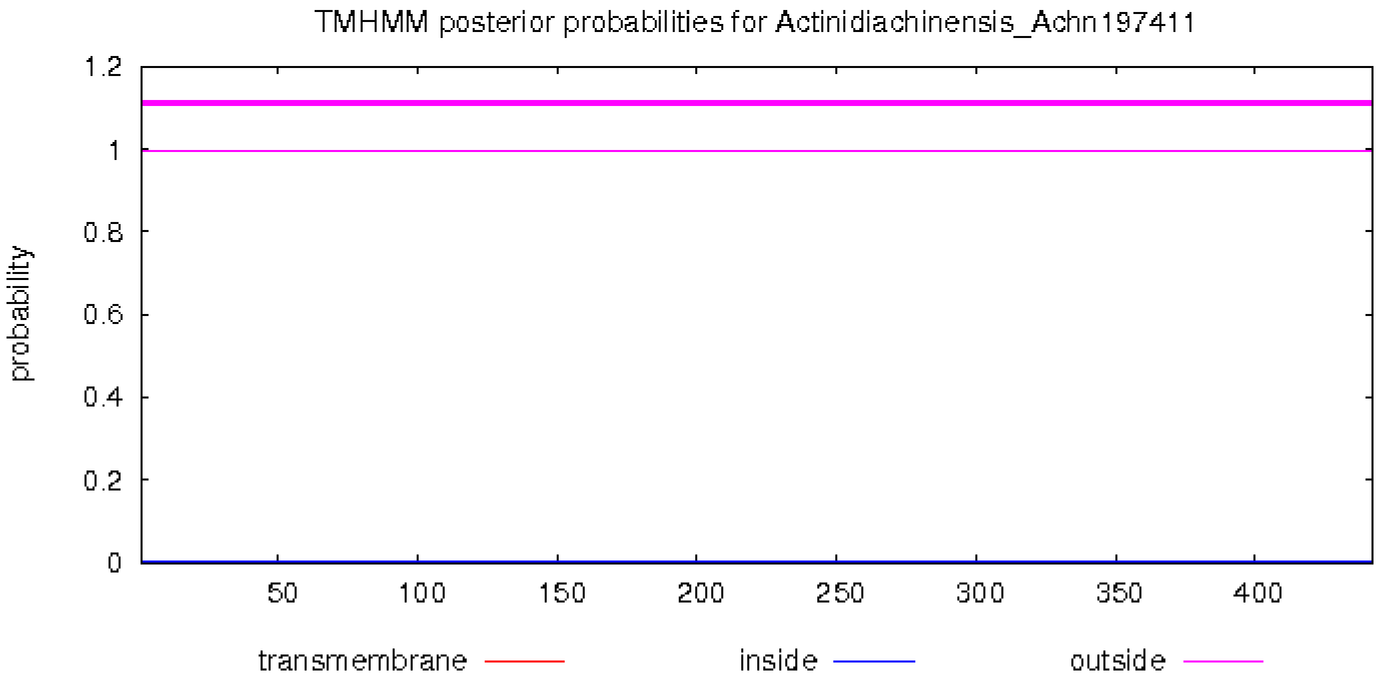

plot in postscript, script for making the plot in gnuplot, data for plot

---

```
# Actinidiachinensis_Achn200291 Length: 261
# Actinidiachinensis_Achn200291 Number of predicted TMHs:  0
# Actinidiachinensis_Achn200291 Exp number of AAs in TMHs: 0.0016
# Actinidiachinensis_Achn200291 Exp number, first 60 AAs:  0.00098
# Actinidiachinensis_Achn200291 Total prob of N-in:        0.09370
Actinidiachinensis_Achn200291   TMHMM2.0        outside      1   261
```

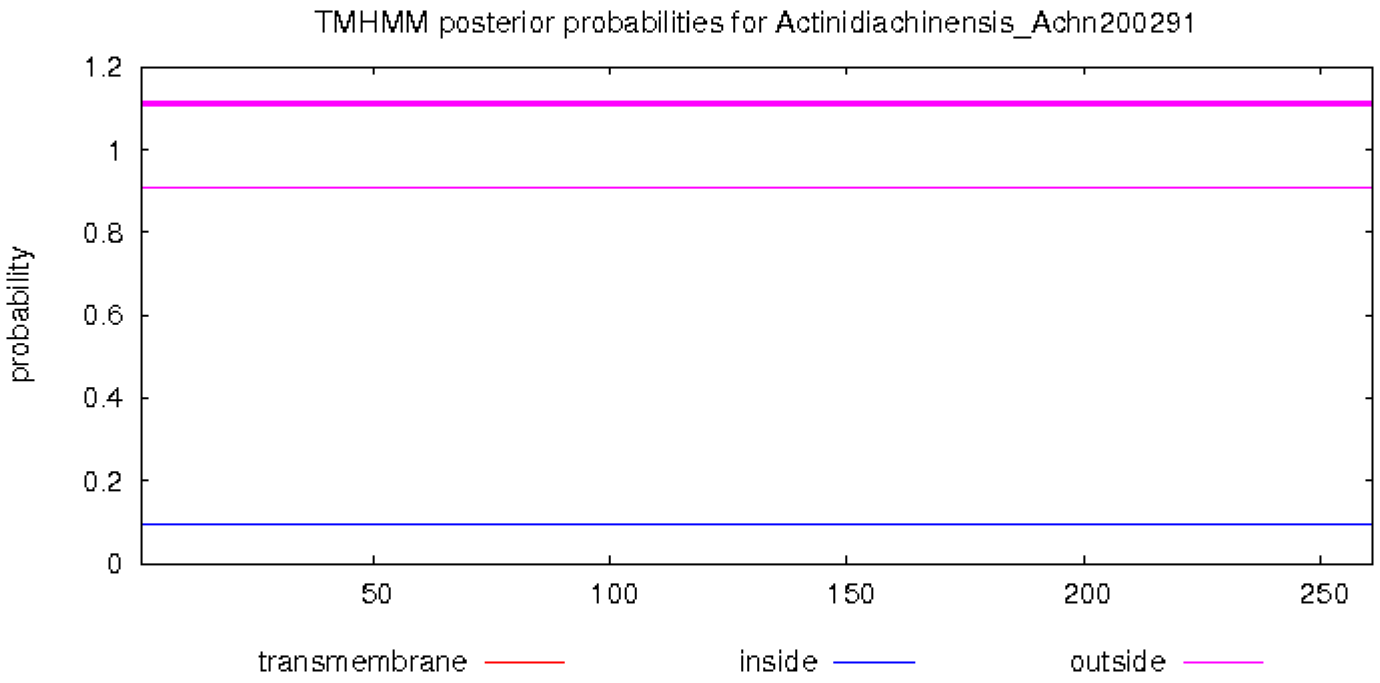

plot in postscript, script for making the plot in gnuplot, data for plot

---

```
# Actinidiachinensis_Achn200301 Length: 236
# Actinidiachinensis_Achn200301 Number of predicted TMHs:  0
# Actinidiachinensis_Achn200301 Exp number of AAs in TMHs: 0.00016
# Actinidiachinensis_Achn200301 Exp number, first 60 AAs:  0
# Actinidiachinensis_Achn200301 Total prob of N-in:        0.01645
Actinidiachinensis_Achn200301   TMHMM2.0        outside      1   236
```

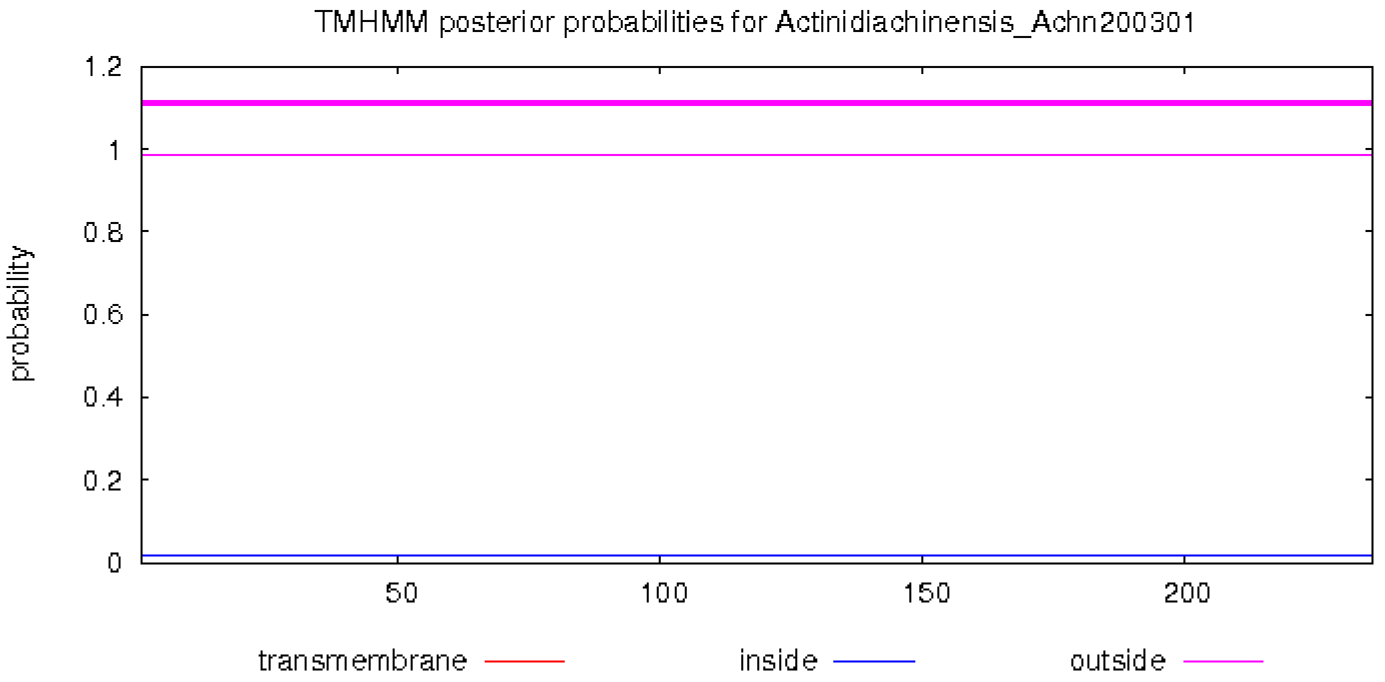

plot in postscript, script for making the plot in gnuplot, data for plot

---

```
# Actinidiachinensis_Achn204171 Length: 387
# Actinidiachinensis_Achn204171 Number of predicted TMHs:  0
# Actinidiachinensis_Achn204171 Exp number of AAs in TMHs: 0
# Actinidiachinensis_Achn204171 Exp number, first 60 AAs:  0
# Actinidiachinensis_Achn204171 Total prob of N-in:        0.00301
Actinidiachinensis_Achn204171   TMHMM2.0        outside      1   387
```

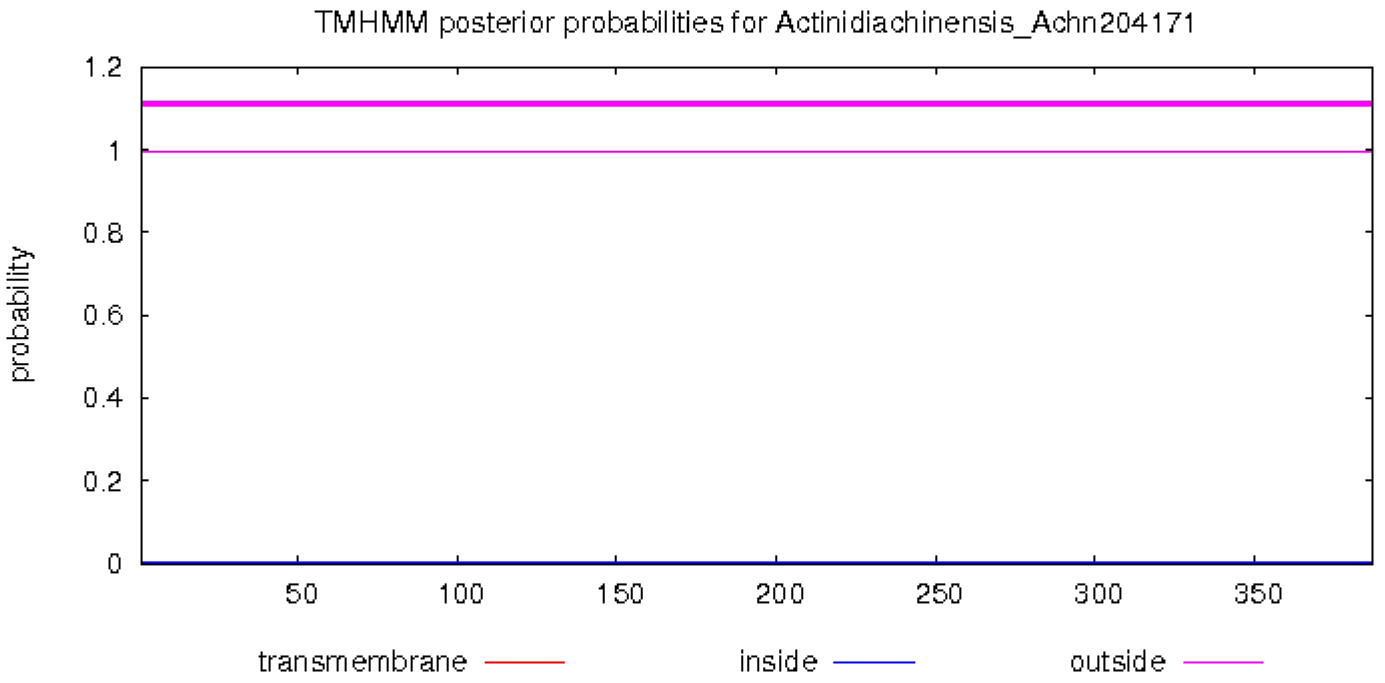

plot in postscript, script for making the plot in gnuplot, data for plot

---

```
# Actinidiachinensis_Achn204181 Length: 549
# Actinidiachinensis_Achn204181 Number of predicted TMHs:  1
# Actinidiachinensis_Achn204181 Exp number of AAs in TMHs: 21.08064
# Actinidiachinensis_Achn204181 Exp number, first 60 AAs:  0
# Actinidiachinensis_Achn204181 Total prob of N-in:        0.00157
Actinidiachinensis_Achn204181   TMHMM2.0        outside      1   519
Actinidiachinensis_Achn204181   TMHMM2.0        TMhelix    520   537
Actinidiachinensis_Achn204181   TMHMM2.0        inside     538   549
```

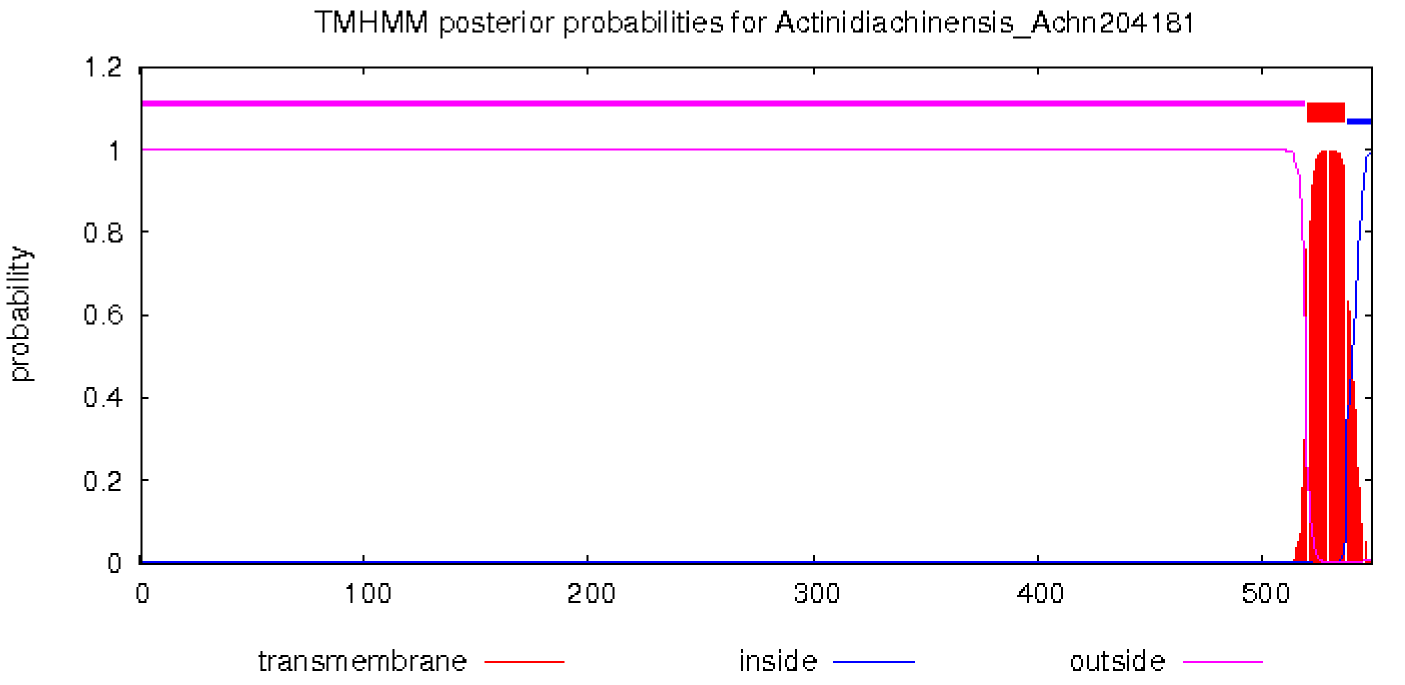

plot in postscript, script for making the plot in gnuplot, data for plot

---

```
# Actinidiachinensis_Achn206771 Length: 181
# Actinidiachinensis_Achn206771 Number of predicted TMHs:  0
# Actinidiachinensis_Achn206771 Exp number of AAs in TMHs: 0.00031
# Actinidiachinensis_Achn206771 Exp number, first 60 AAs:  0.00031
# Actinidiachinensis_Achn206771 Total prob of N-in:        0.36762
Actinidiachinensis_Achn206771   TMHMM2.0        outside      1   181
```

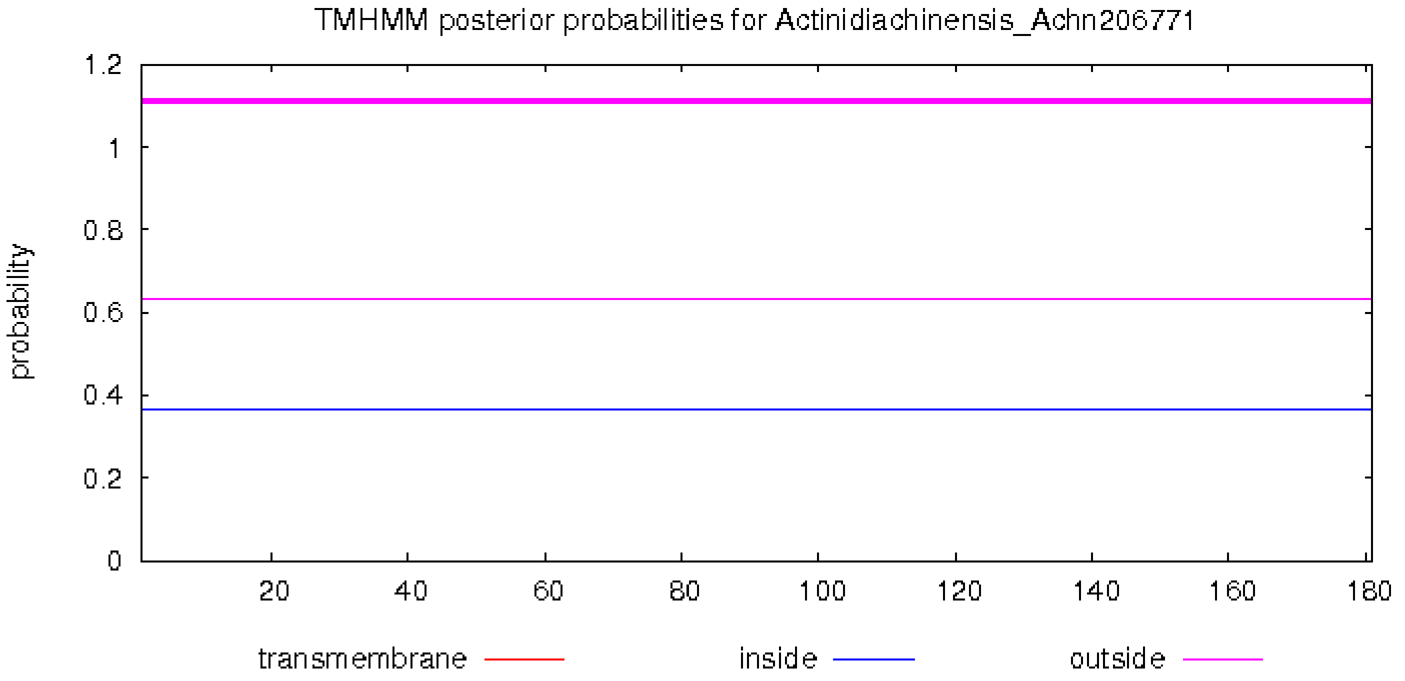

plot in postscript, script for making the plot in gnuplot, data for plot

```
# Actinidiachinensis_Achn207401 Length: 337
# Actinidiachinensis_Achn207401 Number of predicted TMHs:  0
# Actinidiachinensis_Achn207401 Exp number of AAs in TMHs: 0
# Actinidiachinensis_Achn207401 Exp number, first 60 AAs:  0
# Actinidiachinensis_Achn207401 Total prob of N-in:        0.01039
Actinidiachinensis_Achn207401	TMHMM2.0	outside	     1   337
```

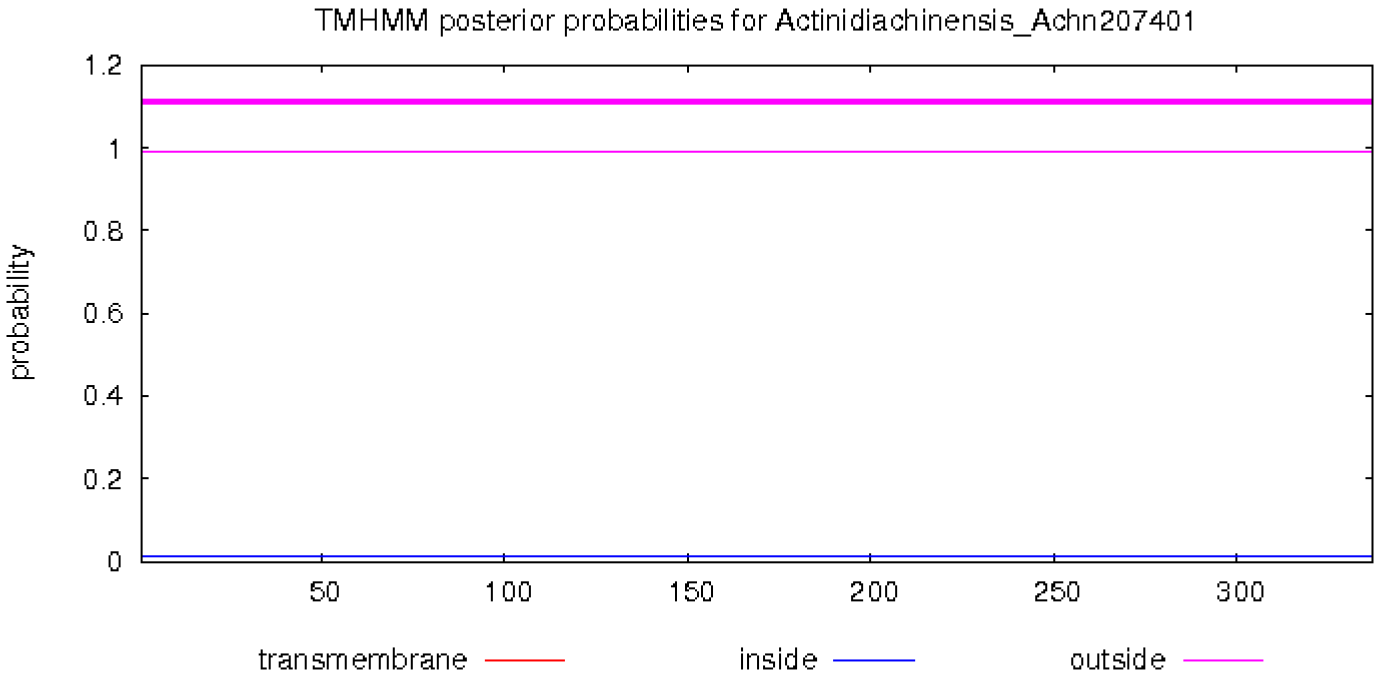

plot in postscript, script for making the plot in gnuplot, data for plot

```
# Actinidiachinensis_Achn208181 Length: 295
# Actinidiachinensis_Achn208181 Number of predicted TMHs:  0
# Actinidiachinensis_Achn208181 Exp number of AAs in TMHs: 0.00186
# Actinidiachinensis_Achn208181 Exp number, first 60 AAs:  0
# Actinidiachinensis_Achn208181 Total prob of N-in:        0.01737
Actinidiachinensis_Achn208181	TMHMM2.0	outside	     1   295
```

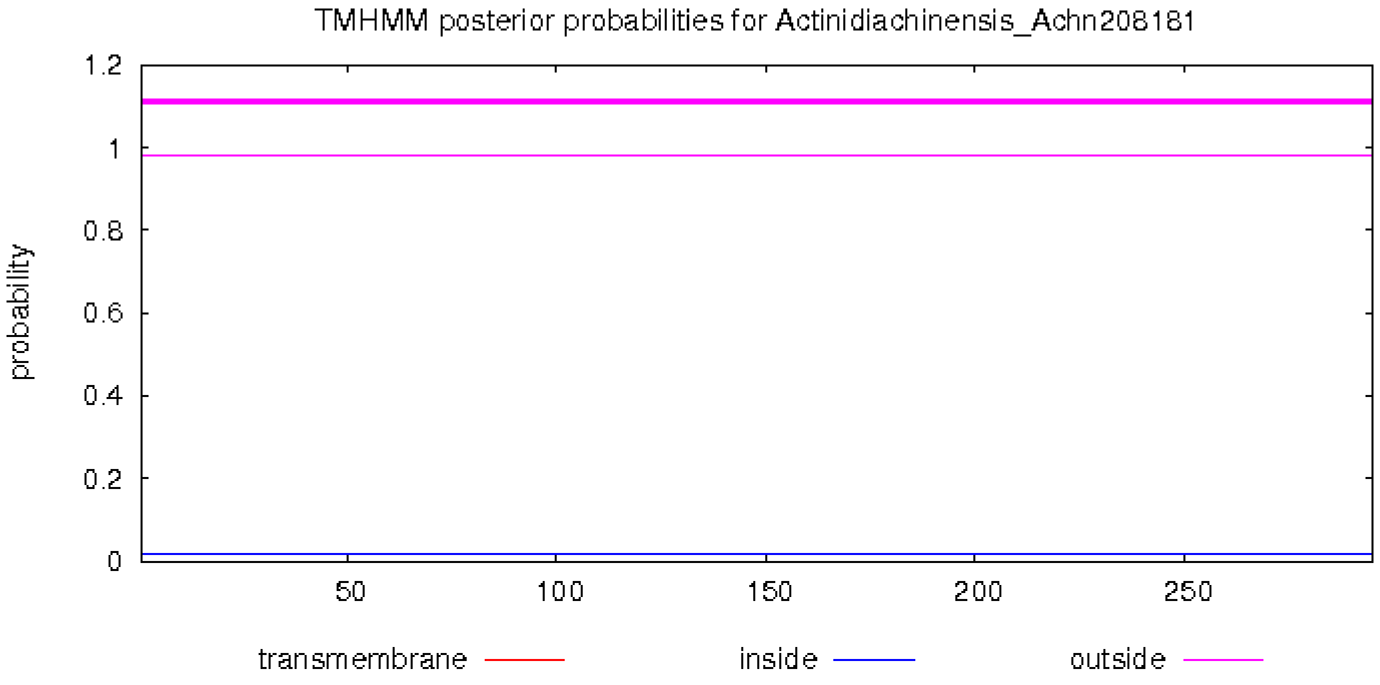

plot in postscript, script for making the plot in gnuplot, data for plot

```
# Actinidiachinensis_Achn210451 Length: 194
# Actinidiachinensis_Achn210451 Number of predicted TMHs:  0
# Actinidiachinensis_Achn210451 Exp number of AAs in TMHs: 0.00661
# Actinidiachinensis_Achn210451 Exp number, first 60 AAs:  0.00658
# Actinidiachinensis_Achn210451 Total prob of N-in:        0.11770
Actinidiachinensis_Achn210451   TMHMM2.0        outside      1   194
```

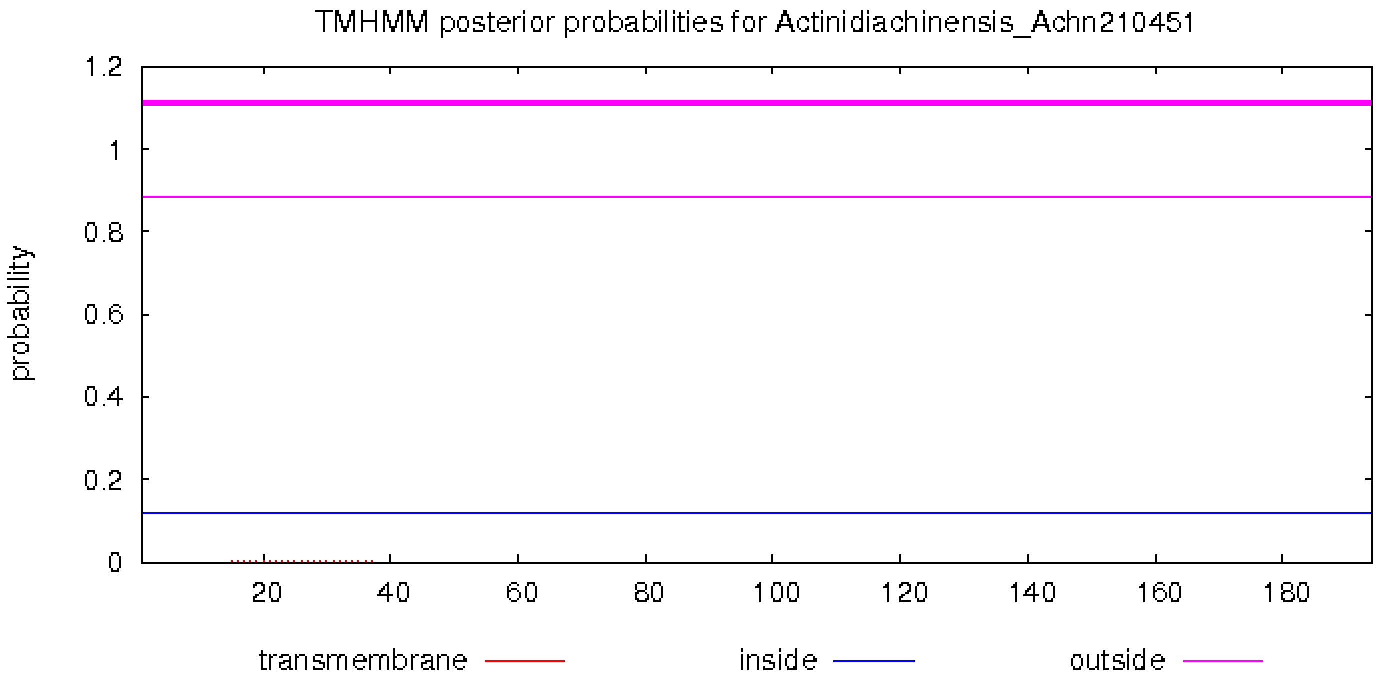

plot in postscript, script for making the plot in gnuplot, data for plot

---

```
# Actinidiachinensis_Achn212001 Length: 179
# Actinidiachinensis_Achn212001 Number of predicted TMHs:  0
# Actinidiachinensis_Achn212001 Exp number of AAs in TMHs: 14.41031
# Actinidiachinensis_Achn212001 Exp number, first 60 AAs:  14.40527
# Actinidiachinensis_Achn212001 Total prob of N-in:        0.78082
# Actinidiachinensis_Achn212001 POSSIBLE N-term signal sequence
Actinidiachinensis_Achn212001   TMHMM2.0        outside      1   179
```

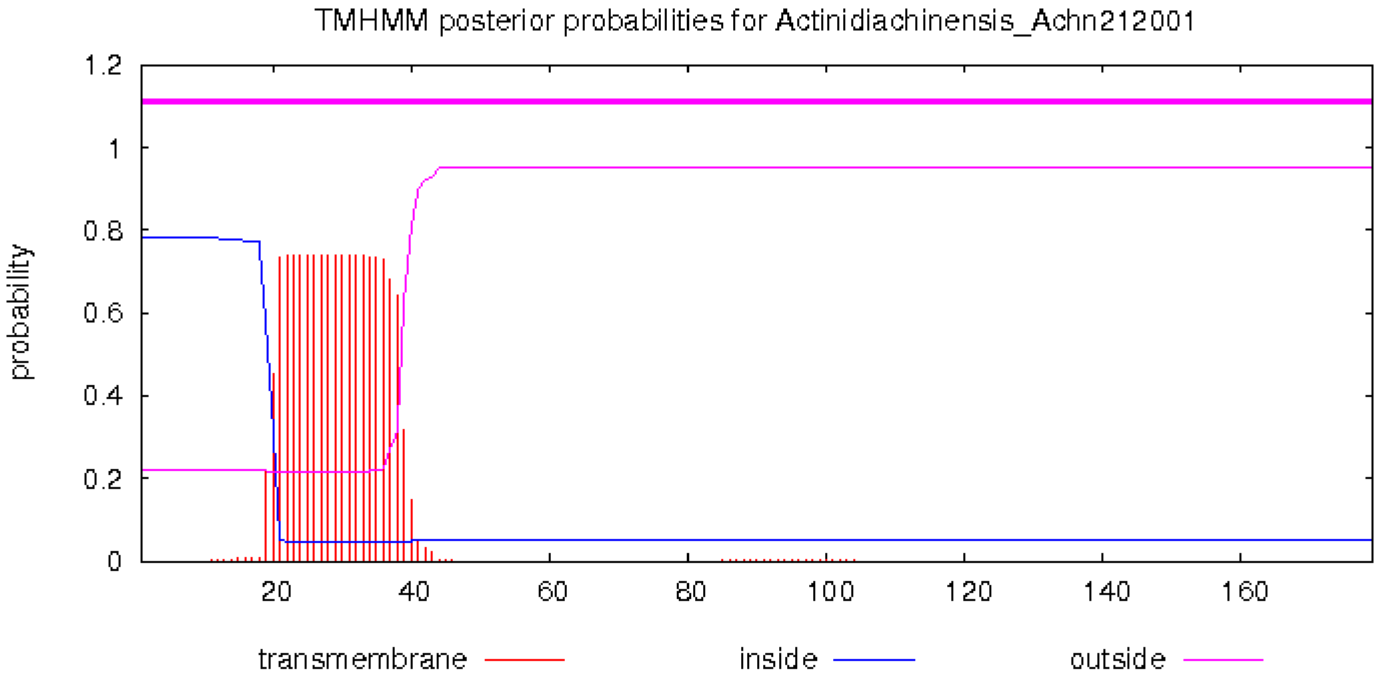

plot in postscript, script for making the plot in gnuplot, data for plot

---

```
# Actinidiachinensis_Achn219111 Length: 731
# Actinidiachinensis_Achn219111 Number of predicted TMHs:  0
# Actinidiachinensis_Achn219111 Exp number of AAs in TMHs: 0.00961999999999999999
# Actinidiachinensis_Achn219111 Exp number, first 60 AAs:  0.00441
# Actinidiachinensis_Achn219111 Total prob of N-in:        0.00044
Actinidiachinensis_Achn219111	TMHMM2.0	outside	     1   731
```

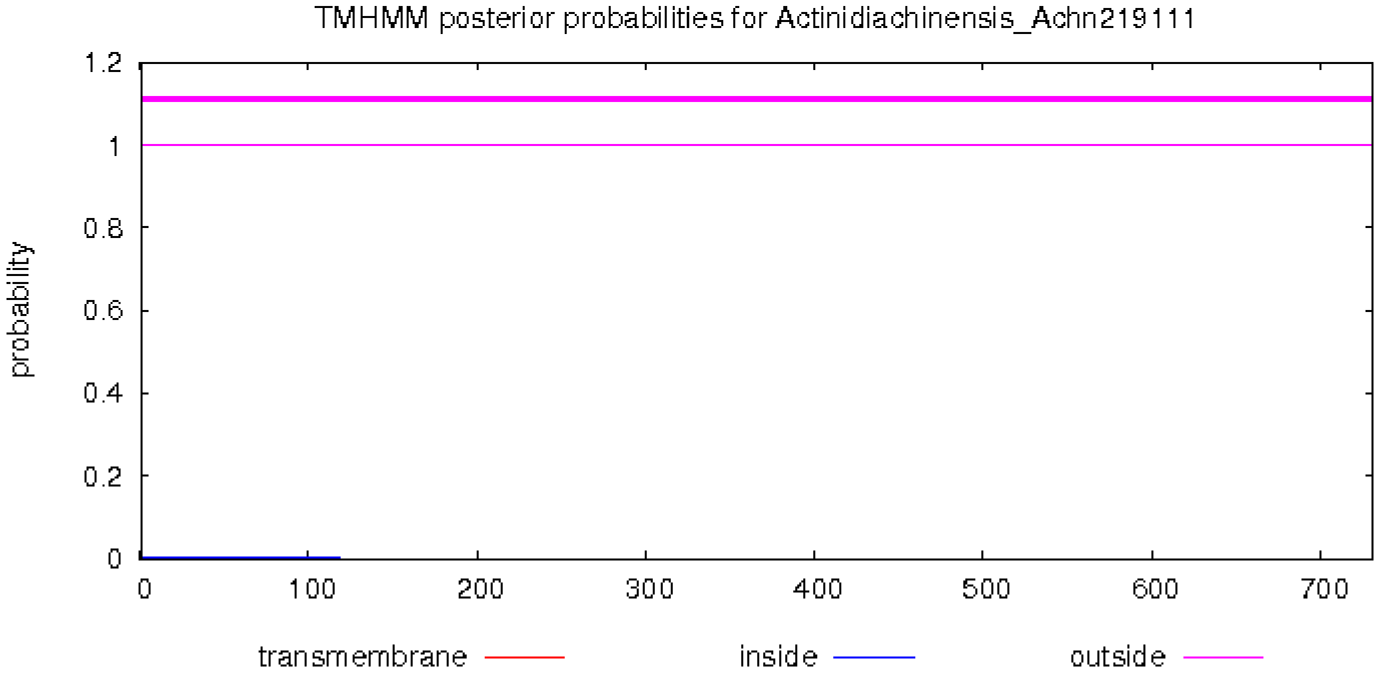

plot in postscript, script for making the plot in gnuplot, data for plot

---

```
# Actinidiachinensis_Achn219211 Length: 382
# Actinidiachinensis_Achn219211 Number of predicted TMHs:  0
# Actinidiachinensis_Achn219211 Exp number of AAs in TMHs: 0.00165
# Actinidiachinensis_Achn219211 Exp number, first 60 AAs:  0
# Actinidiachinensis_Achn219211 Total prob of N-in:        0.00475
Actinidiachinensis_Achn219211	TMHMM2.0	outside	     1   382
```

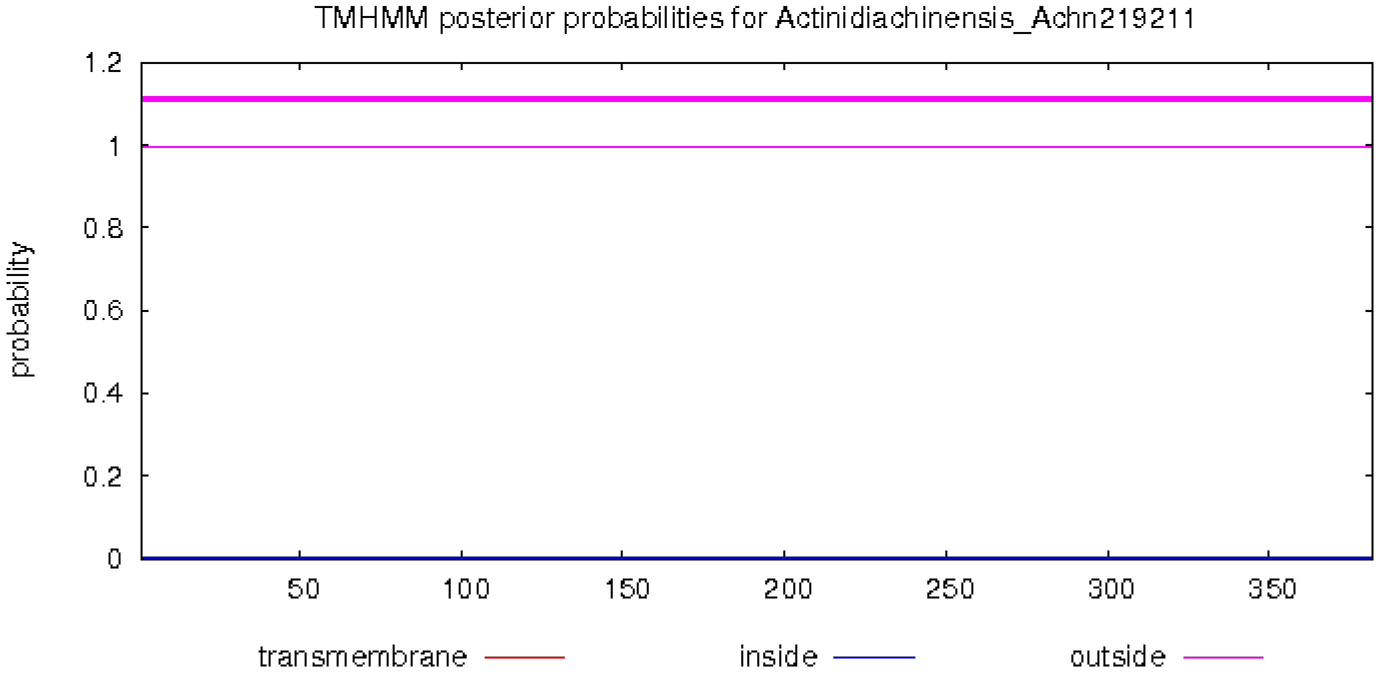

plot in postscript, script for making the plot in gnuplot, data for plot

---

```
# Actinidiachinensis_Achn220201 Length: 291
# Actinidiachinensis_Achn220201 Number of predicted TMHs:  0
# Actinidiachinensis_Achn220201 Exp number of AAs in TMHs: 0.00036
# Actinidiachinensis_Achn220201 Exp number, first 60 AAs:  0.00017
# Actinidiachinensis_Achn220201 Total prob of N-in:        0.00192
Actinidiachinensis_Achn220201   TMHMM2.0        outside      1   291
```

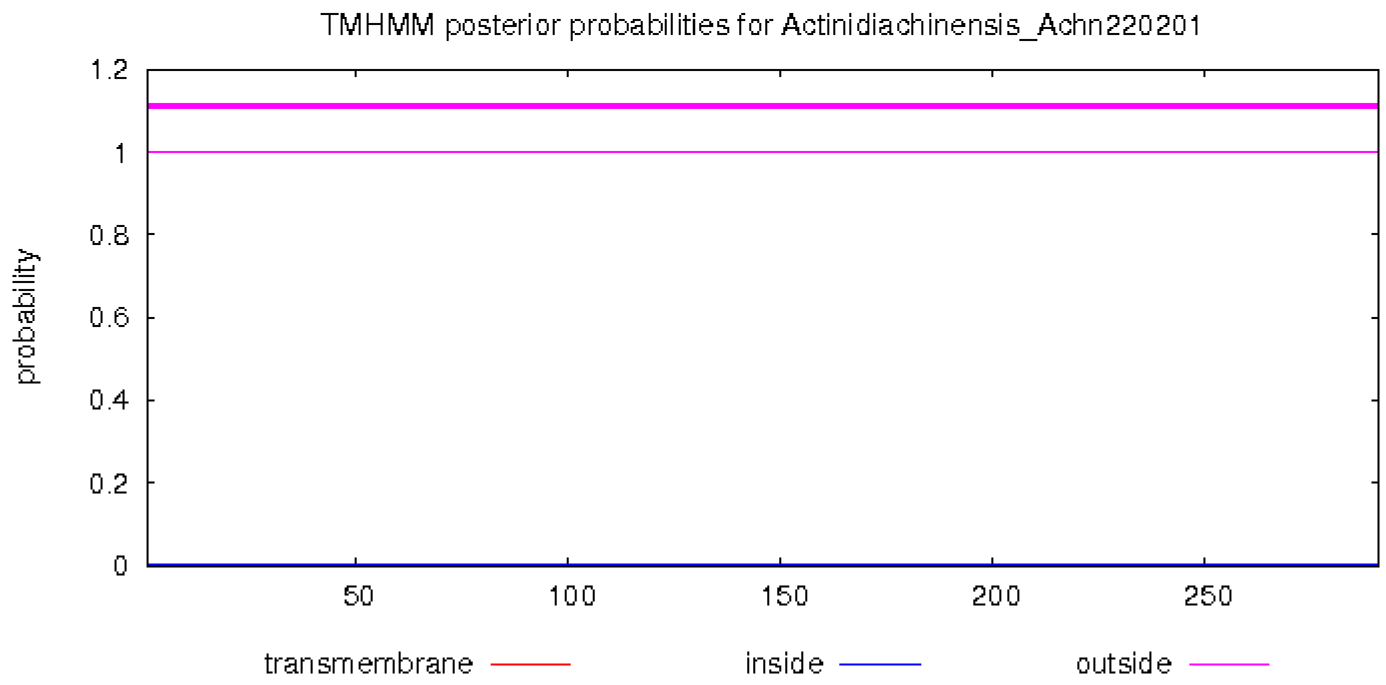

plot in postscript, script for making the plot in gnuplot, data for plot

```
# Actinidiachinensis_Achn220211 Length: 287
# Actinidiachinensis_Achn220211 Number of predicted TMHs:  0
# Actinidiachinensis_Achn220211 Exp number of AAs in TMHs: 0.0127
# Actinidiachinensis_Achn220211 Exp number, first 60 AAs:  0.01212
# Actinidiachinensis_Achn220211 Total prob of N-in:        0.01762
Actinidiachinensis_Achn220211   TMHMM2.0        outside      1   287
```

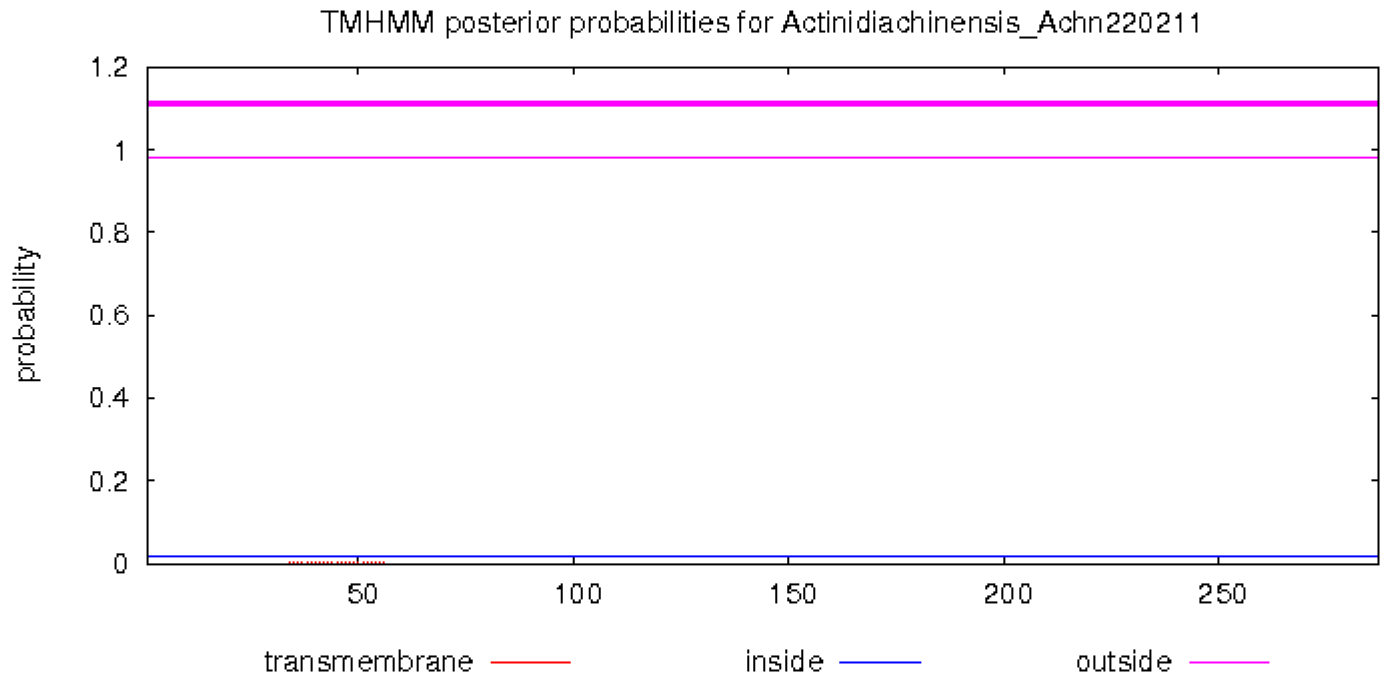

plot in postscript, script for making the plot in gnuplot, data for plot

```
# Actinidiachinensis_Achn221871 Length: 396
# Actinidiachinensis_Achn221871 Number of predicted TMHs:  0
# Actinidiachinensis_Achn221871 Exp number of AAs in TMHs: 0.00036
# Actinidiachinensis_Achn221871 Exp number, first 60 AAs:  0.00017
# Actinidiachinensis_Achn221871 Total prob of N-in:        0.00261
Actinidiachinensis_Achn221871   TMHMM2.0        outside      1   396
```

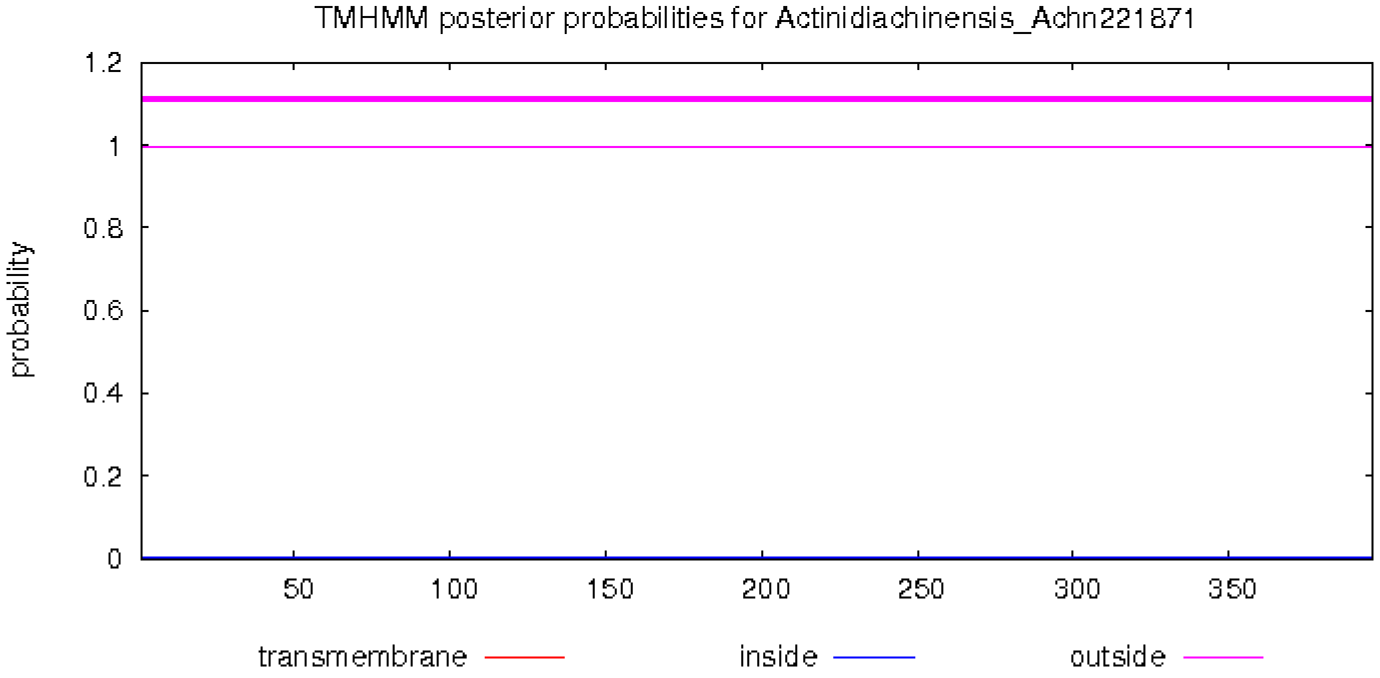

plot in postscript, script for making the plot in gnuplot, data for plot

---

```
# Actinidiachinensis_Achn226931 Length: 324
# Actinidiachinensis_Achn226931 Number of predicted TMHs:  0
# Actinidiachinensis_Achn226931 Exp number of AAs in TMHs: 0.00098
# Actinidiachinensis_Achn226931 Exp number, first 60 AAs:  0.00098
# Actinidiachinensis_Achn226931 Total prob of N-in:        0.02521
Actinidiachinensis_Achn226931   TMHMM2.0        outside      1   324
```

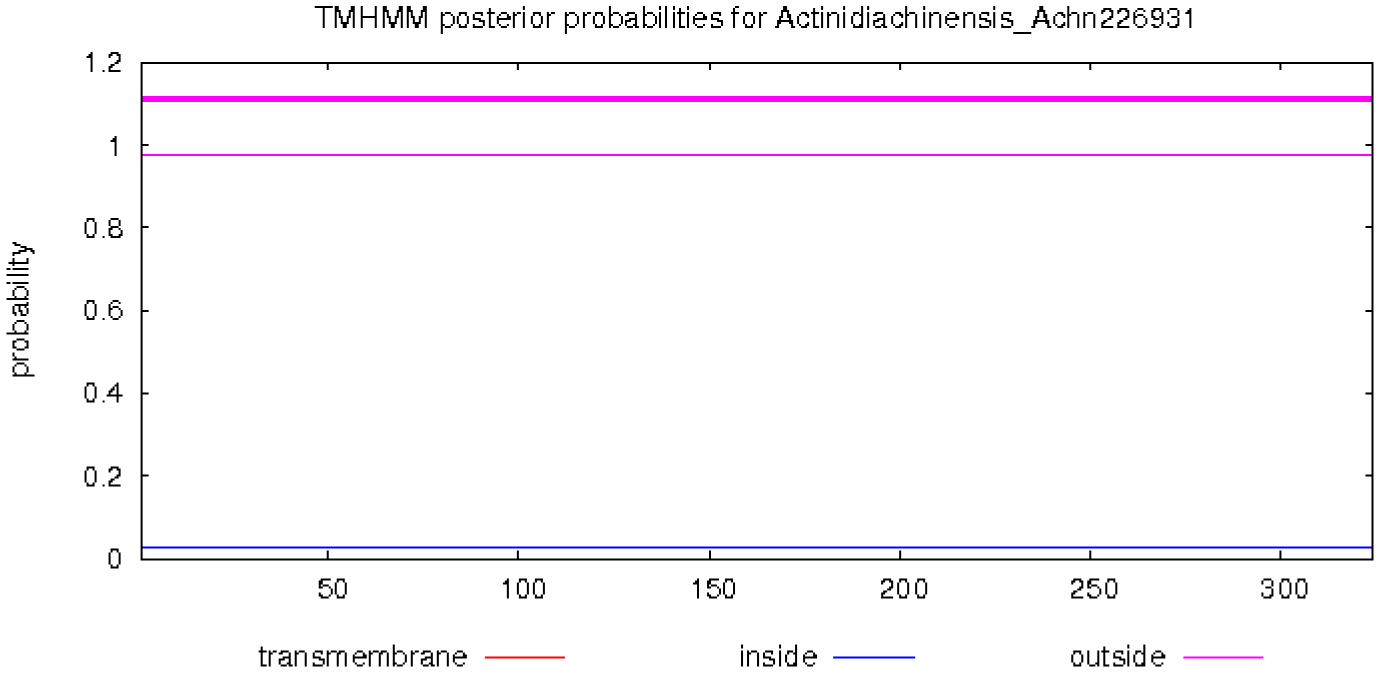

plot in postscript, script for making the plot in gnuplot, data for plot

---

```
# Actinidiachinensis_Achn229031 Length: 319
# Actinidiachinensis_Achn229031 Number of predicted TMHs:  0
# Actinidiachinensis_Achn229031 Exp number of AAs in TMHs: 0.00156
# Actinidiachinensis_Achn229031 Exp number, first 60 AAs:  0
# Actinidiachinensis_Achn229031 Total prob of N-in:        0.05093
Actinidiachinensis_Achn229031   TMHMM2.0        outside      1   319
```

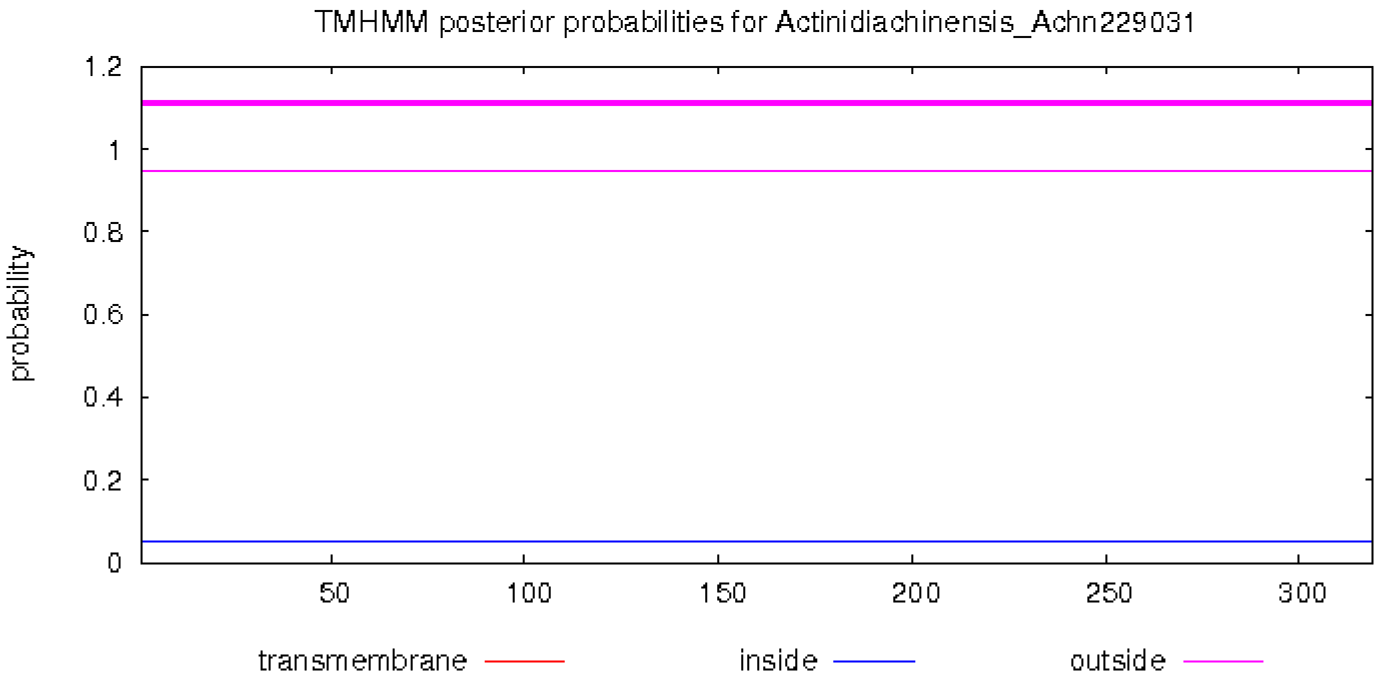

plot in postscript, script for making the plot in gnuplot, data for plot

---

```
# Actinidiachinensis_Achn229061 Length: 221
# Actinidiachinensis_Achn229061 Number of predicted TMHs:  0
# Actinidiachinensis_Achn229061 Exp number of AAs in TMHs: 0
# Actinidiachinensis_Achn229061 Exp number, first 60 AAs:  0
# Actinidiachinensis_Achn229061 Total prob of N-in:        0.10555
Actinidiachinensis_Achn229061   TMHMM2.0        outside      1   221
```

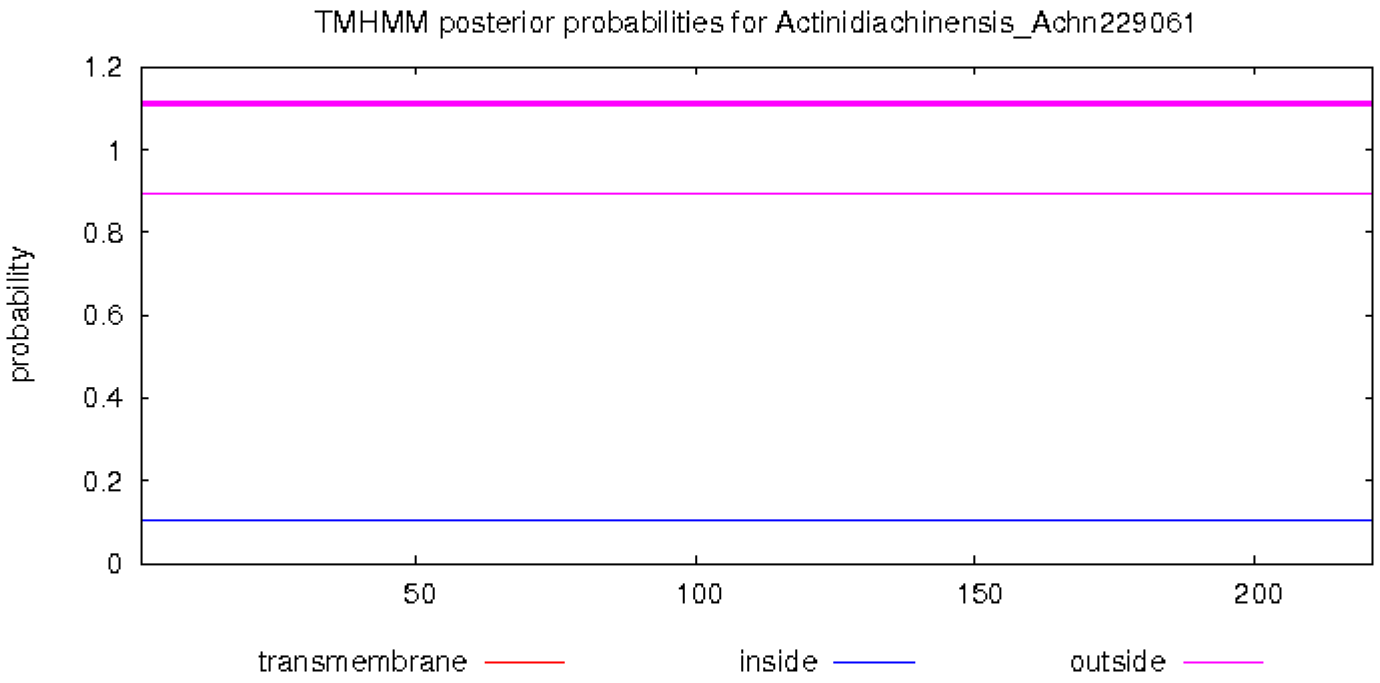

plot in postscript, script for making the plot in gnuplot, data for plot

---

```
# Actinidiachinensis_Achn234861 Length: 367
# Actinidiachinensis_Achn234861 Number of predicted TMHs:  0
# Actinidiachinensis_Achn234861 Exp number of AAs in TMHs: 0.00071
# Actinidiachinensis_Achn234861 Exp number, first 60 AAs:  0
# Actinidiachinensis_Achn234861 Total prob of N-in:        0.03090
Actinidiachinensis_Achn234861   TMHMM2.0        outside      1   367
```

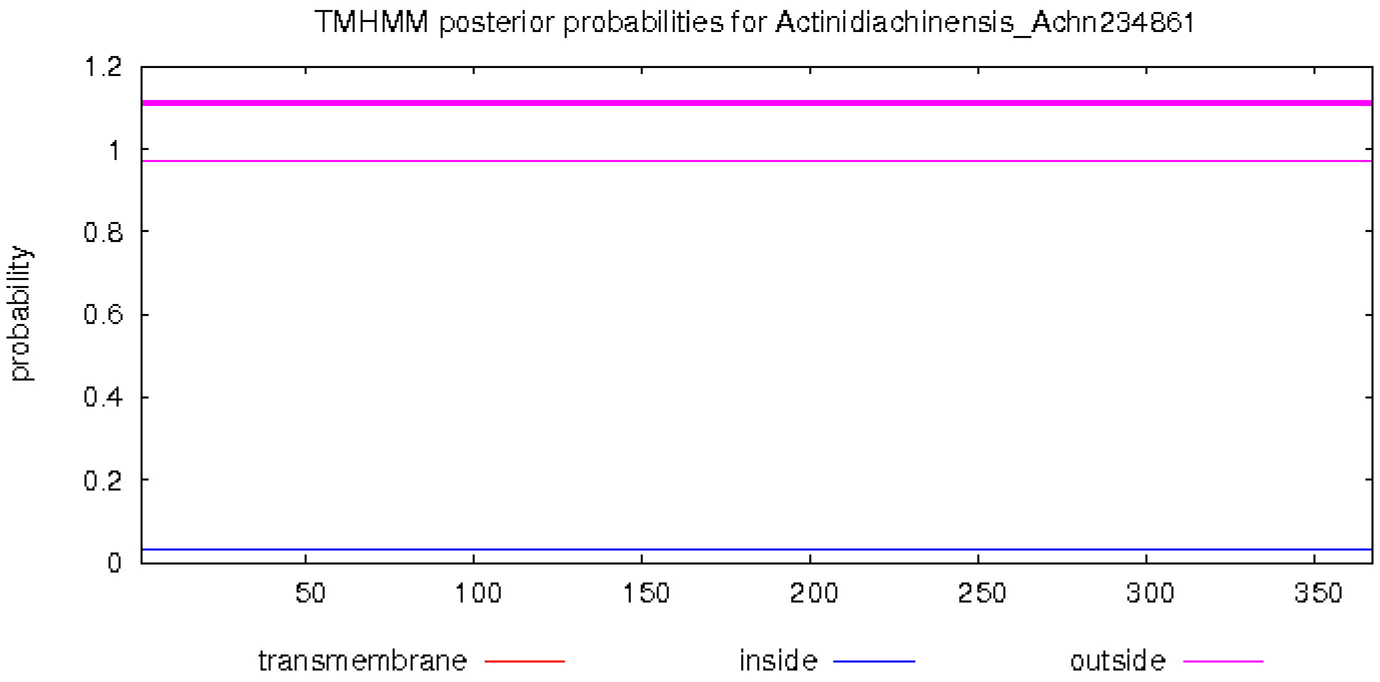

plot in postscript, script for making the plot in gnuplot, data for plot

---

```
# Actinidiachinensis_Achn237291 Length: 341
# Actinidiachinensis_Achn237291 Number of predicted TMHs:  0
# Actinidiachinensis_Achn237291 Exp number of AAs in TMHs: 0
# Actinidiachinensis_Achn237291 Exp number, first 60 AAs:  0
# Actinidiachinensis_Achn237291 Total prob of N-in:        0.00116
Actinidiachinensis_Achn237291   TMHMM2.0        outside      1   341
```

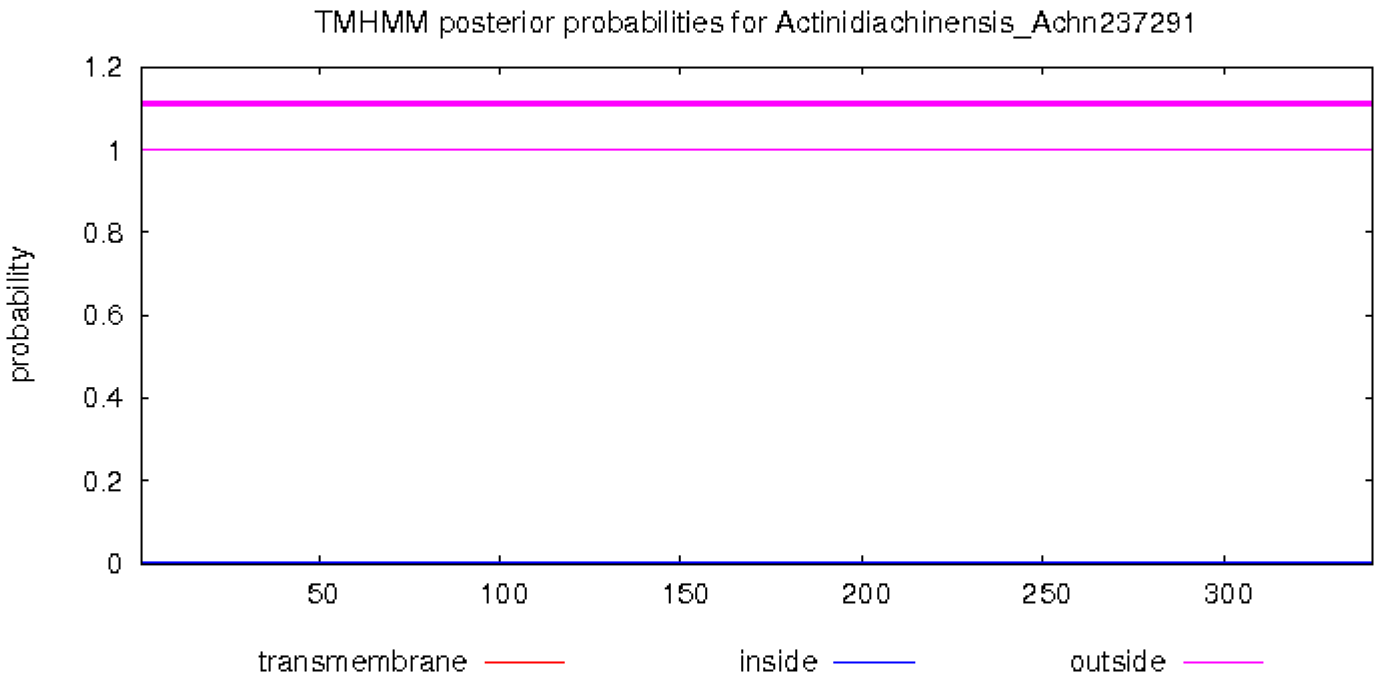

plot in postscript, script for making the plot in gnuplot, data for plot

---

```
# Actinidiachinensis_Achn238591 Length: 307
# Actinidiachinensis_Achn238591 Number of predicted TMHs:  0
# Actinidiachinensis_Achn238591 Exp number of AAs in TMHs: 0
# Actinidiachinensis_Achn238591 Exp number, first 60 AAs:  0
# Actinidiachinensis_Achn238591 Total prob of N-in:        0.00676
Actinidiachinensis_Achn238591   TMHMM2.0        outside      1   307
```

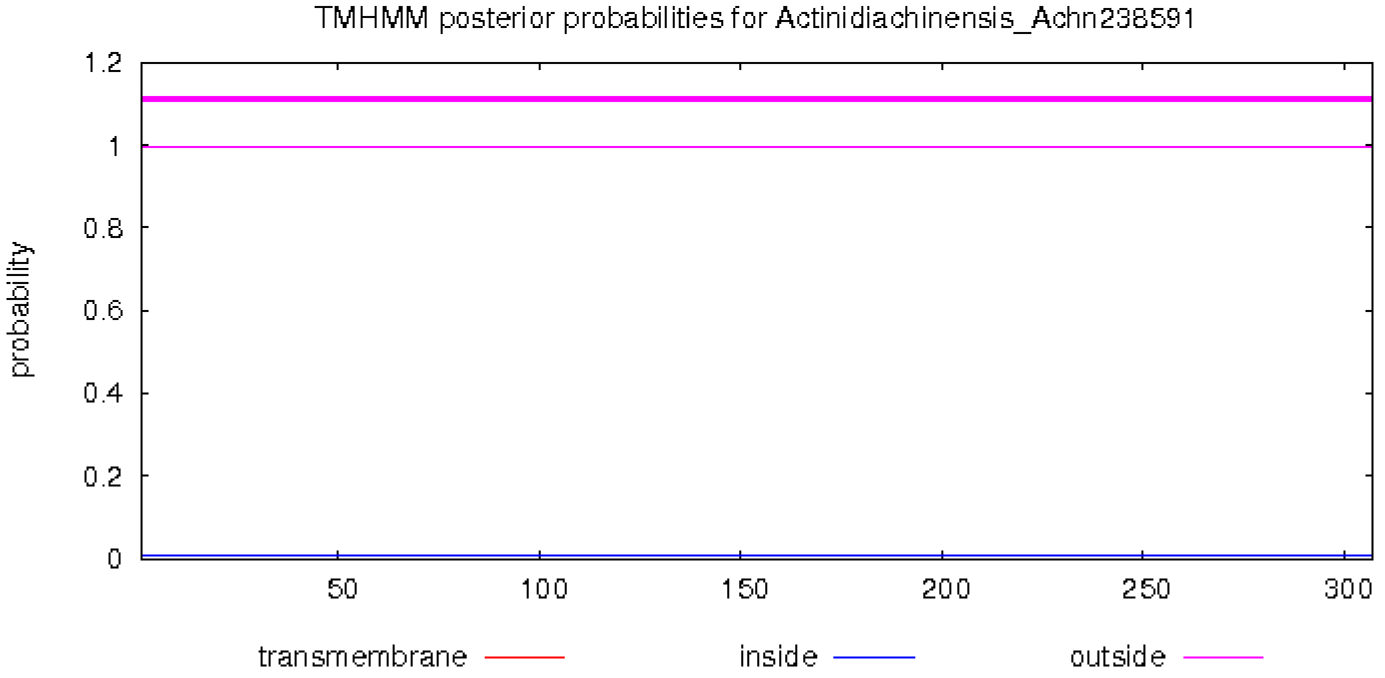

plot in postscript, script for making the plot in gnuplot, data for plot

---

```
# Actinidiachinensis_Achn239781 Length: 307
# Actinidiachinensis_Achn239781 Number of predicted TMHs:  0
# Actinidiachinensis_Achn239781 Exp number of AAs in TMHs: 0.00019
# Actinidiachinensis_Achn239781 Exp number, first 60 AAs:  0
# Actinidiachinensis_Achn239781 Total prob of N-in:        0.00682
Actinidiachinensis_Achn239781   TMHMM2.0        outside      1   307
```

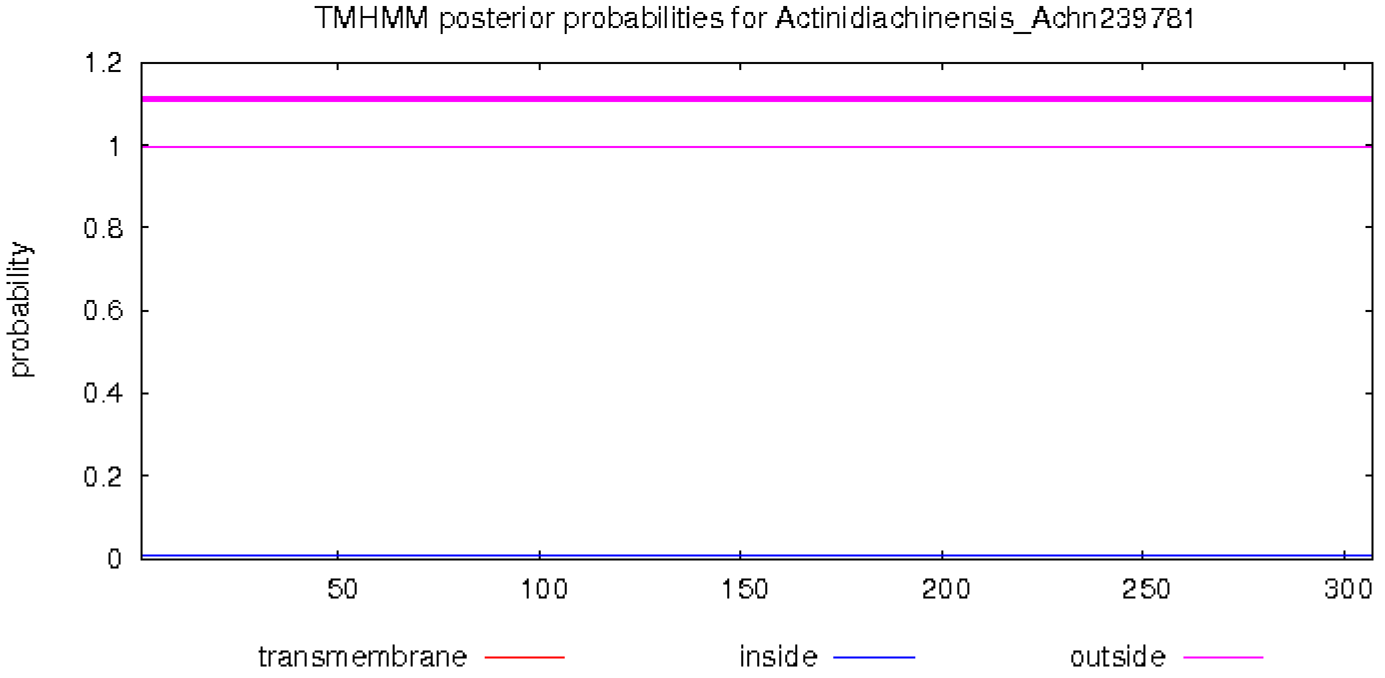

plot in postscript, script for making the plot in gnuplot, data for plot

---

```
# Actinidiachinensis_Achn241171 Length: 388
# Actinidiachinensis_Achn241171 Number of predicted TMHs:  0
# Actinidiachinensis_Achn241171 Exp number of AAs in TMHs: 0
# Actinidiachinensis_Achn241171 Exp number, first 60 AAs:  0
# Actinidiachinensis_Achn241171 Total prob of N-in:        0.00494
Actinidiachinensis_Achn241171   TMHMM2.0        outside      1   388
```

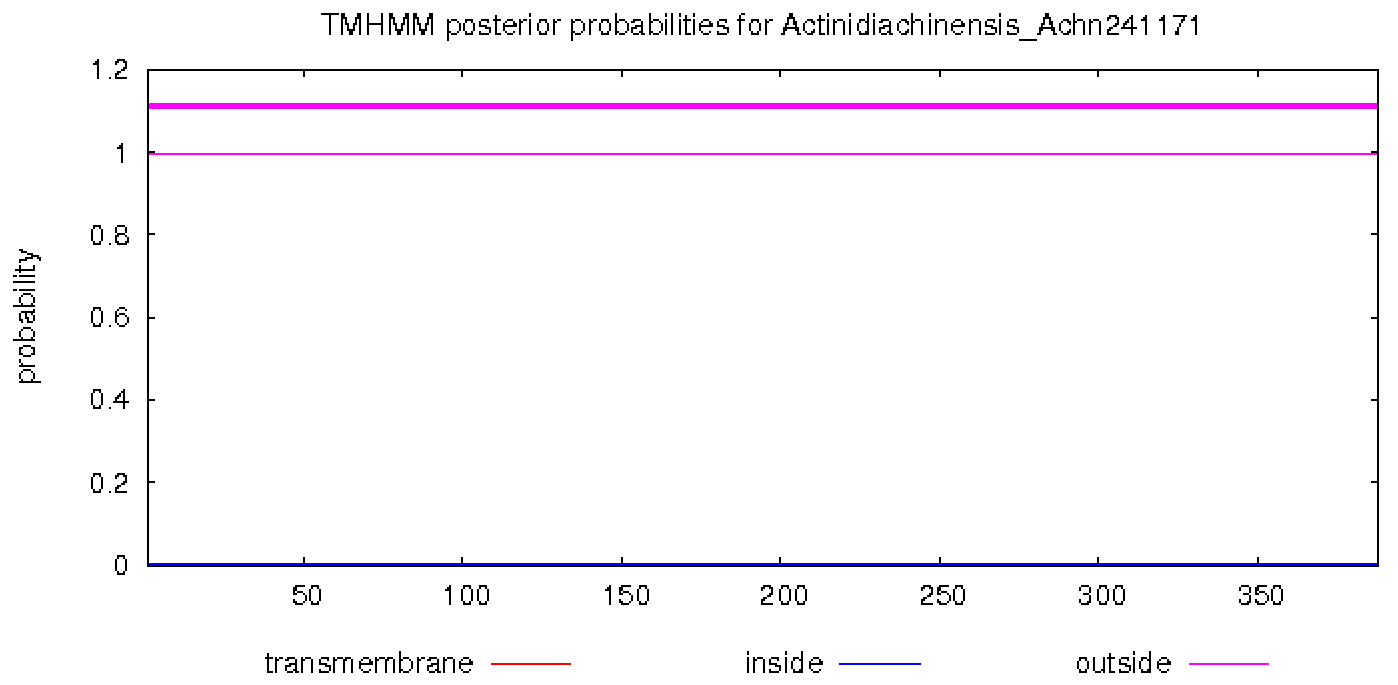

plot in postscript, script for making the plot in gnuplot, data for plot

```
# Actinidiachinensis_Achn241181 Length: 333
# Actinidiachinensis_Achn241181 Number of predicted TMHs:  0
# Actinidiachinensis_Achn241181 Exp number of AAs in TMHs: 0.00023
# Actinidiachinensis_Achn241181 Exp number, first 60 AAs:  0
# Actinidiachinensis_Achn241181 Total prob of N-in:        0.00539
Actinidiachinensis_Achn241181   TMHMM2.0        outside      1   333
```

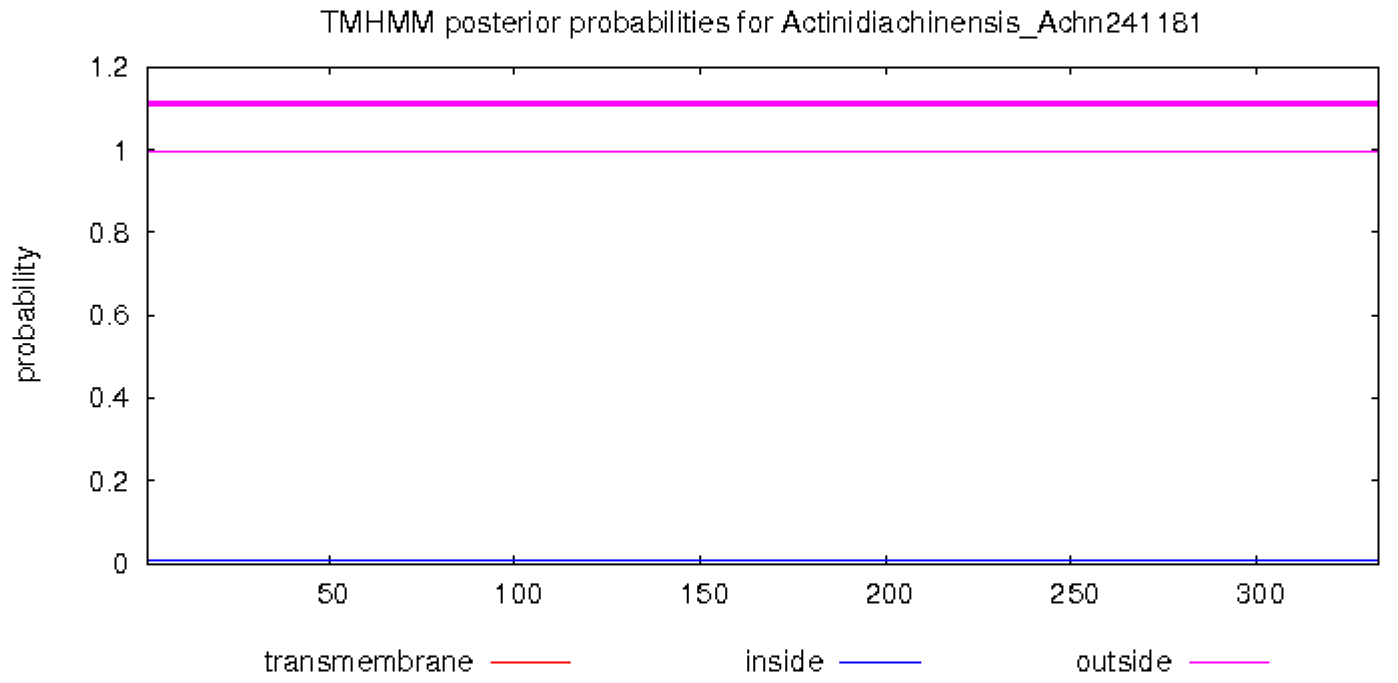

plot in postscript, script for making the plot in gnuplot, data for plot

```
# Actinidiachinensis_Achn244831 Length: 324
# Actinidiachinensis_Achn244831 Number of predicted TMHs:  0
# Actinidiachinensis_Achn244831 Exp number of AAs in TMHs: 0.00315
# Actinidiachinensis_Achn244831 Exp number, first 60 AAs:  0.00296
# Actinidiachinensis_Achn244831 Total prob of N-in:        0.00242
Actinidiachinensis_Achn244831   TMHMM2.0        outside      1   324
```

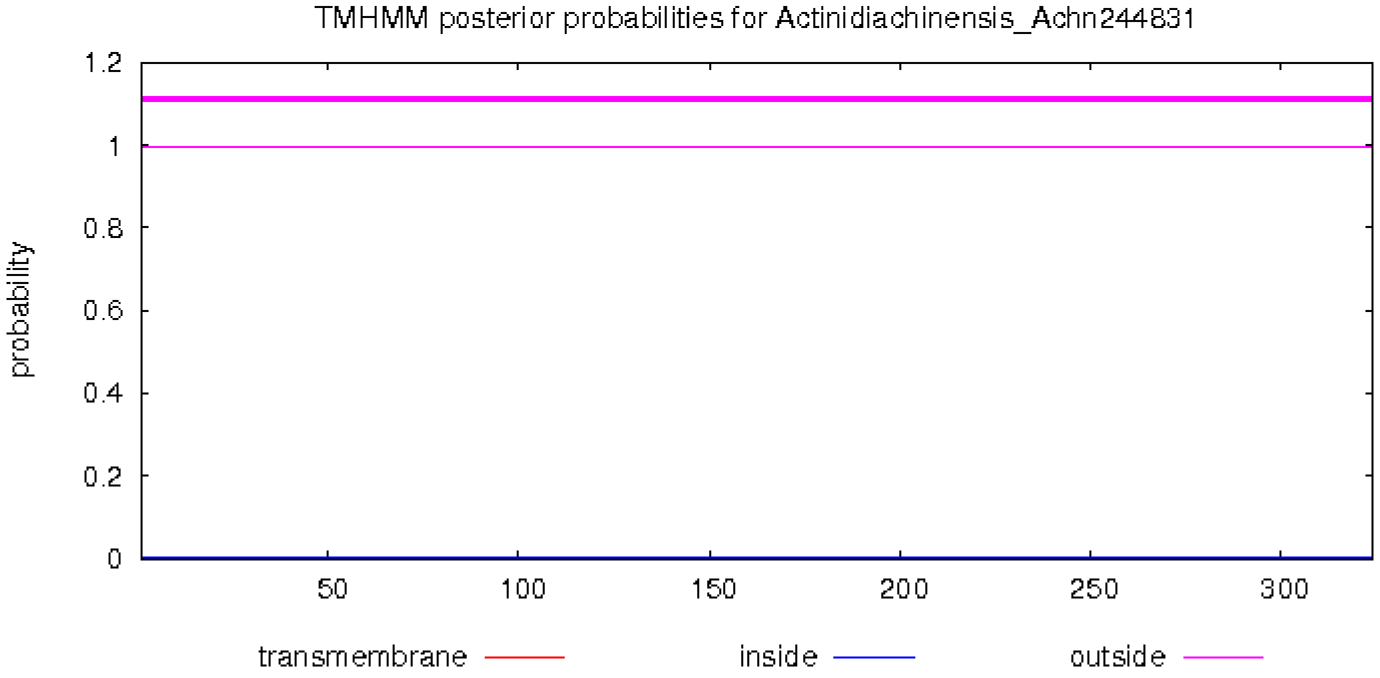

plot in postscript, script for making the plot in gnuplot, data for plot

---

```
# Actinidiachinensis_Achn245061 Length: 340
# Actinidiachinensis_Achn245061 Number of predicted TMHs:  0
# Actinidiachinensis_Achn245061 Exp number of AAs in TMHs: 0
# Actinidiachinensis_Achn245061 Exp number, first 60 AAs:  0
# Actinidiachinensis_Achn245061 Total prob of N-in:        0.01107
Actinidiachinensis_Achn245061   TMHMM2.0        outside      1   340
```

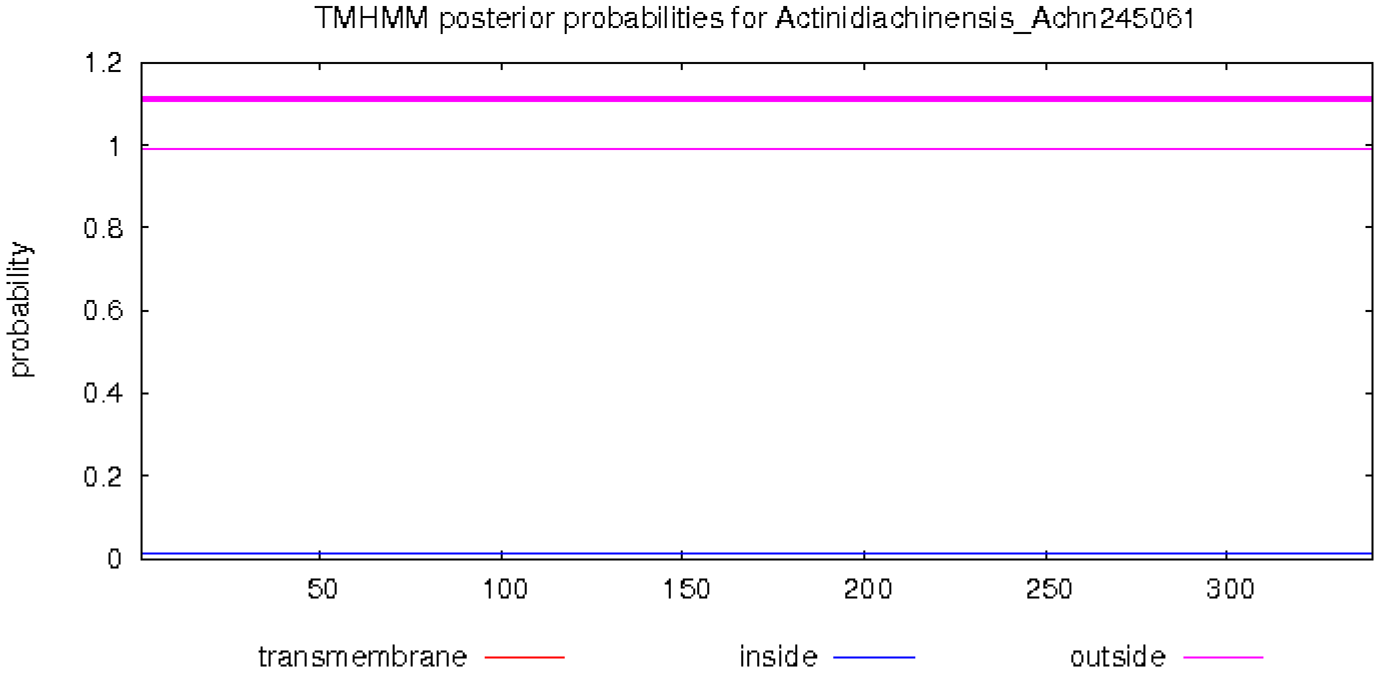

plot in postscript, script for making the plot in gnuplot, data for plot

---

```
# Actinidiachinensis_Achn254041 Length: 340
# Actinidiachinensis_Achn254041 Number of predicted TMHs:  0
# Actinidiachinensis_Achn254041 Exp number of AAs in TMHs: 0
# Actinidiachinensis_Achn254041 Exp number, first 60 AAs:  0
# Actinidiachinensis_Achn254041 Total prob of N-in:        0.00170
Actinidiachinensis_Achn254041   TMHMM2.0        outside      1   340
```

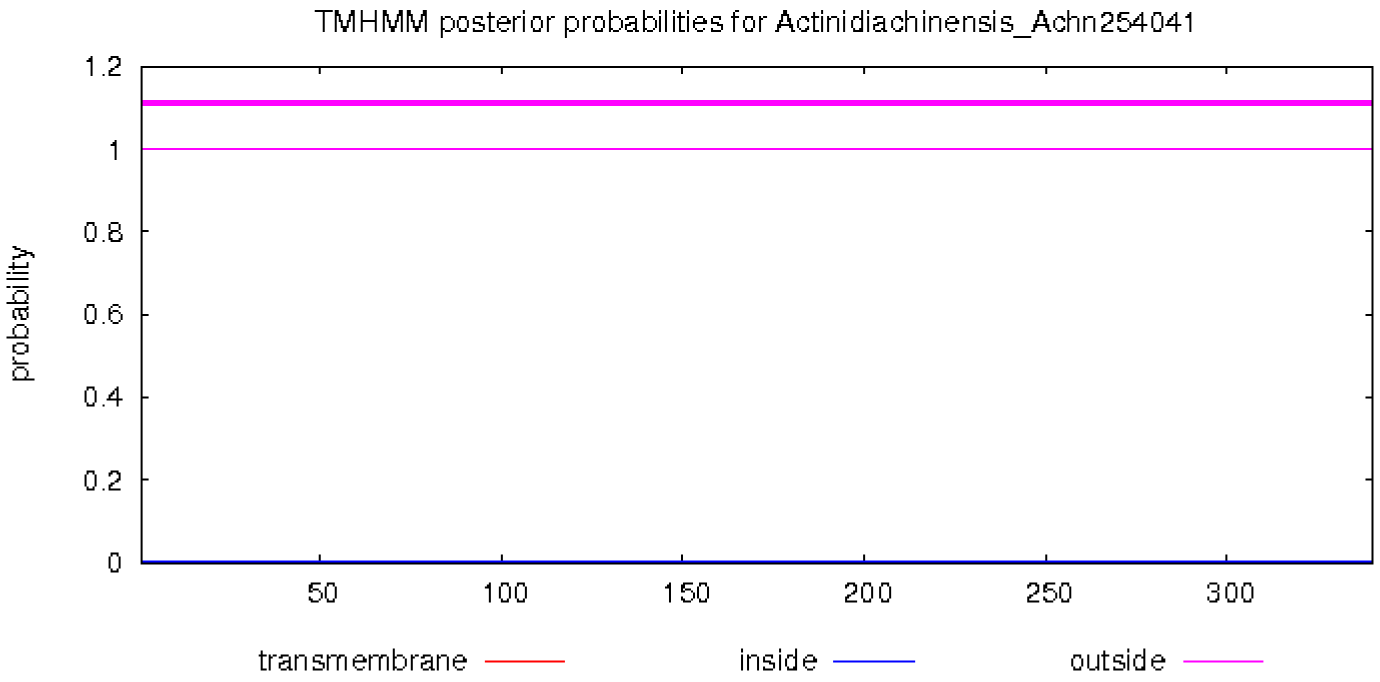

plot in postscript, script for making the plot in gnuplot, data for plot

---

```
# Actinidiachinensis_Achn254051 Length: 198
# Actinidiachinensis_Achn254051 Number of predicted TMHs:  0
# Actinidiachinensis_Achn254051 Exp number of AAs in TMHs: 0.0011
# Actinidiachinensis_Achn254051 Exp number, first 60 AAs:  0.00017
# Actinidiachinensis_Achn254051 Total prob of N-in:        0.05138
Actinidiachinensis_Achn254051   TMHMM2.0        outside      1   198
```

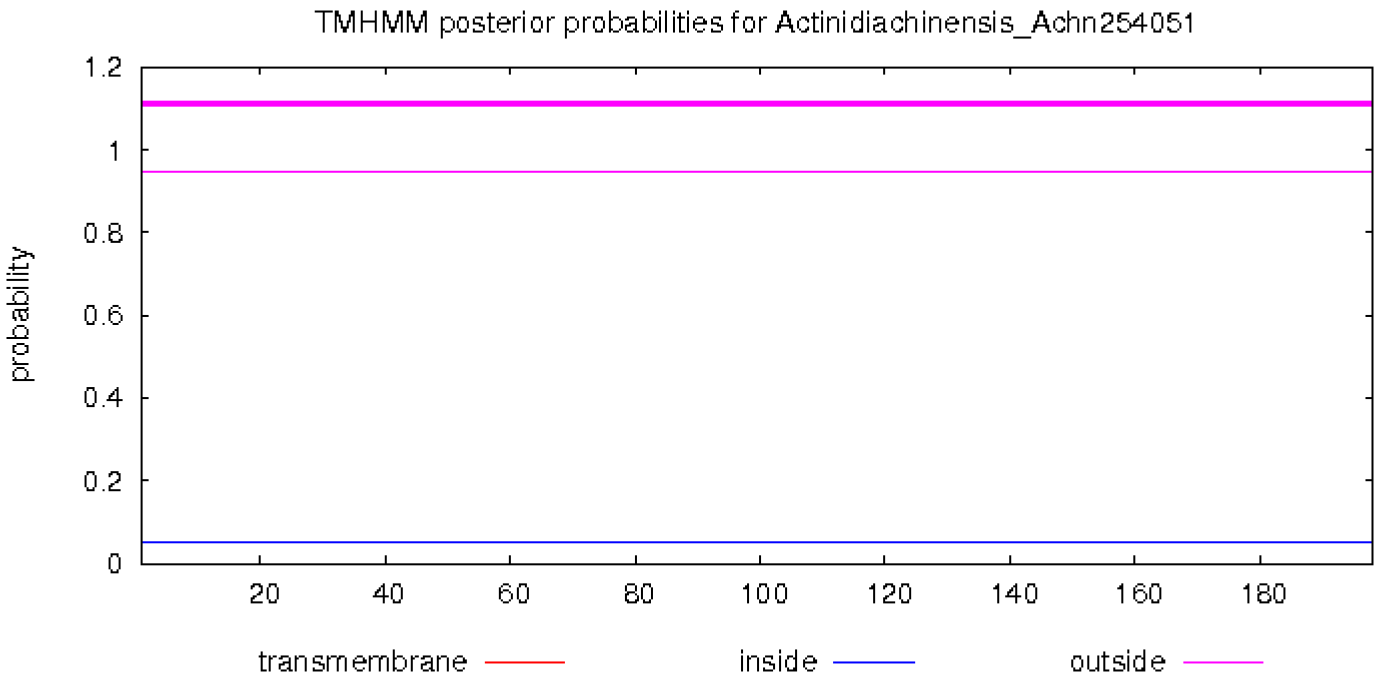

plot in postscript, script for making the plot in gnuplot, data for plot

---

```
# Actinidiachinensis_Achn262621 Length: 323
# Actinidiachinensis_Achn262621 Number of predicted TMHs:  0
# Actinidiachinensis_Achn262621 Exp number of AAs in TMHs: 0.00042
# Actinidiachinensis_Achn262621 Exp number, first 60 AAs:  0
# Actinidiachinensis_Achn262621 Total prob of N-in:        0.02618
Actinidiachinensis_Achn262621   TMHMM2.0        outside      1   323
```

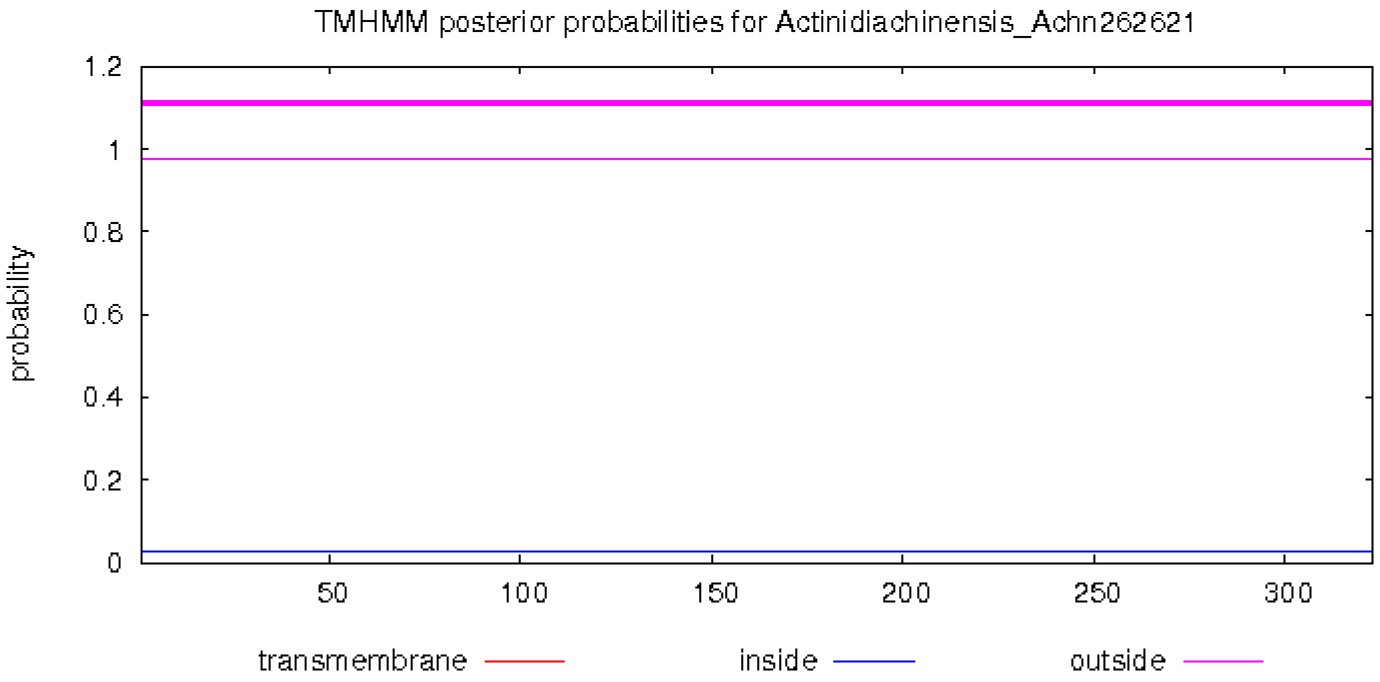

plot in postscript, script for making the plot in gnuplot, data for plot

---

```
# Actinidiachinensis_Achn266441 Length: 563
# Actinidiachinensis_Achn266441 Number of predicted TMHs:  0
# Actinidiachinensis_Achn266441 Exp number of AAs in TMHs: 0.01994
# Actinidiachinensis_Achn266441 Exp number, first 60 AAs:  0.01335
# Actinidiachinensis_Achn266441 Total prob of N-in:        0.00142
Actinidiachinensis_Achn266441   TMHMM2.0        outside      1   563
```

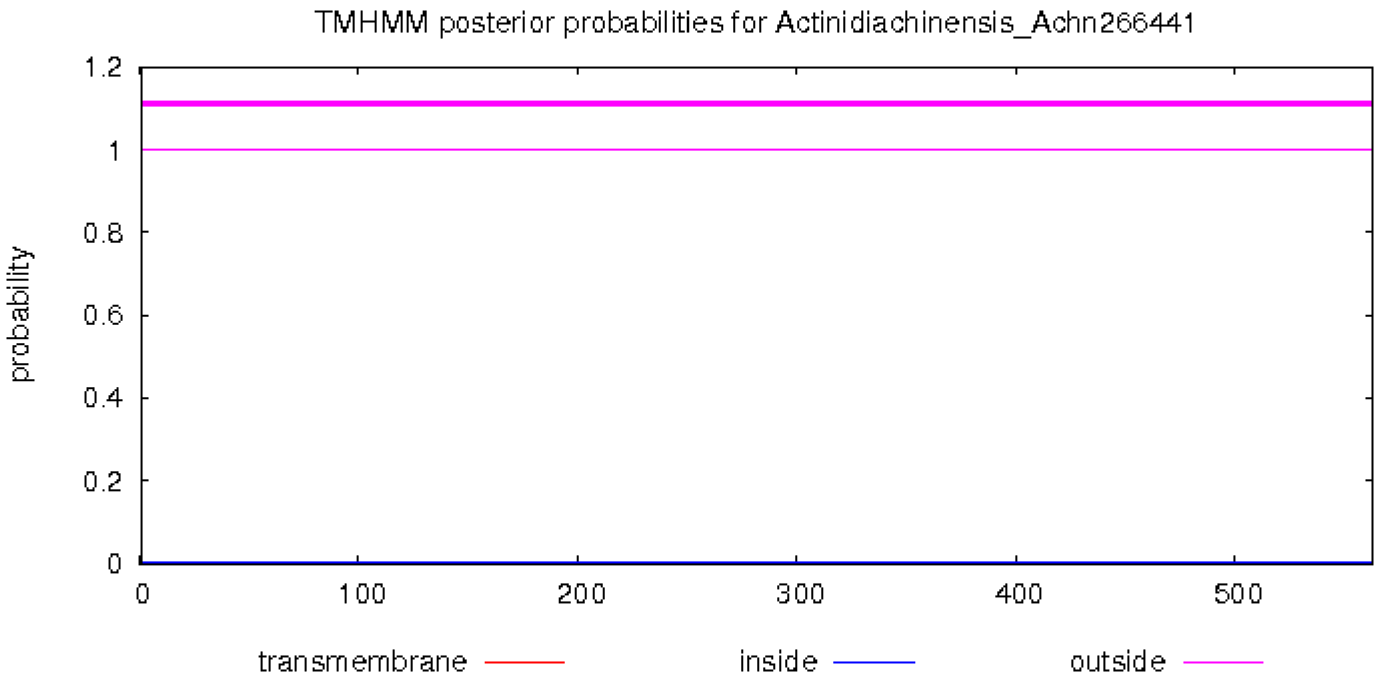

plot in postscript, script for making the plot in gnuplot, data for plot

---

```
# Actinidiachinensis_Achn267671 Length: 519
# Actinidiachinensis_Achn267671 Number of predicted TMHs:  0
# Actinidiachinensis_Achn267671 Exp number of AAs in TMHs: 0.00014
# Actinidiachinensis_Achn267671 Exp number, first 60 AAs:  0
# Actinidiachinensis_Achn267671 Total prob of N-in:        0.00022
Actinidiachinensis_Achn267671   TMHMM2.0        outside      1   519
```

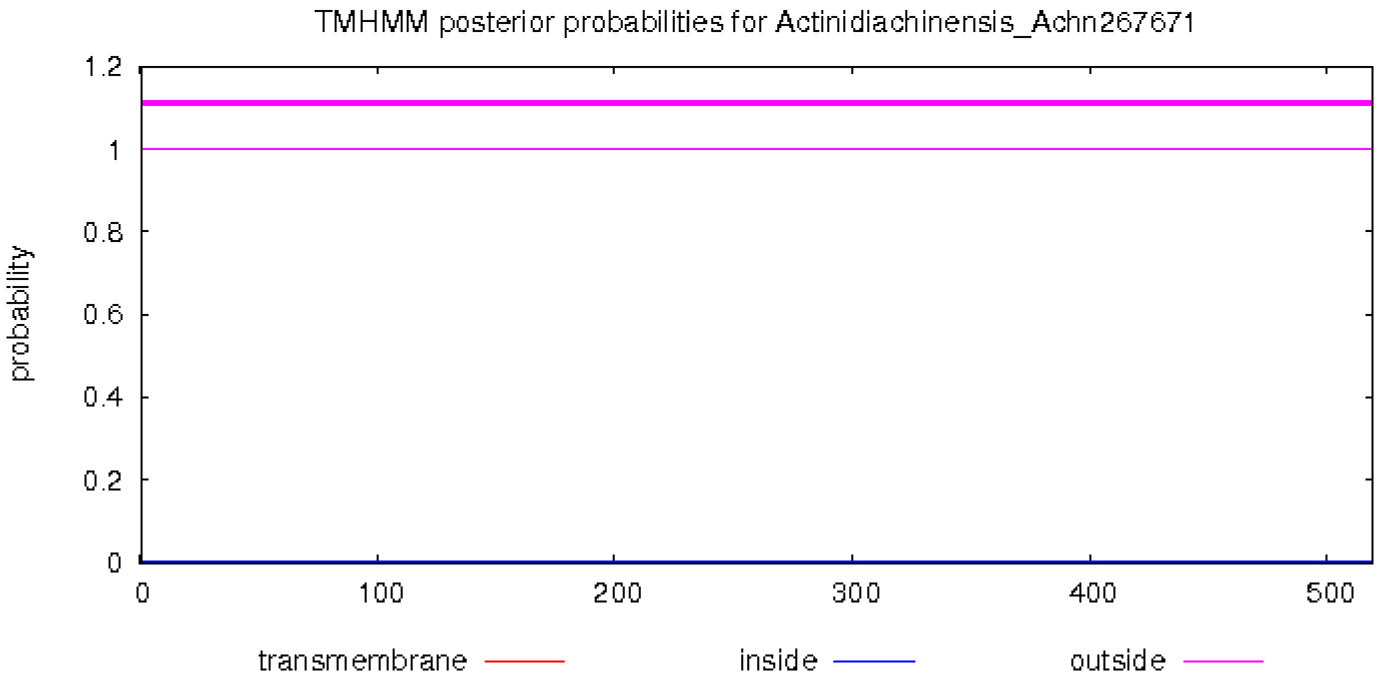

plot in postscript, script for making the plot in gnuplot, data for plot

---

```
# Actinidiachinensis_Achn272561 Length: 391
# Actinidiachinensis_Achn272561 Number of predicted TMHs:  0
# Actinidiachinensis_Achn272561 Exp number of AAs in TMHs: 0.00076
# Actinidiachinensis_Achn272561 Exp number, first 60 AAs:  0.00058
# Actinidiachinensis_Achn272561 Total prob of N-in:        0.00339
Actinidiachinensis_Achn272561   TMHMM2.0        outside      1   391
```

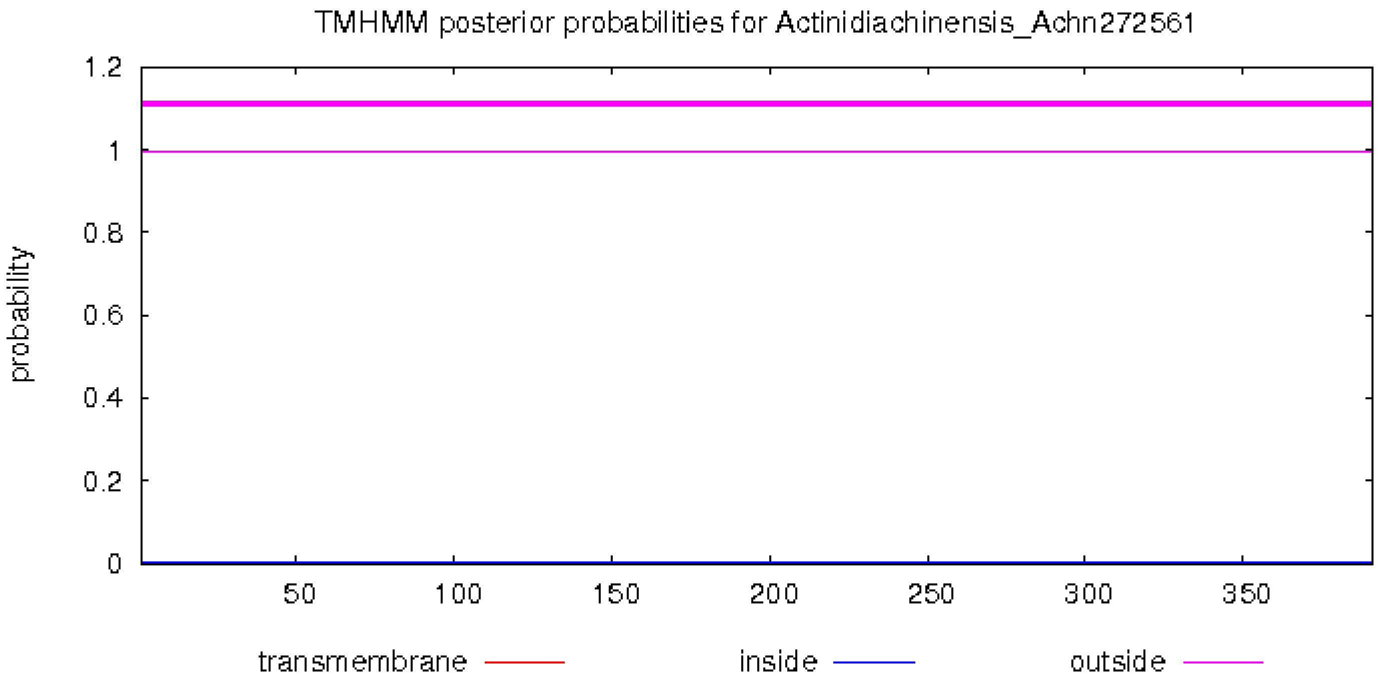

plot in postscript, script for making the plot in gnuplot, data for plot

---

```
# Actinidiachinensis_Achn272571 Length: 480
# Actinidiachinensis_Achn272571 Number of predicted TMHs:  1
# Actinidiachinensis_Achn272571 Exp number of AAs in TMHs: 21.30004
# Actinidiachinensis_Achn272571 Exp number, first 60 AAs:  0.03792
# Actinidiachinensis_Achn272571 Total prob of N-in:        0.00256
Actinidiachinensis_Achn272571   TMHMM2.0        outside      1   450
Actinidiachinensis_Achn272571   TMHMM2.0        TMhelix    451   473
Actinidiachinensis_Achn272571   TMHMM2.0        inside     474   480
```

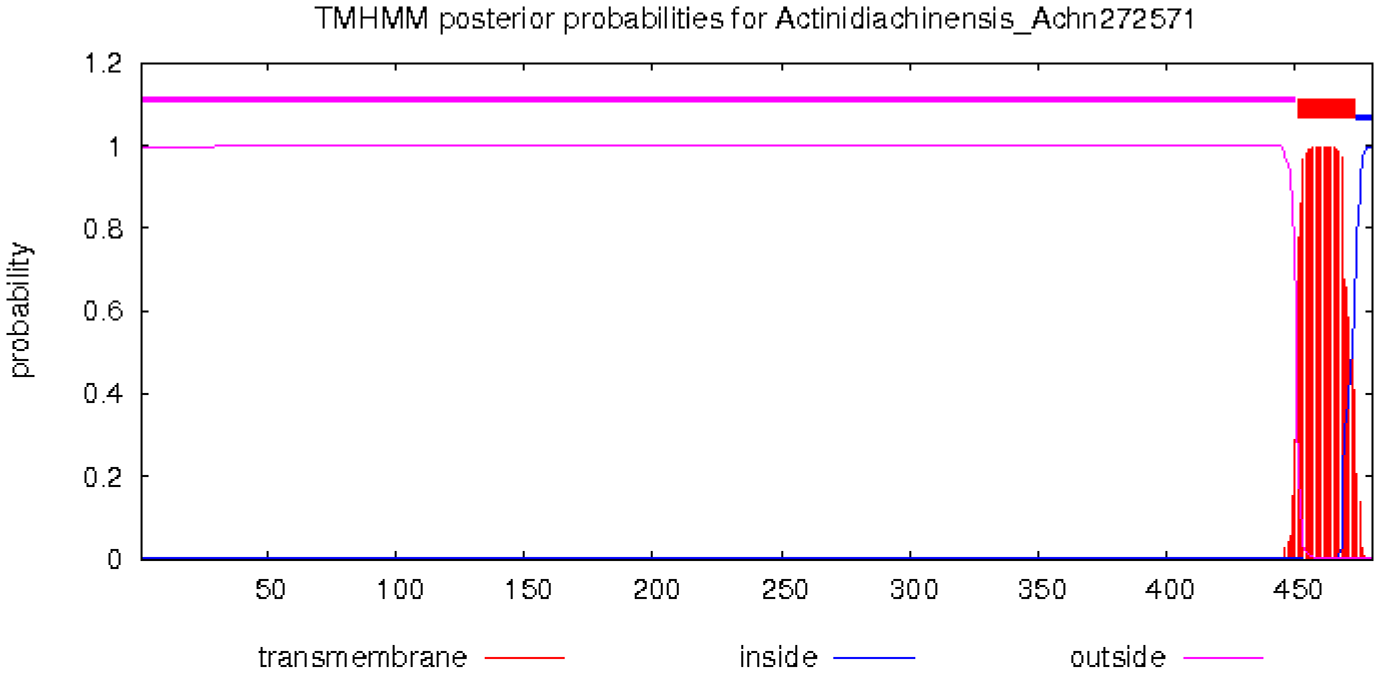

plot in postscript, script for making the plot in gnuplot, data for plot

---

```
# Actinidiachinensis_Achn272901 Length: 366
# Actinidiachinensis_Achn272901 Number of predicted TMHs:  0
# Actinidiachinensis_Achn272901 Exp number of AAs in TMHs: 0.00657
# Actinidiachinensis_Achn272901 Exp number, first 60 AAs:  0.00472
# Actinidiachinensis_Achn272901 Total prob of N-in:        0.01735
Actinidiachinensis_Achn272901   TMHMM2.0        outside      1   366
```

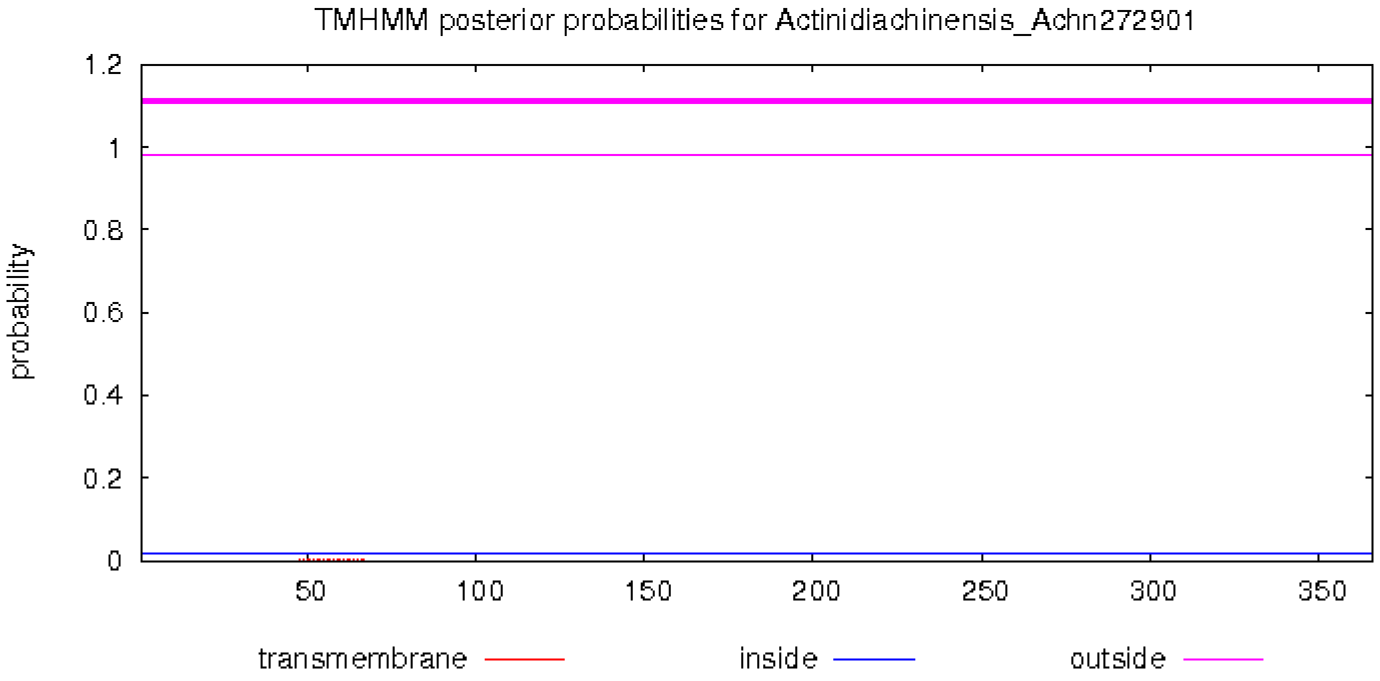

plot in postscript, script for making the plot in gnuplot, data for plot

```
# Actinidiachinensis_Achn274891 Length: 769
# Actinidiachinensis_Achn274891 Number of predicted TMHs:  0
# Actinidiachinensis_Achn274891 Exp number of AAs in TMHs: 14.23252
# Actinidiachinensis_Achn274891 Exp number, first 60 AAs:  5.66766
# Actinidiachinensis_Achn274891 Total prob of N-in:        0.63251
Actinidiachinensis_Achn274891   TMHMM2.0        outside      1   769
```

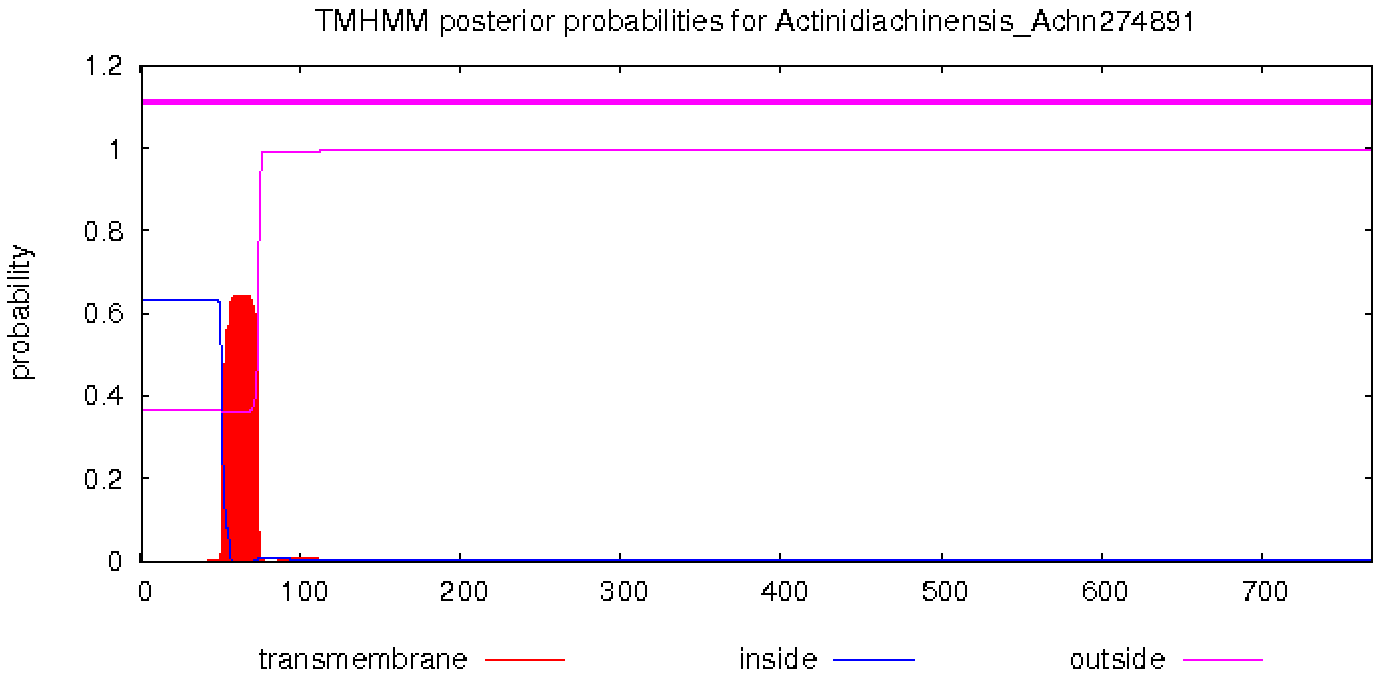

plot in postscript, script for making the plot in gnuplot, data for plot

```
# Actinidiachinensis_Achn276021 Length: 184
# Actinidiachinensis_Achn276021 Number of predicted TMHs:  0
# Actinidiachinensis_Achn276021 Exp number of AAs in TMHs: 0.91539
# Actinidiachinensis_Achn276021 Exp number, first 60 AAs:  0
# Actinidiachinensis_Achn276021 Total prob of N-in:        0.07052
Actinidiachinensis_Achn276021   TMHMM2.0        outside      1   184
```

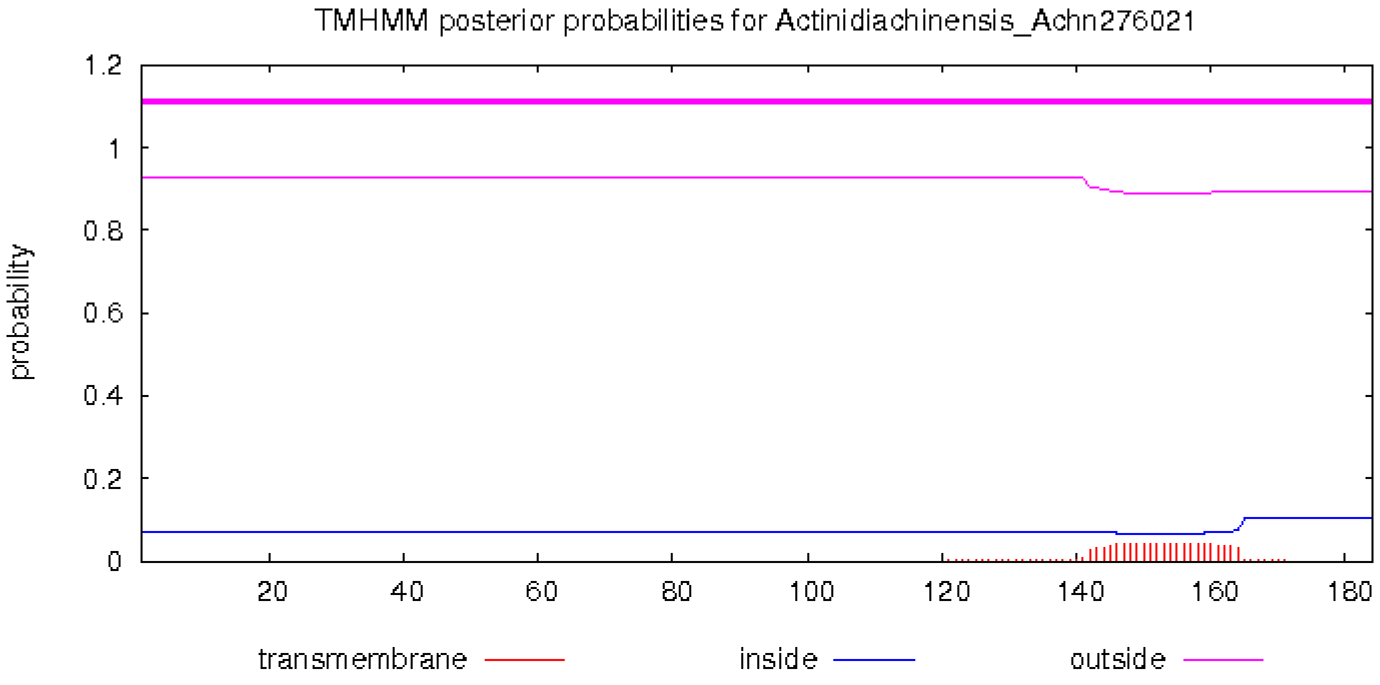

plot in postscript, script for making the plot in gnuplot, data for plot

```
# Actinidiachinensis_Achn276961 Length: 272
# Actinidiachinensis_Achn276961 Number of predicted TMHs:  0
# Actinidiachinensis_Achn276961 Exp number of AAs in TMHs: 0.00017
# Actinidiachinensis_Achn276961 Exp number, first 60 AAs:  0
# Actinidiachinensis_Achn276961 Total prob of N-in:        0.02966
Actinidiachinensis_Achn276961   TMHMM2.0        outside      1   272
```

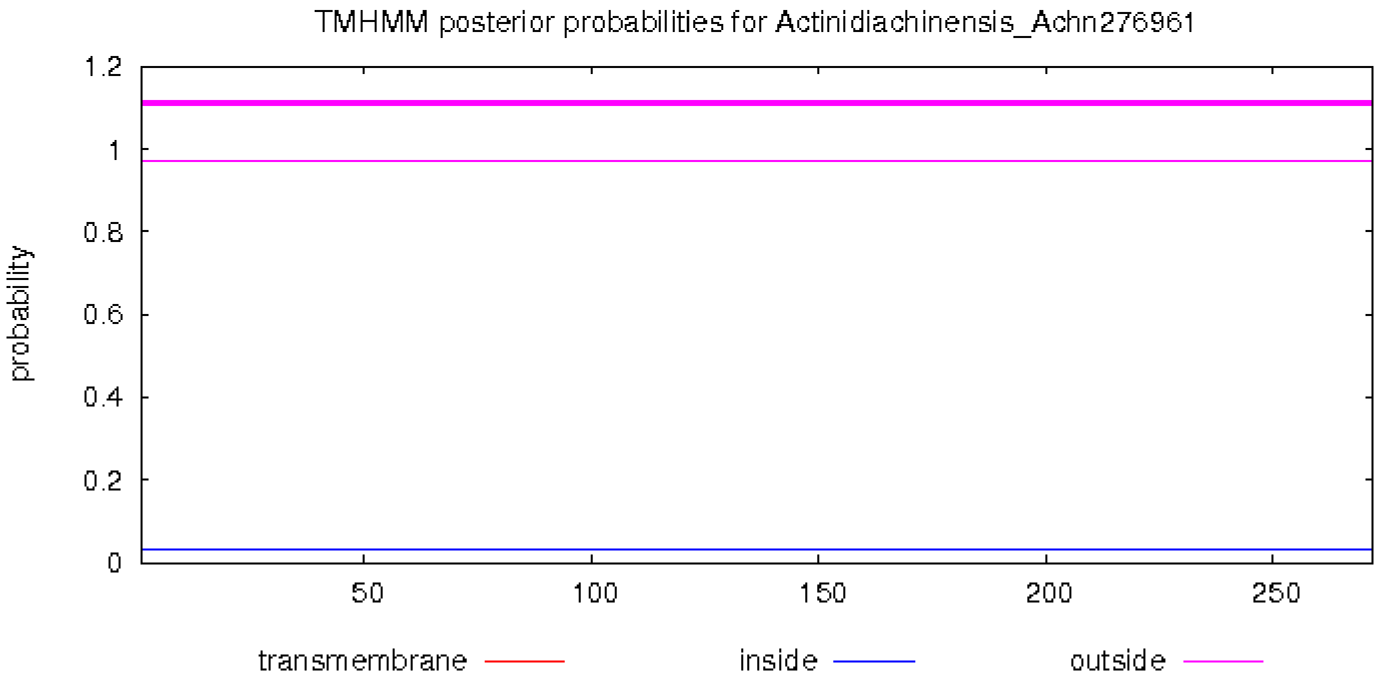

plot in postscript, script for making the plot in gnuplot, data for plot

---

```
# Actinidiachinensis_Achn279951 Length: 193
# Actinidiachinensis_Achn279951 Number of predicted TMHs:  0
# Actinidiachinensis_Achn279951 Exp number of AAs in TMHs: 0.00016
# Actinidiachinensis_Achn279951 Exp number, first 60 AAs:  0
# Actinidiachinensis_Achn279951 Total prob of N-in:        0.06904
Actinidiachinensis_Achn279951   TMHMM2.0        outside      1   193
```

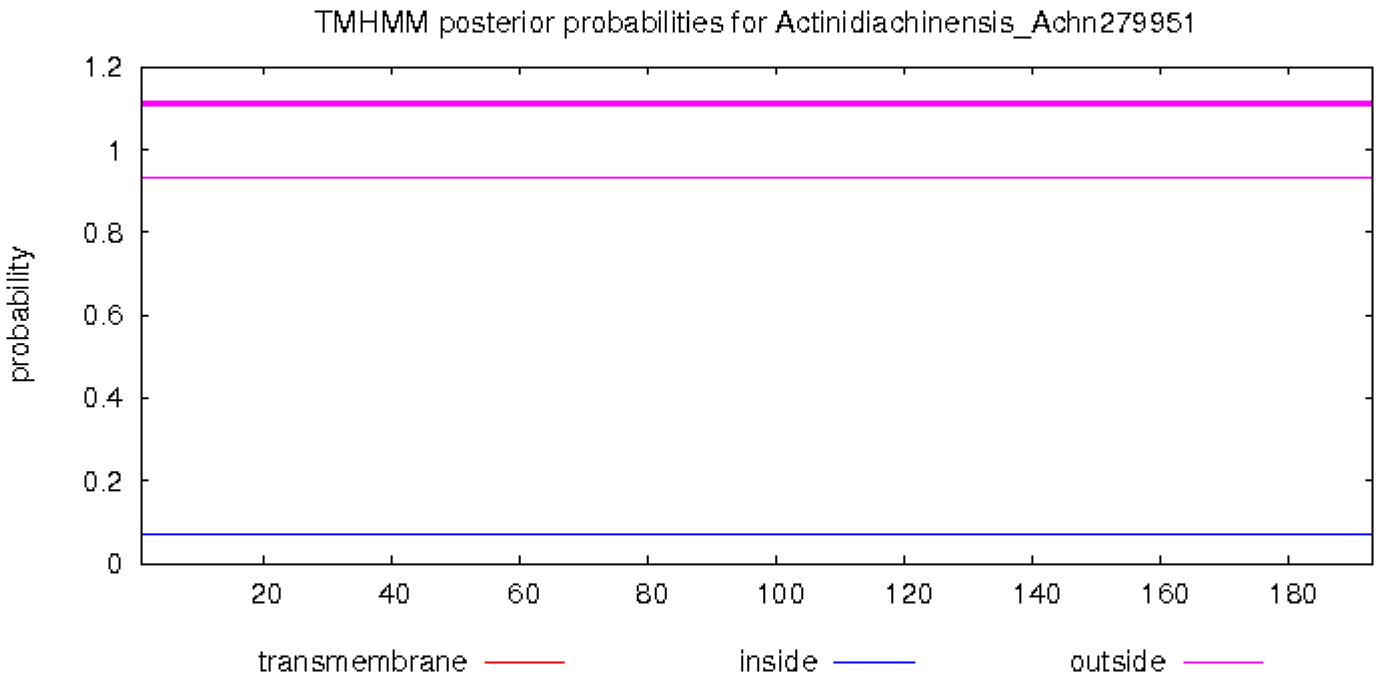

plot in postscript, script for making the plot in gnuplot, data for plot

---

```
# Actinidiachinensis_Achn282481 Length: 326
# Actinidiachinensis_Achn282481 Number of predicted TMHs:  0
# Actinidiachinensis_Achn282481 Exp number of AAs in TMHs: 0.00303
# Actinidiachinensis_Achn282481 Exp number, first 60 AAs:  0
# Actinidiachinensis_Achn282481 Total prob of N-in:        0.00867
Actinidiachinensis_Achn282481   TMHMM2.0        outside      1   326
```

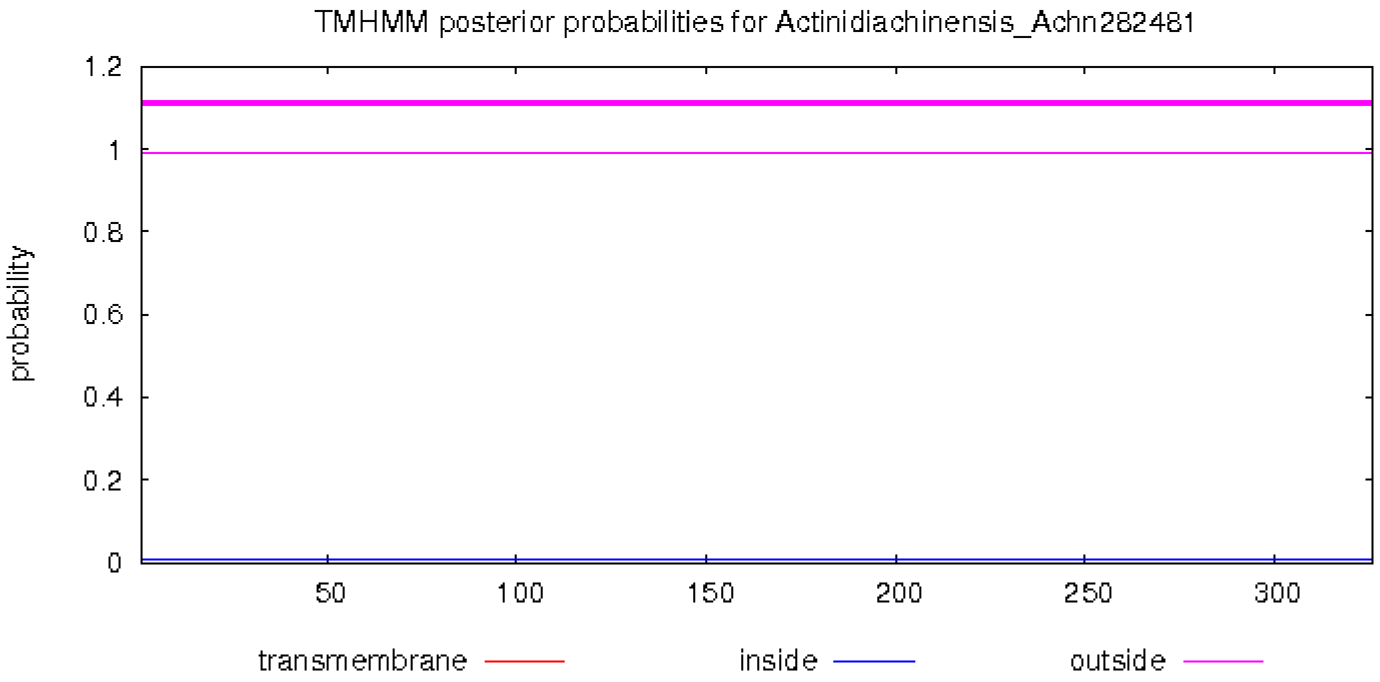

plot in postscript, script for making the plot in gnuplot, data for plot

---

```
# Actinidiachinensis_Achn282651 Length: 339
# Actinidiachinensis_Achn282651 Number of predicted TMHs:  0
# Actinidiachinensis_Achn282651 Exp number of AAs in TMHs: 0.00024
# Actinidiachinensis_Achn282651 Exp number, first 60 AAs:  5e-05
# Actinidiachinensis_Achn282651 Total prob of N-in:        0.02226
Actinidiachinensis_Achn282651   TMHMM2.0        outside      1   339
```

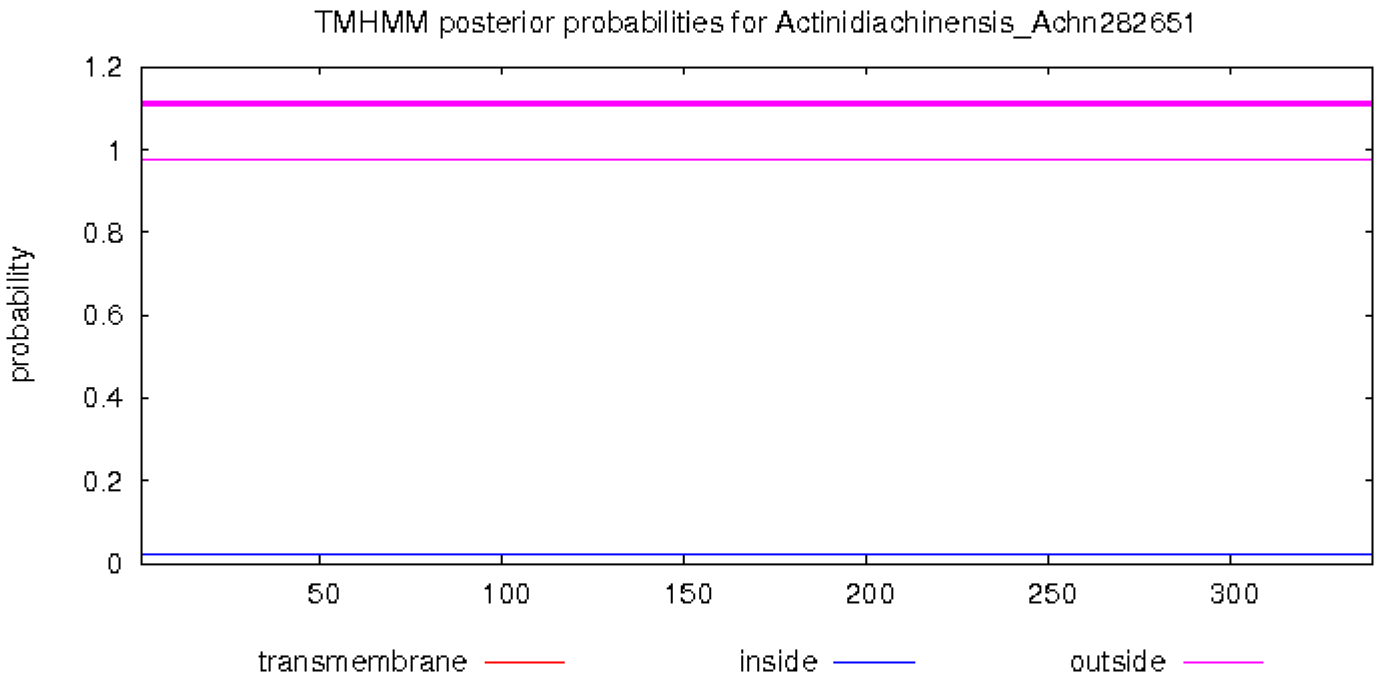

plot in postscript, script for making the plot in gnuplot, data for plot

---

```
# Actinidiachinensis_Achn289231 Length: 439
# Actinidiachinensis_Achn289231 Number of predicted TMHs:  0
# Actinidiachinensis_Achn289231 Exp number of AAs in TMHs: 0.00055
# Actinidiachinensis_Achn289231 Exp number, first 60 AAs:  0.00018
# Actinidiachinensis_Achn289231 Total prob of N-in:        0.00964
Actinidiachinensis_Achn289231	TMHMM2.0	outside	     1   439
```

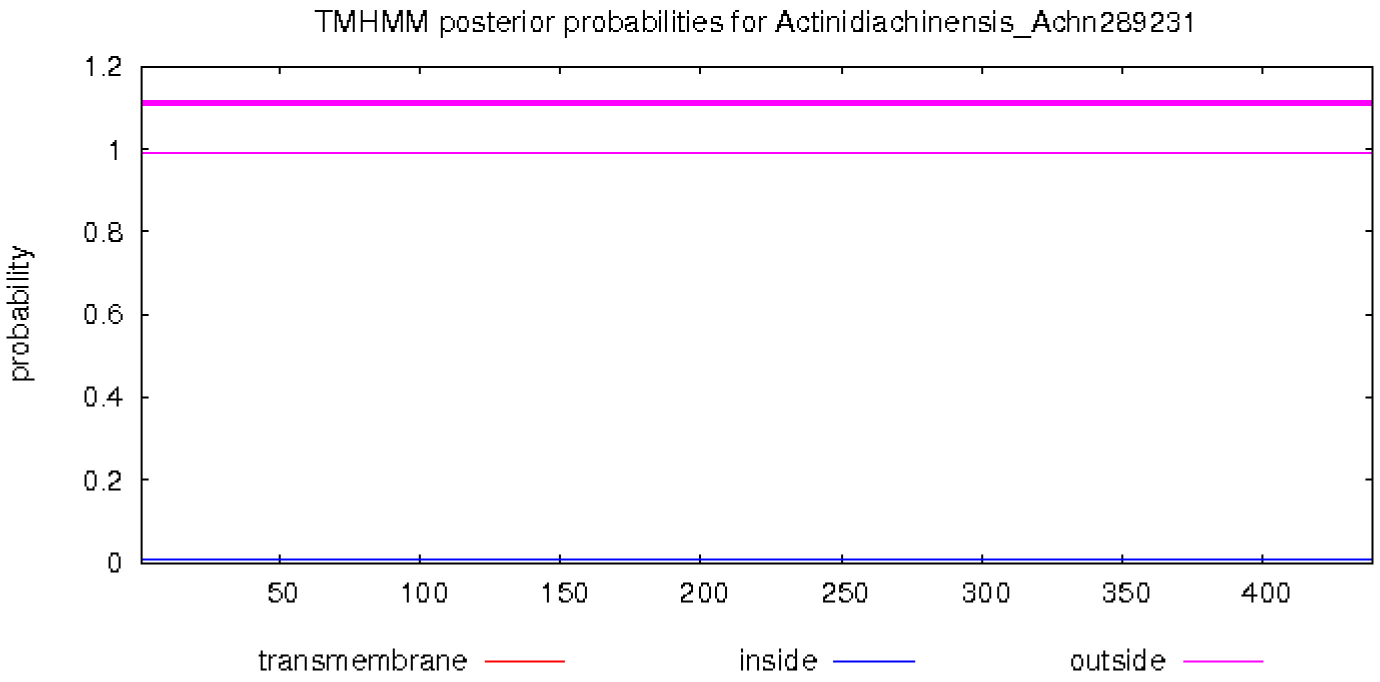

plot in postscript, script for making the plot in gnuplot, data for plot

---

```
# Actinidiachinensis_Achn289291 Length: 330
# Actinidiachinensis_Achn289291 Number of predicted TMHs:  0
# Actinidiachinensis_Achn289291 Exp number of AAs in TMHs: 0
# Actinidiachinensis_Achn289291 Exp number, first 60 AAs:  0
# Actinidiachinensis_Achn289291 Total prob of N-in:        0.00366
Actinidiachinensis_Achn289291	TMHMM2.0	outside	     1   330
```

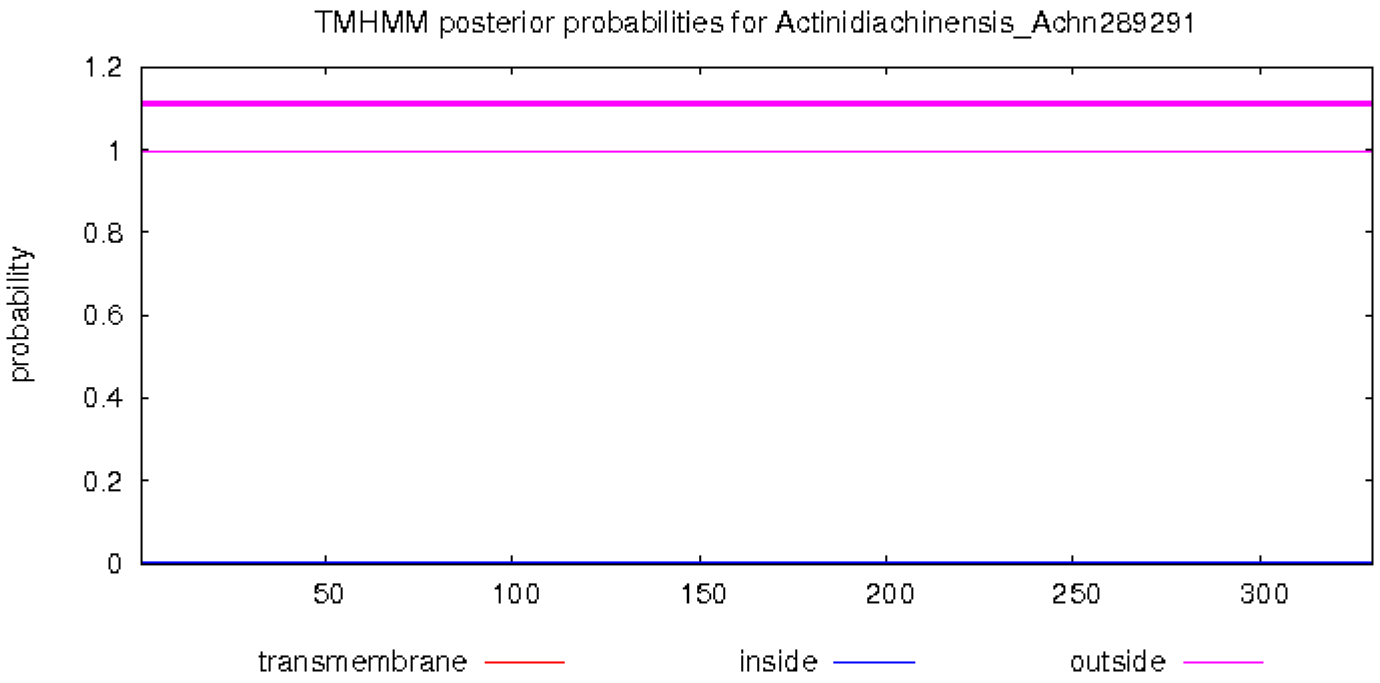

plot in postscript, script for making the plot in gnuplot, data for plot

---

```
# Actinidiachinensis_Achn289301 Length: 321
# Actinidiachinensis_Achn289301 Number of predicted TMHs:  0
# Actinidiachinensis_Achn289301 Exp number of AAs in TMHs: 0.00017
# Actinidiachinensis_Achn289301 Exp number, first 60 AAs:  0.00017
# Actinidiachinensis_Achn289301 Total prob of N-in:        0.02232
Actinidiachinensis_Achn289301   TMHMM2.0        outside      1   321
```

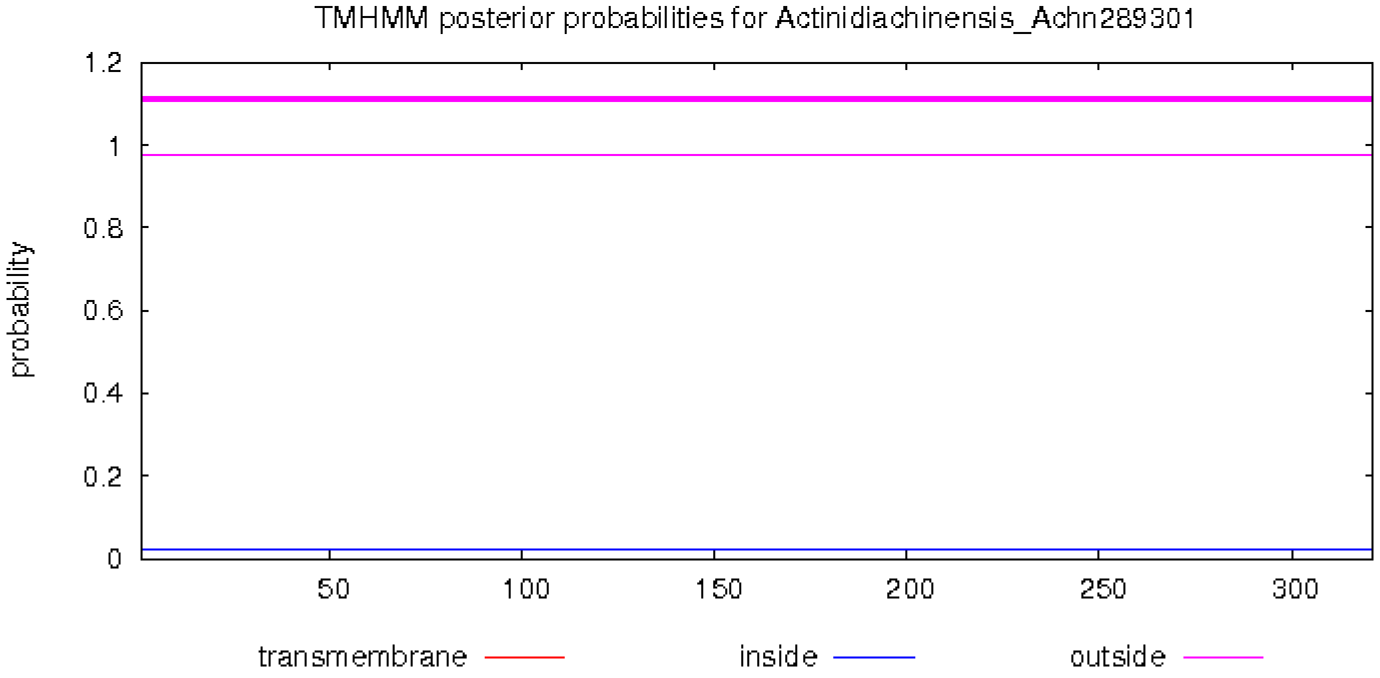

plot in postscript, script for making the plot in gnuplot, data for plot

---

```
# Actinidiachinensis_Achn293411 Length: 265
# Actinidiachinensis_Achn293411 Number of predicted TMHs:  0
# Actinidiachinensis_Achn293411 Exp number of AAs in TMHs: 0.00116
# Actinidiachinensis_Achn293411 Exp number, first 60 AAs:  0
# Actinidiachinensis_Achn293411 Total prob of N-in:        0.01708
Actinidiachinensis_Achn293411   TMHMM2.0        outside      1   265
```

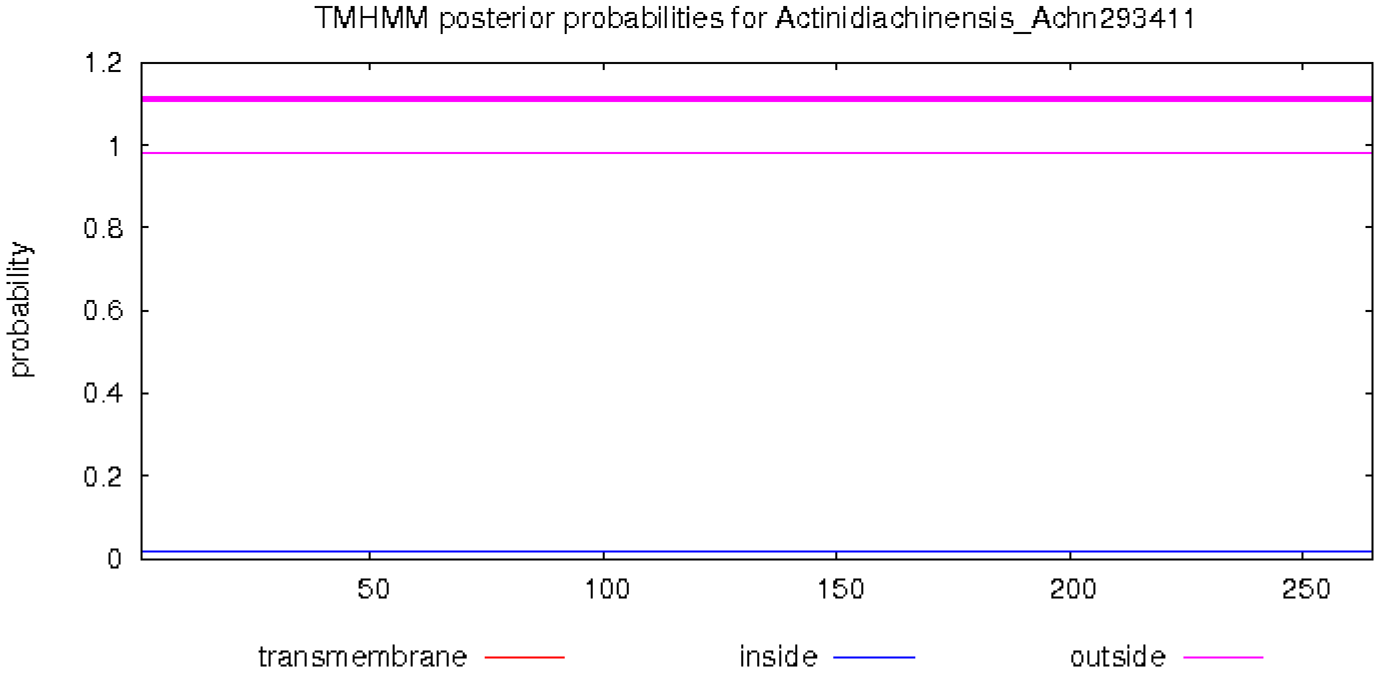

plot in postscript, script for making the plot in gnuplot, data for plot

---

```
# Actinidiachinensis_Achn293491 Length: 345
# Actinidiachinensis_Achn293491 Number of predicted TMHs:  0
# Actinidiachinensis_Achn293491 Exp number of AAs in TMHs: 0
# Actinidiachinensis_Achn293491 Exp number, first 60 AAs:  0
# Actinidiachinensis_Achn293491 Total prob of N-in:        0.02229
Actinidiachinensis_Achn293491	TMHMM2.0	outside	     1   345
```

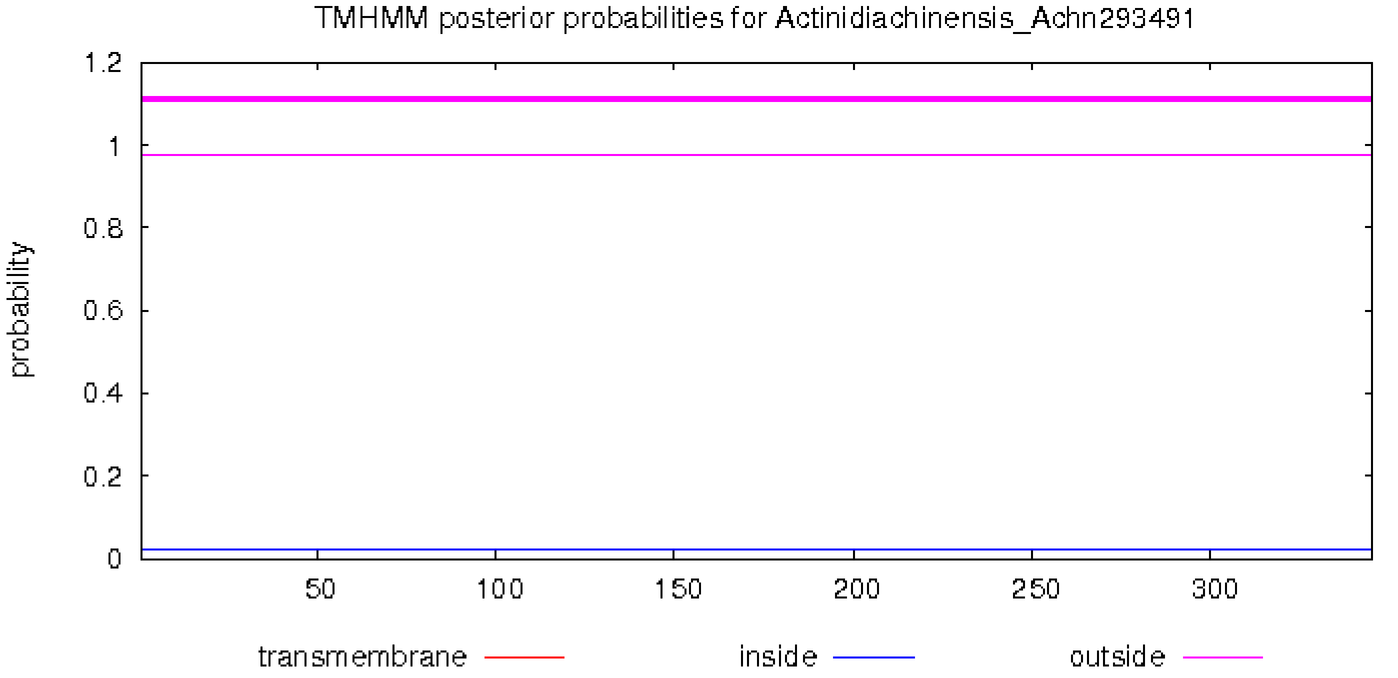

plot in postscript, script for making the plot in gnuplot, data for plot

---

```
# Actinidiachinensis_Achn294661 Length: 404
# Actinidiachinensis_Achn294661 Number of predicted TMHs:  0
# Actinidiachinensis_Achn294661 Exp number of AAs in TMHs: 0.00303
# Actinidiachinensis_Achn294661 Exp number, first 60 AAs:  0.00018
# Actinidiachinensis_Achn294661 Total prob of N-in:        0.01059
Actinidiachinensis_Achn294661	TMHMM2.0	outside	     1   404
```

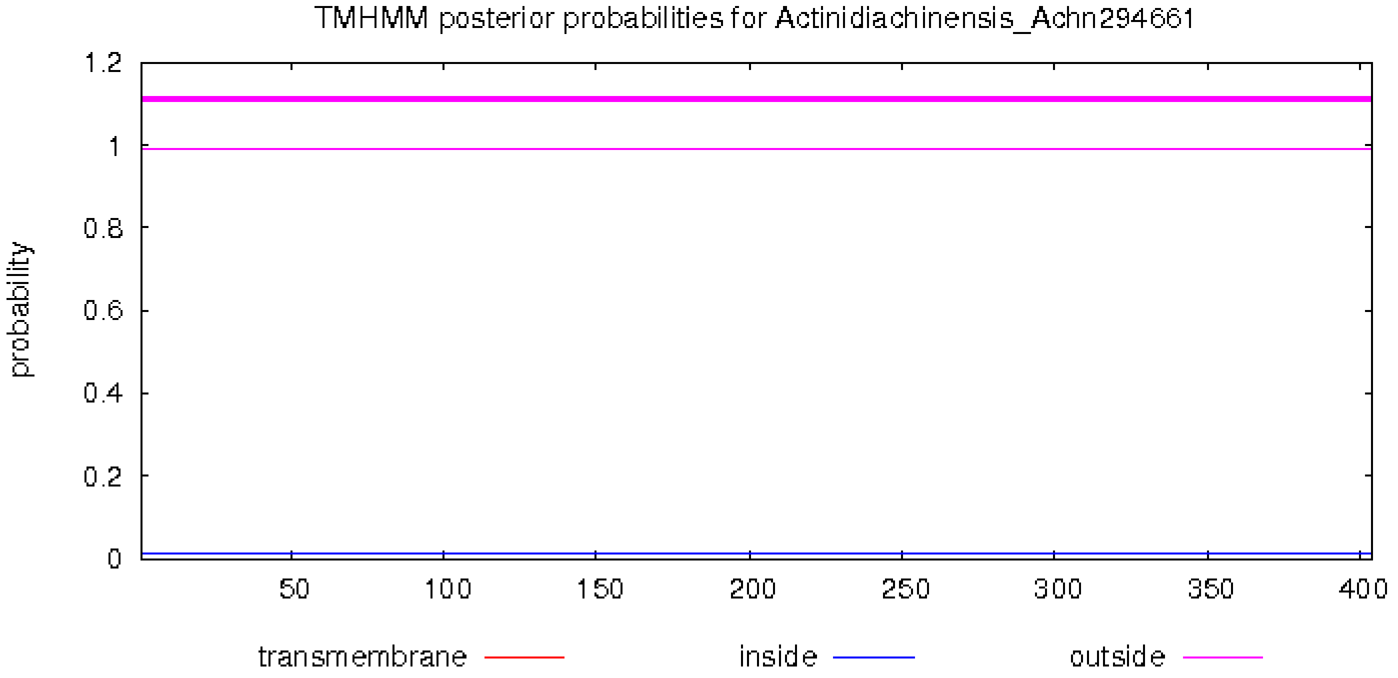

plot in postscript, script for making the plot in gnuplot, data for plot

---

```
# Actinidiachinensis_Achn296141 Length: 238
# Actinidiachinensis_Achn296141 Number of predicted TMHs:  0
# Actinidiachinensis_Achn296141 Exp number of AAs in TMHs: 0.00288
# Actinidiachinensis_Achn296141 Exp number, first 60 AAs:  0.00044
# Actinidiachinensis_Achn296141 Total prob of N-in:        0.15366
Actinidiachinensis_Achn296141	TMHMM2.0	outside	     1   238
```

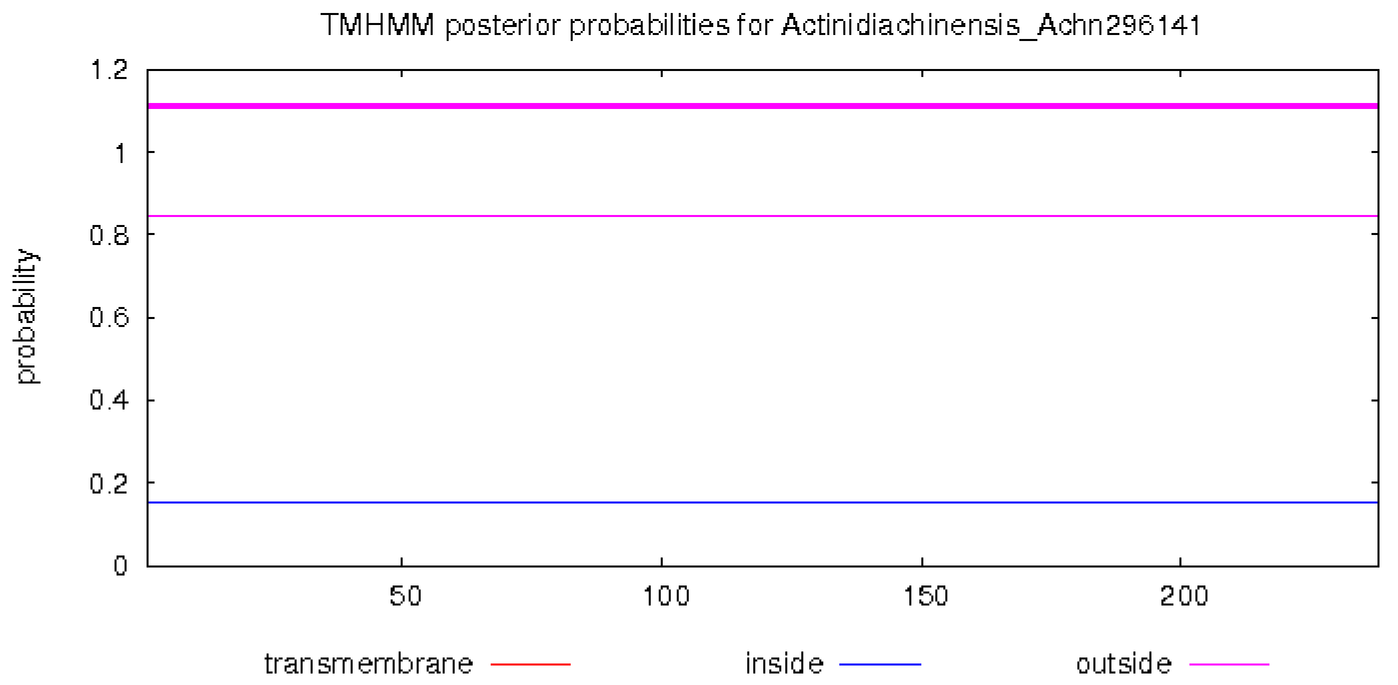

plot in postscript, script for making the plot in gnuplot, data for plot

---

```
# Actinidiachinensis_Achn298161 Length: 397
# Actinidiachinensis_Achn298161 Number of predicted TMHs:  0
# Actinidiachinensis_Achn298161 Exp number of AAs in TMHs: 0.09139
# Actinidiachinensis_Achn298161 Exp number, first 60 AAs:  0
# Actinidiachinensis_Achn298161 Total prob of N-in:        0.00316
Actinidiachinensis_Achn298161	TMHMM2.0	outside	     1   397
```

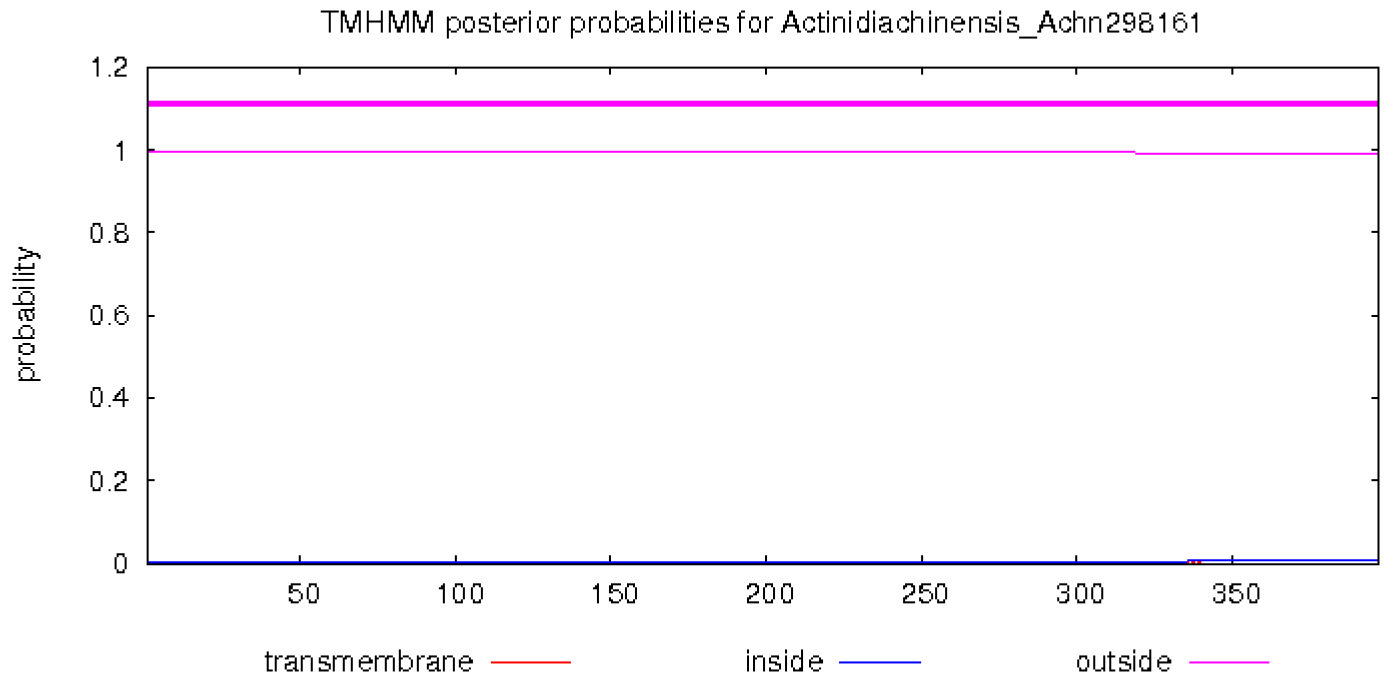

plot in postscript, script for making the plot in gnuplot, data for plot

---

```
# Actinidiachinensis_Achn300931 Length: 240
# Actinidiachinensis_Achn300931 Number of predicted TMHs:  0
# Actinidiachinensis_Achn300931 Exp number of AAs in TMHs: 0.0004
# Actinidiachinensis_Achn300931 Exp number, first 60 AAs:  0
# Actinidiachinensis_Achn300931 Total prob of N-in:        0.00952
Actinidiachinensis_Achn300931   TMHMM2.0        outside      1   240
```

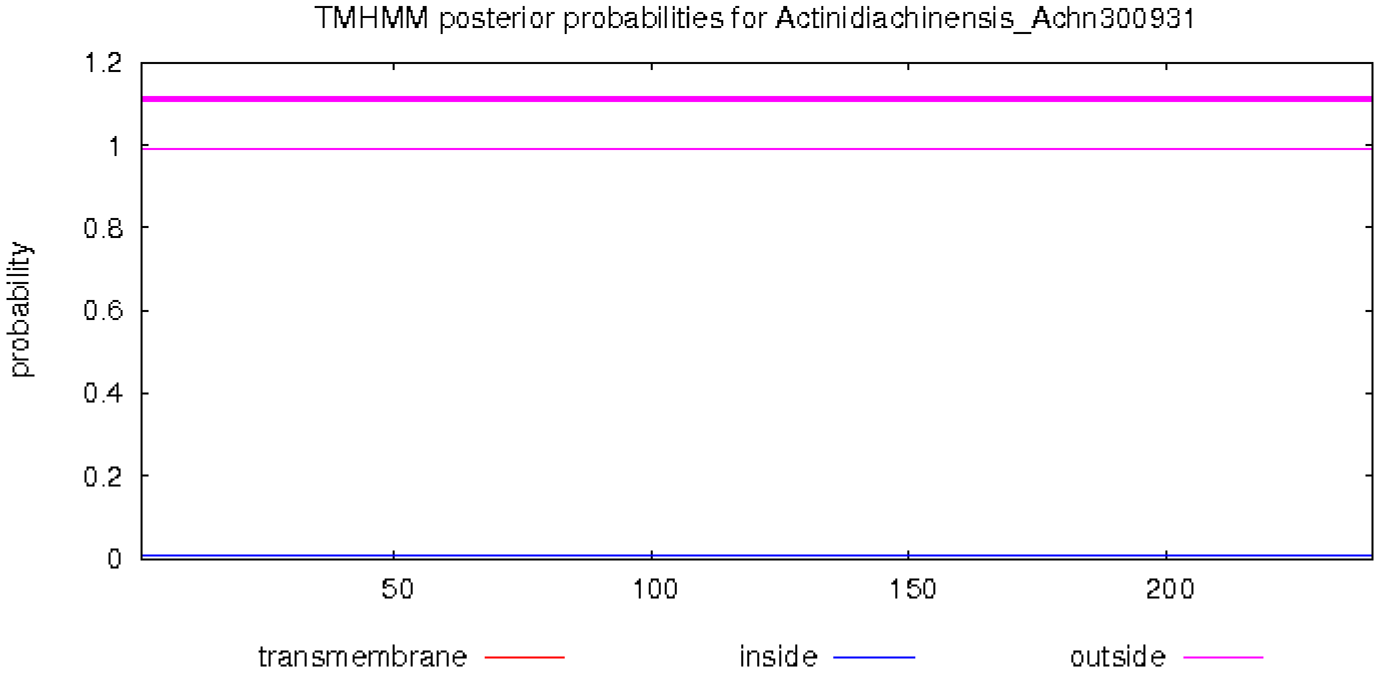

plot in postscript, script for making the plot in gnuplot, data for plot

---

```
# Actinidiachinensis_Achn300981 Length: 204
# Actinidiachinensis_Achn300981 Number of predicted TMHs:  0
# Actinidiachinensis_Achn300981 Exp number of AAs in TMHs: 0.00101
# Actinidiachinensis_Achn300981 Exp number, first 60 AAs:  0.00023
# Actinidiachinensis_Achn300981 Total prob of N-in:        0.13132
Actinidiachinensis_Achn300981   TMHMM2.0        outside      1   204
```

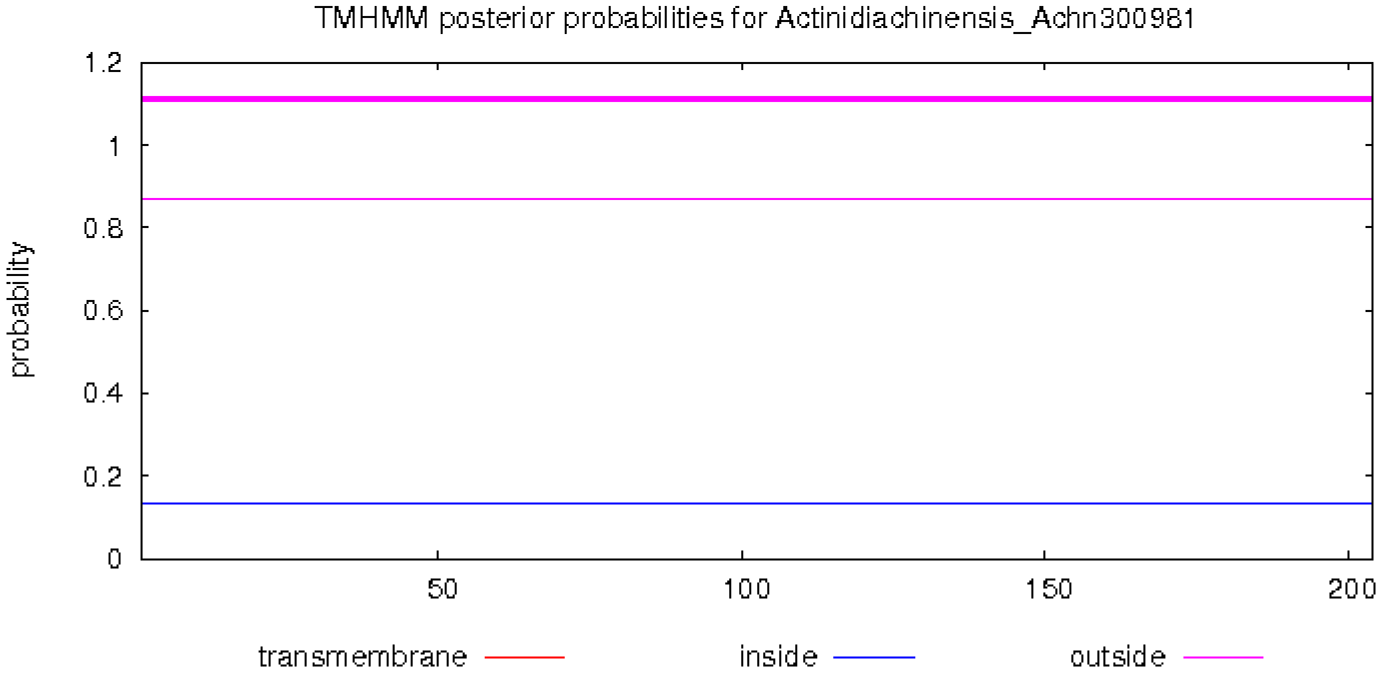

plot in postscript, script for making the plot in gnuplot, data for plot

---

```
# Actinidiachinensis_Achn301081 Length: 314
# Actinidiachinensis_Achn301081 Number of predicted TMHs:  0
# Actinidiachinensis_Achn301081 Exp number of AAs in TMHs: 0.02388
# Actinidiachinensis_Achn301081 Exp number, first 60 AAs:  0.00665
# Actinidiachinensis_Achn301081 Total prob of N-in:        0.02284
Actinidiachinensis_Achn301081   TMHMM2.0        outside      1   314
```

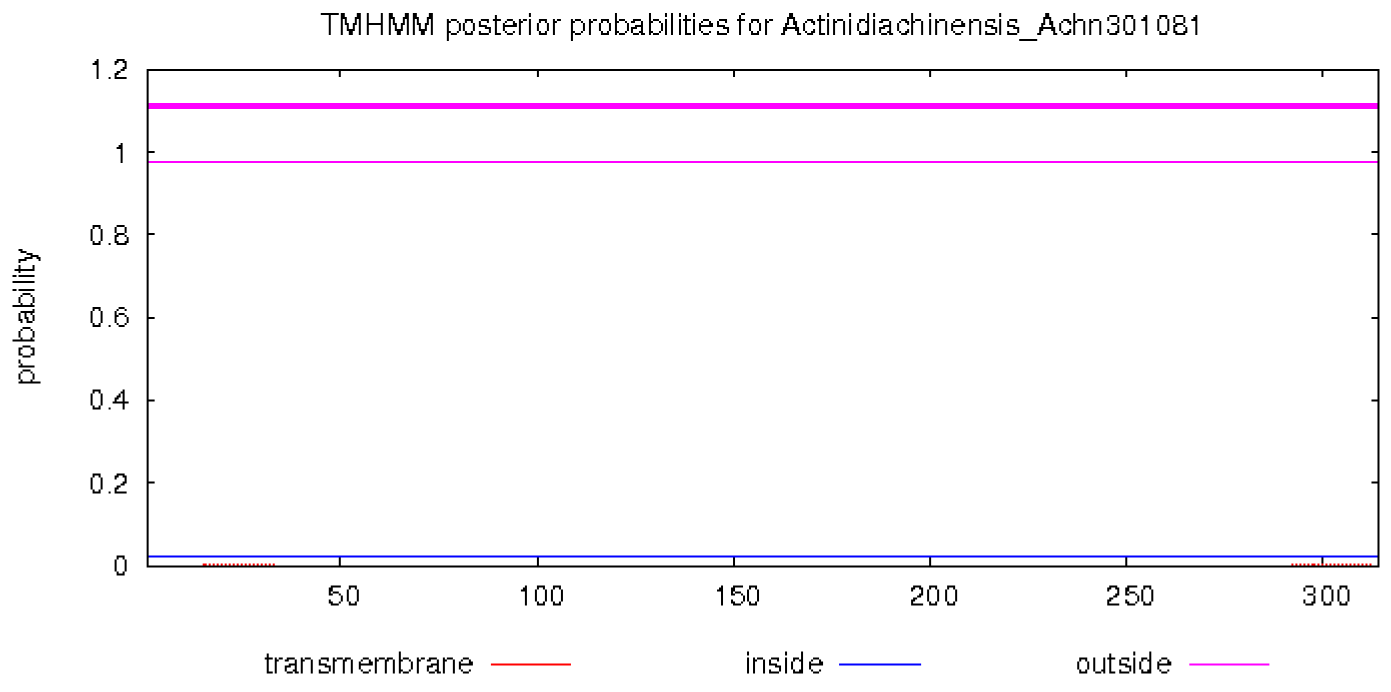

plot in postscript, script for making the plot in gnuplot, data for plot

---

```
# Actinidiachinensis_Achn310421 Length: 438
# Actinidiachinensis_Achn310421 Number of predicted TMHs:  0
# Actinidiachinensis_Achn310421 Exp number of AAs in TMHs: 0
# Actinidiachinensis_Achn310421 Exp number, first 60 AAs:  0
# Actinidiachinensis_Achn310421 Total prob of N-in:        0.00055
Actinidiachinensis_Achn310421   TMHMM2.0        outside      1   438
```

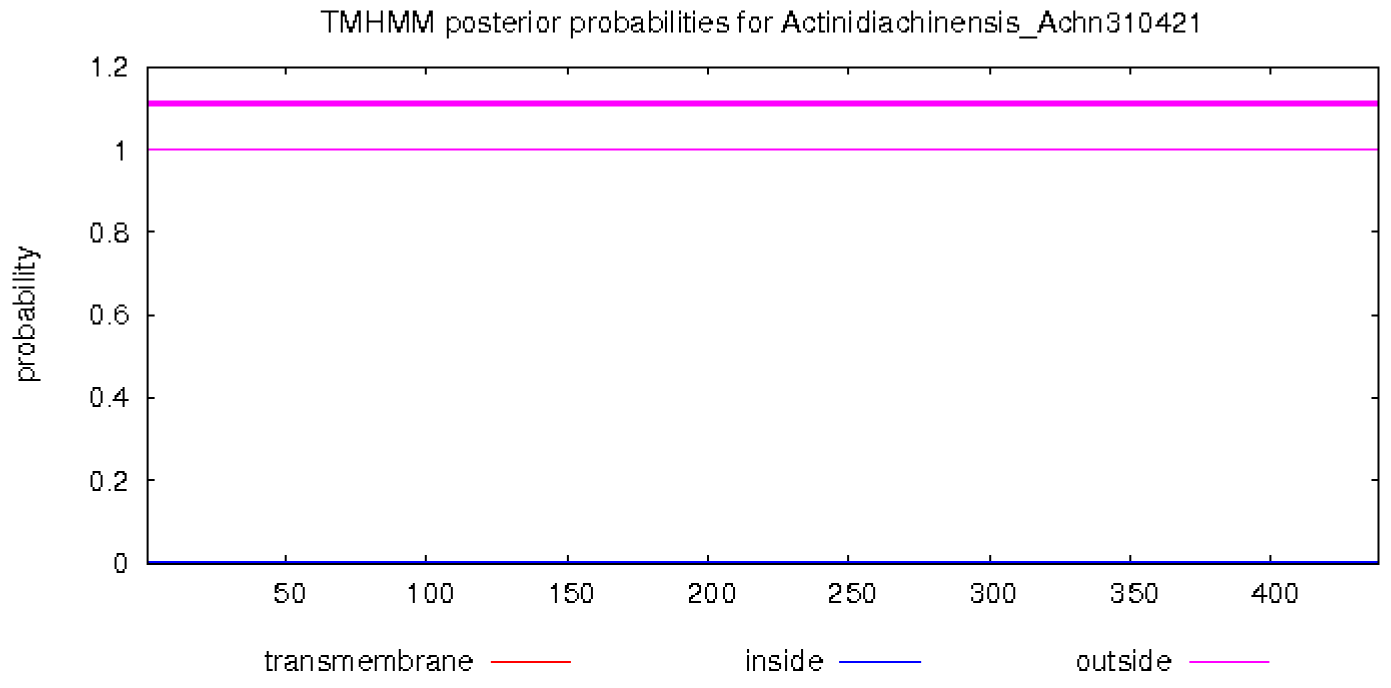

plot in postscript, script for making the plot in gnuplot, data for plot

---

```
# Actinidiachinensis_Achn310791 Length: 305
# Actinidiachinensis_Achn310791 Number of predicted TMHs:  0
# Actinidiachinensis_Achn310791 Exp number of AAs in TMHs: 0.00782
# Actinidiachinensis_Achn310791 Exp number, first 60 AAs:  0
# Actinidiachinensis_Achn310791 Total prob of N-in:        0.10904
Actinidiachinensis_Achn310791   TMHMM2.0        outside      1   305
```

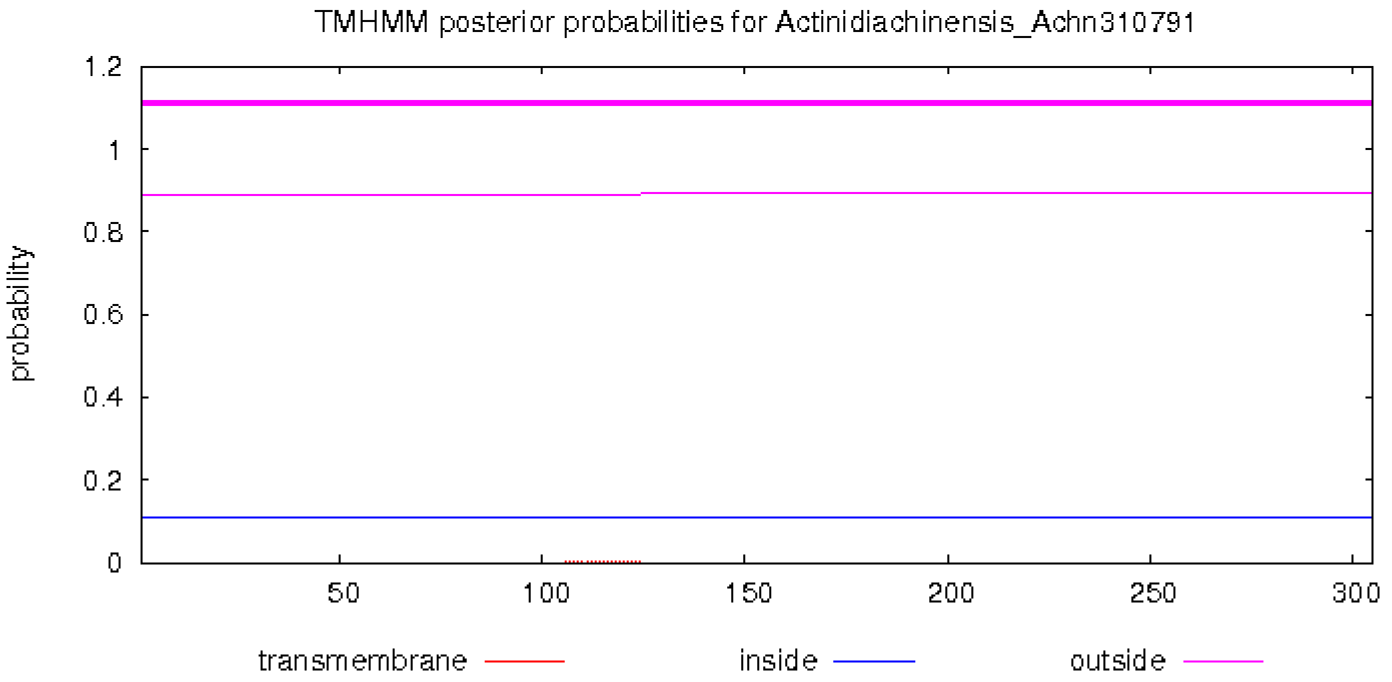

plot in postscript, script for making the plot in gnuplot, data for plot

---

```
# Actinidiachinensis_Achn311471 Length: 337
# Actinidiachinensis_Achn311471 Number of predicted TMHs:  0
# Actinidiachinensis_Achn311471 Exp number of AAs in TMHs: 0.00022
# Actinidiachinensis_Achn311471 Exp number, first 60 AAs:  0
# Actinidiachinensis_Achn311471 Total prob of N-in:        0.00938
Actinidiachinensis_Achn311471   TMHMM2.0        outside      1   337
```

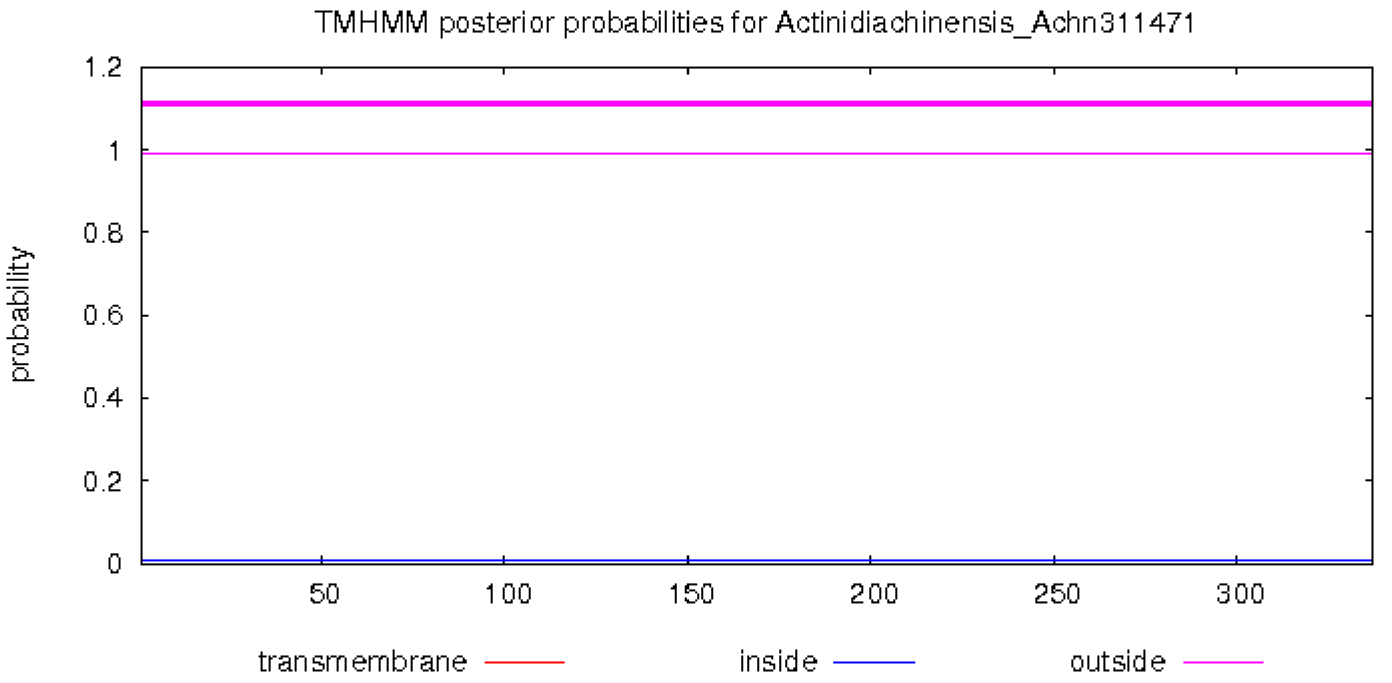

plot in postscript, script for making the plot in gnuplot, data for plot

---

```
# Actinidiachinensis_Achn311531 Length: 394
# Actinidiachinensis_Achn311531 Number of predicted TMHs:  0
# Actinidiachinensis_Achn311531 Exp number of AAs in TMHs: 0.00042
# Actinidiachinensis_Achn311531 Exp number, first 60 AAs:  0
# Actinidiachinensis_Achn311531 Total prob of N-in:        0.00895
Actinidiachinensis_Achn311531   TMHMM2.0        outside      1   394
```

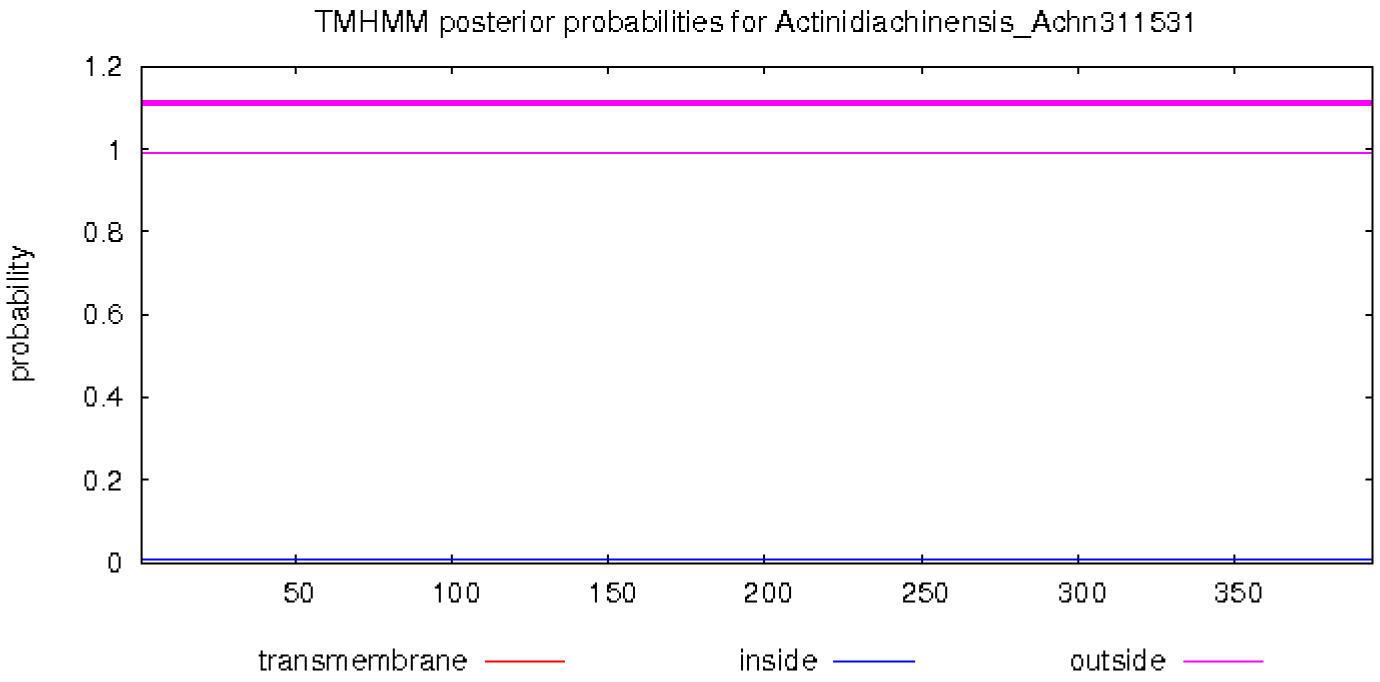

plot in postscript, script for making the plot in gnuplot, data for plot

---

```
# Actinidiachinensis_Achn311801 Length: 234
# Actinidiachinensis_Achn311801 Number of predicted TMHs:  0
# Actinidiachinensis_Achn311801 Exp number of AAs in TMHs: 0.00529
# Actinidiachinensis_Achn311801 Exp number, first 60 AAs:  0
# Actinidiachinensis_Achn311801 Total prob of N-in:        0.24147
Actinidiachinensis_Achn311801   TMHMM2.0        outside      1   234
```

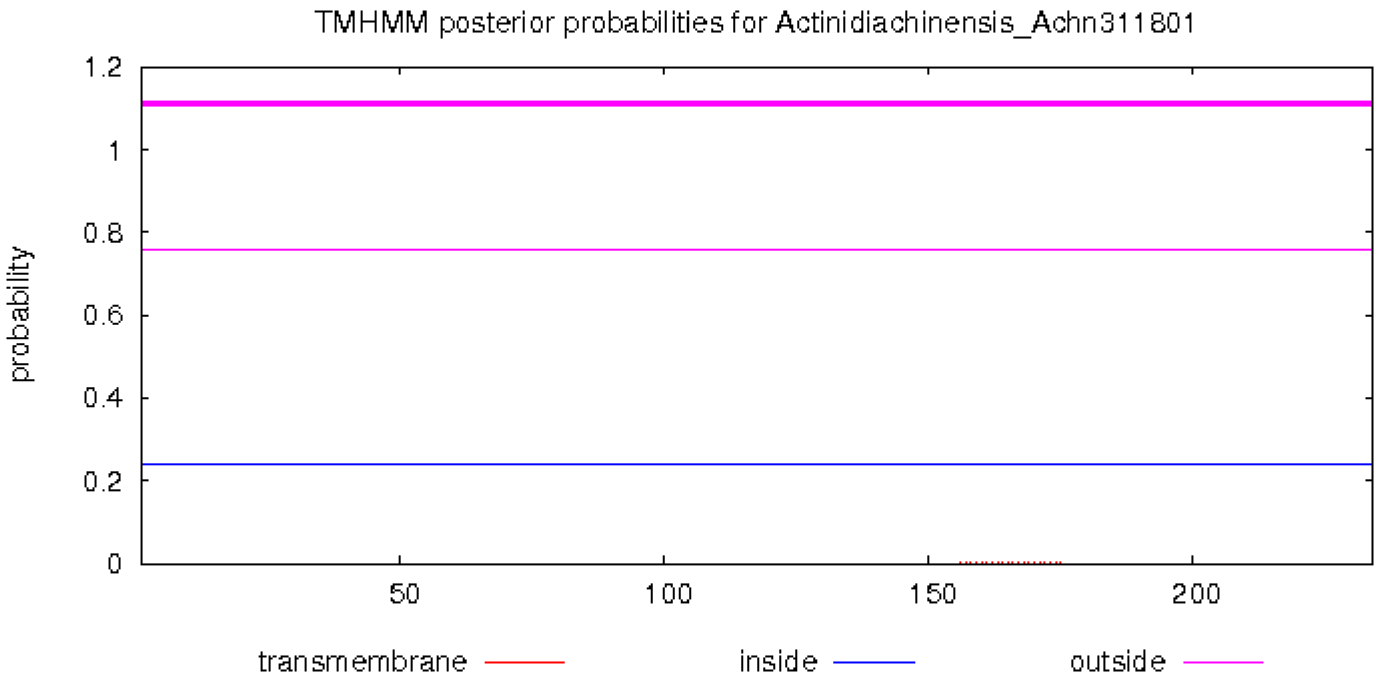

plot in postscript, script for making the plot in gnuplot, data for plot

---

```
# Actinidiachinensis_Achn317511 Length: 345
# Actinidiachinensis_Achn317511 Number of predicted TMHs:  0
# Actinidiachinensis_Achn317511 Exp number of AAs in TMHs: 0.00332
# Actinidiachinensis_Achn317511 Exp number, first 60 AAs:  0.0003
# Actinidiachinensis_Achn317511 Total prob of N-in:        0.01985
Actinidiachinensis_Achn317511   TMHMM2.0        outside      1   345
```

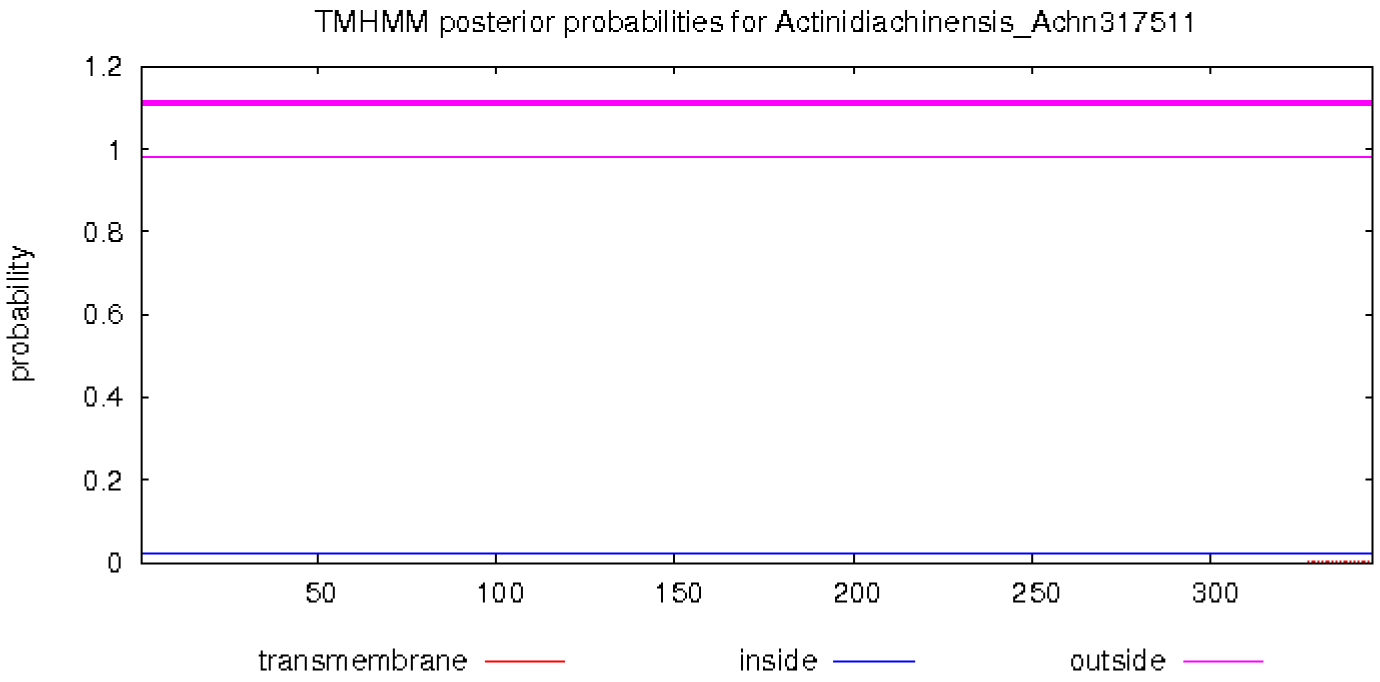

plot in postscript, script for making the plot in gnuplot, data for plot

---

```
# Actinidiachinensis_Achn335281 Length: 1234
# Actinidiachinensis_Achn335281 Number of predicted TMHs:  1
# Actinidiachinensis_Achn335281 Exp number of AAs in TMHs: 41.0376099999999999
# Actinidiachinensis_Achn335281 Exp number, first 60 AAs:  2.46672
# Actinidiachinensis_Achn335281 Total prob of N-in:        0.33810
Actinidiachinensis_Achn335281   TMHMM2.0        inside       1   575
Actinidiachinensis_Achn335281   TMHMM2.0        TMhelix    576   598
Actinidiachinensis_Achn335281   TMHMM2.0        outside    599  1234
```

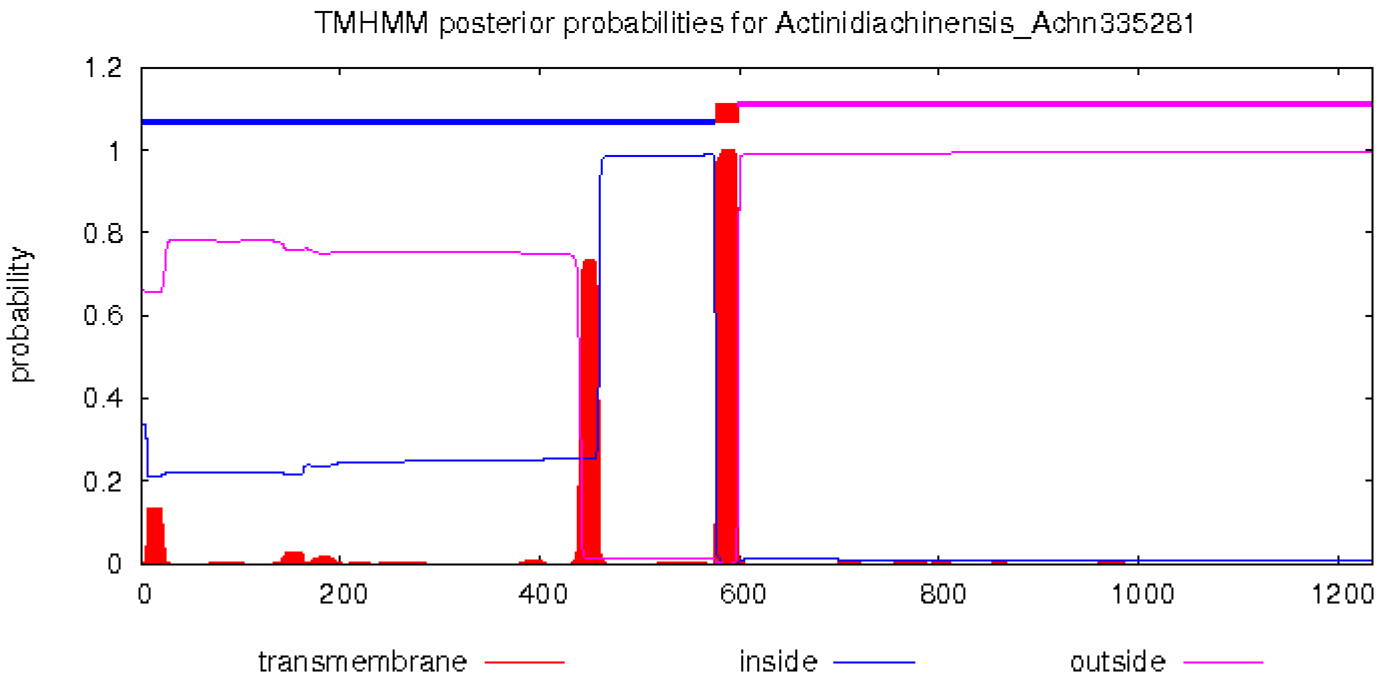

plot in postscript, script for making the plot in gnuplot, data for plot

```
# Actinidiachinensis_Achn335731 Length: 563
# Actinidiachinensis_Achn335731 Number of predicted TMHs:  0
# Actinidiachinensis_Achn335731 Exp number of AAs in TMHs: 0.16741
# Actinidiachinensis_Achn335731 Exp number, first 60 AAs:  0
# Actinidiachinensis_Achn335731 Total prob of N-in:        0.00007
Actinidiachinensis_Achn335731   TMHMM2.0        outside      1   563
```

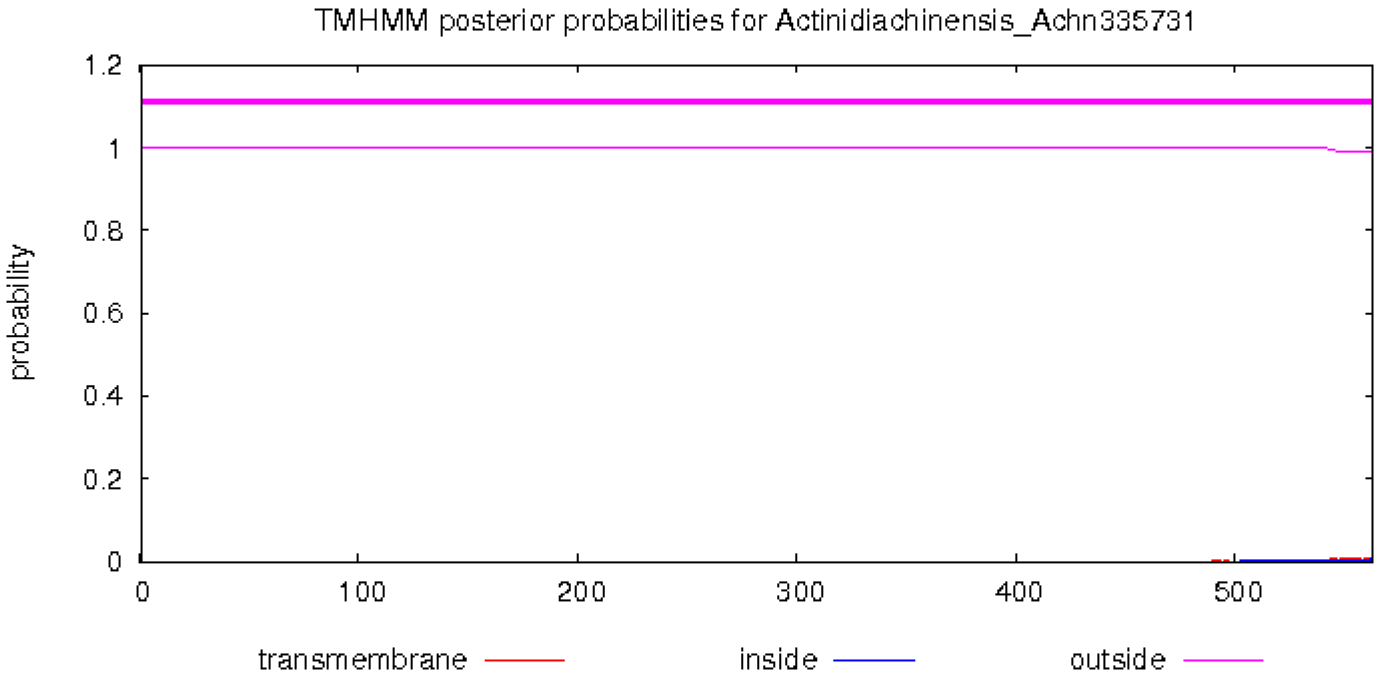

plot in postscript, script for making the plot in gnuplot, data for plot

```
# Actinidiachinensis_Achn341031 Length: 286
# Actinidiachinensis_Achn341031 Number of predicted TMHs:  0
# Actinidiachinensis_Achn341031 Exp number of AAs in TMHs: 0.02841
# Actinidiachinensis_Achn341031 Exp number, first 60 AAs:  0.02841
# Actinidiachinensis_Achn341031 Total prob of N-in:        0.02892
Actinidiachinensis_Achn341031   TMHMM2.0        outside      1   286
```

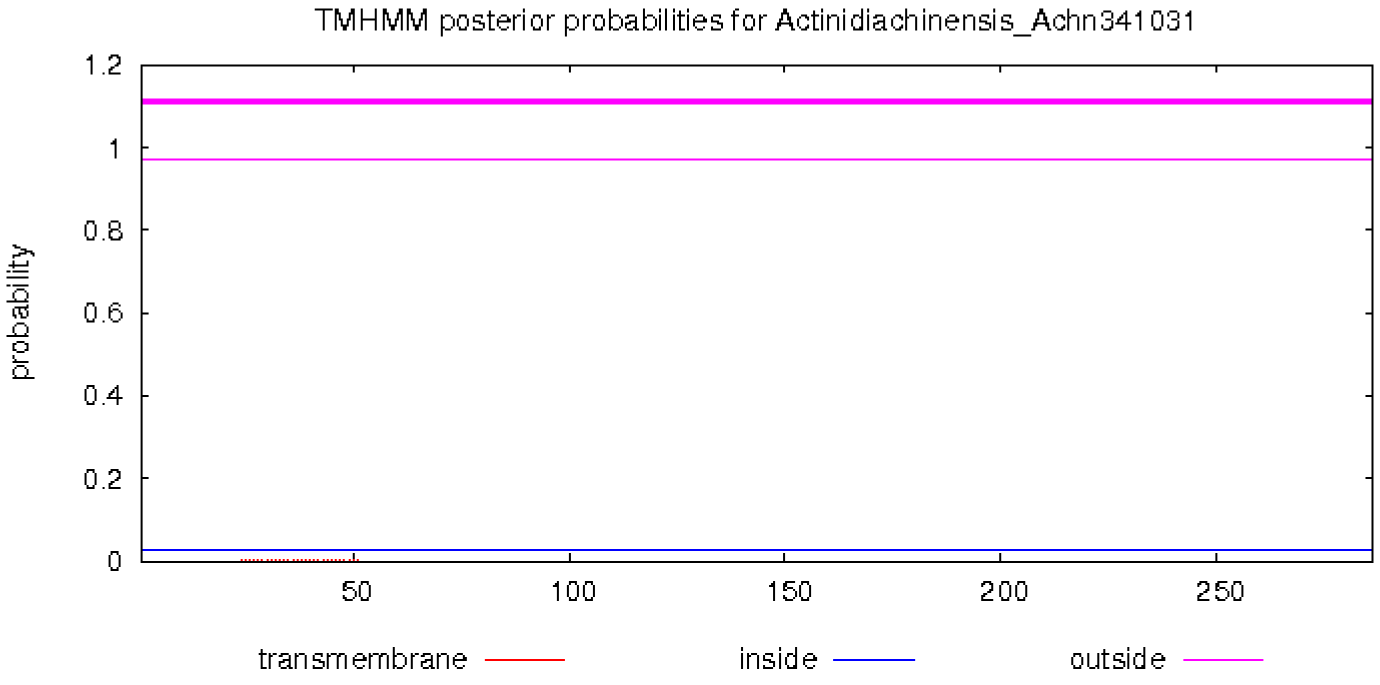

plot in postscript, script for making the plot in gnuplot, data for plot

```
# Actinidiachinensis_Achn343671 Length: 392
# Actinidiachinensis_Achn343671 Number of predicted TMHs:  0
# Actinidiachinensis_Achn343671 Exp number of AAs in TMHs: 0.01352
# Actinidiachinensis_Achn343671 Exp number, first 60 AAs:  0.00403
# Actinidiachinensis_Achn343671 Total prob of N-in:        0.00225
Actinidiachinensis_Achn343671	TMHMM2.0	outside	     1   392
```

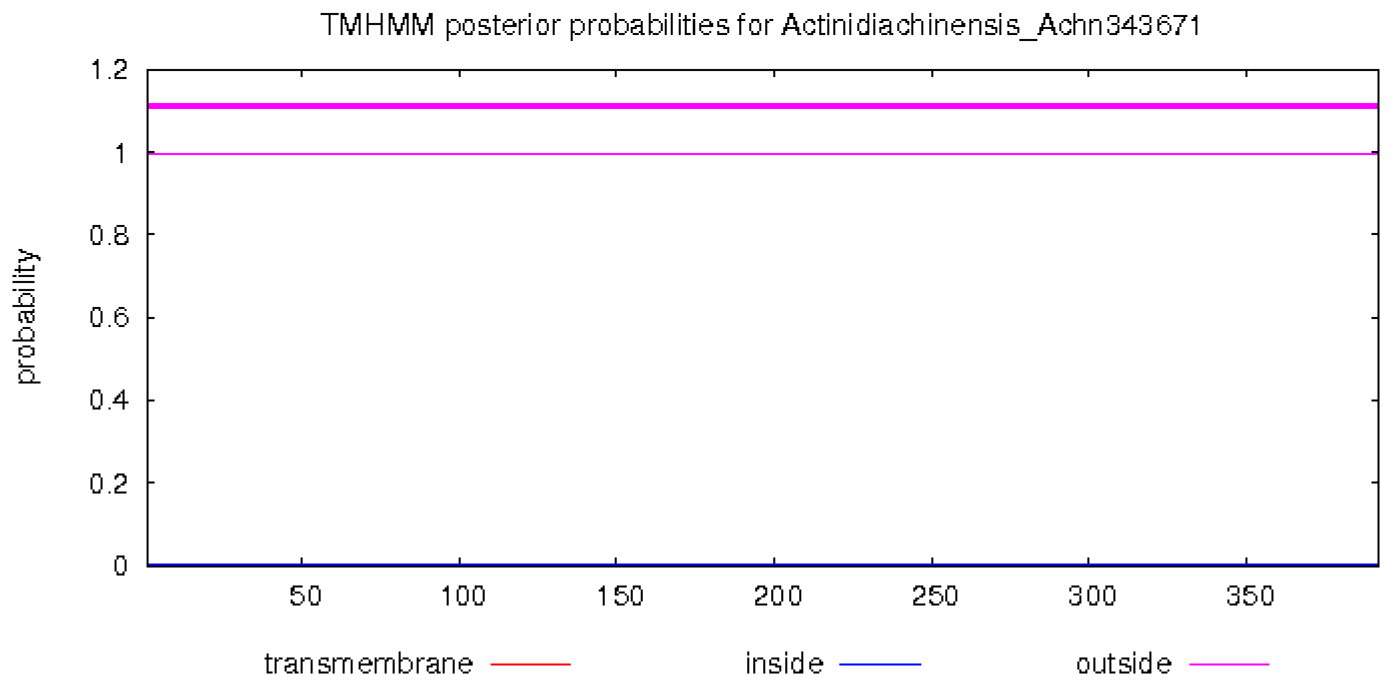

plot in postscript, script for making the plot in gnuplot, data for plot

---

```
# Actinidiachinensis_Achn344701 Length: 337
# Actinidiachinensis_Achn344701 Number of predicted TMHs:  0
# Actinidiachinensis_Achn344701 Exp number of AAs in TMHs: 0.00403
# Actinidiachinensis_Achn344701 Exp number, first 60 AAs:  0.00023
# Actinidiachinensis_Achn344701 Total prob of N-in:        0.00828
Actinidiachinensis_Achn344701	TMHMM2.0	outside	     1   337
```

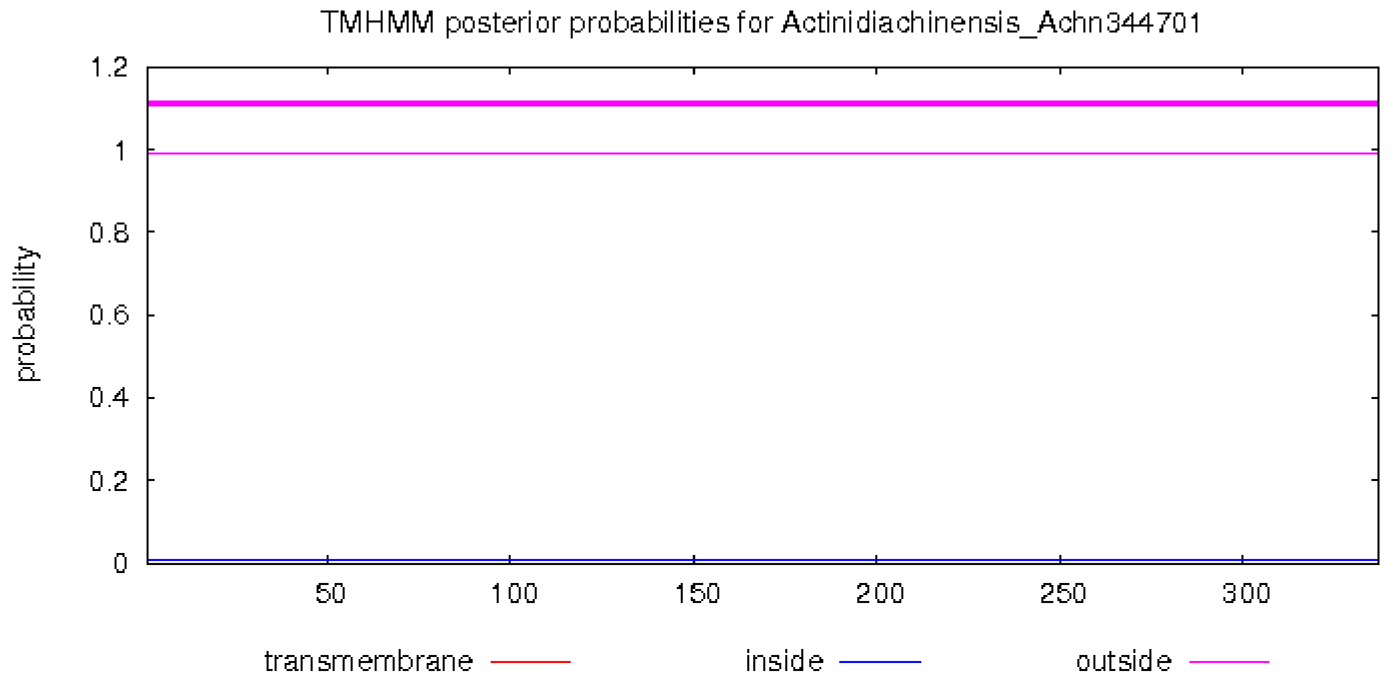

plot in postscript, script for making the plot in gnuplot, data for plot

---

```
# Actinidiachinensis_Achn348191 Length: 242
# Actinidiachinensis_Achn348191 Number of predicted TMHs:  0
# Actinidiachinensis_Achn348191 Exp number of AAs in TMHs: 0.00112
# Actinidiachinensis_Achn348191 Exp number, first 60 AAs:  0.00112
# Actinidiachinensis_Achn348191 Total prob of N-in:        0.02818
Actinidiachinensis_Achn348191	TMHMM2.0	outside	     1   242
```

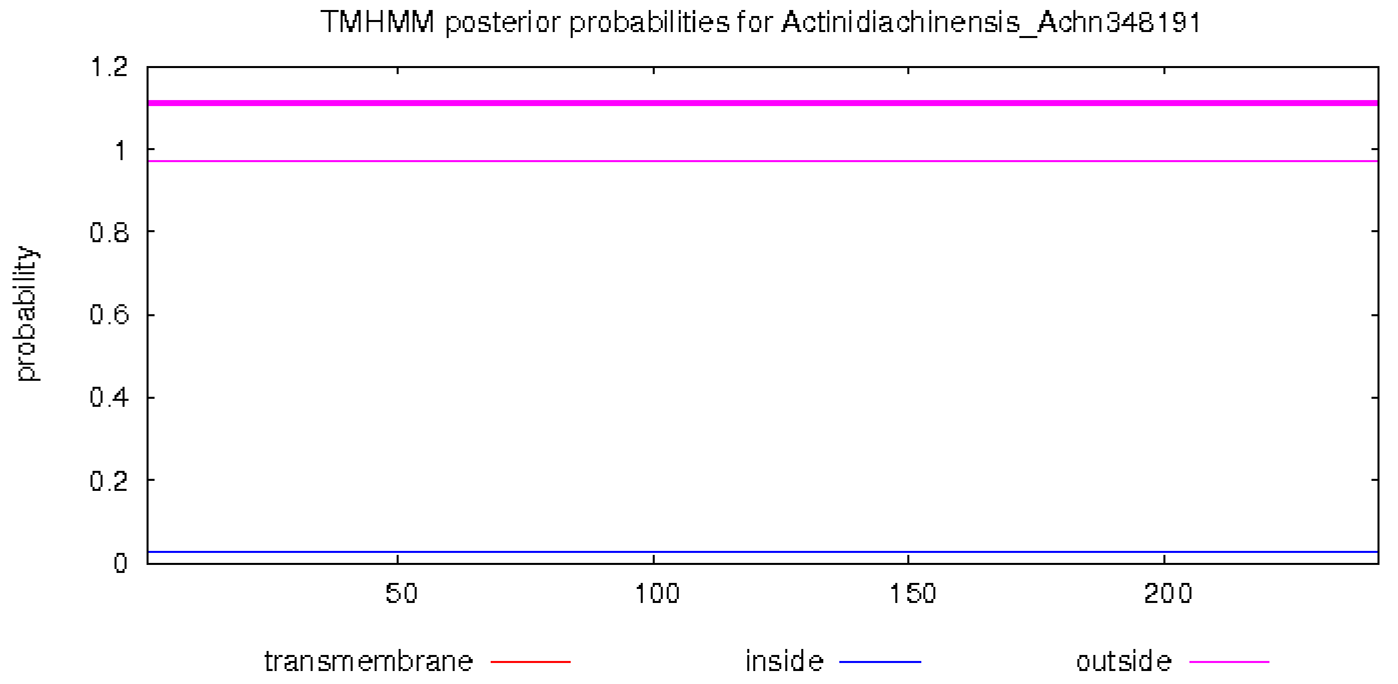

plot in postscript, script for making the plot in gnuplot, data for plot

---

```
# Actinidiachinensis_Achn349691 Length: 248
# Actinidiachinensis_Achn349691 Number of predicted TMHs:  0
# Actinidiachinensis_Achn349691 Exp number of AAs in TMHs: 0.00231
# Actinidiachinensis_Achn349691 Exp number, first 60 AAs:  0
# Actinidiachinensis_Achn349691 Total prob of N-in:        0.06355
Actinidiachinensis_Achn349691	TMHMM2.0	outside	     1   248
```

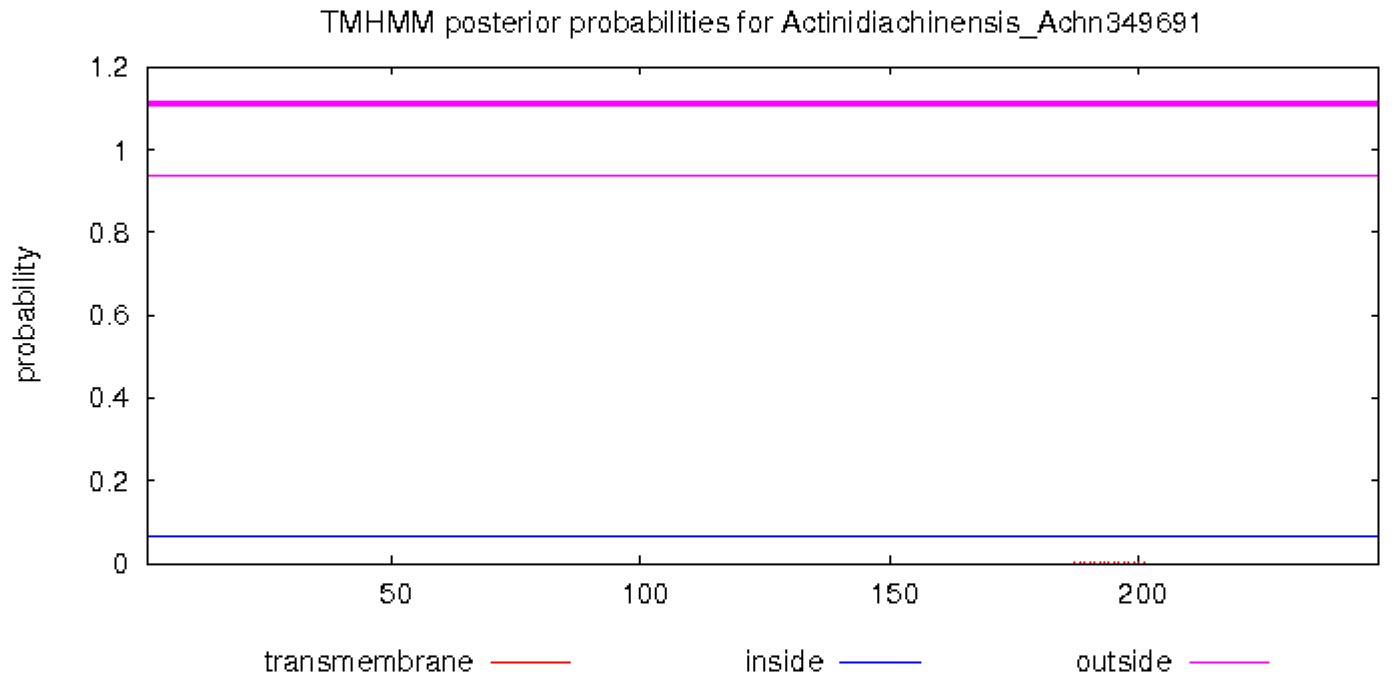

plot in postscript, script for making the plot in gnuplot, data for plot

---

```
# Actinidiachinensis_Achn351611 Length: 268
# Actinidiachinensis_Achn351611 Number of predicted TMHs:  0
# Actinidiachinensis_Achn351611 Exp number of AAs in TMHs: 0.00321
# Actinidiachinensis_Achn351611 Exp number, first 60 AAs:  0
# Actinidiachinensis_Achn351611 Total prob of N-in:        0.04133
Actinidiachinensis_Achn351611   TMHMM2.0        outside      1   268
```

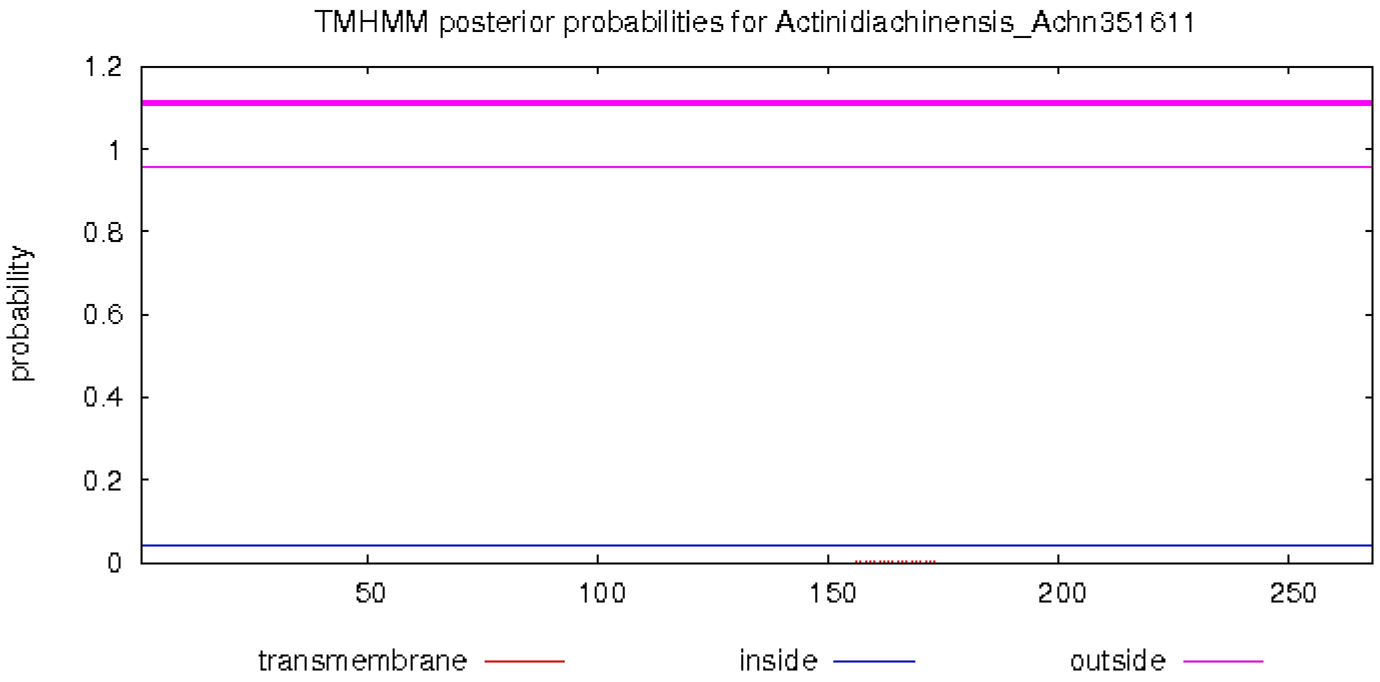

plot in postscript, script for making the plot in gnuplot, data for plot

---

```
# Actinidiachinensis_Achn352721 Length: 291
# Actinidiachinensis_Achn352721 Number of predicted TMHs:  0
# Actinidiachinensis_Achn352721 Exp number of AAs in TMHs: 7e-05
# Actinidiachinensis_Achn352721 Exp number, first 60 AAs:  0
# Actinidiachinensis_Achn352721 Total prob of N-in:        0.07663
Actinidiachinensis_Achn352721   TMHMM2.0        outside      1   291
```

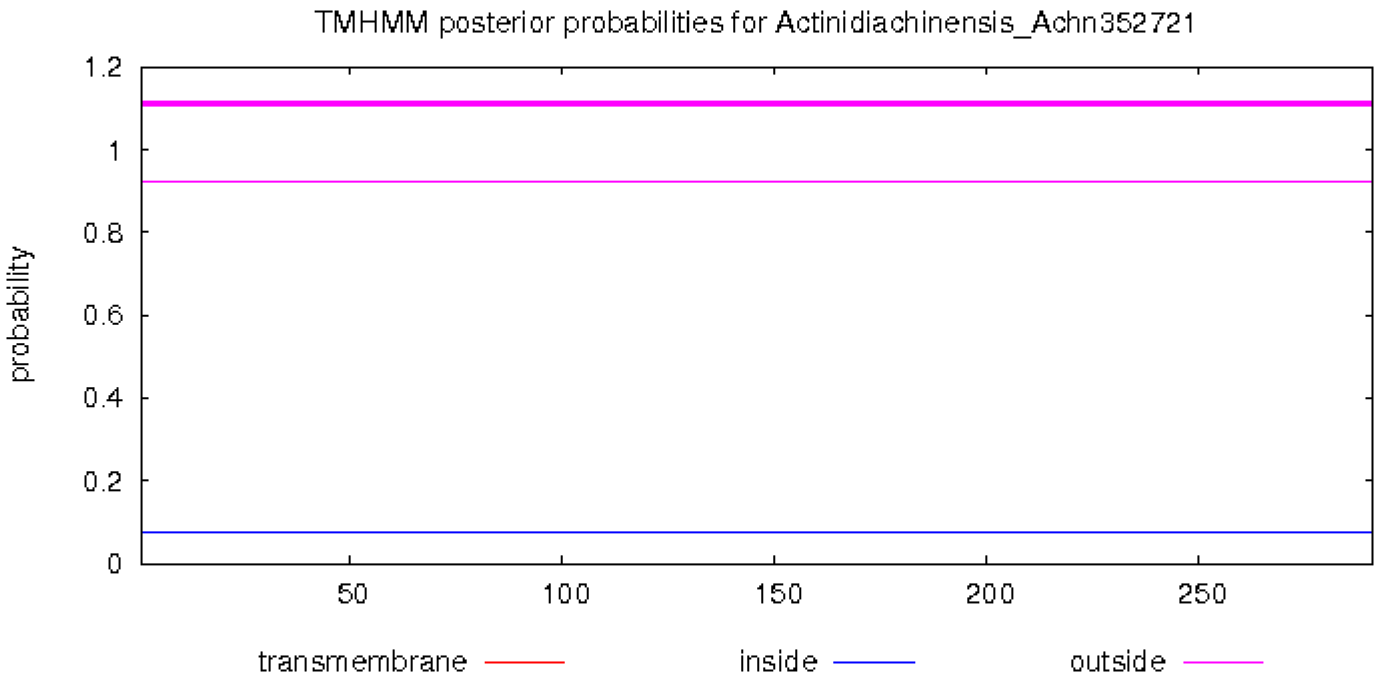

plot in postscript, script for making the plot in gnuplot, data for plot

---

```
# Actinidiachinensis_Achn354611 Length: 311
# Actinidiachinensis_Achn354611 Number of predicted TMHs:  0
# Actinidiachinensis_Achn354611 Exp number of AAs in TMHs: 0.00547
# Actinidiachinensis_Achn354611 Exp number, first 60 AAs:  0
# Actinidiachinensis_Achn354611 Total prob of N-in:        0.00658
Actinidiachinensis_Achn354611   TMHMM2.0        outside      1   311
```

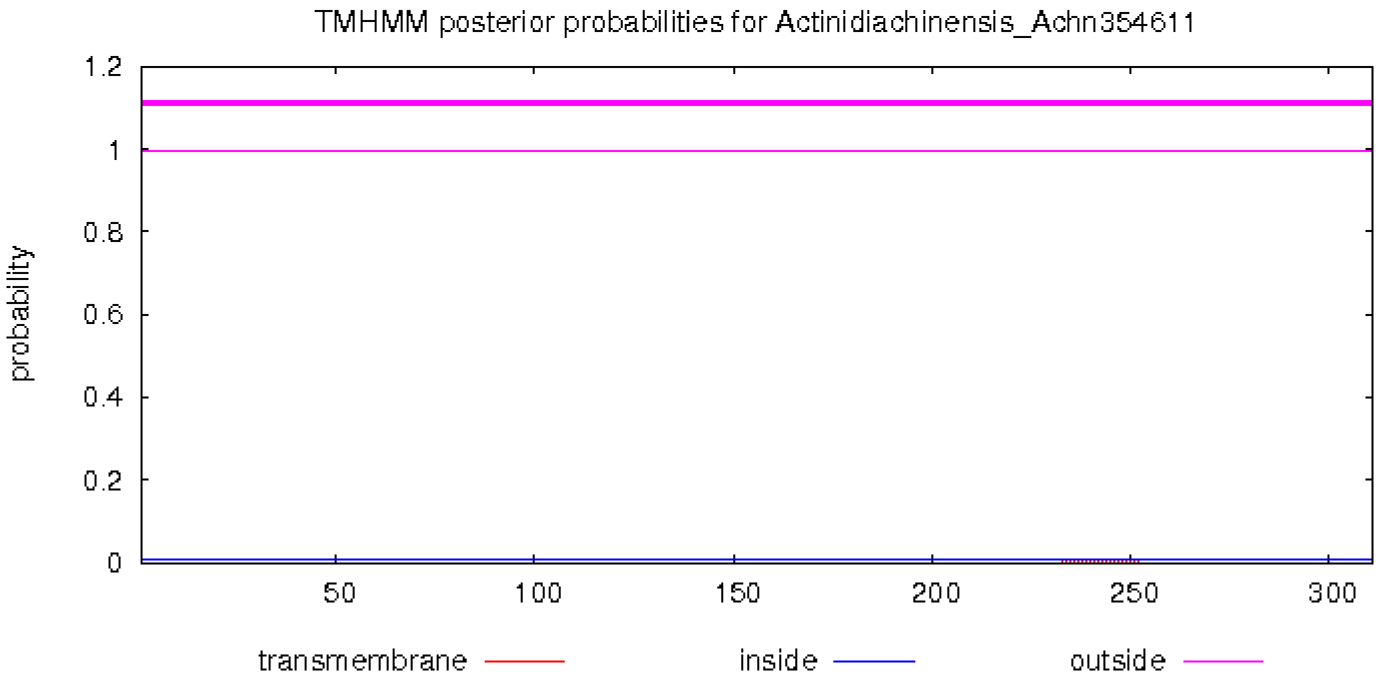

plot in postscript, script for making the plot in gnuplot, data for plot

---

```
# Actinidiachinensis_Achn359751 Length: 258
# Actinidiachinensis_Achn359751 Number of predicted TMHs:  0
# Actinidiachinensis_Achn359751 Exp number of AAs in TMHs: 0
# Actinidiachinensis_Achn359751 Exp number, first 60 AAs:  0
# Actinidiachinensis_Achn359751 Total prob of N-in:        0.01249
Actinidiachinensis_Achn359751   TMHMM2.0        outside      1   258
```

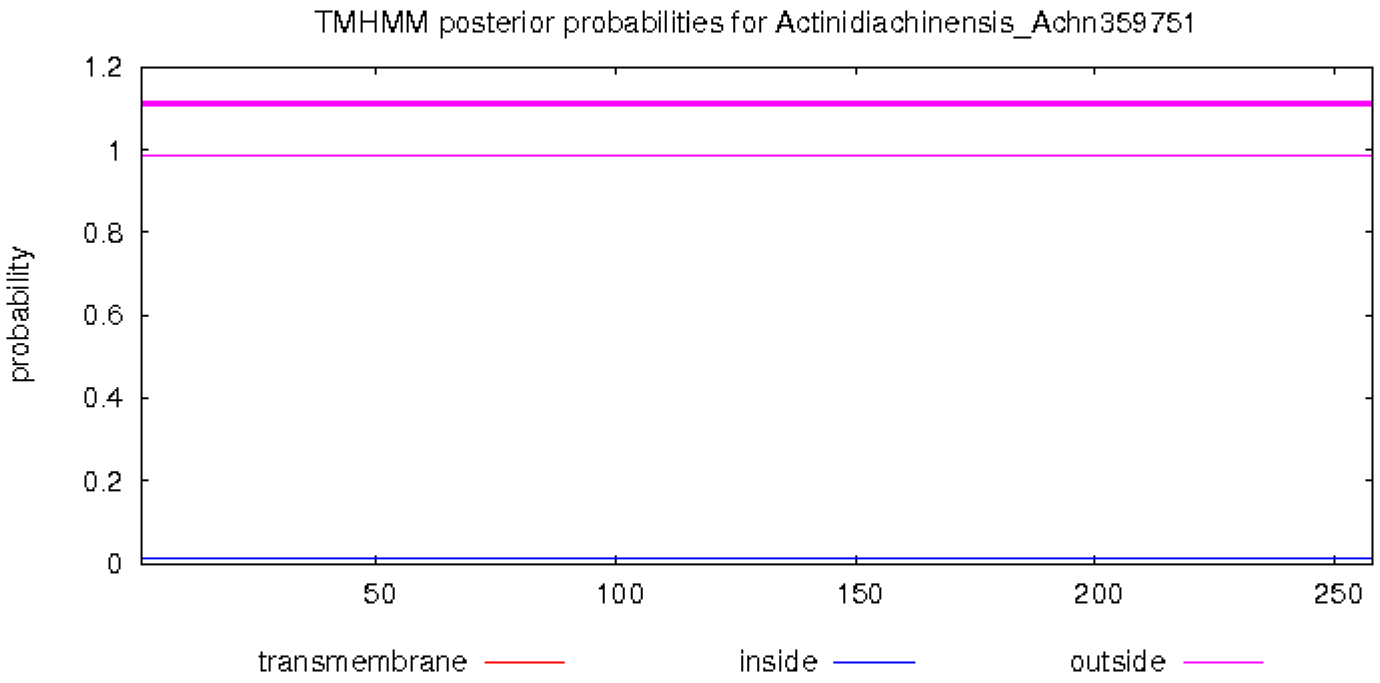

plot in postscript, script for making the plot in gnuplot, data for plot

---

```
# Actinidiachinensis_Achn363171 Length: 286
# Actinidiachinensis_Achn363171 Number of predicted TMHs:  0
# Actinidiachinensis_Achn363171 Exp number of AAs in TMHs: 0.0134
# Actinidiachinensis_Achn363171 Exp number, first 60 AAs:  0.01339
# Actinidiachinensis_Achn363171 Total prob of N-in:        0.01855
Actinidiachinensis_Achn363171	TMHMM2.0	outside	     1   286
```

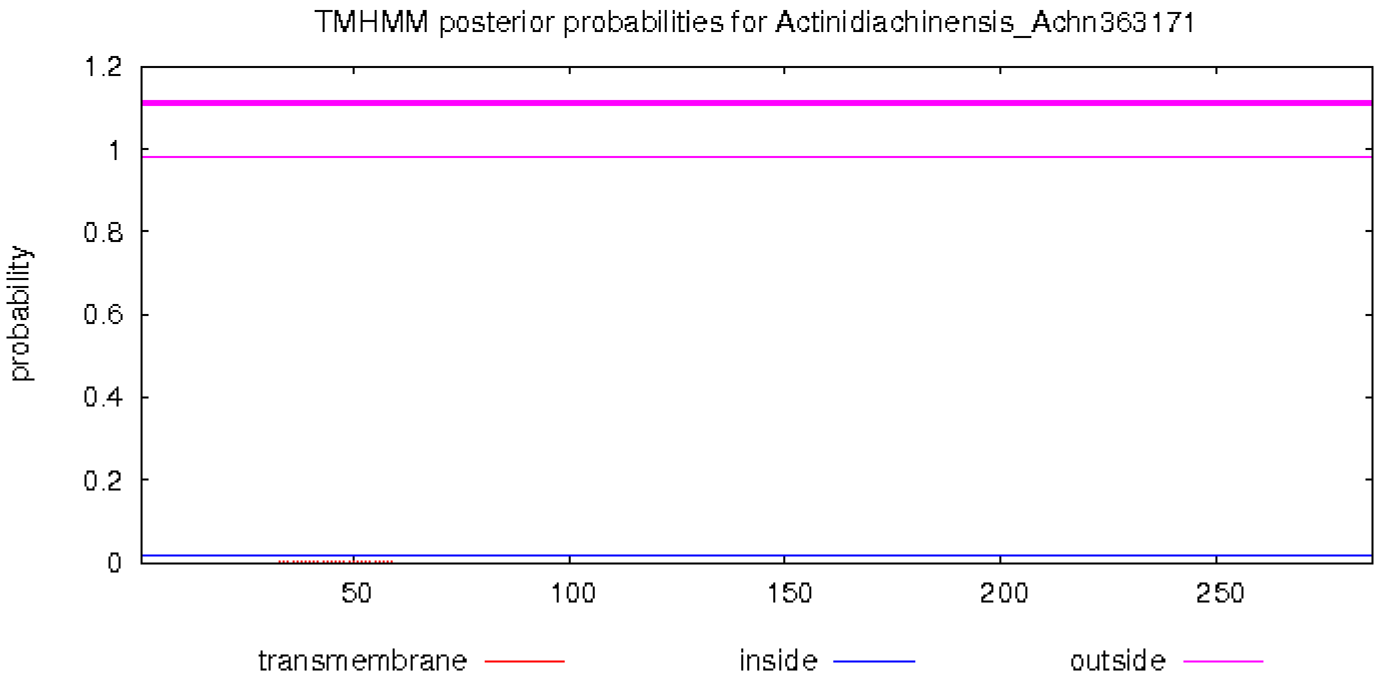

plot in postscript, script for making the plot in gnuplot, data for plot

---

```
# Actinidiachinensis_Achn365331 Length: 307
# Actinidiachinensis_Achn365331 Number of predicted TMHs:  0
# Actinidiachinensis_Achn365331 Exp number of AAs in TMHs: 0
# Actinidiachinensis_Achn365331 Exp number, first 60 AAs:  0
# Actinidiachinensis_Achn365331 Total prob of N-in:        0.00682
Actinidiachinensis_Achn365331	TMHMM2.0	outside	     1   307
```

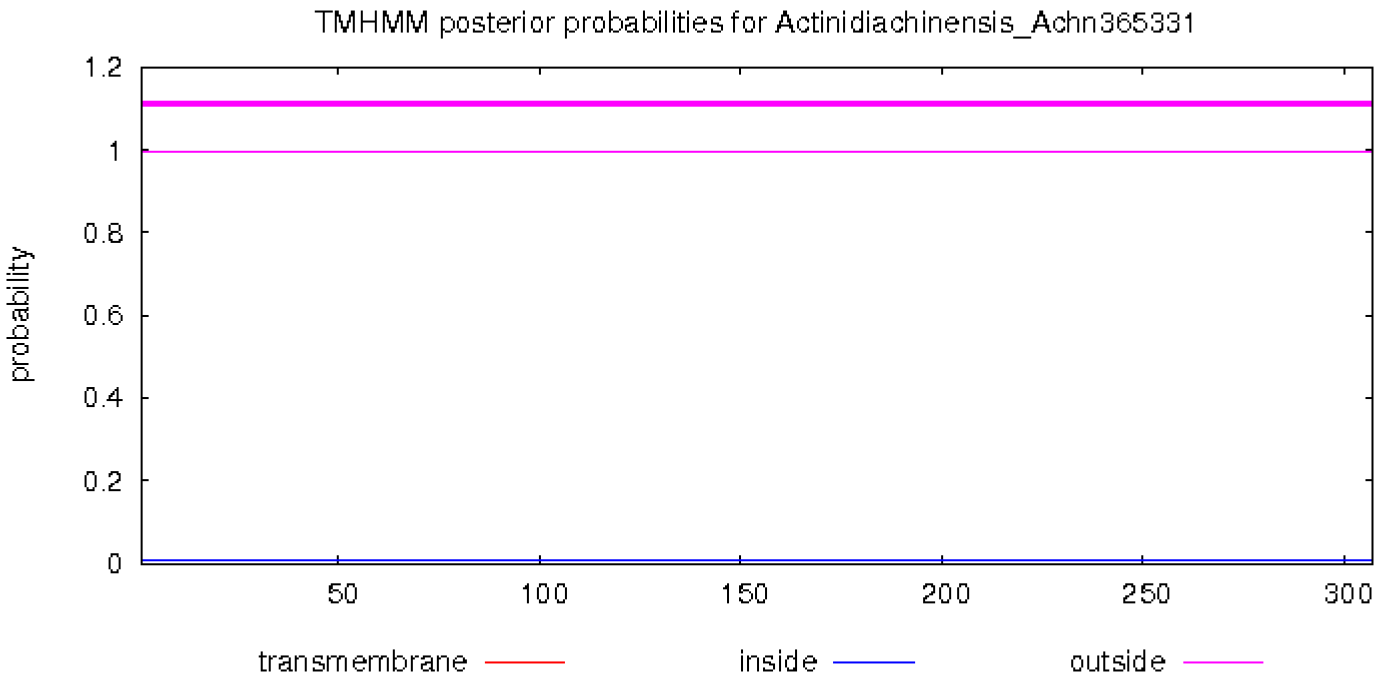

plot in postscript, script for making the plot in gnuplot, data for plot

---

```
# Actinidiachinensis_Achn367821 Length: 236
# Actinidiachinensis_Achn367821 Number of predicted TMHs:  0
# Actinidiachinensis_Achn367821 Exp number of AAs in TMHs: 0.00171
# Actinidiachinensis_Achn367821 Exp number, first 60 AAs:  0.00033
# Actinidiachinensis_Achn367821 Total prob of N-in:        0.11180
Actinidiachinensis_Achn367821   TMHMM2.0        outside      1   236
```

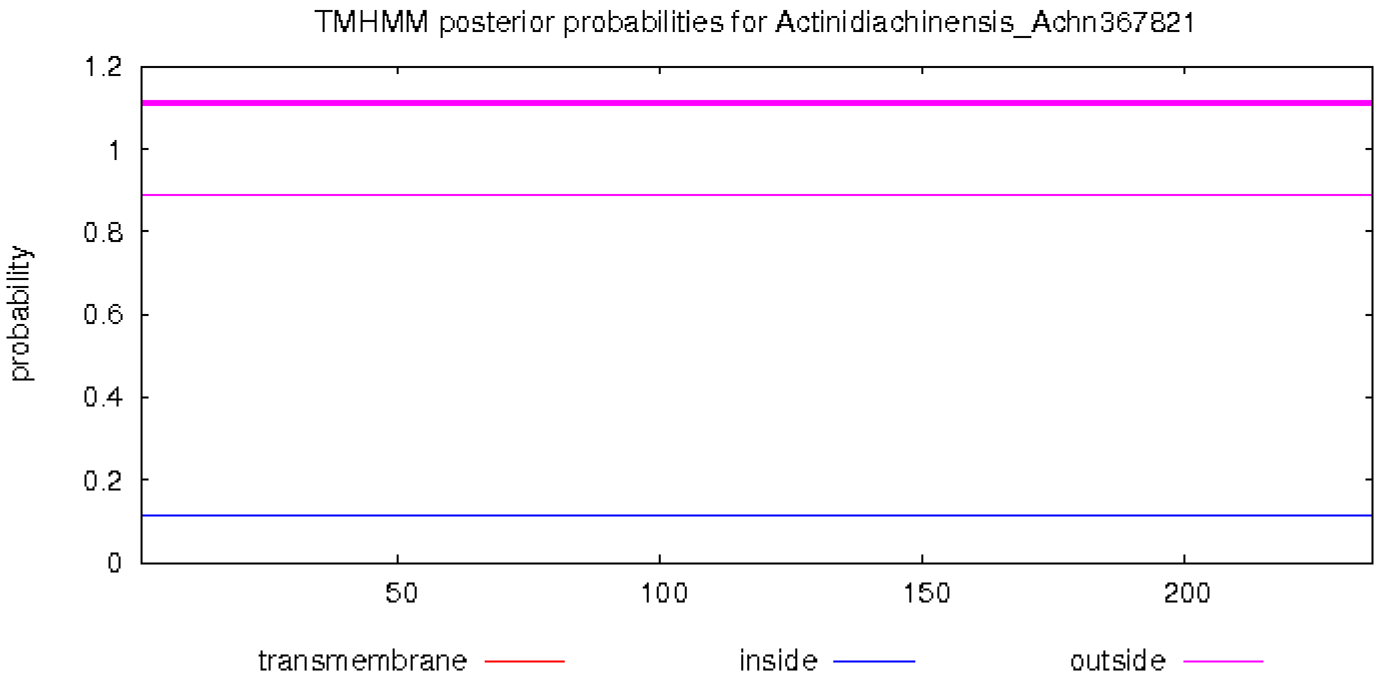

plot in postscript, script for making the plot in gnuplot, data for plot

```
# Actinidiachinensis_Achn374931 Length: 257
# Actinidiachinensis_Achn374931 Number of predicted TMHs:  0
# Actinidiachinensis_Achn374931 Exp number of AAs in TMHs: 0.00125
# Actinidiachinensis_Achn374931 Exp number, first 60 AAs:  0
# Actinidiachinensis_Achn374931 Total prob of N-in:        0.02724
Actinidiachinensis_Achn374931   TMHMM2.0        outside      1   257
```

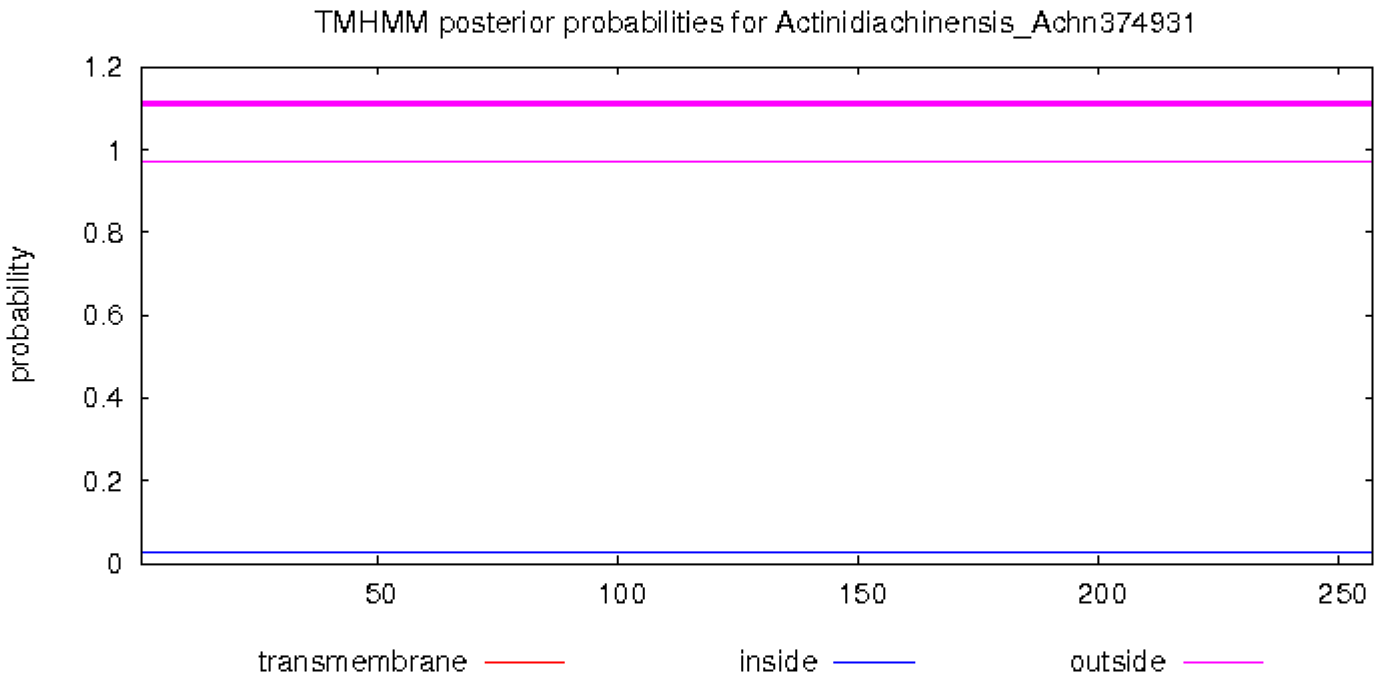

plot in postscript, script for making the plot in gnuplot, data for plot

```
# Actinidiachinensis_Achn377511 Length: 323
# Actinidiachinensis_Achn377511 Number of predicted TMHs:  0
# Actinidiachinensis_Achn377511 Exp number of AAs in TMHs: 0
# Actinidiachinensis_Achn377511 Exp number, first 60 AAs:  0
# Actinidiachinensis_Achn377511 Total prob of N-in:        0.02232
Actinidiachinensis_Achn377511	TMHMM2.0	outside	     1   323
```

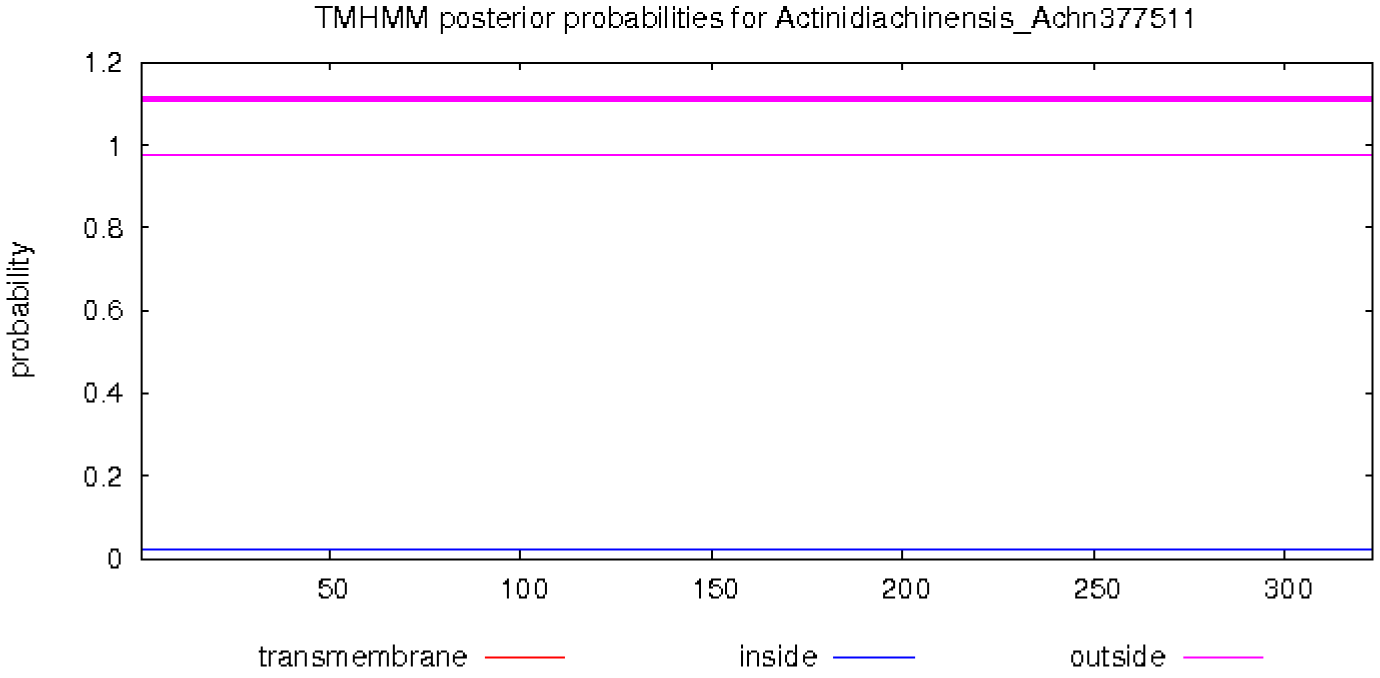

plot in postscript, script for making the plot in gnuplot, data for plot

---

```
# Actinidiachinensis_Achn382311 Length: 285
# Actinidiachinensis_Achn382311 Number of predicted TMHs:  0
# Actinidiachinensis_Achn382311 Exp number of AAs in TMHs: 0.03208
# Actinidiachinensis_Achn382311 Exp number, first 60 AAs:  0.03208
# Actinidiachinensis_Achn382311 Total prob of N-in:        0.01663
Actinidiachinensis_Achn382311	TMHMM2.0	outside	     1   285
```

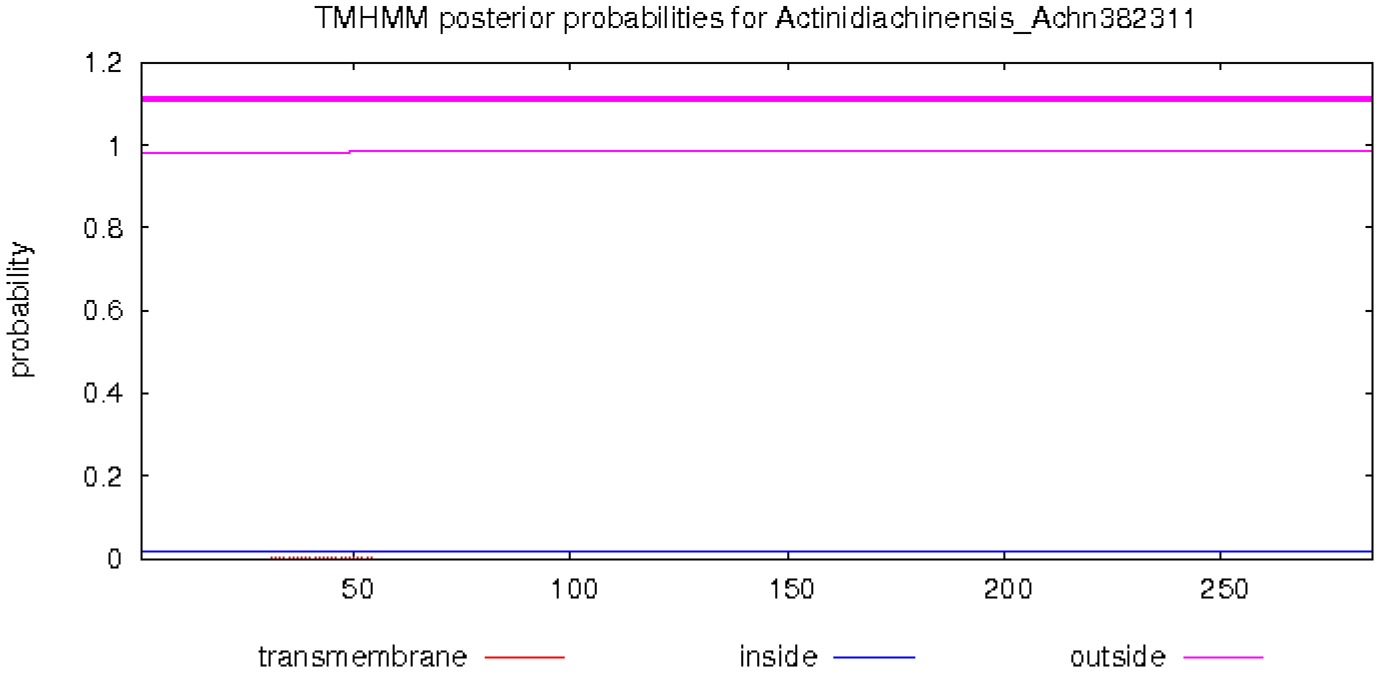

plot in postscript, script for making the plot in gnuplot, data for plot

---

```
# Actinidiachinensis_Achn385691 Length: 244
# Actinidiachinensis_Achn385691 Number of predicted TMHs:  0
# Actinidiachinensis_Achn385691 Exp number of AAs in TMHs: 0
# Actinidiachinensis_Achn385691 Exp number, first 60 AAs:  0
# Actinidiachinensis_Achn385691 Total prob of N-in:        0.11458
Actinidiachinensis_Achn385691   TMHMM2.0        outside      1   244
```

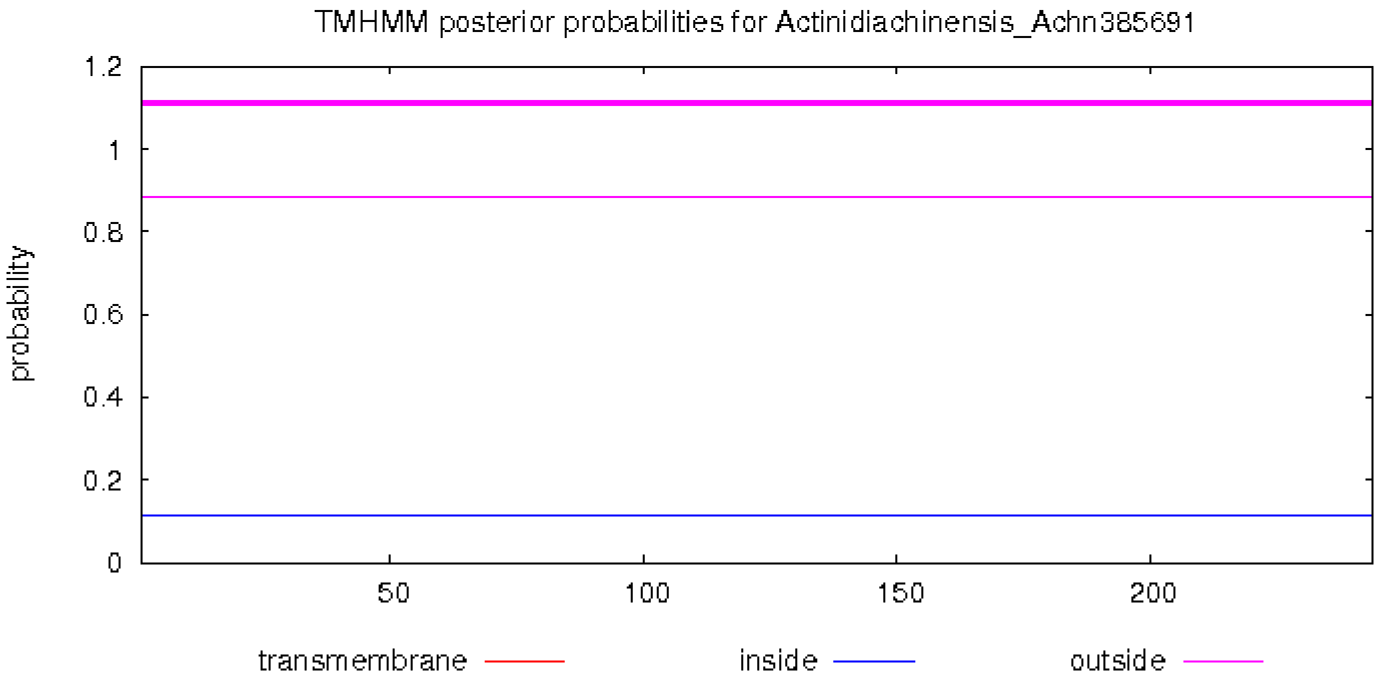

plot in postscript, script for making the plot in gnuplot, data for plot

---

```
# Actinidiachinensis_Achn388561 Length: 650
# Actinidiachinensis_Achn388561 Number of predicted TMHs:  0
# Actinidiachinensis_Achn388561 Exp number of AAs in TMHs: 34.80127
# Actinidiachinensis_Achn388561 Exp number, first 60 AAs:  0
# Actinidiachinensis_Achn388561 Total prob of N-in:        0.00023
Actinidiachinensis_Achn388561   TMHMM2.0        outside      1   650
```

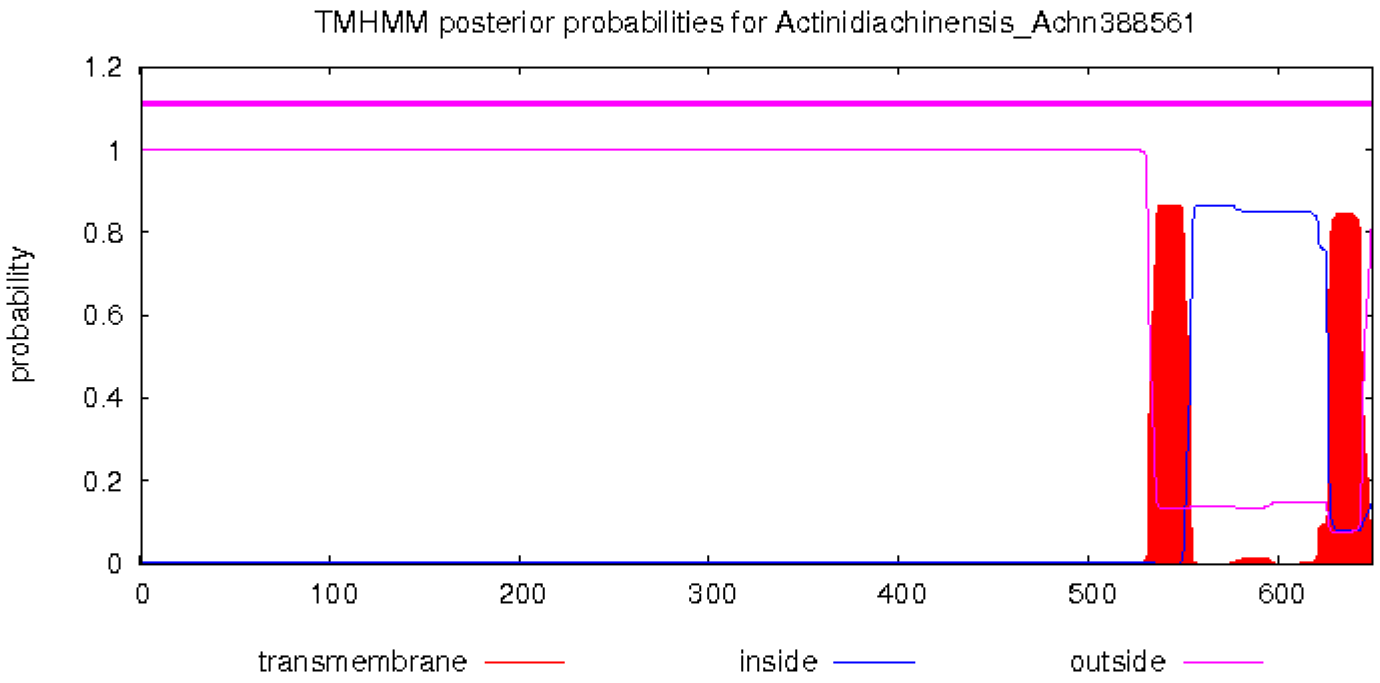

plot in postscript, script for making the plot in gnuplot, data for plot

---

```
# Actinidiachinensis_Achn389881 Length: 598
# Actinidiachinensis_Achn389881 Number of predicted TMHs:  0
# Actinidiachinensis_Achn389881 Exp number of AAs in TMHs: 0.01686
# Actinidiachinensis_Achn389881 Exp number, first 60 AAs:  0.00292
# Actinidiachinensis_Achn389881 Total prob of N-in:        0.00127
Actinidiachinensis_Achn389881   TMHMM2.0        outside      1   598
```

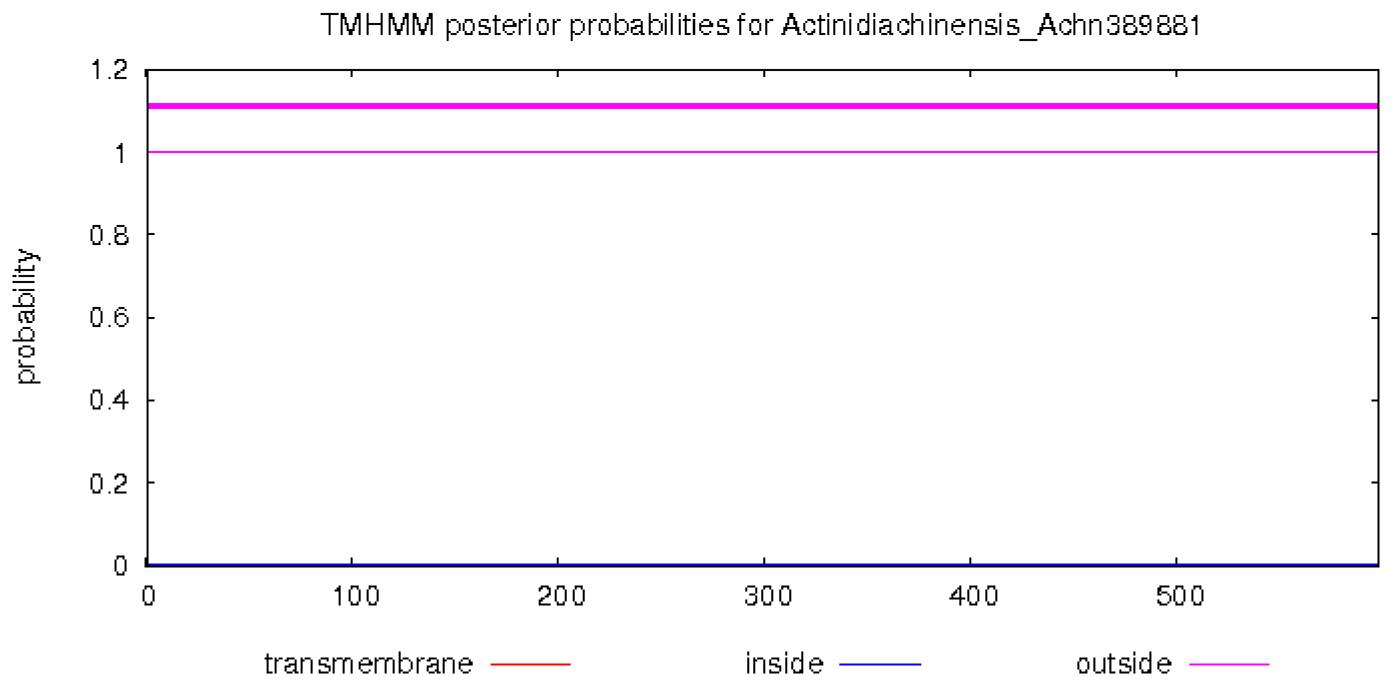

plot in postscript, script for making the plot in gnuplot, data for plot

```
# Aegilopstauschii_EMT00868 Length: 211
# Aegilopstauschii_EMT00868 Number of predicted TMHs:  0
# Aegilopstauschii_EMT00868 Exp number of AAs in TMHs: 0
# Aegilopstauschii_EMT00868 Exp number, first 60 AAs:  0
# Aegilopstauschii_EMT00868 Total prob of N-in:        0.04503
Aegilopstauschii_EMT00868       TMHMM2.0        outside      1   211
```

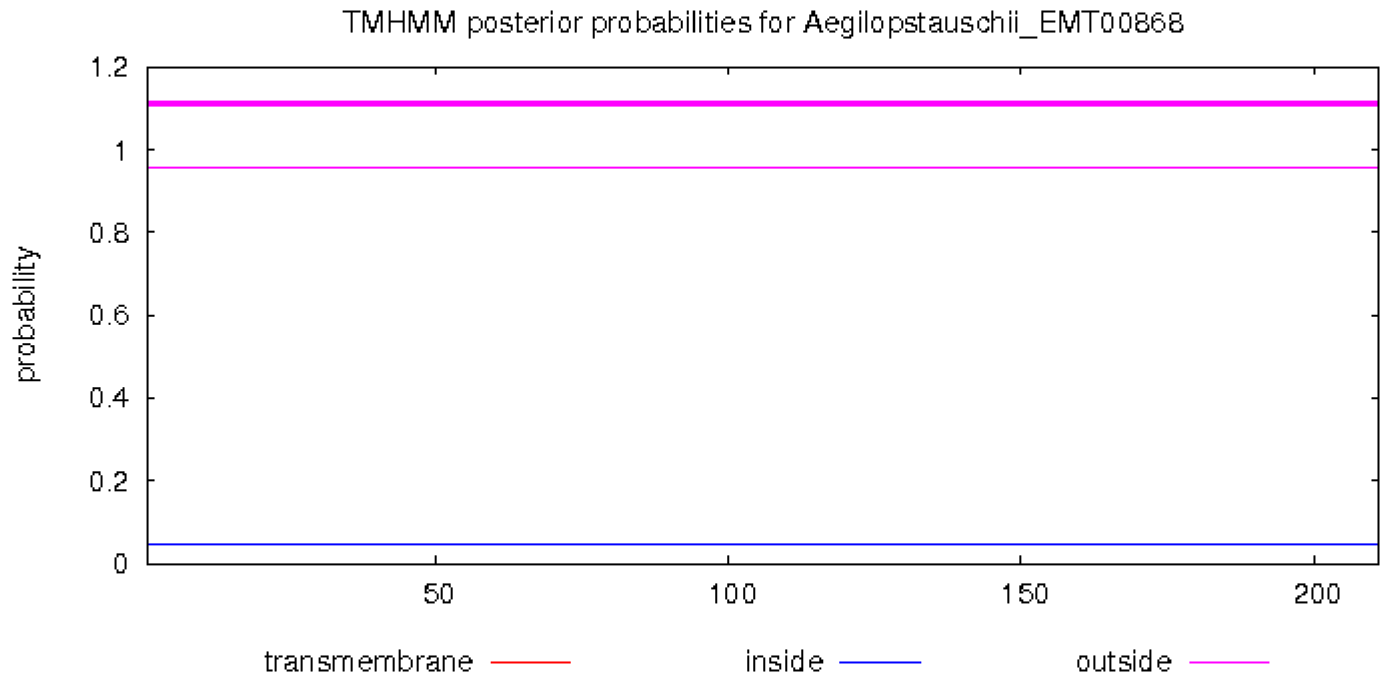

plot in postscript, script for making the plot in gnuplot, data for plot

```
# Aegilopstauschii_EMT01861 Length: 475
# Aegilopstauschii_EMT01861 Number of predicted TMHs:  0
# Aegilopstauschii_EMT01861 Exp number of AAs in TMHs: 0.02338
# Aegilopstauschii_EMT01861 Exp number, first 60 AAs:  0
# Aegilopstauschii_EMT01861 Total prob of N-in:        0.00671
Aegilopstauschii_EMT01861       TMHMM2.0        outside      1   475
```

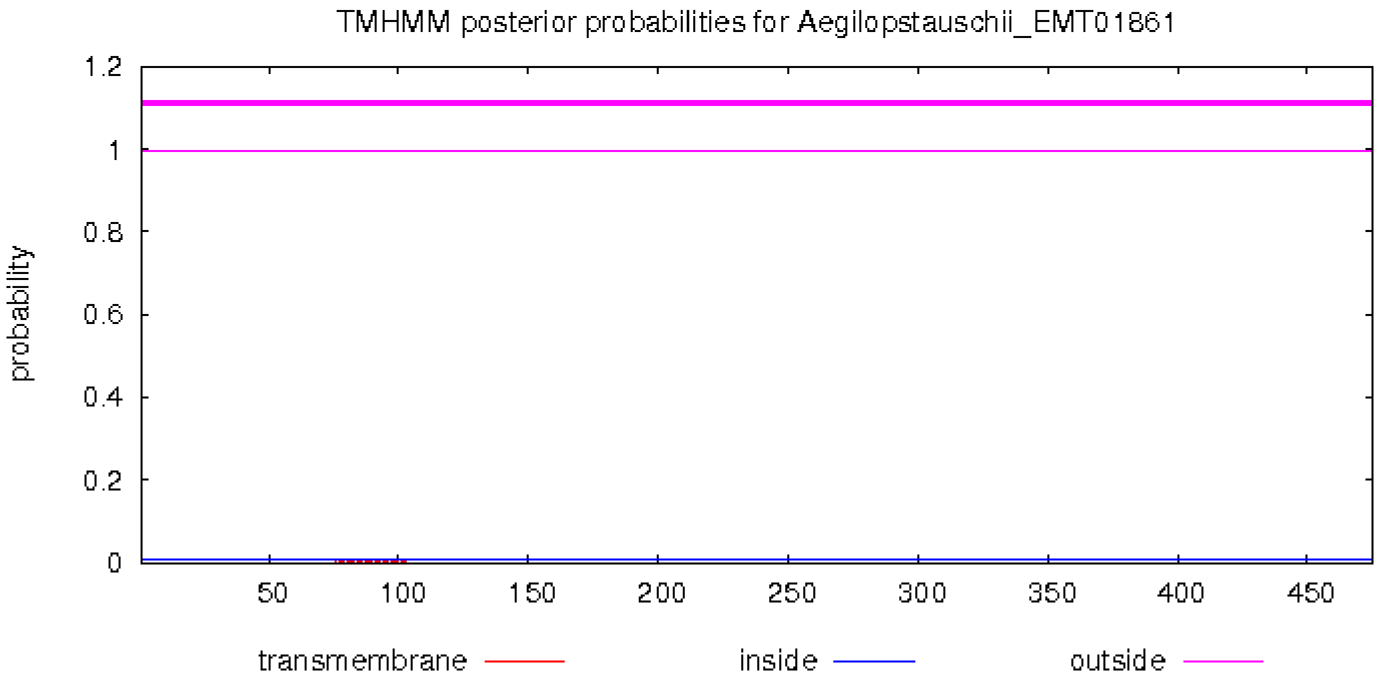

plot in postscript, script for making the plot in gnuplot, data for plot

---

```
# Aegilopstauschii_EMT01908 Length: 271
# Aegilopstauschii_EMT01908 Number of predicted TMHs:  0
# Aegilopstauschii_EMT01908 Exp number of AAs in TMHs: 0.00034
# Aegilopstauschii_EMT01908 Exp number, first 60 AAs:  0.00034
# Aegilopstauschii_EMT01908 Total prob of N-in:        0.00326
Aegilopstauschii_EMT01908       TMHMM2.0        outside      1   271
```

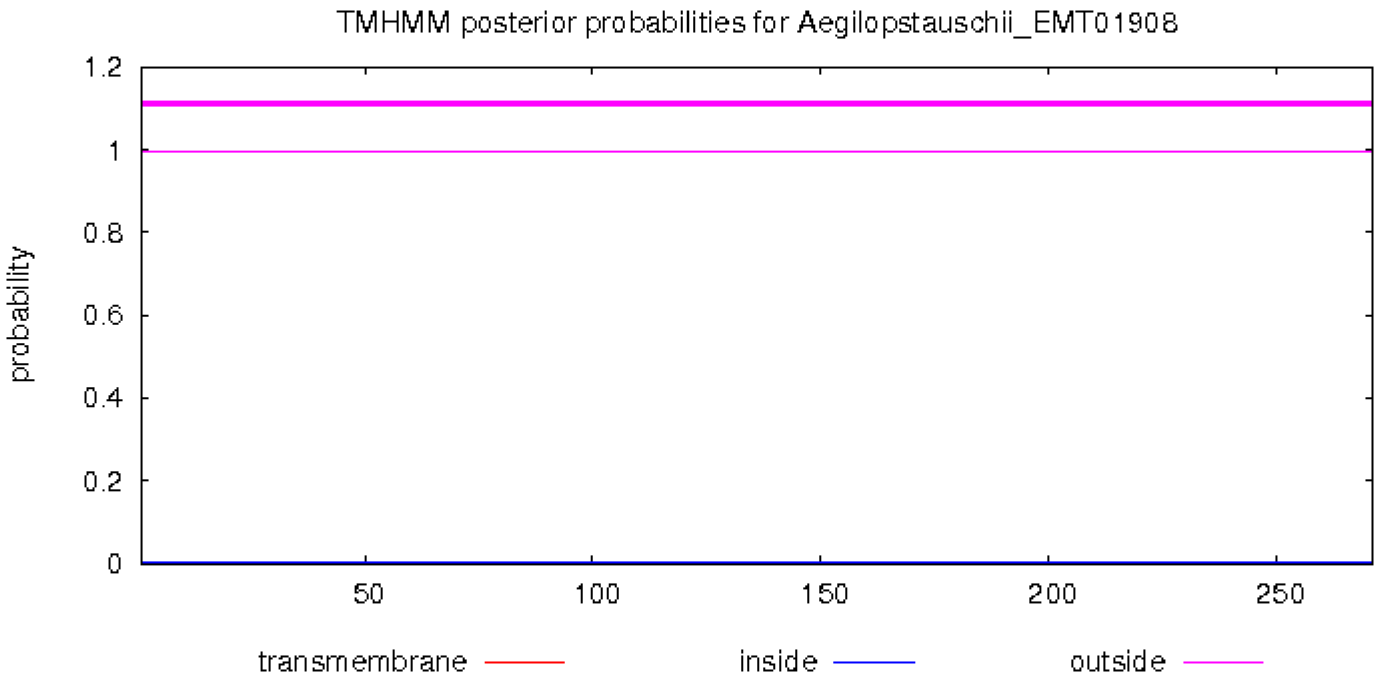

plot in postscript, script for making the plot in gnuplot, data for plot

---

```
# Aegilopstauschii_EMT01994 Length: 308
# Aegilopstauschii_EMT01994 Number of predicted TMHs:  0
# Aegilopstauschii_EMT01994 Exp number of AAs in TMHs: 0.27169
# Aegilopstauschii_EMT01994 Exp number, first 60 AAs:  0.27061
# Aegilopstauschii_EMT01994 Total prob of N-in:        0.02720
Aegilopstauschii_EMT01994	TMHMM2.0	outside	     1   308
```

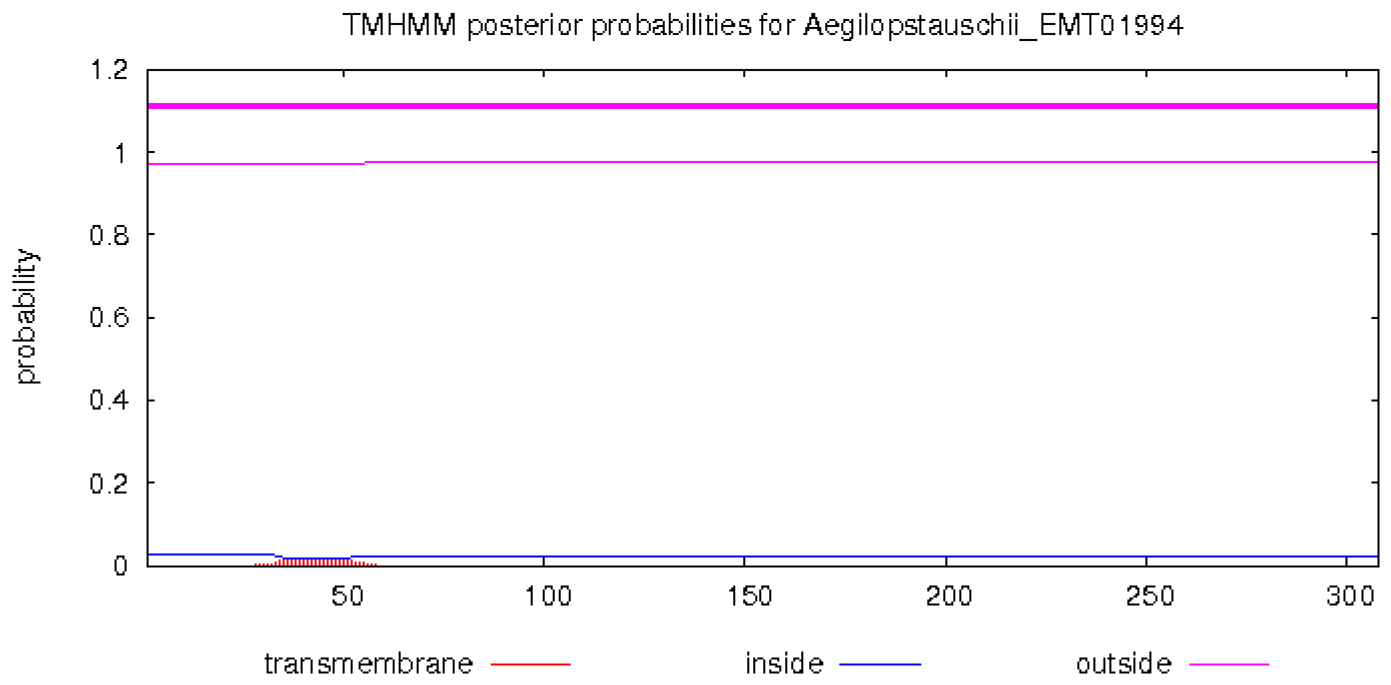

plot in postscript, script for making the plot in gnuplot, data for plot

---

```
# Aegilopstauschii_EMT02241 Length: 228
# Aegilopstauschii_EMT02241 Number of predicted TMHs:  0
# Aegilopstauschii_EMT02241 Exp number of AAs in TMHs: 0
# Aegilopstauschii_EMT02241 Exp number, first 60 AAs:  0
# Aegilopstauschii_EMT02241 Total prob of N-in:        0.00399
Aegilopstauschii_EMT02241	TMHMM2.0	outside	     1   228
```

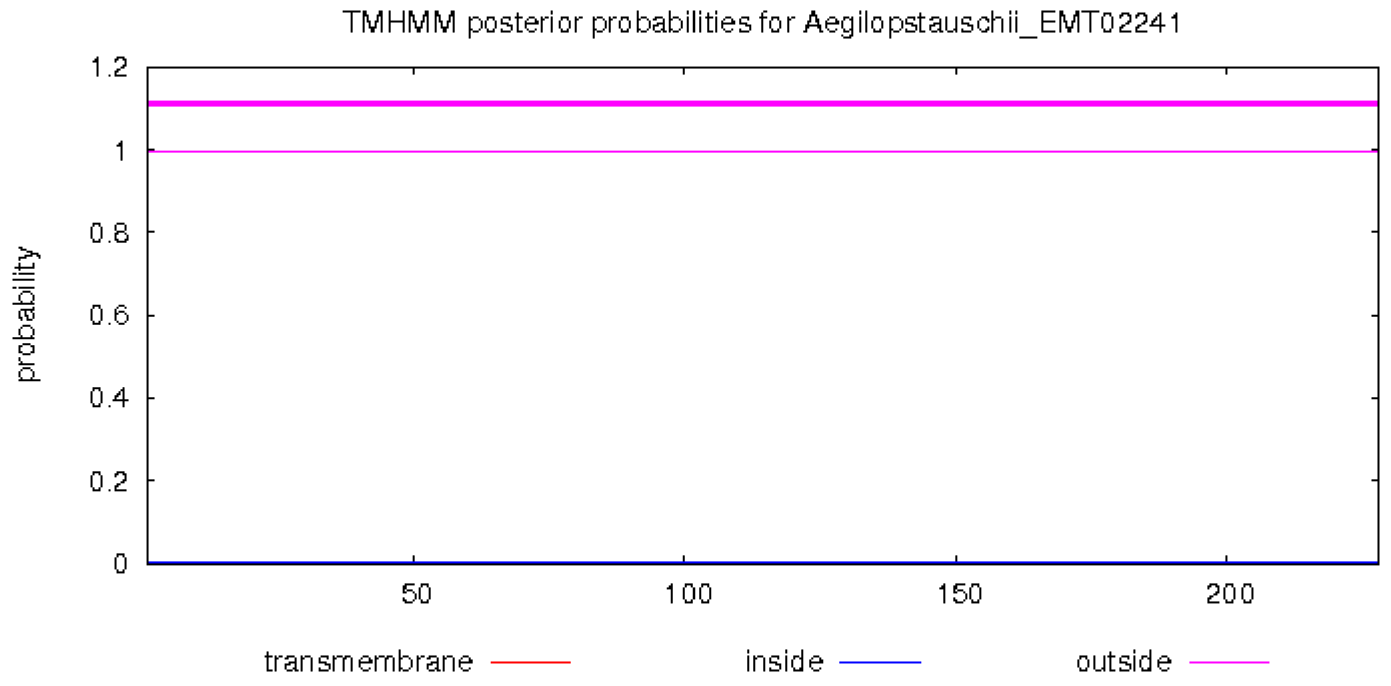

plot in postscript, script for making the plot in gnuplot, data for plot

---

```
# Aegilopstauschii_EMT02809 Length: 290
# Aegilopstauschii_EMT02809 Number of predicted TMHs:  0
# Aegilopstauschii_EMT02809 Exp number of AAs in TMHs: 0.00349
# Aegilopstauschii_EMT02809 Exp number, first 60 AAs:  0.00137
# Aegilopstauschii_EMT02809 Total prob of N-in:        0.17434
Aegilopstauschii_EMT02809	TMHMM2.0	outside	     1   290
```

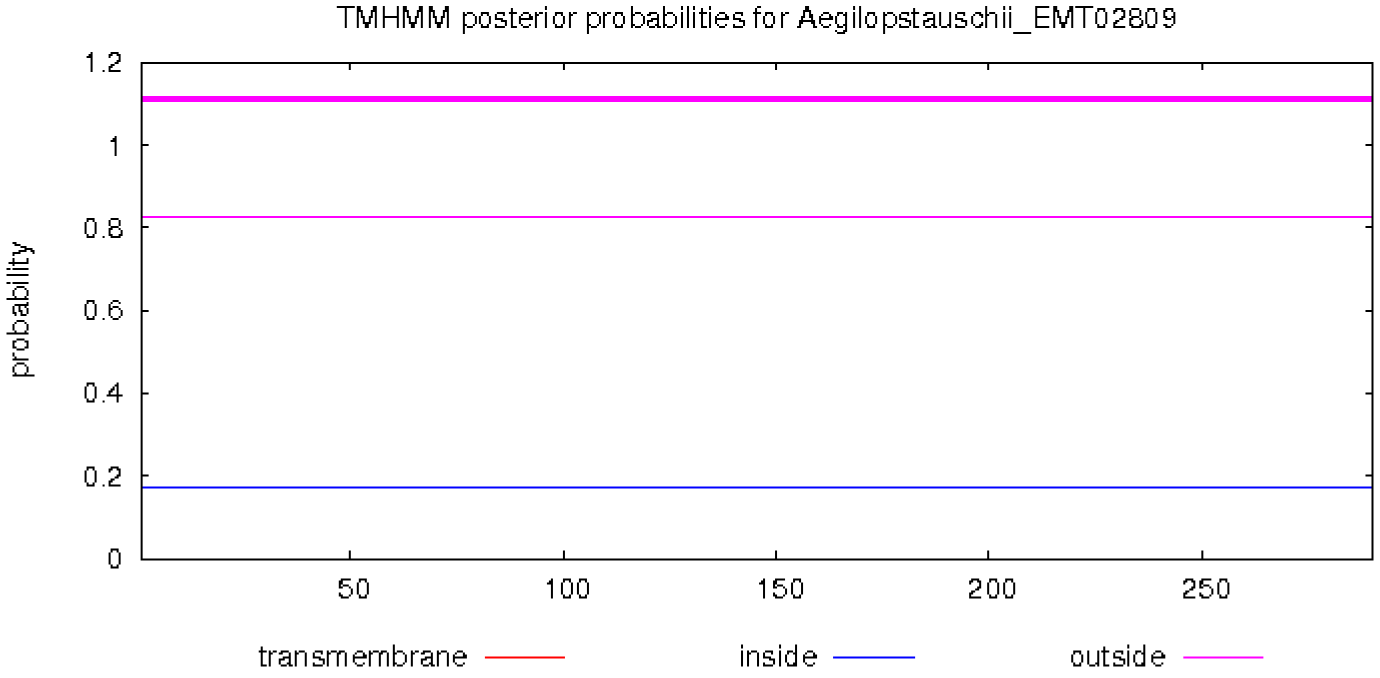

plot in postscript, script for making the plot in gnuplot, data for plot

```
# Aegilopstauschii_EMT03210 Length: 412
# Aegilopstauschii_EMT03210 Number of predicted TMHs:  0
# Aegilopstauschii_EMT03210 Exp number of AAs in TMHs: 0.04757
# Aegilopstauschii_EMT03210 Exp number, first 60 AAs:  0.02705
# Aegilopstauschii_EMT03210 Total prob of N-in:        0.00202
Aegilopstauschii_EMT03210	TMHMM2.0	outside	     1   412
```

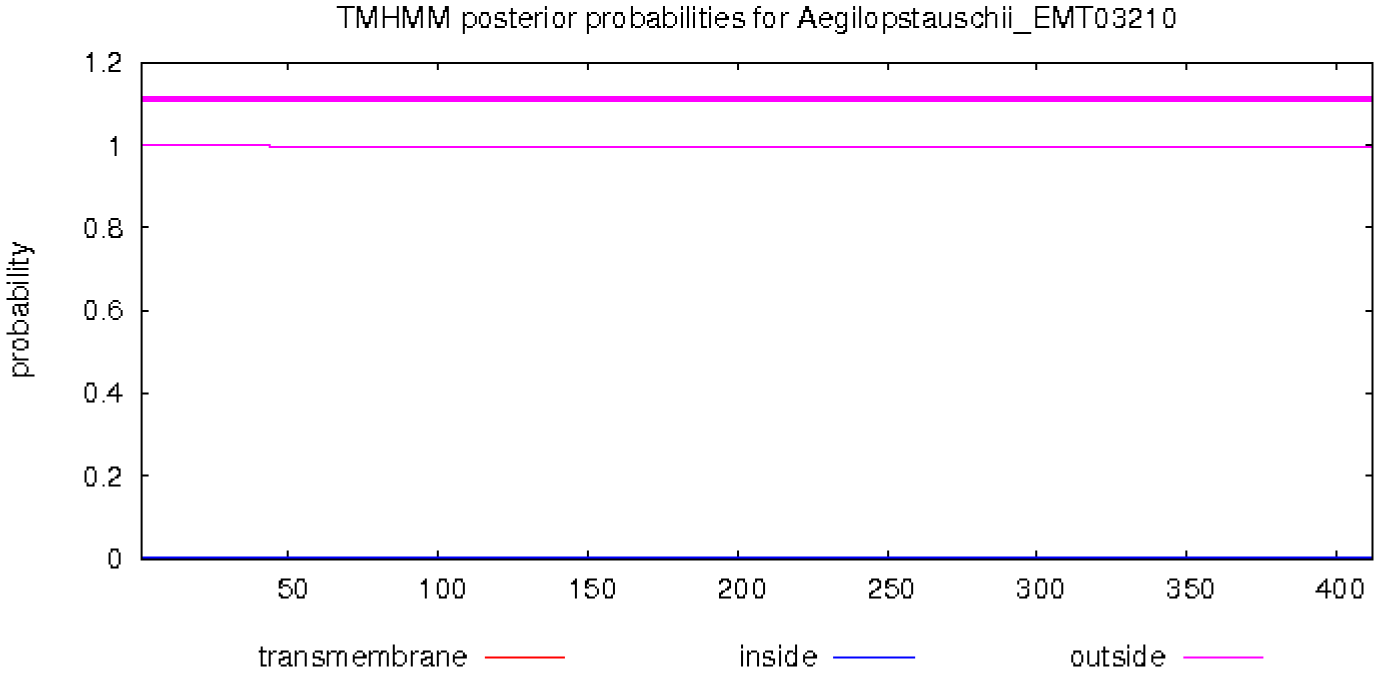

plot in postscript, script for making the plot in gnuplot, data for plot

```
# Aegilopstauschii_EMT03350 Length: 239
# Aegilopstauschii_EMT03350 Number of predicted TMHs:  0
# Aegilopstauschii_EMT03350 Exp number of AAs in TMHs: 0.01656
# Aegilopstauschii_EMT03350 Exp number, first 60 AAs:  0
# Aegilopstauschii_EMT03350 Total prob of N-in:        0.45585
Aegilopstauschii_EMT03350       TMHMM2.0        outside      1   239
```

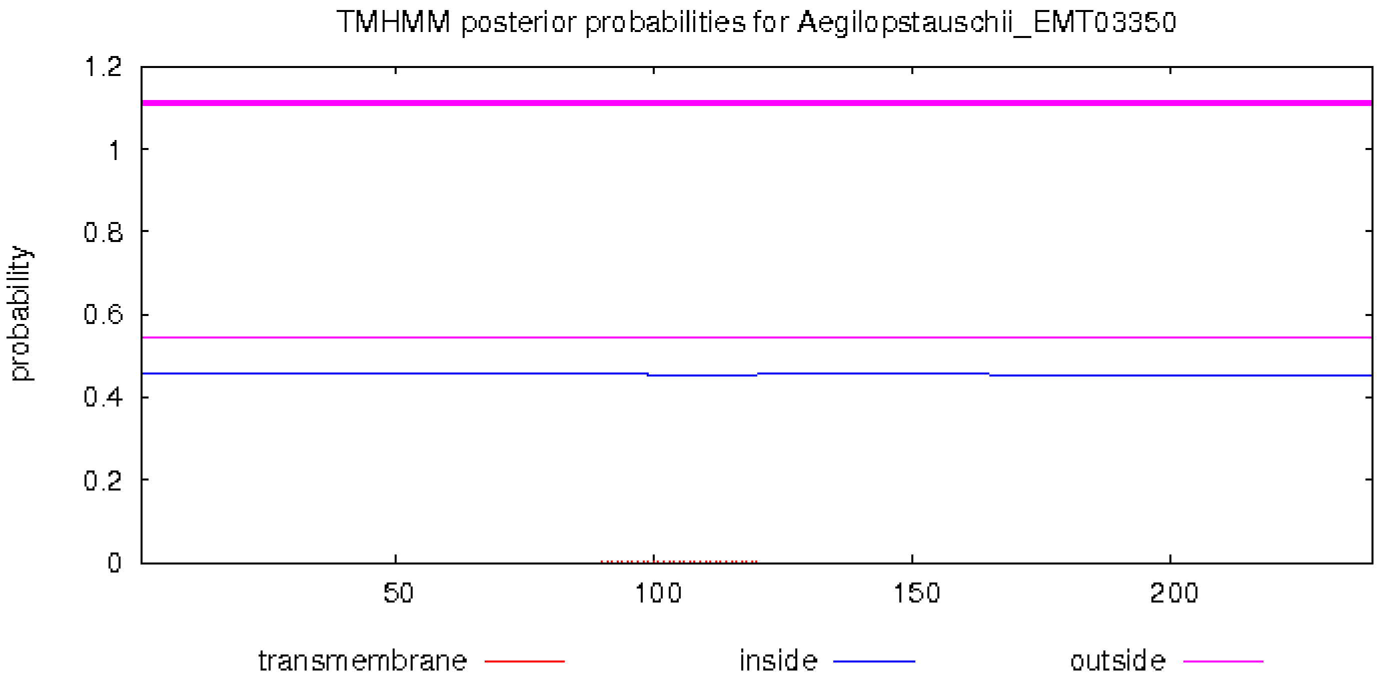

plot in postscript, script for making the plot in gnuplot, data for plot

---

```
# Aegilopstauschii_EMT03425 Length: 514
# Aegilopstauschii_EMT03425 Number of predicted TMHs:  0
# Aegilopstauschii_EMT03425 Exp number of AAs in TMHs: 0.01834
# Aegilopstauschii_EMT03425 Exp number, first 60 AAs:  0.00321
# Aegilopstauschii_EMT03425 Total prob of N-in:        0.00471
Aegilopstauschii_EMT03425       TMHMM2.0        outside      1   514
```

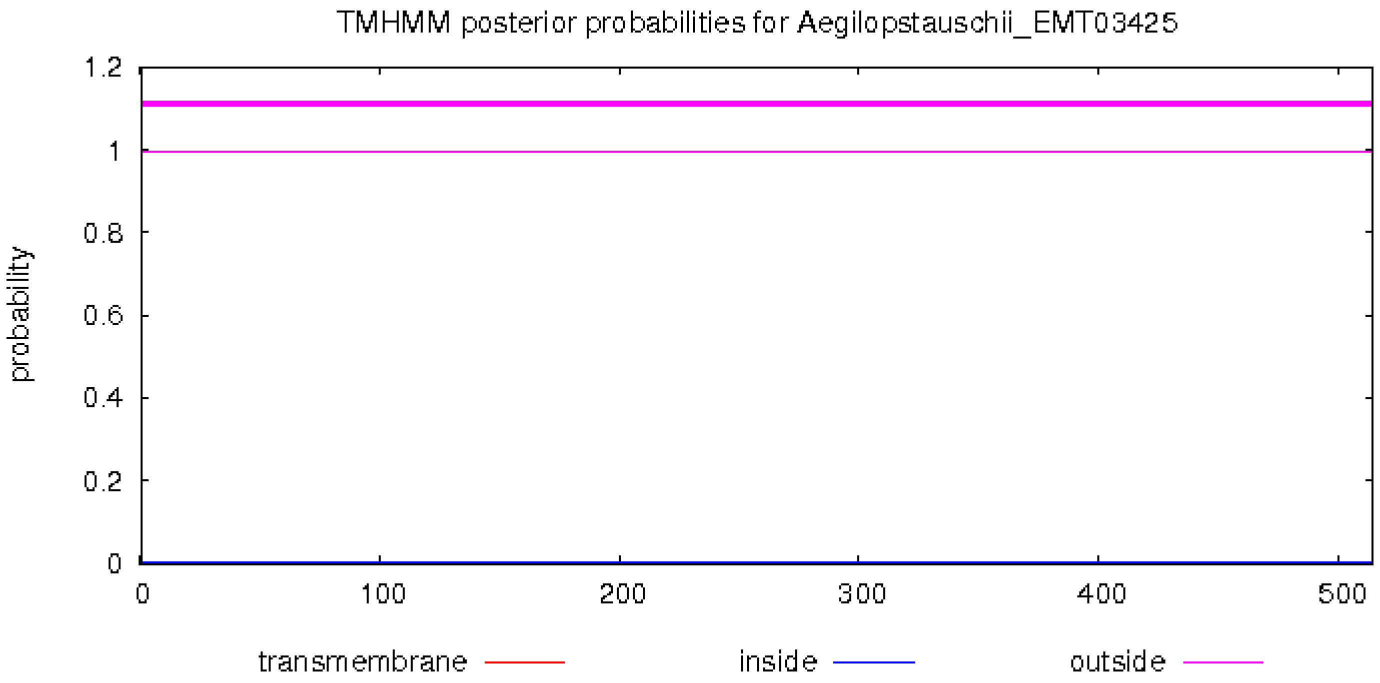

plot in postscript, script for making the plot in gnuplot, data for plot

---

```
# Aegilopstauschii_EMT03563 Length: 387
# Aegilopstauschii_EMT03563 Number of predicted TMHs:  0
# Aegilopstauschii_EMT03563 Exp number of AAs in TMHs: 0.00172
# Aegilopstauschii_EMT03563 Exp number, first 60 AAs:  0
# Aegilopstauschii_EMT03563 Total prob of N-in:        0.02992
Aegilopstauschii_EMT03563       TMHMM2.0        outside      1   387
```

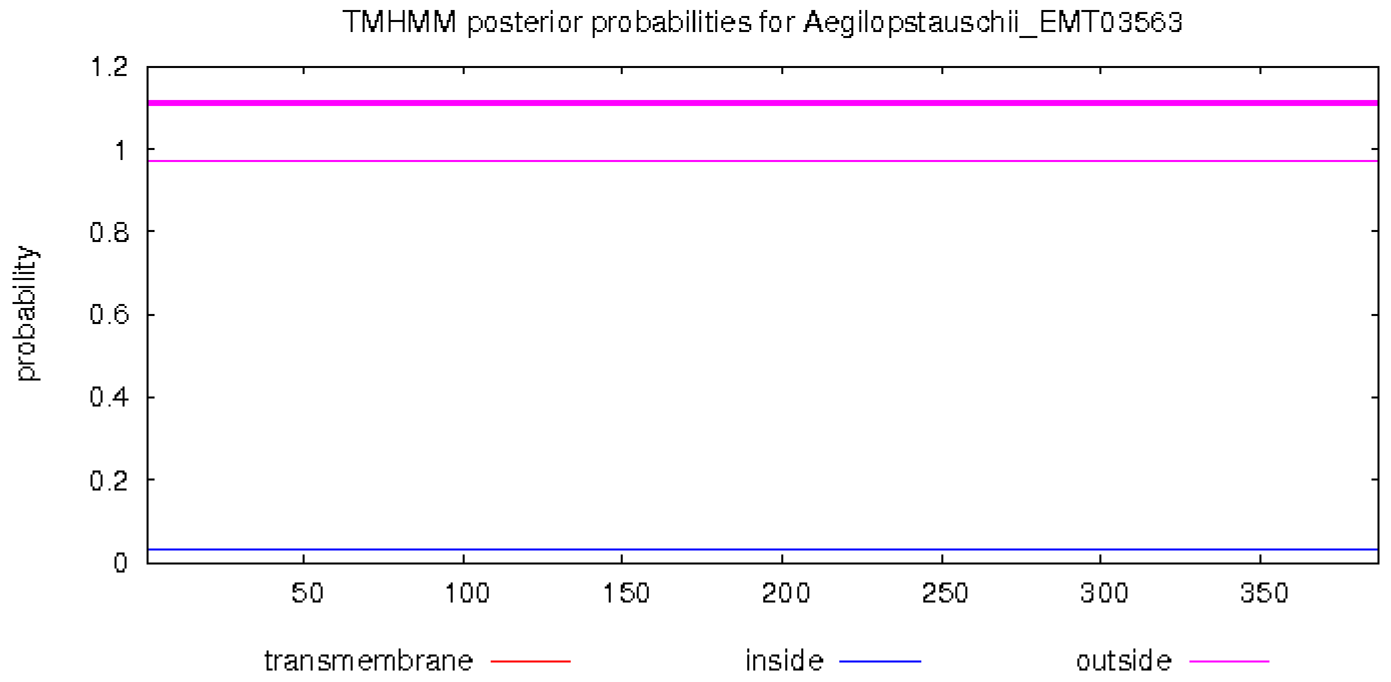

plot in postscript, script for making the plot in gnuplot, data for plot

```
# Aegilopstauschii_EMT03564 Length: 360
# Aegilopstauschii_EMT03564 Number of predicted TMHs:  0
# Aegilopstauschii_EMT03564 Exp number of AAs in TMHs: 0.00031
# Aegilopstauschii_EMT03564 Exp number, first 60 AAs:  0
# Aegilopstauschii_EMT03564 Total prob of N-in:        0.02522
Aegilopstauschii_EMT03564       TMHMM2.0        outside      1   360
```

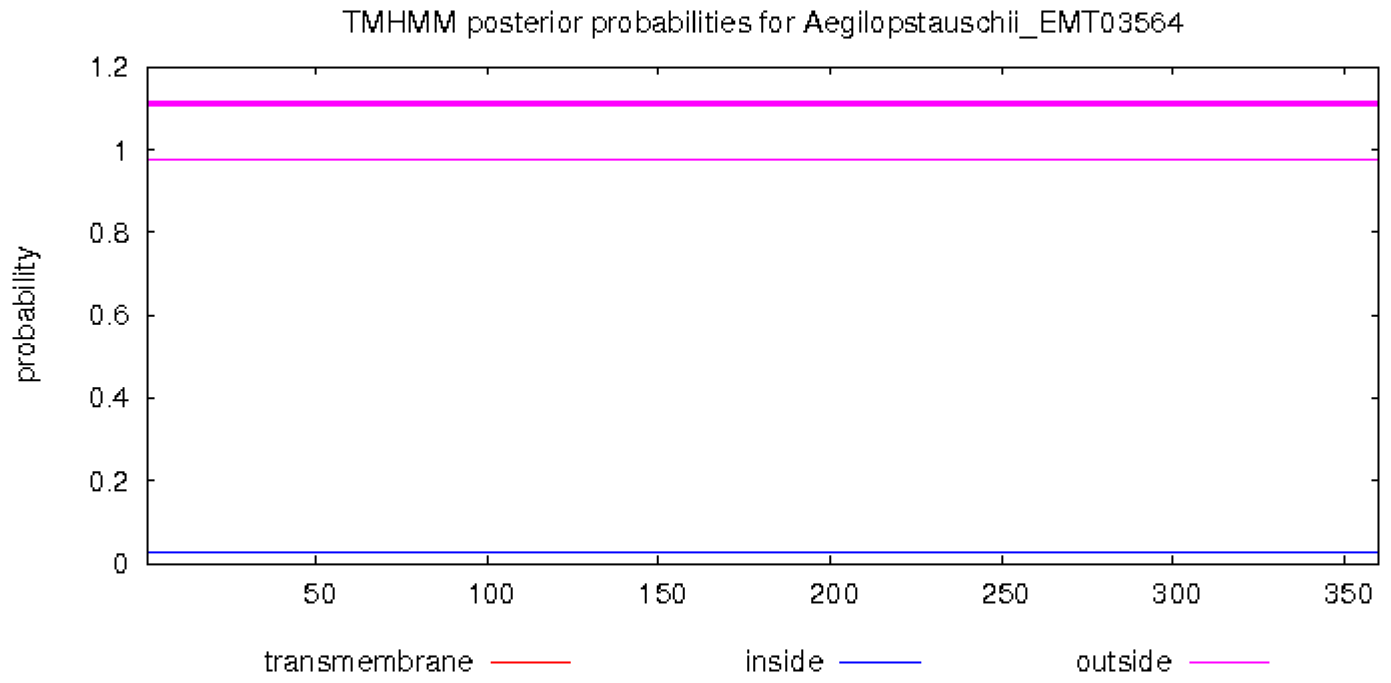

plot in postscript, script for making the plot in gnuplot, data for plot

```
# Aegilopstauschii_EMT03885 Length: 267
# Aegilopstauschii_EMT03885 Number of predicted TMHs:  0
# Aegilopstauschii_EMT03885 Exp number of AAs in TMHs: 0.0002
# Aegilopstauschii_EMT03885 Exp number, first 60 AAs:  0.0002
# Aegilopstauschii_EMT03885 Total prob of N-in:        0.04902
Aegilopstauschii_EMT03885	TMHMM2.0	outside	     1   267
```

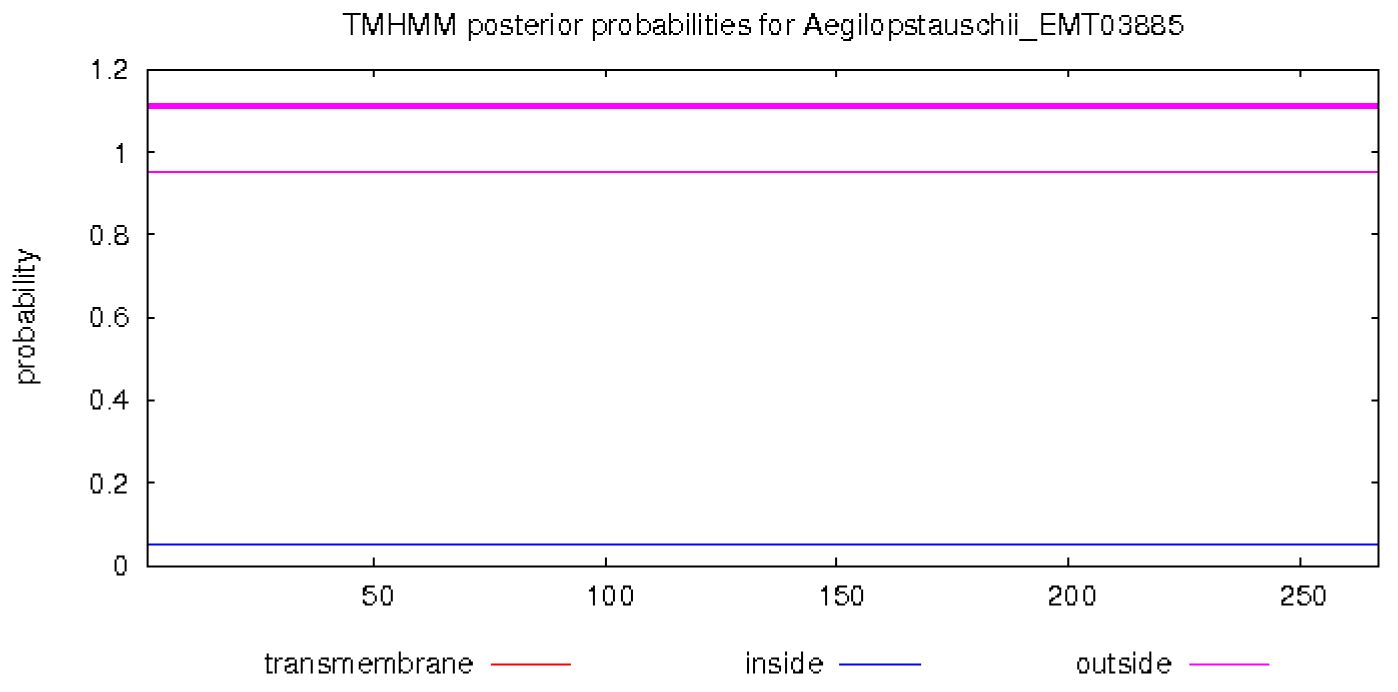

plot in postscript, script for making the plot in gnuplot, data for plot

---

```
# Aegilopstauschii_EMT05112 Length: 206
# Aegilopstauschii_EMT05112 Number of predicted TMHs:  0
# Aegilopstauschii_EMT05112 Exp number of AAs in TMHs: 0.001
# Aegilopstauschii_EMT05112 Exp number, first 60 AAs:  0.0004
# Aegilopstauschii_EMT05112 Total prob of N-in:        0.01851
Aegilopstauschii_EMT05112	TMHMM2.0	outside	     1   206
```

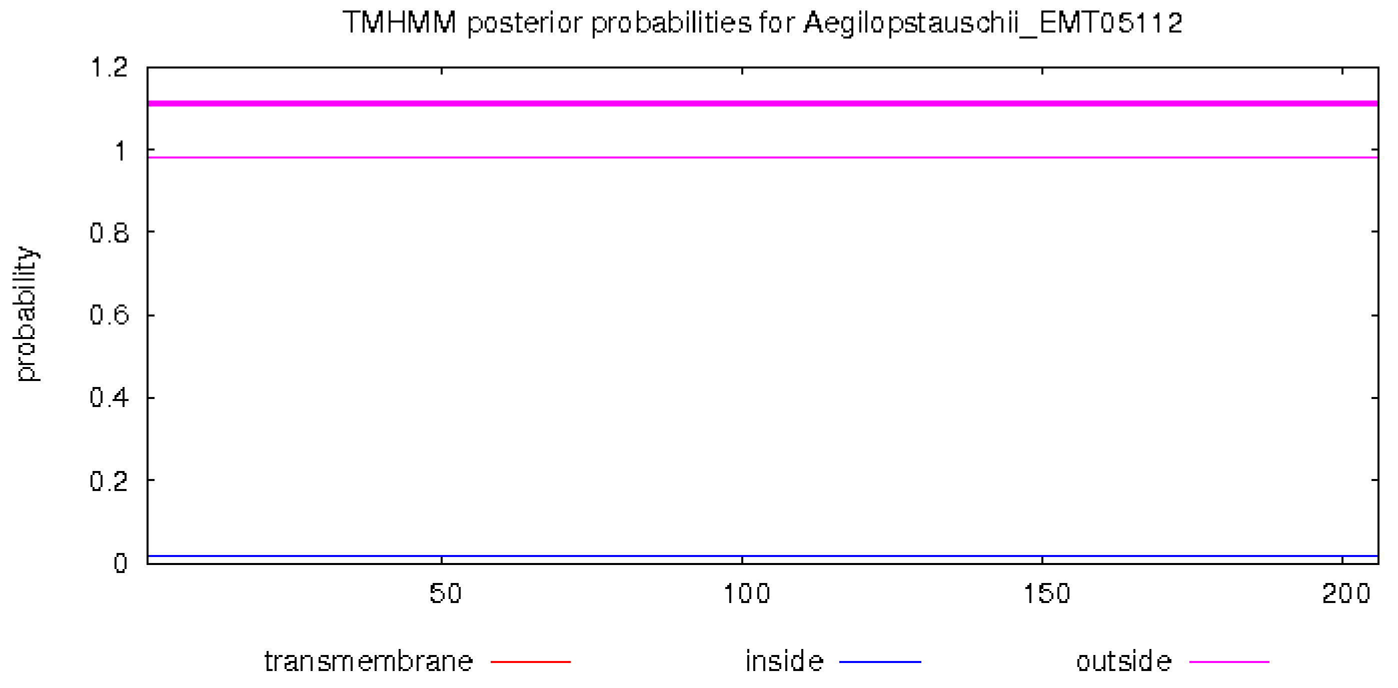

plot in postscript, script for making the plot in gnuplot, data for plot

---

```
# Aegilopstauschii_EMT05113 Length: 58
# Aegilopstauschii_EMT05113 Number of predicted TMHs:  0
# Aegilopstauschii_EMT05113 Exp number of AAs in TMHs: 0
# Aegilopstauschii_EMT05113 Exp number, first 60 AAs:  0
# Aegilopstauschii_EMT05113 Total prob of N-in:        0.06670
Aegilopstauschii_EMT05113	TMHMM2.0	outside	     1    58
```

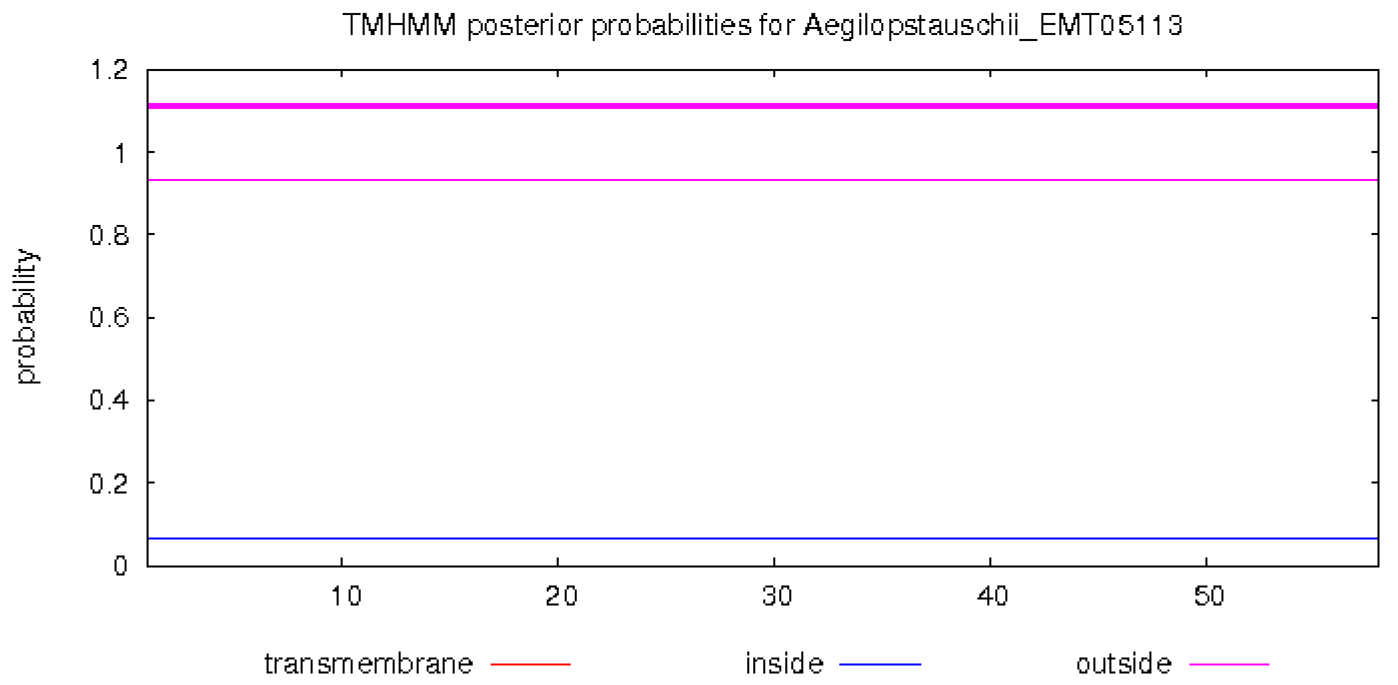

plot in postscript, script for making the plot in gnuplot, data for plot

```
# Aegilopstauschii_EMT05147 Length: 338
# Aegilopstauschii_EMT05147 Number of predicted TMHs:  0
# Aegilopstauschii_EMT05147 Exp number of AAs in TMHs: 0
# Aegilopstauschii_EMT05147 Exp number, first 60 AAs:  0
# Aegilopstauschii_EMT05147 Total prob of N-in:        0.00329
Aegilopstauschii_EMT05147	TMHMM2.0	outside	     1   338
```

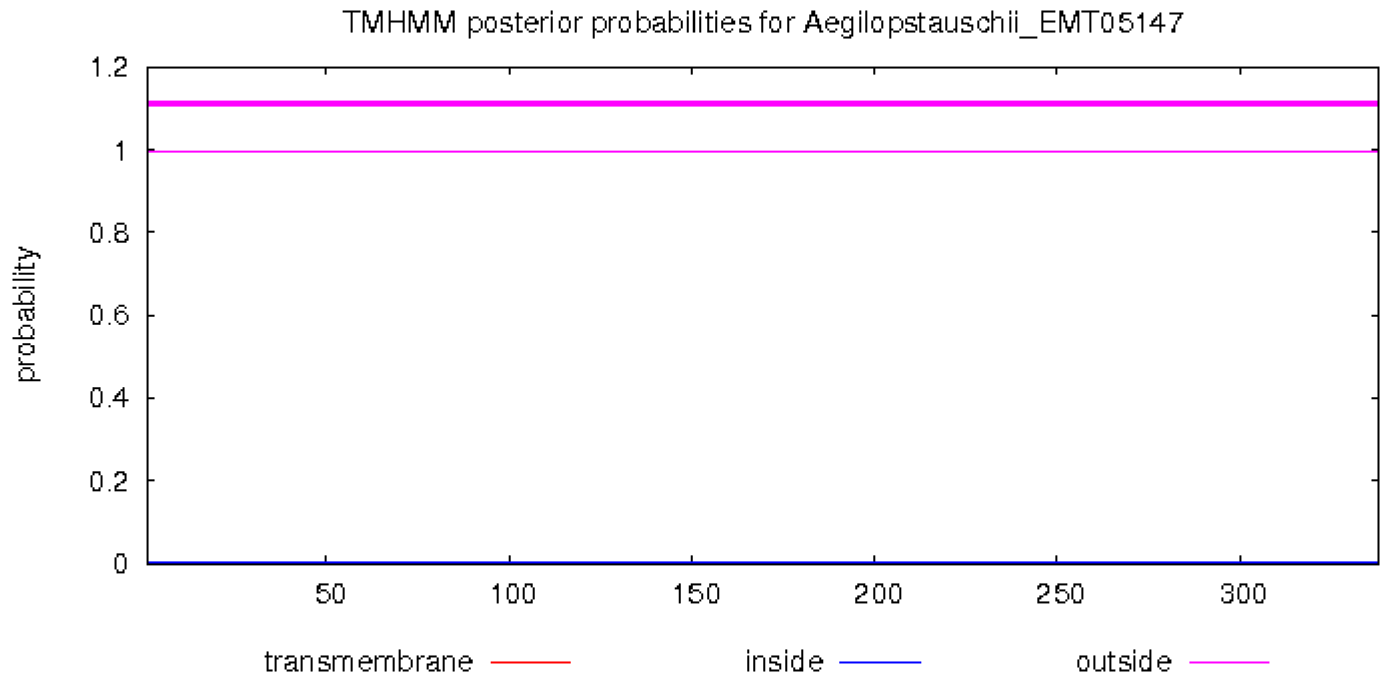

plot in postscript, script for making the plot in gnuplot, data for plot

```
# Aegilopstauschii_EMT05508 Length: 631
# Aegilopstauschii_EMT05508 Number of predicted TMHs:  0
# Aegilopstauschii_EMT05508 Exp number of AAs in TMHs: 0.02327
# Aegilopstauschii_EMT05508 Exp number, first 60 AAs:  0
# Aegilopstauschii_EMT05508 Total prob of N-in:        0.00129
Aegilopstauschii_EMT05508       TMHMM2.0        outside      1   631
```

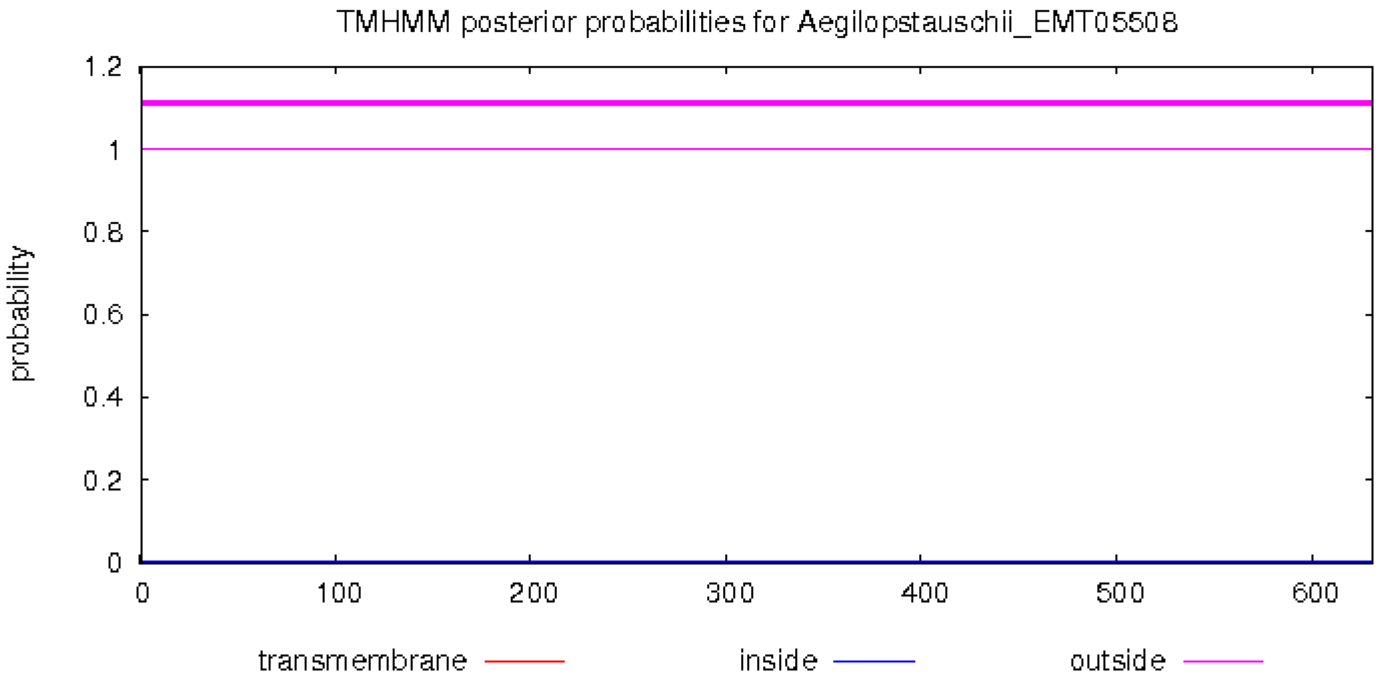

plot in postscript, script for making the plot in gnuplot, data for plot

---

```
# Aegilopstauschii_EMT05947 Length: 360
# Aegilopstauschii_EMT05947 Number of predicted TMHs:  0
# Aegilopstauschii_EMT05947 Exp number of AAs in TMHs: 0.01053
# Aegilopstauschii_EMT05947 Exp number, first 60 AAs:  0.00126
# Aegilopstauschii_EMT05947 Total prob of N-in:        0.02238
Aegilopstauschii_EMT05947       TMHMM2.0        outside      1   360
```

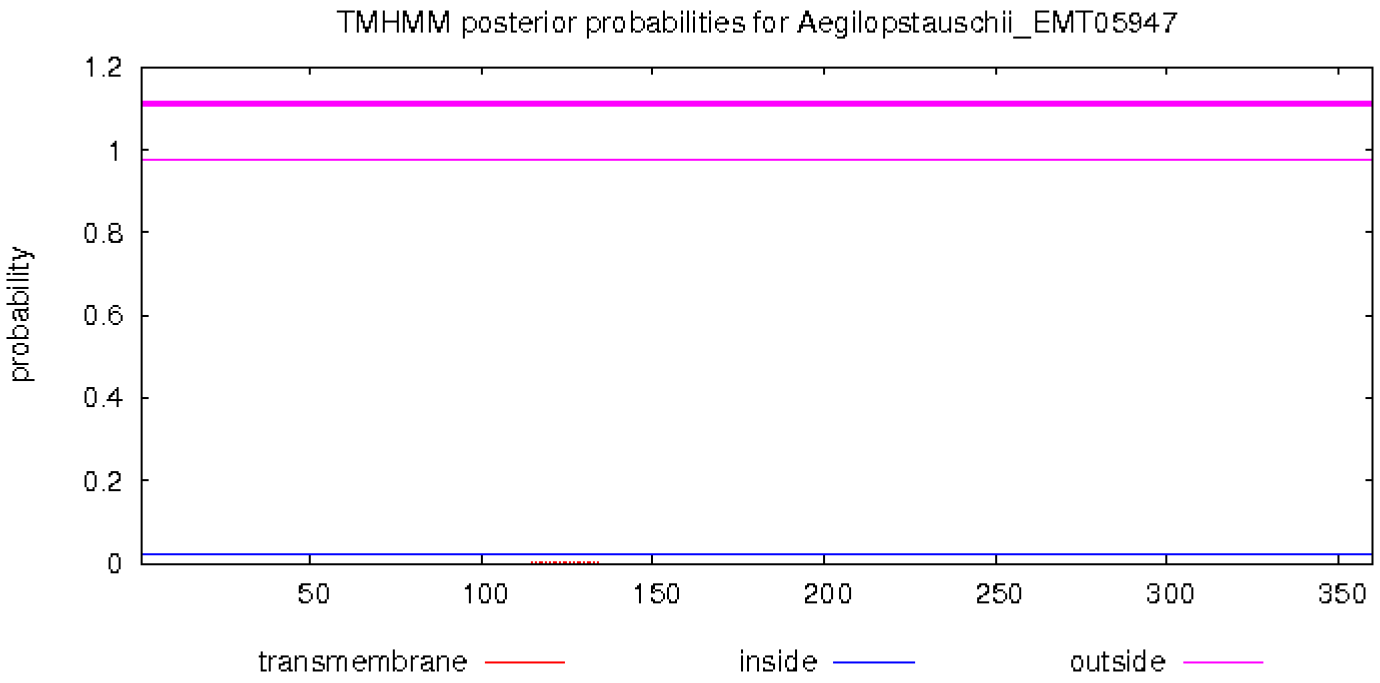

plot in postscript, script for making the plot in gnuplot, data for plot

---

```
# Aegilopstauschii_EMT05948 Length: 375
# Aegilopstauschii_EMT05948 Number of predicted TMHs:  0
# Aegilopstauschii_EMT05948 Exp number of AAs in TMHs: 0.00054
# Aegilopstauschii_EMT05948 Exp number, first 60 AAs:  0
# Aegilopstauschii_EMT05948 Total prob of N-in:        0.00275
Aegilopstauschii_EMT05948       TMHMM2.0        outside      1   375
```

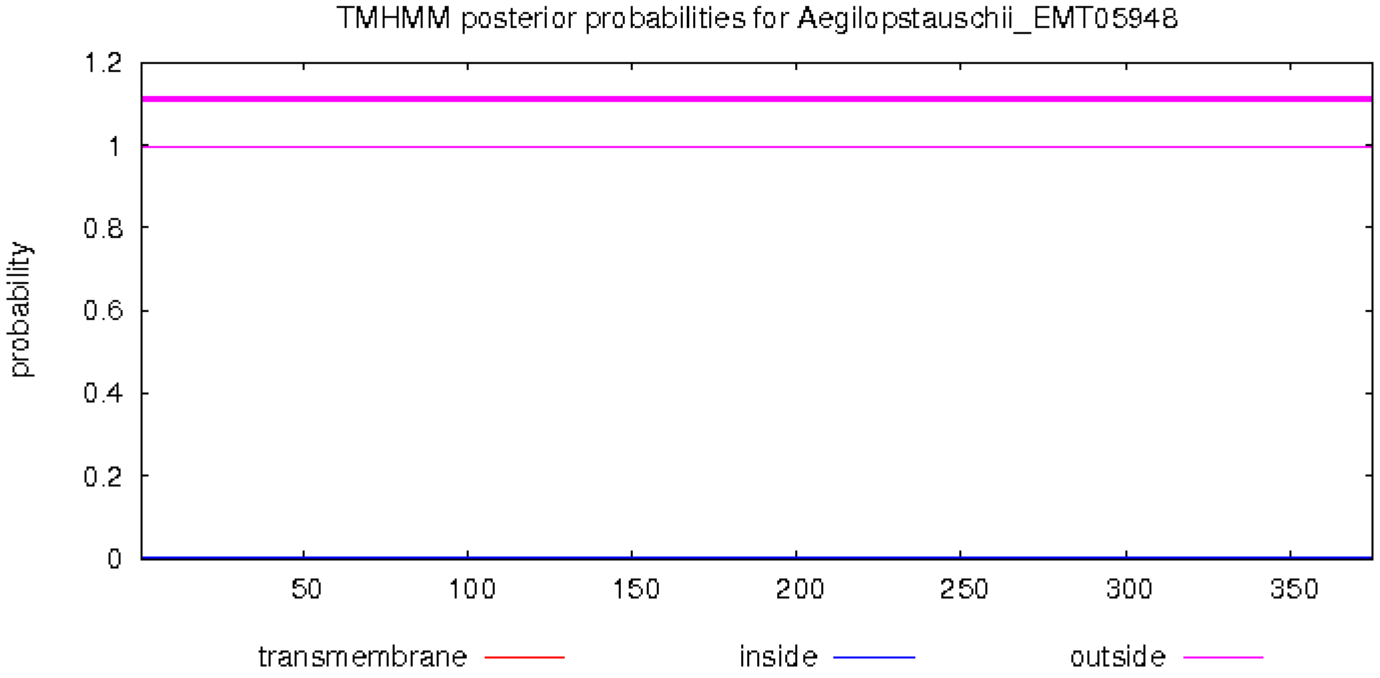

plot in postscript, script for making the plot in gnuplot, data for plot

---

```
# Aegilopstauschii_EMT06396 Length: 627
# Aegilopstauschii_EMT06396 Number of predicted TMHs:  0
# Aegilopstauschii_EMT06396 Exp number of AAs in TMHs: 0.00061
# Aegilopstauschii_EMT06396 Exp number, first 60 AAs:  0
# Aegilopstauschii_EMT06396 Total prob of N-in:        0.00117
Aegilopstauschii_EMT06396       TMHMM2.0        outside      1   627
```

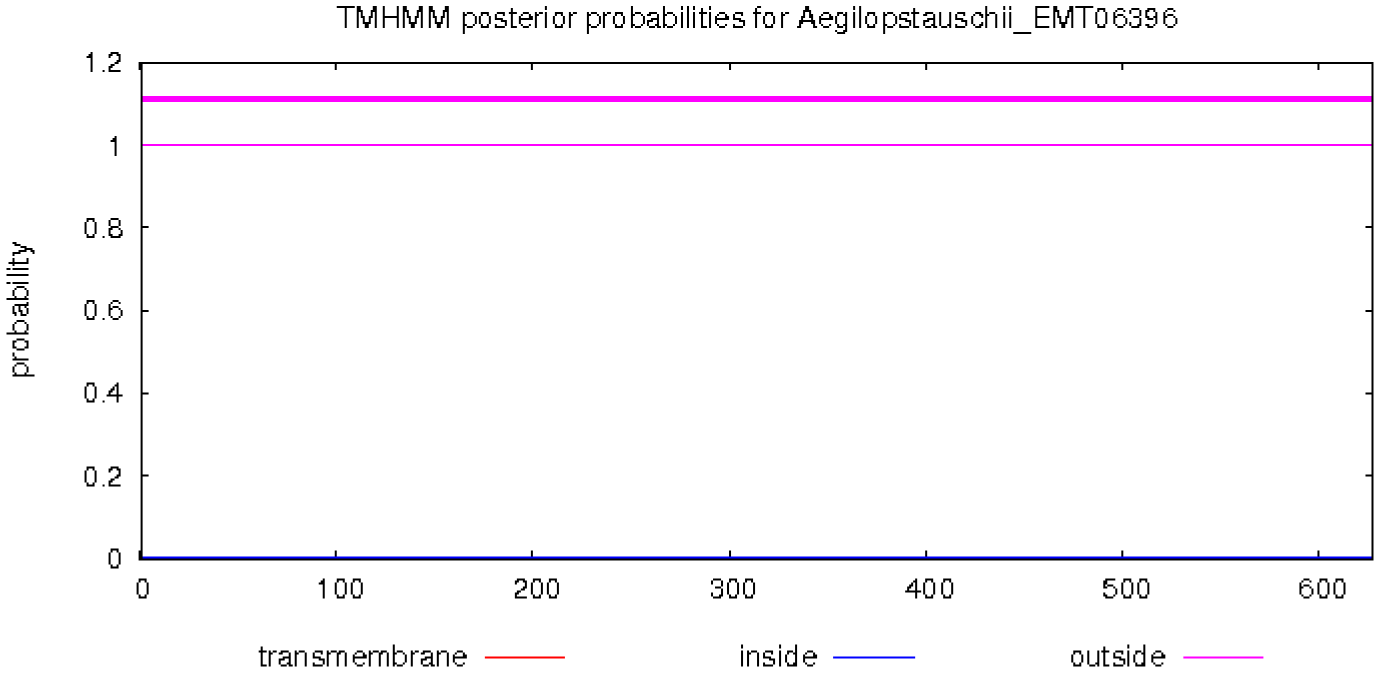

plot in postscript, script for making the plot in gnuplot, data for plot

---

```
# Aegilopstauschii_EMT06613 Length: 406
# Aegilopstauschii_EMT06613 Number of predicted TMHs:  0
# Aegilopstauschii_EMT06613 Exp number of AAs in TMHs: 0.00125
# Aegilopstauschii_EMT06613 Exp number, first 60 AAs:  0
# Aegilopstauschii_EMT06613 Total prob of N-in:        0.00047
Aegilopstauschii_EMT06613       TMHMM2.0        outside      1   406
```

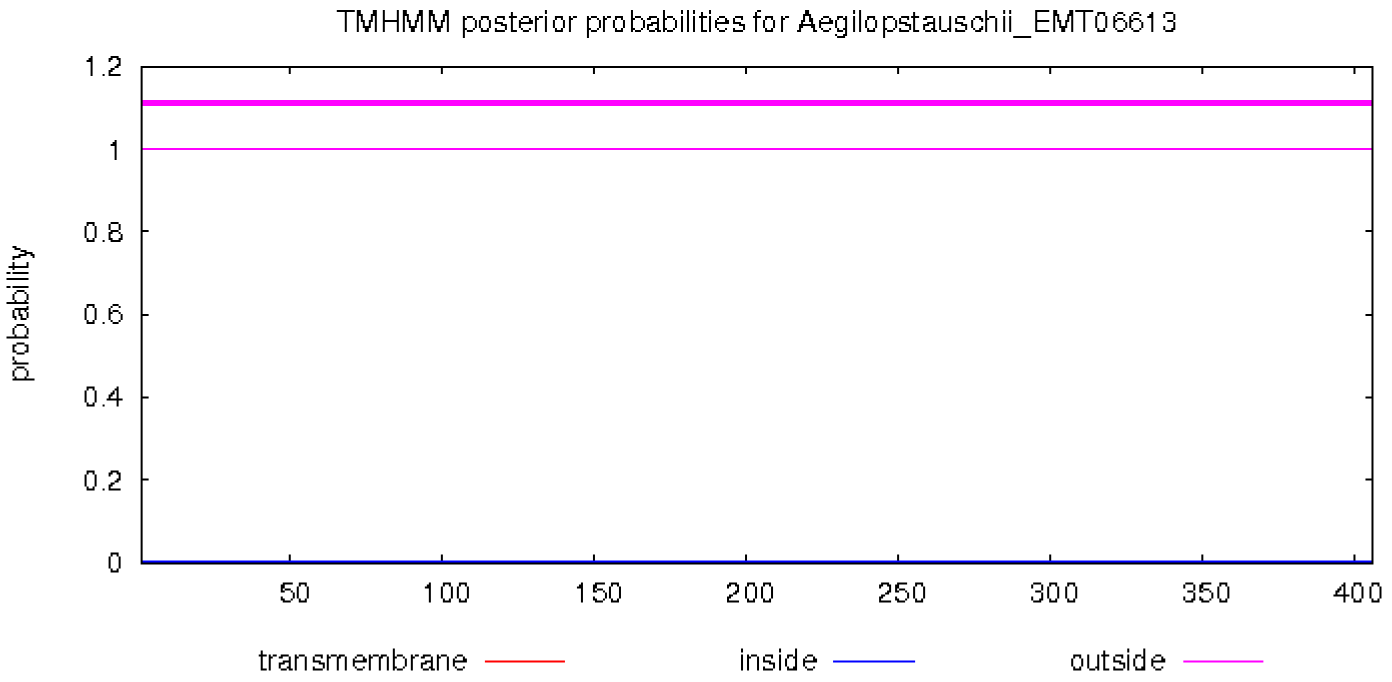

plot in postscript, script for making the plot in gnuplot, data for plot

---

```
# Aegilopstauschii_EMT06951 Length: 233
# Aegilopstauschii_EMT06951 Number of predicted TMHs:  0
# Aegilopstauschii_EMT06951 Exp number of AAs in TMHs: 0.23031
# Aegilopstauschii_EMT06951 Exp number, first 60 AAs:  0.00252
# Aegilopstauschii_EMT06951 Total prob of N-in:        0.13250
Aegilopstauschii_EMT06951       TMHMM2.0        outside      1   233
```

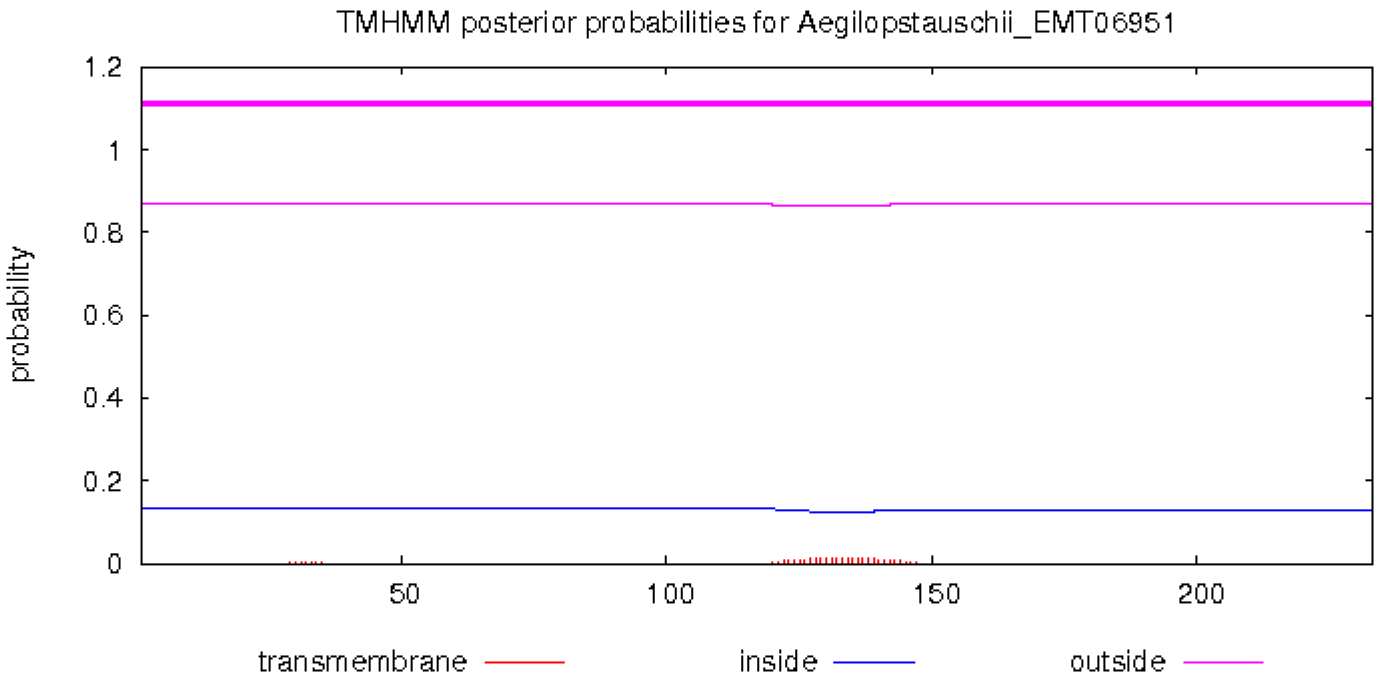

plot in postscript, script for making the plot in gnuplot, data for plot

---

```
# Aegilopstauschii_EMT06992 Length: 341
# Aegilopstauschii_EMT06992 Number of predicted TMHs:  0
# Aegilopstauschii_EMT06992 Exp number of AAs in TMHs: 0.00816
# Aegilopstauschii_EMT06992 Exp number, first 60 AAs:  0
# Aegilopstauschii_EMT06992 Total prob of N-in:        0.02772
Aegilopstauschii_EMT06992       TMHMM2.0        outside      1   341
```

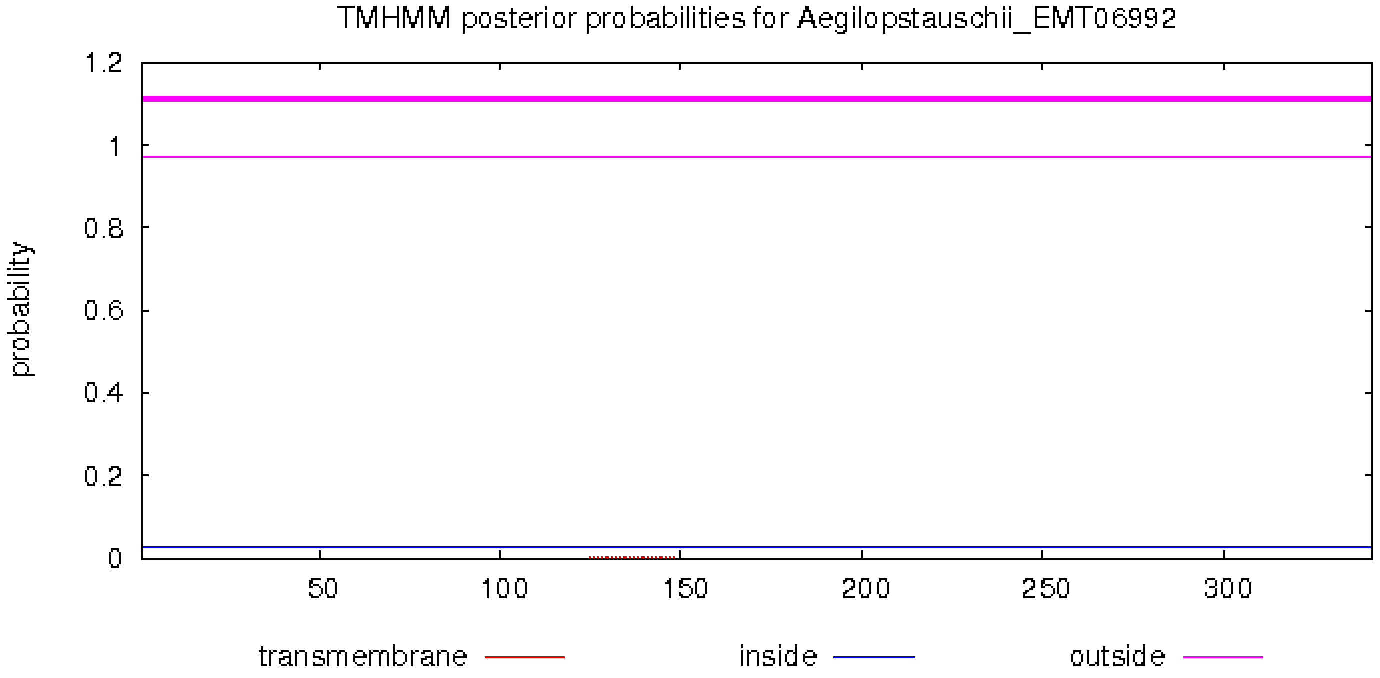

plot in postscript, script for making the plot in gnuplot, data for plot

```
# Aegilopstauschii_EMT07032 Length: 349
# Aegilopstauschii_EMT07032 Number of predicted TMHs:  0
# Aegilopstauschii_EMT07032 Exp number of AAs in TMHs: 0.00033
# Aegilopstauschii_EMT07032 Exp number, first 60 AAs:  0
# Aegilopstauschii_EMT07032 Total prob of N-in:        0.00837
Aegilopstauschii_EMT07032       TMHMM2.0        outside      1   349
```

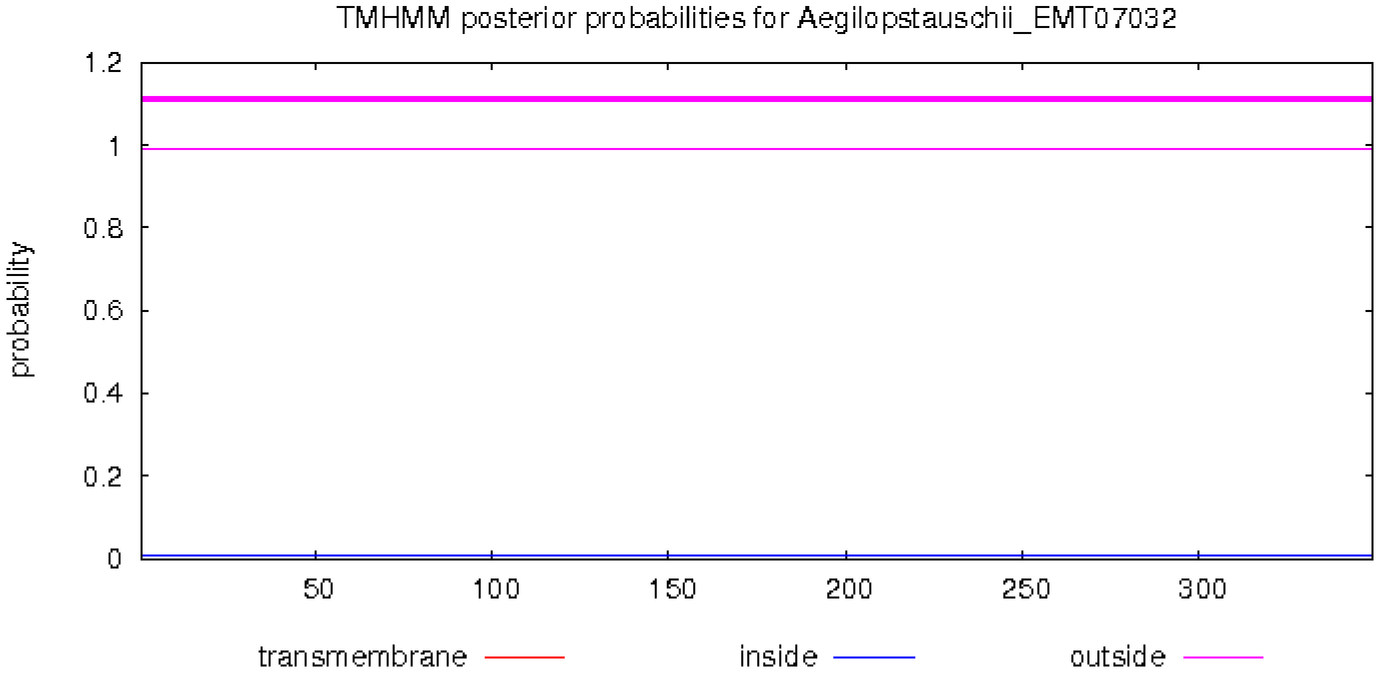

plot in postscript, script for making the plot in gnuplot, data for plot

```
# Aegilopstauschii_EMT07070 Length: 179
# Aegilopstauschii_EMT07070 Number of predicted TMHs:  0
# Aegilopstauschii_EMT07070 Exp number of AAs in TMHs: 0.0017
# Aegilopstauschii_EMT07070 Exp number, first 60 AAs:  0.00157
# Aegilopstauschii_EMT07070 Total prob of N-in:        0.05207
Aegilopstauschii_EMT07070	TMHMM2.0	outside	     1   179
```

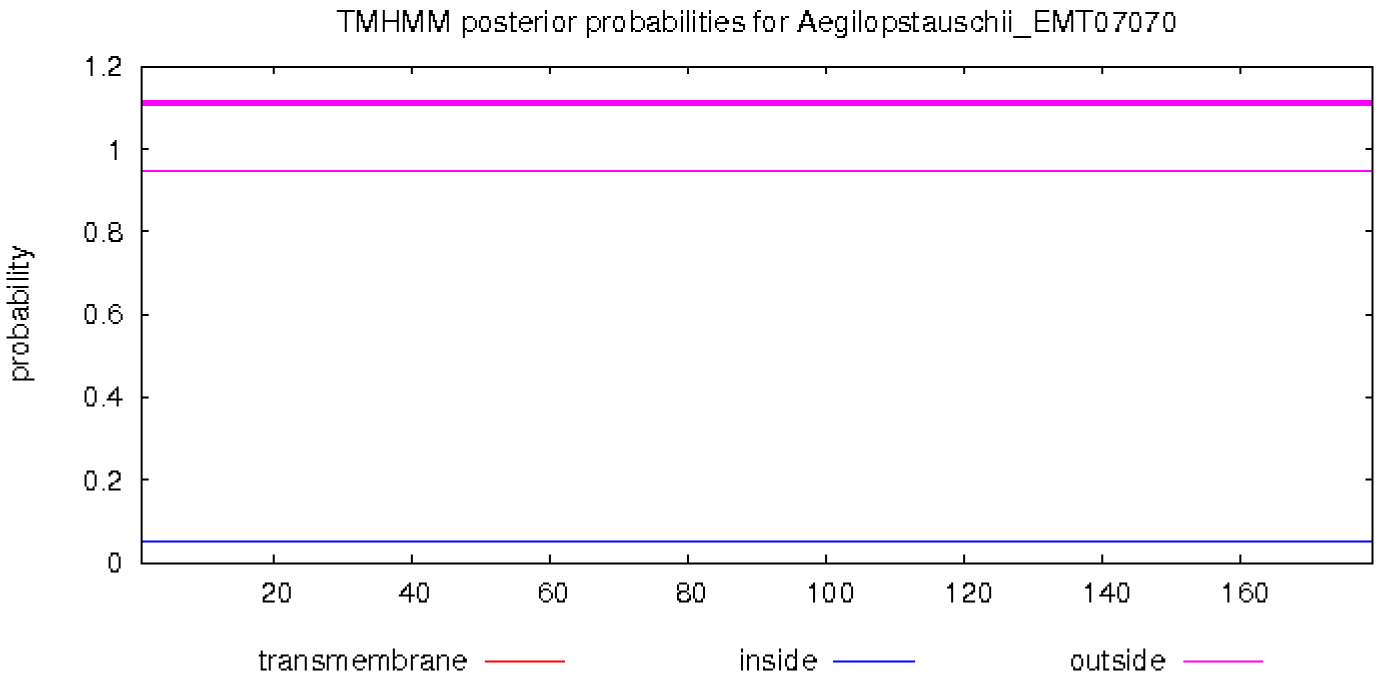

plot in postscript, script for making the plot in gnuplot, data for plot

---

```
# Aegilopstauschii_EMT07151 Length: 411
# Aegilopstauschii_EMT07151 Number of predicted TMHs:  0
# Aegilopstauschii_EMT07151 Exp number of AAs in TMHs: 0.00626
# Aegilopstauschii_EMT07151 Exp number, first 60 AAs:  0.00192
# Aegilopstauschii_EMT07151 Total prob of N-in:        0.00770
Aegilopstauschii_EMT07151	TMHMM2.0	outside	     1   411
```

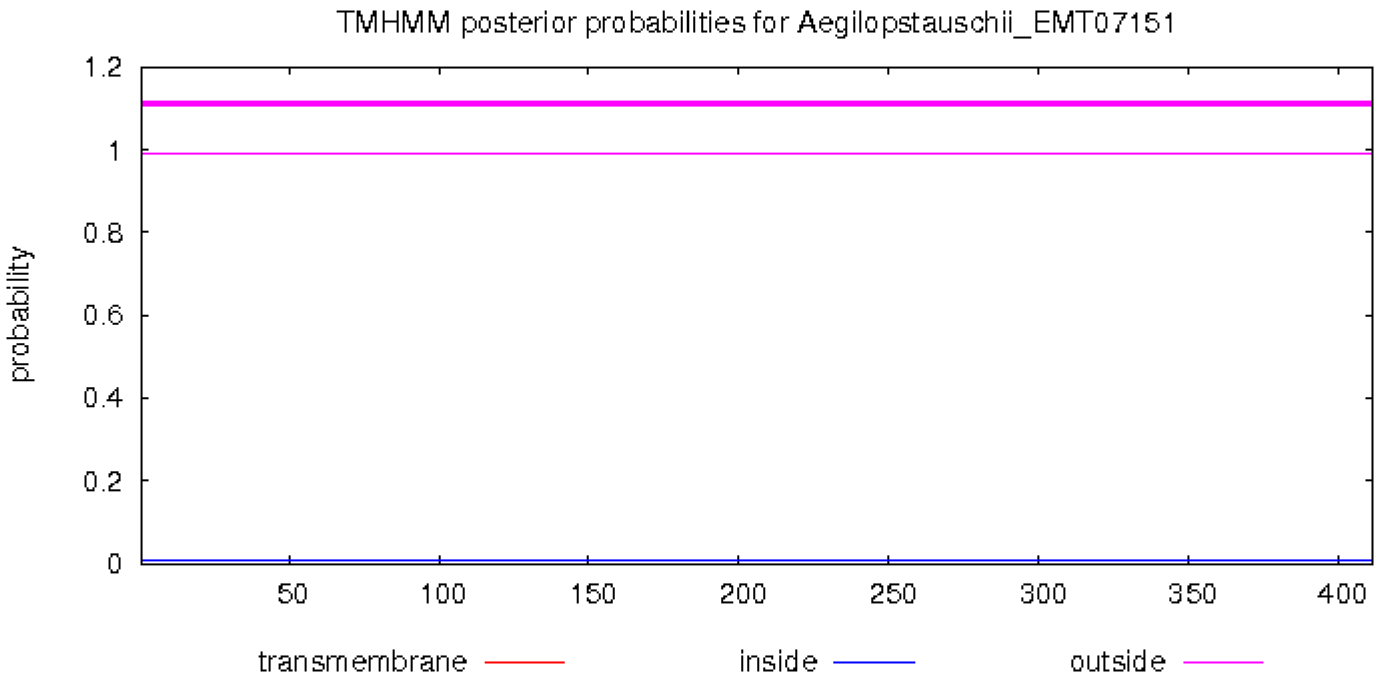

plot in postscript, script for making the plot in gnuplot, data for plot

---

```
# Aegilopstauschii_EMT07371 Length: 333
# Aegilopstauschii_EMT07371 Number of predicted TMHs:  0
# Aegilopstauschii_EMT07371 Exp number of AAs in TMHs: 0.00281
# Aegilopstauschii_EMT07371 Exp number, first 60 AAs:  0
# Aegilopstauschii_EMT07371 Total prob of N-in:        0.00161
Aegilopstauschii_EMT07371       TMHMM2.0        outside      1   333
```

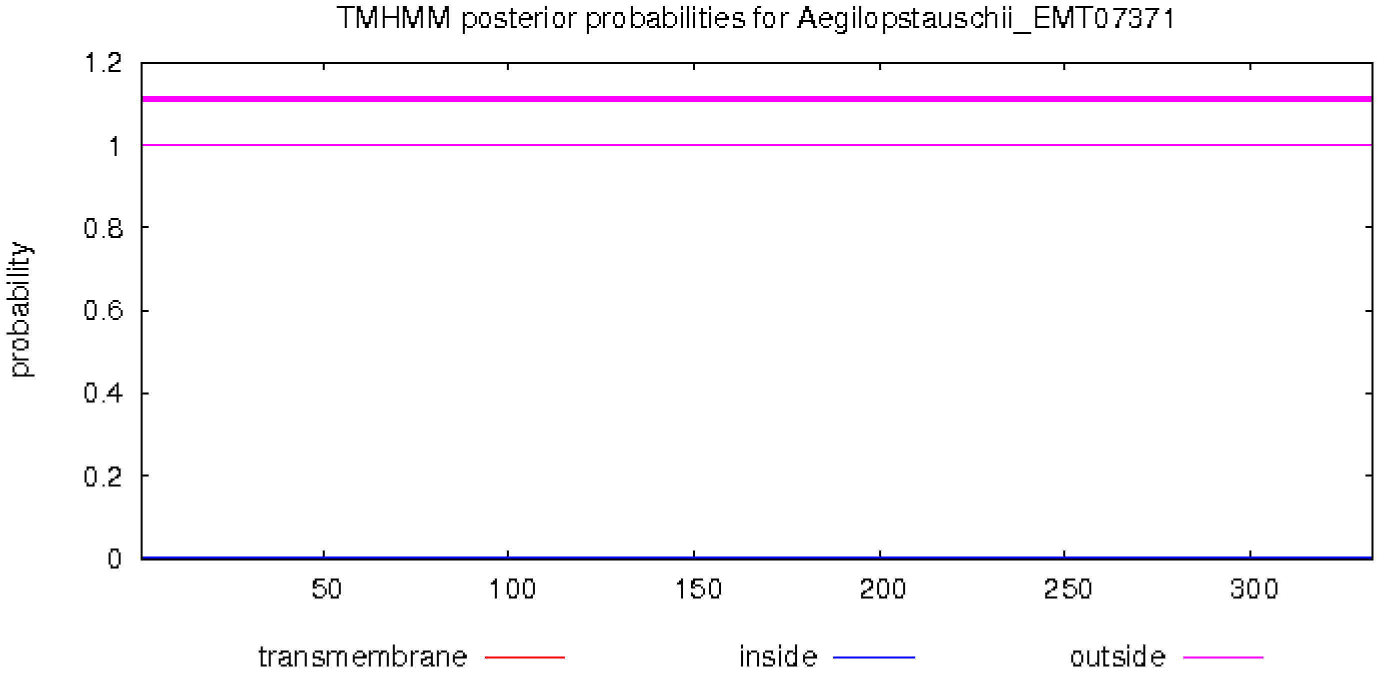

plot in postscript, script for making the plot in gnuplot, data for plot

```
# Aegilopstauschii_EMT07892 Length: 694
# Aegilopstauschii_EMT07892 Number of predicted TMHs:  1
# Aegilopstauschii_EMT07892 Exp number of AAs in TMHs: 21.78468
# Aegilopstauschii_EMT07892 Exp number, first 60 AAs:  0
# Aegilopstauschii_EMT07892 Total prob of N-in:        0.00252
Aegilopstauschii_EMT07892       TMHMM2.0        outside      1   670
Aegilopstauschii_EMT07892       TMHMM2.0        TMhelix    671   693
Aegilopstauschii_EMT07892       TMHMM2.0        inside     694   694
```

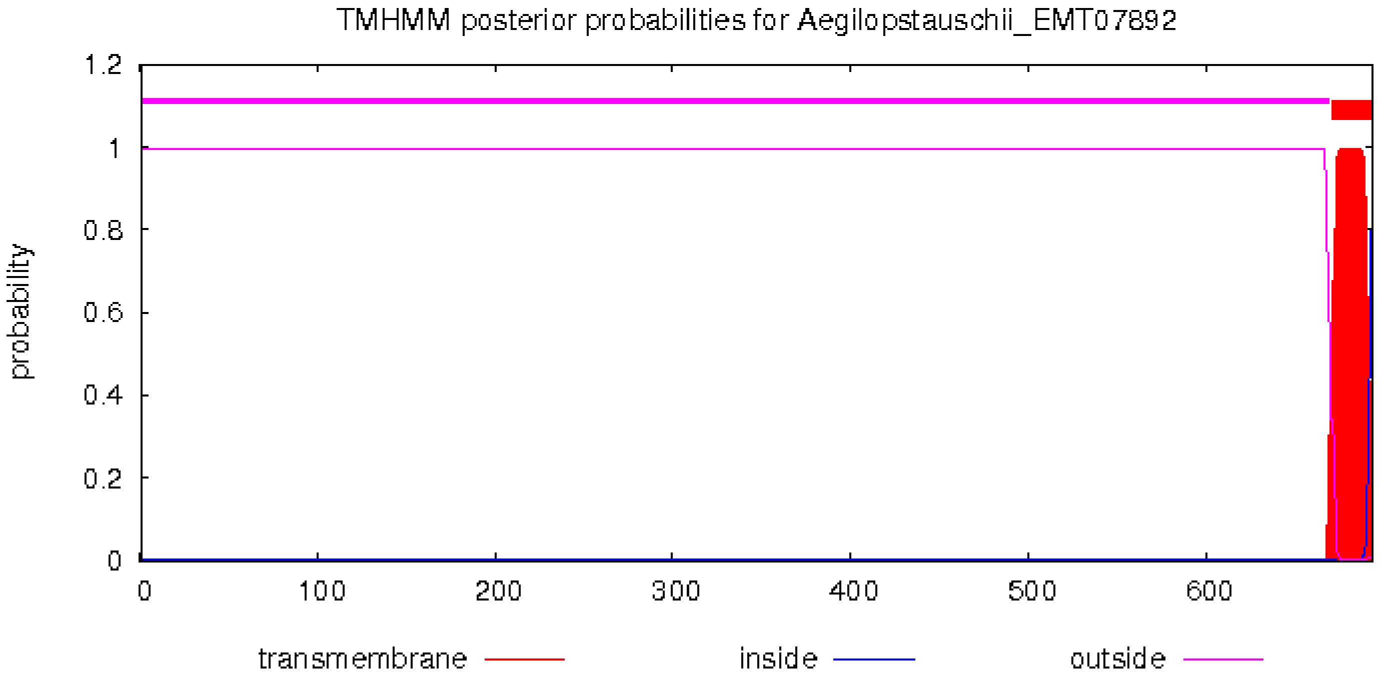

plot in postscript, script for making the plot in gnuplot, data for plot

```
# Aegilopstauschii_EMT08095 Length: 283
# Aegilopstauschii_EMT08095 Number of predicted TMHs:  0
# Aegilopstauschii_EMT08095 Exp number of AAs in TMHs: 0.00056
# Aegilopstauschii_EMT08095 Exp number, first 60 AAs:  0
# Aegilopstauschii_EMT08095 Total prob of N-in:        0.00647
Aegilopstauschii_EMT08095       TMHMM2.0        outside      1   283
```

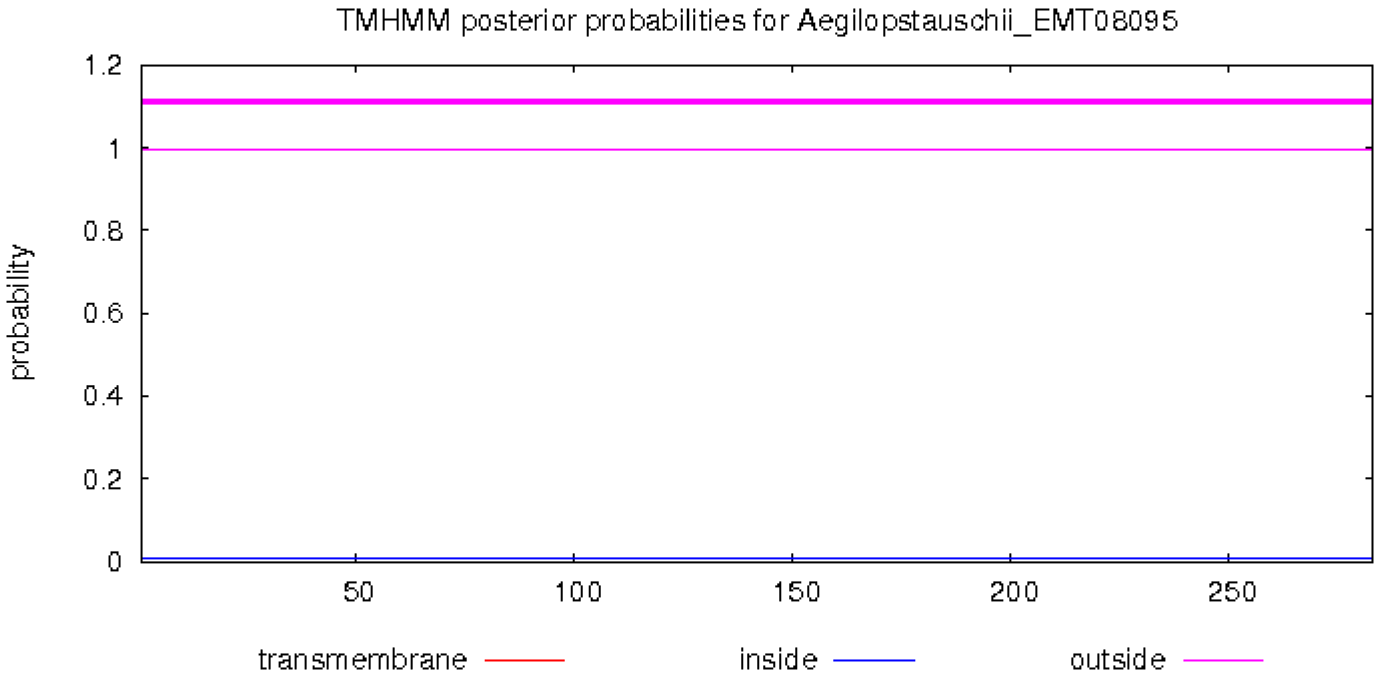

plot in postscript, script for making the plot in gnuplot, data for plot

```
# Aegilopstauschii_EMT08988 Length: 195
# Aegilopstauschii_EMT08988 Number of predicted TMHs:  0
# Aegilopstauschii_EMT08988 Exp number of AAs in TMHs: 0
# Aegilopstauschii_EMT08988 Exp number, first 60 AAs:  0
# Aegilopstauschii_EMT08988 Total prob of N-in:        0.10237
Aegilopstauschii_EMT08988       TMHMM2.0        outside      1   195
```

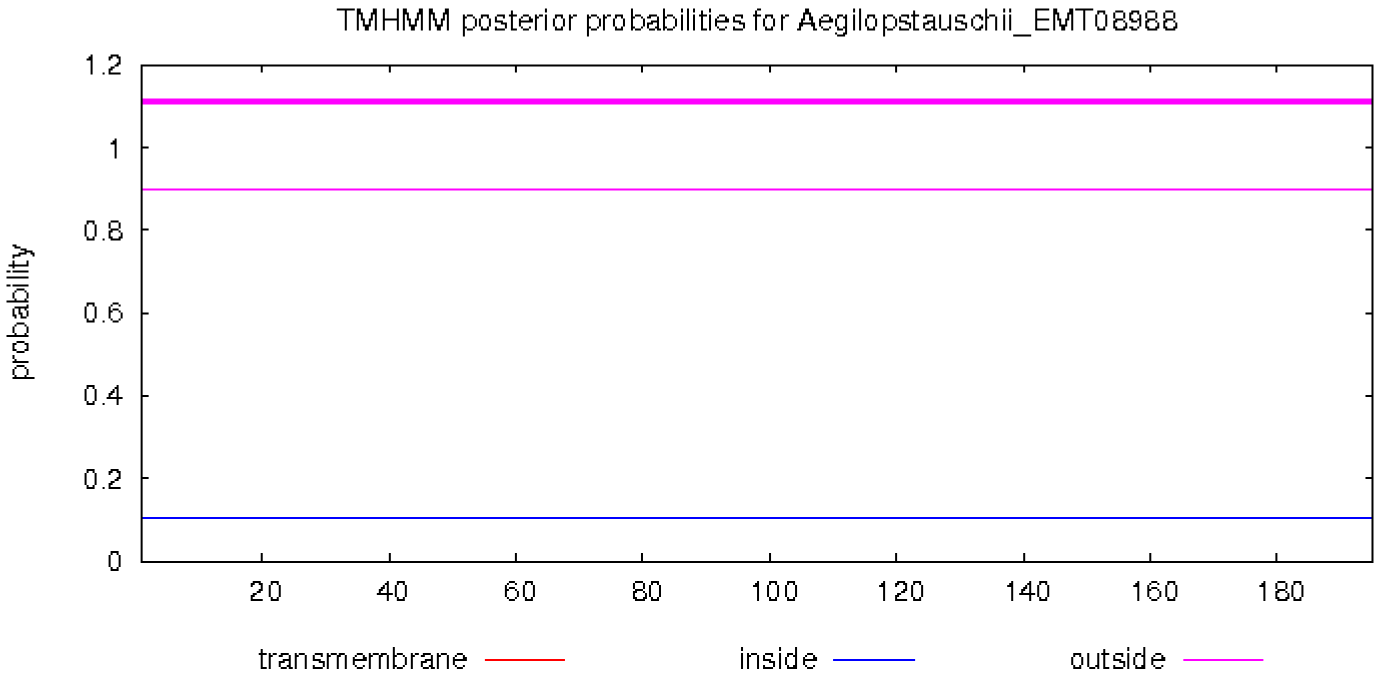

plot in postscript, script for making the plot in gnuplot, data for plot

```
# Aegilopstauschii_EMT10359 Length: 416
# Aegilopstauschii_EMT10359 Number of predicted TMHs:  0
# Aegilopstauschii_EMT10359 Exp number of AAs in TMHs: 0
# Aegilopstauschii_EMT10359 Exp number, first 60 AAs:  0
# Aegilopstauschii_EMT10359 Total prob of N-in:        0.00256
Aegilopstauschii_EMT10359	TMHMM2.0	outside	     1   416
```

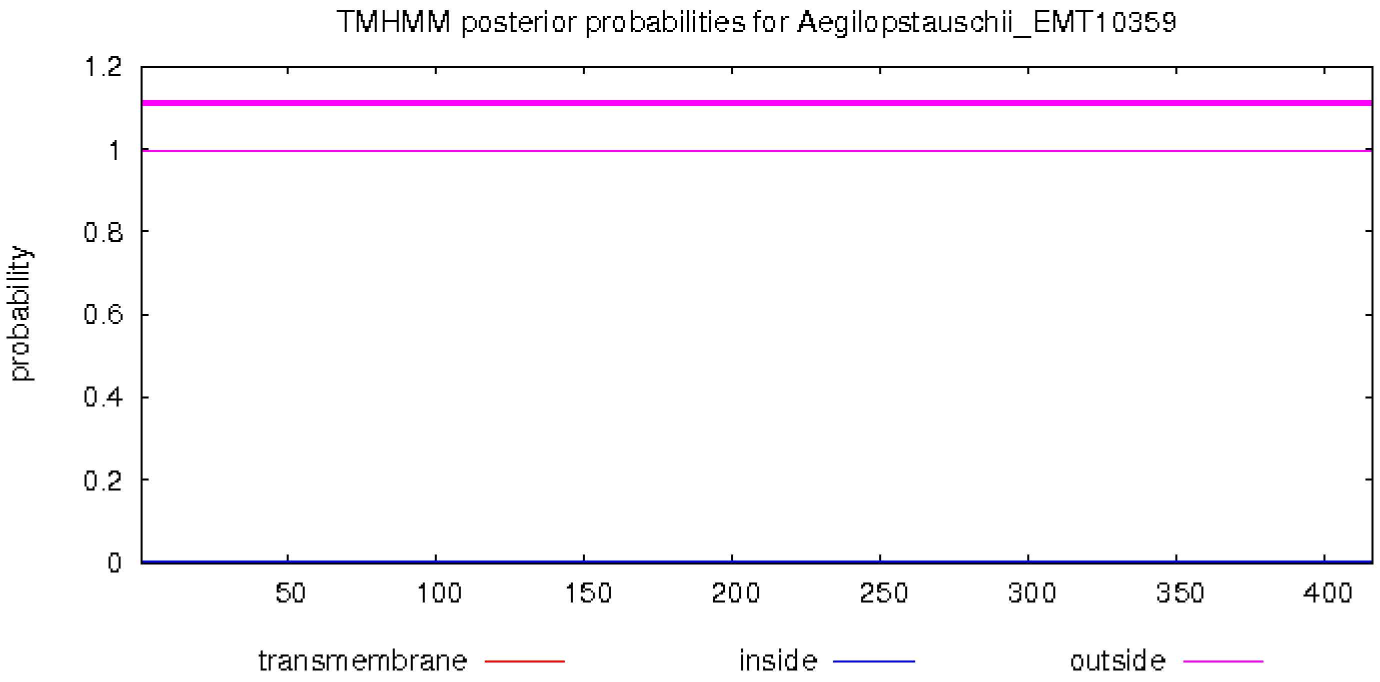

plot in postscript, script for making the plot in gnuplot, data for plot

---

```
# Aegilopstauschii_EMT10902 Length: 257
# Aegilopstauschii_EMT10902 Number of predicted TMHs:  0
# Aegilopstauschii_EMT10902 Exp number of AAs in TMHs: 0.82022
# Aegilopstauschii_EMT10902 Exp number, first 60 AAs:  0.07494
# Aegilopstauschii_EMT10902 Total prob of N-in:        0.03469
Aegilopstauschii_EMT10902	TMHMM2.0	outside	     1   257
```

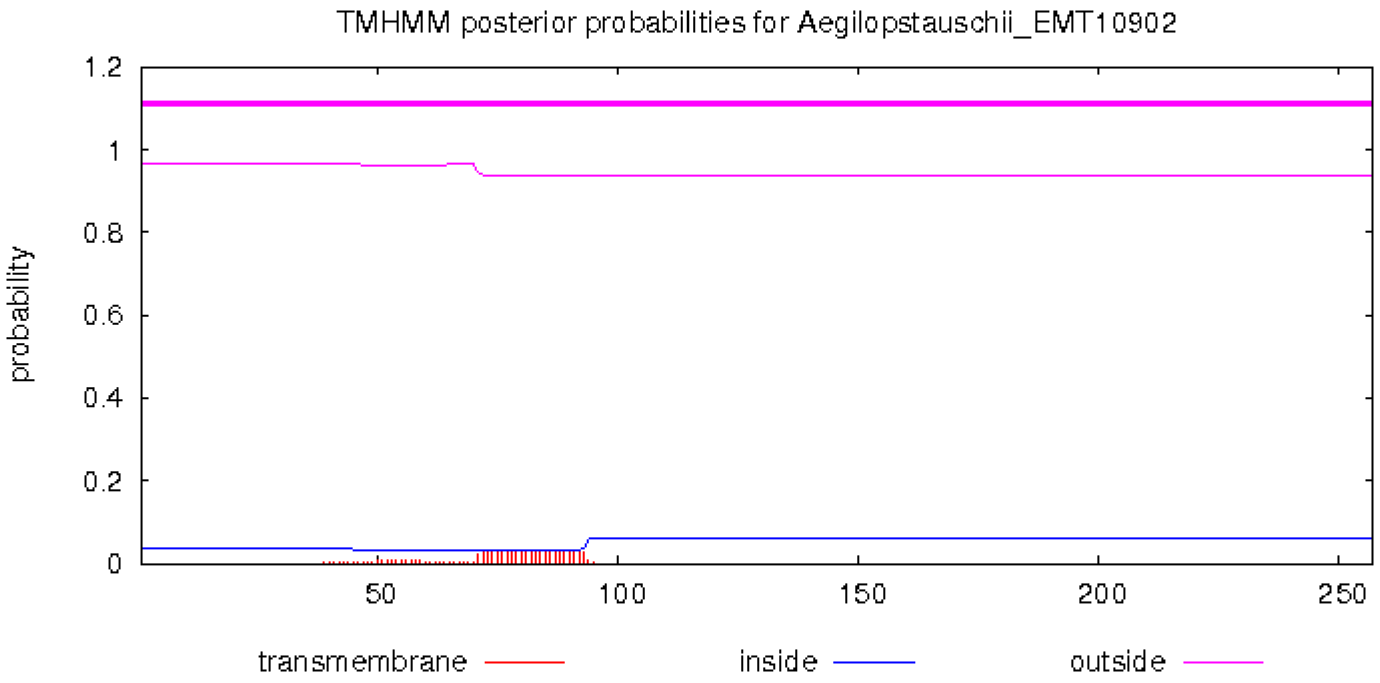

plot in postscript, script for making the plot in gnuplot, data for plot

---

```
# Aegilopstauschii_EMT11023 Length: 403
# Aegilopstauschii_EMT11023 Number of predicted TMHs:  0
# Aegilopstauschii_EMT11023 Exp number of AAs in TMHs: 0.00019
# Aegilopstauschii_EMT11023 Exp number, first 60 AAs:  0
# Aegilopstauschii_EMT11023 Total prob of N-in:        0.00625
Aegilopstauschii_EMT11023	TMHMM2.0	outside	     1   403
```

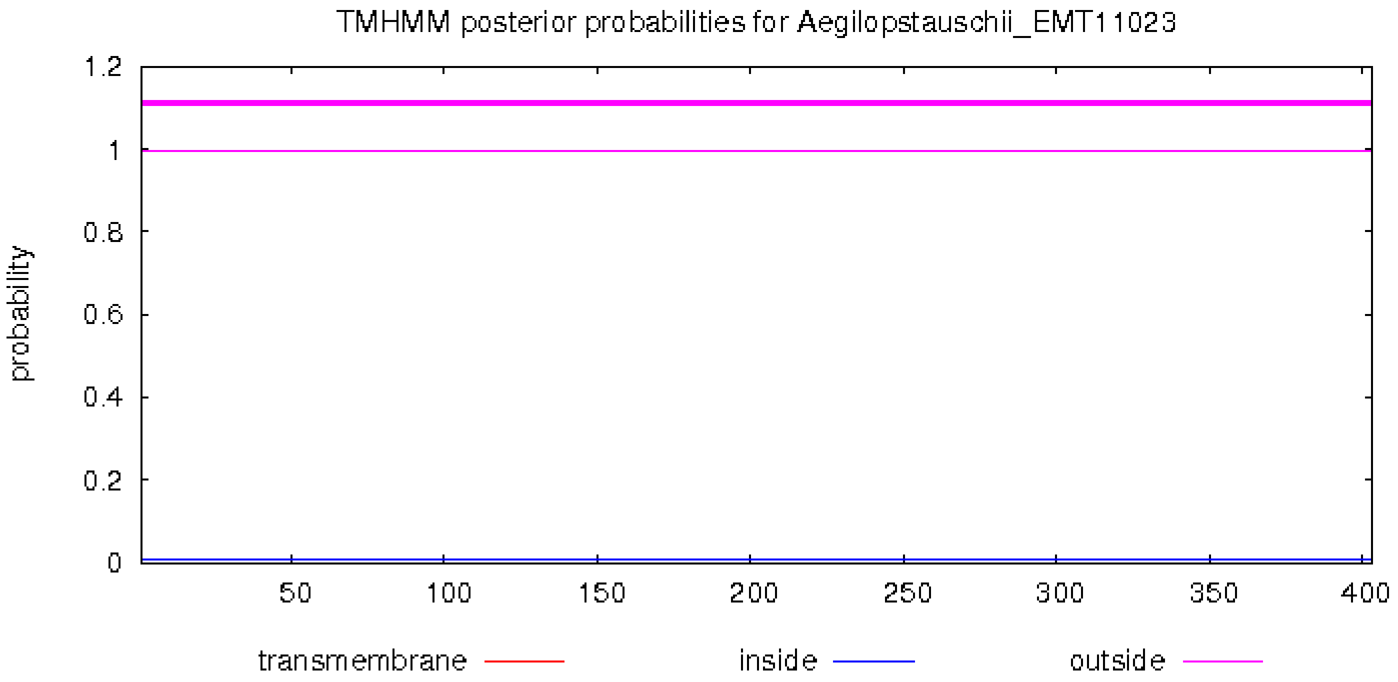

plot in postscript, script for making the plot in gnuplot, data for plot

```
# Aegilopstauschii_EMT11045 Length: 276
# Aegilopstauschii_EMT11045 Number of predicted TMHs:  0
# Aegilopstauschii_EMT11045 Exp number of AAs in TMHs: 0.12064
# Aegilopstauschii_EMT11045 Exp number, first 60 AAs:  0.00012
# Aegilopstauschii_EMT11045 Total prob of N-in:        0.05193
Aegilopstauschii_EMT11045	TMHMM2.0	outside	     1   276
```

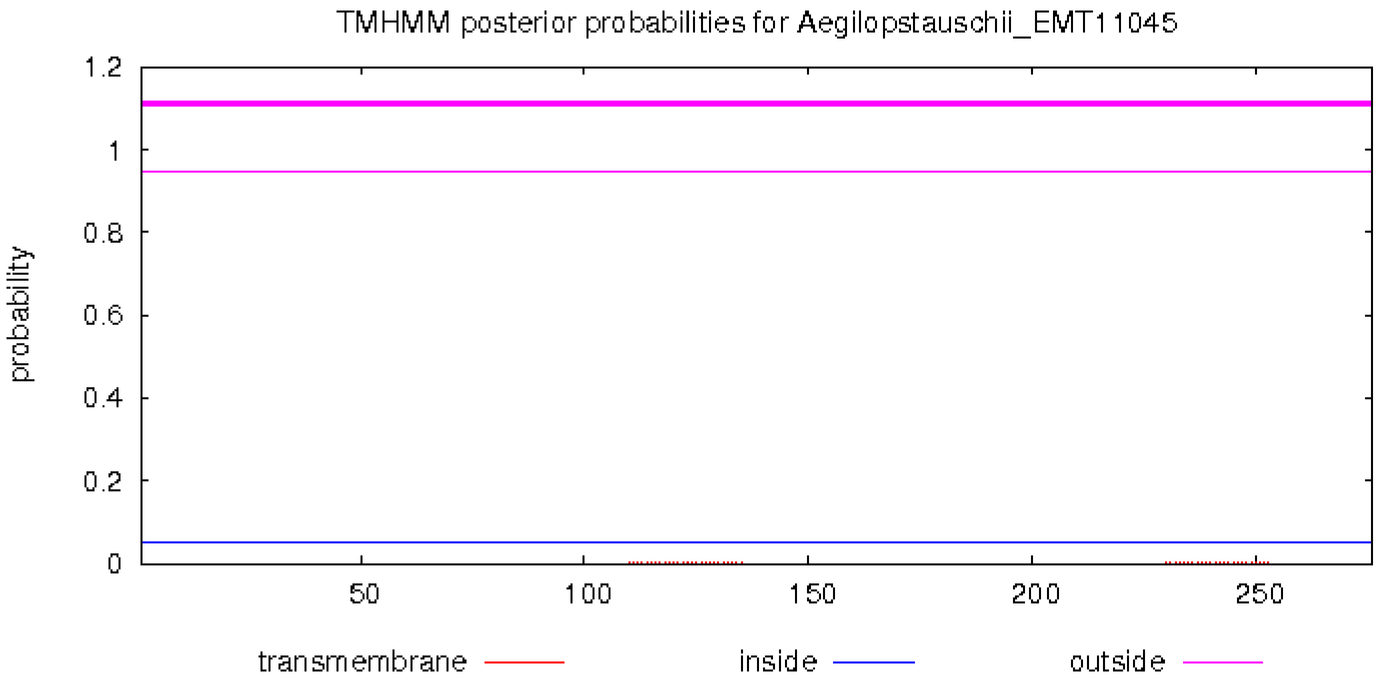

plot in postscript, script for making the plot in gnuplot, data for plot

```
# Aegilopstauschii_EMT11192 Length: 200
# Aegilopstauschii_EMT11192 Number of predicted TMHs:  0
# Aegilopstauschii_EMT11192 Exp number of AAs in TMHs: 0.08666
# Aegilopstauschii_EMT11192 Exp number, first 60 AAs:  0
# Aegilopstauschii_EMT11192 Total prob of N-in:        0.13184
Aegilopstauschii_EMT11192	TMHMM2.0	outside	     1   200
```

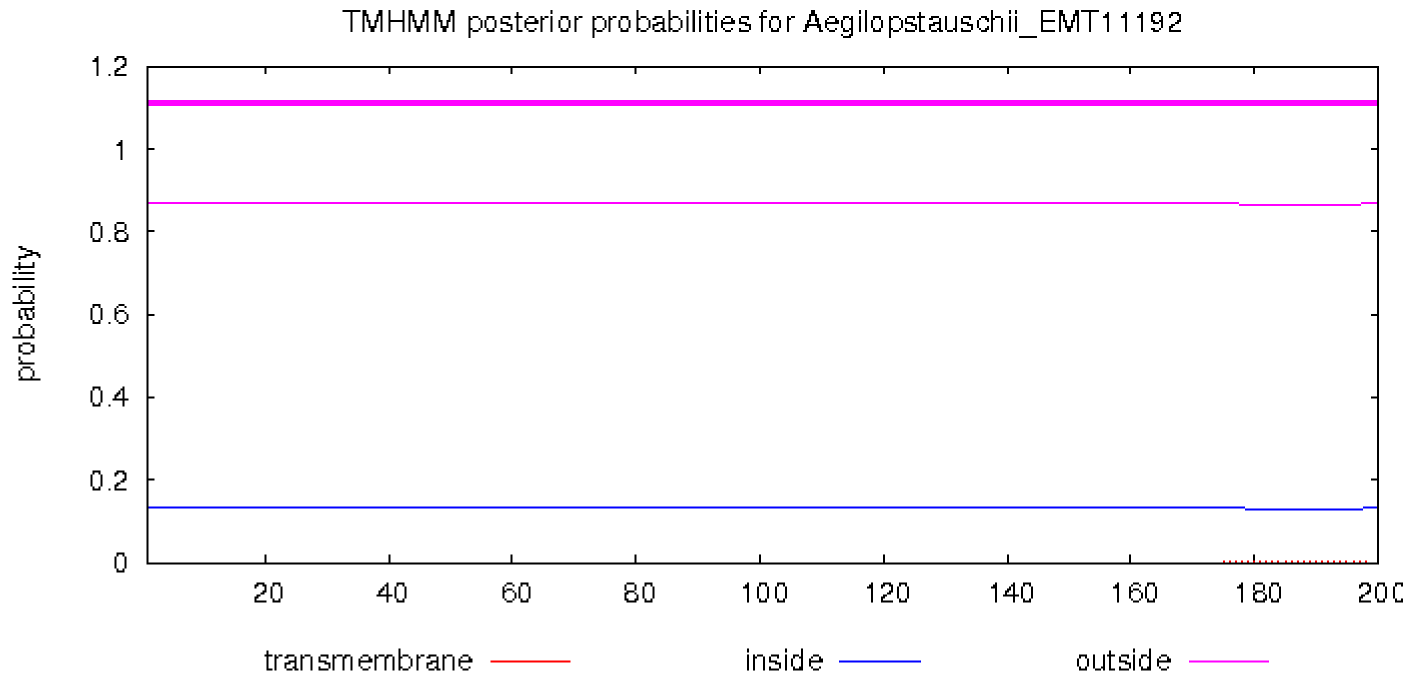

plot in postscript, script for making the plot in gnuplot, data for plot

---

```
# Aegilopstauschii_EMT11879 Length: 395
# Aegilopstauschii_EMT11879 Number of predicted TMHs:  0
# Aegilopstauschii_EMT11879 Exp number of AAs in TMHs: 0.0021
# Aegilopstauschii_EMT11879 Exp number, first 60 AAs:  0
# Aegilopstauschii_EMT11879 Total prob of N-in:        0.00700
Aegilopstauschii_EMT11879	TMHMM2.0	outside	     1   395
```

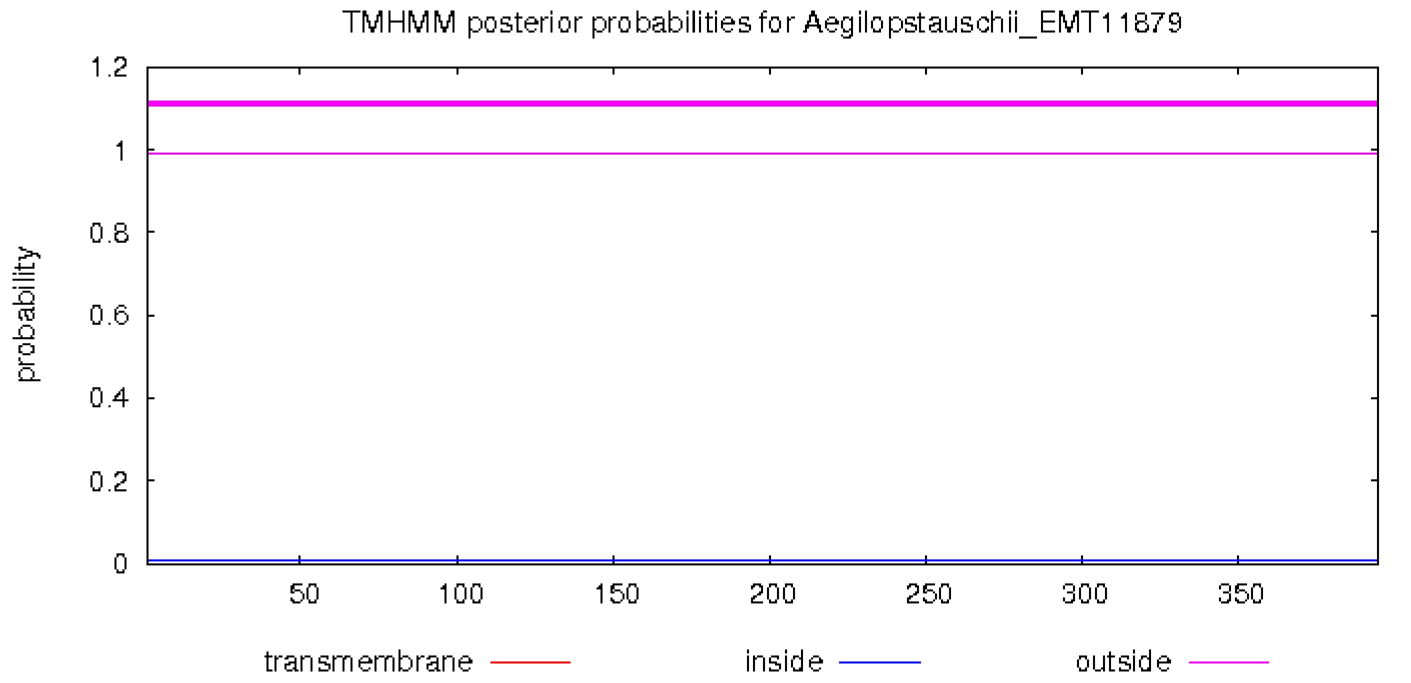

plot in postscript, script for making the plot in gnuplot, data for plot

---

```
# Aegilopstauschii_EMT12034 Length: 253
# Aegilopstauschii_EMT12034 Number of predicted TMHs:  0
# Aegilopstauschii_EMT12034 Exp number of AAs in TMHs: 0.0089
# Aegilopstauschii_EMT12034 Exp number, first 60 AAs:  0.00656
# Aegilopstauschii_EMT12034 Total prob of N-in:        0.04224
Aegilopstauschii_EMT12034       TMHMM2.0        outside      1   253
```

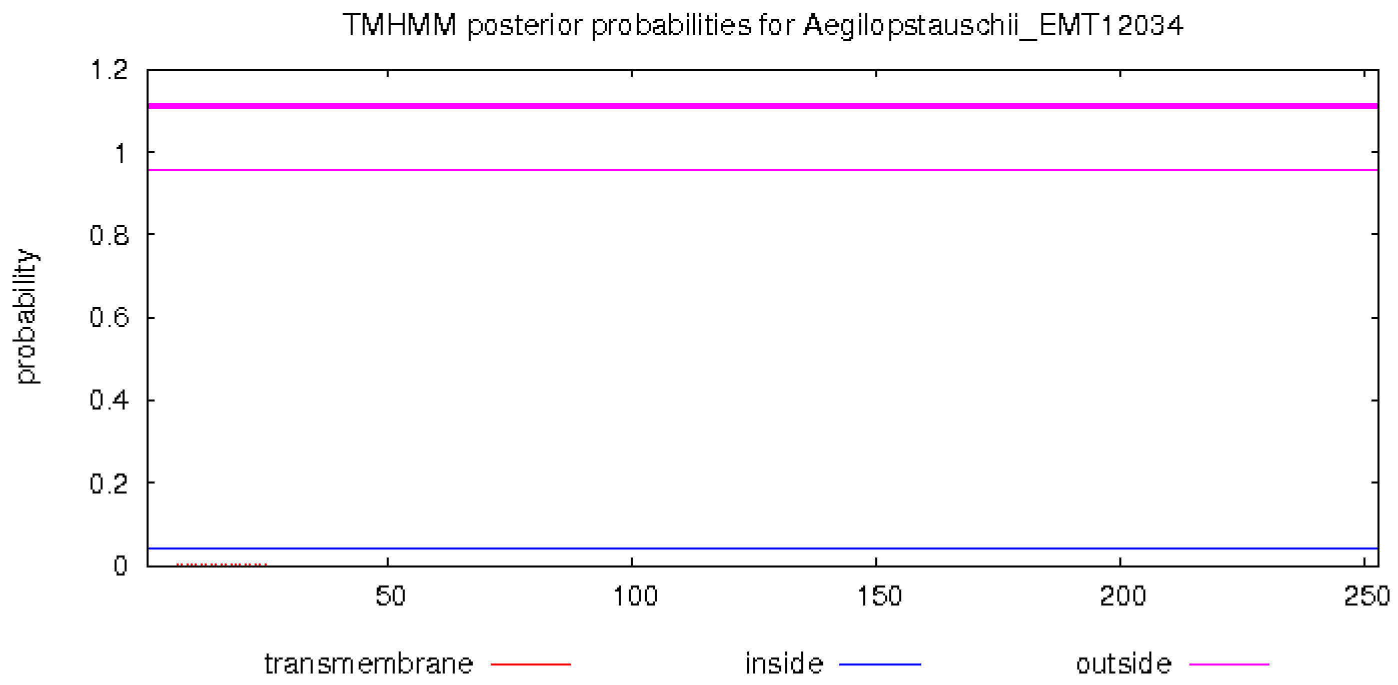

plot in postscript, script for making the plot in gnuplot, data for plot

---

```
# Aegilopstauschii_EMT12583 Length: 182
# Aegilopstauschii_EMT12583 Number of predicted TMHs:  0
# Aegilopstauschii_EMT12583 Exp number of AAs in TMHs: 0.0004
# Aegilopstauschii_EMT12583 Exp number, first 60 AAs:  0.00013
# Aegilopstauschii_EMT12583 Total prob of N-in:        0.02036
Aegilopstauschii_EMT12583       TMHMM2.0        outside      1   182
```

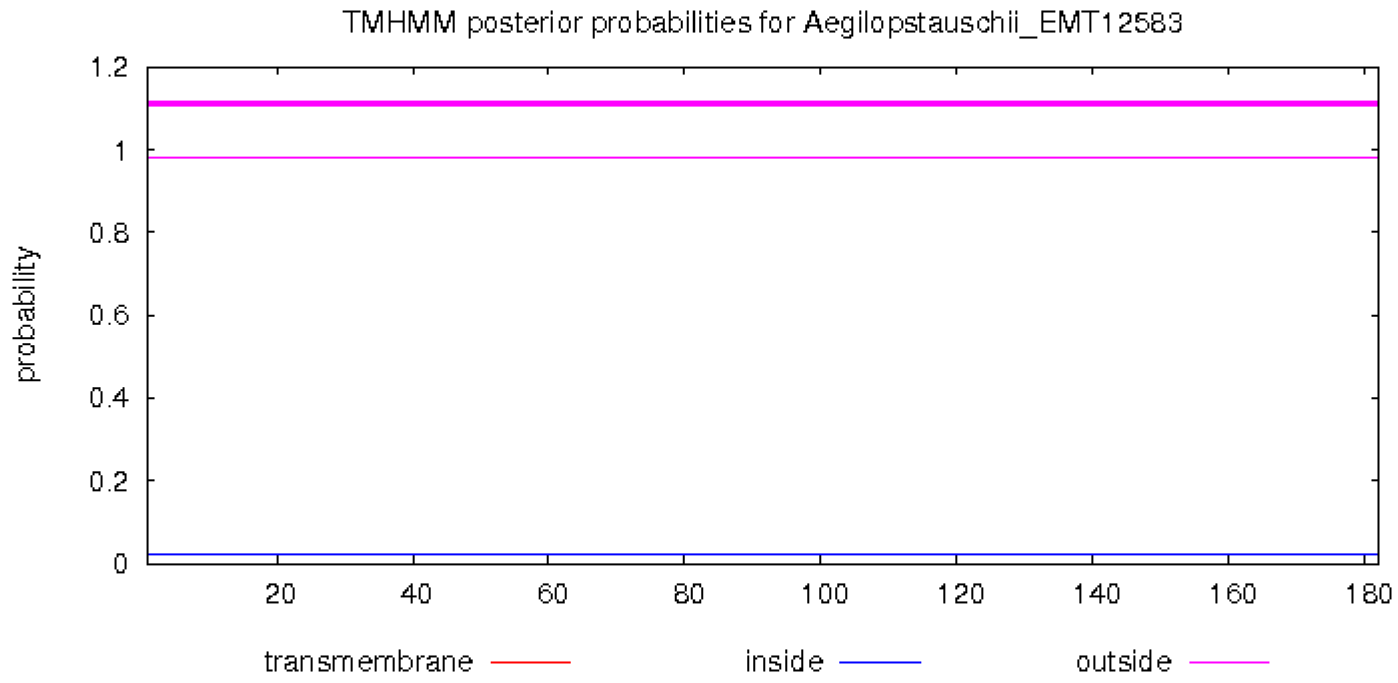

plot in postscript, script for making the plot in gnuplot, data for plot

---

```
# Aegilopstauschii_EMT13314 Length: 297
# Aegilopstauschii_EMT13314 Number of predicted TMHs:  0
# Aegilopstauschii_EMT13314 Exp number of AAs in TMHs: 0.9313
# Aegilopstauschii_EMT13314 Exp number, first 60 AAs:  0.00038
# Aegilopstauschii_EMT13314 Total prob of N-in:        0.05750
Aegilopstauschii_EMT13314	TMHMM2.0	outside	     1   297
```

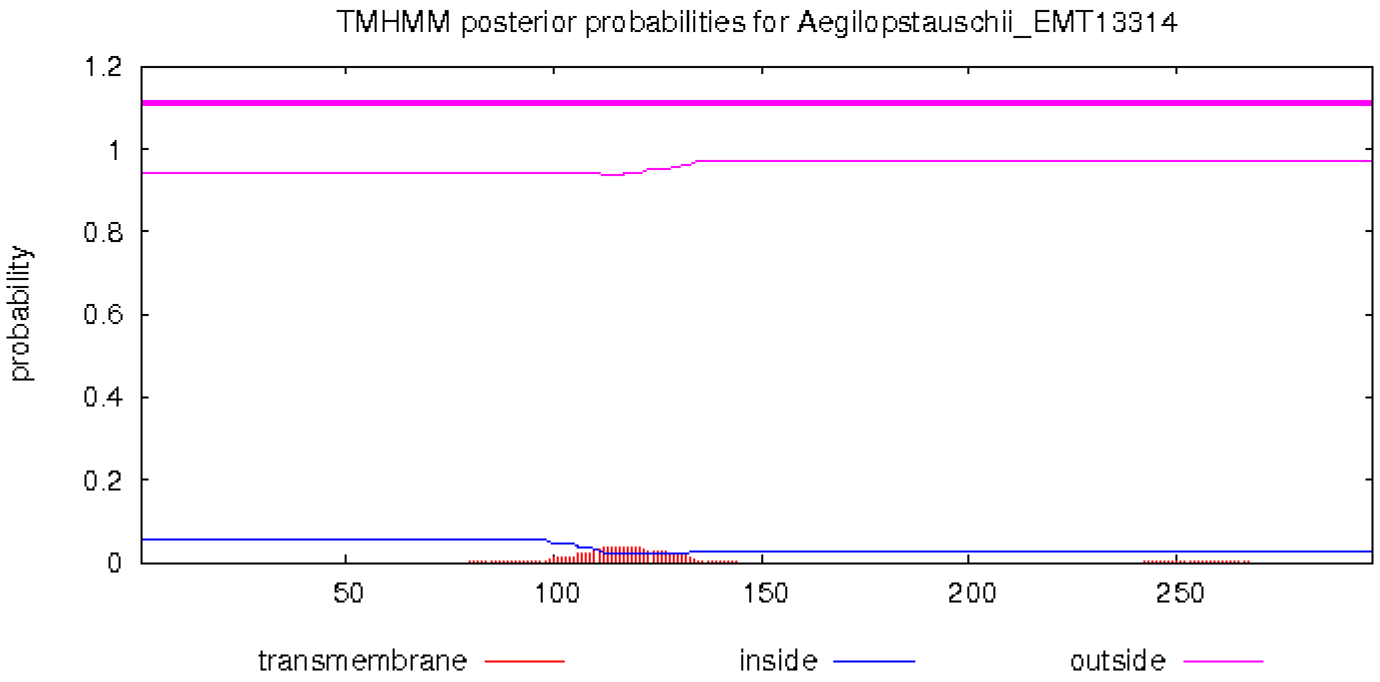

plot in postscript, script for making the plot in gnuplot, data for plot

```
# Aegilopstauschii_EMT13683 Length: 234
# Aegilopstauschii_EMT13683 Number of predicted TMHs:  0
# Aegilopstauschii_EMT13683 Exp number of AAs in TMHs: 0.01824
# Aegilopstauschii_EMT13683 Exp number, first 60 AAs:  0.00226
# Aegilopstauschii_EMT13683 Total prob of N-in:        0.10920
Aegilopstauschii_EMT13683	TMHMM2.0	outside	     1   234
```

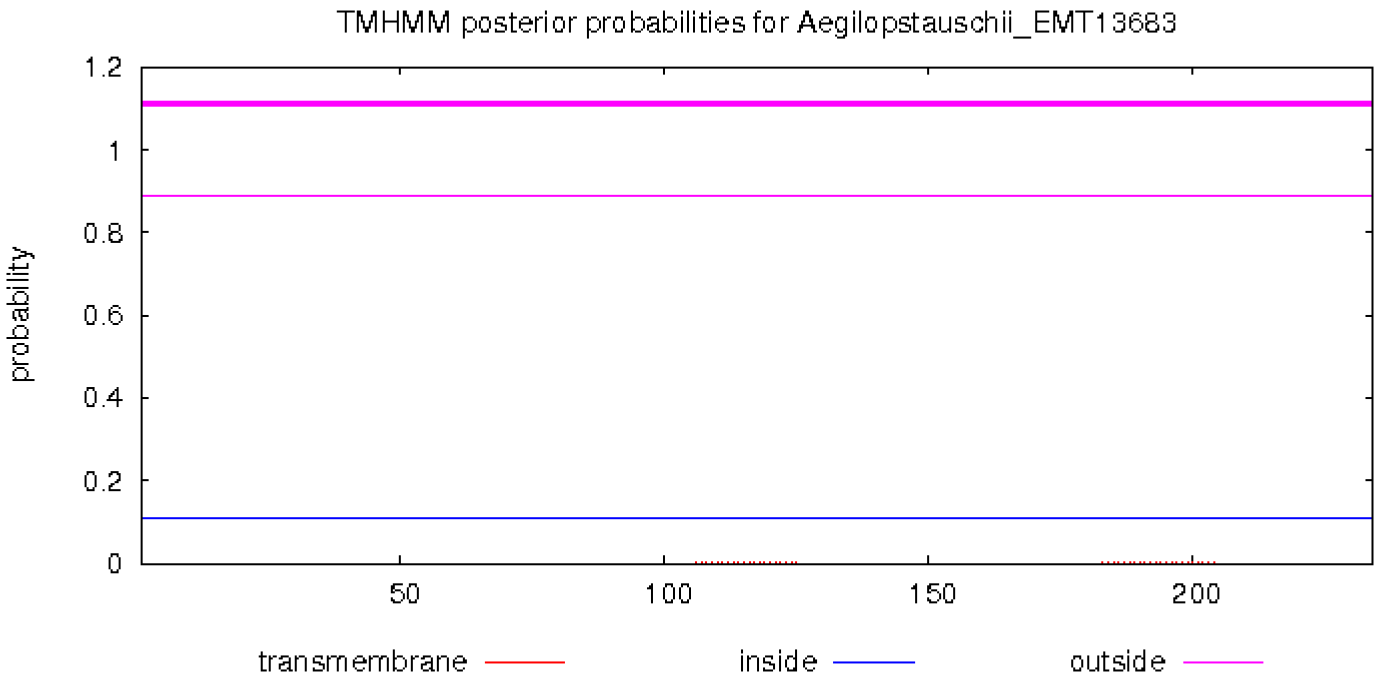

plot in postscript, script for making the plot in gnuplot, data for plot

```
# Aegilopstauschii_EMT13710 Length: 365
# Aegilopstauschii_EMT13710 Number of predicted TMHs:  0
# Aegilopstauschii_EMT13710 Exp number of AAs in TMHs: 0.00022
# Aegilopstauschii_EMT13710 Exp number, first 60 AAs:  0.00022
# Aegilopstauschii_EMT13710 Total prob of N-in:        0.00288
Aegilopstauschii_EMT13710	TMHMM2.0	outside	     1   365
```

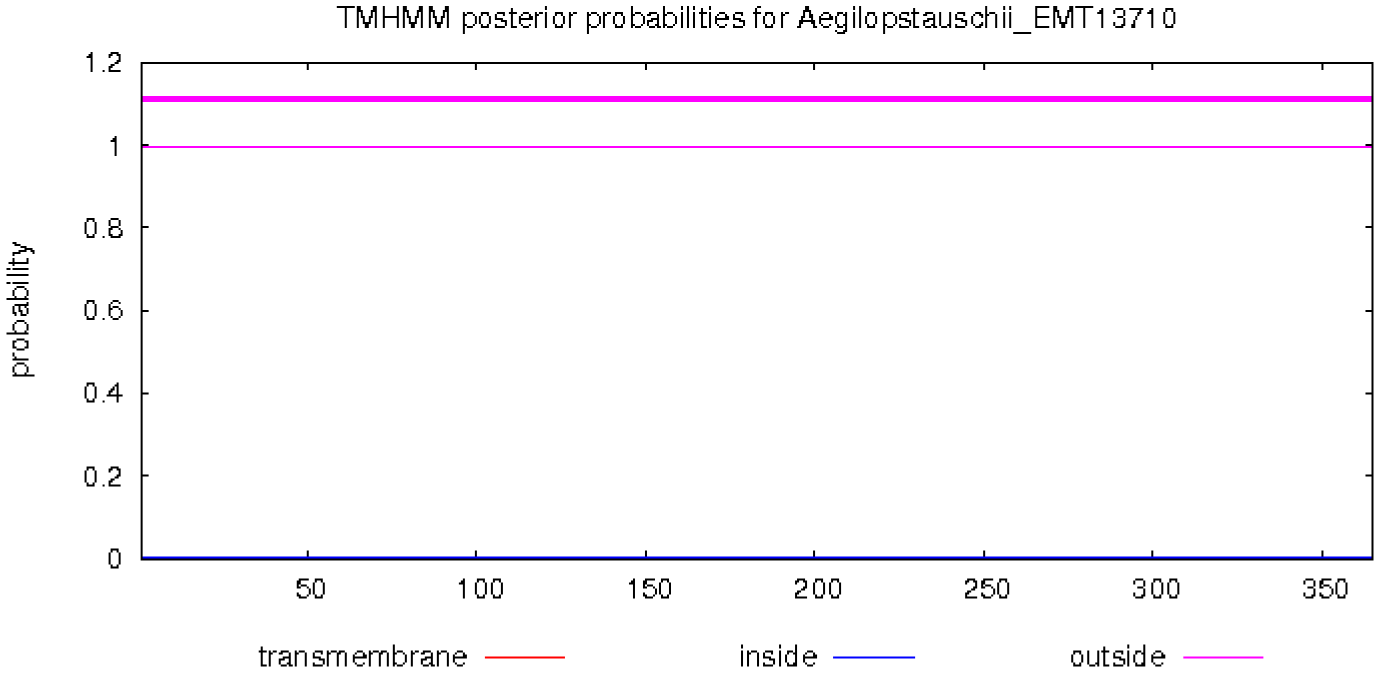

plot in postscript, script for making the plot in gnuplot, data for plot

```
# Aegilopstauschii_EMT13722 Length: 336
# Aegilopstauschii_EMT13722 Number of predicted TMHs:  0
# Aegilopstauschii_EMT13722 Exp number of AAs in TMHs: 0.10895
# Aegilopstauschii_EMT13722 Exp number, first 60 AAs:  0.02128
# Aegilopstauschii_EMT13722 Total prob of N-in:        0.01702
Aegilopstauschii_EMT13722	TMHMM2.0	outside	     1   336
```

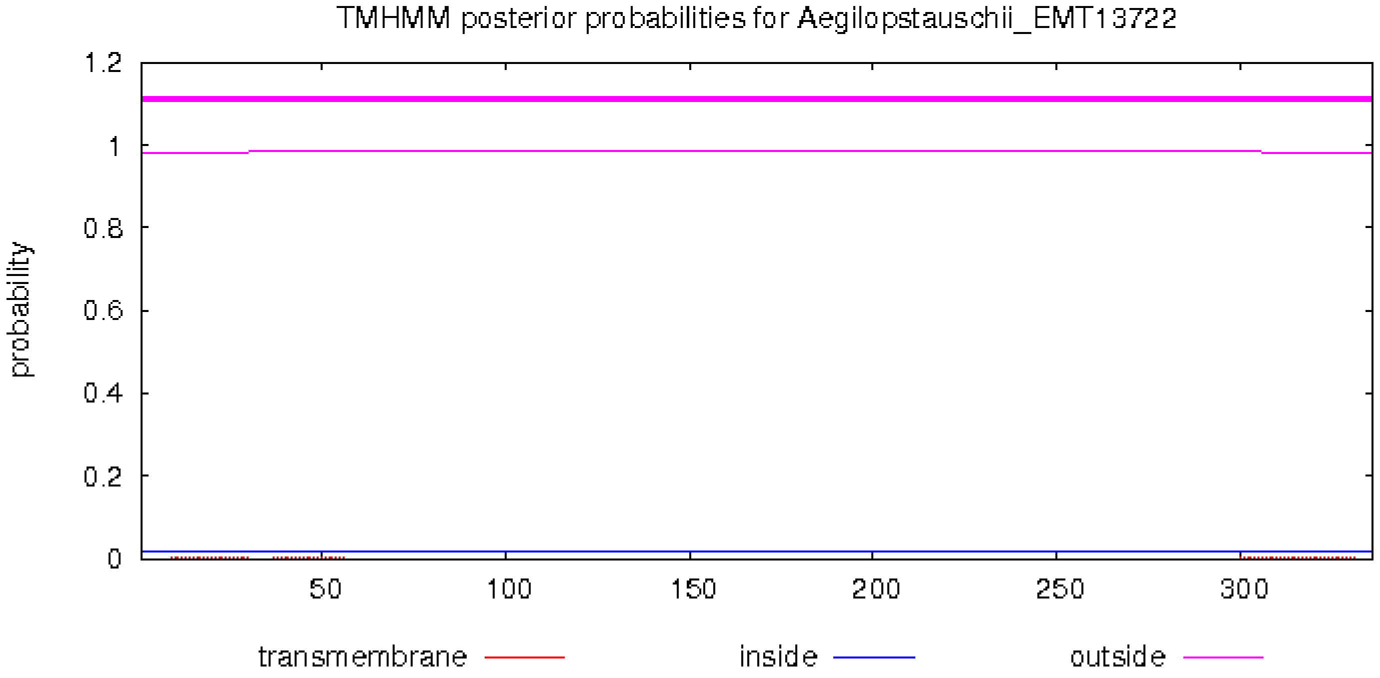

plot in postscript, script for making the plot in gnuplot, data for plot

```
# Aegilopstauschii_EMT13761 Length: 461
# Aegilopstauschii_EMT13761 Number of predicted TMHs:  0
# Aegilopstauschii_EMT13761 Exp number of AAs in TMHs: 0.90989
# Aegilopstauschii_EMT13761 Exp number, first 60 AAs:  0.65776
# Aegilopstauschii_EMT13761 Total prob of N-in:        0.04626
Aegilopstauschii_EMT13761       TMHMM2.0        outside      1   461
```

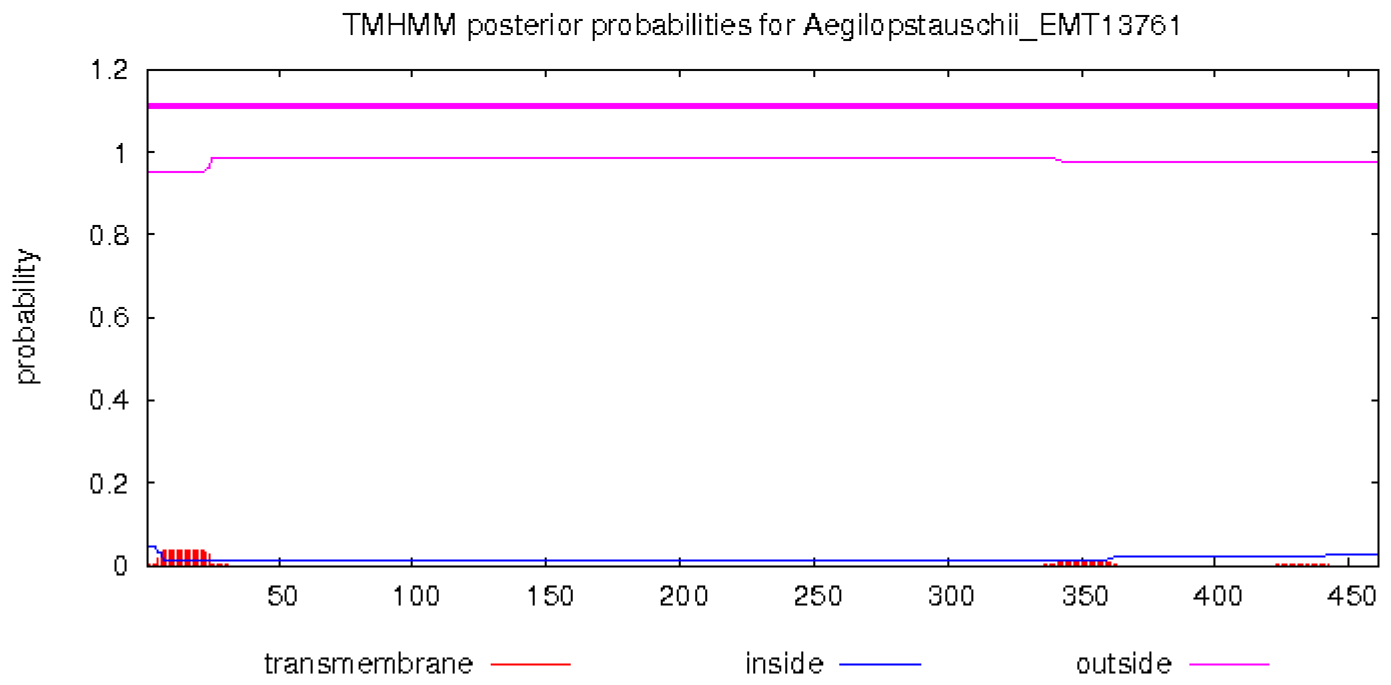

plot in postscript, script for making the plot in gnuplot, data for plot

---

```
# Aegilopstauschii_EMT13975 Length: 256
# Aegilopstauschii_EMT13975 Number of predicted TMHs:  0
# Aegilopstauschii_EMT13975 Exp number of AAs in TMHs: 0.00188
# Aegilopstauschii_EMT13975 Exp number, first 60 AAs:  0
# Aegilopstauschii_EMT13975 Total prob of N-in:        0.01305
Aegilopstauschii_EMT13975       TMHMM2.0        outside      1   256
```

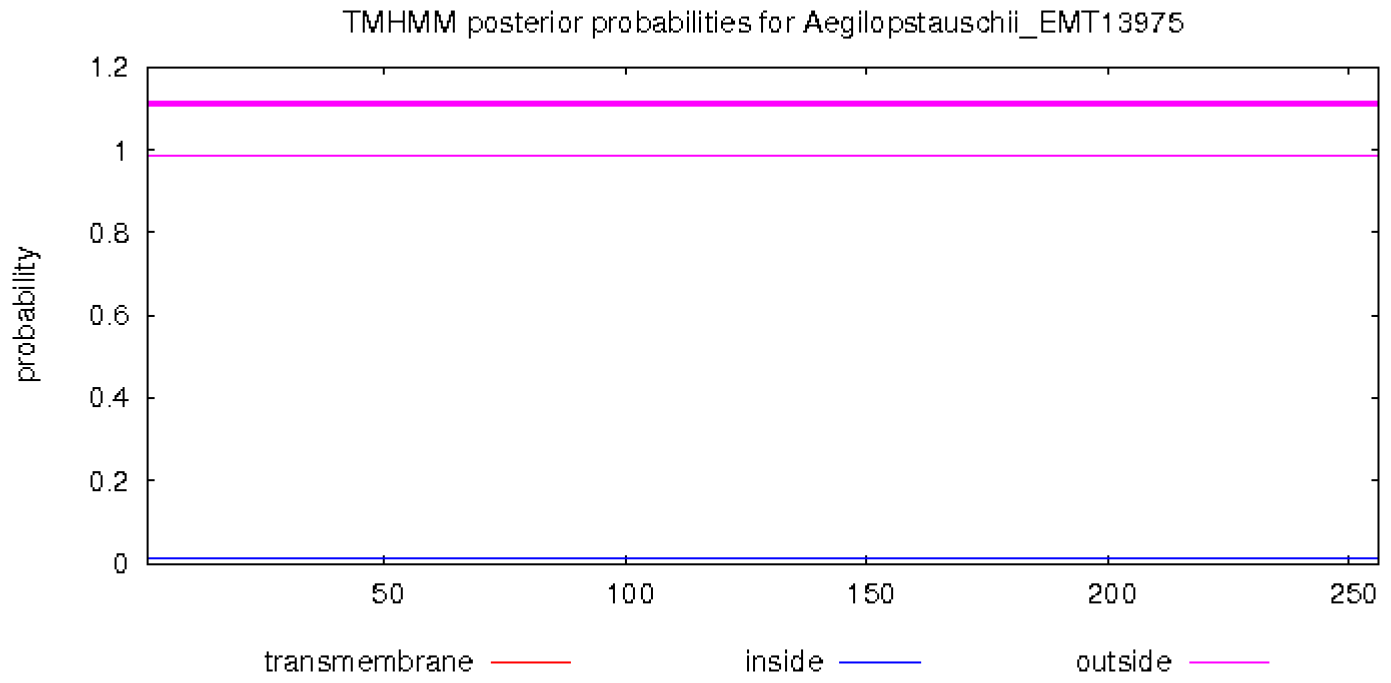

plot in postscript, script for making the plot in gnuplot, data for plot

---

```
# Aegilopstauschii_EMT14094 Length: 402
# Aegilopstauschii_EMT14094 Number of predicted TMHs:  0
# Aegilopstauschii_EMT14094 Exp number of AAs in TMHs: 0.00059
# Aegilopstauschii_EMT14094 Exp number, first 60 AAs:  0
# Aegilopstauschii_EMT14094 Total prob of N-in:        0.00390
Aegilopstauschii_EMT14094       TMHMM2.0        outside      1   402
```

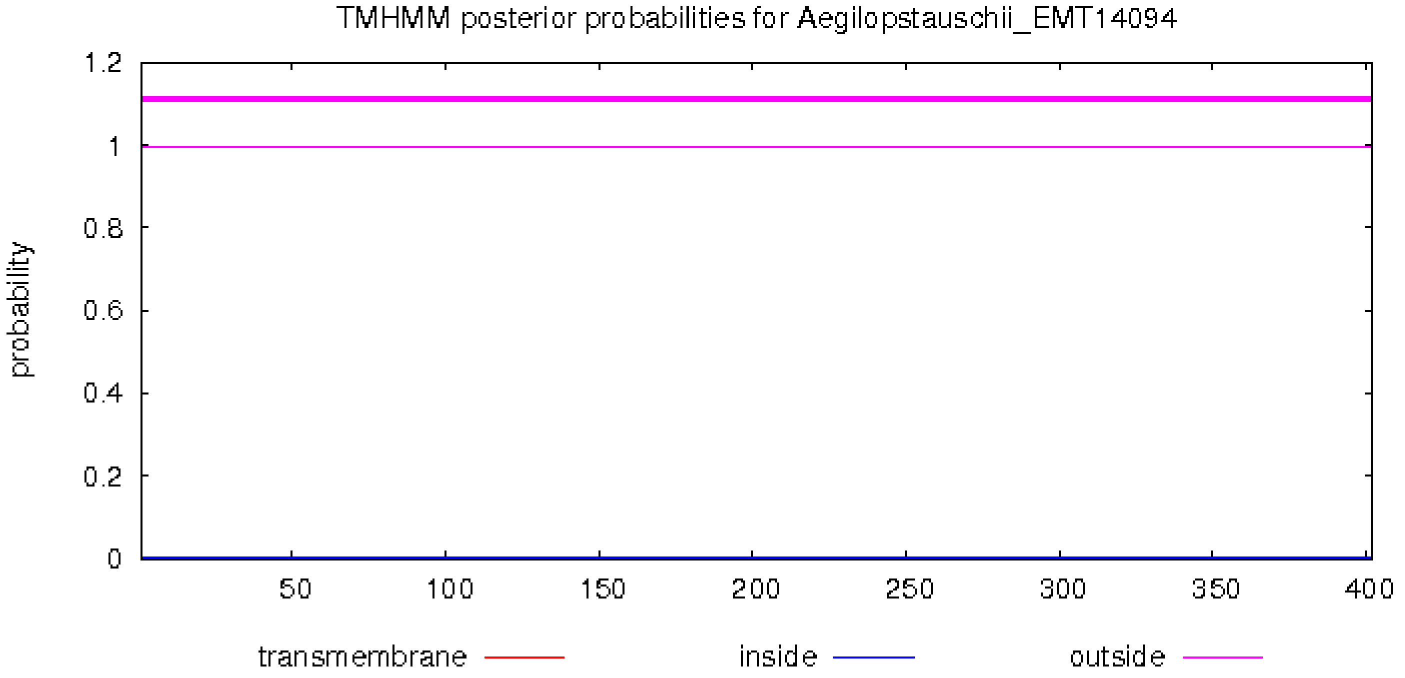

plot in postscript, script for making the plot in gnuplot, data for plot

```
# Aegilopstauschii_EMT14095 Length: 354
# Aegilopstauschii_EMT14095 Number of predicted TMHs:  0
# Aegilopstauschii_EMT14095 Exp number of AAs in TMHs: 0.00063
# Aegilopstauschii_EMT14095 Exp number, first 60 AAs:  0.00016
# Aegilopstauschii_EMT14095 Total prob of N-in:        0.00405
Aegilopstauschii_EMT14095       TMHMM2.0        outside      1   354
```

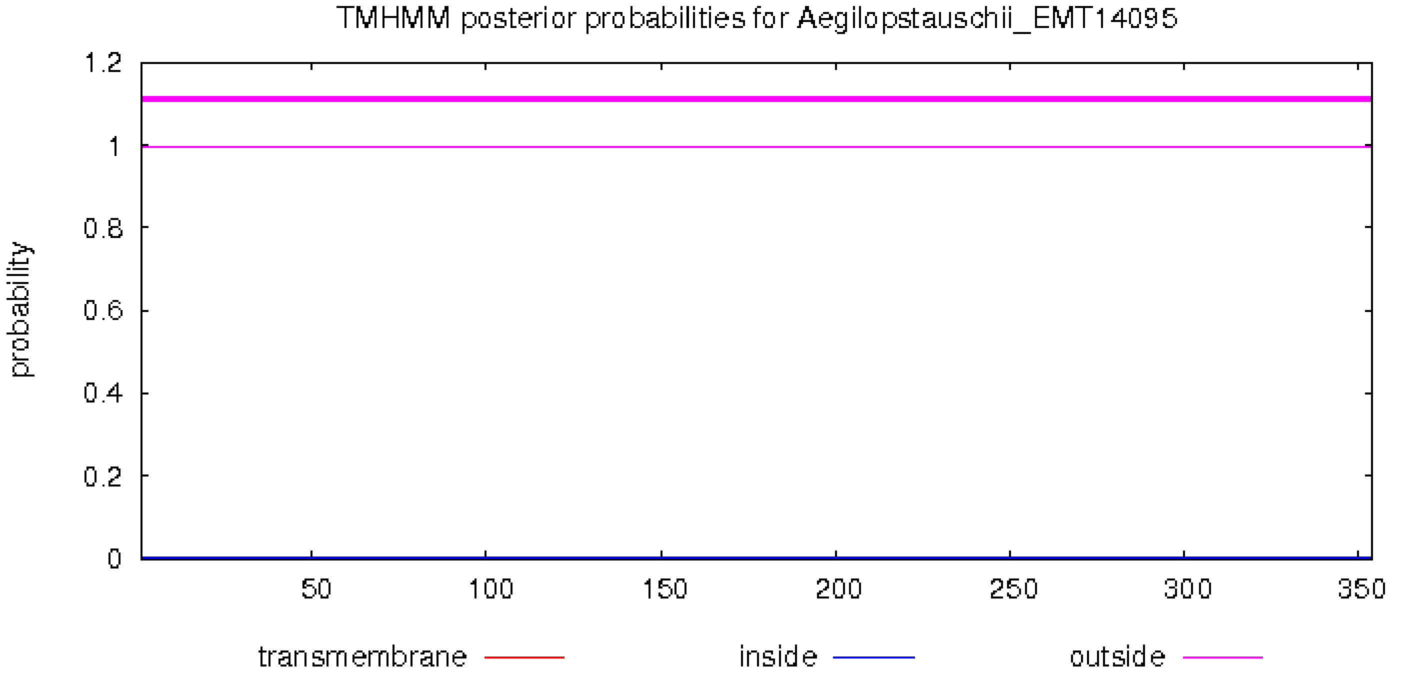

plot in postscript, script for making the plot in gnuplot, data for plot

```
# Aegilopstauschii_EMT14186 Length: 205
# Aegilopstauschii_EMT14186 Number of predicted TMHs:  0
# Aegilopstauschii_EMT14186 Exp number of AAs in TMHs: 0.02475
# Aegilopstauschii_EMT14186 Exp number, first 60 AAs:  0
# Aegilopstauschii_EMT14186 Total prob of N-in:        0.01253
Aegilopstauschii_EMT14186	TMHMM2.0	outside	     1   205
```

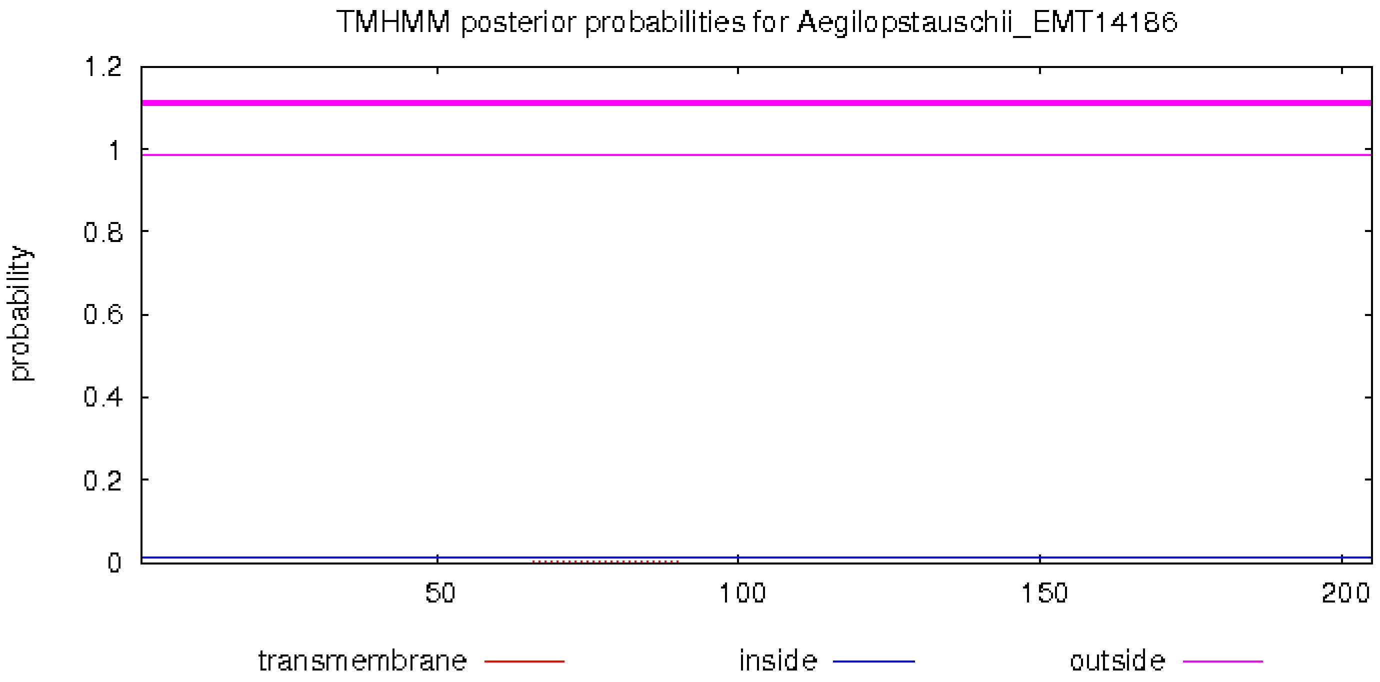

plot in postscript, script for making the plot in gnuplot, data for plot

```
# Aegilopstauschii_EMT14221 Length: 416
# Aegilopstauschii_EMT14221 Number of predicted TMHs:  0
# Aegilopstauschii_EMT14221 Exp number of AAs in TMHs: 0.00675
# Aegilopstauschii_EMT14221 Exp number, first 60 AAs:  0.00588
# Aegilopstauschii_EMT14221 Total prob of N-in:        0.03707
Aegilopstauschii_EMT14221	TMHMM2.0	outside	     1   416
```

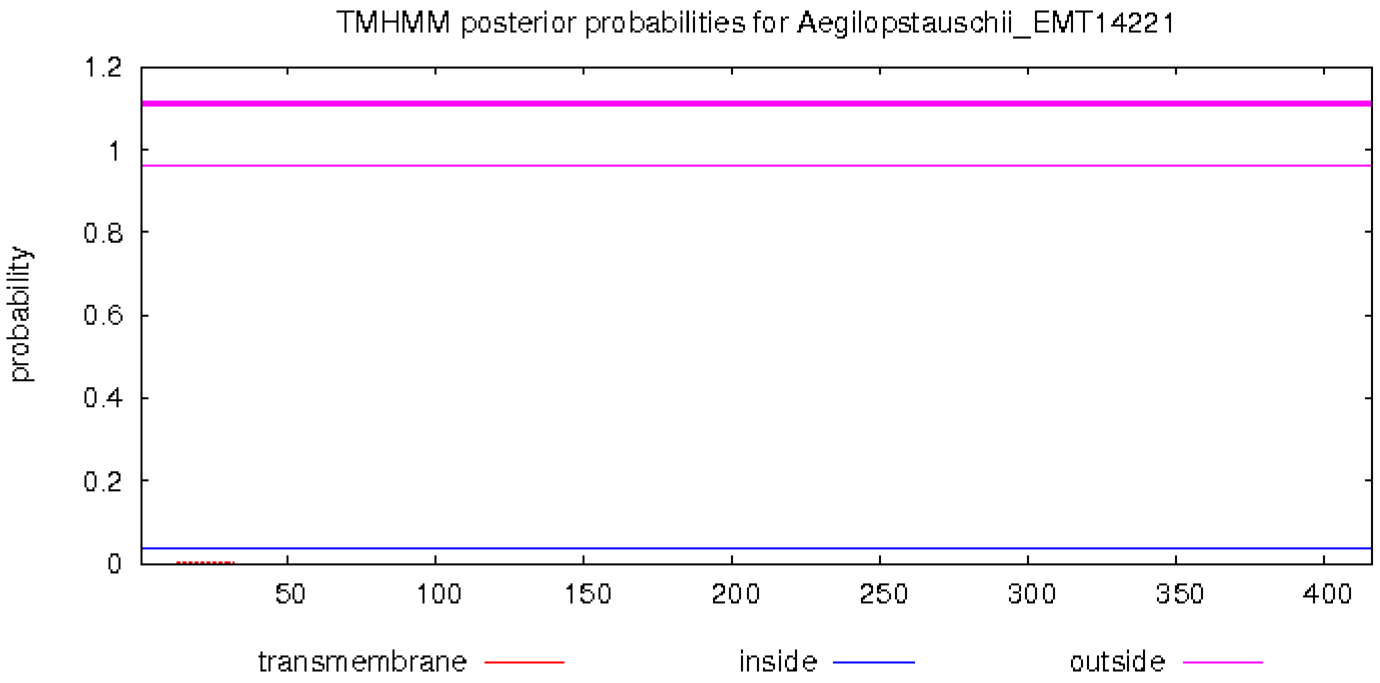

plot in postscript, script for making the plot in gnuplot, data for plot

```
# Aegilopstauschii_EMT14569 Length: 402
# Aegilopstauschii_EMT14569 Number of predicted TMHs:  0
# Aegilopstauschii_EMT14569 Exp number of AAs in TMHs: 0.0002
# Aegilopstauschii_EMT14569 Exp number, first 60 AAs:  0.0002
# Aegilopstauschii_EMT14569 Total prob of N-in:        0.00199
Aegilopstauschii_EMT14569	TMHMM2.0	outside	     1   402
```

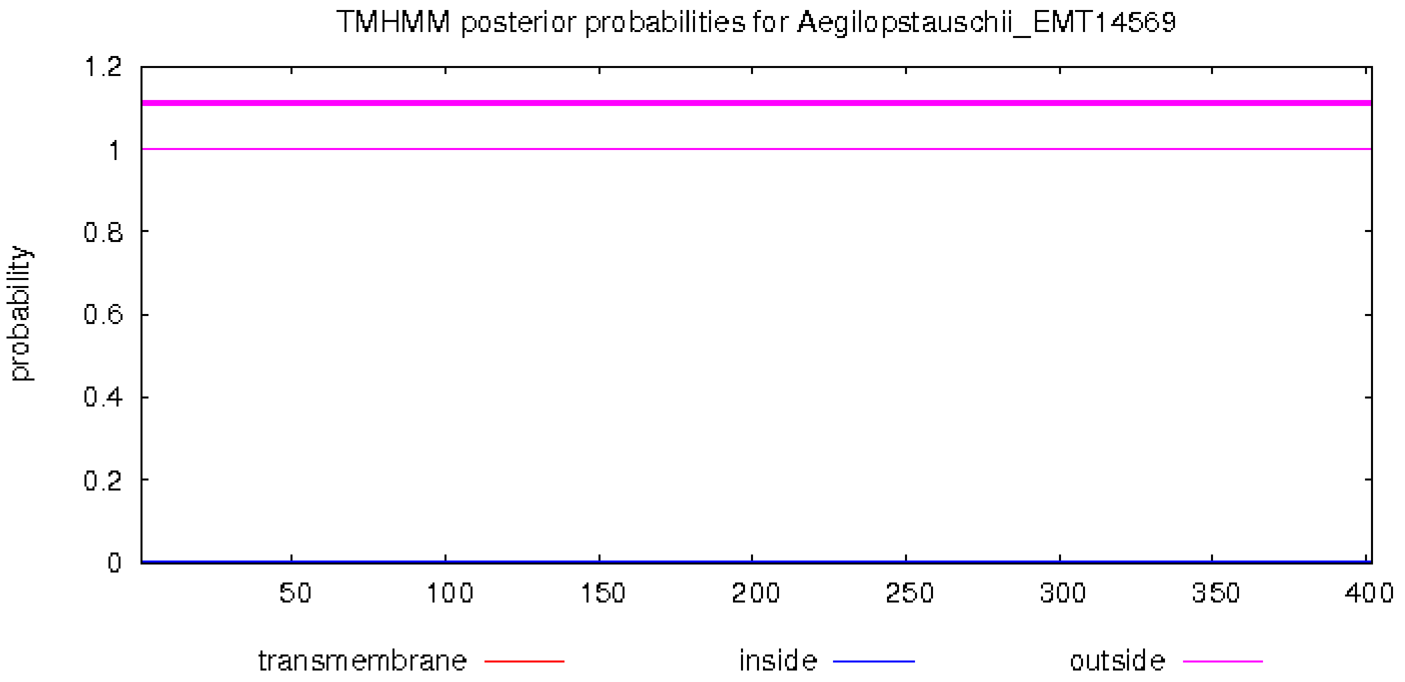

plot in postscript, script for making the plot in gnuplot, data for plot

---

```
# Aegilopstauschii_EMT14807 Length: 291
# Aegilopstauschii_EMT14807 Number of predicted TMHs:  0
# Aegilopstauschii_EMT14807 Exp number of AAs in TMHs: 0.00801
# Aegilopstauschii_EMT14807 Exp number, first 60 AAs:  0
# Aegilopstauschii_EMT14807 Total prob of N-in:        0.02796
Aegilopstauschii_EMT14807	TMHMM2.0	outside	     1   291
```

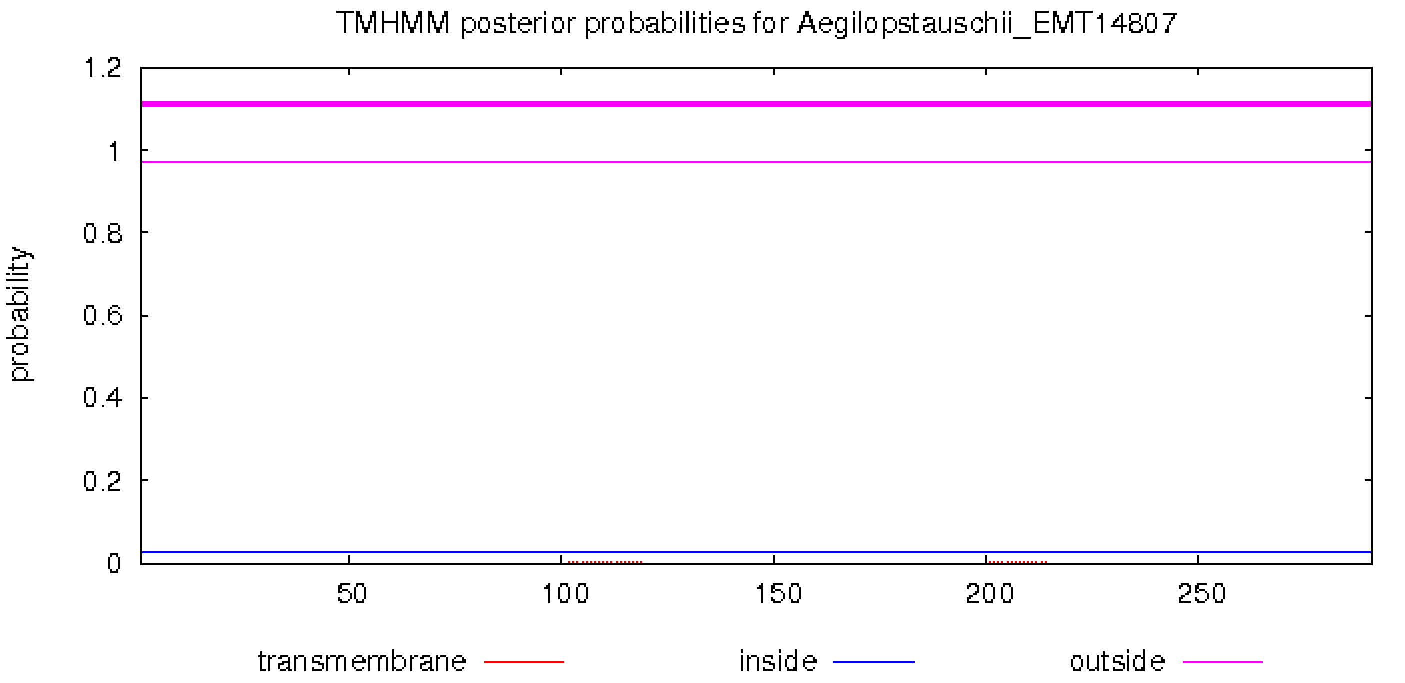

plot in postscript, script for making the plot in gnuplot, data for plot

---

```
# Aegilopstauschii_EMT15405 Length: 248
# Aegilopstauschii_EMT15405 Number of predicted TMHs:  0
# Aegilopstauschii_EMT15405 Exp number of AAs in TMHs: 0
# Aegilopstauschii_EMT15405 Exp number, first 60 AAs:  0
# Aegilopstauschii_EMT15405 Total prob of N-in:        0.00731
Aegilopstauschii_EMT15405       TMHMM2.0        outside      1   248
```

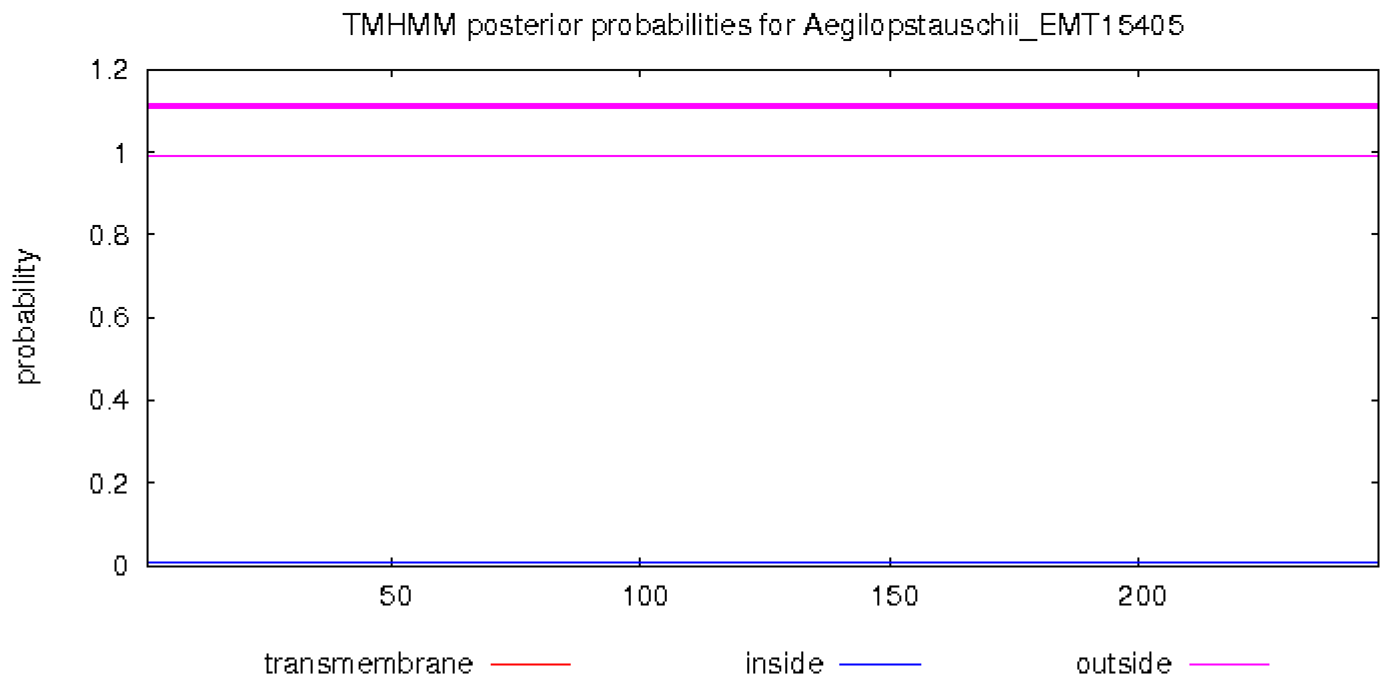

plot in postscript, script for making the plot in gnuplot, data for plot

---

```
# Aegilopstauschii_EMT15453 Length: 283
# Aegilopstauschii_EMT15453 Number of predicted TMHs:  0
# Aegilopstauschii_EMT15453 Exp number of AAs in TMHs: 0
# Aegilopstauschii_EMT15453 Exp number, first 60 AAs:  0
# Aegilopstauschii_EMT15453 Total prob of N-in:        0.05821
Aegilopstauschii_EMT15453       TMHMM2.0        outside      1   283
```

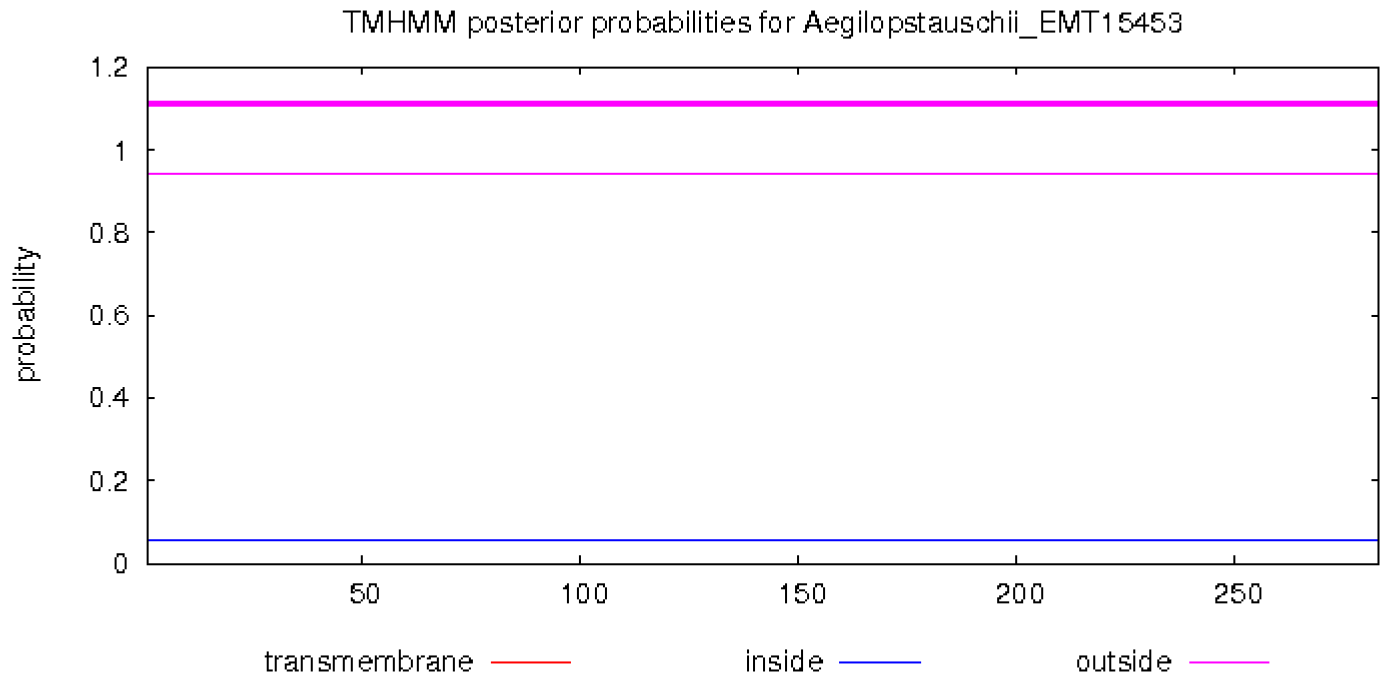

plot in postscript, script for making the plot in gnuplot, data for plot

---

```
# Aegilopstauschii_EMT15468 Length: 439
# Aegilopstauschii_EMT15468 Number of predicted TMHs:  0
# Aegilopstauschii_EMT15468 Exp number of AAs in TMHs: 0
# Aegilopstauschii_EMT15468 Exp number, first 60 AAs:  0
# Aegilopstauschii_EMT15468 Total prob of N-in:        0.00199
Aegilopstauschii_EMT15468       TMHMM2.0        outside      1   439
```

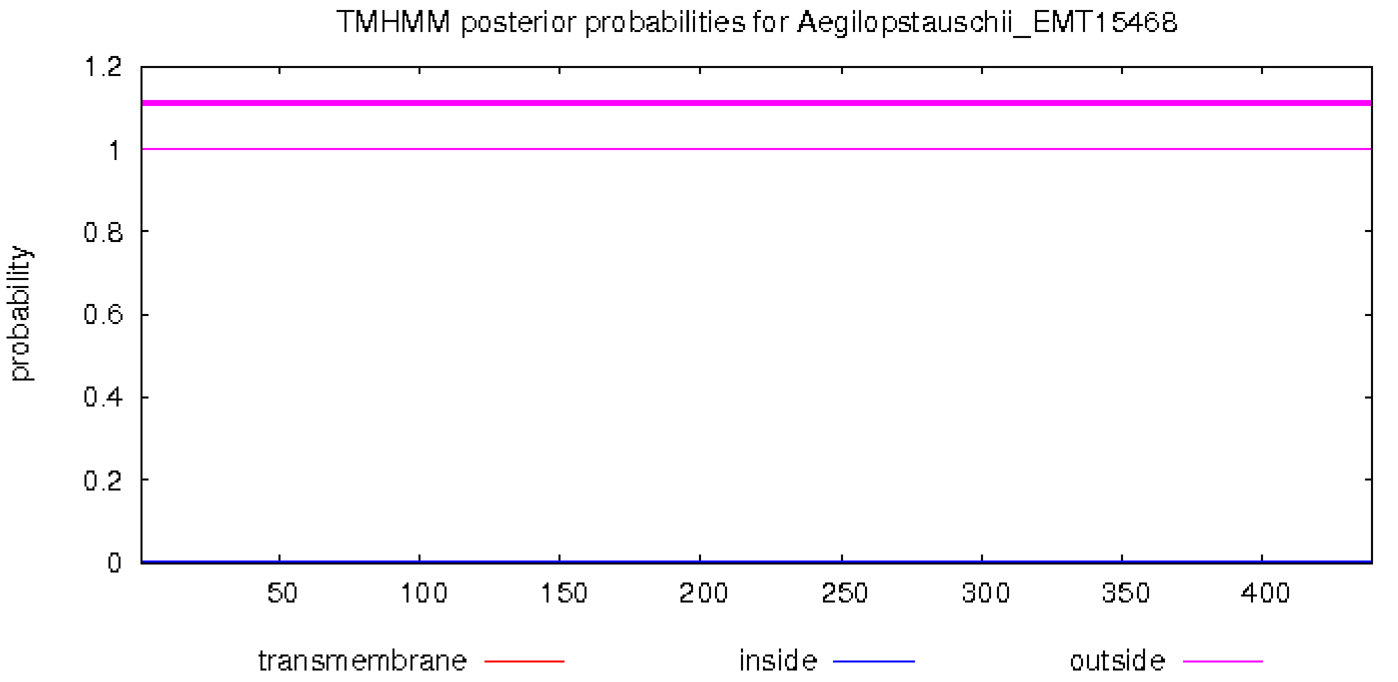

plot in postscript, script for making the plot in gnuplot, data for plot

---

```
# Aegilopstauschii_EMT16679 Length: 302
# Aegilopstauschii_EMT16679 Number of predicted TMHs:  0
# Aegilopstauschii_EMT16679 Exp number of AAs in TMHs: 0.05443
# Aegilopstauschii_EMT16679 Exp number, first 60 AAs:  0
# Aegilopstauschii_EMT16679 Total prob of N-in:        0.05684
Aegilopstauschii_EMT16679       TMHMM2.0        outside      1   302
```

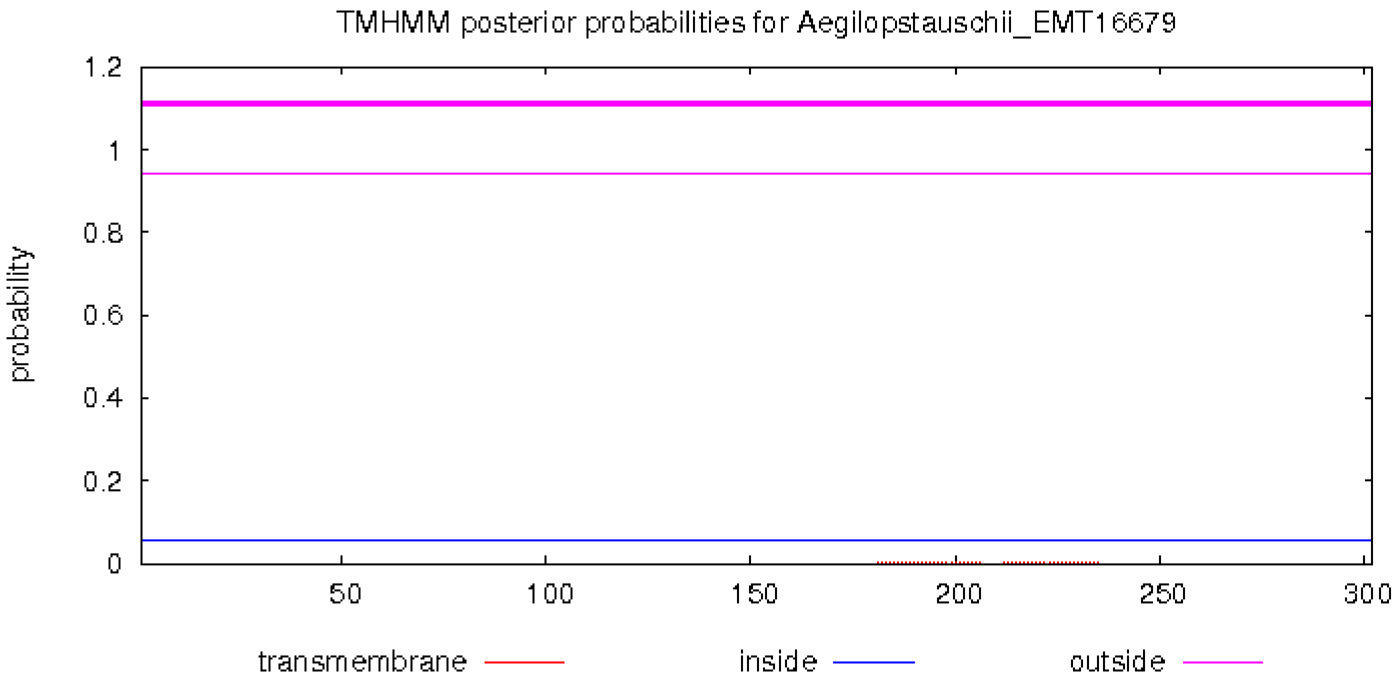

plot in postscript, script for making the plot in gnuplot, data for plot

---

```
# Aegilopstauschii_EMT20495 Length: 303
# Aegilopstauschii_EMT20495 Number of predicted TMHs:  0
# Aegilopstauschii_EMT20495 Exp number of AAs in TMHs: 0.00066
# Aegilopstauschii_EMT20495 Exp number, first 60 AAs:  0
# Aegilopstauschii_EMT20495 Total prob of N-in:        0.01537
Aegilopstauschii_EMT20495       TMHMM2.0        outside      1   303
```

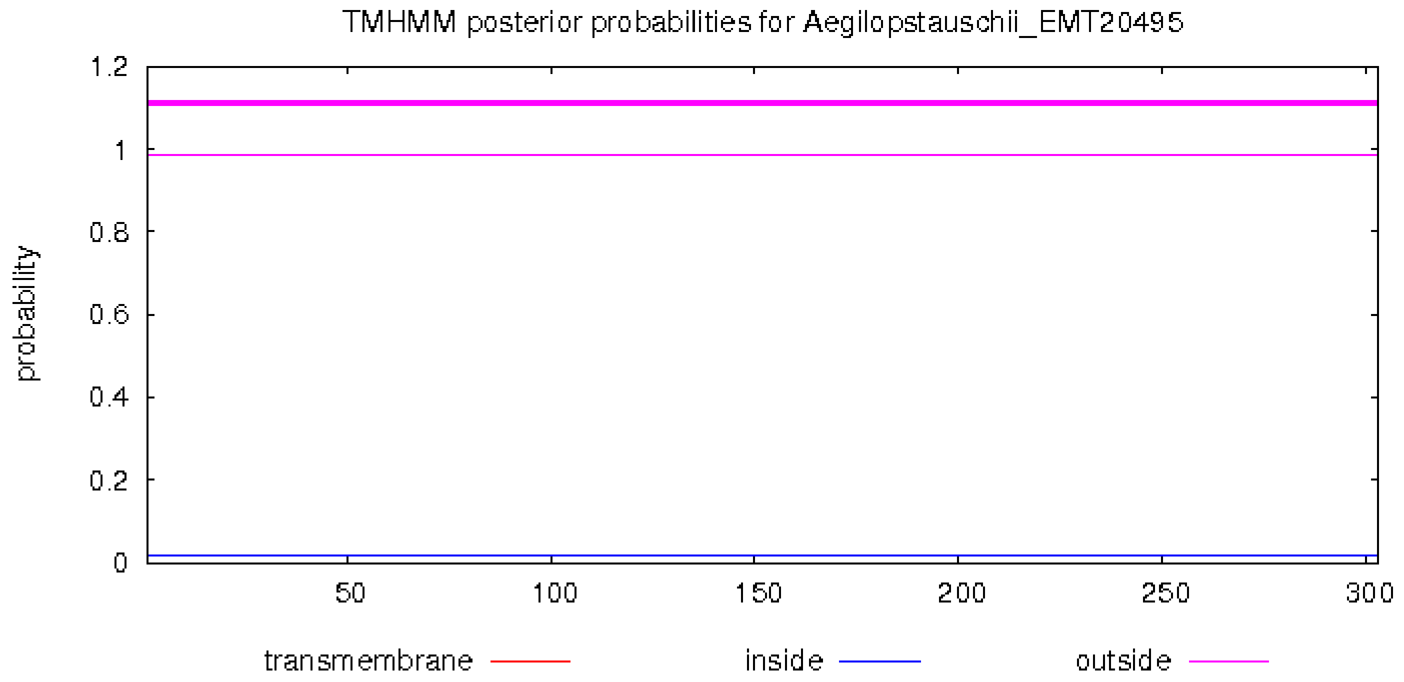

plot in postscript, script for making the plot in gnuplot, data for plot

---

```
# Aegilopstauschii_EMT20593 Length: 365
# Aegilopstauschii_EMT20593 Number of predicted TMHs:  0
# Aegilopstauschii_EMT20593 Exp number of AAs in TMHs: 0.01713
# Aegilopstauschii_EMT20593 Exp number, first 60 AAs:  0
# Aegilopstauschii_EMT20593 Total prob of N-in:        0.01931
Aegilopstauschii_EMT20593       TMHMM2.0        outside      1   365
```

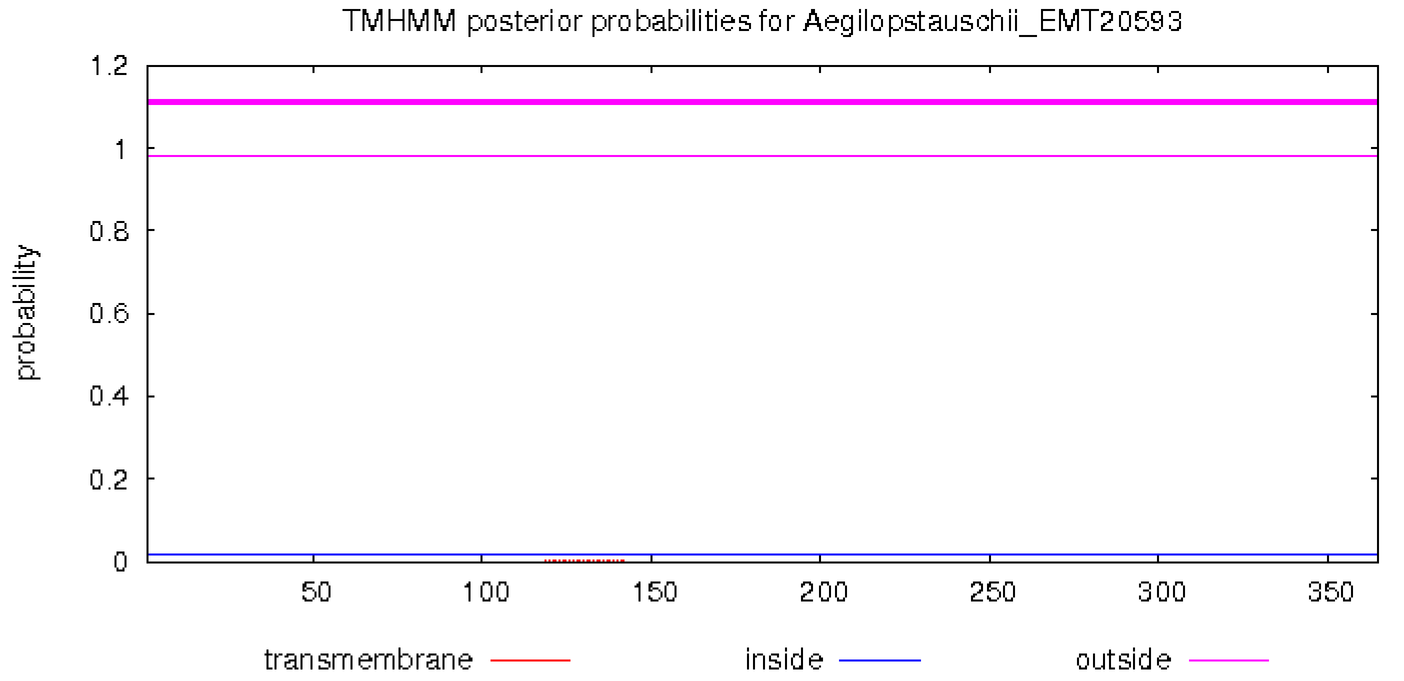

plot in postscript, script for making the plot in gnuplot, data for plot

---

```
# Aegilopstauschii_EMT20647 Length: 279
# Aegilopstauschii_EMT20647 Number of predicted TMHs:  0
# Aegilopstauschii_EMT20647 Exp number of AAs in TMHs: 3.90349
# Aegilopstauschii_EMT20647 Exp number, first 60 AAs:  0
# Aegilopstauschii_EMT20647 Total prob of N-in:        0.01325
Aegilopstauschii_EMT20647	TMHMM2.0	outside	     1   279
```

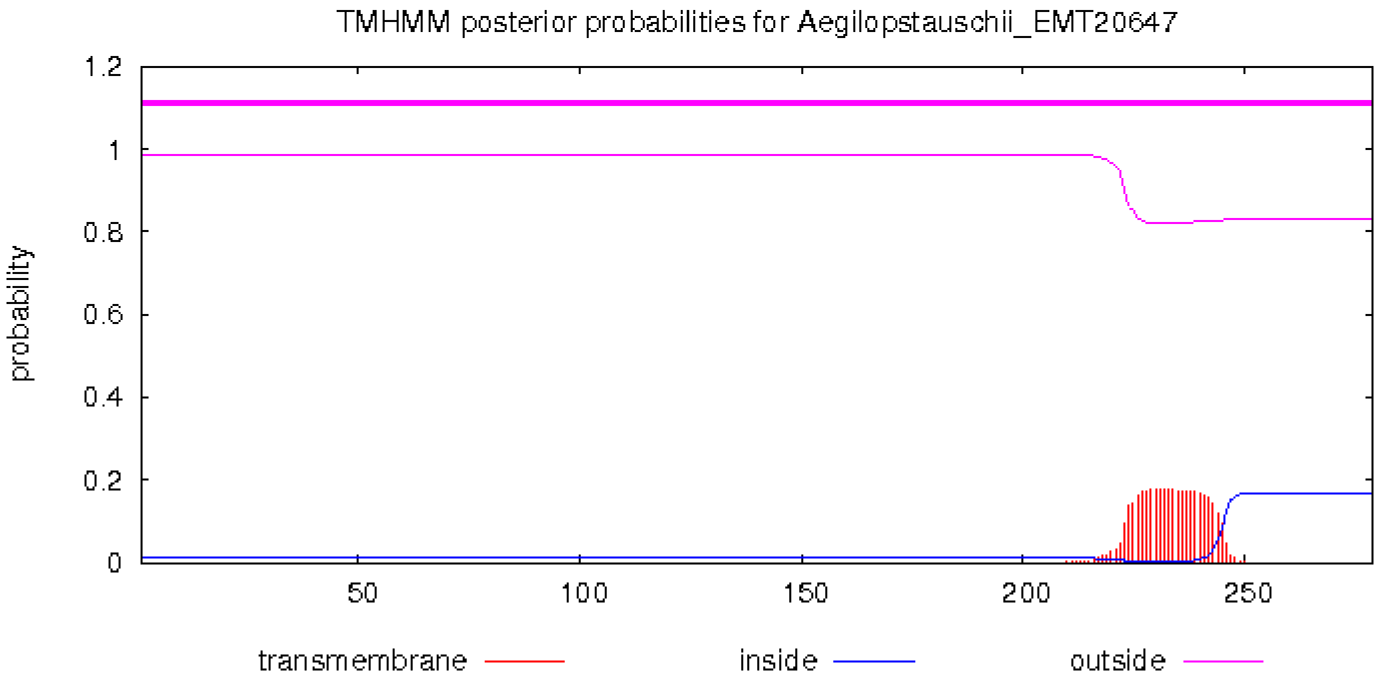

plot in postscript, script for making the plot in gnuplot, data for plot

---

```
# Aegilopstauschii_EMT20851 Length: 240
# Aegilopstauschii_EMT20851 Number of predicted TMHs:  0
# Aegilopstauschii_EMT20851 Exp number of AAs in TMHs: 0.04907
# Aegilopstauschii_EMT20851 Exp number, first 60 AAs:  0.04408
# Aegilopstauschii_EMT20851 Total prob of N-in:        0.04544
Aegilopstauschii_EMT20851	TMHMM2.0	outside	     1   240
```

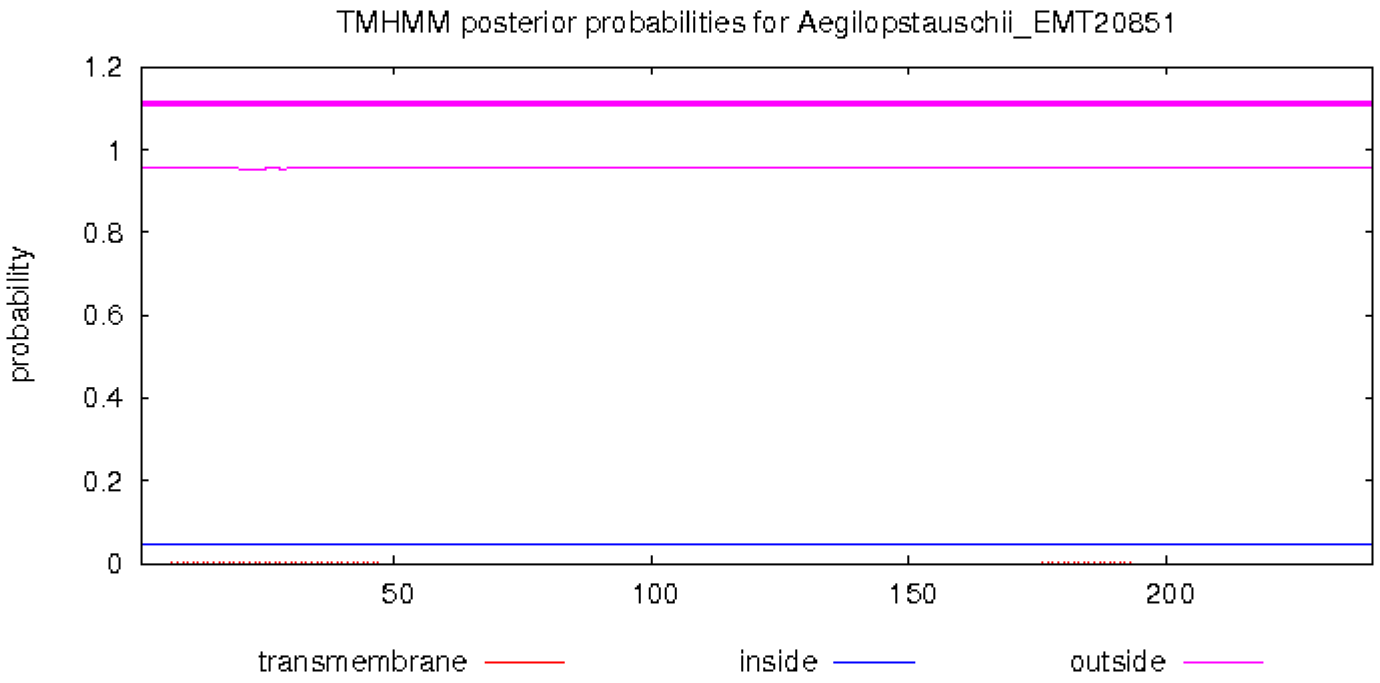

plot in postscript, script for making the plot in gnuplot, data for plot

---

```
# Aegilopstauschii_EMT21053 Length: 504
# Aegilopstauschii_EMT21053 Number of predicted TMHs:  0
# Aegilopstauschii_EMT21053 Exp number of AAs in TMHs: 7.07869
# Aegilopstauschii_EMT21053 Exp number, first 60 AAs:  5.73525
# Aegilopstauschii_EMT21053 Total prob of N-in:        0.16371
Aegilopstauschii_EMT21053	TMHMM2.0	outside	     1   504
```

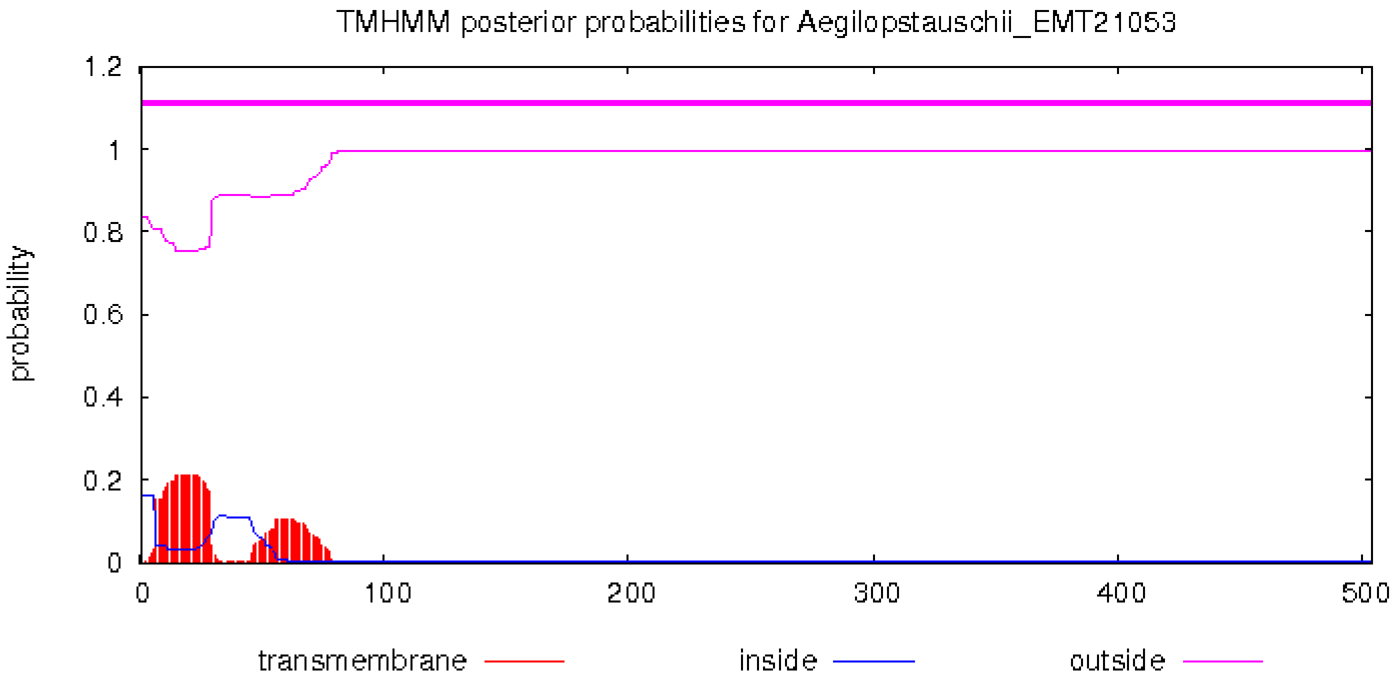

plot in postscript, script for making the plot in gnuplot, data for plot

```
# Aegilopstauschii_EMT21398 Length: 347
# Aegilopstauschii_EMT21398 Number of predicted TMHs:  0
# Aegilopstauschii_EMT21398 Exp number of AAs in TMHs: 0.09892
# Aegilopstauschii_EMT21398 Exp number, first 60 AAs:  0.00118
# Aegilopstauschii_EMT21398 Total prob of N-in:        0.03667
Aegilopstauschii_EMT21398	TMHMM2.0	outside	     1   347
```

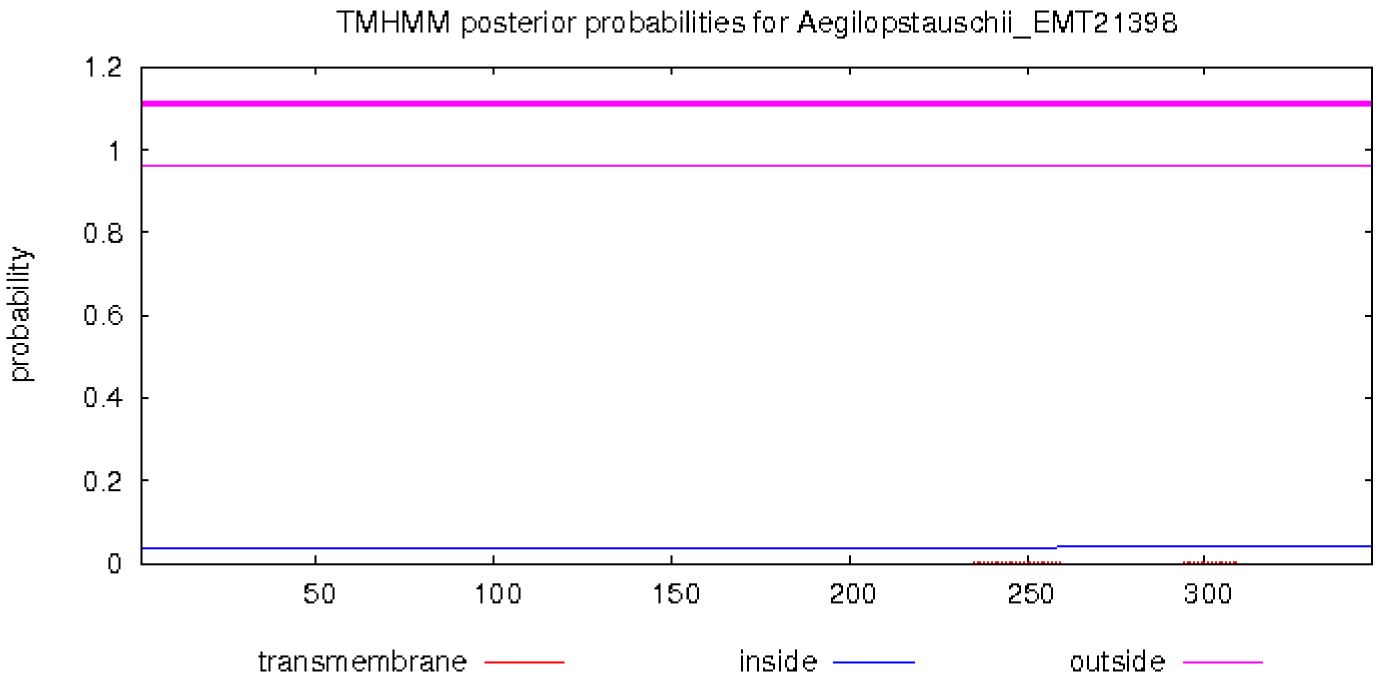

plot in postscript, script for making the plot in gnuplot, data for plot

```
# Aegilopstauschii_EMT21400 Length: 353
# Aegilopstauschii_EMT21400 Number of predicted TMHs:  0
# Aegilopstauschii_EMT21400 Exp number of AAs in TMHs: 0.01872
# Aegilopstauschii_EMT21400 Exp number, first 60 AAs:  0.0004
# Aegilopstauschii_EMT21400 Total prob of N-in:        0.04418
Aegilopstauschii_EMT21400	TMHMM2.0	outside	     1   353
```

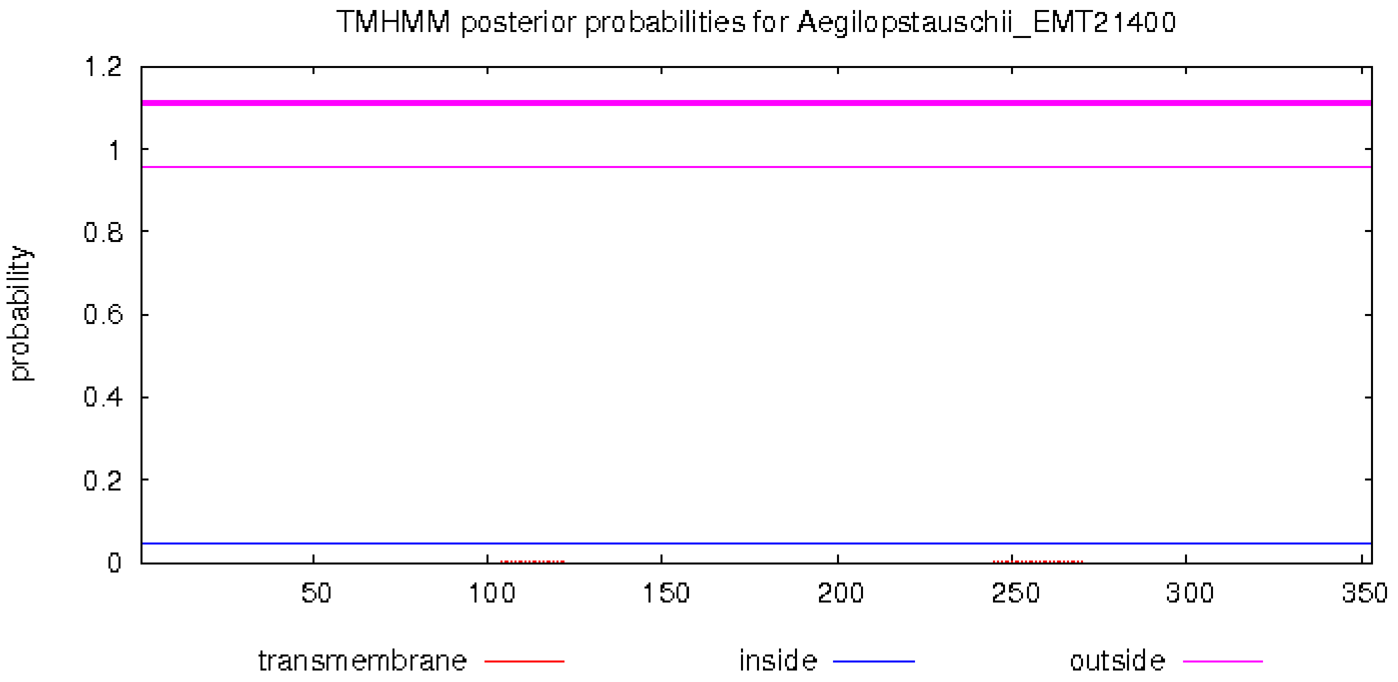

plot in postscript, script for making the plot in gnuplot, data for plot

---

```
# Aegilopstauschii_EMT21589 Length: 344
# Aegilopstauschii_EMT21589 Number of predicted TMHs:  0
# Aegilopstauschii_EMT21589 Exp number of AAs in TMHs: 0.00068
# Aegilopstauschii_EMT21589 Exp number, first 60 AAs:  0.00068
# Aegilopstauschii_EMT21589 Total prob of N-in:        0.00236
Aegilopstauschii_EMT21589	TMHMM2.0	outside	     1   344
```

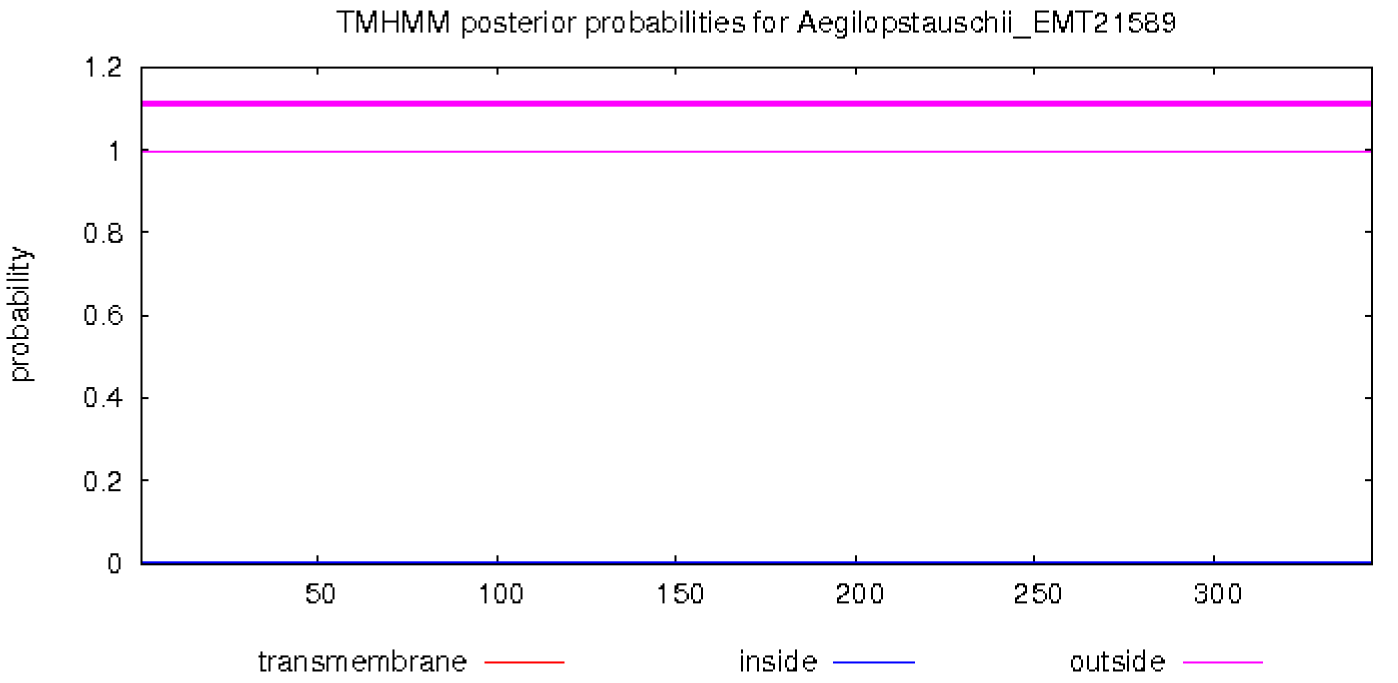

plot in postscript, script for making the plot in gnuplot, data for plot

---

```
# Aegilopstauschii_EMT21626 Length: 336
# Aegilopstauschii_EMT21626 Number of predicted TMHs:  0
# Aegilopstauschii_EMT21626 Exp number of AAs in TMHs: 0
# Aegilopstauschii_EMT21626 Exp number, first 60 AAs:  0
# Aegilopstauschii_EMT21626 Total prob of N-in:        0.00918
Aegilopstauschii_EMT21626       TMHMM2.0        outside      1   336
```

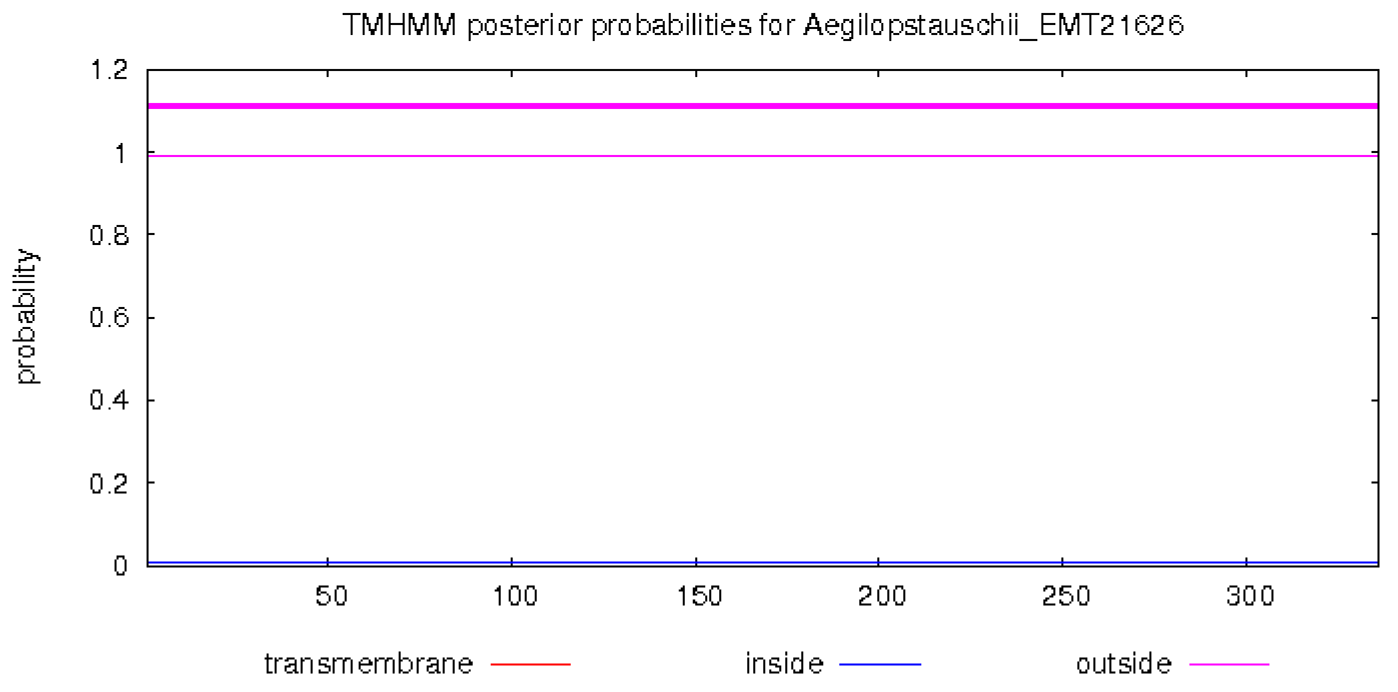

plot in postscript, script for making the plot in gnuplot, data for plot

---

```
# Aegilopstauschii_EMT21706 Length: 1032
# Aegilopstauschii_EMT21706 Number of predicted TMHs:  2
# Aegilopstauschii_EMT21706 Exp number of AAs in TMHs: 38.00929
# Aegilopstauschii_EMT21706 Exp number, first 60 AAs:  0
# Aegilopstauschii_EMT21706 Total prob of N-in:        0.00287
Aegilopstauschii_EMT21706       TMHMM2.0        outside      1   616
Aegilopstauschii_EMT21706       TMHMM2.0        TMhelix    617   639
Aegilopstauschii_EMT21706       TMHMM2.0        inside     640   650
Aegilopstauschii_EMT21706       TMHMM2.0        TMhelix    651   673
Aegilopstauschii_EMT21706       TMHMM2.0        outside    674  1032
```

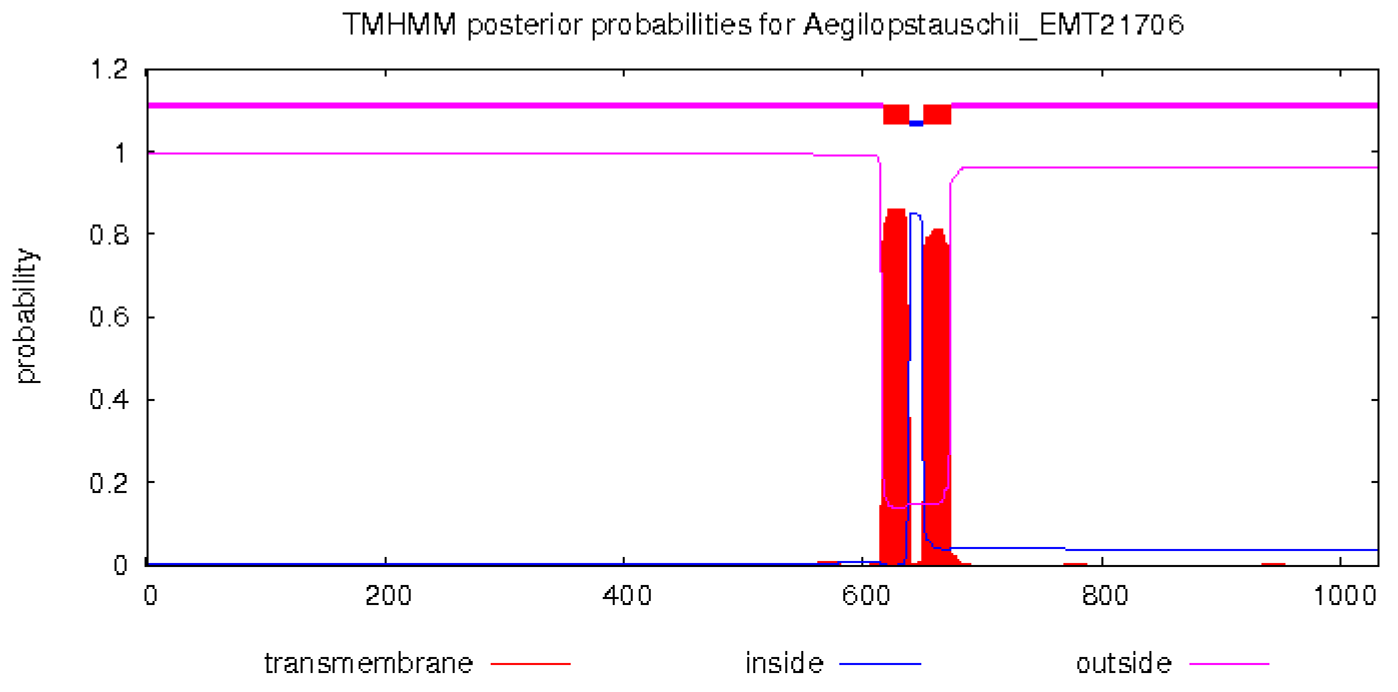

plot in postscript, script for making the plot in gnuplot, data for plot

```
# Aegilopstauschii_EMT22193 Length: 362
# Aegilopstauschii_EMT22193 Number of predicted TMHs:  0
# Aegilopstauschii_EMT22193 Exp number of AAs in TMHs: 0.00159
# Aegilopstauschii_EMT22193 Exp number, first 60 AAs:  2e-05
# Aegilopstauschii_EMT22193 Total prob of N-in:        0.00293
Aegilopstauschii_EMT22193       TMHMM2.0        outside      1   362
```

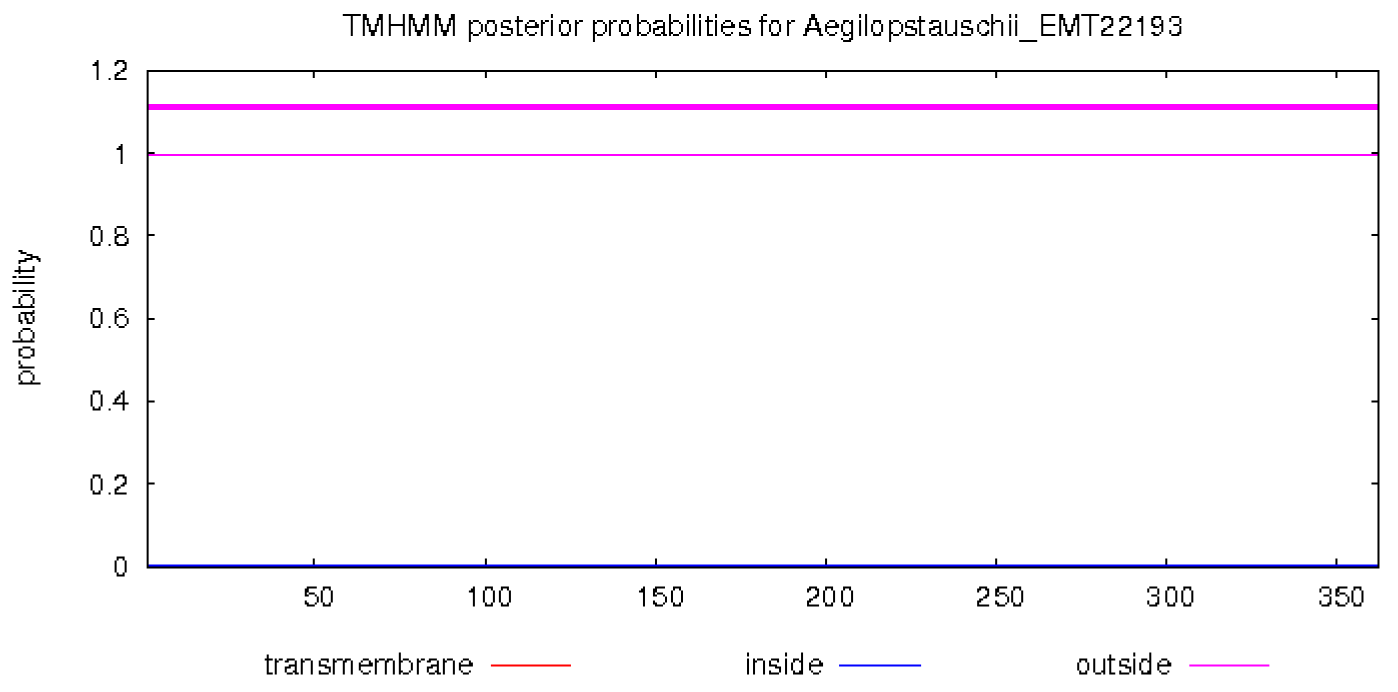

plot in postscript, script for making the plot in gnuplot, data for plot

```
# Aegilopstauschii_EMT22201 Length: 429
# Aegilopstauschii_EMT22201 Number of predicted TMHs:  0
# Aegilopstauschii_EMT22201 Exp number of AAs in TMHs: 0.00018
# Aegilopstauschii_EMT22201 Exp number, first 60 AAs:  0.00018
# Aegilopstauschii_EMT22201 Total prob of N-in:        0.00095
Aegilopstauschii_EMT22201       TMHMM2.0        outside      1   429
```

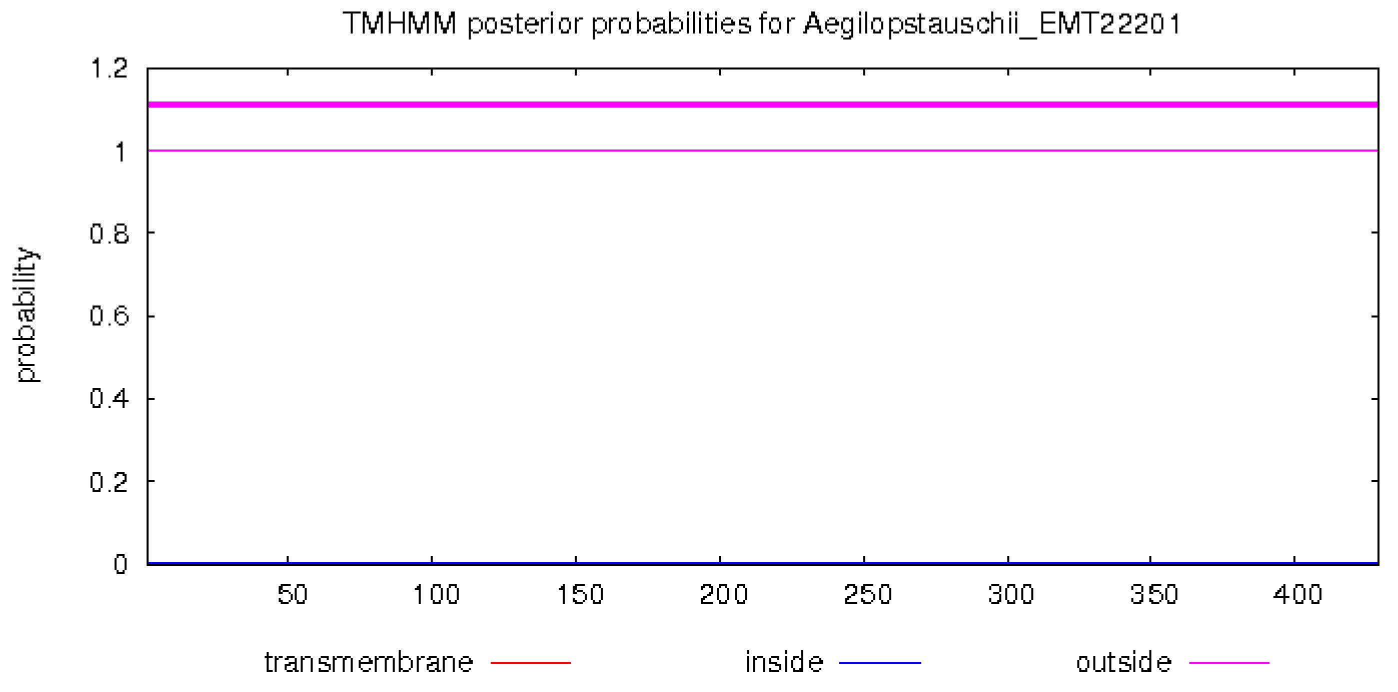

plot in postscript, script for making the plot in gnuplot, data for plot

```
# Aegilopstauschii_EMT22922 Length: 215
# Aegilopstauschii_EMT22922 Number of predicted TMHs:  0
# Aegilopstauschii_EMT22922 Exp number of AAs in TMHs: 0
# Aegilopstauschii_EMT22922 Exp number, first 60 AAs:  0
# Aegilopstauschii_EMT22922 Total prob of N-in:        0.12806
Aegilopstauschii_EMT22922	TMHMM2.0	outside	     1   215
```

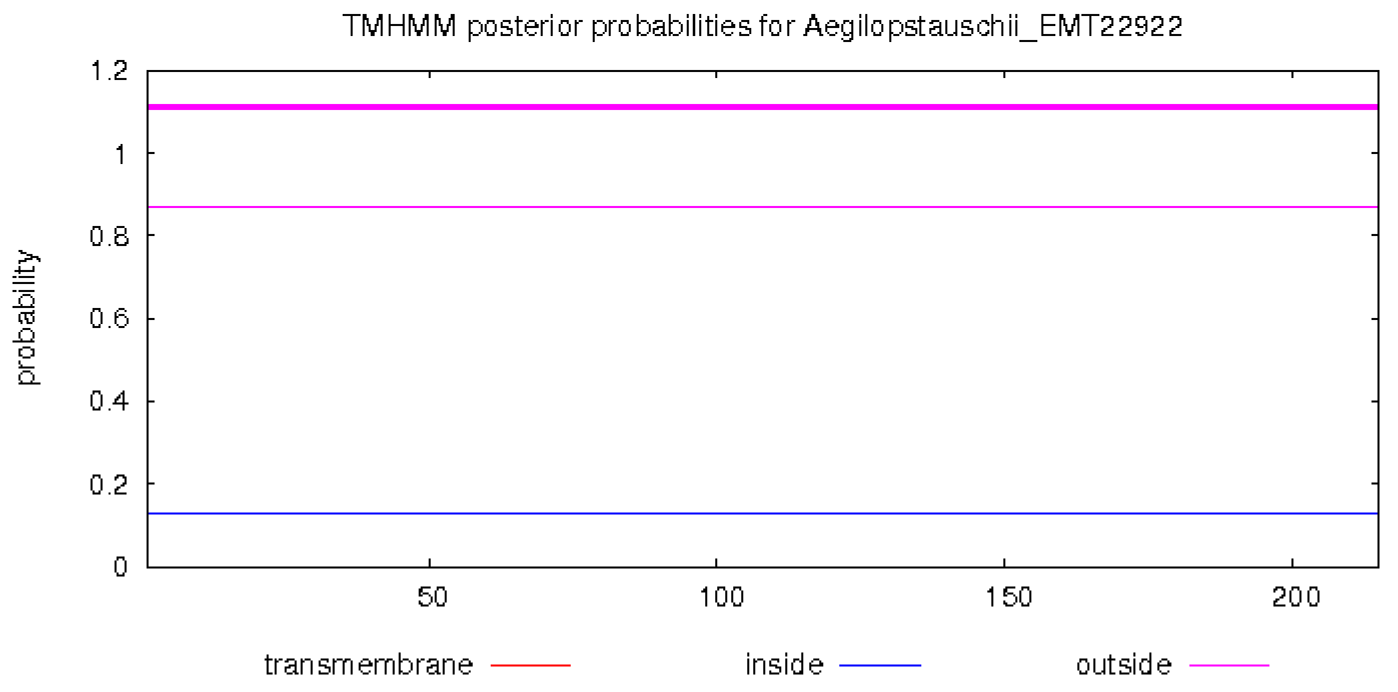

plot in postscript, script for making the plot in gnuplot, data for plot

```
# Aegilopstauschii_EMT23410 Length: 349
# Aegilopstauschii_EMT23410 Number of predicted TMHs:  0
# Aegilopstauschii_EMT23410 Exp number of AAs in TMHs: 0.01274
# Aegilopstauschii_EMT23410 Exp number, first 60 AAs:  0.0005
# Aegilopstauschii_EMT23410 Total prob of N-in:        0.04882
Aegilopstauschii_EMT23410	TMHMM2.0	outside	     1   349
```

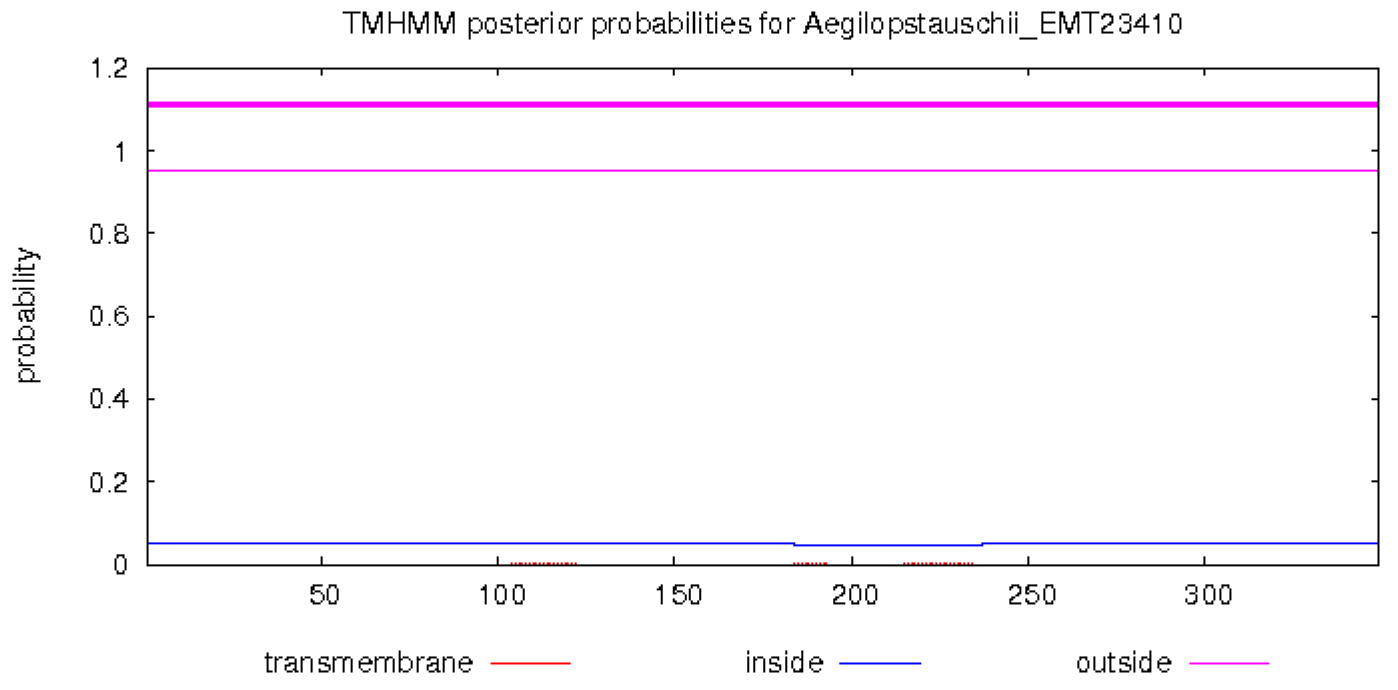

plot in postscript, script for making the plot in gnuplot, data for plot

```
# Aegilopstauschii_EMT23614 Length: 599
# Aegilopstauschii_EMT23614 Number of predicted TMHs:  0
# Aegilopstauschii_EMT23614 Exp number of AAs in TMHs: 0.06545
# Aegilopstauschii_EMT23614 Exp number, first 60 AAs:  0
# Aegilopstauschii_EMT23614 Total prob of N-in:        0.00004
Aegilopstauschii_EMT23614       TMHMM2.0        outside      1   599
```

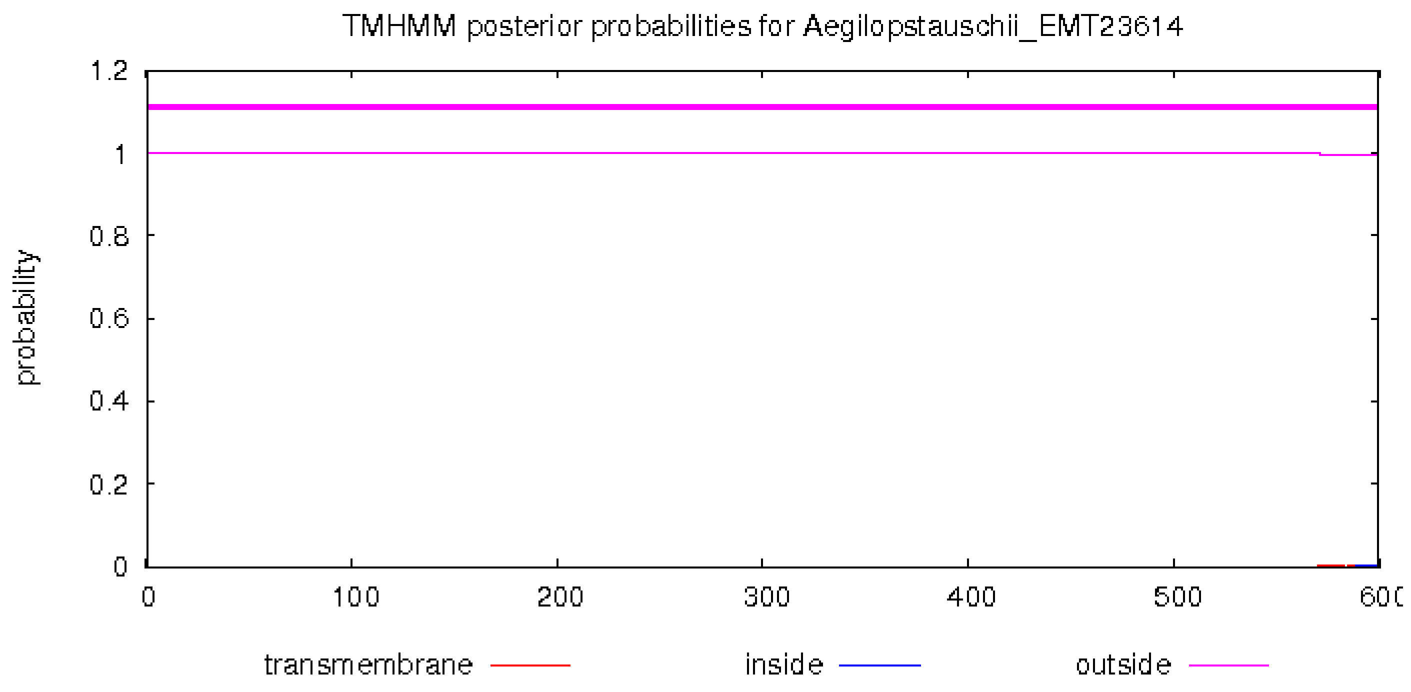

plot in postscript, script for making the plot in gnuplot, data for plot

```
# Aegilopstauschii_EMT24079 Length: 237
# Aegilopstauschii_EMT24079 Number of predicted TMHs:  0
# Aegilopstauschii_EMT24079 Exp number of AAs in TMHs: 0
# Aegilopstauschii_EMT24079 Exp number, first 60 AAs:  0
# Aegilopstauschii_EMT24079 Total prob of N-in:        0.04373
Aegilopstauschii_EMT24079       TMHMM2.0        outside      1   237
```

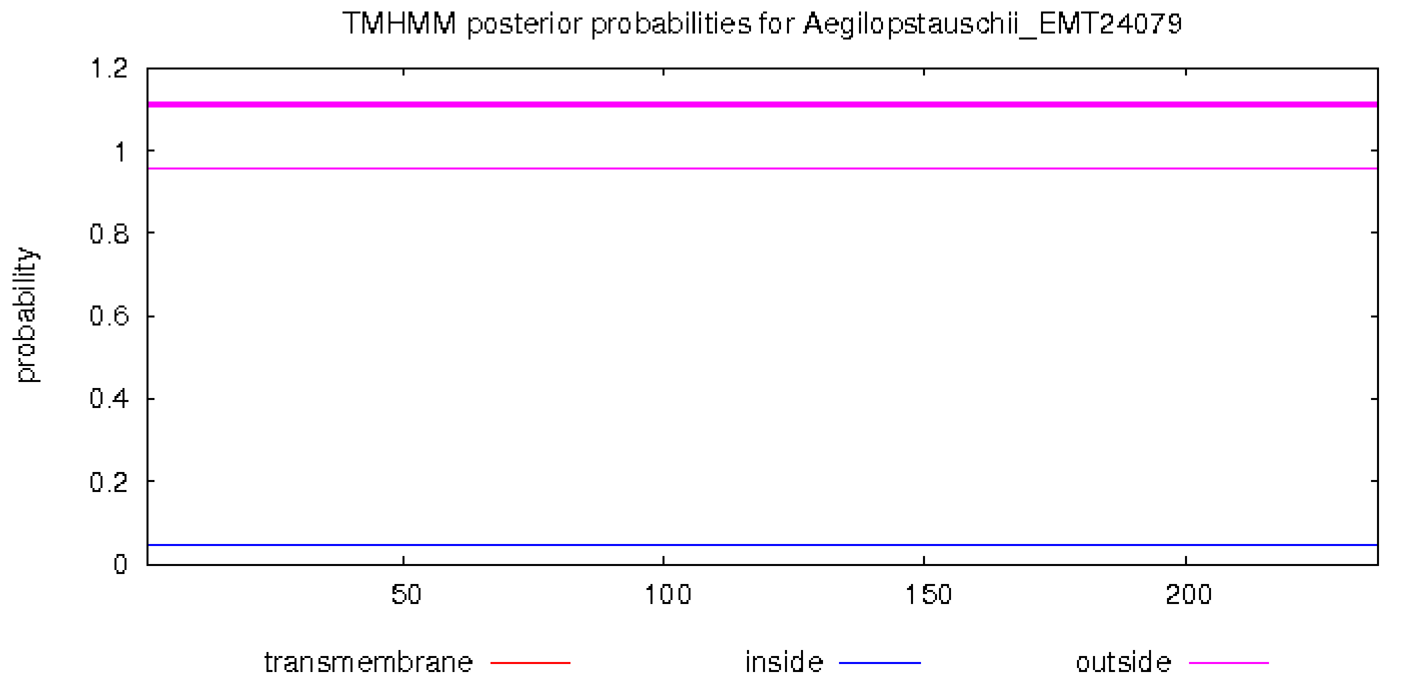

plot in postscript, script for making the plot in gnuplot, data for plot

```
# Aegilopstauschii_EMT24093 Length: 320
# Aegilopstauschii_EMT24093 Number of predicted TMHs:  0
# Aegilopstauschii_EMT24093 Exp number of AAs in TMHs: 0.00095
# Aegilopstauschii_EMT24093 Exp number, first 60 AAs:  0.00055
# Aegilopstauschii_EMT24093 Total prob of N-in:        0.00080
Aegilopstauschii_EMT24093       TMHMM2.0        outside      1   320
```

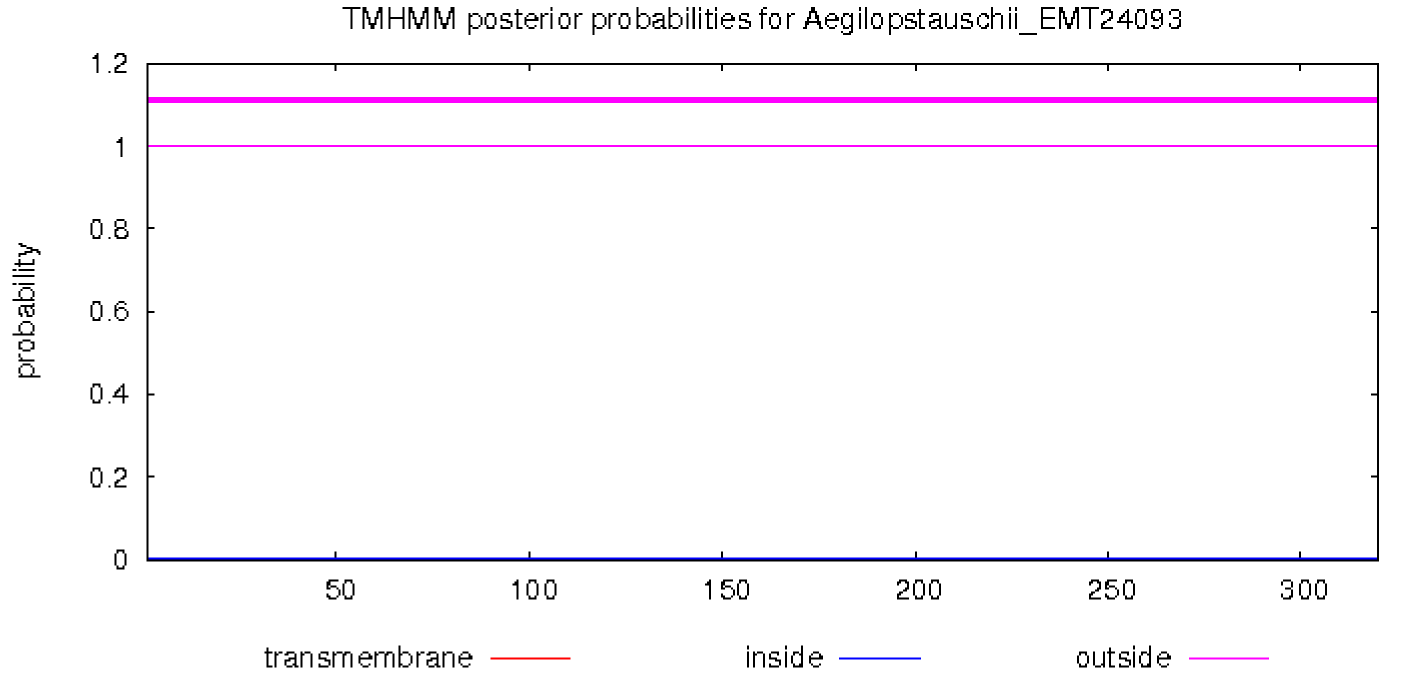

plot in postscript, script for making the plot in gnuplot, data for plot

```
# Aegilopstauschii_EMT24300 Length: 303
# Aegilopstauschii_EMT24300 Number of predicted TMHs:  0
# Aegilopstauschii_EMT24300 Exp number of AAs in TMHs: 0
# Aegilopstauschii_EMT24300 Exp number, first 60 AAs:  0
# Aegilopstauschii_EMT24300 Total prob of N-in:        0.01475
Aegilopstauschii_EMT24300       TMHMM2.0        outside      1   303
```

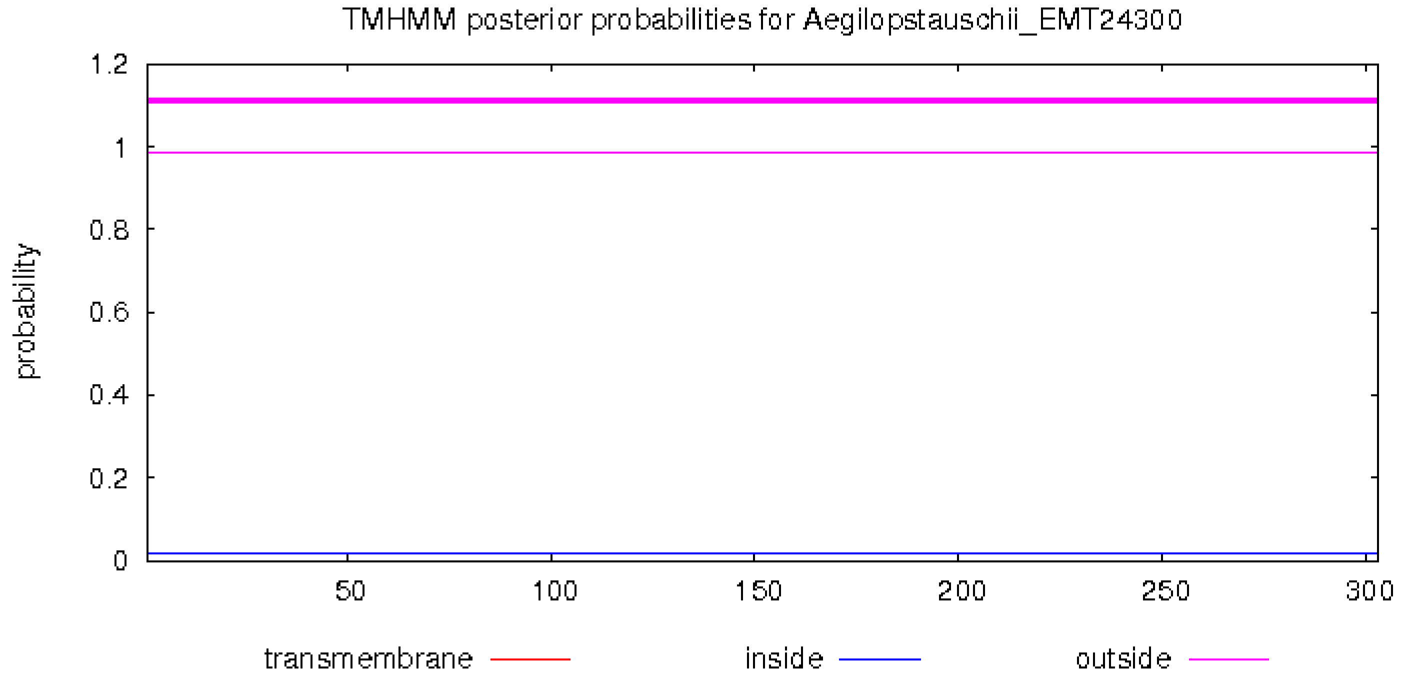

plot in postscript, script for making the plot in gnuplot, data for plot

```
# Aegilopstauschii_EMT24618 Length: 388
# Aegilopstauschii_EMT24618 Number of predicted TMHs:  0
# Aegilopstauschii_EMT24618 Exp number of AAs in TMHs: 0.02338
# Aegilopstauschii_EMT24618 Exp number, first 60 AAs:  0.00018
# Aegilopstauschii_EMT24618 Total prob of N-in:        0.00886
Aegilopstauschii_EMT24618	TMHMM2.0	outside	     1   388
```

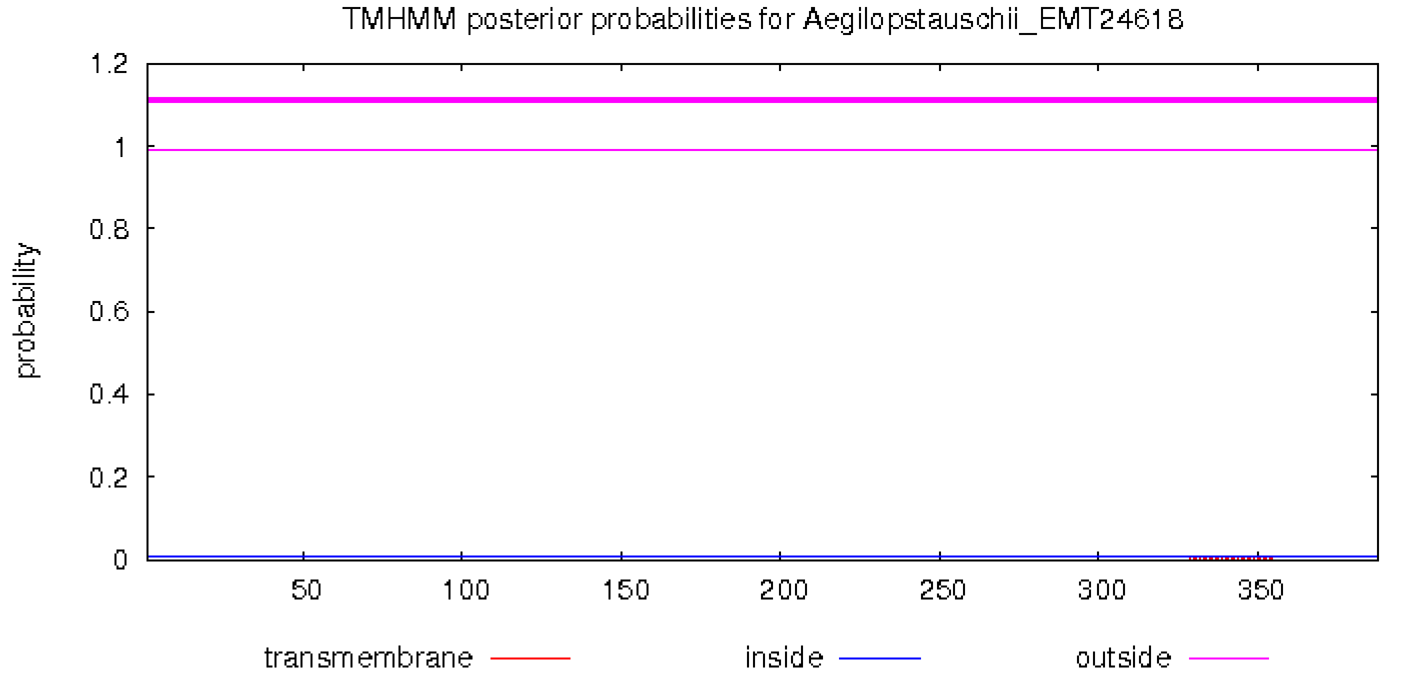

plot in postscript, script for making the plot in gnuplot, data for plot

```
# Aegilopstauschii_EMT24735 Length: 352
# Aegilopstauschii_EMT24735 Number of predicted TMHs:  0
# Aegilopstauschii_EMT24735 Exp number of AAs in TMHs: 0.0002
# Aegilopstauschii_EMT24735 Exp number, first 60 AAs:  0.0002
# Aegilopstauschii_EMT24735 Total prob of N-in:        0.00201
Aegilopstauschii_EMT24735	TMHMM2.0	outside	     1   352
```

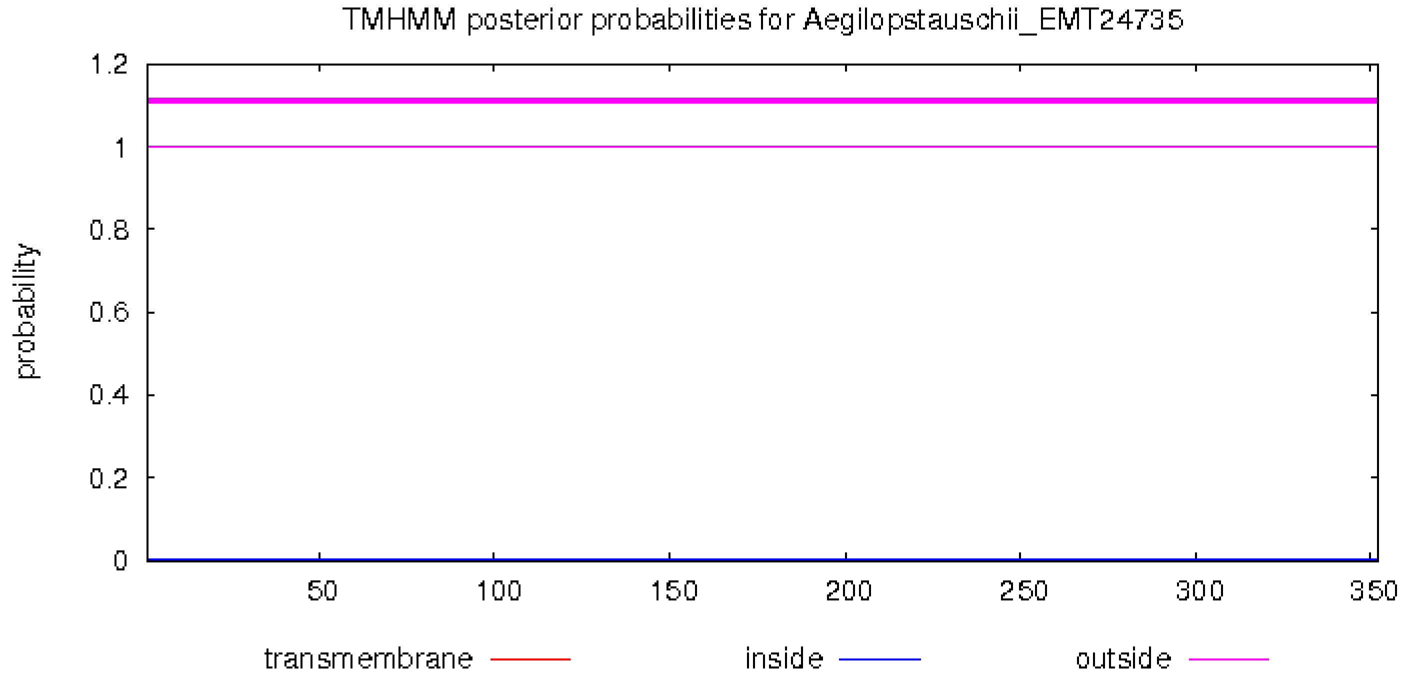

plot in postscript, script for making the plot in gnuplot, data for plot

---

```
# Aegilopstauschii_EMT25084 Length: 342
# Aegilopstauschii_EMT25084 Number of predicted TMHs:  0
# Aegilopstauschii_EMT25084 Exp number of AAs in TMHs: 0.01648
# Aegilopstauschii_EMT25084 Exp number, first 60 AAs:  0.00074
# Aegilopstauschii_EMT25084 Total prob of N-in:        0.01531
Aegilopstauschii_EMT25084       TMHMM2.0        outside      1   342
```

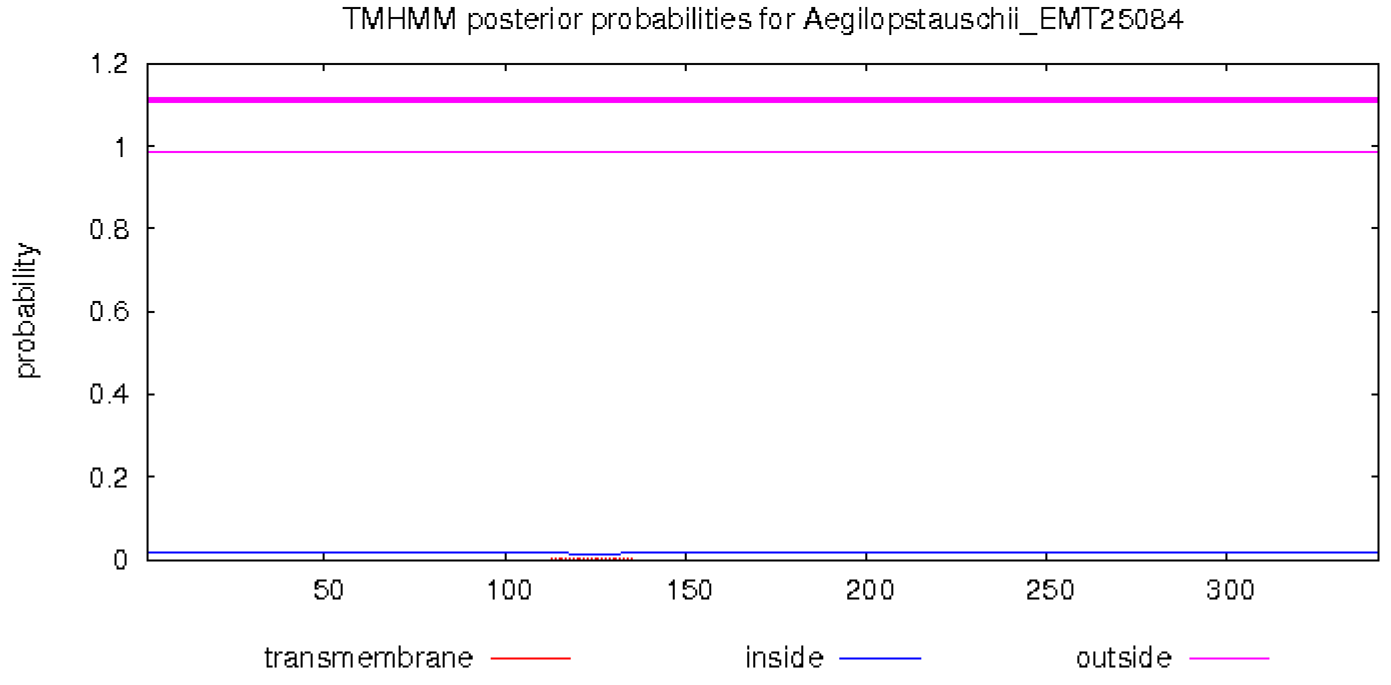

plot in postscript, script for making the plot in gnuplot, data for plot

---

```
# Aegilopstauschii_EMT25749 Length: 329
# Aegilopstauschii_EMT25749 Number of predicted TMHs:  0
# Aegilopstauschii_EMT25749 Exp number of AAs in TMHs: 0.00345
# Aegilopstauschii_EMT25749 Exp number, first 60 AAs:  0.0001
# Aegilopstauschii_EMT25749 Total prob of N-in:        0.02544
Aegilopstauschii_EMT25749       TMHMM2.0        outside      1   329
```

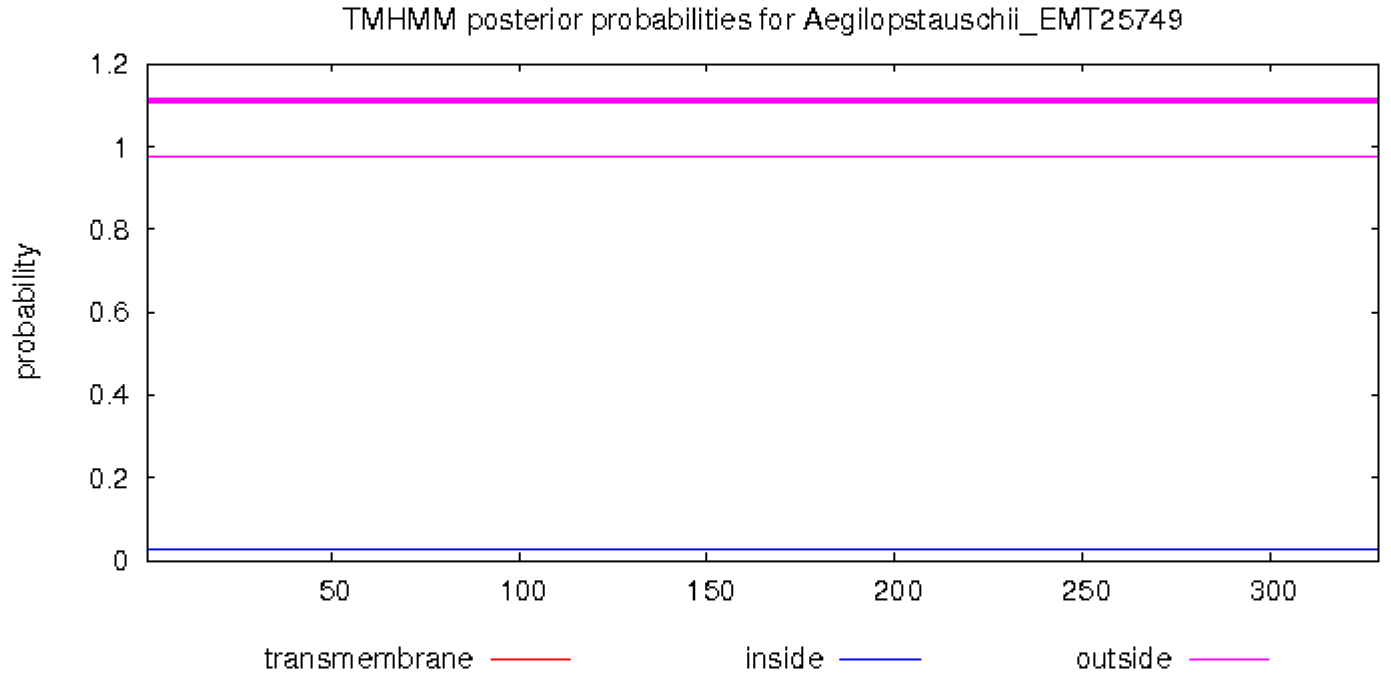

plot in postscript, script for making the plot in gnuplot, data for plot

---

```
# Aegilopstauschii_EMT26194 Length: 396
# Aegilopstauschii_EMT26194 Number of predicted TMHs:  0
# Aegilopstauschii_EMT26194 Exp number of AAs in TMHs: 0.00144
# Aegilopstauschii_EMT26194 Exp number, first 60 AAs:  0
# Aegilopstauschii_EMT26194 Total prob of N-in:        0.00290
Aegilopstauschii_EMT26194	TMHMM2.0	outside	     1   396
```

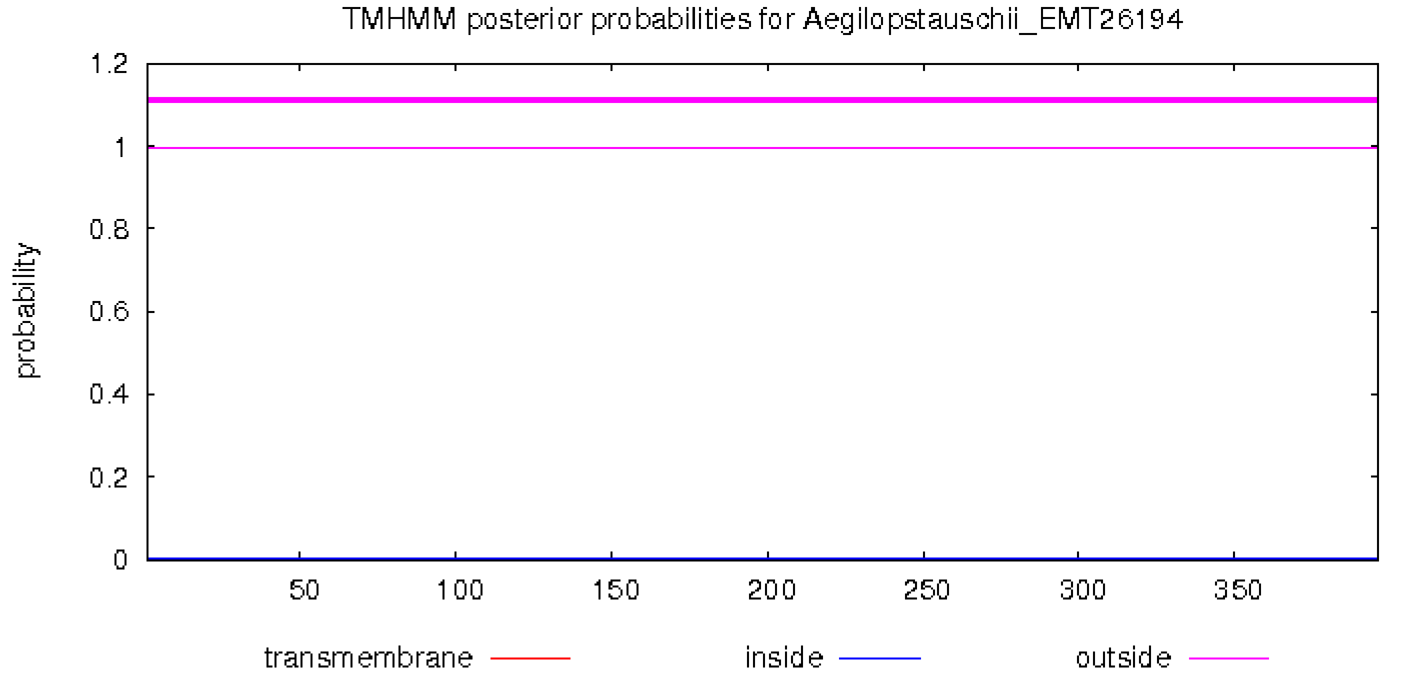

plot in postscript, script for making the plot in gnuplot, data for plot

---

```
# Aegilopstauschii_EMT26627 Length: 350
# Aegilopstauschii_EMT26627 Number of predicted TMHs:  0
# Aegilopstauschii_EMT26627 Exp number of AAs in TMHs: 0.00013
# Aegilopstauschii_EMT26627 Exp number, first 60 AAs:  0.00013
# Aegilopstauschii_EMT26627 Total prob of N-in:        0.00552
Aegilopstauschii_EMT26627	TMHMM2.0	outside	     1   350
```

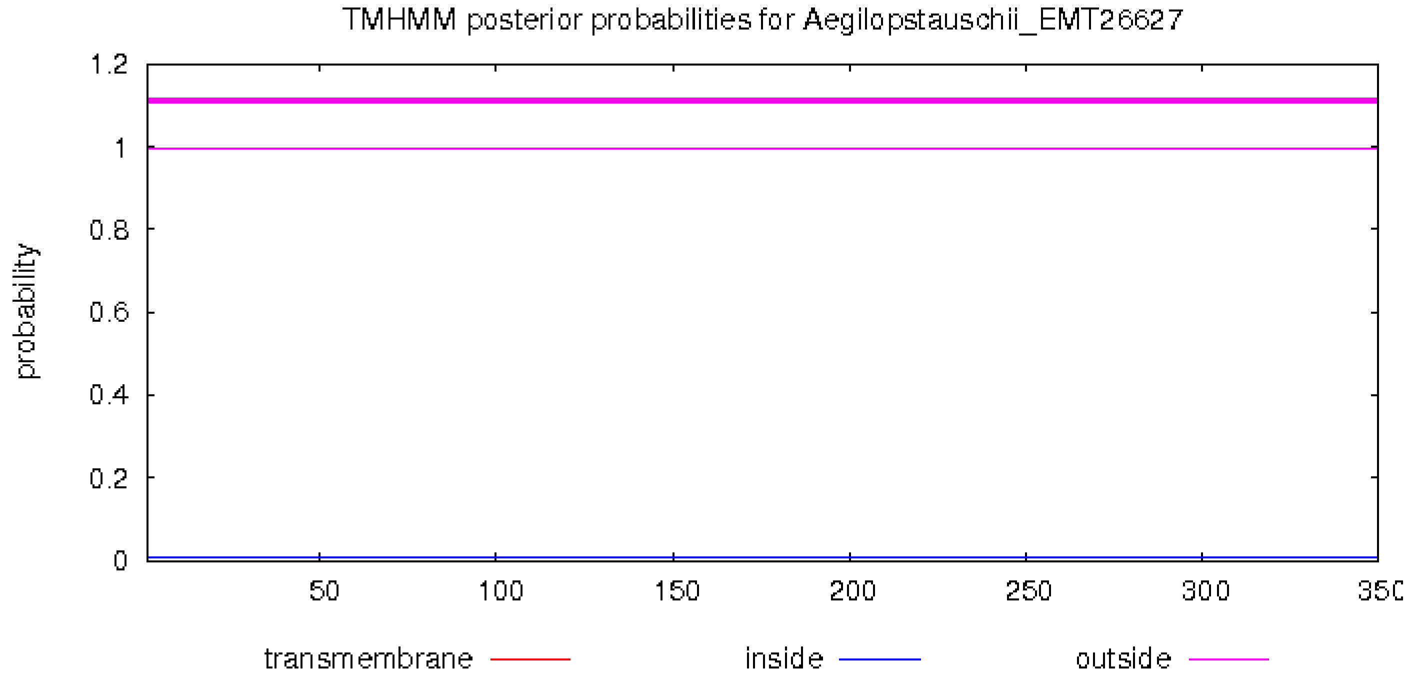

plot in postscript, script for making the plot in gnuplot, data for plot

```
# Aegilopstauschii_EMT26799 Length: 170
# Aegilopstauschii_EMT26799 Number of predicted TMHs:  0
# Aegilopstauschii_EMT26799 Exp number of AAs in TMHs: 0.00178
# Aegilopstauschii_EMT26799 Exp number, first 60 AAs:  0
# Aegilopstauschii_EMT26799 Total prob of N-in:        0.11811
Aegilopstauschii_EMT26799       TMHMM2.0        outside      1   170
```

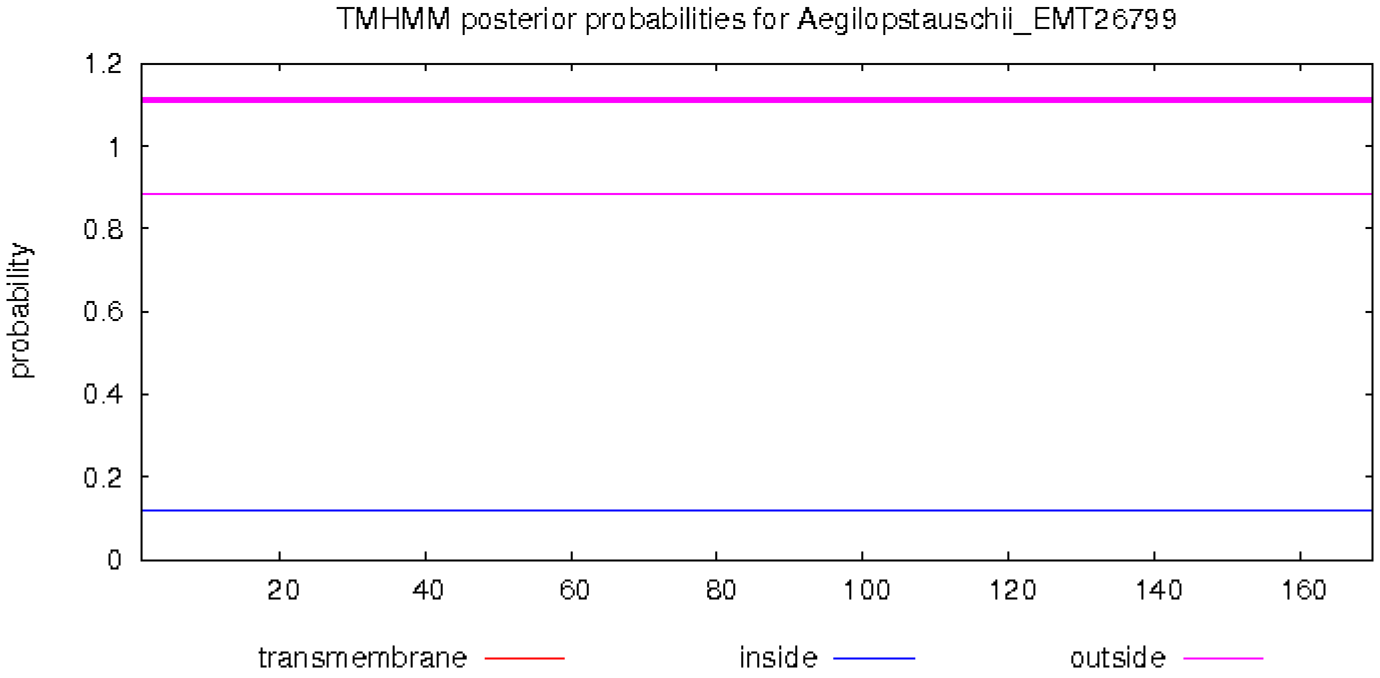

plot in postscript, script for making the plot in gnuplot, data for plot

```
# Aegilopstauschii_EMT27068 Length: 542
# Aegilopstauschii_EMT27068 Number of predicted TMHs:  0
# Aegilopstauschii_EMT27068 Exp number of AAs in TMHs: 0.04171
# Aegilopstauschii_EMT27068 Exp number, first 60 AAs:  0.00496
# Aegilopstauschii_EMT27068 Total prob of N-in:        0.00336
Aegilopstauschii_EMT27068       TMHMM2.0        outside      1   542
```

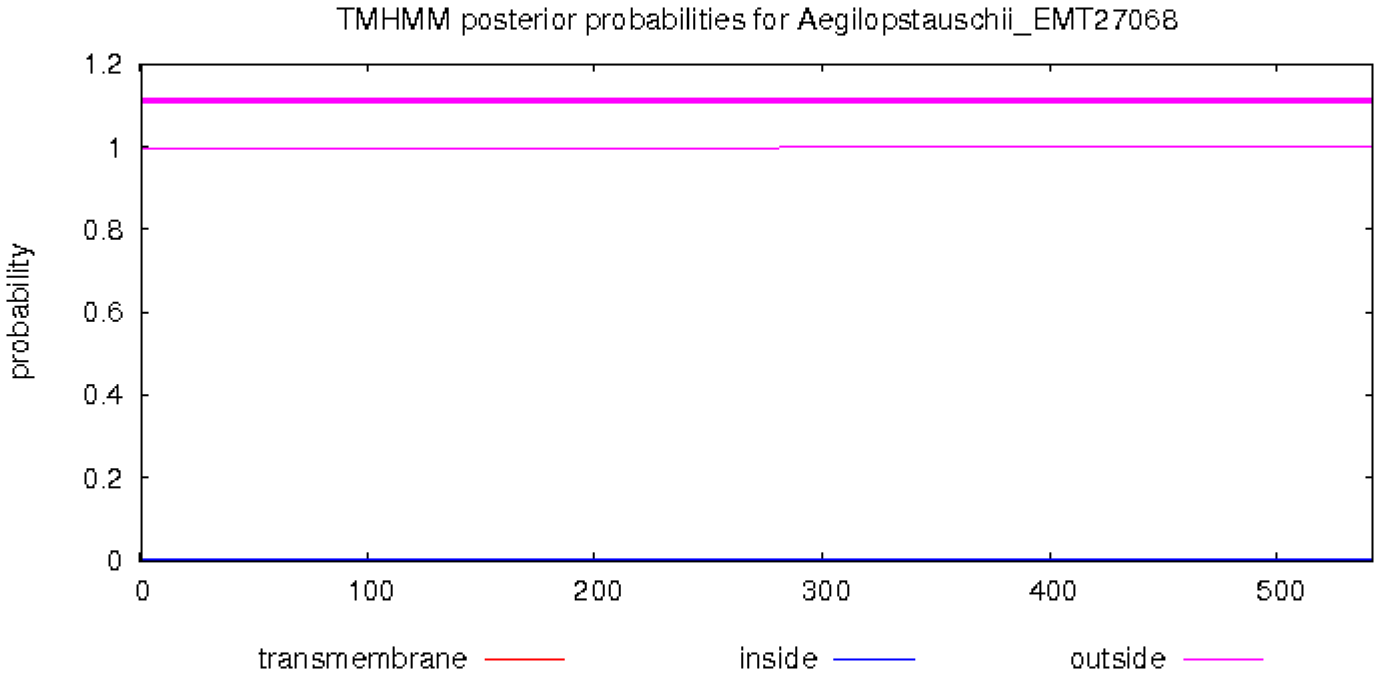

plot in postscript, script for making the plot in gnuplot, data for plot

```
# Aegilopstauschii_EMT27450 Length: 410
# Aegilopstauschii_EMT27450 Number of predicted TMHs:  0
# Aegilopstauschii_EMT27450 Exp number of AAs in TMHs: 0.16338
# Aegilopstauschii_EMT27450 Exp number, first 60 AAs:  0.07691
# Aegilopstauschii_EMT27450 Total prob of N-in:        0.01164
Aegilopstauschii_EMT27450	TMHMM2.0	outside	     1   410
```

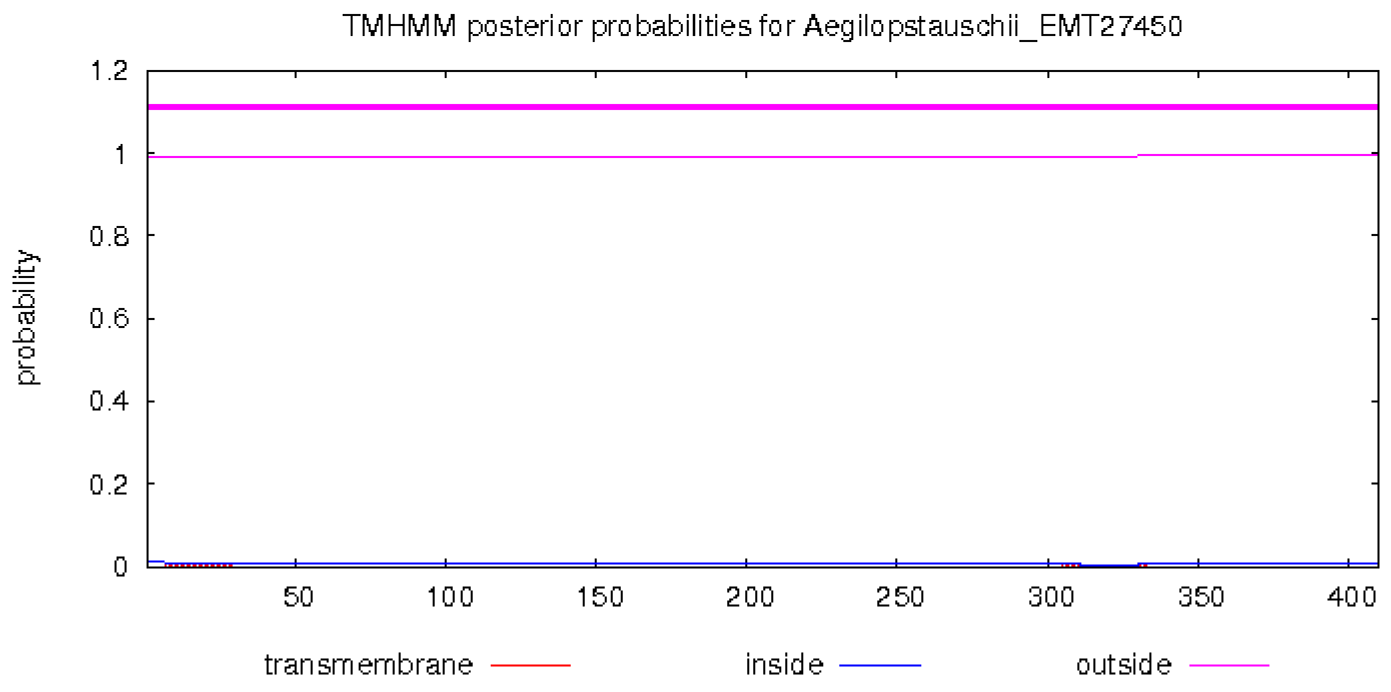

plot in postscript, script for making the plot in gnuplot, data for plot

```
# Aegilopstauschii_EMT27713 Length: 815
# Aegilopstauschii_EMT27713 Number of predicted TMHs:  0
# Aegilopstauschii_EMT27713 Exp number of AAs in TMHs: 8.82860999999999999
# Aegilopstauschii_EMT27713 Exp number, first 60 AAs:  0
# Aegilopstauschii_EMT27713 Total prob of N-in:        0.00479
Aegilopstauschii_EMT27713	TMHMM2.0	outside	     1   815
```

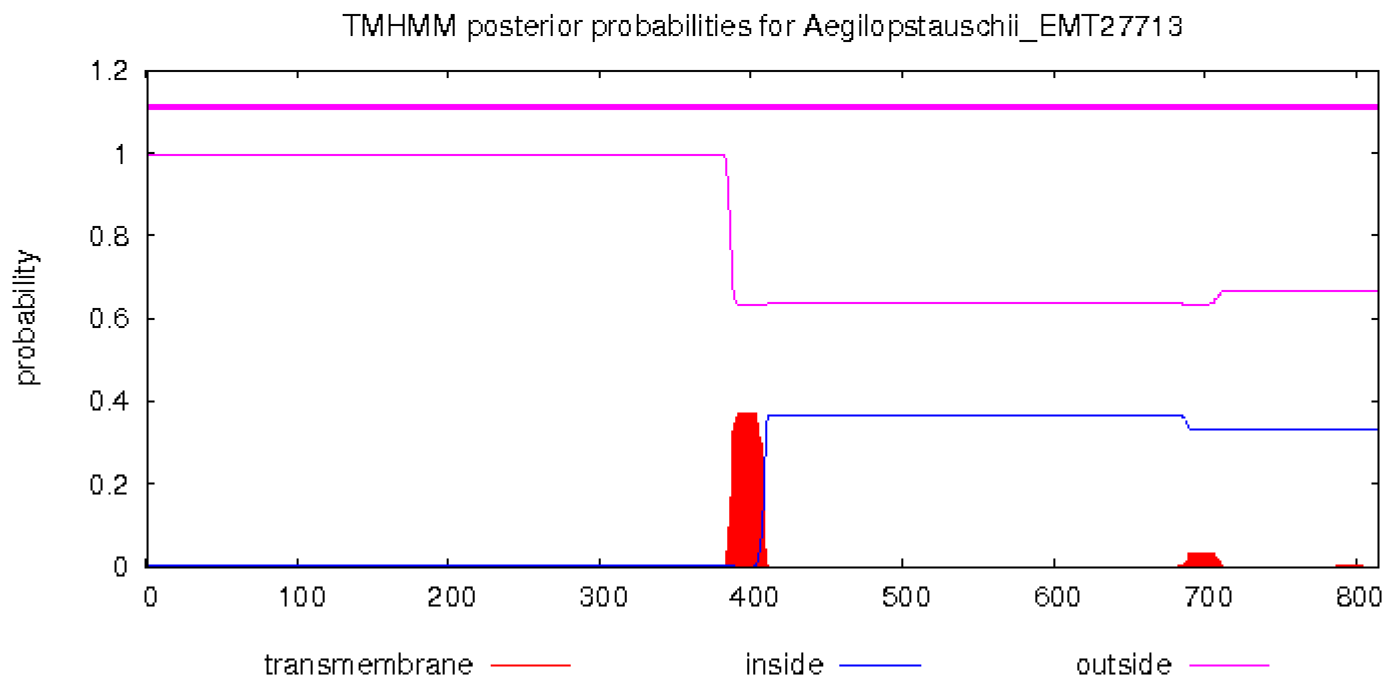

plot in postscript, script for making the plot in gnuplot, data for plot

```
# Aegilopstauschii_EMT27961 Length: 225
# Aegilopstauschii_EMT27961 Number of predicted TMHs:  0
# Aegilopstauschii_EMT27961 Exp number of AAs in TMHs: 0.00362
# Aegilopstauschii_EMT27961 Exp number, first 60 AAs:  0.00041
# Aegilopstauschii_EMT27961 Total prob of N-in:        0.11996
Aegilopstauschii_EMT27961       TMHMM2.0        outside      1   225
```

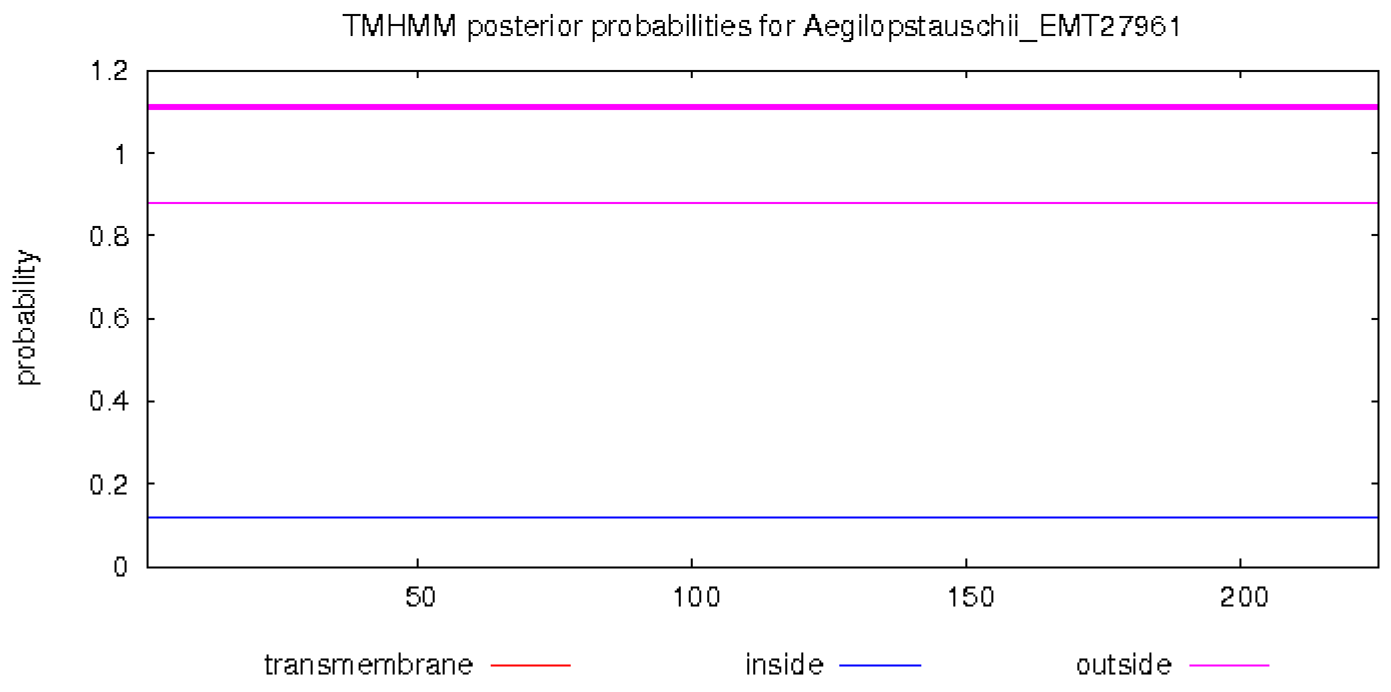

plot in postscript, script for making the plot in gnuplot, data for plot

```
# Aegilopstauschii_EMT28110 Length: 497
# Aegilopstauschii_EMT28110 Number of predicted TMHs:  1
# Aegilopstauschii_EMT28110 Exp number of AAs in TMHs: 20.38259
# Aegilopstauschii_EMT28110 Exp number, first 60 AAs:  0.0625
# Aegilopstauschii_EMT28110 Total prob of N-in:        0.00507
Aegilopstauschii_EMT28110       TMHMM2.0        outside      1   463
Aegilopstauschii_EMT28110       TMHMM2.0        TMhelix    464   486
Aegilopstauschii_EMT28110       TMHMM2.0        inside     487   497
```

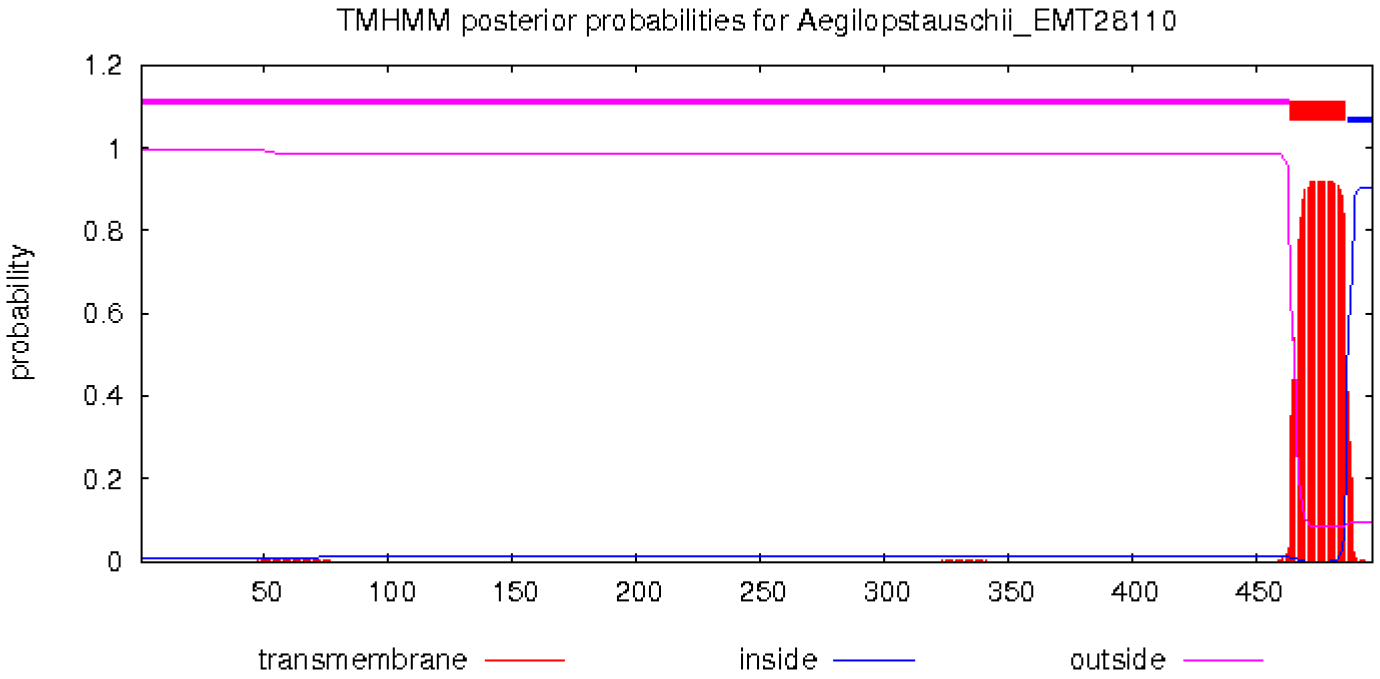

plot in postscript, script for making the plot in gnuplot, data for plot

---

```
# Aegilopstauschii_EMT28157 Length: 347
# Aegilopstauschii_EMT28157 Number of predicted TMHs:  0
# Aegilopstauschii_EMT28157 Exp number of AAs in TMHs: 0.00237
# Aegilopstauschii_EMT28157 Exp number, first 60 AAs:  0.00165
# Aegilopstauschii_EMT28157 Total prob of N-in:        0.01606
Aegilopstauschii_EMT28157       TMHMM2.0        outside      1   347
```

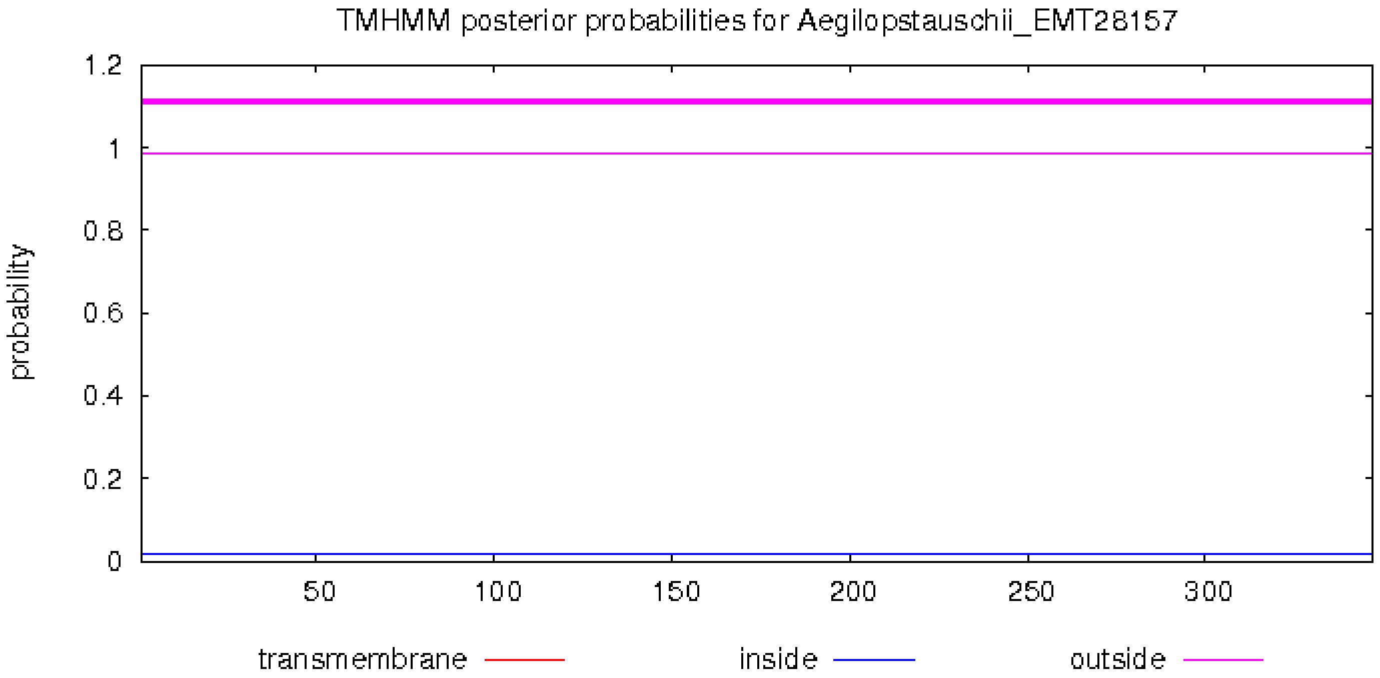

plot in postscript, script for making the plot in gnuplot, data for plot

---

```
# Aegilopstauschii_EMT28257 Length: 381
# Aegilopstauschii_EMT28257 Number of predicted TMHs:  0
# Aegilopstauschii_EMT28257 Exp number of AAs in TMHs: 0.00137
# Aegilopstauschii_EMT28257 Exp number, first 60 AAs:  0.00071
# Aegilopstauschii_EMT28257 Total prob of N-in:        0.01531
Aegilopstauschii_EMT28257       TMHMM2.0        outside      1   381
```

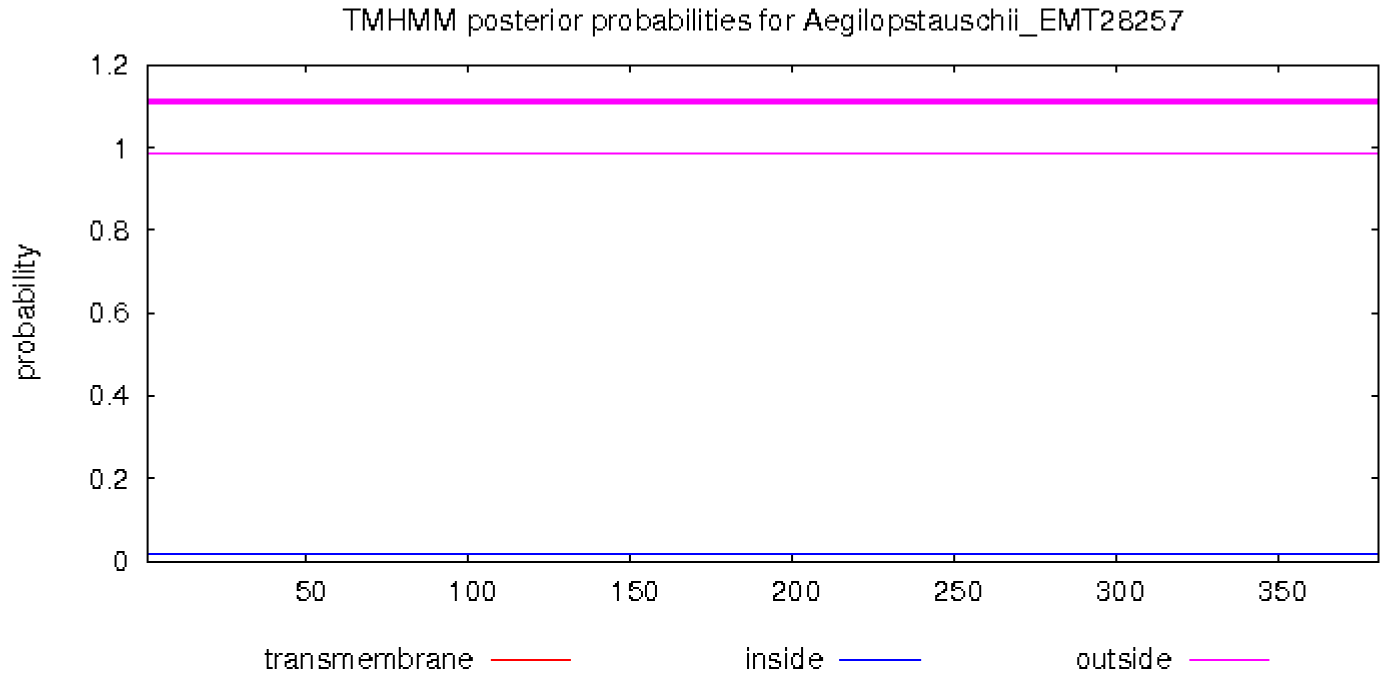

plot in postscript, script for making the plot in gnuplot, data for plot

---

```
# Aegilopstauschii_EMT28295 Length: 349
# Aegilopstauschii_EMT28295 Number of predicted TMHs:  0
# Aegilopstauschii_EMT28295 Exp number of AAs in TMHs: 0.18882
# Aegilopstauschii_EMT28295 Exp number, first 60 AAs:  0.01115
# Aegilopstauschii_EMT28295 Total prob of N-in:        0.02480
Aegilopstauschii_EMT28295       TMHMM2.0        outside      1   349
```

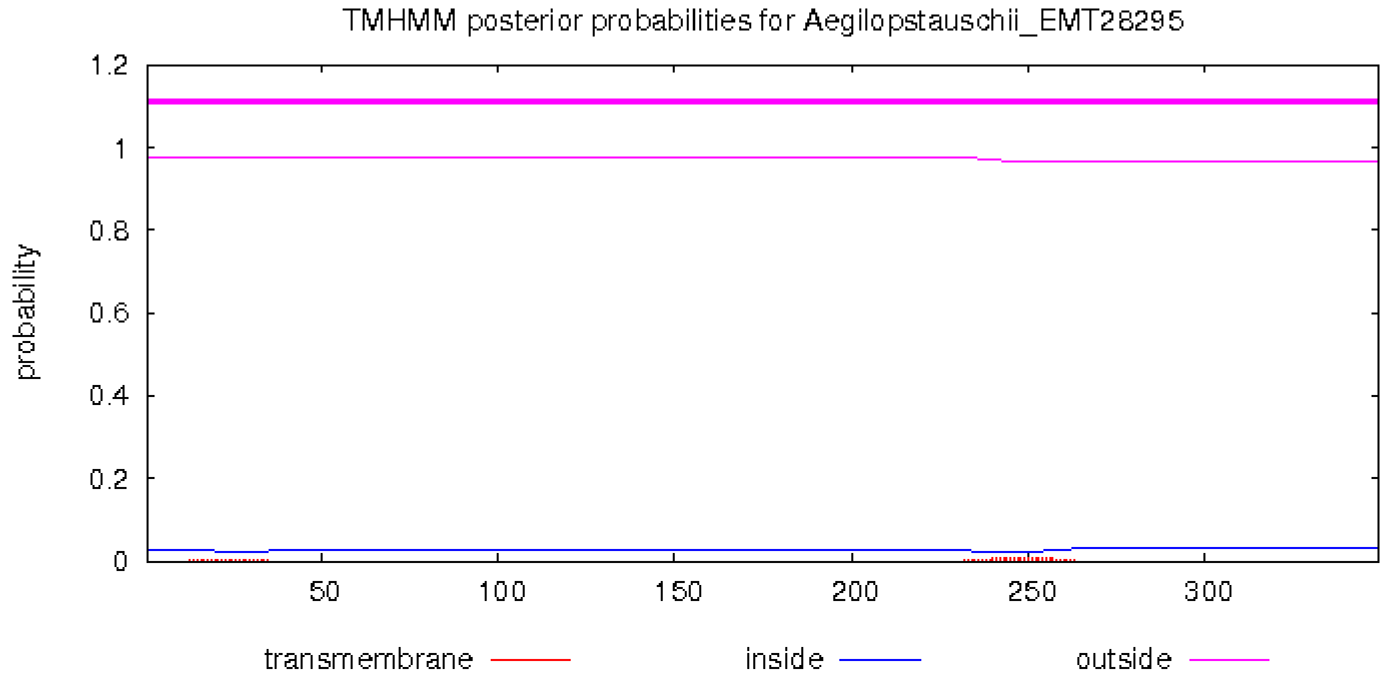

plot in postscript, script for making the plot in gnuplot, data for plot

---

```
# Aegilopstauschii_EMT28787 Length: 456
# Aegilopstauschii_EMT28787 Number of predicted TMHs:  0
# Aegilopstauschii_EMT28787 Exp number of AAs in TMHs: 0.08343
# Aegilopstauschii_EMT28787 Exp number, first 60 AAs:  0
# Aegilopstauschii_EMT28787 Total prob of N-in:        0.02663
Aegilopstauschii_EMT28787       TMHMM2.0        outside      1   456
```

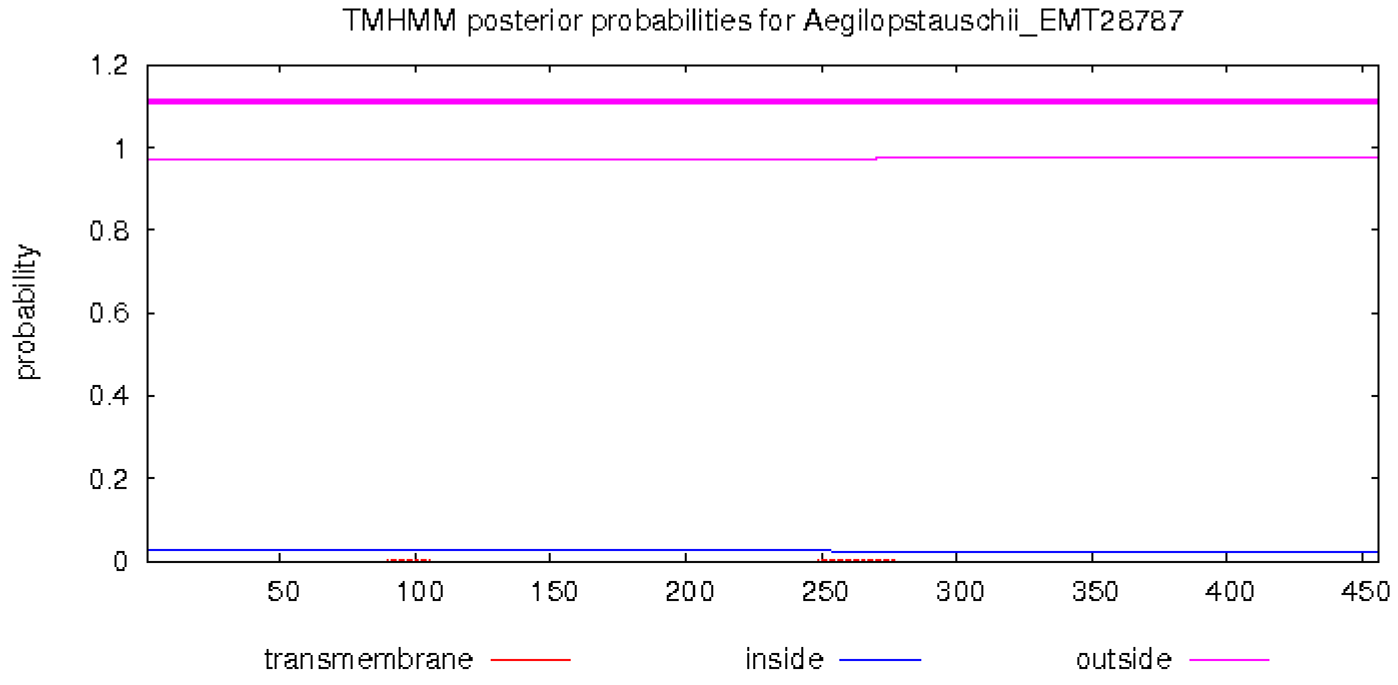

plot in postscript, script for making the plot in gnuplot, data for plot

---

```
# Aegilopstauschii_EMT28823 Length: 488
# Aegilopstauschii_EMT28823 Number of predicted TMHs:  0
# Aegilopstauschii_EMT28823 Exp number of AAs in TMHs: 1.51374
# Aegilopstauschii_EMT28823 Exp number, first 60 AAs:  1.27357
# Aegilopstauschii_EMT28823 Total prob of N-in:        0.05108
Aegilopstauschii_EMT28823       TMHMM2.0        outside      1   488
```

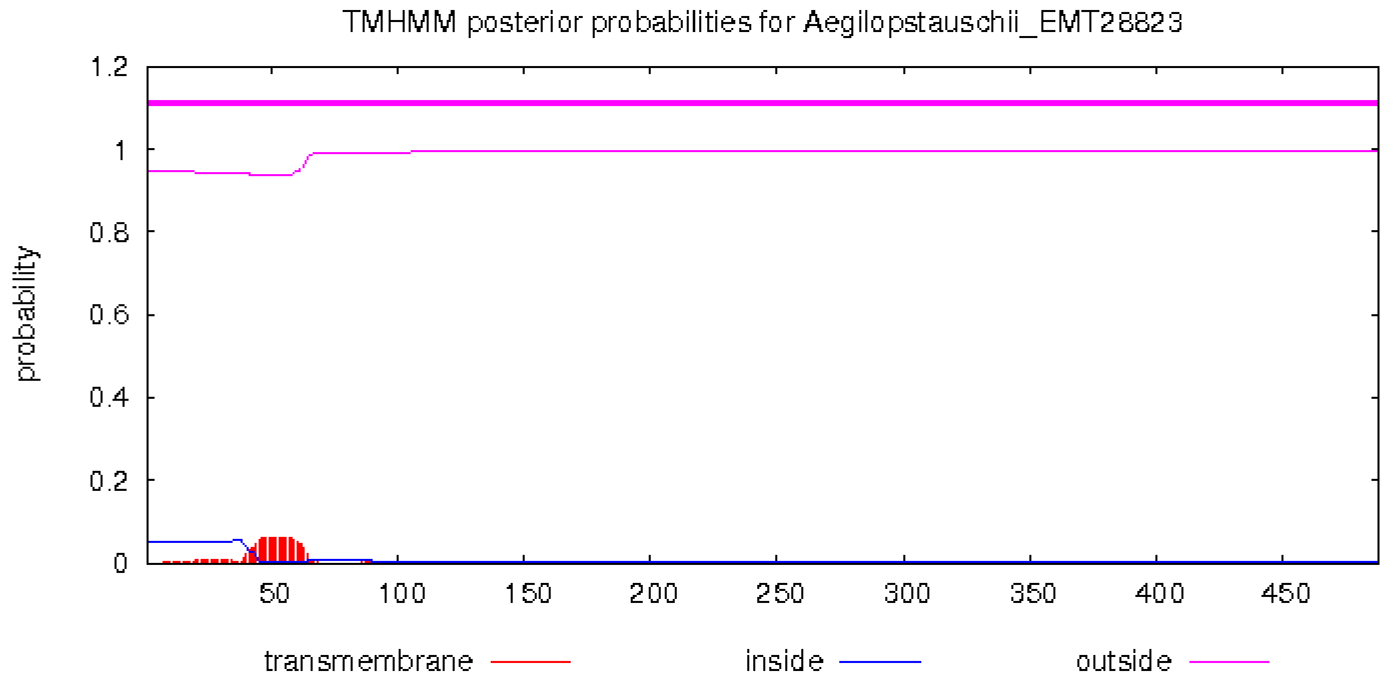

plot in postscript, script for making the plot in gnuplot, data for plot

---

```
# Aegilopstauschii_EMT28859 Length: 358
# Aegilopstauschii_EMT28859 Number of predicted TMHs:  0
# Aegilopstauschii_EMT28859 Exp number of AAs in TMHs: 0.00778
# Aegilopstauschii_EMT28859 Exp number, first 60 AAs:  0.00018
# Aegilopstauschii_EMT28859 Total prob of N-in:        0.01742
Aegilopstauschii_EMT28859       TMHMM2.0        outside      1   358
```

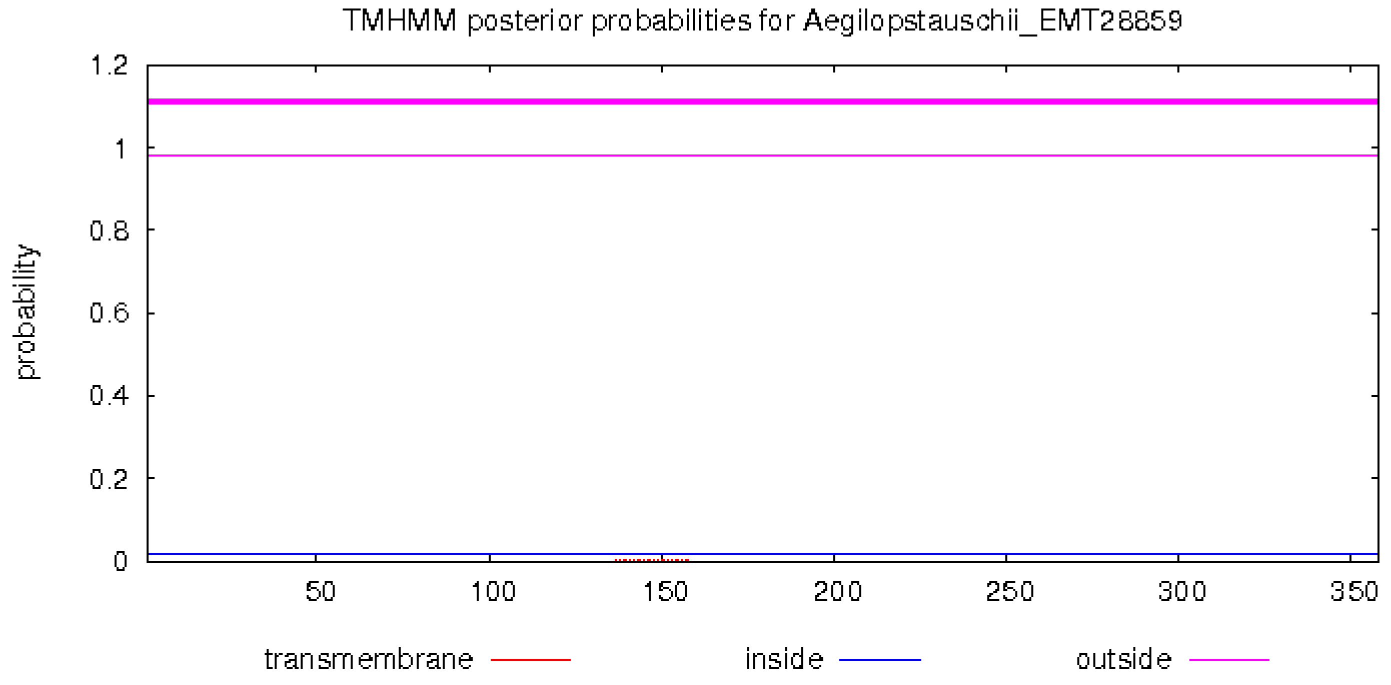

plot in postscript, script for making the plot in gnuplot, data for plot

---

```
# Aegilopstauschii_EMT29234 Length: 312
# Aegilopstauschii_EMT29234 Number of predicted TMHs:  0
# Aegilopstauschii_EMT29234 Exp number of AAs in TMHs: 0.01584
# Aegilopstauschii_EMT29234 Exp number, first 60 AAs:  0.01464
# Aegilopstauschii_EMT29234 Total prob of N-in:        0.02372
Aegilopstauschii_EMT29234	TMHMM2.0	outside	     1   312
```

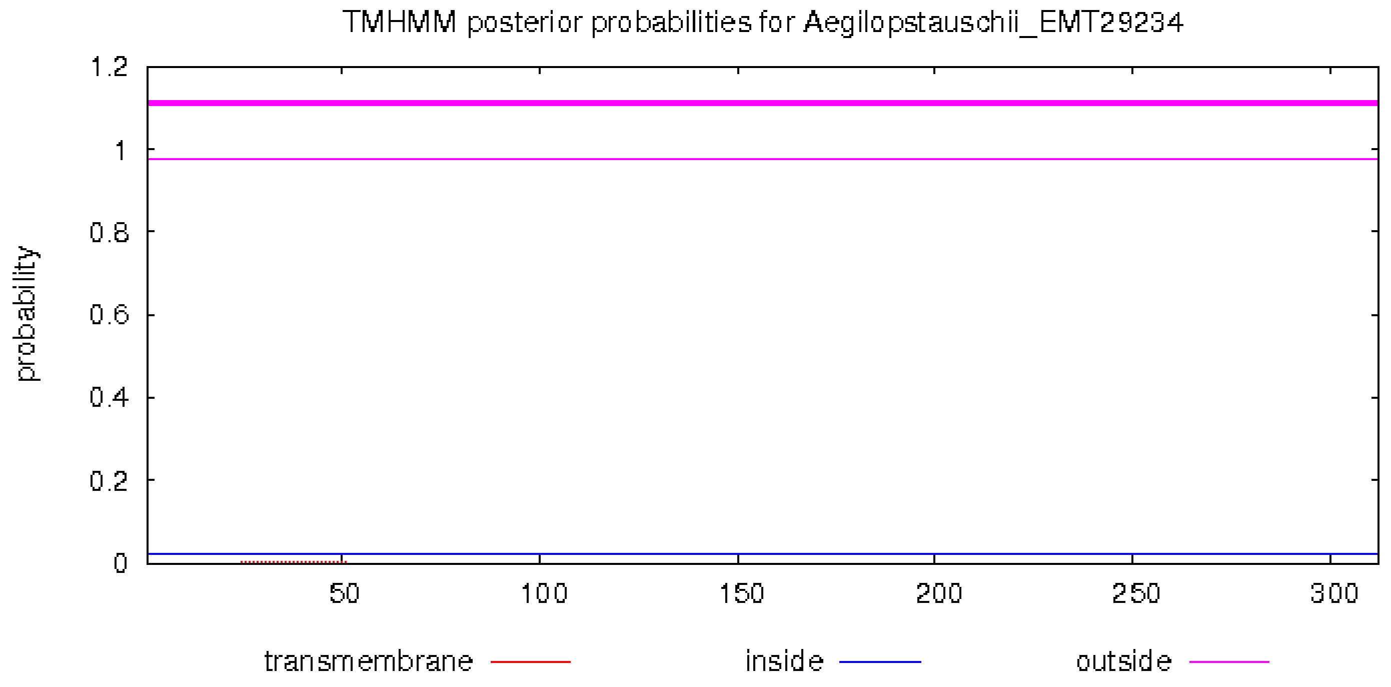

plot in postscript, script for making the plot in gnuplot, data for plot

---

```
# Aegilopstauschii_EMT29472 Length: 236
# Aegilopstauschii_EMT29472 Number of predicted TMHs:  0
# Aegilopstauschii_EMT29472 Exp number of AAs in TMHs: 0.00125
# Aegilopstauschii_EMT29472 Exp number, first 60 AAs:  0.00125
# Aegilopstauschii_EMT29472 Total prob of N-in:        0.00974
Aegilopstauschii_EMT29472	TMHMM2.0	outside	     1   236
```

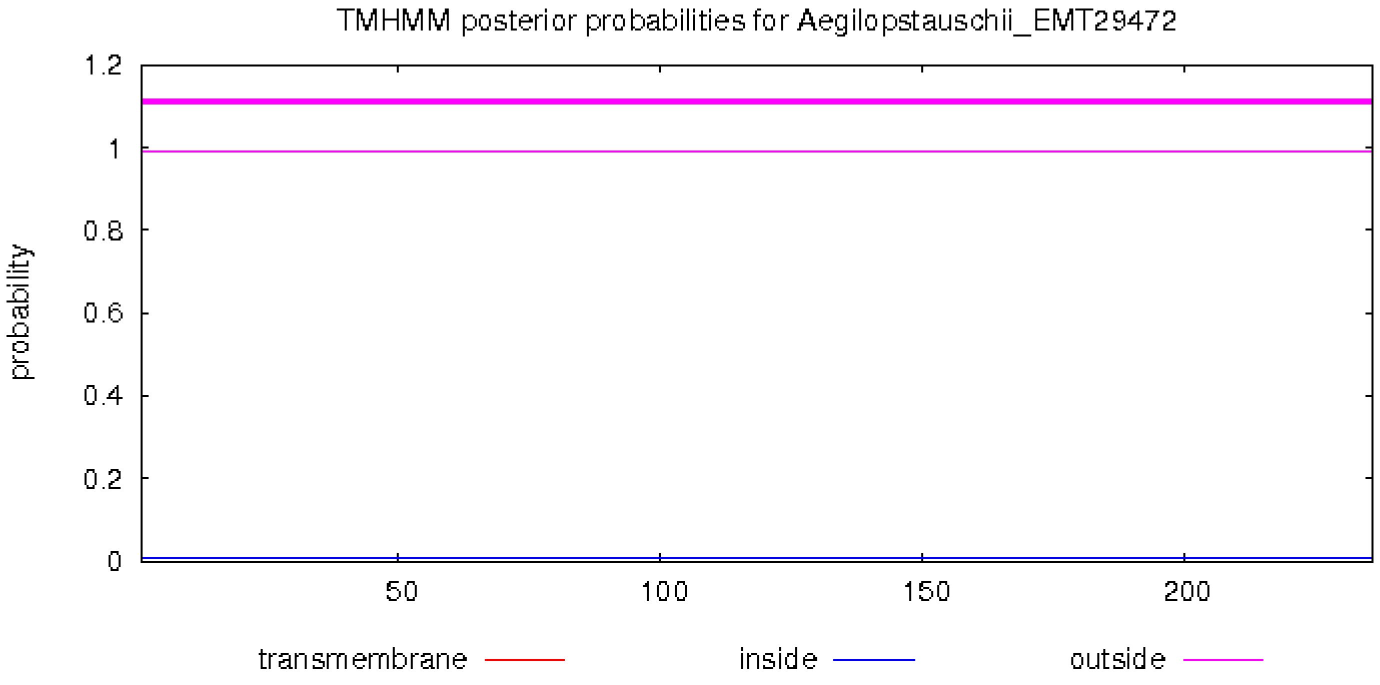

plot in postscript, script for making the plot in gnuplot, data for plot

---

```
# Aegilopstauschii_EMT29816 Length: 300
# Aegilopstauschii_EMT29816 Number of predicted TMHs:  0
# Aegilopstauschii_EMT29816 Exp number of AAs in TMHs: 0.05792
# Aegilopstauschii_EMT29816 Exp number, first 60 AAs:  0
# Aegilopstauschii_EMT29816 Total prob of N-in:        0.01192
Aegilopstauschii_EMT29816	TMHMM2.0	outside	     1   300
```

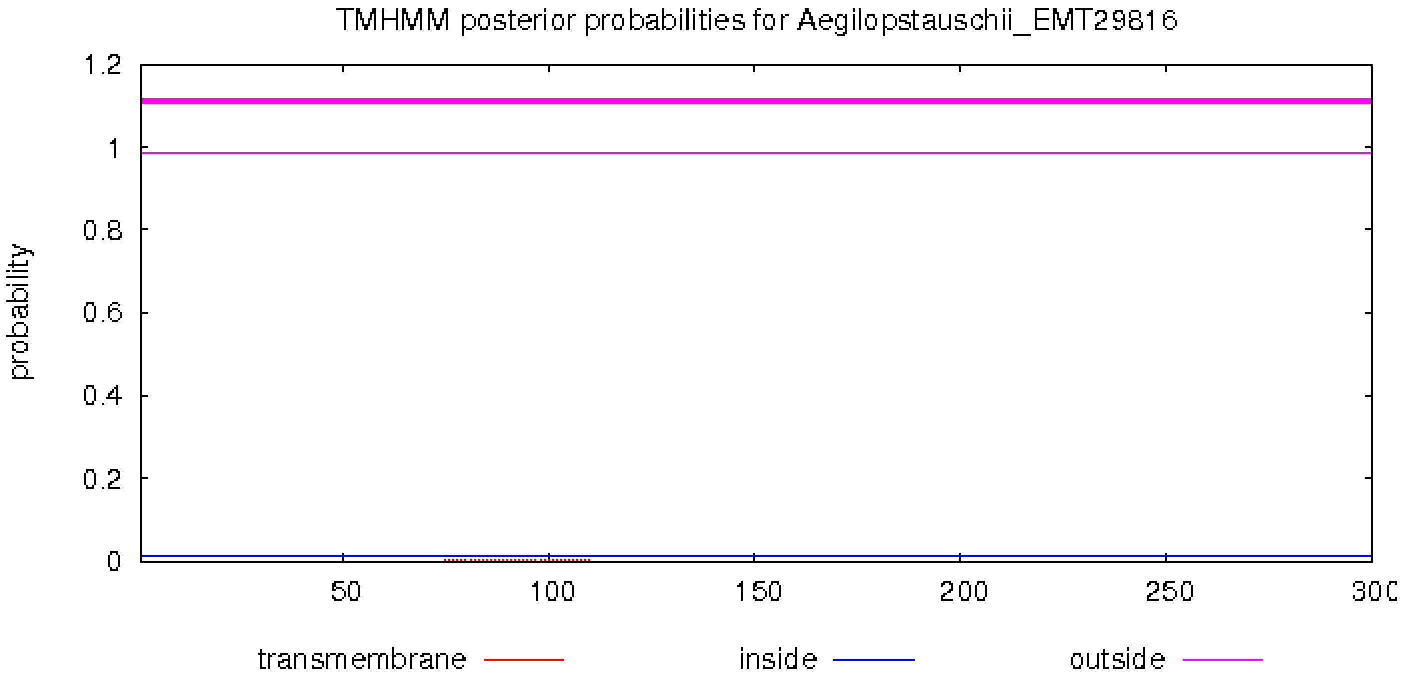

plot in postscript, script for making the plot in gnuplot, data for plot

---

```
# Aegilopstauschii_EMT29965 Length: 308
# Aegilopstauschii_EMT29965 Number of predicted TMHs:  0
# Aegilopstauschii_EMT29965 Exp number of AAs in TMHs: 0.00433
# Aegilopstauschii_EMT29965 Exp number, first 60 AAs:  0.00331
# Aegilopstauschii_EMT29965 Total prob of N-in:        0.00751
Aegilopstauschii_EMT29965	TMHMM2.0	outside	     1   308
```

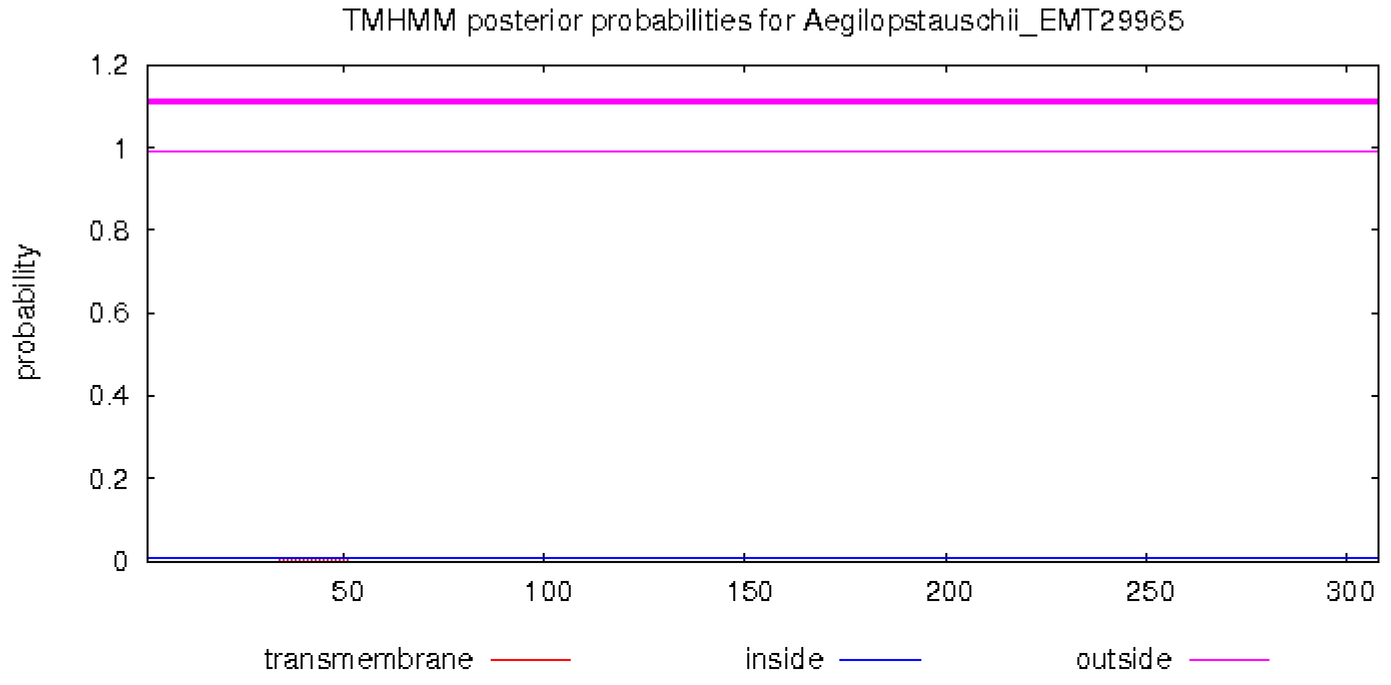

plot in postscript, script for making the plot in gnuplot, data for plot

---

```
# Aegilopstauschii_EMT30422 Length: 328
# Aegilopstauschii_EMT30422 Number of predicted TMHs:  0
# Aegilopstauschii_EMT30422 Exp number of AAs in TMHs: 0.11269
# Aegilopstauschii_EMT30422 Exp number, first 60 AAs:  0.08038
# Aegilopstauschii_EMT30422 Total prob of N-in:        0.04380
Aegilopstauschii_EMT30422	TMHMM2.0	outside	     1   328
```

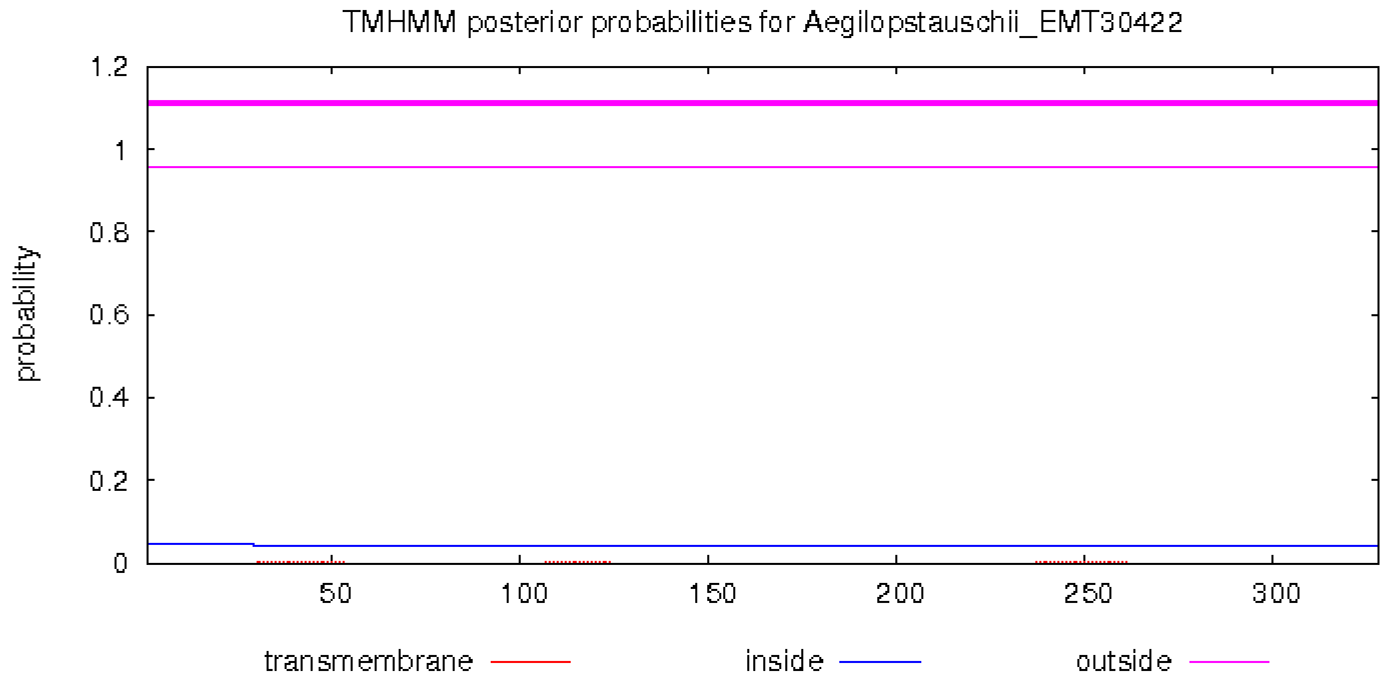

plot in postscript, script for making the plot in gnuplot, data for plot

---

```
# Aegilopstauschii_EMT30514 Length: 274
# Aegilopstauschii_EMT30514 Number of predicted TMHs:  0
# Aegilopstauschii_EMT30514 Exp number of AAs in TMHs: 0.00017
# Aegilopstauschii_EMT30514 Exp number, first 60 AAs:  0
# Aegilopstauschii_EMT30514 Total prob of N-in:        0.00861
Aegilopstauschii_EMT30514	TMHMM2.0	outside	     1   274
```

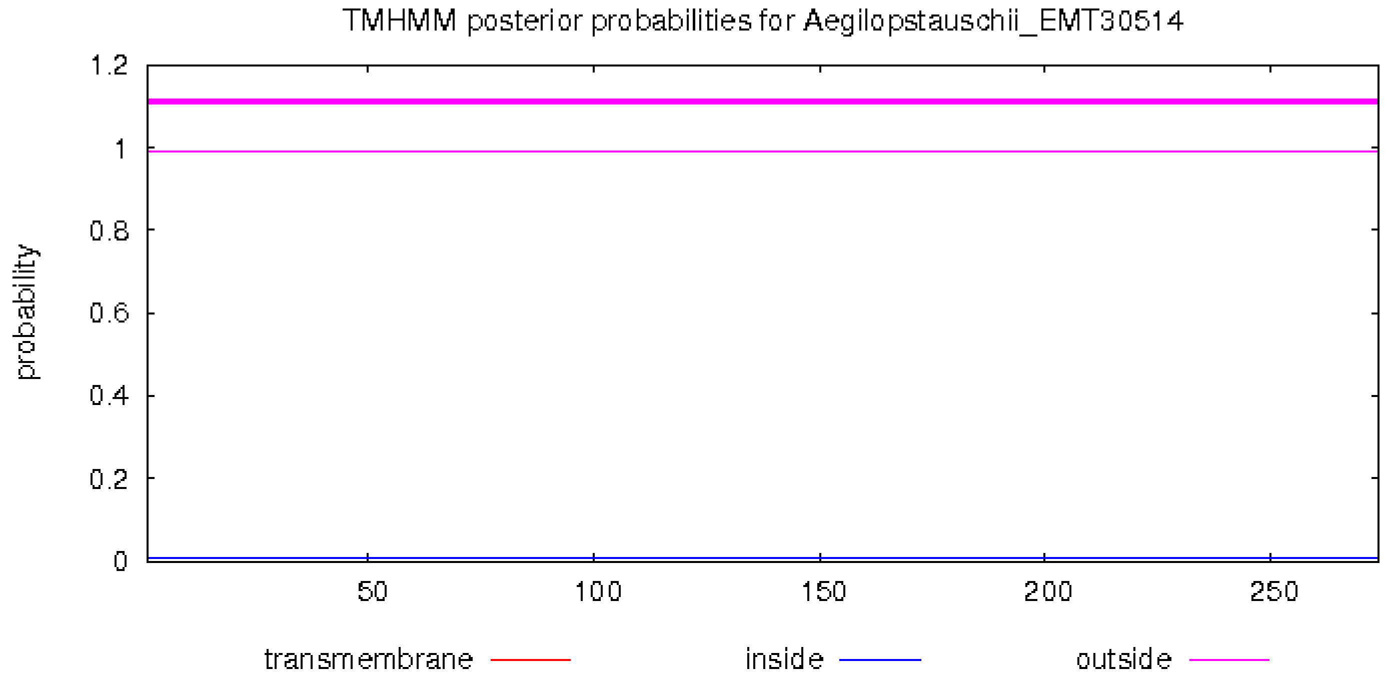

plot in postscript, script for making the plot in gnuplot, data for plot

---

```
# Aegilopstauschii_EMT30661 Length: 371
# Aegilopstauschii_EMT30661 Number of predicted TMHs:  0
# Aegilopstauschii_EMT30661 Exp number of AAs in TMHs: 0
# Aegilopstauschii_EMT30661 Exp number, first 60 AAs:  0
# Aegilopstauschii_EMT30661 Total prob of N-in:        0.01440
Aegilopstauschii_EMT30661       TMHMM2.0        outside      1   371
```

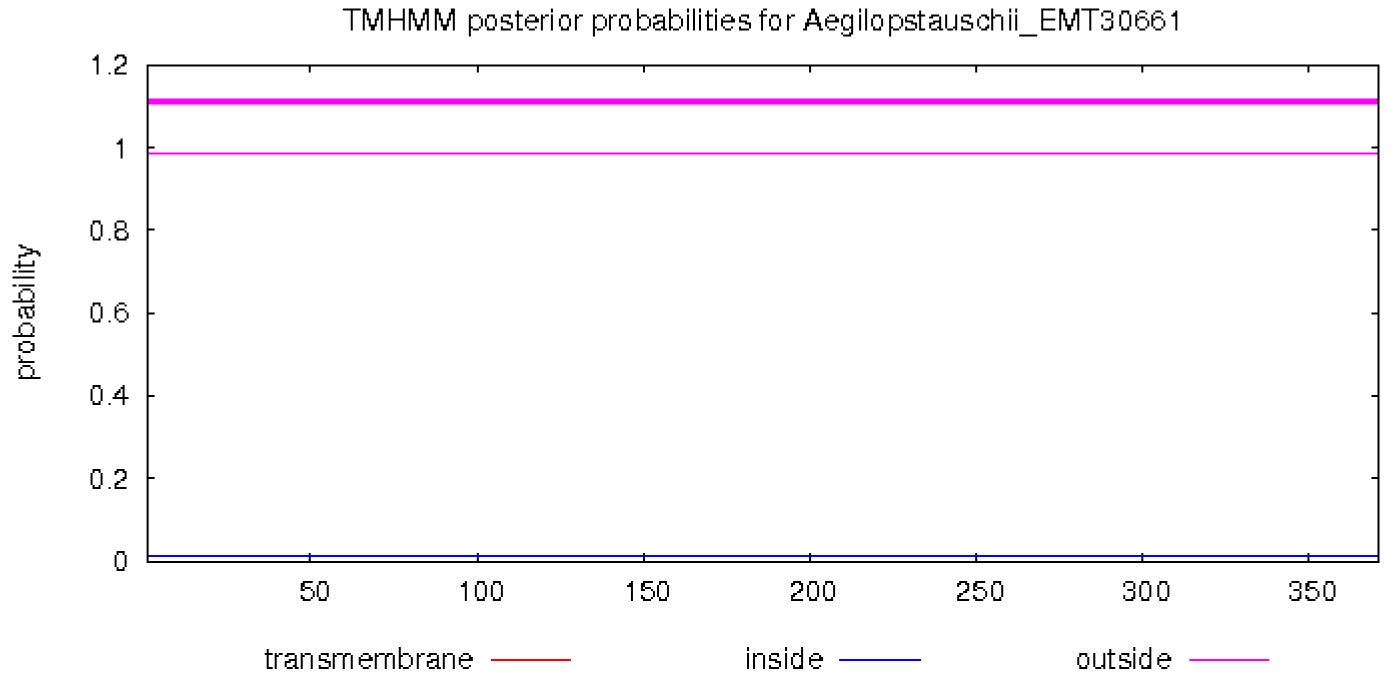

plot in postscript, script for making the plot in gnuplot, data for plot

---

```
# Aegilopstauschii_EMT30898 Length: 428
# Aegilopstauschii_EMT30898 Number of predicted TMHs:  0
# Aegilopstauschii_EMT30898 Exp number of AAs in TMHs: 0.00988
# Aegilopstauschii_EMT30898 Exp number, first 60 AAs:  0
# Aegilopstauschii_EMT30898 Total prob of N-in:        0.00821
Aegilopstauschii_EMT30898       TMHMM2.0        outside      1   428
```

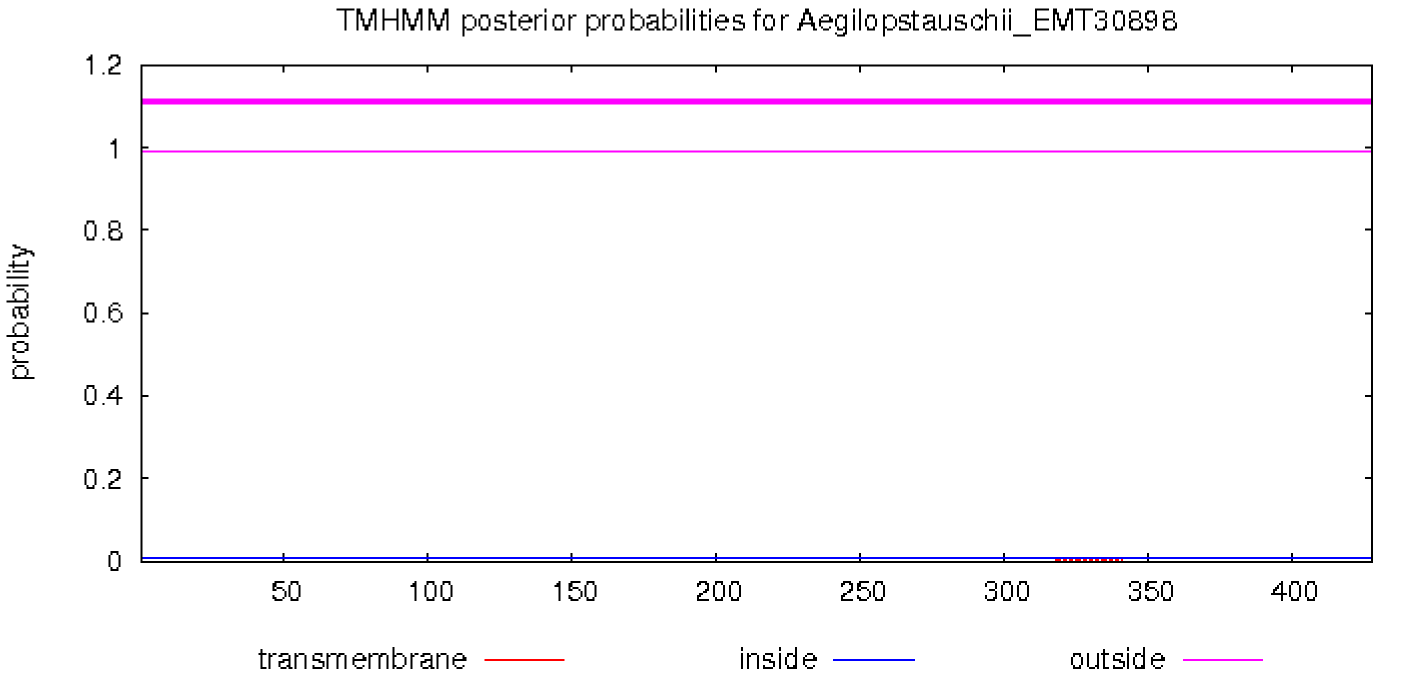

plot in postscript, script for making the plot in gnuplot, data for plot

---

```
# Aegilopstauschii_EMT31805 Length: 300
# Aegilopstauschii_EMT31805 Number of predicted TMHs:  0
# Aegilopstauschii_EMT31805 Exp number of AAs in TMHs: 0.00064
# Aegilopstauschii_EMT31805 Exp number, first 60 AAs:  0
# Aegilopstauschii_EMT31805 Total prob of N-in:        0.06637
Aegilopstauschii_EMT31805       TMHMM2.0        outside      1   300
```

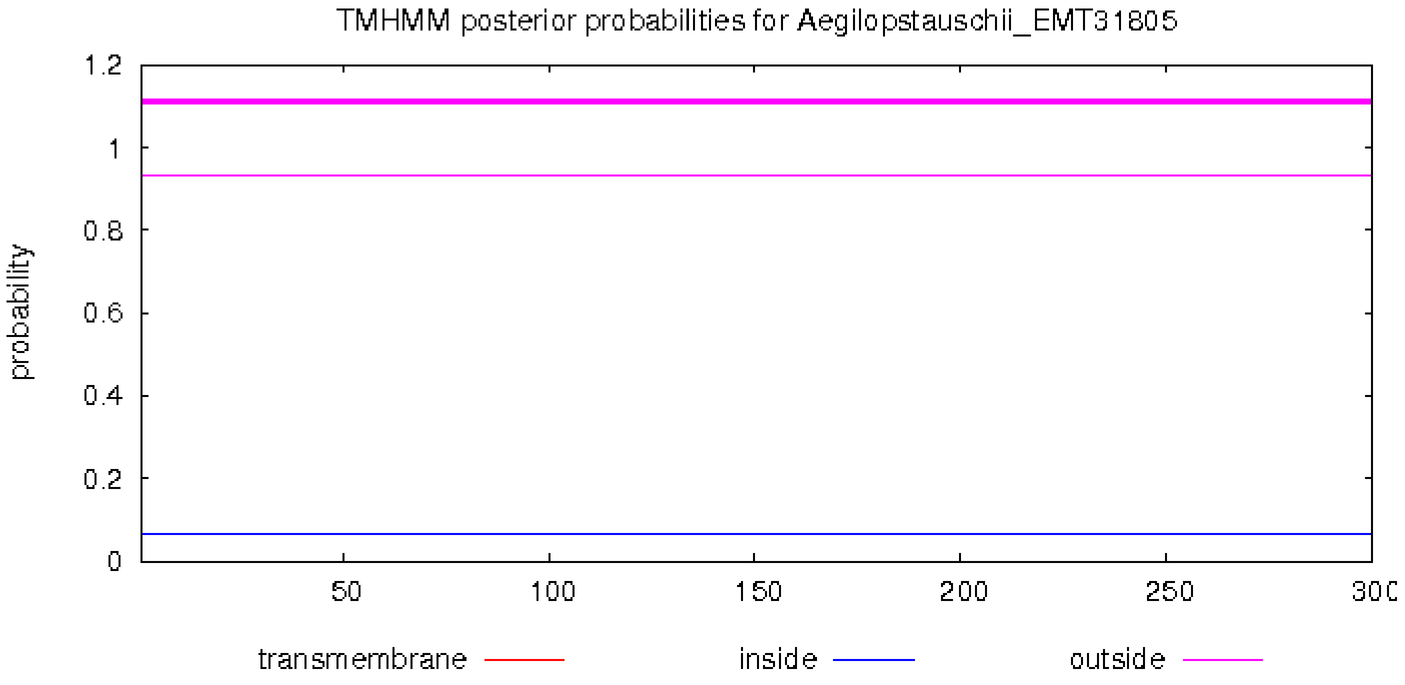

plot in postscript, script for making the plot in gnuplot, data for plot

---

```
# Aegilopstauschii_EMT32184 Length: 410
# Aegilopstauschii_EMT32184 Number of predicted TMHs:  0
# Aegilopstauschii_EMT32184 Exp number of AAs in TMHs: 0.33827
# Aegilopstauschii_EMT32184 Exp number, first 60 AAs:  0.33827
# Aegilopstauschii_EMT32184 Total prob of N-in:        0.01985
Aegilopstauschii_EMT32184       TMHMM2.0        outside      1   410
```

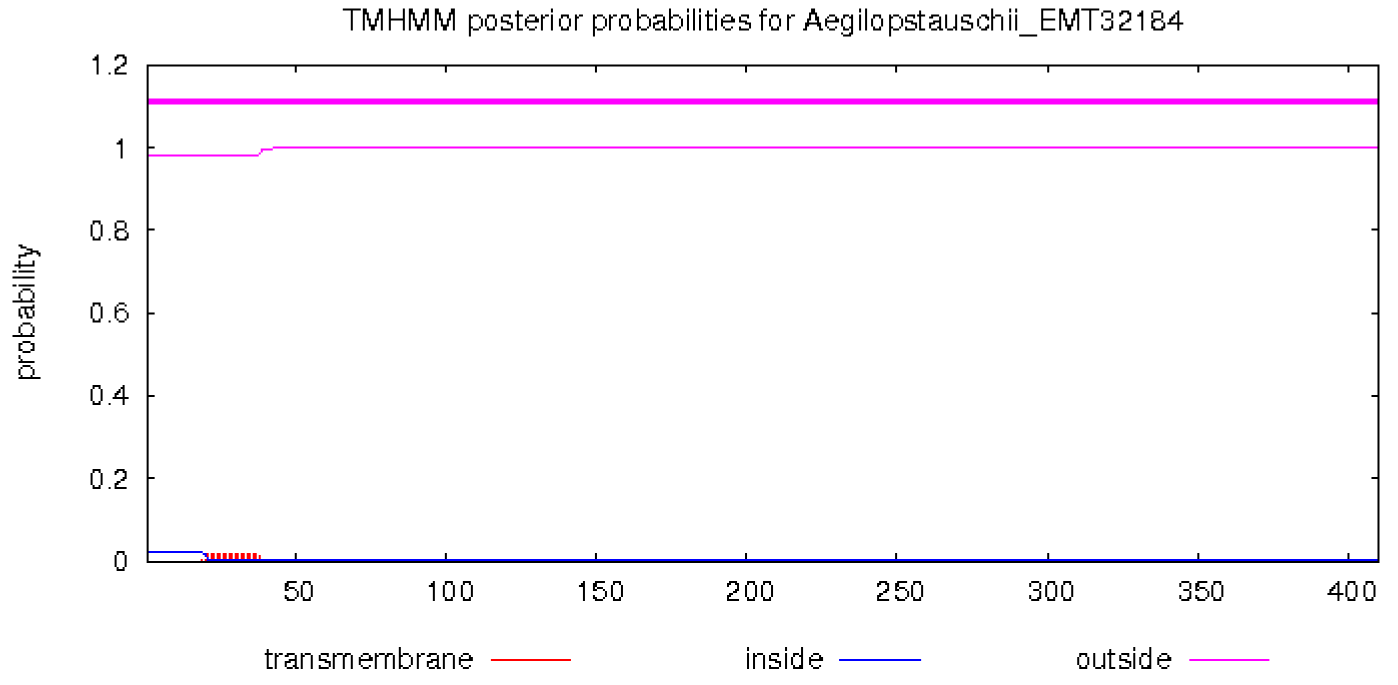

plot in postscript, script for making the plot in gnuplot, data for plot

---

```
# Aegilopstauschii_EMT32450 Length: 315
# Aegilopstauschii_EMT32450 Number of predicted TMHs:  0
# Aegilopstauschii_EMT32450 Exp number of AAs in TMHs: 0.00621
# Aegilopstauschii_EMT32450 Exp number, first 60 AAs:  0
# Aegilopstauschii_EMT32450 Total prob of N-in:        0.03170
Aegilopstauschii_EMT32450       TMHMM2.0        outside      1   315
```

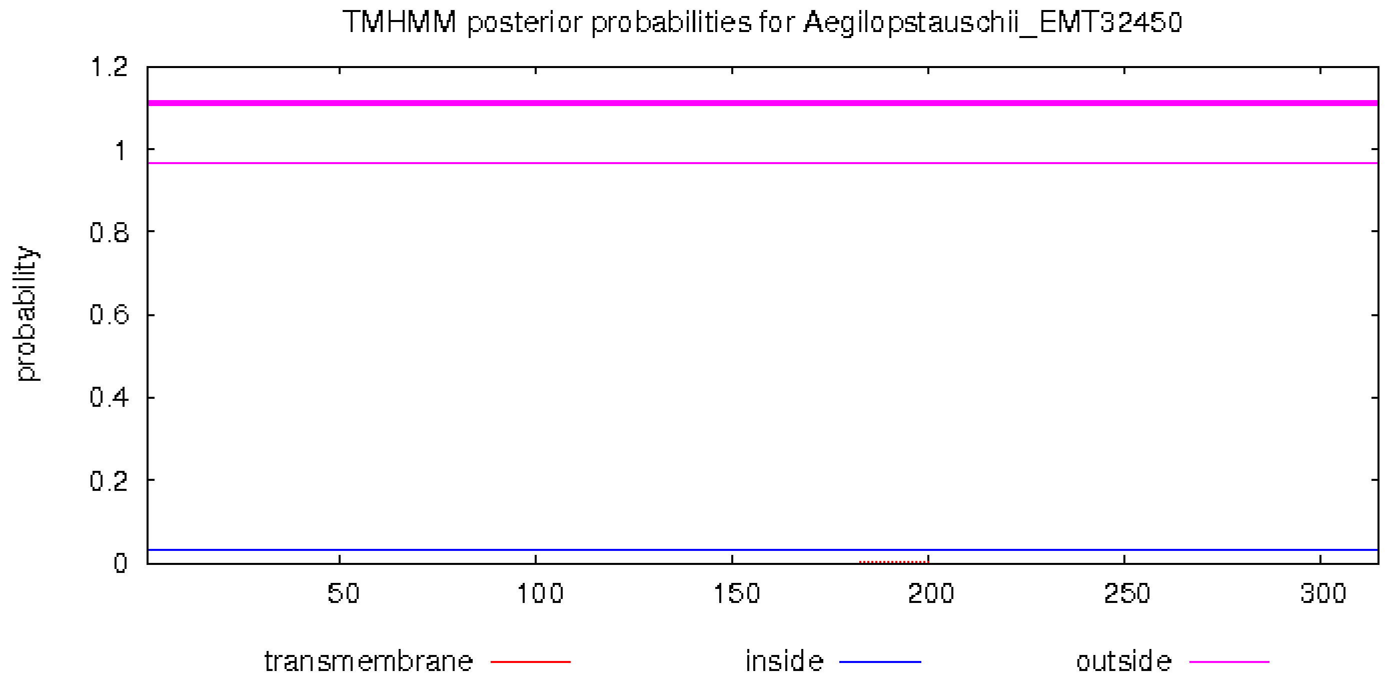

plot in postscript, script for making the plot in gnuplot, data for plot

---

```
# Aegilopstauschii_EMT32451 Length: 307
# Aegilopstauschii_EMT32451 Number of predicted TMHs:  0
# Aegilopstauschii_EMT32451 Exp number of AAs in TMHs: 0.00703
# Aegilopstauschii_EMT32451 Exp number, first 60 AAs:  0.00157
# Aegilopstauschii_EMT32451 Total prob of N-in:        0.04998
Aegilopstauschii_EMT32451       TMHMM2.0        outside      1   307
```

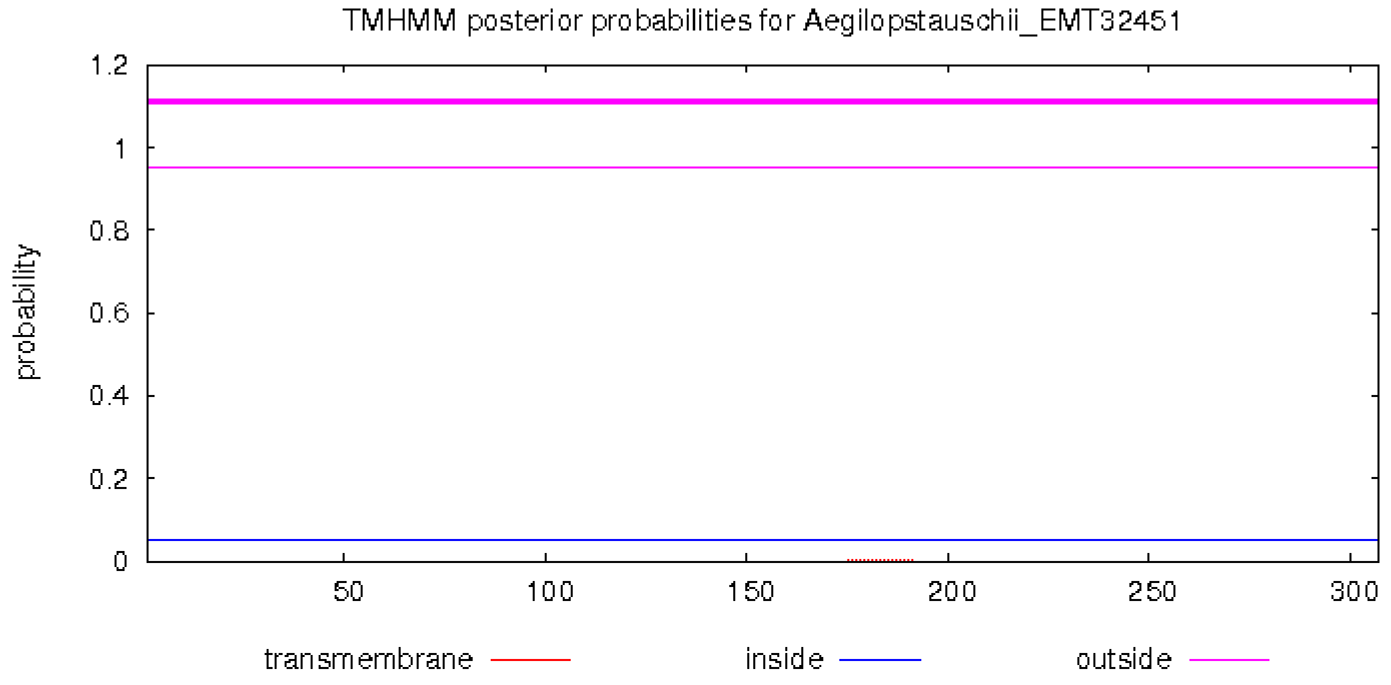

plot in postscript, script for making the plot in gnuplot, data for plot

---

```
# Aegilopstauschii_EMT32771 Length: 395
# Aegilopstauschii_EMT32771 Number of predicted TMHs:  0
# Aegilopstauschii_EMT32771 Exp number of AAs in TMHs: 0.000780000000000000001
# Aegilopstauschii_EMT32771 Exp number, first 60 AAs:  0
# Aegilopstauschii_EMT32771 Total prob of N-in:        0.00726
Aegilopstauschii_EMT32771       TMHMM2.0        outside      1   395
```

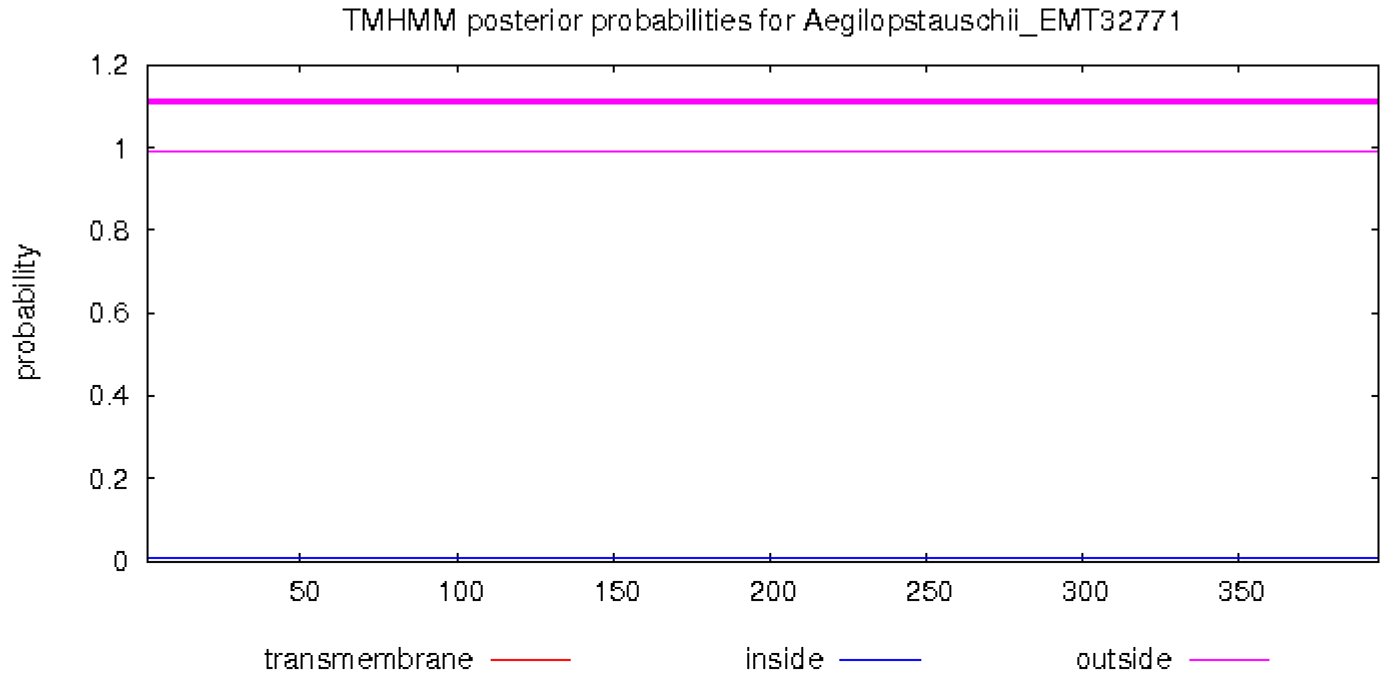

plot in postscript, script for making the plot in gnuplot, data for plot

---

```
# Aegilopstauschii_EMT32772 Length: 411
# Aegilopstauschii_EMT32772 Number of predicted TMHs:  0
# Aegilopstauschii_EMT32772 Exp number of AAs in TMHs: 0.00118
# Aegilopstauschii_EMT32772 Exp number, first 60 AAs:  0
# Aegilopstauschii_EMT32772 Total prob of N-in:        0.00377
Aegilopstauschii_EMT32772       TMHMM2.0        outside      1   411
```

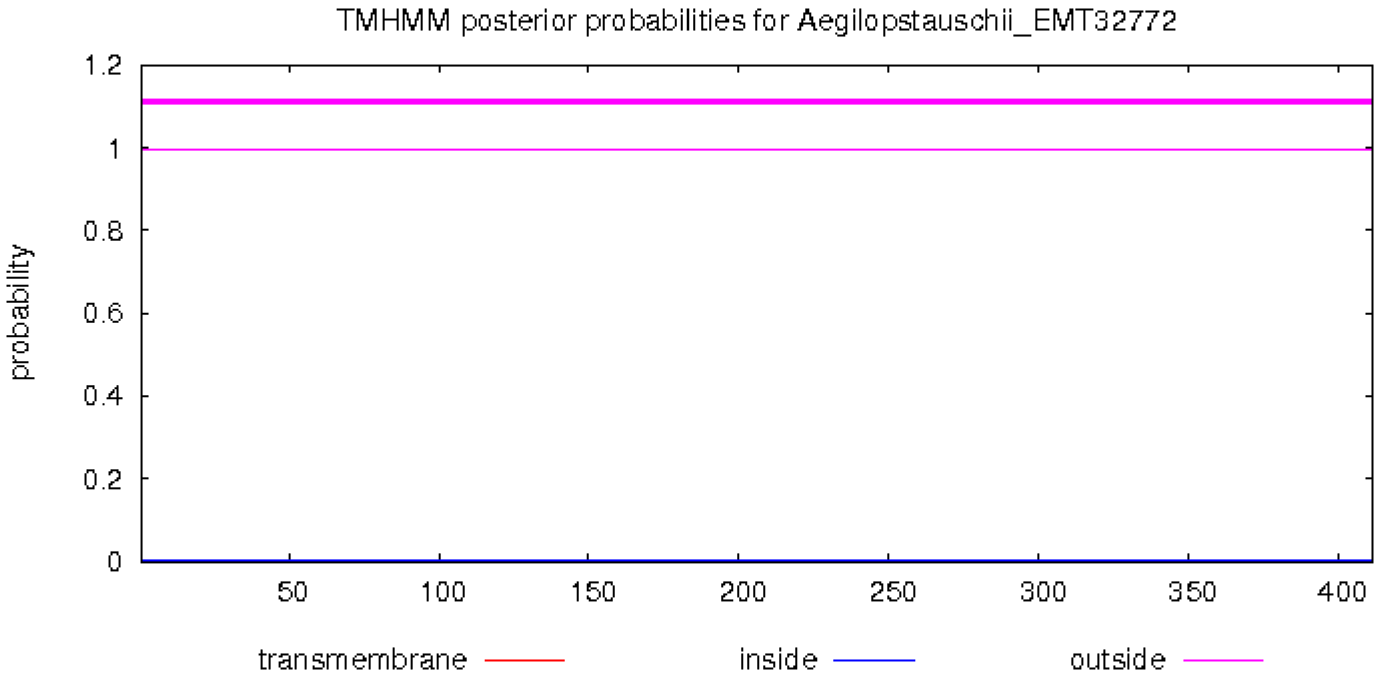

plot in postscript, script for making the plot in gnuplot, data for plot

---

```
# Aegilopstauschii_EMT32940 Length: 205
# Aegilopstauschii_EMT32940 Number of predicted TMHs:  0
# Aegilopstauschii_EMT32940 Exp number of AAs in TMHs: 0.46515
# Aegilopstauschii_EMT32940 Exp number, first 60 AAs:  0.46456
# Aegilopstauschii_EMT32940 Total prob of N-in:        0.13381
Aegilopstauschii_EMT32940       TMHMM2.0        outside      1   205
```

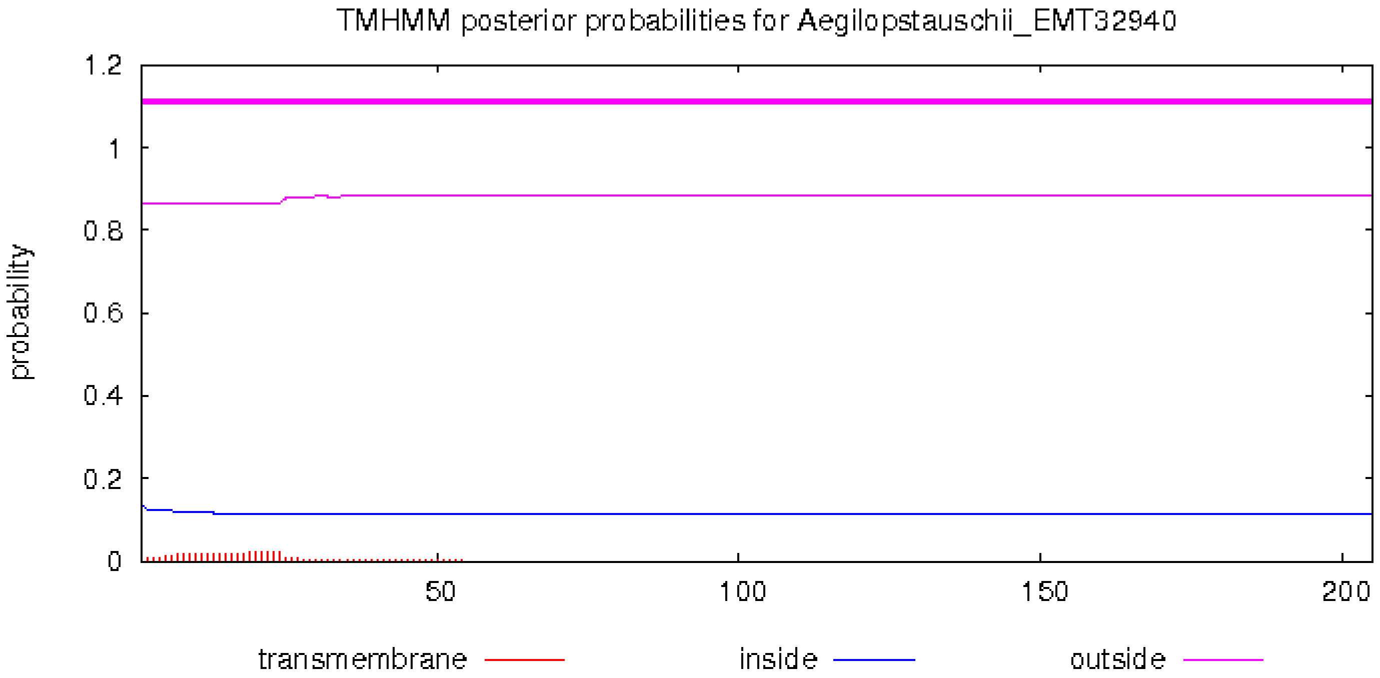

plot in postscript, script for making the plot in gnuplot, data for plot

---

```
# Aegilopstauschii_EMT32999 Length: 303
# Aegilopstauschii_EMT32999 Number of predicted TMHs:  0
# Aegilopstauschii_EMT32999 Exp number of AAs in TMHs: 0.01021
# Aegilopstauschii_EMT32999 Exp number, first 60 AAs:  0.00976
# Aegilopstauschii_EMT32999 Total prob of N-in:        0.06444
Aegilopstauschii_EMT32999       TMHMM2.0        outside      1   303
```

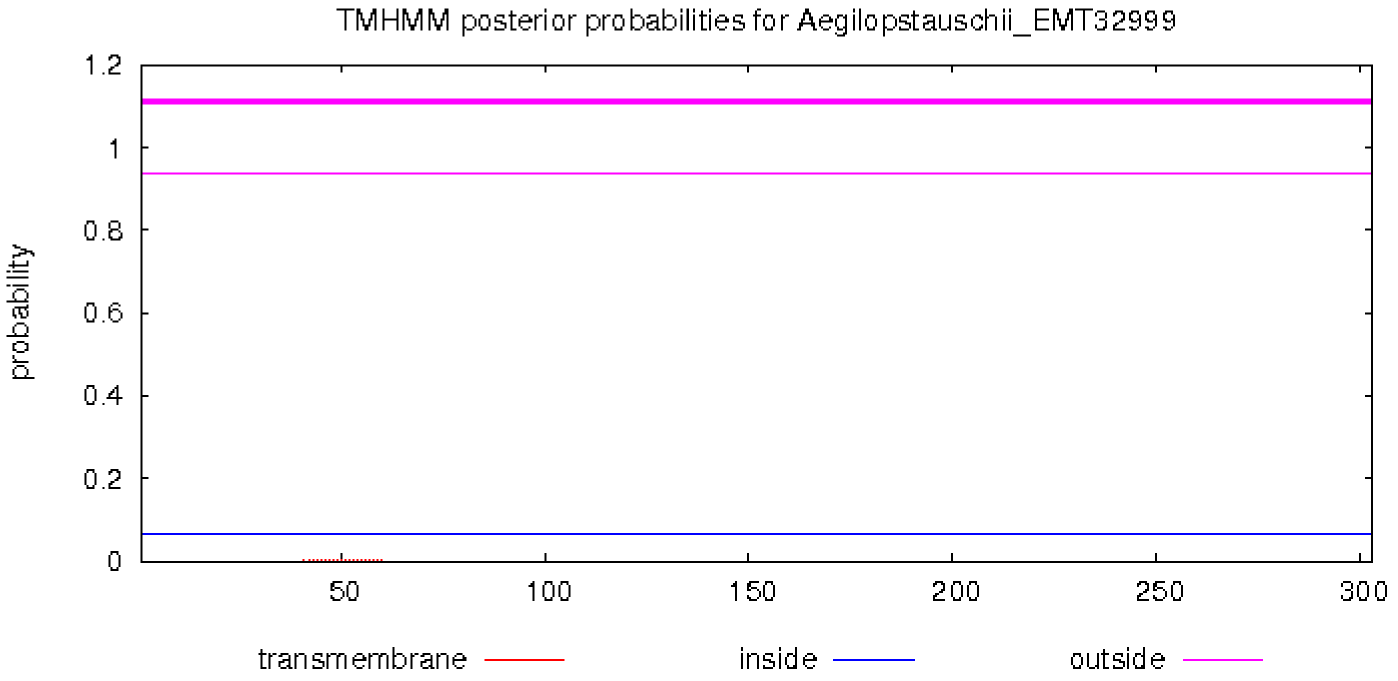

plot in postscript, script for making the plot in gnuplot, data for plot

---

```
# Aegilopstauschii_EMT33003 Length: 408
# Aegilopstauschii_EMT33003 Number of predicted TMHs:  0
# Aegilopstauschii_EMT33003 Exp number of AAs in TMHs: 0.01044
# Aegilopstauschii_EMT33003 Exp number, first 60 AAs:  0
# Aegilopstauschii_EMT33003 Total prob of N-in:        0.00258
Aegilopstauschii_EMT33003       TMHMM2.0        outside      1   408
```

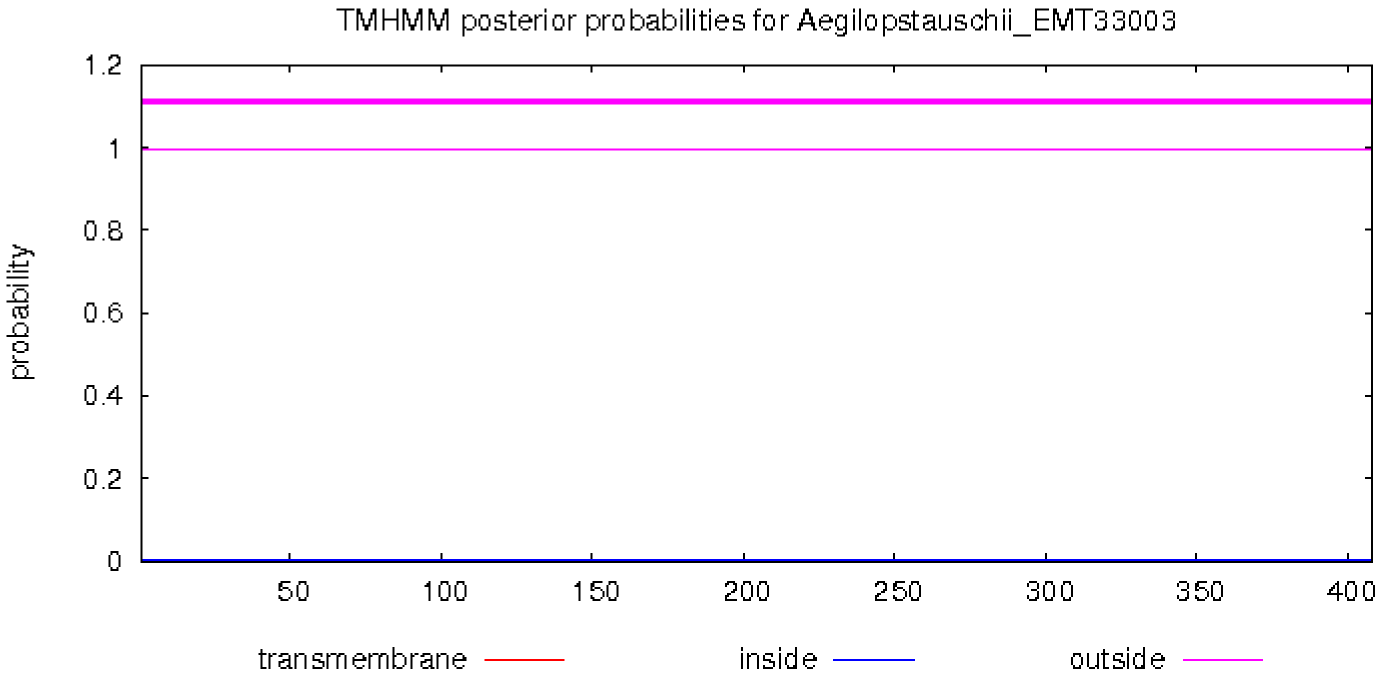

plot in postscript, script for making the plot in gnuplot, data for plot

---

```
# Aegilopstauschii_EMT33279 Length: 217
# Aegilopstauschii_EMT33279 Number of predicted TMHs:  0
# Aegilopstauschii_EMT33279 Exp number of AAs in TMHs: 0
# Aegilopstauschii_EMT33279 Exp number, first 60 AAs:  0
# Aegilopstauschii_EMT33279 Total prob of N-in:        0.00399
Aegilopstauschii_EMT33279       TMHMM2.0        outside      1   217
```

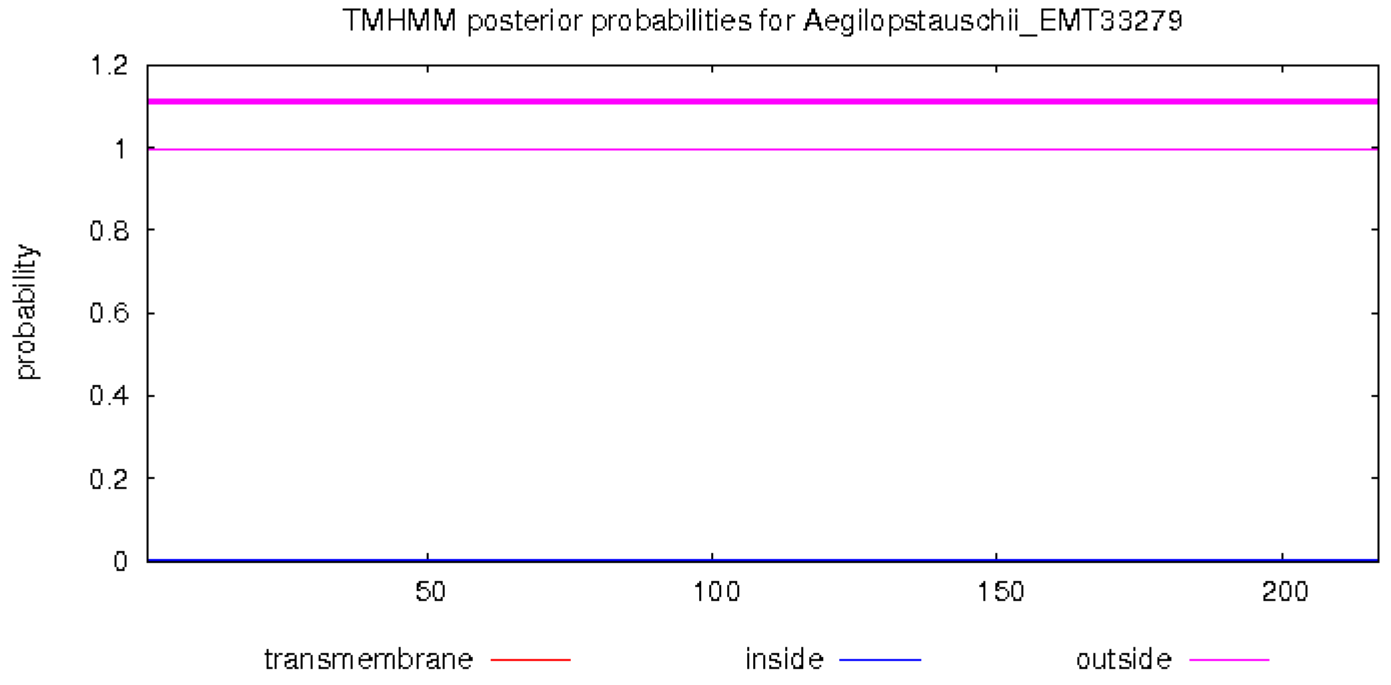

plot in postscript, script for making the plot in gnuplot, data for plot

---

```
# Aegilopstauschii_EMT33280 Length: 228
# Aegilopstauschii_EMT33280 Number of predicted TMHs:  0
# Aegilopstauschii_EMT33280 Exp number of AAs in TMHs: 0.00152
# Aegilopstauschii_EMT33280 Exp number, first 60 AAs:  0
# Aegilopstauschii_EMT33280 Total prob of N-in:        0.00266
Aegilopstauschii_EMT33280       TMHMM2.0        outside      1   228
```

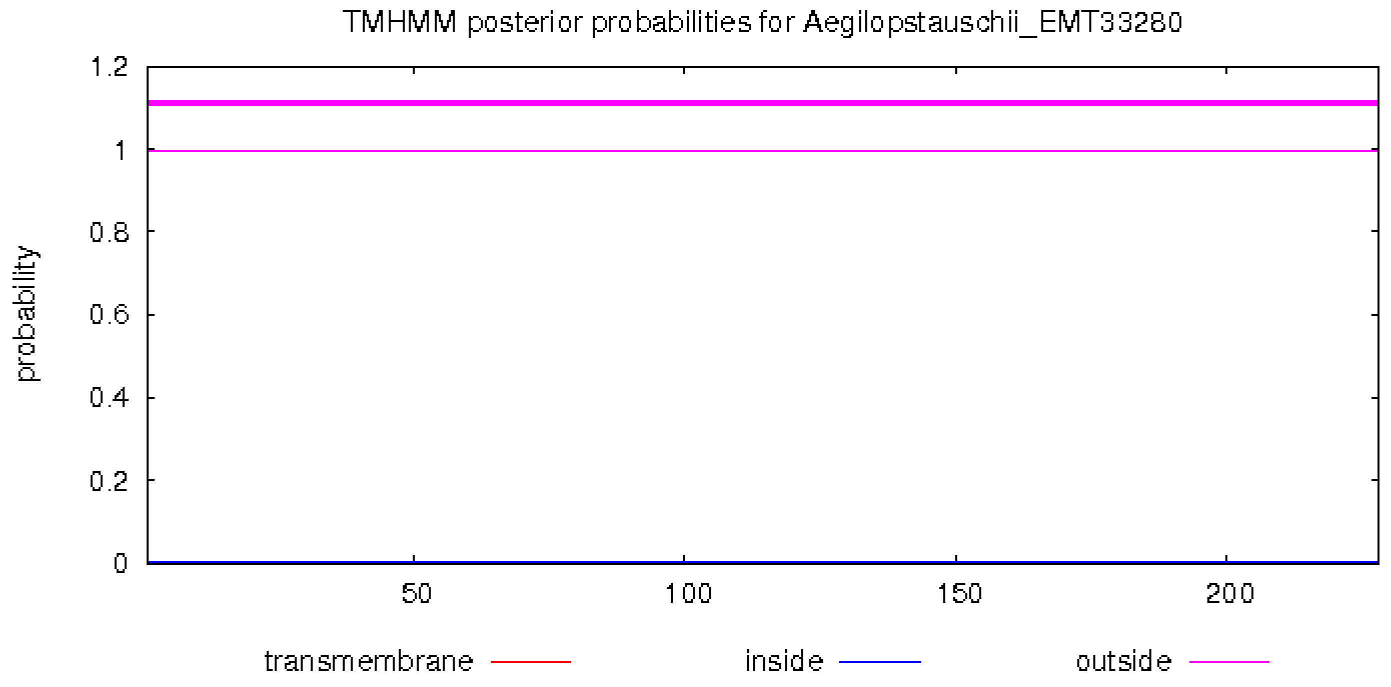

plot in postscript, script for making the plot in gnuplot, data for plot

---

```
# Aegilopstauschii_EMT33580 Length: 330
# Aegilopstauschii_EMT33580 Number of predicted TMHs:  0
# Aegilopstauschii_EMT33580 Exp number of AAs in TMHs: 0.17786
# Aegilopstauschii_EMT33580 Exp number, first 60 AAs:  0.17665
# Aegilopstauschii_EMT33580 Total prob of N-in:        0.06362
Aegilopstauschii_EMT33580       TMHMM2.0        outside      1   330
```

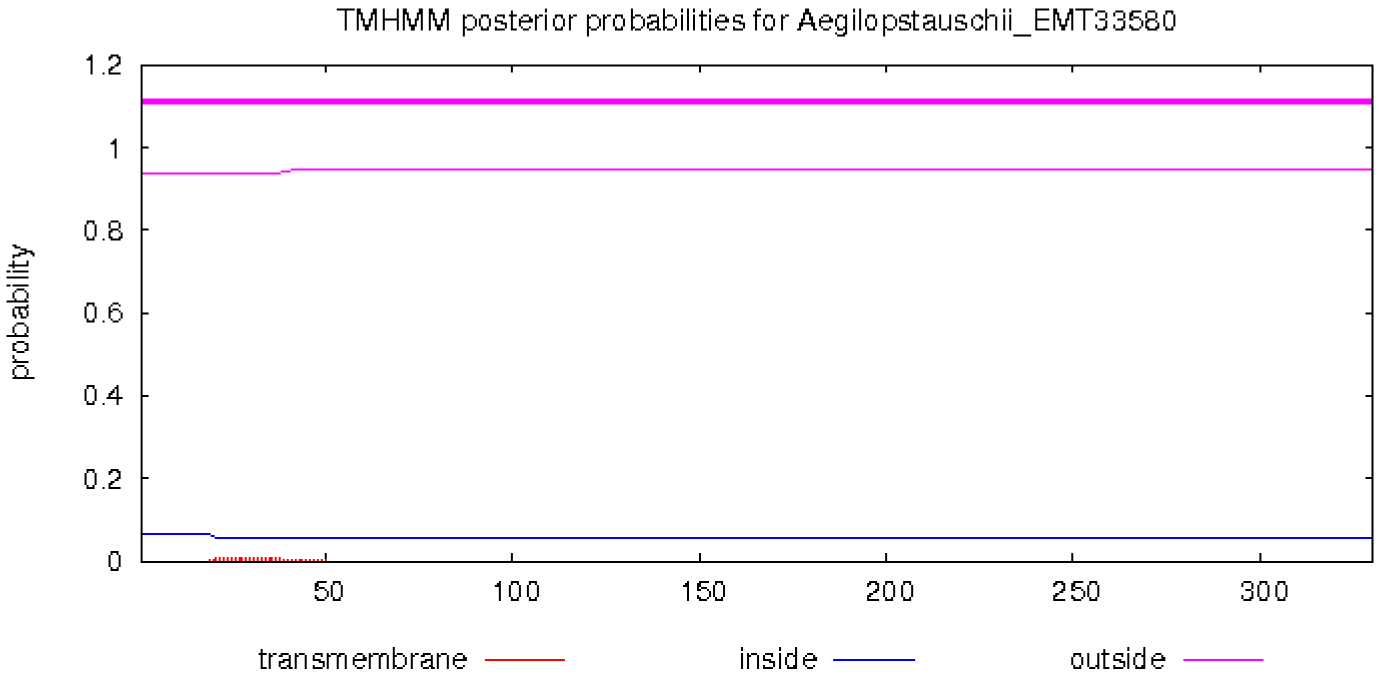

plot in postscript, script for making the plot in gnuplot, data for plot

```
# Aegilopstauschii_EMT33674 Length: 324
# Aegilopstauschii_EMT33674 Number of predicted TMHs:  0
# Aegilopstauschii_EMT33674 Exp number of AAs in TMHs: 0.01362
# Aegilopstauschii_EMT33674 Exp number, first 60 AAs:  0.00056
# Aegilopstauschii_EMT33674 Total prob of N-in:        0.10416
Aegilopstauschii_EMT33674       TMHMM2.0        outside      1   324
```

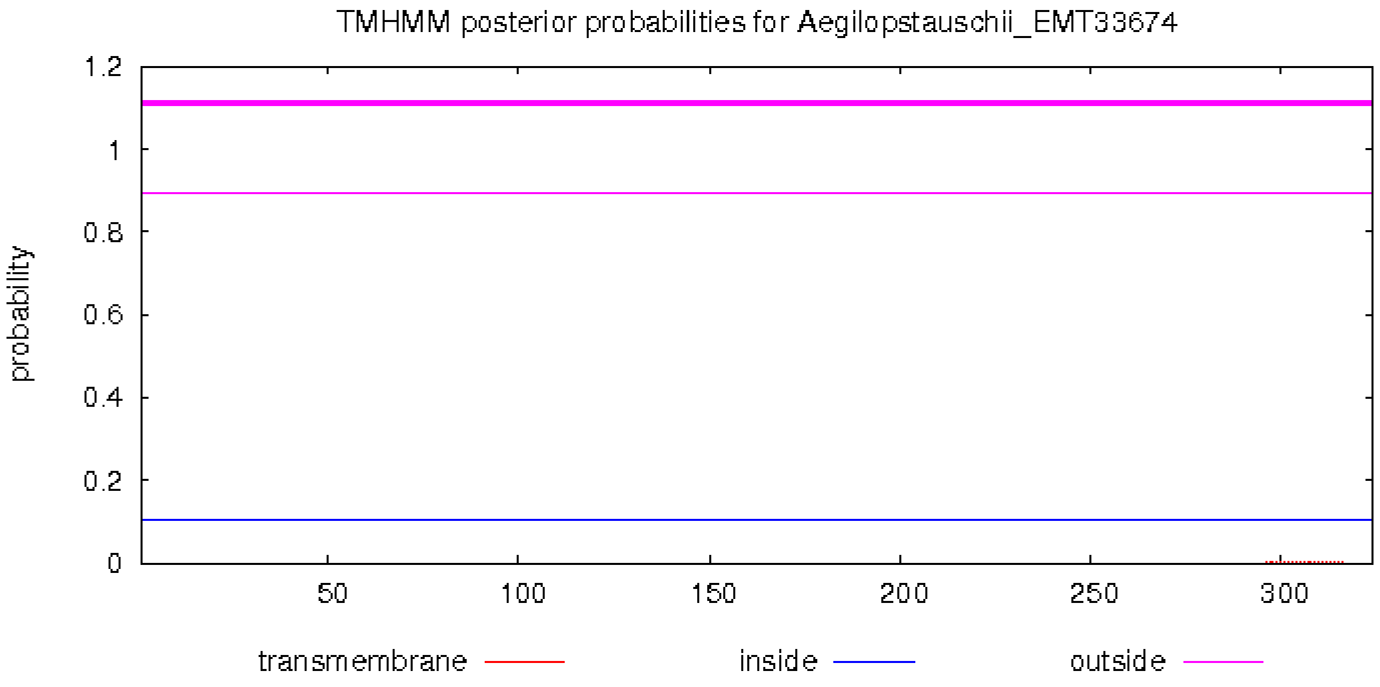

plot in postscript, script for making the plot in gnuplot, data for plot

```
# Aegilopstauschii_EMT33773 Length: 353
# Aegilopstauschii_EMT33773 Number of predicted TMHs:  0
# Aegilopstauschii_EMT33773 Exp number of AAs in TMHs: 0.10525
# Aegilopstauschii_EMT33773 Exp number, first 60 AAs:  0.00112
# Aegilopstauschii_EMT33773 Total prob of N-in:        0.05605
Aegilopstauschii_EMT33773       TMHMM2.0        outside      1   353
```

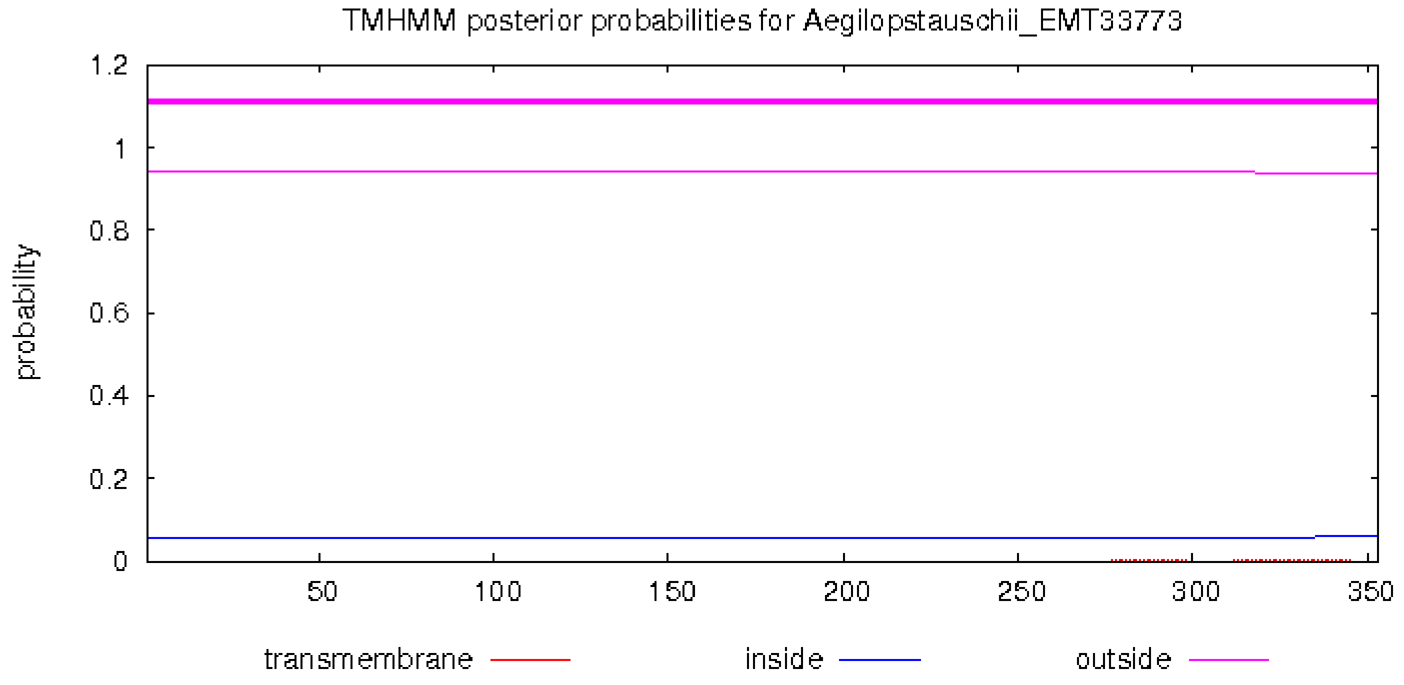

plot in postscript, script for making the plot in gnuplot, data for plot

---

```
# Aegilopstauschii_EMT33859 Length: 648
# Aegilopstauschii_EMT33859 Number of predicted TMHs:  0
# Aegilopstauschii_EMT33859 Exp number of AAs in TMHs: 0.05489
# Aegilopstauschii_EMT33859 Exp number, first 60 AAs:  0.00087
# Aegilopstauschii_EMT33859 Total prob of N-in:        0.00006
Aegilopstauschii_EMT33859       TMHMM2.0        outside      1   648
```

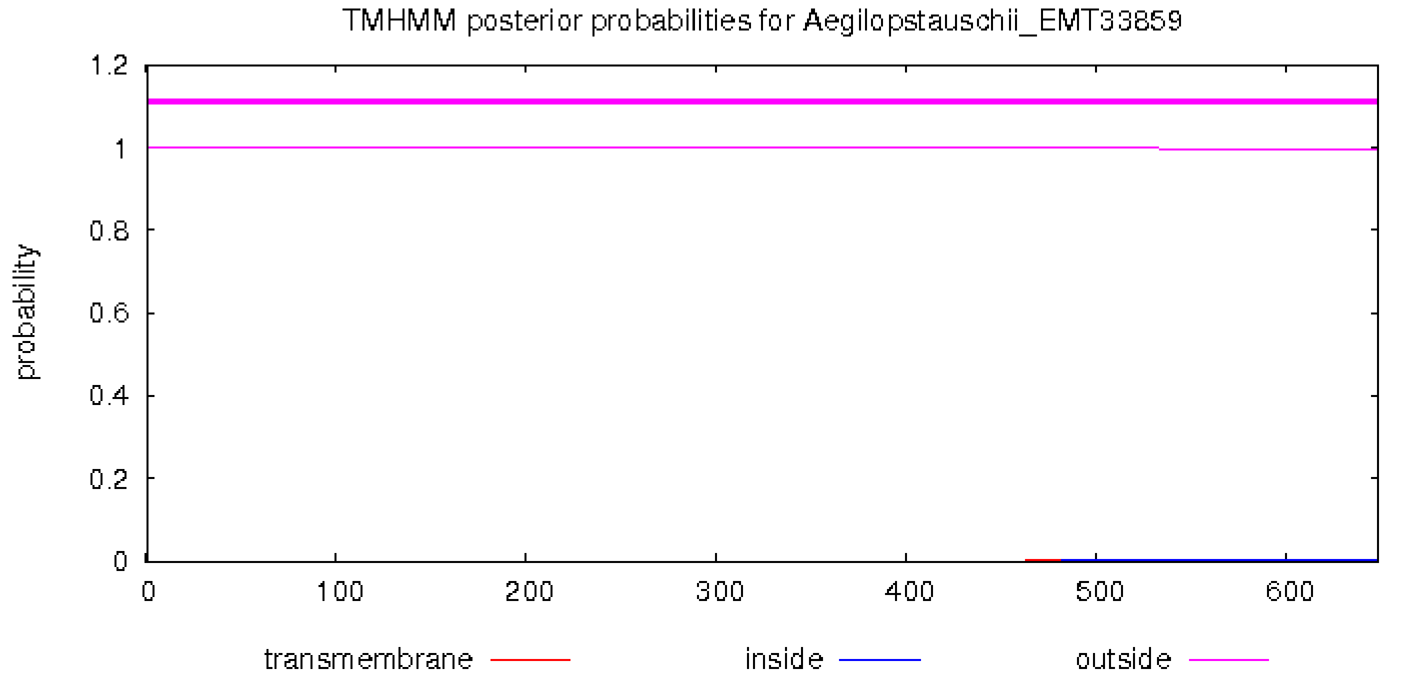

plot in postscript, script for making the plot in gnuplot, data for plot

---

```
# Aethionemaarabicum_AA102G00007 Length: 238
# Aethionemaarabicum_AA102G00007 Number of predicted TMHs:  0
# Aethionemaarabicum_AA102G00007 Exp number of AAs in TMHs: 0.01407
# Aethionemaarabicum_AA102G00007 Exp number, first 60 AAs:  0
# Aethionemaarabicum_AA102G00007 Total prob of N-in:        0.01576
Aethionemaarabicum_AA102G00007  TMHMM2.0        outside      1   238
```

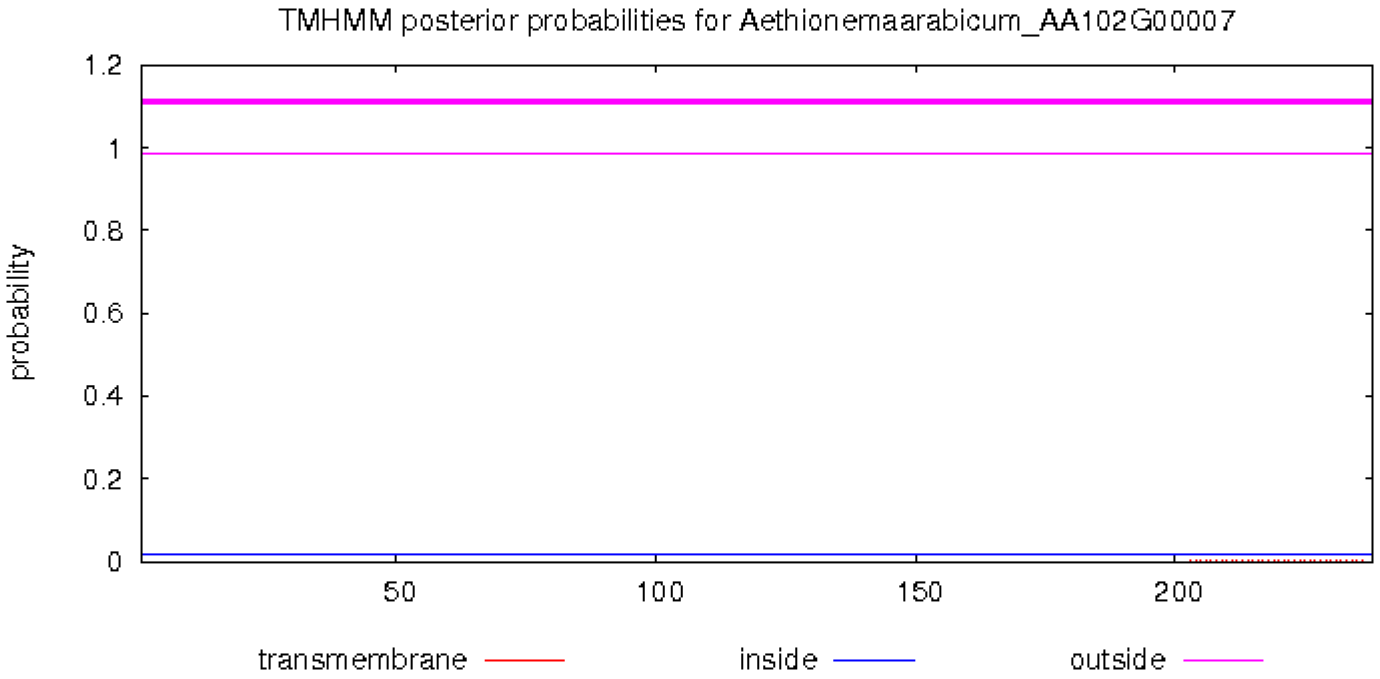

plot in postscript, script for making the plot in gnuplot, data for plot

```
# Aethionemaarabicum_AA102G00073 Length: 283
# Aethionemaarabicum_AA102G00073 Number of predicted TMHs:  1
# Aethionemaarabicum_AA102G00073 Exp number of AAs in TMHs: 21.49455
# Aethionemaarabicum_AA102G00073 Exp number, first 60 AAs:  0.00193
# Aethionemaarabicum_AA102G00073 Total prob of N-in:        0.94863
Aethionemaarabicum_AA102G00073  TMHMM2.0        inside       1   144
Aethionemaarabicum_AA102G00073  TMHMM2.0        TMhelix    145   167
Aethionemaarabicum_AA102G00073  TMHMM2.0        outside    168   283
```

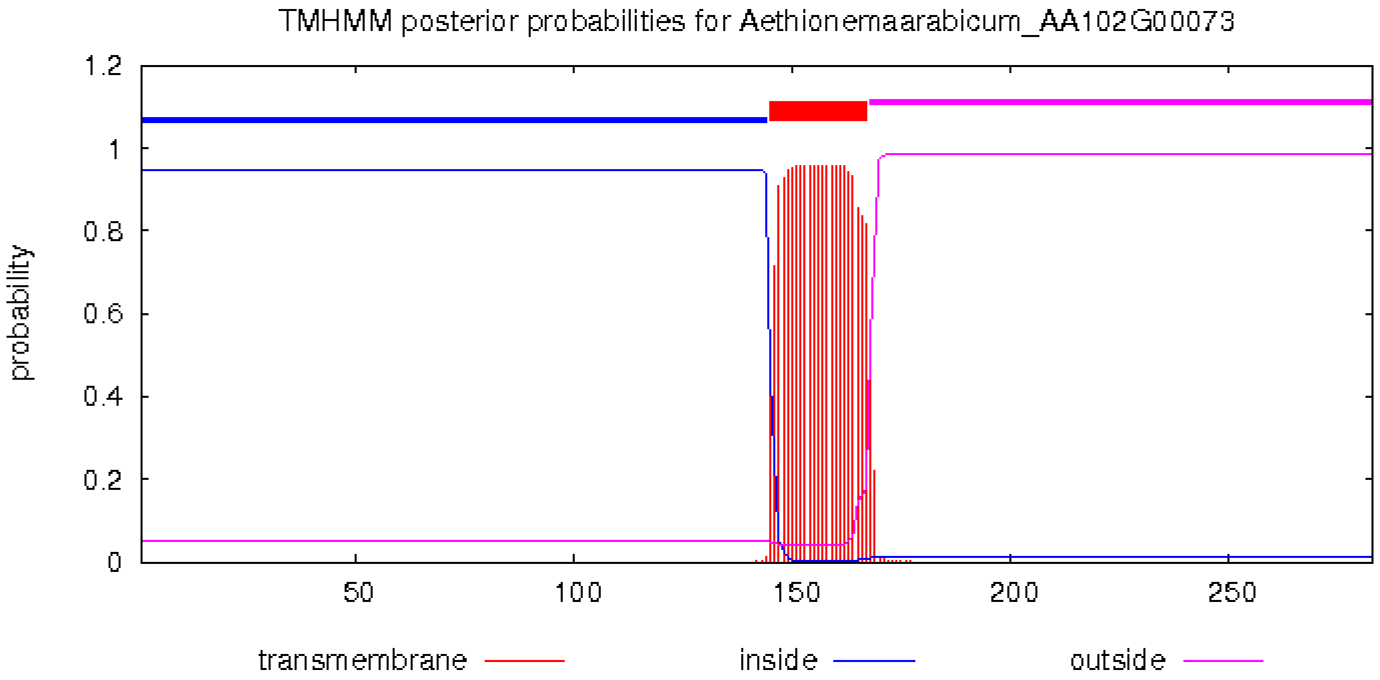

plot in postscript, script for making the plot in gnuplot, data for plot

```
# Aethionemaarabicum_AA10G00196 Length: 642
# Aethionemaarabicum_AA10G00196 Number of predicted TMHs:  0
# Aethionemaarabicum_AA10G00196 Exp number of AAs in TMHs: 0.00089
# Aethionemaarabicum_AA10G00196 Exp number, first 60 AAs:  0.00089
```

```
# Aethionemaarabicum_AA10G00196 Total prob of N-in:        0.00034
Aethionemaarabicum_AA10G00196   TMHMM2.0        outside      1   642
```

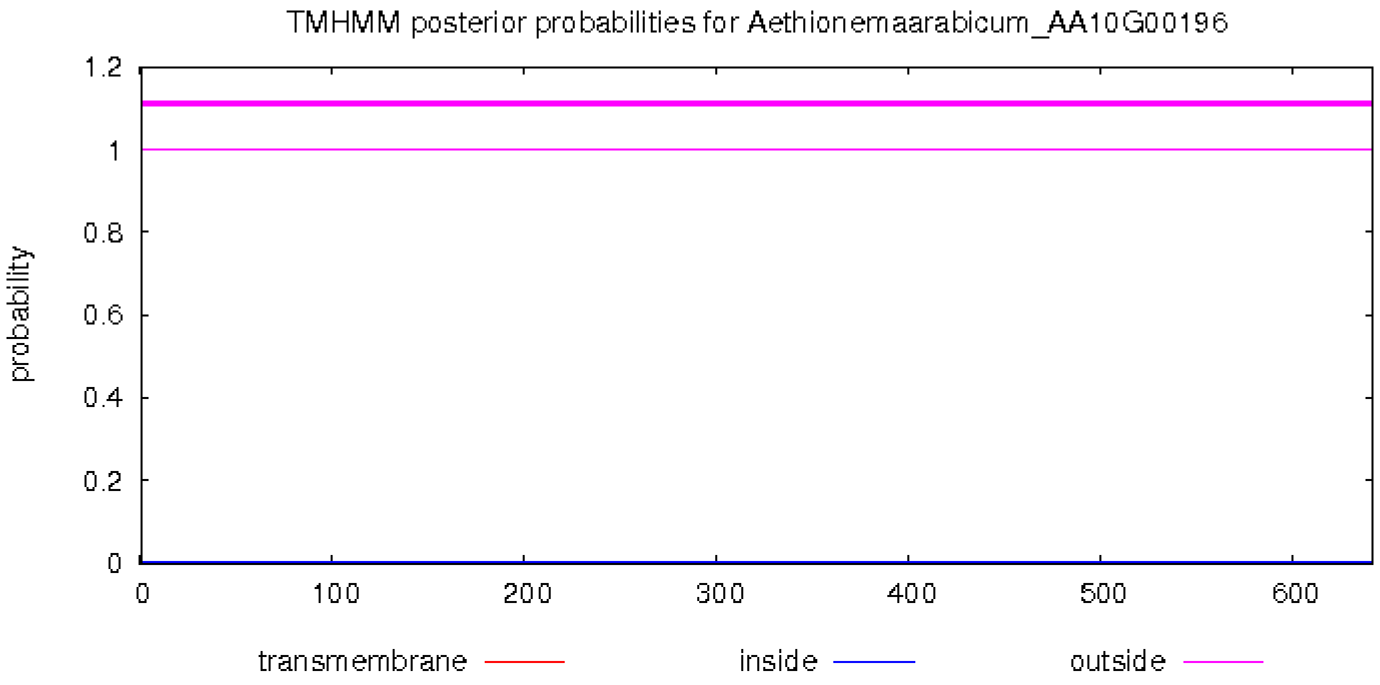

plot in postscript, script for making the plot in gnuplot, data for plot

---

```
# Aethionemaarabicum_AA10G00295 Length: 485
# Aethionemaarabicum_AA10G00295 Number of predicted TMHs:  0
# Aethionemaarabicum_AA10G00295 Exp number of AAs in TMHs: 0.00321
# Aethionemaarabicum_AA10G00295 Exp number, first 60 AAs:  0
# Aethionemaarabicum_AA10G00295 Total prob of N-in:        0.00015
Aethionemaarabicum_AA10G00295   TMHMM2.0        outside      1   485
```

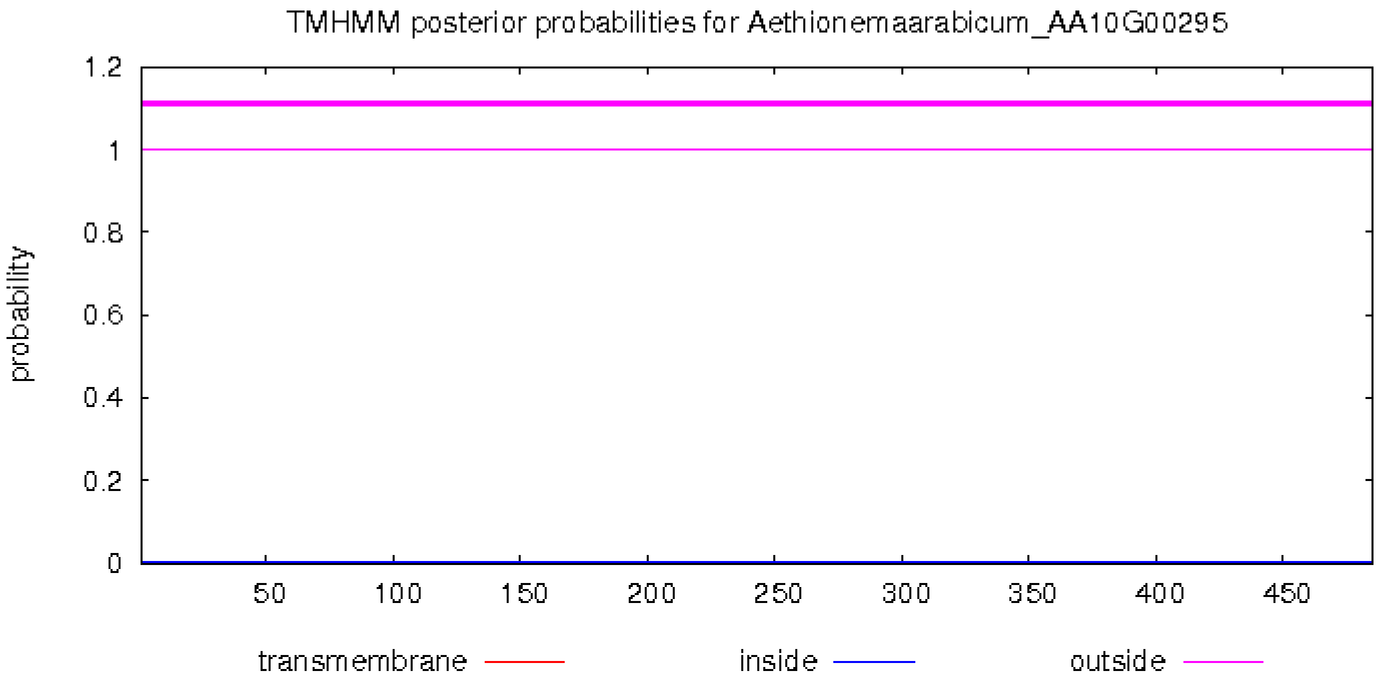

plot in postscript, script for making the plot in gnuplot, data for plot

---

```
# Aethionemaarabicum_AA11G00017 Length: 295
# Aethionemaarabicum_AA11G00017 Number of predicted TMHs:  0
# Aethionemaarabicum_AA11G00017 Exp number of AAs in TMHs: 0.00215
# Aethionemaarabicum_AA11G00017 Exp number, first 60 AAs:  0.00043
```

```
# Aethionemaarabicum_AA11G00017 Total prob of N-in:        0.01462
Aethionemaarabicum_AA11G00017   TMHMM2.0        outside      1   295
```

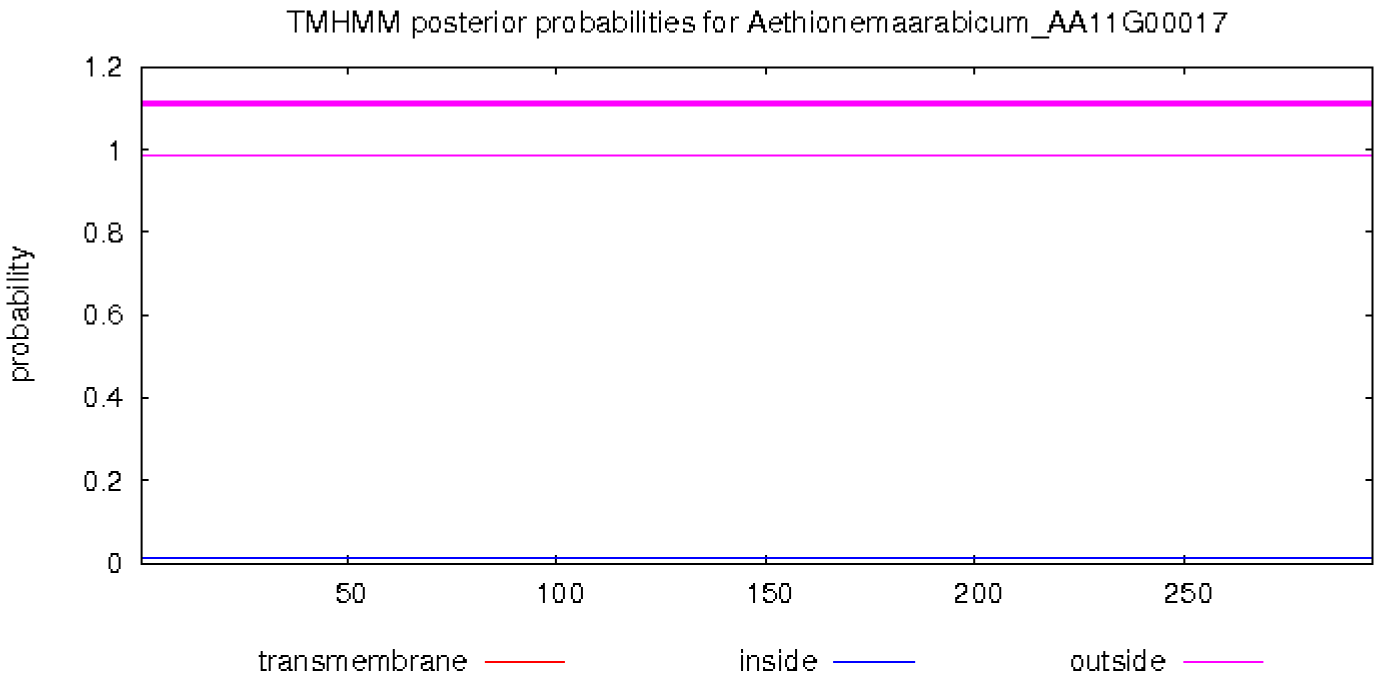

plot in postscript, script for making the plot in gnuplot, data for plot

---

```
# Aethionemaarabicum_AA11G00058 Length: 349
# Aethionemaarabicum_AA11G00058 Number of predicted TMHs:  0
# Aethionemaarabicum_AA11G00058 Exp number of AAs in TMHs: 0.00329
# Aethionemaarabicum_AA11G00058 Exp number, first 60 AAs:  0
# Aethionemaarabicum_AA11G00058 Total prob of N-in:        0.04756
Aethionemaarabicum_AA11G00058   TMHMM2.0        outside      1   349
```

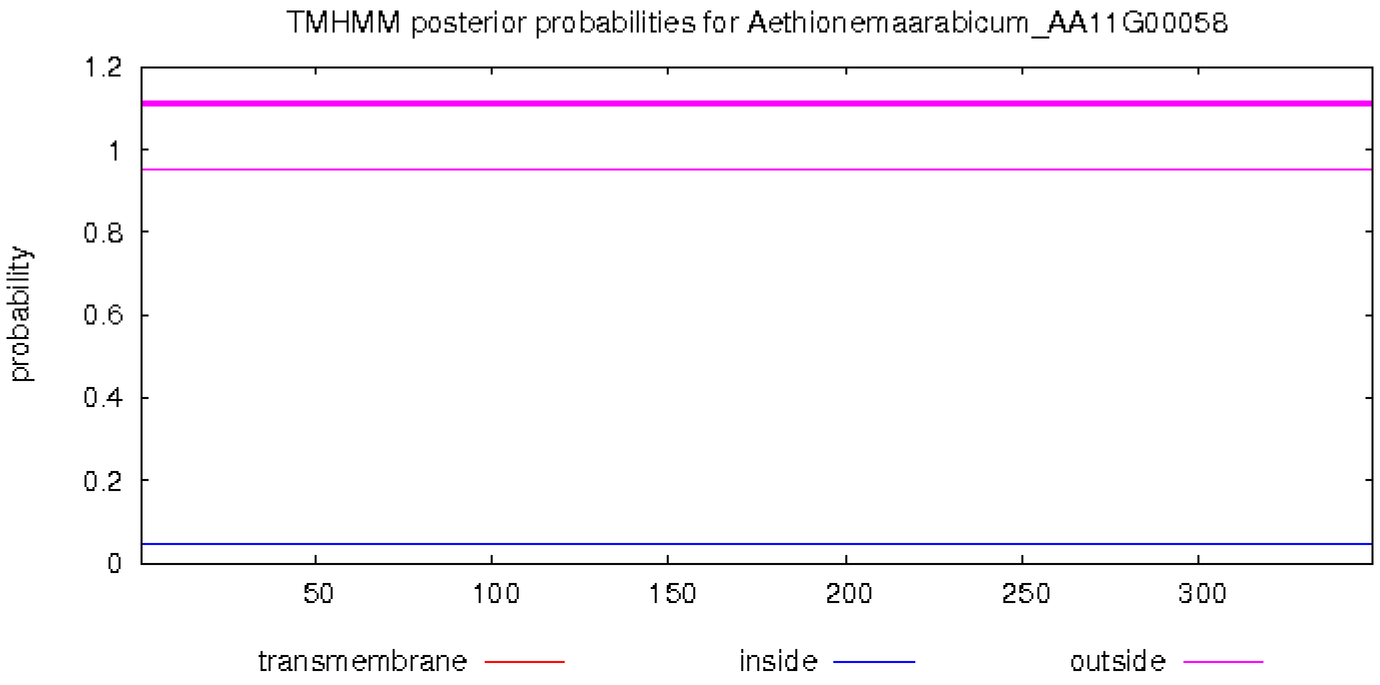

plot in postscript, script for making the plot in gnuplot, data for plot

---

```
# Aethionemaarabicum_AA128G00002 Length: 334
# Aethionemaarabicum_AA128G00002 Number of predicted TMHs:  0
# Aethionemaarabicum_AA128G00002 Exp number of AAs in TMHs: 0.01126
# Aethionemaarabicum_AA128G00002 Exp number, first 60 AAs:  0
```

```
# Aethionemaarabicum_AA128G00002 Total prob of N-in:        0.04472
Aethionemaarabicum_AA128G00002  TMHMM2.0        outside      1   334
```

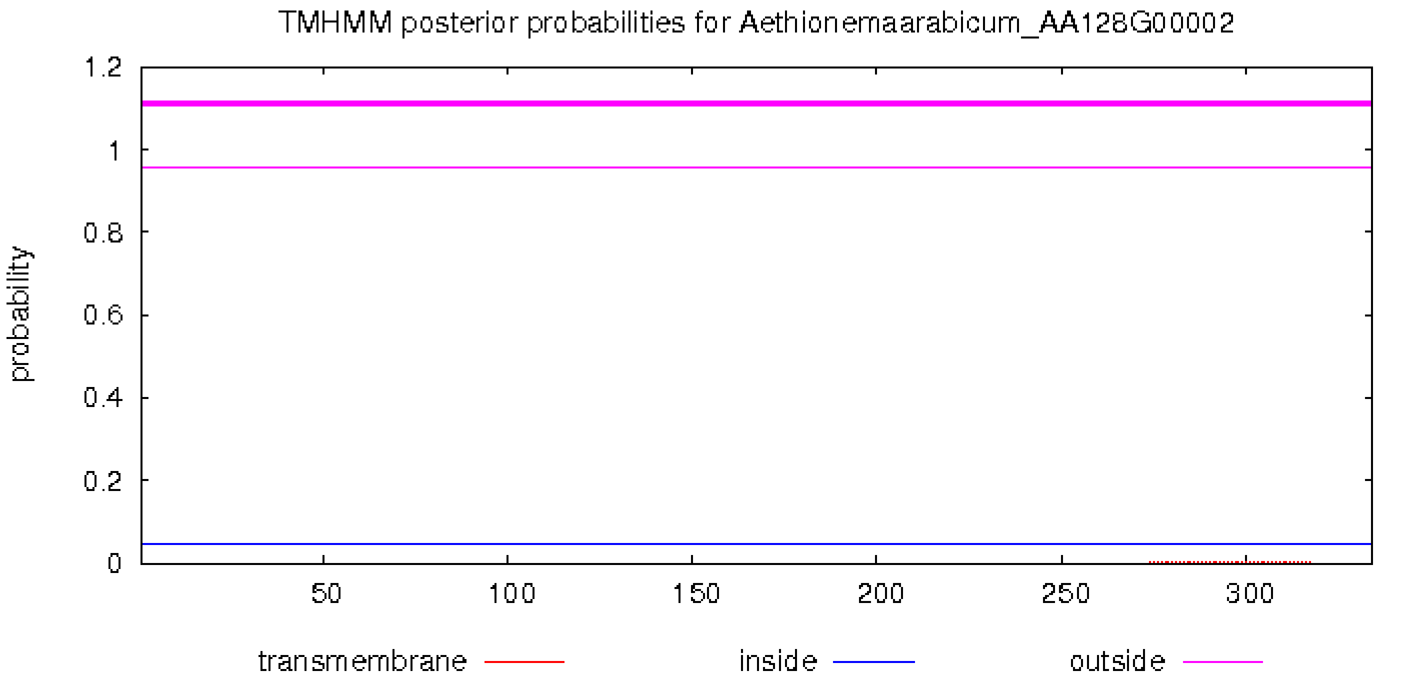

plot in postscript, script for making the plot in gnuplot, data for plot

---

```
# Aethionemaarabicum_AA12G00161 Length: 400
# Aethionemaarabicum_AA12G00161 Number of predicted TMHs:  0
# Aethionemaarabicum_AA12G00161 Exp number of AAs in TMHs: 0.0054
# Aethionemaarabicum_AA12G00161 Exp number, first 60 AAs:  0
# Aethionemaarabicum_AA12G00161 Total prob of N-in:        0.01423
Aethionemaarabicum_AA12G00161   TMHMM2.0        outside      1   400
```

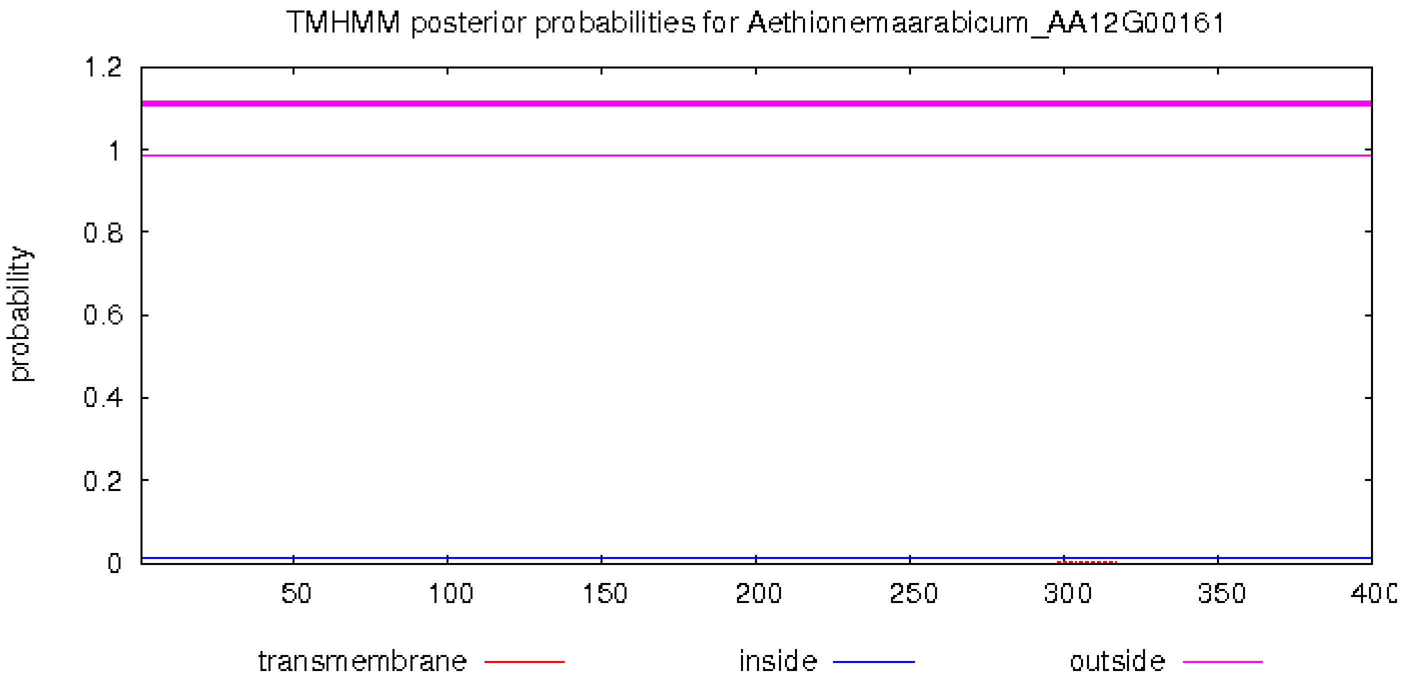

plot in postscript, script for making the plot in gnuplot, data for plot

---

```
# Aethionemaarabicum_AA13G00126 Length: 464
# Aethionemaarabicum_AA13G00126 Number of predicted TMHs:  0
# Aethionemaarabicum_AA13G00126 Exp number of AAs in TMHs: 11.38987
# Aethionemaarabicum_AA13G00126 Exp number, first 60 AAs:  0
```

```
# Aethionemaarabicum_AA13G00126 Total prob of N-in:        0.06589
Aethionemaarabicum_AA13G00126   TMHMM2.0        outside      1   464
```

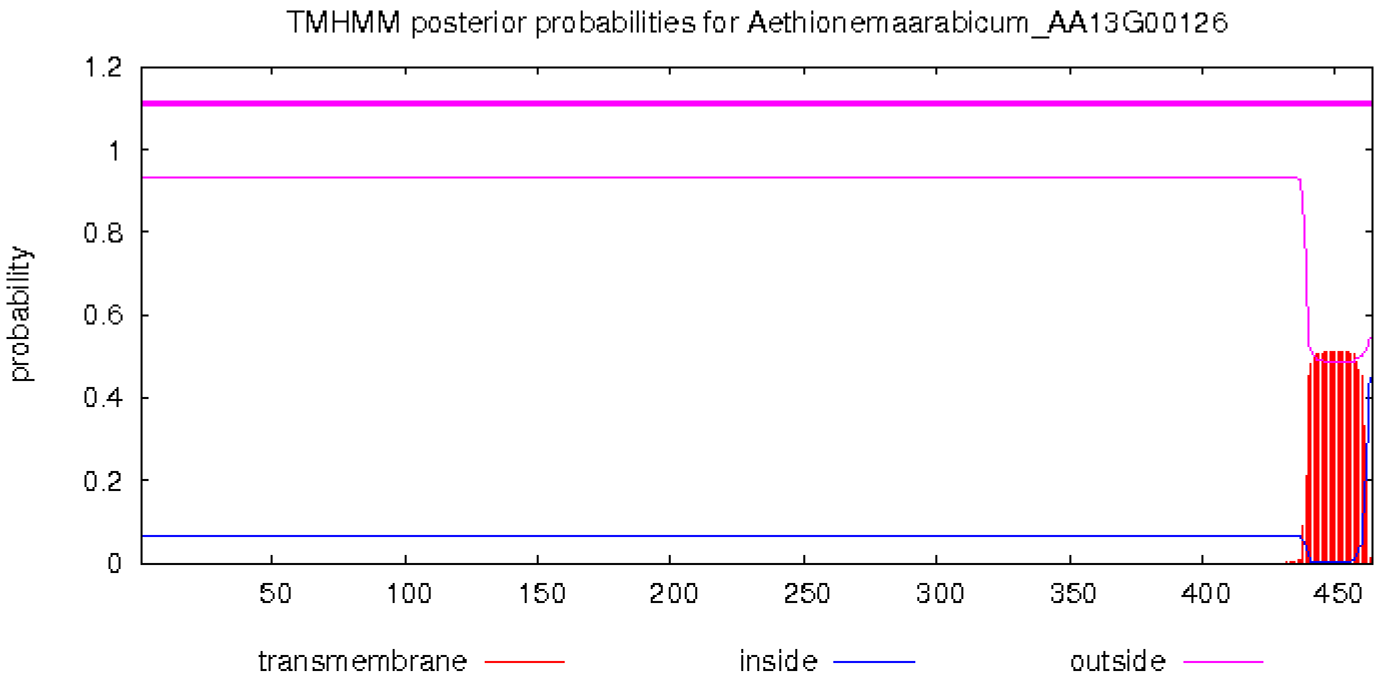

plot in postscript, script for making the plot in gnuplot, data for plot

---

```
# Aethionemaarabicum_AA15G00071 Length: 346
# Aethionemaarabicum_AA15G00071 Number of predicted TMHs:  0
# Aethionemaarabicum_AA15G00071 Exp number of AAs in TMHs: 0.00231
# Aethionemaarabicum_AA15G00071 Exp number, first 60 AAs:  0.00017
# Aethionemaarabicum_AA15G00071 Total prob of N-in:        0.00542
Aethionemaarabicum_AA15G00071   TMHMM2.0        outside      1   346
```

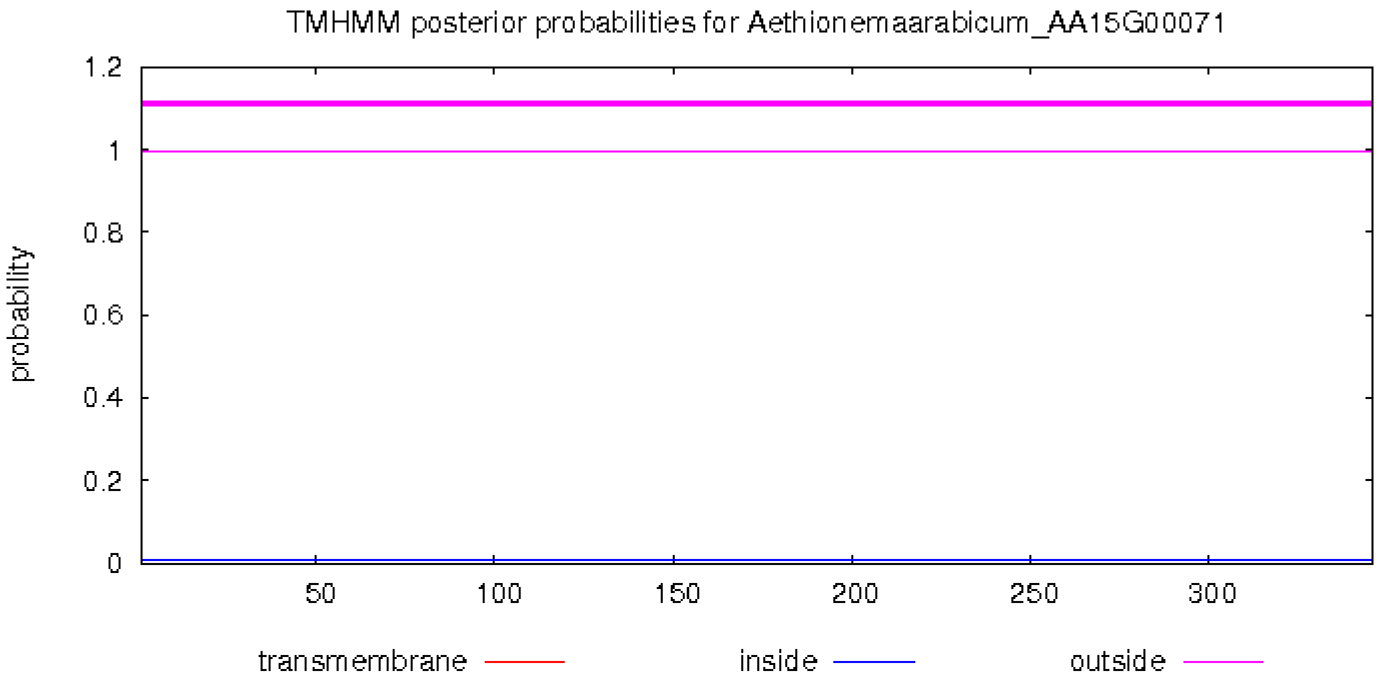

plot in postscript, script for making the plot in gnuplot, data for plot

---

```
# Aethionemaarabicum_AA15G00326 Length: 338
# Aethionemaarabicum_AA15G00326 Number of predicted TMHs:  0
# Aethionemaarabicum_AA15G00326 Exp number of AAs in TMHs: 0.00011
# Aethionemaarabicum_AA15G00326 Exp number, first 60 AAs:  0
```

```
# Aethionemaarabicum_AA15G00326 Total prob of N-in:        0.00154
Aethionemaarabicum_AA15G00326   TMHMM2.0        outside      1   338
```

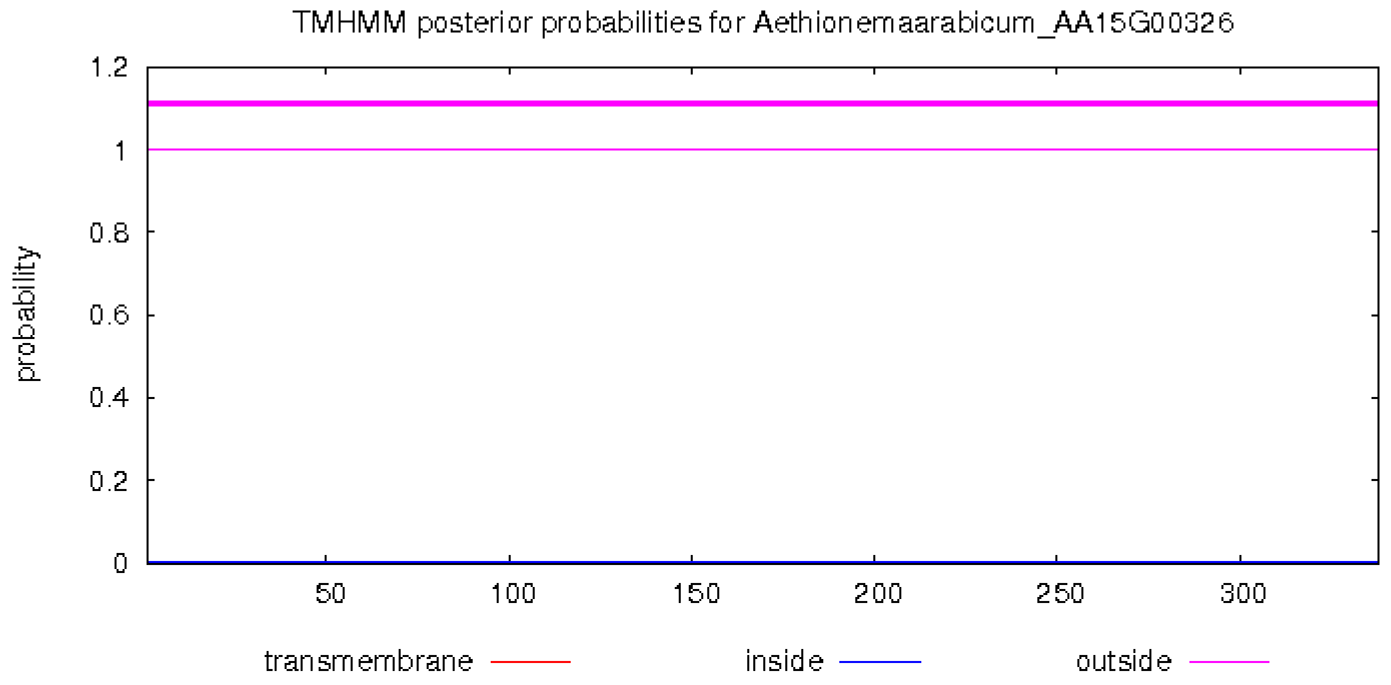

plot in postscript, script for making the plot in gnuplot, data for plot

---

```
# Aethionemaarabicum_AA1G00022 Length: 404
# Aethionemaarabicum_AA1G00022 Number of predicted TMHs:  0
# Aethionemaarabicum_AA1G00022 Exp number of AAs in TMHs: 0.17726
# Aethionemaarabicum_AA1G00022 Exp number, first 60 AAs:  0.06539
# Aethionemaarabicum_AA1G00022 Total prob of N-in:        0.01055
Aethionemaarabicum_AA1G00022    TMHMM2.0        outside      1   404
```

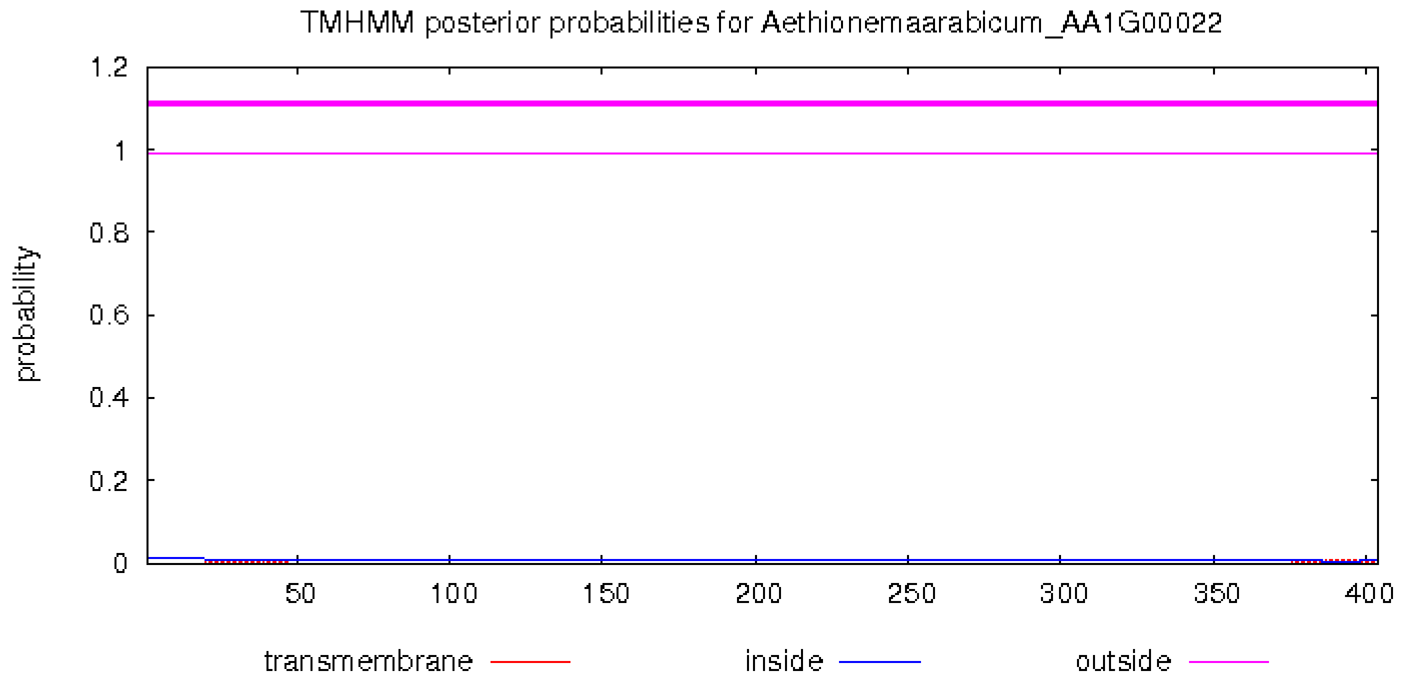

plot in postscript, script for making the plot in gnuplot, data for plot

---

```
# Aethionemaarabicum_AA1G00023 Length: 438
# Aethionemaarabicum_AA1G00023 Number of predicted TMHs:  1
# Aethionemaarabicum_AA1G00023 Exp number of AAs in TMHs: 24.10256
# Aethionemaarabicum_AA1G00023 Exp number, first 60 AAs:  0.00101
```

```
# Aethionemaarabicum_AA1G00023 Total prob of N-in:        0.47385
Aethionemaarabicum_AA1G00023	TMHMM2.0	inside	     1   411
Aethionemaarabicum_AA1G00023	TMHMM2.0	TMhelix	   412   434
Aethionemaarabicum_AA1G00023	TMHMM2.0	outside	   435   438
```

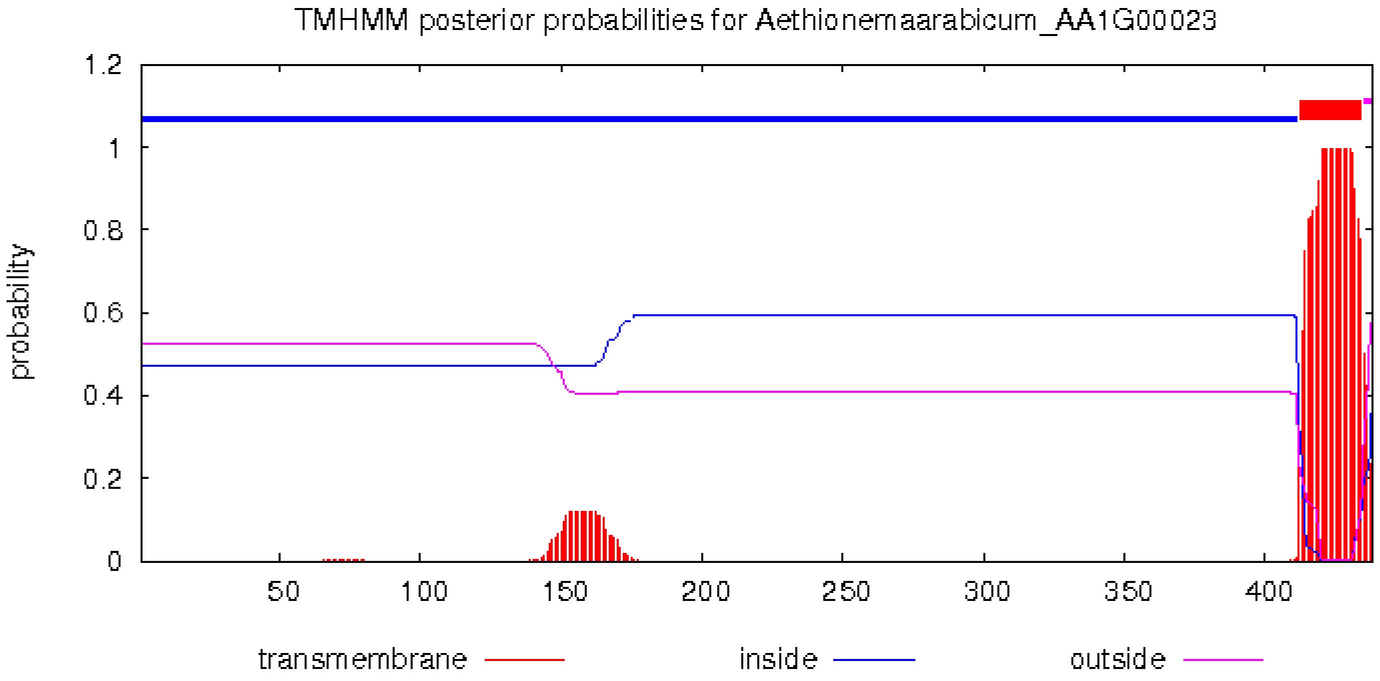

plot in postscript, script for making the plot in gnuplot, data for plot

---

```
# Aethionemaarabicum_AA1G00024 Length: 333
# Aethionemaarabicum_AA1G00024 Number of predicted TMHs:  0
# Aethionemaarabicum_AA1G00024 Exp number of AAs in TMHs: 0.00046
# Aethionemaarabicum_AA1G00024 Exp number, first 60 AAs:  0.00019
# Aethionemaarabicum_AA1G00024 Total prob of N-in:        0.00694
Aethionemaarabicum_AA1G00024	TMHMM2.0	outside	     1   333
```

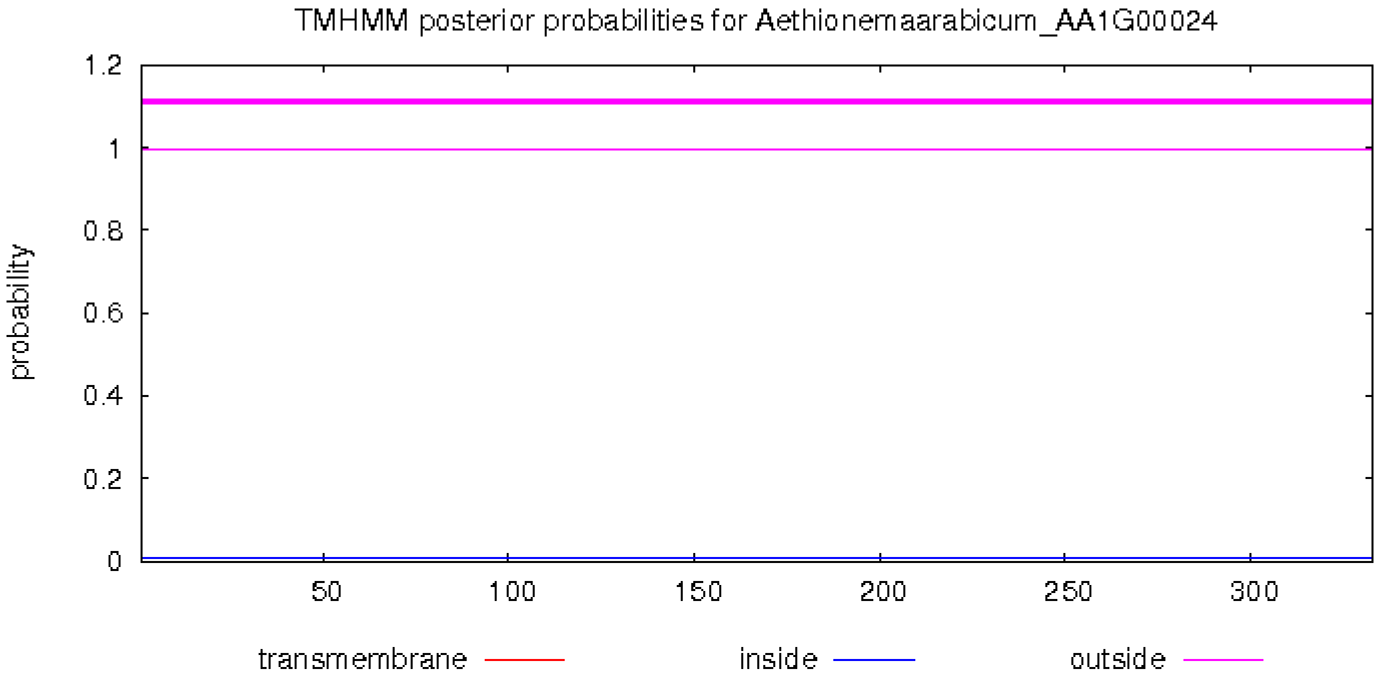

plot in postscript, script for making the plot in gnuplot, data for plot

---

```
# Aethionemaarabicum_AA1G00025 Length: 233
# Aethionemaarabicum_AA1G00025 Number of predicted TMHs:  0
```

```
# Aethionemaarabicum_AA1G00025 Exp number of AAs in TMHs: 0.00015
# Aethionemaarabicum_AA1G00025 Exp number, first 60 AAs:  0
# Aethionemaarabicum_AA1G00025 Total prob of N-in:        0.01753
Aethionemaarabicum_AA1G00025    TMHMM2.0        outside      1   233
```

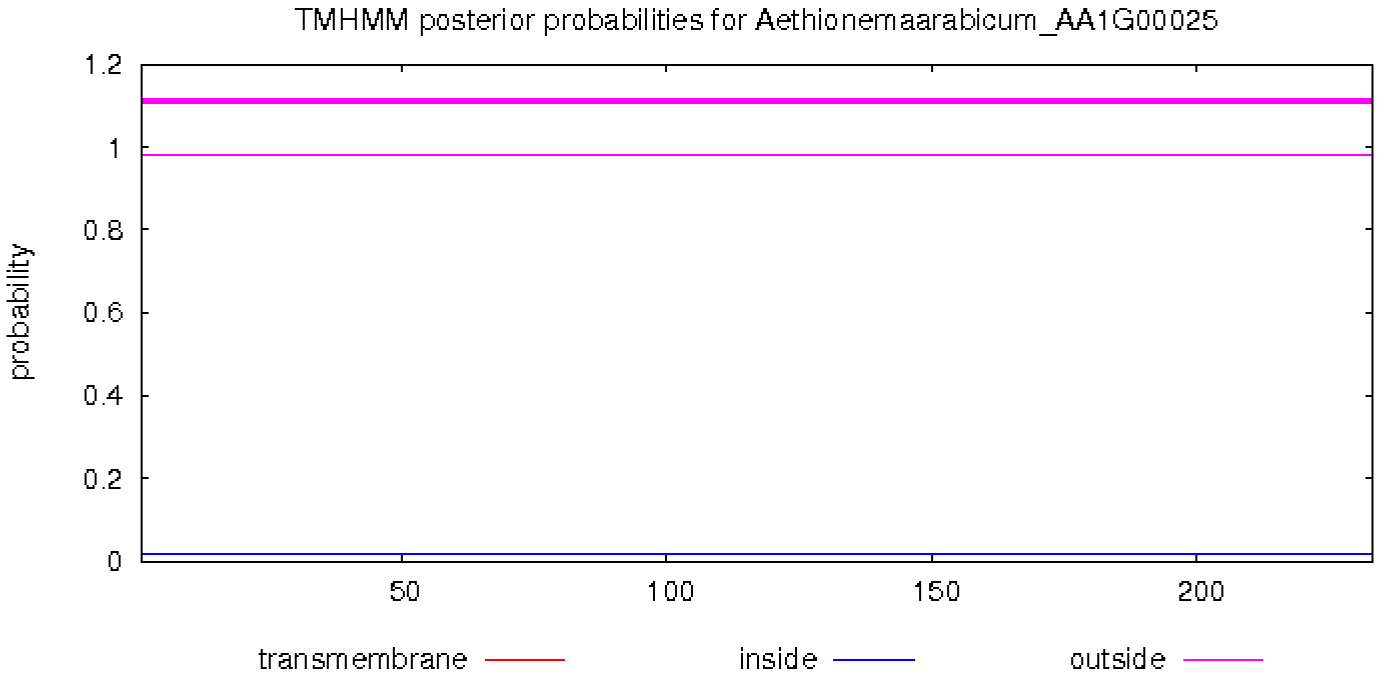

plot in postscript, script for making the plot in gnuplot, data for plot

```
# Aethionemaarabicum_AA1G00027 Length: 459
# Aethionemaarabicum_AA1G00027 Number of predicted TMHs:  1
# Aethionemaarabicum_AA1G00027 Exp number of AAs in TMHs: 21.41952
# Aethionemaarabicum_AA1G00027 Exp number, first 60 AAs:  0.00298
# Aethionemaarabicum_AA1G00027 Total prob of N-in:        0.18474
Aethionemaarabicum_AA1G00027    TMHMM2.0        outside      1   435
Aethionemaarabicum_AA1G00027    TMHMM2.0        TMhelix    436   458
Aethionemaarabicum_AA1G00027    TMHMM2.0        inside     459   459
```

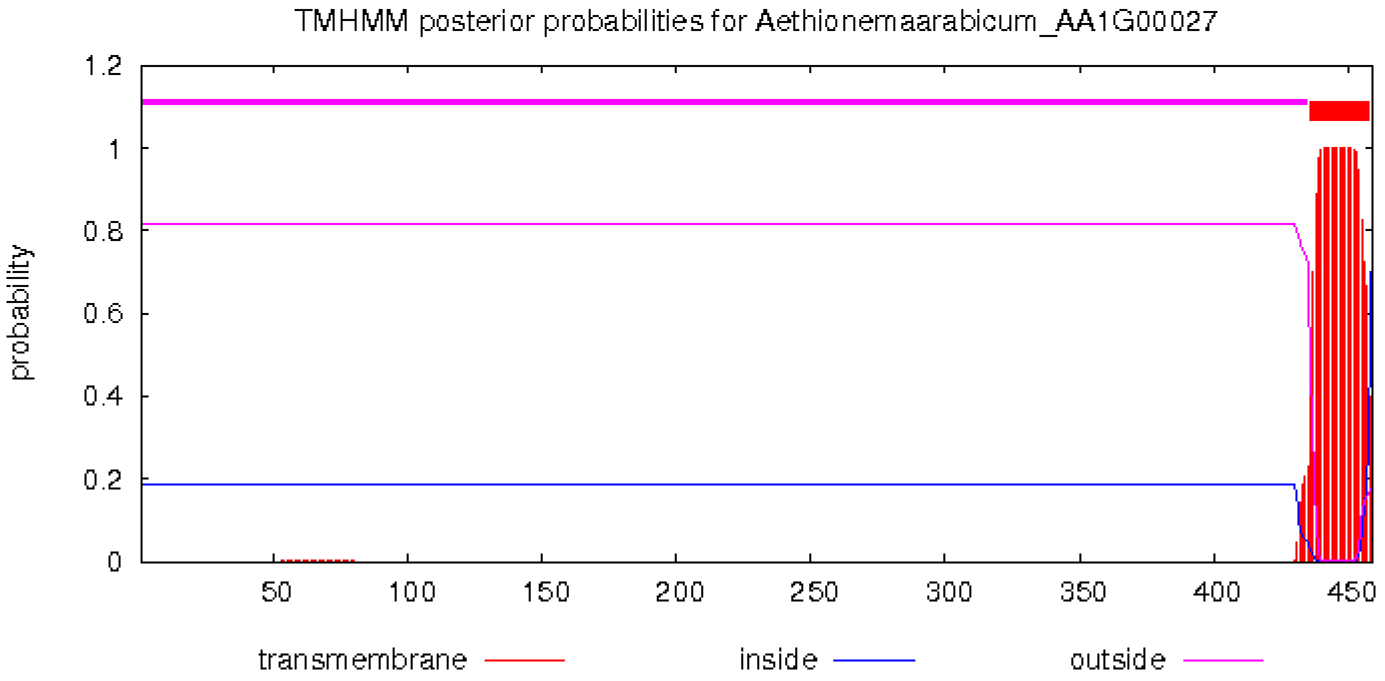

plot in postscript, script for making the plot in gnuplot, data for plot

```
# Aethionemaarabicum_AA1G00029 Length: 390
# Aethionemaarabicum_AA1G00029 Number of predicted TMHs:  0
# Aethionemaarabicum_AA1G00029 Exp number of AAs in TMHs: 0.00054
# Aethionemaarabicum_AA1G00029 Exp number, first 60 AAs:  0.00016
# Aethionemaarabicum_AA1G00029 Total prob of N-in:        0.00307
Aethionemaarabicum_AA1G00029    TMHMM2.0        outside      1   390
```

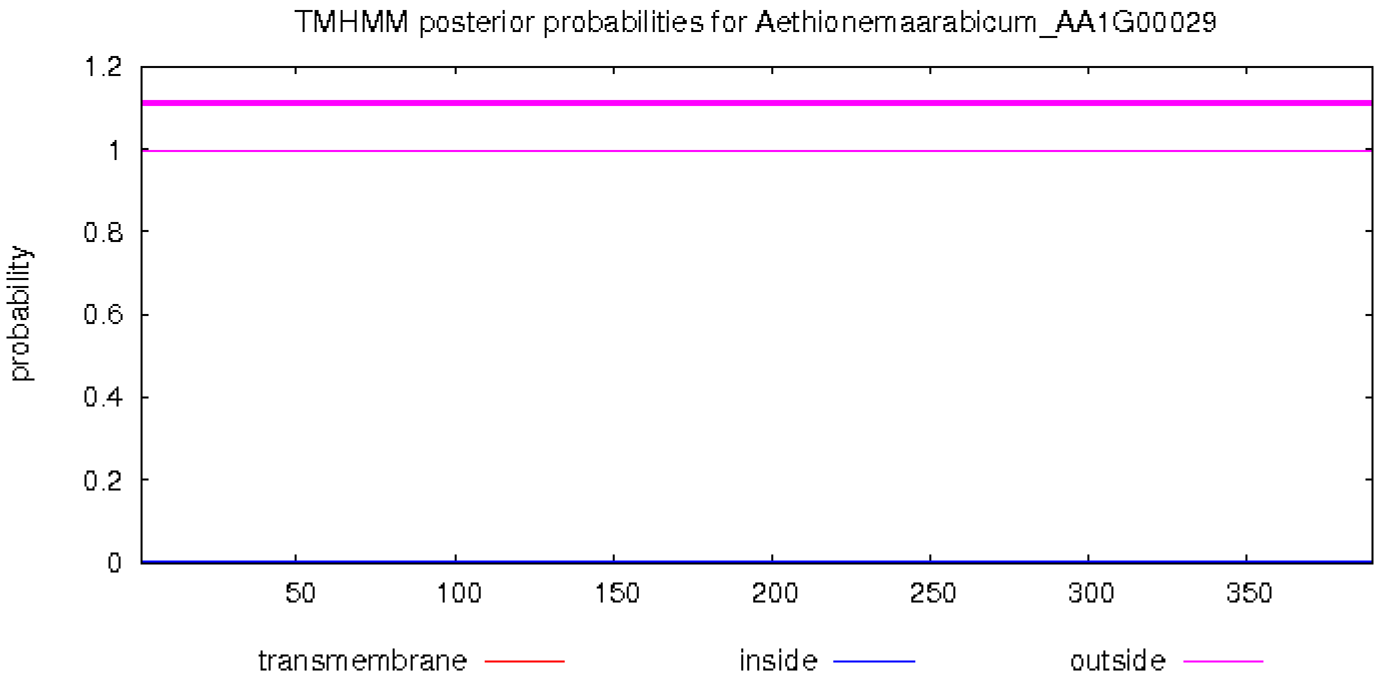

plot in postscript, script for making the plot in gnuplot, data for plot

---

```
# Aethionemaarabicum_AA1G00030 Length: 227
# Aethionemaarabicum_AA1G00030 Number of predicted TMHs:  0
# Aethionemaarabicum_AA1G00030 Exp number of AAs in TMHs: 0.00057
# Aethionemaarabicum_AA1G00030 Exp number, first 60 AAs:  0.00057
# Aethionemaarabicum_AA1G00030 Total prob of N-in:        0.10277
Aethionemaarabicum_AA1G00030    TMHMM2.0        outside      1   227
```

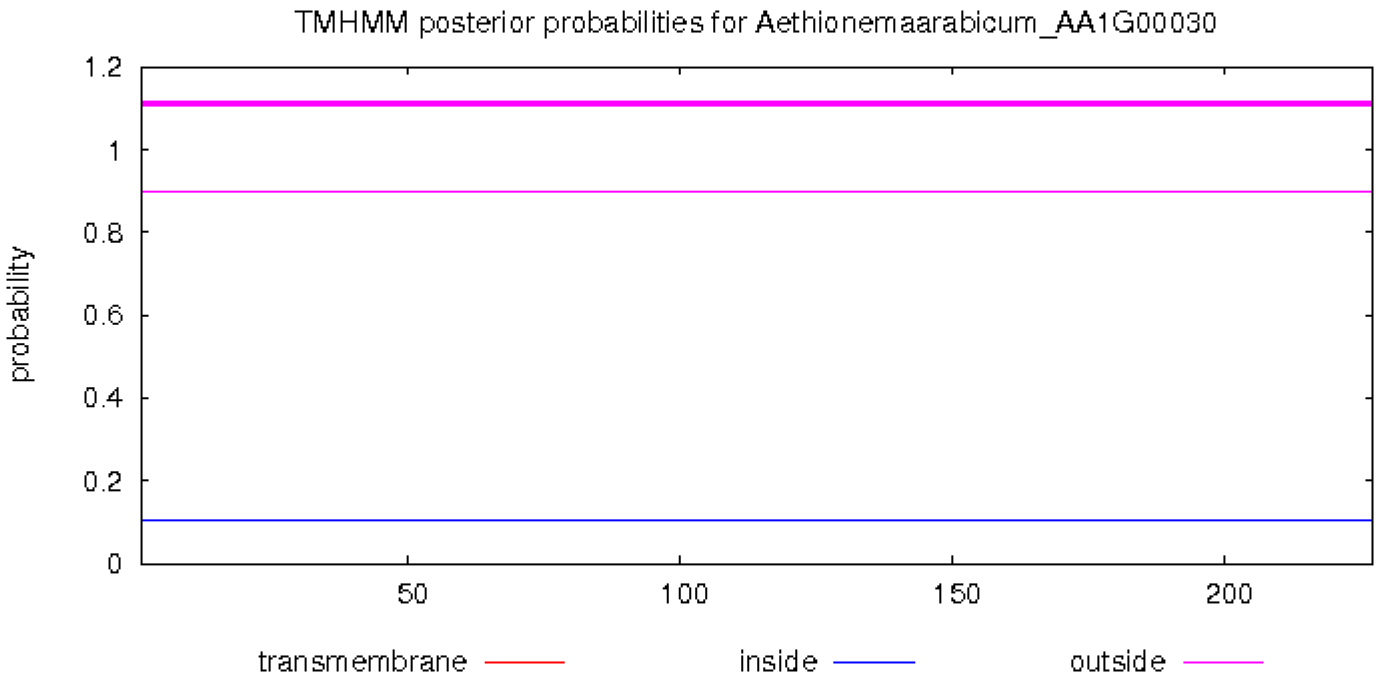

plot in postscript, script for making the plot in gnuplot, data for plot

---

```
# Aethionemaarabicum_AA1G00032 Length: 193
# Aethionemaarabicum_AA1G00032 Number of predicted TMHs:  0
# Aethionemaarabicum_AA1G00032 Exp number of AAs in TMHs: 0.15413
# Aethionemaarabicum_AA1G00032 Exp number, first 60 AAs:  8e-05
# Aethionemaarabicum_AA1G00032 Total prob of N-in:        0.06812
Aethionemaarabicum_AA1G00032    TMHMM2.0        outside      1   193
```

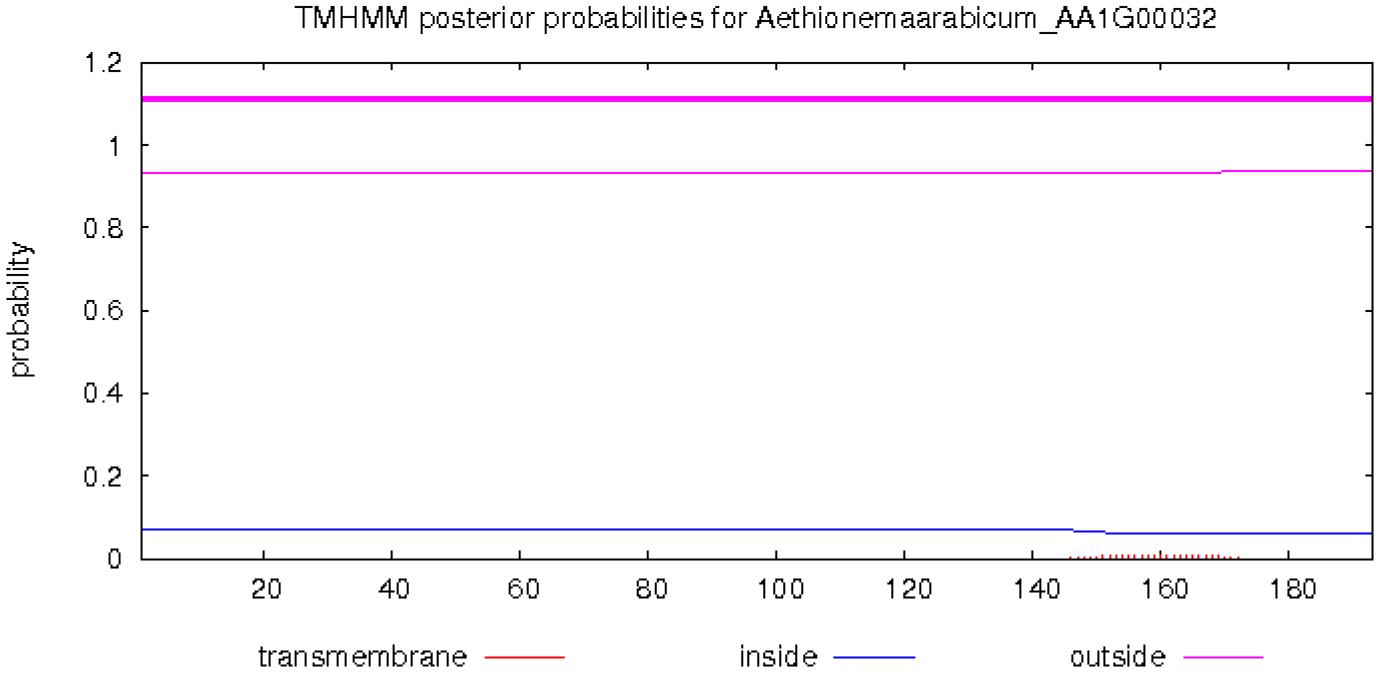

plot in postscript, script for making the plot in gnuplot, data for plot

---

```
# Aethionemaarabicum_AA1G00033 Length: 345
# Aethionemaarabicum_AA1G00033 Number of predicted TMHs:  1
# Aethionemaarabicum_AA1G00033 Exp number of AAs in TMHs: 17.32558
# Aethionemaarabicum_AA1G00033 Exp number, first 60 AAs:  0.00246
# Aethionemaarabicum_AA1G00033 Total prob of N-in:        0.72841
Aethionemaarabicum_AA1G00033    TMHMM2.0        inside       1   323
Aethionemaarabicum_AA1G00033    TMHMM2.0        TMhelix    324   343
Aethionemaarabicum_AA1G00033    TMHMM2.0        outside    344   345
```

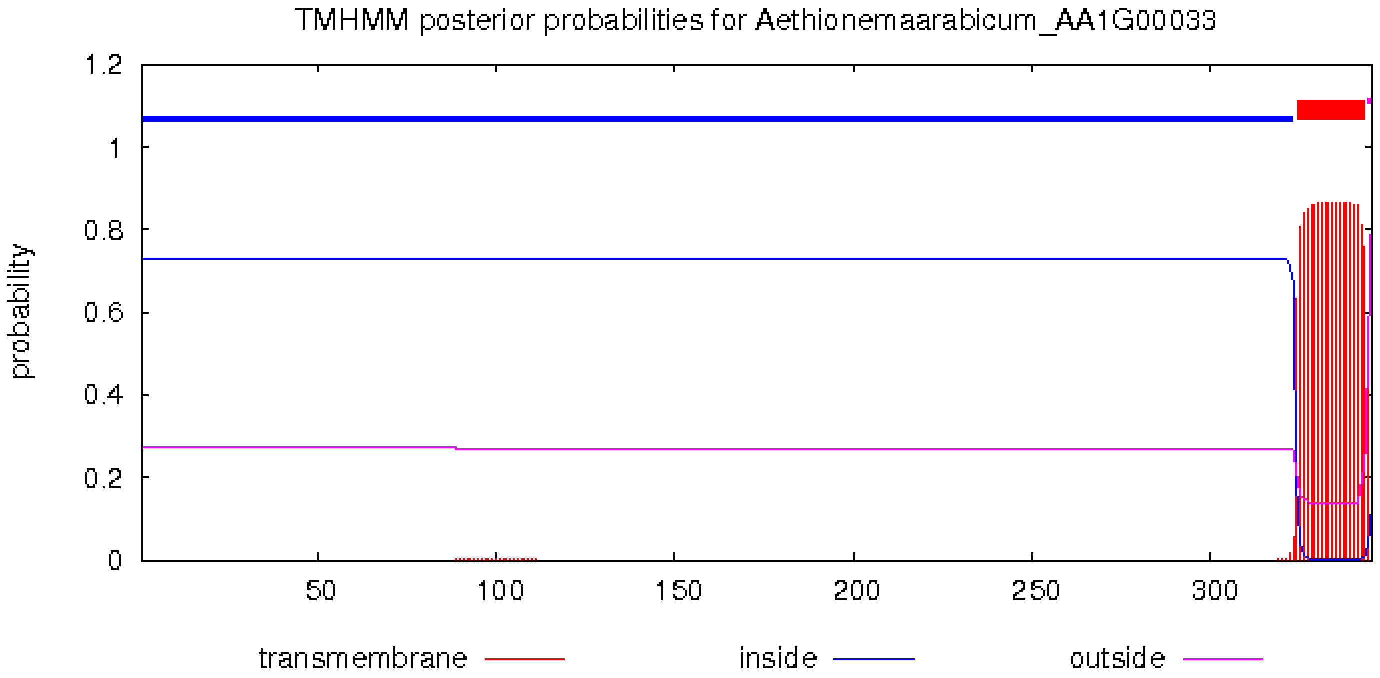

plot in postscript, script for making the plot in gnuplot, data for plot

```
# Aethionemaarabicum_AA1G00095 Length: 304
# Aethionemaarabicum_AA1G00095 Number of predicted TMHs:  0
# Aethionemaarabicum_AA1G00095 Exp number of AAs in TMHs: 6e-05
# Aethionemaarabicum_AA1G00095 Exp number, first 60 AAs:  0
# Aethionemaarabicum_AA1G00095 Total prob of N-in:        0.00234
Aethionemaarabicum_AA1G00095    TMHMM2.0        outside      1   304
```

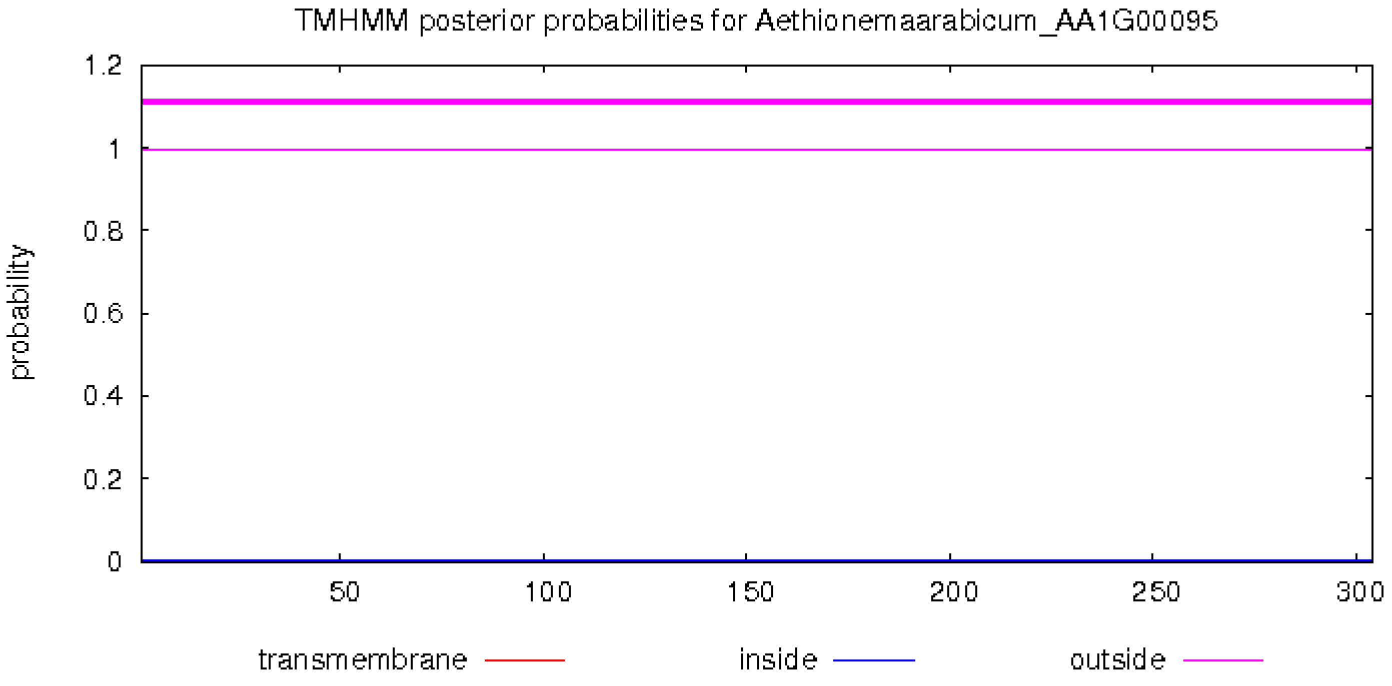

plot in postscript, script for making the plot in gnuplot, data for plot

```
# Aethionemaarabicum_AA204G00012 Length: 199
# Aethionemaarabicum_AA204G00012 Number of predicted TMHs:  0
# Aethionemaarabicum_AA204G00012 Exp number of AAs in TMHs: 0.00284
# Aethionemaarabicum_AA204G00012 Exp number, first 60 AAs:  0.00284
# Aethionemaarabicum_AA204G00012 Total prob of N-in:        0.03609
Aethionemaarabicum_AA204G00012  TMHMM2.0        outside      1   199
```

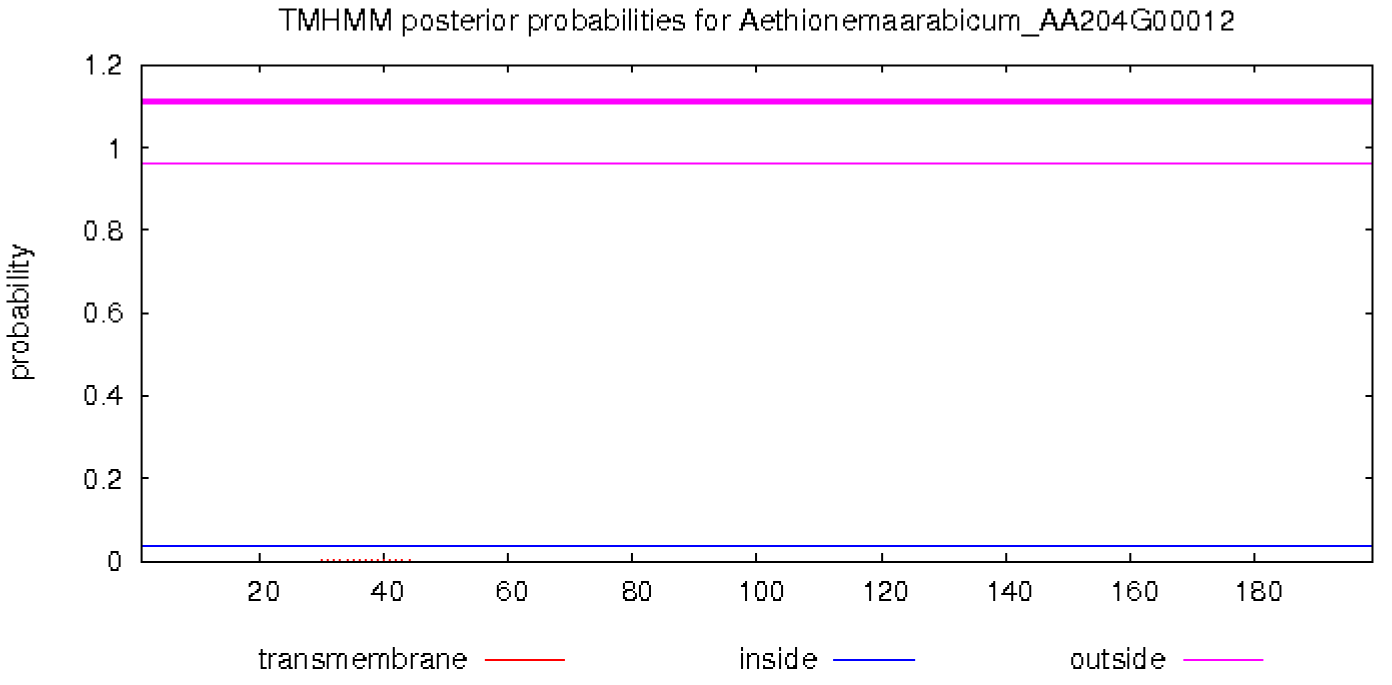

plot in postscript, script for making the plot in gnuplot, data for plot

```
# Aethionemaarabicum_AA2093G00001 Length: 317
# Aethionemaarabicum_AA2093G00001 Number of predicted TMHs:  1
# Aethionemaarabicum_AA2093G00001 Exp number of AAs in TMHs: 20.63075
# Aethionemaarabicum_AA2093G00001 Exp number, first 60 AAs:  0.00034
# Aethionemaarabicum_AA2093G00001 Total prob of N-in:        0.84057
Aethionemaarabicum_AA2093G00001 TMHMM2.0        inside       1   288
Aethionemaarabicum_AA2093G00001 TMHMM2.0        TMhelix    289   311
Aethionemaarabicum_AA2093G00001 TMHMM2.0        outside    312   317
```

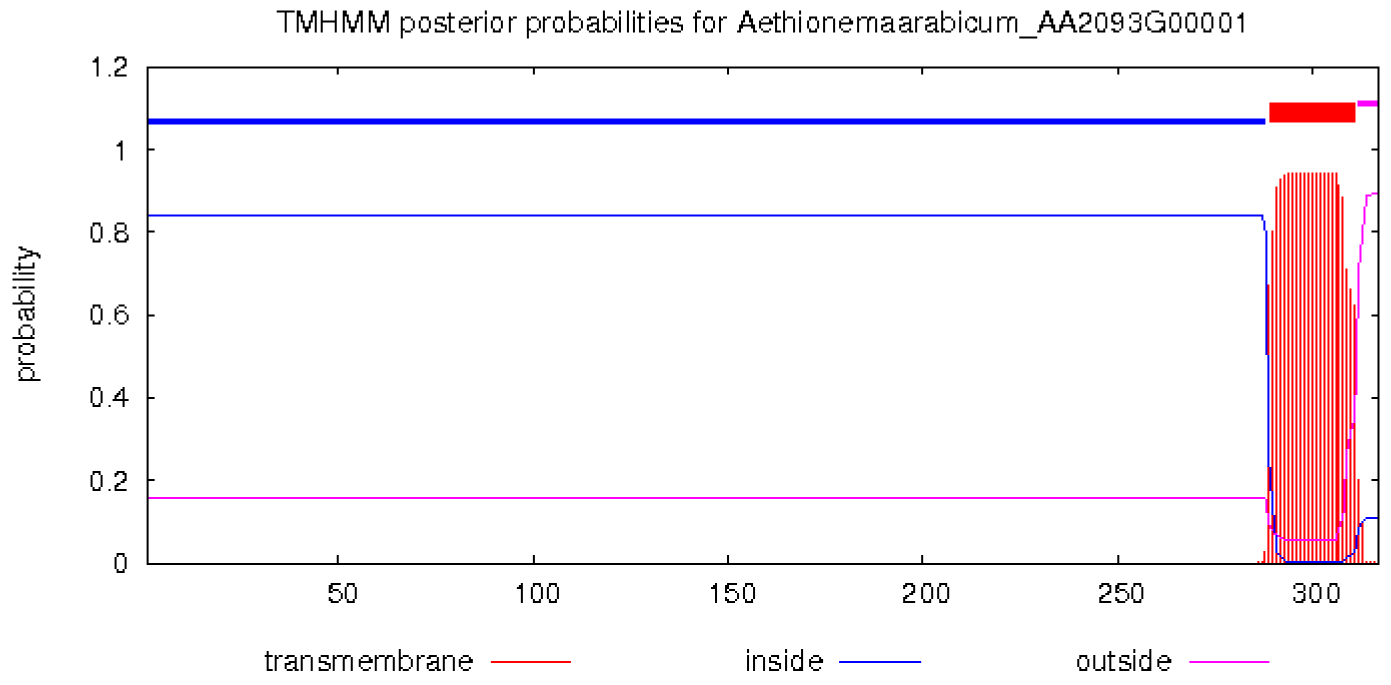

plot in postscript, script for making the plot in gnuplot, data for plot

---

```
# Aethionemaarabicum_AA21G00088 Length: 296
# Aethionemaarabicum_AA21G00088 Number of predicted TMHs:  0
# Aethionemaarabicum_AA21G00088 Exp number of AAs in TMHs: 0.03152
# Aethionemaarabicum_AA21G00088 Exp number, first 60 AAs:  0
# Aethionemaarabicum_AA21G00088 Total prob of N-in:        0.04188
Aethionemaarabicum_AA21G00088   TMHMM2.0        outside      1   296
```

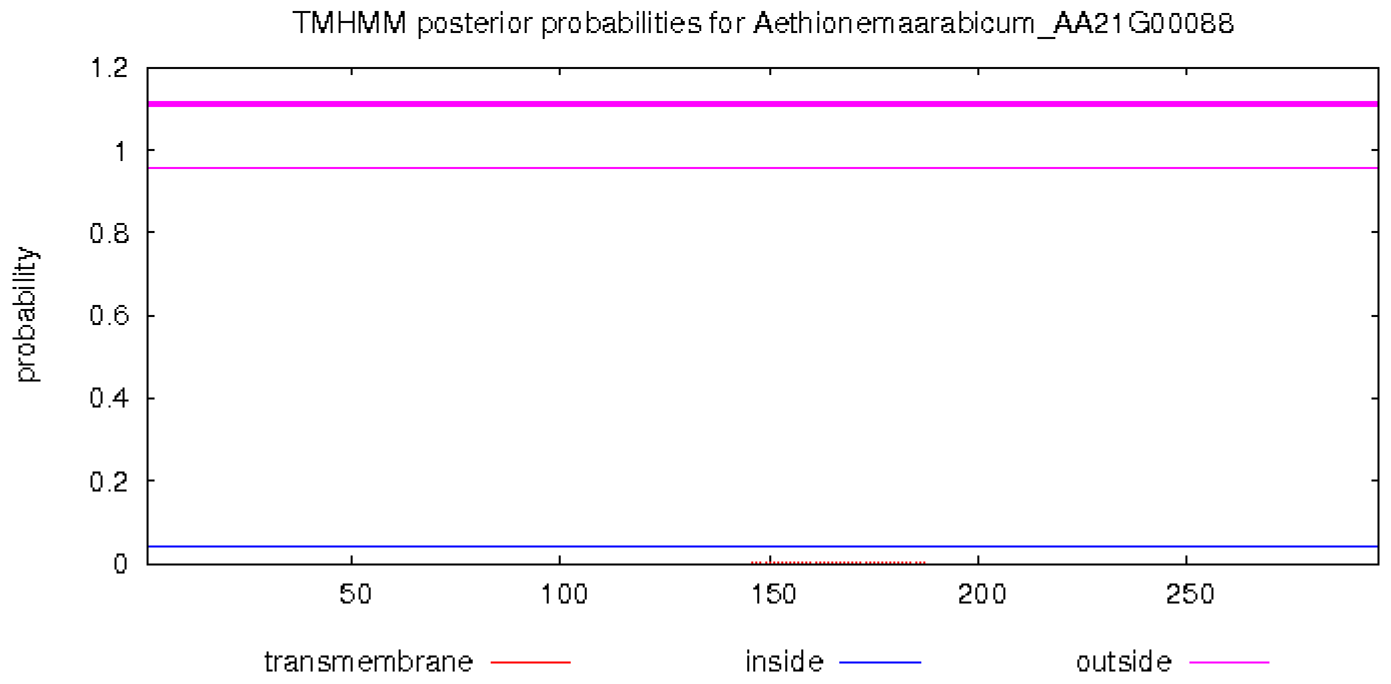

plot in postscript, script for making the plot in gnuplot, data for plot

```
# Aethionemaarabicum_AA21G00405 Length: 348
# Aethionemaarabicum_AA21G00405 Number of predicted TMHs:  0
# Aethionemaarabicum_AA21G00405 Exp number of AAs in TMHs: 0
# Aethionemaarabicum_AA21G00405 Exp number, first 60 AAs:  0
# Aethionemaarabicum_AA21G00405 Total prob of N-in:        0.01094
Aethionemaarabicum_AA21G00405   TMHMM2.0        outside      1   348
```

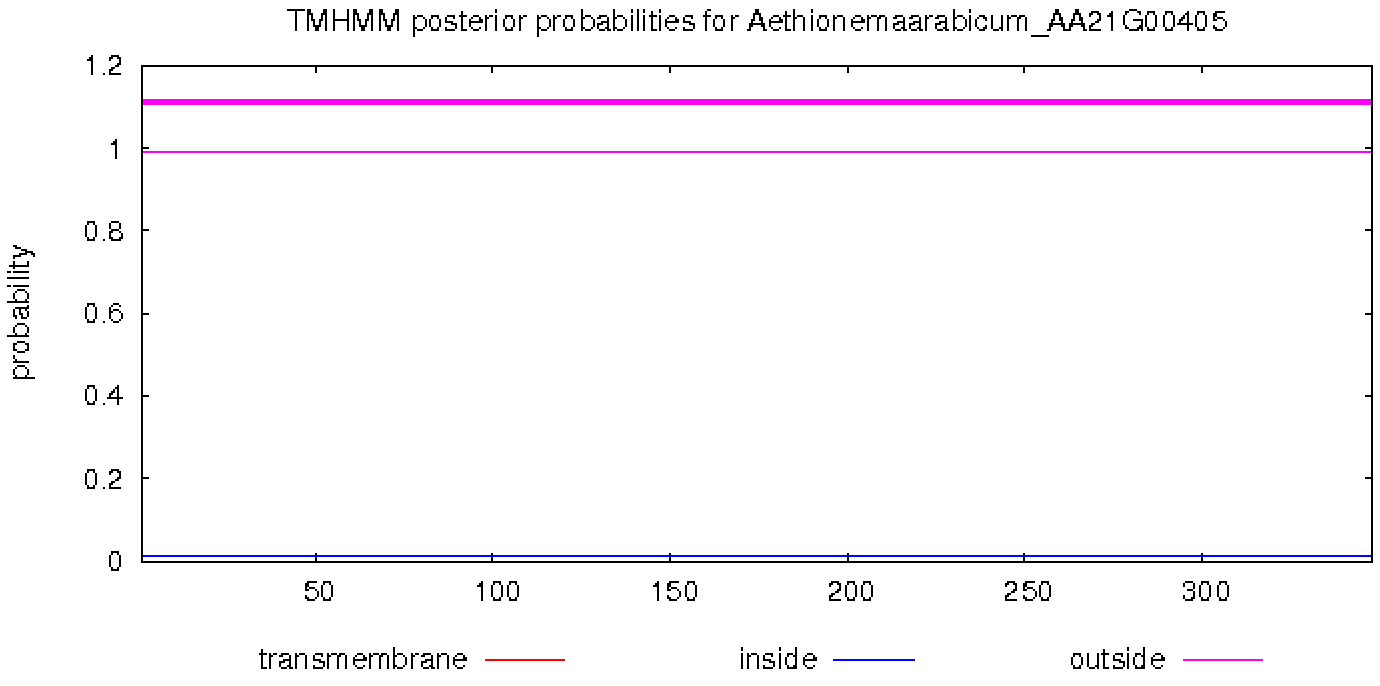

plot in postscript, script for making the plot in gnuplot, data for plot

```
# Aethionemaarabicum_AA24G00045 Length: 342
# Aethionemaarabicum_AA24G00045 Number of predicted TMHs:  0
# Aethionemaarabicum_AA24G00045 Exp number of AAs in TMHs: 16.49524
# Aethionemaarabicum_AA24G00045 Exp number, first 60 AAs:  1.96994
# Aethionemaarabicum_AA24G00045 Total prob of N-in:        0.42642
Aethionemaarabicum_AA24G00045   TMHMM2.0        outside      1   342
```

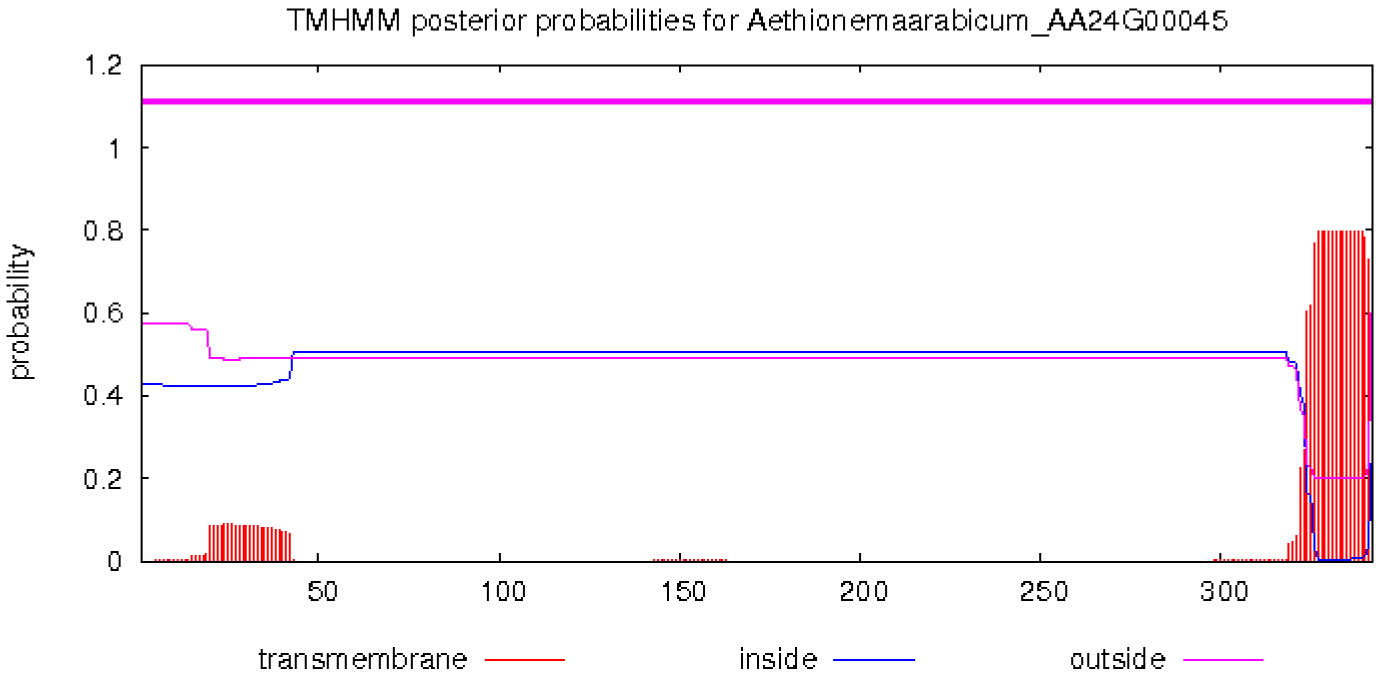

plot in postscript, script for making the plot in gnuplot, data for plot

```
# Aethionemaarabicum_AA26G00012 Length: 357
# Aethionemaarabicum_AA26G00012 Number of predicted TMHs:  0
# Aethionemaarabicum_AA26G00012 Exp number of AAs in TMHs: 0.00348
# Aethionemaarabicum_AA26G00012 Exp number, first 60 AAs:  0
# Aethionemaarabicum_AA26G00012 Total prob of N-in:        0.00677
Aethionemaarabicum_AA26G00012   TMHMM2.0        outside      1   357
```

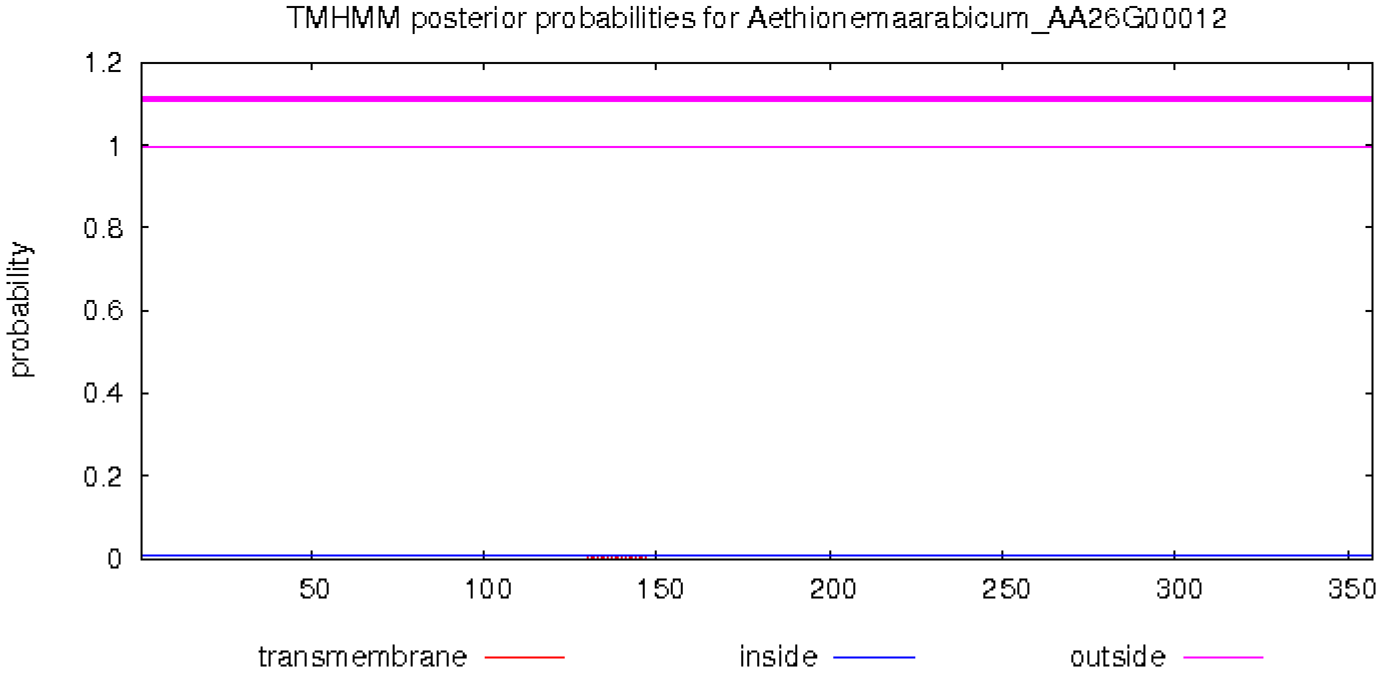

plot in postscript, script for making the plot in gnuplot, data for plot

---

```
# Aethionemaarabicum_AA26G00019 Length: 227
# Aethionemaarabicum_AA26G00019 Number of predicted TMHs:  0
# Aethionemaarabicum_AA26G00019 Exp number of AAs in TMHs: 0.0025
# Aethionemaarabicum_AA26G00019 Exp number, first 60 AAs:  0.00018
# Aethionemaarabicum_AA26G00019 Total prob of N-in:        0.28851
Aethionemaarabicum_AA26G00019   TMHMM2.0        outside      1   227
```

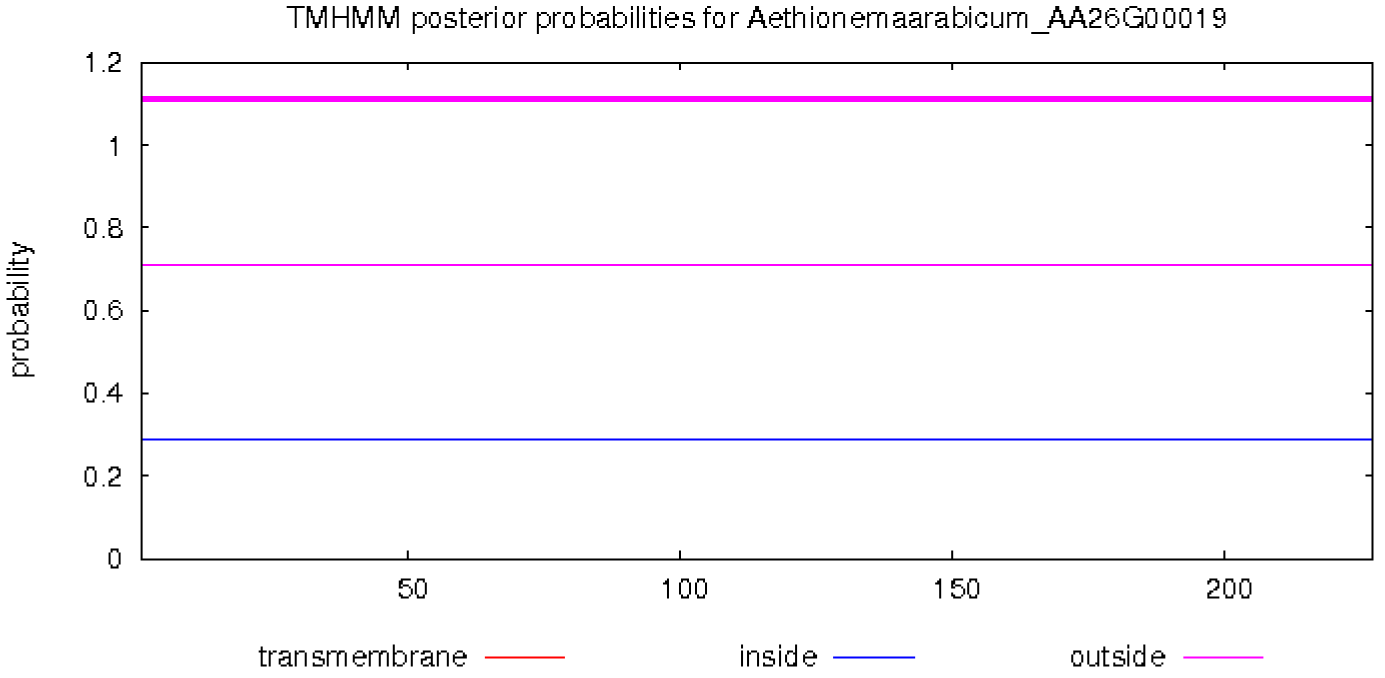

plot in postscript, script for making the plot in gnuplot, data for plot

---

```
# Aethionemaarabicum_AA26G00226 Length: 324
# Aethionemaarabicum_AA26G00226 Number of predicted TMHs:  0
# Aethionemaarabicum_AA26G00226 Exp number of AAs in TMHs: 0.31732
# Aethionemaarabicum_AA26G00226 Exp number, first 60 AAs:  0.00017
# Aethionemaarabicum_AA26G00226 Total prob of N-in:        0.02465
Aethionemaarabicum_AA26G00226   TMHMM2.0        outside      1   324
```

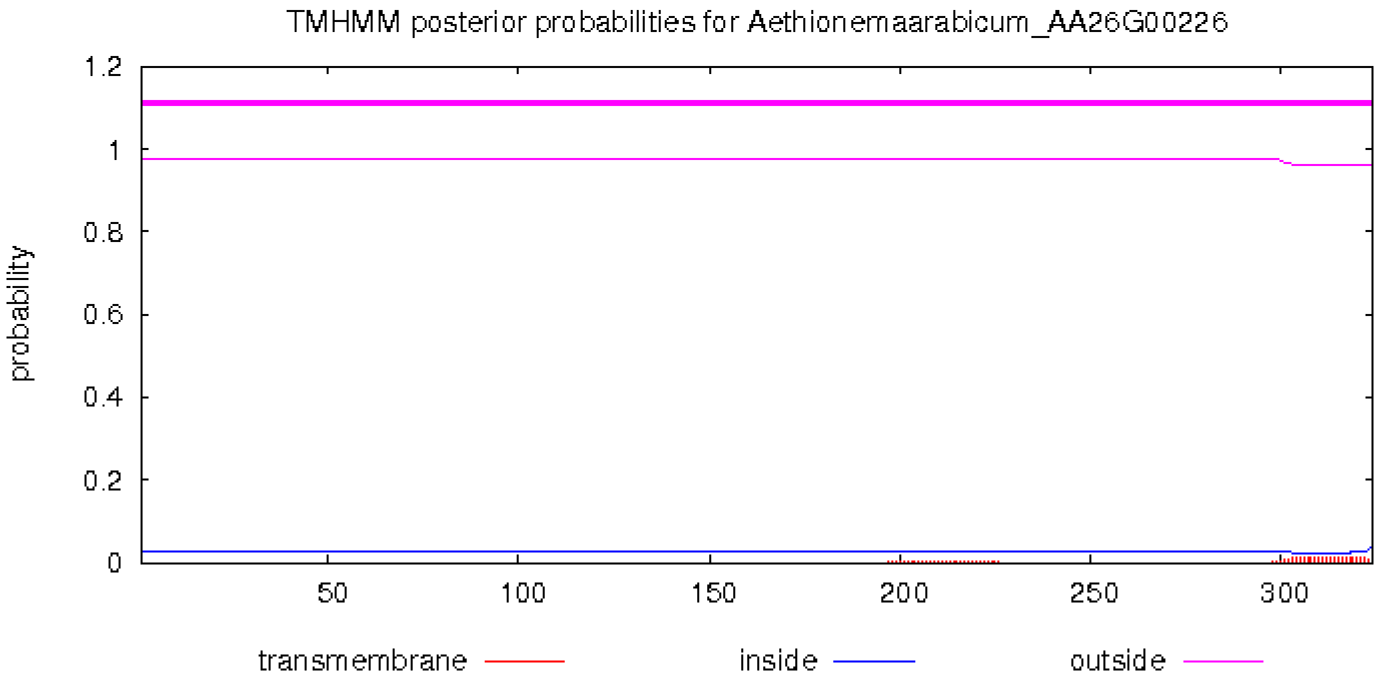

plot in postscript, script for making the plot in gnuplot, data for plot

---

```
# Aethionemaarabicum_AA26G00262 Length: 646
# Aethionemaarabicum_AA26G00262 Number of predicted TMHs:  0
# Aethionemaarabicum_AA26G00262 Exp number of AAs in TMHs: 0.0005
# Aethionemaarabicum_AA26G00262 Exp number, first 60 AAs:  0
# Aethionemaarabicum_AA26G00262 Total prob of N-in:        0.00006
Aethionemaarabicum_AA26G00262   TMHMM2.0        outside      1   646
```

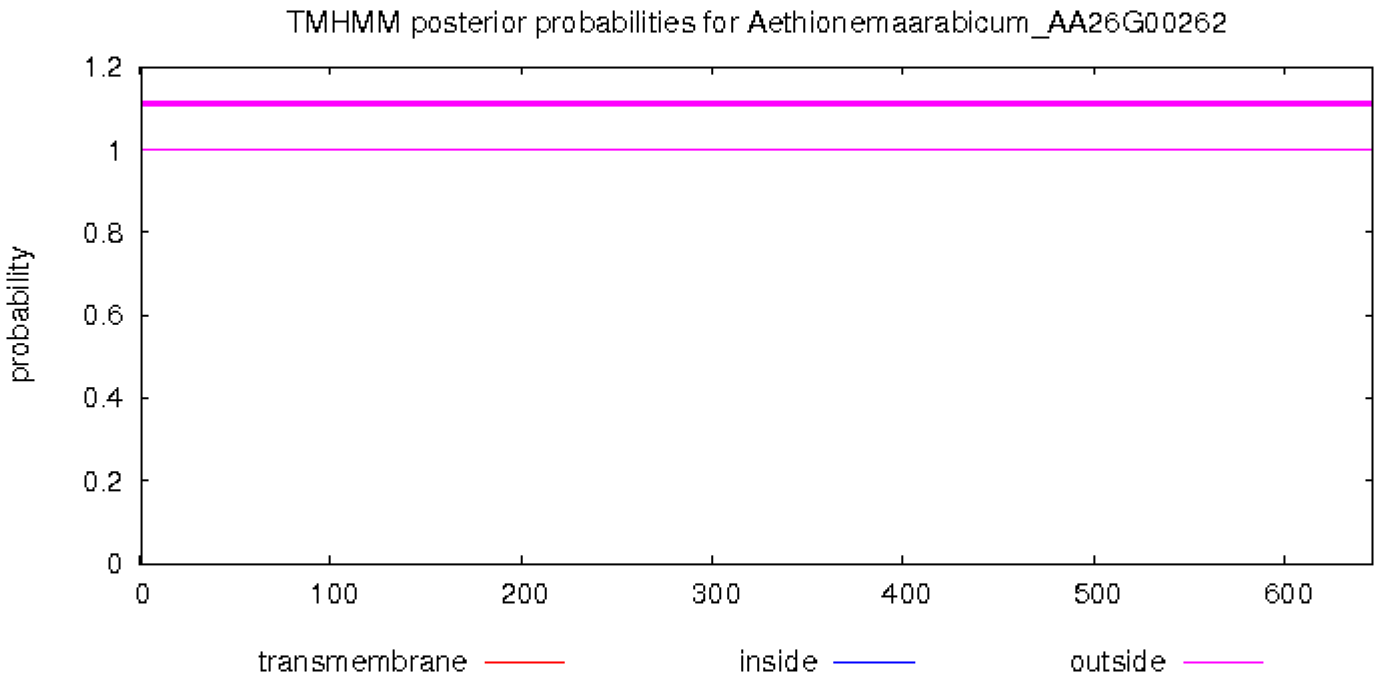

plot in postscript, script for making the plot in gnuplot, data for plot

---

```
# Aethionemaarabicum_AA26G00448 Length: 241
# Aethionemaarabicum_AA26G00448 Number of predicted TMHs:  0
# Aethionemaarabicum_AA26G00448 Exp number of AAs in TMHs: 0
# Aethionemaarabicum_AA26G00448 Exp number, first 60 AAs:  0
# Aethionemaarabicum_AA26G00448 Total prob of N-in:        0.00233
Aethionemaarabicum_AA26G00448   TMHMM2.0        outside      1   241
```

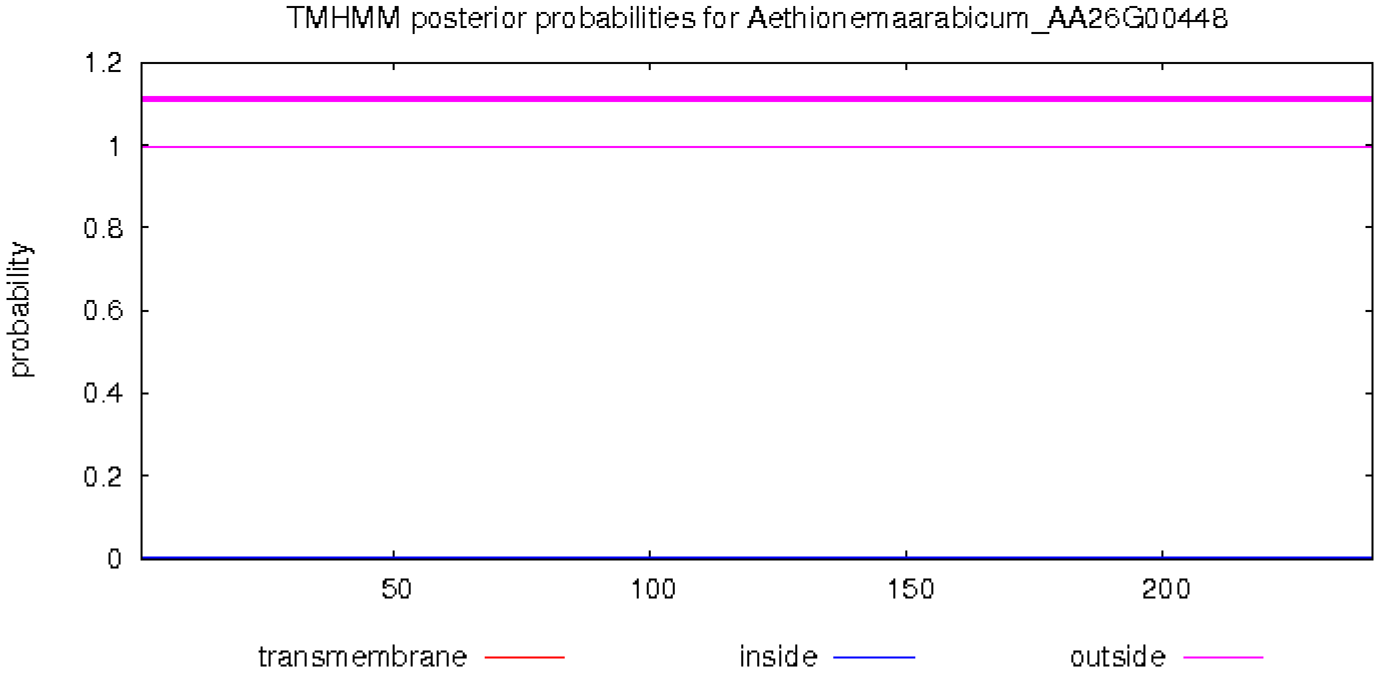

plot in postscript, script for making the plot in gnuplot, data for plot

```
# Aethionemaarabicum_AA26G00510 Length: 311
# Aethionemaarabicum_AA26G00510 Number of predicted TMHs:  0
# Aethionemaarabicum_AA26G00510 Exp number of AAs in TMHs: 0.02199
# Aethionemaarabicum_AA26G00510 Exp number, first 60 AAs:  0
# Aethionemaarabicum_AA26G00510 Total prob of N-in:        0.00408
Aethionemaarabicum_AA26G00510   TMHMM2.0        outside      1   311
```

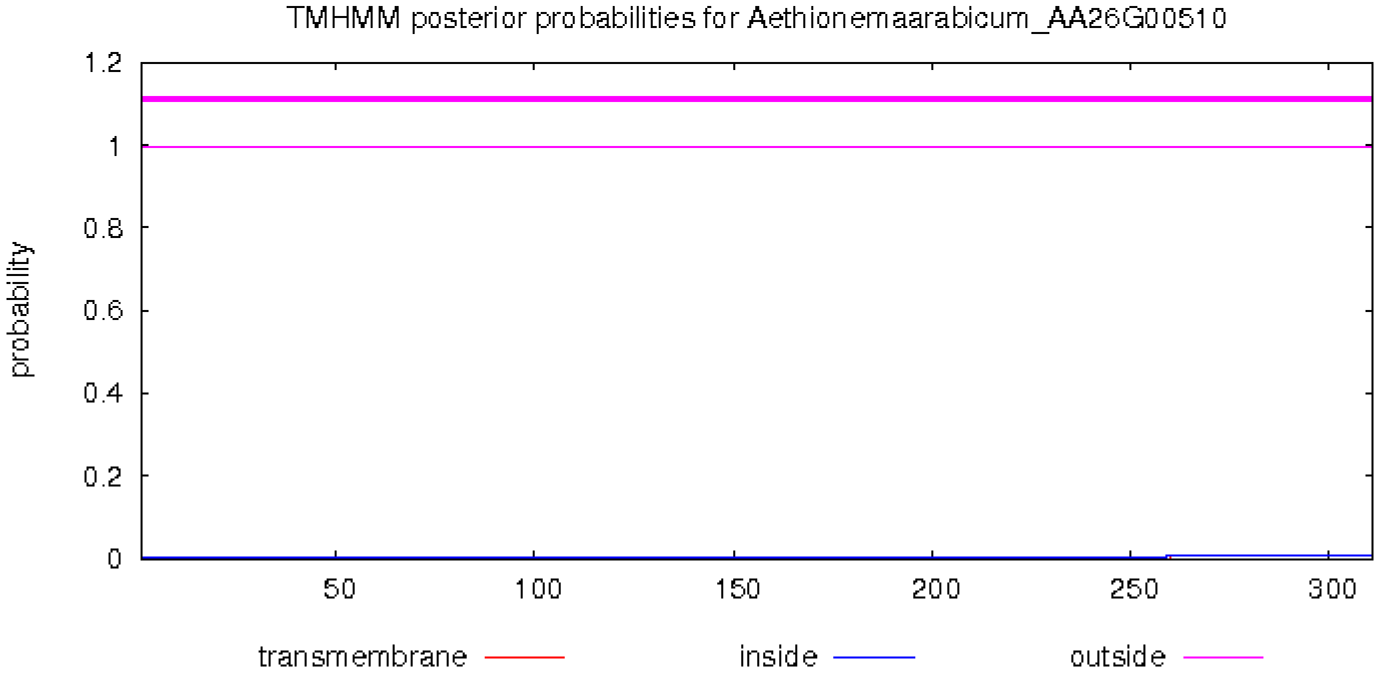

plot in postscript, script for making the plot in gnuplot, data for plot

```
# Aethionemaarabicum_AA30G00135 Length: 336
# Aethionemaarabicum_AA30G00135 Number of predicted TMHs:  0
# Aethionemaarabicum_AA30G00135 Exp number of AAs in TMHs: 0
# Aethionemaarabicum_AA30G00135 Exp number, first 60 AAs:  0
# Aethionemaarabicum_AA30G00135 Total prob of N-in:        0.02127
Aethionemaarabicum_AA30G00135	TMHMM2.0	outside	     1   336
```

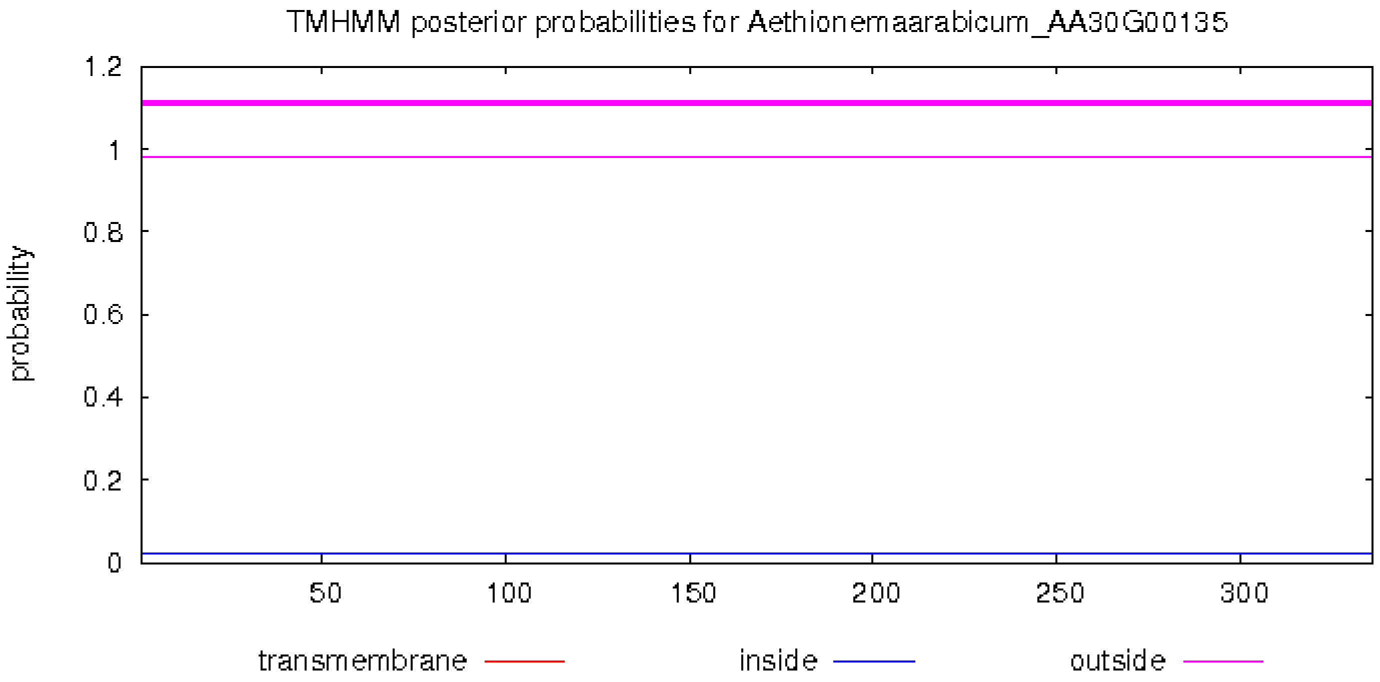

plot in postscript, script for making the plot in gnuplot, data for plot

---

```
# Aethionemaarabicum_AA31G00119 Length: 323
# Aethionemaarabicum_AA31G00119 Number of predicted TMHs:  0
# Aethionemaarabicum_AA31G00119 Exp number of AAs in TMHs: 0
# Aethionemaarabicum_AA31G00119 Exp number, first 60 AAs:  0
# Aethionemaarabicum_AA31G00119 Total prob of N-in:        0.02503
Aethionemaarabicum_AA31G00119	TMHMM2.0	outside	     1   323
```

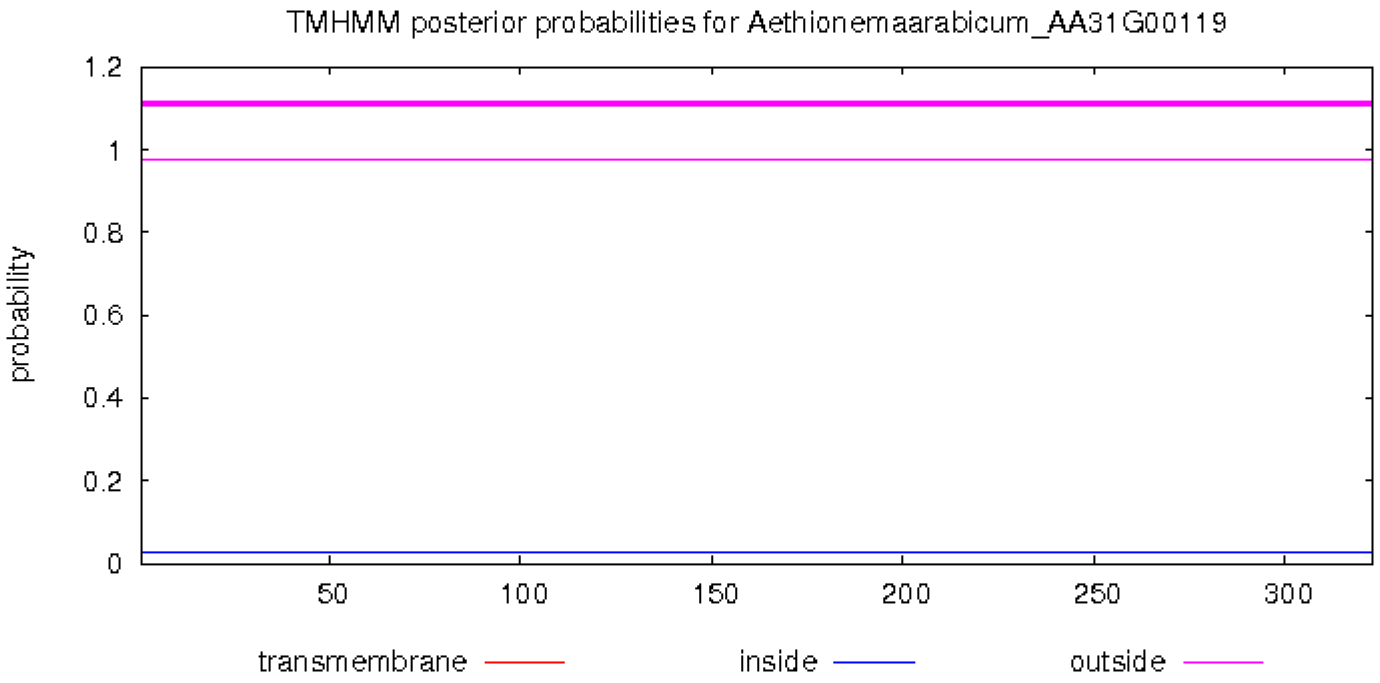

plot in postscript, script for making the plot in gnuplot, data for plot

---

```
# Aethionemaarabicum_AA31G00236 Length: 289
# Aethionemaarabicum_AA31G00236 Number of predicted TMHs:  0
# Aethionemaarabicum_AA31G00236 Exp number of AAs in TMHs: 9.8377
# Aethionemaarabicum_AA31G00236 Exp number, first 60 AAs:  2.32824
# Aethionemaarabicum_AA31G00236 Total prob of N-in:        0.20542
Aethionemaarabicum_AA31G00236   TMHMM2.0        outside      1   289
```

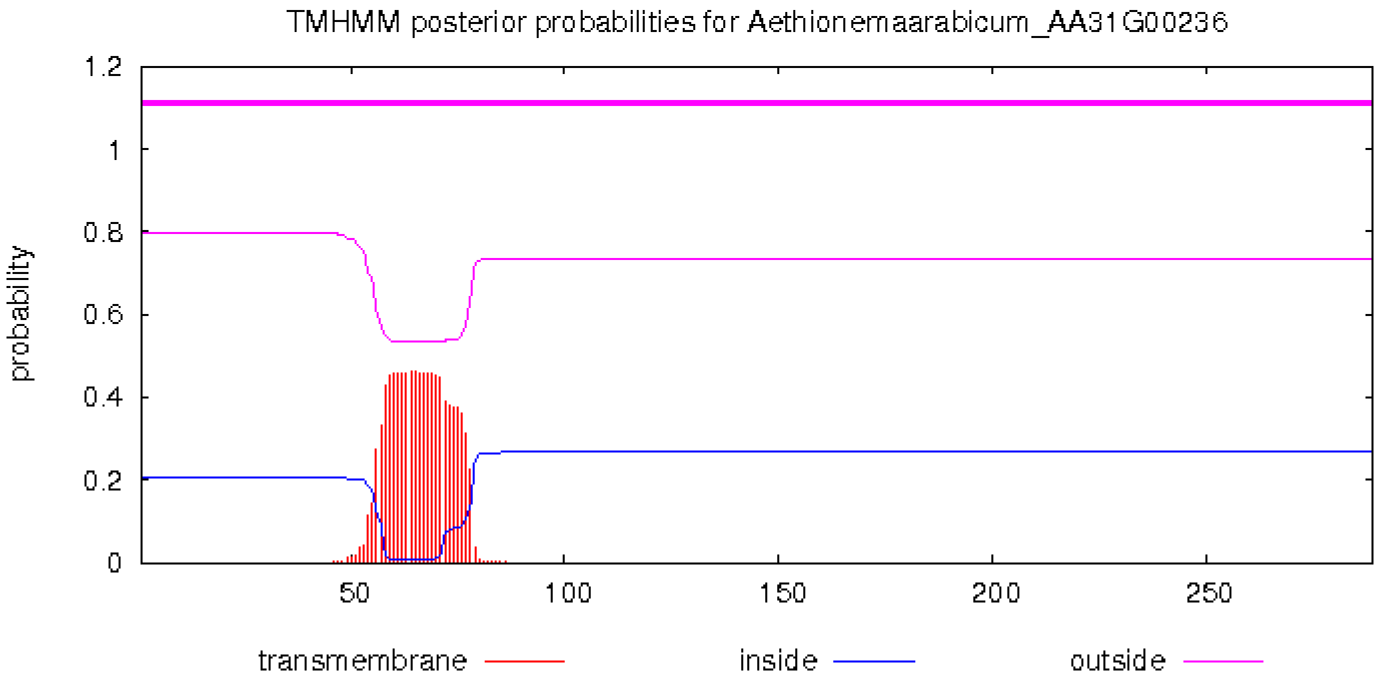

plot in postscript, script for making the plot in gnuplot, data for plot

---

```
# Aethionemaarabicum_AA31G00823 Length: 355
# Aethionemaarabicum_AA31G00823 Number of predicted TMHs:  0
# Aethionemaarabicum_AA31G00823 Exp number of AAs in TMHs: 0.01758
# Aethionemaarabicum_AA31G00823 Exp number, first 60 AAs:  0.01736
# Aethionemaarabicum_AA31G00823 Total prob of N-in:        0.00812
Aethionemaarabicum_AA31G00823   TMHMM2.0        outside      1   355
```

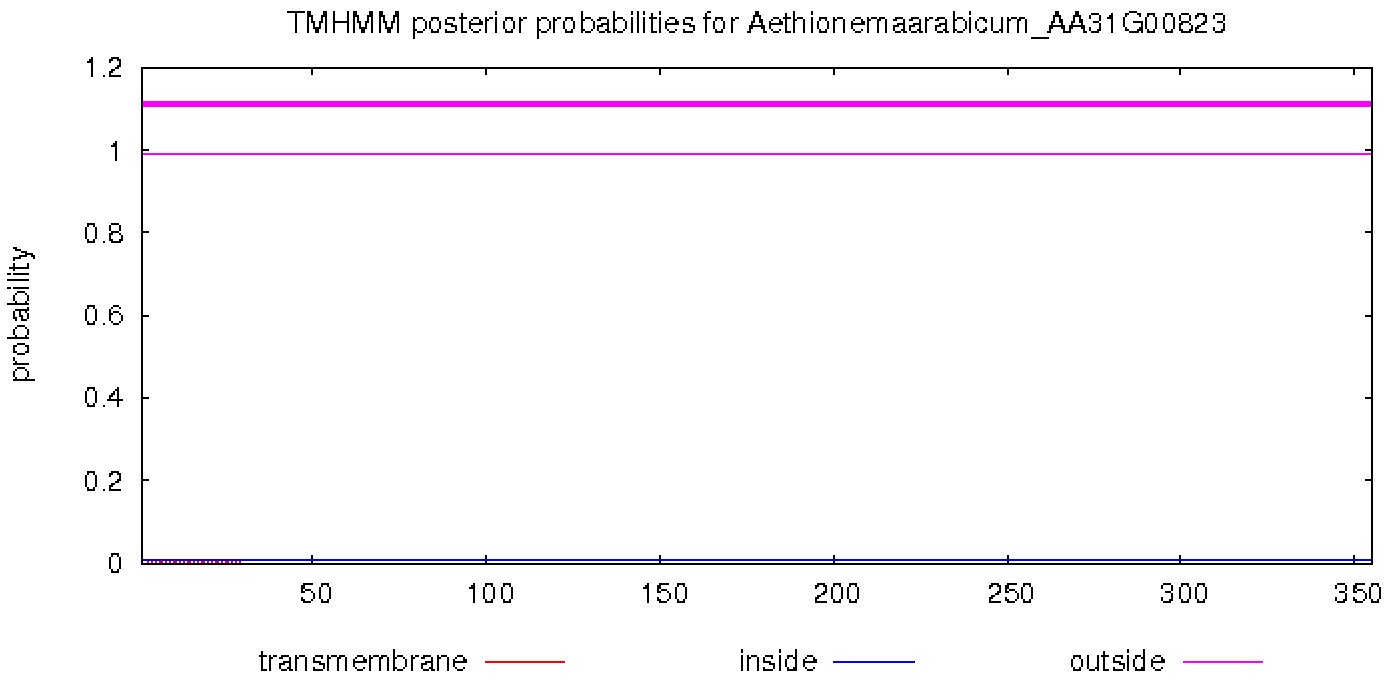

plot in postscript, script for making the plot in gnuplot, data for plot

---

```
# Aethionemaarabicum_AA32G00197 Length: 450
# Aethionemaarabicum_AA32G00197 Number of predicted TMHs:  1
# Aethionemaarabicum_AA32G00197 Exp number of AAs in TMHs: 22.71715
# Aethionemaarabicum_AA32G00197 Exp number, first 60 AAs:  0.00577
# Aethionemaarabicum_AA32G00197 Total prob of N-in:        0.25563
Aethionemaarabicum_AA32G00197   TMHMM2.0        outside      1   423
Aethionemaarabicum_AA32G00197   TMHMM2.0        TMhelix    424   446
Aethionemaarabicum_AA32G00197   TMHMM2.0        inside     447   450
```

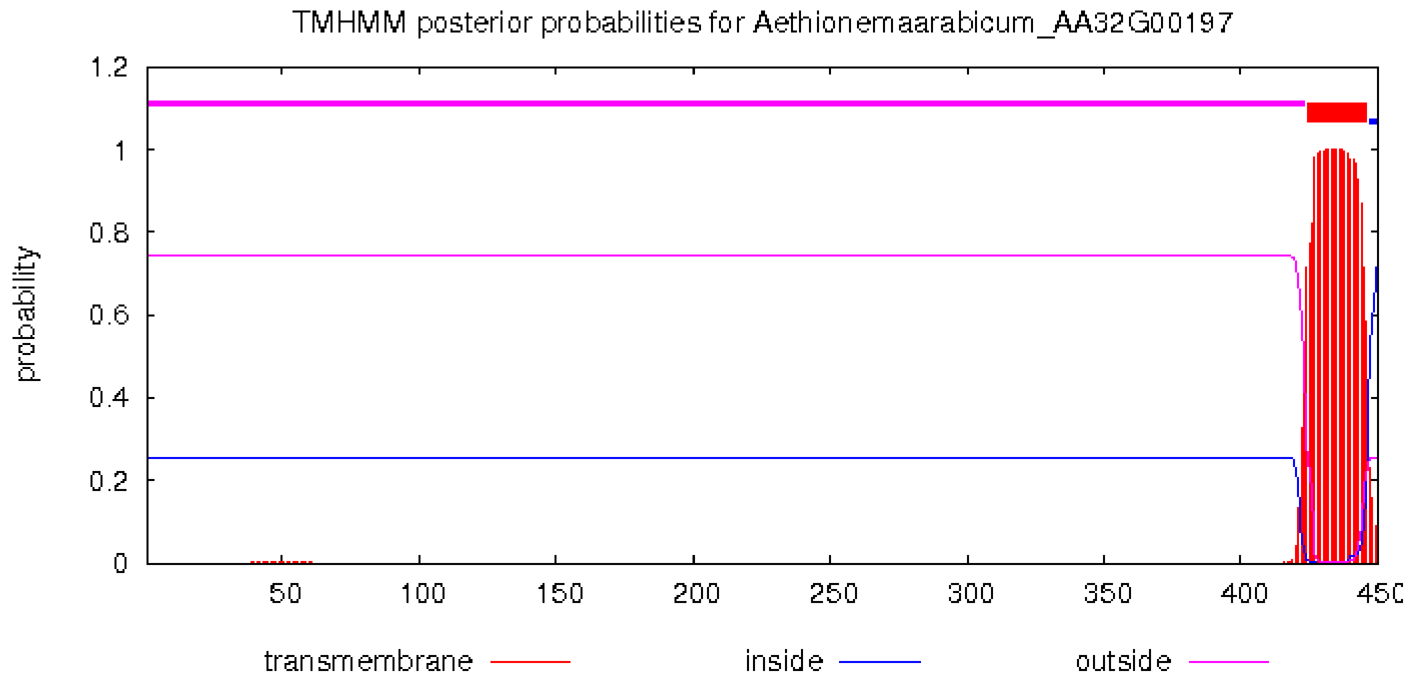

plot in postscript, script for making the plot in gnuplot, data for plot

---

```
# Aethionemaarabicum_AA32G00958 Length: 351
# Aethionemaarabicum_AA32G00958 Number of predicted TMHs:  1
# Aethionemaarabicum_AA32G00958 Exp number of AAs in TMHs: 21.13092
# Aethionemaarabicum_AA32G00958 Exp number, first 60 AAs:  0.10941
# Aethionemaarabicum_AA32G00958 Total prob of N-in:        0.65104
Aethionemaarabicum_AA32G00958   TMHMM2.0        inside       1   321
Aethionemaarabicum_AA32G00958   TMHMM2.0        TMhelix    322   344
Aethionemaarabicum_AA32G00958   TMHMM2.0        outside    345   351
```

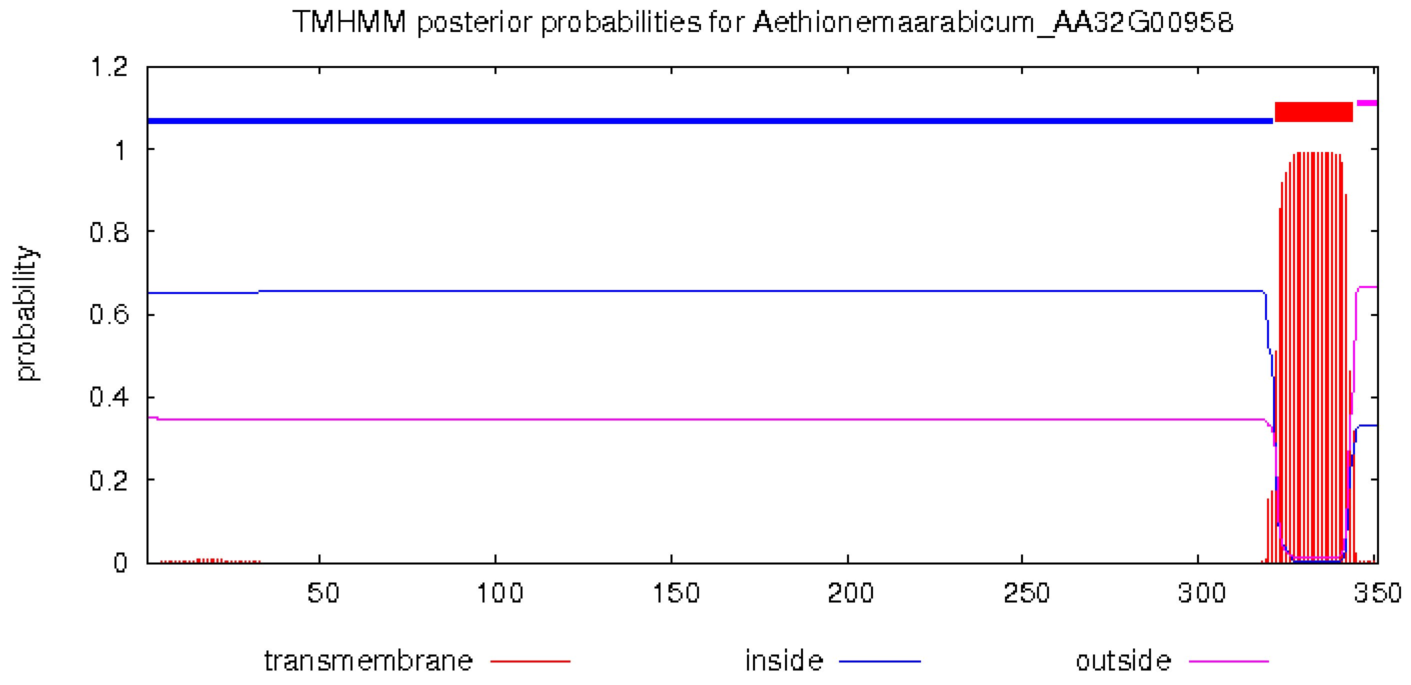

plot in postscript, script for making the plot in gnuplot, data for plot

---

```
# Aethionemaarabicum_AA32G01100 Length: 286
# Aethionemaarabicum_AA32G01100 Number of predicted TMHs:  0
# Aethionemaarabicum_AA32G01100 Exp number of AAs in TMHs: 0.01185
# Aethionemaarabicum_AA32G01100 Exp number, first 60 AAs:  0.0002
# Aethionemaarabicum_AA32G01100 Total prob of N-in:        0.02454
Aethionemaarabicum_AA32G01100   TMHMM2.0        outside      1   286
```

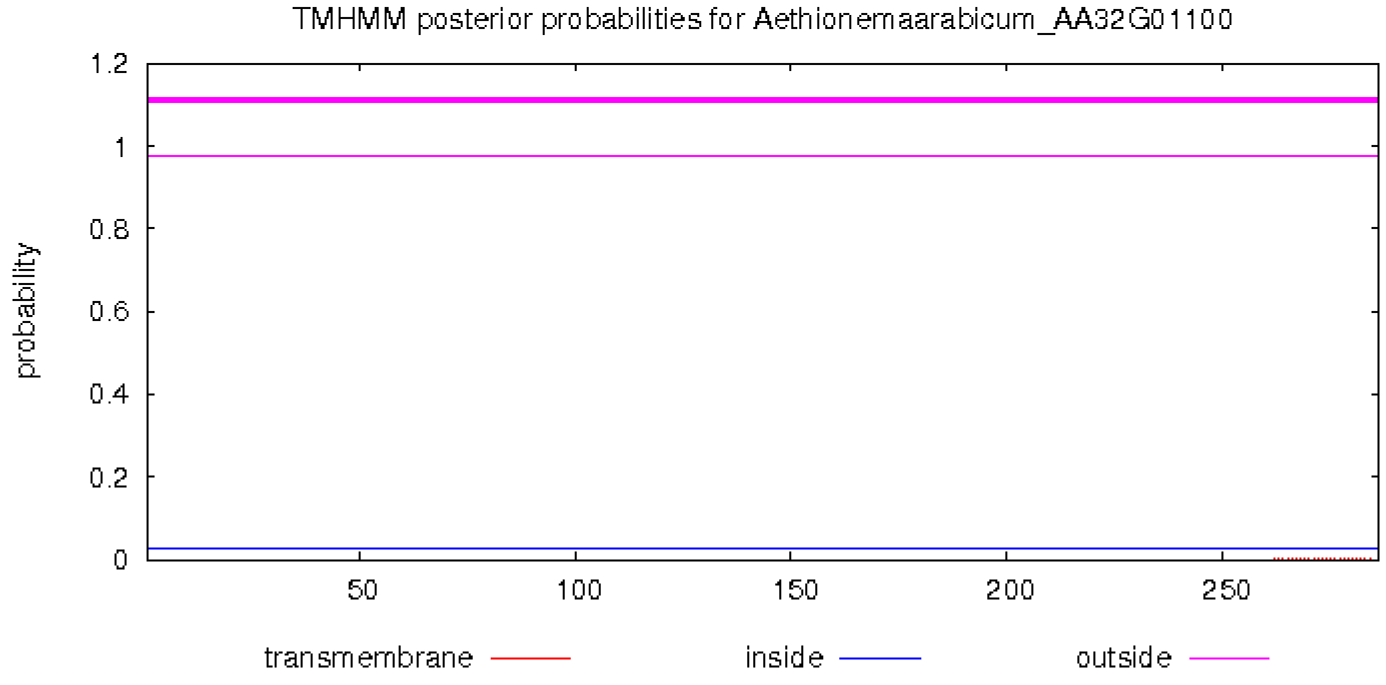

plot in postscript, script for making the plot in gnuplot, data for plot

---

```
# Aethionemaarabicum_AA32G01165 Length: 337
# Aethionemaarabicum_AA32G01165 Number of predicted TMHs:  0
# Aethionemaarabicum_AA32G01165 Exp number of AAs in TMHs: 0.00033
# Aethionemaarabicum_AA32G01165 Exp number, first 60 AAs:  0.00014
# Aethionemaarabicum_AA32G01165 Total prob of N-in:        0.03906
Aethionemaarabicum_AA32G01165   TMHMM2.0        outside      1   337
```

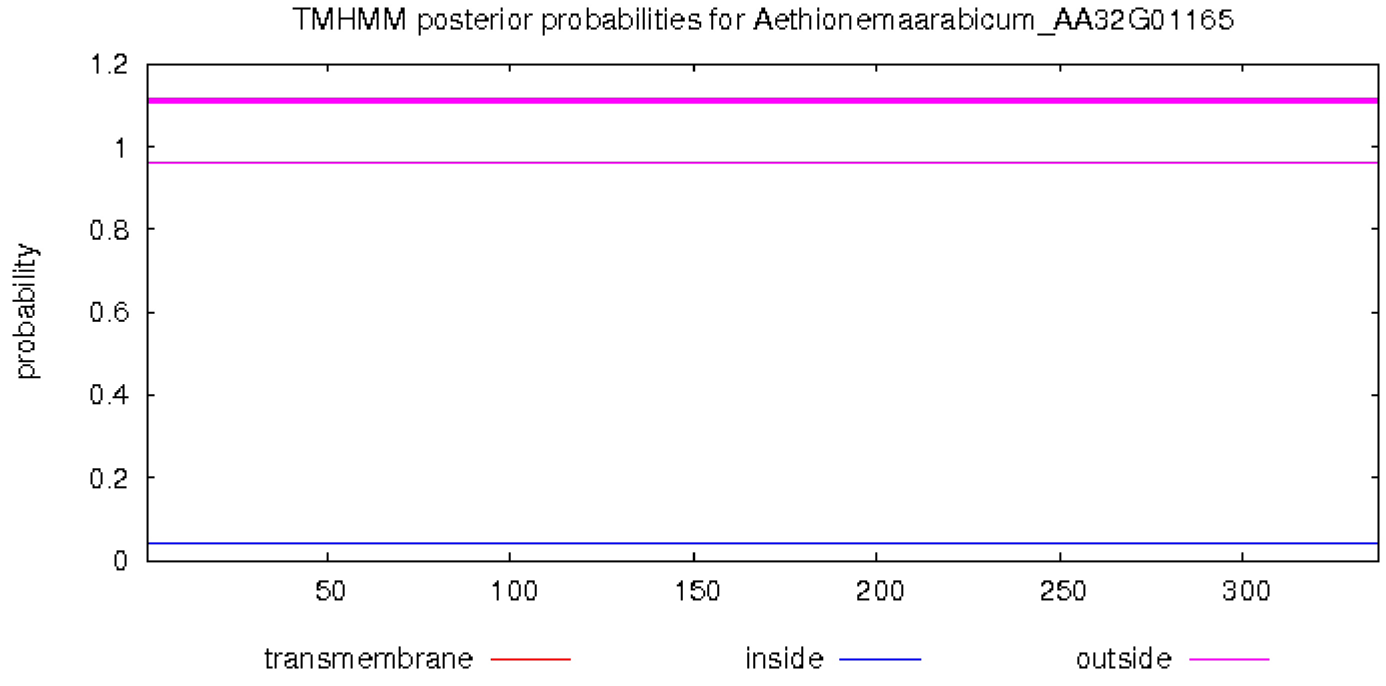

plot in postscript, script for making the plot in gnuplot, data for plot

```
# Aethionemaarabicum_AA33G00018 Length: 279
# Aethionemaarabicum_AA33G00018 Number of predicted TMHs:  0
# Aethionemaarabicum_AA33G00018 Exp number of AAs in TMHs: 0.00126
# Aethionemaarabicum_AA33G00018 Exp number, first 60 AAs:  0.00036
# Aethionemaarabicum_AA33G00018 Total prob of N-in:        0.00769
Aethionemaarabicum_AA33G00018   TMHMM2.0        outside      1   279
```

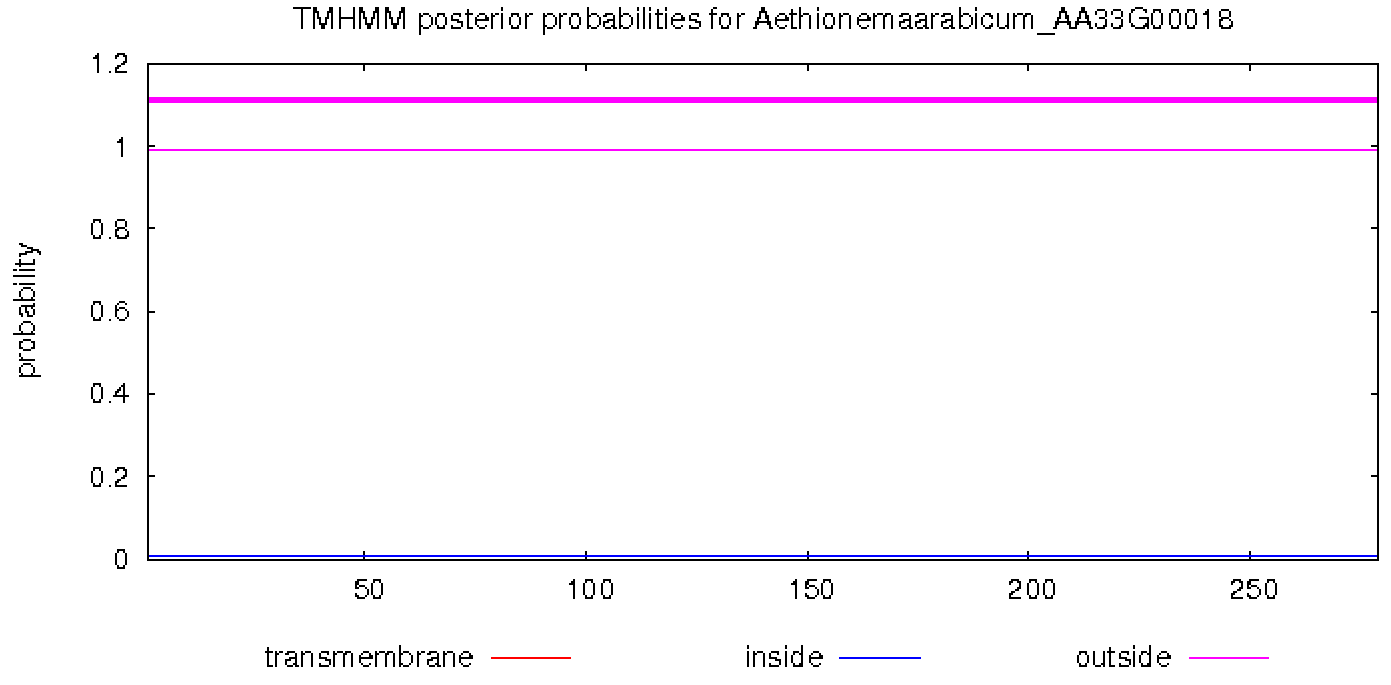

plot in postscript, script for making the plot in gnuplot, data for plot

```
# Aethionemaarabicum_AA33G00102 Length: 327
# Aethionemaarabicum_AA33G00102 Number of predicted TMHs:  0
# Aethionemaarabicum_AA33G00102 Exp number of AAs in TMHs: 0
# Aethionemaarabicum_AA33G00102 Exp number, first 60 AAs:  0
# Aethionemaarabicum_AA33G00102 Total prob of N-in:        0.00291
Aethionemaarabicum_AA33G00102   TMHMM2.0        outside      1   327
```

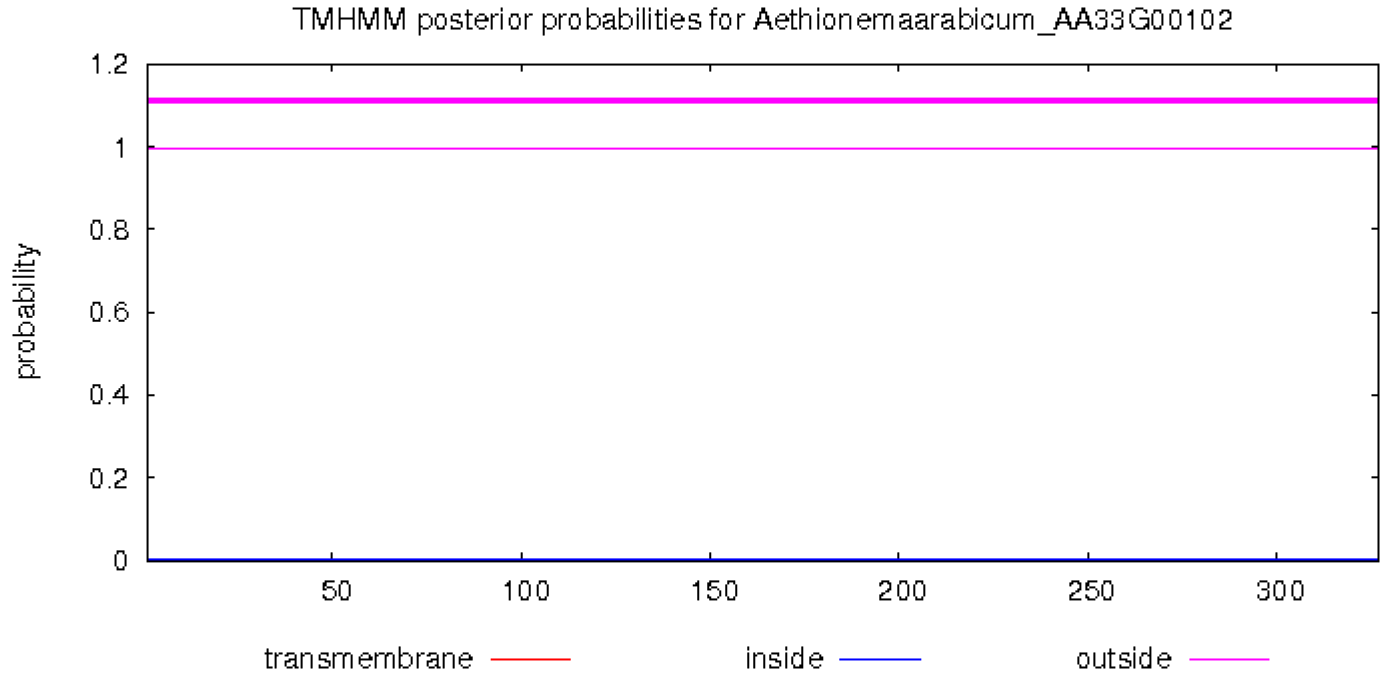

plot in postscript, script for making the plot in gnuplot, data for plot

```
# Aethionemaarabicum_AA361G00005 Length: 303
# Aethionemaarabicum_AA361G00005 Number of predicted TMHs:  0
# Aethionemaarabicum_AA361G00005 Exp number of AAs in TMHs: 0.00058
# Aethionemaarabicum_AA361G00005 Exp number, first 60 AAs:  0.00018
# Aethionemaarabicum_AA361G00005 Total prob of N-in:        0.11844
Aethionemaarabicum_AA361G00005  TMHMM2.0        outside      1   303
```

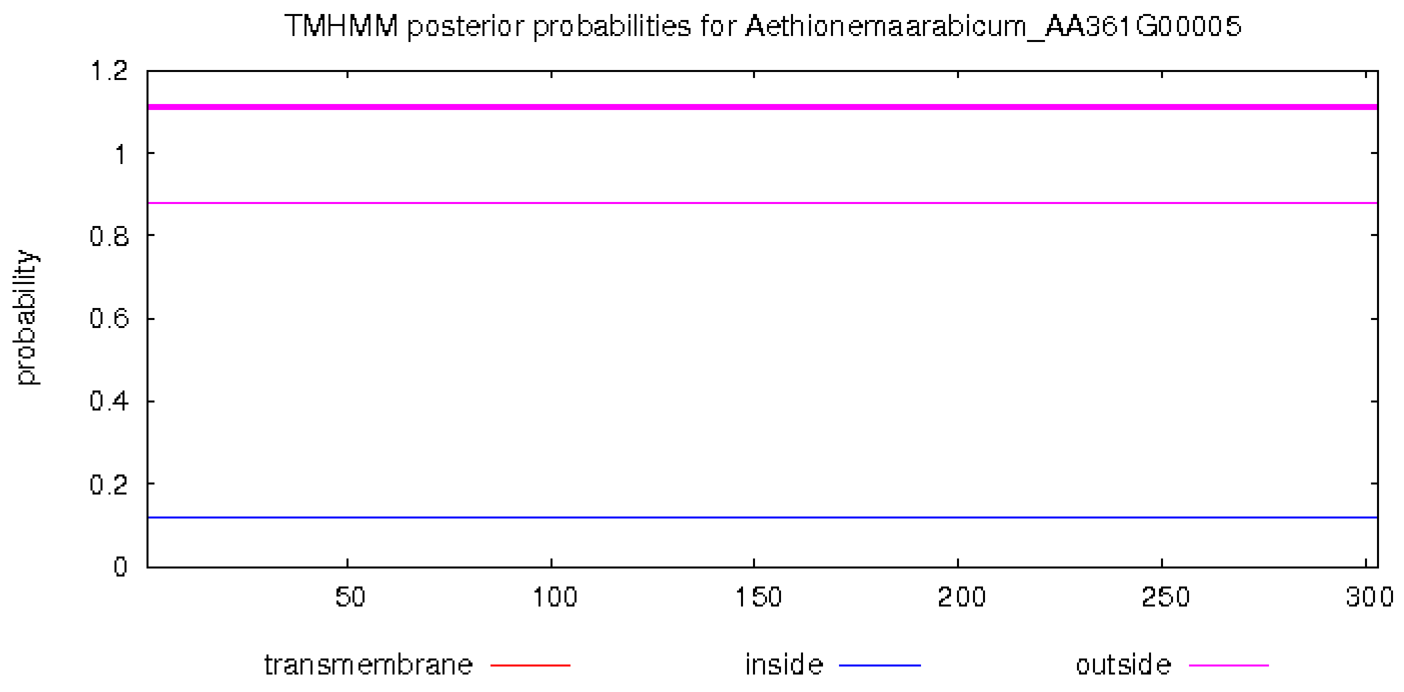

plot in postscript, script for making the plot in gnuplot, data for plot

```
# Aethionemaarabicum_AA39G00465 Length: 514
# Aethionemaarabicum_AA39G00465 Number of predicted TMHs:  1
# Aethionemaarabicum_AA39G00465 Exp number of AAs in TMHs: 22.77306
# Aethionemaarabicum_AA39G00465 Exp number, first 60 AAs:  0.00037
# Aethionemaarabicum_AA39G00465 Total prob of N-in:        0.12279
Aethionemaarabicum_AA39G00465   TMHMM2.0        outside      1   484
Aethionemaarabicum_AA39G00465   TMHMM2.0        TMhelix    485   507
Aethionemaarabicum_AA39G00465   TMHMM2.0        inside     508   514
```

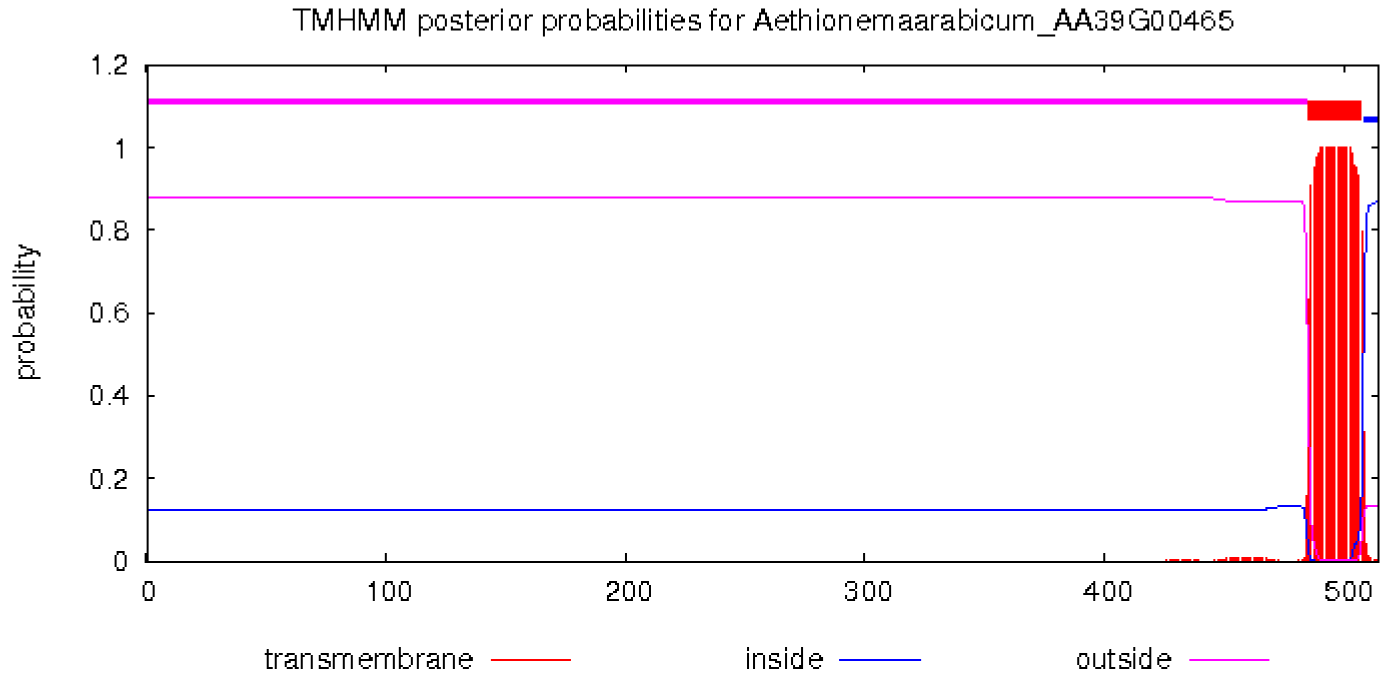

plot in postscript, script for making the plot in gnuplot, data for plot

---

```
# Aethionemaarabicum_AA39G00617 Length: 361
# Aethionemaarabicum_AA39G00617 Number of predicted TMHs:  0
# Aethionemaarabicum_AA39G00617 Exp number of AAs in TMHs: 0
# Aethionemaarabicum_AA39G00617 Exp number, first 60 AAs:  0
# Aethionemaarabicum_AA39G00617 Total prob of N-in:        0.00618
Aethionemaarabicum_AA39G00617   TMHMM2.0        outside      1   361
```

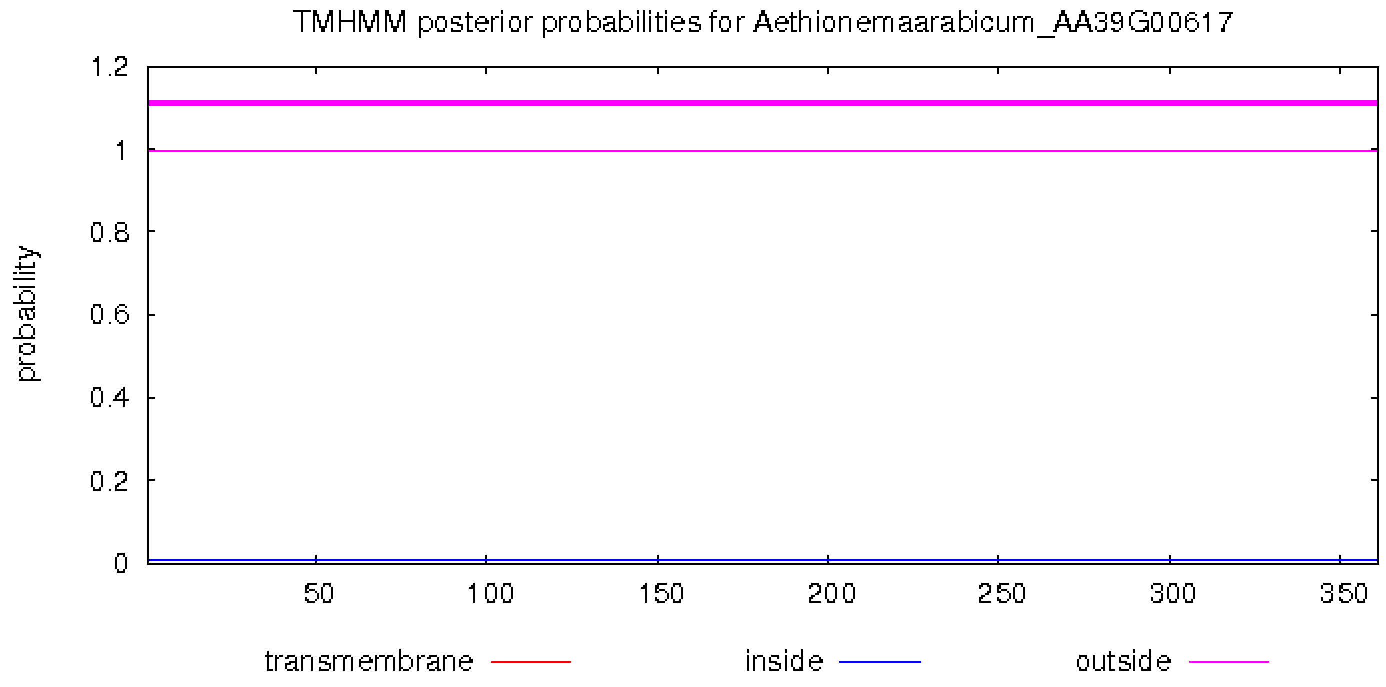

plot in postscript, script for making the plot in gnuplot, data for plot

---

```
# Aethionemaarabicum_AA39G00676 Length: 450
# Aethionemaarabicum_AA39G00676 Number of predicted TMHs:  0
# Aethionemaarabicum_AA39G00676 Exp number of AAs in TMHs: 0.0006
# Aethionemaarabicum_AA39G00676 Exp number, first 60 AAs:  0
# Aethionemaarabicum_AA39G00676 Total prob of N-in:        0.00799
Aethionemaarabicum_AA39G00676   TMHMM2.0        outside      1   450
```

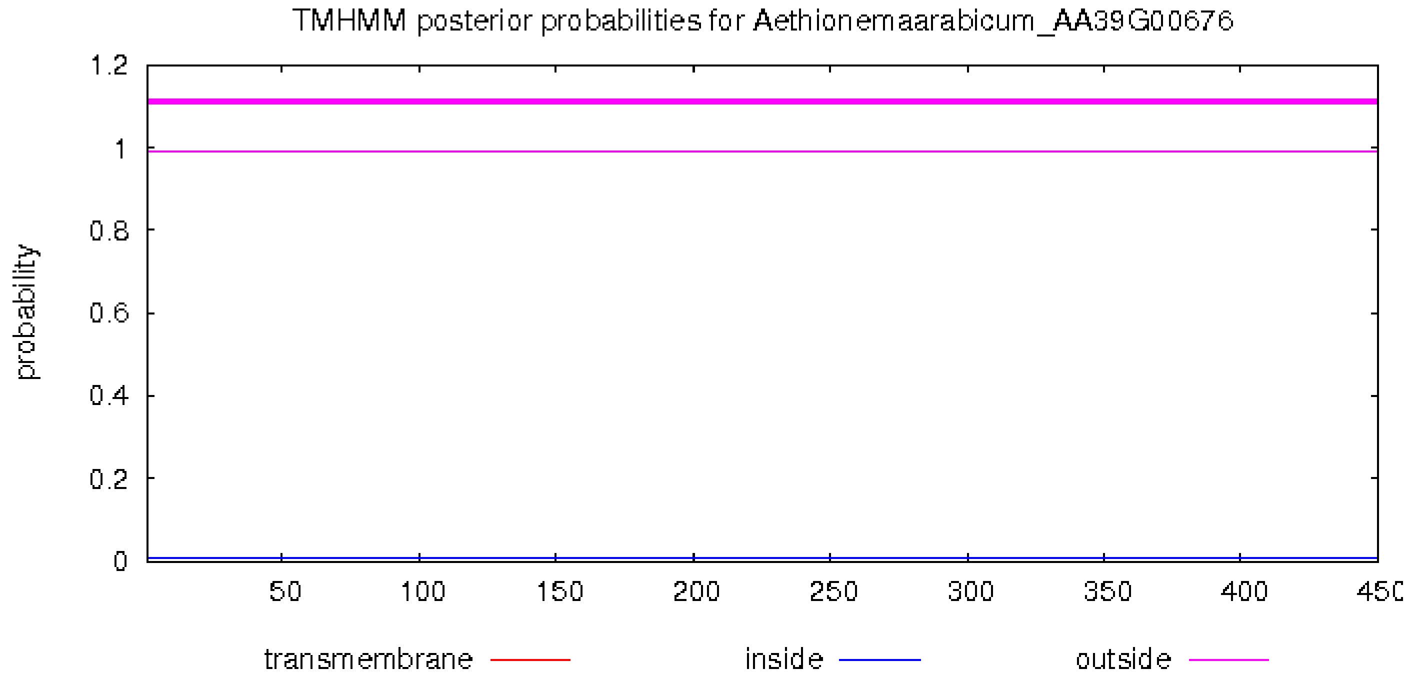

plot in postscript, script for making the plot in gnuplot, data for plot

---

```
# Aethionemaarabicum_AA3G00107 Length: 356
# Aethionemaarabicum_AA3G00107 Number of predicted TMHs:  0
# Aethionemaarabicum_AA3G00107 Exp number of AAs in TMHs: 0
# Aethionemaarabicum_AA3G00107 Exp number, first 60 AAs:  0
# Aethionemaarabicum_AA3G00107 Total prob of N-in:        0.00561
Aethionemaarabicum_AA3G00107	TMHMM2.0	outside	     1   356
```

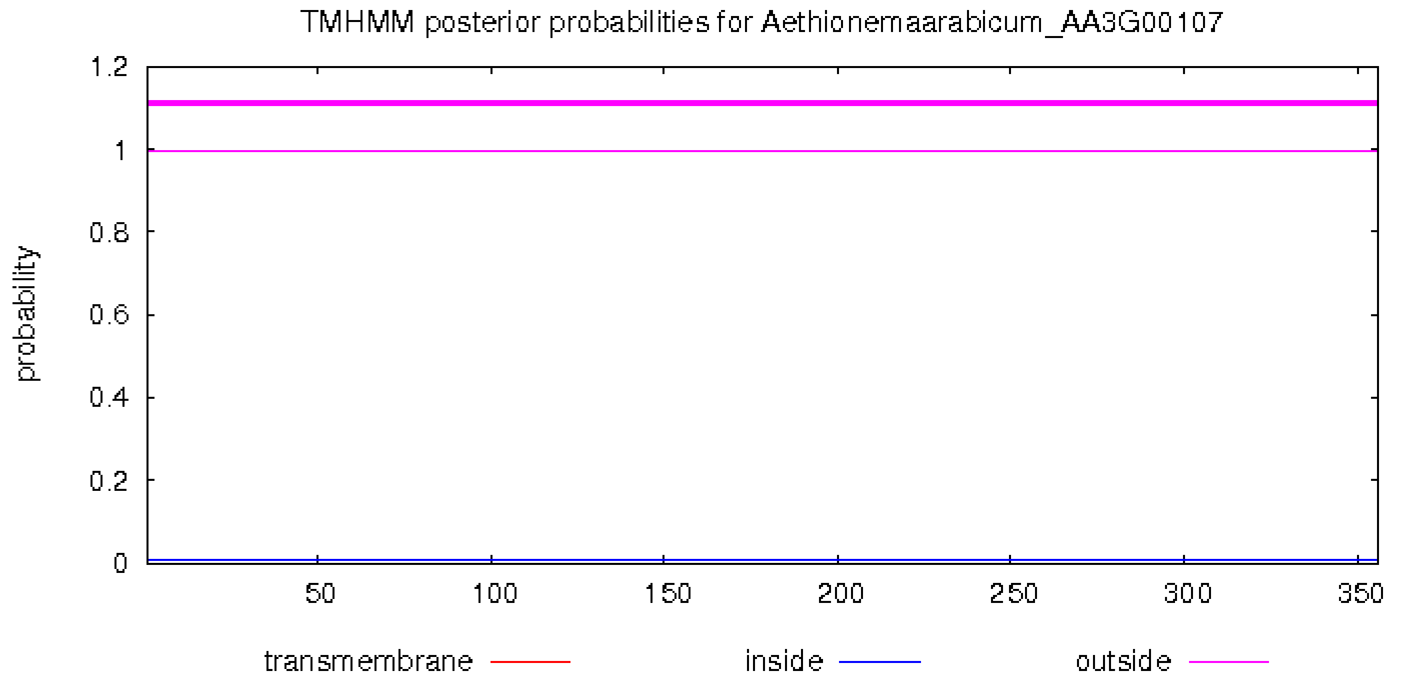

plot in postscript, script for making the plot in gnuplot, data for plot

---

```
# Aethionemaarabicum_AA3G00274 Length: 242
# Aethionemaarabicum_AA3G00274 Number of predicted TMHs:  0
# Aethionemaarabicum_AA3G00274 Exp number of AAs in TMHs: 0.02551
# Aethionemaarabicum_AA3G00274 Exp number, first 60 AAs:  0
# Aethionemaarabicum_AA3G00274 Total prob of N-in:        0.08375
Aethionemaarabicum_AA3G00274	TMHMM2.0	outside	     1   242
```

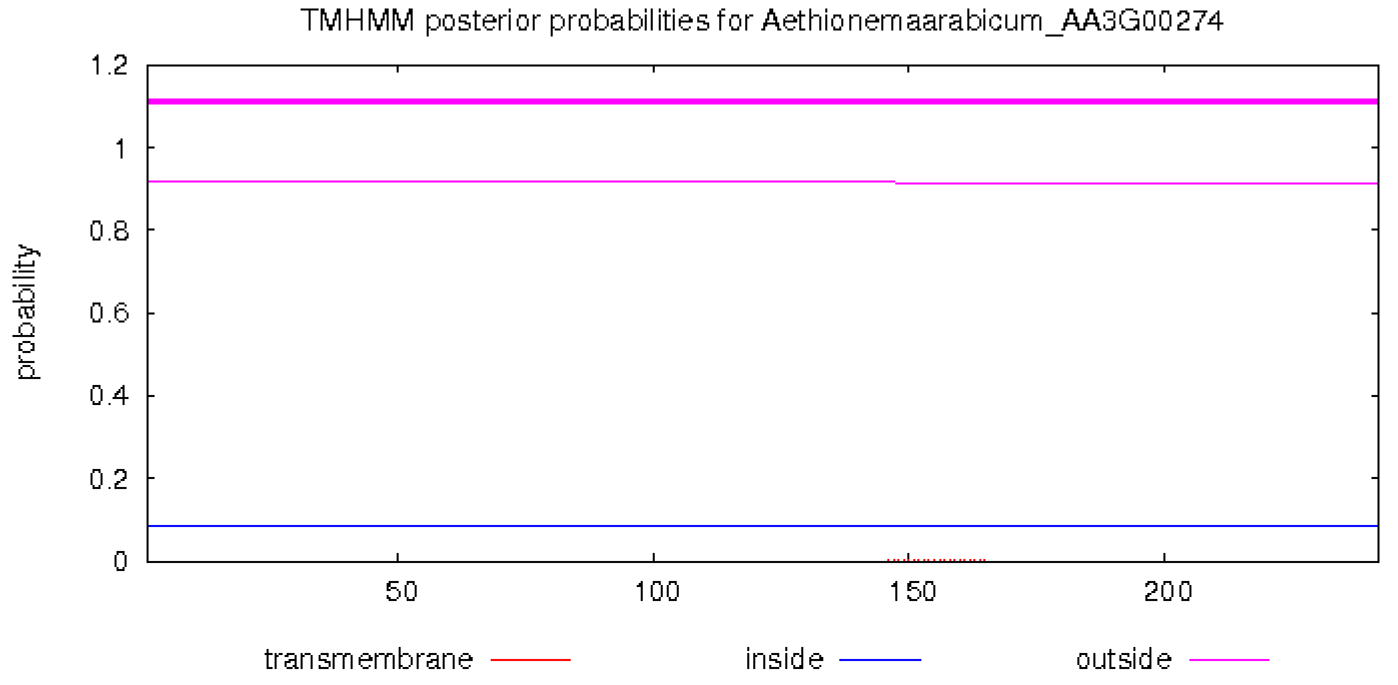

plot in postscript, script for making the plot in gnuplot, data for plot

---

```
# Aethionemaarabicum_AA40G00281 Length: 340
# Aethionemaarabicum_AA40G00281 Number of predicted TMHs:  0
# Aethionemaarabicum_AA40G00281 Exp number of AAs in TMHs: 0.00037
# Aethionemaarabicum_AA40G00281 Exp number, first 60 AAs:  0
# Aethionemaarabicum_AA40G00281 Total prob of N-in:        0.01596
Aethionemaarabicum_AA40G00281   TMHMM2.0        outside      1   340
```

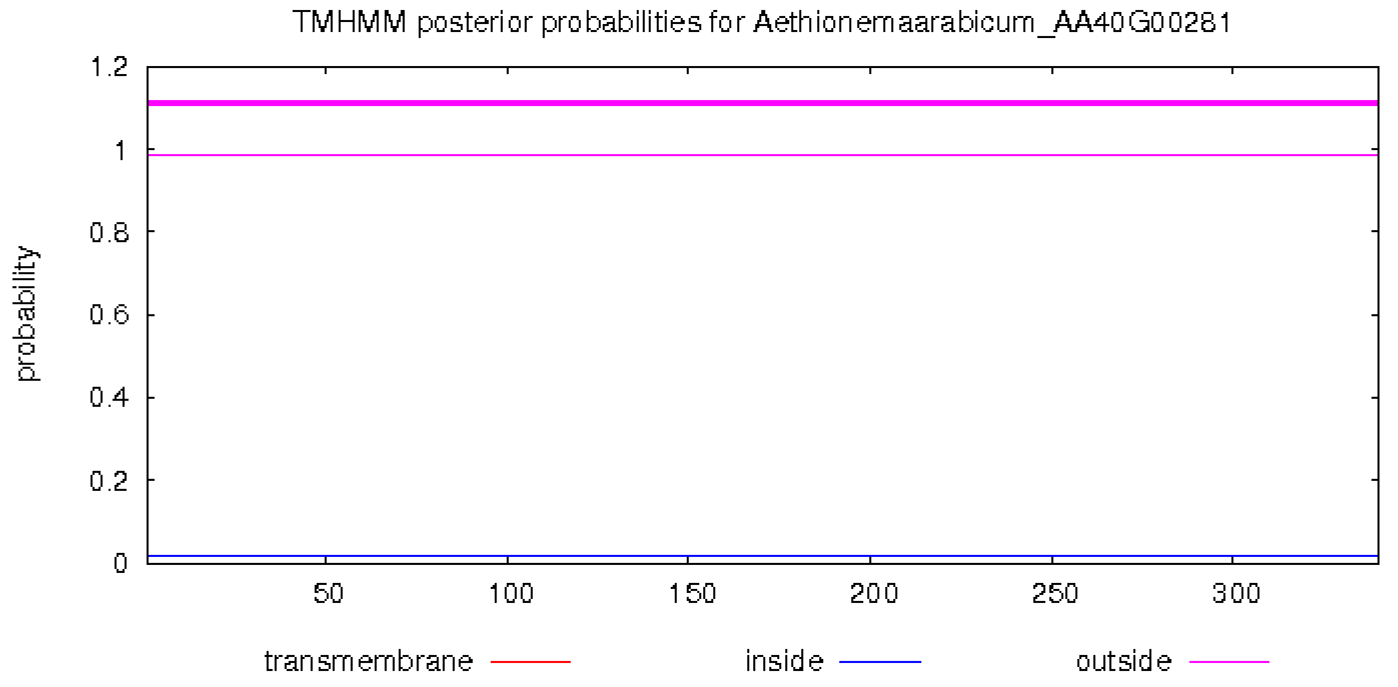

plot in postscript, script for making the plot in gnuplot, data for plot

---

```
# Aethionemaarabicum_AA40G00382 Length: 470
# Aethionemaarabicum_AA40G00382 Number of predicted TMHs:  0
# Aethionemaarabicum_AA40G00382 Exp number of AAs in TMHs: 0
# Aethionemaarabicum_AA40G00382 Exp number, first 60 AAs:  0
# Aethionemaarabicum_AA40G00382 Total prob of N-in:        0.00019
Aethionemaarabicum_AA40G00382   TMHMM2.0        outside      1   470
```

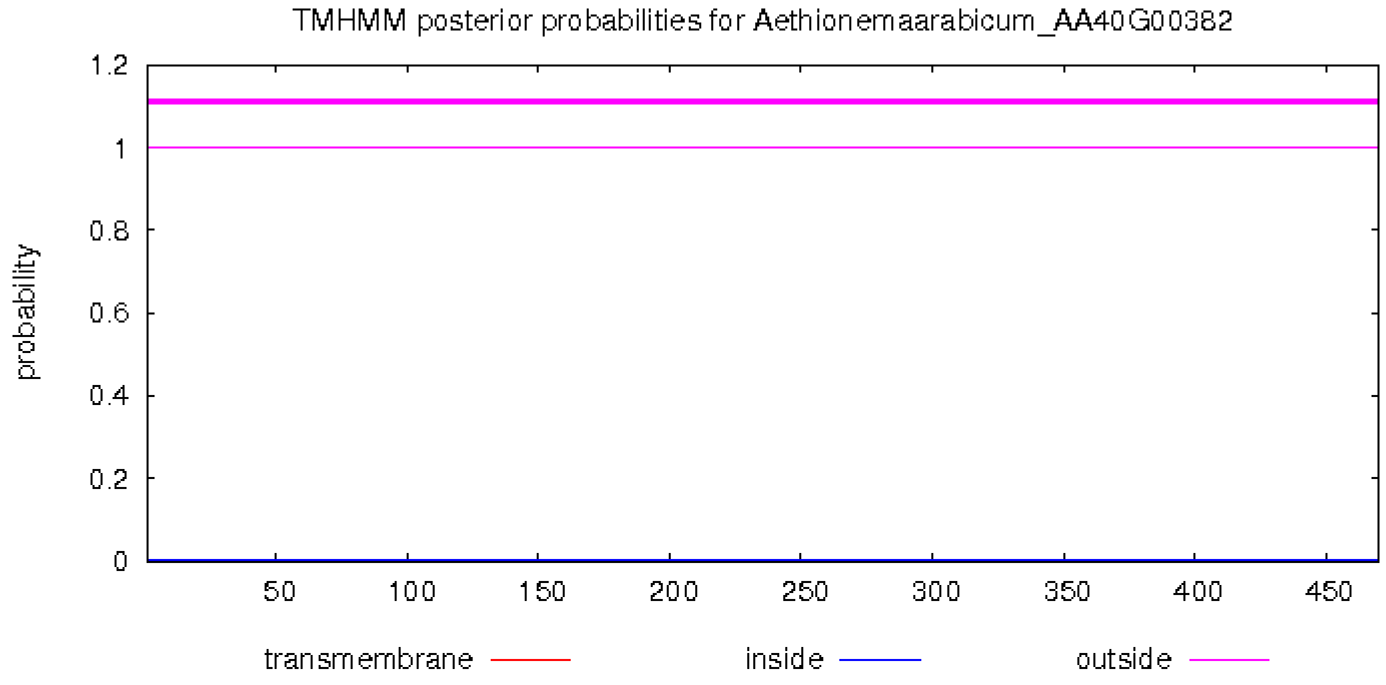

plot in postscript, script for making the plot in gnuplot, data for plot

---

```
# Aethionemaarabicum_AA44G00040 Length: 322
# Aethionemaarabicum_AA44G00040 Number of predicted TMHs:  0
# Aethionemaarabicum_AA44G00040 Exp number of AAs in TMHs: 0
# Aethionemaarabicum_AA44G00040 Exp number, first 60 AAs:  0
# Aethionemaarabicum_AA44G00040 Total prob of N-in:        0.01265
Aethionemaarabicum_AA44G00040	TMHMM2.0	outside	     1   322
```

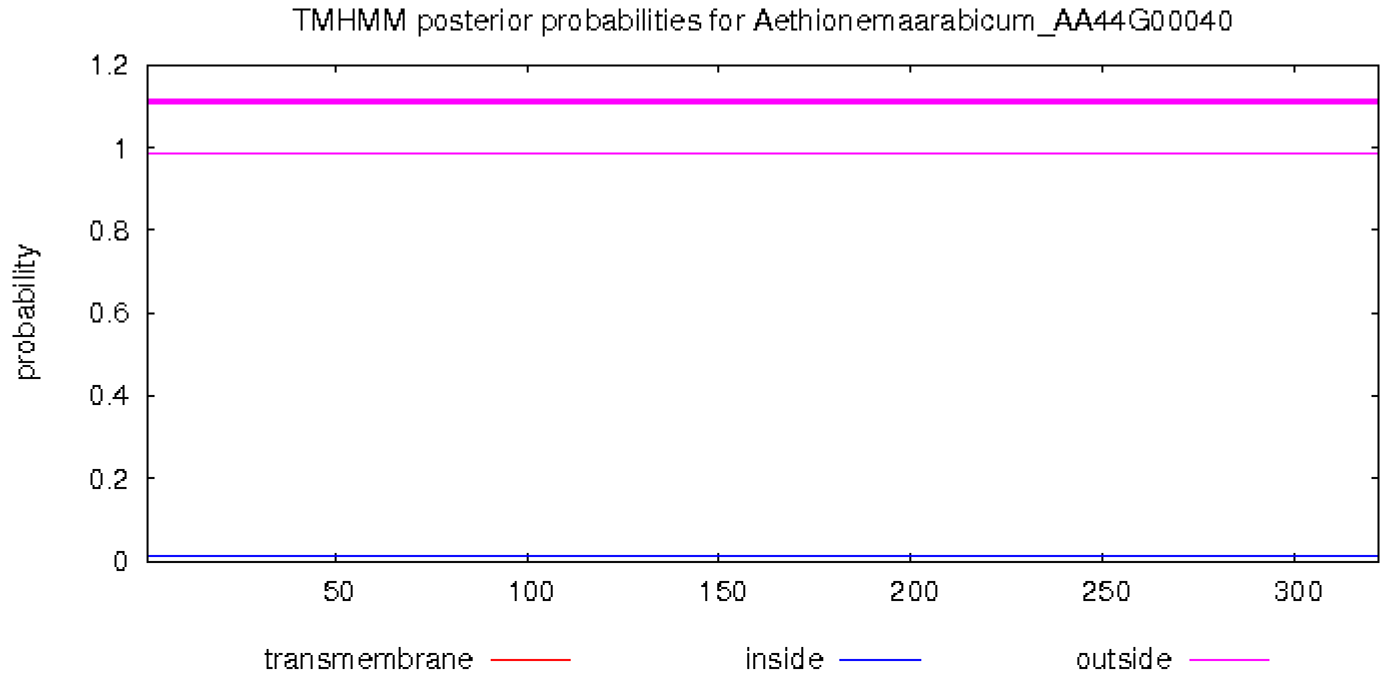

plot in postscript, script for making the plot in gnuplot, data for plot

---

```
# Aethionemaarabicum_AA44G00202 Length: 284
# Aethionemaarabicum_AA44G00202 Number of predicted TMHs:  0
# Aethionemaarabicum_AA44G00202 Exp number of AAs in TMHs: 0.00012
# Aethionemaarabicum_AA44G00202 Exp number, first 60 AAs:  0
# Aethionemaarabicum_AA44G00202 Total prob of N-in:        0.01006
Aethionemaarabicum_AA44G00202	TMHMM2.0	outside	     1   284
```

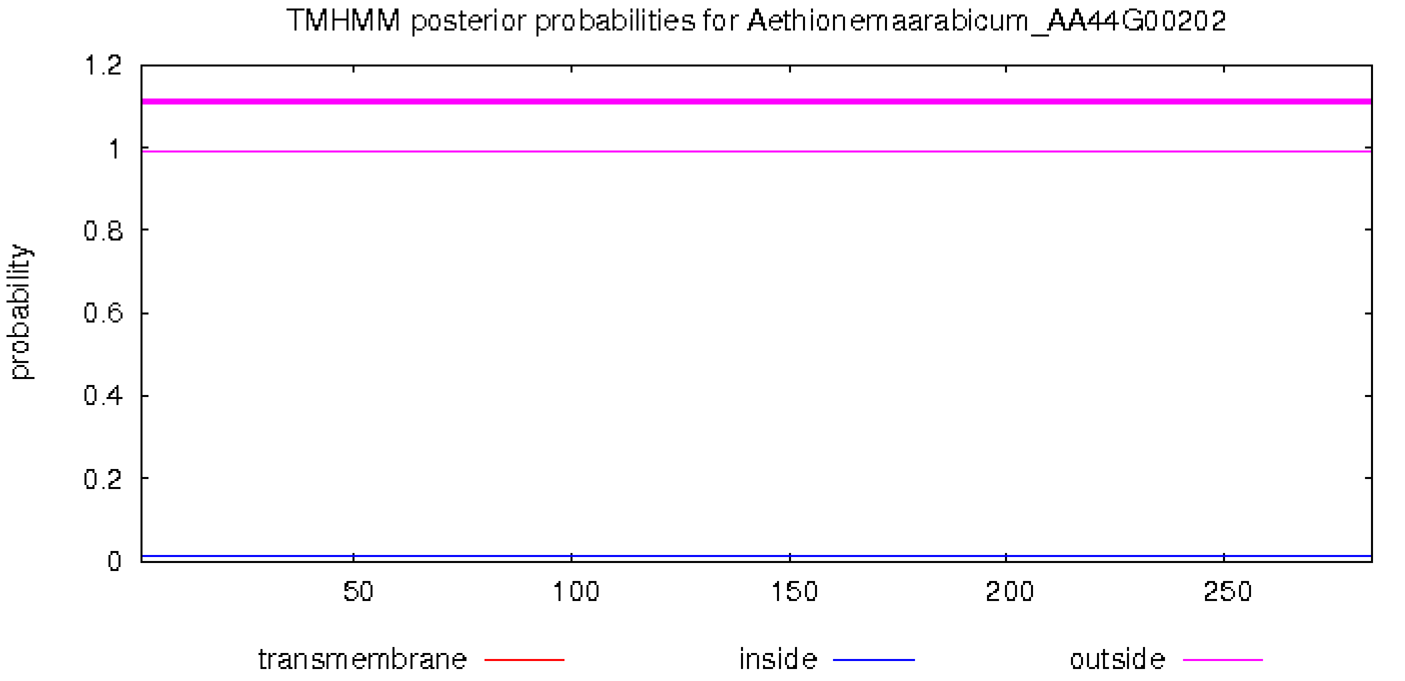

plot in postscript, script for making the plot in gnuplot, data for plot

---

```
# Aethionemaarabicum_AA44G00210 Length: 400
# Aethionemaarabicum_AA44G00210 Number of predicted TMHs:  0
# Aethionemaarabicum_AA44G00210 Exp number of AAs in TMHs: 0.00018
# Aethionemaarabicum_AA44G00210 Exp number, first 60 AAs:  0.00018
# Aethionemaarabicum_AA44G00210 Total prob of N-in:        0.00208
Aethionemaarabicum_AA44G00210   TMHMM2.0        outside      1   400
```

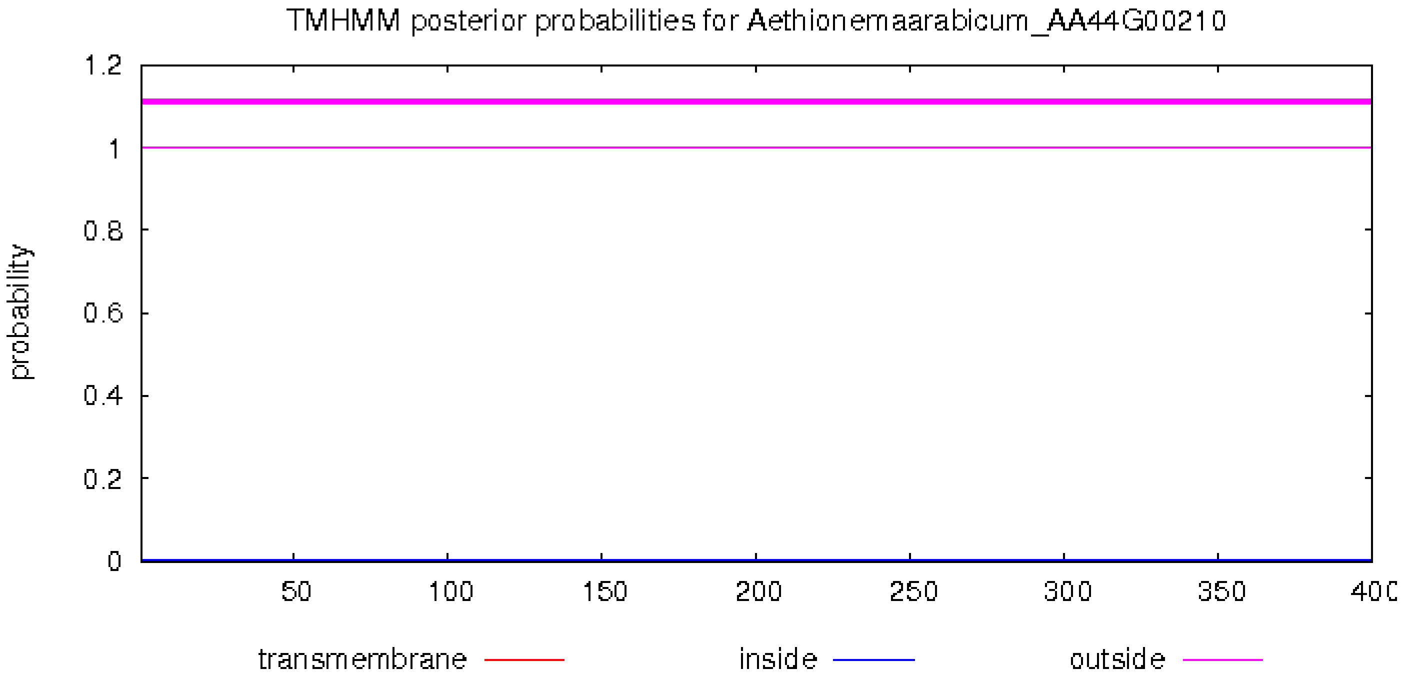

plot in postscript, script for making the plot in gnuplot, data for plot

---

```
# Aethionemaarabicum_AA44G00526 Length: 275
# Aethionemaarabicum_AA44G00526 Number of predicted TMHs:  0
# Aethionemaarabicum_AA44G00526 Exp number of AAs in TMHs: 0.00018
# Aethionemaarabicum_AA44G00526 Exp number, first 60 AAs:  0
# Aethionemaarabicum_AA44G00526 Total prob of N-in:        0.04899
Aethionemaarabicum_AA44G00526   TMHMM2.0        outside      1   275
```

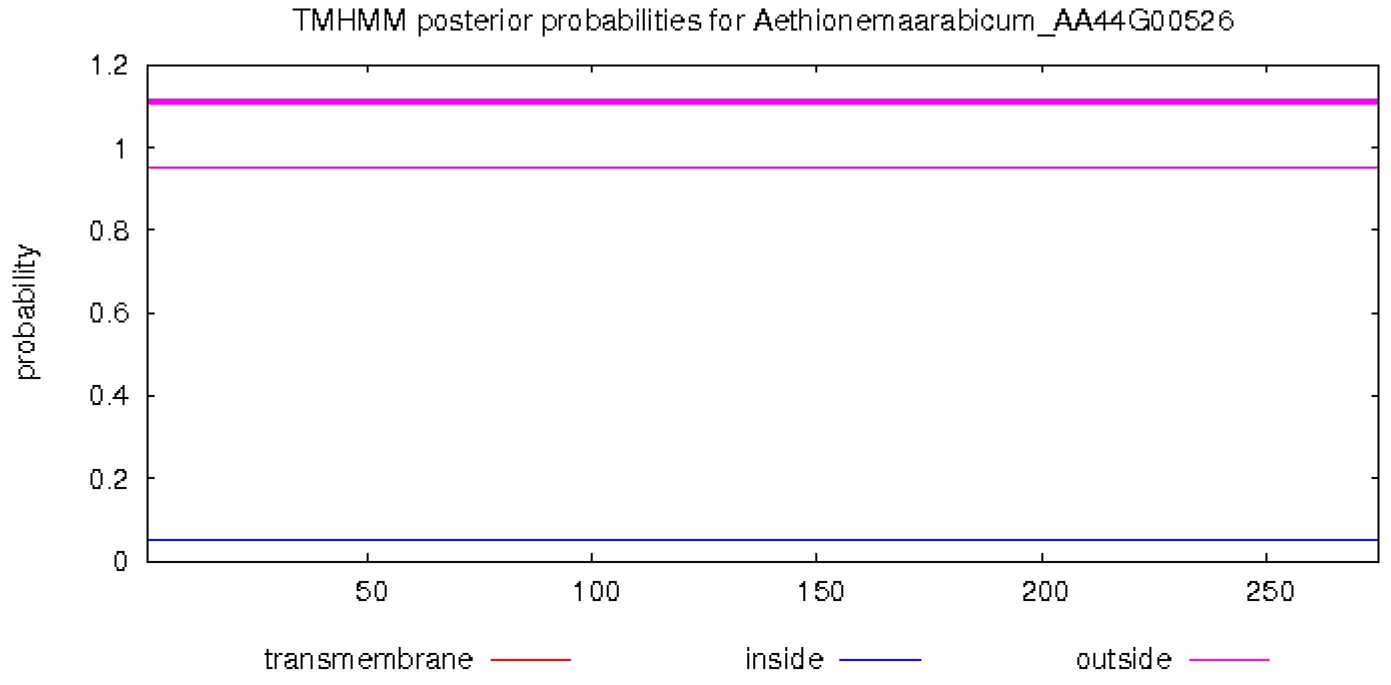

plot in postscript, script for making the plot in gnuplot, data for plot

```
# Aethionemaarabicum_AA44G00721 Length: 372
# Aethionemaarabicum_AA44G00721 Number of predicted TMHs:  0
# Aethionemaarabicum_AA44G00721 Exp number of AAs in TMHs: 0.000820000000000000001
# Aethionemaarabicum_AA44G00721 Exp number, first 60 AAs:  0
# Aethionemaarabicum_AA44G00721 Total prob of N-in:        0.06263
Aethionemaarabicum_AA44G00721   TMHMM2.0        outside      1   372
```

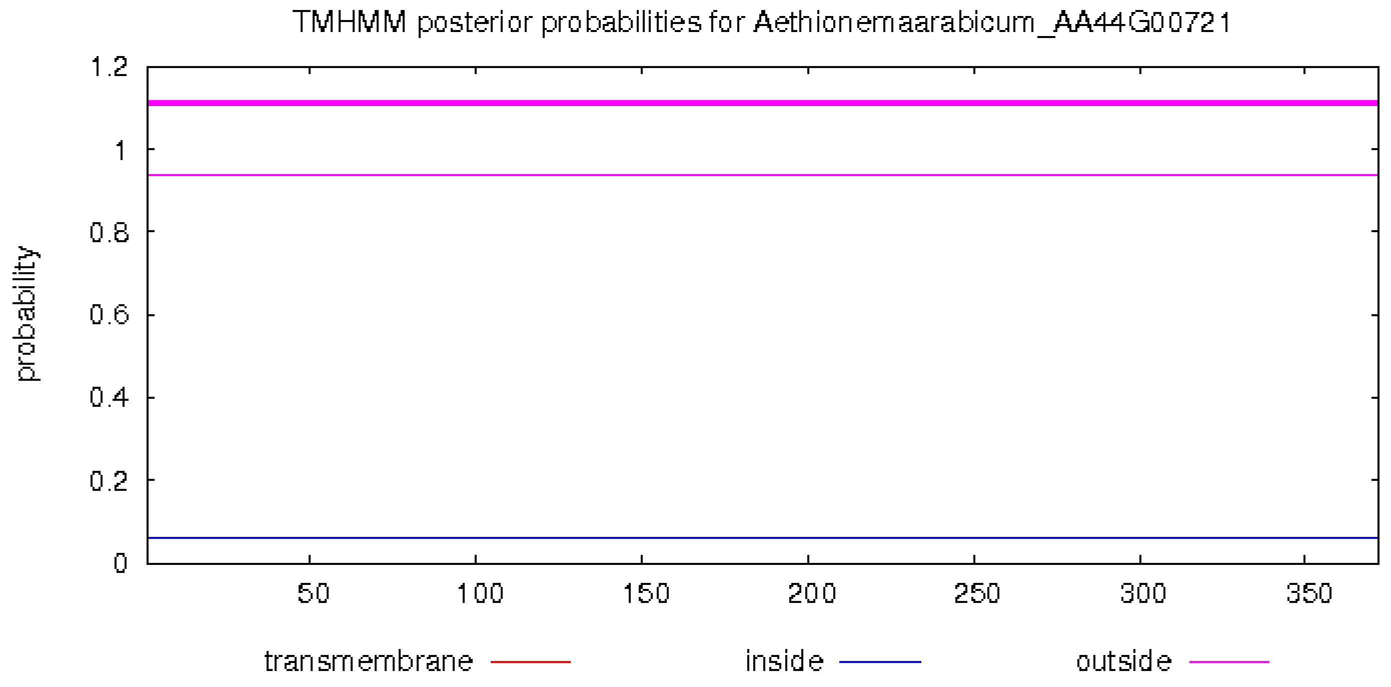

plot in postscript, script for making the plot in gnuplot, data for plot

```
# Aethionemaarabicum_AA47G00012 Length: 242
# Aethionemaarabicum_AA47G00012 Number of predicted TMHs:  0
# Aethionemaarabicum_AA47G00012 Exp number of AAs in TMHs: 0.00115
# Aethionemaarabicum_AA47G00012 Exp number, first 60 AAs:  0
# Aethionemaarabicum_AA47G00012 Total prob of N-in:        0.09201
Aethionemaarabicum_AA47G00012   TMHMM2.0        outside      1   242
```

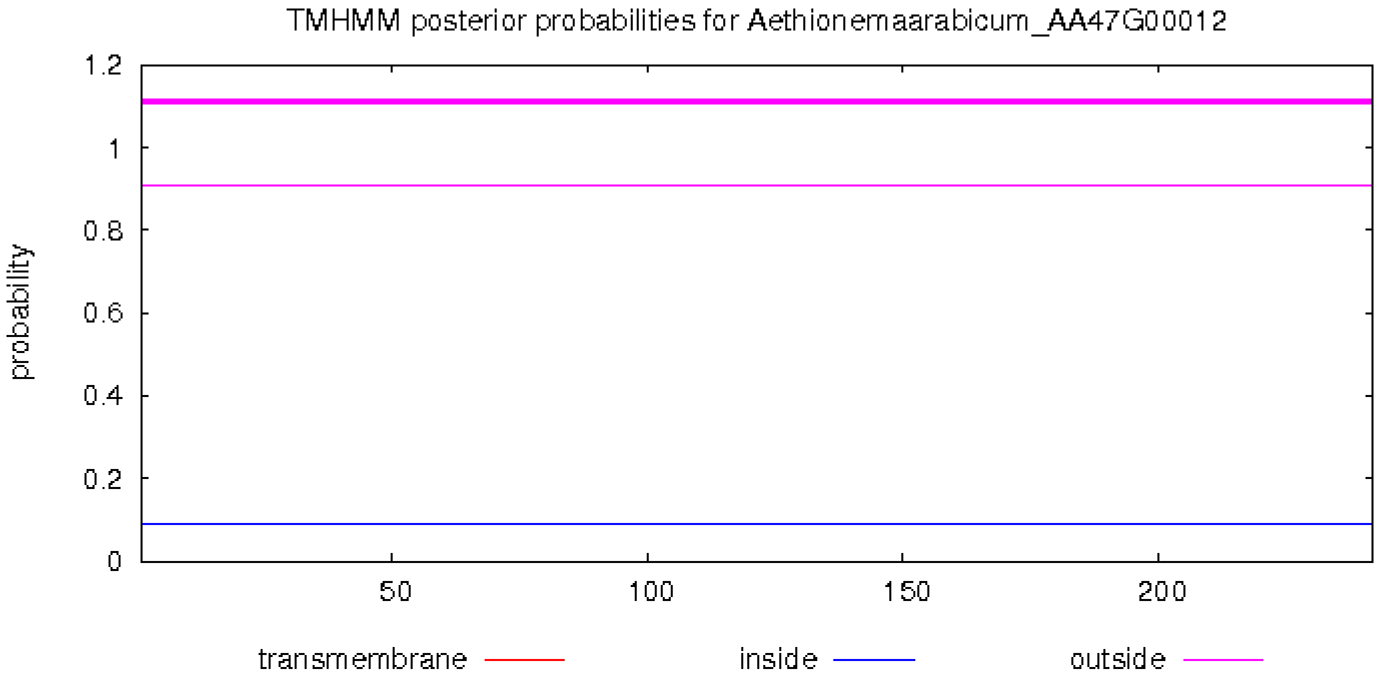

plot in postscript, script for making the plot in gnuplot, data for plot

---

```
# Aethionemaarabicum_AA4G00113 Length: 232
# Aethionemaarabicum_AA4G00113 Number of predicted TMHs:  0
# Aethionemaarabicum_AA4G00113 Exp number of AAs in TMHs: 0.00256
# Aethionemaarabicum_AA4G00113 Exp number, first 60 AAs:  0.00074
# Aethionemaarabicum_AA4G00113 Total prob of N-in:        0.03334
Aethionemaarabicum_AA4G00113    TMHMM2.0        outside      1   232
```

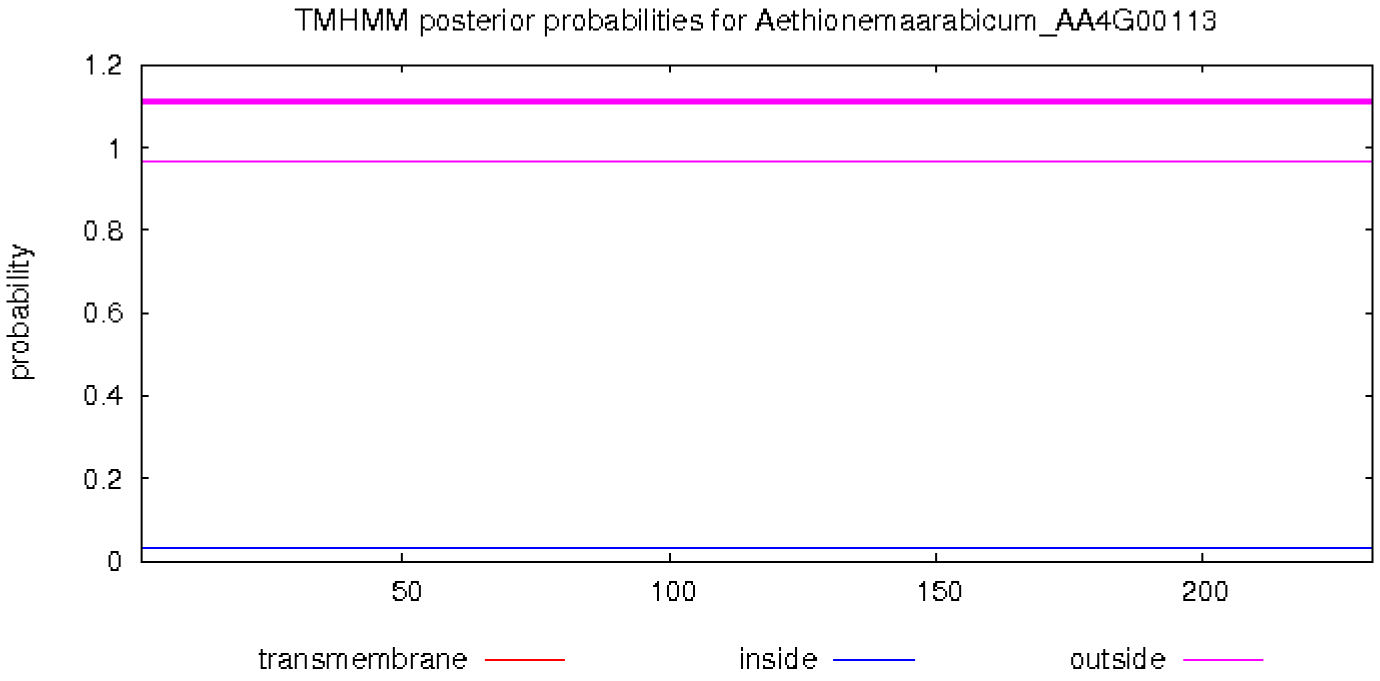

plot in postscript, script for making the plot in gnuplot, data for plot

---

```
# Aethionemaarabicum_AA4G00191 Length: 191
# Aethionemaarabicum_AA4G00191 Number of predicted TMHs:  0
# Aethionemaarabicum_AA4G00191 Exp number of AAs in TMHs: 0.00018
# Aethionemaarabicum_AA4G00191 Exp number, first 60 AAs:  0
# Aethionemaarabicum_AA4G00191 Total prob of N-in:        0.00698
Aethionemaarabicum_AA4G00191    TMHMM2.0        outside      1   191
```

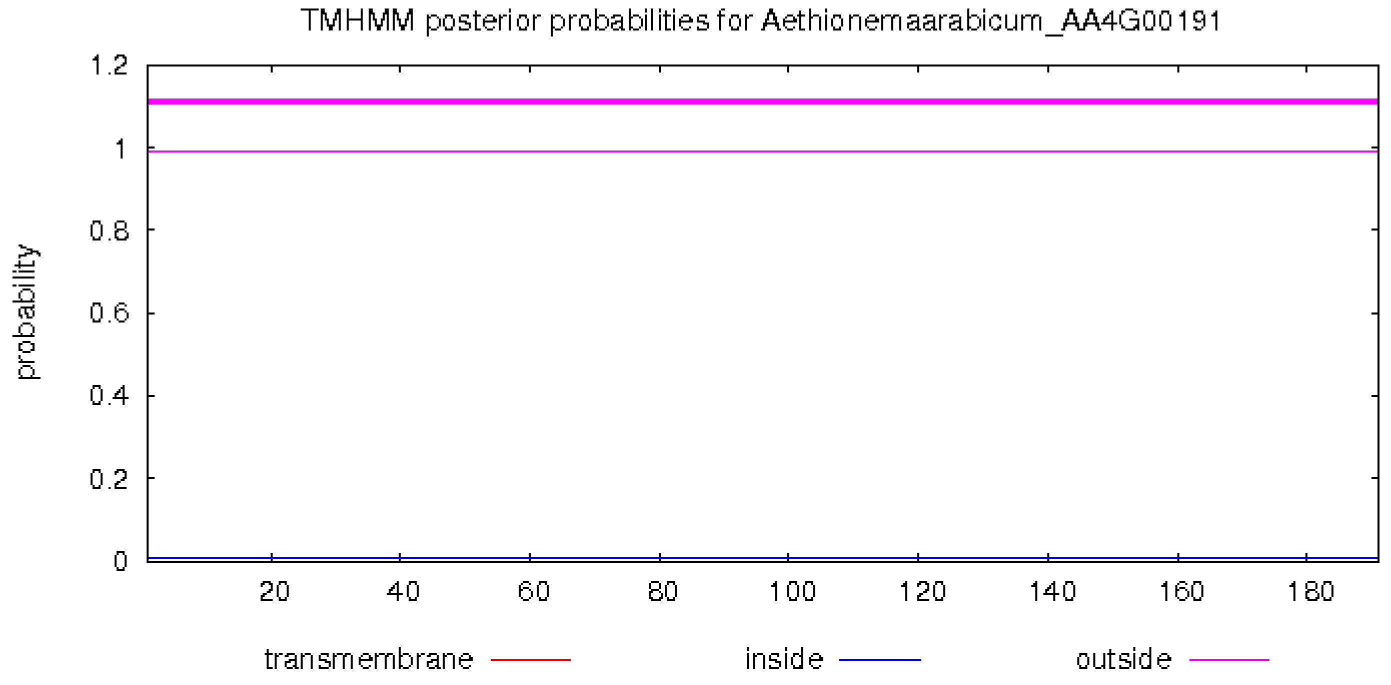

plot in postscript, script for making the plot in gnuplot, data for plot

---

```
# Aethionemaarabicum_AA53G00237 Length: 288
# Aethionemaarabicum_AA53G00237 Number of predicted TMHs:  0
# Aethionemaarabicum_AA53G00237 Exp number of AAs in TMHs: 0
# Aethionemaarabicum_AA53G00237 Exp number, first 60 AAs:  0
# Aethionemaarabicum_AA53G00237 Total prob of N-in:        0.00233
Aethionemaarabicum_AA53G00237   TMHMM2.0        outside      1   288
```

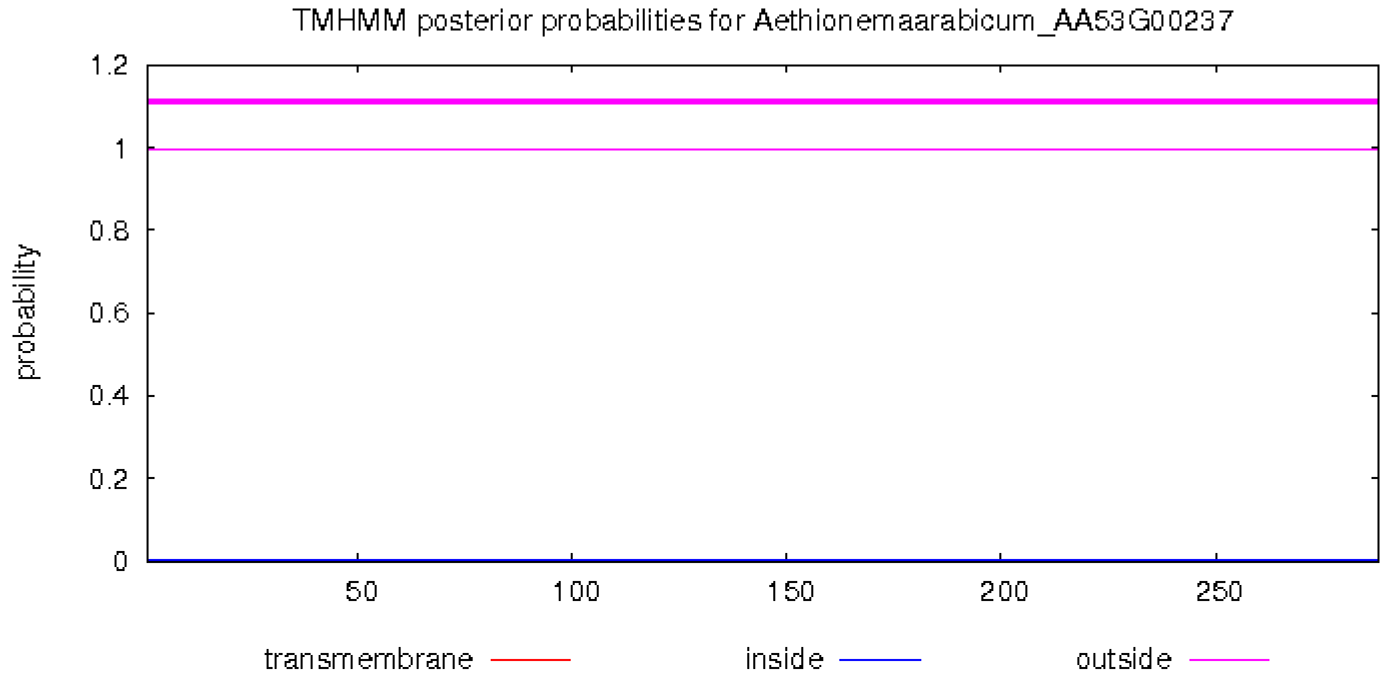

plot in postscript, script for making the plot in gnuplot, data for plot

---

```
# Aethionemaarabicum_AA53G00532 Length: 326
# Aethionemaarabicum_AA53G00532 Number of predicted TMHs:  0
# Aethionemaarabicum_AA53G00532 Exp number of AAs in TMHs: 0.00065
# Aethionemaarabicum_AA53G00532 Exp number, first 60 AAs:  0.00045
# Aethionemaarabicum_AA53G00532 Total prob of N-in:        0.01492
Aethionemaarabicum_AA53G00532   TMHMM2.0        outside      1   326
```

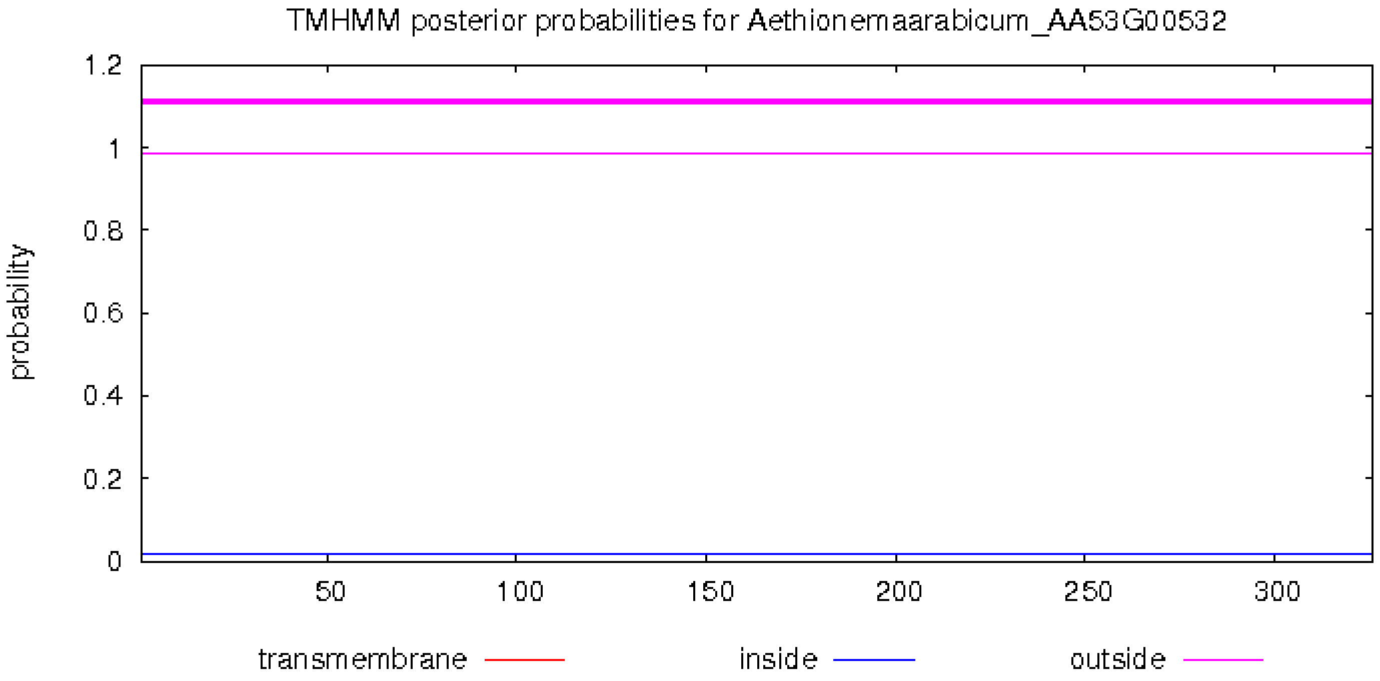

plot in postscript, script for making the plot in gnuplot, data for plot

---

```
# Aethionemaarabicum_AA53G00706 Length: 416
# Aethionemaarabicum_AA53G00706 Number of predicted TMHs:  0
# Aethionemaarabicum_AA53G00706 Exp number of AAs in TMHs: 0.00962
# Aethionemaarabicum_AA53G00706 Exp number, first 60 AAs:  7e-05
# Aethionemaarabicum_AA53G00706 Total prob of N-in:        0.01135
Aethionemaarabicum_AA53G00706   TMHMM2.0        outside      1   416
```

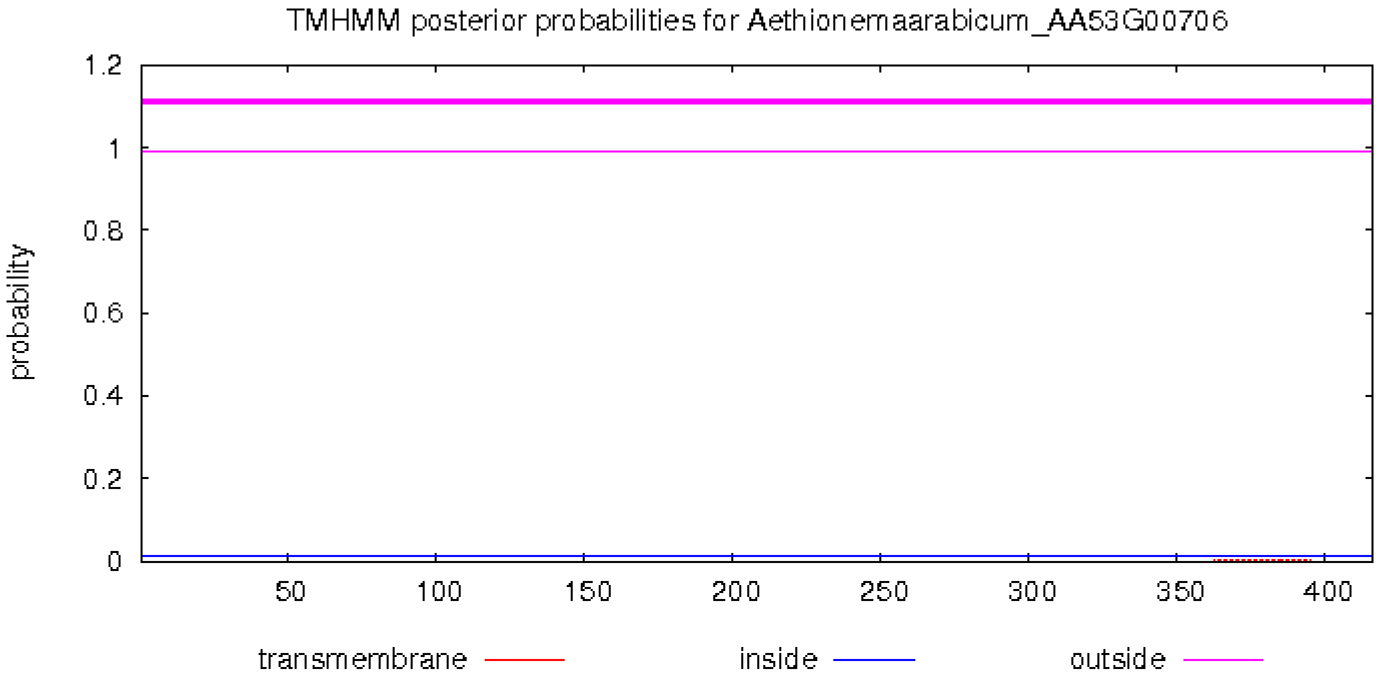

plot in postscript, script for making the plot in gnuplot, data for plot

---

```
# Aethionemaarabicum_AA53G01090 Length: 320
# Aethionemaarabicum_AA53G01090 Number of predicted TMHs:  0
# Aethionemaarabicum_AA53G01090 Exp number of AAs in TMHs: 0.00133
# Aethionemaarabicum_AA53G01090 Exp number, first 60 AAs:  0.00048
# Aethionemaarabicum_AA53G01090 Total prob of N-in:        0.03512
Aethionemaarabicum_AA53G01090   TMHMM2.0        outside      1   320
```

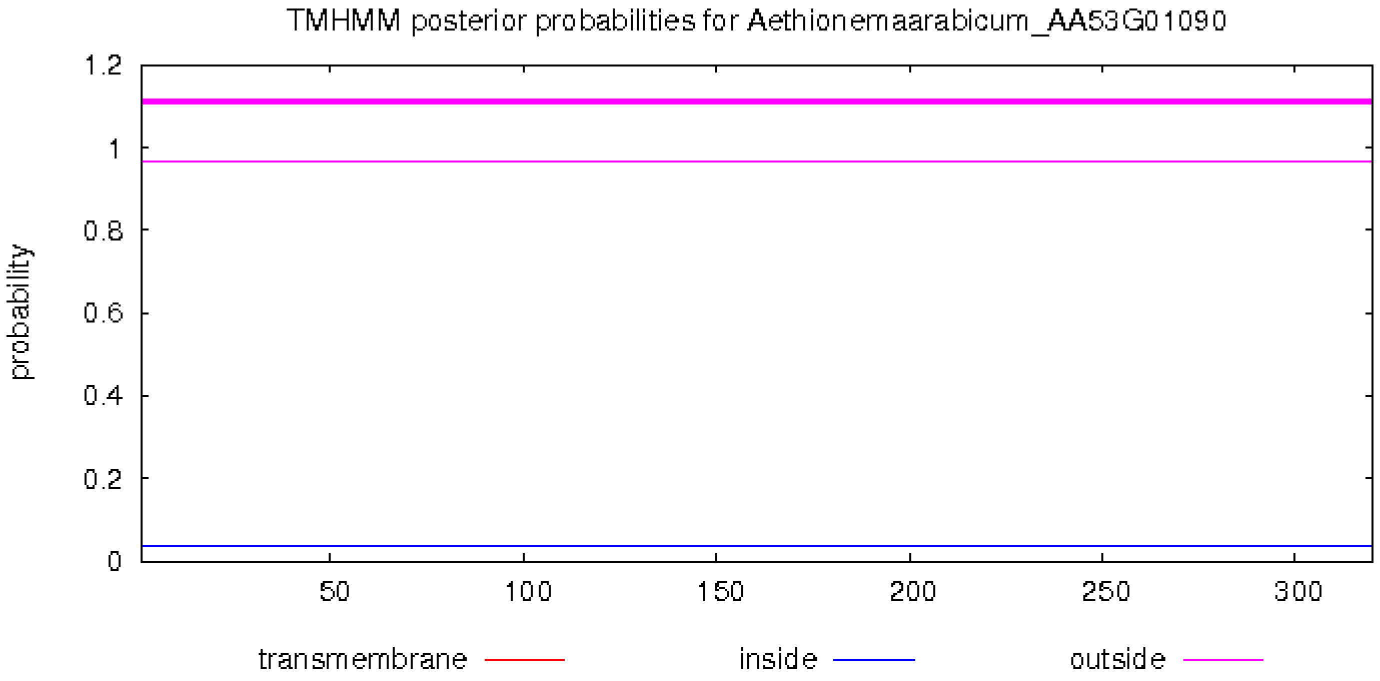

plot in postscript, script for making the plot in gnuplot, data for plot

---

```
# Aethionemaarabicum_AA53G01143 Length: 448
# Aethionemaarabicum_AA53G01143 Number of predicted TMHs:  0
# Aethionemaarabicum_AA53G01143 Exp number of AAs in TMHs: 0
# Aethionemaarabicum_AA53G01143 Exp number, first 60 AAs:  0
# Aethionemaarabicum_AA53G01143 Total prob of N-in:        0.00053
Aethionemaarabicum_AA53G01143   TMHMM2.0        outside      1   448
```

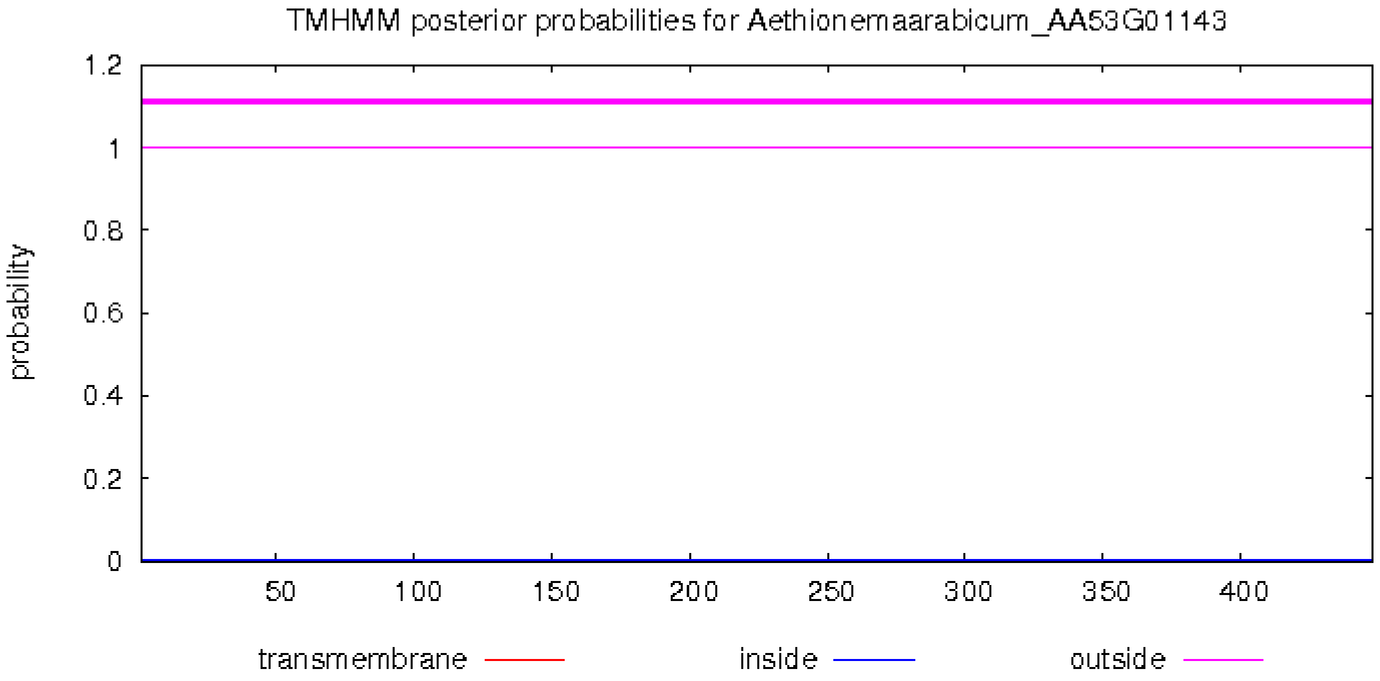

plot in postscript, script for making the plot in gnuplot, data for plot

---

```
# Aethionemaarabicum_AA54G00138 Length: 581
# Aethionemaarabicum_AA54G00138 Number of predicted TMHs:  1
# Aethionemaarabicum_AA54G00138 Exp number of AAs in TMHs: 22.38358
# Aethionemaarabicum_AA54G00138 Exp number, first 60 AAs:  0.01194
# Aethionemaarabicum_AA54G00138 Total prob of N-in:        0.41664
Aethionemaarabicum_AA54G00138   TMHMM2.0        inside       1   551
```

```
Aethionemaarabicum_AA54G00138   TMHMM2.0        TMhelix     552   574
Aethionemaarabicum_AA54G00138   TMHMM2.0        outside     575   581
```

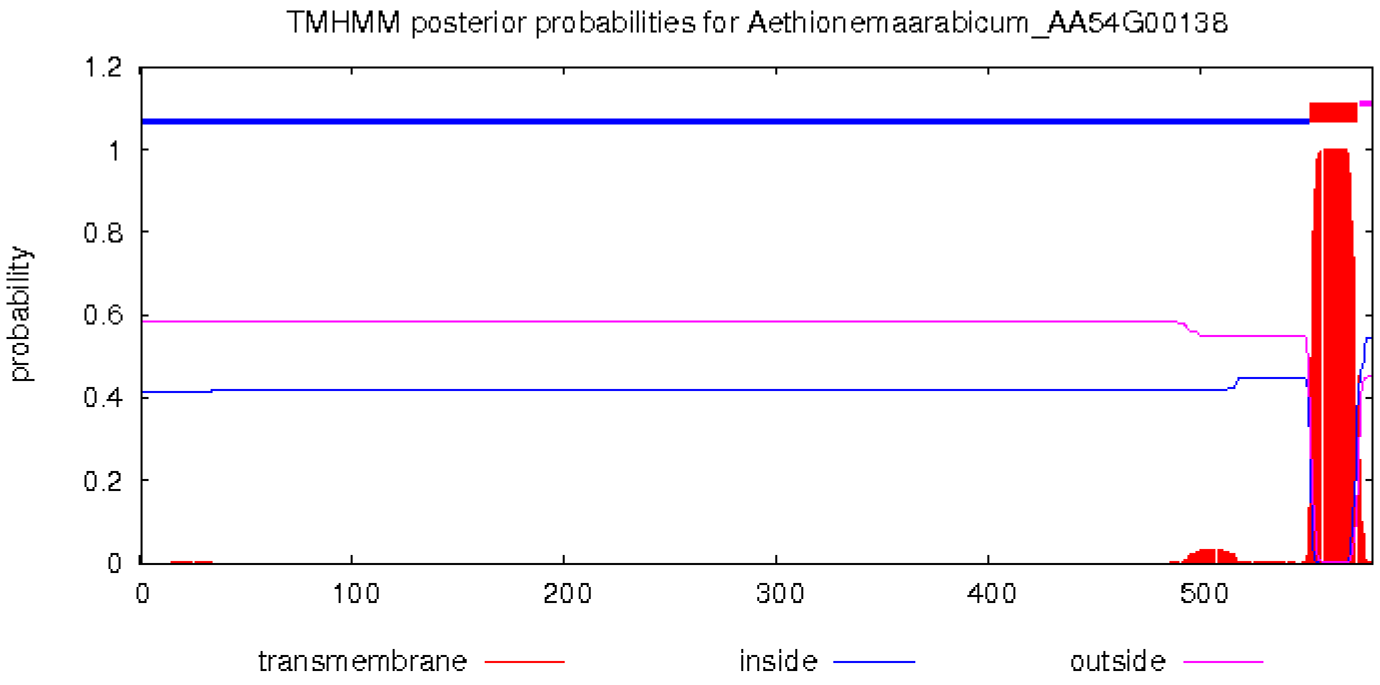

plot in postscript, script for making the plot in gnuplot, data for plot

---

```
# Aethionemaarabicum_AA54G00190 Length: 327
# Aethionemaarabicum_AA54G00190 Number of predicted TMHs:  0
# Aethionemaarabicum_AA54G00190 Exp number of AAs in TMHs: 0.00106
# Aethionemaarabicum_AA54G00190 Exp number, first 60 AAs:  0
# Aethionemaarabicum_AA54G00190 Total prob of N-in:        0.00421
Aethionemaarabicum_AA54G00190   TMHMM2.0        outside      1   327
```

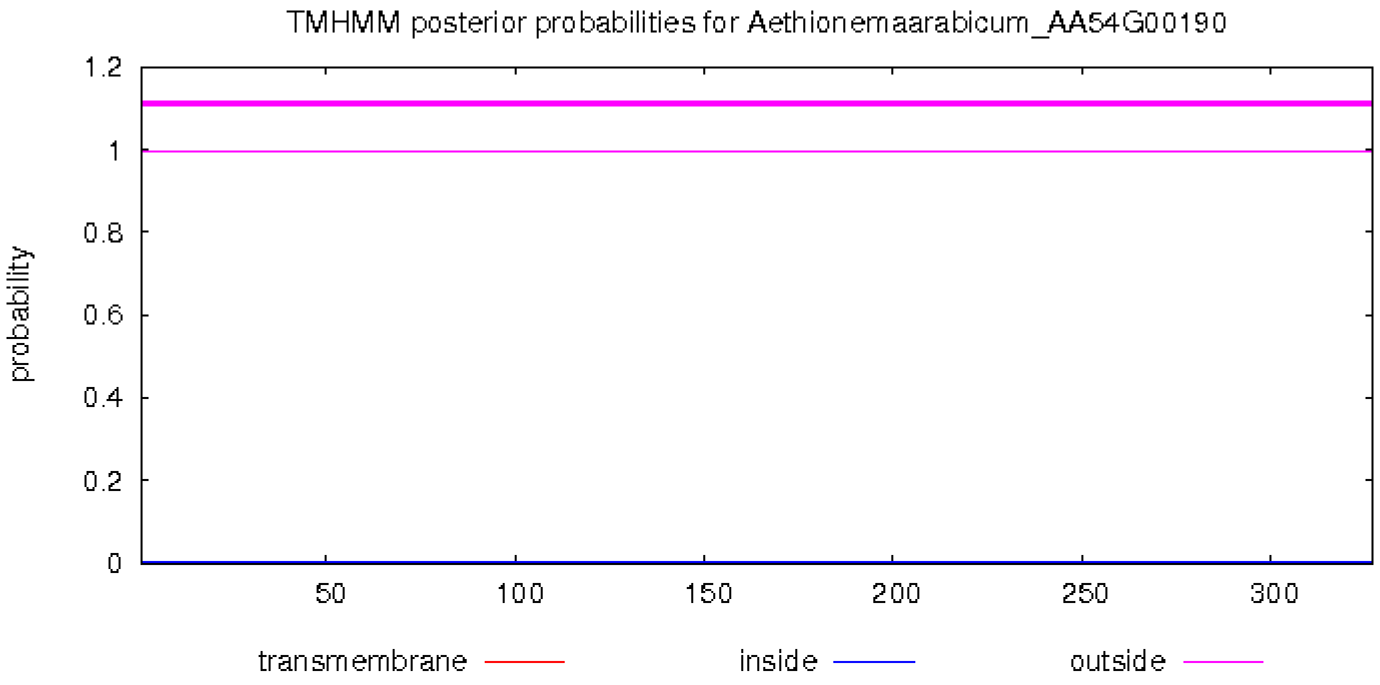

plot in postscript, script for making the plot in gnuplot, data for plot

---

```
# Aethionemaarabicum_AA54G00203 Length: 596
# Aethionemaarabicum_AA54G00203 Number of predicted TMHs:  1
# Aethionemaarabicum_AA54G00203 Exp number of AAs in TMHs: 21.06974
# Aethionemaarabicum_AA54G00203 Exp number, first 60 AAs:  0
```

```
# Aethionemaarabicum_AA54G00203 Total prob of N-in:        0.11423
Aethionemaarabicum_AA54G00203   TMHMM2.0        outside      1   550
Aethionemaarabicum_AA54G00203   TMHMM2.0        TMhelix    551   573
Aethionemaarabicum_AA54G00203   TMHMM2.0        inside     574   596
```

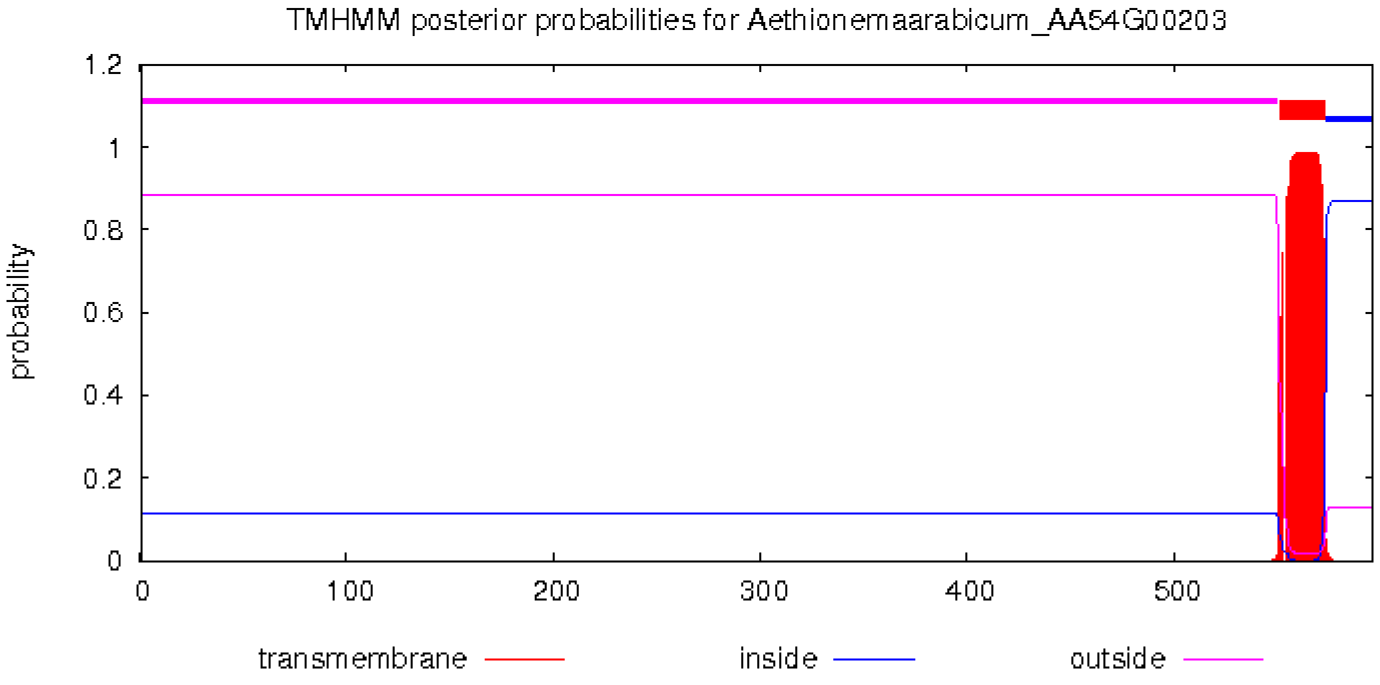

plot in postscript, script for making the plot in gnuplot, data for plot

---

```
# Aethionemaarabicum_AA54G00226 Length: 348
# Aethionemaarabicum_AA54G00226 Number of predicted TMHs:  0
# Aethionemaarabicum_AA54G00226 Exp number of AAs in TMHs: 0.0004
# Aethionemaarabicum_AA54G00226 Exp number, first 60 AAs:  0.00018
# Aethionemaarabicum_AA54G00226 Total prob of N-in:        0.07820
Aethionemaarabicum_AA54G00226   TMHMM2.0        outside      1   348
```

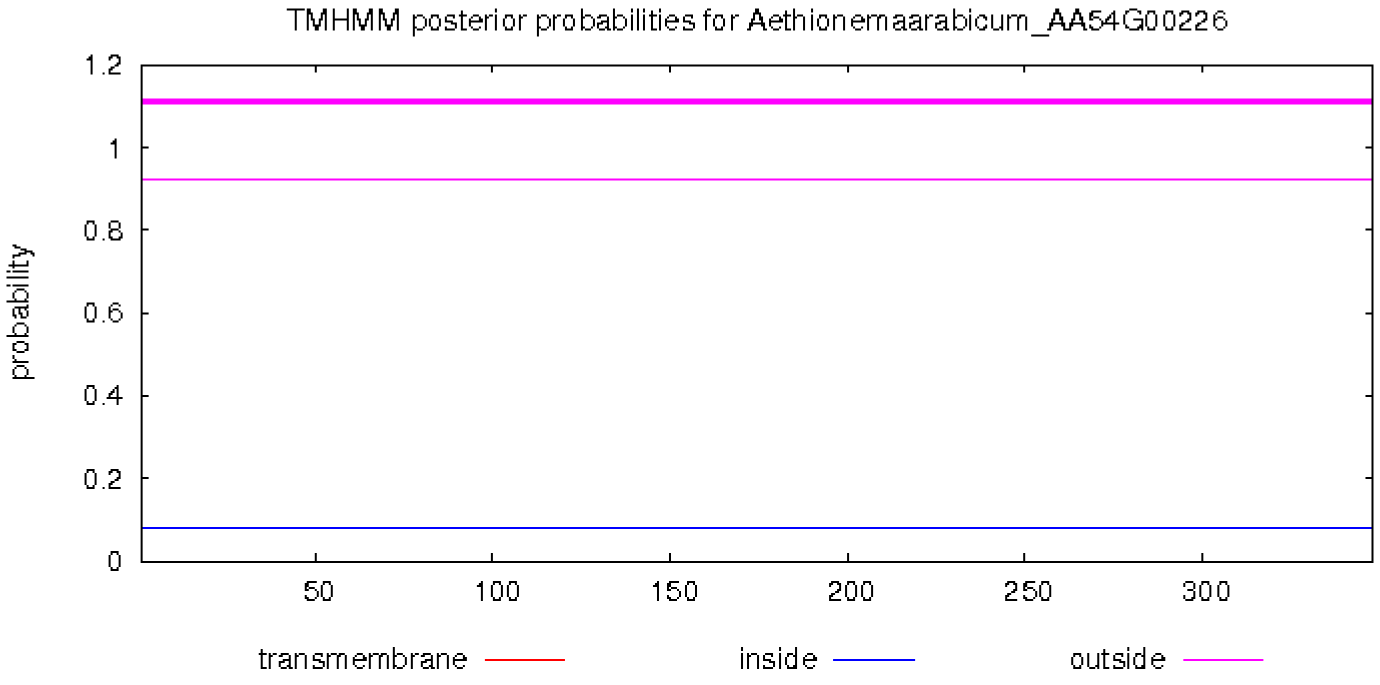

plot in postscript, script for making the plot in gnuplot, data for plot

---

```
# Aethionemaarabicum_AA56G00025 Length: 283
# Aethionemaarabicum_AA56G00025 Number of predicted TMHs:  0
```

```
# Aethionemaarabicum_AA56G00025 Exp number of AAs in TMHs: 0
# Aethionemaarabicum_AA56G00025 Exp number, first 60 AAs:  0
# Aethionemaarabicum_AA56G00025 Total prob of N-in:        0.00244
Aethionemaarabicum_AA56G00025	TMHMM2.0	outside	     1   283
```

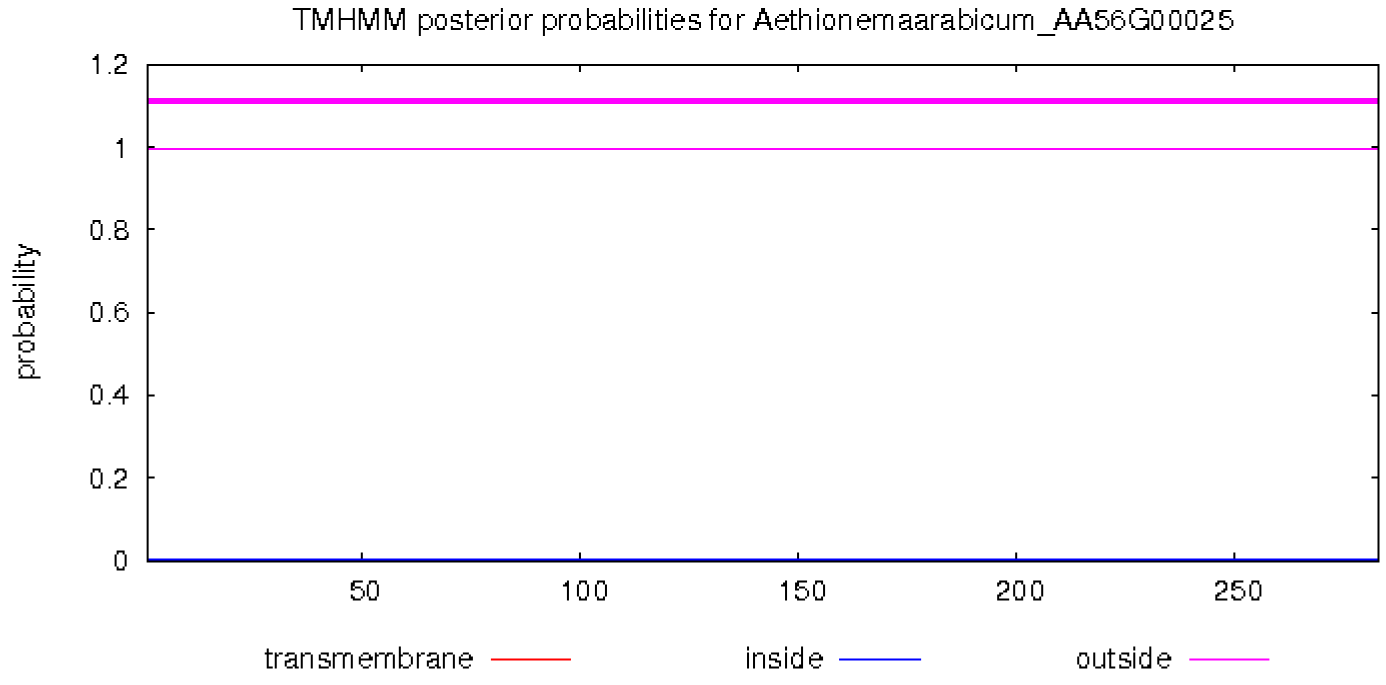

plot in postscript, script for making the plot in gnuplot, data for plot

---

```
# Aethionemaarabicum_AA5G00075 Length: 337
# Aethionemaarabicum_AA5G00075 Number of predicted TMHs:  0
# Aethionemaarabicum_AA5G00075 Exp number of AAs in TMHs: 0.00018
# Aethionemaarabicum_AA5G00075 Exp number, first 60 AAs:  0
# Aethionemaarabicum_AA5G00075 Total prob of N-in:        0.00365
Aethionemaarabicum_AA5G00075	TMHMM2.0	outside	     1   337
```

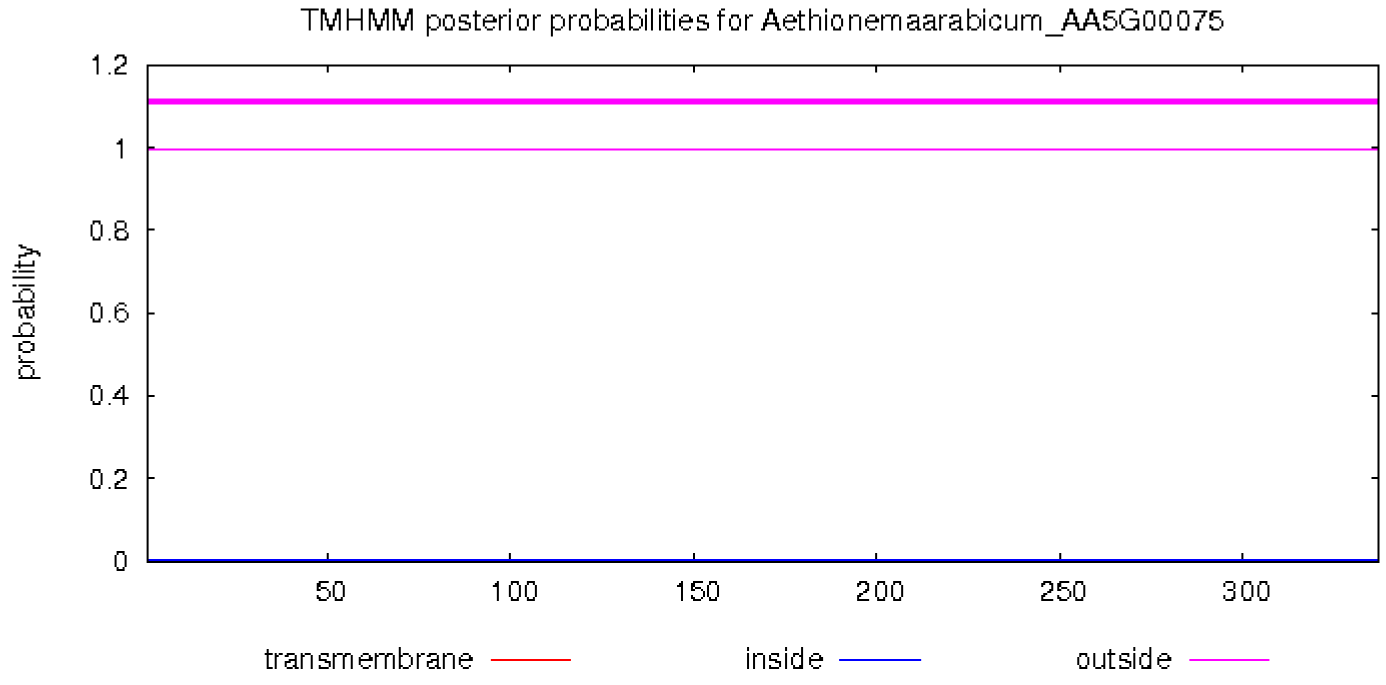

plot in postscript, script for making the plot in gnuplot, data for plot

---

```
# Aethionemaarabicum_AA5G00148 Length: 331
# Aethionemaarabicum_AA5G00148 Number of predicted TMHs:  0
```

```
# Aethionemaarabicum_AA5G00148 Exp number of AAs in TMHs: 0.00042
# Aethionemaarabicum_AA5G00148 Exp number, first 60 AAs:  0
# Aethionemaarabicum_AA5G00148 Total prob of N-in:        0.01099
Aethionemaarabicum_AA5G00148	TMHMM2.0	outside	     1   331
```

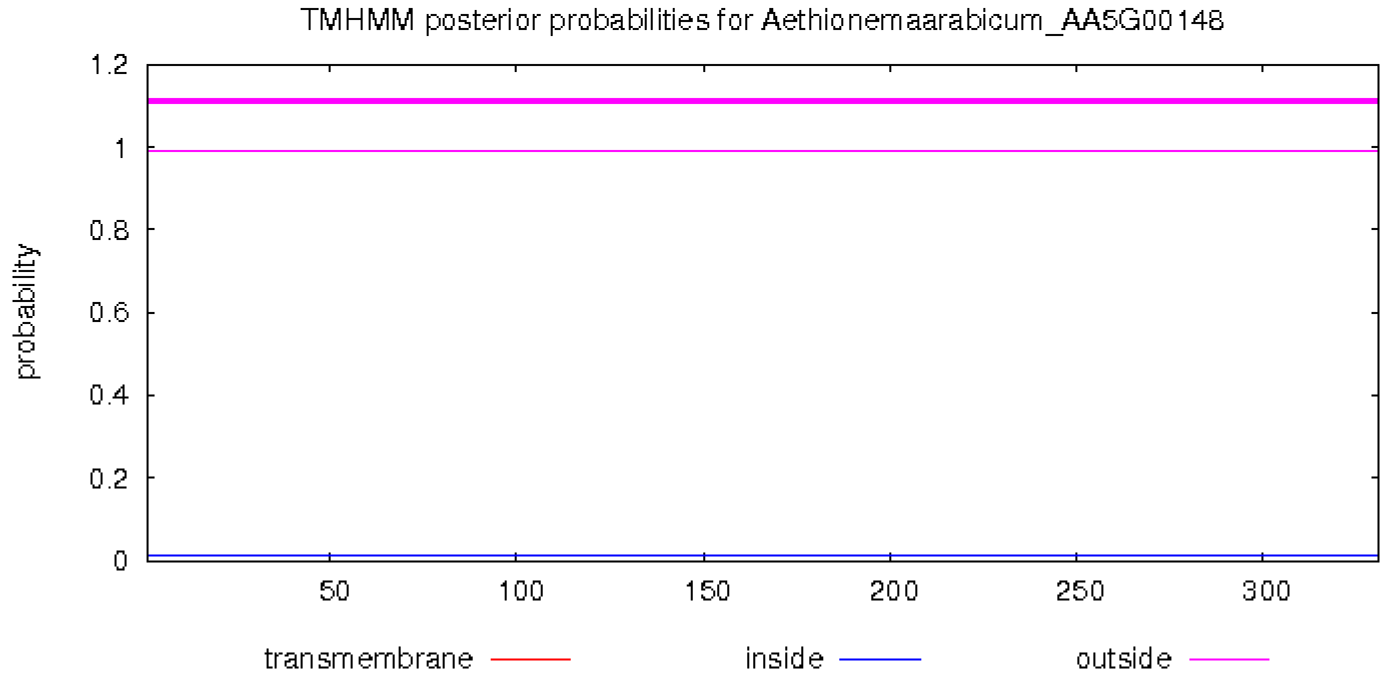

plot in postscript, script for making the plot in gnuplot, data for plot

---

```
# Aethionemaarabicum_AA61G00547 Length: 324
# Aethionemaarabicum_AA61G00547 Number of predicted TMHs:  0
# Aethionemaarabicum_AA61G00547 Exp number of AAs in TMHs: 0.00036
# Aethionemaarabicum_AA61G00547 Exp number, first 60 AAs:  0
# Aethionemaarabicum_AA61G00547 Total prob of N-in:        0.03110
Aethionemaarabicum_AA61G00547	TMHMM2.0	outside	     1   324
```

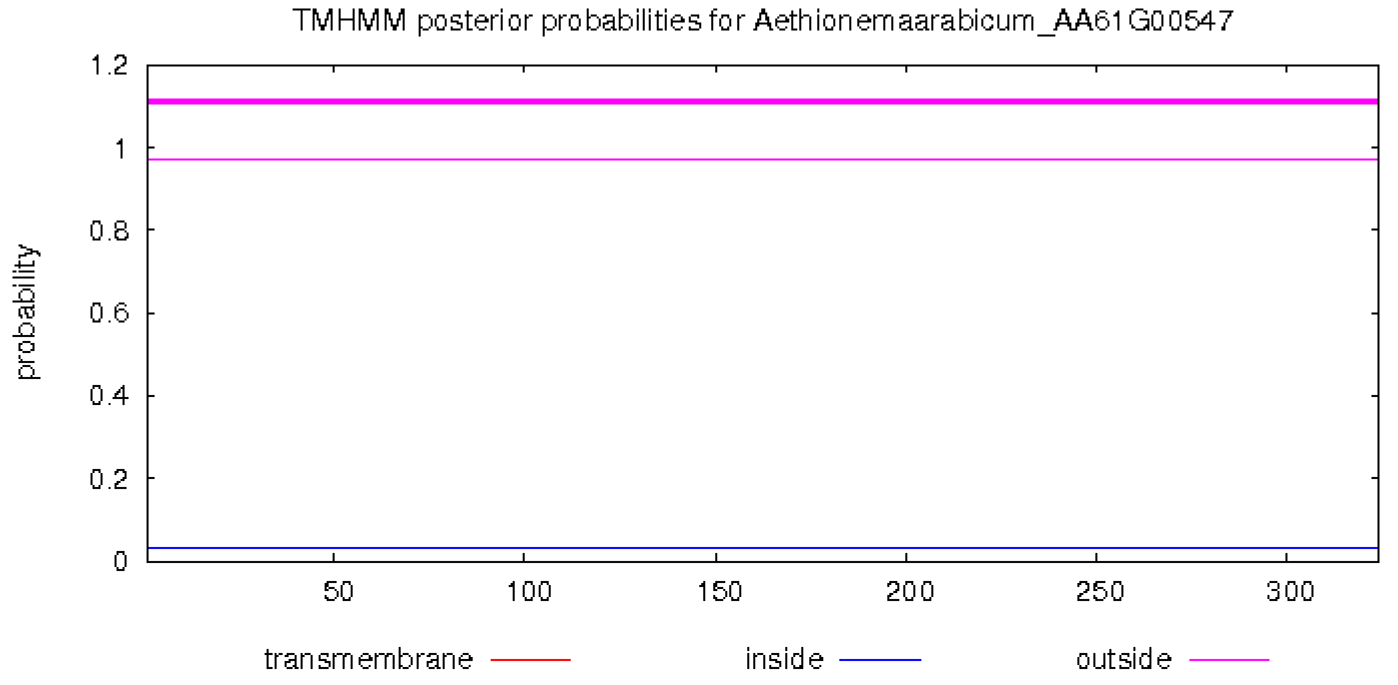

plot in postscript, script for making the plot in gnuplot, data for plot

---

```
# Aethionemaarabicum_AA65G00271 Length: 380
# Aethionemaarabicum_AA65G00271 Number of predicted TMHs:  0
```

```
# Aethionemaarabicum_AA65G00271 Exp number of AAs in TMHs: 0.00018
# Aethionemaarabicum_AA65G00271 Exp number, first 60 AAs:  0
# Aethionemaarabicum_AA65G00271 Total prob of N-in:        0.00201
Aethionemaarabicum_AA65G00271   TMHMM2.0        outside      1   380
```

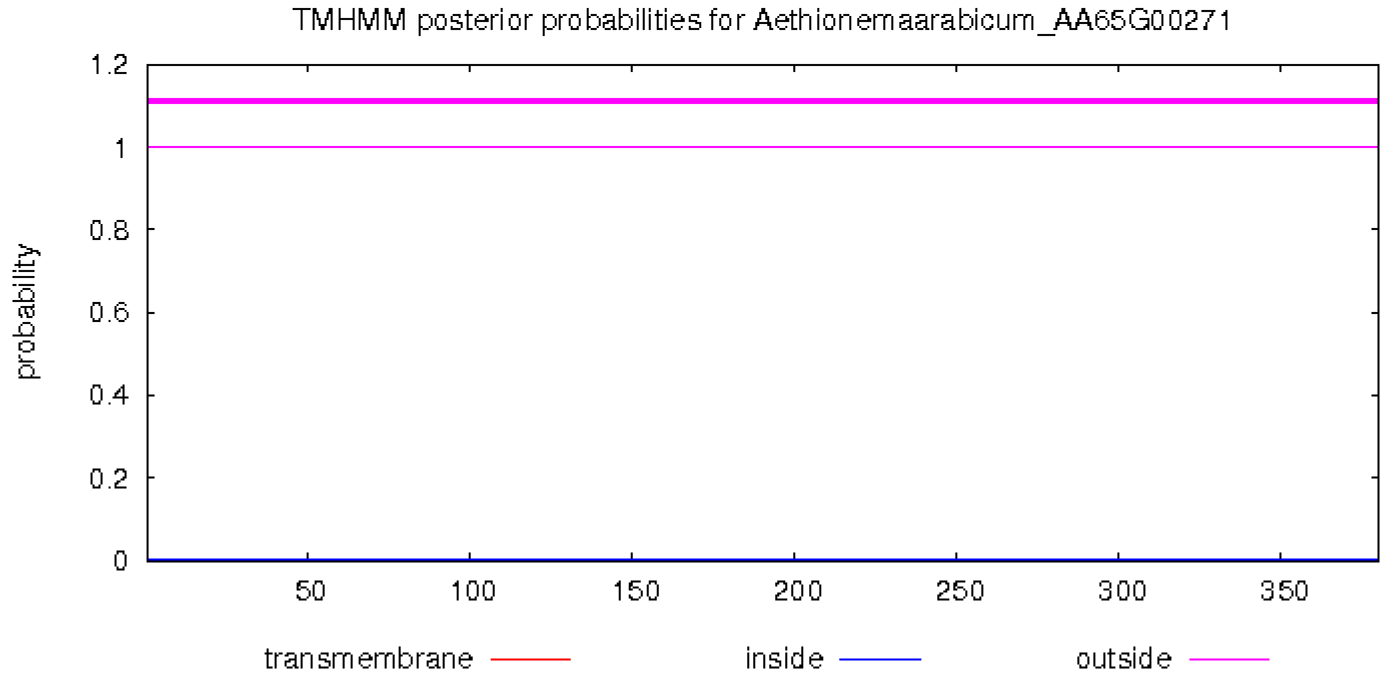

plot in postscript, script for making the plot in gnuplot, data for plot

---

```
# Aethionemaarabicum_AA87G00007 Length: 569
# Aethionemaarabicum_AA87G00007 Number of predicted TMHs:  0
# Aethionemaarabicum_AA87G00007 Exp number of AAs in TMHs: 0.34811
# Aethionemaarabicum_AA87G00007 Exp number, first 60 AAs:  0
# Aethionemaarabicum_AA87G00007 Total prob of N-in:        0.01002
Aethionemaarabicum_AA87G00007   TMHMM2.0        outside      1   569
```

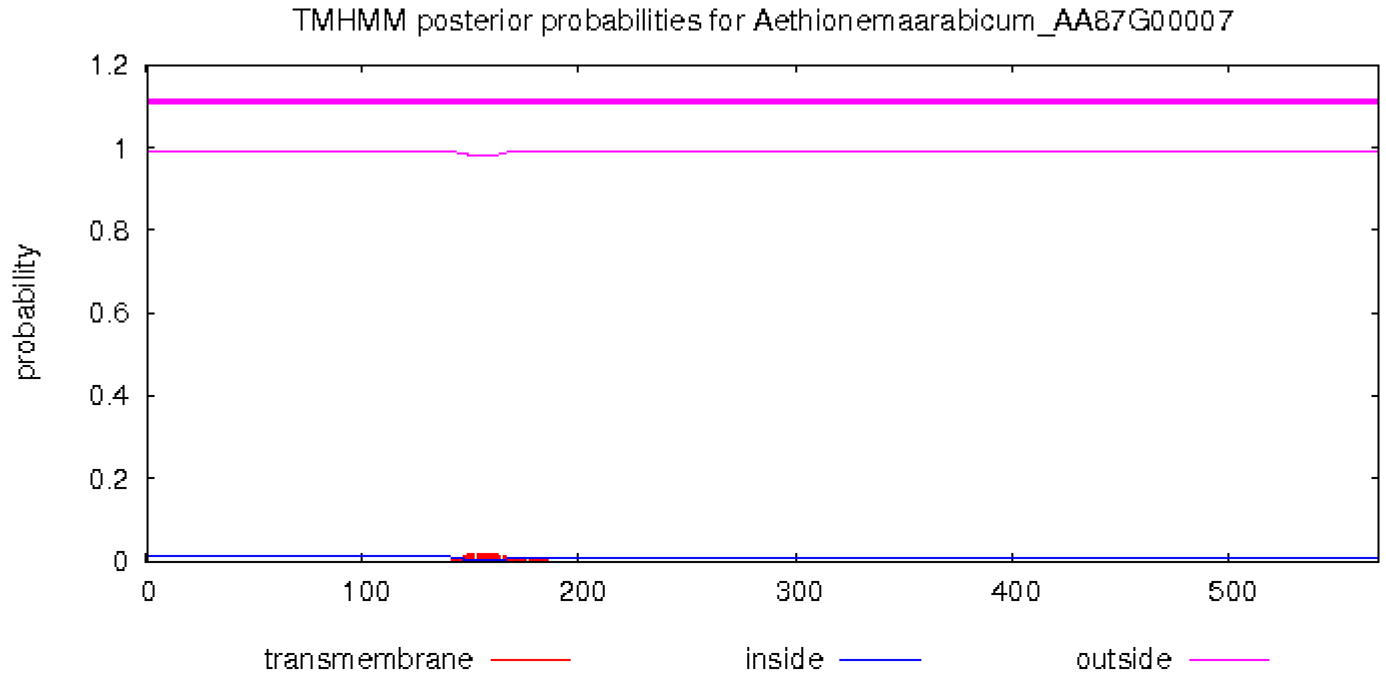

plot in postscript, script for making the plot in gnuplot, data for plot

---

```
# Aethionemaarabicum_AA87G00083 Length: 222
# Aethionemaarabicum_AA87G00083 Number of predicted TMHs:  0
```

```
# Aethionemaarabicum_AA87G00083 Exp number of AAs in TMHs: 0
# Aethionemaarabicum_AA87G00083 Exp number, first 60 AAs:  0
# Aethionemaarabicum_AA87G00083 Total prob of N-in:        0.09709
Aethionemaarabicum_AA87G00083	TMHMM2.0	outside	     1   222
```

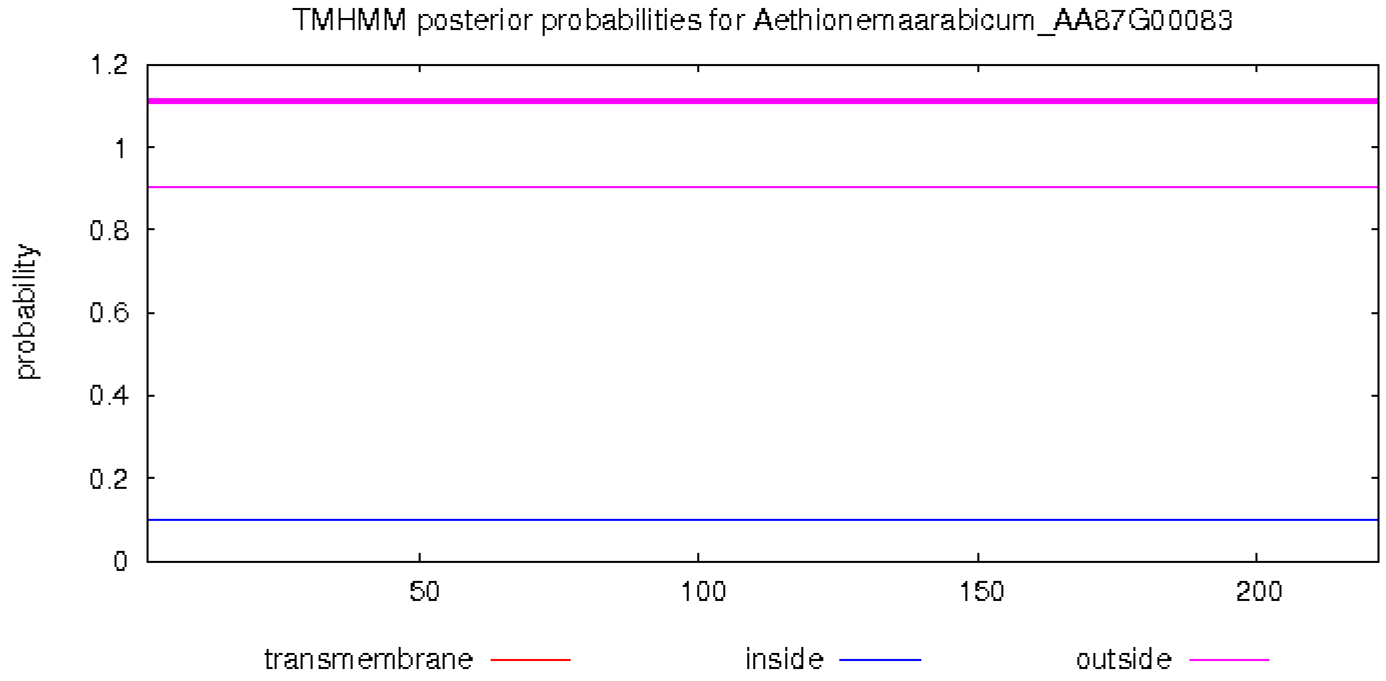

plot in postscript, script for making the plot in gnuplot, data for plot

---

```
# Aethionemaarabicum_AA8G00098 Length: 273
# Aethionemaarabicum_AA8G00098 Number of predicted TMHs:  0
# Aethionemaarabicum_AA8G00098 Exp number of AAs in TMHs: 0
# Aethionemaarabicum_AA8G00098 Exp number, first 60 AAs:  0
# Aethionemaarabicum_AA8G00098 Total prob of N-in:        0.02763
Aethionemaarabicum_AA8G00098	TMHMM2.0	outside	     1   273
```

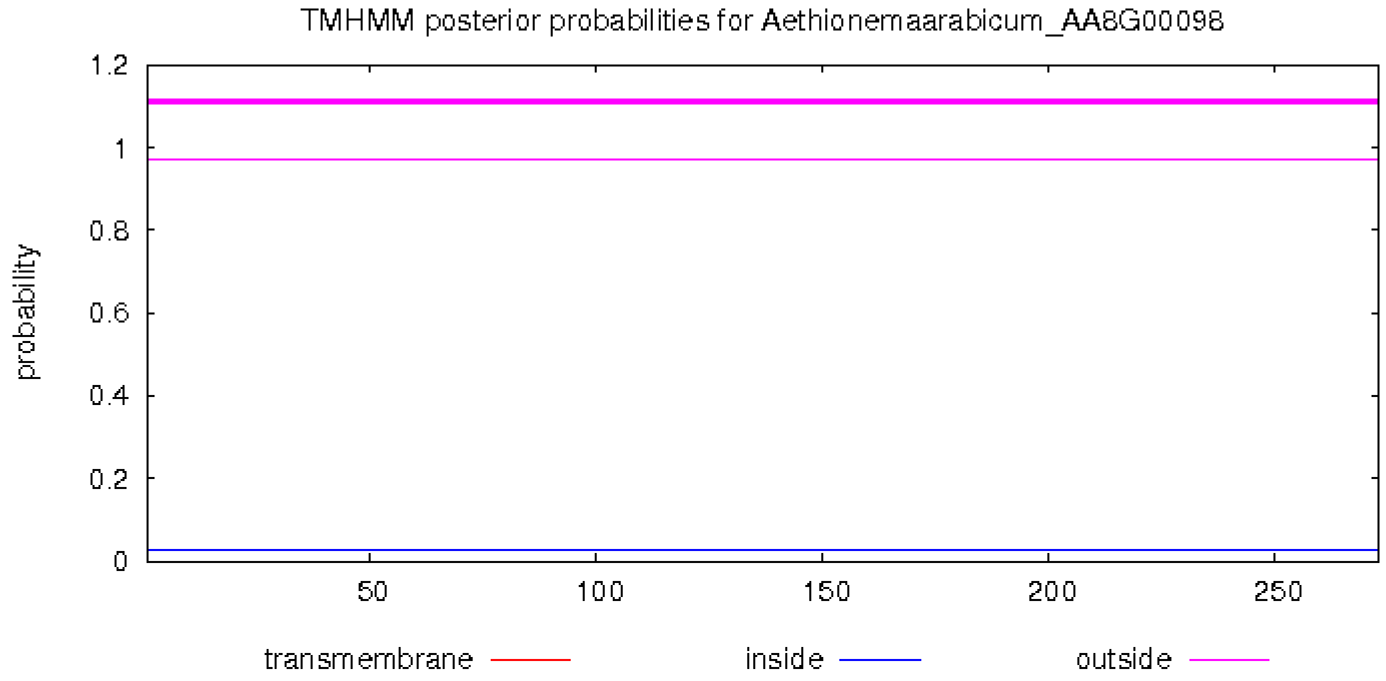

plot in postscript, script for making the plot in gnuplot, data for plot

---

```
# Aethionemaarabicum_AA8G00264 Length: 243
# Aethionemaarabicum_AA8G00264 Number of predicted TMHs:  0
```

```
# Aethionemaarabicum_AA8G00264 Exp number of AAs in TMHs: 0.00035
# Aethionemaarabicum_AA8G00264 Exp number, first 60 AAs:  0
# Aethionemaarabicum_AA8G00264 Total prob of N-in:        0.01097
Aethionemaarabicum_AA8G00264    TMHMM2.0        outside      1   243
```

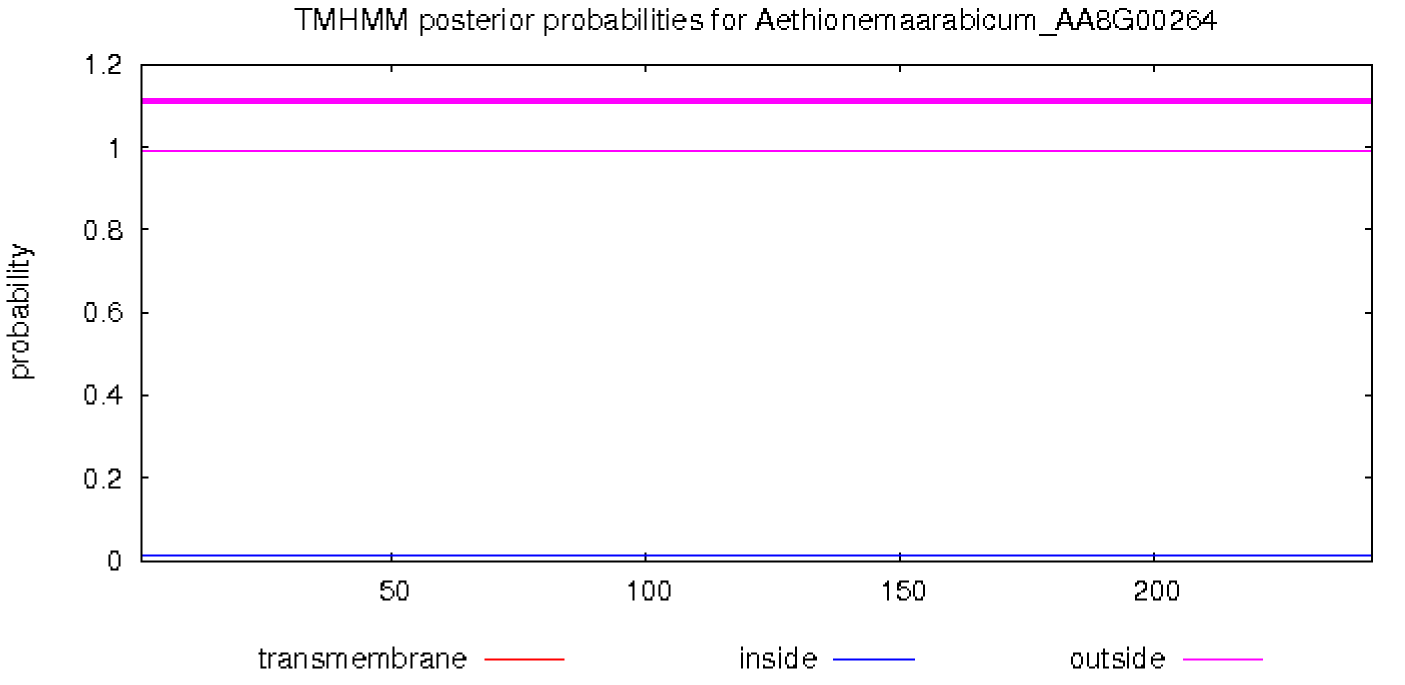

plot in postscript, script for making the plot in gnuplot, data for plot

---

```
# Aethionemaarabicum_AA93G00231 Length: 1013
# Aethionemaarabicum_AA93G00231 Number of predicted TMHs:  1
# Aethionemaarabicum_AA93G00231 Exp number of AAs in TMHs: 23.59873
# Aethionemaarabicum_AA93G00231 Exp number, first 60 AAs:  0.68377
# Aethionemaarabicum_AA93G00231 Total prob of N-in:        0.03071
Aethionemaarabicum_AA93G00231   TMHMM2.0        outside      1   987
Aethionemaarabicum_AA93G00231   TMHMM2.0        TMhelix    988  1010
Aethionemaarabicum_AA93G00231   TMHMM2.0        inside    1011  1013
```

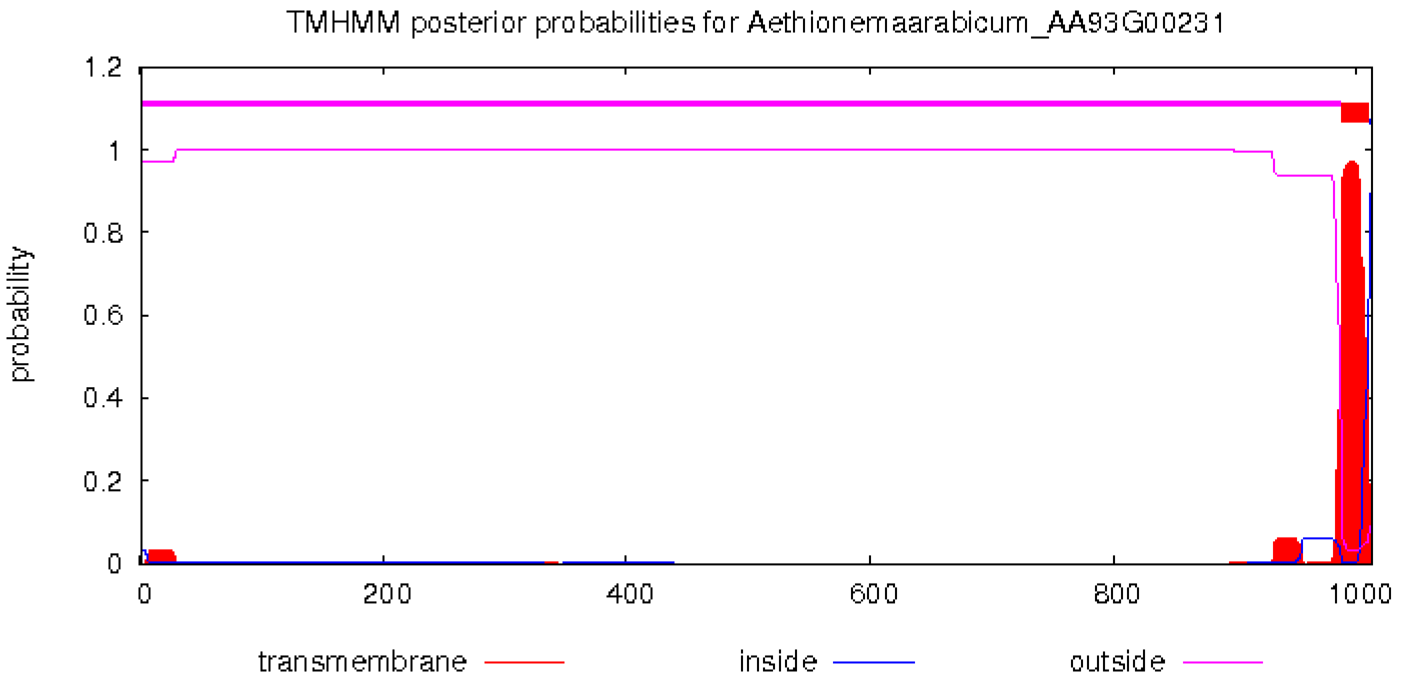

plot in postscript, script for making the plot in gnuplot, data for plot

---

```
# Aethionemaarabicum_AA9G00035 Length: 287
# Aethionemaarabicum_AA9G00035 Number of predicted TMHs:  0
# Aethionemaarabicum_AA9G00035 Exp number of AAs in TMHs: 0.01023
# Aethionemaarabicum_AA9G00035 Exp number, first 60 AAs:  0.00018
# Aethionemaarabicum_AA9G00035 Total prob of N-in:        0.02699
Aethionemaarabicum_AA9G00035    TMHMM2.0        outside      1   287
```

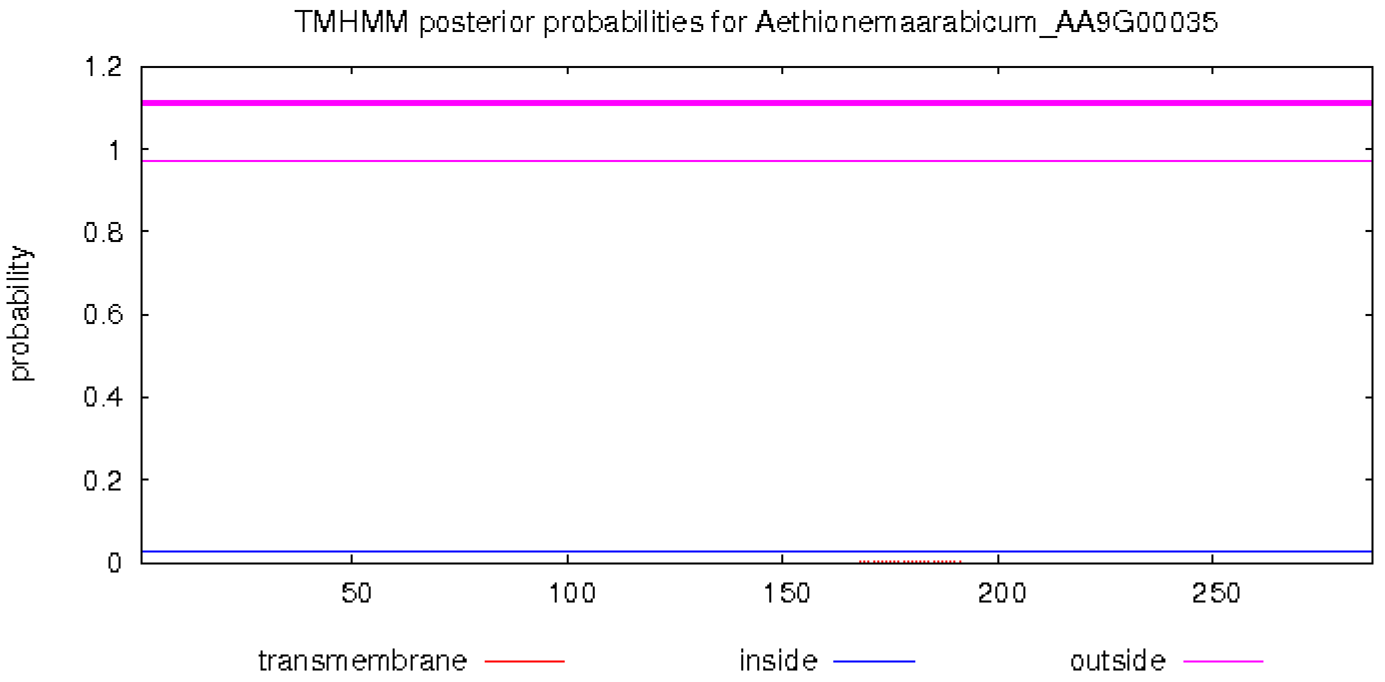

plot in postscript, script for making the plot in gnuplot, data for plot

---

```
# Aethionemaarabicum_AA9G00047 Length: 307
# Aethionemaarabicum_AA9G00047 Number of predicted TMHs:  0
# Aethionemaarabicum_AA9G00047 Exp number of AAs in TMHs: 0.05293
# Aethionemaarabicum_AA9G00047 Exp number, first 60 AAs:  0
# Aethionemaarabicum_AA9G00047 Total prob of N-in:        0.03952
Aethionemaarabicum_AA9G00047    TMHMM2.0        outside      1   307
```

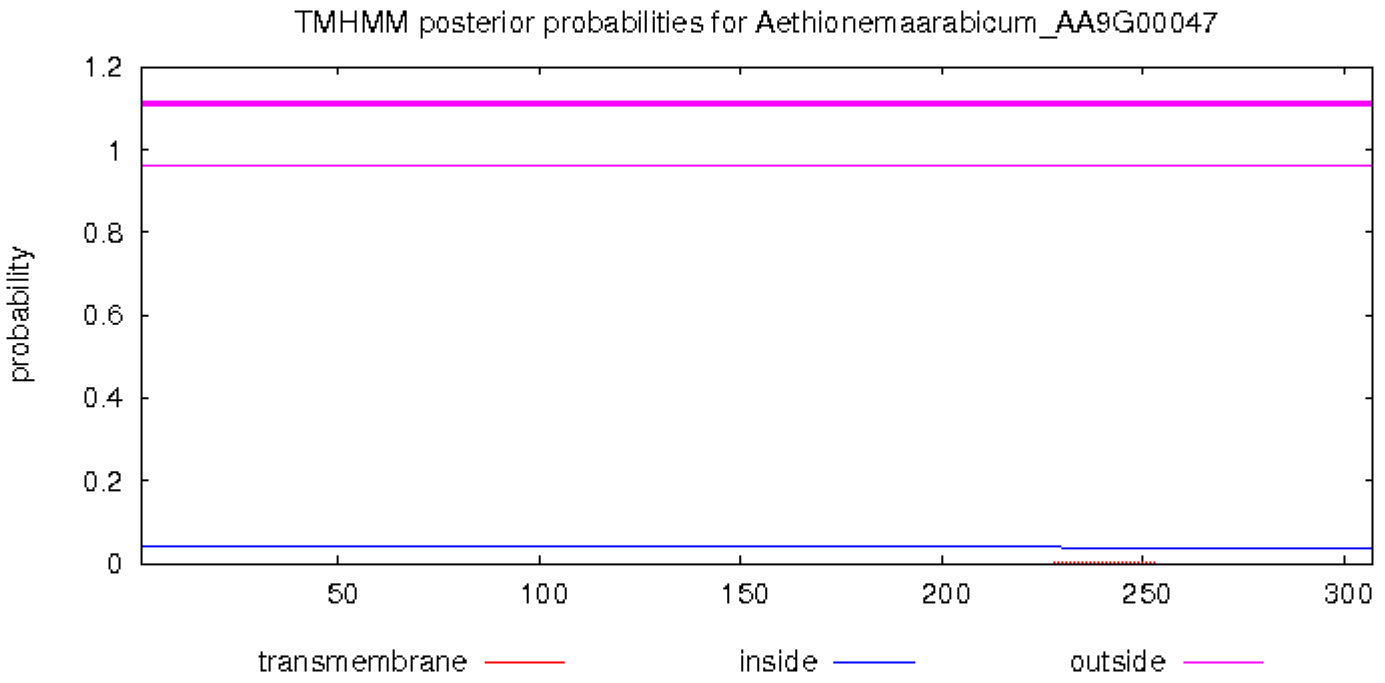

plot in postscript, script for making the plot in gnuplot, data for plot

---

```
# Amaranthushypochondriacus_AHYPO_001248 Length: 299
# Amaranthushypochondriacus_AHYPO_001248 Number of predicted TMHs:  0
# Amaranthushypochondriacus_AHYPO_001248 Exp number of AAs in TMHs: 0.00055
# Amaranthushypochondriacus_AHYPO_001248 Exp number, first 60 AAs:  0
# Amaranthushypochondriacus_AHYPO_001248 Total prob of N-in:        0.09878
Amaranthushypochondriacus_AHYPO_001248  TMHMM2.0        outside      1   299
```

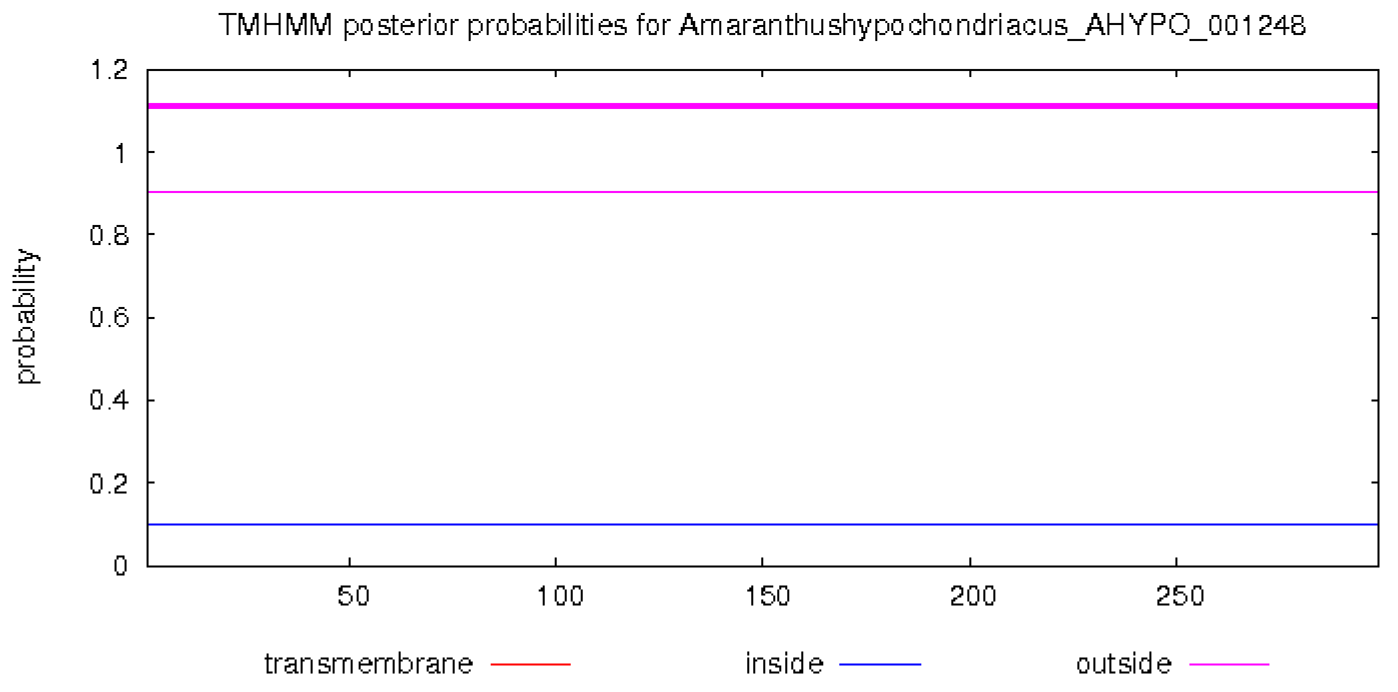

plot in postscript, script for making the plot in gnuplot, data for plot

---

```
# Amaranthushypochondriacus_AHYPO_001663 Length: 307
# Amaranthushypochondriacus_AHYPO_001663 Number of predicted TMHs:  0
# Amaranthushypochondriacus_AHYPO_001663 Exp number of AAs in TMHs: 0.00046
# Amaranthushypochondriacus_AHYPO_001663 Exp number, first 60 AAs:  0
# Amaranthushypochondriacus_AHYPO_001663 Total prob of N-in:        0.05020
Amaranthushypochondriacus_AHYPO_001663  TMHMM2.0        outside      1   307
```

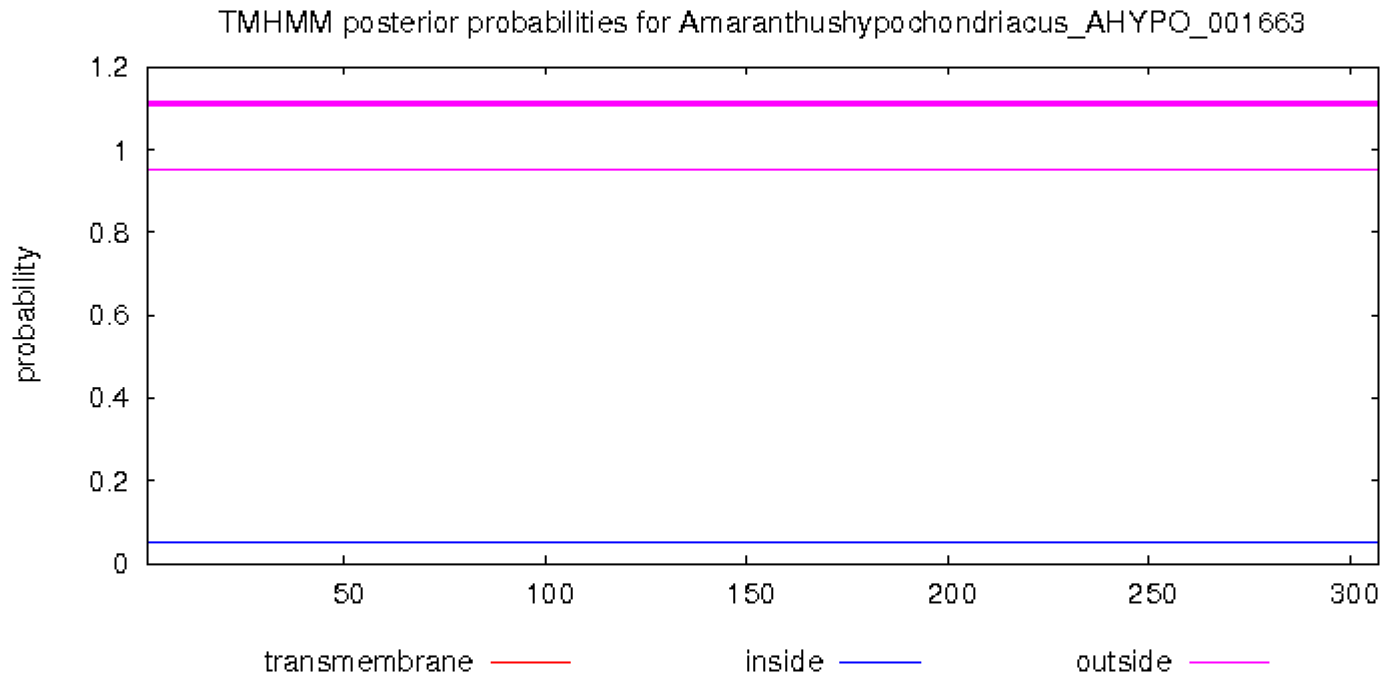

plot in postscript, script for making the plot in gnuplot, data for plot

---

```
# Amaranthushypochondriacus_AHYPO_001938 Length: 333
# Amaranthushypochondriacus_AHYPO_001938 Number of predicted TMHs:  0
# Amaranthushypochondriacus_AHYPO_001938 Exp number of AAs in TMHs: 0.00018
# Amaranthushypochondriacus_AHYPO_001938 Exp number, first 60 AAs:  0
# Amaranthushypochondriacus_AHYPO_001938 Total prob of N-in:        0.01906
Amaranthushypochondriacus_AHYPO_001938  TMHMM2.0        outside      1   333
```

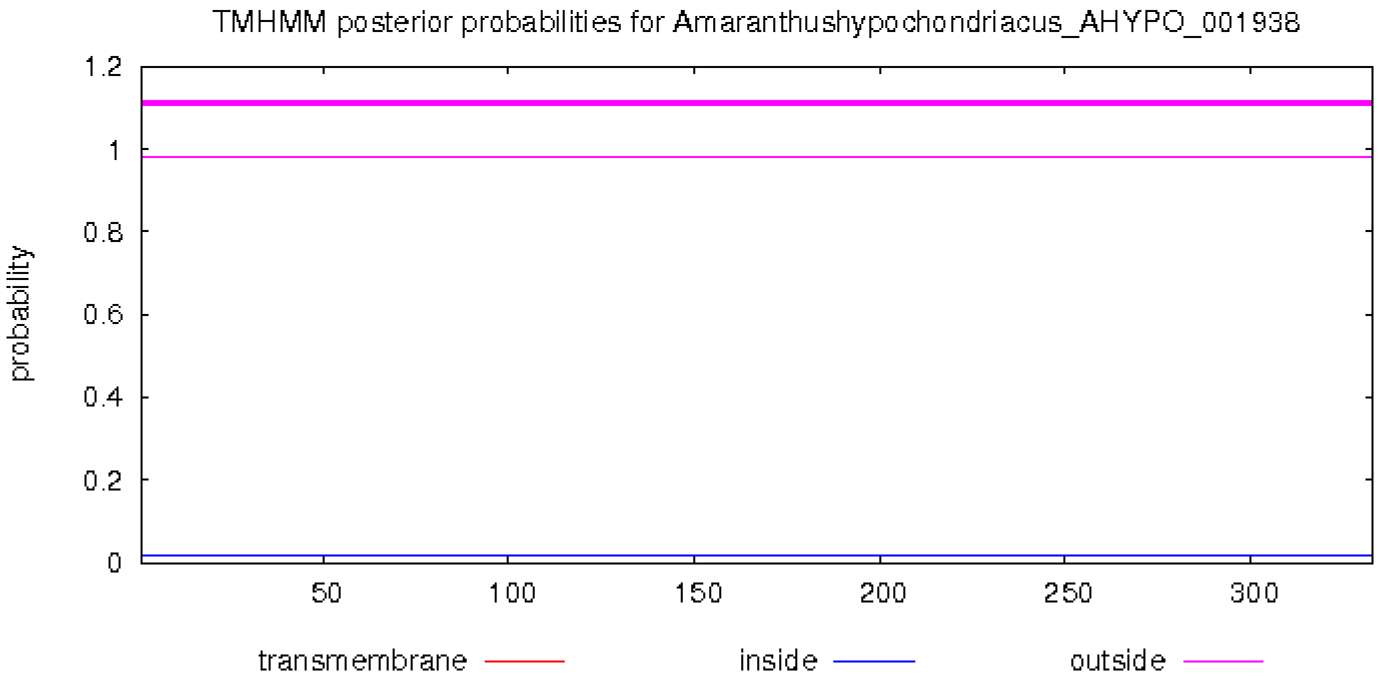

plot in postscript, script for making the plot in gnuplot, data for plot

---

```
# Amaranthushypochondriacus_AHYPO_002189 Length: 478
# Amaranthushypochondriacus_AHYPO_002189 Number of predicted TMHs:  1
# Amaranthushypochondriacus_AHYPO_002189 Exp number of AAs in TMHs: 22.5485
# Amaranthushypochondriacus_AHYPO_002189 Exp number, first 60 AAs:  0
# Amaranthushypochondriacus_AHYPO_002189 Total prob of N-in:        0.00801
Amaranthushypochondriacus_AHYPO_002189  TMHMM2.0        outside      1   445
Amaranthushypochondriacus_AHYPO_002189  TMHMM2.0        TMhelix    446   468
Amaranthushypochondriacus_AHYPO_002189  TMHMM2.0        inside     469   478
```

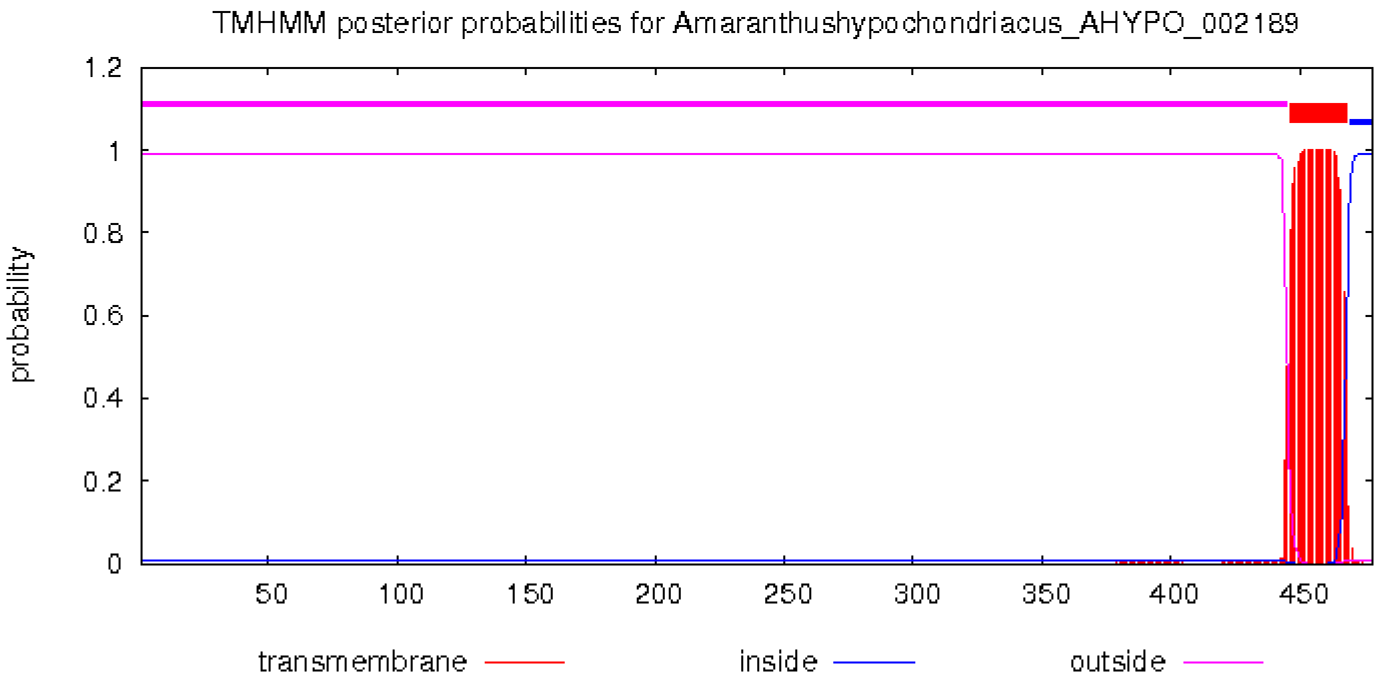

plot in postscript, script for making the plot in gnuplot, data for plot

```
# Amaranthushypochondriacus_AHYPO_002480 Length: 536
# Amaranthushypochondriacus_AHYPO_002480 Number of predicted TMHs:  1
# Amaranthushypochondriacus_AHYPO_002480 Exp number of AAs in TMHs: 20.39743
# Amaranthushypochondriacus_AHYPO_002480 Exp number, first 60 AAs:  0
# Amaranthushypochondriacus_AHYPO_002480 Total prob of N-in:        0.04867
Amaranthushypochondriacus_AHYPO_002480  TMHMM2.0        outside      1   511
Amaranthushypochondriacus_AHYPO_002480  TMHMM2.0        TMhelix    512   534
Amaranthushypochondriacus_AHYPO_002480  TMHMM2.0        inside     535   536
```

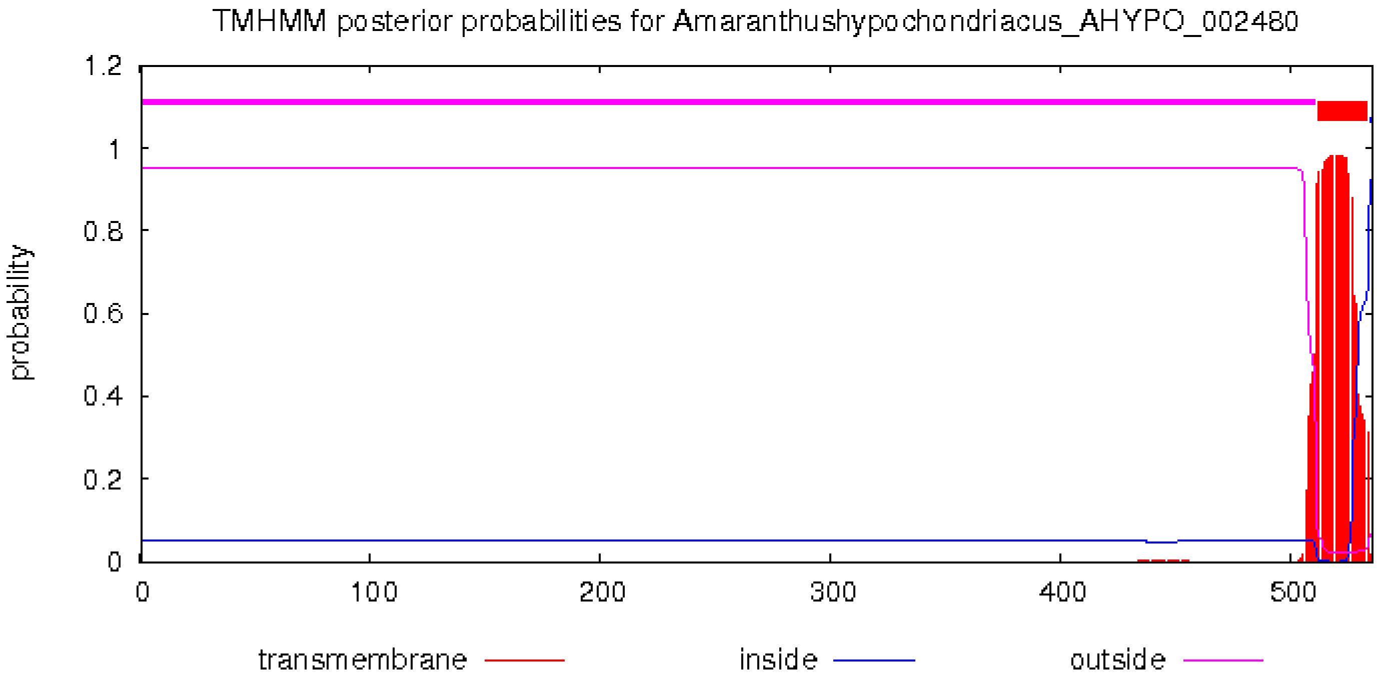

plot in postscript, script for making the plot in gnuplot, data for plot

```
# Amaranthushypochondriacus_AHYPO_003454 Length: 187
# Amaranthushypochondriacus_AHYPO_003454 Number of predicted TMHs:  0
# Amaranthushypochondriacus_AHYPO_003454 Exp number of AAs in TMHs: 5e-05
# Amaranthushypochondriacus_AHYPO_003454 Exp number, first 60 AAs:  2e-05
# Amaranthushypochondriacus_AHYPO_003454 Total prob of N-in:        0.04624
Amaranthushypochondriacus_AHYPO_003454  TMHMM2.0        outside      1   187
```

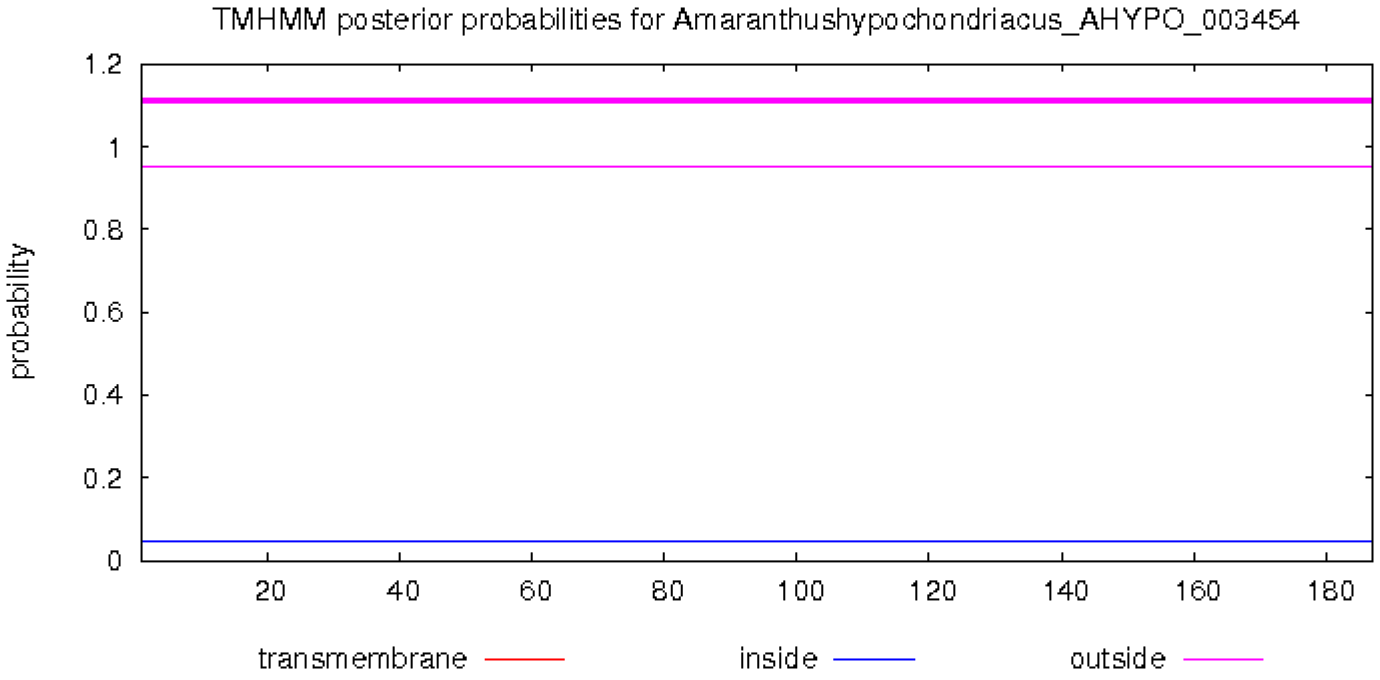

plot in postscript, script for making the plot in gnuplot, data for plot

---

```
# Amaranthushypochondriacus_AHYPO_004169 Length: 64
# Amaranthushypochondriacus_AHYPO_004169 Number of predicted TMHs:  0
# Amaranthushypochondriacus_AHYPO_004169 Exp number of AAs in TMHs: 0.00088
# Amaranthushypochondriacus_AHYPO_004169 Exp number, first 60 AAs:  0.00088
# Amaranthushypochondriacus_AHYPO_004169 Total prob of N-in:        0.50120
Amaranthushypochondriacus_AHYPO_004169  TMHMM2.0        inside       1    64
```

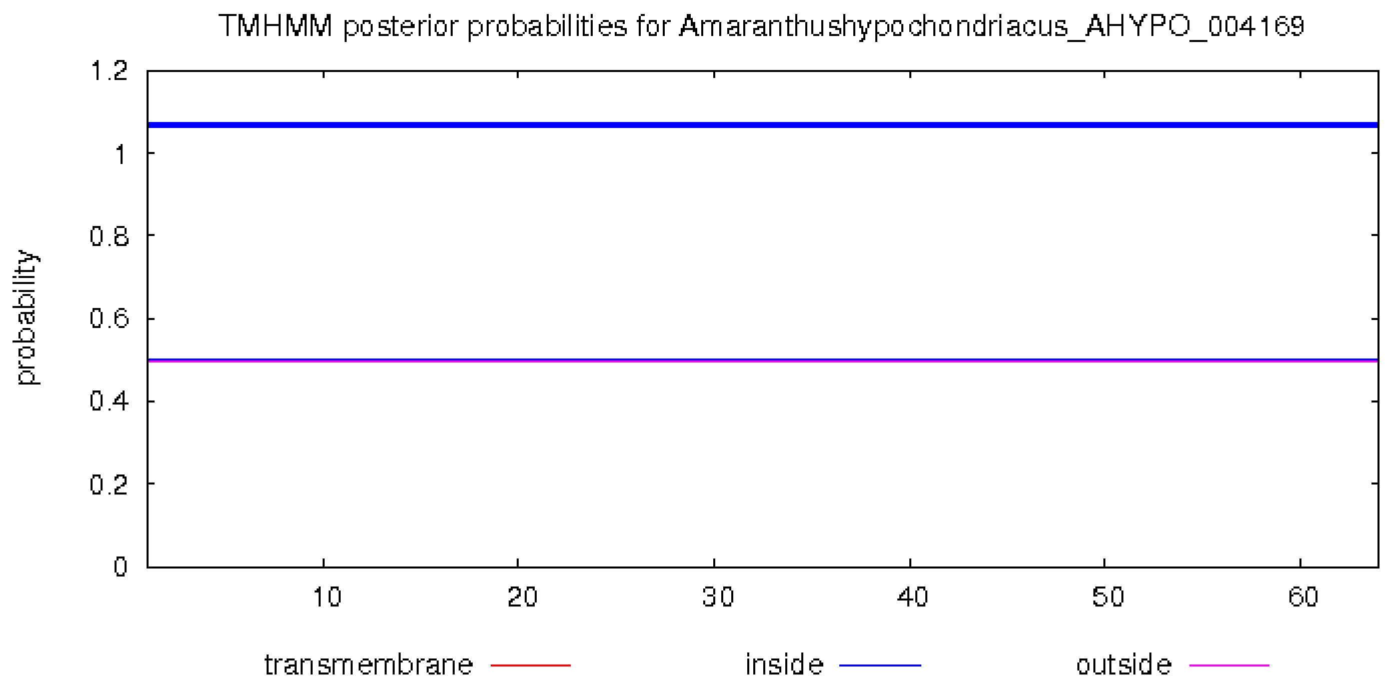

plot in postscript, script for making the plot in gnuplot, data for plot

---

```
# Amaranthushypochondriacus_AHYPO_004861 Length: 290
# Amaranthushypochondriacus_AHYPO_004861 Number of predicted TMHs:  0
# Amaranthushypochondriacus_AHYPO_004861 Exp number of AAs in TMHs: 0.00699
# Amaranthushypochondriacus_AHYPO_004861 Exp number, first 60 AAs:  0
# Amaranthushypochondriacus_AHYPO_004861 Total prob of N-in:        0.05414
Amaranthushypochondriacus_AHYPO_004861  TMHMM2.0        outside      1   290
```

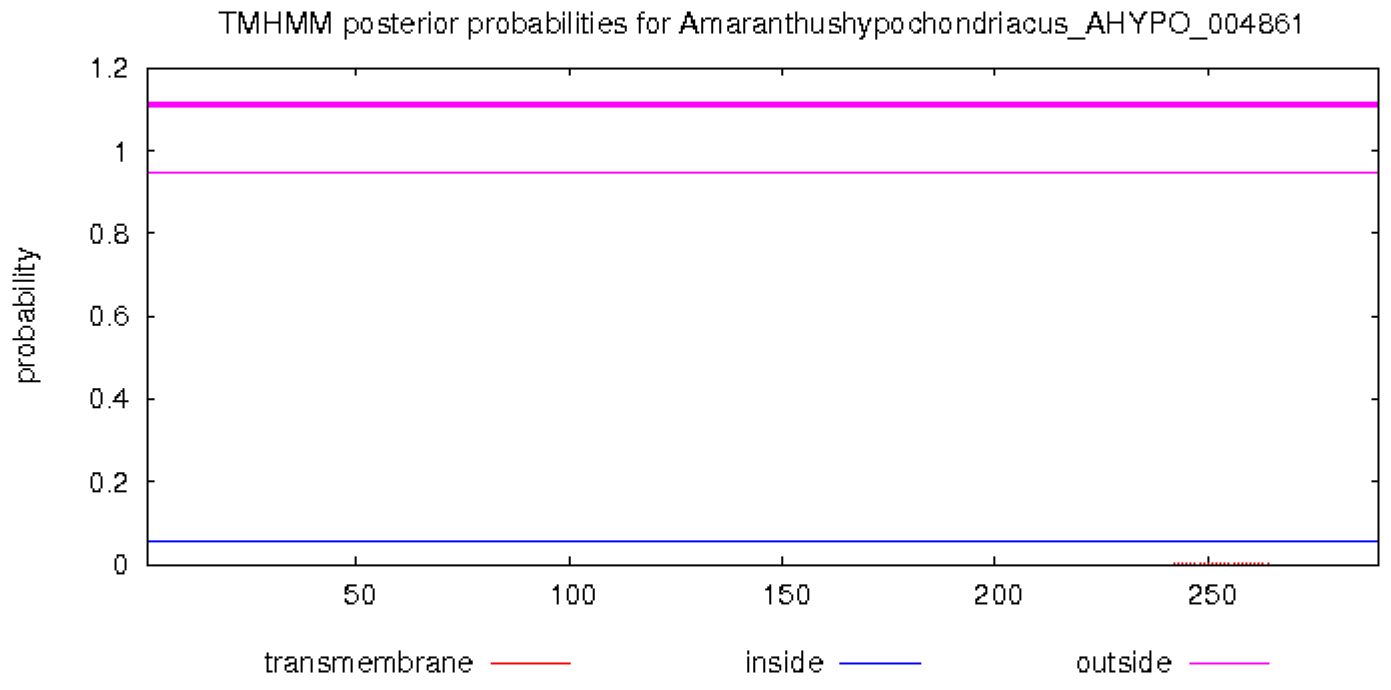

plot in postscript, script for making the plot in gnuplot, data for plot

---

```
# Amaranthushypochondriacus_AHYPO_005582 Length: 444
# Amaranthushypochondriacus_AHYPO_005582 Number of predicted TMHs:  0
# Amaranthushypochondriacus_AHYPO_005582 Exp number of AAs in TMHs: 0
# Amaranthushypochondriacus_AHYPO_005582 Exp number, first 60 AAs:  0
# Amaranthushypochondriacus_AHYPO_005582 Total prob of N-in:        0.00091
Amaranthushypochondriacus_AHYPO_005582  TMHMM2.0        outside      1   444
```

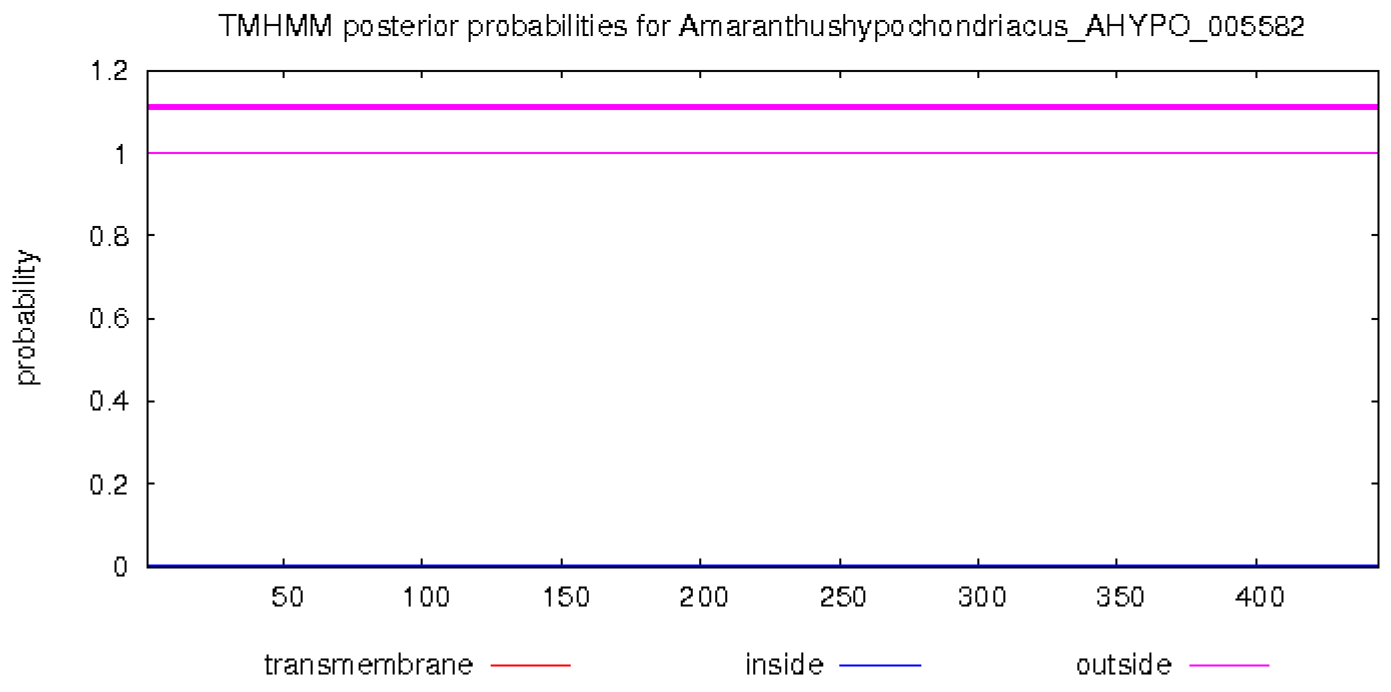

plot in postscript, script for making the plot in gnuplot, data for plot

---

```
# Amaranthushypochondriacus_AHYPO_005721 Length: 241
# Amaranthushypochondriacus_AHYPO_005721 Number of predicted TMHs:  0
# Amaranthushypochondriacus_AHYPO_005721 Exp number of AAs in TMHs: 0.04114
# Amaranthushypochondriacus_AHYPO_005721 Exp number, first 60 AAs:  0
# Amaranthushypochondriacus_AHYPO_005721 Total prob of N-in:        0.02748
Amaranthushypochondriacus_AHYPO_005721  TMHMM2.0        outside      1   241
```

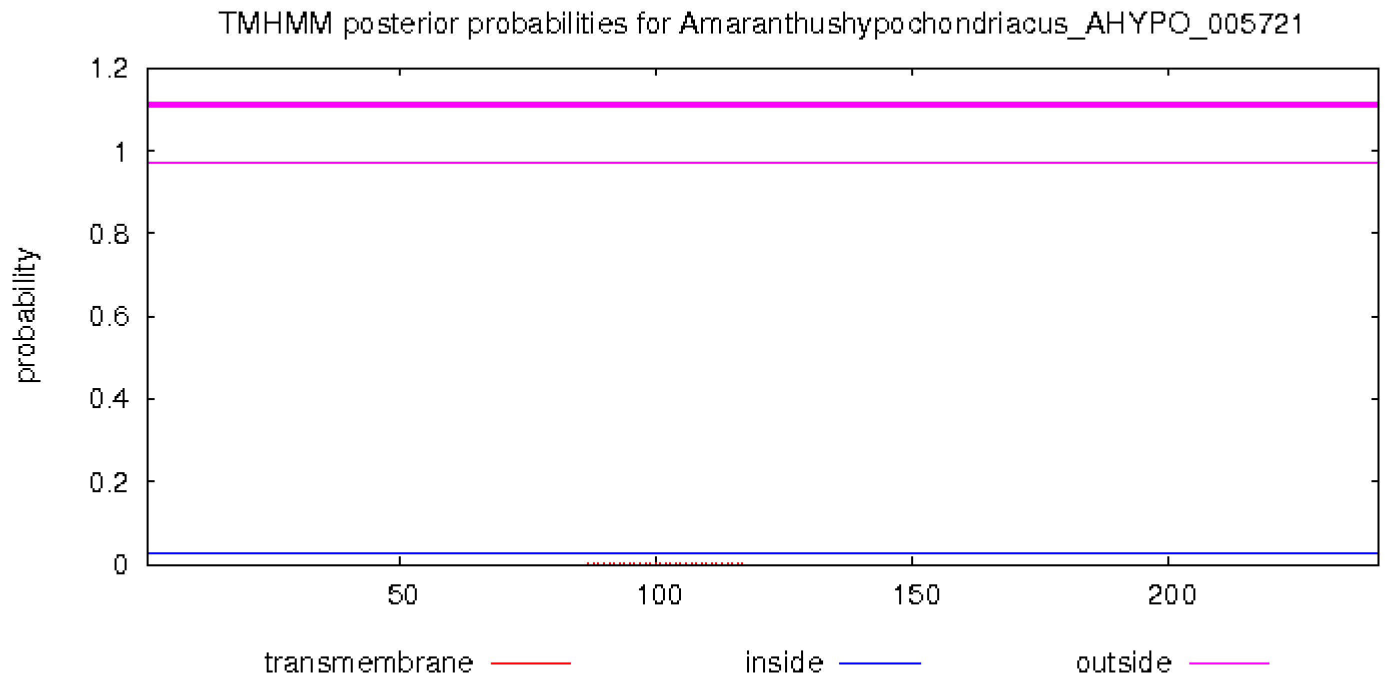

plot in postscript, script for making the plot in gnuplot, data for plot

---

```
# Amaranthushypochondriacus_AHYPO_005917 Length: 286
# Amaranthushypochondriacus_AHYPO_005917 Number of predicted TMHs:  0
# Amaranthushypochondriacus_AHYPO_005917 Exp number of AAs in TMHs: 0.01046
# Amaranthushypochondriacus_AHYPO_005917 Exp number, first 60 AAs:  0
# Amaranthushypochondriacus_AHYPO_005917 Total prob of N-in:        0.01470
Amaranthushypochondriacus_AHYPO_005917  TMHMM2.0        outside      1   286
```

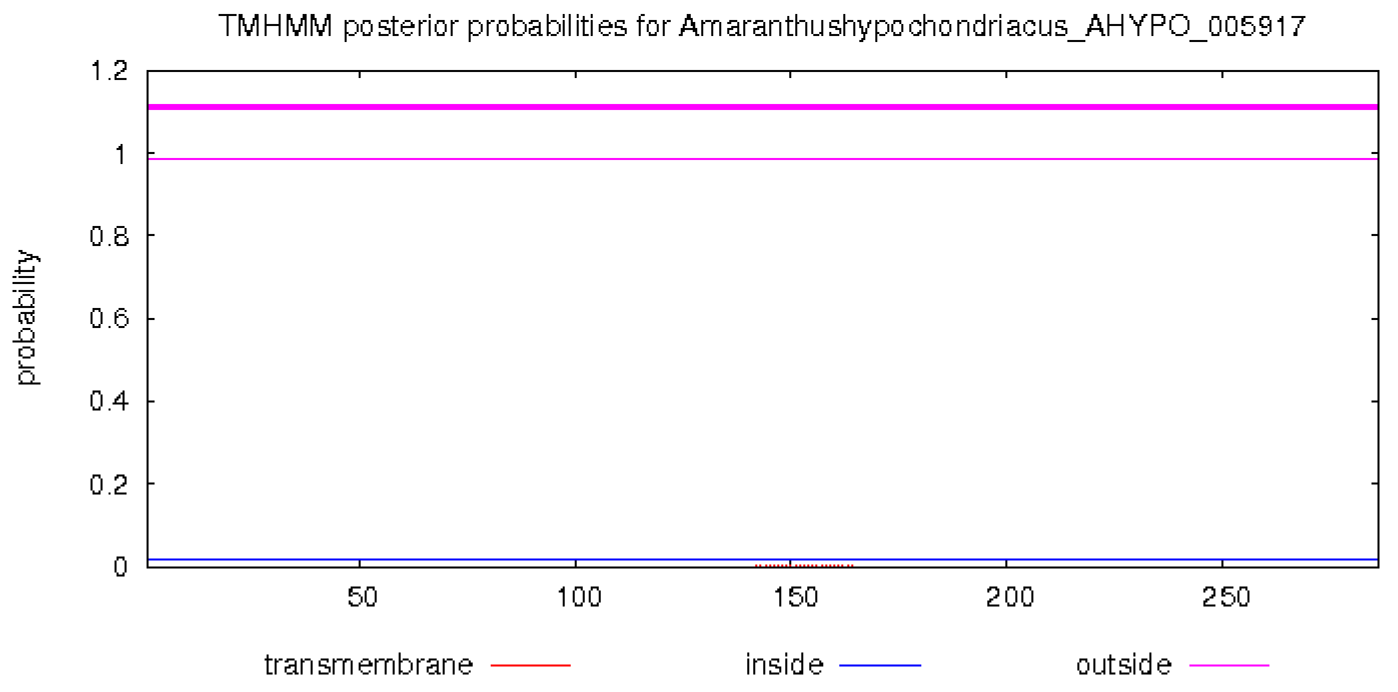

plot in postscript, script for making the plot in gnuplot, data for plot

---

```
# Amaranthushypochondriacus_AHYPO_007548 Length: 260
# Amaranthushypochondriacus_AHYPO_007548 Number of predicted TMHs:  0
# Amaranthushypochondriacus_AHYPO_007548 Exp number of AAs in TMHs: 0
# Amaranthushypochondriacus_AHYPO_007548 Exp number, first 60 AAs:  0
# Amaranthushypochondriacus_AHYPO_007548 Total prob of N-in:        0.05272
Amaranthushypochondriacus_AHYPO_007548  TMHMM2.0        outside      1   260
```

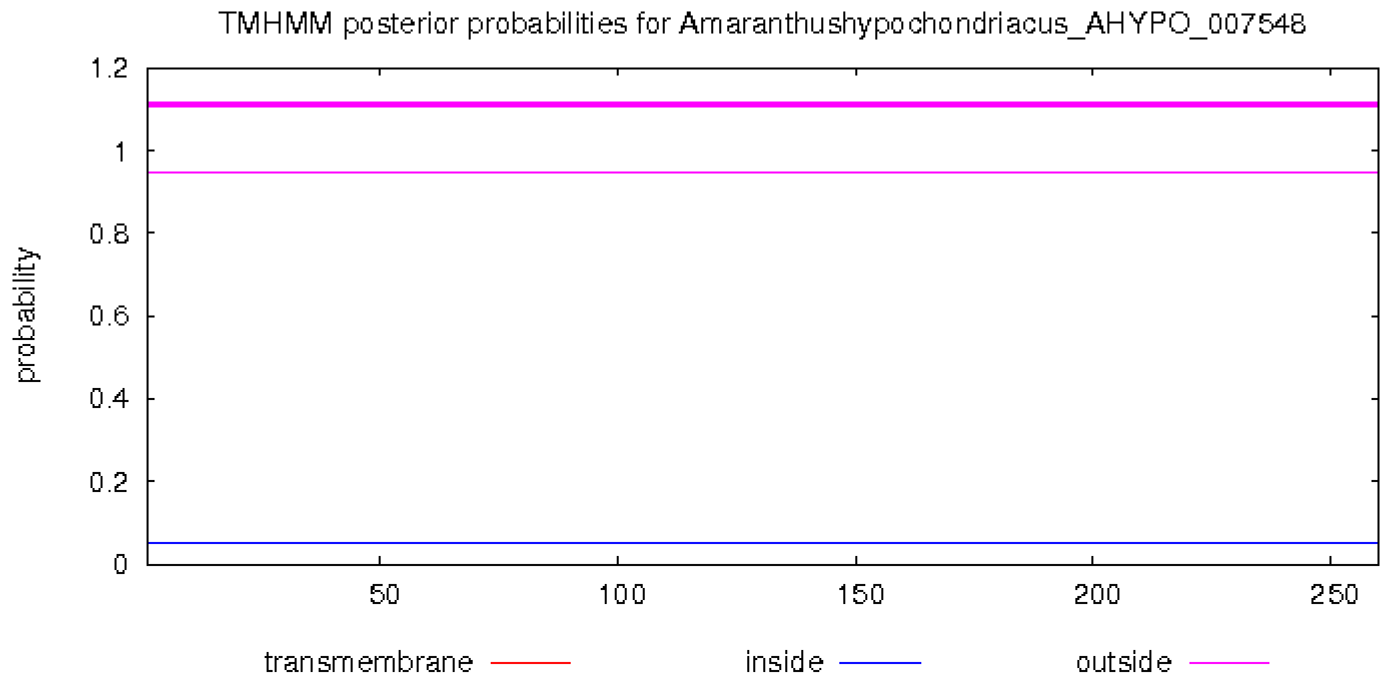

plot in postscript, script for making the plot in gnuplot, data for plot

```
# Amaranthushypochondriacus_AHYPO_007963 Length: 606
# Amaranthushypochondriacus_AHYPO_007963 Number of predicted TMHs:  1
# Amaranthushypochondriacus_AHYPO_007963 Exp number of AAs in TMHs: 22.12433
# Amaranthushypochondriacus_AHYPO_007963 Exp number, first 60 AAs:  0
# Amaranthushypochondriacus_AHYPO_007963 Total prob of N-in:        0.00047
Amaranthushypochondriacus_AHYPO_007963  TMHMM2.0        outside      1   575
Amaranthushypochondriacus_AHYPO_007963  TMHMM2.0        TMhelix    576   598
Amaranthushypochondriacus_AHYPO_007963  TMHMM2.0        inside     599   606
```

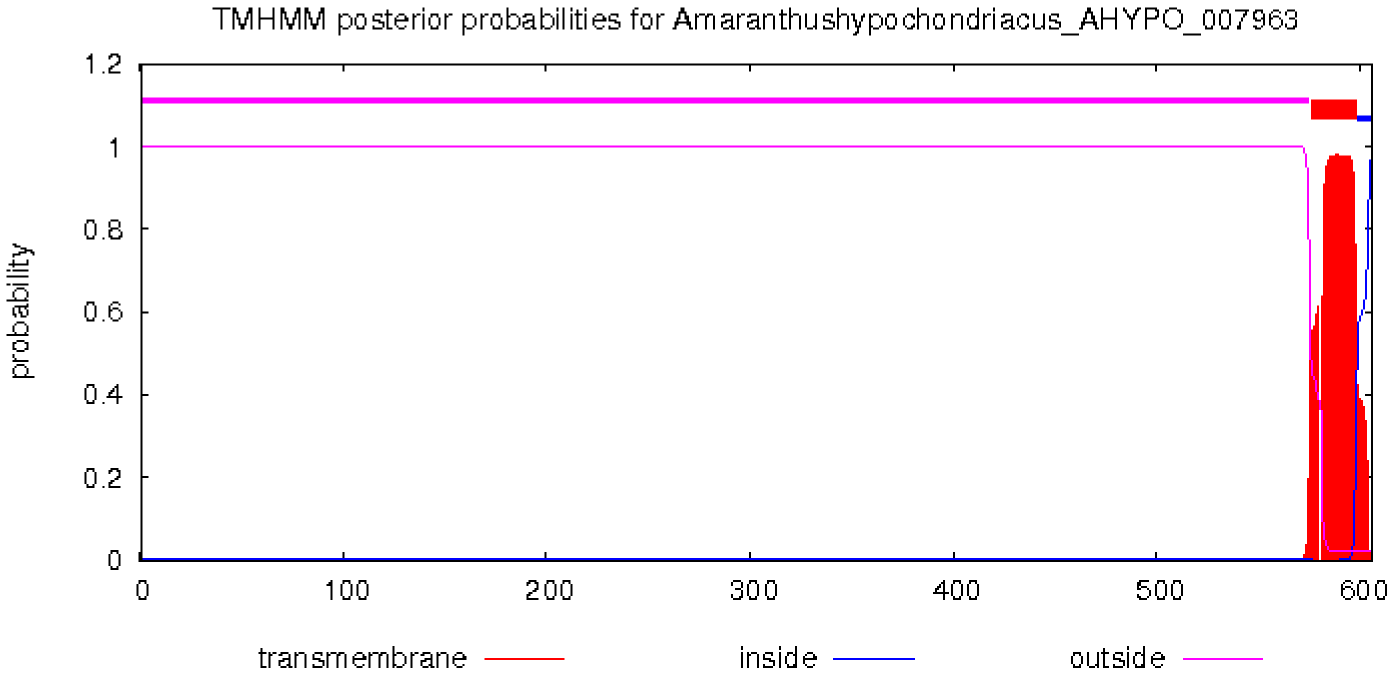

plot in postscript, script for making the plot in gnuplot, data for plot

```
# Amaranthushypochondriacus_AHYPO_008111 Length: 268
# Amaranthushypochondriacus_AHYPO_008111 Number of predicted TMHs:  0
# Amaranthushypochondriacus_AHYPO_008111 Exp number of AAs in TMHs: 0
# Amaranthushypochondriacus_AHYPO_008111 Exp number, first 60 AAs:  0
# Amaranthushypochondriacus_AHYPO_008111 Total prob of N-in:        0.02803
Amaranthushypochondriacus_AHYPO_008111  TMHMM2.0        outside      1   268
```

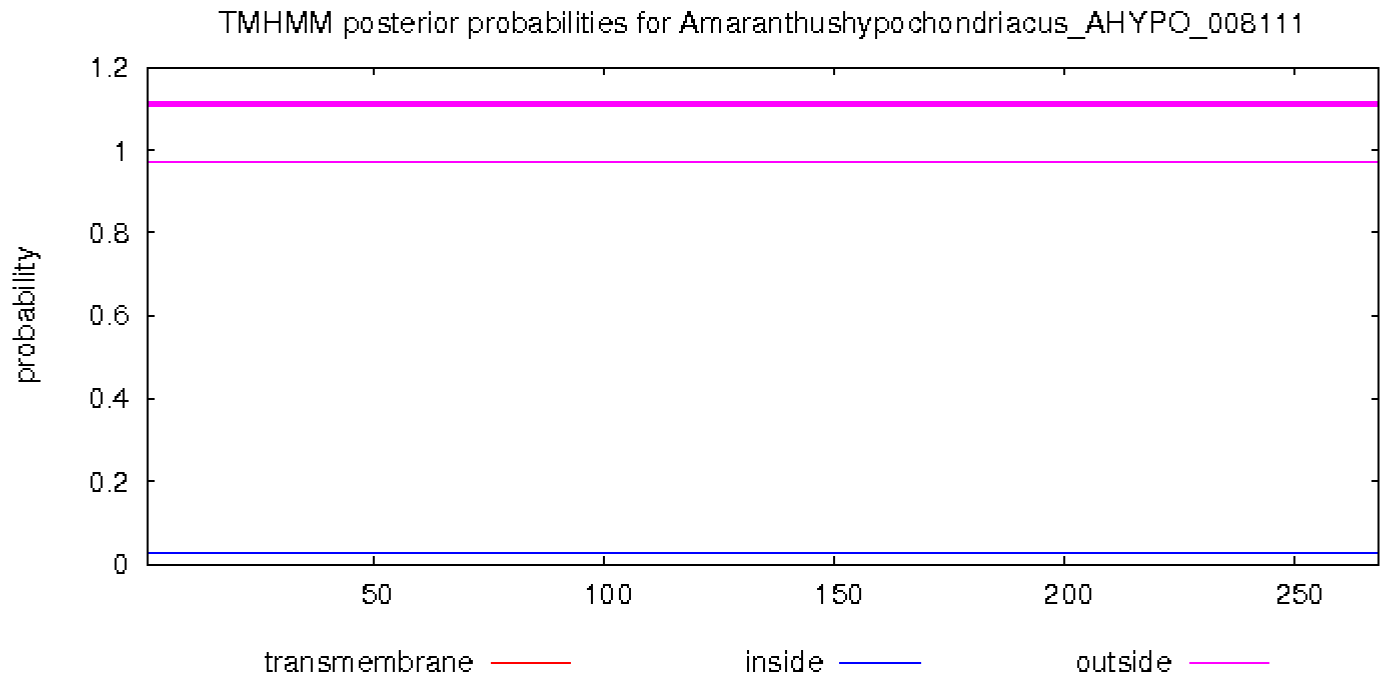

plot in postscript, script for making the plot in gnuplot, data for plot

---

```
# Amaranthushypochondriacus_AHYPO_008532 Length: 310
# Amaranthushypochondriacus_AHYPO_008532 Number of predicted TMHs:  0
# Amaranthushypochondriacus_AHYPO_008532 Exp number of AAs in TMHs: 0
# Amaranthushypochondriacus_AHYPO_008532 Exp number, first 60 AAs:  0
# Amaranthushypochondriacus_AHYPO_008532 Total prob of N-in:        0.00169
Amaranthushypochondriacus_AHYPO_008532  TMHMM2.0        outside      1   310
```

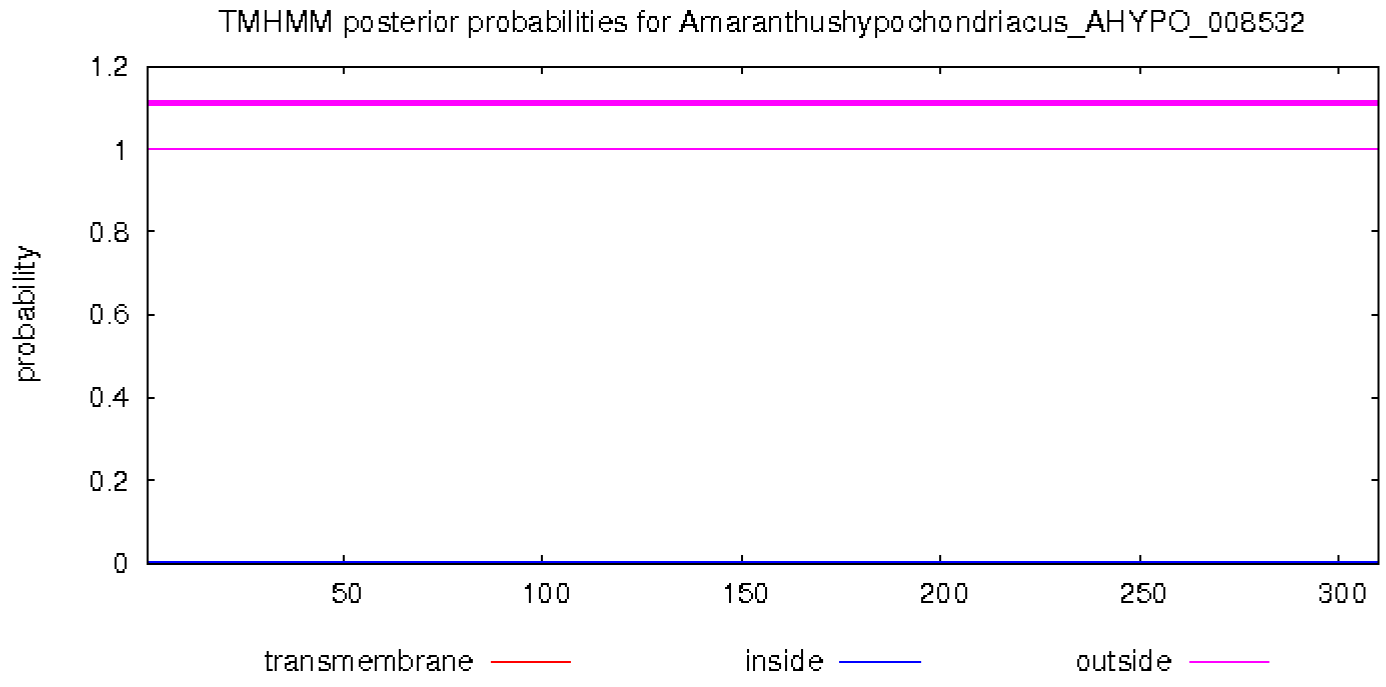

plot in postscript, script for making the plot in gnuplot, data for plot

---

```
# Amaranthushypochondriacus_AHYPO_008586 Length: 176
# Amaranthushypochondriacus_AHYPO_008586 Number of predicted TMHs:  0
# Amaranthushypochondriacus_AHYPO_008586 Exp number of AAs in TMHs: 13.28735
# Amaranthushypochondriacus_AHYPO_008586 Exp number, first 60 AAs:  4.73687
# Amaranthushypochondriacus_AHYPO_008586 Total prob of N-in:        0.00487
Amaranthushypochondriacus_AHYPO_008586  TMHMM2.0        outside      1   176
```

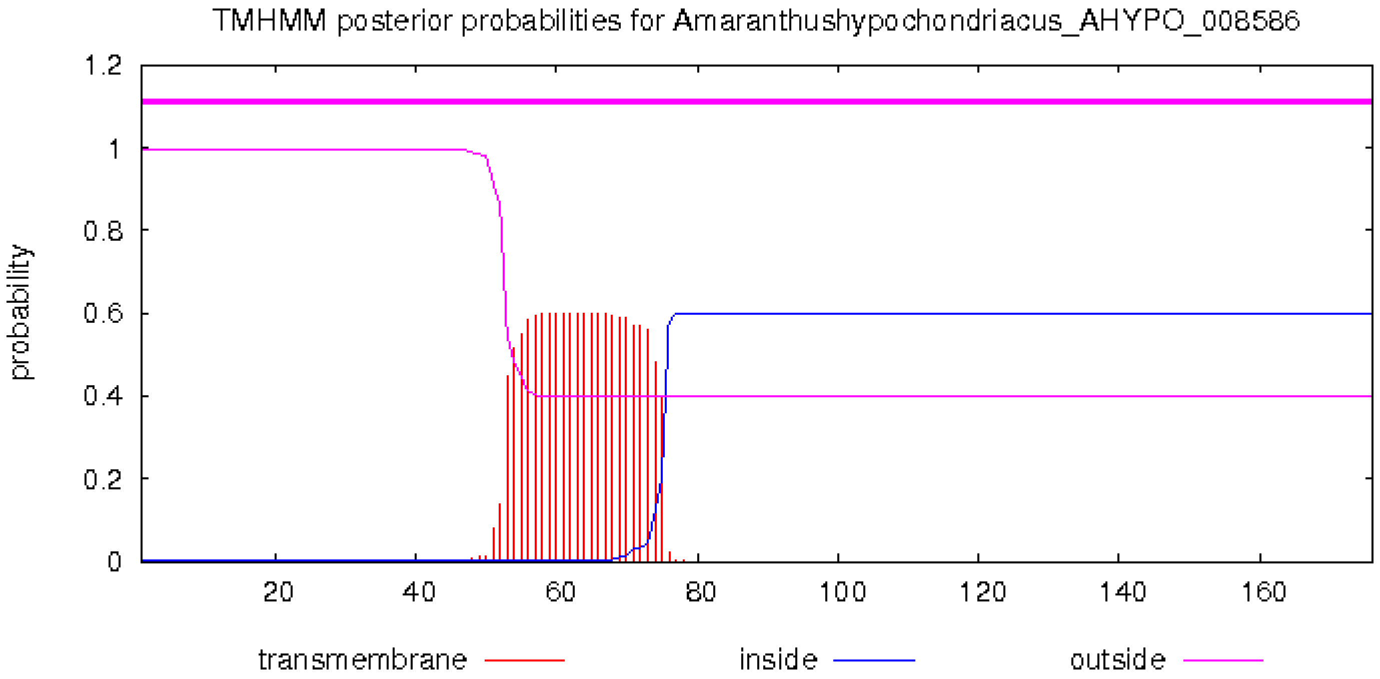

plot in postscript, script for making the plot in gnuplot, data for plot

---

```
# Amaranthushypochondriacus_AHYPO_009365 Length: 620
# Amaranthushypochondriacus_AHYPO_009365 Number of predicted TMHs:  1
# Amaranthushypochondriacus_AHYPO_009365 Exp number of AAs in TMHs: 22.22597
# Amaranthushypochondriacus_AHYPO_009365 Exp number, first 60 AAs:  0
# Amaranthushypochondriacus_AHYPO_009365 Total prob of N-in:        0.00255
Amaranthushypochondriacus_AHYPO_009365  TMHMM2.0        outside      1   595
Amaranthushypochondriacus_AHYPO_009365  TMHMM2.0        TMhelix    596   618
Amaranthushypochondriacus_AHYPO_009365  TMHMM2.0        inside     619   620
```

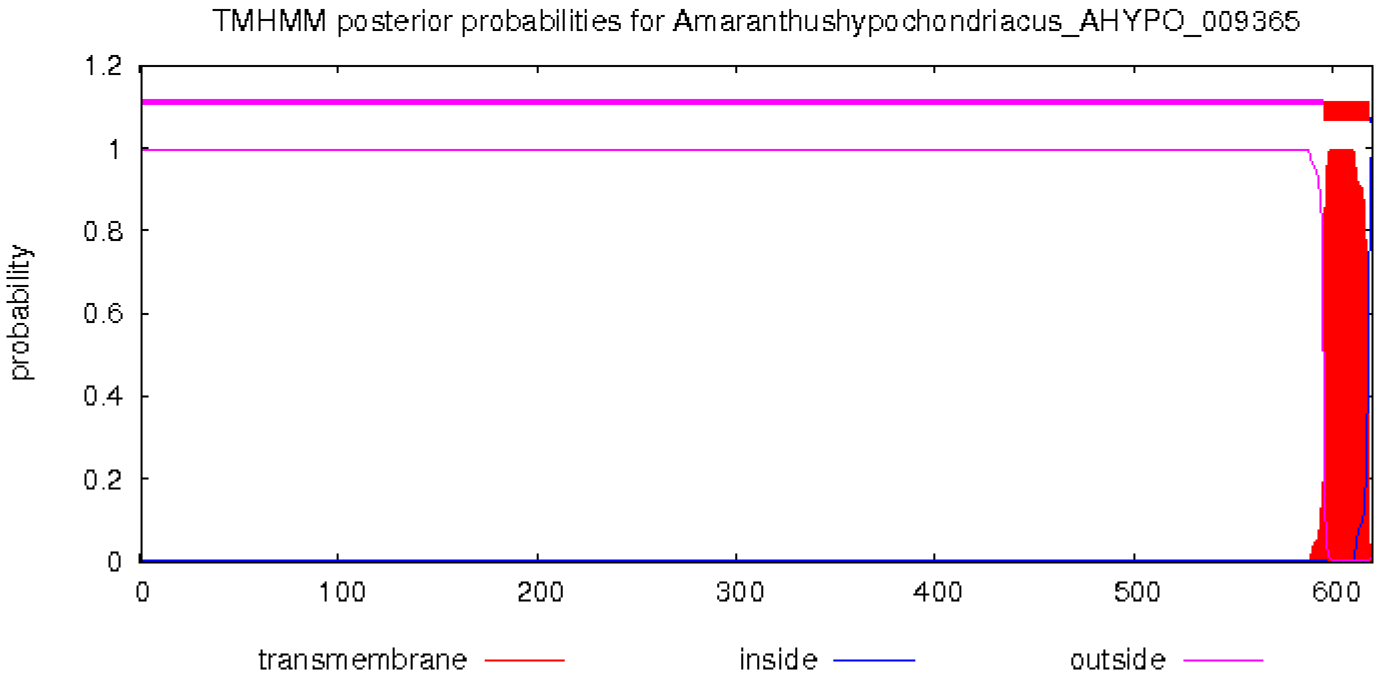

plot in postscript, script for making the plot in gnuplot, data for plot

---

```
# Amaranthushypochondriacus_AHYPO_009543 Length: 515
# Amaranthushypochondriacus_AHYPO_009543 Number of predicted TMHs:  0
# Amaranthushypochondriacus_AHYPO_009543 Exp number of AAs in TMHs: 0.05925
# Amaranthushypochondriacus_AHYPO_009543 Exp number, first 60 AAs:  0
```

```
# Amaranthushypochondriacus_AHYPO_009543 Total prob of N-in:        0.00885
Amaranthushypochondriacus_AHYPO_009543  TMHMM2.0        outside      1   515
```

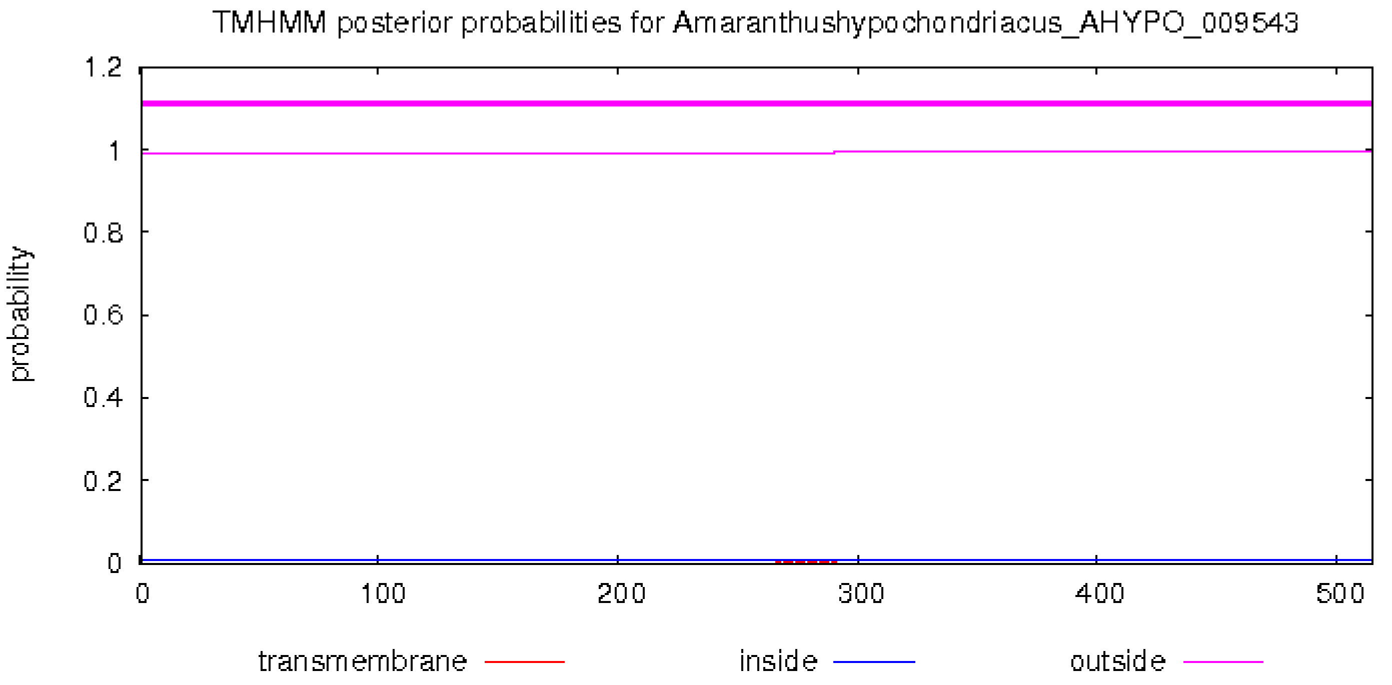

plot in postscript, script for making the plot in gnuplot, data for plot

---

```
# Amaranthushypochondriacus_AHYPO_009690 Length: 355
# Amaranthushypochondriacus_AHYPO_009690 Number of predicted TMHs:  0
# Amaranthushypochondriacus_AHYPO_009690 Exp number of AAs in TMHs: 0.00058
# Amaranthushypochondriacus_AHYPO_009690 Exp number, first 60 AAs:  0.00041
# Amaranthushypochondriacus_AHYPO_009690 Total prob of N-in:        0.00819
Amaranthushypochondriacus_AHYPO_009690  TMHMM2.0        outside      1   355
```

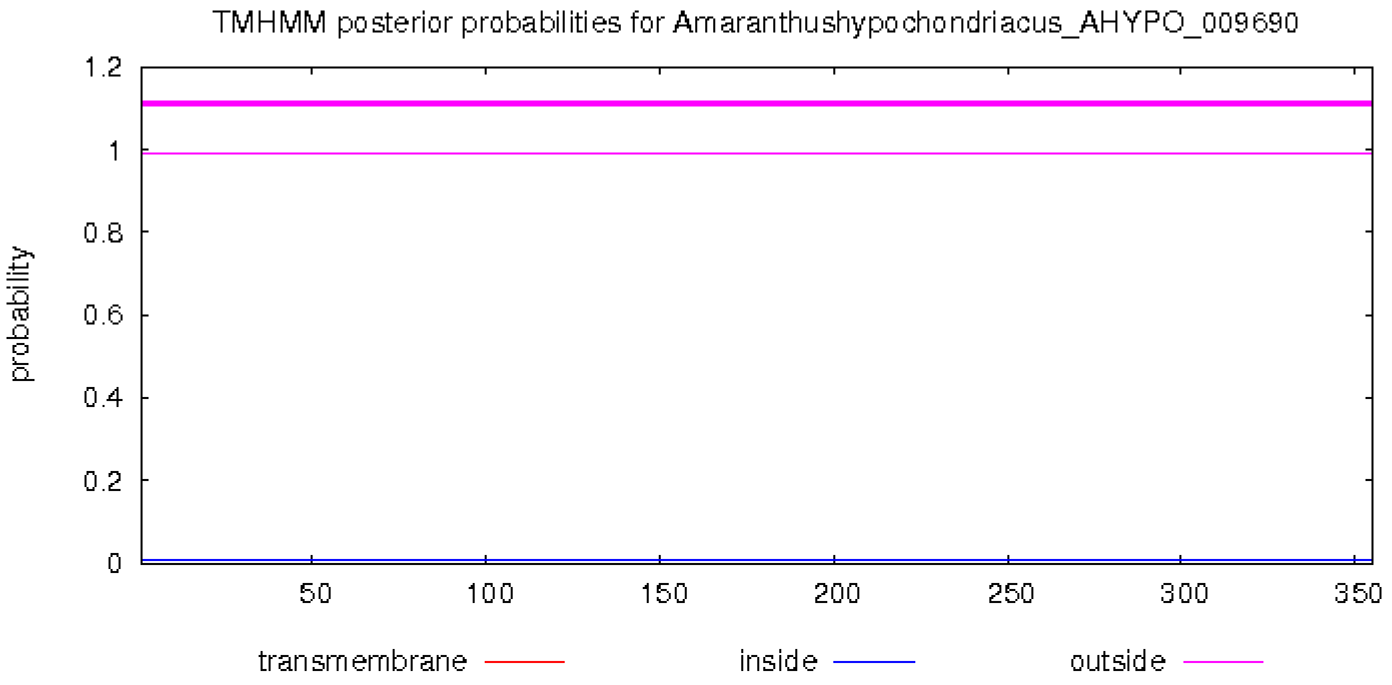

plot in postscript, script for making the plot in gnuplot, data for plot

---

```
# Amaranthushypochondriacus_AHYPO_010449 Length: 330
# Amaranthushypochondriacus_AHYPO_010449 Number of predicted TMHs:  0
# Amaranthushypochondriacus_AHYPO_010449 Exp number of AAs in TMHs: 4e-05
# Amaranthushypochondriacus_AHYPO_010449 Exp number, first 60 AAs:  0
```

```
# Amaranthushypochondriacus_AHYPO_010449 Total prob of N-in:        0.00127
Amaranthushypochondriacus_AHYPO_010449  TMHMM2.0        outside      1   330
```

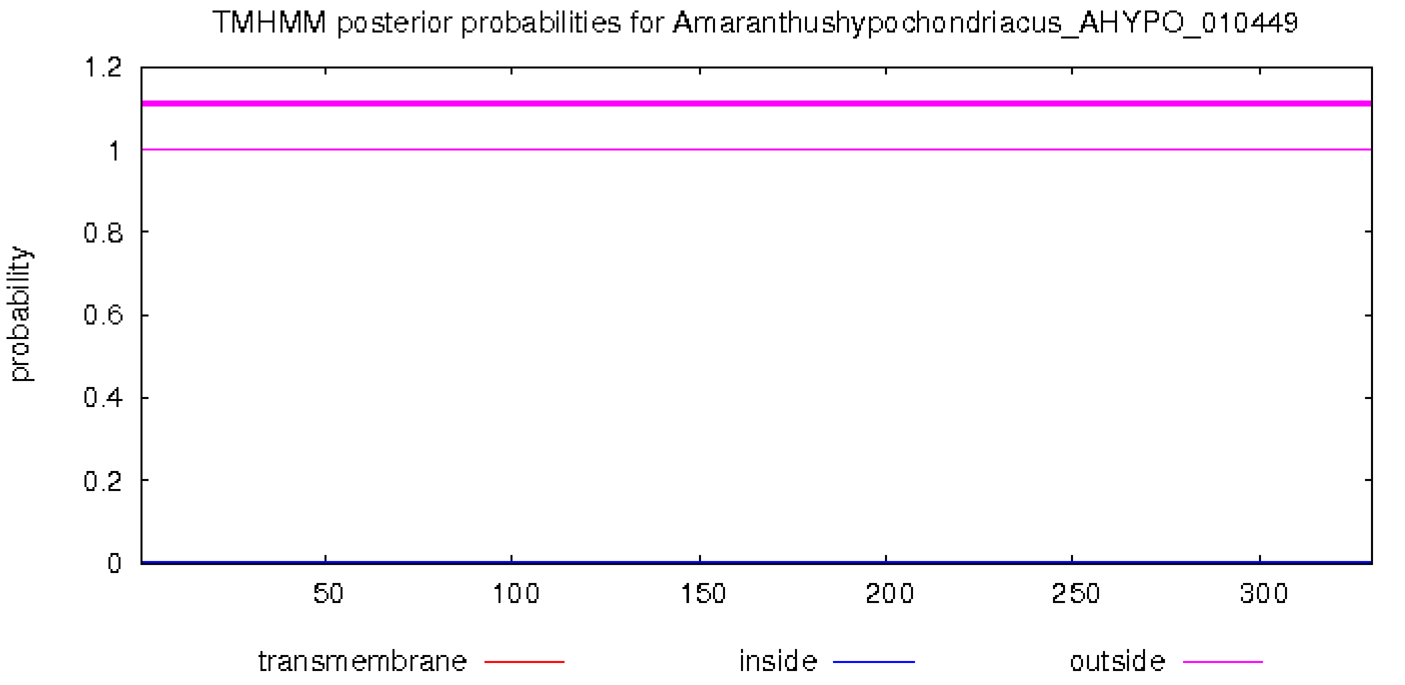

plot in postscript, script for making the plot in gnuplot, data for plot

---

```
# Amaranthushypochondriacus_AHYPO_010500 Length: 350
# Amaranthushypochondriacus_AHYPO_010500 Number of predicted TMHs:  0
# Amaranthushypochondriacus_AHYPO_010500 Exp number of AAs in TMHs: 0.86313
# Amaranthushypochondriacus_AHYPO_010500 Exp number, first 60 AAs:  0.00159
# Amaranthushypochondriacus_AHYPO_010500 Total prob of N-in:        0.01909
Amaranthushypochondriacus_AHYPO_010500  TMHMM2.0        outside      1   350
```

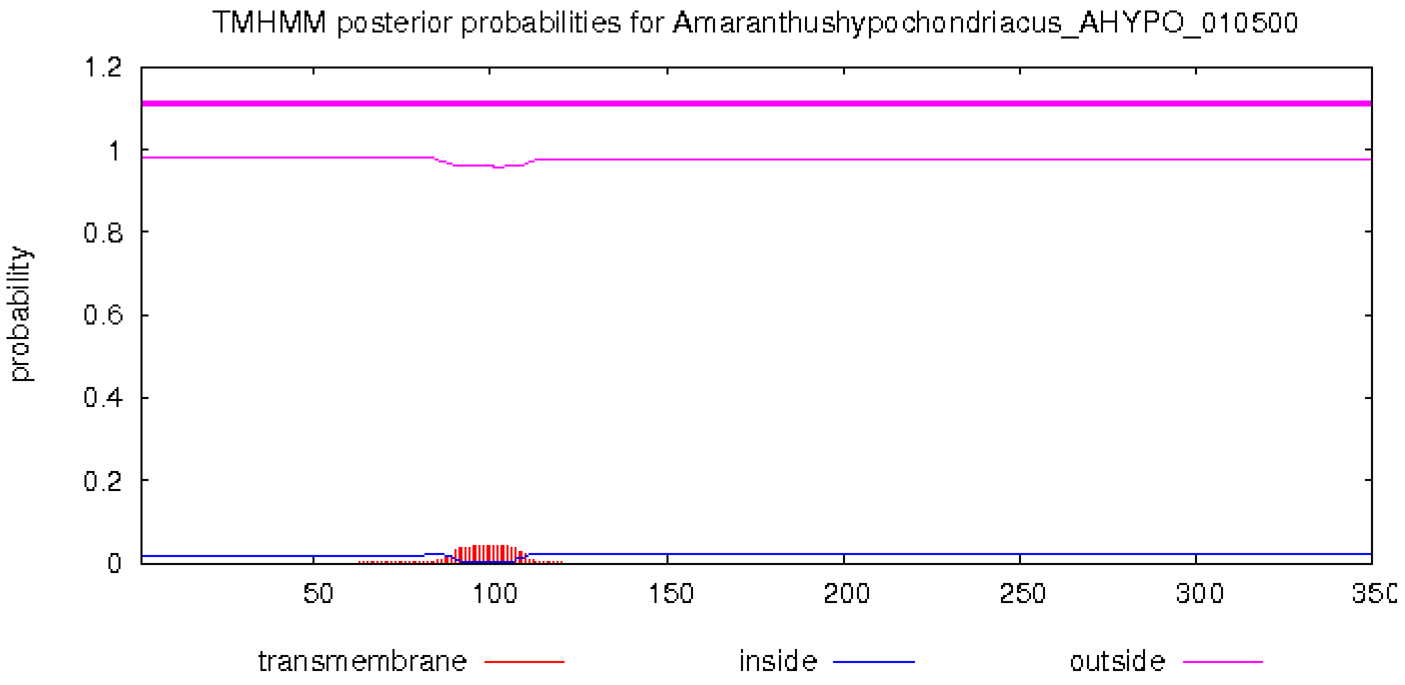

plot in postscript, script for making the plot in gnuplot, data for plot

---

```
# Amaranthushypochondriacus_AHYPO_010646 Length: 213
# Amaranthushypochondriacus_AHYPO_010646 Number of predicted TMHs:  0
# Amaranthushypochondriacus_AHYPO_010646 Exp number of AAs in TMHs: 0.09952
# Amaranthushypochondriacus_AHYPO_010646 Exp number, first 60 AAs:  0
```

```
# Amaranthushypochondriacus_AHYPO_010646 Total prob of N-in:        0.20762
Amaranthushypochondriacus_AHYPO_010646  TMHMM2.0        outside      1   213
```

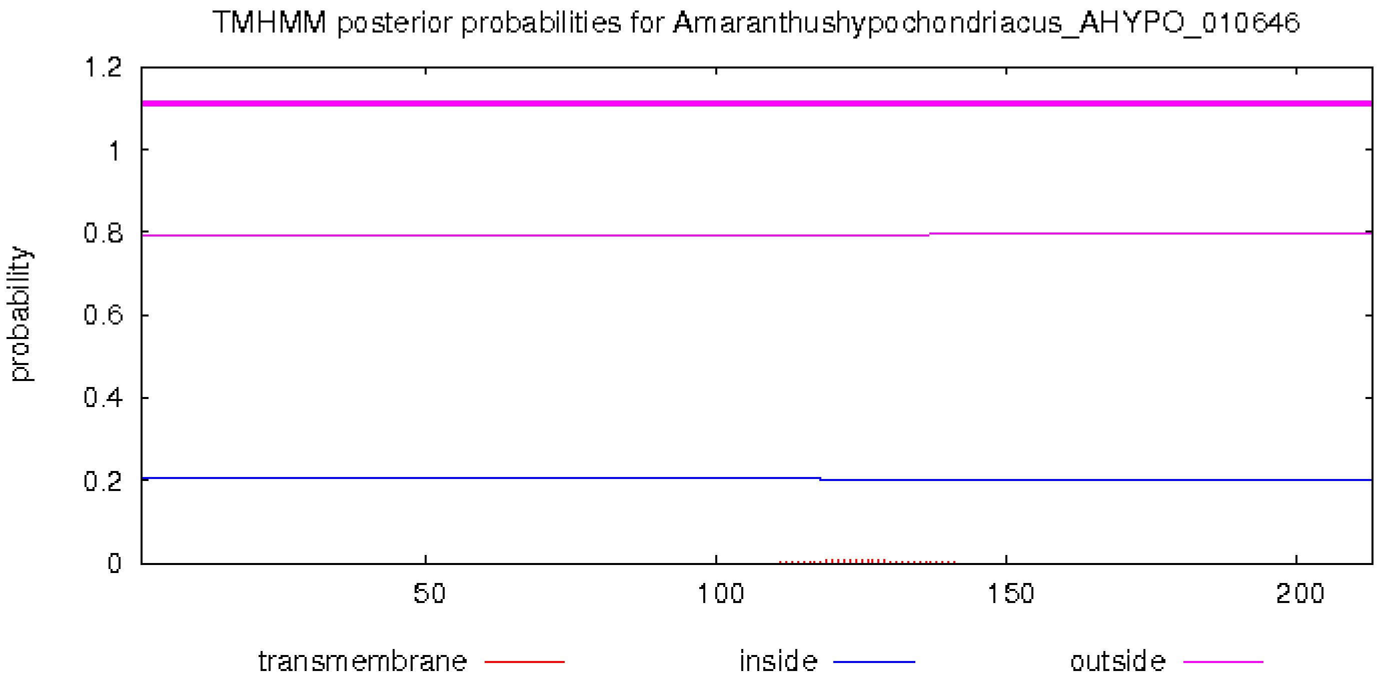

plot in postscript, script for making the plot in gnuplot, data for plot

---

```
# Amaranthushypochondriacus_AHYPO_011135 Length: 357
# Amaranthushypochondriacus_AHYPO_011135 Number of predicted TMHs:  0
# Amaranthushypochondriacus_AHYPO_011135 Exp number of AAs in TMHs: 0
# Amaranthushypochondriacus_AHYPO_011135 Exp number, first 60 AAs:  0
# Amaranthushypochondriacus_AHYPO_011135 Total prob of N-in:        0.00660
Amaranthushypochondriacus_AHYPO_011135  TMHMM2.0        outside      1   357
```

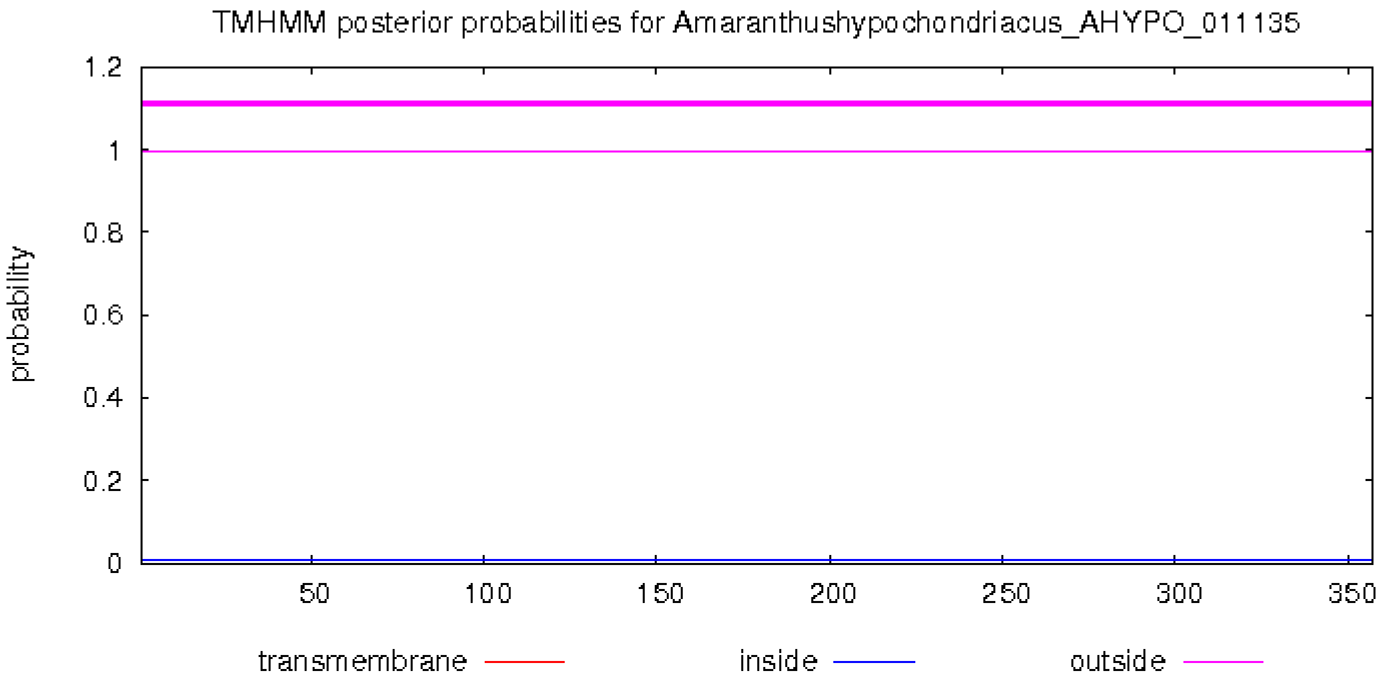

plot in postscript, script for making the plot in gnuplot, data for plot

---

```
# Amaranthushypochondriacus_AHYPO_011586 Length: 359
# Amaranthushypochondriacus_AHYPO_011586 Number of predicted TMHs:  0
# Amaranthushypochondriacus_AHYPO_011586 Exp number of AAs in TMHs: 0
# Amaranthushypochondriacus_AHYPO_011586 Exp number, first 60 AAs:  0
```

Amaranthushypochondriacus_AHYPO_011586 Total prob of N-in: 0.04553
Amaranthushypochondriacus_AHYPO_011586 TMHMM2.0 outside 1 359

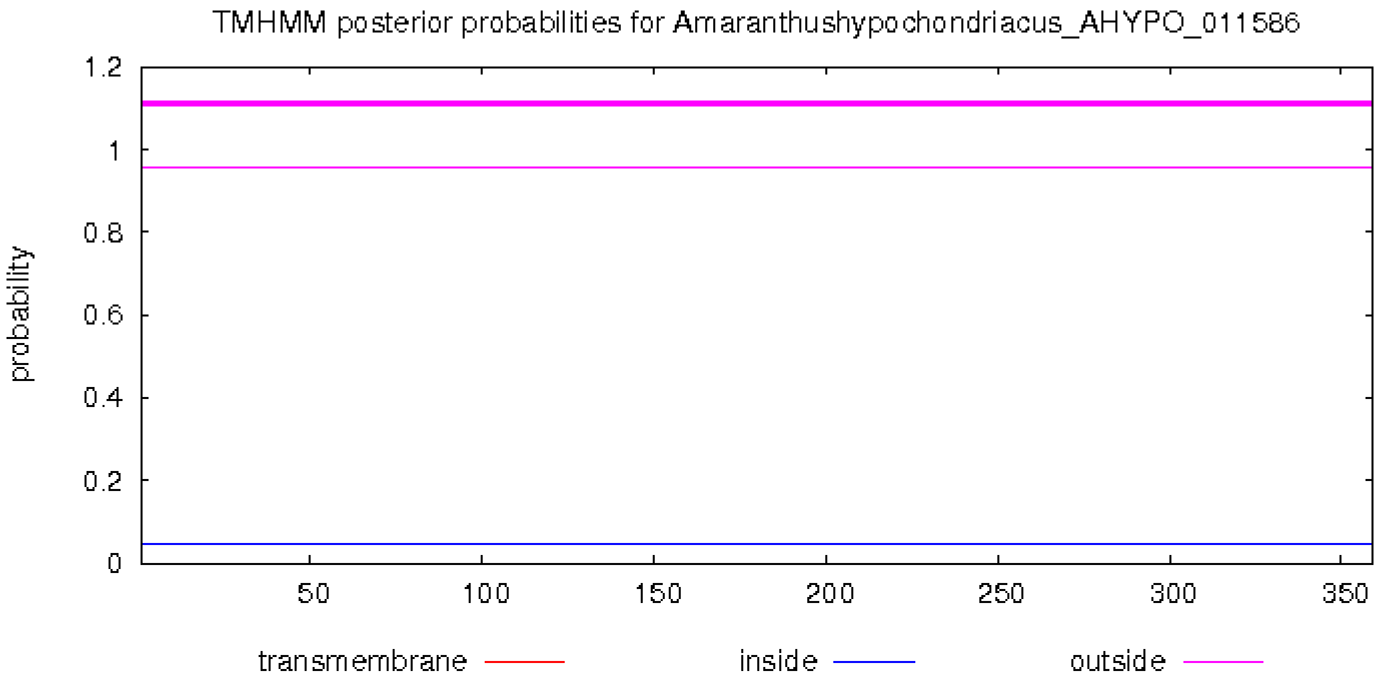

plot in postscript, script for making the plot in gnuplot, data for plot

---

Amaranthushypochondriacus_AHYPO_012201 Length: 128
Amaranthushypochondriacus_AHYPO_012201 Number of predicted TMHs: 0
Amaranthushypochondriacus_AHYPO_012201 Exp number of AAs in TMHs: 0
Amaranthushypochondriacus_AHYPO_012201 Exp number, first 60 AAs: 0
Amaranthushypochondriacus_AHYPO_012201 Total prob of N-in: 0.05799
Amaranthushypochondriacus_AHYPO_012201 TMHMM2.0 outside 1 128

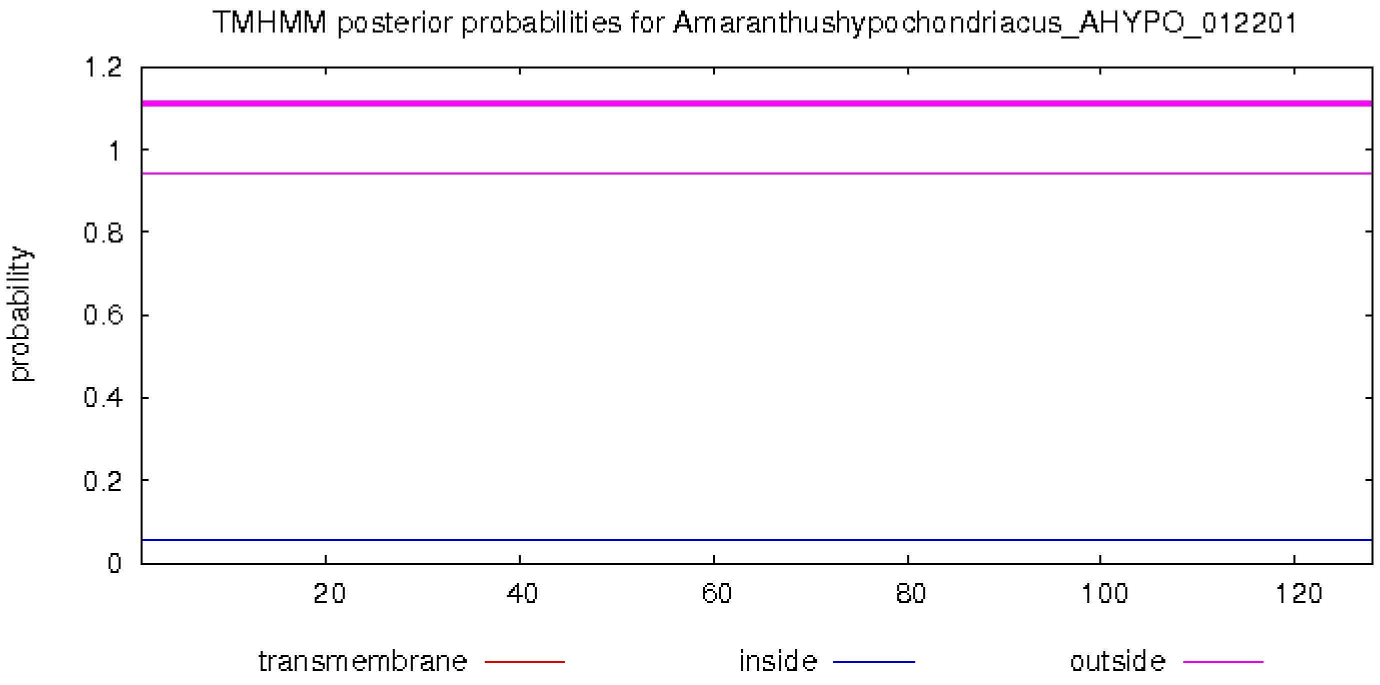

plot in postscript, script for making the plot in gnuplot, data for plot

---

Amaranthushypochondriacus_AHYPO_013086 Length: 279
Amaranthushypochondriacus_AHYPO_013086 Number of predicted TMHs: 0
Amaranthushypochondriacus_AHYPO_013086 Exp number of AAs in TMHs: 0.04507
Amaranthushypochondriacus_AHYPO_013086 Exp number, first 60 AAs: 0.00617

```
# Amaranthushypochondriacus_AHYPO_013086 Total prob of N-in:        0.00711
Amaranthushypochondriacus_AHYPO_013086  TMHMM2.0        outside      1   279
```

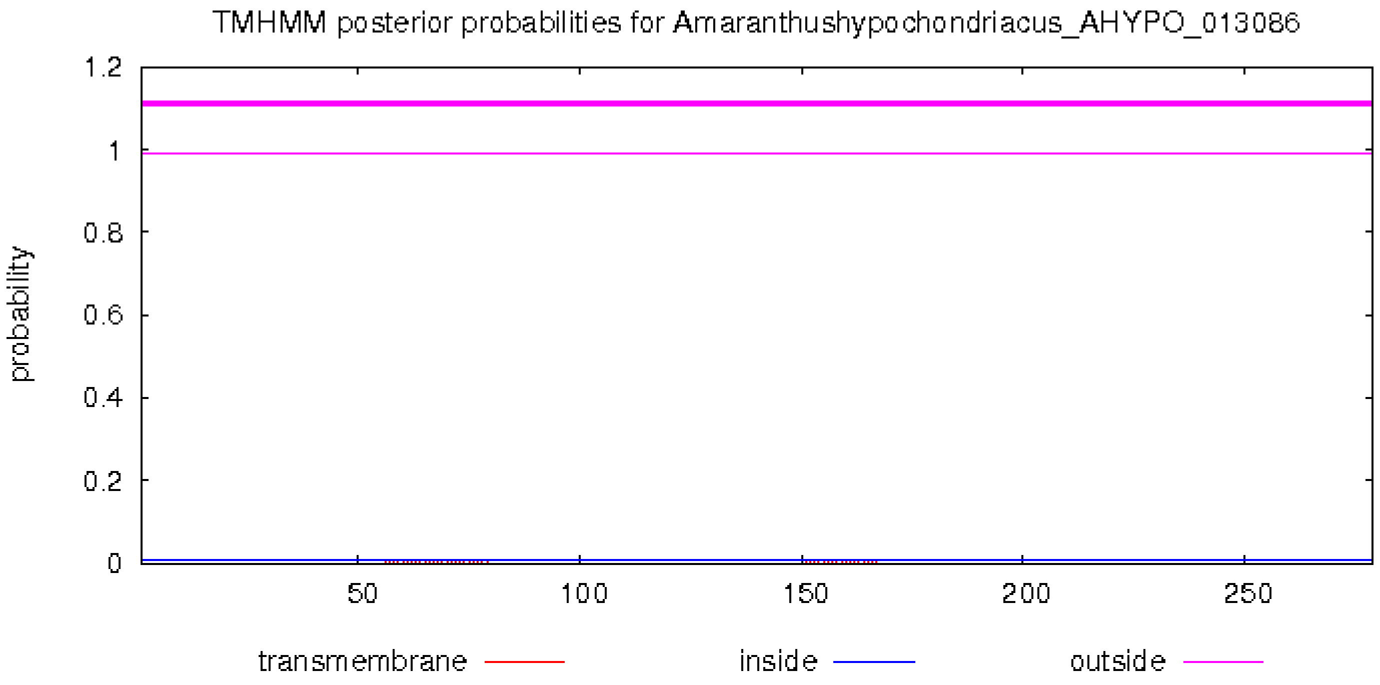

plot in postscript, script for making the plot in gnuplot, data for plot

---

```
# Amaranthushypochondriacus_AHYPO_013259 Length: 227
# Amaranthushypochondriacus_AHYPO_013259 Number of predicted TMHs:  0
# Amaranthushypochondriacus_AHYPO_013259 Exp number of AAs in TMHs: 0
# Amaranthushypochondriacus_AHYPO_013259 Exp number, first 60 AAs:  0
# Amaranthushypochondriacus_AHYPO_013259 Total prob of N-in:        0.01804
Amaranthushypochondriacus_AHYPO_013259  TMHMM2.0        outside      1   227
```

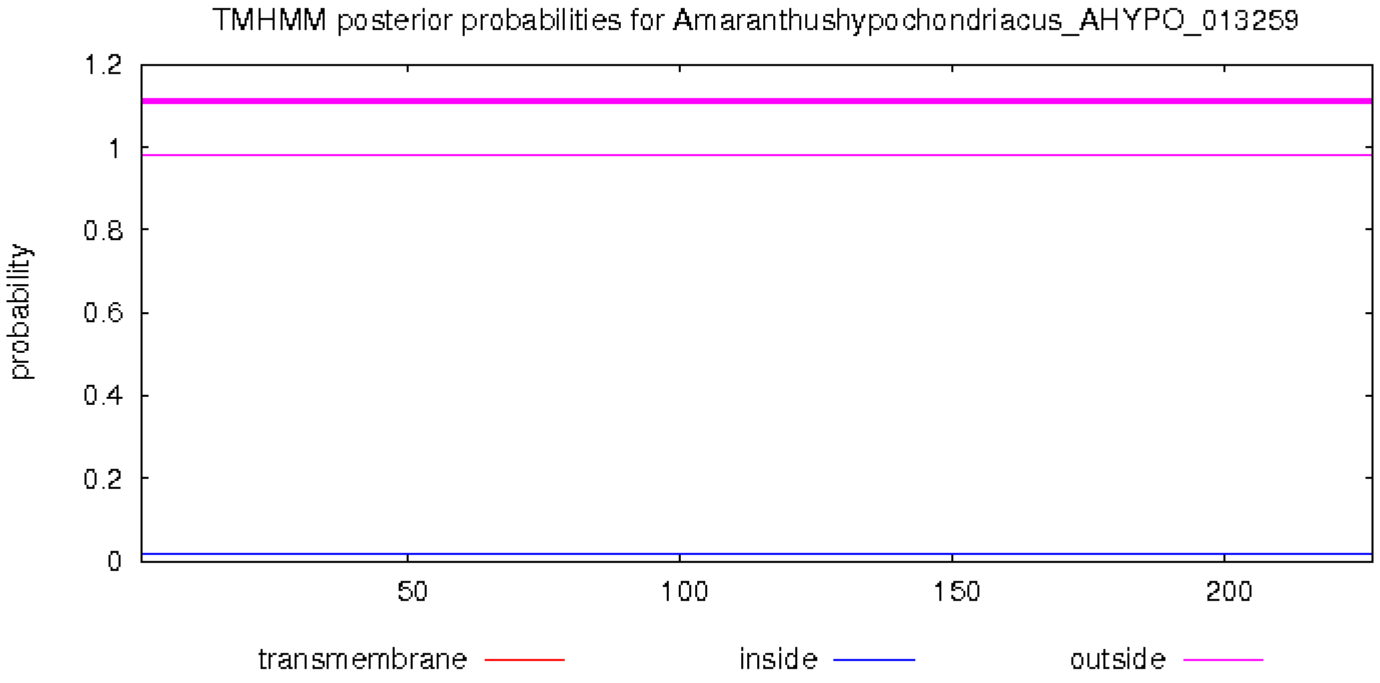

plot in postscript, script for making the plot in gnuplot, data for plot

---

```
# Amaranthushypochondriacus_AHYPO_013998 Length: 351
# Amaranthushypochondriacus_AHYPO_013998 Number of predicted TMHs:  0
# Amaranthushypochondriacus_AHYPO_013998 Exp number of AAs in TMHs: 0.00215
# Amaranthushypochondriacus_AHYPO_013998 Exp number, first 60 AAs:  0
```

```
# Amaranthushypochondriacus_AHYPO_013998 Total prob of N-in:        0.00861
Amaranthushypochondriacus_AHYPO_013998  TMHMM2.0        outside      1   351
```

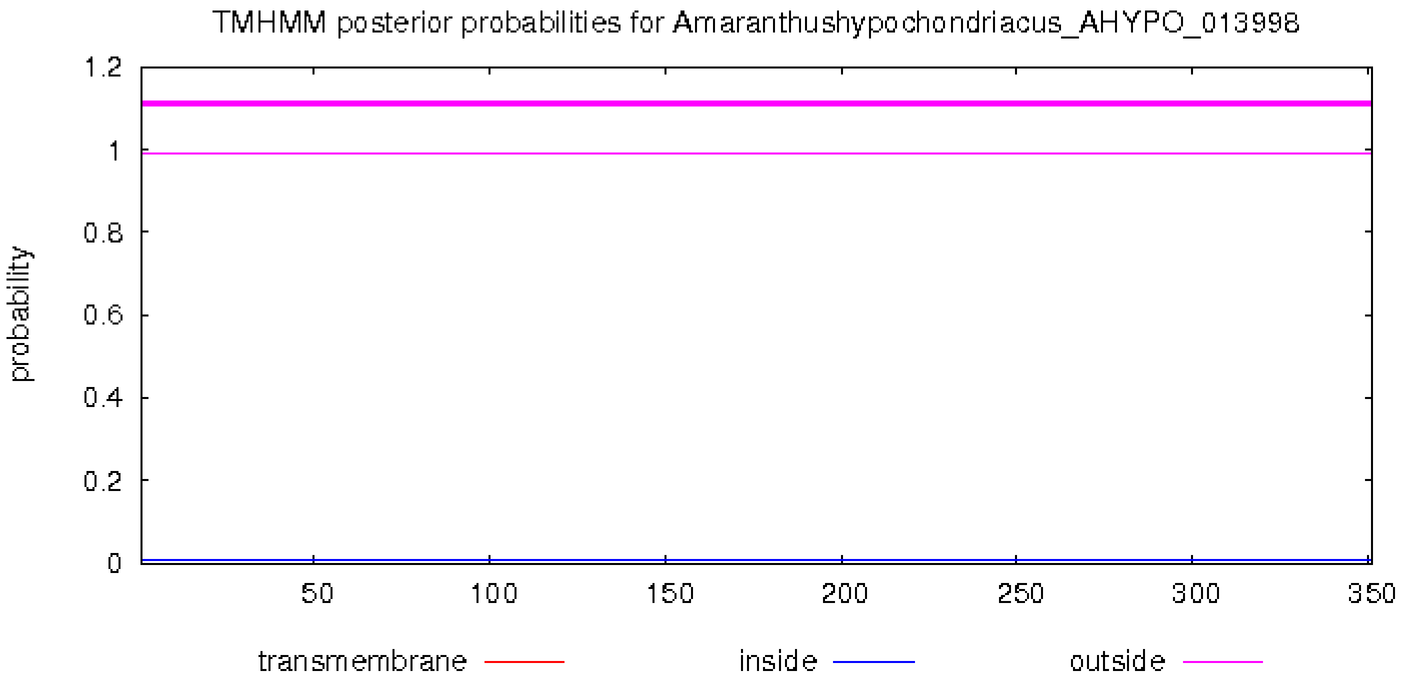

[plot](#) in postscript, [script](#) for making the plot in gnuplot, [data](#) for plot

---

```
# Amaranthushypochondriacus_AHYPO_014151 Length: 545
# Amaranthushypochondriacus_AHYPO_014151 Number of predicted TMHs:  1
# Amaranthushypochondriacus_AHYPO_014151 Exp number of AAs in TMHs: 20.72344
# Amaranthushypochondriacus_AHYPO_014151 Exp number, first 60 AAs:  0.00015
# Amaranthushypochondriacus_AHYPO_014151 Total prob of N-in:        0.08274
Amaranthushypochondriacus_AHYPO_014151  TMHMM2.0        outside      1   520
Amaranthushypochondriacus_AHYPO_014151  TMHMM2.0        TMhelix    521   543
Amaranthushypochondriacus_AHYPO_014151  TMHMM2.0        inside     544   545
```

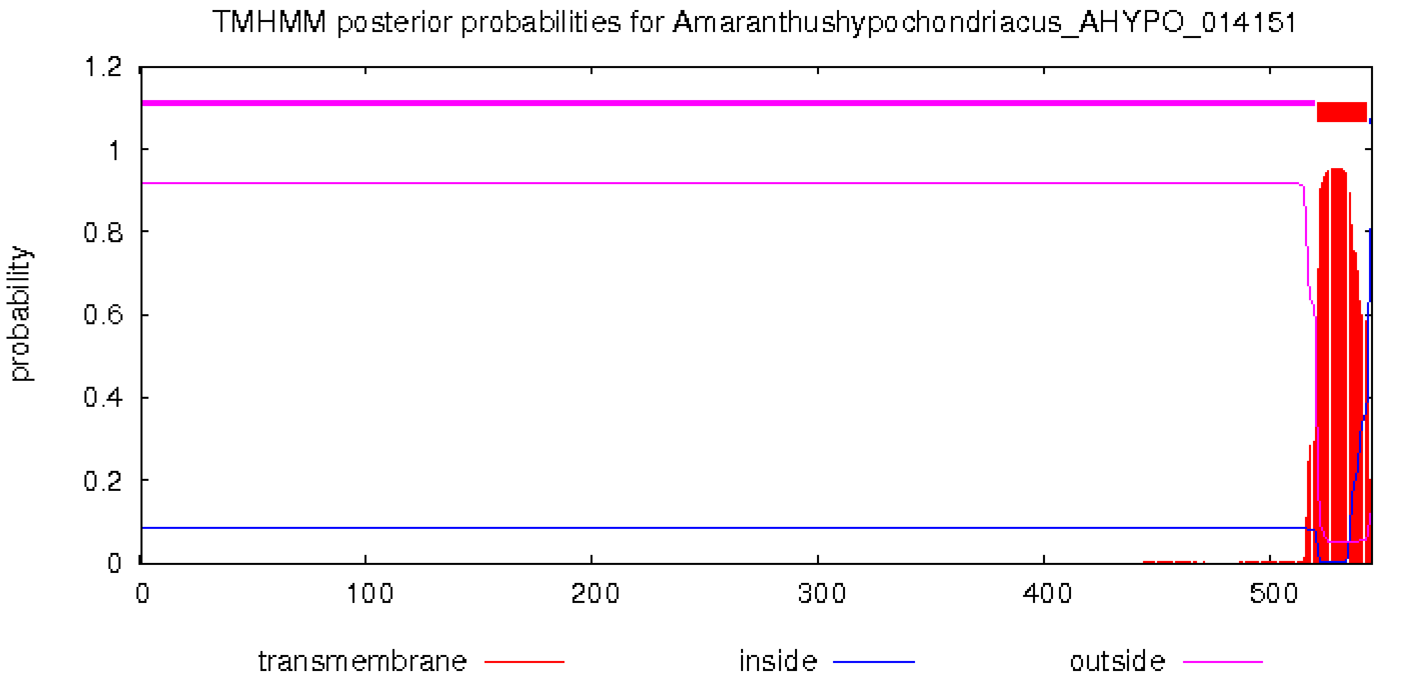

[plot](#) in postscript, [script](#) for making the plot in gnuplot, [data](#) for plot

---

```
# Amaranthushypochondriacus_AHYPO_014758 Length: 233
# Amaranthushypochondriacus_AHYPO_014758 Number of predicted TMHs:  0
```

```
# Amaranthushypochondriacus_AHYPO_014758 Exp number of AAs in TMHs: 0
# Amaranthushypochondriacus_AHYPO_014758 Exp number, first 60 AAs:  0
# Amaranthushypochondriacus_AHYPO_014758 Total prob of N-in:        0.01700
Amaranthushypochondriacus_AHYPO_014758  TMHMM2.0        outside      1   233
```

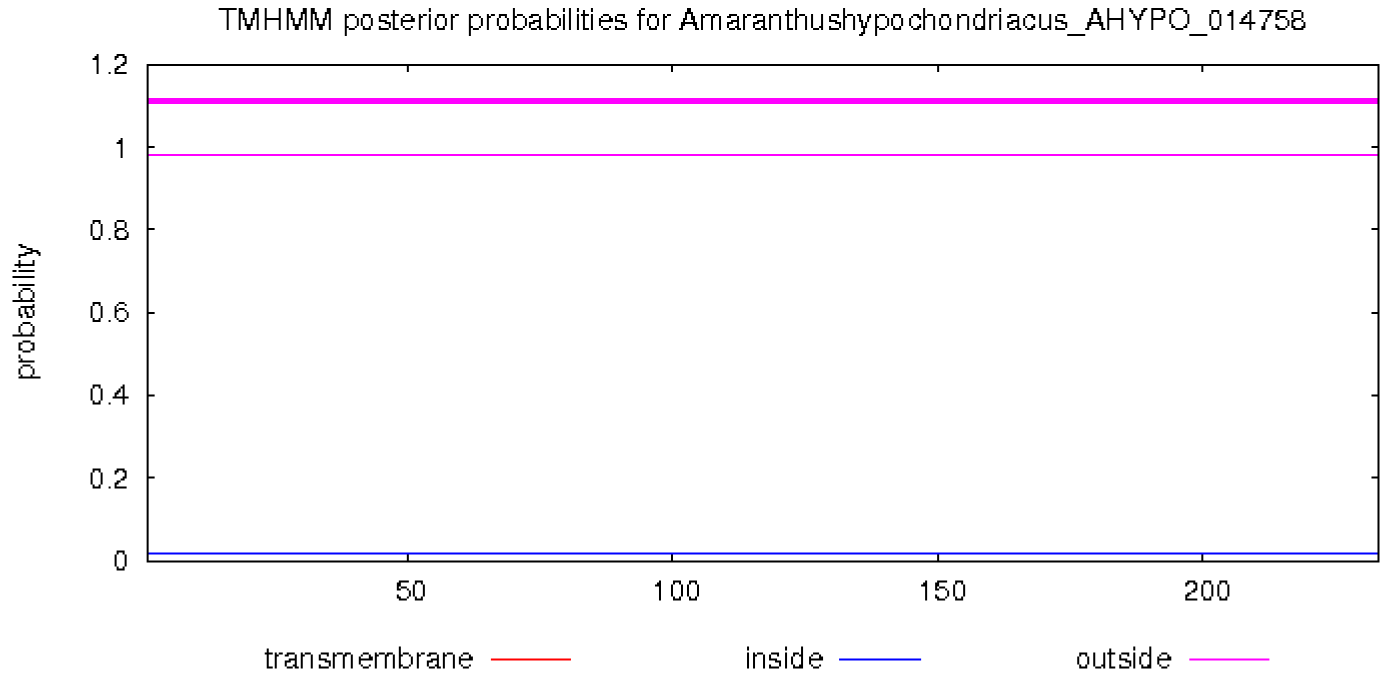

plot in postscript, script for making the plot in gnuplot, data for plot

---

```
# Amaranthushypochondriacus_AHYPO_015602 Length: 266
# Amaranthushypochondriacus_AHYPO_015602 Number of predicted TMHs:  0
# Amaranthushypochondriacus_AHYPO_015602 Exp number of AAs in TMHs: 0.00337
# Amaranthushypochondriacus_AHYPO_015602 Exp number, first 60 AAs:  0
# Amaranthushypochondriacus_AHYPO_015602 Total prob of N-in:        0.05573
Amaranthushypochondriacus_AHYPO_015602  TMHMM2.0        outside      1   266
```

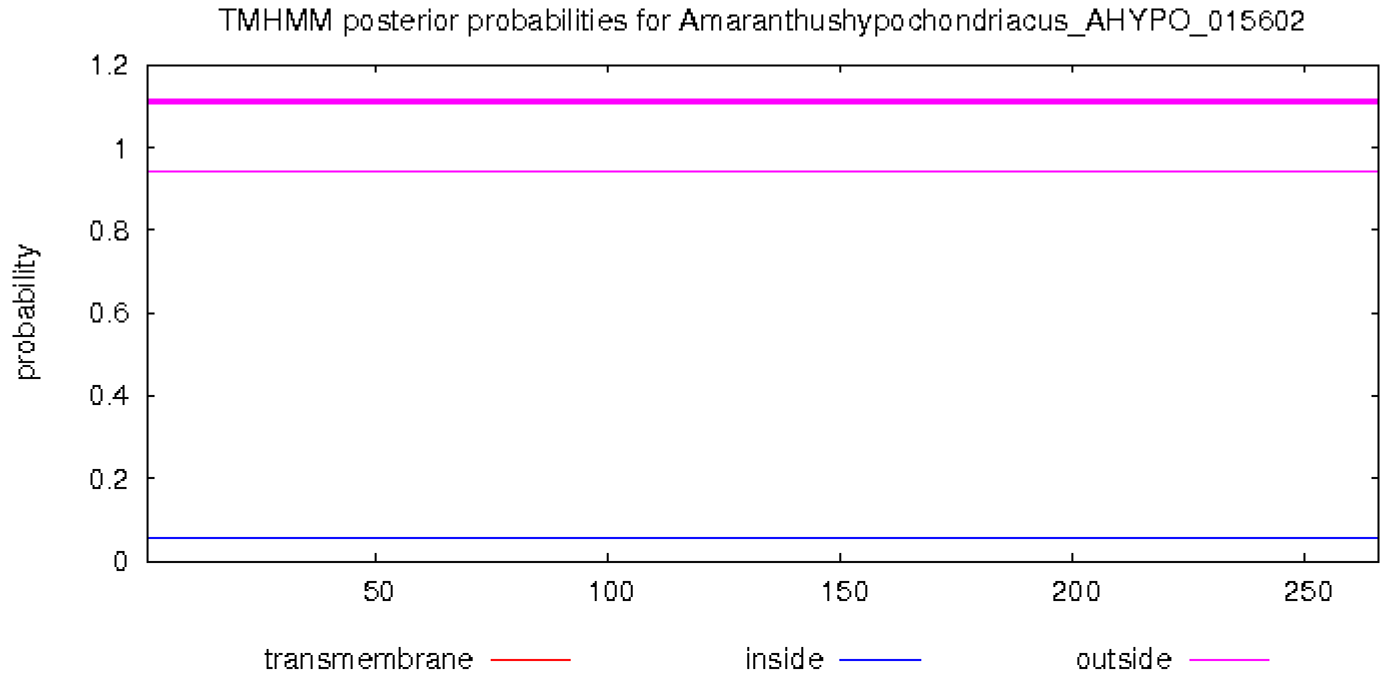

plot in postscript, script for making the plot in gnuplot, data for plot

---

```
# Amaranthushypochondriacus_AHYPO_015615 Length: 309
# Amaranthushypochondriacus_AHYPO_015615 Number of predicted TMHs:  0
```

```
# Amaranthushypochondriacus_AHYPO_015615 Exp number of AAs in TMHs: 0.00069
# Amaranthushypochondriacus_AHYPO_015615 Exp number, first 60 AAs:  0
# Amaranthushypochondriacus_AHYPO_015615 Total prob of N-in:        0.04776
Amaranthushypochondriacus_AHYPO_015615  TMHMM2.0        outside      1   309
```

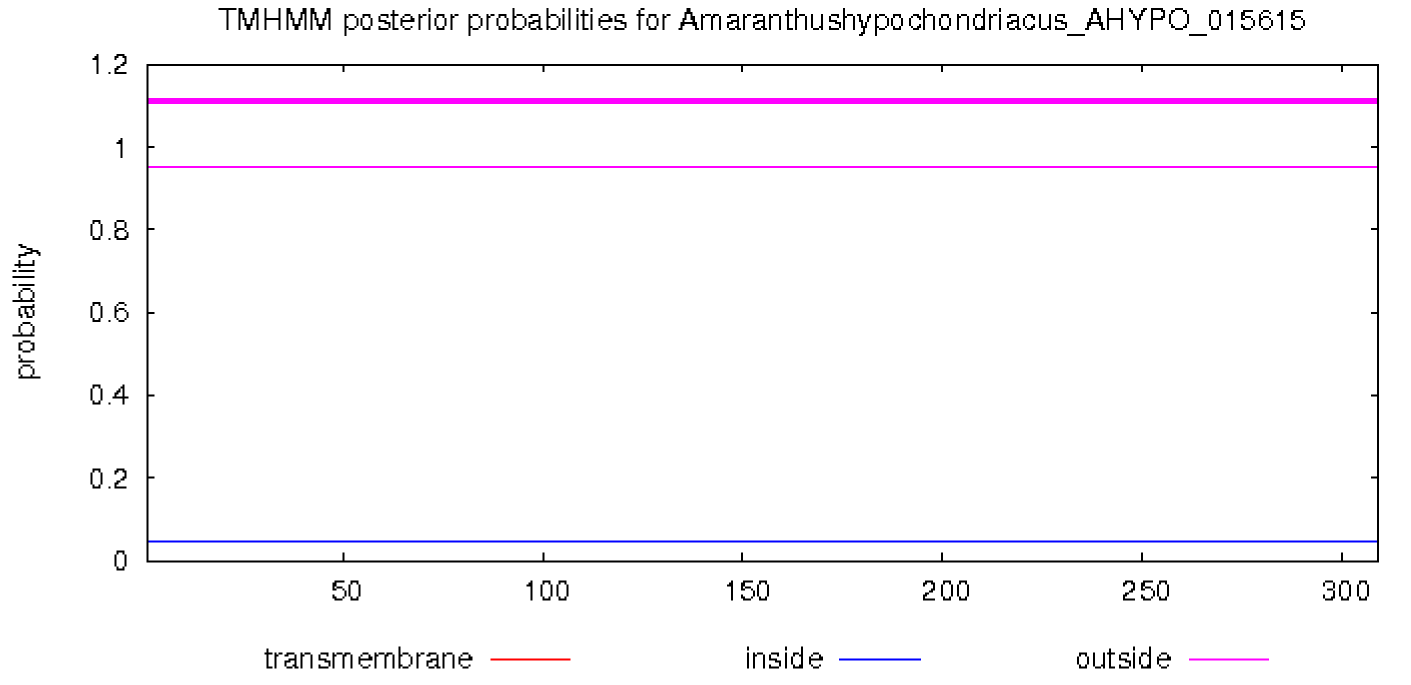

plot in postscript, script for making the plot in gnuplot, data for plot

---

```
# Amaranthushypochondriacus_AHYPO_015940 Length: 302
# Amaranthushypochondriacus_AHYPO_015940 Number of predicted TMHs:  0
# Amaranthushypochondriacus_AHYPO_015940 Exp number of AAs in TMHs: 0.03948
# Amaranthushypochondriacus_AHYPO_015940 Exp number, first 60 AAs:  0.00563
# Amaranthushypochondriacus_AHYPO_015940 Total prob of N-in:        0.04530
Amaranthushypochondriacus_AHYPO_015940  TMHMM2.0        outside      1   302
```

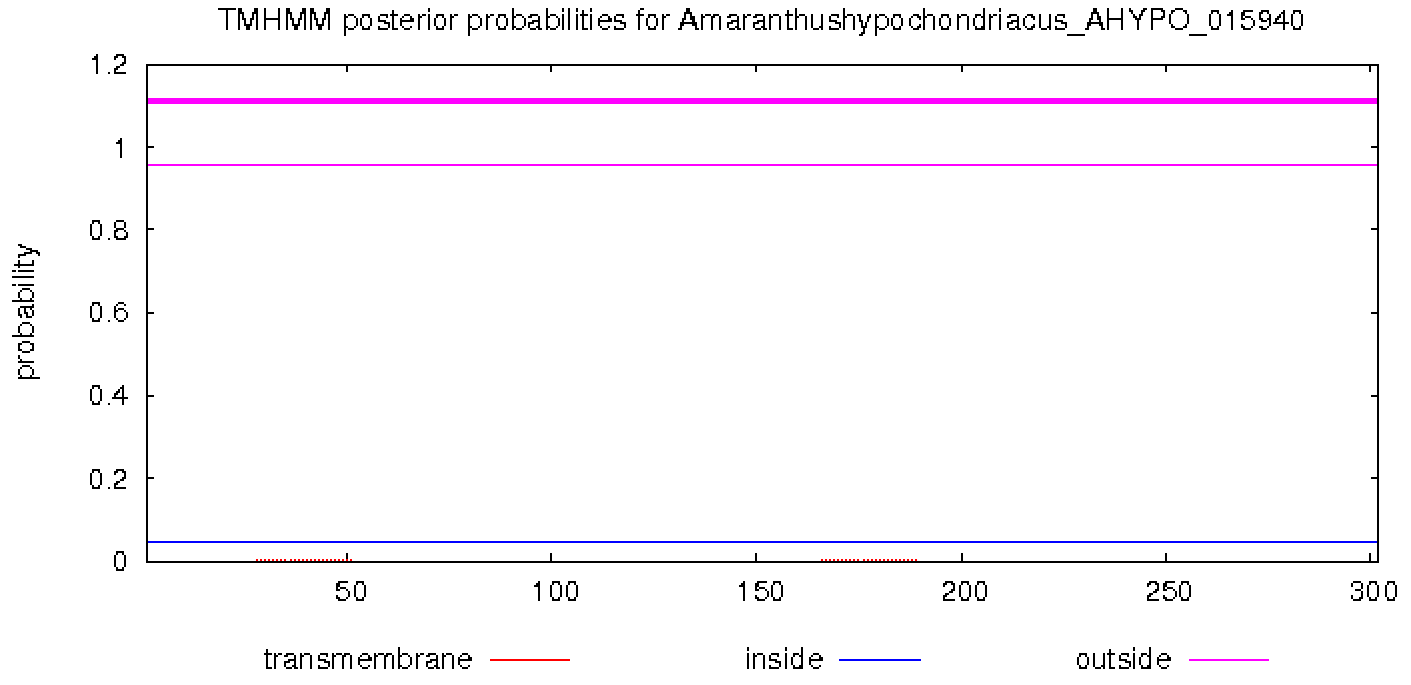

plot in postscript, script for making the plot in gnuplot, data for plot

---

```
# Amaranthushypochondriacus_AHYPO_019273 Length: 451
# Amaranthushypochondriacus_AHYPO_019273 Number of predicted TMHs:  0
```

```
# Amaranthushypochondriacus_AHYPO_019273 Exp number of AAs in TMHs: 0.00038
# Amaranthushypochondriacus_AHYPO_019273 Exp number, first 60 AAs:  0.00018
# Amaranthushypochondriacus_AHYPO_019273 Total prob of N-in:        0.00578
Amaranthushypochondriacus_AHYPO_019273  TMHMM2.0        outside      1   451
```

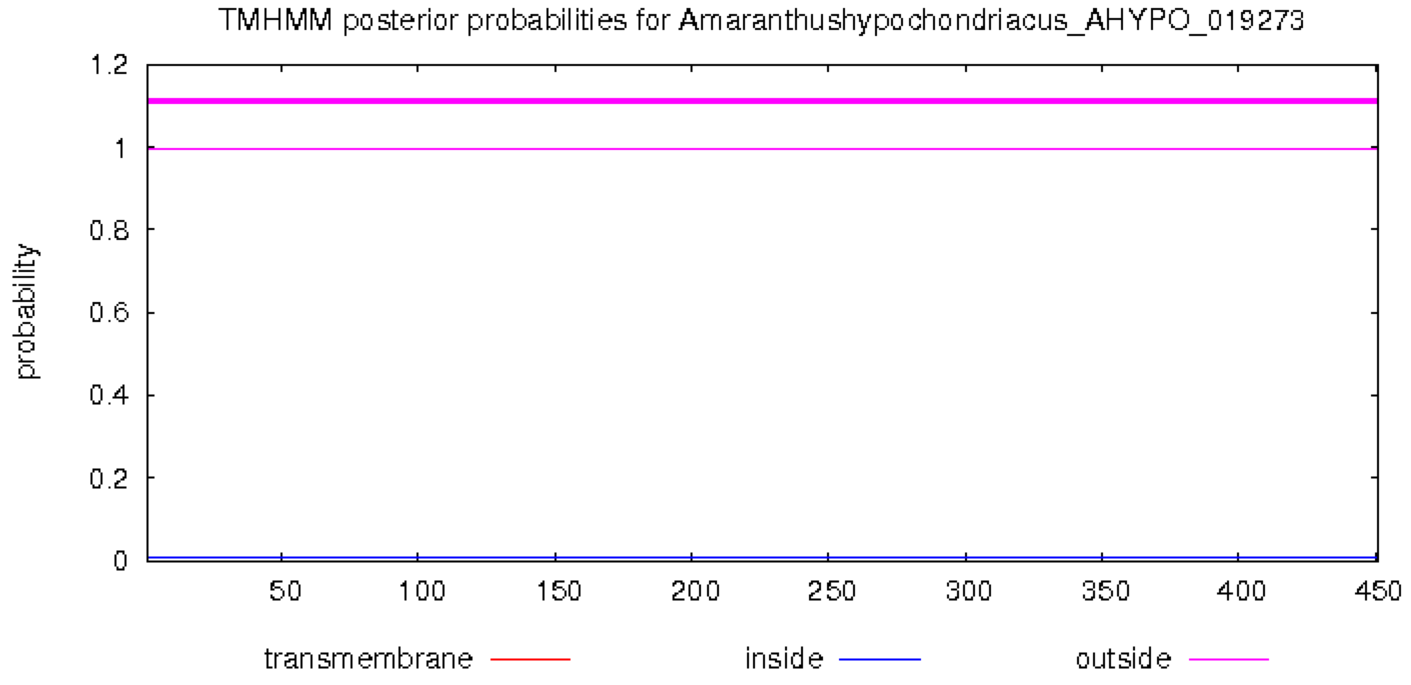

plot in postscript, script for making the plot in gnuplot, data for plot

---

```
# Amaranthushypochondriacus_AHYPO_019388 Length: 276
# Amaranthushypochondriacus_AHYPO_019388 Number of predicted TMHs:  0
# Amaranthushypochondriacus_AHYPO_019388 Exp number of AAs in TMHs: 0
# Amaranthushypochondriacus_AHYPO_019388 Exp number, first 60 AAs:  0
# Amaranthushypochondriacus_AHYPO_019388 Total prob of N-in:        0.09181
Amaranthushypochondriacus_AHYPO_019388  TMHMM2.0        outside      1   276
```

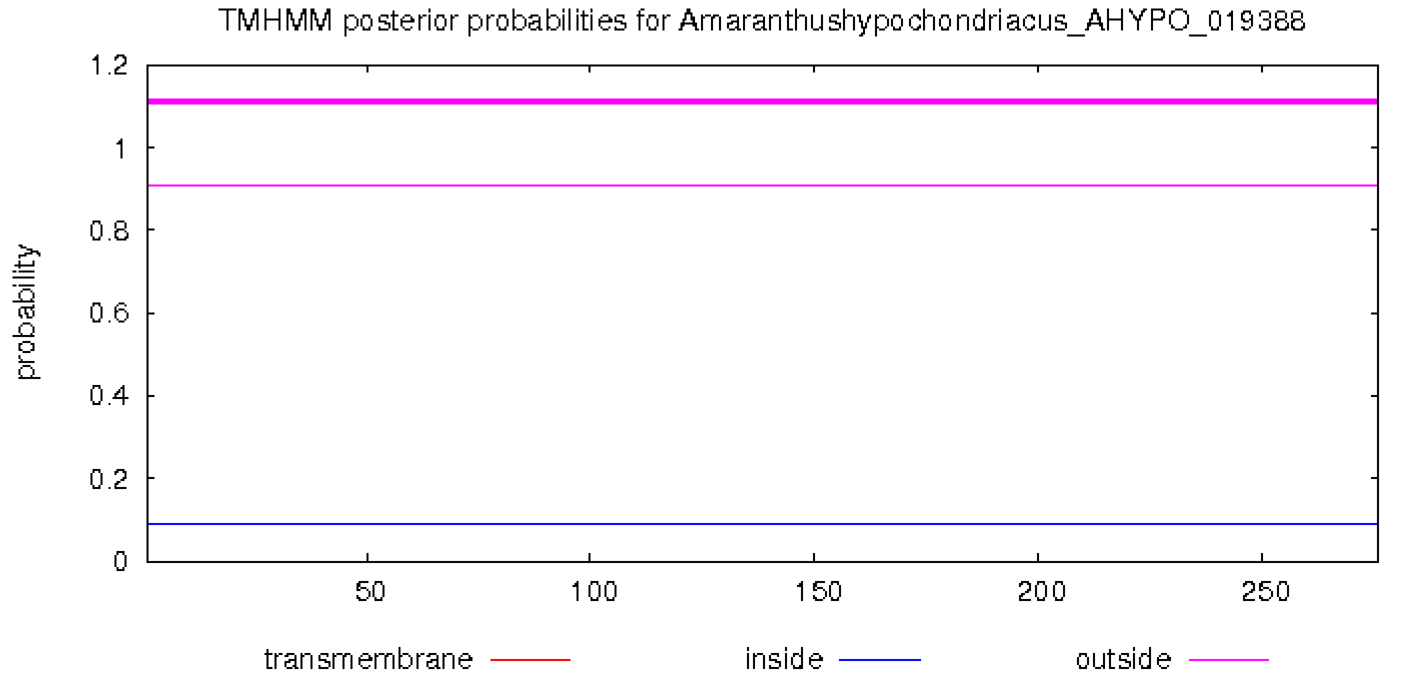

plot in postscript, script for making the plot in gnuplot, data for plot

---

```
# Amaranthushypochondriacus_AHYPO_020247 Length: 197
# Amaranthushypochondriacus_AHYPO_020247 Number of predicted TMHs:  0
```

```
# Amaranthushypochondriacus_AHYPO_020247 Exp number of AAs in TMHs: 0.00364
# Amaranthushypochondriacus_AHYPO_020247 Exp number, first 60 AAs:  0
# Amaranthushypochondriacus_AHYPO_020247 Total prob of N-in:        0.01042
Amaranthushypochondriacus_AHYPO_020247  TMHMM2.0        outside      1   197
```

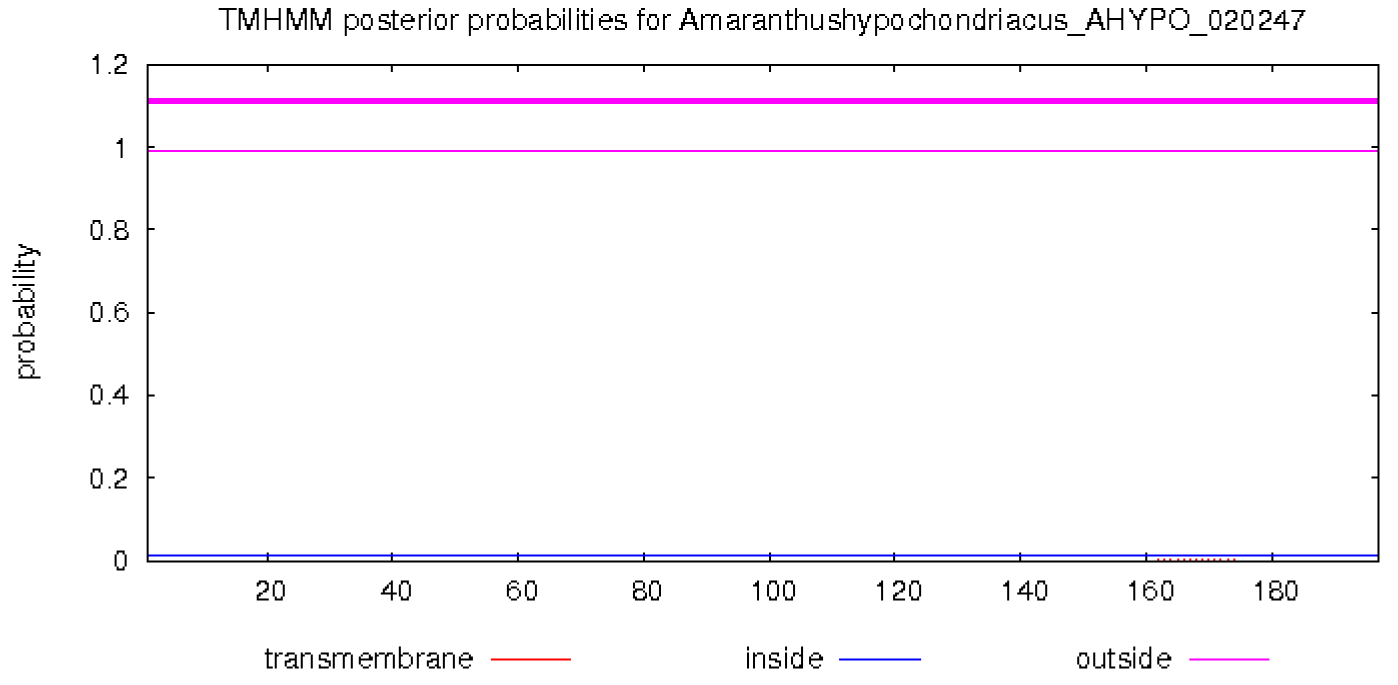

plot in postscript, script for making the plot in gnuplot, data for plot

---

```
# Amaranthushypochondriacus_AHYPO_020702 Length: 406
# Amaranthushypochondriacus_AHYPO_020702 Number of predicted TMHs:  1
# Amaranthushypochondriacus_AHYPO_020702 Exp number of AAs in TMHs: 19.5995
# Amaranthushypochondriacus_AHYPO_020702 Exp number, first 60 AAs:  0.00296
# Amaranthushypochondriacus_AHYPO_020702 Total prob of N-in:        0.60110
Amaranthushypochondriacus_AHYPO_020702  TMHMM2.0        inside       1   385
Amaranthushypochondriacus_AHYPO_020702  TMHMM2.0        TMhelix    386   405
Amaranthushypochondriacus_AHYPO_020702  TMHMM2.0        outside    406   406
```

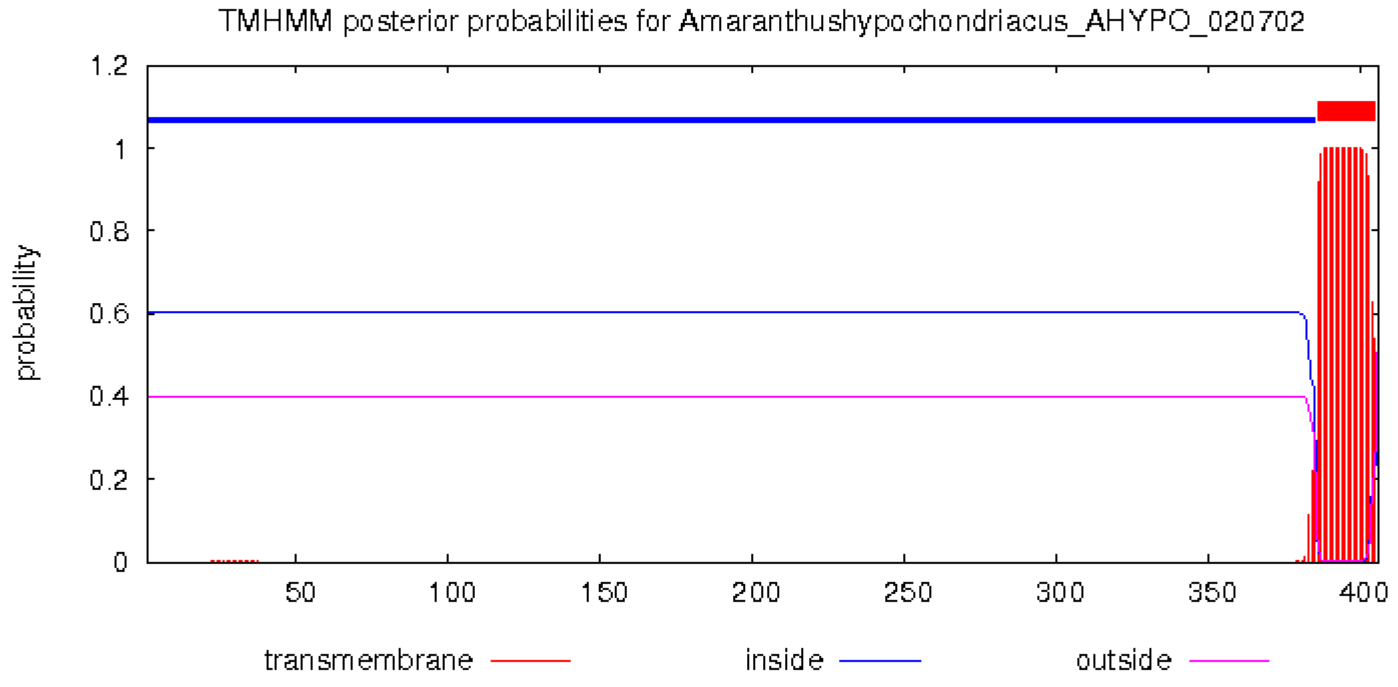

plot in postscript, script for making the plot in gnuplot, data for plot

---

```
# Amaranthushypochondriacus_AHYPO_021403 Length: 197
# Amaranthushypochondriacus_AHYPO_021403 Number of predicted TMHs:  0
# Amaranthushypochondriacus_AHYPO_021403 Exp number of AAs in TMHs: 0.00224
# Amaranthushypochondriacus_AHYPO_021403 Exp number, first 60 AAs:  0
# Amaranthushypochondriacus_AHYPO_021403 Total prob of N-in:        0.04605
Amaranthushypochondriacus_AHYPO_021403  TMHMM2.0        outside      1   197
```

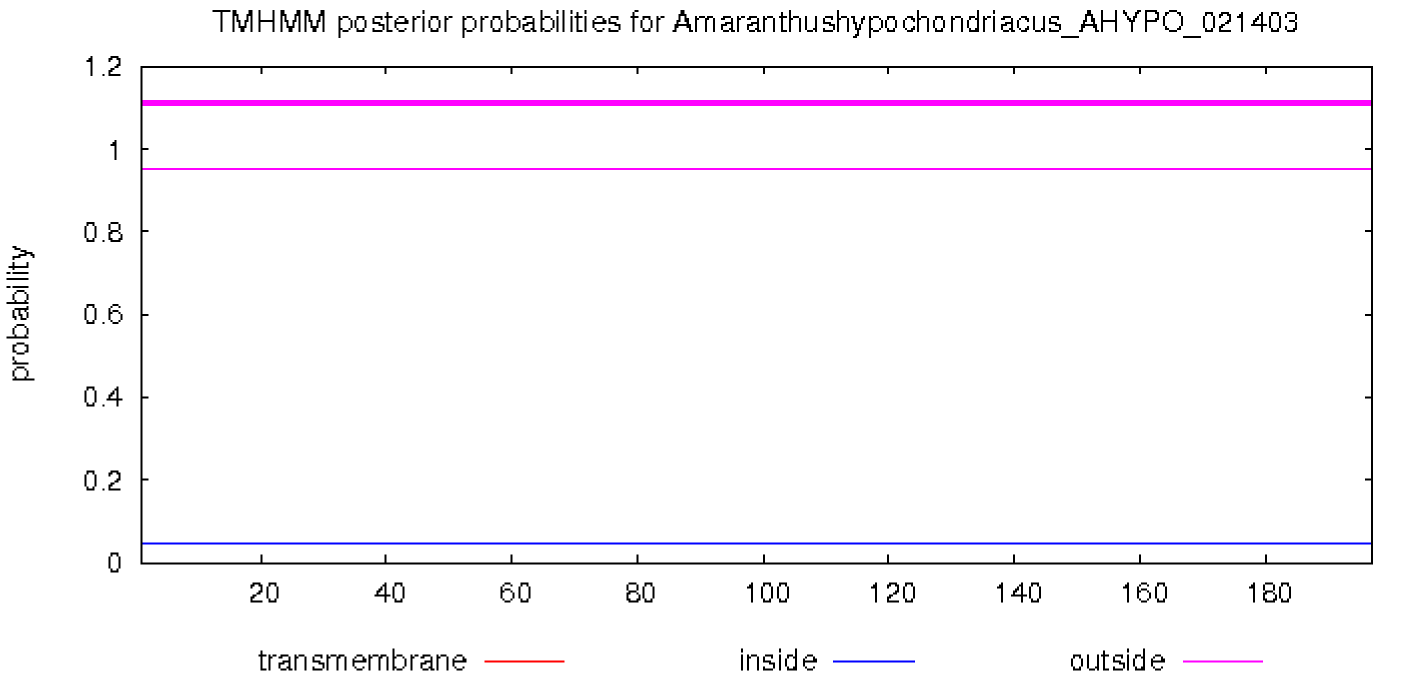

plot in postscript, script for making the plot in gnuplot, data for plot

---

```
# Amborellatrichopoda_evm_27.model.AmTr_v1.0_scaffold00001.192 Length: 251
# Amborellatrichopoda_evm_27.model.AmTr_v1.0_scaffold00001.192 Number of predicted TMHs:  0
# Amborellatrichopoda_evm_27.model.AmTr_v1.0_scaffold00001.192 Exp number of AAs in TMHs: 0.00063
# Amborellatrichopoda_evm_27.model.AmTr_v1.0_scaffold00001.192 Exp number, first 60 AAs:  0.00063
# Amborellatrichopoda_evm_27.model.AmTr_v1.0_scaffold00001.192 Total prob of N-in:        0.00486
Amborellatrichopoda_evm_27.model.AmTr_v1.0_scaffold00001.192    TMHMM2.0        outside      1   251
```

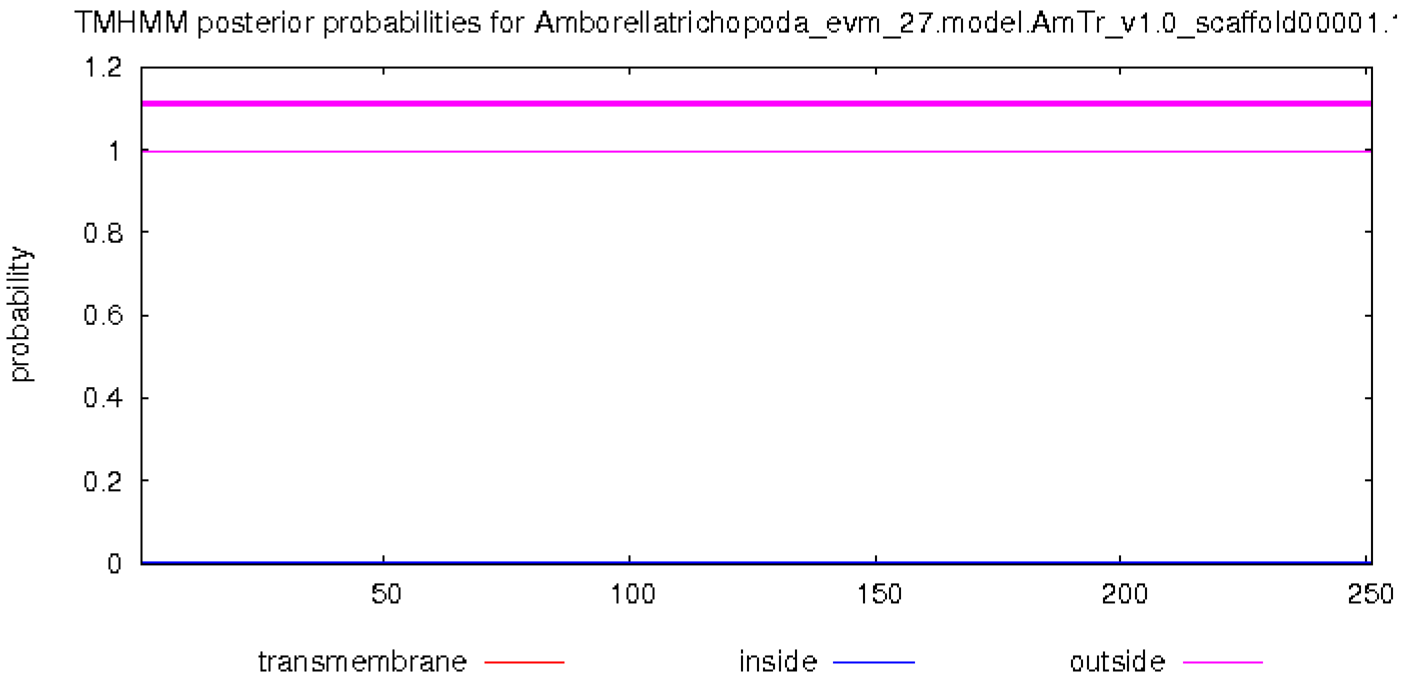

plot in postscript, script for making the plot in gnuplot, data for plot

---

```
# Amborellatrichopoda_evm_27.model.AmTr_v1.0_scaffold00001.280 Length: 267
# Amborellatrichopoda_evm_27.model.AmTr_v1.0_scaffold00001.280 Number of predicted TMHs:  0
# Amborellatrichopoda_evm_27.model.AmTr_v1.0_scaffold00001.280 Exp number of AAs in TMHs: 0.00078
# Amborellatrichopoda_evm_27.model.AmTr_v1.0_scaffold00001.280 Exp number, first 60 AAs:  0.00058
# Amborellatrichopoda_evm_27.model.AmTr_v1.0_scaffold00001.280 Total prob of N-in:        0.00497
Amborellatrichopoda_evm_27.model.AmTr_v1.0_scaffold00001.280    TMHMM2.0        outside      1   267
```

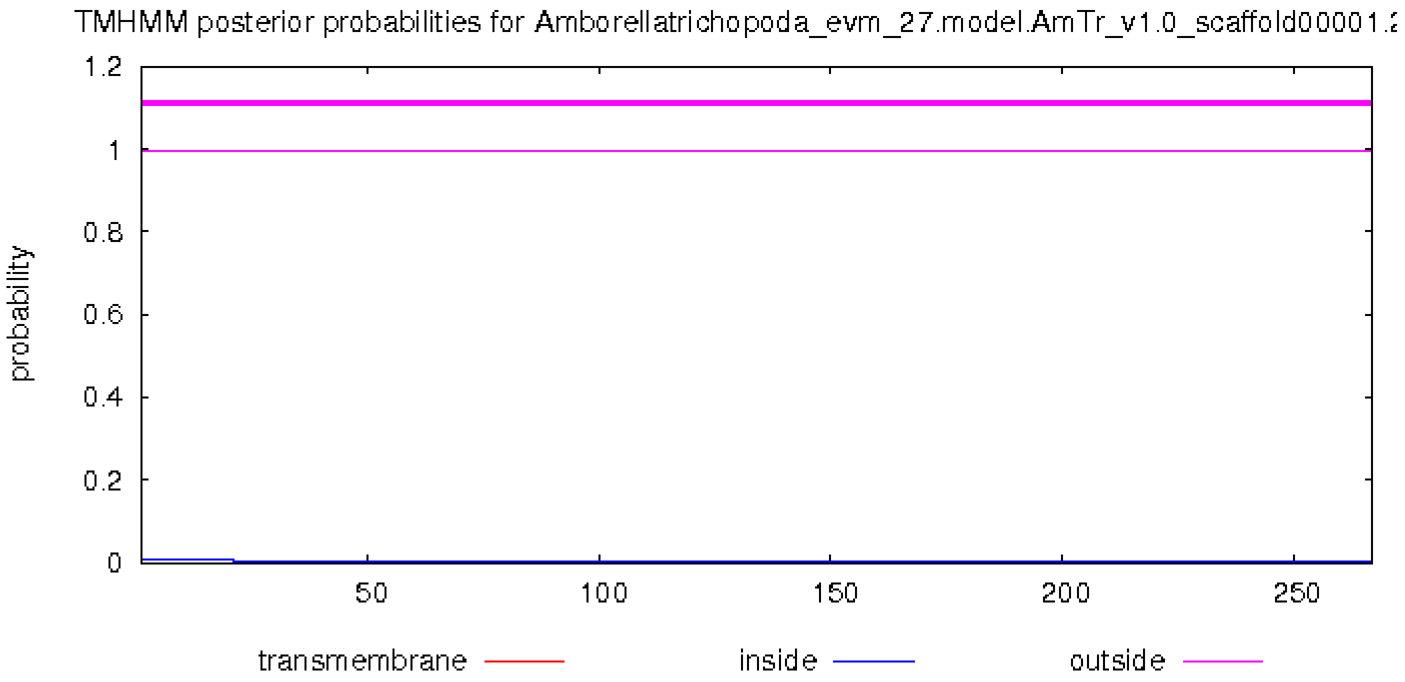

plot in postscript, script for making the plot in gnuplot, data for plot

---

```
# Amborellatrichopoda_evm_27.model.AmTr_v1.0_scaffold00003.302 Length: 349
# Amborellatrichopoda_evm_27.model.AmTr_v1.0_scaffold00003.302 Number of predicted TMHs:  0
# Amborellatrichopoda_evm_27.model.AmTr_v1.0_scaffold00003.302 Exp number of AAs in TMHs: 0.00045
# Amborellatrichopoda_evm_27.model.AmTr_v1.0_scaffold00003.302 Exp number, first 60 AAs:  0.00017
# Amborellatrichopoda_evm_27.model.AmTr_v1.0_scaffold00003.302 Total prob of N-in:        0.01715
Amborellatrichopoda_evm_27.model.AmTr_v1.0_scaffold00003.302    TMHMM2.0        outside      1   349
```

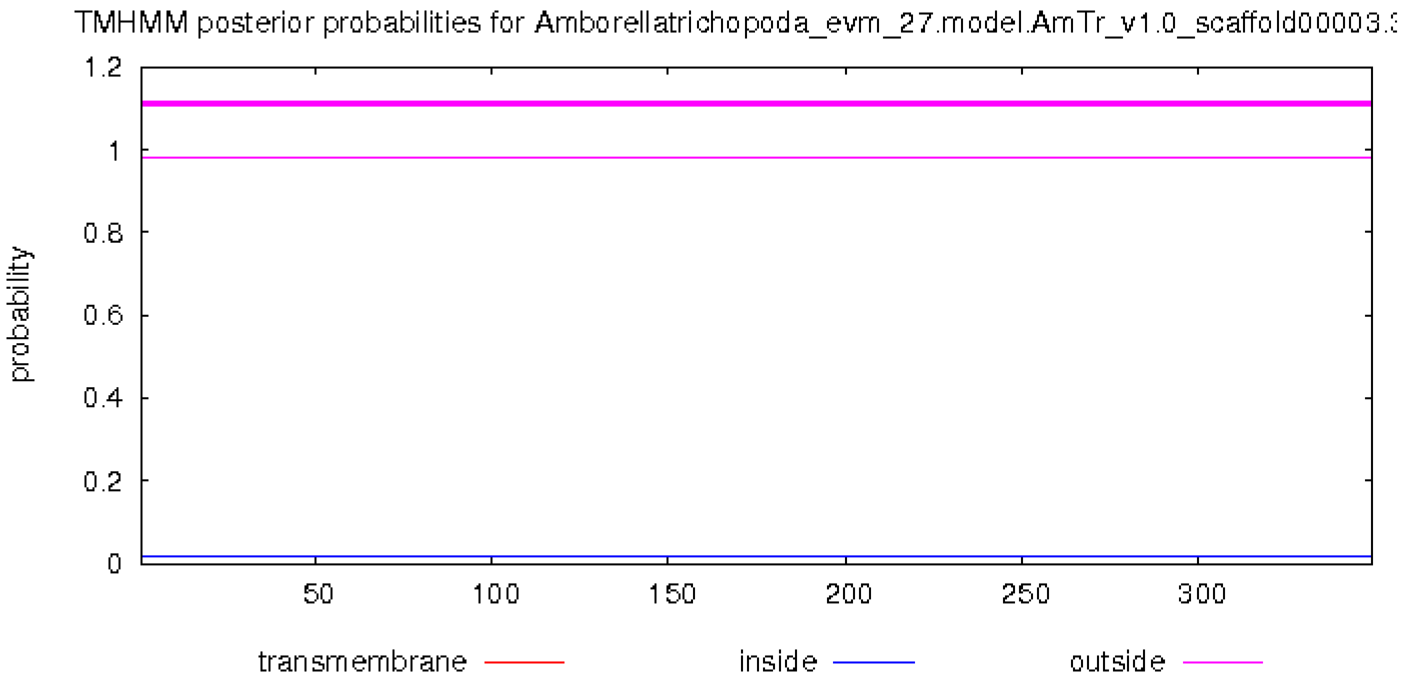

plot in postscript, script for making the plot in gnuplot, data for plot

---

```
# Amborellatrichopoda_evm_27.model.AmTr_v1.0_scaffold00007.33 Length: 361
# Amborellatrichopoda_evm_27.model.AmTr_v1.0_scaffold00007.33 Number of predicted TMHs:  0
# Amborellatrichopoda_evm_27.model.AmTr_v1.0_scaffold00007.33 Exp number of AAs in TMHs: 0
# Amborellatrichopoda_evm_27.model.AmTr_v1.0_scaffold00007.33 Exp number, first 60 AAs:  0
# Amborellatrichopoda_evm_27.model.AmTr_v1.0_scaffold00007.33 Total prob of N-in:        0.00896
Amborellatrichopoda_evm_27.model.AmTr_v1.0_scaffold00007.33     TMHMM2.0        outside      1   361
```

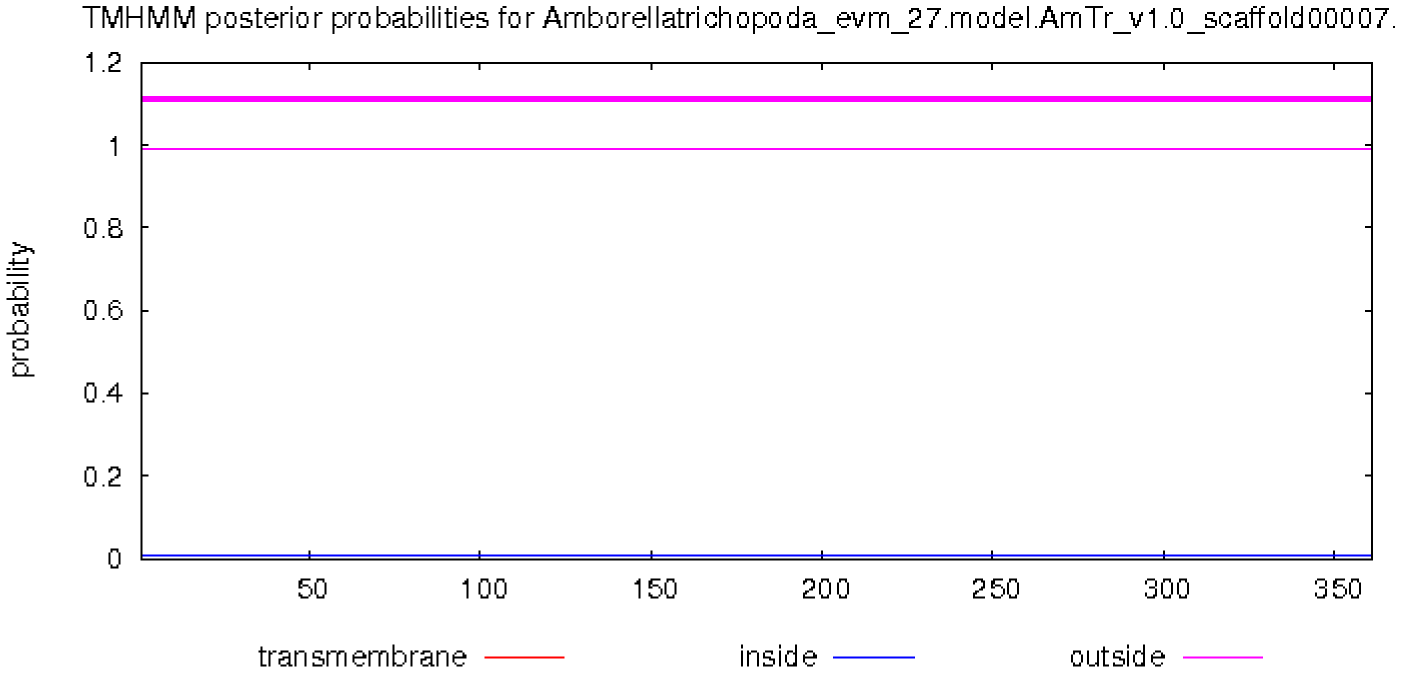

plot in postscript, script for making the plot in gnuplot, data for plot

---

```
# Amborellatrichopoda_evm_27.model.AmTr_v1.0_scaffold00009.309 Length: 291
# Amborellatrichopoda_evm_27.model.AmTr_v1.0_scaffold00009.309 Number of predicted TMHs:  0
# Amborellatrichopoda_evm_27.model.AmTr_v1.0_scaffold00009.309 Exp number of AAs in TMHs: 0.00083
# Amborellatrichopoda_evm_27.model.AmTr_v1.0_scaffold00009.309 Exp number, first 60 AAs:  0.00077
# Amborellatrichopoda_evm_27.model.AmTr_v1.0_scaffold00009.309 Total prob of N-in:        0.00354
Amborellatrichopoda_evm_27.model.AmTr_v1.0_scaffold00009.309    TMHMM2.0        outside      1   291
```

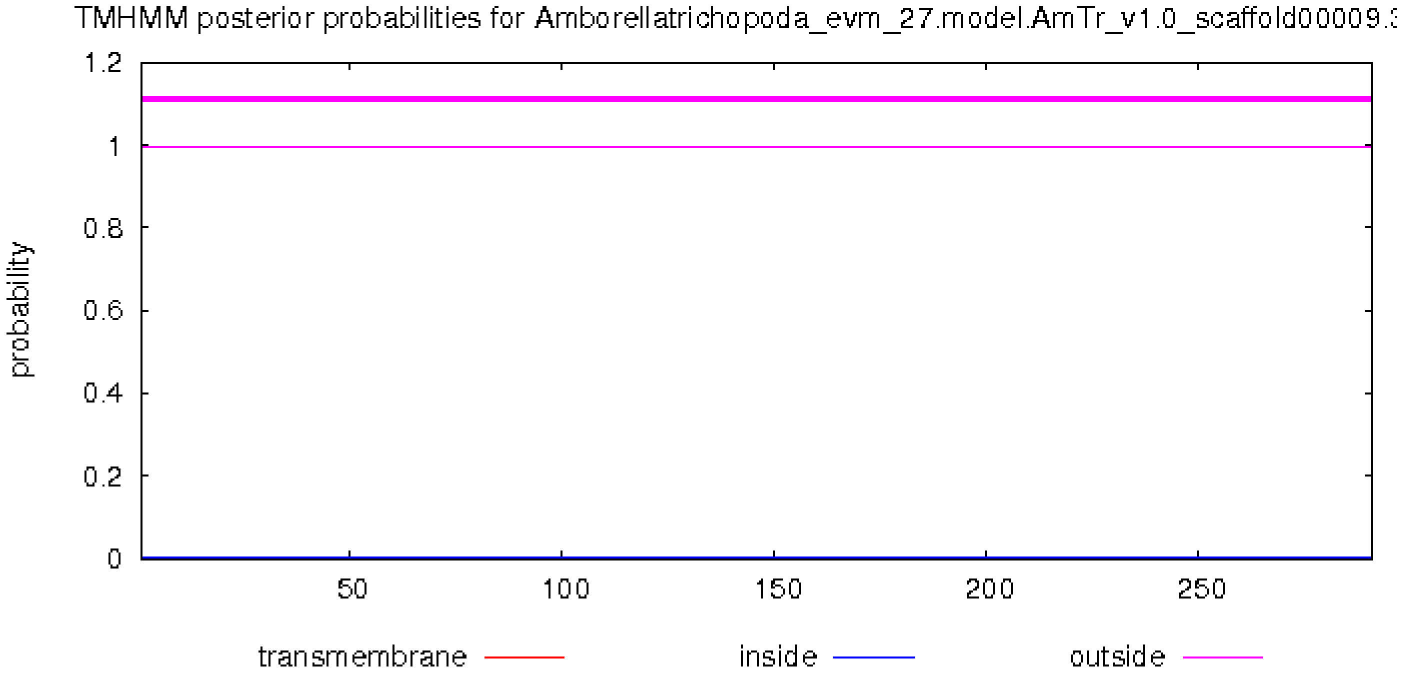

plot in postscript, script for making the plot in gnuplot, data for plot

---

```
# Amborellatrichopoda_evm_27.model.AmTr_v1.0_scaffold00010.432 Length: 345
# Amborellatrichopoda_evm_27.model.AmTr_v1.0_scaffold00010.432 Number of predicted TMHs:  0
# Amborellatrichopoda_evm_27.model.AmTr_v1.0_scaffold00010.432 Exp number of AAs in TMHs: 0.0008
# Amborellatrichopoda_evm_27.model.AmTr_v1.0_scaffold00010.432 Exp number, first 60 AAs:  0.00016
# Amborellatrichopoda_evm_27.model.AmTr_v1.0_scaffold00010.432 Total prob of N-in:        0.02432
Amborellatrichopoda_evm_27.model.AmTr_v1.0_scaffold00010.432	TMHMM2.0	outside	     1   345
```

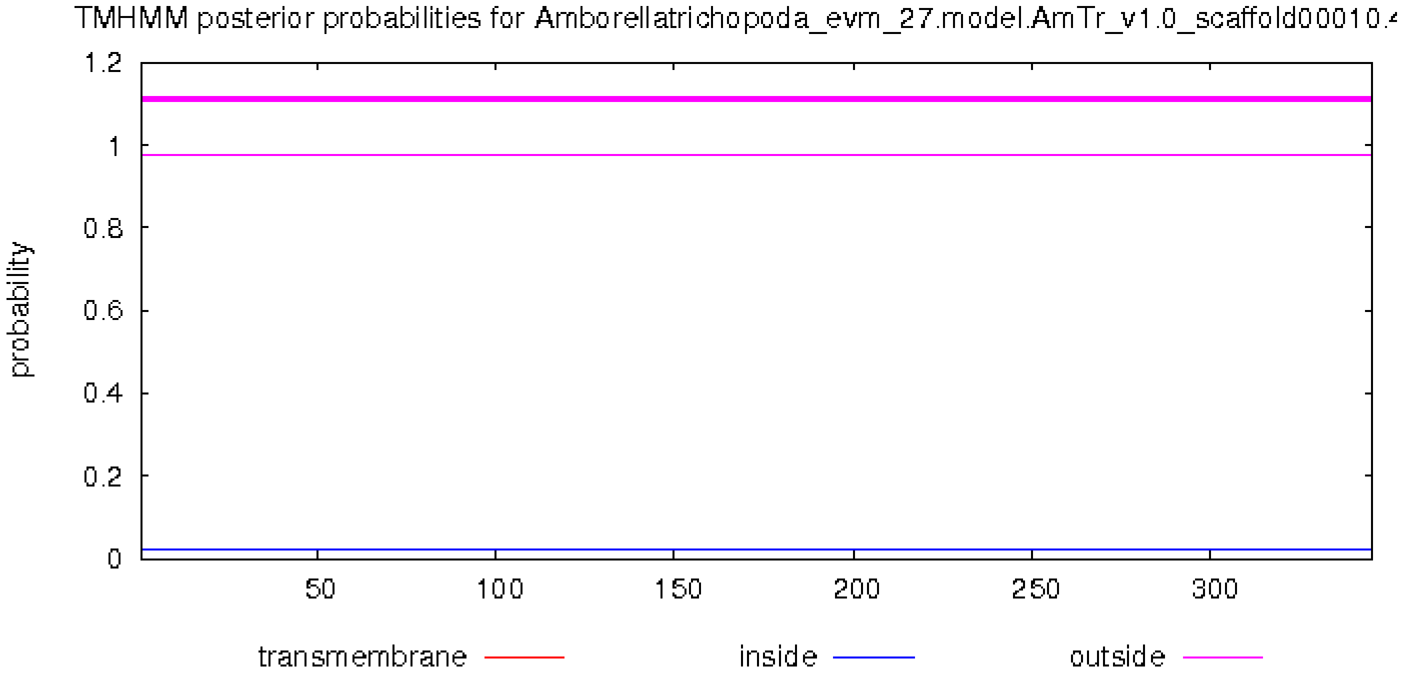

plot in postscript, script for making the plot in gnuplot, data for plot

---

```
# Amborellatrichopoda_evm_27.model.AmTr_v1.0_scaffold00010.523 Length: 379
# Amborellatrichopoda_evm_27.model.AmTr_v1.0_scaffold00010.523 Number of predicted TMHs:  0
# Amborellatrichopoda_evm_27.model.AmTr_v1.0_scaffold00010.523 Exp number of AAs in TMHs: 0
# Amborellatrichopoda_evm_27.model.AmTr_v1.0_scaffold00010.523 Exp number, first 60 AAs:  0
# Amborellatrichopoda_evm_27.model.AmTr_v1.0_scaffold00010.523 Total prob of N-in:        0.00589
Amborellatrichopoda_evm_27.model.AmTr_v1.0_scaffold00010.523	TMHMM2.0	outside	     1   379
```

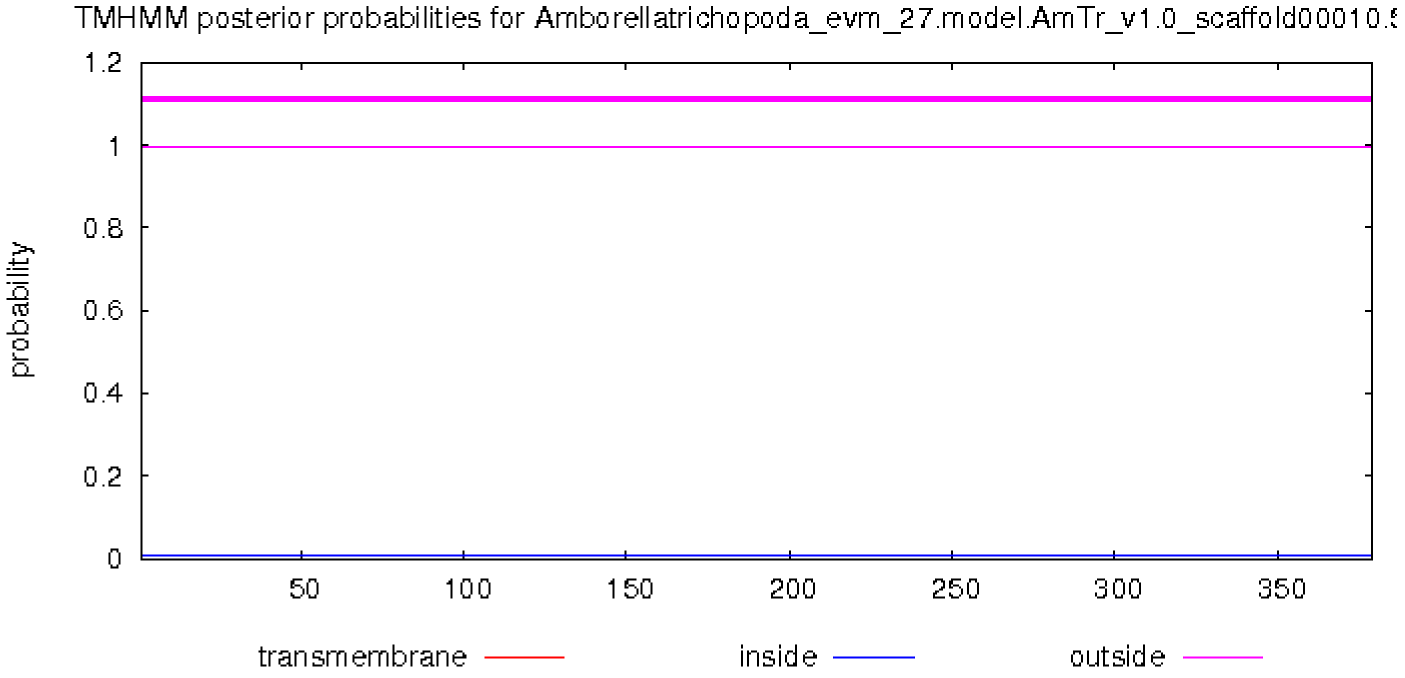

plot in postscript, script for making the plot in gnuplot, data for plot

---

```
# Amborellatrichopoda_evm_27.model.AmTr_v1.0_scaffold00011.156 Length: 318
# Amborellatrichopoda_evm_27.model.AmTr_v1.0_scaffold00011.156 Number of predicted TMHs:  0
# Amborellatrichopoda_evm_27.model.AmTr_v1.0_scaffold00011.156 Exp number of AAs in TMHs: 0
# Amborellatrichopoda_evm_27.model.AmTr_v1.0_scaffold00011.156 Exp number, first 60 AAs:  0
# Amborellatrichopoda_evm_27.model.AmTr_v1.0_scaffold00011.156 Total prob of N-in:        0.00613
Amborellatrichopoda_evm_27.model.AmTr_v1.0_scaffold00011.156	TMHMM2.0	outside	     1   318
```

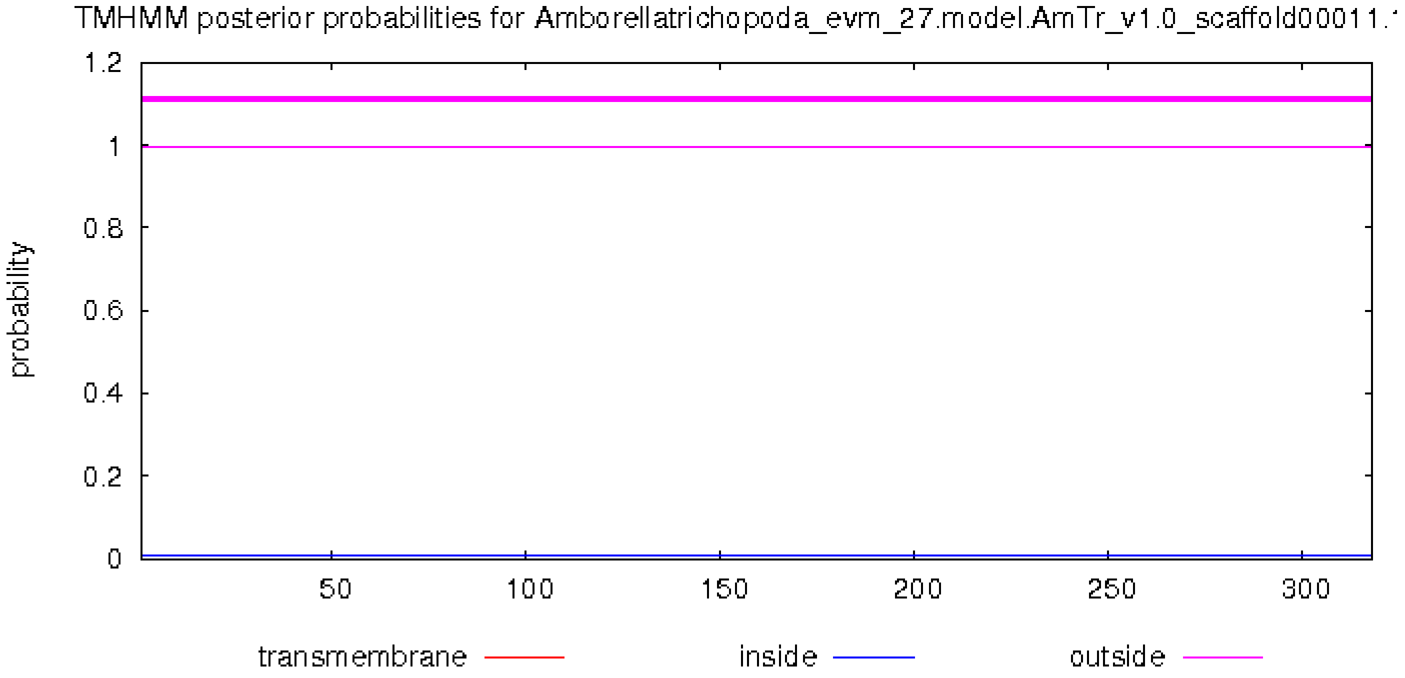

plot in postscript, script for making the plot in gnuplot, data for plot

---

```
# Amborellatrichopoda_evm_27.model.AmTr_v1.0_scaffold00016.162 Length: 234
# Amborellatrichopoda_evm_27.model.AmTr_v1.0_scaffold00016.162 Number of predicted TMHs:  0
# Amborellatrichopoda_evm_27.model.AmTr_v1.0_scaffold00016.162 Exp number of AAs in TMHs: 0.00067
# Amborellatrichopoda_evm_27.model.AmTr_v1.0_scaffold00016.162 Exp number, first 60 AAs:  0.00016
# Amborellatrichopoda_evm_27.model.AmTr_v1.0_scaffold00016.162 Total prob of N-in:        0.10390
Amborellatrichopoda_evm_27.model.AmTr_v1.0_scaffold00016.162	TMHMM2.0	outside	     1   234
```

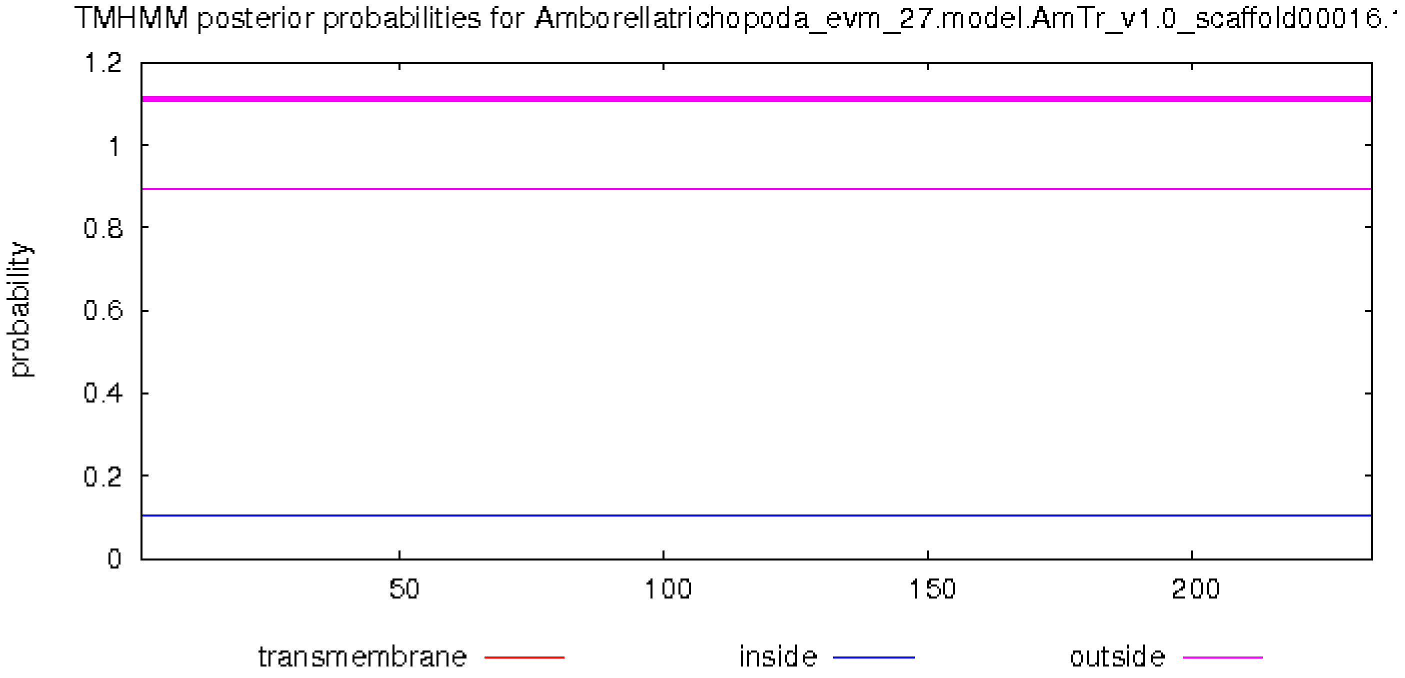

plot in postscript, script for making the plot in gnuplot, data for plot

---

```
# Amborellatrichopoda_evm_27.model.AmTr_v1.0_scaffold00017.277 Length: 188
# Amborellatrichopoda_evm_27.model.AmTr_v1.0_scaffold00017.277 Number of predicted TMHs:  0
# Amborellatrichopoda_evm_27.model.AmTr_v1.0_scaffold00017.277 Exp number of AAs in TMHs: 0.00456
# Amborellatrichopoda_evm_27.model.AmTr_v1.0_scaffold00017.277 Exp number, first 60 AAs:  0.00032
# Amborellatrichopoda_evm_27.model.AmTr_v1.0_scaffold00017.277 Total prob of N-in:        0.02087
Amborellatrichopoda_evm_27.model.AmTr_v1.0_scaffold00017.277	TMHMM2.0	outside	     1   188
```

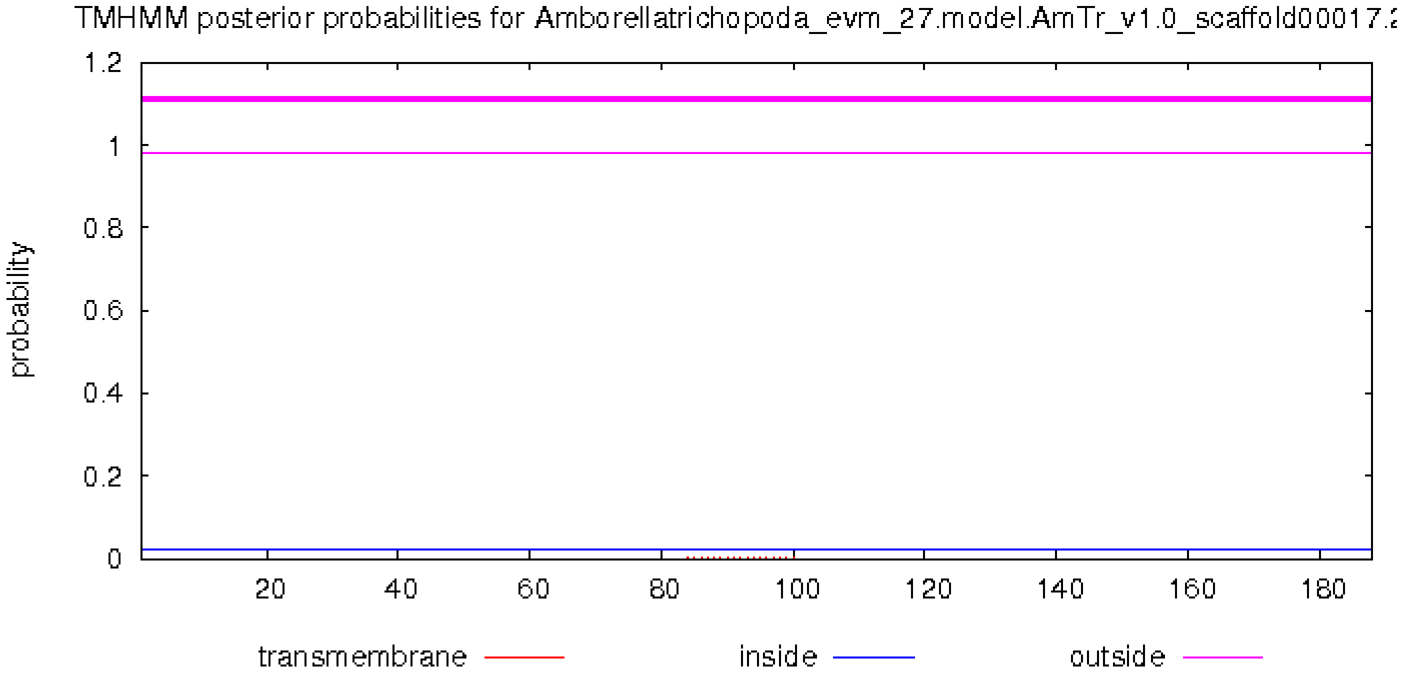

plot in postscript, script for making the plot in gnuplot, data for plot

---

```
# Amborellatrichopoda_evm_27.model.AmTr_v1.0_scaffold00017.45 Length: 281
# Amborellatrichopoda_evm_27.model.AmTr_v1.0_scaffold00017.45 Number of predicted TMHs:  0
# Amborellatrichopoda_evm_27.model.AmTr_v1.0_scaffold00017.45 Exp number of AAs in TMHs: 0
# Amborellatrichopoda_evm_27.model.AmTr_v1.0_scaffold00017.45 Exp number, first 60 AAs:  0
# Amborellatrichopoda_evm_27.model.AmTr_v1.0_scaffold00017.45 Total prob of N-in:        0.01426
Amborellatrichopoda_evm_27.model.AmTr_v1.0_scaffold00017.45	TMHMM2.0	outside	     1   281
```

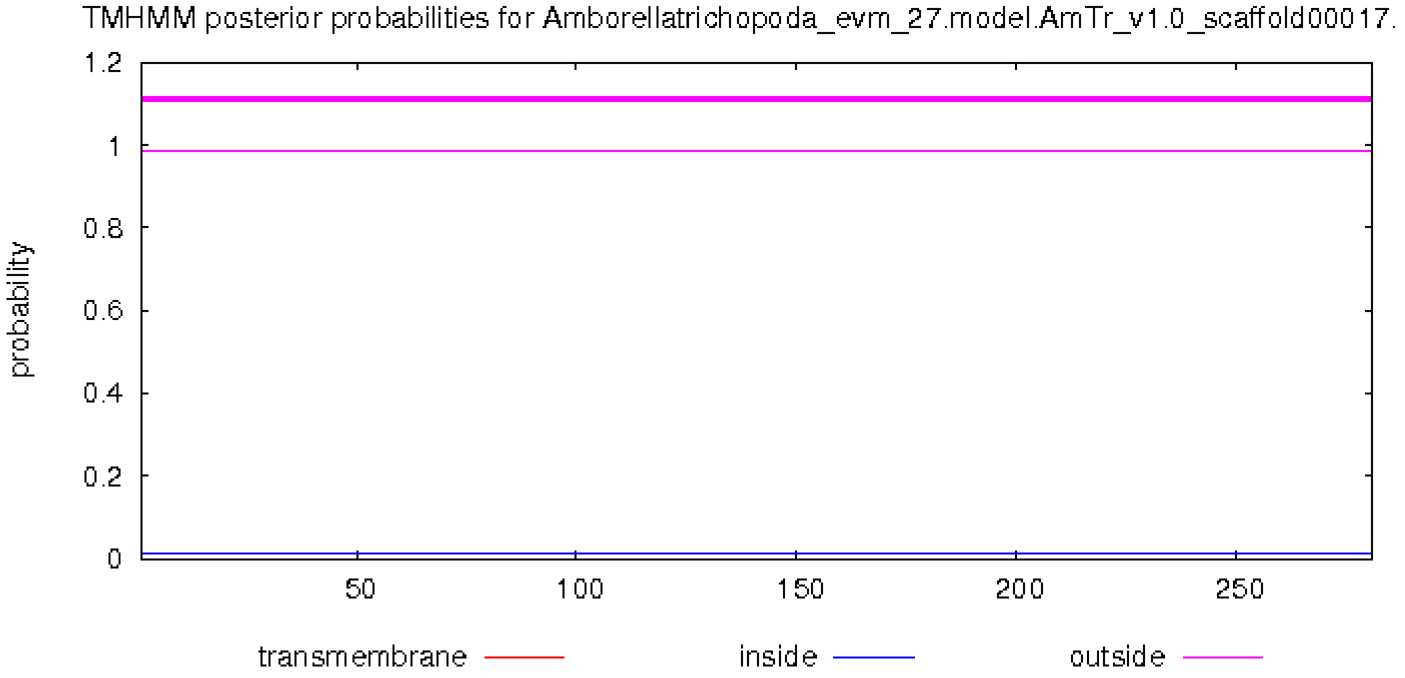

plot in postscript, script for making the plot in gnuplot, data for plot

---

```
# Amborellatrichopoda_evm_27.model.AmTr_v1.0_scaffold00018.24 Length: 177
# Amborellatrichopoda_evm_27.model.AmTr_v1.0_scaffold00018.24 Number of predicted TMHs:  0
# Amborellatrichopoda_evm_27.model.AmTr_v1.0_scaffold00018.24 Exp number of AAs in TMHs: 0.33965
# Amborellatrichopoda_evm_27.model.AmTr_v1.0_scaffold00018.24 Exp number, first 60 AAs:  0
# Amborellatrichopoda_evm_27.model.AmTr_v1.0_scaffold00018.24 Total prob of N-in:        0.02293
Amborellatrichopoda_evm_27.model.AmTr_v1.0_scaffold00018.24     TMHMM2.0        outside      1   177
```

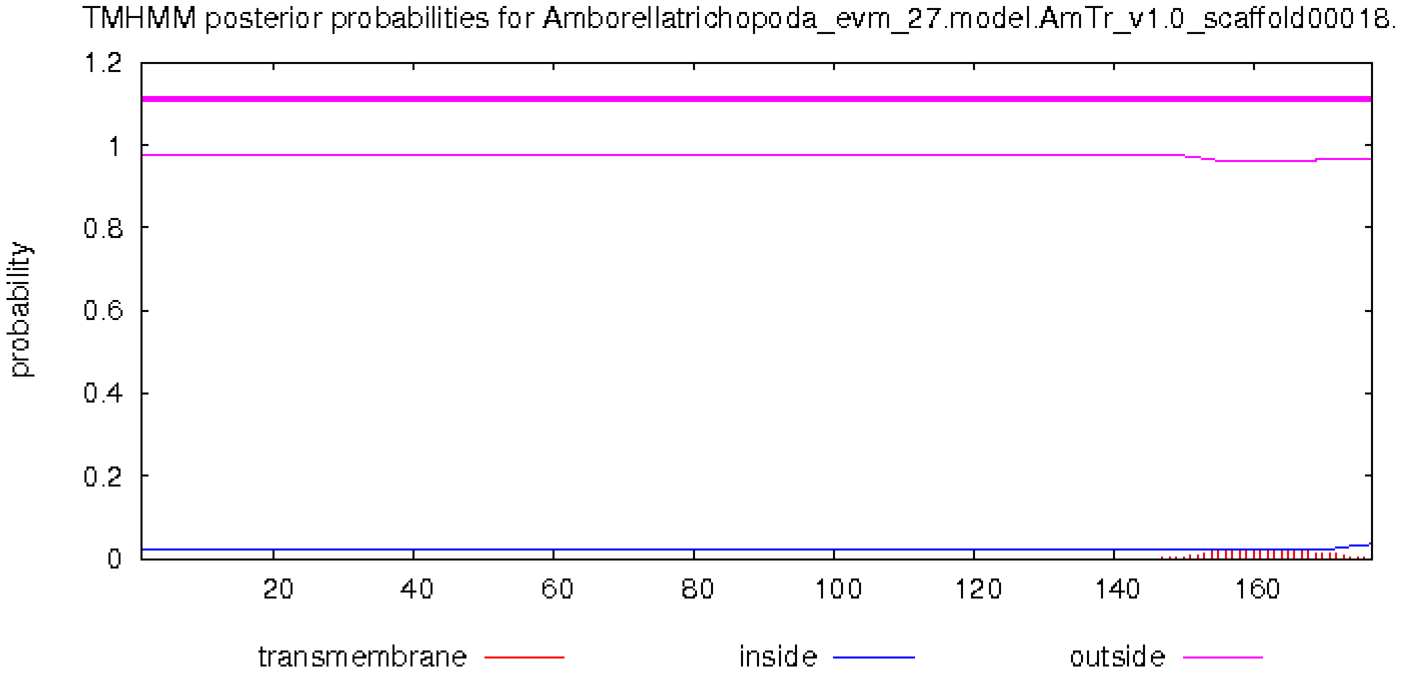

plot in postscript, script for making the plot in gnuplot, data for plot

---

```
# Amborellatrichopoda_evm_27.model.AmTr_v1.0_scaffold00018.27 Length: 567
# Amborellatrichopoda_evm_27.model.AmTr_v1.0_scaffold00018.27 Number of predicted TMHs:  1
# Amborellatrichopoda_evm_27.model.AmTr_v1.0_scaffold00018.27 Exp number of AAs in TMHs: 21.94422
# Amborellatrichopoda_evm_27.model.AmTr_v1.0_scaffold00018.27 Exp number, first 60 AAs:  0
# Amborellatrichopoda_evm_27.model.AmTr_v1.0_scaffold00018.27 Total prob of N-in:        0.00193
Amborellatrichopoda_evm_27.model.AmTr_v1.0_scaffold00018.27     TMHMM2.0        outside      1   543
Amborellatrichopoda_evm_27.model.AmTr_v1.0_scaffold00018.27     TMHMM2.0        TMhelix    544   566
Amborellatrichopoda_evm_27.model.AmTr_v1.0_scaffold00018.27     TMHMM2.0        inside     567   567
```

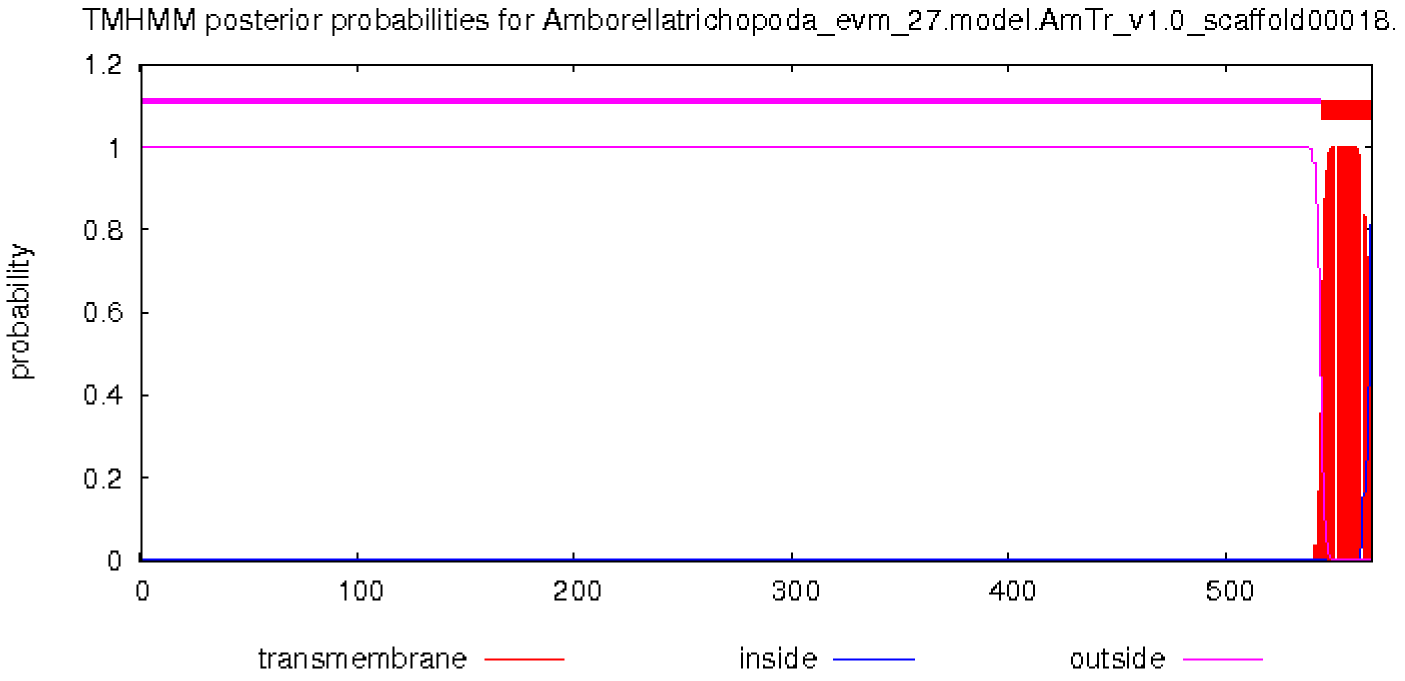

plot in postscript, script for making the plot in gnuplot, data for plot

```
# Amborellatrichopoda_evm_27.model.AmTr_v1.0_scaffold00018.28 Length: 788
# Amborellatrichopoda_evm_27.model.AmTr_v1.0_scaffold00018.28 Number of predicted TMHs:  1
# Amborellatrichopoda_evm_27.model.AmTr_v1.0_scaffold00018.28 Exp number of AAs in TMHs: 20.82463
# Amborellatrichopoda_evm_27.model.AmTr_v1.0_scaffold00018.28 Exp number, first 60 AAs:  0
# Amborellatrichopoda_evm_27.model.AmTr_v1.0_scaffold00018.28 Total prob of N-in:        0.01433
Amborellatrichopoda_evm_27.model.AmTr_v1.0_scaffold00018.28     TMHMM2.0        outside      1   550
Amborellatrichopoda_evm_27.model.AmTr_v1.0_scaffold00018.28     TMHMM2.0        TMhelix    551   573
Amborellatrichopoda_evm_27.model.AmTr_v1.0_scaffold00018.28     TMHMM2.0        inside     574   788
```

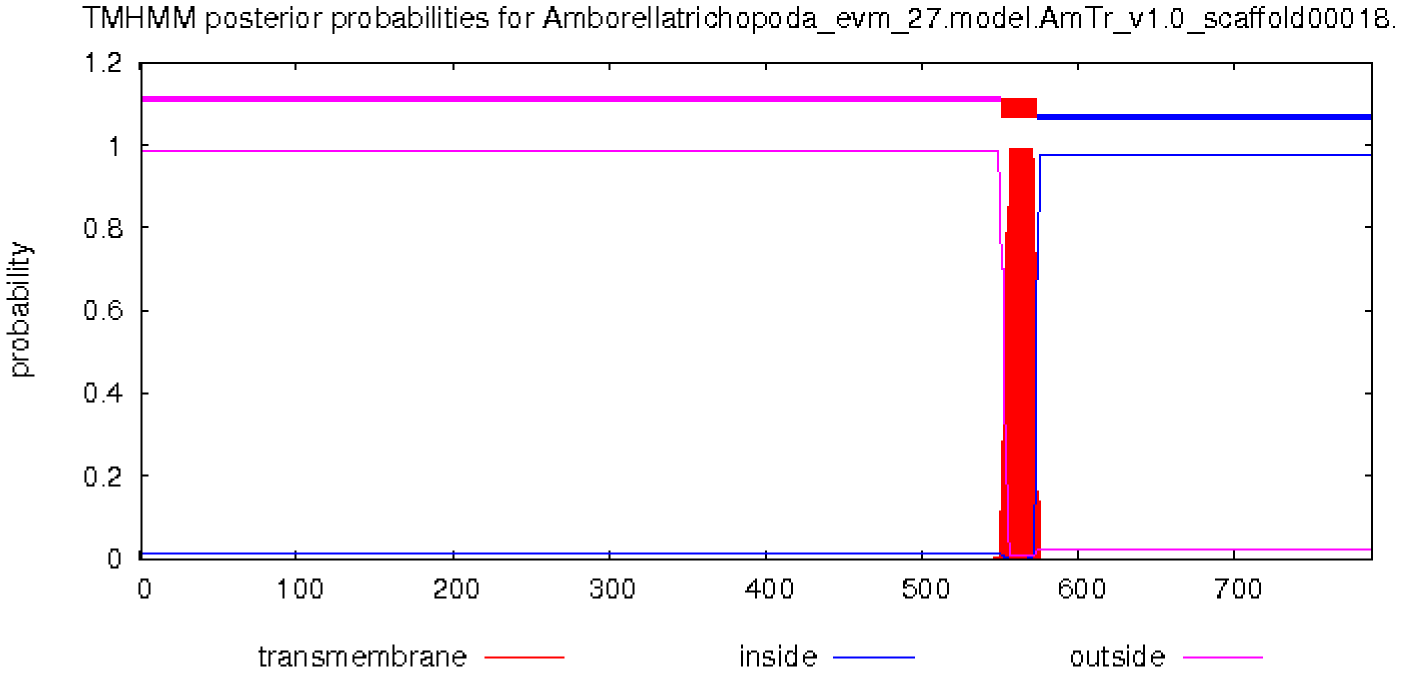

plot in postscript, script for making the plot in gnuplot, data for plot

```
# Amborellatrichopoda_evm_27.model.AmTr_v1.0_scaffold00021.181 Length: 284
# Amborellatrichopoda_evm_27.model.AmTr_v1.0_scaffold00021.181 Number of predicted TMHs:  0
# Amborellatrichopoda_evm_27.model.AmTr_v1.0_scaffold00021.181 Exp number of AAs in TMHs: 0
# Amborellatrichopoda_evm_27.model.AmTr_v1.0_scaffold00021.181 Exp number, first 60 AAs:  0
# Amborellatrichopoda_evm_27.model.AmTr_v1.0_scaffold00021.181 Total prob of N-in:        0.10144
Amborellatrichopoda_evm_27.model.AmTr_v1.0_scaffold00021.181    TMHMM2.0        outside      1   284
```

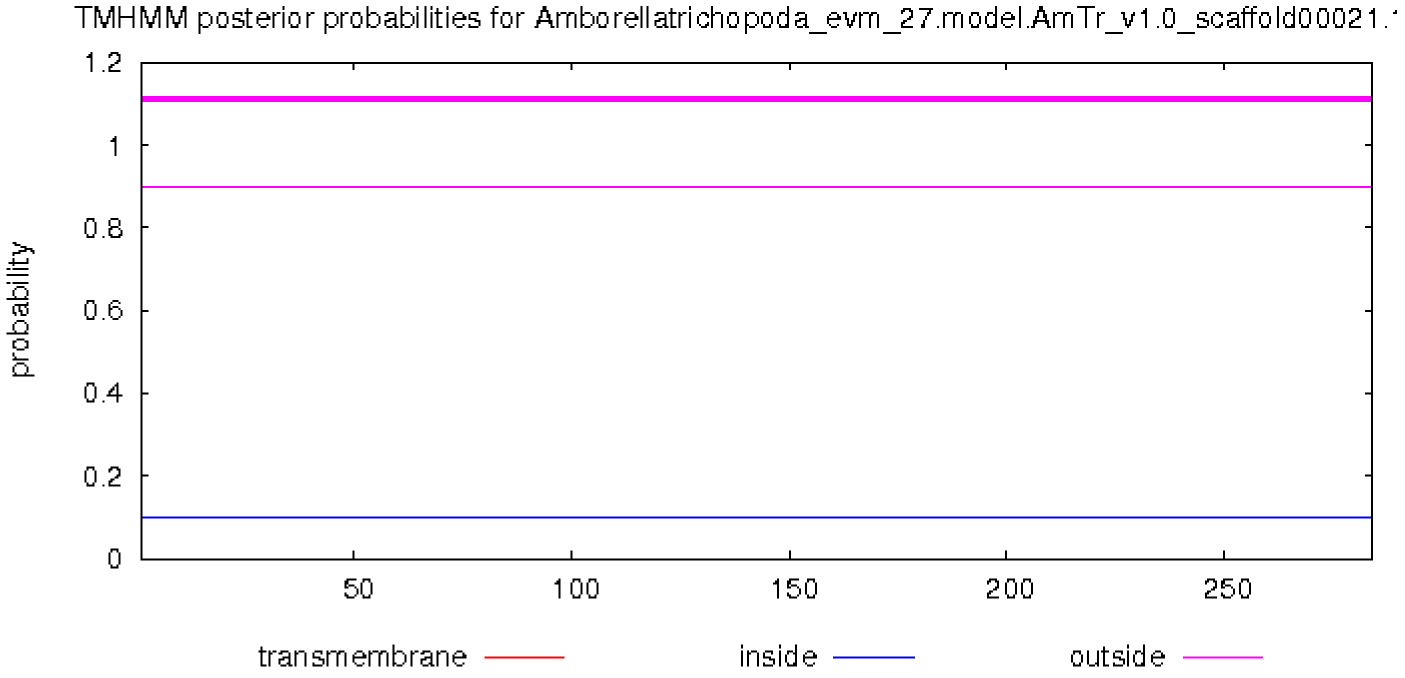

plot in postscript, script for making the plot in gnuplot, data for plot

```
# Amborellatrichopoda_evm_27.model.AmTr_v1.0_scaffold00022.163 Length: 174
# Amborellatrichopoda_evm_27.model.AmTr_v1.0_scaffold00022.163 Number of predicted TMHs:  0
# Amborellatrichopoda_evm_27.model.AmTr_v1.0_scaffold00022.163 Exp number of AAs in TMHs: 0.04469
# Amborellatrichopoda_evm_27.model.AmTr_v1.0_scaffold00022.163 Exp number, first 60 AAs:  0.00363
# Amborellatrichopoda_evm_27.model.AmTr_v1.0_scaffold00022.163 Total prob of N-in:        0.07682
Amborellatrichopoda_evm_27.model.AmTr_v1.0_scaffold00022.163	TMHMM2.0	outside	     1   174
```

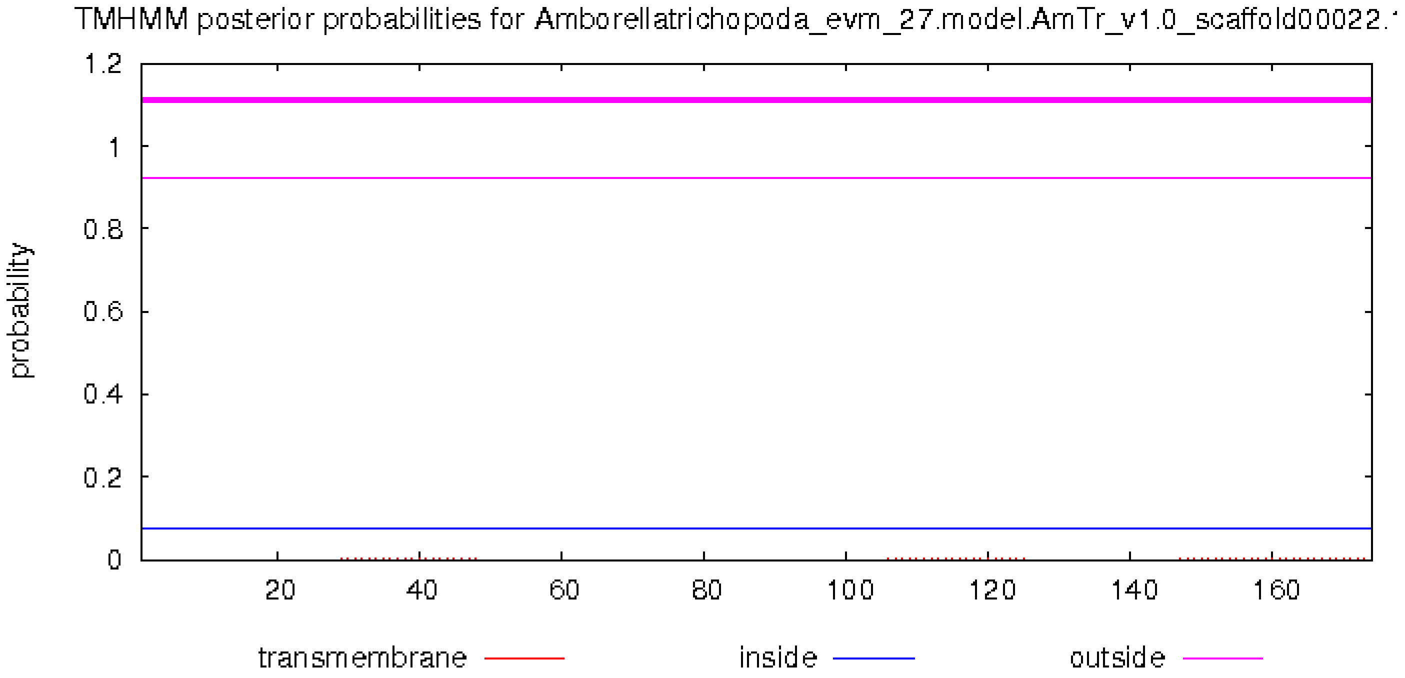

plot in postscript, script for making the plot in gnuplot, data for plot

```
# Amborellatrichopoda_evm_27.model.AmTr_v1.0_scaffold00022.74 Length: 613
# Amborellatrichopoda_evm_27.model.AmTr_v1.0_scaffold00022.74 Number of predicted TMHs:  1
# Amborellatrichopoda_evm_27.model.AmTr_v1.0_scaffold00022.74 Exp number of AAs in TMHs: 23.16969
# Amborellatrichopoda_evm_27.model.AmTr_v1.0_scaffold00022.74 Exp number, first 60 AAs:  0
# Amborellatrichopoda_evm_27.model.AmTr_v1.0_scaffold00022.74 Total prob of N-in:        0.00272
Amborellatrichopoda_evm_27.model.AmTr_v1.0_scaffold00022.74	TMHMM2.0	outside	     1   577
Amborellatrichopoda_evm_27.model.AmTr_v1.0_scaffold00022.74	TMHMM2.0	TMhelix	   578   600
Amborellatrichopoda_evm_27.model.AmTr_v1.0_scaffold00022.74	TMHMM2.0	inside	   601   613
```

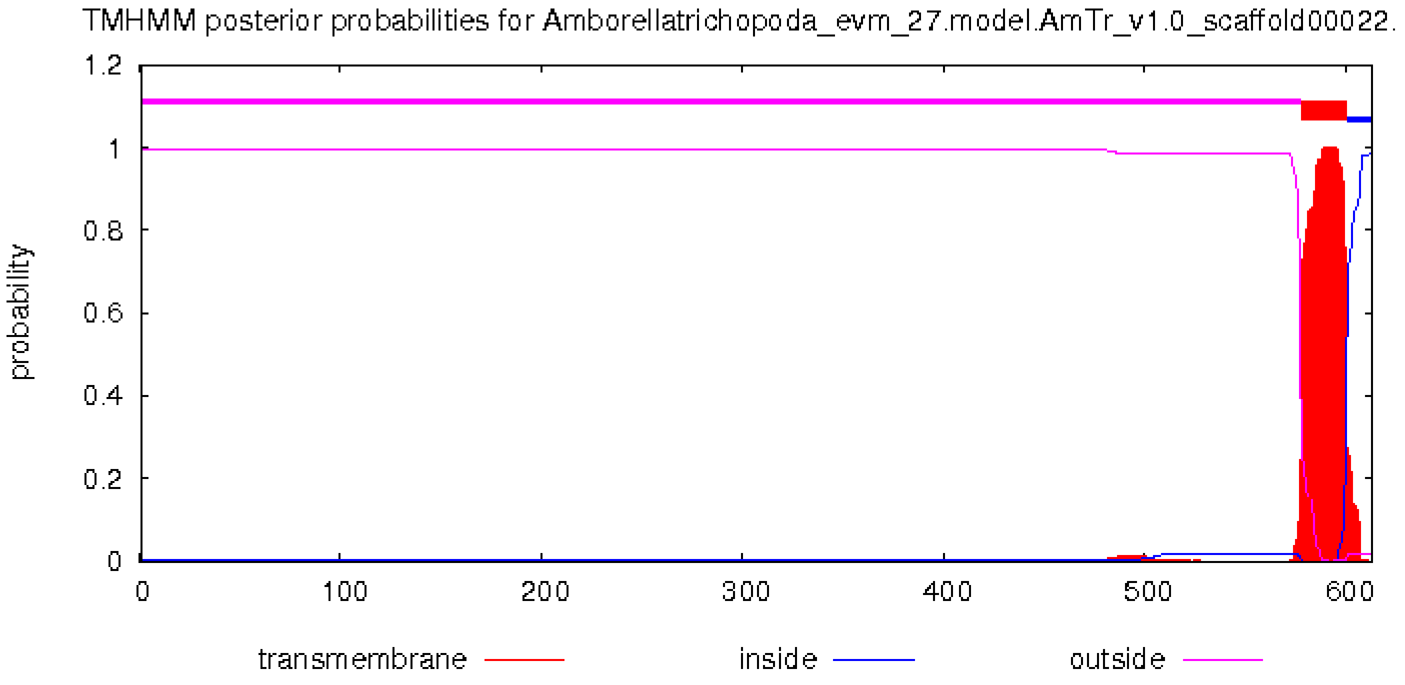

plot in postscript, script for making the plot in gnuplot, data for plot

---

```
# Amborellatrichopoda_evm_27.model.AmTr_v1.0_scaffold00025.209 Length: 316
# Amborellatrichopoda_evm_27.model.AmTr_v1.0_scaffold00025.209 Number of predicted TMHs:  0
# Amborellatrichopoda_evm_27.model.AmTr_v1.0_scaffold00025.209 Exp number of AAs in TMHs: 0.00316
# Amborellatrichopoda_evm_27.model.AmTr_v1.0_scaffold00025.209 Exp number, first 60 AAs:  0
# Amborellatrichopoda_evm_27.model.AmTr_v1.0_scaffold00025.209 Total prob of N-in:        0.00468
Amborellatrichopoda_evm_27.model.AmTr_v1.0_scaffold00025.209	TMHMM2.0	outside	     1   316
```

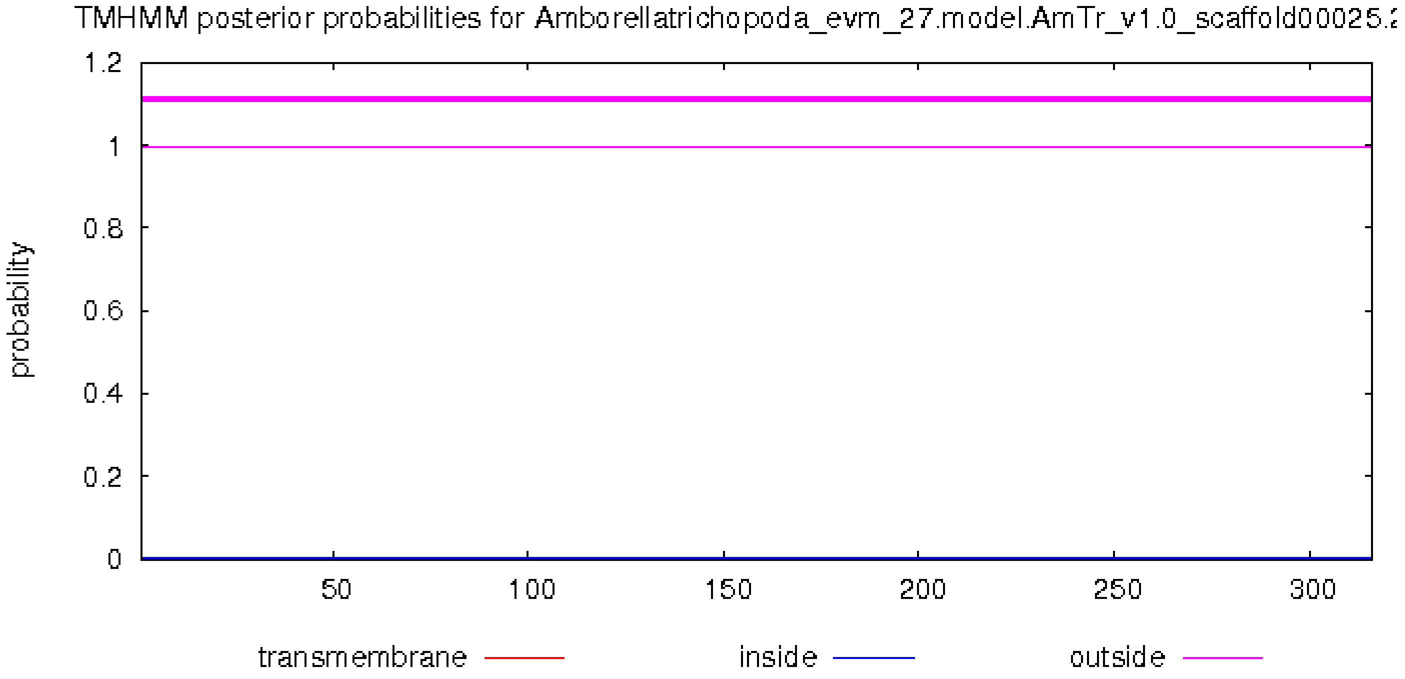

plot in postscript, script for making the plot in gnuplot, data for plot

---

```
# Amborellatrichopoda_evm_27.model.AmTr_v1.0_scaffold00032.87 Length: 297
# Amborellatrichopoda_evm_27.model.AmTr_v1.0_scaffold00032.87 Number of predicted TMHs:  0
# Amborellatrichopoda_evm_27.model.AmTr_v1.0_scaffold00032.87 Exp number of AAs in TMHs: 0.00041
# Amborellatrichopoda_evm_27.model.AmTr_v1.0_scaffold00032.87 Exp number, first 60 AAs:  0
# Amborellatrichopoda_evm_27.model.AmTr_v1.0_scaffold00032.87 Total prob of N-in:        0.03381
Amborellatrichopoda_evm_27.model.AmTr_v1.0_scaffold00032.87	TMHMM2.0	outside	     1   297
```

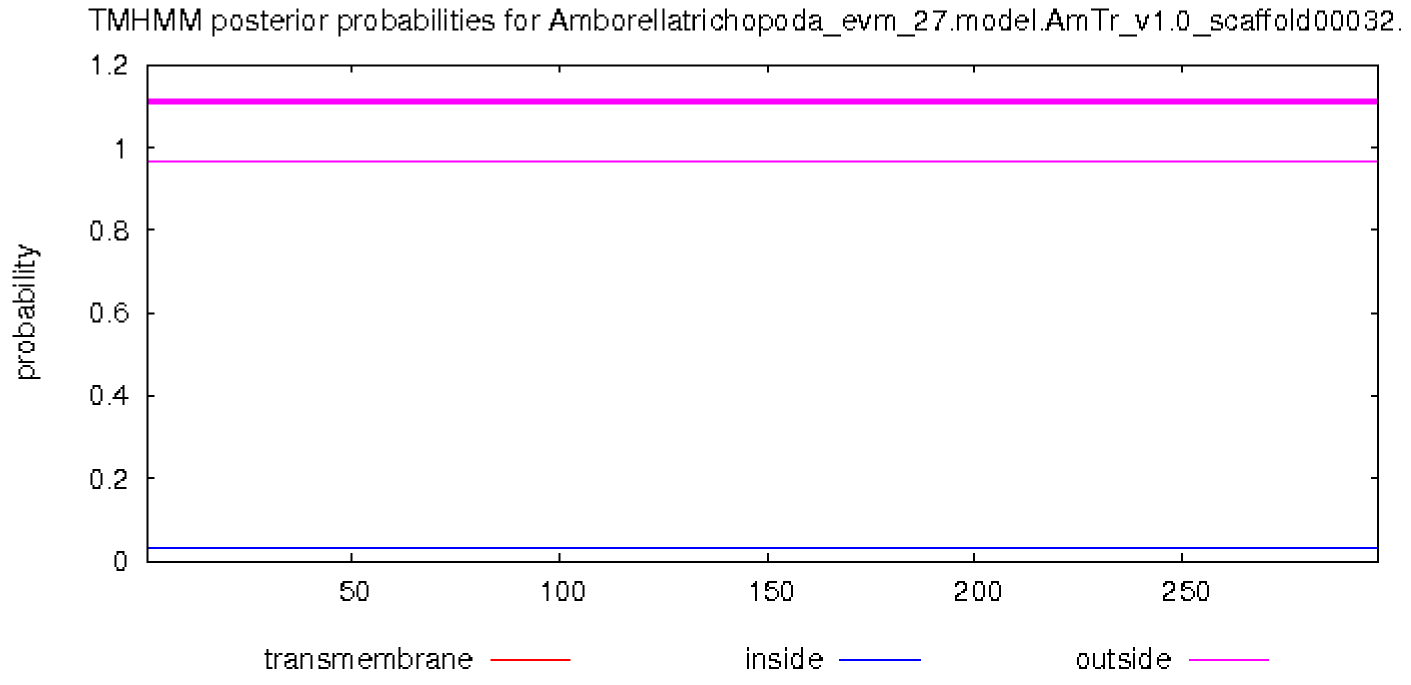

[plot](#) in postscript, [script](#) for making the plot in gnuplot, [data](#) for plot

---

```
# Amborellatrichopoda_evm_27.model.AmTr_v1.0_scaffold00037.62 Length: 333
# Amborellatrichopoda_evm_27.model.AmTr_v1.0_scaffold00037.62 Number of predicted TMHs:  0
# Amborellatrichopoda_evm_27.model.AmTr_v1.0_scaffold00037.62 Exp number of AAs in TMHs: 0.00015
# Amborellatrichopoda_evm_27.model.AmTr_v1.0_scaffold00037.62 Exp number, first 60 AAs:  0
# Amborellatrichopoda_evm_27.model.AmTr_v1.0_scaffold00037.62 Total prob of N-in:        0.00182
Amborellatrichopoda_evm_27.model.AmTr_v1.0_scaffold00037.62     TMHMM2.0        outside      1   333
```

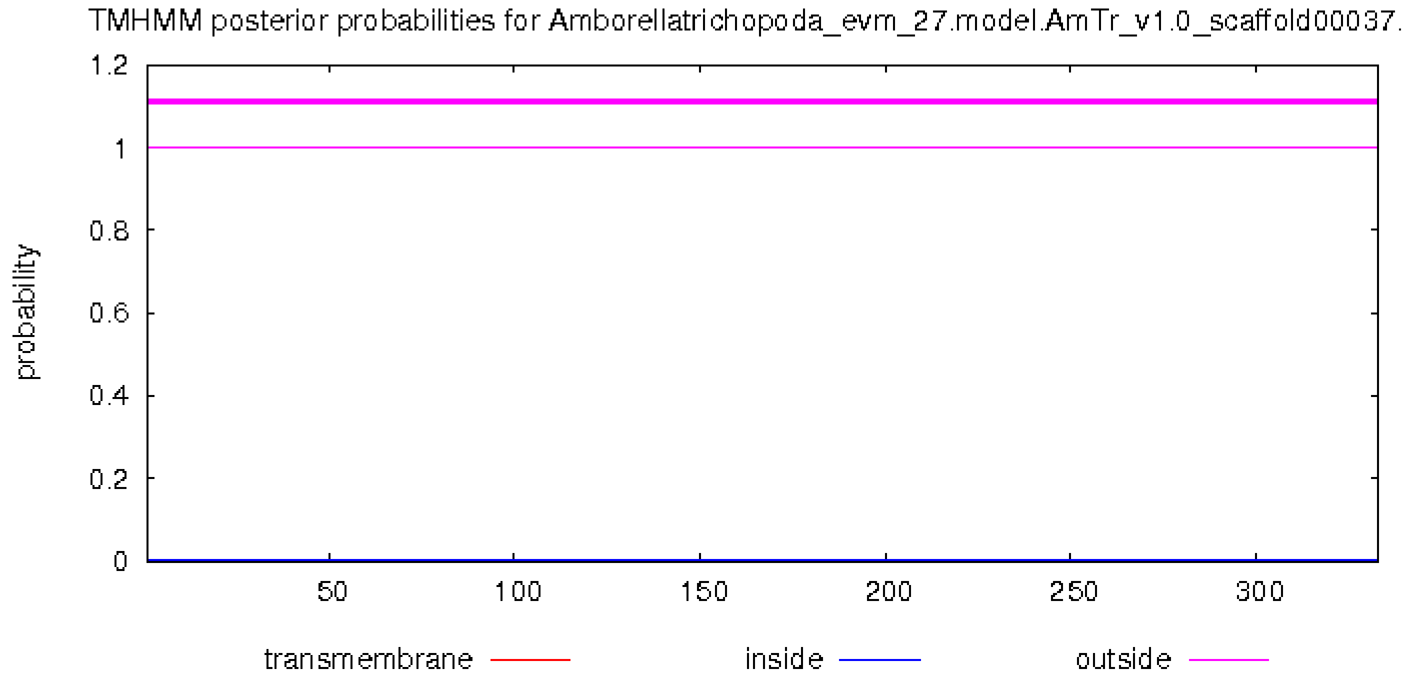

[plot](#) in postscript, [script](#) for making the plot in gnuplot, [data](#) for plot

---

```
# Amborellatrichopoda_evm_27.model.AmTr_v1.0_scaffold00040.86 Length: 258
# Amborellatrichopoda_evm_27.model.AmTr_v1.0_scaffold00040.86 Number of predicted TMHs:  0
# Amborellatrichopoda_evm_27.model.AmTr_v1.0_scaffold00040.86 Exp number of AAs in TMHs: 0.00369
# Amborellatrichopoda_evm_27.model.AmTr_v1.0_scaffold00040.86 Exp number, first 60 AAs:  0
# Amborellatrichopoda_evm_27.model.AmTr_v1.0_scaffold00040.86 Total prob of N-in:        0.00750
Amborellatrichopoda_evm_27.model.AmTr_v1.0_scaffold00040.86     TMHMM2.0        outside      1   258
```

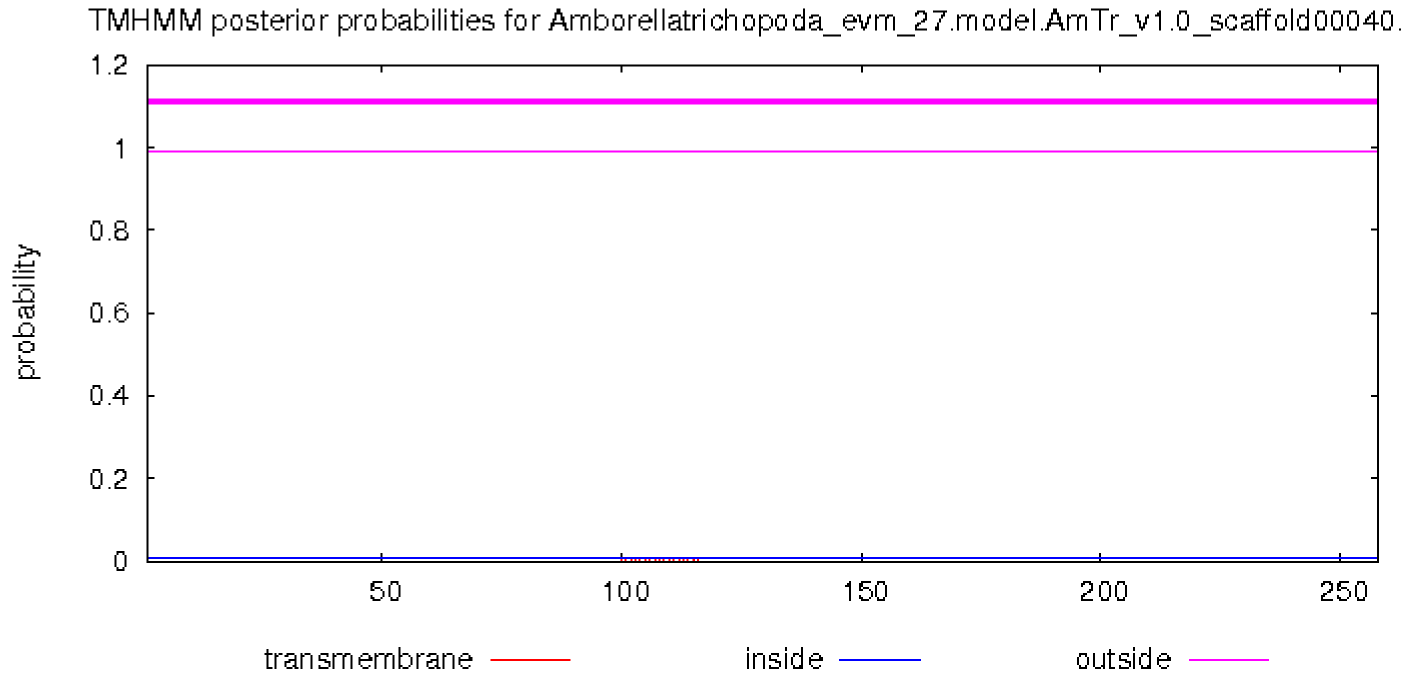

plot in postscript, script for making the plot in gnuplot, data for plot

---

```
# Amborellatrichopoda_evm_27.model.AmTr_v1.0_scaffold00040.87 Length: 260
# Amborellatrichopoda_evm_27.model.AmTr_v1.0_scaffold00040.87 Number of predicted TMHs:  0
# Amborellatrichopoda_evm_27.model.AmTr_v1.0_scaffold00040.87 Exp number of AAs in TMHs: 0.00484
# Amborellatrichopoda_evm_27.model.AmTr_v1.0_scaffold00040.87 Exp number, first 60 AAs:  0.00012
# Amborellatrichopoda_evm_27.model.AmTr_v1.0_scaffold00040.87 Total prob of N-in:        0.02393
Amborellatrichopoda_evm_27.model.AmTr_v1.0_scaffold00040.87	TMHMM2.0	outside	     1   260
```

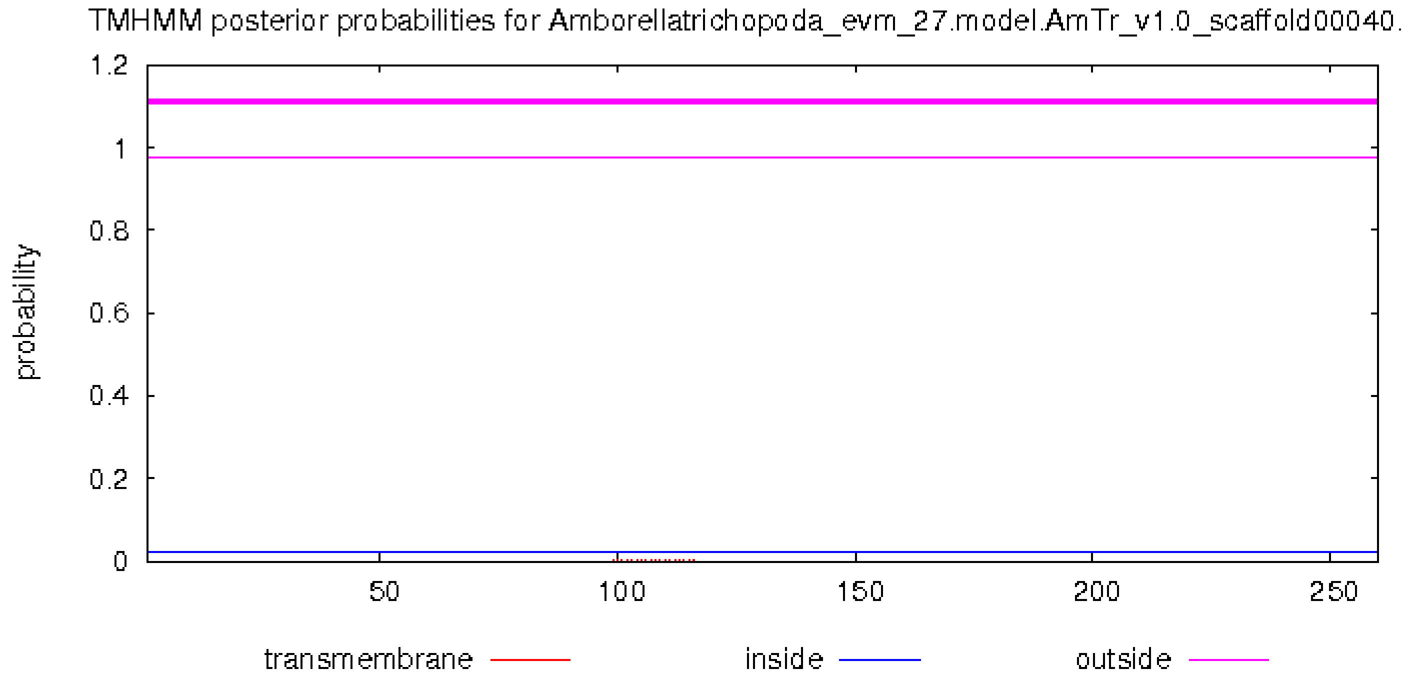

plot in postscript, script for making the plot in gnuplot, data for plot

---

```
# Amborellatrichopoda_evm_27.model.AmTr_v1.0_scaffold00044.29 Length: 188
# Amborellatrichopoda_evm_27.model.AmTr_v1.0_scaffold00044.29 Number of predicted TMHs:  0
# Amborellatrichopoda_evm_27.model.AmTr_v1.0_scaffold00044.29 Exp number of AAs in TMHs: 0
# Amborellatrichopoda_evm_27.model.AmTr_v1.0_scaffold00044.29 Exp number, first 60 AAs:  0
# Amborellatrichopoda_evm_27.model.AmTr_v1.0_scaffold00044.29 Total prob of N-in:        0.03550
Amborellatrichopoda_evm_27.model.AmTr_v1.0_scaffold00044.29	TMHMM2.0	outside	     1   188
```

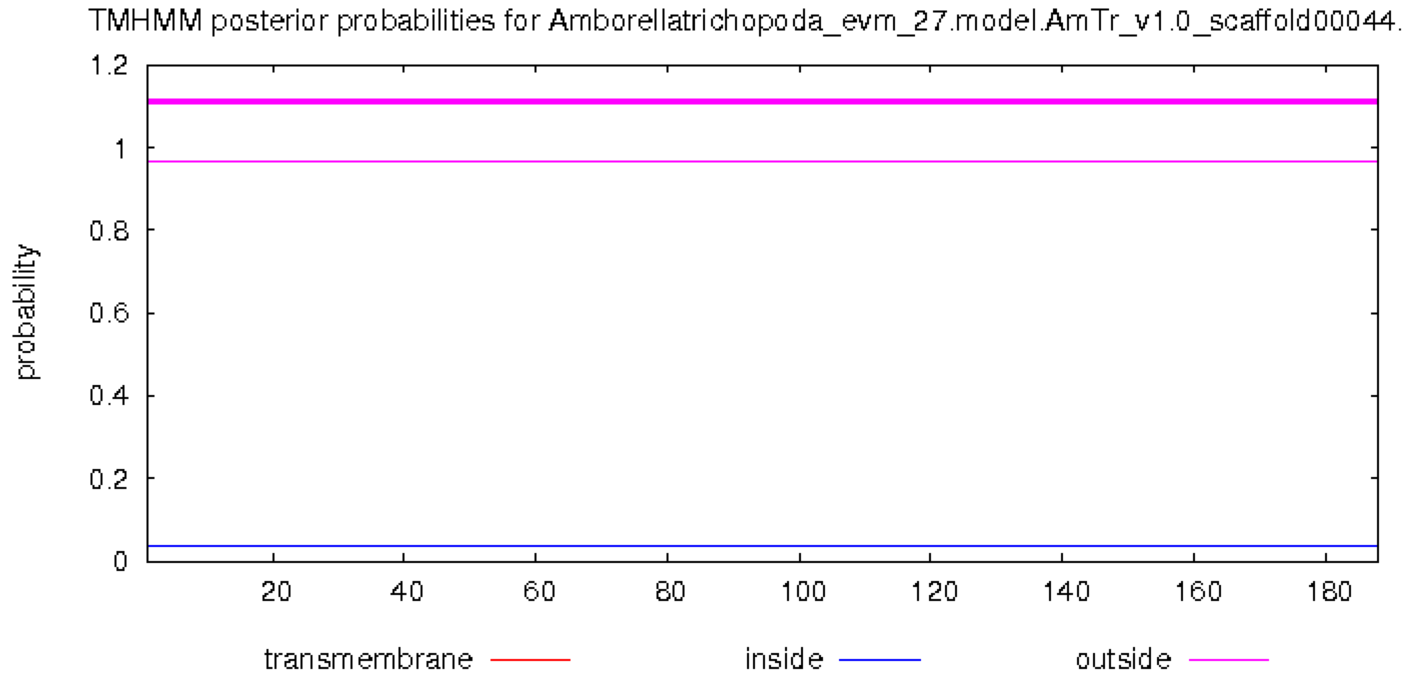

plot in postscript, script for making the plot in gnuplot, data for plot

---

```
# Amborellatrichopoda_evm_27.model.AmTr_v1.0_scaffold00044.30 Length: 809
# Amborellatrichopoda_evm_27.model.AmTr_v1.0_scaffold00044.30 Number of predicted TMHs:  0
# Amborellatrichopoda_evm_27.model.AmTr_v1.0_scaffold00044.30 Exp number of AAs in TMHs: 1.86747
# Amborellatrichopoda_evm_27.model.AmTr_v1.0_scaffold00044.30 Exp number, first 60 AAs:  0
# Amborellatrichopoda_evm_27.model.AmTr_v1.0_scaffold00044.30 Total prob of N-in:        0.00003
Amborellatrichopoda_evm_27.model.AmTr_v1.0_scaffold00044.30     TMHMM2.0        outside      1   809
```

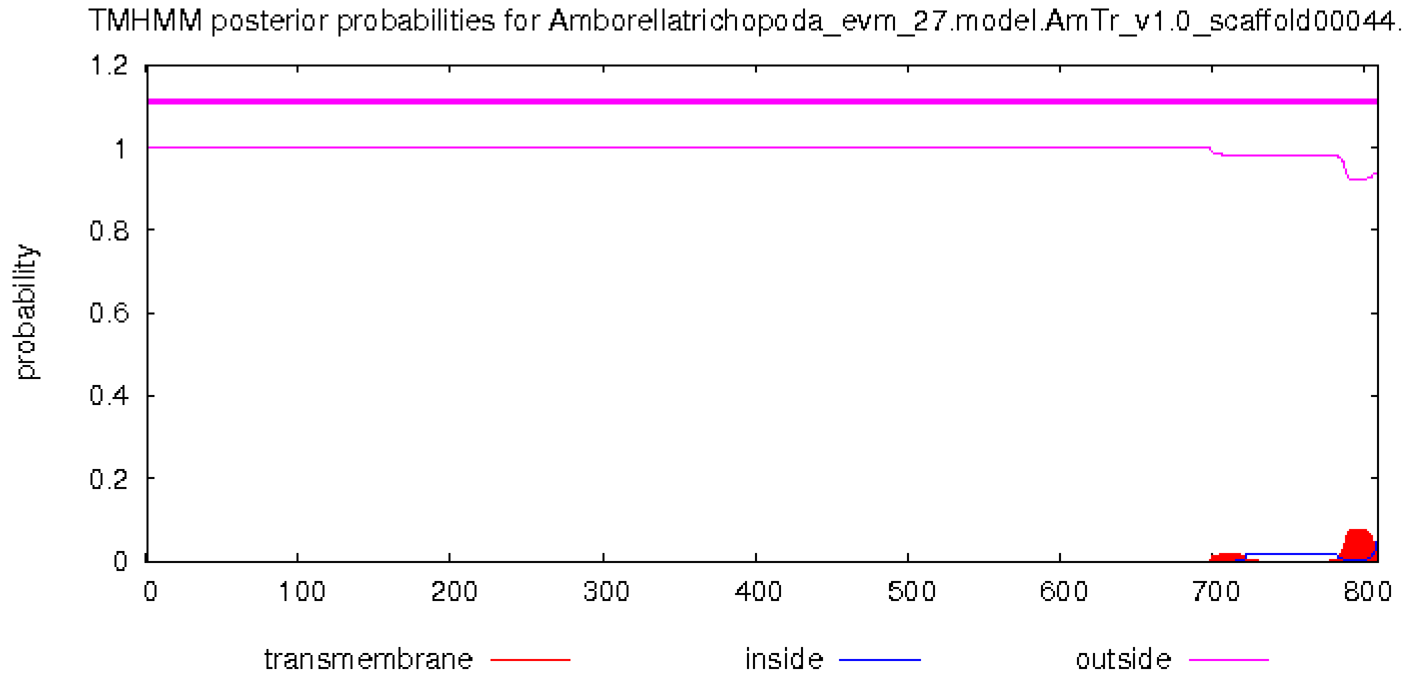

plot in postscript, script for making the plot in gnuplot, data for plot

---

```
# Amborellatrichopoda_evm_27.model.AmTr_v1.0_scaffold00046.19 Length: 351
# Amborellatrichopoda_evm_27.model.AmTr_v1.0_scaffold00046.19 Number of predicted TMHs:  0
# Amborellatrichopoda_evm_27.model.AmTr_v1.0_scaffold00046.19 Exp number of AAs in TMHs: 0.02456
# Amborellatrichopoda_evm_27.model.AmTr_v1.0_scaffold00046.19 Exp number, first 60 AAs:  0
# Amborellatrichopoda_evm_27.model.AmTr_v1.0_scaffold00046.19 Total prob of N-in:        0.00069
Amborellatrichopoda_evm_27.model.AmTr_v1.0_scaffold00046.19     TMHMM2.0        outside      1   351
```

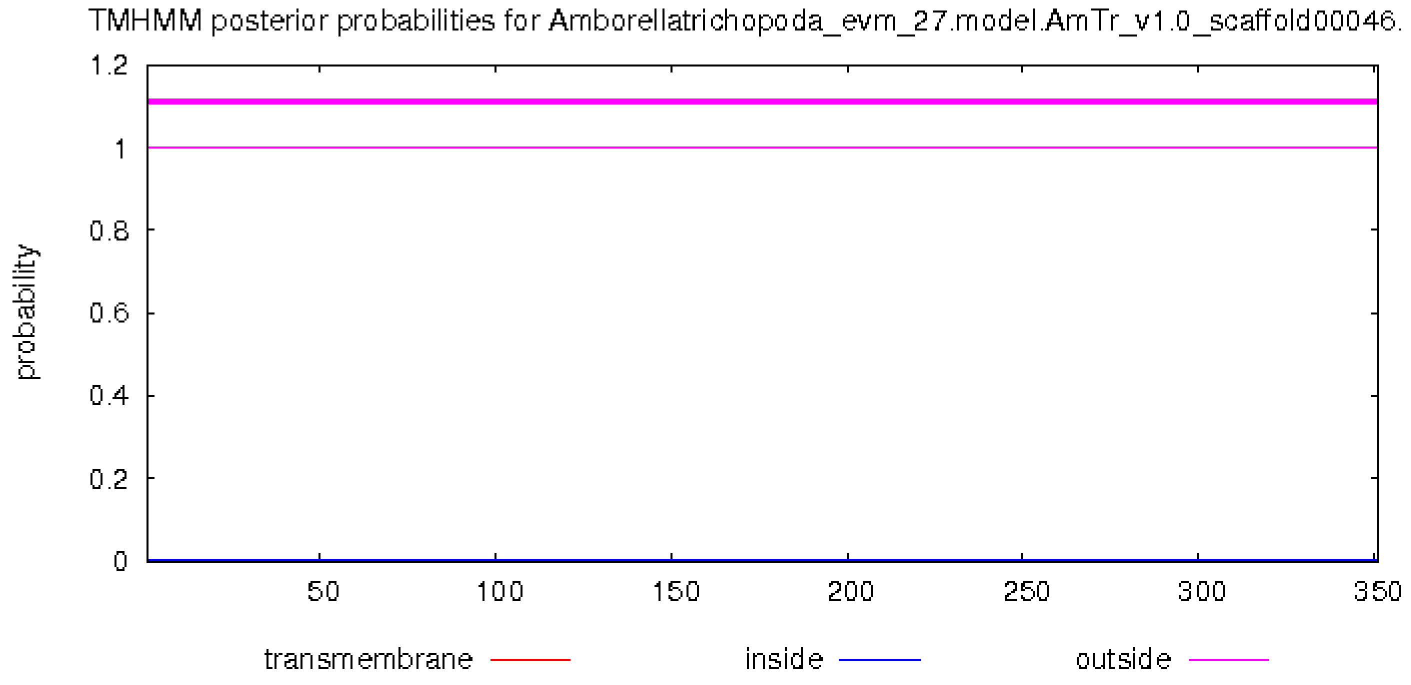

plot in postscript, script for making the plot in gnuplot, data for plot

---

```
# Amborellatrichopoda_evm_27.model.AmTr_v1.0_scaffold00049.251 Length: 354
# Amborellatrichopoda_evm_27.model.AmTr_v1.0_scaffold00049.251 Number of predicted TMHs:  0
# Amborellatrichopoda_evm_27.model.AmTr_v1.0_scaffold00049.251 Exp number of AAs in TMHs: 0.00523
# Amborellatrichopoda_evm_27.model.AmTr_v1.0_scaffold00049.251 Exp number, first 60 AAs:  0
# Amborellatrichopoda_evm_27.model.AmTr_v1.0_scaffold00049.251 Total prob of N-in:        0.02481
Amborellatrichopoda_evm_27.model.AmTr_v1.0_scaffold00049.251	TMHMM2.0	outside	     1   354
```

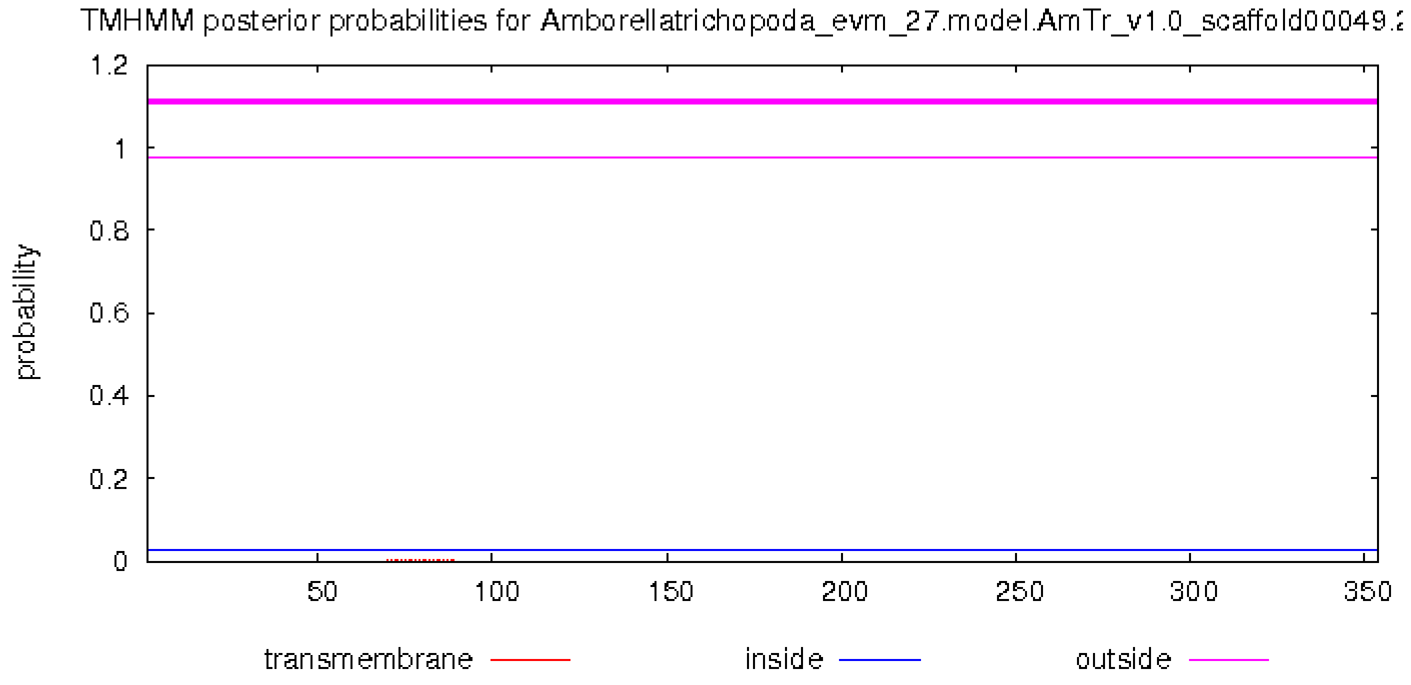

plot in postscript, script for making the plot in gnuplot, data for plot

---

```
# Amborellatrichopoda_evm_27.model.AmTr_v1.0_scaffold00058.98 Length: 336
# Amborellatrichopoda_evm_27.model.AmTr_v1.0_scaffold00058.98 Number of predicted TMHs:  0
# Amborellatrichopoda_evm_27.model.AmTr_v1.0_scaffold00058.98 Exp number of AAs in TMHs: 0.00019
# Amborellatrichopoda_evm_27.model.AmTr_v1.0_scaffold00058.98 Exp number, first 60 AAs:  0
# Amborellatrichopoda_evm_27.model.AmTr_v1.0_scaffold00058.98 Total prob of N-in:        0.00531
Amborellatrichopoda_evm_27.model.AmTr_v1.0_scaffold00058.98	TMHMM2.0	outside	     1   336
```

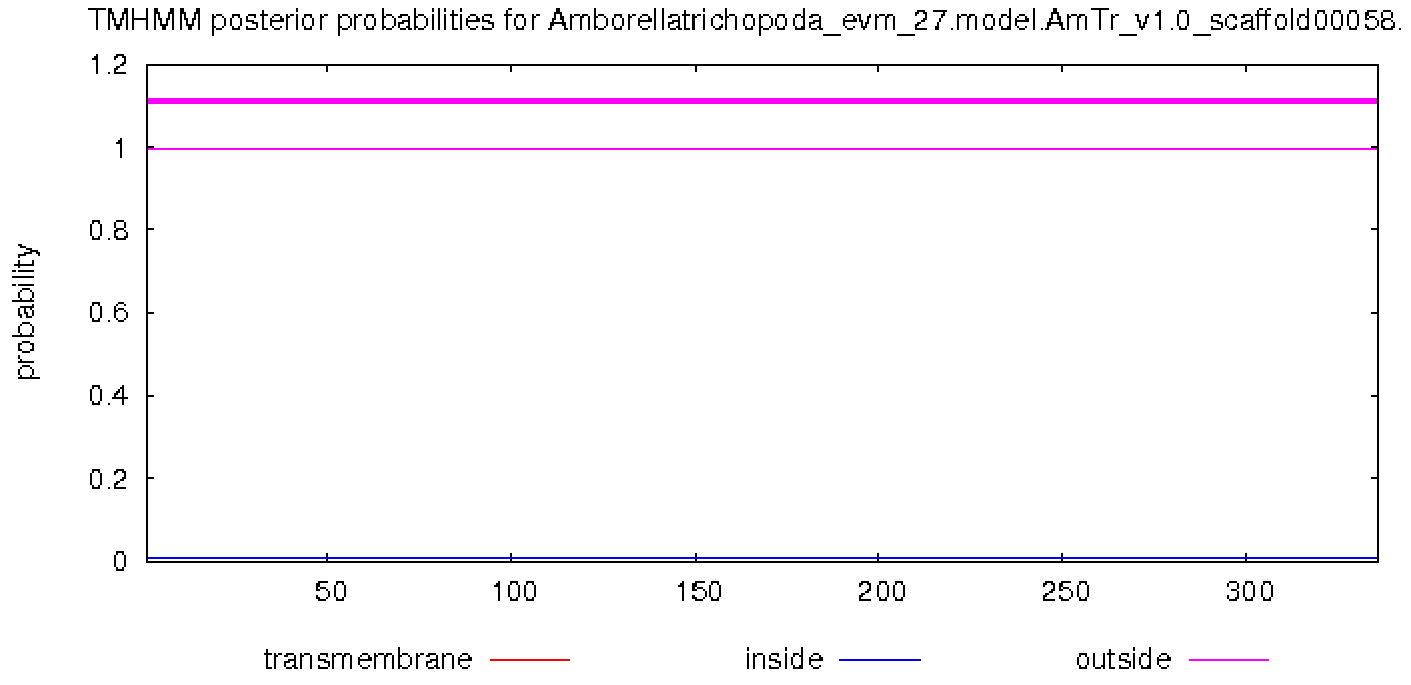

plot in postscript, script for making the plot in gnuplot, data for plot

---

```
# Amborellatrichopoda_evm_27.model.AmTr_v1.0_scaffold00059.206 Length: 166
# Amborellatrichopoda_evm_27.model.AmTr_v1.0_scaffold00059.206 Number of predicted TMHs:  0
# Amborellatrichopoda_evm_27.model.AmTr_v1.0_scaffold00059.206 Exp number of AAs in TMHs: 0.00218
# Amborellatrichopoda_evm_27.model.AmTr_v1.0_scaffold00059.206 Exp number, first 60 AAs:  0.00072
# Amborellatrichopoda_evm_27.model.AmTr_v1.0_scaffold00059.206 Total prob of N-in:        0.13105
Amborellatrichopoda_evm_27.model.AmTr_v1.0_scaffold00059.206    TMHMM2.0        outside      1   166
```

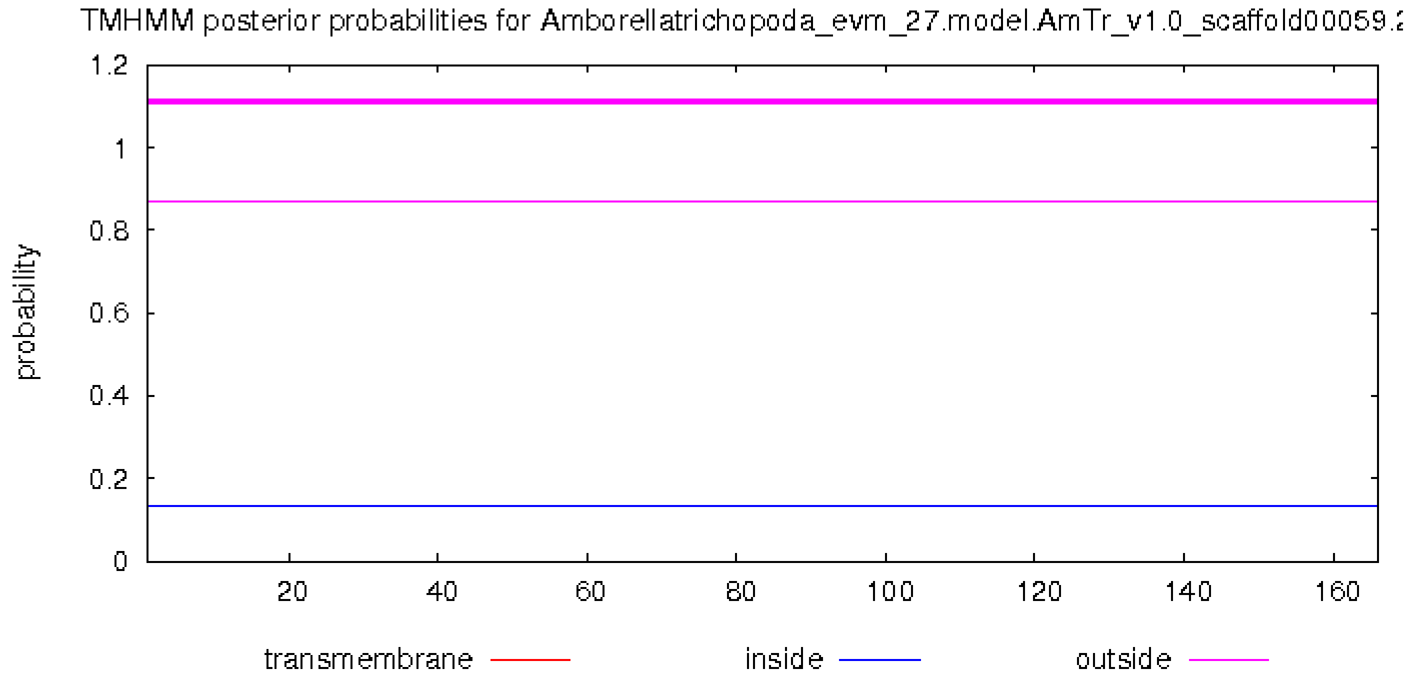

plot in postscript, script for making the plot in gnuplot, data for plot

---

```
# Amborellatrichopoda_evm_27.model.AmTr_v1.0_scaffold00060.14 Length: 404
# Amborellatrichopoda_evm_27.model.AmTr_v1.0_scaffold00060.14 Number of predicted TMHs:  0
# Amborellatrichopoda_evm_27.model.AmTr_v1.0_scaffold00060.14 Exp number of AAs in TMHs: 0
# Amborellatrichopoda_evm_27.model.AmTr_v1.0_scaffold00060.14 Exp number, first 60 AAs:  0
# Amborellatrichopoda_evm_27.model.AmTr_v1.0_scaffold00060.14 Total prob of N-in:        0.00233
Amborellatrichopoda_evm_27.model.AmTr_v1.0_scaffold00060.14     TMHMM2.0        outside      1   404
```

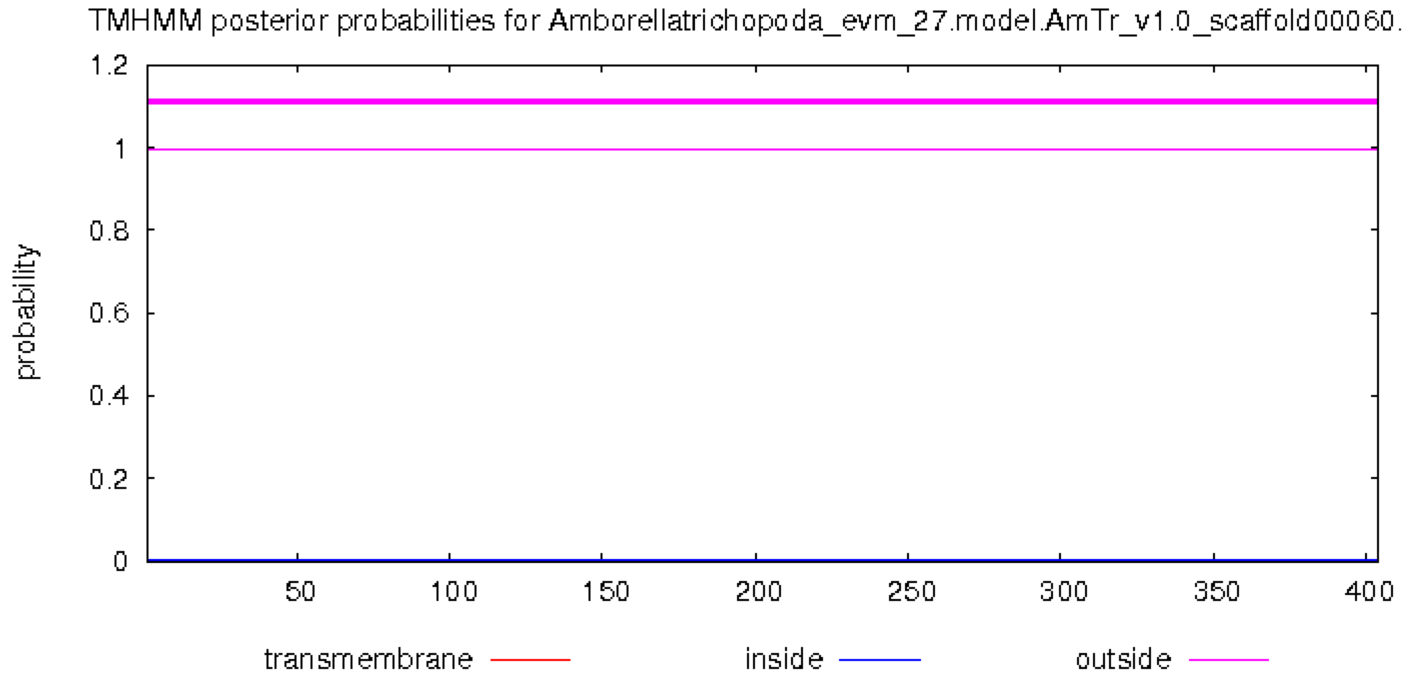

plot in postscript, script for making the plot in gnuplot, data for plot

---

```
# Amborellatrichopoda_evm_27.model.AmTr_v1.0_scaffold00067.40 Length: 246
# Amborellatrichopoda_evm_27.model.AmTr_v1.0_scaffold00067.40 Number of predicted TMHs:  0
# Amborellatrichopoda_evm_27.model.AmTr_v1.0_scaffold00067.40 Exp number of AAs in TMHs: 0
# Amborellatrichopoda_evm_27.model.AmTr_v1.0_scaffold00067.40 Exp number, first 60 AAs:  0
# Amborellatrichopoda_evm_27.model.AmTr_v1.0_scaffold00067.40 Total prob of N-in:        0.01722
Amborellatrichopoda_evm_27.model.AmTr_v1.0_scaffold00067.40     TMHMM2.0        outside      1   246
```

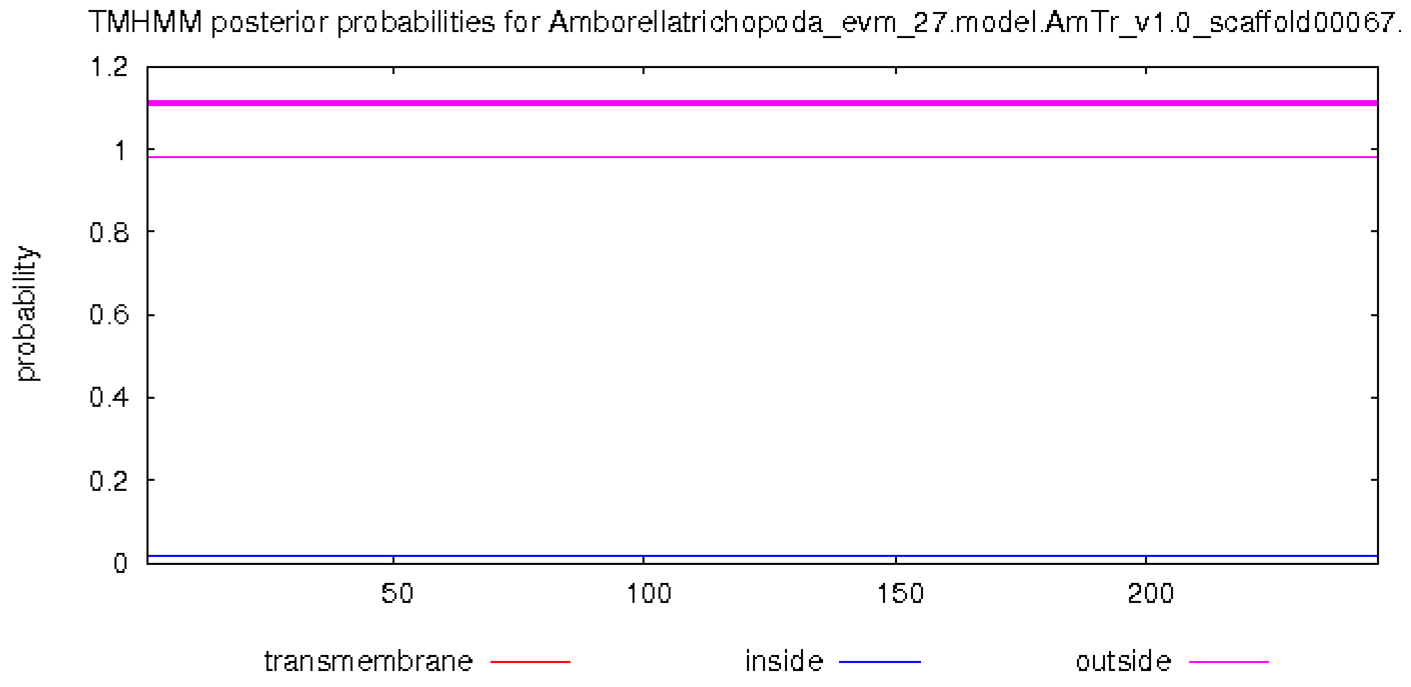

plot in postscript, script for making the plot in gnuplot, data for plot

---

```
# Amborellatrichopoda_evm_27.model.AmTr_v1.0_scaffold00068.186 Length: 333
# Amborellatrichopoda_evm_27.model.AmTr_v1.0_scaffold00068.186 Number of predicted TMHs:  0
# Amborellatrichopoda_evm_27.model.AmTr_v1.0_scaffold00068.186 Exp number of AAs in TMHs: 0.00038
# Amborellatrichopoda_evm_27.model.AmTr_v1.0_scaffold00068.186 Exp number, first 60 AAs:  0
# Amborellatrichopoda_evm_27.model.AmTr_v1.0_scaffold00068.186 Total prob of N-in:        0.01092
Amborellatrichopoda_evm_27.model.AmTr_v1.0_scaffold00068.186    TMHMM2.0        outside      1   333
```

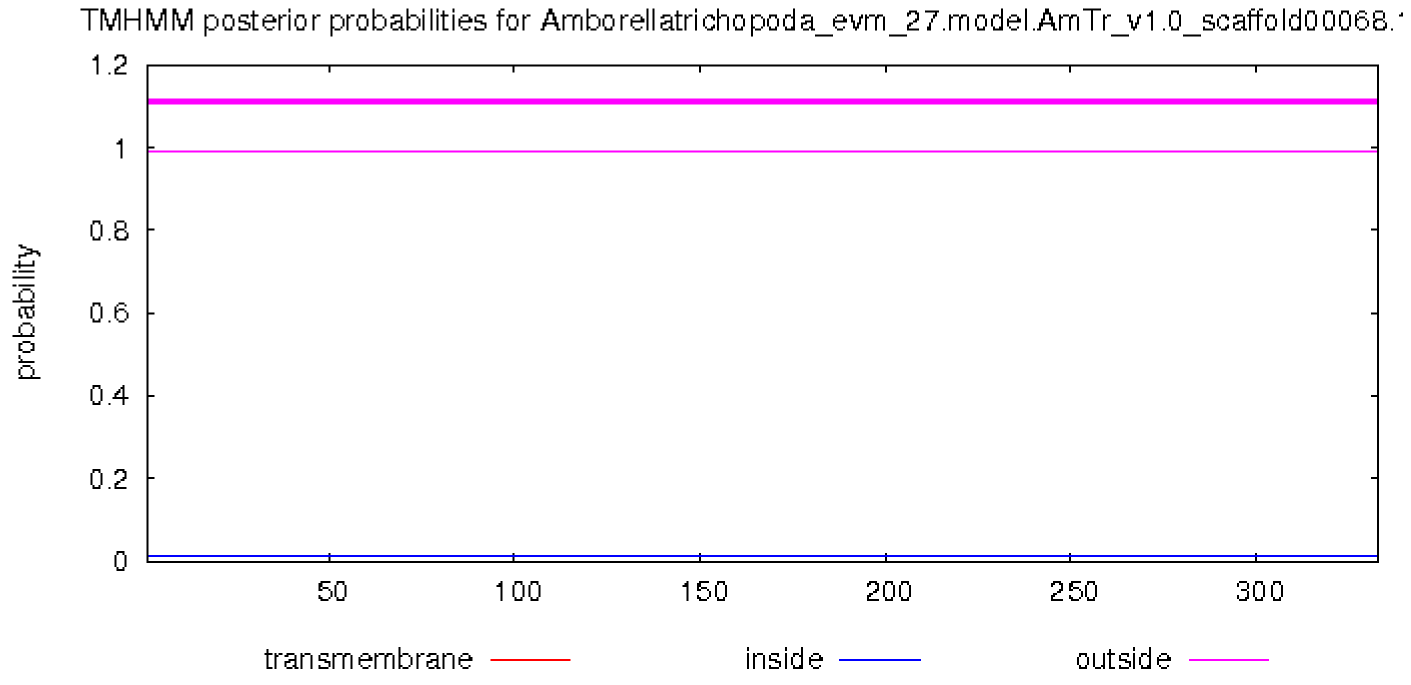

plot in postscript, script for making the plot in gnuplot, data for plot

---

```
# Amborellatrichopoda_evm_27.model.AmTr_v1.0_scaffold00071.30 Length: 588
# Amborellatrichopoda_evm_27.model.AmTr_v1.0_scaffold00071.30 Number of predicted TMHs:  1
# Amborellatrichopoda_evm_27.model.AmTr_v1.0_scaffold00071.30 Exp number of AAs in TMHs: 22.93333
# Amborellatrichopoda_evm_27.model.AmTr_v1.0_scaffold00071.30 Exp number, first 60 AAs:  0
# Amborellatrichopoda_evm_27.model.AmTr_v1.0_scaffold00071.30 Total prob of N-in:        0.01819
Amborellatrichopoda_evm_27.model.AmTr_v1.0_scaffold00071.30     TMHMM2.0        outside      1   553
Amborellatrichopoda_evm_27.model.AmTr_v1.0_scaffold00071.30     TMHMM2.0        TMhelix    554   576
Amborellatrichopoda_evm_27.model.AmTr_v1.0_scaffold00071.30     TMHMM2.0        inside     577   588
```

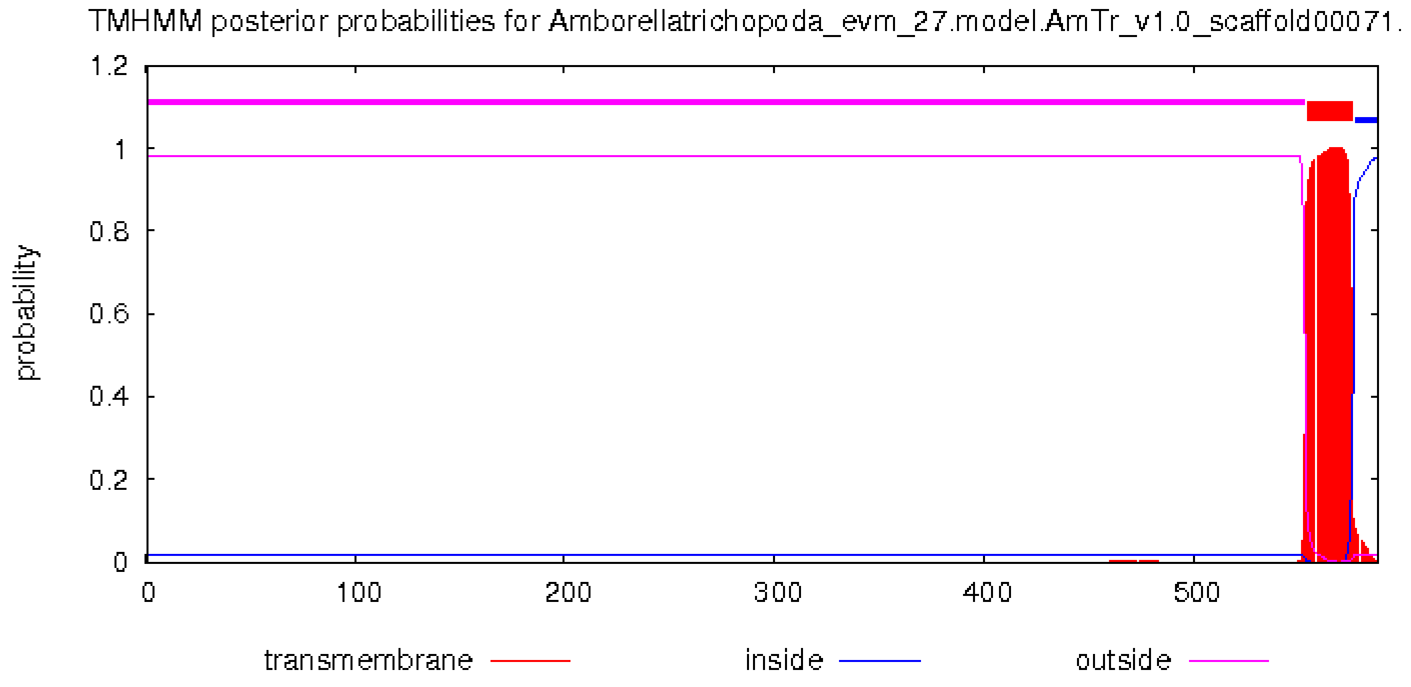

plot in postscript, script for making the plot in gnuplot, data for plot

---

```
# Amborellatrichopoda_evm_27.model.AmTr_v1.0_scaffold00079.33 Length: 315
# Amborellatrichopoda_evm_27.model.AmTr_v1.0_scaffold00079.33 Number of predicted TMHs:  0
# Amborellatrichopoda_evm_27.model.AmTr_v1.0_scaffold00079.33 Exp number of AAs in TMHs: 0.00213
# Amborellatrichopoda_evm_27.model.AmTr_v1.0_scaffold00079.33 Exp number, first 60 AAs:  0
```

```
# Amborellatrichopoda_evm_27.model.AmTr_v1.0_scaffold00079.33 Total prob of N-in:        0.00210
Amborellatrichopoda_evm_27.model.AmTr_v1.0_scaffold00079.33     TMHMM2.0        outside      1   315
```

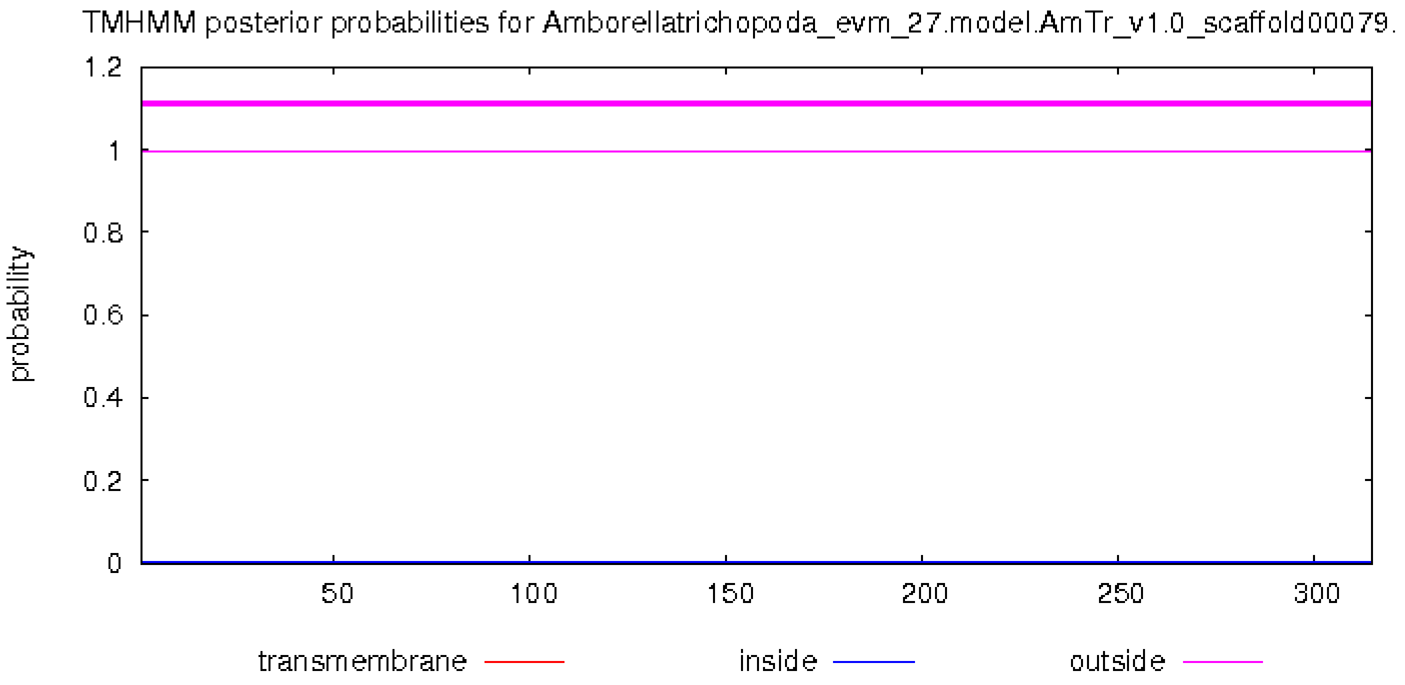

plot in postscript, script for making the plot in gnuplot, data for plot

---

```
# Amborellatrichopoda_evm_27.model.AmTr_v1.0_scaffold00082.23 Length: 568
# Amborellatrichopoda_evm_27.model.AmTr_v1.0_scaffold00082.23 Number of predicted TMHs:  1
# Amborellatrichopoda_evm_27.model.AmTr_v1.0_scaffold00082.23 Exp number of AAs in TMHs: 22.28793
# Amborellatrichopoda_evm_27.model.AmTr_v1.0_scaffold00082.23 Exp number, first 60 AAs:  0.01375
# Amborellatrichopoda_evm_27.model.AmTr_v1.0_scaffold00082.23 Total prob of N-in:        0.01334
Amborellatrichopoda_evm_27.model.AmTr_v1.0_scaffold00082.23     TMHMM2.0        outside      1   530
Amborellatrichopoda_evm_27.model.AmTr_v1.0_scaffold00082.23     TMHMM2.0        TMhelix    531   553
Amborellatrichopoda_evm_27.model.AmTr_v1.0_scaffold00082.23     TMHMM2.0        inside     554   568
```

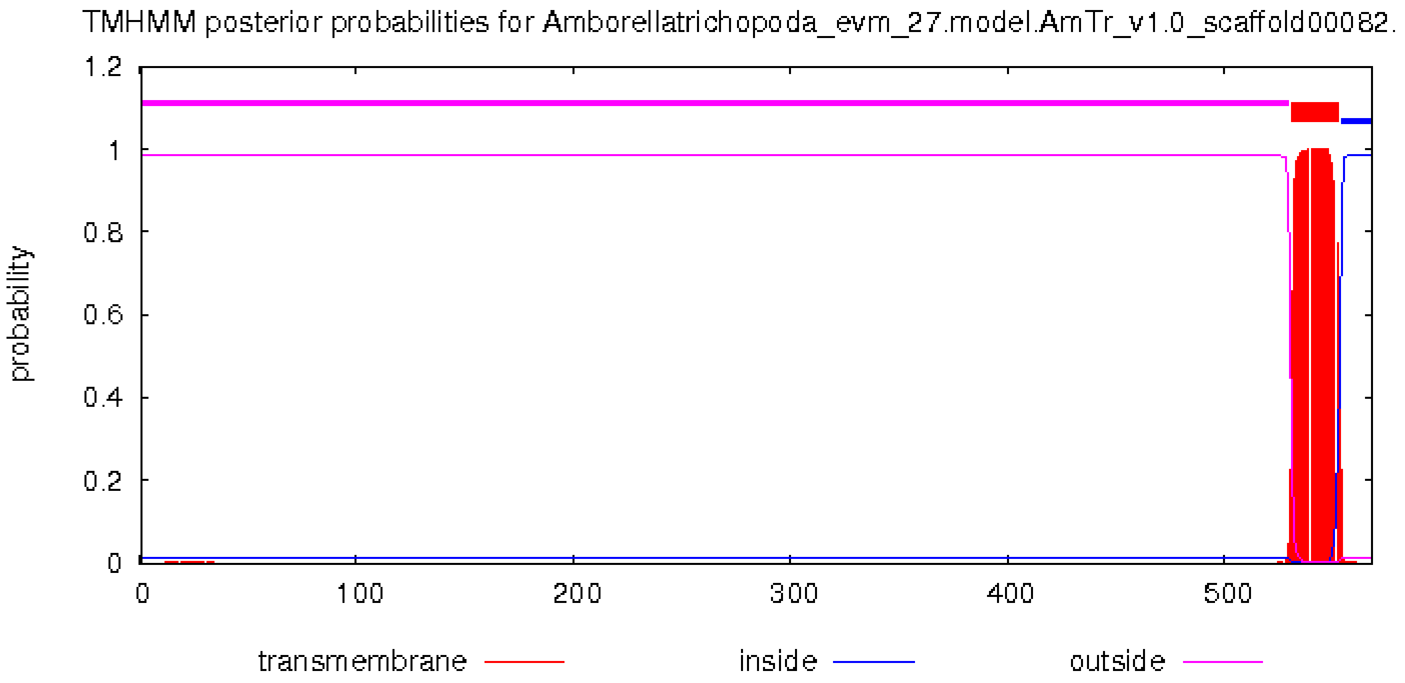

plot in postscript, script for making the plot in gnuplot, data for plot

---

```
# Amborellatrichopoda_evm_27.model.AmTr_v1.0_scaffold00092.111 Length: 320
# Amborellatrichopoda_evm_27.model.AmTr_v1.0_scaffold00092.111 Number of predicted TMHs:  0
```

```
# Amborellatrichopoda_evm_27.model.AmTr_v1.0_scaffold00092.111 Exp number of AAs in TMHs: 0
# Amborellatrichopoda_evm_27.model.AmTr_v1.0_scaffold00092.111 Exp number, first 60 AAs:  0
# Amborellatrichopoda_evm_27.model.AmTr_v1.0_scaffold00092.111 Total prob of N-in:        0.00682
Amborellatrichopoda_evm_27.model.AmTr_v1.0_scaffold00092.111	TMHMM2.0	outside	     1   320
```

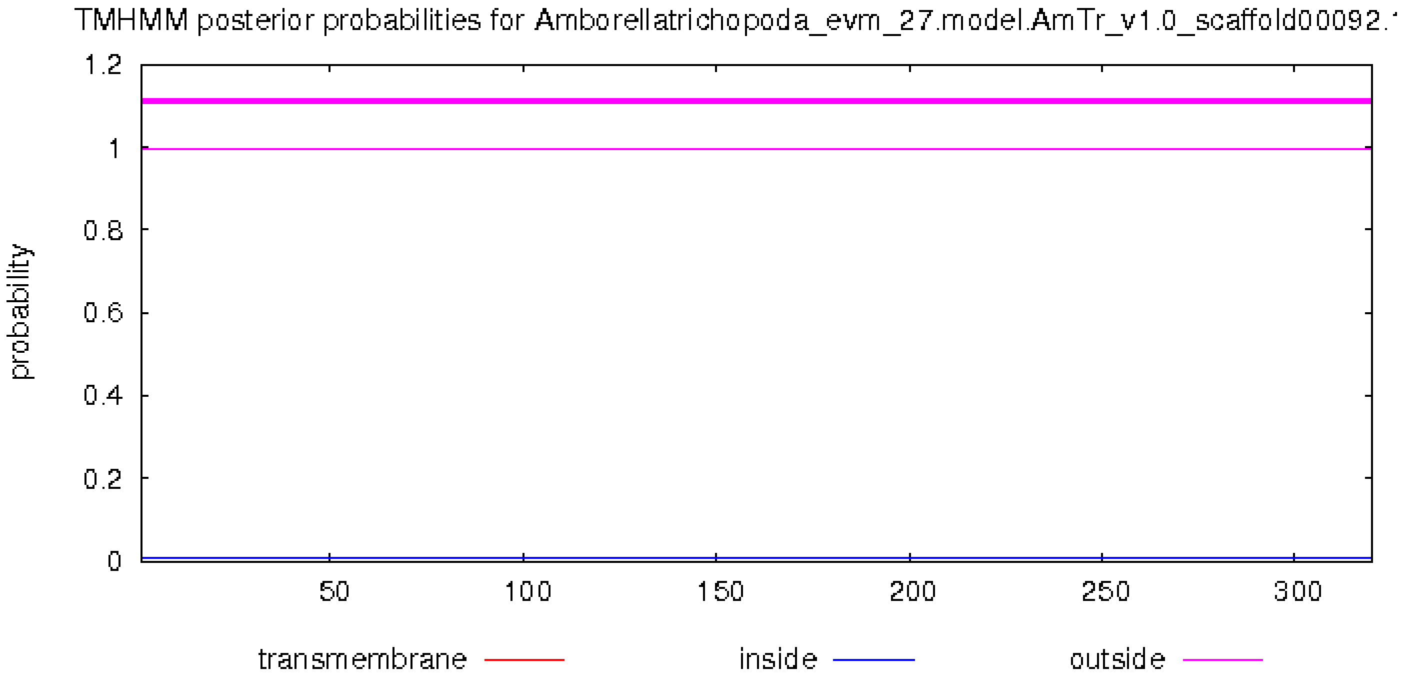

plot in postscript, script for making the plot in gnuplot, data for plot

---

```
# Amborellatrichopoda_evm_27.model.AmTr_v1.0_scaffold00099.117 Length: 359
# Amborellatrichopoda_evm_27.model.AmTr_v1.0_scaffold00099.117 Number of predicted TMHs:  0
# Amborellatrichopoda_evm_27.model.AmTr_v1.0_scaffold00099.117 Exp number of AAs in TMHs: 0
# Amborellatrichopoda_evm_27.model.AmTr_v1.0_scaffold00099.117 Exp number, first 60 AAs:  0
# Amborellatrichopoda_evm_27.model.AmTr_v1.0_scaffold00099.117 Total prob of N-in:        0.00333
Amborellatrichopoda_evm_27.model.AmTr_v1.0_scaffold00099.117	TMHMM2.0	outside	     1   359
```

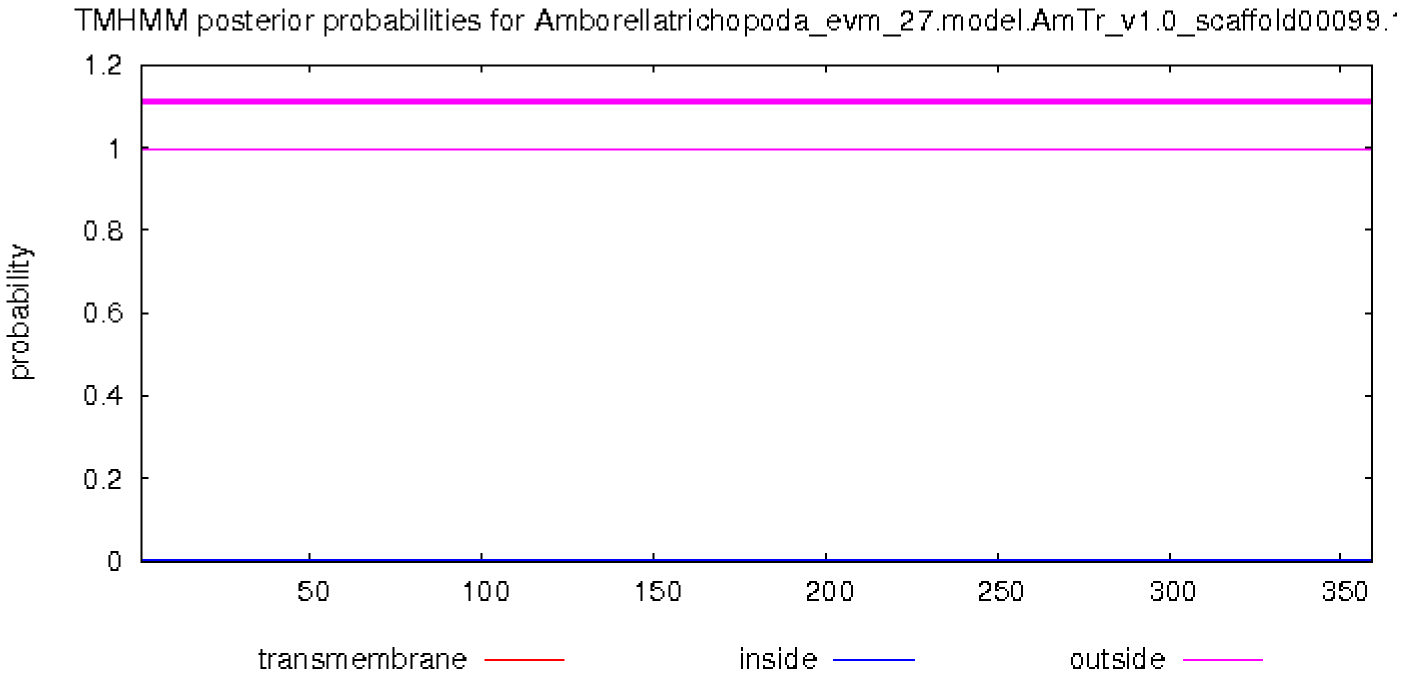

plot in postscript, script for making the plot in gnuplot, data for plot

---

```
# Amborellatrichopoda_evm_27.model.AmTr_v1.0_scaffold00099.164 Length: 322
# Amborellatrichopoda_evm_27.model.AmTr_v1.0_scaffold00099.164 Number of predicted TMHs:  0
```

```
# Amborellatrichopoda_evm_27.model.AmTr_v1.0_scaffold00099.164 Exp number of AAs in TMHs: 0.00053
# Amborellatrichopoda_evm_27.model.AmTr_v1.0_scaffold00099.164 Exp number, first 60 AAs:  0.00026
# Amborellatrichopoda_evm_27.model.AmTr_v1.0_scaffold00099.164 Total prob of N-in:        0.02233
Amborellatrichopoda_evm_27.model.AmTr_v1.0_scaffold00099.164    TMHMM2.0        outside      1   322
```

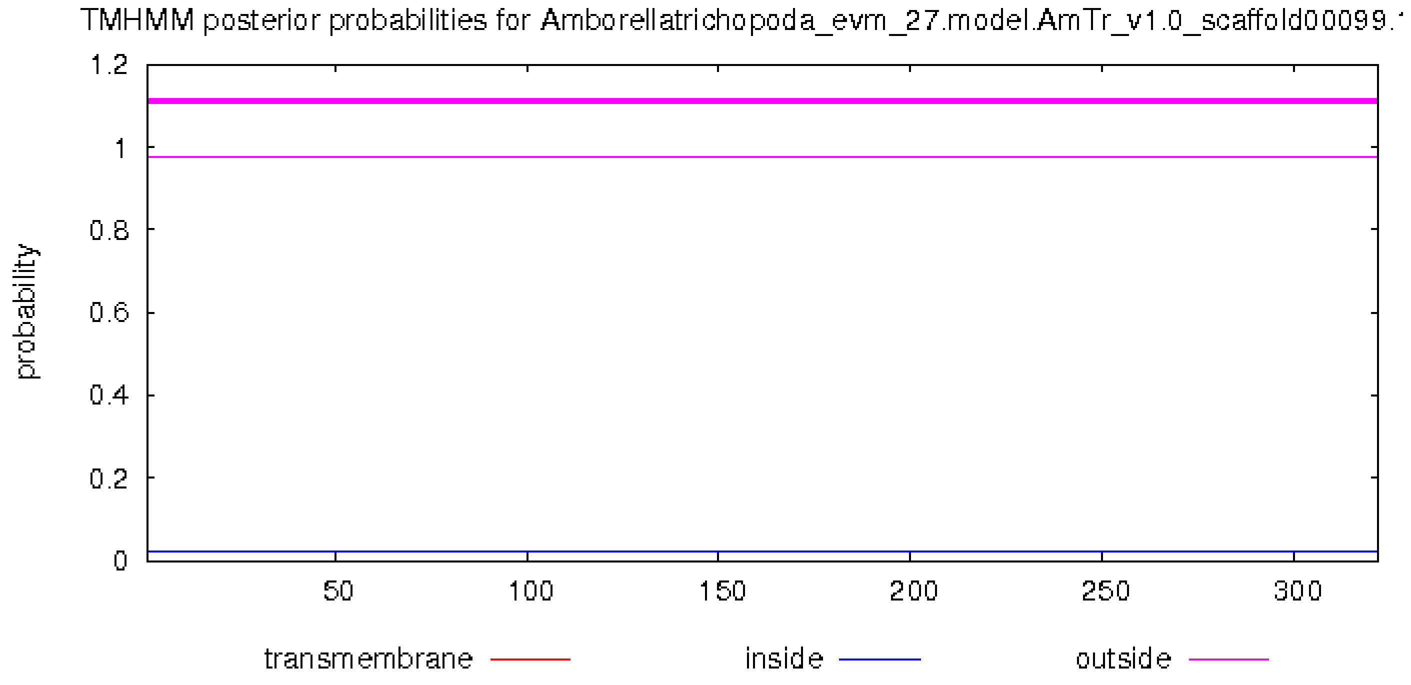

plot in postscript, script for making the plot in gnuplot, data for plot

---

```
# Amborellatrichopoda_evm_27.model.AmTr_v1.0_scaffold00113.16 Length: 553
# Amborellatrichopoda_evm_27.model.AmTr_v1.0_scaffold00113.16 Number of predicted TMHs:  0
# Amborellatrichopoda_evm_27.model.AmTr_v1.0_scaffold00113.16 Exp number of AAs in TMHs: 0.00475
# Amborellatrichopoda_evm_27.model.AmTr_v1.0_scaffold00113.16 Exp number, first 60 AAs:  0.00409
# Amborellatrichopoda_evm_27.model.AmTr_v1.0_scaffold00113.16 Total prob of N-in:        0.00072
Amborellatrichopoda_evm_27.model.AmTr_v1.0_scaffold00113.16     TMHMM2.0        outside      1   553
```

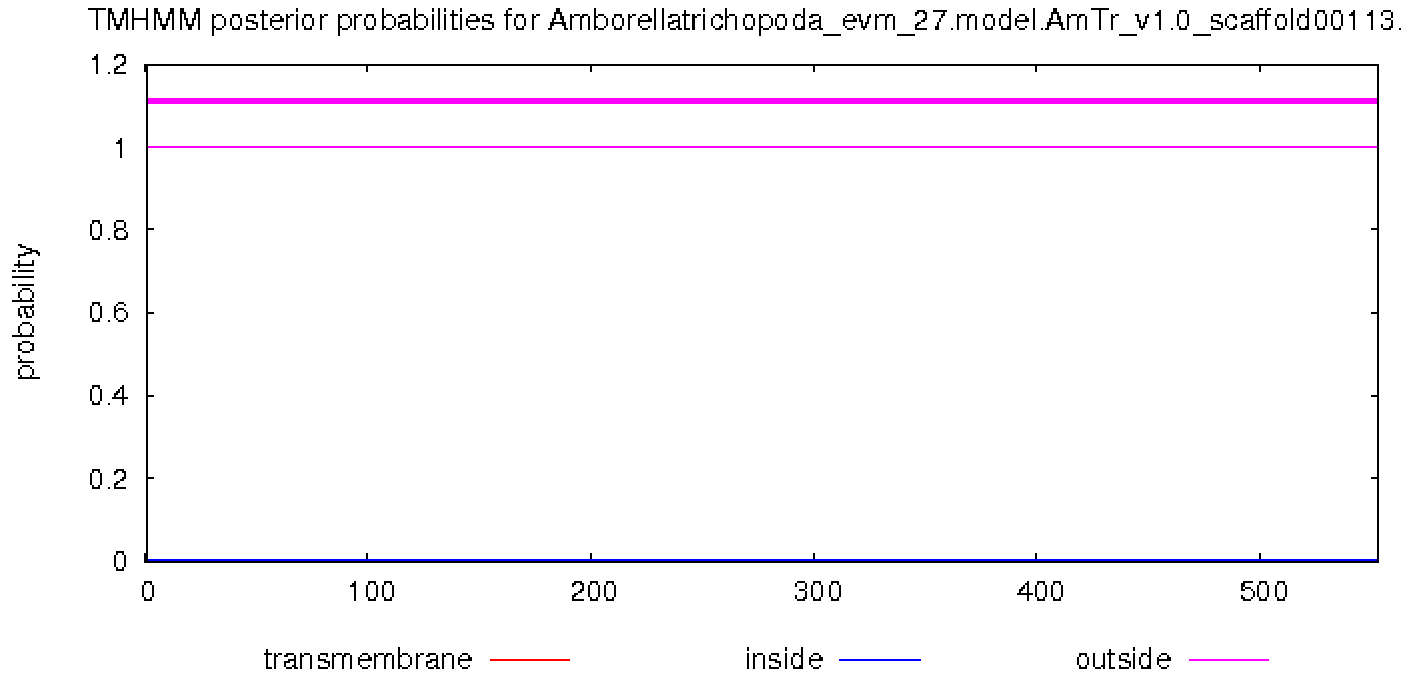

plot in postscript, script for making the plot in gnuplot, data for plot

---

```
# Amborellatrichopoda_evm_27.model.AmTr_v1.0_scaffold00119.12 Length: 314
# Amborellatrichopoda_evm_27.model.AmTr_v1.0_scaffold00119.12 Number of predicted TMHs:  0
```

```
# Amborellatrichopoda_evm_27.model.AmTr_v1.0_scaffold00119.12 Exp number of AAs in TMHs: 0.00143
# Amborellatrichopoda_evm_27.model.AmTr_v1.0_scaffold00119.12 Exp number, first 60 AAs:  0.00143
# Amborellatrichopoda_evm_27.model.AmTr_v1.0_scaffold00119.12 Total prob of N-in:        0.04106
Amborellatrichopoda_evm_27.model.AmTr_v1.0_scaffold00119.12	TMHMM2.0	outside	     1   314
```

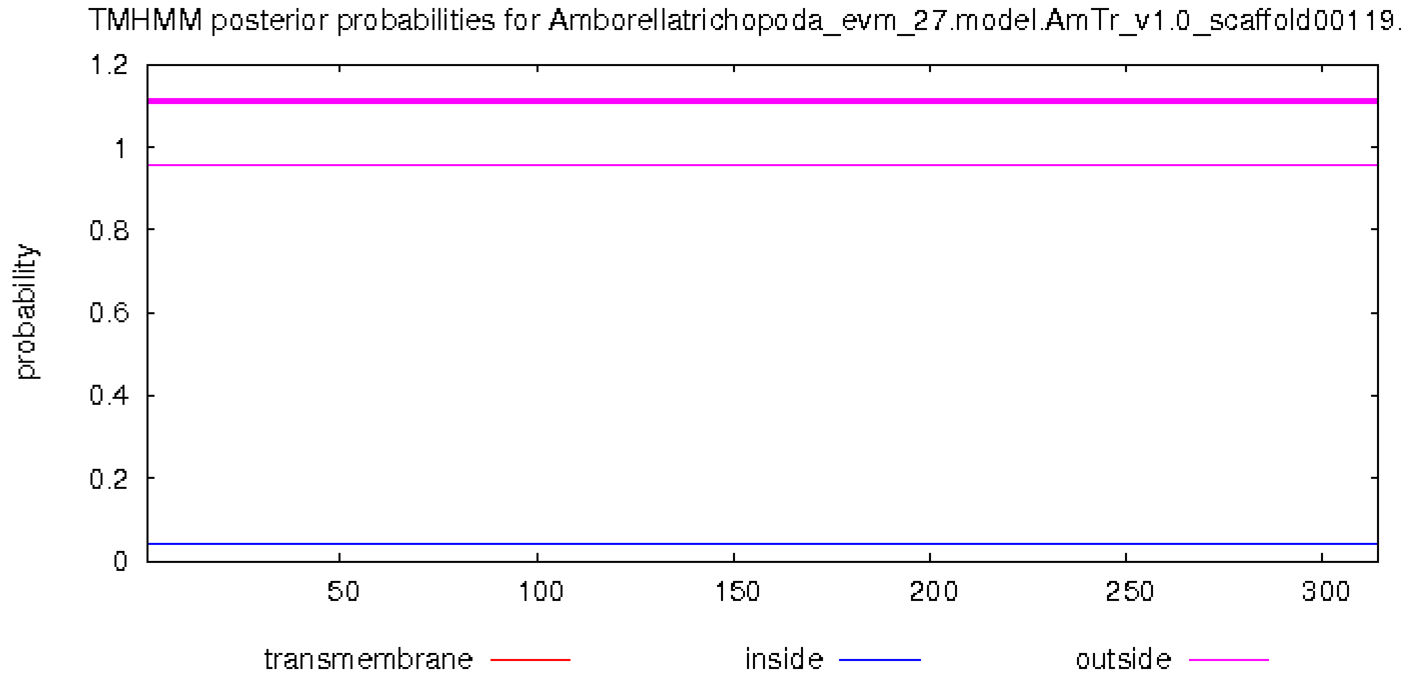

plot in postscript, script for making the plot in gnuplot, data for plot

---

```
# Amborellatrichopoda_evm_27.model.AmTr_v1.0_scaffold00119.21 Length: 357
# Amborellatrichopoda_evm_27.model.AmTr_v1.0_scaffold00119.21 Number of predicted TMHs:  0
# Amborellatrichopoda_evm_27.model.AmTr_v1.0_scaffold00119.21 Exp number of AAs in TMHs: 0.00012
# Amborellatrichopoda_evm_27.model.AmTr_v1.0_scaffold00119.21 Exp number, first 60 AAs:  0
# Amborellatrichopoda_evm_27.model.AmTr_v1.0_scaffold00119.21 Total prob of N-in:        0.00466
Amborellatrichopoda_evm_27.model.AmTr_v1.0_scaffold00119.21	TMHMM2.0	outside	     1   357
```

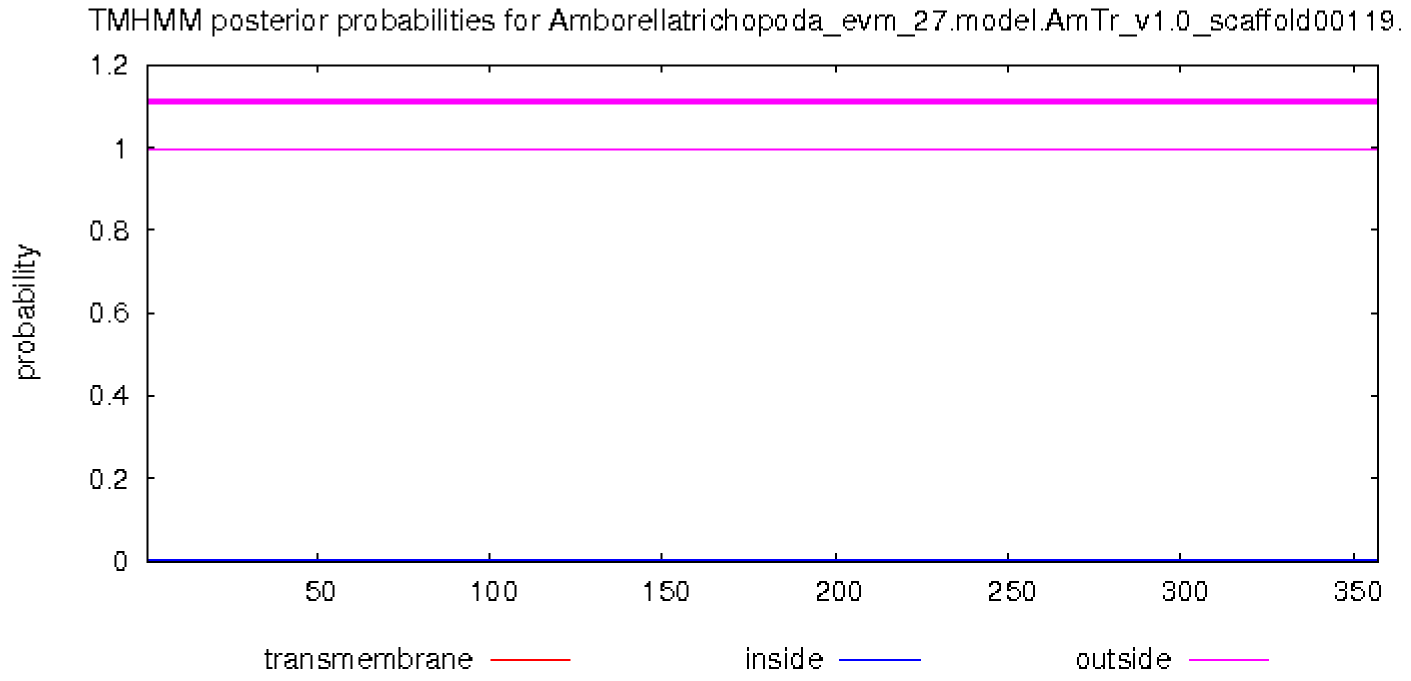

plot in postscript, script for making the plot in gnuplot, data for plot

---

```
# Amborellatrichopoda_evm_27.model.AmTr_v1.0_scaffold00119.25 Length: 400
# Amborellatrichopoda_evm_27.model.AmTr_v1.0_scaffold00119.25 Number of predicted TMHs:  0
```

```
# Amborellatrichopoda_evm_27.model.AmTr_v1.0_scaffold00119.25 Exp number of AAs in TMHs: 0
# Amborellatrichopoda_evm_27.model.AmTr_v1.0_scaffold00119.25 Exp number, first 60 AAs:  0
# Amborellatrichopoda_evm_27.model.AmTr_v1.0_scaffold00119.25 Total prob of N-in:        0.00522
Amborellatrichopoda_evm_27.model.AmTr_v1.0_scaffold00119.25     TMHMM2.0        outside      1   400
```

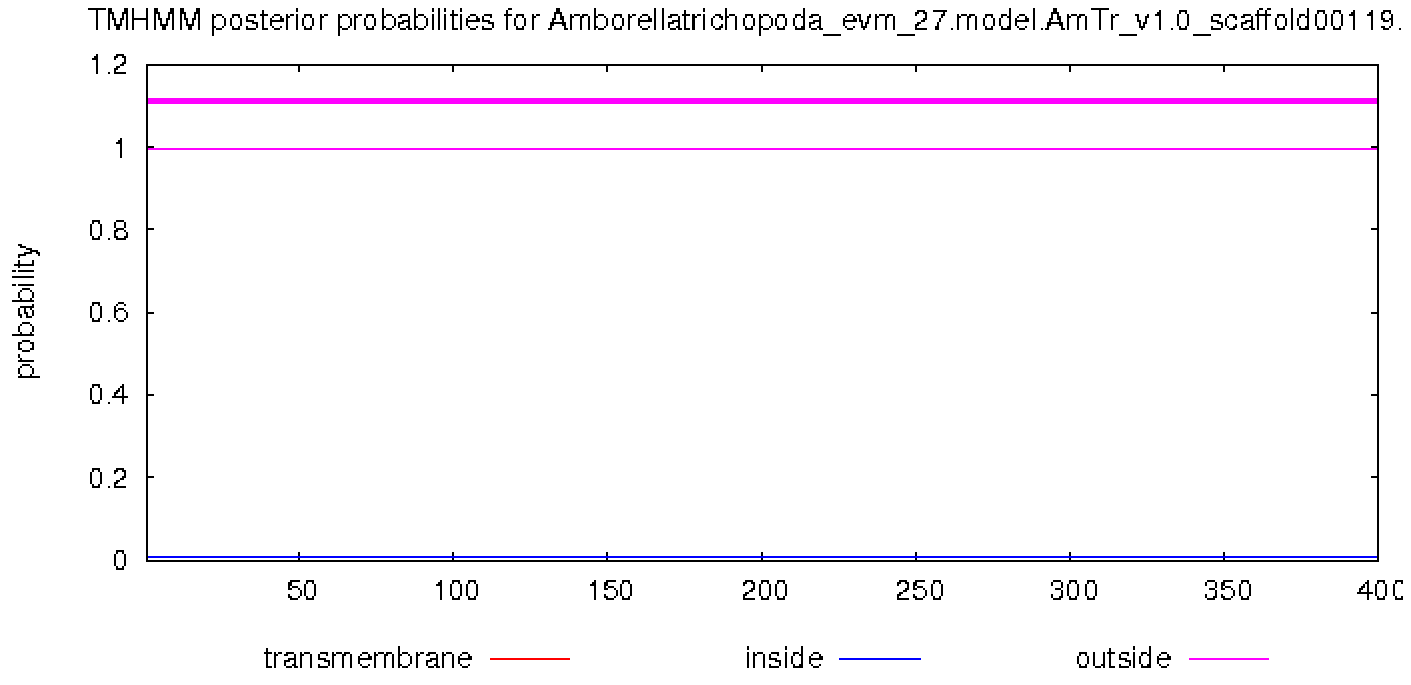

plot in postscript, script for making the plot in gnuplot, data for plot

---

```
# Amborellatrichopoda_evm_27.model.AmTr_v1.0_scaffold00140.39 Length: 637
# Amborellatrichopoda_evm_27.model.AmTr_v1.0_scaffold00140.39 Number of predicted TMHs:  1
# Amborellatrichopoda_evm_27.model.AmTr_v1.0_scaffold00140.39 Exp number of AAs in TMHs: 21.65826
# Amborellatrichopoda_evm_27.model.AmTr_v1.0_scaffold00140.39 Exp number, first 60 AAs:  0
# Amborellatrichopoda_evm_27.model.AmTr_v1.0_scaffold00140.39 Total prob of N-in:        0.00077
Amborellatrichopoda_evm_27.model.AmTr_v1.0_scaffold00140.39     TMHMM2.0        outside      1   601
Amborellatrichopoda_evm_27.model.AmTr_v1.0_scaffold00140.39     TMHMM2.0        TMhelix    602   621
Amborellatrichopoda_evm_27.model.AmTr_v1.0_scaffold00140.39     TMHMM2.0        inside     622   637
```

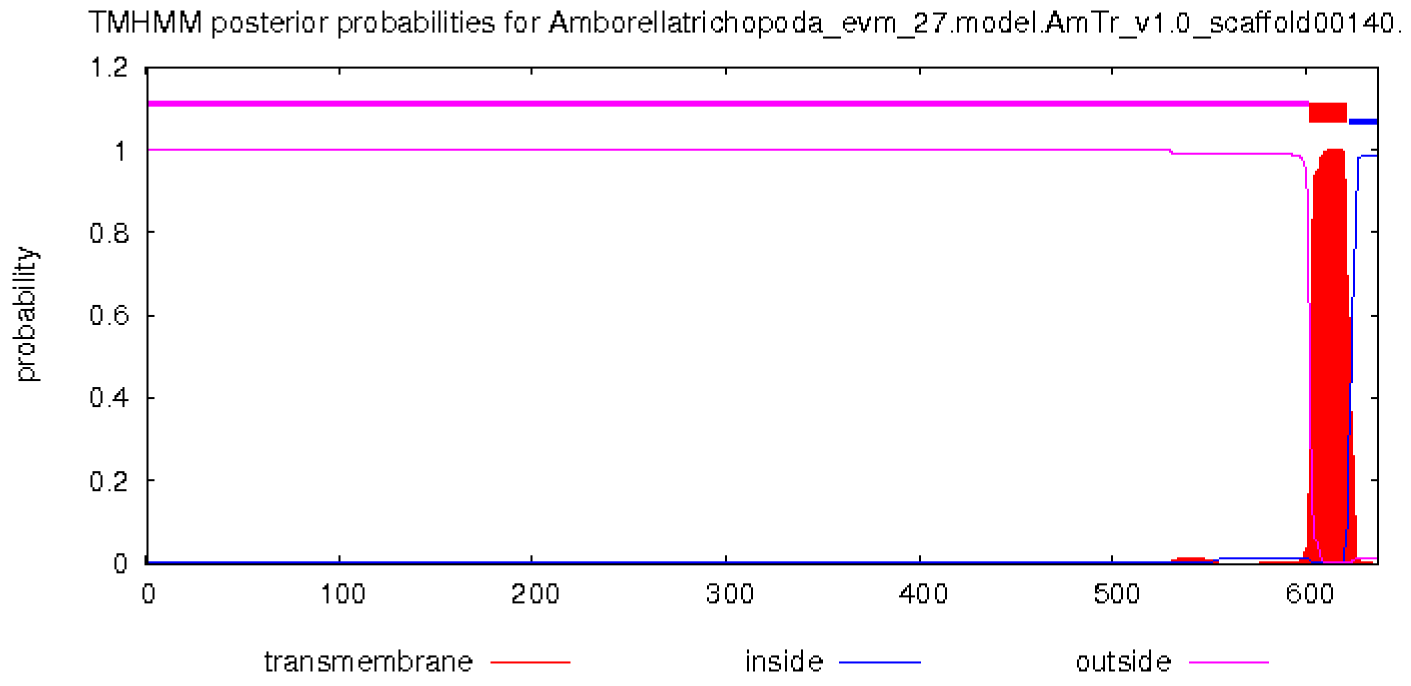

plot in postscript, script for making the plot in gnuplot, data for plot

---

```
# Amborellatrichopoda_evm_27.model.AmTr_v1.0_scaffold00149.46 Length: 358
# Amborellatrichopoda_evm_27.model.AmTr_v1.0_scaffold00149.46 Number of predicted TMHs:  0
# Amborellatrichopoda_evm_27.model.AmTr_v1.0_scaffold00149.46 Exp number of AAs in TMHs: 0
# Amborellatrichopoda_evm_27.model.AmTr_v1.0_scaffold00149.46 Exp number, first 60 AAs:  0
# Amborellatrichopoda_evm_27.model.AmTr_v1.0_scaffold00149.46 Total prob of N-in:        0.00050
Amborellatrichopoda_evm_27.model.AmTr_v1.0_scaffold00149.46     TMHMM2.0        outside      1   358
```

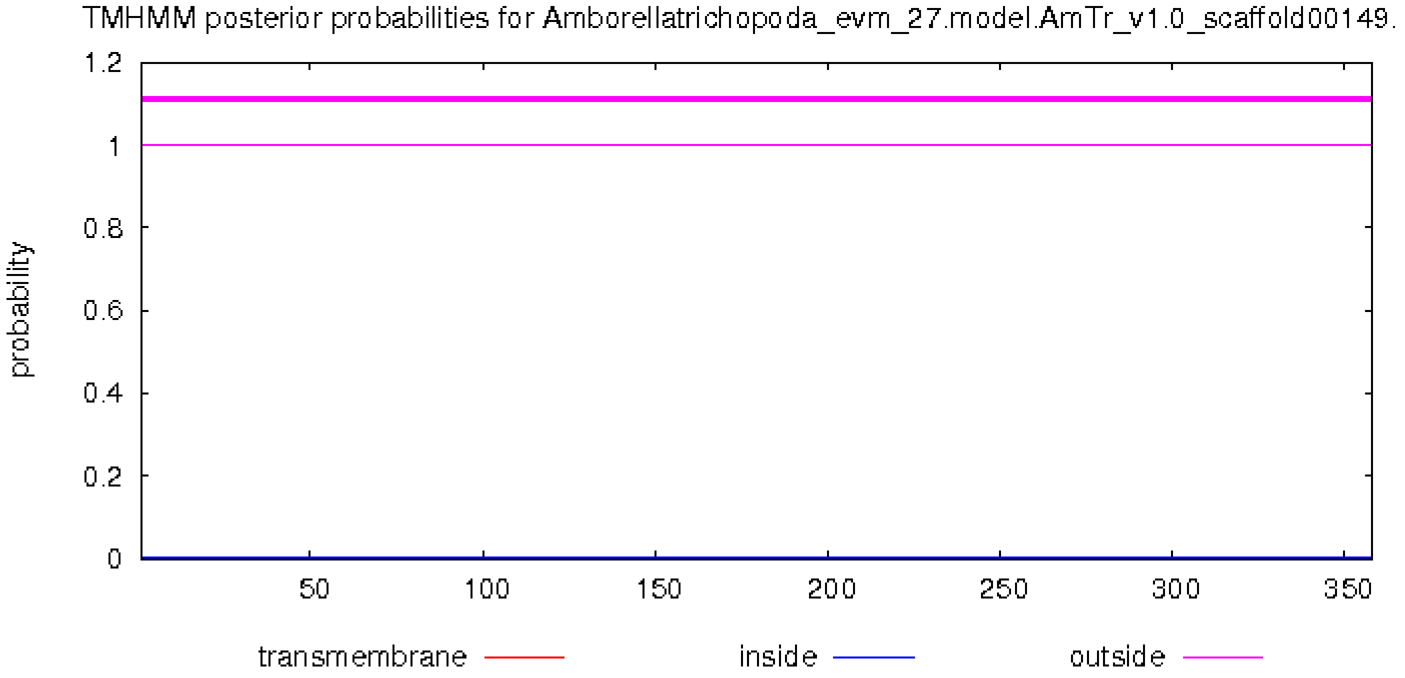

plot in postscript, script for making the plot in gnuplot, data for plot

---

```
# Amborellatrichopoda_evm_27.model.AmTr_v1.0_scaffold02859.1 Length: 310
# Amborellatrichopoda_evm_27.model.AmTr_v1.0_scaffold02859.1 Number of predicted TMHs:  0
# Amborellatrichopoda_evm_27.model.AmTr_v1.0_scaffold02859.1 Exp number of AAs in TMHs: 0
# Amborellatrichopoda_evm_27.model.AmTr_v1.0_scaffold02859.1 Exp number, first 60 AAs:  0
# Amborellatrichopoda_evm_27.model.AmTr_v1.0_scaffold02859.1 Total prob of N-in:        0.02522
Amborellatrichopoda_evm_27.model.AmTr_v1.0_scaffold02859.1      TMHMM2.0        outside      1   310
```

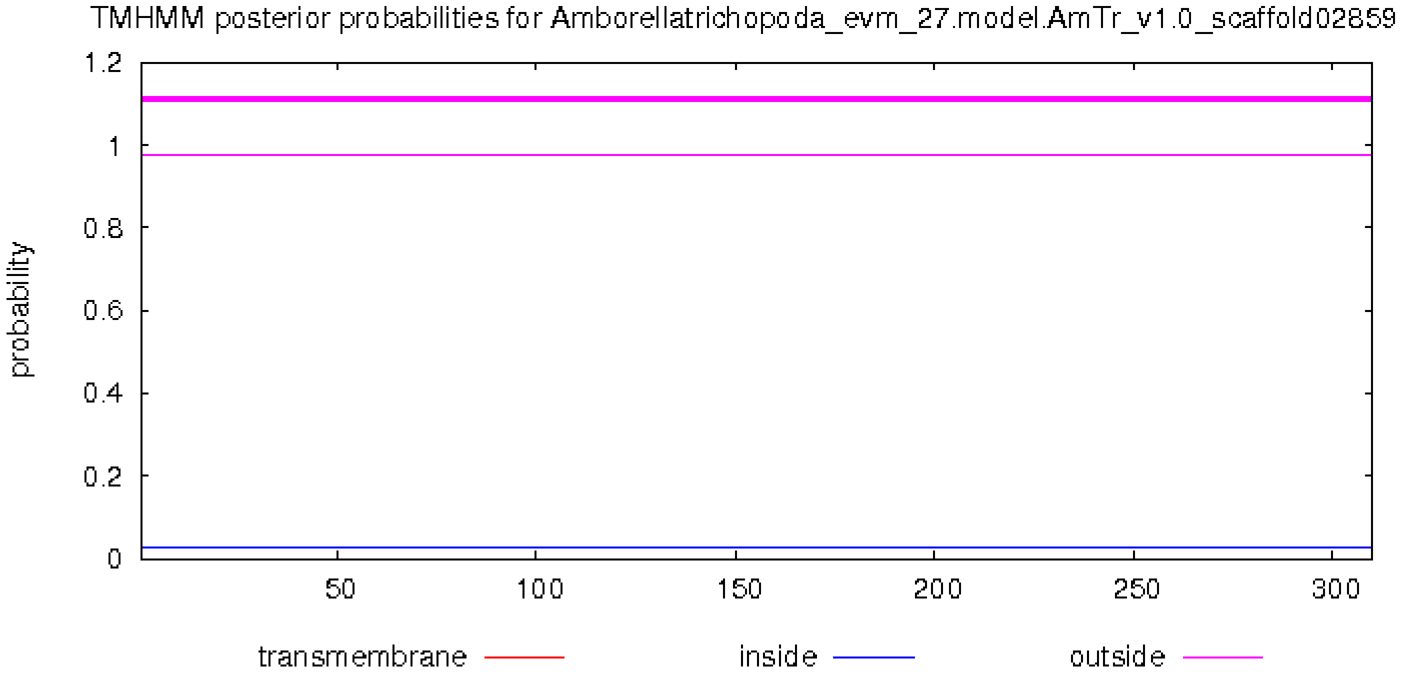

plot in postscript, script for making the plot in gnuplot, data for plot

---

```
# Ananascomosus_Aco000307.1 Length: 320
# Ananascomosus_Aco000307.1 Number of predicted TMHs:  0
# Ananascomosus_Aco000307.1 Exp number of AAs in TMHs: 0.00095
# Ananascomosus_Aco000307.1 Exp number, first 60 AAs:  0.0004
# Ananascomosus_Aco000307.1 Total prob of N-in:        0.00281
Ananascomosus_Aco000307.1       TMHMM2.0        outside      1   320
```

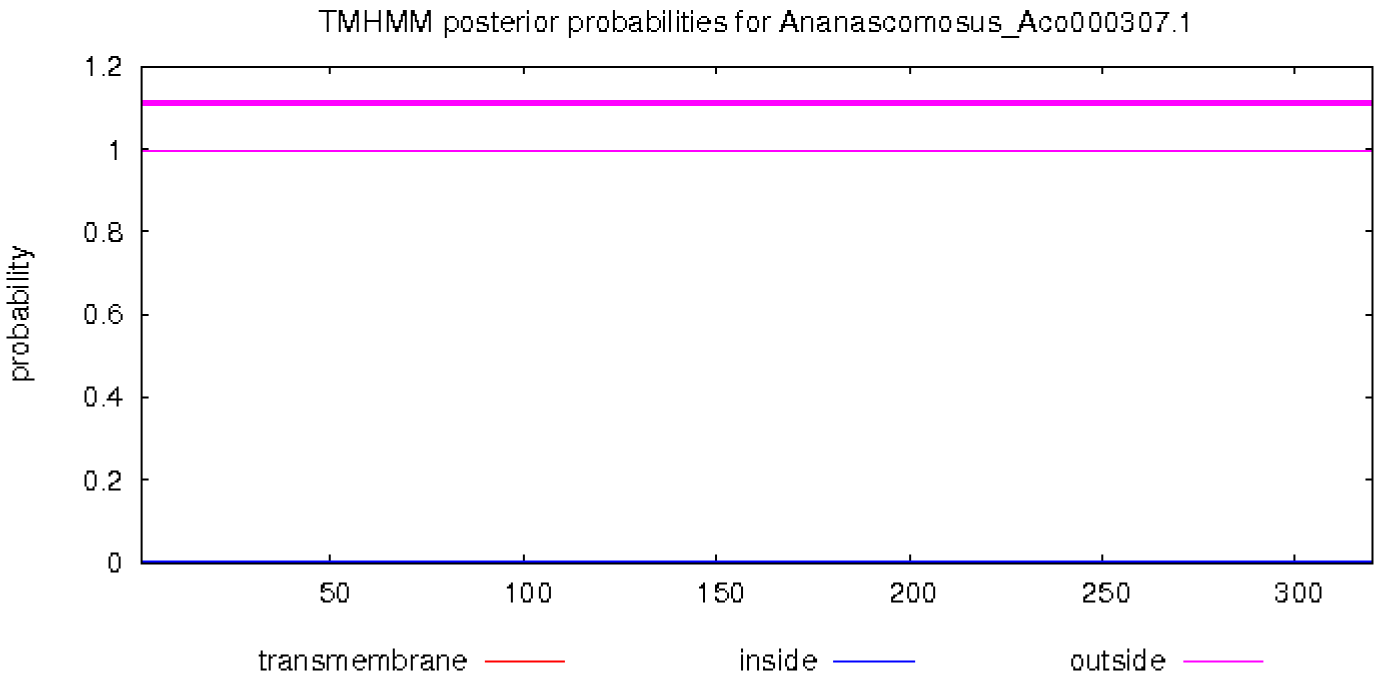

plot in postscript, script for making the plot in gnuplot, data for plot

---

```
# Ananascomosus_Aco000364.1 Length: 304
# Ananascomosus_Aco000364.1 Number of predicted TMHs:  0
# Ananascomosus_Aco000364.1 Exp number of AAs in TMHs: 0.00109
# Ananascomosus_Aco000364.1 Exp number, first 60 AAs:  0
# Ananascomosus_Aco000364.1 Total prob of N-in:        0.03080
Ananascomosus_Aco000364.1       TMHMM2.0        outside      1   304
```

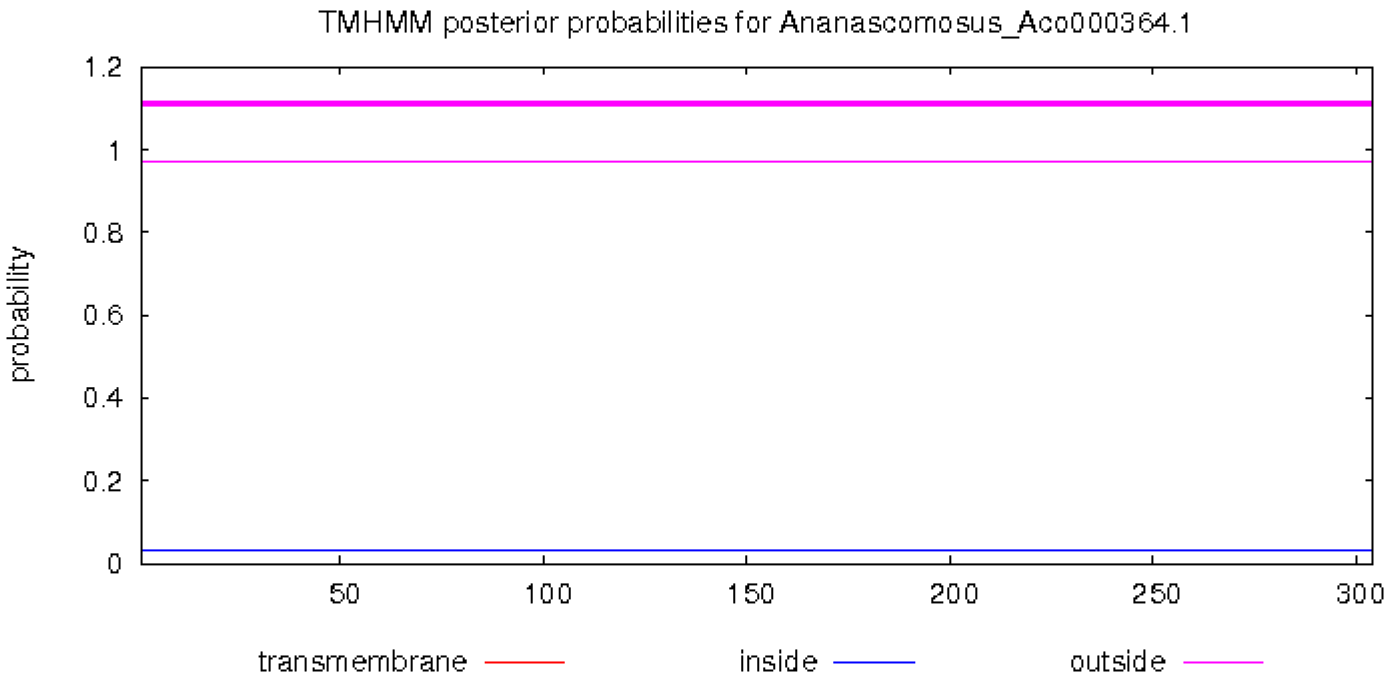

plot in postscript, script for making the plot in gnuplot, data for plot

---

```
# Ananascomosus_Aco000744.1 Length: 320
# Ananascomosus_Aco000744.1 Number of predicted TMHs:  0
# Ananascomosus_Aco000744.1 Exp number of AAs in TMHs: 0.01546
# Ananascomosus_Aco000744.1 Exp number, first 60 AAs:  0.00821
# Ananascomosus_Aco000744.1 Total prob of N-in:        0.01540
Ananascomosus_Aco000744.1	TMHMM2.0	outside	     1   320
```

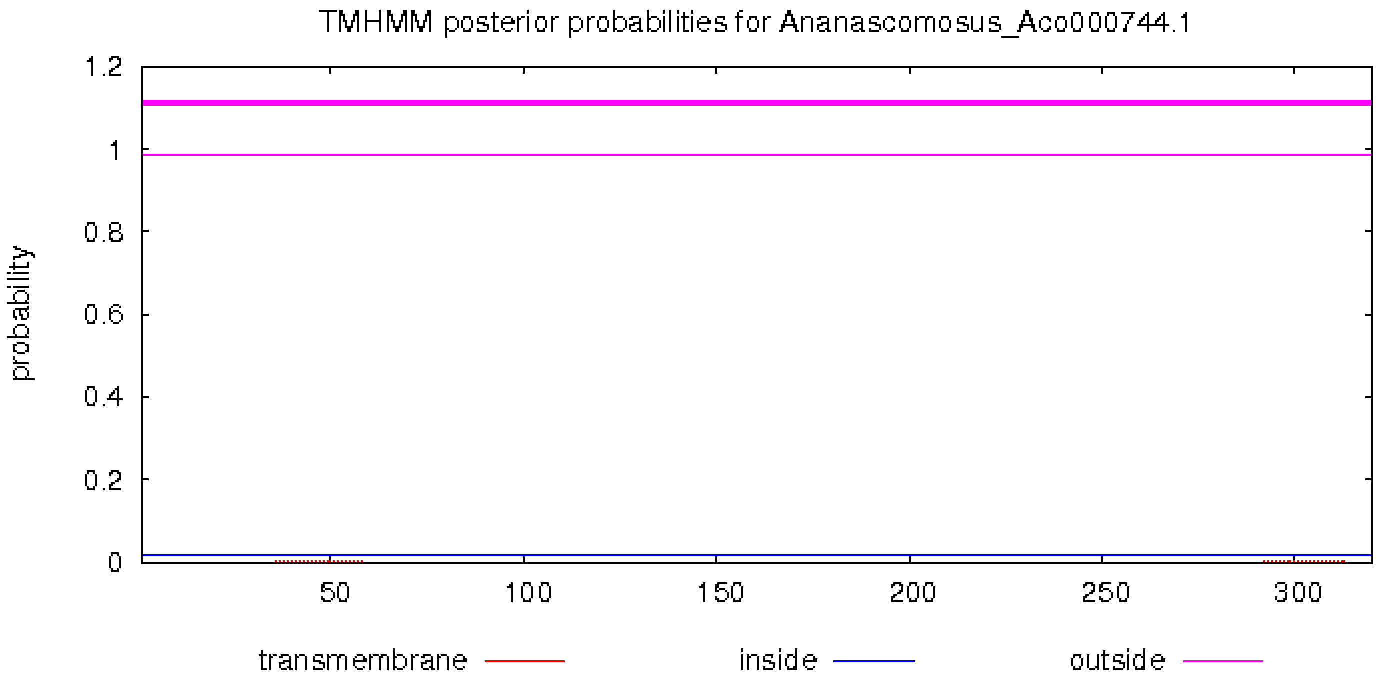

plot in postscript, script for making the plot in gnuplot, data for plot

```
# Ananascomosus_Aco001109.1 Length: 407
# Ananascomosus_Aco001109.1 Number of predicted TMHs:  0
# Ananascomosus_Aco001109.1 Exp number of AAs in TMHs: 0.00037
# Ananascomosus_Aco001109.1 Exp number, first 60 AAs:  0
# Ananascomosus_Aco001109.1 Total prob of N-in:        0.00192
Ananascomosus_Aco001109.1	TMHMM2.0	outside	     1   407
```

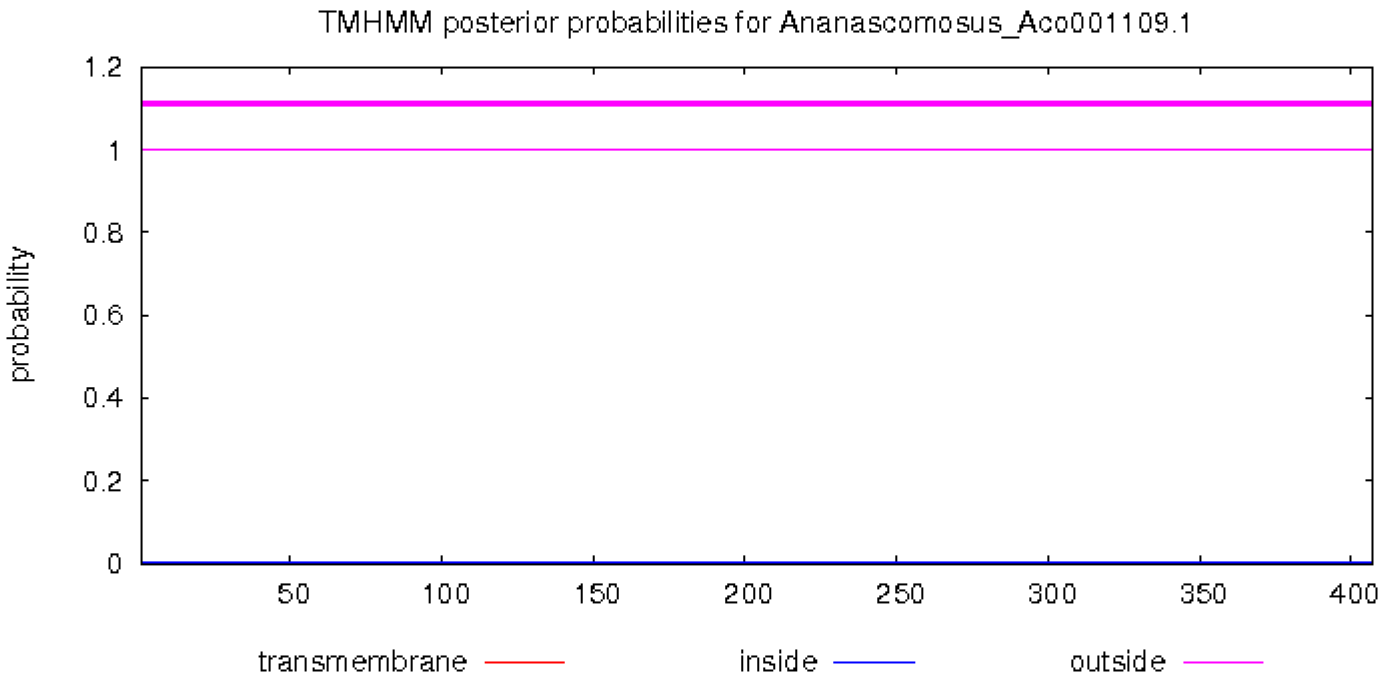

plot in postscript, script for making the plot in gnuplot, data for plot

```
# Ananascomosus_Aco001674.1 Length: 335
# Ananascomosus_Aco001674.1 Number of predicted TMHs:  0
# Ananascomosus_Aco001674.1 Exp number of AAs in TMHs: 0.00212
# Ananascomosus_Aco001674.1 Exp number, first 60 AAs:  0.00061
# Ananascomosus_Aco001674.1 Total prob of N-in:        0.00760
Ananascomosus_Aco001674.1       TMHMM2.0        outside      1   335
```

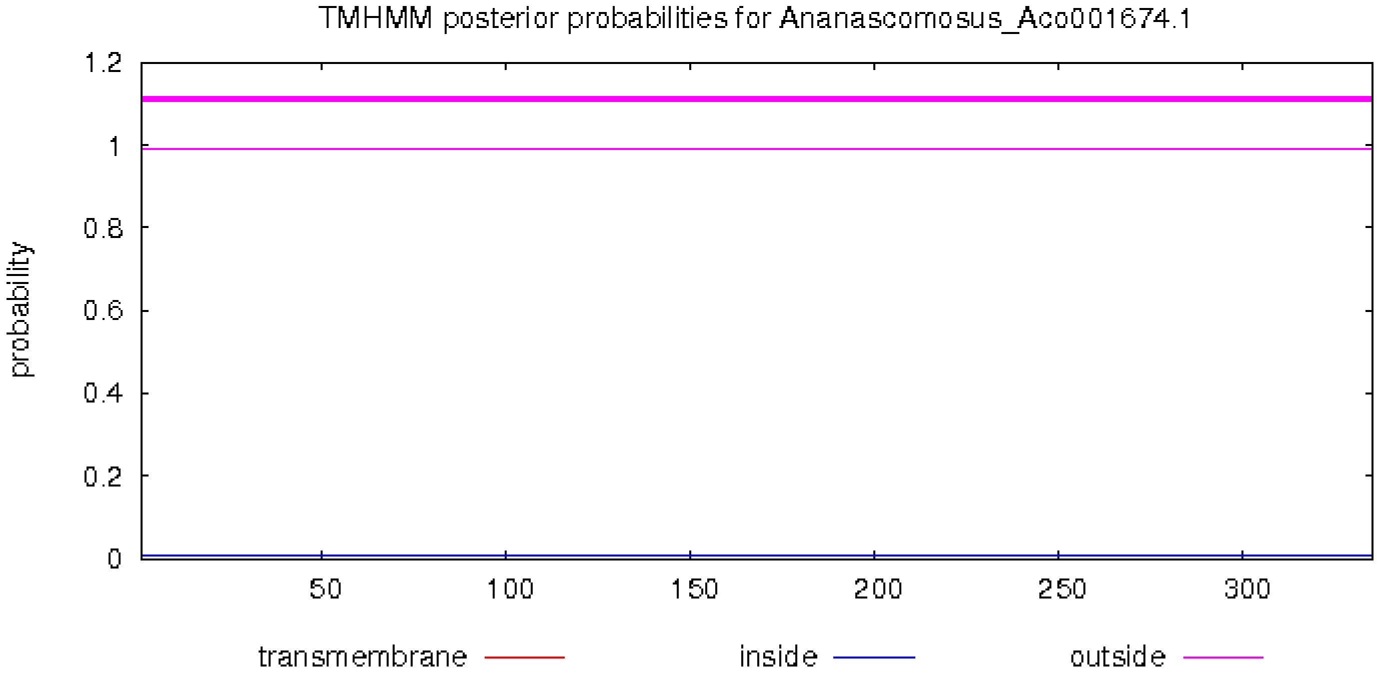

plot in postscript, script for making the plot in gnuplot, data for plot

```
# Ananascomosus_Aco002066.1 Length: 439
# Ananascomosus_Aco002066.1 Number of predicted TMHs:  0
# Ananascomosus_Aco002066.1 Exp number of AAs in TMHs: 0.00984
# Ananascomosus_Aco002066.1 Exp number, first 60 AAs:  0.0011
# Ananascomosus_Aco002066.1 Total prob of N-in:        0.01296
Ananascomosus_Aco002066.1       TMHMM2.0        outside      1   439
```

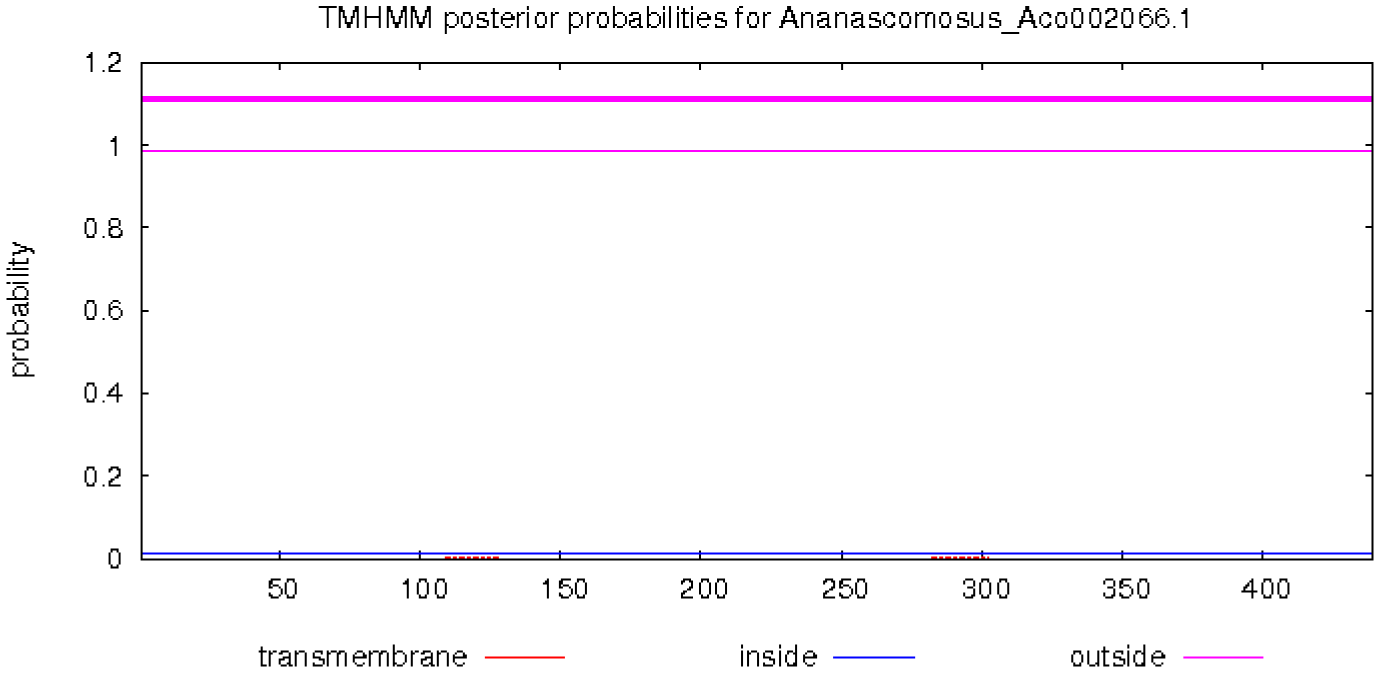

plot in postscript, script for making the plot in gnuplot, data for plot

```
# Ananascomosus_Aco002068.1 Length: 366
# Ananascomosus_Aco002068.1 Number of predicted TMHs:  0
# Ananascomosus_Aco002068.1 Exp number of AAs in TMHs: 0.00943
# Ananascomosus_Aco002068.1 Exp number, first 60 AAs:  0.00149
# Ananascomosus_Aco002068.1 Total prob of N-in:        0.03098
Ananascomosus_Aco002068.1	TMHMM2.0	outside	     1   366
```

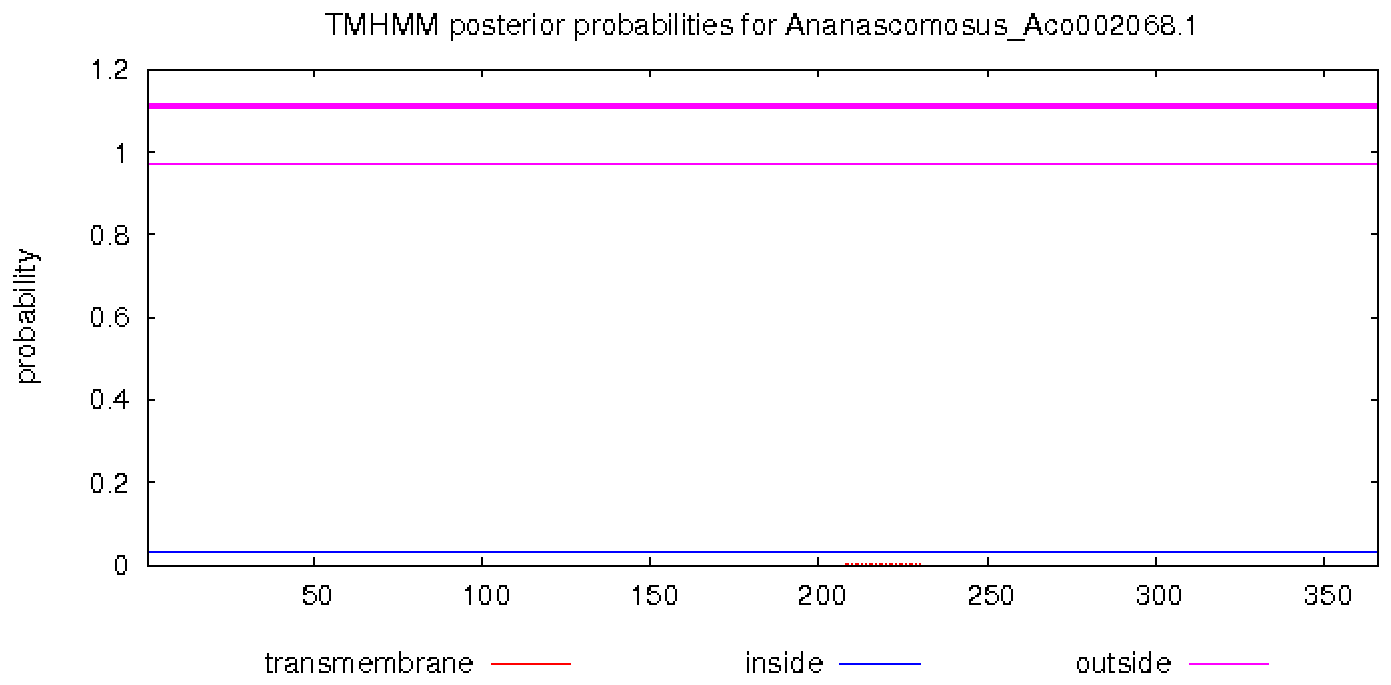

plot in postscript, script for making the plot in gnuplot, data for plot

```
# Ananascomosus_Aco002878.1 Length: 311
# Ananascomosus_Aco002878.1 Number of predicted TMHs:  0
# Ananascomosus_Aco002878.1 Exp number of AAs in TMHs: 0.00016
# Ananascomosus_Aco002878.1 Exp number, first 60 AAs:  0.00016
# Ananascomosus_Aco002878.1 Total prob of N-in:        0.05256
Ananascomosus_Aco002878.1	TMHMM2.0	outside	     1   311
```

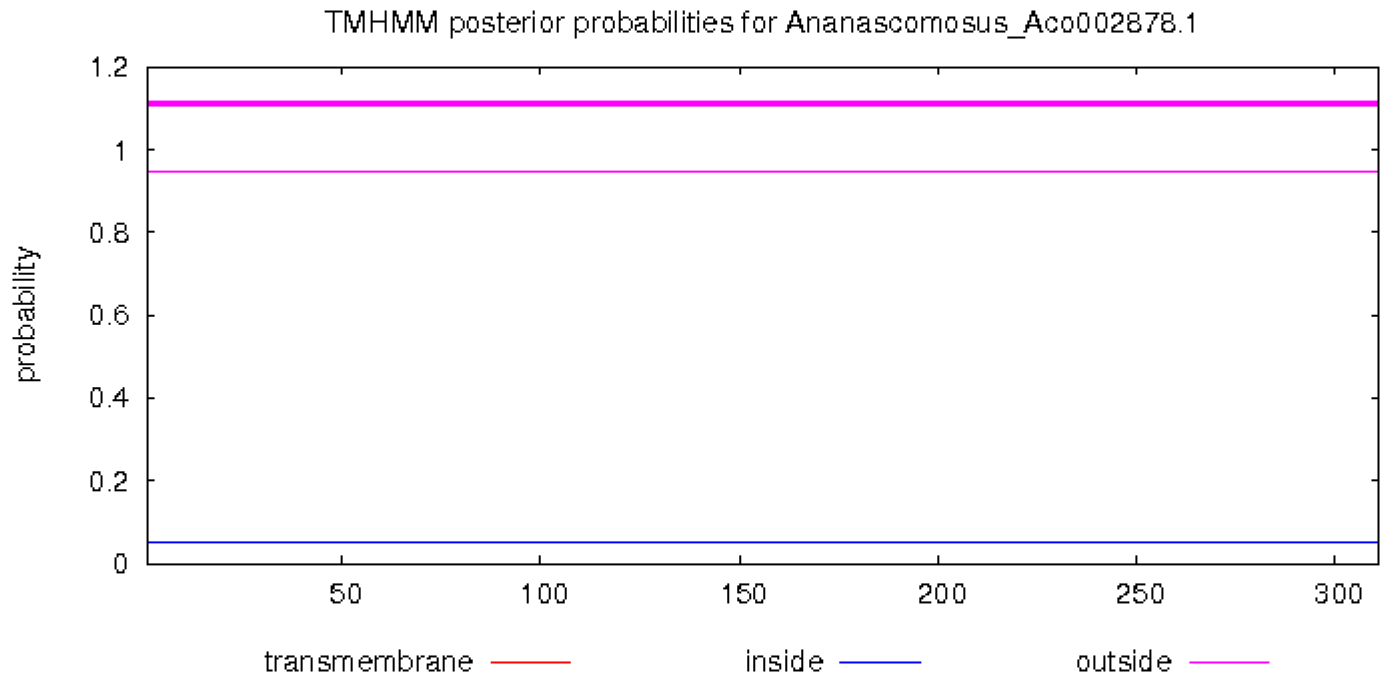

plot in postscript, script for making the plot in gnuplot, data for plot

```
# Ananascomosus_Aco003015.1 Length: 457
# Ananascomosus_Aco003015.1 Number of predicted TMHs:  0
# Ananascomosus_Aco003015.1 Exp number of AAs in TMHs: 0
# Ananascomosus_Aco003015.1 Exp number, first 60 AAs:  0
# Ananascomosus_Aco003015.1 Total prob of N-in:        0.00121
Ananascomosus_Aco003015.1	TMHMM2.0	outside	     1   457
```

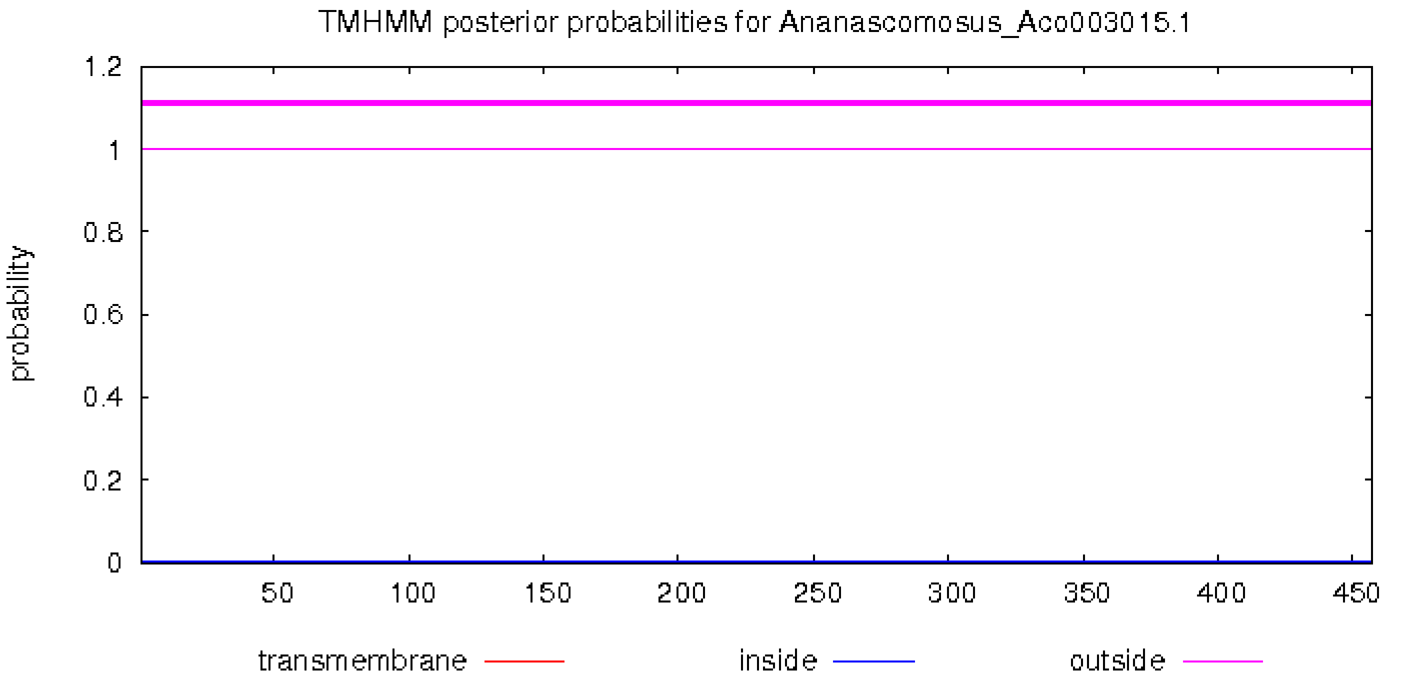

plot in postscript, script for making the plot in gnuplot, data for plot

---

```
# Ananascomosus_Aco003302.1 Length: 200
# Ananascomosus_Aco003302.1 Number of predicted TMHs:  0
# Ananascomosus_Aco003302.1 Exp number of AAs in TMHs: 0.00017
# Ananascomosus_Aco003302.1 Exp number, first 60 AAs:  0
# Ananascomosus_Aco003302.1 Total prob of N-in:        0.01831
Ananascomosus_Aco003302.1	TMHMM2.0	outside	     1   200
```

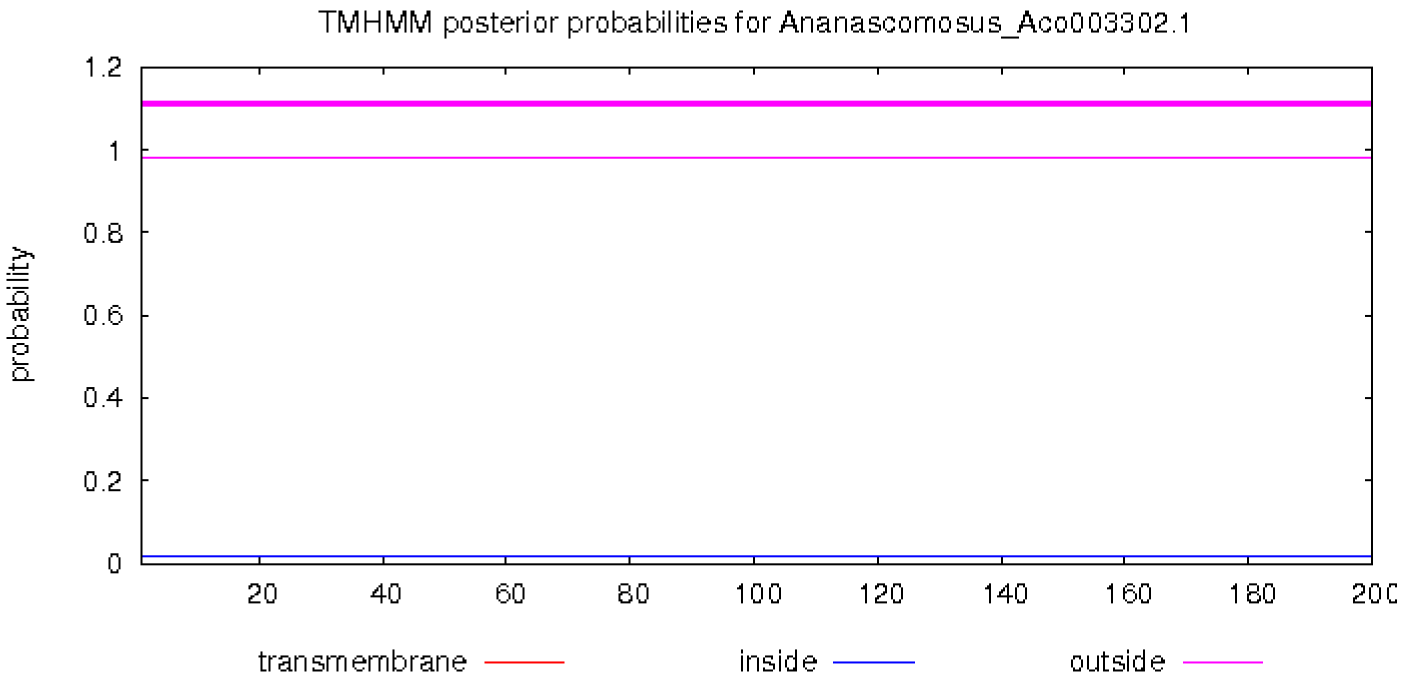

plot in postscript, script for making the plot in gnuplot, data for plot

---

```
# Ananascomosus_Aco003626.1 Length: 234
# Ananascomosus_Aco003626.1 Number of predicted TMHs:  0
# Ananascomosus_Aco003626.1 Exp number of AAs in TMHs: 0.00629
# Ananascomosus_Aco003626.1 Exp number, first 60 AAs:  0.00115
# Ananascomosus_Aco003626.1 Total prob of N-in:        0.12036
Ananascomosus_Aco003626.1	TMHMM2.0	outside	     1   234
```

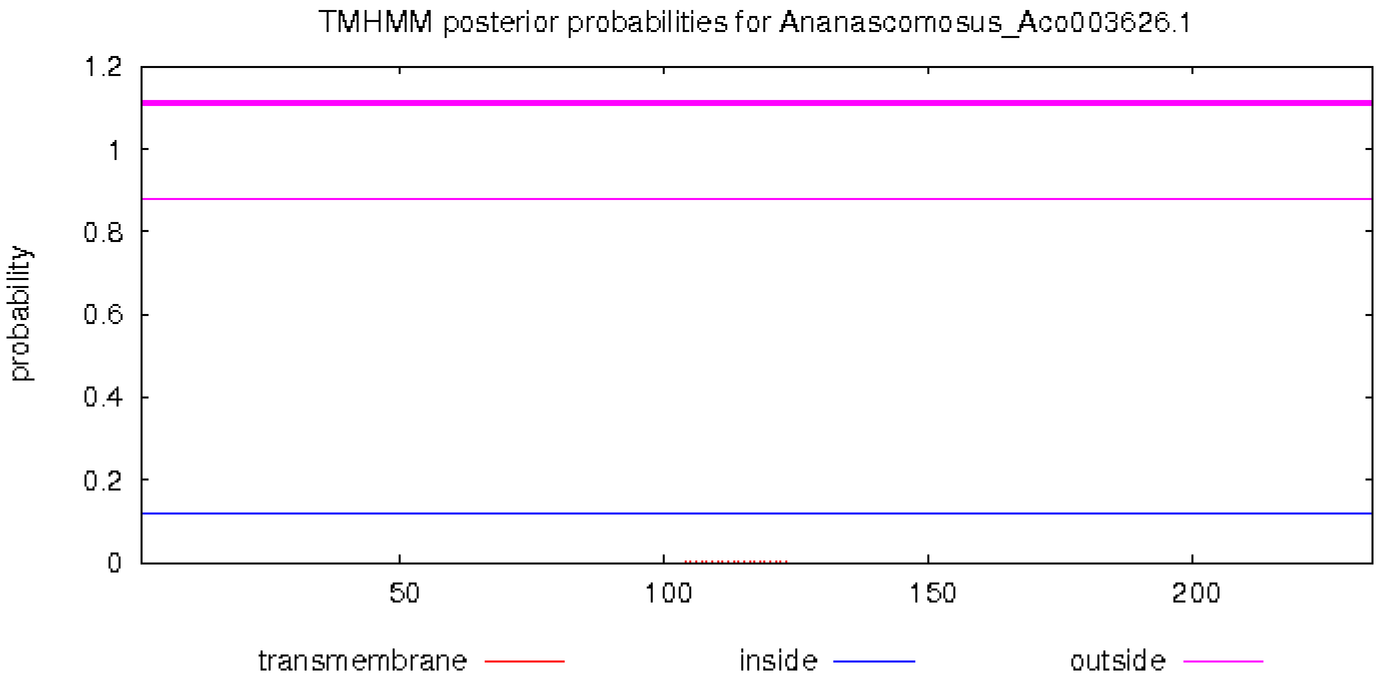

plot in postscript, script for making the plot in gnuplot, data for plot

---

```
# Ananascomosus_Aco003781.1 Length: 345
# Ananascomosus_Aco003781.1 Number of predicted TMHs:  0
# Ananascomosus_Aco003781.1 Exp number of AAs in TMHs: 0
# Ananascomosus_Aco003781.1 Exp number, first 60 AAs:  0
# Ananascomosus_Aco003781.1 Total prob of N-in:        0.00874
Ananascomosus_Aco003781.1	TMHMM2.0	outside	     1   345
```

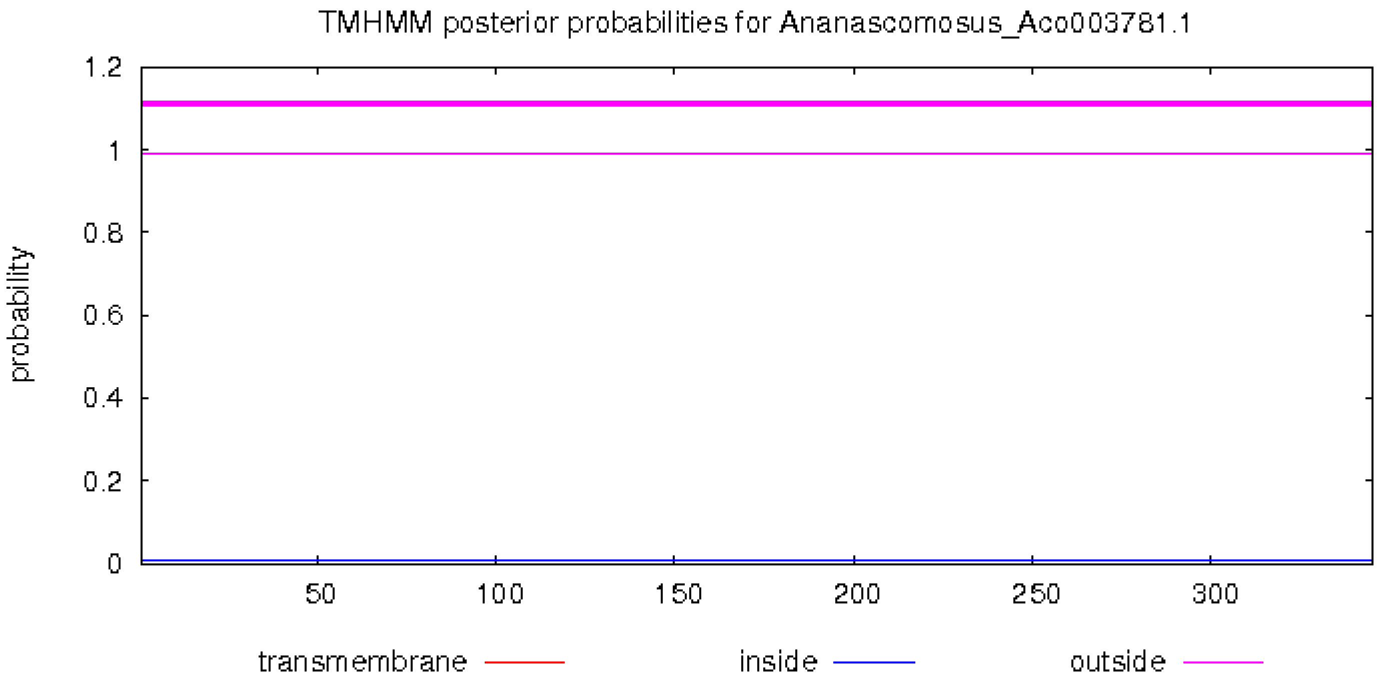

plot in postscript, script for making the plot in gnuplot, data for plot

---

```
# Ananascomosus_Aco004364.1 Length: 323
# Ananascomosus_Aco004364.1 Number of predicted TMHs:  0
# Ananascomosus_Aco004364.1 Exp number of AAs in TMHs: 0
# Ananascomosus_Aco004364.1 Exp number, first 60 AAs:  0
# Ananascomosus_Aco004364.1 Total prob of N-in:        0.03593
Ananascomosus_Aco004364.1       TMHMM2.0        outside      1   323
```

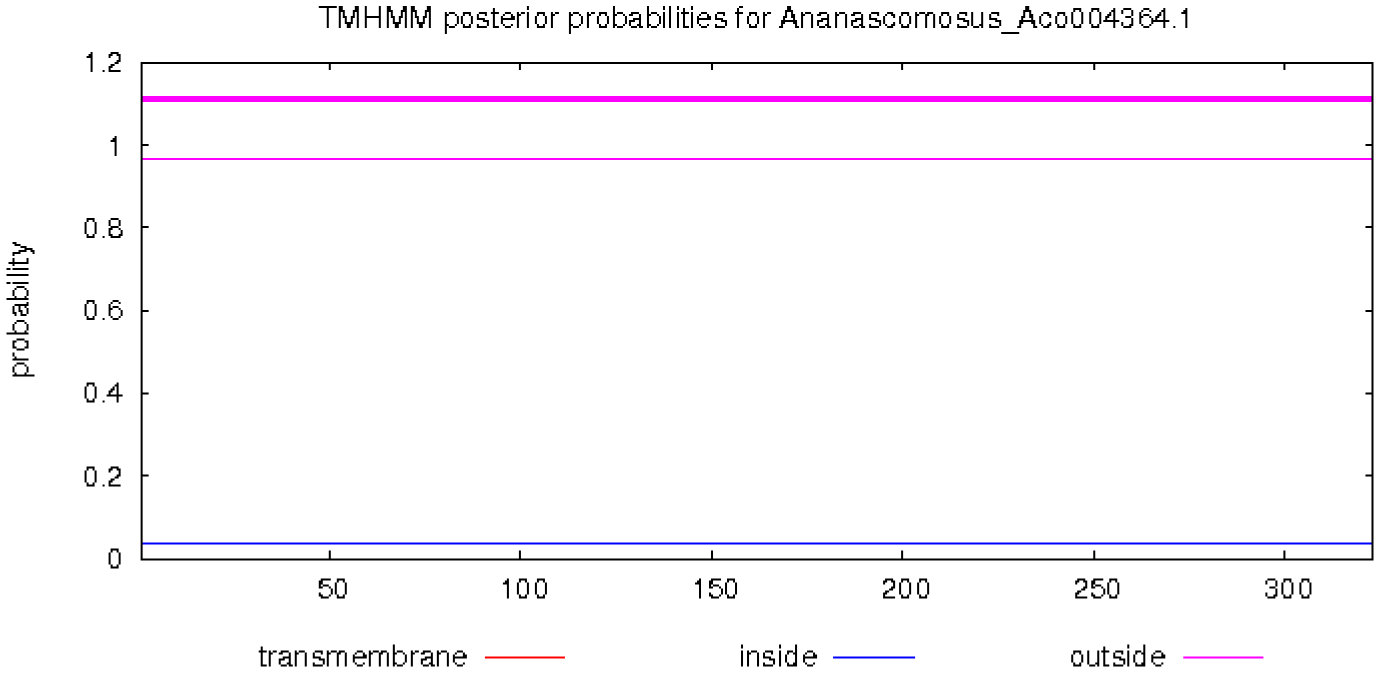

plot in postscript, script for making the plot in gnuplot, data for plot

```
# Ananascomosus_Aco004632.1 Length: 226
# Ananascomosus_Aco004632.1 Number of predicted TMHs:  0
# Ananascomosus_Aco004632.1 Exp number of AAs in TMHs: 0.00033
# Ananascomosus_Aco004632.1 Exp number, first 60 AAs:  0.00033
# Ananascomosus_Aco004632.1 Total prob of N-in:        0.09196
Ananascomosus_Aco004632.1       TMHMM2.0        outside      1   226
```

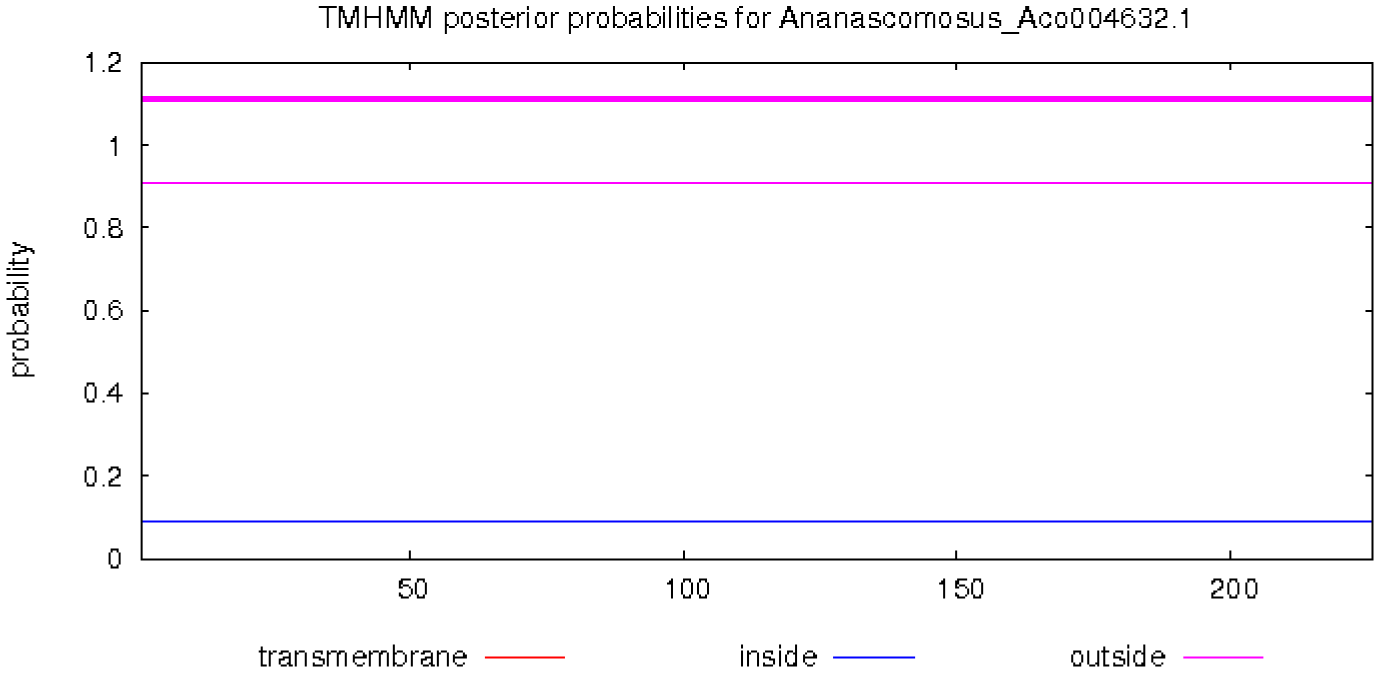

plot in postscript, script for making the plot in gnuplot, data for plot

```
# Ananascomosus_Aco004776.1 Length: 356
# Ananascomosus_Aco004776.1 Number of predicted TMHs:  0
# Ananascomosus_Aco004776.1 Exp number of AAs in TMHs: 0.00833
# Ananascomosus_Aco004776.1 Exp number, first 60 AAs:  0.00283
# Ananascomosus_Aco004776.1 Total prob of N-in:        0.01220
Ananascomosus_Aco004776.1	TMHMM2.0	outside	     1   356
```

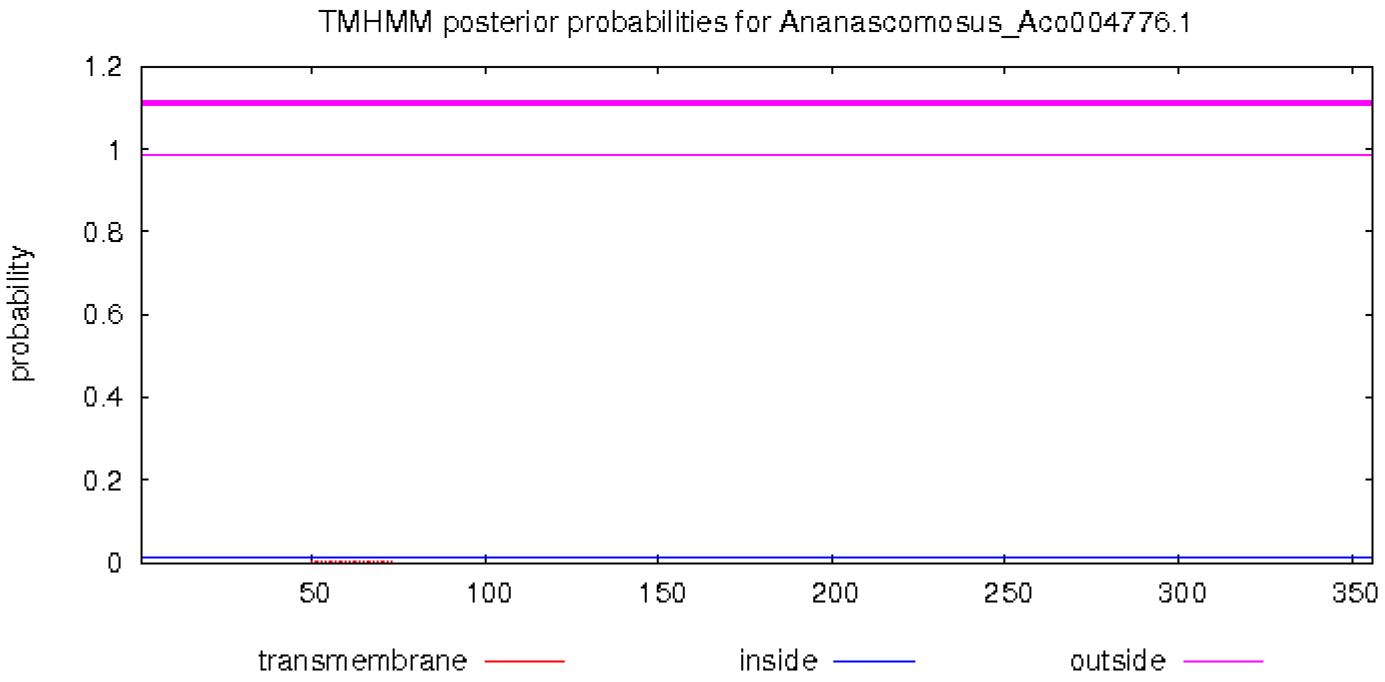

plot in postscript, script for making the plot in gnuplot, data for plot

```
# Ananascomosus_Aco005019.1 Length: 305
# Ananascomosus_Aco005019.1 Number of predicted TMHs:  0
# Ananascomosus_Aco005019.1 Exp number of AAs in TMHs: 0.00046
# Ananascomosus_Aco005019.1 Exp number, first 60 AAs:  0
# Ananascomosus_Aco005019.1 Total prob of N-in:        0.08346
Ananascomosus_Aco005019.1	TMHMM2.0	outside	     1   305
```

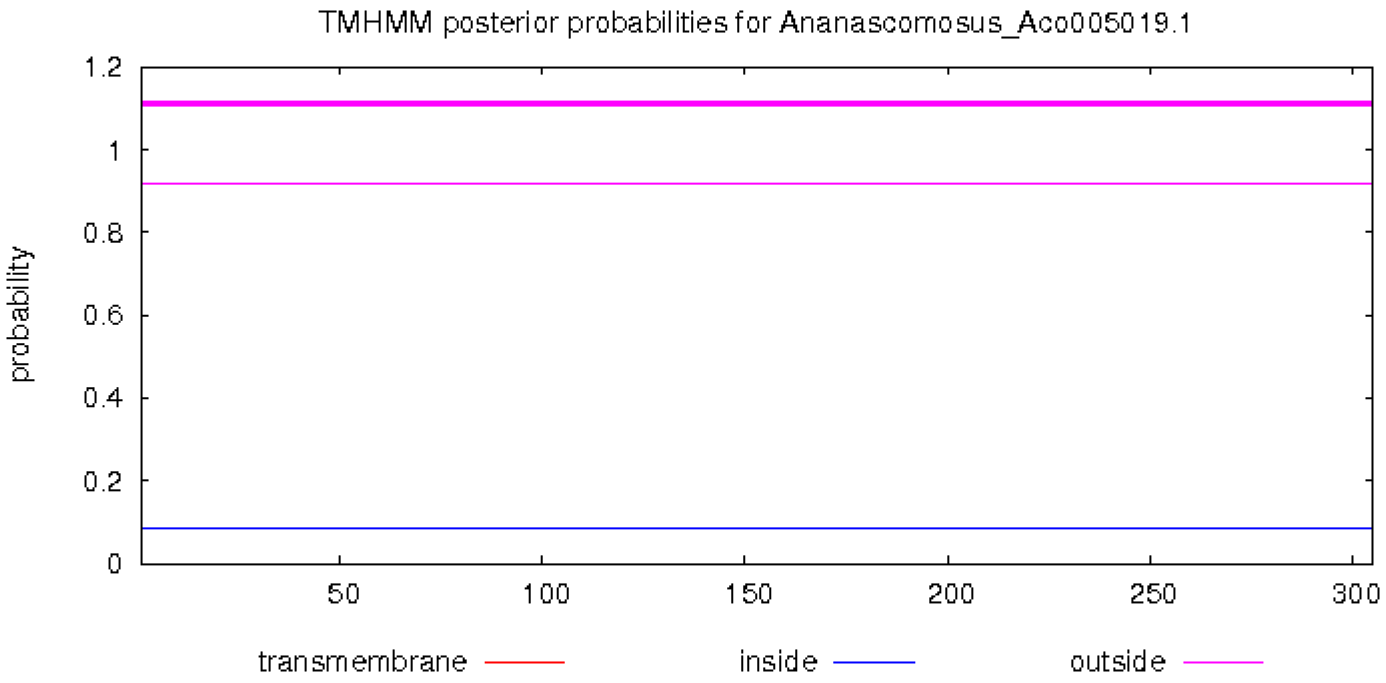

plot in postscript, script for making the plot in gnuplot, data for plot

```
# Ananascomosus_Aco005059.1 Length: 307
# Ananascomosus_Aco005059.1 Number of predicted TMHs:  0
# Ananascomosus_Aco005059.1 Exp number of AAs in TMHs: 0.02127
# Ananascomosus_Aco005059.1 Exp number, first 60 AAs:  0.02126
# Ananascomosus_Aco005059.1 Total prob of N-in:        0.01091
Ananascomosus_Aco005059.1       TMHMM2.0        outside      1   307
```

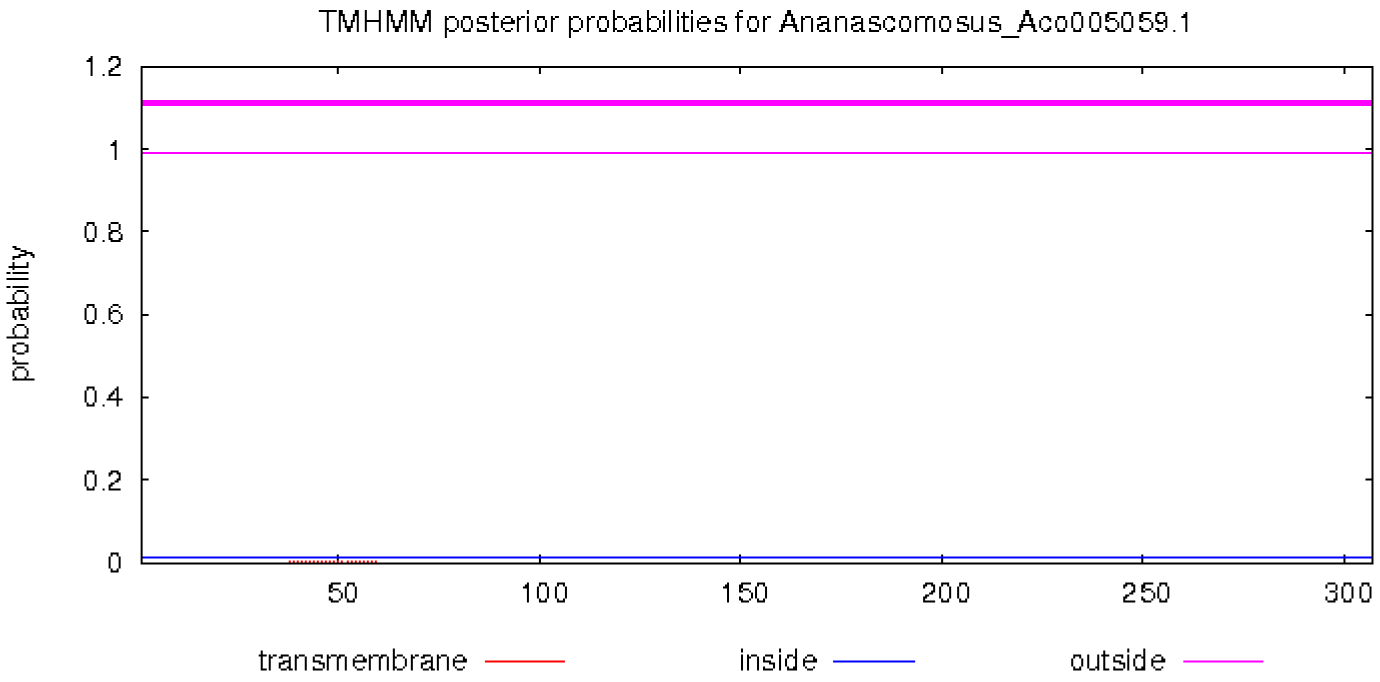

plot in postscript, script for making the plot in gnuplot, data for plot

---

```
# Ananascomosus_Aco006854.1 Length: 644
# Ananascomosus_Aco006854.1 Number of predicted TMHs:  1
# Ananascomosus_Aco006854.1 Exp number of AAs in TMHs: 22.50855
# Ananascomosus_Aco006854.1 Exp number, first 60 AAs:  0
# Ananascomosus_Aco006854.1 Total prob of N-in:        0.00038
Ananascomosus_Aco006854.1       TMHMM2.0        outside      1   612
Ananascomosus_Aco006854.1       TMHMM2.0        TMhelix    613   635
Ananascomosus_Aco006854.1       TMHMM2.0        inside     636   644
```

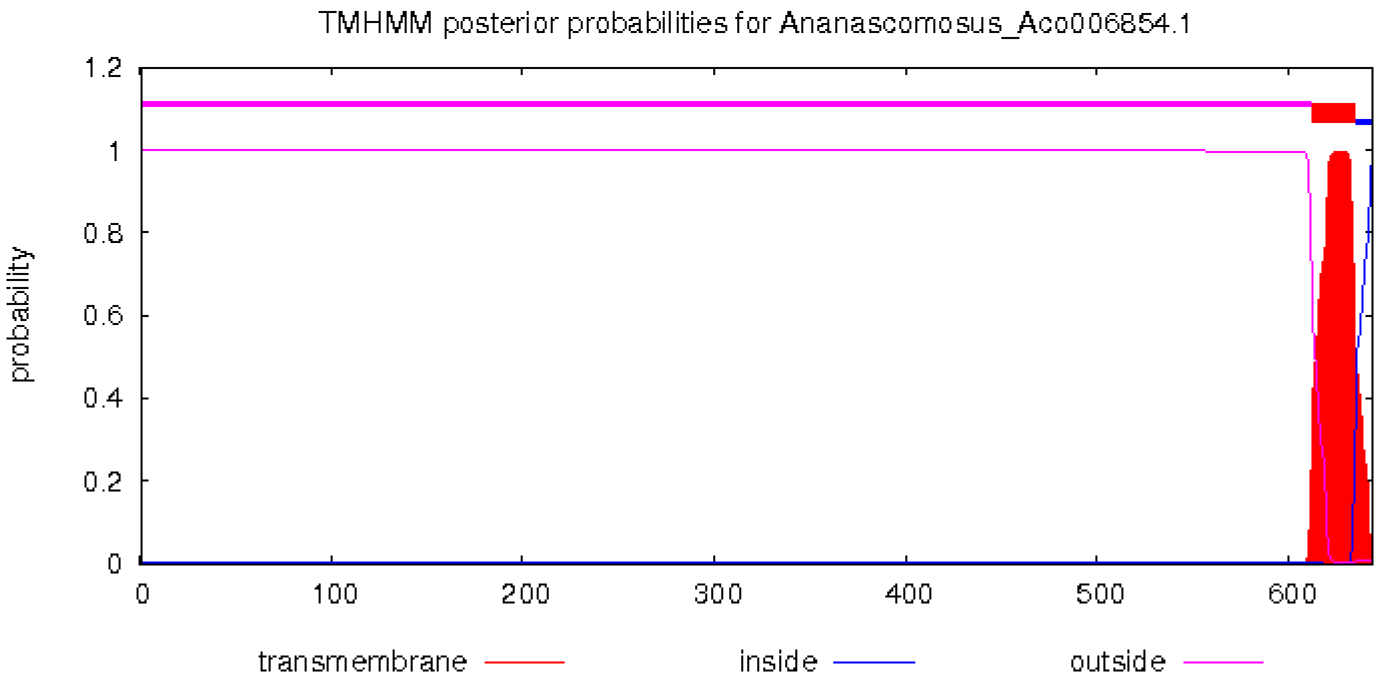

plot in postscript, script for making the plot in gnuplot, data for plot

```
# Ananascomosus_Aco006979.1 Length: 319
# Ananascomosus_Aco006979.1 Number of predicted TMHs:  0
# Ananascomosus_Aco006979.1 Exp number of AAs in TMHs: 0.00054
# Ananascomosus_Aco006979.1 Exp number, first 60 AAs:  0
# Ananascomosus_Aco006979.1 Total prob of N-in:        0.00925
Ananascomosus_Aco006979.1       TMHMM2.0        outside      1   319
```

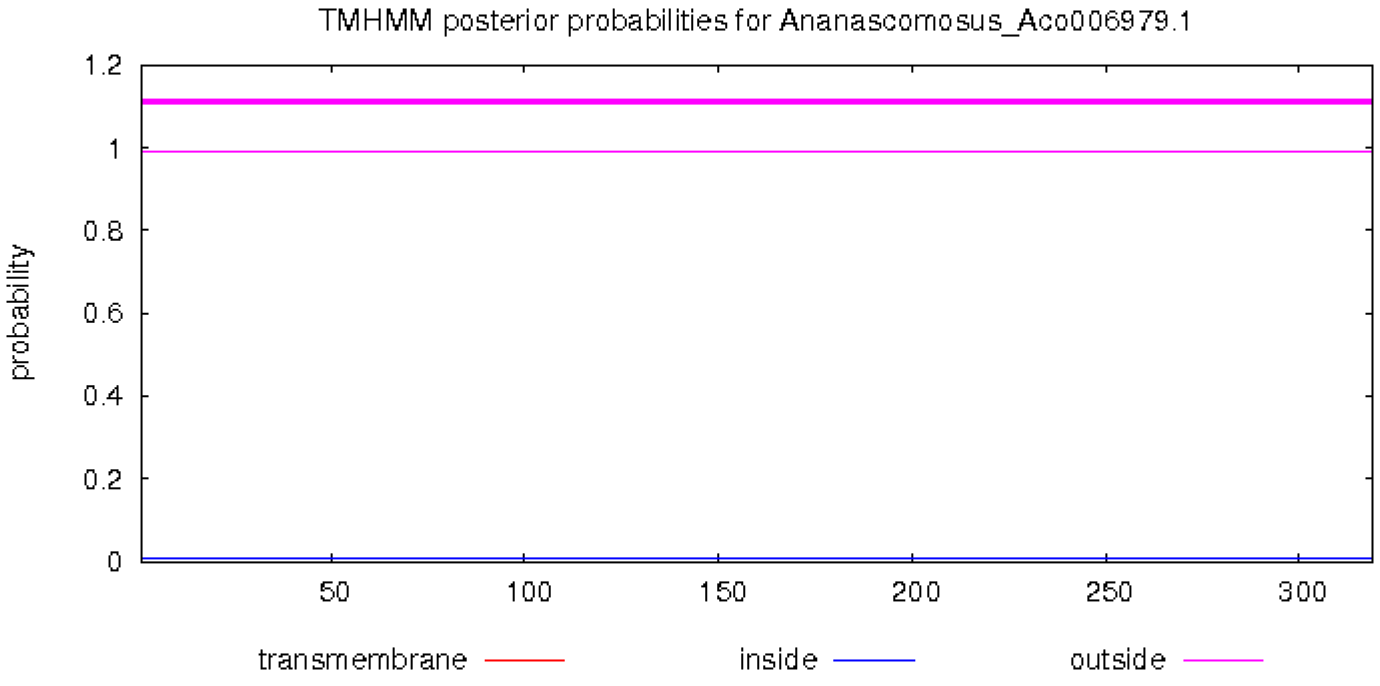

plot in postscript, script for making the plot in gnuplot, data for plot

```
# Ananascomosus_Aco007448.1 Length: 320
# Ananascomosus_Aco007448.1 Number of predicted TMHs:  0
# Ananascomosus_Aco007448.1 Exp number of AAs in TMHs: 0.0124
# Ananascomosus_Aco007448.1 Exp number, first 60 AAs:  0.01188
# Ananascomosus_Aco007448.1 Total prob of N-in:        0.02579
Ananascomosus_Aco007448.1       TMHMM2.0        outside      1   320
```

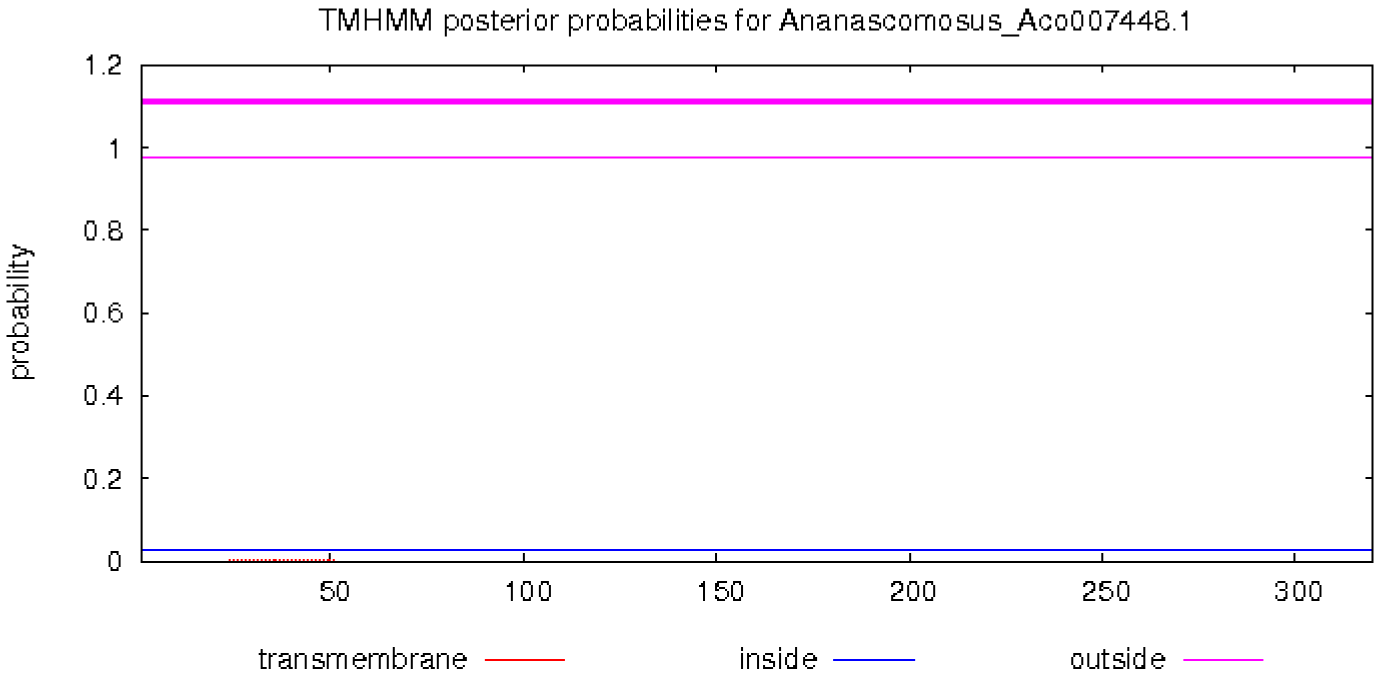

plot in postscript, script for making the plot in gnuplot, data for plot

```
# Ananascomosus_Aco007609.1 Length: 313
# Ananascomosus_Aco007609.1 Number of predicted TMHs:  0
# Ananascomosus_Aco007609.1 Exp number of AAs in TMHs: 0
# Ananascomosus_Aco007609.1 Exp number, first 60 AAs:  0
# Ananascomosus_Aco007609.1 Total prob of N-in:        0.00709
Ananascomosus_Aco007609.1	TMHMM2.0	outside	     1   313
```

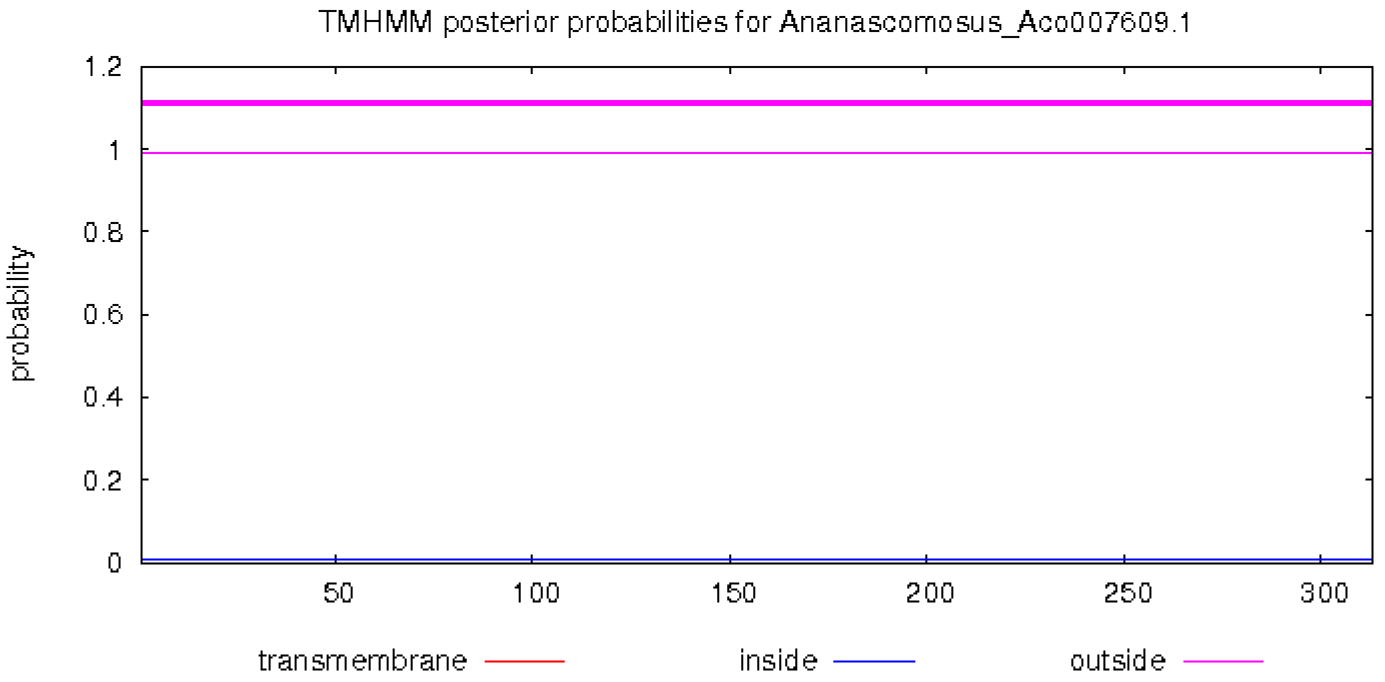

plot in postscript, script for making the plot in gnuplot, data for plot

---

```
# Ananascomosus_Aco007742.1 Length: 285
# Ananascomosus_Aco007742.1 Number of predicted TMHs:  0
# Ananascomosus_Aco007742.1 Exp number of AAs in TMHs: 0.00271
# Ananascomosus_Aco007742.1 Exp number, first 60 AAs:  0
# Ananascomosus_Aco007742.1 Total prob of N-in:        0.01591
Ananascomosus_Aco007742.1	TMHMM2.0	outside	     1   285
```

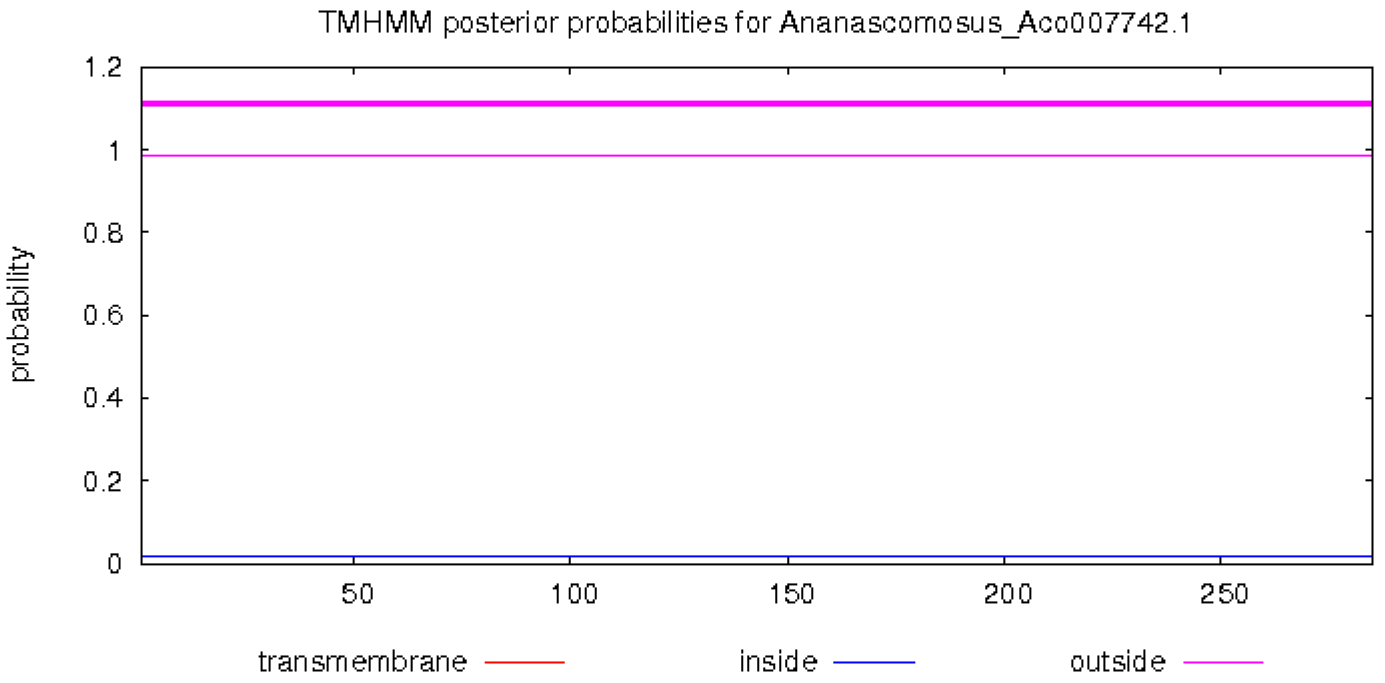

plot in postscript, script for making the plot in gnuplot, data for plot

---

```
# Ananascomosus_Aco007925.1 Length: 201
# Ananascomosus_Aco007925.1 Number of predicted TMHs:  0
# Ananascomosus_Aco007925.1 Exp number of AAs in TMHs: 0.00025
# Ananascomosus_Aco007925.1 Exp number, first 60 AAs:  8e-05
# Ananascomosus_Aco007925.1 Total prob of N-in:        0.02657
Ananascomosus_Aco007925.1       TMHMM2.0        outside      1   201
```

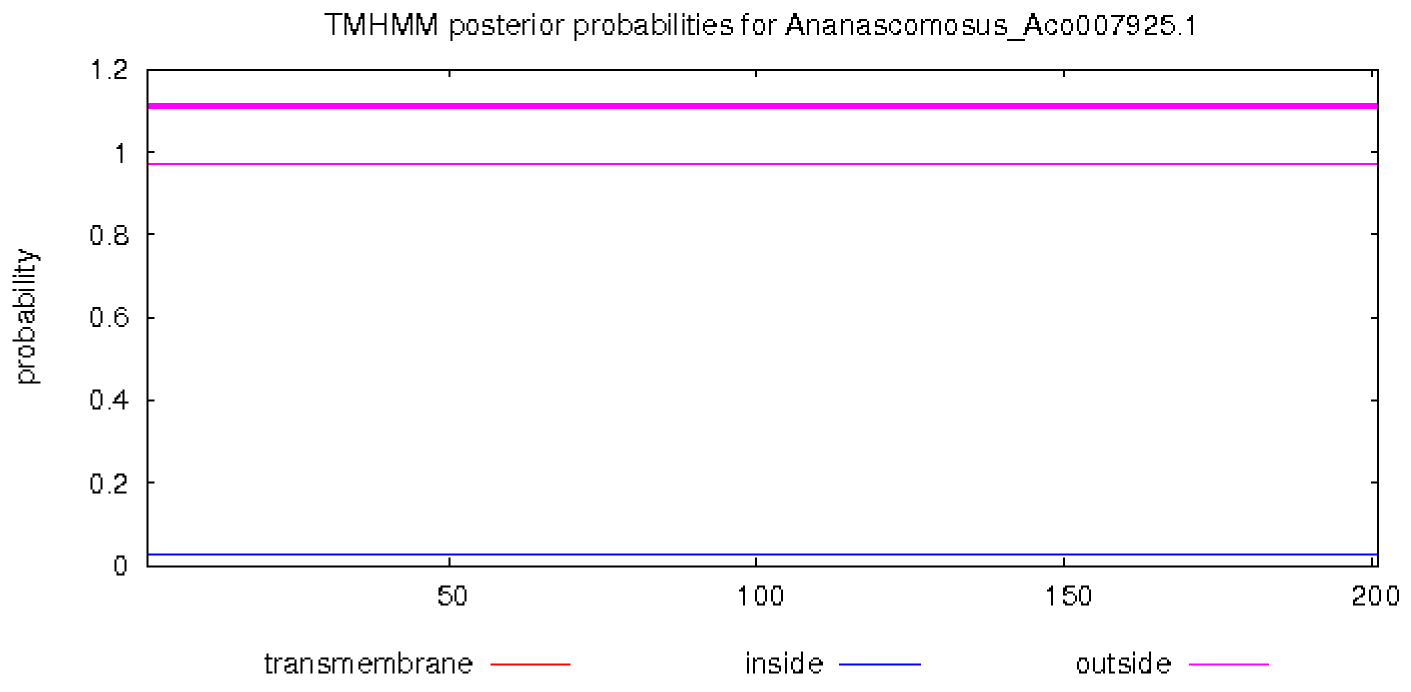

plot in postscript, script for making the plot in gnuplot, data for plot

---

```
# Ananascomosus_Aco008000.1 Length: 336
# Ananascomosus_Aco008000.1 Number of predicted TMHs:  0
# Ananascomosus_Aco008000.1 Exp number of AAs in TMHs: 0.00292
# Ananascomosus_Aco008000.1 Exp number, first 60 AAs:  0.00018
# Ananascomosus_Aco008000.1 Total prob of N-in:        0.01204
Ananascomosus_Aco008000.1       TMHMM2.0        outside      1   336
```

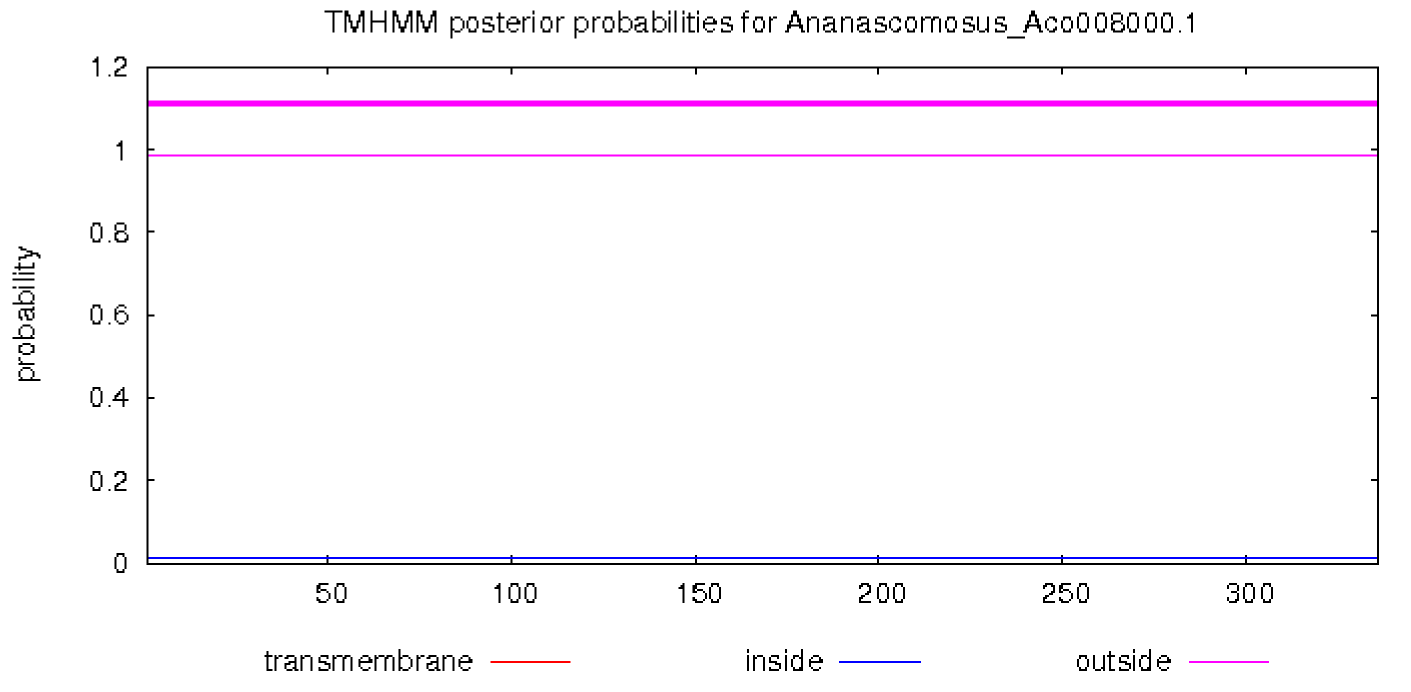

plot in postscript, script for making the plot in gnuplot, data for plot

---

```
# Ananascomosus_Aco008654.1 Length: 320
# Ananascomosus_Aco008654.1 Number of predicted TMHs:  0
# Ananascomosus_Aco008654.1 Exp number of AAs in TMHs: 0.00149
# Ananascomosus_Aco008654.1 Exp number, first 60 AAs:  0
# Ananascomosus_Aco008654.1 Total prob of N-in:        0.06342
Ananascomosus_Aco008654.1       TMHMM2.0        outside      1   320
```

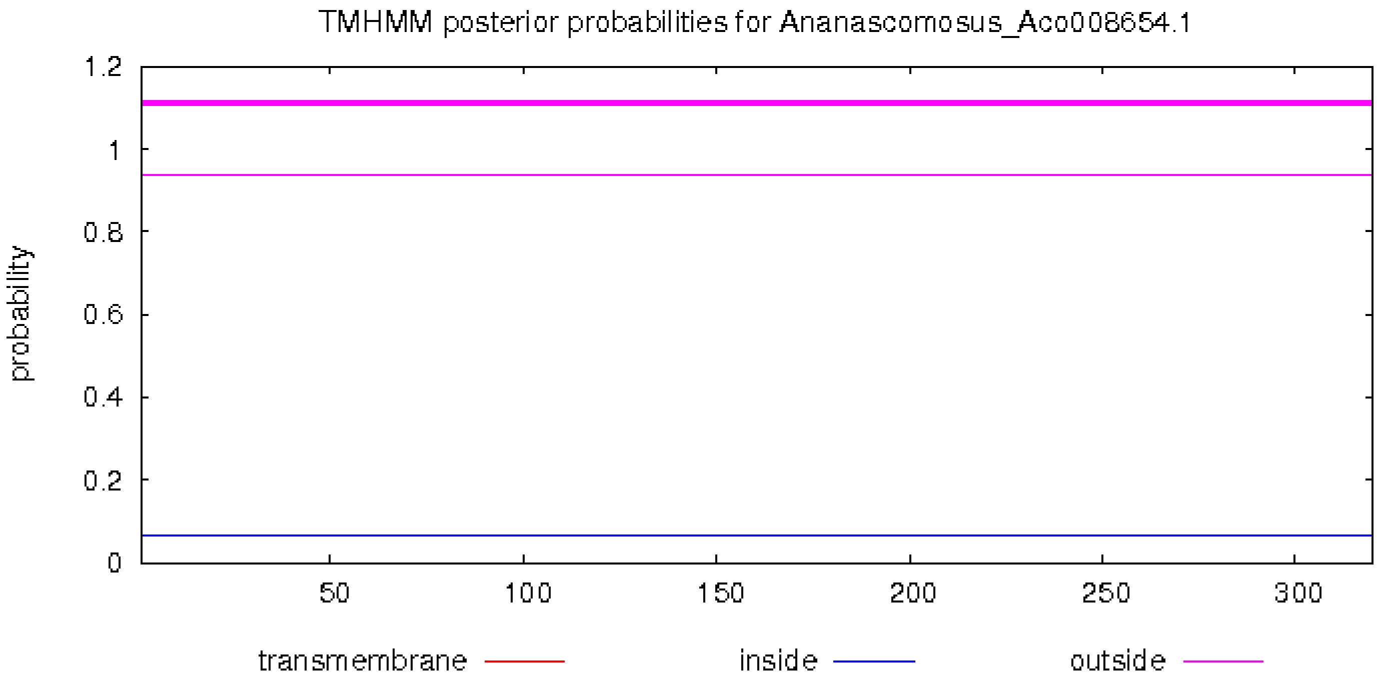

plot in postscript, script for making the plot in gnuplot, data for plot

---

```
# Ananascomosus_Aco009152.1 Length: 284
# Ananascomosus_Aco009152.1 Number of predicted TMHs:  0
# Ananascomosus_Aco009152.1 Exp number of AAs in TMHs: 0.00268
# Ananascomosus_Aco009152.1 Exp number, first 60 AAs:  0.00268
# Ananascomosus_Aco009152.1 Total prob of N-in:        0.01762
Ananascomosus_Aco009152.1       TMHMM2.0        outside      1   284
```

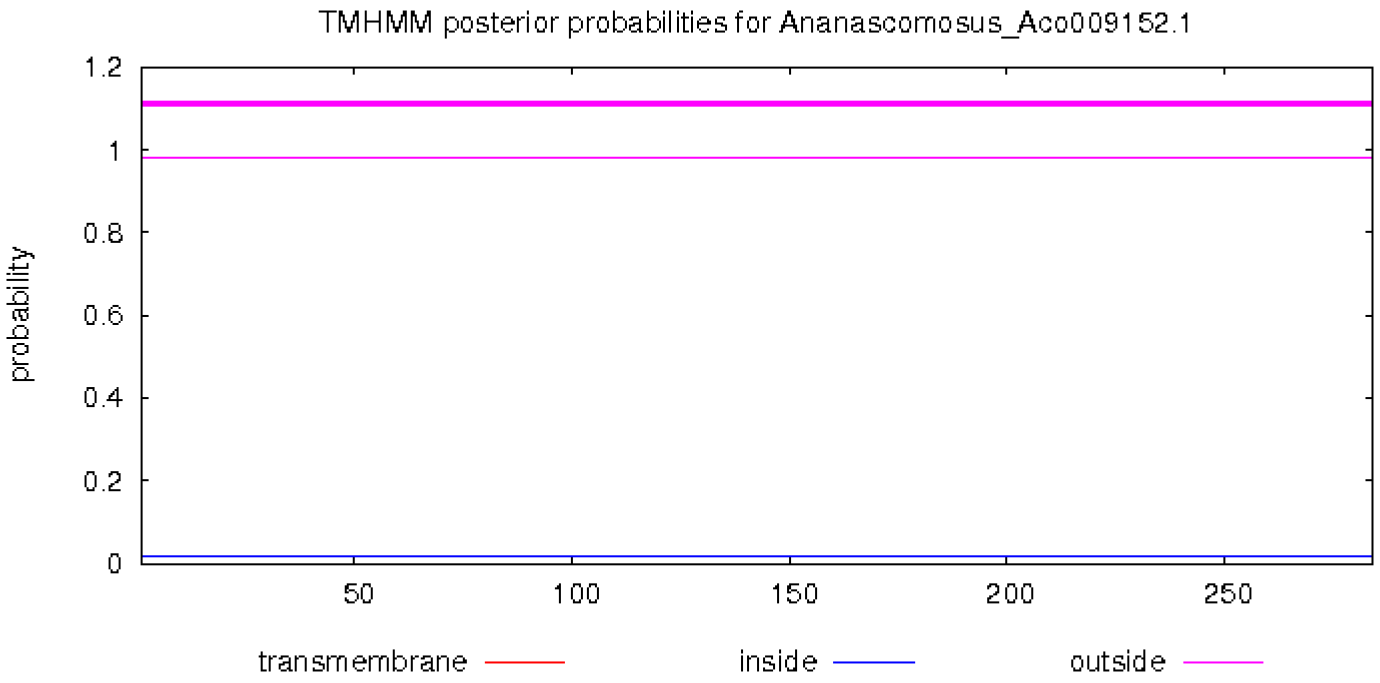

plot in postscript, script for making the plot in gnuplot, data for plot

---

```
# Ananascomosus_Aco010231.1 Length: 673
# Ananascomosus_Aco010231.1 Number of predicted TMHs:  1
# Ananascomosus_Aco010231.1 Exp number of AAs in TMHs: 22.40938
# Ananascomosus_Aco010231.1 Exp number, first 60 AAs:  0.00013
# Ananascomosus_Aco010231.1 Total prob of N-in:        0.00370
Ananascomosus_Aco010231.1	TMHMM2.0	outside	     1   636
Ananascomosus_Aco010231.1	TMHMM2.0	TMhelix	   637   659
Ananascomosus_Aco010231.1	TMHMM2.0	inside	   660   673
```

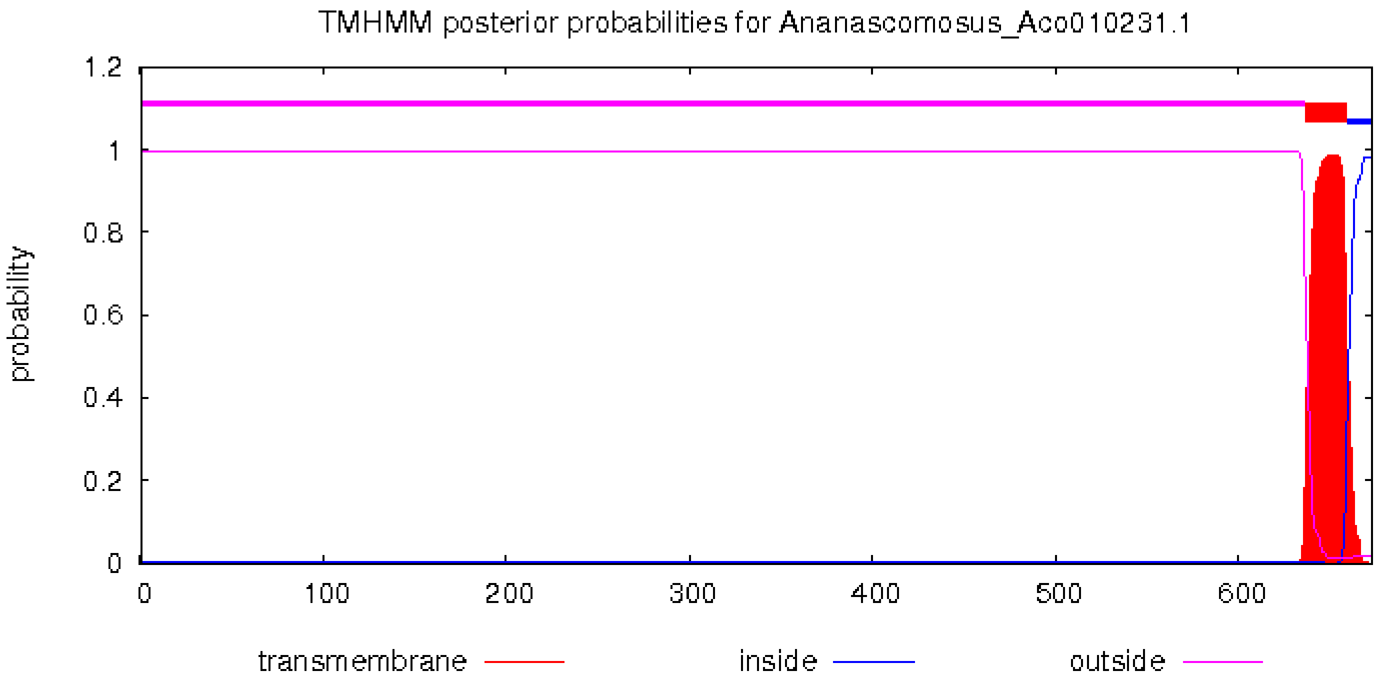

plot in postscript, script for making the plot in gnuplot, data for plot

---

```
# Ananascomosus_Aco010558.1 Length: 715
# Ananascomosus_Aco010558.1 Number of predicted TMHs:  1
# Ananascomosus_Aco010558.1 Exp number of AAs in TMHs: 22.38854
# Ananascomosus_Aco010558.1 Exp number, first 60 AAs:  0
# Ananascomosus_Aco010558.1 Total prob of N-in:        0.00011
Ananascomosus_Aco010558.1	TMHMM2.0	outside	     1   687
Ananascomosus_Aco010558.1	TMHMM2.0	TMhelix	   688   710
Ananascomosus_Aco010558.1	TMHMM2.0	inside	   711   715
```

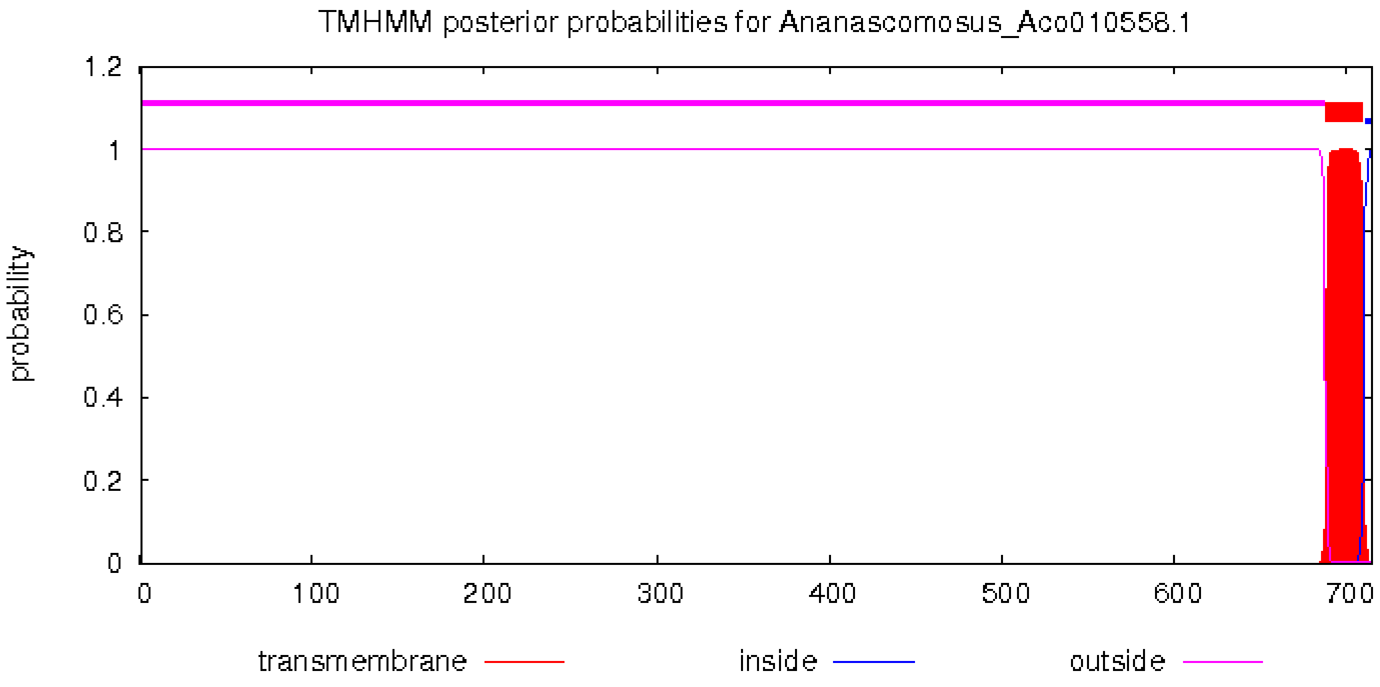

plot in postscript, script for making the plot in gnuplot, data for plot

---

```
# Ananascomosus_Aco011352.1 Length: 367
# Ananascomosus_Aco011352.1 Number of predicted TMHs:  0
# Ananascomosus_Aco011352.1 Exp number of AAs in TMHs: 0.00316
# Ananascomosus_Aco011352.1 Exp number, first 60 AAs:  0.0004
# Ananascomosus_Aco011352.1 Total prob of N-in:        0.02173
Ananascomosus_Aco011352.1	TMHMM2.0	outside	     1   367
```

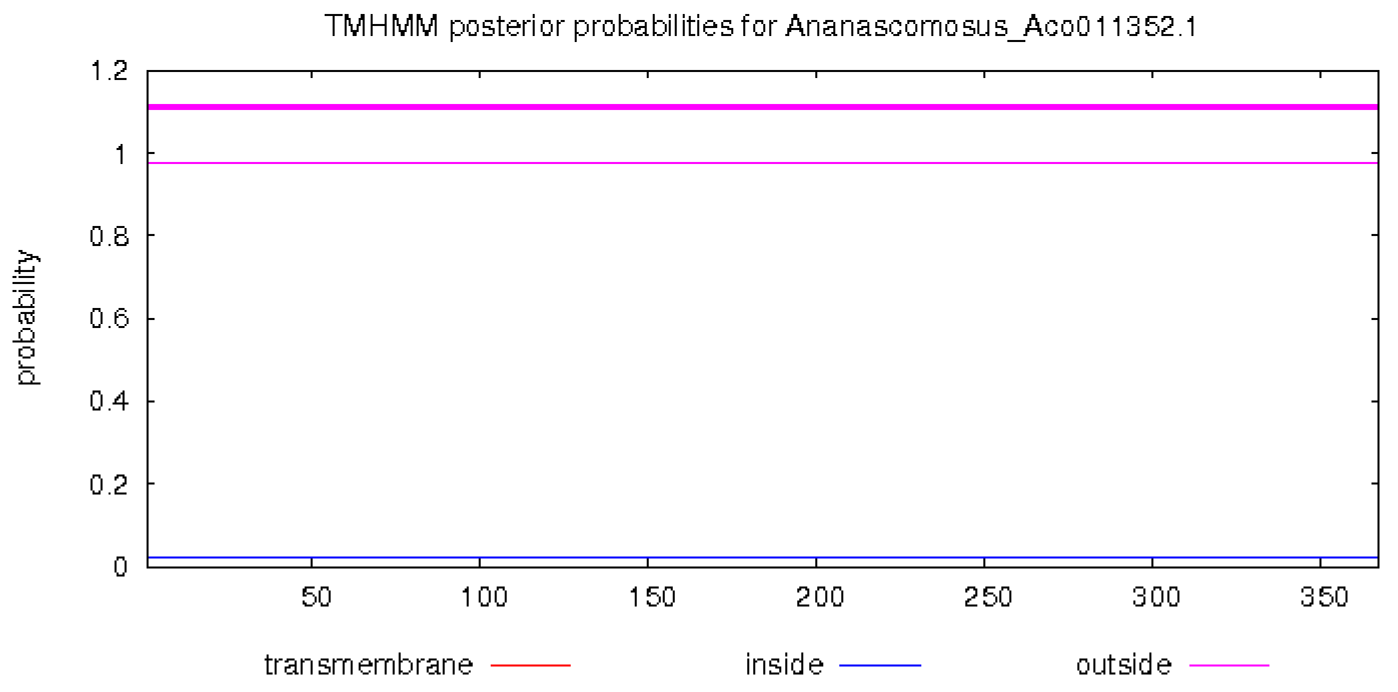

plot in postscript, script for making the plot in gnuplot, data for plot

---

```
# Ananascomosus_Aco011825.1 Length: 288
# Ananascomosus_Aco011825.1 Number of predicted TMHs:  0
# Ananascomosus_Aco011825.1 Exp number of AAs in TMHs: 0.00015
# Ananascomosus_Aco011825.1 Exp number, first 60 AAs:  0.00015
# Ananascomosus_Aco011825.1 Total prob of N-in:        0.03080
Ananascomosus_Aco011825.1	TMHMM2.0	outside	     1   288
```

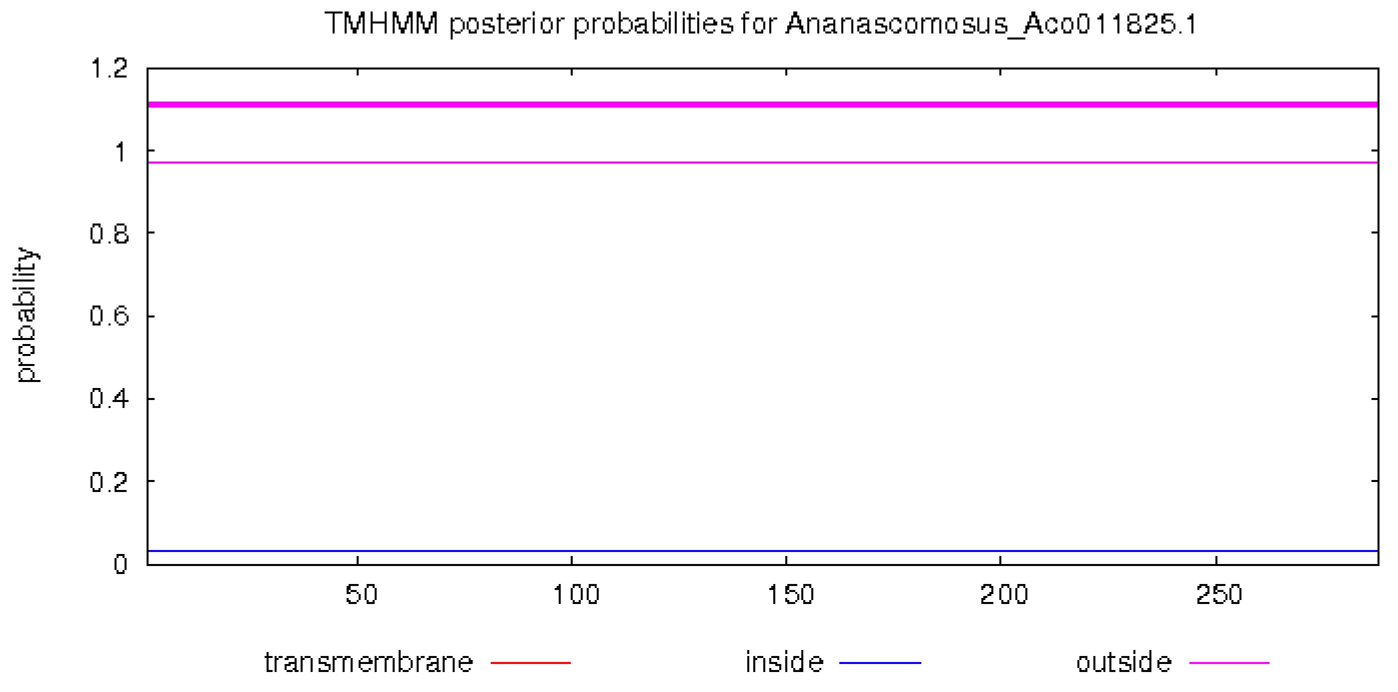

plot in postscript, script for making the plot in gnuplot, data for plot

```
# Ananascomosus_Aco011880.1 Length: 270
# Ananascomosus_Aco011880.1 Number of predicted TMHs:  0
# Ananascomosus_Aco011880.1 Exp number of AAs in TMHs: 0.0005
# Ananascomosus_Aco011880.1 Exp number, first 60 AAs:  0
# Ananascomosus_Aco011880.1 Total prob of N-in:        0.03127
Ananascomosus_Aco011880.1	TMHMM2.0	outside	     1   270
```

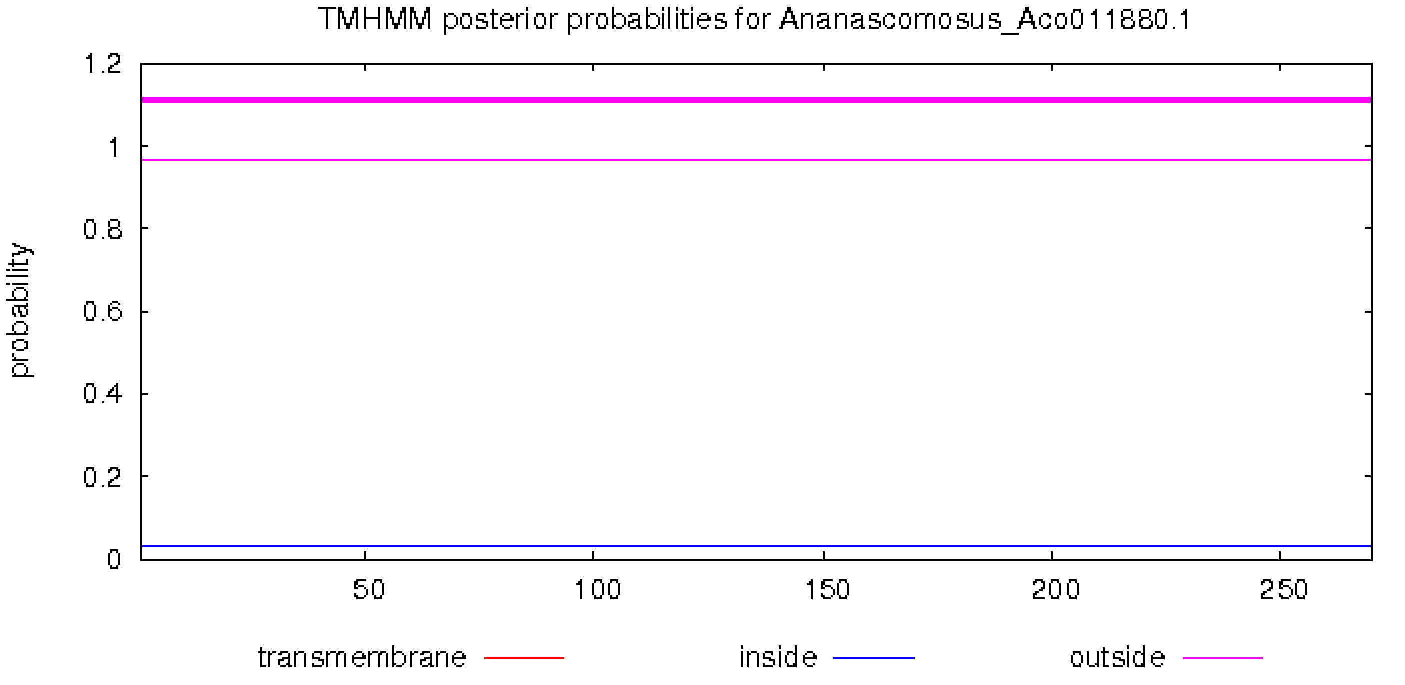

plot in postscript, script for making the plot in gnuplot, data for plot

```
# Ananascomosus_Aco011895.1 Length: 700
# Ananascomosus_Aco011895.1 Number of predicted TMHs:  0
# Ananascomosus_Aco011895.1 Exp number of AAs in TMHs: 2.10938
# Ananascomosus_Aco011895.1 Exp number, first 60 AAs:  0
# Ananascomosus_Aco011895.1 Total prob of N-in:        0.00004
Ananascomosus_Aco011895.1	TMHMM2.0	outside	     1   700
```

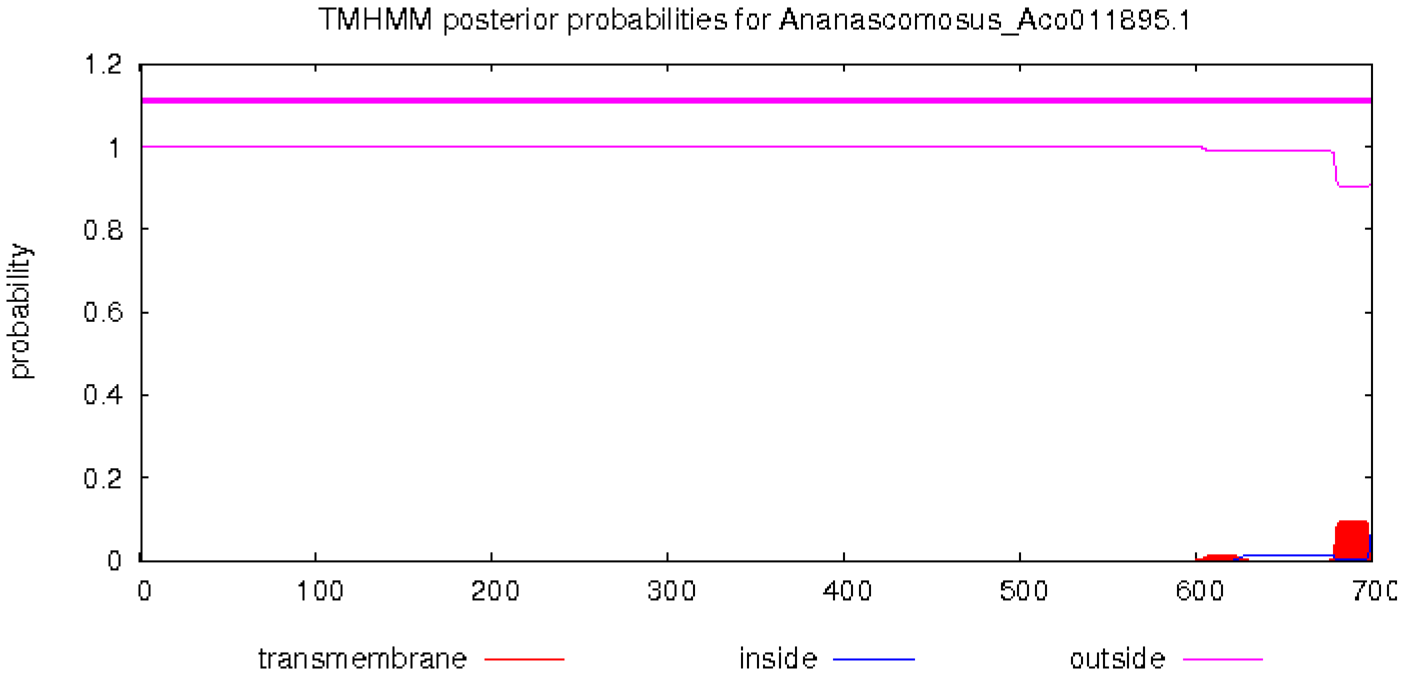

plot in postscript, script for making the plot in gnuplot, data for plot

```
# Ananascomosus_Aco012211.1 Length: 336
# Ananascomosus_Aco012211.1 Number of predicted TMHs:  0
# Ananascomosus_Aco012211.1 Exp number of AAs in TMHs: 0.00881
# Ananascomosus_Aco012211.1 Exp number, first 60 AAs:  0
# Ananascomosus_Aco012211.1 Total prob of N-in:        0.00126
Ananascomosus_Aco012211.1       TMHMM2.0        outside      1   336
```

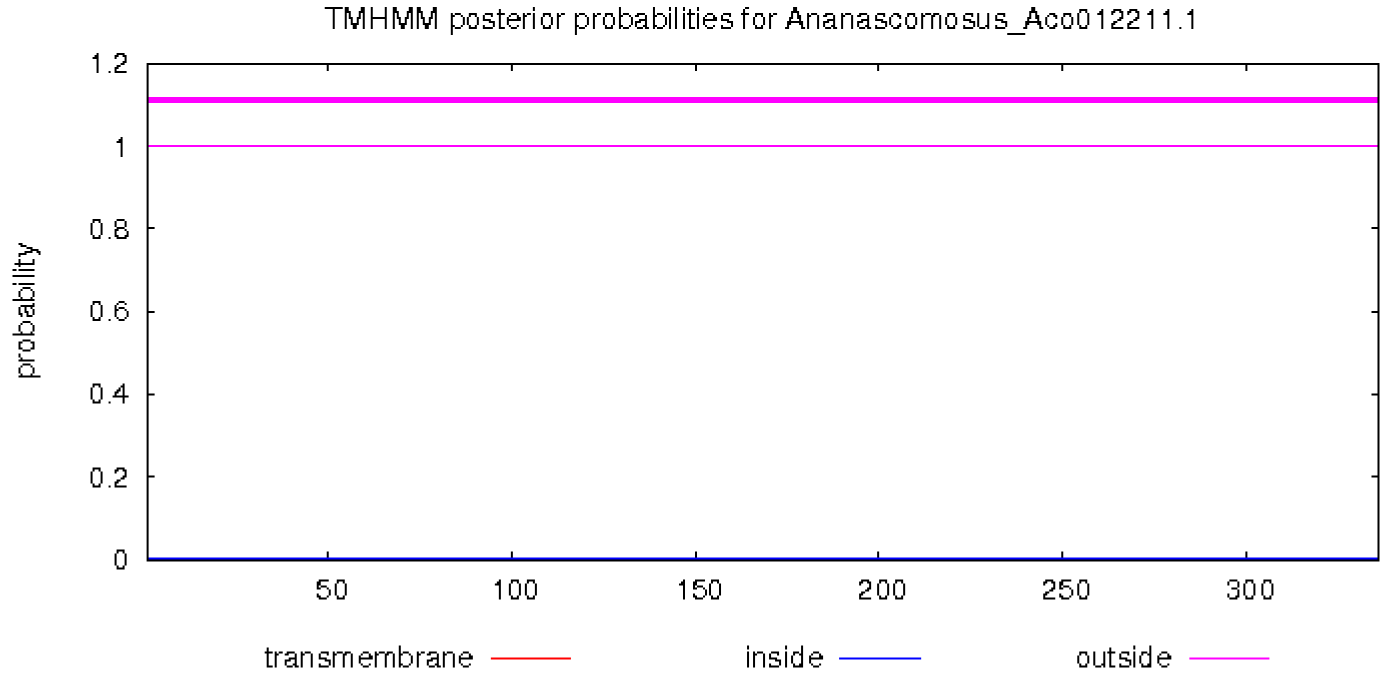

plot in postscript, script for making the plot in gnuplot, data for plot

```
# Ananascomosus_Aco012598.1 Length: 291
# Ananascomosus_Aco012598.1 Number of predicted TMHs:  0
# Ananascomosus_Aco012598.1 Exp number of AAs in TMHs: 0.00074
# Ananascomosus_Aco012598.1 Exp number, first 60 AAs:  0
# Ananascomosus_Aco012598.1 Total prob of N-in:        0.09498
Ananascomosus_Aco012598.1       TMHMM2.0        outside      1   291
```

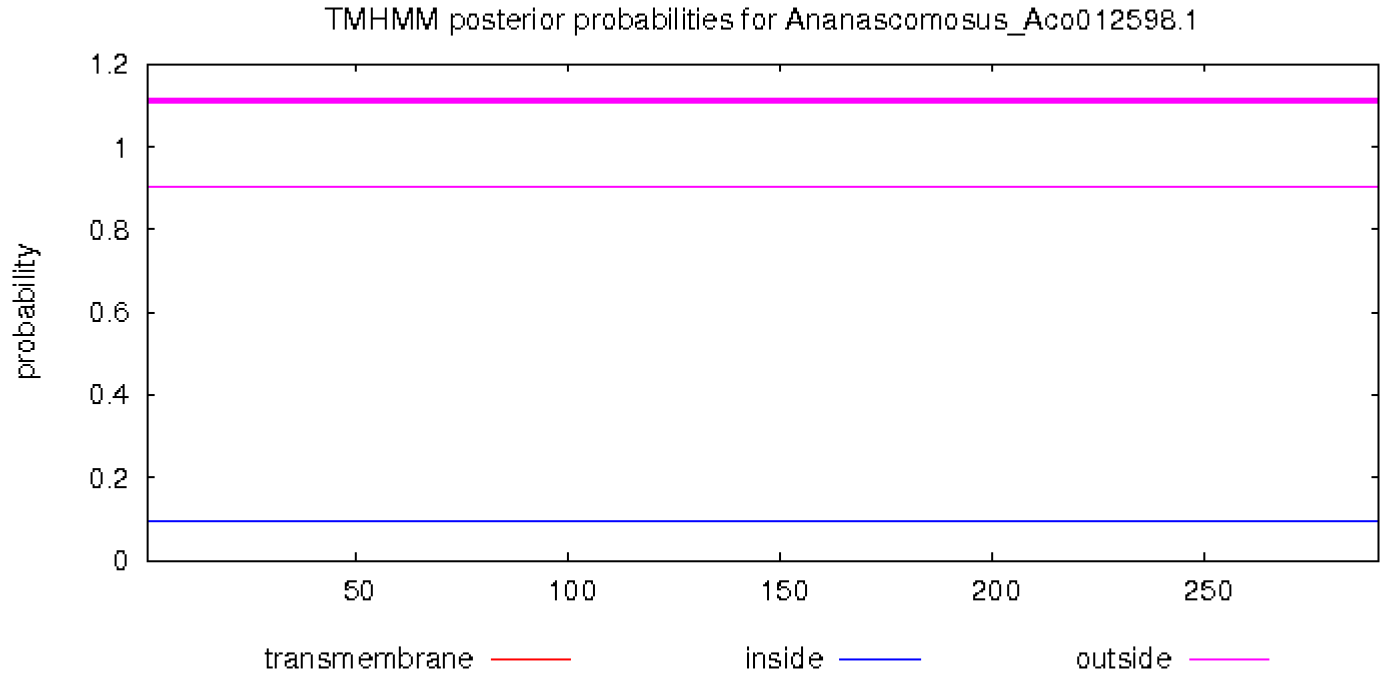

plot in postscript, script for making the plot in gnuplot, data for plot

---

```
# Ananascomosus_Aco012599.1 Length: 284
# Ananascomosus_Aco012599.1 Number of predicted TMHs:  0
# Ananascomosus_Aco012599.1 Exp number of AAs in TMHs: 0
# Ananascomosus_Aco012599.1 Exp number, first 60 AAs:  0
# Ananascomosus_Aco012599.1 Total prob of N-in:        0.01648
Ananascomosus_Aco012599.1       TMHMM2.0        outside      1   284
```

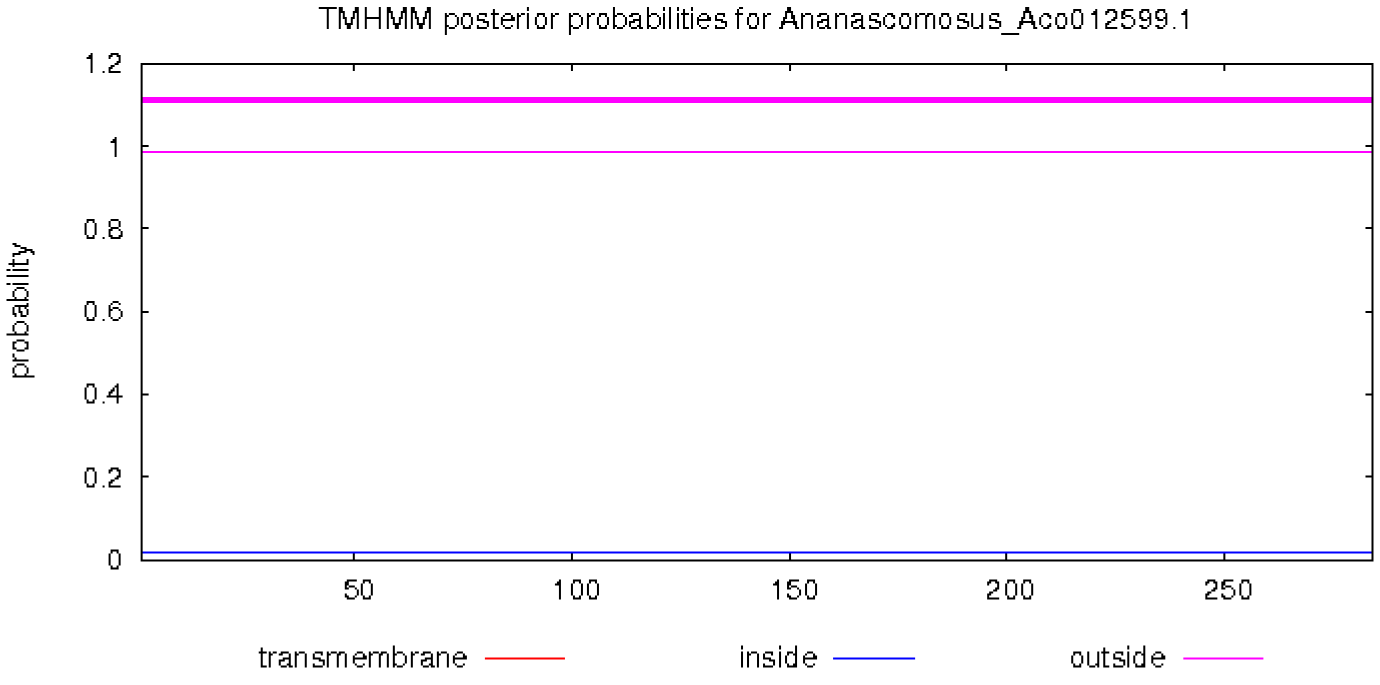

plot in postscript, script for making the plot in gnuplot, data for plot

---

```
# Ananascomosus_Aco012600.1 Length: 387
# Ananascomosus_Aco012600.1 Number of predicted TMHs:  0
# Ananascomosus_Aco012600.1 Exp number of AAs in TMHs: 0.00018
# Ananascomosus_Aco012600.1 Exp number, first 60 AAs:  0
# Ananascomosus_Aco012600.1 Total prob of N-in:        0.00281
Ananascomosus_Aco012600.1       TMHMM2.0        outside      1   387
```

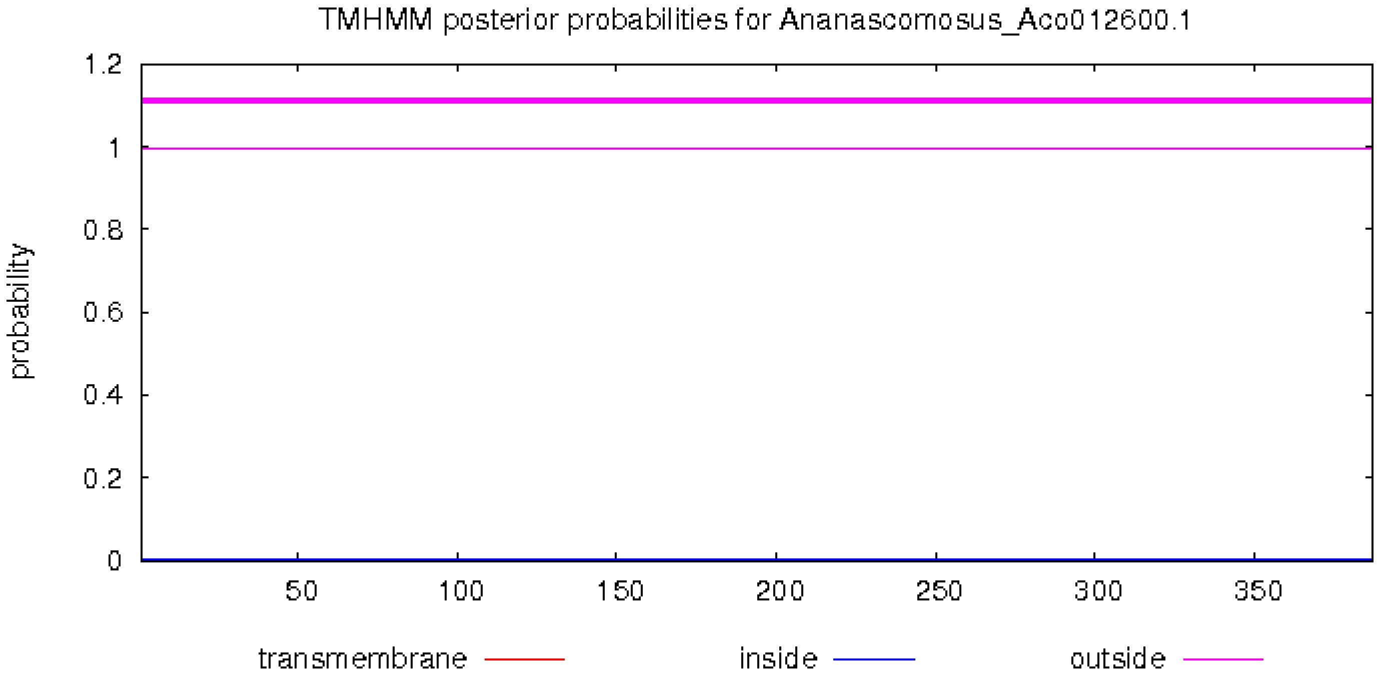

plot in postscript, script for making the plot in gnuplot, data for plot

```
# Ananascomosus_Aco012922.1 Length: 285
# Ananascomosus_Aco012922.1 Number of predicted TMHs:  0
# Ananascomosus_Aco012922.1 Exp number of AAs in TMHs: 0.00126
# Ananascomosus_Aco012922.1 Exp number, first 60 AAs:  0
# Ananascomosus_Aco012922.1 Total prob of N-in:        0.00447
Ananascomosus_Aco012922.1       TMHMM2.0        outside      1   285
```

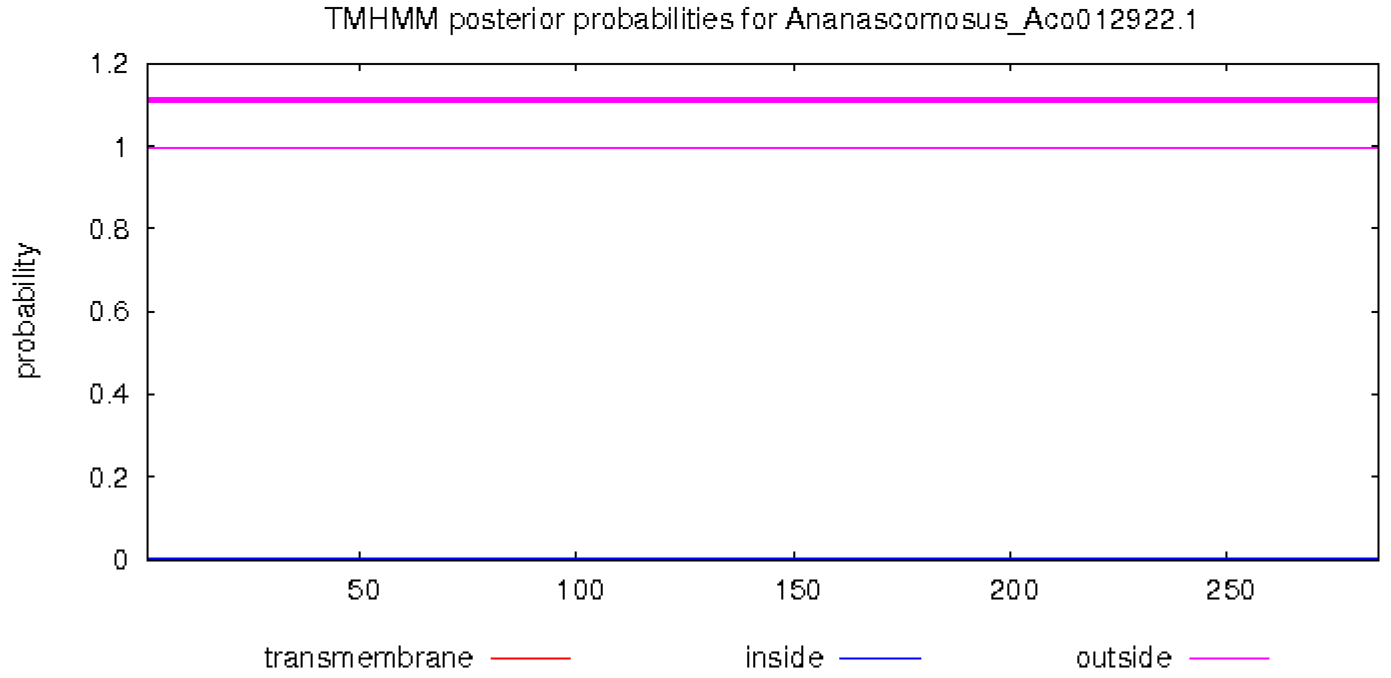

plot in postscript, script for making the plot in gnuplot, data for plot

```
# Ananascomosus_Aco013021.1 Length: 245
# Ananascomosus_Aco013021.1 Number of predicted TMHs:  0
# Ananascomosus_Aco013021.1 Exp number of AAs in TMHs: 0
# Ananascomosus_Aco013021.1 Exp number, first 60 AAs:  0
# Ananascomosus_Aco013021.1 Total prob of N-in:        0.00225
Ananascomosus_Aco013021.1       TMHMM2.0        outside      1   245
```

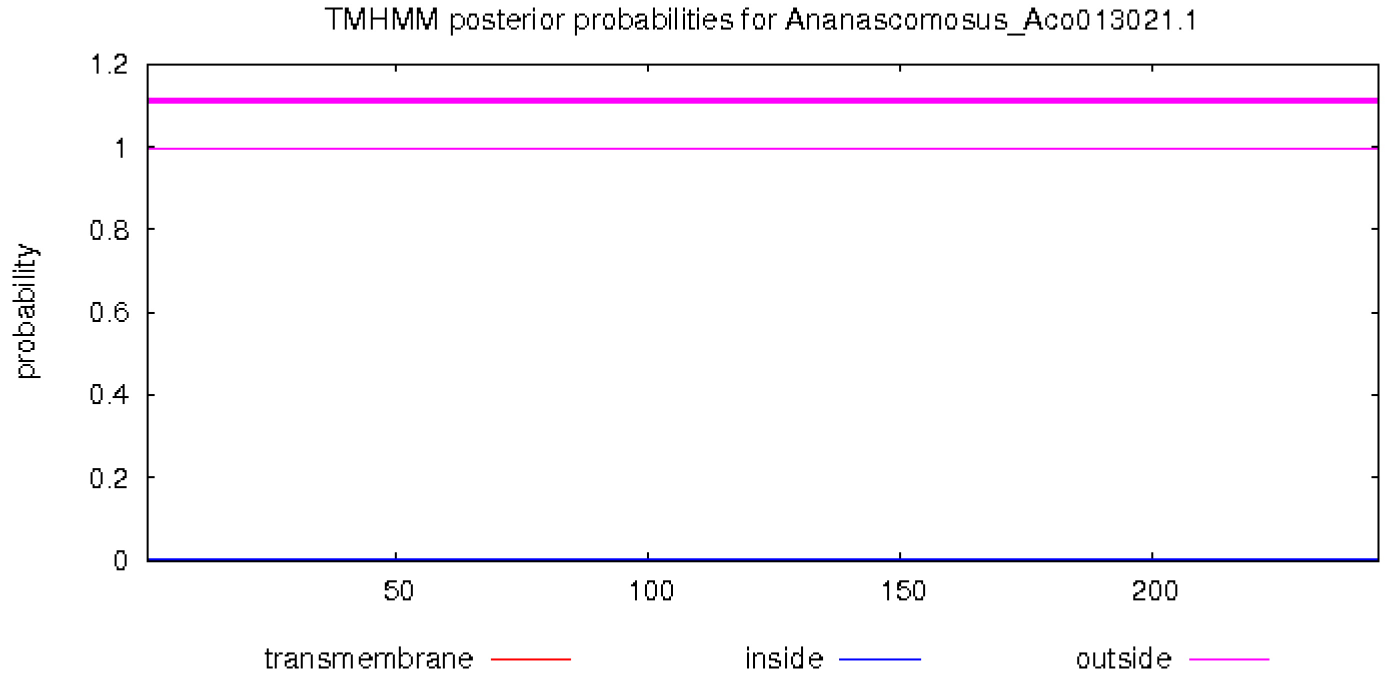

plot in postscript, script for making the plot in gnuplot, data for plot

```
# Ananascomosus_Aco013059.1 Length: 457
# Ananascomosus_Aco013059.1 Number of predicted TMHs:  0
# Ananascomosus_Aco013059.1 Exp number of AAs in TMHs: 0.18082
# Ananascomosus_Aco013059.1 Exp number, first 60 AAs:  0.00066
# Ananascomosus_Aco013059.1 Total prob of N-in:        0.00719
Ananascomosus_Aco013059.1       TMHMM2.0        outside      1   457
```

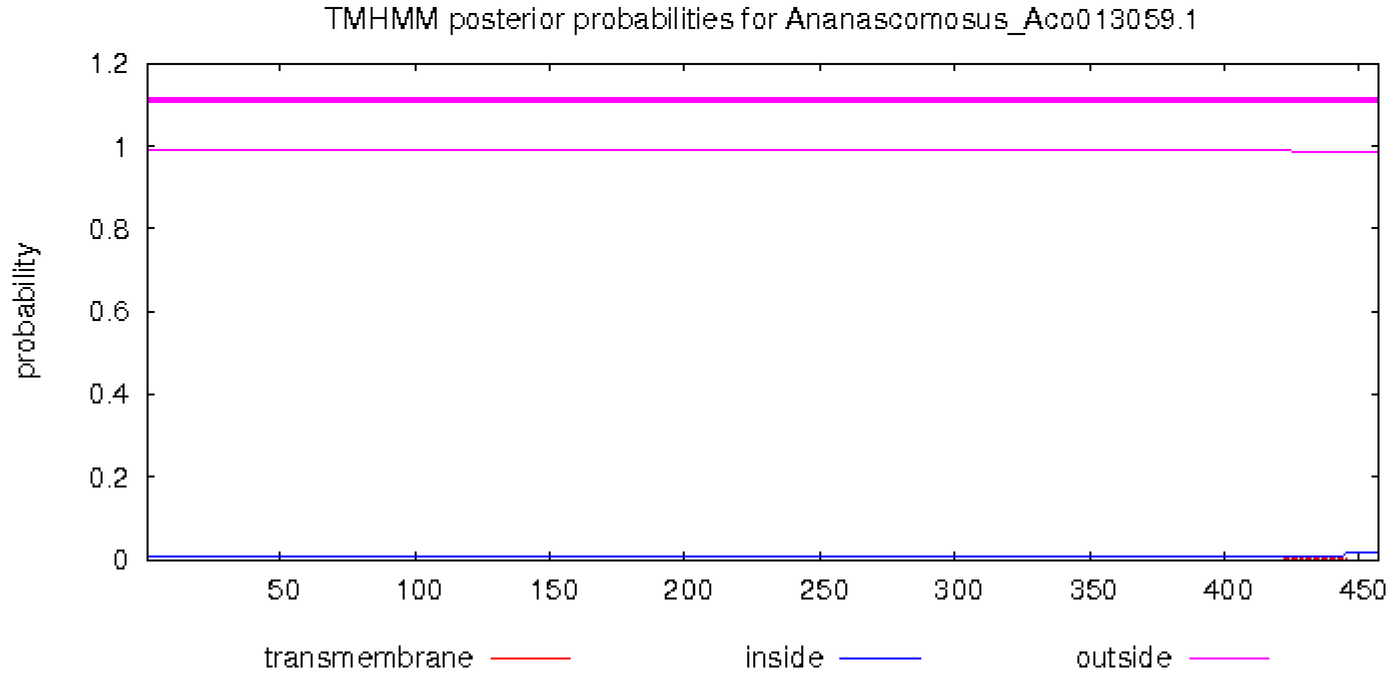

plot in postscript, script for making the plot in gnuplot, data for plot

```
# Ananascomosus_Aco013223.1 Length: 614
# Ananascomosus_Aco013223.1 Number of predicted TMHs:  0
# Ananascomosus_Aco013223.1 Exp number of AAs in TMHs: 0
# Ananascomosus_Aco013223.1 Exp number, first 60 AAs:  0
# Ananascomosus_Aco013223.1 Total prob of N-in:        0.00009
Ananascomosus_Aco013223.1       TMHMM2.0        outside      1   614
```

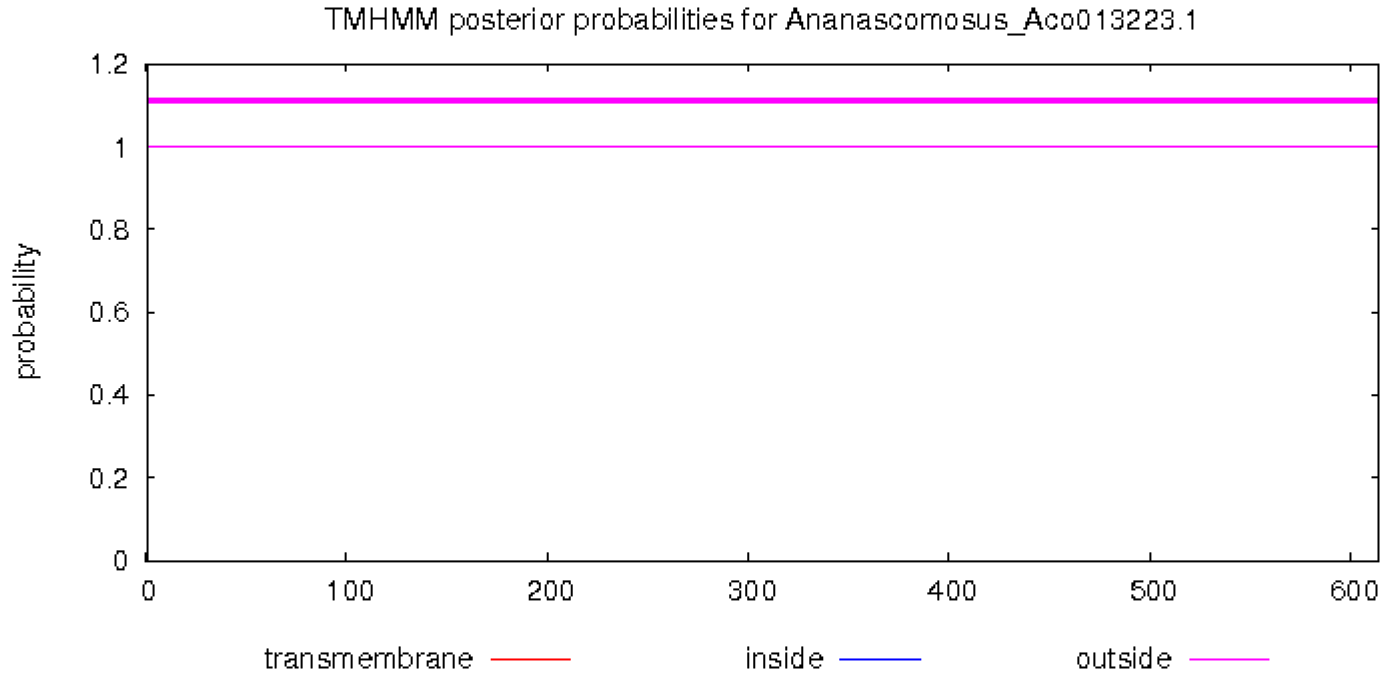

plot in postscript, script for making the plot in gnuplot, data for plot

```
# Ananascomosus_Aco013436.1 Length: 1045
# Ananascomosus_Aco013436.1 Number of predicted TMHs:  0
# Ananascomosus_Aco013436.1 Exp number of AAs in TMHs: 0.00389
# Ananascomosus_Aco013436.1 Exp number, first 60 AAs:  0
# Ananascomosus_Aco013436.1 Total prob of N-in:        0.00001
Ananascomosus_Aco013436.1	TMHMM2.0	outside	     1  1045
```

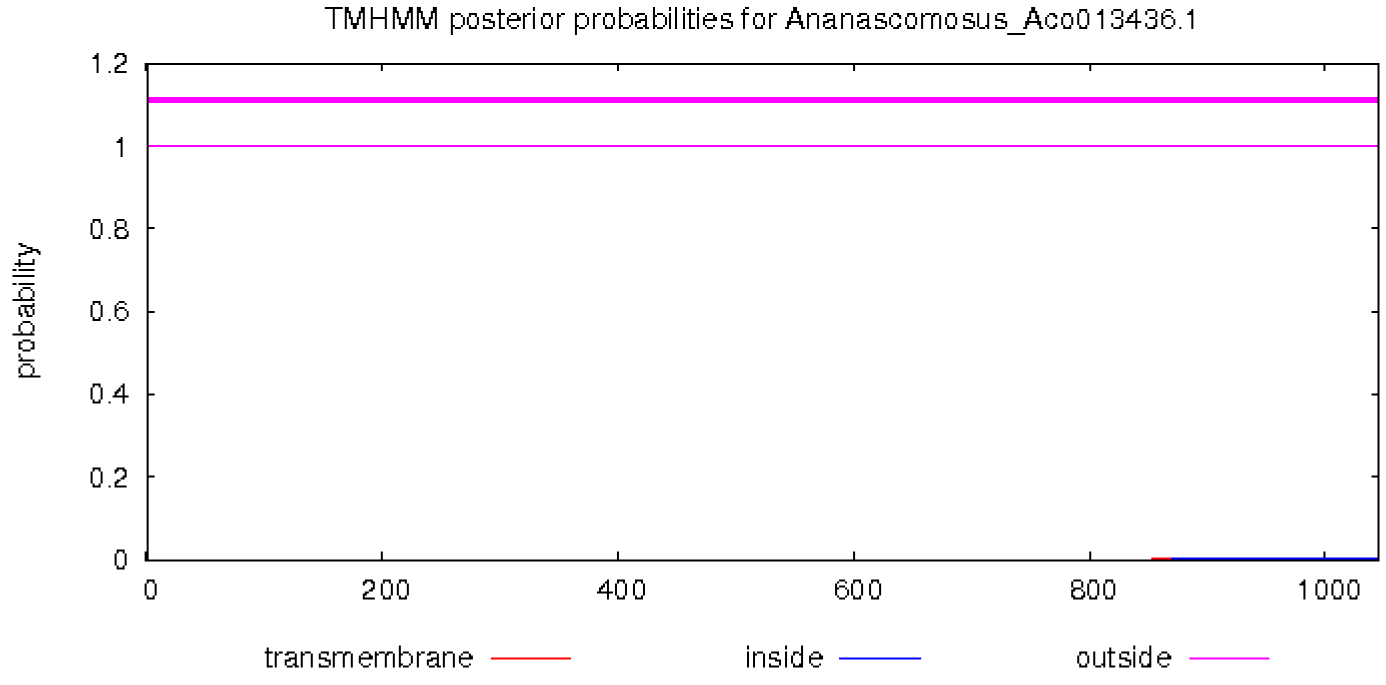

plot in postscript, script for making the plot in gnuplot, data for plot

```
# Ananascomosus_Aco013595.1 Length: 298
# Ananascomosus_Aco013595.1 Number of predicted TMHs:  0
# Ananascomosus_Aco013595.1 Exp number of AAs in TMHs: 0.00181
# Ananascomosus_Aco013595.1 Exp number, first 60 AAs:  0.00112
# Ananascomosus_Aco013595.1 Total prob of N-in:        0.00206
Ananascomosus_Aco013595.1	TMHMM2.0	outside	     1   298
```

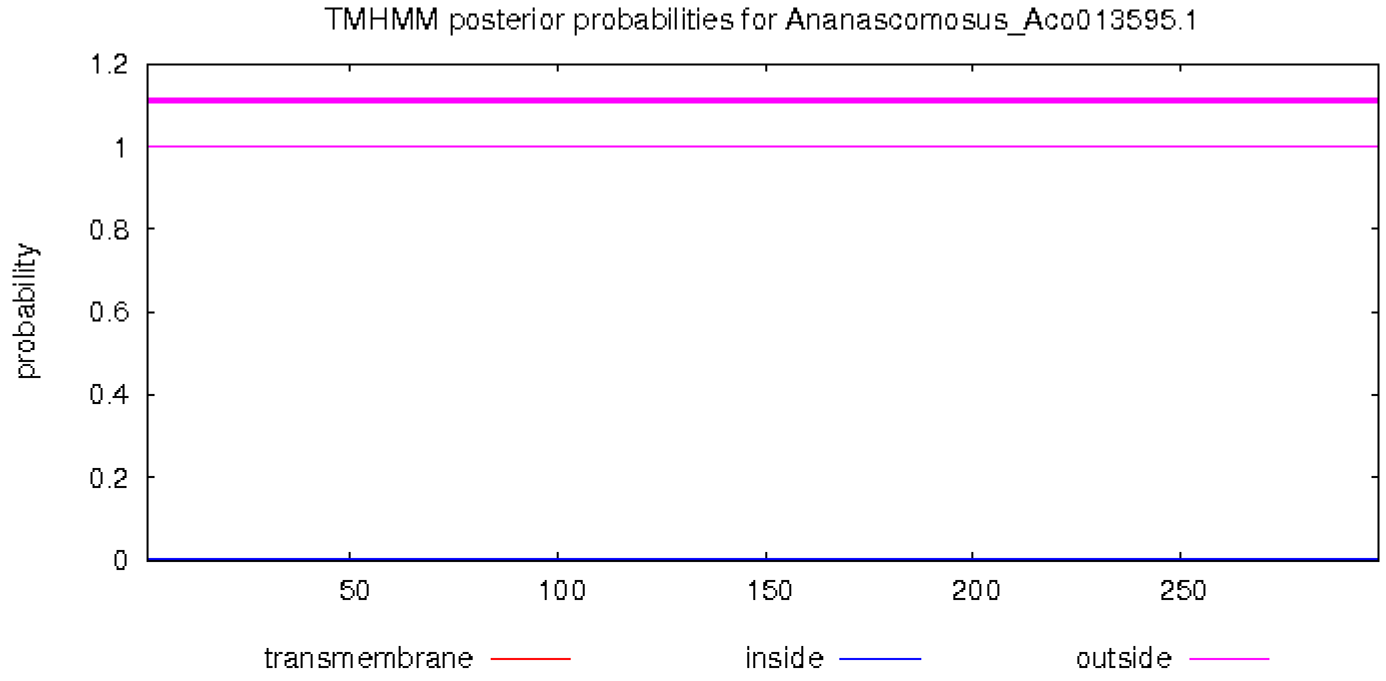

plot in postscript, script for making the plot in gnuplot, data for plot

```
# Ananascomosus_Aco014680.1 Length: 168
# Ananascomosus_Aco014680.1 Number of predicted TMHs:  0
# Ananascomosus_Aco014680.1 Exp number of AAs in TMHs: 0.0019
# Ananascomosus_Aco014680.1 Exp number, first 60 AAs:  0
# Ananascomosus_Aco014680.1 Total prob of N-in:        0.08757
Ananascomosus_Aco014680.1       TMHMM2.0        outside      1   168
```

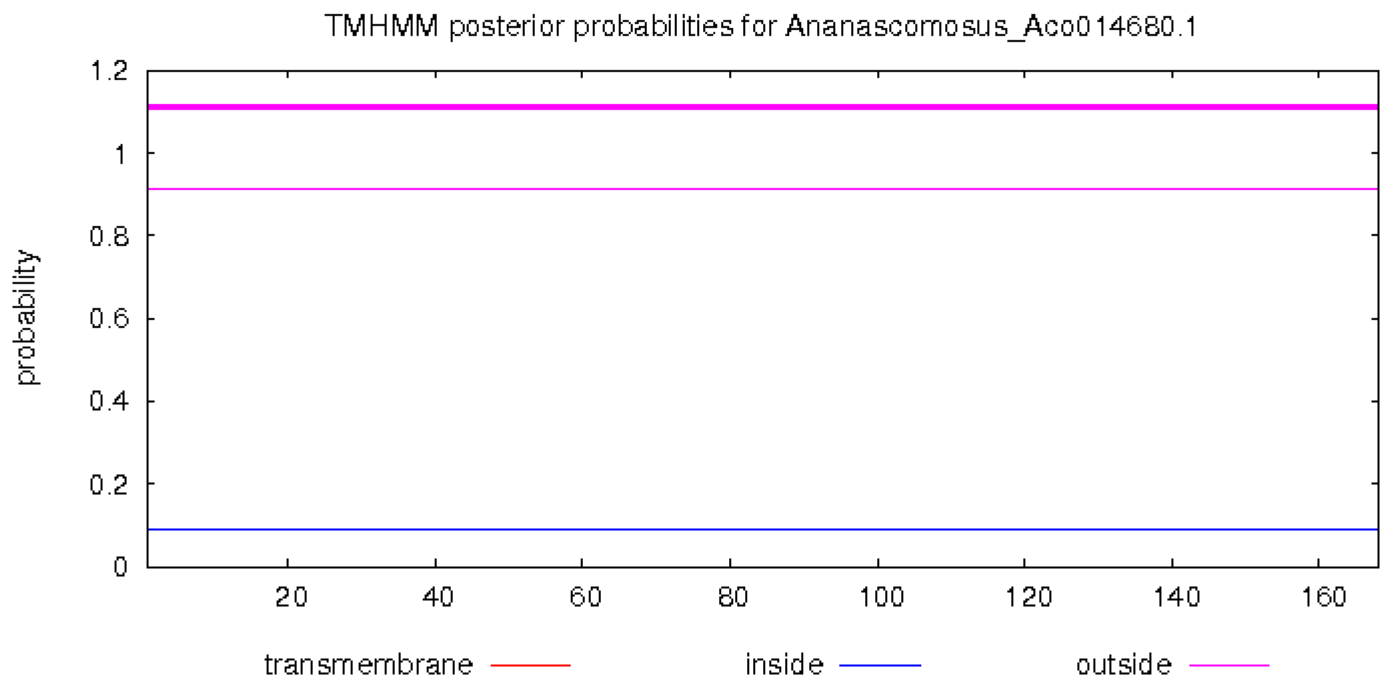

plot in postscript, script for making the plot in gnuplot, data for plot

```
# Ananascomosus_Aco014816.1 Length: 220
# Ananascomosus_Aco014816.1 Number of predicted TMHs:  0
# Ananascomosus_Aco014816.1 Exp number of AAs in TMHs: 0.0016
# Ananascomosus_Aco014816.1 Exp number, first 60 AAs:  0.00116
# Ananascomosus_Aco014816.1 Total prob of N-in:        0.05493
Ananascomosus_Aco014816.1       TMHMM2.0        outside      1   220
```

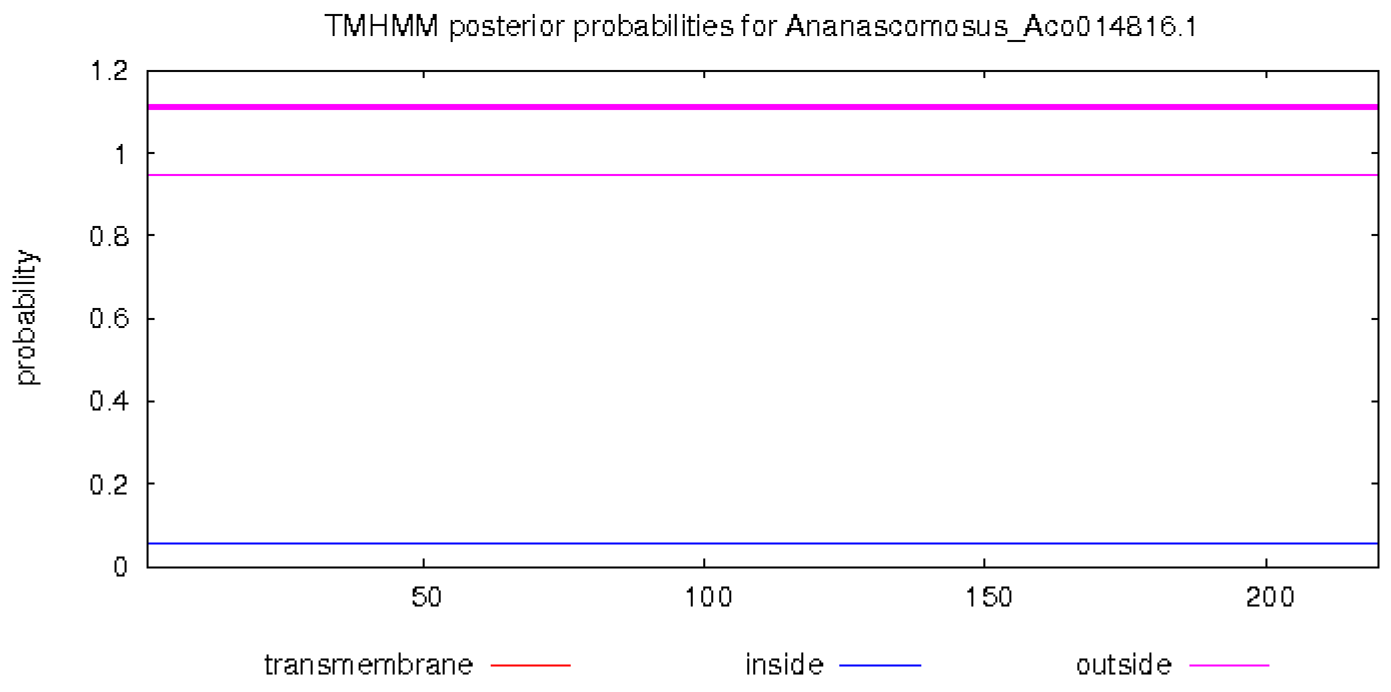

plot in postscript, script for making the plot in gnuplot, data for plot

```
# Ananascomosus_Aco015086.1 Length: 269
# Ananascomosus_Aco015086.1 Number of predicted TMHs:  0
# Ananascomosus_Aco015086.1 Exp number of AAs in TMHs: 0.00103
# Ananascomosus_Aco015086.1 Exp number, first 60 AAs:  5e-05
# Ananascomosus_Aco015086.1 Total prob of N-in:        0.01478
Ananascomosus_Aco015086.1	TMHMM2.0	outside	     1   269
```

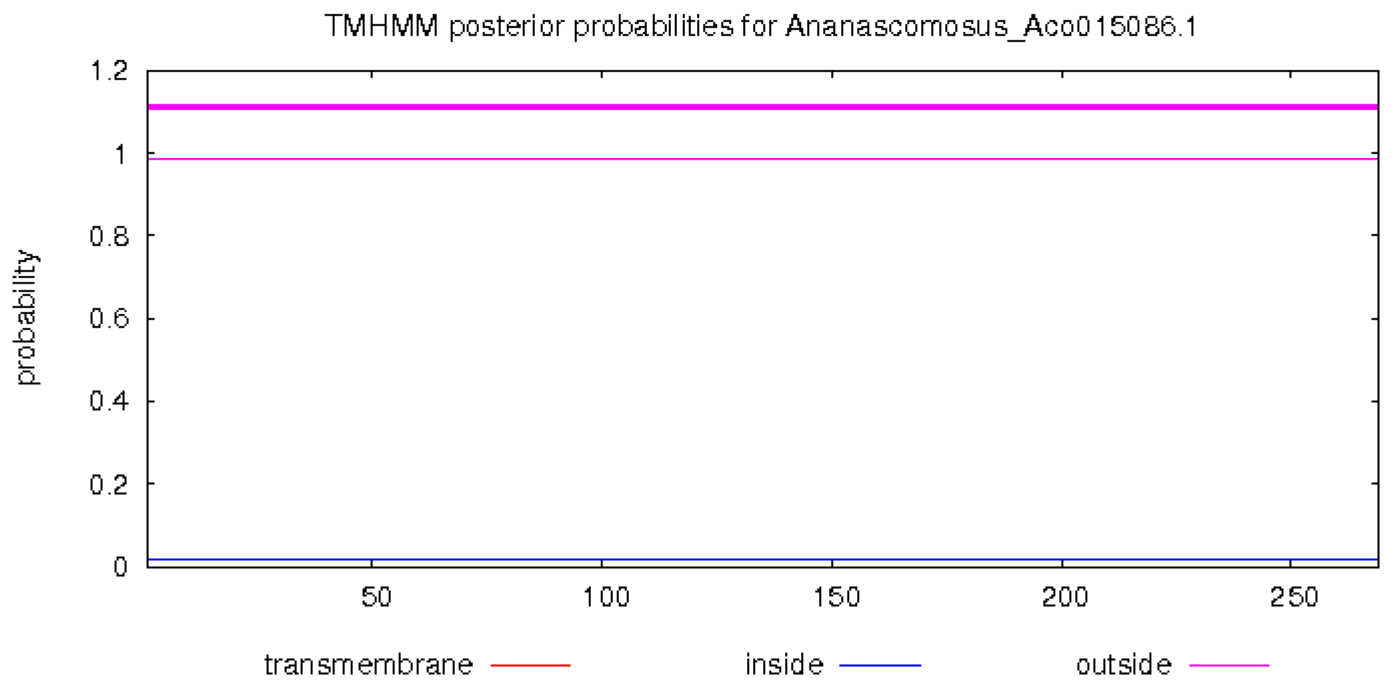

plot in postscript, script for making the plot in gnuplot, data for plot

```
# Ananascomosus_Aco015181.1 Length: 412
# Ananascomosus_Aco015181.1 Number of predicted TMHs:  0
# Ananascomosus_Aco015181.1 Exp number of AAs in TMHs: 0
# Ananascomosus_Aco015181.1 Exp number, first 60 AAs:  0
# Ananascomosus_Aco015181.1 Total prob of N-in:        0.00909
Ananascomosus_Aco015181.1	TMHMM2.0	outside	     1   412
```

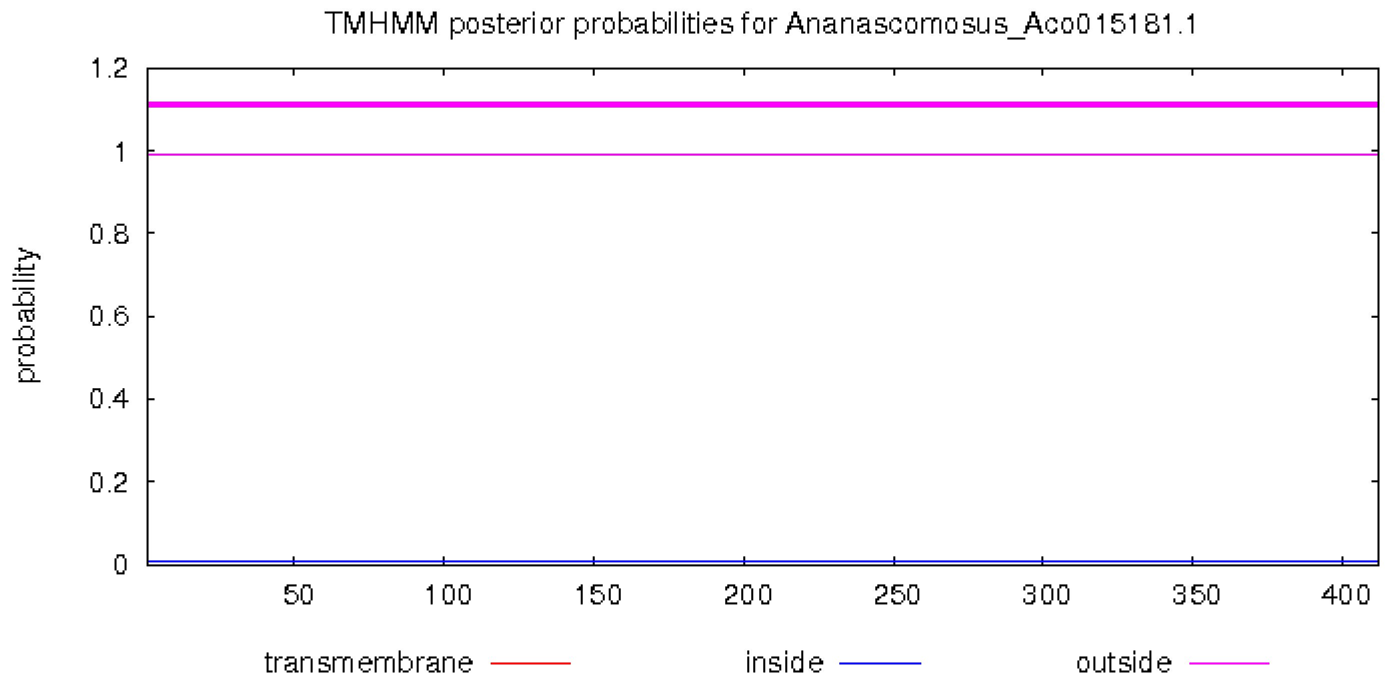

plot in postscript, script for making the plot in gnuplot, data for plot

```
# Ananascomosus_Aco015539.1 Length: 368
# Ananascomosus_Aco015539.1 Number of predicted TMHs:  0
# Ananascomosus_Aco015539.1 Exp number of AAs in TMHs: 0.00021
# Ananascomosus_Aco015539.1 Exp number, first 60 AAs:  0
# Ananascomosus_Aco015539.1 Total prob of N-in:        0.00306
Ananascomosus_Aco015539.1	TMHMM2.0	outside	     1   368
```

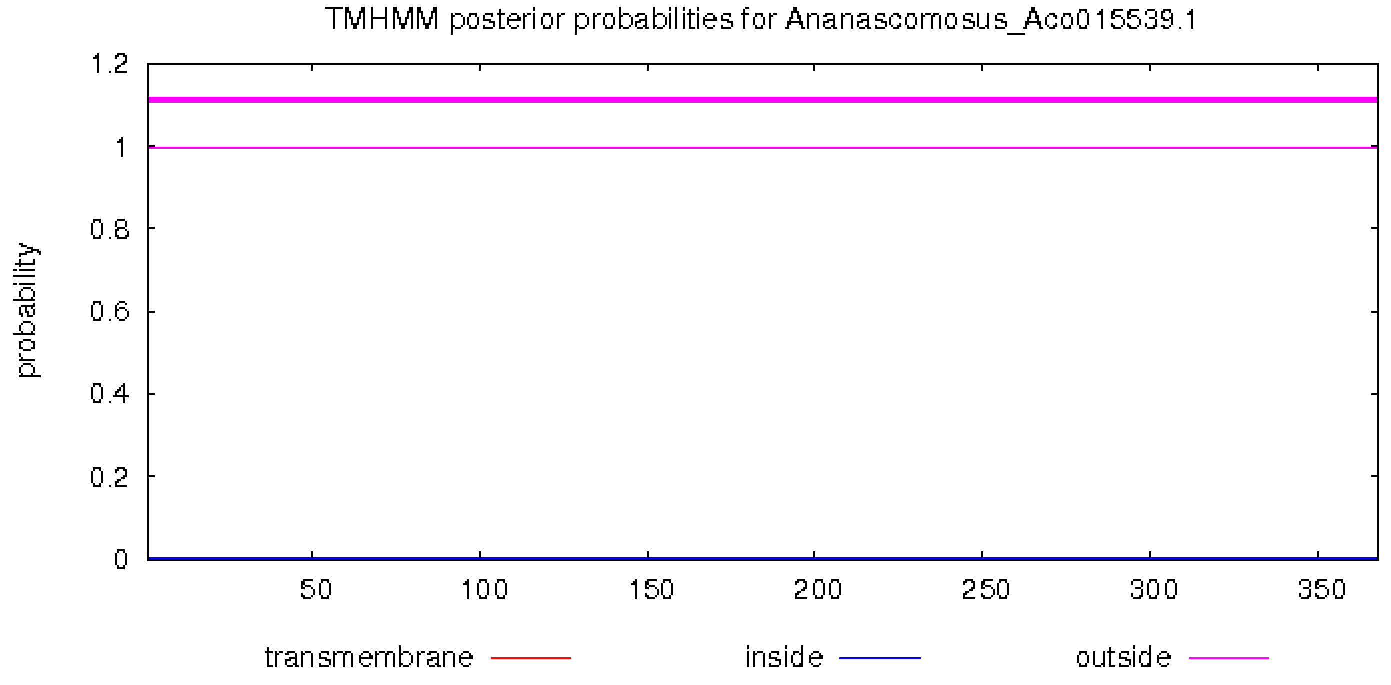

plot in postscript, script for making the plot in gnuplot, data for plot

```
# Ananascomosus_Aco016738.1 Length: 231
# Ananascomosus_Aco016738.1 Number of predicted TMHs:  0
# Ananascomosus_Aco016738.1 Exp number of AAs in TMHs: 0.00499
# Ananascomosus_Aco016738.1 Exp number, first 60 AAs:  0
# Ananascomosus_Aco016738.1 Total prob of N-in:        0.00821
Ananascomosus_Aco016738.1	TMHMM2.0	outside	     1   231
```

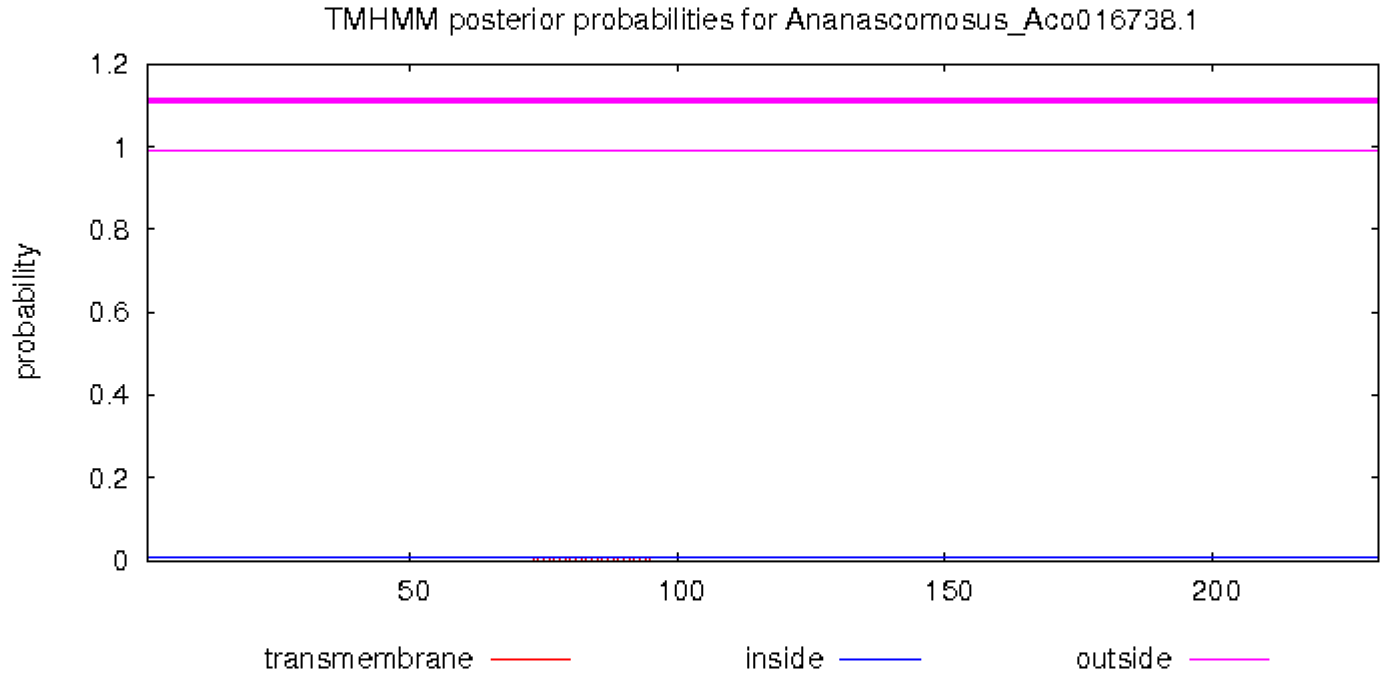

plot in postscript, script for making the plot in gnuplot, data for plot

```
# Ananascomosus_Aco017708.1 Length: 316
# Ananascomosus_Aco017708.1 Number of predicted TMHs:  0
# Ananascomosus_Aco017708.1 Exp number of AAs in TMHs: 0
# Ananascomosus_Aco017708.1 Exp number, first 60 AAs:  0
# Ananascomosus_Aco017708.1 Total prob of N-in:        0.00515
Ananascomosus_Aco017708.1       TMHMM2.0        outside      1   316
```

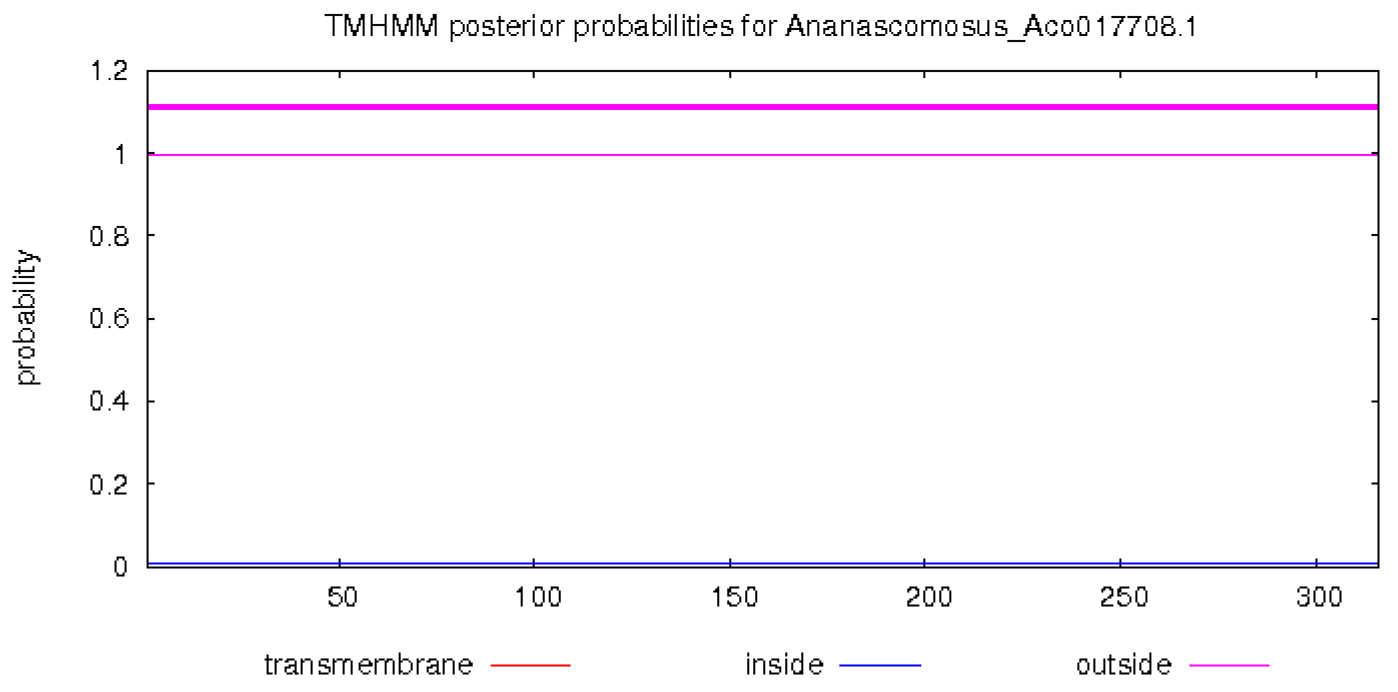

plot in postscript, script for making the plot in gnuplot, data for plot

```
# Ananascomosus_Aco018062.1 Length: 274
# Ananascomosus_Aco018062.1 Number of predicted TMHs:  0
# Ananascomosus_Aco018062.1 Exp number of AAs in TMHs: 0.02058
# Ananascomosus_Aco018062.1 Exp number, first 60 AAs:  0
# Ananascomosus_Aco018062.1 Total prob of N-in:        0.07797
Ananascomosus_Aco018062.1       TMHMM2.0        outside      1   274
```

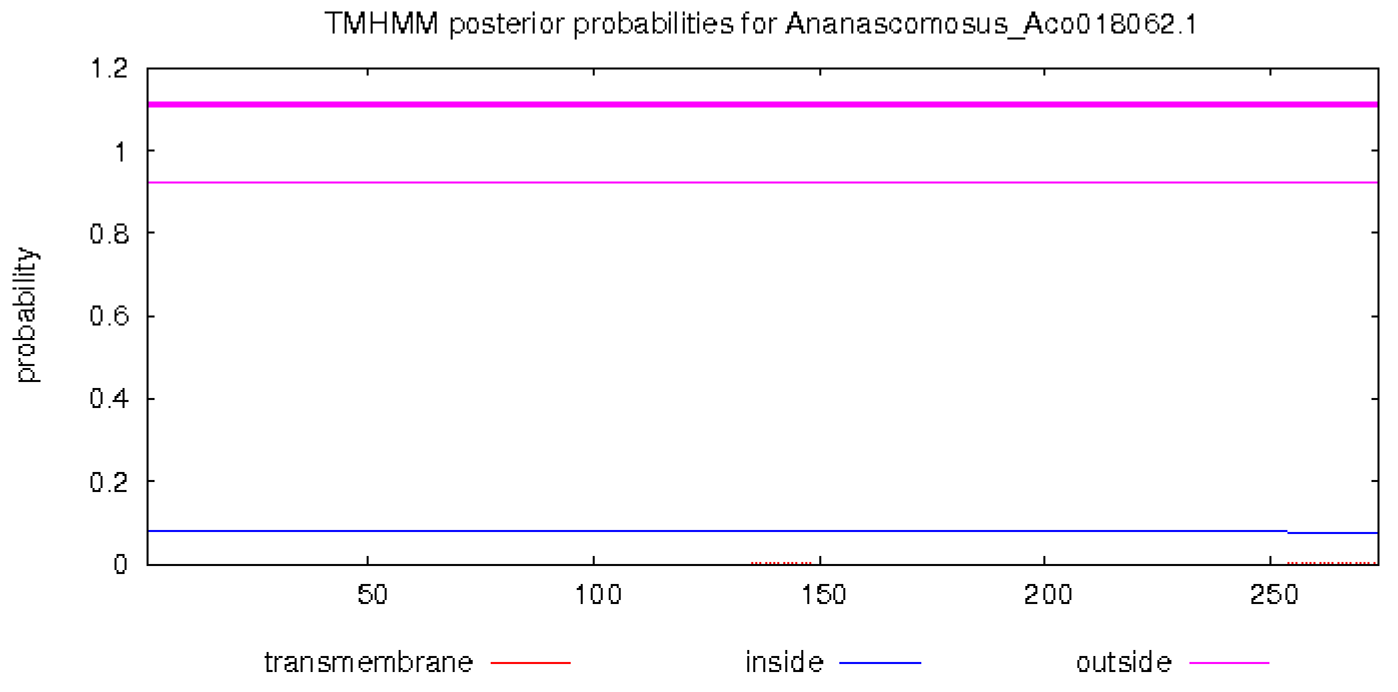

plot in postscript, script for making the plot in gnuplot, data for plot

```
# Ananascomosus_Aco018555.1 Length: 694
# Ananascomosus_Aco018555.1 Number of predicted TMHs:  1
# Ananascomosus_Aco018555.1 Exp number of AAs in TMHs: 22.85137
# Ananascomosus_Aco018555.1 Exp number, first 60 AAs:  0.23176
# Ananascomosus_Aco018555.1 Total prob of N-in:        0.03300
Ananascomosus_Aco018555.1       TMHMM2.0        outside      1   658
Ananascomosus_Aco018555.1       TMHMM2.0        TMhelix    659   681
Ananascomosus_Aco018555.1       TMHMM2.0        inside     682   694
```

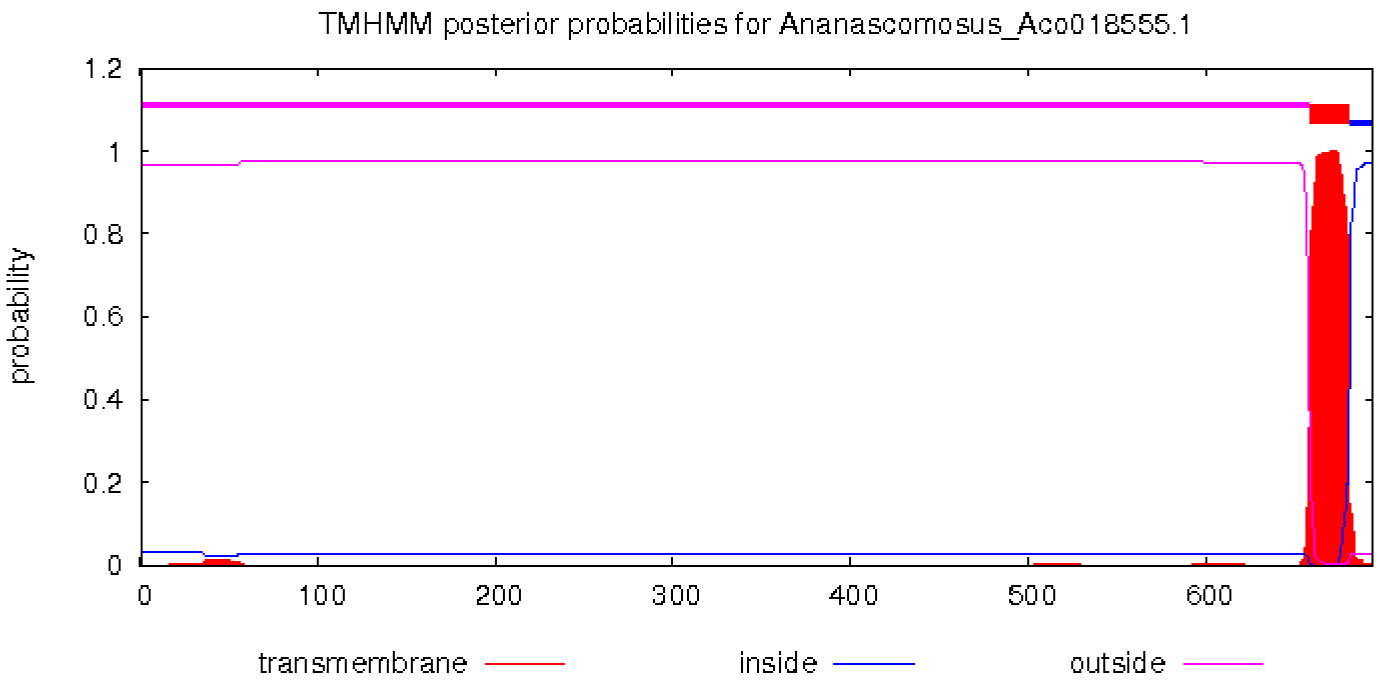

plot in postscript, script for making the plot in gnuplot, data for plot

```
# Ananascomosus_Aco018659.1 Length: 334
# Ananascomosus_Aco018659.1 Number of predicted TMHs:  0
# Ananascomosus_Aco018659.1 Exp number of AAs in TMHs: 0.00187
# Ananascomosus_Aco018659.1 Exp number, first 60 AAs:  0
# Ananascomosus_Aco018659.1 Total prob of N-in:        0.04600
Ananascomosus_Aco018659.1       TMHMM2.0        outside      1   334
```

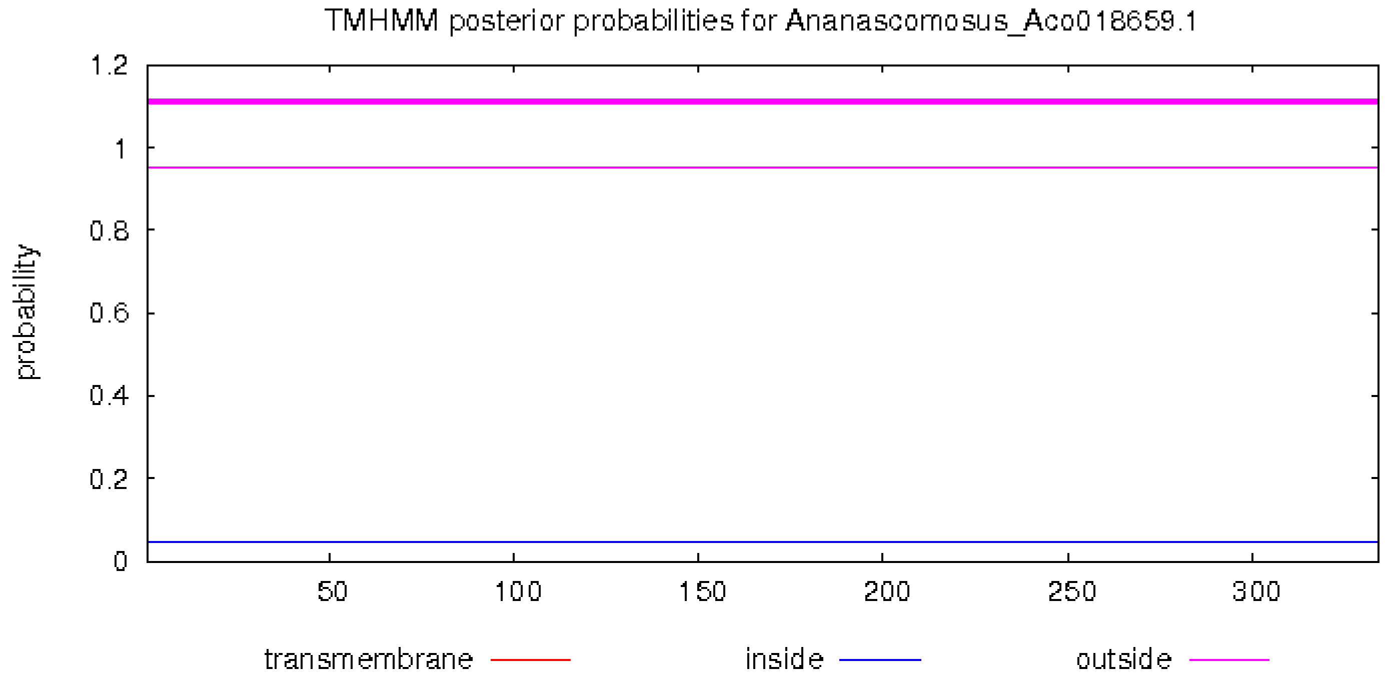

plot in postscript, script for making the plot in gnuplot, data for plot

---

```
# Ananascomosus_Aco018846.1 Length: 721
# Ananascomosus_Aco018846.1 Number of predicted TMHs:  0
# Ananascomosus_Aco018846.1 Exp number of AAs in TMHs: 0.12792
# Ananascomosus_Aco018846.1 Exp number, first 60 AAs:  0
# Ananascomosus_Aco018846.1 Total prob of N-in:        0.00008
Ananascomosus_Aco018846.1       TMHMM2.0        outside      1   721
```

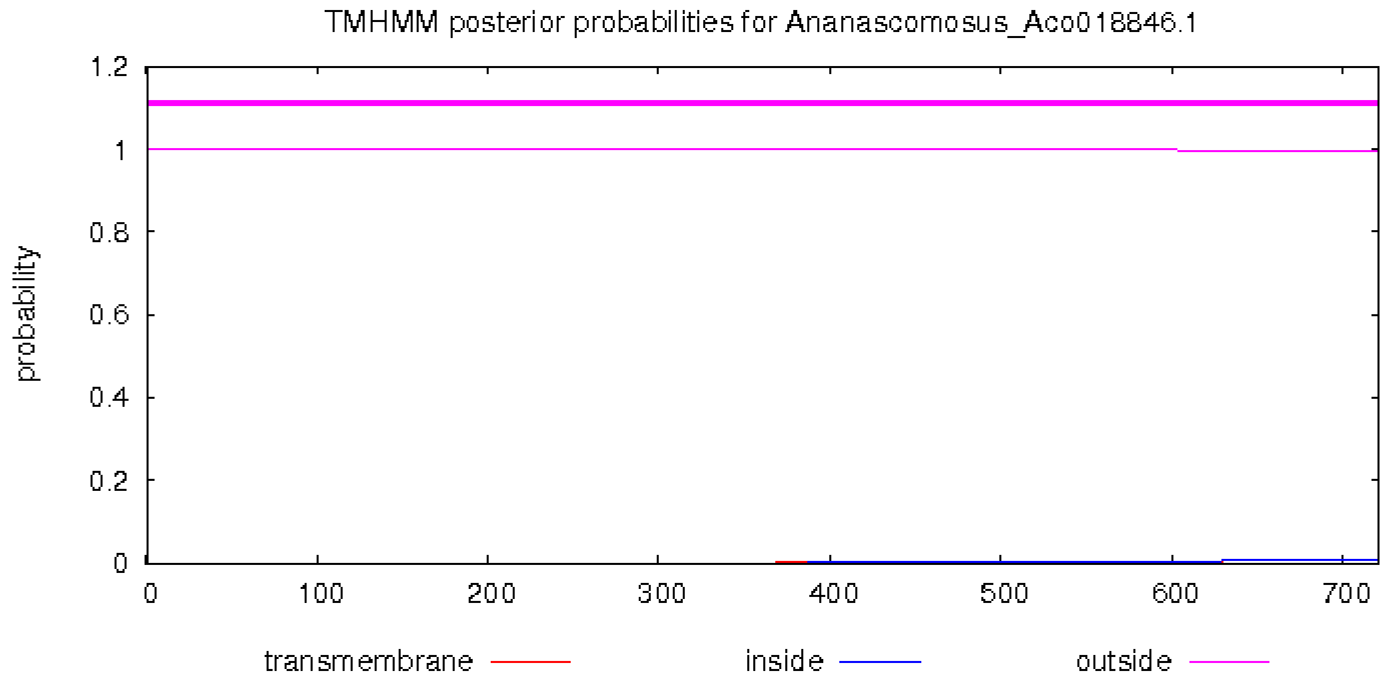

plot in postscript, script for making the plot in gnuplot, data for plot

---

```
# Ananascomosus_Aco019352.1 Length: 490
# Ananascomosus_Aco019352.1 Number of predicted TMHs:  1
# Ananascomosus_Aco019352.1 Exp number of AAs in TMHs: 27.75968
# Ananascomosus_Aco019352.1 Exp number, first 60 AAs:  4.54166
# Ananascomosus_Aco019352.1 Total prob of N-in:        0.72219
Ananascomosus_Aco019352.1       TMHMM2.0        inside       1    60
```

```
Ananascomosus_Aco019352.1       TMHMM2.0        TMhelix      61    83
Ananascomosus_Aco019352.1       TMHMM2.0        outside      84   490
```

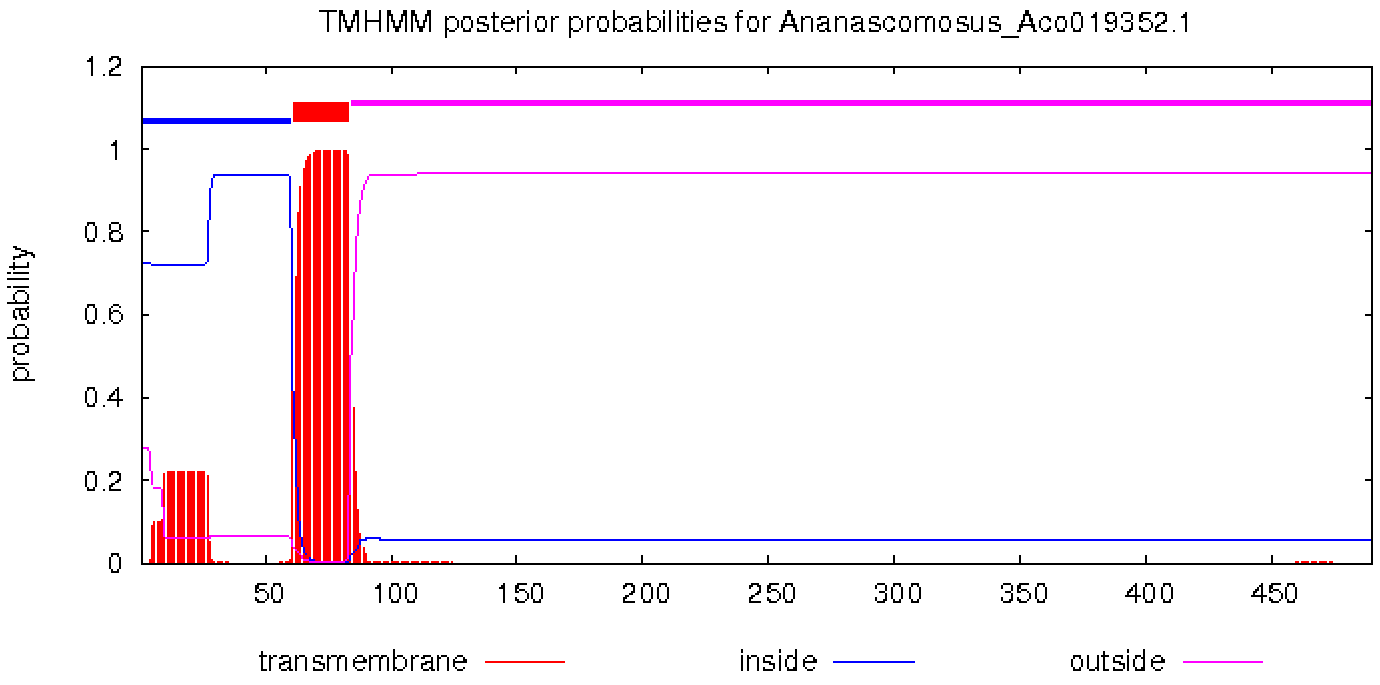

plot in postscript, script for making the plot in gnuplot, data for plot

---

```
# Ananascomosus_Aco019379.1 Length: 293
# Ananascomosus_Aco019379.1 Number of predicted TMHs:  0
# Ananascomosus_Aco019379.1 Exp number of AAs in TMHs: 0.00266
# Ananascomosus_Aco019379.1 Exp number, first 60 AAs:  0.00258
# Ananascomosus_Aco019379.1 Total prob of N-in:        0.00429
Ananascomosus_Aco019379.1       TMHMM2.0        outside      1   293
```

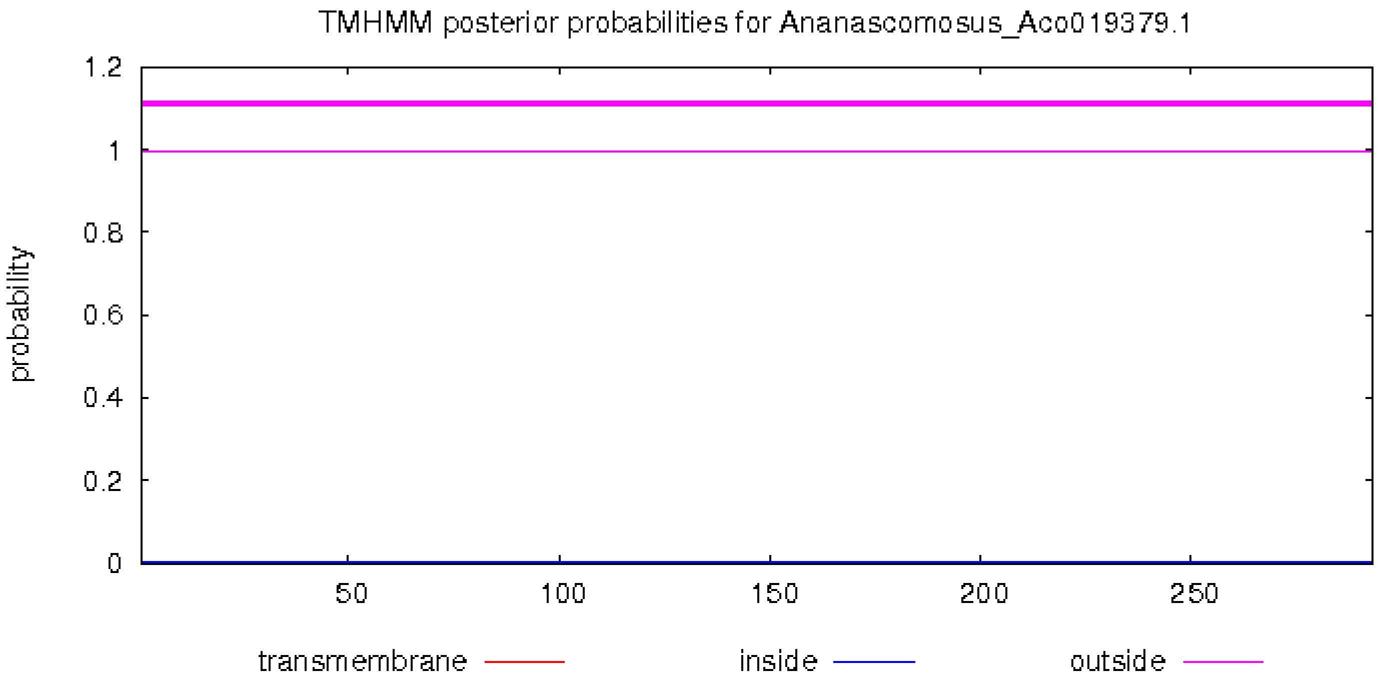

plot in postscript, script for making the plot in gnuplot, data for plot

---

```
# Ananascomosus_Aco020033.1 Length: 326
# Ananascomosus_Aco020033.1 Number of predicted TMHs:  0
# Ananascomosus_Aco020033.1 Exp number of AAs in TMHs: 0.00438
# Ananascomosus_Aco020033.1 Exp number, first 60 AAs:  0
```

```
# Ananascomosus_Aco020033.1 Total prob of N-in:        0.00369
Ananascomosus_Aco020033.1       TMHMM2.0        outside      1   326
```

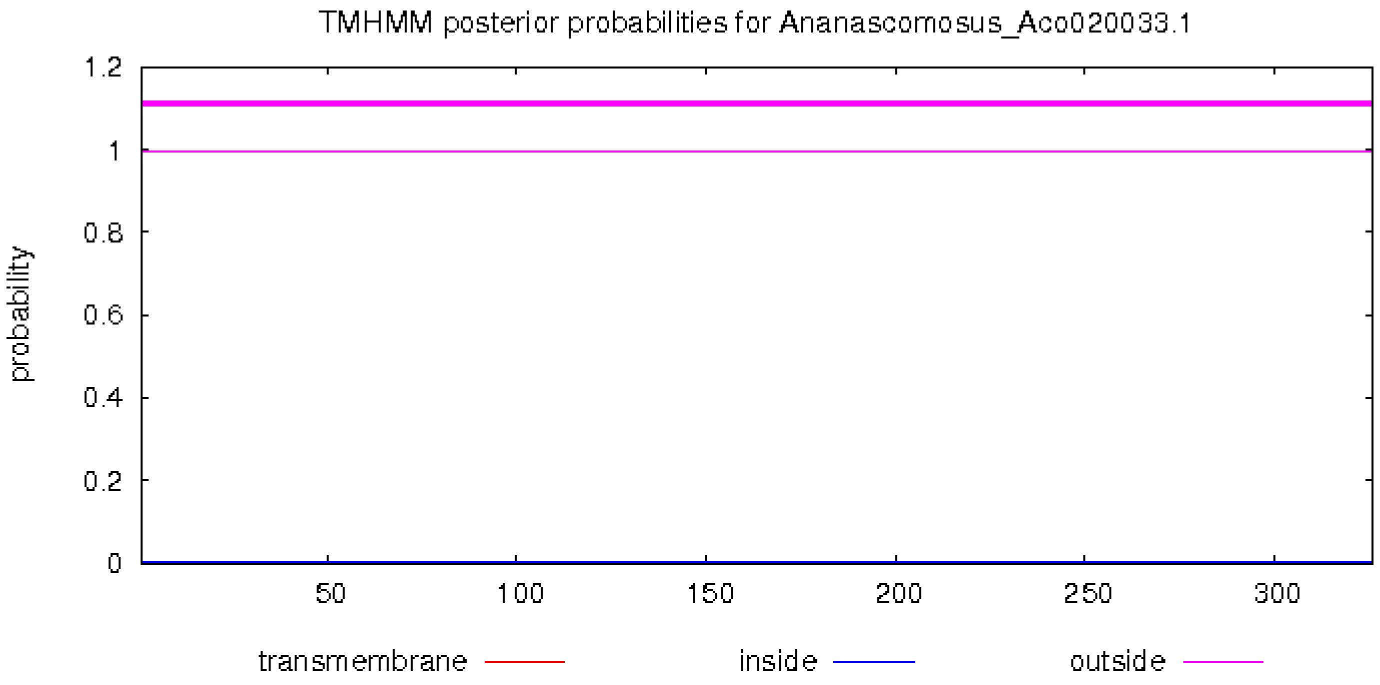

plot in postscript, script for making the plot in gnuplot, data for plot

---

```
# Ananascomosus_Aco020094.1 Length: 361
# Ananascomosus_Aco020094.1 Number of predicted TMHs:  0
# Ananascomosus_Aco020094.1 Exp number of AAs in TMHs: 0.07517
# Ananascomosus_Aco020094.1 Exp number, first 60 AAs:  0.0004
# Ananascomosus_Aco020094.1 Total prob of N-in:        0.03950
Ananascomosus_Aco020094.1       TMHMM2.0        outside      1   361
```

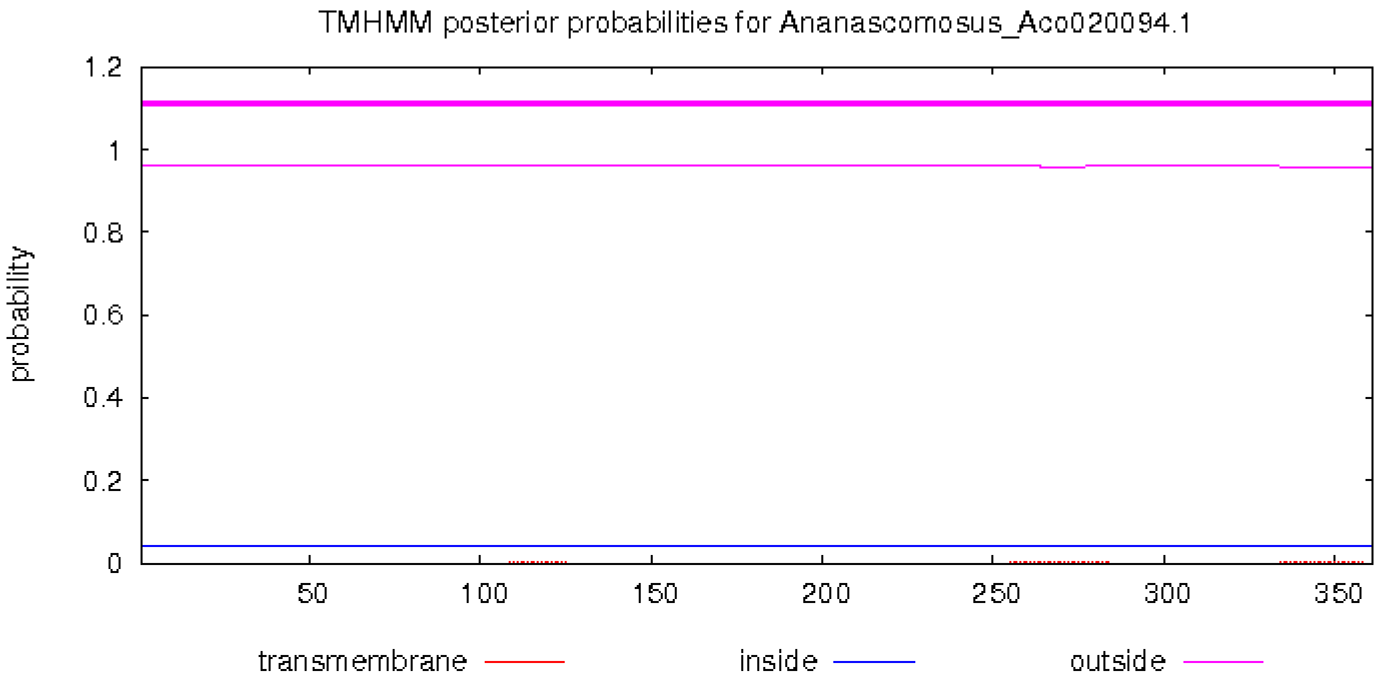

plot in postscript, script for making the plot in gnuplot, data for plot

---

```
# Ananascomosus_Aco020562.1 Length: 466
# Ananascomosus_Aco020562.1 Number of predicted TMHs:  1
# Ananascomosus_Aco020562.1 Exp number of AAs in TMHs: 21.04168
# Ananascomosus_Aco020562.1 Exp number, first 60 AAs:  0.05276
```

```
# Ananascomosus_Aco020562.1 Total prob of N-in:        0.13437
Ananascomosus_Aco020562.1       TMHMM2.0        outside      1   431
Ananascomosus_Aco020562.1       TMHMM2.0        TMhelix    432   450
Ananascomosus_Aco020562.1       TMHMM2.0        inside     451   466
```

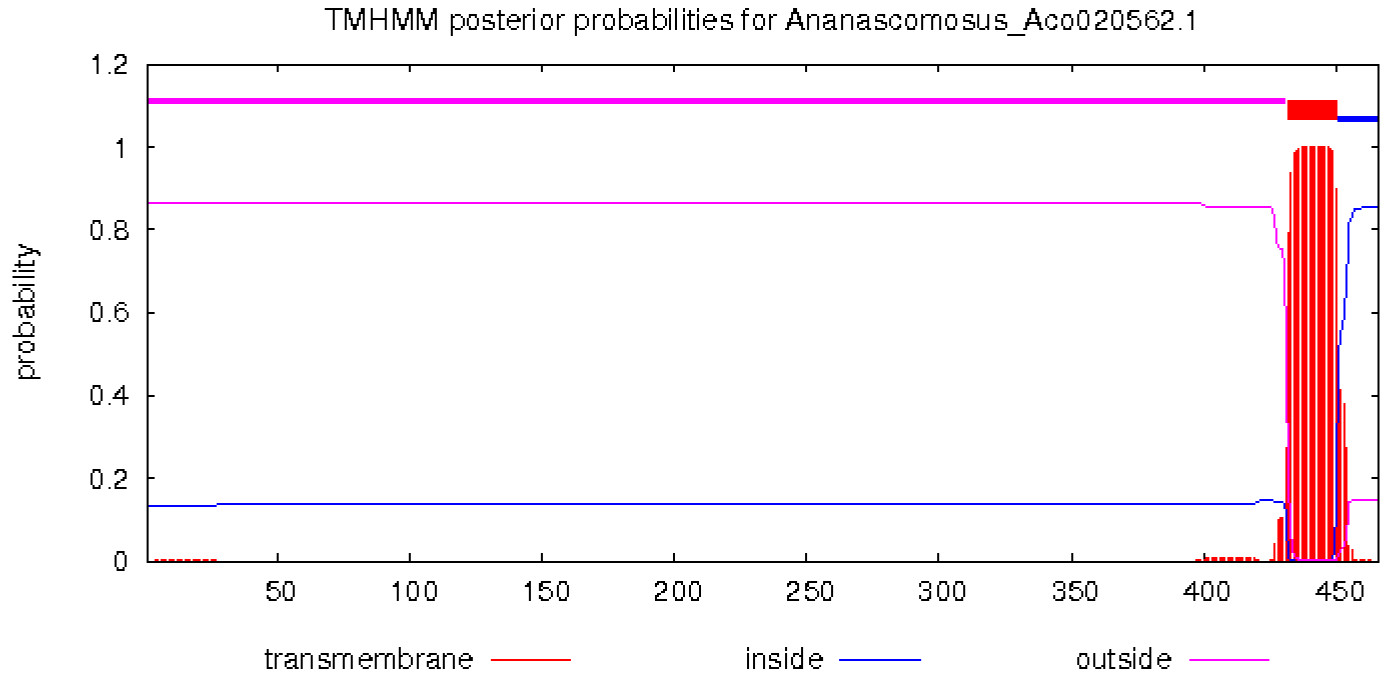

plot in postscript, script for making the plot in gnuplot, data for plot

---

```
# Ananascomosus_Aco021379.1 Length: 297
# Ananascomosus_Aco021379.1 Number of predicted TMHs:  0
# Ananascomosus_Aco021379.1 Exp number of AAs in TMHs: 0.00554
# Ananascomosus_Aco021379.1 Exp number, first 60 AAs:  0.00176
# Ananascomosus_Aco021379.1 Total prob of N-in:        0.10347
Ananascomosus_Aco021379.1       TMHMM2.0        outside      1   297
```

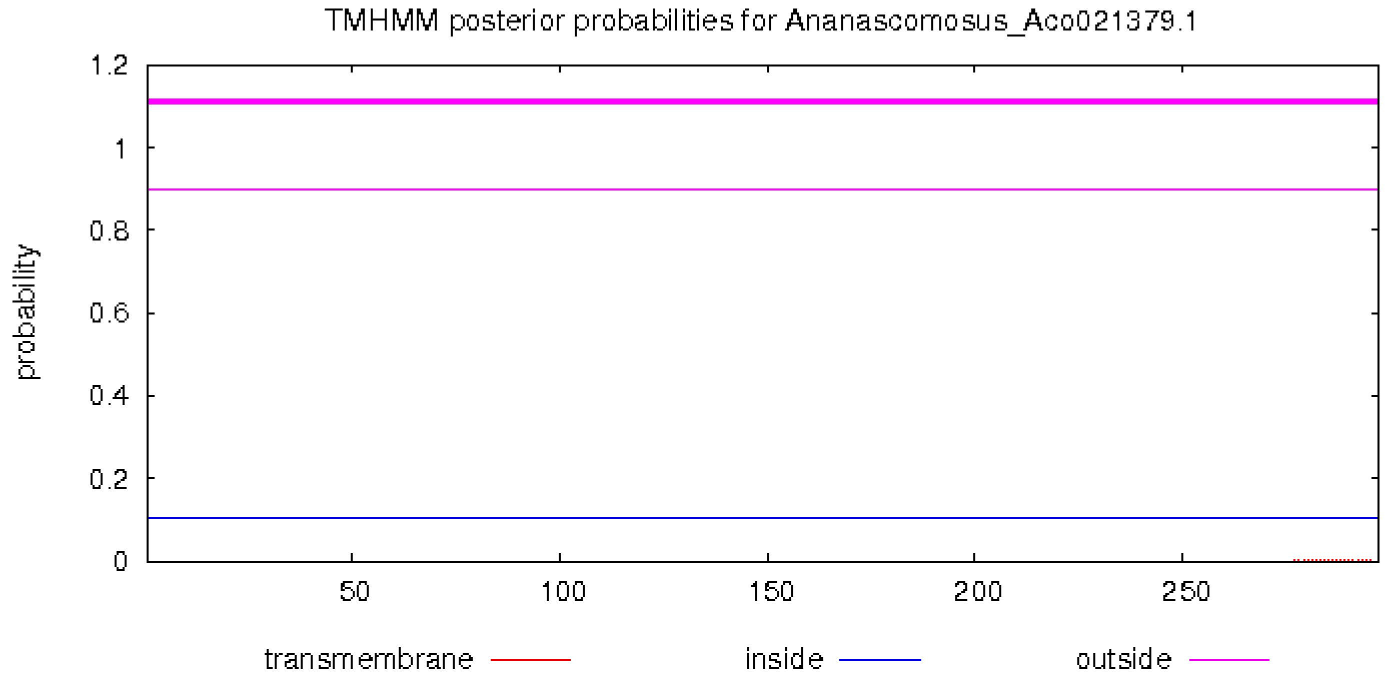

plot in postscript, script for making the plot in gnuplot, data for plot

---

```
# Ananascomosus_Aco022043.1 Length: 345
# Ananascomosus_Aco022043.1 Number of predicted TMHs:  0
```

```
# Ananascomosus_Aco022043.1 Exp number of AAs in TMHs: 0.00018
# Ananascomosus_Aco022043.1 Exp number, first 60 AAs:  0
# Ananascomosus_Aco022043.1 Total prob of N-in:        0.00192
Ananascomosus_Aco022043.1       TMHMM2.0        outside      1   345
```

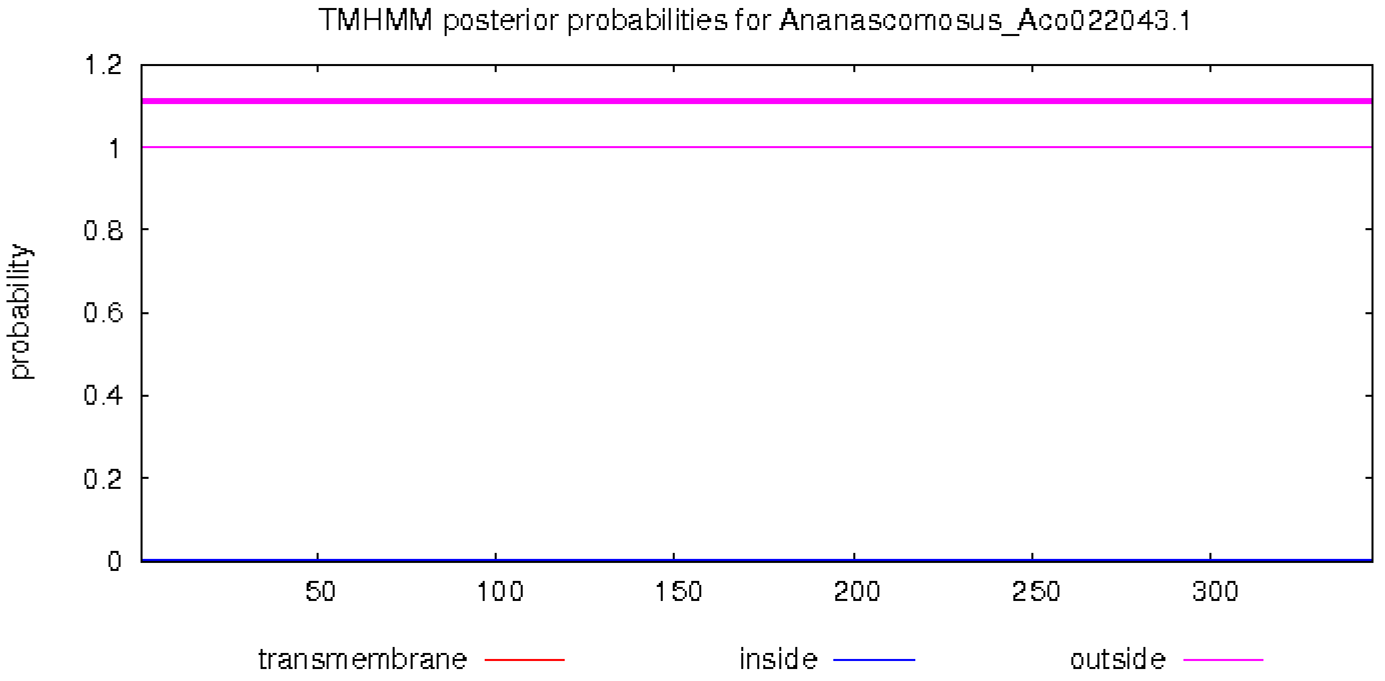

plot in postscript, script for making the plot in gnuplot, data for plot

---

```
# Ananascomosus_Aco023285.1 Length: 284
# Ananascomosus_Aco023285.1 Number of predicted TMHs:  0
# Ananascomosus_Aco023285.1 Exp number of AAs in TMHs: 0.0025
# Ananascomosus_Aco023285.1 Exp number, first 60 AAs:  0.00019
# Ananascomosus_Aco023285.1 Total prob of N-in:        0.14173
Ananascomosus_Aco023285.1       TMHMM2.0        outside      1   284
```

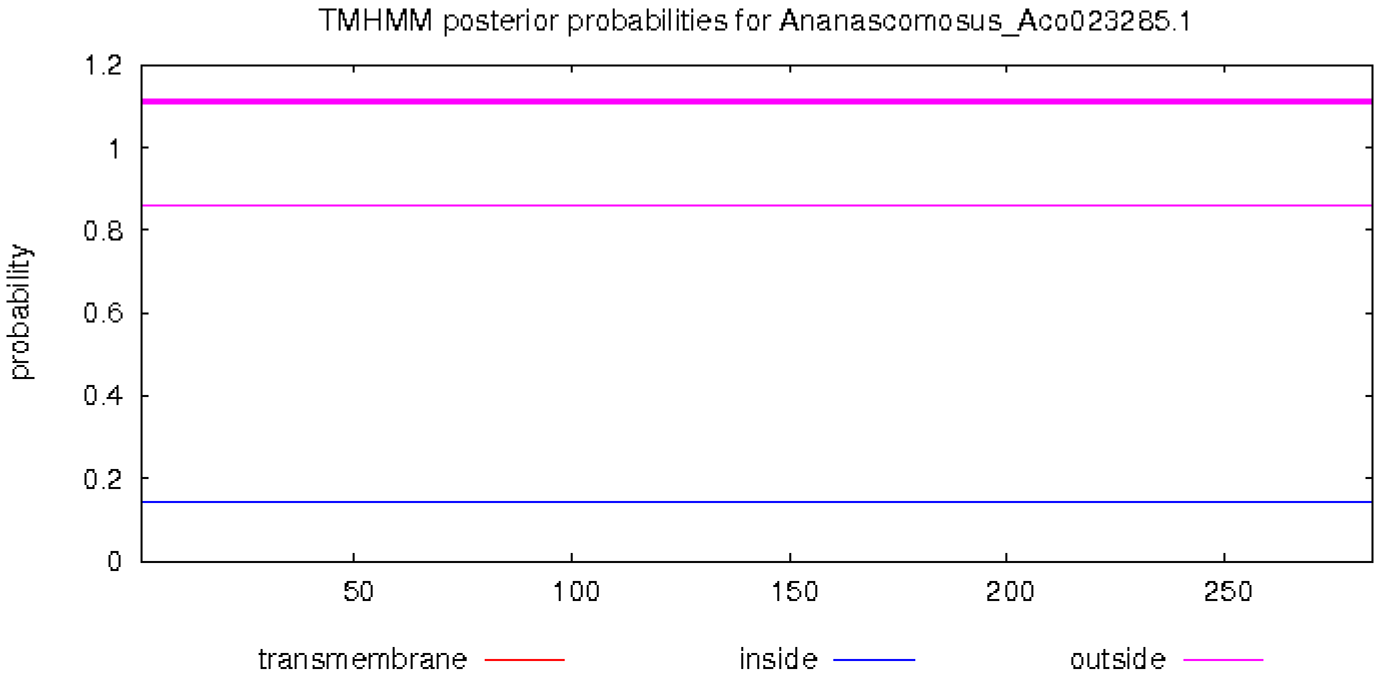

plot in postscript, script for making the plot in gnuplot, data for plot

---

```
# Ananascomosus_Aco023288.1 Length: 348
# Ananascomosus_Aco023288.1 Number of predicted TMHs:  0
```

```
# Ananascomosus_Aco023288.1 Exp number of AAs in TMHs: 0.00062
# Ananascomosus_Aco023288.1 Exp number, first 60 AAs:  0.00023
# Ananascomosus_Aco023288.1 Total prob of N-in:        0.00149
Ananascomosus_Aco023288.1	TMHMM2.0	outside	     1   348
```

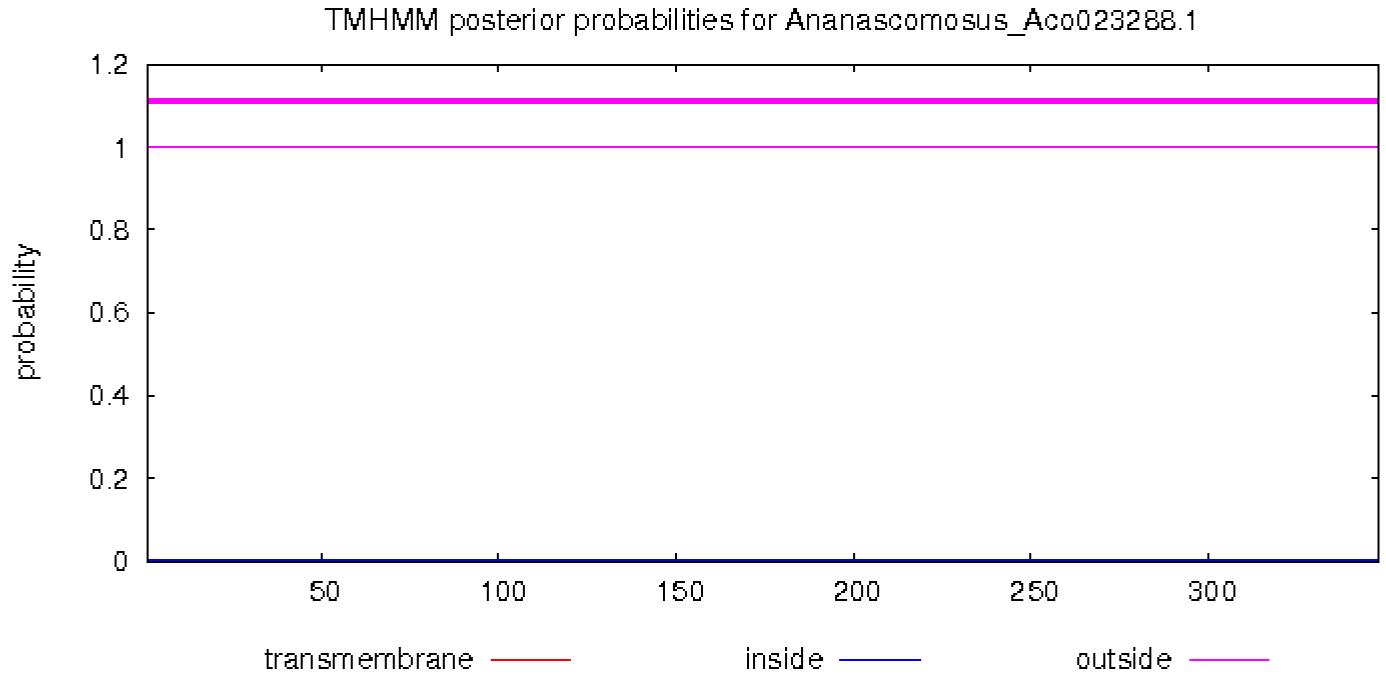

plot in postscript, script for making the plot in gnuplot, data for plot

---

```
# Ananascomosus_Aco023290.1 Length: 372
# Ananascomosus_Aco023290.1 Number of predicted TMHs:  0
# Ananascomosus_Aco023290.1 Exp number of AAs in TMHs: 0.00163
# Ananascomosus_Aco023290.1 Exp number, first 60 AAs:  0
# Ananascomosus_Aco023290.1 Total prob of N-in:        0.00614
Ananascomosus_Aco023290.1	TMHMM2.0	outside	     1   372
```

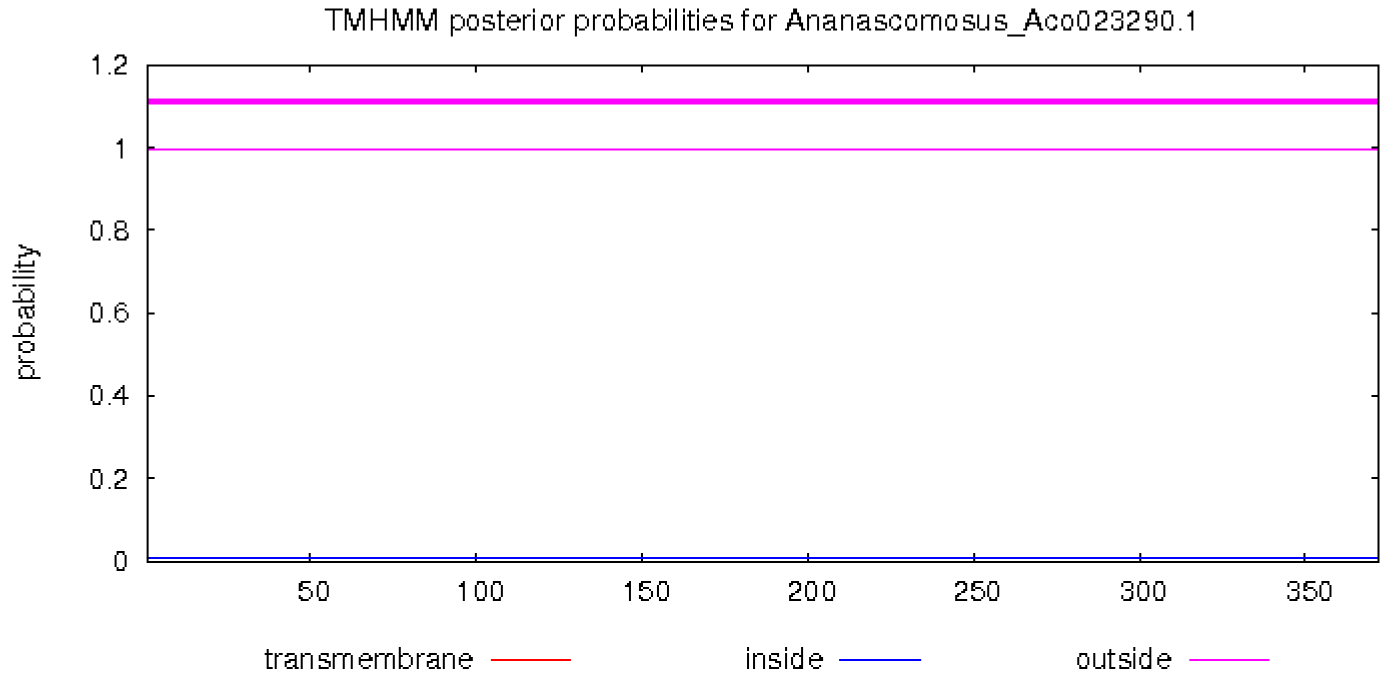

plot in postscript, script for making the plot in gnuplot, data for plot

---

```
# Ananascomosus_Aco023481.1 Length: 392
# Ananascomosus_Aco023481.1 Number of predicted TMHs:  0
```

```
# Ananascomosus_Aco023481.1 Exp number of AAs in TMHs: 0.00018
# Ananascomosus_Aco023481.1 Exp number, first 60 AAs:  0
# Ananascomosus_Aco023481.1 Total prob of N-in:        0.00538
Ananascomosus_Aco023481.1	TMHMM2.0	outside	     1   392
```

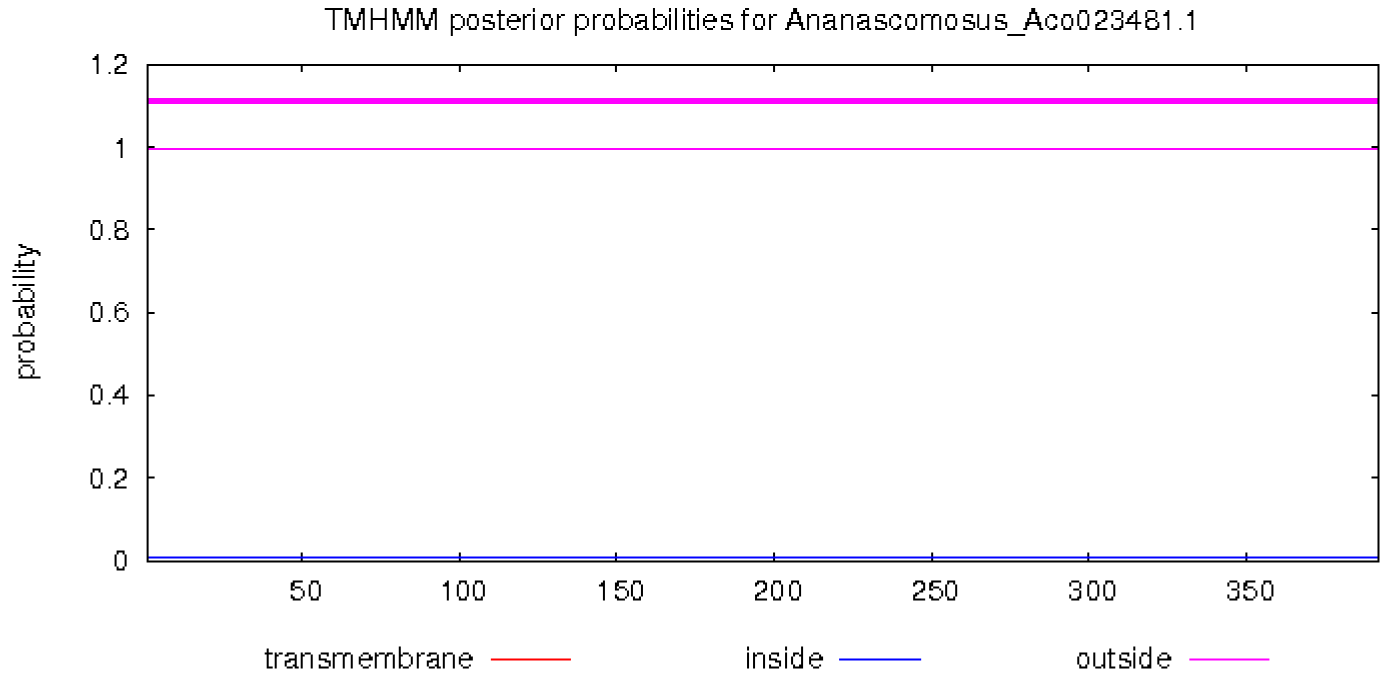

plot in postscript, script for making the plot in gnuplot, data for plot

---

```
# Ananascomosus_Aco024095.1 Length: 465
# Ananascomosus_Aco024095.1 Number of predicted TMHs:  0
# Ananascomosus_Aco024095.1 Exp number of AAs in TMHs: 0.00589
# Ananascomosus_Aco024095.1 Exp number, first 60 AAs:  0
# Ananascomosus_Aco024095.1 Total prob of N-in:        0.00492
Ananascomosus_Aco024095.1	TMHMM2.0	outside	     1   465
```

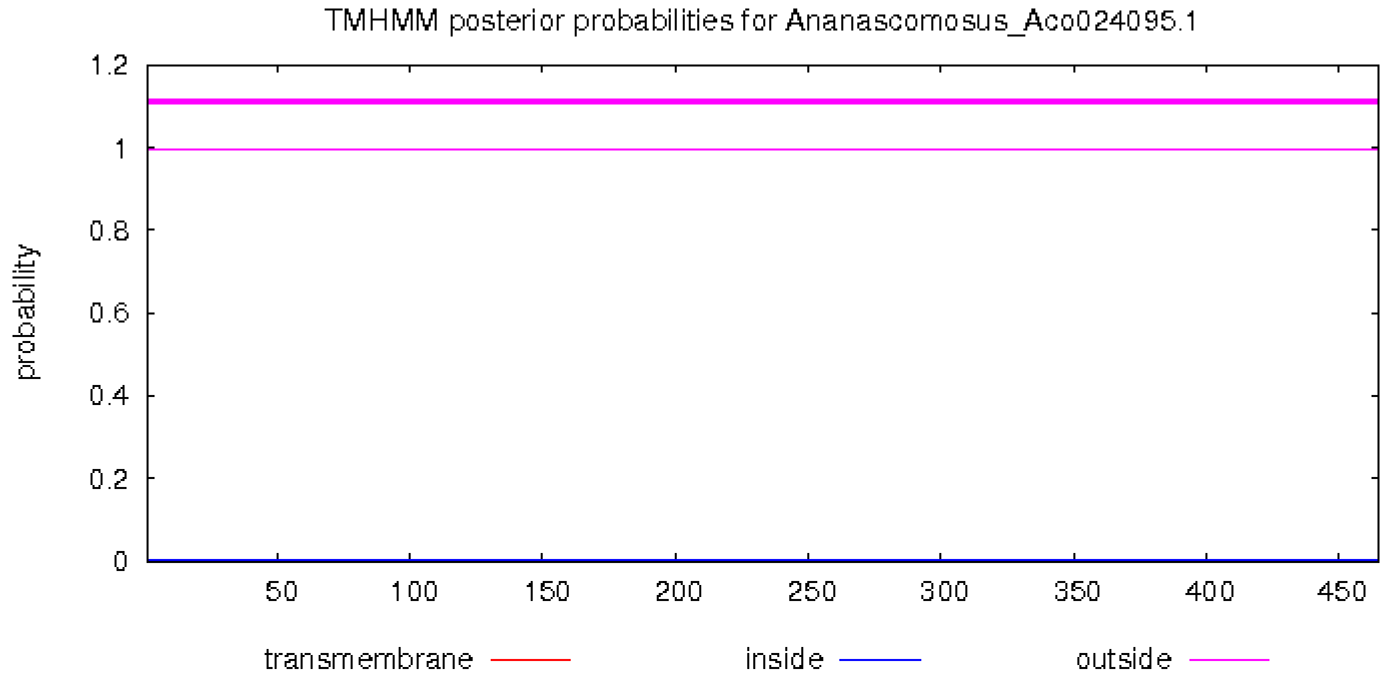

plot in postscript, script for making the plot in gnuplot, data for plot

---

```
# Ananascomosus_Aco027001.1 Length: 717
# Ananascomosus_Aco027001.1 Number of predicted TMHs:  0
```

```
# Ananascomosus_Aco027001.1 Exp number of AAs in TMHs: 2.11473
# Ananascomosus_Aco027001.1 Exp number, first 60 AAs:  0.00715
# Ananascomosus_Aco027001.1 Total prob of N-in:        0.00080
Ananascomosus_Aco027001.1       TMHMM2.0        outside      1   717
```

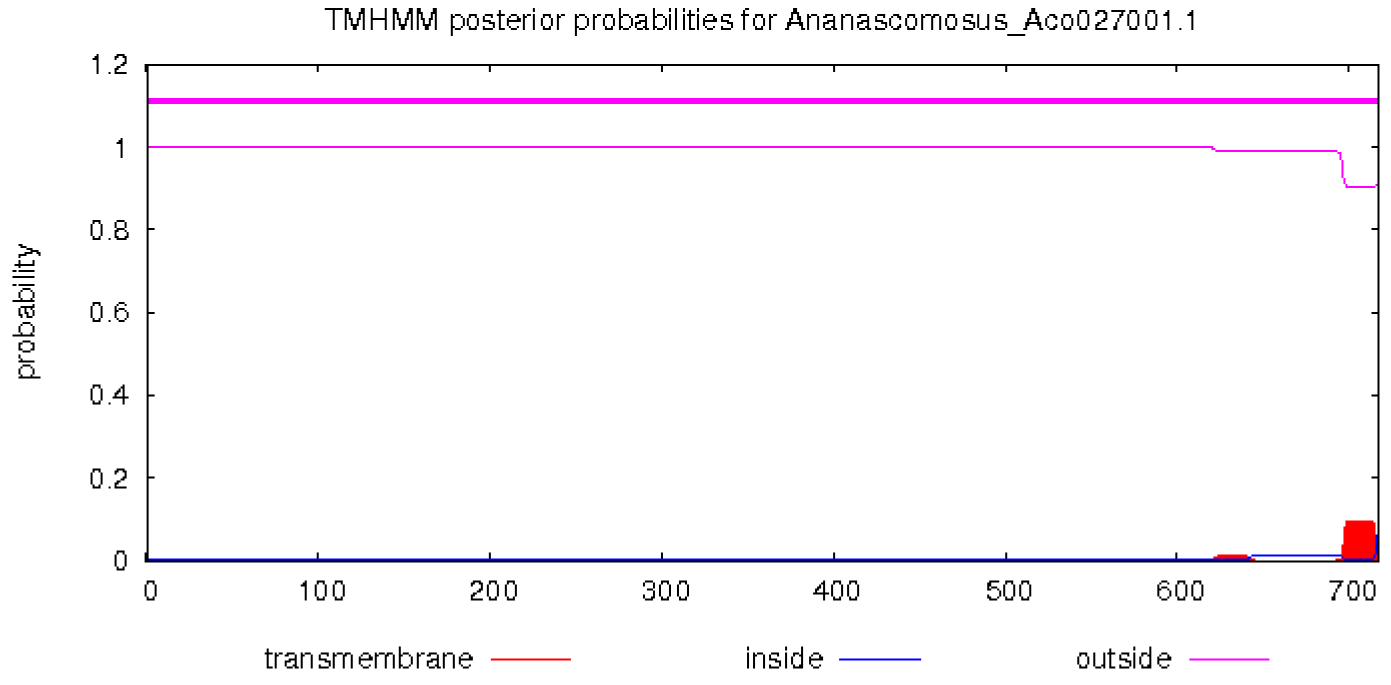

plot in postscript, script for making the plot in gnuplot, data for plot

---

```
# Ananascomosus_Aco028749.1 Length: 362
# Ananascomosus_Aco028749.1 Number of predicted TMHs:  0
# Ananascomosus_Aco028749.1 Exp number of AAs in TMHs: 0.00042
# Ananascomosus_Aco028749.1 Exp number, first 60 AAs:  0
# Ananascomosus_Aco028749.1 Total prob of N-in:        0.01950
Ananascomosus_Aco028749.1       TMHMM2.0        outside      1   362
```

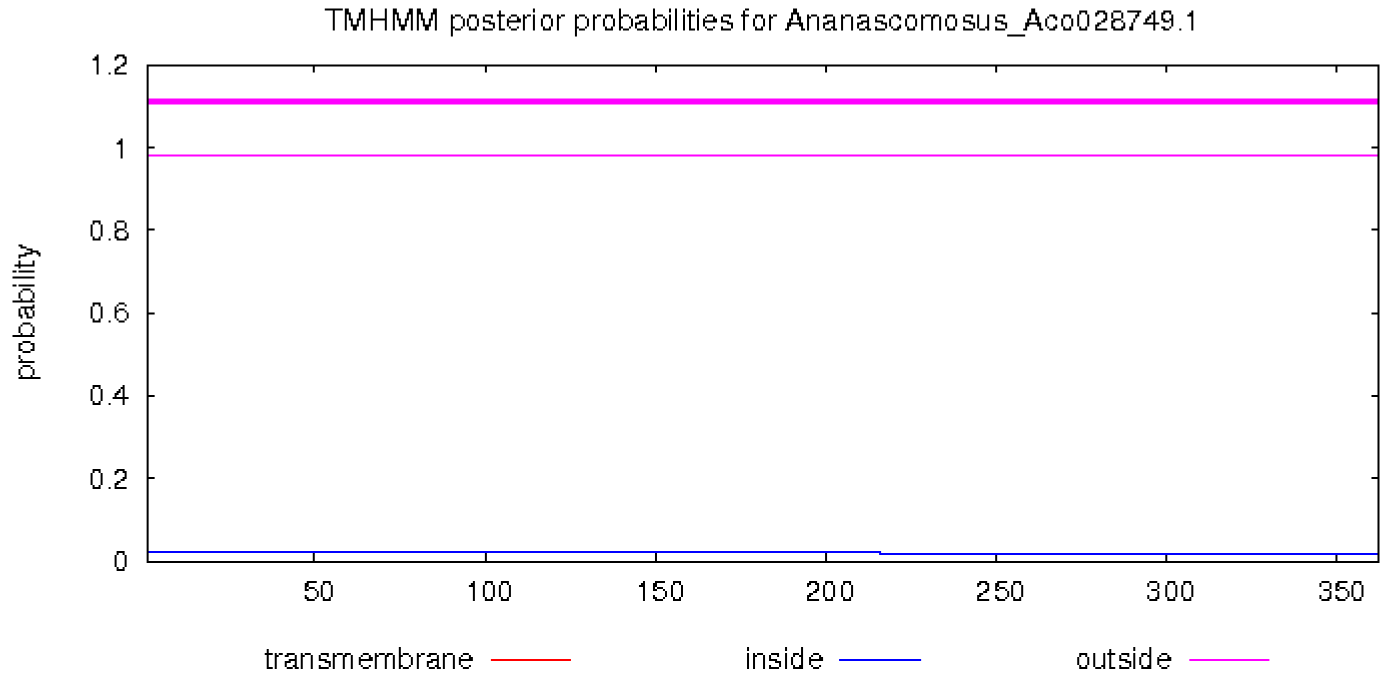

plot in postscript, script for making the plot in gnuplot, data for plot

---

```
# Ananascomosus_Aco029272.1 Length: 469
# Ananascomosus_Aco029272.1 Number of predicted TMHs:  0
```

```
# Ananascomosus_Aco029272.1 Exp number of AAs in TMHs: 0.02773
# Ananascomosus_Aco029272.1 Exp number, first 60 AAs:  0.00147
# Ananascomosus_Aco029272.1 Total prob of N-in:        0.00999
Ananascomosus_Aco029272.1	TMHMM2.0	outside	     1   469
```

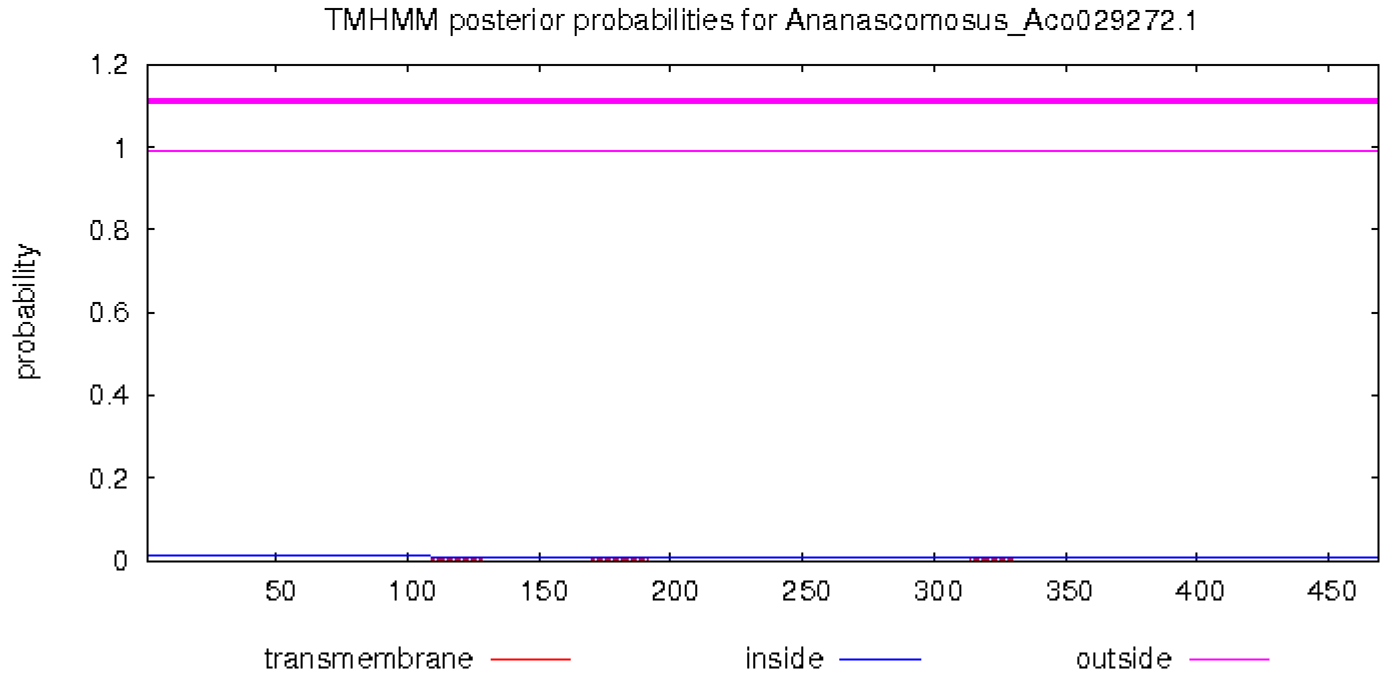

plot in postscript, script for making the plot in gnuplot, data for plot

---

```
# Ananascomosus_Aco031295.1 Length: 319
# Ananascomosus_Aco031295.1 Number of predicted TMHs:  0
# Ananascomosus_Aco031295.1 Exp number of AAs in TMHs: 0.00044
# Ananascomosus_Aco031295.1 Exp number, first 60 AAs:  0
# Ananascomosus_Aco031295.1 Total prob of N-in:        0.05000
Ananascomosus_Aco031295.1	TMHMM2.0	outside	     1   319
```

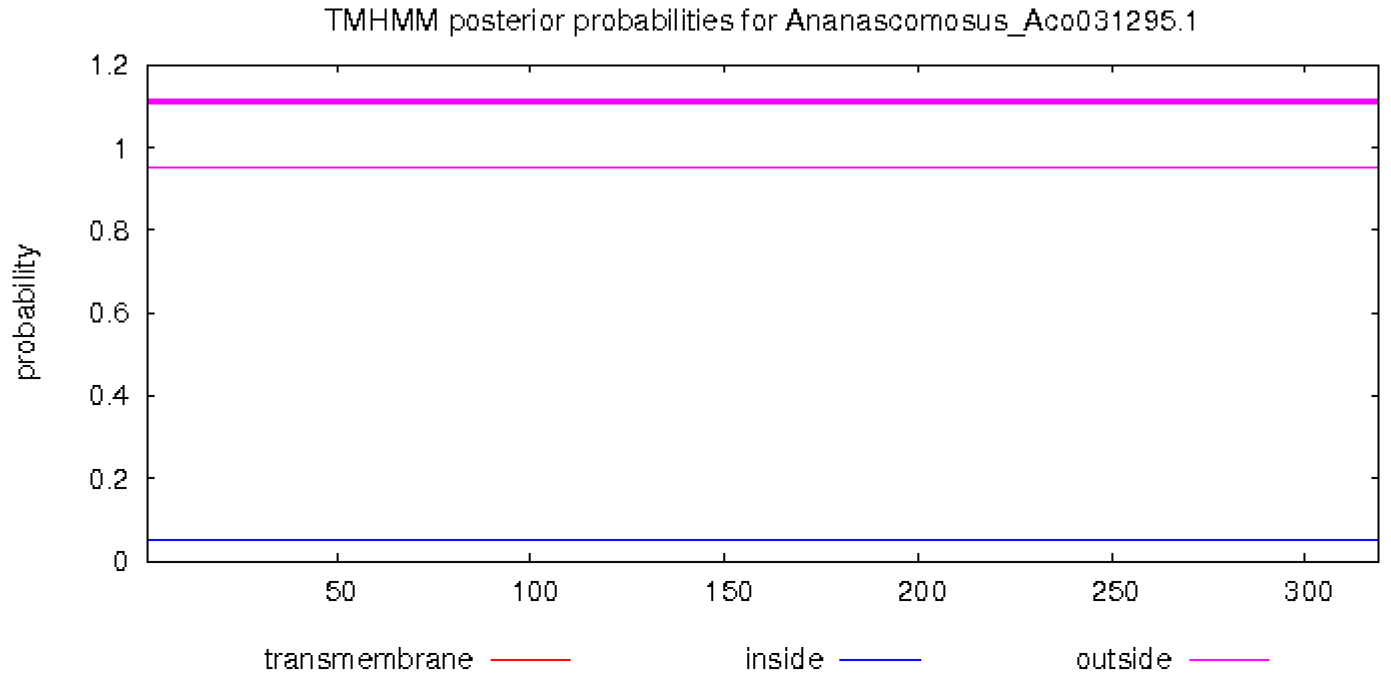

plot in postscript, script for making the plot in gnuplot, data for plot

---

```
# Ananascomosus_Aco031345.1 Length: 311
# Ananascomosus_Aco031345.1 Number of predicted TMHs:  0
```

```
# Ananascomosus_Aco031345.1 Exp number of AAs in TMHs: 0.0238
# Ananascomosus_Aco031345.1 Exp number, first 60 AAs:  0
# Ananascomosus_Aco031345.1 Total prob of N-in:        0.04712
Ananascomosus_Aco031345.1       TMHMM2.0        outside      1   311
```

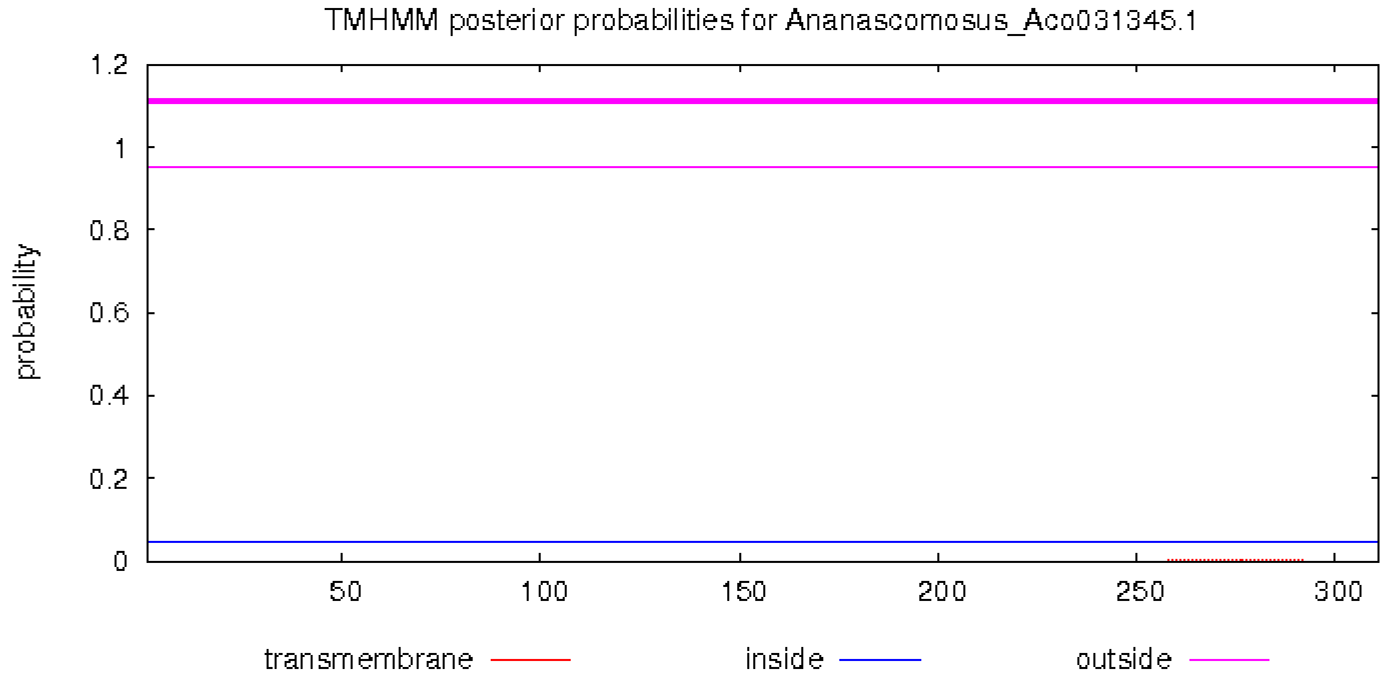

plot in postscript, script for making the plot in gnuplot, data for plot

---

```
# Aquilegiacoerulea_Aqcoe1G029900.1.p Length: 398
# Aquilegiacoerulea_Aqcoe1G029900.1.p Number of predicted TMHs:  0
# Aquilegiacoerulea_Aqcoe1G029900.1.p Exp number of AAs in TMHs: 0.00083
# Aquilegiacoerulea_Aqcoe1G029900.1.p Exp number, first 60 AAs:  0.00011
# Aquilegiacoerulea_Aqcoe1G029900.1.p Total prob of N-in:        0.01973
Aquilegiacoerulea_Aqcoe1G029900.1.p     TMHMM2.0        outside      1   398
```

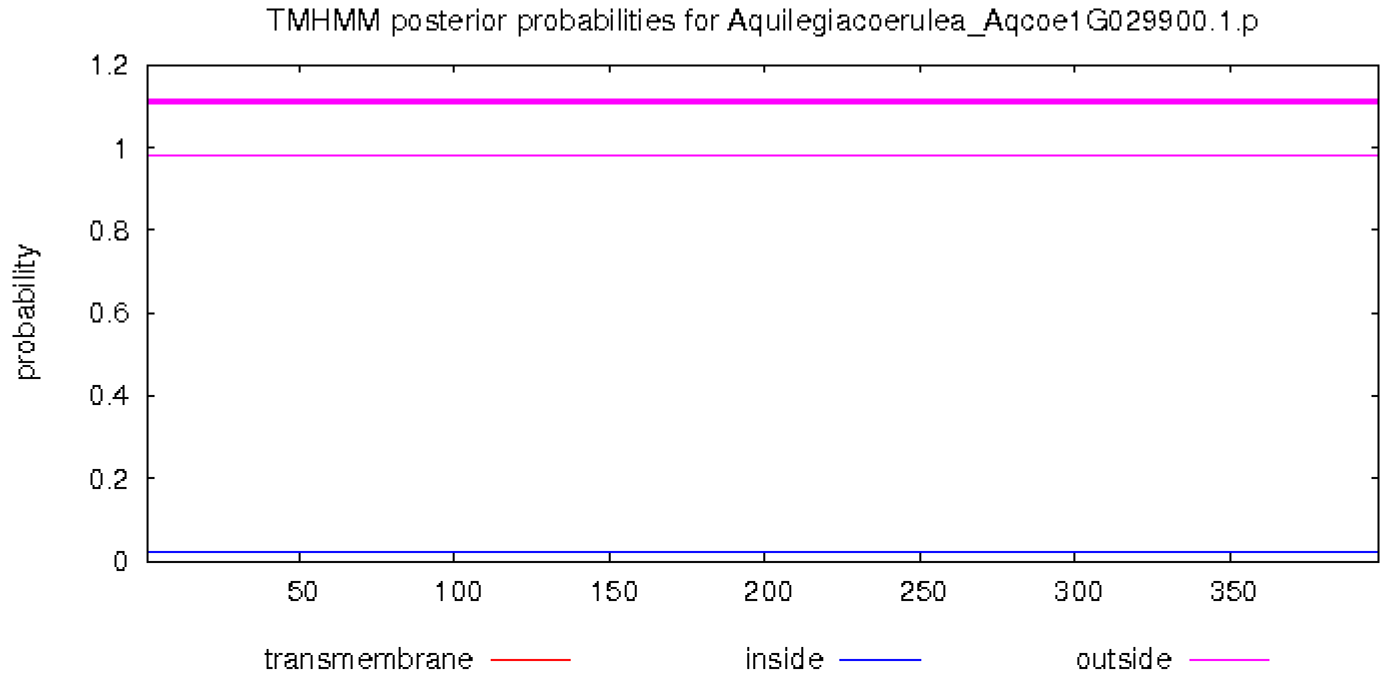

plot in postscript, script for making the plot in gnuplot, data for plot

---

```
# Aquilegiacoerulea_Aqcoe1G058100.1.p Length: 469
# Aquilegiacoerulea_Aqcoe1G058100.1.p Number of predicted TMHs:  0
```

```
# Aquilegiacoerulea_Aqcoe1G058100.1.p Exp number of AAs in TMHs: 0.00473
# Aquilegiacoerulea_Aqcoe1G058100.1.p Exp number, first 60 AAs:  0.00284
# Aquilegiacoerulea_Aqcoe1G058100.1.p Total prob of N-in:        0.05740
Aquilegiacoerulea_Aqcoe1G058100.1.p	TMHMM2.0	outside	     1   469
```

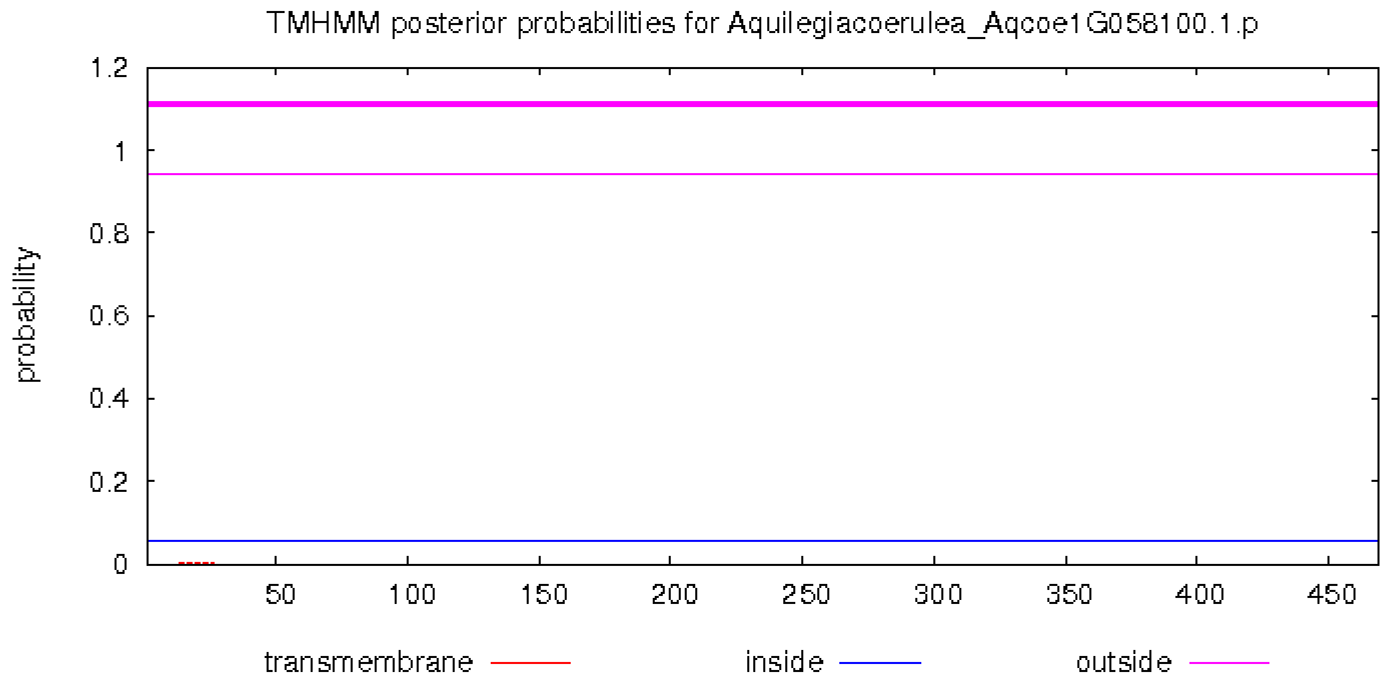

plot in postscript, script for making the plot in gnuplot, data for plot

---

```
# Aquilegiacoerulea_Aqcoe1G110100.1.p Length: 510
# Aquilegiacoerulea_Aqcoe1G110100.1.p Number of predicted TMHs:  0
# Aquilegiacoerulea_Aqcoe1G110100.1.p Exp number of AAs in TMHs: 0.0262
# Aquilegiacoerulea_Aqcoe1G110100.1.p Exp number, first 60 AAs:  0.0014
# Aquilegiacoerulea_Aqcoe1G110100.1.p Total prob of N-in:        0.02130
Aquilegiacoerulea_Aqcoe1G110100.1.p	TMHMM2.0	outside	     1   510
```

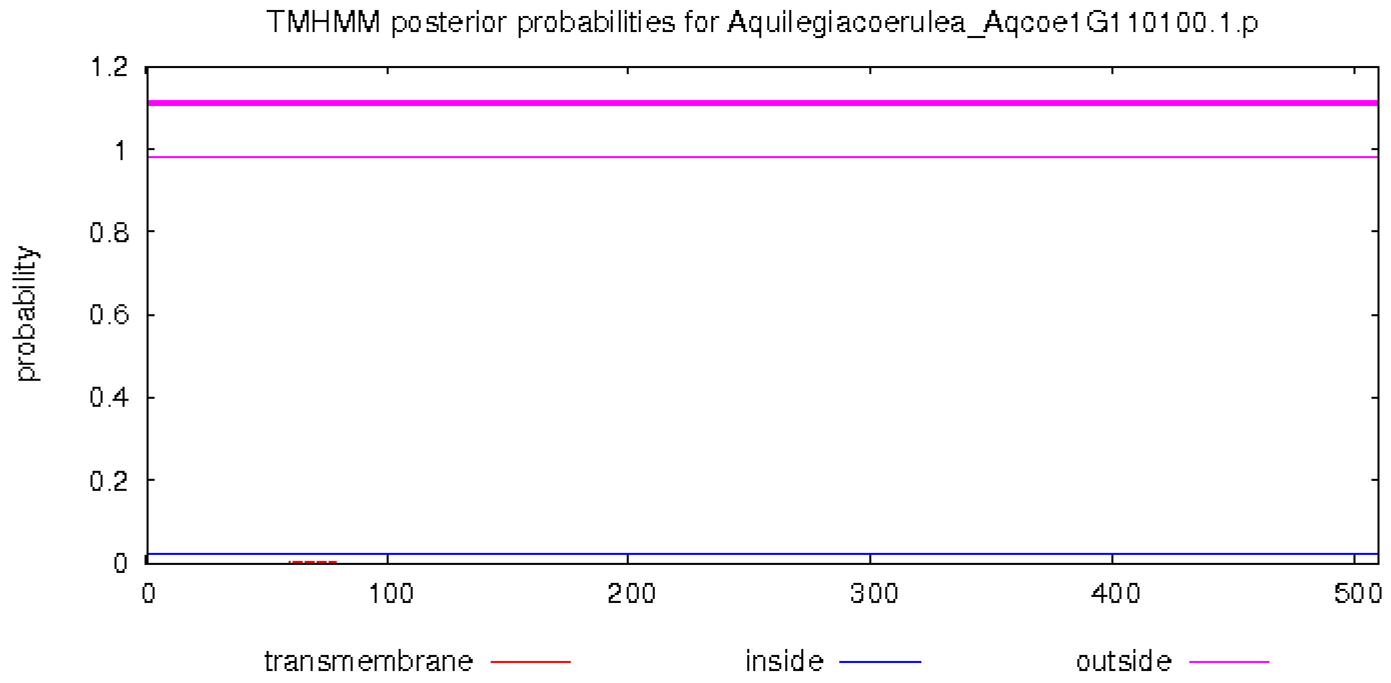

plot in postscript, script for making the plot in gnuplot, data for plot

---

```
# Aquilegiacoerulea_Aqcoe1G126700.1.p Length: 544
# Aquilegiacoerulea_Aqcoe1G126700.1.p Number of predicted TMHs:  1
```

```
# Aquilegiacoerulea_Aqcoe1G126700.1.p Exp number of AAs in TMHs: 20.95987
# Aquilegiacoerulea_Aqcoe1G126700.1.p Exp number, first 60 AAs:  0.00038
# Aquilegiacoerulea_Aqcoe1G126700.1.p Total prob of N-in:        0.00458
Aquilegiacoerulea_Aqcoe1G126700.1.p     TMHMM2.0        outside      1   512
Aquilegiacoerulea_Aqcoe1G126700.1.p     TMHMM2.0        TMhelix    513   535
Aquilegiacoerulea_Aqcoe1G126700.1.p     TMHMM2.0        inside     536   544
```

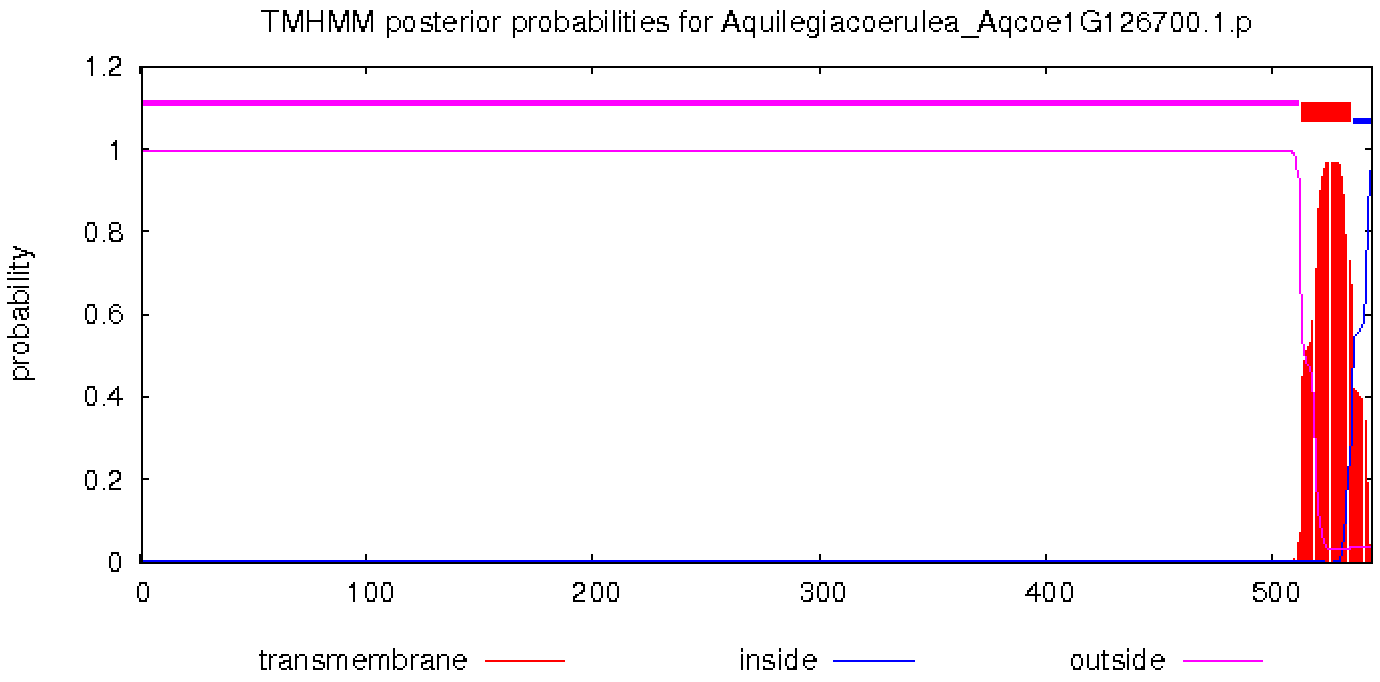

plot in postscript, script for making the plot in gnuplot, data for plot

---

```
# Aquilegiacoerulea_Aqcoe1G154800.1.p Length: 334
# Aquilegiacoerulea_Aqcoe1G154800.1.p Number of predicted TMHs:  0
# Aquilegiacoerulea_Aqcoe1G154800.1.p Exp number of AAs in TMHs: 0.00083
# Aquilegiacoerulea_Aqcoe1G154800.1.p Exp number, first 60 AAs:  0
# Aquilegiacoerulea_Aqcoe1G154800.1.p Total prob of N-in:        0.01147
Aquilegiacoerulea_Aqcoe1G154800.1.p     TMHMM2.0        outside      1   334
```

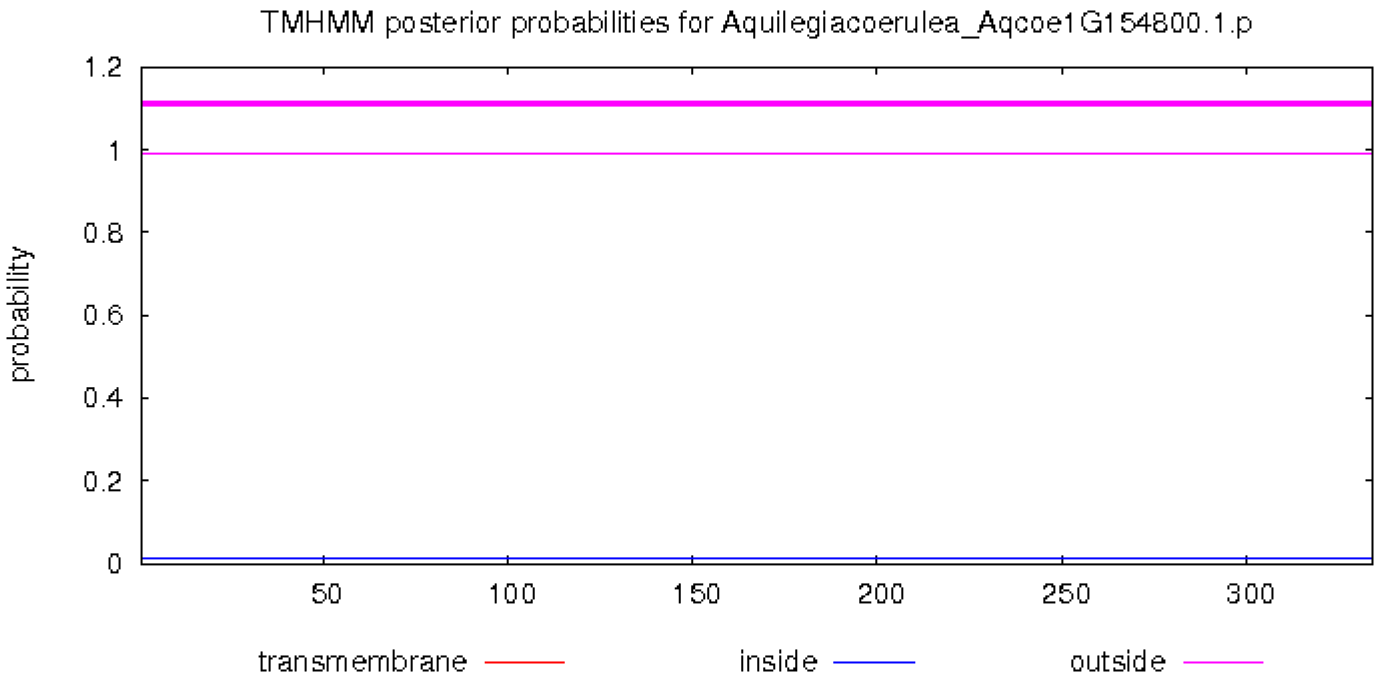

plot in postscript, script for making the plot in gnuplot, data for plot

---

```
# Aquilegiacoerulea_Aqcoe1G177900.1.p Length: 312
# Aquilegiacoerulea_Aqcoe1G177900.1.p Number of predicted TMHs:  0
# Aquilegiacoerulea_Aqcoe1G177900.1.p Exp number of AAs in TMHs: 0.00054
# Aquilegiacoerulea_Aqcoe1G177900.1.p Exp number, first 60 AAs:  0
# Aquilegiacoerulea_Aqcoe1G177900.1.p Total prob of N-in:        0.03076
Aquilegiacoerulea_Aqcoe1G177900.1.p     TMHMM2.0        outside      1   312
```

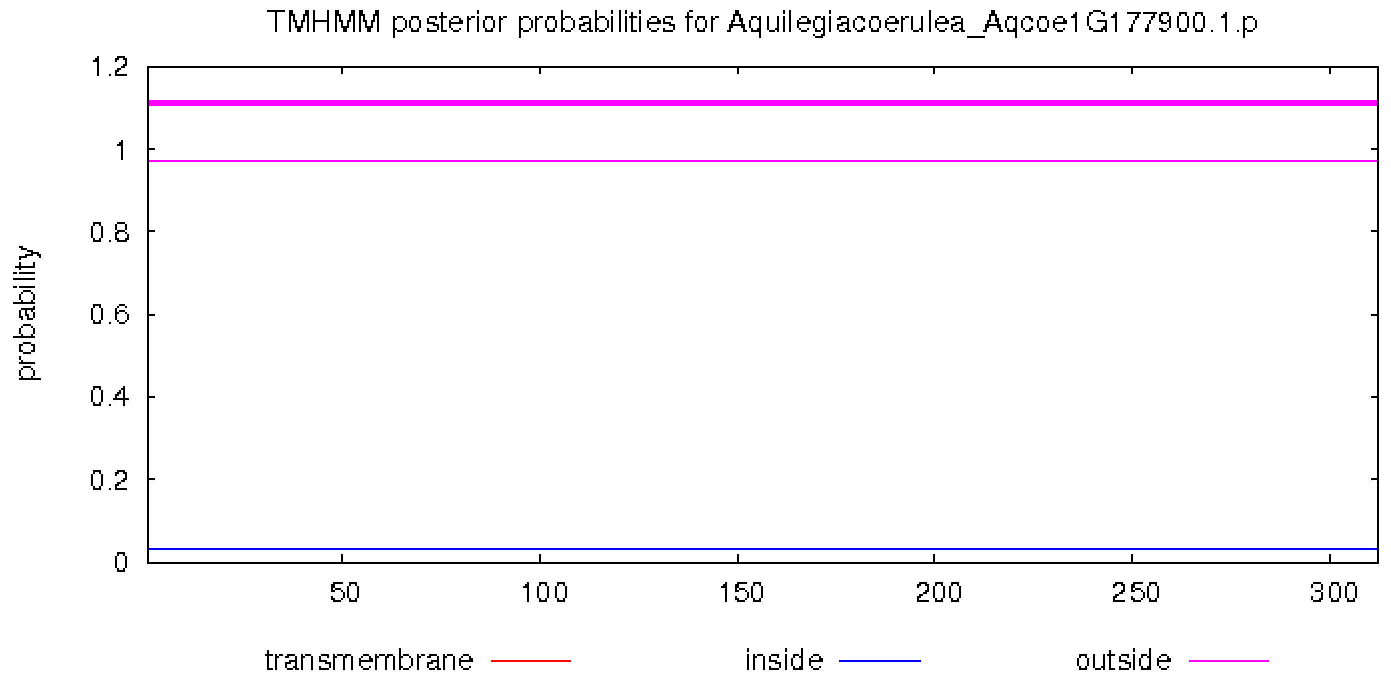

plot in postscript, script for making the plot in gnuplot, data for plot

---

```
# Aquilegiacoerulea_Aqcoe1G182600.1.p Length: 490
# Aquilegiacoerulea_Aqcoe1G182600.1.p Number of predicted TMHs:  0
# Aquilegiacoerulea_Aqcoe1G182600.1.p Exp number of AAs in TMHs: 0.00035
# Aquilegiacoerulea_Aqcoe1G182600.1.p Exp number, first 60 AAs:  0
# Aquilegiacoerulea_Aqcoe1G182600.1.p Total prob of N-in:        0.01321
Aquilegiacoerulea_Aqcoe1G182600.1.p     TMHMM2.0        outside      1   490
```

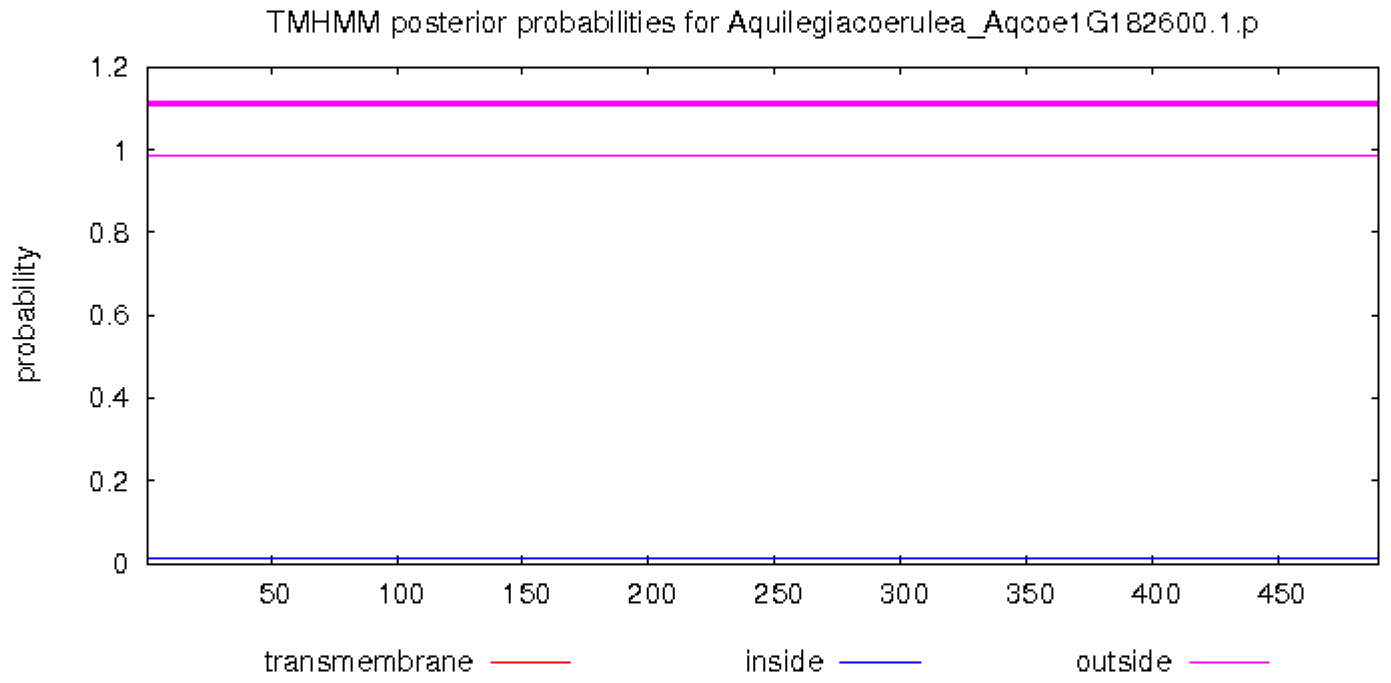

plot in postscript, script for making the plot in gnuplot, data for plot

---

```
# Aquilegiacoerulea_Aqcoe1G251800.1.p Length: 288
# Aquilegiacoerulea_Aqcoe1G251800.1.p Number of predicted TMHs:  0
# Aquilegiacoerulea_Aqcoe1G251800.1.p Exp number of AAs in TMHs: 0.00274
# Aquilegiacoerulea_Aqcoe1G251800.1.p Exp number, first 60 AAs:  0
# Aquilegiacoerulea_Aqcoe1G251800.1.p Total prob of N-in:        0.07373
Aquilegiacoerulea_Aqcoe1G251800.1.p     TMHMM2.0        outside      1   288
```

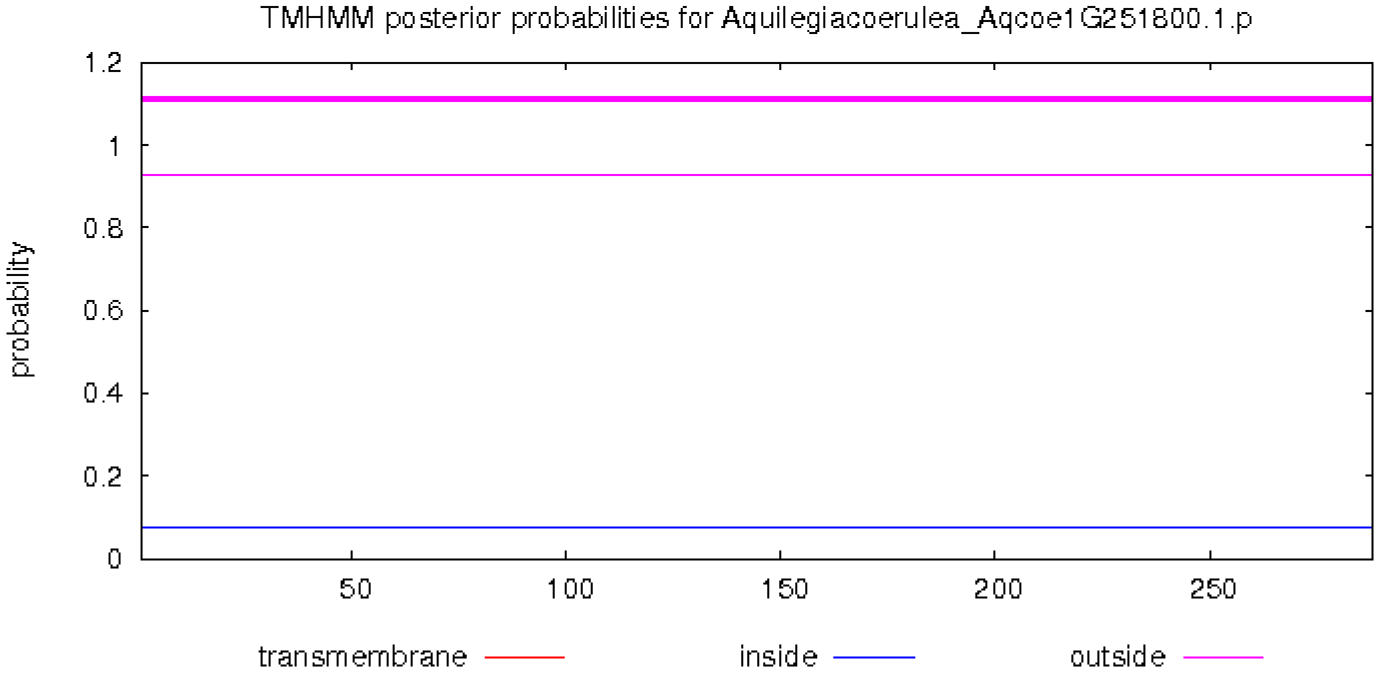

plot in postscript, script for making the plot in gnuplot, data for plot

---

```
# Aquilegiacoerulea_Aqcoe1G321200.1.p Length: 330
# Aquilegiacoerulea_Aqcoe1G321200.1.p Number of predicted TMHs:  0
# Aquilegiacoerulea_Aqcoe1G321200.1.p Exp number of AAs in TMHs: 0.00037
# Aquilegiacoerulea_Aqcoe1G321200.1.p Exp number, first 60 AAs:  0
# Aquilegiacoerulea_Aqcoe1G321200.1.p Total prob of N-in:        0.00899
Aquilegiacoerulea_Aqcoe1G321200.1.p     TMHMM2.0        outside      1   330
```

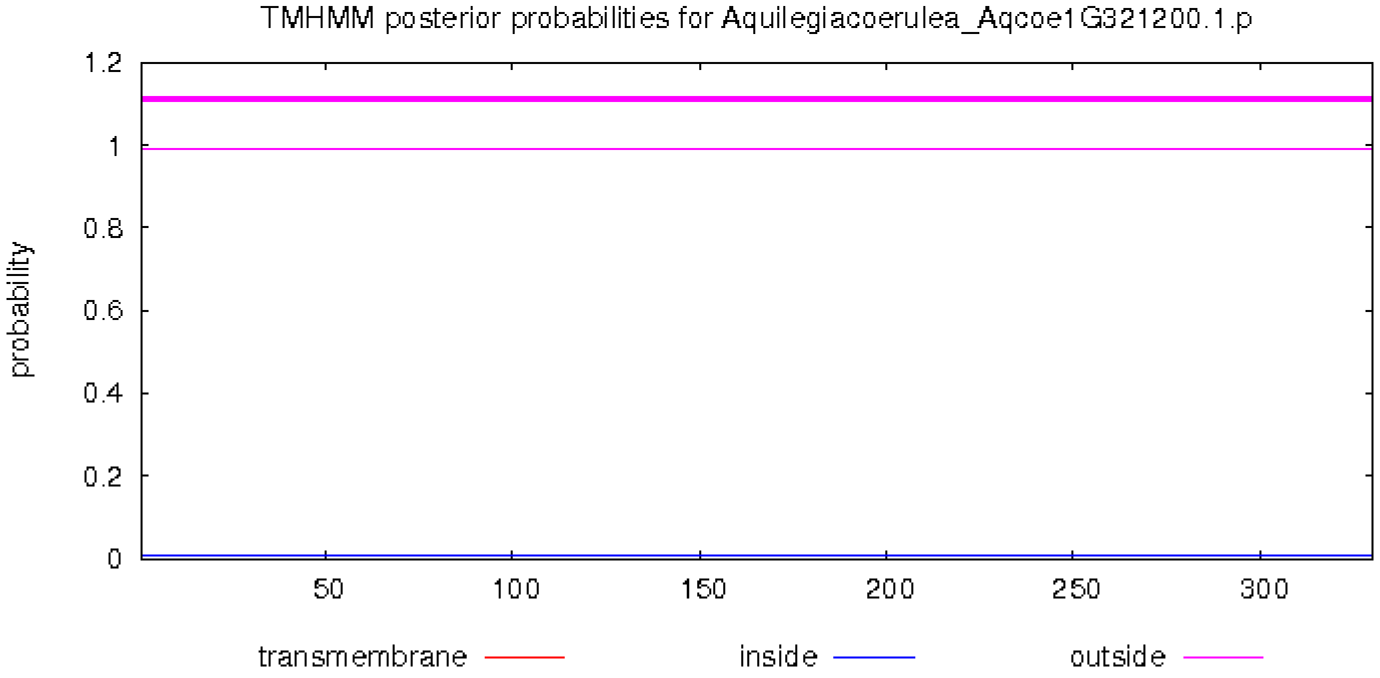

plot in postscript, script for making the plot in gnuplot, data for plot

---

```
# Aquilegiacoerulea_Aqcoe1G321300.1.p Length: 343
# Aquilegiacoerulea_Aqcoe1G321300.1.p Number of predicted TMHs:  0
# Aquilegiacoerulea_Aqcoe1G321300.1.p Exp number of AAs in TMHs: 0
# Aquilegiacoerulea_Aqcoe1G321300.1.p Exp number, first 60 AAs:  0
# Aquilegiacoerulea_Aqcoe1G321300.1.p Total prob of N-in:        0.01113
Aquilegiacoerulea_Aqcoe1G321300.1.p     TMHMM2.0        outside      1   343
```

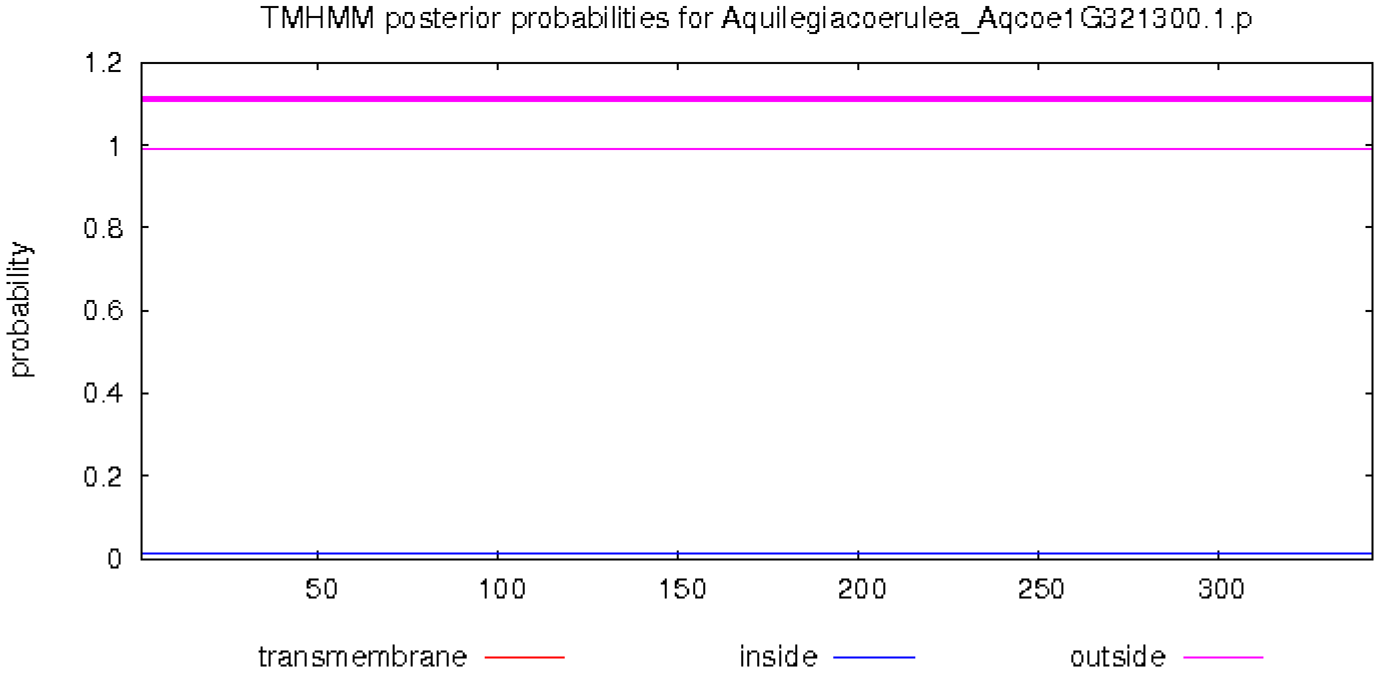

plot in postscript, script for making the plot in gnuplot, data for plot

---

```
# Aquilegiacoerulea_Aqcoe1G351500.1.p Length: 489
# Aquilegiacoerulea_Aqcoe1G351500.1.p Number of predicted TMHs:  0
# Aquilegiacoerulea_Aqcoe1G351500.1.p Exp number of AAs in TMHs: 0.00037
# Aquilegiacoerulea_Aqcoe1G351500.1.p Exp number, first 60 AAs:  7e-05
# Aquilegiacoerulea_Aqcoe1G351500.1.p Total prob of N-in:        0.00023
Aquilegiacoerulea_Aqcoe1G351500.1.p     TMHMM2.0        outside      1   489
```

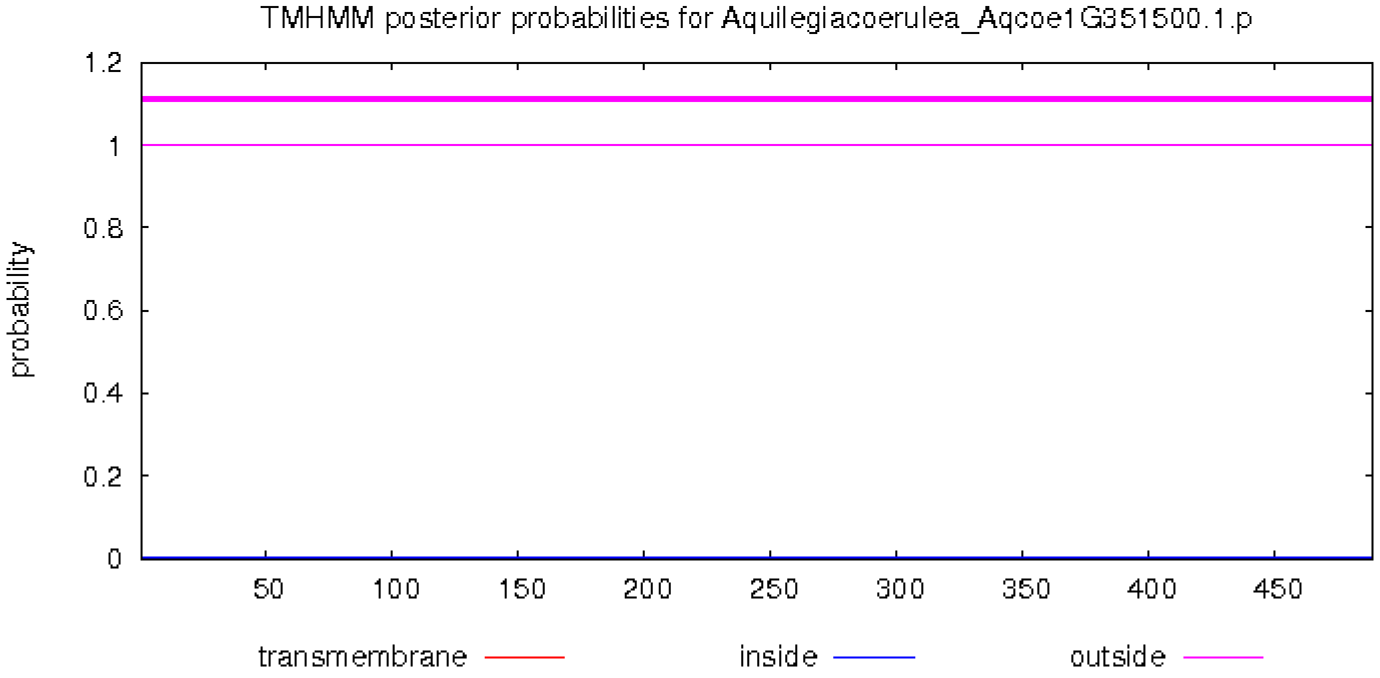

plot in postscript, script for making the plot in gnuplot, data for plot

---

```
# Aquilegiacoerulea_Aqcoe1G372100.1.p Length: 543
# Aquilegiacoerulea_Aqcoe1G372100.1.p Number of predicted TMHs:  1
# Aquilegiacoerulea_Aqcoe1G372100.1.p Exp number of AAs in TMHs: 20.35644
# Aquilegiacoerulea_Aqcoe1G372100.1.p Exp number, first 60 AAs:  0.00028
# Aquilegiacoerulea_Aqcoe1G372100.1.p Total prob of N-in:        0.00050
Aquilegiacoerulea_Aqcoe1G372100.1.p     TMHMM2.0        outside      1   523
Aquilegiacoerulea_Aqcoe1G372100.1.p     TMHMM2.0        TMhelix    524   541
Aquilegiacoerulea_Aqcoe1G372100.1.p     TMHMM2.0        inside     542   543
```

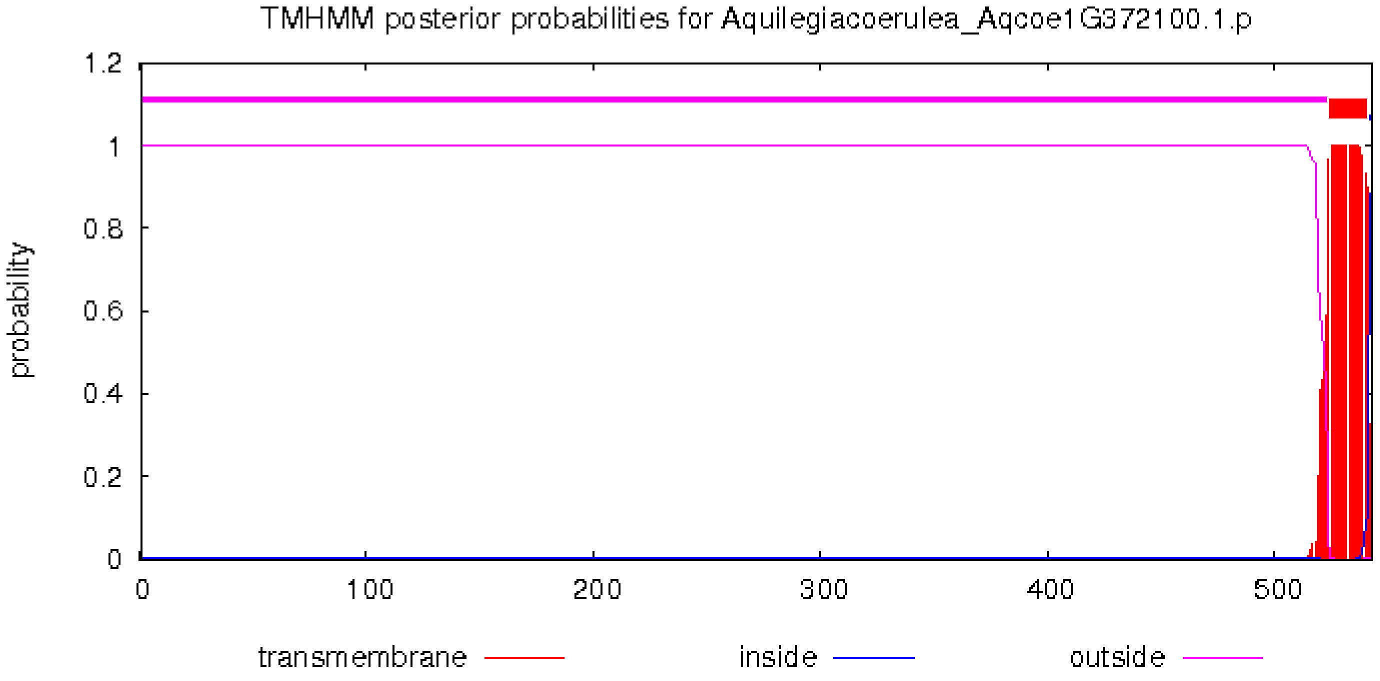

plot in postscript, script for making the plot in gnuplot, data for plot

---

```
# Aquilegiacoerulea_Aqcoe1G373200.1.p Length: 246
# Aquilegiacoerulea_Aqcoe1G373200.1.p Number of predicted TMHs:  0
# Aquilegiacoerulea_Aqcoe1G373200.1.p Exp number of AAs in TMHs: 0.00045
# Aquilegiacoerulea_Aqcoe1G373200.1.p Exp number, first 60 AAs:  0.00012
# Aquilegiacoerulea_Aqcoe1G373200.1.p Total prob of N-in:        0.00247
Aquilegiacoerulea_Aqcoe1G373200.1.p     TMHMM2.0        outside      1   246
```

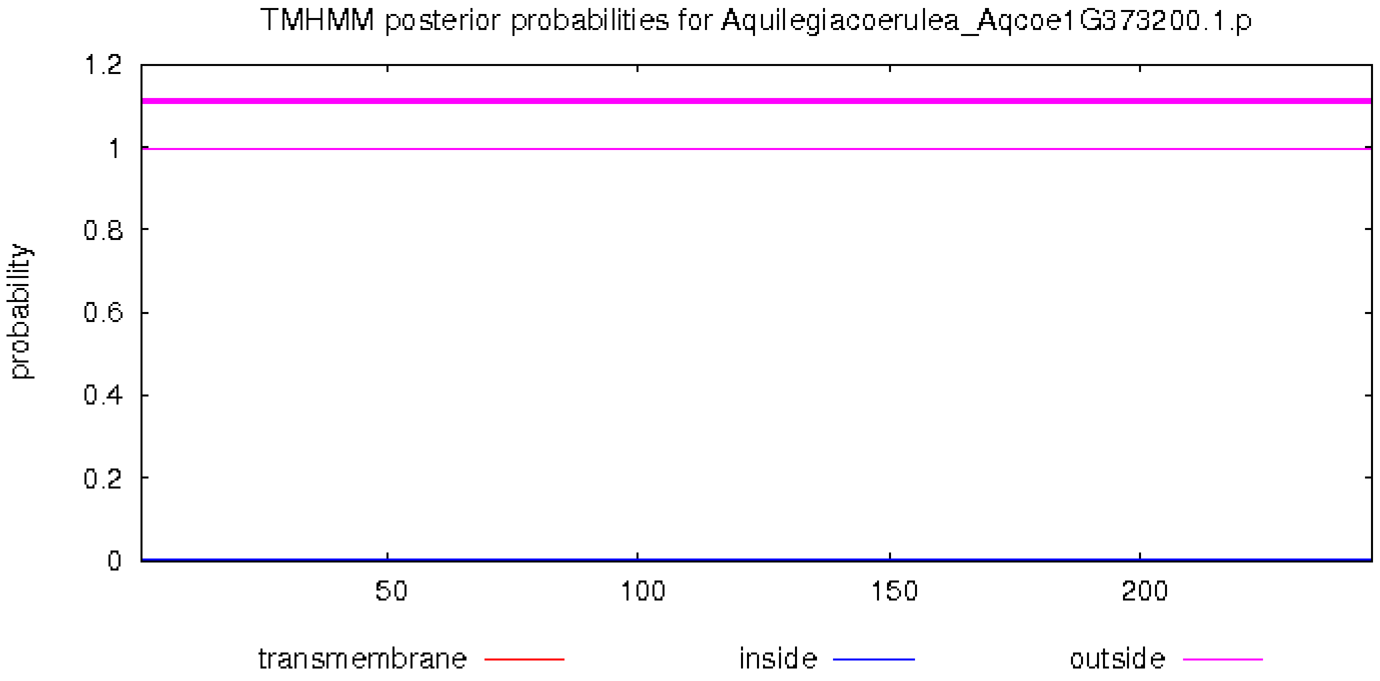

plot in postscript, script for making the plot in gnuplot, data for plot

```
# Aquilegiacoerulea_Aqcoe1G373300.1.p Length: 413
# Aquilegiacoerulea_Aqcoe1G373300.1.p Number of predicted TMHs:  0
# Aquilegiacoerulea_Aqcoe1G373300.1.p Exp number of AAs in TMHs: 0.24249
# Aquilegiacoerulea_Aqcoe1G373300.1.p Exp number, first 60 AAs:  0.24214
# Aquilegiacoerulea_Aqcoe1G373300.1.p Total prob of N-in:        0.02688
Aquilegiacoerulea_Aqcoe1G373300.1.p     TMHMM2.0        outside      1   413
```

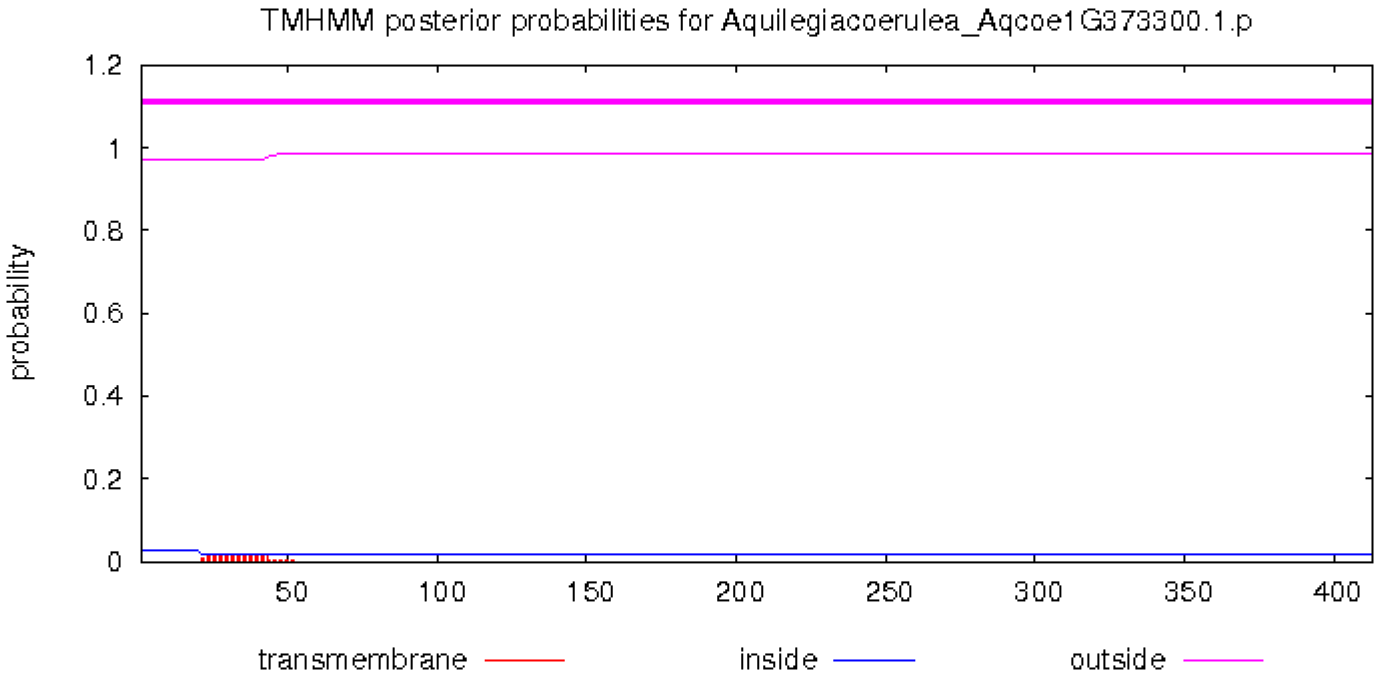

plot in postscript, script for making the plot in gnuplot, data for plot

```
# Aquilegiacoerulea_Aqcoe1G373400.1.p Length: 566
# Aquilegiacoerulea_Aqcoe1G373400.1.p Number of predicted TMHs:  1
# Aquilegiacoerulea_Aqcoe1G373400.1.p Exp number of AAs in TMHs: 20.36488
# Aquilegiacoerulea_Aqcoe1G373400.1.p Exp number, first 60 AAs:  0.00019
# Aquilegiacoerulea_Aqcoe1G373400.1.p Total prob of N-in:        0.00040
Aquilegiacoerulea_Aqcoe1G373400.1.p     TMHMM2.0        outside      1   546
Aquilegiacoerulea_Aqcoe1G373400.1.p     TMHMM2.0        TMhelix    547   564
Aquilegiacoerulea_Aqcoe1G373400.1.p     TMHMM2.0        inside     565   566
```

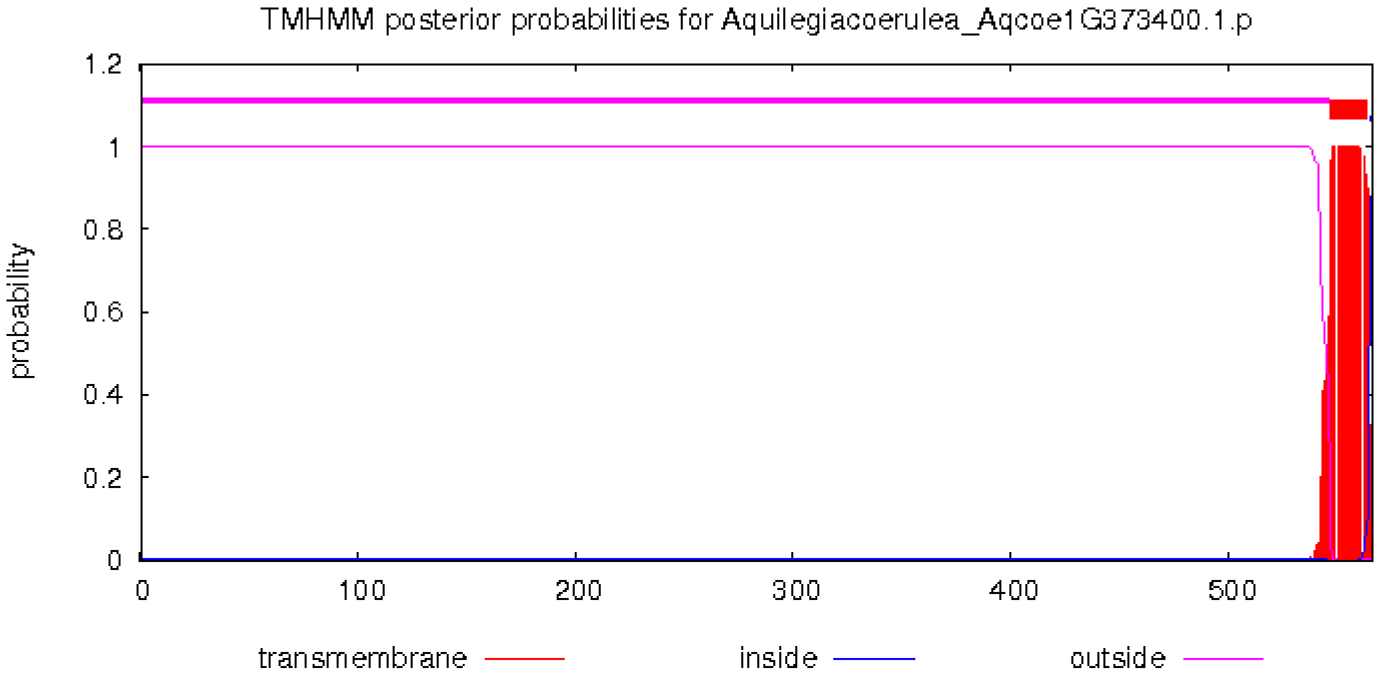

plot in postscript, script for making the plot in gnuplot, data for plot

```
# Aquilegiacoerulea_Aqcoe1G398000.1.p Length: 244
# Aquilegiacoerulea_Aqcoe1G398000.1.p Number of predicted TMHs:  0
# Aquilegiacoerulea_Aqcoe1G398000.1.p Exp number of AAs in TMHs: 9e-05
# Aquilegiacoerulea_Aqcoe1G398000.1.p Exp number, first 60 AAs:  0
# Aquilegiacoerulea_Aqcoe1G398000.1.p Total prob of N-in:        0.00471
Aquilegiacoerulea_Aqcoe1G398000.1.p     TMHMM2.0        outside      1   244
```

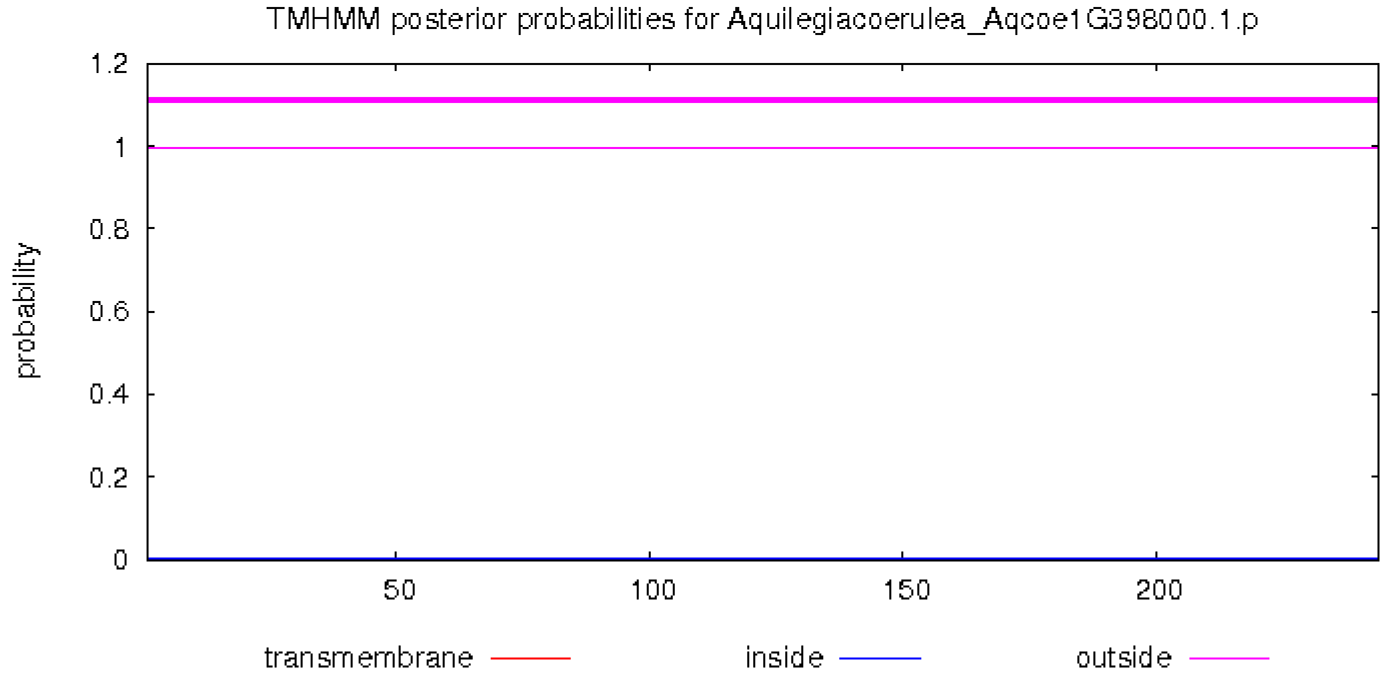

plot in postscript, script for making the plot in gnuplot, data for plot

```
# Aquilegiacoerulea_Aqcoe1G439000.1.p Length: 275
# Aquilegiacoerulea_Aqcoe1G439000.1.p Number of predicted TMHs:  0
# Aquilegiacoerulea_Aqcoe1G439000.1.p Exp number of AAs in TMHs: 0
# Aquilegiacoerulea_Aqcoe1G439000.1.p Exp number, first 60 AAs:  0
# Aquilegiacoerulea_Aqcoe1G439000.1.p Total prob of N-in:        0.00632
Aquilegiacoerulea_Aqcoe1G439000.1.p     TMHMM2.0        outside      1   275
```

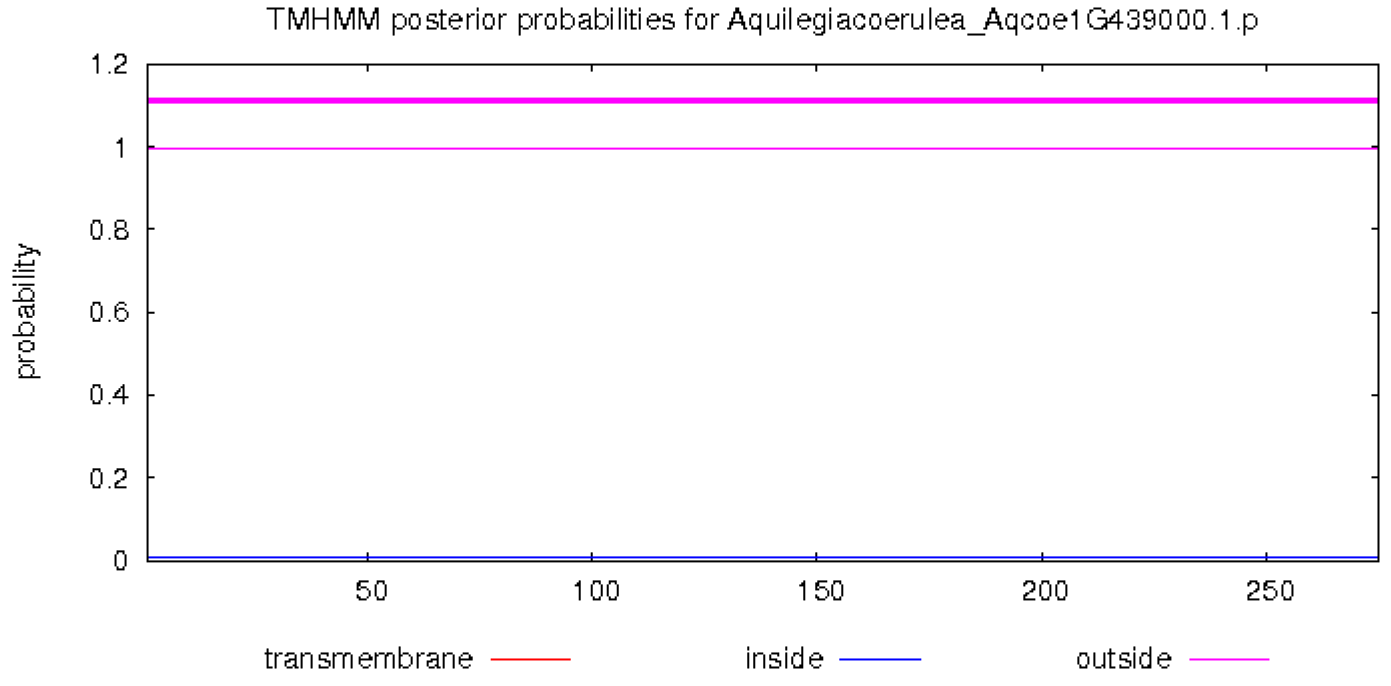

plot in postscript, script for making the plot in gnuplot, data for plot

```
# Aquilegiacoerulea_Aqcoe1G457300.1.p Length: 461
# Aquilegiacoerulea_Aqcoe1G457300.1.p Number of predicted TMHs:  0
# Aquilegiacoerulea_Aqcoe1G457300.1.p Exp number of AAs in TMHs: 0.02894
# Aquilegiacoerulea_Aqcoe1G457300.1.p Exp number, first 60 AAs:  0.02764
# Aquilegiacoerulea_Aqcoe1G457300.1.p Total prob of N-in:        0.01362
Aquilegiacoerulea_Aqcoe1G457300.1.p     TMHMM2.0        outside      1   461
```

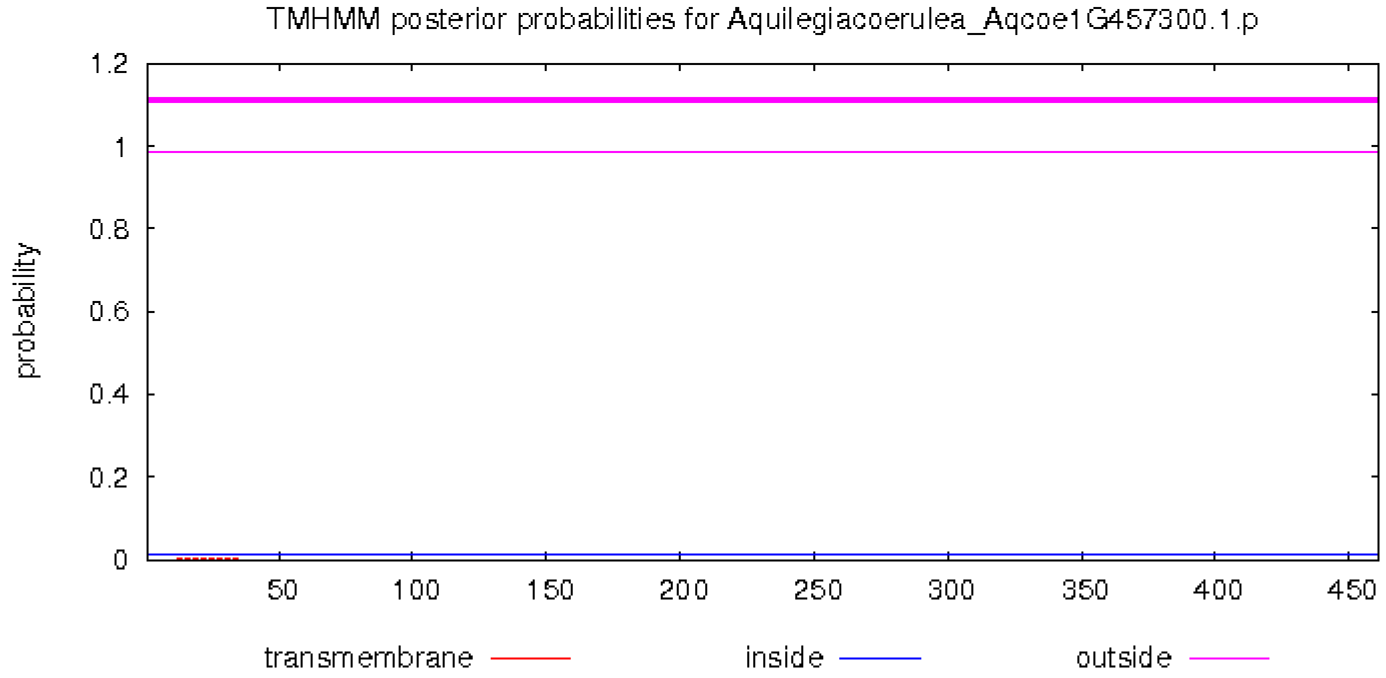

plot in postscript, script for making the plot in gnuplot, data for plot

```
# Aquilegiacoerulea_Aqcoe1G463400.1.p Length: 461
# Aquilegiacoerulea_Aqcoe1G463400.1.p Number of predicted TMHs:  0
# Aquilegiacoerulea_Aqcoe1G463400.1.p Exp number of AAs in TMHs: 0.02931
# Aquilegiacoerulea_Aqcoe1G463400.1.p Exp number, first 60 AAs:  0.02764
# Aquilegiacoerulea_Aqcoe1G463400.1.p Total prob of N-in:        0.01363
Aquilegiacoerulea_Aqcoe1G463400.1.p     TMHMM2.0        outside      1   461
```

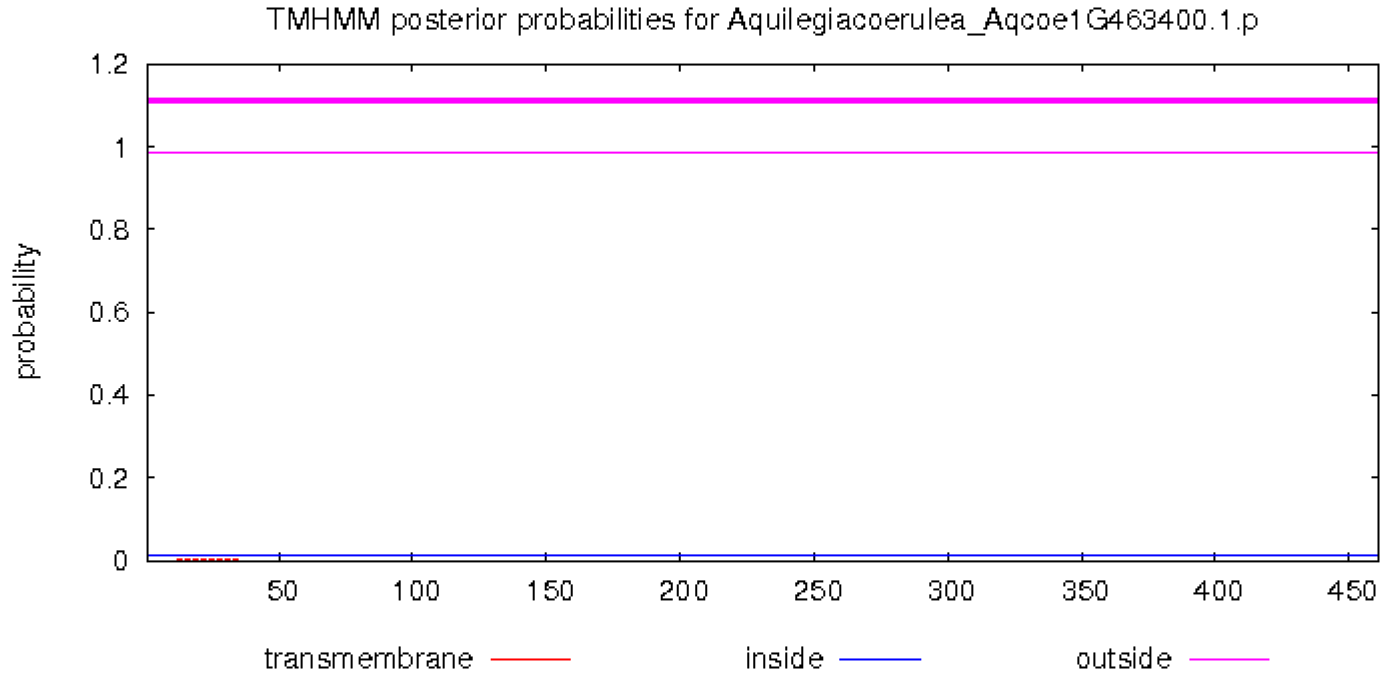

plot in postscript, script for making the plot in gnuplot, data for plot

---

```
# Aquilegiacoerulea_Aqcoe1G496900.1.p Length: 357
# Aquilegiacoerulea_Aqcoe1G496900.1.p Number of predicted TMHs:  0
# Aquilegiacoerulea_Aqcoe1G496900.1.p Exp number of AAs in TMHs: 0.00042
# Aquilegiacoerulea_Aqcoe1G496900.1.p Exp number, first 60 AAs:  0.00042
# Aquilegiacoerulea_Aqcoe1G496900.1.p Total prob of N-in:        0.02562
Aquilegiacoerulea_Aqcoe1G496900.1.p     TMHMM2.0        outside      1   357
```

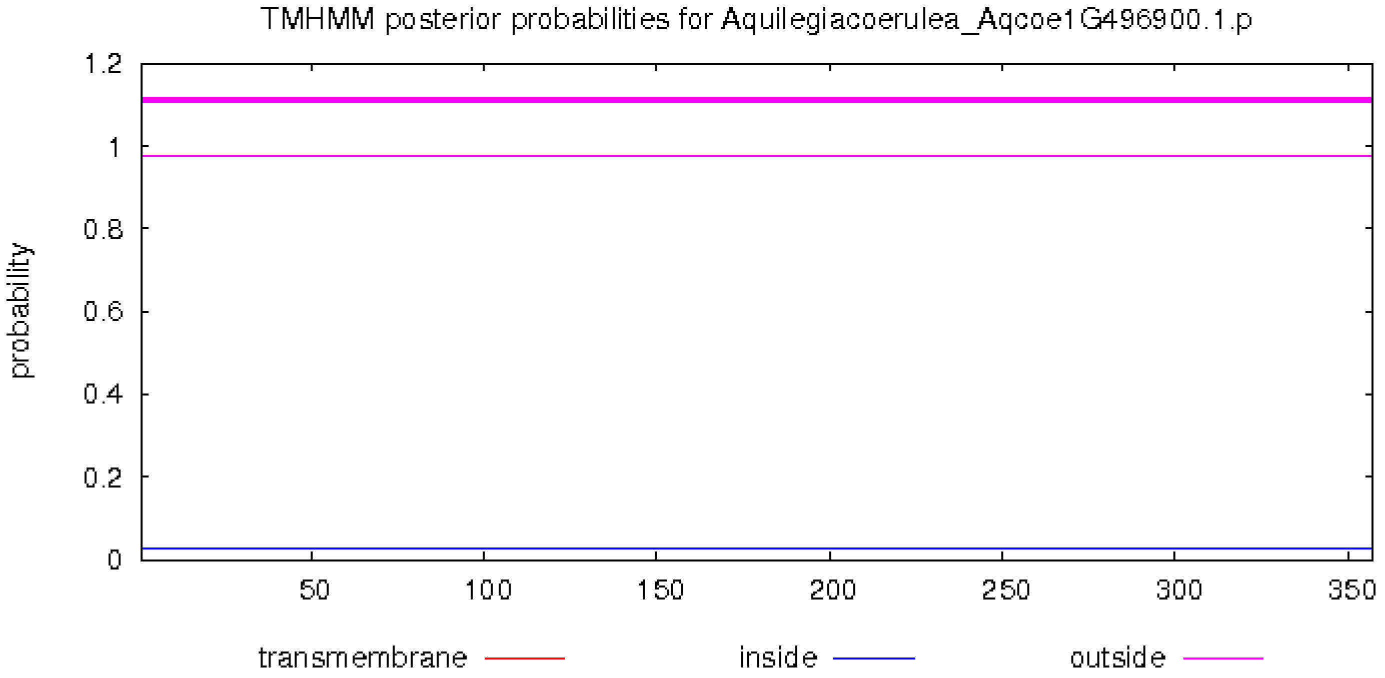

plot in postscript, script for making the plot in gnuplot, data for plot

---

```
# Aquilegiacoerulea_Aqcoe2G001300.1.p Length: 444
# Aquilegiacoerulea_Aqcoe2G001300.1.p Number of predicted TMHs:  0
# Aquilegiacoerulea_Aqcoe2G001300.1.p Exp number of AAs in TMHs: 0.00043
# Aquilegiacoerulea_Aqcoe2G001300.1.p Exp number, first 60 AAs:  0
# Aquilegiacoerulea_Aqcoe2G001300.1.p Total prob of N-in:        0.00380
Aquilegiacoerulea_Aqcoe2G001300.1.p     TMHMM2.0        outside      1   444
```

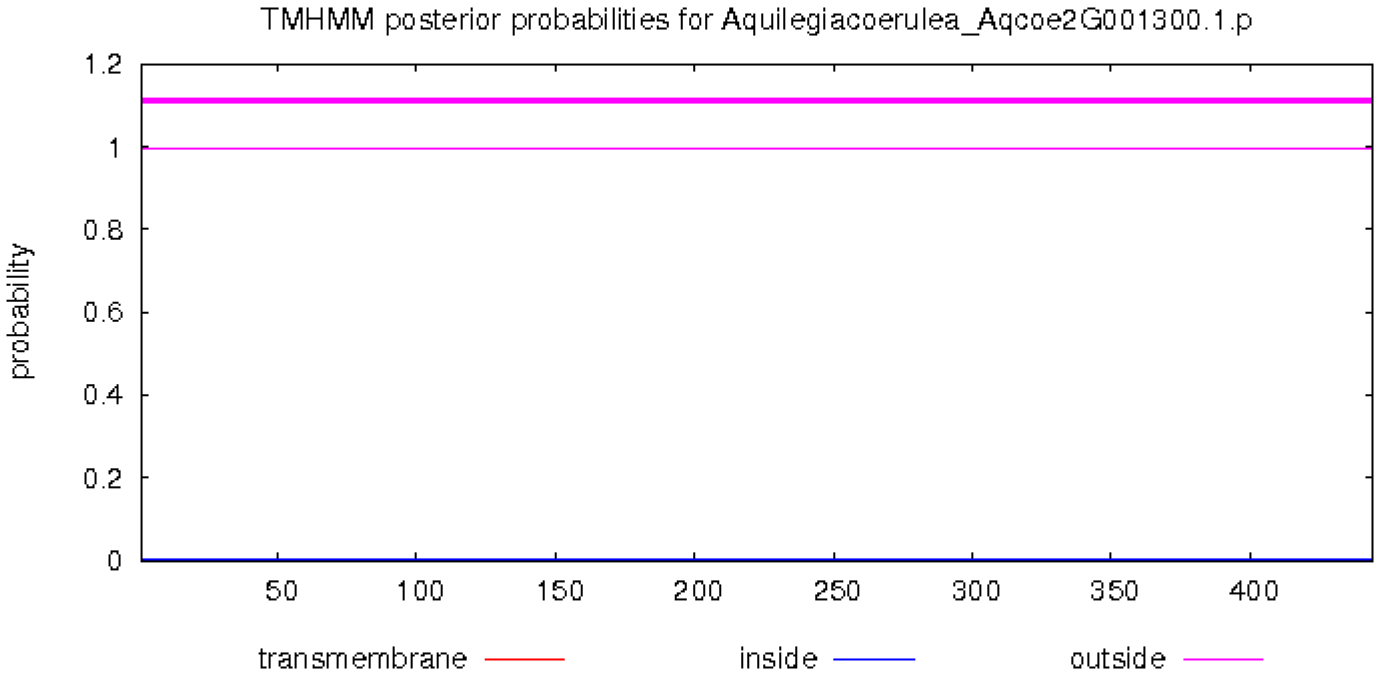

plot in postscript, script for making the plot in gnuplot, data for plot

---

```
# Aquilegiacoerulea_Aqcoe2G001600.1.p Length: 306
# Aquilegiacoerulea_Aqcoe2G001600.1.p Number of predicted TMHs:  0
# Aquilegiacoerulea_Aqcoe2G001600.1.p Exp number of AAs in TMHs: 0.00164
# Aquilegiacoerulea_Aqcoe2G001600.1.p Exp number, first 60 AAs:  0.00127
# Aquilegiacoerulea_Aqcoe2G001600.1.p Total prob of N-in:        0.05680
Aquilegiacoerulea_Aqcoe2G001600.1.p	TMHMM2.0	outside	     1   306
```

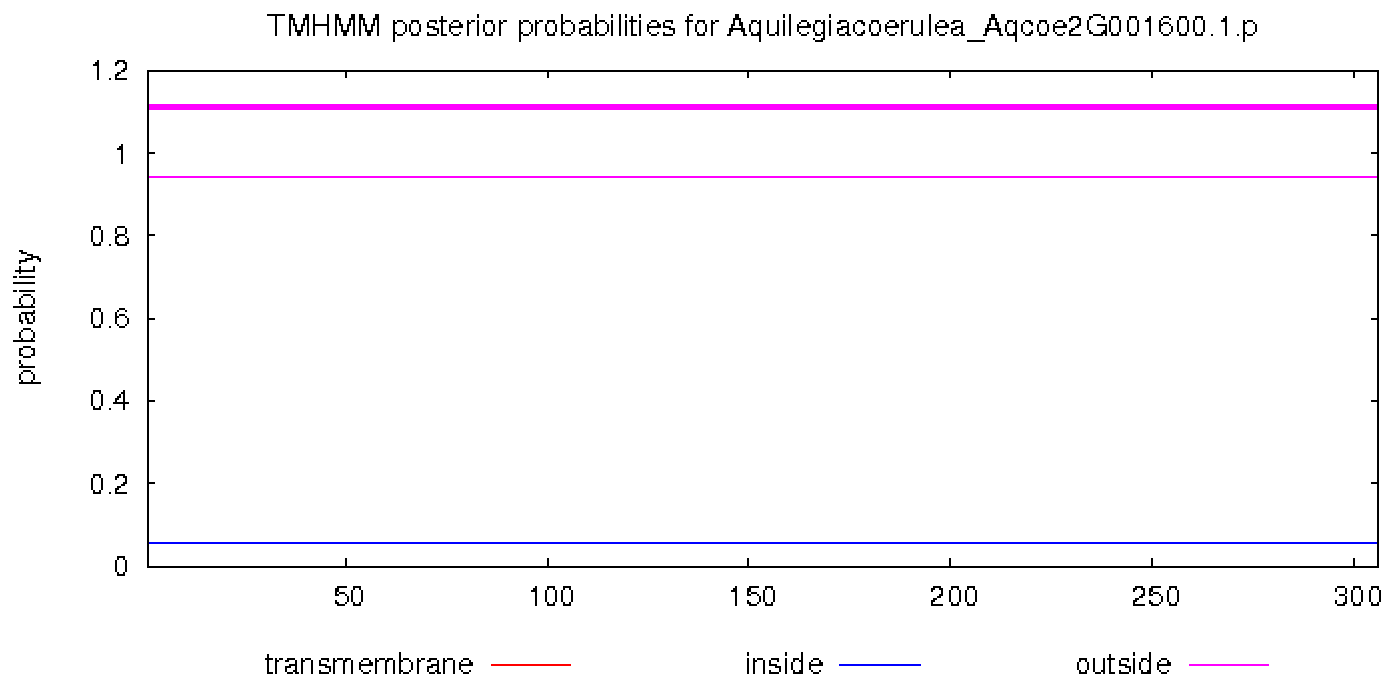

plot in postscript, script for making the plot in gnuplot, data for plot

---

```
# Aquilegiacoerulea_Aqcoe2G158200.1.p Length: 446
# Aquilegiacoerulea_Aqcoe2G158200.1.p Number of predicted TMHs:  1
# Aquilegiacoerulea_Aqcoe2G158200.1.p Exp number of AAs in TMHs: 22.71052
# Aquilegiacoerulea_Aqcoe2G158200.1.p Exp number, first 60 AAs:  0.00129
# Aquilegiacoerulea_Aqcoe2G158200.1.p Total prob of N-in:        0.00949
Aquilegiacoerulea_Aqcoe2G158200.1.p	TMHMM2.0	outside	     1   412
Aquilegiacoerulea_Aqcoe2G158200.1.p	TMHMM2.0	TMhelix	   413   435
Aquilegiacoerulea_Aqcoe2G158200.1.p	TMHMM2.0	inside	   436   446
```

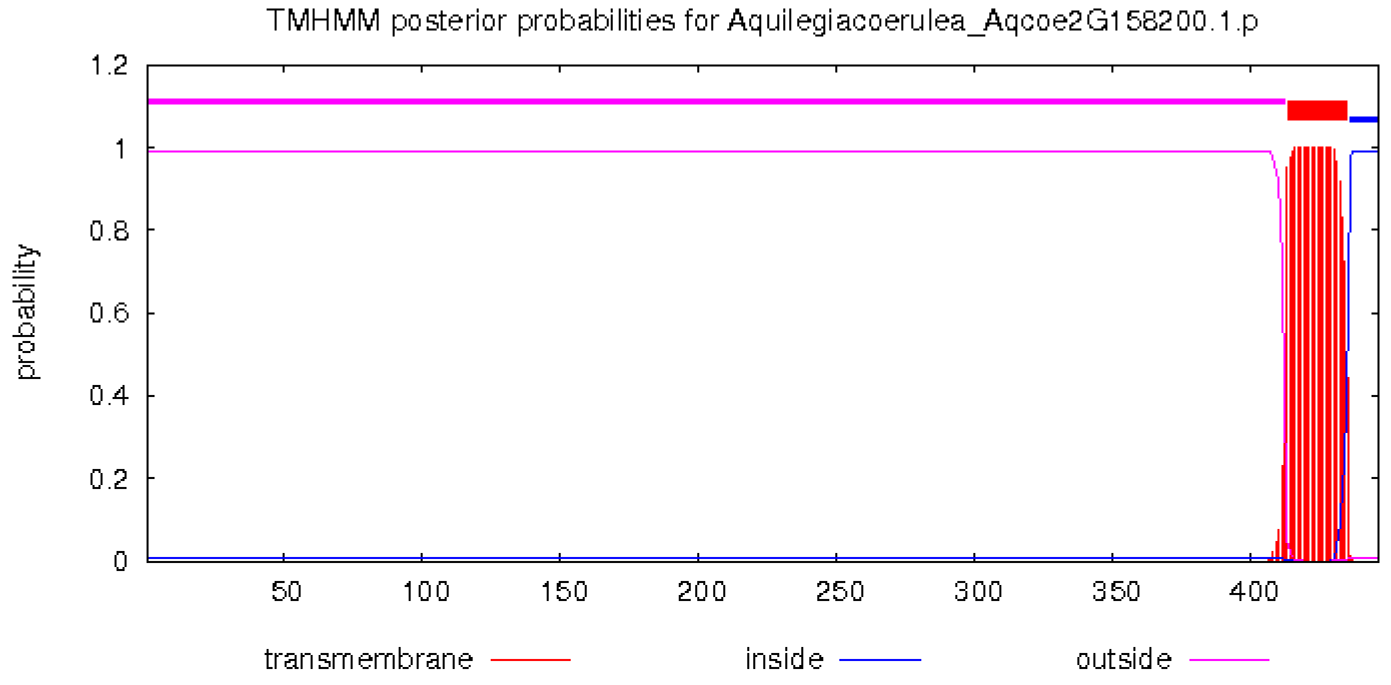

plot in postscript, script for making the plot in gnuplot, data for plot

---

```
# Aquilegiacoerulea_Aqcoe2G306500.1.p Length: 278
# Aquilegiacoerulea_Aqcoe2G306500.1.p Number of predicted TMHs:  0
# Aquilegiacoerulea_Aqcoe2G306500.1.p Exp number of AAs in TMHs: 0.001
# Aquilegiacoerulea_Aqcoe2G306500.1.p Exp number, first 60 AAs:  0.00067
# Aquilegiacoerulea_Aqcoe2G306500.1.p Total prob of N-in:        0.01039
Aquilegiacoerulea_Aqcoe2G306500.1.p     TMHMM2.0        outside      1   278
```

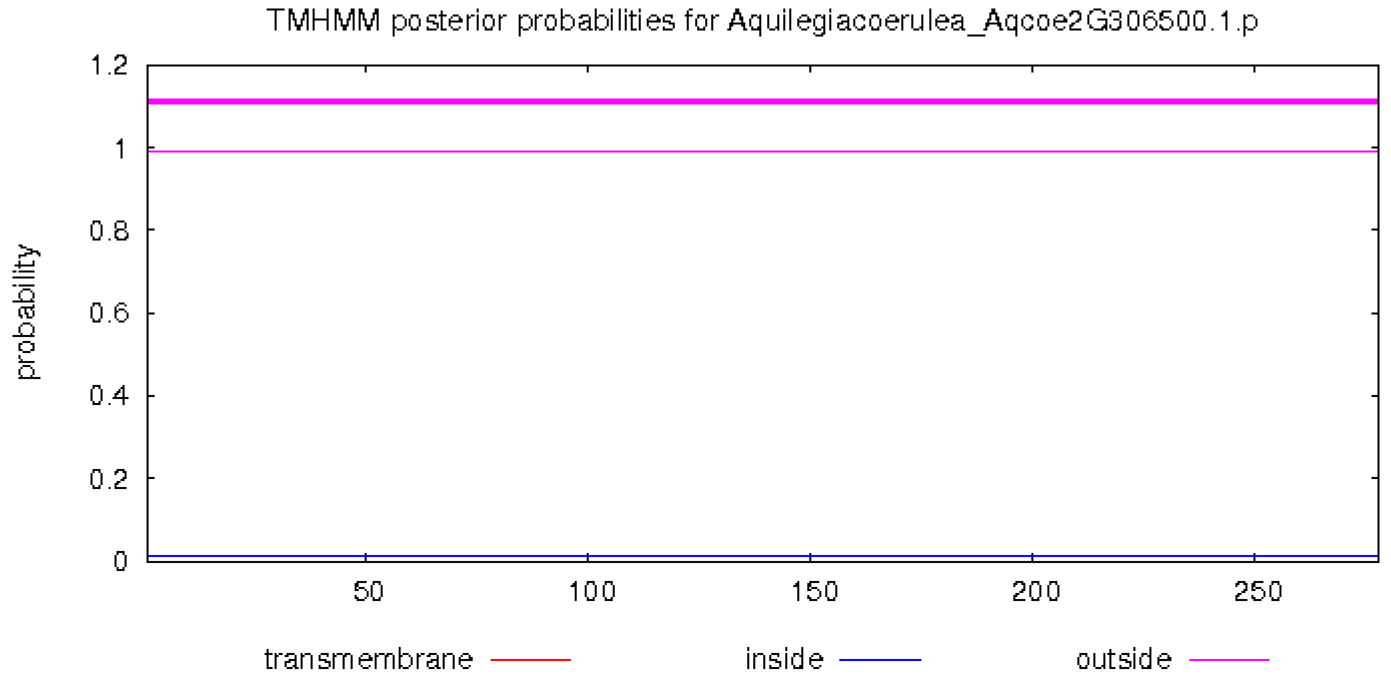

plot in postscript, script for making the plot in gnuplot, data for plot

---

```
# Aquilegiacoerulea_Aqcoe2G308700.1.p Length: 273
# Aquilegiacoerulea_Aqcoe2G308700.1.p Number of predicted TMHs:  0
# Aquilegiacoerulea_Aqcoe2G308700.1.p Exp number of AAs in TMHs: 0.00123
# Aquilegiacoerulea_Aqcoe2G308700.1.p Exp number, first 60 AAs:  0.00117
# Aquilegiacoerulea_Aqcoe2G308700.1.p Total prob of N-in:        0.02200
Aquilegiacoerulea_Aqcoe2G308700.1.p     TMHMM2.0        outside      1   273
```

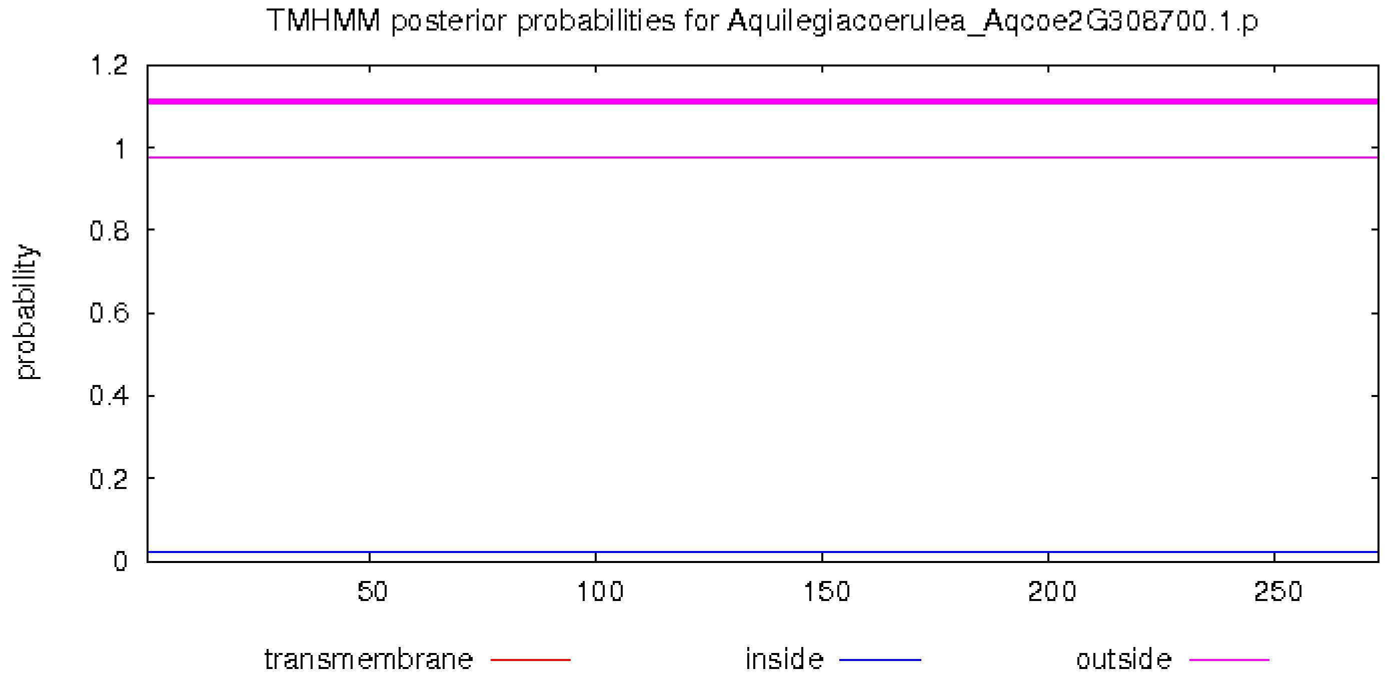

plot in postscript, script for making the plot in gnuplot, data for plot

---

```
# Aquilegiacoerulea_Aqcoe2G312600.1.p Length: 321
# Aquilegiacoerulea_Aqcoe2G312600.1.p Number of predicted TMHs:  0
# Aquilegiacoerulea_Aqcoe2G312600.1.p Exp number of AAs in TMHs: 0.20383
# Aquilegiacoerulea_Aqcoe2G312600.1.p Exp number, first 60 AAs:  0.00033
# Aquilegiacoerulea_Aqcoe2G312600.1.p Total prob of N-in:        0.08215
Aquilegiacoerulea_Aqcoe2G312600.1.p     TMHMM2.0        outside      1   321
```

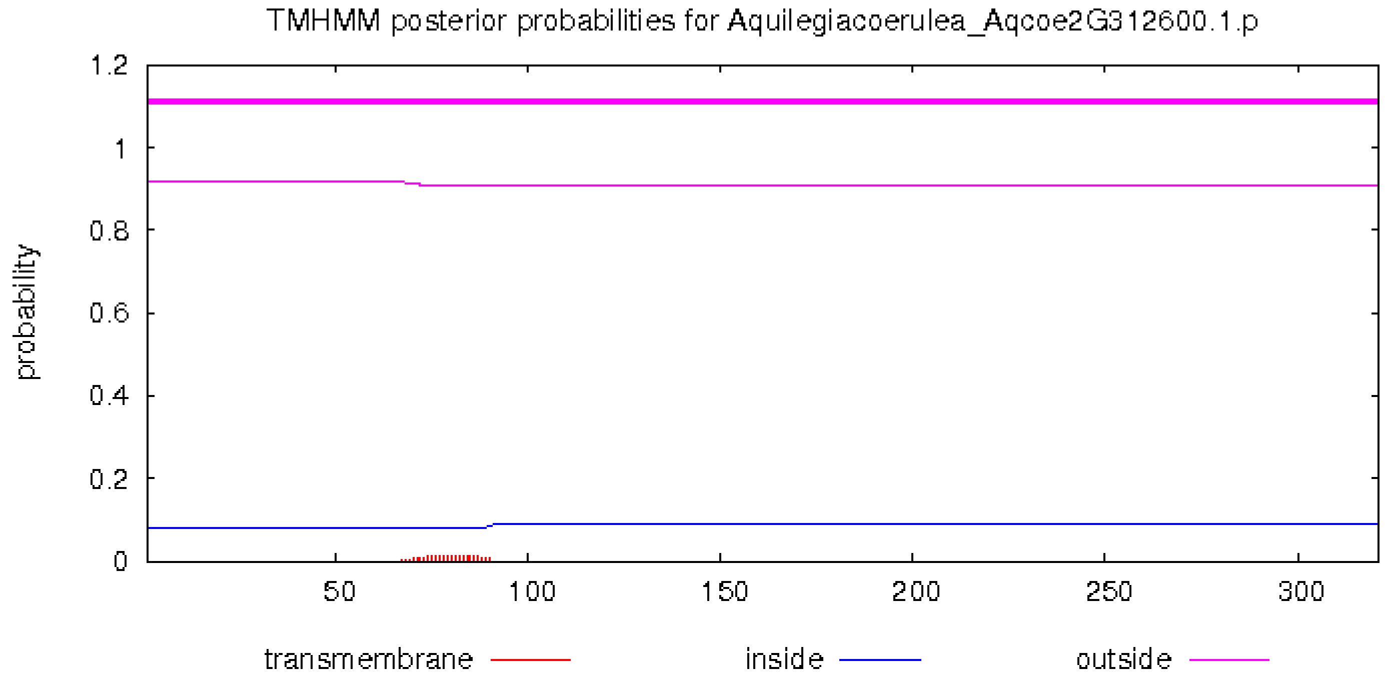

plot in postscript, script for making the plot in gnuplot, data for plot

---

```
# Aquilegiacoerulea_Aqcoe2G366500.1.p Length: 334
# Aquilegiacoerulea_Aqcoe2G366500.1.p Number of predicted TMHs:  0
# Aquilegiacoerulea_Aqcoe2G366500.1.p Exp number of AAs in TMHs: 0.00035
# Aquilegiacoerulea_Aqcoe2G366500.1.p Exp number, first 60 AAs:  0
# Aquilegiacoerulea_Aqcoe2G366500.1.p Total prob of N-in:        0.08983
Aquilegiacoerulea_Aqcoe2G366500.1.p     TMHMM2.0        outside      1   334
```

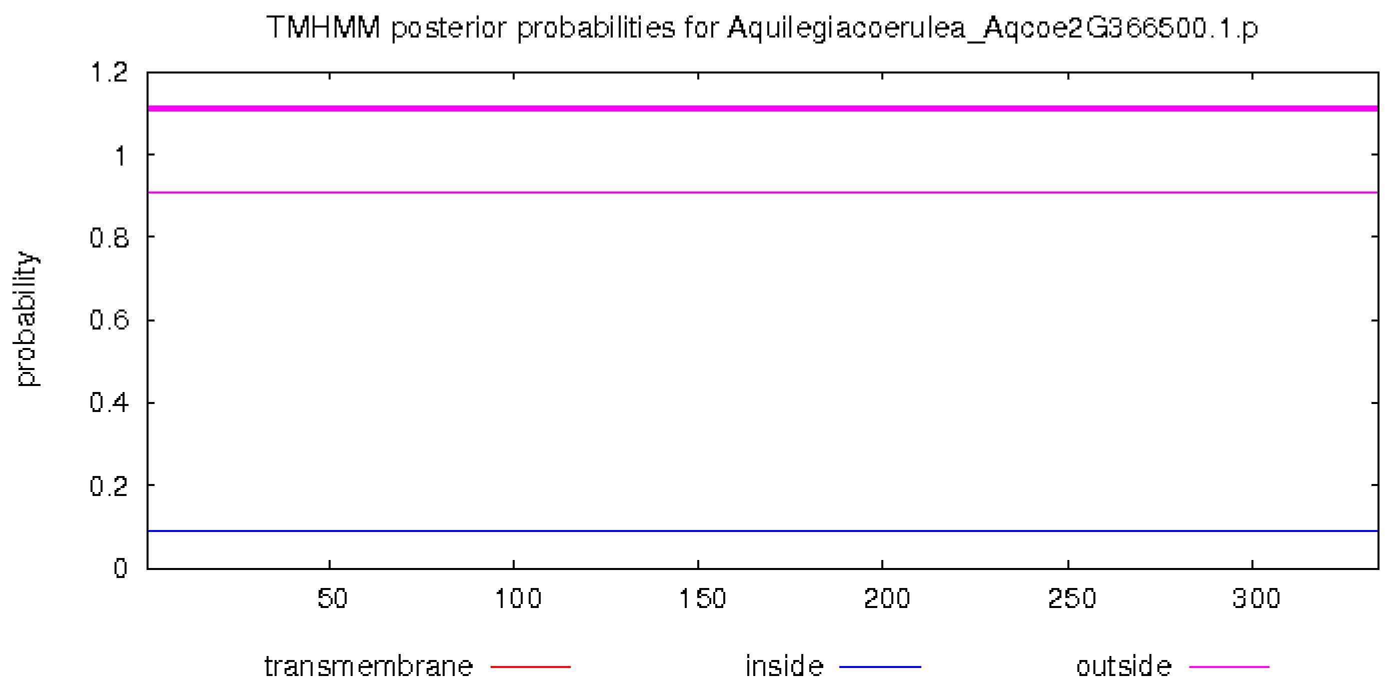

plot in postscript, script for making the plot in gnuplot, data for plot

---

```
# Aquilegiacoerulea_Aqcoe2G375500.1.p Length: 286
# Aquilegiacoerulea_Aqcoe2G375500.1.p Number of predicted TMHs:  0
# Aquilegiacoerulea_Aqcoe2G375500.1.p Exp number of AAs in TMHs: 0.0124
# Aquilegiacoerulea_Aqcoe2G375500.1.p Exp number, first 60 AAs:  0
# Aquilegiacoerulea_Aqcoe2G375500.1.p Total prob of N-in:        0.02336
Aquilegiacoerulea_Aqcoe2G375500.1.p     TMHMM2.0        outside      1   286
```

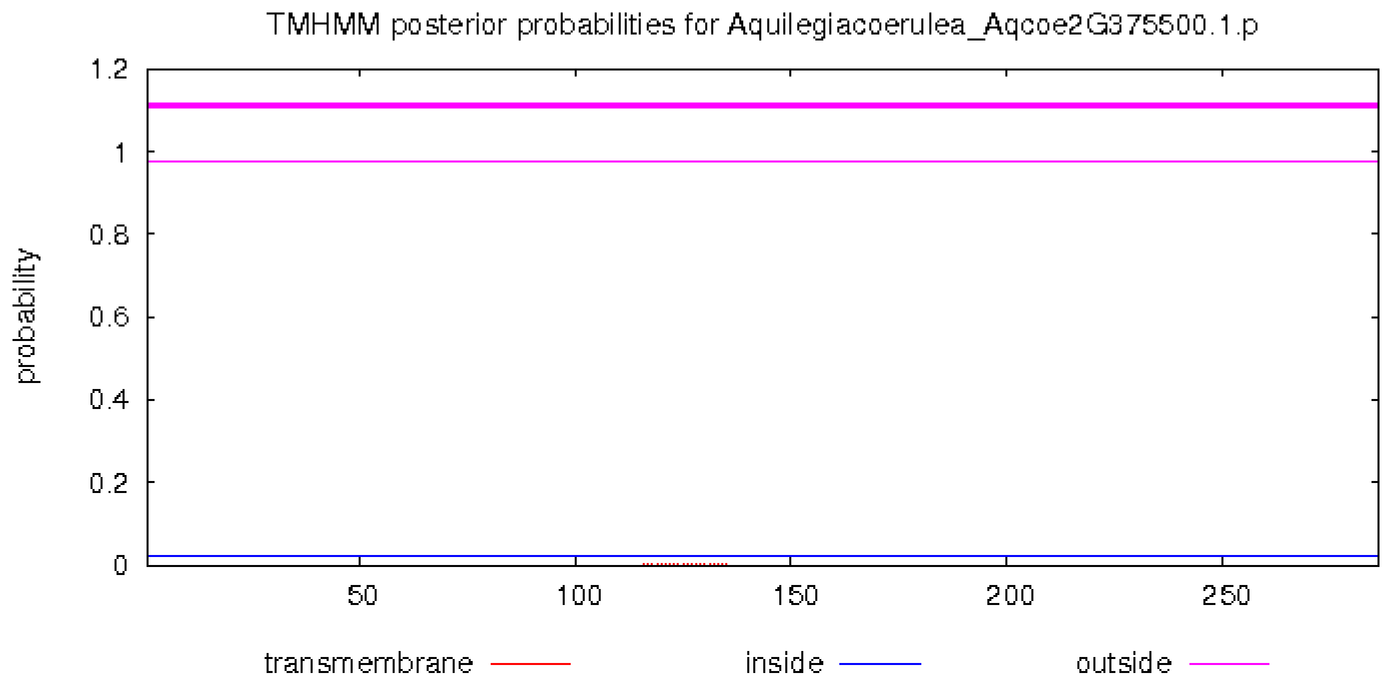

plot in postscript, script for making the plot in gnuplot, data for plot

---

```
# Aquilegiacoerulea_Aqcoe3G037800.1.p Length: 302
# Aquilegiacoerulea_Aqcoe3G037800.1.p Number of predicted TMHs:  0
# Aquilegiacoerulea_Aqcoe3G037800.1.p Exp number of AAs in TMHs: 0.01004
# Aquilegiacoerulea_Aqcoe3G037800.1.p Exp number, first 60 AAs:  0
# Aquilegiacoerulea_Aqcoe3G037800.1.p Total prob of N-in:        0.00364
Aquilegiacoerulea_Aqcoe3G037800.1.p     TMHMM2.0        outside      1   302
```

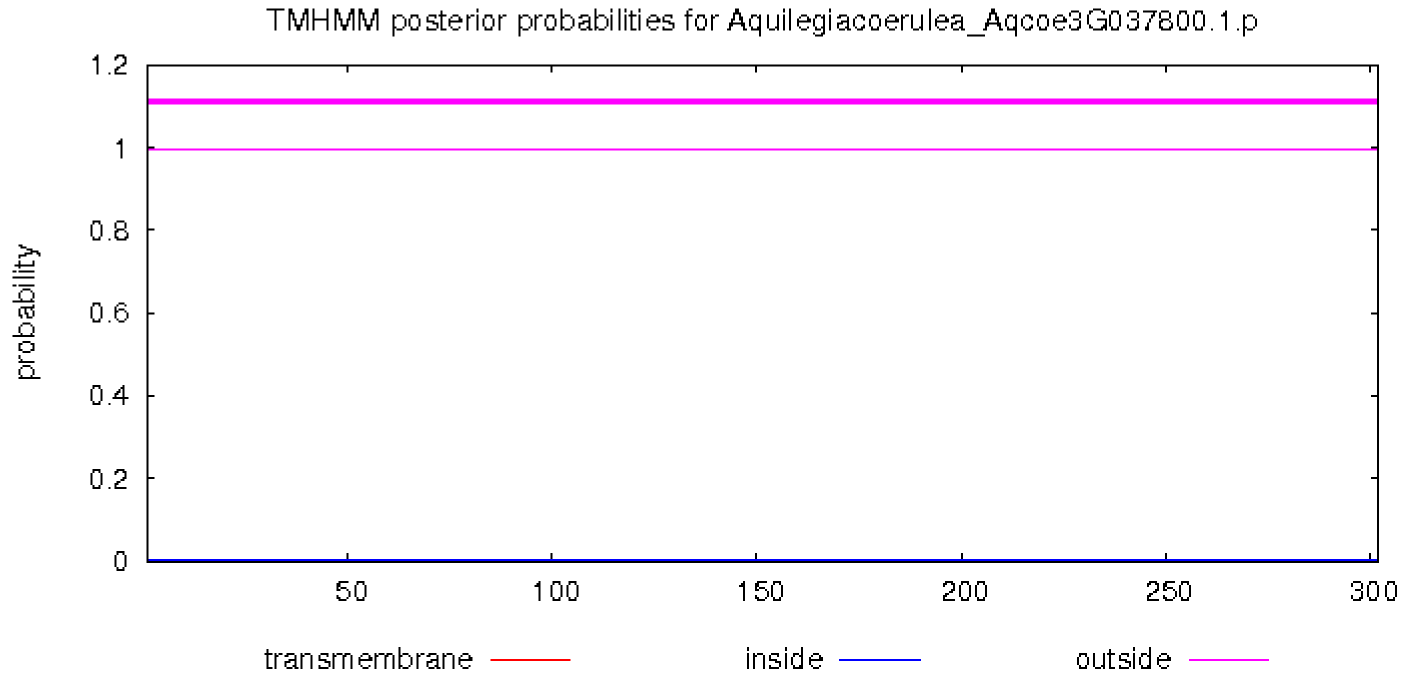

plot in postscript, script for making the plot in gnuplot, data for plot

```
# Aquilegiacoerulea_Aqcoe3G079400.1.p Length: 363
# Aquilegiacoerulea_Aqcoe3G079400.1.p Number of predicted TMHs:  0
# Aquilegiacoerulea_Aqcoe3G079400.1.p Exp number of AAs in TMHs: 0.00145
# Aquilegiacoerulea_Aqcoe3G079400.1.p Exp number, first 60 AAs:  0
# Aquilegiacoerulea_Aqcoe3G079400.1.p Total prob of N-in:        0.03235
Aquilegiacoerulea_Aqcoe3G079400.1.p     TMHMM2.0        outside      1   363
```

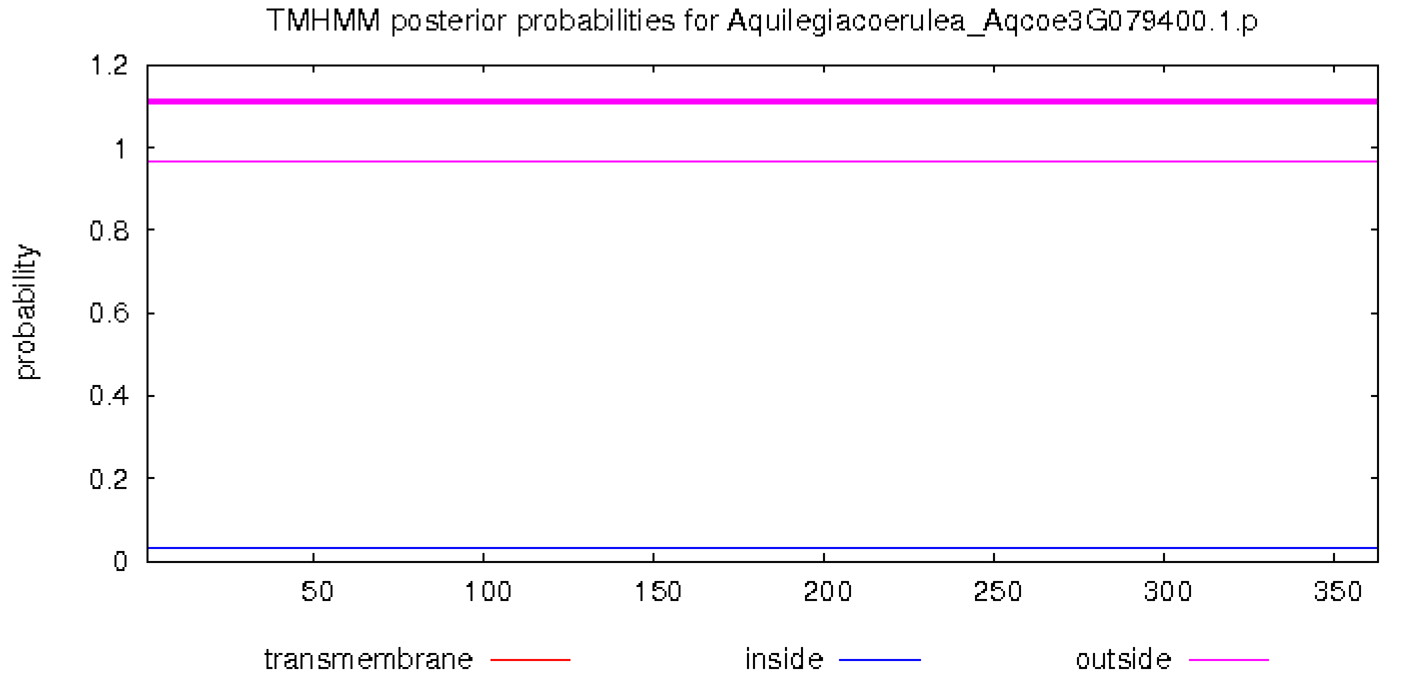

plot in postscript, script for making the plot in gnuplot, data for plot

```
# Aquilegiacoerulea_Aqcoe3G130600.1.p Length: 377
# Aquilegiacoerulea_Aqcoe3G130600.1.p Number of predicted TMHs:  0
# Aquilegiacoerulea_Aqcoe3G130600.1.p Exp number of AAs in TMHs: 0
# Aquilegiacoerulea_Aqcoe3G130600.1.p Exp number, first 60 AAs:  0
# Aquilegiacoerulea_Aqcoe3G130600.1.p Total prob of N-in:        0.00660
Aquilegiacoerulea_Aqcoe3G130600.1.p     TMHMM2.0        outside      1   377
```

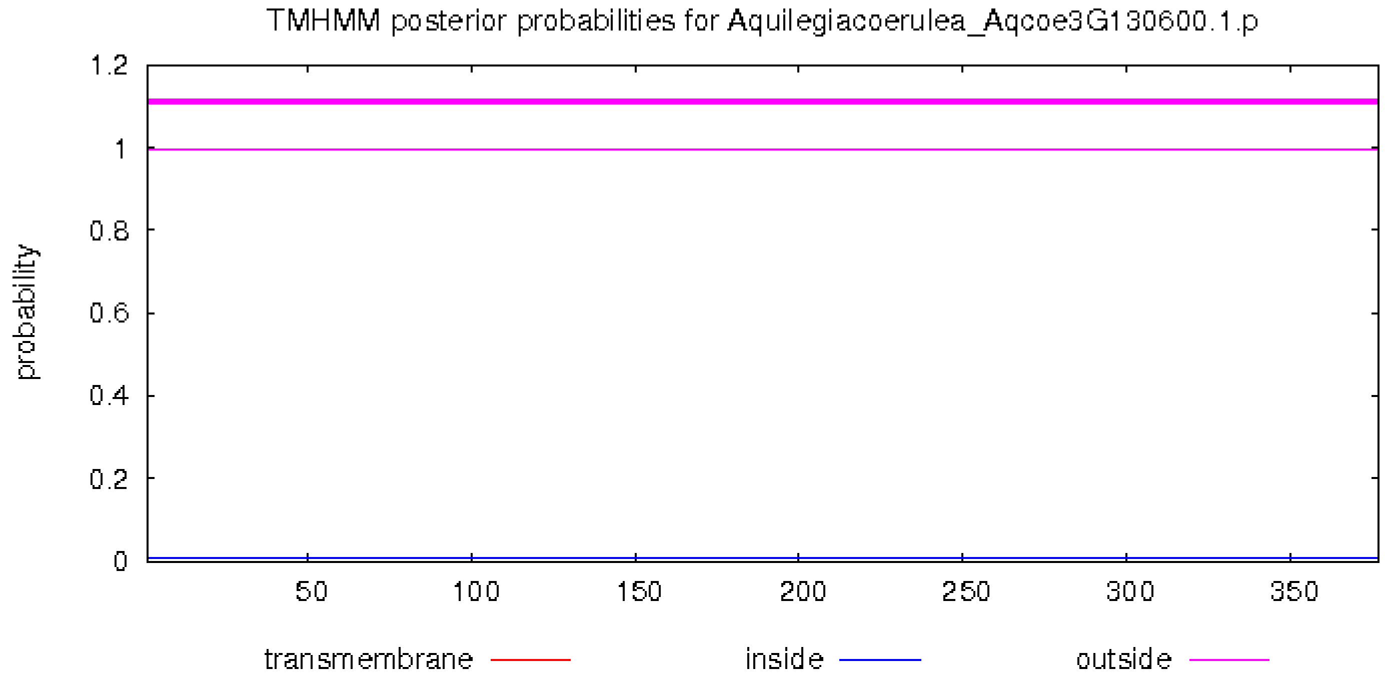

plot in postscript, script for making the plot in gnuplot, data for plot

---

```
# Aquilegiacoerulea_Aqcoe3G136600.1.p Length: 396
# Aquilegiacoerulea_Aqcoe3G136600.1.p Number of predicted TMHs:  0
# Aquilegiacoerulea_Aqcoe3G136600.1.p Exp number of AAs in TMHs: 0.17821
# Aquilegiacoerulea_Aqcoe3G136600.1.p Exp number, first 60 AAs:  0
# Aquilegiacoerulea_Aqcoe3G136600.1.p Total prob of N-in:        0.10928
Aquilegiacoerulea_Aqcoe3G136600.1.p     TMHMM2.0        outside      1   396
```

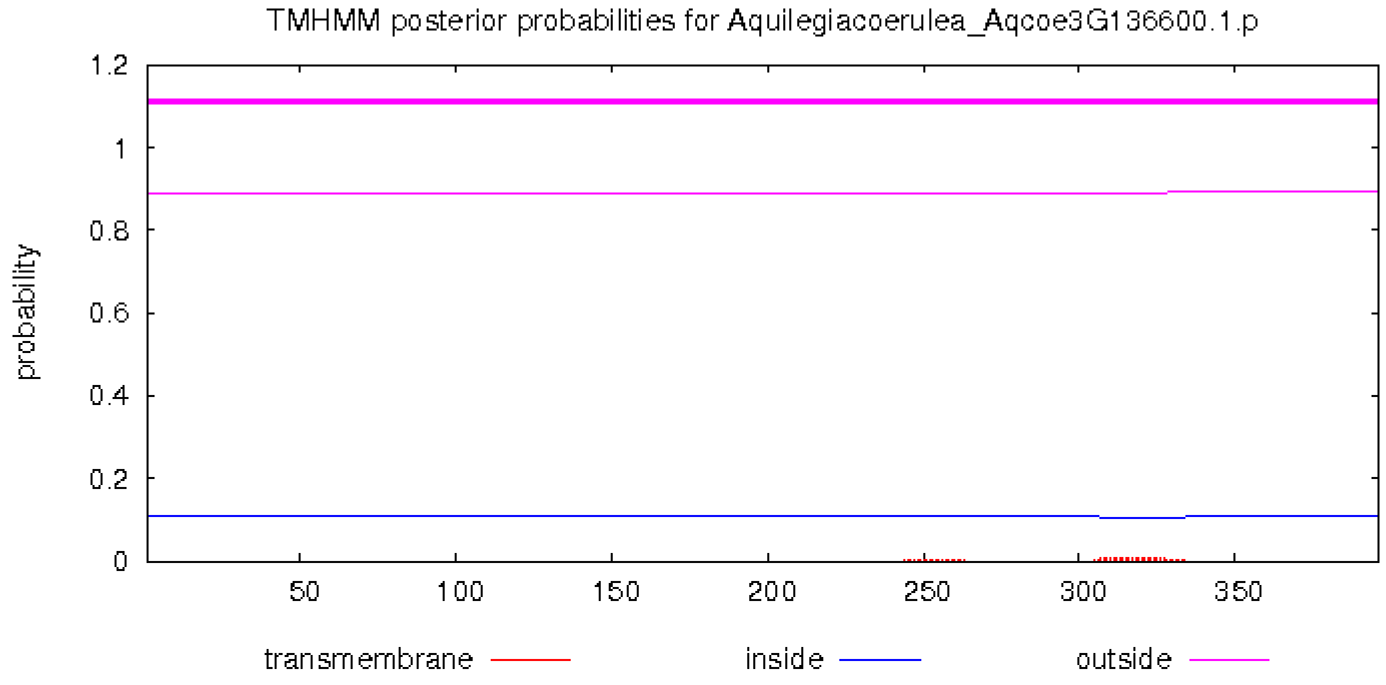

plot in postscript, script for making the plot in gnuplot, data for plot

---

```
# Aquilegiacoerulea_Aqcoe3G142100.1.p Length: 428
# Aquilegiacoerulea_Aqcoe3G142100.1.p Number of predicted TMHs:  0
# Aquilegiacoerulea_Aqcoe3G142100.1.p Exp number of AAs in TMHs: 0.64093
# Aquilegiacoerulea_Aqcoe3G142100.1.p Exp number, first 60 AAs:  0
# Aquilegiacoerulea_Aqcoe3G142100.1.p Total prob of N-in:        0.03699
Aquilegiacoerulea_Aqcoe3G142100.1.p     TMHMM2.0        outside      1   428
```

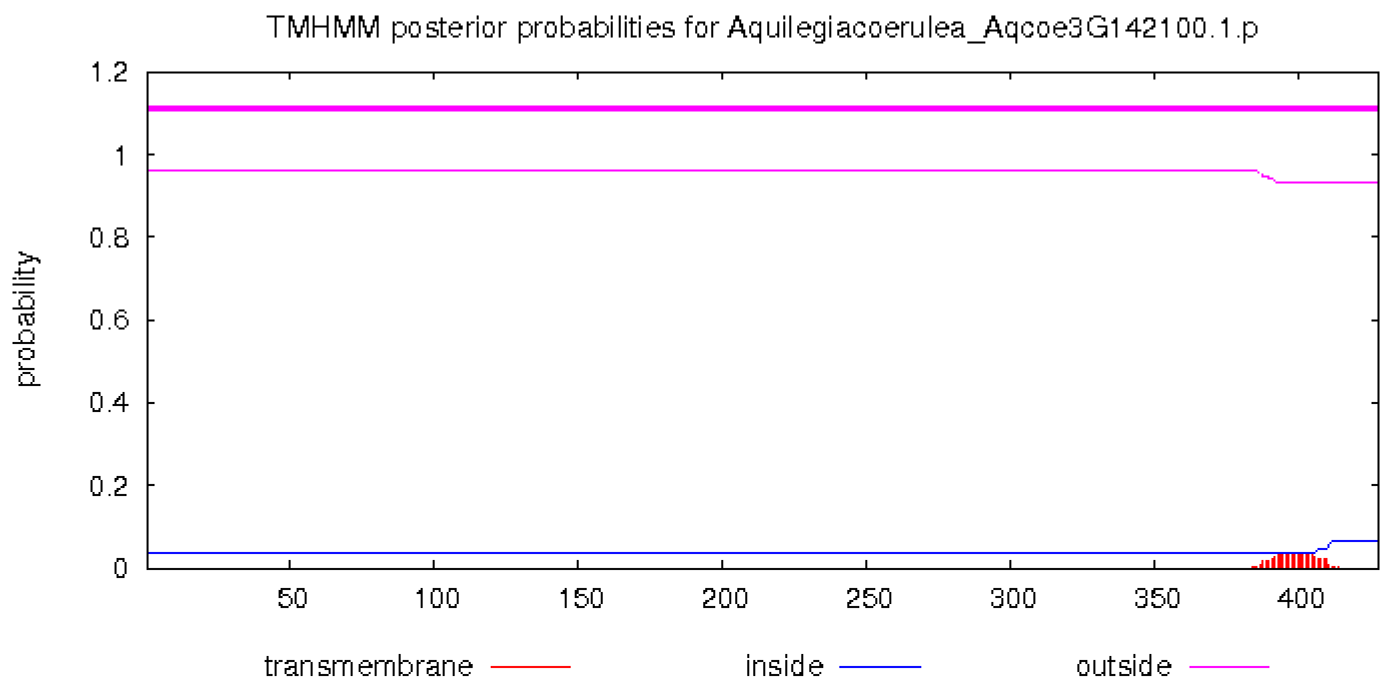

plot in postscript, script for making the plot in gnuplot, data for plot

---

```
# Aquilegiacoerulea_Aqcoe3G179900.1.p Length: 397
# Aquilegiacoerulea_Aqcoe3G179900.1.p Number of predicted TMHs:  0
# Aquilegiacoerulea_Aqcoe3G179900.1.p Exp number of AAs in TMHs: 0.00029
# Aquilegiacoerulea_Aqcoe3G179900.1.p Exp number, first 60 AAs:  0.00012
# Aquilegiacoerulea_Aqcoe3G179900.1.p Total prob of N-in:        0.00744
Aquilegiacoerulea_Aqcoe3G179900.1.p	TMHMM2.0	outside	     1   397
```

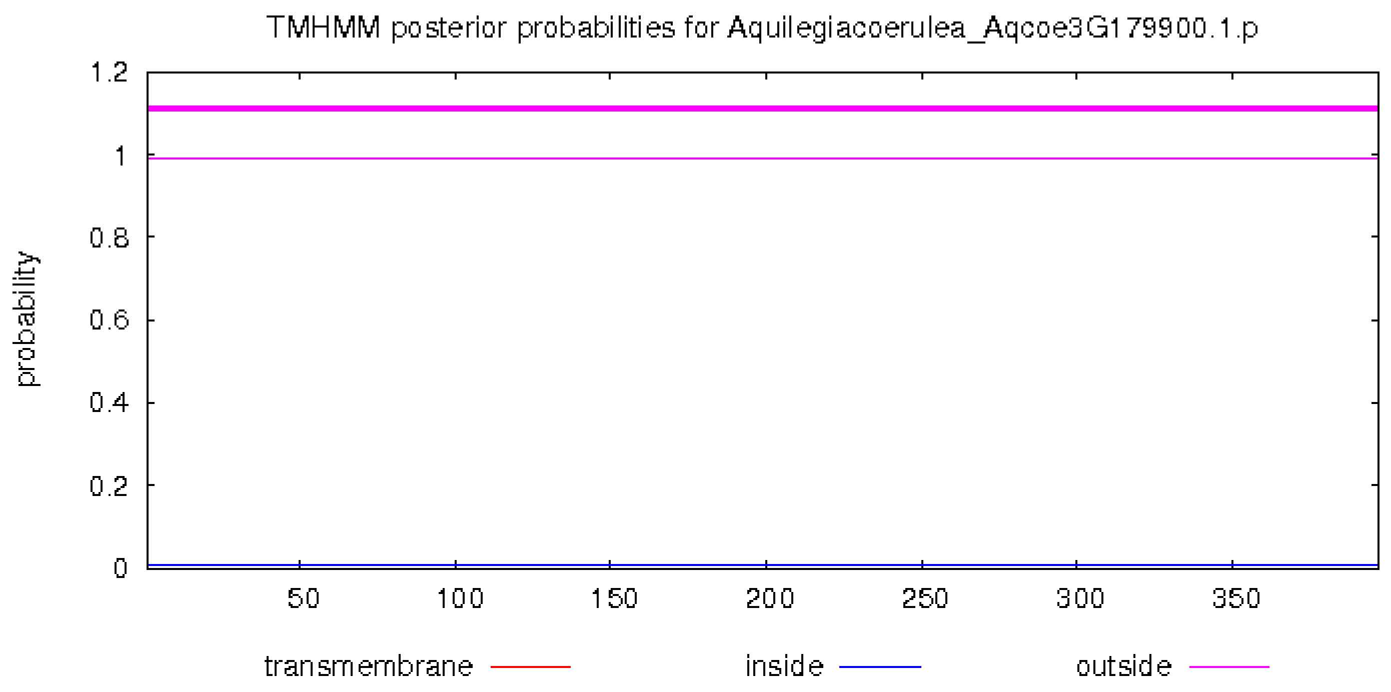

plot in postscript, script for making the plot in gnuplot, data for plot

---

```
# Aquilegiacoerulea_Aqcoe3G199000.1.p Length: 221
# Aquilegiacoerulea_Aqcoe3G199000.1.p Number of predicted TMHs:  0
# Aquilegiacoerulea_Aqcoe3G199000.1.p Exp number of AAs in TMHs: 0
# Aquilegiacoerulea_Aqcoe3G199000.1.p Exp number, first 60 AAs:  0
# Aquilegiacoerulea_Aqcoe3G199000.1.p Total prob of N-in:        0.08488
Aquilegiacoerulea_Aqcoe3G199000.1.p	TMHMM2.0	outside	     1   221
```

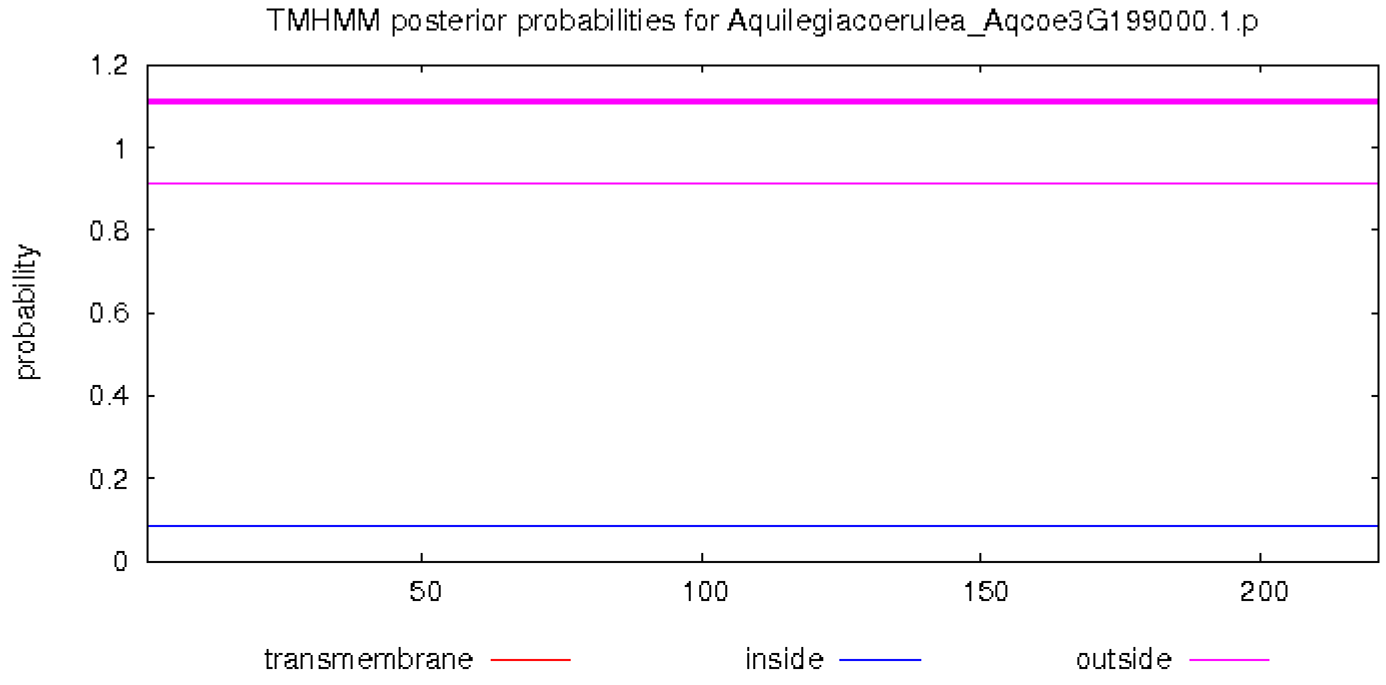

plot in postscript, script for making the plot in gnuplot, data for plot

---

```
# Aquilegiacoerulea_Aqcoe3G236400.1.p Length: 371
# Aquilegiacoerulea_Aqcoe3G236400.1.p Number of predicted TMHs:  0
# Aquilegiacoerulea_Aqcoe3G236400.1.p Exp number of AAs in TMHs: 0.00011
# Aquilegiacoerulea_Aqcoe3G236400.1.p Exp number, first 60 AAs:  0
# Aquilegiacoerulea_Aqcoe3G236400.1.p Total prob of N-in:        0.00101
Aquilegiacoerulea_Aqcoe3G236400.1.p     TMHMM2.0        outside      1   371
```

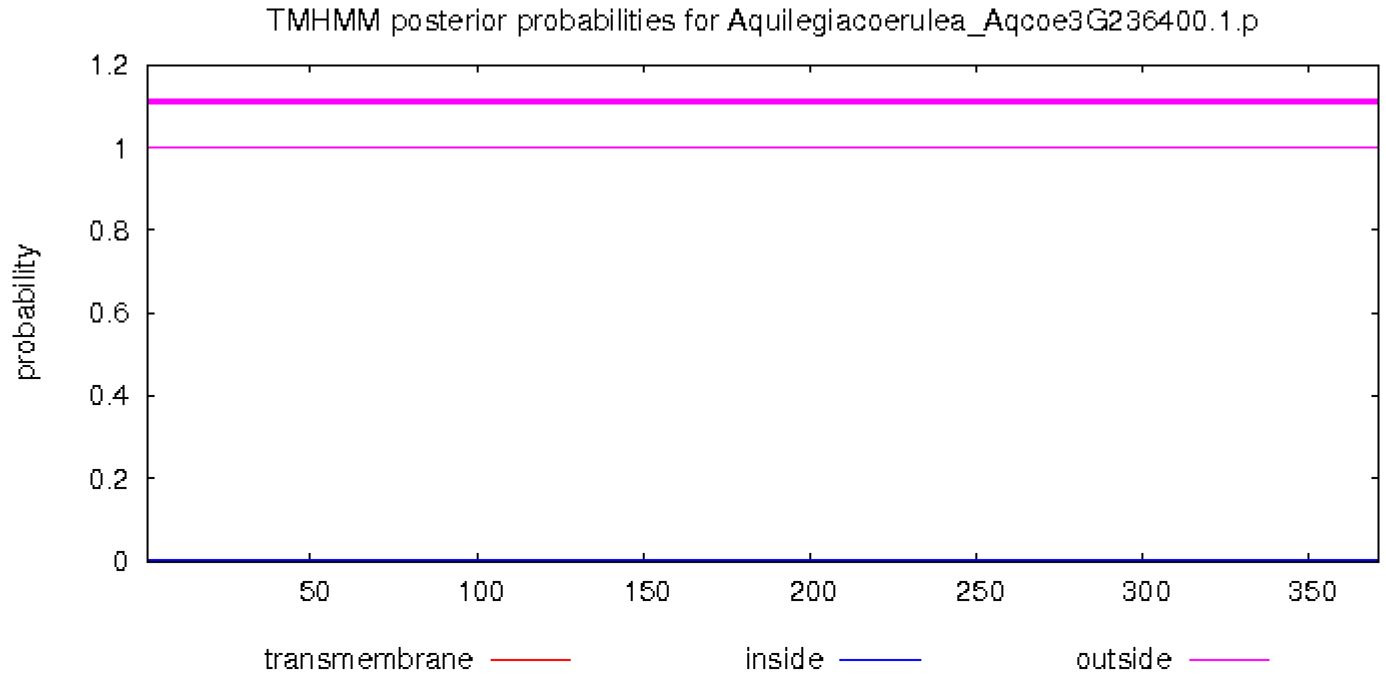

plot in postscript, script for making the plot in gnuplot, data for plot

---

```
# Aquilegiacoerulea_Aqcoe3G337400.1.p Length: 402
# Aquilegiacoerulea_Aqcoe3G337400.1.p Number of predicted TMHs:  0
# Aquilegiacoerulea_Aqcoe3G337400.1.p Exp number of AAs in TMHs: 0.00055
# Aquilegiacoerulea_Aqcoe3G337400.1.p Exp number, first 60 AAs:  0.00018
# Aquilegiacoerulea_Aqcoe3G337400.1.p Total prob of N-in:        0.02465
Aquilegiacoerulea_Aqcoe3G337400.1.p     TMHMM2.0        outside      1   402
```

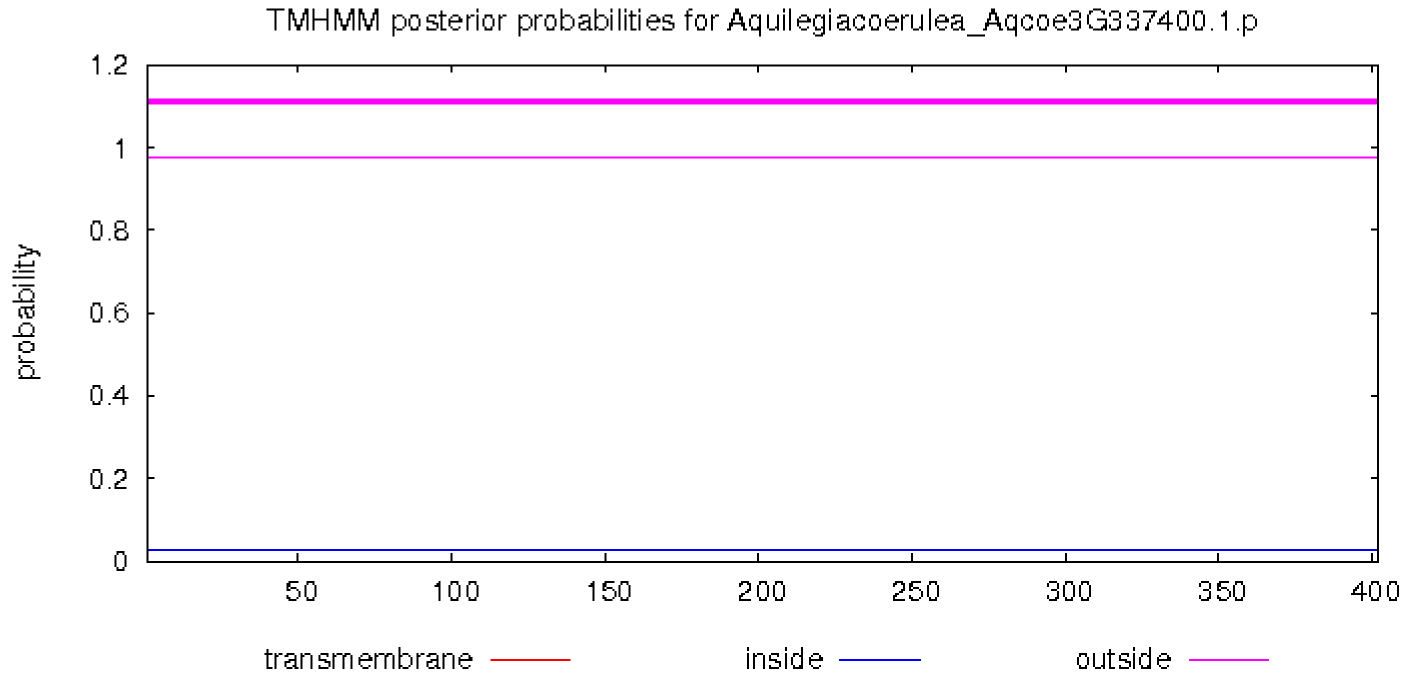

plot in postscript, script for making the plot in gnuplot, data for plot

---

```
# Aquilegiacoerulea_Aqcoe3G345800.1.p Length: 375
# Aquilegiacoerulea_Aqcoe3G345800.1.p Number of predicted TMHs:  0
# Aquilegiacoerulea_Aqcoe3G345800.1.p Exp number of AAs in TMHs: 0.00013
# Aquilegiacoerulea_Aqcoe3G345800.1.p Exp number, first 60 AAs:  0
# Aquilegiacoerulea_Aqcoe3G345800.1.p Total prob of N-in:        0.01265
Aquilegiacoerulea_Aqcoe3G345800.1.p     TMHMM2.0        outside      1   375
```

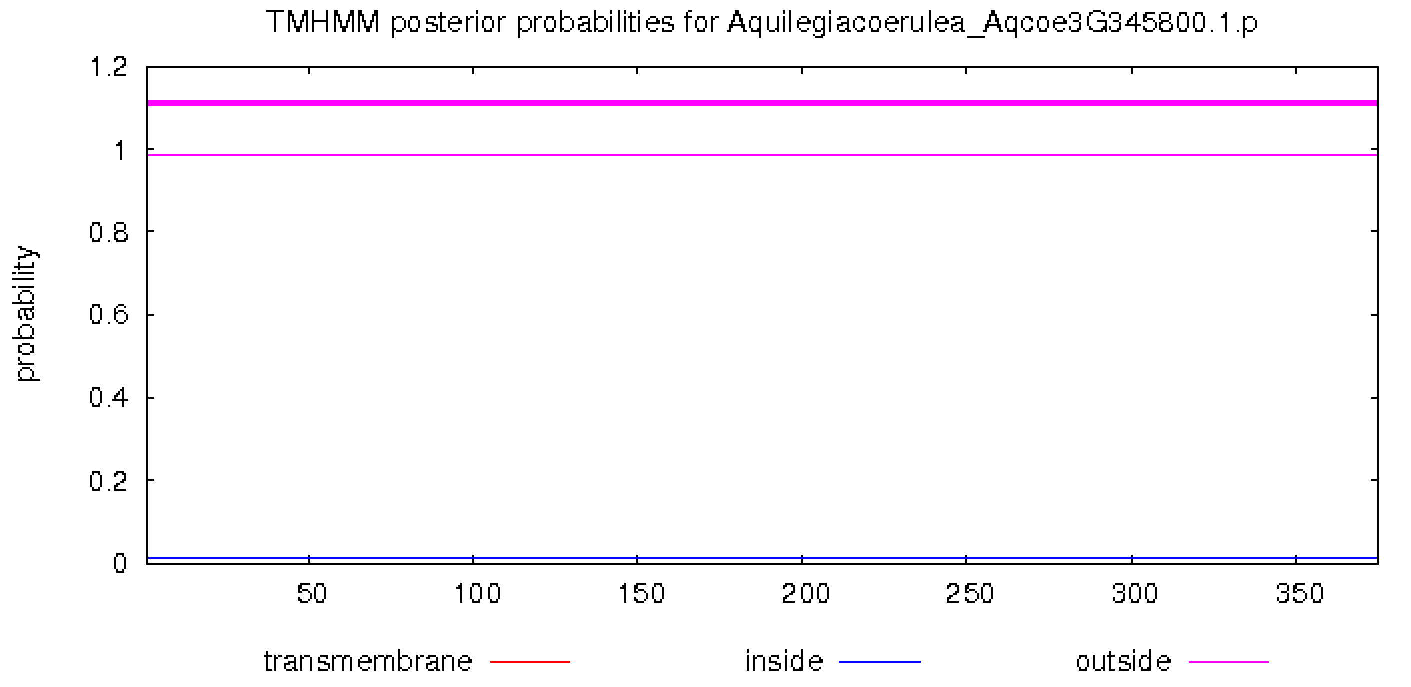

plot in postscript, script for making the plot in gnuplot, data for plot

---

```
# Aquilegiacoerulea_Aqcoe3G345900.1.p Length: 224
# Aquilegiacoerulea_Aqcoe3G345900.1.p Number of predicted TMHs:  0
# Aquilegiacoerulea_Aqcoe3G345900.1.p Exp number of AAs in TMHs: 0.00047
# Aquilegiacoerulea_Aqcoe3G345900.1.p Exp number, first 60 AAs:  0.00012
# Aquilegiacoerulea_Aqcoe3G345900.1.p Total prob of N-in:        0.03981
Aquilegiacoerulea_Aqcoe3G345900.1.p     TMHMM2.0        outside      1   224
```

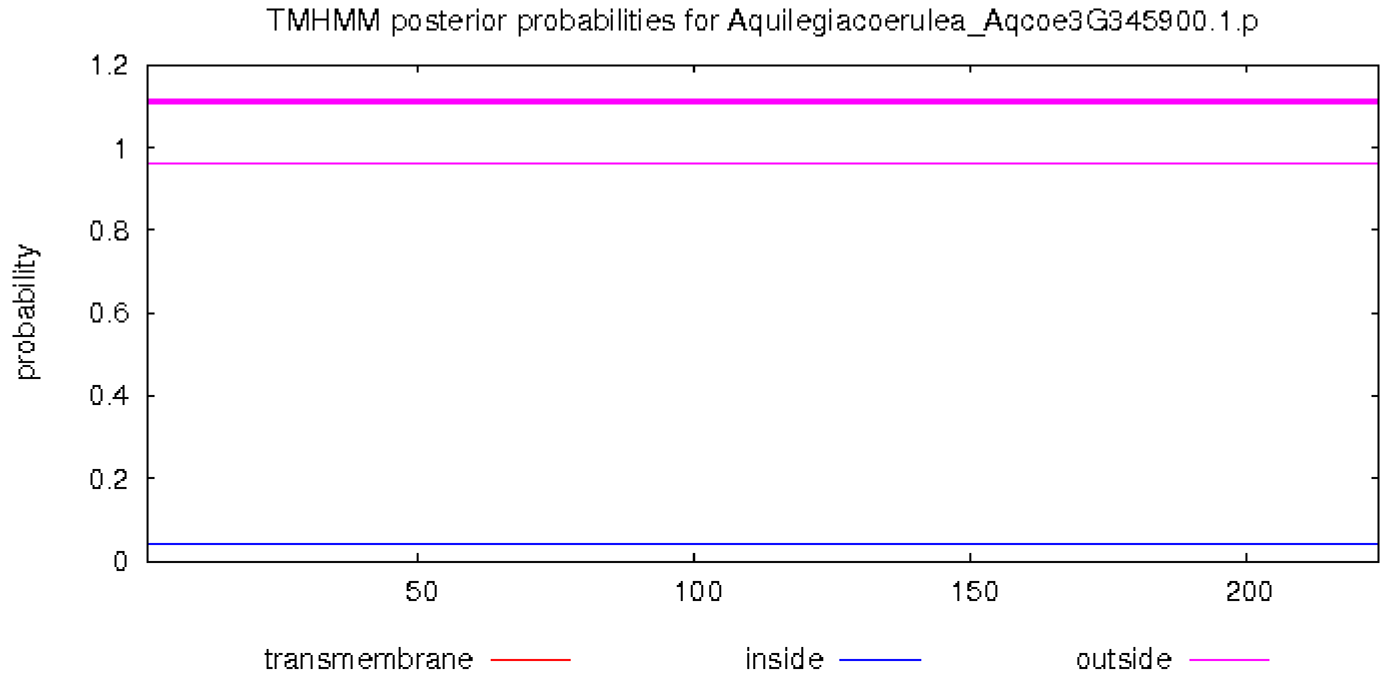

plot in postscript, script for making the plot in gnuplot, data for plot

---

```
# Aquilegiacoerulea_Aqcoe3G361700.1.p Length: 196
# Aquilegiacoerulea_Aqcoe3G361700.1.p Number of predicted TMHs:  0
# Aquilegiacoerulea_Aqcoe3G361700.1.p Exp number of AAs in TMHs: 0
# Aquilegiacoerulea_Aqcoe3G361700.1.p Exp number, first 60 AAs:  0
# Aquilegiacoerulea_Aqcoe3G361700.1.p Total prob of N-in:        0.04350
Aquilegiacoerulea_Aqcoe3G361700.1.p     TMHMM2.0        outside      1   196
```

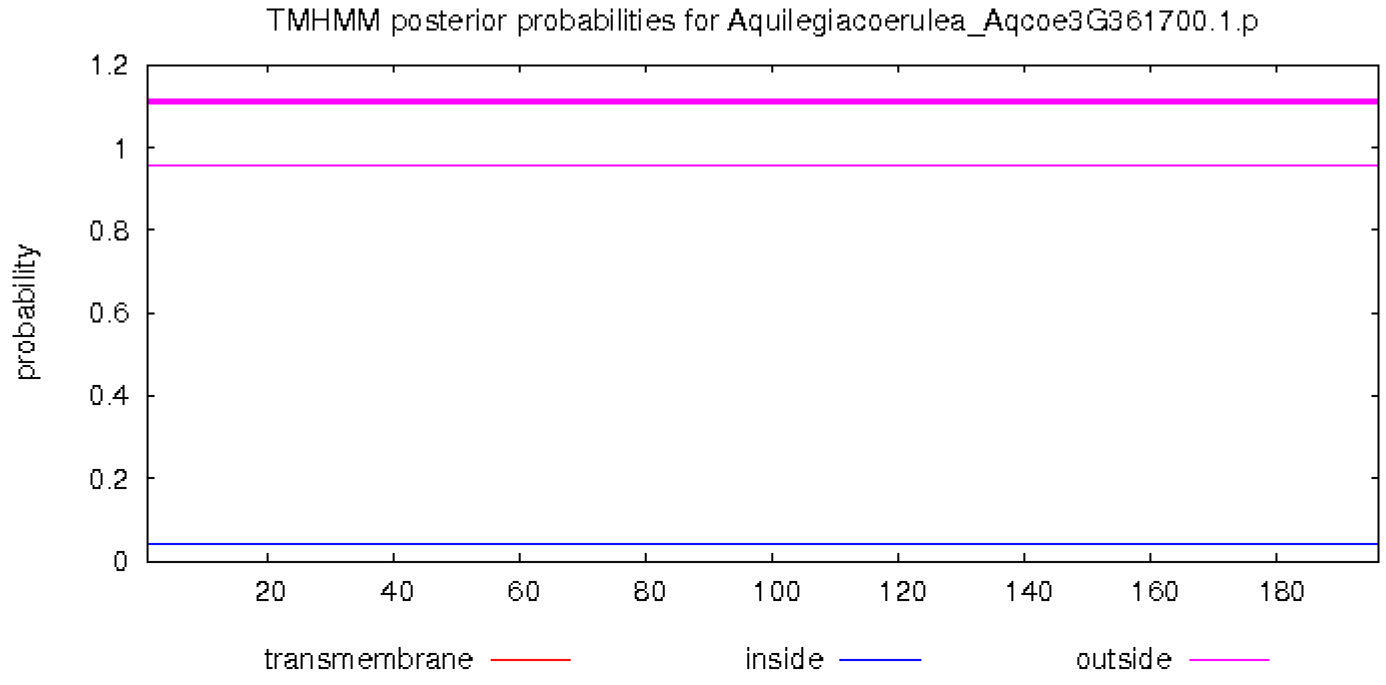

plot in postscript, script for making the plot in gnuplot, data for plot

---

```
# Aquilegiacoerulea_Aqcoe3G368500.1.p Length: 357
# Aquilegiacoerulea_Aqcoe3G368500.1.p Number of predicted TMHs:  0
# Aquilegiacoerulea_Aqcoe3G368500.1.p Exp number of AAs in TMHs: 0.00305
# Aquilegiacoerulea_Aqcoe3G368500.1.p Exp number, first 60 AAs:  0.00305
# Aquilegiacoerulea_Aqcoe3G368500.1.p Total prob of N-in:        0.04116
Aquilegiacoerulea_Aqcoe3G368500.1.p     TMHMM2.0        outside      1   357
```

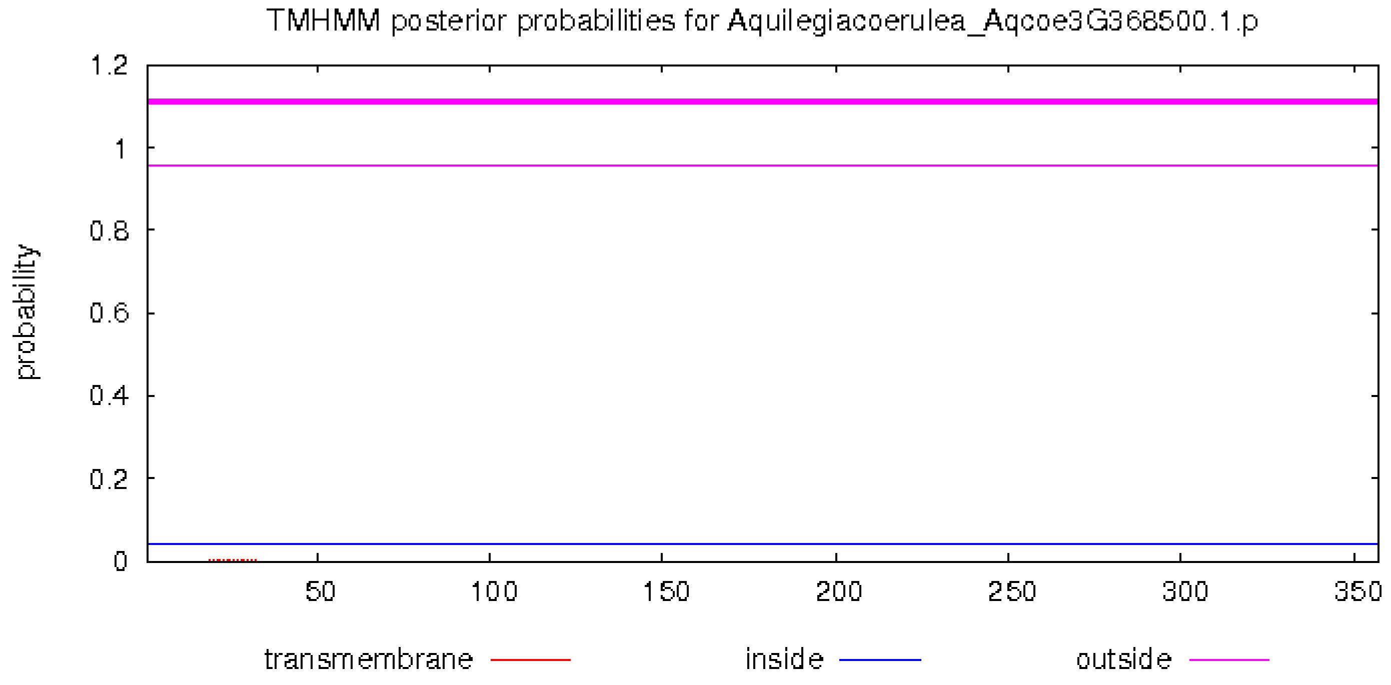

plot in postscript, script for making the plot in gnuplot, data for plot

```
# Aquilegiacoerulea_Aqcoe3G396000.1.p Length: 495
# Aquilegiacoerulea_Aqcoe3G396000.1.p Number of predicted TMHs:  0
# Aquilegiacoerulea_Aqcoe3G396000.1.p Exp number of AAs in TMHs: 0.00058
# Aquilegiacoerulea_Aqcoe3G396000.1.p Exp number, first 60 AAs:  0
# Aquilegiacoerulea_Aqcoe3G396000.1.p Total prob of N-in:        0.00214
Aquilegiacoerulea_Aqcoe3G396000.1.p     TMHMM2.0        outside      1   495
```

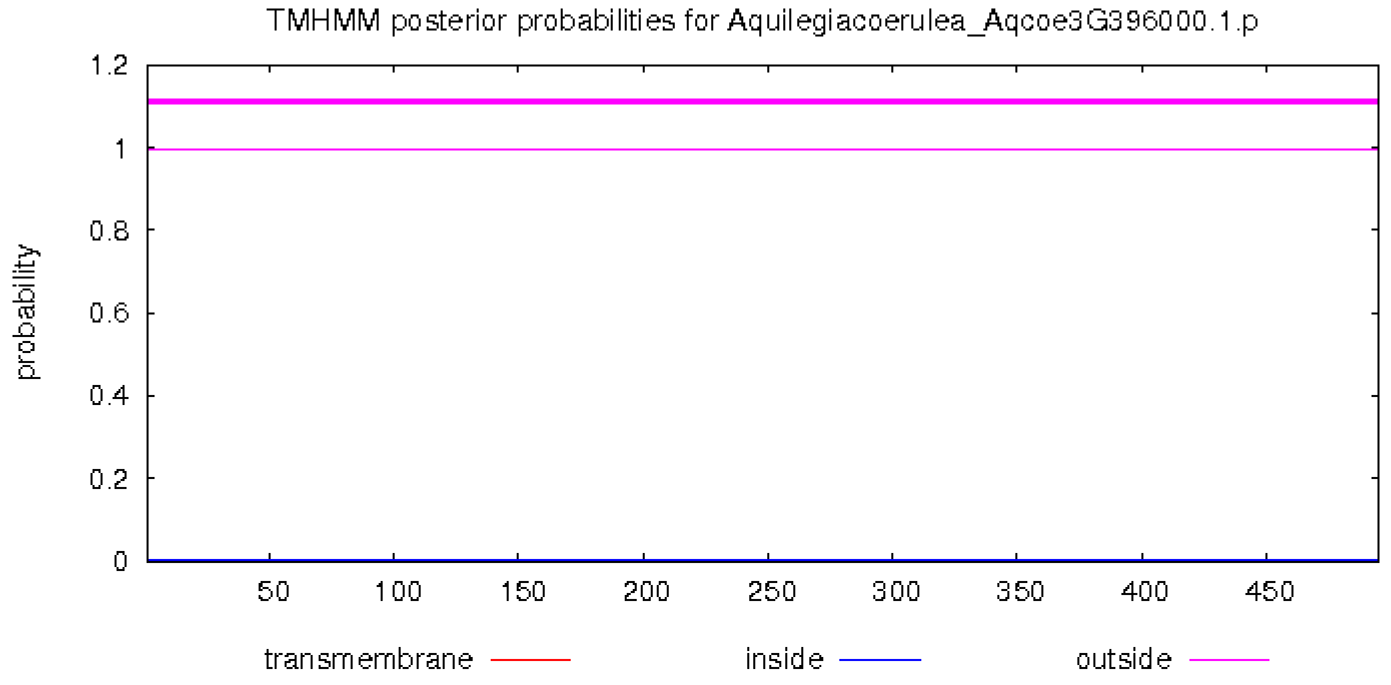

plot in postscript, script for making the plot in gnuplot, data for plot

```
# Aquilegiacoerulea_Aqcoe3G424400.1.p Length: 238
# Aquilegiacoerulea_Aqcoe3G424400.1.p Number of predicted TMHs:  0
# Aquilegiacoerulea_Aqcoe3G424400.1.p Exp number of AAs in TMHs: 0
# Aquilegiacoerulea_Aqcoe3G424400.1.p Exp number, first 60 AAs:  0
# Aquilegiacoerulea_Aqcoe3G424400.1.p Total prob of N-in:        0.00216
Aquilegiacoerulea_Aqcoe3G424400.1.p     TMHMM2.0        outside      1   238
```

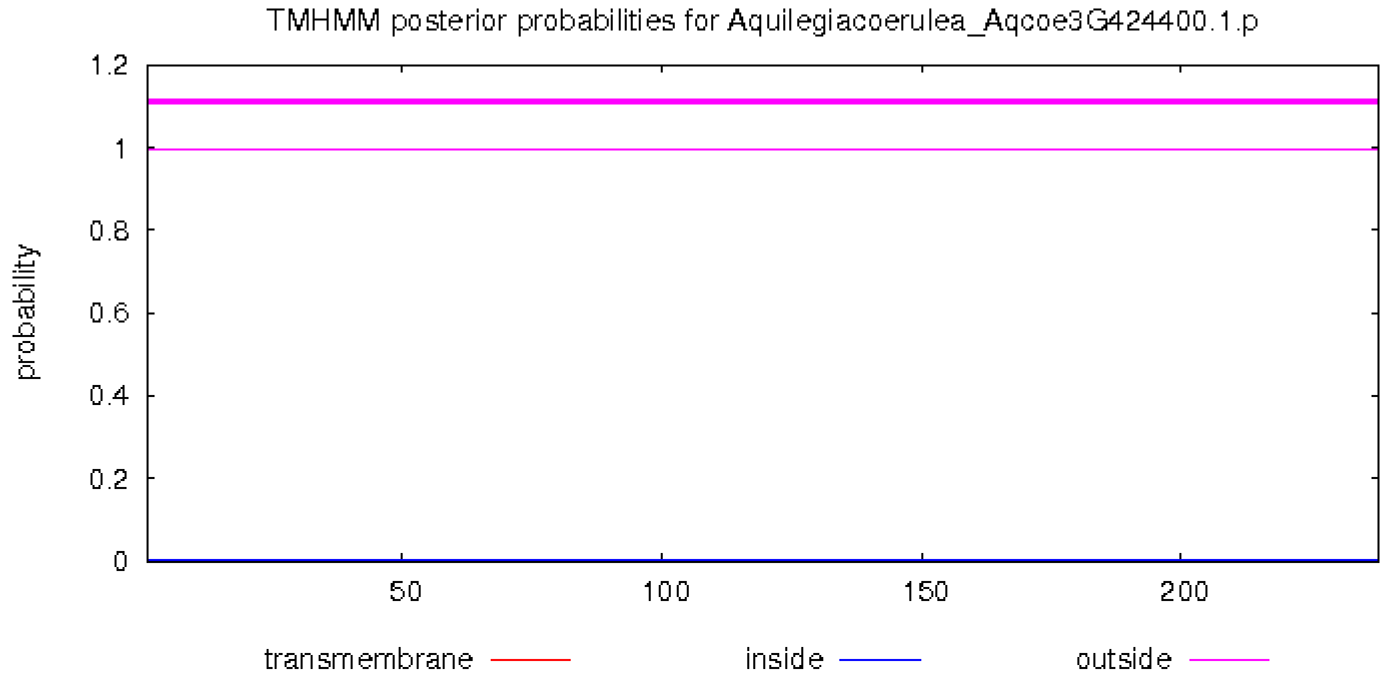

plot in postscript, script for making the plot in gnuplot, data for plot

---

```
# Aquilegiacoerulea_Aqcoe3G424500.1.p Length: 674
# Aquilegiacoerulea_Aqcoe3G424500.1.p Number of predicted TMHs:  0
# Aquilegiacoerulea_Aqcoe3G424500.1.p Exp number of AAs in TMHs: 5.85372
# Aquilegiacoerulea_Aqcoe3G424500.1.p Exp number, first 60 AAs:  0
# Aquilegiacoerulea_Aqcoe3G424500.1.p Total prob of N-in:        0.00011
Aquilegiacoerulea_Aqcoe3G424500.1.p     TMHMM2.0        outside      1   674
```

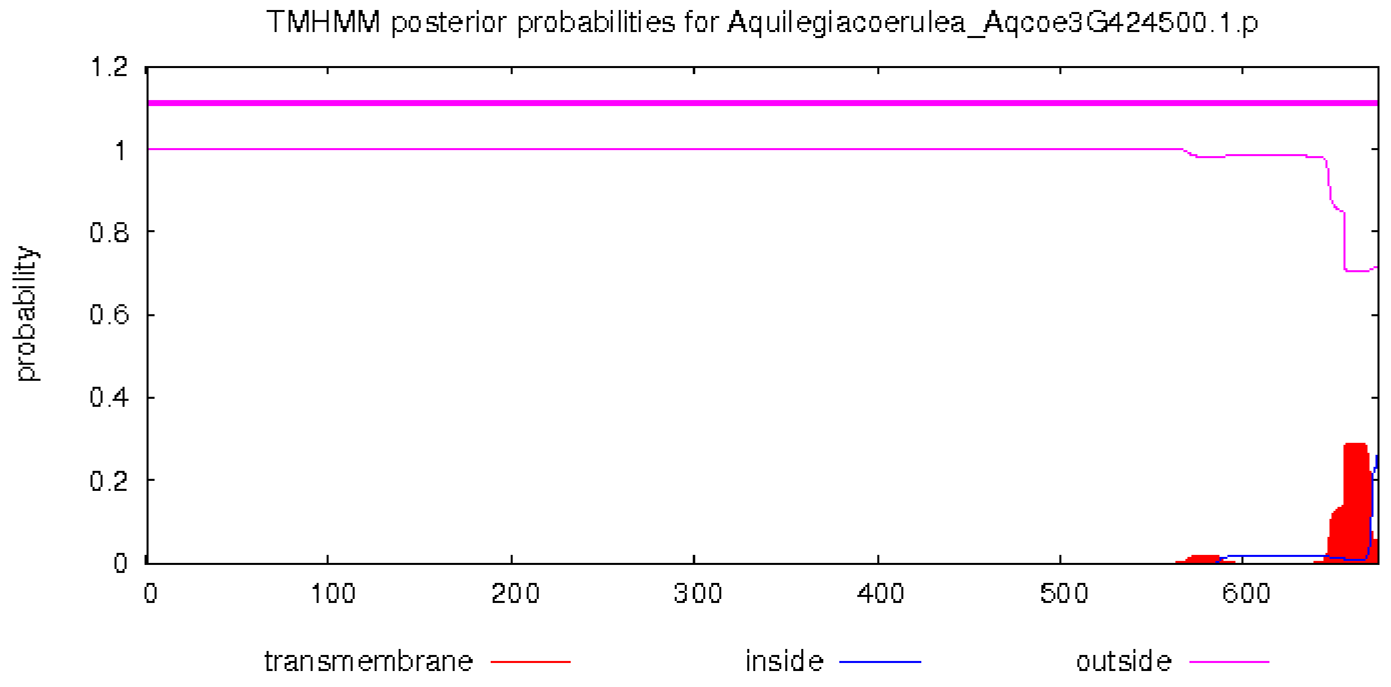

plot in postscript, script for making the plot in gnuplot, data for plot

---

```
# Aquilegiacoerulea_Aqcoe4G076900.1.p Length: 290
# Aquilegiacoerulea_Aqcoe4G076900.1.p Number of predicted TMHs:  0
# Aquilegiacoerulea_Aqcoe4G076900.1.p Exp number of AAs in TMHs: 0.00016
# Aquilegiacoerulea_Aqcoe4G076900.1.p Exp number, first 60 AAs:  0
# Aquilegiacoerulea_Aqcoe4G076900.1.p Total prob of N-in:        0.14592
Aquilegiacoerulea_Aqcoe4G076900.1.p     TMHMM2.0        outside      1   290
```

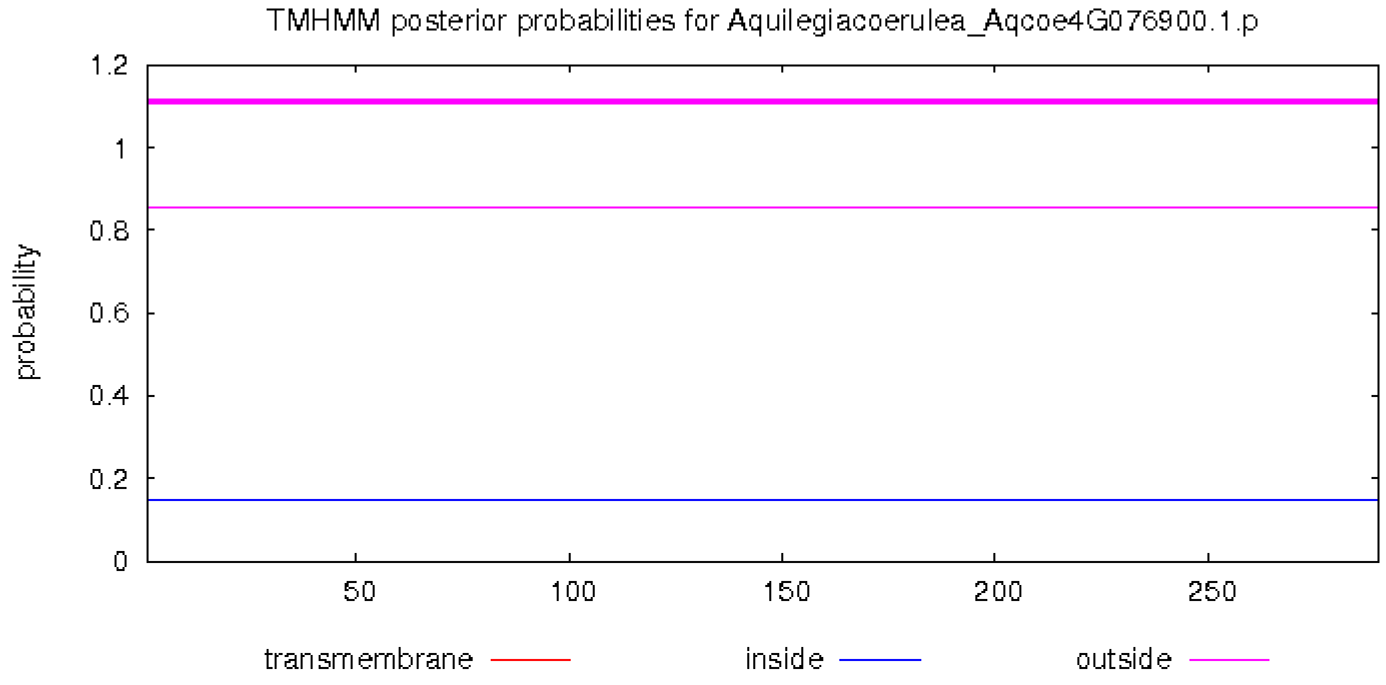

plot in postscript, script for making the plot in gnuplot, data for plot

```
# Aquilegiacoerulea_Aqcoe4G090300.1.p Length: 363
# Aquilegiacoerulea_Aqcoe4G090300.1.p Number of predicted TMHs:  0
# Aquilegiacoerulea_Aqcoe4G090300.1.p Exp number of AAs in TMHs: 0.00066
# Aquilegiacoerulea_Aqcoe4G090300.1.p Exp number, first 60 AAs:  0
# Aquilegiacoerulea_Aqcoe4G090300.1.p Total prob of N-in:        0.00227
Aquilegiacoerulea_Aqcoe4G090300.1.p     TMHMM2.0        outside      1   363
```

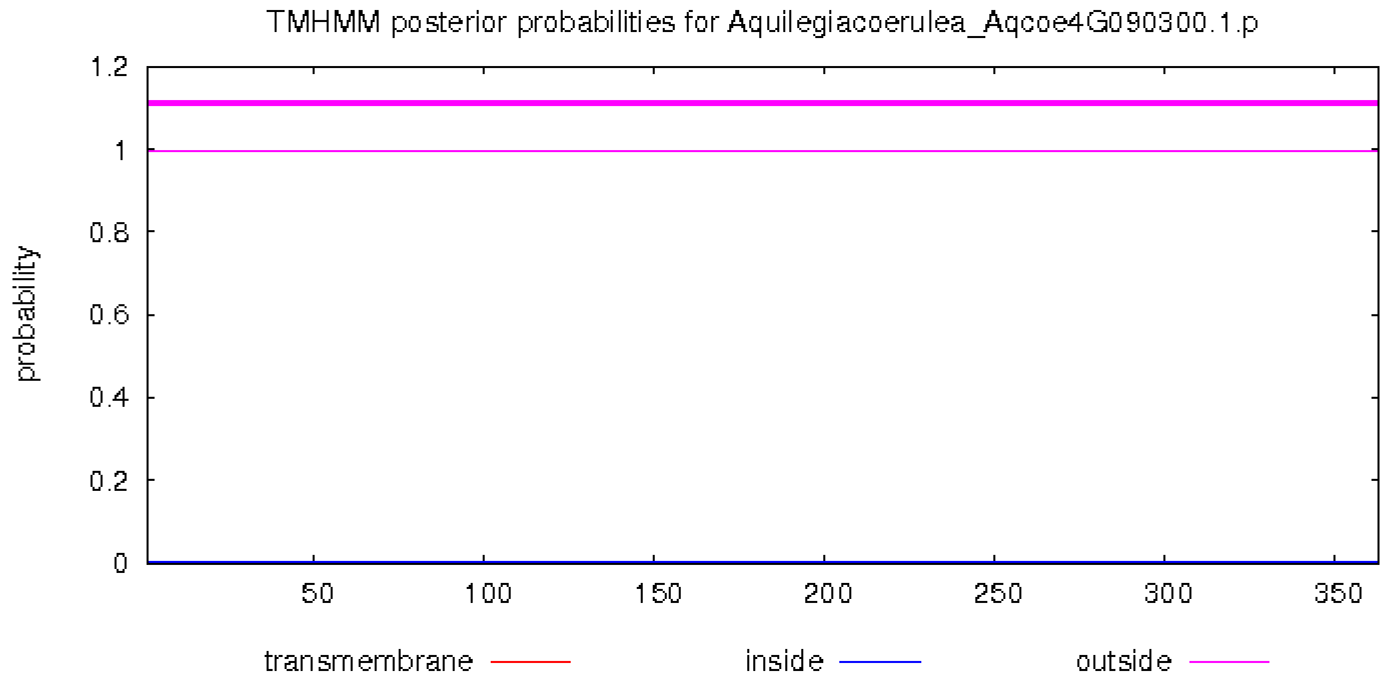

plot in postscript, script for making the plot in gnuplot, data for plot

```
# Aquilegiacoerulea_Aqcoe4G132100.1.p Length: 231
# Aquilegiacoerulea_Aqcoe4G132100.1.p Number of predicted TMHs:  0
# Aquilegiacoerulea_Aqcoe4G132100.1.p Exp number of AAs in TMHs: 0.00104
# Aquilegiacoerulea_Aqcoe4G132100.1.p Exp number, first 60 AAs:  0.00018
# Aquilegiacoerulea_Aqcoe4G132100.1.p Total prob of N-in:        0.11407
Aquilegiacoerulea_Aqcoe4G132100.1.p     TMHMM2.0        outside      1   231
```

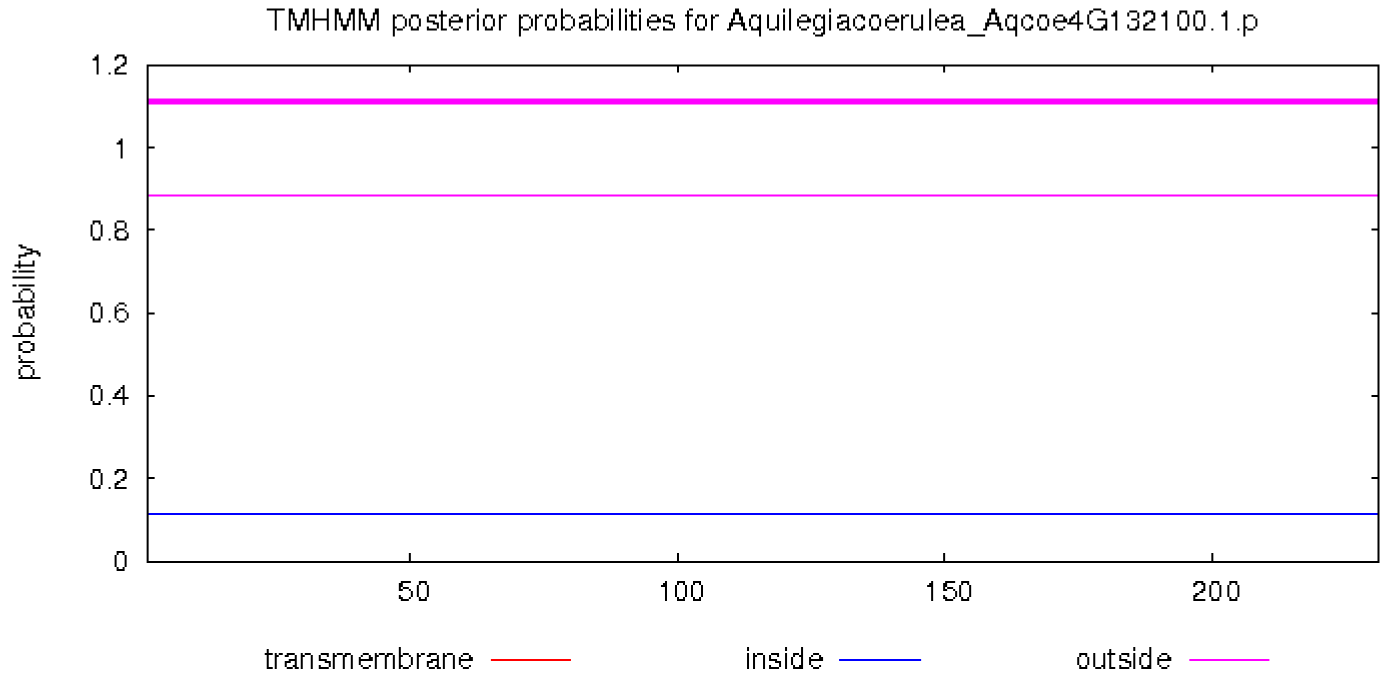

plot in postscript, script for making the plot in gnuplot, data for plot

---

```
# Aquilegiacoerulea_Aqcoe4G152500.1.p Length: 346
# Aquilegiacoerulea_Aqcoe4G152500.1.p Number of predicted TMHs:  0
# Aquilegiacoerulea_Aqcoe4G152500.1.p Exp number of AAs in TMHs: 0.00019
# Aquilegiacoerulea_Aqcoe4G152500.1.p Exp number, first 60 AAs:  0.00019
# Aquilegiacoerulea_Aqcoe4G152500.1.p Total prob of N-in:        0.01544
Aquilegiacoerulea_Aqcoe4G152500.1.p     TMHMM2.0        outside      1   346
```

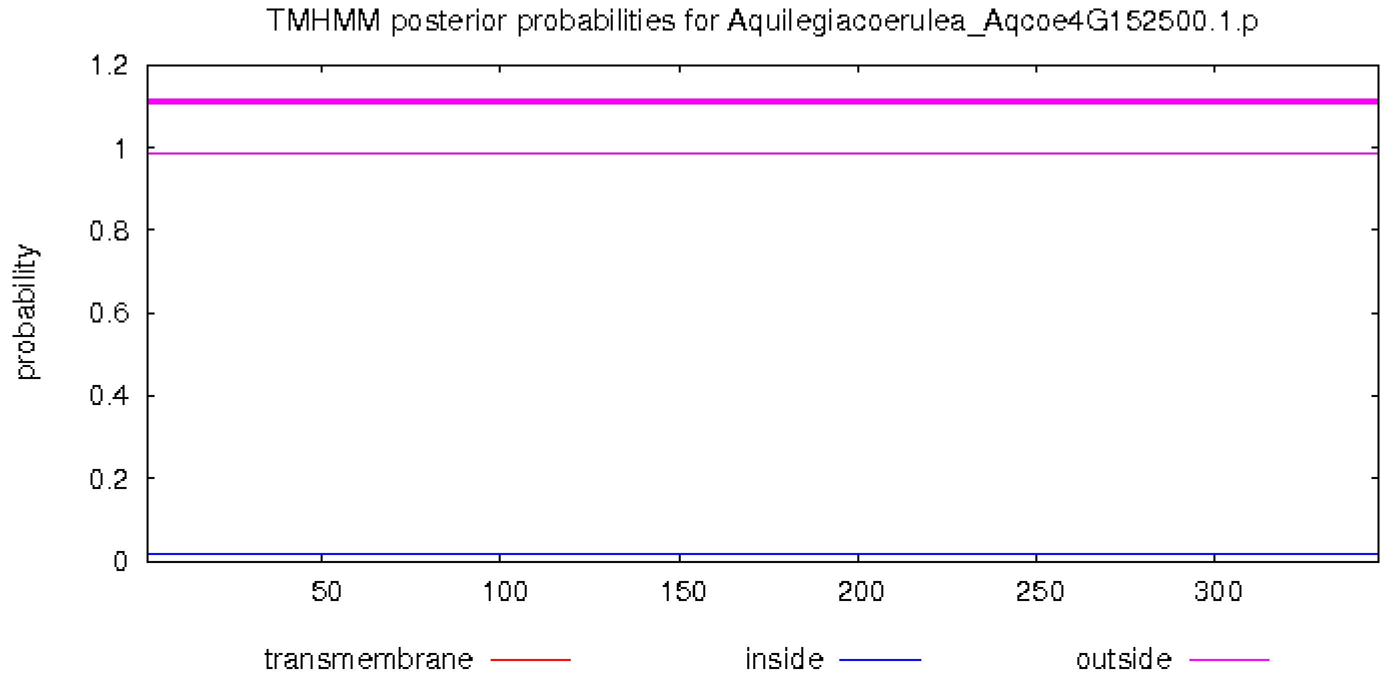

plot in postscript, script for making the plot in gnuplot, data for plot

---

```
# Aquilegiacoerulea_Aqcoe5G024200.1.p Length: 269
# Aquilegiacoerulea_Aqcoe5G024200.1.p Number of predicted TMHs:  0
# Aquilegiacoerulea_Aqcoe5G024200.1.p Exp number of AAs in TMHs: 0.00018
# Aquilegiacoerulea_Aqcoe5G024200.1.p Exp number, first 60 AAs:  0
# Aquilegiacoerulea_Aqcoe5G024200.1.p Total prob of N-in:        0.02513
Aquilegiacoerulea_Aqcoe5G024200.1.p     TMHMM2.0        outside      1   269
```

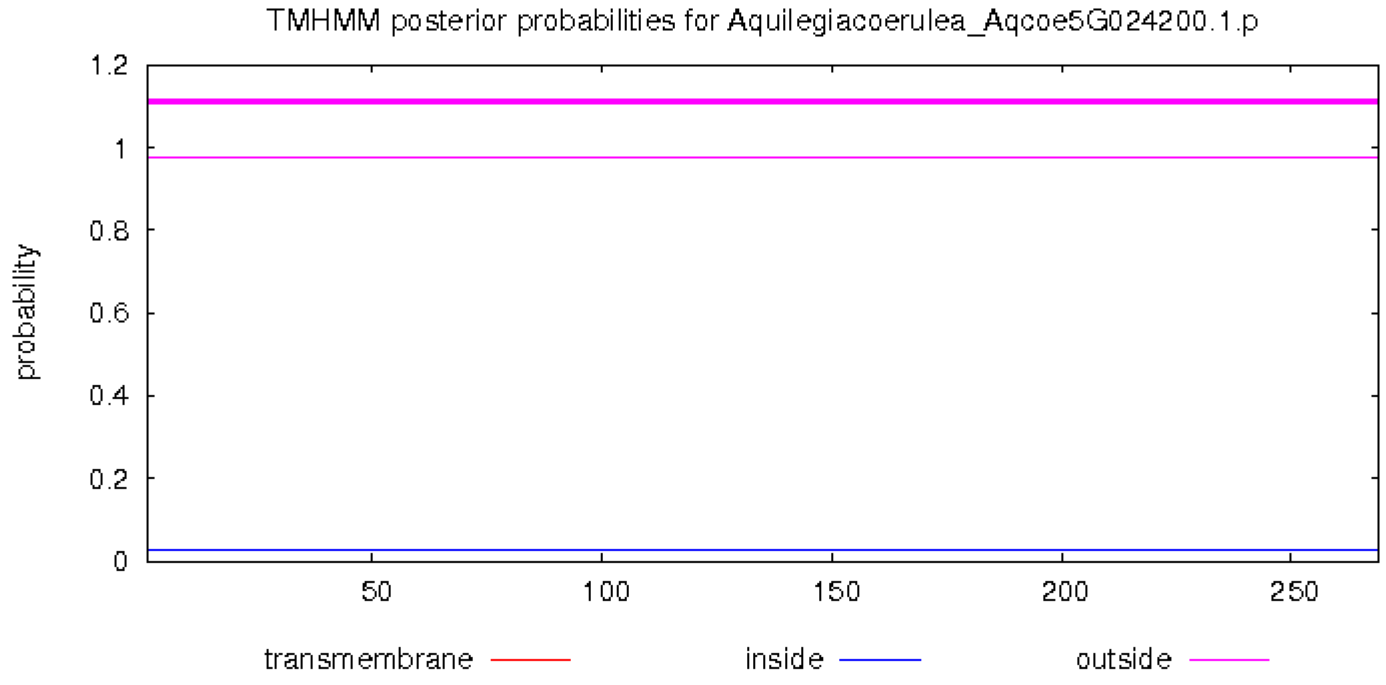

plot in postscript, script for making the plot in gnuplot, data for plot

---

```
# Aquilegiacoerulea_Aqcoe5G084800.1.p Length: 368
# Aquilegiacoerulea_Aqcoe5G084800.1.p Number of predicted TMHs:  0
# Aquilegiacoerulea_Aqcoe5G084800.1.p Exp number of AAs in TMHs: 0.00713
# Aquilegiacoerulea_Aqcoe5G084800.1.p Exp number, first 60 AAs:  0.00019
# Aquilegiacoerulea_Aqcoe5G084800.1.p Total prob of N-in:        0.00986
Aquilegiacoerulea_Aqcoe5G084800.1.p     TMHMM2.0        outside      1   368
```

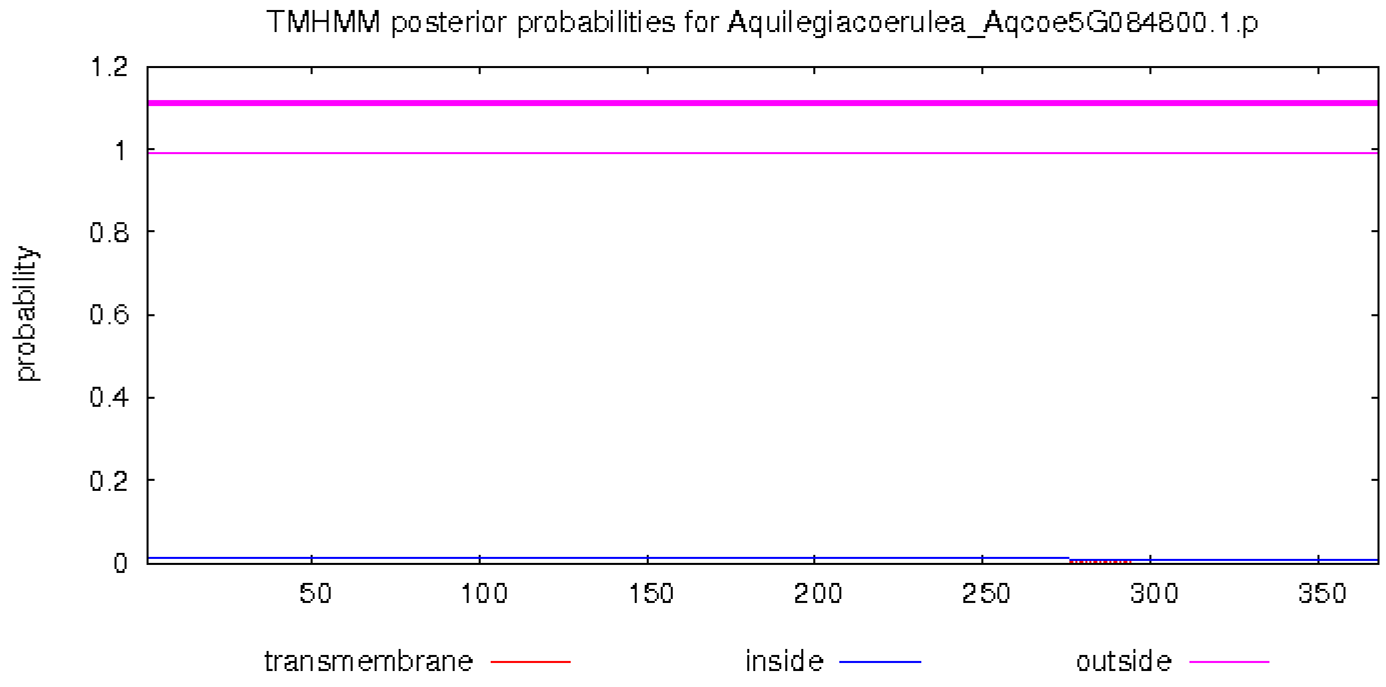

plot in postscript, script for making the plot in gnuplot, data for plot

---

```
# Aquilegiacoerulea_Aqcoe5G095700.1.p Length: 279
# Aquilegiacoerulea_Aqcoe5G095700.1.p Number of predicted TMHs:  0
# Aquilegiacoerulea_Aqcoe5G095700.1.p Exp number of AAs in TMHs: 0.00054
# Aquilegiacoerulea_Aqcoe5G095700.1.p Exp number, first 60 AAs:  0.00017
# Aquilegiacoerulea_Aqcoe5G095700.1.p Total prob of N-in:        0.07514
Aquilegiacoerulea_Aqcoe5G095700.1.p     TMHMM2.0        outside      1   279
```

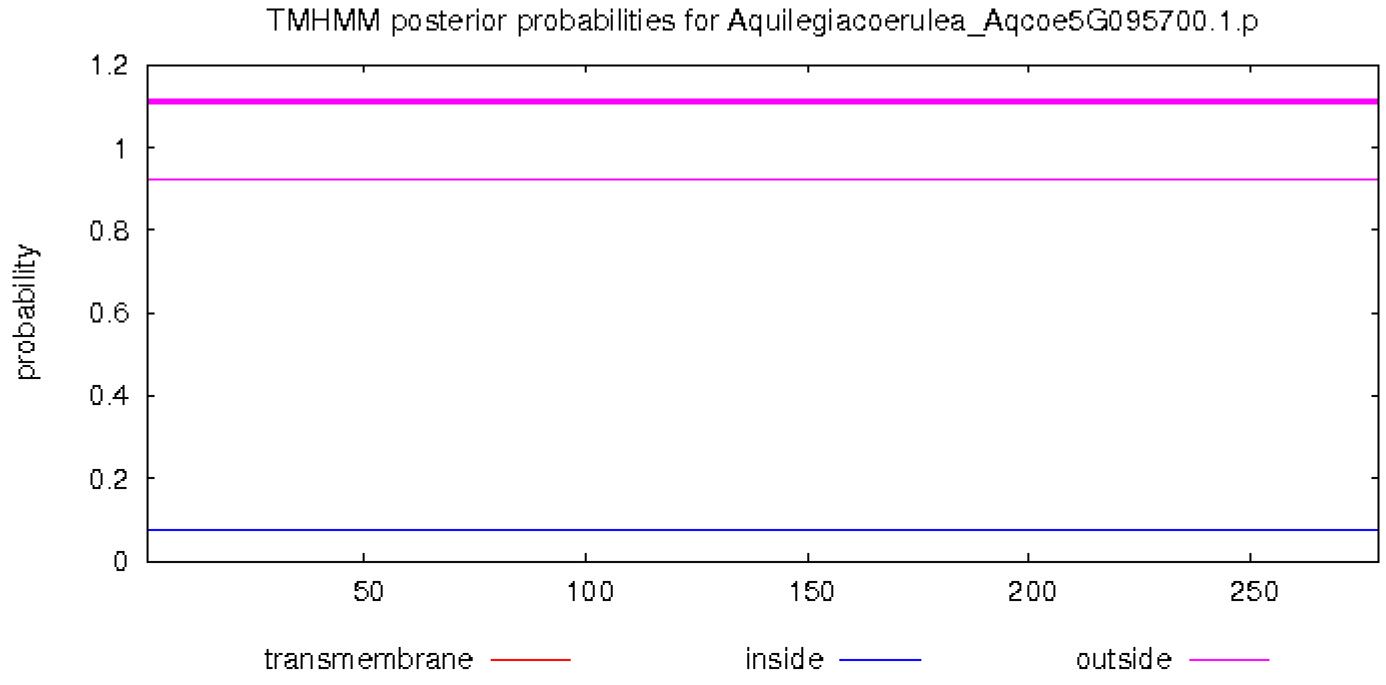

plot in postscript, script for making the plot in gnuplot, data for plot

---

```
# Aquilegiacoerulea_Aqcoe5G095800.1.p Length: 320
# Aquilegiacoerulea_Aqcoe5G095800.1.p Number of predicted TMHs:  0
# Aquilegiacoerulea_Aqcoe5G095800.1.p Exp number of AAs in TMHs: 0.00019
# Aquilegiacoerulea_Aqcoe5G095800.1.p Exp number, first 60 AAs:  0
# Aquilegiacoerulea_Aqcoe5G095800.1.p Total prob of N-in:        0.00421
Aquilegiacoerulea_Aqcoe5G095800.1.p     TMHMM2.0        outside      1   320
```

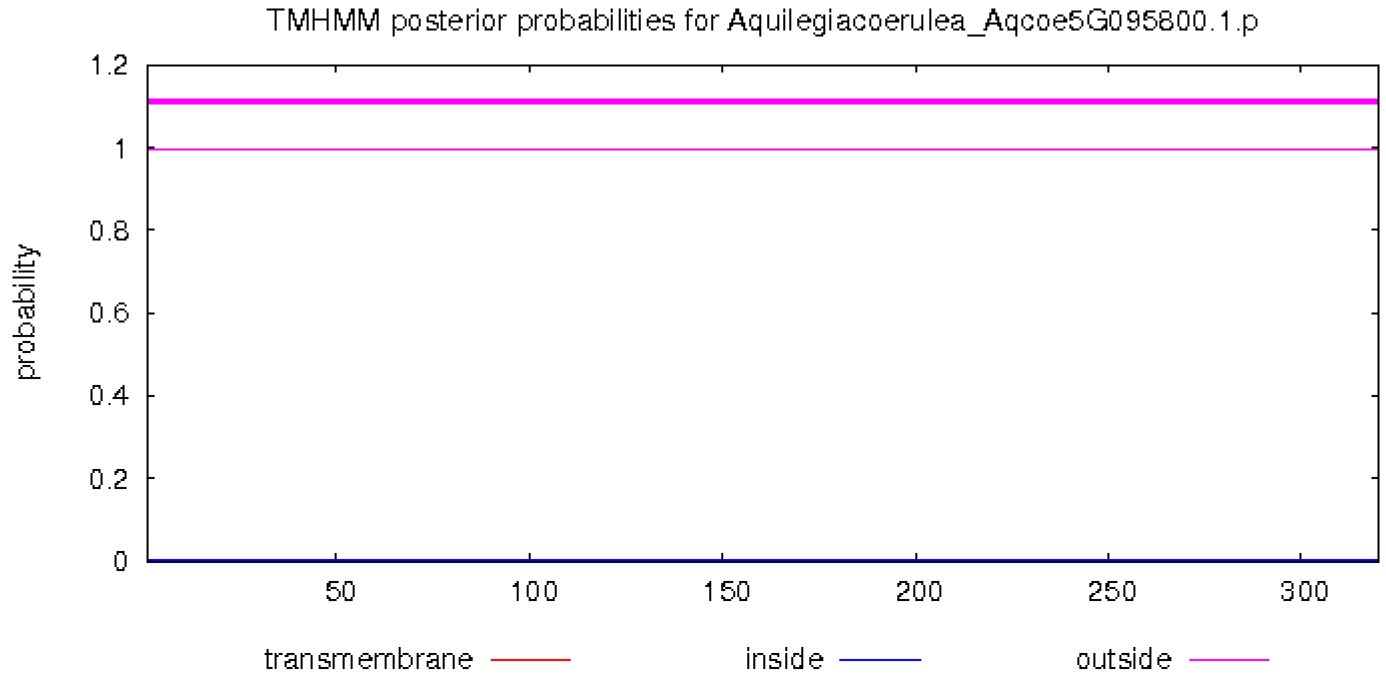

plot in postscript, script for making the plot in gnuplot, data for plot

---

```
# Aquilegiacoerulea_Aqcoe5G106600.1.p Length: 618
# Aquilegiacoerulea_Aqcoe5G106600.1.p Number of predicted TMHs:  1
# Aquilegiacoerulea_Aqcoe5G106600.1.p Exp number of AAs in TMHs: 25.33317
# Aquilegiacoerulea_Aqcoe5G106600.1.p Exp number, first 60 AAs:  0.00165
# Aquilegiacoerulea_Aqcoe5G106600.1.p Total prob of N-in:        0.22727
Aquilegiacoerulea_Aqcoe5G106600.1.p     TMHMM2.0        outside      1   582
```

```
Aquilegiacoerulea_Aqcoe5G106600.1.p     TMHMM2.0        TMhelix     583    605
Aquilegiacoerulea_Aqcoe5G106600.1.p     TMHMM2.0        inside      606    618
```

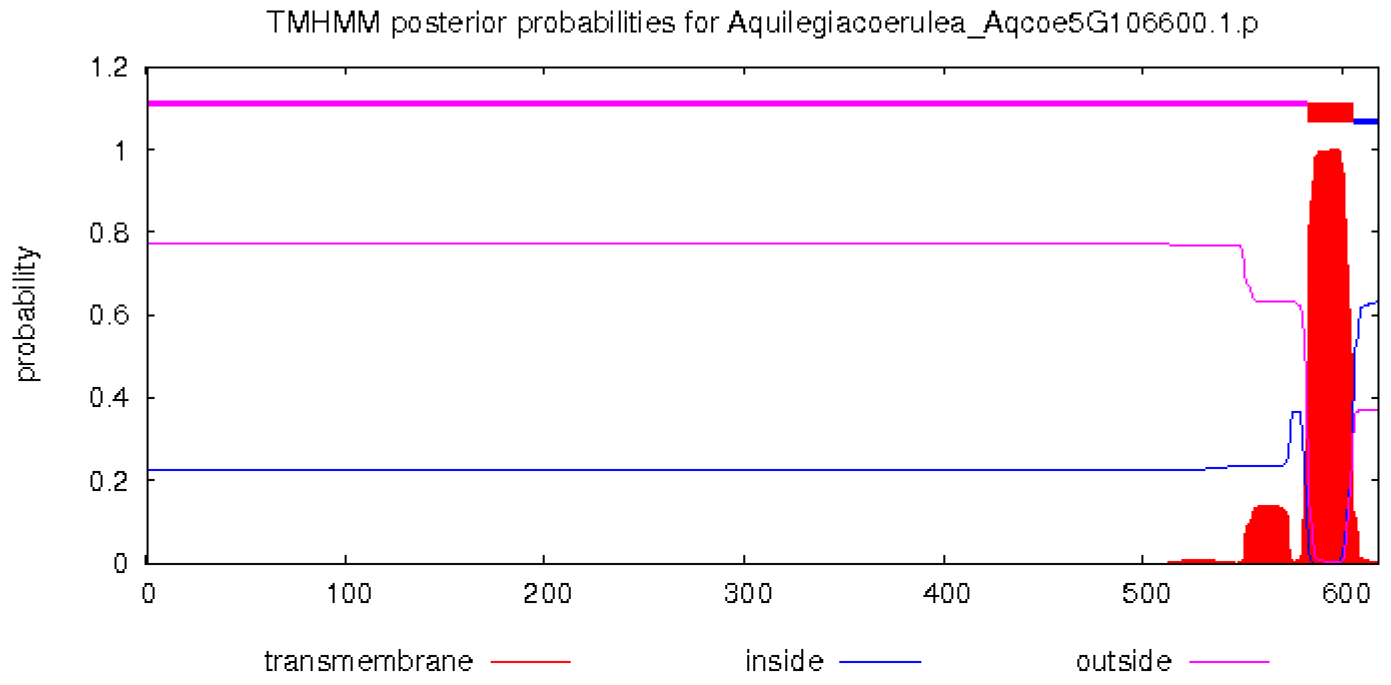

plot in postscript, script for making the plot in gnuplot, data for plot

---

```
# Aquilegiacoerulea_Aqcoe5G113900.1.p Length: 363
# Aquilegiacoerulea_Aqcoe5G113900.1.p Number of predicted TMHs:  0
# Aquilegiacoerulea_Aqcoe5G113900.1.p Exp number of AAs in TMHs: 0.00018
# Aquilegiacoerulea_Aqcoe5G113900.1.p Exp number, first 60 AAs:  0.00018
# Aquilegiacoerulea_Aqcoe5G113900.1.p Total prob of N-in:        0.00226
Aquilegiacoerulea_Aqcoe5G113900.1.p     TMHMM2.0        outside      1   363
```

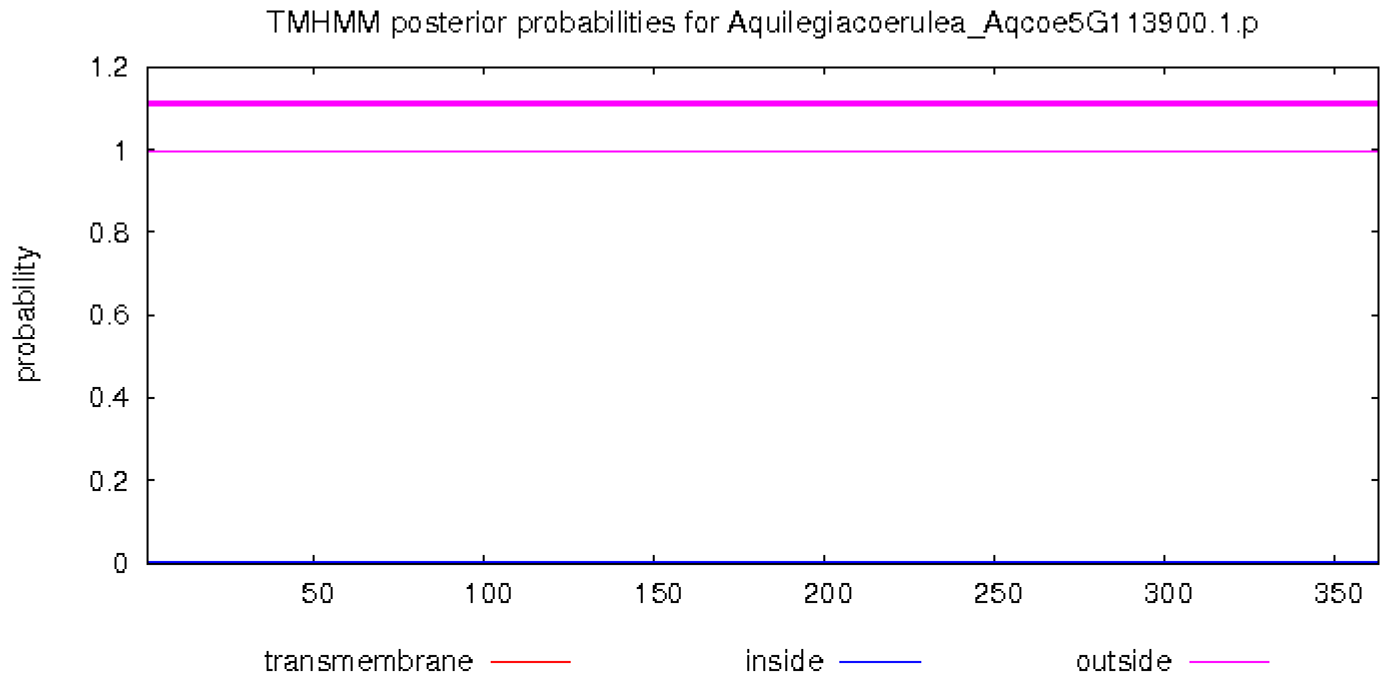

plot in postscript, script for making the plot in gnuplot, data for plot

---

```
# Aquilegiacoerulea_Aqcoe5G177100.1.p Length: 327
# Aquilegiacoerulea_Aqcoe5G177100.1.p Number of predicted TMHs:  0
# Aquilegiacoerulea_Aqcoe5G177100.1.p Exp number of AAs in TMHs: 0
# Aquilegiacoerulea_Aqcoe5G177100.1.p Exp number, first 60 AAs:  0
```

```
# Aquilegiacoerulea_Aqcoe5G177100.1.p Total prob of N-in:        0.00835
Aquilegiacoerulea_Aqcoe5G177100.1.p     TMHMM2.0        outside      1   327
```

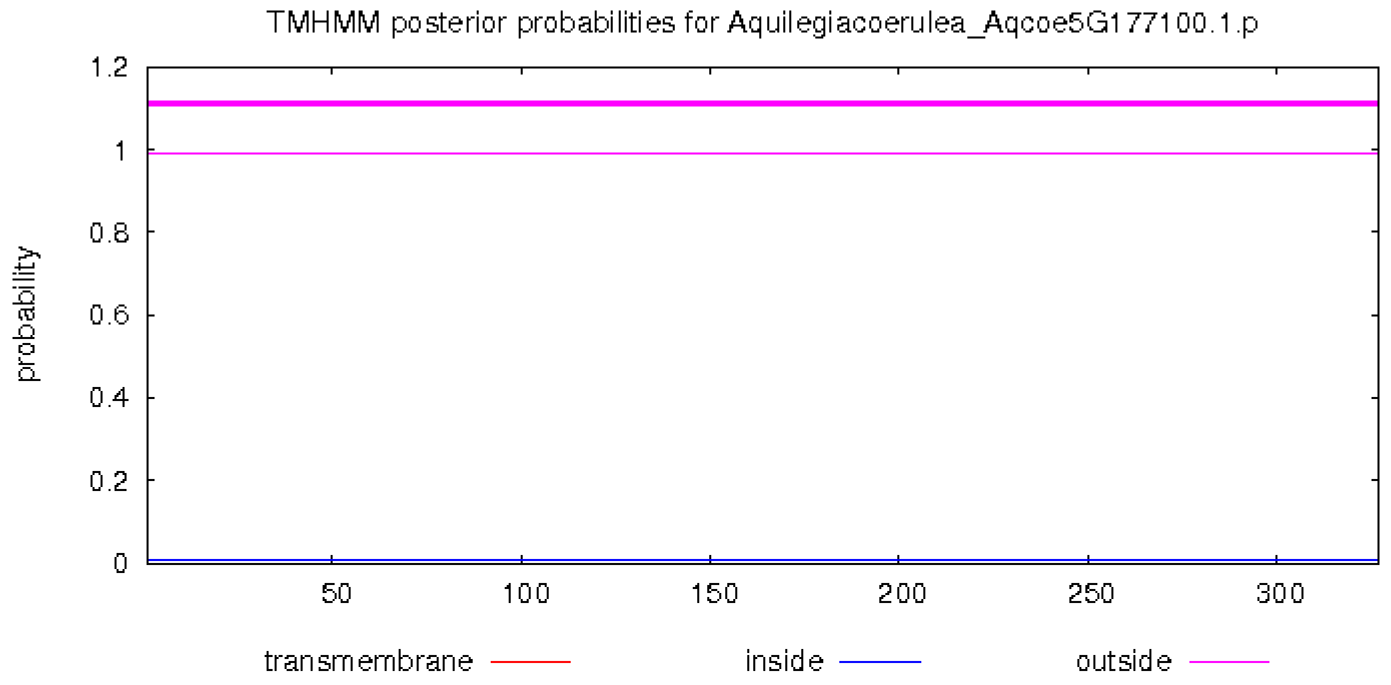

plot in postscript, script for making the plot in gnuplot, data for plot

---

```
# Aquilegiacoerulea_Aqcoe5G198100.1.p Length: 196
# Aquilegiacoerulea_Aqcoe5G198100.1.p Number of predicted TMHs:  0
# Aquilegiacoerulea_Aqcoe5G198100.1.p Exp number of AAs in TMHs: 0.01491
# Aquilegiacoerulea_Aqcoe5G198100.1.p Exp number, first 60 AAs:  0.01452
# Aquilegiacoerulea_Aqcoe5G198100.1.p Total prob of N-in:        0.02036
Aquilegiacoerulea_Aqcoe5G198100.1.p     TMHMM2.0        outside      1   196
```

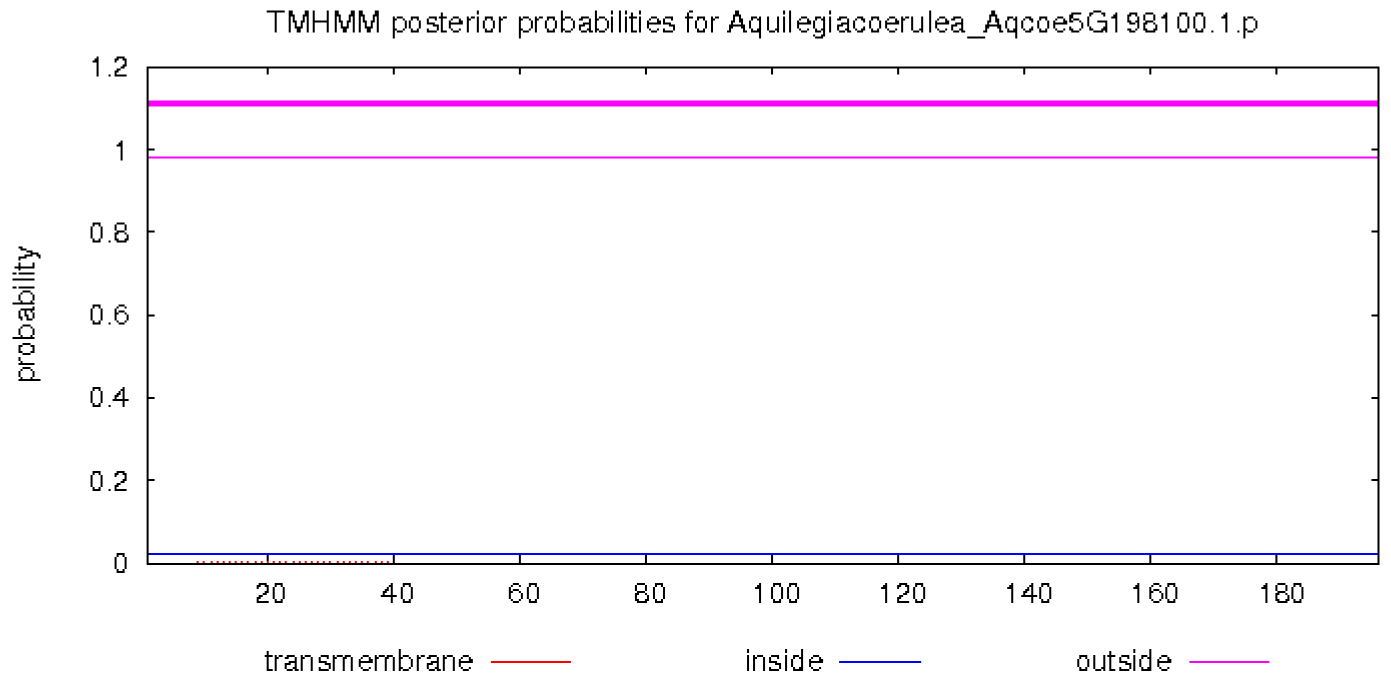

plot in postscript, script for making the plot in gnuplot, data for plot

---

```
# Aquilegiacoerulea_Aqcoe5G198200.1.p Length: 195
# Aquilegiacoerulea_Aqcoe5G198200.1.p Number of predicted TMHs:  0
# Aquilegiacoerulea_Aqcoe5G198200.1.p Exp number of AAs in TMHs: 0.06155
# Aquilegiacoerulea_Aqcoe5G198200.1.p Exp number, first 60 AAs:  0.05333
```

```
# Aquilegiacoerulea_Aqcoe5G198200.1.p Total prob of N-in:        0.00643
Aquilegiacoerulea_Aqcoe5G198200.1.p     TMHMM2.0        outside      1   195
```

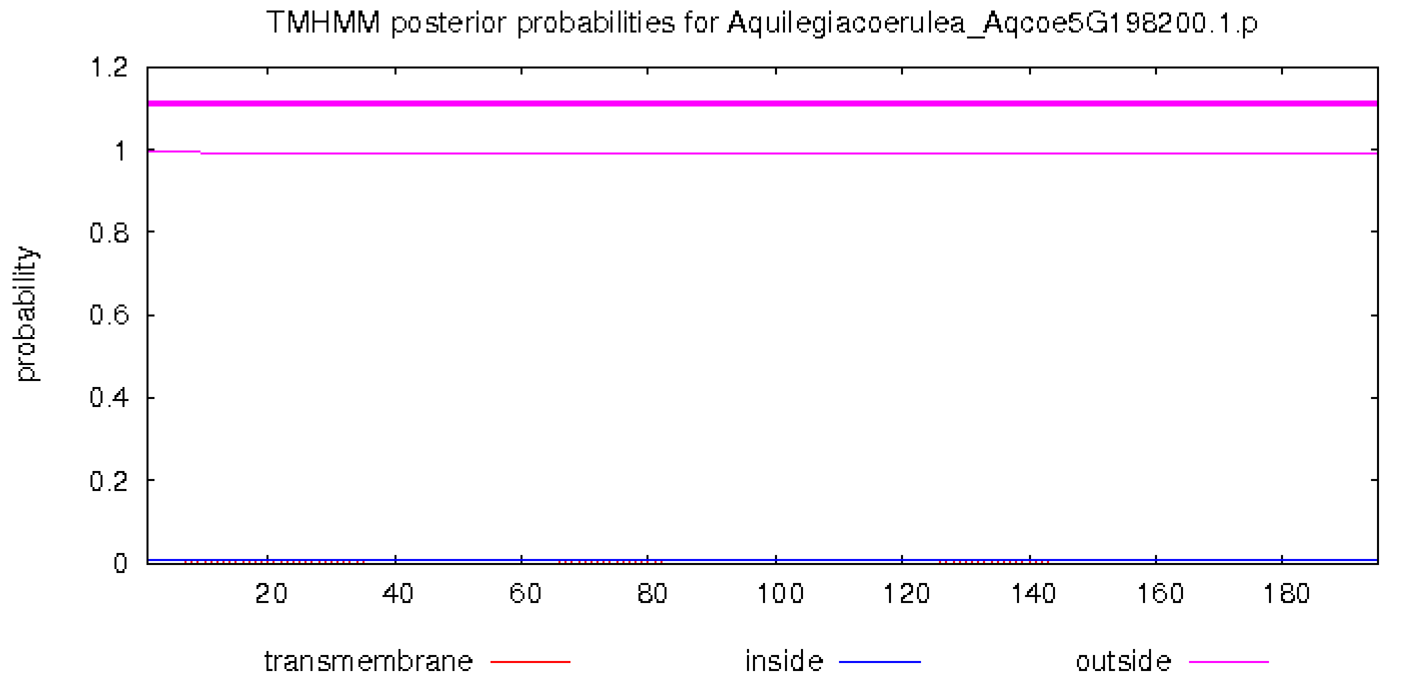

plot in postscript, script for making the plot in gnuplot, data for plot

---

```
# Aquilegiacoerulea_Aqcoe5G225900.1.p Length: 600
# Aquilegiacoerulea_Aqcoe5G225900.1.p Number of predicted TMHs:  0
# Aquilegiacoerulea_Aqcoe5G225900.1.p Exp number of AAs in TMHs: 0.0128
# Aquilegiacoerulea_Aqcoe5G225900.1.p Exp number, first 60 AAs:  0.0006
# Aquilegiacoerulea_Aqcoe5G225900.1.p Total prob of N-in:        0.00342
Aquilegiacoerulea_Aqcoe5G225900.1.p     TMHMM2.0        outside      1   600
```

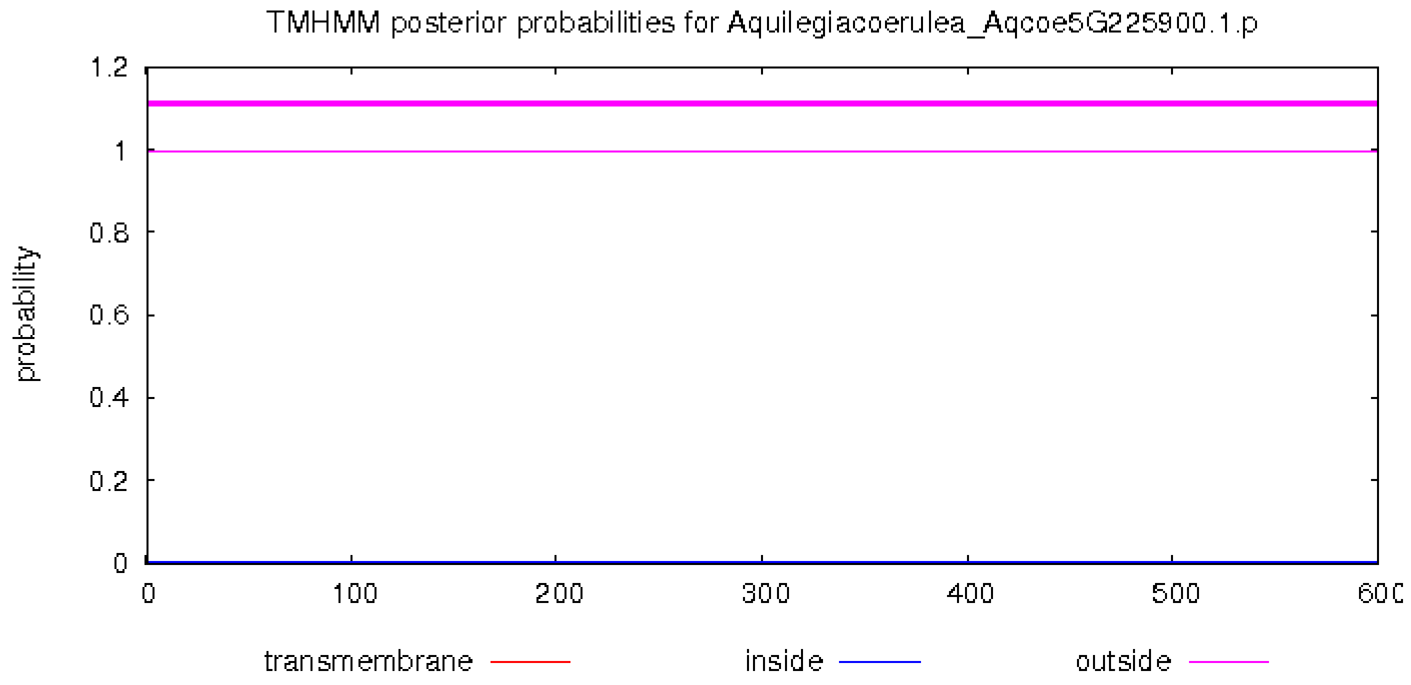

plot in postscript, script for making the plot in gnuplot, data for plot

---

```
# Aquilegiacoerulea_Aqcoe5G233600.1.p Length: 195
# Aquilegiacoerulea_Aqcoe5G233600.1.p Number of predicted TMHs:  0
# Aquilegiacoerulea_Aqcoe5G233600.1.p Exp number of AAs in TMHs: 0.10339
# Aquilegiacoerulea_Aqcoe5G233600.1.p Exp number, first 60 AAs:  0.00094
```

```
# Aquilegiacoerulea_Aqcoe5G233600.1.p Total prob of N-in:        0.04275
Aquilegiacoerulea_Aqcoe5G233600.1.p     TMHMM2.0        outside      1   195
```

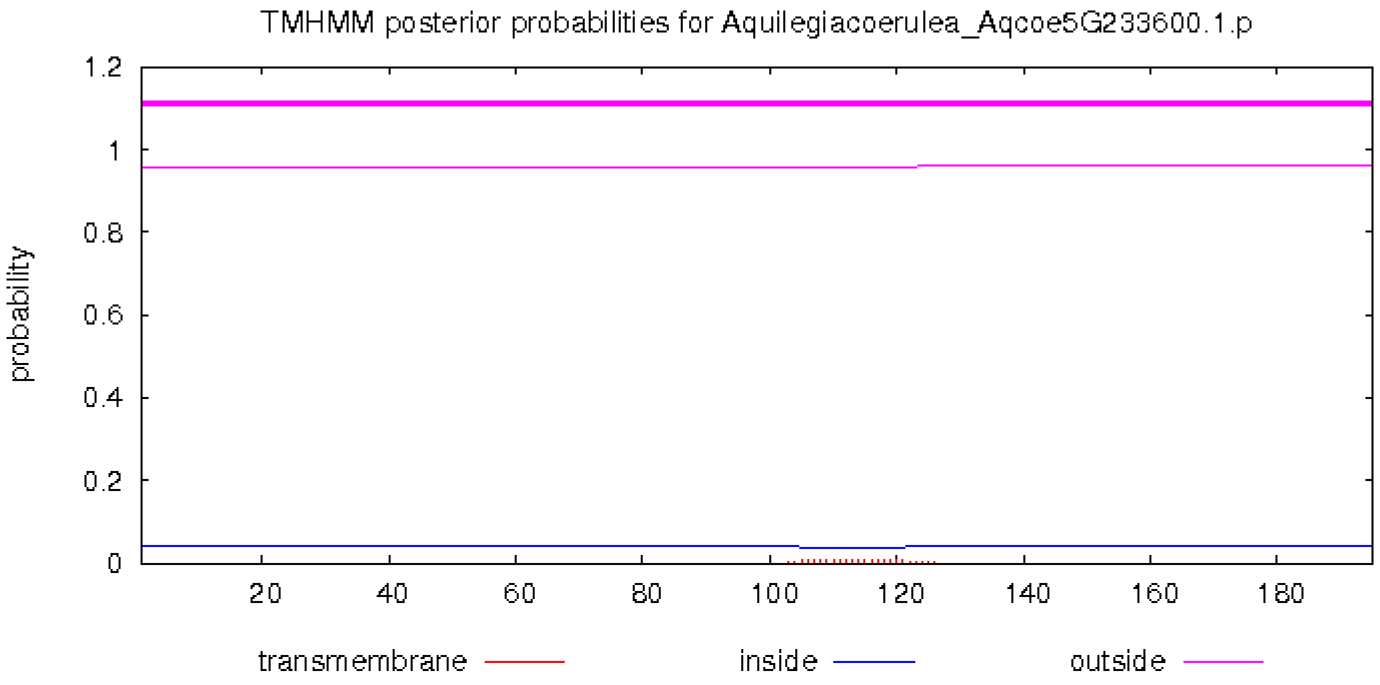

plot in postscript, script for making the plot in gnuplot, data for plot

---

```
# Aquilegiacoerulea_Aqcoe5G234900.1.p Length: 671
# Aquilegiacoerulea_Aqcoe5G234900.1.p Number of predicted TMHs:  1
# Aquilegiacoerulea_Aqcoe5G234900.1.p Exp number of AAs in TMHs: 22.88165
# Aquilegiacoerulea_Aqcoe5G234900.1.p Exp number, first 60 AAs:  0.00161
# Aquilegiacoerulea_Aqcoe5G234900.1.p Total prob of N-in:        0.00046
Aquilegiacoerulea_Aqcoe5G234900.1.p     TMHMM2.0        outside      1   636
Aquilegiacoerulea_Aqcoe5G234900.1.p     TMHMM2.0        TMhelix    637   659
Aquilegiacoerulea_Aqcoe5G234900.1.p     TMHMM2.0        inside     660   671
```

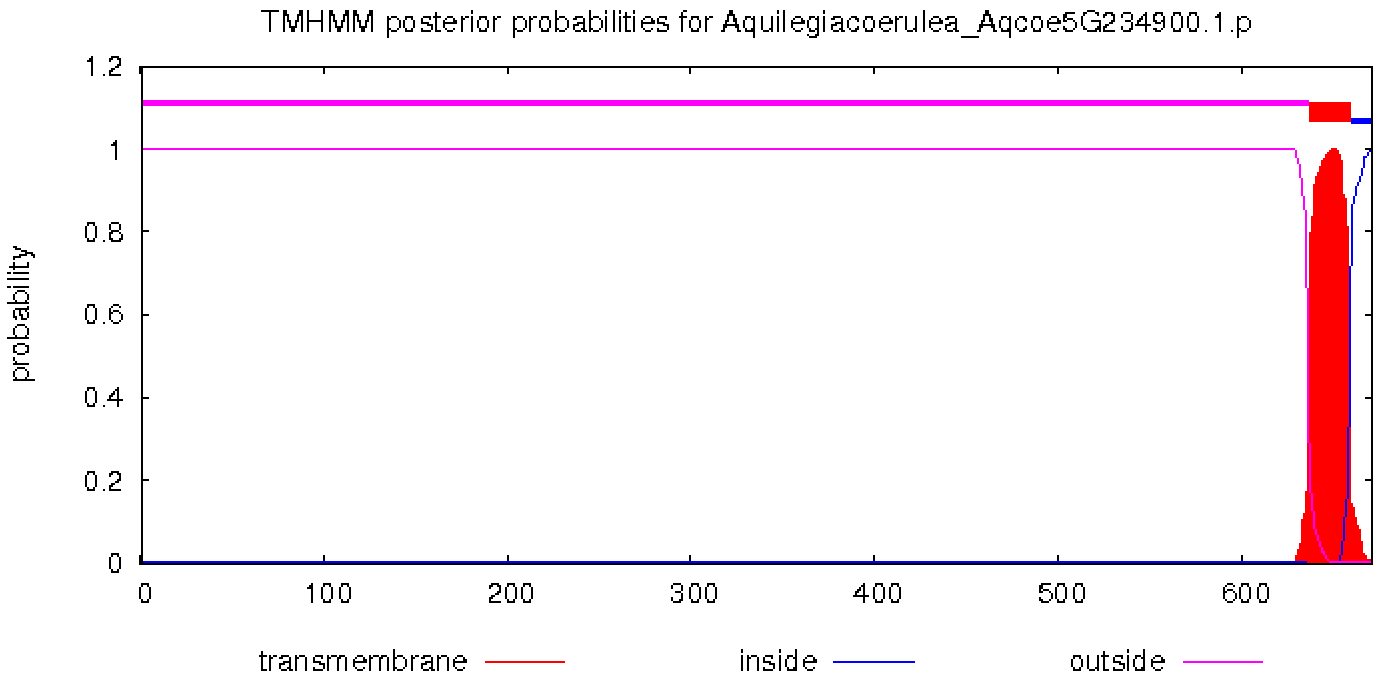

plot in postscript, script for making the plot in gnuplot, data for plot

---

```
# Aquilegiacoerulea_Aqcoe5G240700.1.p Length: 516
# Aquilegiacoerulea_Aqcoe5G240700.1.p Number of predicted TMHs:  0
```

```
# Aquilegiacoerulea_Aqcoe5G240700.1.p Exp number of AAs in TMHs: 0.0033
# Aquilegiacoerulea_Aqcoe5G240700.1.p Exp number, first 60 AAs:  0.00118
# Aquilegiacoerulea_Aqcoe5G240700.1.p Total prob of N-in:        0.00235
Aquilegiacoerulea_Aqcoe5G240700.1.p     TMHMM2.0        outside      1   516
```

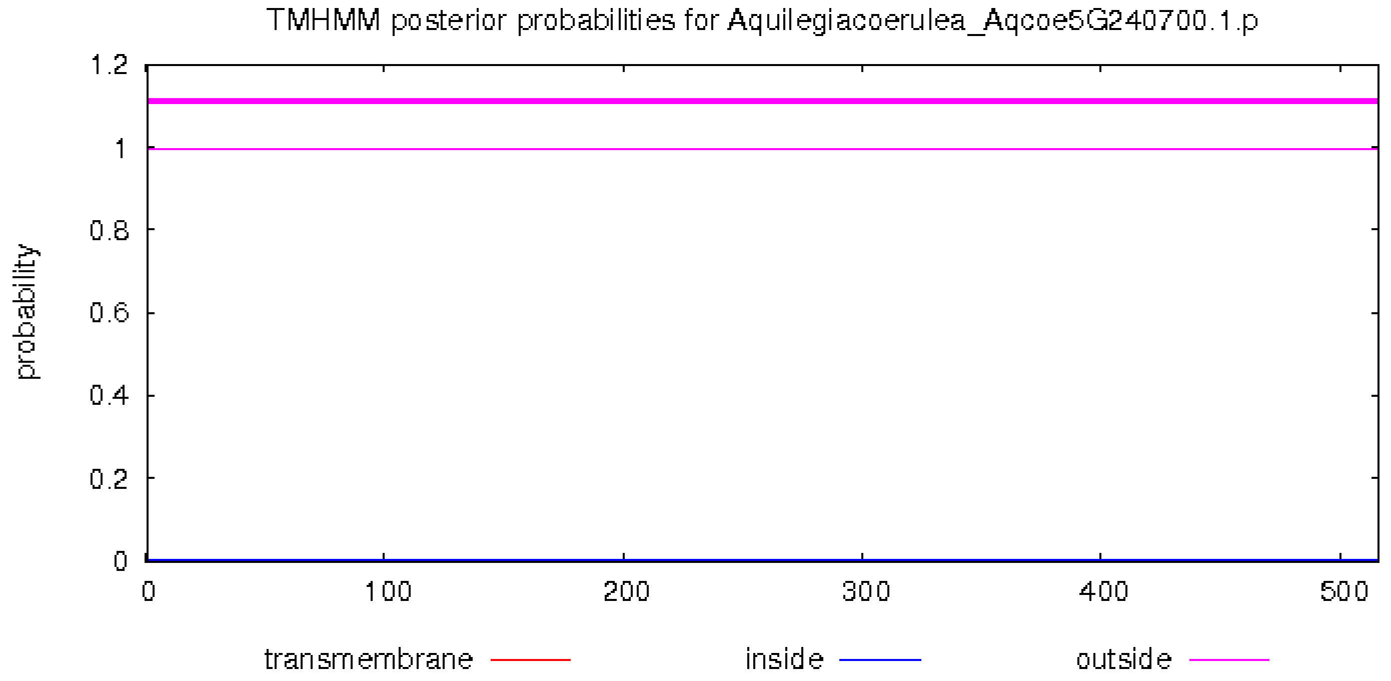

plot in postscript, script for making the plot in gnuplot, data for plot

---

```
# Aquilegiacoerulea_Aqcoe5G304500.1.p Length: 318
# Aquilegiacoerulea_Aqcoe5G304500.1.p Number of predicted TMHs:  0
# Aquilegiacoerulea_Aqcoe5G304500.1.p Exp number of AAs in TMHs: 0
# Aquilegiacoerulea_Aqcoe5G304500.1.p Exp number, first 60 AAs:  0
# Aquilegiacoerulea_Aqcoe5G304500.1.p Total prob of N-in:        0.00572
Aquilegiacoerulea_Aqcoe5G304500.1.p     TMHMM2.0        outside      1   318
```

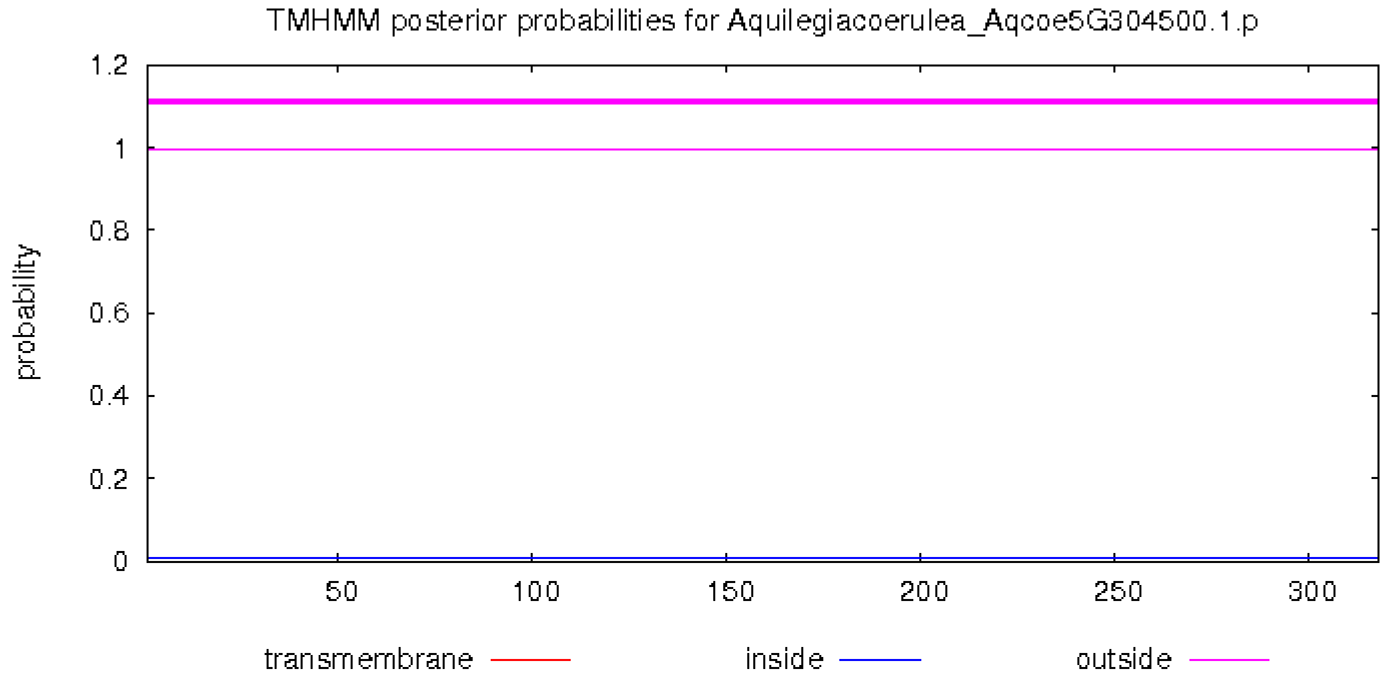

plot in postscript, script for making the plot in gnuplot, data for plot

---

```
# Aquilegiacoerulea_Aqcoe5G344500.1.p Length: 246
# Aquilegiacoerulea_Aqcoe5G344500.1.p Number of predicted TMHs:  0
```

```
# Aquilegiacoerulea_Aqcoe5G344500.1.p Exp number of AAs in TMHs: 0.00018
# Aquilegiacoerulea_Aqcoe5G344500.1.p Exp number, first 60 AAs:  0.00018
# Aquilegiacoerulea_Aqcoe5G344500.1.p Total prob of N-in:        0.09703
Aquilegiacoerulea_Aqcoe5G344500.1.p     TMHMM2.0        outside      1   246
```

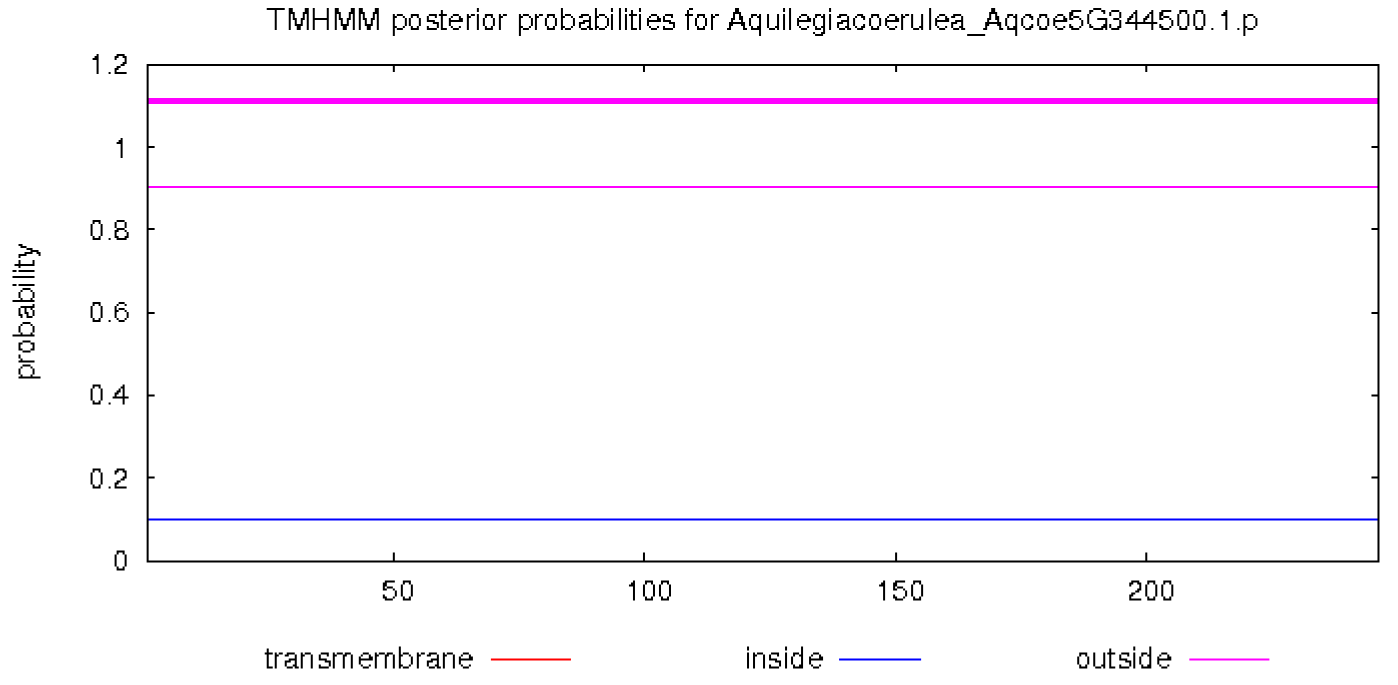

plot in postscript, script for making the plot in gnuplot, data for plot

```
# Aquilegiacoerulea_Aqcoe5G470900.1.p Length: 397
# Aquilegiacoerulea_Aqcoe5G470900.1.p Number of predicted TMHs:  0
# Aquilegiacoerulea_Aqcoe5G470900.1.p Exp number of AAs in TMHs: 0
# Aquilegiacoerulea_Aqcoe5G470900.1.p Exp number, first 60 AAs:  0
# Aquilegiacoerulea_Aqcoe5G470900.1.p Total prob of N-in:        0.00565
Aquilegiacoerulea_Aqcoe5G470900.1.p     TMHMM2.0        outside      1   397
```

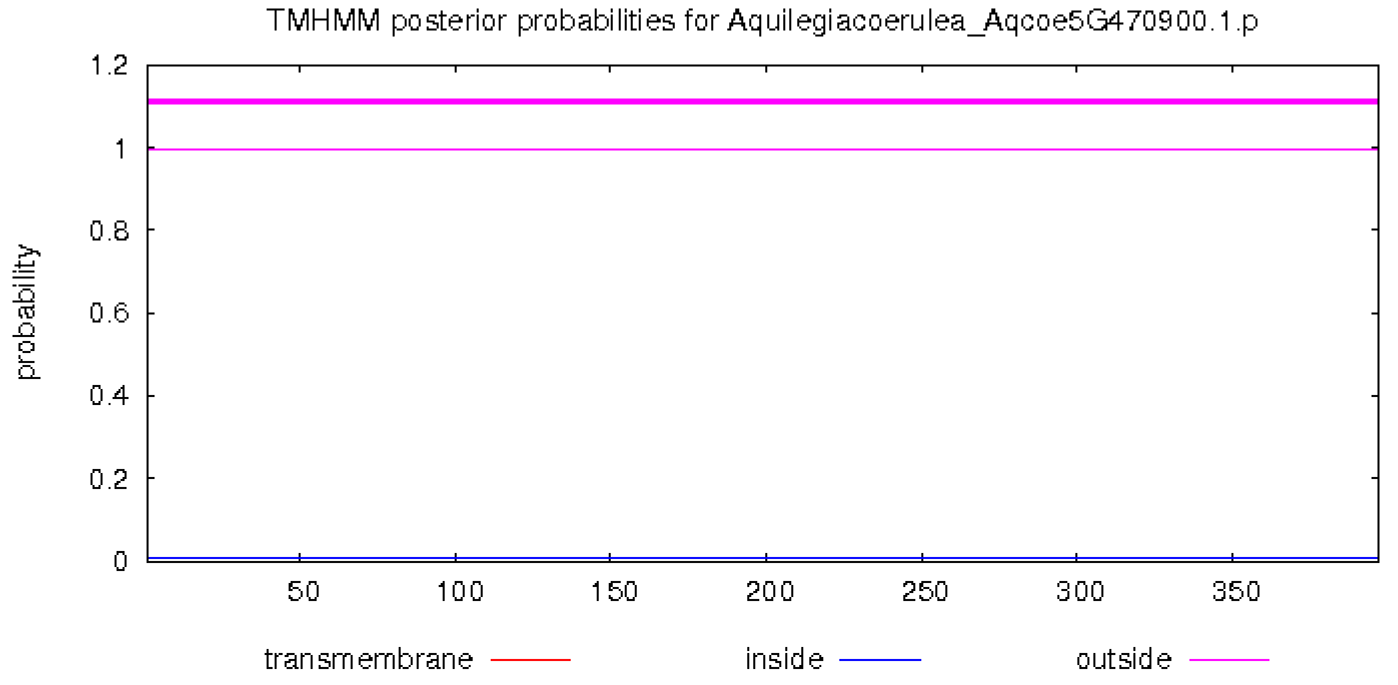

plot in postscript, script for making the plot in gnuplot, data for plot

```
# Aquilegiacoerulea_Aqcoe6G001700.1.p Length: 373
# Aquilegiacoerulea_Aqcoe6G001700.1.p Number of predicted TMHs:  0
```

```
# Aquilegiacoerulea_Aqcoe6G001700.1.p Exp number of AAs in TMHs: 0.0027
# Aquilegiacoerulea_Aqcoe6G001700.1.p Exp number, first 60 AAs:  0.00048
# Aquilegiacoerulea_Aqcoe6G001700.1.p Total prob of N-in:        0.02661
Aquilegiacoerulea_Aqcoe6G001700.1.p     TMHMM2.0        outside      1   373
```

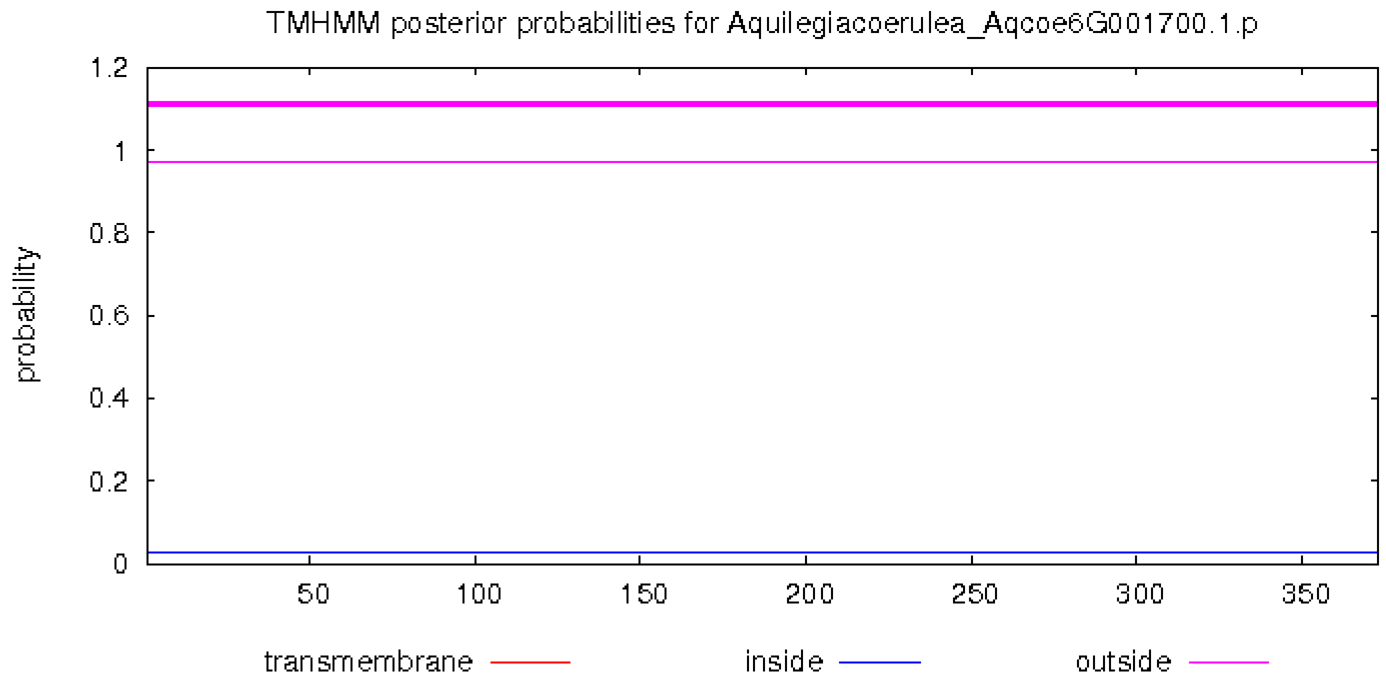

plot in postscript, script for making the plot in gnuplot, data for plot

---

```
# Aquilegiacoerulea_Aqcoe6G003200.1.p Length: 368
# Aquilegiacoerulea_Aqcoe6G003200.1.p Number of predicted TMHs:  0
# Aquilegiacoerulea_Aqcoe6G003200.1.p Exp number of AAs in TMHs: 0
# Aquilegiacoerulea_Aqcoe6G003200.1.p Exp number, first 60 AAs:  0
# Aquilegiacoerulea_Aqcoe6G003200.1.p Total prob of N-in:        0.00308
Aquilegiacoerulea_Aqcoe6G003200.1.p     TMHMM2.0        outside      1   368
```

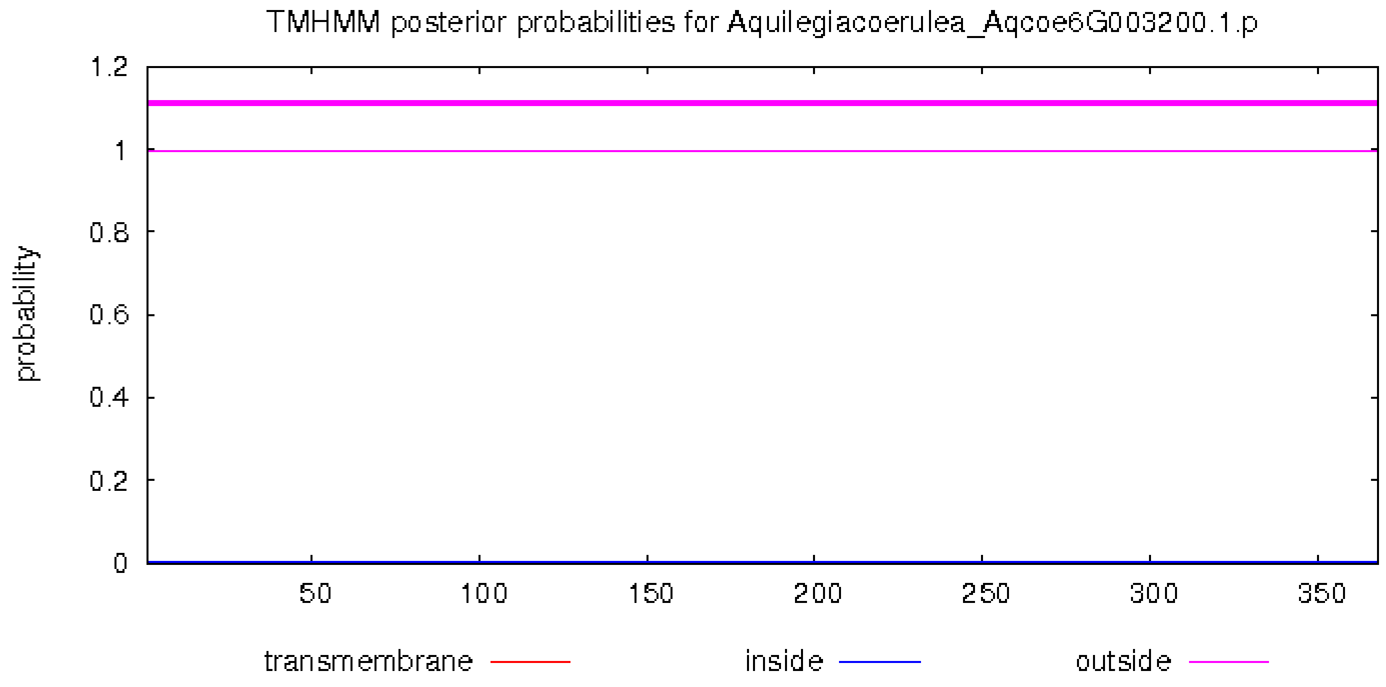

plot in postscript, script for making the plot in gnuplot, data for plot

---

```
# Aquilegiacoerulea_Aqcoe6G137000.1.p Length: 290
# Aquilegiacoerulea_Aqcoe6G137000.1.p Number of predicted TMHs:  0
```

```
# Aquilegiacoerulea_Aqcoe6G137000.1.p Exp number of AAs in TMHs: 0.00817
# Aquilegiacoerulea_Aqcoe6G137000.1.p Exp number, first 60 AAs:  0
# Aquilegiacoerulea_Aqcoe6G137000.1.p Total prob of N-in:        0.04933
Aquilegiacoerulea_Aqcoe6G137000.1.p     TMHMM2.0        outside      1   290
```

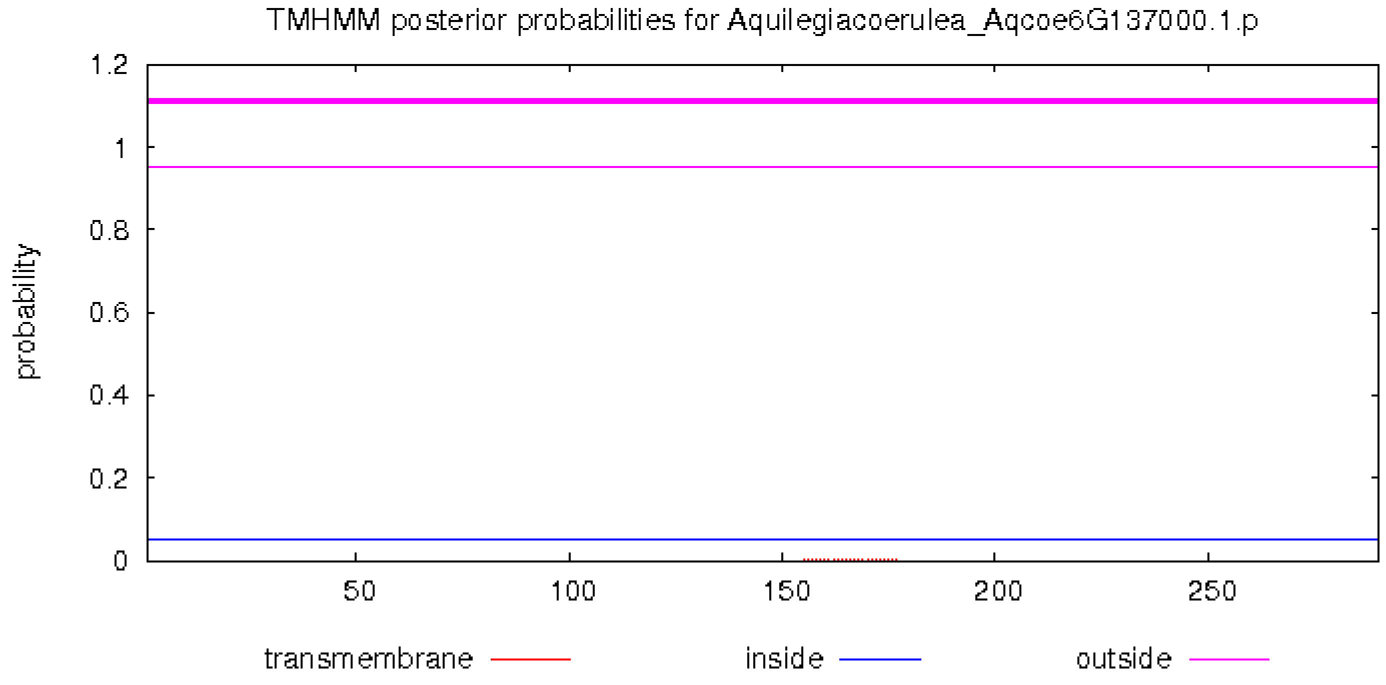

plot in postscript, script for making the plot in gnuplot, data for plot

---

```
# Aquilegiacoerulea_Aqcoe6G165500.1.p Length: 395
# Aquilegiacoerulea_Aqcoe6G165500.1.p Number of predicted TMHs:  0
# Aquilegiacoerulea_Aqcoe6G165500.1.p Exp number of AAs in TMHs: 0.00334
# Aquilegiacoerulea_Aqcoe6G165500.1.p Exp number, first 60 AAs:  0.00085
# Aquilegiacoerulea_Aqcoe6G165500.1.p Total prob of N-in:        0.00180
Aquilegiacoerulea_Aqcoe6G165500.1.p     TMHMM2.0        outside      1   395
```

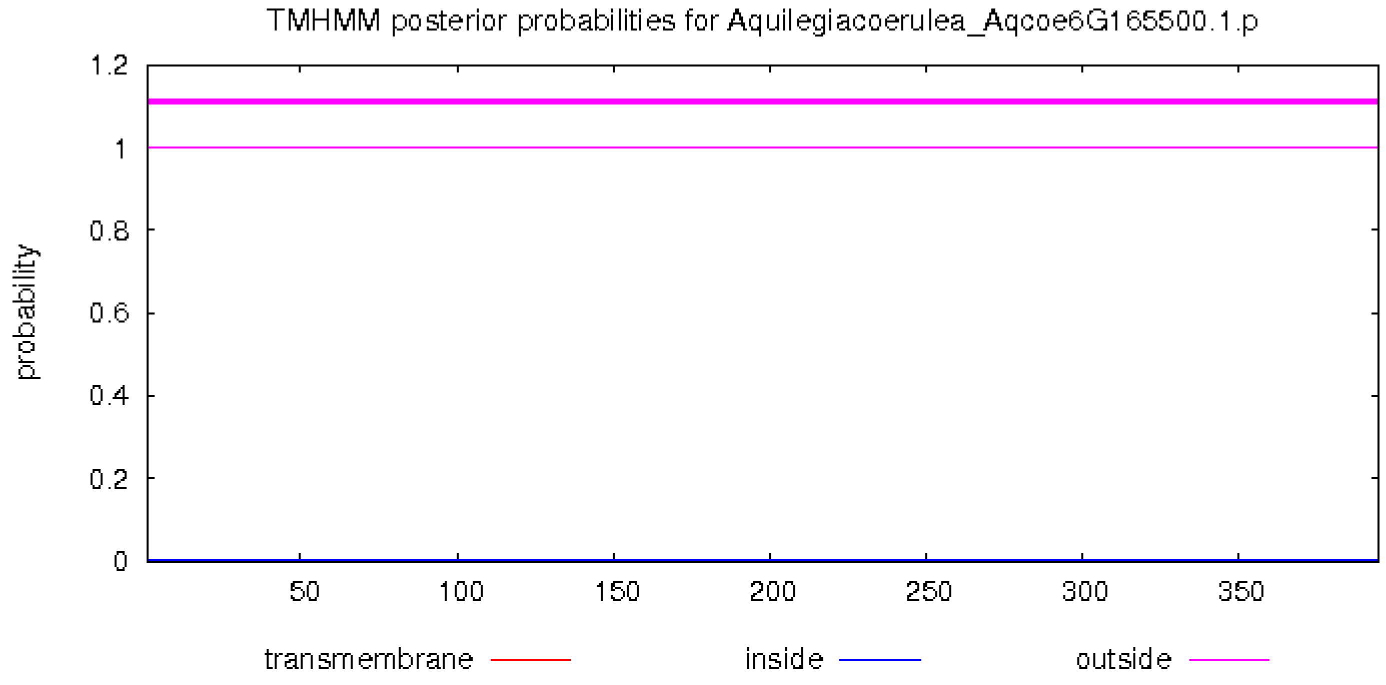

plot in postscript, script for making the plot in gnuplot, data for plot

---

```
# Aquilegiacoerulea_Aqcoe6G193500.1.p Length: 286
# Aquilegiacoerulea_Aqcoe6G193500.1.p Number of predicted TMHs:  0
```

```
# Aquilegiacoerulea_Aqcoe6G193500.1.p Exp number of AAs in TMHs: 0.00268
# Aquilegiacoerulea_Aqcoe6G193500.1.p Exp number, first 60 AAs:  0.00267
# Aquilegiacoerulea_Aqcoe6G193500.1.p Total prob of N-in:        0.03731
Aquilegiacoerulea_Aqcoe6G193500.1.p     TMHMM2.0        outside      1   286
```

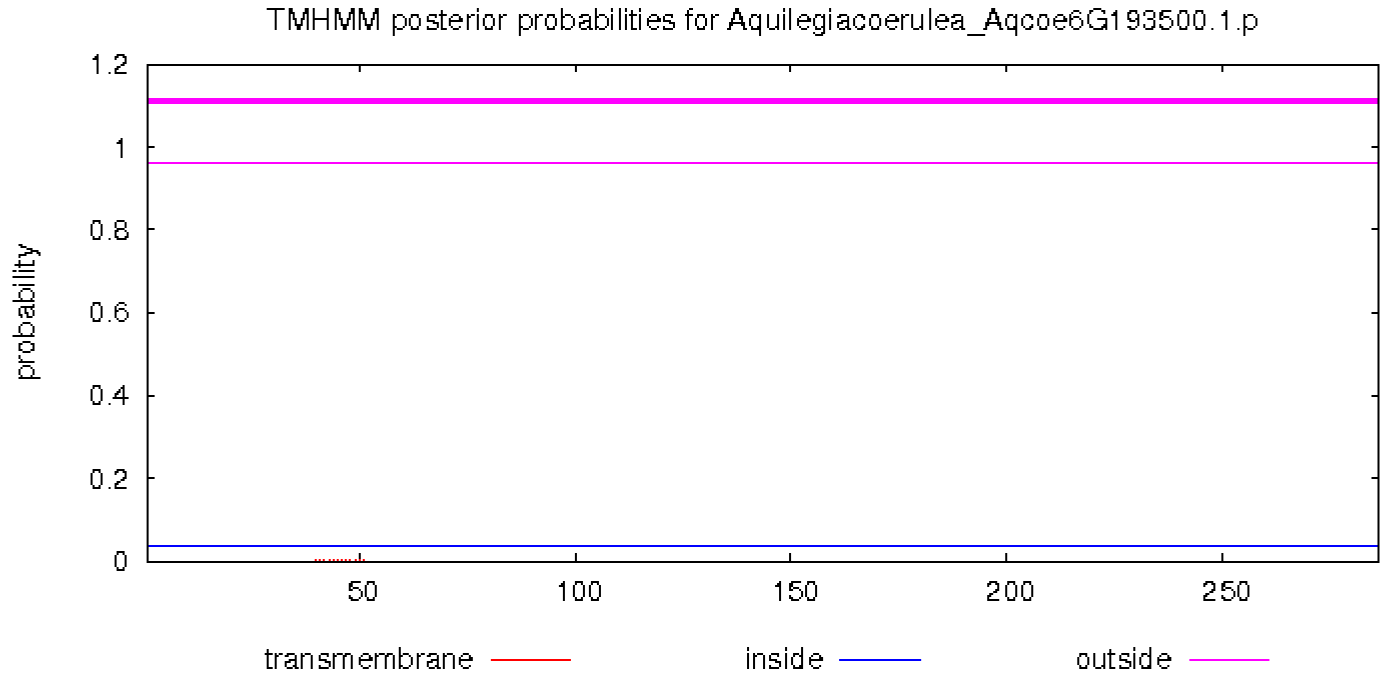

plot in postscript, script for making the plot in gnuplot, data for plot

```
# Aquilegiacoerulea_Aqcoe6G244500.1.p Length: 334
# Aquilegiacoerulea_Aqcoe6G244500.1.p Number of predicted TMHs:  0
# Aquilegiacoerulea_Aqcoe6G244500.1.p Exp number of AAs in TMHs: 0
# Aquilegiacoerulea_Aqcoe6G244500.1.p Exp number, first 60 AAs:  0
# Aquilegiacoerulea_Aqcoe6G244500.1.p Total prob of N-in:        0.00257
Aquilegiacoerulea_Aqcoe6G244500.1.p     TMHMM2.0        outside      1   334
```

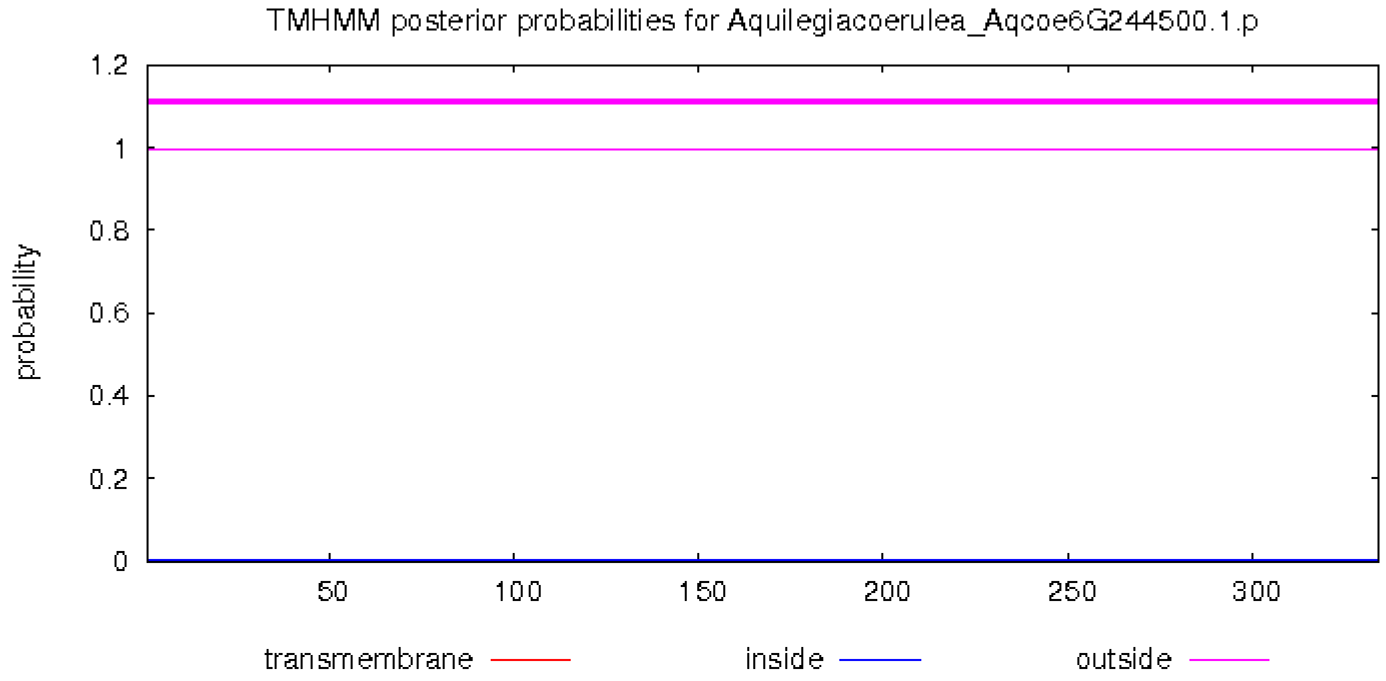

plot in postscript, script for making the plot in gnuplot, data for plot

```
# Aquilegiacoerulea_Aqcoe7G033600.1.p Length: 305
# Aquilegiacoerulea_Aqcoe7G033600.1.p Number of predicted TMHs:  0
```

```
# Aquilegiacoerulea_Aqcoe7G033600.1.p Exp number of AAs in TMHs: 0.04238
# Aquilegiacoerulea_Aqcoe7G033600.1.p Exp number, first 60 AAs:  0.00559
# Aquilegiacoerulea_Aqcoe7G033600.1.p Total prob of N-in:        0.05652
Aquilegiacoerulea_Aqcoe7G033600.1.p     TMHMM2.0        outside      1   305
```

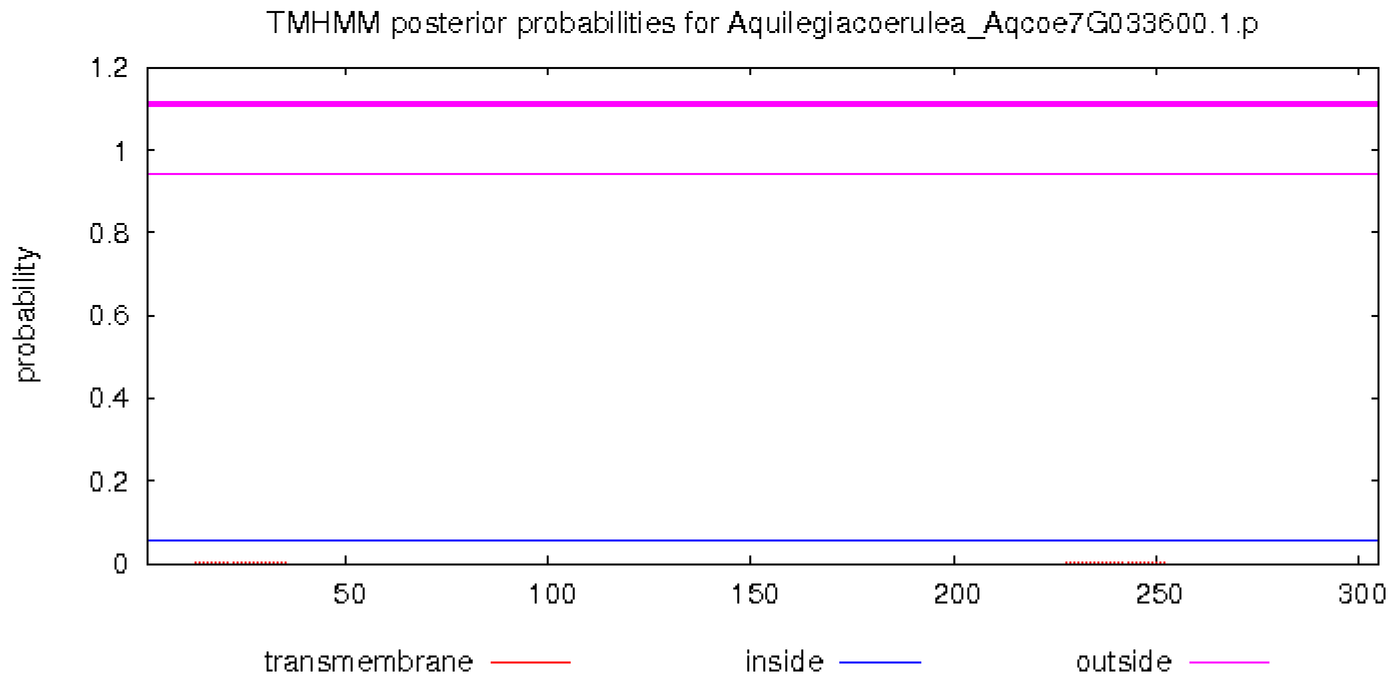

plot in postscript, script for making the plot in gnuplot, data for plot

---

```
# Aquilegiacoerulea_Aqcoe7G078200.1.p Length: 358
# Aquilegiacoerulea_Aqcoe7G078200.1.p Number of predicted TMHs:  0
# Aquilegiacoerulea_Aqcoe7G078200.1.p Exp number of AAs in TMHs: 0
# Aquilegiacoerulea_Aqcoe7G078200.1.p Exp number, first 60 AAs:  0
# Aquilegiacoerulea_Aqcoe7G078200.1.p Total prob of N-in:        0.00384
Aquilegiacoerulea_Aqcoe7G078200.1.p     TMHMM2.0        outside      1   358
```

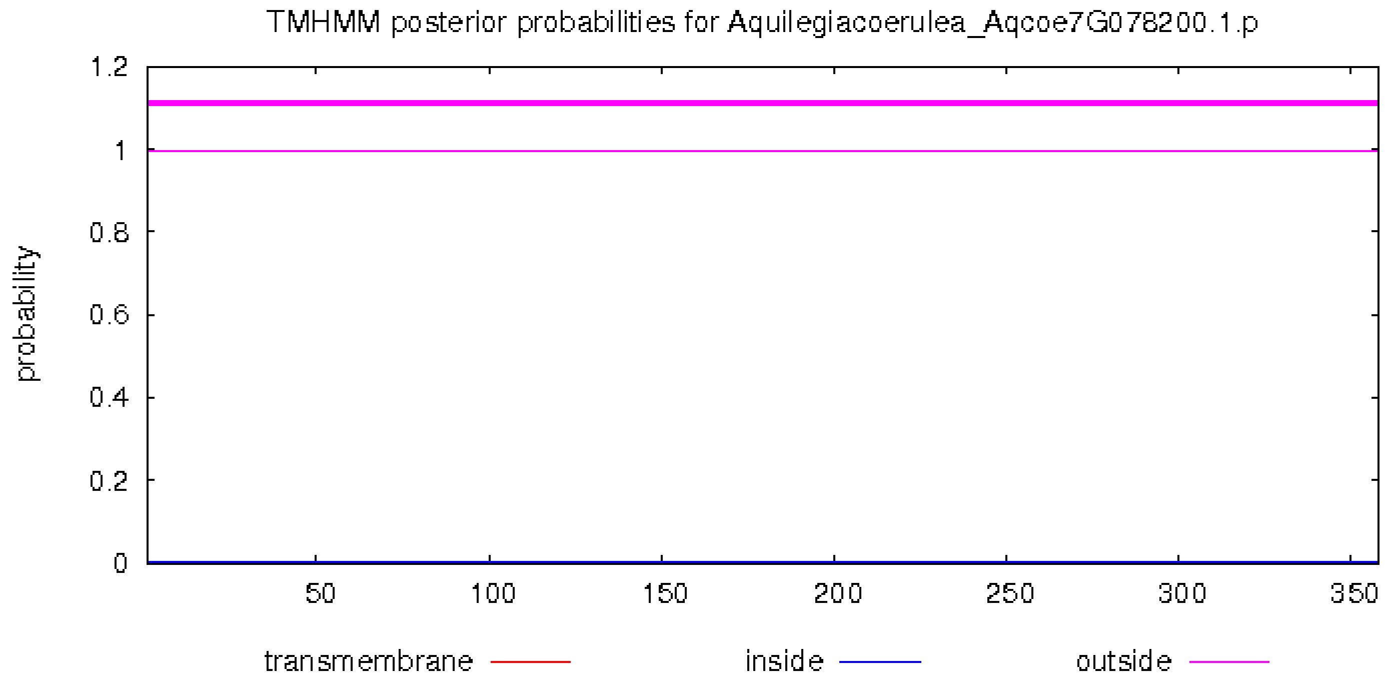

plot in postscript, script for making the plot in gnuplot, data for plot

---

```
# Aquilegiacoerulea_Aqcoe7G145000.1.p Length: 472
# Aquilegiacoerulea_Aqcoe7G145000.1.p Number of predicted TMHs:  0
```

```
# Aquilegiacoerulea_Aqcoe7G145000.1.p Exp number of AAs in TMHs: 0.00188
# Aquilegiacoerulea_Aqcoe7G145000.1.p Exp number, first 60 AAs:  0
# Aquilegiacoerulea_Aqcoe7G145000.1.p Total prob of N-in:        0.00577
Aquilegiacoerulea_Aqcoe7G145000.1.p     TMHMM2.0        outside      1   472
```

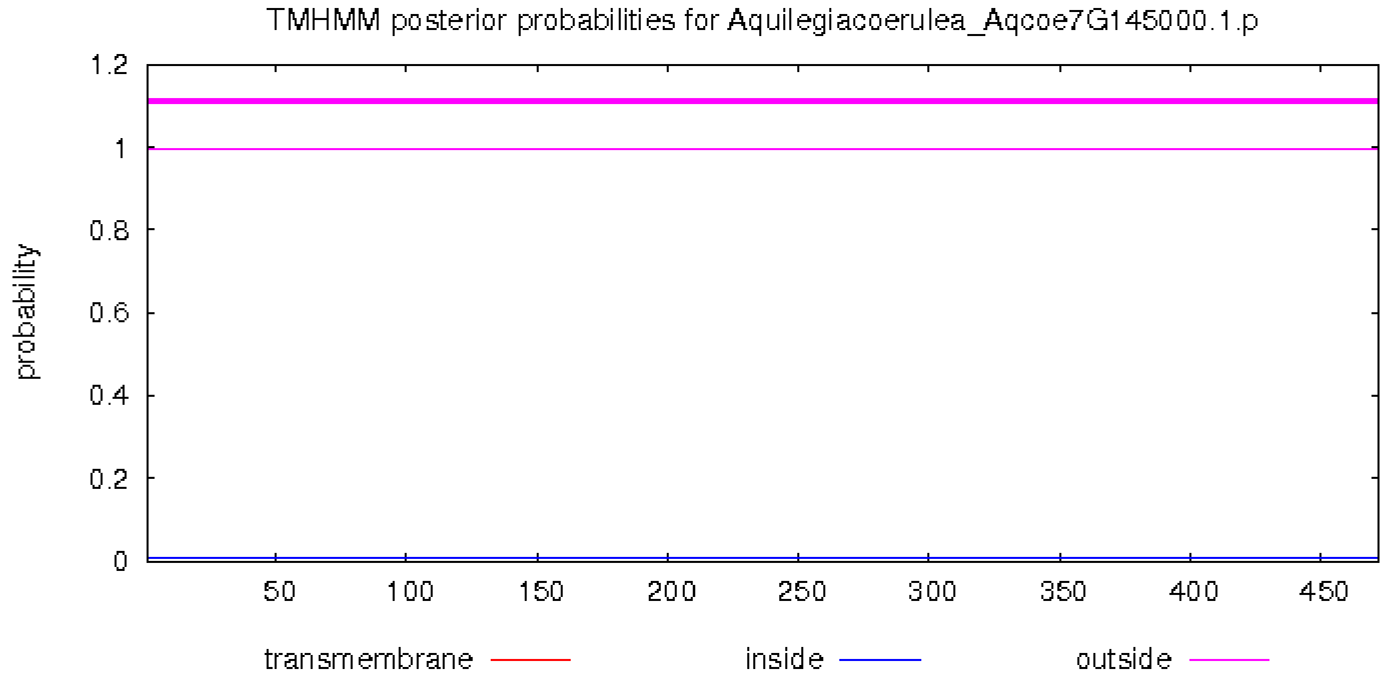

plot in postscript, script for making the plot in gnuplot, data for plot

---

```
# Aquilegiacoerulea_Aqcoe7G292300.1.p Length: 319
# Aquilegiacoerulea_Aqcoe7G292300.1.p Number of predicted TMHs:  0
# Aquilegiacoerulea_Aqcoe7G292300.1.p Exp number of AAs in TMHs: 0.00197
# Aquilegiacoerulea_Aqcoe7G292300.1.p Exp number, first 60 AAs:  0
# Aquilegiacoerulea_Aqcoe7G292300.1.p Total prob of N-in:        0.03285
Aquilegiacoerulea_Aqcoe7G292300.1.p     TMHMM2.0        outside      1   319
```

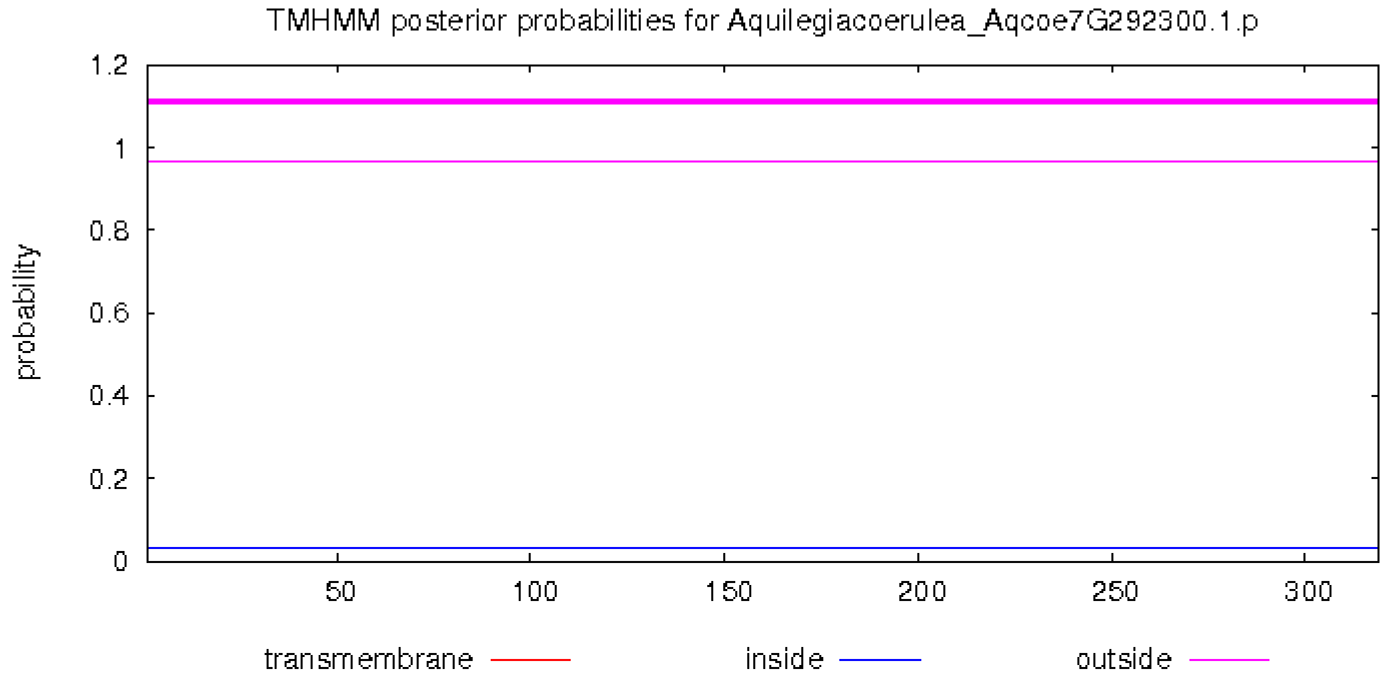

plot in postscript, script for making the plot in gnuplot, data for plot

---

```
# Aquilegiacoerulea_Aqcoe7G342900.1.p Length: 327
# Aquilegiacoerulea_Aqcoe7G342900.1.p Number of predicted TMHs:  0
```

```
# Aquilegiacoerulea_Aqcoe7G342900.1.p Exp number of AAs in TMHs: 0.00113
# Aquilegiacoerulea_Aqcoe7G342900.1.p Exp number, first 60 AAs:  0.00113
# Aquilegiacoerulea_Aqcoe7G342900.1.p Total prob of N-in:        0.01540
Aquilegiacoerulea_Aqcoe7G342900.1.p     TMHMM2.0        outside      1   327
```

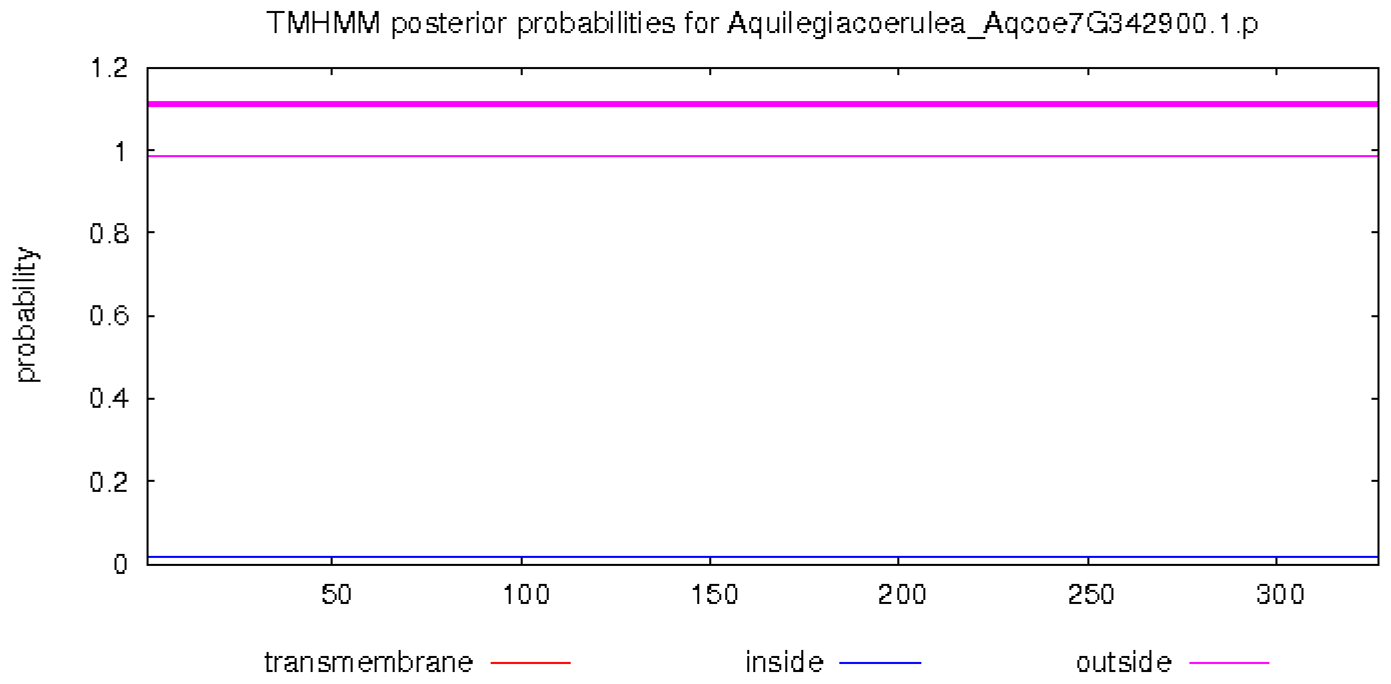

plot in postscript, script for making the plot in gnuplot, data for plot

---

```
# Aquilegiacoerulea_Aqcoe7G346600.1.p Length: 240
# Aquilegiacoerulea_Aqcoe7G346600.1.p Number of predicted TMHs:  0
# Aquilegiacoerulea_Aqcoe7G346600.1.p Exp number of AAs in TMHs: 0.00118
# Aquilegiacoerulea_Aqcoe7G346600.1.p Exp number, first 60 AAs:  0.00066
# Aquilegiacoerulea_Aqcoe7G346600.1.p Total prob of N-in:        0.03699
Aquilegiacoerulea_Aqcoe7G346600.1.p     TMHMM2.0        outside      1   240
```

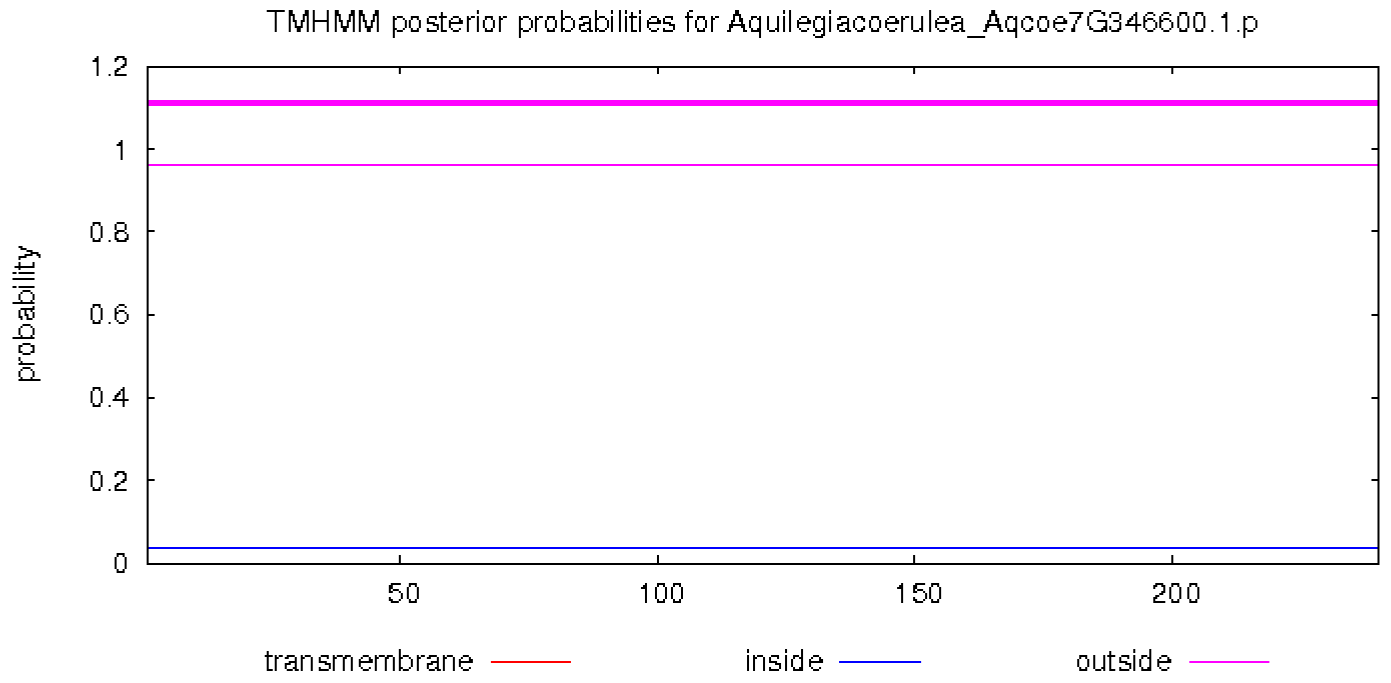

plot in postscript, script for making the plot in gnuplot, data for plot

---

```
# Aquilegiacoerulea_Aqcoe7G377900.1.p Length: 573
# Aquilegiacoerulea_Aqcoe7G377900.1.p Number of predicted TMHs:  1
```

```
# Aquilegiacoerulea_Aqcoe7G377900.1.p Exp number of AAs in TMHs: 21.78976
# Aquilegiacoerulea_Aqcoe7G377900.1.p Exp number, first 60 AAs:  0
# Aquilegiacoerulea_Aqcoe7G377900.1.p Total prob of N-in:        0.00029
Aquilegiacoerulea_Aqcoe7G377900.1.p     TMHMM2.0        outside      1   545
Aquilegiacoerulea_Aqcoe7G377900.1.p     TMHMM2.0        TMhelix    546   568
Aquilegiacoerulea_Aqcoe7G377900.1.p     TMHMM2.0        inside     569   573
```

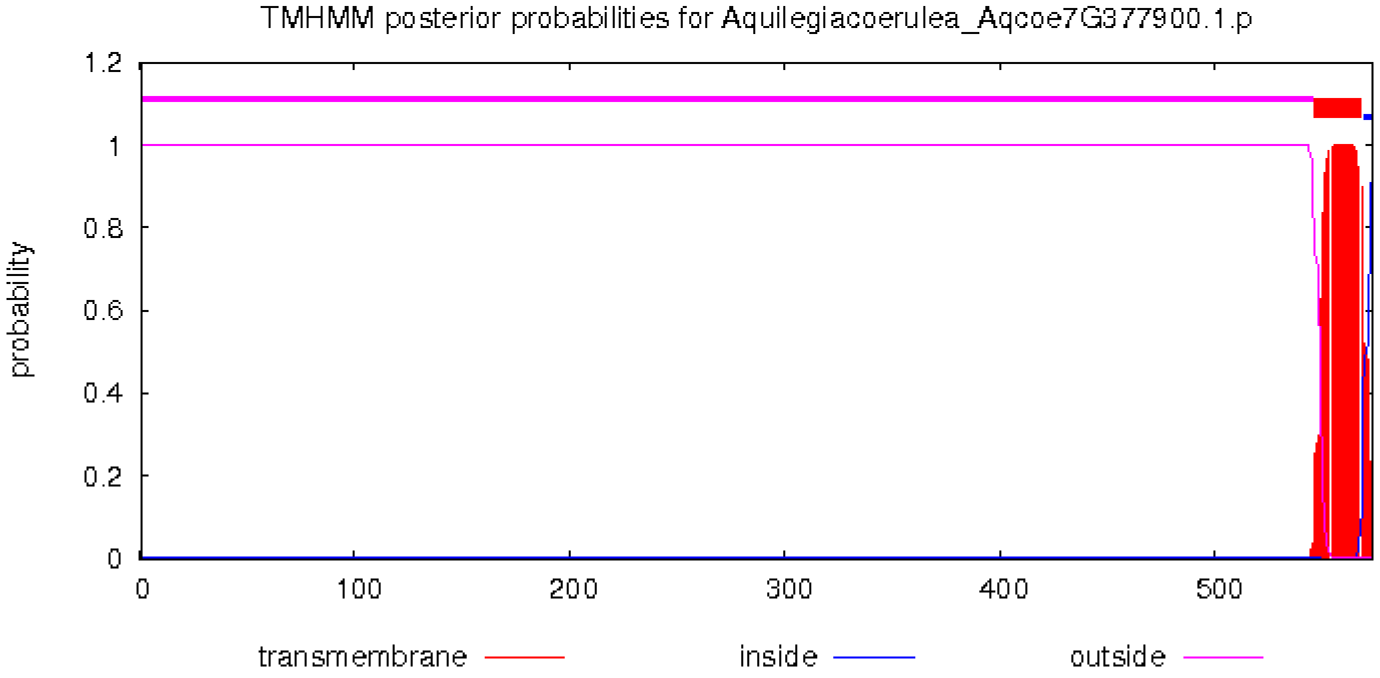

plot in postscript, script for making the plot in gnuplot, data for plot

---

```
# Aquilegiacoerulea_Aqcoe7G378100.1.p Length: 579
# Aquilegiacoerulea_Aqcoe7G378100.1.p Number of predicted TMHs:  1
# Aquilegiacoerulea_Aqcoe7G378100.1.p Exp number of AAs in TMHs: 21.78957
# Aquilegiacoerulea_Aqcoe7G378100.1.p Exp number, first 60 AAs:  0
# Aquilegiacoerulea_Aqcoe7G378100.1.p Total prob of N-in:        0.00025
Aquilegiacoerulea_Aqcoe7G378100.1.p     TMHMM2.0        outside      1   551
Aquilegiacoerulea_Aqcoe7G378100.1.p     TMHMM2.0        TMhelix    552   574
Aquilegiacoerulea_Aqcoe7G378100.1.p     TMHMM2.0        inside     575   579
```

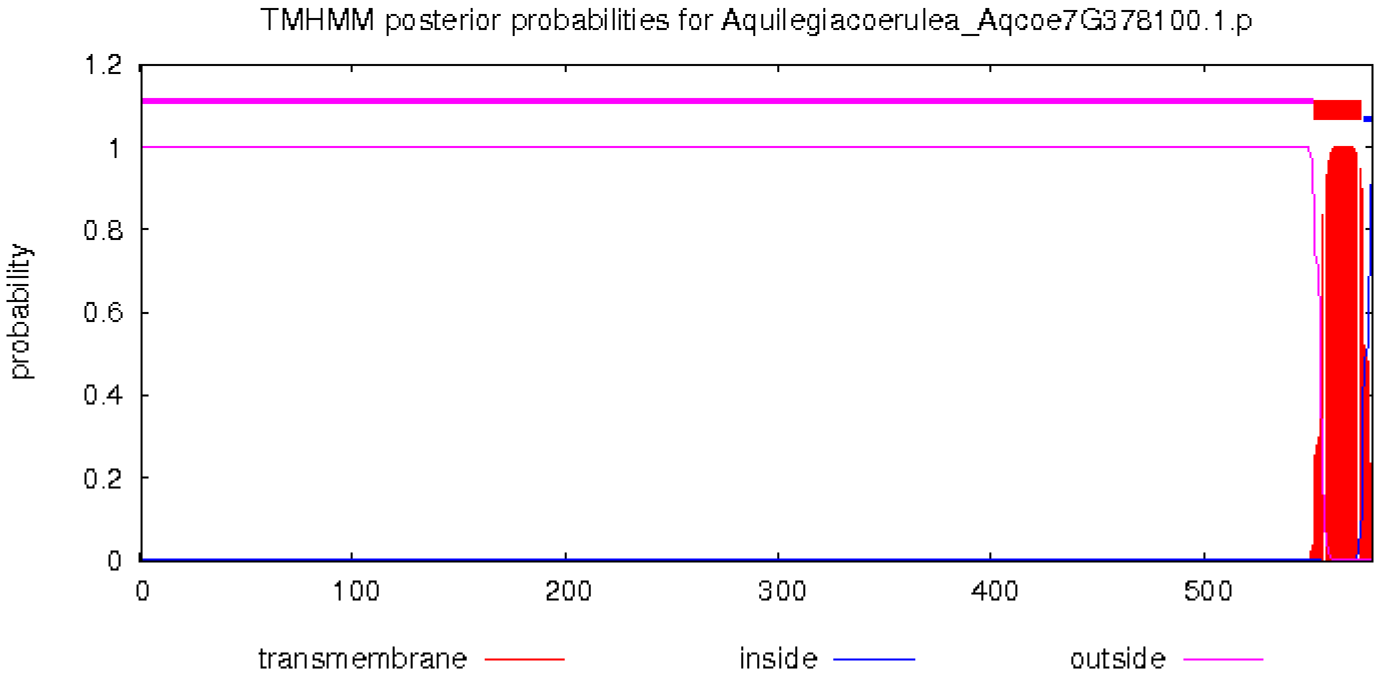

plot in postscript, script for making the plot in gnuplot, data for plot

```
# Arabidopsishalleri_Araha.0070s0003.1.p Length: 332
# Arabidopsishalleri_Araha.0070s0003.1.p Number of predicted TMHs:  0
# Arabidopsishalleri_Araha.0070s0003.1.p Exp number of AAs in TMHs: 0.00046
# Arabidopsishalleri_Araha.0070s0003.1.p Exp number, first 60 AAs:  0
# Arabidopsishalleri_Araha.0070s0003.1.p Total prob of N-in:        0.00807
Arabidopsishalleri_Araha.0070s0003.1.p  TMHMM2.0        outside      1   332
```

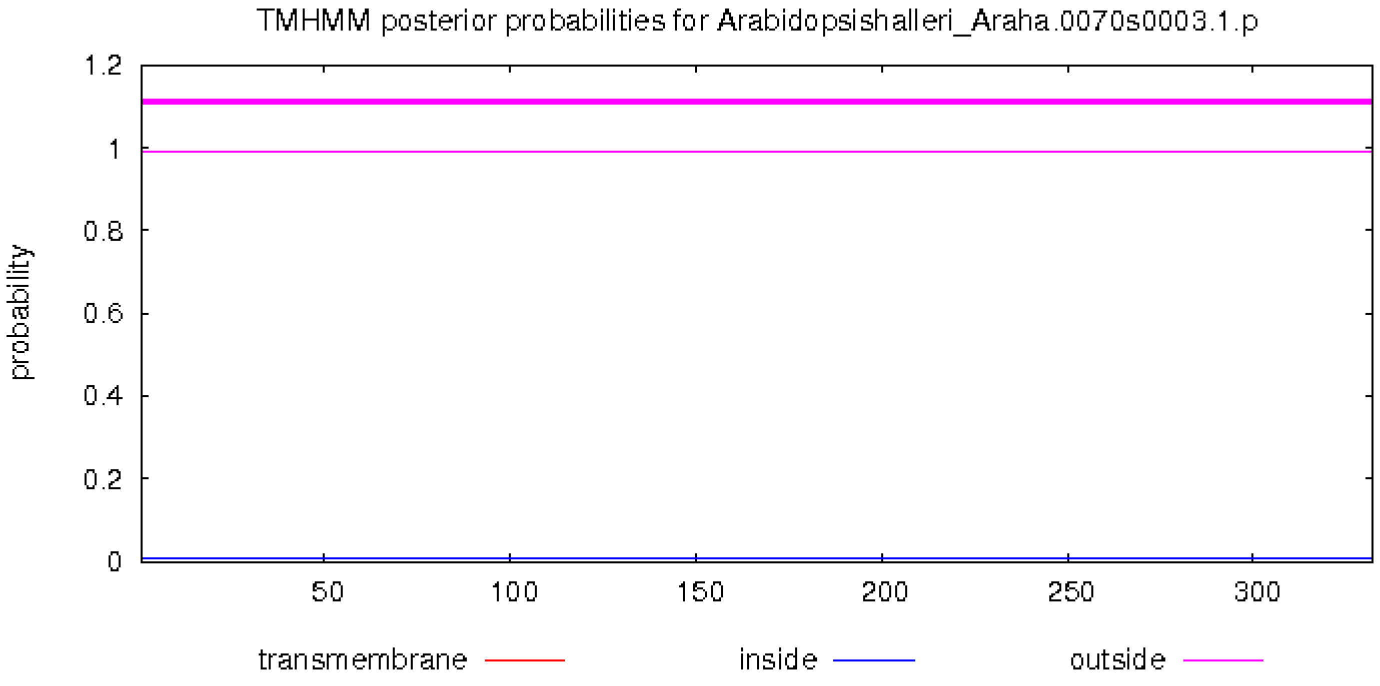

plot in postscript, script for making the plot in gnuplot, data for plot

```
# Arabidopsishalleri_Araha.0129s0005.1.p Length: 214
# Arabidopsishalleri_Araha.0129s0005.1.p Number of predicted TMHs:  0
# Arabidopsishalleri_Araha.0129s0005.1.p Exp number of AAs in TMHs: 0.52306
# Arabidopsishalleri_Araha.0129s0005.1.p Exp number, first 60 AAs:  0.09263
# Arabidopsishalleri_Araha.0129s0005.1.p Total prob of N-in:        0.17858
Arabidopsishalleri_Araha.0129s0005.1.p  TMHMM2.0        outside      1   214
```

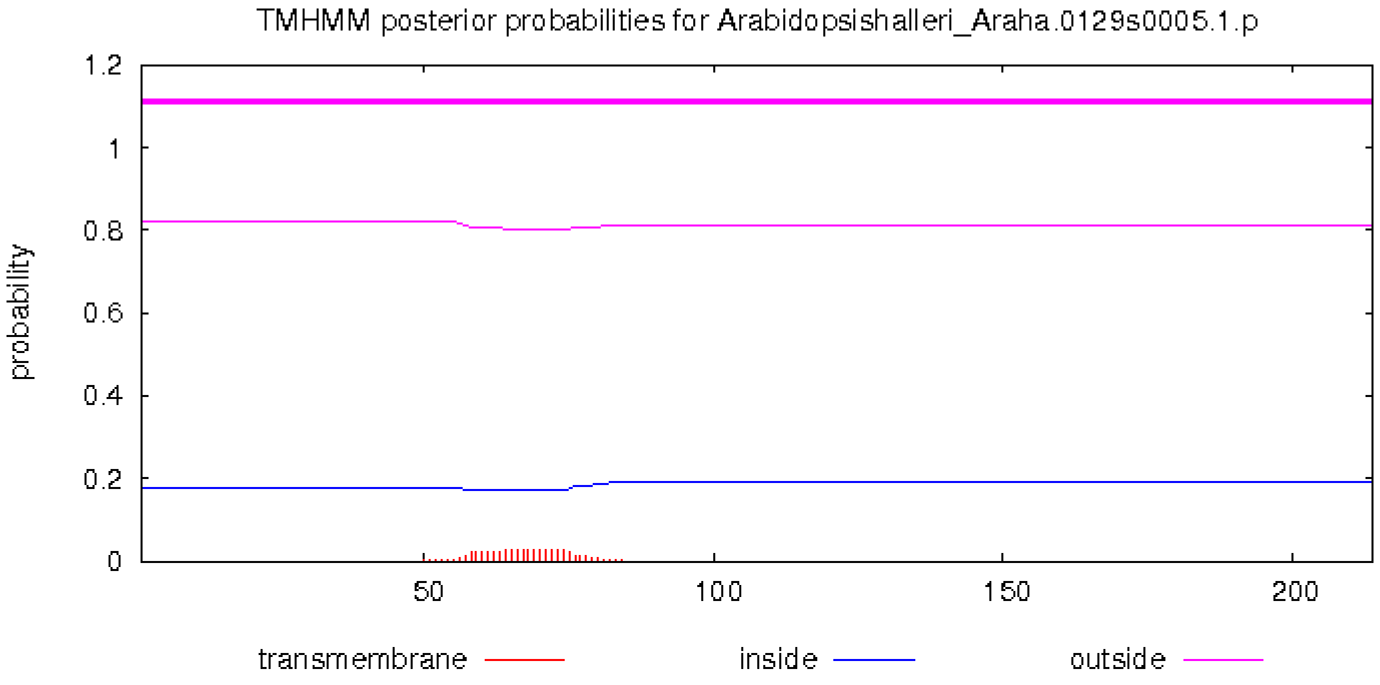

plot in postscript, script for making the plot in gnuplot, data for plot

```
# Arabidopsishalleri_Araha.0173s0016.1.p Length: 274
# Arabidopsishalleri_Araha.0173s0016.1.p Number of predicted TMHs:  0
# Arabidopsishalleri_Araha.0173s0016.1.p Exp number of AAs in TMHs: 0.00051
# Arabidopsishalleri_Araha.0173s0016.1.p Exp number, first 60 AAs:  0
# Arabidopsishalleri_Araha.0173s0016.1.p Total prob of N-in:        0.01653
Arabidopsishalleri_Araha.0173s0016.1.p  TMHMM2.0        outside      1   274
```

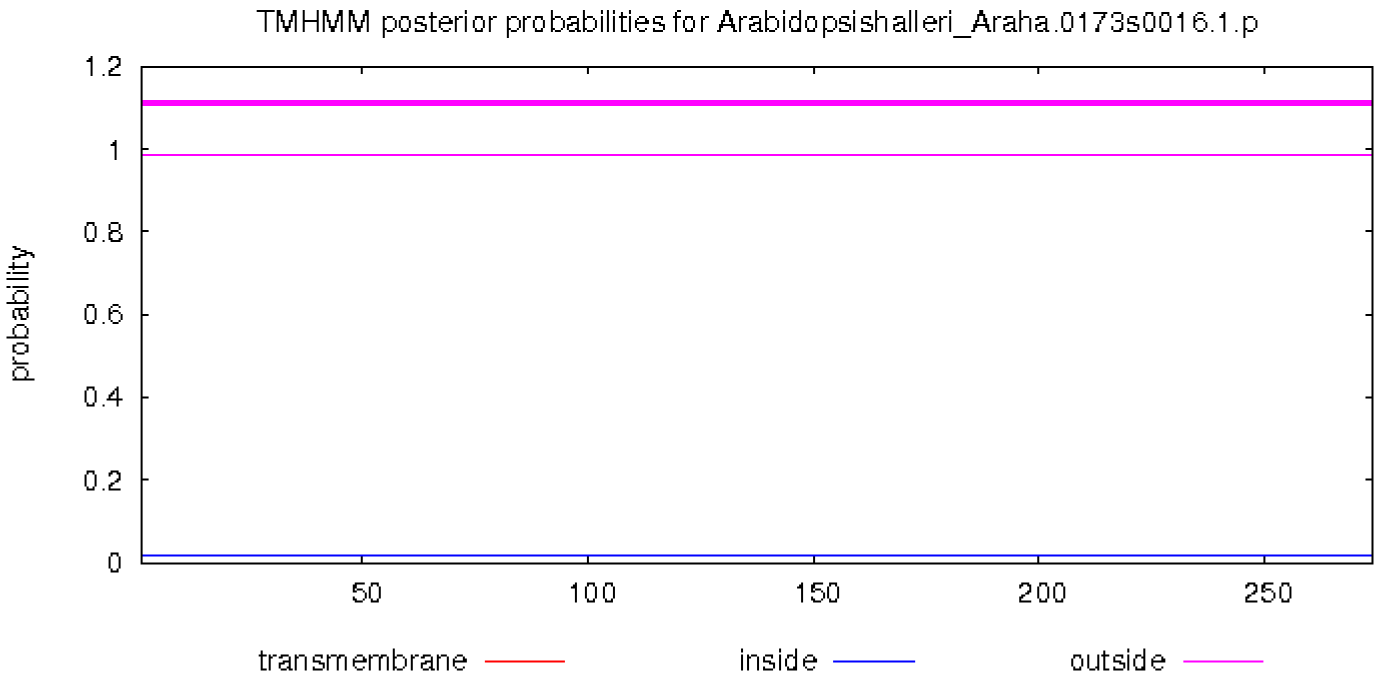

plot in postscript, script for making the plot in gnuplot, data for plot

---

```
# Arabidopsishalleri_Araha.0299s0007.1.p Length: 188
# Arabidopsishalleri_Araha.0299s0007.1.p Number of predicted TMHs:  0
# Arabidopsishalleri_Araha.0299s0007.1.p Exp number of AAs in TMHs: 0
# Arabidopsishalleri_Araha.0299s0007.1.p Exp number, first 60 AAs:  0
# Arabidopsishalleri_Araha.0299s0007.1.p Total prob of N-in:        0.00724
Arabidopsishalleri_Araha.0299s0007.1.p  TMHMM2.0        outside      1   188
```

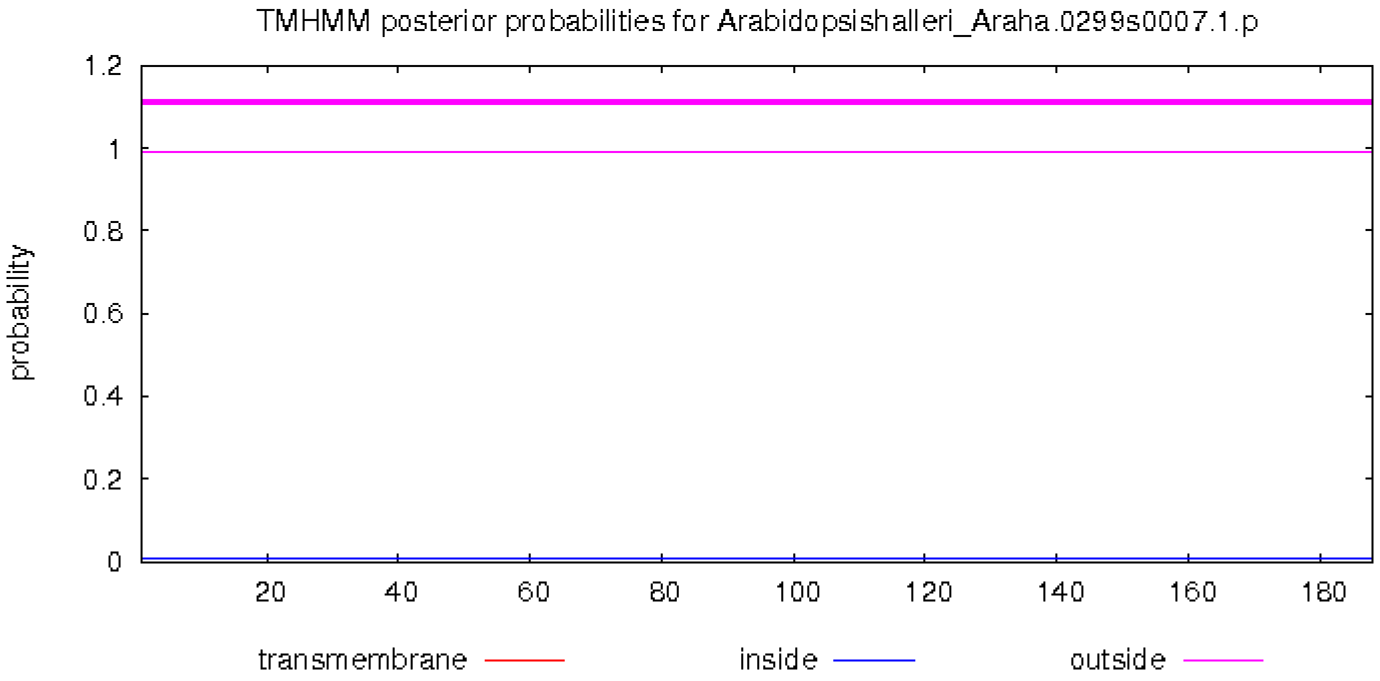

plot in postscript, script for making the plot in gnuplot, data for plot

---

```
# Arabidopsishalleri_Araha.0650s0016.1.p Length: 342
# Arabidopsishalleri_Araha.0650s0016.1.p Number of predicted TMHs:  0
# Arabidopsishalleri_Araha.0650s0016.1.p Exp number of AAs in TMHs: 0.00018
# Arabidopsishalleri_Araha.0650s0016.1.p Exp number, first 60 AAs:  0
# Arabidopsishalleri_Araha.0650s0016.1.p Total prob of N-in:        0.00322
Arabidopsishalleri_Araha.0650s0016.1.p  TMHMM2.0        outside      1   342
```

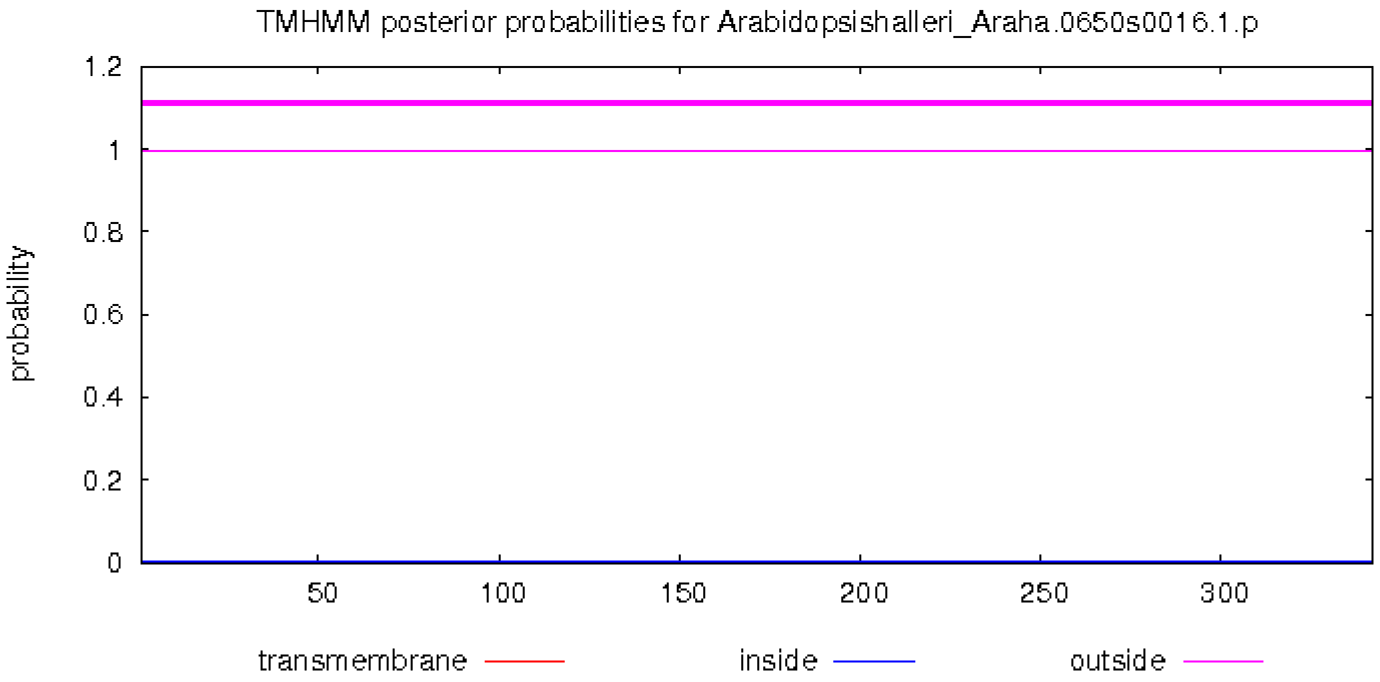

plot in postscript, script for making the plot in gnuplot, data for plot

---

```
# Arabidopsishalleri_Araha.0709s0001.1.p Length: 361
# Arabidopsishalleri_Araha.0709s0001.1.p Number of predicted TMHs:  0
# Arabidopsishalleri_Araha.0709s0001.1.p Exp number of AAs in TMHs: 0
# Arabidopsishalleri_Araha.0709s0001.1.p Exp number, first 60 AAs:  0
# Arabidopsishalleri_Araha.0709s0001.1.p Total prob of N-in:        0.00988
Arabidopsishalleri_Araha.0709s0001.1.p  TMHMM2.0        outside      1   361
```

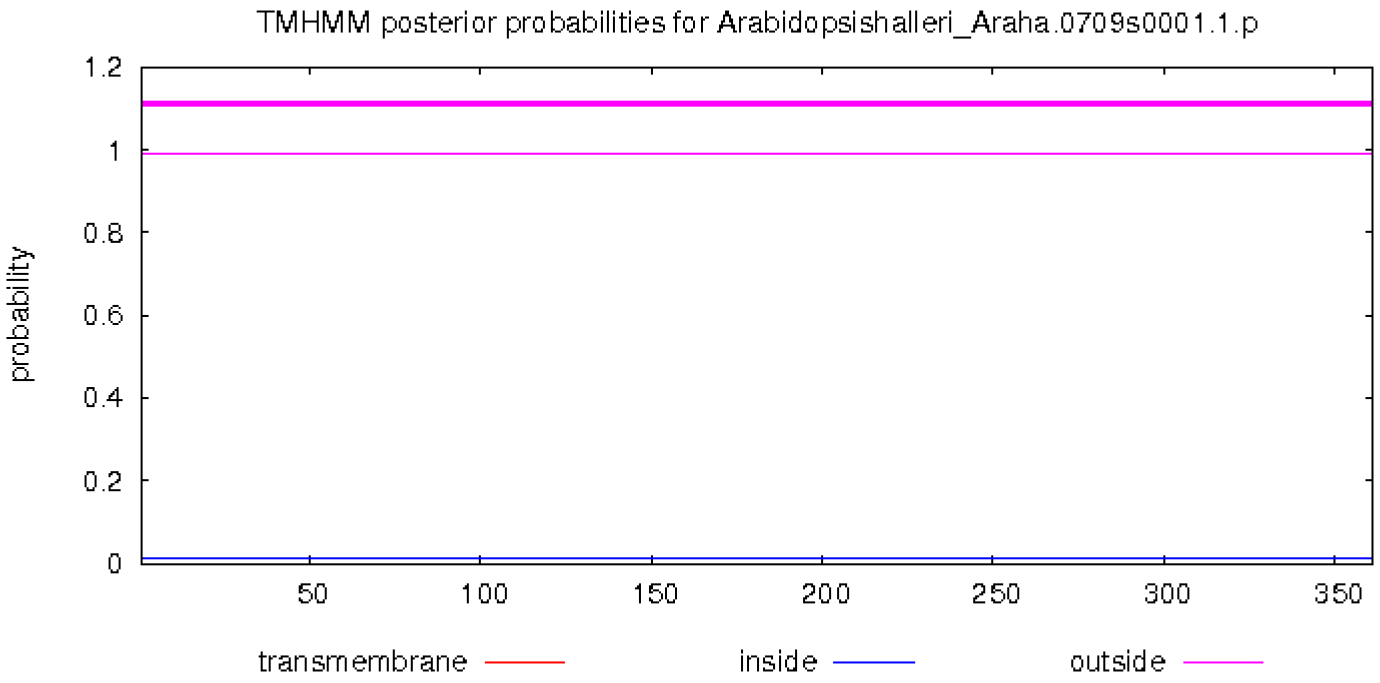

plot in postscript, script for making the plot in gnuplot, data for plot

---

```
# Arabidopsishalleri_Araha.10341s0001.1.p Length: 331
# Arabidopsishalleri_Araha.10341s0001.1.p Number of predicted TMHs:  1
# Arabidopsishalleri_Araha.10341s0001.1.p Exp number of AAs in TMHs: 19.53476
# Arabidopsishalleri_Araha.10341s0001.1.p Exp number, first 60 AAs:  0.15012
# Arabidopsishalleri_Araha.10341s0001.1.p Total prob of N-in:        0.42628
Arabidopsishalleri_Araha.10341s0001.1.p TMHMM2.0        inside       1   312
Arabidopsishalleri_Araha.10341s0001.1.p TMHMM2.0        TMhelix    313   330
Arabidopsishalleri_Araha.10341s0001.1.p TMHMM2.0        outside    331   331
```

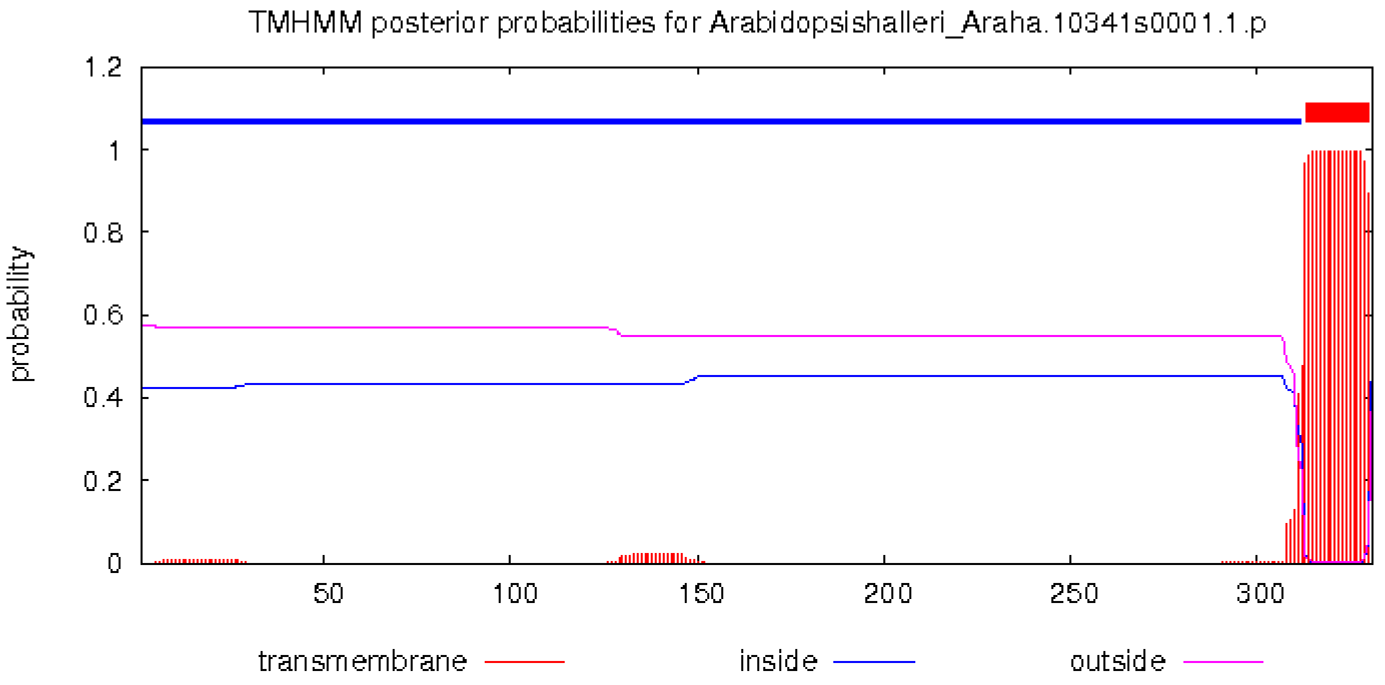

plot in postscript, script for making the plot in gnuplot, data for plot

---

```
# Arabidopsishalleri_Araha.10656s0016.1.p Length: 294
# Arabidopsishalleri_Araha.10656s0016.1.p Number of predicted TMHs:  0
# Arabidopsishalleri_Araha.10656s0016.1.p Exp number of AAs in TMHs: 0.00038
# Arabidopsishalleri_Araha.10656s0016.1.p Exp number, first 60 AAs:  0
# Arabidopsishalleri_Araha.10656s0016.1.p Total prob of N-in:        0.07606
Arabidopsishalleri_Araha.10656s0016.1.p TMHMM2.0        outside      1   294
```

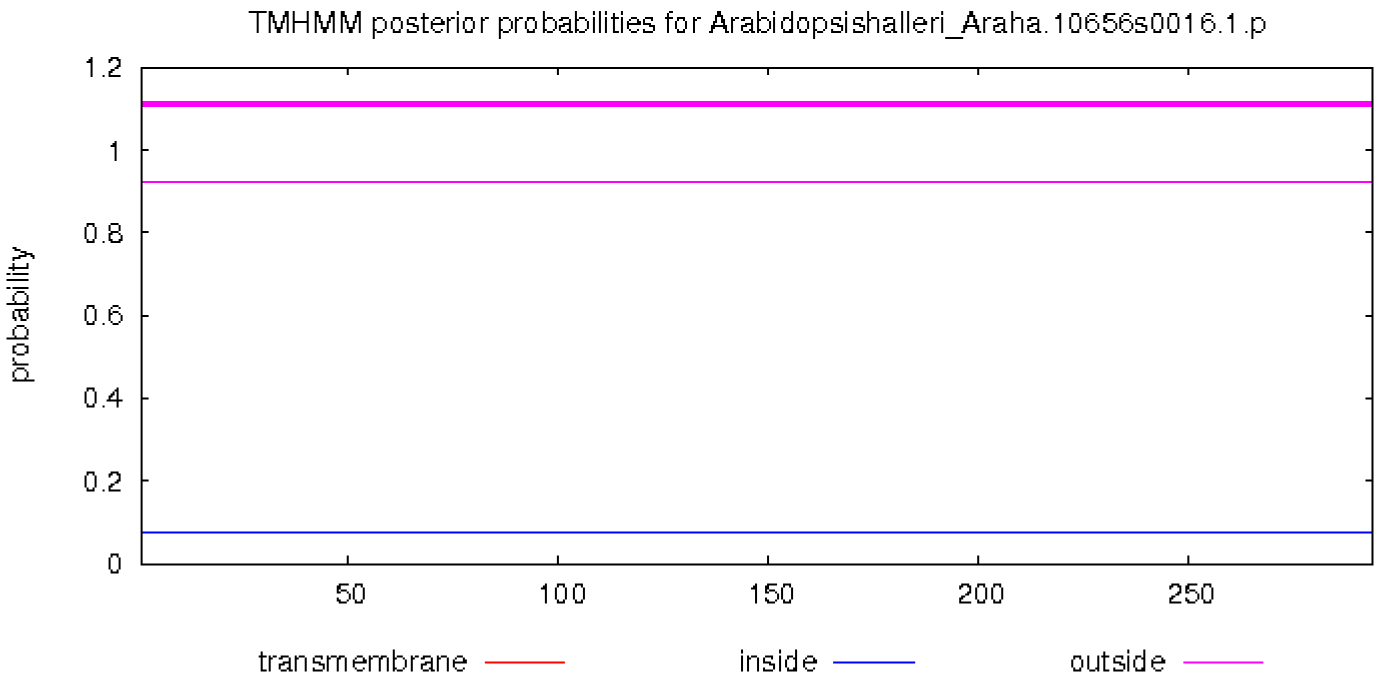

plot in postscript, script for making the plot in gnuplot, data for plot

```
# Arabidopsishalleri_Araha.1084s0026.1.p Length: 289
# Arabidopsishalleri_Araha.1084s0026.1.p Number of predicted TMHs:  0
# Arabidopsishalleri_Araha.1084s0026.1.p Exp number of AAs in TMHs: 0.0007
# Arabidopsishalleri_Araha.1084s0026.1.p Exp number, first 60 AAs:  0
# Arabidopsishalleri_Araha.1084s0026.1.p Total prob of N-in:        0.06162
Arabidopsishalleri_Araha.1084s0026.1.p  TMHMM2.0        outside      1   289
```

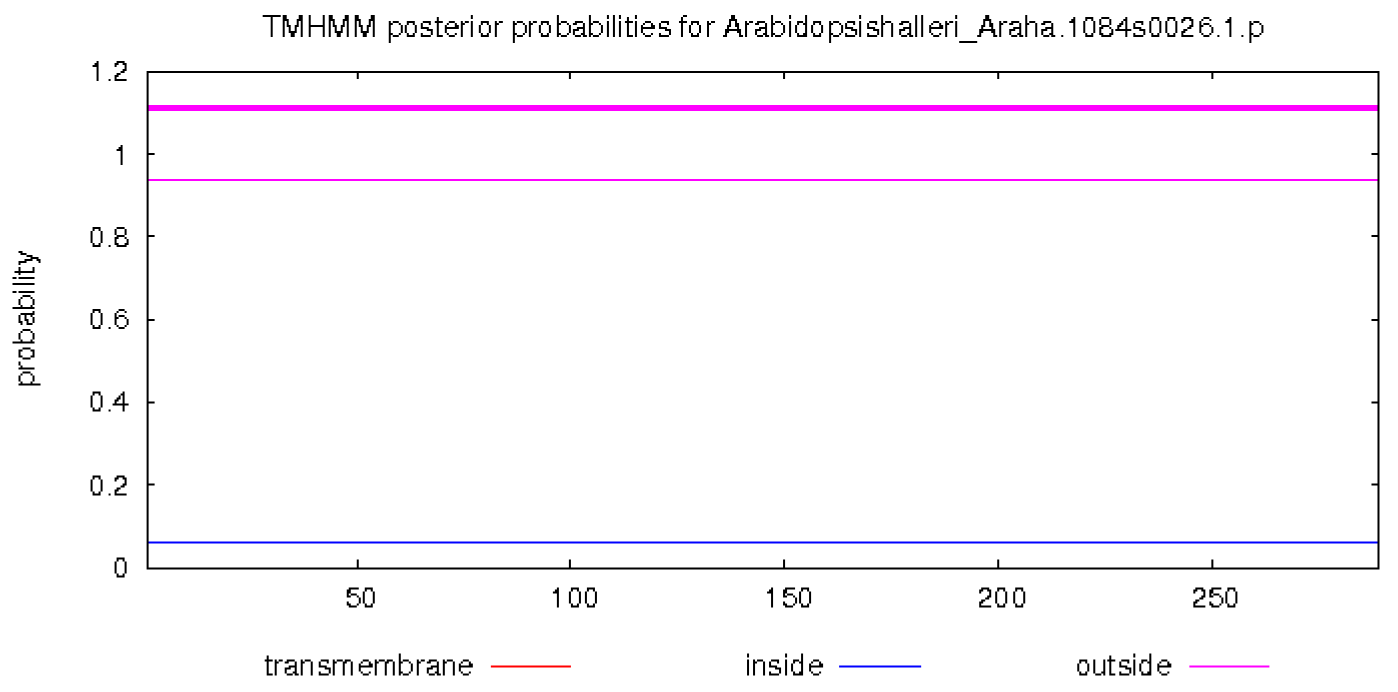

plot in postscript, script for making the plot in gnuplot, data for plot

```
# Arabidopsishalleri_Araha.1146s0008.1.p Length: 415
# Arabidopsishalleri_Araha.1146s0008.1.p Number of predicted TMHs:  1
# Arabidopsishalleri_Araha.1146s0008.1.p Exp number of AAs in TMHs: 22.5503
# Arabidopsishalleri_Araha.1146s0008.1.p Exp number, first 60 AAs:  0.00056
# Arabidopsishalleri_Araha.1146s0008.1.p Total prob of N-in:        0.17082
Arabidopsishalleri_Araha.1146s0008.1.p  TMHMM2.0        outside      1   386
Arabidopsishalleri_Araha.1146s0008.1.p  TMHMM2.0        TMhelix    387   409
Arabidopsishalleri_Araha.1146s0008.1.p  TMHMM2.0        inside     410   415
```

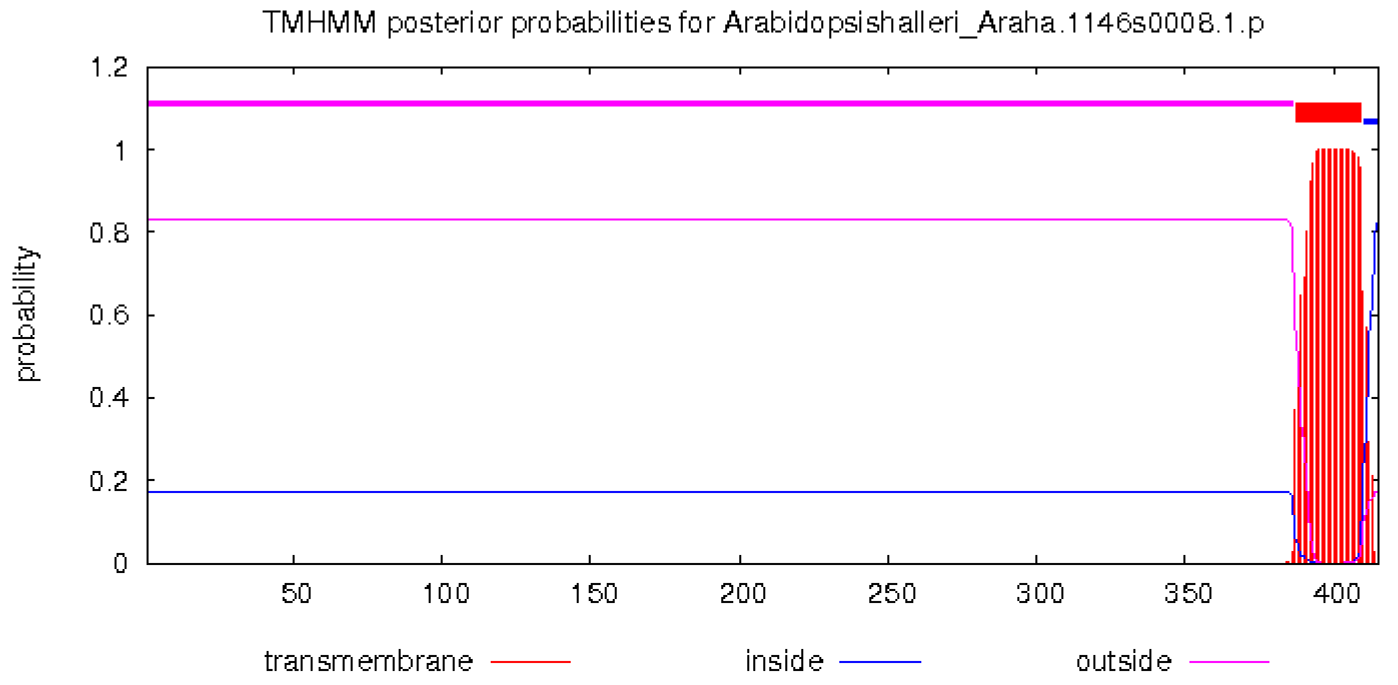

plot in postscript, script for making the plot in gnuplot, data for plot

```
# Arabidopsishalleri_Araha.1146s0009.1.p Length: 479
# Arabidopsishalleri_Araha.1146s0009.1.p Number of predicted TMHs:  0
# Arabidopsishalleri_Araha.1146s0009.1.p Exp number of AAs in TMHs: 4.72707
# Arabidopsishalleri_Araha.1146s0009.1.p Exp number, first 60 AAs:  0
# Arabidopsishalleri_Araha.1146s0009.1.p Total prob of N-in:        0.00896
Arabidopsishalleri_Araha.1146s0009.1.p  TMHMM2.0        outside      1   479
```

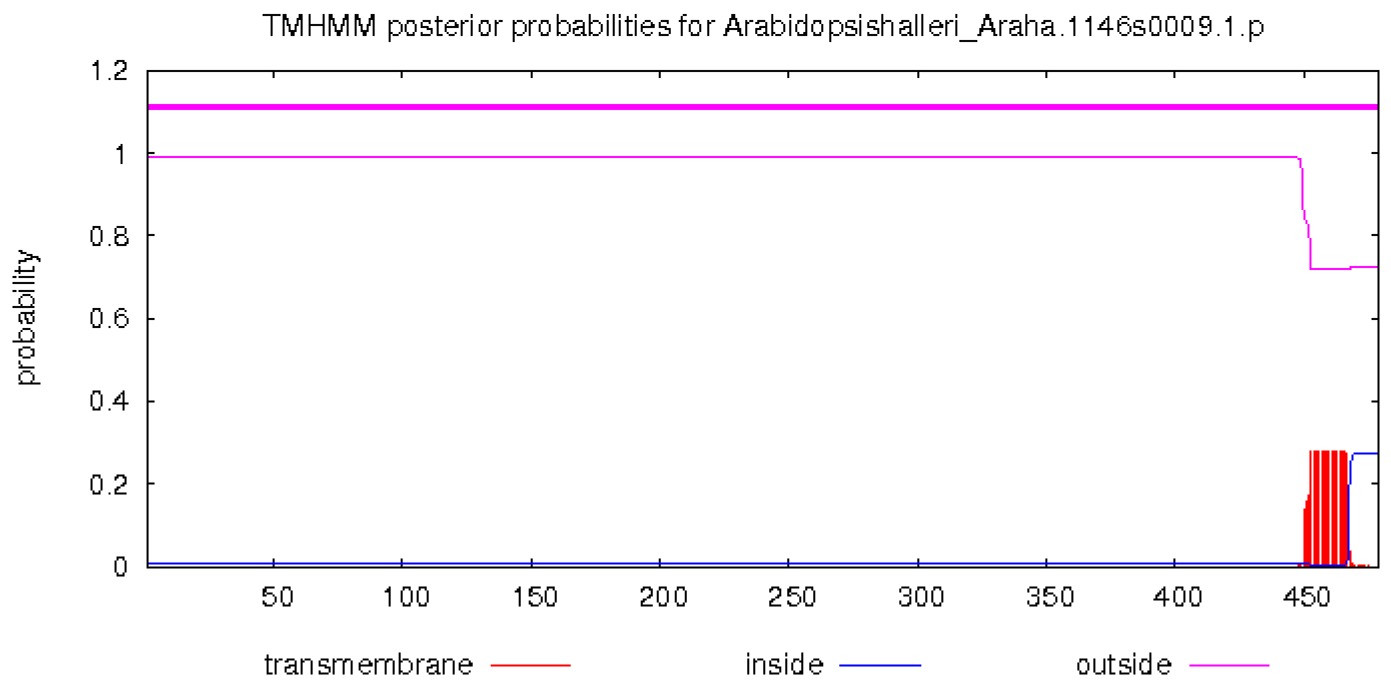

plot in postscript, script for making the plot in gnuplot, data for plot

```
# Arabidopsishalleri_Araha.1212s0002.1.p Length: 461
# Arabidopsishalleri_Araha.1212s0002.1.p Number of predicted TMHs:  0
# Arabidopsishalleri_Araha.1212s0002.1.p Exp number of AAs in TMHs: 13.51889
# Arabidopsishalleri_Araha.1212s0002.1.p Exp number, first 60 AAs:  0.00367
# Arabidopsishalleri_Araha.1212s0002.1.p Total prob of N-in:        0.01106
Arabidopsishalleri_Araha.1212s0002.1.p  TMHMM2.0        outside      1   461
```

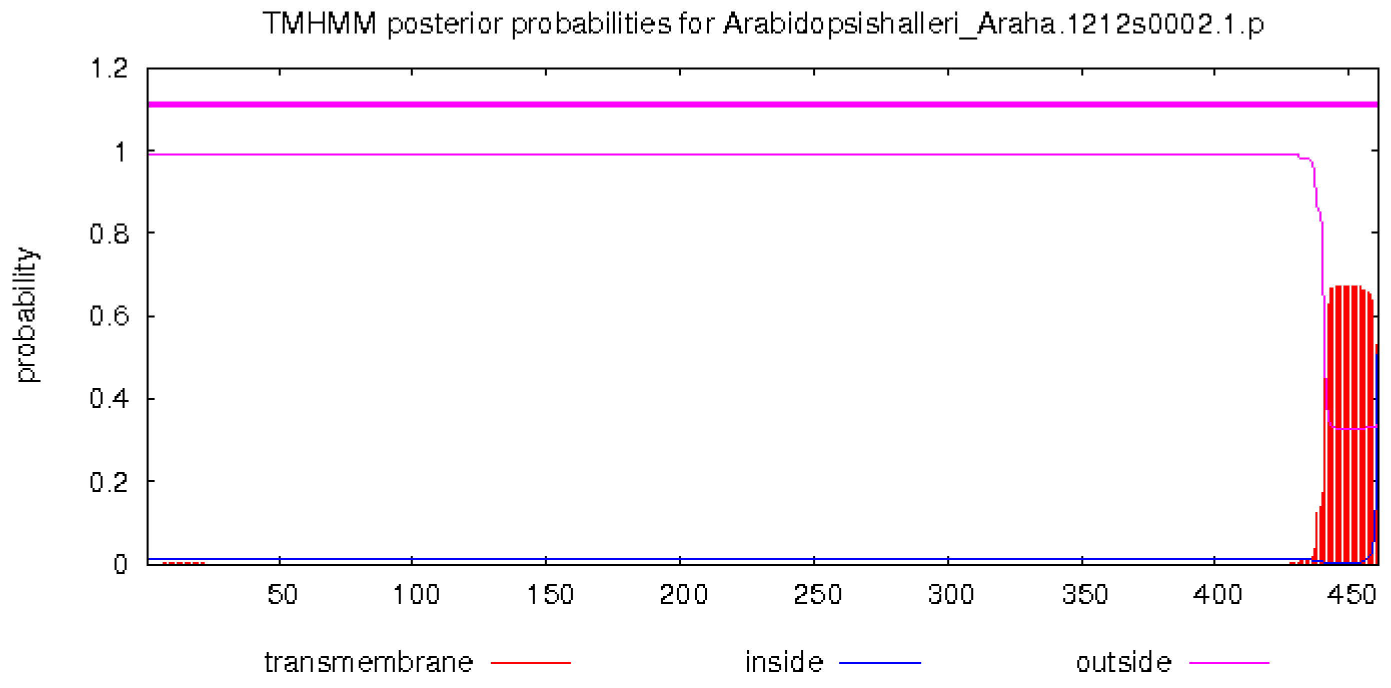

plot in postscript, script for making the plot in gnuplot, data for plot

---

```
# Arabidopsishalleri_Araha.13692s0015.1.p Length: 378
# Arabidopsishalleri_Araha.13692s0015.1.p Number of predicted TMHs:  0
# Arabidopsishalleri_Araha.13692s0015.1.p Exp number of AAs in TMHs: 0.00018
# Arabidopsishalleri_Araha.13692s0015.1.p Exp number, first 60 AAs:  0.00018
# Arabidopsishalleri_Araha.13692s0015.1.p Total prob of N-in:        0.00693
Arabidopsishalleri_Araha.13692s0015.1.p TMHMM2.0        outside      1   378
```

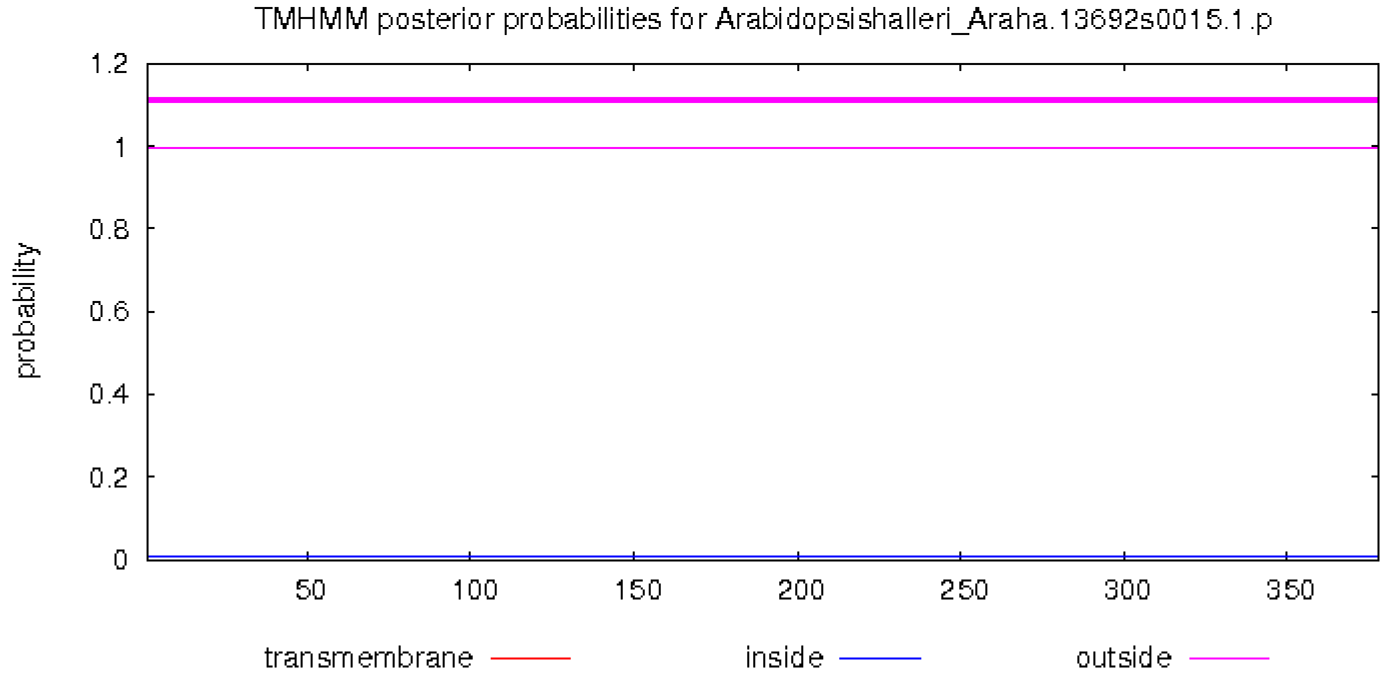

plot in postscript, script for making the plot in gnuplot, data for plot

---

```
# Arabidopsishalleri_Araha.13695s0002.1.p Length: 568
# Arabidopsishalleri_Araha.13695s0002.1.p Number of predicted TMHs:  1
# Arabidopsishalleri_Araha.13695s0002.1.p Exp number of AAs in TMHs: 21.3576
# Arabidopsishalleri_Araha.13695s0002.1.p Exp number, first 60 AAs:  0
# Arabidopsishalleri_Araha.13695s0002.1.p Total prob of N-in:        0.02412
Arabidopsishalleri_Araha.13695s0002.1.p TMHMM2.0        outside      1   542
Arabidopsishalleri_Araha.13695s0002.1.p TMHMM2.0        TMhelix    543   565
Arabidopsishalleri_Araha.13695s0002.1.p TMHMM2.0        inside     566   568
```

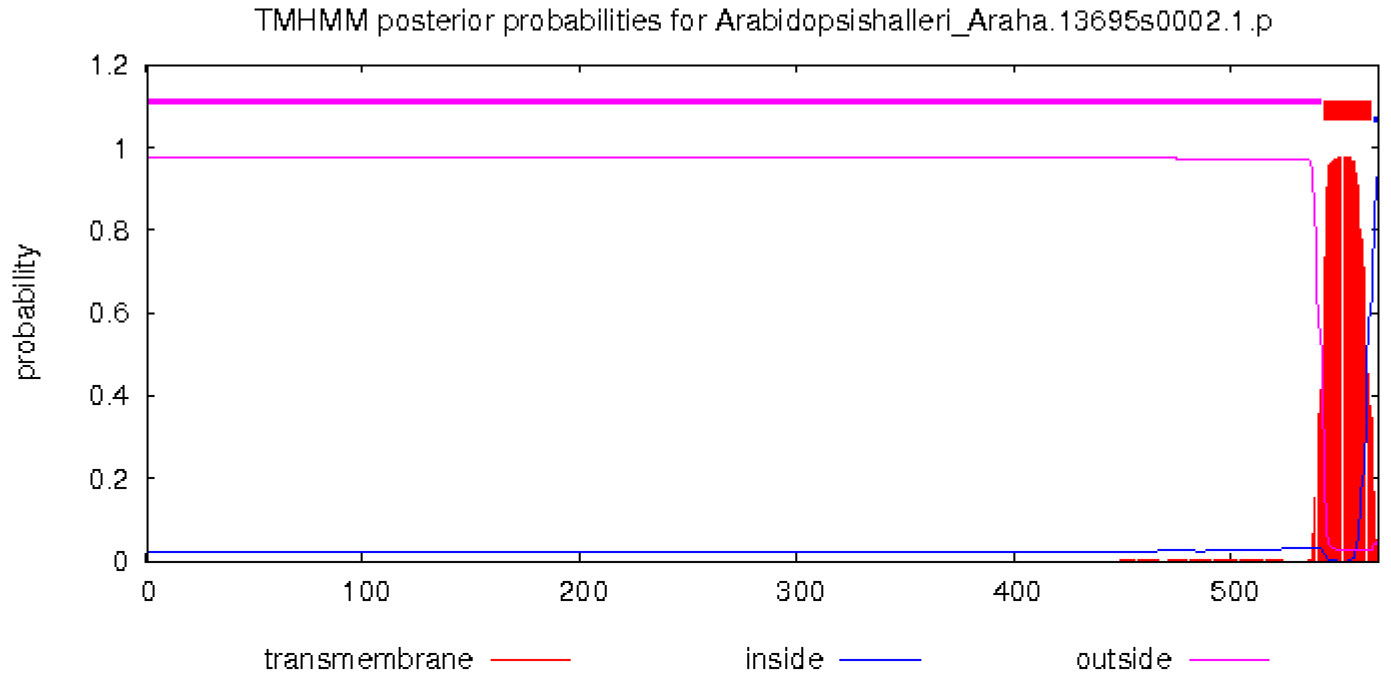

plot in postscript, script for making the plot in gnuplot, data for plot

---

```
# Arabidopsishalleri_Araha.1448s0011.1.p Length: 336
# Arabidopsishalleri_Araha.1448s0011.1.p Number of predicted TMHs:  0
# Arabidopsishalleri_Araha.1448s0011.1.p Exp number of AAs in TMHs: 0.00022
# Arabidopsishalleri_Araha.1448s0011.1.p Exp number, first 60 AAs:  0
# Arabidopsishalleri_Araha.1448s0011.1.p Total prob of N-in:        0.01238
Arabidopsishalleri_Araha.1448s0011.1.p  TMHMM2.0        outside      1   336
```

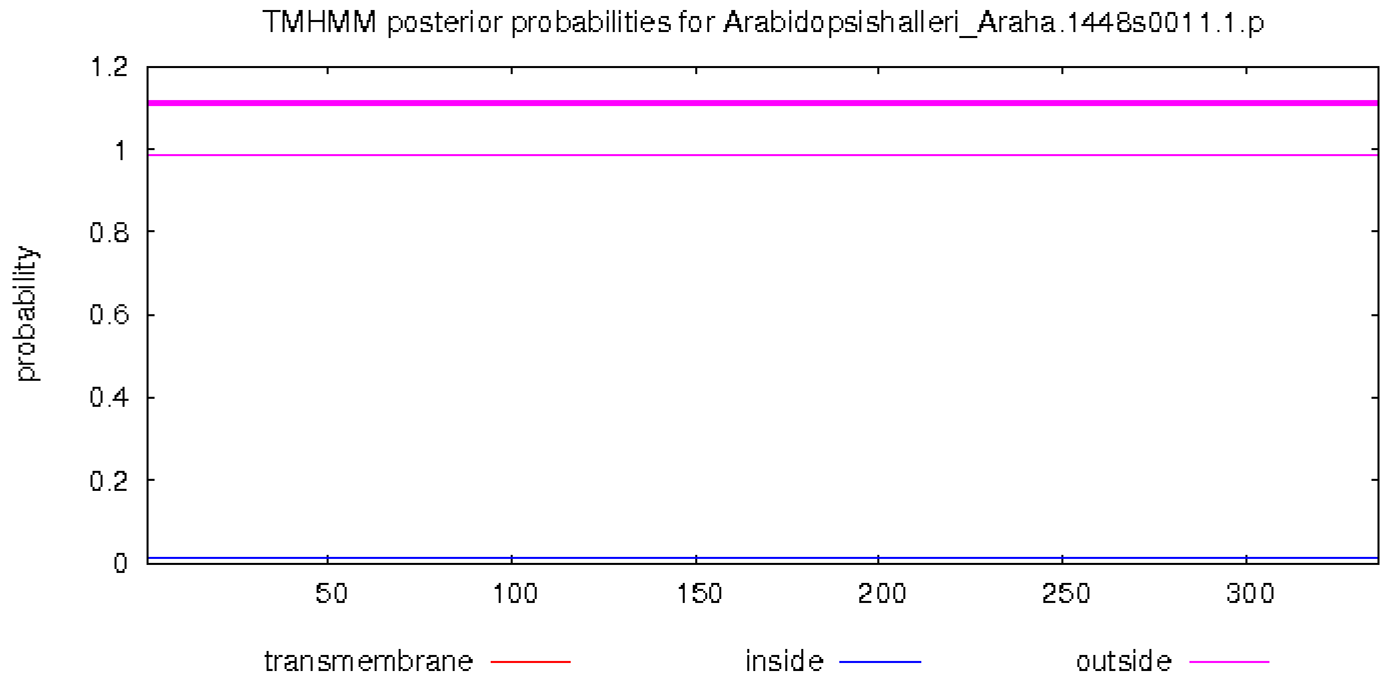

plot in postscript, script for making the plot in gnuplot, data for plot

---

```
# Arabidopsishalleri_Araha.16028s0003.1.p Length: 304
# Arabidopsishalleri_Araha.16028s0003.1.p Number of predicted TMHs:  0
# Arabidopsishalleri_Araha.16028s0003.1.p Exp number of AAs in TMHs: 0.00183
# Arabidopsishalleri_Araha.16028s0003.1.p Exp number, first 60 AAs:  0
# Arabidopsishalleri_Araha.16028s0003.1.p Total prob of N-in:        0.02125
Arabidopsishalleri_Araha.16028s0003.1.p TMHMM2.0        outside      1   304
```

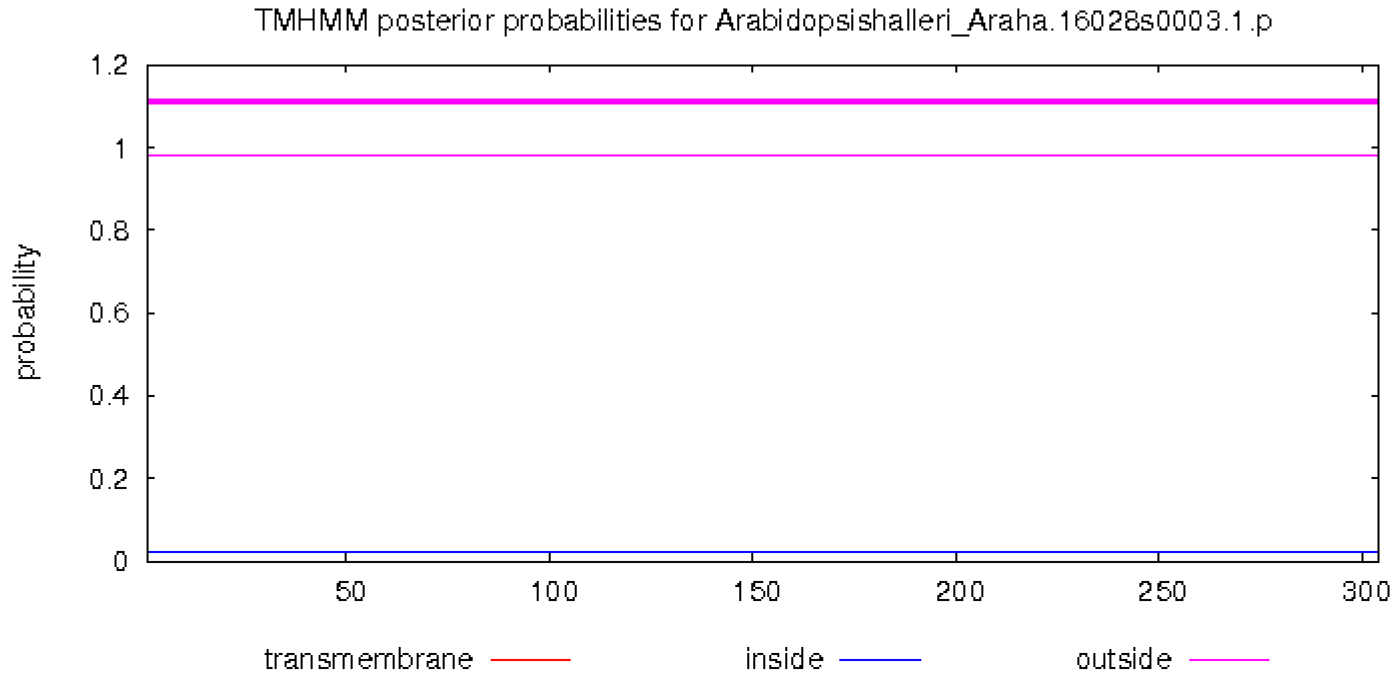

plot in postscript, script for making the plot in gnuplot, data for plot

---

```
# Arabidopsishalleri_Araha.16094s0001.1.p Length: 469
# Arabidopsishalleri_Araha.16094s0001.1.p Number of predicted TMHs:  1
# Arabidopsishalleri_Araha.16094s0001.1.p Exp number of AAs in TMHs: 19.46787
# Arabidopsishalleri_Araha.16094s0001.1.p Exp number, first 60 AAs:  0.00017
# Arabidopsishalleri_Araha.16094s0001.1.p Total prob of N-in:        0.23451
Arabidopsishalleri_Araha.16094s0001.1.p TMHMM2.0        outside      1   440
Arabidopsishalleri_Araha.16094s0001.1.p TMHMM2.0        TMhelix    441   463
Arabidopsishalleri_Araha.16094s0001.1.p TMHMM2.0        inside     464   469
```

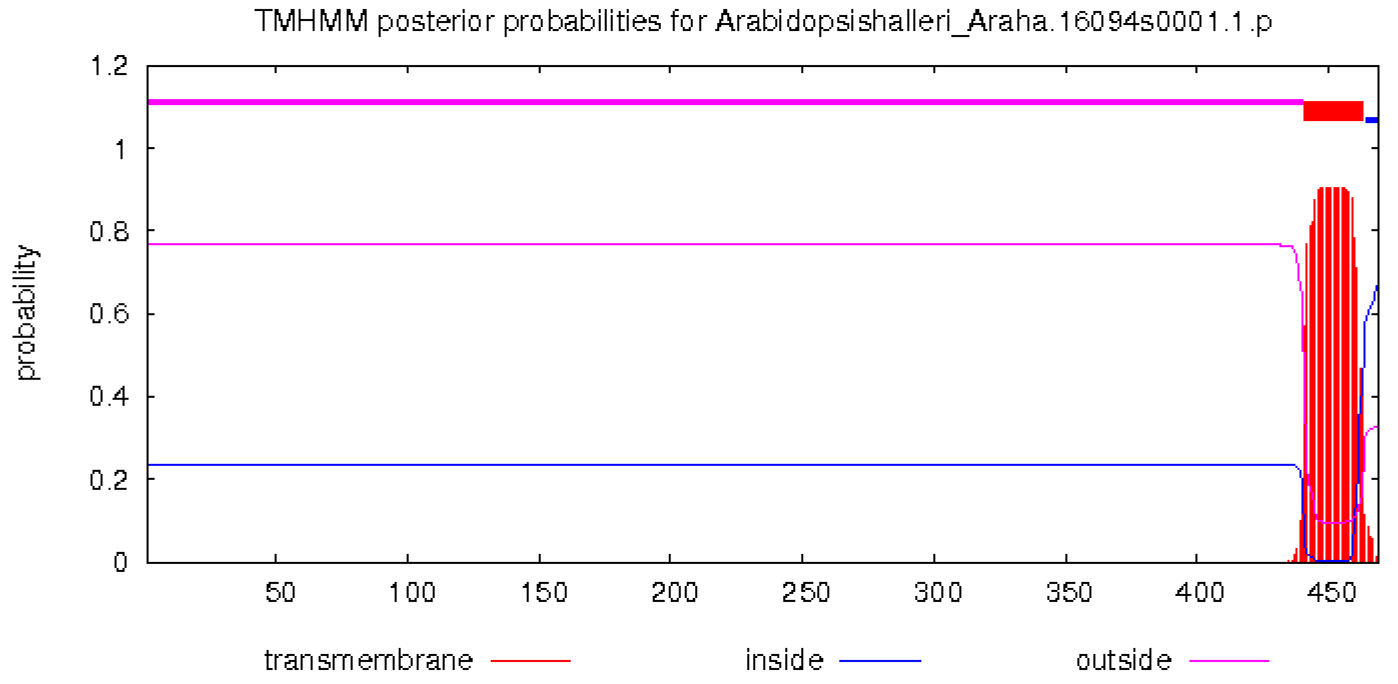

plot in postscript, script for making the plot in gnuplot, data for plot

---

```
# Arabidopsishalleri_Araha.16225s0003.1.p Length: 365
# Arabidopsishalleri_Araha.16225s0003.1.p Number of predicted TMHs:  0
# Arabidopsishalleri_Araha.16225s0003.1.p Exp number of AAs in TMHs: 0
# Arabidopsishalleri_Araha.16225s0003.1.p Exp number, first 60 AAs:  0
```

```
# Arabidopsishalleri_Araha.16225s0003.1.p Total prob of N-in:        0.00588
Arabidopsishalleri_Araha.16225s0003.1.p TMHMM2.0        outside      1   365
```

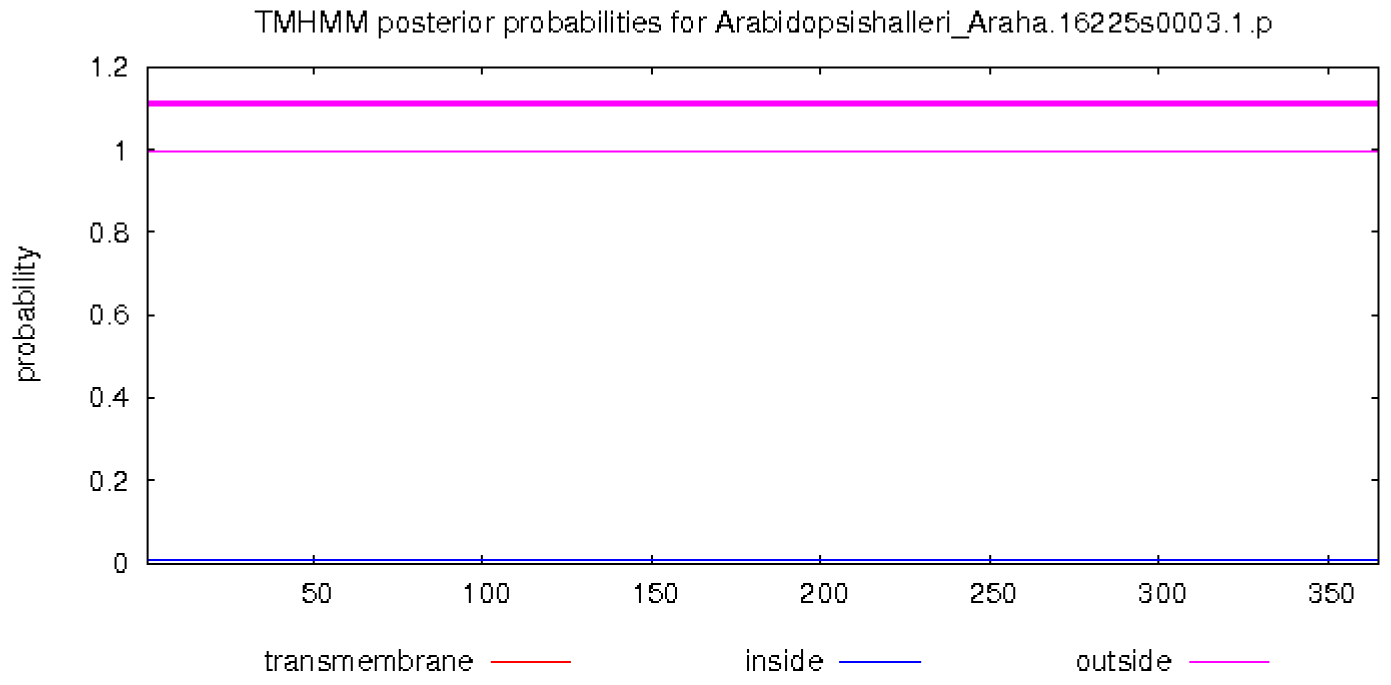

plot in postscript, script for making the plot in gnuplot, data for plot

---

```
# Arabidopsishalleri_Araha.16261s0010.1.p Length: 295
# Arabidopsishalleri_Araha.16261s0010.1.p Number of predicted TMHs:  0
# Arabidopsishalleri_Araha.16261s0010.1.p Exp number of AAs in TMHs: 12.08994
# Arabidopsishalleri_Araha.16261s0010.1.p Exp number, first 60 AAs:  0
# Arabidopsishalleri_Araha.16261s0010.1.p Total prob of N-in:        0.18651
Arabidopsishalleri_Araha.16261s0010.1.p TMHMM2.0        outside      1   295
```

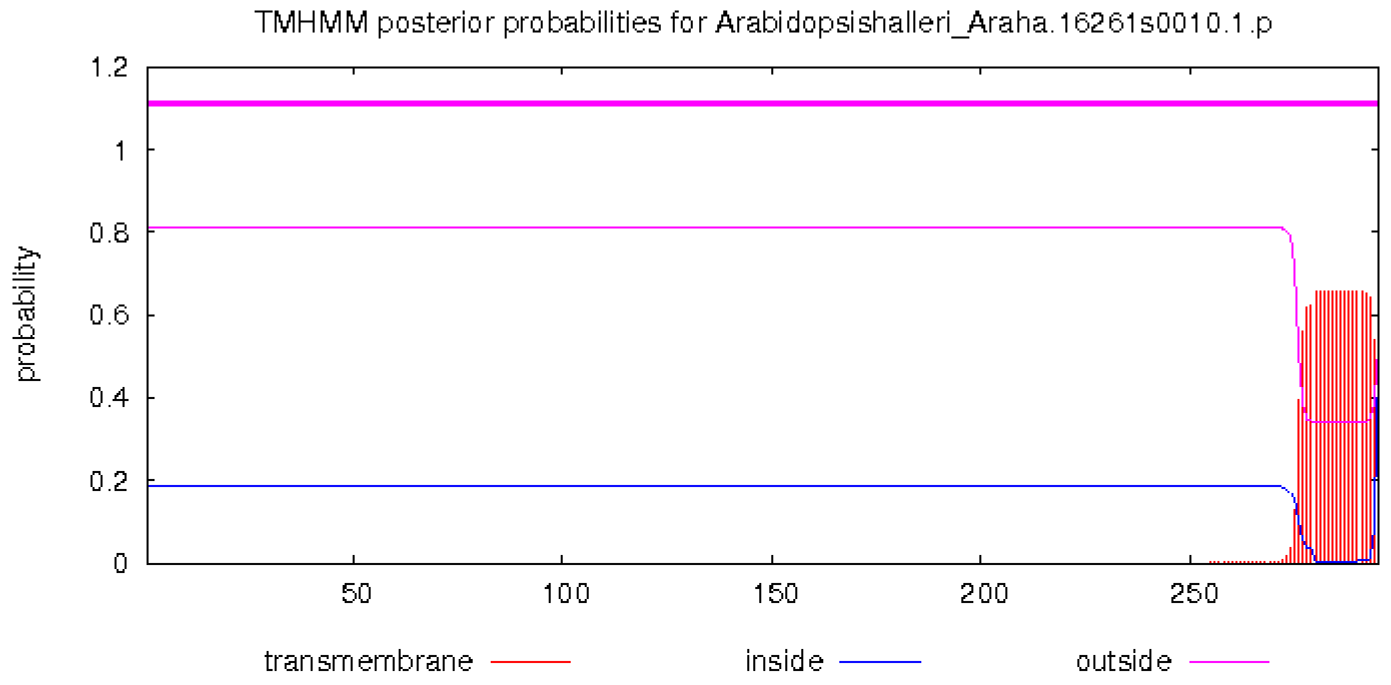

plot in postscript, script for making the plot in gnuplot, data for plot

---

```
# Arabidopsishalleri_Araha.17797s0002.1.p Length: 326
# Arabidopsishalleri_Araha.17797s0002.1.p Number of predicted TMHs:  0
# Arabidopsishalleri_Araha.17797s0002.1.p Exp number of AAs in TMHs: 0
# Arabidopsishalleri_Araha.17797s0002.1.p Exp number, first 60 AAs:  0
```

```
# Arabidopsishalleri_Araha.17797s0002.1.p Total prob of N-in:        0.01705
Arabidopsishalleri_Araha.17797s0002.1.p TMHMM2.0        outside      1   326
```

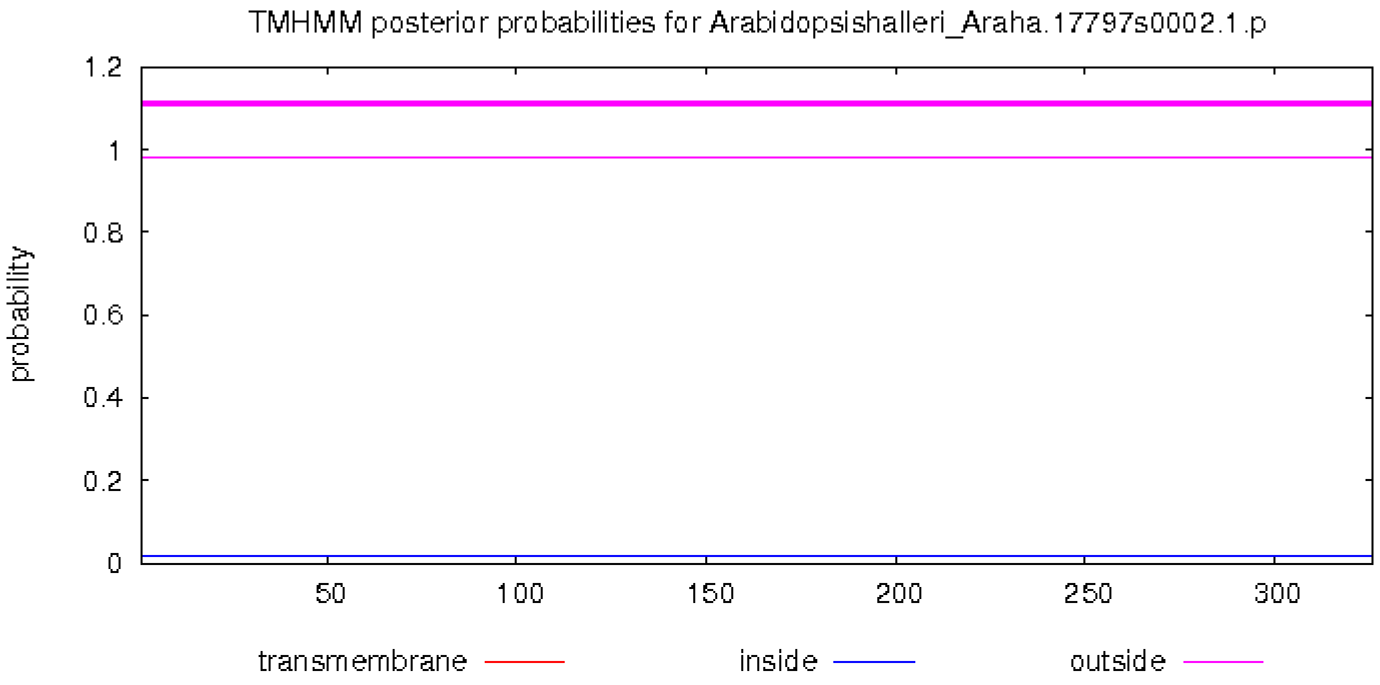

plot in postscript, script for making the plot in gnuplot, data for plot

---

```
# Arabidopsishalleri_Araha.18637s0005.1.p Length: 266
# Arabidopsishalleri_Araha.18637s0005.1.p Number of predicted TMHs:  0
# Arabidopsishalleri_Araha.18637s0005.1.p Exp number of AAs in TMHs: 0.00021
# Arabidopsishalleri_Araha.18637s0005.1.p Exp number, first 60 AAs:  0
# Arabidopsishalleri_Araha.18637s0005.1.p Total prob of N-in:        0.07434
Arabidopsishalleri_Araha.18637s0005.1.p TMHMM2.0        outside      1   266
```

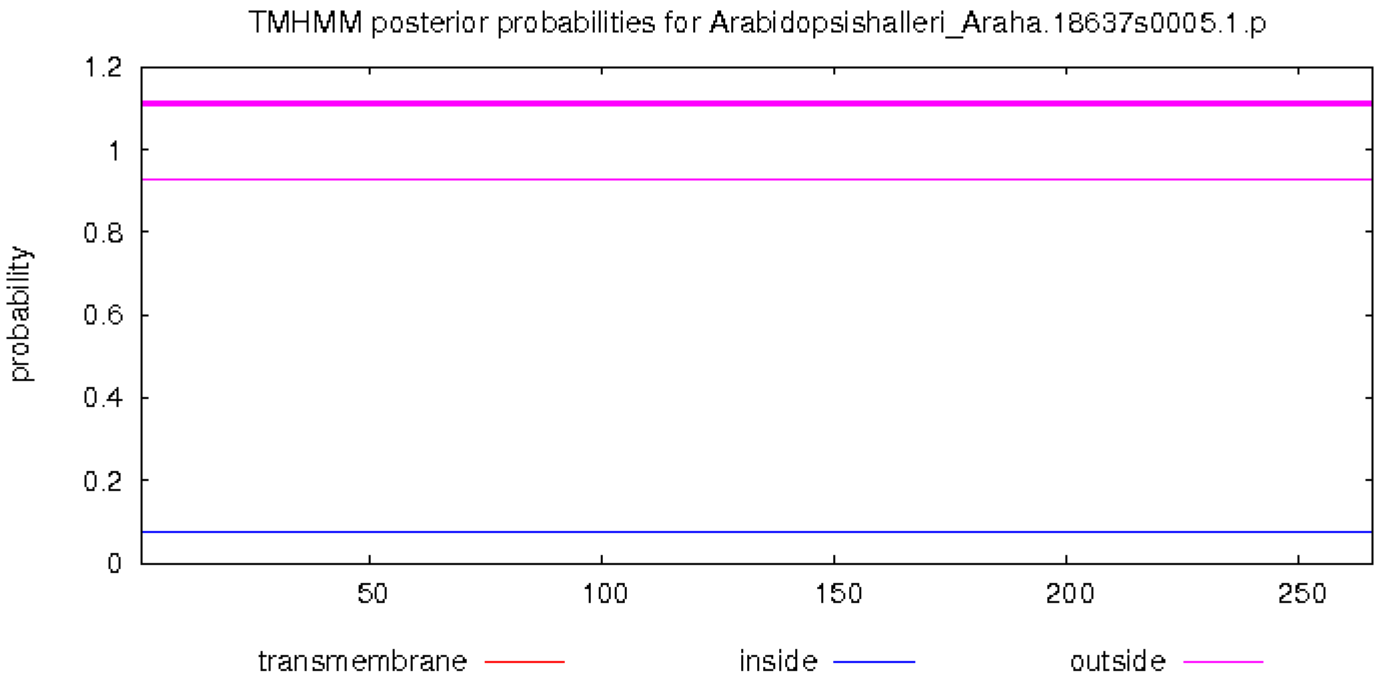

plot in postscript, script for making the plot in gnuplot, data for plot

---

```
# Arabidopsishalleri_Araha.19157s0013.1.p Length: 512
# Arabidopsishalleri_Araha.19157s0013.1.p Number of predicted TMHs:  1
# Arabidopsishalleri_Araha.19157s0013.1.p Exp number of AAs in TMHs: 21.11533
# Arabidopsishalleri_Araha.19157s0013.1.p Exp number, first 60 AAs:  0
```

```
# Arabidopsishalleri_Araha.19157s0013.1.p Total prob of N-in:        0.14705
Arabidopsishalleri_Araha.19157s0013.1.p TMHMM2.0        outside      1   488
Arabidopsishalleri_Araha.19157s0013.1.p TMHMM2.0        TMhelix    489   511
Arabidopsishalleri_Araha.19157s0013.1.p TMHMM2.0        inside     512   512
```

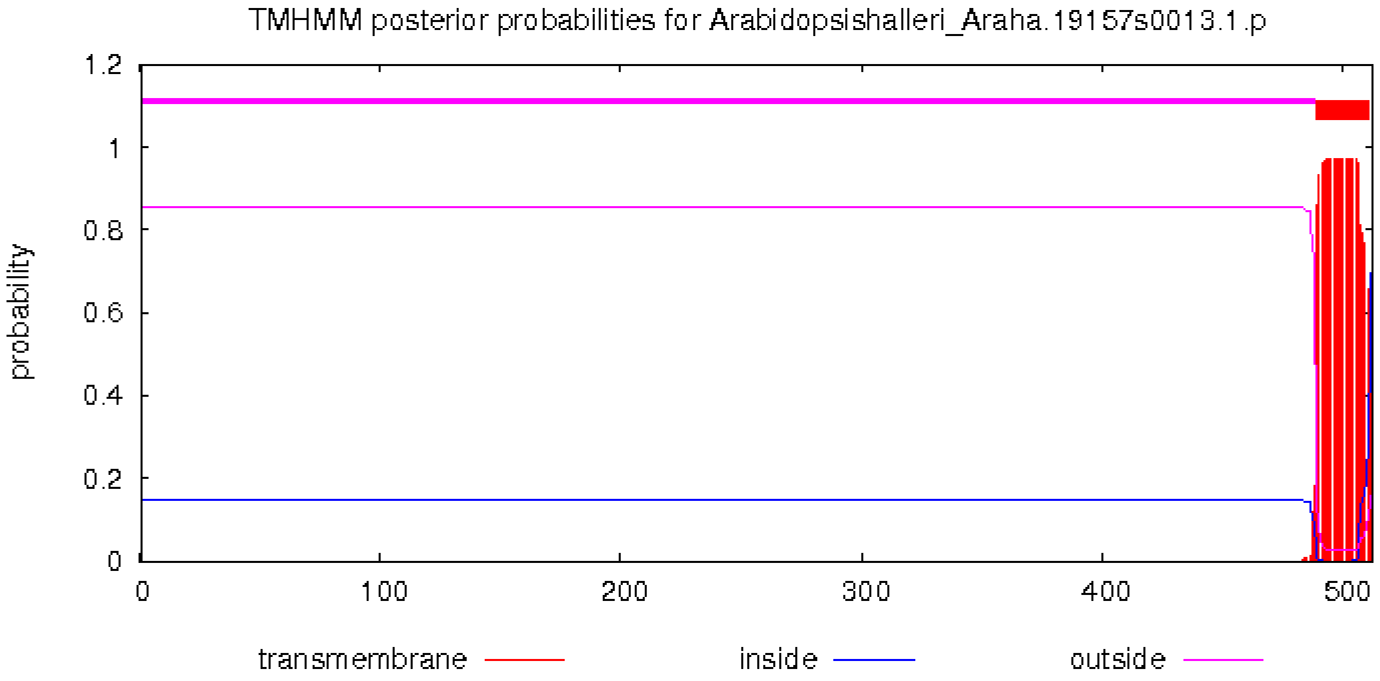

plot in postscript, script for making the plot in gnuplot, data for plot

---

```
# Arabidopsishalleri_Araha.19457s0003.1.p Length: 324
# Arabidopsishalleri_Araha.19457s0003.1.p Number of predicted TMHs:  0
# Arabidopsishalleri_Araha.19457s0003.1.p Exp number of AAs in TMHs: 0.00064
# Arabidopsishalleri_Araha.19457s0003.1.p Exp number, first 60 AAs:  0.00041
# Arabidopsishalleri_Araha.19457s0003.1.p Total prob of N-in:        0.01633
Arabidopsishalleri_Araha.19457s0003.1.p TMHMM2.0        outside      1   324
```

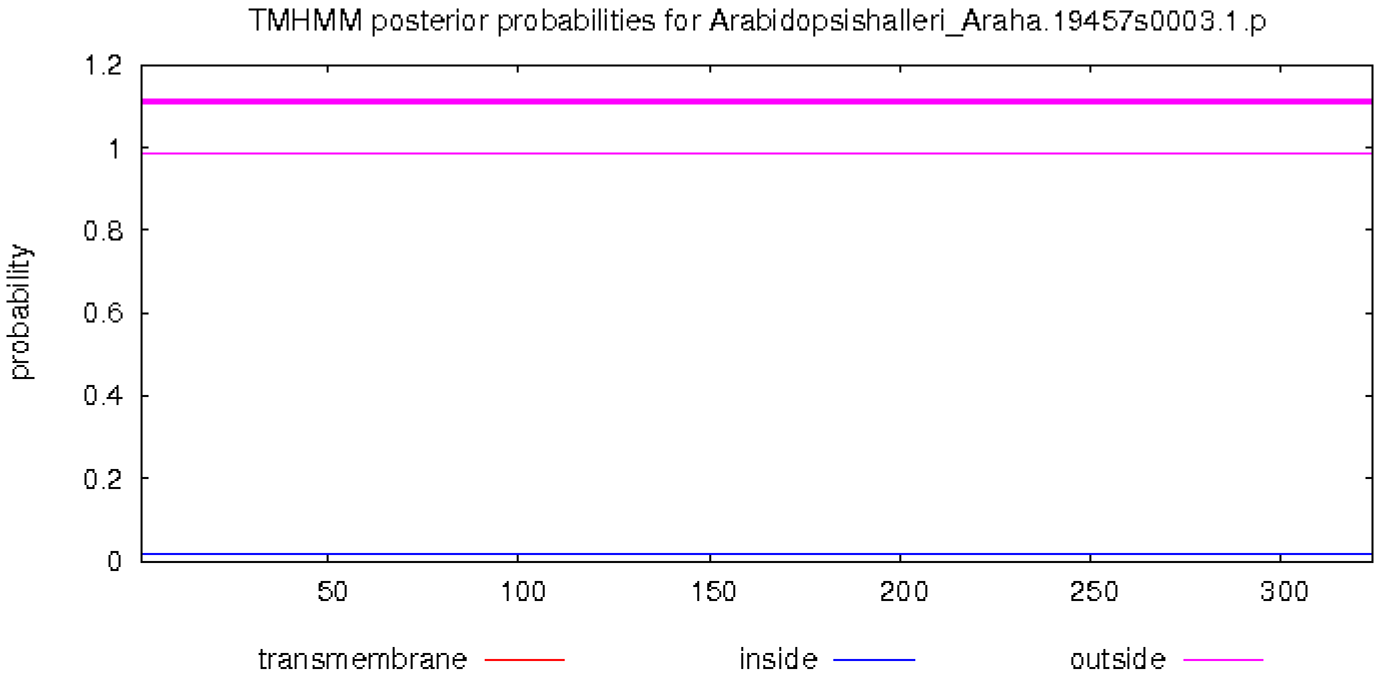

plot in postscript, script for making the plot in gnuplot, data for plot

---

```
# Arabidopsishalleri_Araha.20405s0001.1.p Length: 209
# Arabidopsishalleri_Araha.20405s0001.1.p Number of predicted TMHs:  0
```

```
# Arabidopsishalleri_Araha.20405s0001.1.p Exp number of AAs in TMHs: 0
# Arabidopsishalleri_Araha.20405s0001.1.p Exp number, first 60 AAs:  0
# Arabidopsishalleri_Araha.20405s0001.1.p Total prob of N-in:        0.02222
Arabidopsishalleri_Araha.20405s0001.1.p TMHMM2.0        outside      1   209
```

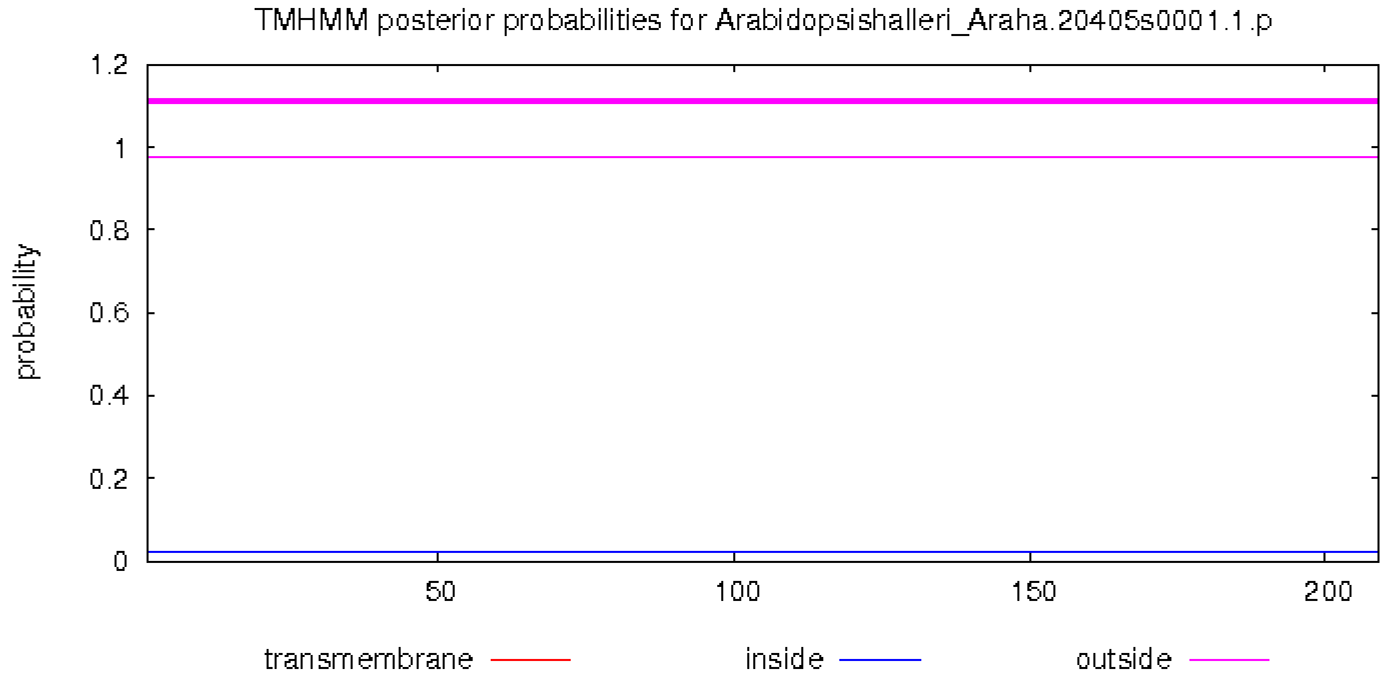

plot in postscript, script for making the plot in gnuplot, data for plot

---

```
# Arabidopsishalleri_Araha.21058s0001.1.p Length: 217
# Arabidopsishalleri_Araha.21058s0001.1.p Number of predicted TMHs:  0
# Arabidopsishalleri_Araha.21058s0001.1.p Exp number of AAs in TMHs: 0.17847
# Arabidopsishalleri_Araha.21058s0001.1.p Exp number, first 60 AAs:  0
# Arabidopsishalleri_Araha.21058s0001.1.p Total prob of N-in:        0.10359
Arabidopsishalleri_Araha.21058s0001.1.p TMHMM2.0        outside      1   217
```

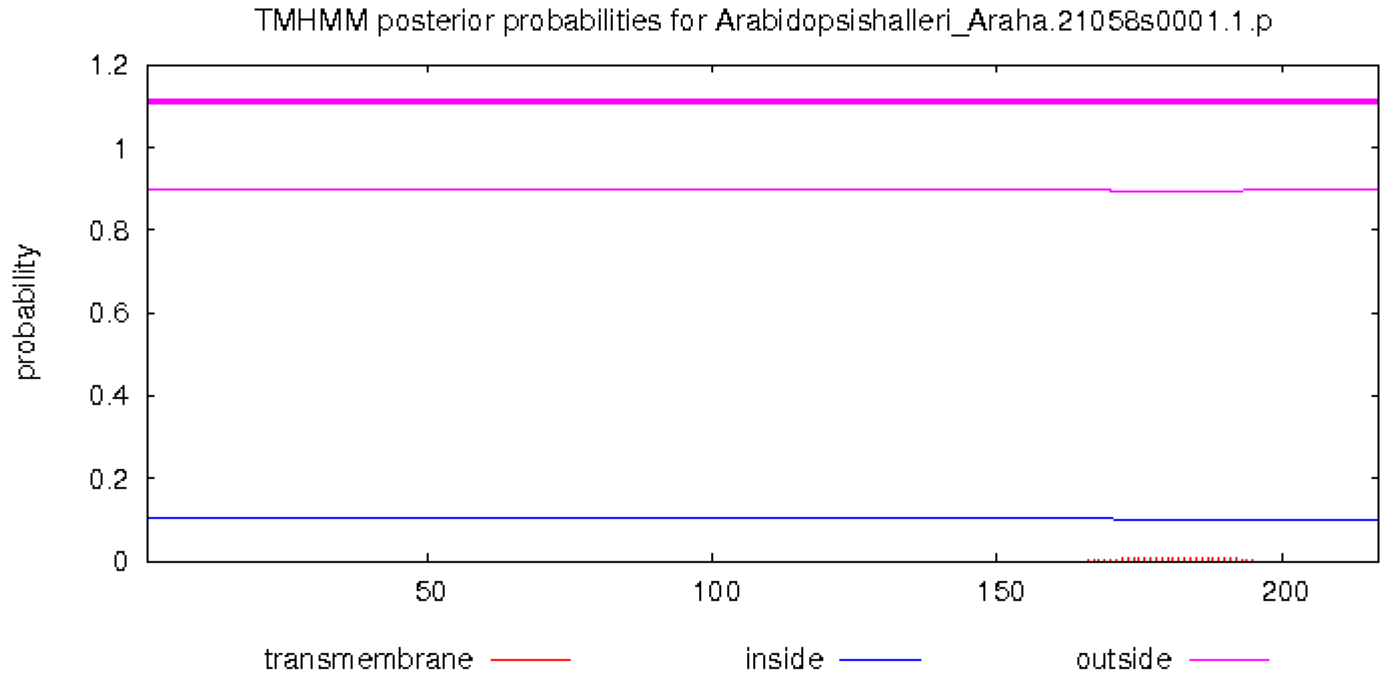

plot in postscript, script for making the plot in gnuplot, data for plot

---

```
# Arabidopsishalleri_Araha.21283s0004.1.p Length: 363
# Arabidopsishalleri_Araha.21283s0004.1.p Number of predicted TMHs:  0
```

```
# Arabidopsishalleri_Araha.21283s0004.1.p Exp number of AAs in TMHs: 0.00018
# Arabidopsishalleri_Araha.21283s0004.1.p Exp number, first 60 AAs:  0.00018
# Arabidopsishalleri_Araha.21283s0004.1.p Total prob of N-in:        0.00434
Arabidopsishalleri_Araha.21283s0004.1.p TMHMM2.0        outside      1   363
```

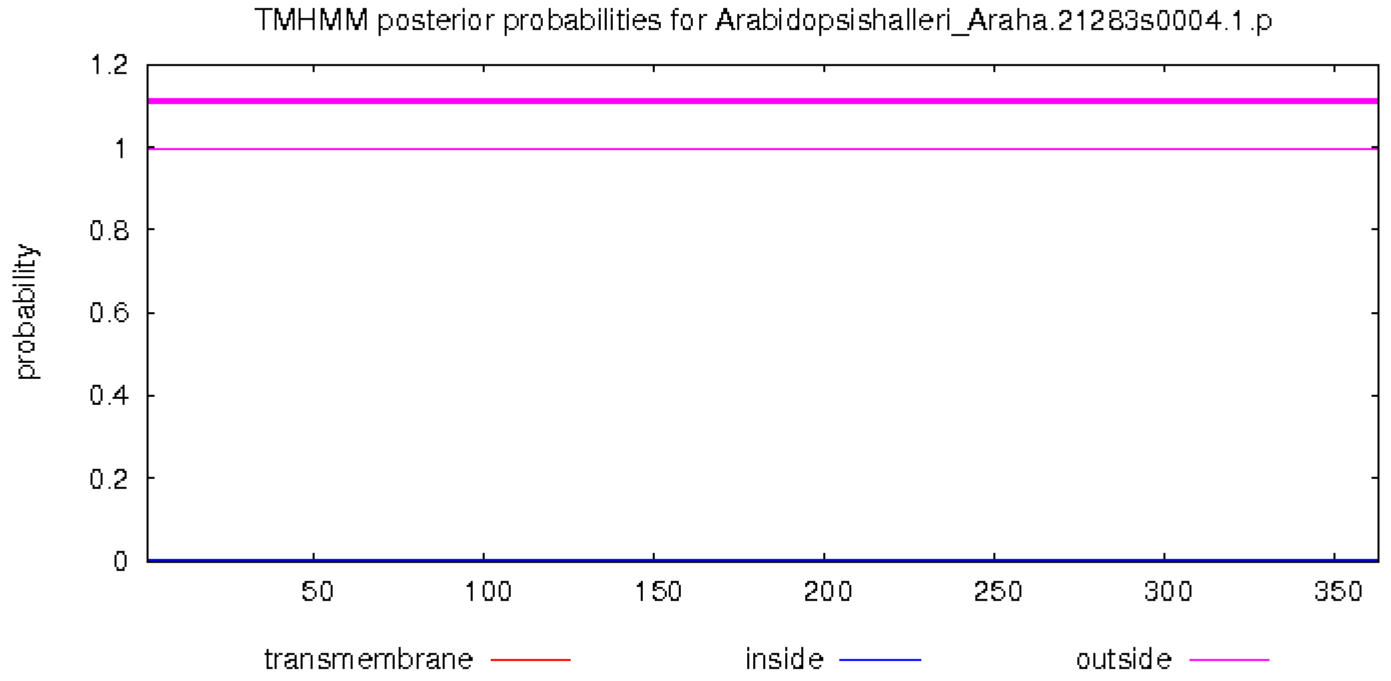

plot in postscript, script for making the plot in gnuplot, data for plot

---

```
# Arabidopsishalleri_Araha.21478s0011.1.p Length: 371
# Arabidopsishalleri_Araha.21478s0011.1.p Number of predicted TMHs:  0
# Arabidopsishalleri_Araha.21478s0011.1.p Exp number of AAs in TMHs: 0.00235
# Arabidopsishalleri_Araha.21478s0011.1.p Exp number, first 60 AAs:  0.00096
# Arabidopsishalleri_Araha.21478s0011.1.p Total prob of N-in:        0.00767
Arabidopsishalleri_Araha.21478s0011.1.p TMHMM2.0        outside      1   371
```

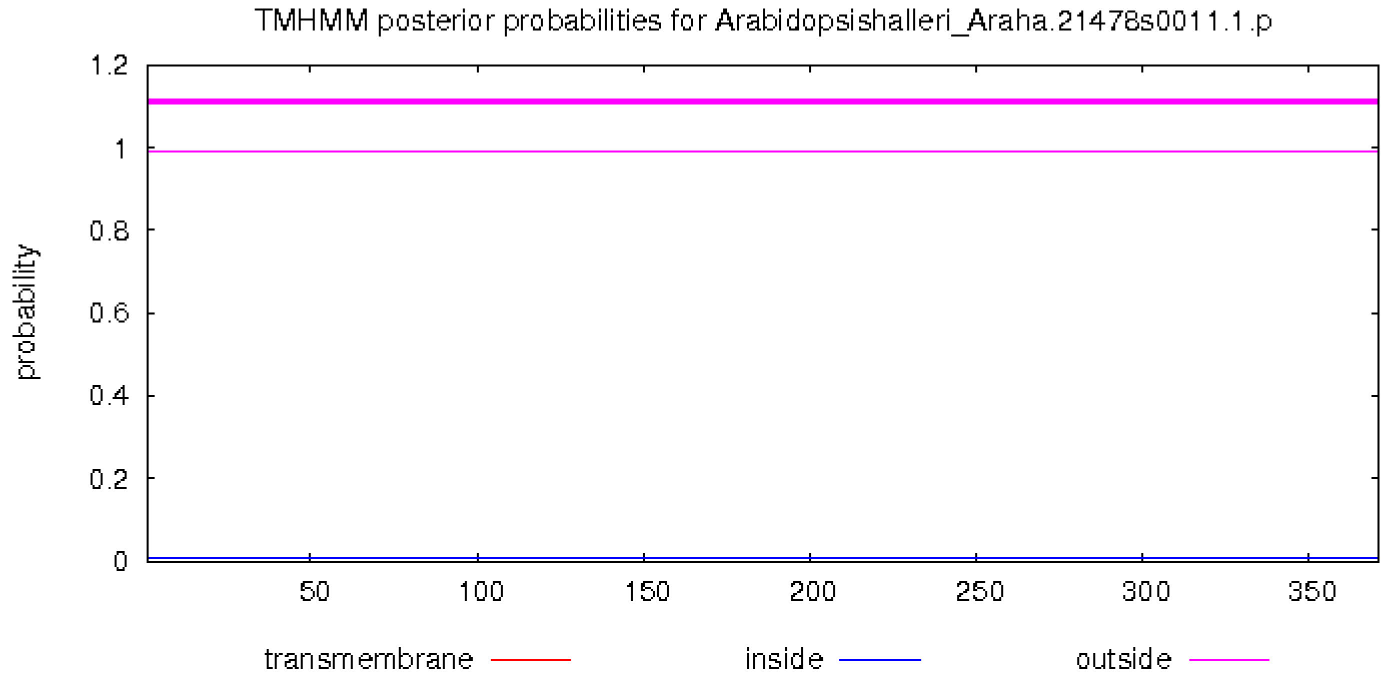

plot in postscript, script for making the plot in gnuplot, data for plot

---

```
# Arabidopsishalleri_Araha.21527s0003.1.p Length: 211
# Arabidopsishalleri_Araha.21527s0003.1.p Number of predicted TMHs:  0
```

```
# Arabidopsishalleri_Araha.21527s0003.1.p Exp number of AAs in TMHs: 0.03866
# Arabidopsishalleri_Araha.21527s0003.1.p Exp number, first 60 AAs:  0.0014
# Arabidopsishalleri_Araha.21527s0003.1.p Total prob of N-in:        0.01768
Arabidopsishalleri_Araha.21527s0003.1.p TMHMM2.0        outside      1   211
```

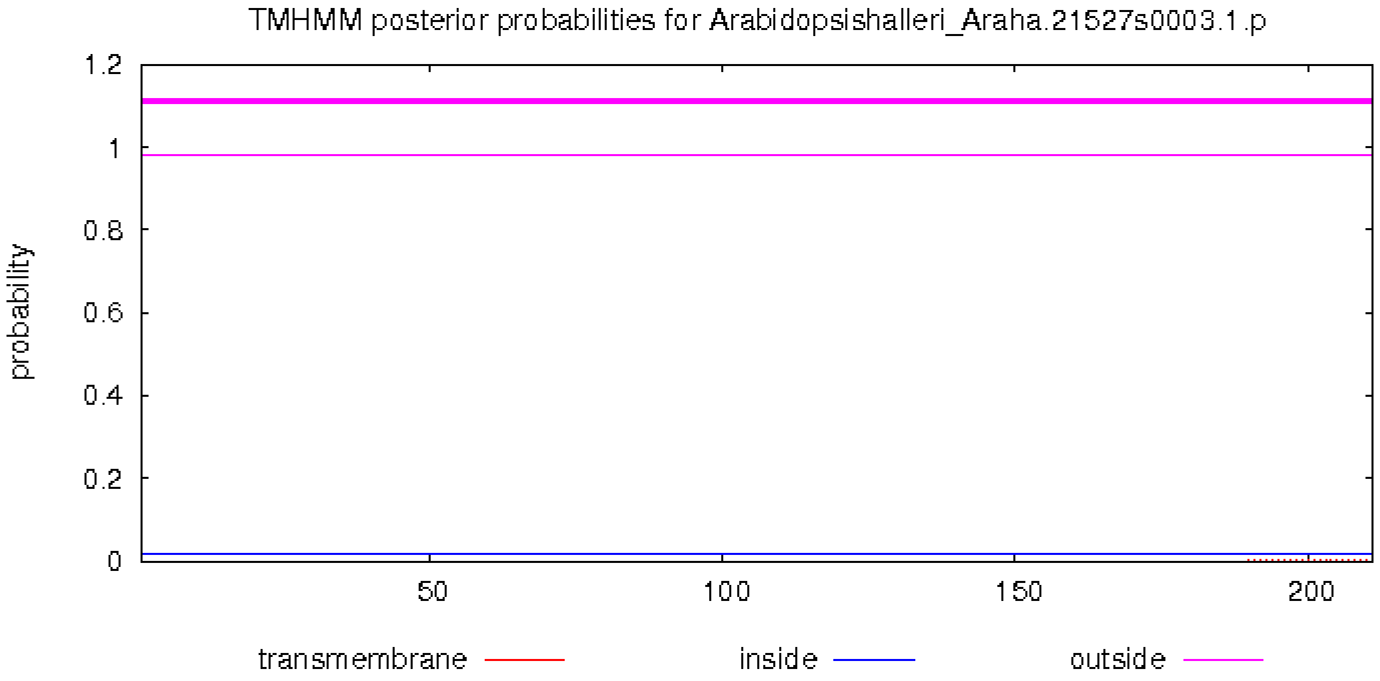

plot in postscript, script for making the plot in gnuplot, data for plot

---

```
# Arabidopsishalleri_Araha.23250s0004.1.p Length: 220
# Arabidopsishalleri_Araha.23250s0004.1.p Number of predicted TMHs:  0
# Arabidopsishalleri_Araha.23250s0004.1.p Exp number of AAs in TMHs: 0.06946
# Arabidopsishalleri_Araha.23250s0004.1.p Exp number, first 60 AAs:  0.00032
# Arabidopsishalleri_Araha.23250s0004.1.p Total prob of N-in:        0.12270
Arabidopsishalleri_Araha.23250s0004.1.p TMHMM2.0        outside      1   220
```

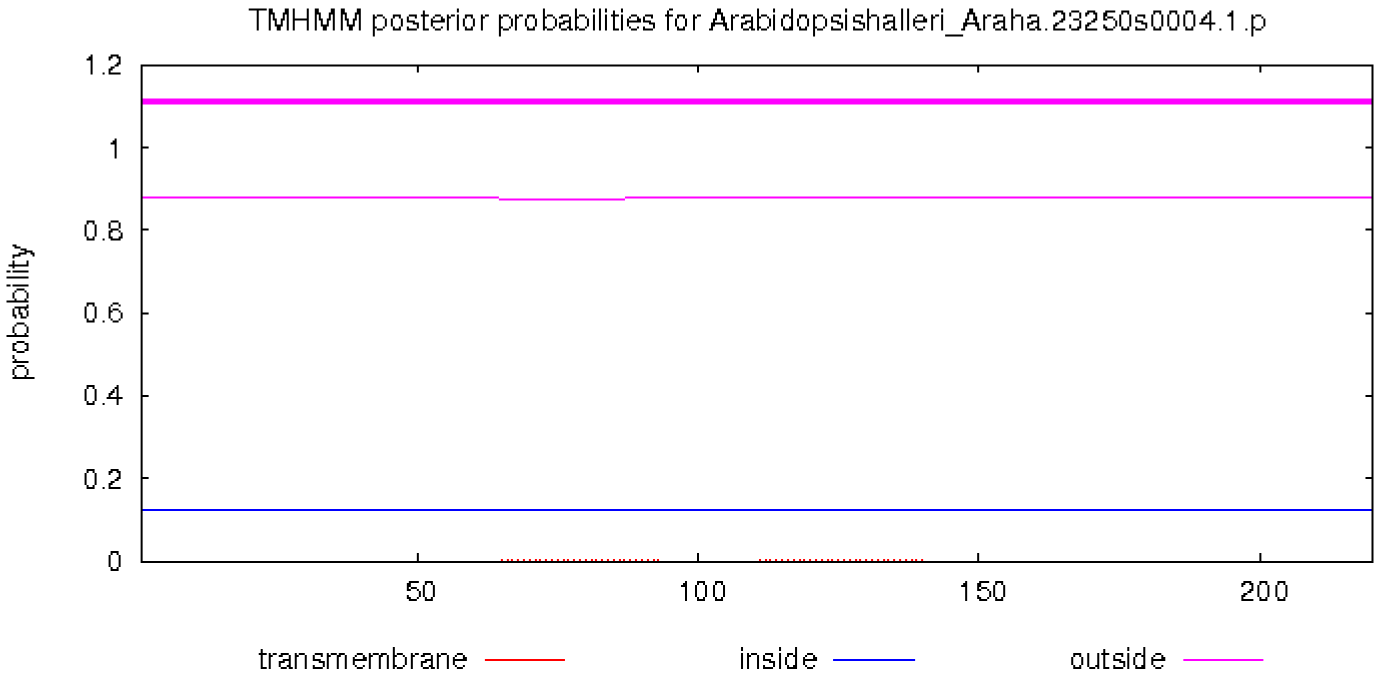

plot in postscript, script for making the plot in gnuplot, data for plot

---

```
# Arabidopsishalleri_Araha.23250s0005.1.p Length: 194
# Arabidopsishalleri_Araha.23250s0005.1.p Number of predicted TMHs:  0
```

```
# Arabidopsishalleri_Araha.23250s0005.1.p Exp number of AAs in TMHs: 0
# Arabidopsishalleri_Araha.23250s0005.1.p Exp number, first 60 AAs:  0
# Arabidopsishalleri_Araha.23250s0005.1.p Total prob of N-in:        0.01990
Arabidopsishalleri_Araha.23250s0005.1.p TMHMM2.0        outside      1   194
```

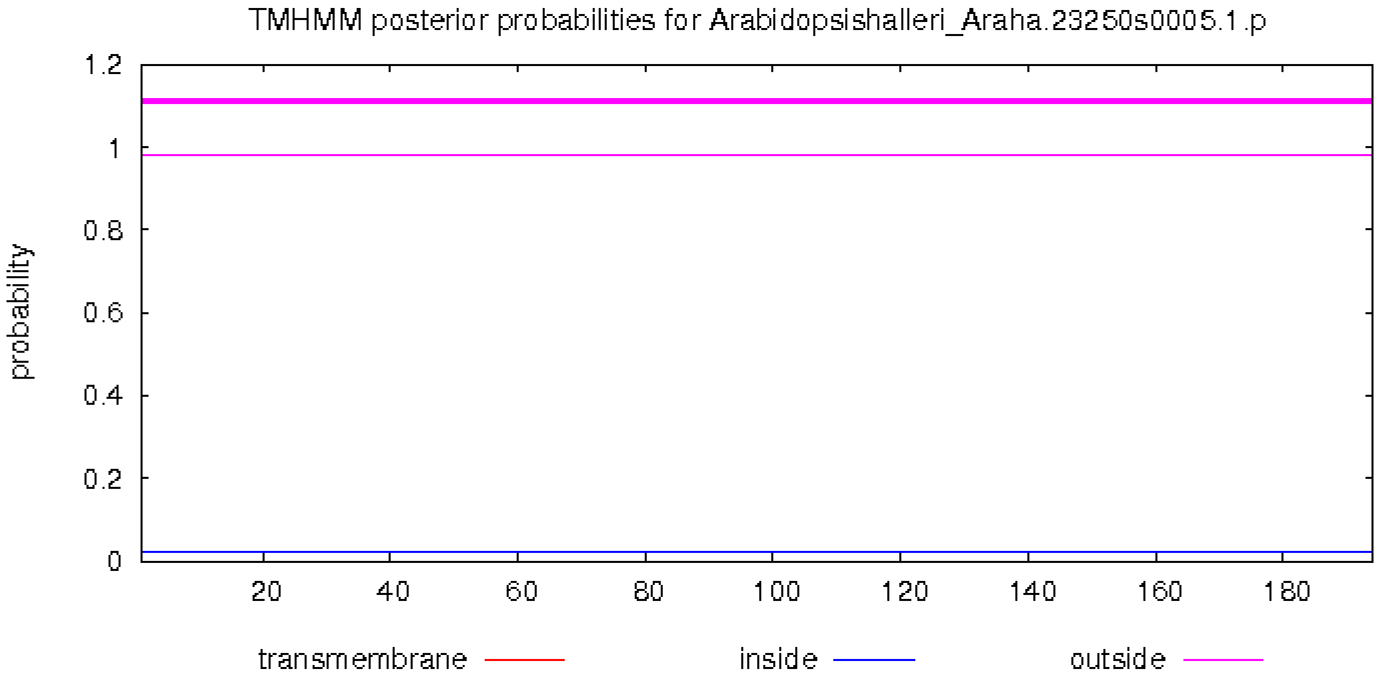

plot in postscript, script for making the plot in gnuplot, data for plot

---

```
# Arabidopsishalleri_Araha.24359s0001.1.p Length: 356
# Arabidopsishalleri_Araha.24359s0001.1.p Number of predicted TMHs:  1
# Arabidopsishalleri_Araha.24359s0001.1.p Exp number of AAs in TMHs: 20.45571
# Arabidopsishalleri_Araha.24359s0001.1.p Exp number, first 60 AAs:  0.02057
# Arabidopsishalleri_Araha.24359s0001.1.p Total prob of N-in:        0.04297
Arabidopsishalleri_Araha.24359s0001.1.p TMHMM2.0        outside      1   331
Arabidopsishalleri_Araha.24359s0001.1.p TMHMM2.0        TMhelix    332   351
Arabidopsishalleri_Araha.24359s0001.1.p TMHMM2.0        inside     352   356
```

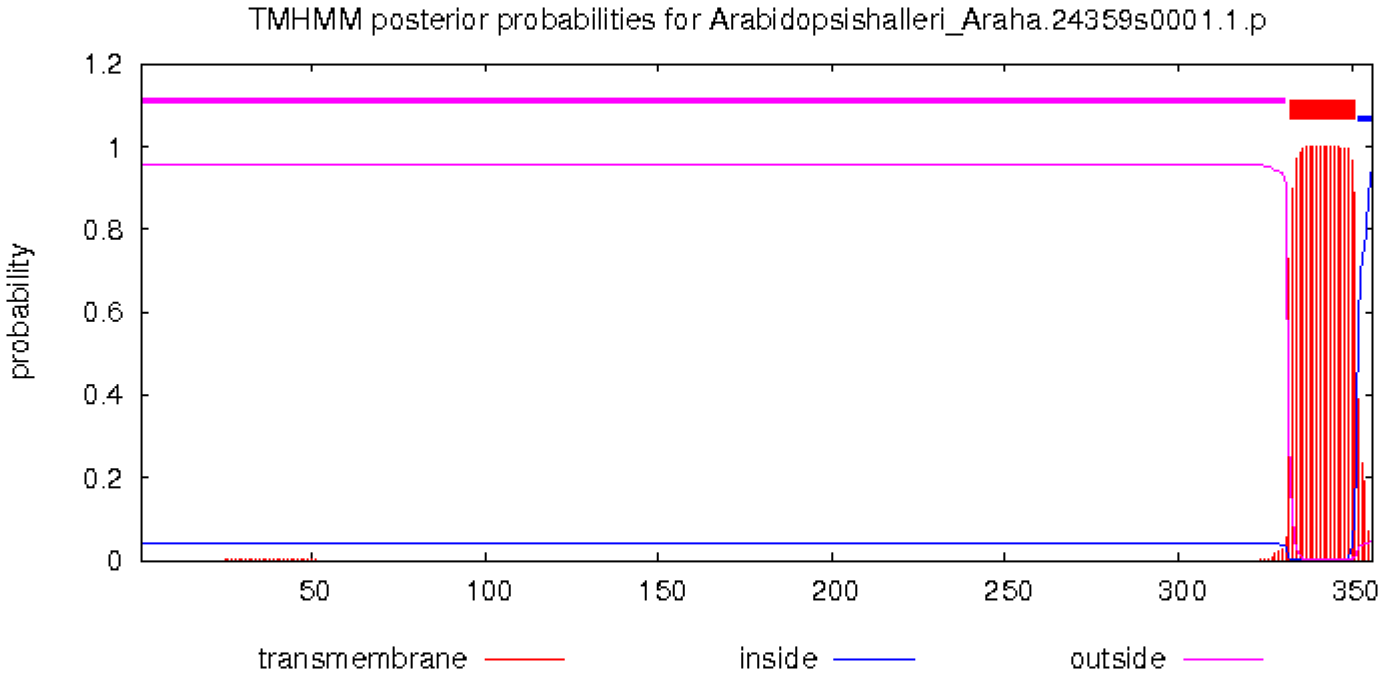

plot in postscript, script for making the plot in gnuplot, data for plot

---

```
# Arabidopsishalleri_Araha.24857s0004.1.p Length: 302
# Arabidopsishalleri_Araha.24857s0004.1.p Number of predicted TMHs:  0
# Arabidopsishalleri_Araha.24857s0004.1.p Exp number of AAs in TMHs: 0.00162
# Arabidopsishalleri_Araha.24857s0004.1.p Exp number, first 60 AAs:  0
# Arabidopsishalleri_Araha.24857s0004.1.p Total prob of N-in:        0.06560
Arabidopsishalleri_Araha.24857s0004.1.p TMHMM2.0        outside      1   302
```

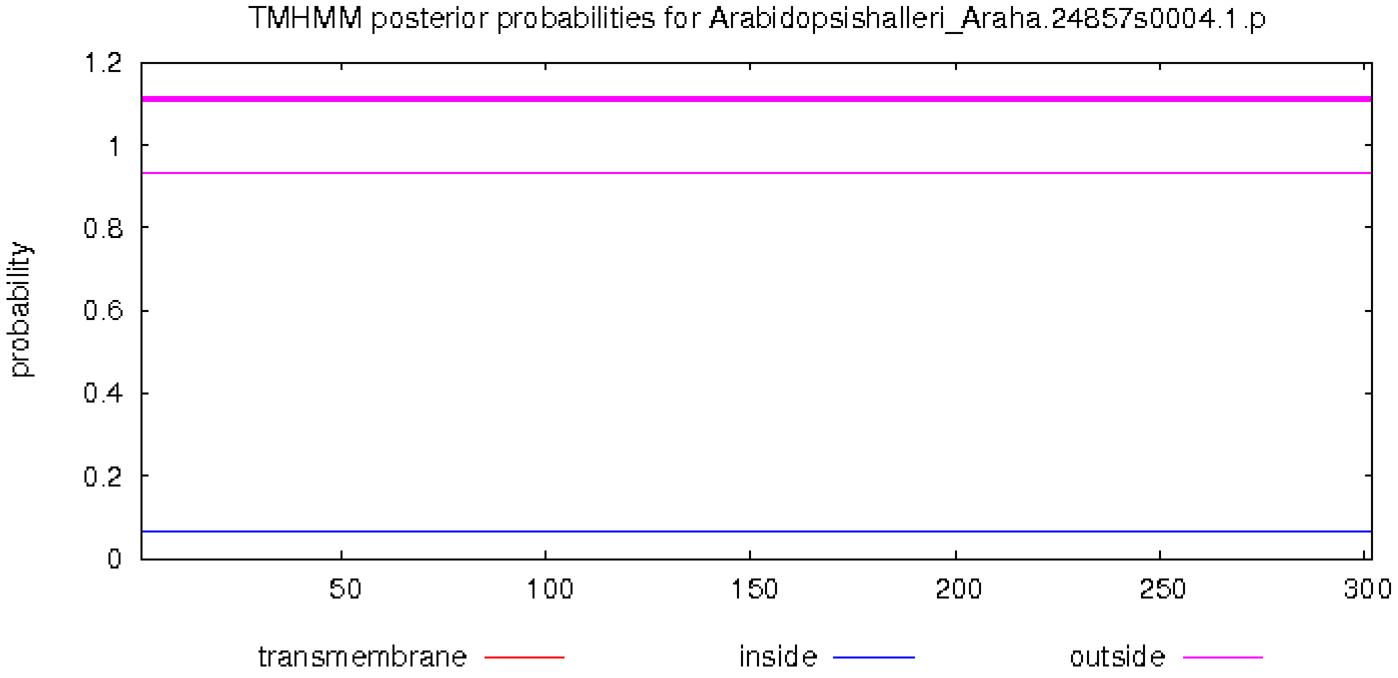

plot in postscript, script for making the plot in gnuplot, data for plot

---

```
# Arabidopsishalleri_Araha.25334s0001.1.p Length: 288
# Arabidopsishalleri_Araha.25334s0001.1.p Number of predicted TMHs:  0
# Arabidopsishalleri_Araha.25334s0001.1.p Exp number of AAs in TMHs: 0.00099
# Arabidopsishalleri_Araha.25334s0001.1.p Exp number, first 60 AAs:  0.00082
# Arabidopsishalleri_Araha.25334s0001.1.p Total prob of N-in:        0.06340
Arabidopsishalleri_Araha.25334s0001.1.p TMHMM2.0        outside      1   288
```

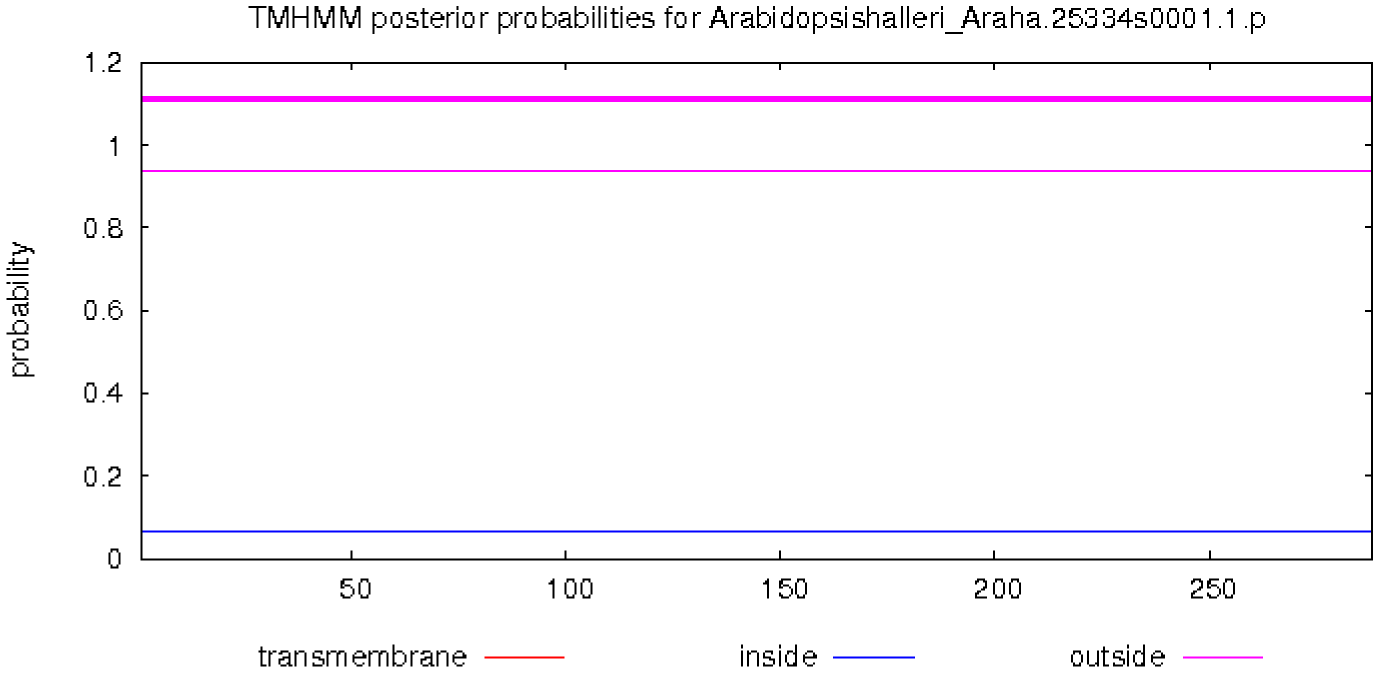

plot in postscript, script for making the plot in gnuplot, data for plot

---

```
# Arabidopsishalleri_Araha.27238s0003.1.p Length: 320
# Arabidopsishalleri_Araha.27238s0003.1.p Number of predicted TMHs:  0
# Arabidopsishalleri_Araha.27238s0003.1.p Exp number of AAs in TMHs: 0.01084
# Arabidopsishalleri_Araha.27238s0003.1.p Exp number, first 60 AAs:  0.00021
# Arabidopsishalleri_Araha.27238s0003.1.p Total prob of N-in:        0.00901
Arabidopsishalleri_Araha.27238s0003.1.p TMHMM2.0        outside      1   320
```

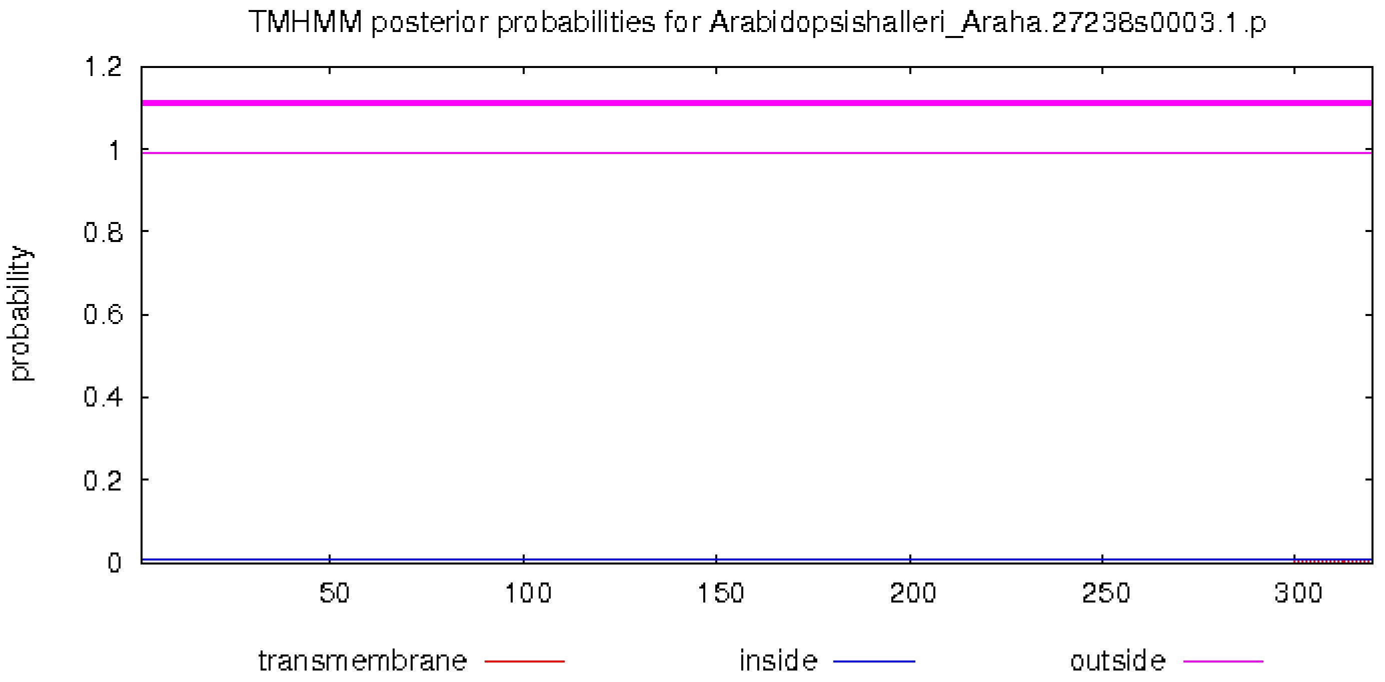

plot in postscript, script for making the plot in gnuplot, data for plot

---

```
# Arabidopsishalleri_Araha.3034s0046.1.p Length: 352
# Arabidopsishalleri_Araha.3034s0046.1.p Number of predicted TMHs:  0
# Arabidopsishalleri_Araha.3034s0046.1.p Exp number of AAs in TMHs: 0.0012
# Arabidopsishalleri_Araha.3034s0046.1.p Exp number, first 60 AAs:  0
# Arabidopsishalleri_Araha.3034s0046.1.p Total prob of N-in:        0.00052
Arabidopsishalleri_Araha.3034s0046.1.p  TMHMM2.0        outside      1   352
```

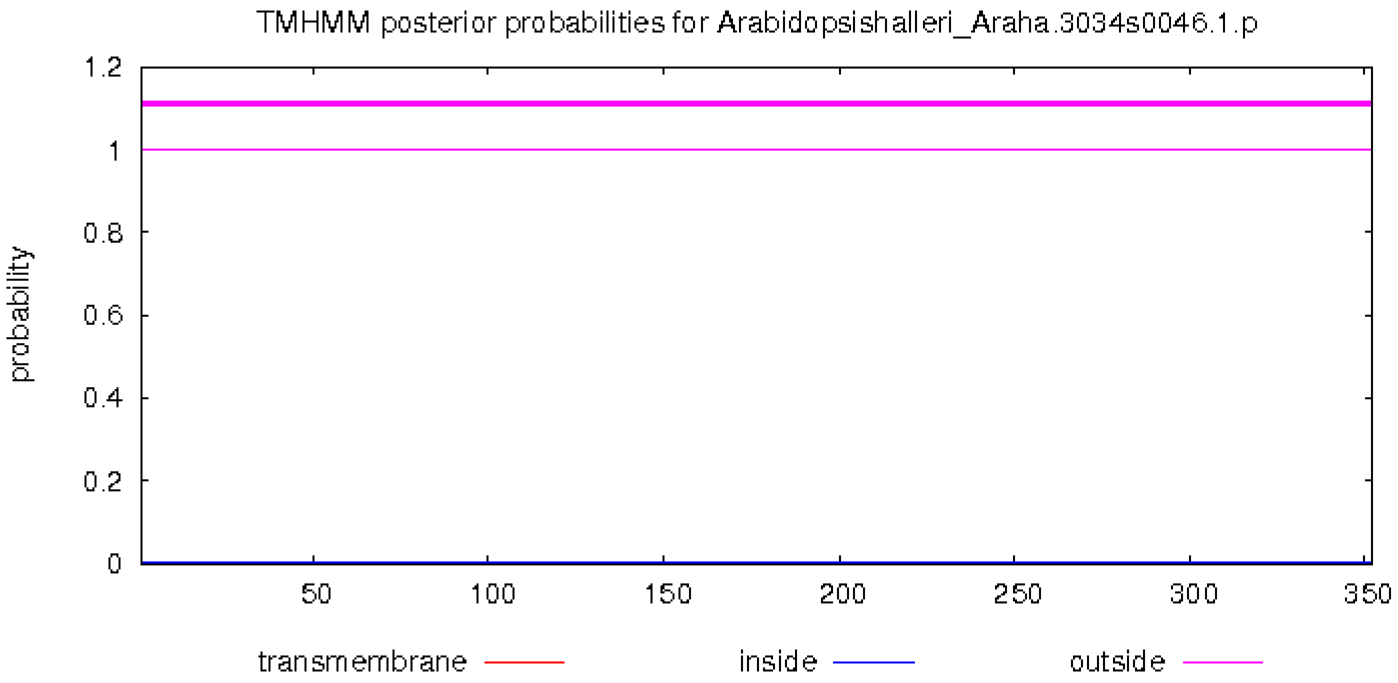

plot in postscript, script for making the plot in gnuplot, data for plot

---

```
# Arabidopsishalleri_Araha.3105s0045.1.p Length: 206
# Arabidopsishalleri_Araha.3105s0045.1.p Number of predicted TMHs:  0
# Arabidopsishalleri_Araha.3105s0045.1.p Exp number of AAs in TMHs: 1.14411
# Arabidopsishalleri_Araha.3105s0045.1.p Exp number, first 60 AAs:  0.88721
# Arabidopsishalleri_Araha.3105s0045.1.p Total prob of N-in:        0.15773
Arabidopsishalleri_Araha.3105s0045.1.p  TMHMM2.0        outside      1   206
```

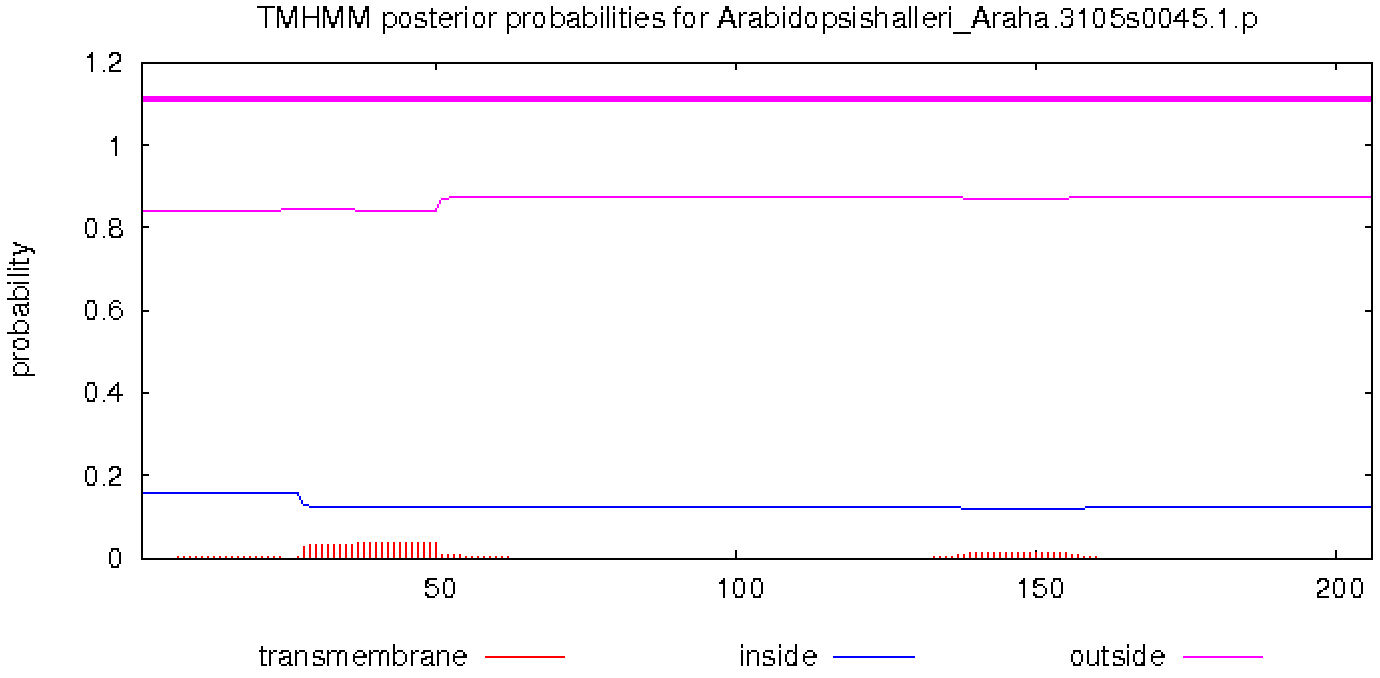

plot in postscript, script for making the plot in gnuplot, data for plot

---

```
# Arabidopsishalleri_Araha.3134s0014.1.p Length: 270
# Arabidopsishalleri_Araha.3134s0014.1.p Number of predicted TMHs:  0
# Arabidopsishalleri_Araha.3134s0014.1.p Exp number of AAs in TMHs: 0.00107
# Arabidopsishalleri_Araha.3134s0014.1.p Exp number, first 60 AAs:  0
# Arabidopsishalleri_Araha.3134s0014.1.p Total prob of N-in:        0.02694
Arabidopsishalleri_Araha.3134s0014.1.p  TMHMM2.0        outside      1   270
```

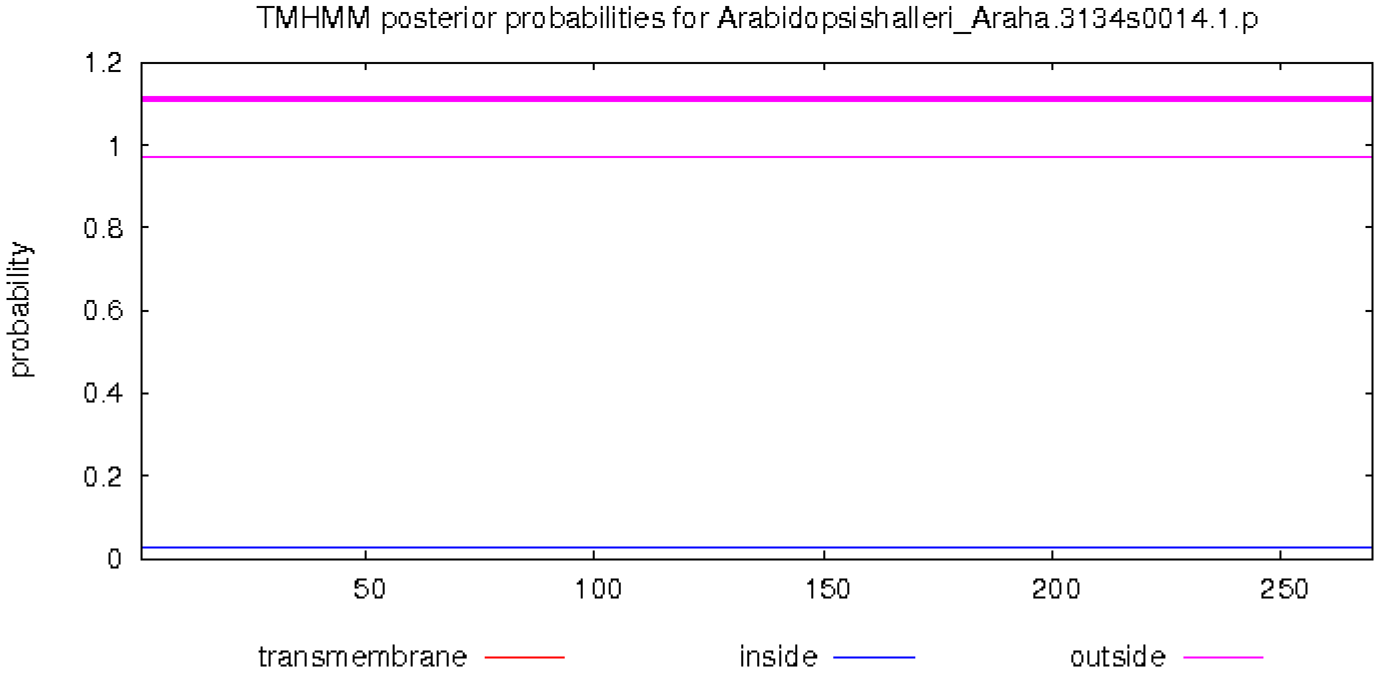

plot in postscript, script for making the plot in gnuplot, data for plot

---

```
# Arabidopsishalleri_Araha.32489s0002.1.p Length: 304
# Arabidopsishalleri_Araha.32489s0002.1.p Number of predicted TMHs:  0
# Arabidopsishalleri_Araha.32489s0002.1.p Exp number of AAs in TMHs: 0.00336
# Arabidopsishalleri_Araha.32489s0002.1.p Exp number, first 60 AAs:  0
# Arabidopsishalleri_Araha.32489s0002.1.p Total prob of N-in:        0.02264
Arabidopsishalleri_Araha.32489s0002.1.p TMHMM2.0        outside      1   304
```

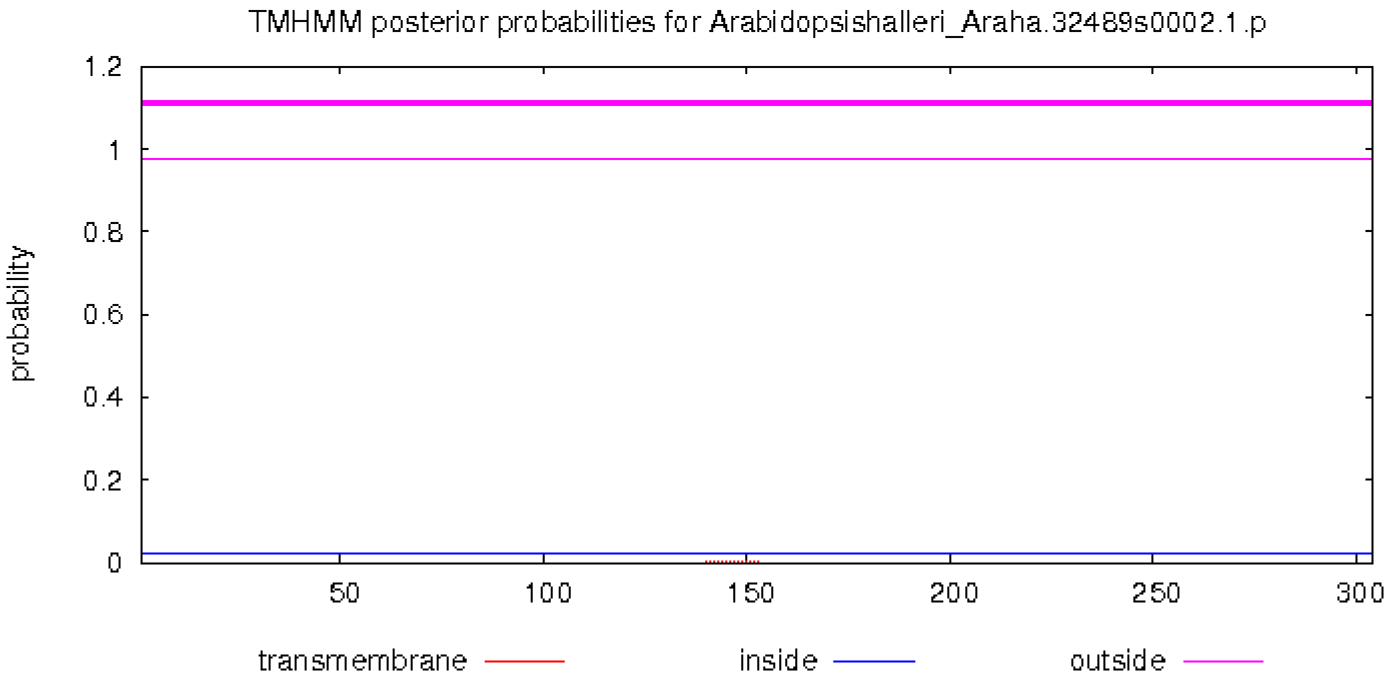

plot in postscript, script for making the plot in gnuplot, data for plot

---

```
# Arabidopsishalleri_Araha.3353s0007.1.p Length: 288
# Arabidopsishalleri_Araha.3353s0007.1.p Number of predicted TMHs:  0
# Arabidopsishalleri_Araha.3353s0007.1.p Exp number of AAs in TMHs: 0.00173
# Arabidopsishalleri_Araha.3353s0007.1.p Exp number, first 60 AAs:  0.00062
# Arabidopsishalleri_Araha.3353s0007.1.p Total prob of N-in:        0.00331
Arabidopsishalleri_Araha.3353s0007.1.p  TMHMM2.0        outside      1   288
```

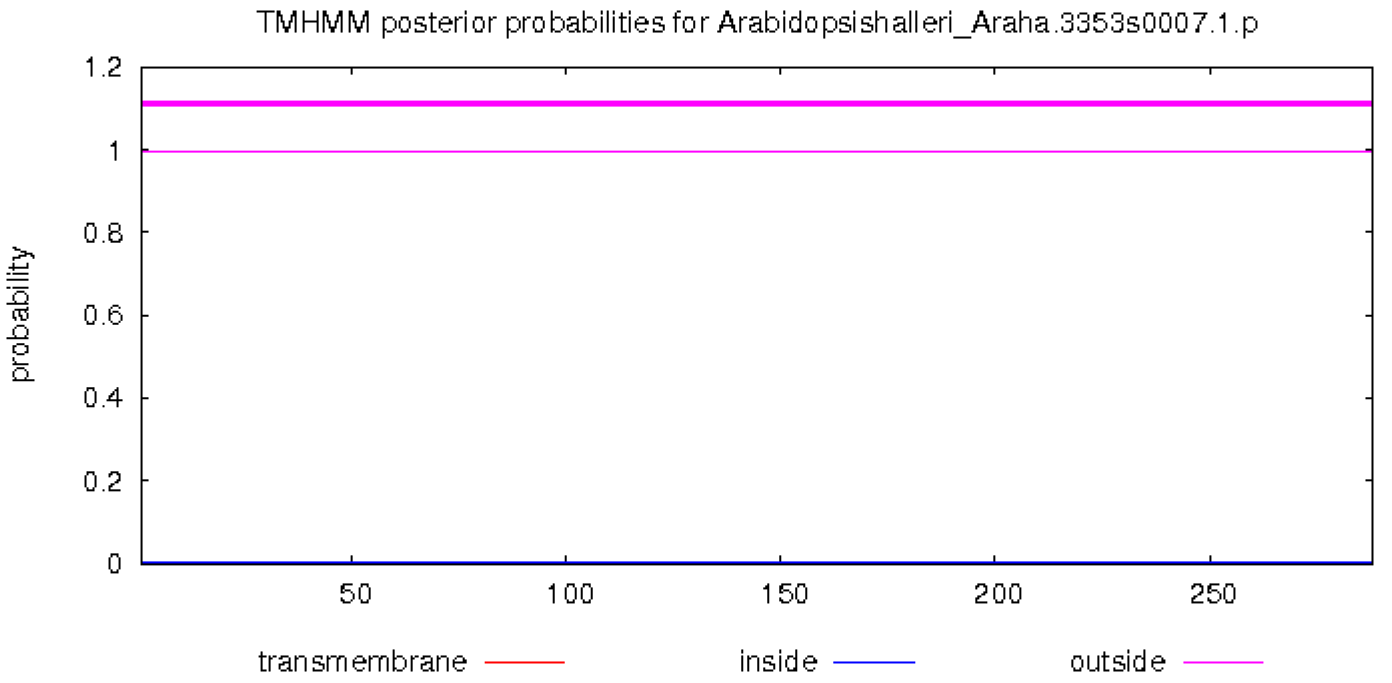

plot in postscript, script for making the plot in gnuplot, data for plot

---

```
# Arabidopsishalleri_Araha.3365s0004.1.p Length: 301
# Arabidopsishalleri_Araha.3365s0004.1.p Number of predicted TMHs:  0
# Arabidopsishalleri_Araha.3365s0004.1.p Exp number of AAs in TMHs: 0.00079
# Arabidopsishalleri_Araha.3365s0004.1.p Exp number, first 60 AAs:  0
# Arabidopsishalleri_Araha.3365s0004.1.p Total prob of N-in:        0.10318
Arabidopsishalleri_Araha.3365s0004.1.p  TMHMM2.0        outside      1   301
```

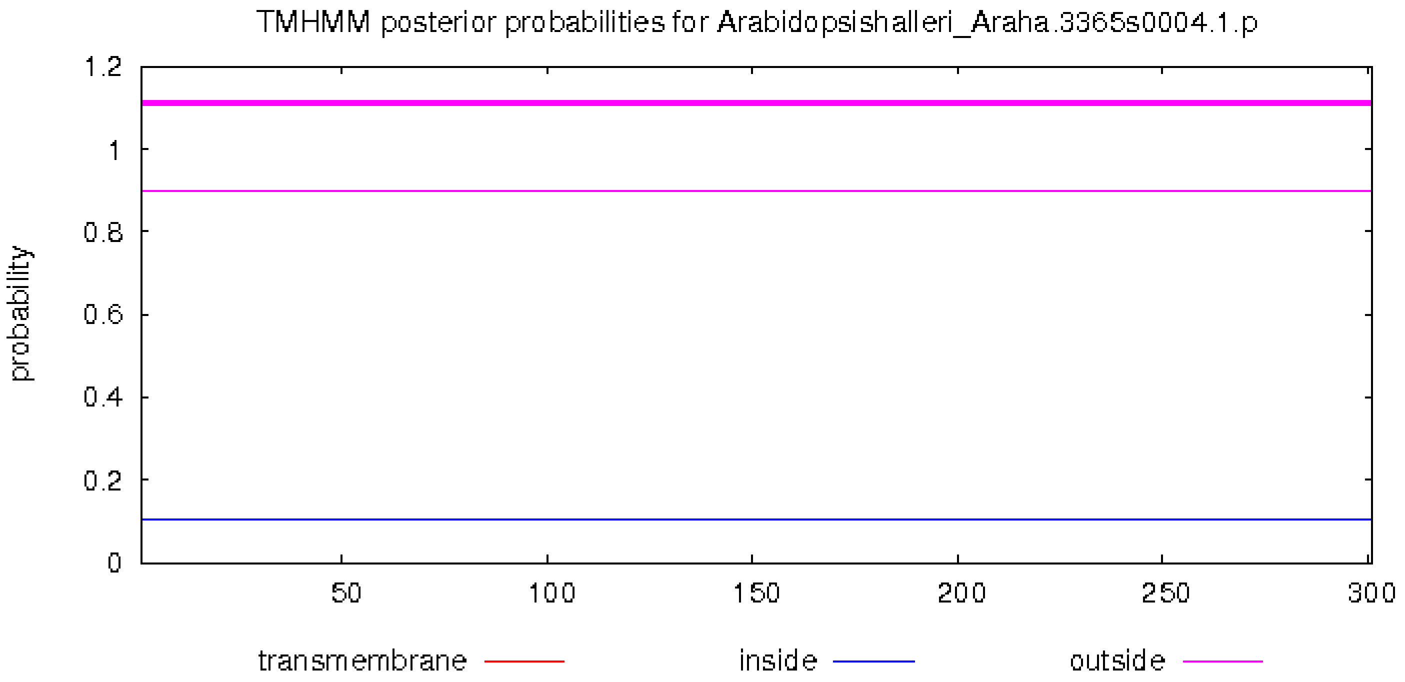

plot in postscript, script for making the plot in gnuplot, data for plot

---

```
# Arabidopsishalleri_Araha.3365s0007.1.p Length: 349
# Arabidopsishalleri_Araha.3365s0007.1.p Number of predicted TMHs:  0
# Arabidopsishalleri_Araha.3365s0007.1.p Exp number of AAs in TMHs: 0
# Arabidopsishalleri_Araha.3365s0007.1.p Exp number, first 60 AAs:  0
# Arabidopsishalleri_Araha.3365s0007.1.p Total prob of N-in:        0.00603
Arabidopsishalleri_Araha.3365s0007.1.p  TMHMM2.0        outside      1   349
```

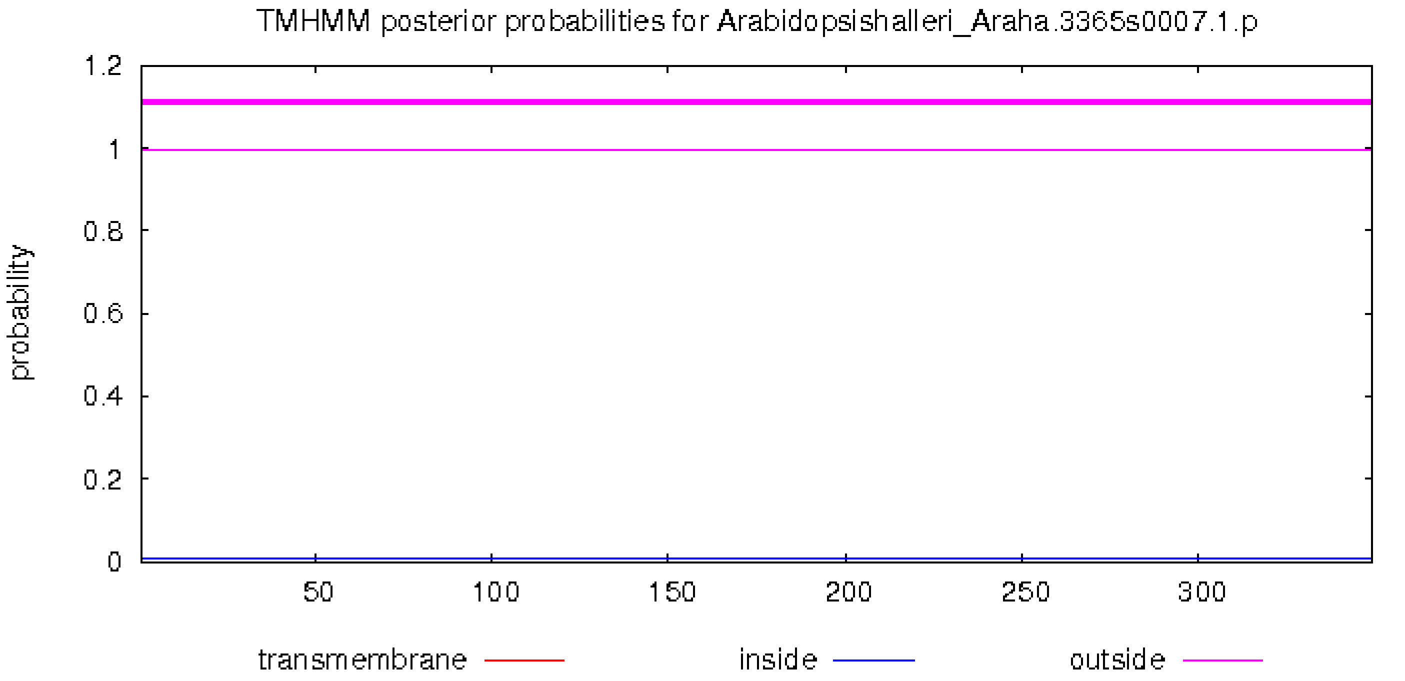

plot in postscript, script for making the plot in gnuplot, data for plot

---

```
# Arabidopsishalleri_Araha.34065s0002.1.p Length: 339
# Arabidopsishalleri_Araha.34065s0002.1.p Number of predicted TMHs:  0
# Arabidopsishalleri_Araha.34065s0002.1.p Exp number of AAs in TMHs: 0.00033
# Arabidopsishalleri_Araha.34065s0002.1.p Exp number, first 60 AAs:  0.00014
# Arabidopsishalleri_Araha.34065s0002.1.p Total prob of N-in:        0.00801
Arabidopsishalleri_Araha.34065s0002.1.p TMHMM2.0        outside      1   339
```

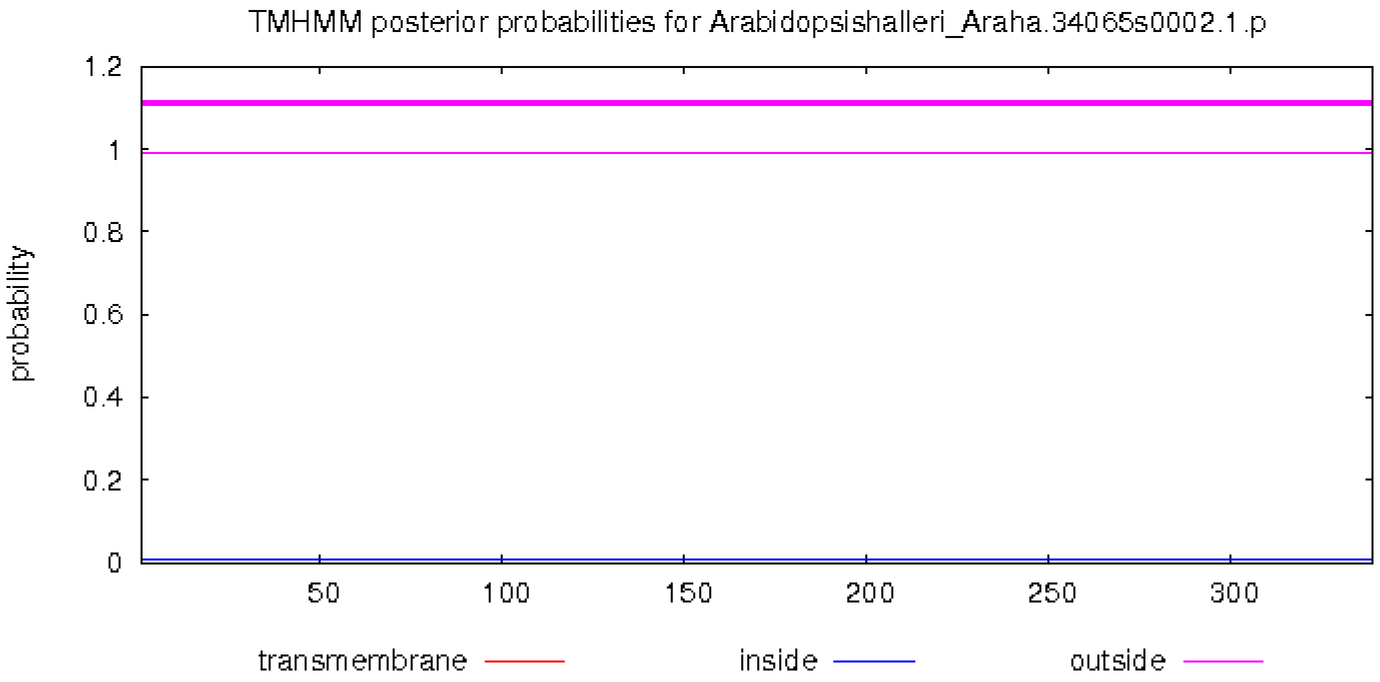

plot in postscript, script for making the plot in gnuplot, data for plot

---

```
# Arabidopsishalleri_Araha.34090s0001.1.p Length: 318
# Arabidopsishalleri_Araha.34090s0001.1.p Number of predicted TMHs:  0
# Arabidopsishalleri_Araha.34090s0001.1.p Exp number of AAs in TMHs: 0
# Arabidopsishalleri_Araha.34090s0001.1.p Exp number, first 60 AAs:  0
# Arabidopsishalleri_Araha.34090s0001.1.p Total prob of N-in:        0.00236
Arabidopsishalleri_Araha.34090s0001.1.p TMHMM2.0        outside      1   318
```

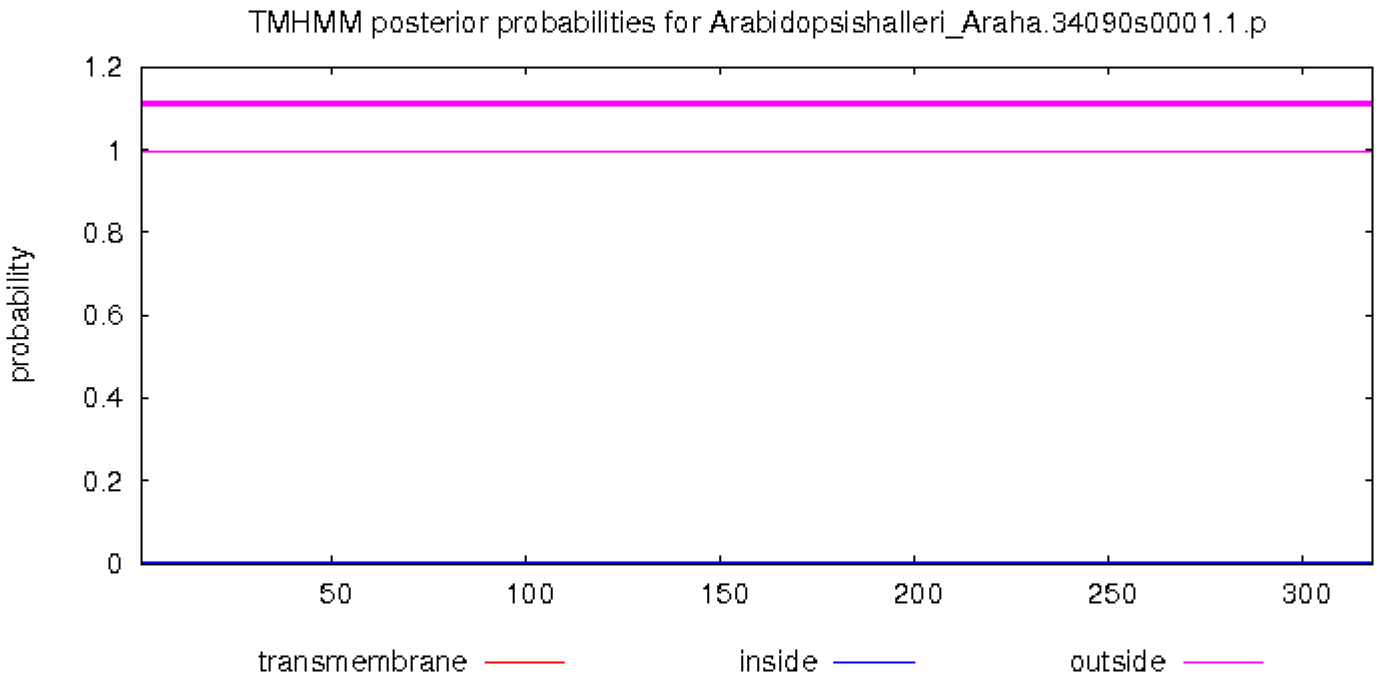

plot in postscript, script for making the plot in gnuplot, data for plot

---

```
# Arabidopsishalleri_Araha.3440s0004.1.p Length: 360
# Arabidopsishalleri_Araha.3440s0004.1.p Number of predicted TMHs:  0
# Arabidopsishalleri_Araha.3440s0004.1.p Exp number of AAs in TMHs: 0
# Arabidopsishalleri_Araha.3440s0004.1.p Exp number, first 60 AAs:  0
# Arabidopsishalleri_Araha.3440s0004.1.p Total prob of N-in:        0.00786
Arabidopsishalleri_Araha.3440s0004.1.p  TMHMM2.0        outside      1   360
```

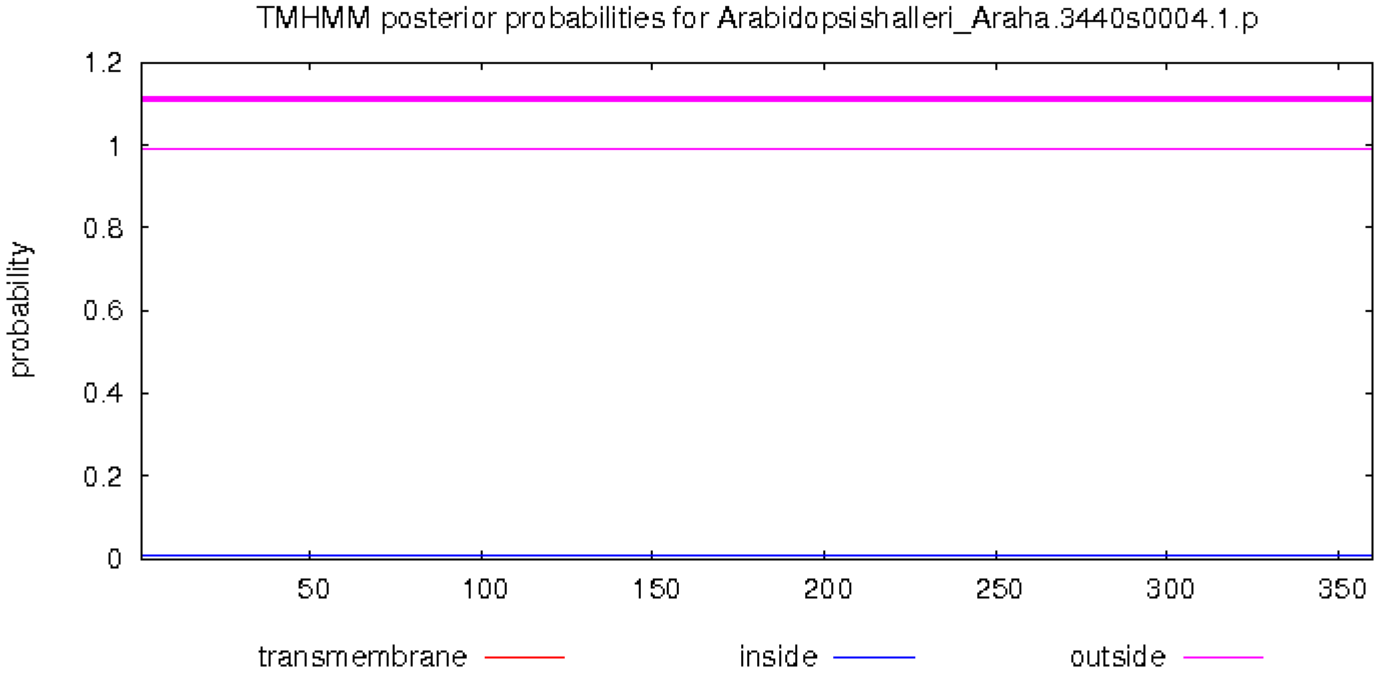

plot in postscript, script for making the plot in gnuplot, data for plot

```
# Arabidopsishalleri_Araha.3440s0005.1.p Length: 167
# Arabidopsishalleri_Araha.3440s0005.1.p Number of predicted TMHs:  0
# Arabidopsishalleri_Araha.3440s0005.1.p Exp number of AAs in TMHs: 0
# Arabidopsishalleri_Araha.3440s0005.1.p Exp number, first 60 AAs:  0
# Arabidopsishalleri_Araha.3440s0005.1.p Total prob of N-in:        0.11776
Arabidopsishalleri_Araha.3440s0005.1.p  TMHMM2.0        outside      1   167
```

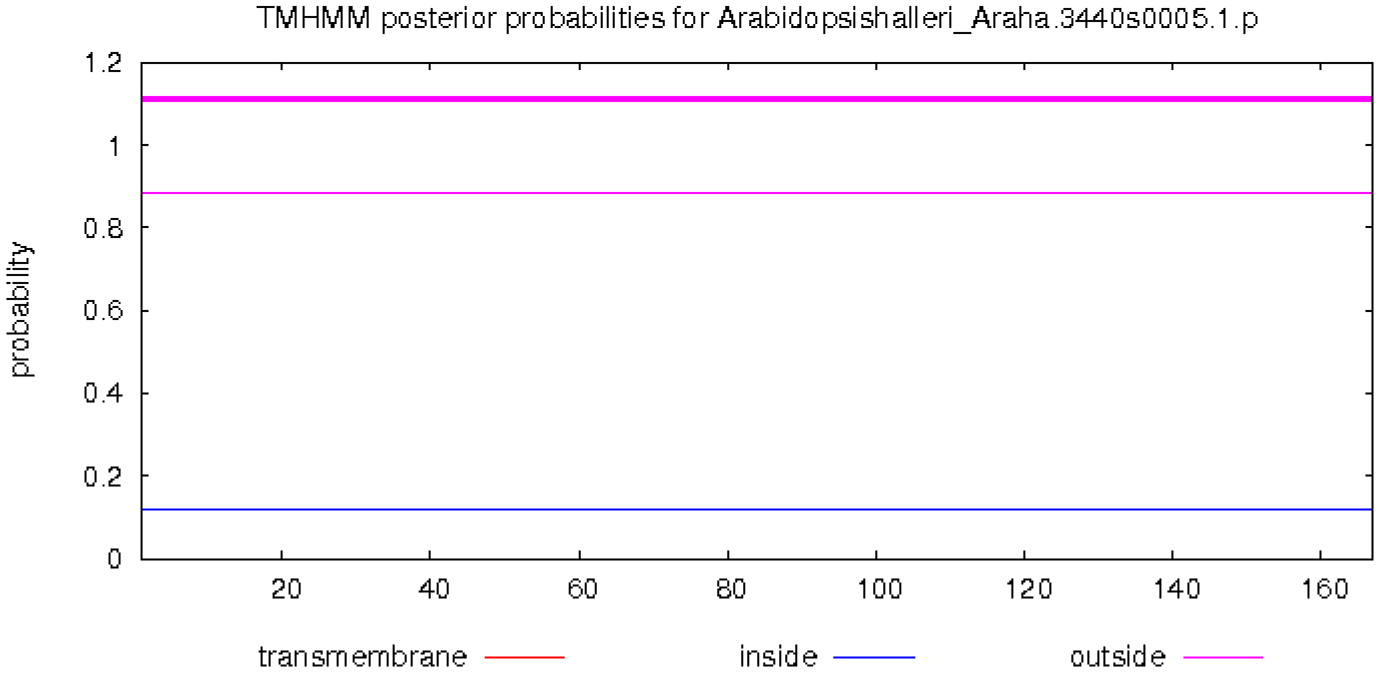

plot in postscript, script for making the plot in gnuplot, data for plot

```
# Arabidopsishalleri_Araha.3538s0001.1.p Length: 136
# Arabidopsishalleri_Araha.3538s0001.1.p Number of predicted TMHs:  0
# Arabidopsishalleri_Araha.3538s0001.1.p Exp number of AAs in TMHs: 0
# Arabidopsishalleri_Araha.3538s0001.1.p Exp number, first 60 AAs:  0
# Arabidopsishalleri_Araha.3538s0001.1.p Total prob of N-in:        0.01735
Arabidopsishalleri_Araha.3538s0001.1.p  TMHMM2.0        outside      1   136
```

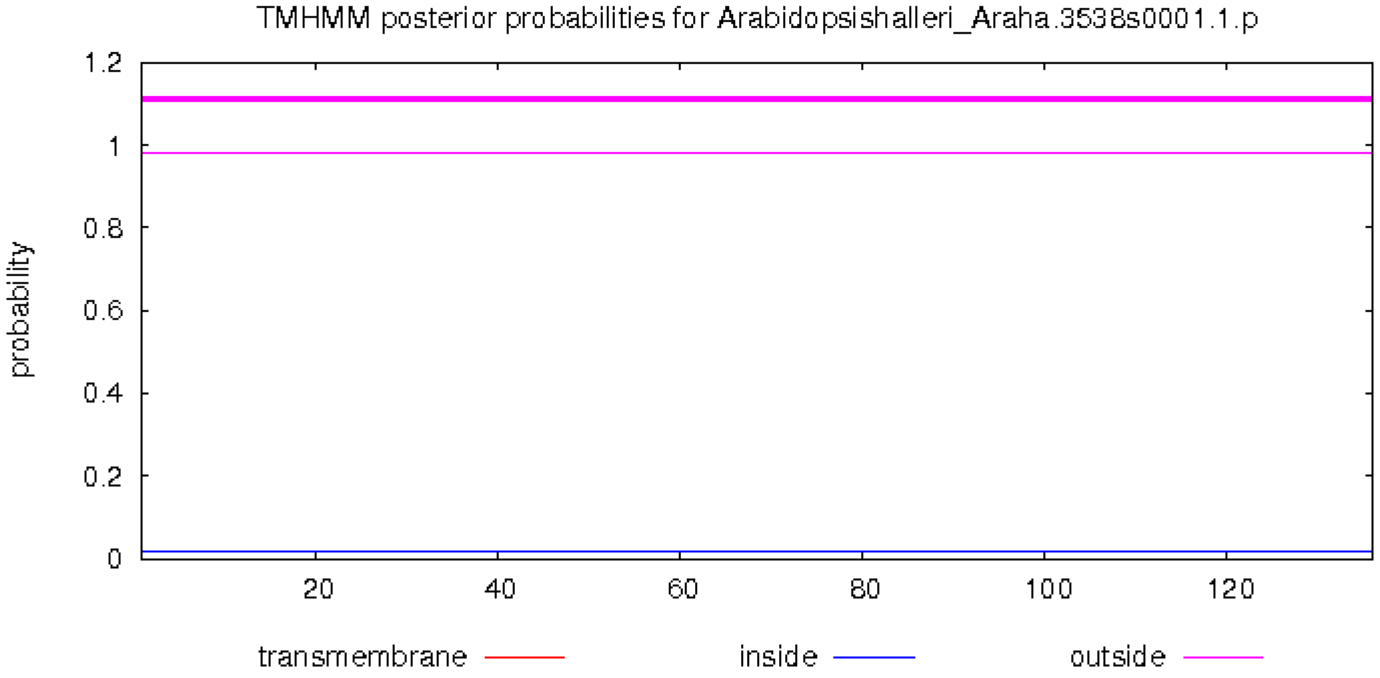

plot in postscript, script for making the plot in gnuplot, data for plot

---

```
# Arabidopsishalleri_Araha.35673s0001.1.p Length: 368
# Arabidopsishalleri_Araha.35673s0001.1.p Number of predicted TMHs:  0
# Arabidopsishalleri_Araha.35673s0001.1.p Exp number of AAs in TMHs: 0.00018
# Arabidopsishalleri_Araha.35673s0001.1.p Exp number, first 60 AAs:  0
# Arabidopsishalleri_Araha.35673s0001.1.p Total prob of N-in:        0.02665
Arabidopsishalleri_Araha.35673s0001.1.p TMHMM2.0        outside      1   368
```

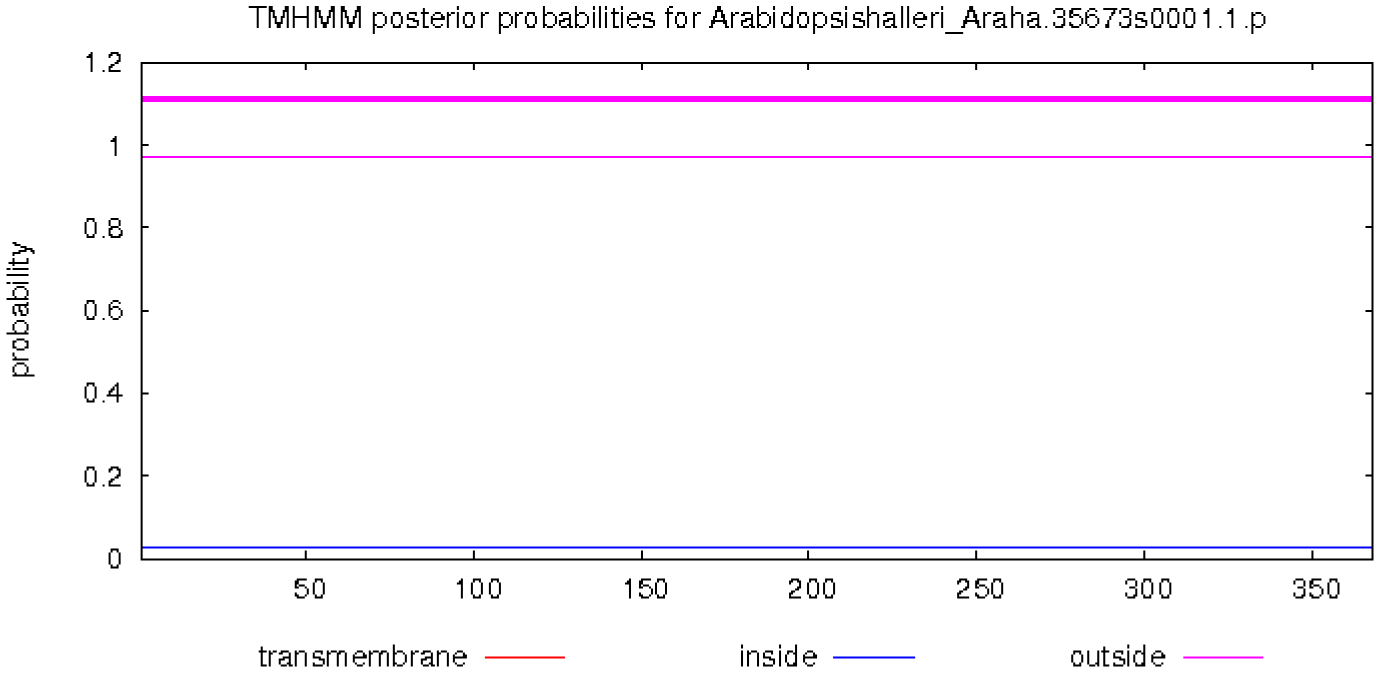

plot in postscript, script for making the plot in gnuplot, data for plot

---

```
# Arabidopsishalleri_Araha.3578s0006.1.p Length: 432
# Arabidopsishalleri_Araha.3578s0006.1.p Number of predicted TMHs:  0
# Arabidopsishalleri_Araha.3578s0006.1.p Exp number of AAs in TMHs: 0.00731
# Arabidopsishalleri_Araha.3578s0006.1.p Exp number, first 60 AAs:  0.00134
# Arabidopsishalleri_Araha.3578s0006.1.p Total prob of N-in:        0.03659
Arabidopsishalleri_Araha.3578s0006.1.p  TMHMM2.0        outside      1   432
```

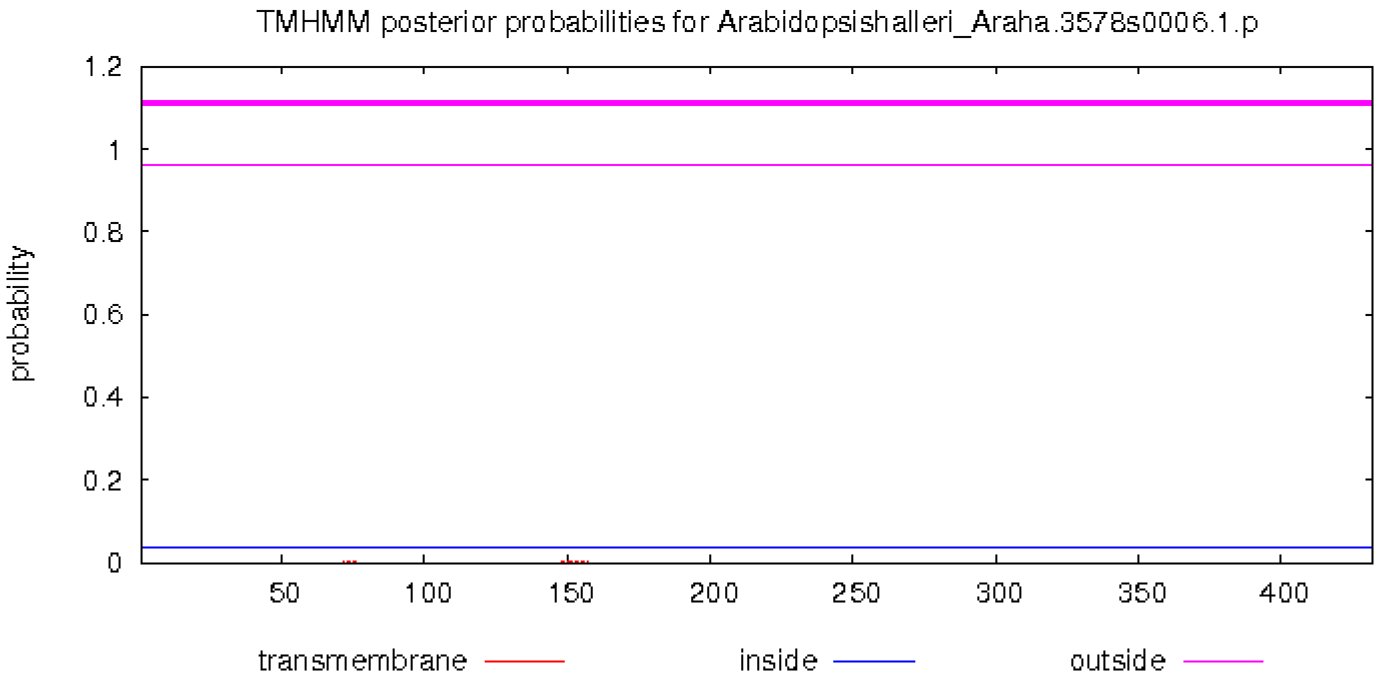

plot in postscript, script for making the plot in gnuplot, data for plot

---

```
# Arabidopsishalleri_Araha.37102s0001.1.p Length: 459
# Arabidopsishalleri_Araha.37102s0001.1.p Number of predicted TMHs:  0
# Arabidopsishalleri_Araha.37102s0001.1.p Exp number of AAs in TMHs: 0.0002
# Arabidopsishalleri_Araha.37102s0001.1.p Exp number, first 60 AAs:  0
# Arabidopsishalleri_Araha.37102s0001.1.p Total prob of N-in:        0.00265
Arabidopsishalleri_Araha.37102s0001.1.p TMHMM2.0        outside      1   459
```

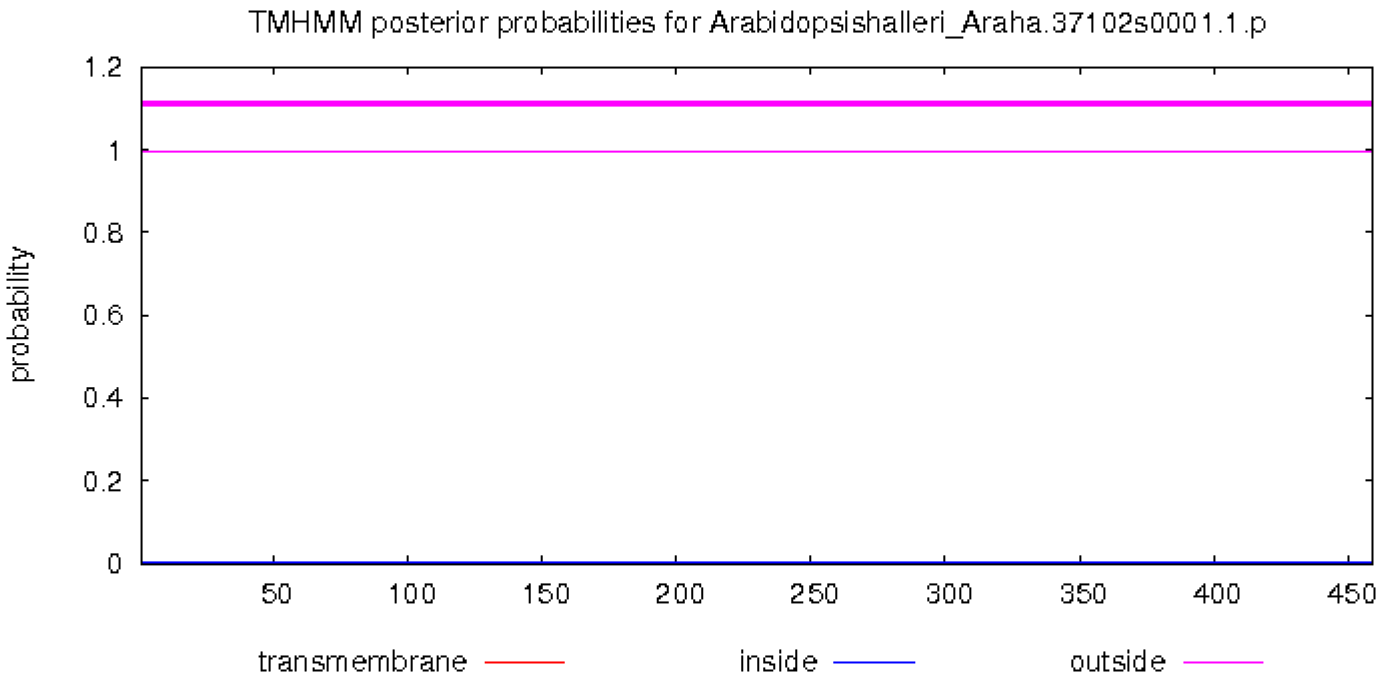

plot in postscript, script for making the plot in gnuplot, data for plot

---

```
# Arabidopsishalleri_Araha.3726s0007.1.p Length: 317
# Arabidopsishalleri_Araha.3726s0007.1.p Number of predicted TMHs:  0
# Arabidopsishalleri_Araha.3726s0007.1.p Exp number of AAs in TMHs: 0.00073
# Arabidopsishalleri_Araha.3726s0007.1.p Exp number, first 60 AAs:  0
# Arabidopsishalleri_Araha.3726s0007.1.p Total prob of N-in:        0.03050
Arabidopsishalleri_Araha.3726s0007.1.p  TMHMM2.0        outside      1   317
```

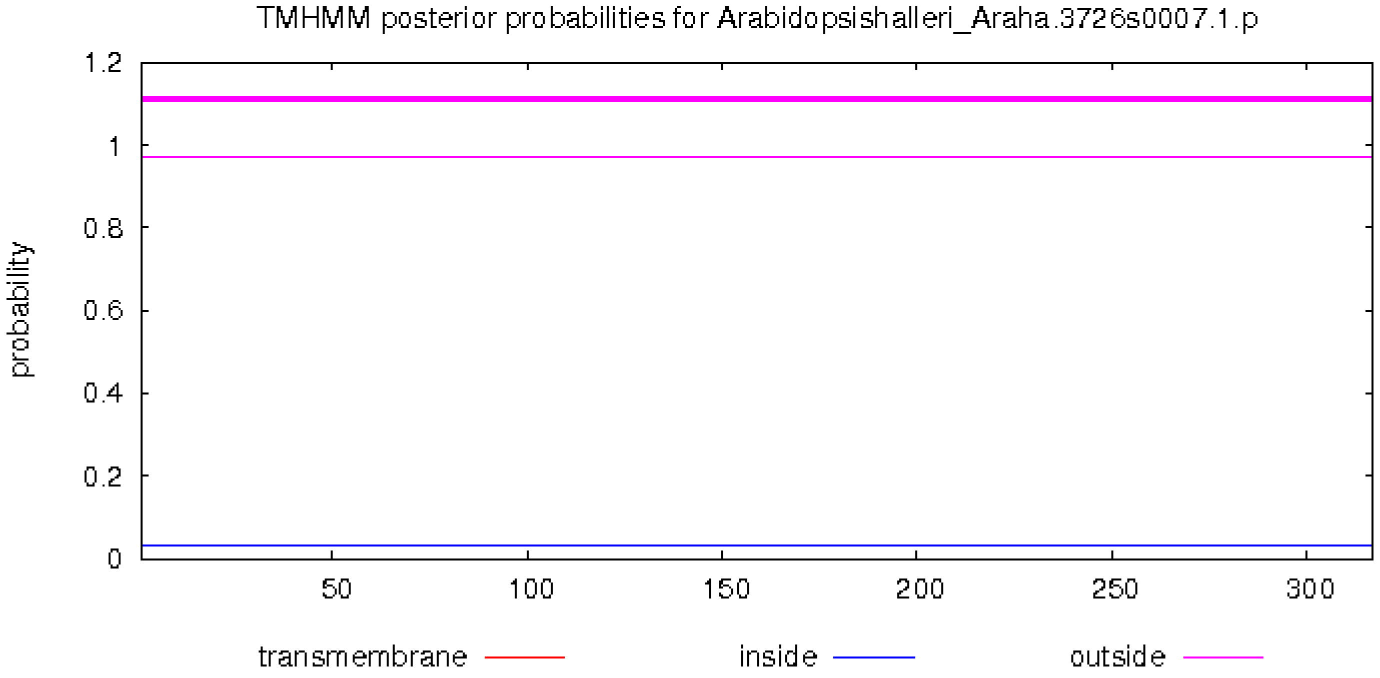

plot in postscript, script for making the plot in gnuplot, data for plot

```
# Arabidopsishalleri_Araha.37771s0001.1.p Length: 240
# Arabidopsishalleri_Araha.37771s0001.1.p Number of predicted TMHs:  0
# Arabidopsishalleri_Araha.37771s0001.1.p Exp number of AAs in TMHs: 0.00016
# Arabidopsishalleri_Araha.37771s0001.1.p Exp number, first 60 AAs:  0
# Arabidopsishalleri_Araha.37771s0001.1.p Total prob of N-in:        0.14354
Arabidopsishalleri_Araha.37771s0001.1.p TMHMM2.0        outside      1   240
```

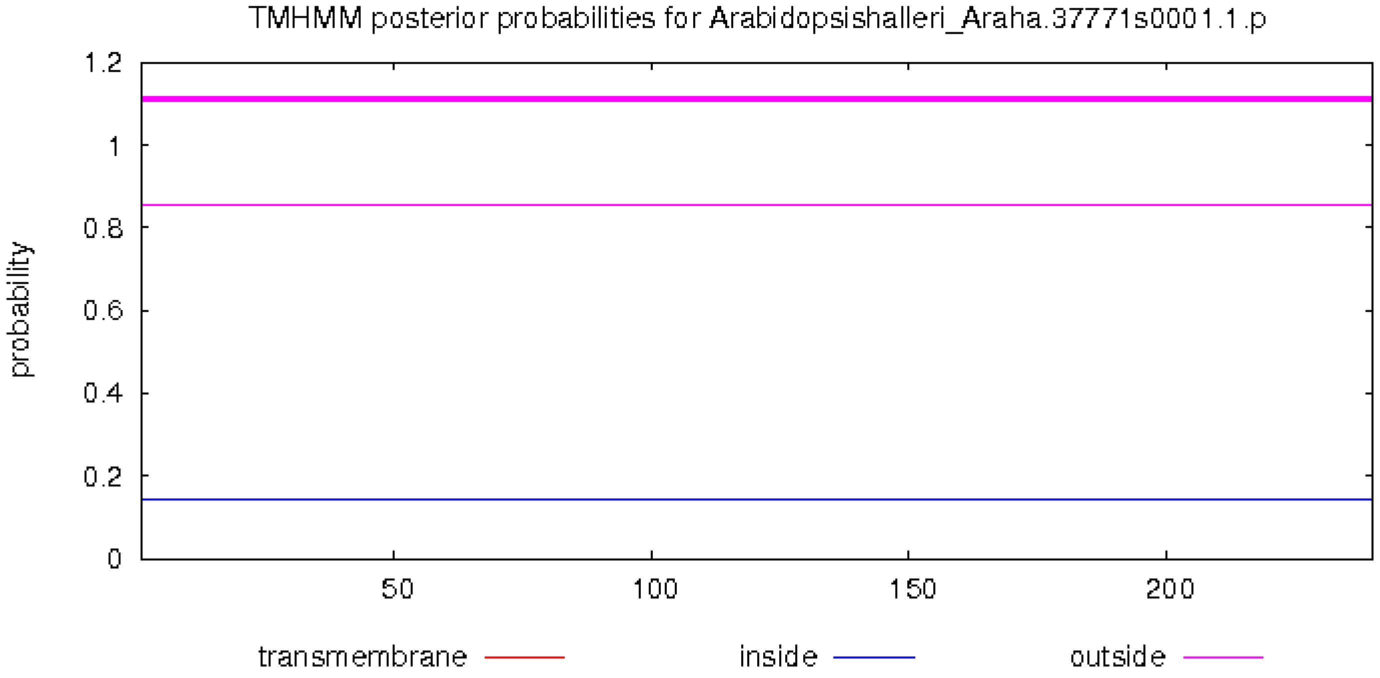

plot in postscript, script for making the plot in gnuplot, data for plot

```
# Arabidopsishalleri_Araha.40634s0001.1.p Length: 366
# Arabidopsishalleri_Araha.40634s0001.1.p Number of predicted TMHs:  0
# Arabidopsishalleri_Araha.40634s0001.1.p Exp number of AAs in TMHs: 0.00021
# Arabidopsishalleri_Araha.40634s0001.1.p Exp number, first 60 AAs:  0
# Arabidopsishalleri_Araha.40634s0001.1.p Total prob of N-in:        0.01714
Arabidopsishalleri_Araha.40634s0001.1.p TMHMM2.0        outside      1   366
```

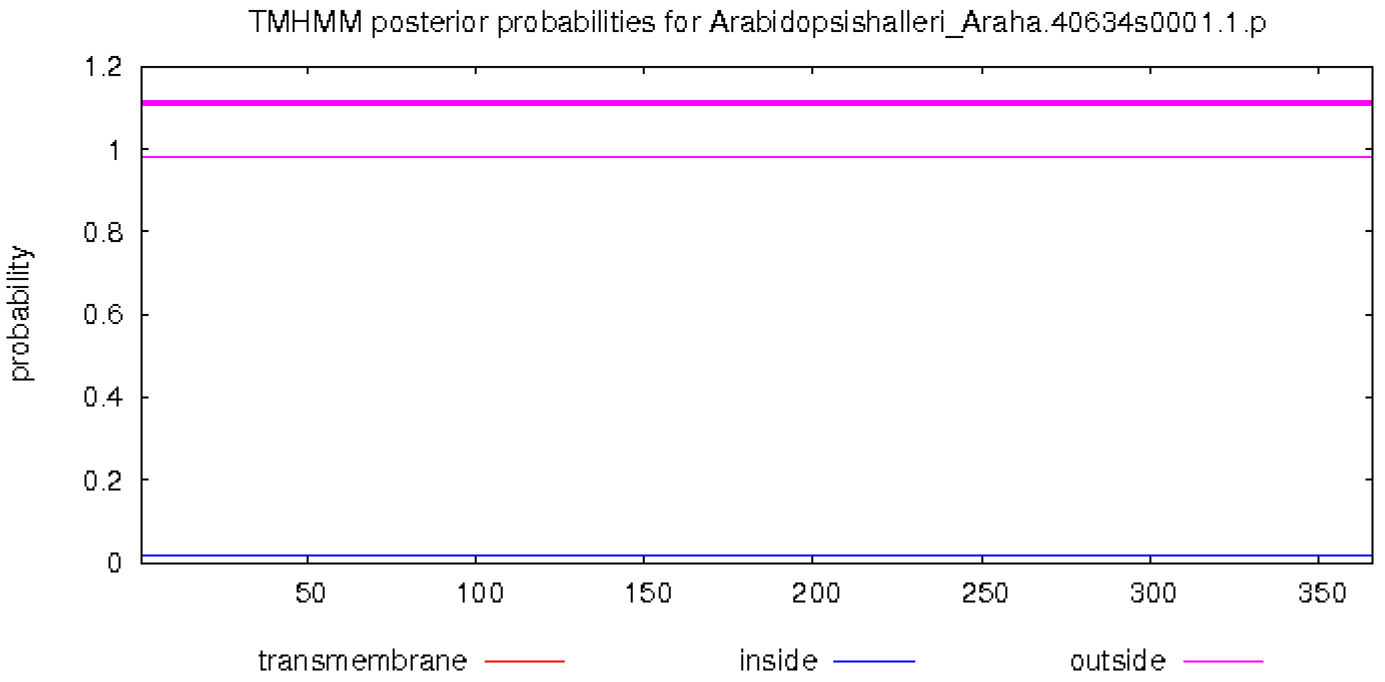

plot in postscript, script for making the plot in gnuplot, data for plot

```
# Arabidopsishalleri_Araha.41818s0002.1.p Length: 222
# Arabidopsishalleri_Araha.41818s0002.1.p Number of predicted TMHs:  0
# Arabidopsishalleri_Araha.41818s0002.1.p Exp number of AAs in TMHs: 0.05033
# Arabidopsishalleri_Araha.41818s0002.1.p Exp number, first 60 AAs:  0.00019
# Arabidopsishalleri_Araha.41818s0002.1.p Total prob of N-in:        0.19441
Arabidopsishalleri_Araha.41818s0002.1.p TMHMM2.0        outside      1   222
```

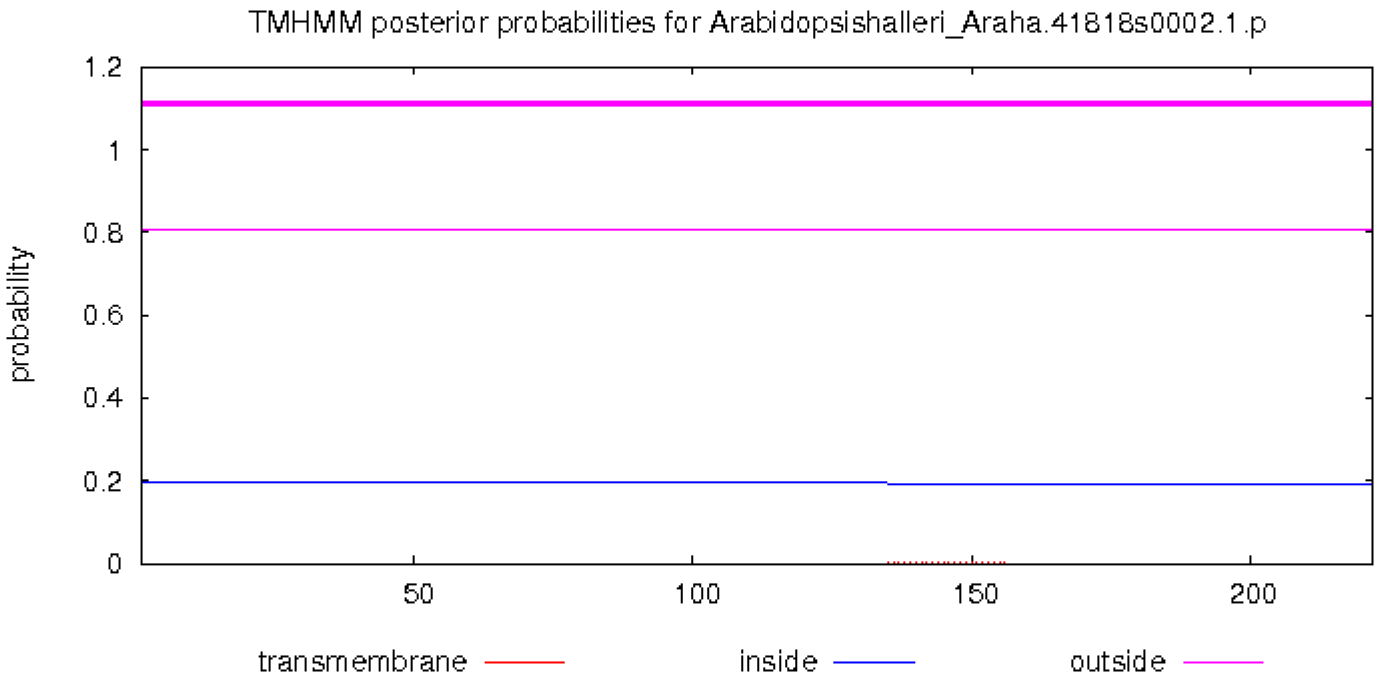

plot in postscript, script for making the plot in gnuplot, data for plot

```
# Arabidopsishalleri_Araha.43003s0010.1.p Length: 239
# Arabidopsishalleri_Araha.43003s0010.1.p Number of predicted TMHs:  0
# Arabidopsishalleri_Araha.43003s0010.1.p Exp number of AAs in TMHs: 0.00717
# Arabidopsishalleri_Araha.43003s0010.1.p Exp number, first 60 AAs:  0.00021
# Arabidopsishalleri_Araha.43003s0010.1.p Total prob of N-in:        0.02537
Arabidopsishalleri_Araha.43003s0010.1.p TMHMM2.0        outside      1   239
```

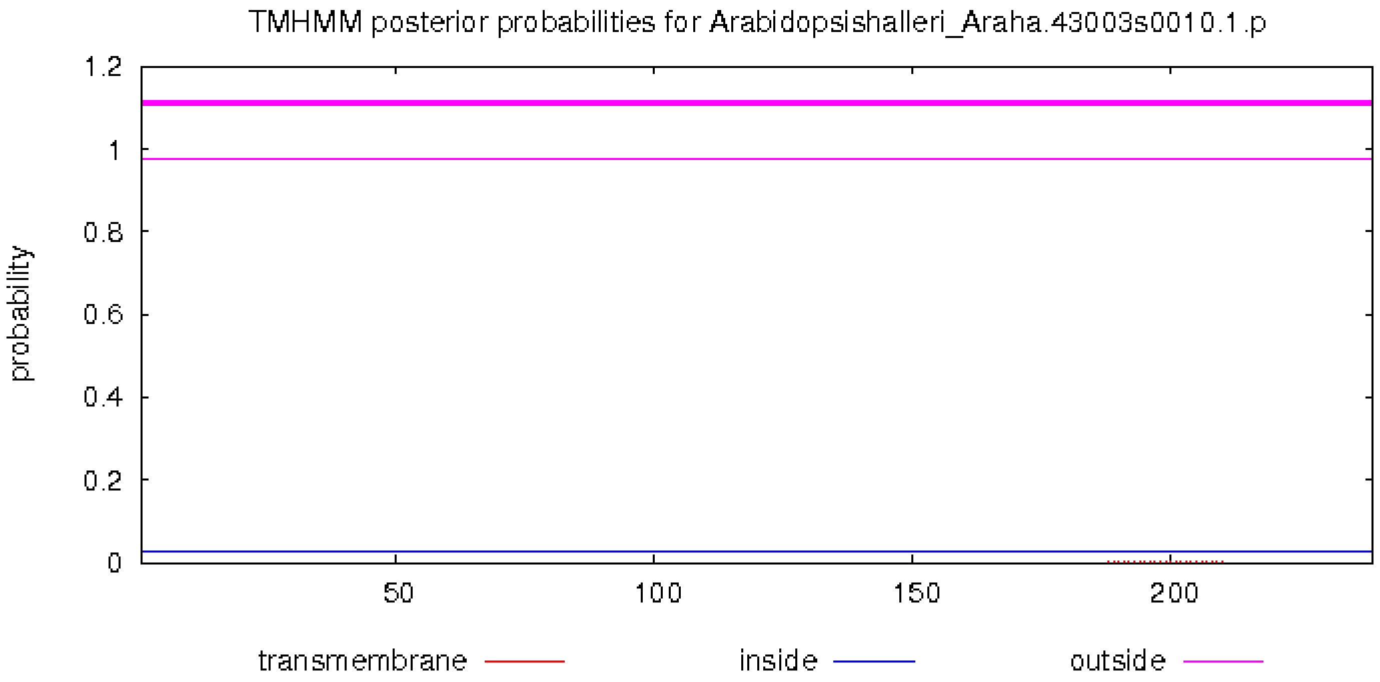

plot in postscript, script for making the plot in gnuplot, data for plot

---

```
# Arabidopsishalleri_Araha.4353s0004.1.p Length: 193
# Arabidopsishalleri_Araha.4353s0004.1.p Number of predicted TMHs:  0
# Arabidopsishalleri_Araha.4353s0004.1.p Exp number of AAs in TMHs: 0.00226
# Arabidopsishalleri_Araha.4353s0004.1.p Exp number, first 60 AAs:  0
# Arabidopsishalleri_Araha.4353s0004.1.p Total prob of N-in:        0.12753
Arabidopsishalleri_Araha.4353s0004.1.p  TMHMM2.0        outside      1   193
```

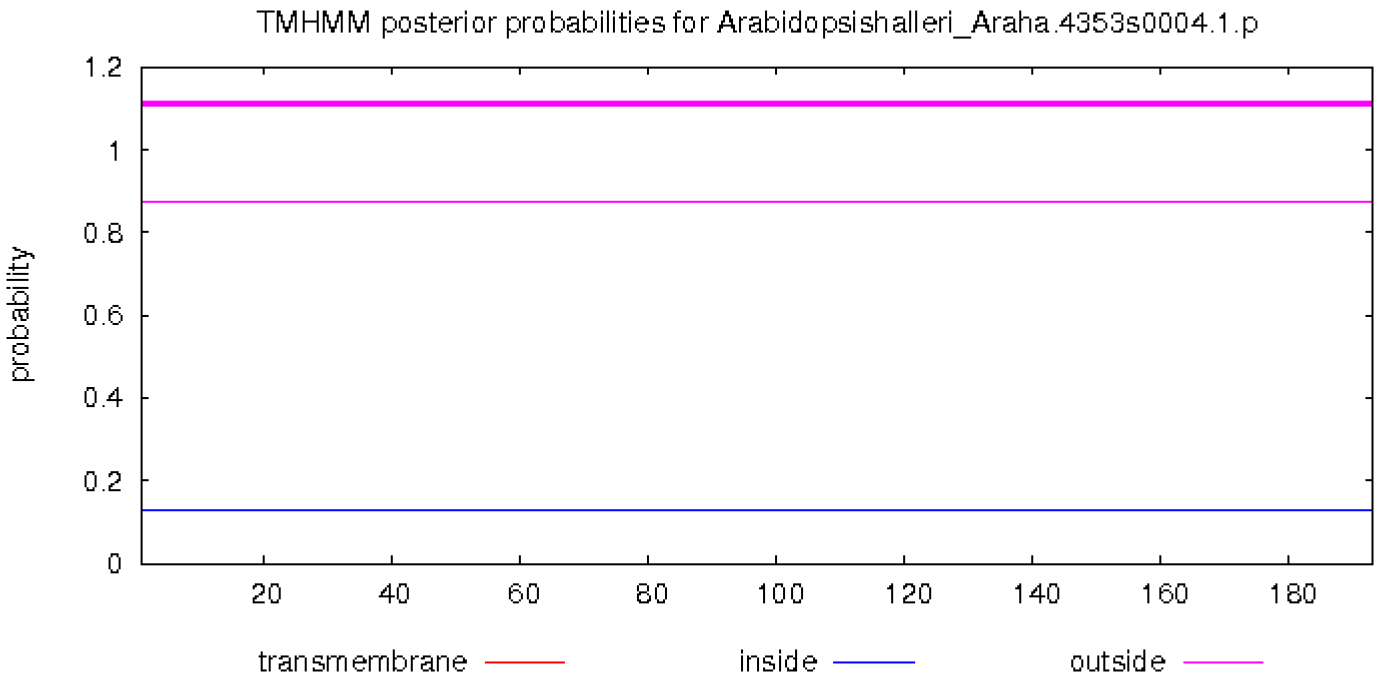

plot in postscript, script for making the plot in gnuplot, data for plot

---

```
# Arabidopsishalleri_Araha.4375s0001.1.p Length: 241
# Arabidopsishalleri_Araha.4375s0001.1.p Number of predicted TMHs:  0
# Arabidopsishalleri_Araha.4375s0001.1.p Exp number of AAs in TMHs: 0.02015
# Arabidopsishalleri_Araha.4375s0001.1.p Exp number, first 60 AAs:  0.02015
# Arabidopsishalleri_Araha.4375s0001.1.p Total prob of N-in:        0.01115
Arabidopsishalleri_Araha.4375s0001.1.p  TMHMM2.0        outside      1   241
```

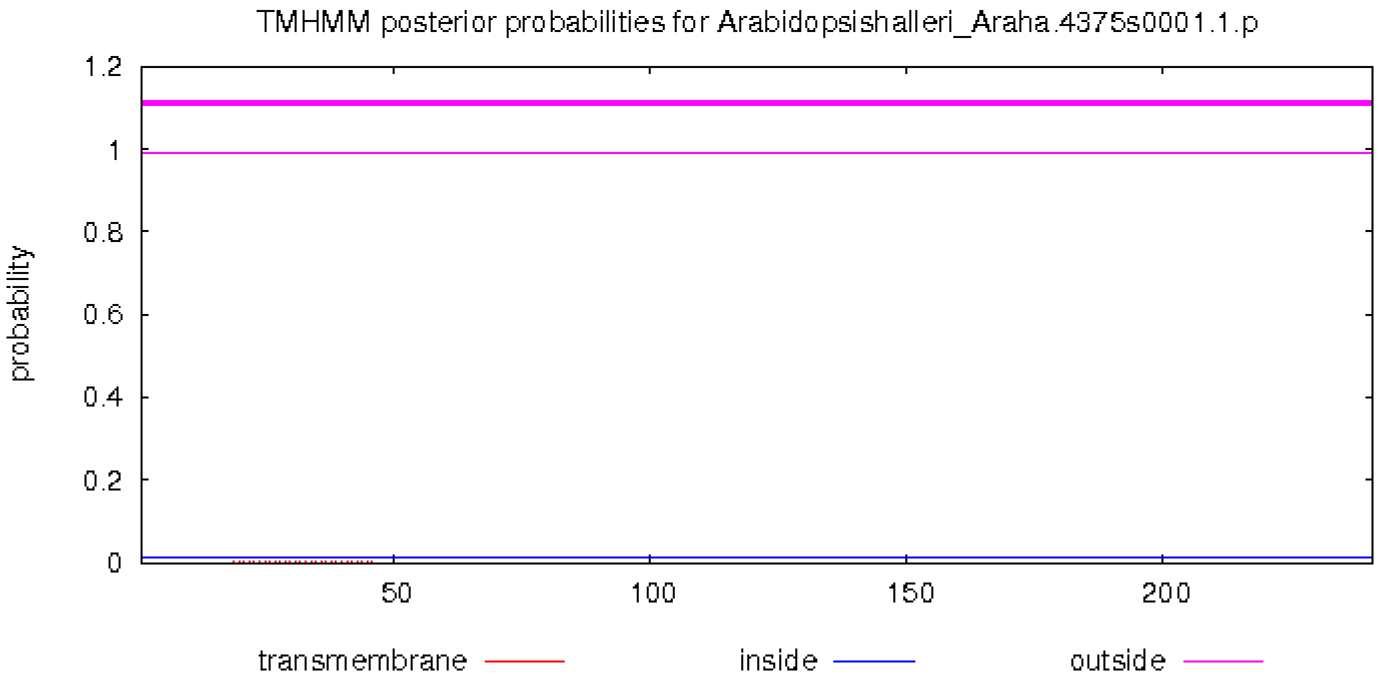

plot in postscript, script for making the plot in gnuplot, data for plot

```
# Arabidopsishalleri_Araha.4414s0025.1.p Length: 349
# Arabidopsishalleri_Araha.4414s0025.1.p Number of predicted TMHs:  0
# Arabidopsishalleri_Araha.4414s0025.1.p Exp number of AAs in TMHs: 0.00256
# Arabidopsishalleri_Araha.4414s0025.1.p Exp number, first 60 AAs:  0
# Arabidopsishalleri_Araha.4414s0025.1.p Total prob of N-in:        0.00215
Arabidopsishalleri_Araha.4414s0025.1.p  TMHMM2.0        outside      1   349
```

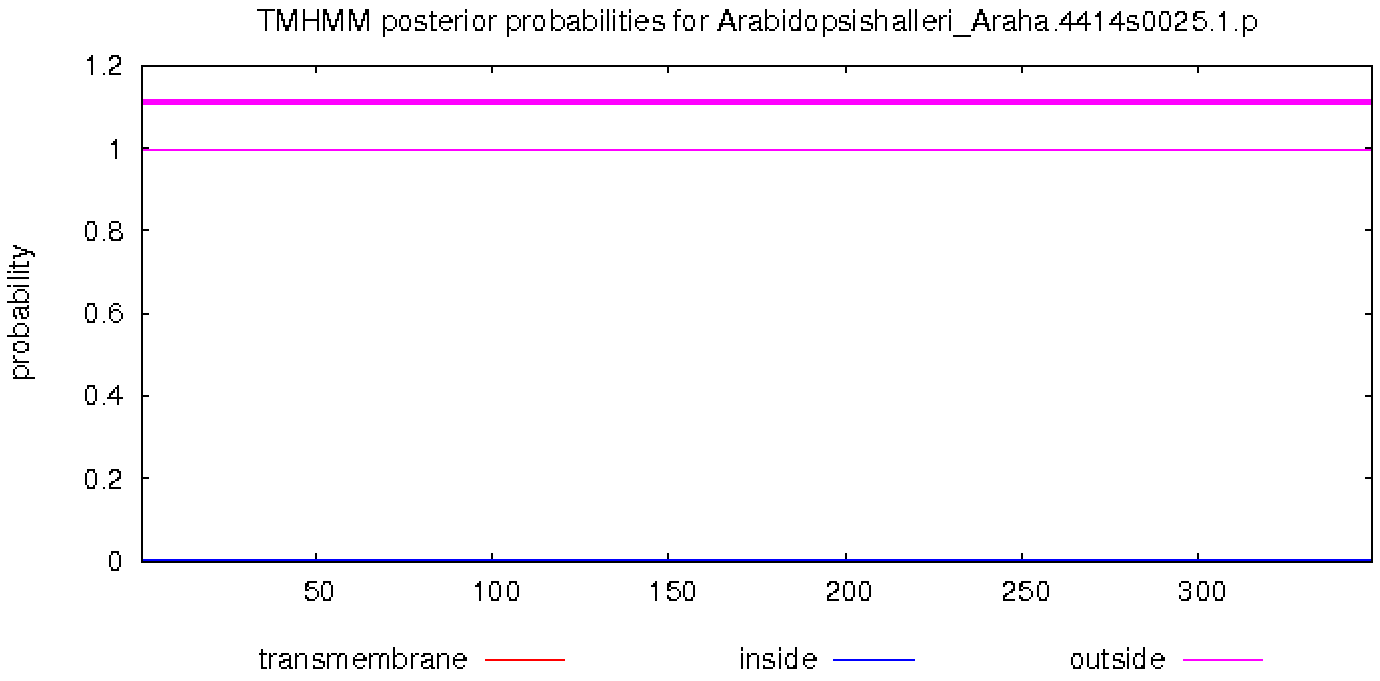

plot in postscript, script for making the plot in gnuplot, data for plot

```
# Arabidopsishalleri_Araha.46415s0001.1.p Length: 344
# Arabidopsishalleri_Araha.46415s0001.1.p Number of predicted TMHs:  0
# Arabidopsishalleri_Araha.46415s0001.1.p Exp number of AAs in TMHs: 0.00032
# Arabidopsishalleri_Araha.46415s0001.1.p Exp number, first 60 AAs:  0
# Arabidopsishalleri_Araha.46415s0001.1.p Total prob of N-in:        0.02679
Arabidopsishalleri_Araha.46415s0001.1.p TMHMM2.0        outside      1   344
```

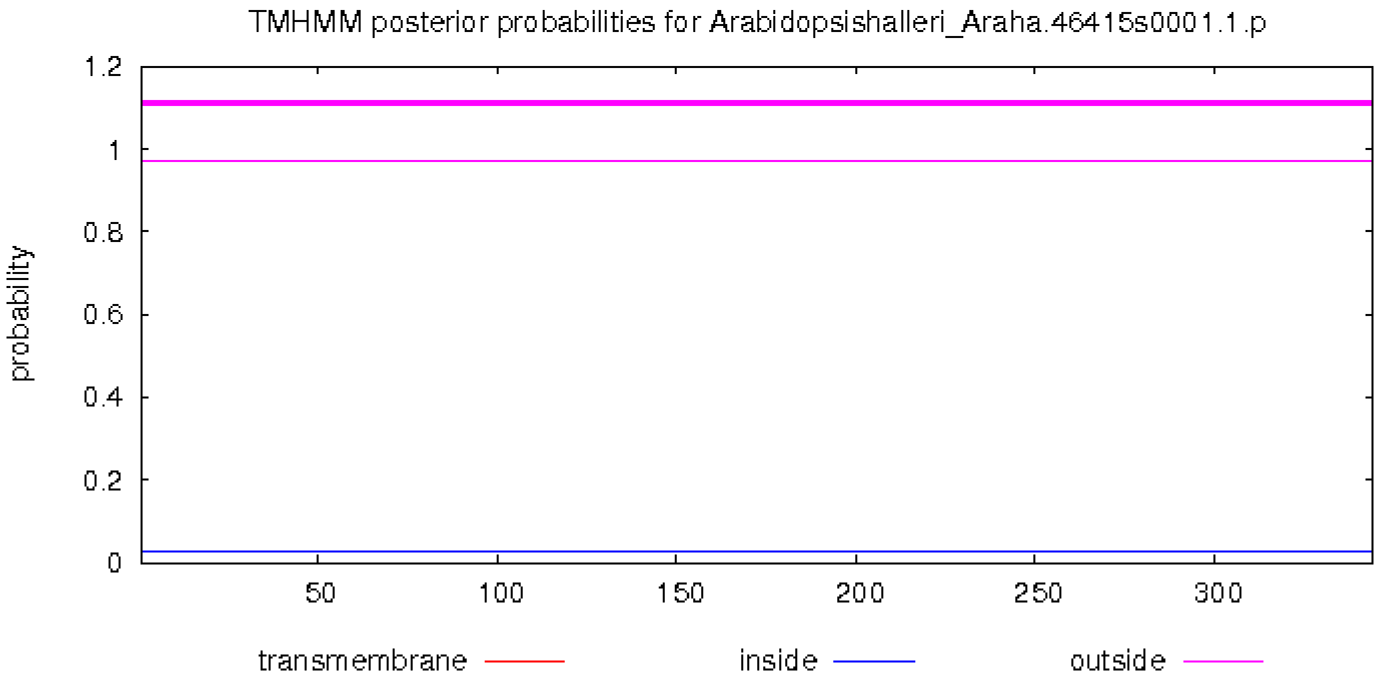

plot in postscript, script for making the plot in gnuplot, data for plot

---

```
# Arabidopsishalleri_Araha.4814s0003.1.p Length: 272
# Arabidopsishalleri_Araha.4814s0003.1.p Number of predicted TMHs:  0
# Arabidopsishalleri_Araha.4814s0003.1.p Exp number of AAs in TMHs: 0.041
# Arabidopsishalleri_Araha.4814s0003.1.p Exp number, first 60 AAs:  0
# Arabidopsishalleri_Araha.4814s0003.1.p Total prob of N-in:        0.22563
Arabidopsishalleri_Araha.4814s0003.1.p  TMHMM2.0        outside      1   272
```

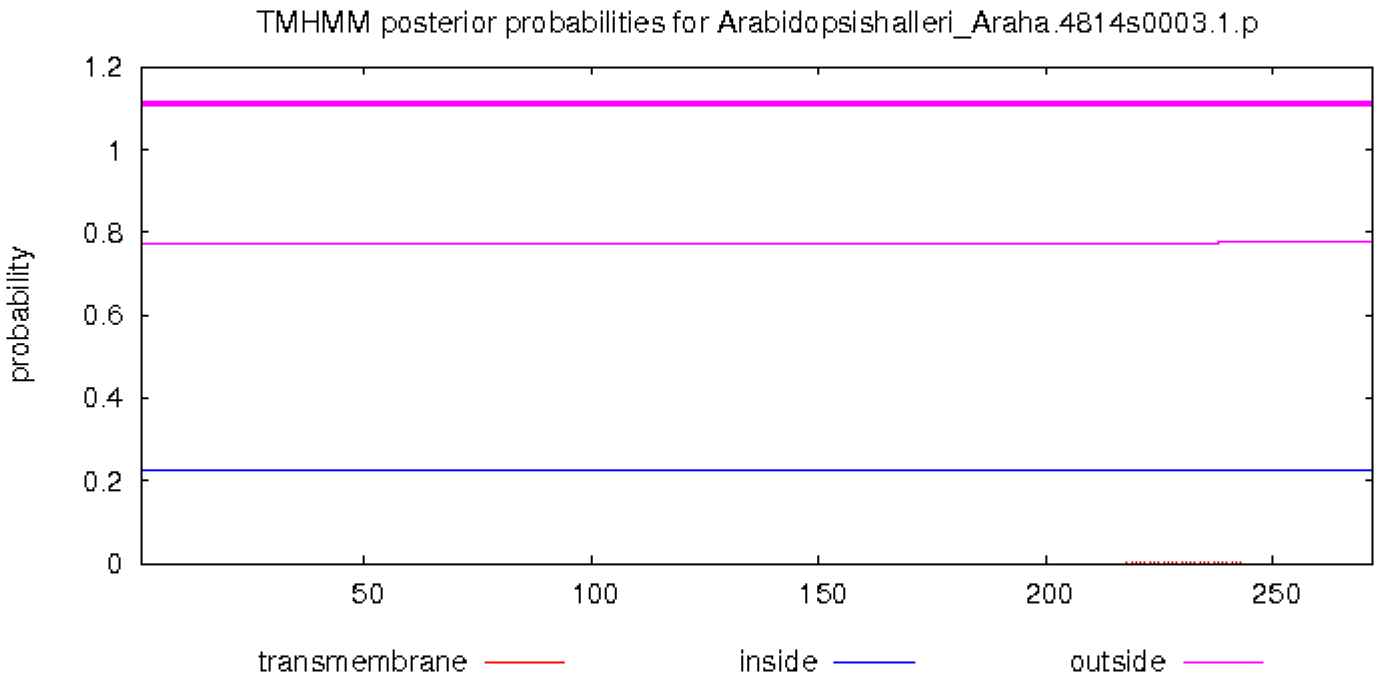

plot in postscript, script for making the plot in gnuplot, data for plot

---

```
# Arabidopsishalleri_Araha.4954s0010.1.p Length: 390
# Arabidopsishalleri_Araha.4954s0010.1.p Number of predicted TMHs:  0
# Arabidopsishalleri_Araha.4954s0010.1.p Exp number of AAs in TMHs: 0.01201
# Arabidopsishalleri_Araha.4954s0010.1.p Exp number, first 60 AAs:  0
# Arabidopsishalleri_Araha.4954s0010.1.p Total prob of N-in:        0.00416
Arabidopsishalleri_Araha.4954s0010.1.p  TMHMM2.0        outside      1   390
```

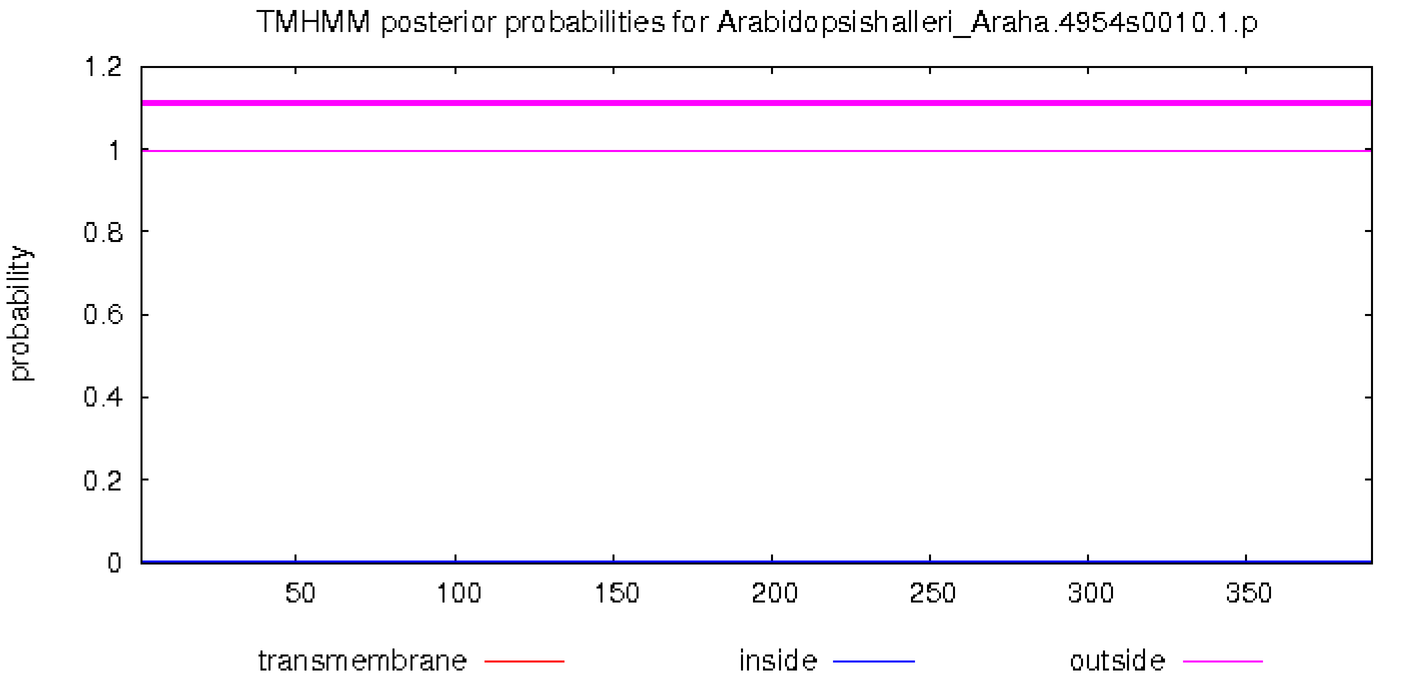

plot in postscript, script for making the plot in gnuplot, data for plot

---

```
# Arabidopsishalleri_Araha.4954s0011.1.p Length: 351
# Arabidopsishalleri_Araha.4954s0011.1.p Number of predicted TMHs:  0
# Arabidopsishalleri_Araha.4954s0011.1.p Exp number of AAs in TMHs: 0.14963
# Arabidopsishalleri_Araha.4954s0011.1.p Exp number, first 60 AAs:  0.00037
# Arabidopsishalleri_Araha.4954s0011.1.p Total prob of N-in:        0.11350
Arabidopsishalleri_Araha.4954s0011.1.p  TMHMM2.0        outside      1   351
```

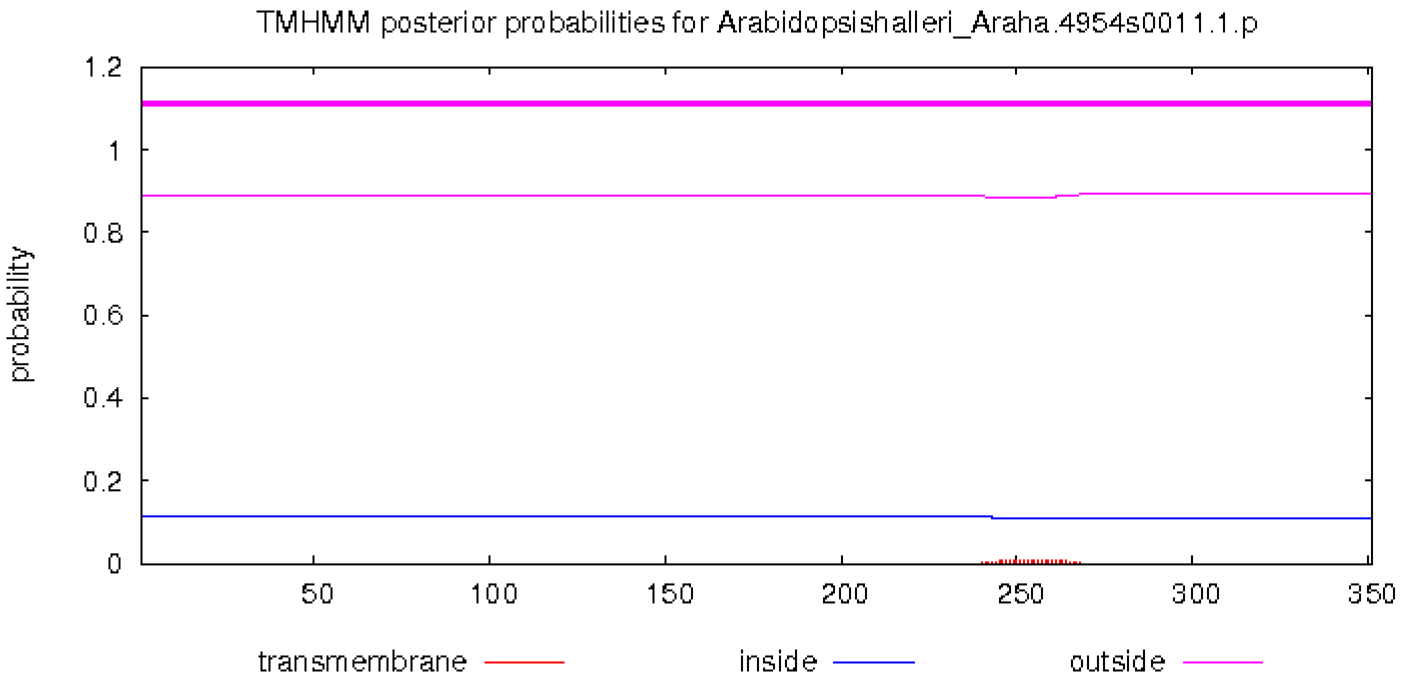

plot in postscript, script for making the plot in gnuplot, data for plot

---

```
# Arabidopsishalleri_Araha.4954s0012.1.p Length: 560
# Arabidopsishalleri_Araha.4954s0012.1.p Number of predicted TMHs:  1
# Arabidopsishalleri_Araha.4954s0012.1.p Exp number of AAs in TMHs: 21.58638
# Arabidopsishalleri_Araha.4954s0012.1.p Exp number, first 60 AAs:  0
# Arabidopsishalleri_Araha.4954s0012.1.p Total prob of N-in:        0.01245
Arabidopsishalleri_Araha.4954s0012.1.p  TMHMM2.0        outside      1   533
Arabidopsishalleri_Araha.4954s0012.1.p  TMHMM2.0        TMhelix    534   556
Arabidopsishalleri_Araha.4954s0012.1.p  TMHMM2.0        inside     557   560
```

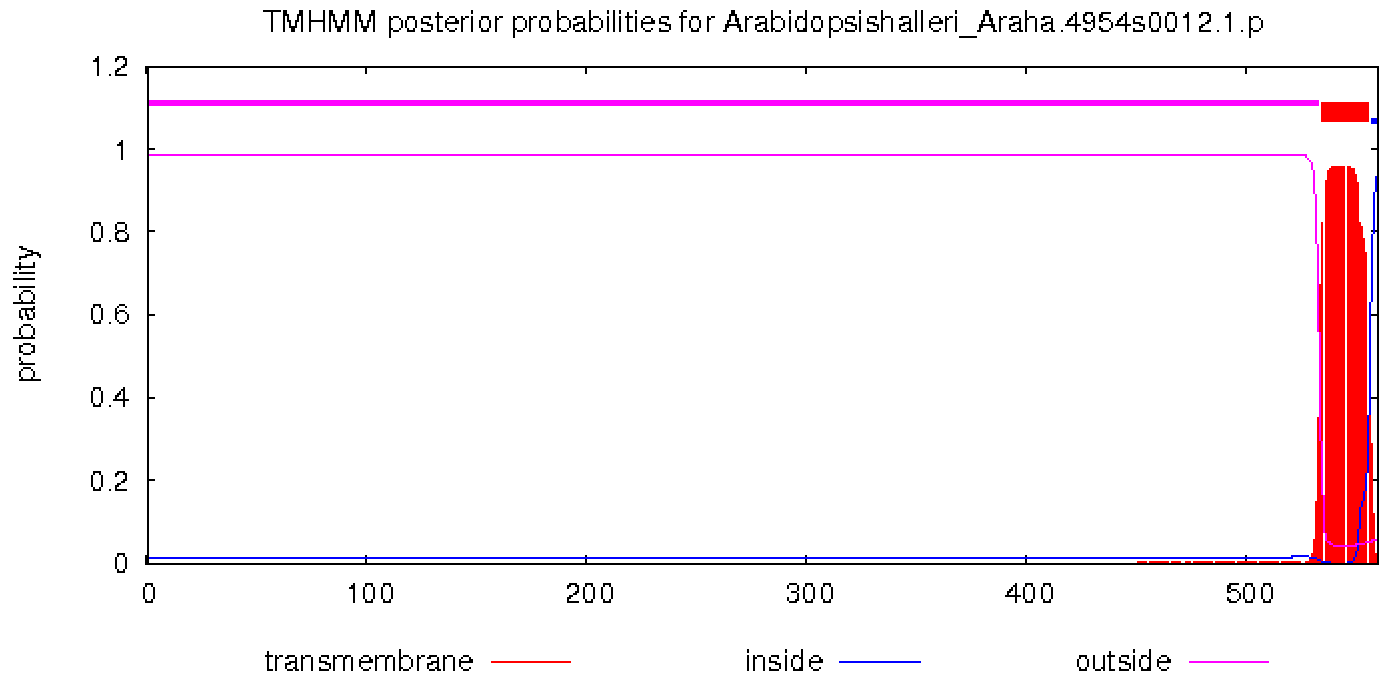

plot in postscript, script for making the plot in gnuplot, data for plot

---

```
# Arabidopsishalleri_Araha.4956s0002.1.p Length: 292
# Arabidopsishalleri_Araha.4956s0002.1.p Number of predicted TMHs:  0
# Arabidopsishalleri_Araha.4956s0002.1.p Exp number of AAs in TMHs: 0
# Arabidopsishalleri_Araha.4956s0002.1.p Exp number, first 60 AAs:  0
# Arabidopsishalleri_Araha.4956s0002.1.p Total prob of N-in:        0.00215
Arabidopsishalleri_Araha.4956s0002.1.p  TMHMM2.0        outside      1   292
```

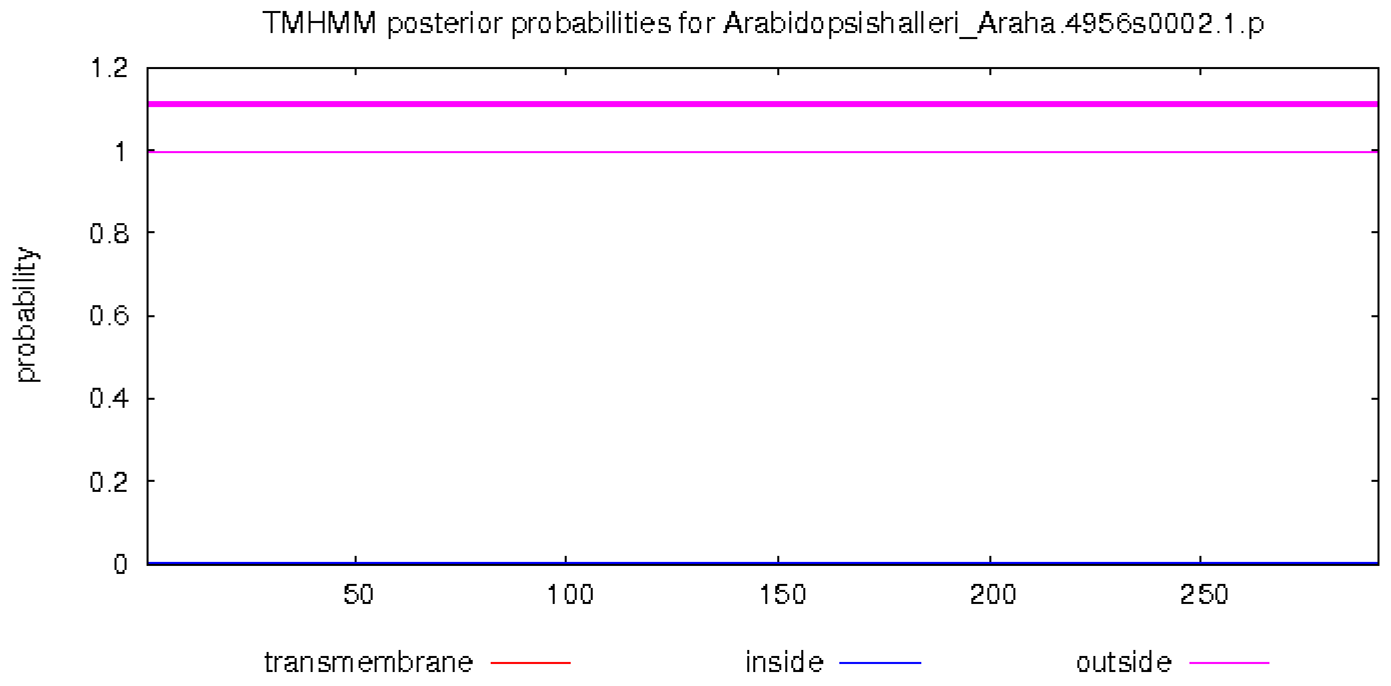

plot in postscript, script for making the plot in gnuplot, data for plot

```
# Arabidopsishalleri_Araha.5052s0042.1.p Length: 373
# Arabidopsishalleri_Araha.5052s0042.1.p Number of predicted TMHs:  0
# Arabidopsishalleri_Araha.5052s0042.1.p Exp number of AAs in TMHs: 0.00107
# Arabidopsishalleri_Araha.5052s0042.1.p Exp number, first 60 AAs:  0.00074
# Arabidopsishalleri_Araha.5052s0042.1.p Total prob of N-in:        0.00642
Arabidopsishalleri_Araha.5052s0042.1.p  TMHMM2.0        outside      1   373
```

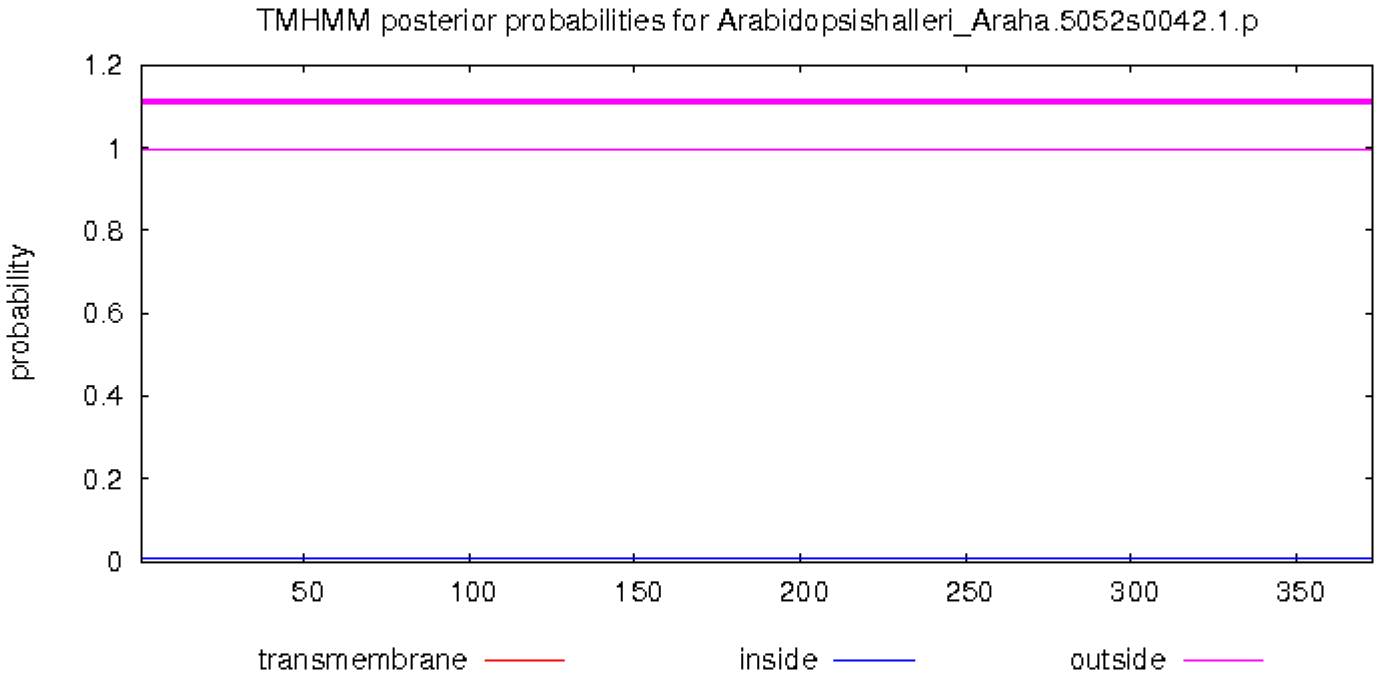

plot in postscript, script for making the plot in gnuplot, data for plot

```
# Arabidopsishalleri_Araha.5099s0006.1.p Length: 280
# Arabidopsishalleri_Araha.5099s0006.1.p Number of predicted TMHs:  0
# Arabidopsishalleri_Araha.5099s0006.1.p Exp number of AAs in TMHs: 4e-05
# Arabidopsishalleri_Araha.5099s0006.1.p Exp number, first 60 AAs:  0
# Arabidopsishalleri_Araha.5099s0006.1.p Total prob of N-in:        0.00261
Arabidopsishalleri_Araha.5099s0006.1.p  TMHMM2.0        outside      1   280
```

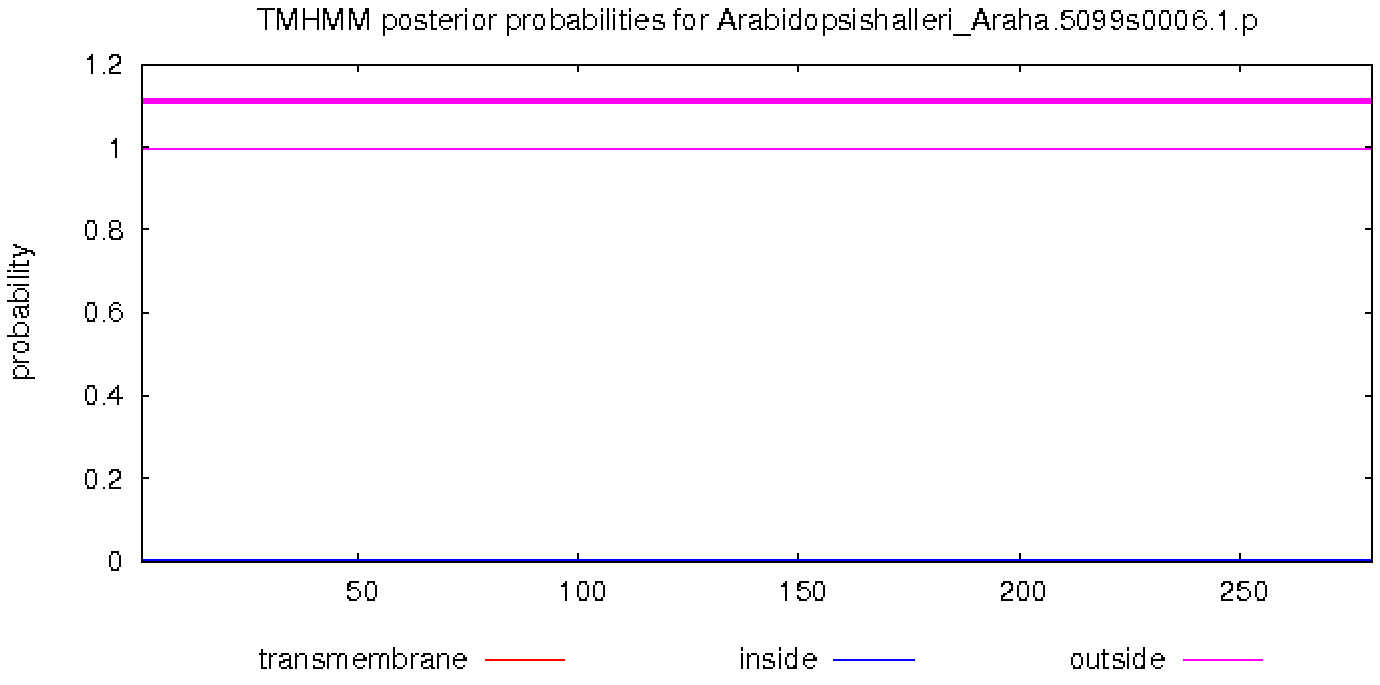

plot in postscript, script for making the plot in gnuplot, data for plot

```
# Arabidopsishalleri_Araha.5101s0001.1.p Length: 412
# Arabidopsishalleri_Araha.5101s0001.1.p Number of predicted TMHs:  0
# Arabidopsishalleri_Araha.5101s0001.1.p Exp number of AAs in TMHs: 0.01409
# Arabidopsishalleri_Araha.5101s0001.1.p Exp number, first 60 AAs:  0
# Arabidopsishalleri_Araha.5101s0001.1.p Total prob of N-in:        0.00930
Arabidopsishalleri_Araha.5101s0001.1.p  TMHMM2.0        outside      1   412
```

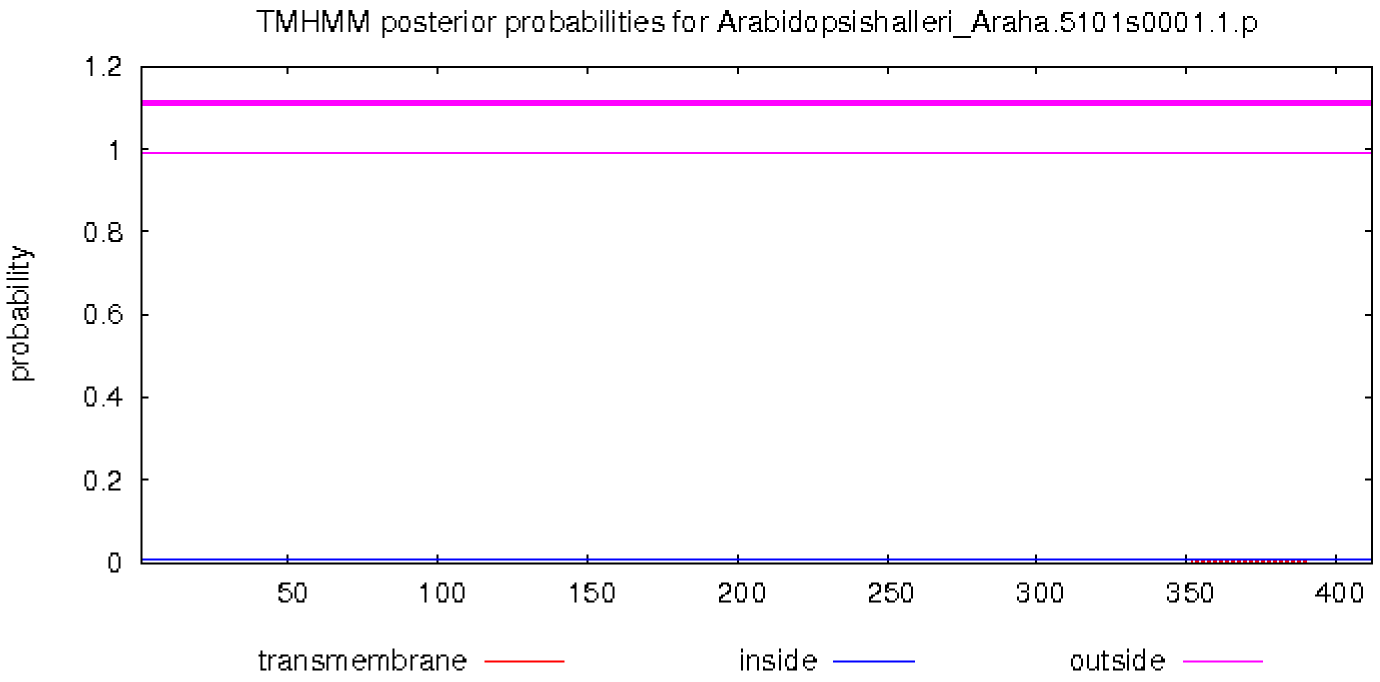

plot in postscript, script for making the plot in gnuplot, data for plot

---

```
# Arabidopsishalleri_Araha.55234s0001.1.p Length: 212
# Arabidopsishalleri_Araha.55234s0001.1.p Number of predicted TMHs:  0
# Arabidopsishalleri_Araha.55234s0001.1.p Exp number of AAs in TMHs: 0.00268
# Arabidopsishalleri_Araha.55234s0001.1.p Exp number, first 60 AAs:  0.00265
# Arabidopsishalleri_Araha.55234s0001.1.p Total prob of N-in:        0.32613
Arabidopsishalleri_Araha.55234s0001.1.p TMHMM2.0        outside      1   212
```

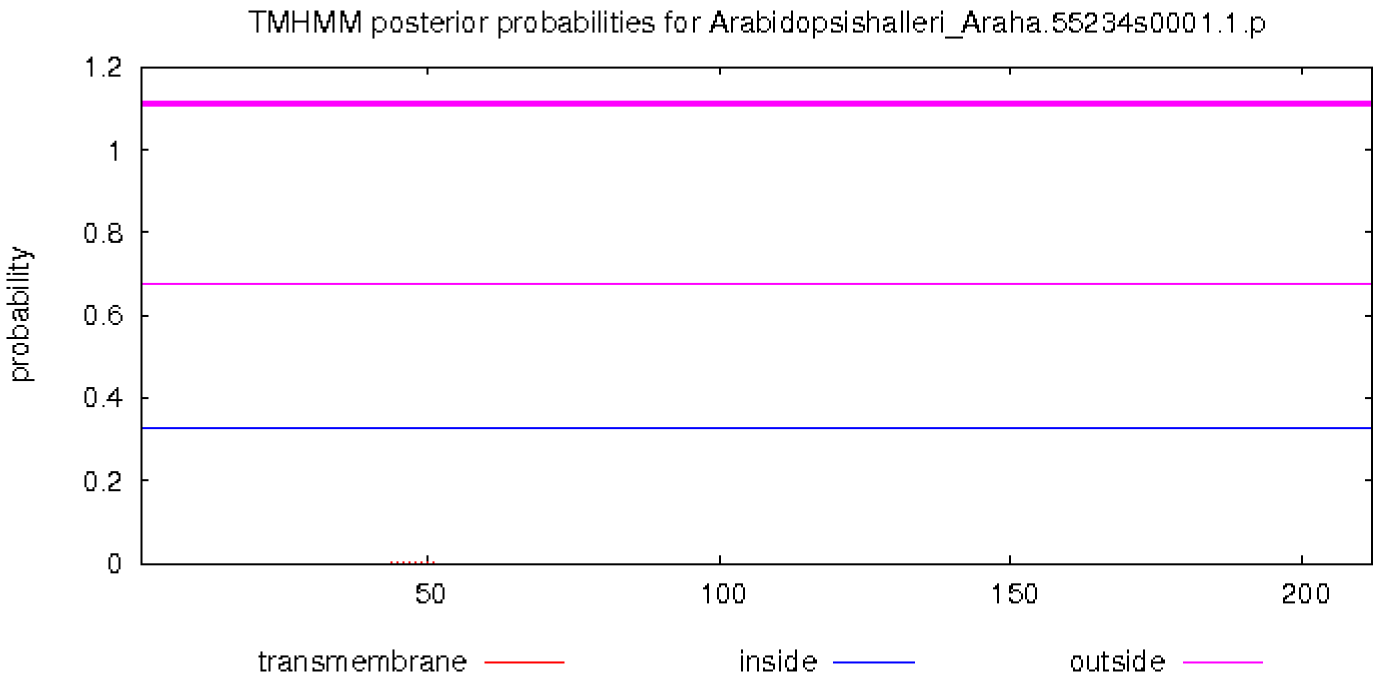

plot in postscript, script for making the plot in gnuplot, data for plot

---

```
# Arabidopsishalleri_Araha.5579s0003.1.p Length: 402
# Arabidopsishalleri_Araha.5579s0003.1.p Number of predicted TMHs:  0
# Arabidopsishalleri_Araha.5579s0003.1.p Exp number of AAs in TMHs: 0.0001
# Arabidopsishalleri_Araha.5579s0003.1.p Exp number, first 60 AAs:  0
# Arabidopsishalleri_Araha.5579s0003.1.p Total prob of N-in:        0.00841
Arabidopsishalleri_Araha.5579s0003.1.p  TMHMM2.0        outside      1   402
```

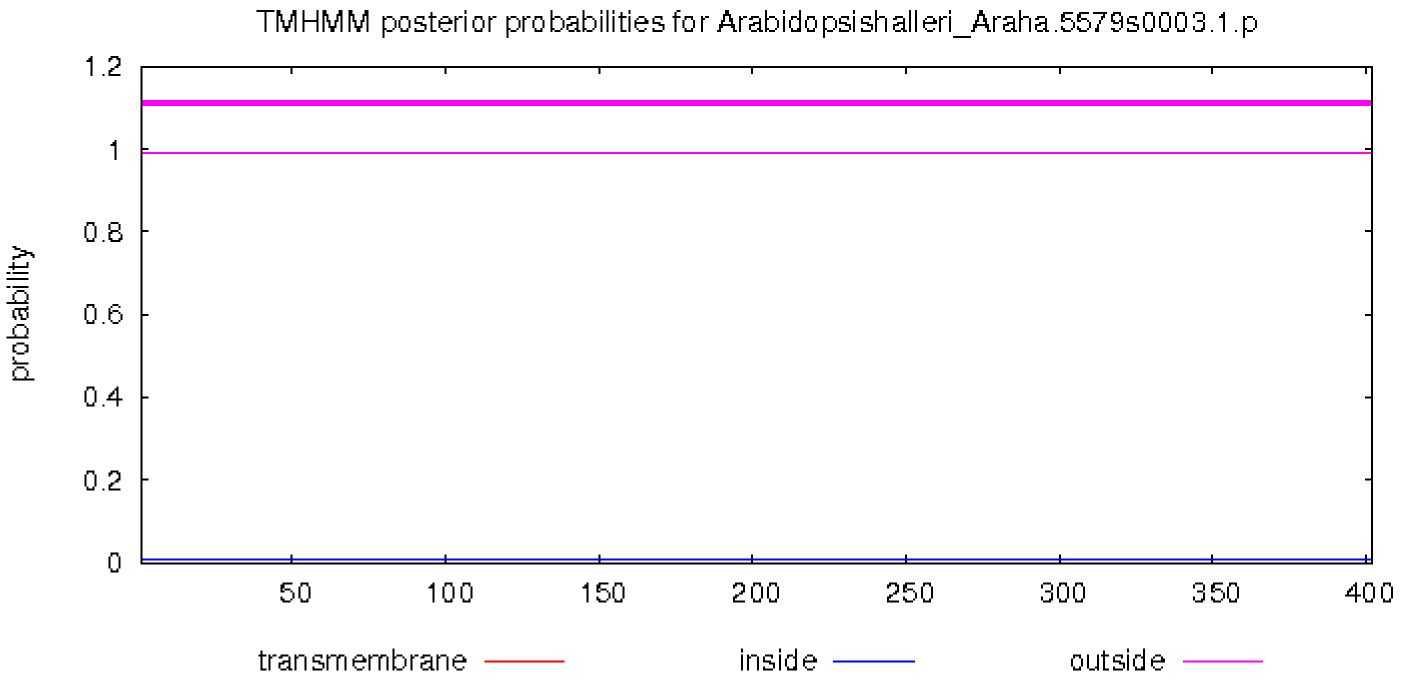

plot in postscript, script for making the plot in gnuplot, data for plot

---

```
# Arabidopsishalleri_Araha.5599s0009.1.p Length: 325
# Arabidopsishalleri_Araha.5599s0009.1.p Number of predicted TMHs:  0
# Arabidopsishalleri_Araha.5599s0009.1.p Exp number of AAs in TMHs: 0.00041
# Arabidopsishalleri_Araha.5599s0009.1.p Exp number, first 60 AAs:  0
# Arabidopsishalleri_Araha.5599s0009.1.p Total prob of N-in:        0.16498
Arabidopsishalleri_Araha.5599s0009.1.p  TMHMM2.0        outside      1   325
```

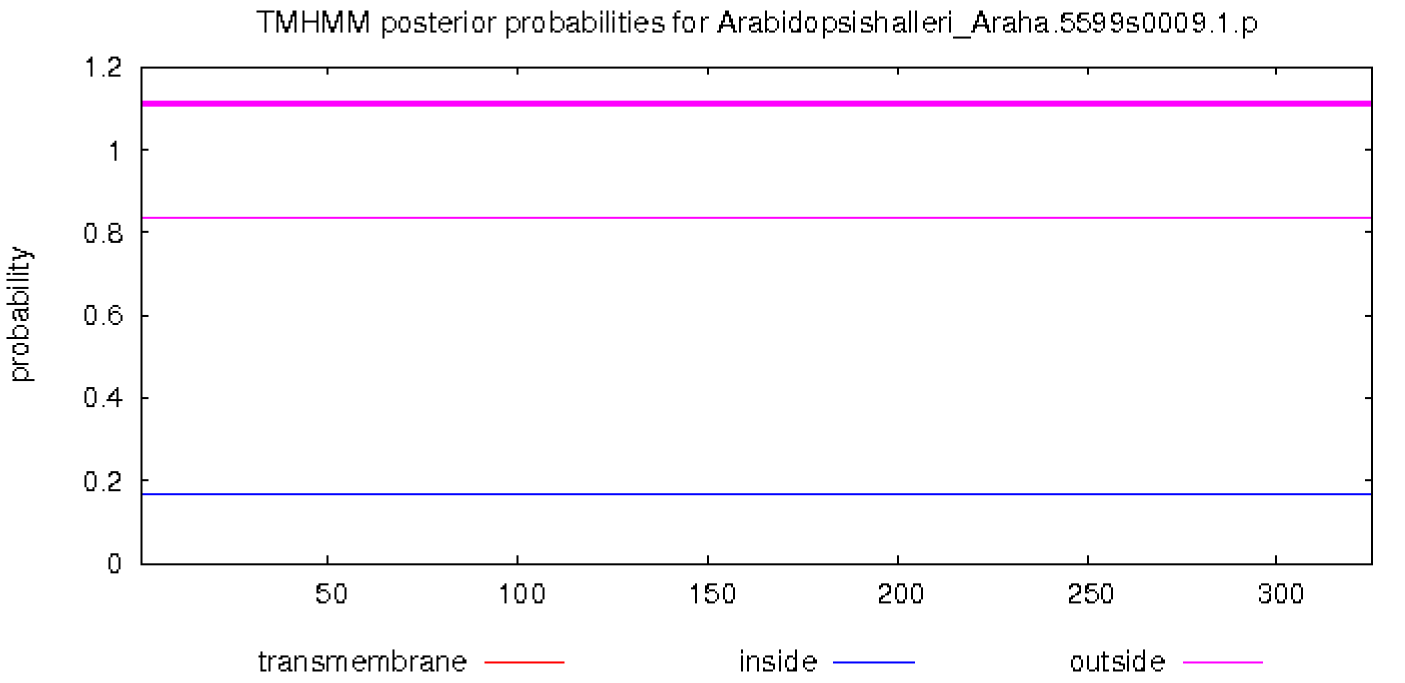

plot in postscript, script for making the plot in gnuplot, data for plot

---

```
# Arabidopsishalleri_Araha.57496s0006.1.p Length: 143
# Arabidopsishalleri_Araha.57496s0006.1.p Number of predicted TMHs:  0
# Arabidopsishalleri_Araha.57496s0006.1.p Exp number of AAs in TMHs: 0.00061
# Arabidopsishalleri_Araha.57496s0006.1.p Exp number, first 60 AAs:  0.00061
# Arabidopsishalleri_Araha.57496s0006.1.p Total prob of N-in:        0.08591
Arabidopsishalleri_Araha.57496s0006.1.p TMHMM2.0        outside      1   143
```

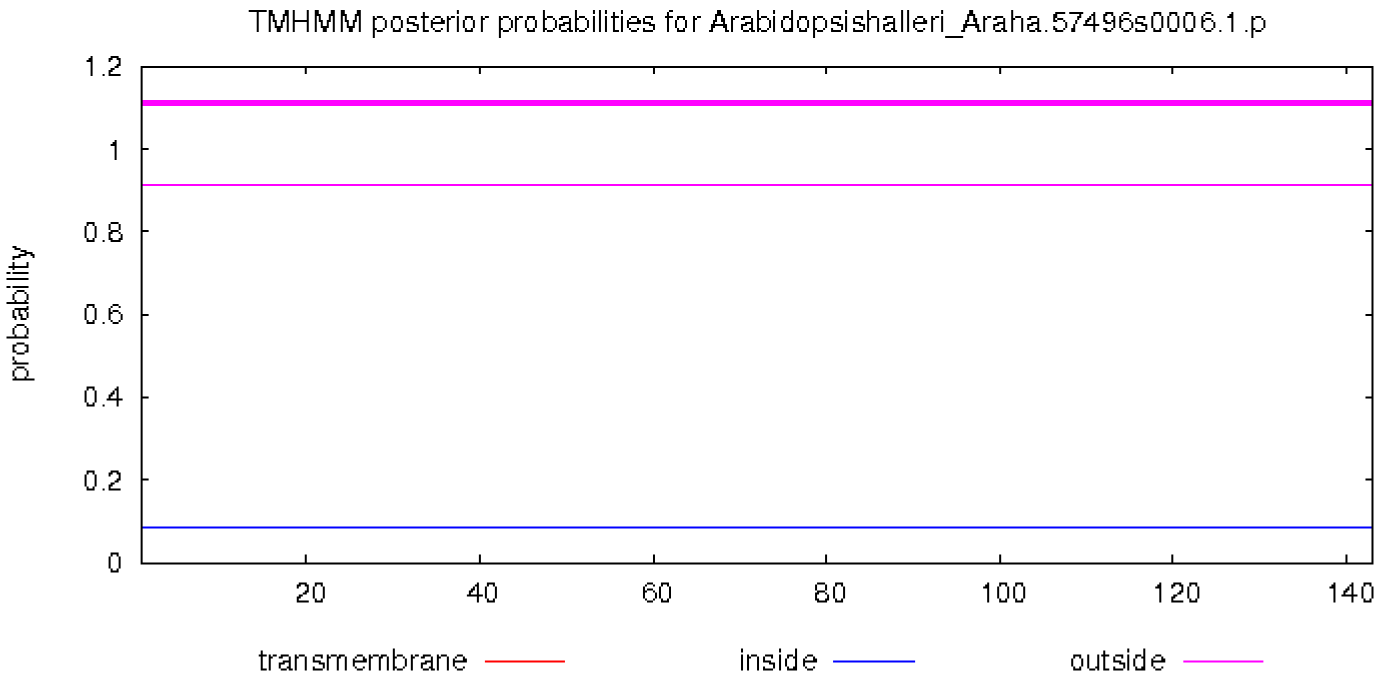

plot in postscript, script for making the plot in gnuplot, data for plot

```
# Arabidopsishalleri_Araha.5892s0003.1.p Length: 209
# Arabidopsishalleri_Araha.5892s0003.1.p Number of predicted TMHs:  0
# Arabidopsishalleri_Araha.5892s0003.1.p Exp number of AAs in TMHs: 0.0207
# Arabidopsishalleri_Araha.5892s0003.1.p Exp number, first 60 AAs:  0.02051
# Arabidopsishalleri_Araha.5892s0003.1.p Total prob of N-in:        0.03925
Arabidopsishalleri_Araha.5892s0003.1.p  TMHMM2.0        outside      1   209
```

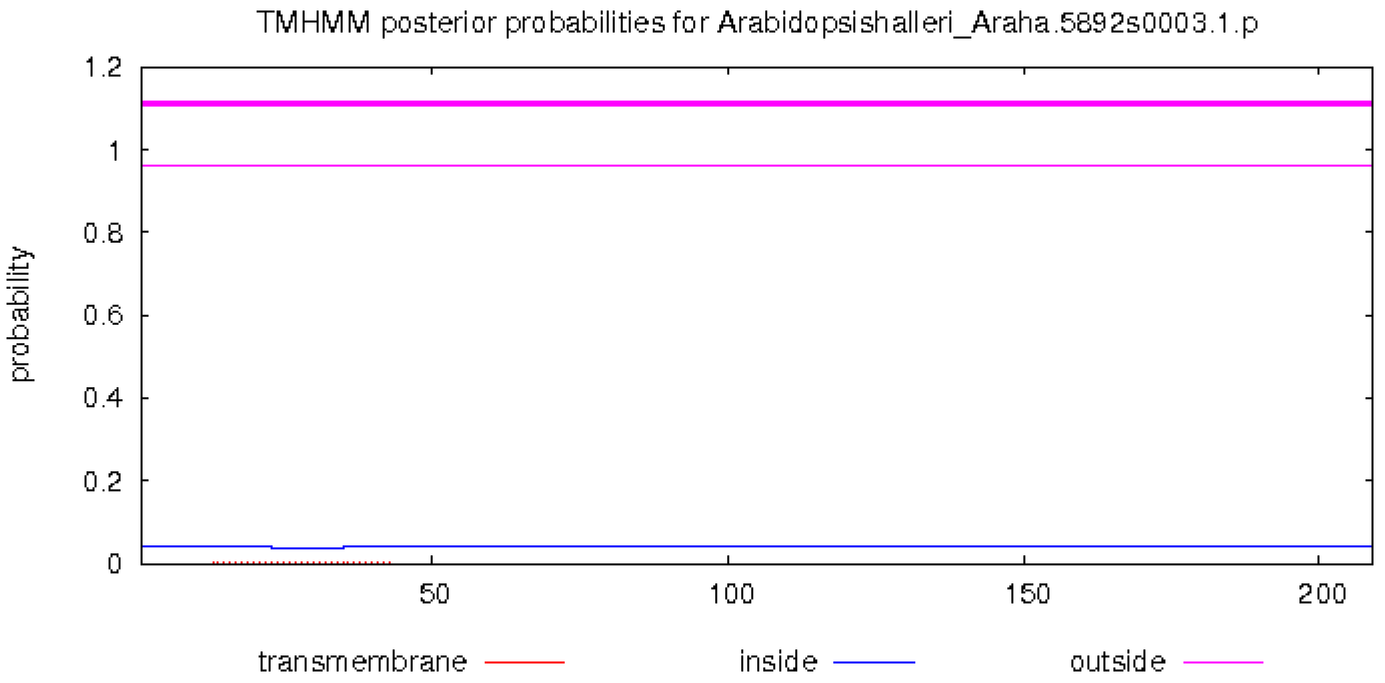

plot in postscript, script for making the plot in gnuplot, data for plot

```
# Arabidopsishalleri_Araha.59992s0001.1.p Length: 246
# Arabidopsishalleri_Araha.59992s0001.1.p Number of predicted TMHs:  0
# Arabidopsishalleri_Araha.59992s0001.1.p Exp number of AAs in TMHs: 0
# Arabidopsishalleri_Araha.59992s0001.1.p Exp number, first 60 AAs:  0
# Arabidopsishalleri_Araha.59992s0001.1.p Total prob of N-in:        0.00222
Arabidopsishalleri_Araha.59992s0001.1.p TMHMM2.0        outside      1   246
```

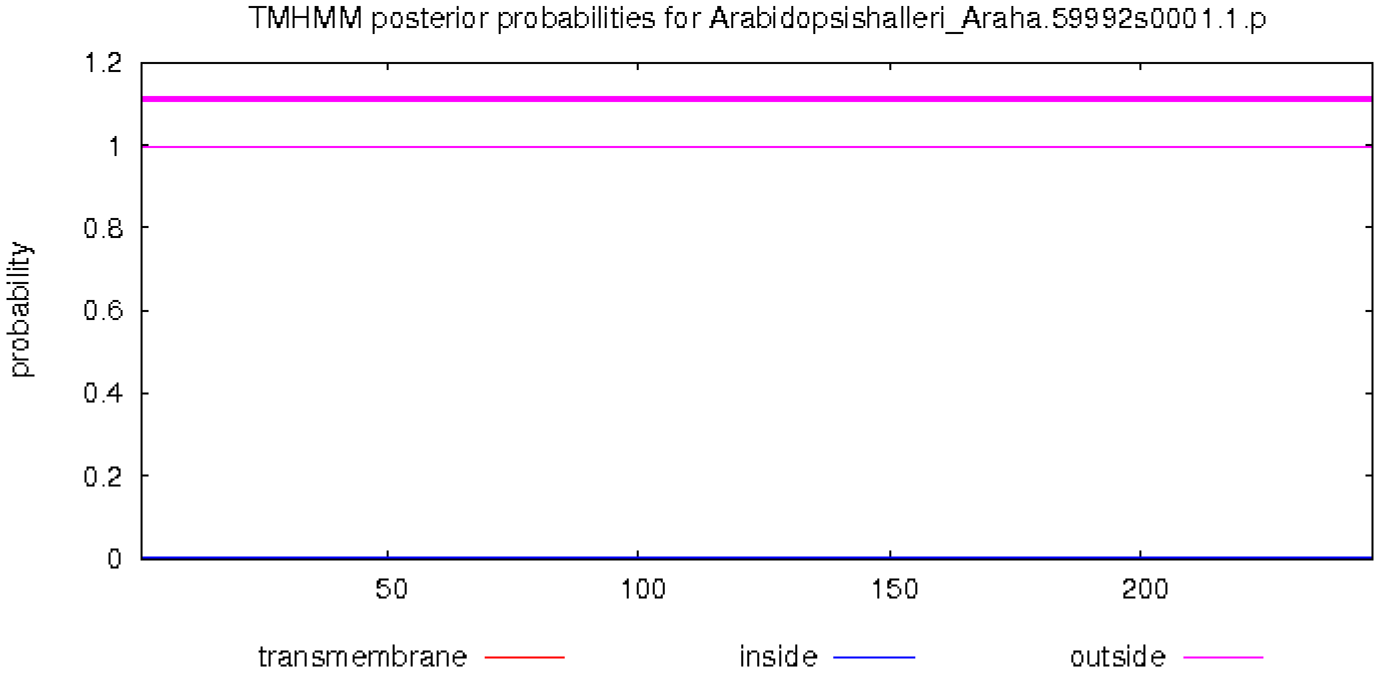

plot in postscript, script for making the plot in gnuplot, data for plot

```
# Arabidopsishalleri_Araha.6161s0007.1.p Length: 162
# Arabidopsishalleri_Araha.6161s0007.1.p Number of predicted TMHs:  0
# Arabidopsishalleri_Araha.6161s0007.1.p Exp number of AAs in TMHs: 0.00134
# Arabidopsishalleri_Araha.6161s0007.1.p Exp number, first 60 AAs:  0
# Arabidopsishalleri_Araha.6161s0007.1.p Total prob of N-in:        0.34507
Arabidopsishalleri_Araha.6161s0007.1.p  TMHMM2.0        outside      1   162
```

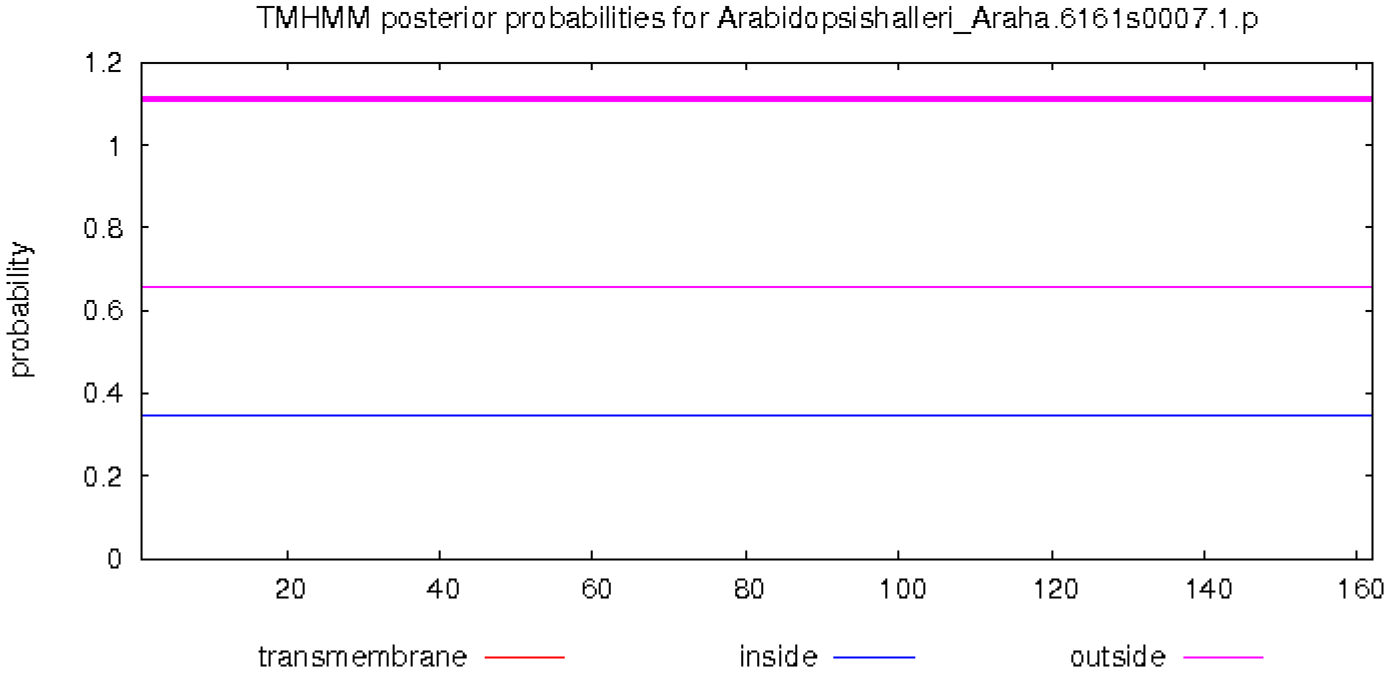

plot in postscript, script for making the plot in gnuplot, data for plot

```
# Arabidopsishalleri_Araha.61839s0001.1.p Length: 334
# Arabidopsishalleri_Araha.61839s0001.1.p Number of predicted TMHs:  0
# Arabidopsishalleri_Araha.61839s0001.1.p Exp number of AAs in TMHs: 0.04277
# Arabidopsishalleri_Araha.61839s0001.1.p Exp number, first 60 AAs:  0
# Arabidopsishalleri_Araha.61839s0001.1.p Total prob of N-in:        0.03572
Arabidopsishalleri_Araha.61839s0001.1.p TMHMM2.0        outside      1   334
```

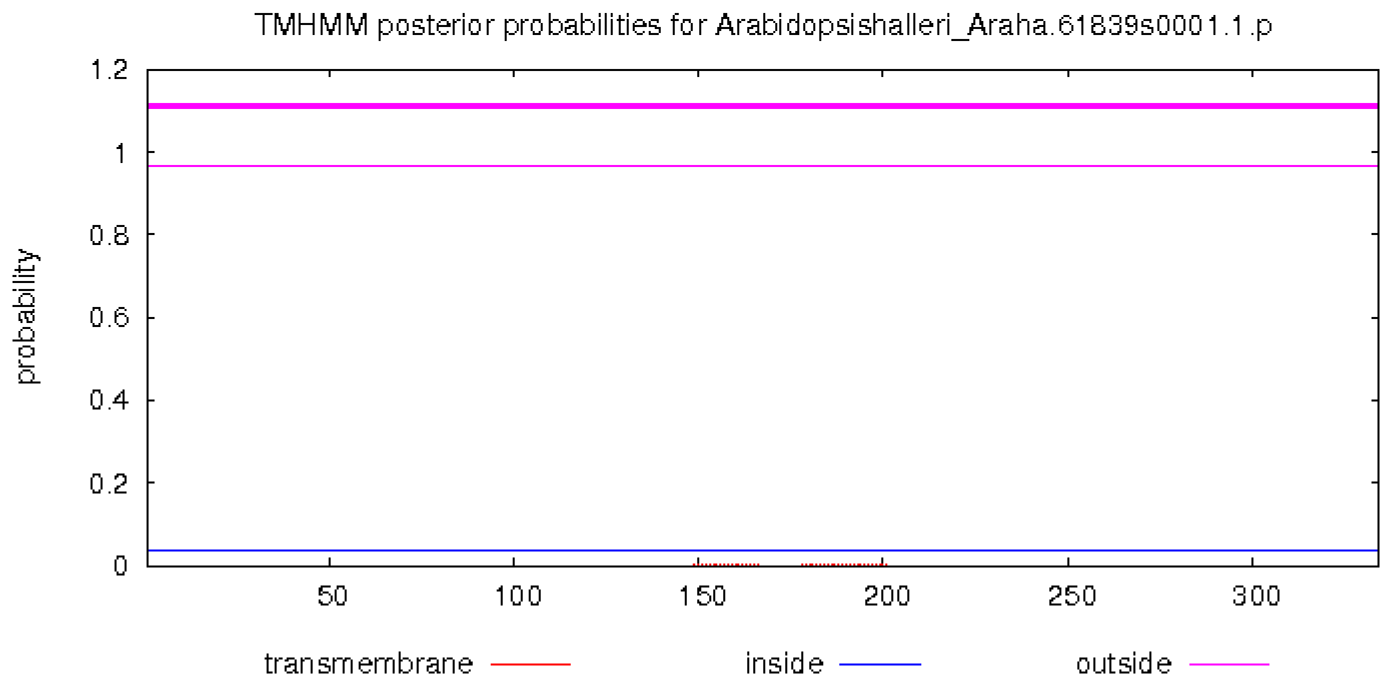

plot in postscript, script for making the plot in gnuplot, data for plot

---

```
# Arabidopsishalleri_Araha.6475s0002.1.p Length: 278
# Arabidopsishalleri_Araha.6475s0002.1.p Number of predicted TMHs:  0
# Arabidopsishalleri_Araha.6475s0002.1.p Exp number of AAs in TMHs: 0.00123
# Arabidopsishalleri_Araha.6475s0002.1.p Exp number, first 60 AAs:  0
# Arabidopsishalleri_Araha.6475s0002.1.p Total prob of N-in:        0.00447
Arabidopsishalleri_Araha.6475s0002.1.p  TMHMM2.0        outside      1   278
```

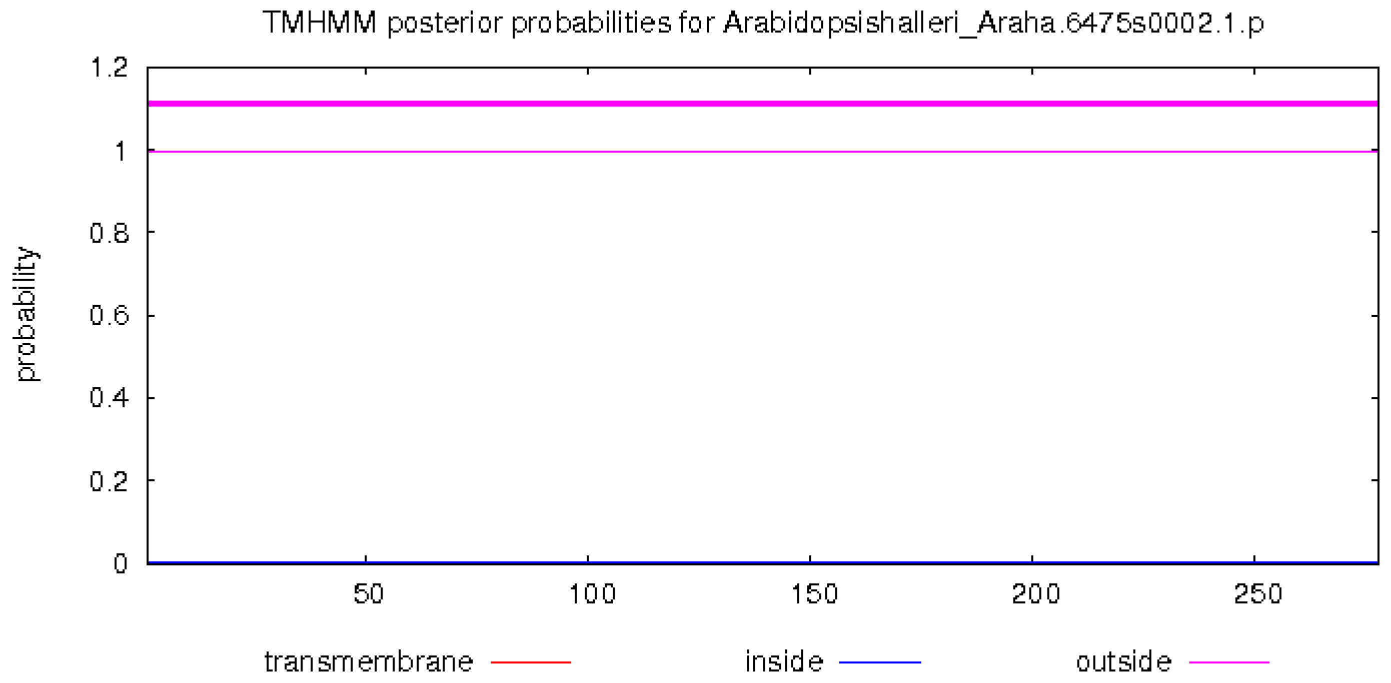

plot in postscript, script for making the plot in gnuplot, data for plot

---

```
# Arabidopsishalleri_Araha.65799s0001.1.p Length: 573
# Arabidopsishalleri_Araha.65799s0001.1.p Number of predicted TMHs:  1
# Arabidopsishalleri_Araha.65799s0001.1.p Exp number of AAs in TMHs: 22.91479
# Arabidopsishalleri_Araha.65799s0001.1.p Exp number, first 60 AAs:  0.00929
# Arabidopsishalleri_Araha.65799s0001.1.p Total prob of N-in:        0.13243
Arabidopsishalleri_Araha.65799s0001.1.p TMHMM2.0        outside      1   545
Arabidopsishalleri_Araha.65799s0001.1.p TMHMM2.0        TMhelix    546   568
Arabidopsishalleri_Araha.65799s0001.1.p TMHMM2.0        inside     569   573
```

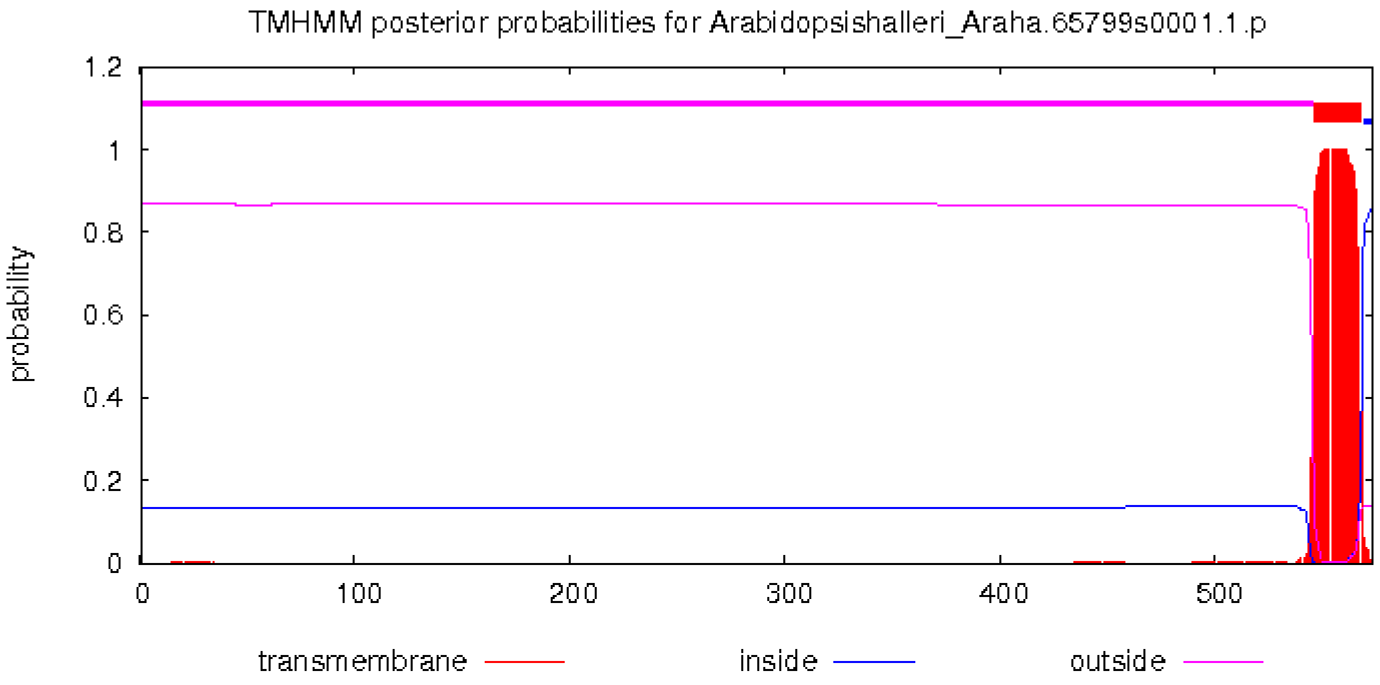

plot in postscript, script for making the plot in gnuplot, data for plot

---

```
# Arabidopsishalleri_Araha.6755s0003.1.p Length: 174
# Arabidopsishalleri_Araha.6755s0003.1.p Number of predicted TMHs:  0
# Arabidopsishalleri_Araha.6755s0003.1.p Exp number of AAs in TMHs: 0.1783
# Arabidopsishalleri_Araha.6755s0003.1.p Exp number, first 60 AAs:  0.17177
# Arabidopsishalleri_Araha.6755s0003.1.p Total prob of N-in:        0.34423
Arabidopsishalleri_Araha.6755s0003.1.p  TMHMM2.0        outside      1   174
```

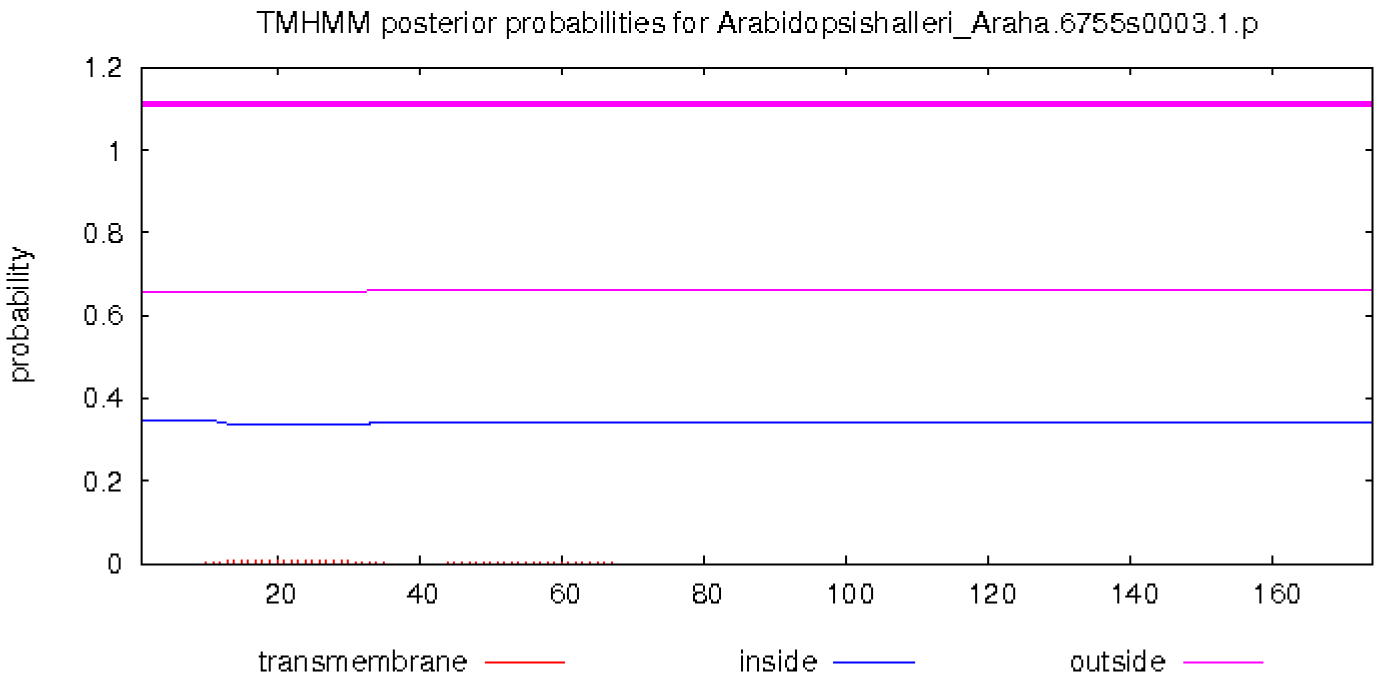

plot in postscript, script for making the plot in gnuplot, data for plot

```
# Arabidopsishalleri_Araha.68360s0001.1.p Length: 153
# Arabidopsishalleri_Araha.68360s0001.1.p Number of predicted TMHs:  0
# Arabidopsishalleri_Araha.68360s0001.1.p Exp number of AAs in TMHs: 0.00046
# Arabidopsishalleri_Araha.68360s0001.1.p Exp number, first 60 AAs:  0.00046
# Arabidopsishalleri_Araha.68360s0001.1.p Total prob of N-in:        0.14409
Arabidopsishalleri_Araha.68360s0001.1.p TMHMM2.0        outside      1   153
```

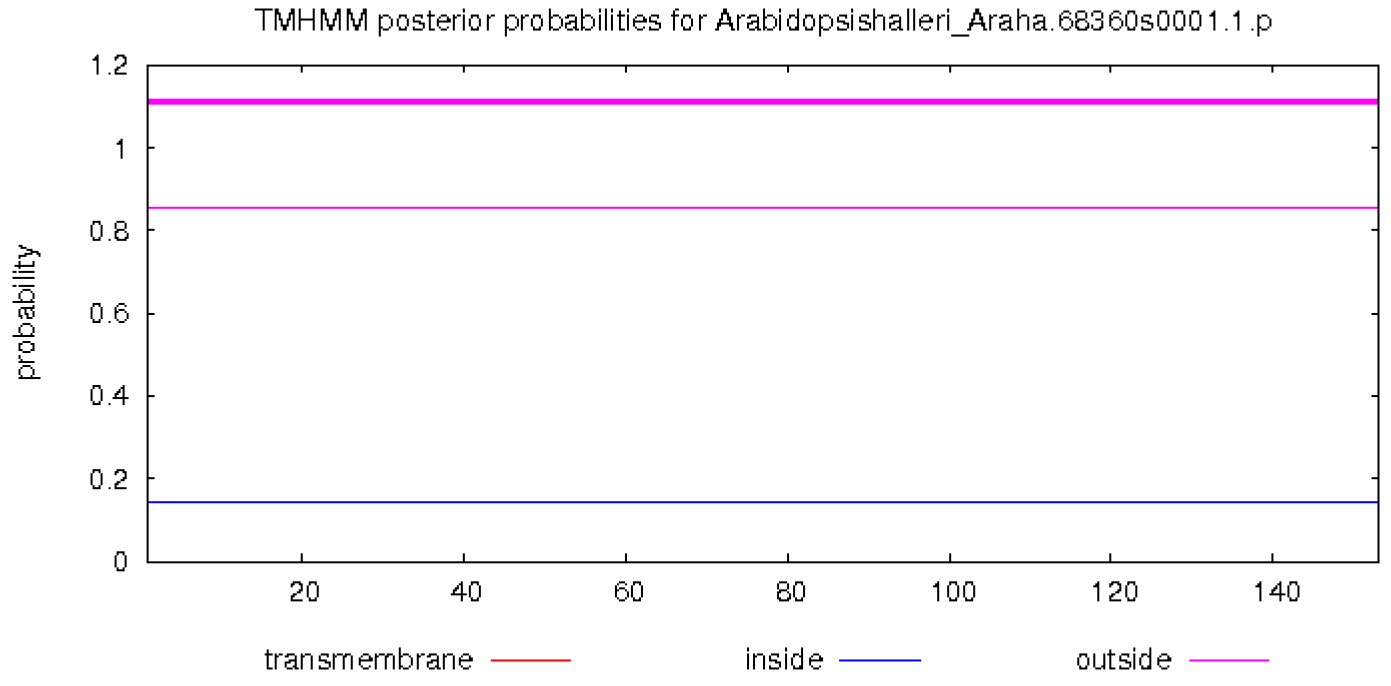

plot in postscript, script for making the plot in gnuplot, data for plot

```
# Arabidopsishalleri_Araha.7513s0013.1.p Length: 463
# Arabidopsishalleri_Araha.7513s0013.1.p Number of predicted TMHs:  0
# Arabidopsishalleri_Araha.7513s0013.1.p Exp number of AAs in TMHs: 0.00073
# Arabidopsishalleri_Araha.7513s0013.1.p Exp number, first 60 AAs:  0
# Arabidopsishalleri_Araha.7513s0013.1.p Total prob of N-in:        0.00022
Arabidopsishalleri_Araha.7513s0013.1.p  TMHMM2.0        outside      1   463
```

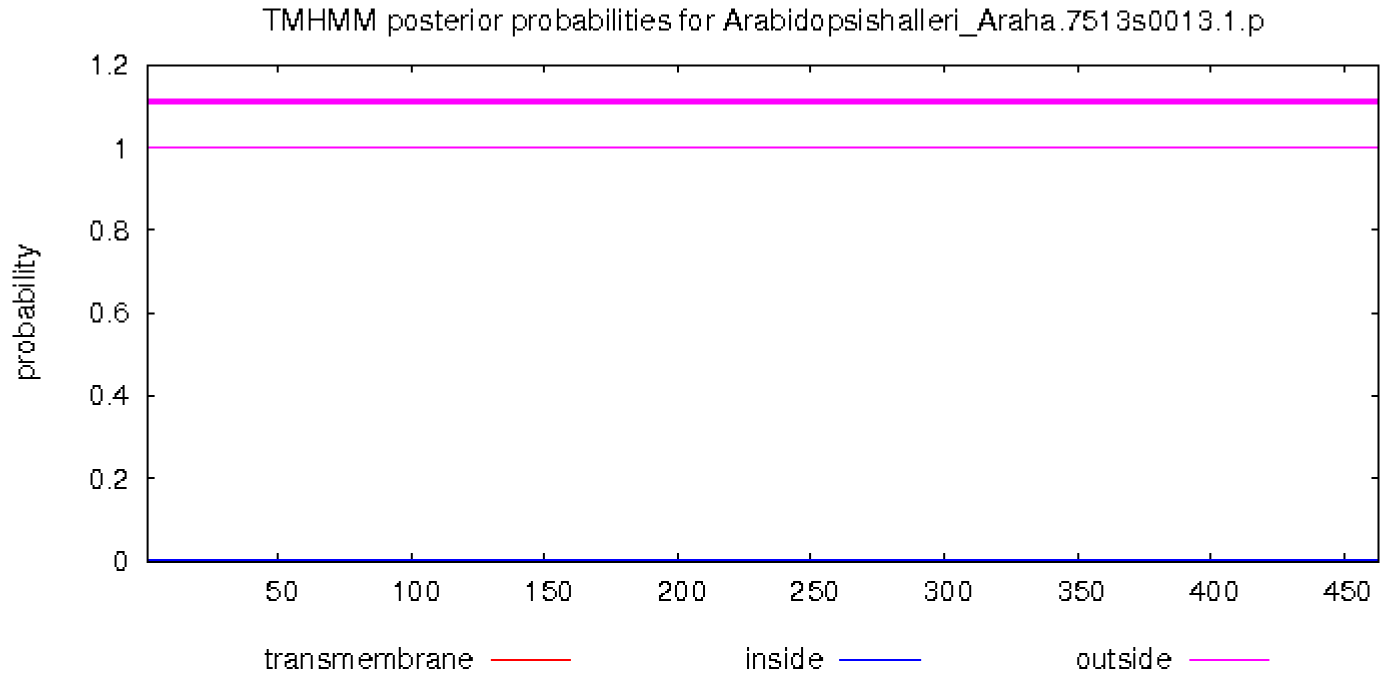

plot in postscript, script for making the plot in gnuplot, data for plot

```
# Arabidopsishalleri_Araha.7640s0010.1.p Length: 283
# Arabidopsishalleri_Araha.7640s0010.1.p Number of predicted TMHs:  0
# Arabidopsishalleri_Araha.7640s0010.1.p Exp number of AAs in TMHs: 0.00019
# Arabidopsishalleri_Araha.7640s0010.1.p Exp number, first 60 AAs:  0
# Arabidopsishalleri_Araha.7640s0010.1.p Total prob of N-in:        0.01024
Arabidopsishalleri_Araha.7640s0010.1.p  TMHMM2.0        outside      1   283
```

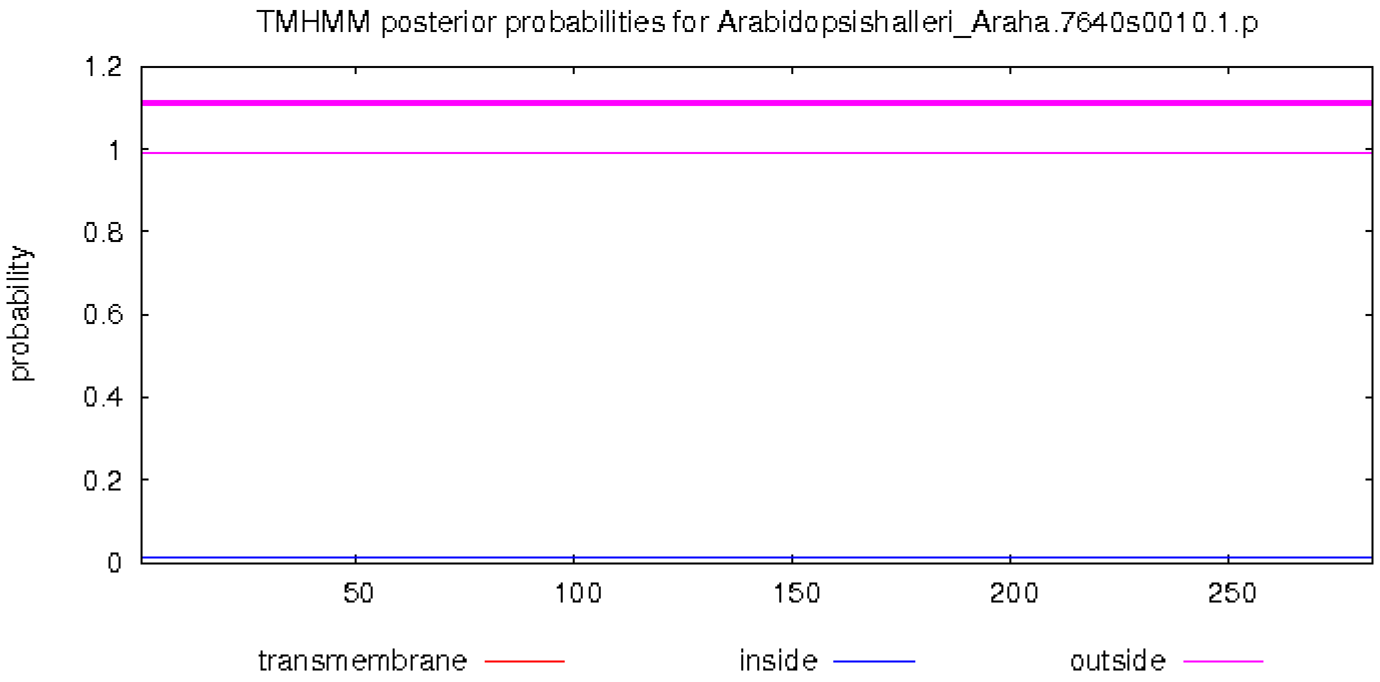

plot in postscript, script for making the plot in gnuplot, data for plot

---

```
# Arabidopsishalleri_Araha.8686s0001.1.p Length: 256
# Arabidopsishalleri_Araha.8686s0001.1.p Number of predicted TMHs:  0
# Arabidopsishalleri_Araha.8686s0001.1.p Exp number of AAs in TMHs: 0.0002
# Arabidopsishalleri_Araha.8686s0001.1.p Exp number, first 60 AAs:  0
# Arabidopsishalleri_Araha.8686s0001.1.p Total prob of N-in:        0.11032
Arabidopsishalleri_Araha.8686s0001.1.p  TMHMM2.0        outside      1   256
```

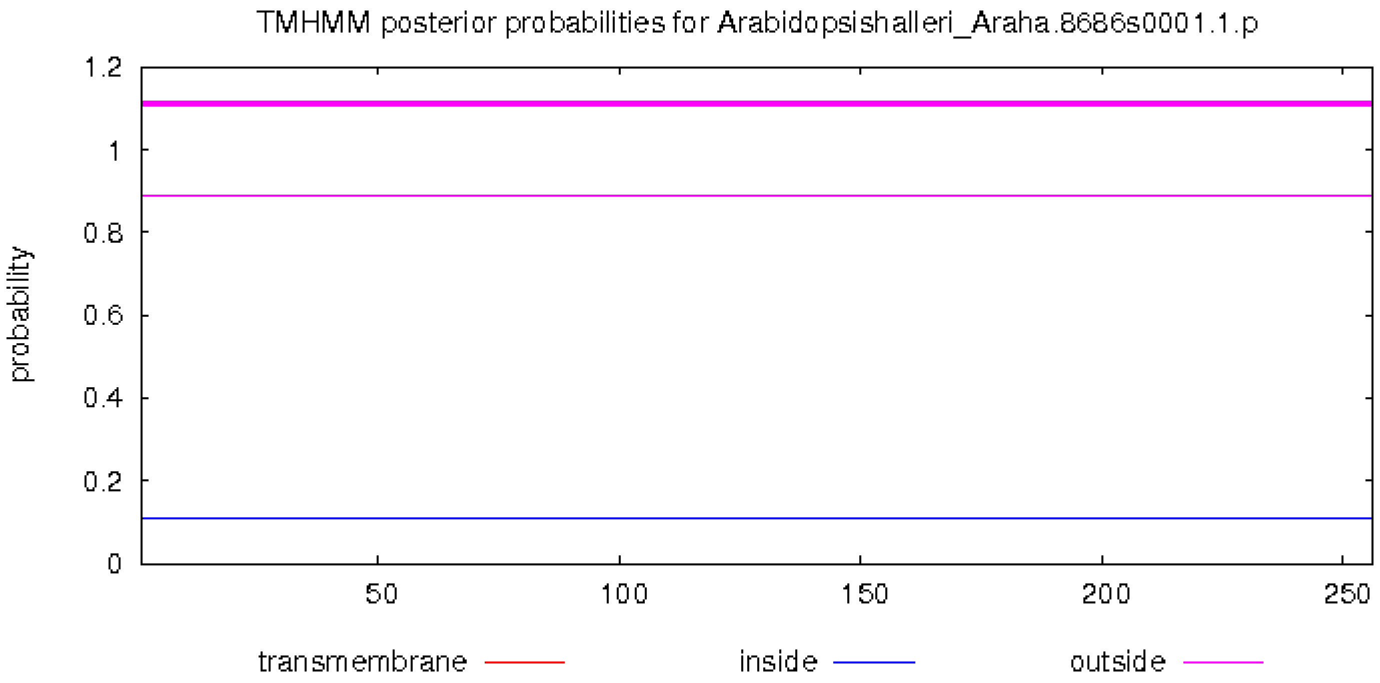

plot in postscript, script for making the plot in gnuplot, data for plot

---

```
# Arabidopsishalleri_Araha.8689s0023.1.p Length: 334
# Arabidopsishalleri_Araha.8689s0023.1.p Number of predicted TMHs:  0
# Arabidopsishalleri_Araha.8689s0023.1.p Exp number of AAs in TMHs: 0
# Arabidopsishalleri_Araha.8689s0023.1.p Exp number, first 60 AAs:  0
# Arabidopsishalleri_Araha.8689s0023.1.p Total prob of N-in:        0.00862
Arabidopsishalleri_Araha.8689s0023.1.p  TMHMM2.0        outside      1   334
```

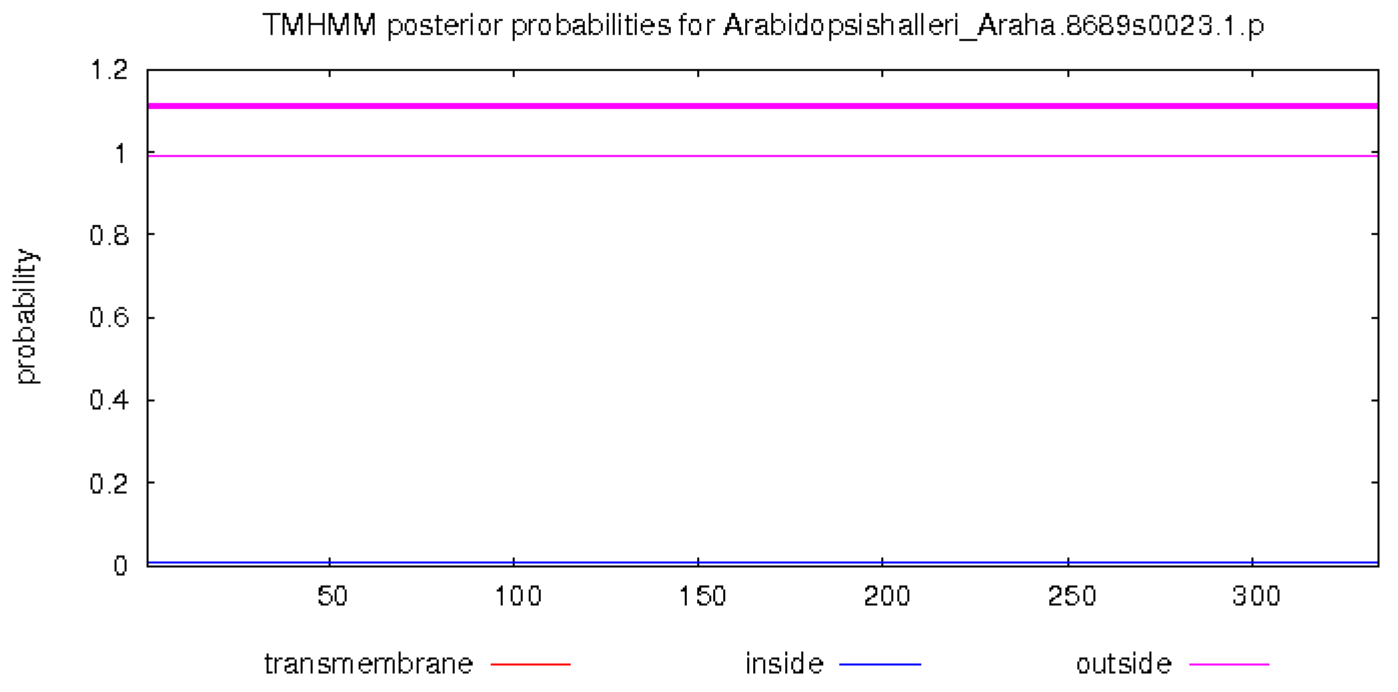

plot in postscript, script for making the plot in gnuplot, data for plot

```
# Arabidopsishalleri_Araha.8824s0002.1.p Length: 305
# Arabidopsishalleri_Araha.8824s0002.1.p Number of predicted TMHs:  0
# Arabidopsishalleri_Araha.8824s0002.1.p Exp number of AAs in TMHs: 0
# Arabidopsishalleri_Araha.8824s0002.1.p Exp number, first 60 AAs:  0
# Arabidopsishalleri_Araha.8824s0002.1.p Total prob of N-in:        0.00496
Arabidopsishalleri_Araha.8824s0002.1.p  TMHMM2.0        outside      1   305
```

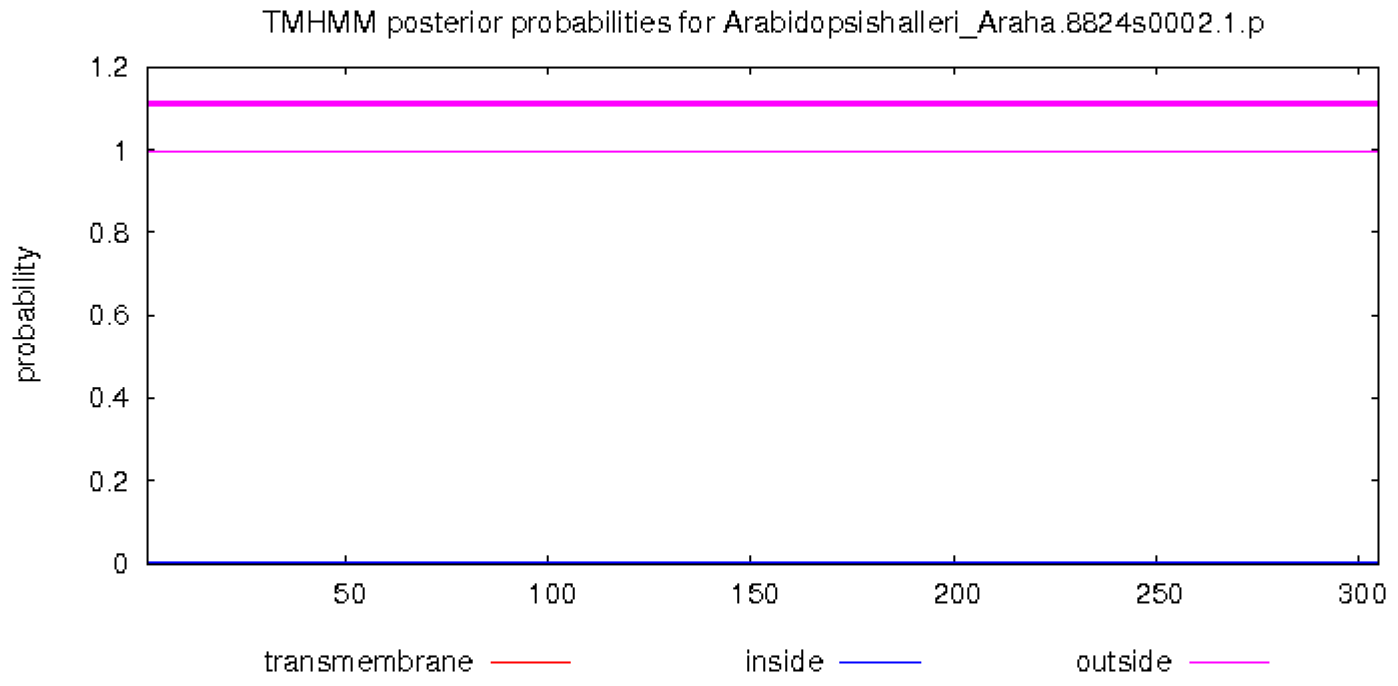

plot in postscript, script for making the plot in gnuplot, data for plot

```
# Arabidopsishalleri_Araha.8989s0001.1.p Length: 202
# Arabidopsishalleri_Araha.8989s0001.1.p Number of predicted TMHs:  0
# Arabidopsishalleri_Araha.8989s0001.1.p Exp number of AAs in TMHs: 0
# Arabidopsishalleri_Araha.8989s0001.1.p Exp number, first 60 AAs:  0
# Arabidopsishalleri_Araha.8989s0001.1.p Total prob of N-in:        0.09537
Arabidopsishalleri_Araha.8989s0001.1.p  TMHMM2.0        outside      1   202
```

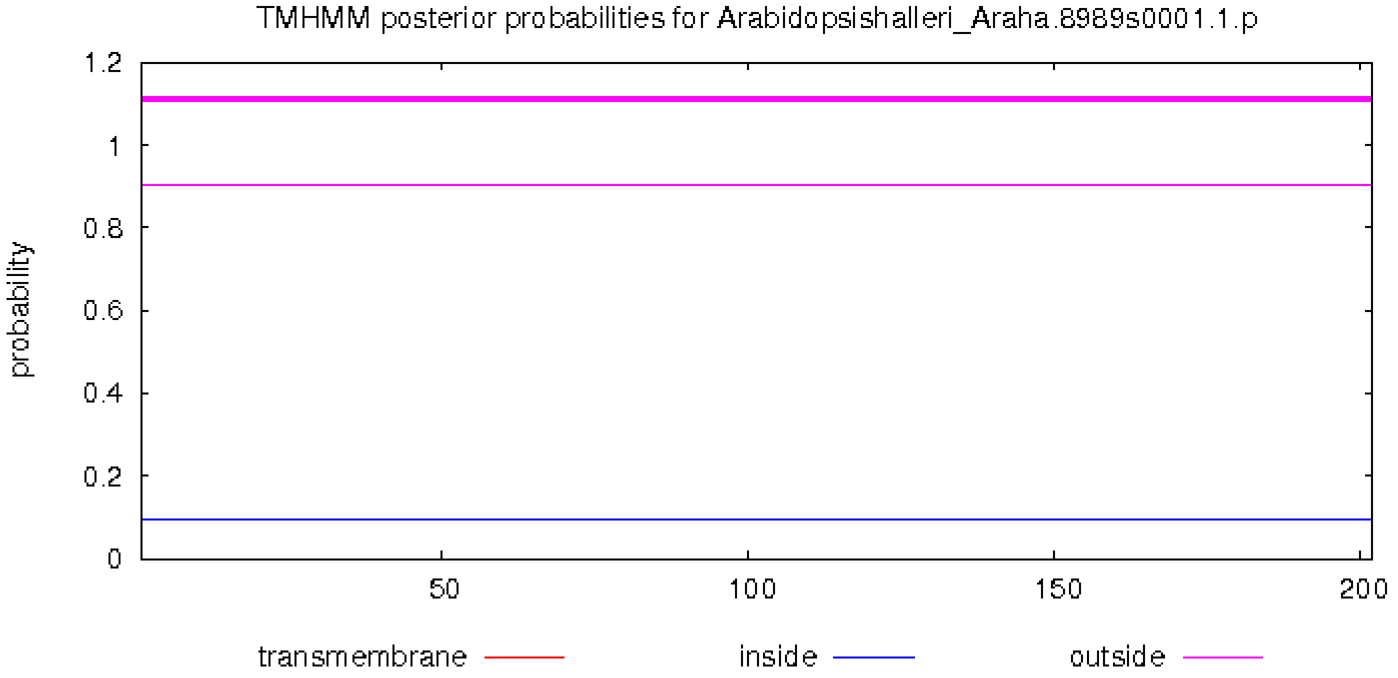

plot in postscript, script for making the plot in gnuplot, data for plot

---

```
# Arabidopsishalleri_Araha.9027s0002.1.p Length: 654
# Arabidopsishalleri_Araha.9027s0002.1.p Number of predicted TMHs:  1
# Arabidopsishalleri_Araha.9027s0002.1.p Exp number of AAs in TMHs: 20.08116
# Arabidopsishalleri_Araha.9027s0002.1.p Exp number, first 60 AAs:  0.00039
# Arabidopsishalleri_Araha.9027s0002.1.p Total prob of N-in:        0.06177
Arabidopsishalleri_Araha.9027s0002.1.p  TMHMM2.0        outside      1   611
Arabidopsishalleri_Araha.9027s0002.1.p  TMHMM2.0        TMhelix    612   631
Arabidopsishalleri_Araha.9027s0002.1.p  TMHMM2.0        inside     632   654
```

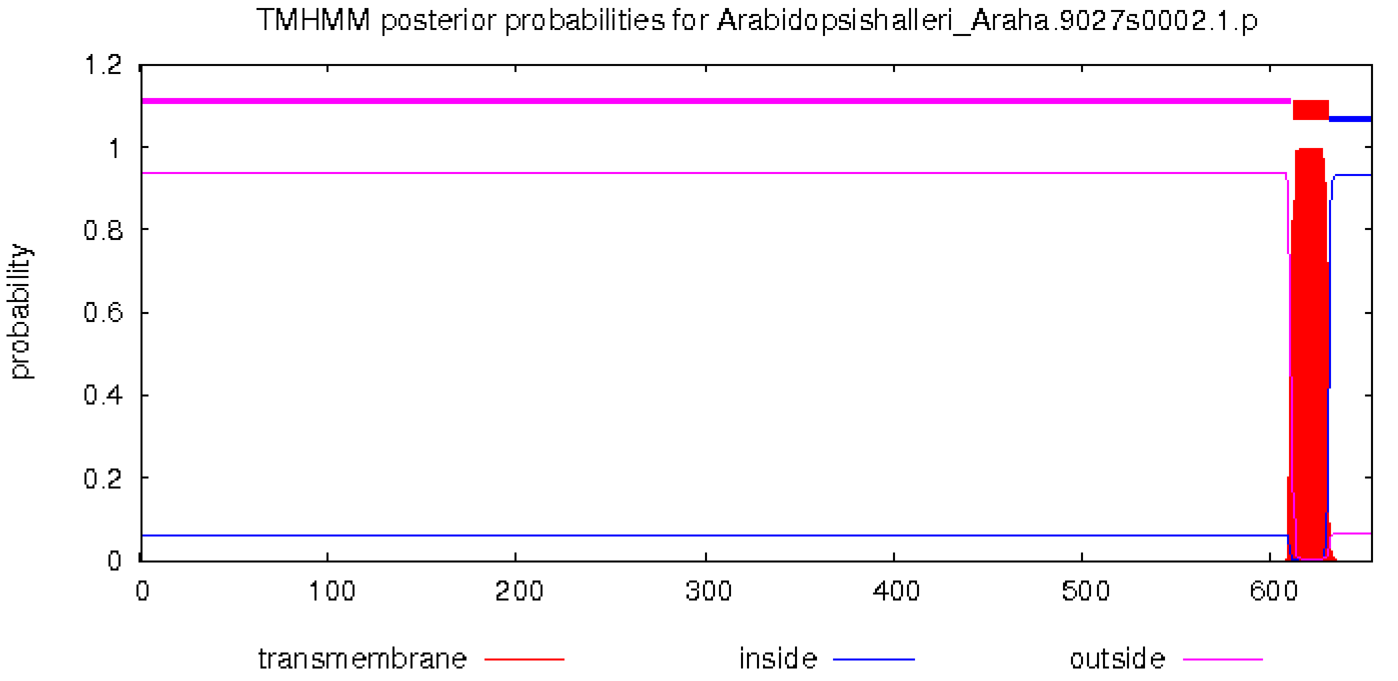

plot in postscript, script for making the plot in gnuplot, data for plot

```
# Arabidopsishalleri_Araha.9424s0001.1.p Length: 258
# Arabidopsishalleri_Araha.9424s0001.1.p Number of predicted TMHs:  0
# Arabidopsishalleri_Araha.9424s0001.1.p Exp number of AAs in TMHs: 0.01304
# Arabidopsishalleri_Araha.9424s0001.1.p Exp number, first 60 AAs:  0.00014
# Arabidopsishalleri_Araha.9424s0001.1.p Total prob of N-in:        0.03103
Arabidopsishalleri_Araha.9424s0001.1.p  TMHMM2.0        outside      1   258
```

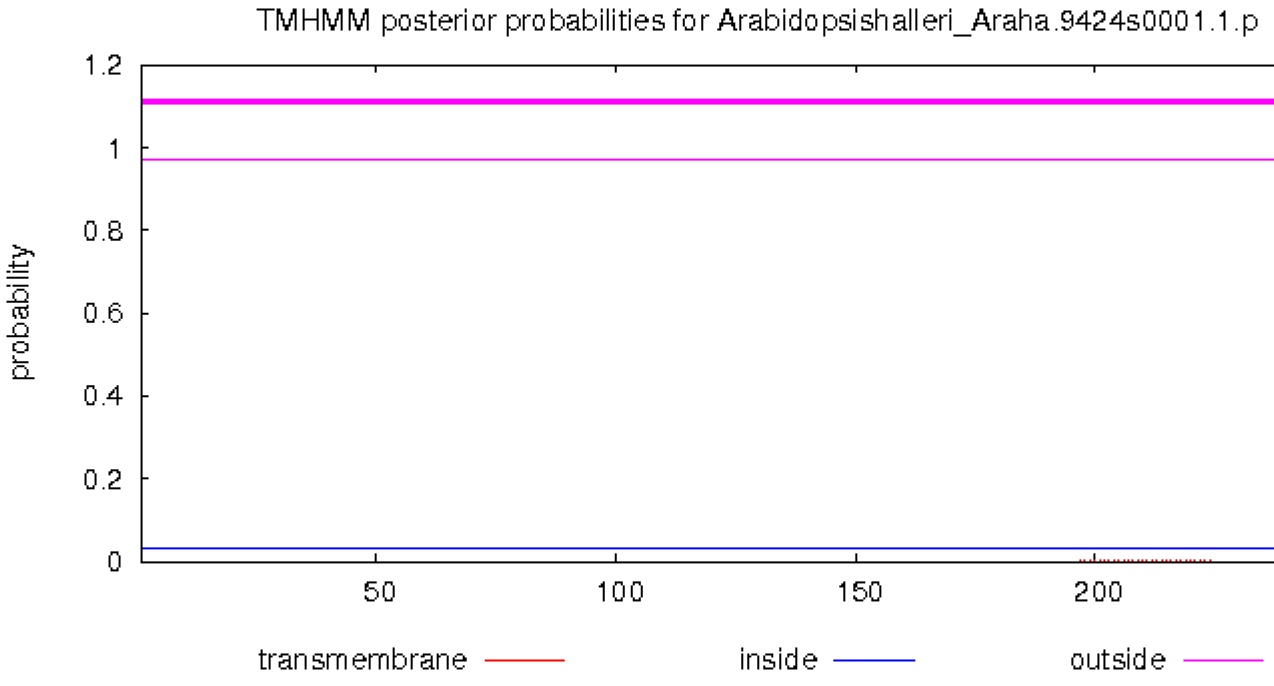

plot in postscript, script for making the plot in gnuplot, data for plot

```
# Arabidopsishalleri_Araha.9431s0002.1.p Length: 402
# Arabidopsishalleri_Araha.9431s0002.1.p Number of predicted TMHs:  0
# Arabidopsishalleri_Araha.9431s0002.1.p Exp number of AAs in TMHs: 0.00018
# Arabidopsishalleri_Araha.9431s0002.1.p Exp number, first 60 AAs:  0
# Arabidopsishalleri_Araha.9431s0002.1.p Total prob of N-in:        0.00184
Arabidopsishalleri_Araha.9431s0002.1.p  TMHMM2.0        outside      1   402
```

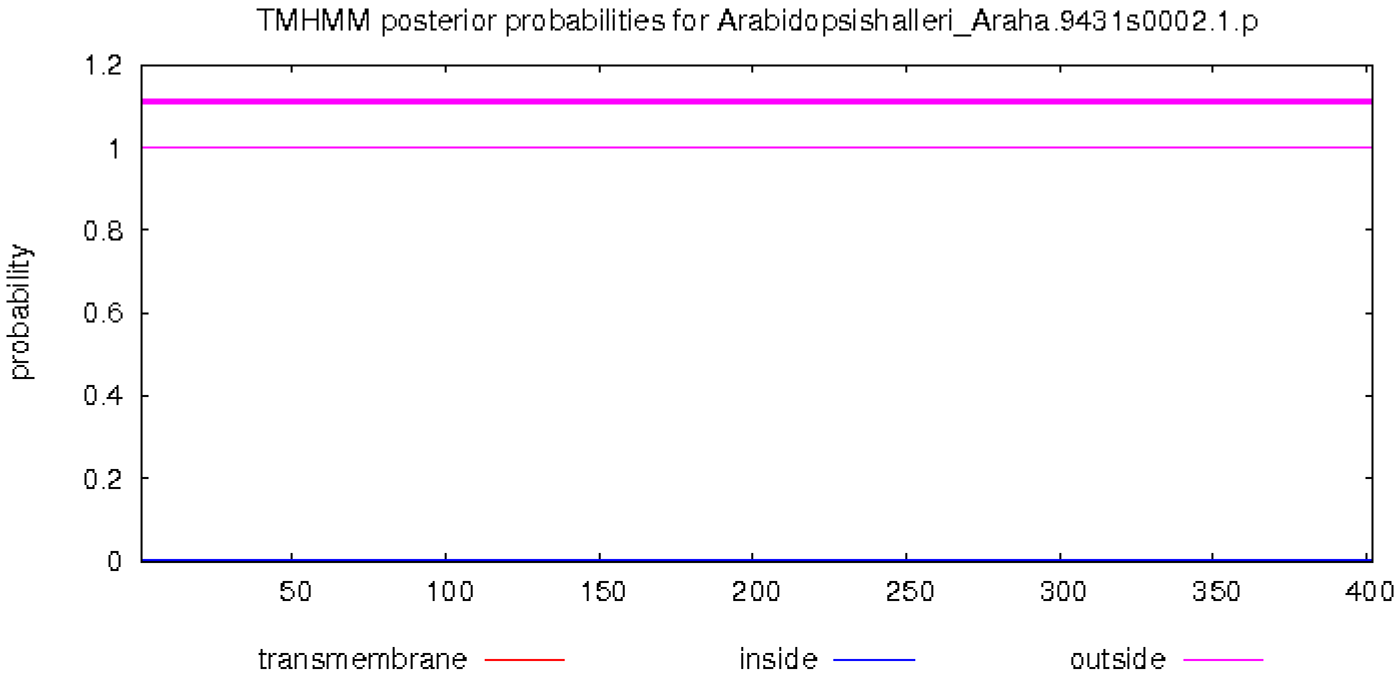

plot in postscript, script for making the plot in gnuplot, data for plot

```
# Arabidopsishalleri_Araha.9570s0009.1.p Length: 316
# Arabidopsishalleri_Araha.9570s0009.1.p Number of predicted TMHs:  0
# Arabidopsishalleri_Araha.9570s0009.1.p Exp number of AAs in TMHs: 0.02863
# Arabidopsishalleri_Araha.9570s0009.1.p Exp number, first 60 AAs:  0
# Arabidopsishalleri_Araha.9570s0009.1.p Total prob of N-in:        0.00394
Arabidopsishalleri_Araha.9570s0009.1.p  TMHMM2.0        outside      1   316
```

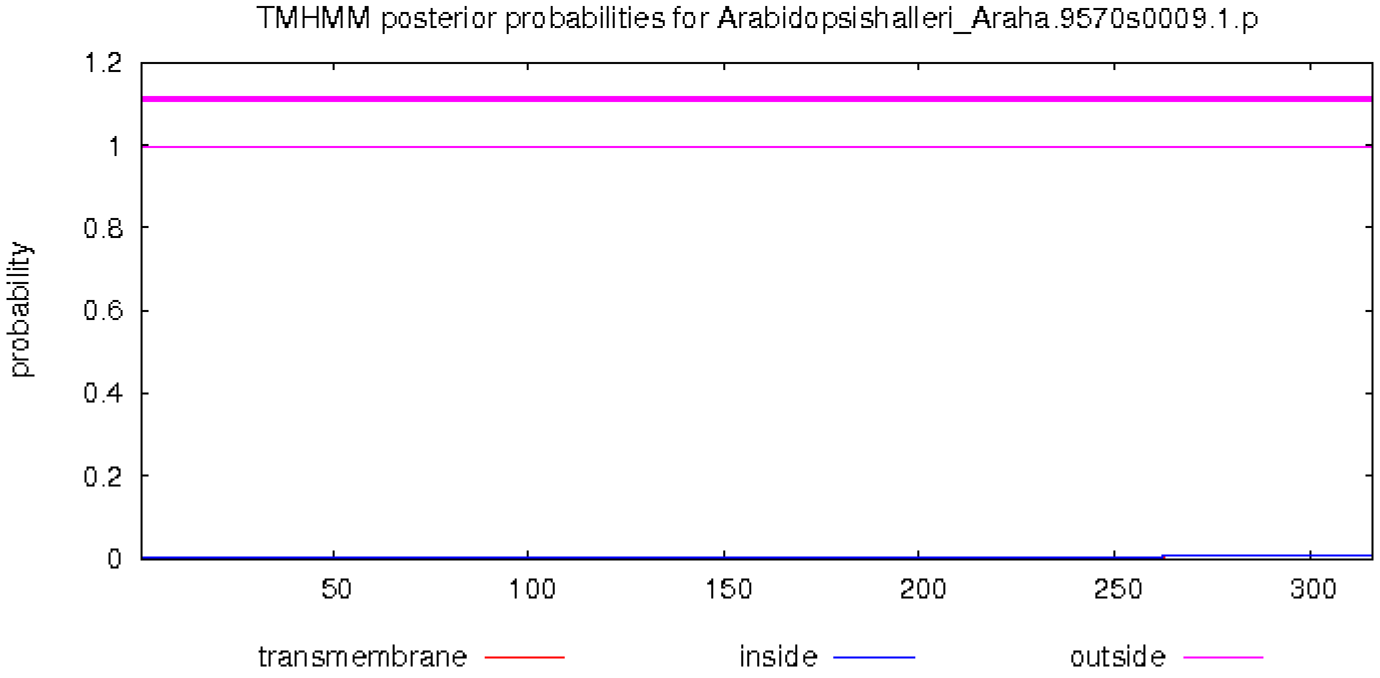

plot in postscript, script for making the plot in gnuplot, data for plot

---

```
# Arabidopsishalleri_Araha.9776s0006.1.p Length: 156
# Arabidopsishalleri_Araha.9776s0006.1.p Number of predicted TMHs:  0
# Arabidopsishalleri_Araha.9776s0006.1.p Exp number of AAs in TMHs: 1.11377
# Arabidopsishalleri_Araha.9776s0006.1.p Exp number, first 60 AAs:  0
# Arabidopsishalleri_Araha.9776s0006.1.p Total prob of N-in:        0.64142
Arabidopsishalleri_Araha.9776s0006.1.p  TMHMM2.0        inside       1   156
```

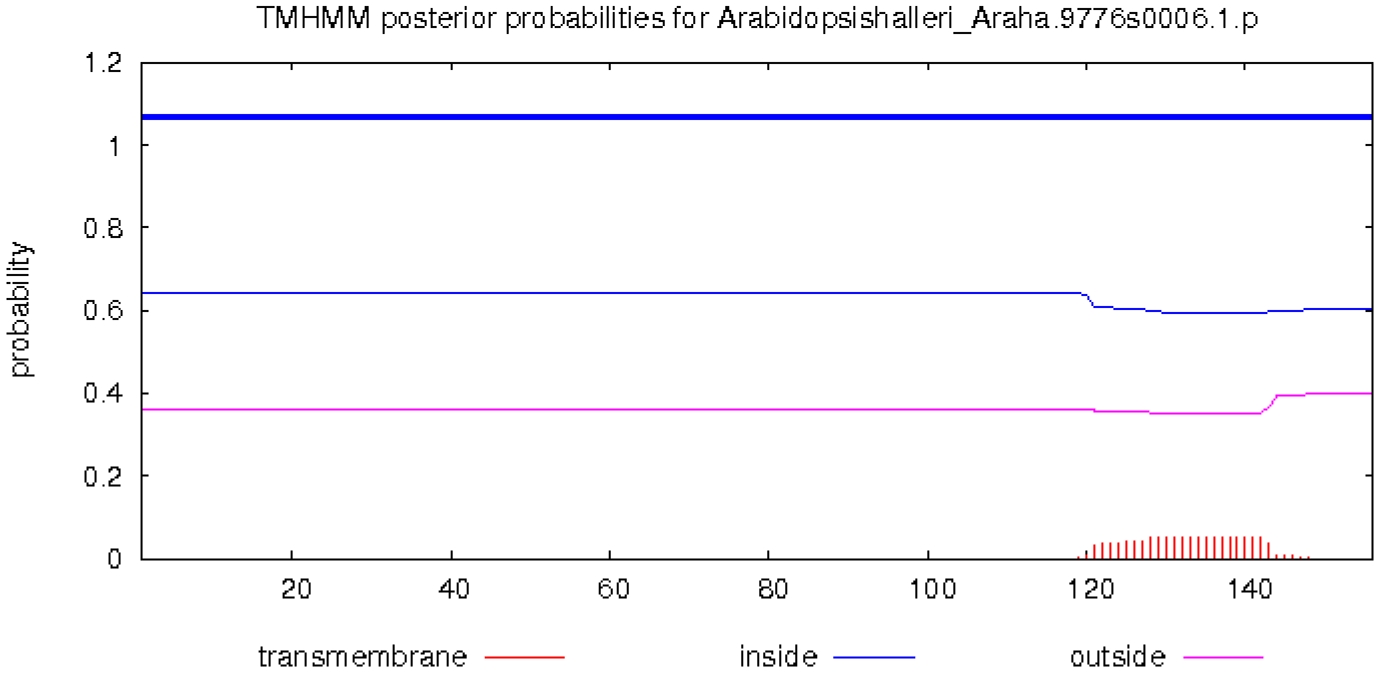

plot in postscript, script for making the plot in gnuplot, data for plot

---

```
# Arabidopsishalleri_Araha.9836s0005.1.p Length: 241
# Arabidopsishalleri_Araha.9836s0005.1.p Number of predicted TMHs:  0
# Arabidopsishalleri_Araha.9836s0005.1.p Exp number of AAs in TMHs: 0
# Arabidopsishalleri_Araha.9836s0005.1.p Exp number, first 60 AAs:  0
# Arabidopsishalleri_Araha.9836s0005.1.p Total prob of N-in:        0.03866
Arabidopsishalleri_Araha.9836s0005.1.p  TMHMM2.0        outside      1   241
```

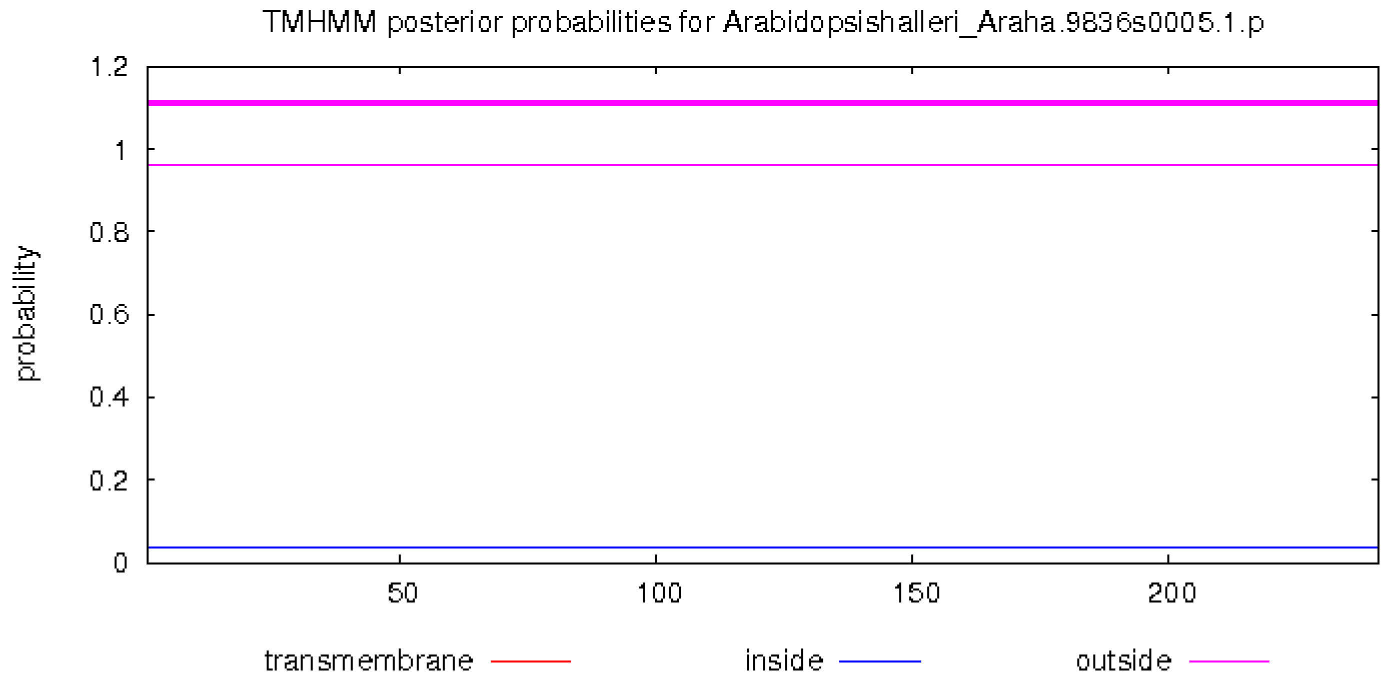

plot in postscript, script for making the plot in gnuplot, data for plot

---

```
# Arabidopsislyrata_311334 Length: 377
# Arabidopsislyrata_311334 Number of predicted TMHs:  0
# Arabidopsislyrata_311334 Exp number of AAs in TMHs: 0.0004
# Arabidopsislyrata_311334 Exp number, first 60 AAs:  0
# Arabidopsislyrata_311334 Total prob of N-in:        0.00584
Arabidopsislyrata_311334        TMHMM2.0        outside      1   377
```

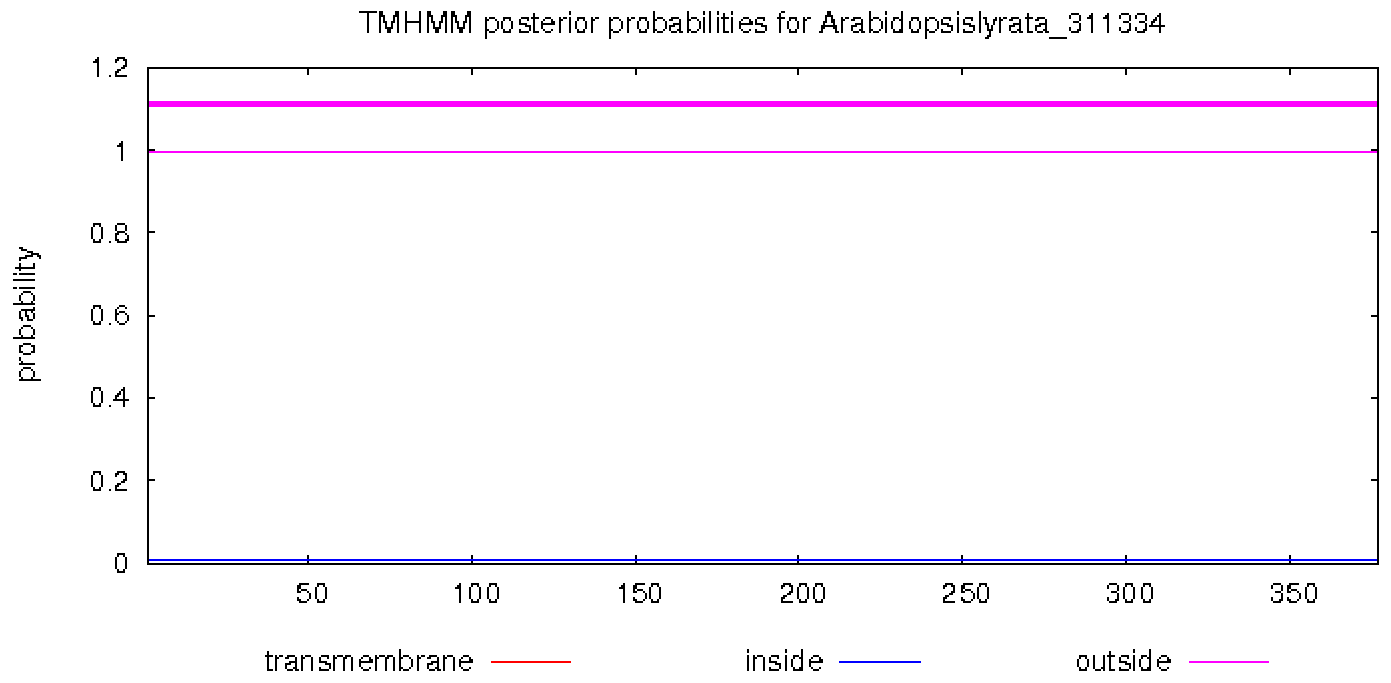

plot in postscript, script for making the plot in gnuplot, data for plot

---

```
# Arabidopsislyrata_311447 Length: 271
# Arabidopsislyrata_311447 Number of predicted TMHs:  0
# Arabidopsislyrata_311447 Exp number of AAs in TMHs: 0.000900000000000000001
# Arabidopsislyrata_311447 Exp number, first 60 AAs:  0.000750000000000000001
# Arabidopsislyrata_311447 Total prob of N-in:        0.02251
Arabidopsislyrata_311447        TMHMM2.0        outside      1   271
```

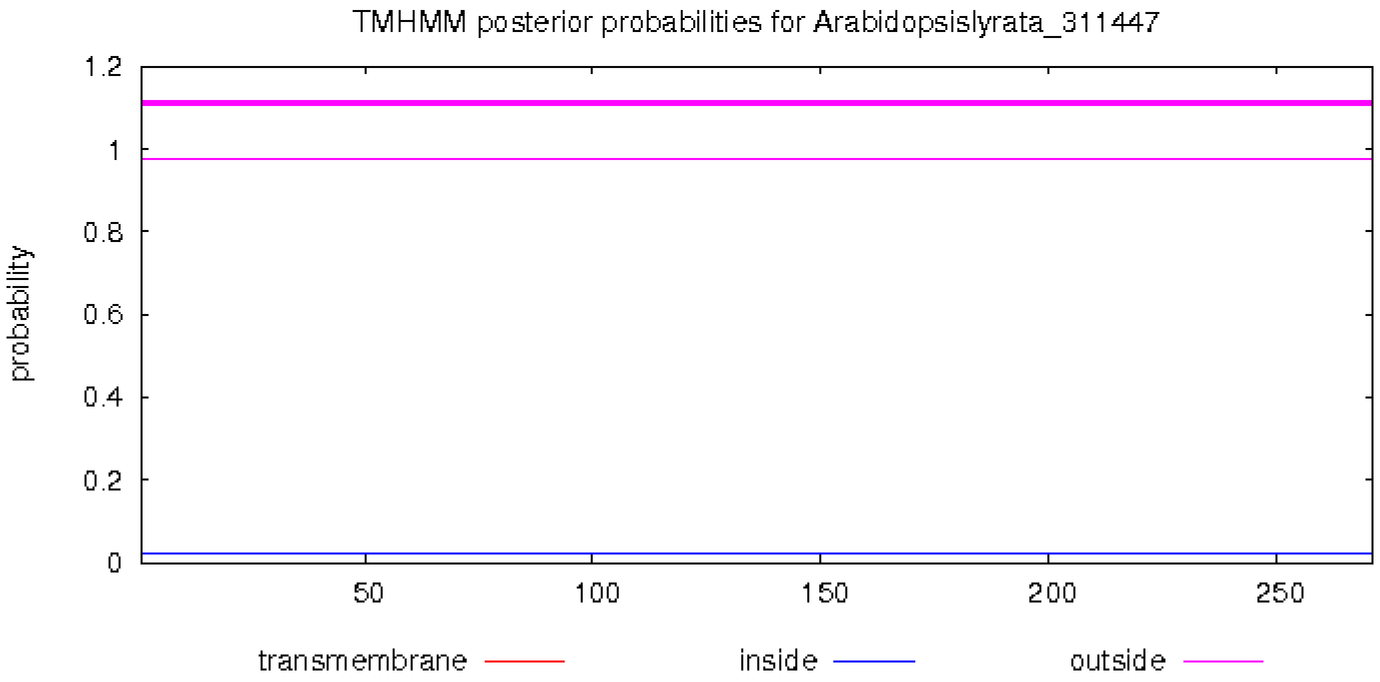

plot in postscript, script for making the plot in gnuplot, data for plot

---

```
# Arabidopsislyrata_313821 Length: 281
# Arabidopsislyrata_313821 Number of predicted TMHs:  0
# Arabidopsislyrata_313821 Exp number of AAs in TMHs: 0
# Arabidopsislyrata_313821 Exp number, first 60 AAs:  0
# Arabidopsislyrata_313821 Total prob of N-in:        0.00252
Arabidopsislyrata_313821        TMHMM2.0        outside      1   281
```

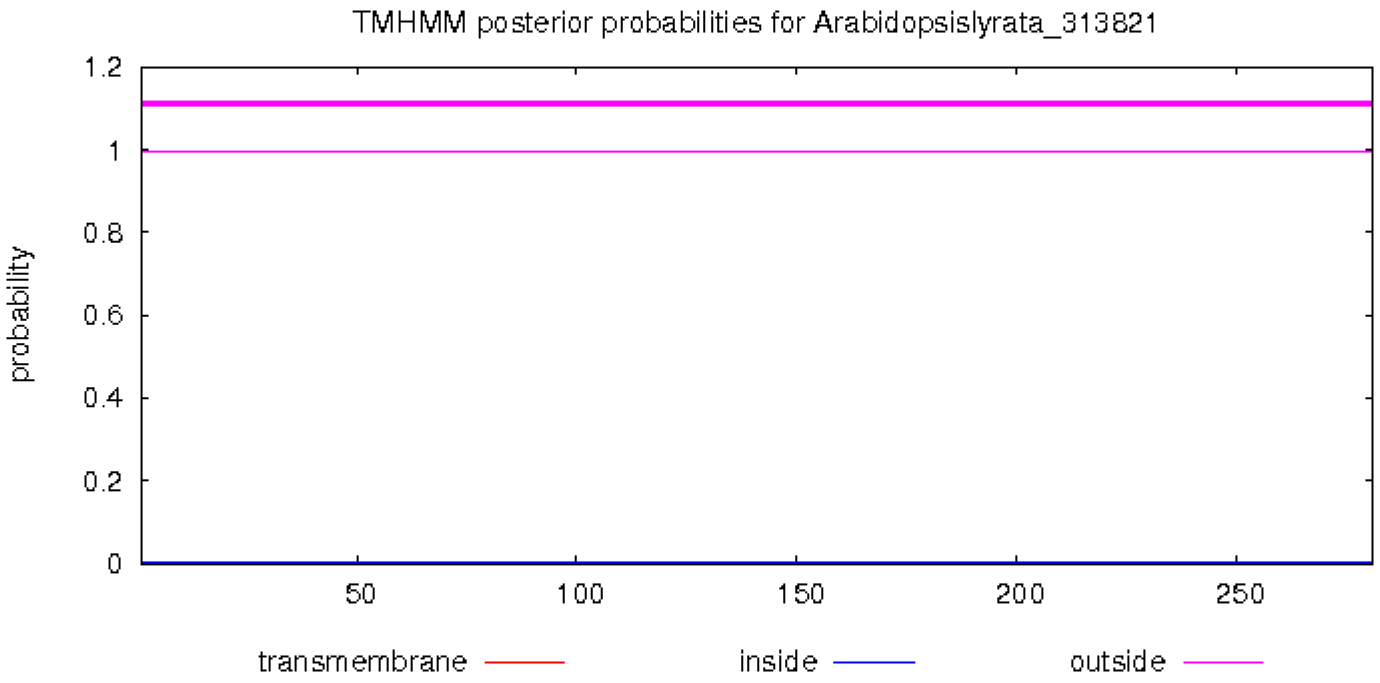

plot in postscript, script for making the plot in gnuplot, data for plot

---

```
# Arabidopsislyrata_313839 Length: 363
# Arabidopsislyrata_313839 Number of predicted TMHs:  0
# Arabidopsislyrata_313839 Exp number of AAs in TMHs: 0.00021
# Arabidopsislyrata_313839 Exp number, first 60 AAs:  0
# Arabidopsislyrata_313839 Total prob of N-in:        0.01778
Arabidopsislyrata_313839        TMHMM2.0        outside      1   363
```

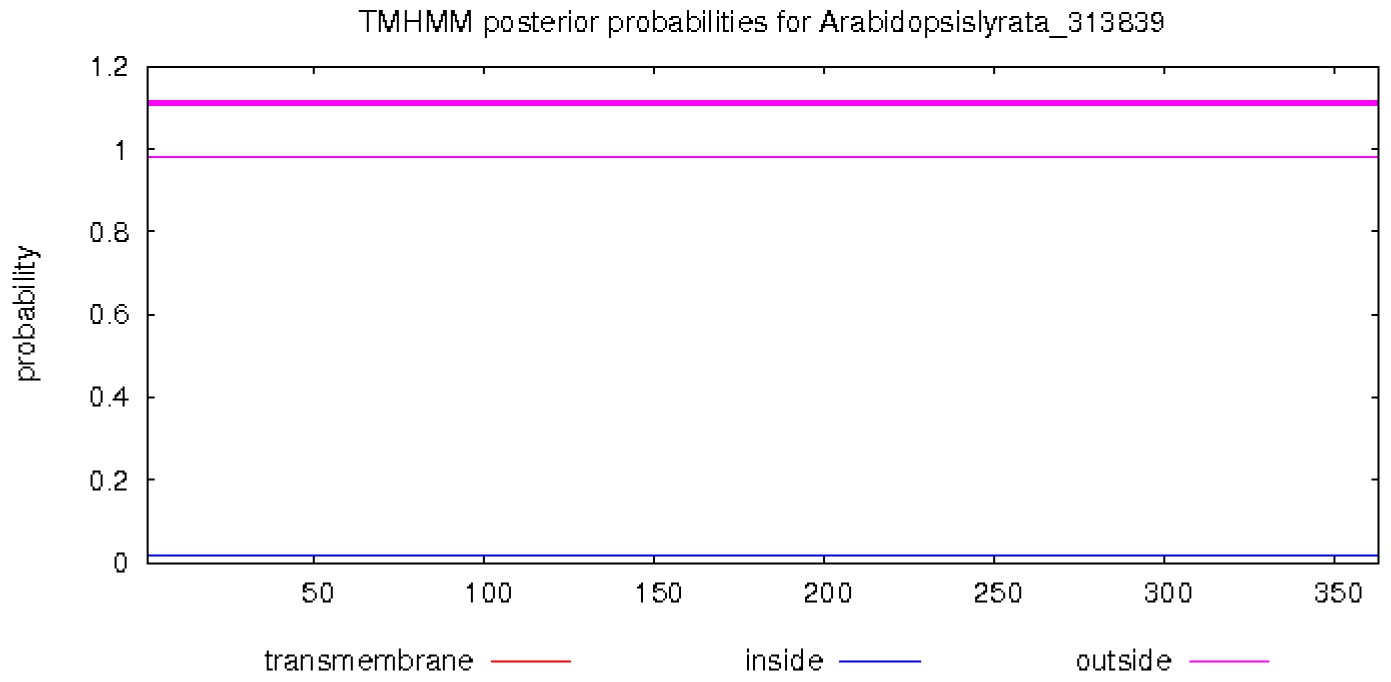

plot in postscript, script for making the plot in gnuplot, data for plot

```
# Arabidopsislyrata_313859 Length: 647
# Arabidopsislyrata_313859 Number of predicted TMHs:  1
# Arabidopsislyrata_313859 Exp number of AAs in TMHs: 19.45473
# Arabidopsislyrata_313859 Exp number, first 60 AAs:  0.0004
# Arabidopsislyrata_313859 Total prob of N-in:        0.03007
Arabidopsislyrata_313859        TMHMM2.0        outside      1   604
Arabidopsislyrata_313859        TMHMM2.0        TMhelix    605   624
Arabidopsislyrata_313859        TMHMM2.0        inside     625   647
```

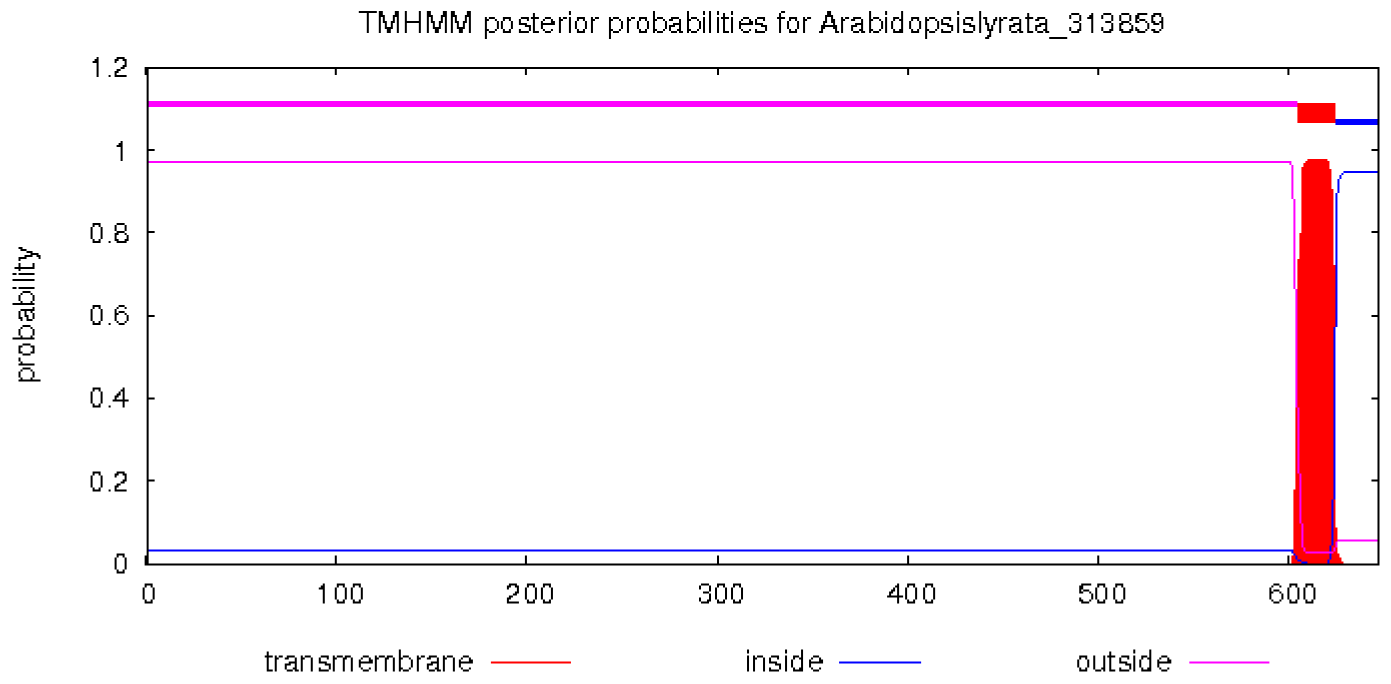

plot in postscript, script for making the plot in gnuplot, data for plot

```
# Arabidopsislyrata_315244 Length: 326
# Arabidopsislyrata_315244 Number of predicted TMHs:  0
# Arabidopsislyrata_315244 Exp number of AAs in TMHs: 0.00042
# Arabidopsislyrata_315244 Exp number, first 60 AAs:  0.00019
# Arabidopsislyrata_315244 Total prob of N-in:        0.01101
Arabidopsislyrata_315244	TMHMM2.0	outside	     1   326
```

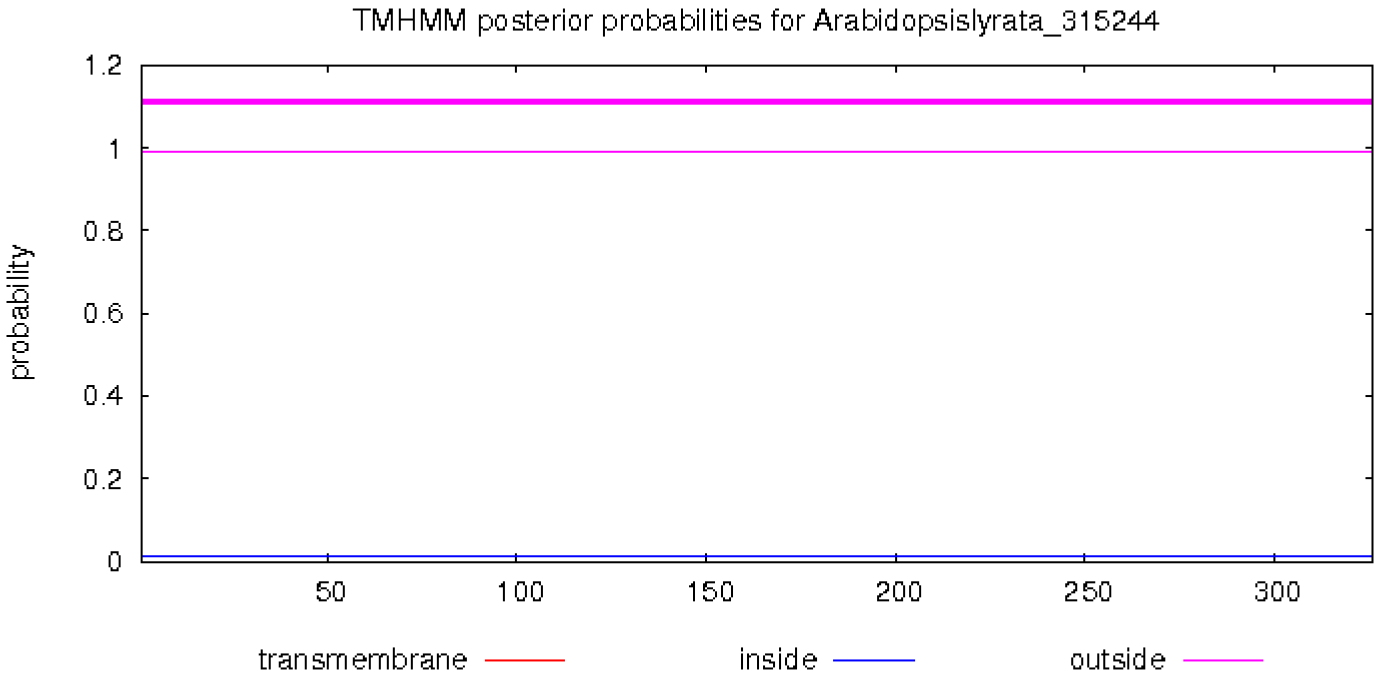

plot in postscript, script for making the plot in gnuplot, data for plot

```
# Arabidopsislyrata_316163 Length: 326
# Arabidopsislyrata_316163 Number of predicted TMHs:  0
# Arabidopsislyrata_316163 Exp number of AAs in TMHs: 0.00027
# Arabidopsislyrata_316163 Exp number, first 60 AAs:  0
# Arabidopsislyrata_316163 Total prob of N-in:        0.01706
Arabidopsislyrata_316163	TMHMM2.0	outside	     1   326
```

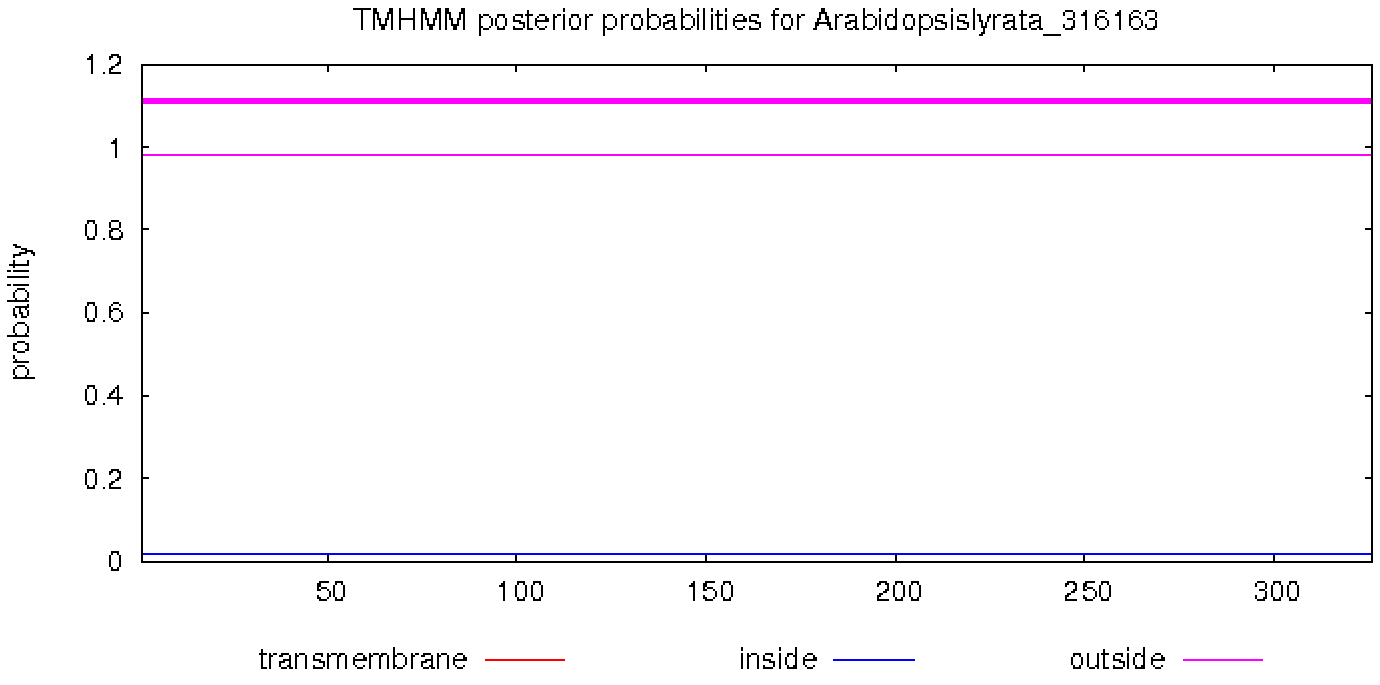

plot in postscript, script for making the plot in gnuplot, data for plot

```
# Arabidopsislyrata_317848 Length: 454
# Arabidopsislyrata_317848 Number of predicted TMHs:  0
# Arabidopsislyrata_317848 Exp number of AAs in TMHs: 0.05049
# Arabidopsislyrata_317848 Exp number, first 60 AAs:  0.03387
# Arabidopsislyrata_317848 Total prob of N-in:        0.00387
Arabidopsislyrata_317848	TMHMM2.0	outside	     1   454
```

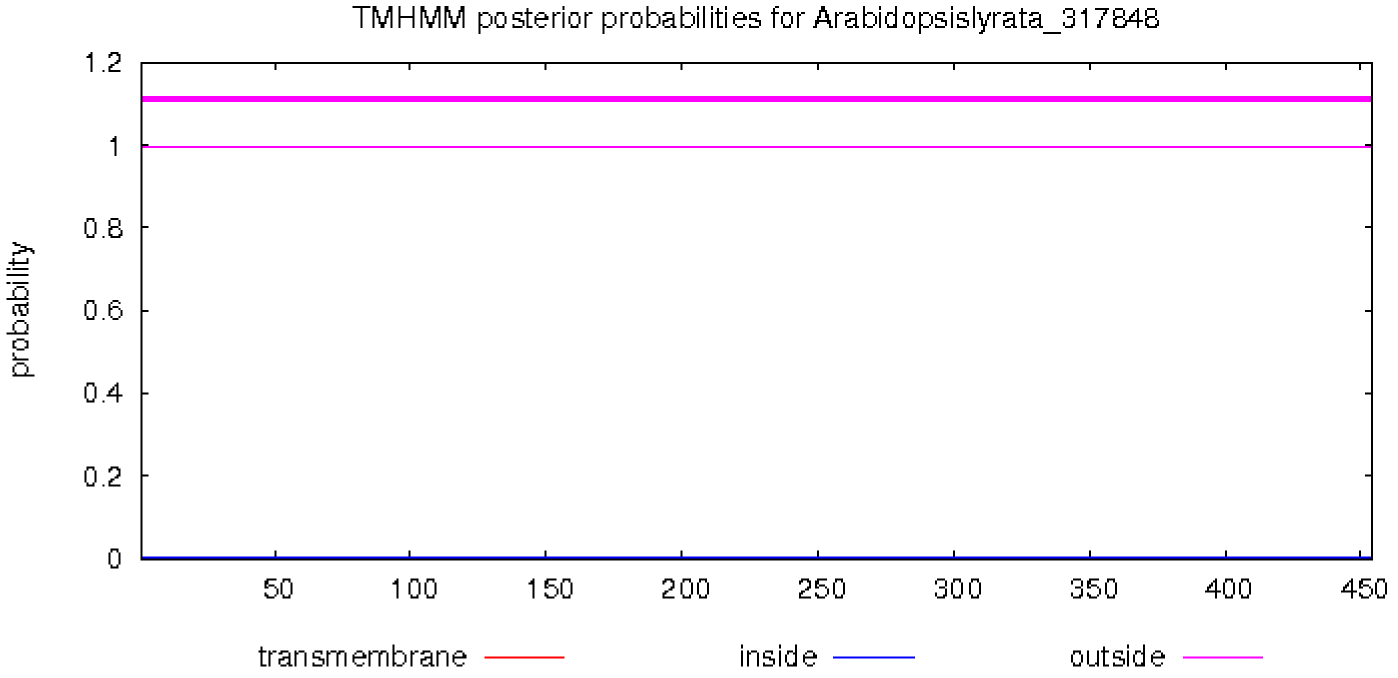

plot in postscript, script for making the plot in gnuplot, data for plot

---

```
# Arabidopsislyrata_324164 Length: 167
# Arabidopsislyrata_324164 Number of predicted TMHs:  0
# Arabidopsislyrata_324164 Exp number of AAs in TMHs: 0.00057
# Arabidopsislyrata_324164 Exp number, first 60 AAs:  0.00038
# Arabidopsislyrata_324164 Total prob of N-in:        0.05987
Arabidopsislyrata_324164	TMHMM2.0	outside	     1   167
```

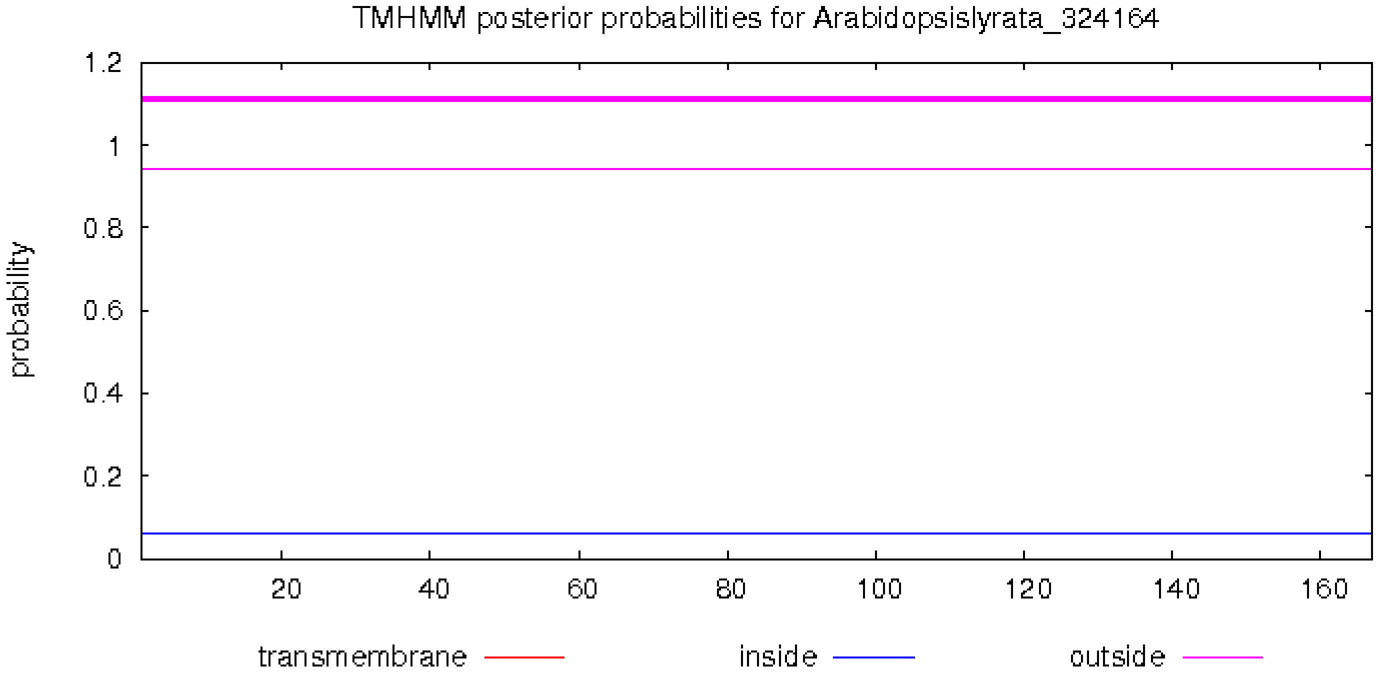

plot in postscript, script for making the plot in gnuplot, data for plot

---

```
# Arabidopsislyrata_325871 Length: 206
# Arabidopsislyrata_325871 Number of predicted TMHs:  0
# Arabidopsislyrata_325871 Exp number of AAs in TMHs: 1.47037
# Arabidopsislyrata_325871 Exp number, first 60 AAs:  1.13341
# Arabidopsislyrata_325871 Total prob of N-in:        0.21293
Arabidopsislyrata_325871	TMHMM2.0	outside	     1   206
```

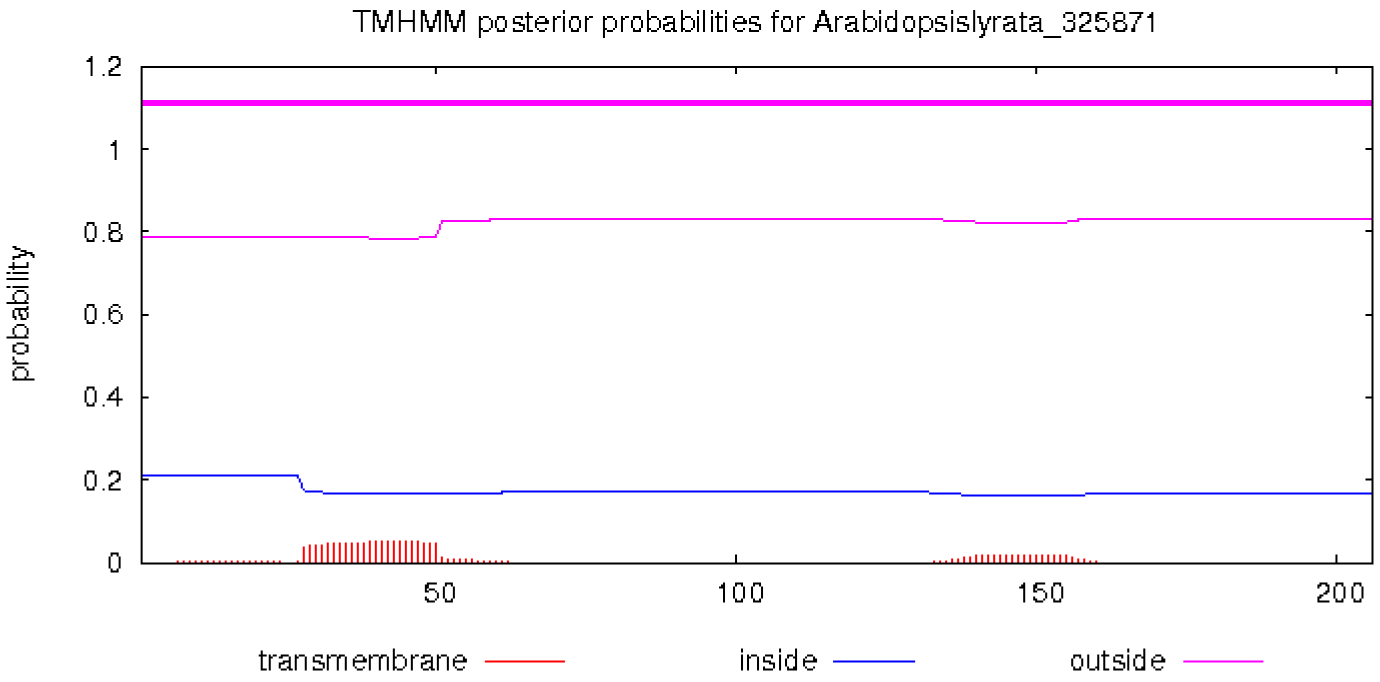

plot in postscript, script for making the plot in gnuplot, data for plot

---

```
# Arabidopsislyrata_326242 Length: 334
# Arabidopsislyrata_326242 Number of predicted TMHs:  0
# Arabidopsislyrata_326242 Exp number of AAs in TMHs: 0
# Arabidopsislyrata_326242 Exp number, first 60 AAs:  0
# Arabidopsislyrata_326242 Total prob of N-in:        0.00862
Arabidopsislyrata_326242	TMHMM2.0	outside	     1   334
```

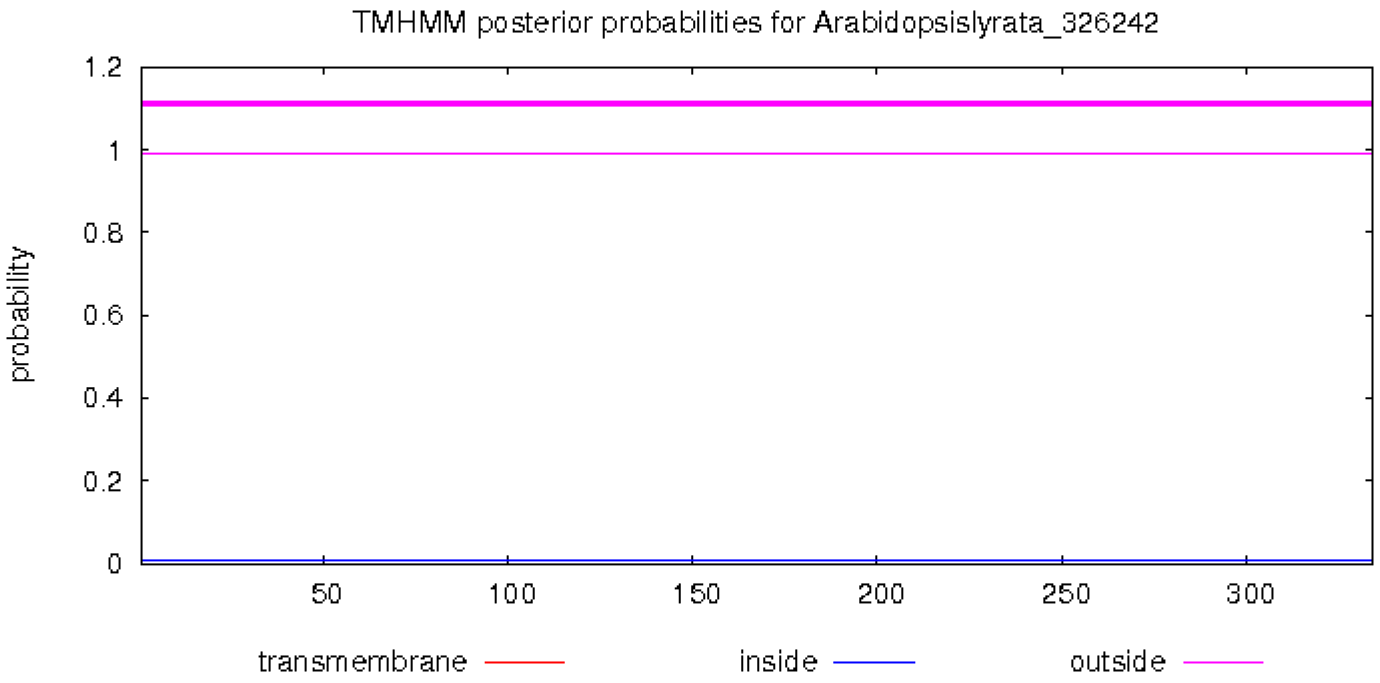

plot in postscript, script for making the plot in gnuplot, data for plot

---

```
# Arabidopsislyrata_326748 Length: 461
# Arabidopsislyrata_326748 Number of predicted TMHs:  0
# Arabidopsislyrata_326748 Exp number of AAs in TMHs: 11.6196
# Arabidopsislyrata_326748 Exp number, first 60 AAs:  0.00367
# Arabidopsislyrata_326748 Total prob of N-in:        0.01108
Arabidopsislyrata_326748	TMHMM2.0	outside	     1   461
```

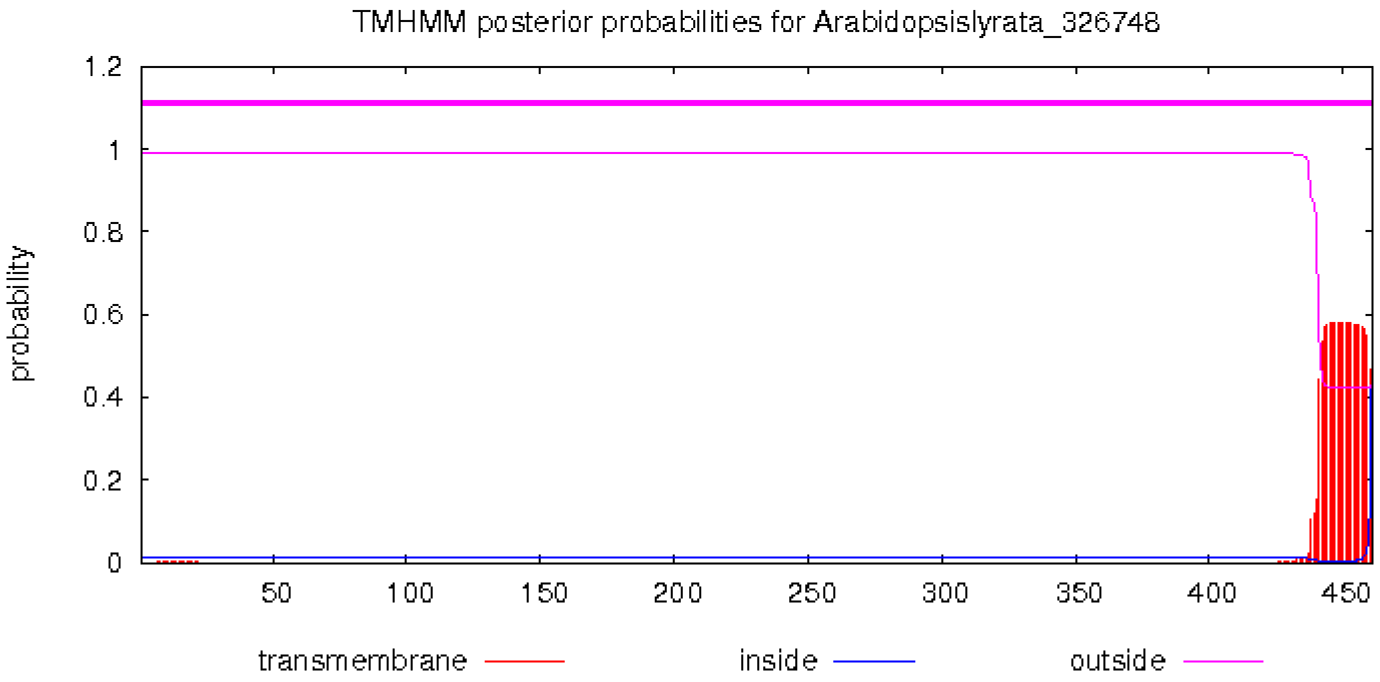

plot in postscript, script for making the plot in gnuplot, data for plot

---

```
# Arabidopsislyrata_327634 Length: 411
# Arabidopsislyrata_327634 Number of predicted TMHs:  0
# Arabidopsislyrata_327634 Exp number of AAs in TMHs: 0.00037
# Arabidopsislyrata_327634 Exp number, first 60 AAs:  0.00037
# Arabidopsislyrata_327634 Total prob of N-in:        0.00850
Arabidopsislyrata_327634	TMHMM2.0	outside	     1   411
```

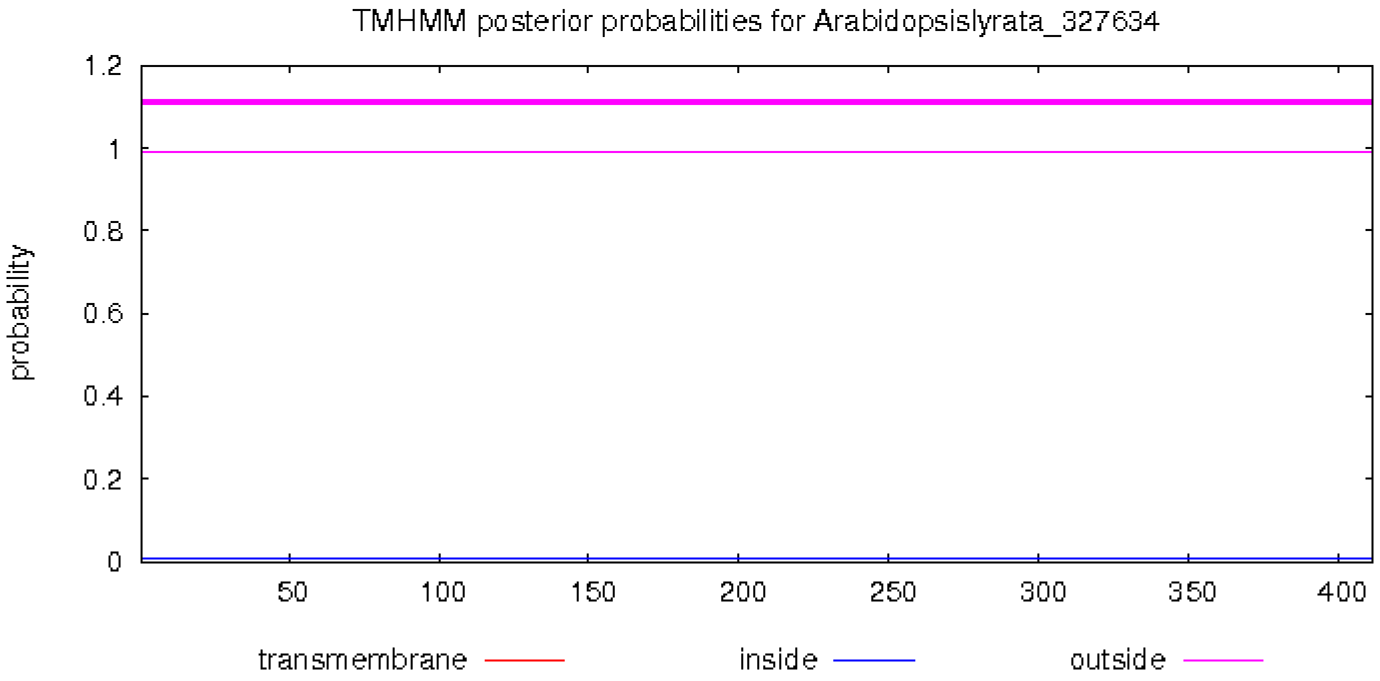

plot in postscript, script for making the plot in gnuplot, data for plot

---

```
# Arabidopsislyrata_328786 Length: 497
# Arabidopsislyrata_328786 Number of predicted TMHs:  0
# Arabidopsislyrata_328786 Exp number of AAs in TMHs: 5.29975
# Arabidopsislyrata_328786 Exp number, first 60 AAs:  0
# Arabidopsislyrata_328786 Total prob of N-in:        0.19077
Arabidopsislyrata_328786        TMHMM2.0        outside      1   497
```

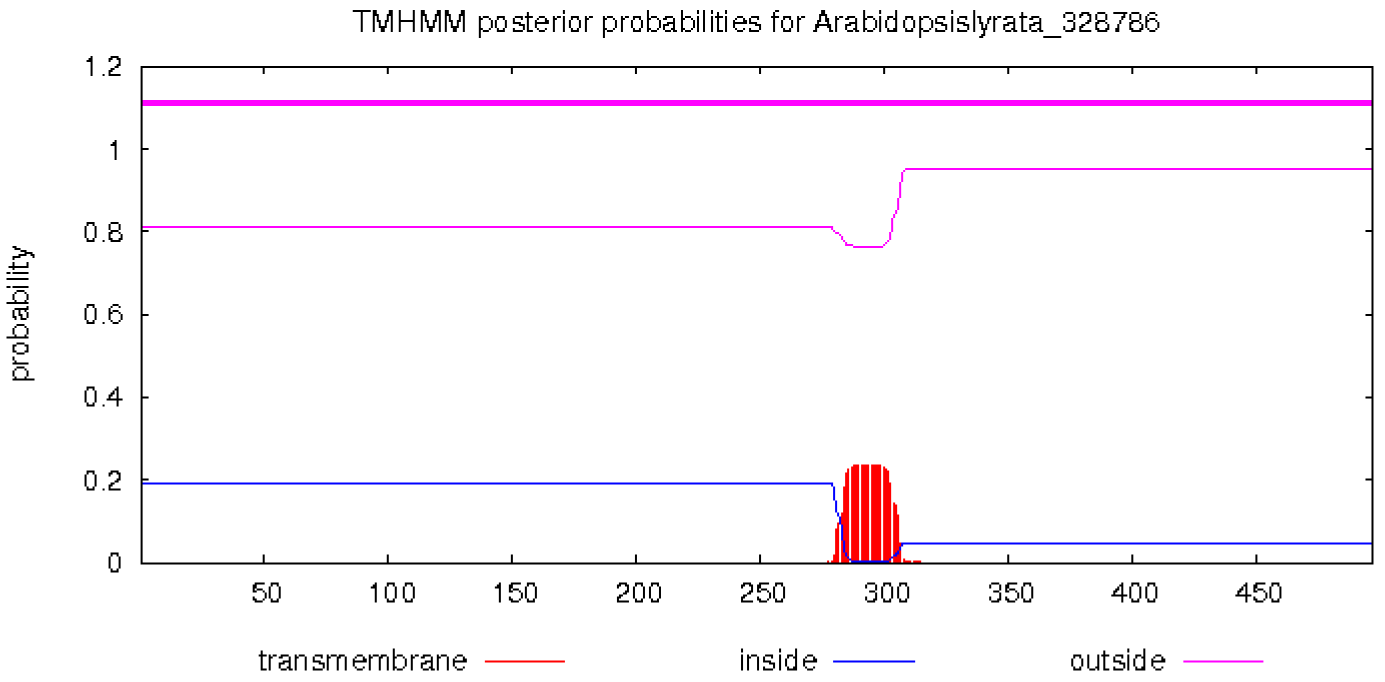

plot in postscript, script for making the plot in gnuplot, data for plot

---

```
# Arabidopsislyrata_332471 Length: 349
# Arabidopsislyrata_332471 Number of predicted TMHs:  0
# Arabidopsislyrata_332471 Exp number of AAs in TMHs: 0.00149
# Arabidopsislyrata_332471 Exp number, first 60 AAs:  0
# Arabidopsislyrata_332471 Total prob of N-in:        0.00195
Arabidopsislyrata_332471        TMHMM2.0        outside      1   349
```

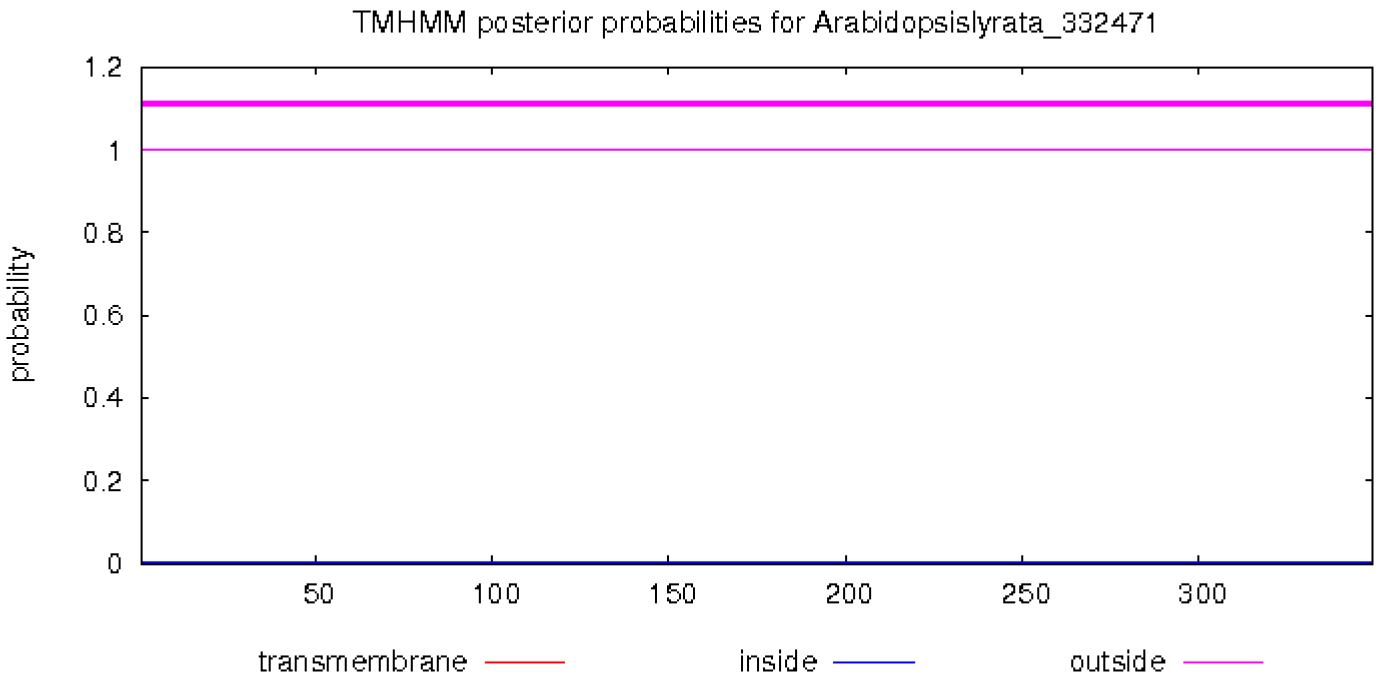

plot in postscript, script for making the plot in gnuplot, data for plot

---

```
# Arabidopsislyrata_333554 Length: 465
# Arabidopsislyrata_333554 Number of predicted TMHs:  1
# Arabidopsislyrata_333554 Exp number of AAs in TMHs: 20.03779
# Arabidopsislyrata_333554 Exp number, first 60 AAs:  0.01072
# Arabidopsislyrata_333554 Total prob of N-in:        0.37090
Arabidopsislyrata_333554        TMHMM2.0        outside      1   446
Arabidopsislyrata_333554        TMHMM2.0        TMhelix    447   464
Arabidopsislyrata_333554        TMHMM2.0        inside     465   465
```

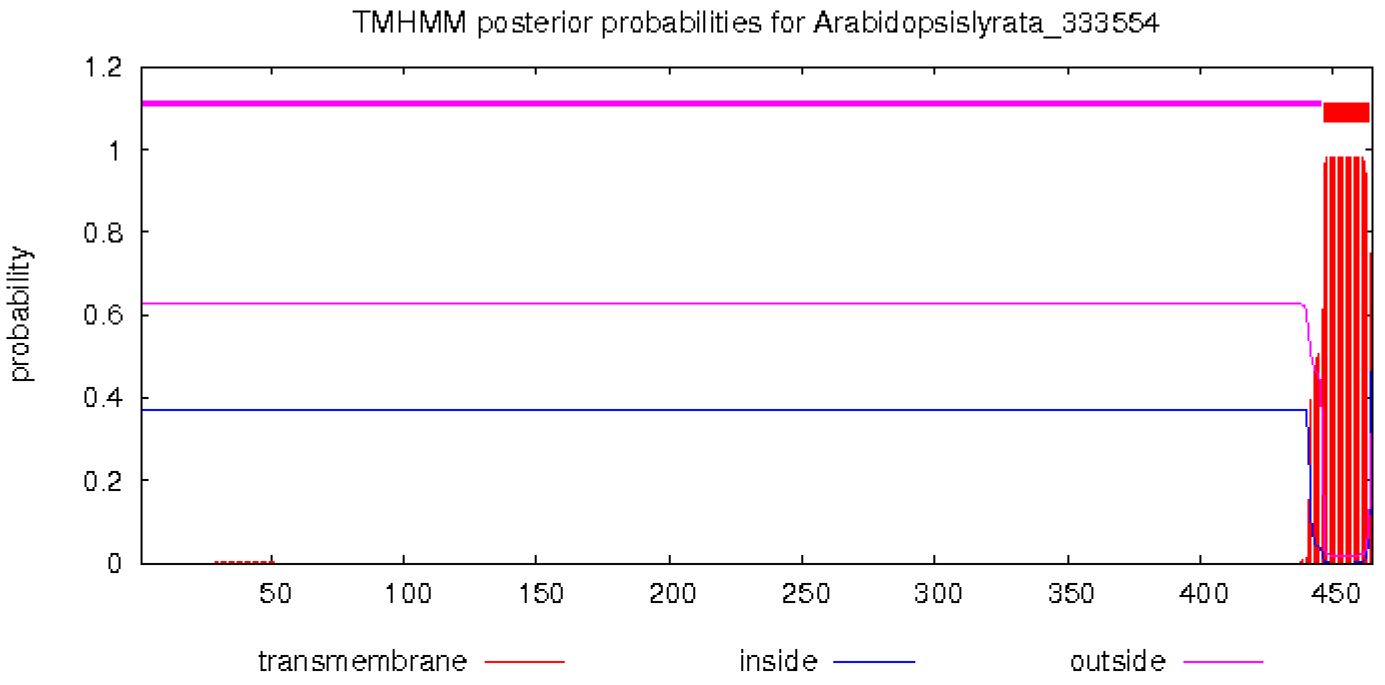

plot in postscript, script for making the plot in gnuplot, data for plot

```
# Arabidopsislyrata_333558 Length: 216
# Arabidopsislyrata_333558 Number of predicted TMHs:  0
# Arabidopsislyrata_333558 Exp number of AAs in TMHs: 0.00103
# Arabidopsislyrata_333558 Exp number, first 60 AAs:  0
# Arabidopsislyrata_333558 Total prob of N-in:        0.04406
Arabidopsislyrata_333558        TMHMM2.0        outside      1   216
```

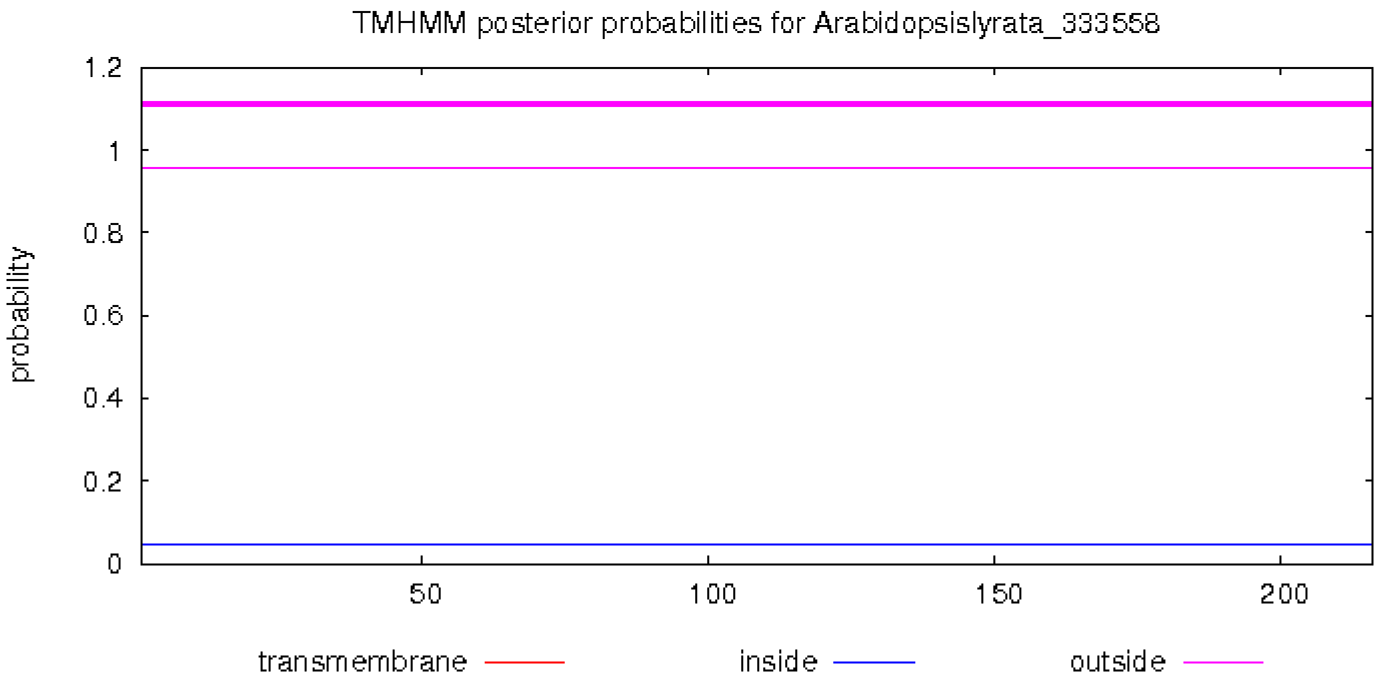

plot in postscript, script for making the plot in gnuplot, data for plot

```
# Arabidopsislyrata_335709 Length: 416
# Arabidopsislyrata_335709 Number of predicted TMHs:  0
# Arabidopsislyrata_335709 Exp number of AAs in TMHs: 0.00017
# Arabidopsislyrata_335709 Exp number, first 60 AAs:  0
# Arabidopsislyrata_335709 Total prob of N-in:        0.01181
Arabidopsislyrata_335709        TMHMM2.0        outside      1   416
```

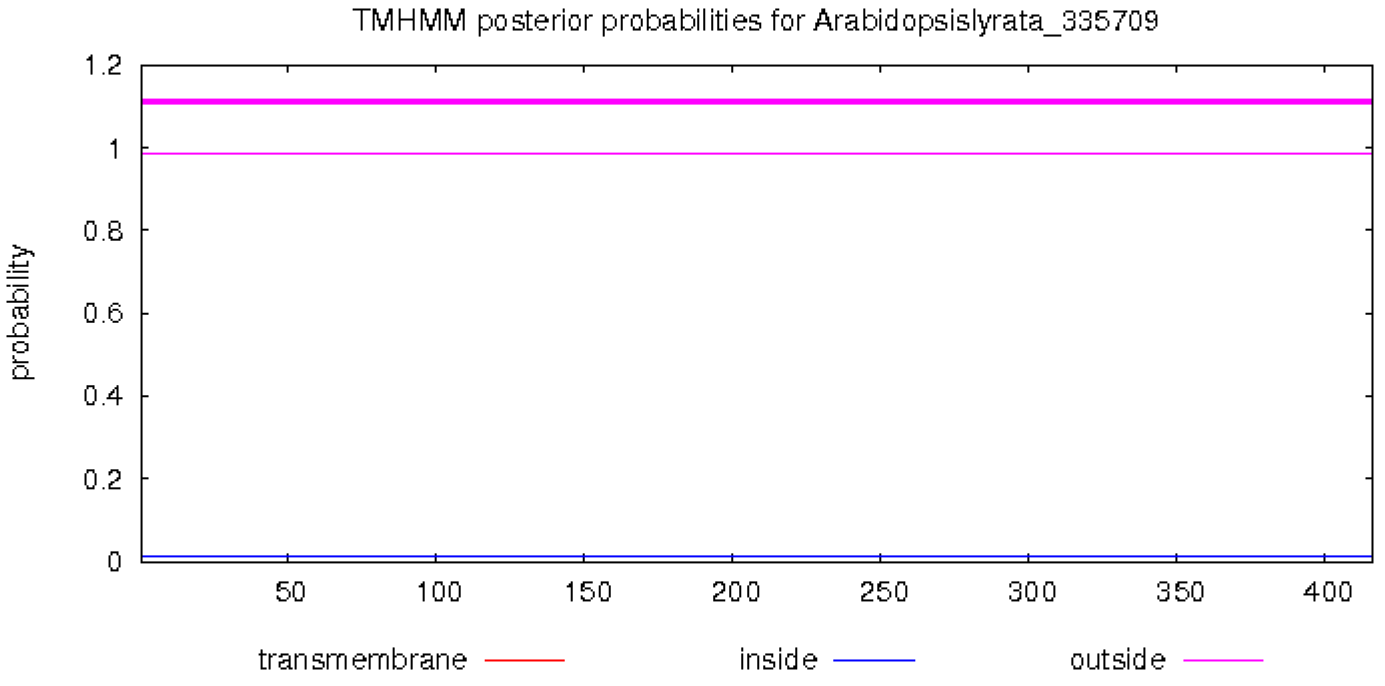

plot in postscript, script for making the plot in gnuplot, data for plot

```
# Arabidopsislyrata_336483 Length: 547
# Arabidopsislyrata_336483 Number of predicted TMHs:  1
# Arabidopsislyrata_336483 Exp number of AAs in TMHs: 21.46181
# Arabidopsislyrata_336483 Exp number, first 60 AAs:  0.0075
# Arabidopsislyrata_336483 Total prob of N-in:        0.10032
Arabidopsislyrata_336483        TMHMM2.0        outside      1   517
Arabidopsislyrata_336483        TMHMM2.0        TMhelix    518   540
Arabidopsislyrata_336483        TMHMM2.0        inside     541   547
```

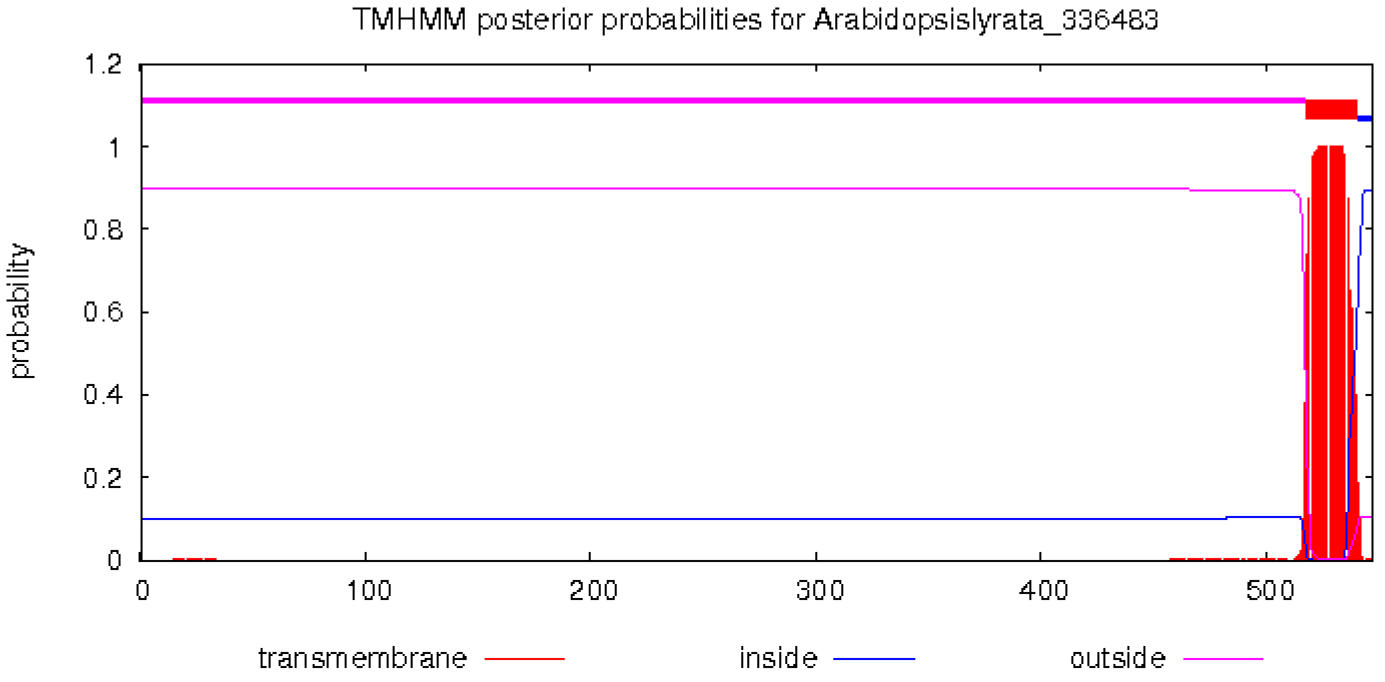

plot in postscript, script for making the plot in gnuplot, data for plot

```
# Arabidopsislyrata_338342 Length: 631
# Arabidopsislyrata_338342 Number of predicted TMHs:  0
# Arabidopsislyrata_338342 Exp number of AAs in TMHs: 0.00714
# Arabidopsislyrata_338342 Exp number, first 60 AAs:  0
# Arabidopsislyrata_338342 Total prob of N-in:        0.00168
Arabidopsislyrata_338342        TMHMM2.0        outside      1   631
```

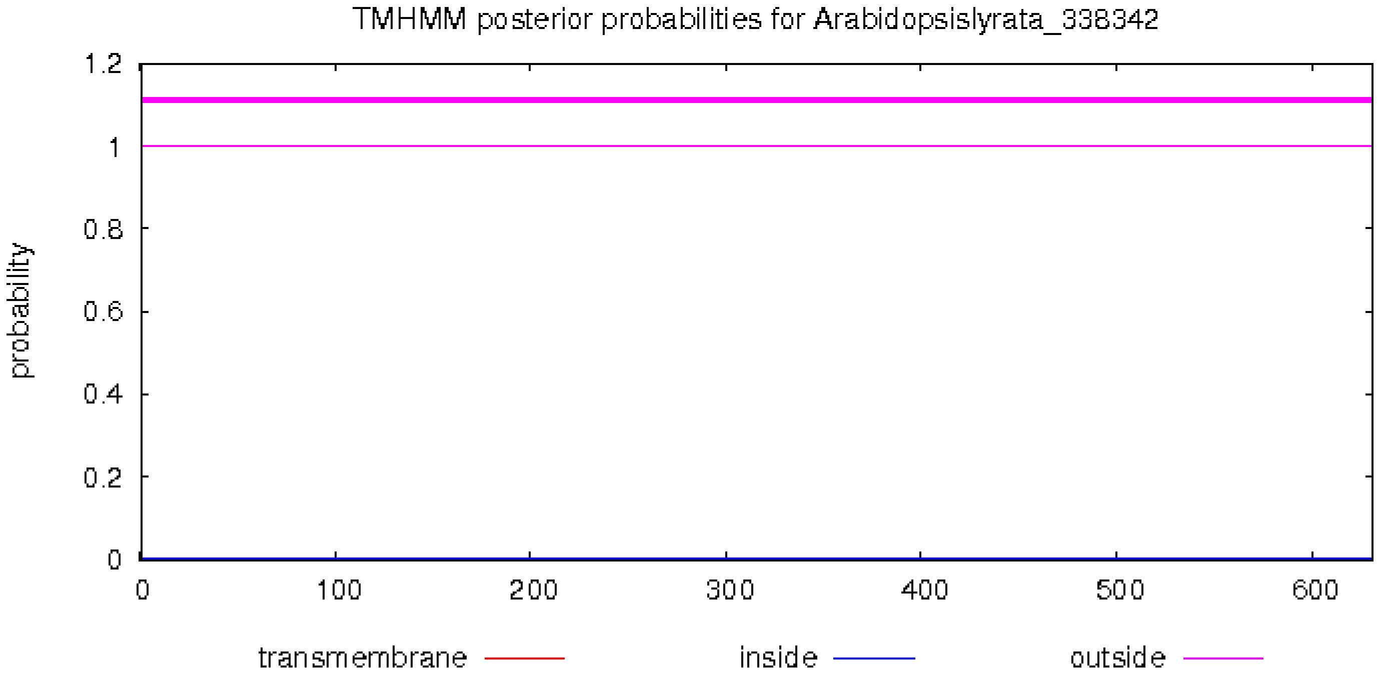

plot in postscript, script for making the plot in gnuplot, data for plot

```
# Arabidopsislyrata_340623 Length: 463
# Arabidopsislyrata_340623 Number of predicted TMHs:  1
# Arabidopsislyrata_340623 Exp number of AAs in TMHs: 22.28898
# Arabidopsislyrata_340623 Exp number, first 60 AAs:  0.00033
# Arabidopsislyrata_340623 Total prob of N-in:        0.21320
Arabidopsislyrata_340623        TMHMM2.0        outside      1   439
Arabidopsislyrata_340623        TMHMM2.0        TMhelix    440   462
Arabidopsislyrata_340623        TMHMM2.0        inside     463   463
```

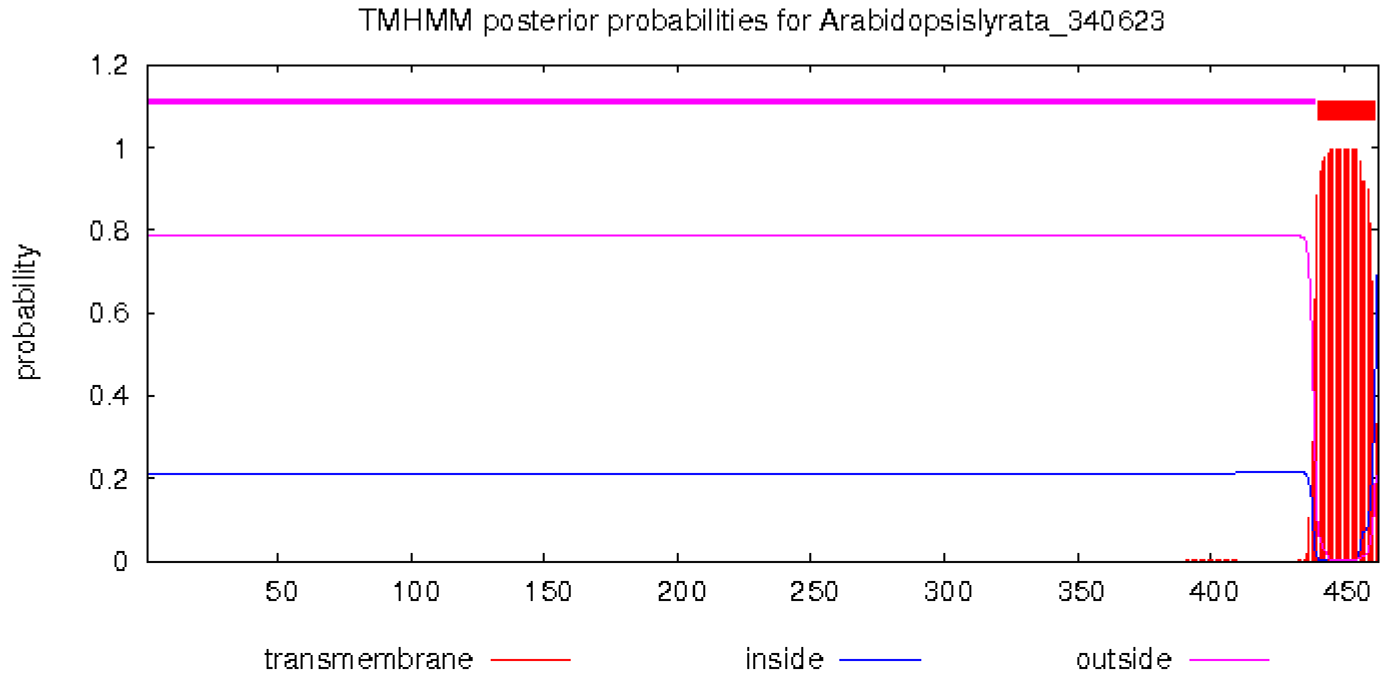

plot in postscript, script for making the plot in gnuplot, data for plot

---

```
# Arabidopsislyrata_344010 Length: 319
# Arabidopsislyrata_344010 Number of predicted TMHs:  0
# Arabidopsislyrata_344010 Exp number of AAs in TMHs: 0.02926
# Arabidopsislyrata_344010 Exp number, first 60 AAs:  0
# Arabidopsislyrata_344010 Total prob of N-in:        0.07202
Arabidopsislyrata_344010        TMHMM2.0        outside      1   319
```

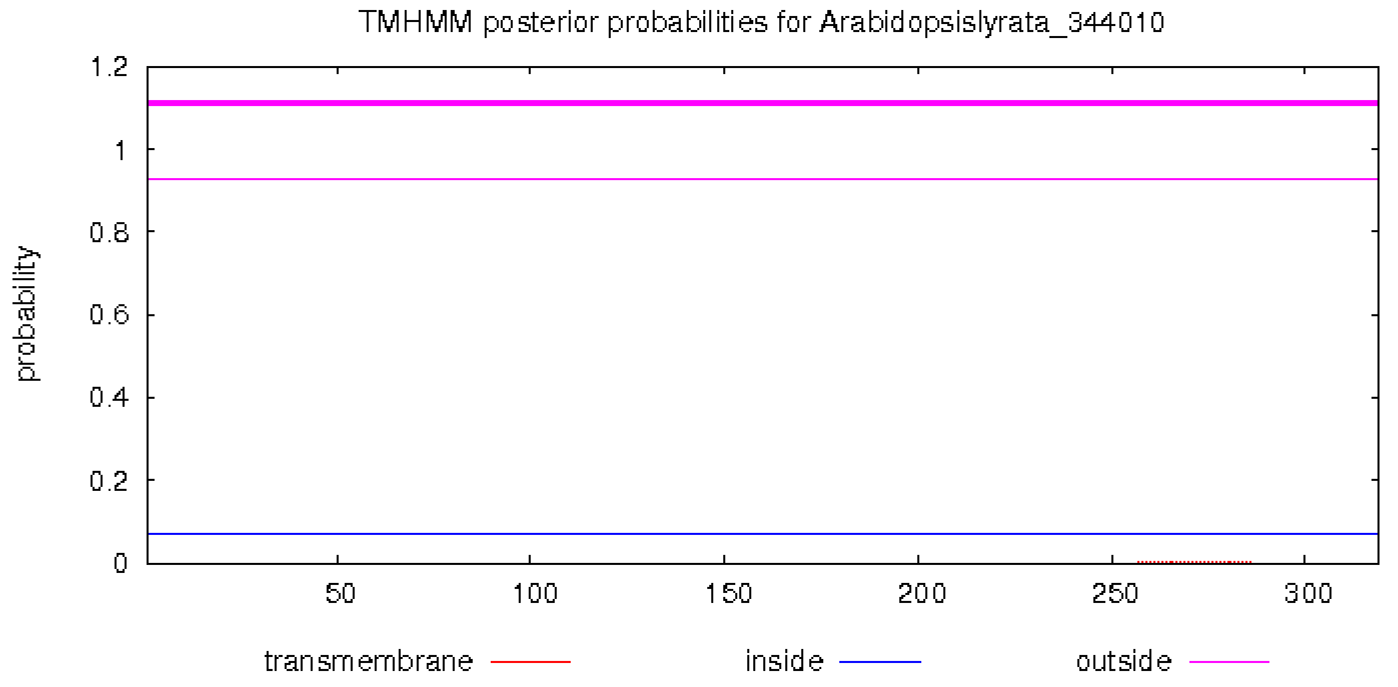

plot in postscript, script for making the plot in gnuplot, data for plot

---

```
# Arabidopsislyrata_347510 Length: 331
# Arabidopsislyrata_347510 Number of predicted TMHs:  1
# Arabidopsislyrata_347510 Exp number of AAs in TMHs: 18.48051
# Arabidopsislyrata_347510 Exp number, first 60 AAs:  0.10228
# Arabidopsislyrata_347510 Total prob of N-in:        0.28495
Arabidopsislyrata_347510        TMHMM2.0        outside      1   312
```

```
Arabidopsislyrata_347510	TMHMM2.0	TMhelix	313	330
Arabidopsislyrata_347510	TMHMM2.0	inside	331	331
```

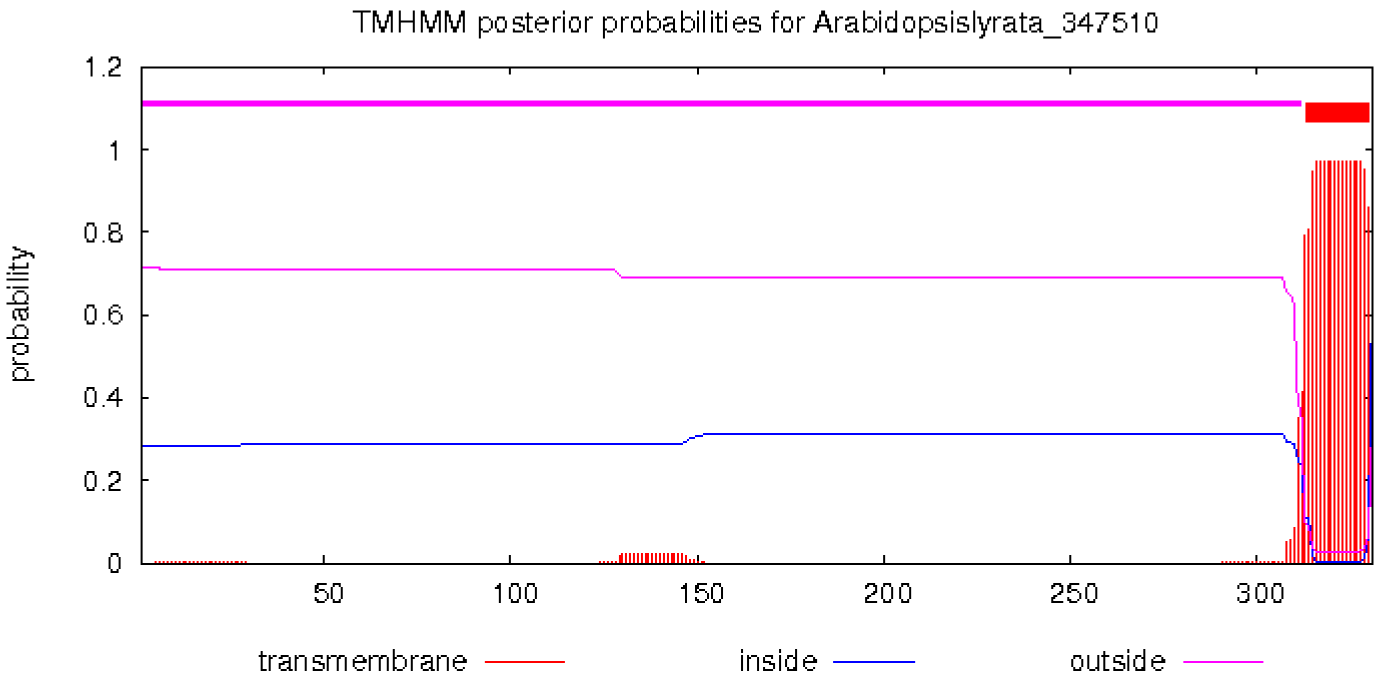

plot in postscript, script for making the plot in gnuplot, data for plot

---

```
# Arabidopsislyrata_347836 Length: 535
# Arabidopsislyrata_347836 Number of predicted TMHs:  0
# Arabidopsislyrata_347836 Exp number of AAs in TMHs: 0.00562
# Arabidopsislyrata_347836 Exp number, first 60 AAs:  0.00046
# Arabidopsislyrata_347836 Total prob of N-in:        0.00062
Arabidopsislyrata_347836	TMHMM2.0	outside	1	535
```

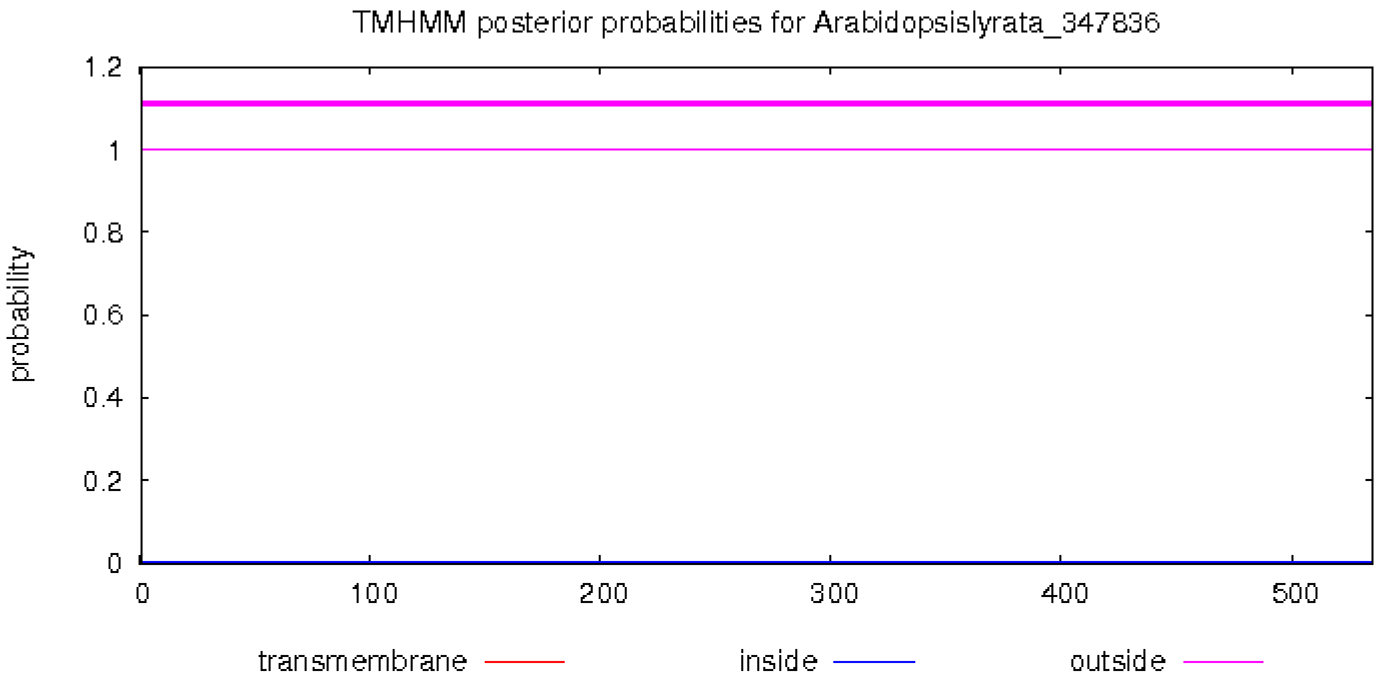

plot in postscript, script for making the plot in gnuplot, data for plot

---

```
# Arabidopsislyrata_352687 Length: 525
# Arabidopsislyrata_352687 Number of predicted TMHs:  1
# Arabidopsislyrata_352687 Exp number of AAs in TMHs: 20.81442
# Arabidopsislyrata_352687 Exp number, first 60 AAs:  0.00018
```

```
# Arabidopsislyrata_352687 Total prob of N-in:        0.06024
Arabidopsislyrata_352687        TMHMM2.0        outside      1   501
Arabidopsislyrata_352687        TMHMM2.0        TMhelix    502   524
Arabidopsislyrata_352687        TMHMM2.0        inside     525   525
```

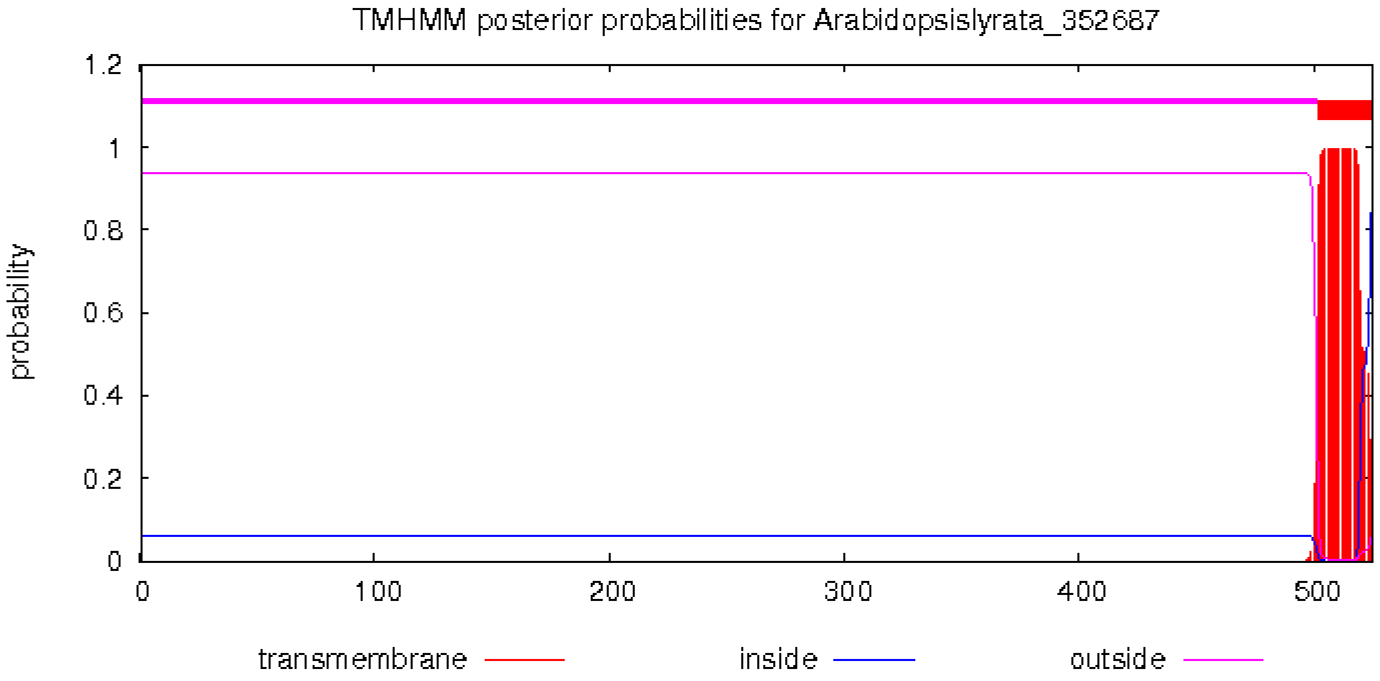

plot in postscript, script for making the plot in gnuplot, data for plot

---

```
# Arabidopsislyrata_355644 Length: 328
# Arabidopsislyrata_355644 Number of predicted TMHs:  0
# Arabidopsislyrata_355644 Exp number of AAs in TMHs: 0.00197
# Arabidopsislyrata_355644 Exp number, first 60 AAs:  0.00054
# Arabidopsislyrata_355644 Total prob of N-in:        0.02885
Arabidopsislyrata_355644        TMHMM2.0        outside      1   328
```

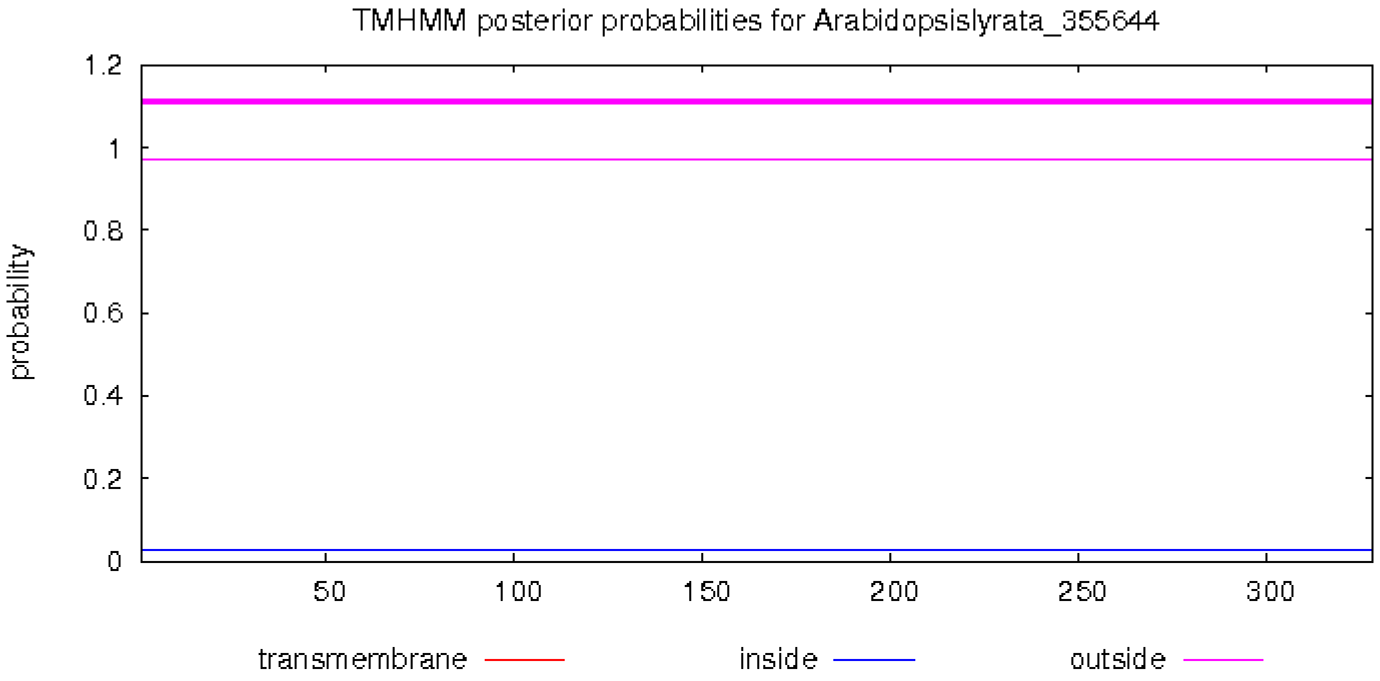

plot in postscript, script for making the plot in gnuplot, data for plot

---

```
# Arabidopsislyrata_357570 Length: 333
# Arabidopsislyrata_357570 Number of predicted TMHs:  0
```

```
# Arabidopsislyrata_357570 Exp number of AAs in TMHs: 0.00019
# Arabidopsislyrata_357570 Exp number, first 60 AAs:  0
# Arabidopsislyrata_357570 Total prob of N-in:        0.04374
Arabidopsislyrata_357570        TMHMM2.0        outside      1   333
```

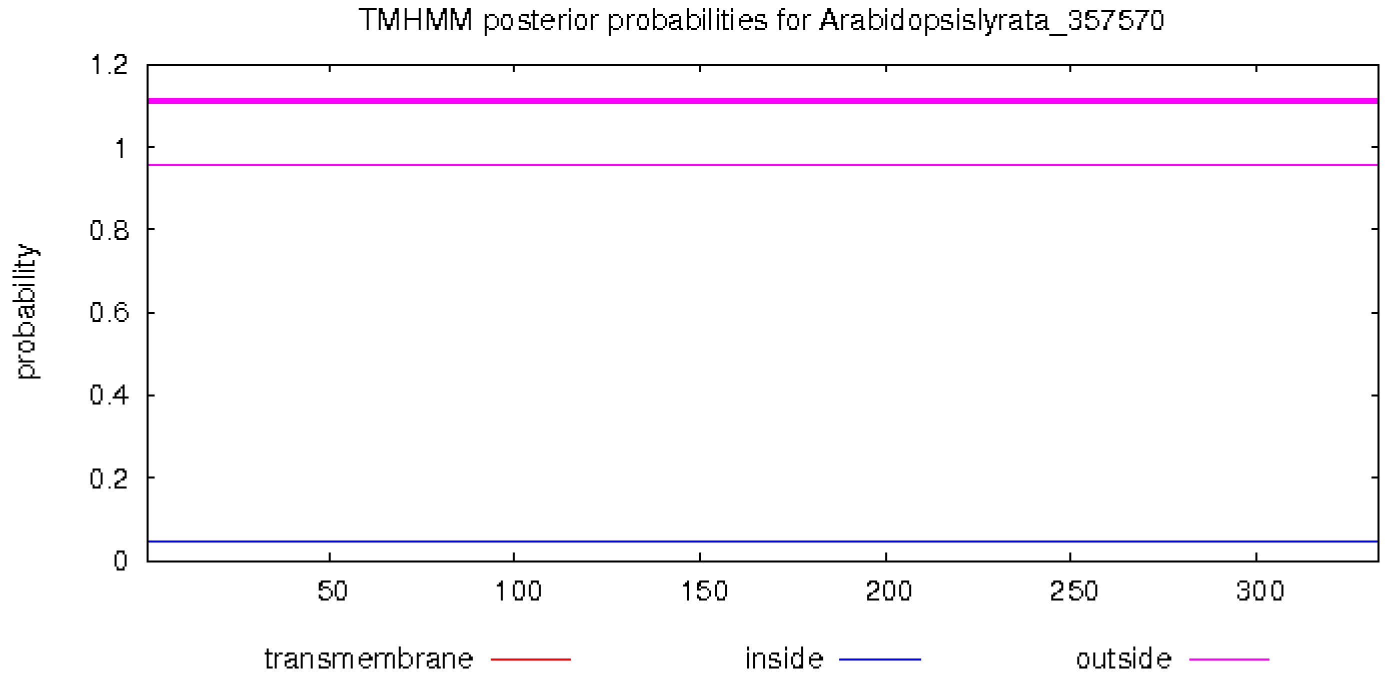

plot in postscript, script for making the plot in gnuplot, data for plot

---

```
# Arabidopsislyrata_357630 Length: 184
# Arabidopsislyrata_357630 Number of predicted TMHs:  0
# Arabidopsislyrata_357630 Exp number of AAs in TMHs: 0.0033
# Arabidopsislyrata_357630 Exp number, first 60 AAs:  0.0023
# Arabidopsislyrata_357630 Total prob of N-in:        0.06953
Arabidopsislyrata_357630        TMHMM2.0        outside      1   184
```

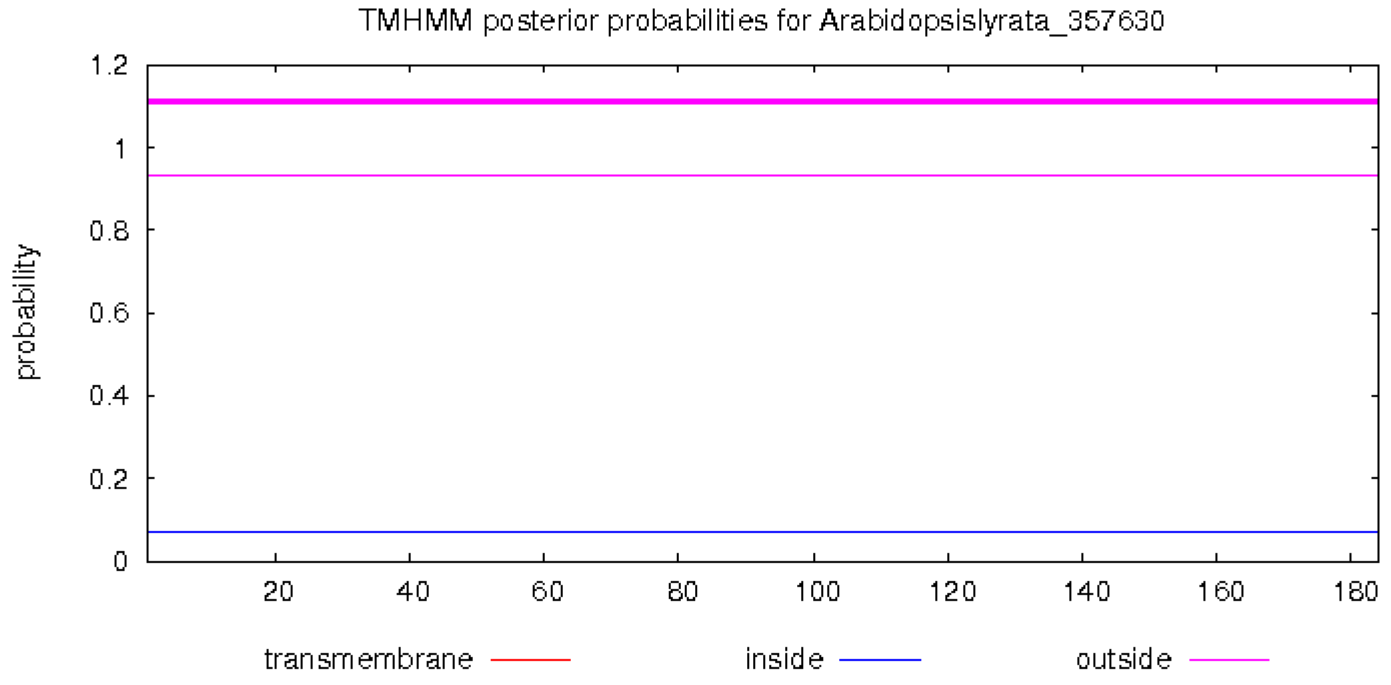

plot in postscript, script for making the plot in gnuplot, data for plot

---

```
# Arabidopsislyrata_358128 Length: 251
# Arabidopsislyrata_358128 Number of predicted TMHs:  0
```

```
# Arabidopsislyrata_358128 Exp number of AAs in TMHs: 0
# Arabidopsislyrata_358128 Exp number, first 60 AAs:  0
# Arabidopsislyrata_358128 Total prob of N-in:        0.12275
Arabidopsislyrata_358128        TMHMM2.0        outside      1   251
```

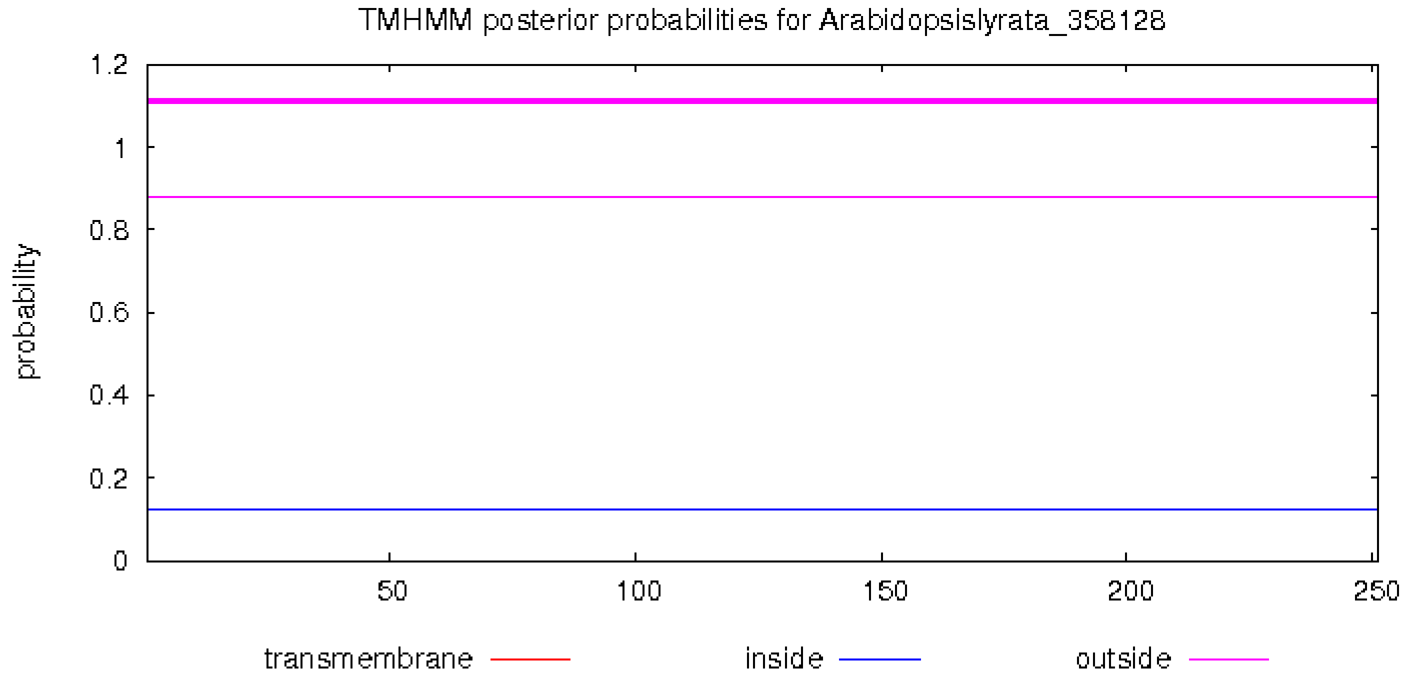

plot in postscript, script for making the plot in gnuplot, data for plot

---

```
# Arabidopsislyrata_471376 Length: 395
# Arabidopsislyrata_471376 Number of predicted TMHs:  0
# Arabidopsislyrata_471376 Exp number of AAs in TMHs: 0.00018
# Arabidopsislyrata_471376 Exp number, first 60 AAs:  0
# Arabidopsislyrata_471376 Total prob of N-in:        0.00167
Arabidopsislyrata_471376        TMHMM2.0        outside      1   395
```

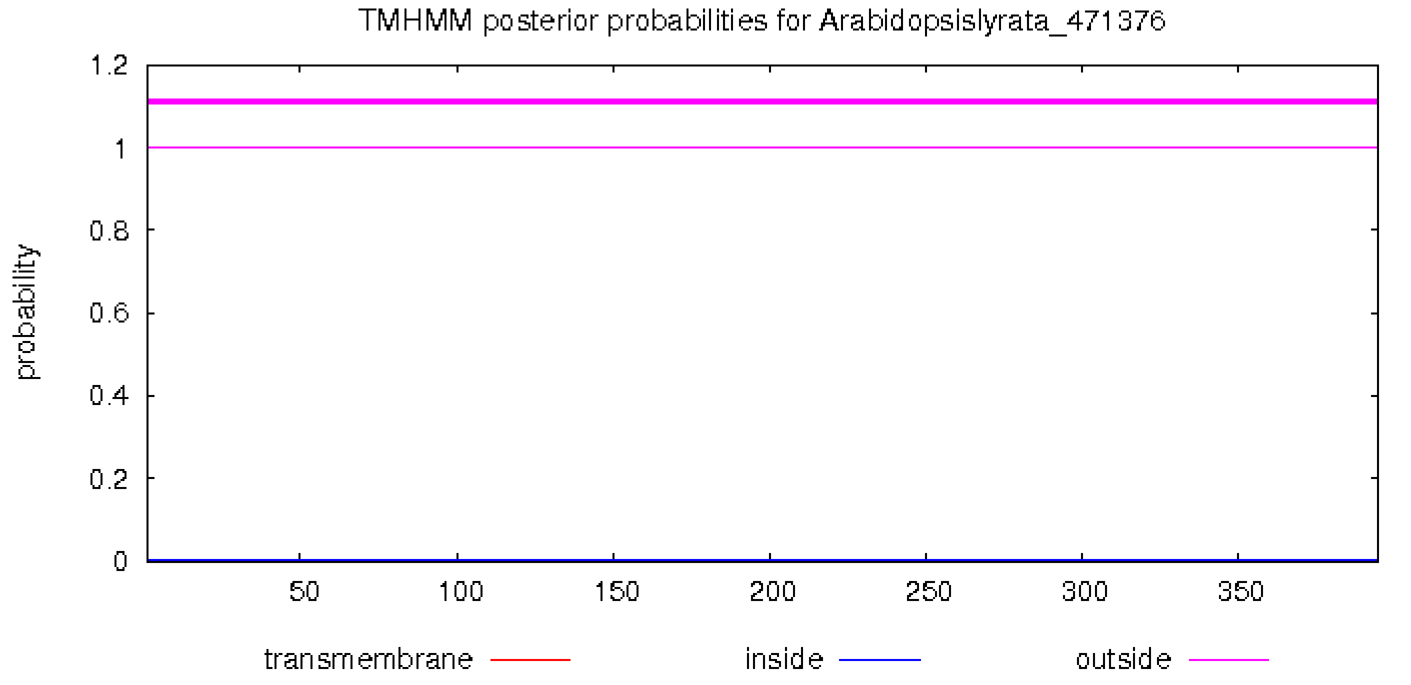

plot in postscript, script for making the plot in gnuplot, data for plot

---

```
# Arabidopsislyrata_473471 Length: 533
# Arabidopsislyrata_473471 Number of predicted TMHs:  1
```

```
# Arabidopsislyrata_473471 Exp number of AAs in TMHs: 21.1973
# Arabidopsislyrata_473471 Exp number, first 60 AAs:  0.0463
# Arabidopsislyrata_473471 Total prob of N-in:        0.66700
Arabidopsislyrata_473471	TMHMM2.0	inside	     1   503
Arabidopsislyrata_473471	TMHMM2.0	TMhelix	   504   526
Arabidopsislyrata_473471	TMHMM2.0	outside	   527   533
```

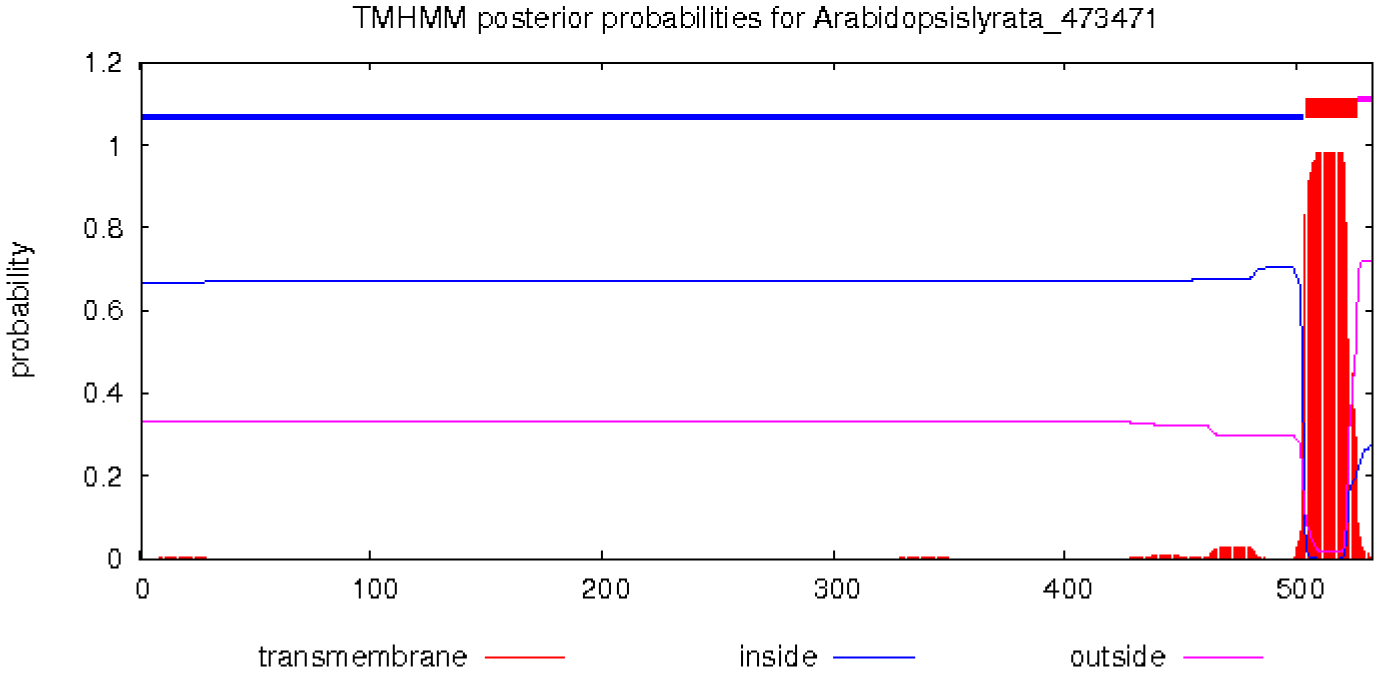

plot in postscript, script for making the plot in gnuplot, data for plot

---

```
# Arabidopsislyrata_473504 Length: 305
# Arabidopsislyrata_473504 Number of predicted TMHs:  0
# Arabidopsislyrata_473504 Exp number of AAs in TMHs: 0
# Arabidopsislyrata_473504 Exp number, first 60 AAs:  0
# Arabidopsislyrata_473504 Total prob of N-in:        0.00496
Arabidopsislyrata_473504	TMHMM2.0	outside	     1   305
```

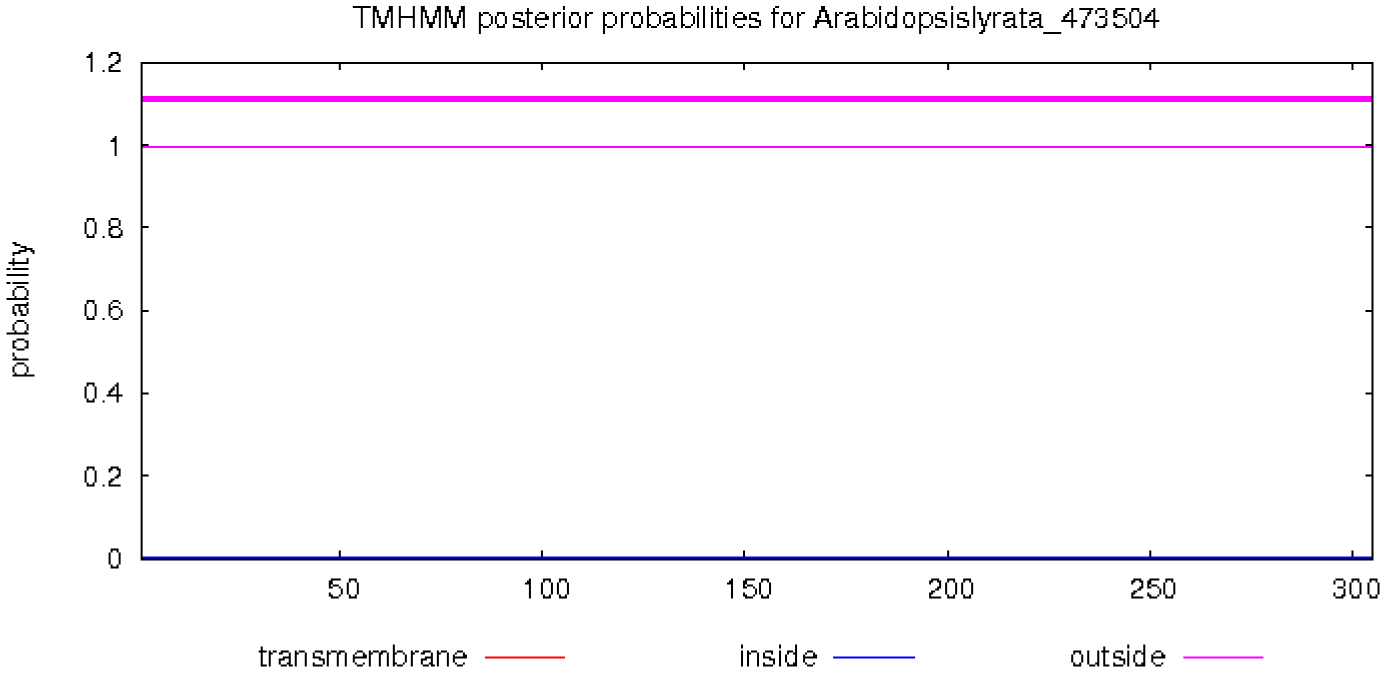

plot in postscript, script for making the plot in gnuplot, data for plot

---

```
# Arabidopsislyrata_473571 Length: 573
# Arabidopsislyrata_473571 Number of predicted TMHs:  1
# Arabidopsislyrata_473571 Exp number of AAs in TMHs: 22.8481
# Arabidopsislyrata_473571 Exp number, first 60 AAs:  0.00833
# Arabidopsislyrata_473571 Total prob of N-in:        0.13285
Arabidopsislyrata_473571        TMHMM2.0        outside      1   545
Arabidopsislyrata_473571        TMHMM2.0        TMhelix    546   568
Arabidopsislyrata_473571        TMHMM2.0        inside     569   573
```

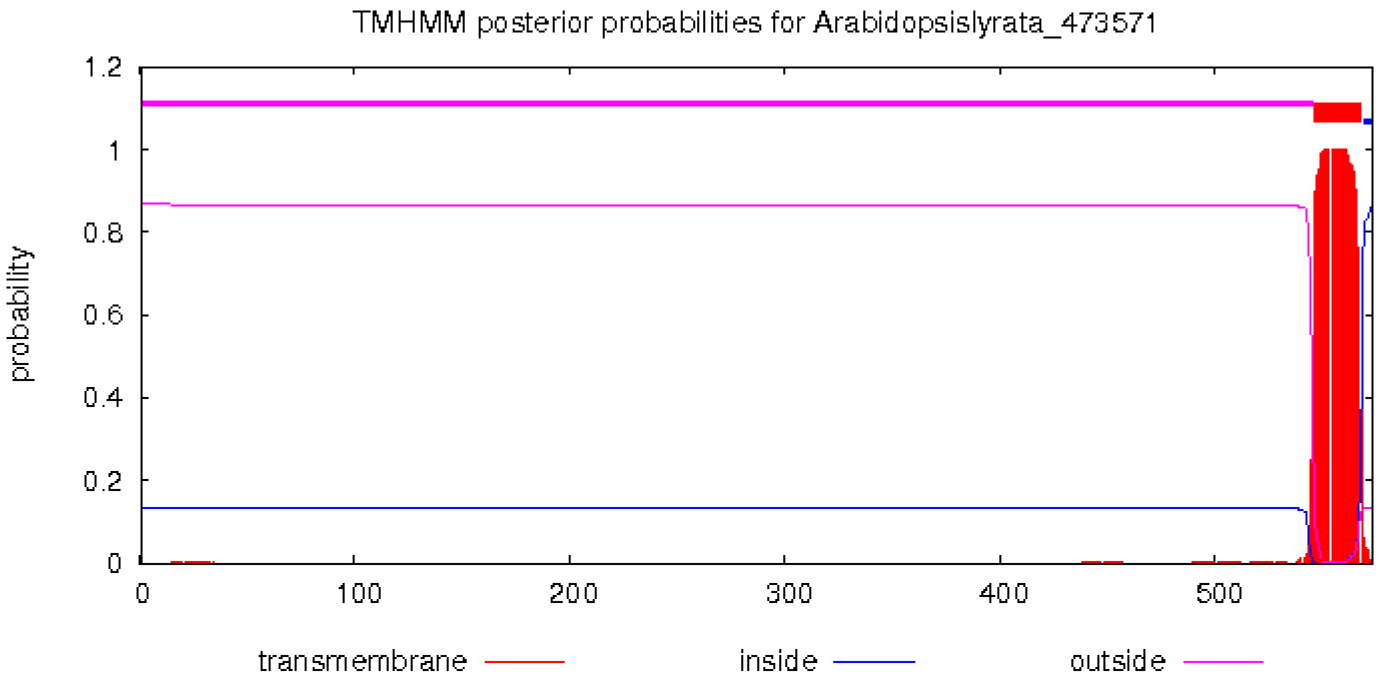

plot in postscript, script for making the plot in gnuplot, data for plot

---

```
# Arabidopsislyrata_474412 Length: 320
# Arabidopsislyrata_474412 Number of predicted TMHs:  0
# Arabidopsislyrata_474412 Exp number of AAs in TMHs: 0.00088
# Arabidopsislyrata_474412 Exp number, first 60 AAs:  0
# Arabidopsislyrata_474412 Total prob of N-in:        0.01102
Arabidopsislyrata_474412        TMHMM2.0        outside      1   320
```

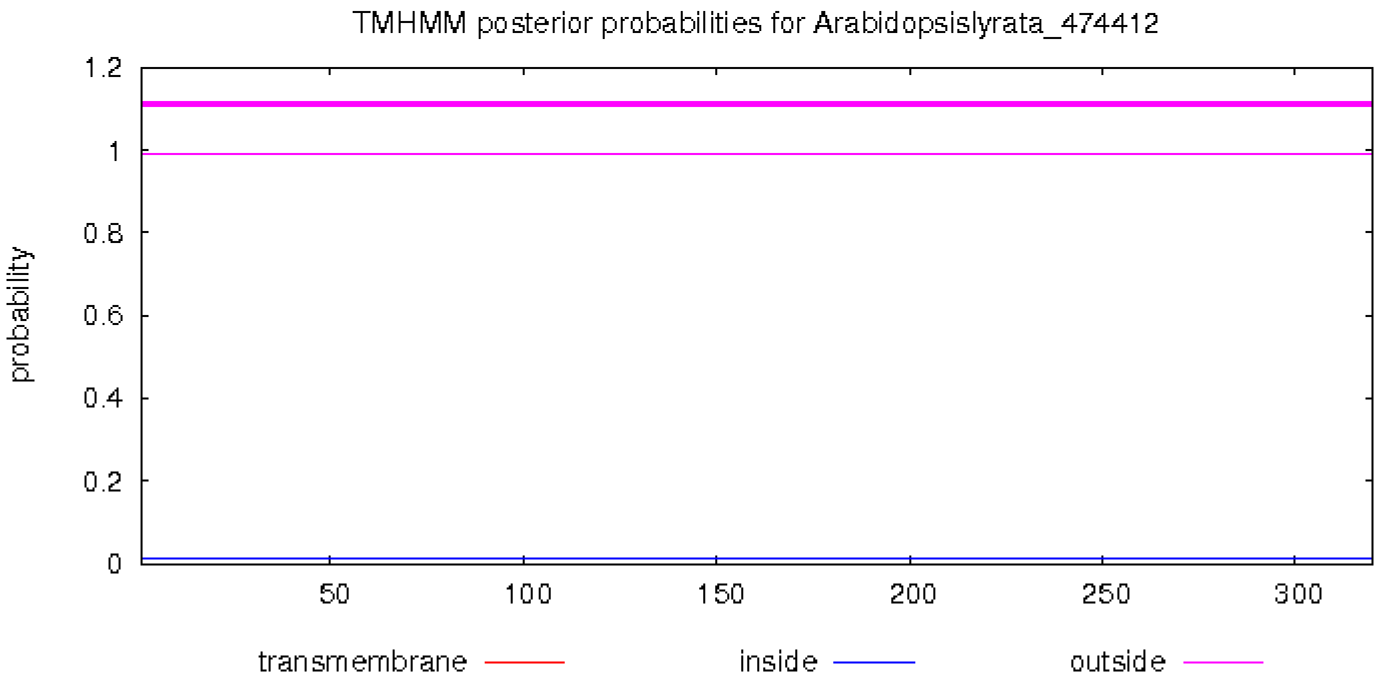

plot in postscript, script for making the plot in gnuplot, data for plot

```
# Arabidopsislyrata_474413 Length: 320
# Arabidopsislyrata_474413 Number of predicted TMHs:  0
# Arabidopsislyrata_474413 Exp number of AAs in TMHs: 0.01073
# Arabidopsislyrata_474413 Exp number, first 60 AAs:  0.00021
# Arabidopsislyrata_474413 Total prob of N-in:        0.00901
Arabidopsislyrata_474413        TMHMM2.0        outside      1   320
```

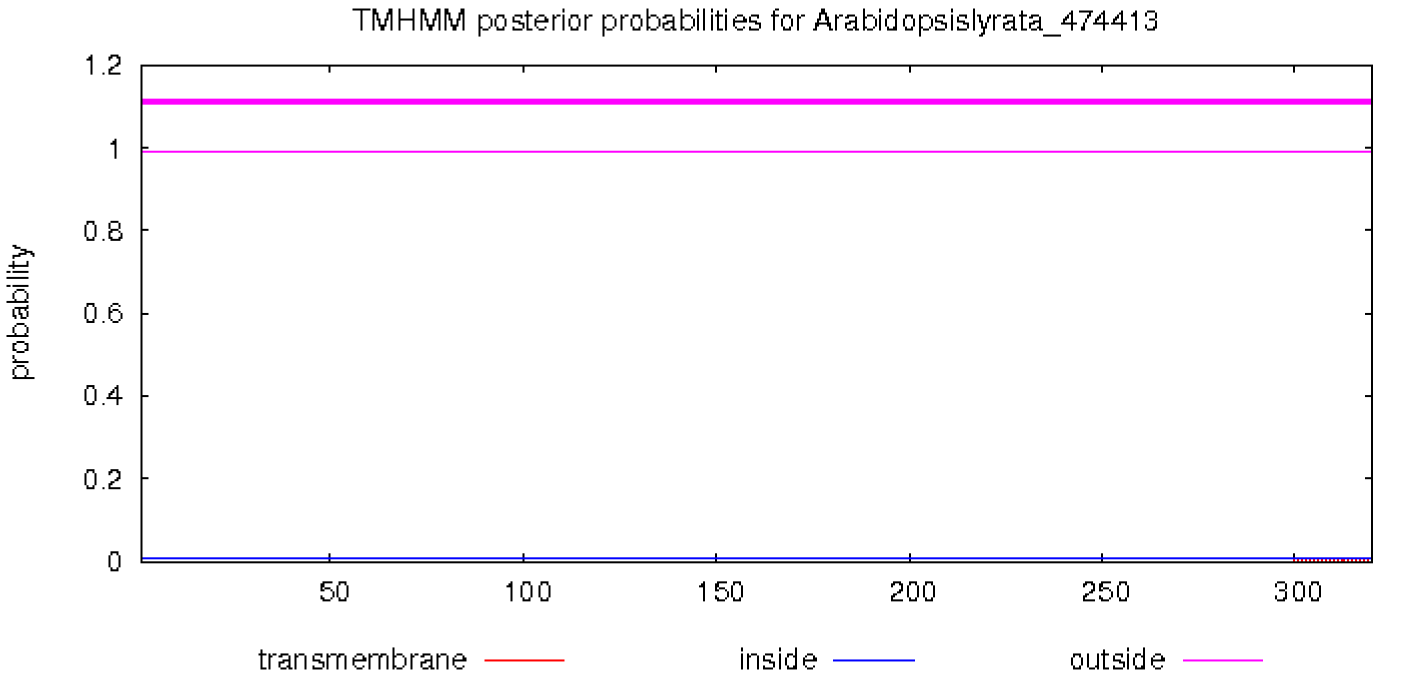

plot in postscript, script for making the plot in gnuplot, data for plot

```
# Arabidopsislyrata_474694 Length: 301
# Arabidopsislyrata_474694 Number of predicted TMHs:  0
# Arabidopsislyrata_474694 Exp number of AAs in TMHs: 0
# Arabidopsislyrata_474694 Exp number, first 60 AAs:  0
# Arabidopsislyrata_474694 Total prob of N-in:        0.00142
Arabidopsislyrata_474694        TMHMM2.0        outside      1   301
```

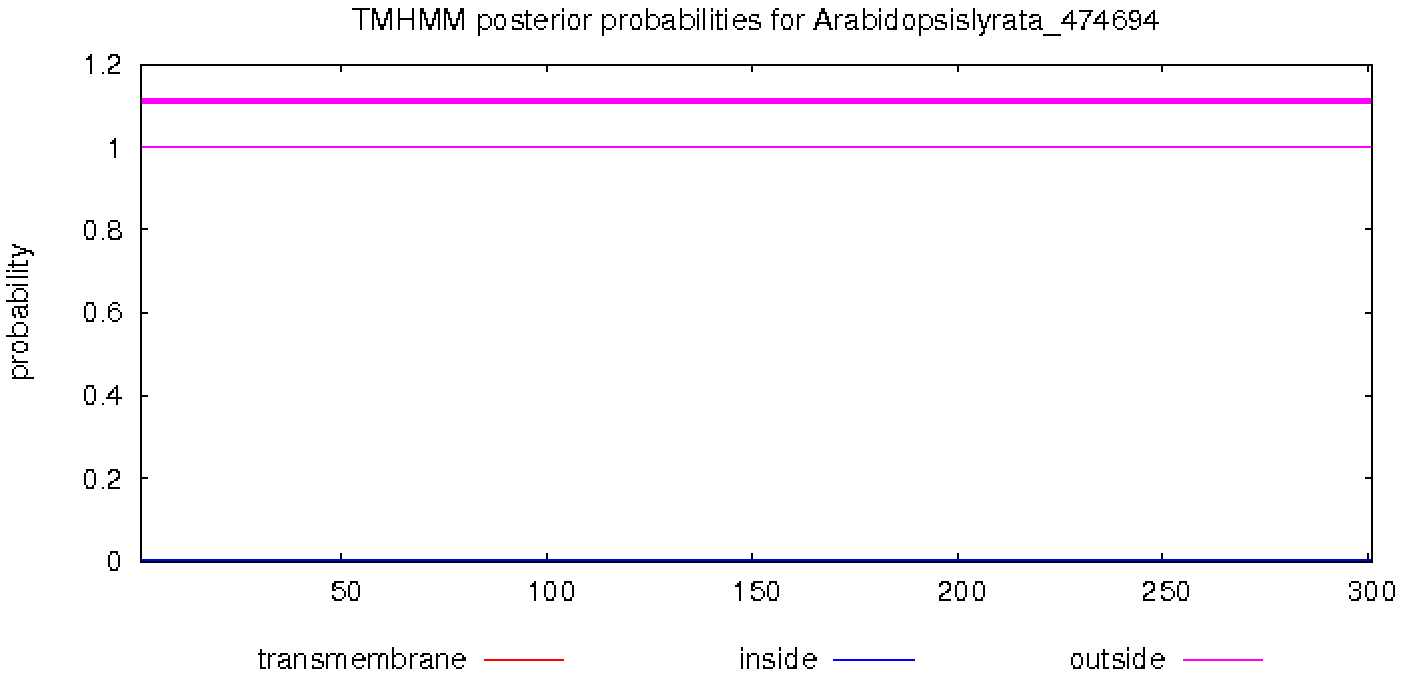

plot in postscript, script for making the plot in gnuplot, data for plot

```
# Arabidopsislyrata_475082 Length: 402
# Arabidopsislyrata_475082 Number of predicted TMHs:  0
# Arabidopsislyrata_475082 Exp number of AAs in TMHs: 0.00018
# Arabidopsislyrata_475082 Exp number, first 60 AAs:  0
# Arabidopsislyrata_475082 Total prob of N-in:        0.00184
Arabidopsislyrata_475082        TMHMM2.0        outside      1   402
```

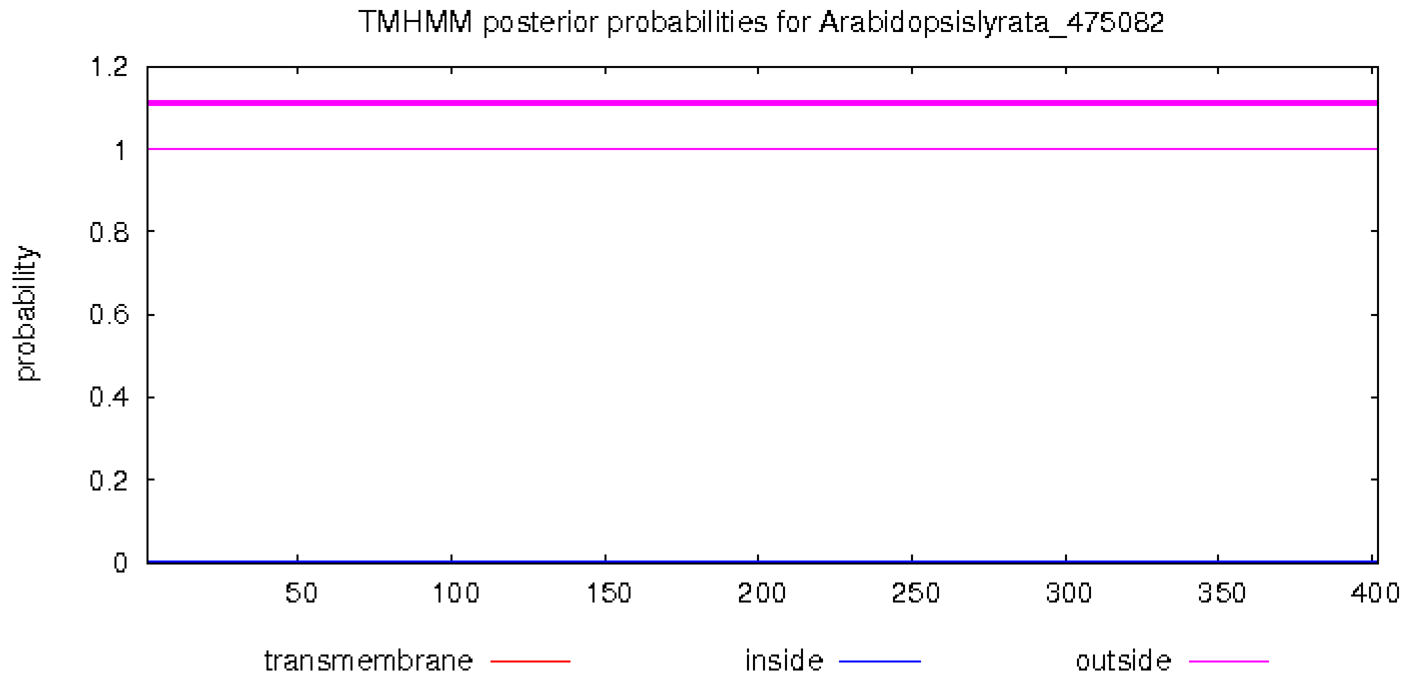

plot in postscript, script for making the plot in gnuplot, data for plot

---

```
# Arabidopsislyrata_475292 Length: 318
# Arabidopsislyrata_475292 Number of predicted TMHs:  0
# Arabidopsislyrata_475292 Exp number of AAs in TMHs: 0.00054
# Arabidopsislyrata_475292 Exp number, first 60 AAs:  0
# Arabidopsislyrata_475292 Total prob of N-in:        0.00861
Arabidopsislyrata_475292        TMHMM2.0        outside      1   318
```

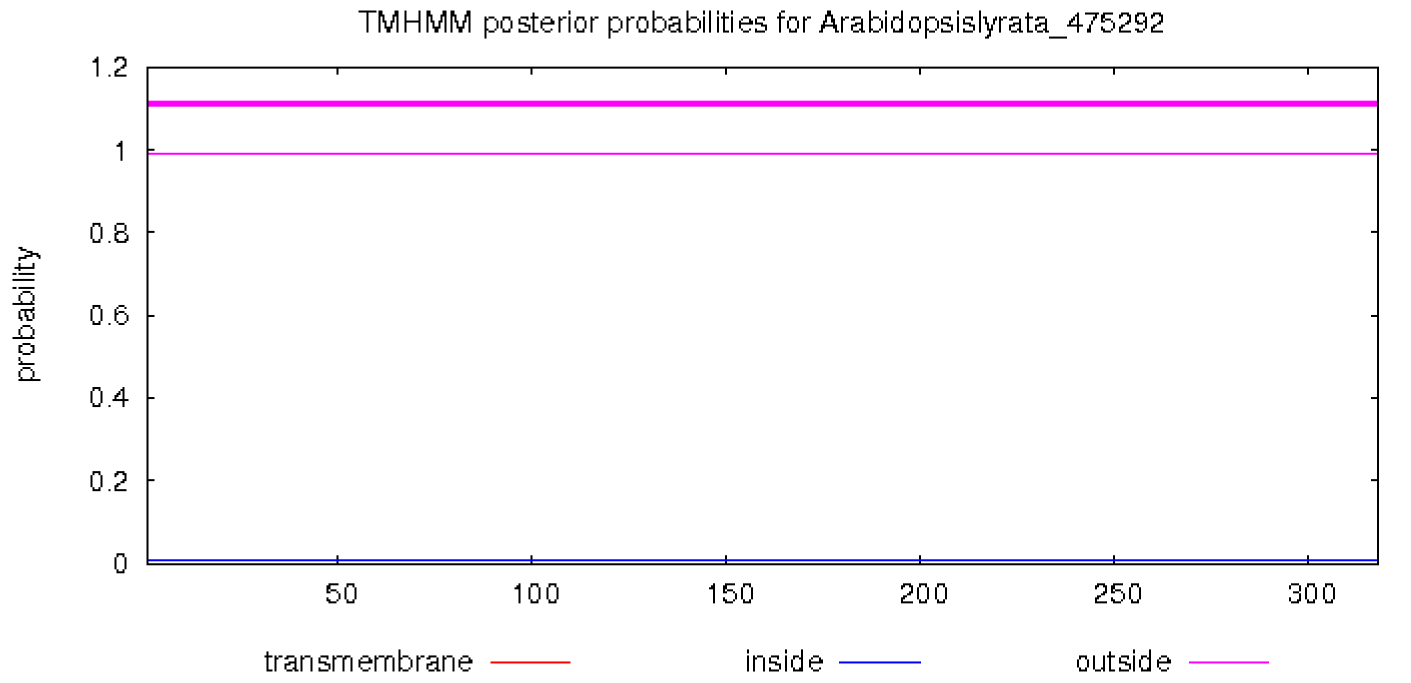

plot in postscript, script for making the plot in gnuplot, data for plot

---

```
# Arabidopsislyrata_475296 Length: 174
# Arabidopsislyrata_475296 Number of predicted TMHs:  0
# Arabidopsislyrata_475296 Exp number of AAs in TMHs: 0.19491
# Arabidopsislyrata_475296 Exp number, first 60 AAs:  0.18723
# Arabidopsislyrata_475296 Total prob of N-in:        0.50191
Arabidopsislyrata_475296	TMHMM2.0	outside	     1   174
```

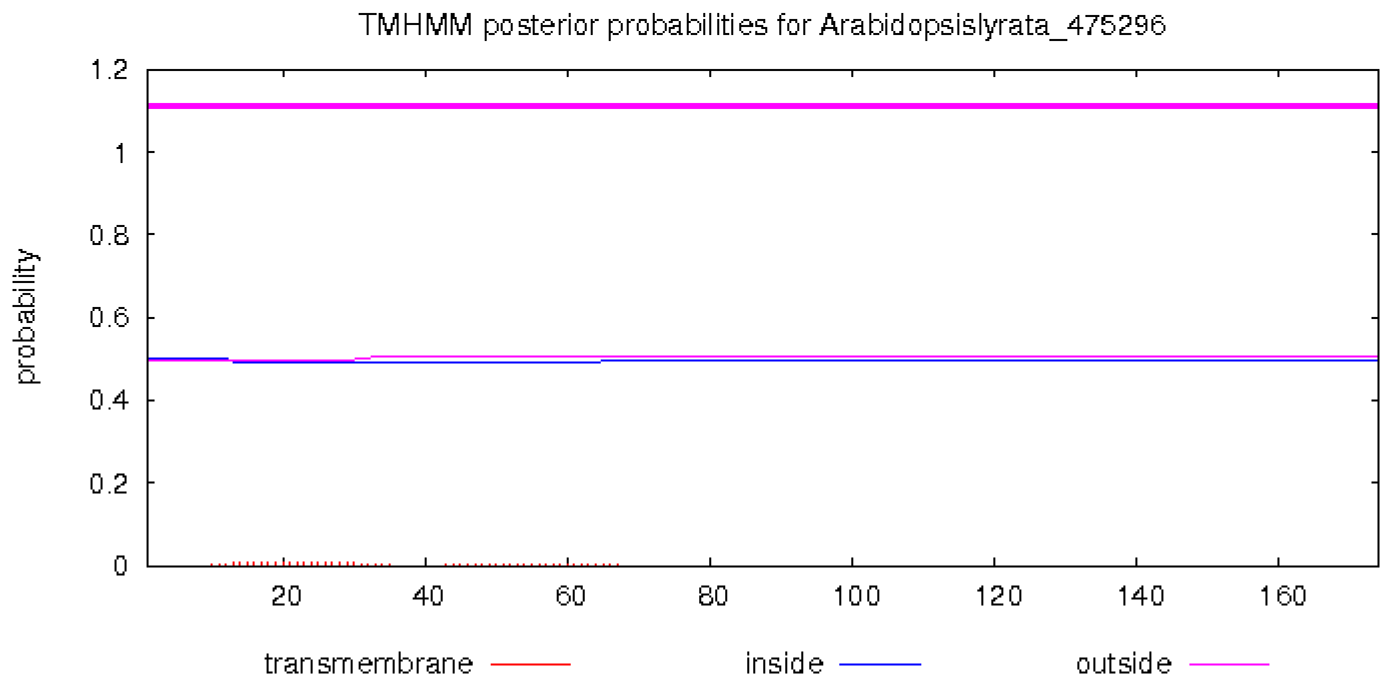

plot in postscript, script for making the plot in gnuplot, data for plot

```
# Arabidopsislyrata_476076 Length: 270
# Arabidopsislyrata_476076 Number of predicted TMHs:  0
# Arabidopsislyrata_476076 Exp number of AAs in TMHs: 0.00092
# Arabidopsislyrata_476076 Exp number, first 60 AAs:  0
# Arabidopsislyrata_476076 Total prob of N-in:        0.02694
Arabidopsislyrata_476076	TMHMM2.0	outside	     1   270
```

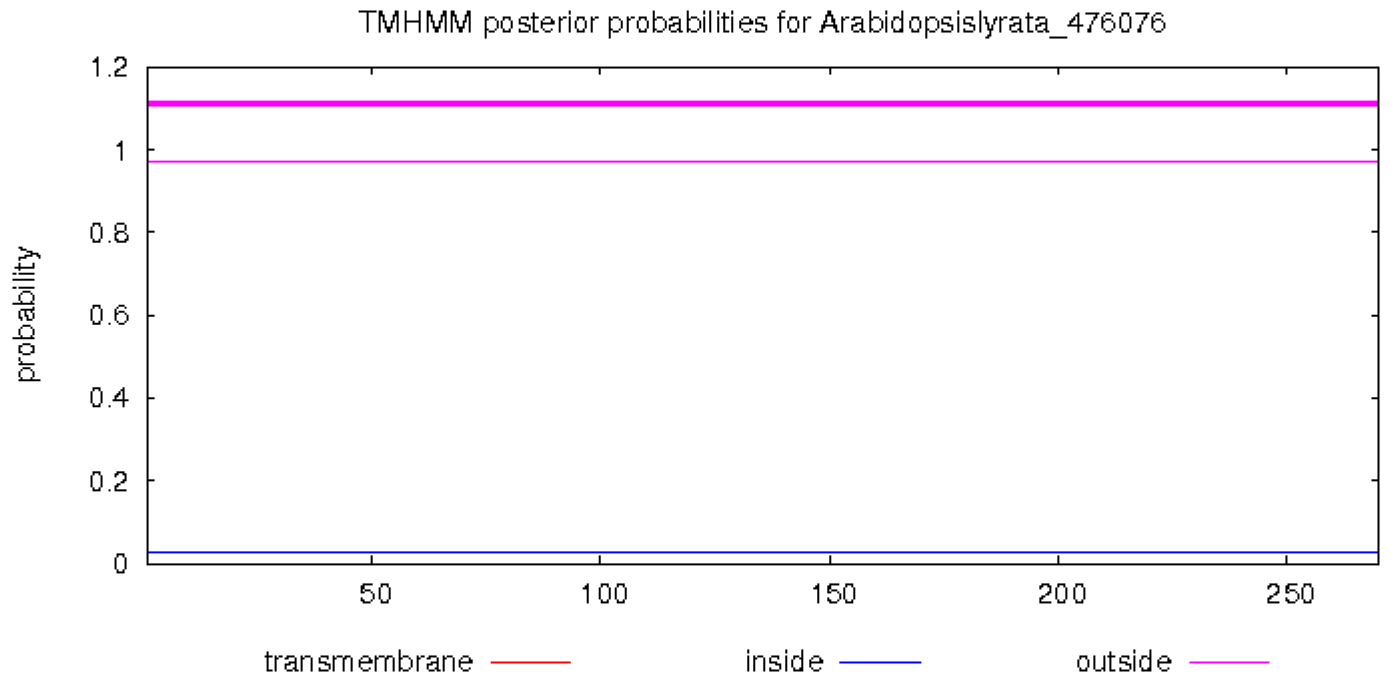

plot in postscript, script for making the plot in gnuplot, data for plot

```
# Arabidopsislyrata_476799 Length: 335
# Arabidopsislyrata_476799 Number of predicted TMHs:  0
# Arabidopsislyrata_476799 Exp number of AAs in TMHs: 0.01211
# Arabidopsislyrata_476799 Exp number, first 60 AAs:  0.00993
# Arabidopsislyrata_476799 Total prob of N-in:        0.00506
Arabidopsislyrata_476799	TMHMM2.0	outside	     1   335
```

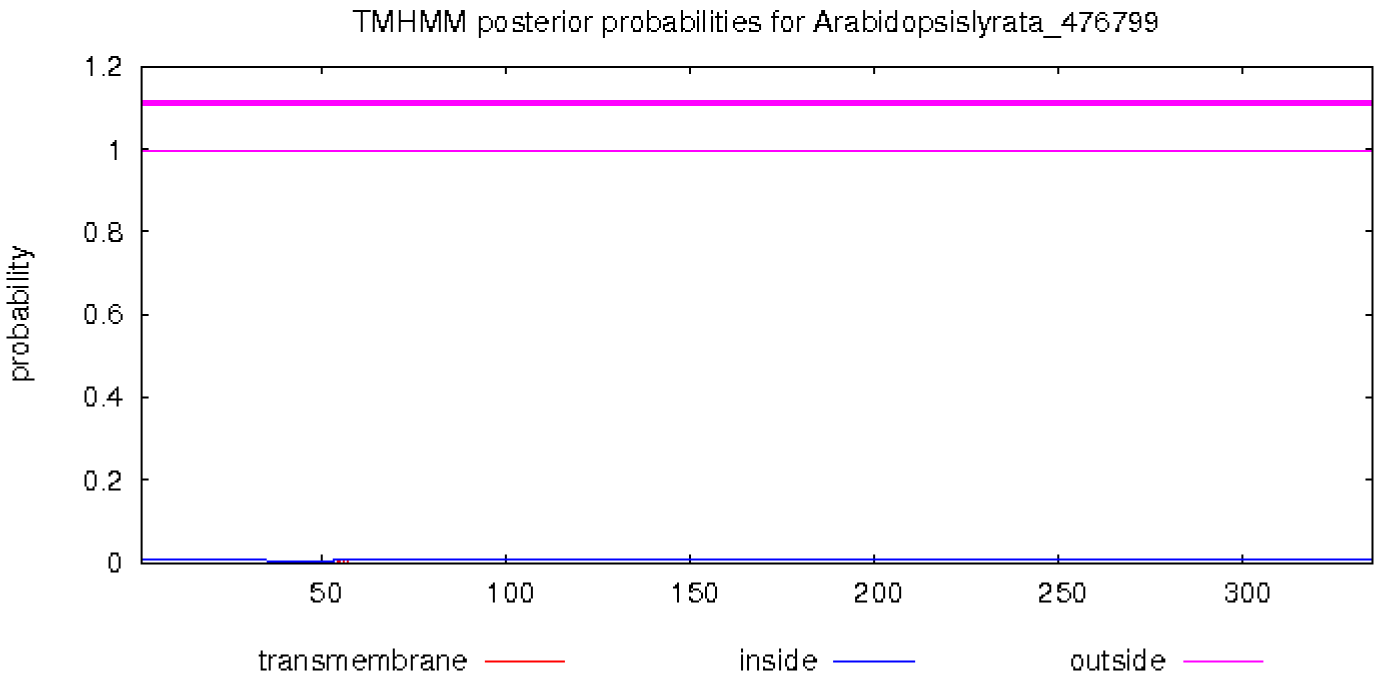

plot in postscript, script for making the plot in gnuplot, data for plot

```
# Arabidopsislyrata_477665 Length: 342
# Arabidopsislyrata_477665 Number of predicted TMHs:  0
# Arabidopsislyrata_477665 Exp number of AAs in TMHs: 0.00033
# Arabidopsislyrata_477665 Exp number, first 60 AAs:  0.00014
# Arabidopsislyrata_477665 Total prob of N-in:        0.00772
Arabidopsislyrata_477665	TMHMM2.0	outside	     1   342
```

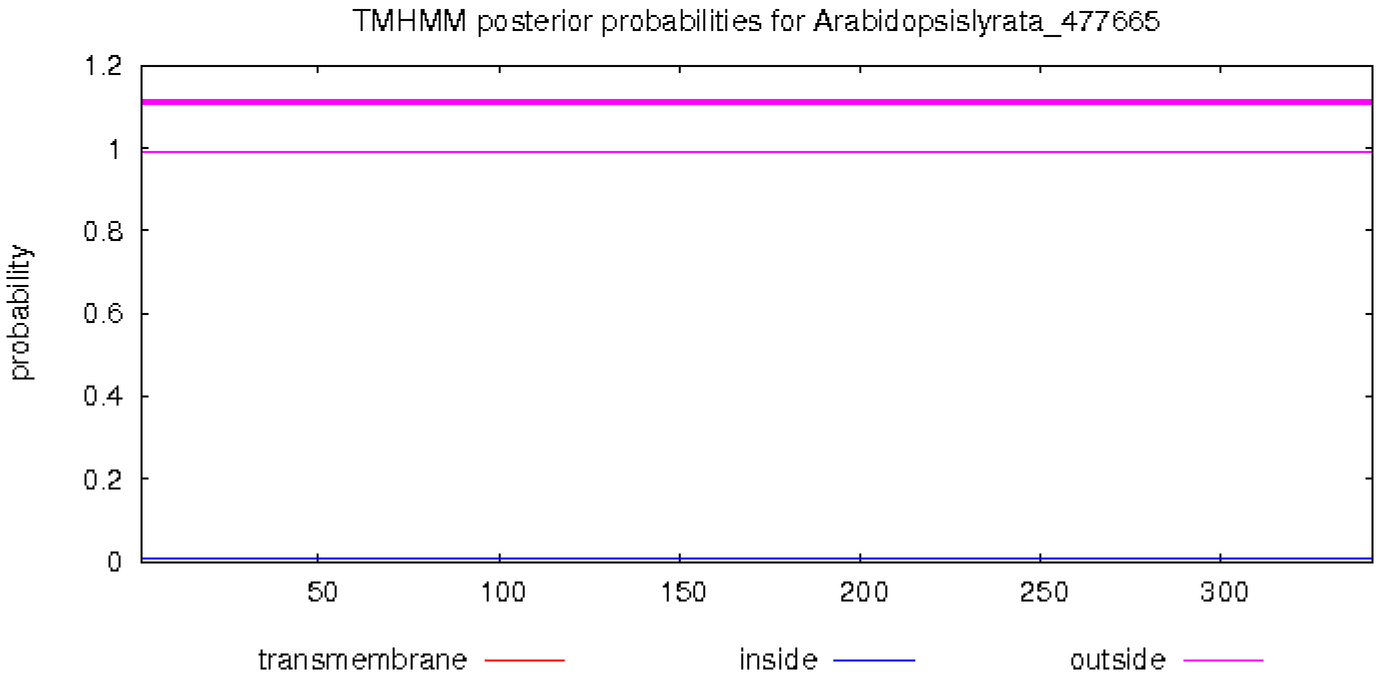

plot in postscript, script for making the plot in gnuplot, data for plot

```
# Arabidopsislyrata_477666 Length: 361
# Arabidopsislyrata_477666 Number of predicted TMHs:  0
# Arabidopsislyrata_477666 Exp number of AAs in TMHs: 0
# Arabidopsislyrata_477666 Exp number, first 60 AAs:  0
# Arabidopsislyrata_477666 Total prob of N-in:        0.00988
Arabidopsislyrata_477666        TMHMM2.0        outside      1   361
```

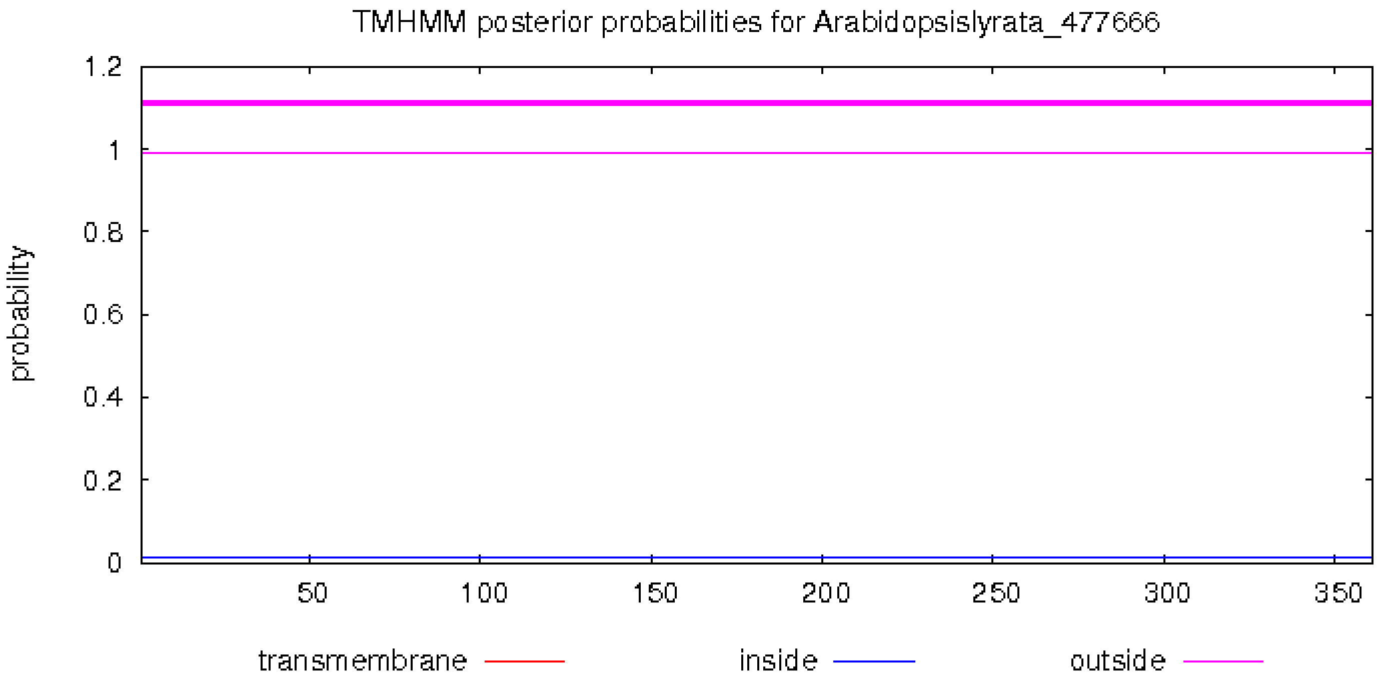

plot in postscript, script for making the plot in gnuplot, data for plot

---

```
# Arabidopsislyrata_478382 Length: 451
# Arabidopsislyrata_478382 Number of predicted TMHs:  0
# Arabidopsislyrata_478382 Exp number of AAs in TMHs: 0.25542
# Arabidopsislyrata_478382 Exp number, first 60 AAs:  0.23602
# Arabidopsislyrata_478382 Total prob of N-in:        0.01459
Arabidopsislyrata_478382        TMHMM2.0        outside      1   451
```

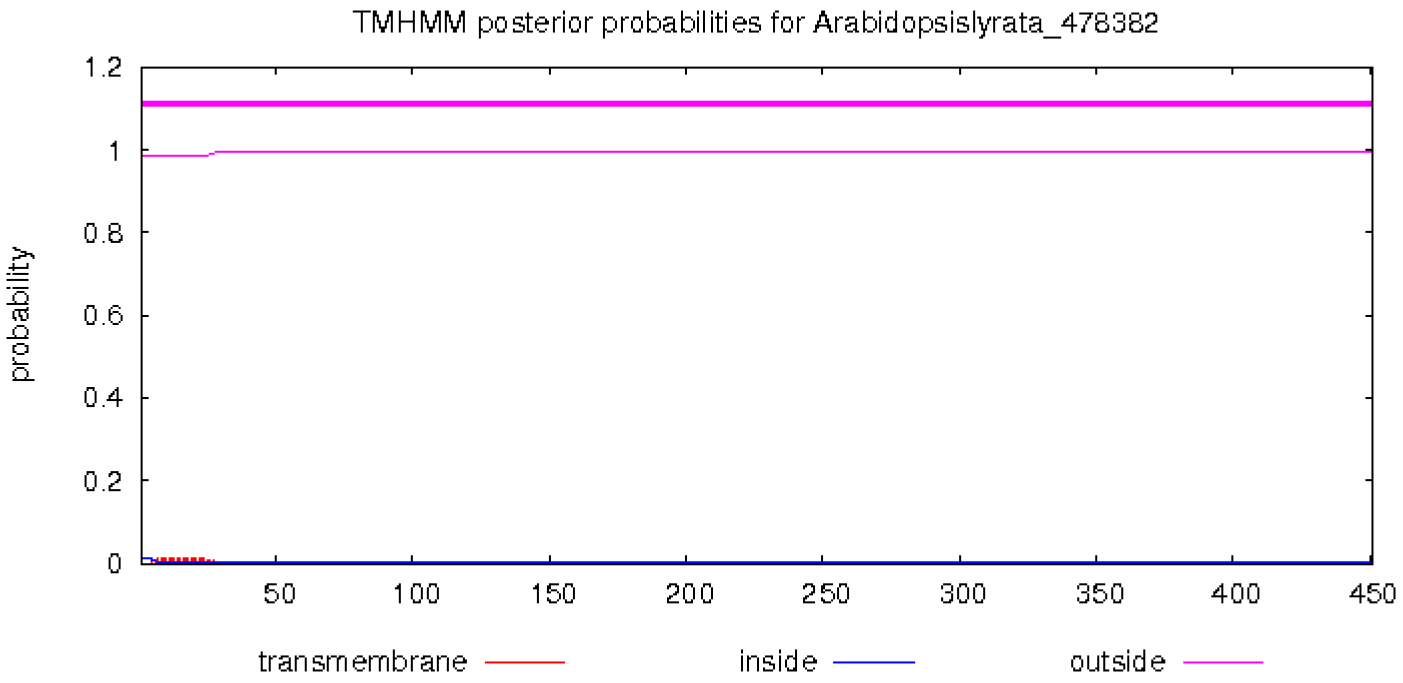

plot in postscript, script for making the plot in gnuplot, data for plot

---

```
# Arabidopsislyrata_478384 Length: 560
# Arabidopsislyrata_478384 Number of predicted TMHs:  1
# Arabidopsislyrata_478384 Exp number of AAs in TMHs: 21.58527
# Arabidopsislyrata_478384 Exp number, first 60 AAs:  0
# Arabidopsislyrata_478384 Total prob of N-in:        0.00959
Arabidopsislyrata_478384	TMHMM2.0	outside	     1   533
Arabidopsislyrata_478384	TMHMM2.0	TMhelix	   534   556
Arabidopsislyrata_478384	TMHMM2.0	inside	   557   560
```

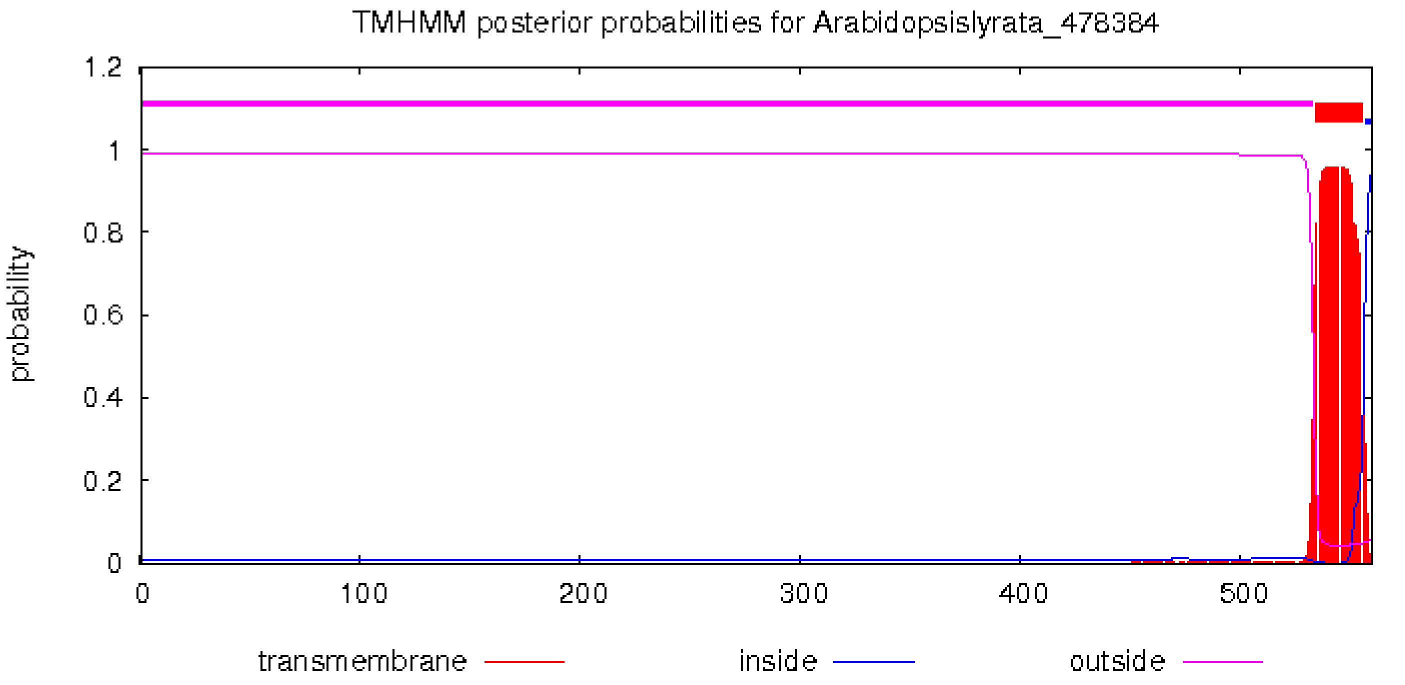

plot in postscript, script for making the plot in gnuplot, data for plot

---

```
# Arabidopsislyrata_478652 Length: 303
# Arabidopsislyrata_478652 Number of predicted TMHs:  0
# Arabidopsislyrata_478652 Exp number of AAs in TMHs: 0.00162
# Arabidopsislyrata_478652 Exp number, first 60 AAs:  0
# Arabidopsislyrata_478652 Total prob of N-in:        0.06484
Arabidopsislyrata_478652	TMHMM2.0	outside	     1   303
```

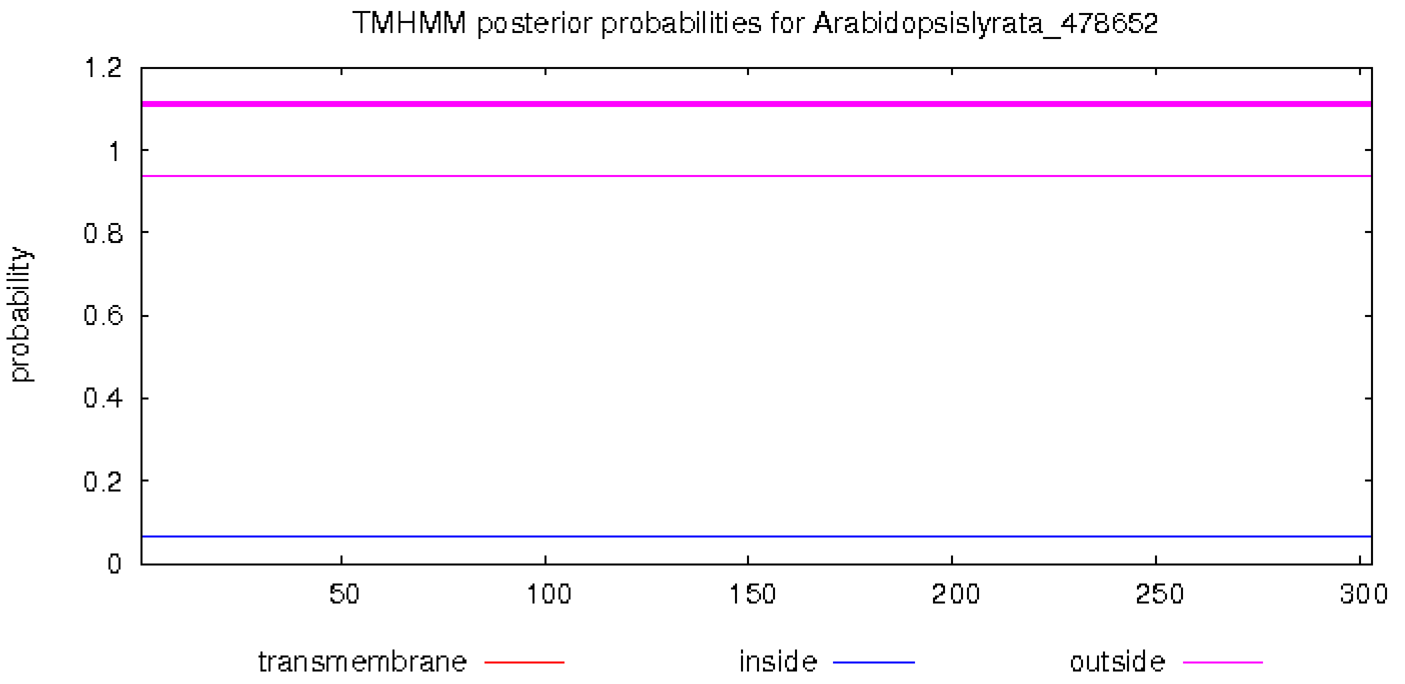

plot in postscript, script for making the plot in gnuplot, data for plot

```
# Arabidopsislyrata_478949 Length: 310
# Arabidopsislyrata_478949 Number of predicted TMHs:  0
# Arabidopsislyrata_478949 Exp number of AAs in TMHs: 0.35233
# Arabidopsislyrata_478949 Exp number, first 60 AAs:  0.0002
# Arabidopsislyrata_478949 Total prob of N-in:        0.01074
Arabidopsislyrata_478949        TMHMM2.0        outside      1   310
```

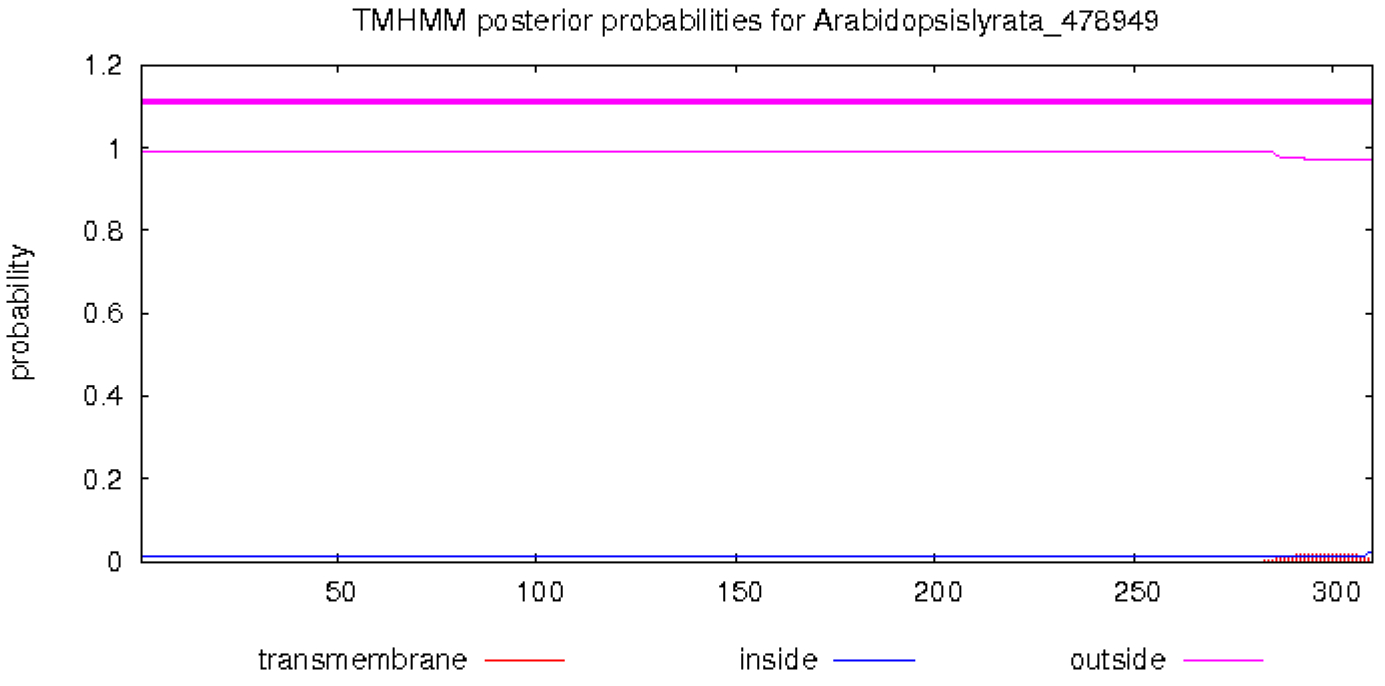

plot in postscript, script for making the plot in gnuplot, data for plot

```
# Arabidopsislyrata_478996 Length: 317
# Arabidopsislyrata_478996 Number of predicted TMHs:  0
# Arabidopsislyrata_478996 Exp number of AAs in TMHs: 0
# Arabidopsislyrata_478996 Exp number, first 60 AAs:  0
# Arabidopsislyrata_478996 Total prob of N-in:        0.00239
Arabidopsislyrata_478996        TMHMM2.0        outside      1   317
```

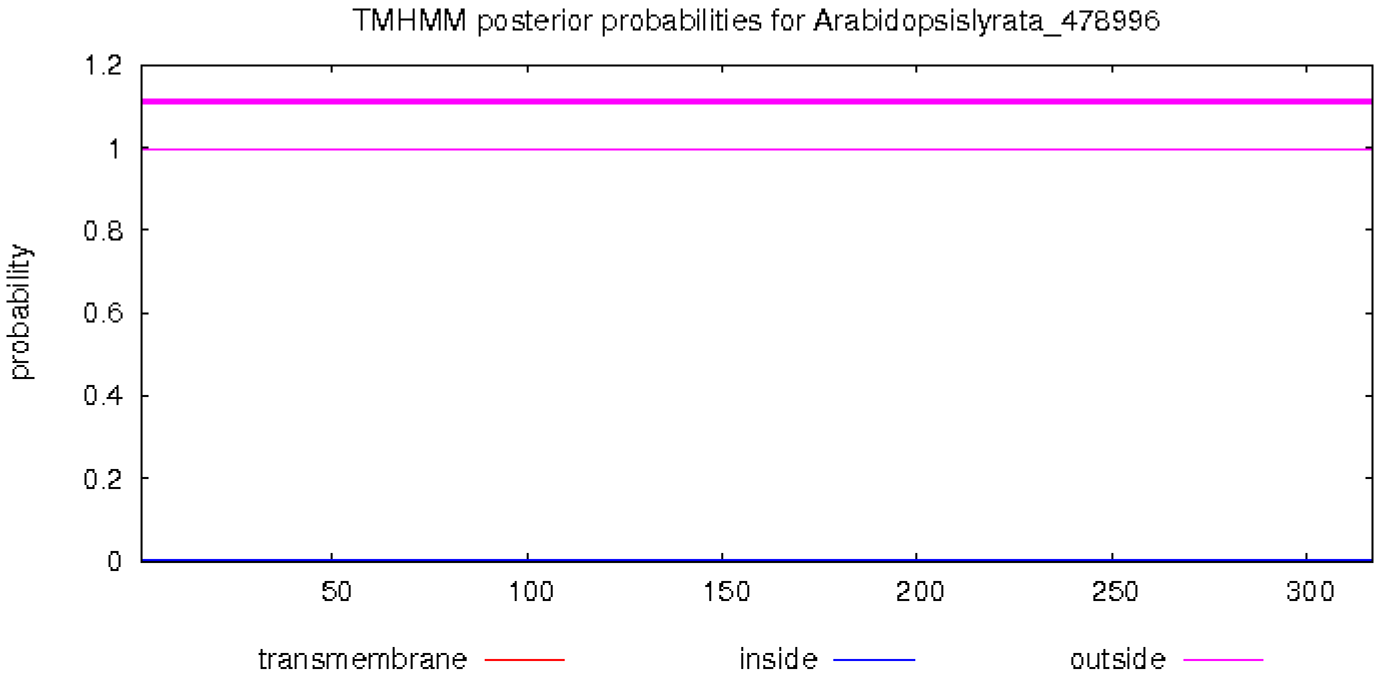

plot in postscript, script for making the plot in gnuplot, data for plot

```
# Arabidopsislyrata_478997 Length: 363
# Arabidopsislyrata_478997 Number of predicted TMHs:  0
# Arabidopsislyrata_478997 Exp number of AAs in TMHs: 0.00194
# Arabidopsislyrata_478997 Exp number, first 60 AAs:  0.00017
# Arabidopsislyrata_478997 Total prob of N-in:        0.00418
Arabidopsislyrata_478997        TMHMM2.0        outside      1   363
```

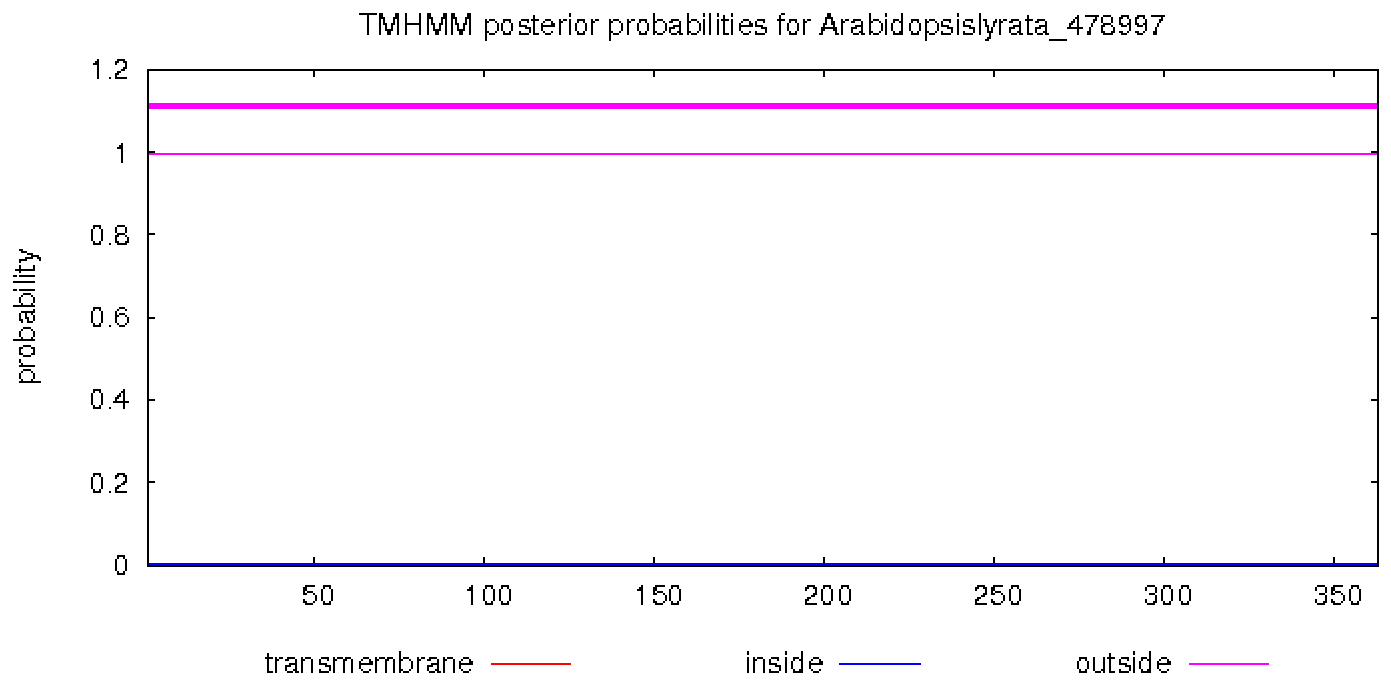

plot in postscript, script for making the plot in gnuplot, data for plot

---

```
# Arabidopsislyrata_480590 Length: 278
# Arabidopsislyrata_480590 Number of predicted TMHs:  0
# Arabidopsislyrata_480590 Exp number of AAs in TMHs: 0.00123
# Arabidopsislyrata_480590 Exp number, first 60 AAs:  0
# Arabidopsislyrata_480590 Total prob of N-in:        0.00447
Arabidopsislyrata_480590        TMHMM2.0        outside      1   278
```

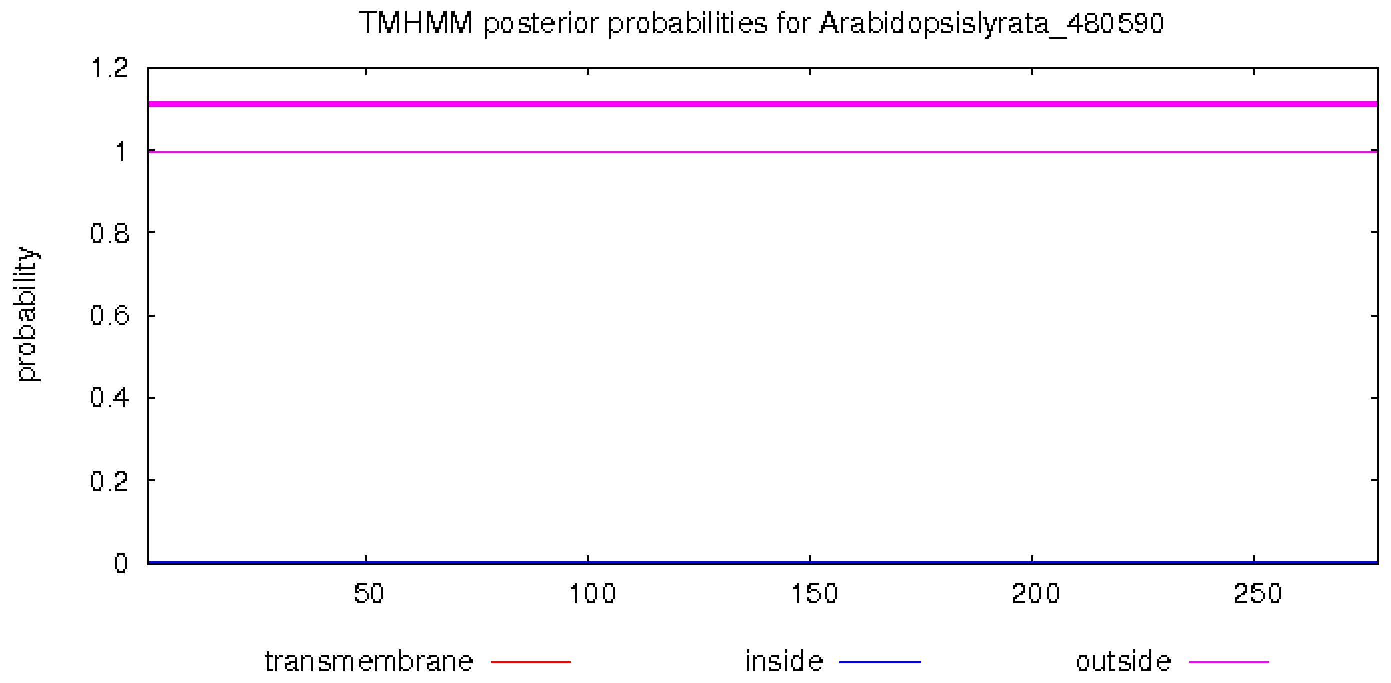

plot in postscript, script for making the plot in gnuplot, data for plot

---

```
# Arabidopsislyrata_481629 Length: 333
# Arabidopsislyrata_481629 Number of predicted TMHs:  0
# Arabidopsislyrata_481629 Exp number of AAs in TMHs: 10.24245
# Arabidopsislyrata_481629 Exp number, first 60 AAs:  0.05294
# Arabidopsislyrata_481629 Total prob of N-in:        0.31538
Arabidopsislyrata_481629	TMHMM2.0	outside	     1   333
```

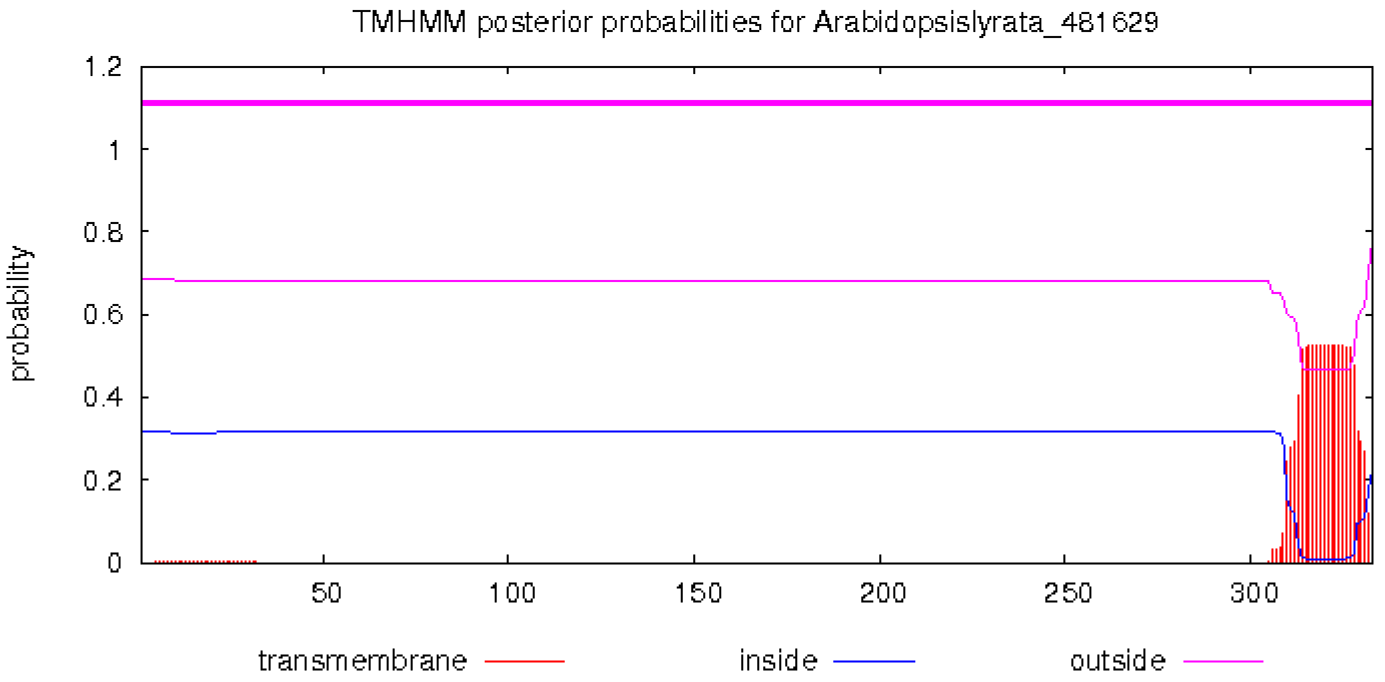

plot in postscript, script for making the plot in gnuplot, data for plot

---

```
# Arabidopsislyrata_482294 Length: 258
# Arabidopsislyrata_482294 Number of predicted TMHs:  0
# Arabidopsislyrata_482294 Exp number of AAs in TMHs: 0.02005
# Arabidopsislyrata_482294 Exp number, first 60 AAs:  0
# Arabidopsislyrata_482294 Total prob of N-in:        0.17346
Arabidopsislyrata_482294	TMHMM2.0	outside	     1   258
```

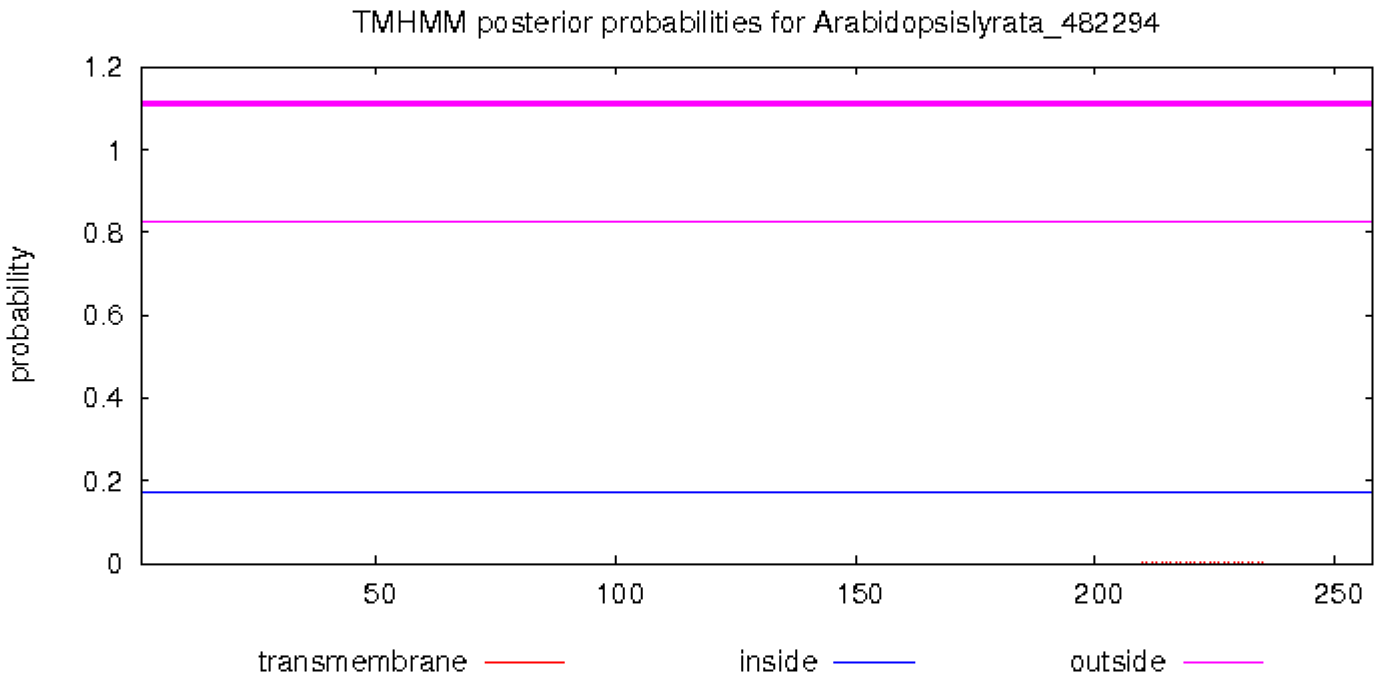

plot in postscript, script for making the plot in gnuplot, data for plot

---

```
# Arabidopsislyrata_483400 Length: 273
# Arabidopsislyrata_483400 Number of predicted TMHs:  0
# Arabidopsislyrata_483400 Exp number of AAs in TMHs: 0.0005
# Arabidopsislyrata_483400 Exp number, first 60 AAs:  0
# Arabidopsislyrata_483400 Total prob of N-in:        0.01674
Arabidopsislyrata_483400        TMHMM2.0        outside      1   273
```

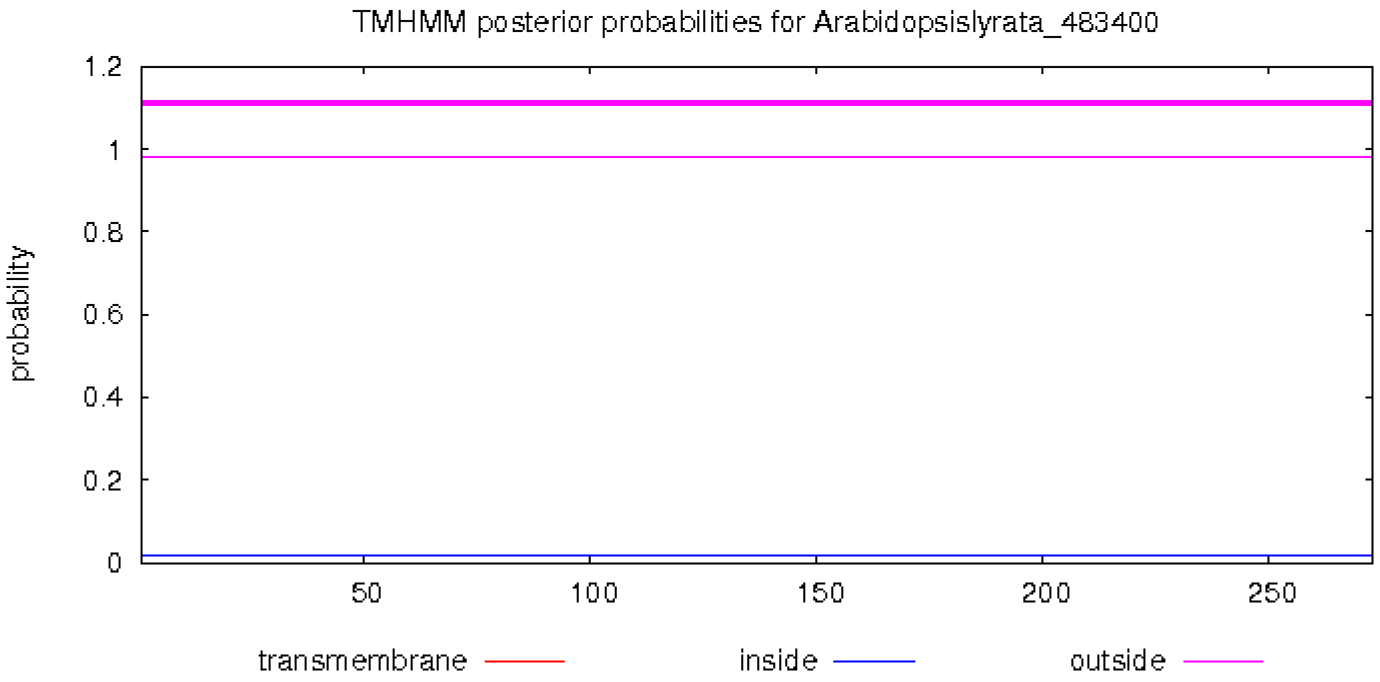

plot in postscript, script for making the plot in gnuplot, data for plot

---

```
# Arabidopsislyrata_484160 Length: 375
# Arabidopsislyrata_484160 Number of predicted TMHs:  0
# Arabidopsislyrata_484160 Exp number of AAs in TMHs: 0.00538
# Arabidopsislyrata_484160 Exp number, first 60 AAs:  0
# Arabidopsislyrata_484160 Total prob of N-in:        0.01384
Arabidopsislyrata_484160        TMHMM2.0        outside      1   375
```

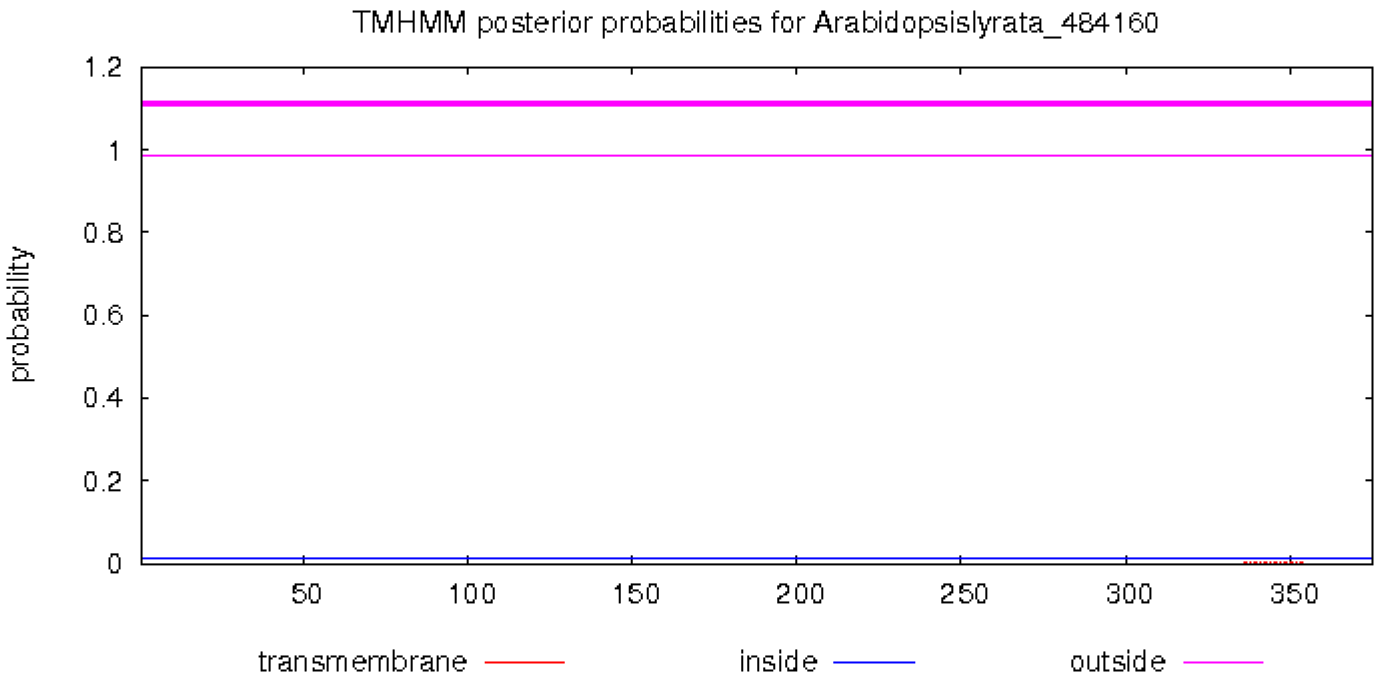

plot in postscript, script for making the plot in gnuplot, data for plot

---

```
# Arabidopsislyrata_484673 Length: 322
# Arabidopsislyrata_484673 Number of predicted TMHs:  0
# Arabidopsislyrata_484673 Exp number of AAs in TMHs: 0.02816
# Arabidopsislyrata_484673 Exp number, first 60 AAs:  0.00137
# Arabidopsislyrata_484673 Total prob of N-in:        0.04037
Arabidopsislyrata_484673        TMHMM2.0        outside      1   322
```

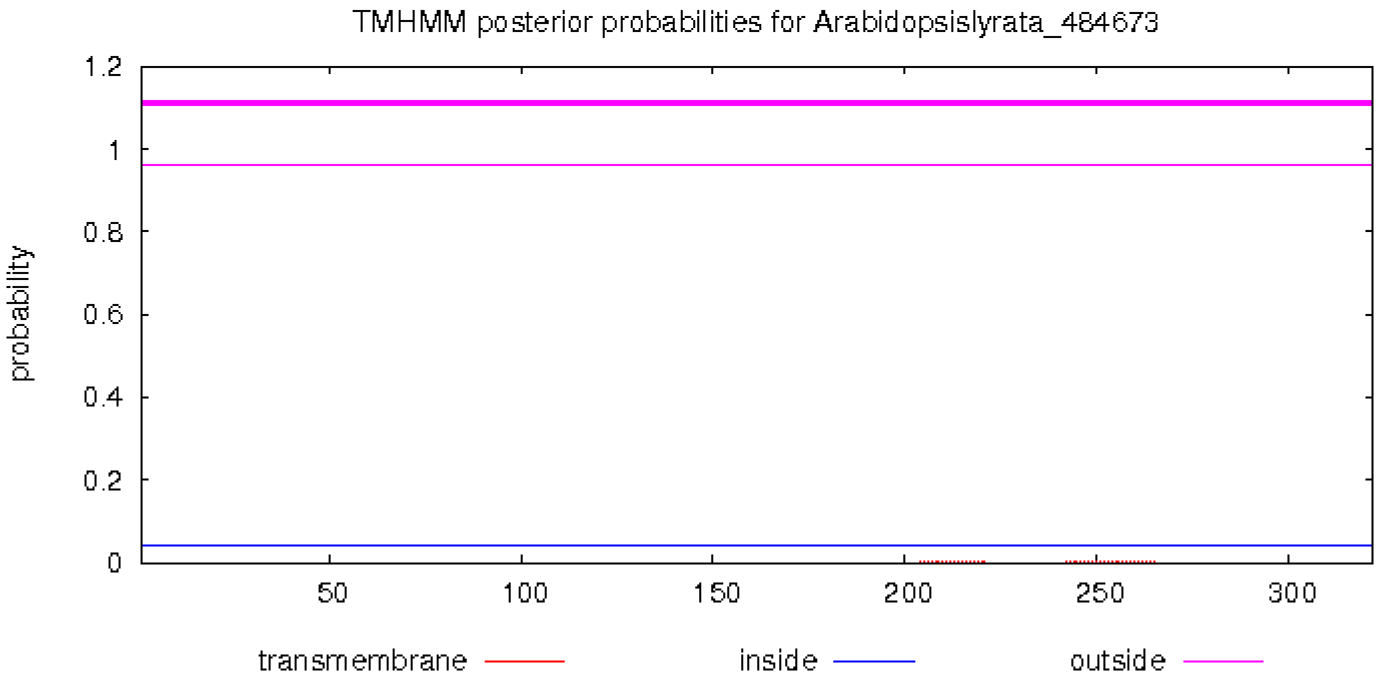

plot in postscript, script for making the plot in gnuplot, data for plot

```
# Arabidopsislyrata_486095 Length: 301
# Arabidopsislyrata_486095 Number of predicted TMHs:  0
# Arabidopsislyrata_486095 Exp number of AAs in TMHs: 0.00281
# Arabidopsislyrata_486095 Exp number, first 60 AAs:  0.00038
# Arabidopsislyrata_486095 Total prob of N-in:        0.21215
Arabidopsislyrata_486095        TMHMM2.0        outside      1   301
```

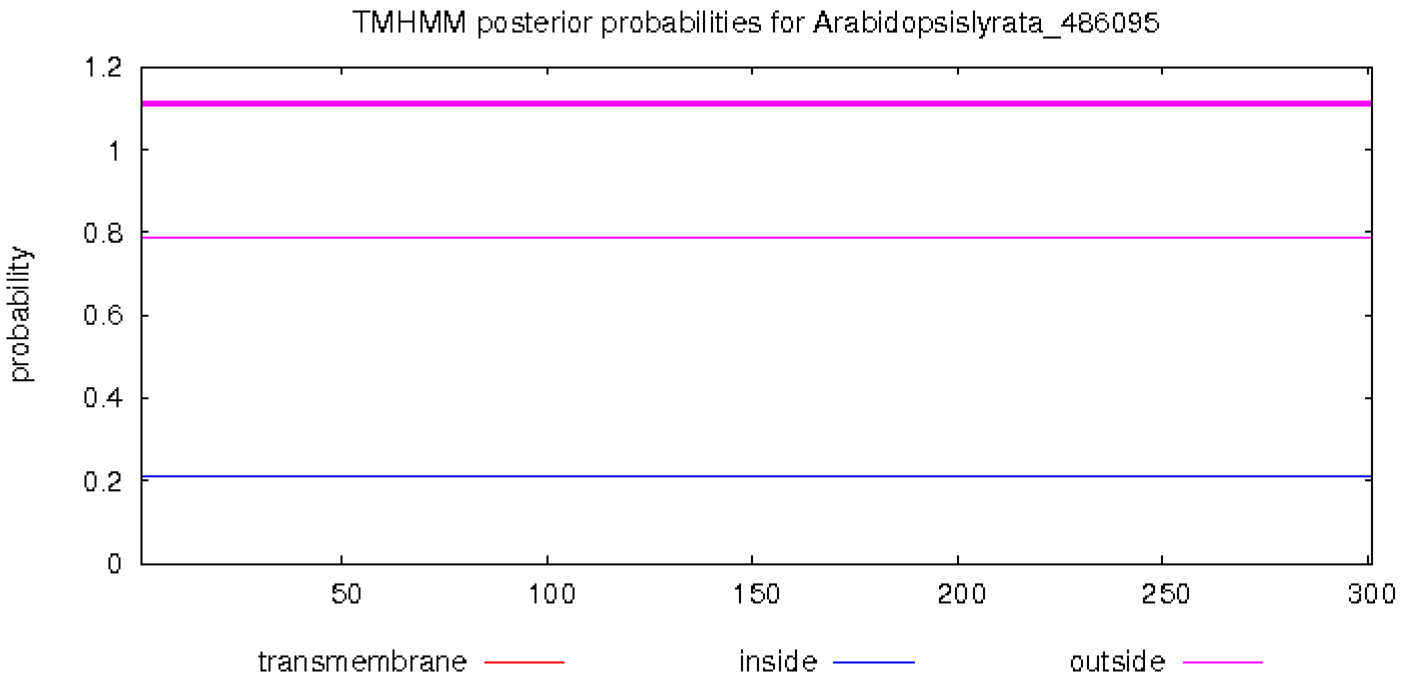

plot in postscript, script for making the plot in gnuplot, data for plot

```
# Arabidopsislyrata_487602 Length: 332
# Arabidopsislyrata_487602 Number of predicted TMHs:  0
# Arabidopsislyrata_487602 Exp number of AAs in TMHs: 0.0002
# Arabidopsislyrata_487602 Exp number, first 60 AAs:  0
# Arabidopsislyrata_487602 Total prob of N-in:        0.00807
Arabidopsislyrata_487602	TMHMM2.0	outside	     1   332
```

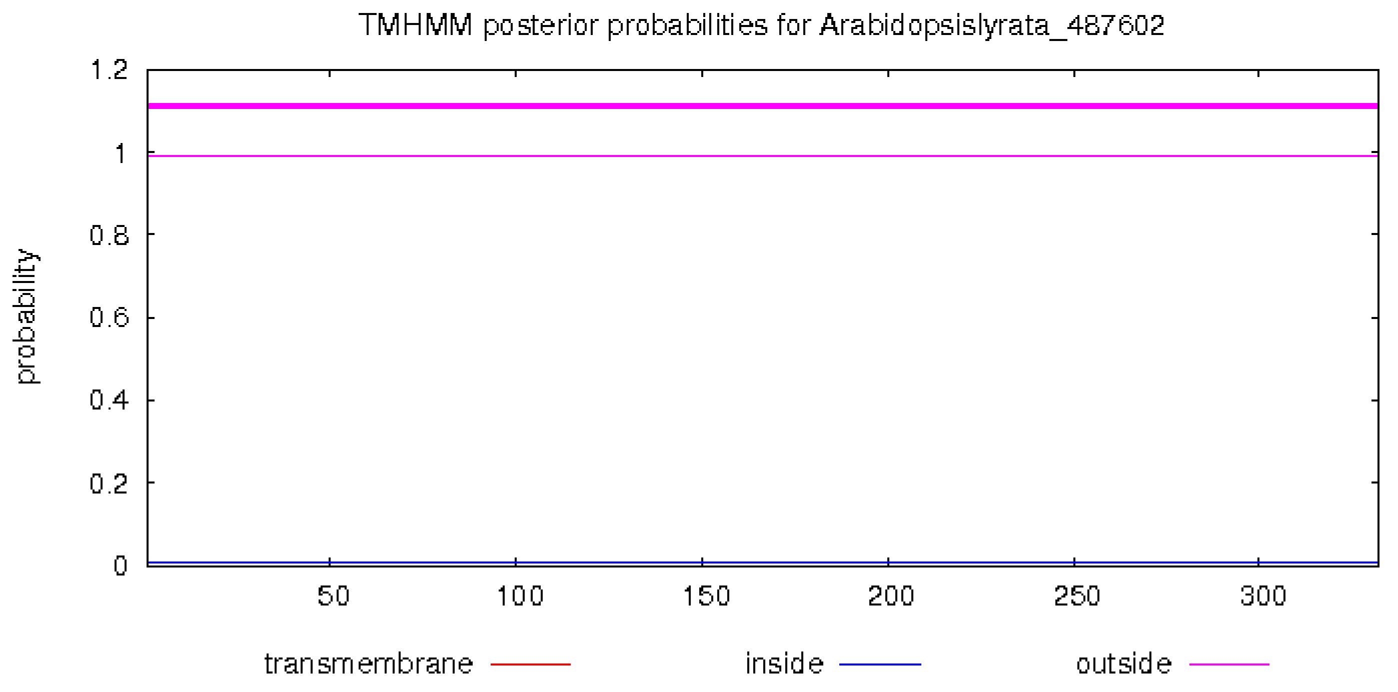

plot in postscript, script for making the plot in gnuplot, data for plot

---

```
# Arabidopsislyrata_487745 Length: 411
# Arabidopsislyrata_487745 Number of predicted TMHs:  0
# Arabidopsislyrata_487745 Exp number of AAs in TMHs: 0.00018
# Arabidopsislyrata_487745 Exp number, first 60 AAs:  0.00018
# Arabidopsislyrata_487745 Total prob of N-in:        0.00286
Arabidopsislyrata_487745	TMHMM2.0	outside	     1   411
```

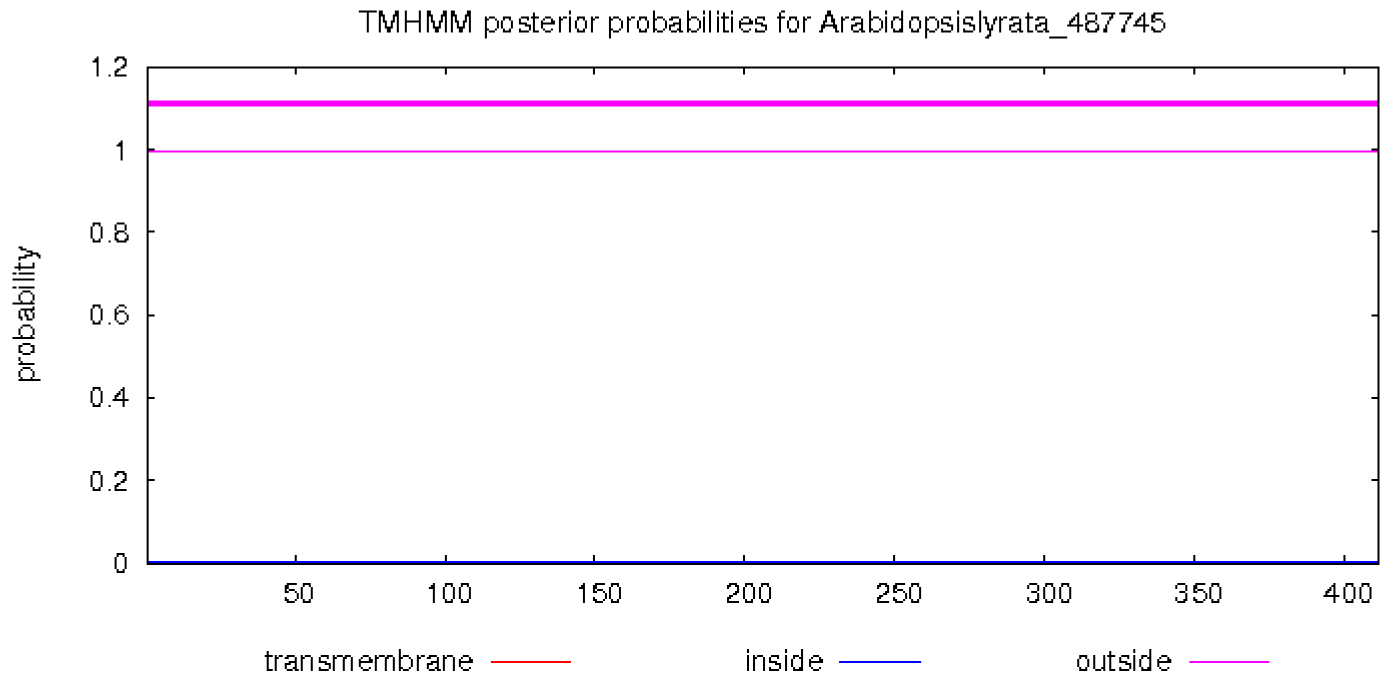

plot in postscript, script for making the plot in gnuplot, data for plot

---

```
# Arabidopsislyrata_488590 Length: 476
# Arabidopsislyrata_488590 Number of predicted TMHs:  0
# Arabidopsislyrata_488590 Exp number of AAs in TMHs: 0.00079
# Arabidopsislyrata_488590 Exp number, first 60 AAs:  0
# Arabidopsislyrata_488590 Total prob of N-in:        0.00018
Arabidopsislyrata_488590	TMHMM2.0	outside	     1   476
```

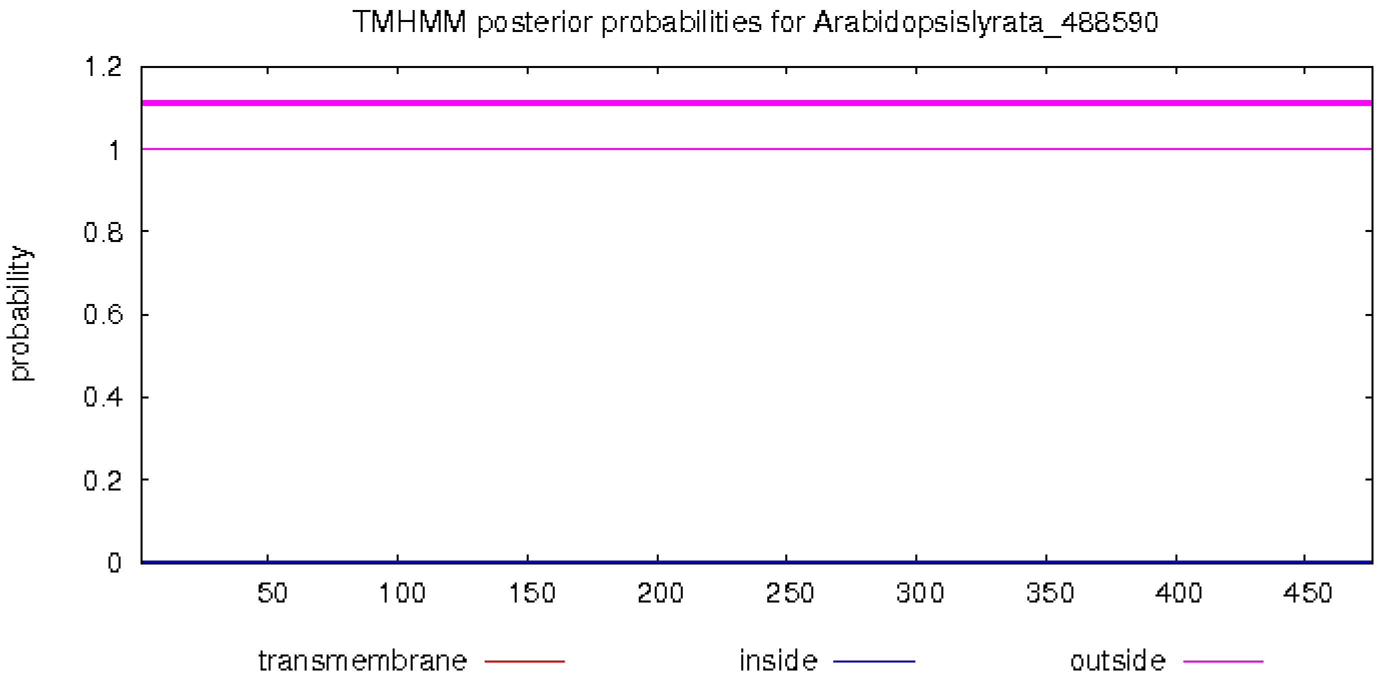

plot in postscript, script for making the plot in gnuplot, data for plot

```
# Arabidopsislyrata_488703 Length: 143
# Arabidopsislyrata_488703 Number of predicted TMHs:  0
# Arabidopsislyrata_488703 Exp number of AAs in TMHs: 0.00061
# Arabidopsislyrata_488703 Exp number, first 60 AAs:  0.00061
# Arabidopsislyrata_488703 Total prob of N-in:        0.08591
Arabidopsislyrata_488703	TMHMM2.0	outside	     1   143
```

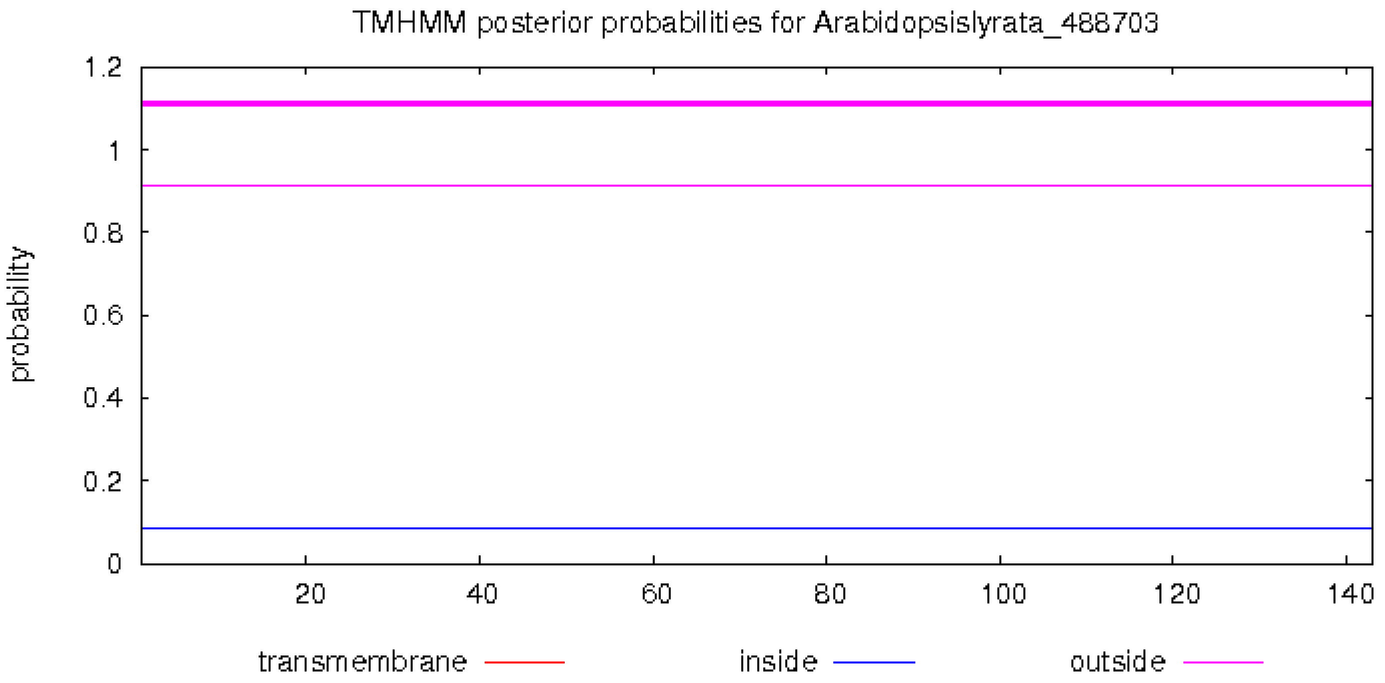

plot in postscript, script for making the plot in gnuplot, data for plot

```
# Arabidopsislyrata_489094 Length: 235
# Arabidopsislyrata_489094 Number of predicted TMHs:  0
# Arabidopsislyrata_489094 Exp number of AAs in TMHs: 0.00016
# Arabidopsislyrata_489094 Exp number, first 60 AAs:  0.00016
# Arabidopsislyrata_489094 Total prob of N-in:        0.04813
Arabidopsislyrata_489094	TMHMM2.0	outside	     1   235
```

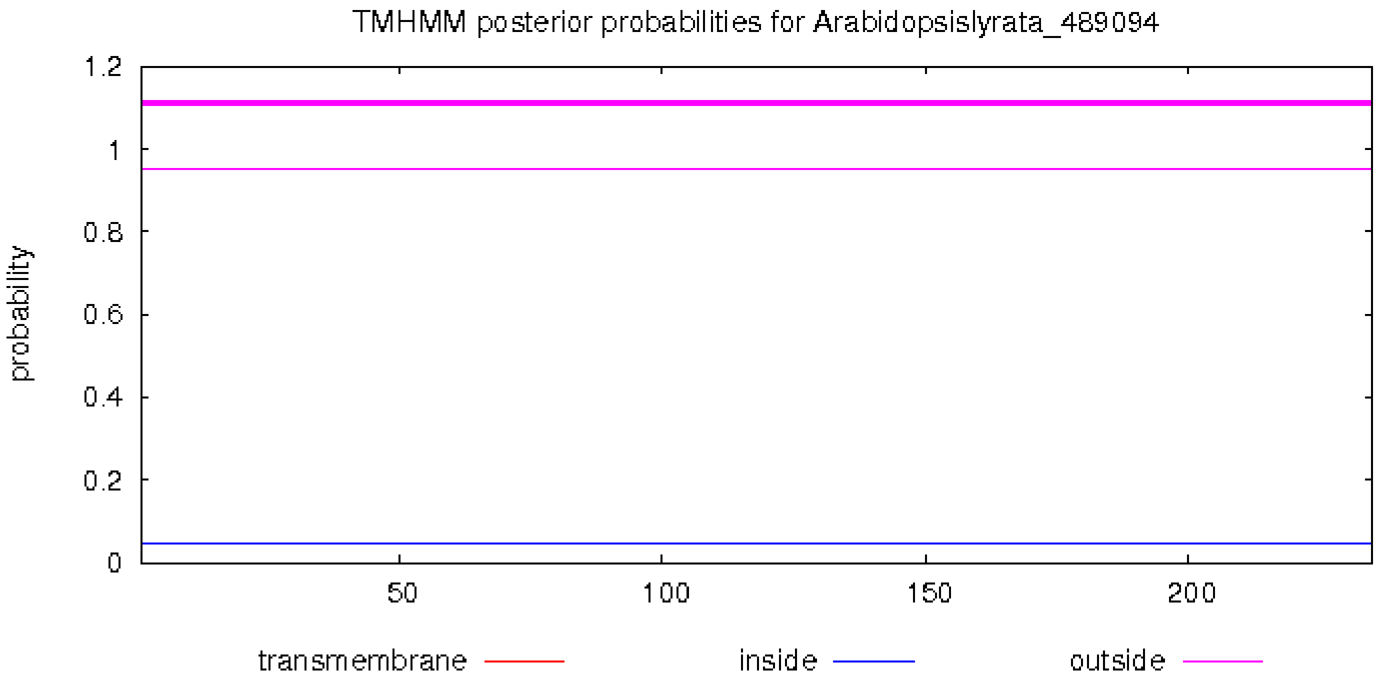

plot in postscript, script for making the plot in gnuplot, data for plot

```
# Arabidopsislyrata_491017 Length: 376
# Arabidopsislyrata_491017 Number of predicted TMHs:  0
# Arabidopsislyrata_491017 Exp number of AAs in TMHs: 0
# Arabidopsislyrata_491017 Exp number, first 60 AAs:  0
# Arabidopsislyrata_491017 Total prob of N-in:        0.01203
Arabidopsislyrata_491017	TMHMM2.0	outside	     1   376
```

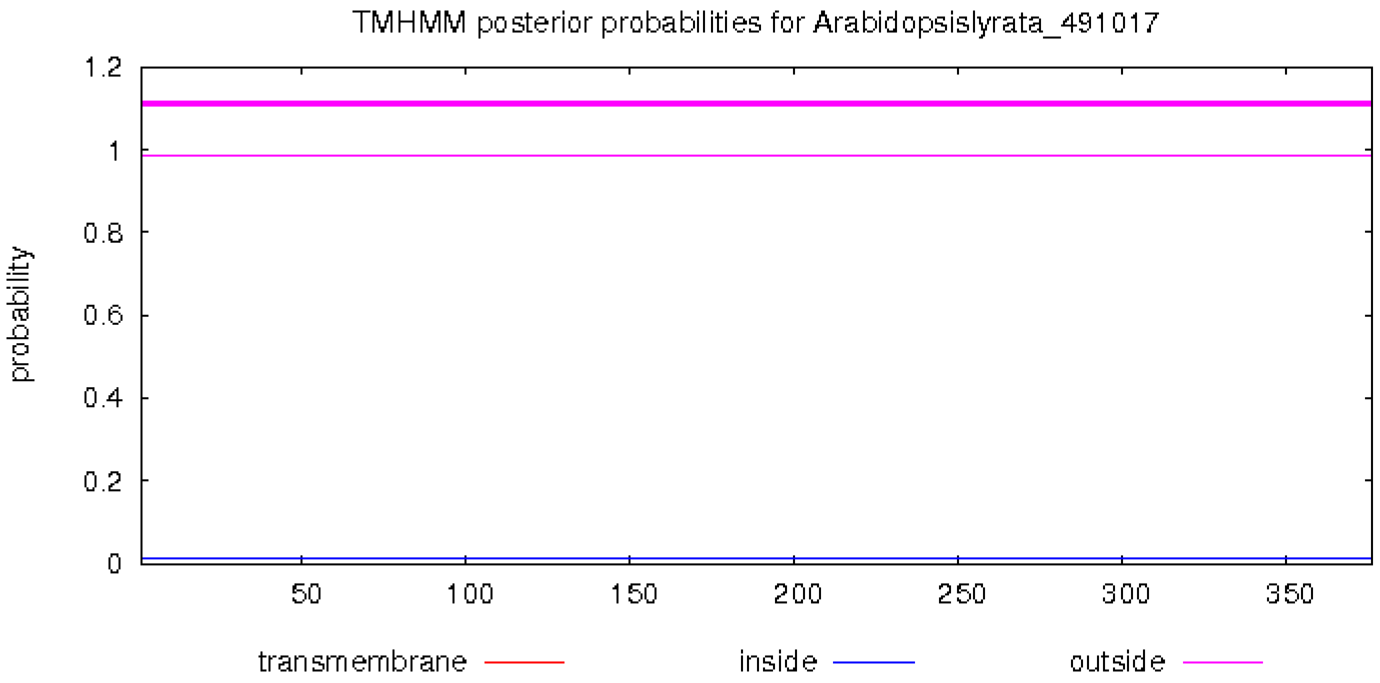

plot in postscript, script for making the plot in gnuplot, data for plot

```
# Arabidopsislyrata_491088 Length: 507
# Arabidopsislyrata_491088 Number of predicted TMHs:  1
# Arabidopsislyrata_491088 Exp number of AAs in TMHs: 22.1354
# Arabidopsislyrata_491088 Exp number, first 60 AAs:  0
# Arabidopsislyrata_491088 Total prob of N-in:        0.43811
Arabidopsislyrata_491088        TMHMM2.0        outside      1   483
Arabidopsislyrata_491088        TMHMM2.0        TMhelix    484   506
Arabidopsislyrata_491088        TMHMM2.0        inside     507   507
```

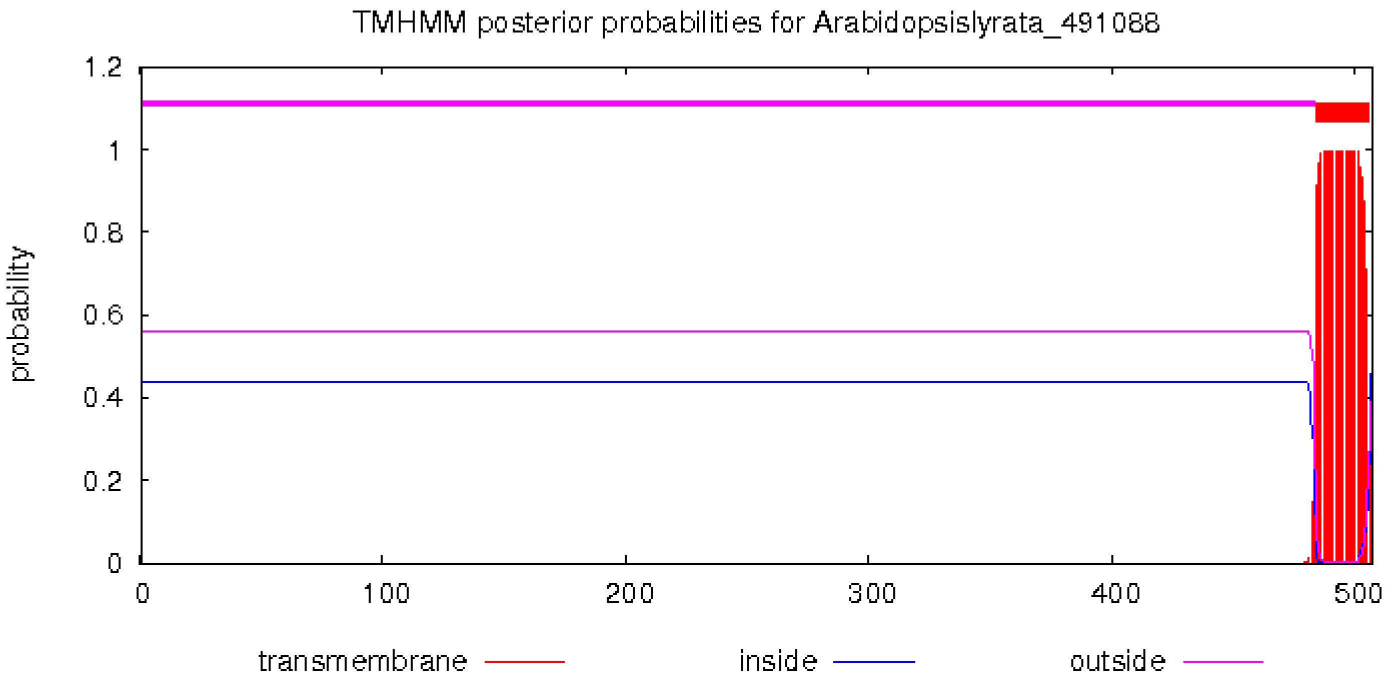

plot in postscript, script for making the plot in gnuplot, data for plot

---

```
# Arabidopsislyrata_491912 Length: 349
# Arabidopsislyrata_491912 Number of predicted TMHs:  0
# Arabidopsislyrata_491912 Exp number of AAs in TMHs: 0
# Arabidopsislyrata_491912 Exp number, first 60 AAs:  0
# Arabidopsislyrata_491912 Total prob of N-in:        0.00613
Arabidopsislyrata_491912        TMHMM2.0        outside      1   349
```

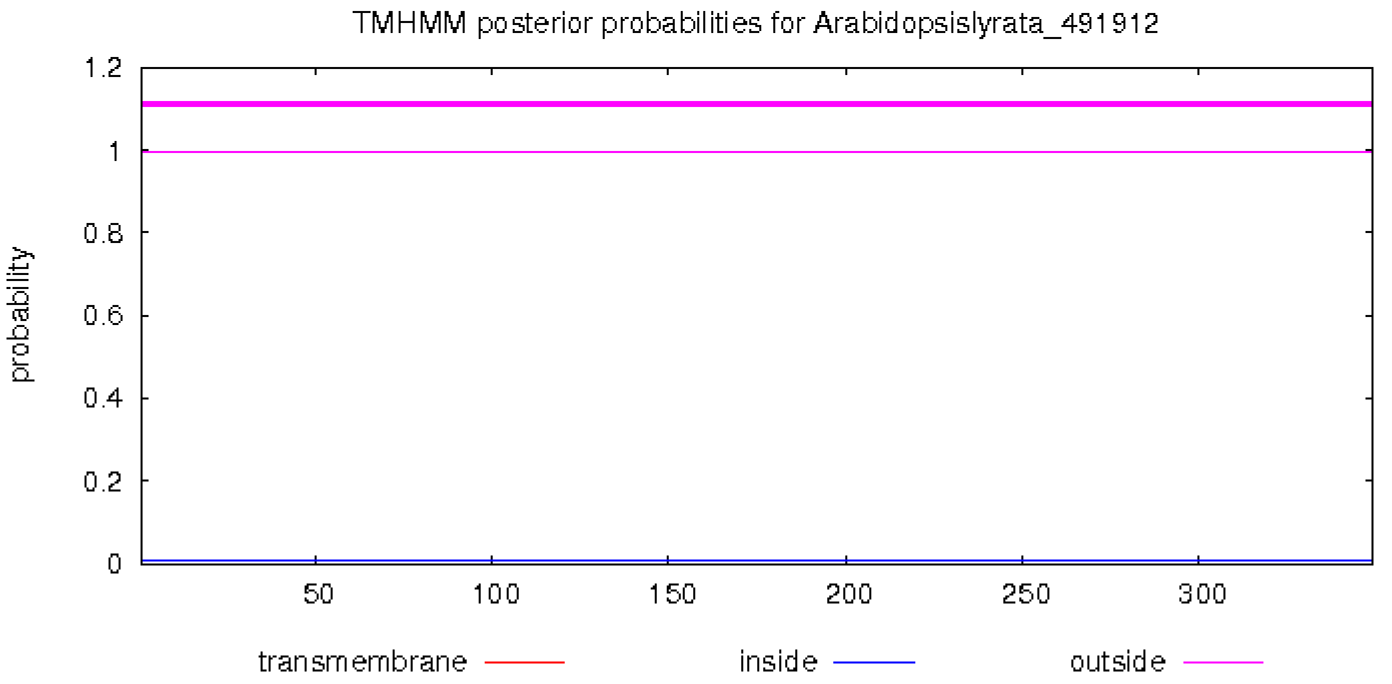

plot in postscript, script for making the plot in gnuplot, data for plot

```
# Arabidopsislyrata_492058 Length: 314
# Arabidopsislyrata_492058 Number of predicted TMHs:  0
# Arabidopsislyrata_492058 Exp number of AAs in TMHs: 0.00137
# Arabidopsislyrata_492058 Exp number, first 60 AAs:  0.00037
# Arabidopsislyrata_492058 Total prob of N-in:        0.01786
Arabidopsislyrata_492058	TMHMM2.0	outside	     1   314
```

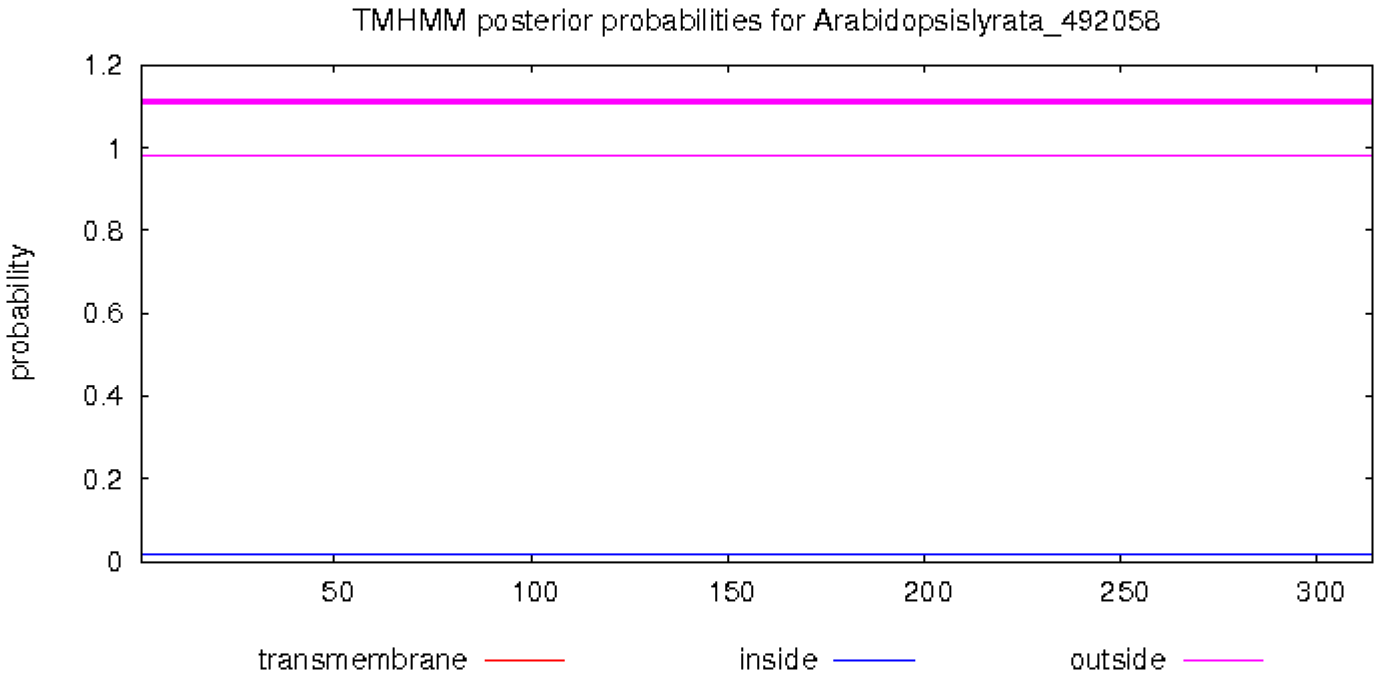

plot in postscript, script for making the plot in gnuplot, data for plot

```
# Arabidopsislyrata_494028 Length: 288
# Arabidopsislyrata_494028 Number of predicted TMHs:  0
# Arabidopsislyrata_494028 Exp number of AAs in TMHs: 0.00102
# Arabidopsislyrata_494028 Exp number, first 60 AAs:  0.00082
# Arabidopsislyrata_494028 Total prob of N-in:        0.06341
Arabidopsislyrata_494028	TMHMM2.0	outside	     1   288
```

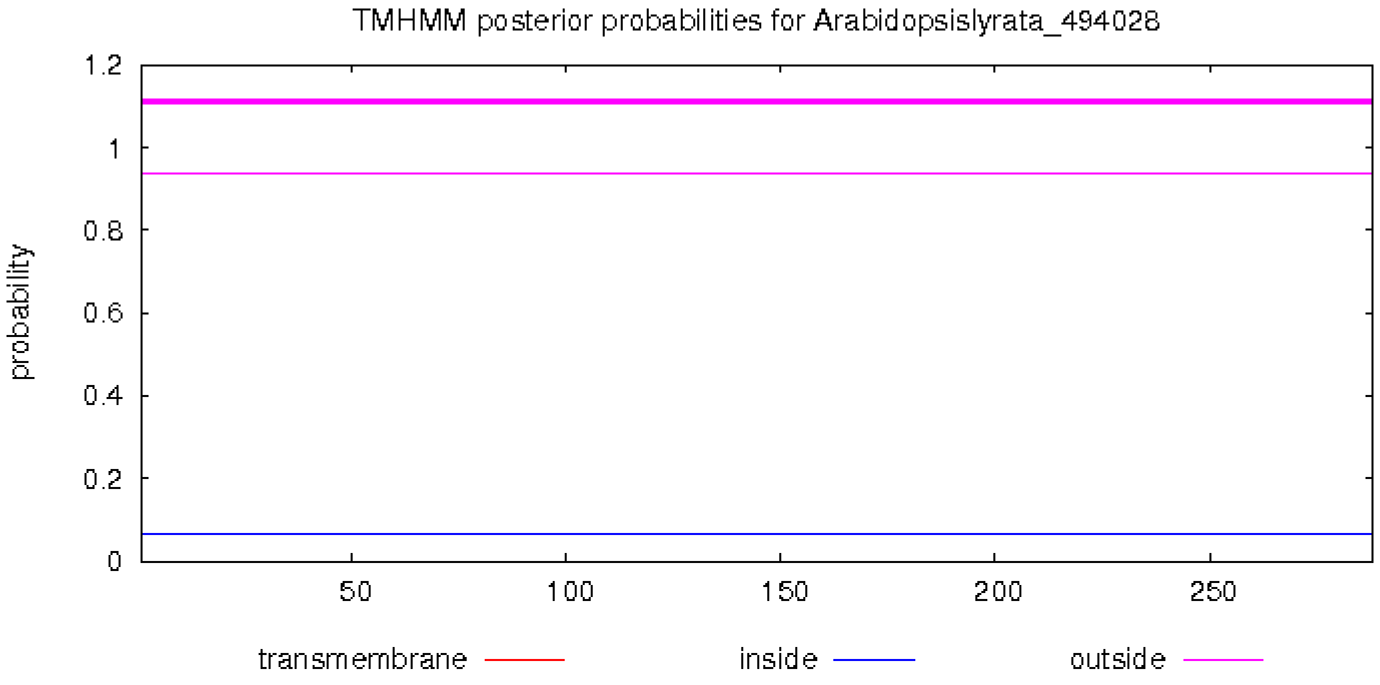

plot in postscript, script for making the plot in gnuplot, data for plot

```
# Arabidopsislyrata_494319 Length: 292
# Arabidopsislyrata_494319 Number of predicted TMHs:  0
# Arabidopsislyrata_494319 Exp number of AAs in TMHs: 0
# Arabidopsislyrata_494319 Exp number, first 60 AAs:  0
# Arabidopsislyrata_494319 Total prob of N-in:        0.00215
Arabidopsislyrata_494319        TMHMM2.0        outside      1   292
```

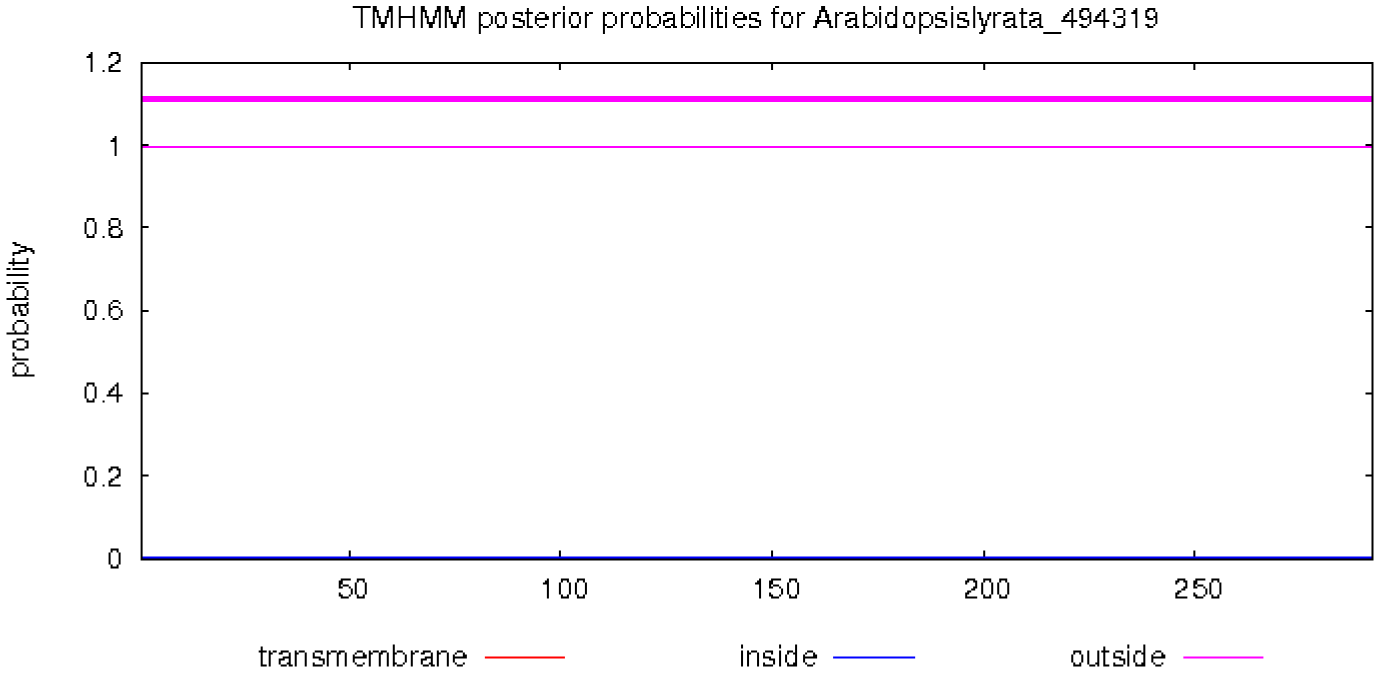

plot in postscript, script for making the plot in gnuplot, data for plot

```
# Arabidopsislyrata_495144 Length: 193
# Arabidopsislyrata_495144 Number of predicted TMHs:  0
# Arabidopsislyrata_495144 Exp number of AAs in TMHs: 0.00177
# Arabidopsislyrata_495144 Exp number, first 60 AAs:  0
# Arabidopsislyrata_495144 Total prob of N-in:        0.12752
Arabidopsislyrata_495144        TMHMM2.0        outside      1   193
```

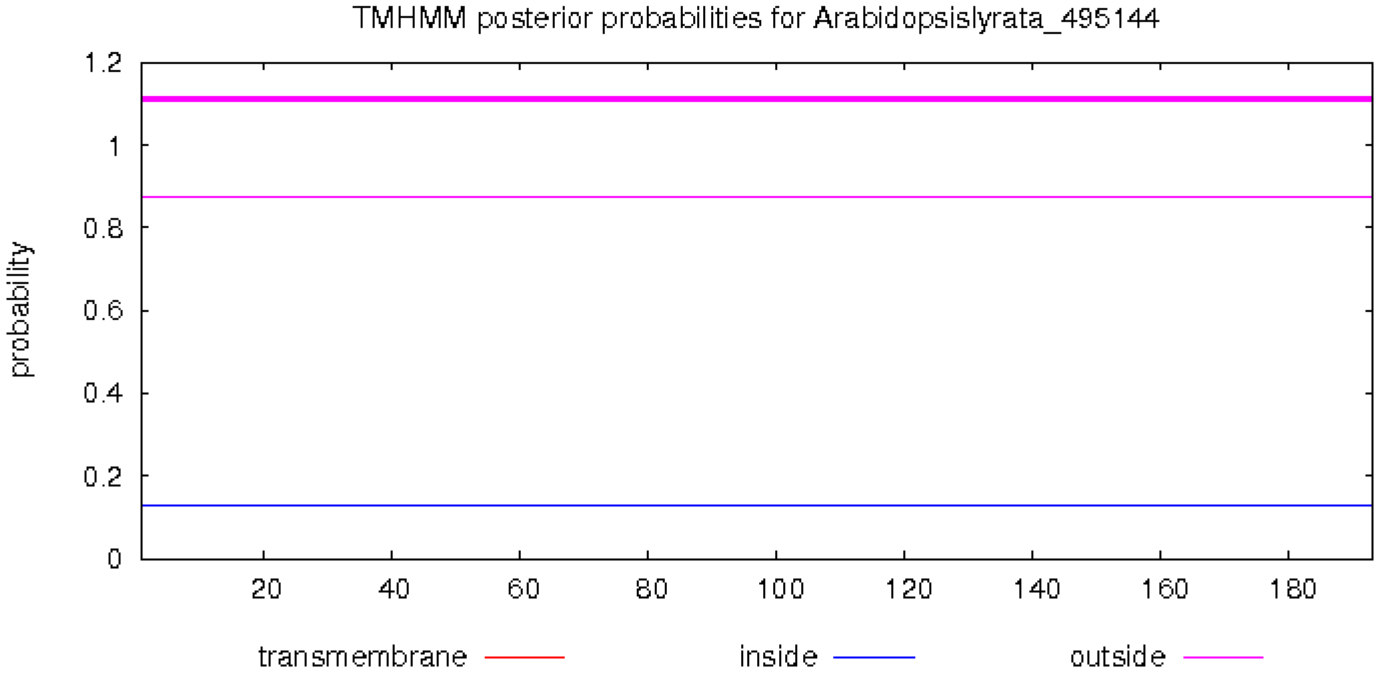

plot in postscript, script for making the plot in gnuplot, data for plot

```
# Arabidopsislyrata_495753 Length: 390
# Arabidopsislyrata_495753 Number of predicted TMHs:  0
# Arabidopsislyrata_495753 Exp number of AAs in TMHs: 0.10083
# Arabidopsislyrata_495753 Exp number, first 60 AAs:  0
# Arabidopsislyrata_495753 Total prob of N-in:        0.03499
Arabidopsislyrata_495753        TMHMM2.0        outside      1   390
```

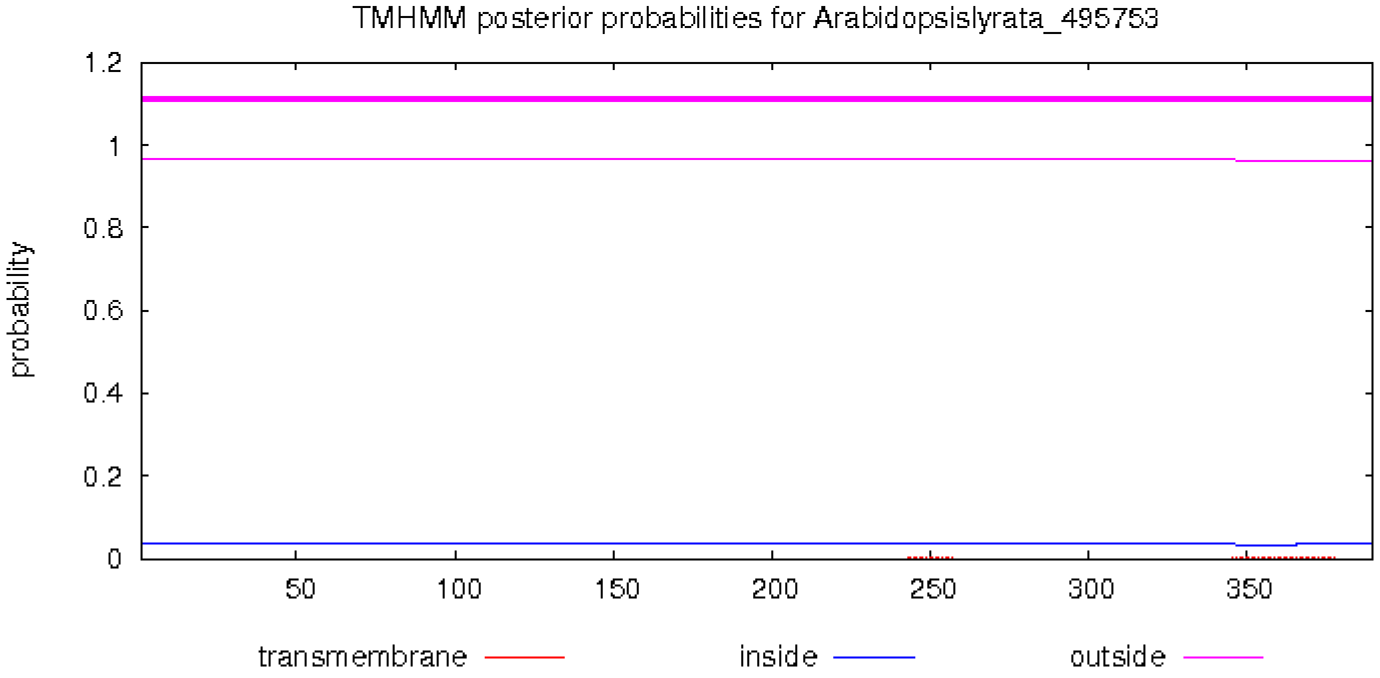

plot in postscript, script for making the plot in gnuplot, data for plot

---

```
# Arabidopsislyrata_496302 Length: 336
# Arabidopsislyrata_496302 Number of predicted TMHs:  0
# Arabidopsislyrata_496302 Exp number of AAs in TMHs: 0
# Arabidopsislyrata_496302 Exp number, first 60 AAs:  0
# Arabidopsislyrata_496302 Total prob of N-in:        0.01237
Arabidopsislyrata_496302        TMHMM2.0        outside      1   336
```

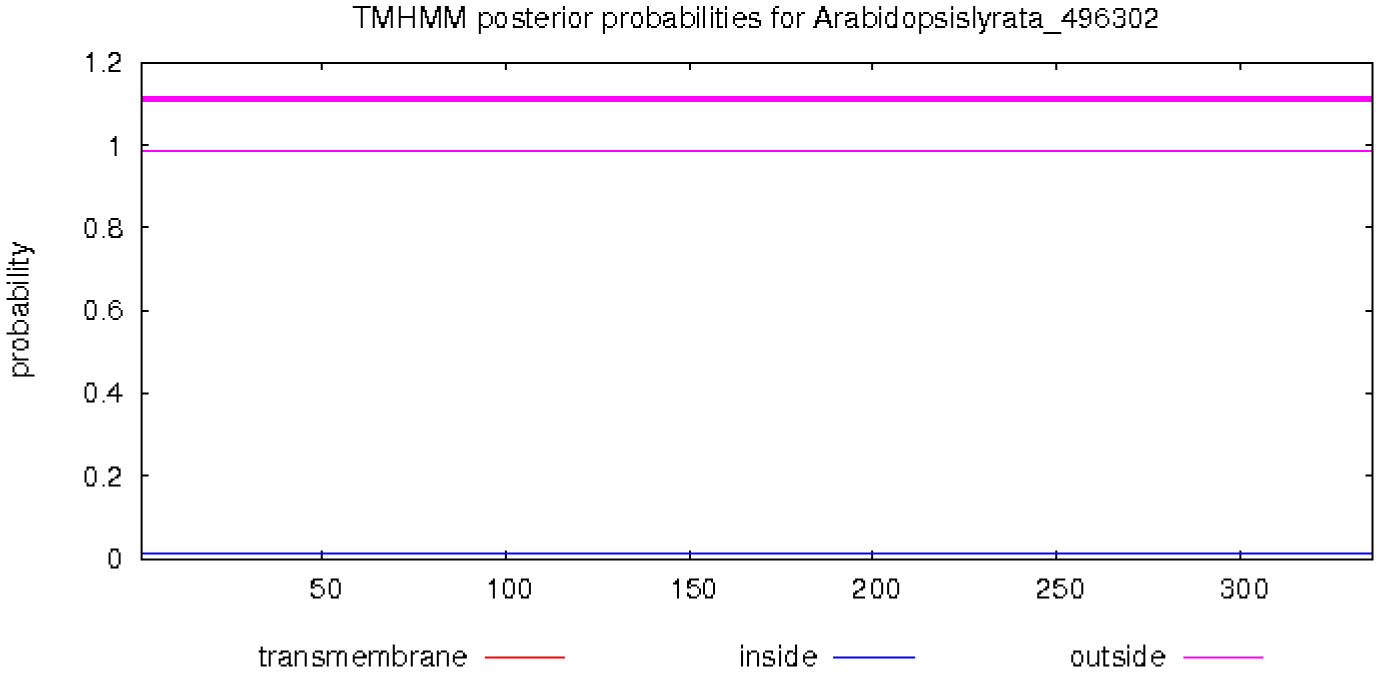

plot in postscript, script for making the plot in gnuplot, data for plot

---

```
# Arabidopsislyrata_496601 Length: 363
# Arabidopsislyrata_496601 Number of predicted TMHs:  0
# Arabidopsislyrata_496601 Exp number of AAs in TMHs: 0.00018
# Arabidopsislyrata_496601 Exp number, first 60 AAs:  0.00018
# Arabidopsislyrata_496601 Total prob of N-in:        0.00434
Arabidopsislyrata_496601        TMHMM2.0        outside      1   363
```

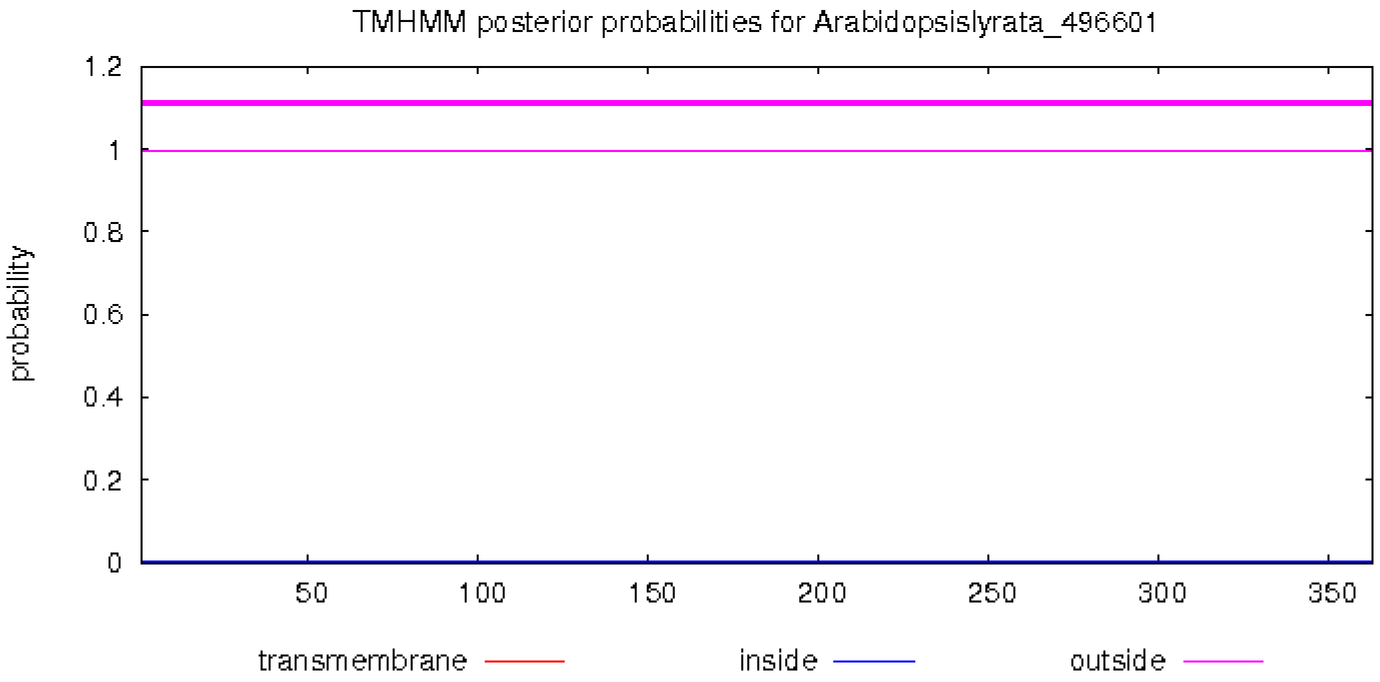

plot in postscript, script for making the plot in gnuplot, data for plot

---

```
# Arabidopsislyrata_496665 Length: 188
# Arabidopsislyrata_496665 Number of predicted TMHs:  0
# Arabidopsislyrata_496665 Exp number of AAs in TMHs: 0
# Arabidopsislyrata_496665 Exp number, first 60 AAs:  0
# Arabidopsislyrata_496665 Total prob of N-in:        0.00724
Arabidopsislyrata_496665        TMHMM2.0        outside      1   188
```

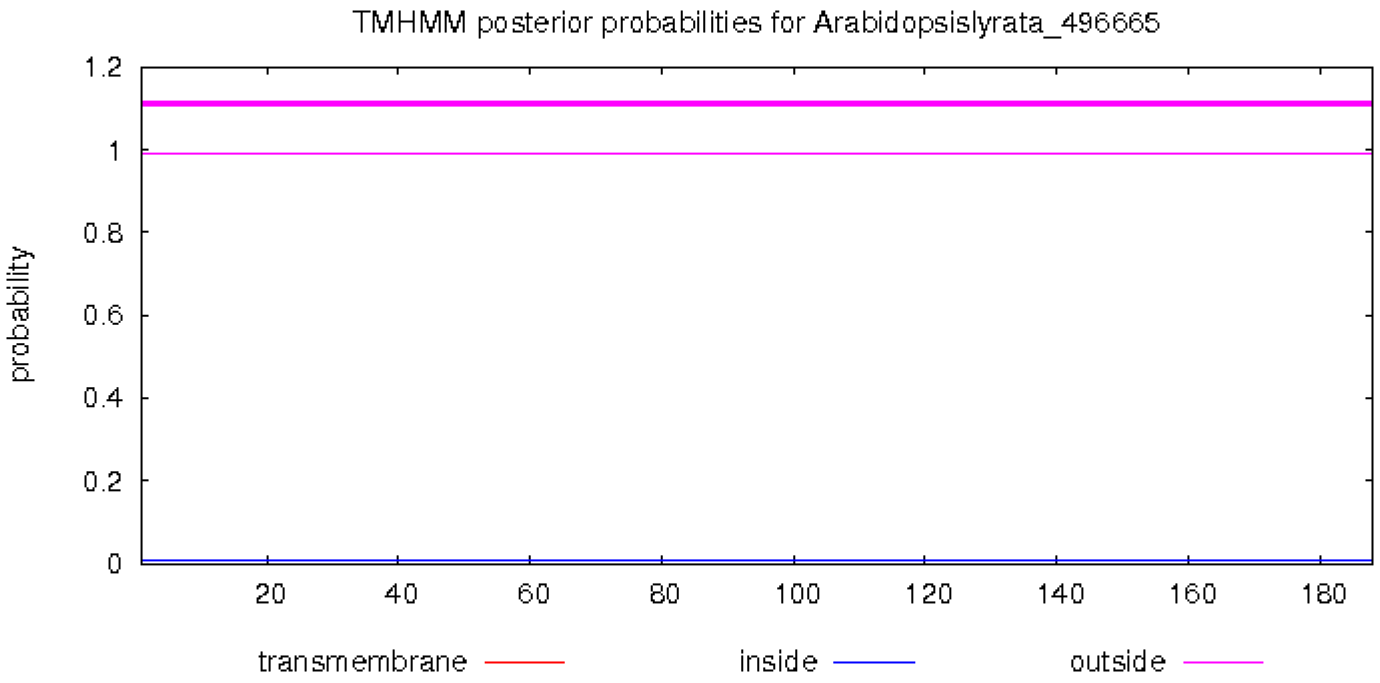

plot in postscript, script for making the plot in gnuplot, data for plot

---

```
# Arabidopsislyrata_496976 Length: 288
# Arabidopsislyrata_496976 Number of predicted TMHs:  0
# Arabidopsislyrata_496976 Exp number of AAs in TMHs: 0.00056
# Arabidopsislyrata_496976 Exp number, first 60 AAs:  0
# Arabidopsislyrata_496976 Total prob of N-in:        0.05613
Arabidopsislyrata_496976        TMHMM2.0        outside      1   288
```

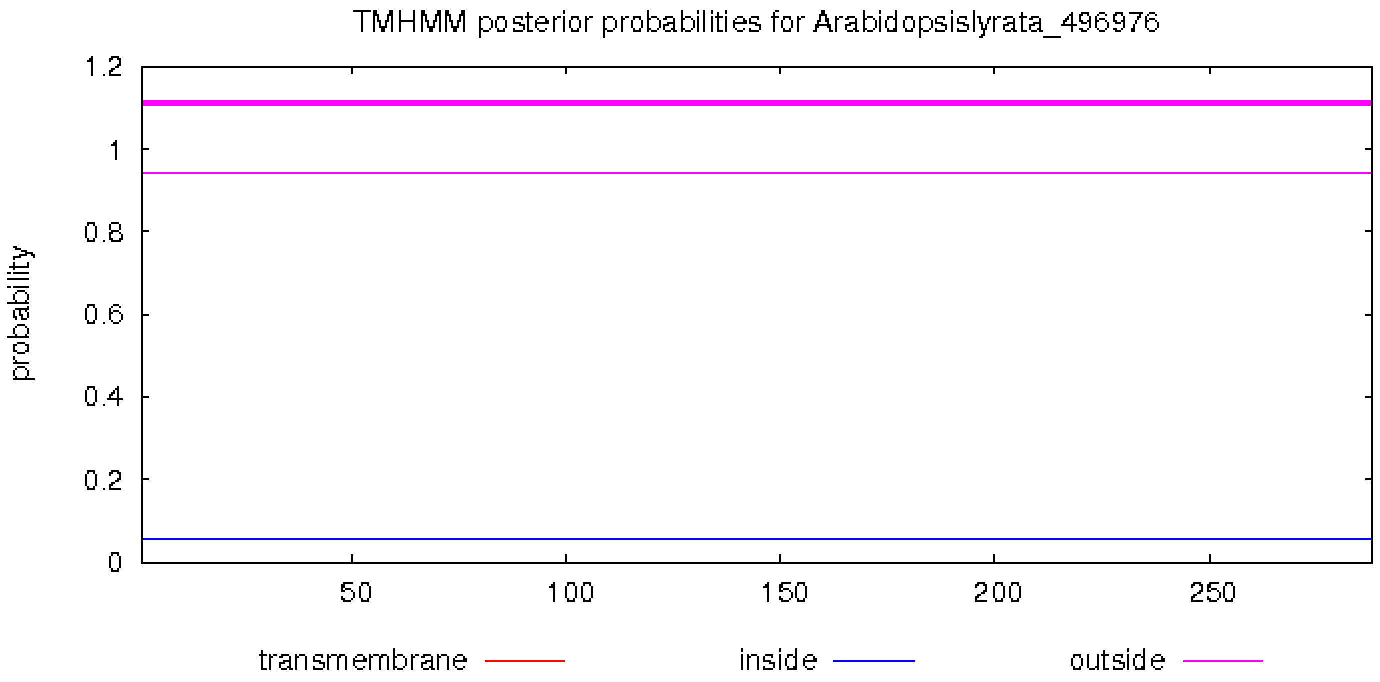

plot in postscript, script for making the plot in gnuplot, data for plot

---

```
# Arabidopsislyrata_857774 Length: 303
# Arabidopsislyrata_857774 Number of predicted TMHs:  0
# Arabidopsislyrata_857774 Exp number of AAs in TMHs: 0.00083
# Arabidopsislyrata_857774 Exp number, first 60 AAs:  0
# Arabidopsislyrata_857774 Total prob of N-in:        0.10089
Arabidopsislyrata_857774        TMHMM2.0        outside      1   303
```

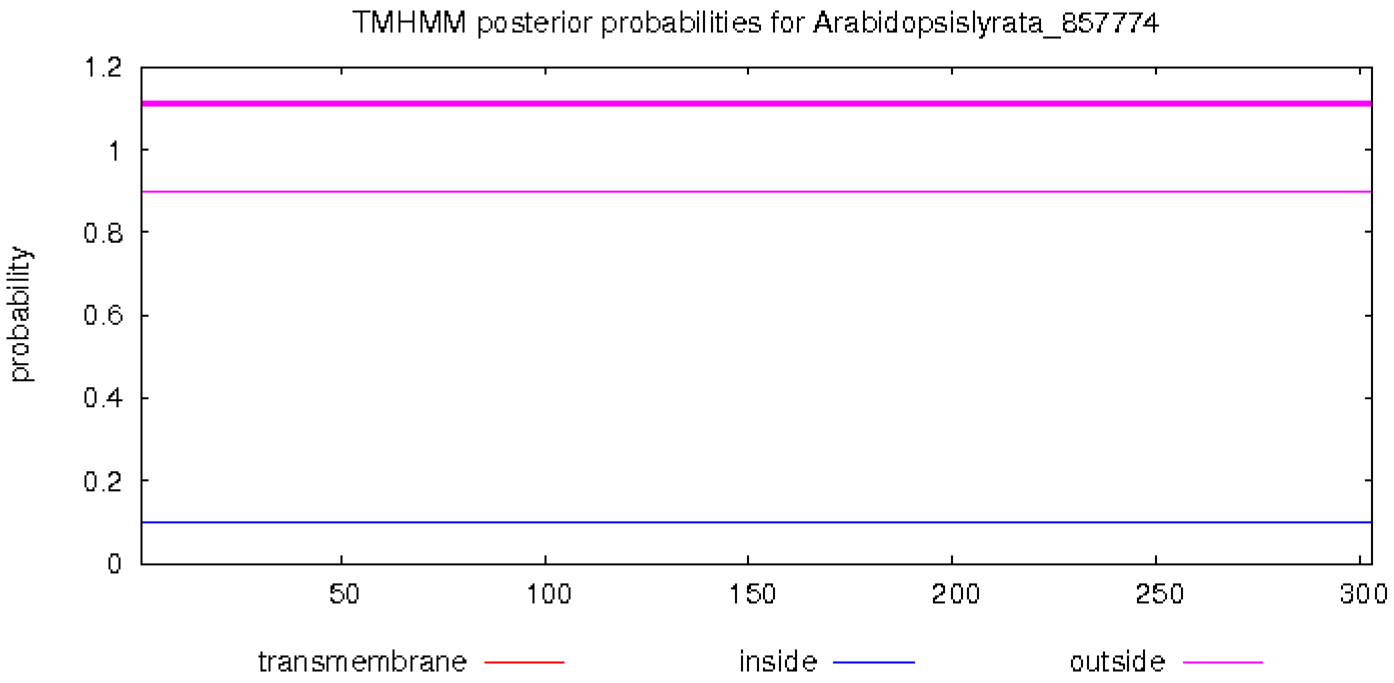

plot in postscript, script for making the plot in gnuplot, data for plot

---

```
# Arabidopsislyrata_858896 Length: 261
# Arabidopsislyrata_858896 Number of predicted TMHs:  0
# Arabidopsislyrata_858896 Exp number of AAs in TMHs: 0.0002
# Arabidopsislyrata_858896 Exp number, first 60 AAs:  0
# Arabidopsislyrata_858896 Total prob of N-in:        0.00313
Arabidopsislyrata_858896        TMHMM2.0        outside      1   261
```

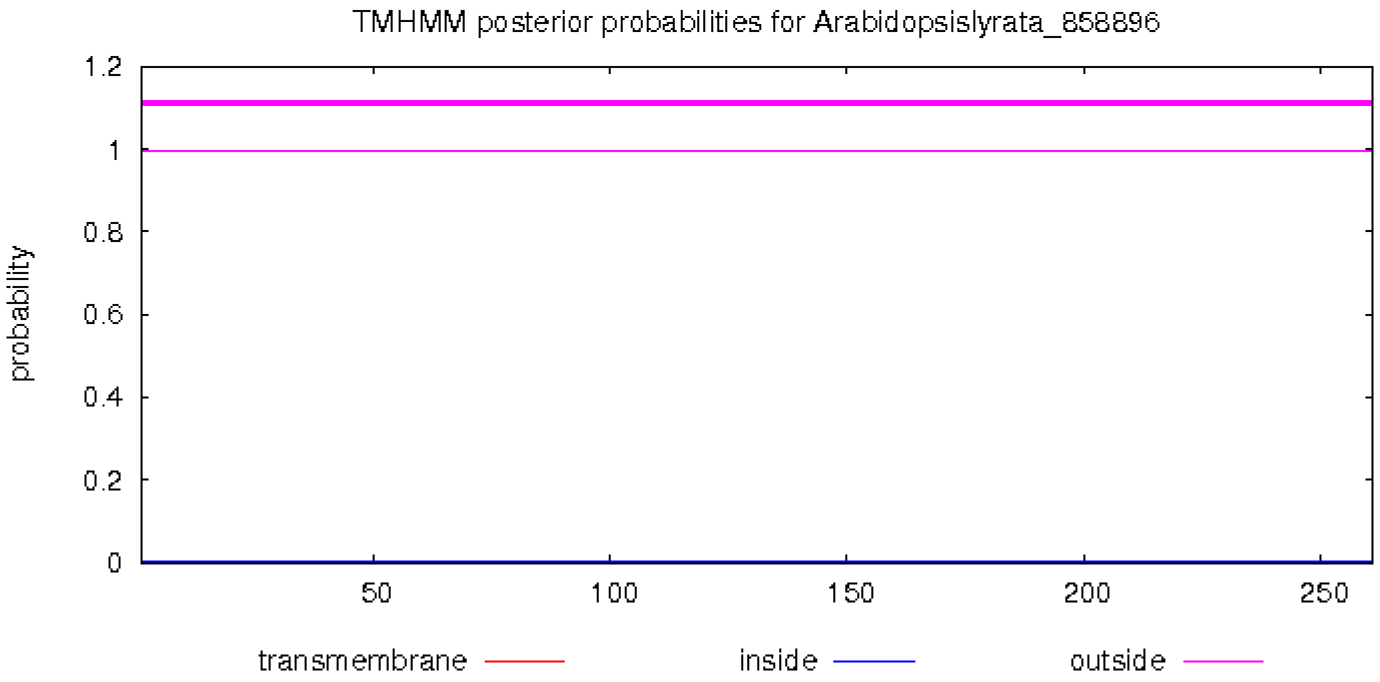

plot in postscript, script for making the plot in gnuplot, data for plot

---

```
# Arabidopsislyrata_860042 Length: 209
# Arabidopsislyrata_860042 Number of predicted TMHs:  0
# Arabidopsislyrata_860042 Exp number of AAs in TMHs: 0.00056
# Arabidopsislyrata_860042 Exp number, first 60 AAs:  0.00038
# Arabidopsislyrata_860042 Total prob of N-in:        0.03876
Arabidopsislyrata_860042        TMHMM2.0        outside      1   209
```

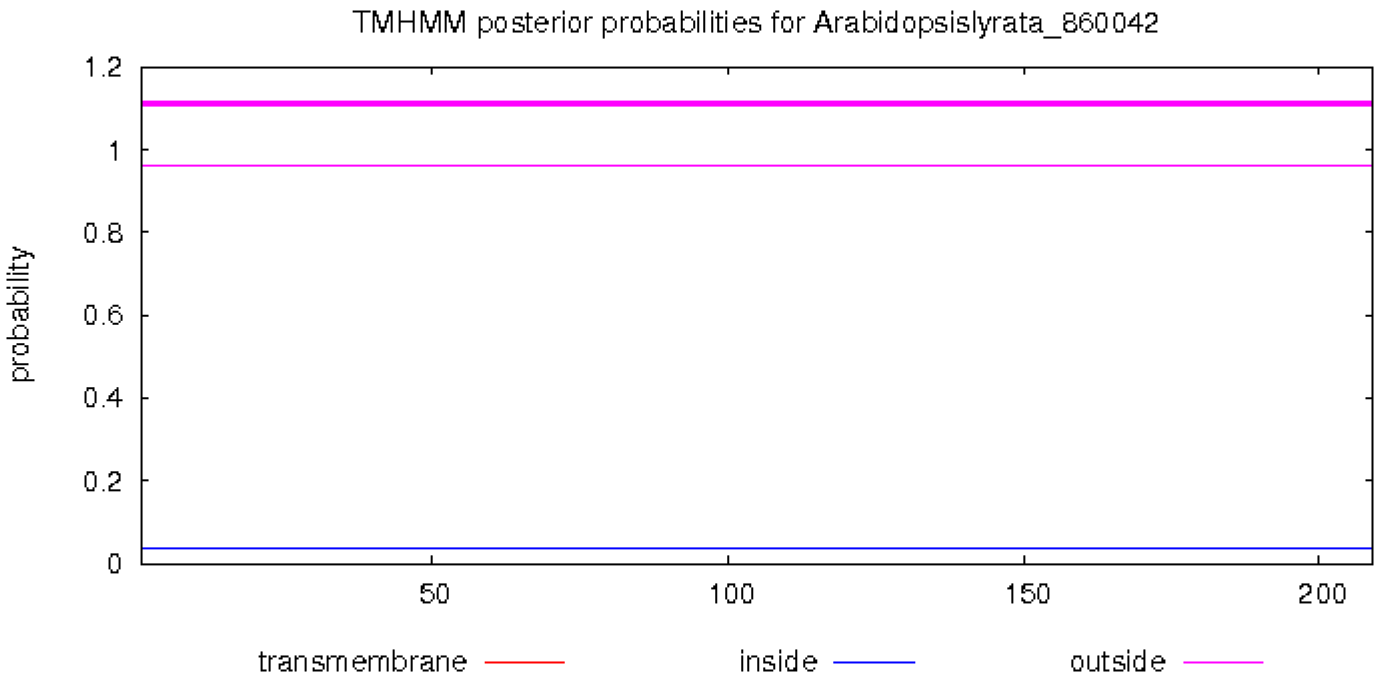

plot in postscript, script for making the plot in gnuplot, data for plot

---

```
# Arabidopsislyrata_860234 Length: 325
# Arabidopsislyrata_860234 Number of predicted TMHs:  0
# Arabidopsislyrata_860234 Exp number of AAs in TMHs: 0.00054
# Arabidopsislyrata_860234 Exp number, first 60 AAs:  0
# Arabidopsislyrata_860234 Total prob of N-in:        0.01936
Arabidopsislyrata_860234        TMHMM2.0        outside      1   325
```

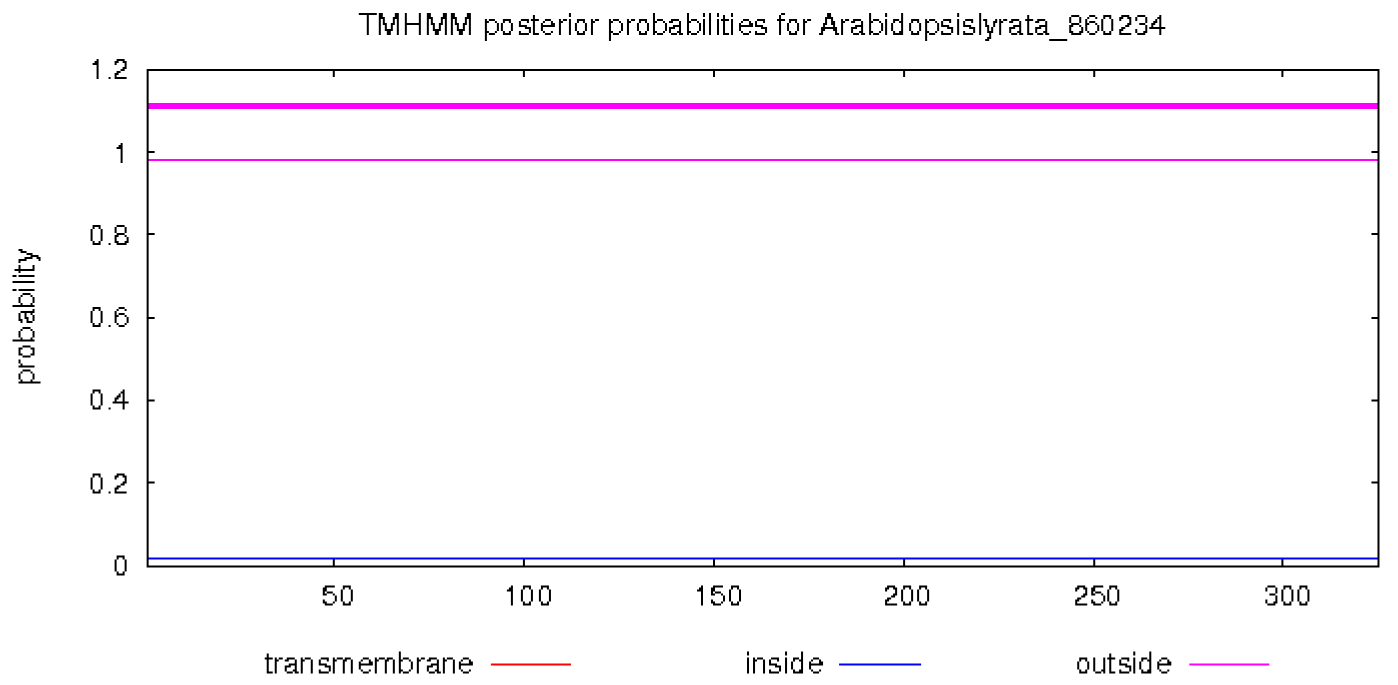

plot in postscript, script for making the plot in gnuplot, data for plot

---

```
# Arabidopsislyrata_861842 Length: 365
# Arabidopsislyrata_861842 Number of predicted TMHs:  0
# Arabidopsislyrata_861842 Exp number of AAs in TMHs: 0.00022
# Arabidopsislyrata_861842 Exp number, first 60 AAs:  0
# Arabidopsislyrata_861842 Total prob of N-in:        0.03298
Arabidopsislyrata_861842        TMHMM2.0        outside      1   365
```

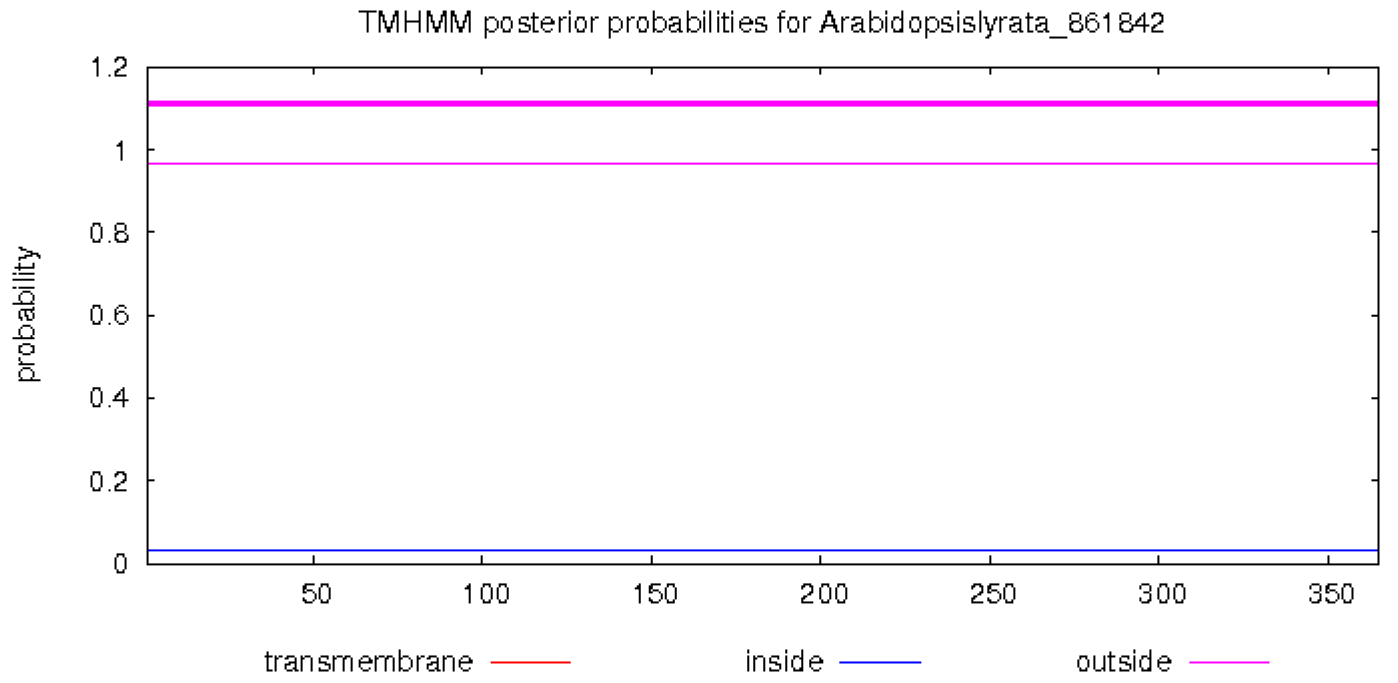

plot in postscript, script for making the plot in gnuplot, data for plot

---

```
# Arabidopsislyrata_863655 Length: 342
# Arabidopsislyrata_863655 Number of predicted TMHs:  0
# Arabidopsislyrata_863655 Exp number of AAs in TMHs: 0
# Arabidopsislyrata_863655 Exp number, first 60 AAs:  0
# Arabidopsislyrata_863655 Total prob of N-in:        0.00431
Arabidopsislyrata_863655        TMHMM2.0        outside      1   342
```

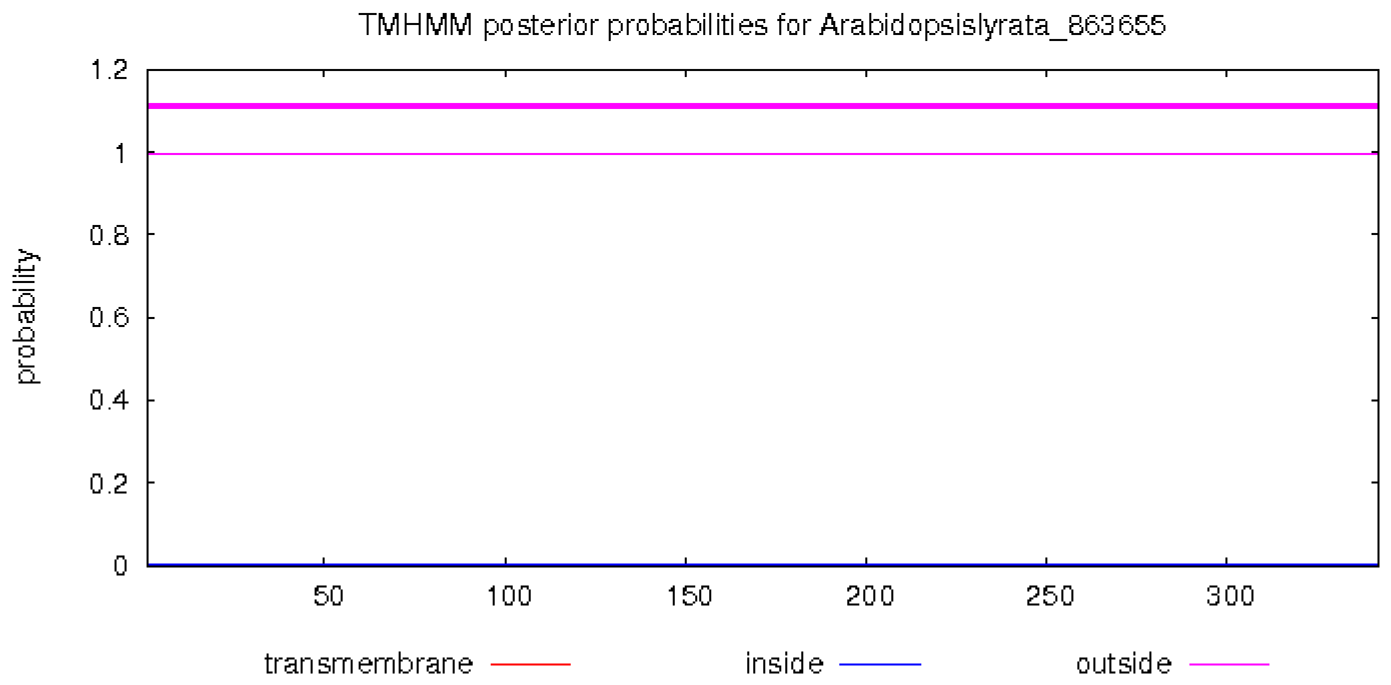

plot in postscript, script for making the plot in gnuplot, data for plot

---

```
# Arabidopsislyrata_865684 Length: 232
# Arabidopsislyrata_865684 Number of predicted TMHs:  0
# Arabidopsislyrata_865684 Exp number of AAs in TMHs: 0.02054
# Arabidopsislyrata_865684 Exp number, first 60 AAs:  0.02054
# Arabidopsislyrata_865684 Total prob of N-in:        0.01236
Arabidopsislyrata_865684        TMHMM2.0        outside      1   232
```

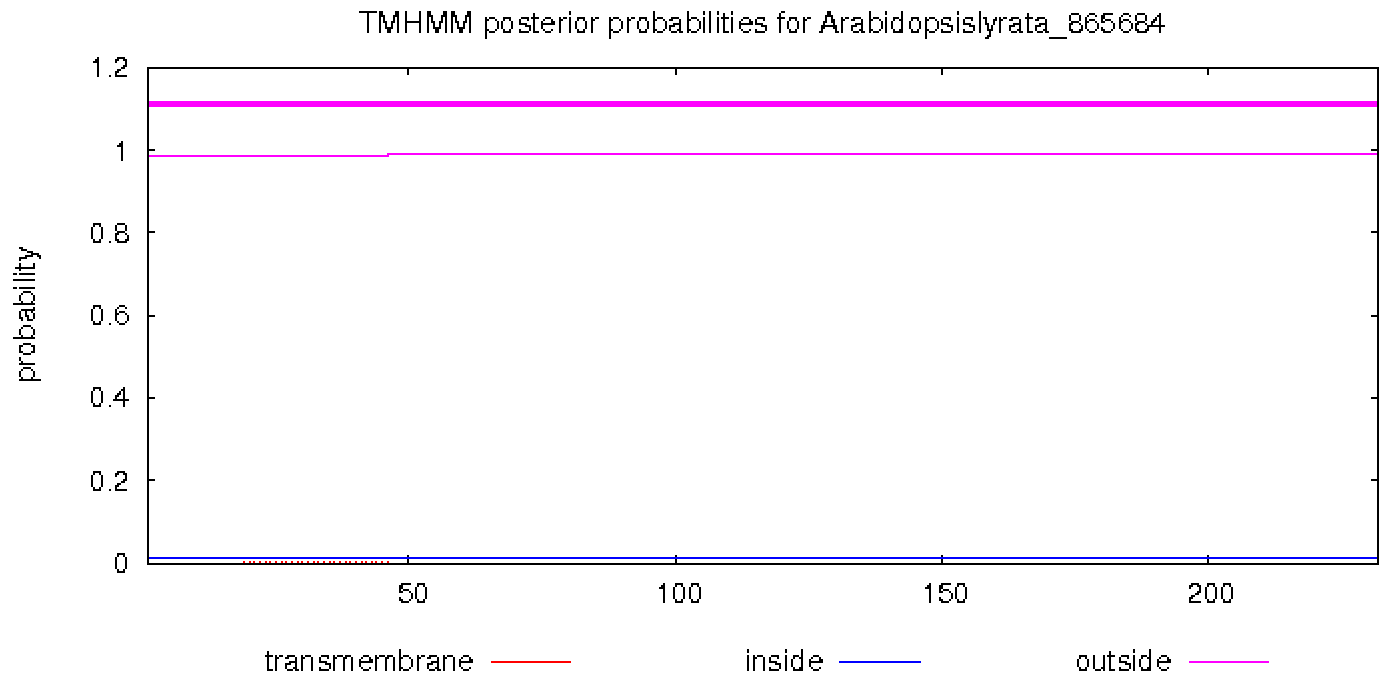

plot in postscript, script for making the plot in gnuplot, data for plot

---

```
# Arabidopsislyrata_866071 Length: 376
# Arabidopsislyrata_866071 Number of predicted TMHs:  0
# Arabidopsislyrata_866071 Exp number of AAs in TMHs: 0.03056
# Arabidopsislyrata_866071 Exp number, first 60 AAs:  0.00042
# Arabidopsislyrata_866071 Total prob of N-in:        0.10725
Arabidopsislyrata_866071	TMHMM2.0	outside	     1   376
```

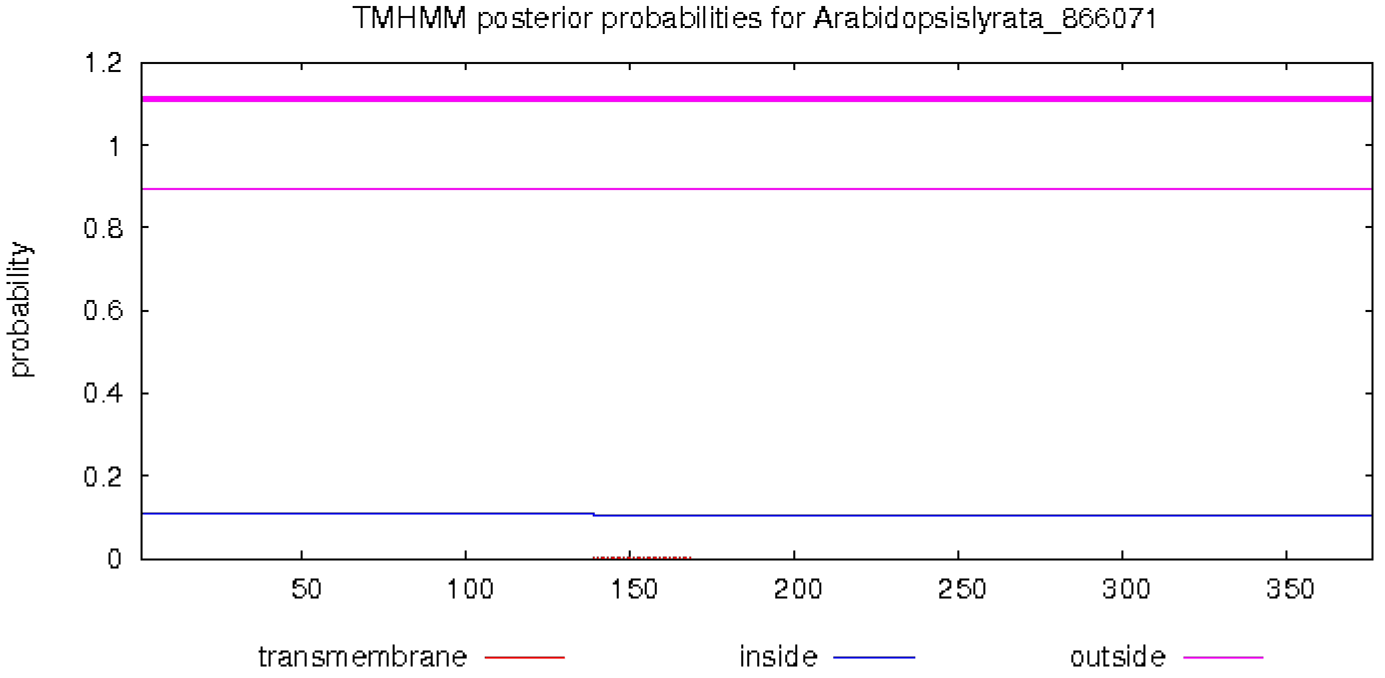

plot in postscript, script for making the plot in gnuplot, data for plot

---

```
# Arabidopsislyrata_871410 Length: 357
# Arabidopsislyrata_871410 Number of predicted TMHs:  0
# Arabidopsislyrata_871410 Exp number of AAs in TMHs: 0.00017
# Arabidopsislyrata_871410 Exp number, first 60 AAs:  0
# Arabidopsislyrata_871410 Total prob of N-in:        0.01769
Arabidopsislyrata_871410	TMHMM2.0	outside	     1   357
```

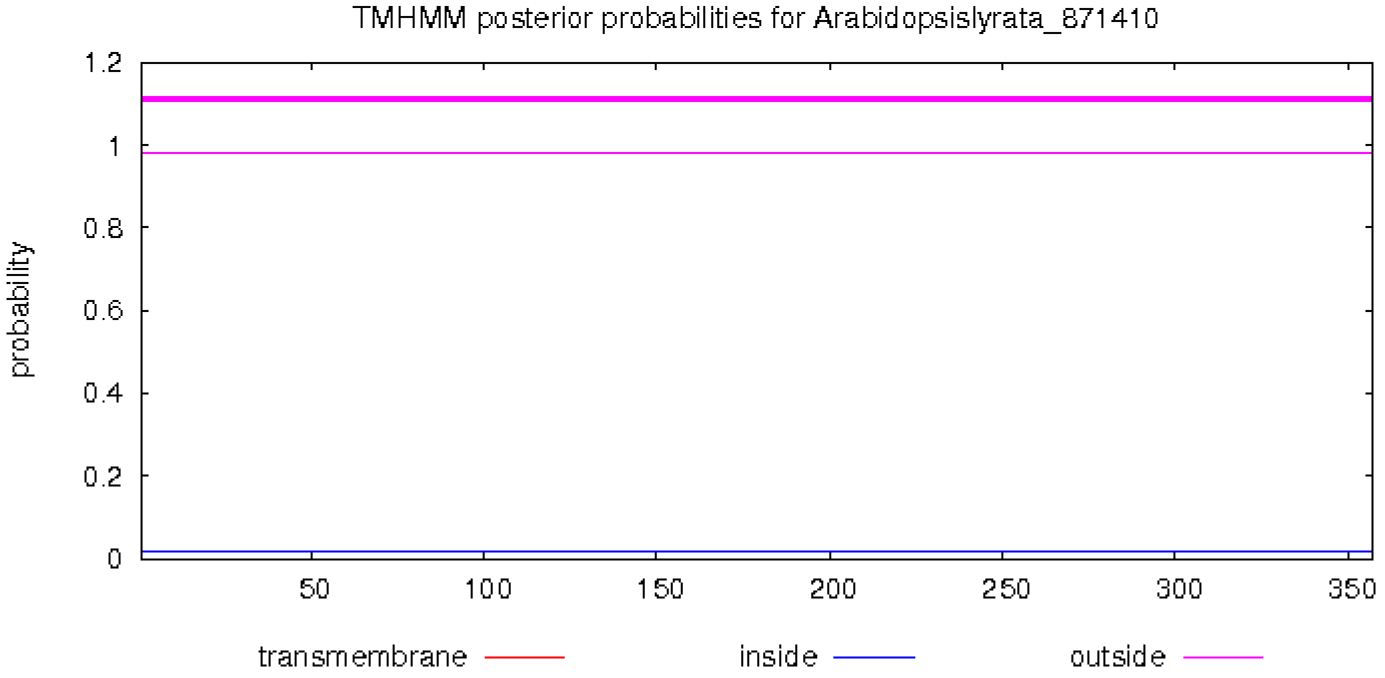

plot in postscript, script for making the plot in gnuplot, data for plot

---

```
# Arabidopsislyrata_871619 Length: 481
# Arabidopsislyrata_871619 Number of predicted TMHs:  0
# Arabidopsislyrata_871619 Exp number of AAs in TMHs: 0.00072
# Arabidopsislyrata_871619 Exp number, first 60 AAs:  0
# Arabidopsislyrata_871619 Total prob of N-in:        0.00017
Arabidopsislyrata_871619        TMHMM2.0        outside      1   481
```

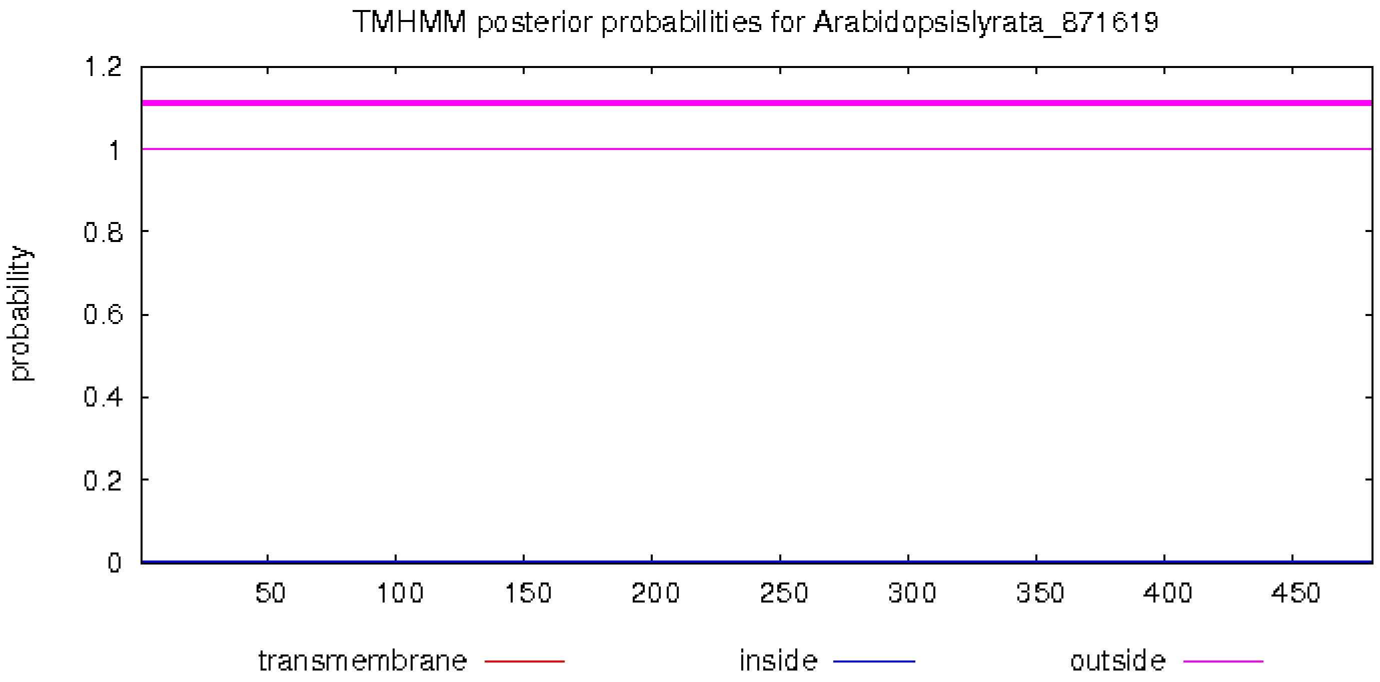

plot in postscript, script for making the plot in gnuplot, data for plot

---

```
# Arabidopsislyrata_873225 Length: 246
# Arabidopsislyrata_873225 Number of predicted TMHs:  0
# Arabidopsislyrata_873225 Exp number of AAs in TMHs: 0
# Arabidopsislyrata_873225 Exp number, first 60 AAs:  0
# Arabidopsislyrata_873225 Total prob of N-in:        0.00222
Arabidopsislyrata_873225        TMHMM2.0        outside      1   246
```

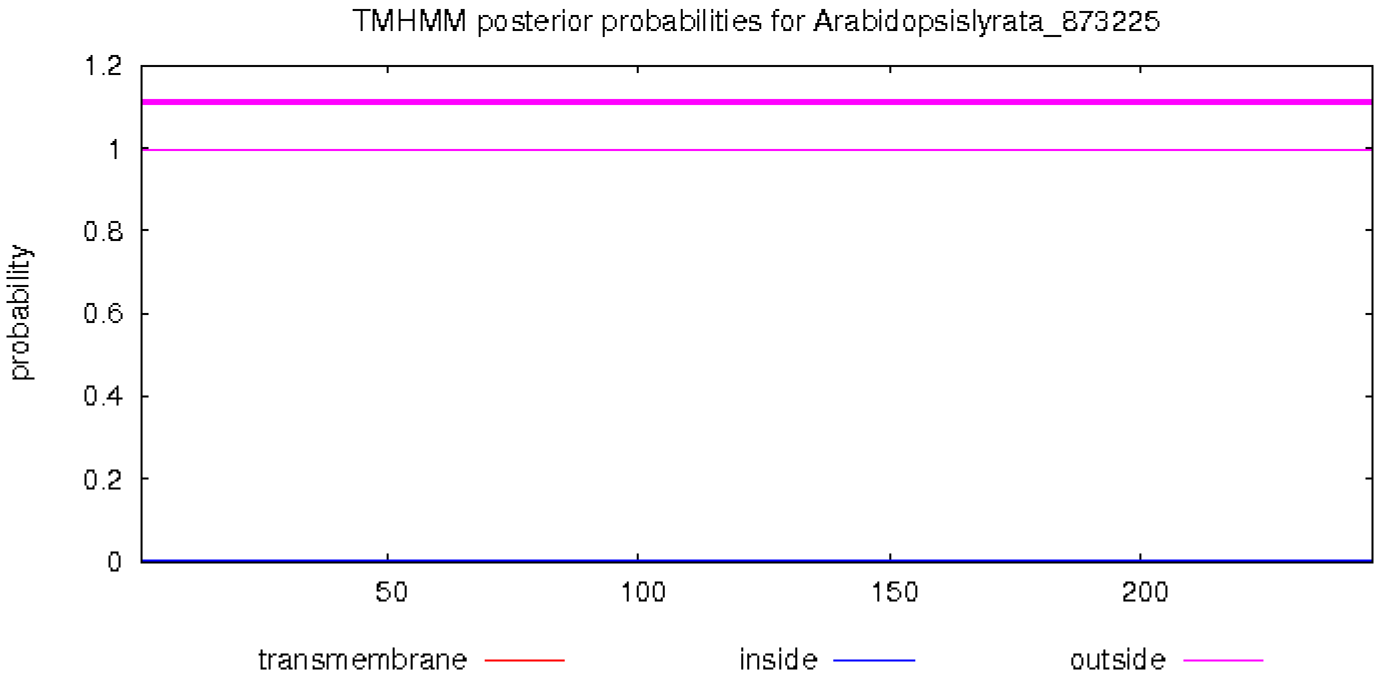

plot in postscript, script for making the plot in gnuplot, data for plot

---

```
# Arabidopsislyrata_873301 Length: 314
# Arabidopsislyrata_873301 Number of predicted TMHs:  0
# Arabidopsislyrata_873301 Exp number of AAs in TMHs: 0.02441
# Arabidopsislyrata_873301 Exp number, first 60 AAs:  0
# Arabidopsislyrata_873301 Total prob of N-in:        0.00403
Arabidopsislyrata_873301	TMHMM2.0	outside	     1   314
```

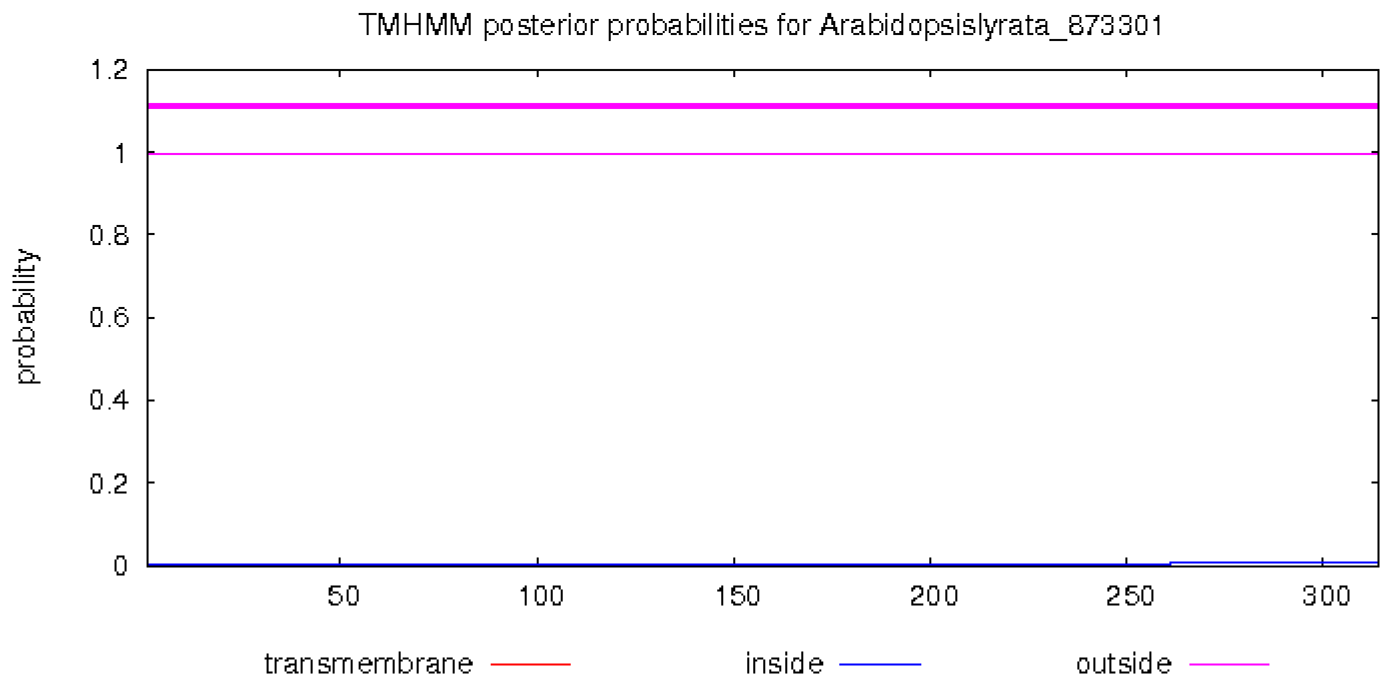

plot in postscript, script for making the plot in gnuplot, data for plot

---

```
# Arabidopsislyrata_875131 Length: 365
# Arabidopsislyrata_875131 Number of predicted TMHs:  0
# Arabidopsislyrata_875131 Exp number of AAs in TMHs: 0
# Arabidopsislyrata_875131 Exp number, first 60 AAs:  0
# Arabidopsislyrata_875131 Total prob of N-in:        0.00588
Arabidopsislyrata_875131	TMHMM2.0	outside	     1   365
```

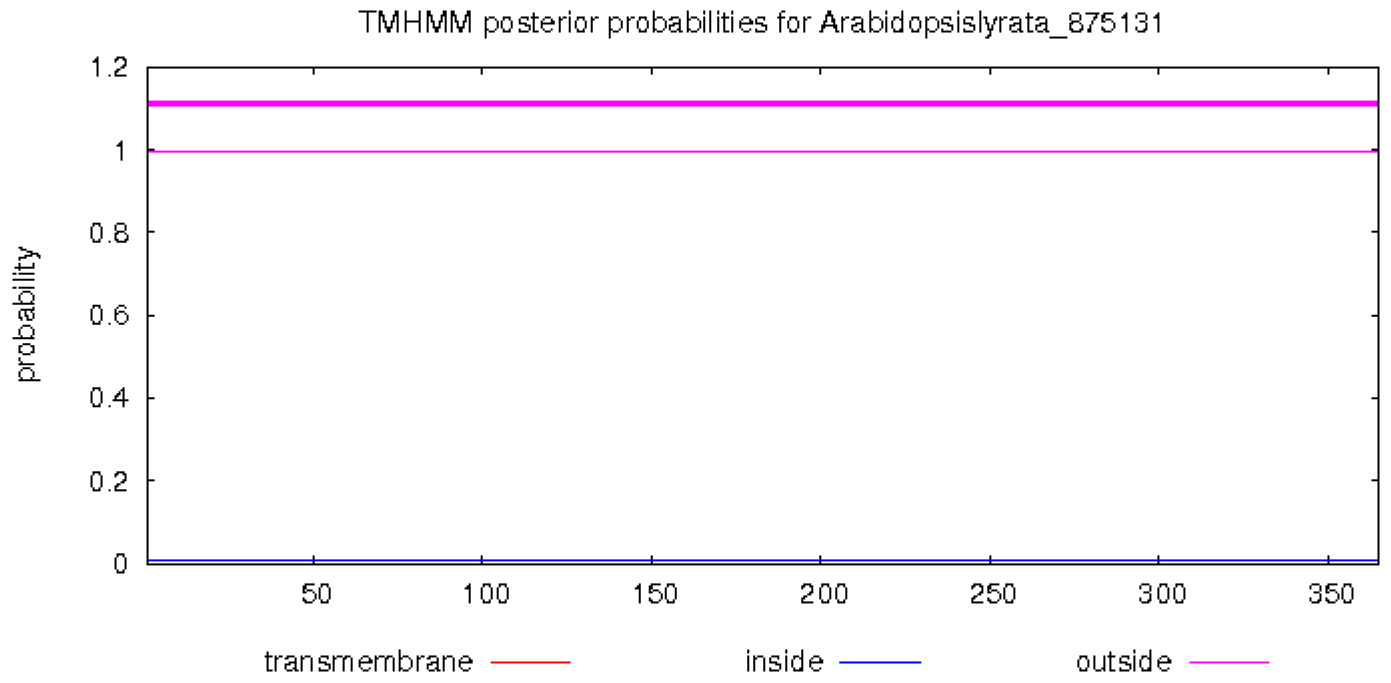

plot in postscript, script for making the plot in gnuplot, data for plot

---

```
# Arabidopsislyrata_876543 Length: 632
# Arabidopsislyrata_876543 Number of predicted TMHs:  0
# Arabidopsislyrata_876543 Exp number of AAs in TMHs: 1.49087
# Arabidopsislyrata_876543 Exp number, first 60 AAs:  0
# Arabidopsislyrata_876543 Total prob of N-in:        0.00009
Arabidopsislyrata_876543	TMHMM2.0	outside	     1   632
```

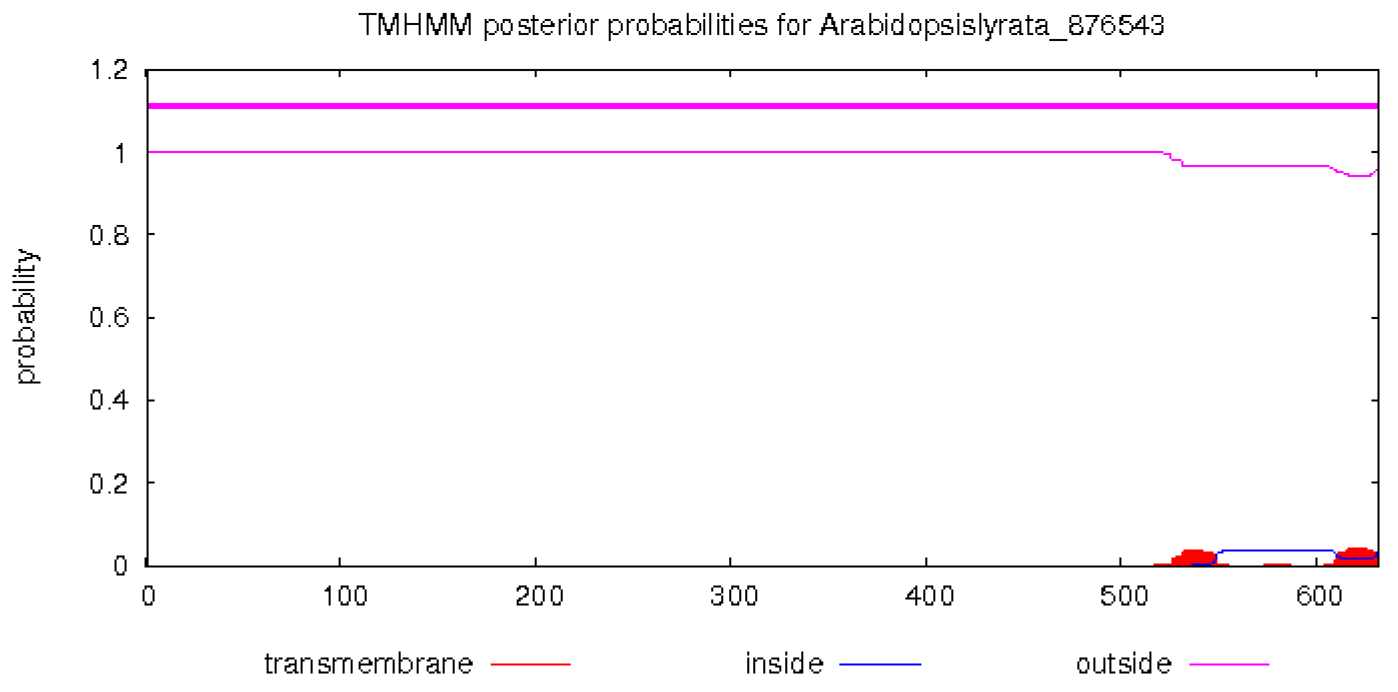

plot in postscript, script for making the plot in gnuplot, data for plot

```
# Arabidopsislyrata_878368 Length: 199
# Arabidopsislyrata_878368 Number of predicted TMHs:  0
# Arabidopsislyrata_878368 Exp number of AAs in TMHs: 0
# Arabidopsislyrata_878368 Exp number, first 60 AAs:  0
# Arabidopsislyrata_878368 Total prob of N-in:        0.08711
Arabidopsislyrata_878368	TMHMM2.0	outside	     1   199
```

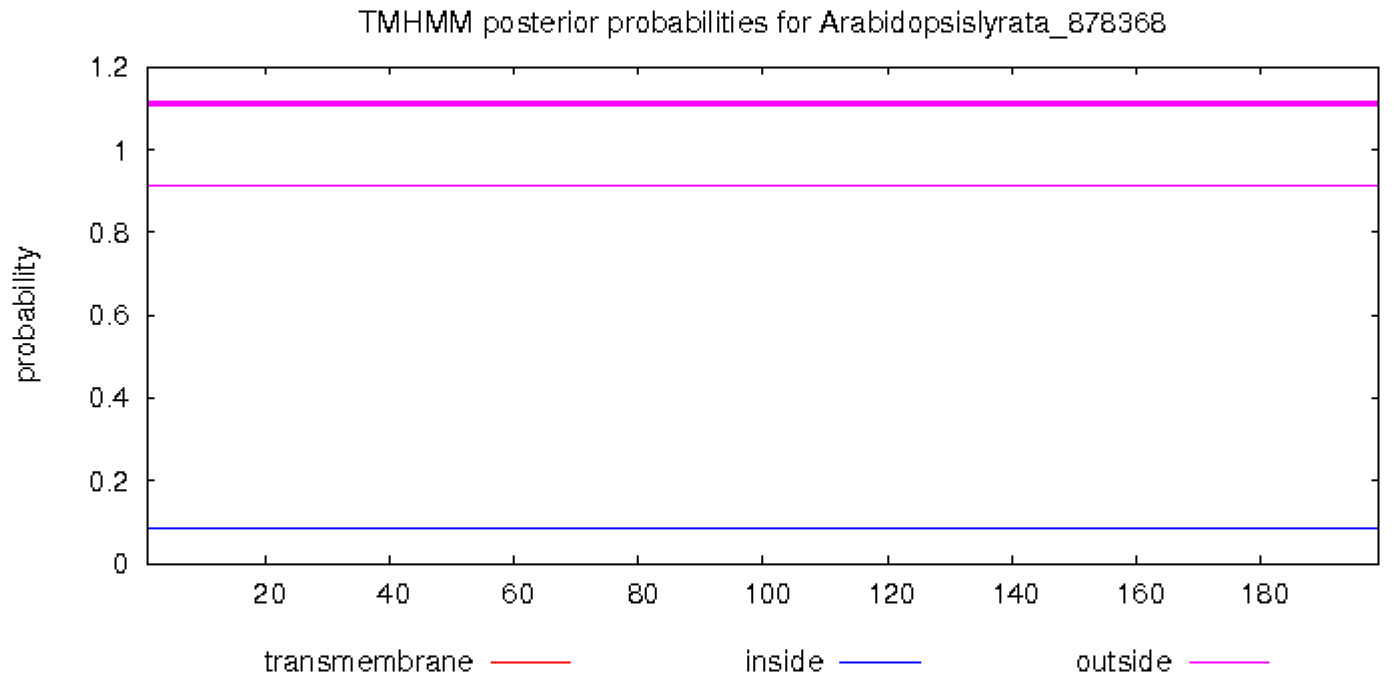

plot in postscript, script for making the plot in gnuplot, data for plot

```
# Arabidopsislyrata_878370 Length: 360
# Arabidopsislyrata_878370 Number of predicted TMHs:  0
# Arabidopsislyrata_878370 Exp number of AAs in TMHs: 0
# Arabidopsislyrata_878370 Exp number, first 60 AAs:  0
# Arabidopsislyrata_878370 Total prob of N-in:        0.00786
Arabidopsislyrata_878370        TMHMM2.0        outside      1   360
```

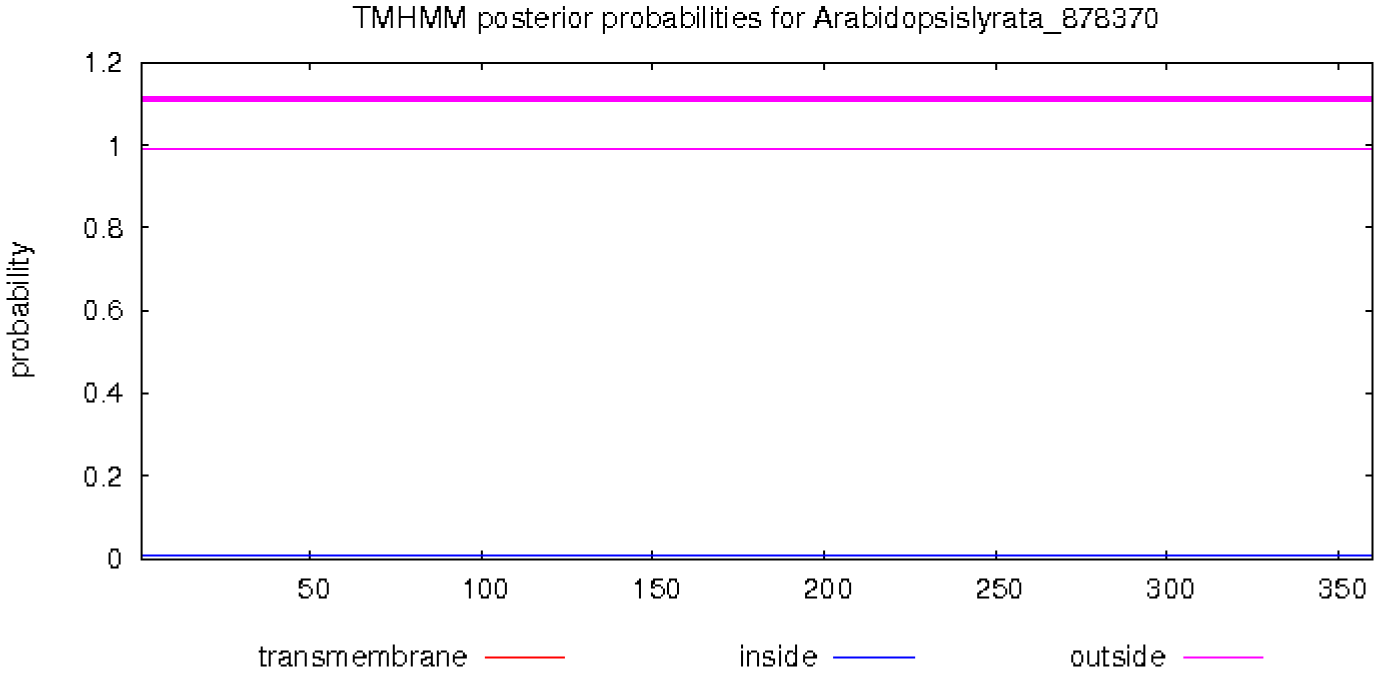

plot in postscript, script for making the plot in gnuplot, data for plot

```
# Arabidopsislyrata_881355 Length: 192
# Arabidopsislyrata_881355 Number of predicted TMHs:  0
# Arabidopsislyrata_881355 Exp number of AAs in TMHs: 0.00018
# Arabidopsislyrata_881355 Exp number, first 60 AAs:  0
# Arabidopsislyrata_881355 Total prob of N-in:        0.05713
Arabidopsislyrata_881355        TMHMM2.0        outside      1   192
```

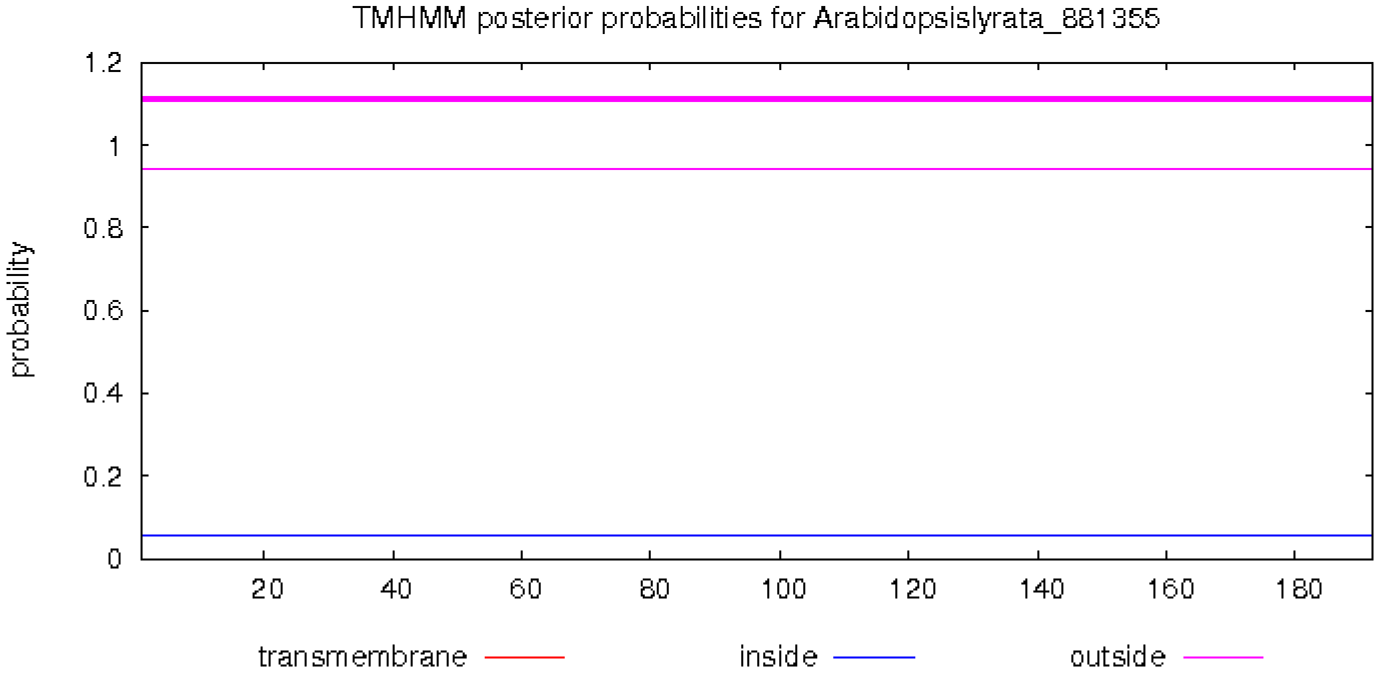

plot in postscript, script for making the plot in gnuplot, data for plot

```
# Arabidopsislyrata_883225 Length: 318
# Arabidopsislyrata_883225 Number of predicted TMHs:  0
# Arabidopsislyrata_883225 Exp number of AAs in TMHs: 0.0007
# Arabidopsislyrata_883225 Exp number, first 60 AAs:  0
# Arabidopsislyrata_883225 Total prob of N-in:        0.02490
Arabidopsislyrata_883225        TMHMM2.0        outside      1   318
```

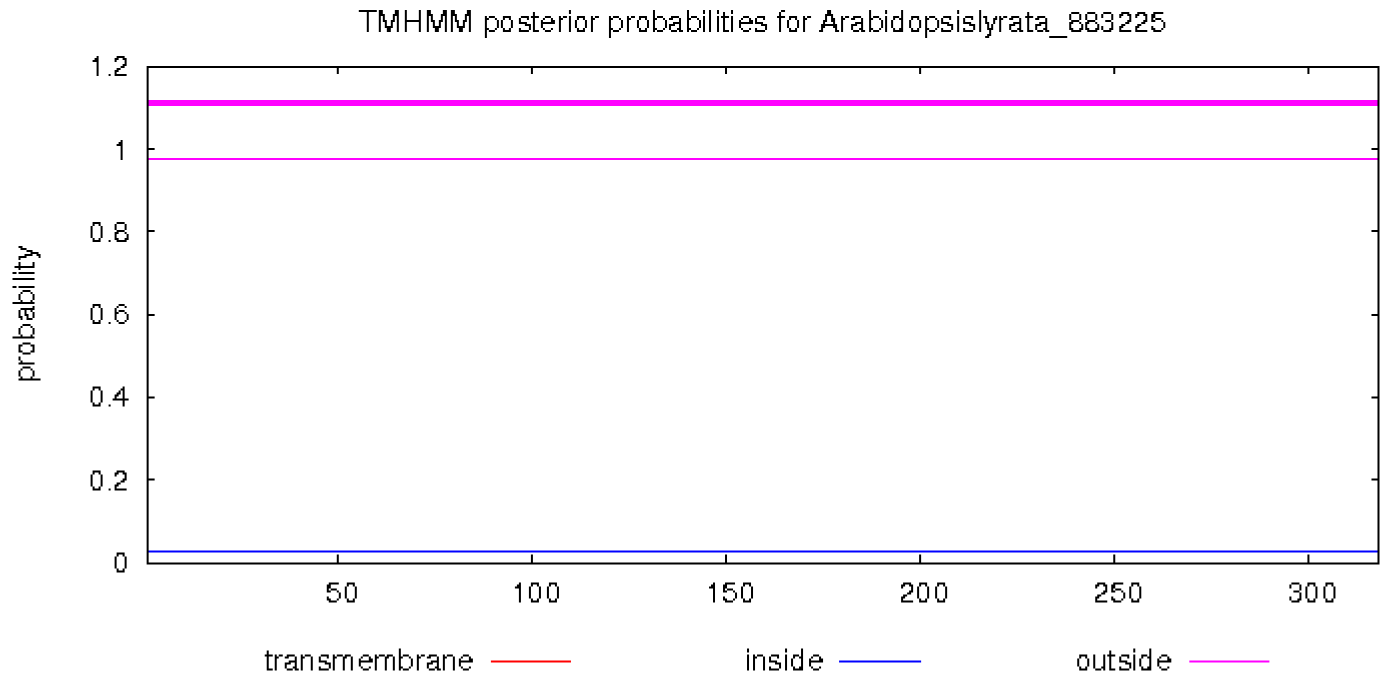

plot in postscript, script for making the plot in gnuplot, data for plot

---

```
# Arabidopsislyrata_885551 Length: 369
# Arabidopsislyrata_885551 Number of predicted TMHs:  0
# Arabidopsislyrata_885551 Exp number of AAs in TMHs: 0.01237
# Arabidopsislyrata_885551 Exp number, first 60 AAs:  0
# Arabidopsislyrata_885551 Total prob of N-in:        0.02229
Arabidopsislyrata_885551        TMHMM2.0        outside      1   369
```

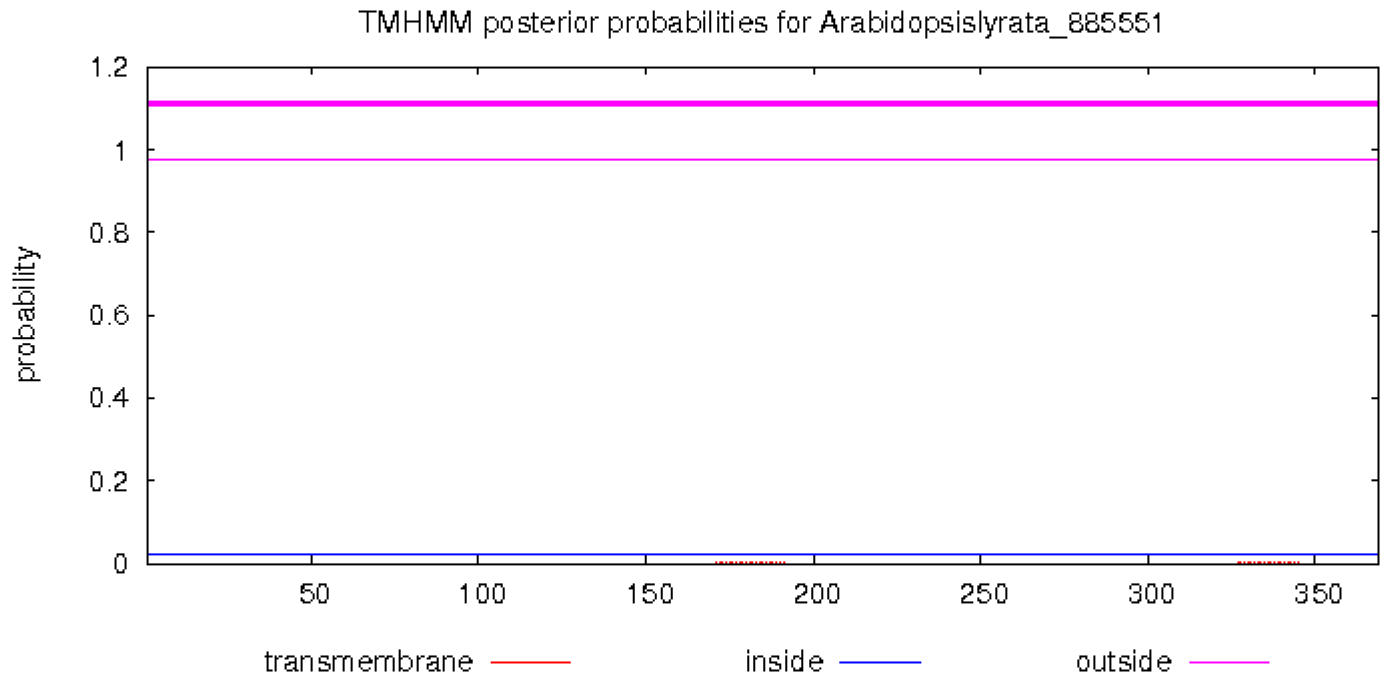

plot in postscript, script for making the plot in gnuplot, data for plot

---

```
# Arabidopsislyrata_886649 Length: 269
# Arabidopsislyrata_886649 Number of predicted TMHs:  0
# Arabidopsislyrata_886649 Exp number of AAs in TMHs: 0.00017
# Arabidopsislyrata_886649 Exp number, first 60 AAs:  0
# Arabidopsislyrata_886649 Total prob of N-in:        0.01477
Arabidopsislyrata_886649        TMHMM2.0        outside      1   269
```

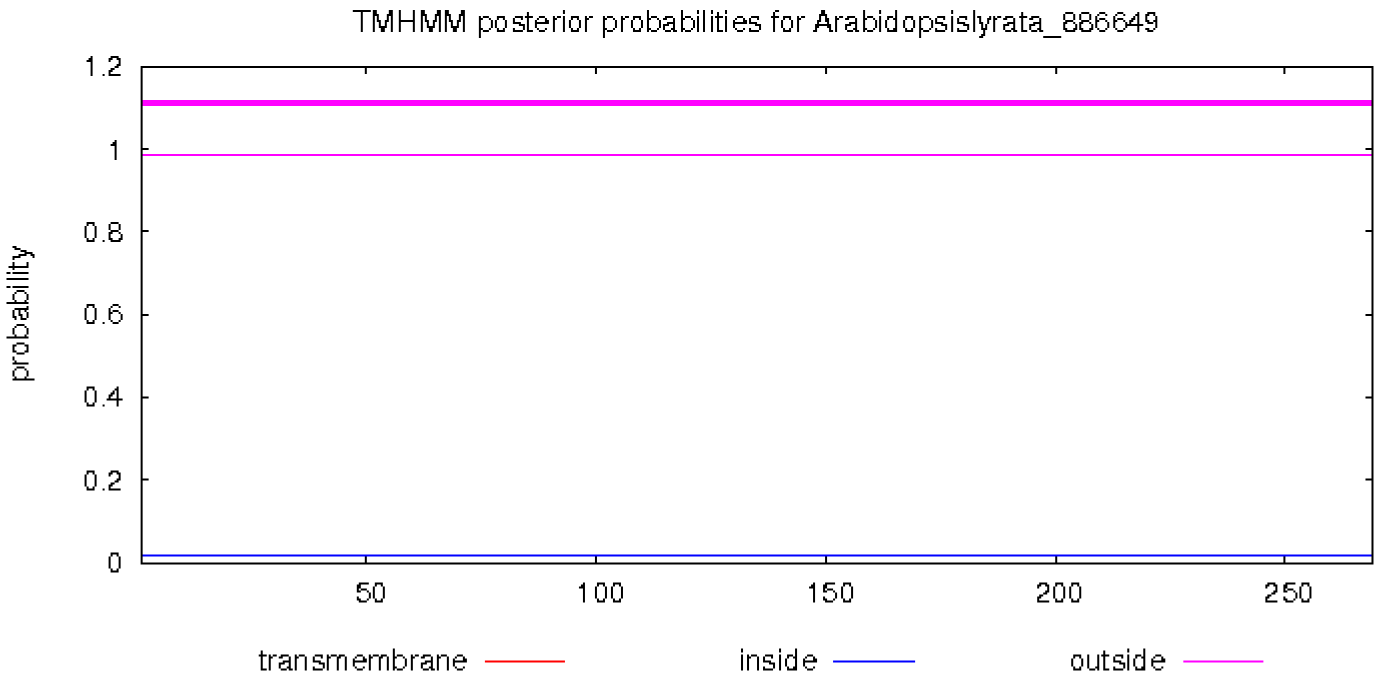

plot in postscript, script for making the plot in gnuplot, data for plot

```
# Arabidopsislyrata_887272 Length: 295
# Arabidopsislyrata_887272 Number of predicted TMHs:  0
# Arabidopsislyrata_887272 Exp number of AAs in TMHs: 0.00137
# Arabidopsislyrata_887272 Exp number, first 60 AAs:  0.00044
# Arabidopsislyrata_887272 Total prob of N-in:        0.00303
Arabidopsislyrata_887272        TMHMM2.0        outside      1   295
```

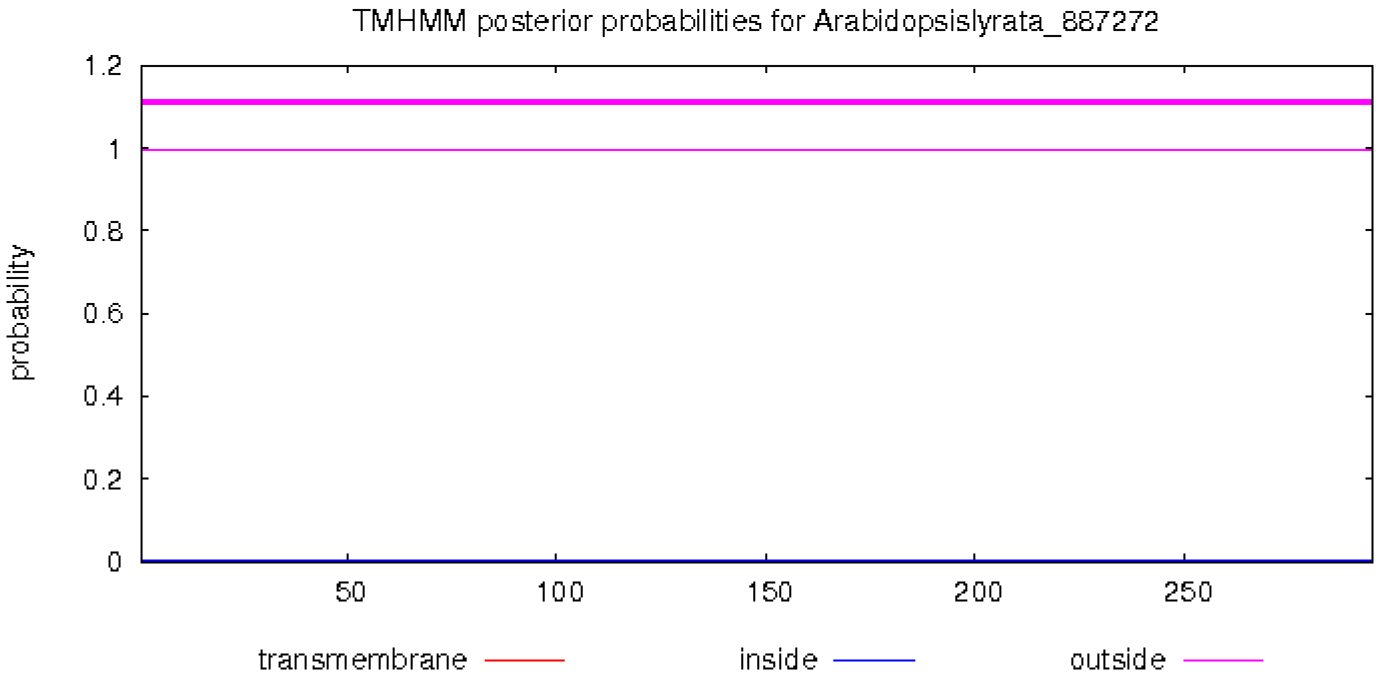

plot in postscript, script for making the plot in gnuplot, data for plot

```
# Arabidopsislyrata_887359 Length: 201
# Arabidopsislyrata_887359 Number of predicted TMHs:  0
# Arabidopsislyrata_887359 Exp number of AAs in TMHs: 0
# Arabidopsislyrata_887359 Exp number, first 60 AAs:  0
# Arabidopsislyrata_887359 Total prob of N-in:        0.11312
Arabidopsislyrata_887359	TMHMM2.0	outside	     1   201
```

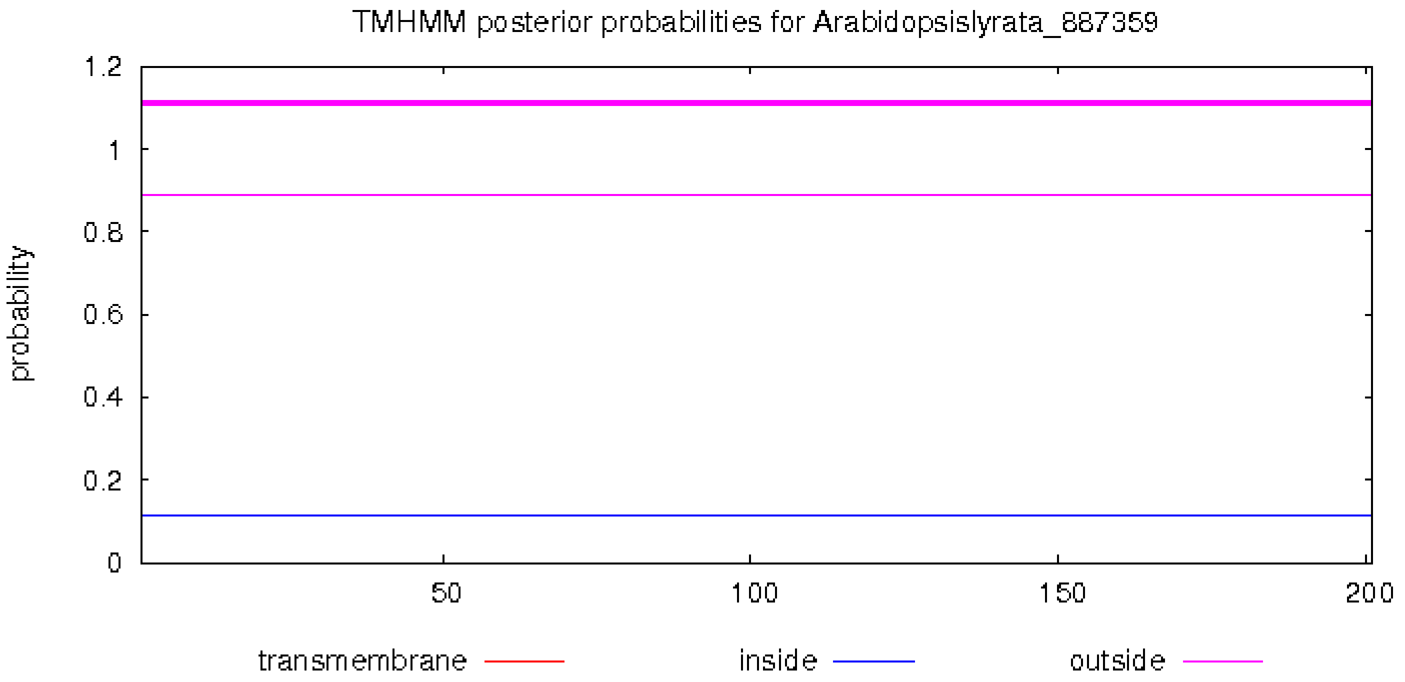

plot in postscript, script for making the plot in gnuplot, data for plot

---

```
# Arabidopsislyrata_887556 Length: 357
# Arabidopsislyrata_887556 Number of predicted TMHs:  0
# Arabidopsislyrata_887556 Exp number of AAs in TMHs: 0.00037
# Arabidopsislyrata_887556 Exp number, first 60 AAs:  0.00021
# Arabidopsislyrata_887556 Total prob of N-in:        0.00457
Arabidopsislyrata_887556	TMHMM2.0	outside	     1   357
```

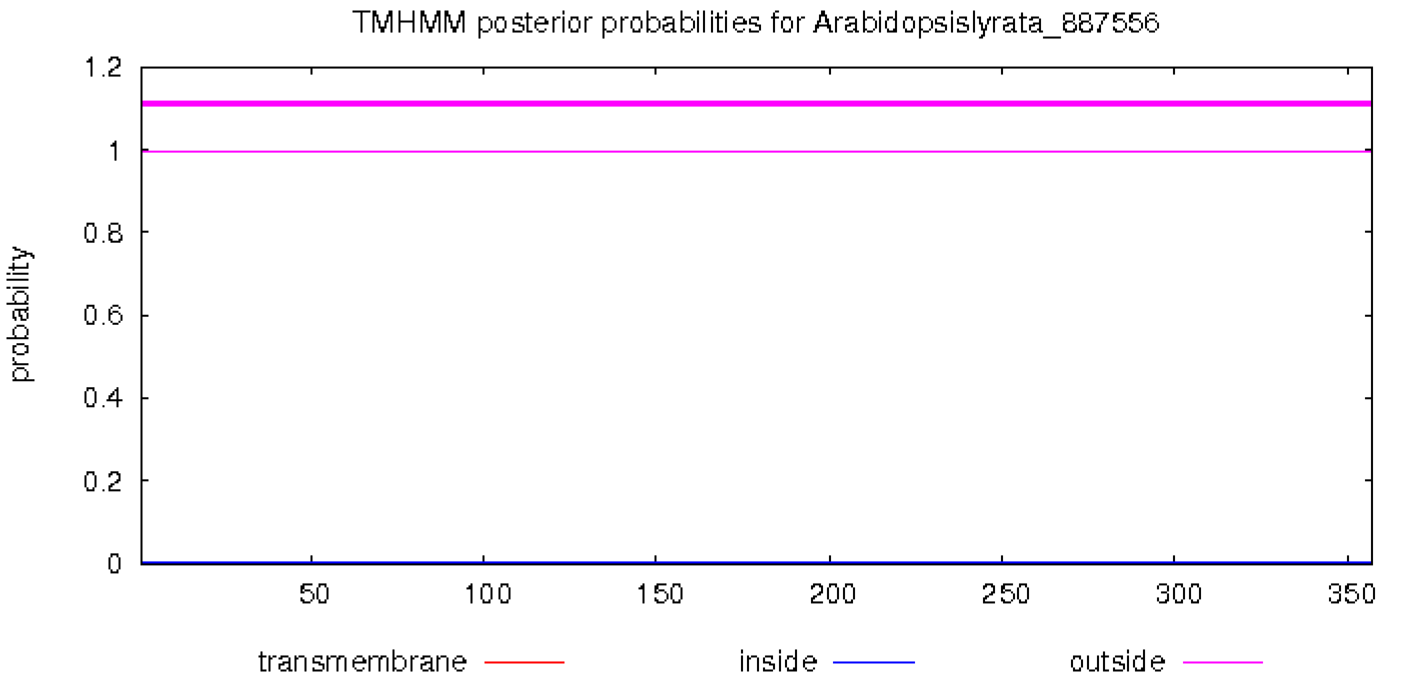

plot in postscript, script for making the plot in gnuplot, data for plot

---

```
# Arabidopsislyrata_890145 Length: 459
# Arabidopsislyrata_890145 Number of predicted TMHs:  0
# Arabidopsislyrata_890145 Exp number of AAs in TMHs: 0.0002
# Arabidopsislyrata_890145 Exp number, first 60 AAs:  0
# Arabidopsislyrata_890145 Total prob of N-in:        0.00266
Arabidopsislyrata_890145        TMHMM2.0        outside      1   459
```

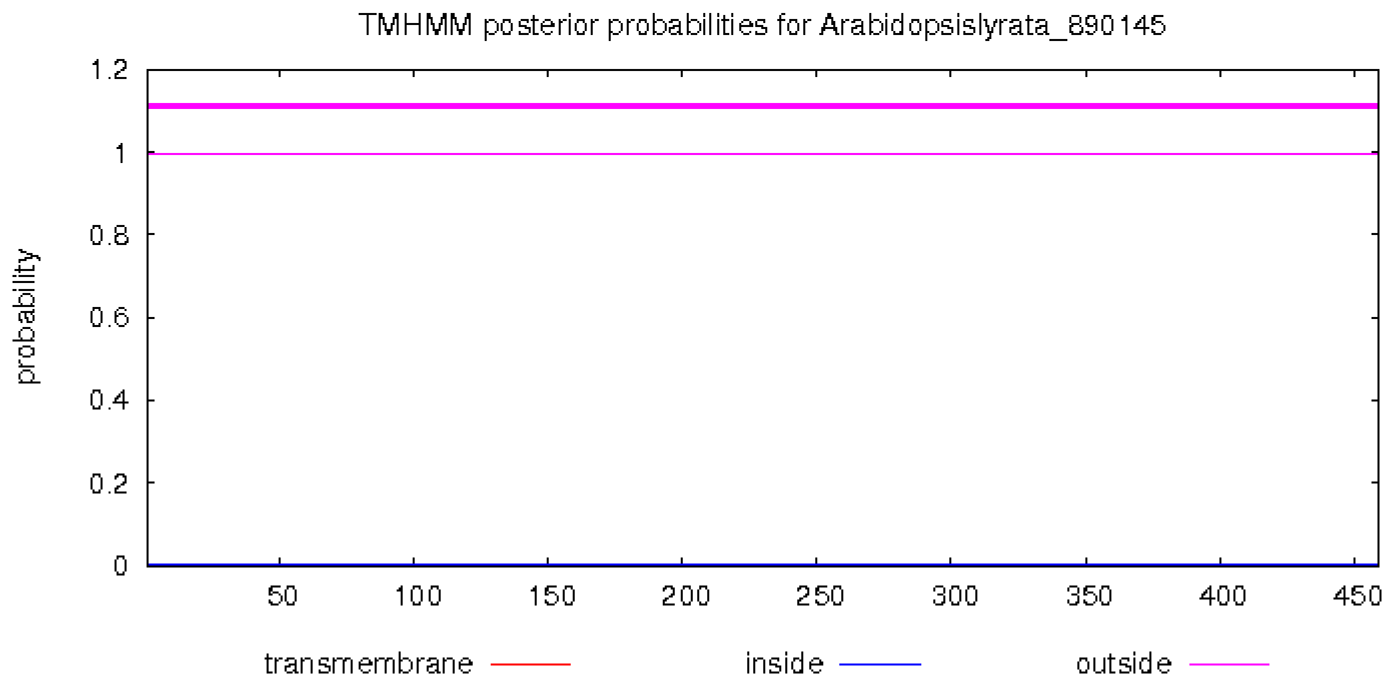

plot in postscript, script for making the plot in gnuplot, data for plot

---

```
# Arabidopsislyrata_890389 Length: 316
# Arabidopsislyrata_890389 Number of predicted TMHs:  0
# Arabidopsislyrata_890389 Exp number of AAs in TMHs: 0.00021
# Arabidopsislyrata_890389 Exp number, first 60 AAs:  0
# Arabidopsislyrata_890389 Total prob of N-in:        0.04323
Arabidopsislyrata_890389        TMHMM2.0        outside      1   316
```

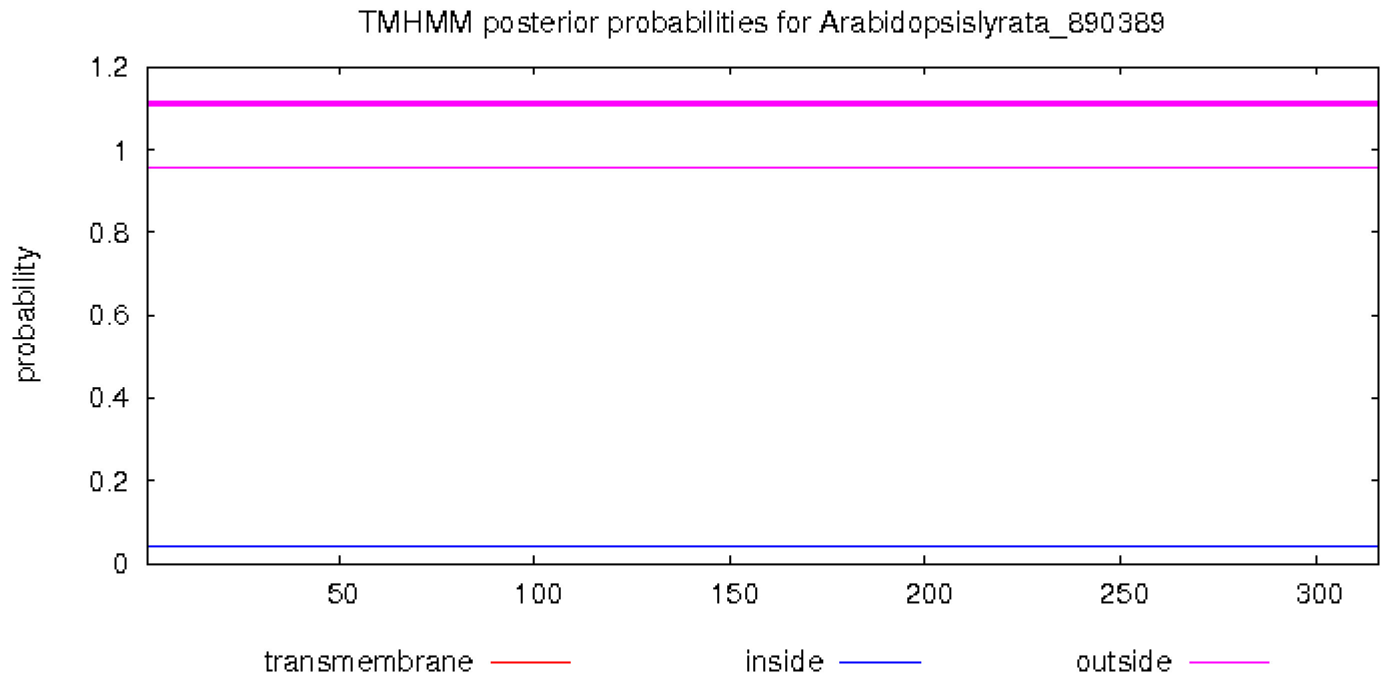

plot in postscript, script for making the plot in gnuplot, data for plot

---

```
# Arabidopsislyrata_893515 Length: 333
# Arabidopsislyrata_893515 Number of predicted TMHs:  0
# Arabidopsislyrata_893515 Exp number of AAs in TMHs: 0.00396
# Arabidopsislyrata_893515 Exp number, first 60 AAs:  0
# Arabidopsislyrata_893515 Total prob of N-in:        0.02372
Arabidopsislyrata_893515        TMHMM2.0        outside      1   333
```

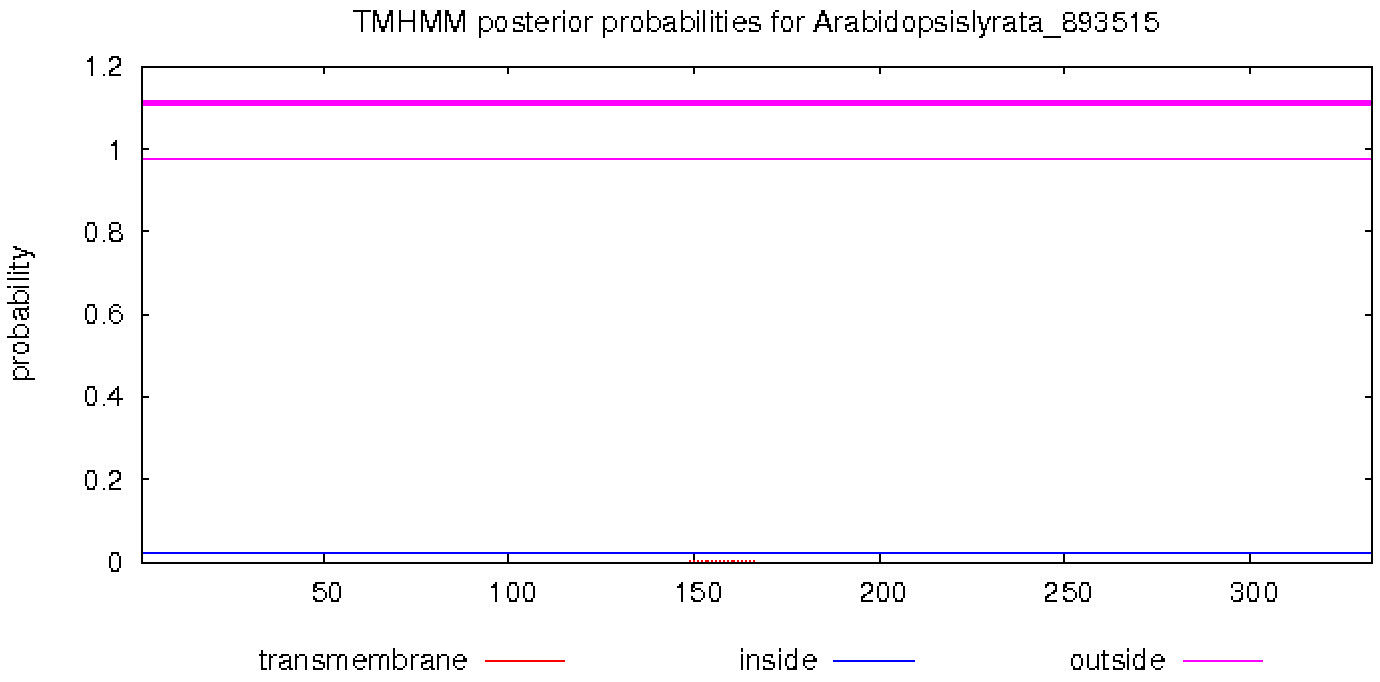

plot in postscript, script for making the plot in gnuplot, data for plot

---

```
# Arabidopsislyrata_893532 Length: 313
# Arabidopsislyrata_893532 Number of predicted TMHs:  0
# Arabidopsislyrata_893532 Exp number of AAs in TMHs: 0.00663
# Arabidopsislyrata_893532 Exp number, first 60 AAs:  0
# Arabidopsislyrata_893532 Total prob of N-in:        0.08703
Arabidopsislyrata_893532        TMHMM2.0        outside      1   313
```

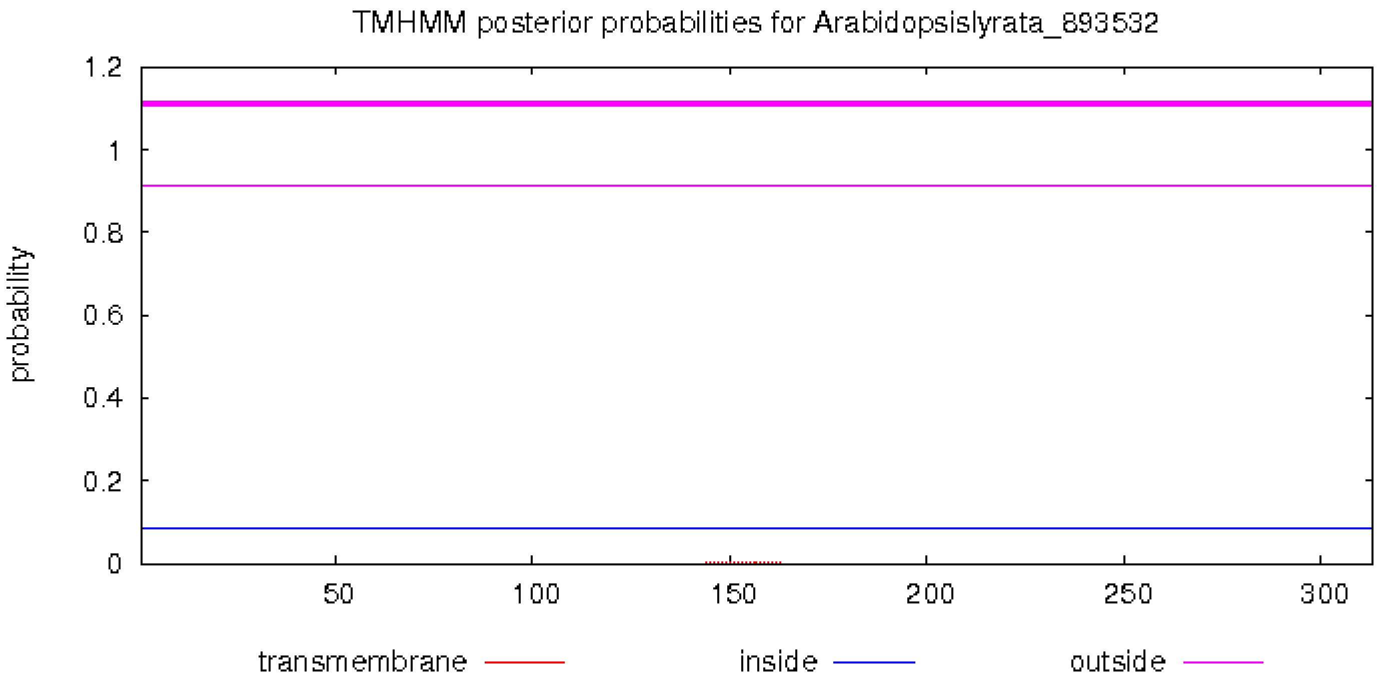

plot in postscript, script for making the plot in gnuplot, data for plot

---

```
# Arabidopsislyrata_895634 Length: 250
# Arabidopsislyrata_895634 Number of predicted TMHs:  0
# Arabidopsislyrata_895634 Exp number of AAs in TMHs: 0
# Arabidopsislyrata_895634 Exp number, first 60 AAs:  0
# Arabidopsislyrata_895634 Total prob of N-in:        0.03470
Arabidopsislyrata_895634        TMHMM2.0        outside      1   250
```

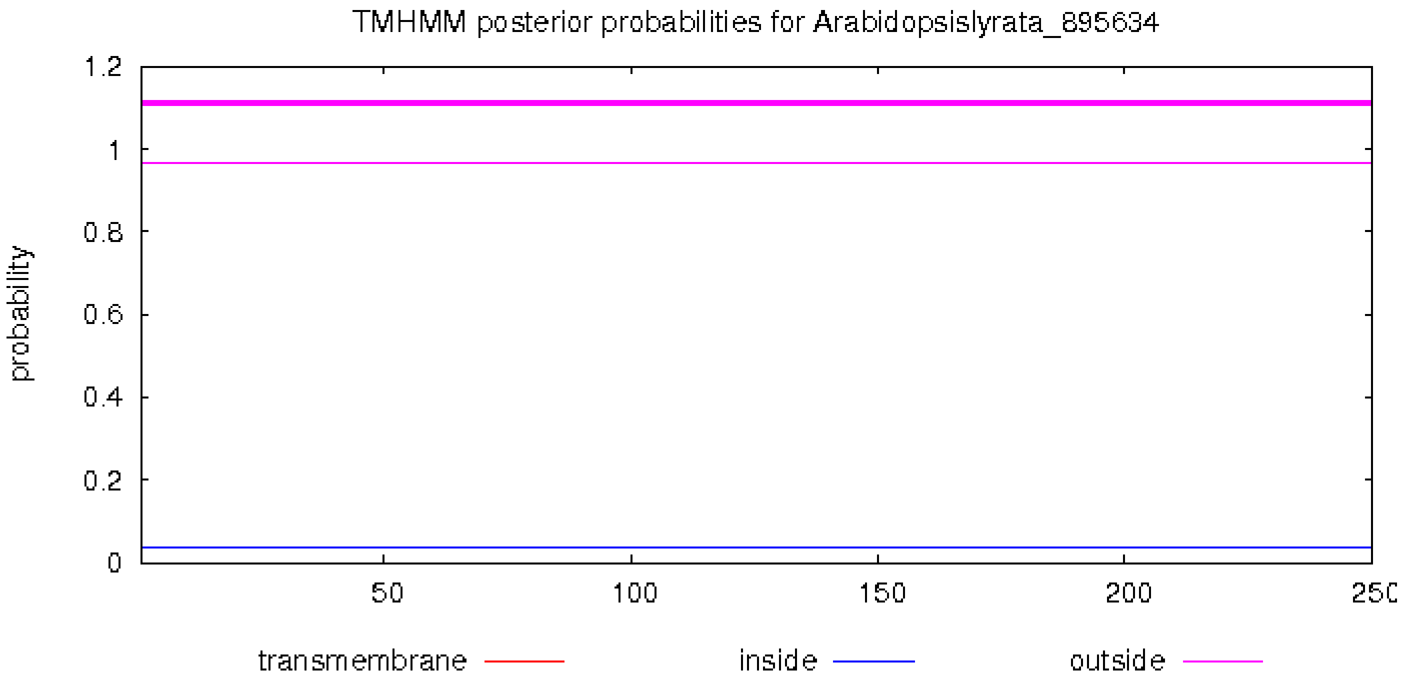

plot in postscript, script for making the plot in gnuplot, data for plot

---

```
# Arabidopsislyrata_895868 Length: 372
# Arabidopsislyrata_895868 Number of predicted TMHs:  0
# Arabidopsislyrata_895868 Exp number of AAs in TMHs: 0.0034
# Arabidopsislyrata_895868 Exp number, first 60 AAs:  0.00096
# Arabidopsislyrata_895868 Total prob of N-in:        0.00758
Arabidopsislyrata_895868        TMHMM2.0        outside      1   372
```

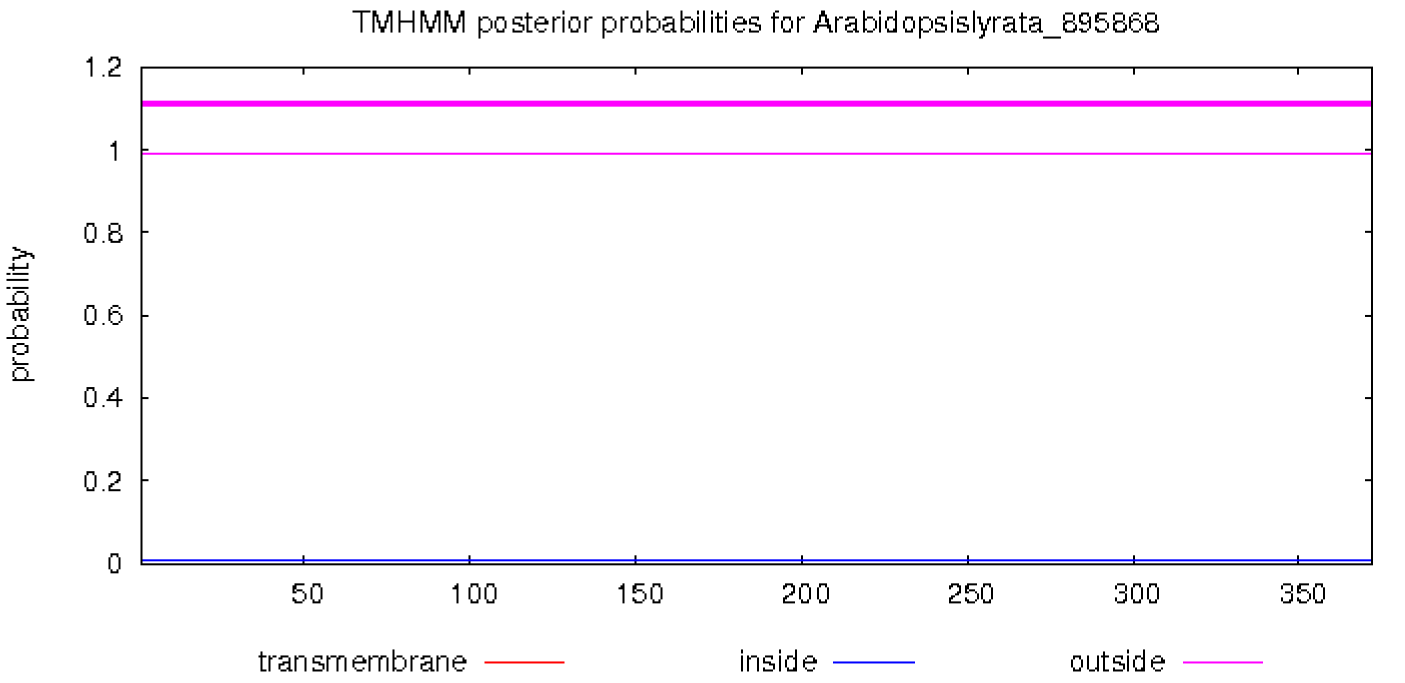

plot in postscript, script for making the plot in gnuplot, data for plot

---

```
# Arabidopsislyrata_897508 Length: 280
# Arabidopsislyrata_897508 Number of predicted TMHs:  0
# Arabidopsislyrata_897508 Exp number of AAs in TMHs: 0.0002
# Arabidopsislyrata_897508 Exp number, first 60 AAs:  0
# Arabidopsislyrata_897508 Total prob of N-in:        0.06378
Arabidopsislyrata_897508	TMHMM2.0	outside	     1   280
```

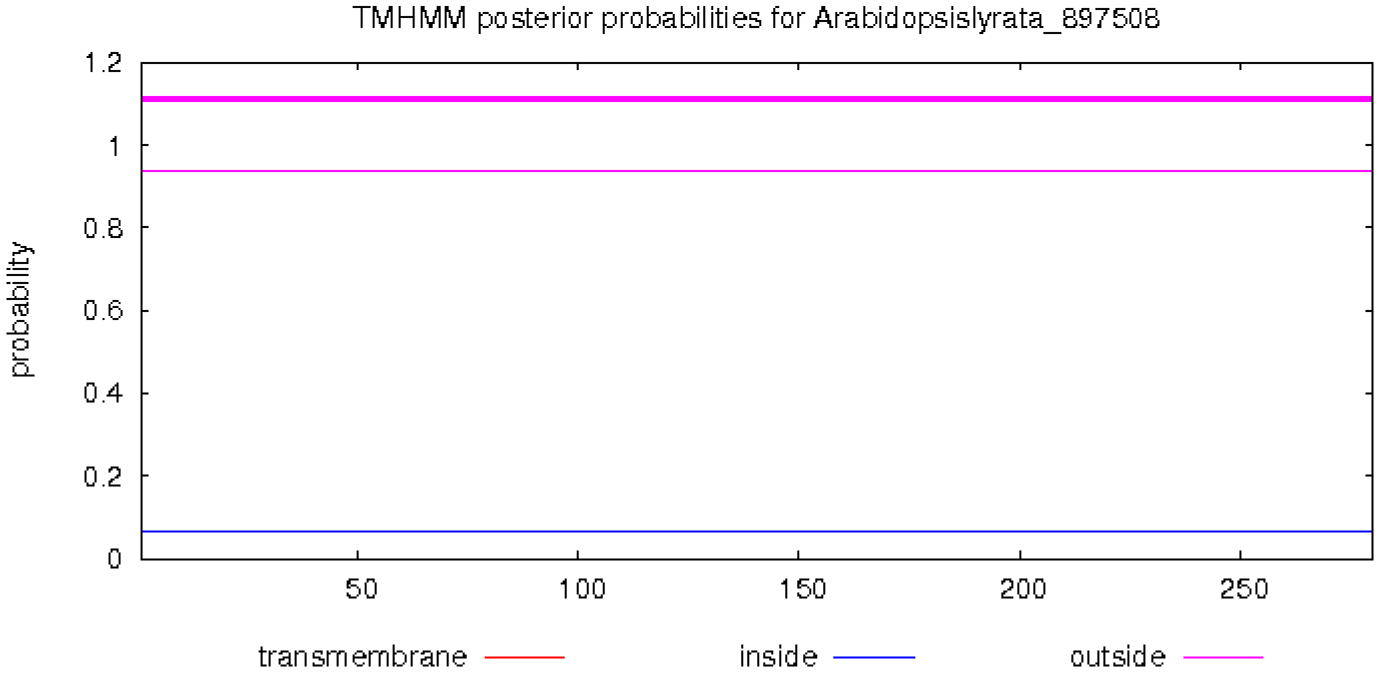

plot in postscript, script for making the plot in gnuplot, data for plot

---

```
# Arabidopsislyrata_901406 Length: 89
# Arabidopsislyrata_901406 Number of predicted TMHs:  0
# Arabidopsislyrata_901406 Exp number of AAs in TMHs: 0.04533
# Arabidopsislyrata_901406 Exp number, first 60 AAs:  0.01263
# Arabidopsislyrata_901406 Total prob of N-in:        0.74795
Arabidopsislyrata_901406	TMHMM2.0	inside	     1    89
```

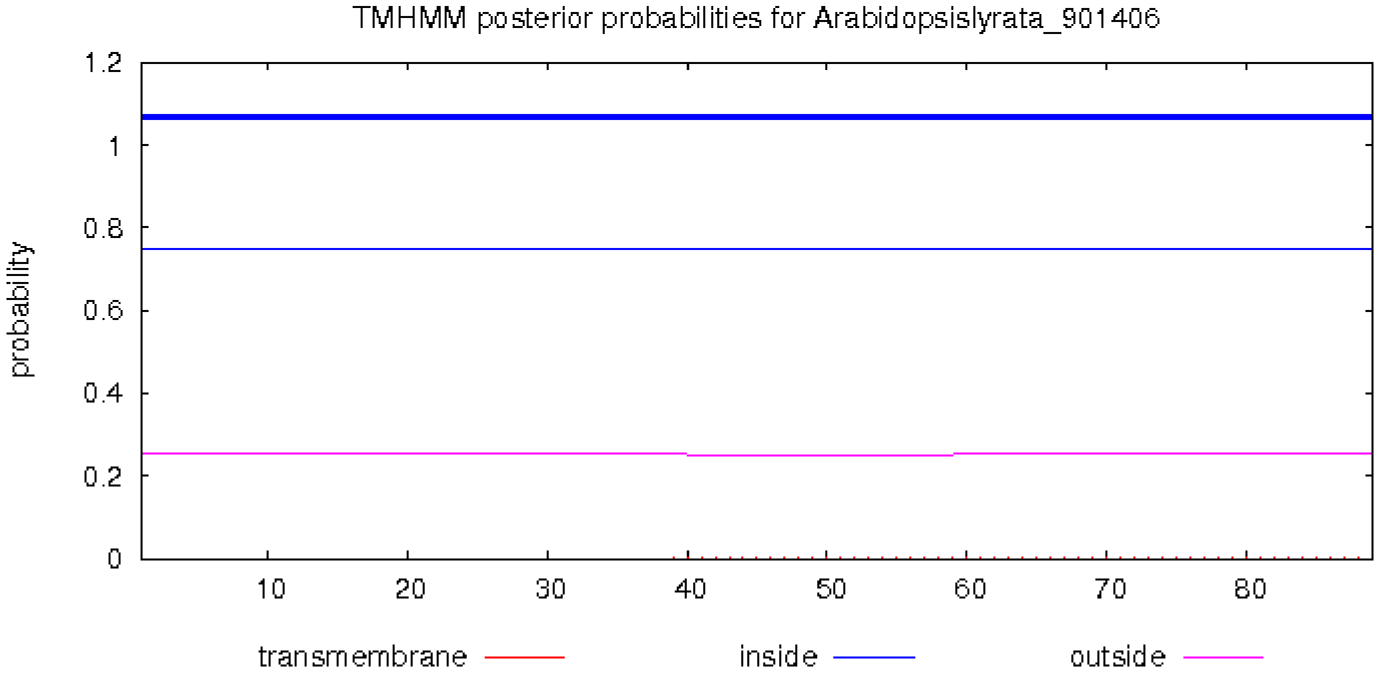

plot in postscript, script for making the plot in gnuplot, data for plot

---

```
# Arabidopsislyrata_901407 Length: 89
# Arabidopsislyrata_901407 Number of predicted TMHs:  0
# Arabidopsislyrata_901407 Exp number of AAs in TMHs: 0.04533
# Arabidopsislyrata_901407 Exp number, first 60 AAs:  0.01263
# Arabidopsislyrata_901407 Total prob of N-in:        0.74795
Arabidopsislyrata_901407	TMHMM2.0	inside	     1    89
```

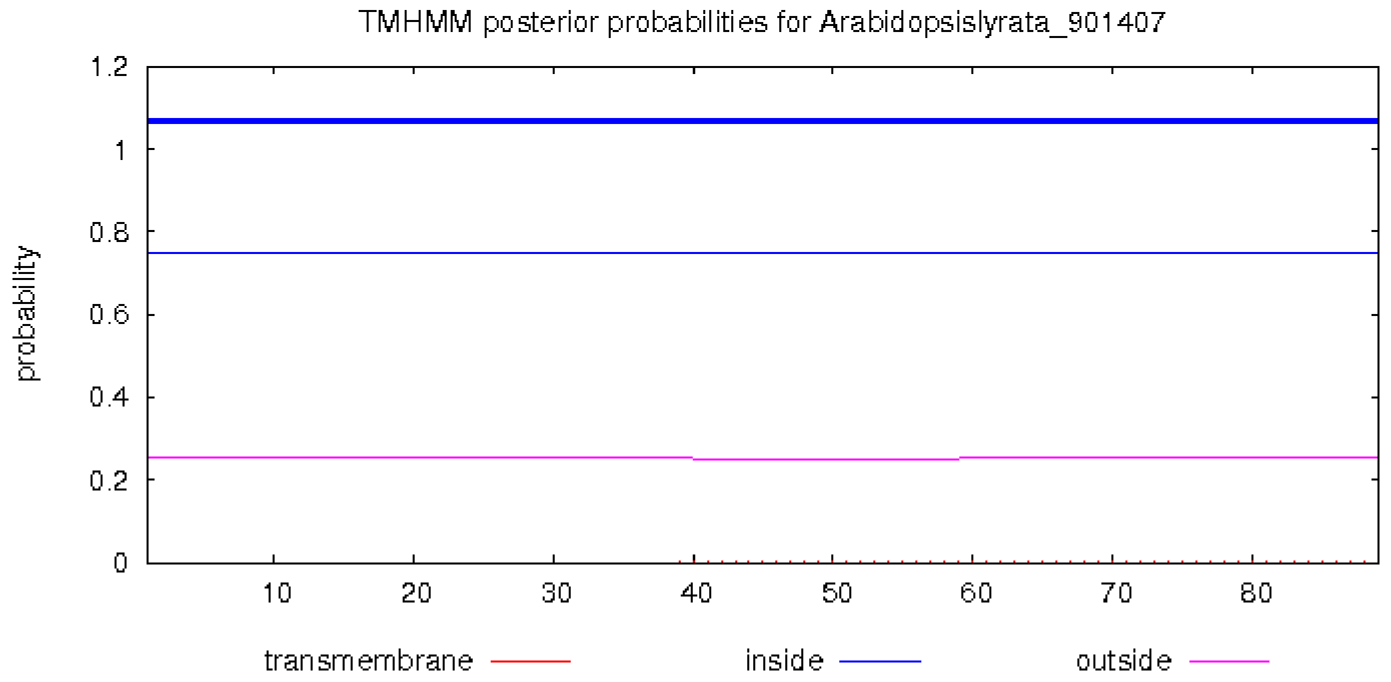

plot in postscript, script for making the plot in gnuplot, data for plot

---

```
# Arabidopsislyrata_901535 Length: 283
# Arabidopsislyrata_901535 Number of predicted TMHs:  0
# Arabidopsislyrata_901535 Exp number of AAs in TMHs: 0.00019
# Arabidopsislyrata_901535 Exp number, first 60 AAs:  0
# Arabidopsislyrata_901535 Total prob of N-in:        0.06440
Arabidopsislyrata_901535	TMHMM2.0	outside	     1   283
```

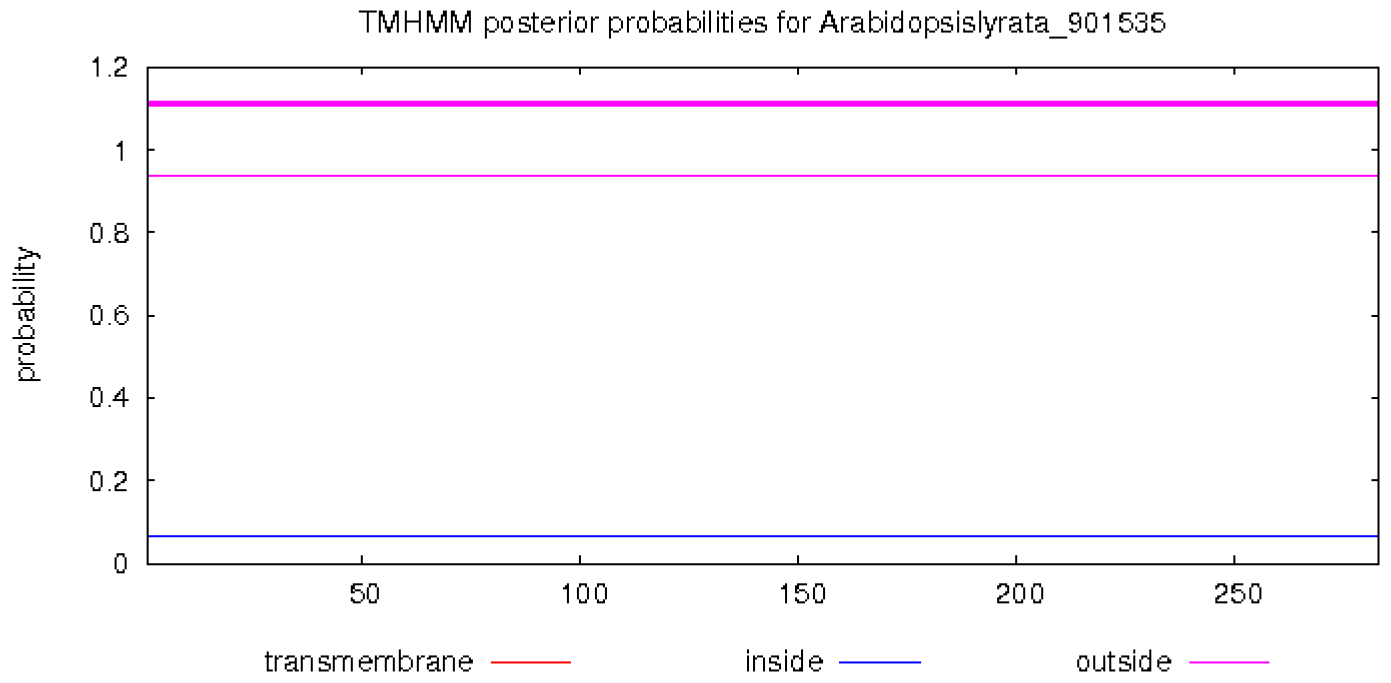

plot in postscript, script for making the plot in gnuplot, data for plot

---

```
# Arabidopsislyrata_901538 Length: 323
# Arabidopsislyrata_901538 Number of predicted TMHs:  0
# Arabidopsislyrata_901538 Exp number of AAs in TMHs: 0.00019
# Arabidopsislyrata_901538 Exp number, first 60 AAs:  0
# Arabidopsislyrata_901538 Total prob of N-in:        0.06842
Arabidopsislyrata_901538	TMHMM2.0	outside	     1   323
```

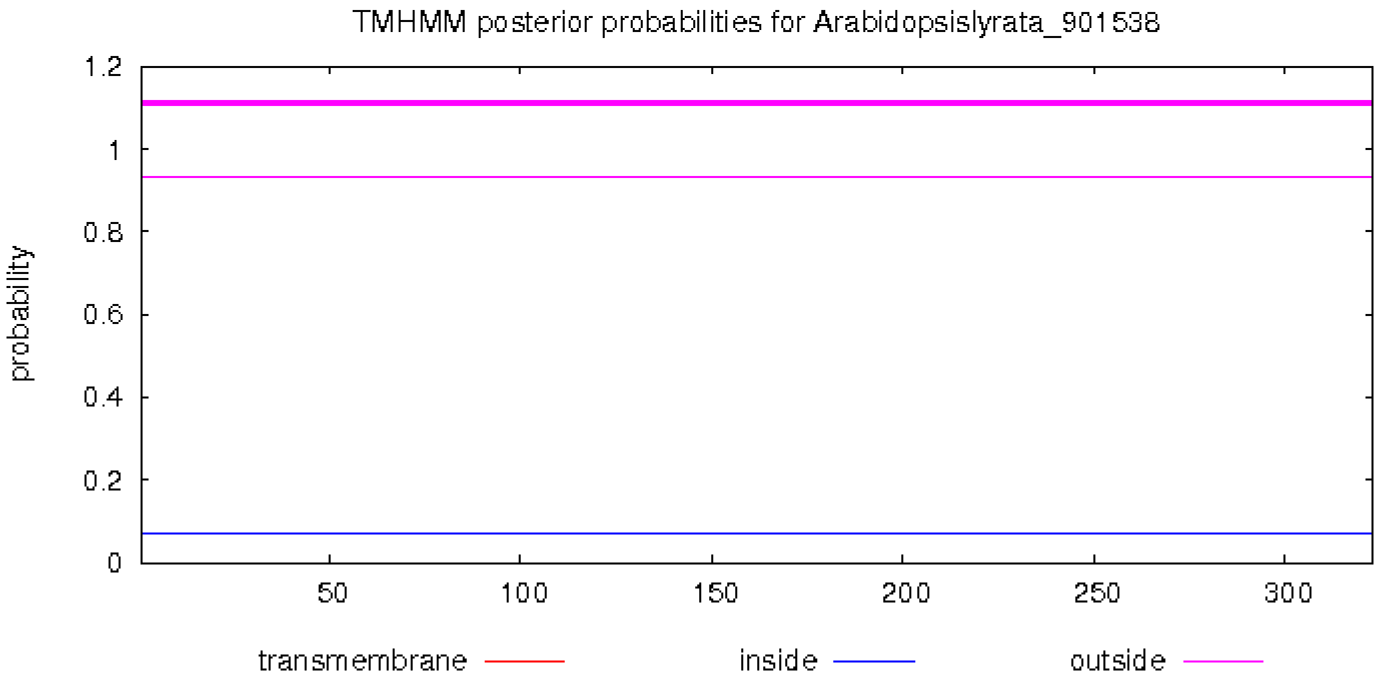

plot in postscript, script for making the plot in gnuplot, data for plot

```
# Arabidopsislyrata_904148 Length: 355
# Arabidopsislyrata_904148 Number of predicted TMHs:  0
# Arabidopsislyrata_904148 Exp number of AAs in TMHs: 0.00106
# Arabidopsislyrata_904148 Exp number, first 60 AAs:  0
# Arabidopsislyrata_904148 Total prob of N-in:        0.01809
Arabidopsislyrata_904148	TMHMM2.0	outside	     1   355
```

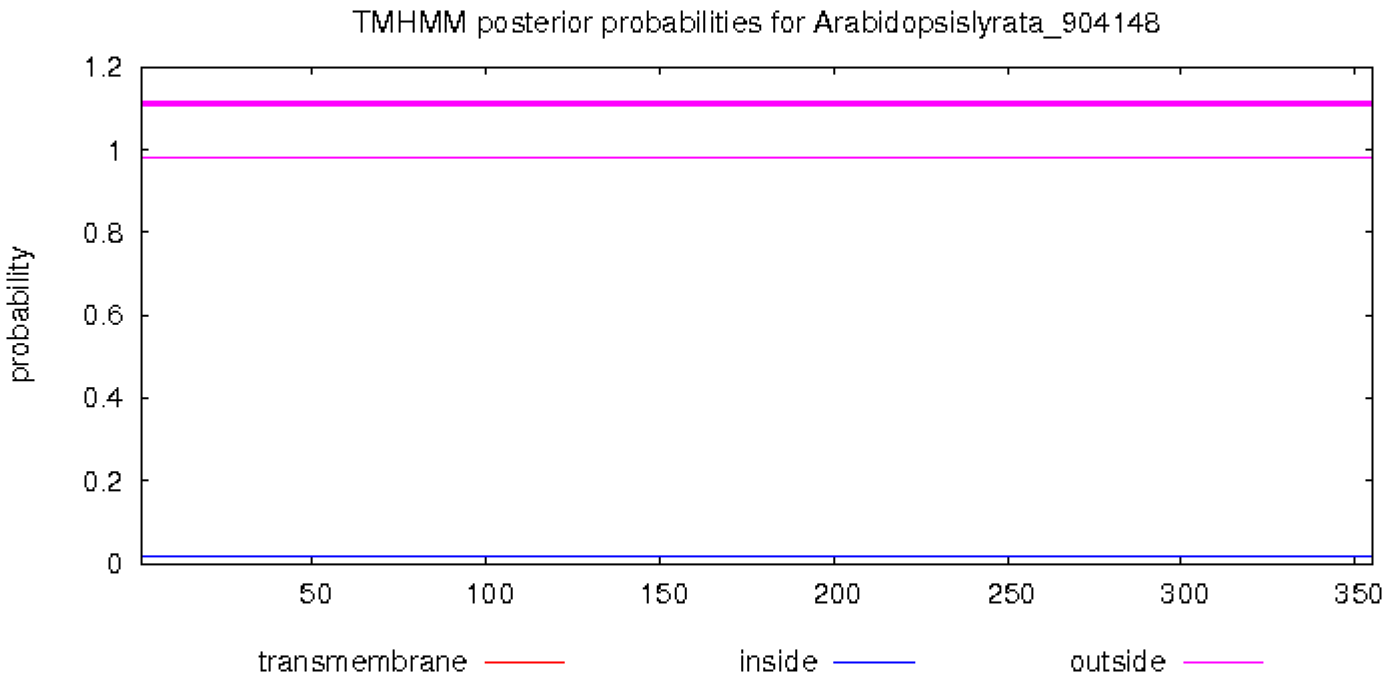

plot in postscript, script for making the plot in gnuplot, data for plot

```
# Arabidopsislyrata_904950 Length: 194
# Arabidopsislyrata_904950 Number of predicted TMHs:  0
# Arabidopsislyrata_904950 Exp number of AAs in TMHs: 0
# Arabidopsislyrata_904950 Exp number, first 60 AAs:  0
# Arabidopsislyrata_904950 Total prob of N-in:        0.01990
Arabidopsislyrata_904950        TMHMM2.0        outside      1   194
```

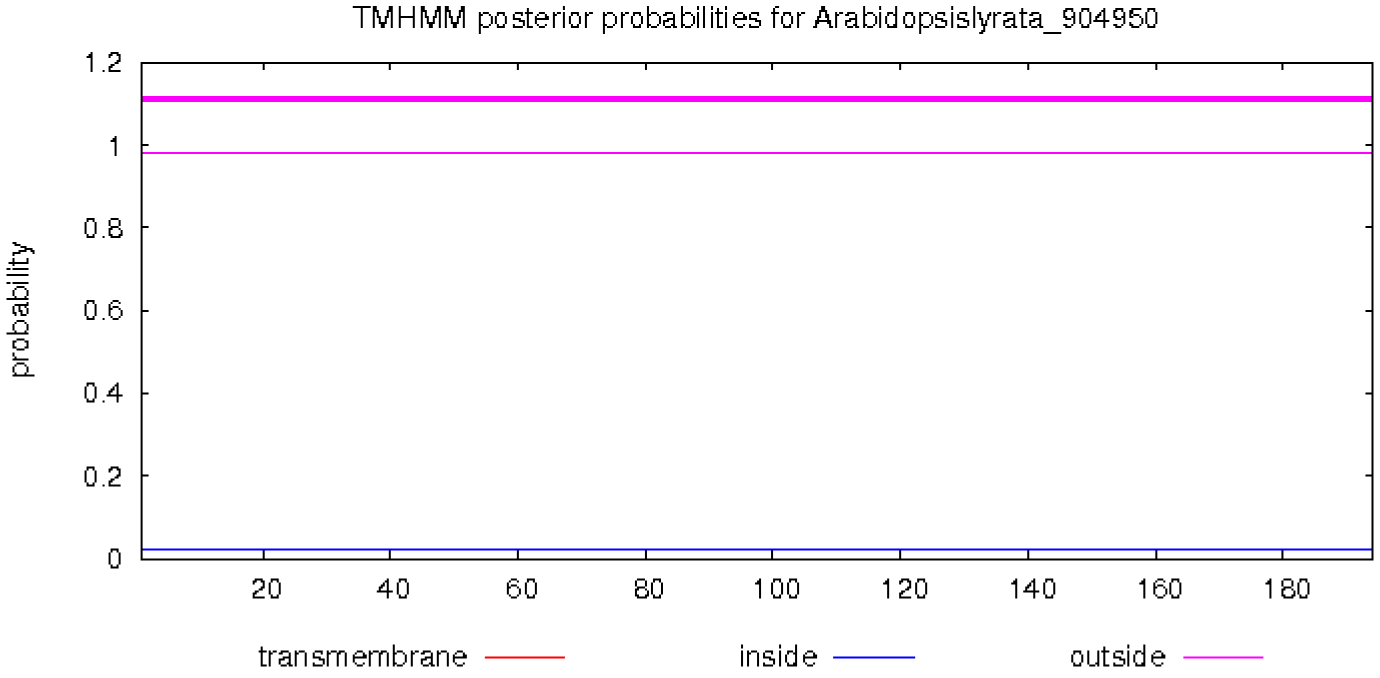

plot in postscript, script for making the plot in gnuplot, data for plot

```
# Arabidopsislyrata_905851 Length: 97
# Arabidopsislyrata_905851 Number of predicted TMHs:  0
# Arabidopsislyrata_905851 Exp number of AAs in TMHs: 0.10658
# Arabidopsislyrata_905851 Exp number, first 60 AAs:  0.10441
# Arabidopsislyrata_905851 Total prob of N-in:        0.24668
Arabidopsislyrata_905851        TMHMM2.0        outside      1    97
```

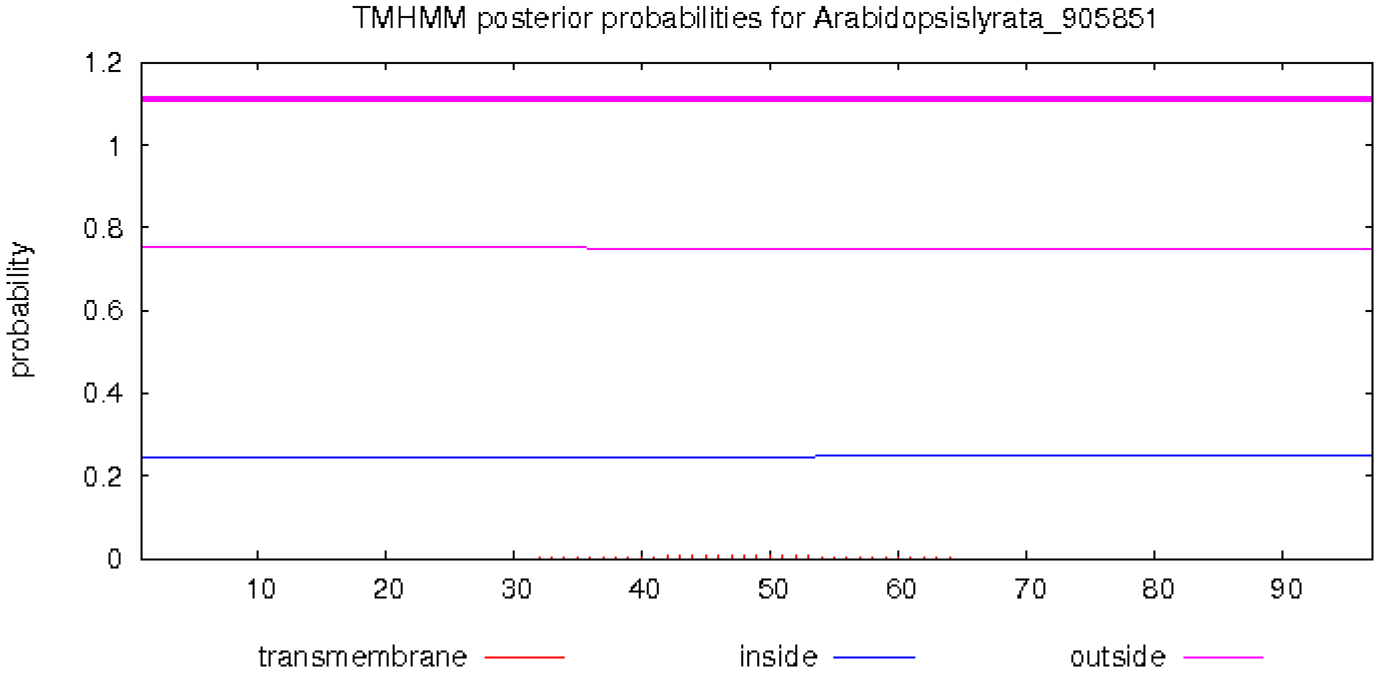

plot in postscript, script for making the plot in gnuplot, data for plot

```
# Arabidopsislyrata_905966 Length: 170
# Arabidopsislyrata_905966 Number of predicted TMHs:  0
# Arabidopsislyrata_905966 Exp number of AAs in TMHs: 0.23424
# Arabidopsislyrata_905966 Exp number, first 60 AAs:  0
# Arabidopsislyrata_905966 Total prob of N-in:        0.33633
Arabidopsislyrata_905966        TMHMM2.0        outside      1   170
```

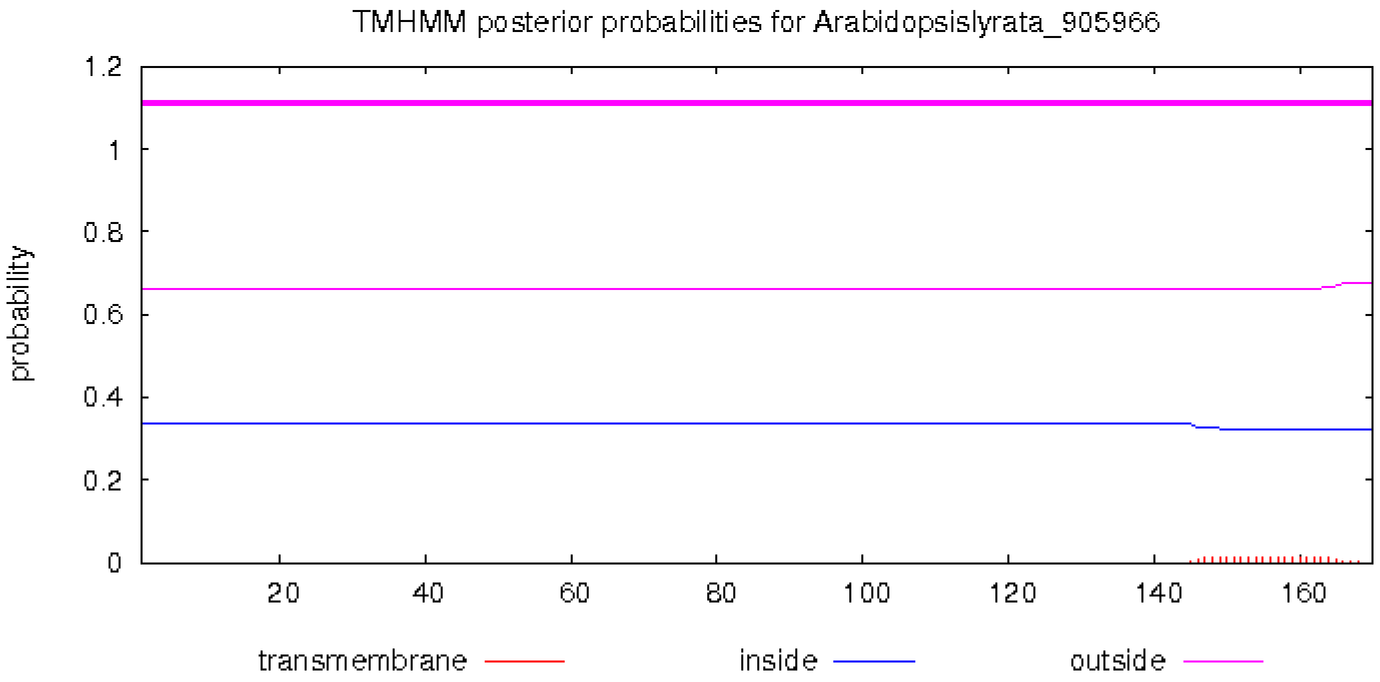

plot in postscript, script for making the plot in gnuplot, data for plot

---

```
# Arabidopsislyrata_906244 Length: 469
# Arabidopsislyrata_906244 Number of predicted TMHs:  1
# Arabidopsislyrata_906244 Exp number of AAs in TMHs: 20.23484
# Arabidopsislyrata_906244 Exp number, first 60 AAs:  0
# Arabidopsislyrata_906244 Total prob of N-in:        0.13532
Arabidopsislyrata_906244        TMHMM2.0        outside      1   438
Arabidopsislyrata_906244        TMHMM2.0        TMhelix    439   461
Arabidopsislyrata_906244        TMHMM2.0        inside     462   469
```

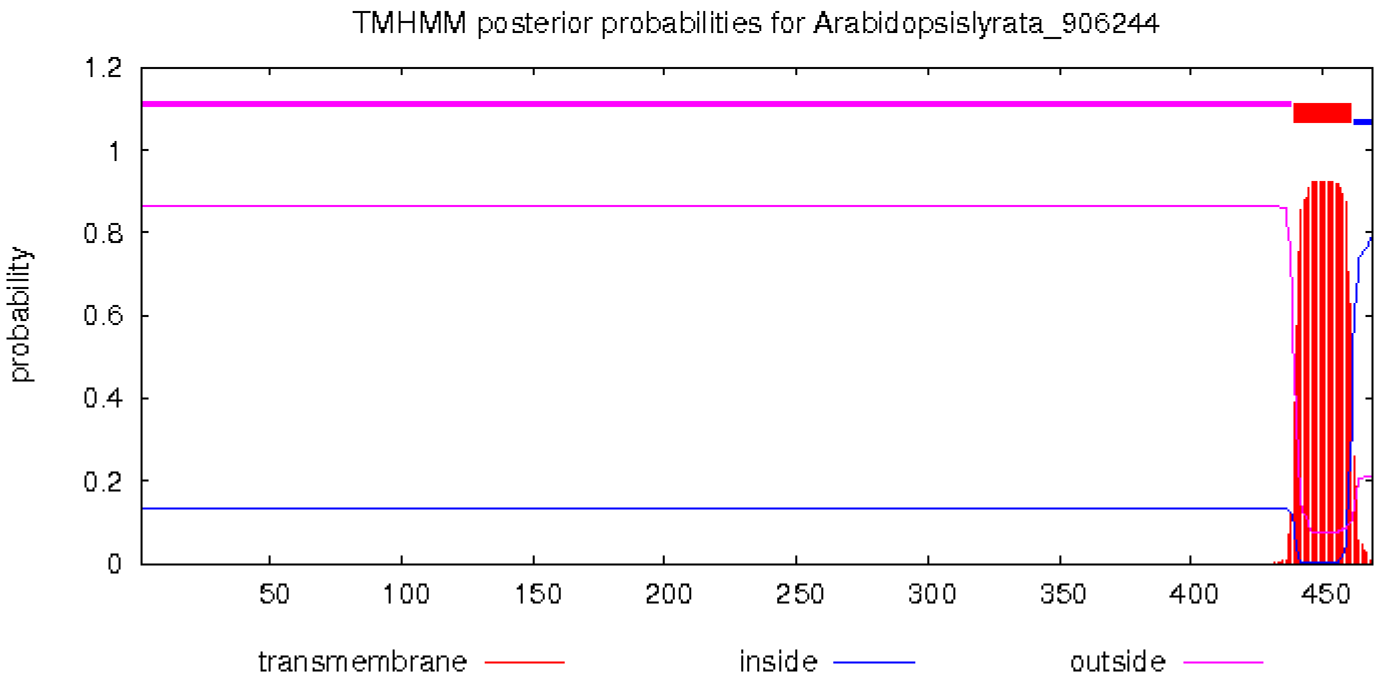

plot in postscript, script for making the plot in gnuplot, data for plot

```
# Arabidopsislyrata_907751 Length: 332
# Arabidopsislyrata_907751 Number of predicted TMHs:  0
# Arabidopsislyrata_907751 Exp number of AAs in TMHs: 7e-05
# Arabidopsislyrata_907751 Exp number, first 60 AAs:  0
# Arabidopsislyrata_907751 Total prob of N-in:        0.02844
Arabidopsislyrata_907751        TMHMM2.0        outside      1   332
```

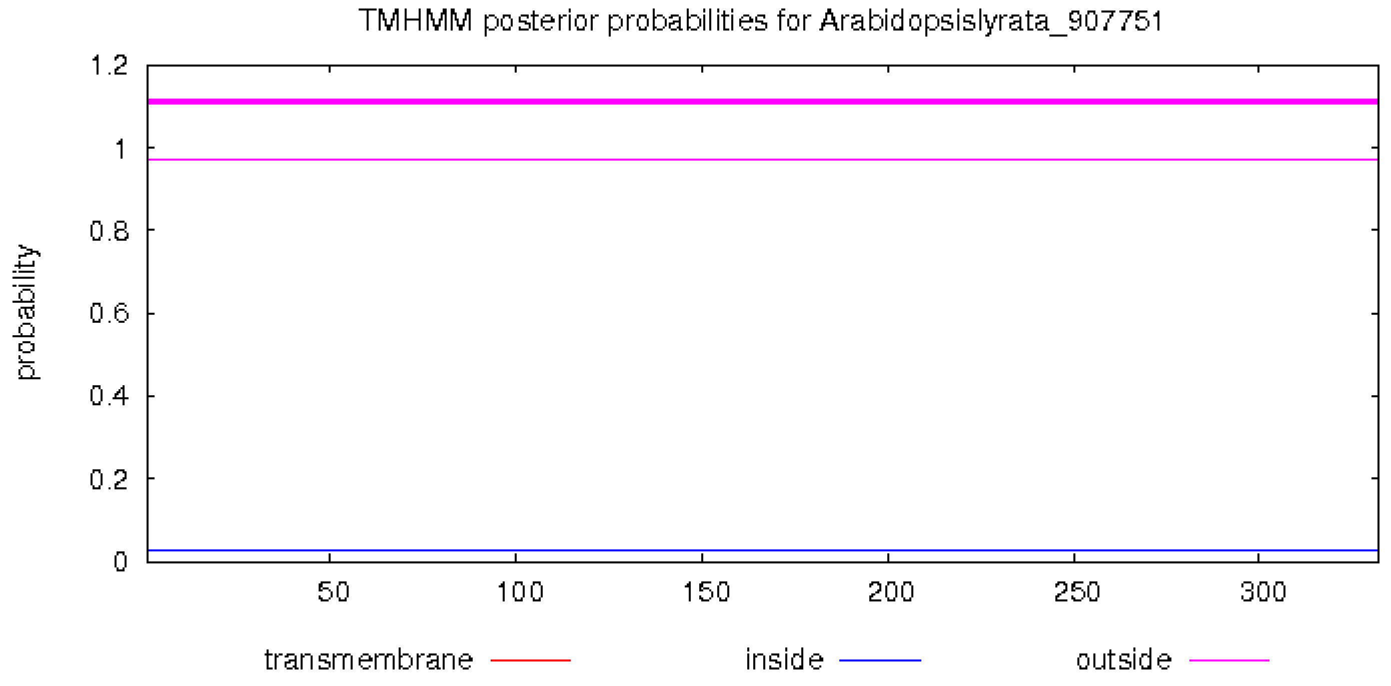

plot in postscript, script for making the plot in gnuplot, data for plot

```
# Arabidopsislyrata_908325 Length: 568
# Arabidopsislyrata_908325 Number of predicted TMHs:  1
# Arabidopsislyrata_908325 Exp number of AAs in TMHs: 22.25087
# Arabidopsislyrata_908325 Exp number, first 60 AAs:  0
# Arabidopsislyrata_908325 Total prob of N-in:        0.05361
Arabidopsislyrata_908325        TMHMM2.0        outside      1   543
Arabidopsislyrata_908325        TMHMM2.0        TMhelix    544   566
Arabidopsislyrata_908325        TMHMM2.0        inside     567   568
```

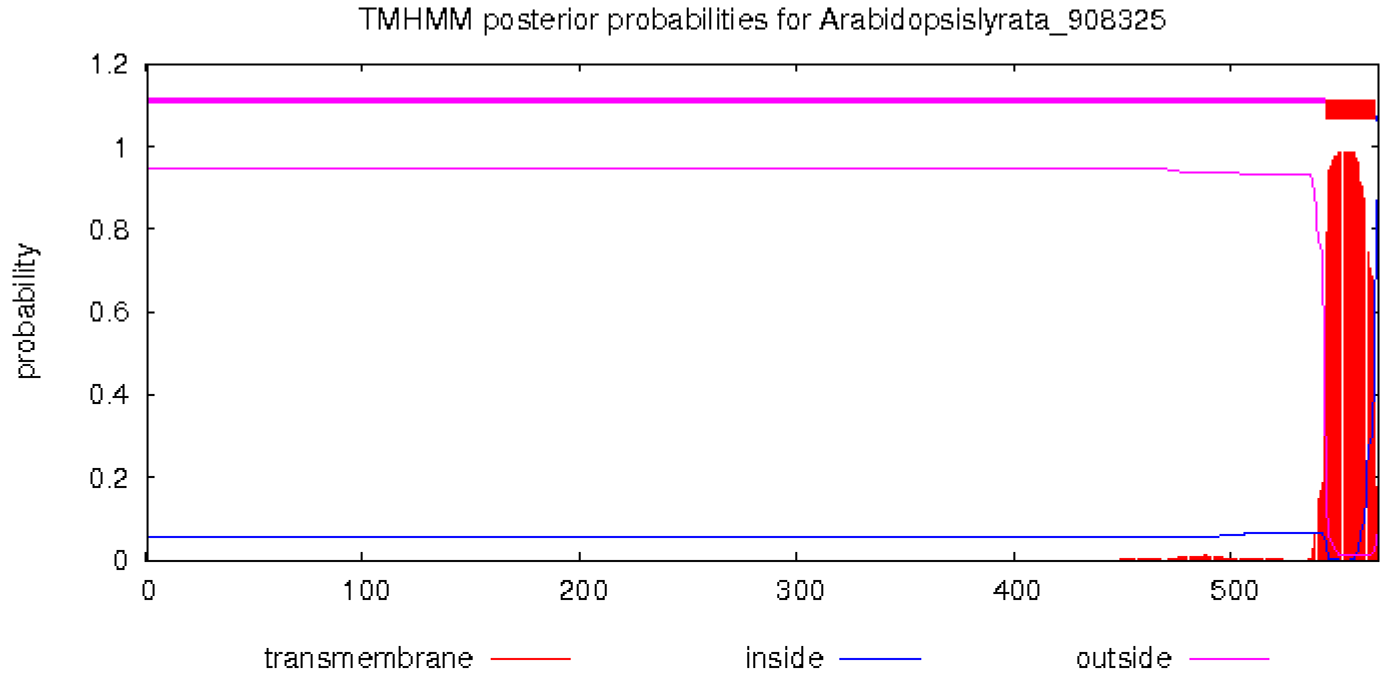

plot in postscript, script for making the plot in gnuplot, data for plot

---

```
# Arabidopsislyrata_908859 Length: 283
# Arabidopsislyrata_908859 Number of predicted TMHs:  0
# Arabidopsislyrata_908859 Exp number of AAs in TMHs: 0.00019
# Arabidopsislyrata_908859 Exp number, first 60 AAs:  0
# Arabidopsislyrata_908859 Total prob of N-in:        0.01024
Arabidopsislyrata_908859        TMHMM2.0        outside      1   283
```

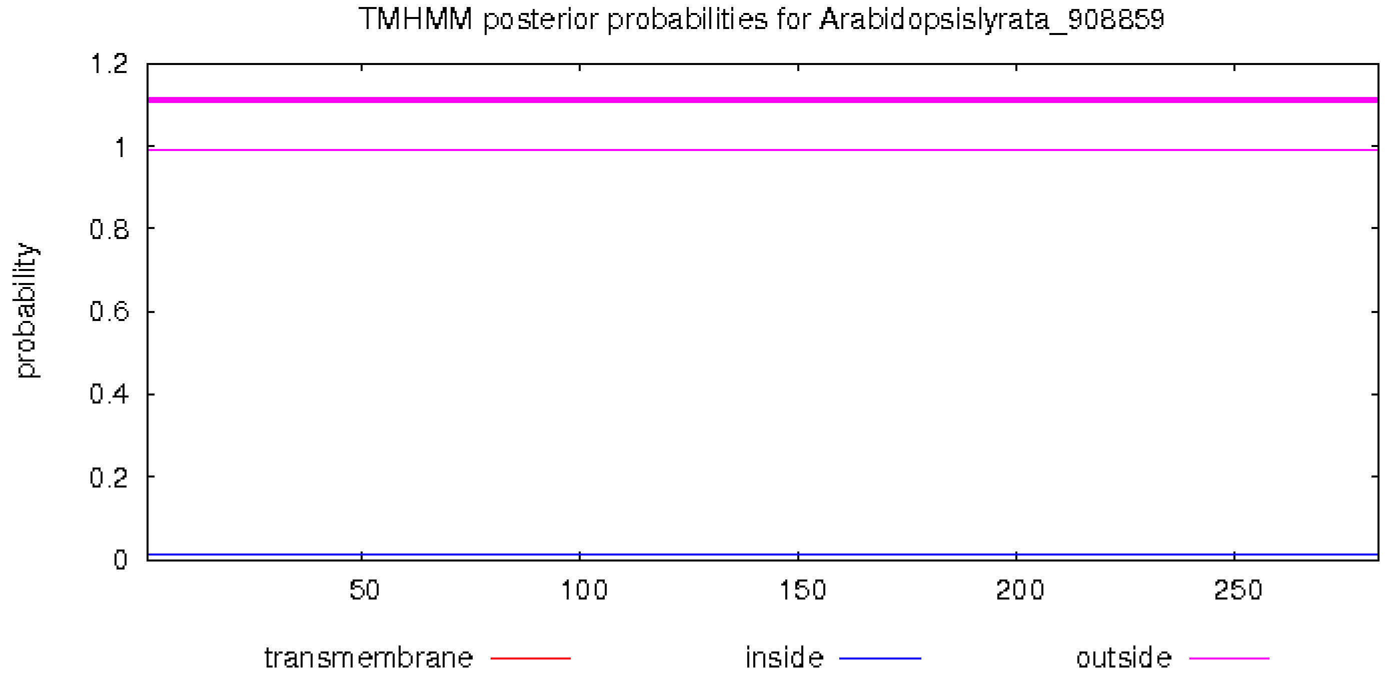

plot in postscript, script for making the plot in gnuplot, data for plot

---

```
# Arabidopsislyrata_909271 Length: 255
# Arabidopsislyrata_909271 Number of predicted TMHs:  0
# Arabidopsislyrata_909271 Exp number of AAs in TMHs: 0.0002
# Arabidopsislyrata_909271 Exp number, first 60 AAs:  0
# Arabidopsislyrata_909271 Total prob of N-in:        0.10746
Arabidopsislyrata_909271        TMHMM2.0        outside      1   255
```

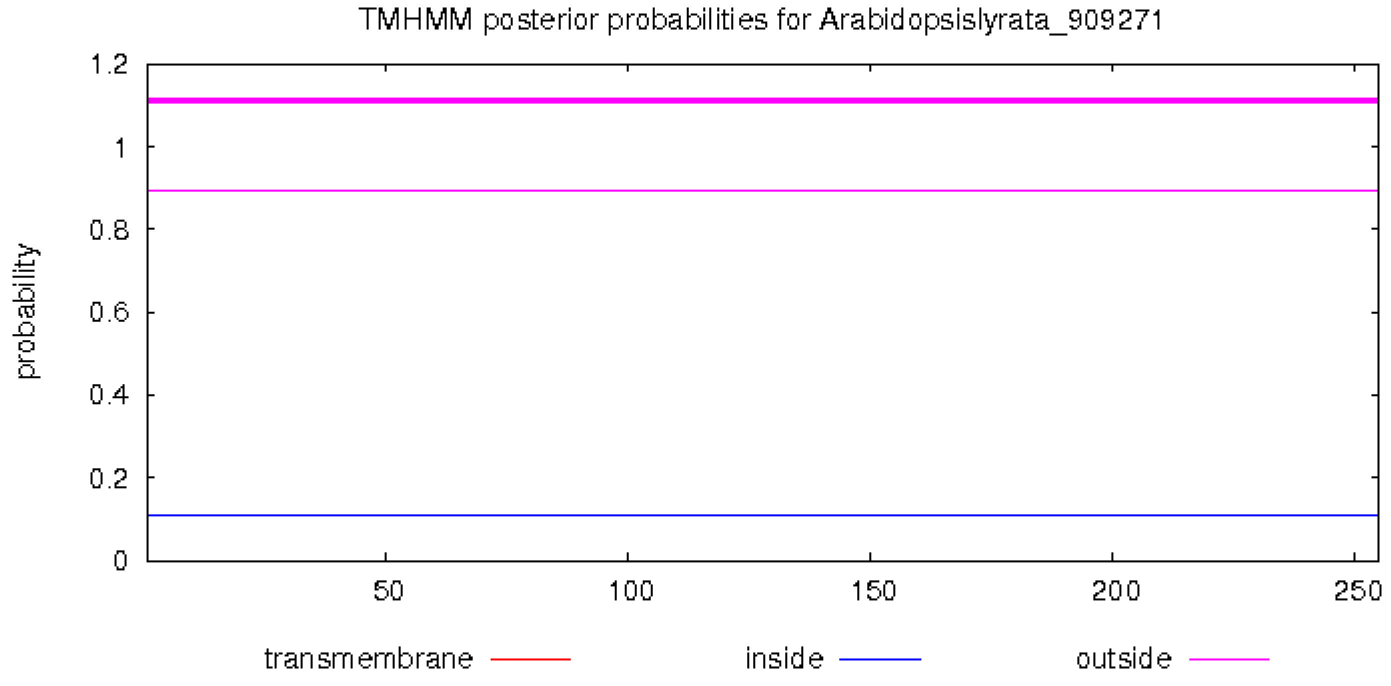

plot in postscript, script for making the plot in gnuplot, data for plot

---

```
# Arabidopsislyrata_910218 Length: 333
# Arabidopsislyrata_910218 Number of predicted TMHs:  1
# Arabidopsislyrata_910218 Exp number of AAs in TMHs: 17.29877
# Arabidopsislyrata_910218 Exp number, first 60 AAs:  0.20095
# Arabidopsislyrata_910218 Total prob of N-in:        0.61560
Arabidopsislyrata_910218	TMHMM2.0	inside	     1   313
Arabidopsislyrata_910218	TMHMM2.0	TMhelix	   314   331
Arabidopsislyrata_910218	TMHMM2.0	outside	   332   333
```

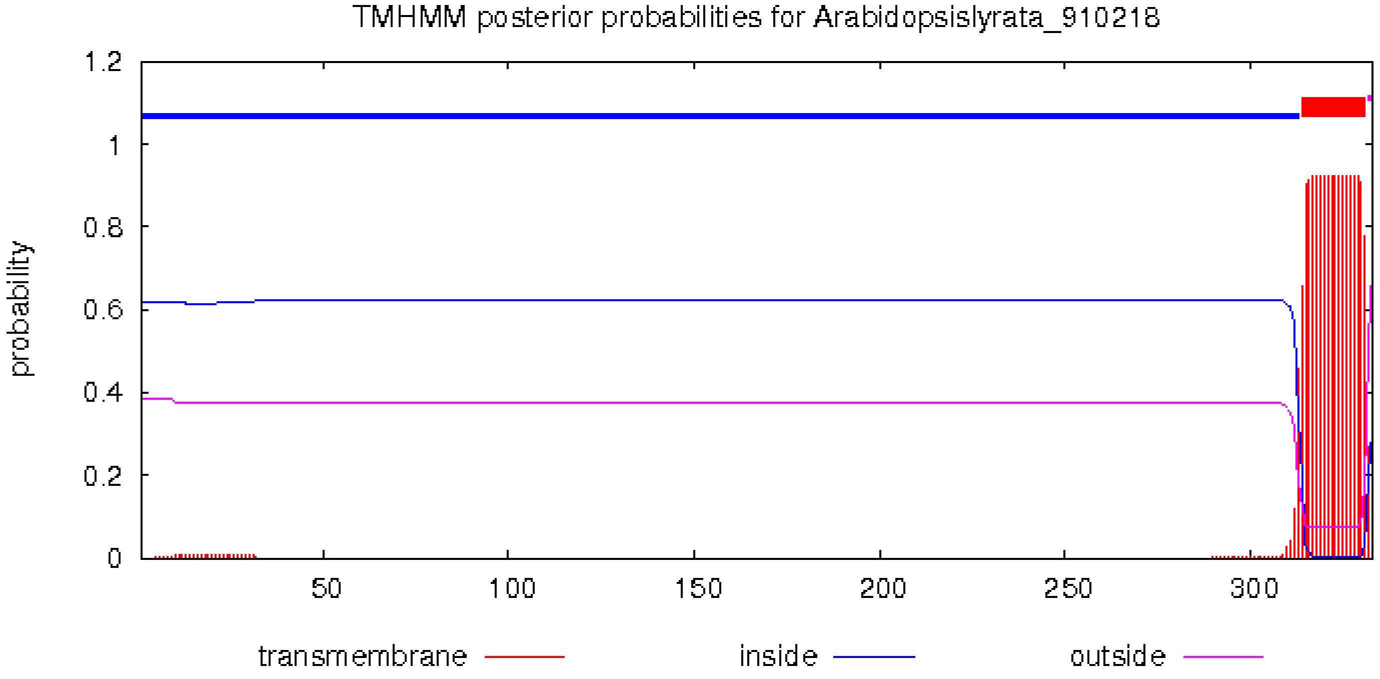

plot in postscript, script for making the plot in gnuplot, data for plot

---

```
# Arabidopsislyrata_912076 Length: 480
# Arabidopsislyrata_912076 Number of predicted TMHs:  0
# Arabidopsislyrata_912076 Exp number of AAs in TMHs: 1.77334
# Arabidopsislyrata_912076 Exp number, first 60 AAs:  0
# Arabidopsislyrata_912076 Total prob of N-in:        0.01174
Arabidopsislyrata_912076	TMHMM2.0	outside	     1   480
```

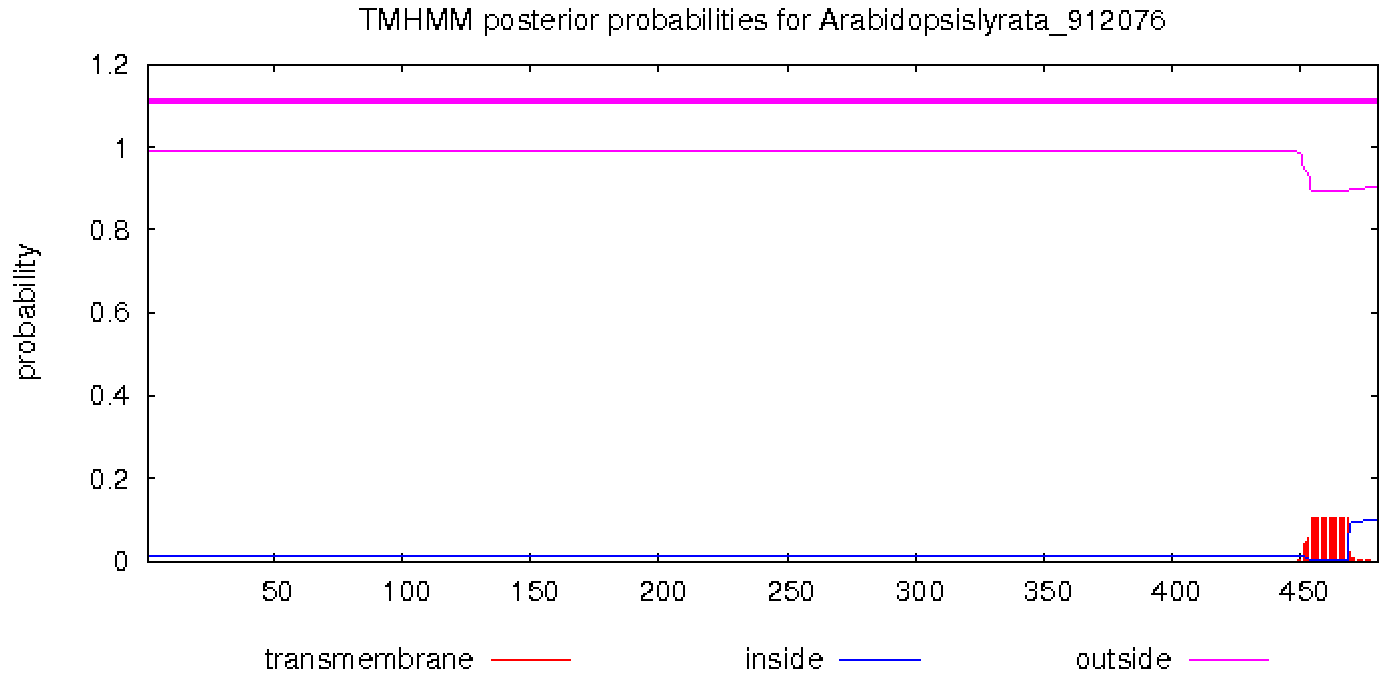

plot in postscript, script for making the plot in gnuplot, data for plot

---

```
# Arabidopsislyrata_918048 Length: 222
# Arabidopsislyrata_918048 Number of predicted TMHs:  0
# Arabidopsislyrata_918048 Exp number of AAs in TMHs: 0.00036
# Arabidopsislyrata_918048 Exp number, first 60 AAs:  0
# Arabidopsislyrata_918048 Total prob of N-in:        0.02678
Arabidopsislyrata_918048        TMHMM2.0        outside      1   222
```

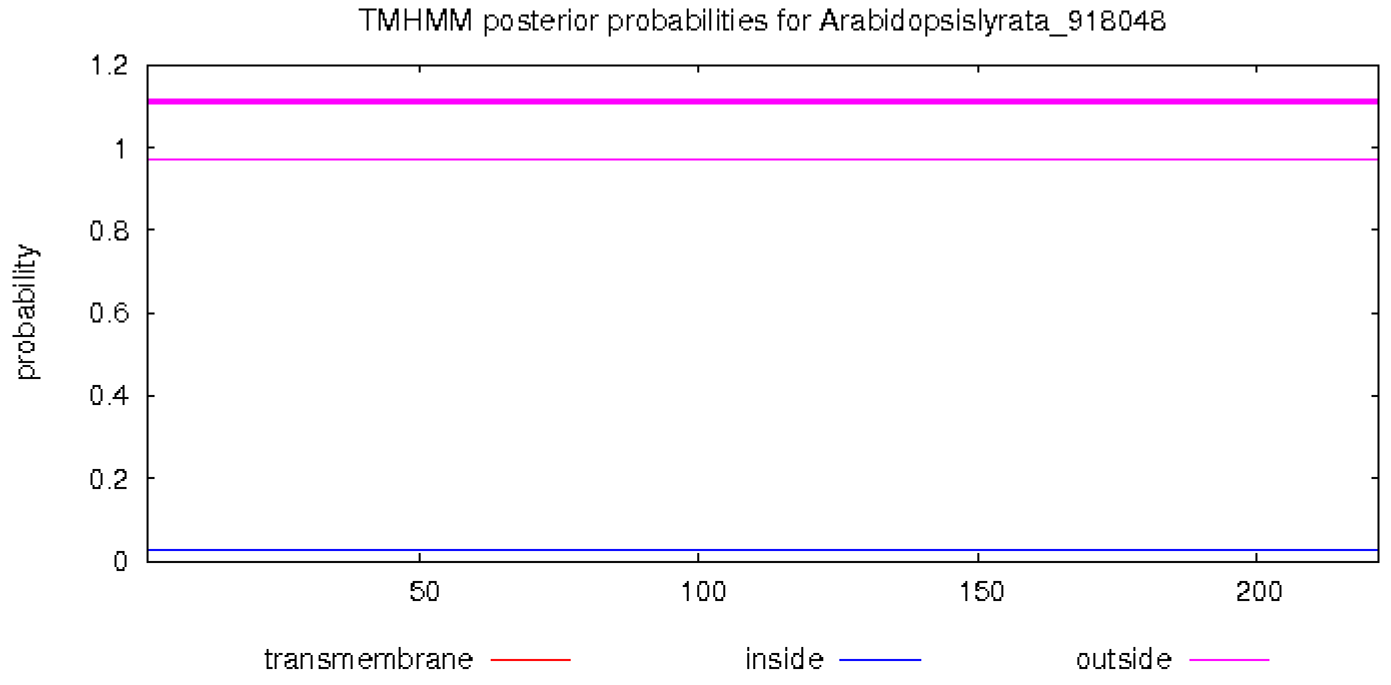

plot in postscript, script for making the plot in gnuplot, data for plot

---

```
# Arabidopsislyrata_918808 Length: 133
# Arabidopsislyrata_918808 Number of predicted TMHs:  0
# Arabidopsislyrata_918808 Exp number of AAs in TMHs: 0.00213
# Arabidopsislyrata_918808 Exp number, first 60 AAs:  0.00213
# Arabidopsislyrata_918808 Total prob of N-in:        0.41638
Arabidopsislyrata_918808        TMHMM2.0        outside      1   133
```

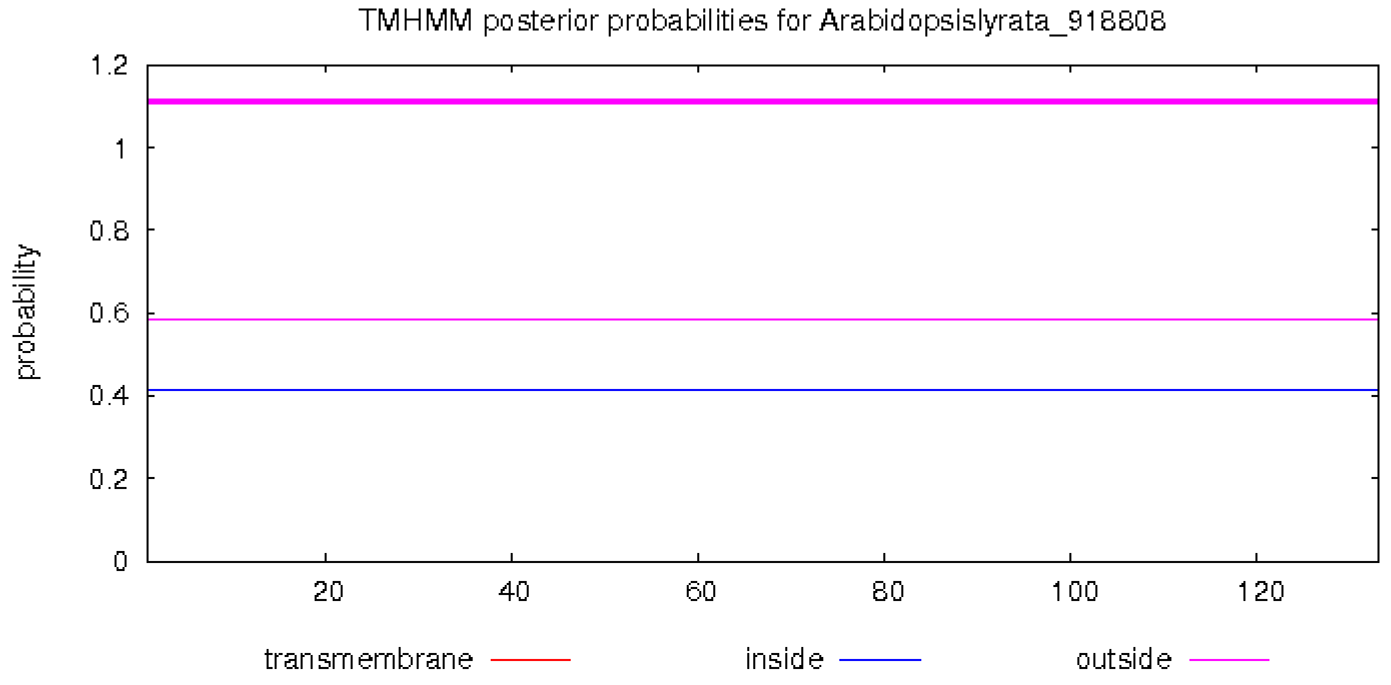

plot in postscript, script for making the plot in gnuplot, data for plot

---

```
# Arabidopsislyrata_919094 Length: 293
# Arabidopsislyrata_919094 Number of predicted TMHs:  0
# Arabidopsislyrata_919094 Exp number of AAs in TMHs: 0
# Arabidopsislyrata_919094 Exp number, first 60 AAs:  0
# Arabidopsislyrata_919094 Total prob of N-in:        0.09943
Arabidopsislyrata_919094        TMHMM2.0        outside      1   293
```

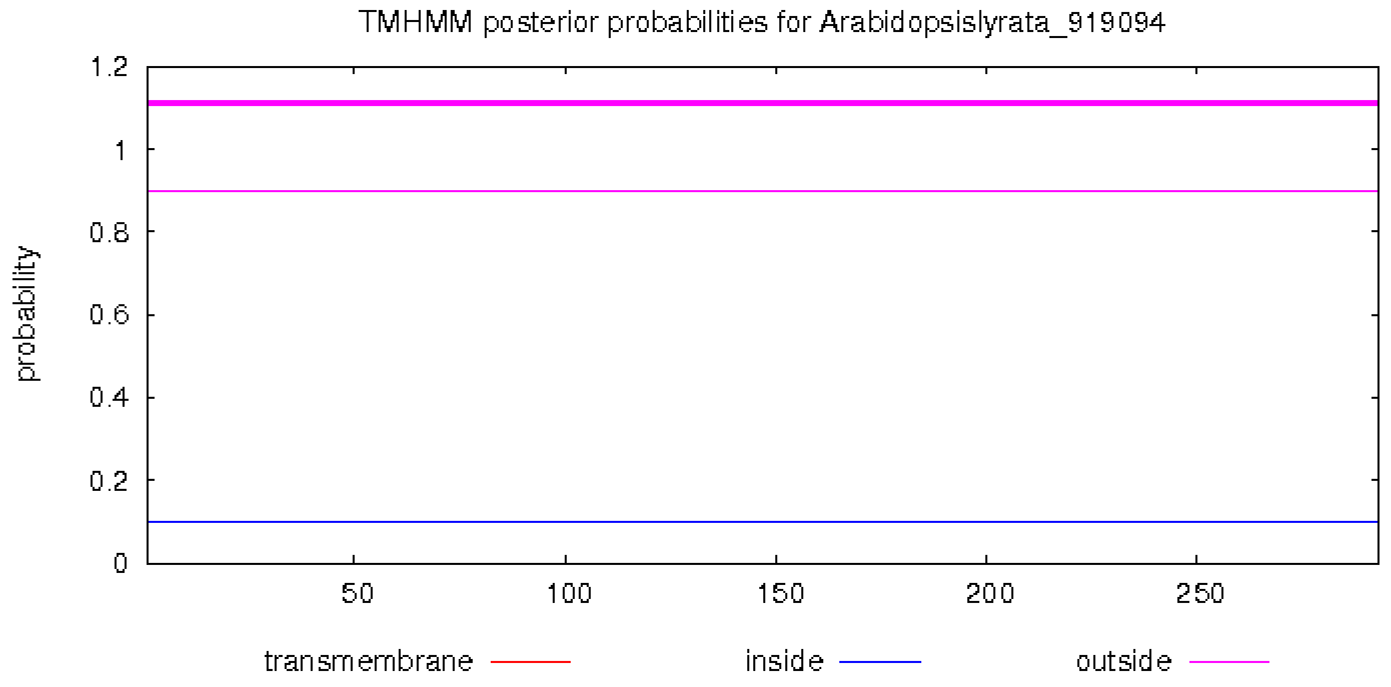

plot in postscript, script for making the plot in gnuplot, data for plot

---

```
# Arabidopsislyrata_919155 Length: 282
# Arabidopsislyrata_919155 Number of predicted TMHs:  0
# Arabidopsislyrata_919155 Exp number of AAs in TMHs: 0
# Arabidopsislyrata_919155 Exp number, first 60 AAs:  0
# Arabidopsislyrata_919155 Total prob of N-in:        0.07380
Arabidopsislyrata_919155        TMHMM2.0        outside      1   282
```

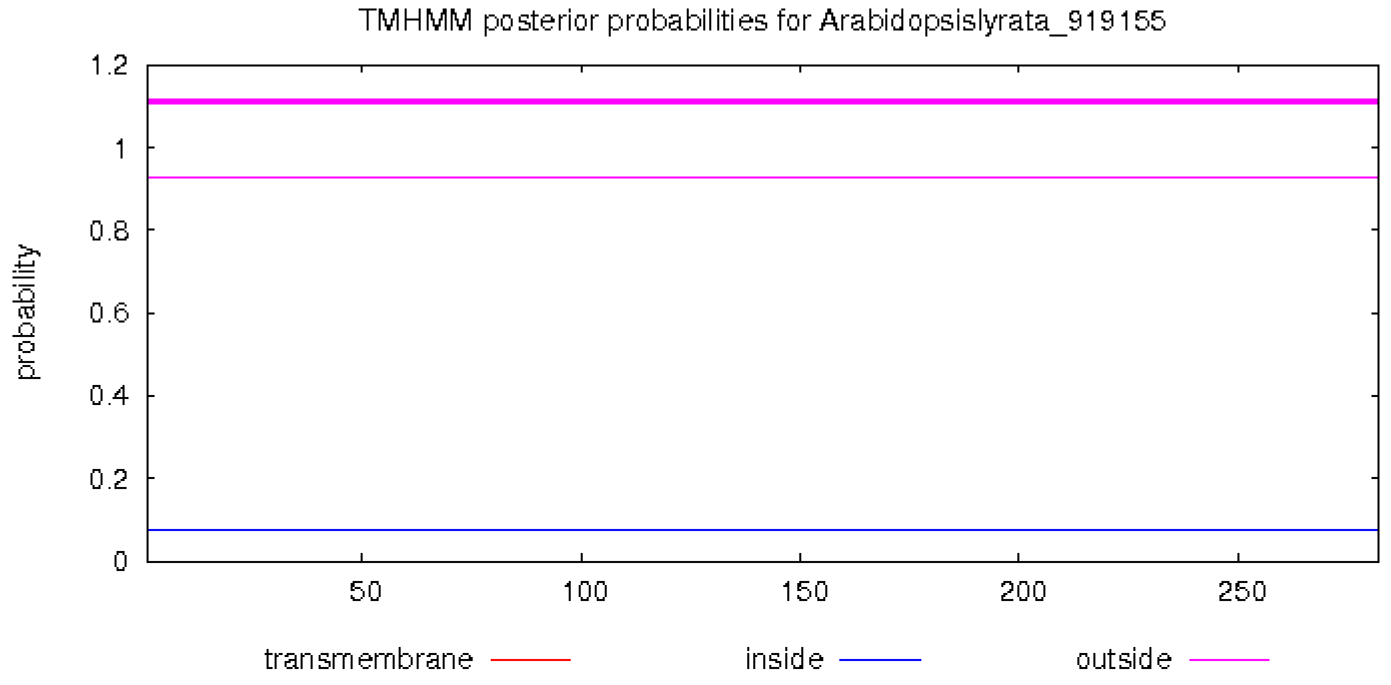

plot in postscript, script for making the plot in gnuplot, data for plot

```
# Arabidopsisthaliana_AT1G01010.1 Length: 429
# Arabidopsisthaliana_AT1G01010.1 Number of predicted TMHs:  1
# Arabidopsisthaliana_AT1G01010.1 Exp number of AAs in TMHs: 18.92056
# Arabidopsisthaliana_AT1G01010.1 Exp number, first 60 AAs:  0.00036
# Arabidopsisthaliana_AT1G01010.1 Total prob of N-in:        0.14970
Arabidopsisthaliana_AT1G01010.1 TMHMM2.0        outside      1   410
Arabidopsisthaliana_AT1G01010.1 TMHMM2.0        TMhelix    411   428
Arabidopsisthaliana_AT1G01010.1 TMHMM2.0        inside     429   429
```

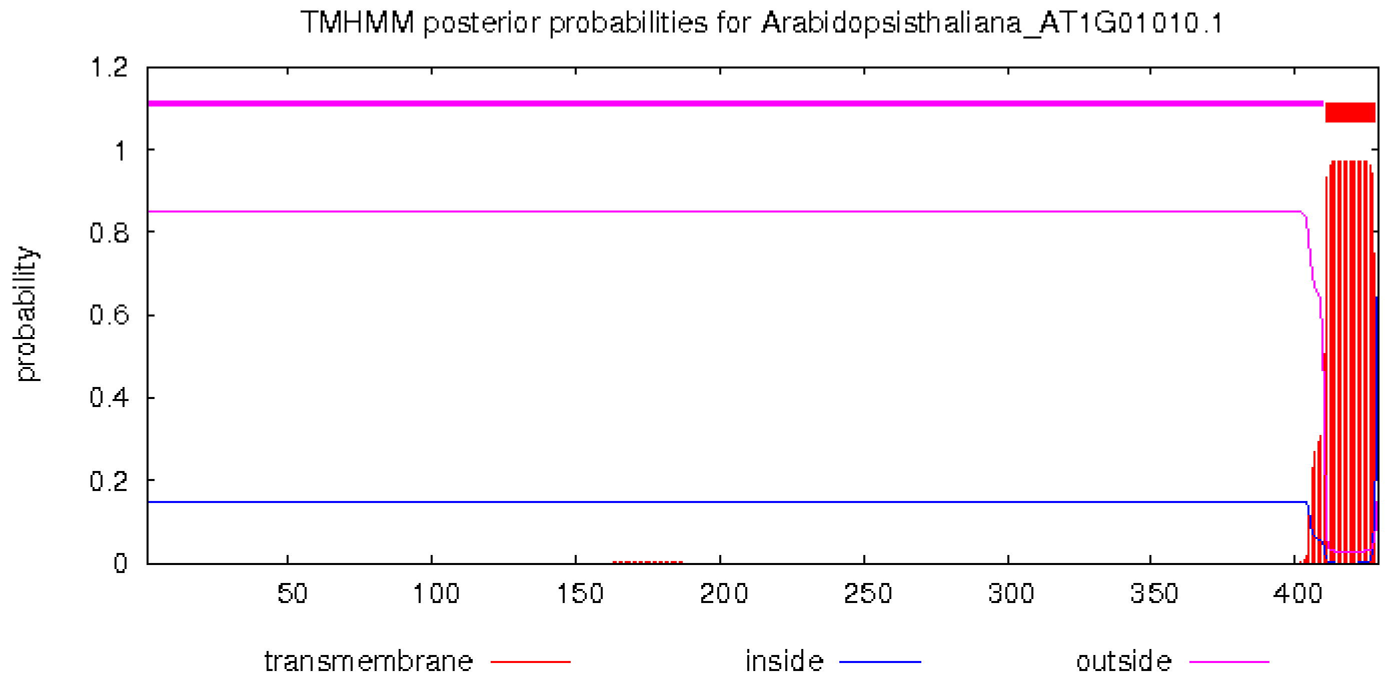

plot in postscript, script for making the plot in gnuplot, data for plot

```
# Arabidopsisthaliana_AT1G01720.1 Length: 289
# Arabidopsisthaliana_AT1G01720.1 Number of predicted TMHs:  0
# Arabidopsisthaliana_AT1G01720.1 Exp number of AAs in TMHs: 0.00163
# Arabidopsisthaliana_AT1G01720.1 Exp number, first 60 AAs:  0.00062
```

```
# Arabidopsisthaliana_AT1G01720.1 Total prob of N-in:        0.00327
Arabidopsisthaliana_AT1G01720.1 TMHMM2.0        outside      1   289
```

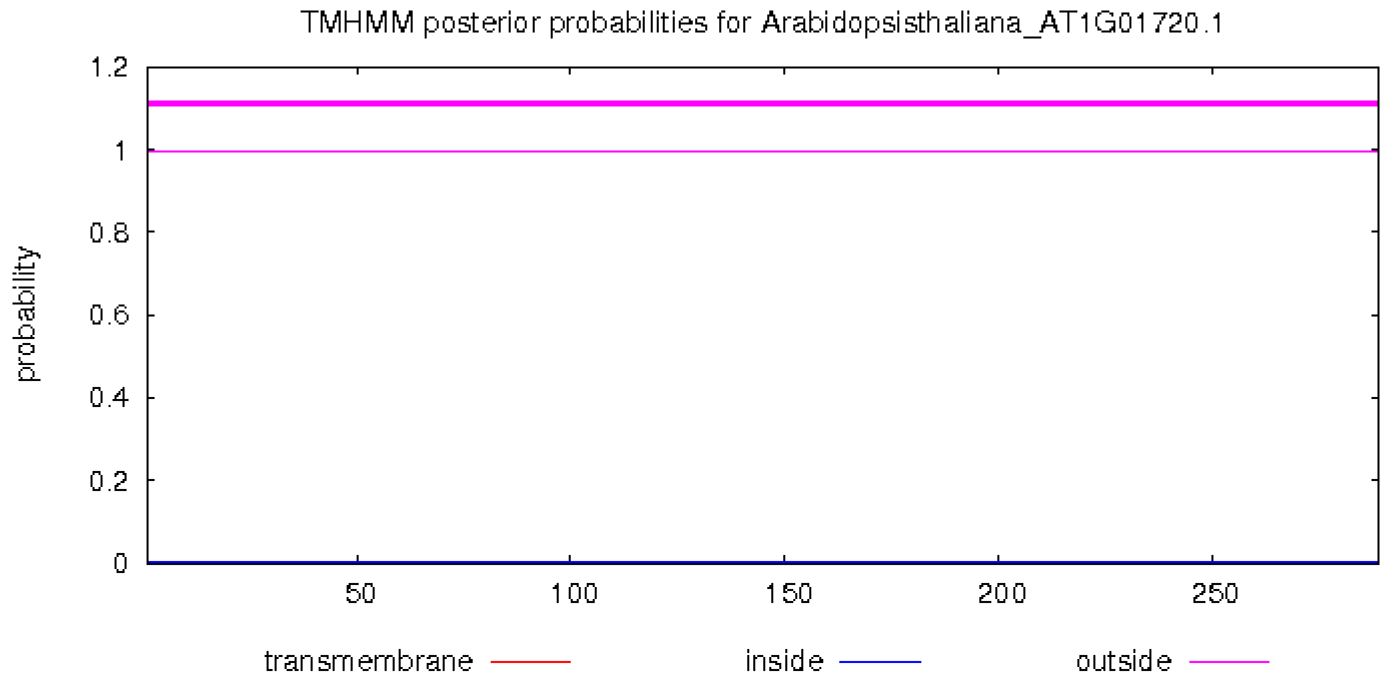

plot in postscript, script for making the plot in gnuplot, data for plot

---

```
# Arabidopsisthaliana_AT1G02210.1 Length: 87
# Arabidopsisthaliana_AT1G02210.1 Number of predicted TMHs:  0
# Arabidopsisthaliana_AT1G02210.1 Exp number of AAs in TMHs: 0.00616
# Arabidopsisthaliana_AT1G02210.1 Exp number, first 60 AAs:  0.00048
# Arabidopsisthaliana_AT1G02210.1 Total prob of N-in:        0.08528
Arabidopsisthaliana_AT1G02210.1 TMHMM2.0        outside      1    87
```

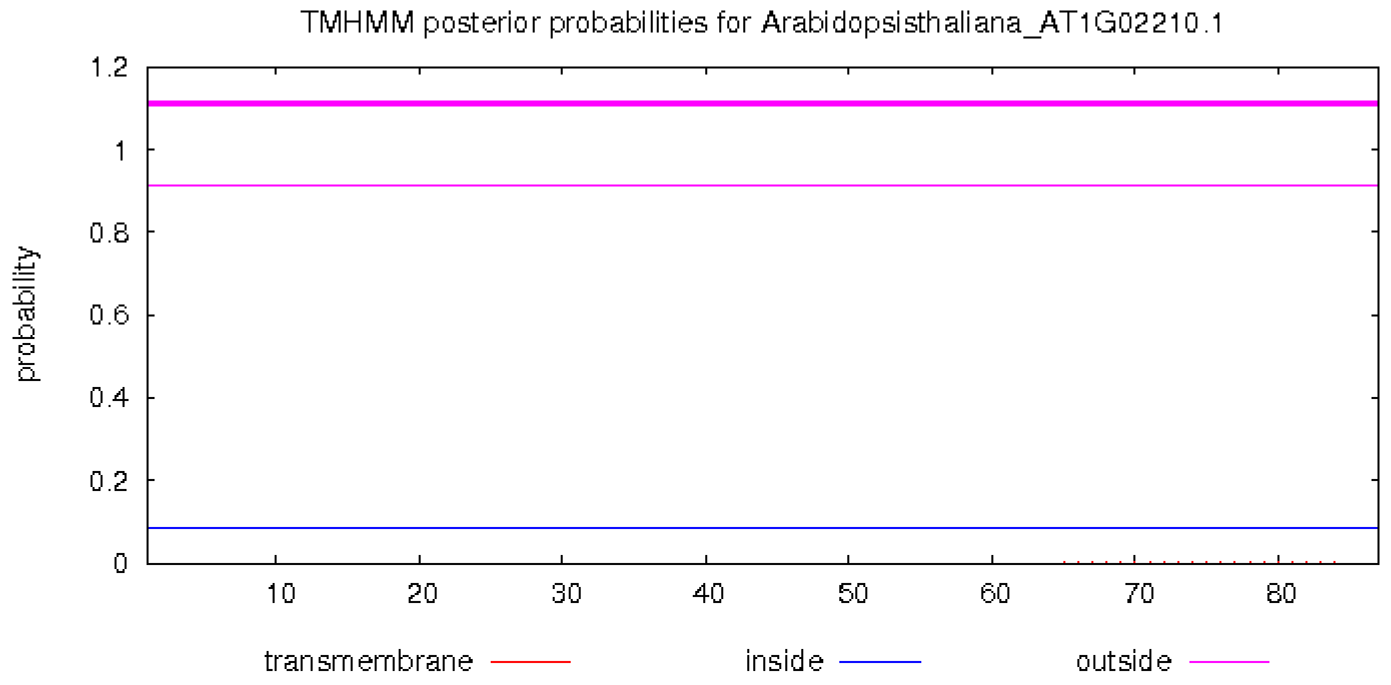

plot in postscript, script for making the plot in gnuplot, data for plot

---

```
# Arabidopsisthaliana_AT1G02220.1 Length: 394
# Arabidopsisthaliana_AT1G02220.1 Number of predicted TMHs:  0
# Arabidopsisthaliana_AT1G02220.1 Exp number of AAs in TMHs: 0.00021
# Arabidopsisthaliana_AT1G02220.1 Exp number, first 60 AAs:  0
```

```
# Arabidopsisthaliana_AT1G02220.1 Total prob of N-in:        0.00353
Arabidopsisthaliana_AT1G02220.1 TMHMM2.0        outside      1   394
```

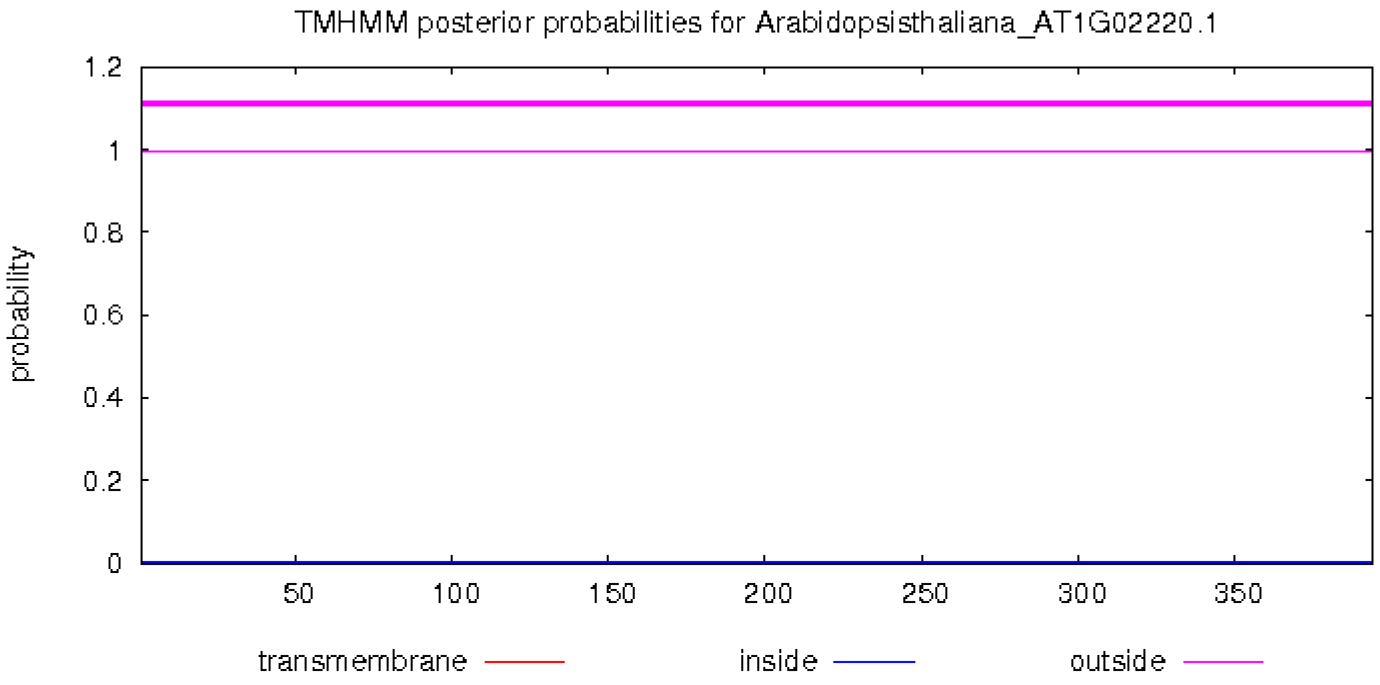

plot in postscript, script for making the plot in gnuplot, data for plot

---

```
# Arabidopsisthaliana_AT1G02230.1 Length: 359
# Arabidopsisthaliana_AT1G02230.1 Number of predicted TMHs:  0
# Arabidopsisthaliana_AT1G02230.1 Exp number of AAs in TMHs: 0
# Arabidopsisthaliana_AT1G02230.1 Exp number, first 60 AAs:  0
# Arabidopsisthaliana_AT1G02230.1 Total prob of N-in:        0.00880
Arabidopsisthaliana_AT1G02230.1 TMHMM2.0        outside      1   359
```

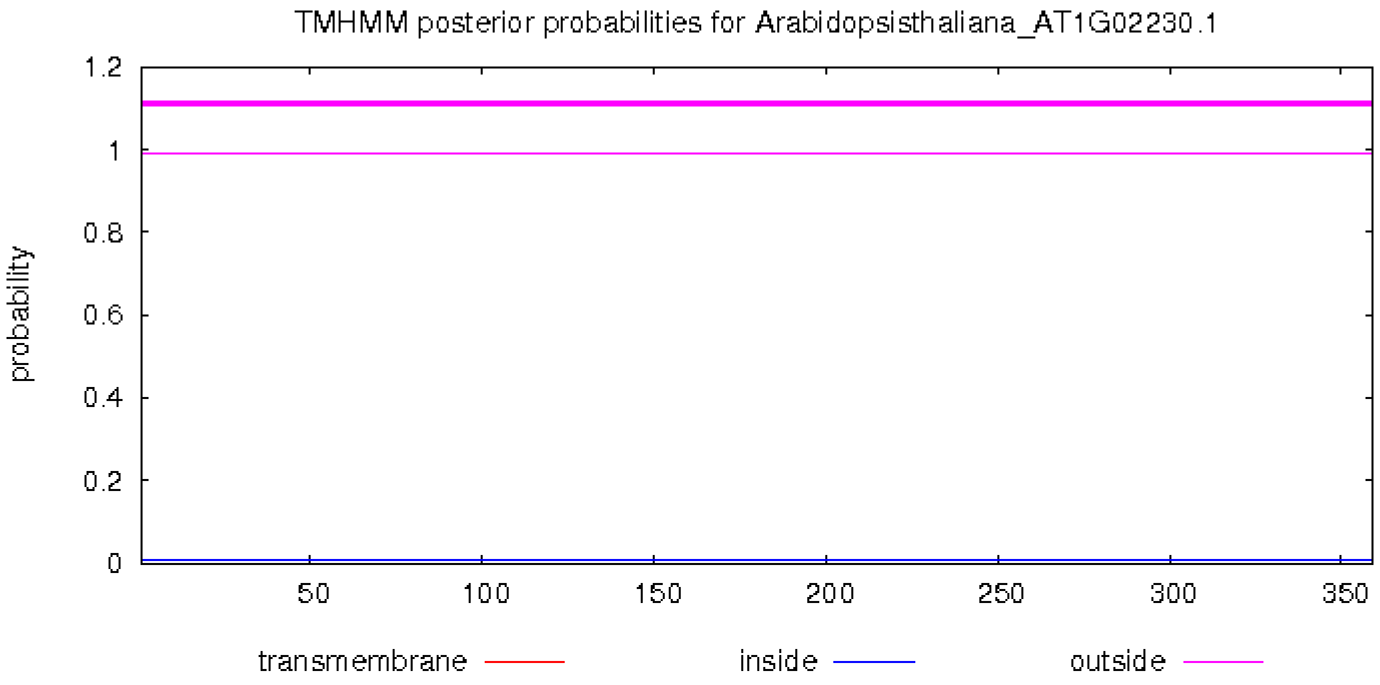

plot in postscript, script for making the plot in gnuplot, data for plot

---

```
# Arabidopsisthaliana_AT1G02250.1 Length: 362
# Arabidopsisthaliana_AT1G02250.1 Number of predicted TMHs:  0
# Arabidopsisthaliana_AT1G02250.1 Exp number of AAs in TMHs: 0
# Arabidopsisthaliana_AT1G02250.1 Exp number, first 60 AAs:  0
```

```
# Arabidopsisthaliana_AT1G02250.1 Total prob of N-in:        0.01095
Arabidopsisthaliana_AT1G02250.1 TMHMM2.0        outside      1   362
```

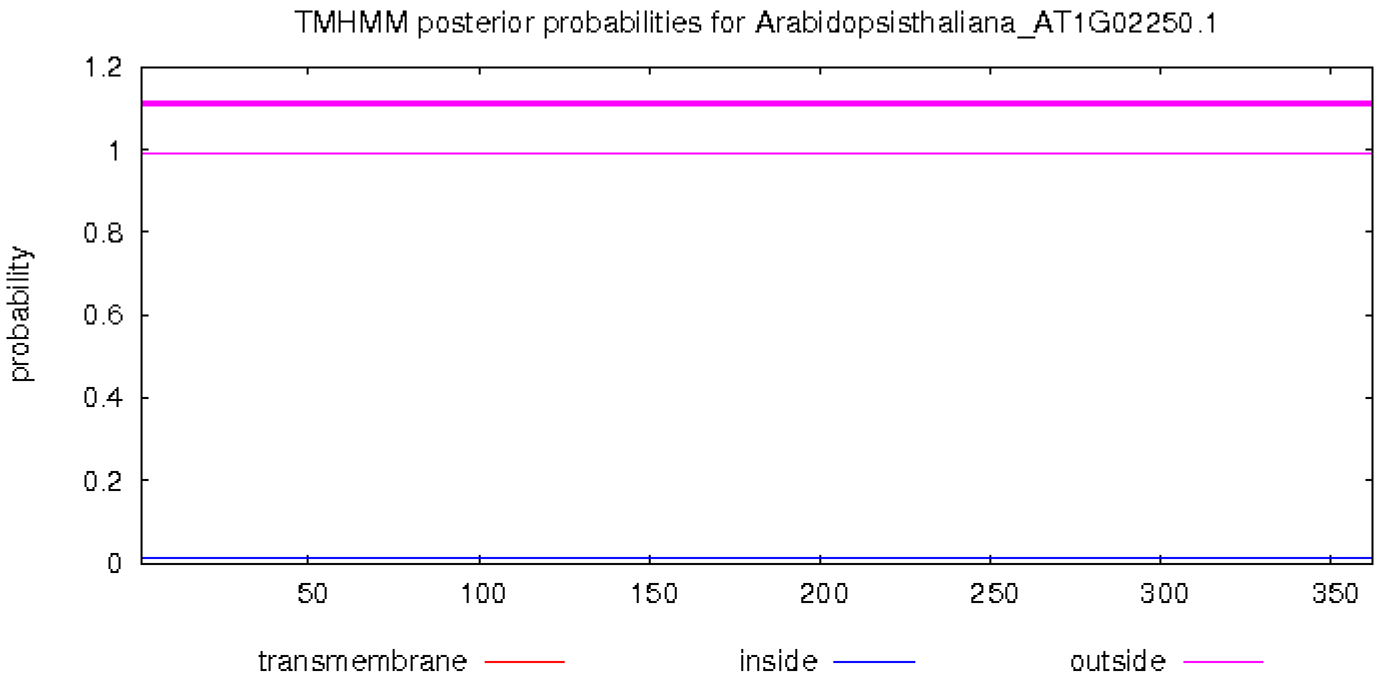

plot in postscript, script for making the plot in gnuplot, data for plot

---

```
# Arabidopsisthaliana_AT1G03490.1 Length: 281
# Arabidopsisthaliana_AT1G03490.1 Number of predicted TMHs:  0
# Arabidopsisthaliana_AT1G03490.1 Exp number of AAs in TMHs: 0.00054
# Arabidopsisthaliana_AT1G03490.1 Exp number, first 60 AAs:  0.00054
# Arabidopsisthaliana_AT1G03490.1 Total prob of N-in:        0.01319
Arabidopsisthaliana_AT1G03490.1 TMHMM2.0        outside      1   281
```

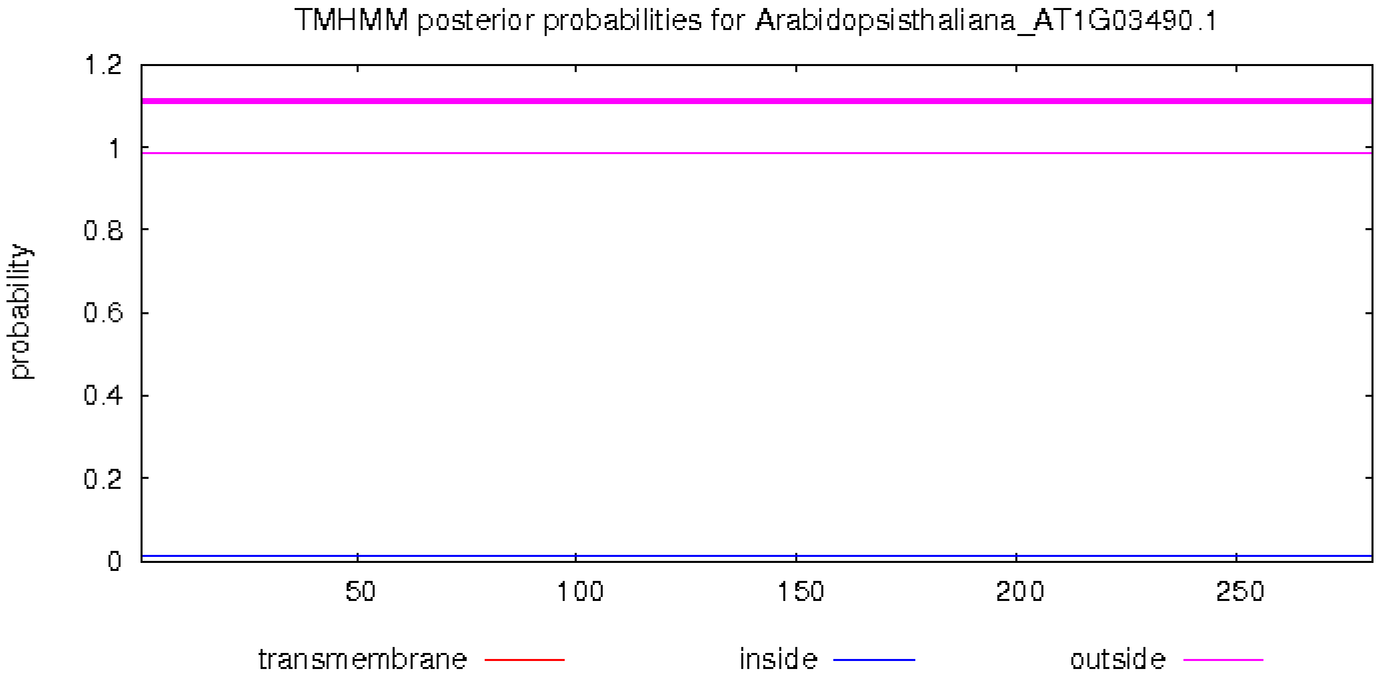

plot in postscript, script for making the plot in gnuplot, data for plot

---

```
# Arabidopsisthaliana_AT1G12260.1 Length: 395
# Arabidopsisthaliana_AT1G12260.1 Number of predicted TMHs:  0
# Arabidopsisthaliana_AT1G12260.1 Exp number of AAs in TMHs: 0.00018
# Arabidopsisthaliana_AT1G12260.1 Exp number, first 60 AAs:  0
```

```
# Arabidopsisthaliana_AT1G12260.1 Total prob of N-in:        0.00167
Arabidopsisthaliana_AT1G12260.1 TMHMM2.0        outside      1   395
```

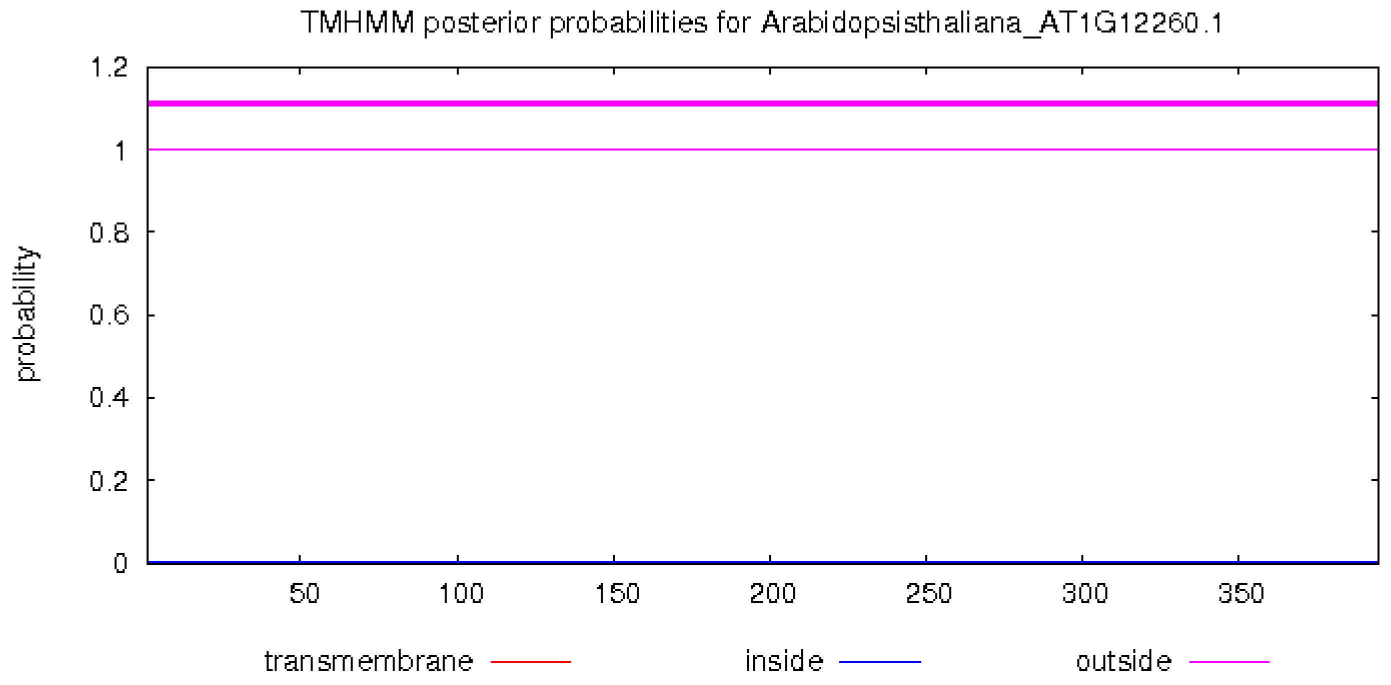

plot in postscript, script for making the plot in gnuplot, data for plot

---

```
# Arabidopsisthaliana_AT1G19040.1 Length: 172
# Arabidopsisthaliana_AT1G19040.1 Number of predicted TMHs:  0
# Arabidopsisthaliana_AT1G19040.1 Exp number of AAs in TMHs: 0.02368
# Arabidopsisthaliana_AT1G19040.1 Exp number, first 60 AAs:  0
# Arabidopsisthaliana_AT1G19040.1 Total prob of N-in:        0.09882
Arabidopsisthaliana_AT1G19040.1 TMHMM2.0        outside      1   172
```

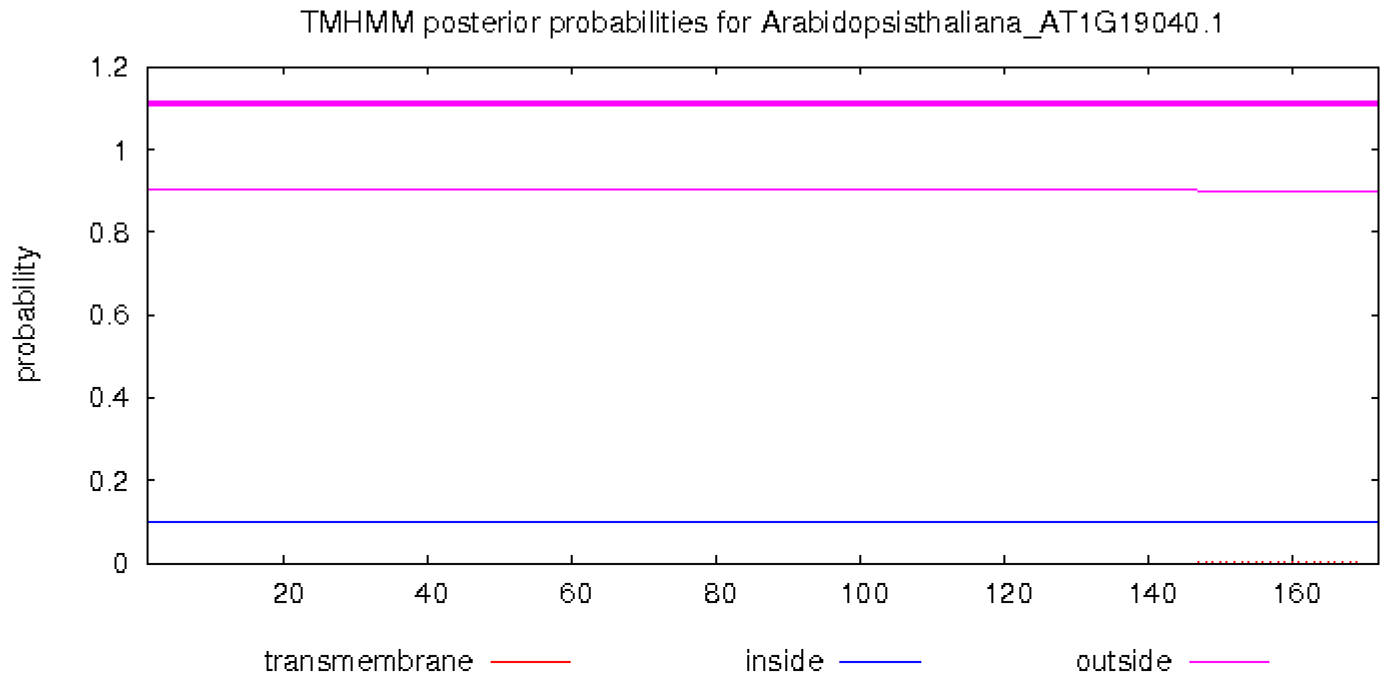

plot in postscript, script for making the plot in gnuplot, data for plot

---

```
# Arabidopsisthaliana_AT1G25580.1 Length: 449
# Arabidopsisthaliana_AT1G25580.1 Number of predicted TMHs:  0
# Arabidopsisthaliana_AT1G25580.1 Exp number of AAs in TMHs: 0.0002
# Arabidopsisthaliana_AT1G25580.1 Exp number, first 60 AAs:  0
```

```
# Arabidopsisthaliana_AT1G25580.1 Total prob of N-in:        0.00300
Arabidopsisthaliana_AT1G25580.1 TMHMM2.0        outside      1   449
```

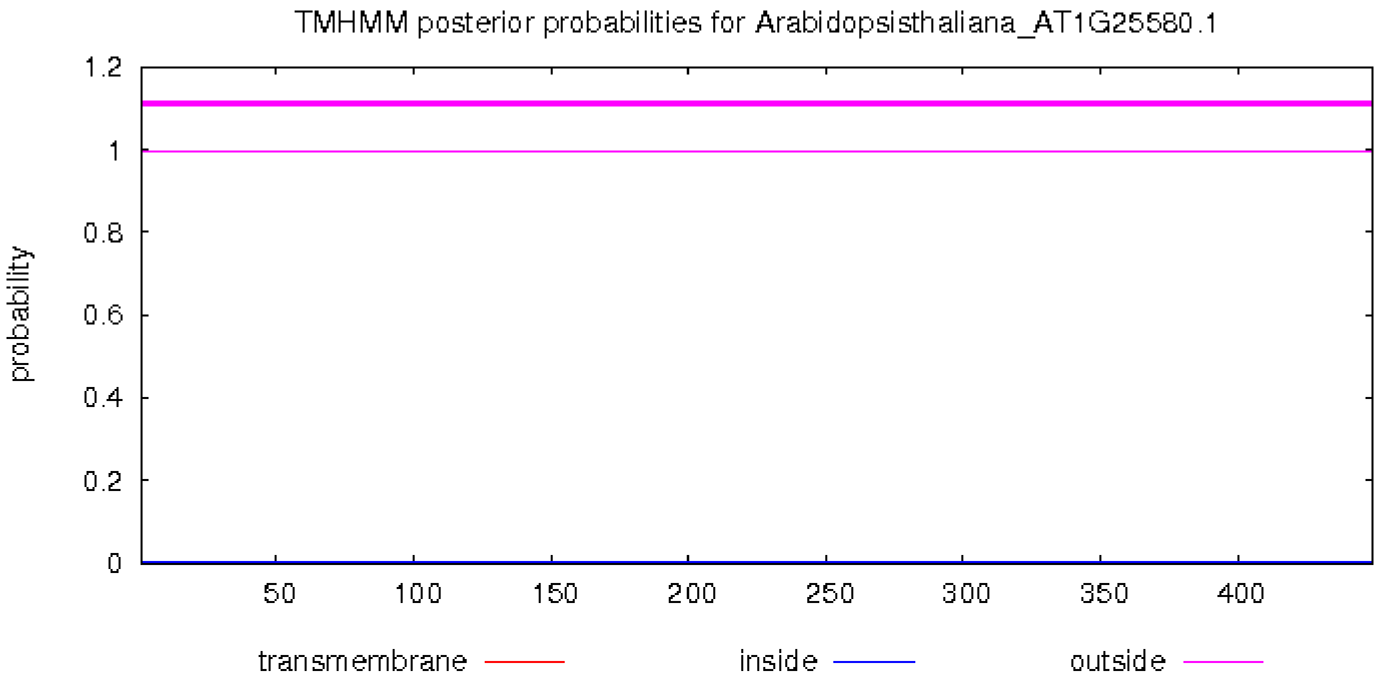

plot in postscript, script for making the plot in gnuplot, data for plot

---

```
# Arabidopsisthaliana_AT1G26870.1 Length: 425
# Arabidopsisthaliana_AT1G26870.1 Number of predicted TMHs:  0
# Arabidopsisthaliana_AT1G26870.1 Exp number of AAs in TMHs: 0.00018
# Arabidopsisthaliana_AT1G26870.1 Exp number, first 60 AAs:  0
# Arabidopsisthaliana_AT1G26870.1 Total prob of N-in:        0.00283
Arabidopsisthaliana_AT1G26870.1 TMHMM2.0        outside      1   425
```

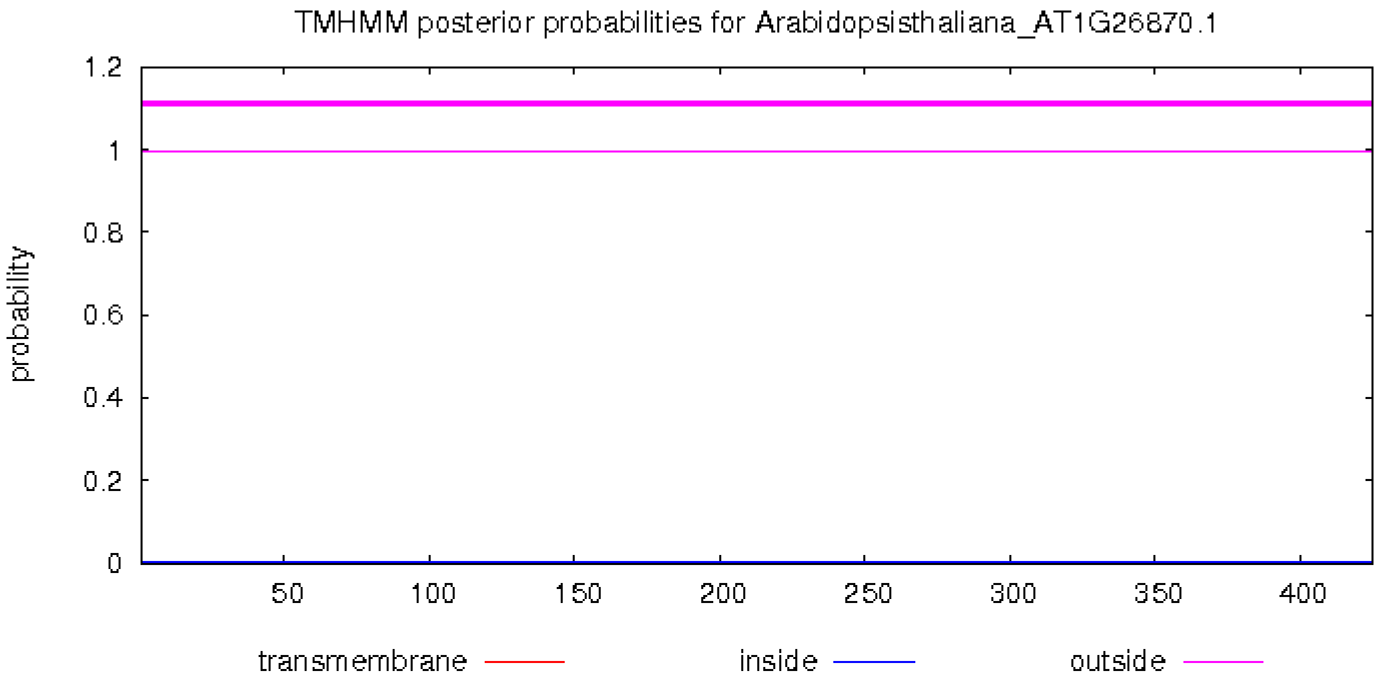

plot in postscript, script for making the plot in gnuplot, data for plot

---

```
# Arabidopsisthaliana_AT1G28470.1 Length: 314
# Arabidopsisthaliana_AT1G28470.1 Number of predicted TMHs:  0
# Arabidopsisthaliana_AT1G28470.1 Exp number of AAs in TMHs: 0.0002
# Arabidopsisthaliana_AT1G28470.1 Exp number, first 60 AAs:  0
```

```
# Arabidopsisthaliana_AT1G28470.1 Total prob of N-in:        0.04427
Arabidopsisthaliana_AT1G28470.1 TMHMM2.0        outside      1   314
```

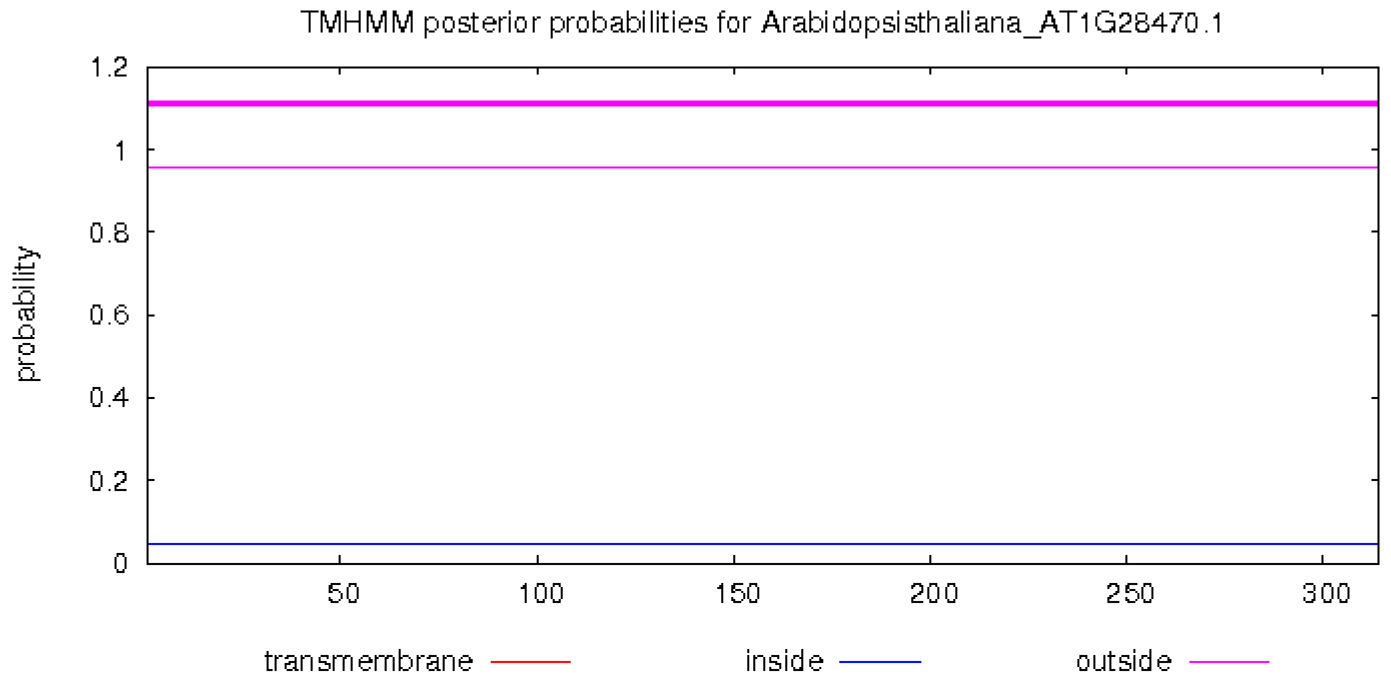

plot in postscript, script for making the plot in gnuplot, data for plot

---

```
# Arabidopsisthaliana_AT1G32510.1 Length: 283
# Arabidopsisthaliana_AT1G32510.1 Number of predicted TMHs:  0
# Arabidopsisthaliana_AT1G32510.1 Exp number of AAs in TMHs: 0
# Arabidopsisthaliana_AT1G32510.1 Exp number, first 60 AAs:  0
# Arabidopsisthaliana_AT1G32510.1 Total prob of N-in:        0.00125
Arabidopsisthaliana_AT1G32510.1 TMHMM2.0        outside      1   283
```

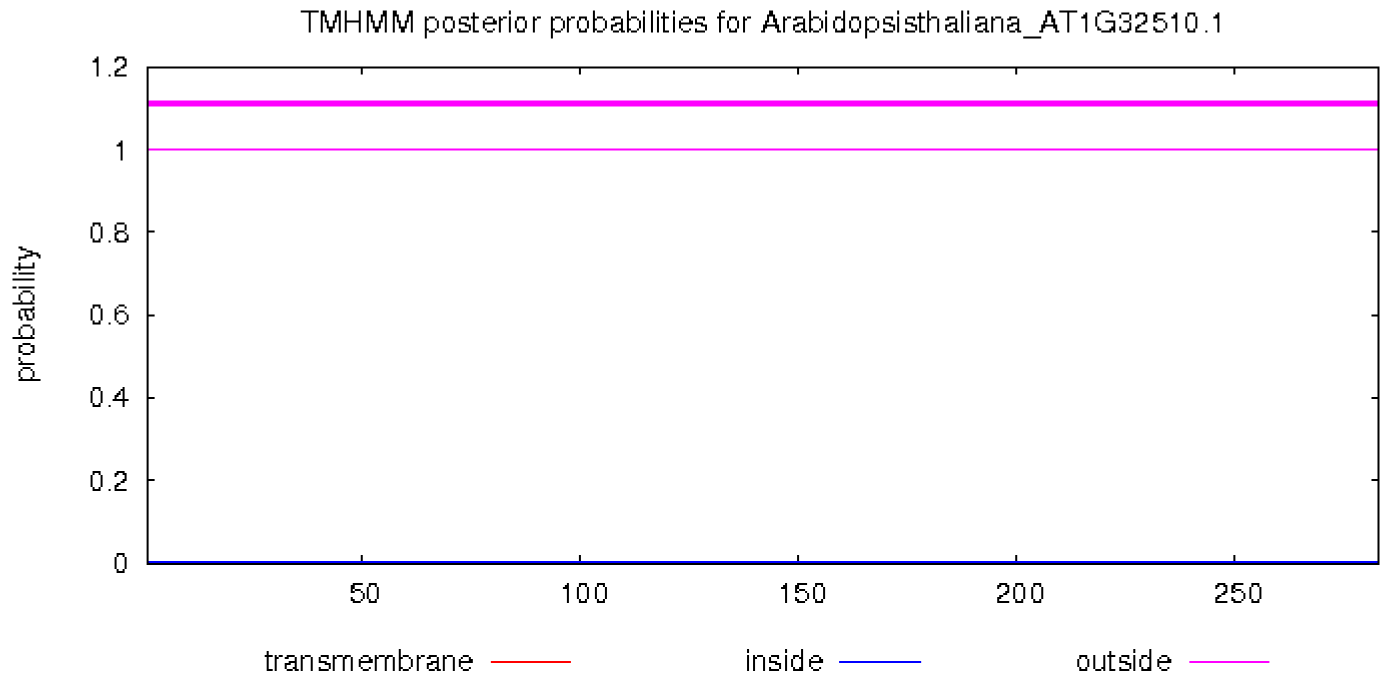

plot in postscript, script for making the plot in gnuplot, data for plot

---

```
# Arabidopsisthaliana_AT1G32770.1 Length: 358
# Arabidopsisthaliana_AT1G32770.1 Number of predicted TMHs:  0
# Arabidopsisthaliana_AT1G32770.1 Exp number of AAs in TMHs: 0
# Arabidopsisthaliana_AT1G32770.1 Exp number, first 60 AAs:  0
```

```
# Arabidopsisthaliana_AT1G32770.1 Total prob of N-in:        0.01890
Arabidopsisthaliana_AT1G32770.1 TMHMM2.0        outside      1   358
```

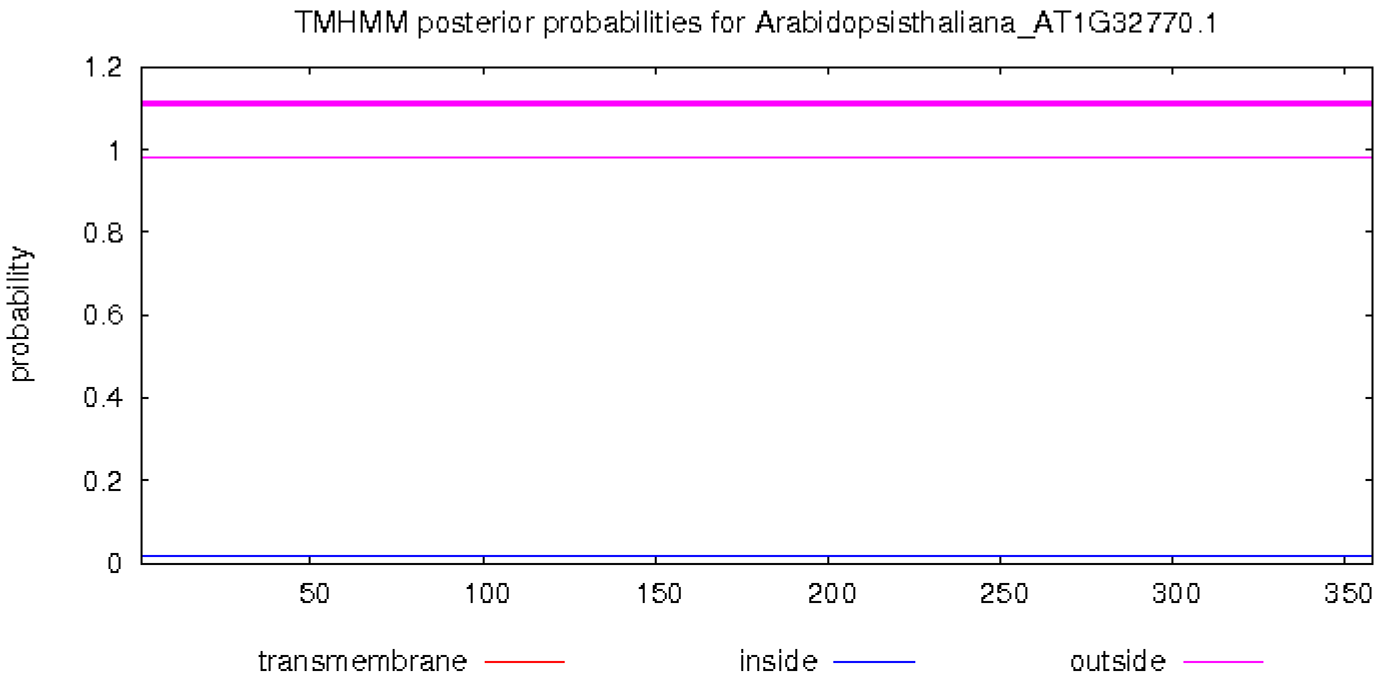

plot in postscript, script for making the plot in gnuplot, data for plot

```
# Arabidopsisthaliana_AT1G32870.1 Length: 528
# Arabidopsisthaliana_AT1G32870.1 Number of predicted TMHs:  1
# Arabidopsisthaliana_AT1G32870.1 Exp number of AAs in TMHs: 22.2032
# Arabidopsisthaliana_AT1G32870.1 Exp number, first 60 AAs:  0.09193
# Arabidopsisthaliana_AT1G32870.1 Total prob of N-in:        0.87431
Arabidopsisthaliana_AT1G32870.1 TMHMM2.0        inside       1   497
Arabidopsisthaliana_AT1G32870.1 TMHMM2.0        TMhelix    498   520
Arabidopsisthaliana_AT1G32870.1 TMHMM2.0        outside    521   528
```

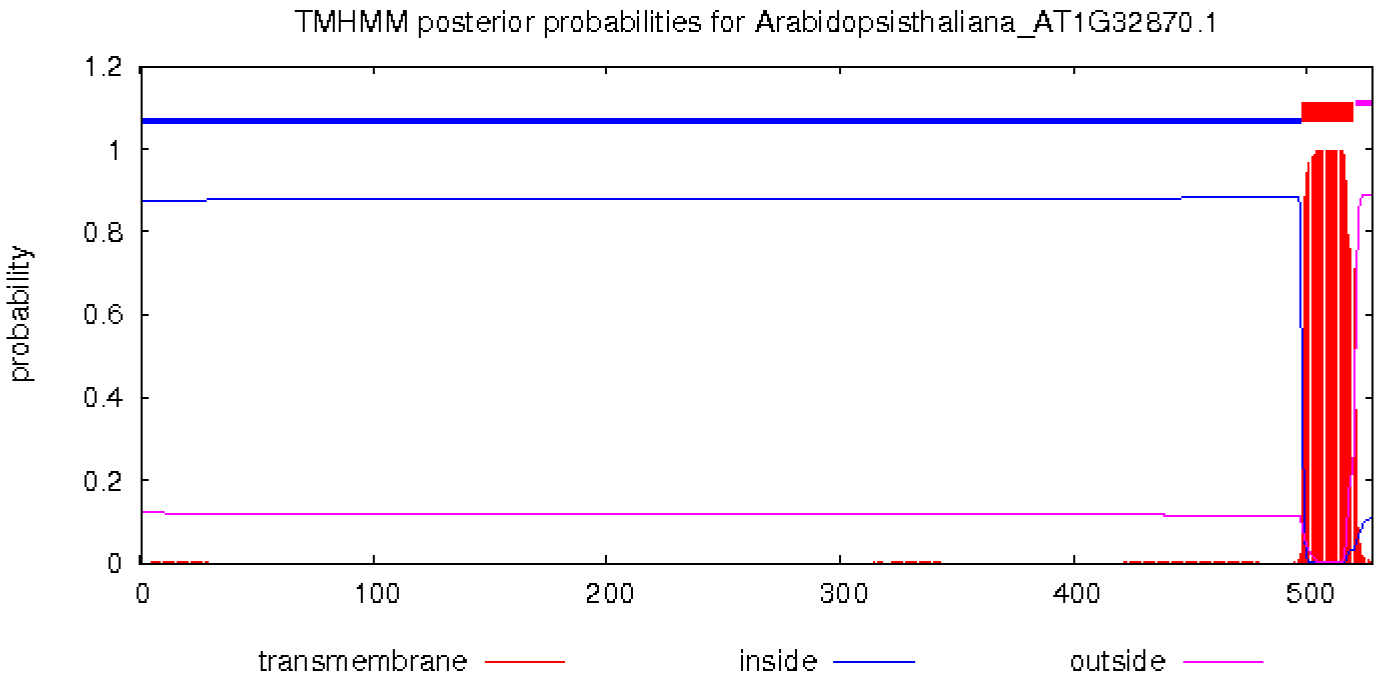

plot in postscript, script for making the plot in gnuplot, data for plot

```
# Arabidopsisthaliana_AT1G33060.1 Length: 648
# Arabidopsisthaliana_AT1G33060.1 Number of predicted TMHs:  1
```

```
# Arabidopsisthaliana_AT1G33060.1 Exp number of AAs in TMHs: 18.06541
# Arabidopsisthaliana_AT1G33060.1 Exp number, first 60 AAs:  0.00031
# Arabidopsisthaliana_AT1G33060.1 Total prob of N-in:        0.02901
Arabidopsisthaliana_AT1G33060.1 TMHMM2.0        outside      1   605
Arabidopsisthaliana_AT1G33060.1 TMHMM2.0        TMhelix    606   625
Arabidopsisthaliana_AT1G33060.1 TMHMM2.0        inside     626   648
```

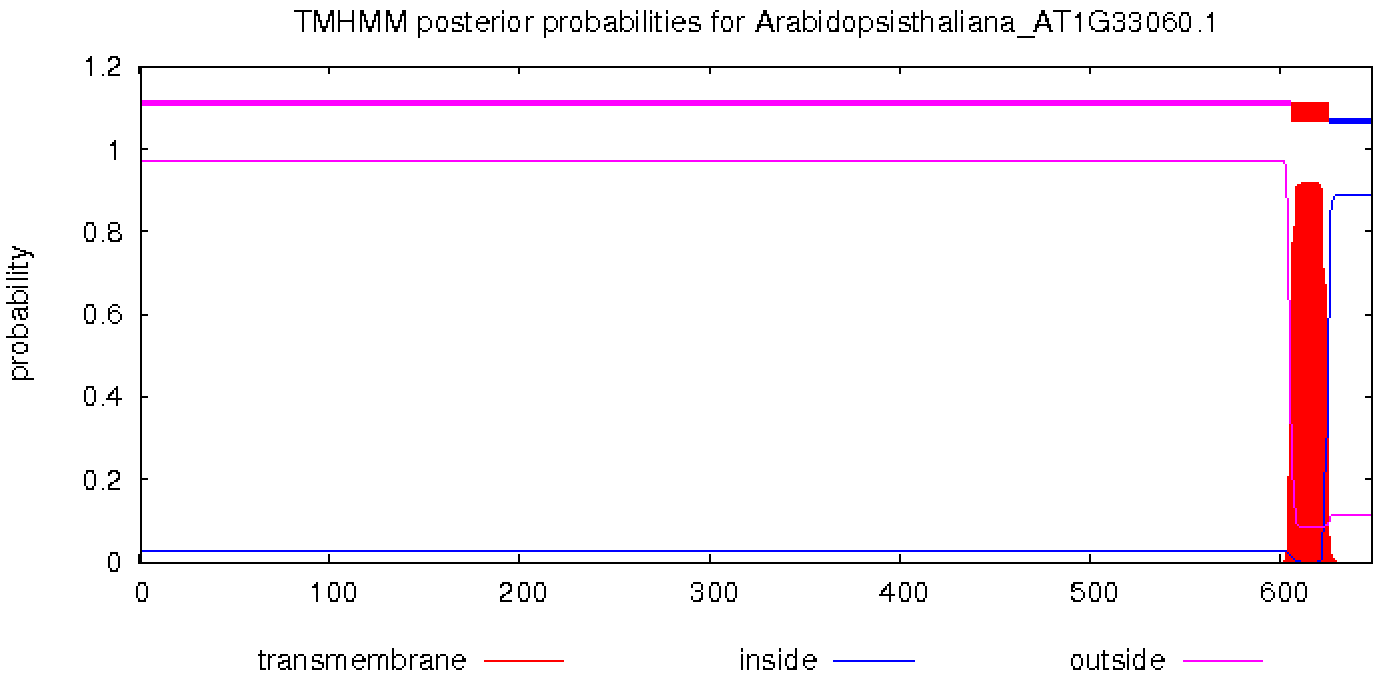

plot in postscript, script for making the plot in gnuplot, data for plot

---

```
# Arabidopsisthaliana_AT1G33280.1 Length: 305
# Arabidopsisthaliana_AT1G33280.1 Number of predicted TMHs:  0
# Arabidopsisthaliana_AT1G33280.1 Exp number of AAs in TMHs: 0
# Arabidopsisthaliana_AT1G33280.1 Exp number, first 60 AAs:  0
# Arabidopsisthaliana_AT1G33280.1 Total prob of N-in:        0.00496
Arabidopsisthaliana_AT1G33280.1 TMHMM2.0        outside      1   305
```

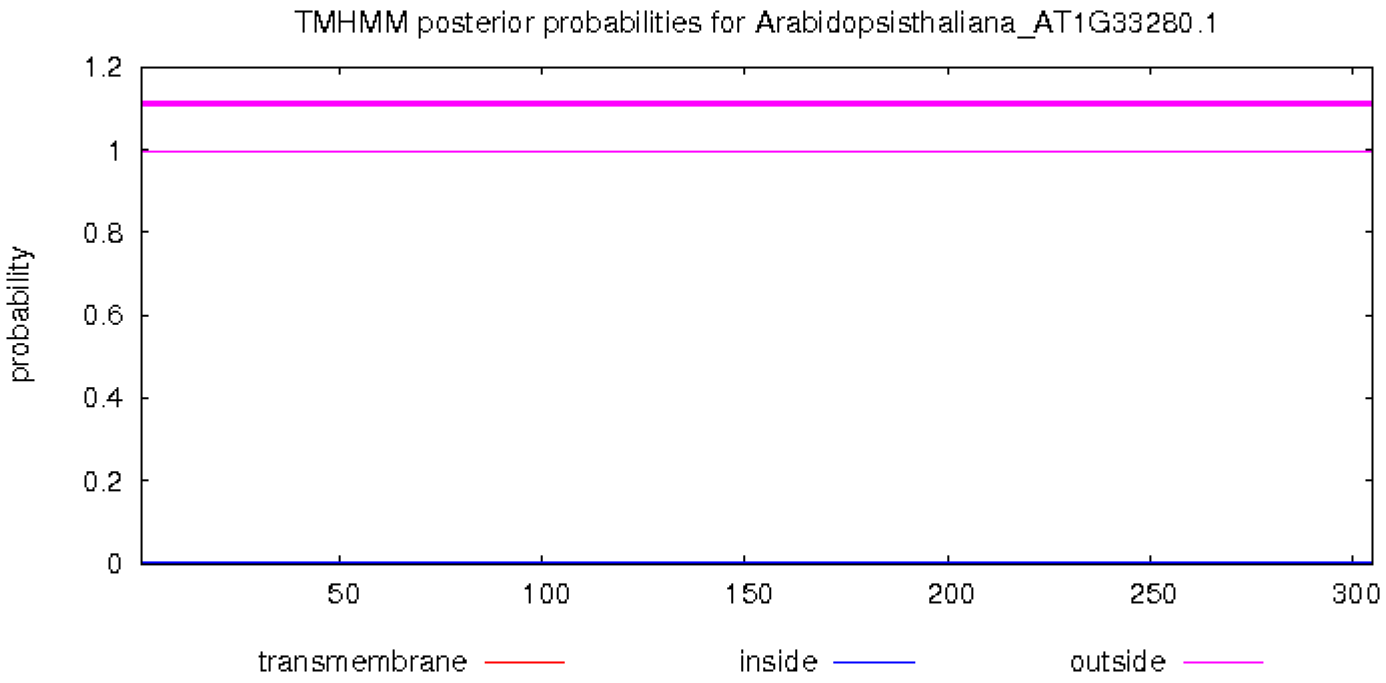

plot in postscript, script for making the plot in gnuplot, data for plot

---

```
# Arabidopsisthaliana_AT1G34180.1 Length: 564
# Arabidopsisthaliana_AT1G34180.1 Number of predicted TMHs:  1
# Arabidopsisthaliana_AT1G34180.1 Exp number of AAs in TMHs: 21.30483
# Arabidopsisthaliana_AT1G34180.1 Exp number, first 60 AAs:  0.00487
# Arabidopsisthaliana_AT1G34180.1 Total prob of N-in:        0.43607
Arabidopsisthaliana_AT1G34180.1 TMHMM2.0        inside       1   534
Arabidopsisthaliana_AT1G34180.1 TMHMM2.0        TMhelix    535   557
Arabidopsisthaliana_AT1G34180.1 TMHMM2.0        outside    558   564
```

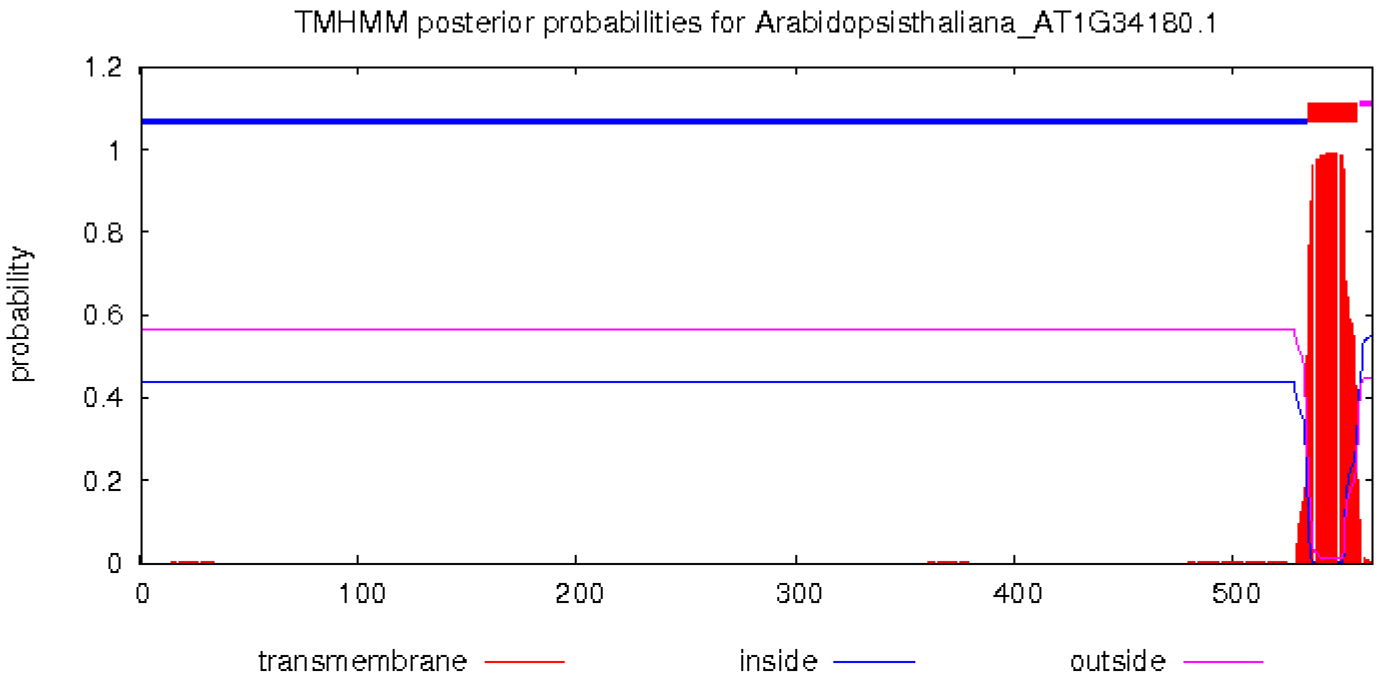

plot in postscript, script for making the plot in gnuplot, data for plot

---

```
# Arabidopsisthaliana_AT1G34190.1 Length: 557
# Arabidopsisthaliana_AT1G34190.1 Number of predicted TMHs:  1
# Arabidopsisthaliana_AT1G34190.1 Exp number of AAs in TMHs: 21.67526
# Arabidopsisthaliana_AT1G34190.1 Exp number, first 60 AAs:  0.01068
# Arabidopsisthaliana_AT1G34190.1 Total prob of N-in:        0.14224
Arabidopsisthaliana_AT1G34190.1 TMHMM2.0        outside      1   525
Arabidopsisthaliana_AT1G34190.1 TMHMM2.0        TMhelix    526   548
Arabidopsisthaliana_AT1G34190.1 TMHMM2.0        inside     549   557
```

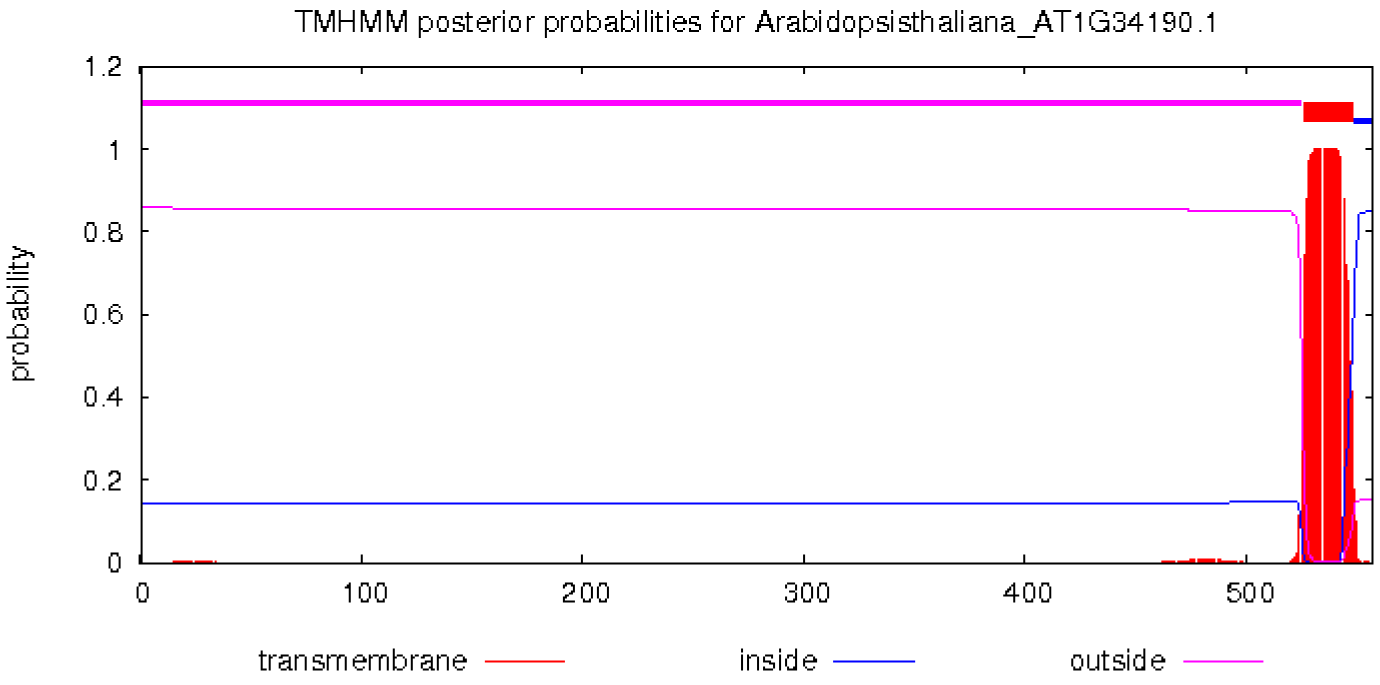

plot in postscript, script for making the plot in gnuplot, data for plot

```
# Arabidopsisthaliana_AT1G52880.1 Length: 320
# Arabidopsisthaliana_AT1G52880.1 Number of predicted TMHs:  0
# Arabidopsisthaliana_AT1G52880.1 Exp number of AAs in TMHs: 0.00067
# Arabidopsisthaliana_AT1G52880.1 Exp number, first 60 AAs:  0
# Arabidopsisthaliana_AT1G52880.1 Total prob of N-in:        0.00713
Arabidopsisthaliana_AT1G52880.1 TMHMM2.0        outside      1   320
```

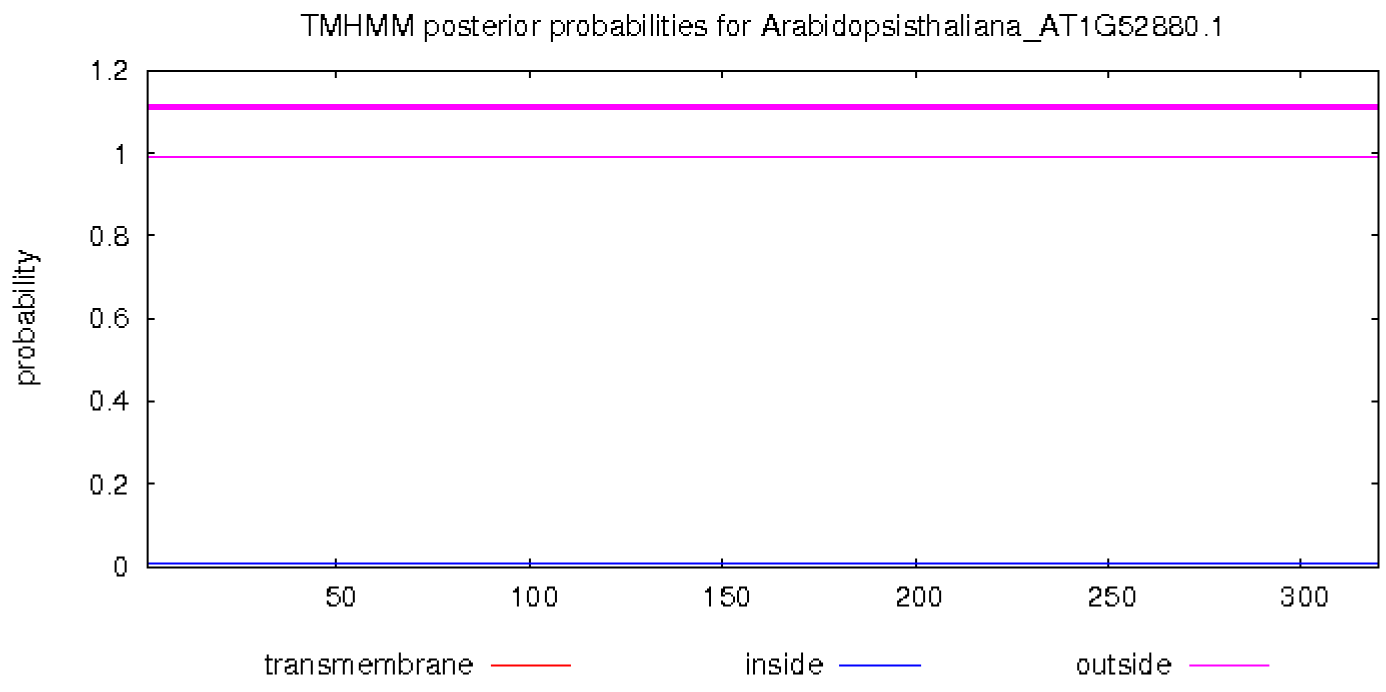

plot in postscript, script for making the plot in gnuplot, data for plot

```
# Arabidopsisthaliana_AT1G52890.1 Length: 317
# Arabidopsisthaliana_AT1G52890.1 Number of predicted TMHs:  0
# Arabidopsisthaliana_AT1G52890.1 Exp number of AAs in TMHs: 0.00139
# Arabidopsisthaliana_AT1G52890.1 Exp number, first 60 AAs:  0.00022
# Arabidopsisthaliana_AT1G52890.1 Total prob of N-in:        0.01188
Arabidopsisthaliana_AT1G52890.1 TMHMM2.0        outside      1   317
```

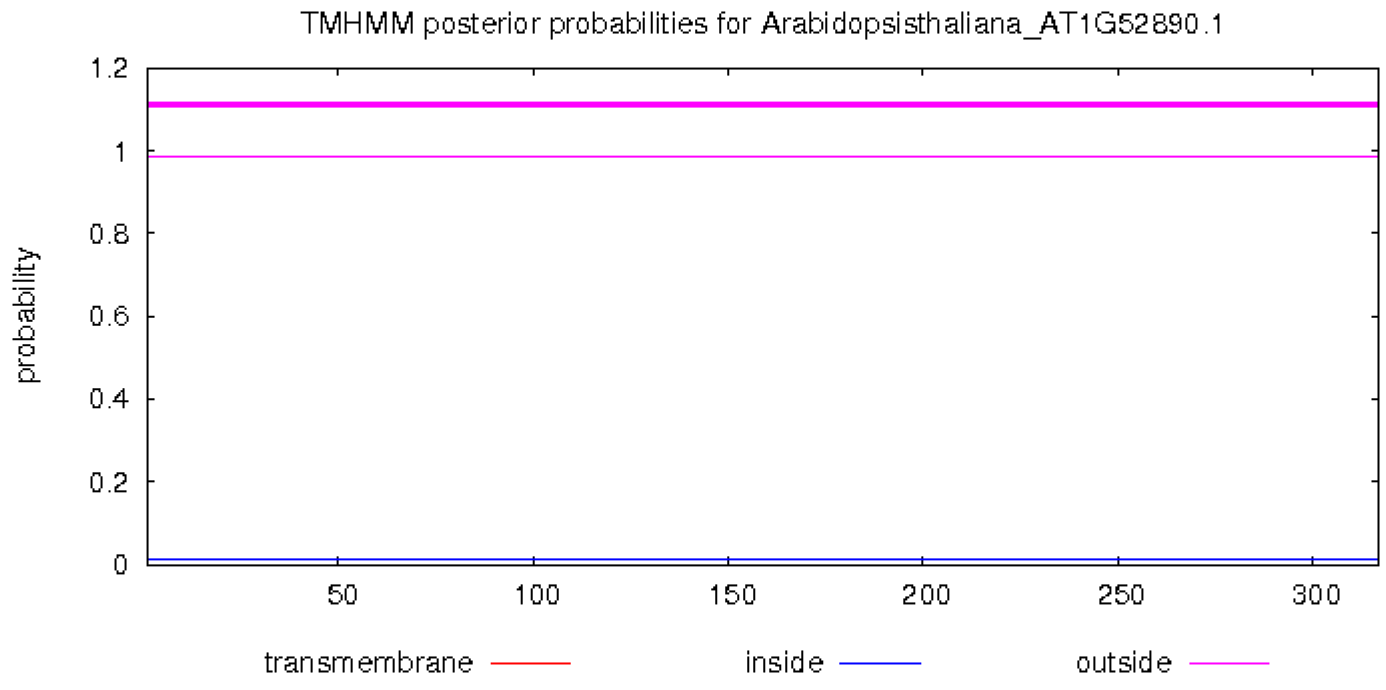

plot in postscript, script for making the plot in gnuplot, data for plot

```
# Arabidopsisthaliana_AT1G54330.1 Length: 301
# Arabidopsisthaliana_AT1G54330.1 Number of predicted TMHs:  0
# Arabidopsisthaliana_AT1G54330.1 Exp number of AAs in TMHs: 0
# Arabidopsisthaliana_AT1G54330.1 Exp number, first 60 AAs:  0
# Arabidopsisthaliana_AT1G54330.1 Total prob of N-in:        0.00142
Arabidopsisthaliana_AT1G54330.1 TMHMM2.0        outside      1   301
```

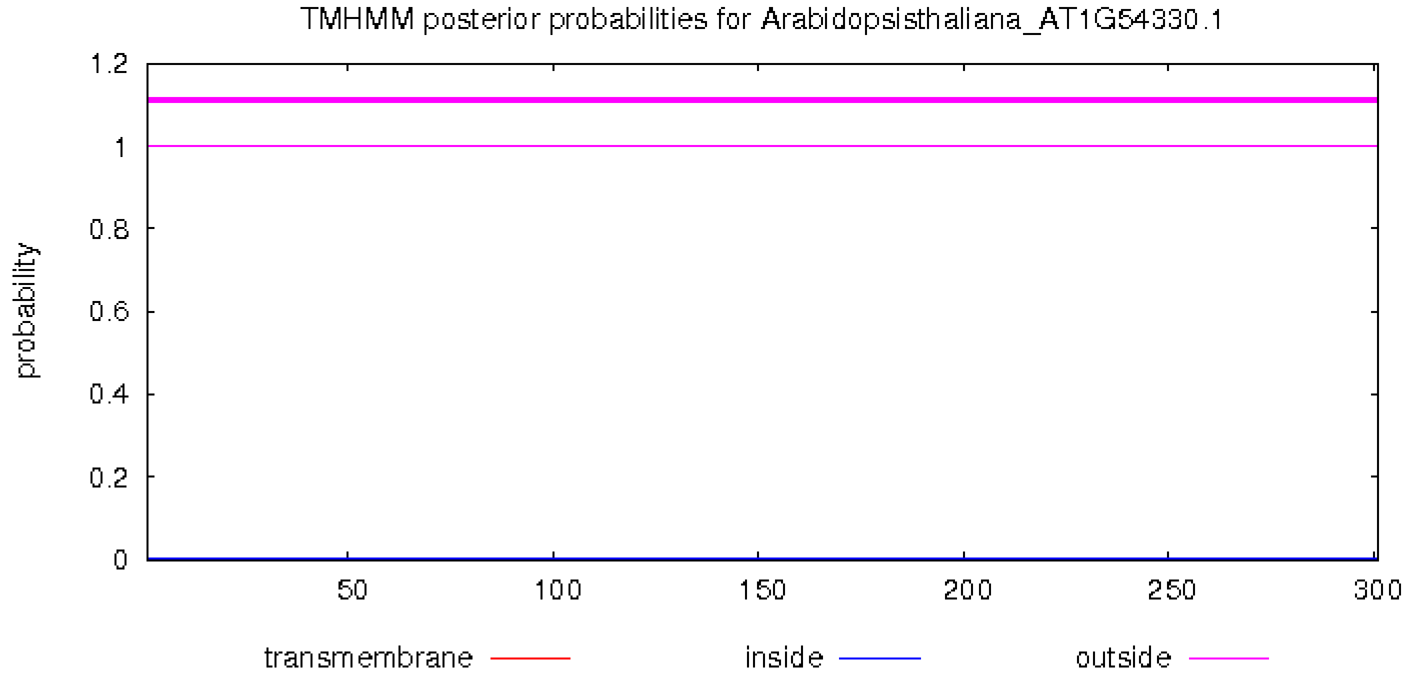

plot in postscript, script for making the plot in gnuplot, data for plot

```
# Arabidopsisthaliana_AT1G56010.1 Length: 257
# Arabidopsisthaliana_AT1G56010.1 Number of predicted TMHs:  0
# Arabidopsisthaliana_AT1G56010.1 Exp number of AAs in TMHs: 0.00233
# Arabidopsisthaliana_AT1G56010.1 Exp number, first 60 AAs:  0.00103
# Arabidopsisthaliana_AT1G56010.1 Total prob of N-in:        0.06993
Arabidopsisthaliana_AT1G56010.1 TMHMM2.0        outside      1   257
```

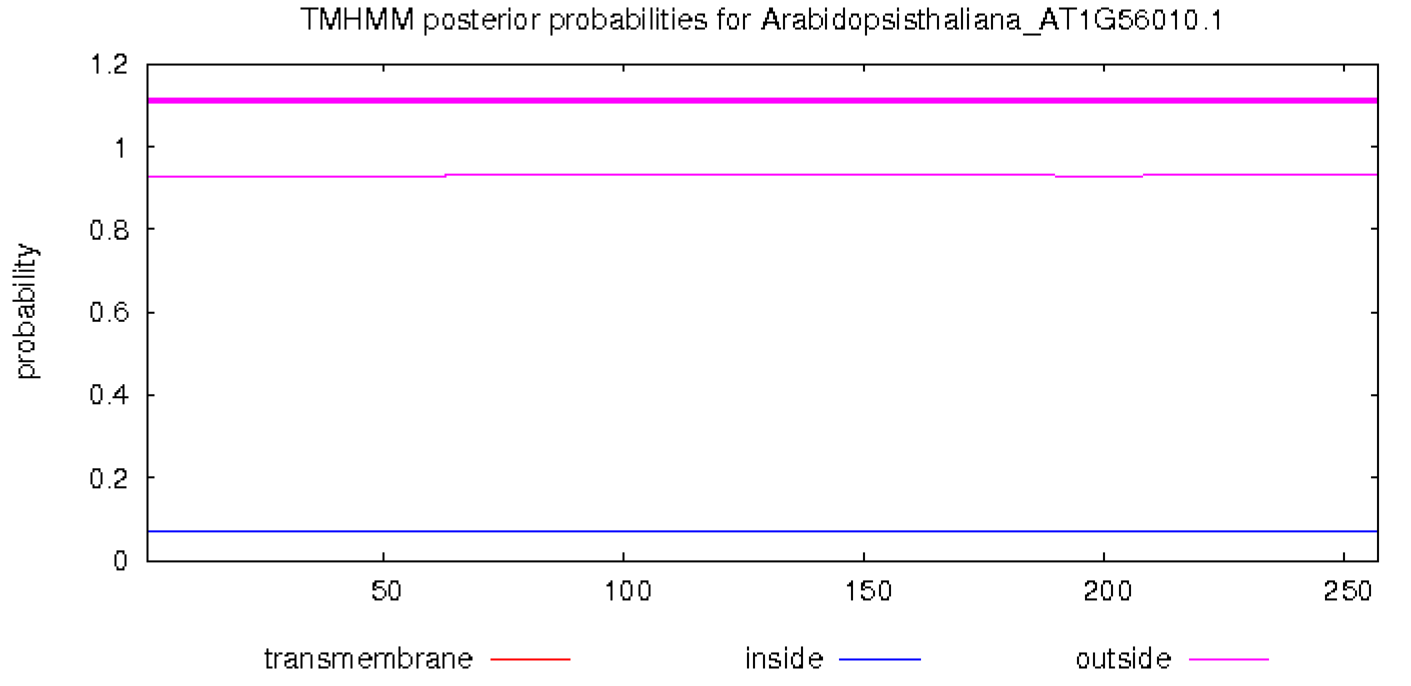

plot in postscript, script for making the plot in gnuplot, data for plot

```
# Arabidopsisthaliana_AT1G60240.1 Length: 175
# Arabidopsisthaliana_AT1G60240.1 Number of predicted TMHs:  0
# Arabidopsisthaliana_AT1G60240.1 Exp number of AAs in TMHs: 0.0194
# Arabidopsisthaliana_AT1G60240.1 Exp number, first 60 AAs:  0.0159
# Arabidopsisthaliana_AT1G60240.1 Total prob of N-in:        0.45714
Arabidopsisthaliana_AT1G60240.1 TMHMM2.0        outside      1   175
```

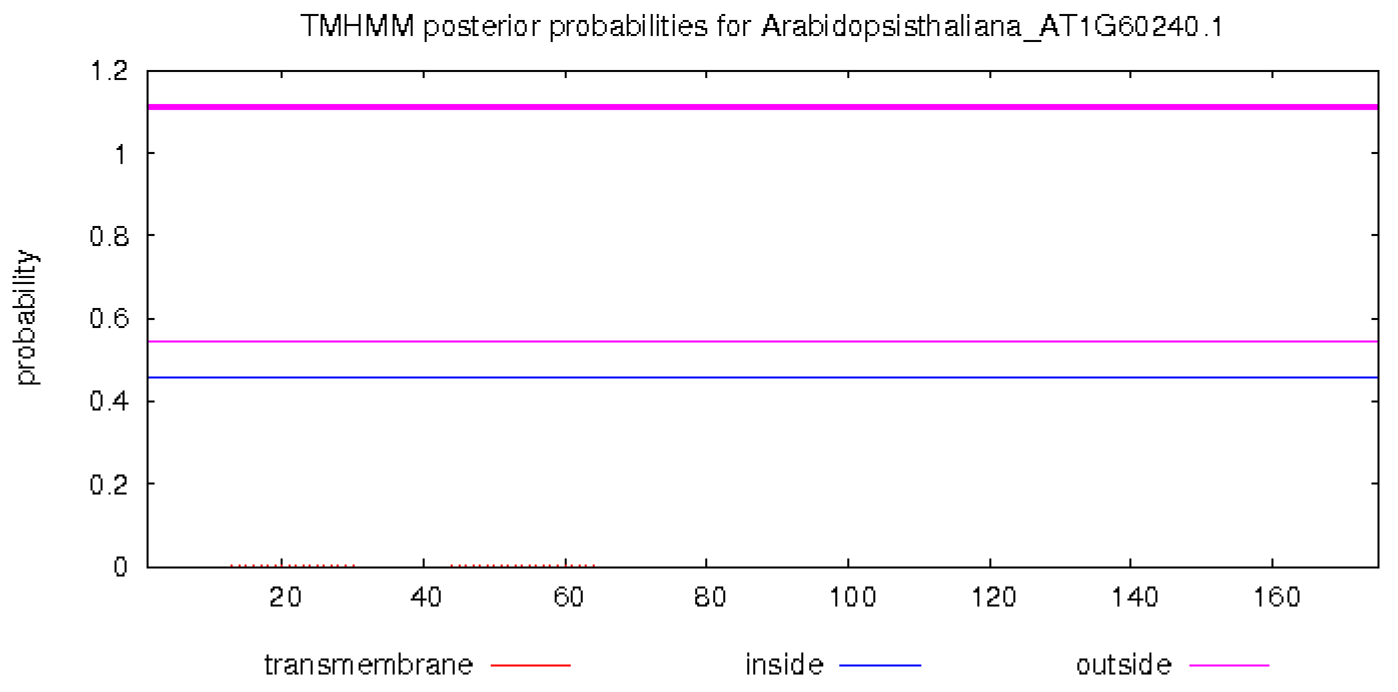

plot in postscript, script for making the plot in gnuplot, data for plot

```
# Arabidopsisthaliana_AT1G60280.1 Length: 347
# Arabidopsisthaliana_AT1G60280.1 Number of predicted TMHs:  0
# Arabidopsisthaliana_AT1G60280.1 Exp number of AAs in TMHs: 0.00124
# Arabidopsisthaliana_AT1G60280.1 Exp number, first 60 AAs:  0
# Arabidopsisthaliana_AT1G60280.1 Total prob of N-in:        0.03566
Arabidopsisthaliana_AT1G60280.1 TMHMM2.0        outside      1   347
```

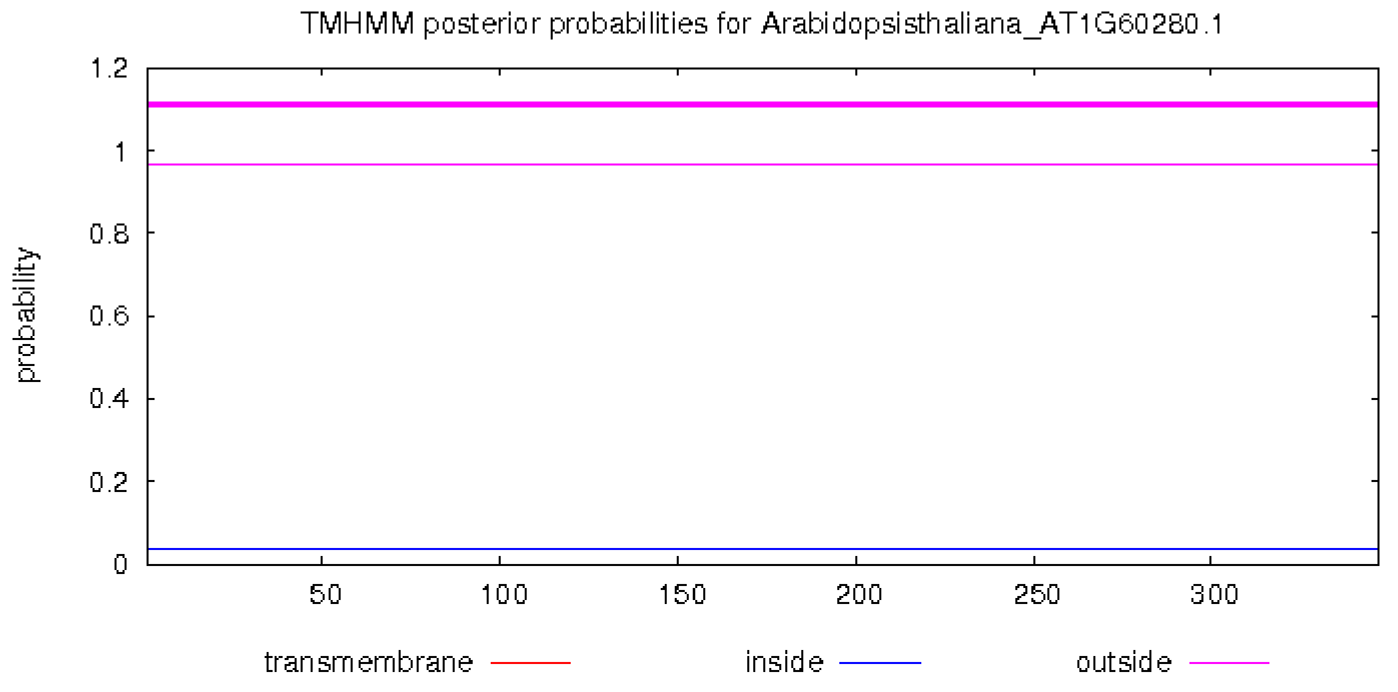

plot in postscript, script for making the plot in gnuplot, data for plot

```
# Arabidopsisthaliana_AT1G60300.1 Length: 322
# Arabidopsisthaliana_AT1G60300.1 Number of predicted TMHs:  0
# Arabidopsisthaliana_AT1G60300.1 Exp number of AAs in TMHs: 0.0002
# Arabidopsisthaliana_AT1G60300.1 Exp number, first 60 AAs:  0
# Arabidopsisthaliana_AT1G60300.1 Total prob of N-in:        0.02518
Arabidopsisthaliana_AT1G60300.1 TMHMM2.0        outside      1   322
```

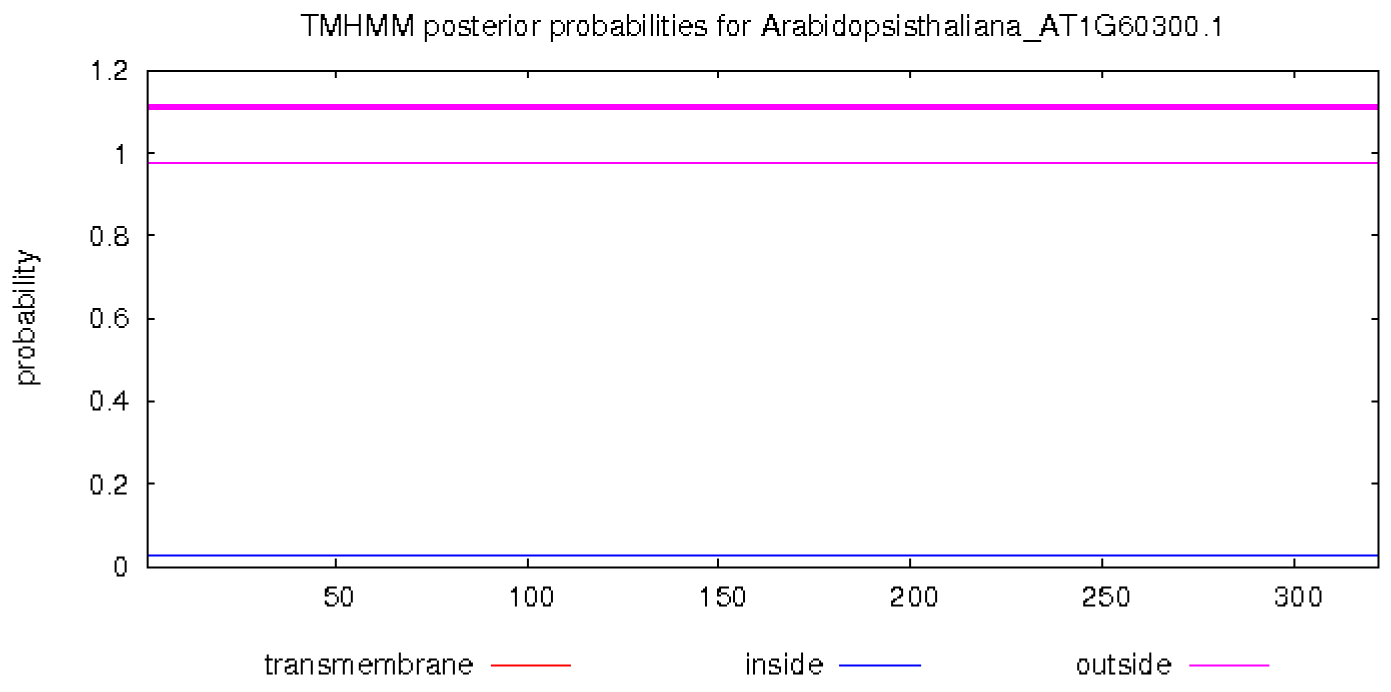

plot in postscript, script for making the plot in gnuplot, data for plot

```
# Arabidopsisthaliana_AT1G60340.1 Length: 371
# Arabidopsisthaliana_AT1G60340.1 Number of predicted TMHs:  0
# Arabidopsisthaliana_AT1G60340.1 Exp number of AAs in TMHs: 0.00819
# Arabidopsisthaliana_AT1G60340.1 Exp number, first 60 AAs:  0
# Arabidopsisthaliana_AT1G60340.1 Total prob of N-in:        0.03246
Arabidopsisthaliana_AT1G60340.1 TMHMM2.0        outside      1   371
```

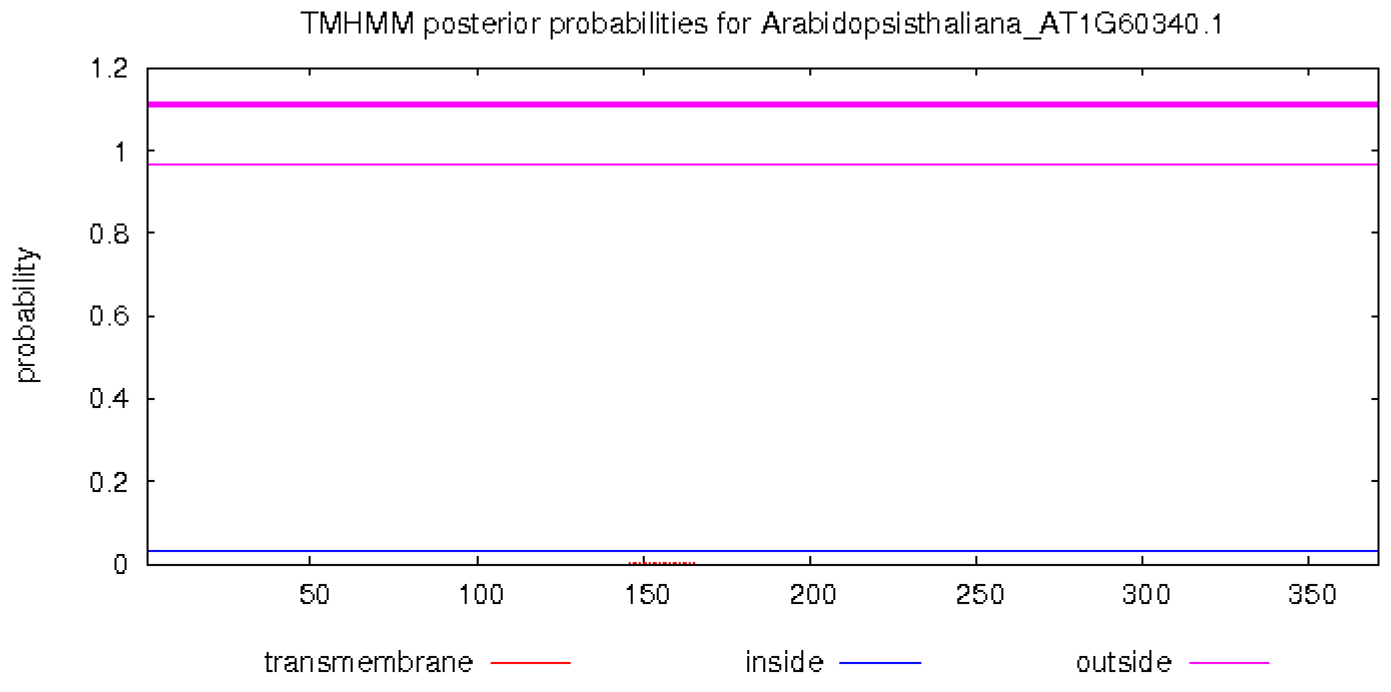

plot in postscript, script for making the plot in gnuplot, data for plot

```
# Arabidopsisthaliana_AT1G60350.1 Length: 320
# Arabidopsisthaliana_AT1G60350.1 Number of predicted TMHs:  0
# Arabidopsisthaliana_AT1G60350.1 Exp number of AAs in TMHs: 0.00032
# Arabidopsisthaliana_AT1G60350.1 Exp number, first 60 AAs:  0
# Arabidopsisthaliana_AT1G60350.1 Total prob of N-in:        0.00470
Arabidopsisthaliana_AT1G60350.1 TMHMM2.0        outside      1   320
```

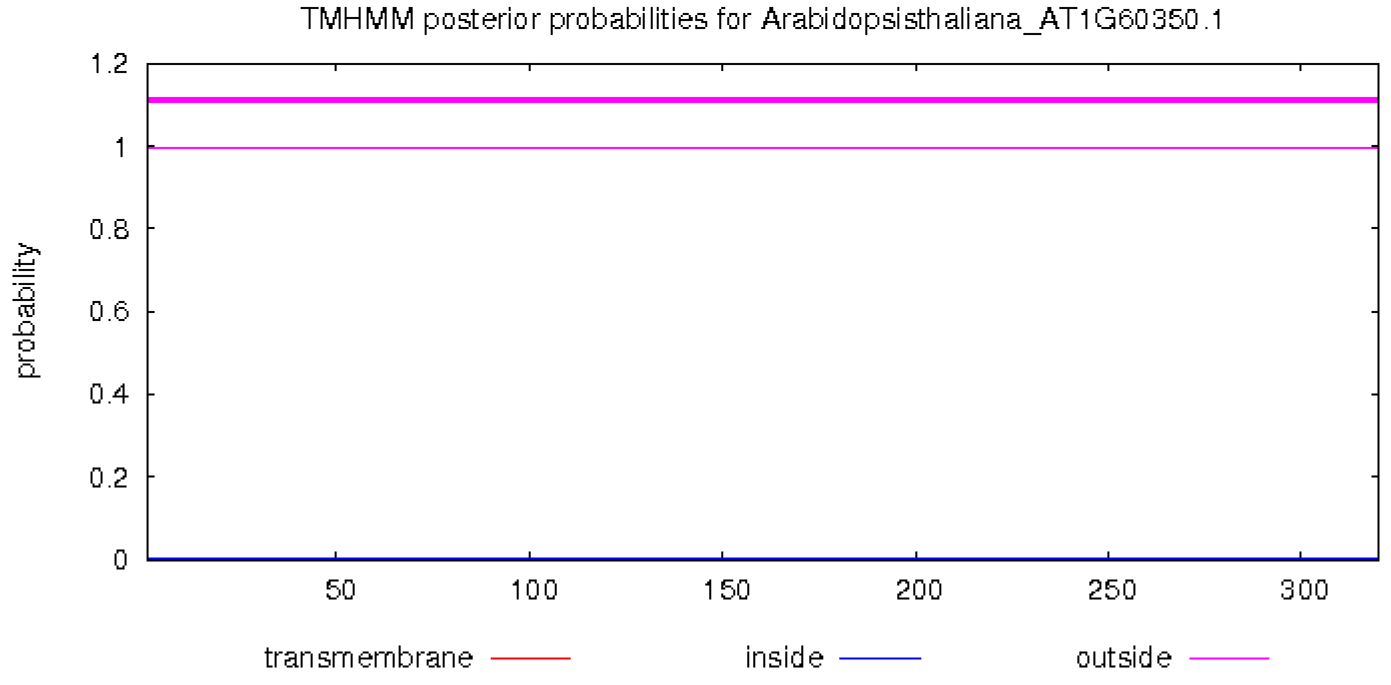

plot in postscript, script for making the plot in gnuplot, data for plot

```
# Arabidopsisthaliana_AT1G60380.1 Length: 318
# Arabidopsisthaliana_AT1G60380.1 Number of predicted TMHs:  0
# Arabidopsisthaliana_AT1G60380.1 Exp number of AAs in TMHs: 0.00301
# Arabidopsisthaliana_AT1G60380.1 Exp number, first 60 AAs:  0.00092
# Arabidopsisthaliana_AT1G60380.1 Total prob of N-in:        0.01600
Arabidopsisthaliana_AT1G60380.1 TMHMM2.0        outside      1   318
```

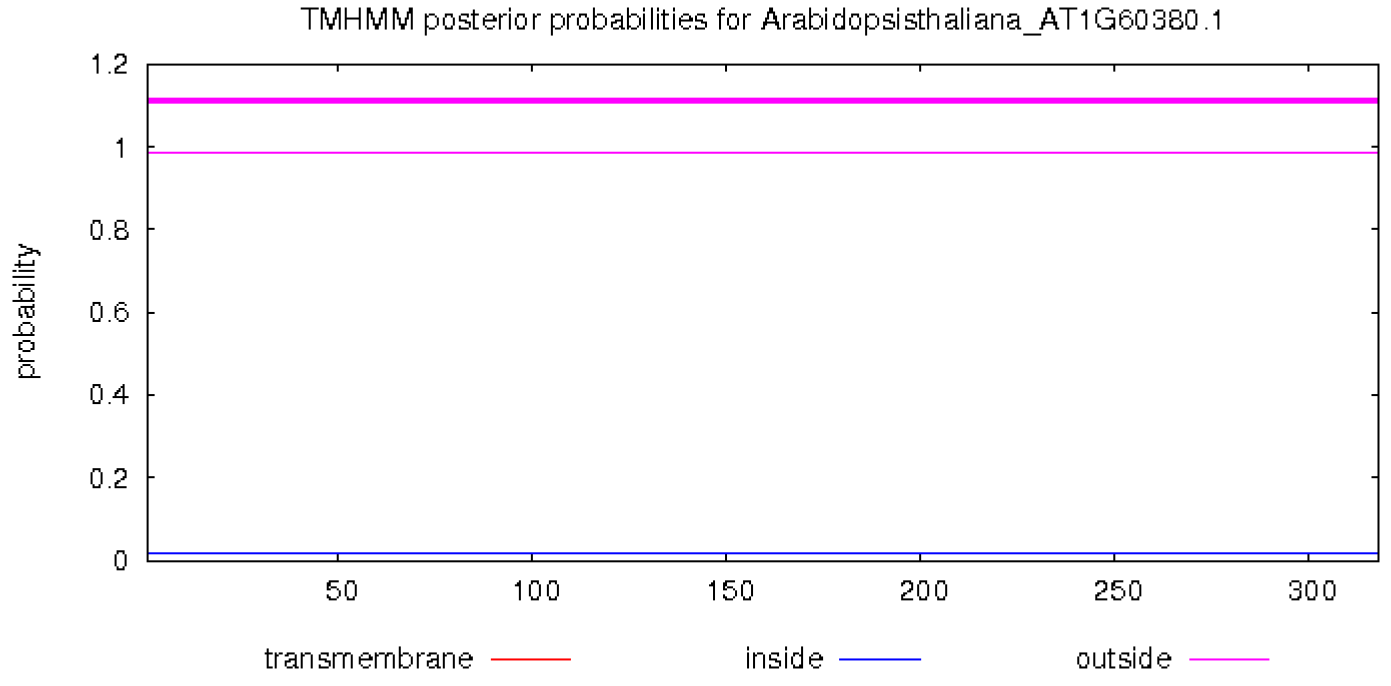

plot in postscript, script for making the plot in gnuplot, data for plot

```
# Arabidopsisthaliana_AT1G61110.1 Length: 323
# Arabidopsisthaliana_AT1G61110.1 Number of predicted TMHs:  0
# Arabidopsisthaliana_AT1G61110.1 Exp number of AAs in TMHs: 0.00022
# Arabidopsisthaliana_AT1G61110.1 Exp number, first 60 AAs:  0
# Arabidopsisthaliana_AT1G61110.1 Total prob of N-in:        0.01141
Arabidopsisthaliana_AT1G61110.1 TMHMM2.0        outside      1   323
```

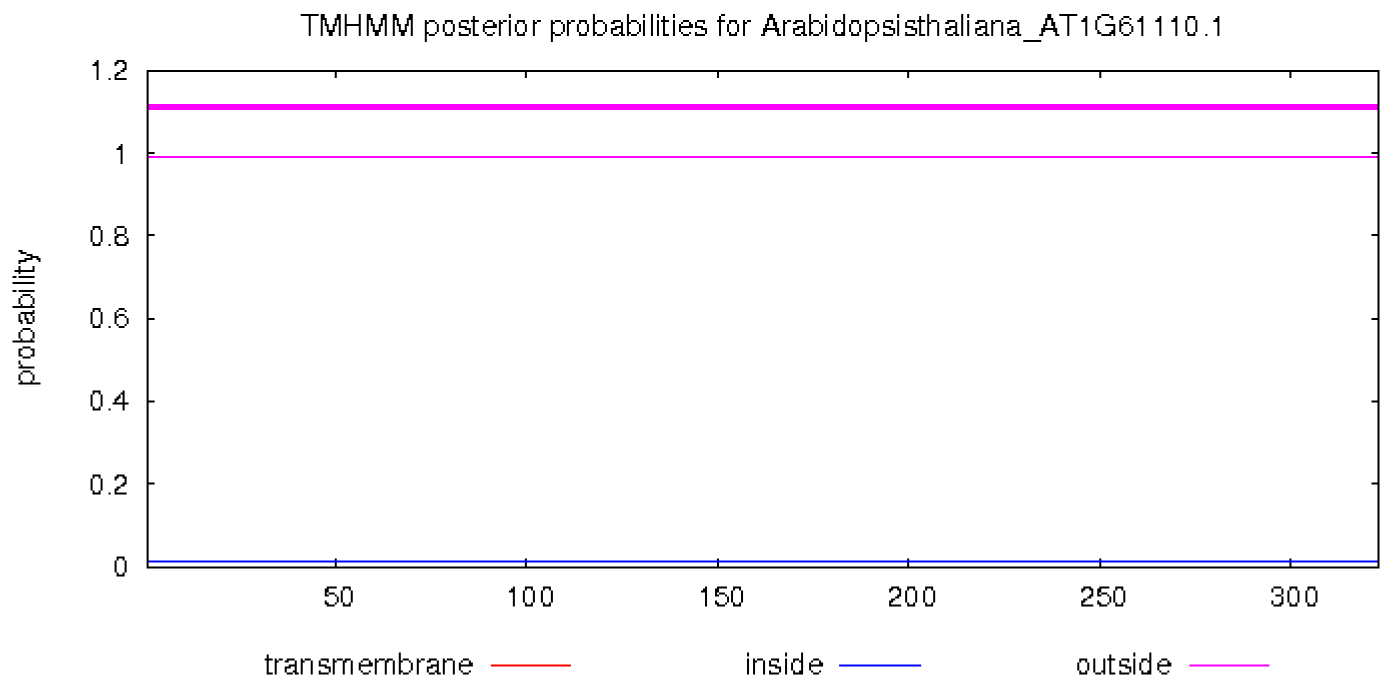

plot in postscript, script for making the plot in gnuplot, data for plot

```
# Arabidopsisthaliana_AT1G62700.1 Length: 394
# Arabidopsisthaliana_AT1G62700.1 Number of predicted TMHs:  0
# Arabidopsisthaliana_AT1G62700.1 Exp number of AAs in TMHs: 0.00018
# Arabidopsisthaliana_AT1G62700.1 Exp number, first 60 AAs:  0
# Arabidopsisthaliana_AT1G62700.1 Total prob of N-in:        0.00189
Arabidopsisthaliana_AT1G62700.1 TMHMM2.0        outside      1   394
```

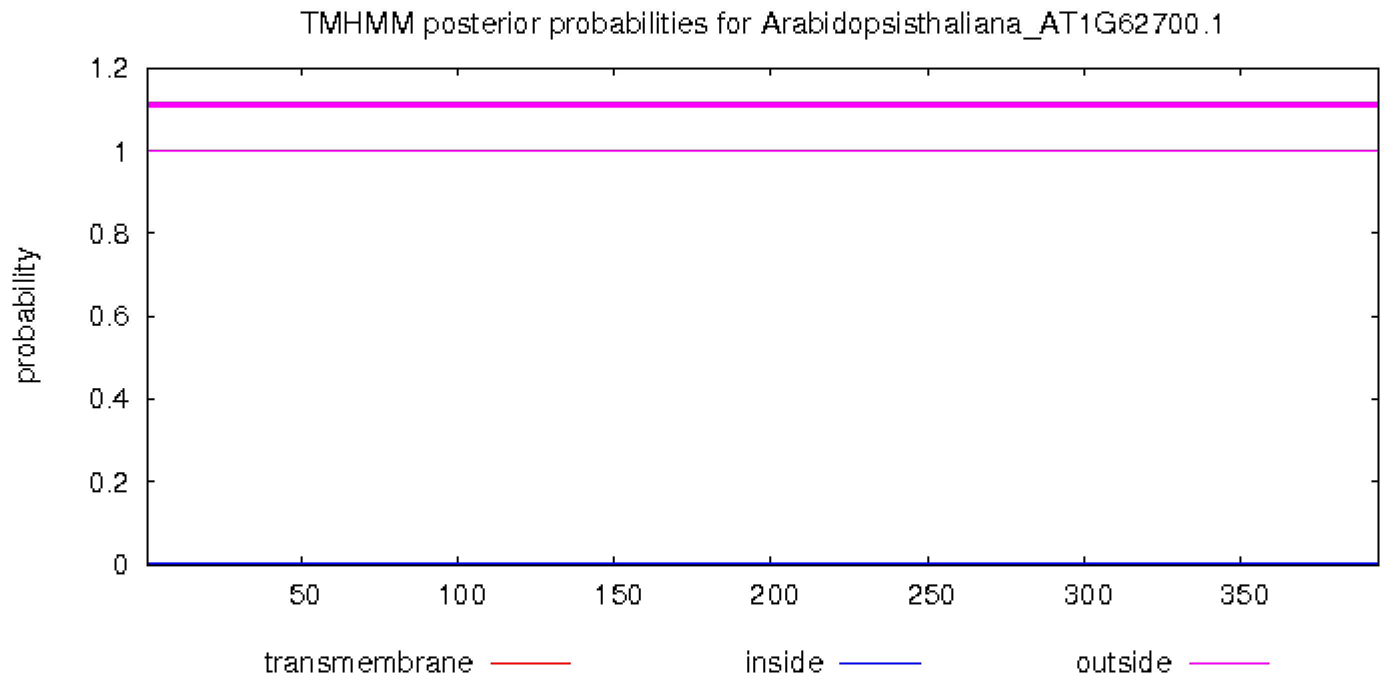

plot in postscript, script for making the plot in gnuplot, data for plot

```
# Arabidopsisthaliana_AT1G64100.2 Length: 806
# Arabidopsisthaliana_AT1G64100.2 Number of predicted TMHs:  0
# Arabidopsisthaliana_AT1G64100.2 Exp number of AAs in TMHs: 0.15548
# Arabidopsisthaliana_AT1G64100.2 Exp number, first 60 AAs:  0
# Arabidopsisthaliana_AT1G64100.2 Total prob of N-in:        0.00559
Arabidopsisthaliana_AT1G64100.2 TMHMM2.0        outside      1   806
```

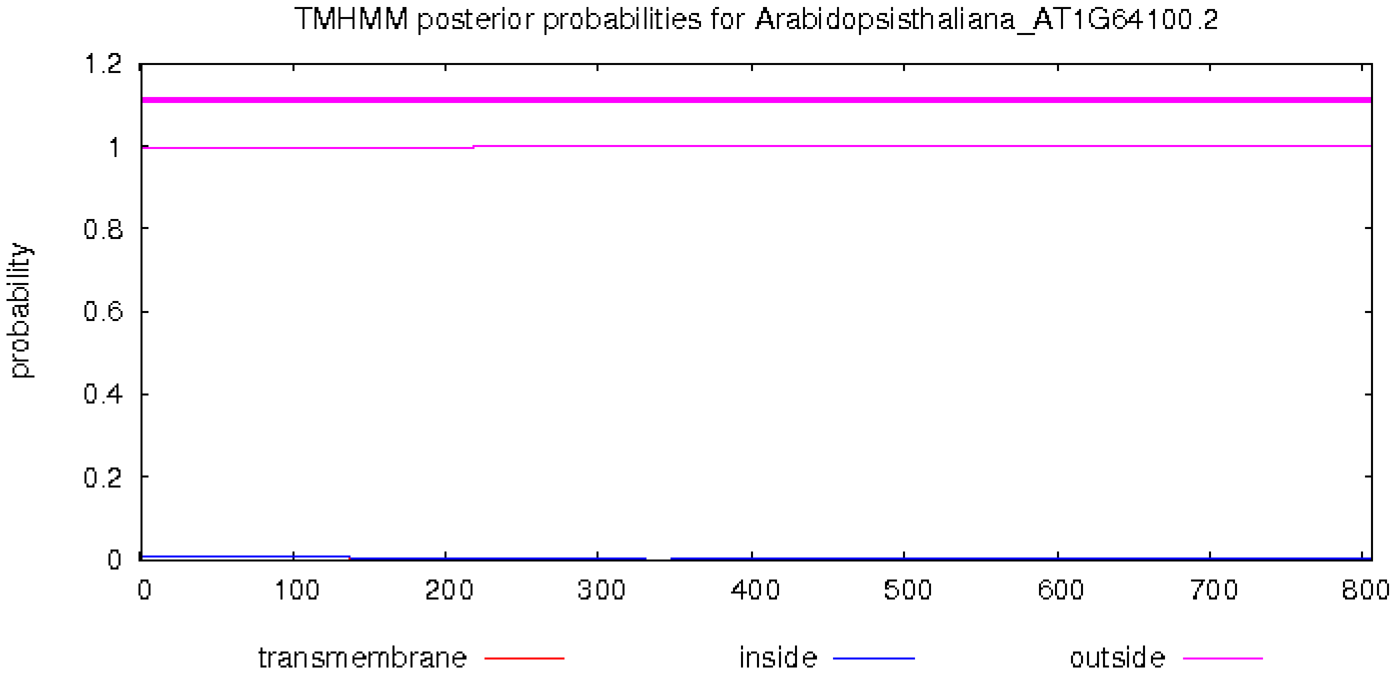

plot in postscript, script for making the plot in gnuplot, data for plot

```
# Arabidopsisthaliana_AT1G64105.1 Length: 161
# Arabidopsisthaliana_AT1G64105.1 Number of predicted TMHs:  0
# Arabidopsisthaliana_AT1G64105.1 Exp number of AAs in TMHs: 0.00764
# Arabidopsisthaliana_AT1G64105.1 Exp number, first 60 AAs:  0.00052
# Arabidopsisthaliana_AT1G64105.1 Total prob of N-in:        0.22416
Arabidopsisthaliana_AT1G64105.1 TMHMM2.0        outside      1   161
```

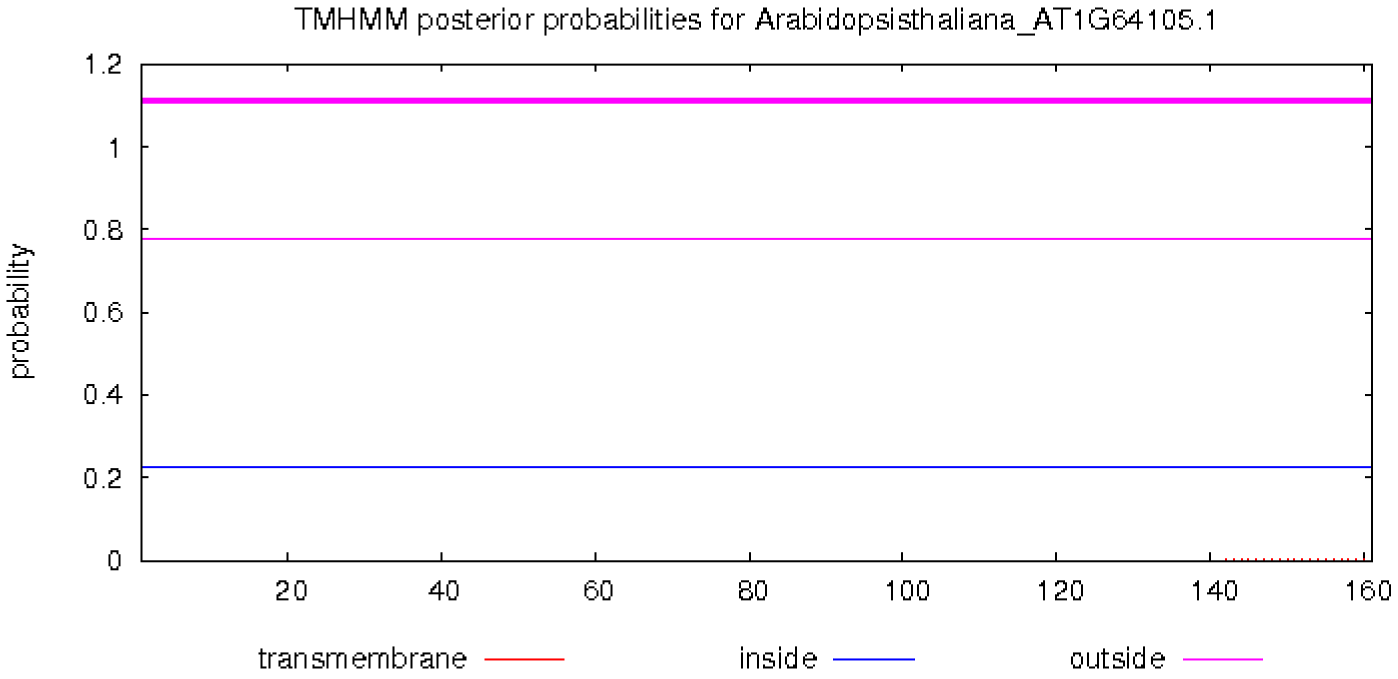

plot in postscript, script for making the plot in gnuplot, data for plot

```
# Arabidopsisthaliana_AT1G65910.1 Length: 631
# Arabidopsisthaliana_AT1G65910.1 Number of predicted TMHs:  0
# Arabidopsisthaliana_AT1G65910.1 Exp number of AAs in TMHs: 1.87306
# Arabidopsisthaliana_AT1G65910.1 Exp number, first 60 AAs:  0
# Arabidopsisthaliana_AT1G65910.1 Total prob of N-in:        0.00008
Arabidopsisthaliana_AT1G65910.1 TMHMM2.0        outside      1   631
```

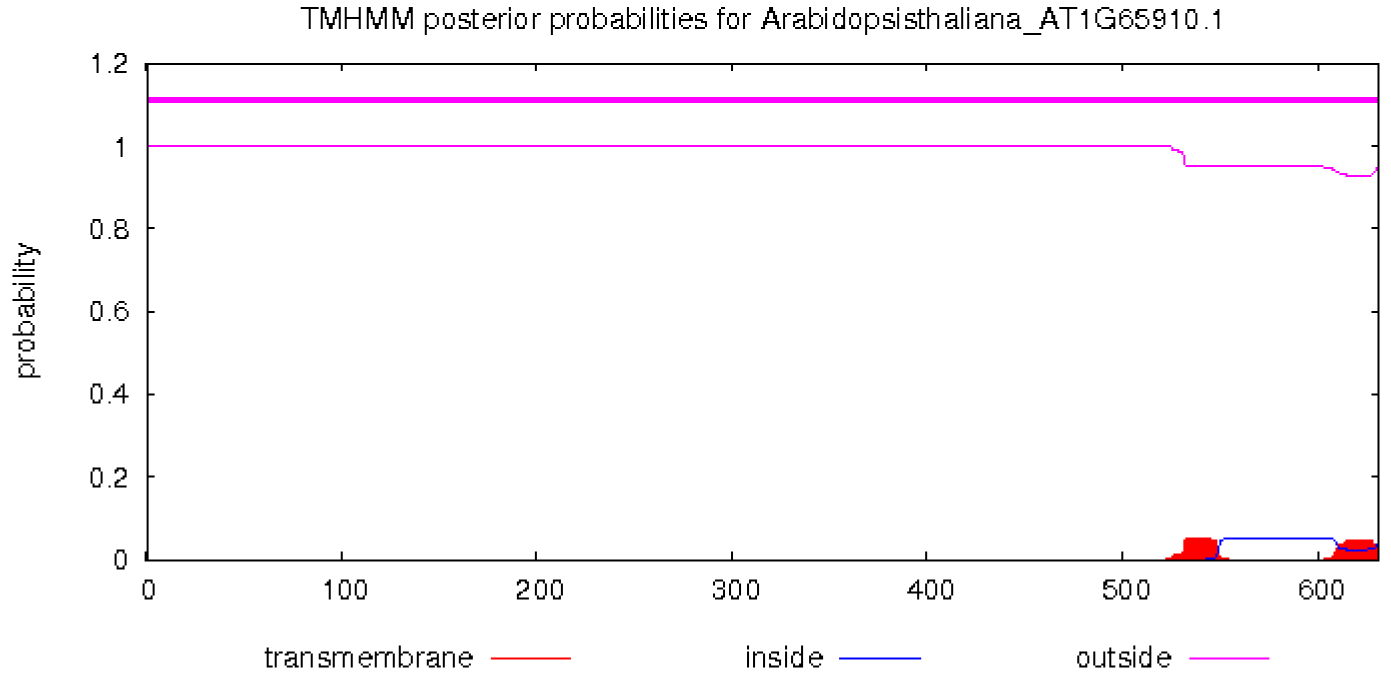

plot in postscript, script for making the plot in gnuplot, data for plot

```
# Arabidopsisthaliana_AT1G69490.1 Length: 268
# Arabidopsisthaliana_AT1G69490.1 Number of predicted TMHs:  0
# Arabidopsisthaliana_AT1G69490.1 Exp number of AAs in TMHs: 0.00053
# Arabidopsisthaliana_AT1G69490.1 Exp number, first 60 AAs:  0
# Arabidopsisthaliana_AT1G69490.1 Total prob of N-in:        0.03841
Arabidopsisthaliana_AT1G69490.1 TMHMM2.0        outside      1   268
```

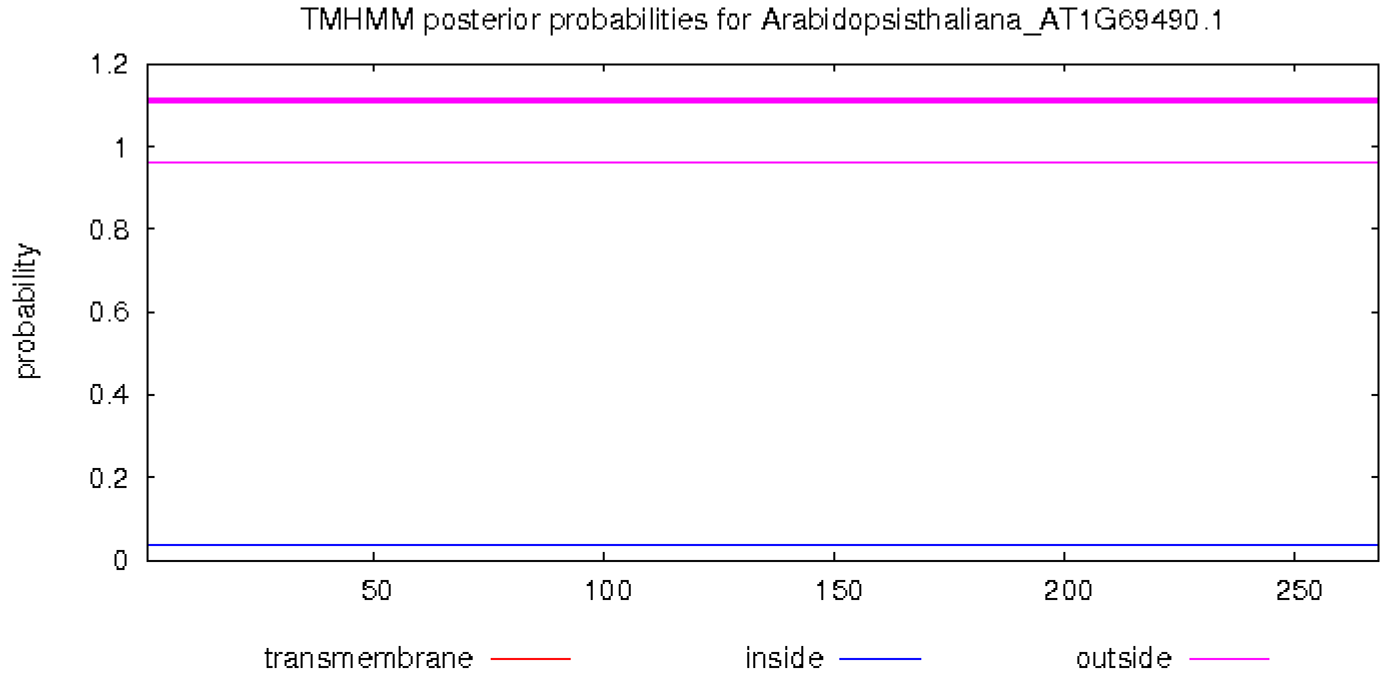

plot in postscript, script for making the plot in gnuplot, data for plot

```
# Arabidopsisthaliana_AT1G71930.1 Length: 324
# Arabidopsisthaliana_AT1G71930.1 Number of predicted TMHs:  0
# Arabidopsisthaliana_AT1G71930.1 Exp number of AAs in TMHs: 0
# Arabidopsisthaliana_AT1G71930.1 Exp number, first 60 AAs:  0
# Arabidopsisthaliana_AT1G71930.1 Total prob of N-in:        0.01747
Arabidopsisthaliana_AT1G71930.1 TMHMM2.0        outside      1   324
```

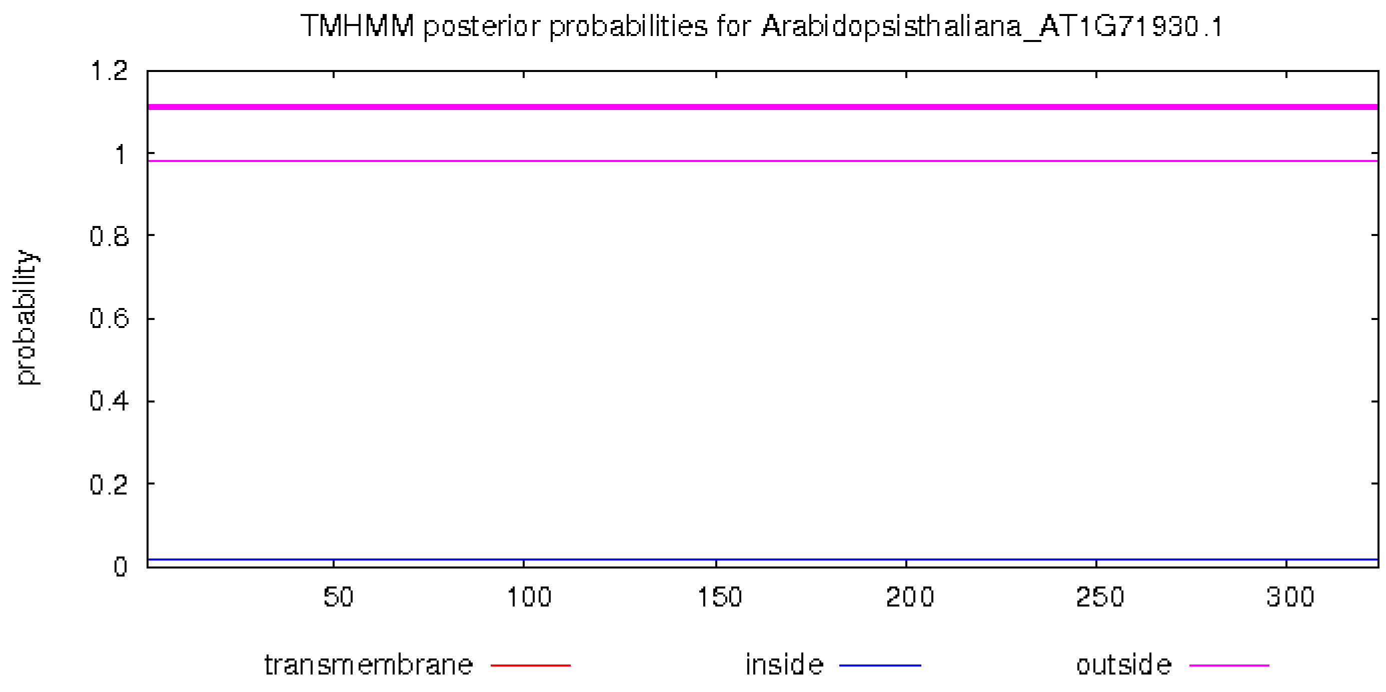

plot in postscript, script for making the plot in gnuplot, data for plot

```
# Arabidopsisthaliana_AT1G76420.1 Length: 334
# Arabidopsisthaliana_AT1G76420.1 Number of predicted TMHs:  0
# Arabidopsisthaliana_AT1G76420.1 Exp number of AAs in TMHs: 0.01157
# Arabidopsisthaliana_AT1G76420.1 Exp number, first 60 AAs:  0.00996
# Arabidopsisthaliana_AT1G76420.1 Total prob of N-in:        0.00510
Arabidopsisthaliana_AT1G76420.1 TMHMM2.0        outside      1   334
```

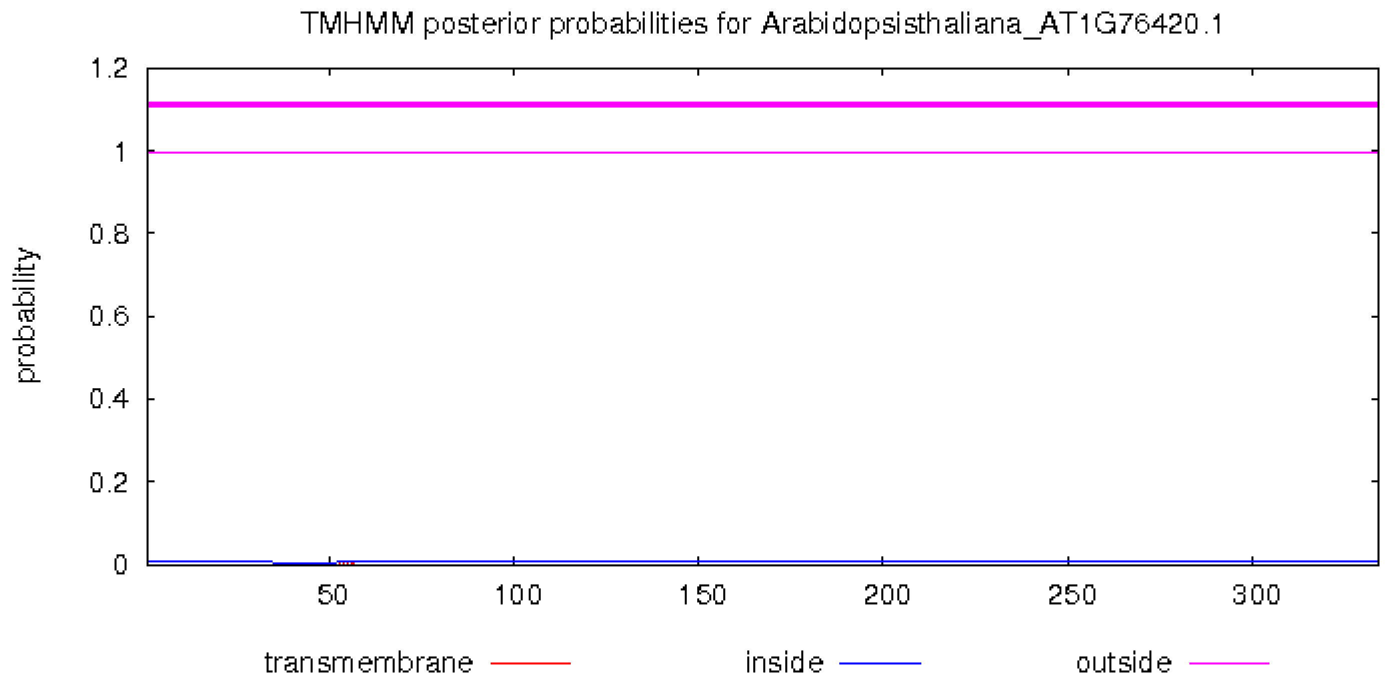

plot in postscript, script for making the plot in gnuplot, data for plot

```
# Arabidopsisthaliana_AT1G77450.1 Length: 253
# Arabidopsisthaliana_AT1G77450.1 Number of predicted TMHs:  0
# Arabidopsisthaliana_AT1G77450.1 Exp number of AAs in TMHs: 7e-05
# Arabidopsisthaliana_AT1G77450.1 Exp number, first 60 AAs:  0
# Arabidopsisthaliana_AT1G77450.1 Total prob of N-in:        0.02696
Arabidopsisthaliana_AT1G77450.1 TMHMM2.0        outside      1   253
```

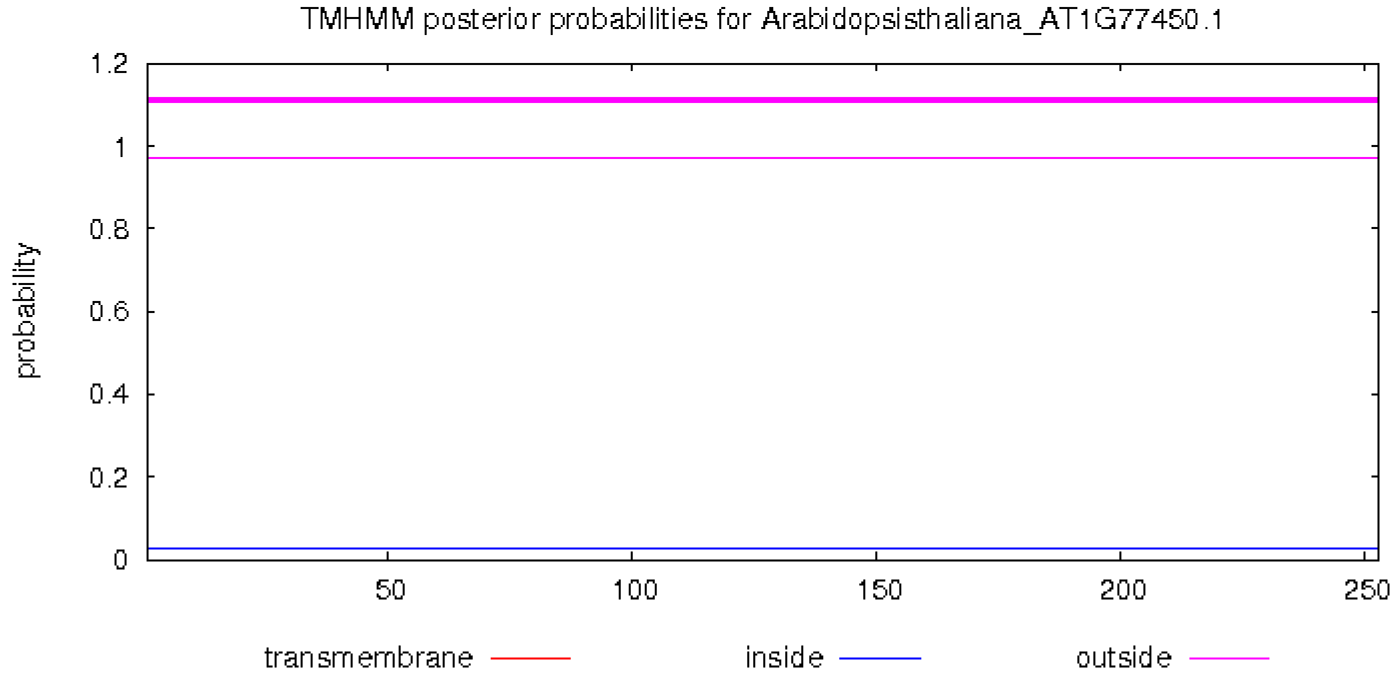

plot in postscript, script for making the plot in gnuplot, data for plot

```
# Arabidopsisthaliana_AT1G79580.1 Length: 371
# Arabidopsisthaliana_AT1G79580.1 Number of predicted TMHs:  0
# Arabidopsisthaliana_AT1G79580.1 Exp number of AAs in TMHs: 0.00209
# Arabidopsisthaliana_AT1G79580.1 Exp number, first 60 AAs:  0.00096
# Arabidopsisthaliana_AT1G79580.1 Total prob of N-in:        0.00767
Arabidopsisthaliana_AT1G79580.1 TMHMM2.0        outside      1   371
```

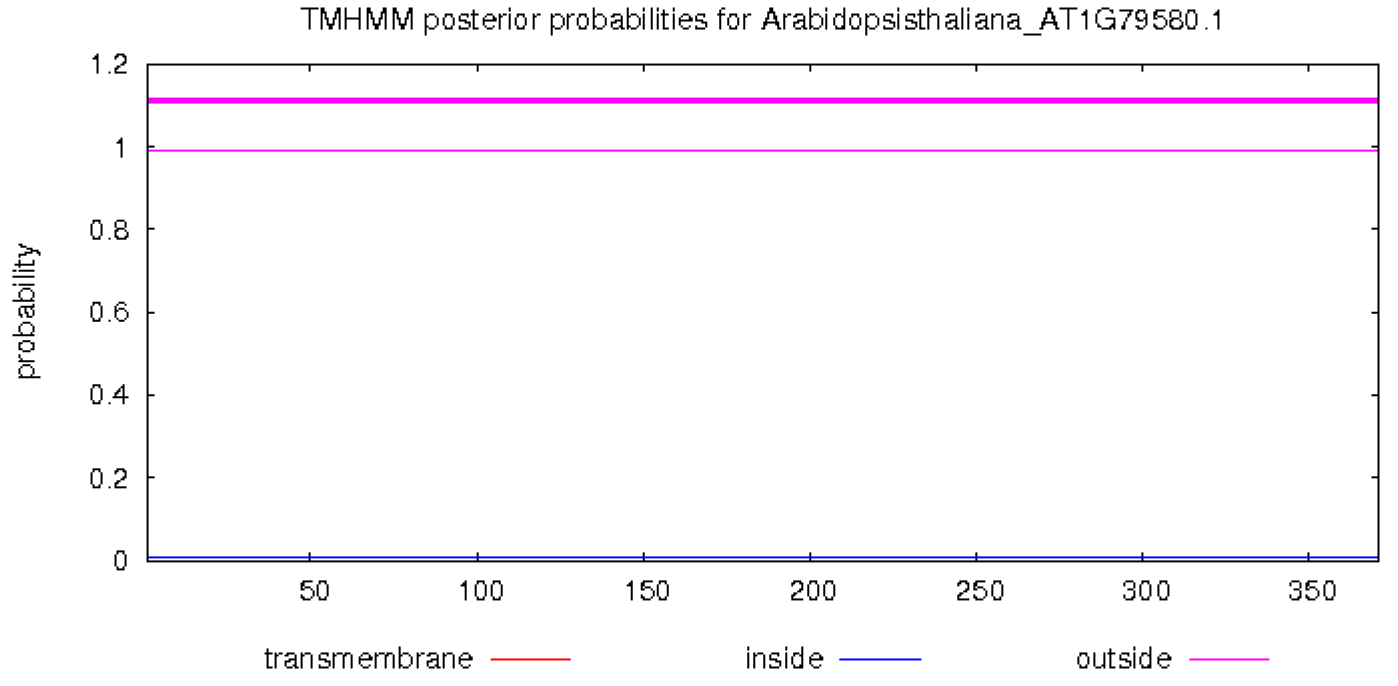

plot in postscript, script for making the plot in gnuplot, data for plot

---

```
# Arabidopsisthaliana_AT2G02450.1 Length: 379
# Arabidopsisthaliana_AT2G02450.1 Number of predicted TMHs:  0
# Arabidopsisthaliana_AT2G02450.1 Exp number of AAs in TMHs: 0.01421
# Arabidopsisthaliana_AT2G02450.1 Exp number, first 60 AAs:  0
# Arabidopsisthaliana_AT2G02450.1 Total prob of N-in:        0.01355
Arabidopsisthaliana_AT2G02450.1 TMHMM2.0        outside      1   379
```

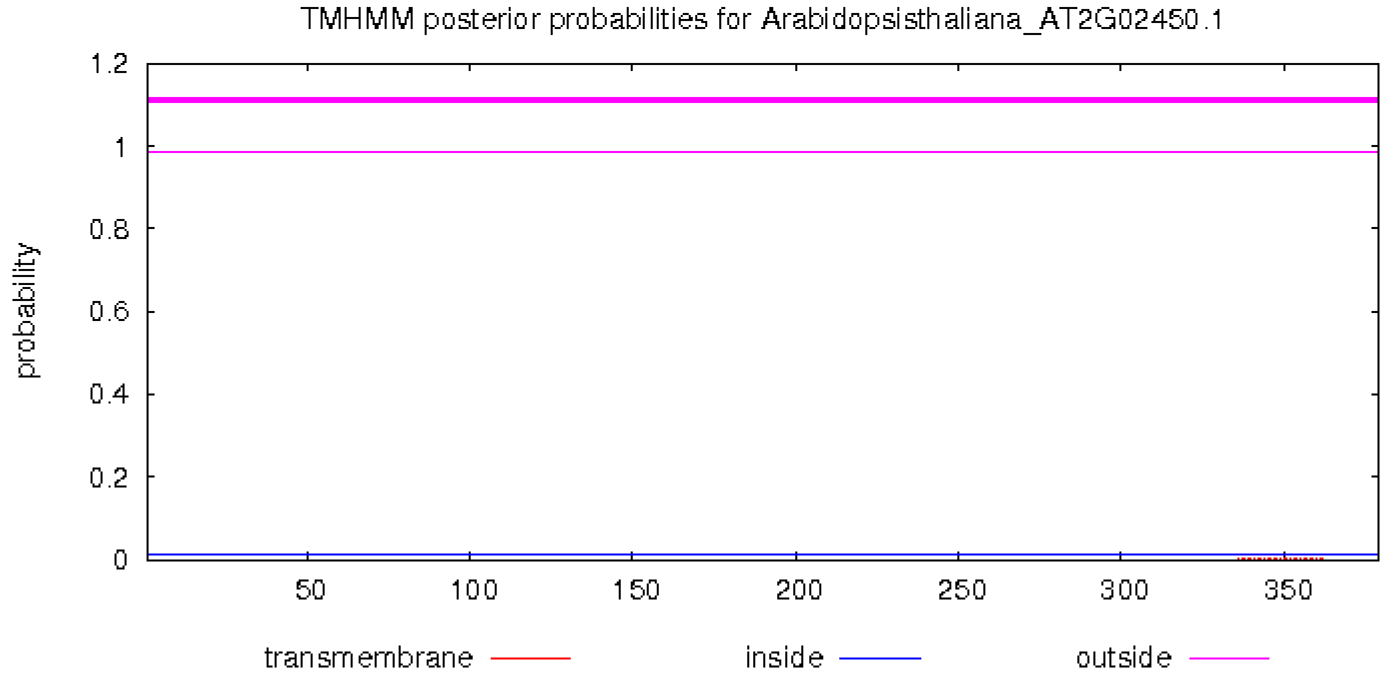

plot in postscript, script for making the plot in gnuplot, data for plot

---

```
# Arabidopsisthaliana_AT2G17040.1 Length: 276
# Arabidopsisthaliana_AT2G17040.1 Number of predicted TMHs:  0
# Arabidopsisthaliana_AT2G17040.1 Exp number of AAs in TMHs: 0.00081
# Arabidopsisthaliana_AT2G17040.1 Exp number, first 60 AAs:  0
# Arabidopsisthaliana_AT2G17040.1 Total prob of N-in:        0.00577
Arabidopsisthaliana_AT2G17040.1 TMHMM2.0        outside      1   276
```

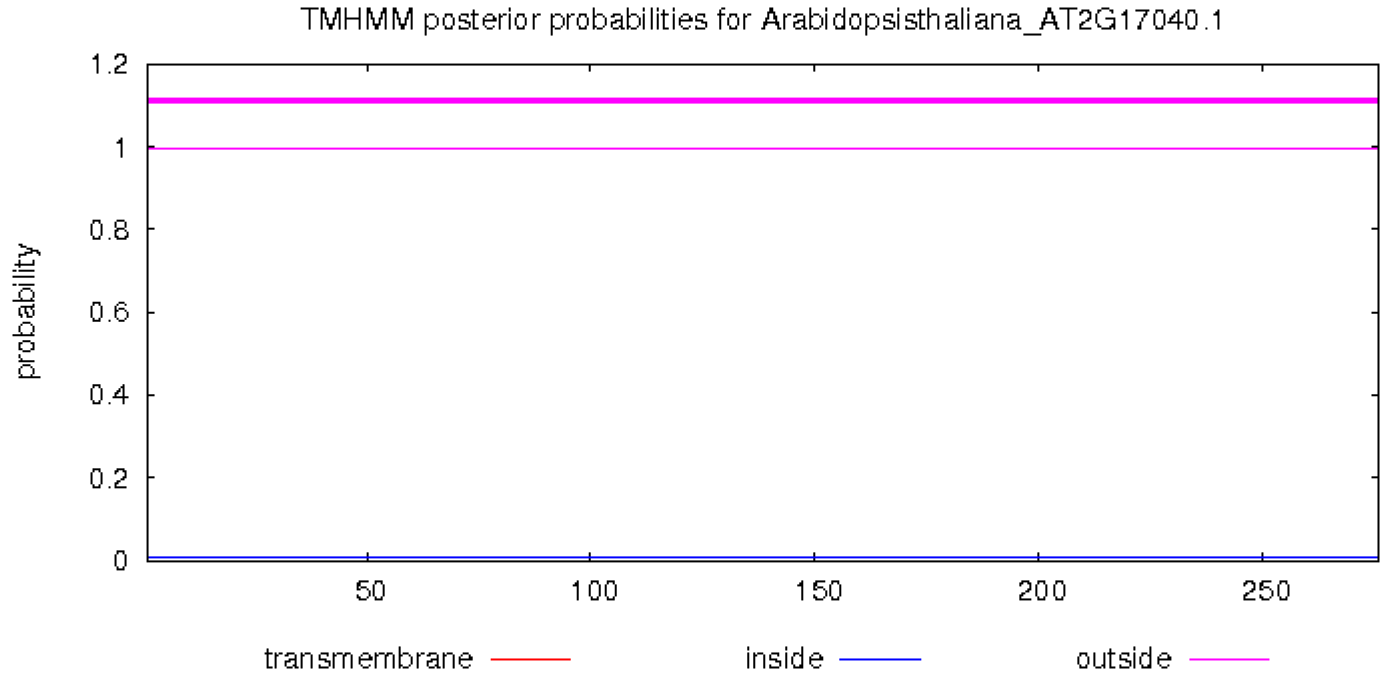

plot in postscript, script for making the plot in gnuplot, data for plot

---

```
# Arabidopsisthaliana_AT2G18060.1 Length: 365
# Arabidopsisthaliana_AT2G18060.1 Number of predicted TMHs:  0
# Arabidopsisthaliana_AT2G18060.1 Exp number of AAs in TMHs: 0
# Arabidopsisthaliana_AT2G18060.1 Exp number, first 60 AAs:  0
# Arabidopsisthaliana_AT2G18060.1 Total prob of N-in:        0.00392
Arabidopsisthaliana_AT2G18060.1 TMHMM2.0        outside      1   365
```

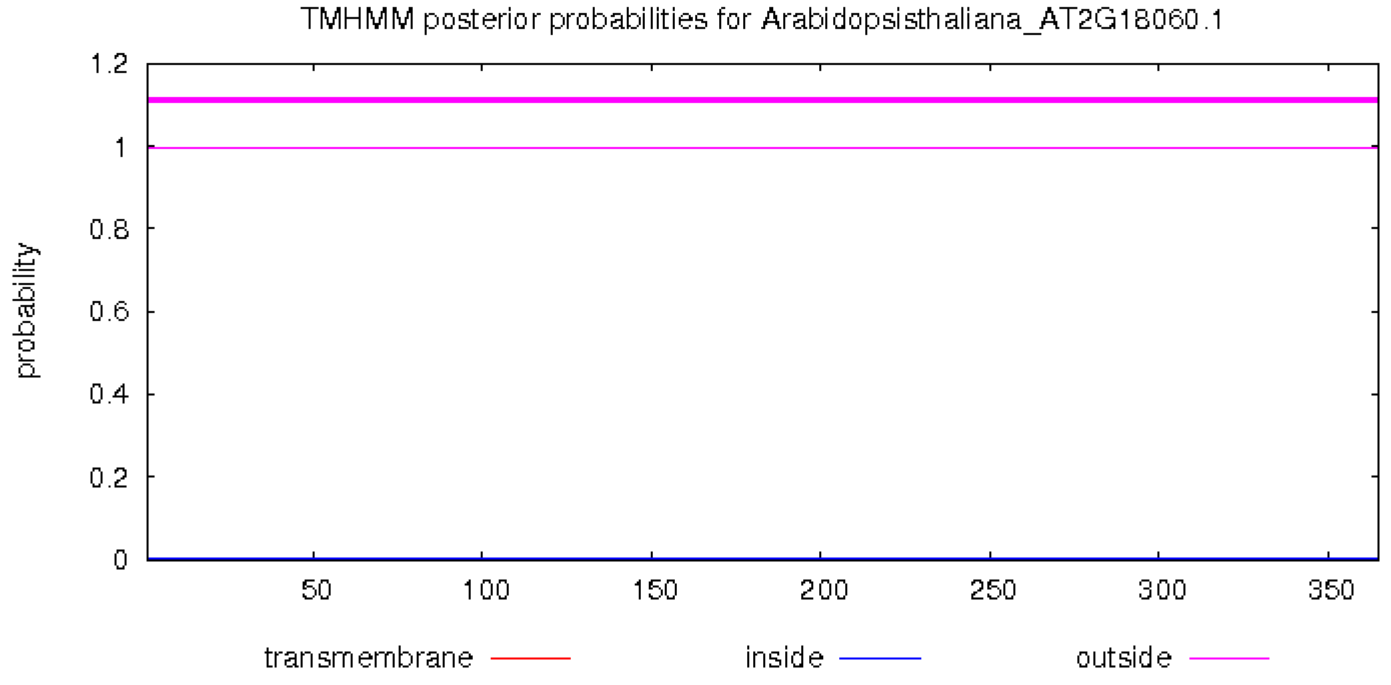

plot in postscript, script for making the plot in gnuplot, data for plot

---

```
# Arabidopsisthaliana_AT2G24430.1 Length: 316
# Arabidopsisthaliana_AT2G24430.1 Number of predicted TMHs:  0
# Arabidopsisthaliana_AT2G24430.1 Exp number of AAs in TMHs: 0.01127
# Arabidopsisthaliana_AT2G24430.1 Exp number, first 60 AAs:  0
# Arabidopsisthaliana_AT2G24430.1 Total prob of N-in:        0.06226
Arabidopsisthaliana_AT2G24430.1 TMHMM2.0        outside      1   316
```

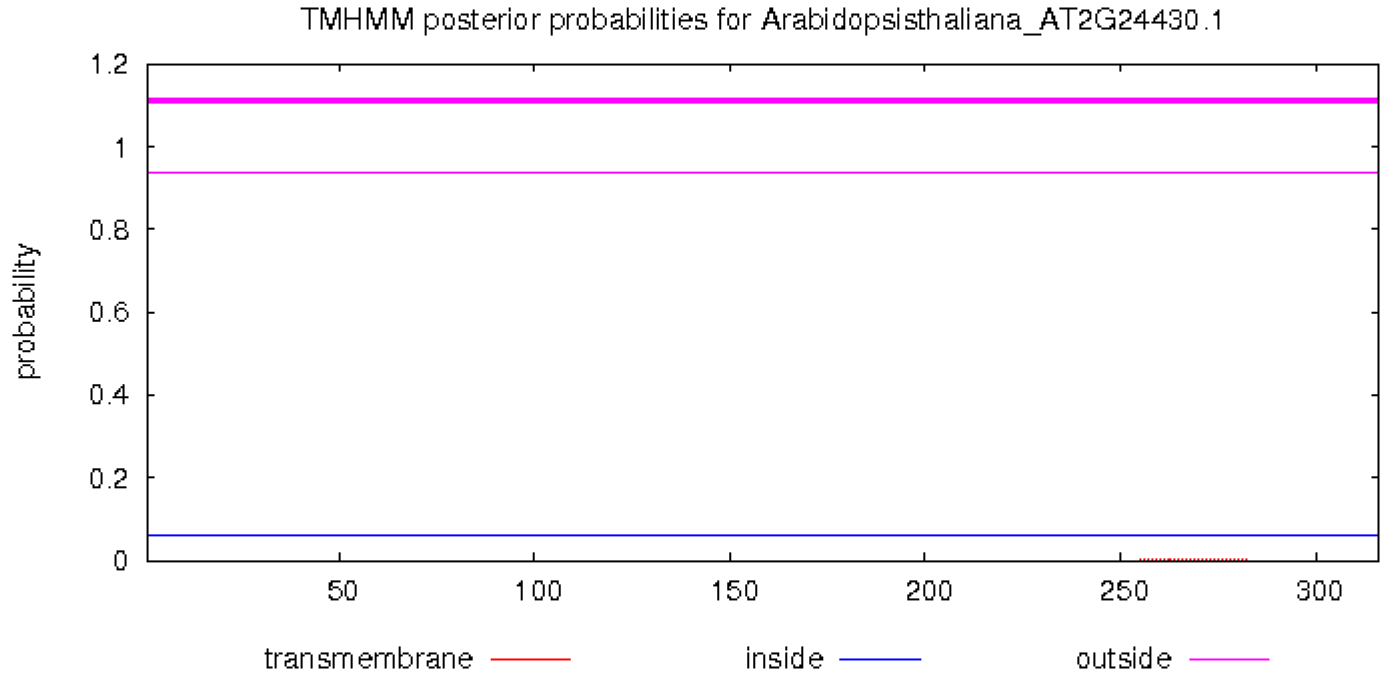

plot in postscript, script for making the plot in gnuplot, data for plot

```
# Arabidopsisthaliana_AT2G27300.1 Length: 335
# Arabidopsisthaliana_AT2G27300.1 Number of predicted TMHs:  1
# Arabidopsisthaliana_AT2G27300.1 Exp number of AAs in TMHs: 21.14018
# Arabidopsisthaliana_AT2G27300.1 Exp number, first 60 AAs:  0.09115
# Arabidopsisthaliana_AT2G27300.1 Total prob of N-in:        0.46458
Arabidopsisthaliana_AT2G27300.1 TMHMM2.0        outside      1   312
Arabidopsisthaliana_AT2G27300.1 TMHMM2.0        TMhelix    313   332
Arabidopsisthaliana_AT2G27300.1 TMHMM2.0        inside     333   335
```

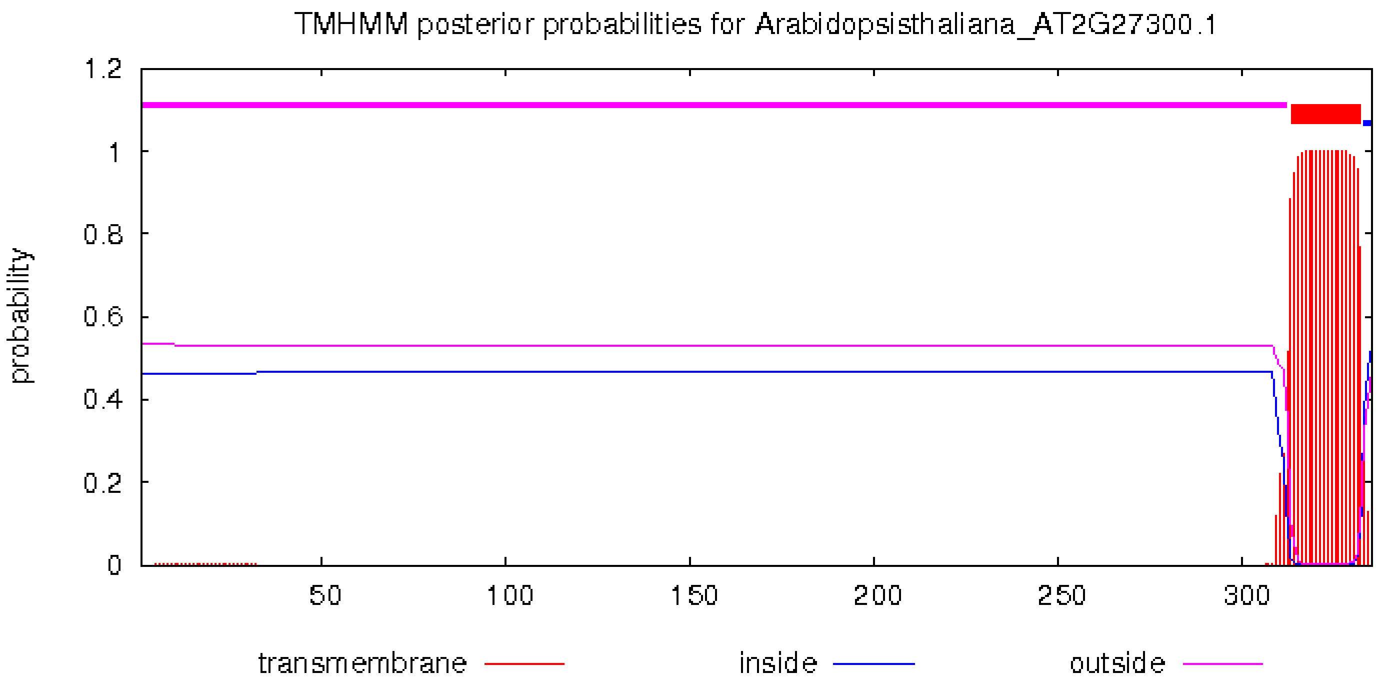

plot in postscript, script for making the plot in gnuplot, data for plot

```
# Arabidopsisthaliana_AT2G33480.1 Length: 268
# Arabidopsisthaliana_AT2G33480.1 Number of predicted TMHs:  0
# Arabidopsisthaliana_AT2G33480.1 Exp number of AAs in TMHs: 0.02564
# Arabidopsisthaliana_AT2G33480.1 Exp number, first 60 AAs:  0
# Arabidopsisthaliana_AT2G33480.1 Total prob of N-in:        0.23506
Arabidopsisthaliana_AT2G33480.1 TMHMM2.0        outside      1   268
```

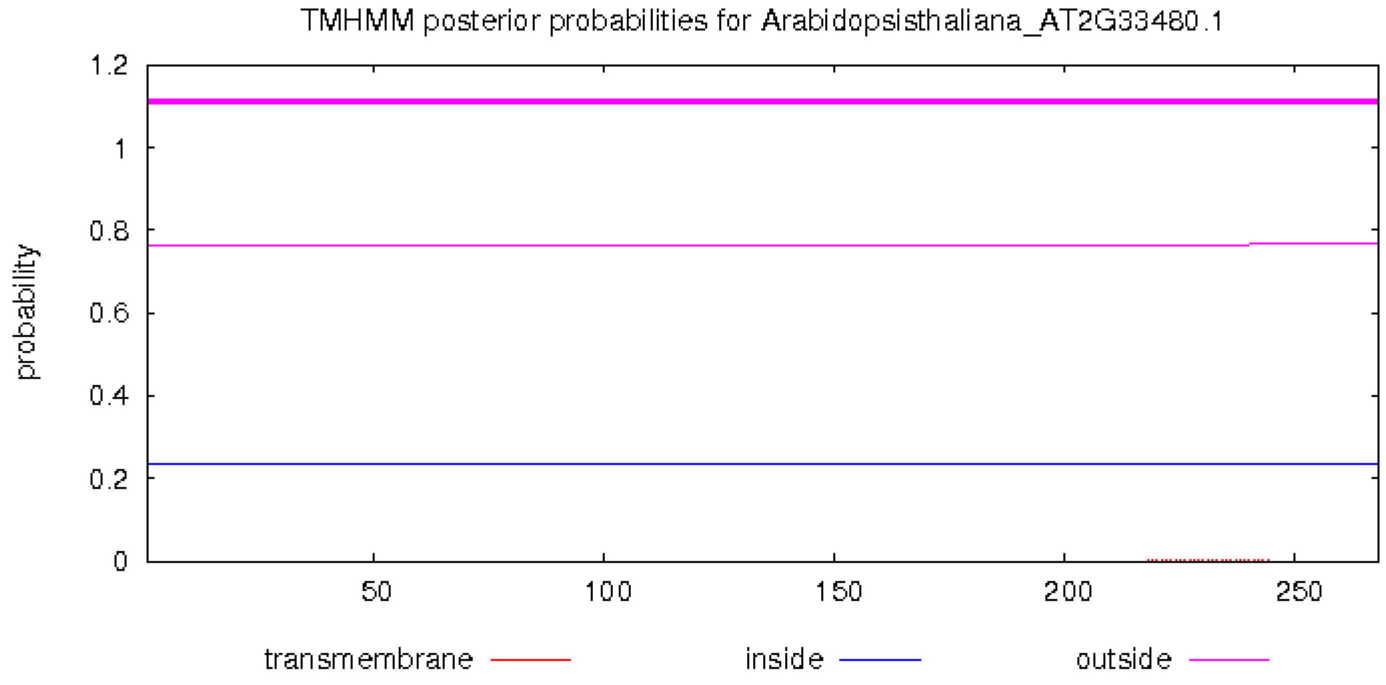

plot in postscript, script for making the plot in gnuplot, data for plot

---

```
# Arabidopsisthaliana_AT2G43000.1 Length: 275
# Arabidopsisthaliana_AT2G43000.1 Number of predicted TMHs:  0
# Arabidopsisthaliana_AT2G43000.1 Exp number of AAs in TMHs: 0.00045
# Arabidopsisthaliana_AT2G43000.1 Exp number, first 60 AAs:  0
# Arabidopsisthaliana_AT2G43000.1 Total prob of N-in:        0.01633
Arabidopsisthaliana_AT2G43000.1 TMHMM2.0        outside      1   275
```

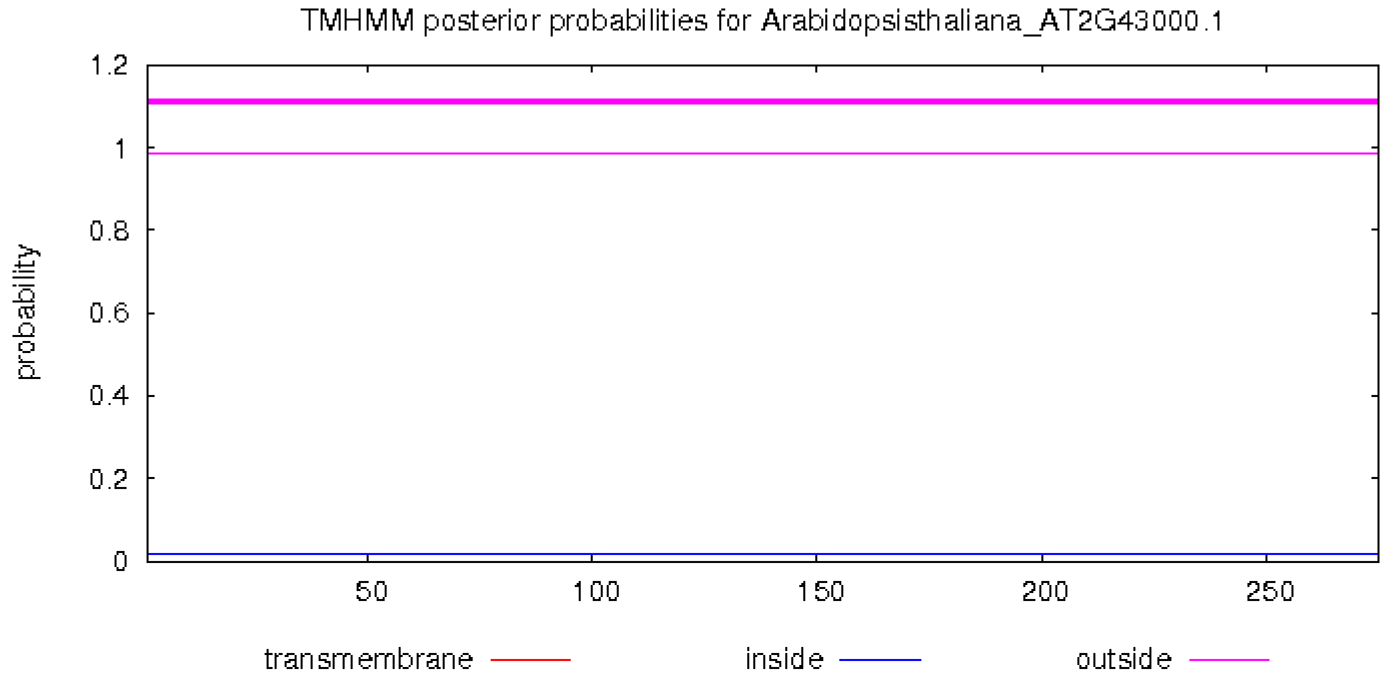

plot in postscript, script for making the plot in gnuplot, data for plot

---

```
# Arabidopsisthaliana_AT2G46770.1 Length: 365
# Arabidopsisthaliana_AT2G46770.1 Number of predicted TMHs:  0
# Arabidopsisthaliana_AT2G46770.1 Exp number of AAs in TMHs: 0.00018
# Arabidopsisthaliana_AT2G46770.1 Exp number, first 60 AAs:  0
# Arabidopsisthaliana_AT2G46770.1 Total prob of N-in:        0.01205
Arabidopsisthaliana_AT2G46770.1 TMHMM2.0        outside      1   365
```

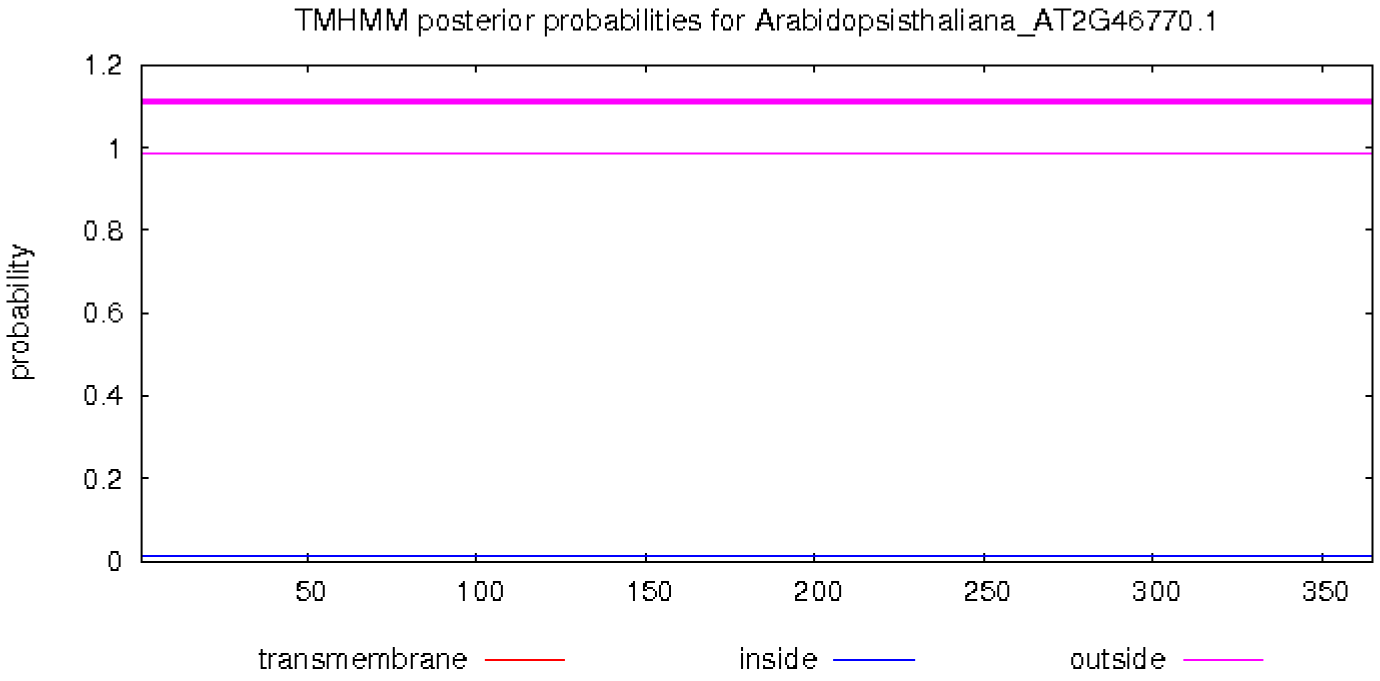

plot in postscript, script for making the plot in gnuplot, data for plot

---

```
# Arabidopsisthaliana_AT3G01600.1 Length: 370
# Arabidopsisthaliana_AT3G01600.1 Number of predicted TMHs:  0
# Arabidopsisthaliana_AT3G01600.1 Exp number of AAs in TMHs: 0.00018
# Arabidopsisthaliana_AT3G01600.1 Exp number, first 60 AAs:  0
# Arabidopsisthaliana_AT3G01600.1 Total prob of N-in:        0.02833
Arabidopsisthaliana_AT3G01600.1 TMHMM2.0        outside      1   370
```

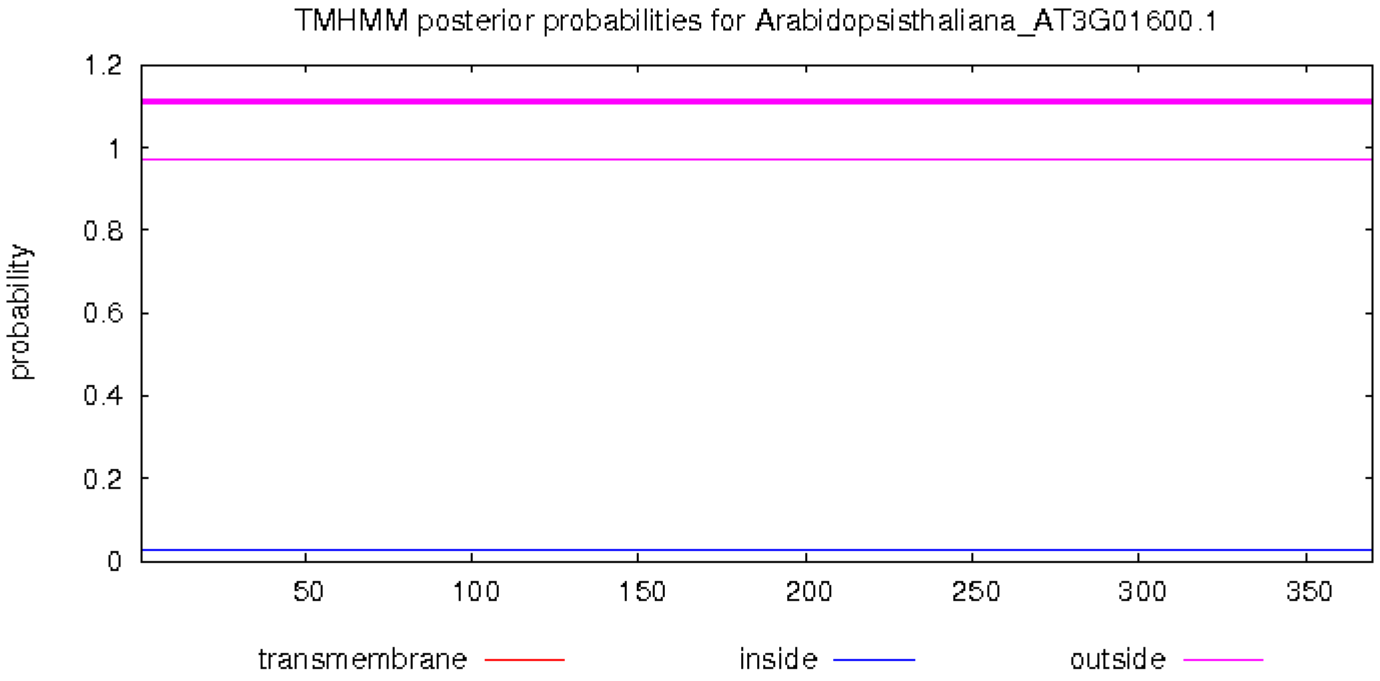

plot in postscript, script for making the plot in gnuplot, data for plot

---

```
# Arabidopsisthaliana_AT3G03200.1 Length: 479
# Arabidopsisthaliana_AT3G03200.1 Number of predicted TMHs:  0
# Arabidopsisthaliana_AT3G03200.1 Exp number of AAs in TMHs: 0.00101
# Arabidopsisthaliana_AT3G03200.1 Exp number, first 60 AAs:  0
# Arabidopsisthaliana_AT3G03200.1 Total prob of N-in:        0.00016
Arabidopsisthaliana_AT3G03200.1 TMHMM2.0        outside      1   479
```

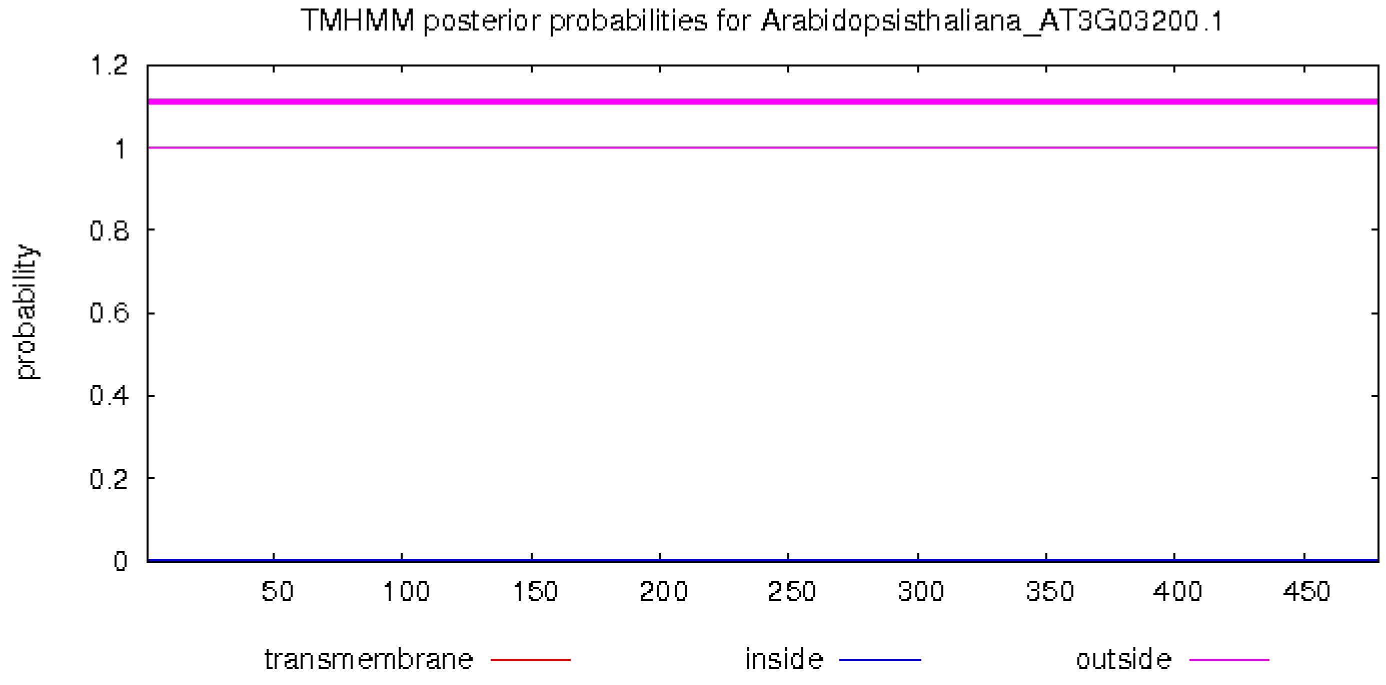

plot in postscript, script for making the plot in gnuplot, data for plot

---

```
# Arabidopsisthaliana_AT3G04060.1 Length: 338
# Arabidopsisthaliana_AT3G04060.1 Number of predicted TMHs:  0
# Arabidopsisthaliana_AT3G04060.1 Exp number of AAs in TMHs: 0.00072
# Arabidopsisthaliana_AT3G04060.1 Exp number, first 60 AAs:  0.00014
# Arabidopsisthaliana_AT3G04060.1 Total prob of N-in:        0.00813
Arabidopsisthaliana_AT3G04060.1 TMHMM2.0        outside      1   338
```

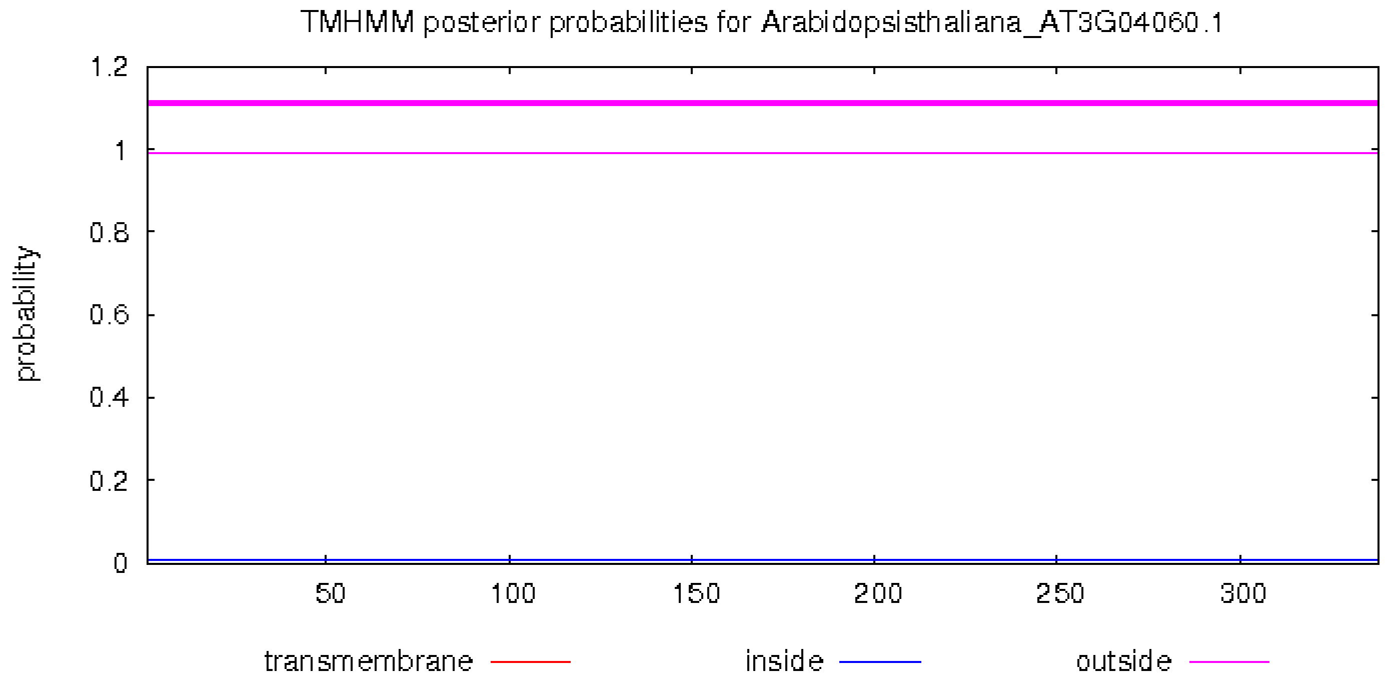

plot in postscript, script for making the plot in gnuplot, data for plot

---

```
# Arabidopsisthaliana_AT3G04070.1 Length: 359
# Arabidopsisthaliana_AT3G04070.1 Number of predicted TMHs:  0
# Arabidopsisthaliana_AT3G04070.1 Exp number of AAs in TMHs: 0
# Arabidopsisthaliana_AT3G04070.1 Exp number, first 60 AAs:  0
# Arabidopsisthaliana_AT3G04070.1 Total prob of N-in:        0.01033
Arabidopsisthaliana_AT3G04070.1 TMHMM2.0        outside      1   359
```

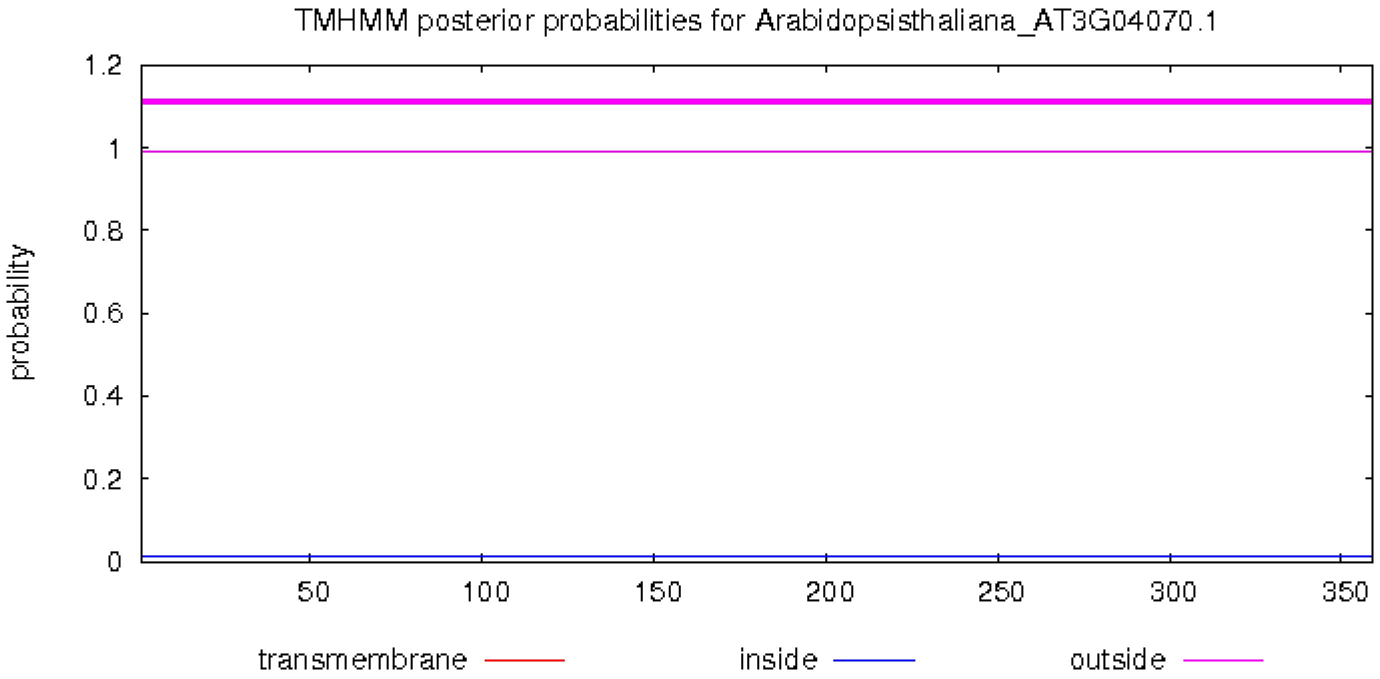

plot in postscript, script for making the plot in gnuplot, data for plot

```
# Arabidopsisthaliana_AT3G04410.1 Length: 76
# Arabidopsisthaliana_AT3G04410.1 Number of predicted TMHs:  0
# Arabidopsisthaliana_AT3G04410.1 Exp number of AAs in TMHs: 0
# Arabidopsisthaliana_AT3G04410.1 Exp number, first 60 AAs:  0
# Arabidopsisthaliana_AT3G04410.1 Total prob of N-in:        0.19322
Arabidopsisthaliana_AT3G04410.1 TMHMM2.0        outside      1    76
```

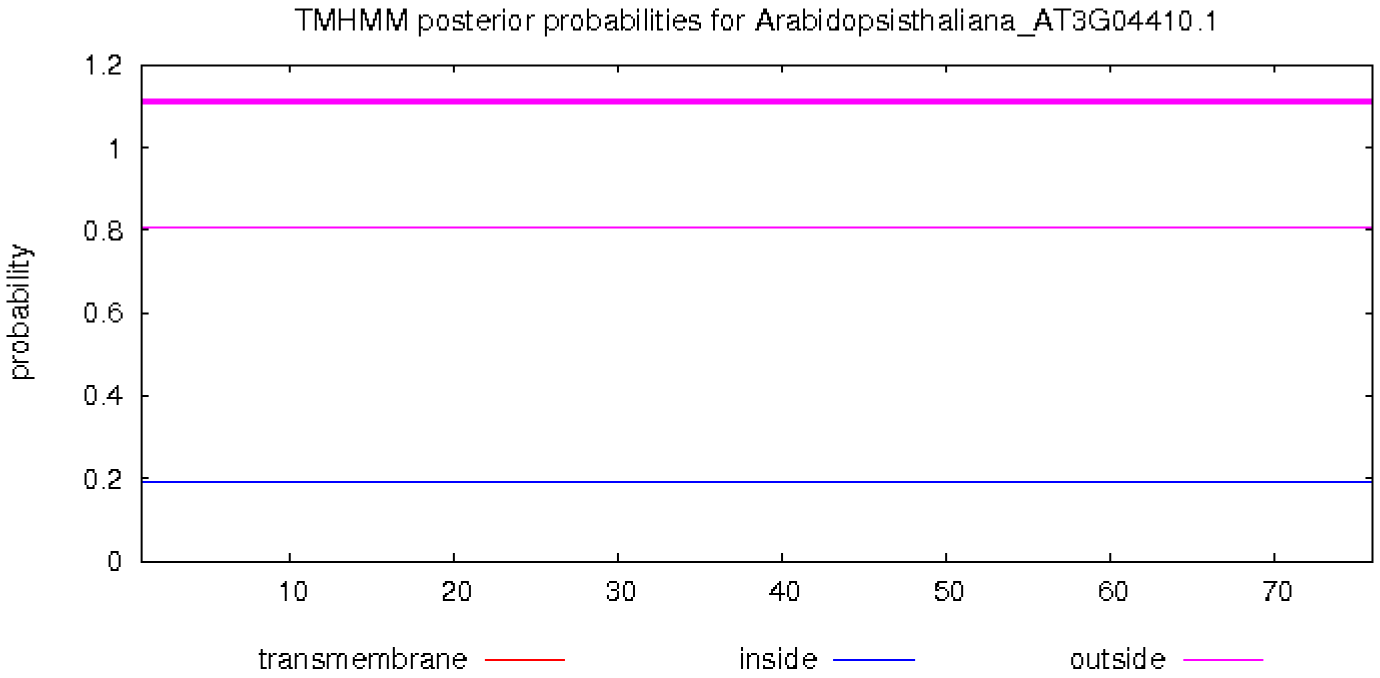

plot in postscript, script for making the plot in gnuplot, data for plot

```
# Arabidopsisthaliana_AT3G04420.1 Length: 342
# Arabidopsisthaliana_AT3G04420.1 Number of predicted TMHs:  0
# Arabidopsisthaliana_AT3G04420.1 Exp number of AAs in TMHs: 0.00041
# Arabidopsisthaliana_AT3G04420.1 Exp number, first 60 AAs:  0
# Arabidopsisthaliana_AT3G04420.1 Total prob of N-in:        0.00913
Arabidopsisthaliana_AT3G04420.1 TMHMM2.0        outside      1   342
```

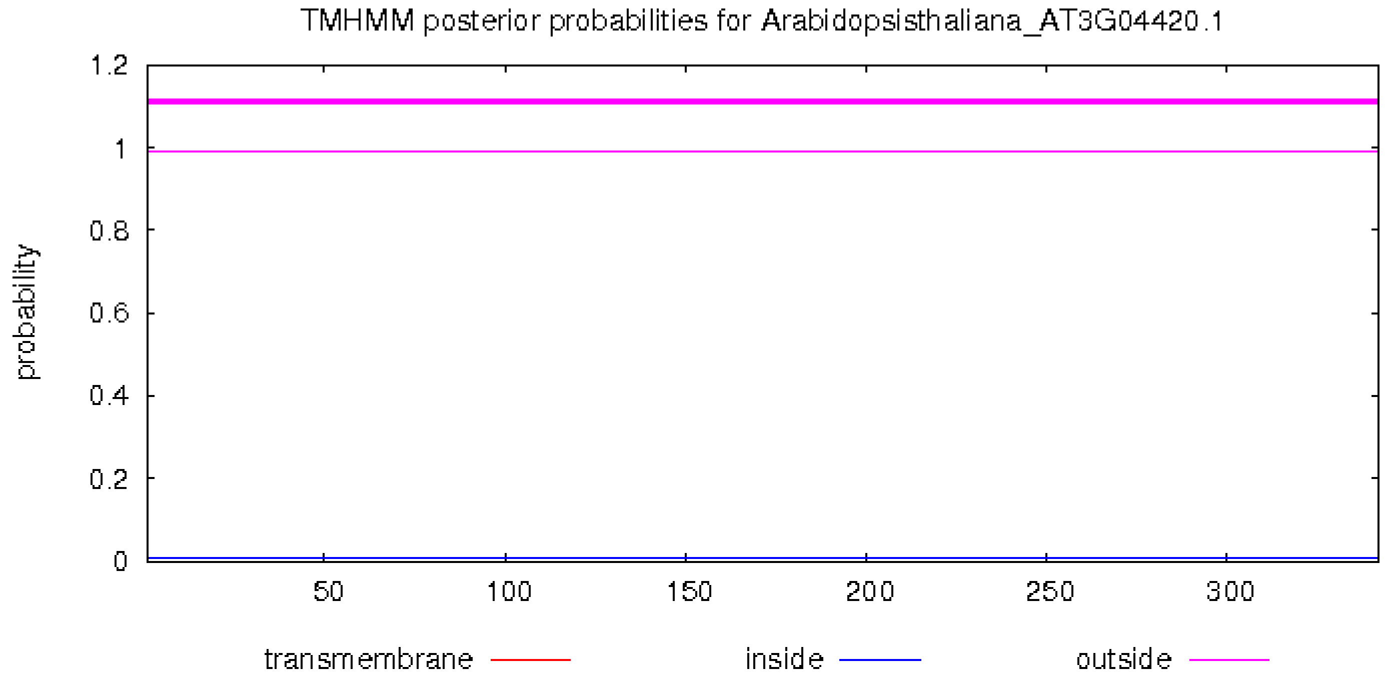

plot in postscript, script for making the plot in gnuplot, data for plot

---

```
# Arabidopsisthaliana_AT3G04430.1 Length: 198
# Arabidopsisthaliana_AT3G04430.1 Number of predicted TMHs:  0
# Arabidopsisthaliana_AT3G04430.1 Exp number of AAs in TMHs: 0
# Arabidopsisthaliana_AT3G04430.1 Exp number, first 60 AAs:  0
# Arabidopsisthaliana_AT3G04430.1 Total prob of N-in:        0.09828
Arabidopsisthaliana_AT3G04430.1 TMHMM2.0        outside      1   198
```

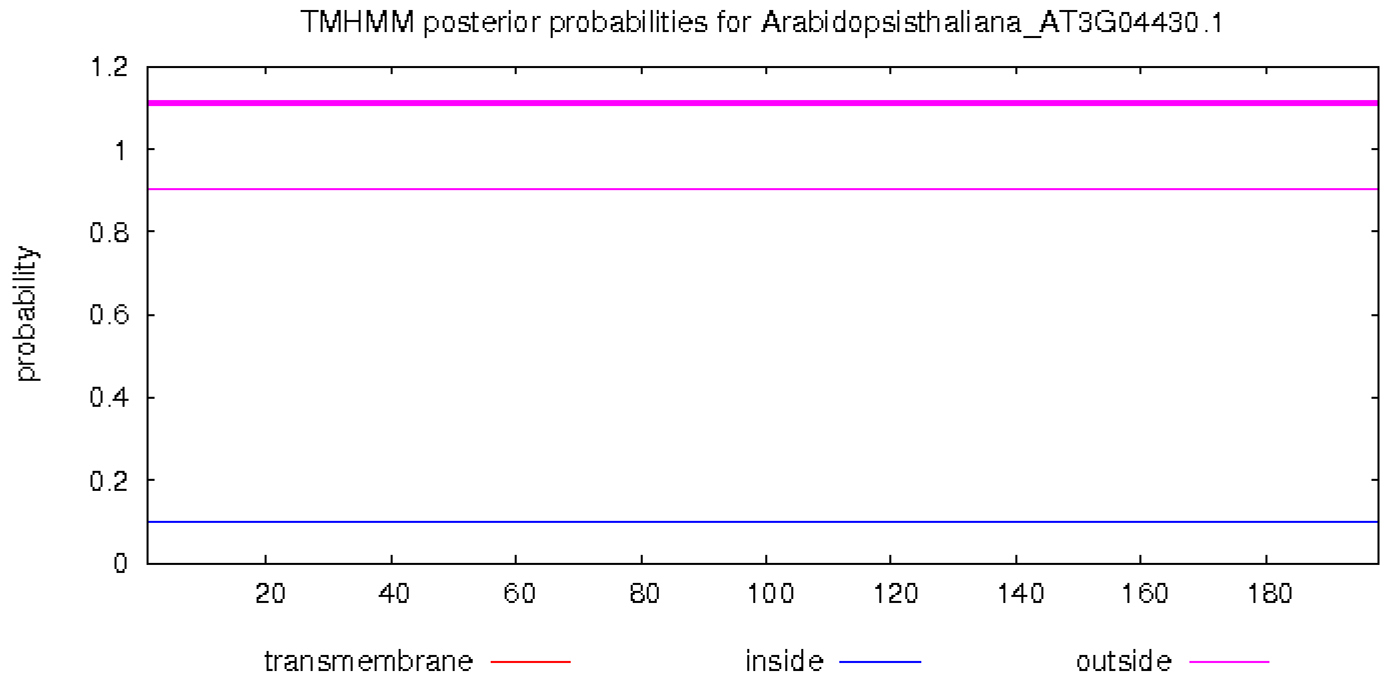

plot in postscript, script for making the plot in gnuplot, data for plot

---

```
# Arabidopsisthaliana_AT3G10480.1 Length: 447
# Arabidopsisthaliana_AT3G10480.1 Number of predicted TMHs:  0
# Arabidopsisthaliana_AT3G10480.1 Exp number of AAs in TMHs: 0.07621
# Arabidopsisthaliana_AT3G10480.1 Exp number, first 60 AAs:  0.03391
# Arabidopsisthaliana_AT3G10480.1 Total prob of N-in:        0.00417
Arabidopsisthaliana_AT3G10480.1 TMHMM2.0        outside      1   447
```

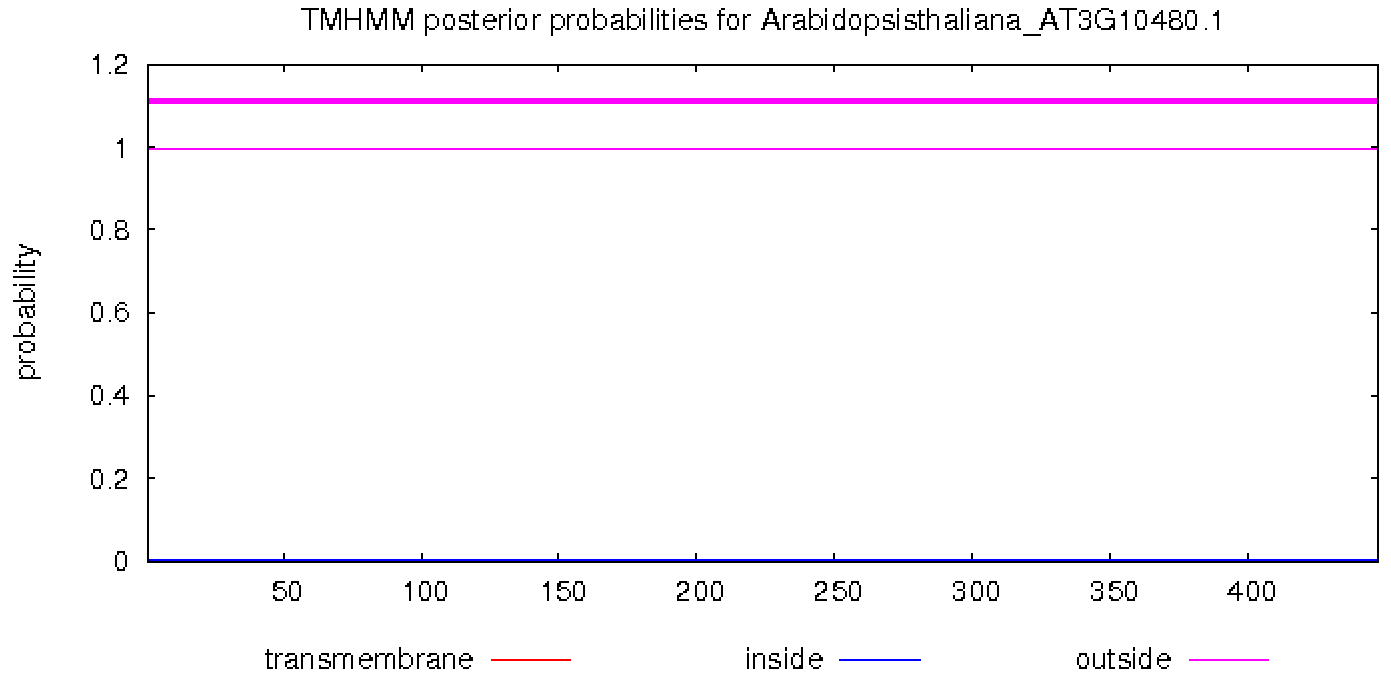

plot in postscript, script for making the plot in gnuplot, data for plot

---

```
# Arabidopsisthaliana_AT3G10490.1 Length: 238
# Arabidopsisthaliana_AT3G10490.1 Number of predicted TMHs:  0
# Arabidopsisthaliana_AT3G10490.1 Exp number of AAs in TMHs: 0.30069
# Arabidopsisthaliana_AT3G10490.1 Exp number, first 60 AAs:  0.30036
# Arabidopsisthaliana_AT3G10490.1 Total prob of N-in:        0.08167
Arabidopsisthaliana_AT3G10490.1 TMHMM2.0        outside      1   238
```

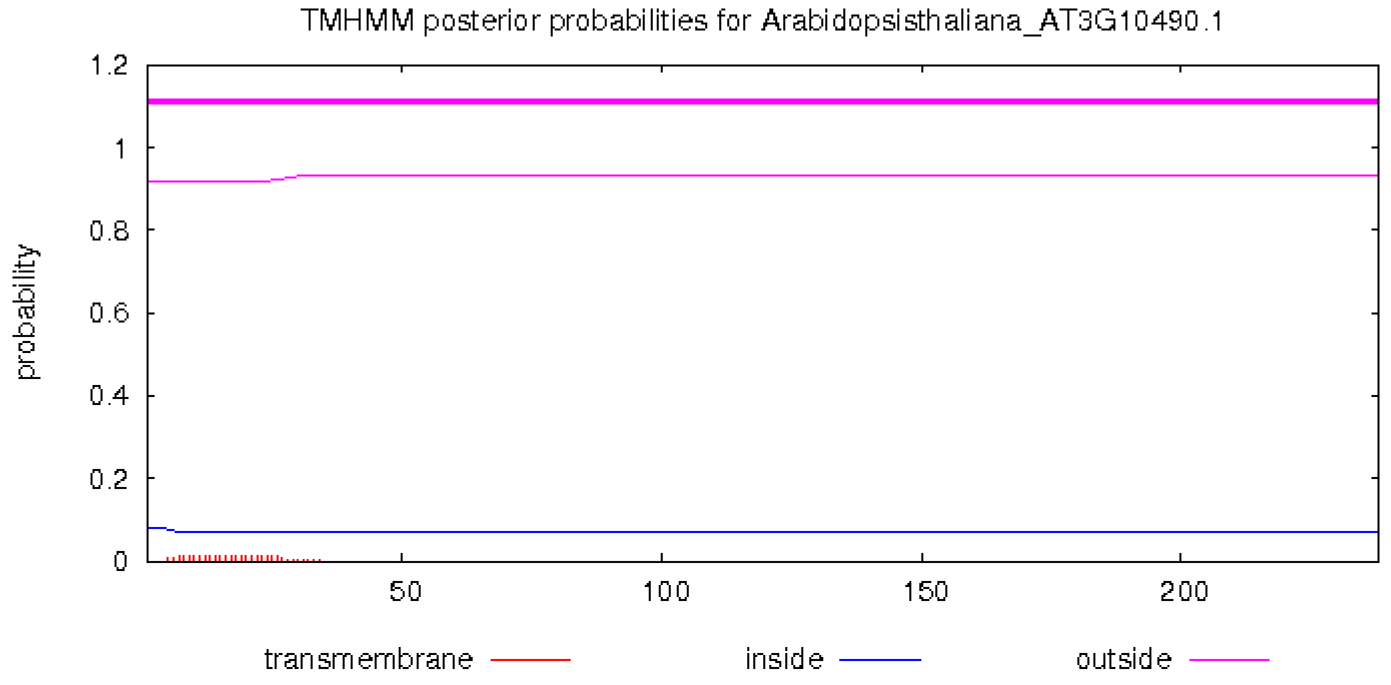

plot in postscript, script for making the plot in gnuplot, data for plot

---

```
# Arabidopsisthaliana_AT3G10500.1 Length: 549
# Arabidopsisthaliana_AT3G10500.1 Number of predicted TMHs:  1
# Arabidopsisthaliana_AT3G10500.1 Exp number of AAs in TMHs: 22.24214
# Arabidopsisthaliana_AT3G10500.1 Exp number, first 60 AAs:  0
# Arabidopsisthaliana_AT3G10500.1 Total prob of N-in:        0.00266
Arabidopsisthaliana_AT3G10500.1 TMHMM2.0        outside      1   522
```

```
Arabidopsisthaliana_AT3G10500.1 TMHMM2.0        TMhelix    523   545
Arabidopsisthaliana_AT3G10500.1 TMHMM2.0        inside     546   549
```

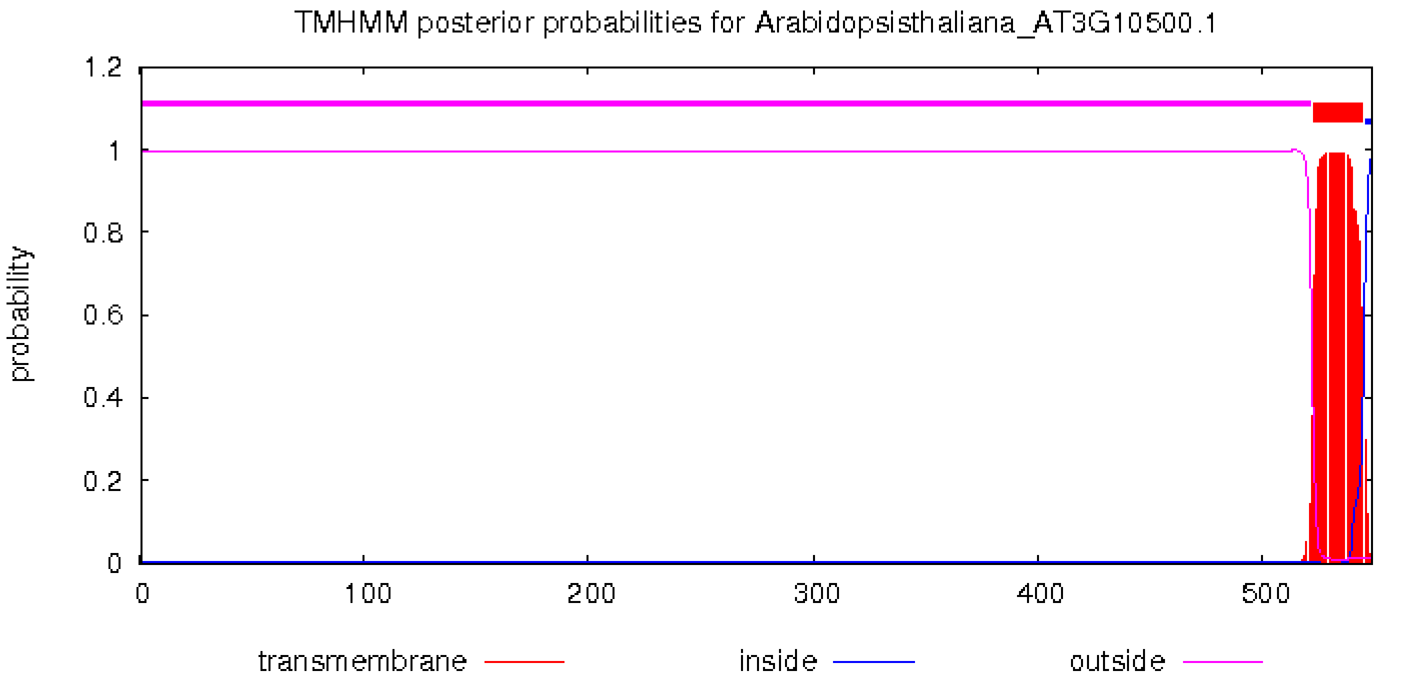

plot in postscript, script for making the plot in gnuplot, data for plot

---

```
# Arabidopsisthaliana_AT3G12910.1 Length: 303
# Arabidopsisthaliana_AT3G12910.1 Number of predicted TMHs:  0
# Arabidopsisthaliana_AT3G12910.1 Exp number of AAs in TMHs: 0.00063
# Arabidopsisthaliana_AT3G12910.1 Exp number, first 60 AAs:  0
# Arabidopsisthaliana_AT3G12910.1 Total prob of N-in:        0.04394
Arabidopsisthaliana_AT3G12910.1 TMHMM2.0        outside      1   303
```

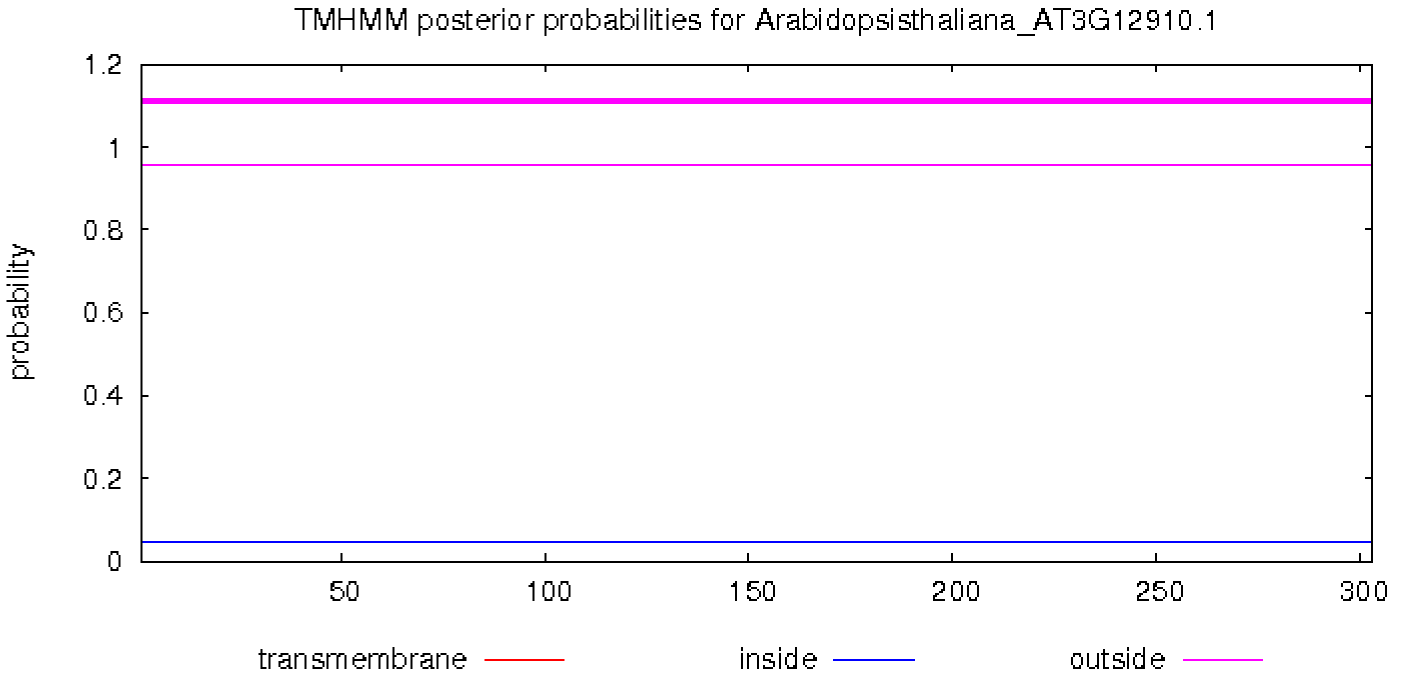

plot in postscript, script for making the plot in gnuplot, data for plot

---

```
# Arabidopsisthaliana_AT3G12977.1 Length: 279
# Arabidopsisthaliana_AT3G12977.1 Number of predicted TMHs:  0
# Arabidopsisthaliana_AT3G12977.1 Exp number of AAs in TMHs: 0.00034
# Arabidopsisthaliana_AT3G12977.1 Exp number, first 60 AAs:  0
```

```
# Arabidopsisthaliana_AT3G12977.1 Total prob of N-in:        0.07803
Arabidopsisthaliana_AT3G12977.1 TMHMM2.0        outside      1   279
```

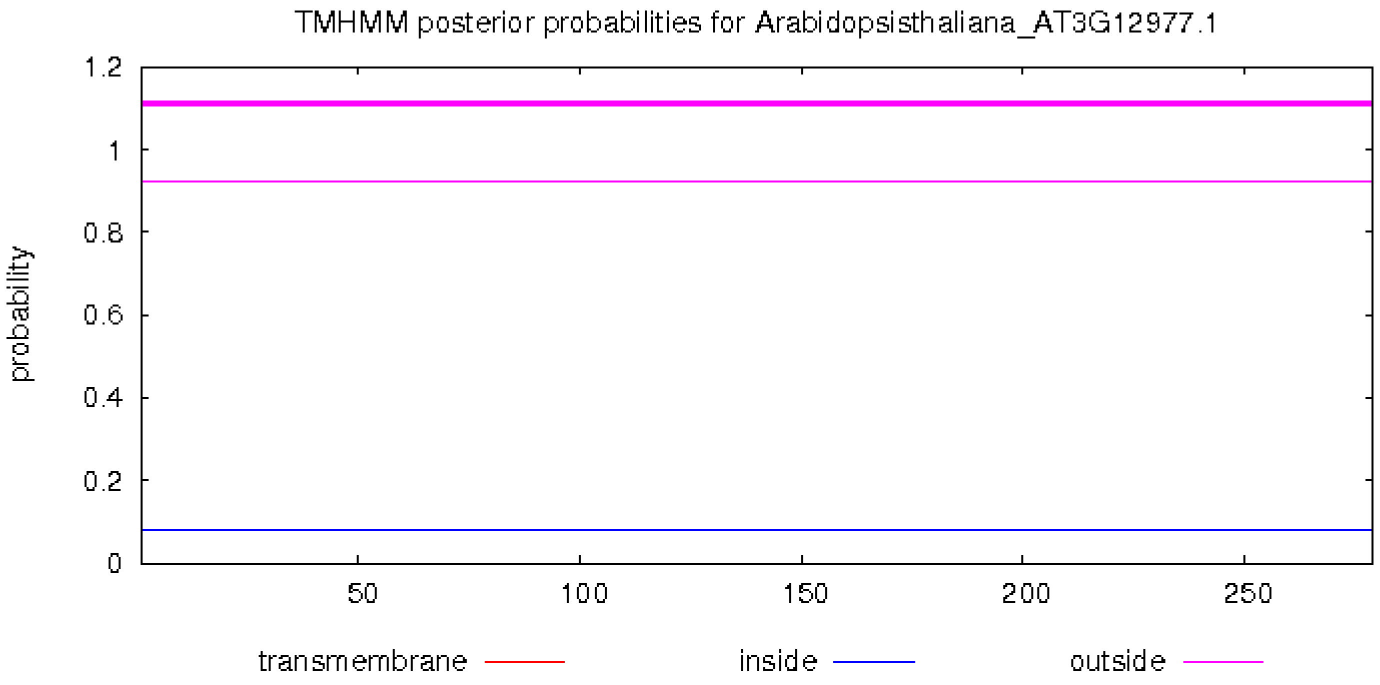

plot in postscript, script for making the plot in gnuplot, data for plot

---

```
# Arabidopsisthaliana_AT3G15170.1 Length: 310
# Arabidopsisthaliana_AT3G15170.1 Number of predicted TMHs:  0
# Arabidopsisthaliana_AT3G15170.1 Exp number of AAs in TMHs: 0.0419
# Arabidopsisthaliana_AT3G15170.1 Exp number, first 60 AAs:  0.0002
# Arabidopsisthaliana_AT3G15170.1 Total prob of N-in:        0.01089
Arabidopsisthaliana_AT3G15170.1 TMHMM2.0        outside      1   310
```

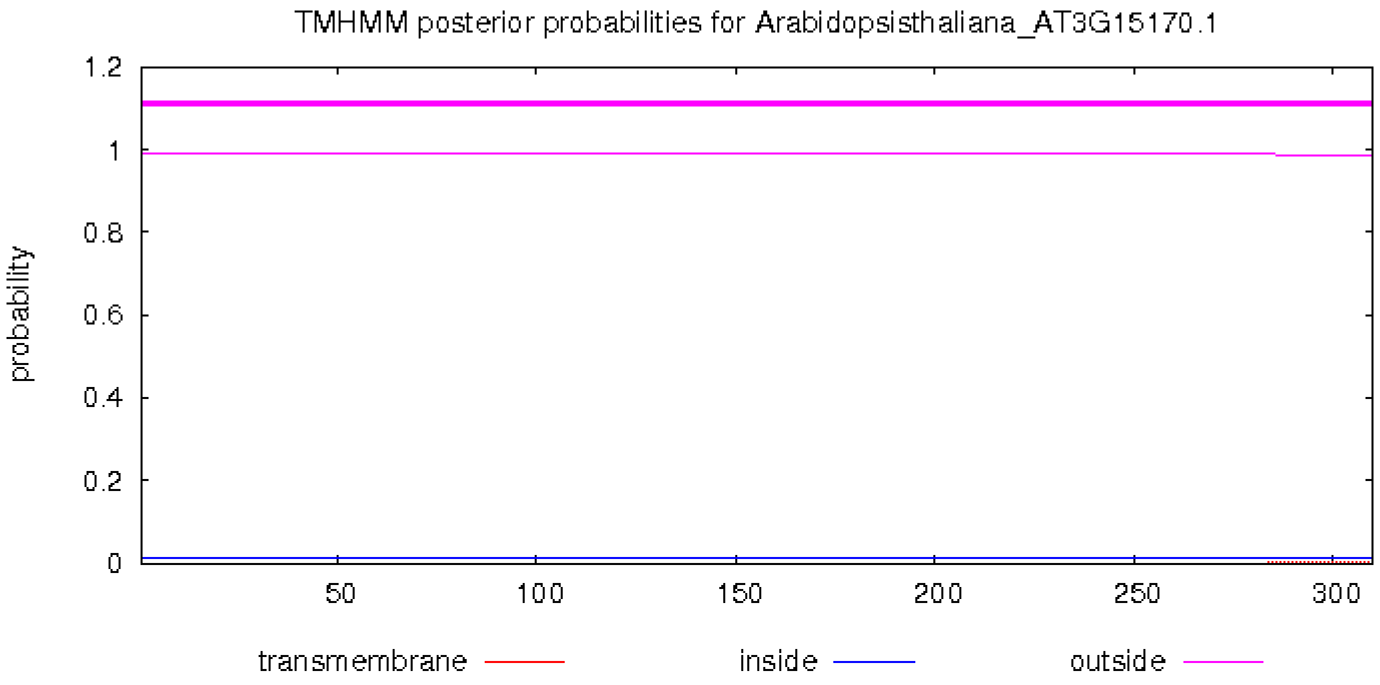

plot in postscript, script for making the plot in gnuplot, data for plot

---

```
# Arabidopsisthaliana_AT3G15500.1 Length: 317
# Arabidopsisthaliana_AT3G15500.1 Number of predicted TMHs:  0
# Arabidopsisthaliana_AT3G15500.1 Exp number of AAs in TMHs: 0
# Arabidopsisthaliana_AT3G15500.1 Exp number, first 60 AAs:  0
```

```
# Arabidopsisthaliana_AT3G15500.1 Total prob of N-in:        0.00239
Arabidopsisthaliana_AT3G15500.1 TMHMM2.0        outside      1   317
```

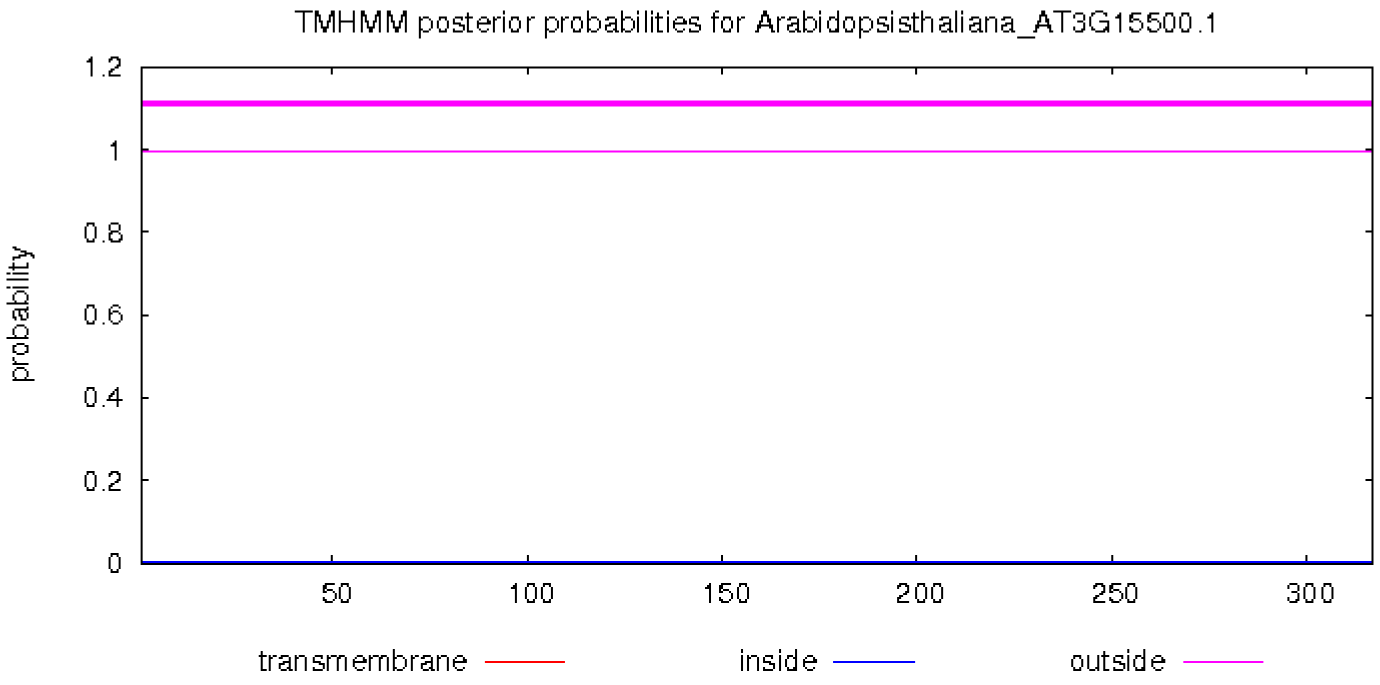

plot in postscript, script for making the plot in gnuplot, data for plot

---

```
# Arabidopsisthaliana_AT3G15510.1 Length: 364
# Arabidopsisthaliana_AT3G15510.1 Number of predicted TMHs:  0
# Arabidopsisthaliana_AT3G15510.1 Exp number of AAs in TMHs: 0.00161
# Arabidopsisthaliana_AT3G15510.1 Exp number, first 60 AAs:  0.00017
# Arabidopsisthaliana_AT3G15510.1 Total prob of N-in:        0.00412
Arabidopsisthaliana_AT3G15510.1 TMHMM2.0        outside      1   364
```

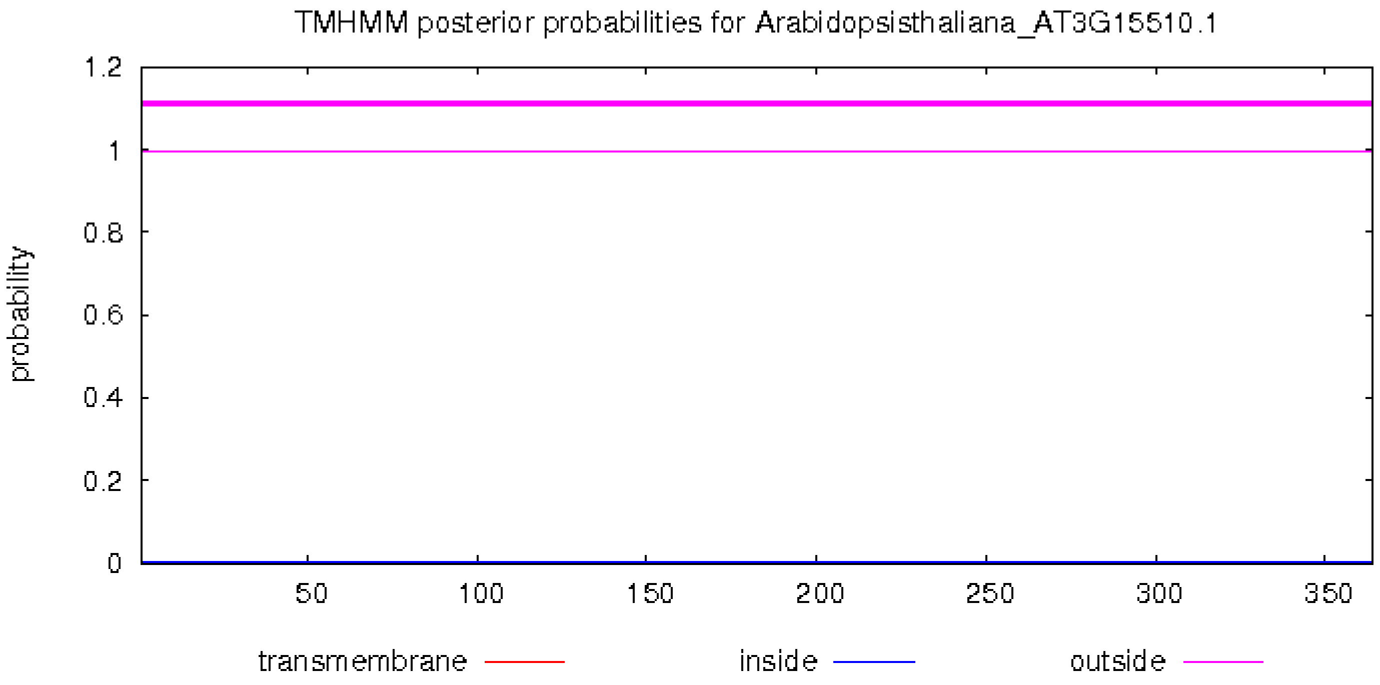

plot in postscript, script for making the plot in gnuplot, data for plot

---

```
# Arabidopsisthaliana_AT3G17730.1 Length: 246
# Arabidopsisthaliana_AT3G17730.1 Number of predicted TMHs:  0
# Arabidopsisthaliana_AT3G17730.1 Exp number of AAs in TMHs: 0
# Arabidopsisthaliana_AT3G17730.1 Exp number, first 60 AAs:  0
```

```
# Arabidopsisthaliana_AT3G17730.1 Total prob of N-in:        0.00222
Arabidopsisthaliana_AT3G17730.1 TMHMM2.0        outside      1   246
```

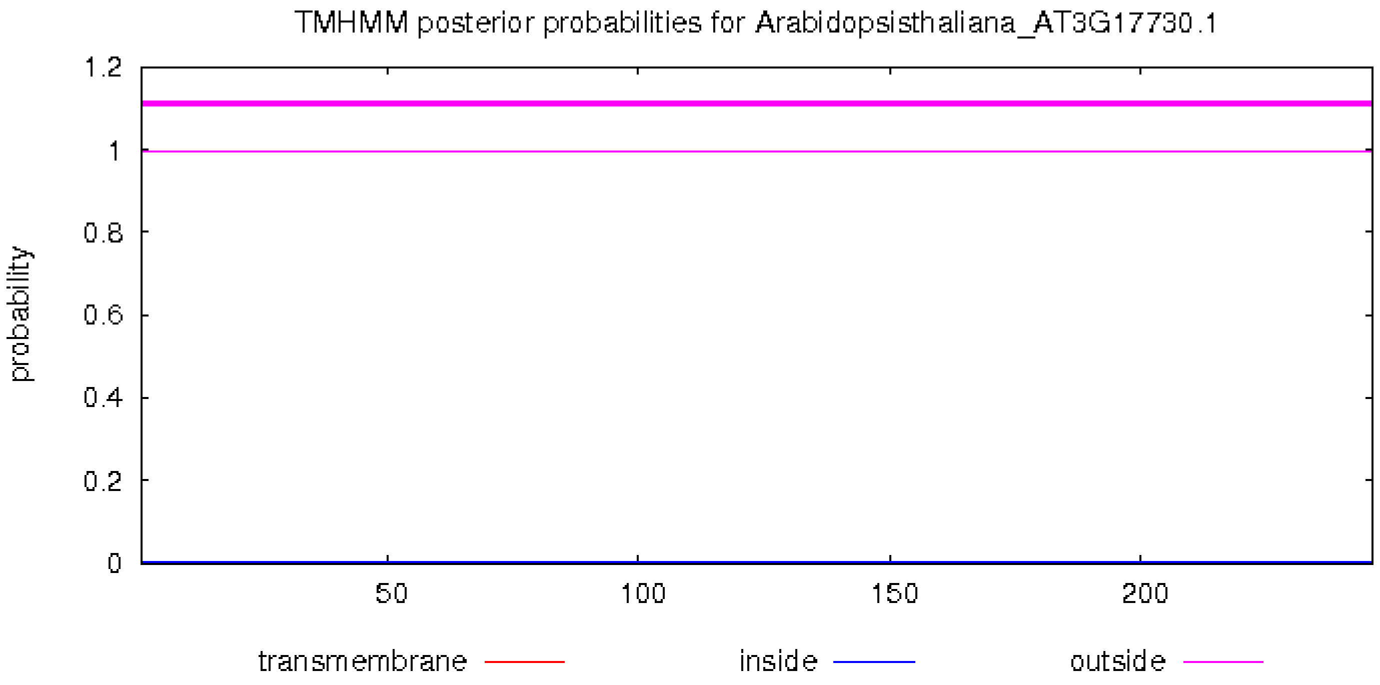

[plot](#) in postscript, [script](#) for making the plot in gnuplot, [data](#) for plot

---

```
# Arabidopsisthaliana_AT3G18400.1 Length: 314
# Arabidopsisthaliana_AT3G18400.1 Number of predicted TMHs:  0
# Arabidopsisthaliana_AT3G18400.1 Exp number of AAs in TMHs: 0.06056
# Arabidopsisthaliana_AT3G18400.1 Exp number, first 60 AAs:  0
# Arabidopsisthaliana_AT3G18400.1 Total prob of N-in:        0.00404
Arabidopsisthaliana_AT3G18400.1 TMHMM2.0        outside      1   314
```

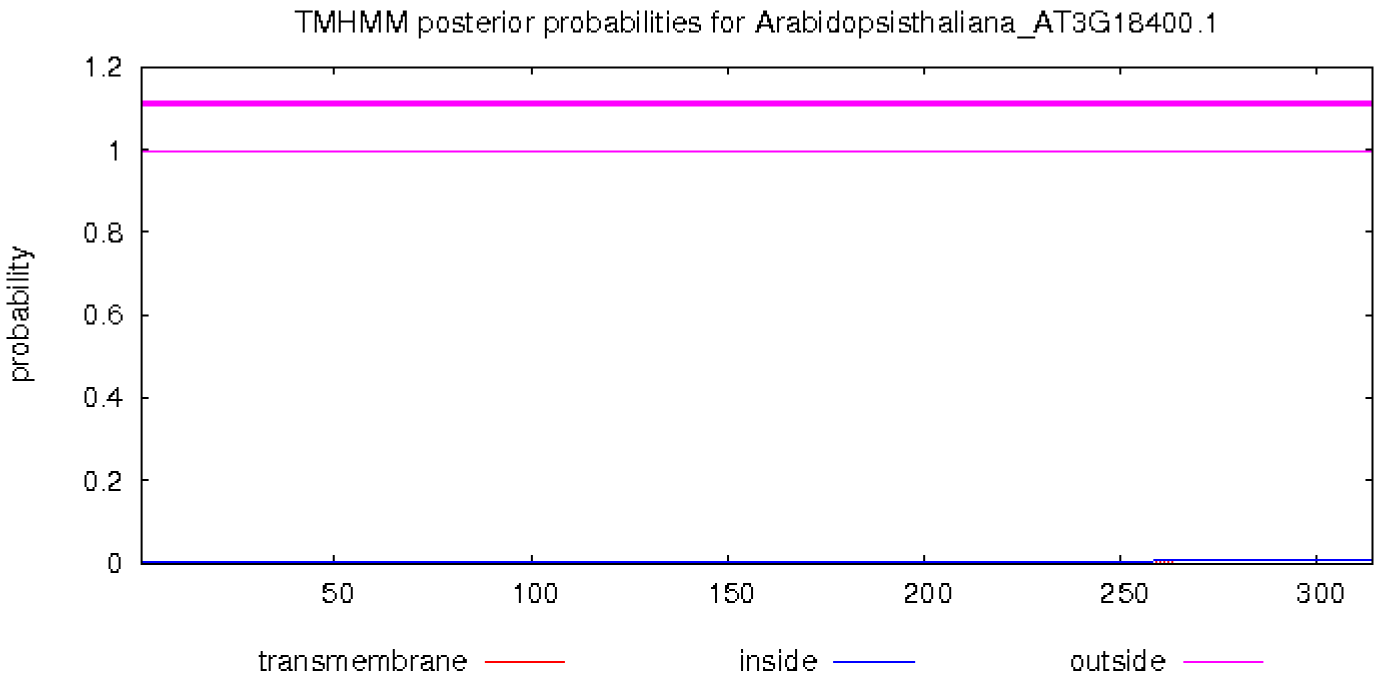

[plot](#) in postscript, [script](#) for making the plot in gnuplot, [data](#) for plot

---

```
# Arabidopsisthaliana_AT3G29035.1 Length: 318
# Arabidopsisthaliana_AT3G29035.1 Number of predicted TMHs:  0
# Arabidopsisthaliana_AT3G29035.1 Exp number of AAs in TMHs: 0.00906
# Arabidopsisthaliana_AT3G29035.1 Exp number, first 60 AAs:  0.00104
```

```
# Arabidopsisthaliana_AT3G29035.1 Total prob of N-in:        0.04482
Arabidopsisthaliana_AT3G29035.1 TMHMM2.0        outside      1   318
```

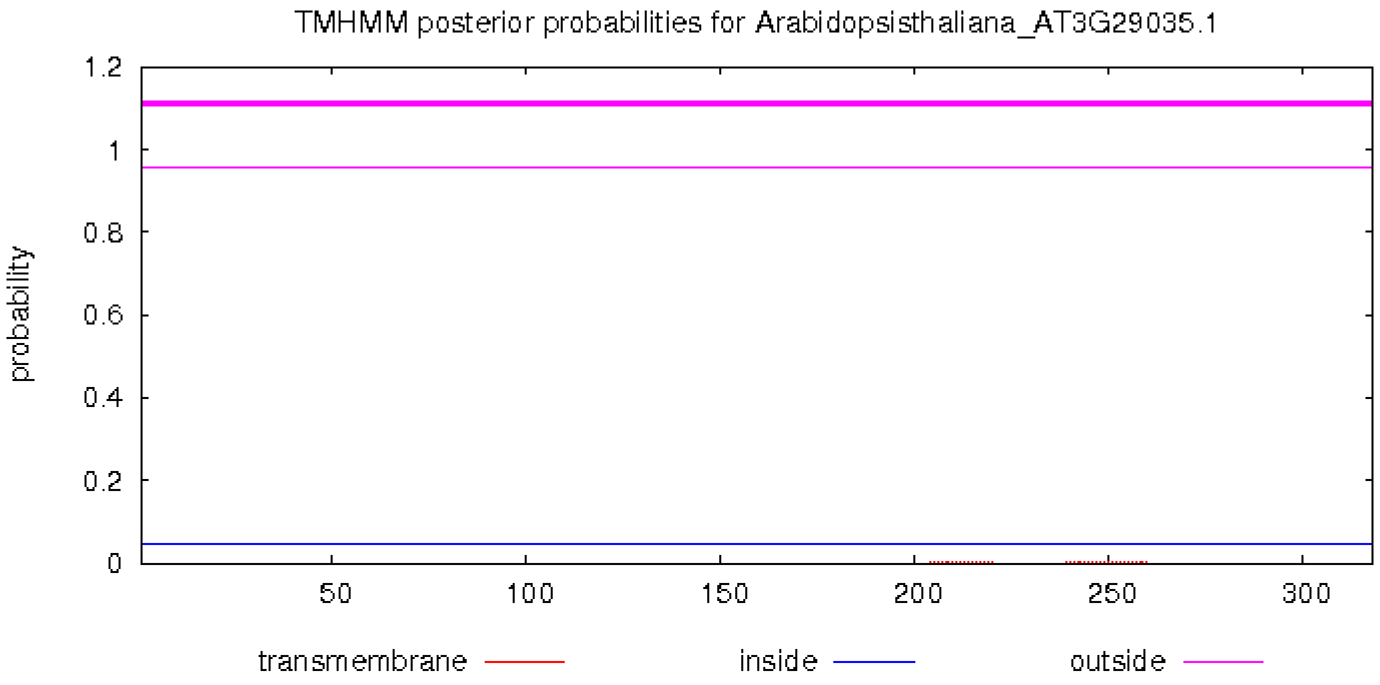

plot in postscript, script for making the plot in gnuplot, data for plot

---

```
# Arabidopsisthaliana_AT3G44290.1 Length: 335
# Arabidopsisthaliana_AT3G44290.1 Number of predicted TMHs:  1
# Arabidopsisthaliana_AT3G44290.1 Exp number of AAs in TMHs: 18.6581
# Arabidopsisthaliana_AT3G44290.1 Exp number, first 60 AAs:  0.28355
# Arabidopsisthaliana_AT3G44290.1 Total prob of N-in:        0.06993
Arabidopsisthaliana_AT3G44290.1 TMHMM2.0        outside      1   316
Arabidopsisthaliana_AT3G44290.1 TMHMM2.0        TMhelix    317   334
Arabidopsisthaliana_AT3G44290.1 TMHMM2.0        inside     335   335
```

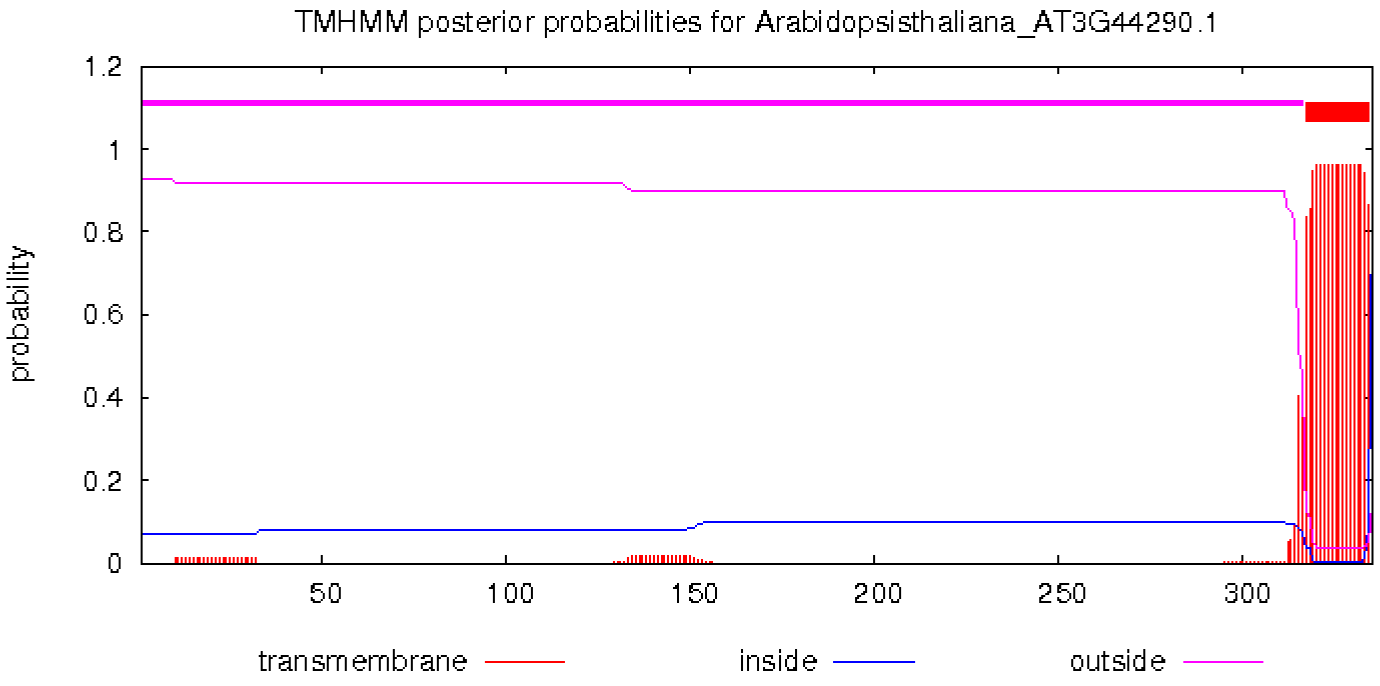

plot in postscript, script for making the plot in gnuplot, data for plot

---

```
# Arabidopsisthaliana_AT3G44350.1 Length: 228
# Arabidopsisthaliana_AT3G44350.1 Number of predicted TMHs:  0
```

```
# Arabidopsisthaliana_AT3G44350.1 Exp number of AAs in TMHs: 0.025
# Arabidopsisthaliana_AT3G44350.1 Exp number, first 60 AAs:  0.025
# Arabidopsisthaliana_AT3G44350.1 Total prob of N-in:        0.01579
Arabidopsisthaliana_AT3G44350.1 TMHMM2.0        outside      1   228
```

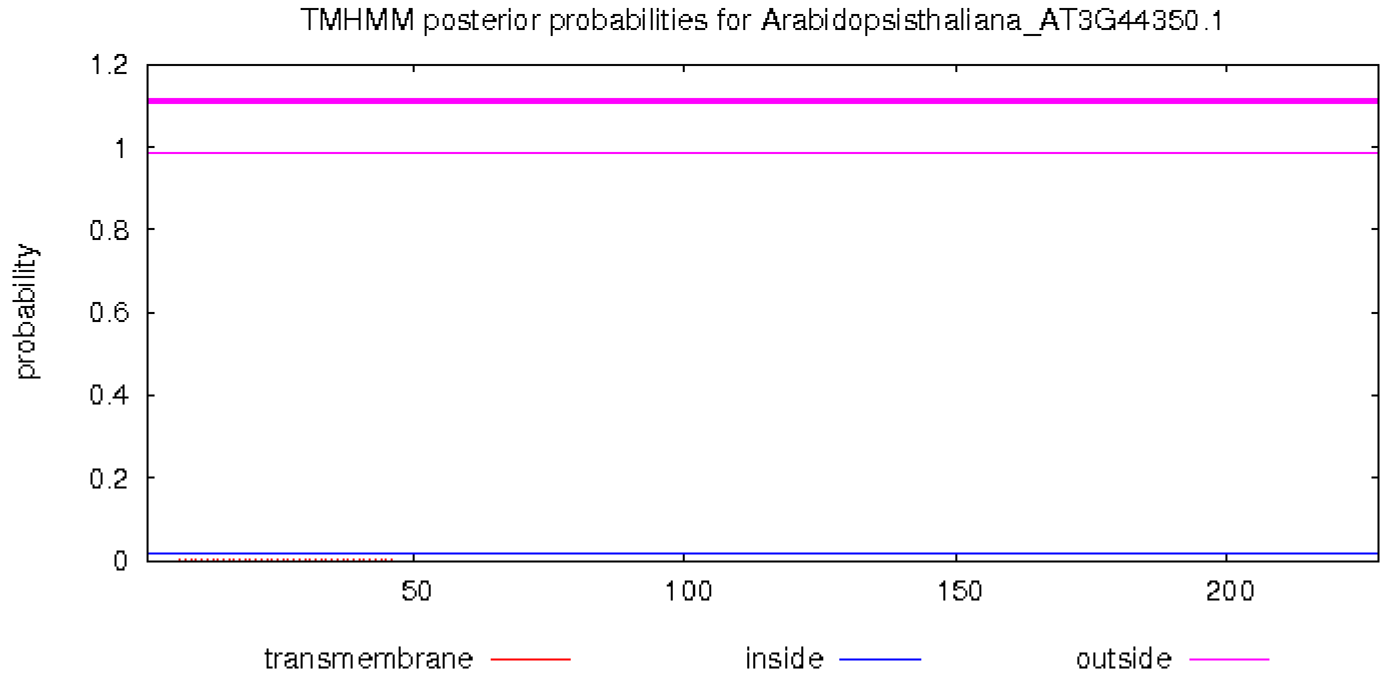

plot in postscript, script for making the plot in gnuplot, data for plot

---

```
# Arabidopsisthaliana_AT3G49530.1 Length: 469
# Arabidopsisthaliana_AT3G49530.1 Number of predicted TMHs:  0
# Arabidopsisthaliana_AT3G49530.1 Exp number of AAs in TMHs: 16.10925
# Arabidopsisthaliana_AT3G49530.1 Exp number, first 60 AAs:  0.00018
# Arabidopsisthaliana_AT3G49530.1 Total prob of N-in:        0.10453
Arabidopsisthaliana_AT3G49530.1 TMHMM2.0        outside      1   469
```

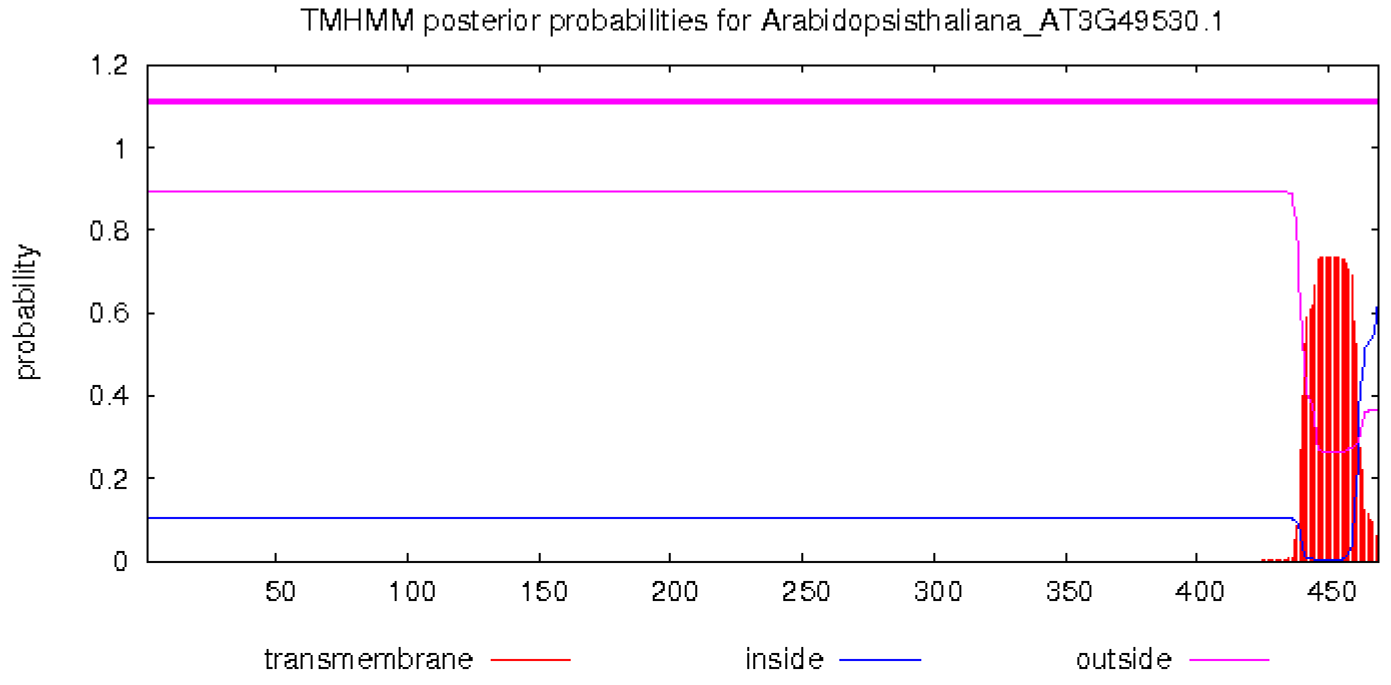

plot in postscript, script for making the plot in gnuplot, data for plot

---

```
# Arabidopsisthaliana_AT3G55210.1 Length: 280
# Arabidopsisthaliana_AT3G55210.1 Number of predicted TMHs:  0
```

```
# Arabidopsisthaliana_AT3G55210.1 Exp number of AAs in TMHs: 0.00057
# Arabidopsisthaliana_AT3G55210.1 Exp number, first 60 AAs:  0.00017
# Arabidopsisthaliana_AT3G55210.1 Total prob of N-in:        0.01719
Arabidopsisthaliana_AT3G55210.1 TMHMM2.0        outside      1   280
```

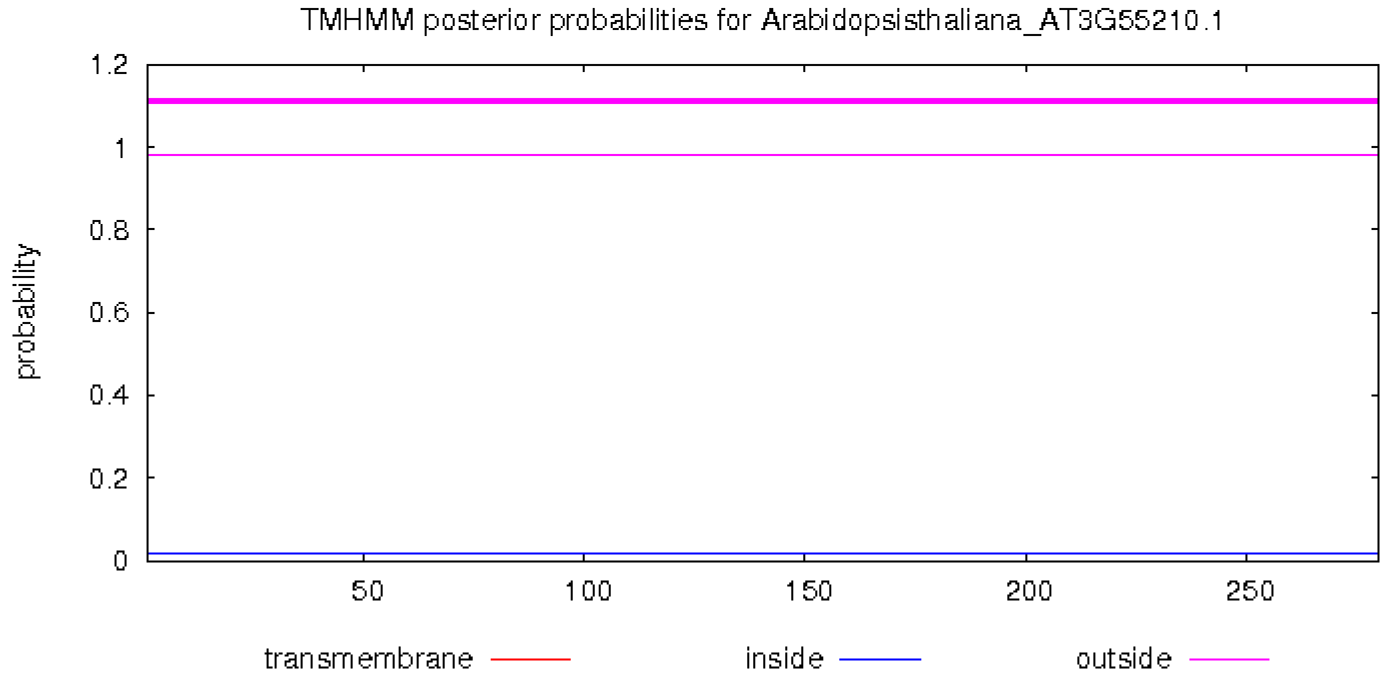

plot in postscript, script for making the plot in gnuplot, data for plot

```
# Arabidopsisthaliana_AT3G56520.1 Length: 175
# Arabidopsisthaliana_AT3G56520.1 Number of predicted TMHs:  0
# Arabidopsisthaliana_AT3G56520.1 Exp number of AAs in TMHs: 0.00095
# Arabidopsisthaliana_AT3G56520.1 Exp number, first 60 AAs:  0.0002
# Arabidopsisthaliana_AT3G56520.1 Total prob of N-in:        0.05747
Arabidopsisthaliana_AT3G56520.1 TMHMM2.0        outside      1   175
```

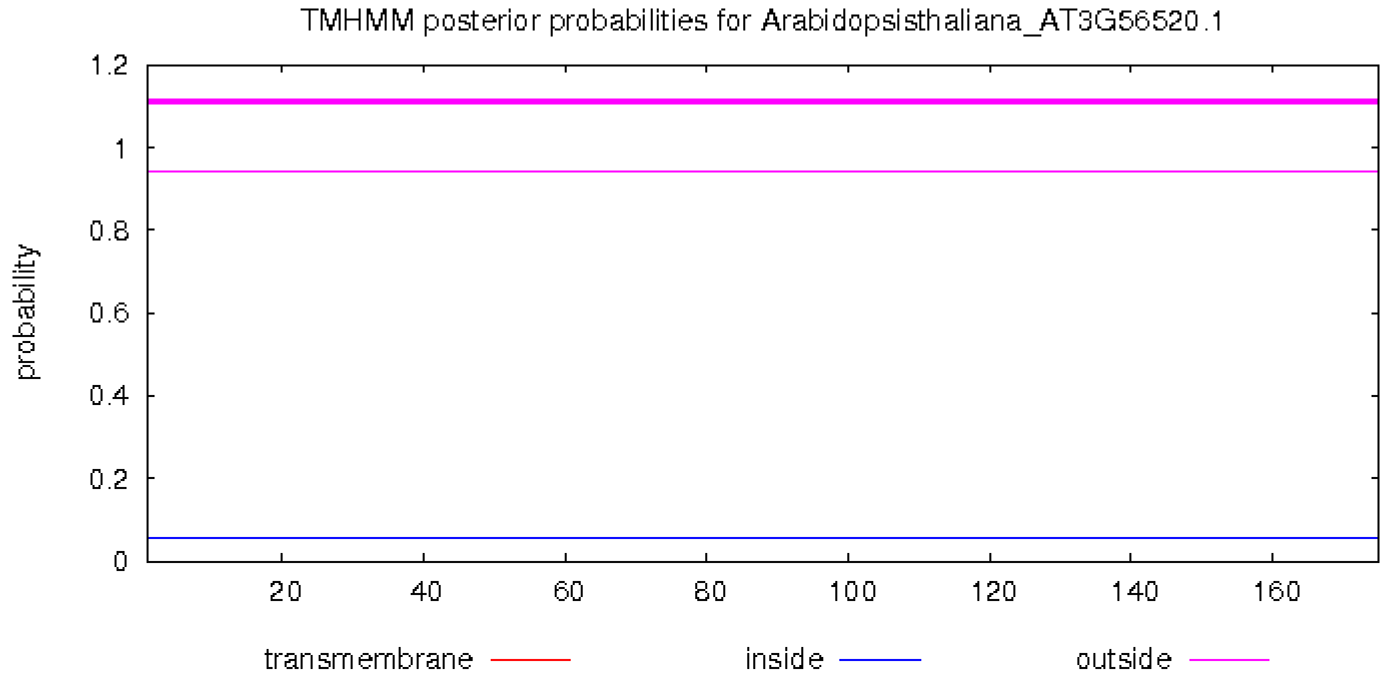

plot in postscript, script for making the plot in gnuplot, data for plot

```
# Arabidopsisthaliana_AT3G56530.1 Length: 319
# Arabidopsisthaliana_AT3G56530.1 Number of predicted TMHs:  0
```

```
# Arabidopsisthaliana_AT3G56530.1 Exp number of AAs in TMHs: 0.02344
# Arabidopsisthaliana_AT3G56530.1 Exp number, first 60 AAs:  0.00033
# Arabidopsisthaliana_AT3G56530.1 Total prob of N-in:        0.16760
Arabidopsisthaliana_AT3G56530.1 TMHMM2.0        outside      1   319
```

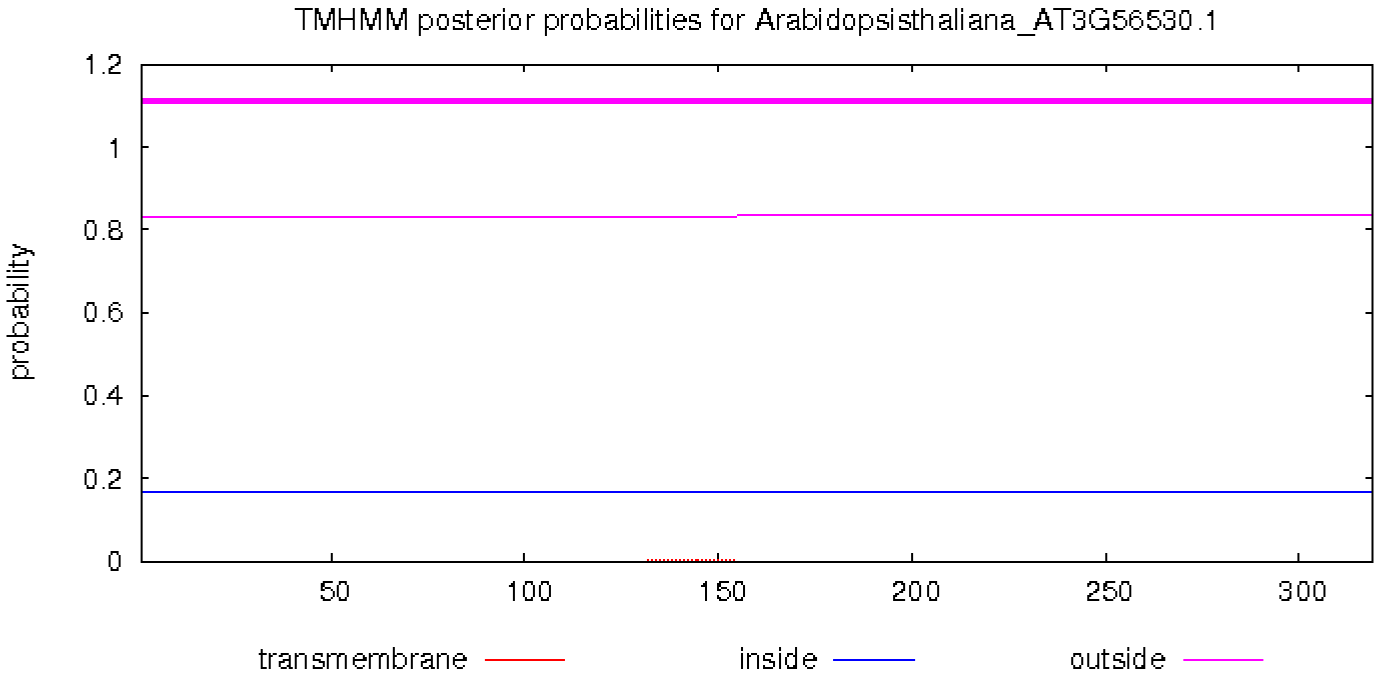

plot in postscript, script for making the plot in gnuplot, data for plot

---

```
# Arabidopsisthaliana_AT3G56560.1 Length: 228
# Arabidopsisthaliana_AT3G56560.1 Number of predicted TMHs:  0
# Arabidopsisthaliana_AT3G56560.1 Exp number of AAs in TMHs: 0.00037
# Arabidopsisthaliana_AT3G56560.1 Exp number, first 60 AAs:  0.00011
# Arabidopsisthaliana_AT3G56560.1 Total prob of N-in:        0.01718
Arabidopsisthaliana_AT3G56560.1 TMHMM2.0        outside      1   228
```

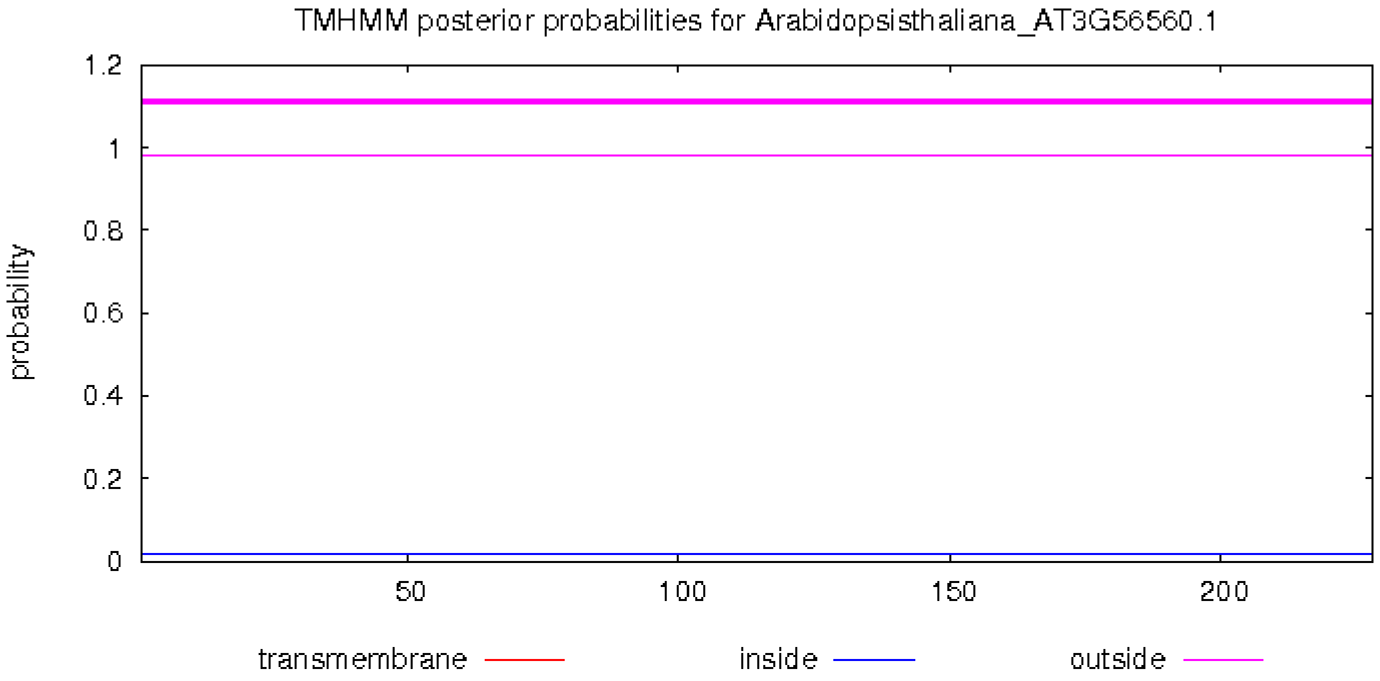

plot in postscript, script for making the plot in gnuplot, data for plot

---

```
# Arabidopsisthaliana_AT3G61910.1 Length: 334
# Arabidopsisthaliana_AT3G61910.1 Number of predicted TMHs:  0
```

```
# Arabidopsisthaliana_AT3G61910.1 Exp number of AAs in TMHs: 0
# Arabidopsisthaliana_AT3G61910.1 Exp number, first 60 AAs:  0
# Arabidopsisthaliana_AT3G61910.1 Total prob of N-in:        0.02755
Arabidopsisthaliana_AT3G61910.1 TMHMM2.0        outside      1   334
```

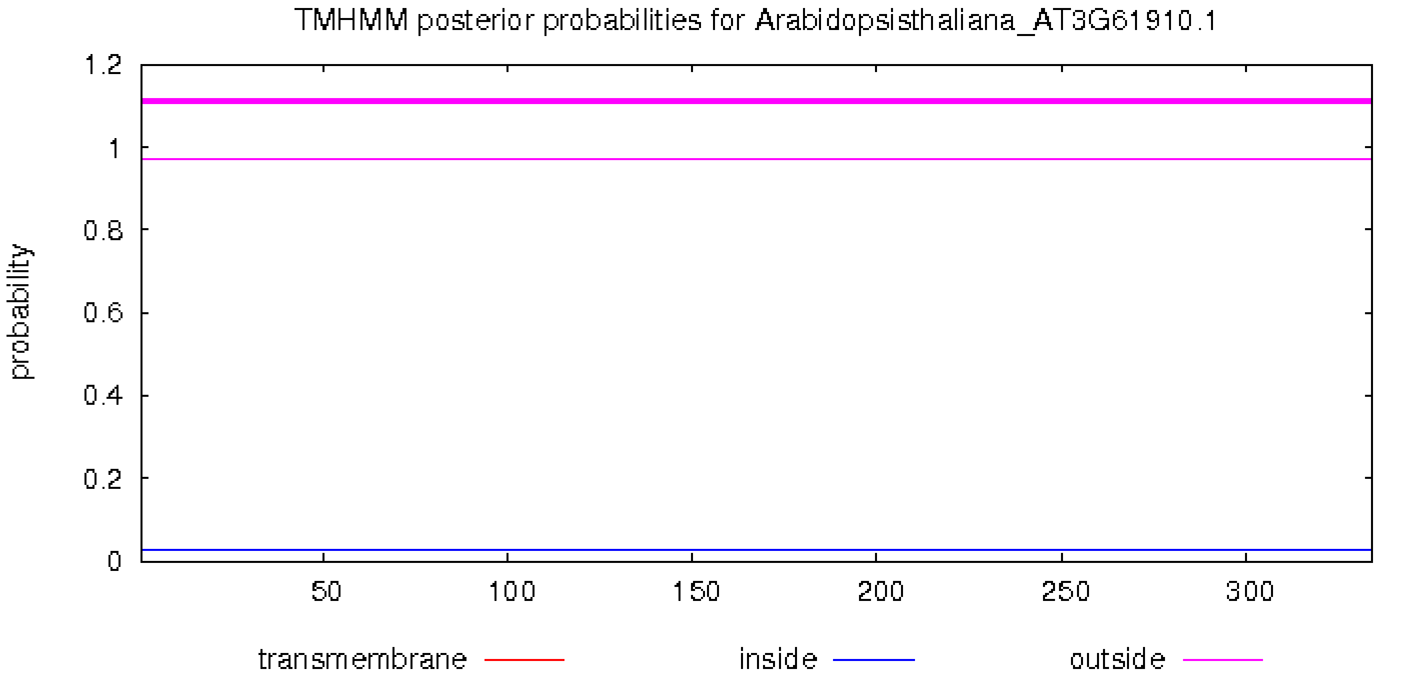

plot in postscript, script for making the plot in gnuplot, data for plot

---

```
# Arabidopsisthaliana_AT4G01520.1 Length: 302
# Arabidopsisthaliana_AT4G01520.1 Number of predicted TMHs:  0
# Arabidopsisthaliana_AT4G01520.1 Exp number of AAs in TMHs: 0.00037
# Arabidopsisthaliana_AT4G01520.1 Exp number, first 60 AAs:  0
# Arabidopsisthaliana_AT4G01520.1 Total prob of N-in:        0.03258
Arabidopsisthaliana_AT4G01520.1 TMHMM2.0        outside      1   302
```

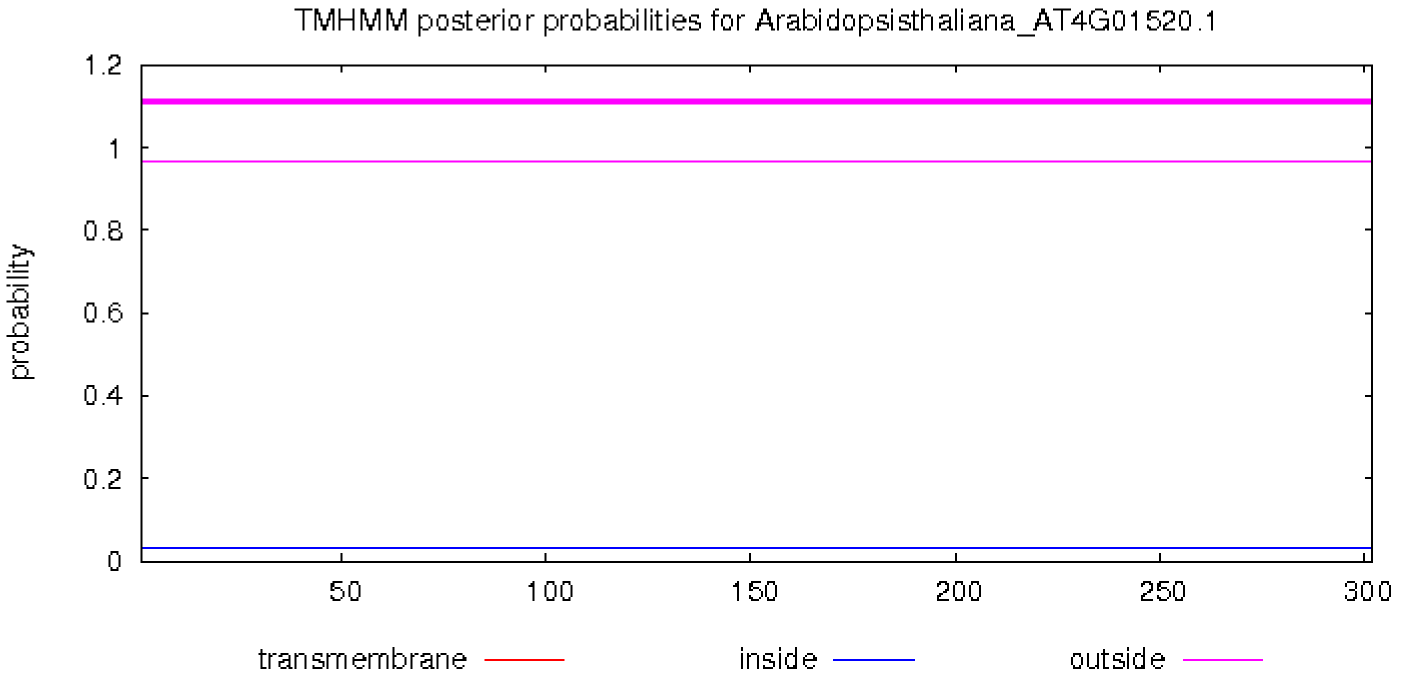

plot in postscript, script for making the plot in gnuplot, data for plot

---

```
# Arabidopsisthaliana_AT4G01540.1 Length: 473
# Arabidopsisthaliana_AT4G01540.1 Number of predicted TMHs:  1
```

```
# Arabidopsisthaliana_AT4G01540.1 Exp number of AAs in TMHs: 17.25648
# Arabidopsisthaliana_AT4G01540.1 Exp number, first 60 AAs:  0.00018
# Arabidopsisthaliana_AT4G01540.1 Total prob of N-in:        0.06422
Arabidopsisthaliana_AT4G01540.1 TMHMM2.0        outside      1   445
Arabidopsisthaliana_AT4G01540.1 TMHMM2.0        TMhelix    446   468
Arabidopsisthaliana_AT4G01540.1 TMHMM2.0        inside     469   473
```

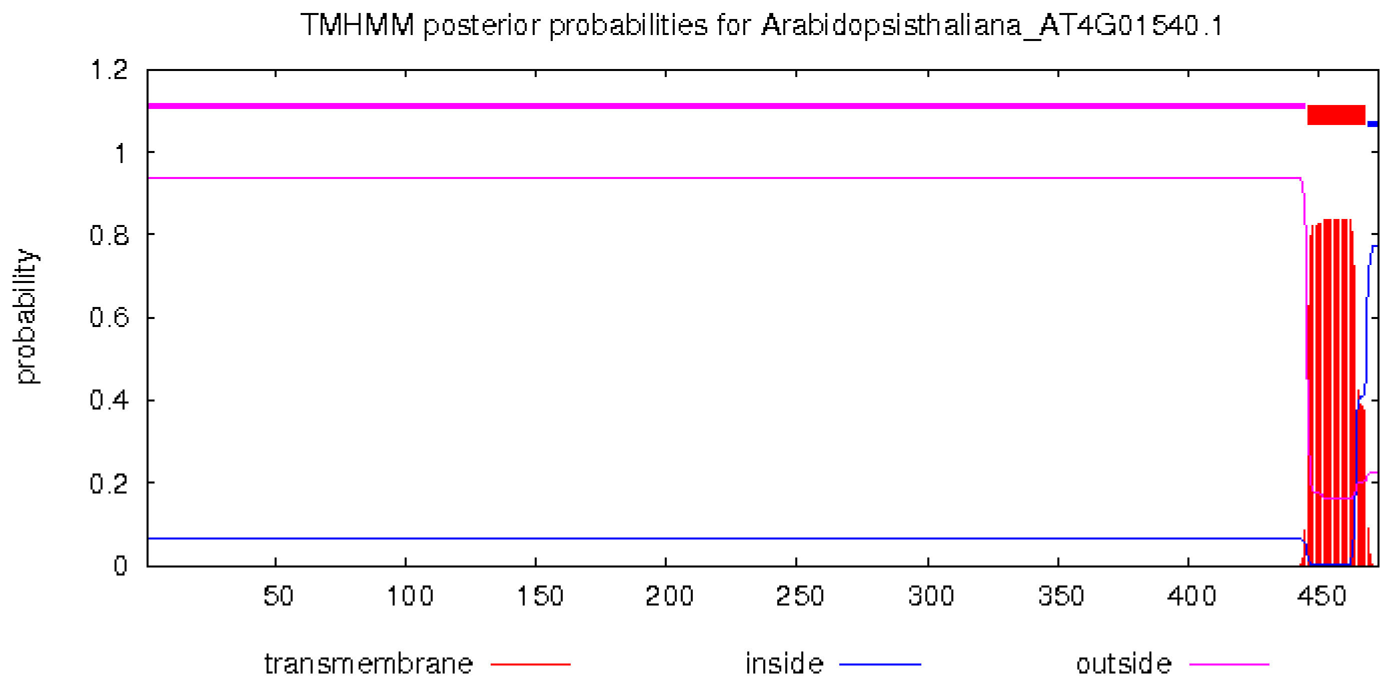

plot in postscript, script for making the plot in gnuplot, data for plot

---

```
# Arabidopsisthaliana_AT4G01550.1 Length: 457
# Arabidopsisthaliana_AT4G01550.1 Number of predicted TMHs:  1
# Arabidopsisthaliana_AT4G01550.1 Exp number of AAs in TMHs: 22.65639
# Arabidopsisthaliana_AT4G01550.1 Exp number, first 60 AAs:  0.00059
# Arabidopsisthaliana_AT4G01550.1 Total prob of N-in:        0.17007
Arabidopsisthaliana_AT4G01550.1 TMHMM2.0        outside      1   420
Arabidopsisthaliana_AT4G01550.1 TMHMM2.0        TMhelix    421   443
Arabidopsisthaliana_AT4G01550.1 TMHMM2.0        inside     444   457
```

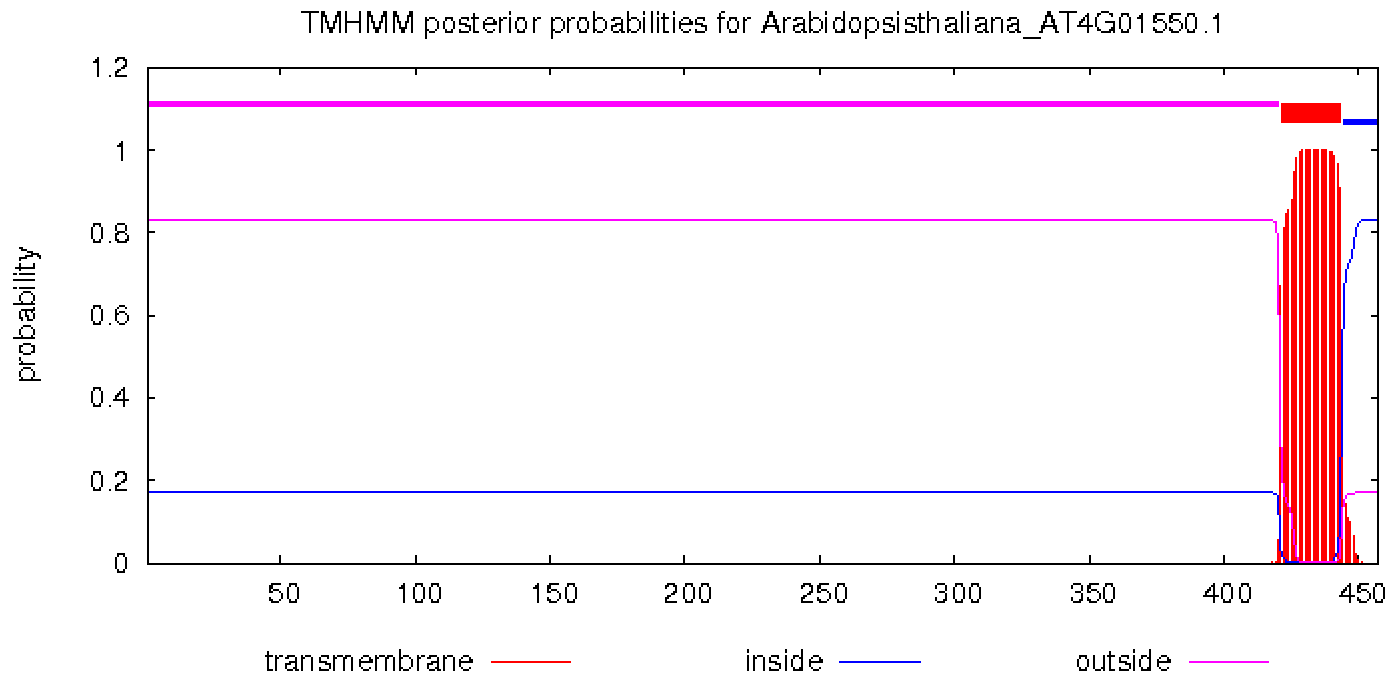

plot in postscript, script for making the plot in gnuplot, data for plot

```
# Arabidopsisthaliana_AT4G10350.1 Length: 341
# Arabidopsisthaliana_AT4G10350.1 Number of predicted TMHs:  0
# Arabidopsisthaliana_AT4G10350.1 Exp number of AAs in TMHs: 0
# Arabidopsisthaliana_AT4G10350.1 Exp number, first 60 AAs:  0
# Arabidopsisthaliana_AT4G10350.1 Total prob of N-in:        0.00436
Arabidopsisthaliana_AT4G10350.1 TMHMM2.0        outside      1   341
```

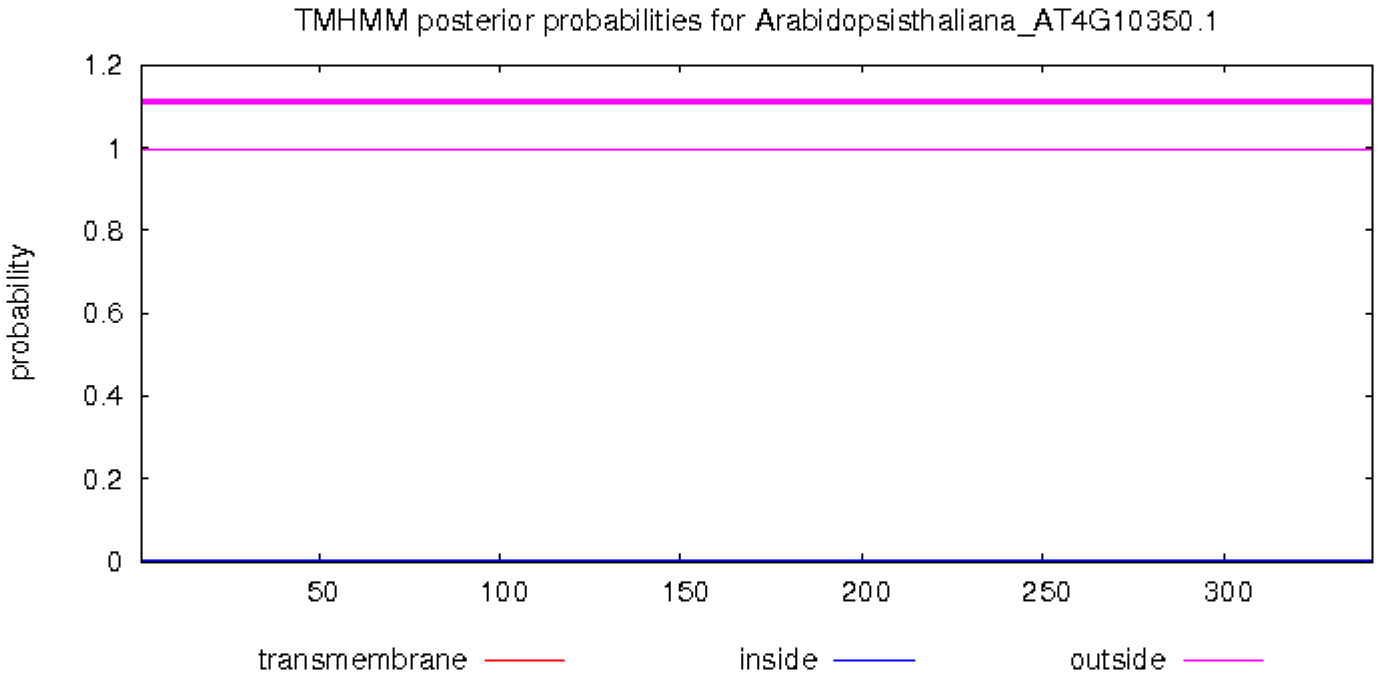

plot in postscript, script for making the plot in gnuplot, data for plot

```
# Arabidopsisthaliana_AT4G17980.1 Length: 262
# Arabidopsisthaliana_AT4G17980.1 Number of predicted TMHs:  0
# Arabidopsisthaliana_AT4G17980.1 Exp number of AAs in TMHs: 0
# Arabidopsisthaliana_AT4G17980.1 Exp number, first 60 AAs:  0
# Arabidopsisthaliana_AT4G17980.1 Total prob of N-in:        0.00344
Arabidopsisthaliana_AT4G17980.1 TMHMM2.0        outside      1   262
```

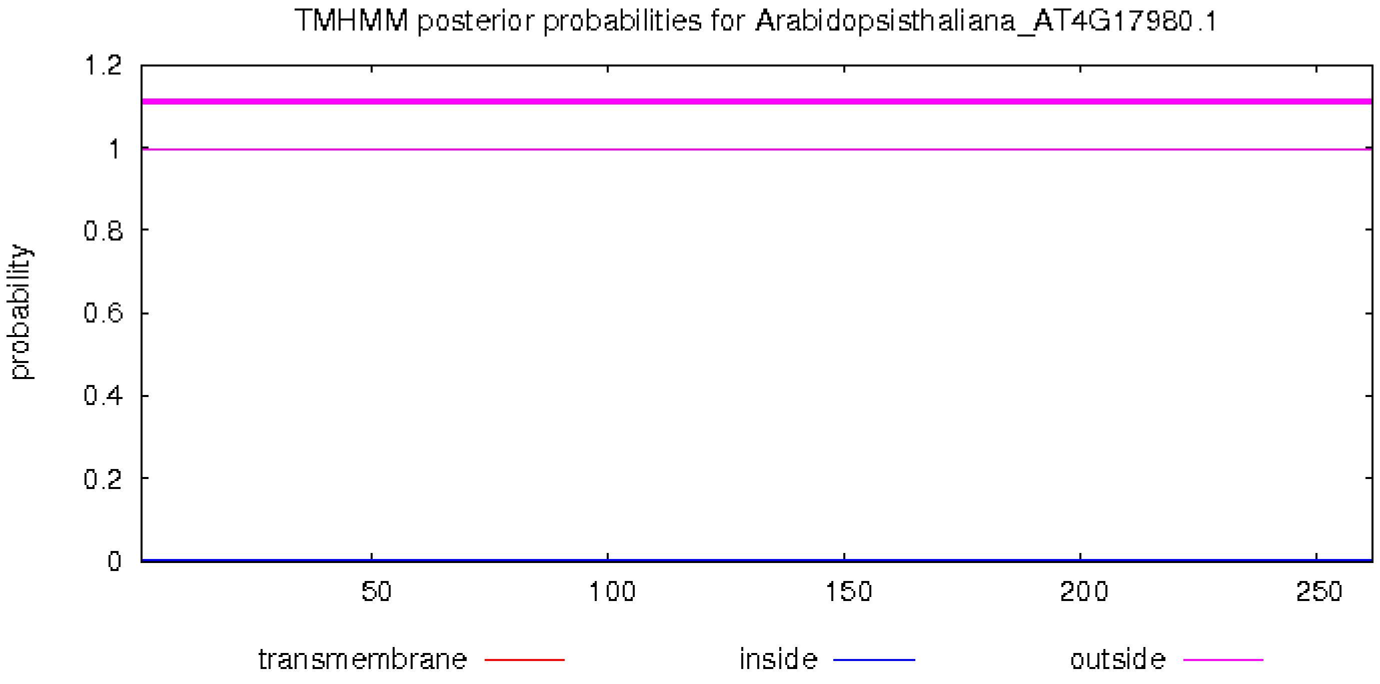

plot in postscript, script for making the plot in gnuplot, data for plot

```
# Arabidopsisthaliana_AT4G27410.2 Length: 297
# Arabidopsisthaliana_AT4G27410.2 Number of predicted TMHs:  0
# Arabidopsisthaliana_AT4G27410.2 Exp number of AAs in TMHs: 0.0025
# Arabidopsisthaliana_AT4G27410.2 Exp number, first 60 AAs:  0.00041
# Arabidopsisthaliana_AT4G27410.2 Total prob of N-in:        0.02200
Arabidopsisthaliana_AT4G27410.2 TMHMM2.0        outside      1   297
```

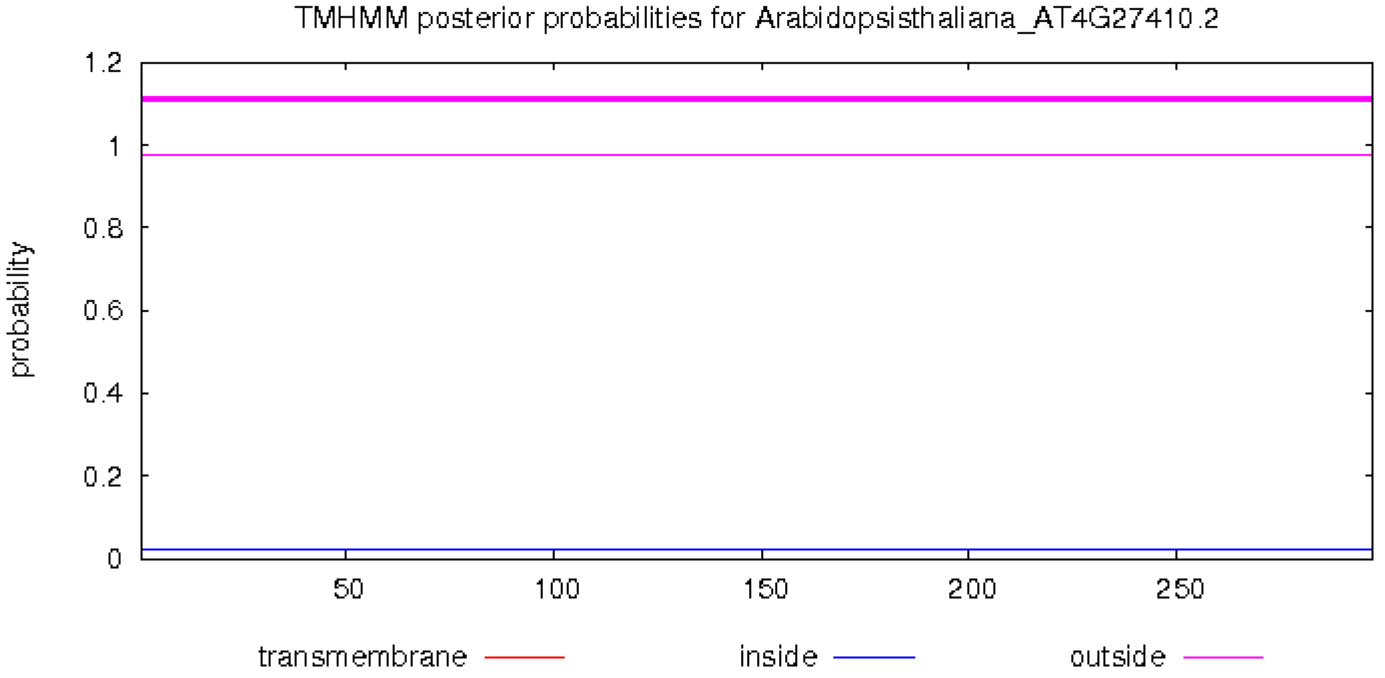

plot in postscript, script for making the plot in gnuplot, data for plot

---

```
# Arabidopsisthaliana_AT4G28500.1 Length: 305
# Arabidopsisthaliana_AT4G28500.1 Number of predicted TMHs:  0
# Arabidopsisthaliana_AT4G28500.1 Exp number of AAs in TMHs: 0.00158
# Arabidopsisthaliana_AT4G28500.1 Exp number, first 60 AAs:  0.0002
# Arabidopsisthaliana_AT4G28500.1 Total prob of N-in:        0.09868
Arabidopsisthaliana_AT4G28500.1 TMHMM2.0        outside      1   305
```

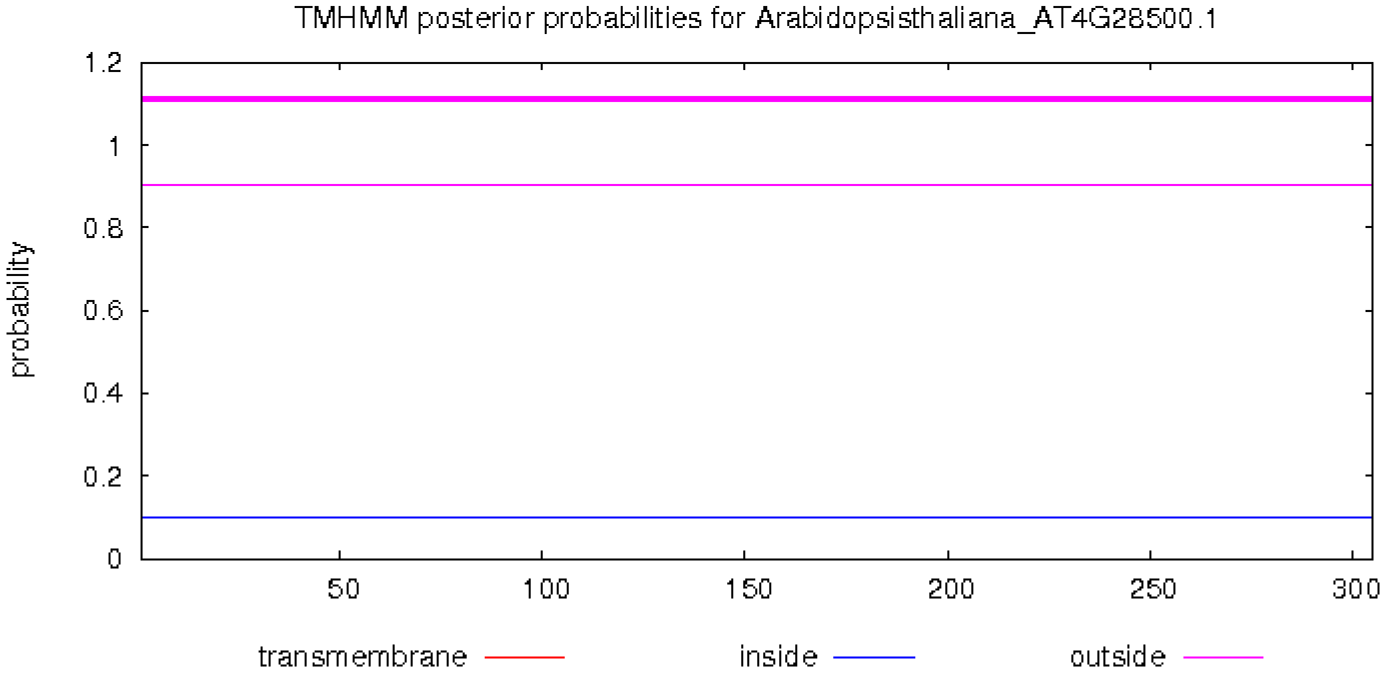

plot in postscript, script for making the plot in gnuplot, data for plot

---

```
# Arabidopsisthaliana_AT4G28530.1 Length: 352
# Arabidopsisthaliana_AT4G28530.1 Number of predicted TMHs:  0
# Arabidopsisthaliana_AT4G28530.1 Exp number of AAs in TMHs: 0
# Arabidopsisthaliana_AT4G28530.1 Exp number, first 60 AAs:  0
# Arabidopsisthaliana_AT4G28530.1 Total prob of N-in:        0.00590
Arabidopsisthaliana_AT4G28530.1 TMHMM2.0        outside      1   352
```

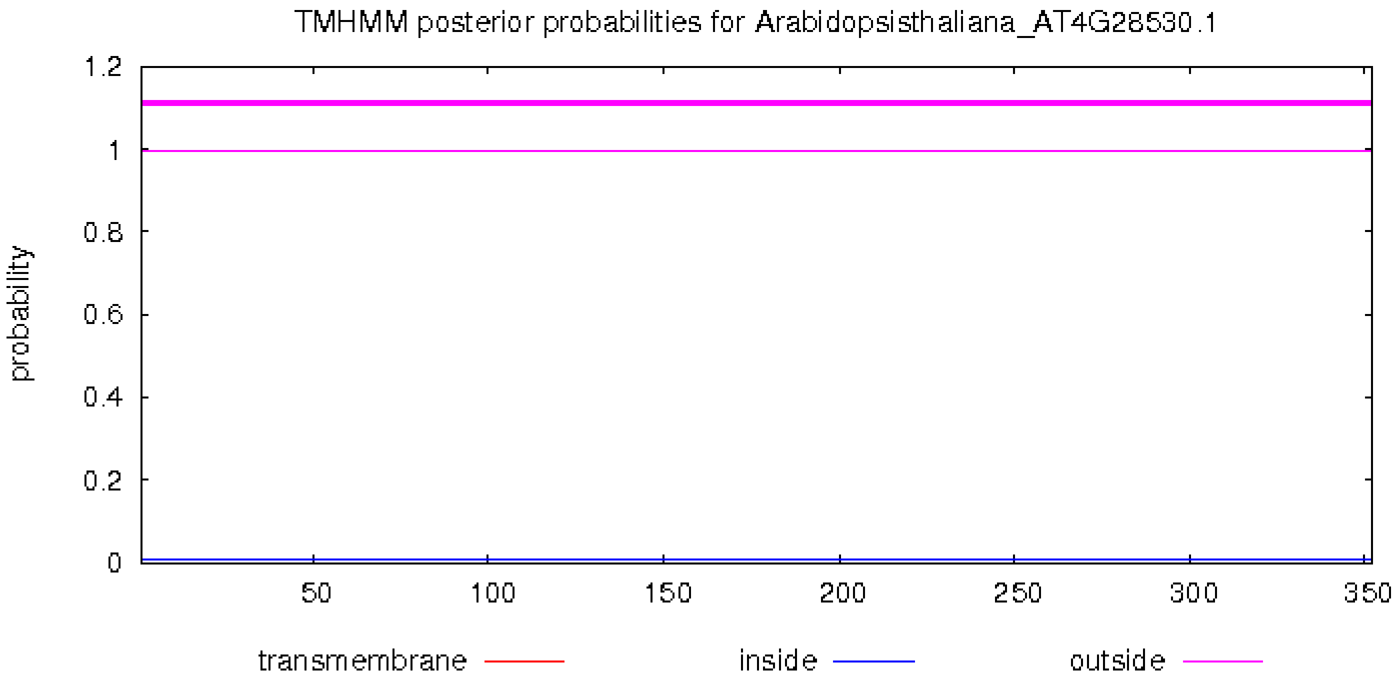

plot in postscript, script for making the plot in gnuplot, data for plot

---

```
# Arabidopsisthaliana_AT4G29230.1 Length: 498
# Arabidopsisthaliana_AT4G29230.1 Number of predicted TMHs:  0
# Arabidopsisthaliana_AT4G29230.1 Exp number of AAs in TMHs: 5.29036
# Arabidopsisthaliana_AT4G29230.1 Exp number, first 60 AAs:  0
# Arabidopsisthaliana_AT4G29230.1 Total prob of N-in:        0.19084
Arabidopsisthaliana_AT4G29230.1 TMHMM2.0        outside      1   498
```

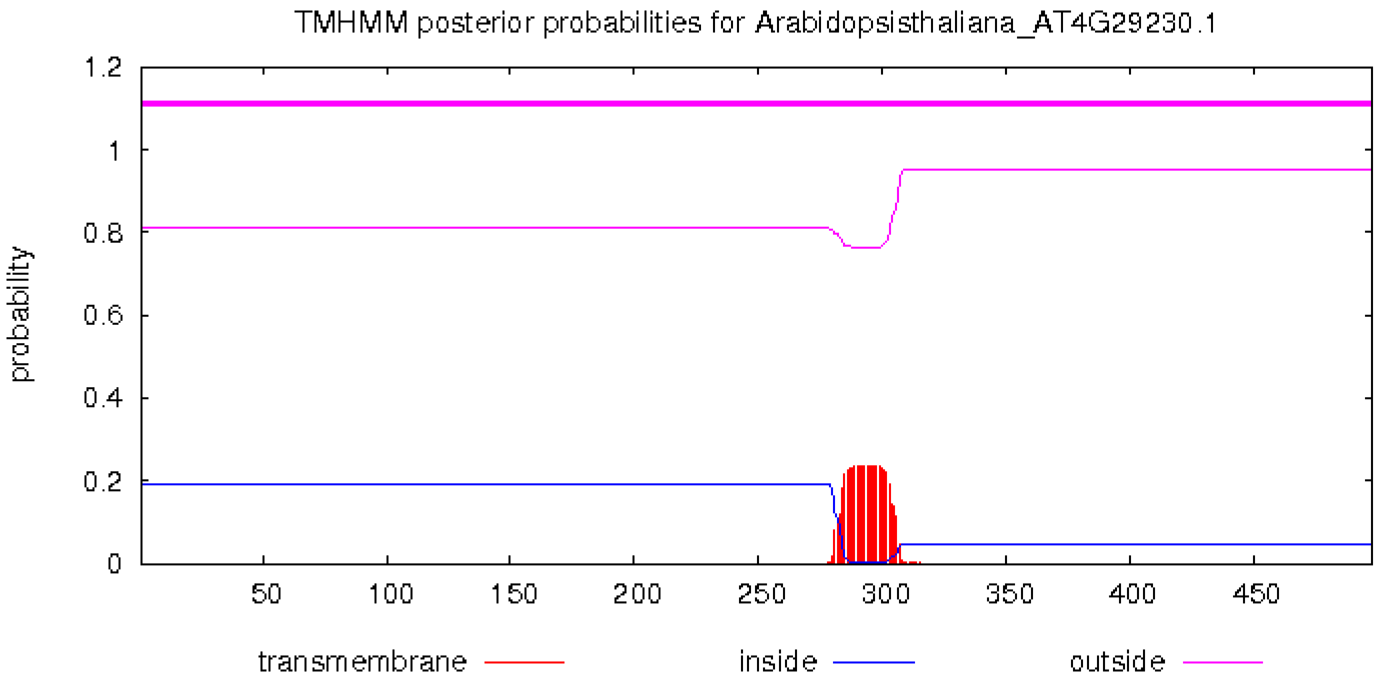

plot in postscript, script for making the plot in gnuplot, data for plot

---

```
# Arabidopsisthaliana_AT4G35580.1 Length: 512
# Arabidopsisthaliana_AT4G35580.1 Number of predicted TMHs:  1
# Arabidopsisthaliana_AT4G35580.1 Exp number of AAs in TMHs: 21.35486
# Arabidopsisthaliana_AT4G35580.1 Exp number, first 60 AAs:  0
# Arabidopsisthaliana_AT4G35580.1 Total prob of N-in:        0.14711
Arabidopsisthaliana_AT4G35580.1 TMHMM2.0        outside      1   488
Arabidopsisthaliana_AT4G35580.1 TMHMM2.0        TMhelix    489   511
Arabidopsisthaliana_AT4G35580.1 TMHMM2.0        inside     512   512
```

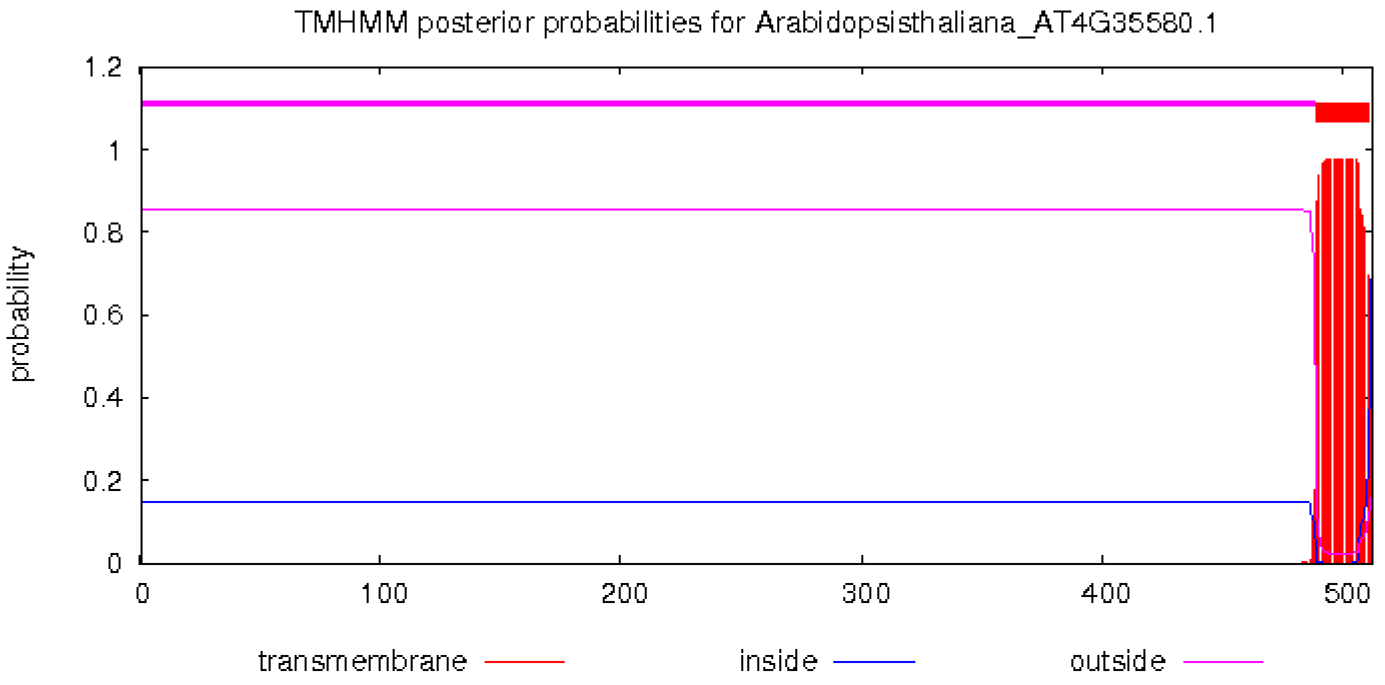

plot in postscript, script for making the plot in gnuplot, data for plot

---

```
# Arabidopsisthaliana_AT4G36160.1 Length: 377
# Arabidopsisthaliana_AT4G36160.1 Number of predicted TMHs:  0
# Arabidopsisthaliana_AT4G36160.1 Exp number of AAs in TMHs: 0
# Arabidopsisthaliana_AT4G36160.1 Exp number, first 60 AAs:  0
# Arabidopsisthaliana_AT4G36160.1 Total prob of N-in:        0.01252
Arabidopsisthaliana_AT4G36160.1 TMHMM2.0        outside      1   377
```

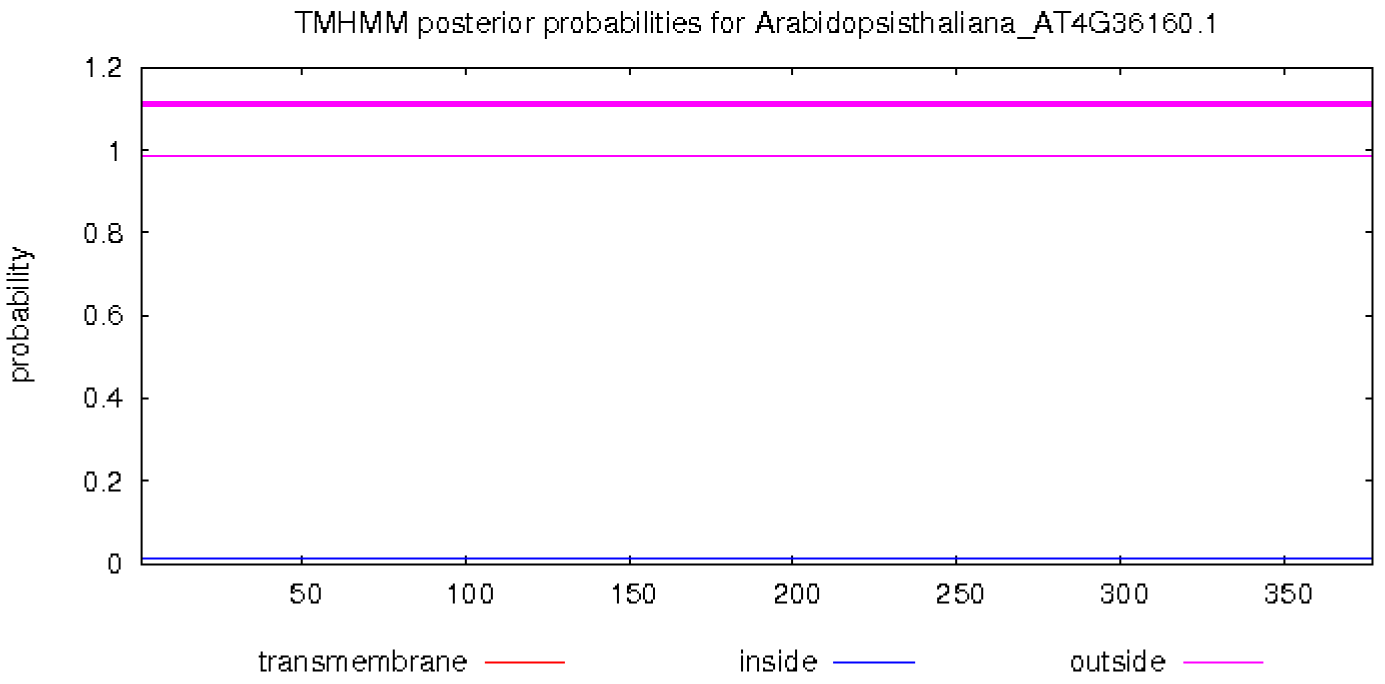

plot in postscript, script for making the plot in gnuplot, data for plot

```
# Arabidopsisthaliana_AT5G04400.1 Length: 395
# Arabidopsisthaliana_AT5G04400.1 Number of predicted TMHs:  0
# Arabidopsisthaliana_AT5G04400.1 Exp number of AAs in TMHs: 5.85993
# Arabidopsisthaliana_AT5G04400.1 Exp number, first 60 AAs:  0.04778
# Arabidopsisthaliana_AT5G04400.1 Total prob of N-in:        0.25349
Arabidopsisthaliana_AT5G04400.1 TMHMM2.0        outside      1   395
```

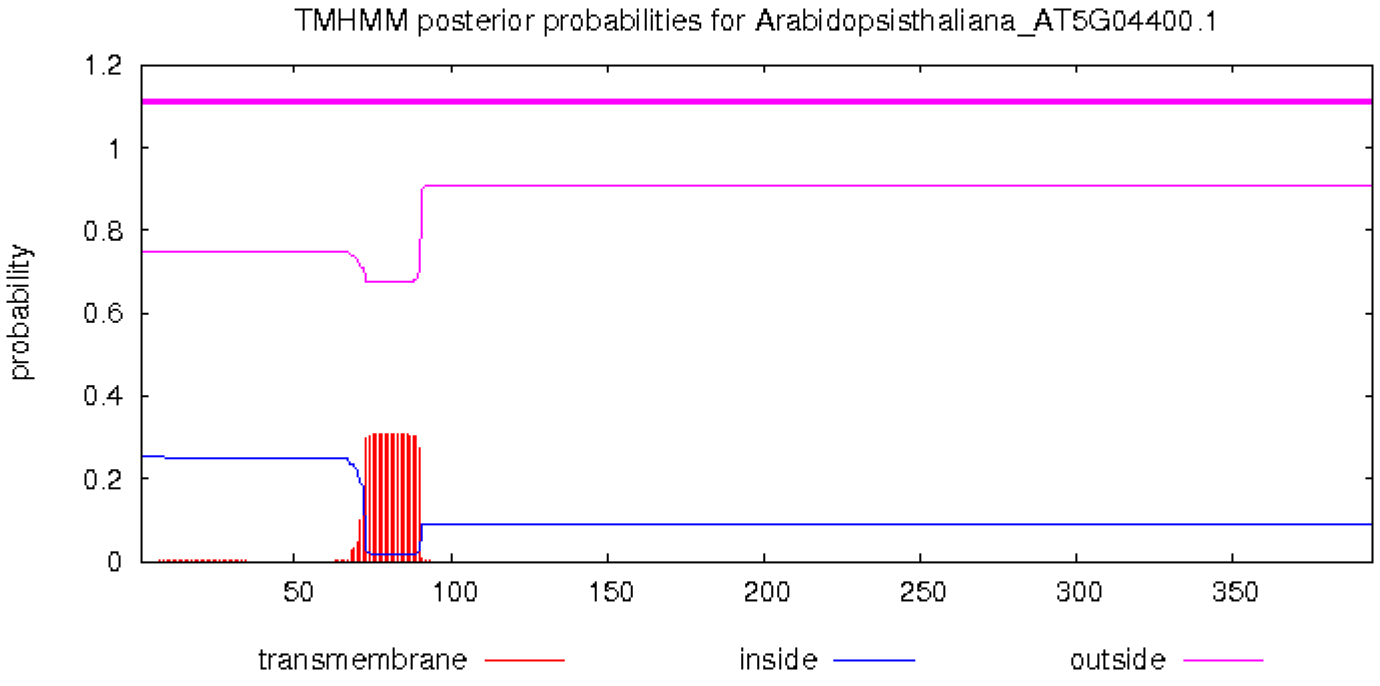

plot in postscript, script for making the plot in gnuplot, data for plot

```
# Arabidopsisthaliana_AT5G04410.1 Length: 567
# Arabidopsisthaliana_AT5G04410.1 Number of predicted TMHs:  1
# Arabidopsisthaliana_AT5G04410.1 Exp number of AAs in TMHs: 21.61122
# Arabidopsisthaliana_AT5G04410.1 Exp number, first 60 AAs:  0
# Arabidopsisthaliana_AT5G04410.1 Total prob of N-in:        0.01265
Arabidopsisthaliana_AT5G04410.1 TMHMM2.0        outside      1   541
Arabidopsisthaliana_AT5G04410.1 TMHMM2.0        TMhelix    542   564
Arabidopsisthaliana_AT5G04410.1 TMHMM2.0        inside     565   567
```

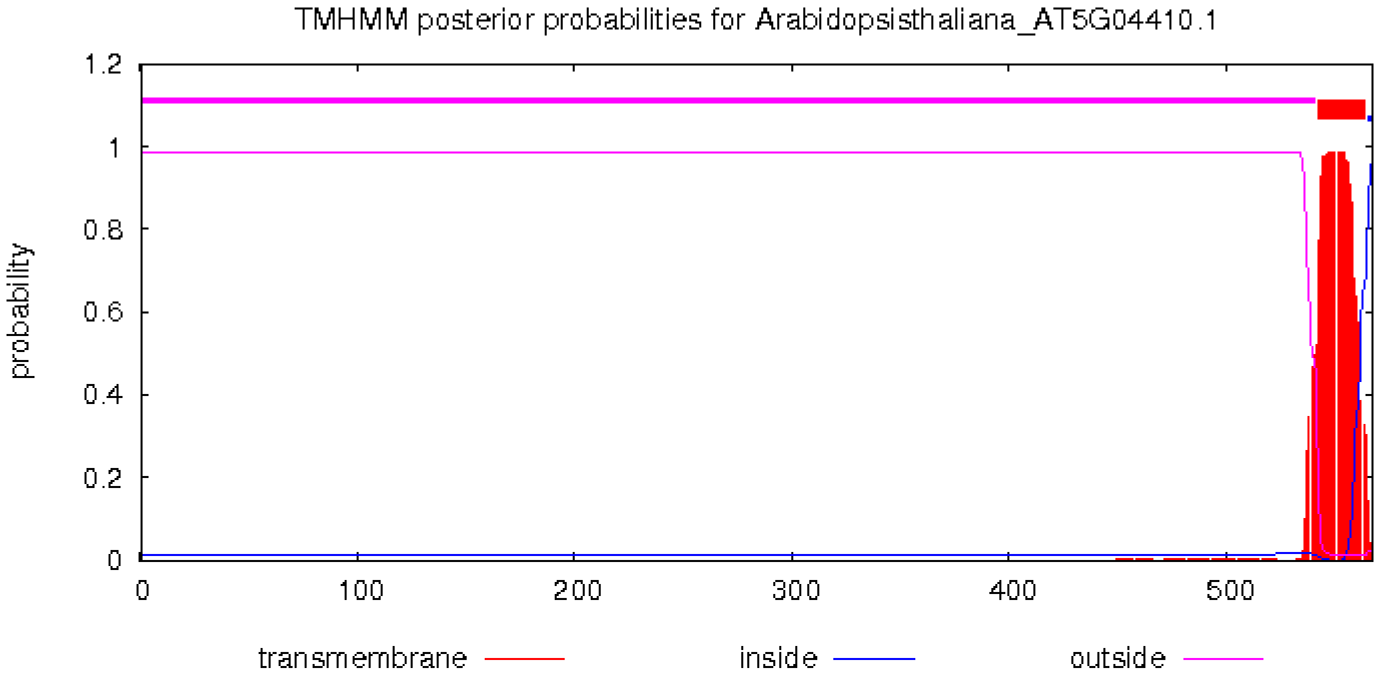

plot in postscript, script for making the plot in gnuplot, data for plot

```
# Arabidopsisthaliana_AT5G07680.1 Length: 329
# Arabidopsisthaliana_AT5G07680.1 Number of predicted TMHs:  0
# Arabidopsisthaliana_AT5G07680.1 Exp number of AAs in TMHs: 0.0002
# Arabidopsisthaliana_AT5G07680.1 Exp number, first 60 AAs:  0
# Arabidopsisthaliana_AT5G07680.1 Total prob of N-in:        0.00837
Arabidopsisthaliana_AT5G07680.1 TMHMM2.0        outside      1   329
```

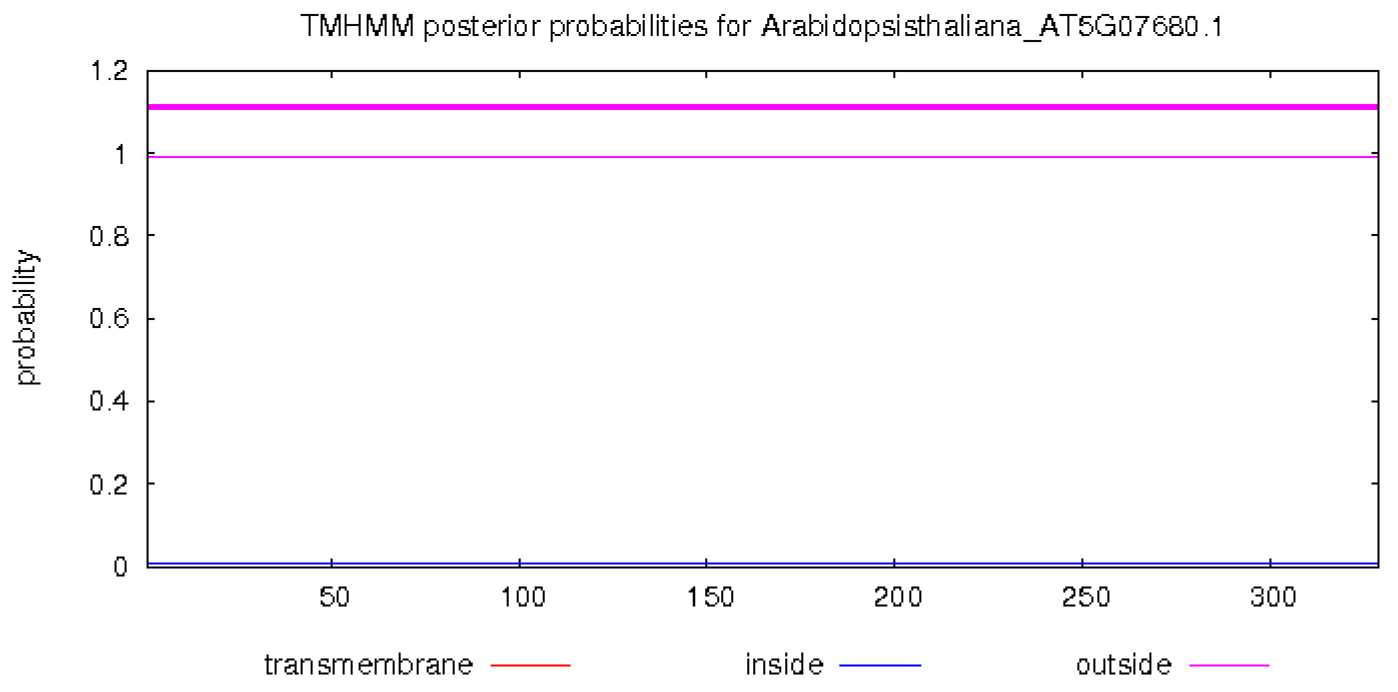

plot in postscript, script for making the plot in gnuplot, data for plot

```
# Arabidopsisthaliana_AT5G08790.1 Length: 283
# Arabidopsisthaliana_AT5G08790.1 Number of predicted TMHs:  0
# Arabidopsisthaliana_AT5G08790.1 Exp number of AAs in TMHs: 0.00019
# Arabidopsisthaliana_AT5G08790.1 Exp number, first 60 AAs:  0
# Arabidopsisthaliana_AT5G08790.1 Total prob of N-in:        0.00901
Arabidopsisthaliana_AT5G08790.1 TMHMM2.0        outside      1   283
```

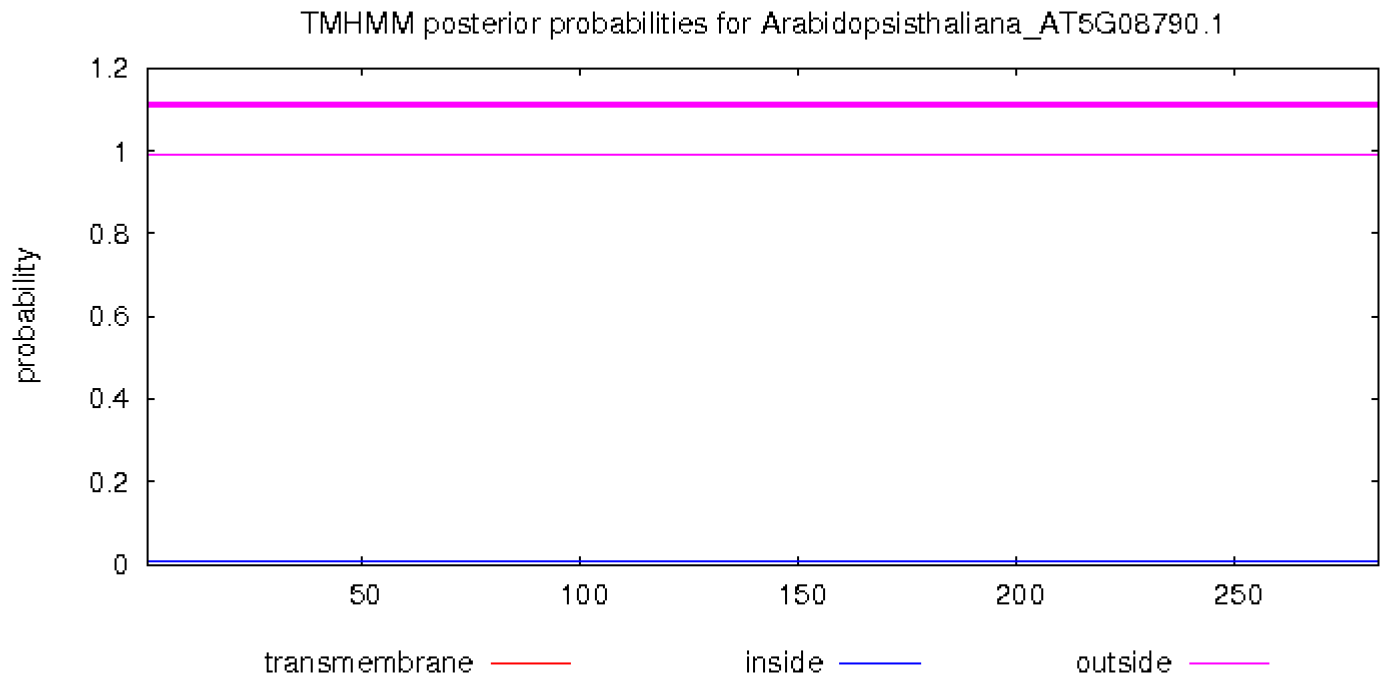

plot in postscript, script for making the plot in gnuplot, data for plot

---

```
# Arabidopsisthaliana_AT5G09330.1 Length: 489
# Arabidopsisthaliana_AT5G09330.1 Number of predicted TMHs:  0
# Arabidopsisthaliana_AT5G09330.1 Exp number of AAs in TMHs: 0.00018
# Arabidopsisthaliana_AT5G09330.1 Exp number, first 60 AAs:  0.00018
# Arabidopsisthaliana_AT5G09330.1 Total prob of N-in:        0.00194
Arabidopsisthaliana_AT5G09330.1 TMHMM2.0        outside      1   489
```

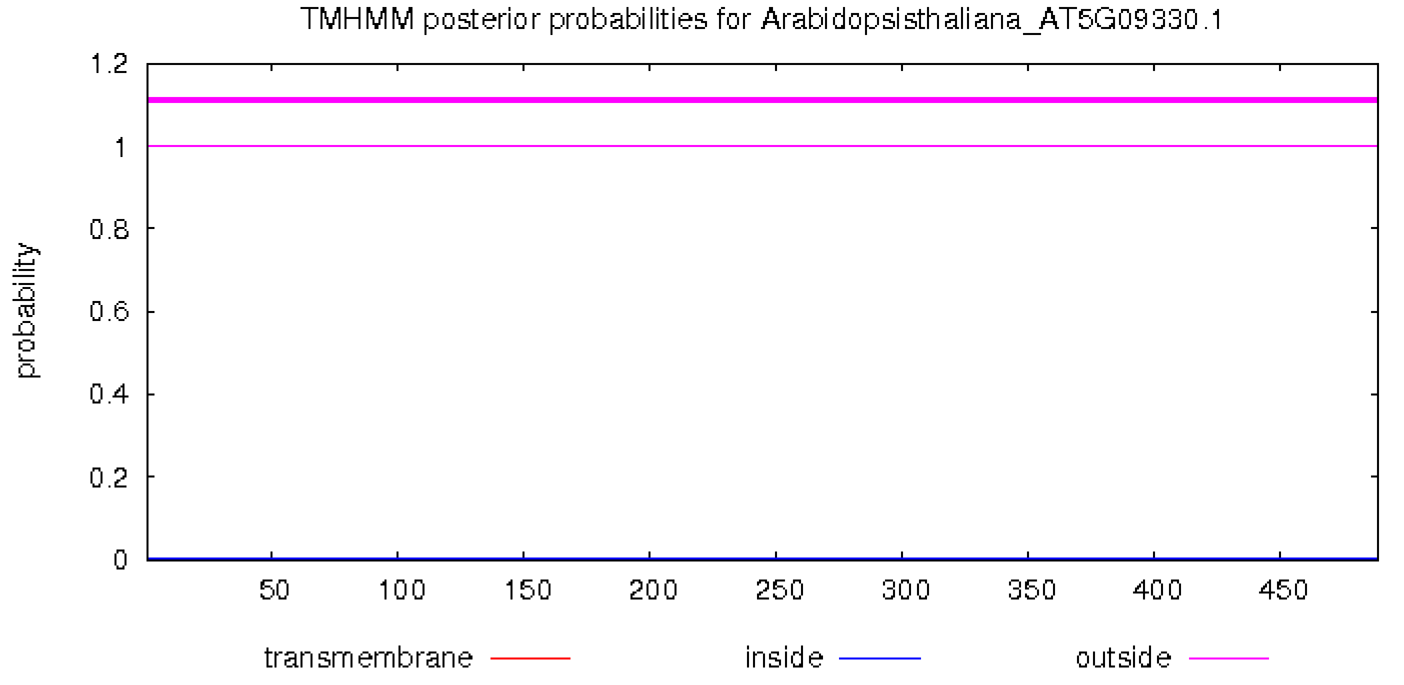

plot in postscript, script for making the plot in gnuplot, data for plot

---

```
# Arabidopsisthaliana_AT5G13180.1 Length: 252
# Arabidopsisthaliana_AT5G13180.1 Number of predicted TMHs:  0
# Arabidopsisthaliana_AT5G13180.1 Exp number of AAs in TMHs: 0.0002
# Arabidopsisthaliana_AT5G13180.1 Exp number, first 60 AAs:  0
# Arabidopsisthaliana_AT5G13180.1 Total prob of N-in:        0.09729
Arabidopsisthaliana_AT5G13180.1 TMHMM2.0        outside      1   252
```

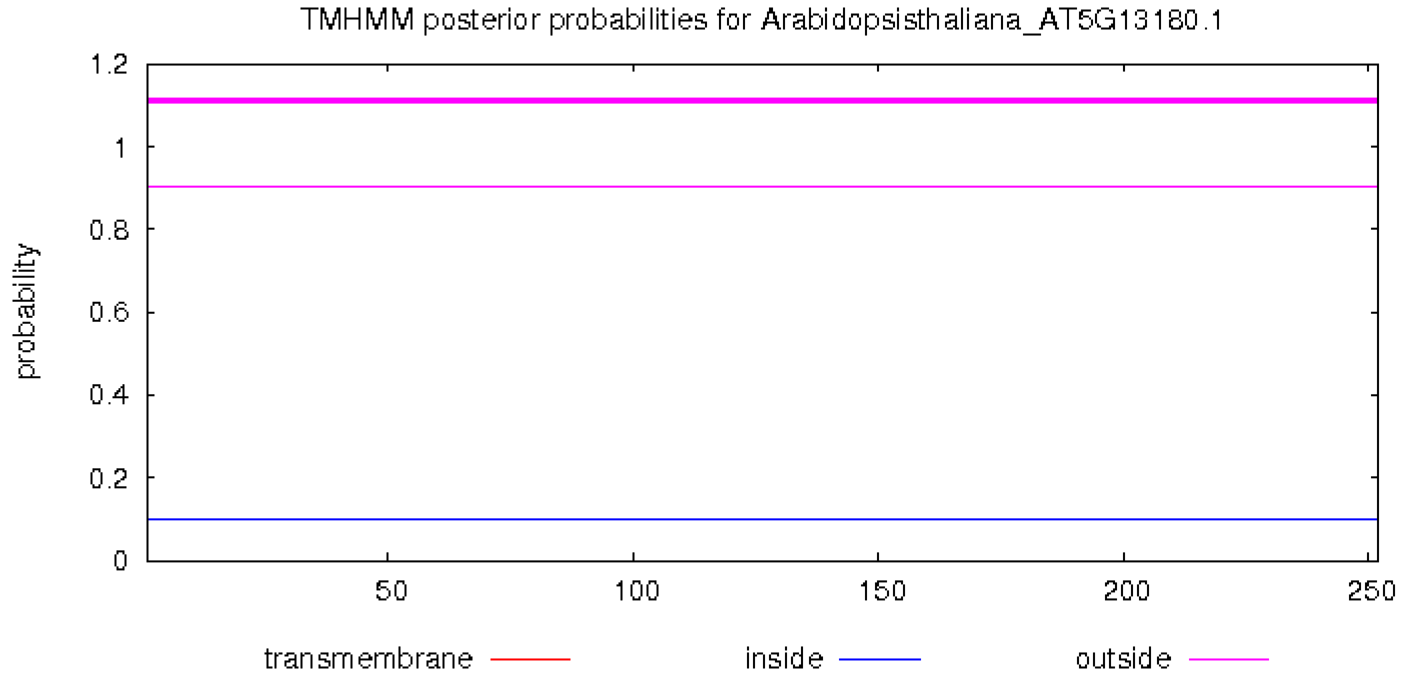

plot in postscript, script for making the plot in gnuplot, data for plot

```
# Arabidopsisthaliana_AT5G14000.1 Length: 206
# Arabidopsisthaliana_AT5G14000.1 Number of predicted TMHs:  0
# Arabidopsisthaliana_AT5G14000.1 Exp number of AAs in TMHs: 1.13214
# Arabidopsisthaliana_AT5G14000.1 Exp number, first 60 AAs:  1.06702
# Arabidopsisthaliana_AT5G14000.1 Total prob of N-in:        0.20507
Arabidopsisthaliana_AT5G14000.1 TMHMM2.0        outside      1   206
```

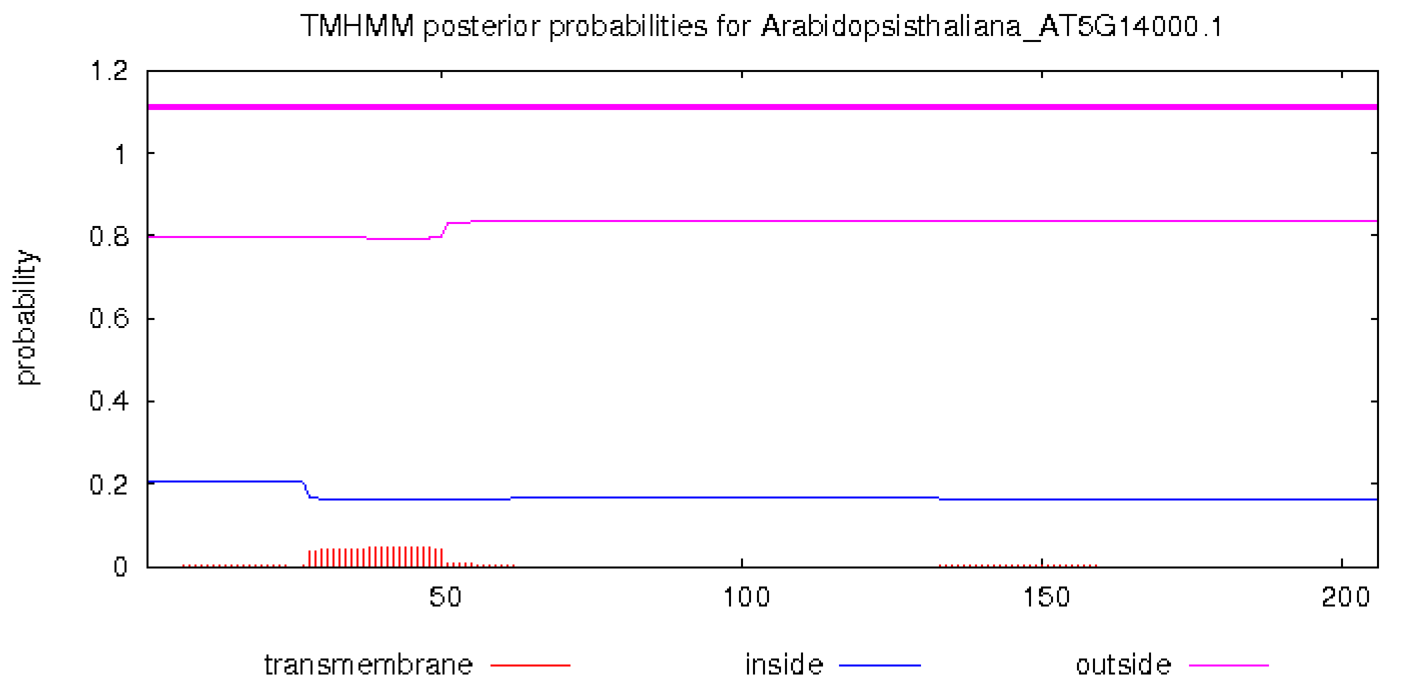

plot in postscript, script for making the plot in gnuplot, data for plot

```
# Arabidopsisthaliana_AT5G14490.1 Length: 350
# Arabidopsisthaliana_AT5G14490.1 Number of predicted TMHs:  0
# Arabidopsisthaliana_AT5G14490.1 Exp number of AAs in TMHs: 0.00024
# Arabidopsisthaliana_AT5G14490.1 Exp number, first 60 AAs:  0.00024
# Arabidopsisthaliana_AT5G14490.1 Total prob of N-in:        0.04301
Arabidopsisthaliana_AT5G14490.1 TMHMM2.0        outside      1   350
```

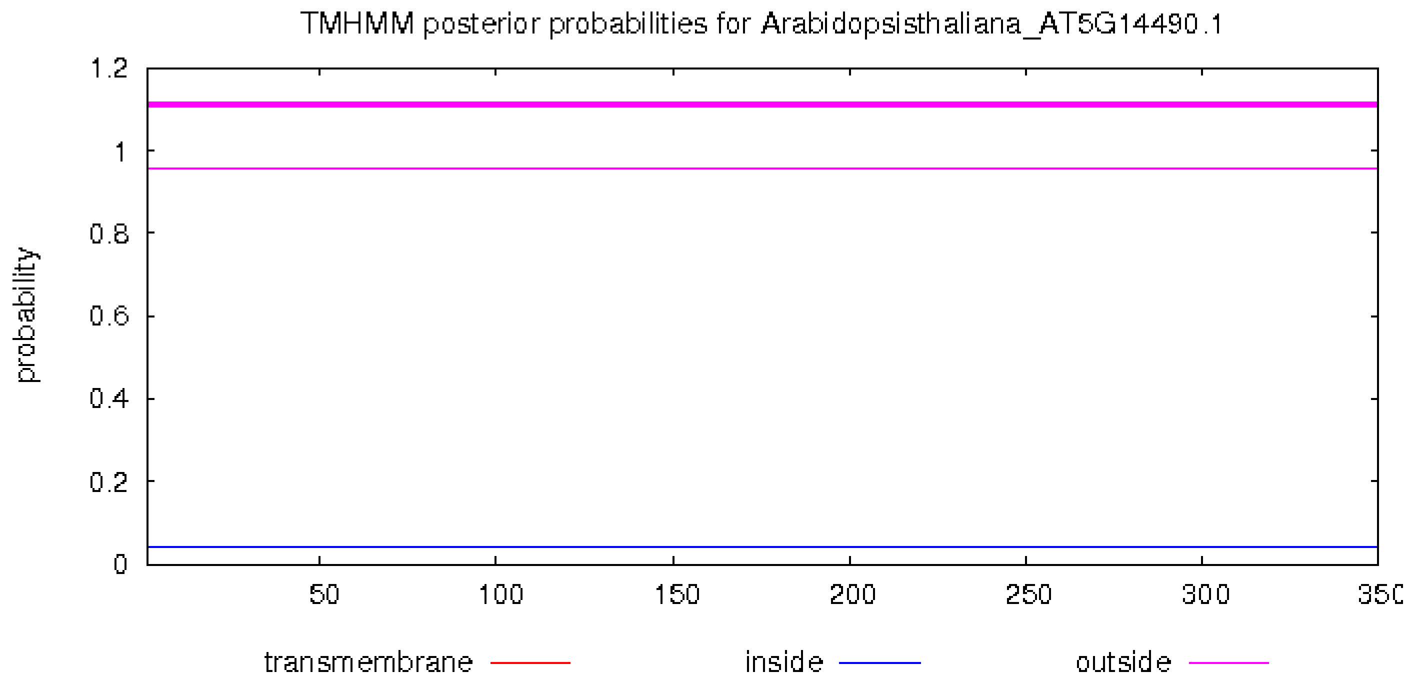

plot in postscript, script for making the plot in gnuplot, data for plot

---

```
# Arabidopsisthaliana_AT5G17260.1 Length: 476
# Arabidopsisthaliana_AT5G17260.1 Number of predicted TMHs:  0
# Arabidopsisthaliana_AT5G17260.1 Exp number of AAs in TMHs: 0.00079
# Arabidopsisthaliana_AT5G17260.1 Exp number, first 60 AAs:  0
# Arabidopsisthaliana_AT5G17260.1 Total prob of N-in:        0.00018
Arabidopsisthaliana_AT5G17260.1 TMHMM2.0        outside      1   476
```

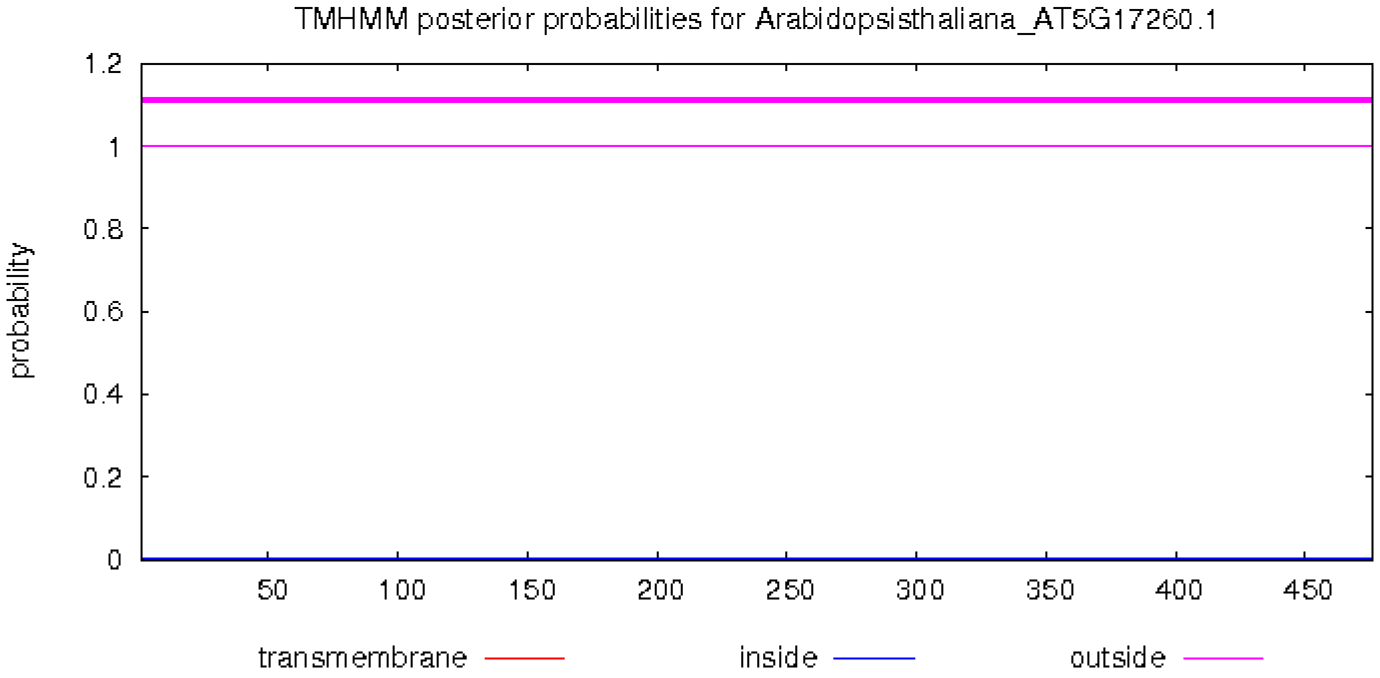

plot in postscript, script for making the plot in gnuplot, data for plot

---

```
# Arabidopsisthaliana_AT5G18037.1 Length: 71
# Arabidopsisthaliana_AT5G18037.1 Number of predicted TMHs:  0
# Arabidopsisthaliana_AT5G18037.1 Exp number of AAs in TMHs: 0.04902
# Arabidopsisthaliana_AT5G18037.1 Exp number, first 60 AAs:  0.04902
# Arabidopsisthaliana_AT5G18037.1 Total prob of N-in:        0.59825
Arabidopsisthaliana_AT5G18037.1 TMHMM2.0        inside       1    71
```

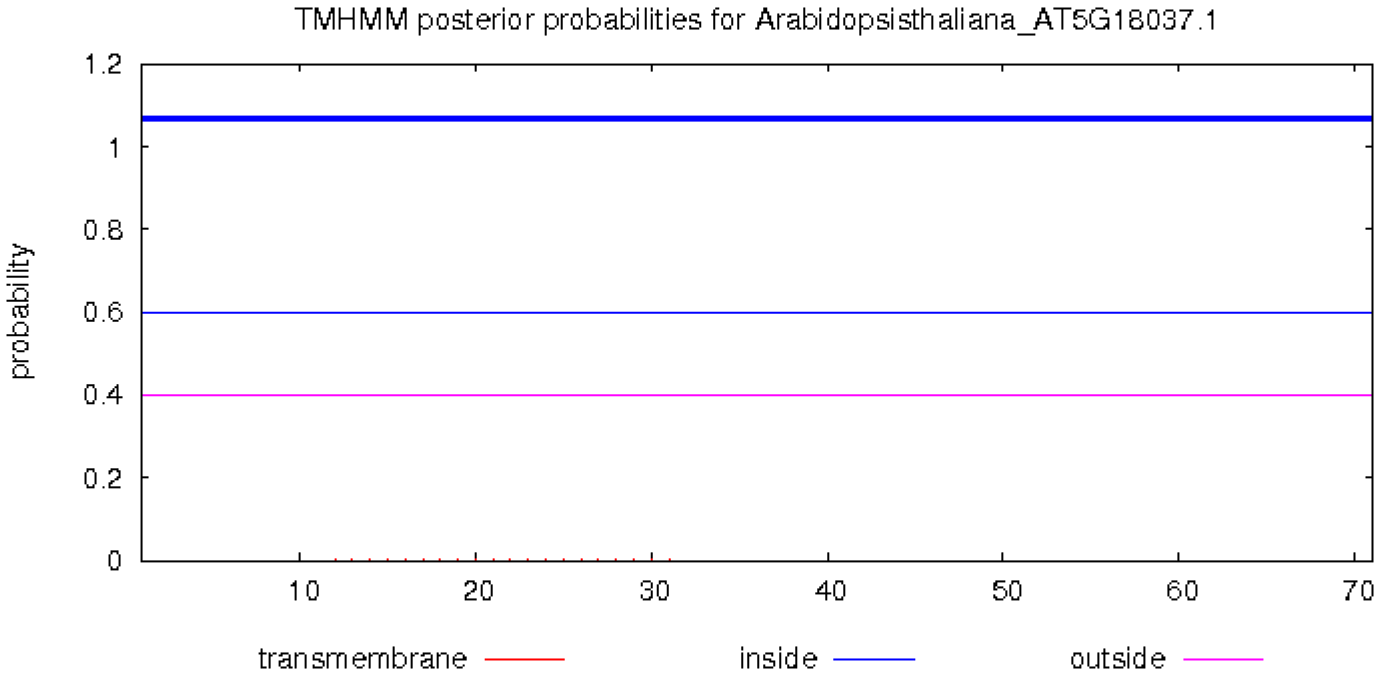

plot in postscript, script for making the plot in gnuplot, data for plot

```
# Arabidopsisthaliana_AT5G18270.1 Length: 335
# Arabidopsisthaliana_AT5G18270.1 Number of predicted TMHs:  0
# Arabidopsisthaliana_AT5G18270.1 Exp number of AAs in TMHs: 0.00017
# Arabidopsisthaliana_AT5G18270.1 Exp number, first 60 AAs:  0
# Arabidopsisthaliana_AT5G18270.1 Total prob of N-in:        0.00852
Arabidopsisthaliana_AT5G18270.1 TMHMM2.0        outside      1   335
```

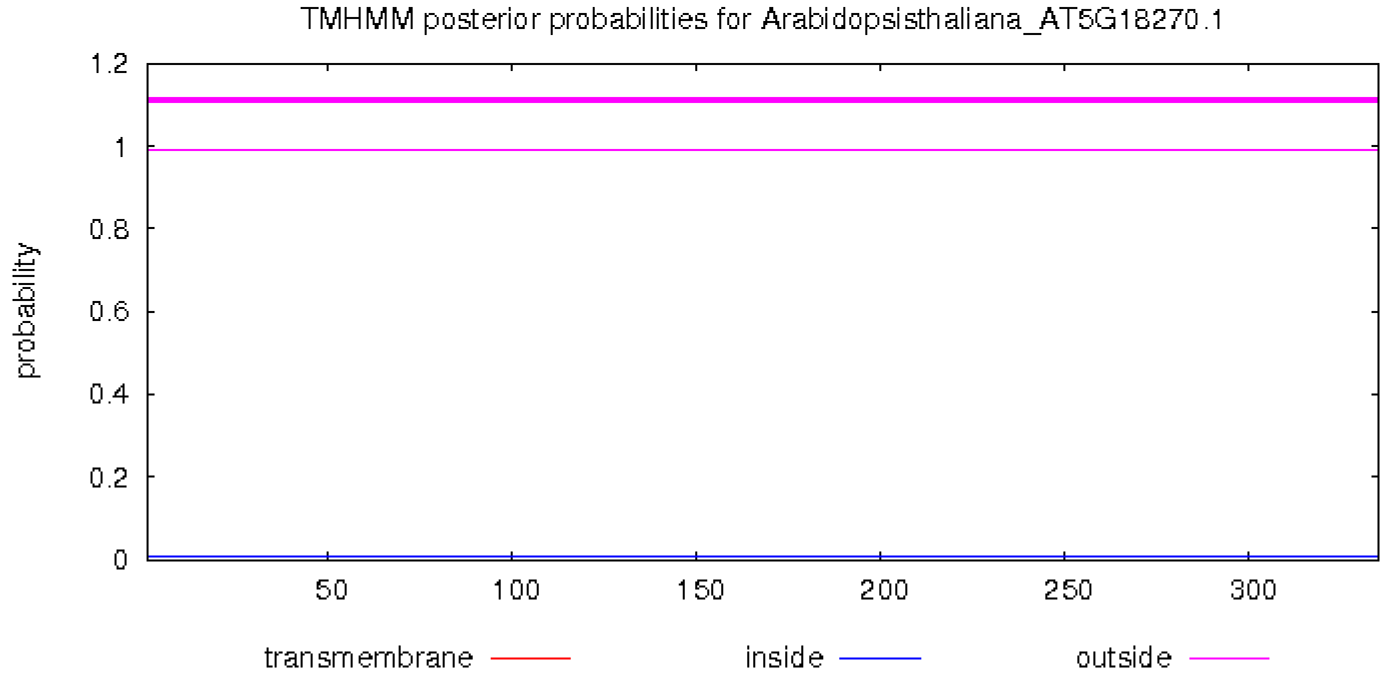

plot in postscript, script for making the plot in gnuplot, data for plot

```
# Arabidopsisthaliana_AT5G18300.1 Length: 147
# Arabidopsisthaliana_AT5G18300.1 Number of predicted TMHs:  0
# Arabidopsisthaliana_AT5G18300.1 Exp number of AAs in TMHs: 0.0004
# Arabidopsisthaliana_AT5G18300.1 Exp number, first 60 AAs:  0.0004
# Arabidopsisthaliana_AT5G18300.1 Total prob of N-in:        0.08829
Arabidopsisthaliana_AT5G18300.1 TMHMM2.0        outside      1   147
```

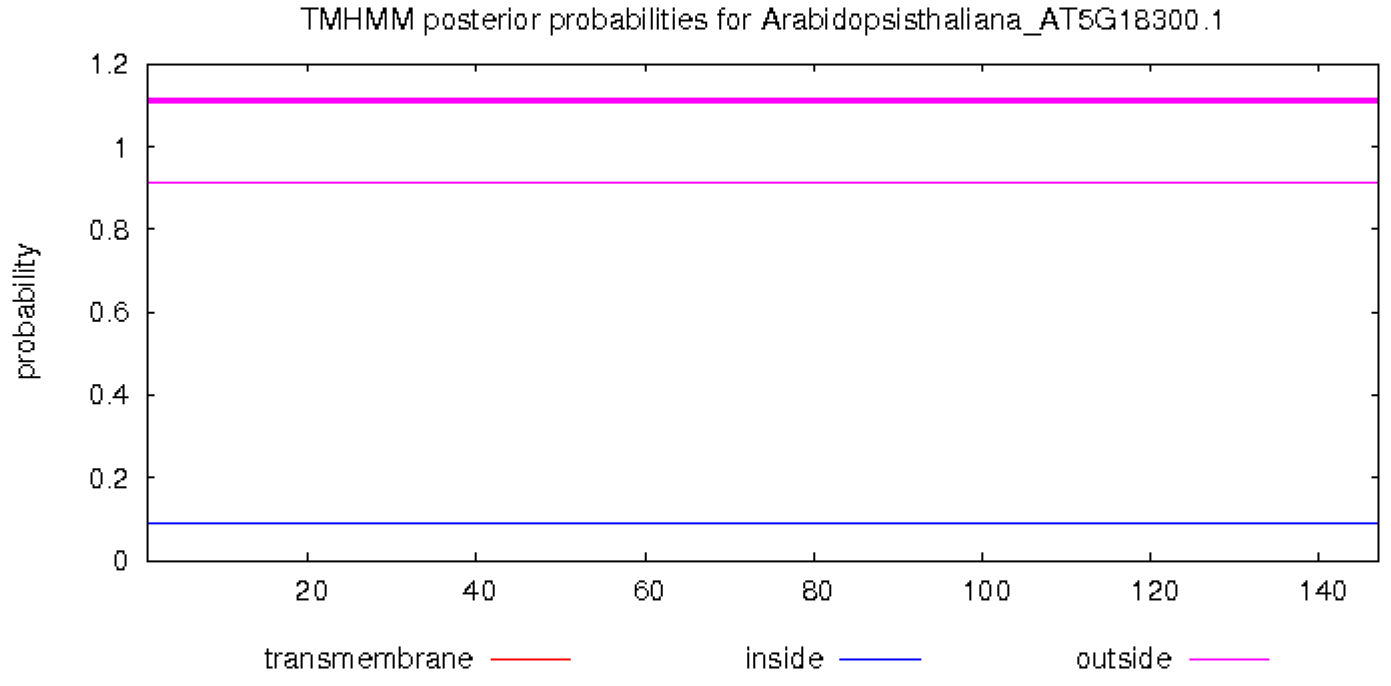

plot in postscript, script for making the plot in gnuplot, data for plot

---

```
# Arabidopsisthaliana_AT5G22290.1 Length: 340
# Arabidopsisthaliana_AT5G22290.1 Number of predicted TMHs:  0
# Arabidopsisthaliana_AT5G22290.1 Exp number of AAs in TMHs: 11.62916
# Arabidopsisthaliana_AT5G22290.1 Exp number, first 60 AAs:  0.03215
# Arabidopsisthaliana_AT5G22290.1 Total prob of N-in:        0.29616
Arabidopsisthaliana_AT5G22290.1 TMHMM2.0        outside      1   340
```

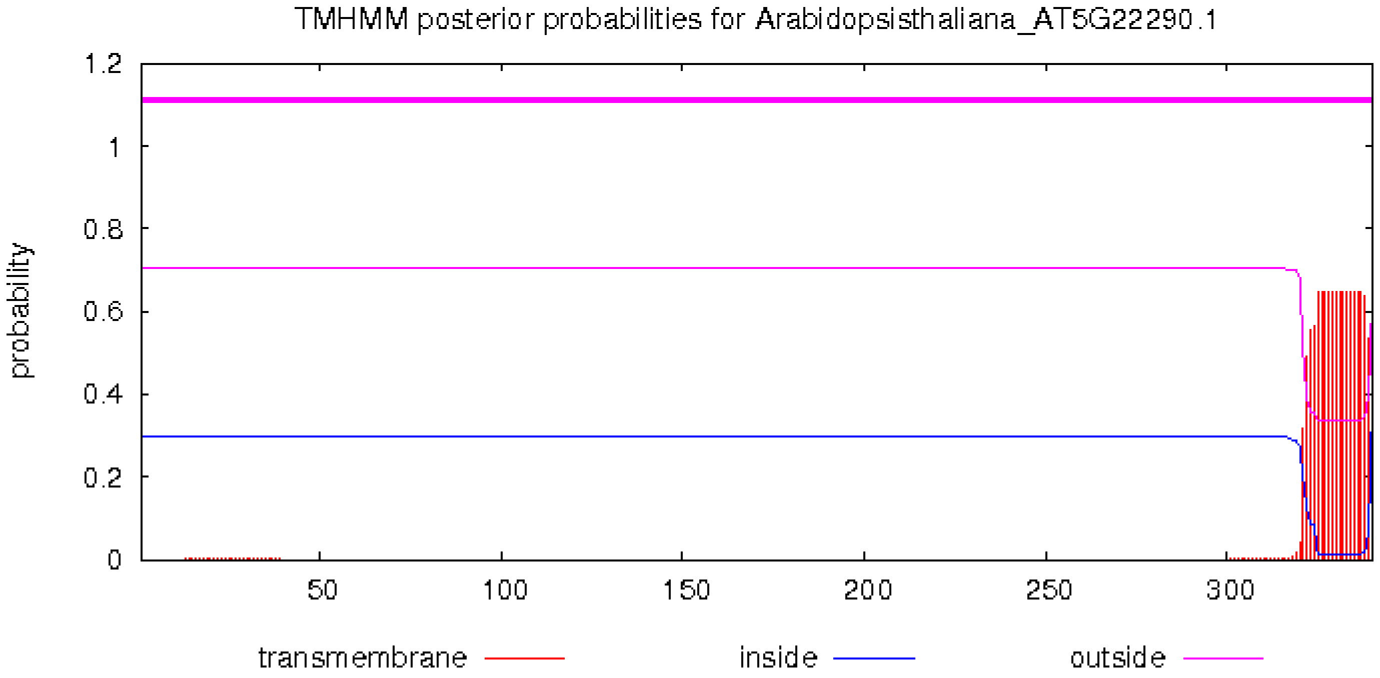

plot in postscript, script for making the plot in gnuplot, data for plot

---

```
# Arabidopsisthaliana_AT5G22380.1 Length: 235
# Arabidopsisthaliana_AT5G22380.1 Number of predicted TMHs:  0
# Arabidopsisthaliana_AT5G22380.1 Exp number of AAs in TMHs: 0
# Arabidopsisthaliana_AT5G22380.1 Exp number, first 60 AAs:  0
# Arabidopsisthaliana_AT5G22380.1 Total prob of N-in:        0.04879
Arabidopsisthaliana_AT5G22380.1 TMHMM2.0        outside      1   235
```

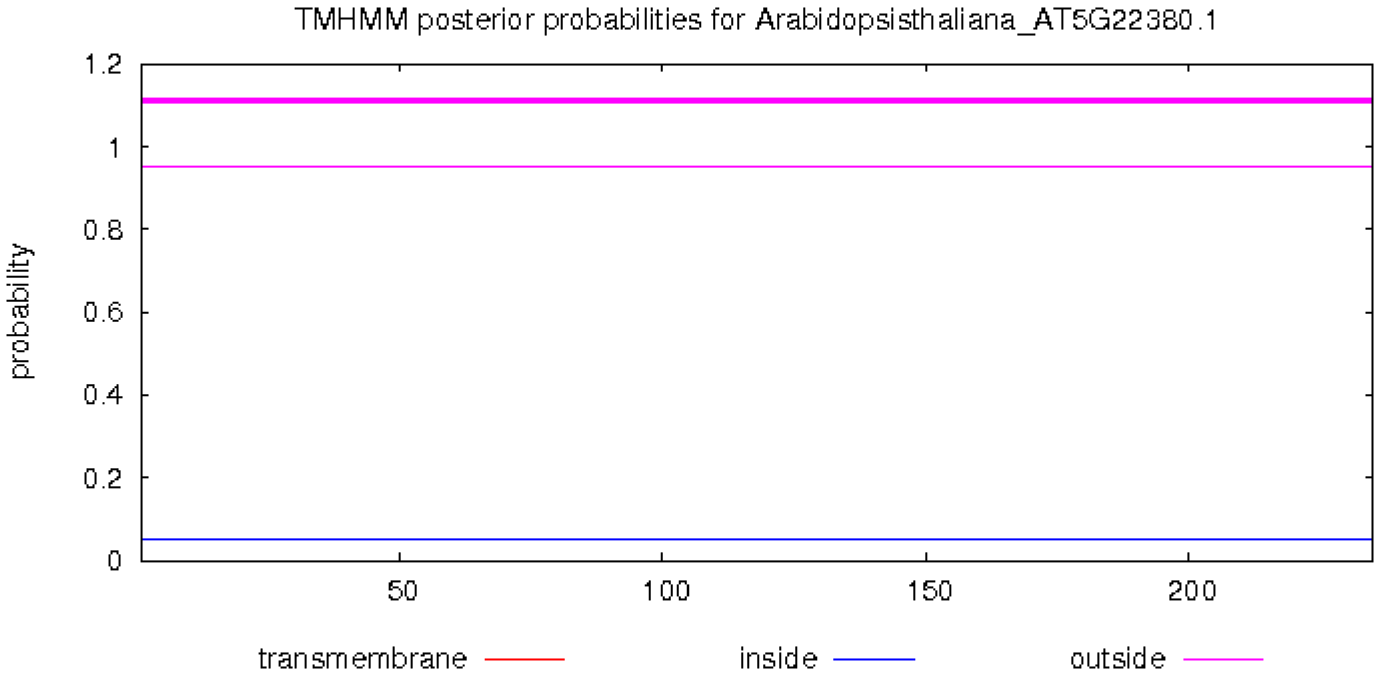

plot in postscript, script for making the plot in gnuplot, data for plot

```
# Arabidopsisthaliana_AT5G24590.2 Length: 451
# Arabidopsisthaliana_AT5G24590.2 Number of predicted TMHs:  0
# Arabidopsisthaliana_AT5G24590.2 Exp number of AAs in TMHs: 11.57938
# Arabidopsisthaliana_AT5G24590.2 Exp number, first 60 AAs:  0.00368
# Arabidopsisthaliana_AT5G24590.2 Total prob of N-in:        0.01400
Arabidopsisthaliana_AT5G24590.2 TMHMM2.0        outside      1   451
```

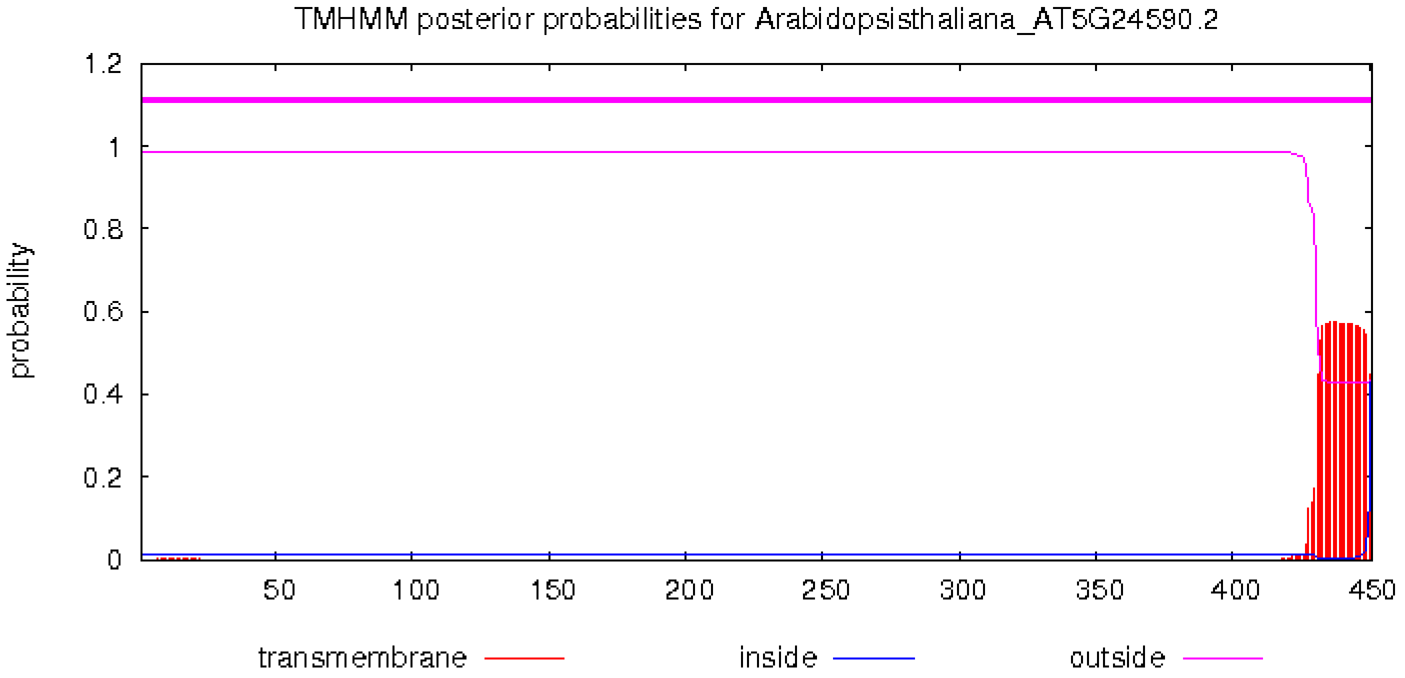

plot in postscript, script for making the plot in gnuplot, data for plot

```
# Arabidopsisthaliana_AT5G39610.1 Length: 285
# Arabidopsisthaliana_AT5G39610.1 Number of predicted TMHs:  0
# Arabidopsisthaliana_AT5G39610.1 Exp number of AAs in TMHs: 0.0008
# Arabidopsisthaliana_AT5G39610.1 Exp number, first 60 AAs:  0.00063
# Arabidopsisthaliana_AT5G39610.1 Total prob of N-in:        0.04952
Arabidopsisthaliana_AT5G39610.1 TMHMM2.0        outside      1   285
```

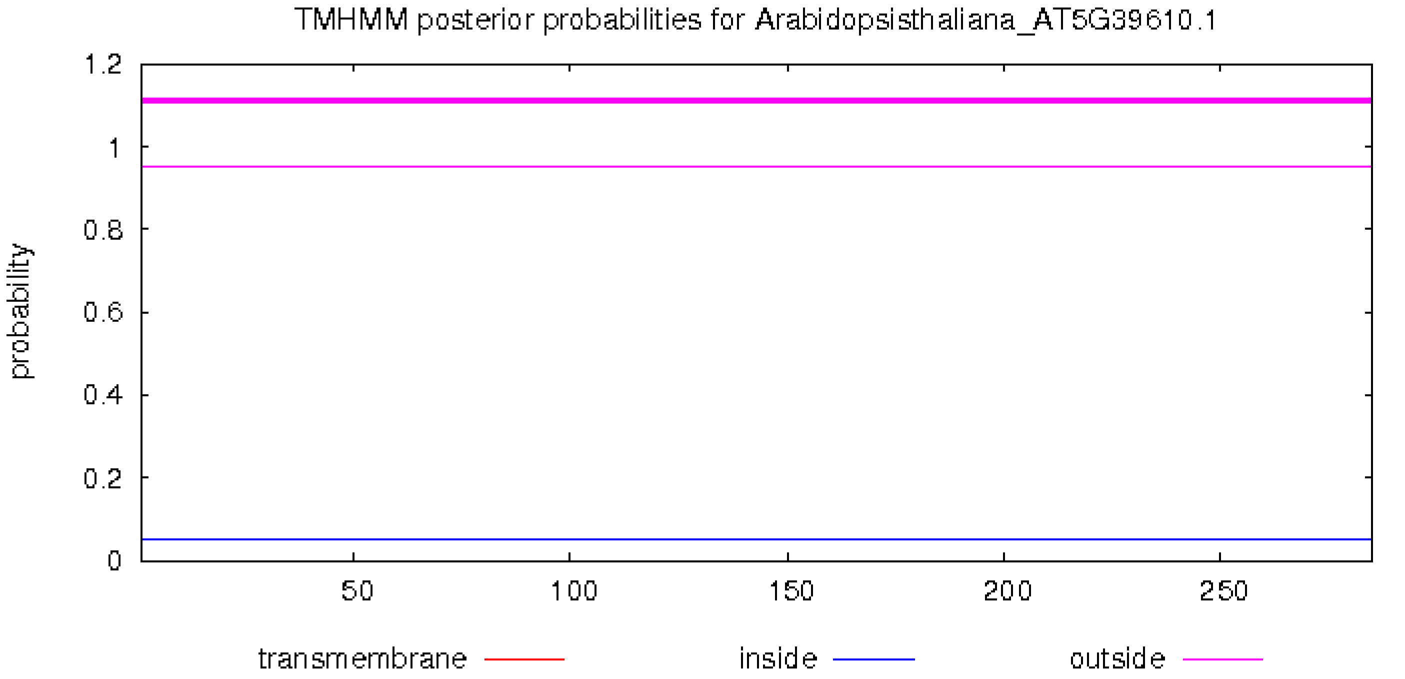

plot in postscript, script for making the plot in gnuplot, data for plot

---

```
# Arabidopsisthaliana_AT5G39690.1 Length: 294
# Arabidopsisthaliana_AT5G39690.1 Number of predicted TMHs:  0
# Arabidopsisthaliana_AT5G39690.1 Exp number of AAs in TMHs: 0.01945
# Arabidopsisthaliana_AT5G39690.1 Exp number, first 60 AAs:  0.01457
# Arabidopsisthaliana_AT5G39690.1 Total prob of N-in:        0.11119
Arabidopsisthaliana_AT5G39690.1 TMHMM2.0        outside      1   294
```

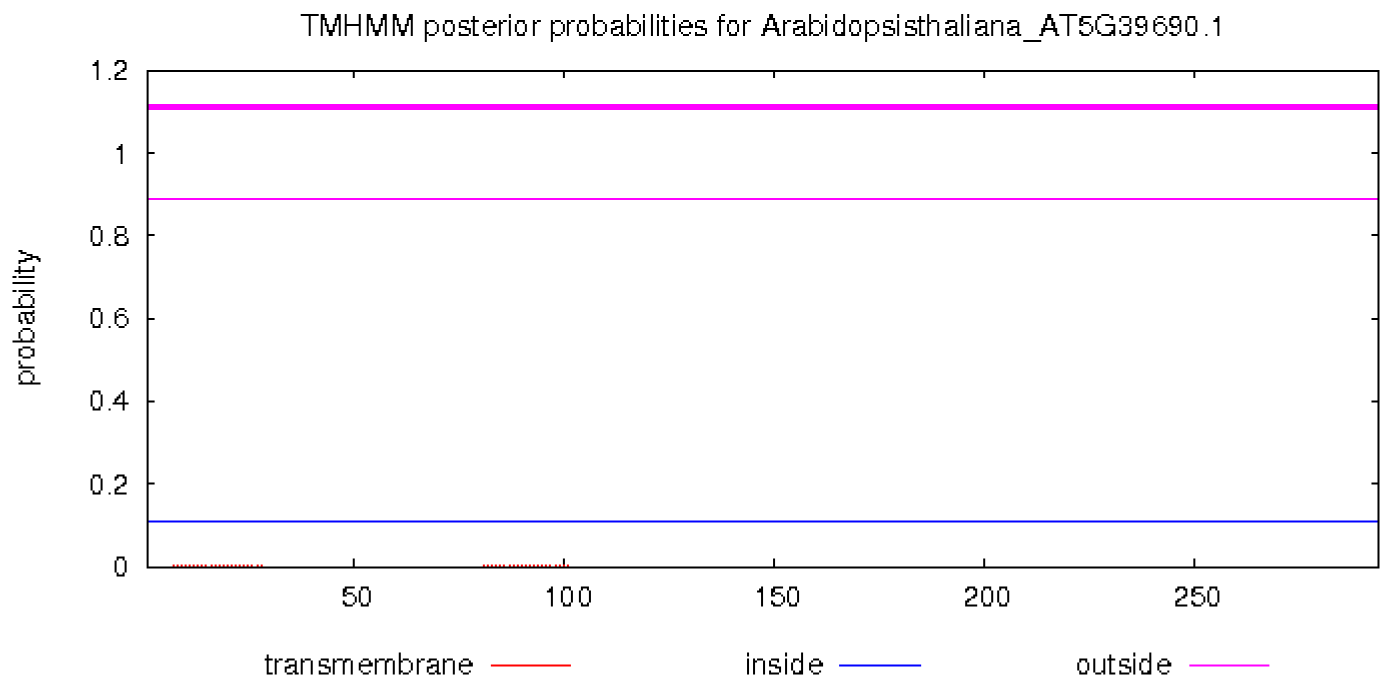

plot in postscript, script for making the plot in gnuplot, data for plot

---

```
# Arabidopsisthaliana_AT5G39820.1 Length: 337
# Arabidopsisthaliana_AT5G39820.1 Number of predicted TMHs:  0
# Arabidopsisthaliana_AT5G39820.1 Exp number of AAs in TMHs: 0.00098
# Arabidopsisthaliana_AT5G39820.1 Exp number, first 60 AAs:  0
# Arabidopsisthaliana_AT5G39820.1 Total prob of N-in:        0.00854
Arabidopsisthaliana_AT5G39820.1 TMHMM2.0        outside      1   337
```

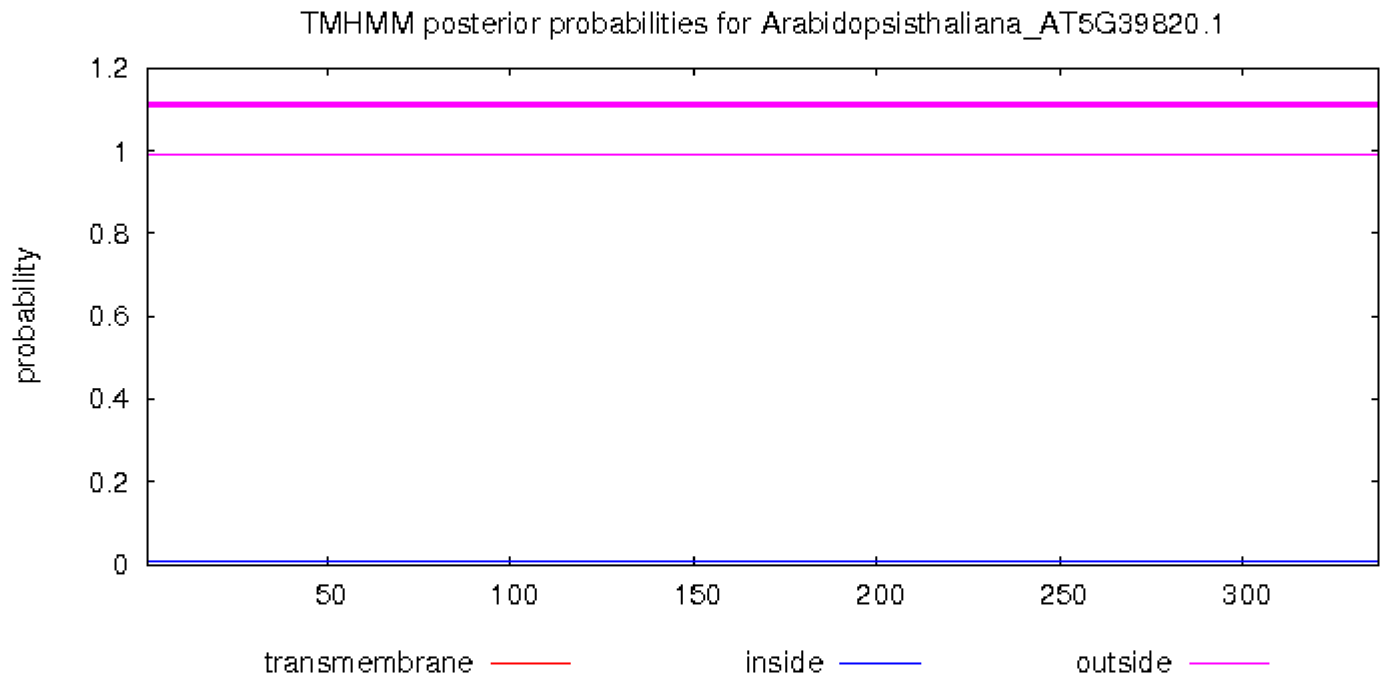

plot in postscript, script for making the plot in gnuplot, data for plot

```
# Arabidopsisthaliana_AT5G41090.1 Length: 212
# Arabidopsisthaliana_AT5G41090.1 Number of predicted TMHs:  0
# Arabidopsisthaliana_AT5G41090.1 Exp number of AAs in TMHs: 0.03024
# Arabidopsisthaliana_AT5G41090.1 Exp number, first 60 AAs:  0.03024
# Arabidopsisthaliana_AT5G41090.1 Total prob of N-in:        0.03616
Arabidopsisthaliana_AT5G41090.1 TMHMM2.0        outside      1   212
```

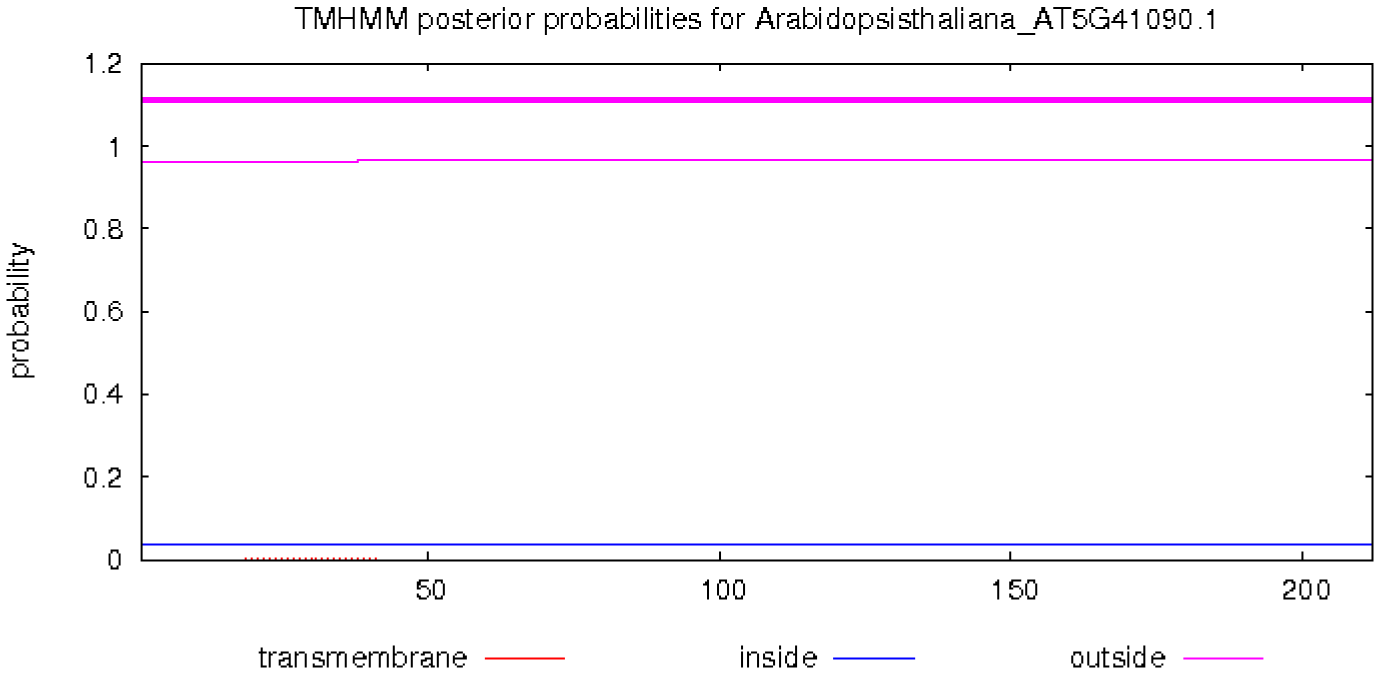

plot in postscript, script for making the plot in gnuplot, data for plot

```
# Arabidopsisthaliana_AT5G46590.1 Length: 292
# Arabidopsisthaliana_AT5G46590.1 Number of predicted TMHs:  0
# Arabidopsisthaliana_AT5G46590.1 Exp number of AAs in TMHs: 0.00015
# Arabidopsisthaliana_AT5G46590.1 Exp number, first 60 AAs:  0
# Arabidopsisthaliana_AT5G46590.1 Total prob of N-in:        0.00215
Arabidopsisthaliana_AT5G46590.1 TMHMM2.0        outside      1   292
```

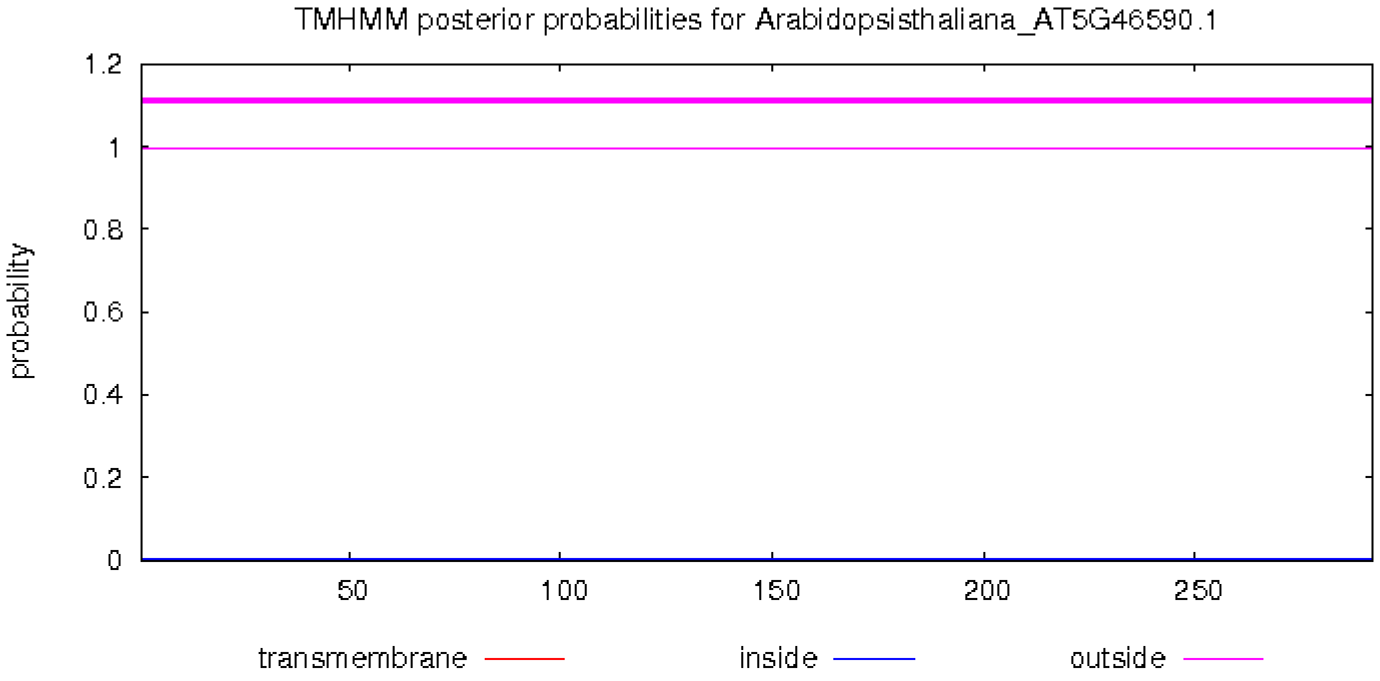

plot in postscript, script for making the plot in gnuplot, data for plot

```
# Arabidopsisthaliana_AT5G50820.1 Length: 193
# Arabidopsisthaliana_AT5G50820.1 Number of predicted TMHs:  0
# Arabidopsisthaliana_AT5G50820.1 Exp number of AAs in TMHs: 0.00249
# Arabidopsisthaliana_AT5G50820.1 Exp number, first 60 AAs:  0
# Arabidopsisthaliana_AT5G50820.1 Total prob of N-in:        0.12754
Arabidopsisthaliana_AT5G50820.1 TMHMM2.0        outside      1   193
```

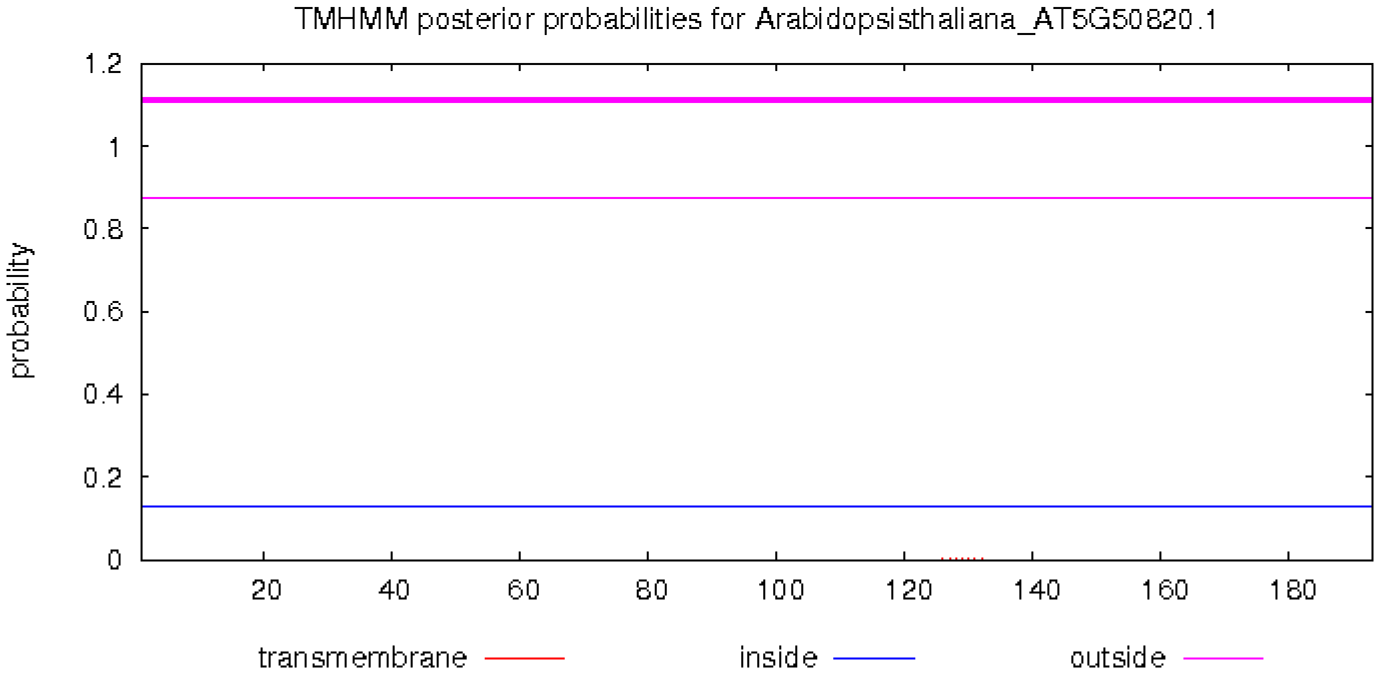

plot in postscript, script for making the plot in gnuplot, data for plot

```
# Arabidopsisthaliana_AT5G53950.1 Length: 375
# Arabidopsisthaliana_AT5G53950.1 Number of predicted TMHs:  0
# Arabidopsisthaliana_AT5G53950.1 Exp number of AAs in TMHs: 0.01309
# Arabidopsisthaliana_AT5G53950.1 Exp number, first 60 AAs:  0
# Arabidopsisthaliana_AT5G53950.1 Total prob of N-in:        0.01680
Arabidopsisthaliana_AT5G53950.1 TMHMM2.0        outside      1   375
```

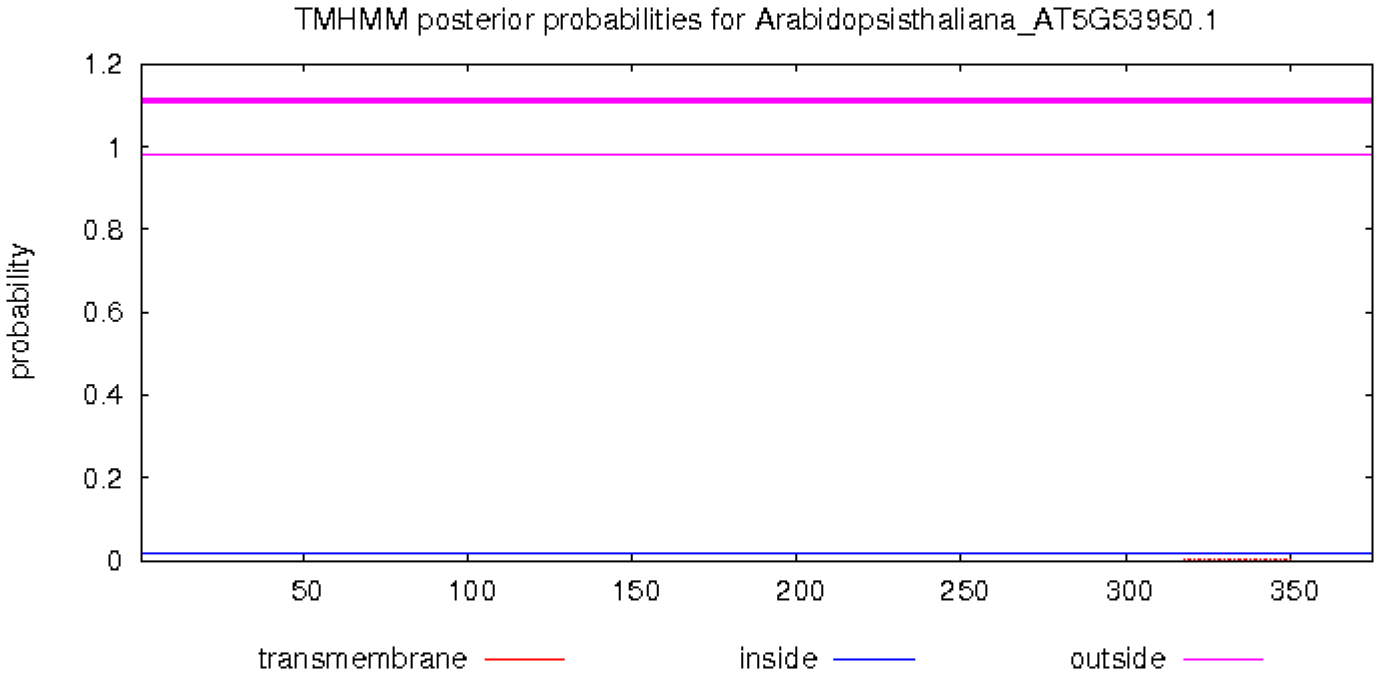

plot in postscript, script for making the plot in gnuplot, data for plot

```
# Arabidopsisthaliana_AT5G56620.1 Length: 386
# Arabidopsisthaliana_AT5G56620.1 Number of predicted TMHs:  0
# Arabidopsisthaliana_AT5G56620.1 Exp number of AAs in TMHs: 0.11796
# Arabidopsisthaliana_AT5G56620.1 Exp number, first 60 AAs:  0
# Arabidopsisthaliana_AT5G56620.1 Total prob of N-in:        0.03375
Arabidopsisthaliana_AT5G56620.1 TMHMM2.0        outside      1   386
```

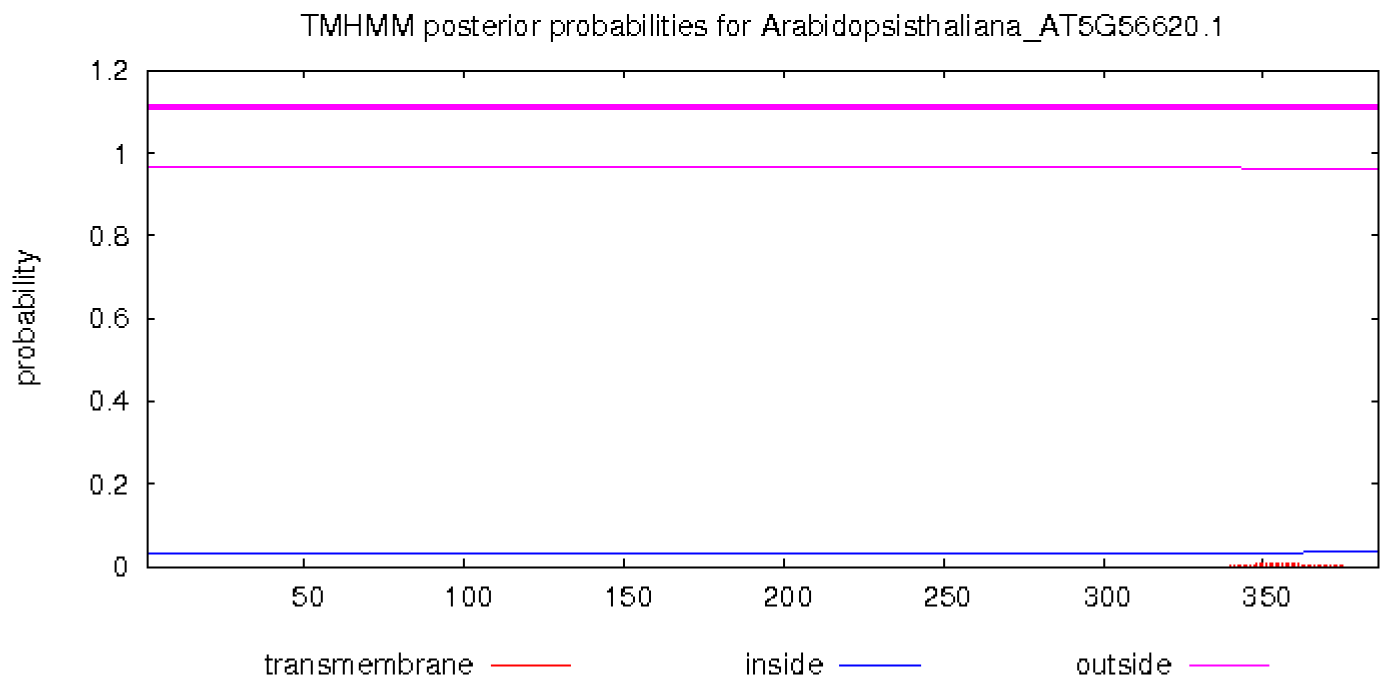

plot in postscript, script for making the plot in gnuplot, data for plot

```
# Arabidopsisthaliana_AT5G61430.1 Length: 336
# Arabidopsisthaliana_AT5G61430.1 Number of predicted TMHs:  0
# Arabidopsisthaliana_AT5G61430.1 Exp number of AAs in TMHs: 0
# Arabidopsisthaliana_AT5G61430.1 Exp number, first 60 AAs:  0
# Arabidopsisthaliana_AT5G61430.1 Total prob of N-in:        0.01237
Arabidopsisthaliana_AT5G61430.1 TMHMM2.0        outside      1   336
```

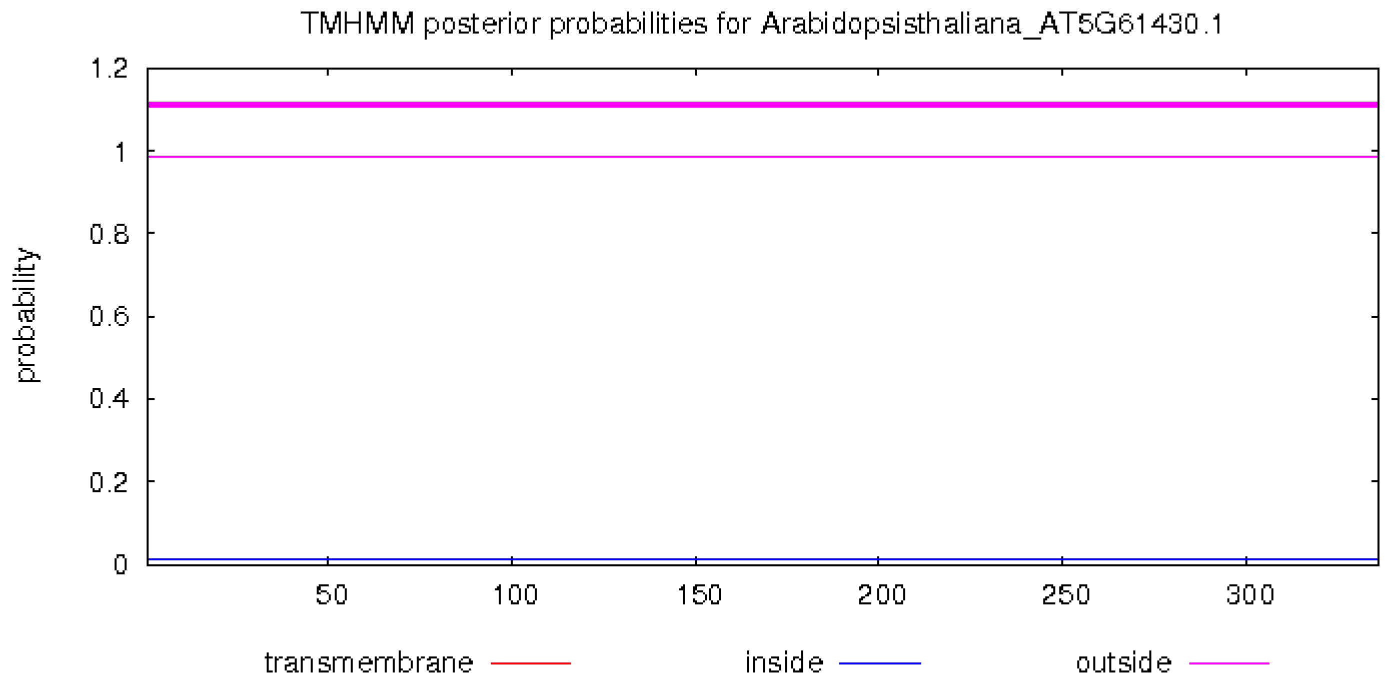

plot in postscript, script for making the plot in gnuplot, data for plot

---

```
# Arabidopsisthaliana_AT5G62380.1 Length: 348
# Arabidopsisthaliana_AT5G62380.1 Number of predicted TMHs:  0
# Arabidopsisthaliana_AT5G62380.1 Exp number of AAs in TMHs: 0.00149
# Arabidopsisthaliana_AT5G62380.1 Exp number, first 60 AAs:  0
# Arabidopsisthaliana_AT5G62380.1 Total prob of N-in:        0.00218
Arabidopsisthaliana_AT5G62380.1 TMHMM2.0        outside      1   348
```

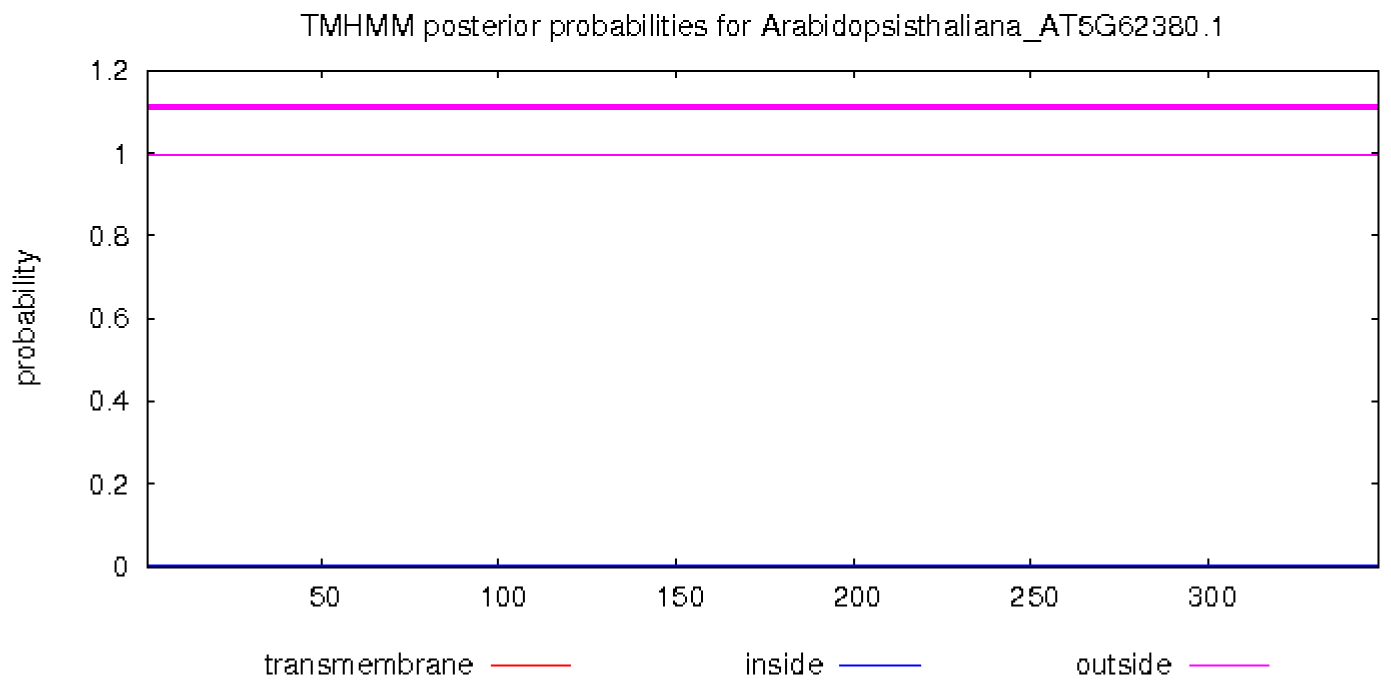

plot in postscript, script for making the plot in gnuplot, data for plot

---

```
# Arabidopsisthaliana_AT5G63790.1 Length: 312
# Arabidopsisthaliana_AT5G63790.1 Number of predicted TMHs:  0
# Arabidopsisthaliana_AT5G63790.1 Exp number of AAs in TMHs: 0
# Arabidopsisthaliana_AT5G63790.1 Exp number, first 60 AAs:  0
# Arabidopsisthaliana_AT5G63790.1 Total prob of N-in:        0.00352
Arabidopsisthaliana_AT5G63790.1 TMHMM2.0        outside      1   312
```

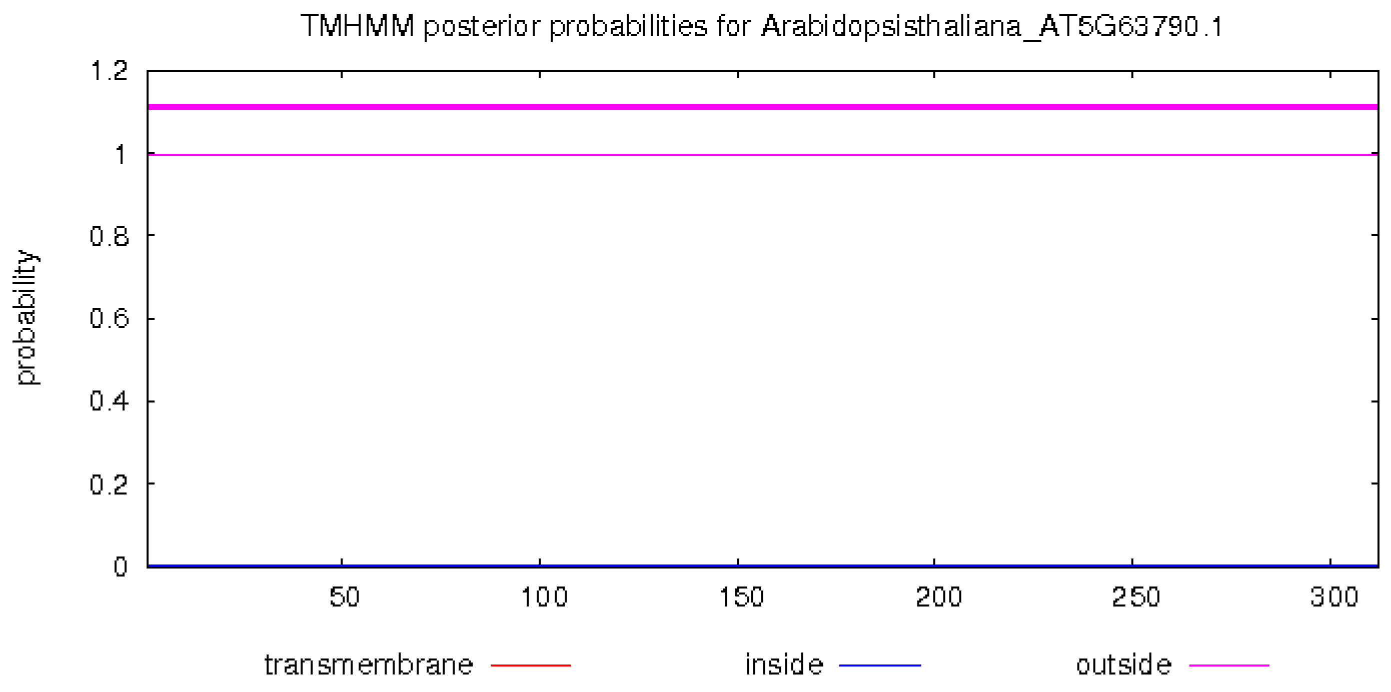

plot in postscript, script for making the plot in gnuplot, data for plot

```
# Arabidopsisthaliana_AT5G64060.1 Length: 356
# Arabidopsisthaliana_AT5G64060.1 Number of predicted TMHs:  0
# Arabidopsisthaliana_AT5G64060.1 Exp number of AAs in TMHs: 0.00018
# Arabidopsisthaliana_AT5G64060.1 Exp number, first 60 AAs:  0.00018
# Arabidopsisthaliana_AT5G64060.1 Total prob of N-in:        0.00483
Arabidopsisthaliana_AT5G64060.1 TMHMM2.0        outside      1   356
```

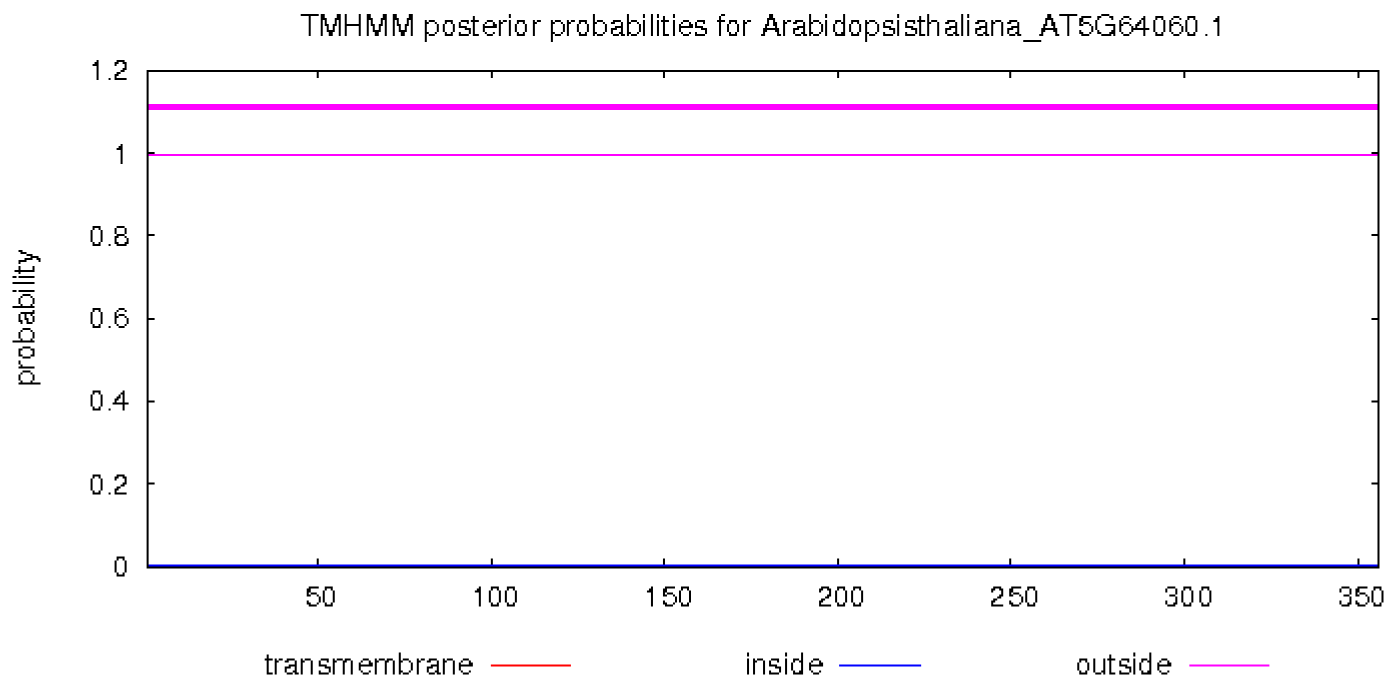

plot in postscript, script for making the plot in gnuplot, data for plot

```
# Arabidopsisthaliana_AT5G64530.1 Length: 187
# Arabidopsisthaliana_AT5G64530.1 Number of predicted TMHs:  0
# Arabidopsisthaliana_AT5G64530.1 Exp number of AAs in TMHs: 0
# Arabidopsisthaliana_AT5G64530.1 Exp number, first 60 AAs:  0
# Arabidopsisthaliana_AT5G64530.1 Total prob of N-in:        0.00733
Arabidopsisthaliana_AT5G64530.1 TMHMM2.0        outside      1   187
```

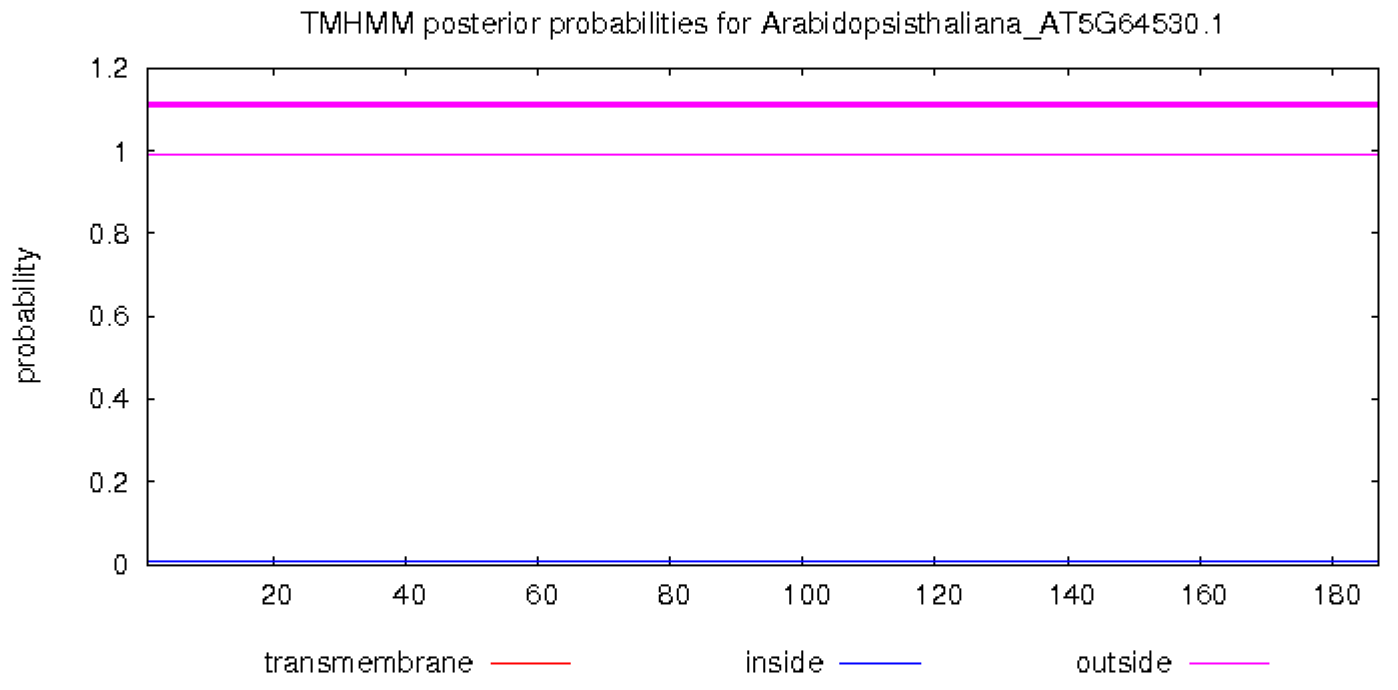

plot in postscript, script for making the plot in gnuplot, data for plot

```
# Arabidopsisthaliana_AT5G66300.1 Length: 292
# Arabidopsisthaliana_AT5G66300.1 Number of predicted TMHs:  0
# Arabidopsisthaliana_AT5G66300.1 Exp number of AAs in TMHs: 0.00113
# Arabidopsisthaliana_AT5G66300.1 Exp number, first 60 AAs:  0
# Arabidopsisthaliana_AT5G66300.1 Total prob of N-in:        0.09441
Arabidopsisthaliana_AT5G66300.1 TMHMM2.0        outside      1   292
```

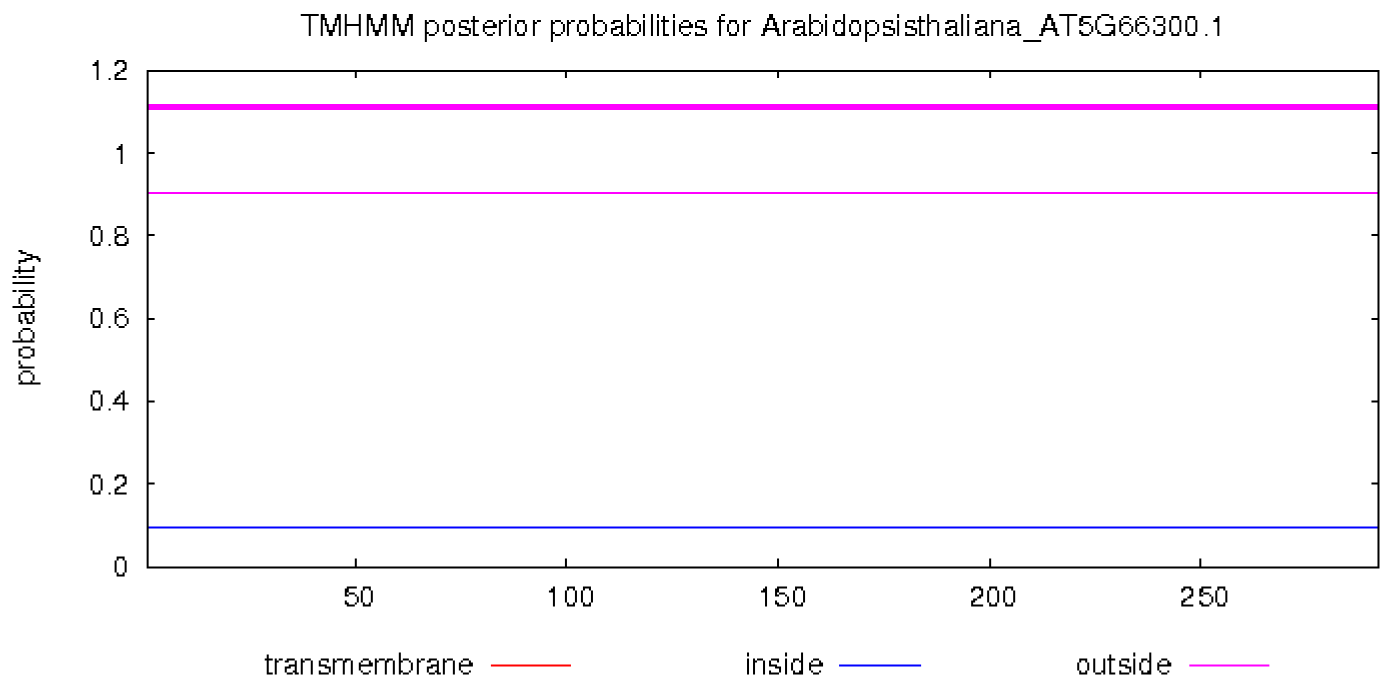

plot in postscript, script for making the plot in gnuplot, data for plot

```
# Arabisalpina_KFK22641.1 Length: 196
# Arabisalpina_KFK22641.1 Number of predicted TMHs:  0
# Arabisalpina_KFK22641.1 Exp number of AAs in TMHs: 0.00699
# Arabisalpina_KFK22641.1 Exp number, first 60 AAs:  0.00645
# Arabisalpina_KFK22641.1 Total prob of N-in:        0.15970
Arabisalpina_KFK22641.1 TMHMM2.0        outside      1   196
```

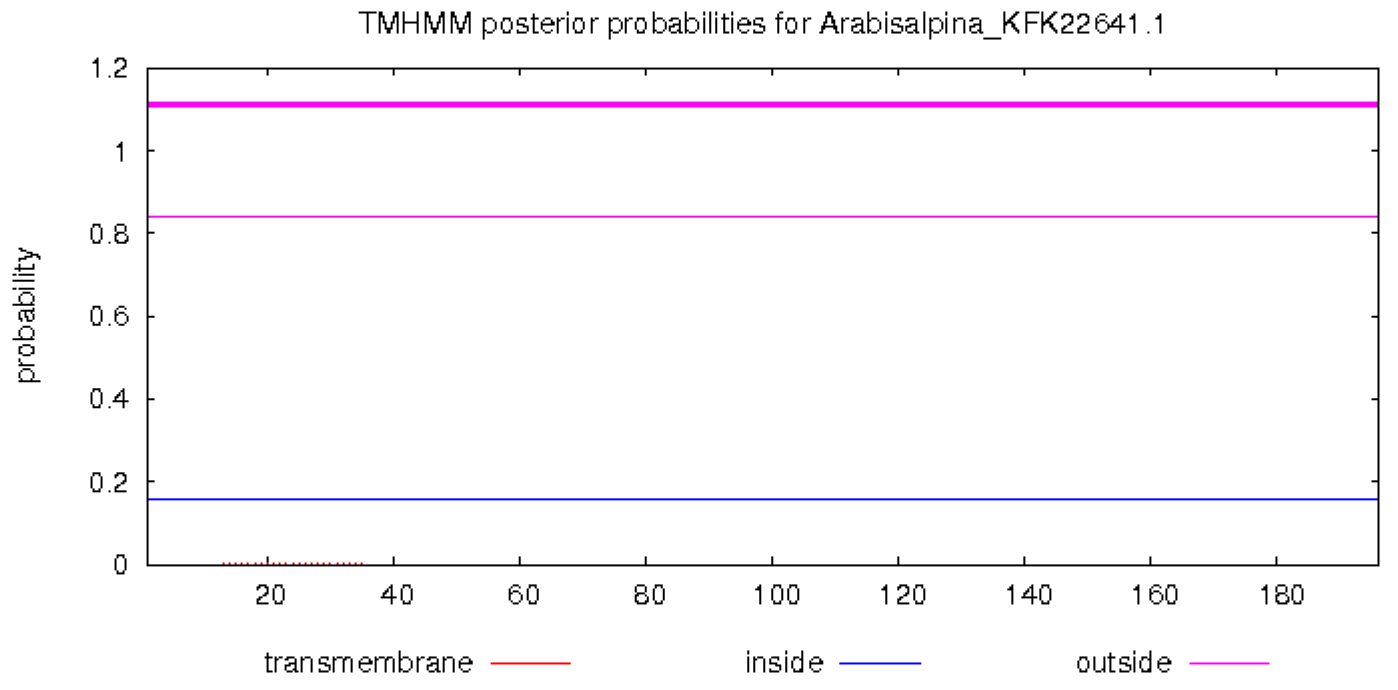

plot in postscript, script for making the plot in gnuplot, data for plot

```
# Arabisalpina_KFK22783.1 Length: 280
# Arabisalpina_KFK22783.1 Number of predicted TMHs:  0
# Arabisalpina_KFK22783.1 Exp number of AAs in TMHs: 0
# Arabisalpina_KFK22783.1 Exp number, first 60 AAs:  0
# Arabisalpina_KFK22783.1 Total prob of N-in:        0.06657
Arabisalpina_KFK22783.1 TMHMM2.0        outside      1   280
```

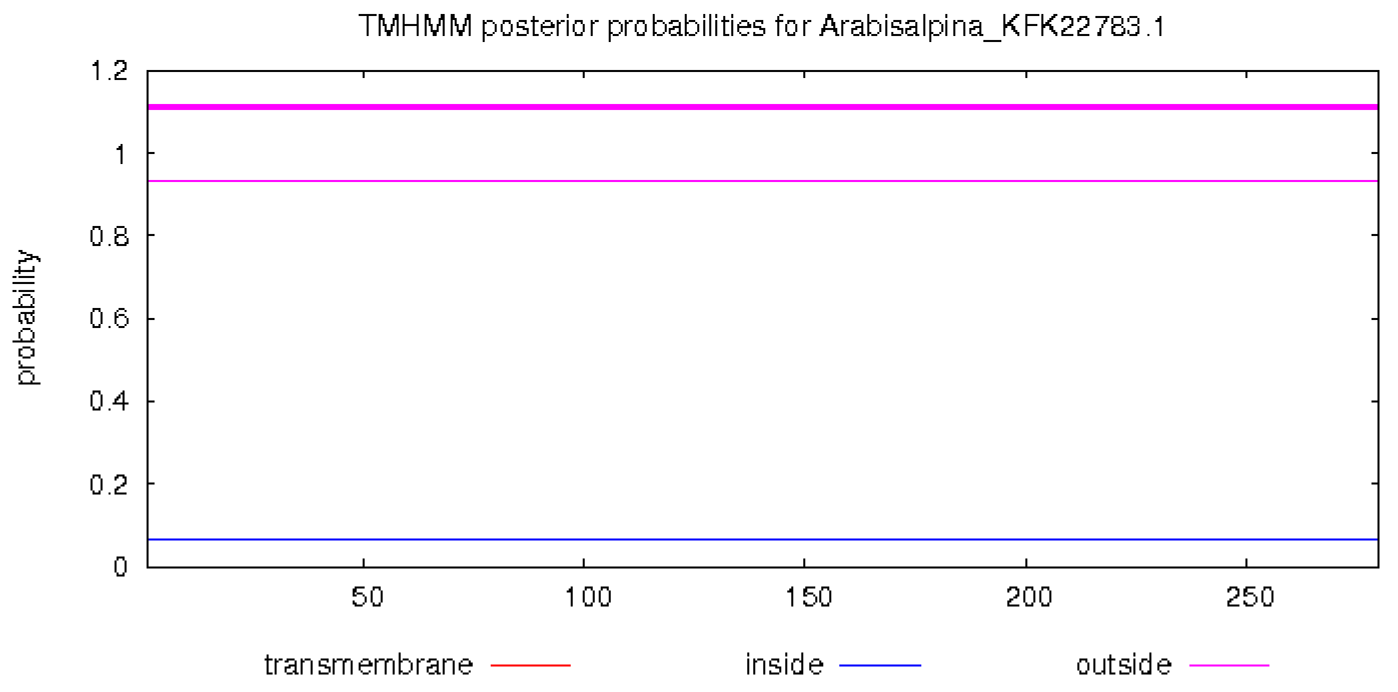

plot in postscript, script for making the plot in gnuplot, data for plot

```
# Arabisalpina_KFK23146.1 Length: 236
# Arabisalpina_KFK23146.1 Number of predicted TMHs:  0
# Arabisalpina_KFK23146.1 Exp number of AAs in TMHs: 0.00035
# Arabisalpina_KFK23146.1 Exp number, first 60 AAs:  0
# Arabisalpina_KFK23146.1 Total prob of N-in:        0.02278
Arabisalpina_KFK23146.1 TMHMM2.0        outside      1   236
```

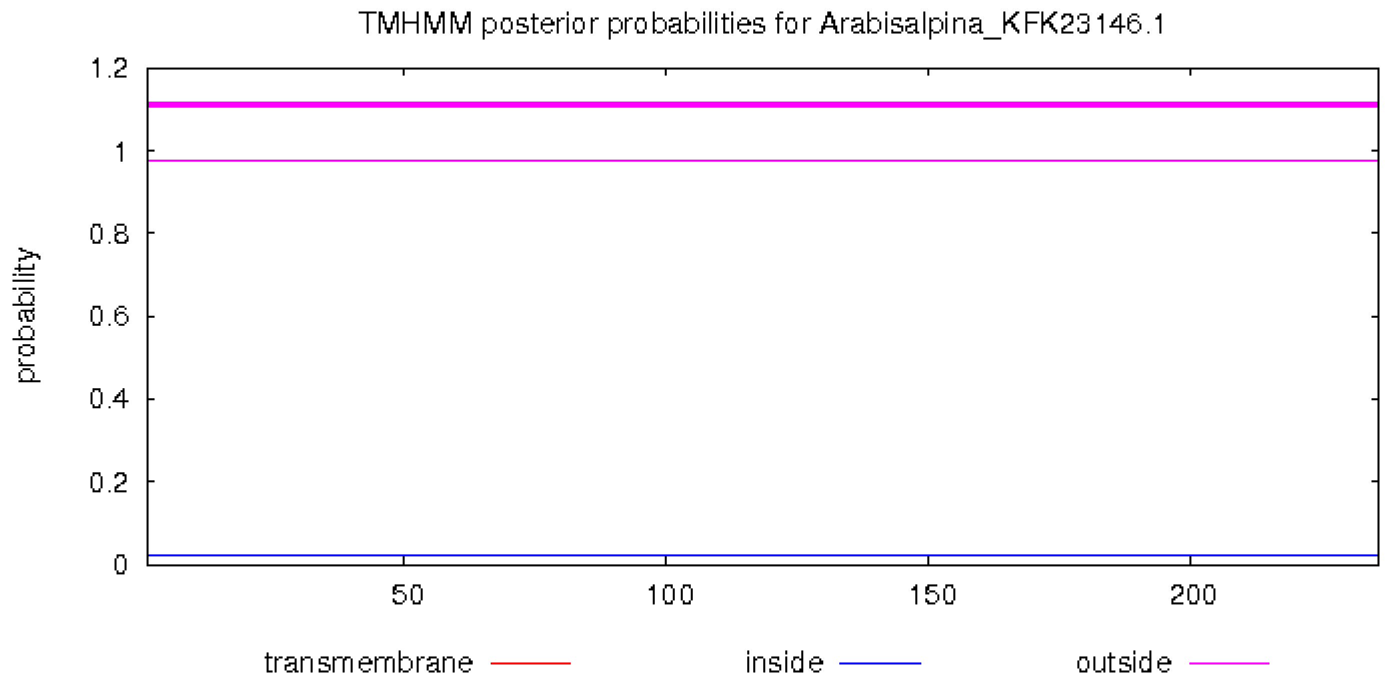

plot in postscript, script for making the plot in gnuplot, data for plot

```
# Arabisalpina_KFK24130.1 Length: 245
# Arabisalpina_KFK24130.1 Number of predicted TMHs:  0
# Arabisalpina_KFK24130.1 Exp number of AAs in TMHs: 0.00019
# Arabisalpina_KFK24130.1 Exp number, first 60 AAs:  0
# Arabisalpina_KFK24130.1 Total prob of N-in:        0.04525
Arabisalpina_KFK24130.1 TMHMM2.0        outside      1   245
```

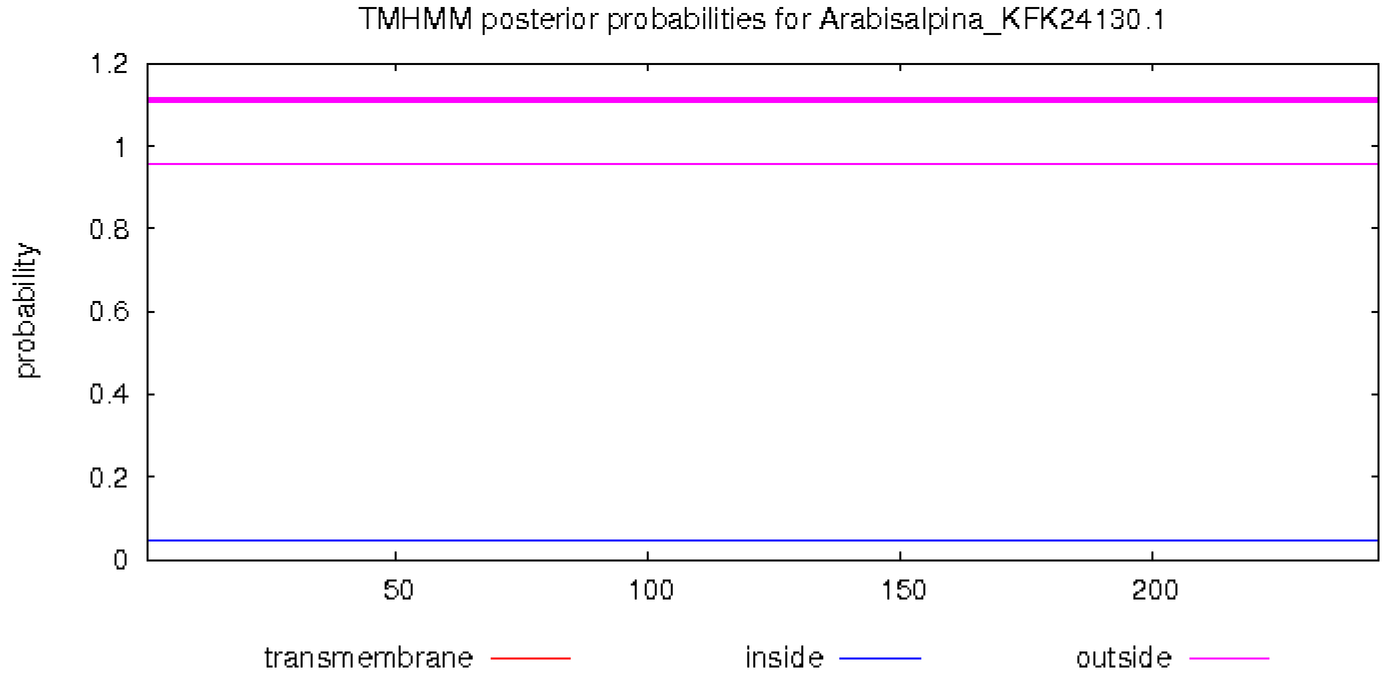

plot in postscript, script for making the plot in gnuplot, data for plot

```
# Arabisalpina_KFK24890.1 Length: 371
# Arabisalpina_KFK24890.1 Number of predicted TMHs:  0
# Arabisalpina_KFK24890.1 Exp number of AAs in TMHs: 0.00033
# Arabisalpina_KFK24890.1 Exp number, first 60 AAs:  0
# Arabisalpina_KFK24890.1 Total prob of N-in:        0.02034
Arabisalpina_KFK24890.1 TMHMM2.0        outside      1   371
```

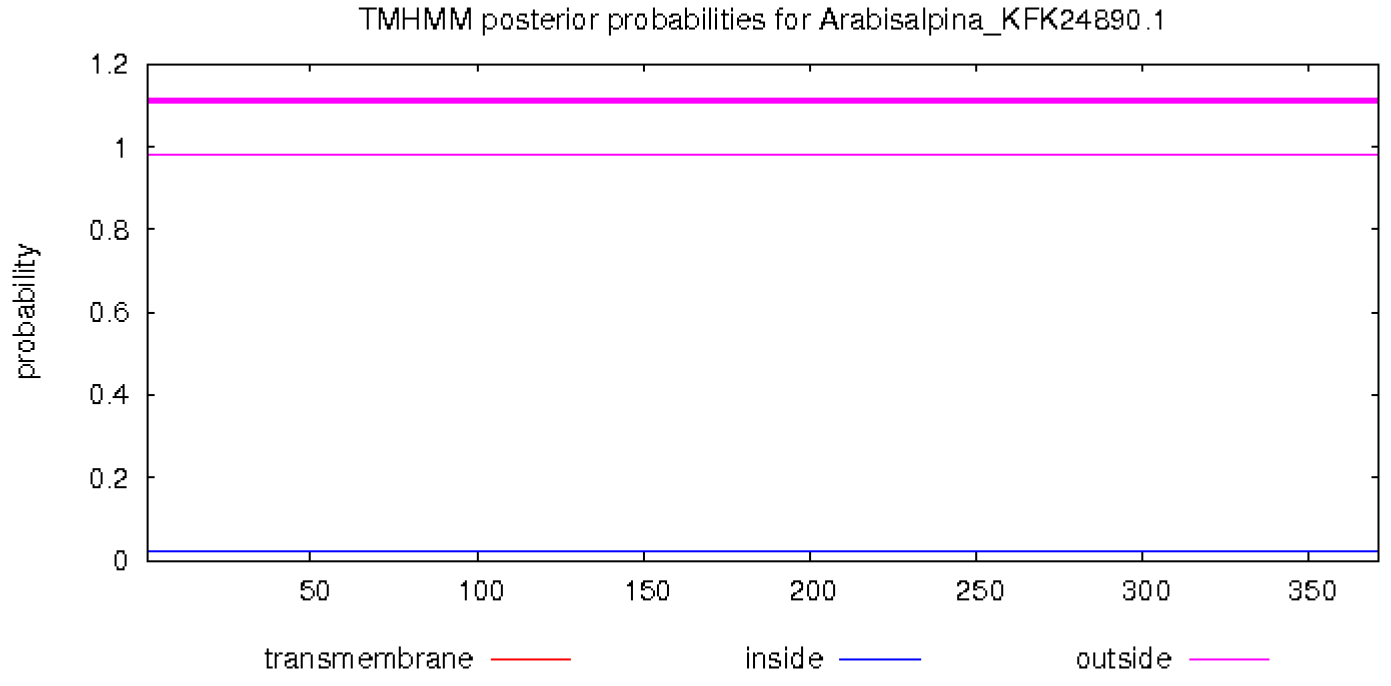

plot in postscript, script for making the plot in gnuplot, data for plot

```
# Arabisalpina_KFK24891.1 Length: 580
# Arabisalpina_KFK24891.1 Number of predicted TMHs:  1
# Arabisalpina_KFK24891.1 Exp number of AAs in TMHs: 21.32113
# Arabisalpina_KFK24891.1 Exp number, first 60 AAs:  0
# Arabisalpina_KFK24891.1 Total prob of N-in:        0.01559
Arabisalpina_KFK24891.1 TMHMM2.0        outside      1   554
Arabisalpina_KFK24891.1 TMHMM2.0        TMhelix    555   577
Arabisalpina_KFK24891.1 TMHMM2.0        inside     578   580
```

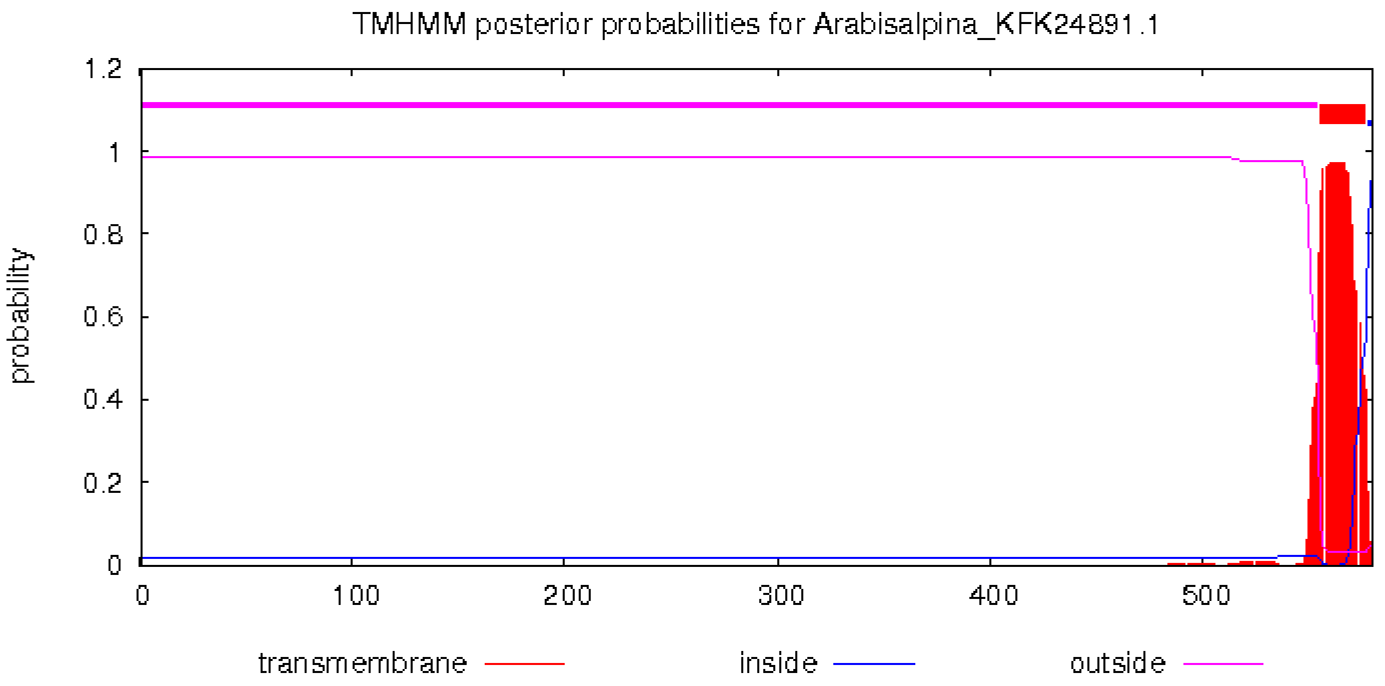

plot in postscript, script for making the plot in gnuplot, data for plot

```
# Arabisalpina_KFK25161.1 Length: 320
# Arabisalpina_KFK25161.1 Number of predicted TMHs:  0
# Arabisalpina_KFK25161.1 Exp number of AAs in TMHs: 8e-05
# Arabisalpina_KFK25161.1 Exp number, first 60 AAs:  0
# Arabisalpina_KFK25161.1 Total prob of N-in:        0.00716
Arabisalpina_KFK25161.1 TMHMM2.0        outside      1   320
```

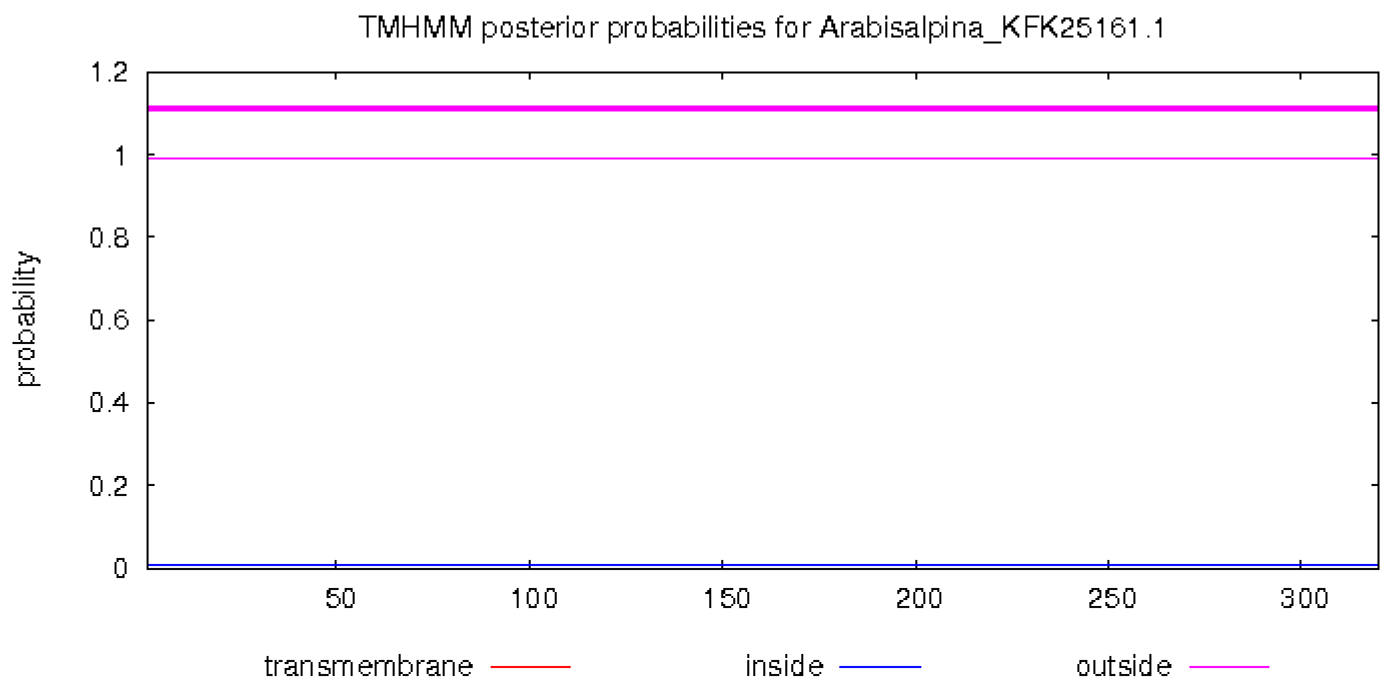

plot in postscript, script for making the plot in gnuplot, data for plot

```
# Arabisalpina_KFK25187.1 Length: 287
# Arabisalpina_KFK25187.1 Number of predicted TMHs:  0
# Arabisalpina_KFK25187.1 Exp number of AAs in TMHs: 0.05495
# Arabisalpina_KFK25187.1 Exp number, first 60 AAs:  0.05476
# Arabisalpina_KFK25187.1 Total prob of N-in:        0.03538
Arabisalpina_KFK25187.1 TMHMM2.0        outside      1   287
```

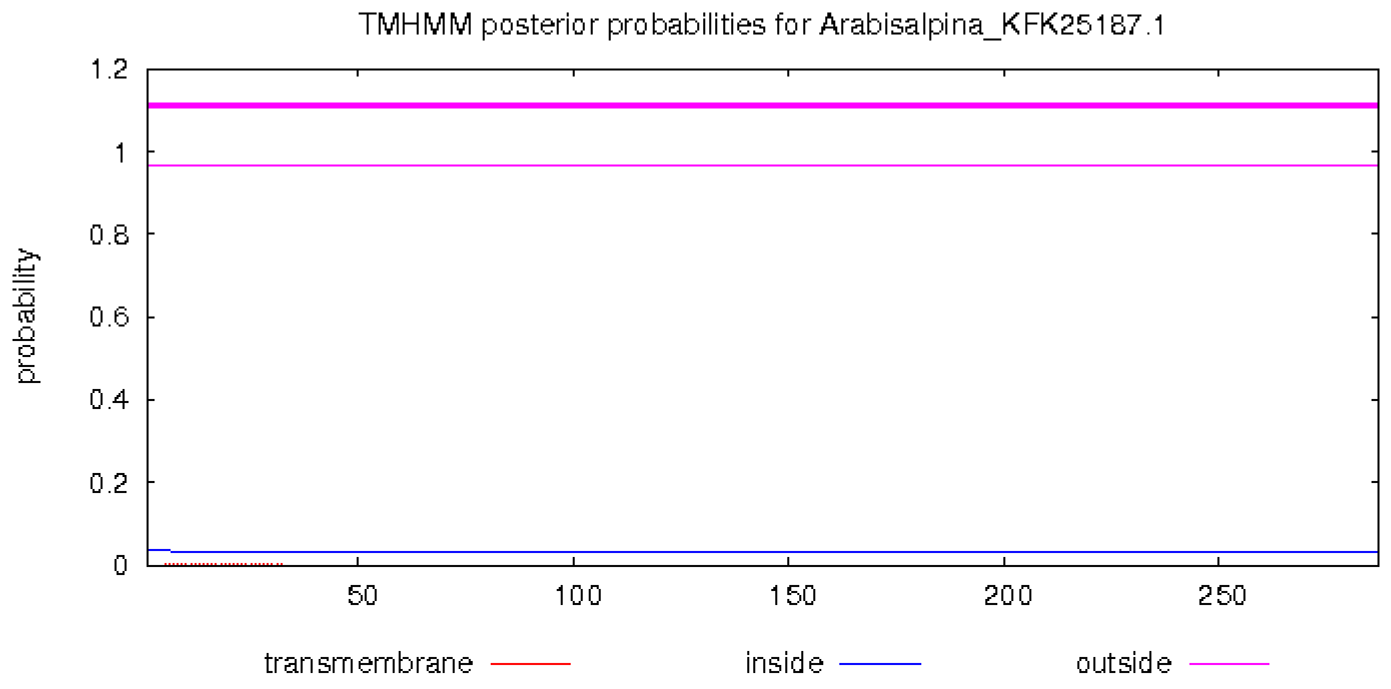

plot in postscript, script for making the plot in gnuplot, data for plot

```
# Arabisalpina_KFK25233.1 Length: 284
# Arabisalpina_KFK25233.1 Number of predicted TMHs:  0
# Arabisalpina_KFK25233.1 Exp number of AAs in TMHs: 0.00019
# Arabisalpina_KFK25233.1 Exp number, first 60 AAs:  0
# Arabisalpina_KFK25233.1 Total prob of N-in:        0.01009
Arabisalpina_KFK25233.1 TMHMM2.0        outside      1   284
```

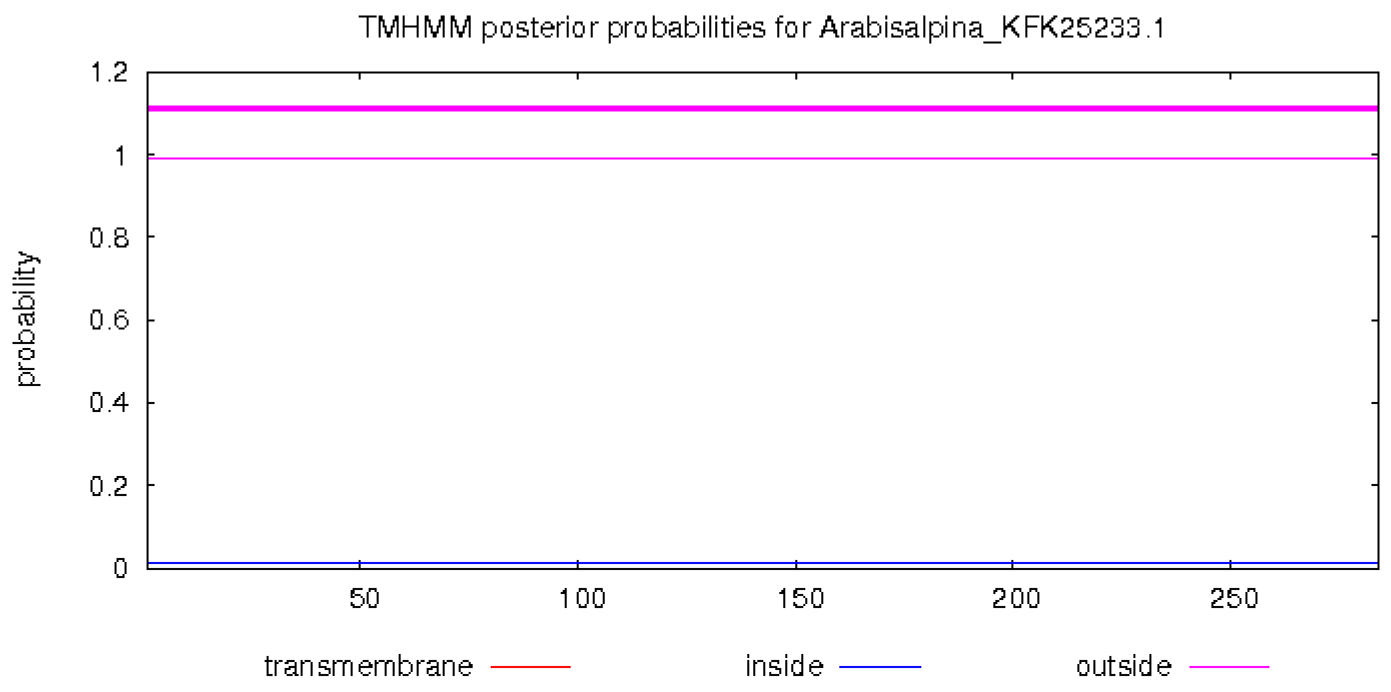

plot in postscript, script for making the plot in gnuplot, data for plot

---

```
# Arabisalpina_KFK25634.1 Length: 194
# Arabisalpina_KFK25634.1 Number of predicted TMHs:  0
# Arabisalpina_KFK25634.1 Exp number of AAs in TMHs: 0.45767
# Arabisalpina_KFK25634.1 Exp number, first 60 AAs:  0.2649
# Arabisalpina_KFK25634.1 Total prob of N-in:        0.03285
Arabisalpina_KFK25634.1 TMHMM2.0        outside      1   194
```

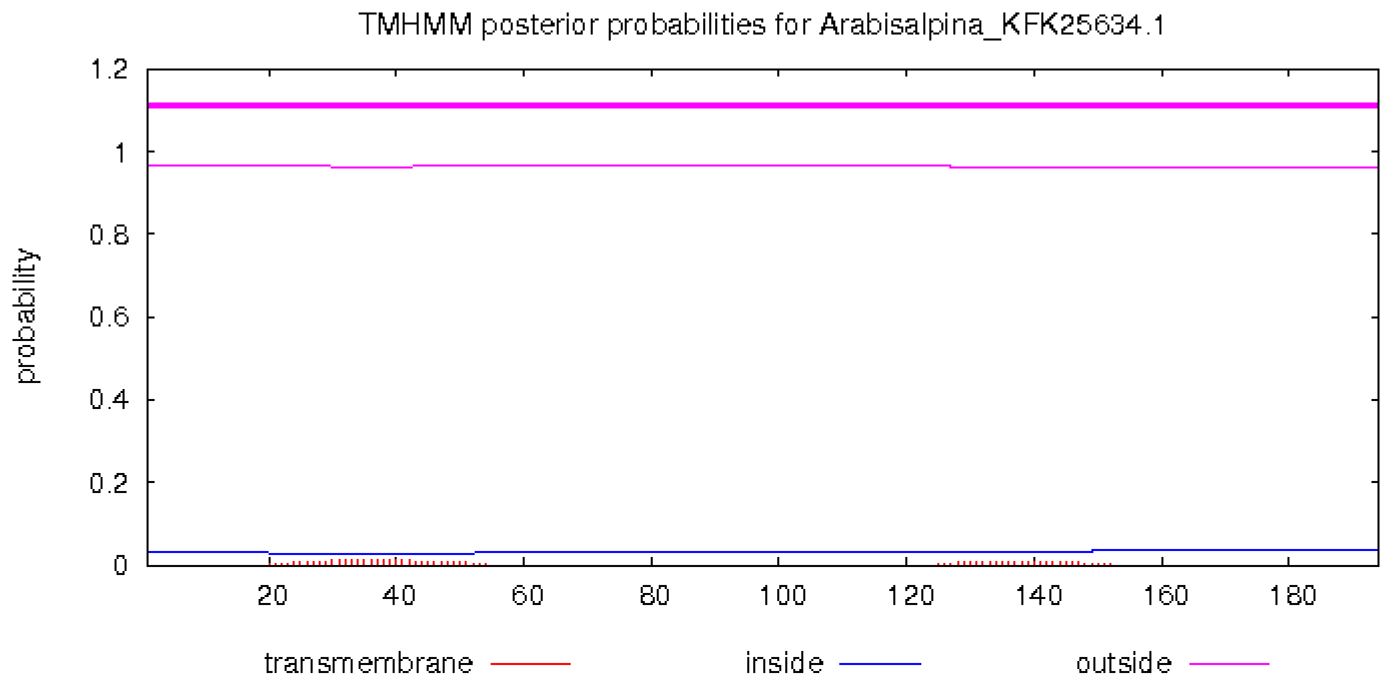

plot in postscript, script for making the plot in gnuplot, data for plot

---

```
# Arabisalpina_KFK25688.1 Length: 609
# Arabisalpina_KFK25688.1 Number of predicted TMHs:  0
# Arabisalpina_KFK25688.1 Exp number of AAs in TMHs: 0.28145
# Arabisalpina_KFK25688.1 Exp number, first 60 AAs:  0
# Arabisalpina_KFK25688.1 Total prob of N-in:        0.01663
Arabisalpina_KFK25688.1 TMHMM2.0        outside      1   609
```

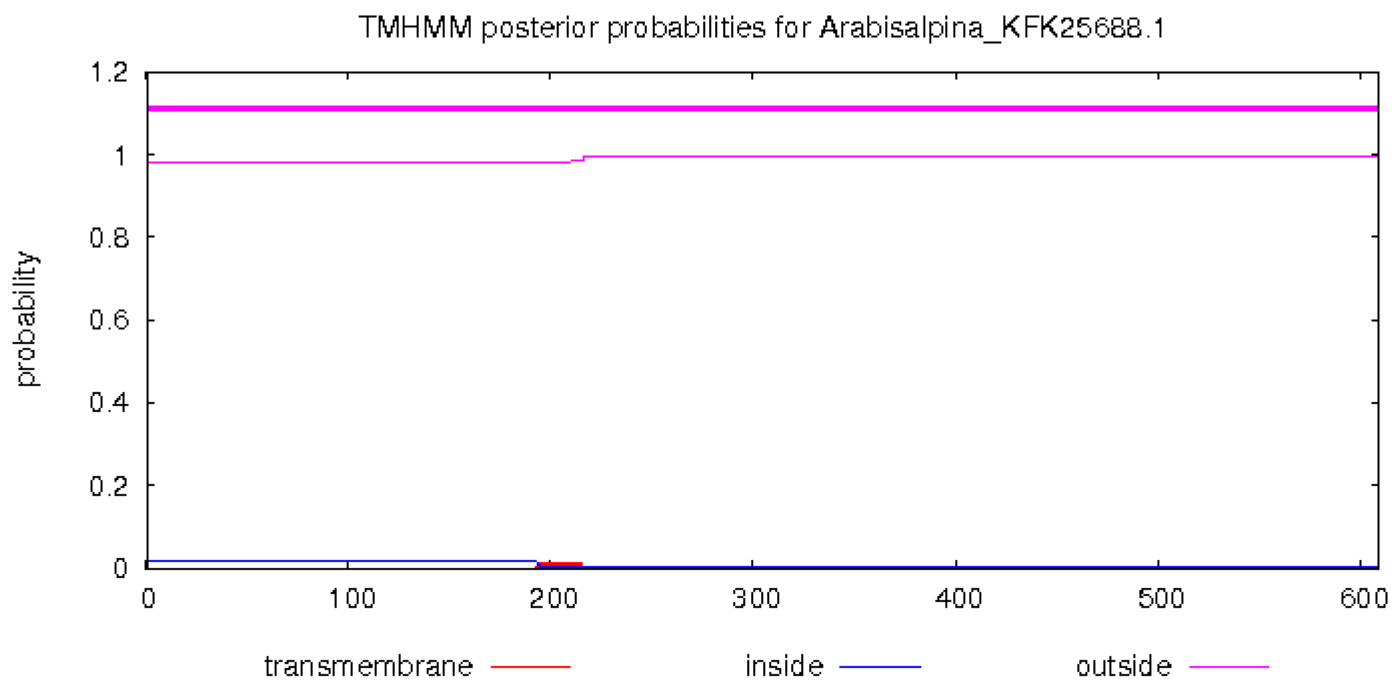

plot in postscript, script for making the plot in gnuplot, data for plot

---

```
# Arabisalpina_KFK26065.1 Length: 338
# Arabisalpina_KFK26065.1 Number of predicted TMHs:  0
# Arabisalpina_KFK26065.1 Exp number of AAs in TMHs: 0.00041
# Arabisalpina_KFK26065.1 Exp number, first 60 AAs:  0
# Arabisalpina_KFK26065.1 Total prob of N-in:        0.01215
Arabisalpina_KFK26065.1 TMHMM2.0        outside      1   338
```

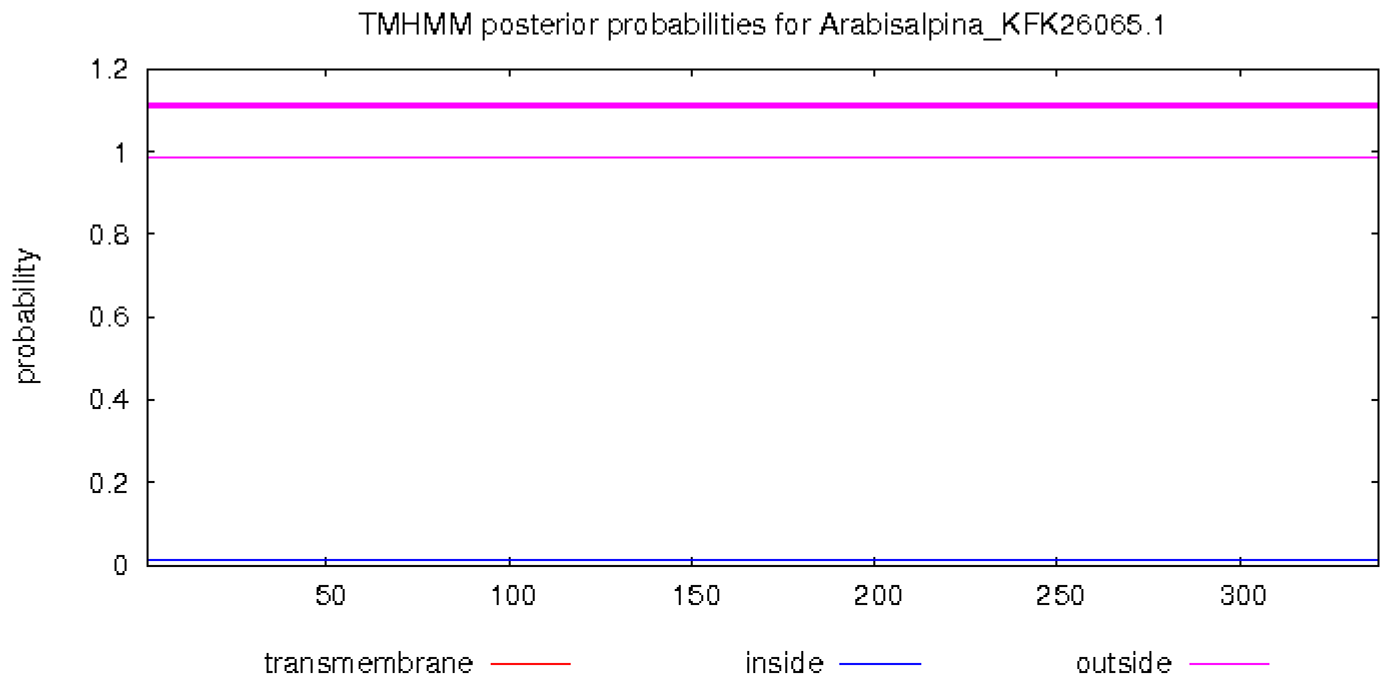

plot in postscript, script for making the plot in gnuplot, data for plot

---

```
# Arabisalpina_KFK26331.1 Length: 342
# Arabisalpina_KFK26331.1 Number of predicted TMHs:  0
# Arabisalpina_KFK26331.1 Exp number of AAs in TMHs: 11.11197
# Arabisalpina_KFK26331.1 Exp number, first 60 AAs:  0.02289
# Arabisalpina_KFK26331.1 Total prob of N-in:        0.15315
Arabisalpina_KFK26331.1 TMHMM2.0        outside      1   342
```

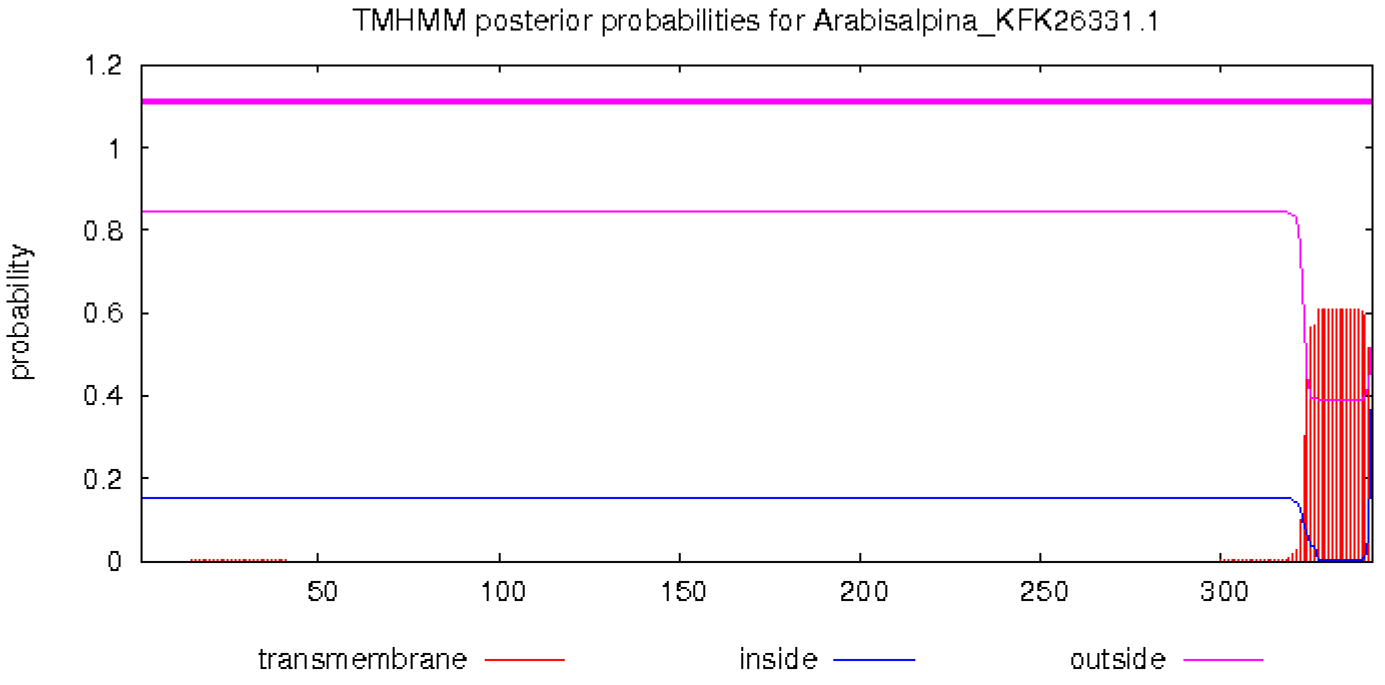

plot in postscript, script for making the plot in gnuplot, data for plot

---

```
# Arabisalpina_KFK26711.1 Length: 188
# Arabisalpina_KFK26711.1 Number of predicted TMHs:  0
# Arabisalpina_KFK26711.1 Exp number of AAs in TMHs: 0.00066
# Arabisalpina_KFK26711.1 Exp number, first 60 AAs:  0
# Arabisalpina_KFK26711.1 Total prob of N-in:        0.05974
Arabisalpina_KFK26711.1 TMHMM2.0        outside      1   188
```

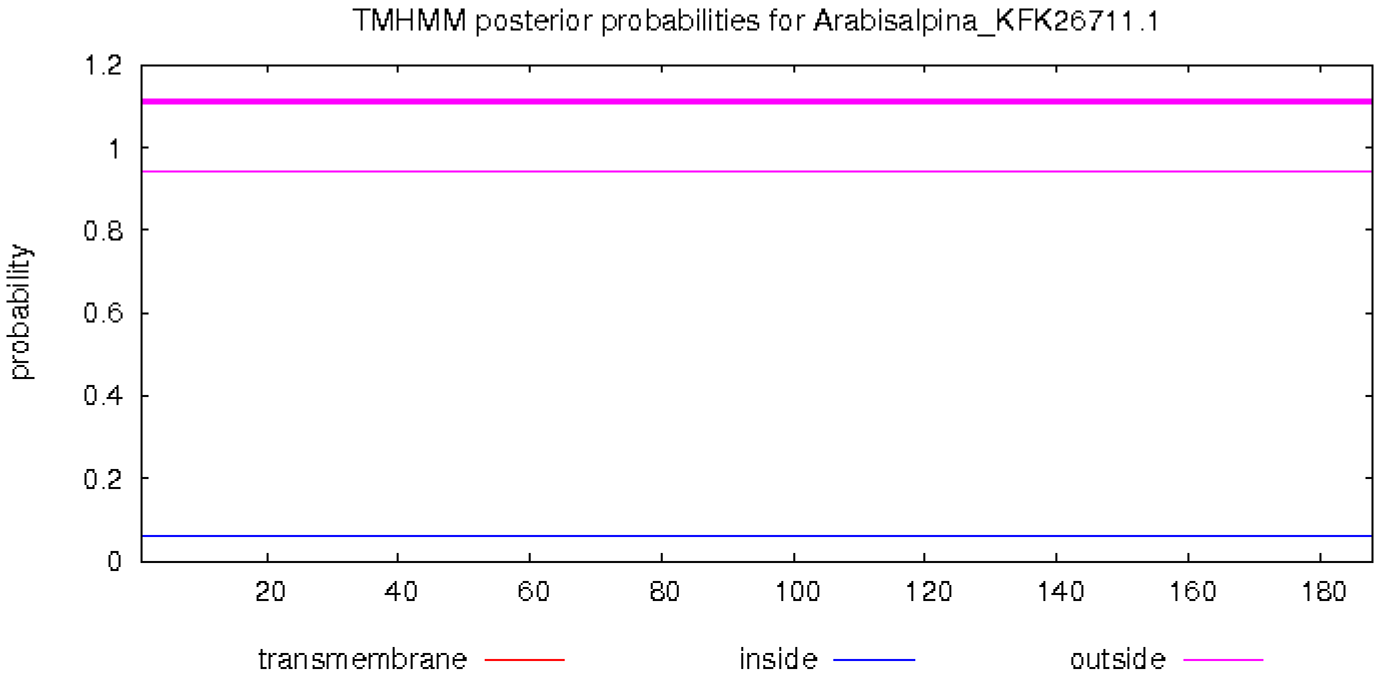

plot in postscript, script for making the plot in gnuplot, data for plot

---

```
# Arabisalpina_KFK26712.1 Length: 189
# Arabisalpina_KFK26712.1 Number of predicted TMHs:  0
# Arabisalpina_KFK26712.1 Exp number of AAs in TMHs: 0.00069
# Arabisalpina_KFK26712.1 Exp number, first 60 AAs:  0
# Arabisalpina_KFK26712.1 Total prob of N-in:        0.05904
Arabisalpina_KFK26712.1 TMHMM2.0        outside      1   189
```

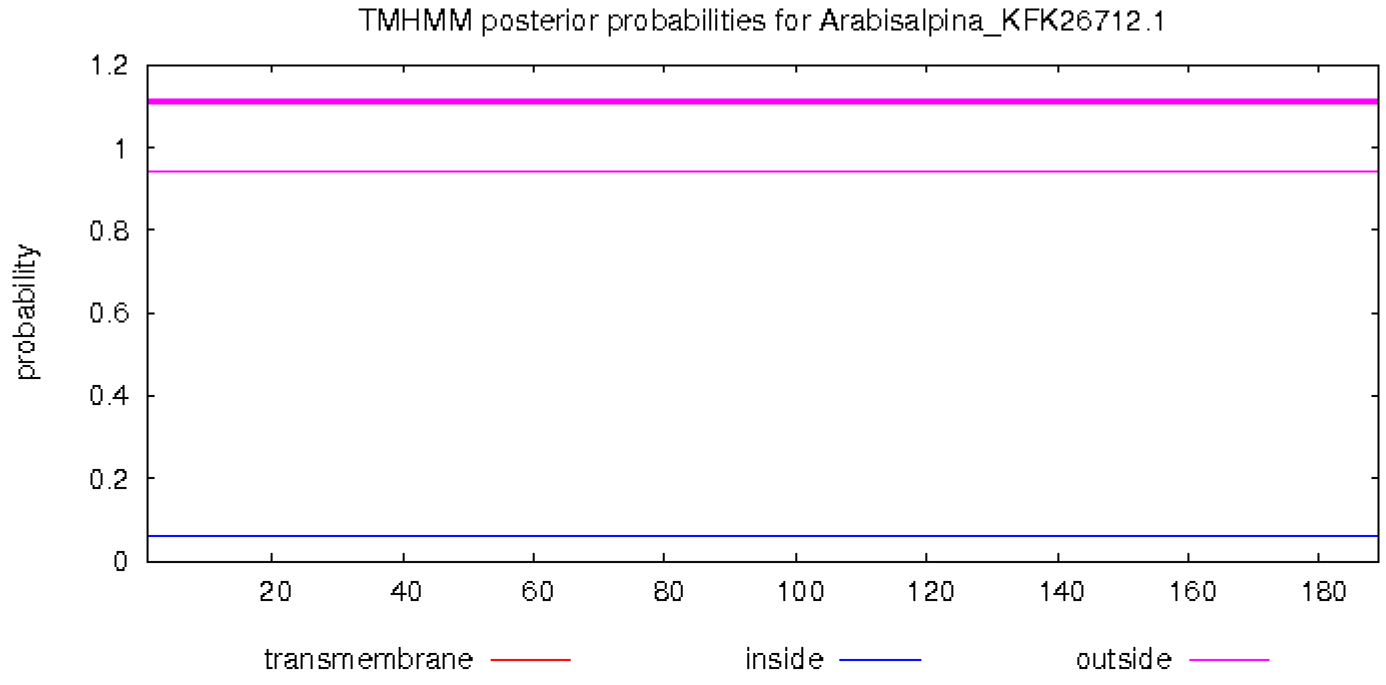

plot in postscript, script for making the plot in gnuplot, data for plot

---

```
# Arabisalpina_KFK27011.1 Length: 368
# Arabisalpina_KFK27011.1 Number of predicted TMHs:  0
# Arabisalpina_KFK27011.1 Exp number of AAs in TMHs: 0.01199
# Arabisalpina_KFK27011.1 Exp number, first 60 AAs:  0
# Arabisalpina_KFK27011.1 Total prob of N-in:        0.02135
Arabisalpina_KFK27011.1 TMHMM2.0        outside      1   368
```

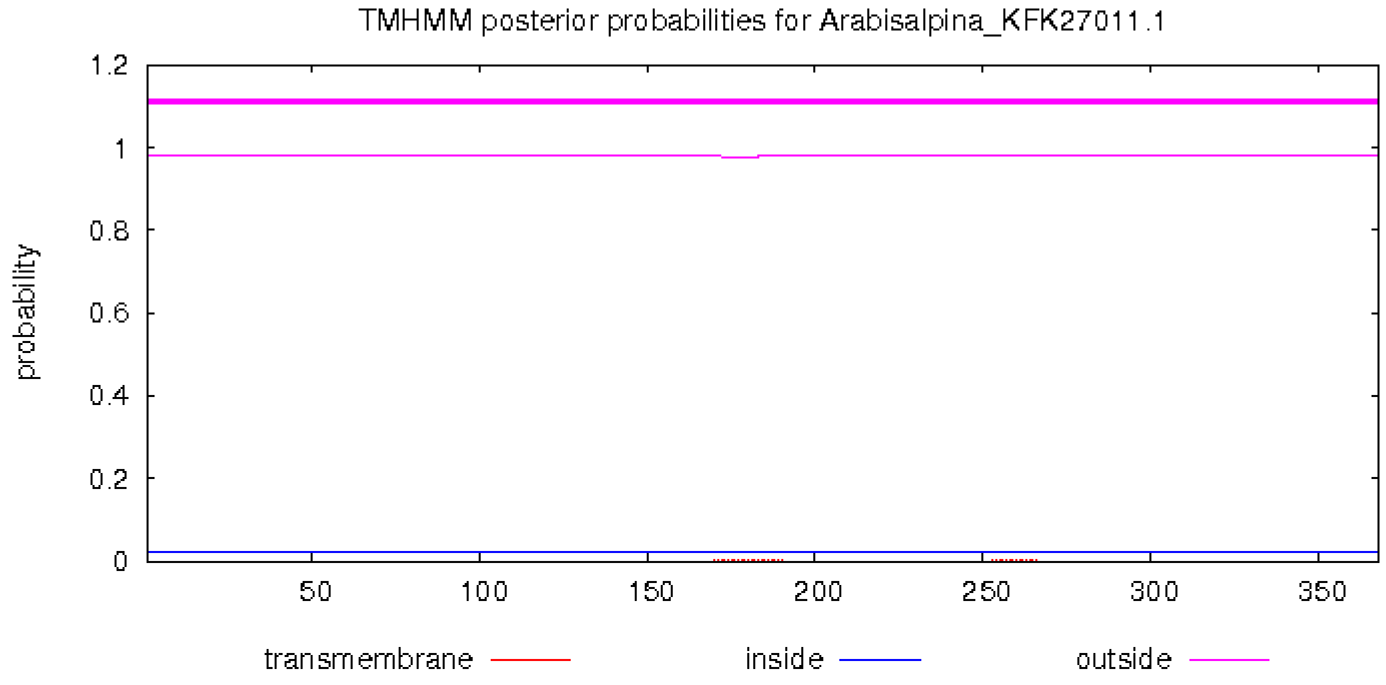

plot in postscript, script for making the plot in gnuplot, data for plot

---

```
# Arabisalpina_KFK27422.1 Length: 357
# Arabisalpina_KFK27422.1 Number of predicted TMHs:  0
# Arabisalpina_KFK27422.1 Exp number of AAs in TMHs: 0.0004
# Arabisalpina_KFK27422.1 Exp number, first 60 AAs:  0
# Arabisalpina_KFK27422.1 Total prob of N-in:        0.01249
Arabisalpina_KFK27422.1 TMHMM2.0        outside      1   357
```

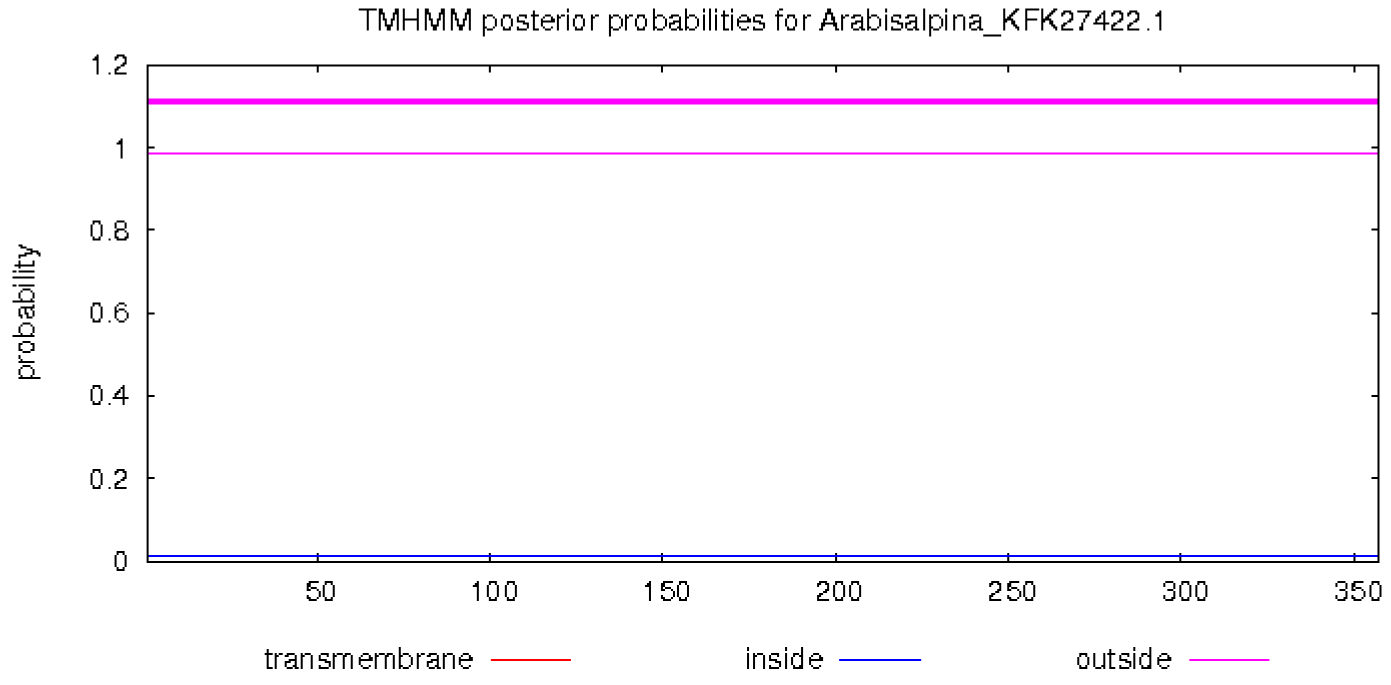

plot in postscript, script for making the plot in gnuplot, data for plot

---

```
# Arabisalpina_KFK27438.1 Length: 245
# Arabisalpina_KFK27438.1 Number of predicted TMHs:  0
# Arabisalpina_KFK27438.1 Exp number of AAs in TMHs: 0.00056
# Arabisalpina_KFK27438.1 Exp number, first 60 AAs:  0.0005
# Arabisalpina_KFK27438.1 Total prob of N-in:        0.04448
Arabisalpina_KFK27438.1 TMHMM2.0        outside      1   245
```

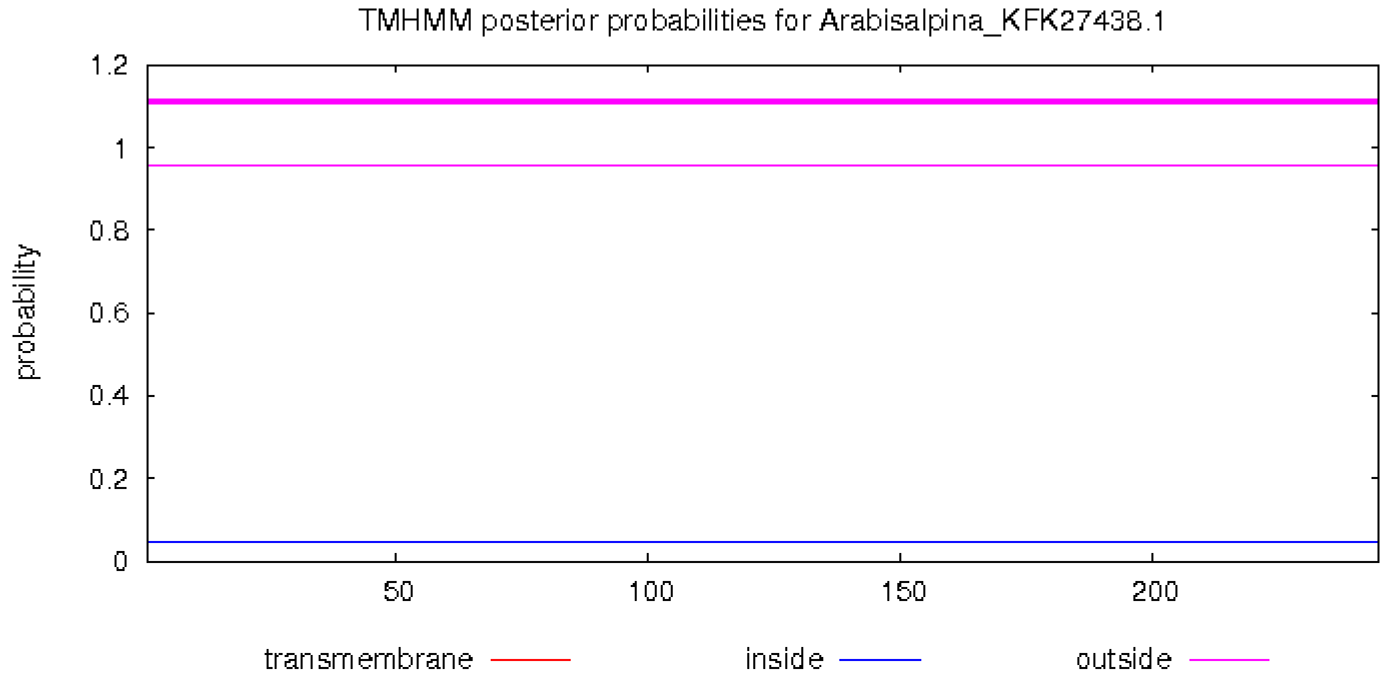

plot in postscript, script for making the plot in gnuplot, data for plot

---

```
# Arabisalpina_KFK27441.1 Length: 356
# Arabisalpina_KFK27441.1 Number of predicted TMHs:  0
# Arabisalpina_KFK27441.1 Exp number of AAs in TMHs: 0.00019
# Arabisalpina_KFK27441.1 Exp number, first 60 AAs:  0
# Arabisalpina_KFK27441.1 Total prob of N-in:        0.01263
Arabisalpina_KFK27441.1 TMHMM2.0        outside      1   356
```

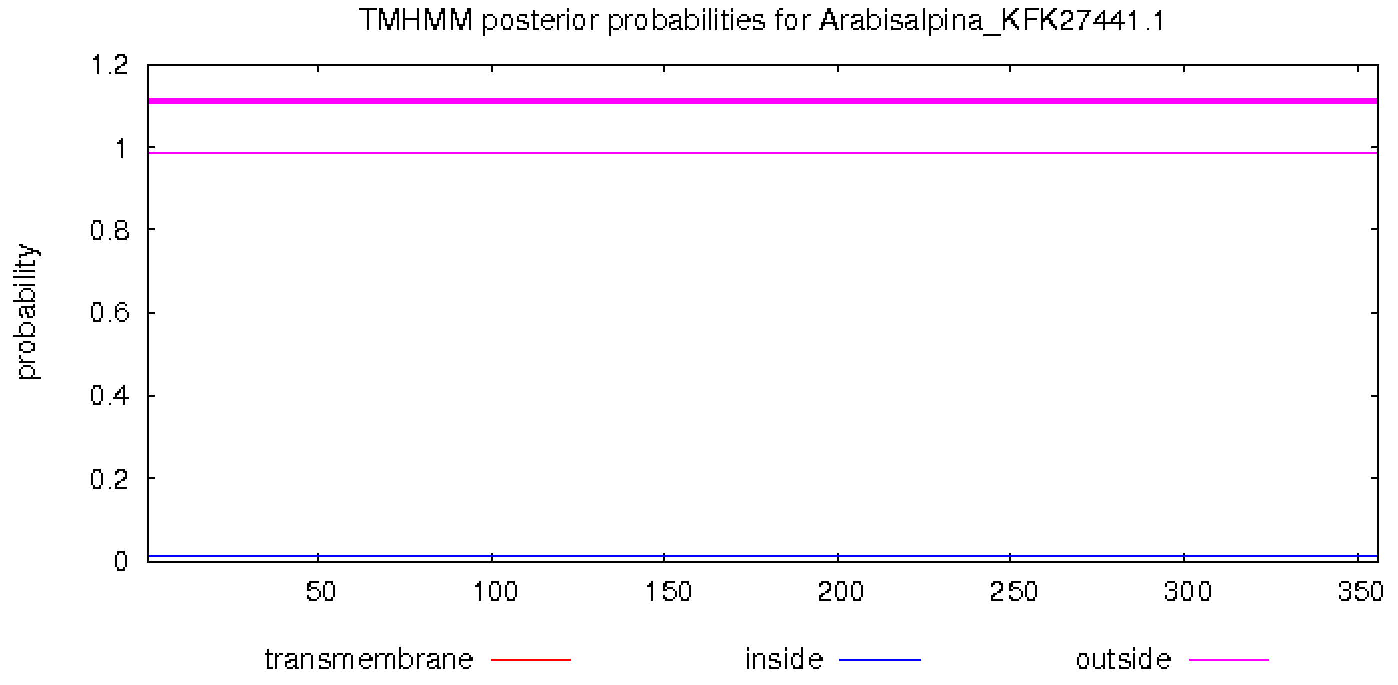

plot in postscript, script for making the plot in gnuplot, data for plot

```
# Arabisalpina_KFK27735.1 Length: 464
# Arabisalpina_KFK27735.1 Number of predicted TMHs:  1
# Arabisalpina_KFK27735.1 Exp number of AAs in TMHs: 22.10464
# Arabisalpina_KFK27735.1 Exp number, first 60 AAs:  0.00477
# Arabisalpina_KFK27735.1 Total prob of N-in:        0.03185
Arabisalpina_KFK27735.1 TMHMM2.0        outside      1   440
Arabisalpina_KFK27735.1 TMHMM2.0        TMhelix    441   463
Arabisalpina_KFK27735.1 TMHMM2.0        inside     464   464
```

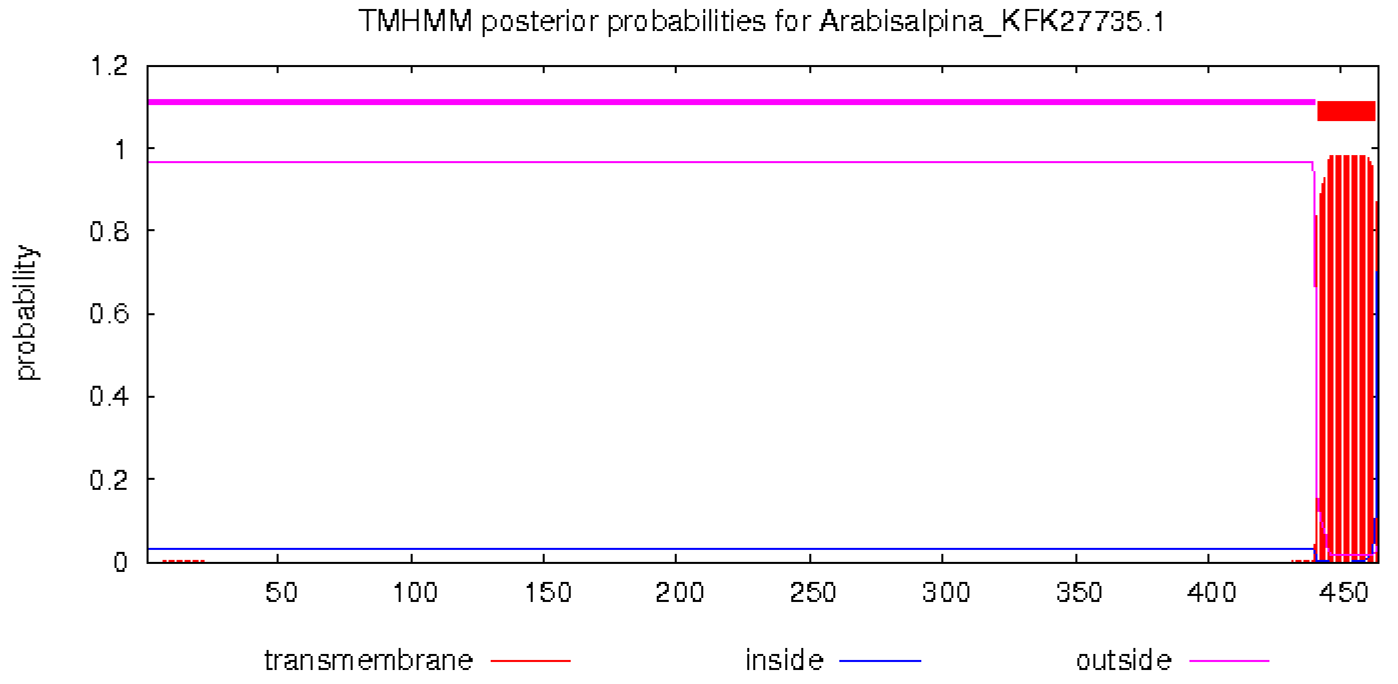

plot in postscript, script for making the plot in gnuplot, data for plot

```
# Arabisalpina_KFK27900.1 Length: 336
# Arabisalpina_KFK27900.1 Number of predicted TMHs:  0
# Arabisalpina_KFK27900.1 Exp number of AAs in TMHs: 0
# Arabisalpina_KFK27900.1 Exp number, first 60 AAs:  0
```

```
# Arabisalpina_KFK27900.1 Total prob of N-in:        0.01252
Arabisalpina_KFK27900.1 TMHMM2.0        outside      1   336
```

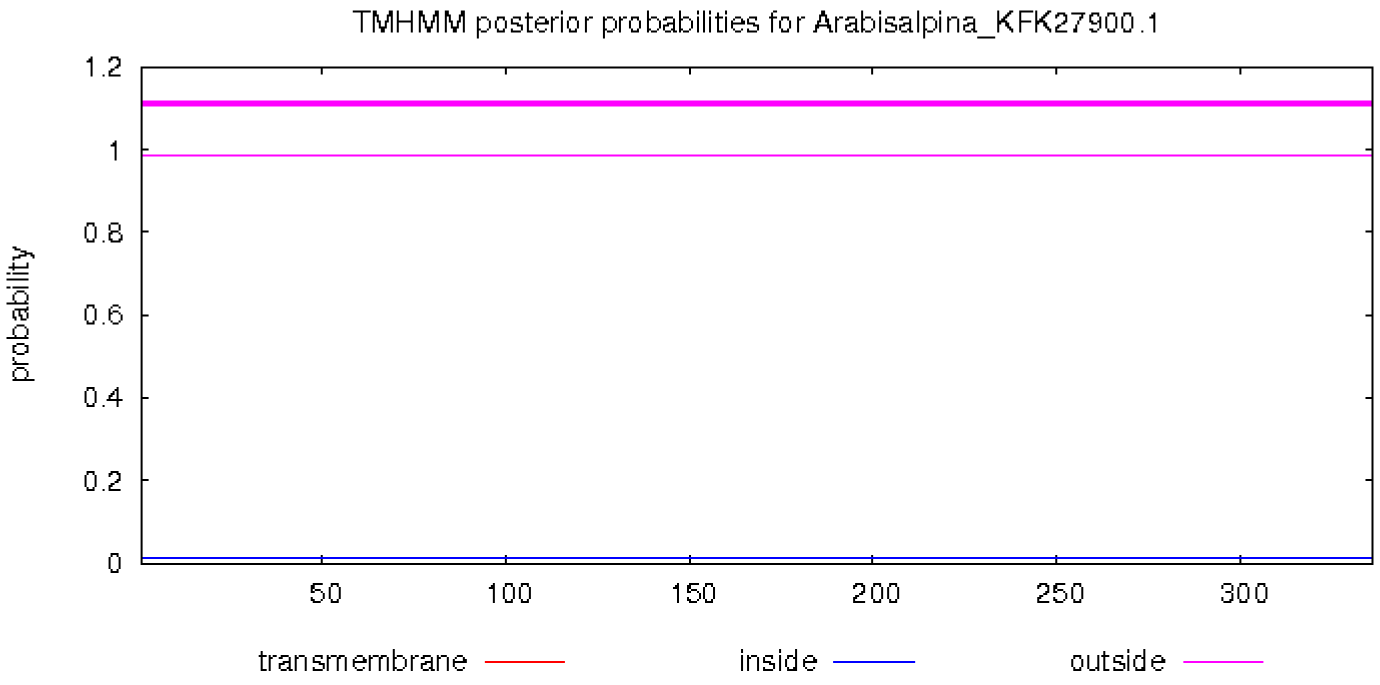

plot in postscript, script for making the plot in gnuplot, data for plot

---

```
# Arabisalpina_KFK28073.1 Length: 264
# Arabisalpina_KFK28073.1 Number of predicted TMHs:  0
# Arabisalpina_KFK28073.1 Exp number of AAs in TMHs: 0.00017
# Arabisalpina_KFK28073.1 Exp number, first 60 AAs:  0
# Arabisalpina_KFK28073.1 Total prob of N-in:        0.01285
Arabisalpina_KFK28073.1 TMHMM2.0        outside      1   264
```

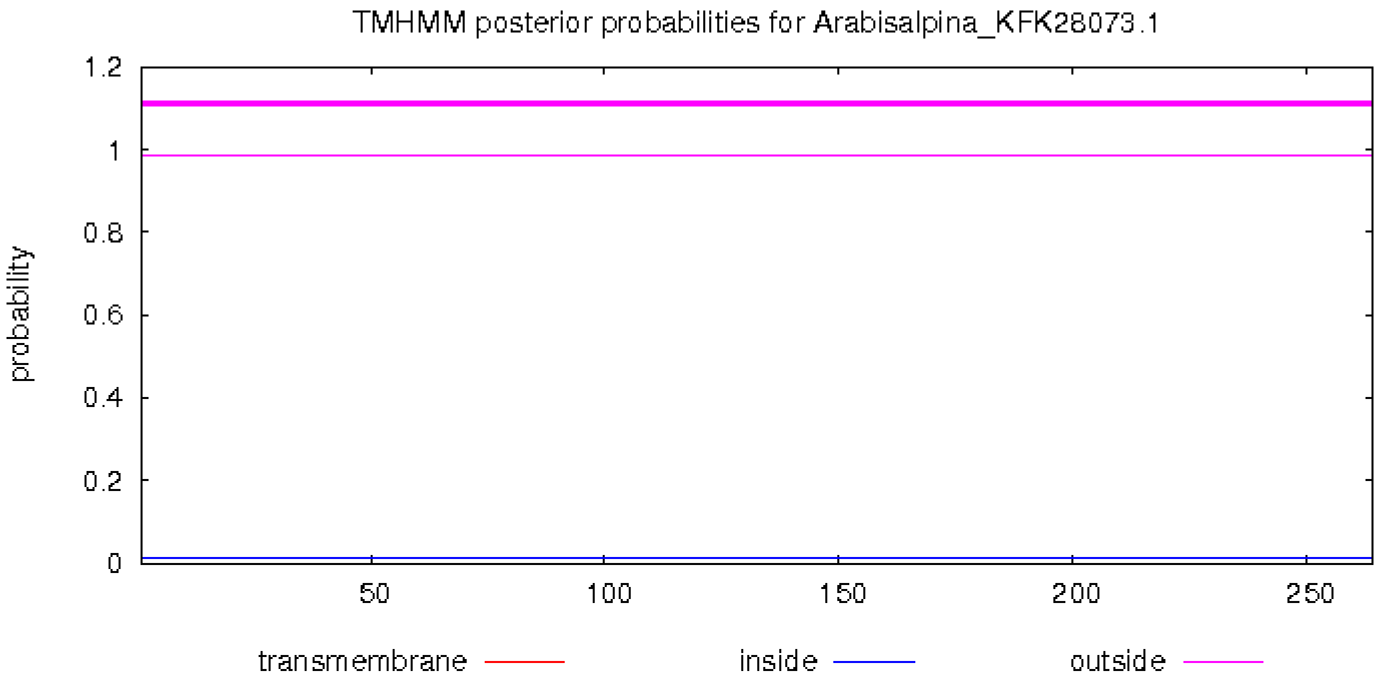

plot in postscript, script for making the plot in gnuplot, data for plot

---

```
# Arabisalpina_KFK28146.1 Length: 186
# Arabisalpina_KFK28146.1 Number of predicted TMHs:  0
# Arabisalpina_KFK28146.1 Exp number of AAs in TMHs: 0.00014
# Arabisalpina_KFK28146.1 Exp number, first 60 AAs:  0
```

```
# Arabisalpina_KFK28146.1 Total prob of N-in:        0.00743
Arabisalpina_KFK28146.1 TMHMM2.0        outside      1   186
```

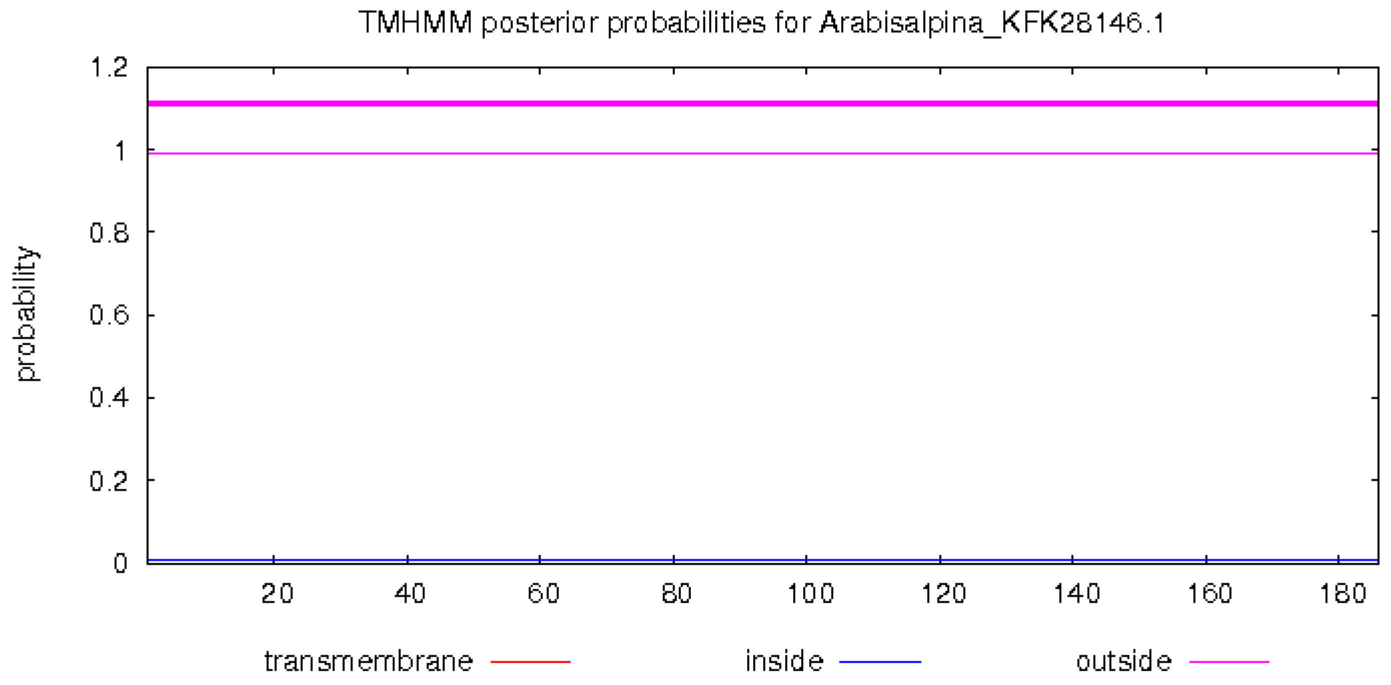

plot in postscript, script for making the plot in gnuplot, data for plot

---

```
# Arabisalpina_KFK28483.1 Length: 289
# Arabisalpina_KFK28483.1 Number of predicted TMHs:  0
# Arabisalpina_KFK28483.1 Exp number of AAs in TMHs: 0
# Arabisalpina_KFK28483.1 Exp number, first 60 AAs:  0
# Arabisalpina_KFK28483.1 Total prob of N-in:        0.00230
Arabisalpina_KFK28483.1 TMHMM2.0        outside      1   289
```

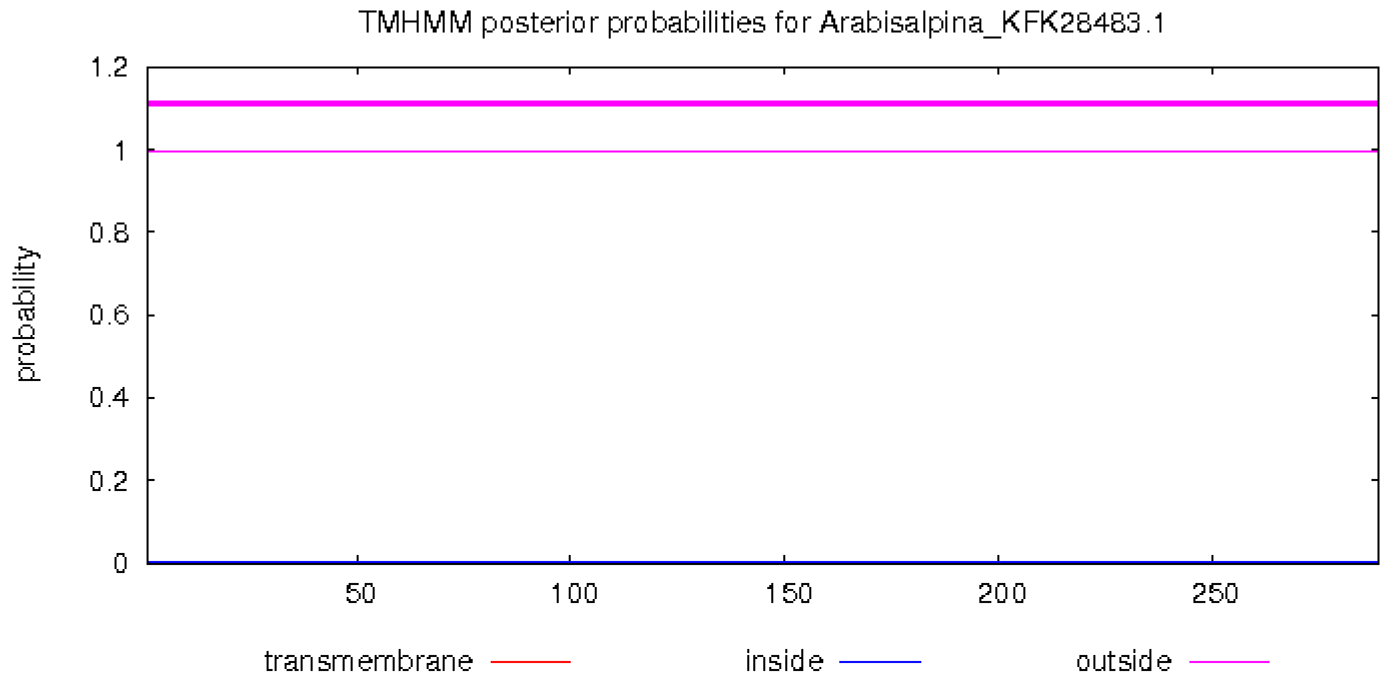

plot in postscript, script for making the plot in gnuplot, data for plot

---

```
# Arabisalpina_KFK29255.1 Length: 294
# Arabisalpina_KFK29255.1 Number of predicted TMHs:  0
# Arabisalpina_KFK29255.1 Exp number of AAs in TMHs: 0.0006
# Arabisalpina_KFK29255.1 Exp number, first 60 AAs:  0
```

```
# Arabisalpina_KFK29255.1 Total prob of N-in:        0.02333
Arabisalpina_KFK29255.1 TMHMM2.0        outside      1   294
```

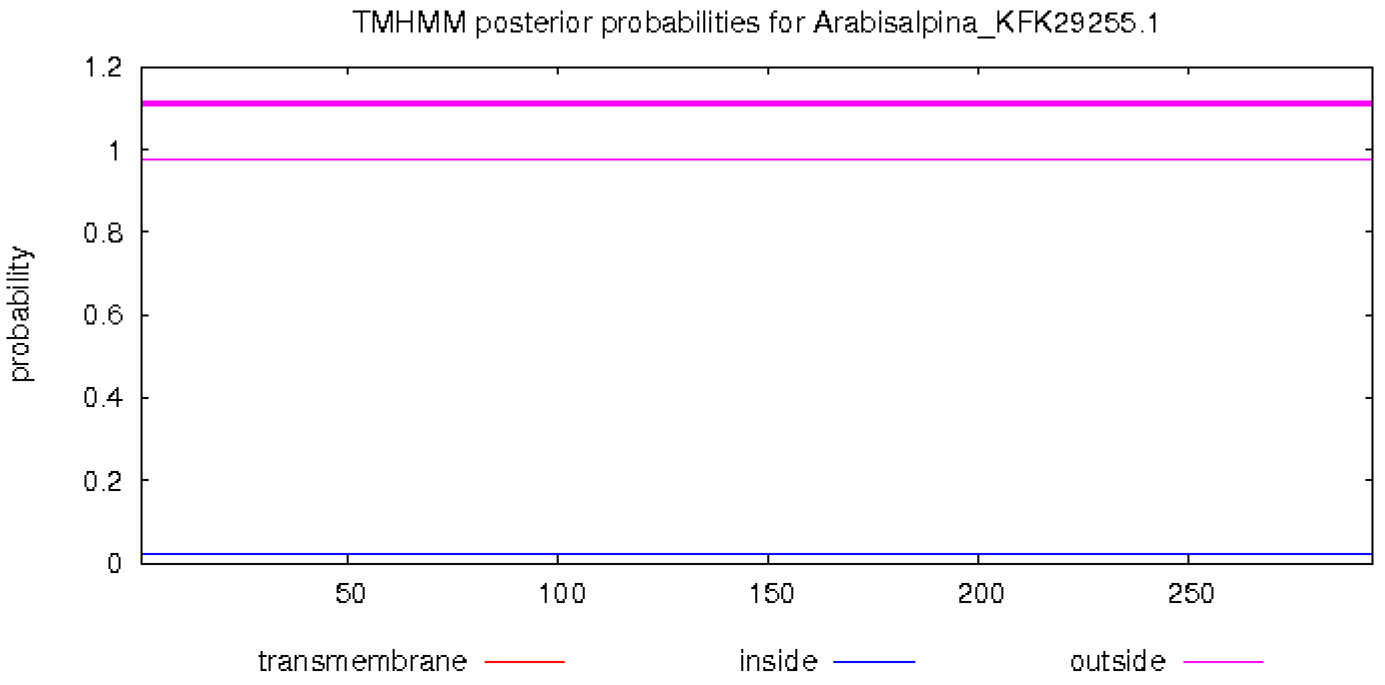

plot in postscript, script for making the plot in gnuplot, data for plot

---

```
# Arabisalpina_KFK29384.1 Length: 305
# Arabisalpina_KFK29384.1 Number of predicted TMHs:  0
# Arabisalpina_KFK29384.1 Exp number of AAs in TMHs: 0.00255
# Arabisalpina_KFK29384.1 Exp number, first 60 AAs:  0
# Arabisalpina_KFK29384.1 Total prob of N-in:        0.10079
Arabisalpina_KFK29384.1 TMHMM2.0        outside      1   305
```

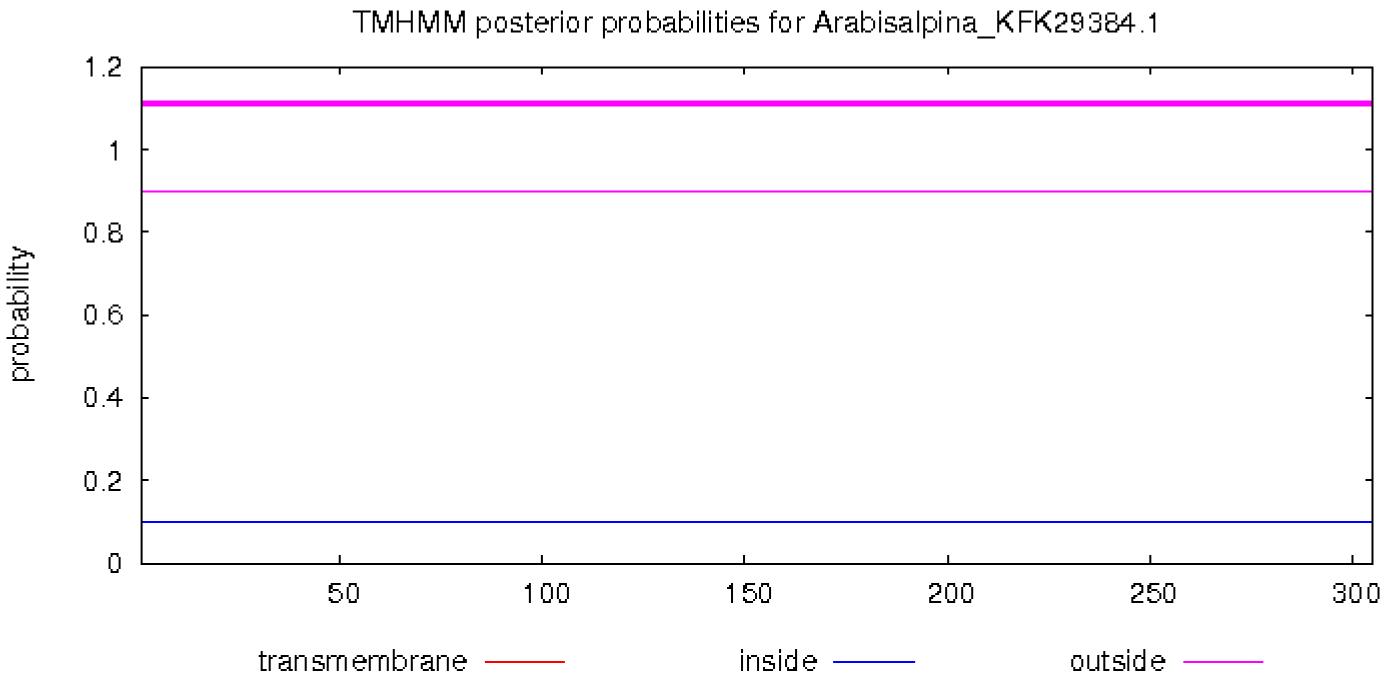

plot in postscript, script for making the plot in gnuplot, data for plot

---

```
# Arabisalpina_KFK29397.1 Length: 343
# Arabisalpina_KFK29397.1 Number of predicted TMHs:  0
# Arabisalpina_KFK29397.1 Exp number of AAs in TMHs: 0.00015
# Arabisalpina_KFK29397.1 Exp number, first 60 AAs:  0
```

```
# Arabisalpina_KFK29397.1 Total prob of N-in:        0.00633
Arabisalpina_KFK29397.1 TMHMM2.0        outside      1   343
```

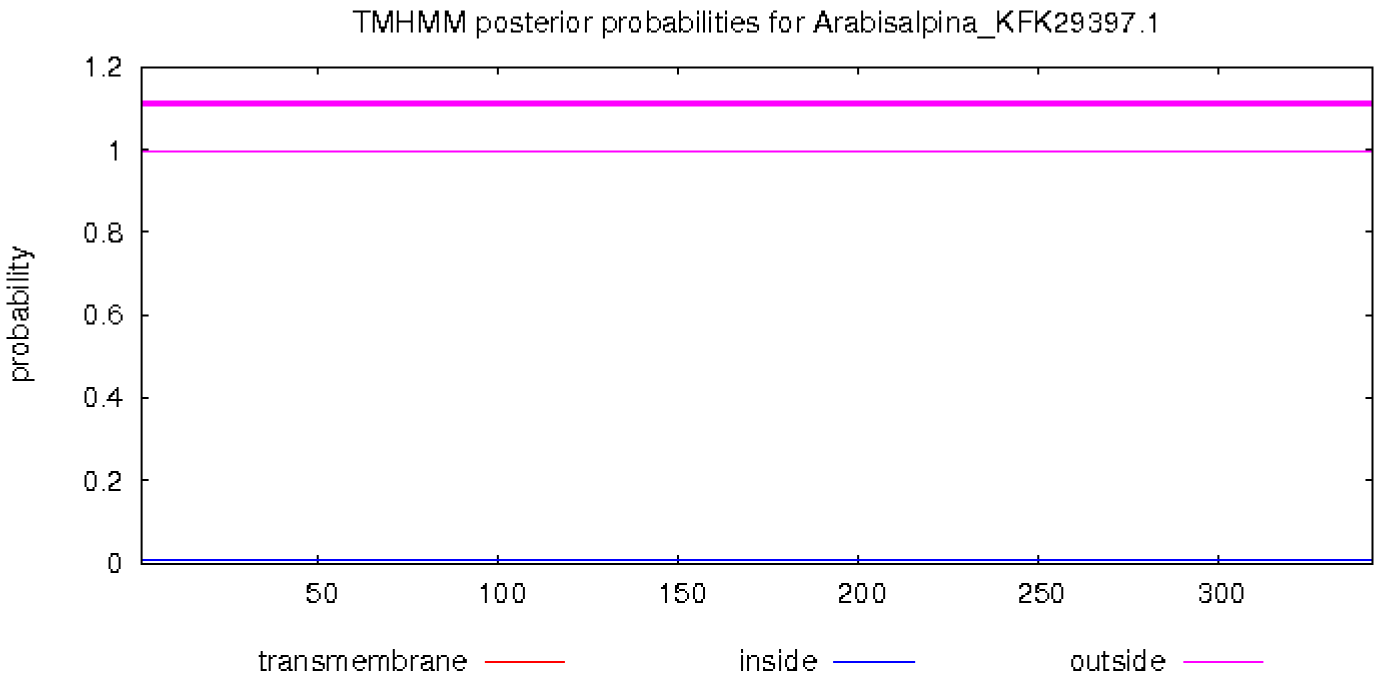

plot in postscript, script for making the plot in gnuplot, data for plot

---

```
# Arabisalpina_KFK30695.1 Length: 429
# Arabisalpina_KFK30695.1 Number of predicted TMHs:  1
# Arabisalpina_KFK30695.1 Exp number of AAs in TMHs: 18.56467
# Arabisalpina_KFK30695.1 Exp number, first 60 AAs:  0.00113
# Arabisalpina_KFK30695.1 Total prob of N-in:        0.12589
Arabisalpina_KFK30695.1 TMHMM2.0        outside      1   408
Arabisalpina_KFK30695.1 TMHMM2.0        TMhelix    409   426
Arabisalpina_KFK30695.1 TMHMM2.0        inside     427   429
```

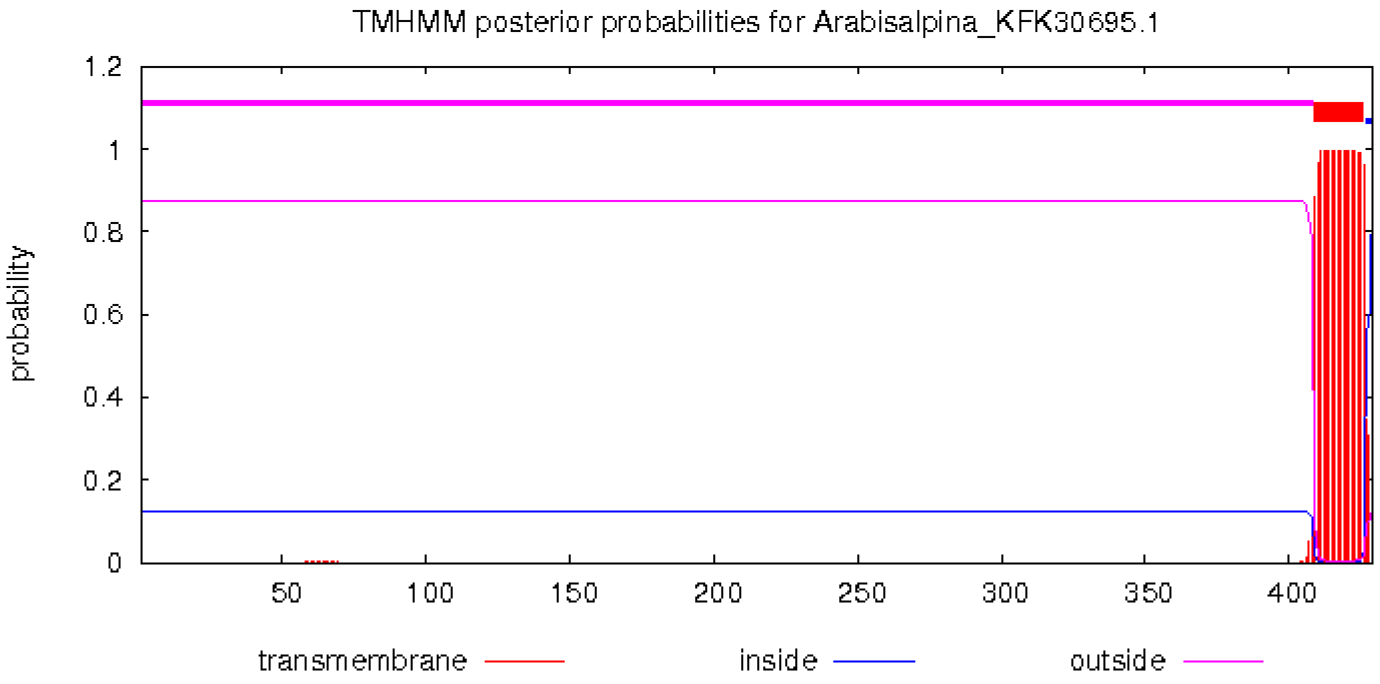

plot in postscript, script for making the plot in gnuplot, data for plot

---

```
# Arabisalpina_KFK30696.1 Length: 418
# Arabisalpina_KFK30696.1 Number of predicted TMHs:  1
```

```
# Arabisalpina_KFK30696.1 Exp number of AAs in TMHs: 21.8984
# Arabisalpina_KFK30696.1 Exp number, first 60 AAs:  0.0002
# Arabisalpina_KFK30696.1 Total prob of N-in:        0.71843
Arabisalpina_KFK30696.1 TMHMM2.0        inside       1   394
Arabisalpina_KFK30696.1 TMHMM2.0        TMhelix    395   417
Arabisalpina_KFK30696.1 TMHMM2.0        outside    418   418
```

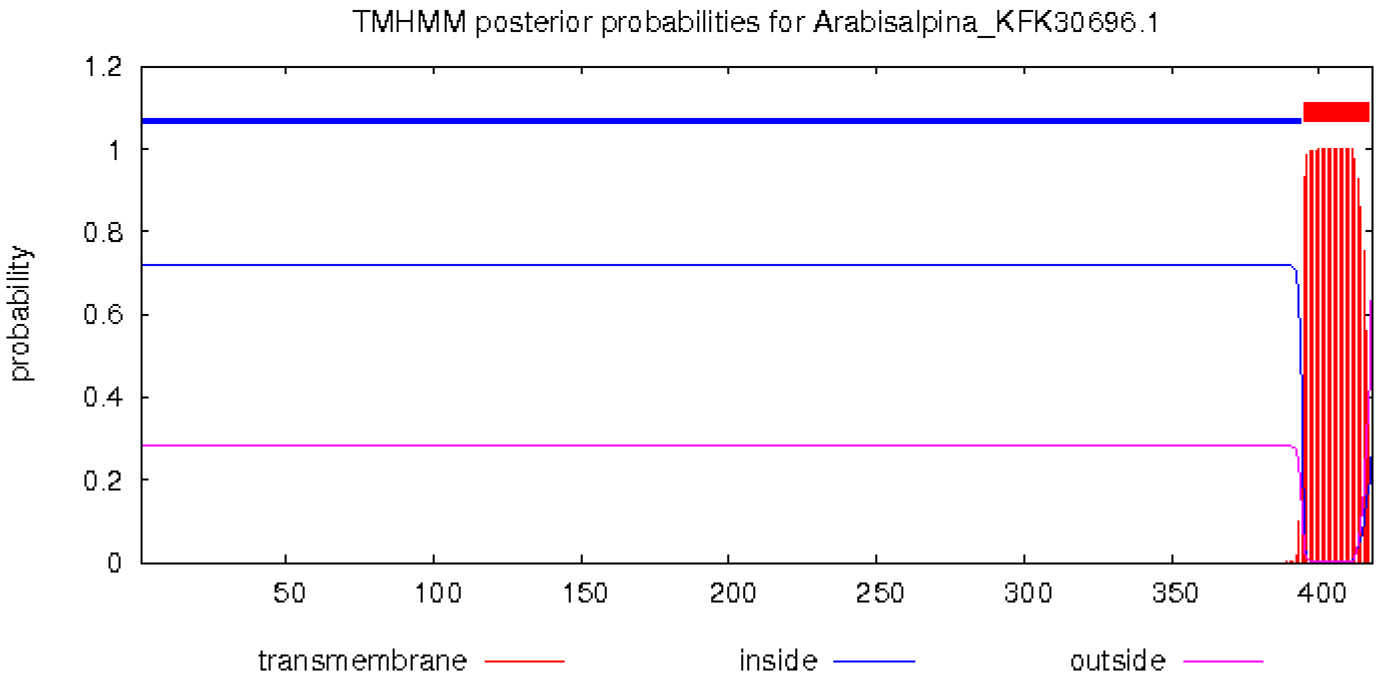

plot in postscript, script for making the plot in gnuplot, data for plot

---

```
# Arabisalpina_KFK30743.1 Length: 621
# Arabisalpina_KFK30743.1 Number of predicted TMHs:  0
# Arabisalpina_KFK30743.1 Exp number of AAs in TMHs: 0
# Arabisalpina_KFK30743.1 Exp number, first 60 AAs:  0
# Arabisalpina_KFK30743.1 Total prob of N-in:        0.00054
Arabisalpina_KFK30743.1 TMHMM2.0        outside      1   621
```

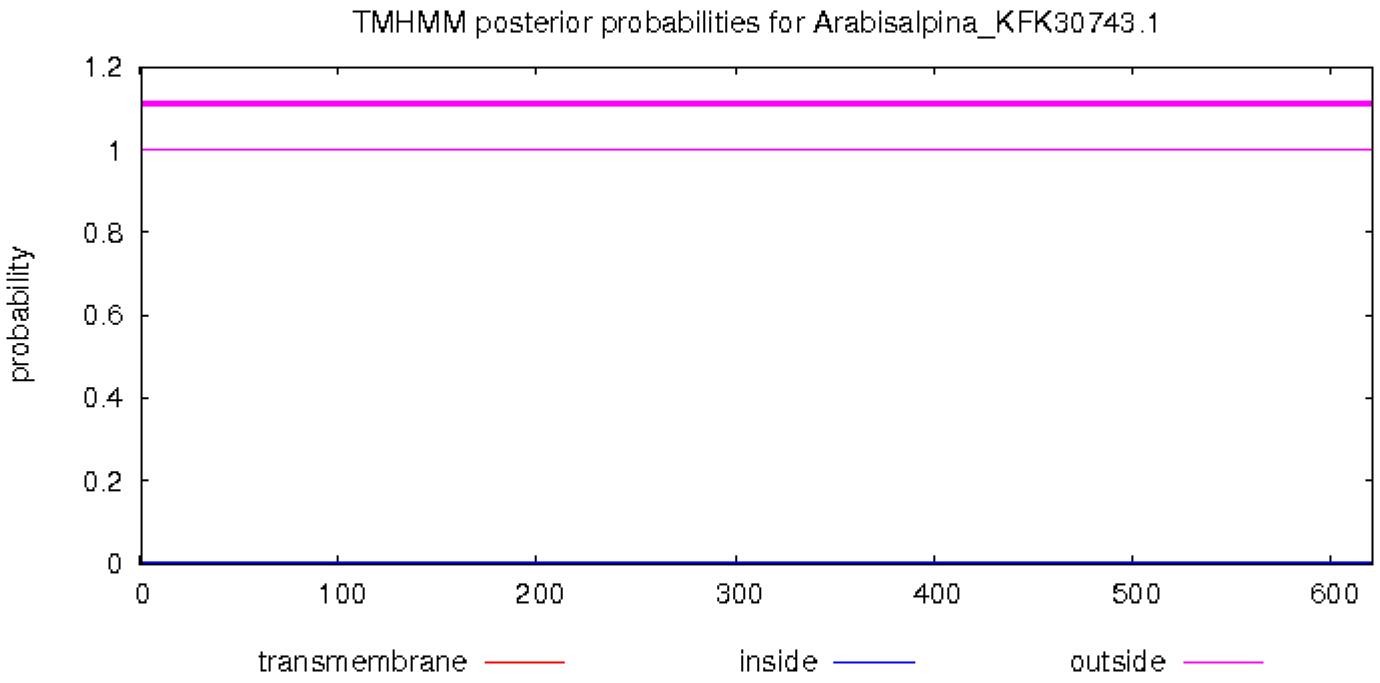

plot in postscript, script for making the plot in gnuplot, data for plot

---

```
# Arabisalpina_KFK31456.1 Length: 246
# Arabisalpina_KFK31456.1 Number of predicted TMHs:  0
# Arabisalpina_KFK31456.1 Exp number of AAs in TMHs: 0
# Arabisalpina_KFK31456.1 Exp number, first 60 AAs:  0
# Arabisalpina_KFK31456.1 Total prob of N-in:        0.00383
Arabisalpina_KFK31456.1 TMHMM2.0        outside      1   246
```

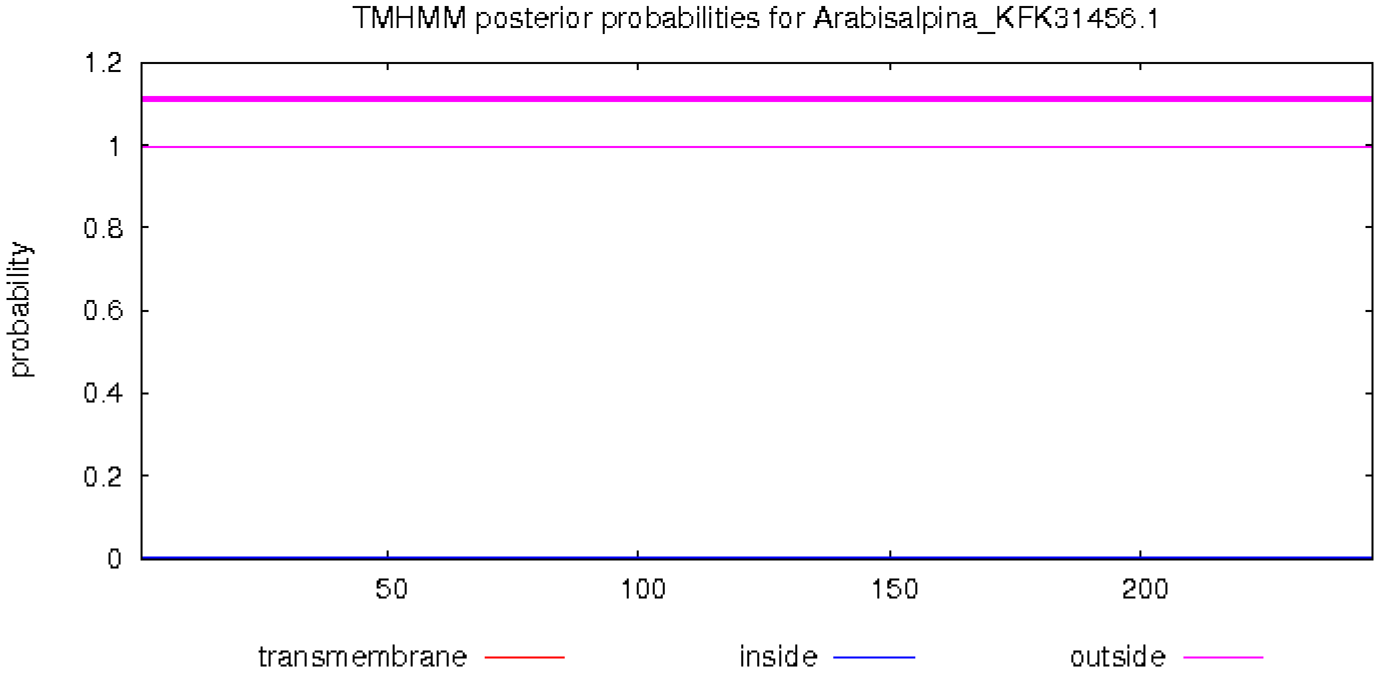

plot in postscript, script for making the plot in gnuplot, data for plot

```
# Arabisalpina_KFK31989.1 Length: 340
# Arabisalpina_KFK31989.1 Number of predicted TMHs:  0
# Arabisalpina_KFK31989.1 Exp number of AAs in TMHs: 0
# Arabisalpina_KFK31989.1 Exp number, first 60 AAs:  0
# Arabisalpina_KFK31989.1 Total prob of N-in:        0.00619
Arabisalpina_KFK31989.1 TMHMM2.0        outside      1   340
```

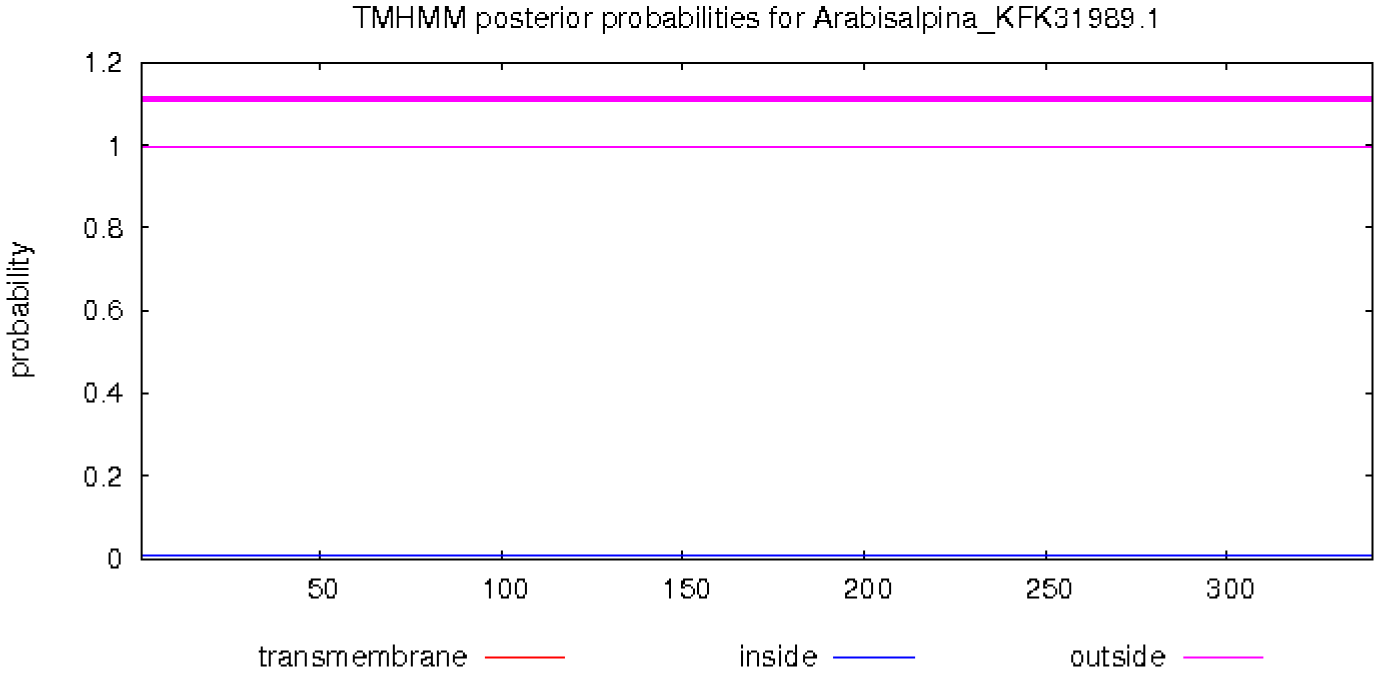

plot in postscript, script for making the plot in gnuplot, data for plot

```
# Arabisalpina_KFK32634.1 Length: 317
# Arabisalpina_KFK32634.1 Number of predicted TMHs:  0
# Arabisalpina_KFK32634.1 Exp number of AAs in TMHs: 0.02484
# Arabisalpina_KFK32634.1 Exp number, first 60 AAs:  0
# Arabisalpina_KFK32634.1 Total prob of N-in:        0.08994
Arabisalpina_KFK32634.1 TMHMM2.0        outside      1   317
```

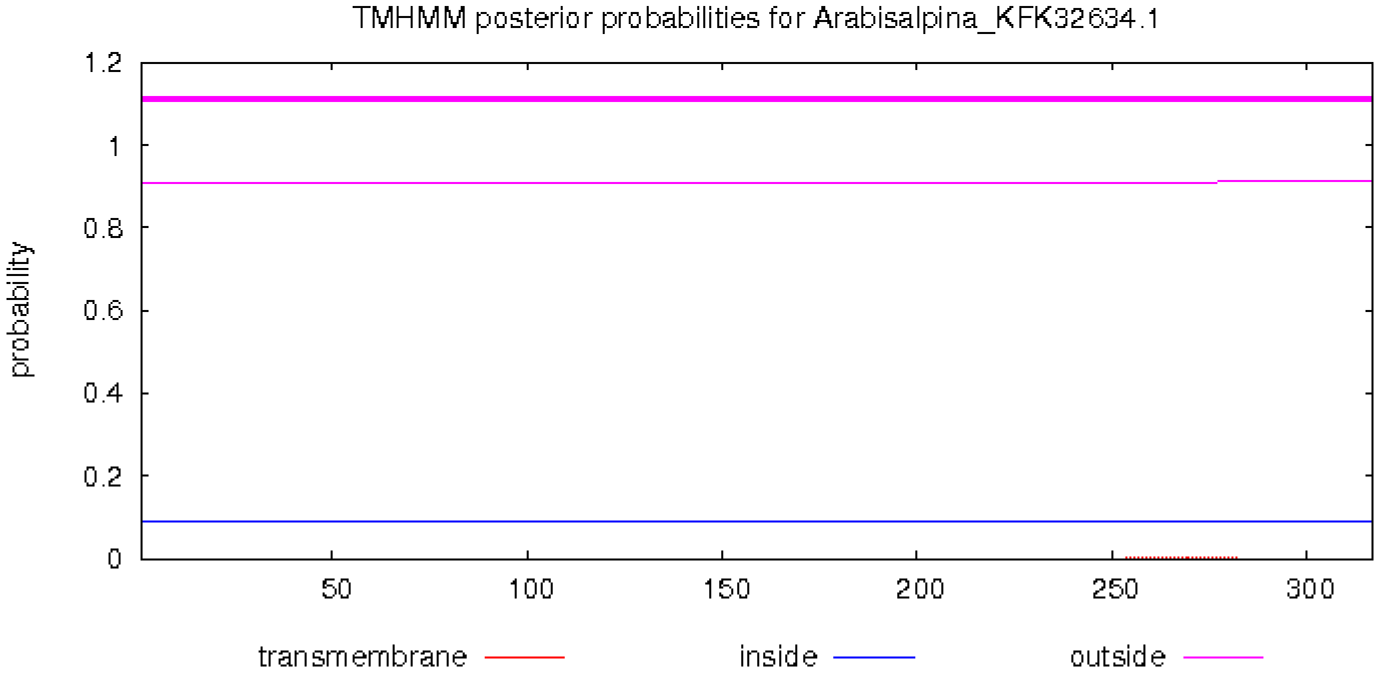

plot in postscript, script for making the plot in gnuplot, data for plot

---

```
# Arabisalpina_KFK33032.1 Length: 211
# Arabisalpina_KFK33032.1 Number of predicted TMHs:  0
# Arabisalpina_KFK33032.1 Exp number of AAs in TMHs: 0.00188
# Arabisalpina_KFK33032.1 Exp number, first 60 AAs:  0
# Arabisalpina_KFK33032.1 Total prob of N-in:        0.03847
Arabisalpina_KFK33032.1 TMHMM2.0        outside      1   211
```

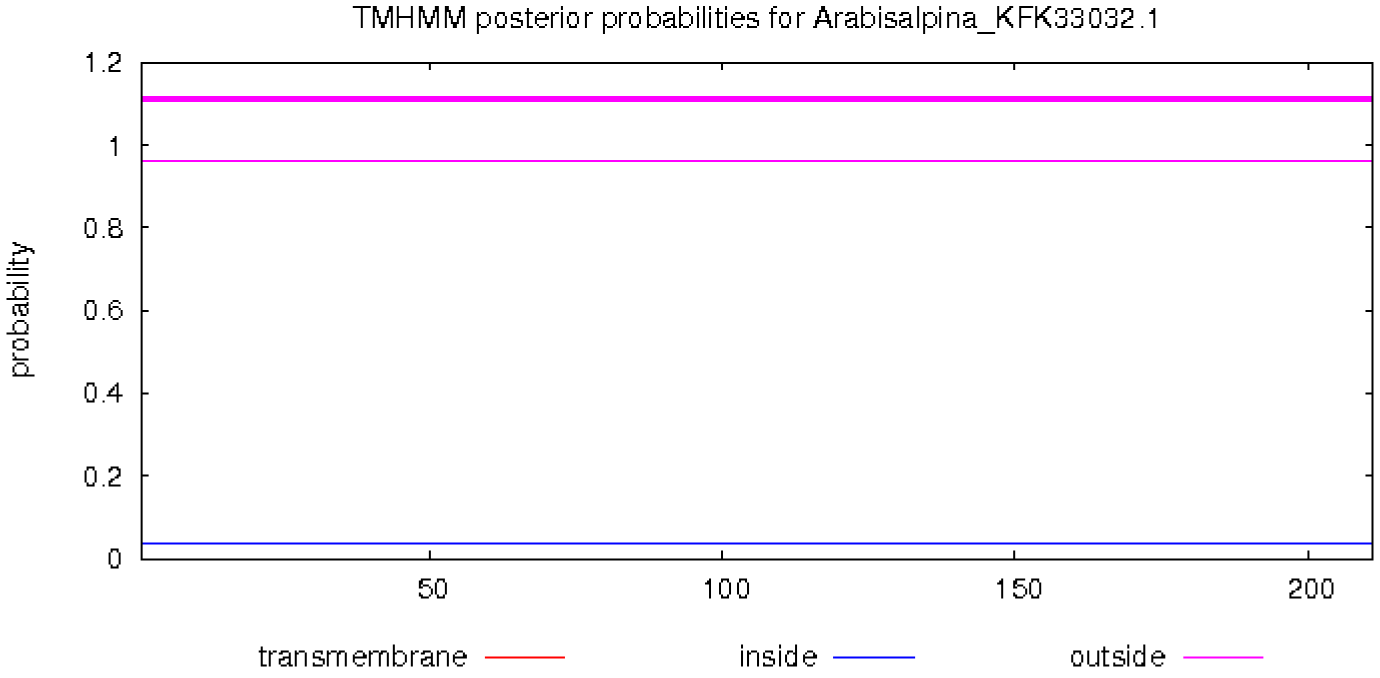

plot in postscript, script for making the plot in gnuplot, data for plot

---

```
# Arabisalpina_KFK33194.1 Length: 282
# Arabisalpina_KFK33194.1 Number of predicted TMHs:  0
# Arabisalpina_KFK33194.1 Exp number of AAs in TMHs: 4e-05
# Arabisalpina_KFK33194.1 Exp number, first 60 AAs:  0
# Arabisalpina_KFK33194.1 Total prob of N-in:        0.03517
Arabisalpina_KFK33194.1 TMHMM2.0        outside      1   282
```

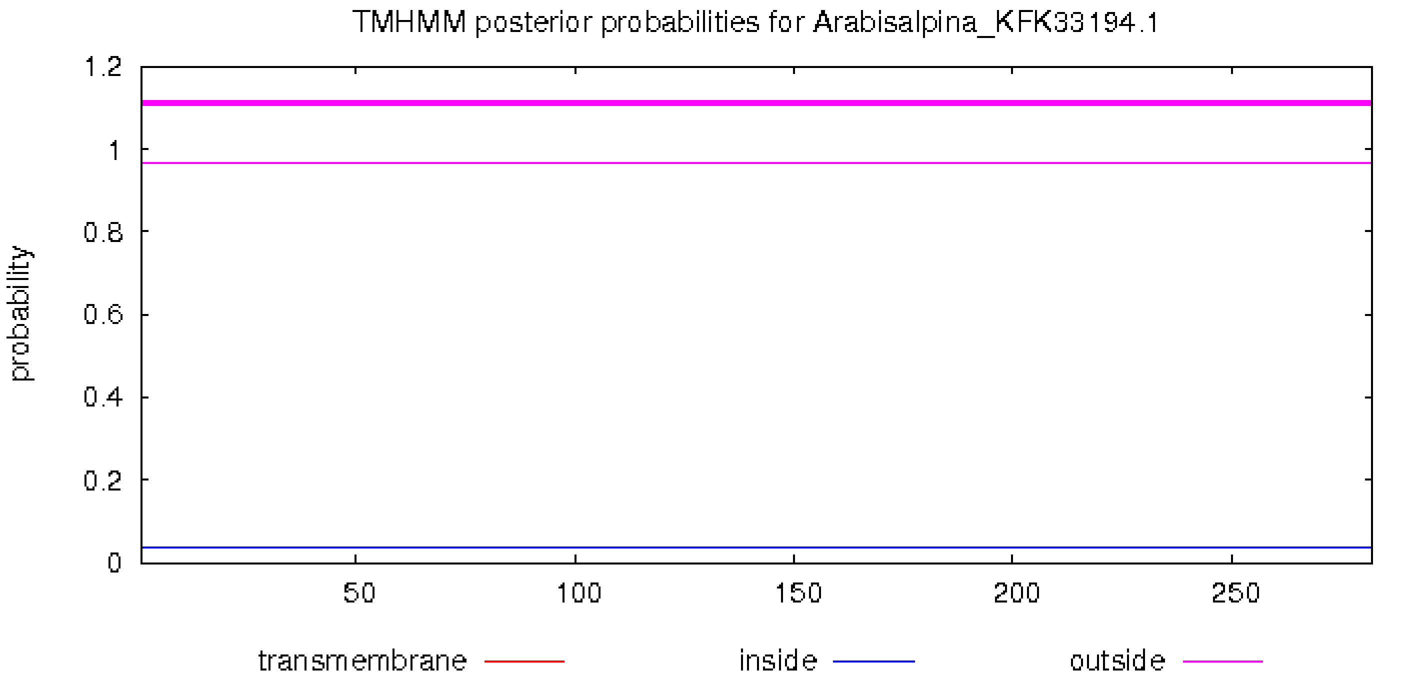

plot in postscript, script for making the plot in gnuplot, data for plot

---

```
# Arabisalpina_KFK33442.1 Length: 401
# Arabisalpina_KFK33442.1 Number of predicted TMHs:  0
# Arabisalpina_KFK33442.1 Exp number of AAs in TMHs: 0.17387
# Arabisalpina_KFK33442.1 Exp number, first 60 AAs:  0
# Arabisalpina_KFK33442.1 Total prob of N-in:        0.06186
Arabisalpina_KFK33442.1 TMHMM2.0        outside      1   401
```

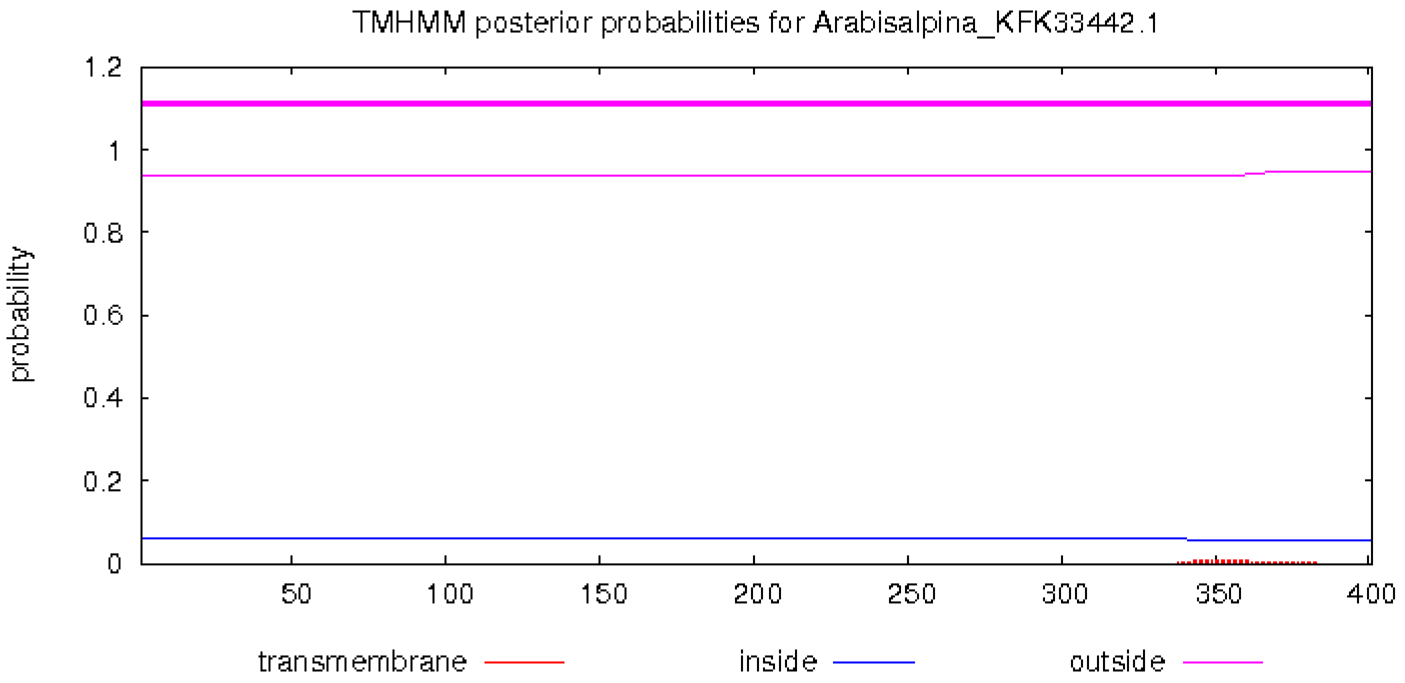

plot in postscript, script for making the plot in gnuplot, data for plot

---

```
# Arabisalpina_KFK33777.1 Length: 309
# Arabisalpina_KFK33777.1 Number of predicted TMHs:  0
# Arabisalpina_KFK33777.1 Exp number of AAs in TMHs: 0.02058
# Arabisalpina_KFK33777.1 Exp number, first 60 AAs:  0.00033
# Arabisalpina_KFK33777.1 Total prob of N-in:        0.03749
Arabisalpina_KFK33777.1 TMHMM2.0        outside      1   309
```

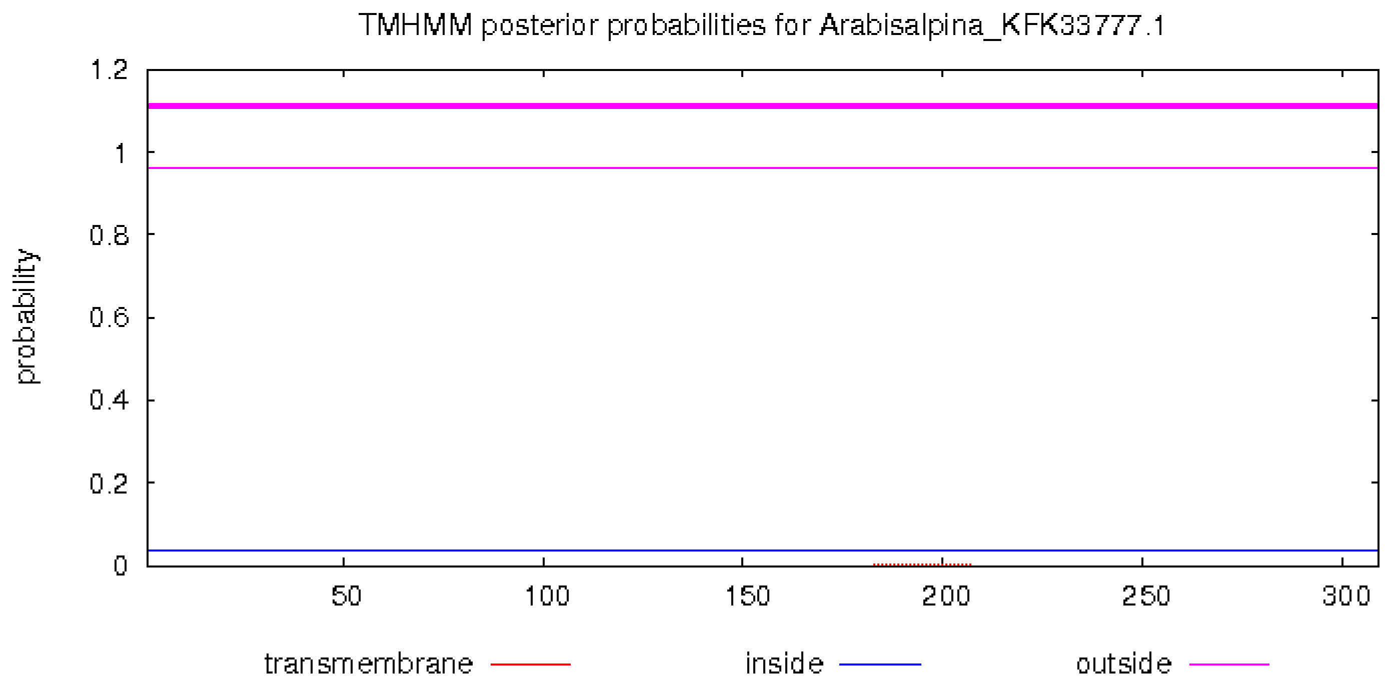

plot in postscript, script for making the plot in gnuplot, data for plot

---

```
# Arabisalpina_KFK33874.1 Length: 339
# Arabisalpina_KFK33874.1 Number of predicted TMHs:  1
# Arabisalpina_KFK33874.1 Exp number of AAs in TMHs: 17.8966
# Arabisalpina_KFK33874.1 Exp number, first 60 AAs:  0.02128
# Arabisalpina_KFK33874.1 Total prob of N-in:        0.30263
Arabisalpina_KFK33874.1 TMHMM2.0        outside      1   320
Arabisalpina_KFK33874.1 TMHMM2.0        TMhelix    321   338
Arabisalpina_KFK33874.1 TMHMM2.0        inside     339   339
```

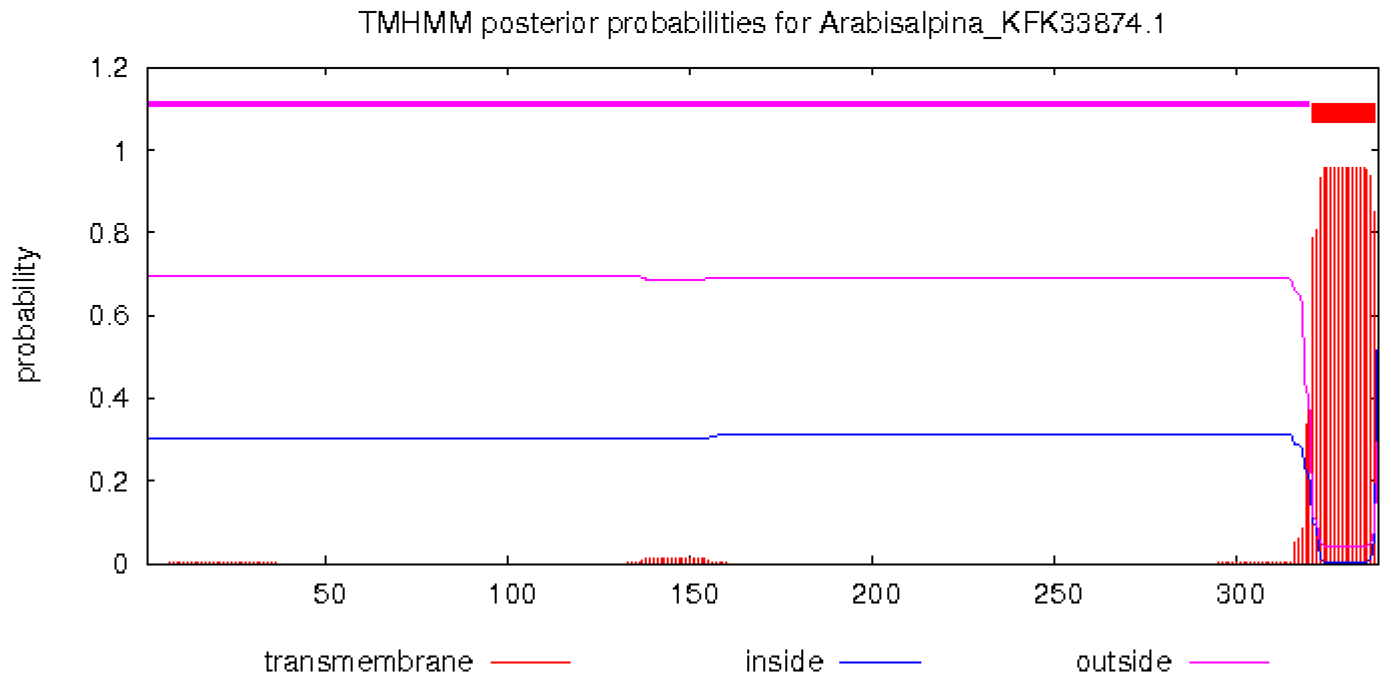

plot in postscript, script for making the plot in gnuplot, data for plot

```
# Arabisalpina_KFK33878.1 Length: 244
# Arabisalpina_KFK33878.1 Number of predicted TMHs:  0
# Arabisalpina_KFK33878.1 Exp number of AAs in TMHs: 0.03131
# Arabisalpina_KFK33878.1 Exp number, first 60 AAs:  0.03131
# Arabisalpina_KFK33878.1 Total prob of N-in:        0.00830
Arabisalpina_KFK33878.1 TMHMM2.0        outside      1   244
```

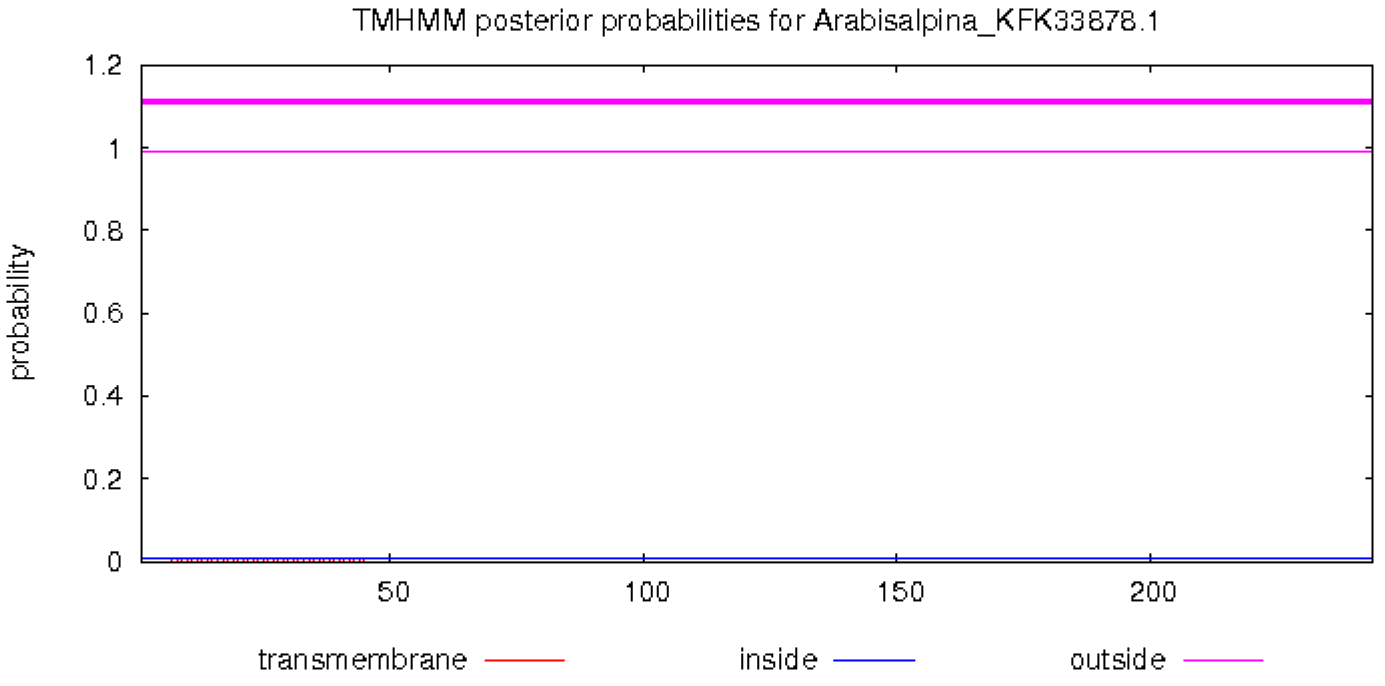

plot in postscript, script for making the plot in gnuplot, data for plot

```
# Arabisalpina_KFK34231.1 Length: 458
# Arabisalpina_KFK34231.1 Number of predicted TMHs:  0
# Arabisalpina_KFK34231.1 Exp number of AAs in TMHs: 18.87024
# Arabisalpina_KFK34231.1 Exp number, first 60 AAs:  0.00015
# Arabisalpina_KFK34231.1 Total prob of N-in:        0.04365
Arabisalpina_KFK34231.1 TMHMM2.0        outside      1   458
```

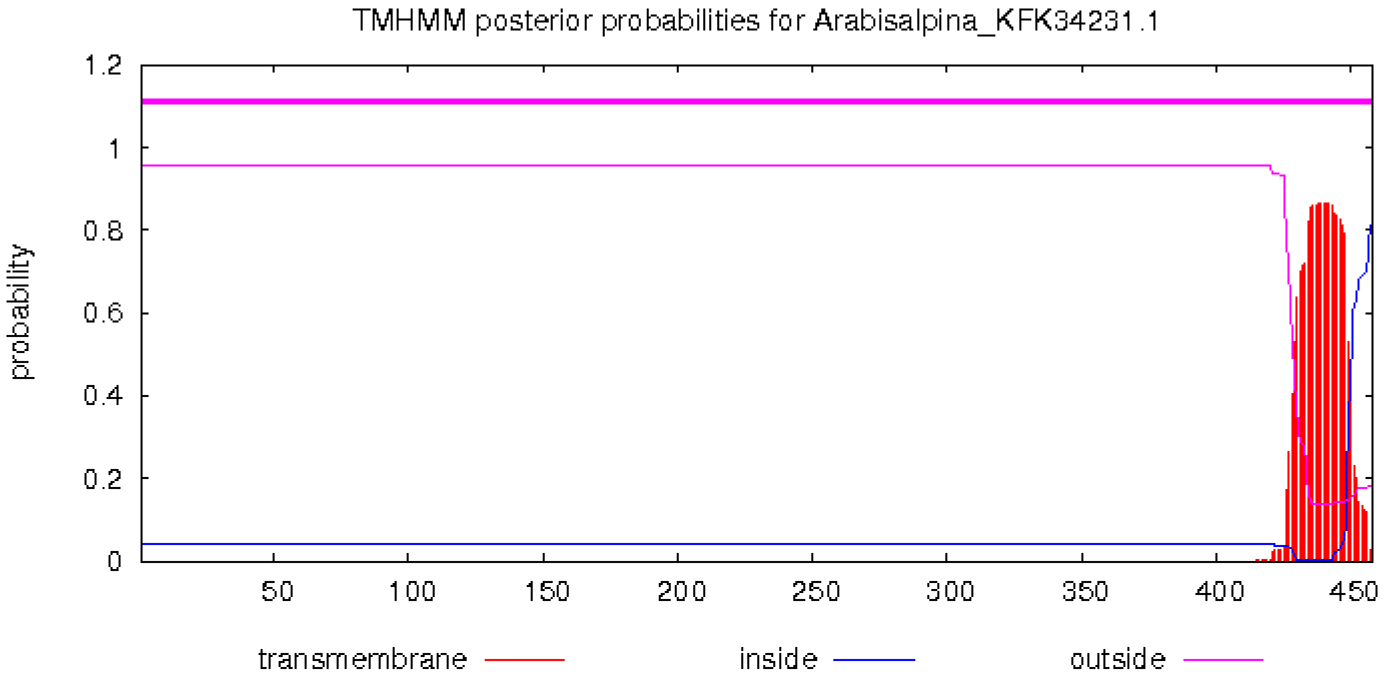

plot in postscript, script for making the plot in gnuplot, data for plot

```
# Arabisalpina_KFK35377.1 Length: 335
# Arabisalpina_KFK35377.1 Number of predicted TMHs:  0
# Arabisalpina_KFK35377.1 Exp number of AAs in TMHs: 0.00014
# Arabisalpina_KFK35377.1 Exp number, first 60 AAs:  0
# Arabisalpina_KFK35377.1 Total prob of N-in:        0.02993
Arabisalpina_KFK35377.1 TMHMM2.0        outside      1   335
```

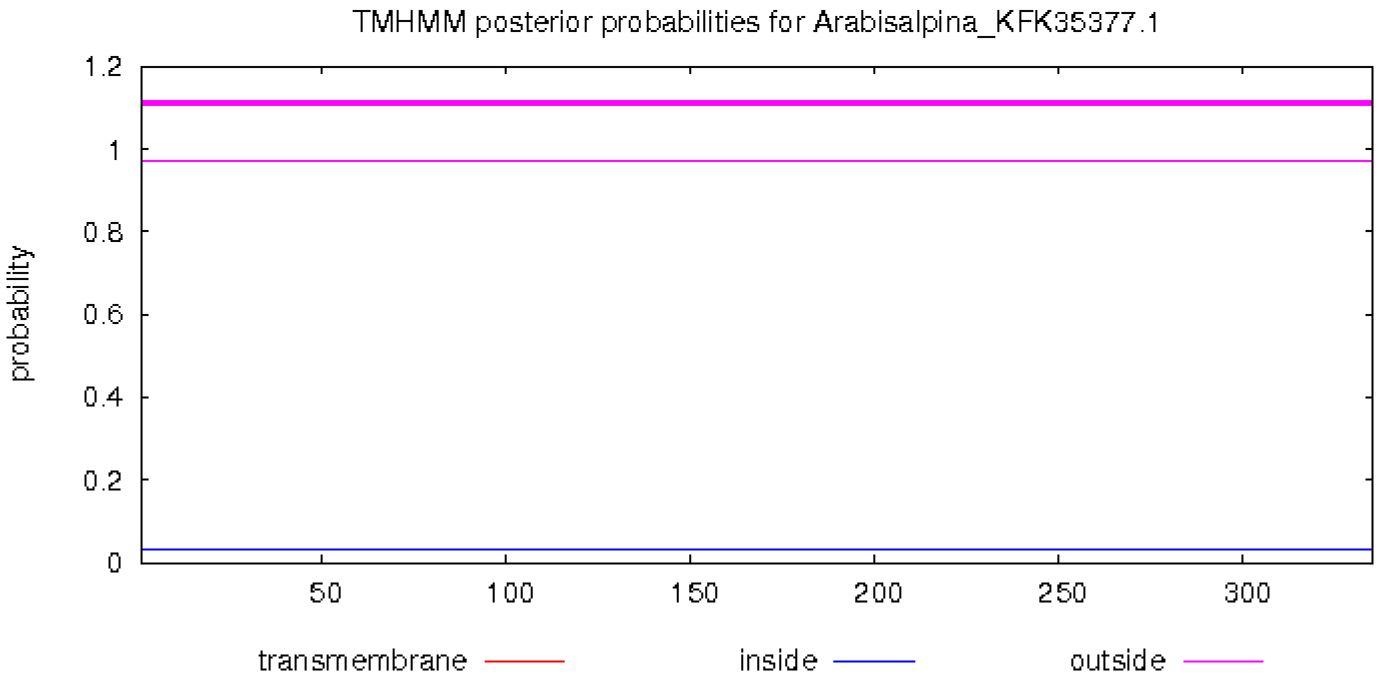

plot in postscript, script for making the plot in gnuplot, data for plot

---

```
# Arabisalpina_KFK35527.1 Length: 312
# Arabisalpina_KFK35527.1 Number of predicted TMHs:  0
# Arabisalpina_KFK35527.1 Exp number of AAs in TMHs: 0
# Arabisalpina_KFK35527.1 Exp number, first 60 AAs:  0
# Arabisalpina_KFK35527.1 Total prob of N-in:        0.00124
Arabisalpina_KFK35527.1 TMHMM2.0        outside      1   312
```

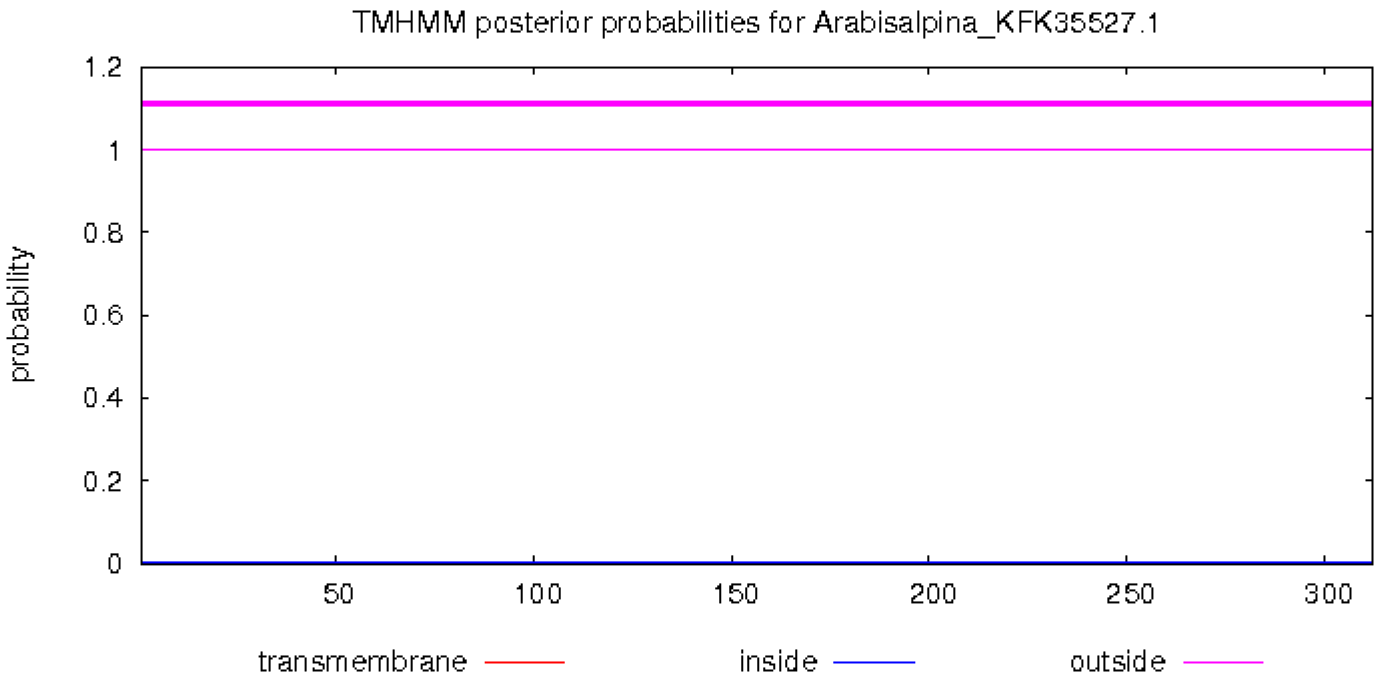

plot in postscript, script for making the plot in gnuplot, data for plot

---

```
# Arabisalpina_KFK35543.1 Length: 319
# Arabisalpina_KFK35543.1 Number of predicted TMHs:  0
# Arabisalpina_KFK35543.1 Exp number of AAs in TMHs: 0.00284
# Arabisalpina_KFK35543.1 Exp number, first 60 AAs:  0
# Arabisalpina_KFK35543.1 Total prob of N-in:        0.01804
Arabisalpina_KFK35543.1 TMHMM2.0        outside      1   319
```

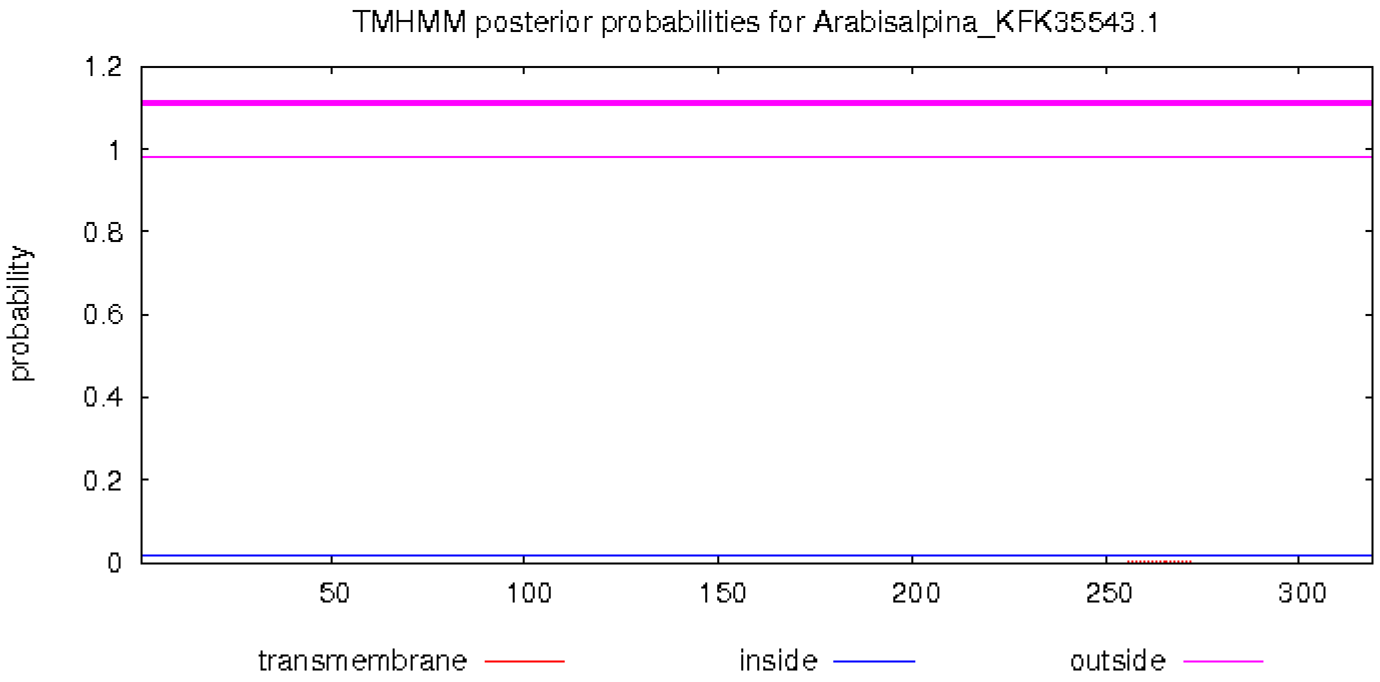

plot in postscript, script for making the plot in gnuplot, data for plot

---

```
# Arabisalpina_KFK35760.1 Length: 311
# Arabisalpina_KFK35760.1 Number of predicted TMHs:  0
# Arabisalpina_KFK35760.1 Exp number of AAs in TMHs: 0.00895
# Arabisalpina_KFK35760.1 Exp number, first 60 AAs:  0.00015
# Arabisalpina_KFK35760.1 Total prob of N-in:        0.01008
Arabisalpina_KFK35760.1 TMHMM2.0        outside      1   311
```

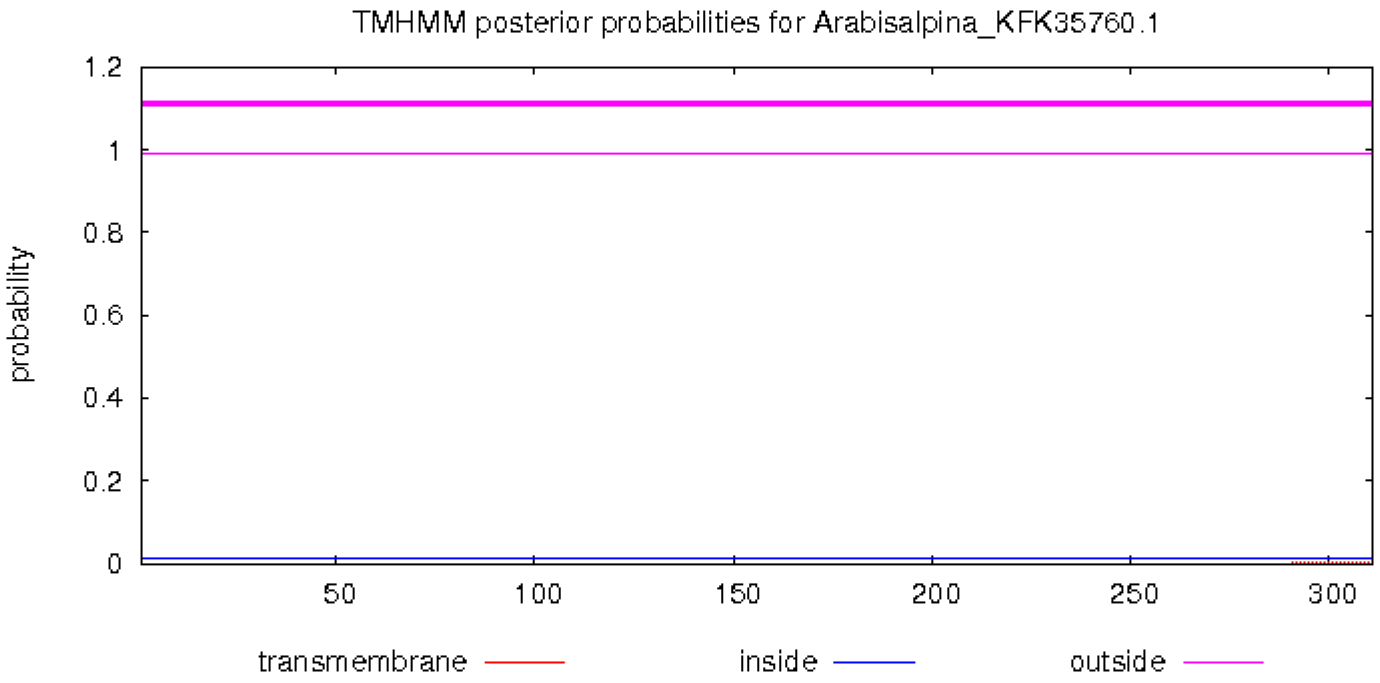

plot in postscript, script for making the plot in gnuplot, data for plot

---

```
# Arabisalpina_KFK35761.1 Length: 280
# Arabisalpina_KFK35761.1 Number of predicted TMHs:  0
# Arabisalpina_KFK35761.1 Exp number of AAs in TMHs: 0.00131
# Arabisalpina_KFK35761.1 Exp number, first 60 AAs:  0.00032
# Arabisalpina_KFK35761.1 Total prob of N-in:        0.01185
Arabisalpina_KFK35761.1 TMHMM2.0        outside      1   280
```

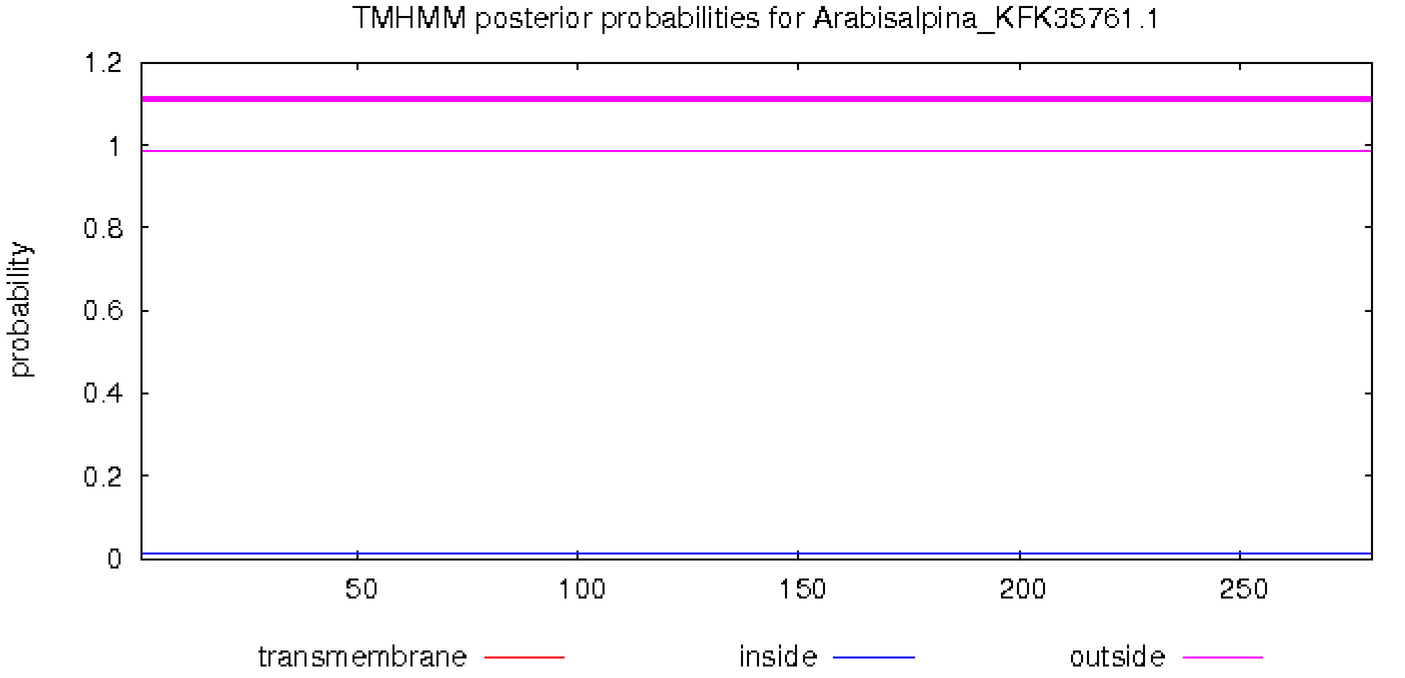

plot in postscript, script for making the plot in gnuplot, data for plot

```
# Arabisalpina_KFK37142.1 Length: 250
# Arabisalpina_KFK37142.1 Number of predicted TMHs:  0
# Arabisalpina_KFK37142.1 Exp number of AAs in TMHs: 0
# Arabisalpina_KFK37142.1 Exp number, first 60 AAs:  0
# Arabisalpina_KFK37142.1 Total prob of N-in:        0.05369
Arabisalpina_KFK37142.1 TMHMM2.0        outside      1   250
```

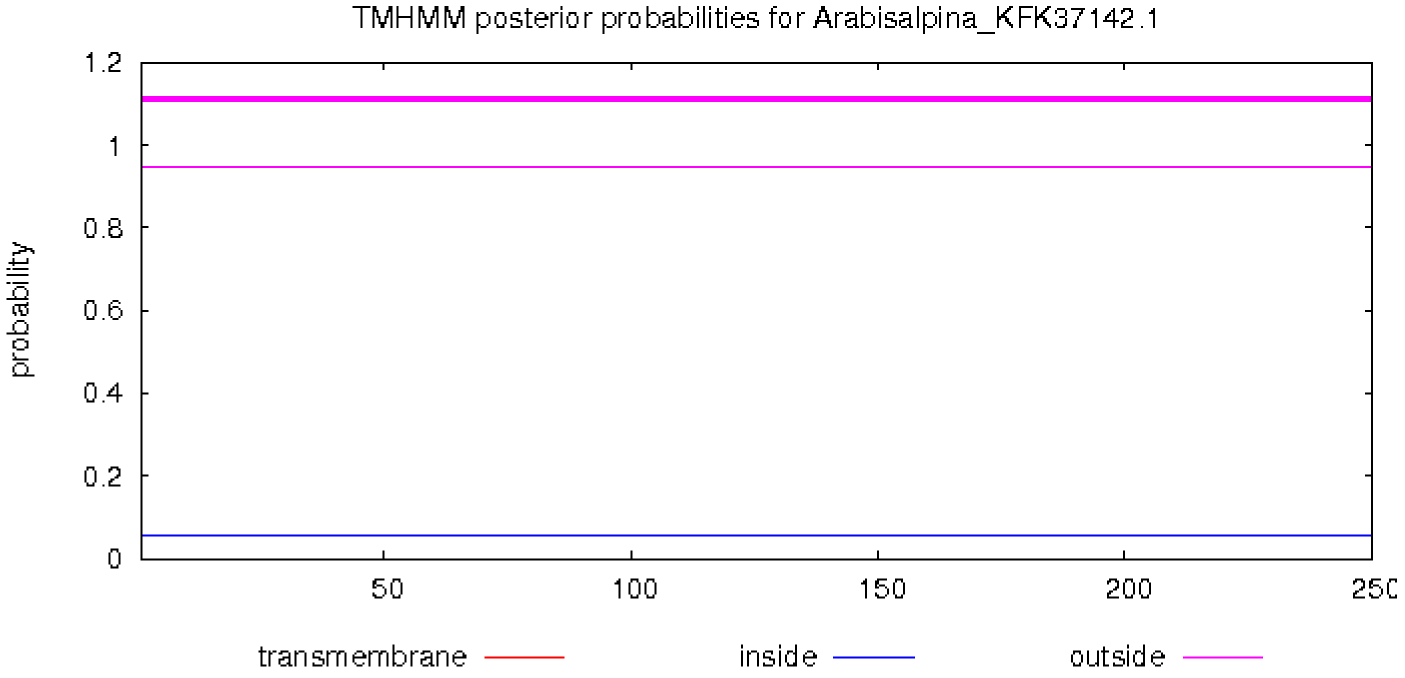

plot in postscript, script for making the plot in gnuplot, data for plot

```
# Arabisalpina_KFK37442.1 Length: 355
# Arabisalpina_KFK37442.1 Number of predicted TMHs:  0
# Arabisalpina_KFK37442.1 Exp number of AAs in TMHs: 0
# Arabisalpina_KFK37442.1 Exp number, first 60 AAs:  0
# Arabisalpina_KFK37442.1 Total prob of N-in:        0.01008
Arabisalpina_KFK37442.1 TMHMM2.0        outside      1   355
```

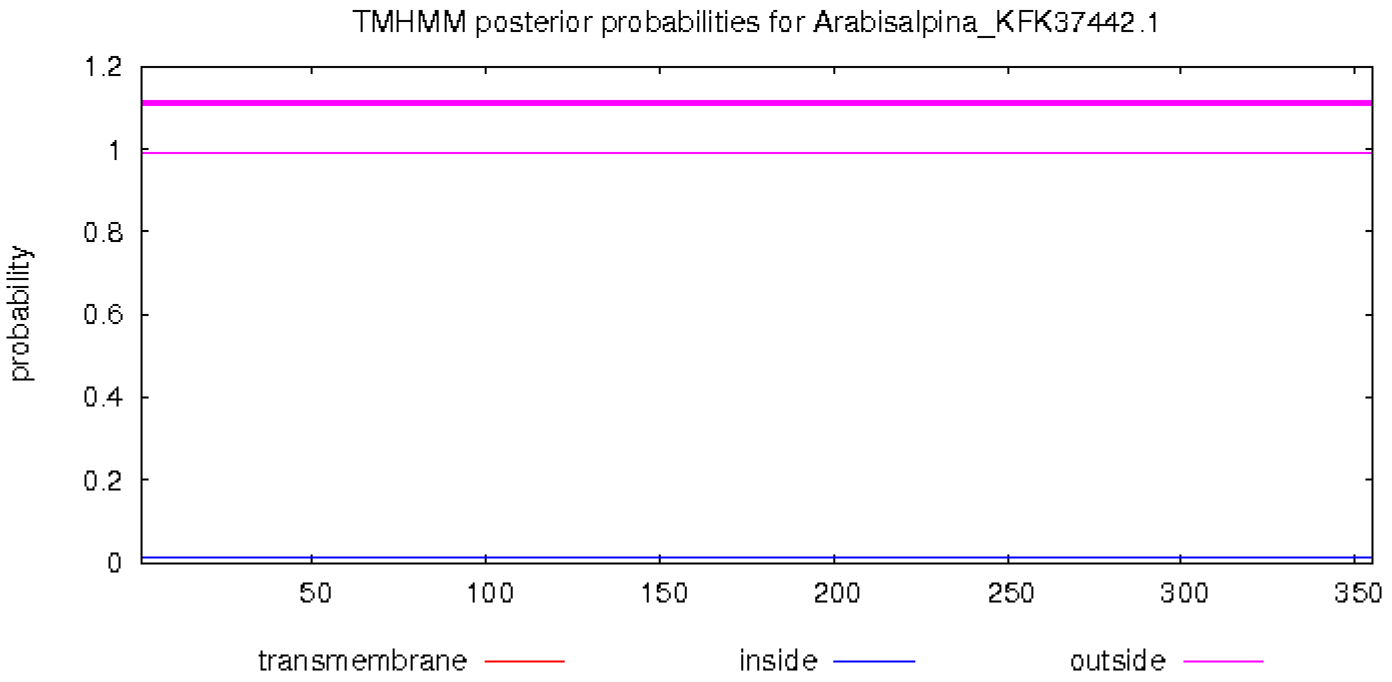

plot in postscript, script for making the plot in gnuplot, data for plot

---

```
# Arabisalpina_KFK37625.1 Length: 365
# Arabisalpina_KFK37625.1 Number of predicted TMHs:  0
# Arabisalpina_KFK37625.1 Exp number of AAs in TMHs: 0.00089
# Arabisalpina_KFK37625.1 Exp number, first 60 AAs:  0
# Arabisalpina_KFK37625.1 Total prob of N-in:        0.02839
Arabisalpina_KFK37625.1 TMHMM2.0        outside      1   365
```

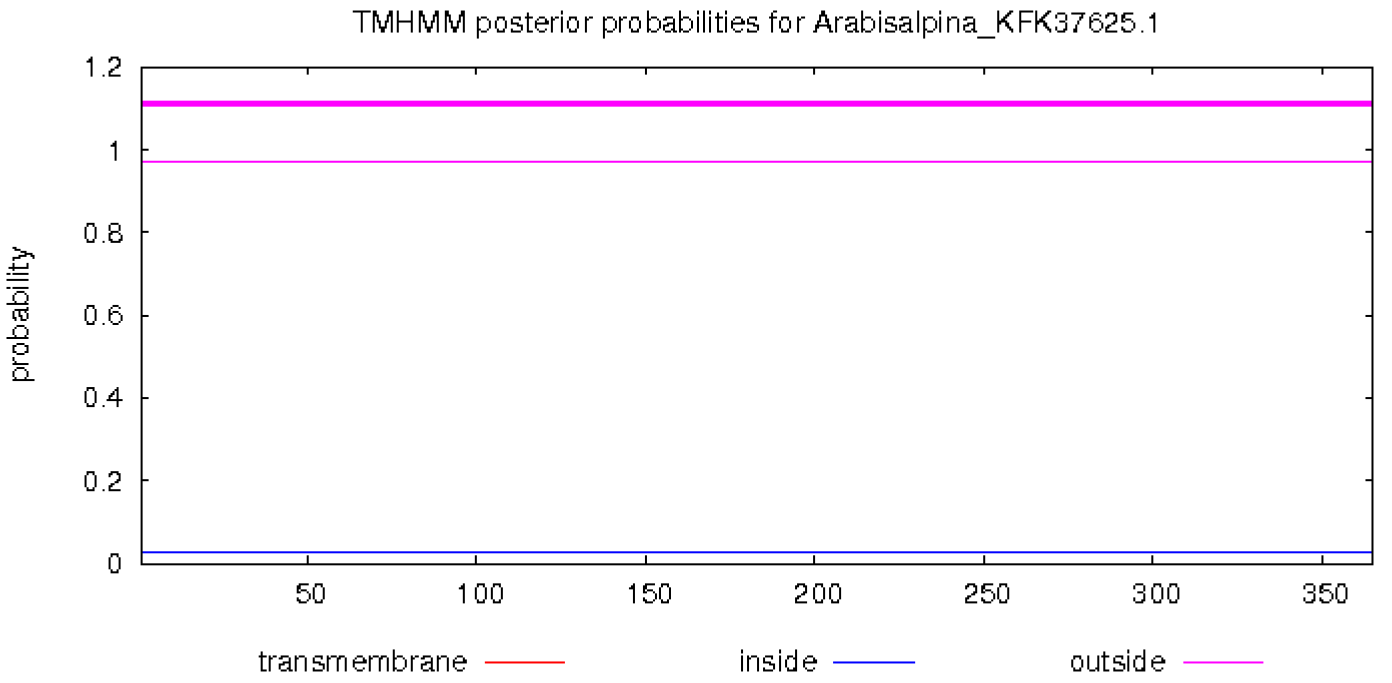

plot in postscript, script for making the plot in gnuplot, data for plot

---

```
# Arabisalpina_KFK37763.1 Length: 405
# Arabisalpina_KFK37763.1 Number of predicted TMHs:  0
# Arabisalpina_KFK37763.1 Exp number of AAs in TMHs: 0
# Arabisalpina_KFK37763.1 Exp number, first 60 AAs:  0
# Arabisalpina_KFK37763.1 Total prob of N-in:        0.00036
Arabisalpina_KFK37763.1 TMHMM2.0        outside      1   405
```

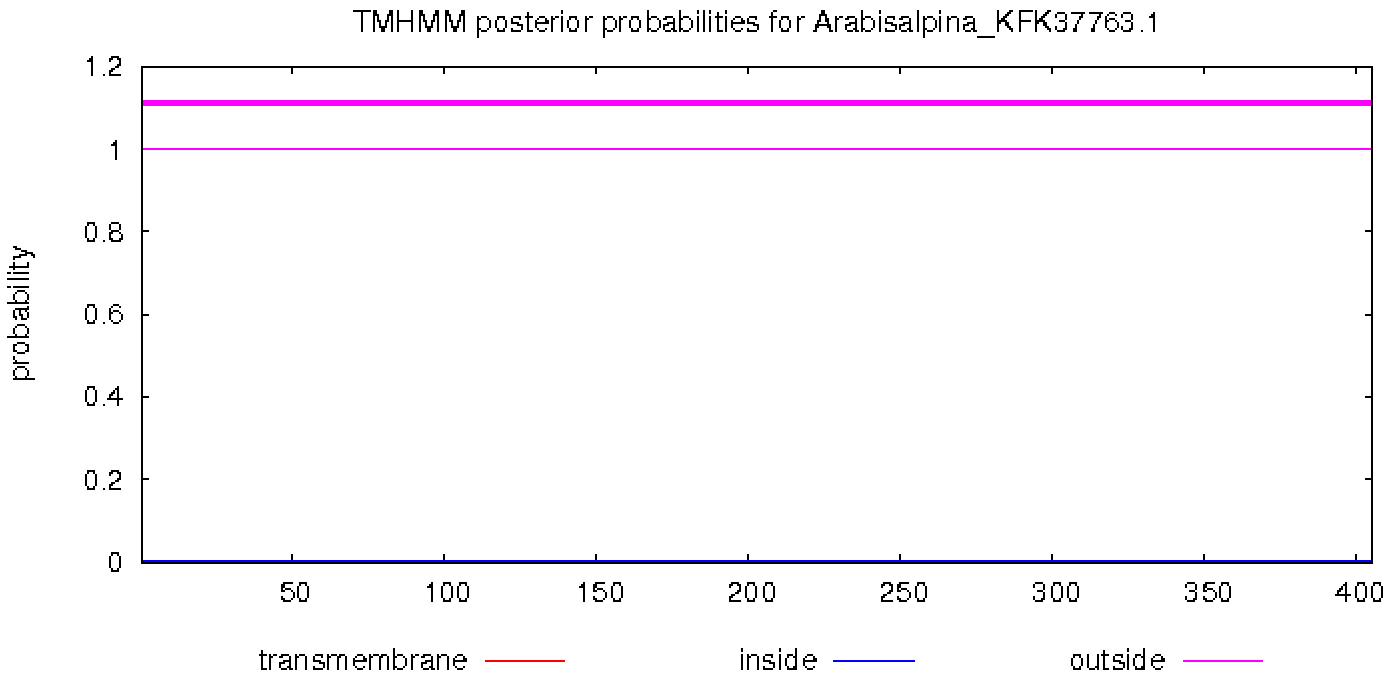

plot in postscript, script for making the plot in gnuplot, data for plot

---

```
# Arabisalpina_KFK37841.1 Length: 325
# Arabisalpina_KFK37841.1 Number of predicted TMHs:  0
# Arabisalpina_KFK37841.1 Exp number of AAs in TMHs: 0.00085
# Arabisalpina_KFK37841.1 Exp number, first 60 AAs:  0
# Arabisalpina_KFK37841.1 Total prob of N-in:        0.00995
Arabisalpina_KFK37841.1 TMHMM2.0        outside      1   325
```

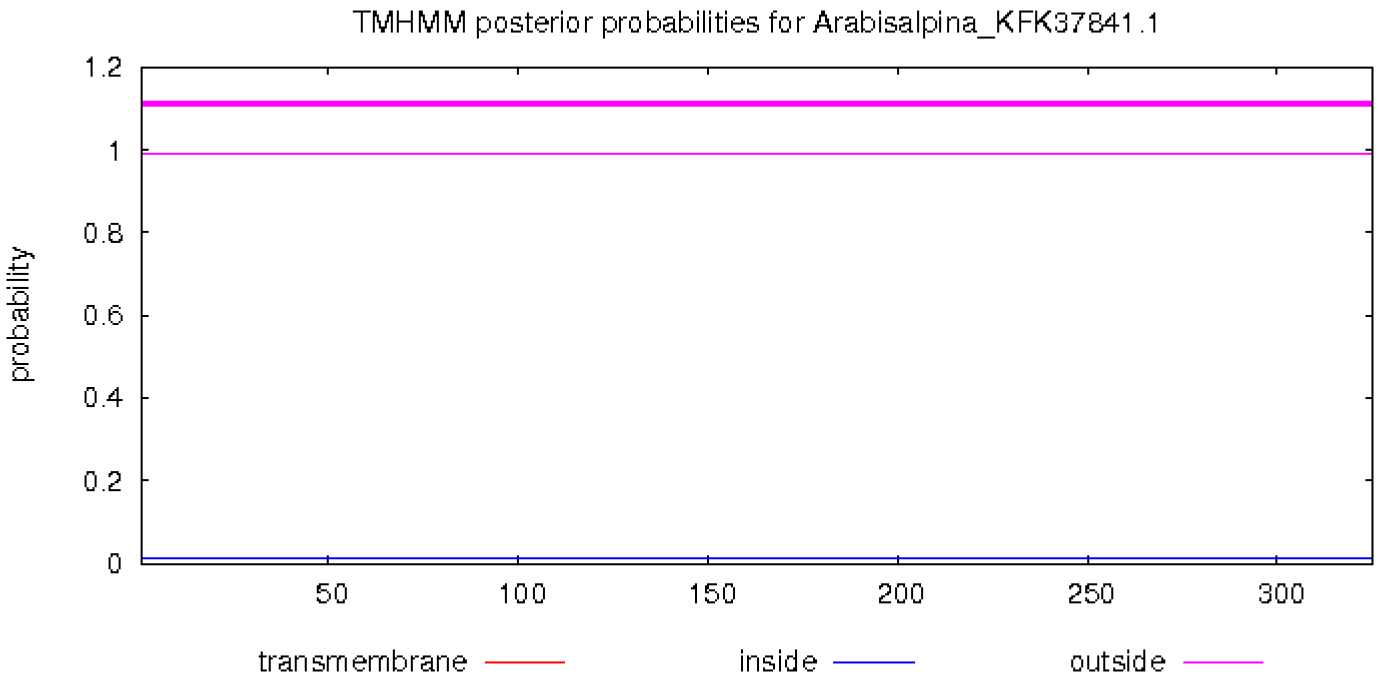

plot in postscript, script for making the plot in gnuplot, data for plot

---

```
# Arabisalpina_KFK37842.1 Length: 354
# Arabisalpina_KFK37842.1 Number of predicted TMHs:  0
# Arabisalpina_KFK37842.1 Exp number of AAs in TMHs: 0
# Arabisalpina_KFK37842.1 Exp number, first 60 AAs:  0
# Arabisalpina_KFK37842.1 Total prob of N-in:        0.01048
Arabisalpina_KFK37842.1 TMHMM2.0        outside      1   354
```

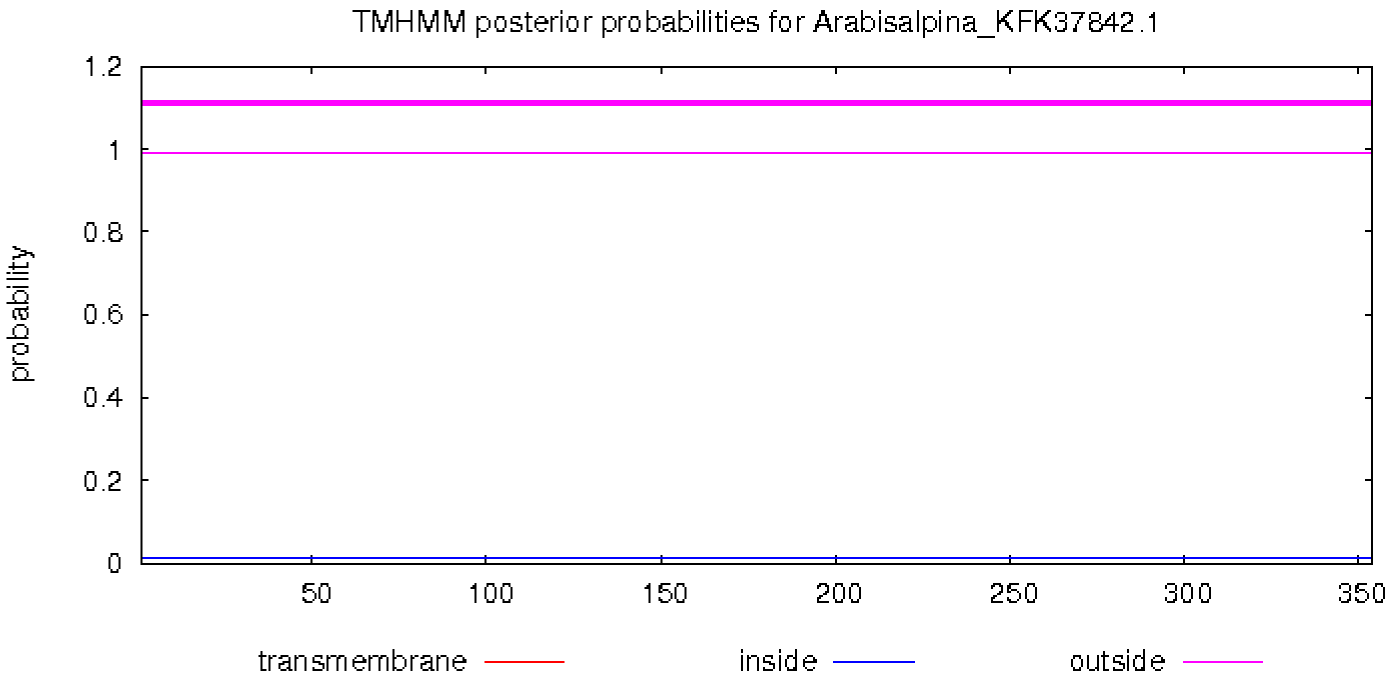

plot in postscript, script for making the plot in gnuplot, data for plot

---

```
# Arabisalpina_KFK38436.1 Length: 467
# Arabisalpina_KFK38436.1 Number of predicted TMHs:  0
# Arabisalpina_KFK38436.1 Exp number of AAs in TMHs: 0.02342
# Arabisalpina_KFK38436.1 Exp number, first 60 AAs:  0.017
# Arabisalpina_KFK38436.1 Total prob of N-in:        0.00224
Arabisalpina_KFK38436.1 TMHMM2.0        outside      1   467
```

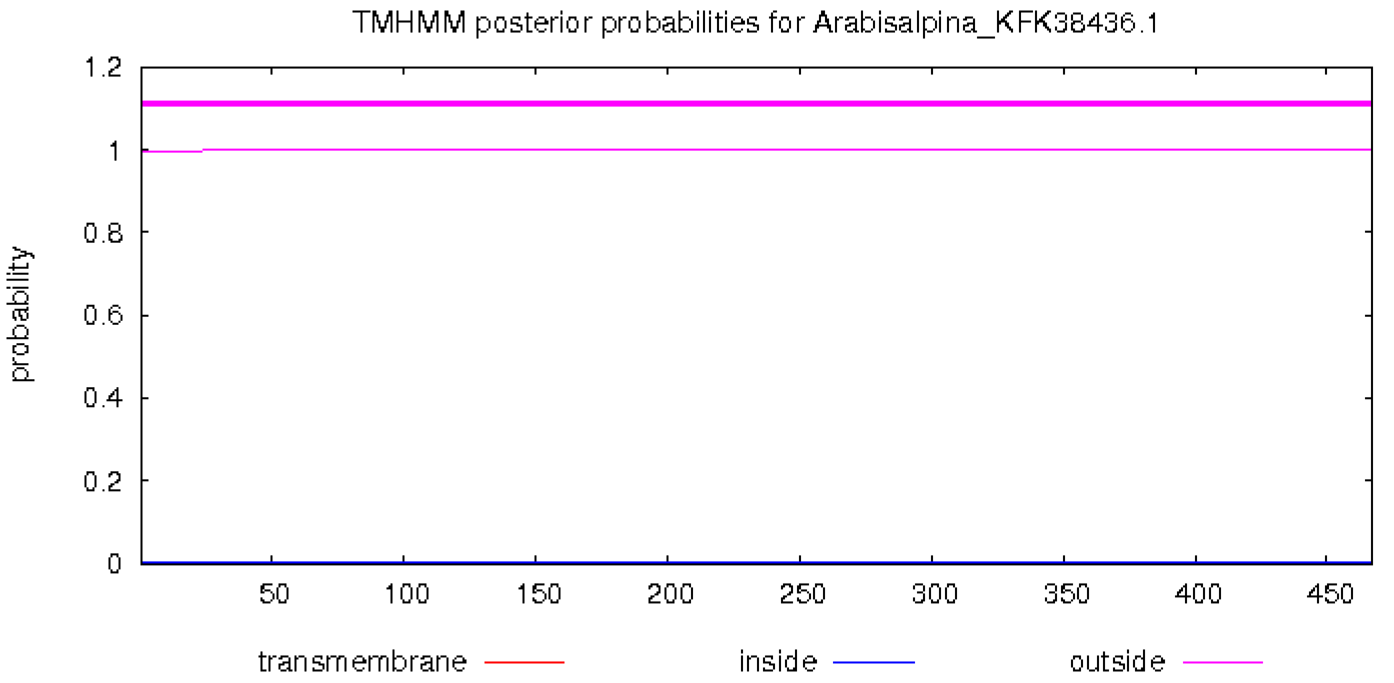

plot in postscript, script for making the plot in gnuplot, data for plot

---

```
# Arabisalpina_KFK38437.1 Length: 470
# Arabisalpina_KFK38437.1 Number of predicted TMHs:  0
# Arabisalpina_KFK38437.1 Exp number of AAs in TMHs: 0.0234
# Arabisalpina_KFK38437.1 Exp number, first 60 AAs:  0.017
# Arabisalpina_KFK38437.1 Total prob of N-in:        0.00218
Arabisalpina_KFK38437.1 TMHMM2.0        outside      1   470
```

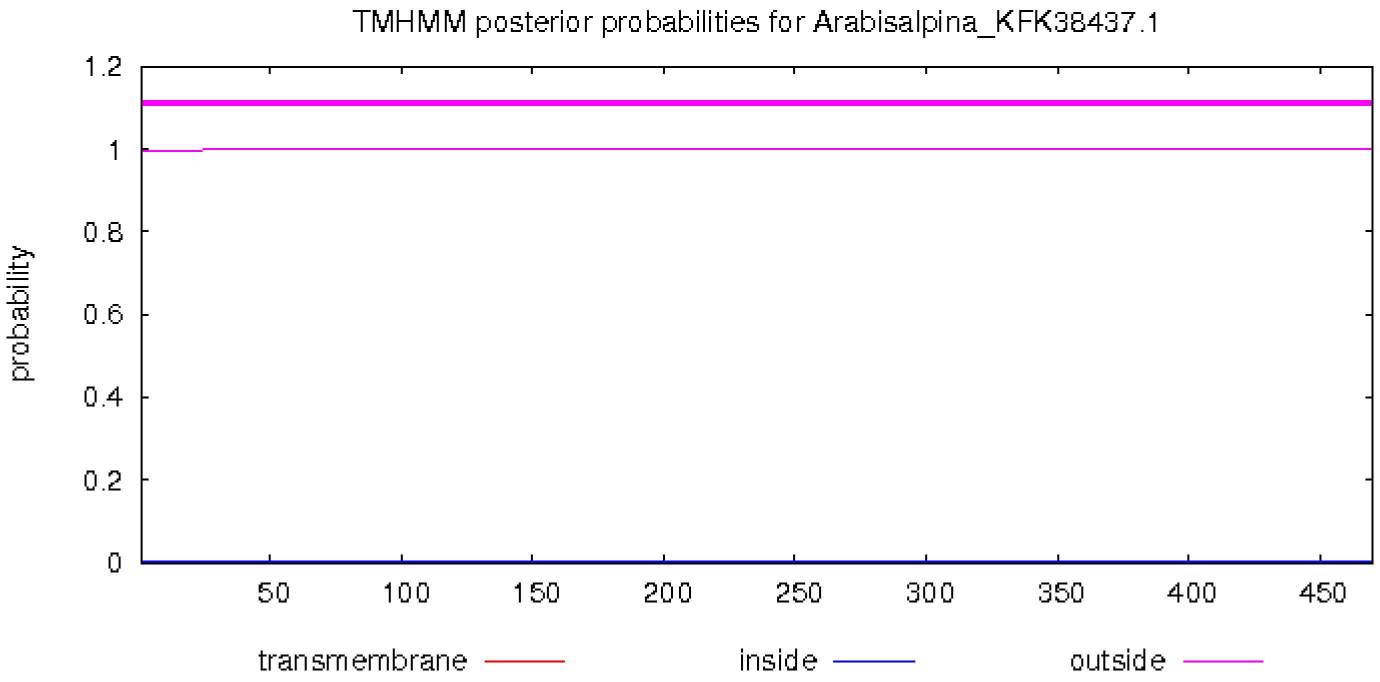

plot in postscript, script for making the plot in gnuplot, data for plot

---

```
# Arabisalpina_KFK38438.1 Length: 558
# Arabisalpina_KFK38438.1 Number of predicted TMHs:  1
# Arabisalpina_KFK38438.1 Exp number of AAs in TMHs: 21.29415
# Arabisalpina_KFK38438.1 Exp number, first 60 AAs:  0
# Arabisalpina_KFK38438.1 Total prob of N-in:        0.00108
Arabisalpina_KFK38438.1 TMHMM2.0        outside      1   532
Arabisalpina_KFK38438.1 TMHMM2.0        TMhelix    533   555
Arabisalpina_KFK38438.1 TMHMM2.0        inside     556   558
```

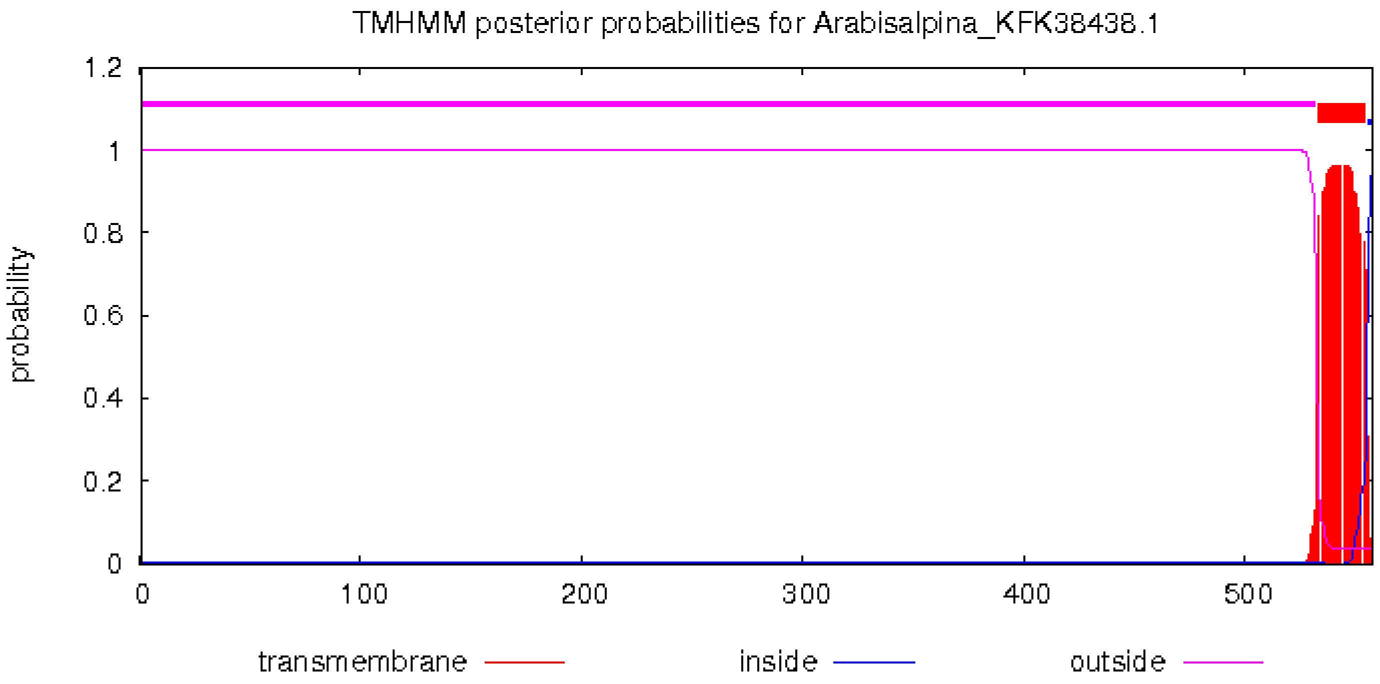

plot in postscript, script for making the plot in gnuplot, data for plot

```
# Arabisalpina_KFK38633.1 Length: 306
# Arabisalpina_KFK38633.1 Number of predicted TMHs:  0
# Arabisalpina_KFK38633.1 Exp number of AAs in TMHs: 0.00347
# Arabisalpina_KFK38633.1 Exp number, first 60 AAs:  0
# Arabisalpina_KFK38633.1 Total prob of N-in:        0.00652
Arabisalpina_KFK38633.1 TMHMM2.0        outside      1   306
```

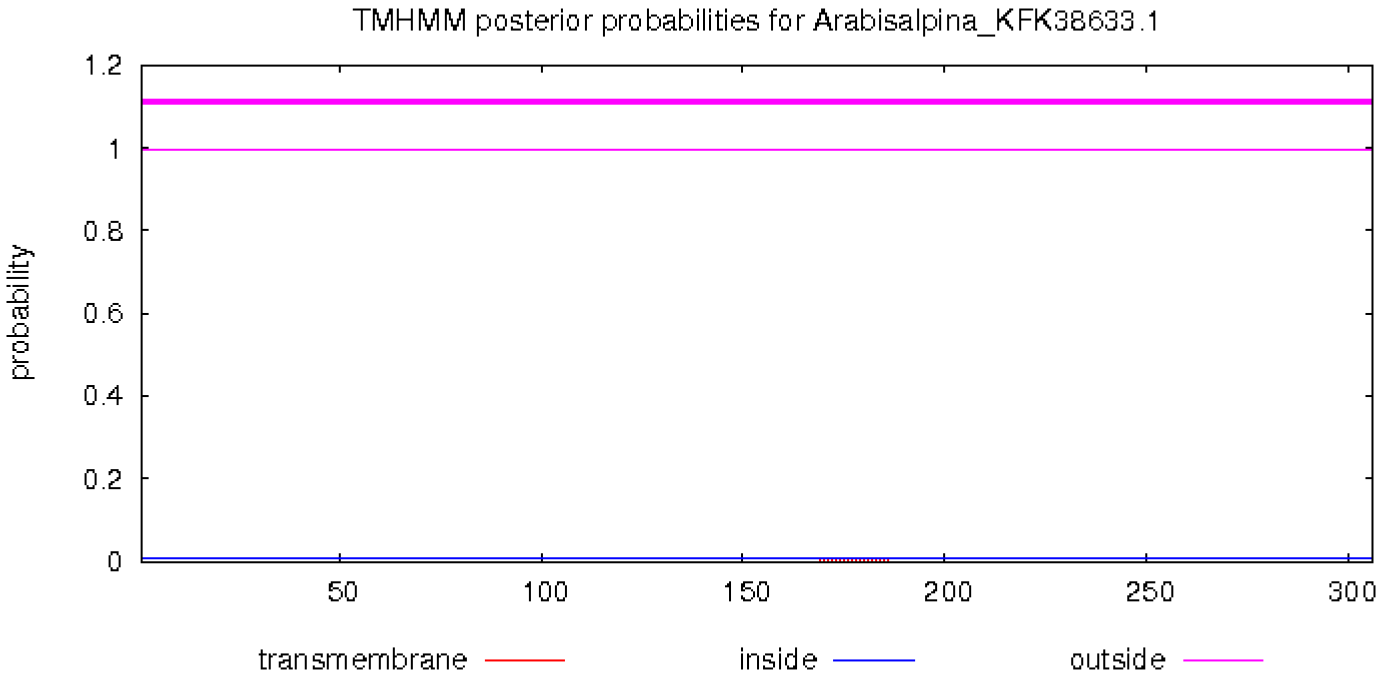

plot in postscript, script for making the plot in gnuplot, data for plot

```
# Arabisalpina_KFK38638.1 Length: 278
# Arabisalpina_KFK38638.1 Number of predicted TMHs:  0
# Arabisalpina_KFK38638.1 Exp number of AAs in TMHs: 0.00011
# Arabisalpina_KFK38638.1 Exp number, first 60 AAs:  0
# Arabisalpina_KFK38638.1 Total prob of N-in:        0.07421
Arabisalpina_KFK38638.1 TMHMM2.0        outside      1   278
```

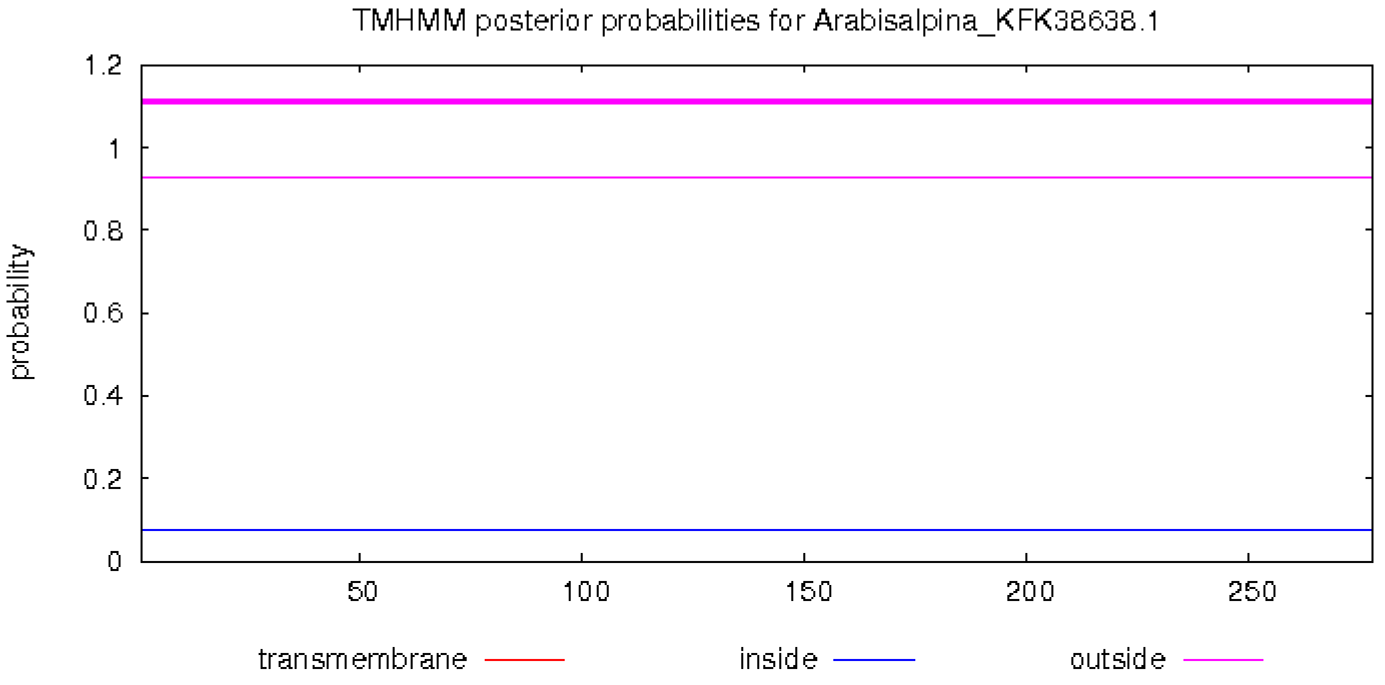

plot in postscript, script for making the plot in gnuplot, data for plot

```
# Arabisalpina_KFK38892.1 Length: 328
# Arabisalpina_KFK38892.1 Number of predicted TMHs:  0
# Arabisalpina_KFK38892.1 Exp number of AAs in TMHs: 0.00106
# Arabisalpina_KFK38892.1 Exp number, first 60 AAs:  0
# Arabisalpina_KFK38892.1 Total prob of N-in:        0.00354
Arabisalpina_KFK38892.1 TMHMM2.0        outside      1   328
```

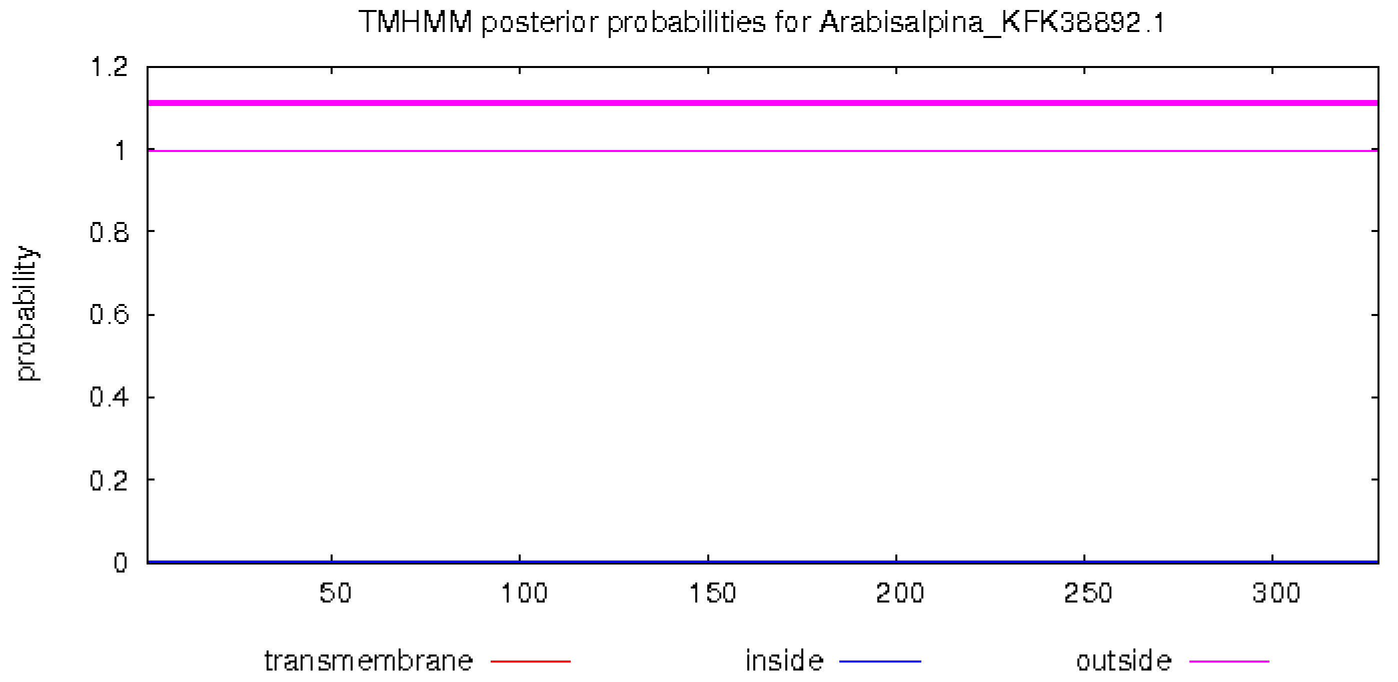

plot in postscript, script for making the plot in gnuplot, data for plot

---

```
# Arabisalpina_KFK38893.1 Length: 342
# Arabisalpina_KFK38893.1 Number of predicted TMHs:  0
# Arabisalpina_KFK38893.1 Exp number of AAs in TMHs: 0.00061
# Arabisalpina_KFK38893.1 Exp number, first 60 AAs:  0.00017
# Arabisalpina_KFK38893.1 Total prob of N-in:        0.00538
Arabisalpina_KFK38893.1 TMHMM2.0        outside      1   342
```

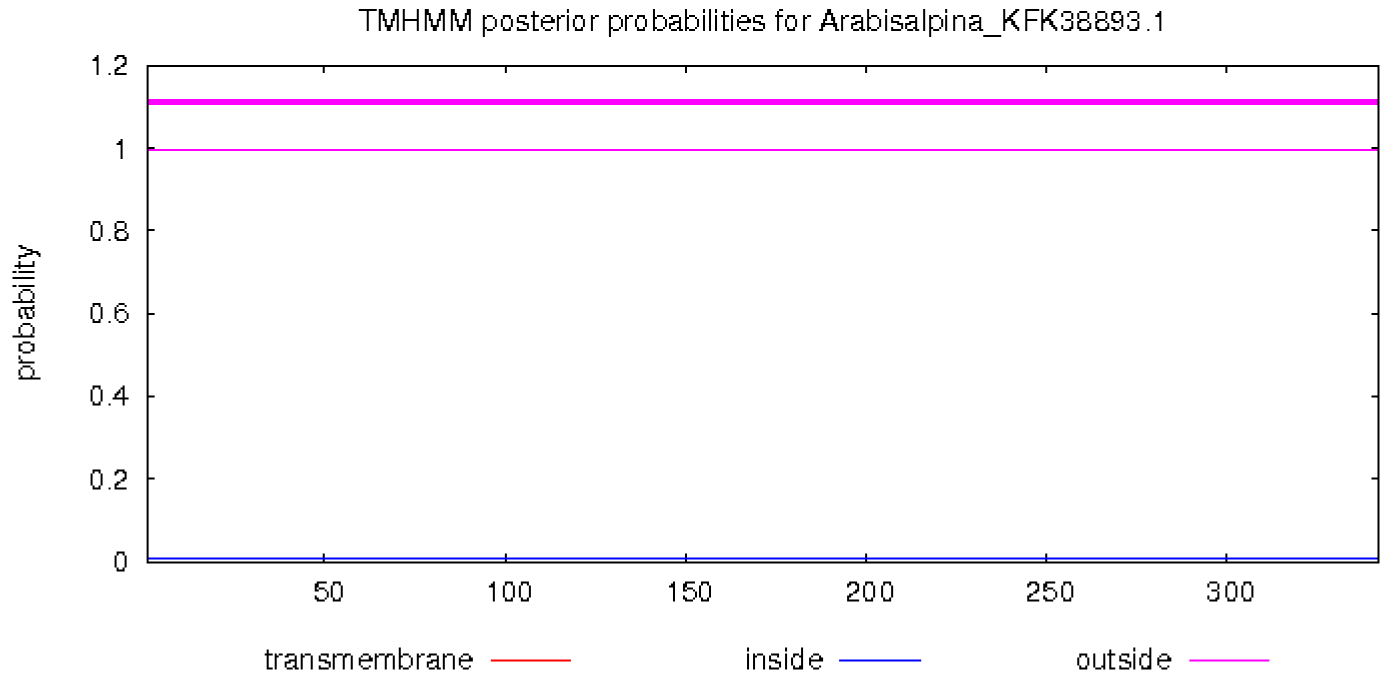

plot in postscript, script for making the plot in gnuplot, data for plot

---

```
# Arabisalpina_KFK39102.1 Length: 244
# Arabisalpina_KFK39102.1 Number of predicted TMHs:  0
# Arabisalpina_KFK39102.1 Exp number of AAs in TMHs: 0
# Arabisalpina_KFK39102.1 Exp number, first 60 AAs:  0
# Arabisalpina_KFK39102.1 Total prob of N-in:        0.00228
Arabisalpina_KFK39102.1 TMHMM2.0        outside      1   244
```

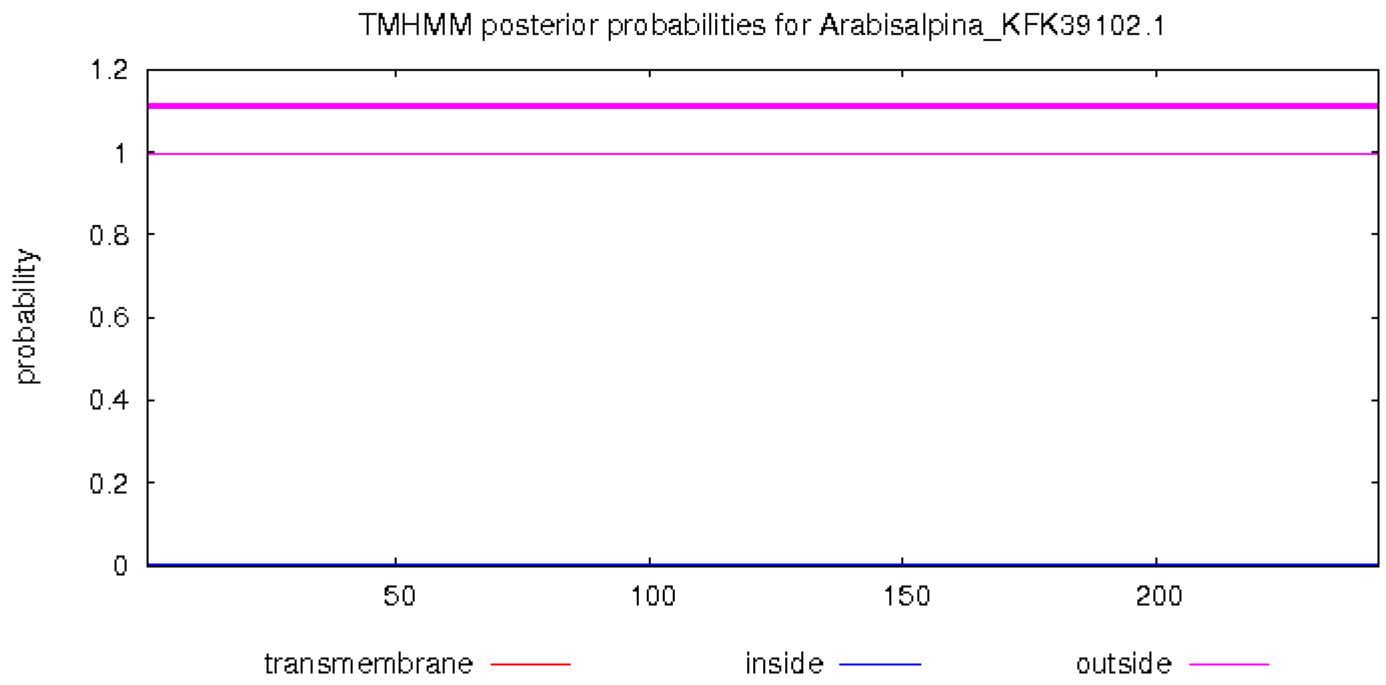

plot in postscript, script for making the plot in gnuplot, data for plot

```
# Arabisalpina_KFK40091.1 Length: 285
# Arabisalpina_KFK40091.1 Number of predicted TMHs:  0
# Arabisalpina_KFK40091.1 Exp number of AAs in TMHs: 0.00167
# Arabisalpina_KFK40091.1 Exp number, first 60 AAs:  0.00022
# Arabisalpina_KFK40091.1 Total prob of N-in:        0.00778
Arabisalpina_KFK40091.1 TMHMM2.0        outside      1   285
```

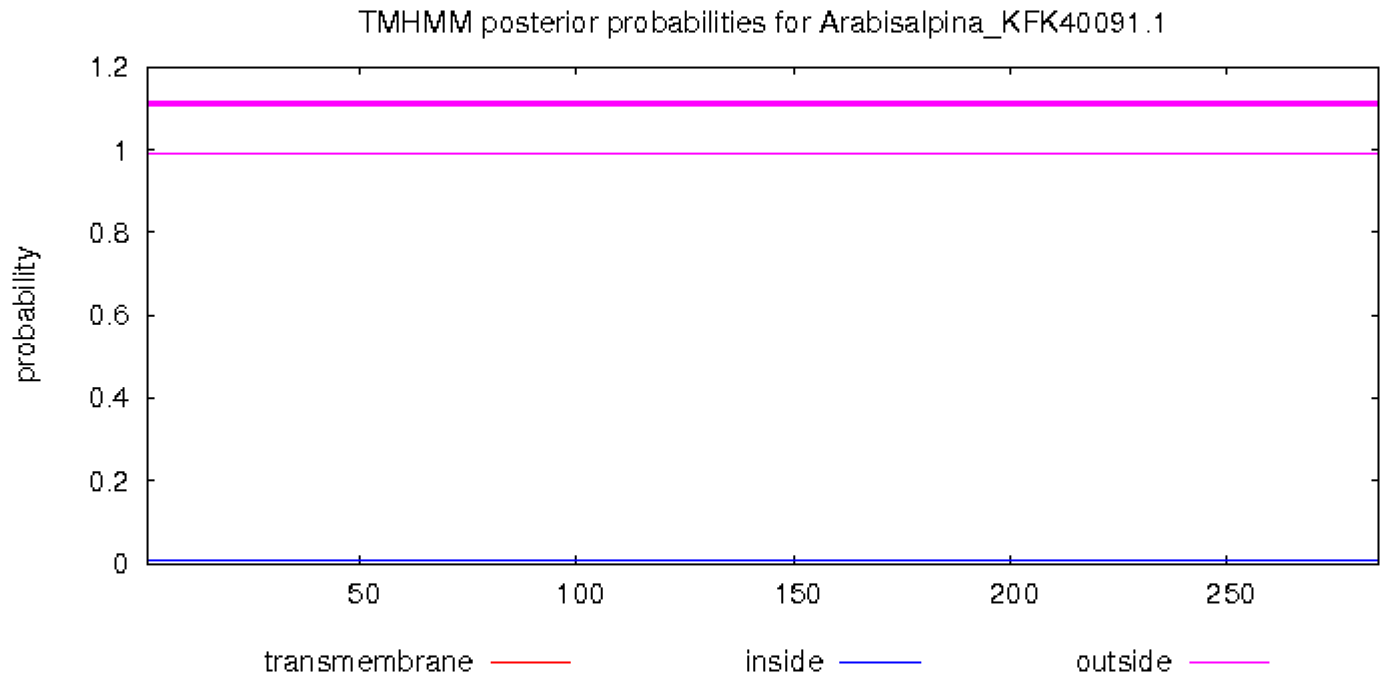

plot in postscript, script for making the plot in gnuplot, data for plot

```
# Arabisalpina_KFK41373.1 Length: 272
# Arabisalpina_KFK41373.1 Number of predicted TMHs:  0
# Arabisalpina_KFK41373.1 Exp number of AAs in TMHs: 0.00171
# Arabisalpina_KFK41373.1 Exp number, first 60 AAs:  0
# Arabisalpina_KFK41373.1 Total prob of N-in:        0.02718
Arabisalpina_KFK41373.1 TMHMM2.0        outside      1   272
```

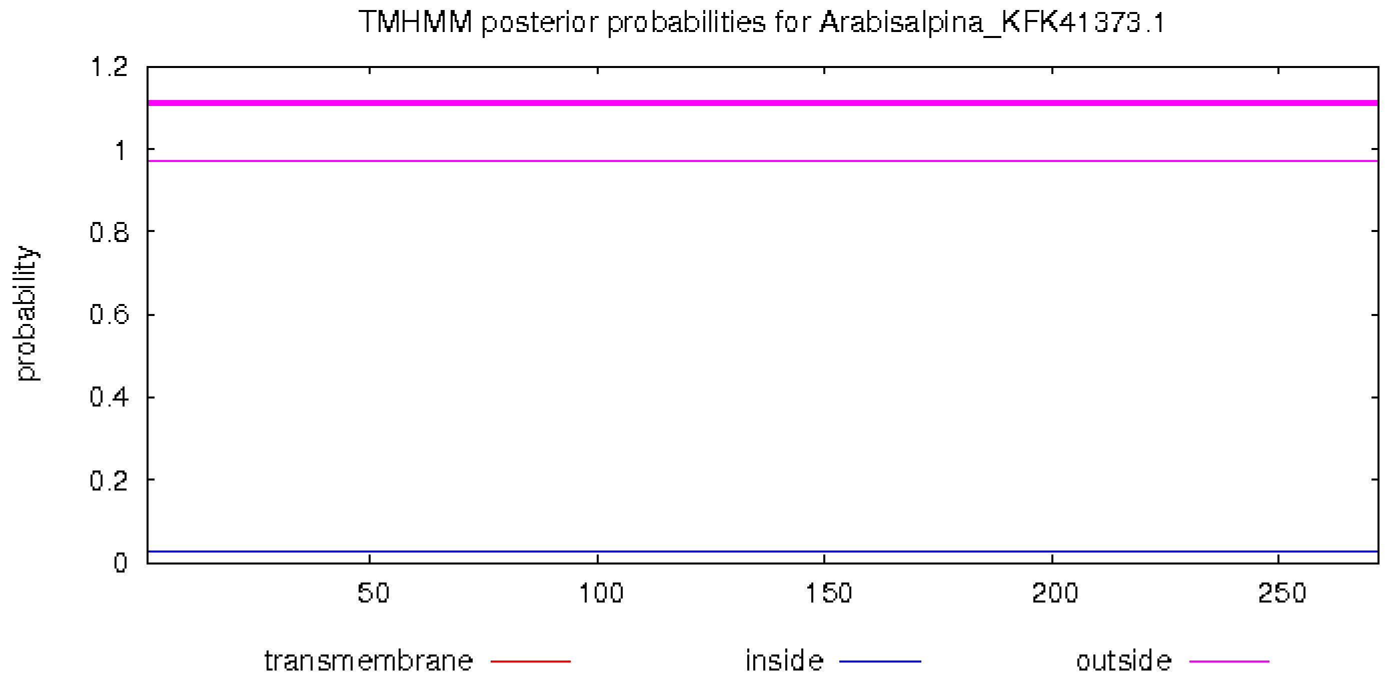

plot in postscript, script for making the plot in gnuplot, data for plot

```
# Arabisalpina_KFK41460.1 Length: 559
# Arabisalpina_KFK41460.1 Number of predicted TMHs:  0
# Arabisalpina_KFK41460.1 Exp number of AAs in TMHs: 0.00583
# Arabisalpina_KFK41460.1 Exp number, first 60 AAs:  0
# Arabisalpina_KFK41460.1 Total prob of N-in:        0.00057
Arabisalpina_KFK41460.1 TMHMM2.0        outside      1   559
```

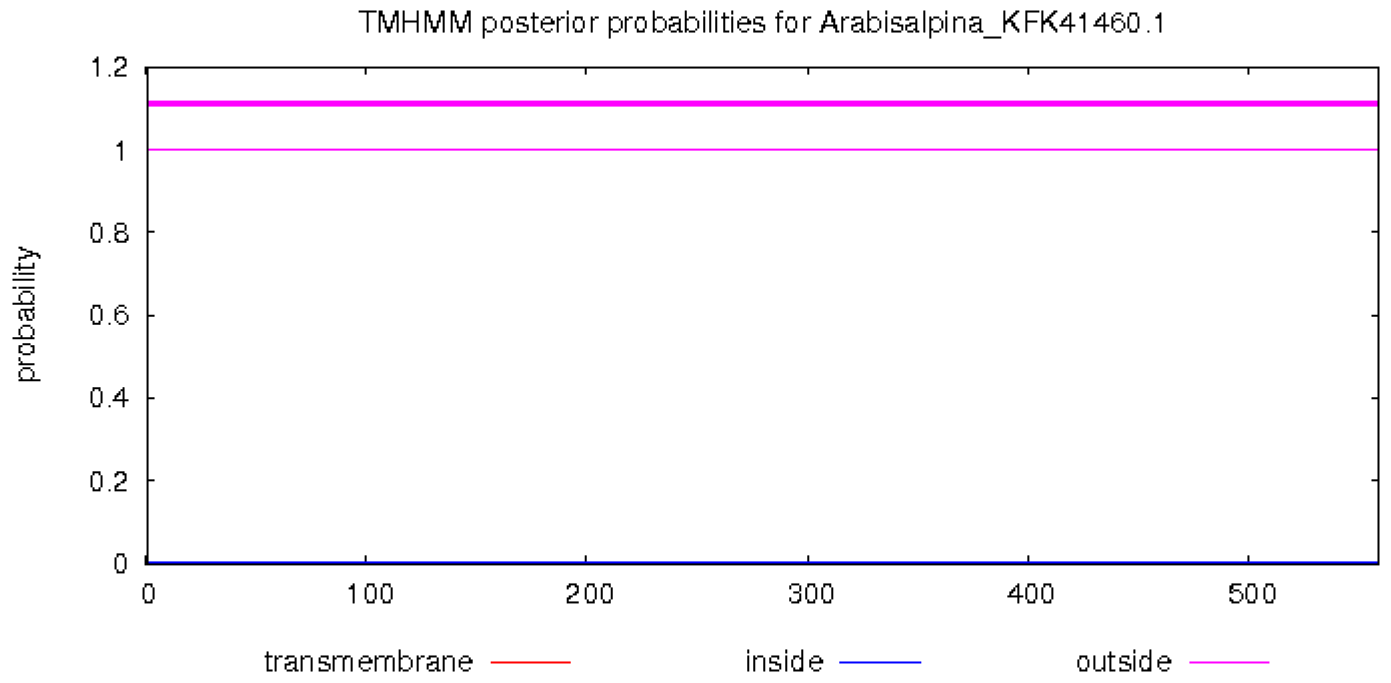

plot in postscript, script for making the plot in gnuplot, data for plot

```
# Arabisalpina_KFK42062.1 Length: 334
# Arabisalpina_KFK42062.1 Number of predicted TMHs:  0
# Arabisalpina_KFK42062.1 Exp number of AAs in TMHs: 0.672270000000000001
# Arabisalpina_KFK42062.1 Exp number, first 60 AAs:  0.05796
# Arabisalpina_KFK42062.1 Total prob of N-in:        0.02664
Arabisalpina_KFK42062.1 TMHMM2.0        outside      1   334
```

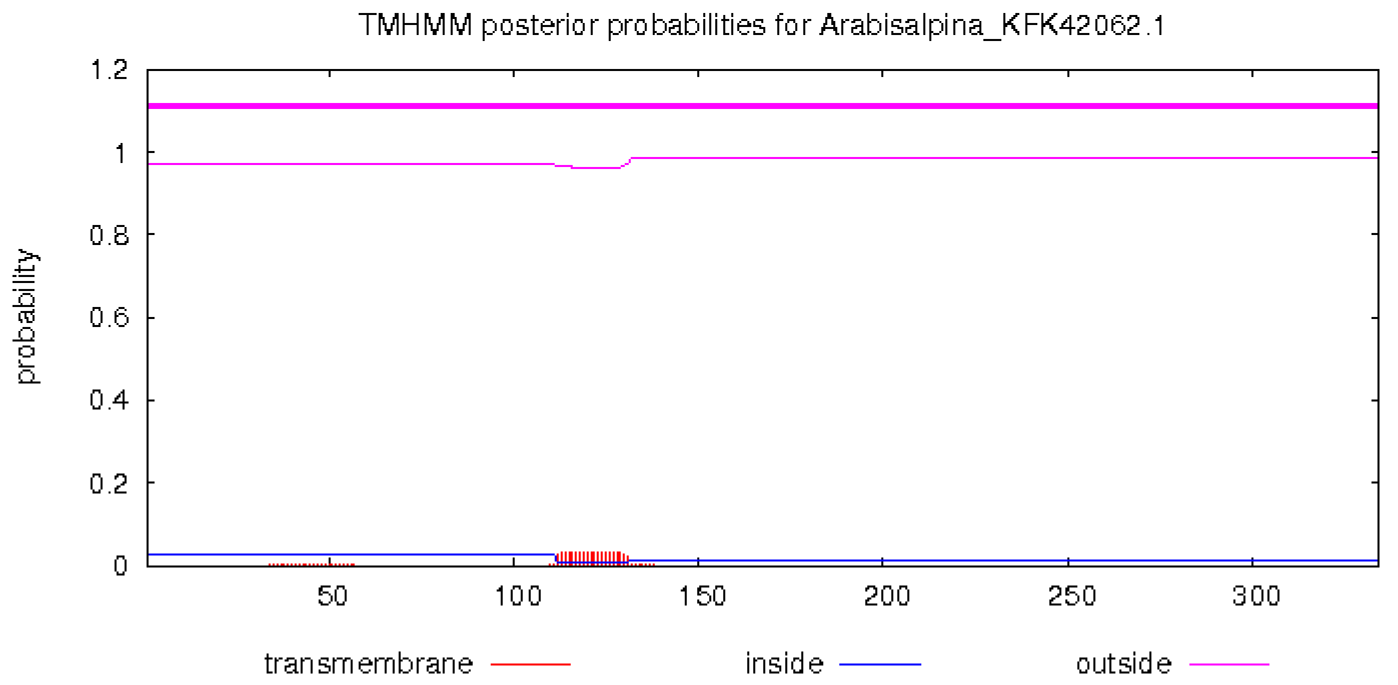

plot in postscript, script for making the plot in gnuplot, data for plot

```
# Arabisalpina_KFK42139.1 Length: 264
# Arabisalpina_KFK42139.1 Number of predicted TMHs:  0
# Arabisalpina_KFK42139.1 Exp number of AAs in TMHs: 0.00105
# Arabisalpina_KFK42139.1 Exp number, first 60 AAs:  0.00072
# Arabisalpina_KFK42139.1 Total prob of N-in:        0.04312
Arabisalpina_KFK42139.1 TMHMM2.0        outside      1   264
```

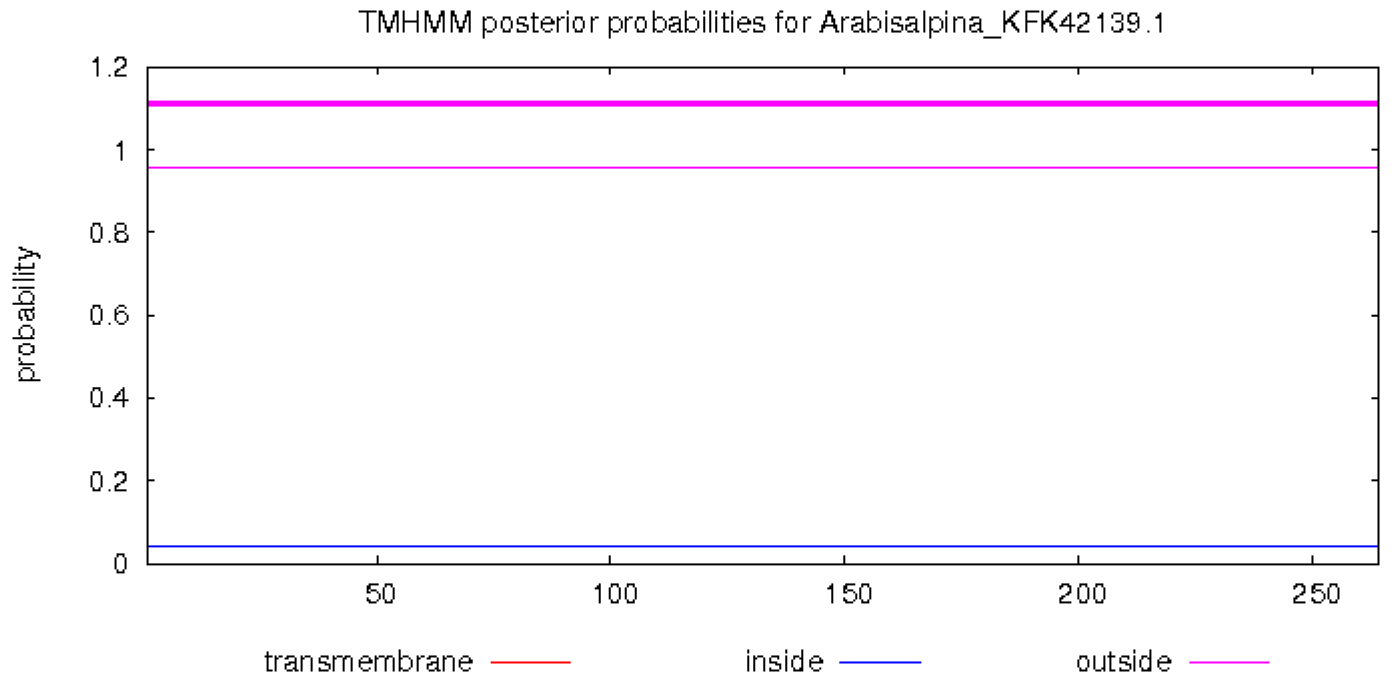

plot in postscript, script for making the plot in gnuplot, data for plot

```
# Arabisalpina_KFK42489.1 Length: 570
# Arabisalpina_KFK42489.1 Number of predicted TMHs:  0
# Arabisalpina_KFK42489.1 Exp number of AAs in TMHs: 0.00174
# Arabisalpina_KFK42489.1 Exp number, first 60 AAs:  0
# Arabisalpina_KFK42489.1 Total prob of N-in:        0.00286
Arabisalpina_KFK42489.1 TMHMM2.0        outside      1   570
```

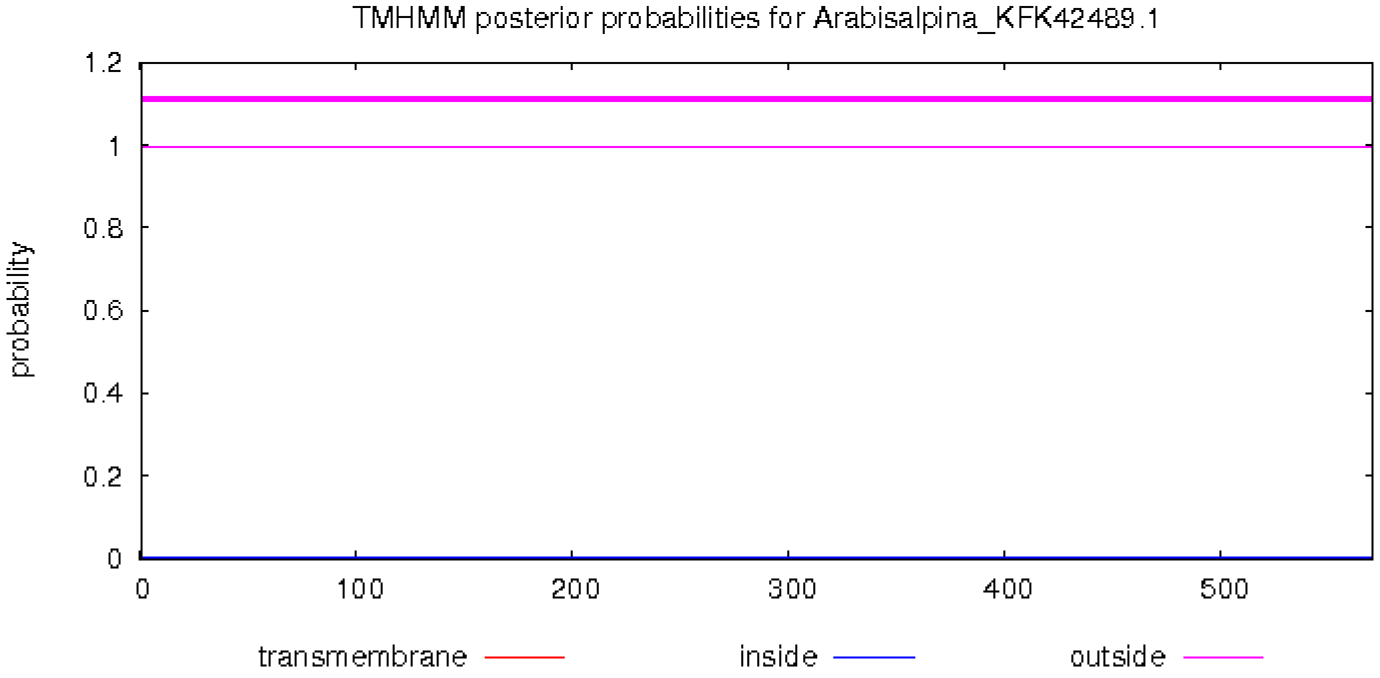

plot in postscript, script for making the plot in gnuplot, data for plot

---

```
# Arabisalpina_KFK42490.1 Length: 271
# Arabisalpina_KFK42490.1 Number of predicted TMHs:  0
# Arabisalpina_KFK42490.1 Exp number of AAs in TMHs: 0.02202
# Arabisalpina_KFK42490.1 Exp number, first 60 AAs:  0
# Arabisalpina_KFK42490.1 Total prob of N-in:        0.03594
Arabisalpina_KFK42490.1 TMHMM2.0        outside      1   271
```

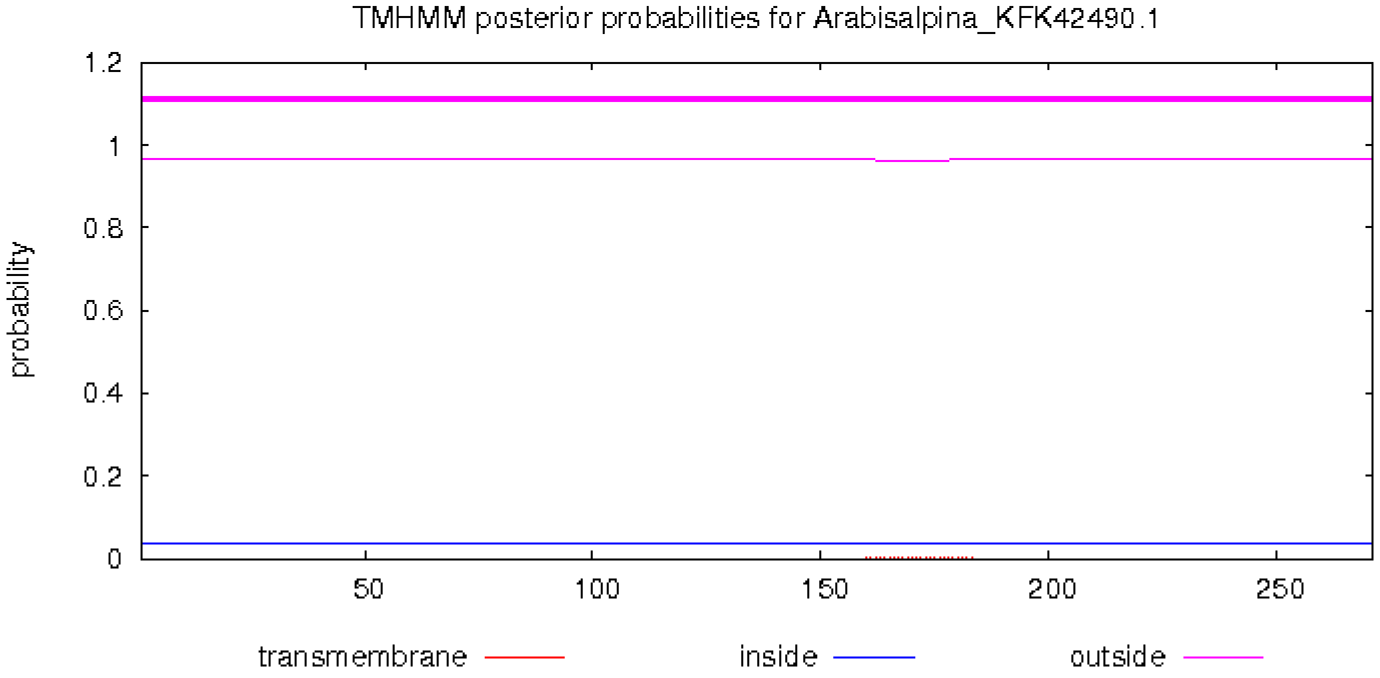

plot in postscript, script for making the plot in gnuplot, data for plot

---

```
# Arabisalpina_KFK42491.1 Length: 424
# Arabisalpina_KFK42491.1 Number of predicted TMHs:  1
# Arabisalpina_KFK42491.1 Exp number of AAs in TMHs: 23.02073
# Arabisalpina_KFK42491.1 Exp number, first 60 AAs:  0.00051
# Arabisalpina_KFK42491.1 Total prob of N-in:        0.24188
Arabisalpina_KFK42491.1 TMHMM2.0        outside      1   400
Arabisalpina_KFK42491.1 TMHMM2.0        TMhelix    401   423
Arabisalpina_KFK42491.1 TMHMM2.0        inside     424   424
```

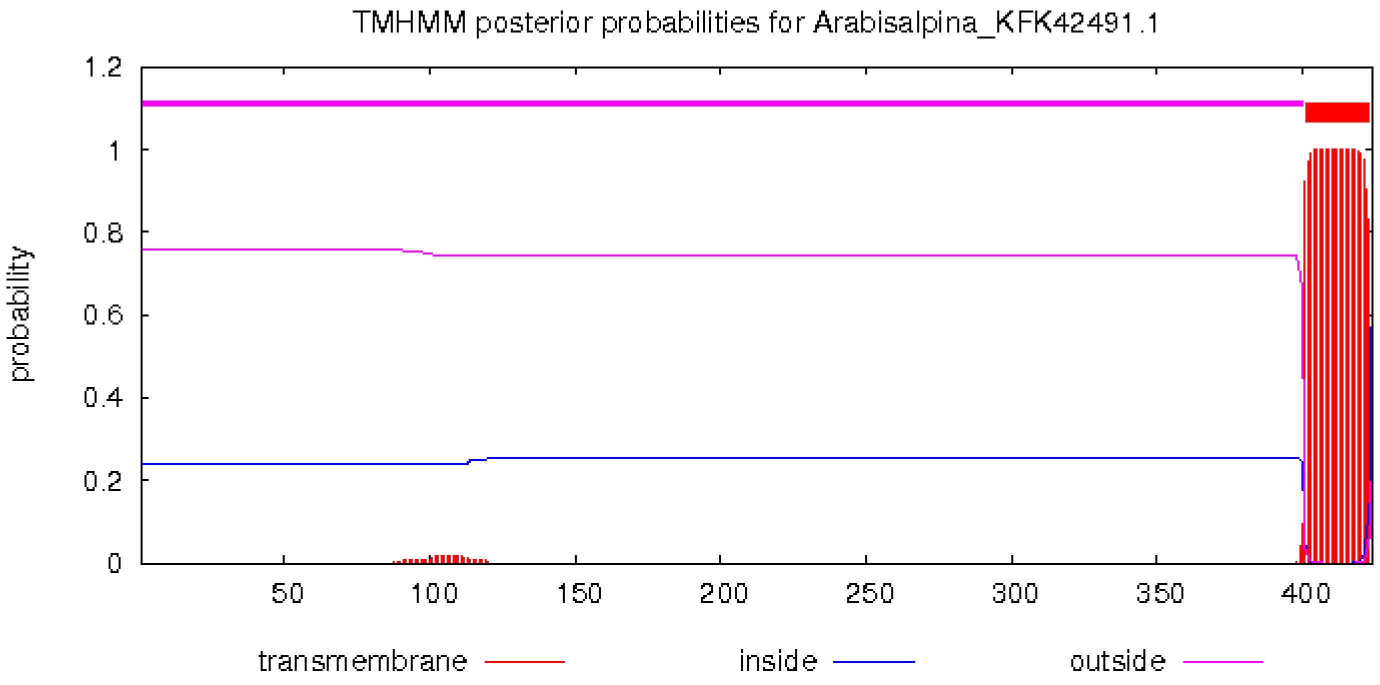

plot in postscript, script for making the plot in gnuplot, data for plot

---

```
# Arabisalpina_KFK42525.1 Length: 293
# Arabisalpina_KFK42525.1 Number of predicted TMHs:  0
# Arabisalpina_KFK42525.1 Exp number of AAs in TMHs: 0.00697
# Arabisalpina_KFK42525.1 Exp number, first 60 AAs:  0
# Arabisalpina_KFK42525.1 Total prob of N-in:        0.05143
Arabisalpina_KFK42525.1 TMHMM2.0        outside      1   293
```

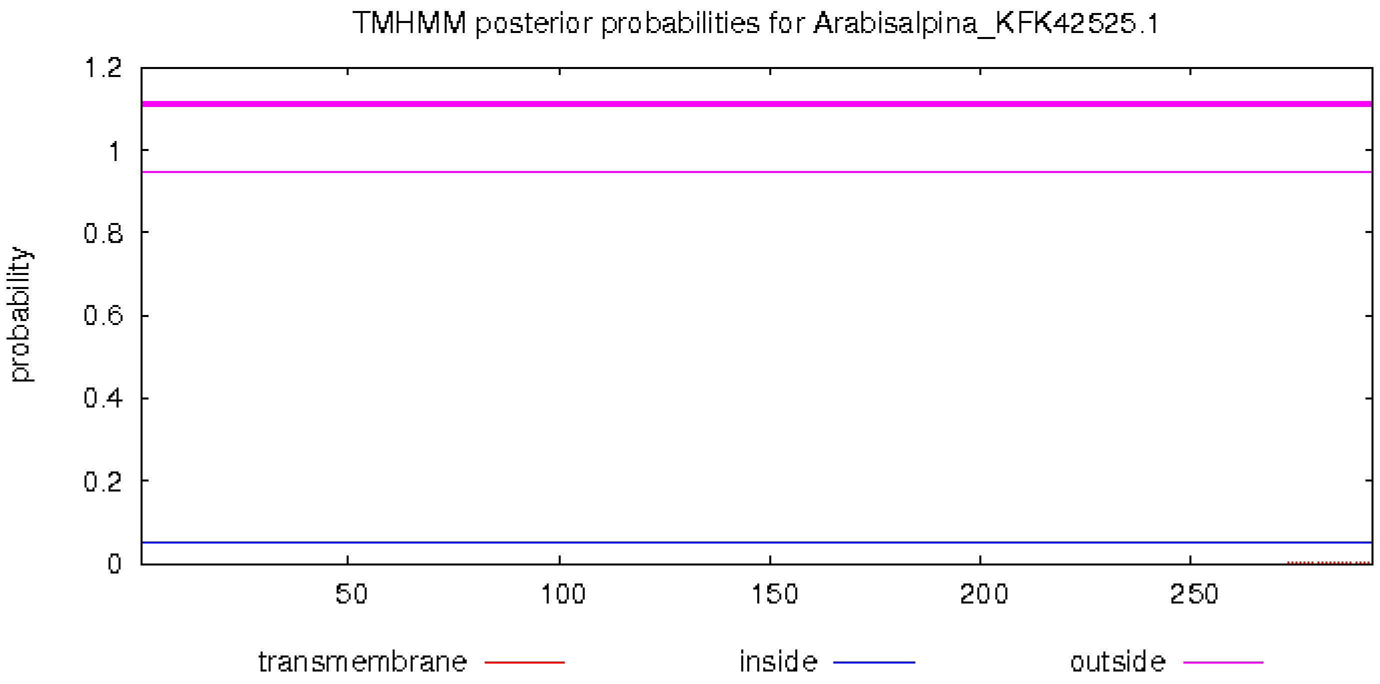

plot in postscript, script for making the plot in gnuplot, data for plot

```
# Arabisalpina_KFK42526.1 Length: 364
# Arabisalpina_KFK42526.1 Number of predicted TMHs:  0
# Arabisalpina_KFK42526.1 Exp number of AAs in TMHs: 0
# Arabisalpina_KFK42526.1 Exp number, first 60 AAs:  0
# Arabisalpina_KFK42526.1 Total prob of N-in:        0.00396
Arabisalpina_KFK42526.1 TMHMM2.0        outside      1   364
```

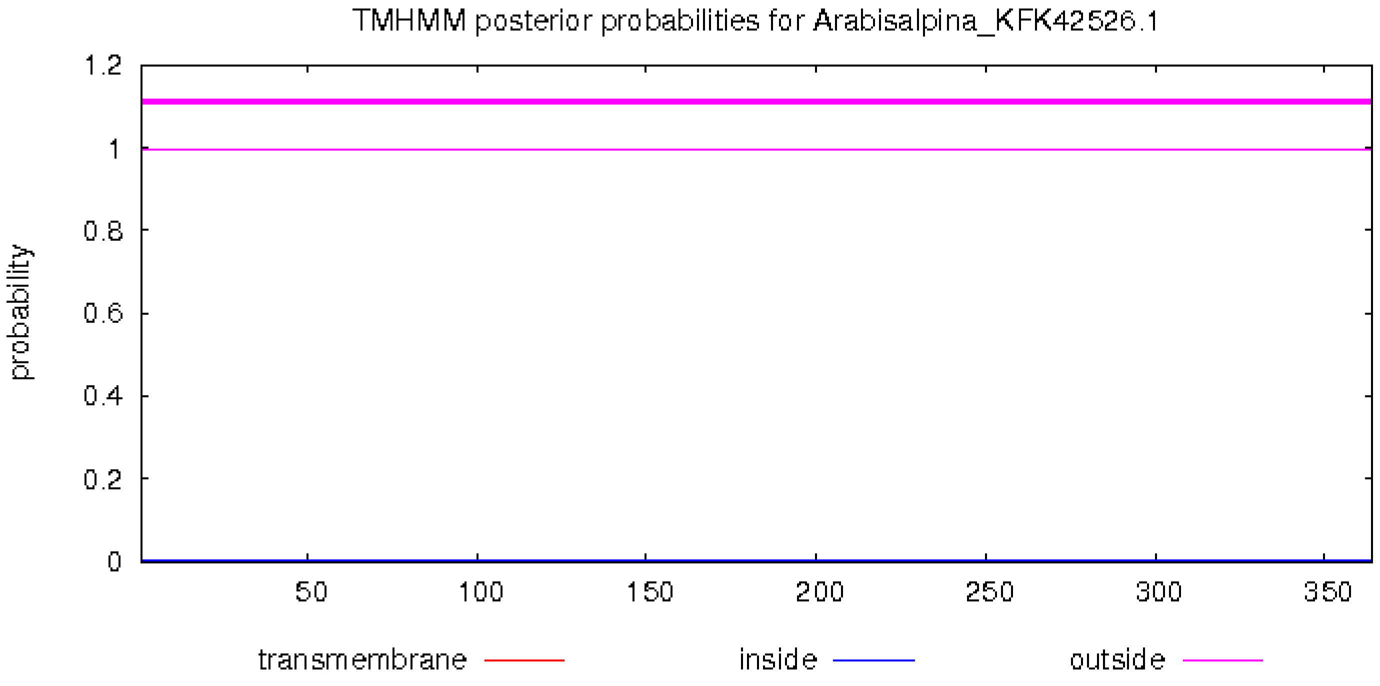

plot in postscript, script for making the plot in gnuplot, data for plot

```
# Arabisalpina_KFK42547.1 Length: 296
# Arabisalpina_KFK42547.1 Number of predicted TMHs:  0
# Arabisalpina_KFK42547.1 Exp number of AAs in TMHs: 0.00051
# Arabisalpina_KFK42547.1 Exp number, first 60 AAs:  0.0002
# Arabisalpina_KFK42547.1 Total prob of N-in:        0.00301
Arabisalpina_KFK42547.1 TMHMM2.0        outside      1   296
```

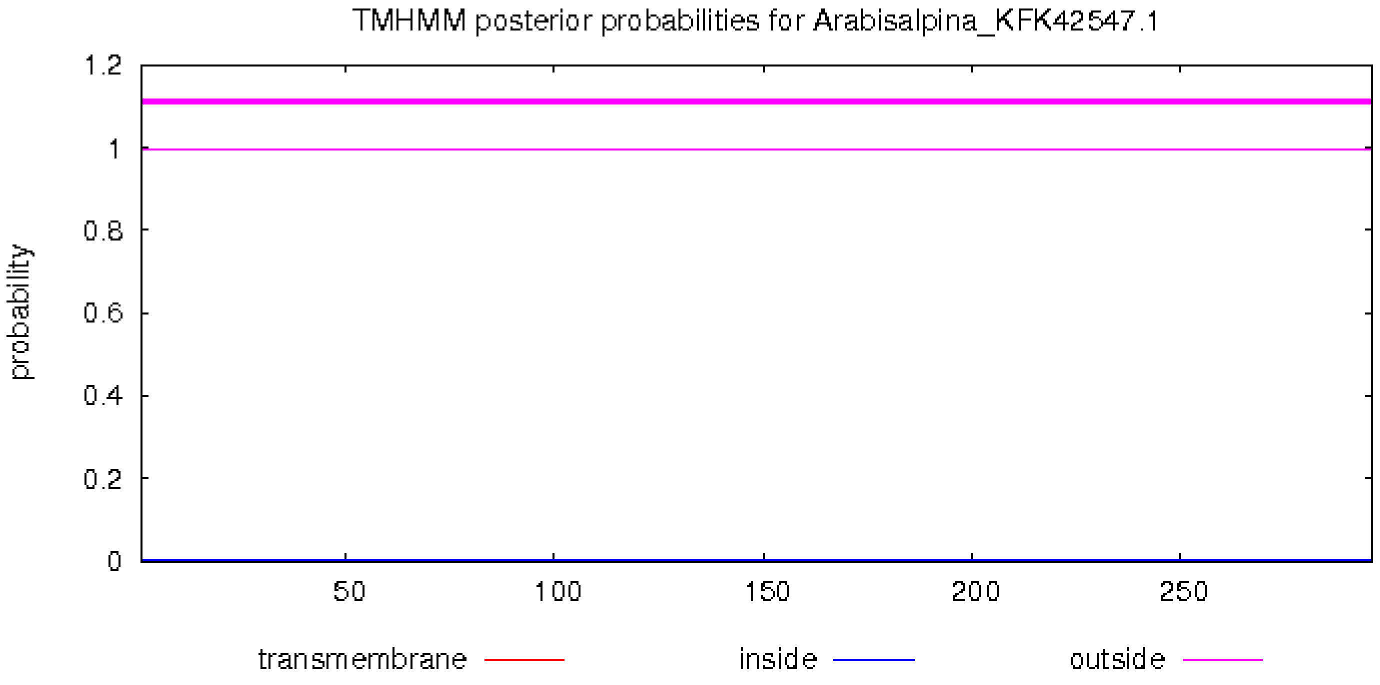

plot in postscript, script for making the plot in gnuplot, data for plot

```
# Arabisalpina_KFK42739.1 Length: 566
# Arabisalpina_KFK42739.1 Number of predicted TMHs:  0
# Arabisalpina_KFK42739.1 Exp number of AAs in TMHs: 0.004
# Arabisalpina_KFK42739.1 Exp number, first 60 AAs:  0
# Arabisalpina_KFK42739.1 Total prob of N-in:        0.00138
Arabisalpina_KFK42739.1 TMHMM2.0        outside      1   566
```

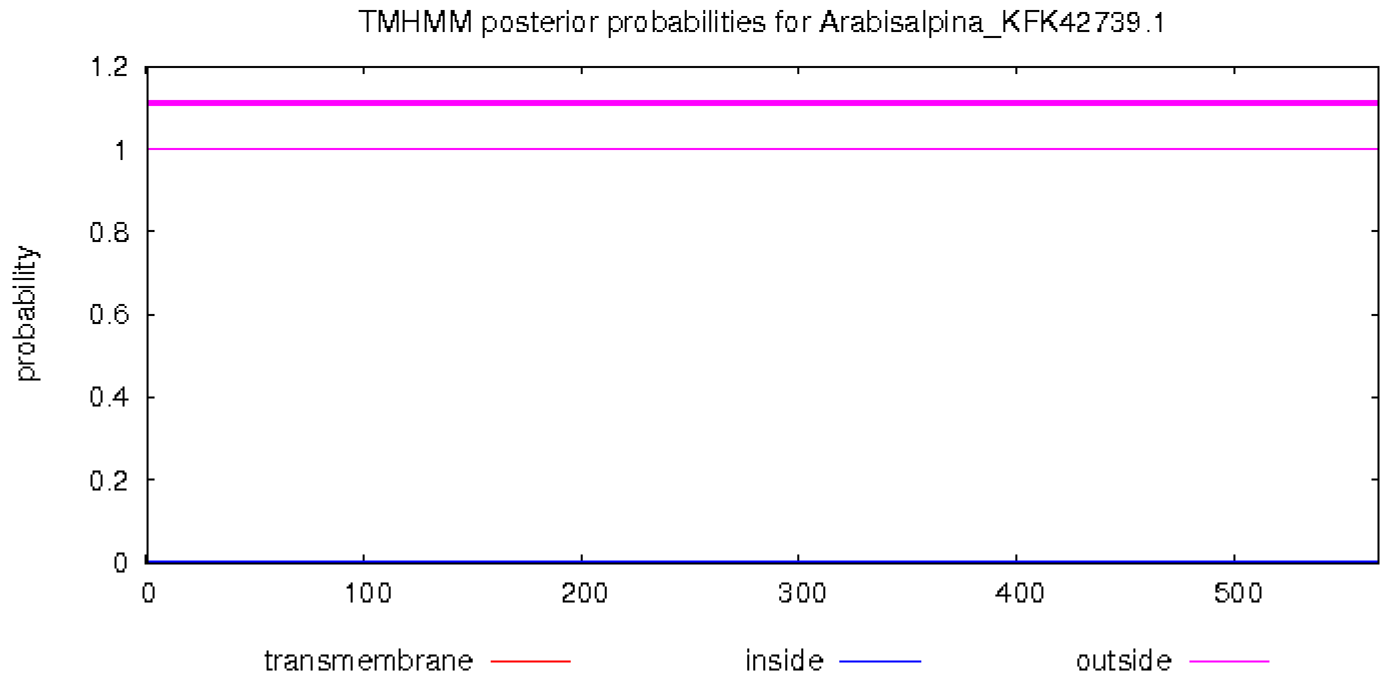

plot in postscript, script for making the plot in gnuplot, data for plot

---

```
# Arabisalpina_KFK42926.1 Length: 324
# Arabisalpina_KFK42926.1 Number of predicted TMHs:  0
# Arabisalpina_KFK42926.1 Exp number of AAs in TMHs: 0.00017
# Arabisalpina_KFK42926.1 Exp number, first 60 AAs:  0.00017
# Arabisalpina_KFK42926.1 Total prob of N-in:        0.01128
Arabisalpina_KFK42926.1 TMHMM2.0        outside      1   324
```

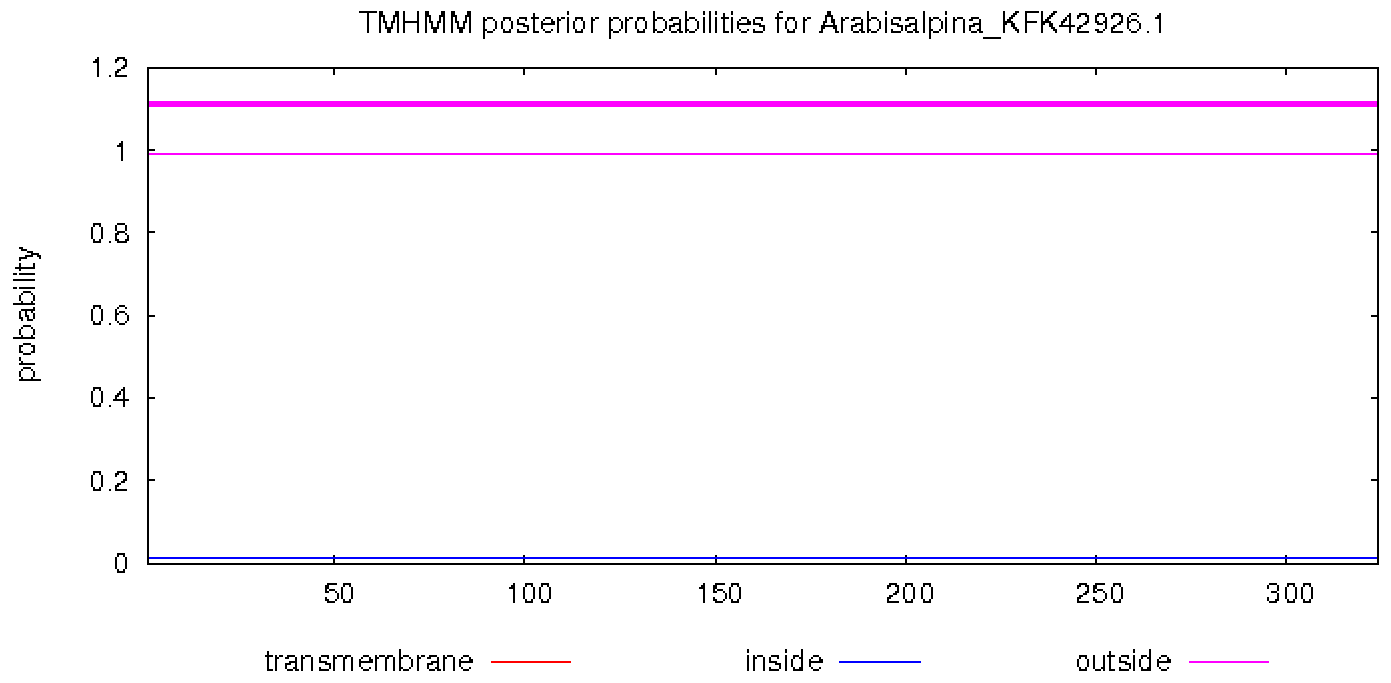

plot in postscript, script for making the plot in gnuplot, data for plot

---

```
# Arabisalpina_KFK44551.1 Length: 458
# Arabisalpina_KFK44551.1 Number of predicted TMHs:  0
# Arabisalpina_KFK44551.1 Exp number of AAs in TMHs: 0.00312
# Arabisalpina_KFK44551.1 Exp number, first 60 AAs:  0
# Arabisalpina_KFK44551.1 Total prob of N-in:        0.00685
Arabisalpina_KFK44551.1 TMHMM2.0        outside      1   458
```

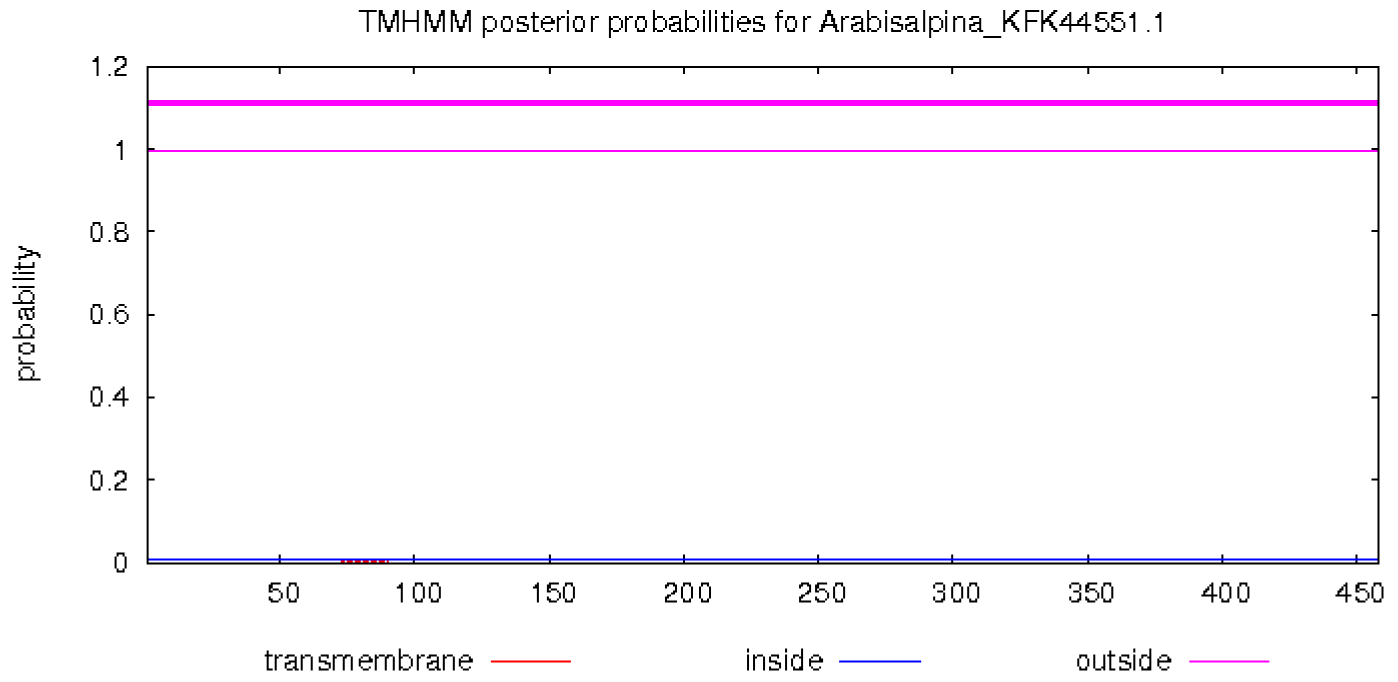

plot in postscript, script for making the plot in gnuplot, data for plot

---

```
# Arabisalpina_KFK44718.1 Length: 311
# Arabisalpina_KFK44718.1 Number of predicted TMHs:  0
# Arabisalpina_KFK44718.1 Exp number of AAs in TMHs: 0.00034
# Arabisalpina_KFK44718.1 Exp number, first 60 AAs:  0
# Arabisalpina_KFK44718.1 Total prob of N-in:        0.04588
Arabisalpina_KFK44718.1 TMHMM2.0        outside      1   311
```

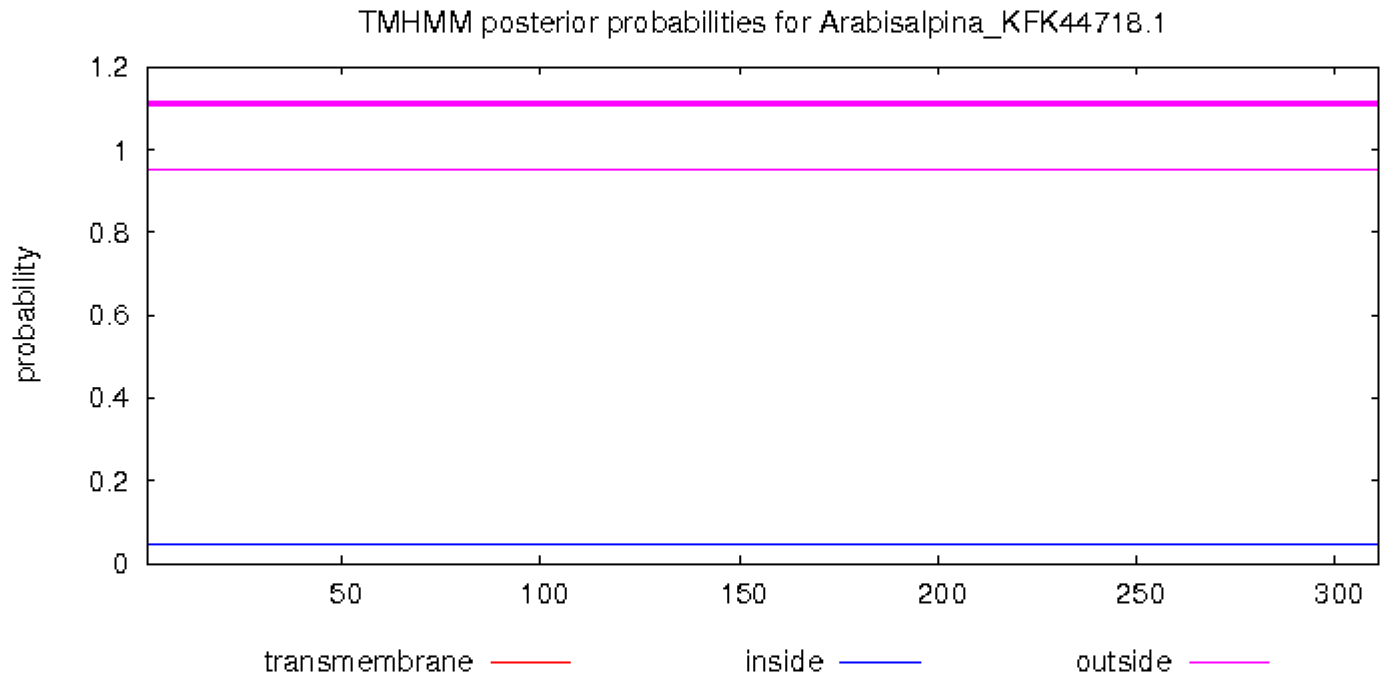

plot in postscript, script for making the plot in gnuplot, data for plot

---

```
# Arabisalpina_KFK44992.1 Length: 238
# Arabisalpina_KFK44992.1 Number of predicted TMHs:  0
# Arabisalpina_KFK44992.1 Exp number of AAs in TMHs: 0
# Arabisalpina_KFK44992.1 Exp number, first 60 AAs:  0
# Arabisalpina_KFK44992.1 Total prob of N-in:        0.00367
Arabisalpina_KFK44992.1 TMHMM2.0        outside      1   238
```

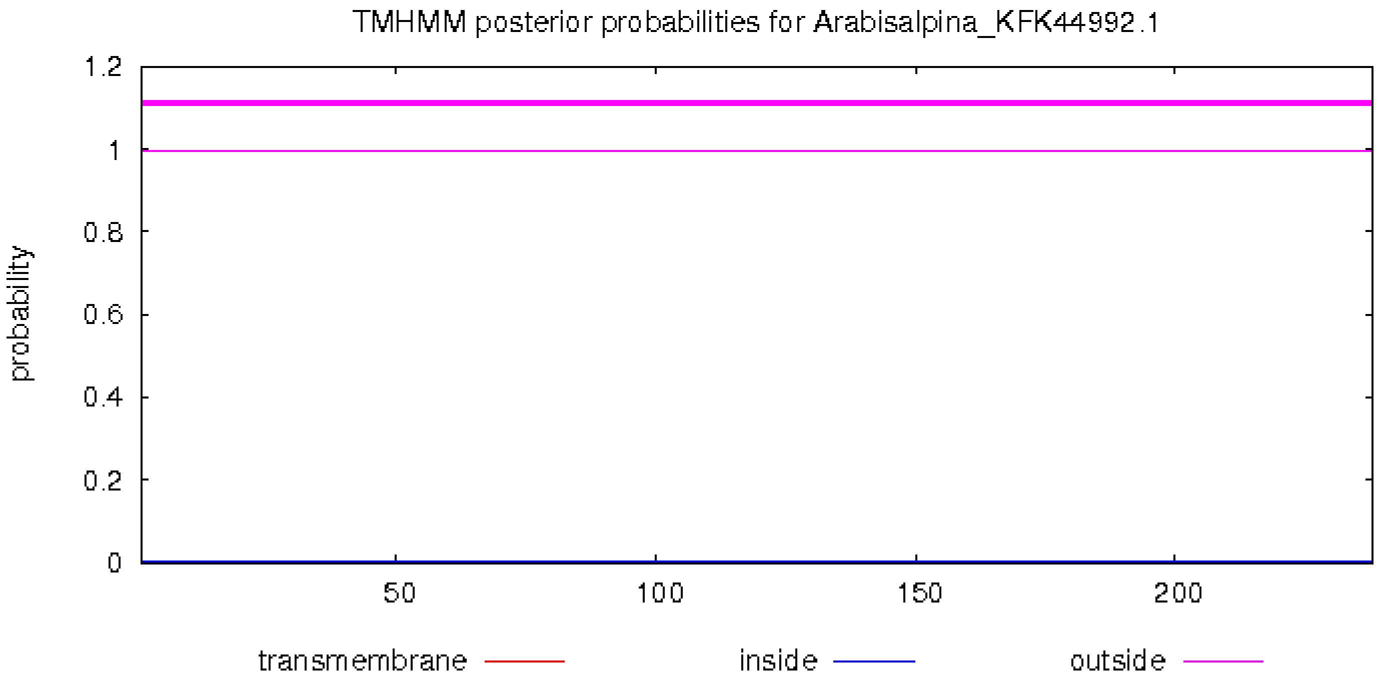

plot in postscript, script for making the plot in gnuplot, data for plot

---

```
# Arabisalpina_KFK45025.1 Length: 338
# Arabisalpina_KFK45025.1 Number of predicted TMHs:  0
# Arabisalpina_KFK45025.1 Exp number of AAs in TMHs: 0
# Arabisalpina_KFK45025.1 Exp number, first 60 AAs:  0
# Arabisalpina_KFK45025.1 Total prob of N-in:        0.02014
Arabisalpina_KFK45025.1 TMHMM2.0        outside      1   338
```

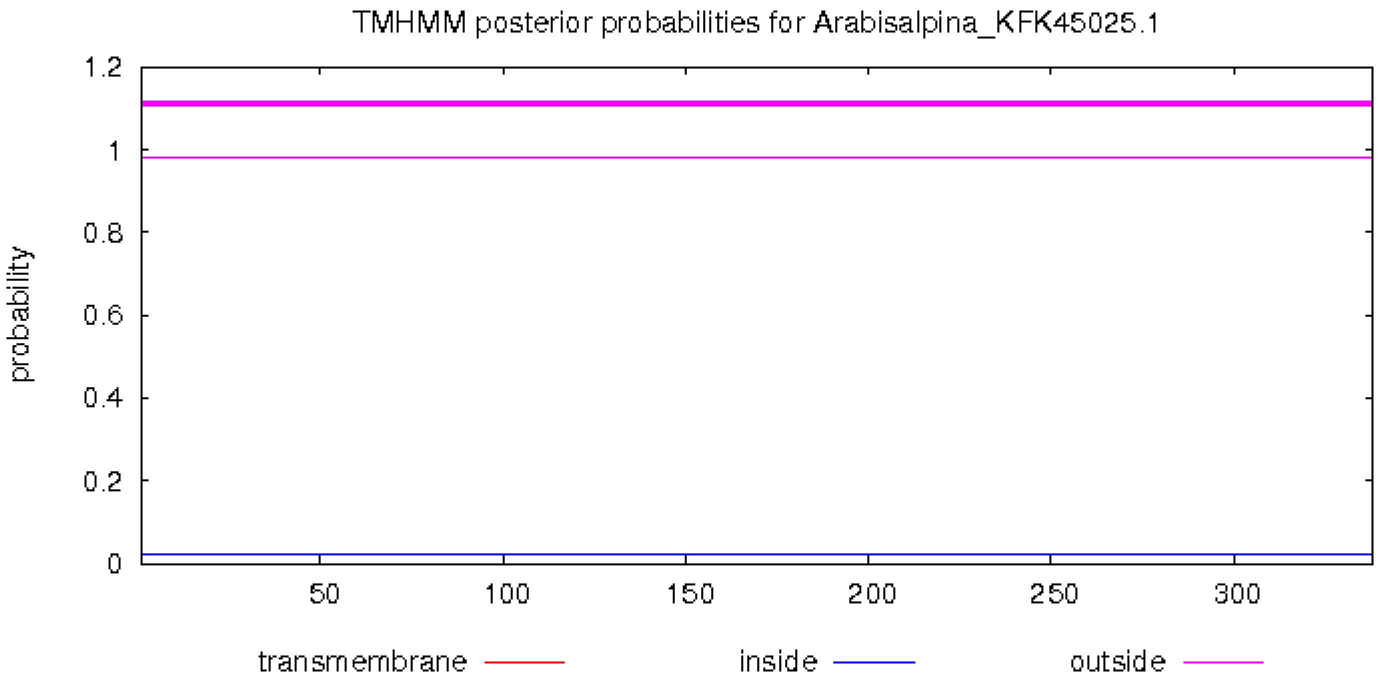

plot in postscript, script for making the plot in gnuplot, data for plot

---

```
# Arabisalpina_KFK45026.1 Length: 342
# Arabisalpina_KFK45026.1 Number of predicted TMHs:  0
# Arabisalpina_KFK45026.1 Exp number of AAs in TMHs: 0
# Arabisalpina_KFK45026.1 Exp number, first 60 AAs:  0
# Arabisalpina_KFK45026.1 Total prob of N-in:        0.01918
Arabisalpina_KFK45026.1 TMHMM2.0        outside      1   342
```

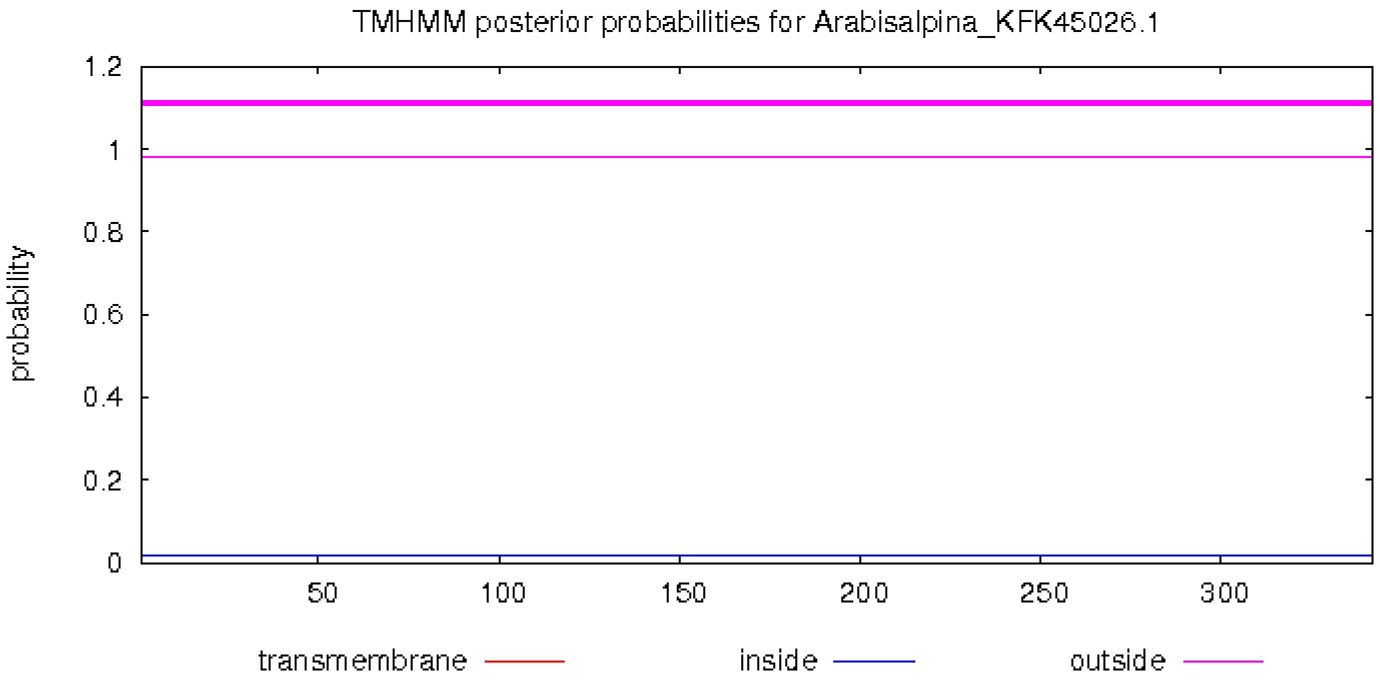

plot in postscript, script for making the plot in gnuplot, data for plot

---

```
# Arabisalpina_KFK45049.1 Length: 649
# Arabisalpina_KFK45049.1 Number of predicted TMHs:  1
# Arabisalpina_KFK45049.1 Exp number of AAs in TMHs: 20.61963
# Arabisalpina_KFK45049.1 Exp number, first 60 AAs:  0
# Arabisalpina_KFK45049.1 Total prob of N-in:        0.04365
Arabisalpina_KFK45049.1 TMHMM2.0        outside      1   603
Arabisalpina_KFK45049.1 TMHMM2.0        TMhelix    604   626
Arabisalpina_KFK45049.1 TMHMM2.0        inside     627   649
```

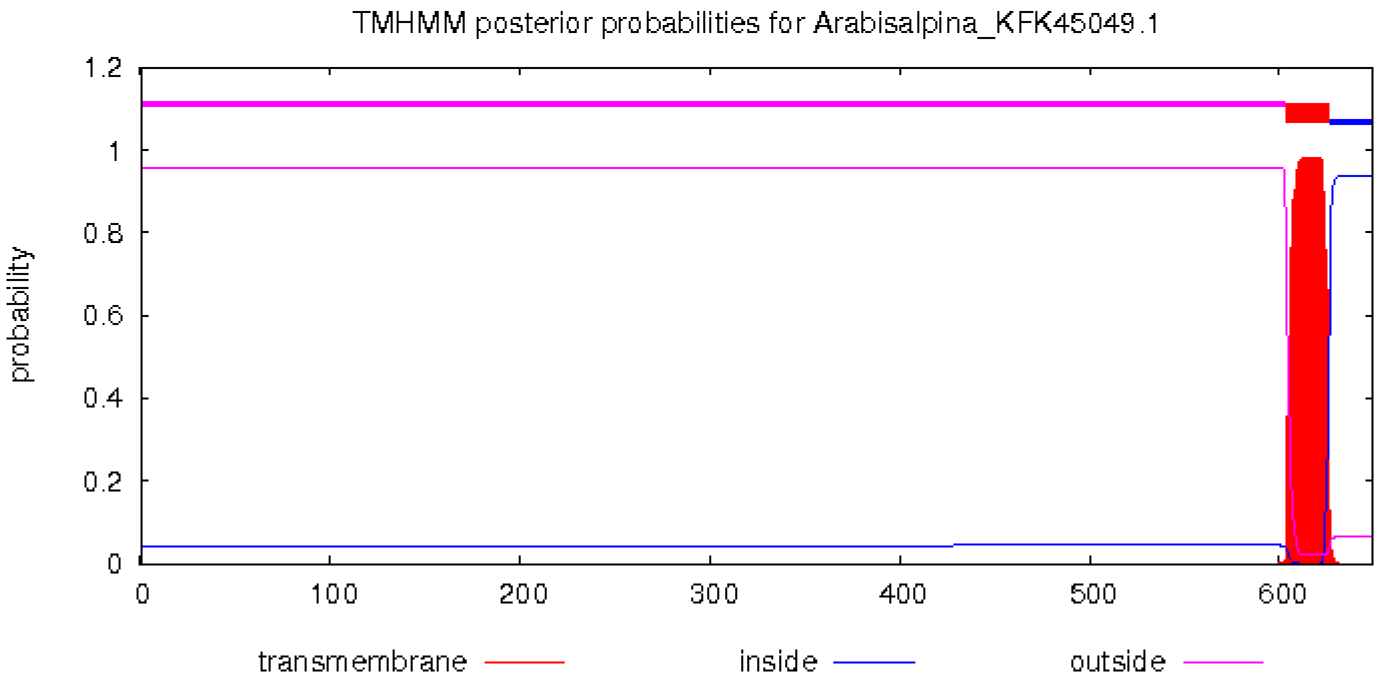

plot in postscript, script for making the plot in gnuplot, data for plot

```
# Arabisalpina_KFK45050.1 Length: 650
# Arabisalpina_KFK45050.1 Number of predicted TMHs:  1
# Arabisalpina_KFK45050.1 Exp number of AAs in TMHs: 20.61844
# Arabisalpina_KFK45050.1 Exp number, first 60 AAs:  0
# Arabisalpina_KFK45050.1 Total prob of N-in:        0.04314
Arabisalpina_KFK45050.1 TMHMM2.0        outside      1   604
Arabisalpina_KFK45050.1 TMHMM2.0        TMhelix    605   627
Arabisalpina_KFK45050.1 TMHMM2.0        inside     628   650
```

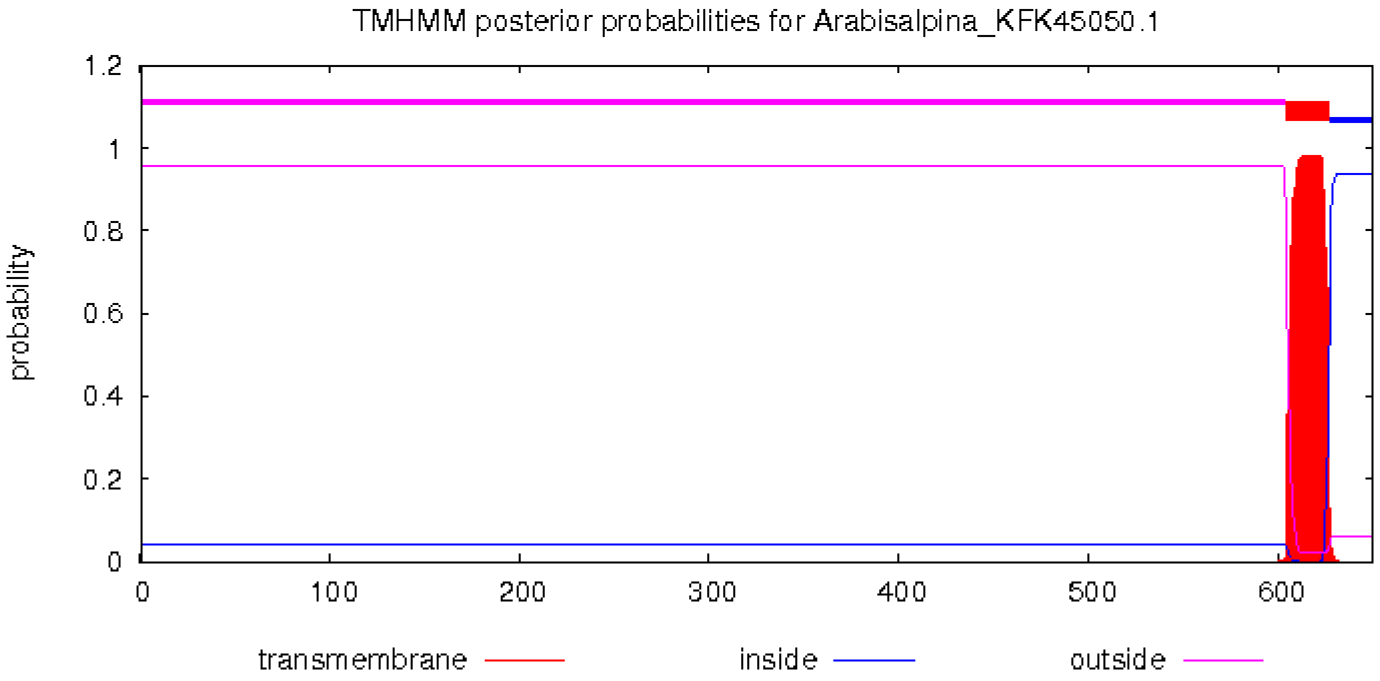

plot in postscript, script for making the plot in gnuplot, data for plot

```
# Arabisalpina_KFK45065.1 Length: 312
# Arabisalpina_KFK45065.1 Number of predicted TMHs:  0
# Arabisalpina_KFK45065.1 Exp number of AAs in TMHs: 0
# Arabisalpina_KFK45065.1 Exp number, first 60 AAs:  0
# Arabisalpina_KFK45065.1 Total prob of N-in:        0.00455
Arabisalpina_KFK45065.1 TMHMM2.0        outside      1   312
```

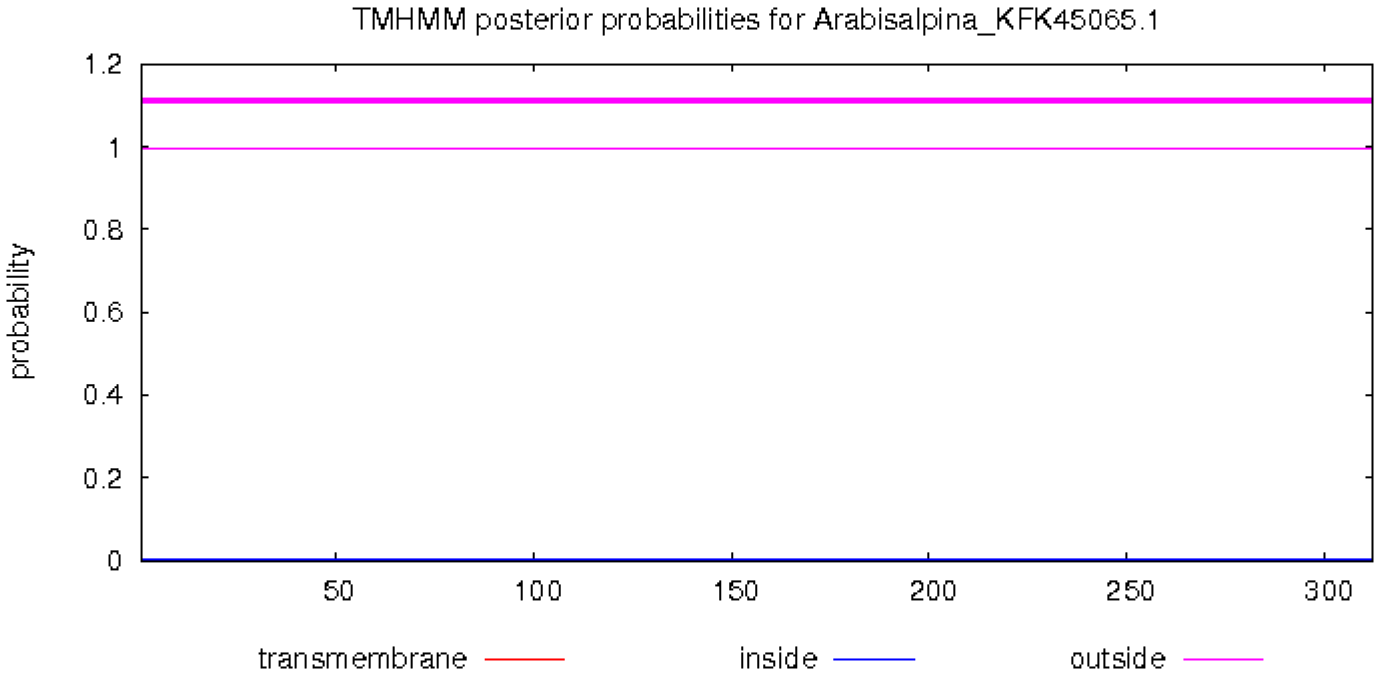

plot in postscript, script for making the plot in gnuplot, data for plot

```
# Arabisalpina_KFK45122.1 Length: 570
# Arabisalpina_KFK45122.1 Number of predicted TMHs:  1
# Arabisalpina_KFK45122.1 Exp number of AAs in TMHs: 23.25345
# Arabisalpina_KFK45122.1 Exp number, first 60 AAs:  0.00088
# Arabisalpina_KFK45122.1 Total prob of N-in:        0.23017
Arabisalpina_KFK45122.1 TMHMM2.0        outside      1   542
Arabisalpina_KFK45122.1 TMHMM2.0        TMhelix    543   565
Arabisalpina_KFK45122.1 TMHMM2.0        inside     566   570
```

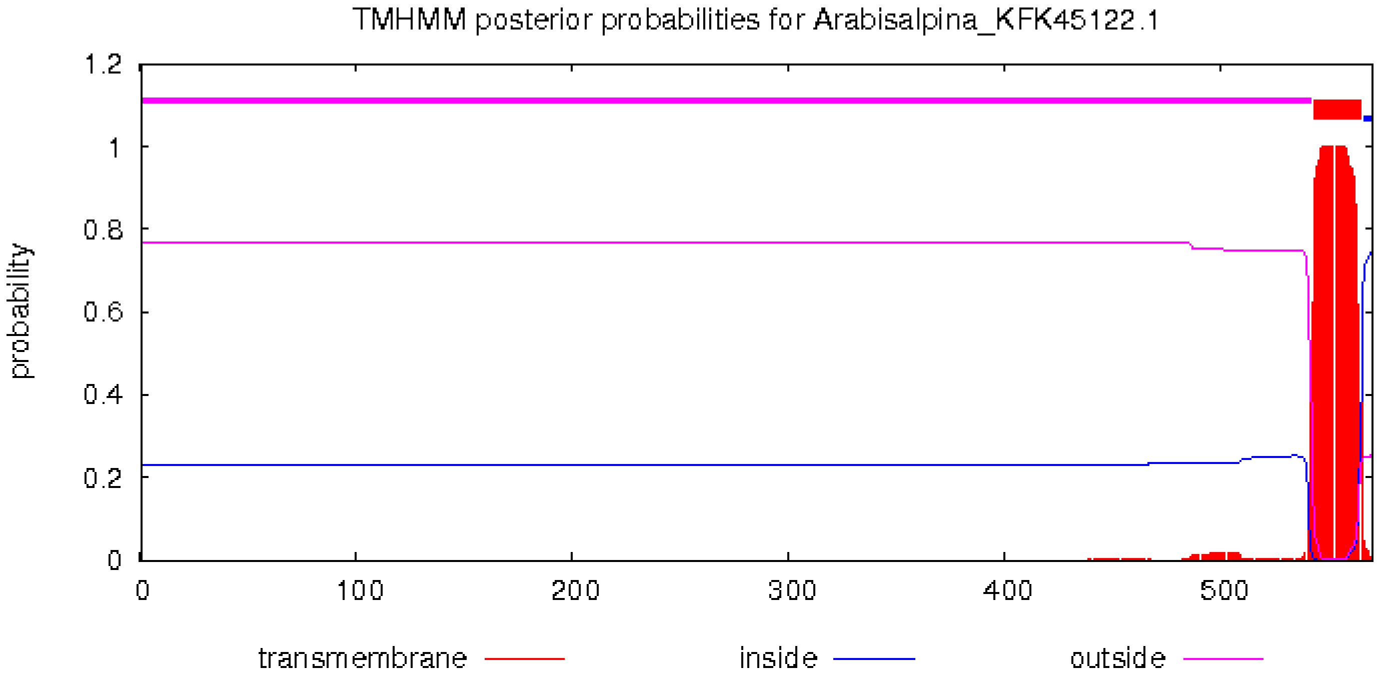

plot in postscript, script for making the plot in gnuplot, data for plot

```
# Arabisalpina_KFK45123.1 Length: 575
# Arabisalpina_KFK45123.1 Number of predicted TMHs:  1
# Arabisalpina_KFK45123.1 Exp number of AAs in TMHs: 21.51088
# Arabisalpina_KFK45123.1 Exp number, first 60 AAs:  0.02775
# Arabisalpina_KFK45123.1 Total prob of N-in:        0.20449
Arabisalpina_KFK45123.1 TMHMM2.0        outside      1   547
Arabisalpina_KFK45123.1 TMHMM2.0        TMhelix    548   570
Arabisalpina_KFK45123.1 TMHMM2.0        inside     571   575
```

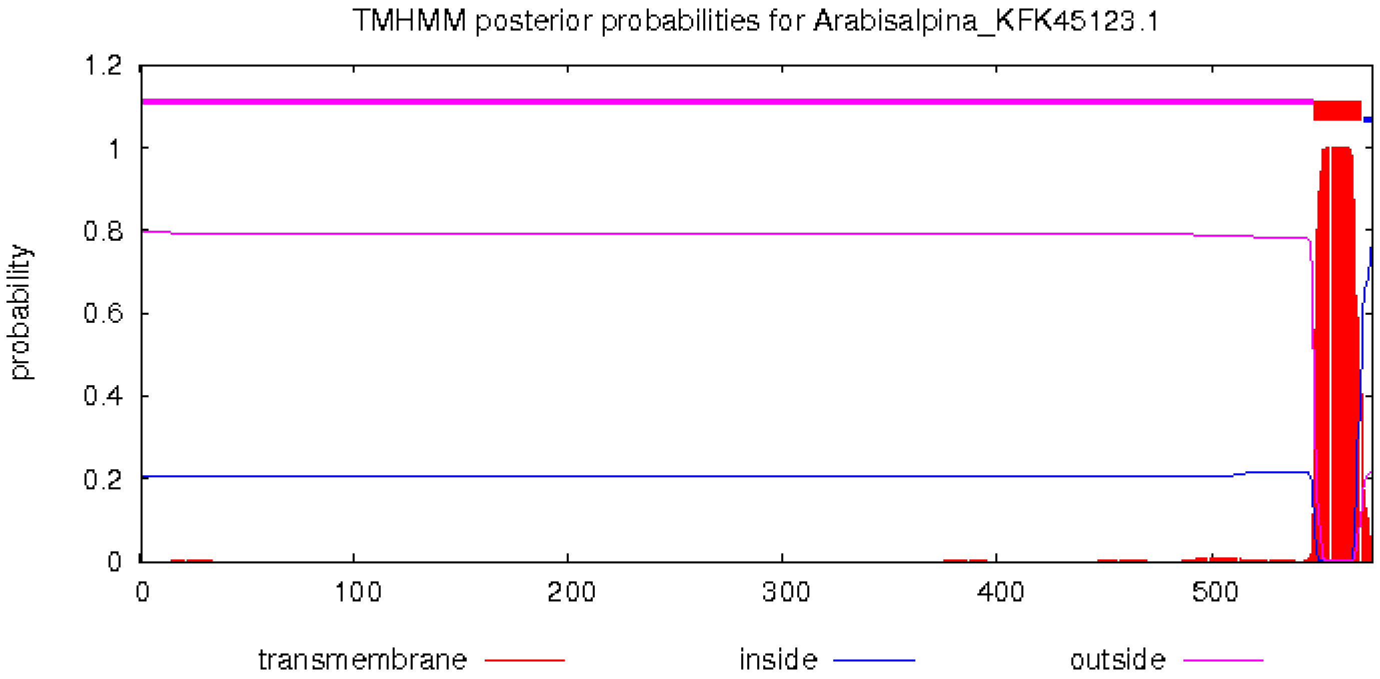

plot in postscript, script for making the plot in gnuplot, data for plot

```
# Arachisduranensis_Aradu.08GFU Length: 367
# Arachisduranensis_Aradu.08GFU Number of predicted TMHs:  0
# Arachisduranensis_Aradu.08GFU Exp number of AAs in TMHs: 0.0002
# Arachisduranensis_Aradu.08GFU Exp number, first 60 AAs:  0
# Arachisduranensis_Aradu.08GFU Total prob of N-in:        0.02786
Arachisduranensis_Aradu.08GFU   TMHMM2.0        outside      1   367
```

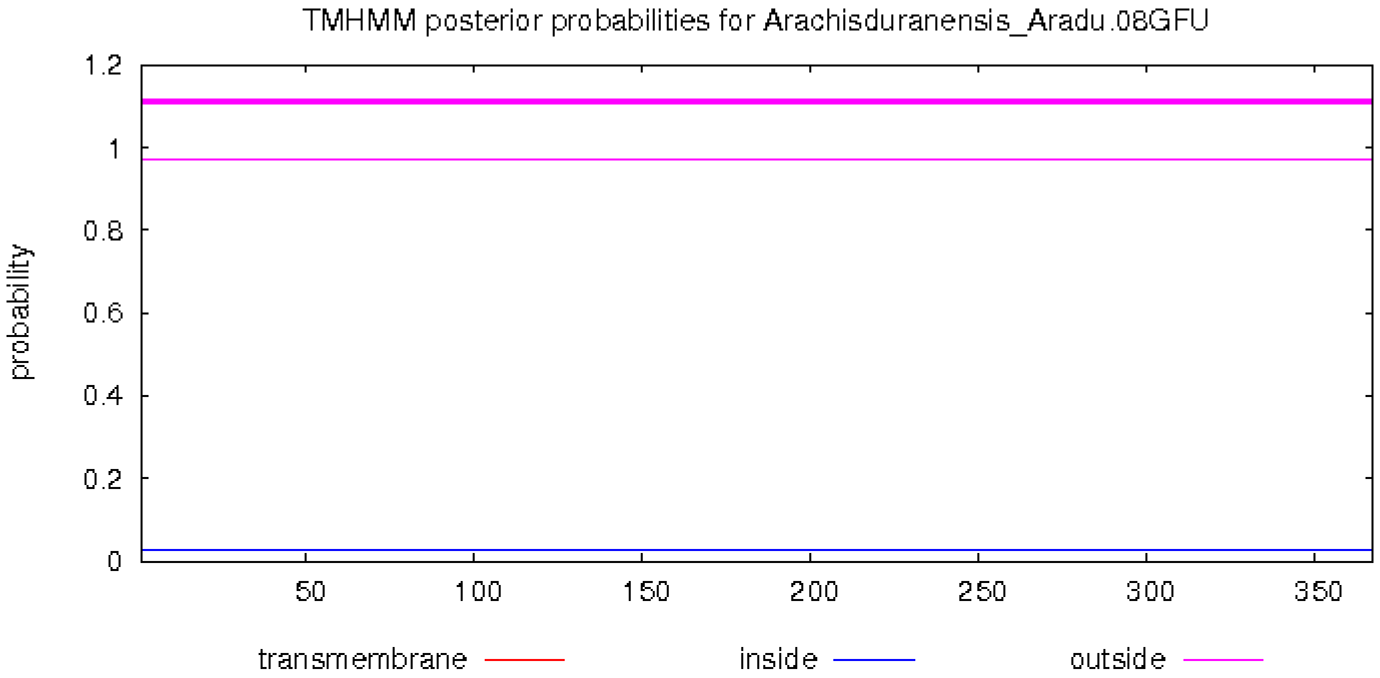

plot in postscript, script for making the plot in gnuplot, data for plot

```
# Arachisduranensis_Aradu.08TAH Length: 229
# Arachisduranensis_Aradu.08TAH Number of predicted TMHs:  0
# Arachisduranensis_Aradu.08TAH Exp number of AAs in TMHs: 8.13563
# Arachisduranensis_Aradu.08TAH Exp number, first 60 AAs:  8.13151
# Arachisduranensis_Aradu.08TAH Total prob of N-in:        0.41576
Arachisduranensis_Aradu.08TAH   TMHMM2.0        outside      1   229
```

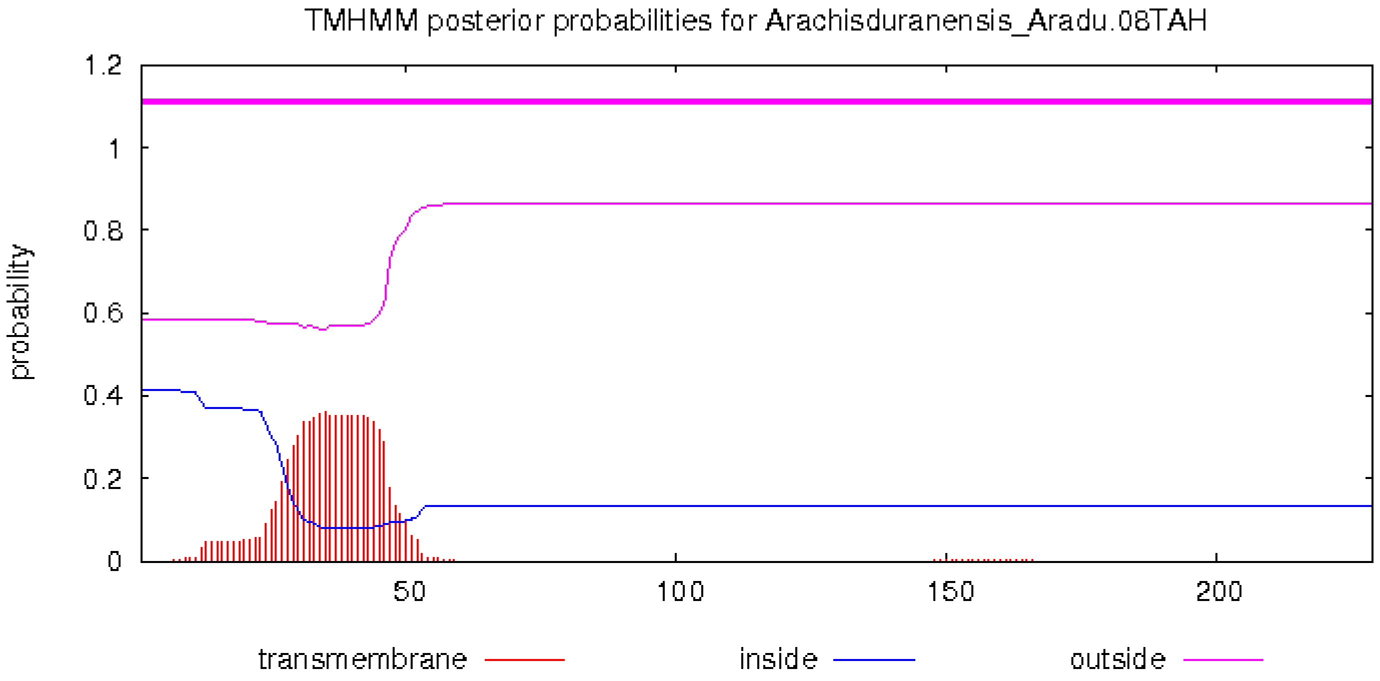

plot in postscript, script for making the plot in gnuplot, data for plot

---

```
# Arachisduranensis_Aradu.0MJ0X Length: 384
# Arachisduranensis_Aradu.0MJ0X Number of predicted TMHs:  0
# Arachisduranensis_Aradu.0MJ0X Exp number of AAs in TMHs: 0
# Arachisduranensis_Aradu.0MJ0X Exp number, first 60 AAs:  0
# Arachisduranensis_Aradu.0MJ0X Total prob of N-in:        0.00372
Arachisduranensis_Aradu.0MJ0X	TMHMM2.0	outside	     1   384
```

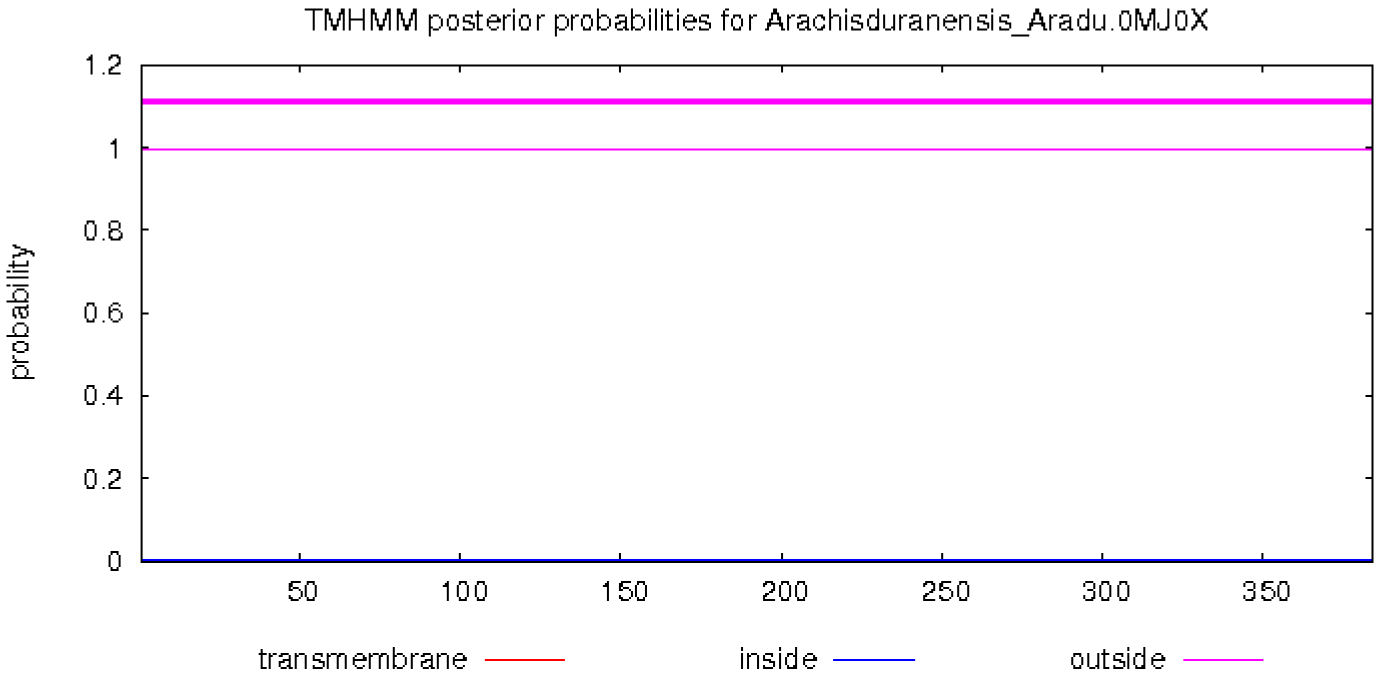

plot in postscript, script for making the plot in gnuplot, data for plot

---

```
# Arachisduranensis_Aradu.13D06 Length: 396
# Arachisduranensis_Aradu.13D06 Number of predicted TMHs:  0
# Arachisduranensis_Aradu.13D06 Exp number of AAs in TMHs: 0.00056
# Arachisduranensis_Aradu.13D06 Exp number, first 60 AAs:  0
# Arachisduranensis_Aradu.13D06 Total prob of N-in:        0.00565
Arachisduranensis_Aradu.13D06	TMHMM2.0	outside	     1   396
```

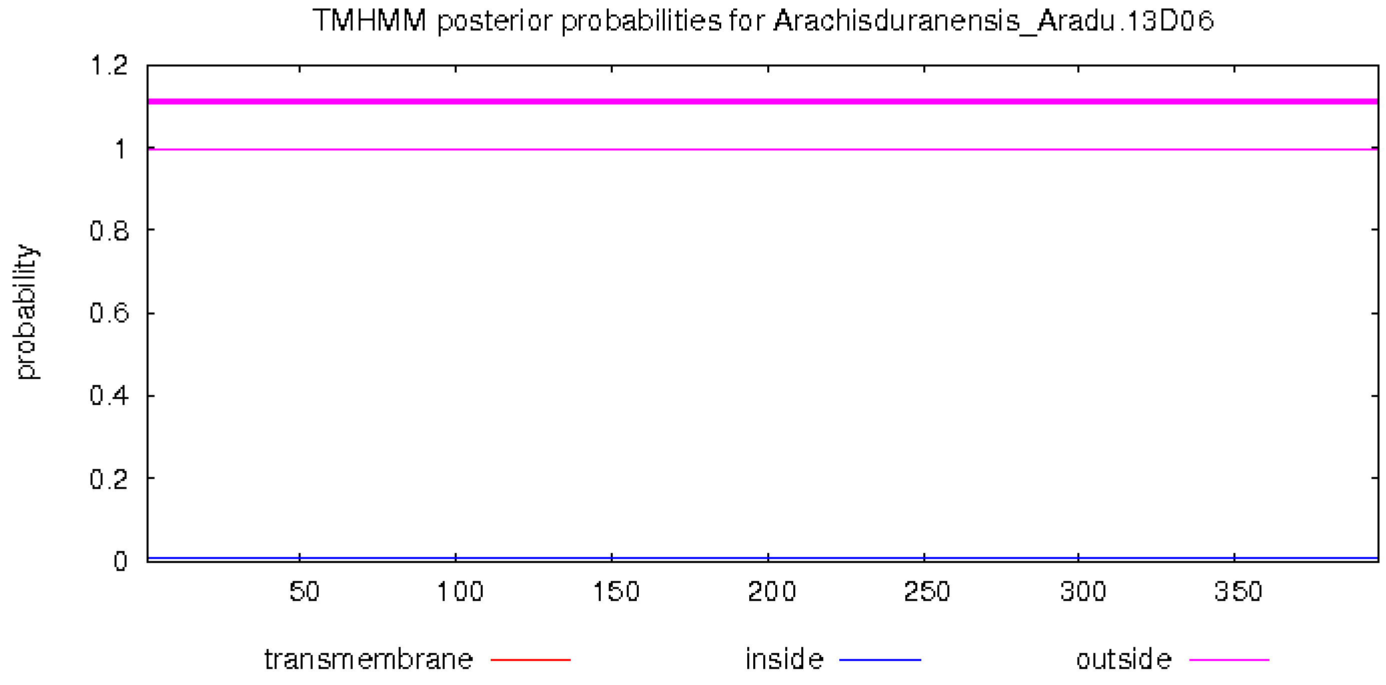

plot in postscript, script for making the plot in gnuplot, data for plot

---

```
# Arachisduranensis_Aradu.15JI0 Length: 150
# Arachisduranensis_Aradu.15JI0 Number of predicted TMHs:  0
# Arachisduranensis_Aradu.15JI0 Exp number of AAs in TMHs: 0.00107
# Arachisduranensis_Aradu.15JI0 Exp number, first 60 AAs:  0.00107
# Arachisduranensis_Aradu.15JI0 Total prob of N-in:        0.16363
Arachisduranensis_Aradu.15JI0   TMHMM2.0        outside      1   150
```

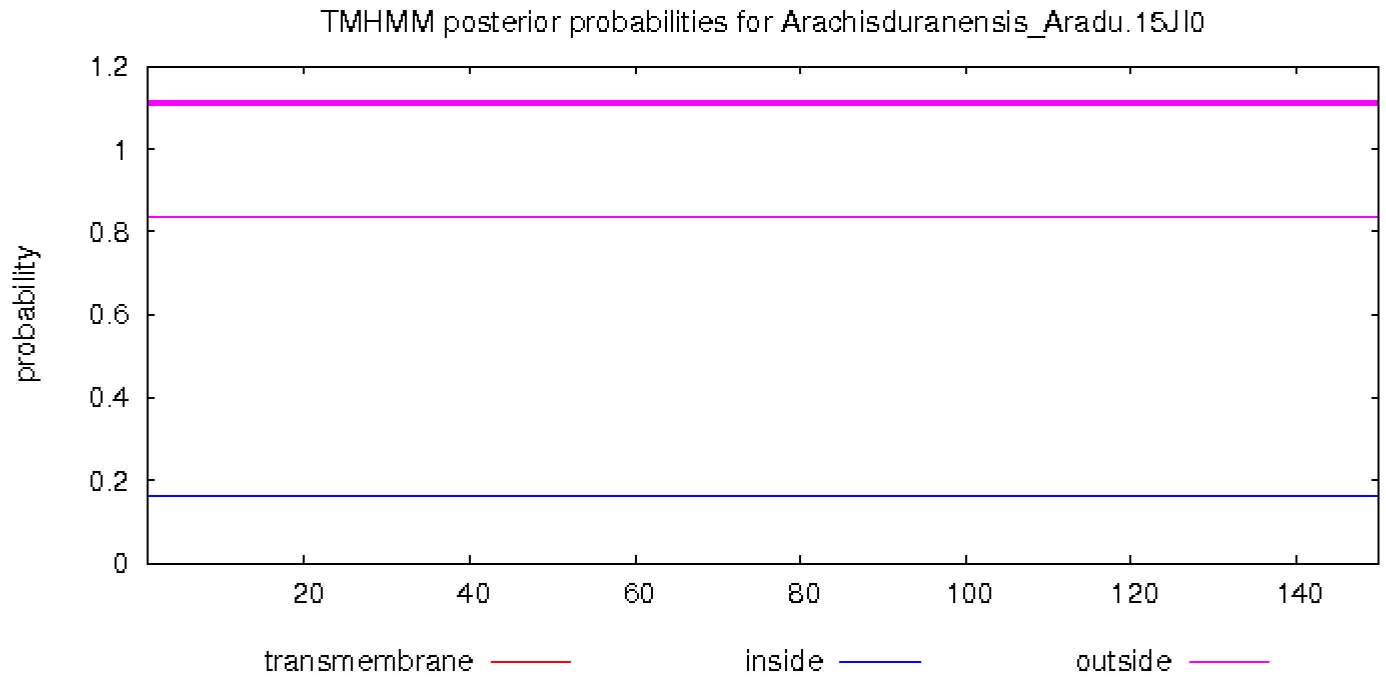

plot in postscript, script for making the plot in gnuplot, data for plot

---

```
# Arachisduranensis_Aradu.15QQT Length: 135
# Arachisduranensis_Aradu.15QQT Number of predicted TMHs:  0
# Arachisduranensis_Aradu.15QQT Exp number of AAs in TMHs: 0.00283
# Arachisduranensis_Aradu.15QQT Exp number, first 60 AAs:  0
# Arachisduranensis_Aradu.15QQT Total prob of N-in:        0.11046
Arachisduranensis_Aradu.15QQT   TMHMM2.0        outside      1   135
```

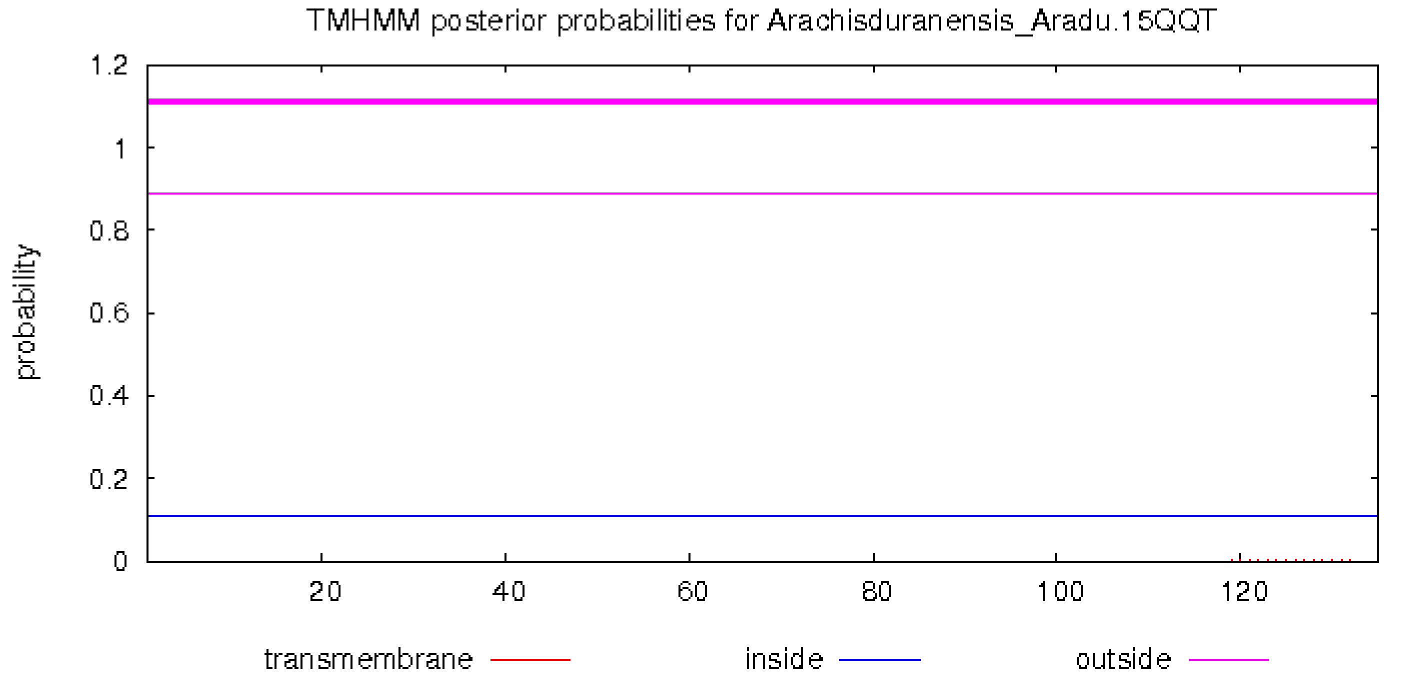

plot in postscript, script for making the plot in gnuplot, data for plot

---

```
# Arachisduranensis_Aradu.1AJ4F Length: 350
# Arachisduranensis_Aradu.1AJ4F Number of predicted TMHs:  0
# Arachisduranensis_Aradu.1AJ4F Exp number of AAs in TMHs: 0.00015
# Arachisduranensis_Aradu.1AJ4F Exp number, first 60 AAs:  0
# Arachisduranensis_Aradu.1AJ4F Total prob of N-in:        0.01024
Arachisduranensis_Aradu.1AJ4F	TMHMM2.0	outside	     1   350
```

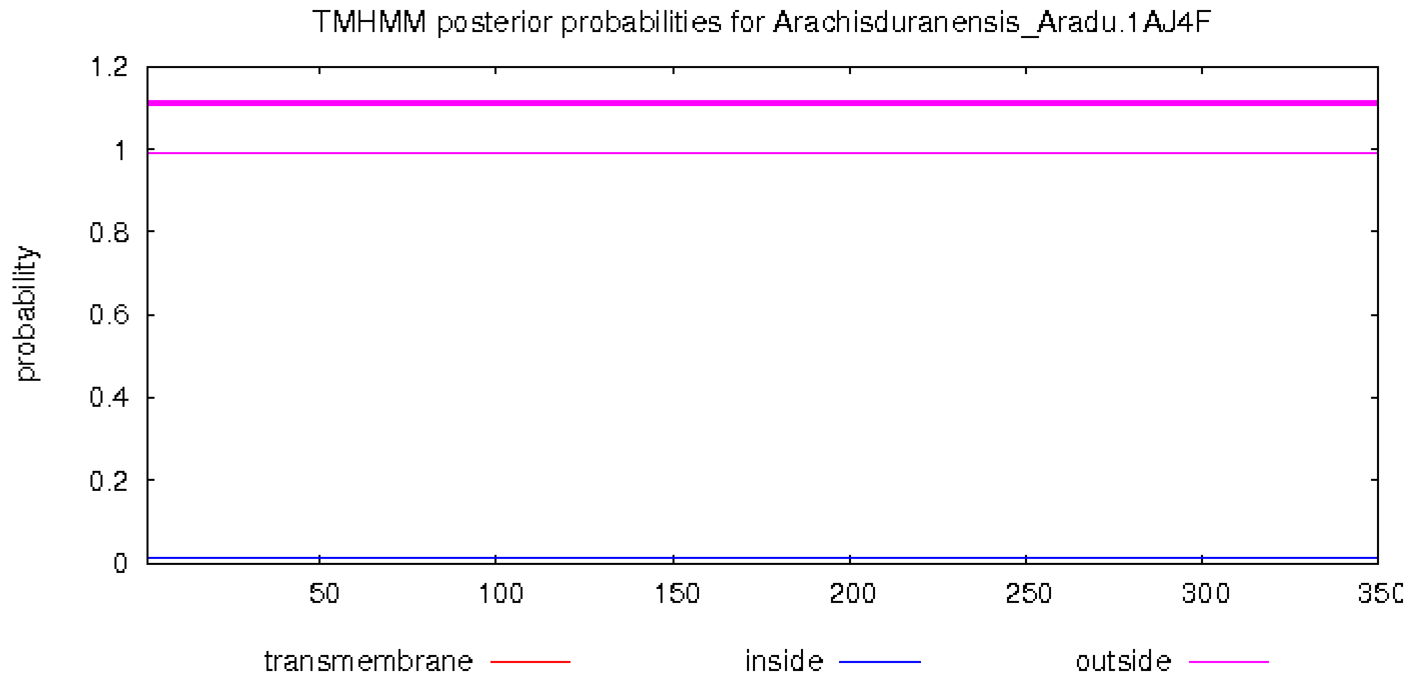

plot in postscript, script for making the plot in gnuplot, data for plot

---

```
# Arachisduranensis_Aradu.215DG Length: 322
# Arachisduranensis_Aradu.215DG Number of predicted TMHs:  0
# Arachisduranensis_Aradu.215DG Exp number of AAs in TMHs: 0.10675
# Arachisduranensis_Aradu.215DG Exp number, first 60 AAs:  0
# Arachisduranensis_Aradu.215DG Total prob of N-in:        0.09915
Arachisduranensis_Aradu.215DG	TMHMM2.0	outside	     1   322
```

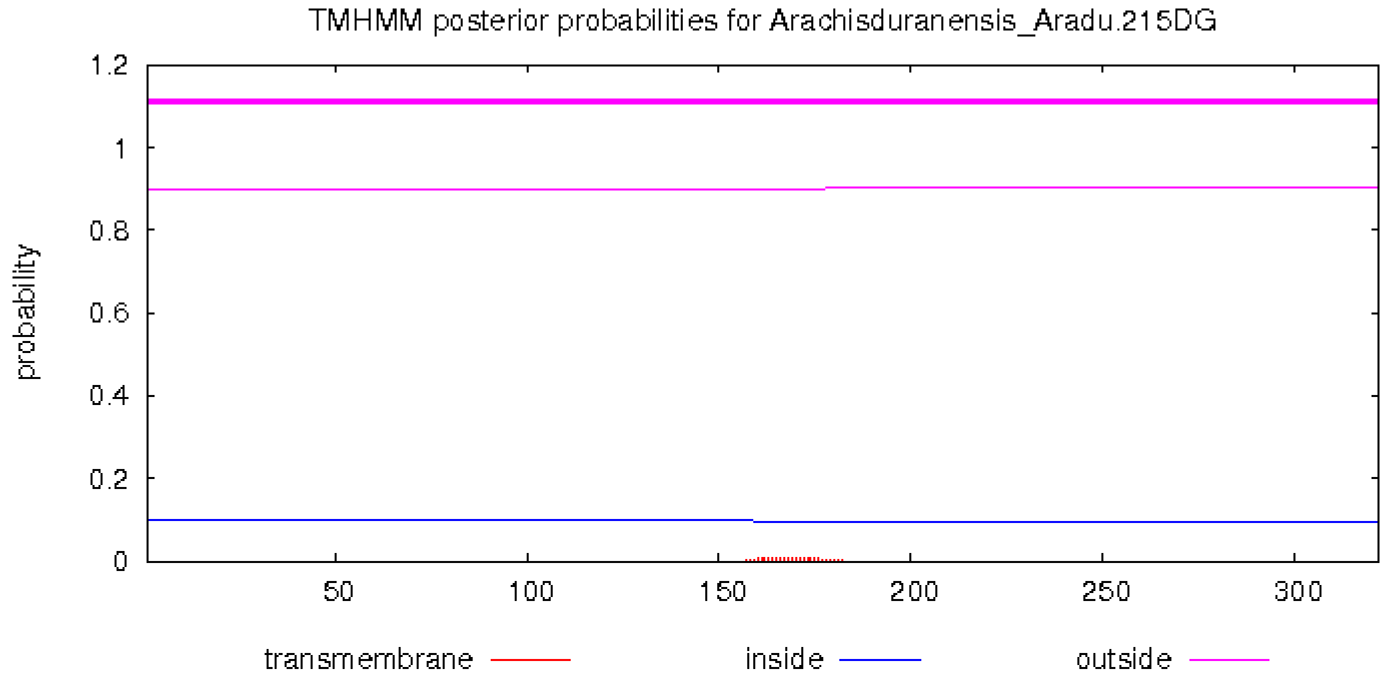

plot in postscript, script for making the plot in gnuplot, data for plot

---

```
# Arachisduranensis_Aradu.22647 Length: 274
# Arachisduranensis_Aradu.22647 Number of predicted TMHs:  0
# Arachisduranensis_Aradu.22647 Exp number of AAs in TMHs: 0.00531
# Arachisduranensis_Aradu.22647 Exp number, first 60 AAs:  0.0045
# Arachisduranensis_Aradu.22647 Total prob of N-in:        0.03674
Arachisduranensis_Aradu.22647   TMHMM2.0        outside      1   274
```

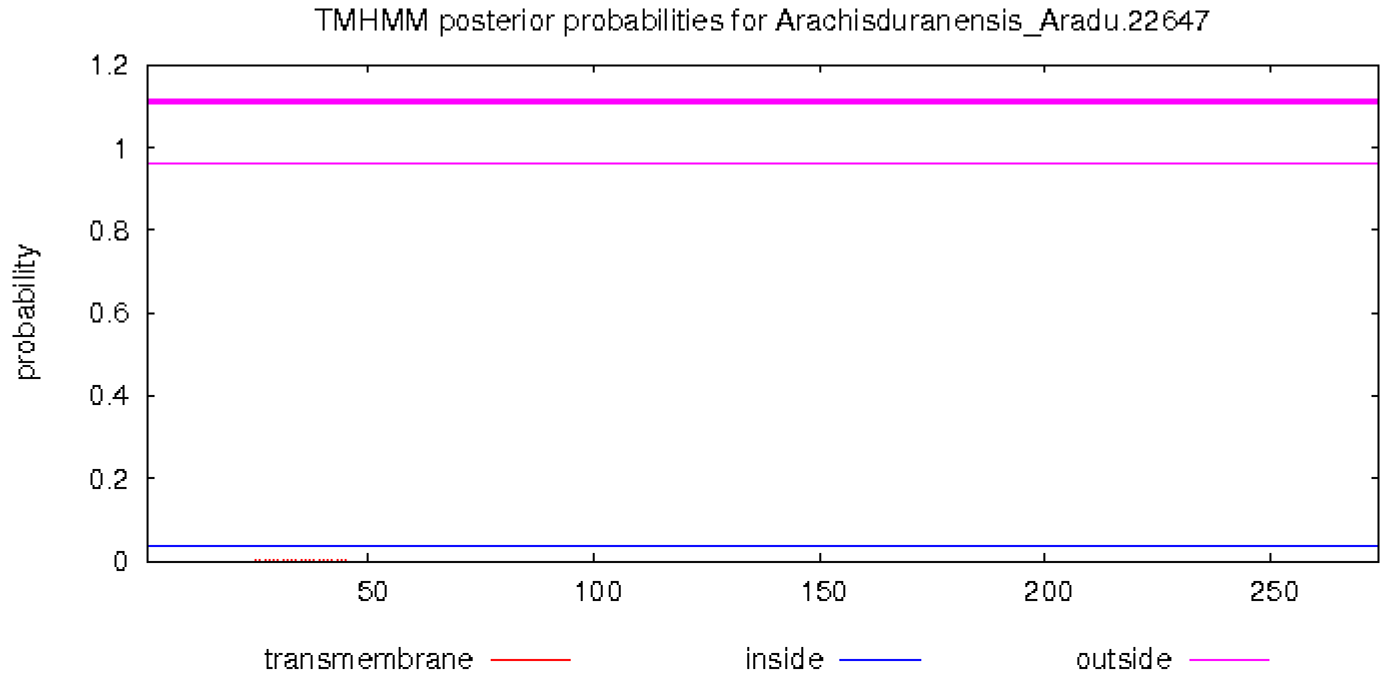

plot in postscript, script for making the plot in gnuplot, data for plot

---

```
# Arachisduranensis_Aradu.30S8W Length: 288
# Arachisduranensis_Aradu.30S8W Number of predicted TMHs:  0
# Arachisduranensis_Aradu.30S8W Exp number of AAs in TMHs: 0
# Arachisduranensis_Aradu.30S8W Exp number, first 60 AAs:  0
# Arachisduranensis_Aradu.30S8W Total prob of N-in:        0.00895
Arachisduranensis_Aradu.30S8W   TMHMM2.0        outside      1   288
```

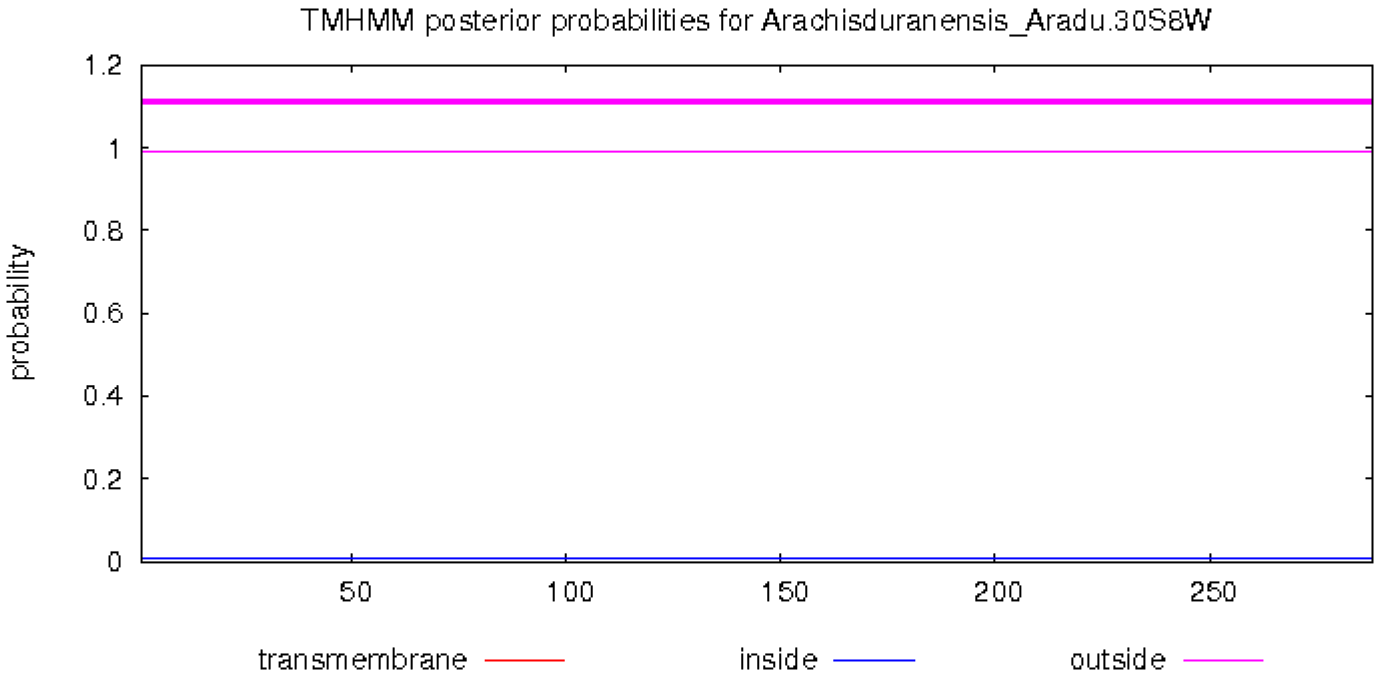

plot in postscript, script for making the plot in gnuplot, data for plot

---

```
# Arachisduranensis_Aradu.3R7A3 Length: 481
# Arachisduranensis_Aradu.3R7A3 Number of predicted TMHs:  0
# Arachisduranensis_Aradu.3R7A3 Exp number of AAs in TMHs: 6.88598
# Arachisduranensis_Aradu.3R7A3 Exp number, first 60 AAs:  6.88551
# Arachisduranensis_Aradu.3R7A3 Total prob of N-in:        0.33931
Arachisduranensis_Aradu.3R7A3   TMHMM2.0        outside      1   481
```

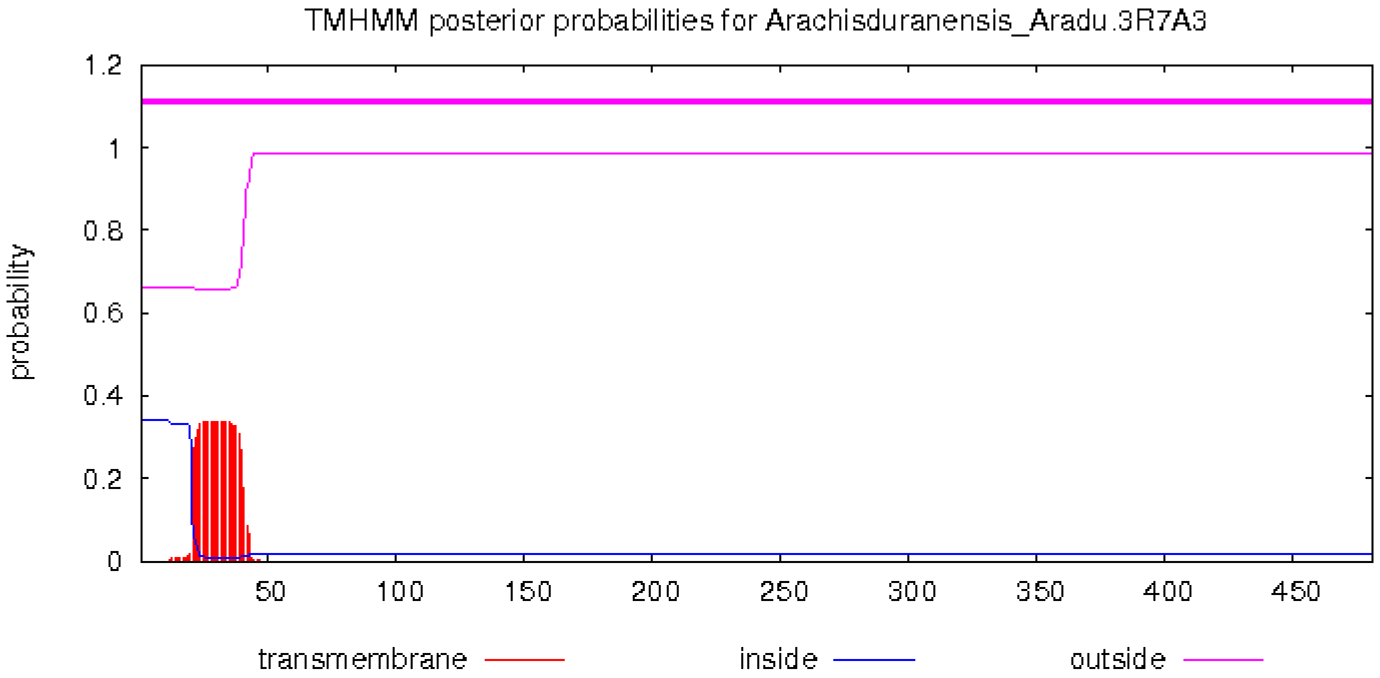

plot in postscript, script for making the plot in gnuplot, data for plot

---

```
# Arachisduranensis_Aradu.46U1T Length: 251
# Arachisduranensis_Aradu.46U1T Number of predicted TMHs:  0
# Arachisduranensis_Aradu.46U1T Exp number of AAs in TMHs: 0
# Arachisduranensis_Aradu.46U1T Exp number, first 60 AAs:  0
# Arachisduranensis_Aradu.46U1T Total prob of N-in:        0.02407
Arachisduranensis_Aradu.46U1T   TMHMM2.0        outside      1   251
```

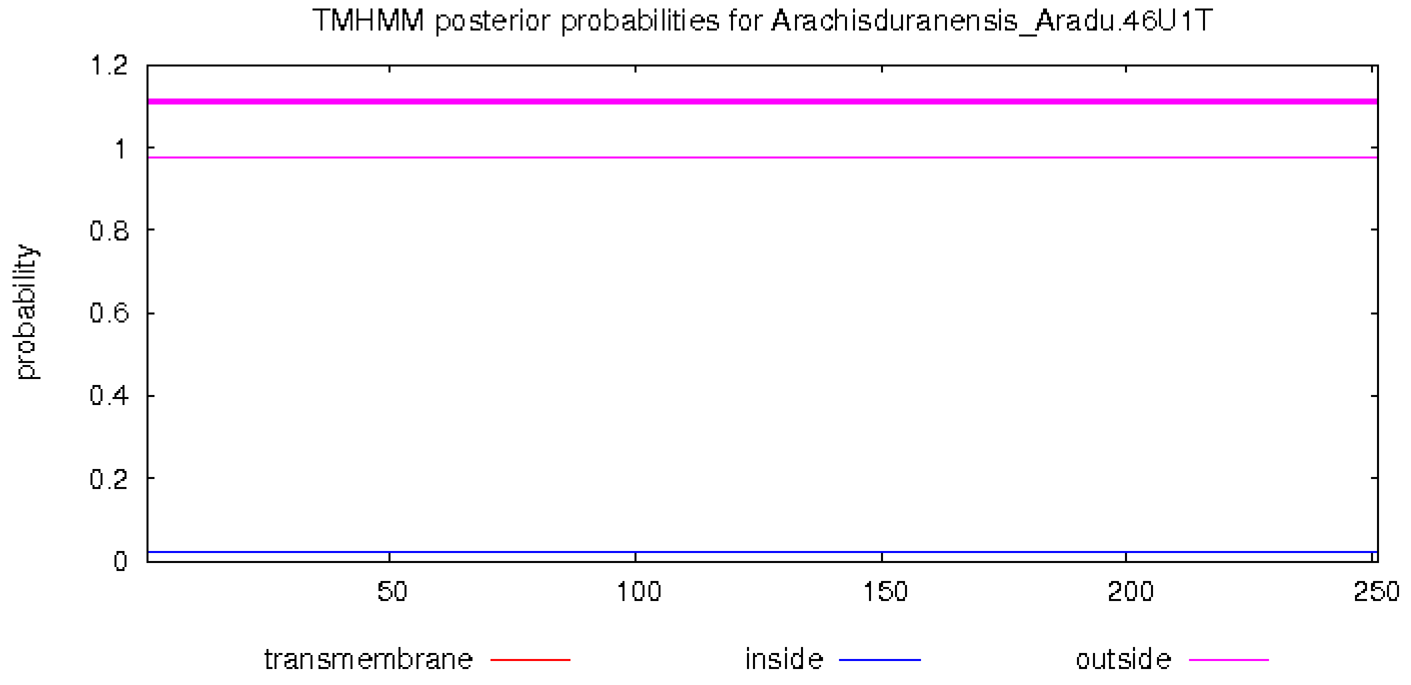

plot in postscript, script for making the plot in gnuplot, data for plot

---

```
# Arachisduranensis_Aradu.47JQU Length: 321
# Arachisduranensis_Aradu.47JQU Number of predicted TMHs:  0
# Arachisduranensis_Aradu.47JQU Exp number of AAs in TMHs: 0
# Arachisduranensis_Aradu.47JQU Exp number, first 60 AAs:  0
# Arachisduranensis_Aradu.47JQU Total prob of N-in:        0.02448
Arachisduranensis_Aradu.47JQU   TMHMM2.0        outside      1   321
```

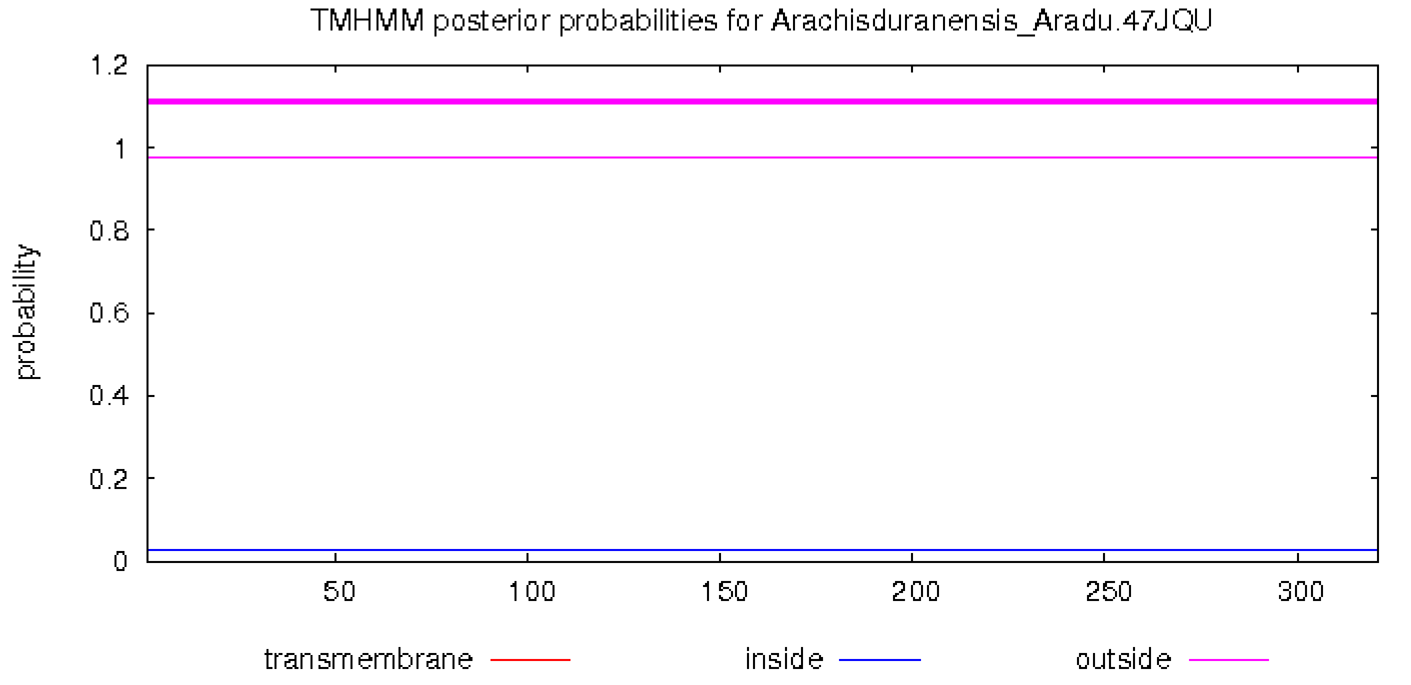

plot in postscript, script for making the plot in gnuplot, data for plot

---

```
# Arachisduranensis_Aradu.4RJ0E Length: 355
# Arachisduranensis_Aradu.4RJ0E Number of predicted TMHs:  0
# Arachisduranensis_Aradu.4RJ0E Exp number of AAs in TMHs: 0.00026
# Arachisduranensis_Aradu.4RJ0E Exp number, first 60 AAs:  0.00026
# Arachisduranensis_Aradu.4RJ0E Total prob of N-in:        0.00142
Arachisduranensis_Aradu.4RJ0E   TMHMM2.0        outside      1   355
```

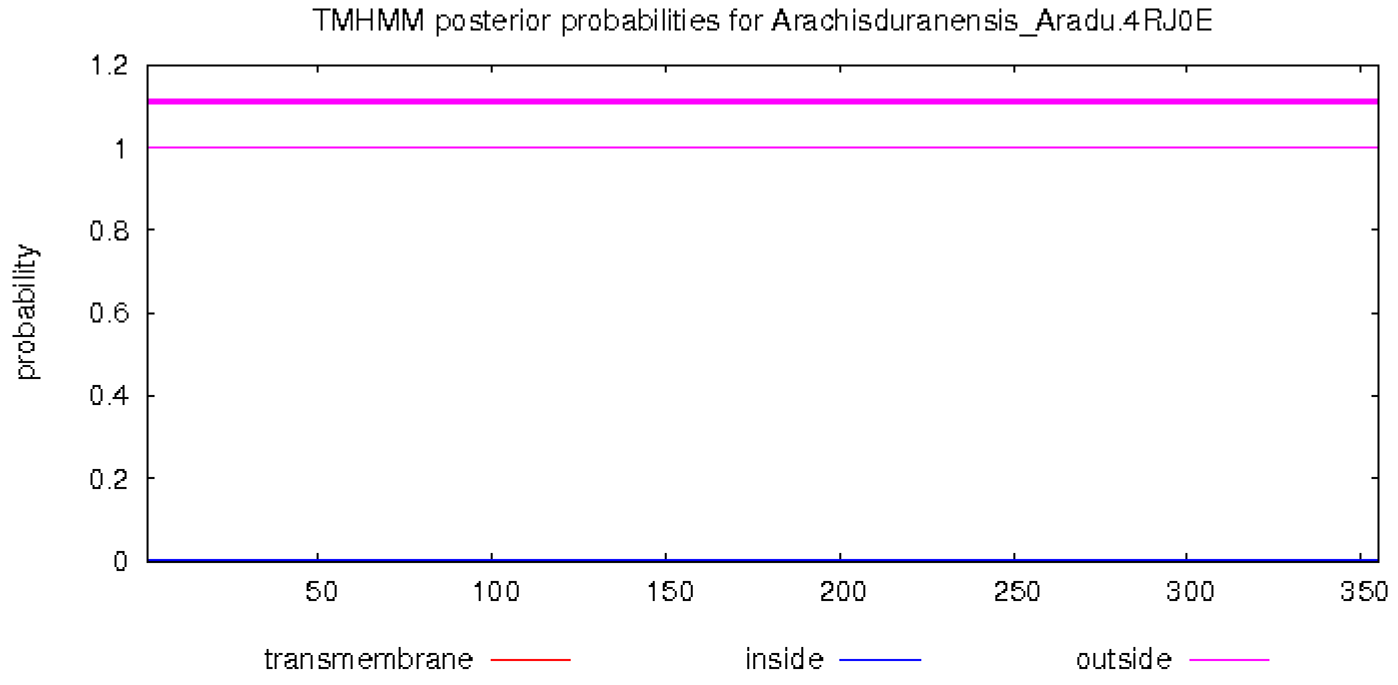

plot in postscript, script for making the plot in gnuplot, data for plot

---

```
# Arachisduranensis_Aradu.58D1A Length: 193
# Arachisduranensis_Aradu.58D1A Number of predicted TMHs:  0
# Arachisduranensis_Aradu.58D1A Exp number of AAs in TMHs: 0.00169
# Arachisduranensis_Aradu.58D1A Exp number, first 60 AAs:  0.00146
# Arachisduranensis_Aradu.58D1A Total prob of N-in:        0.28253
Arachisduranensis_Aradu.58D1A   TMHMM2.0        outside      1   193
```

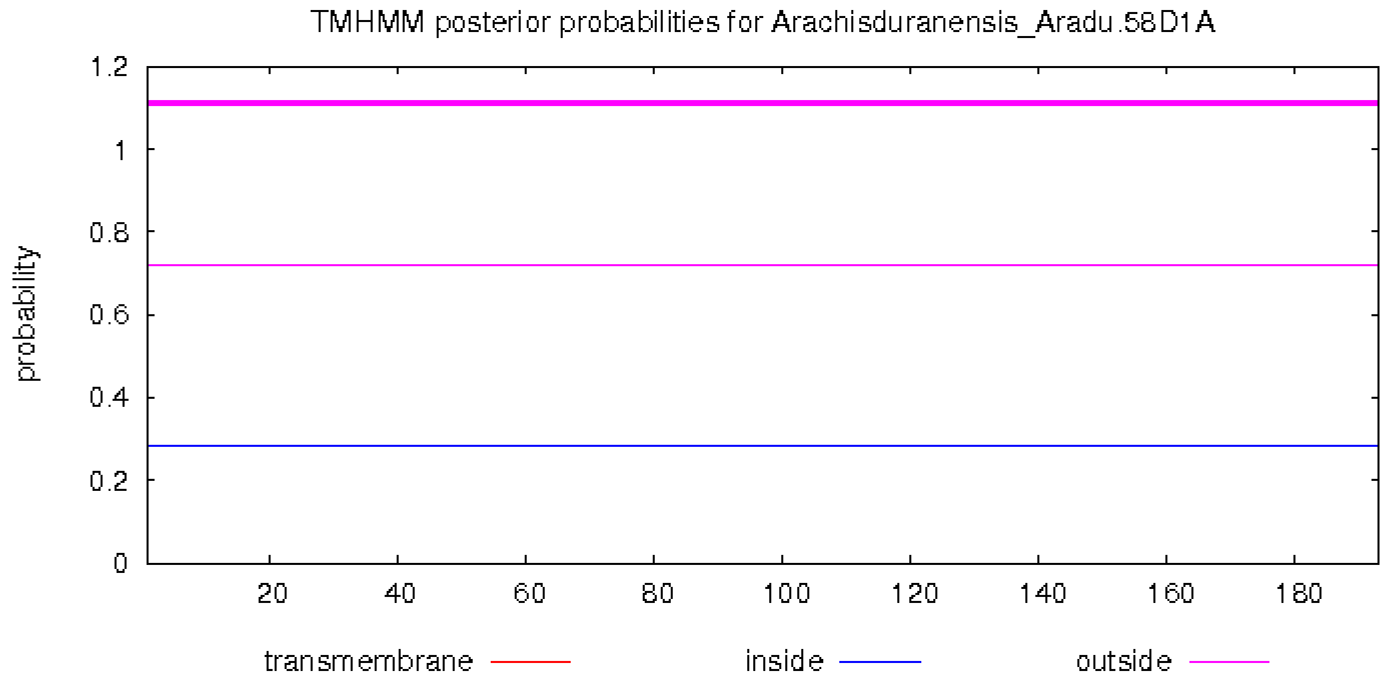

plot in postscript, script for making the plot in gnuplot, data for plot

---

```
# Arachisduranensis_Aradu.5D5JN Length: 592
# Arachisduranensis_Aradu.5D5JN Number of predicted TMHs:  1
# Arachisduranensis_Aradu.5D5JN Exp number of AAs in TMHs: 22.56383
# Arachisduranensis_Aradu.5D5JN Exp number, first 60 AAs:  0.01596
# Arachisduranensis_Aradu.5D5JN Total prob of N-in:        0.03107
Arachisduranensis_Aradu.5D5JN   TMHMM2.0        outside      1   568
```

```
Arachisduranensis_Aradu.5D5JN   TMHMM2.0        TMhelix    569   591
Arachisduranensis_Aradu.5D5JN   TMHMM2.0        inside     592   592
```

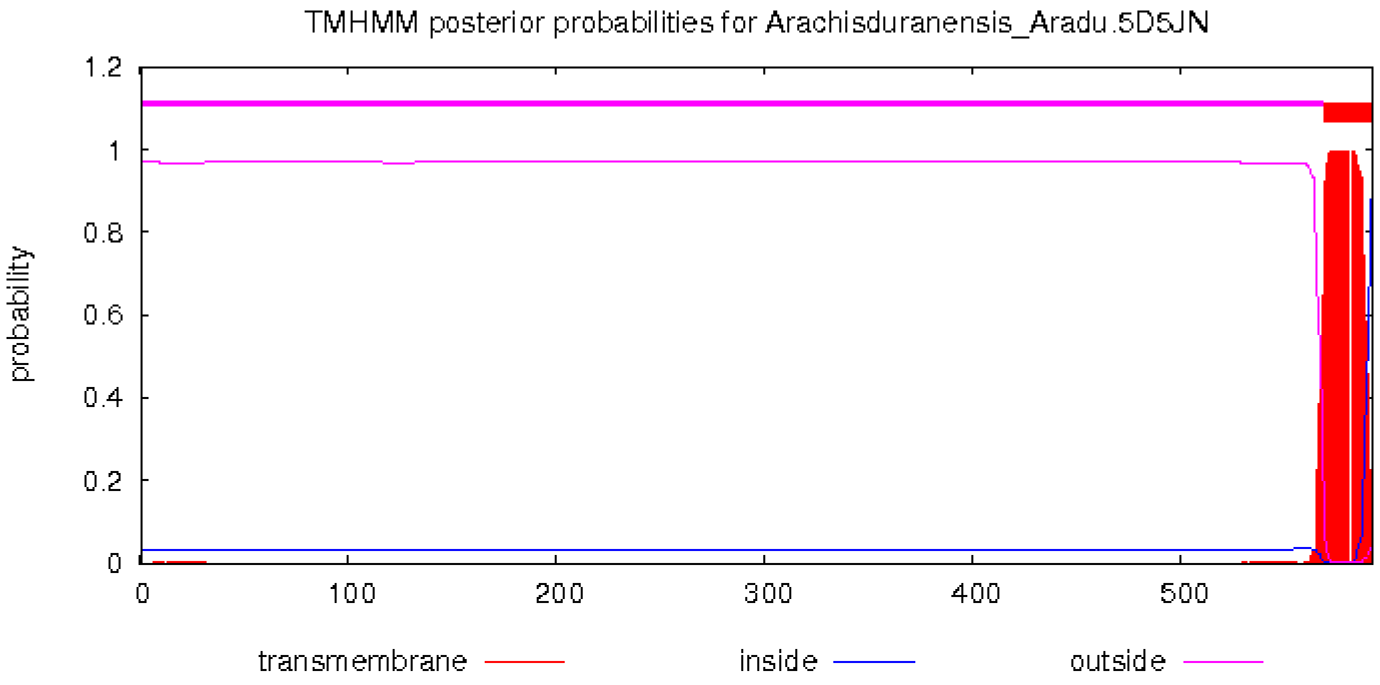

plot in postscript, script for making the plot in gnuplot, data for plot

---

```
# Arachisduranensis_Aradu.60U13 Length: 374
# Arachisduranensis_Aradu.60U13 Number of predicted TMHs:  0
# Arachisduranensis_Aradu.60U13 Exp number of AAs in TMHs: 0.07383
# Arachisduranensis_Aradu.60U13 Exp number, first 60 AAs:  0
# Arachisduranensis_Aradu.60U13 Total prob of N-in:        0.04442
Arachisduranensis_Aradu.60U13   TMHMM2.0        outside      1   374
```

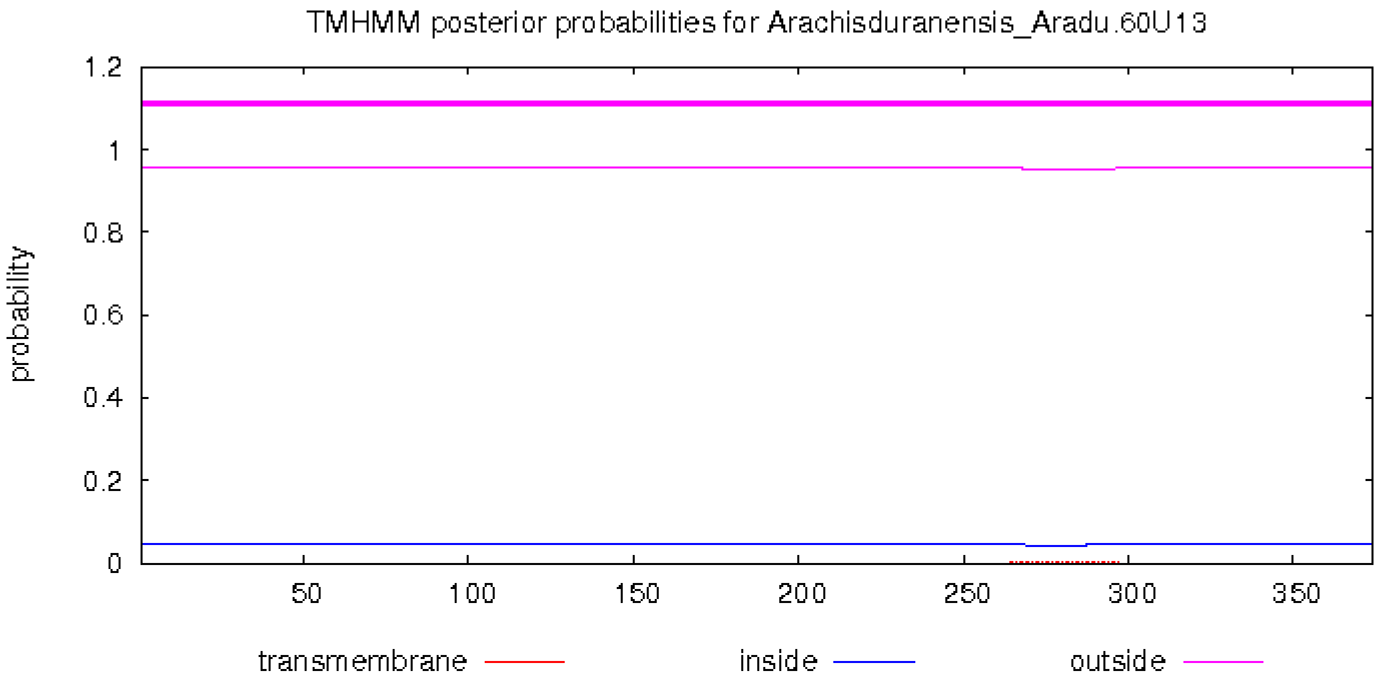

plot in postscript, script for making the plot in gnuplot, data for plot

---

```
# Arachisduranensis_Aradu.66XRP Length: 318
# Arachisduranensis_Aradu.66XRP Number of predicted TMHs:  0
# Arachisduranensis_Aradu.66XRP Exp number of AAs in TMHs: 0.00016
# Arachisduranensis_Aradu.66XRP Exp number, first 60 AAs:  0.00015
```

```
# Arachisduranensis_Aradu.66XRP Total prob of N-in:        0.03216
Arachisduranensis_Aradu.66XRP   TMHMM2.0        outside      1   318
```

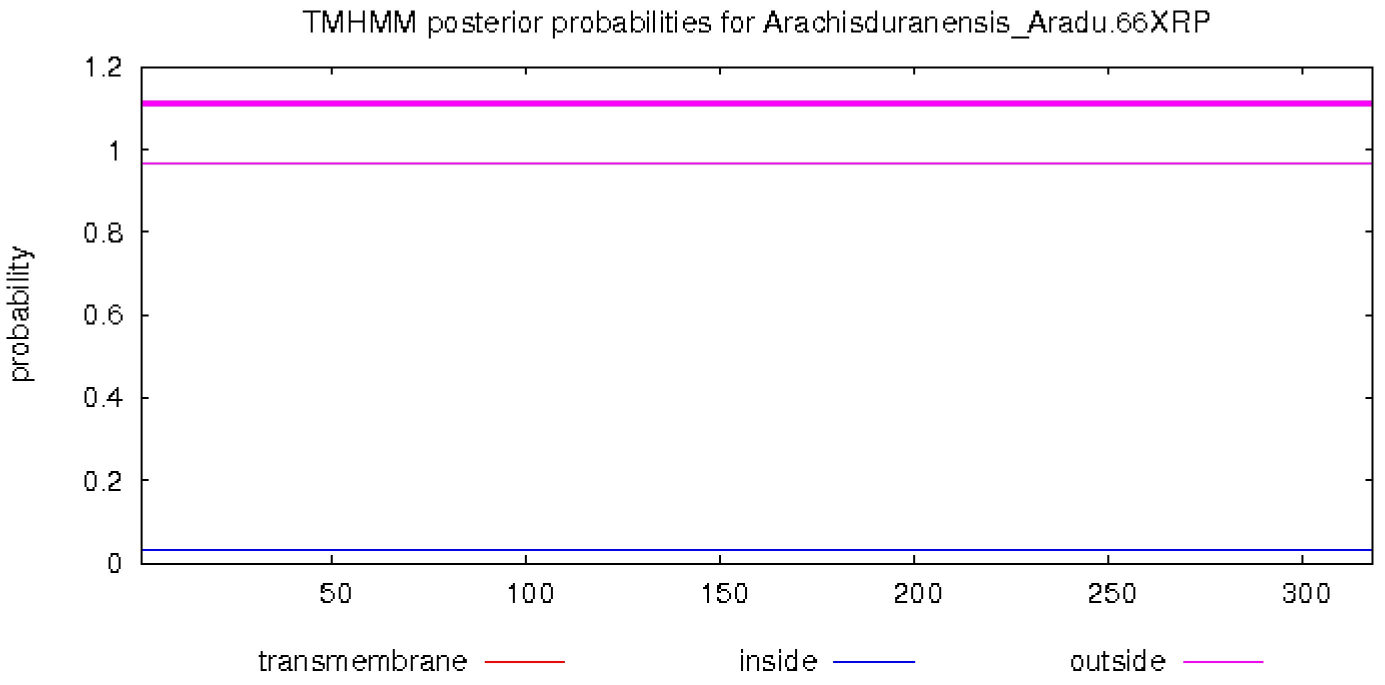

plot in postscript, script for making the plot in gnuplot, data for plot

---

```
# Arachisduranensis_Aradu.6H4PP Length: 230
# Arachisduranensis_Aradu.6H4PP Number of predicted TMHs:  0
# Arachisduranensis_Aradu.6H4PP Exp number of AAs in TMHs: 0.0863
# Arachisduranensis_Aradu.6H4PP Exp number, first 60 AAs:  0.08583
# Arachisduranensis_Aradu.6H4PP Total prob of N-in:        0.05783
Arachisduranensis_Aradu.6H4PP   TMHMM2.0        outside      1   230
```

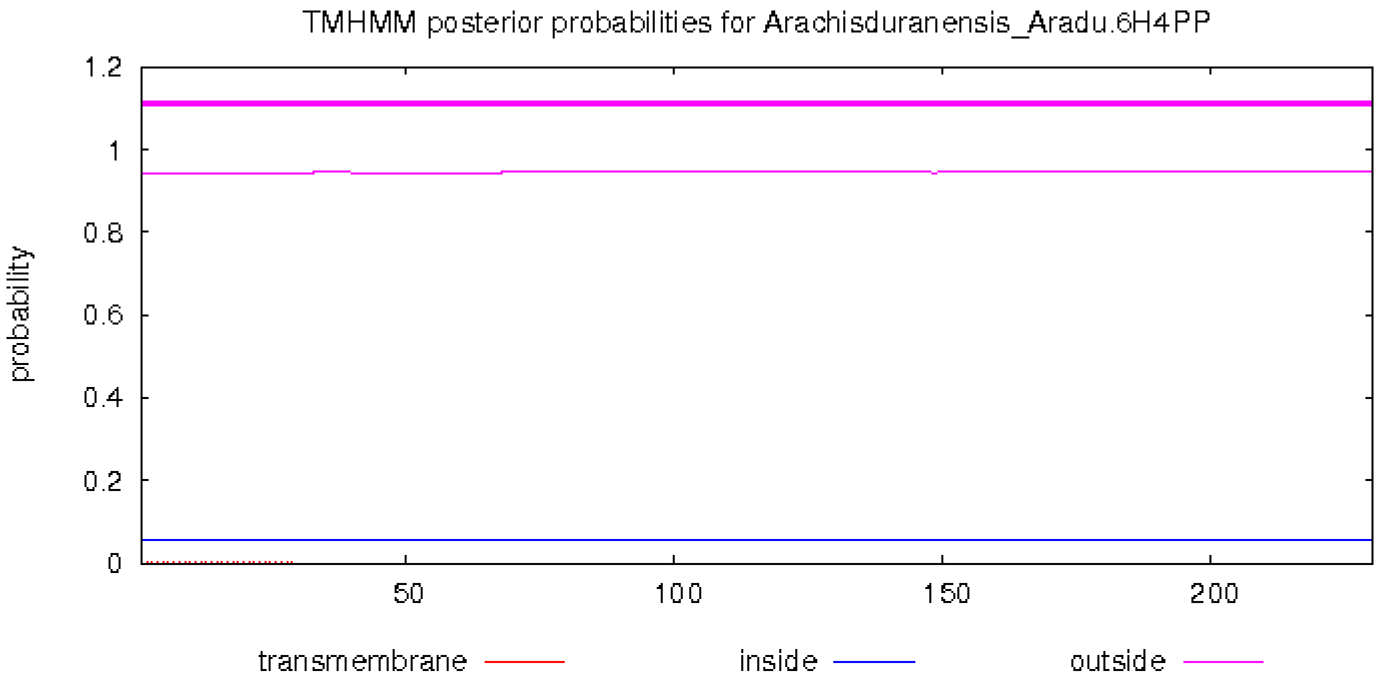

plot in postscript, script for making the plot in gnuplot, data for plot

---

```
# Arachisduranensis_Aradu.79PL2 Length: 211
# Arachisduranensis_Aradu.79PL2 Number of predicted TMHs:  0
# Arachisduranensis_Aradu.79PL2 Exp number of AAs in TMHs: 0.00224
# Arachisduranensis_Aradu.79PL2 Exp number, first 60 AAs:  0.00224
```

```
# Arachisduranensis_Aradu.79PL2 Total prob of N-in:        0.15080
Arachisduranensis_Aradu.79PL2   TMHMM2.0        outside      1   211
```

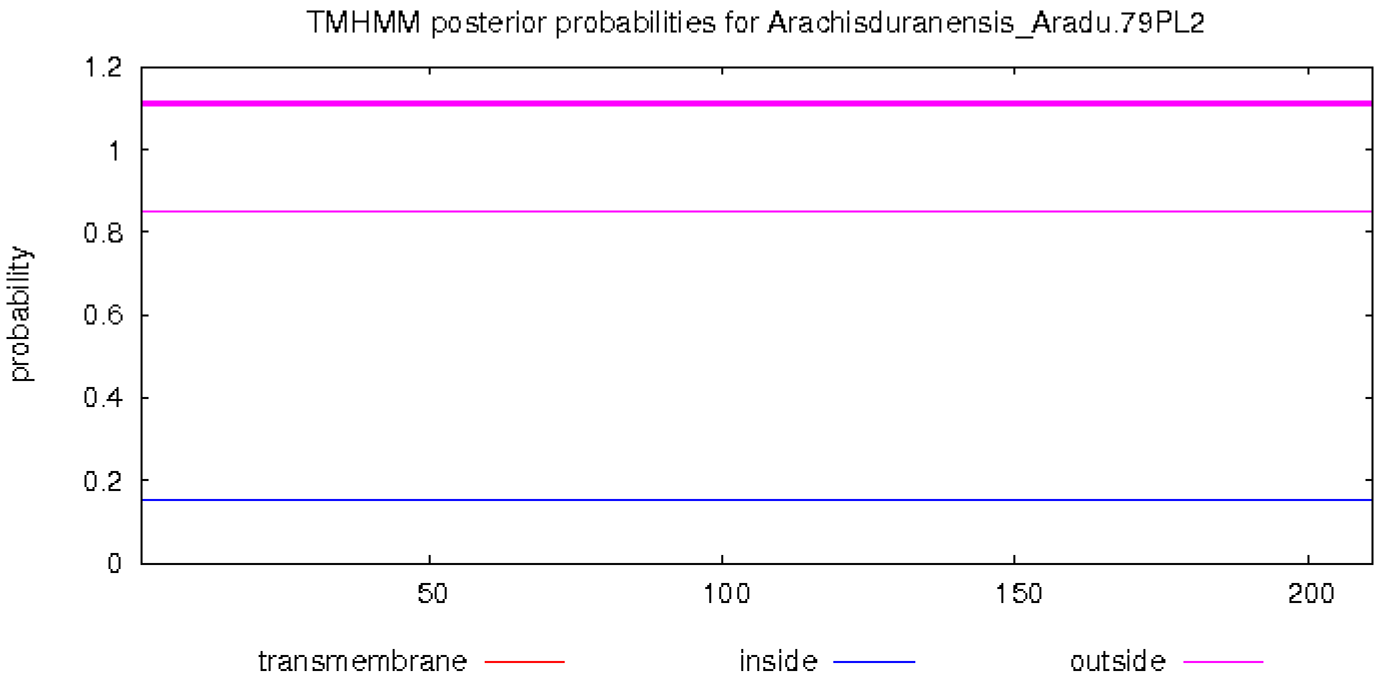

plot in postscript, script for making the plot in gnuplot, data for plot

---

```
# Arachisduranensis_Aradu.7NI41 Length: 286
# Arachisduranensis_Aradu.7NI41 Number of predicted TMHs:  0
# Arachisduranensis_Aradu.7NI41 Exp number of AAs in TMHs: 0.00021
# Arachisduranensis_Aradu.7NI41 Exp number, first 60 AAs:  0
# Arachisduranensis_Aradu.7NI41 Total prob of N-in:        0.05800
Arachisduranensis_Aradu.7NI41   TMHMM2.0        outside      1   286
```

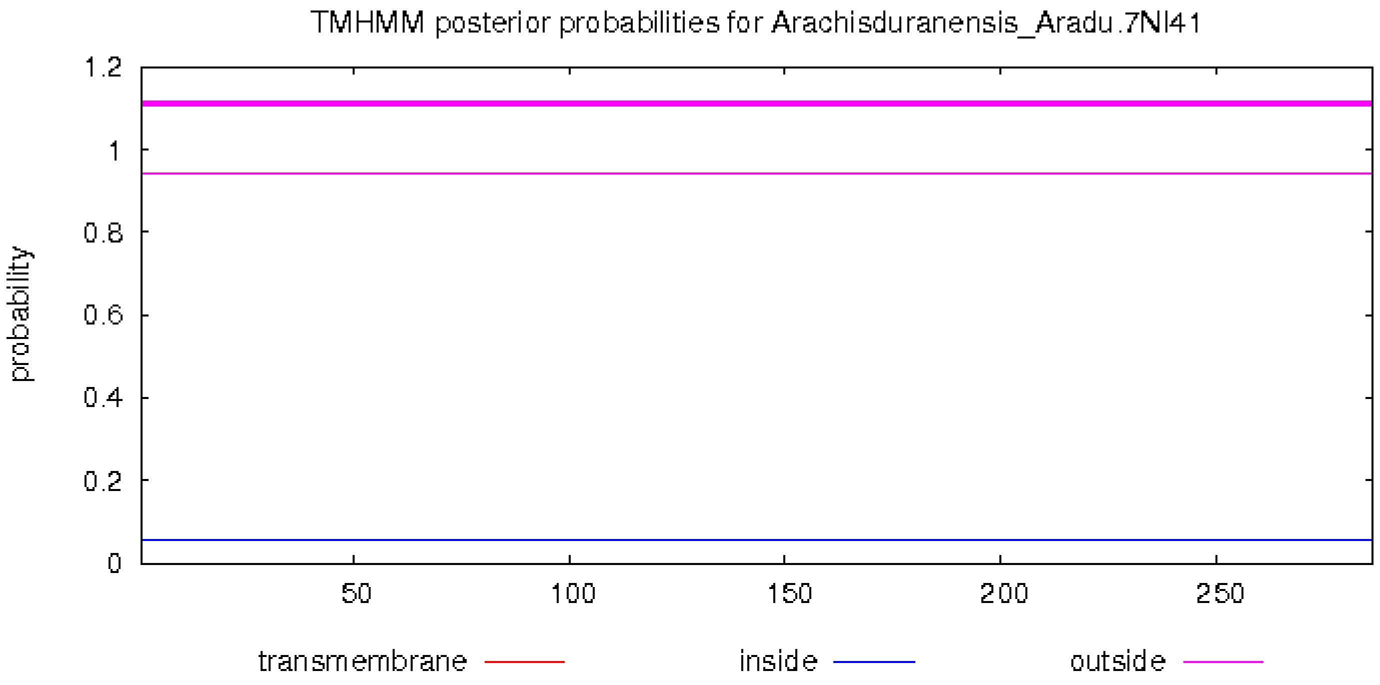

plot in postscript, script for making the plot in gnuplot, data for plot

---

```
# Arachisduranensis_Aradu.7X5EV Length: 328
# Arachisduranensis_Aradu.7X5EV Number of predicted TMHs:  0
# Arachisduranensis_Aradu.7X5EV Exp number of AAs in TMHs: 0.00058
# Arachisduranensis_Aradu.7X5EV Exp number, first 60 AAs:  0.00058
```

```
# Arachisduranensis_Aradu.7X5EV Total prob of N-in:        0.01760
Arachisduranensis_Aradu.7X5EV   TMHMM2.0        outside      1   328
```

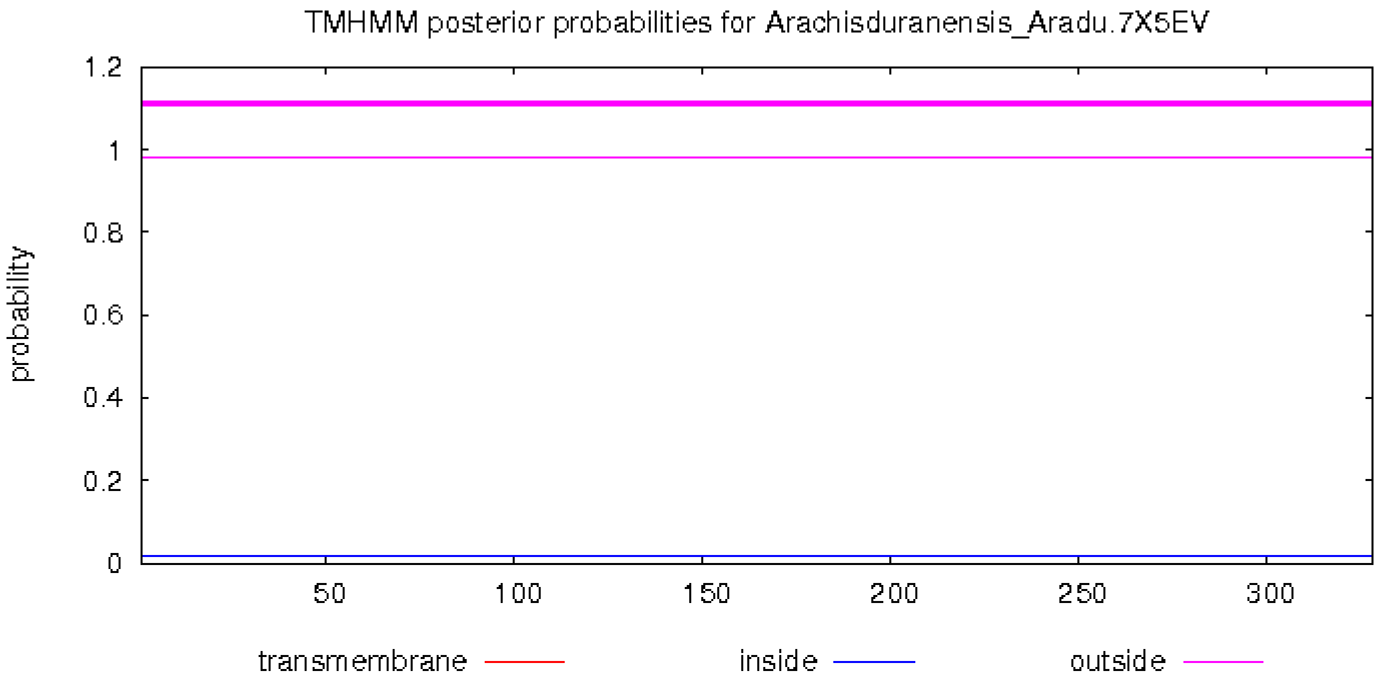

plot in postscript, script for making the plot in gnuplot, data for plot

---

```
# Arachisduranensis_Aradu.895HT Length: 61
# Arachisduranensis_Aradu.895HT Number of predicted TMHs:  0
# Arachisduranensis_Aradu.895HT Exp number of AAs in TMHs: 0.00019
# Arachisduranensis_Aradu.895HT Exp number, first 60 AAs:  0.00019
# Arachisduranensis_Aradu.895HT Total prob of N-in:        0.13435
Arachisduranensis_Aradu.895HT   TMHMM2.0        outside      1    61
```

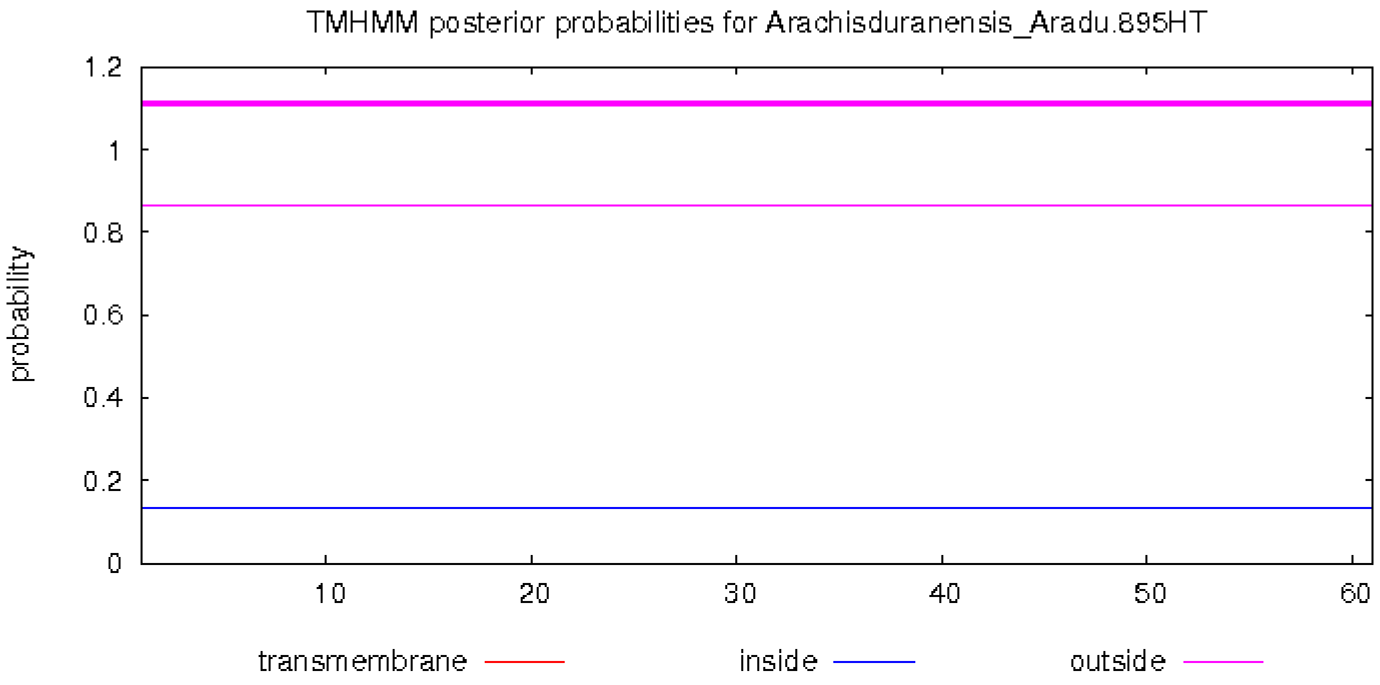

plot in postscript, script for making the plot in gnuplot, data for plot

---

```
# Arachisduranensis_Aradu.8Q7DY Length: 313
# Arachisduranensis_Aradu.8Q7DY Number of predicted TMHs:  0
# Arachisduranensis_Aradu.8Q7DY Exp number of AAs in TMHs: 0.00019
# Arachisduranensis_Aradu.8Q7DY Exp number, first 60 AAs:  0
```

```
# Arachisduranensis_Aradu.8Q7DY Total prob of N-in:        0.00712
Arachisduranensis_Aradu.8Q7DY   TMHMM2.0        outside      1   313
```

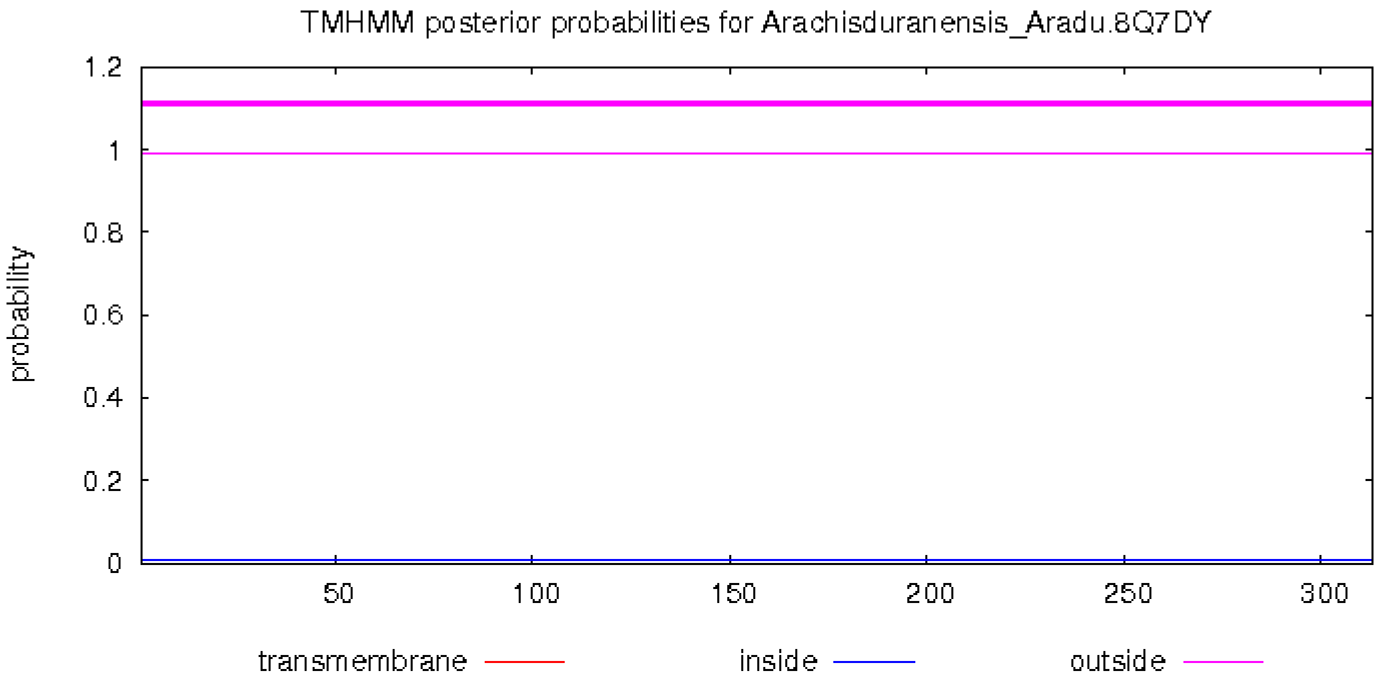

plot in postscript, script for making the plot in gnuplot, data for plot

---

```
# Arachisduranensis_Aradu.9FF24 Length: 583
# Arachisduranensis_Aradu.9FF24 Number of predicted TMHs:  2
# Arachisduranensis_Aradu.9FF24 Exp number of AAs in TMHs: 39.30286
# Arachisduranensis_Aradu.9FF24 Exp number, first 60 AAs:  0
# Arachisduranensis_Aradu.9FF24 Total prob of N-in:        0.00357
Arachisduranensis_Aradu.9FF24   TMHMM2.0        outside      1   530
Arachisduranensis_Aradu.9FF24   TMHMM2.0        TMhelix    531   553
Arachisduranensis_Aradu.9FF24   TMHMM2.0        inside     554   557
Arachisduranensis_Aradu.9FF24   TMHMM2.0        TMhelix    558   580
Arachisduranensis_Aradu.9FF24   TMHMM2.0        outside    581   583
```

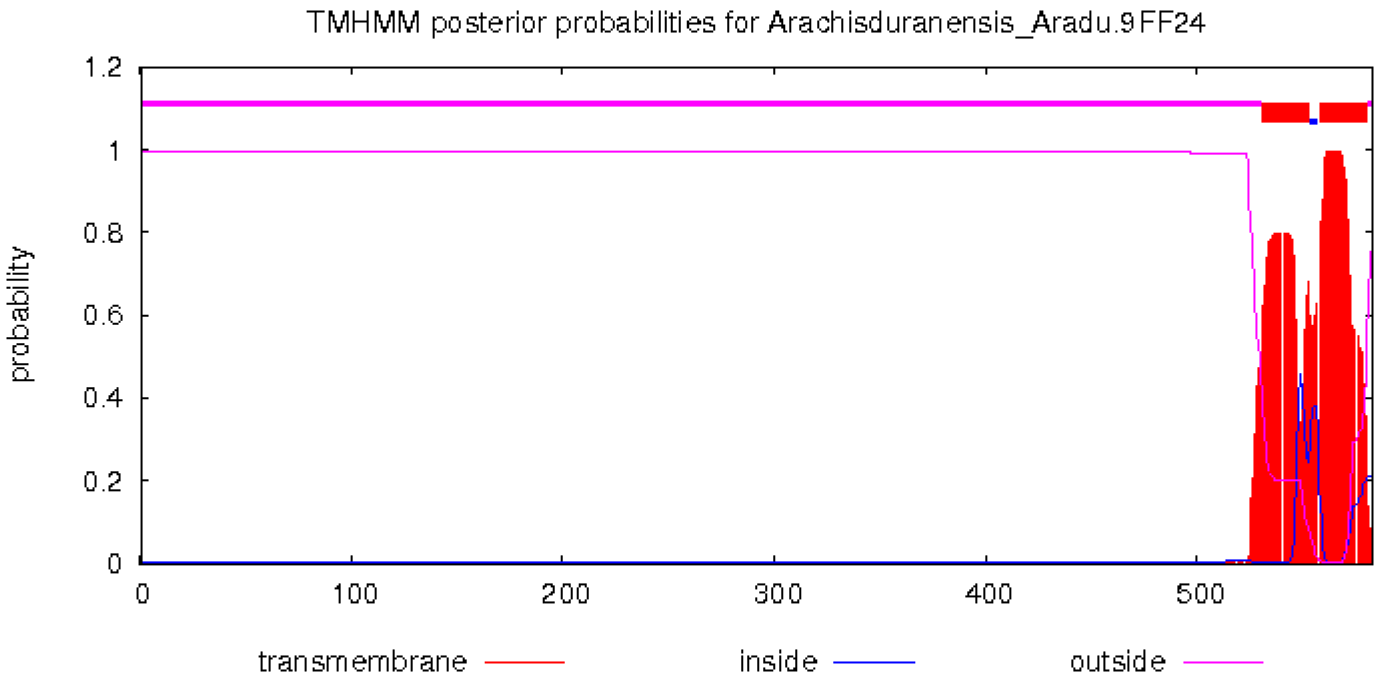

plot in postscript, script for making the plot in gnuplot, data for plot

---

```
# Arachisduranensis_Aradu.9T4H8 Length: 228
# Arachisduranensis_Aradu.9T4H8 Number of predicted TMHs:  0
# Arachisduranensis_Aradu.9T4H8 Exp number of AAs in TMHs: 0.47776
# Arachisduranensis_Aradu.9T4H8 Exp number, first 60 AAs:  0.01589
# Arachisduranensis_Aradu.9T4H8 Total prob of N-in:        0.04383
Arachisduranensis_Aradu.9T4H8   TMHMM2.0        outside      1   228
```

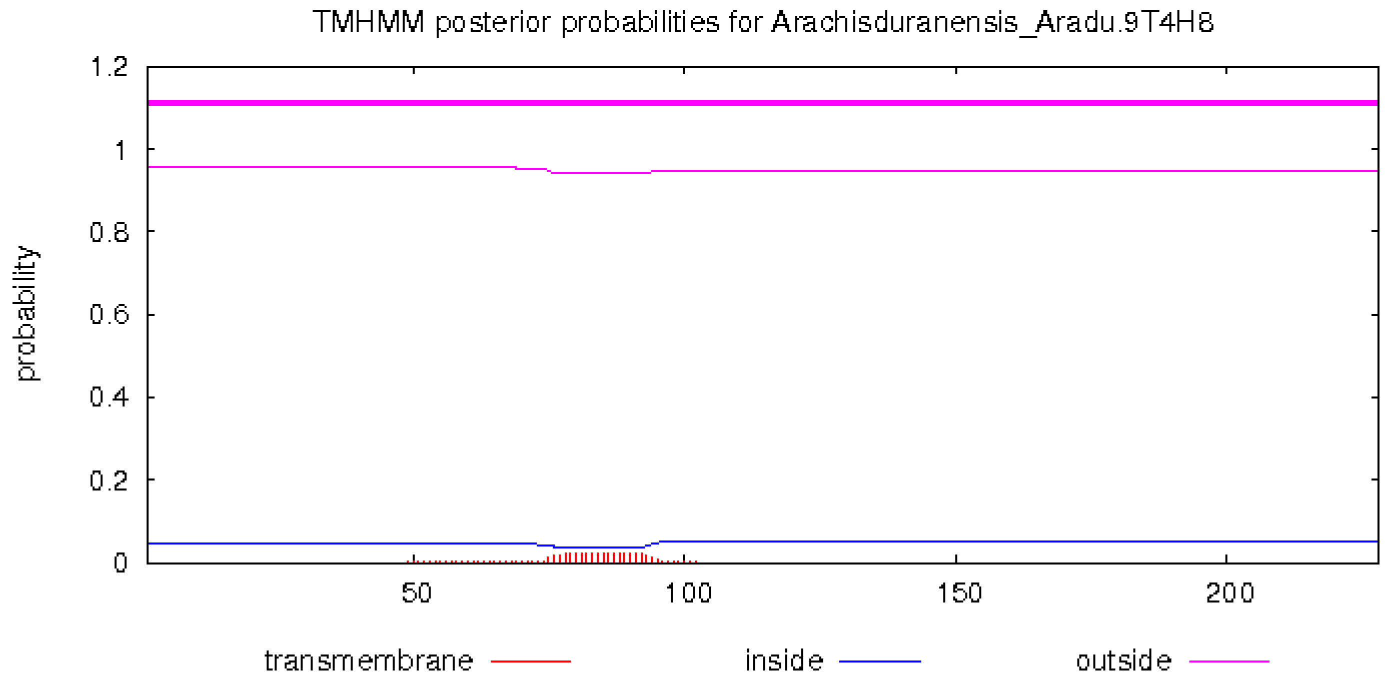

plot in postscript, script for making the plot in gnuplot, data for plot

---

```
# Arachisduranensis_Aradu.9Y6NH Length: 432
# Arachisduranensis_Aradu.9Y6NH Number of predicted TMHs:  0
# Arachisduranensis_Aradu.9Y6NH Exp number of AAs in TMHs: 0.00382
# Arachisduranensis_Aradu.9Y6NH Exp number, first 60 AAs:  0
# Arachisduranensis_Aradu.9Y6NH Total prob of N-in:        0.00256
Arachisduranensis_Aradu.9Y6NH   TMHMM2.0        outside      1   432
```

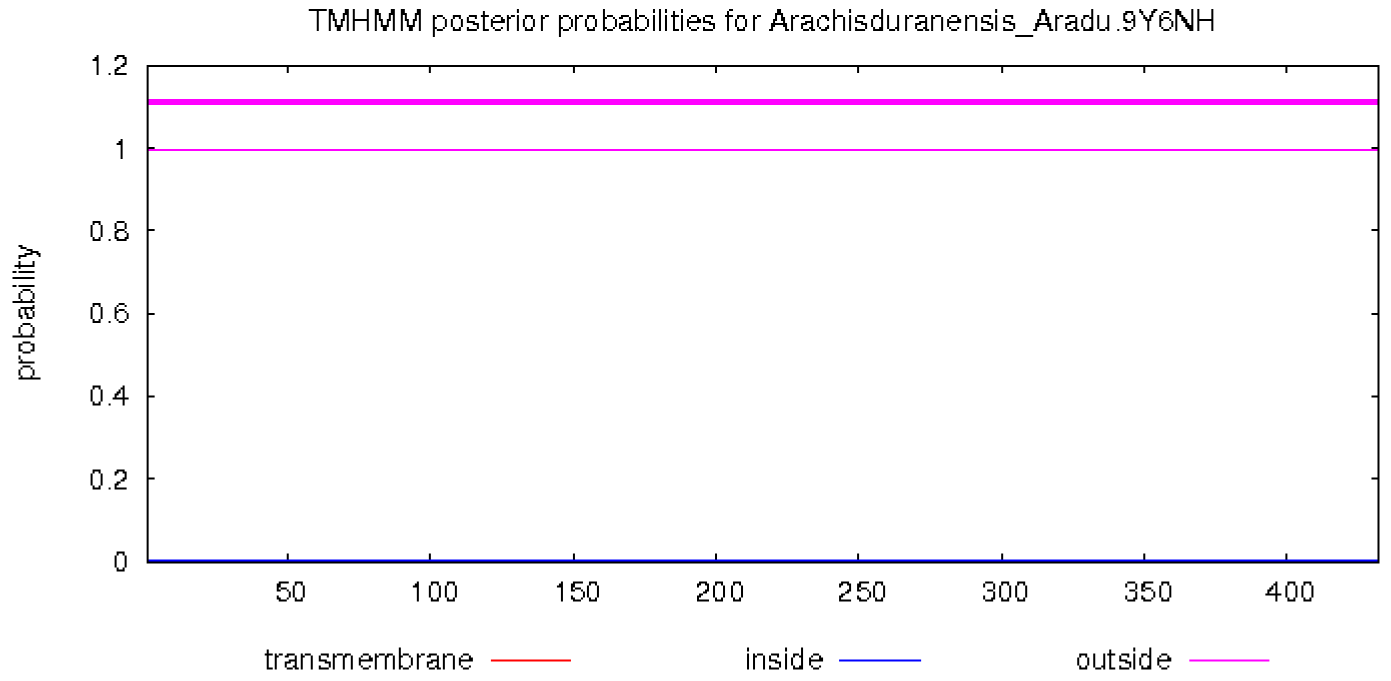

plot in postscript, script for making the plot in gnuplot, data for plot

---

```
# Arachisduranensis_Aradu.AF9FZ Length: 373
# Arachisduranensis_Aradu.AF9FZ Number of predicted TMHs:  0
# Arachisduranensis_Aradu.AF9FZ Exp number of AAs in TMHs: 0.28084
# Arachisduranensis_Aradu.AF9FZ Exp number, first 60 AAs:  0
# Arachisduranensis_Aradu.AF9FZ Total prob of N-in:        0.01766
Arachisduranensis_Aradu.AF9FZ   TMHMM2.0        outside      1   373
```

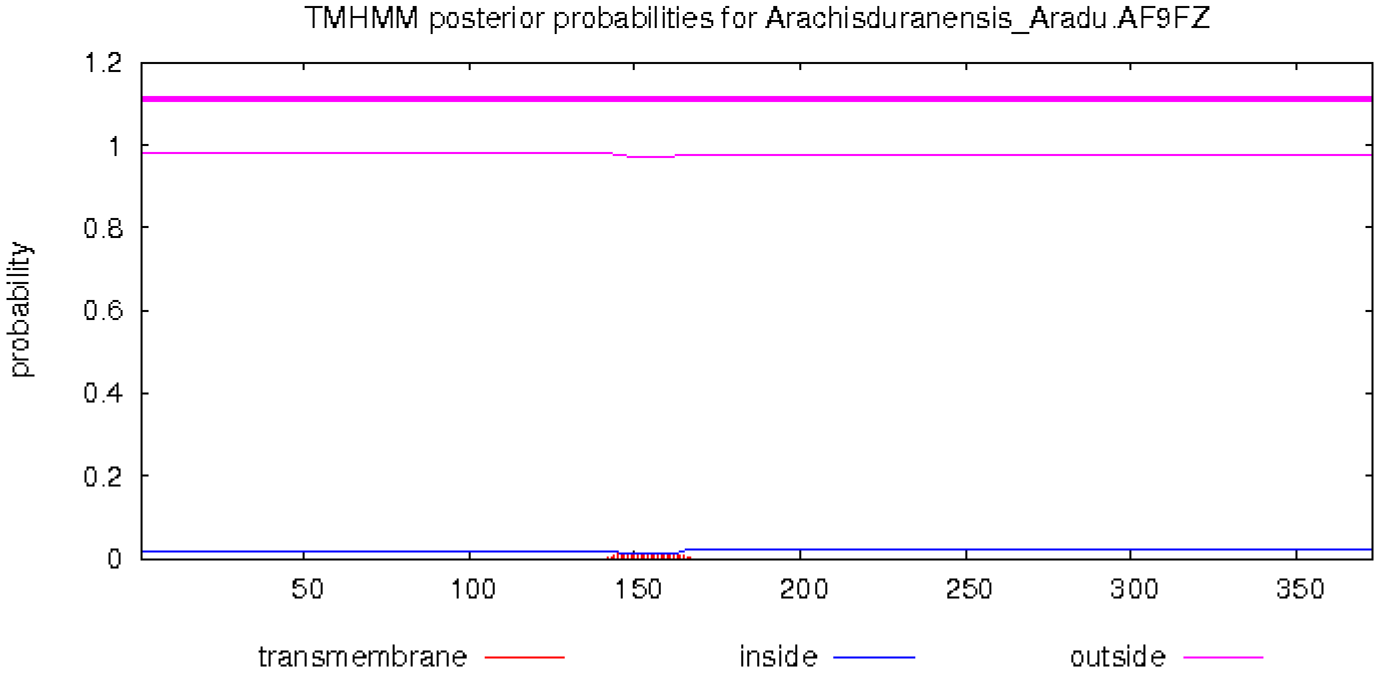

plot in postscript, script for making the plot in gnuplot, data for plot

---

```
# Arachisduranensis_Aradu.B5XXI Length: 405
# Arachisduranensis_Aradu.B5XXI Number of predicted TMHs:  0
# Arachisduranensis_Aradu.B5XXI Exp number of AAs in TMHs: 0.00327
# Arachisduranensis_Aradu.B5XXI Exp number, first 60 AAs:  0
# Arachisduranensis_Aradu.B5XXI Total prob of N-in:        0.02860
Arachisduranensis_Aradu.B5XXI   TMHMM2.0        outside      1   405
```

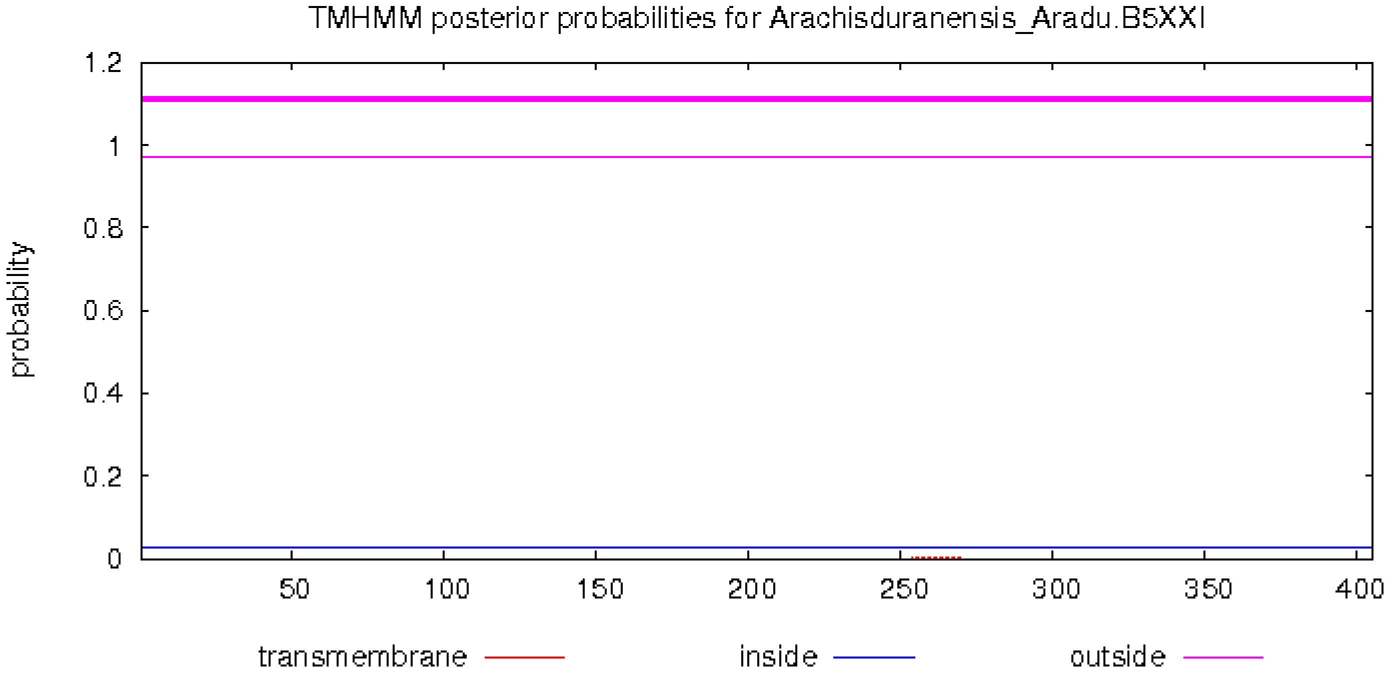

plot in postscript, script for making the plot in gnuplot, data for plot

---

```
# Arachisduranensis_Aradu.BPK98 Length: 463
# Arachisduranensis_Aradu.BPK98 Number of predicted TMHs:  1
# Arachisduranensis_Aradu.BPK98 Exp number of AAs in TMHs: 19.13534
# Arachisduranensis_Aradu.BPK98 Exp number, first 60 AAs:  0
# Arachisduranensis_Aradu.BPK98 Total prob of N-in:        0.08353
Arachisduranensis_Aradu.BPK98   TMHMM2.0        outside      1   439
Arachisduranensis_Aradu.BPK98   TMHMM2.0        TMhelix    440   462
Arachisduranensis_Aradu.BPK98   TMHMM2.0        inside     463   463
```

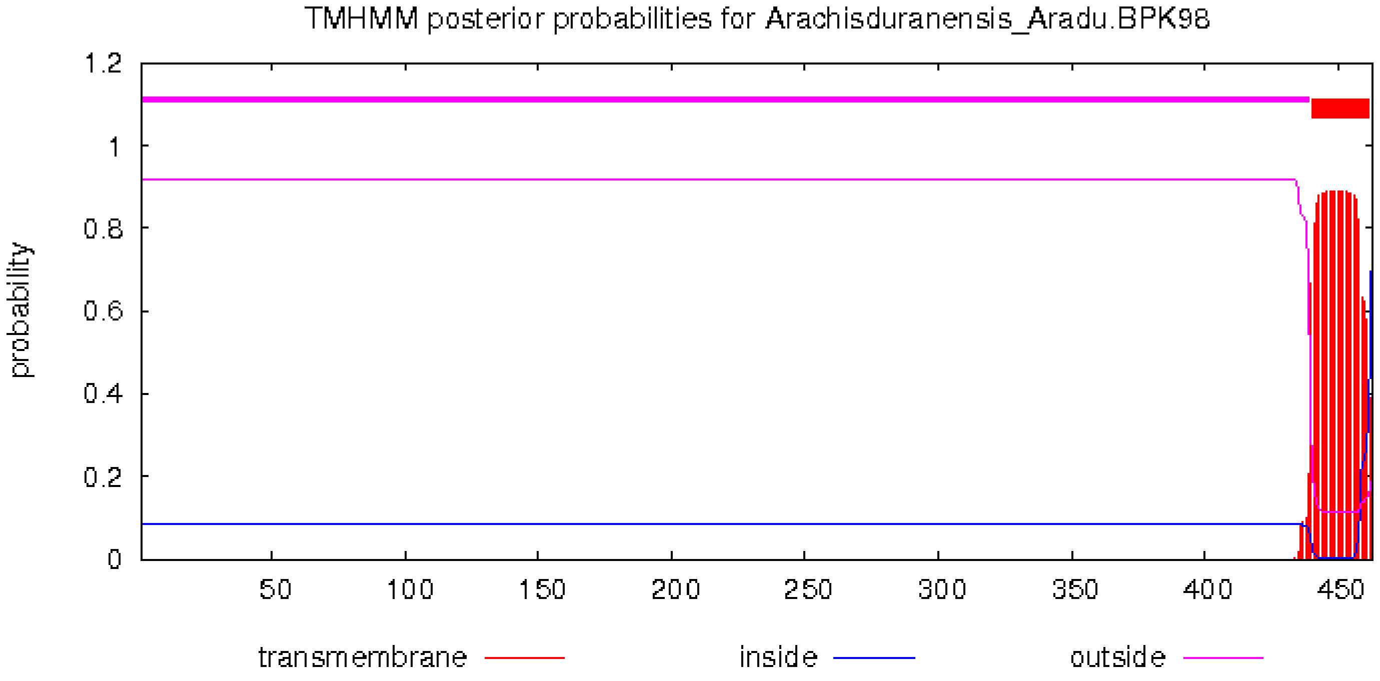

plot in postscript, script for making the plot in gnuplot, data for plot

---

```
# Arachisduranensis_Aradu.BS3JU Length: 300
# Arachisduranensis_Aradu.BS3JU Number of predicted TMHs:  0
# Arachisduranensis_Aradu.BS3JU Exp number of AAs in TMHs: 0.00056
# Arachisduranensis_Aradu.BS3JU Exp number, first 60 AAs:  0
# Arachisduranensis_Aradu.BS3JU Total prob of N-in:        0.07003
Arachisduranensis_Aradu.BS3JU   TMHMM2.0        outside      1   300
```

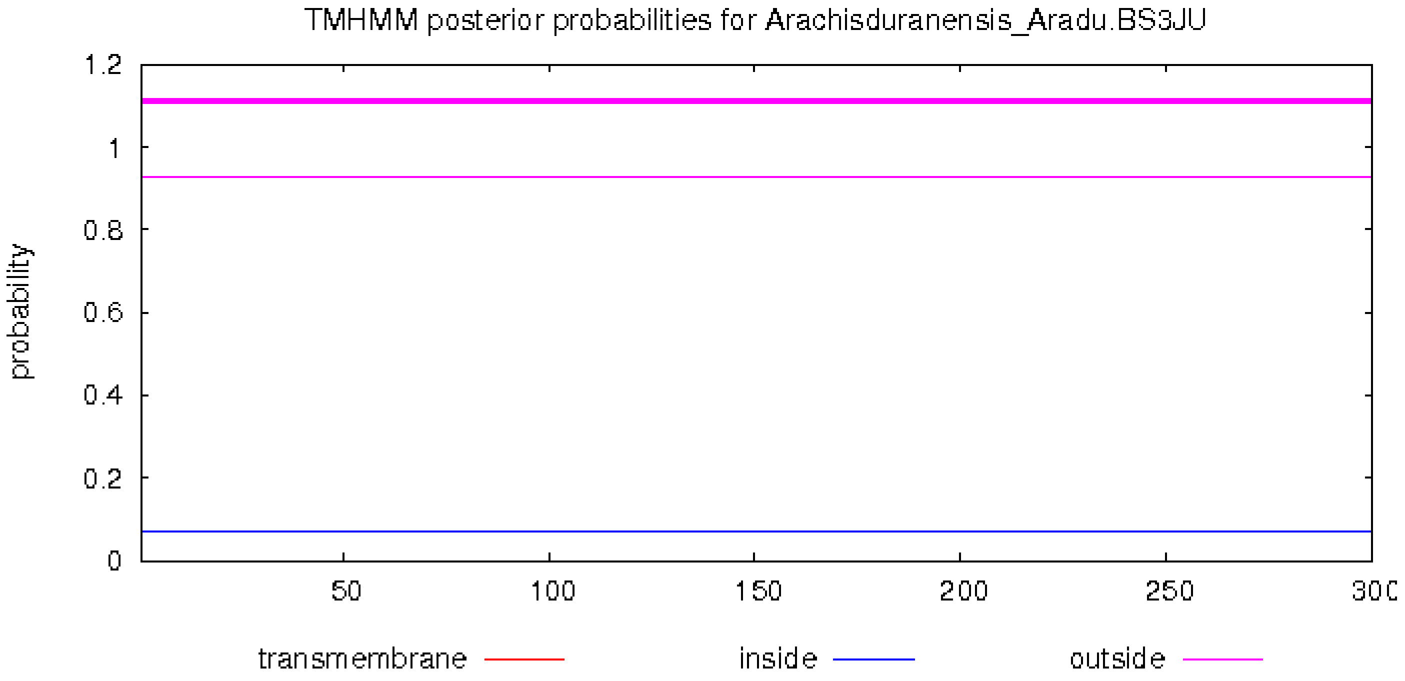

plot in postscript, script for making the plot in gnuplot, data for plot

```
# Arachisduranensis_Aradu.C1Q0A Length: 372
# Arachisduranensis_Aradu.C1Q0A Number of predicted TMHs:  1
# Arachisduranensis_Aradu.C1Q0A Exp number of AAs in TMHs: 22.63751
# Arachisduranensis_Aradu.C1Q0A Exp number, first 60 AAs:  0.00223
# Arachisduranensis_Aradu.C1Q0A Total prob of N-in:        0.03025
Arachisduranensis_Aradu.C1Q0A   TMHMM2.0        outside      1   332
Arachisduranensis_Aradu.C1Q0A   TMHMM2.0        TMhelix    333   355
Arachisduranensis_Aradu.C1Q0A   TMHMM2.0        inside     356   372
```

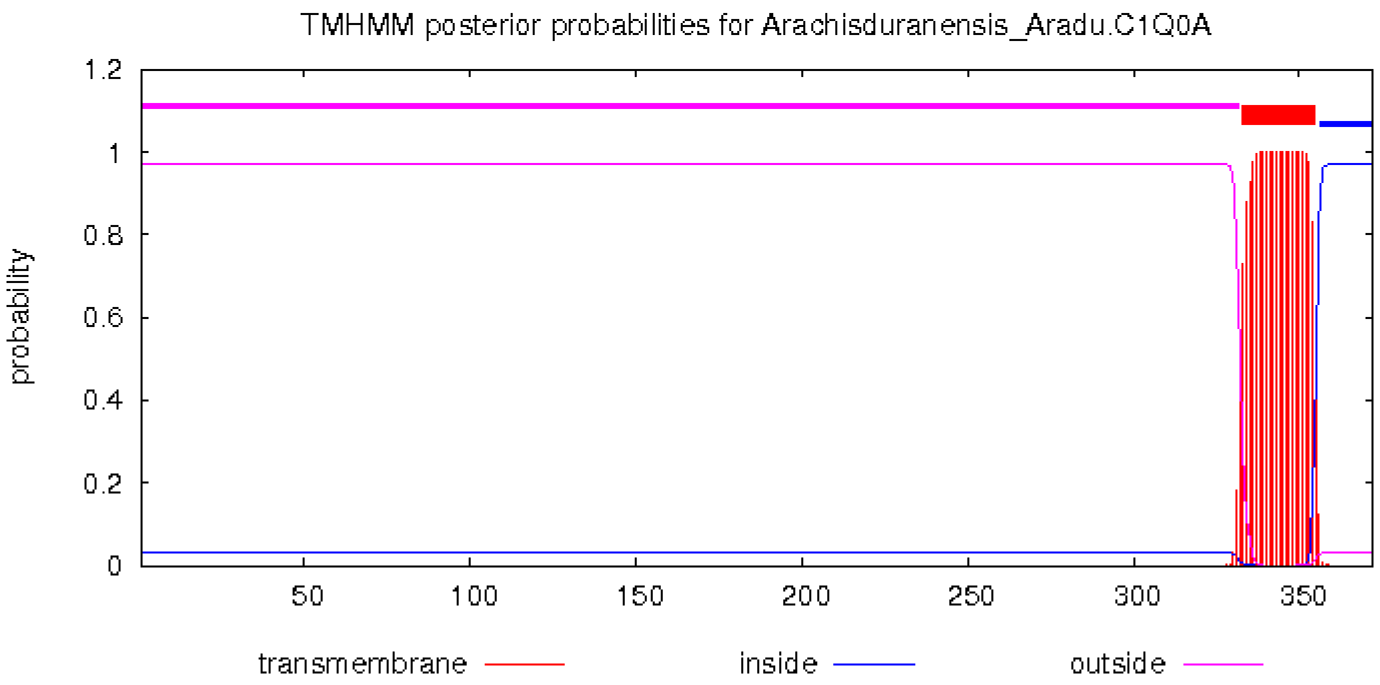

plot in postscript, script for making the plot in gnuplot, data for plot

```
# Arachisduranensis_Aradu.DII8L Length: 245
# Arachisduranensis_Aradu.DII8L Number of predicted TMHs:  0
# Arachisduranensis_Aradu.DII8L Exp number of AAs in TMHs: 0.00563
# Arachisduranensis_Aradu.DII8L Exp number, first 60 AAs:  0
# Arachisduranensis_Aradu.DII8L Total prob of N-in:        0.03537
Arachisduranensis_Aradu.DII8L   TMHMM2.0        outside      1   245
```

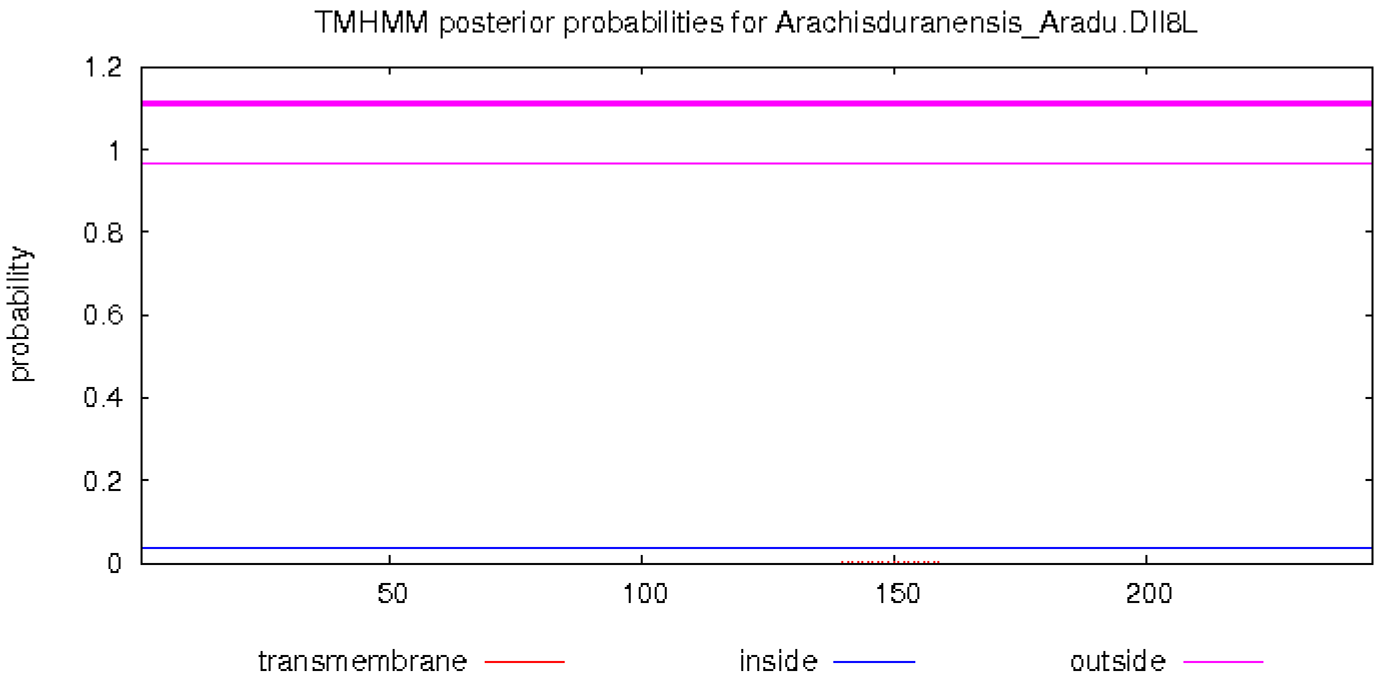

plot in postscript, script for making the plot in gnuplot, data for plot

```
# Arachisduranensis_Aradu.DQR3M Length: 347
# Arachisduranensis_Aradu.DQR3M Number of predicted TMHs:  0
# Arachisduranensis_Aradu.DQR3M Exp number of AAs in TMHs: 0.00023
# Arachisduranensis_Aradu.DQR3M Exp number, first 60 AAs:  0.00023
# Arachisduranensis_Aradu.DQR3M Total prob of N-in:        0.00453
Arachisduranensis_Aradu.DQR3M   TMHMM2.0        outside      1   347
```

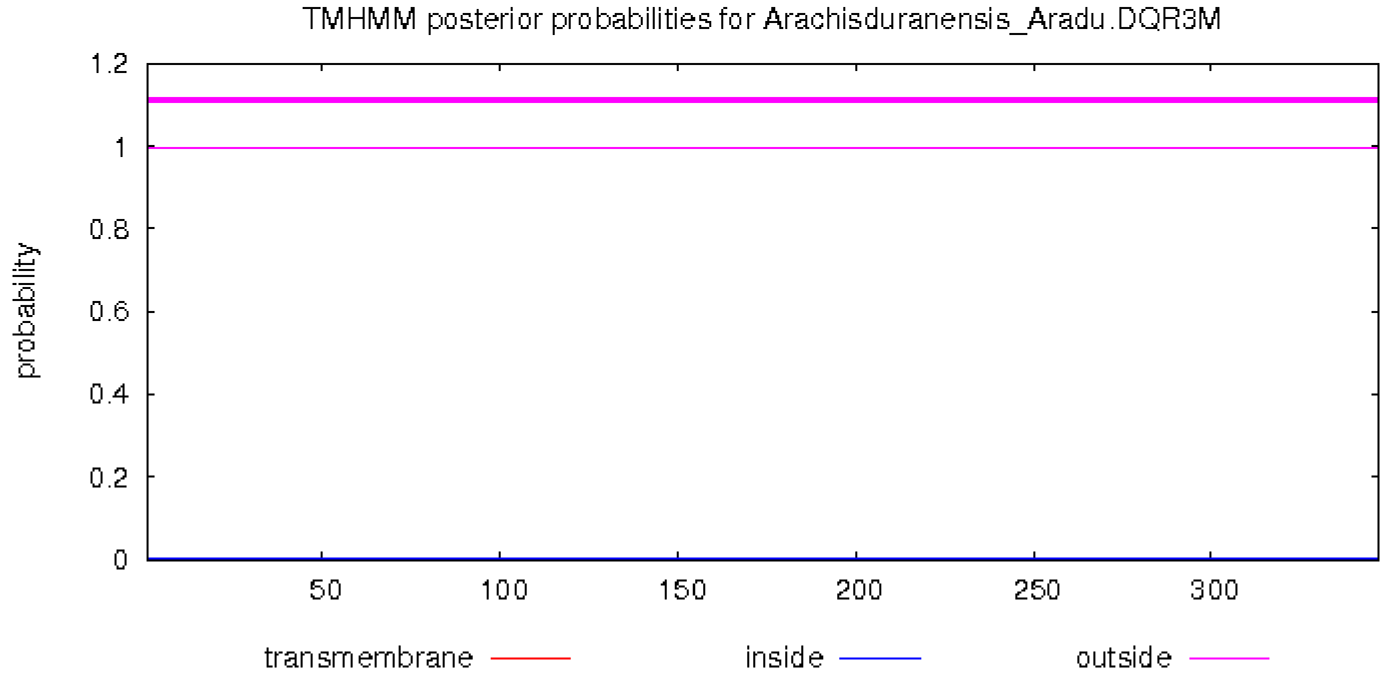

plot in postscript, script for making the plot in gnuplot, data for plot

```
# Arachisduranensis_Aradu.EP425 Length: 367
# Arachisduranensis_Aradu.EP425 Number of predicted TMHs:  0
# Arachisduranensis_Aradu.EP425 Exp number of AAs in TMHs: 0.00257
# Arachisduranensis_Aradu.EP425 Exp number, first 60 AAs:  0.00161
# Arachisduranensis_Aradu.EP425 Total prob of N-in:        0.01110
Arachisduranensis_Aradu.EP425   TMHMM2.0        outside      1   367
```

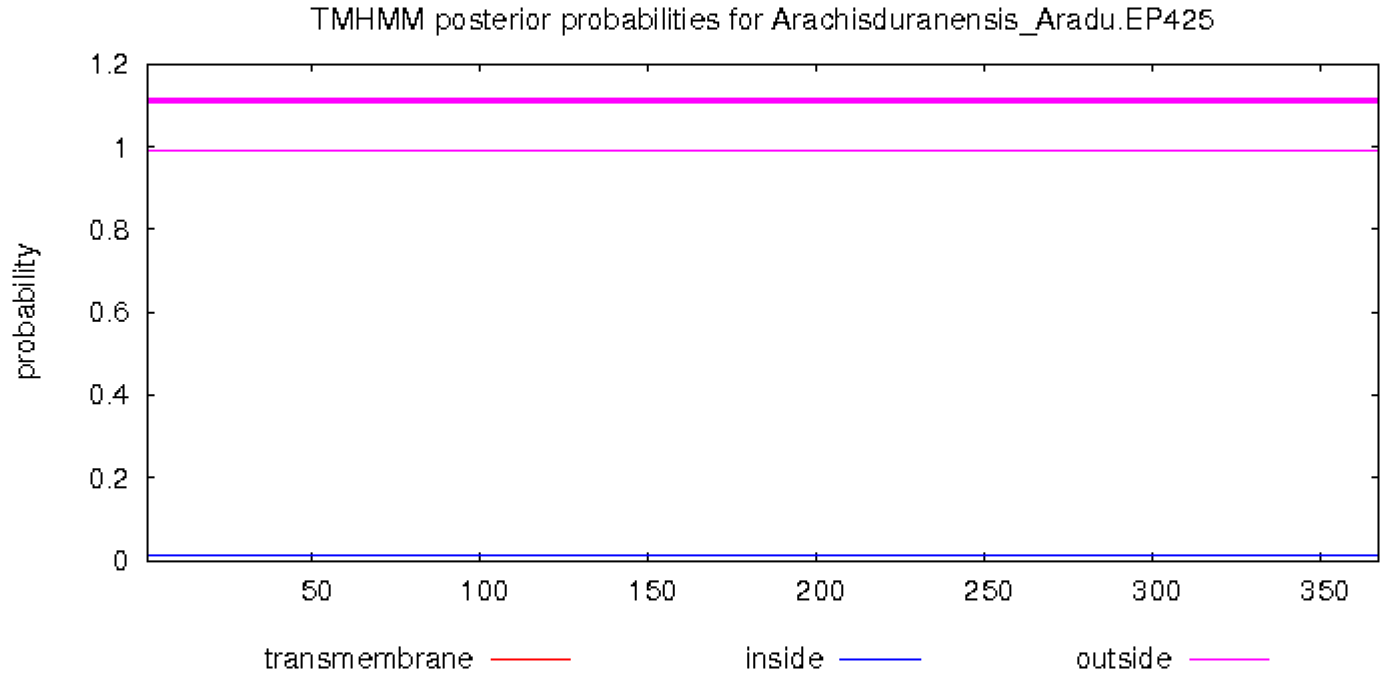

plot in postscript, script for making the plot in gnuplot, data for plot

```
# Arachisduranensis_Aradu.ETZ8K Length: 356
# Arachisduranensis_Aradu.ETZ8K Number of predicted TMHs:  0
# Arachisduranensis_Aradu.ETZ8K Exp number of AAs in TMHs: 0
# Arachisduranensis_Aradu.ETZ8K Exp number, first 60 AAs:  0
# Arachisduranensis_Aradu.ETZ8K Total prob of N-in:        0.00074
Arachisduranensis_Aradu.ETZ8K	TMHMM2.0	outside	     1   356
```

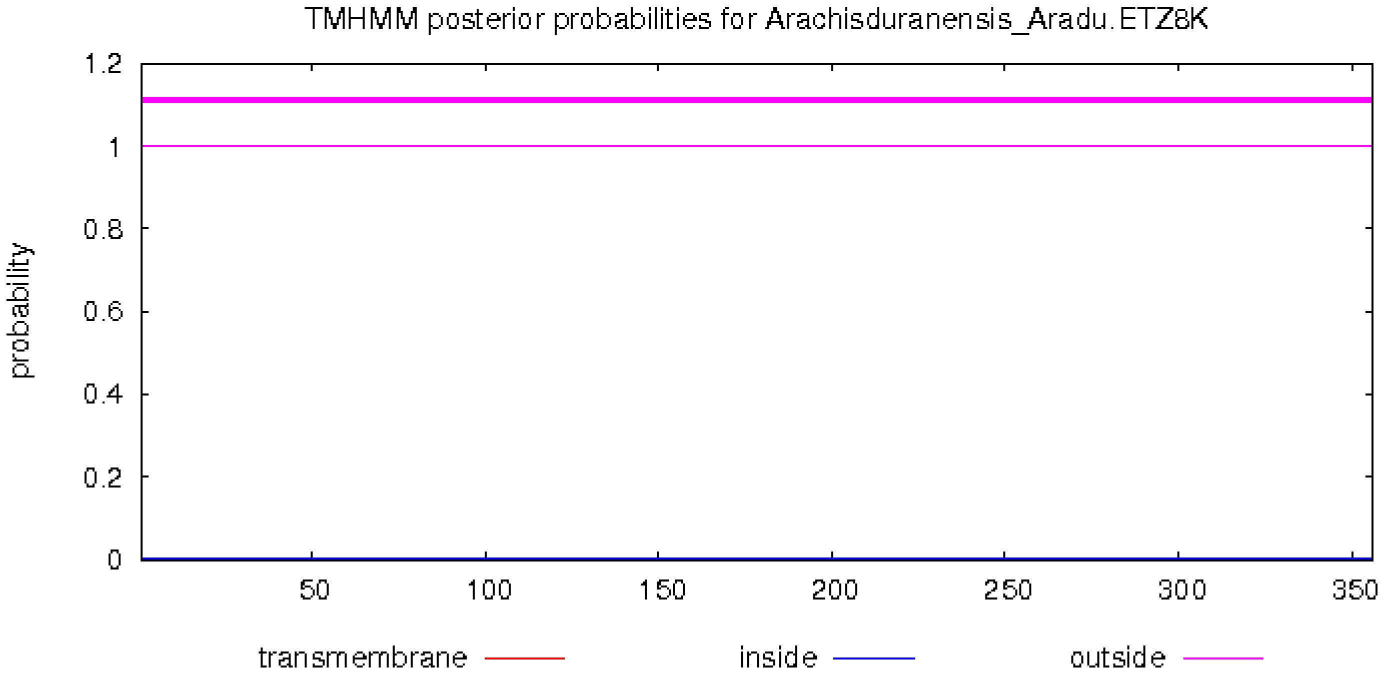

plot in postscript, script for making the plot in gnuplot, data for plot

```
# Arachisduranensis_Aradu.F2DT2 Length: 360
# Arachisduranensis_Aradu.F2DT2 Number of predicted TMHs:  0
# Arachisduranensis_Aradu.F2DT2 Exp number of AAs in TMHs: 0.00123
# Arachisduranensis_Aradu.F2DT2 Exp number, first 60 AAs:  0
# Arachisduranensis_Aradu.F2DT2 Total prob of N-in:        0.00639
Arachisduranensis_Aradu.F2DT2	TMHMM2.0	outside	     1   360
```

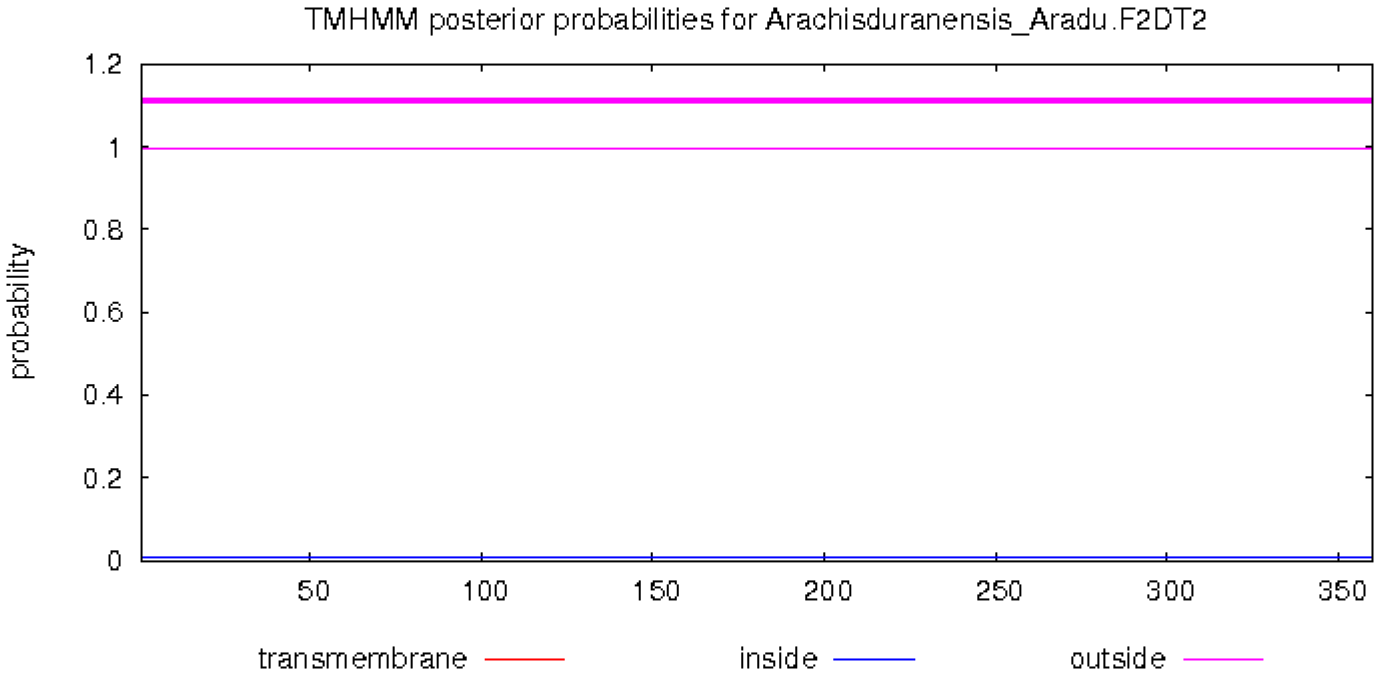

plot in postscript, script for making the plot in gnuplot, data for plot

```
# Arachisduranensis_Aradu.F48KW Length: 557
# Arachisduranensis_Aradu.F48KW Number of predicted TMHs:  1
# Arachisduranensis_Aradu.F48KW Exp number of AAs in TMHs: 22.16109
# Arachisduranensis_Aradu.F48KW Exp number, first 60 AAs:  0.00049
# Arachisduranensis_Aradu.F48KW Total prob of N-in:        0.00517
Arachisduranensis_Aradu.F48KW   TMHMM2.0        outside      1   528
Arachisduranensis_Aradu.F48KW   TMHMM2.0        TMhelix    529   551
Arachisduranensis_Aradu.F48KW   TMHMM2.0        inside     552   557
```

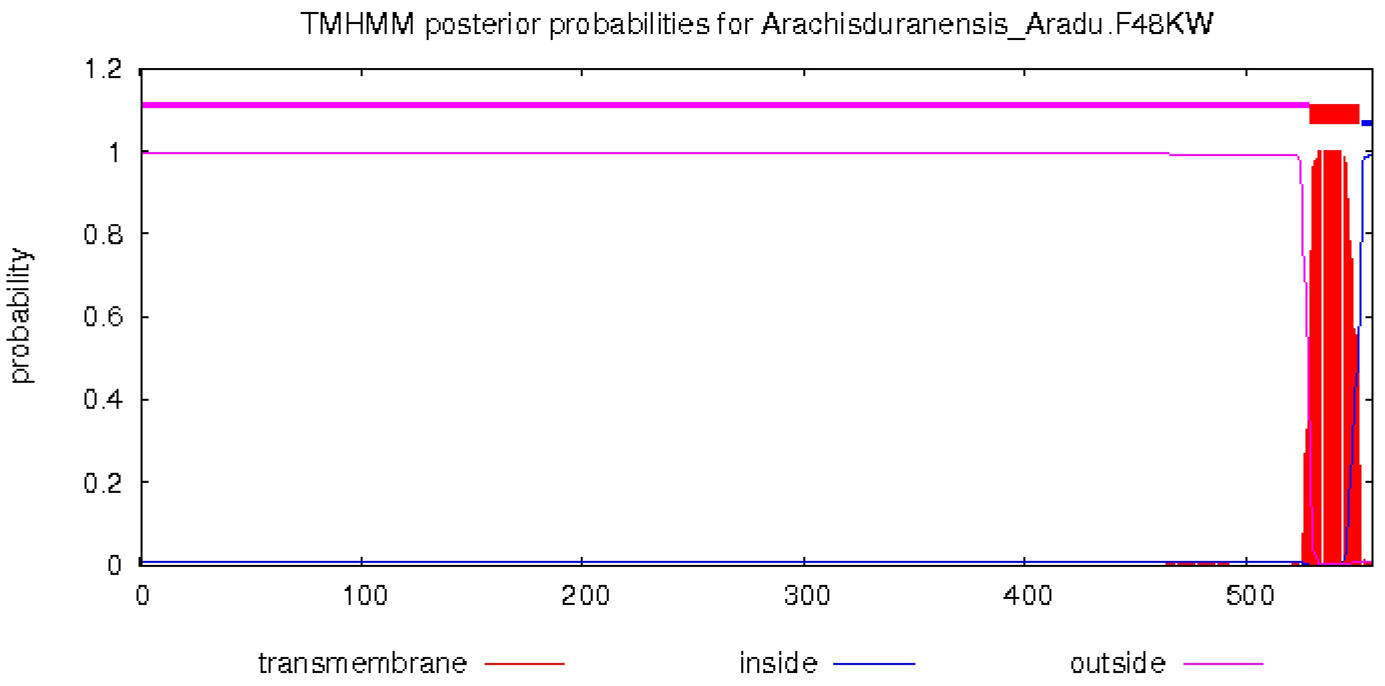

plot in postscript, script for making the plot in gnuplot, data for plot

```
# Arachisduranensis_Aradu.F6Z4G Length: 330
# Arachisduranensis_Aradu.F6Z4G Number of predicted TMHs:  0
# Arachisduranensis_Aradu.F6Z4G Exp number of AAs in TMHs: 0.00143
# Arachisduranensis_Aradu.F6Z4G Exp number, first 60 AAs:  0
# Arachisduranensis_Aradu.F6Z4G Total prob of N-in:        0.01303
Arachisduranensis_Aradu.F6Z4G   TMHMM2.0        outside      1   330
```

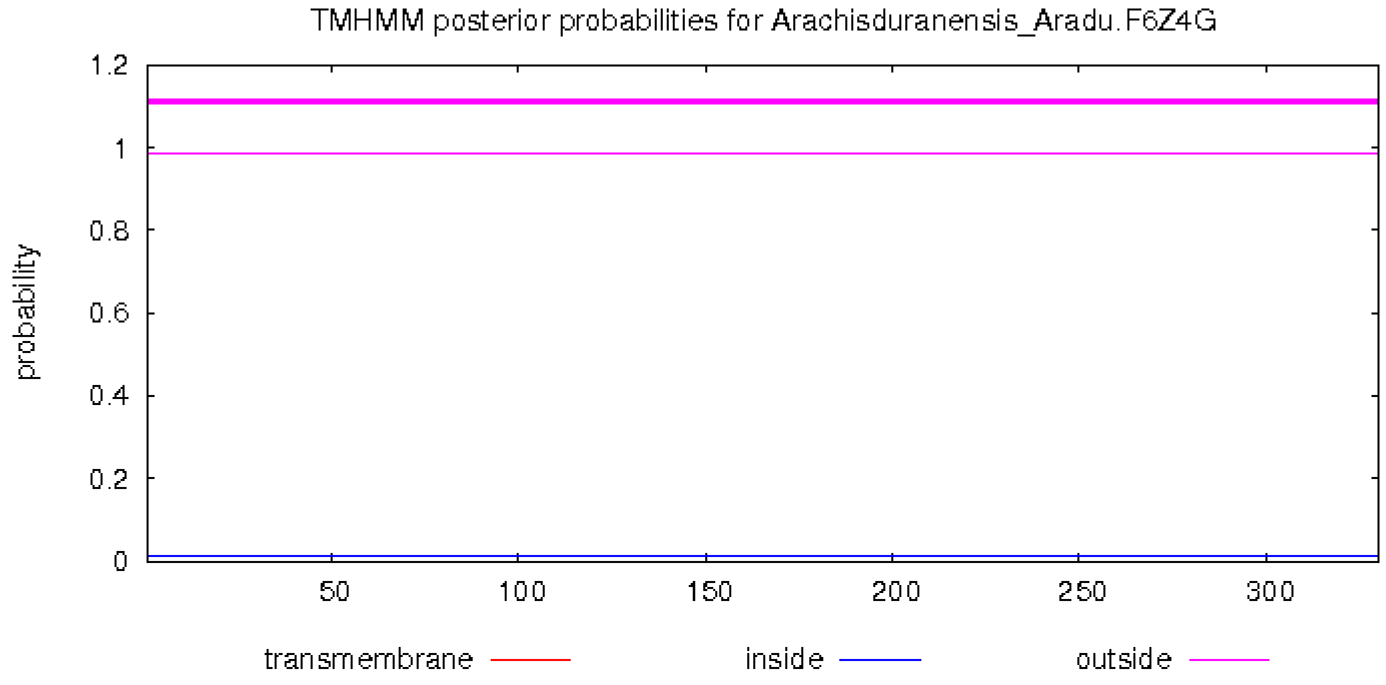

plot in postscript, script for making the plot in gnuplot, data for plot

---

```
# Arachisduranensis_Aradu.F8VRL Length: 382
# Arachisduranensis_Aradu.F8VRL Number of predicted TMHs:  0
# Arachisduranensis_Aradu.F8VRL Exp number of AAs in TMHs: 0.00053
# Arachisduranensis_Aradu.F8VRL Exp number, first 60 AAs:  0
# Arachisduranensis_Aradu.F8VRL Total prob of N-in:        0.02089
Arachisduranensis_Aradu.F8VRL	TMHMM2.0	outside	     1   382
```

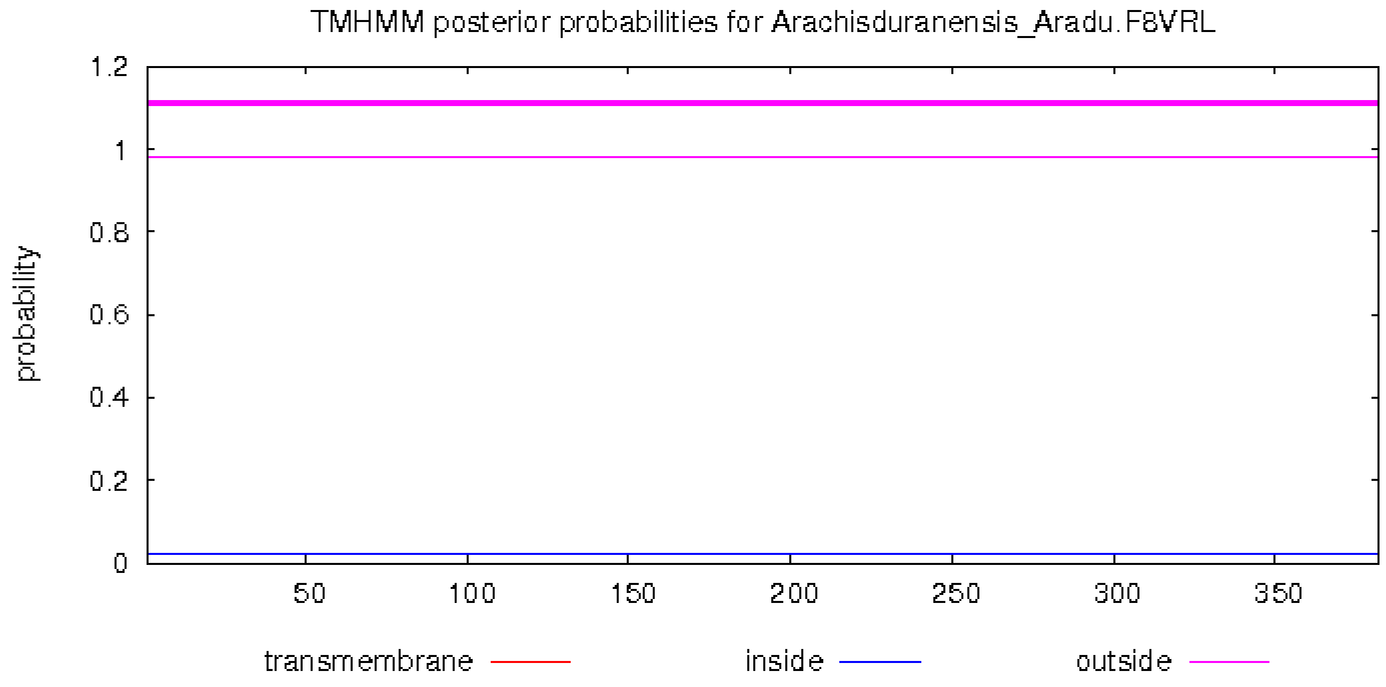

plot in postscript, script for making the plot in gnuplot, data for plot

---

```
# Arachisduranensis_Aradu.H2YS3 Length: 226
# Arachisduranensis_Aradu.H2YS3 Number of predicted TMHs:  0
# Arachisduranensis_Aradu.H2YS3 Exp number of AAs in TMHs: 0.04274
# Arachisduranensis_Aradu.H2YS3 Exp number, first 60 AAs:  0.04274
# Arachisduranensis_Aradu.H2YS3 Total prob of N-in:        0.02624
Arachisduranensis_Aradu.H2YS3	TMHMM2.0	outside	     1   226
```

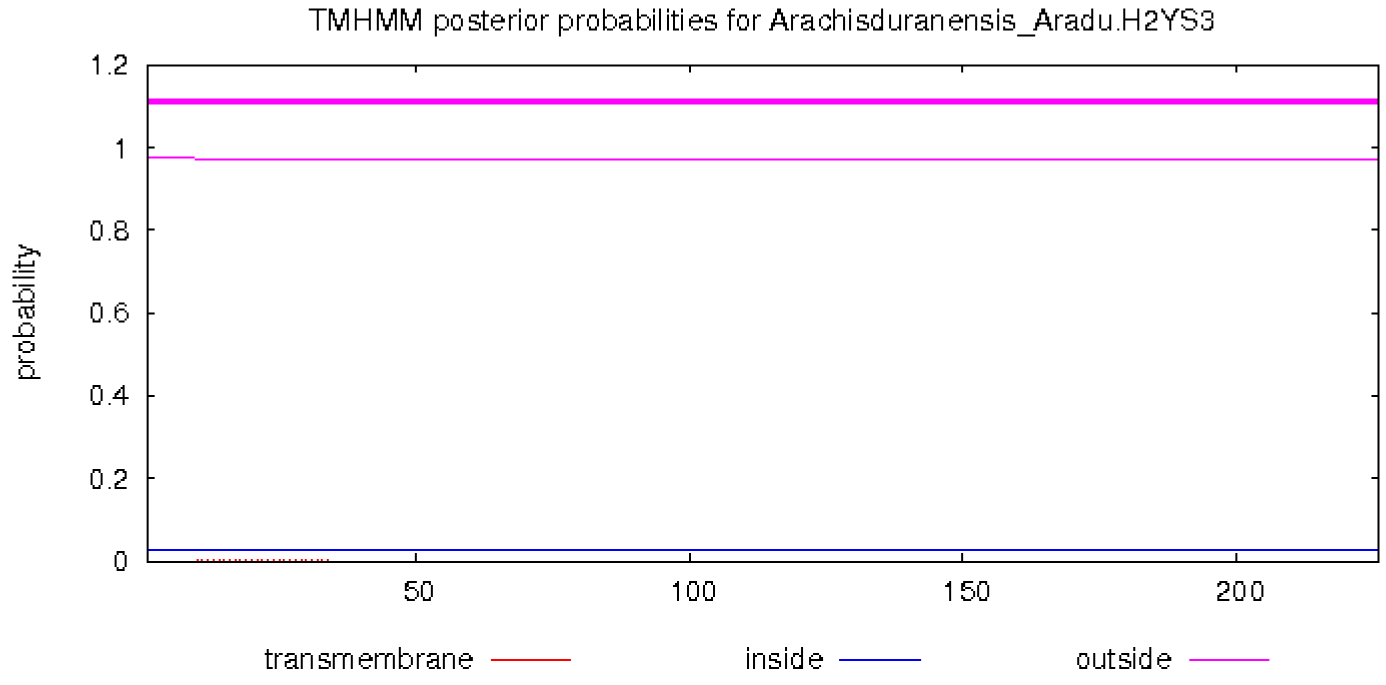

plot in postscript, script for making the plot in gnuplot, data for plot

---

```
# Arachisduranensis_Aradu.H5KV7 Length: 317
# Arachisduranensis_Aradu.H5KV7 Number of predicted TMHs:  0
# Arachisduranensis_Aradu.H5KV7 Exp number of AAs in TMHs: 0.0426
# Arachisduranensis_Aradu.H5KV7 Exp number, first 60 AAs:  0.0426
# Arachisduranensis_Aradu.H5KV7 Total prob of N-in:        0.00960
Arachisduranensis_Aradu.H5KV7   TMHMM2.0        outside      1   317
```

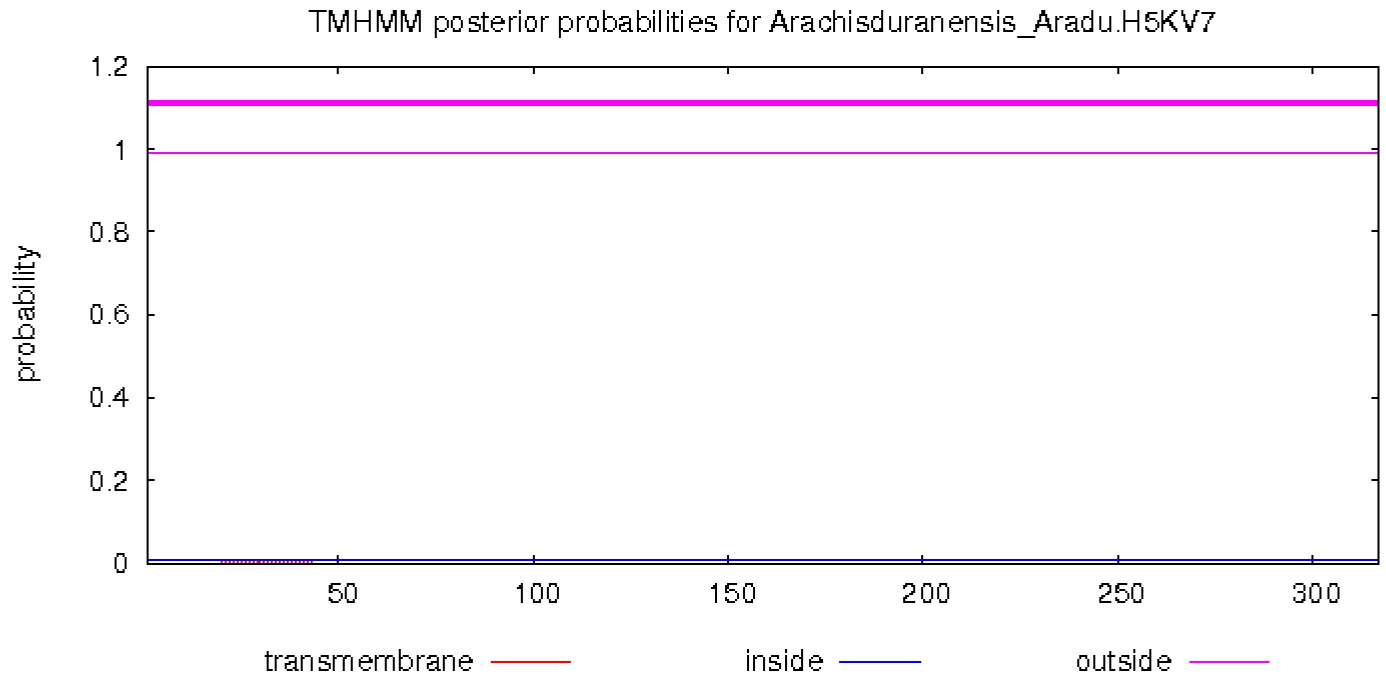

plot in postscript, script for making the plot in gnuplot, data for plot

---

```
# Arachisduranensis_Aradu.JV7AK Length: 451
# Arachisduranensis_Aradu.JV7AK Number of predicted TMHs:  0
# Arachisduranensis_Aradu.JV7AK Exp number of AAs in TMHs: 0.00022
# Arachisduranensis_Aradu.JV7AK Exp number, first 60 AAs:  0
# Arachisduranensis_Aradu.JV7AK Total prob of N-in:        0.00319
Arachisduranensis_Aradu.JV7AK   TMHMM2.0        outside      1   451
```

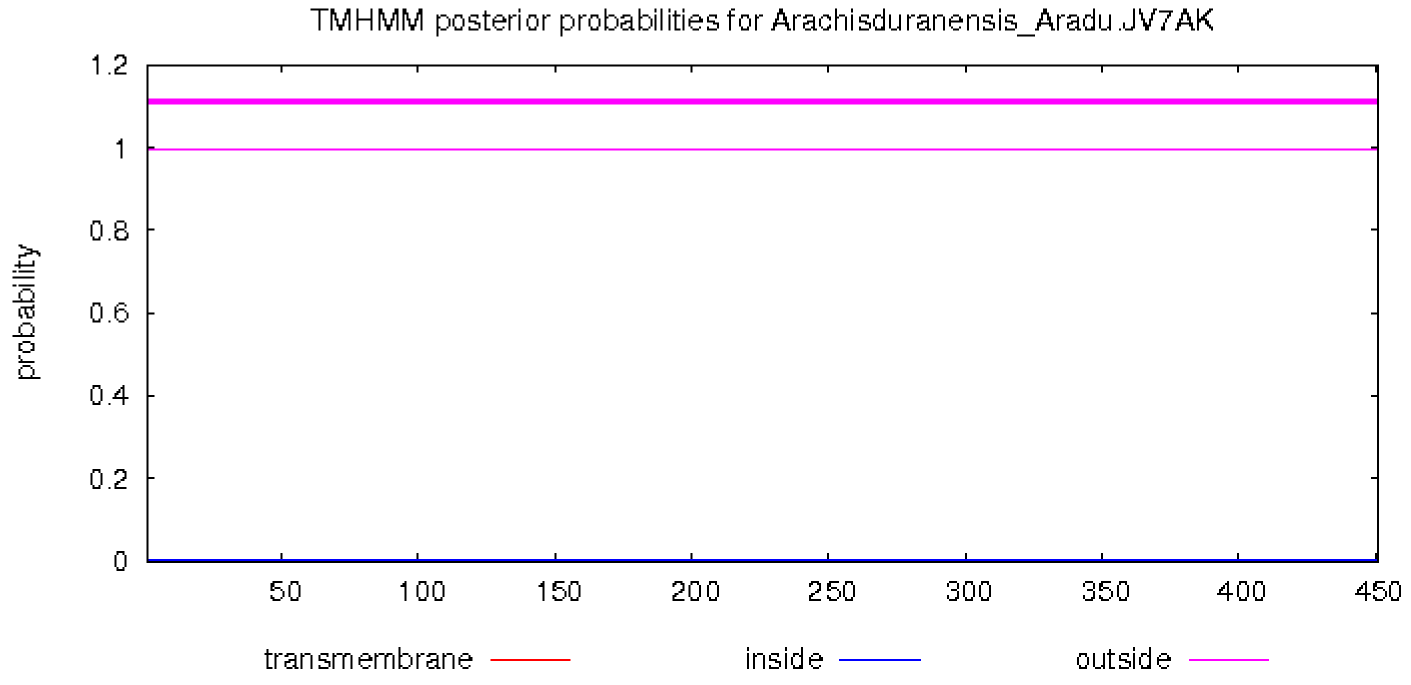

plot in postscript, script for making the plot in gnuplot, data for plot

---

```
# Arachisduranensis_Aradu.JZK1S Length: 95
# Arachisduranensis_Aradu.JZK1S Number of predicted TMHs:  0
# Arachisduranensis_Aradu.JZK1S Exp number of AAs in TMHs: 0
# Arachisduranensis_Aradu.JZK1S Exp number, first 60 AAs:  0
# Arachisduranensis_Aradu.JZK1S Total prob of N-in:        0.19052
Arachisduranensis_Aradu.JZK1S   TMHMM2.0        outside      1    95
```

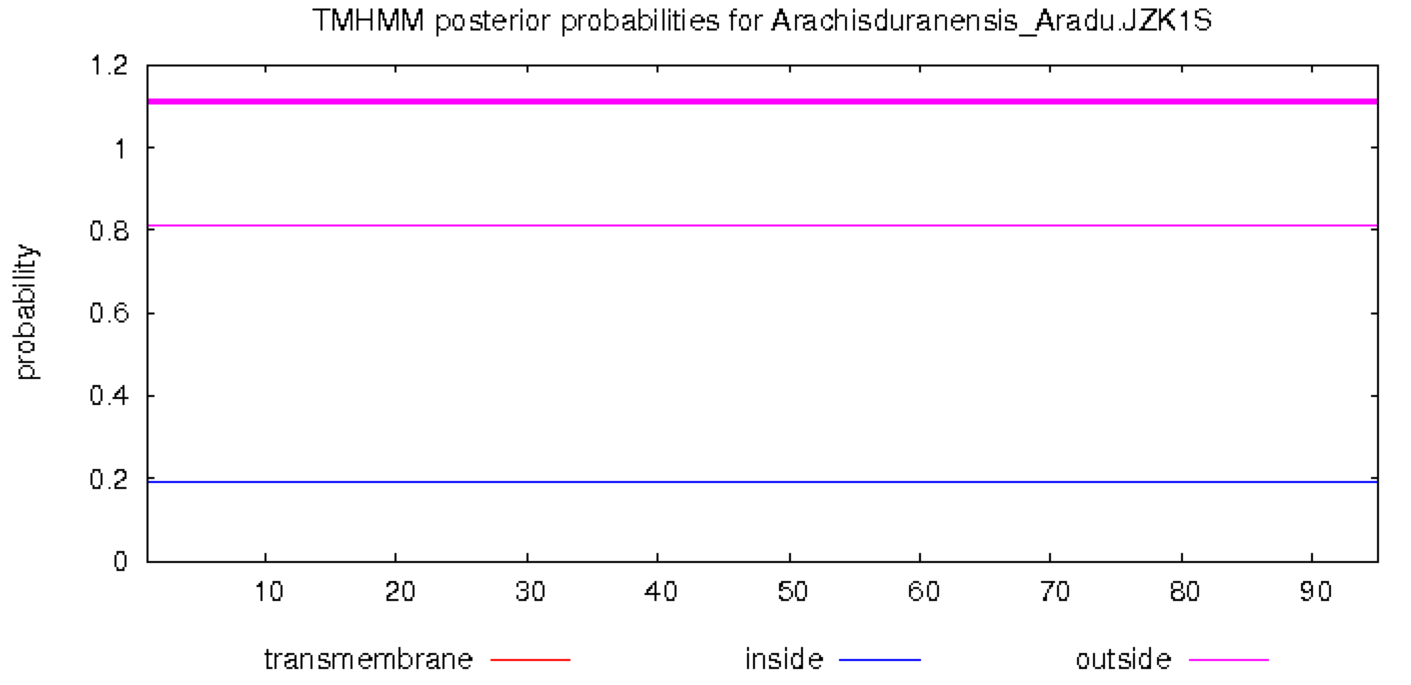

plot in postscript, script for making the plot in gnuplot, data for plot

---

```
# Arachisduranensis_Aradu.K2UJH Length: 681
# Arachisduranensis_Aradu.K2UJH Number of predicted TMHs:  1
# Arachisduranensis_Aradu.K2UJH Exp number of AAs in TMHs: 22.60254
# Arachisduranensis_Aradu.K2UJH Exp number, first 60 AAs:  0.01789
# Arachisduranensis_Aradu.K2UJH Total prob of N-in:        0.00632
Arachisduranensis_Aradu.K2UJH   TMHMM2.0        outside      1   651
```

```
Arachisduranensis_Aradu.K2UJH   TMHMM2.0        TMhelix    652   674
Arachisduranensis_Aradu.K2UJH   TMHMM2.0        inside     675   681
```

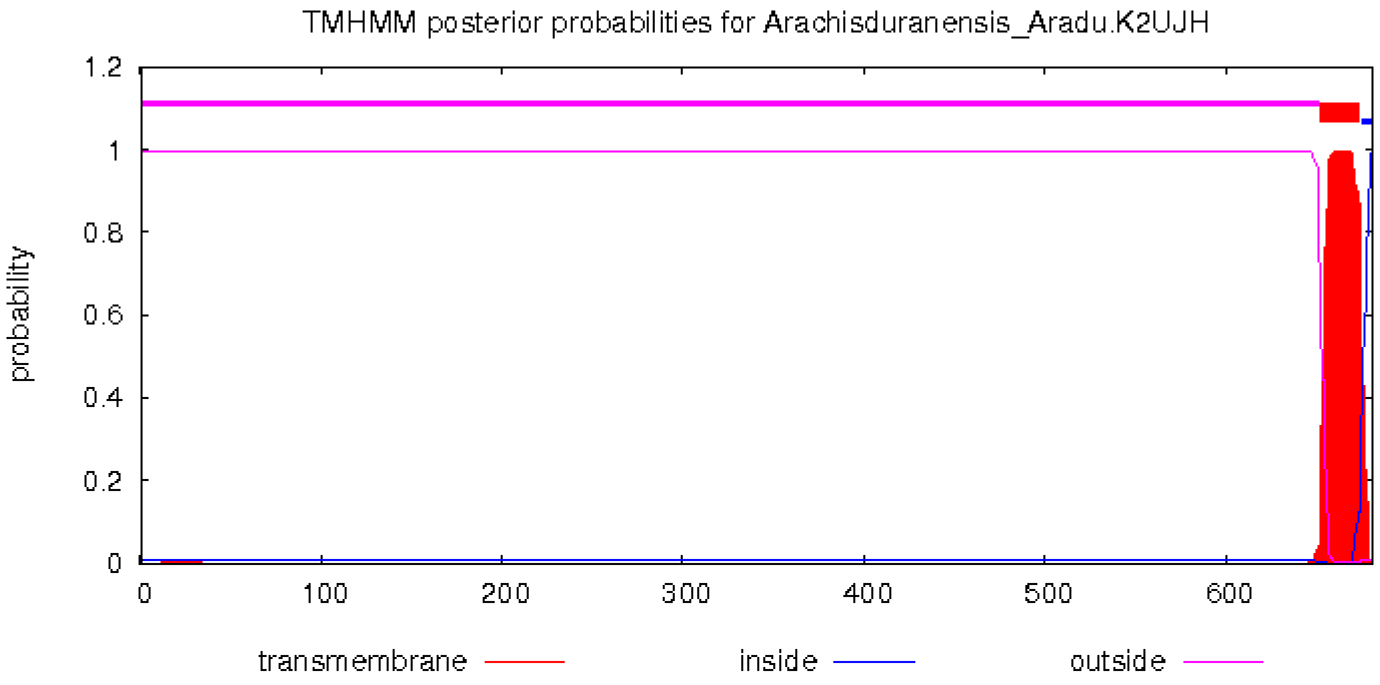

plot in postscript, script for making the plot in gnuplot, data for plot

---

```
# Arachisduranensis_Aradu.KF8UQ Length: 184
# Arachisduranensis_Aradu.KF8UQ Number of predicted TMHs:  0
# Arachisduranensis_Aradu.KF8UQ Exp number of AAs in TMHs: 0.00079
# Arachisduranensis_Aradu.KF8UQ Exp number, first 60 AAs:  0.00015
# Arachisduranensis_Aradu.KF8UQ Total prob of N-in:        0.11476
Arachisduranensis_Aradu.KF8UQ   TMHMM2.0        outside      1   184
```

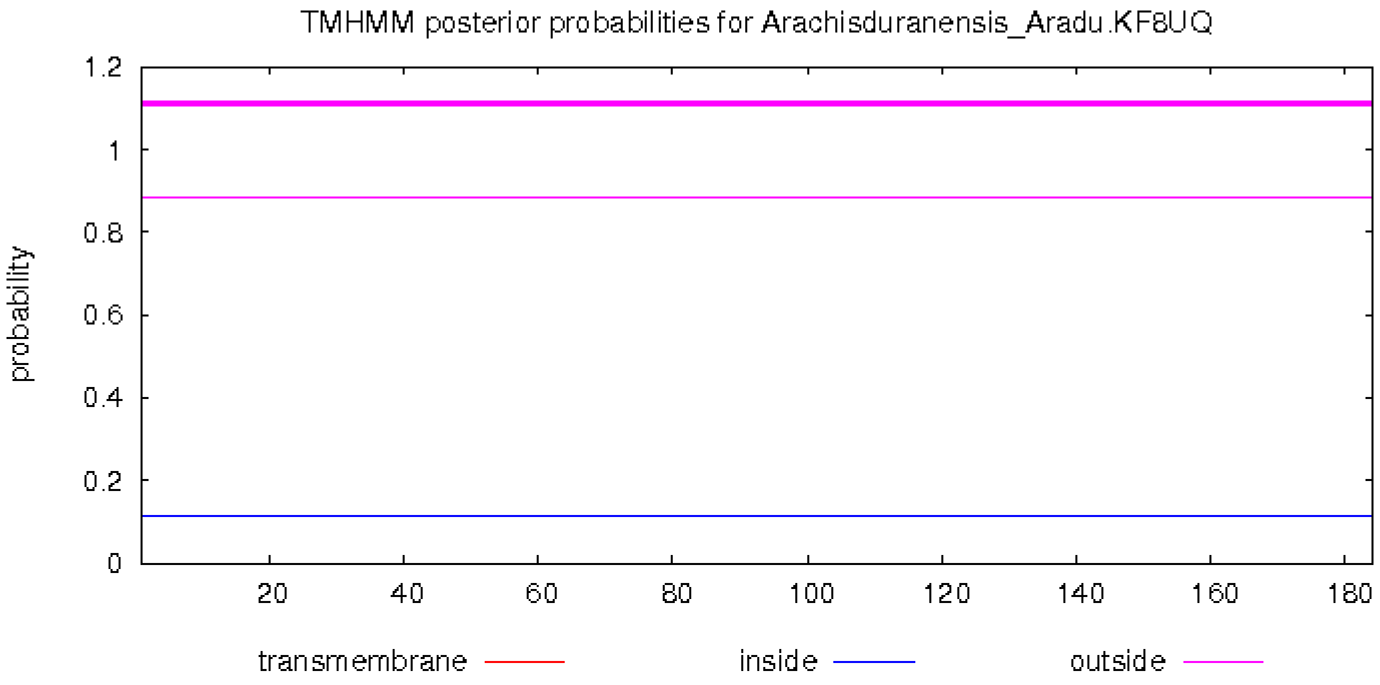

plot in postscript, script for making the plot in gnuplot, data for plot

---

```
# Arachisduranensis_Aradu.L3QY1 Length: 331
# Arachisduranensis_Aradu.L3QY1 Number of predicted TMHs:  0
# Arachisduranensis_Aradu.L3QY1 Exp number of AAs in TMHs: 0.0036
# Arachisduranensis_Aradu.L3QY1 Exp number, first 60 AAs:  0
```

```
# Arachisduranensis_Aradu.L3QY1 Total prob of N-in:        0.00086
Arachisduranensis_Aradu.L3QY1   TMHMM2.0        outside      1   331
```

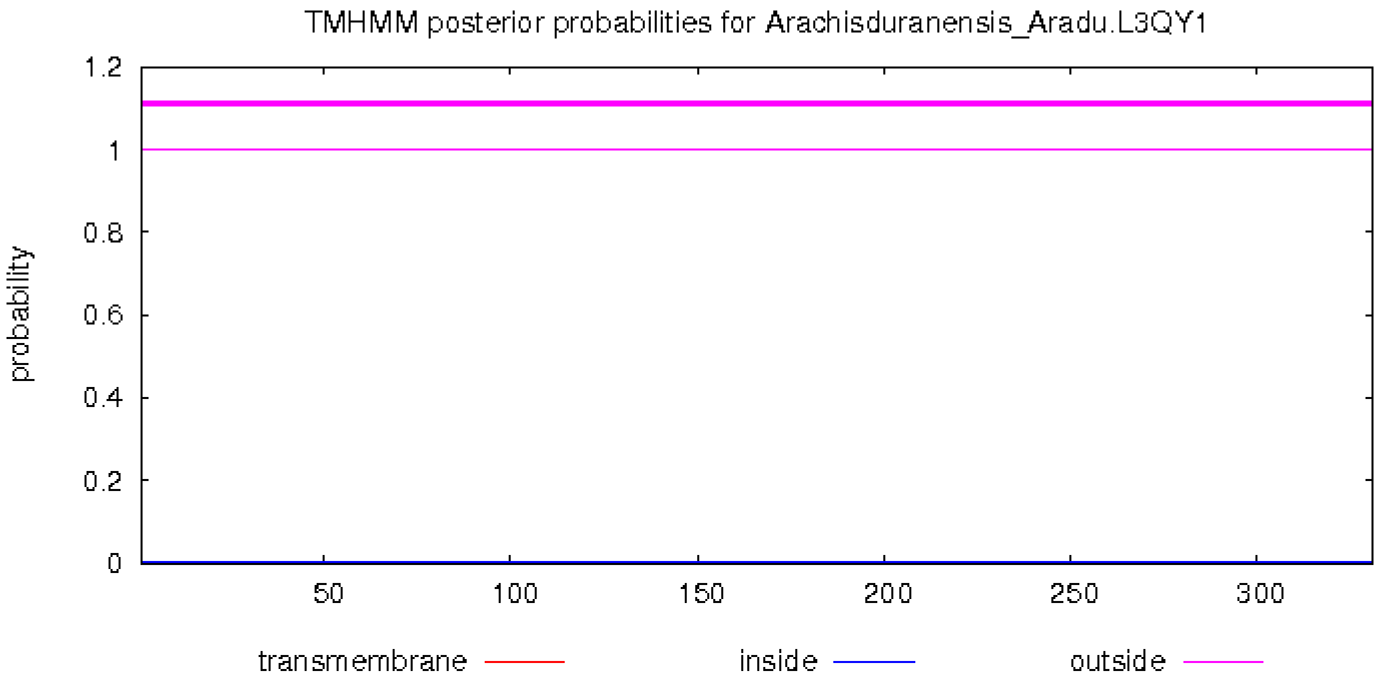

plot in postscript, script for making the plot in gnuplot, data for plot

---

```
# Arachisduranensis_Aradu.L6S7Y Length: 246
# Arachisduranensis_Aradu.L6S7Y Number of predicted TMHs:  0
# Arachisduranensis_Aradu.L6S7Y Exp number of AAs in TMHs: 0
# Arachisduranensis_Aradu.L6S7Y Exp number, first 60 AAs:  0
# Arachisduranensis_Aradu.L6S7Y Total prob of N-in:        0.01847
Arachisduranensis_Aradu.L6S7Y   TMHMM2.0        outside      1   246
```

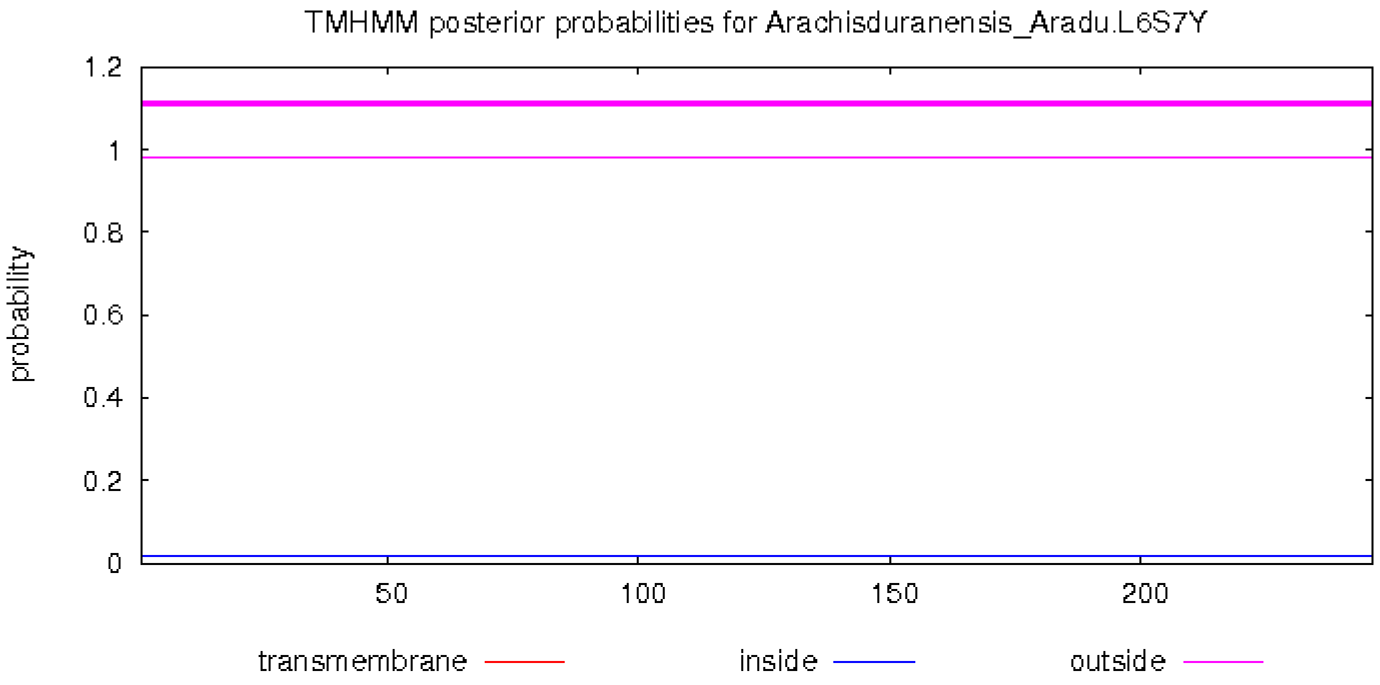

plot in postscript, script for making the plot in gnuplot, data for plot

---

```
# Arachisduranensis_Aradu.L8SVN Length: 286
# Arachisduranensis_Aradu.L8SVN Number of predicted TMHs:  0
# Arachisduranensis_Aradu.L8SVN Exp number of AAs in TMHs: 0.01244
# Arachisduranensis_Aradu.L8SVN Exp number, first 60 AAs:  0.0002
```

```
# Arachisduranensis_Aradu.L8SVN Total prob of N-in:        0.02098
Arachisduranensis_Aradu.L8SVN   TMHMM2.0        outside      1   286
```

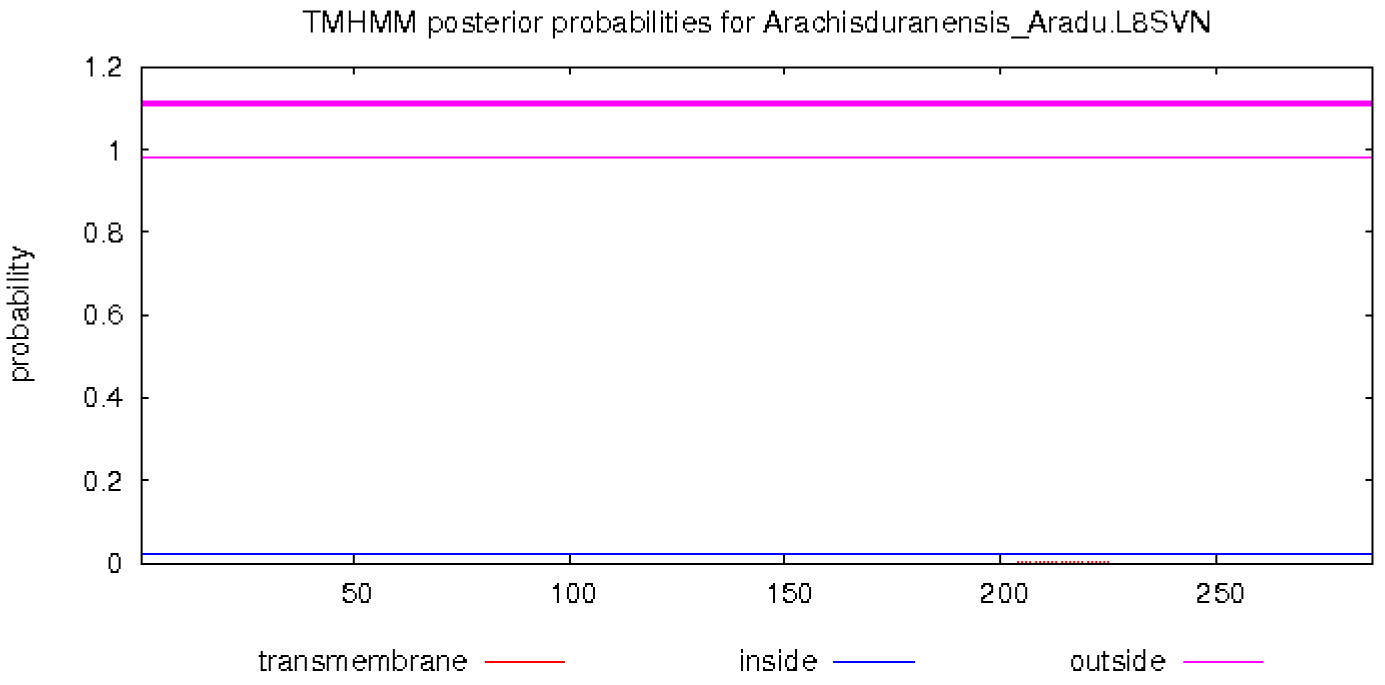

[plot](#) in postscript, [script](#) for making the plot in gnuplot, [data](#) for plot

---

```
# Arachisduranensis_Aradu.LG4RX Length: 218
# Arachisduranensis_Aradu.LG4RX Number of predicted TMHs:  0
# Arachisduranensis_Aradu.LG4RX Exp number of AAs in TMHs: 0.70946
# Arachisduranensis_Aradu.LG4RX Exp number, first 60 AAs:  0.00123
# Arachisduranensis_Aradu.LG4RX Total prob of N-in:        0.15033
Arachisduranensis_Aradu.LG4RX   TMHMM2.0        outside      1   218
```

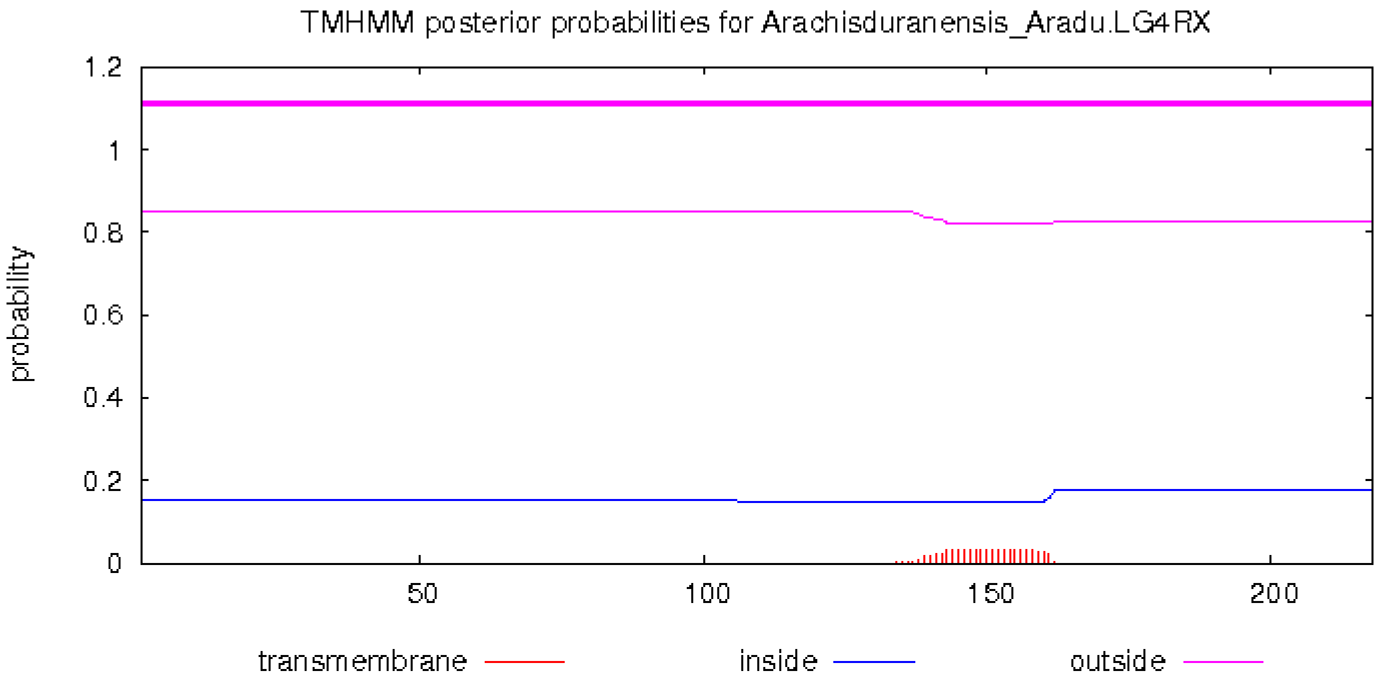

[plot](#) in postscript, [script](#) for making the plot in gnuplot, [data](#) for plot

---

```
# Arachisduranensis_Aradu.LZ0D8 Length: 351
# Arachisduranensis_Aradu.LZ0D8 Number of predicted TMHs:  0
# Arachisduranensis_Aradu.LZ0D8 Exp number of AAs in TMHs: 0.00019
# Arachisduranensis_Aradu.LZ0D8 Exp number, first 60 AAs:  0
```

```
# Arachisduranensis_Aradu.LZ0D8 Total prob of N-in:        0.01247
Arachisduranensis_Aradu.LZ0D8   TMHMM2.0        outside      1   351
```

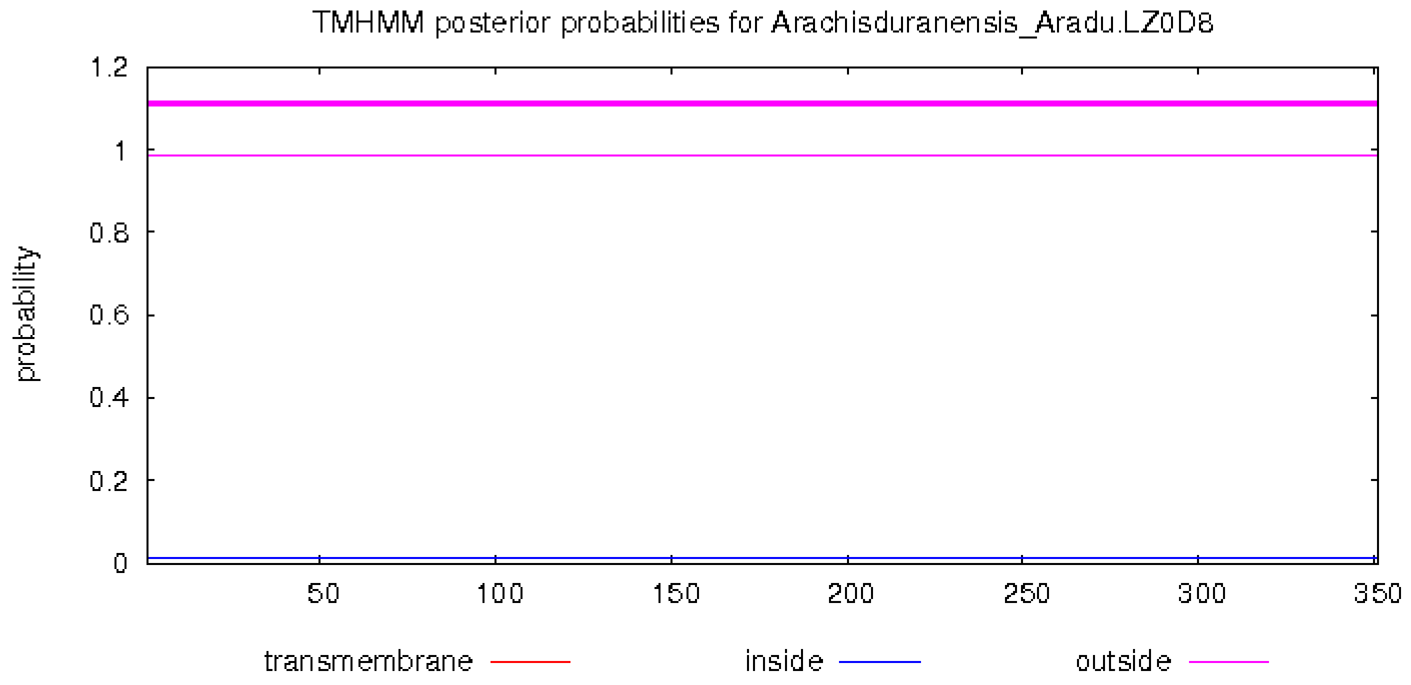

plot in postscript, script for making the plot in gnuplot, data for plot

---

```
# Arachisduranensis_Aradu.M7213 Length: 324
# Arachisduranensis_Aradu.M7213 Number of predicted TMHs:  0
# Arachisduranensis_Aradu.M7213 Exp number of AAs in TMHs: 0.0013
# Arachisduranensis_Aradu.M7213 Exp number, first 60 AAs:  0
# Arachisduranensis_Aradu.M7213 Total prob of N-in:        0.00298
Arachisduranensis_Aradu.M7213   TMHMM2.0        outside      1   324
```

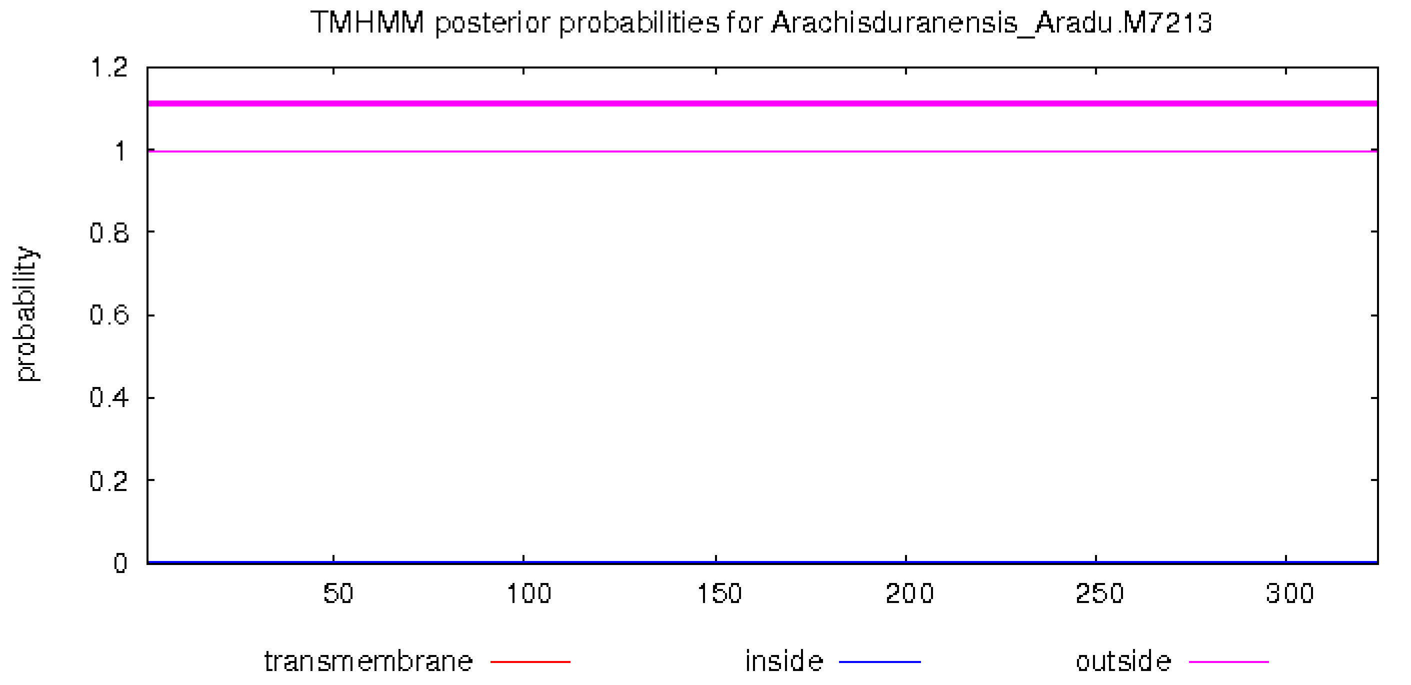

plot in postscript, script for making the plot in gnuplot, data for plot

---

```
# Arachisduranensis_Aradu.M8PFR Length: 425
# Arachisduranensis_Aradu.M8PFR Number of predicted TMHs:  0
# Arachisduranensis_Aradu.M8PFR Exp number of AAs in TMHs: 0.01185
# Arachisduranensis_Aradu.M8PFR Exp number, first 60 AAs:  0.00087
```

```
# Arachisduranensis_Aradu.M8PFR Total prob of N-in:        0.00457
Arachisduranensis_Aradu.M8PFR   TMHMM2.0        outside      1   425
```

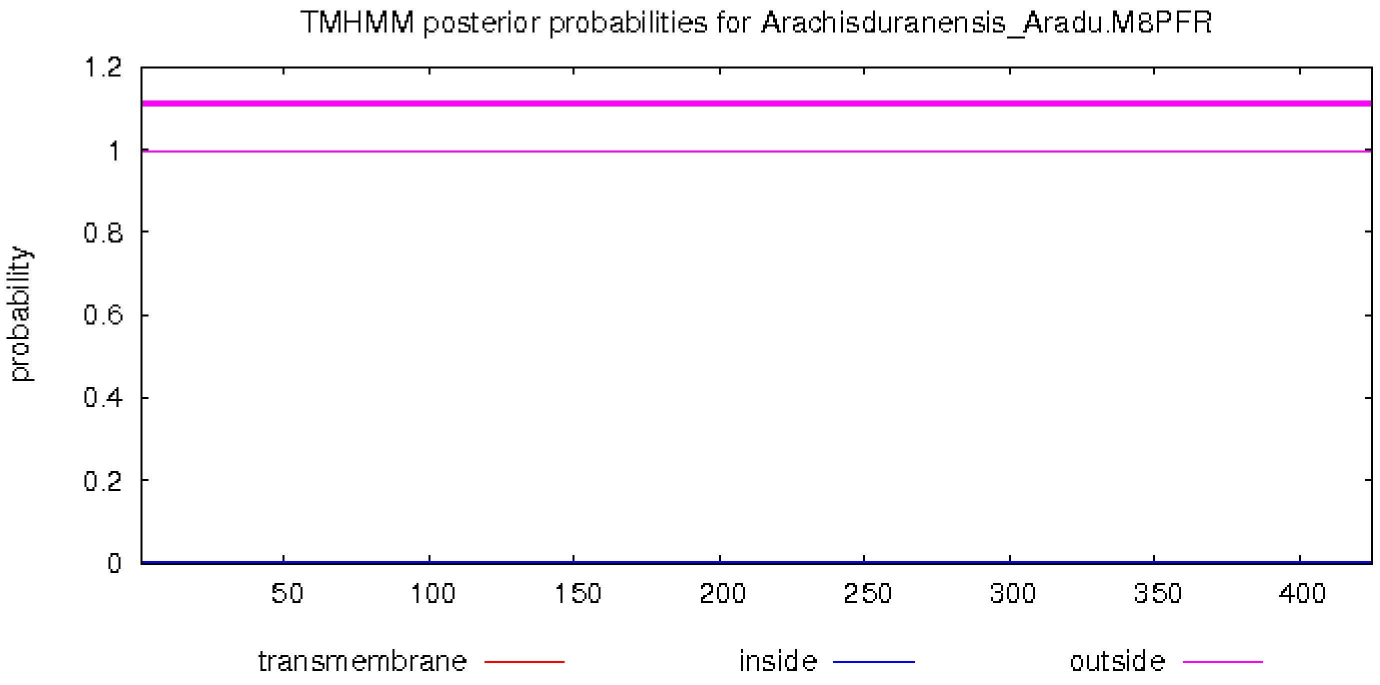

plot in postscript, script for making the plot in gnuplot, data for plot

---

```
# Arachisduranensis_Aradu.M9GL4 Length: 308
# Arachisduranensis_Aradu.M9GL4 Number of predicted TMHs:  0
# Arachisduranensis_Aradu.M9GL4 Exp number of AAs in TMHs: 0.00118
# Arachisduranensis_Aradu.M9GL4 Exp number, first 60 AAs:  0
# Arachisduranensis_Aradu.M9GL4 Total prob of N-in:        0.04432
Arachisduranensis_Aradu.M9GL4   TMHMM2.0        outside      1   308
```

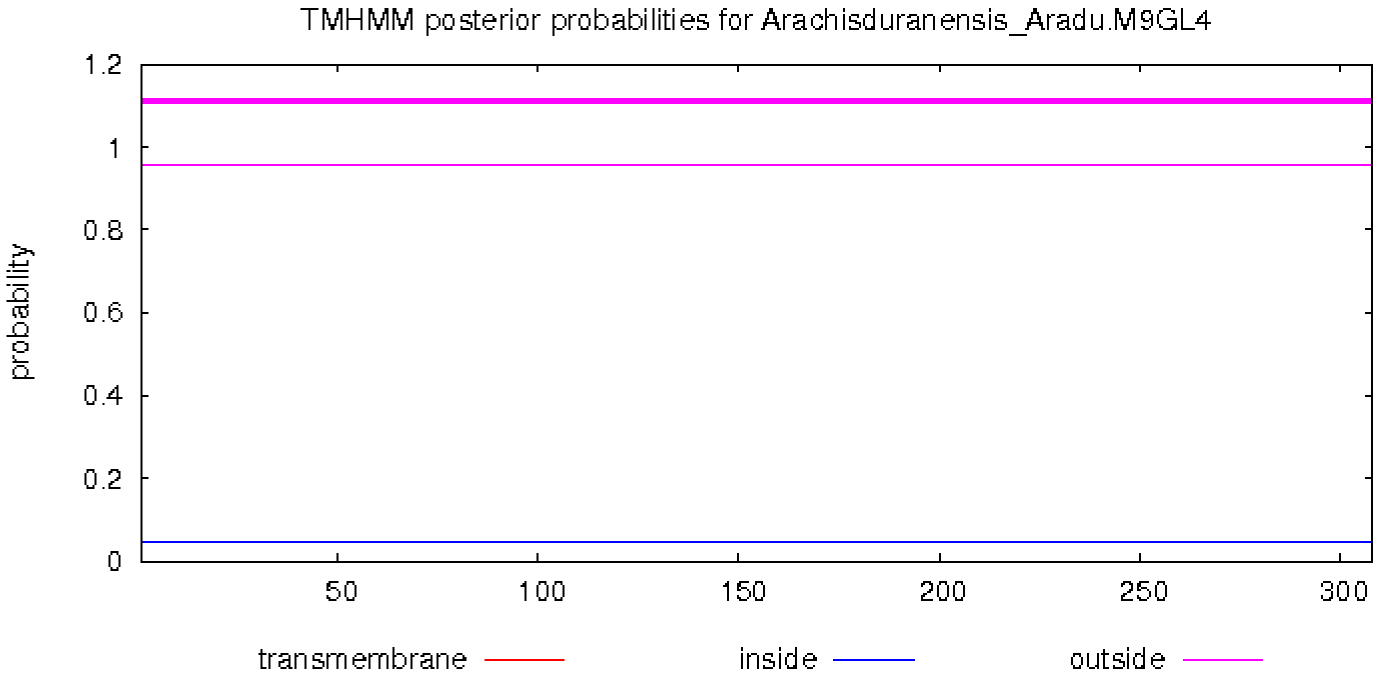

plot in postscript, script for making the plot in gnuplot, data for plot

---

```
# Arachisduranensis_Aradu.N8F6V Length: 363
# Arachisduranensis_Aradu.N8F6V Number of predicted TMHs:  0
# Arachisduranensis_Aradu.N8F6V Exp number of AAs in TMHs: 0.03935
# Arachisduranensis_Aradu.N8F6V Exp number, first 60 AAs:  0.03935
```

```
# Arachisduranensis_Aradu.N8F6V Total prob of N-in:        0.01846
Arachisduranensis_Aradu.N8F6V   TMHMM2.0        outside      1   363
```

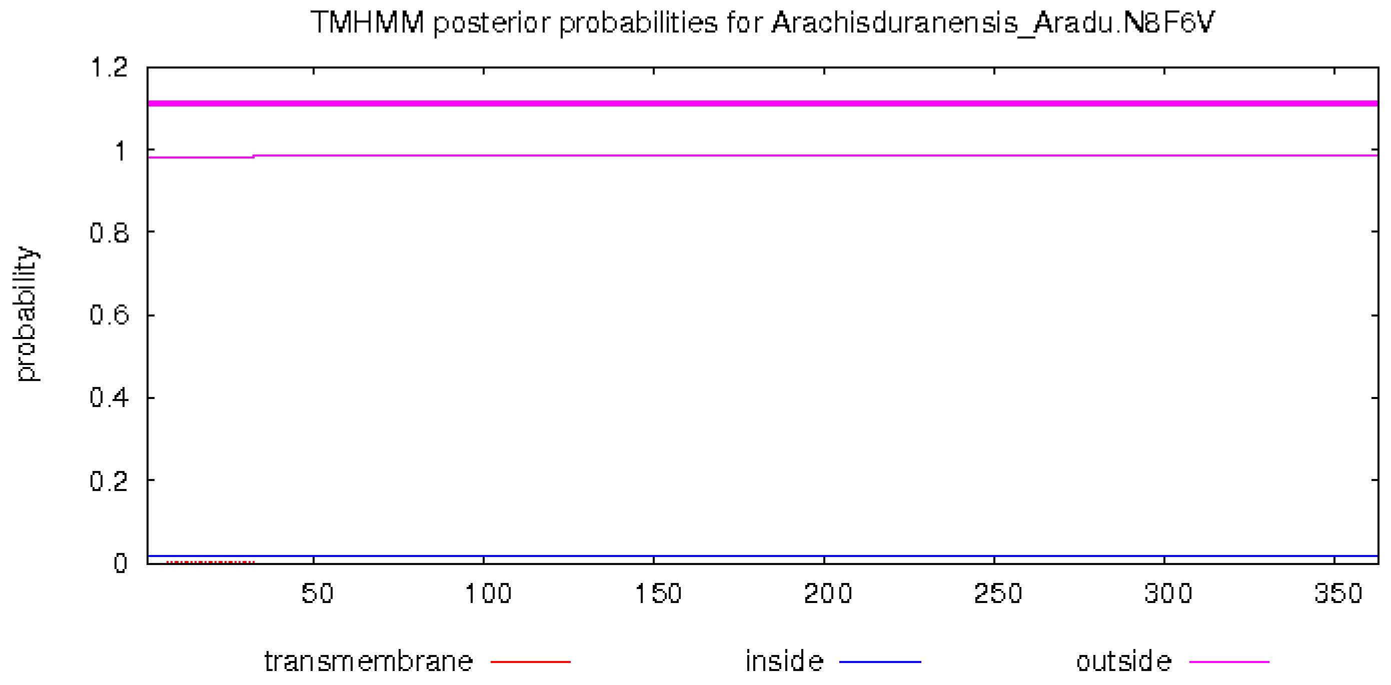

plot in postscript, script for making the plot in gnuplot, data for plot

---

```
# Arachisduranensis_Aradu.N8MU8 Length: 362
# Arachisduranensis_Aradu.N8MU8 Number of predicted TMHs:  0
# Arachisduranensis_Aradu.N8MU8 Exp number of AAs in TMHs: 0.00422
# Arachisduranensis_Aradu.N8MU8 Exp number, first 60 AAs:  0
# Arachisduranensis_Aradu.N8MU8 Total prob of N-in:        0.00208
Arachisduranensis_Aradu.N8MU8   TMHMM2.0        outside      1   362
```

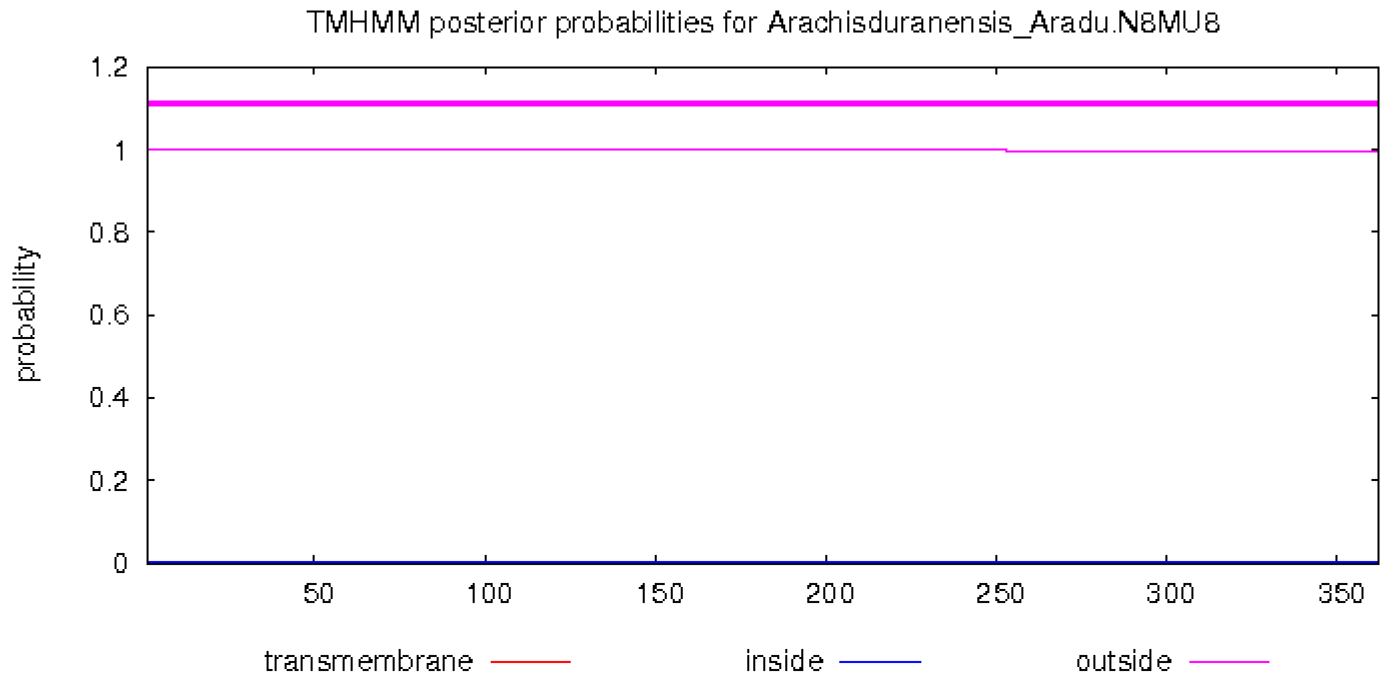

plot in postscript, script for making the plot in gnuplot, data for plot

---

```
# Arachisduranensis_Aradu.NEU1C Length: 255
# Arachisduranensis_Aradu.NEU1C Number of predicted TMHs:  0
# Arachisduranensis_Aradu.NEU1C Exp number of AAs in TMHs: 0.00043
# Arachisduranensis_Aradu.NEU1C Exp number, first 60 AAs:  0
```

```
# Arachisduranensis_Aradu.NEU1C Total prob of N-in:        0.02367
Arachisduranensis_Aradu.NEU1C   TMHMM2.0        outside      1   255
```

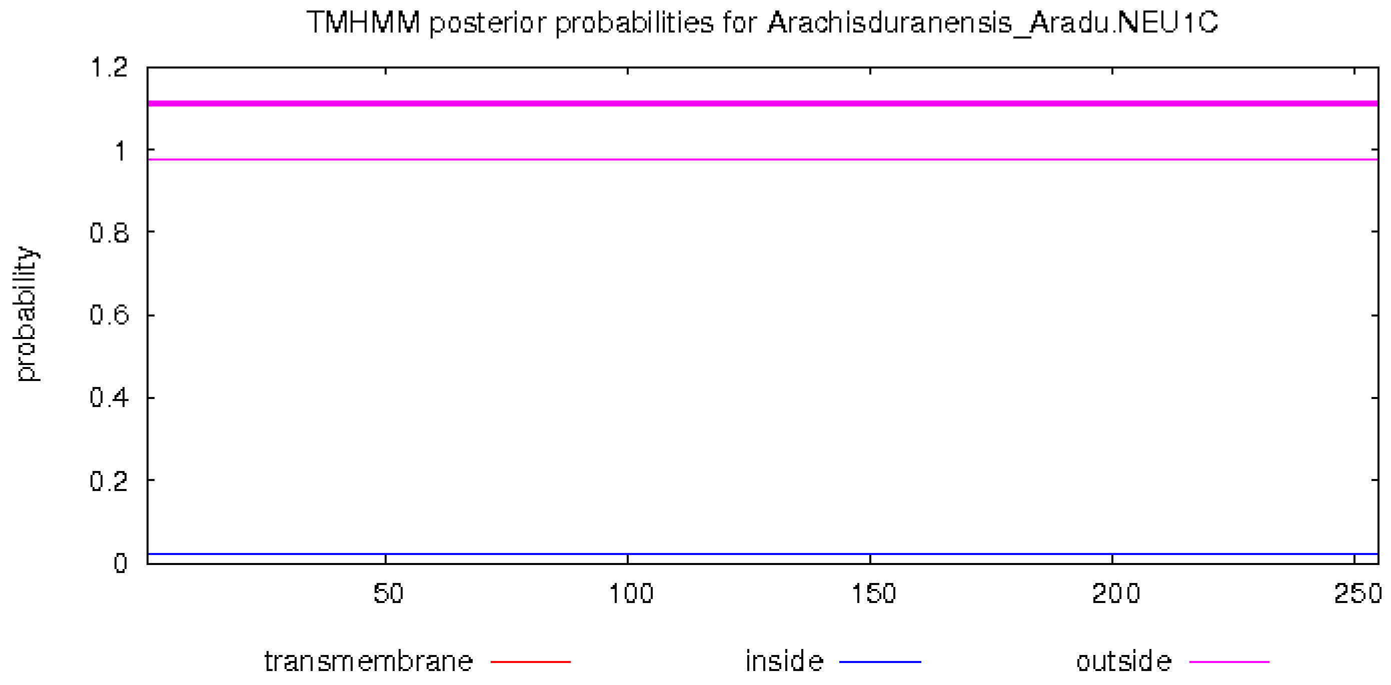

plot in postscript, script for making the plot in gnuplot, data for plot

---

```
# Arachisduranensis_Aradu.R9F07 Length: 463
# Arachisduranensis_Aradu.R9F07 Number of predicted TMHs:  0
# Arachisduranensis_Aradu.R9F07 Exp number of AAs in TMHs: 0.00085
# Arachisduranensis_Aradu.R9F07 Exp number, first 60 AAs:  0
# Arachisduranensis_Aradu.R9F07 Total prob of N-in:        0.00181
Arachisduranensis_Aradu.R9F07   TMHMM2.0        outside      1   463
```

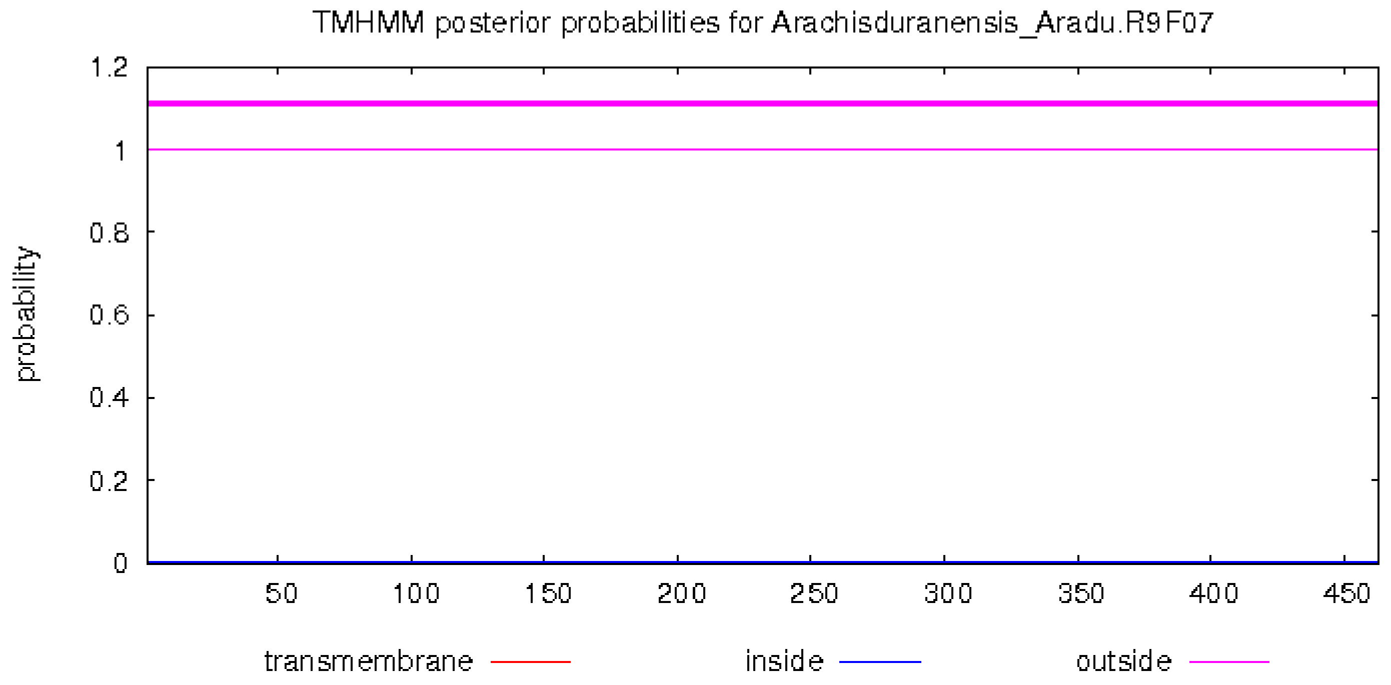

plot in postscript, script for making the plot in gnuplot, data for plot

---

```
# Arachisduranensis_Aradu.RP61F Length: 306
# Arachisduranensis_Aradu.RP61F Number of predicted TMHs:  0
# Arachisduranensis_Aradu.RP61F Exp number of AAs in TMHs: 0.15674
# Arachisduranensis_Aradu.RP61F Exp number, first 60 AAs:  0
```

```
# Arachisduranensis_Aradu.RP61F Total prob of N-in:        0.03073
Arachisduranensis_Aradu.RP61F   TMHMM2.0        outside      1   306
```

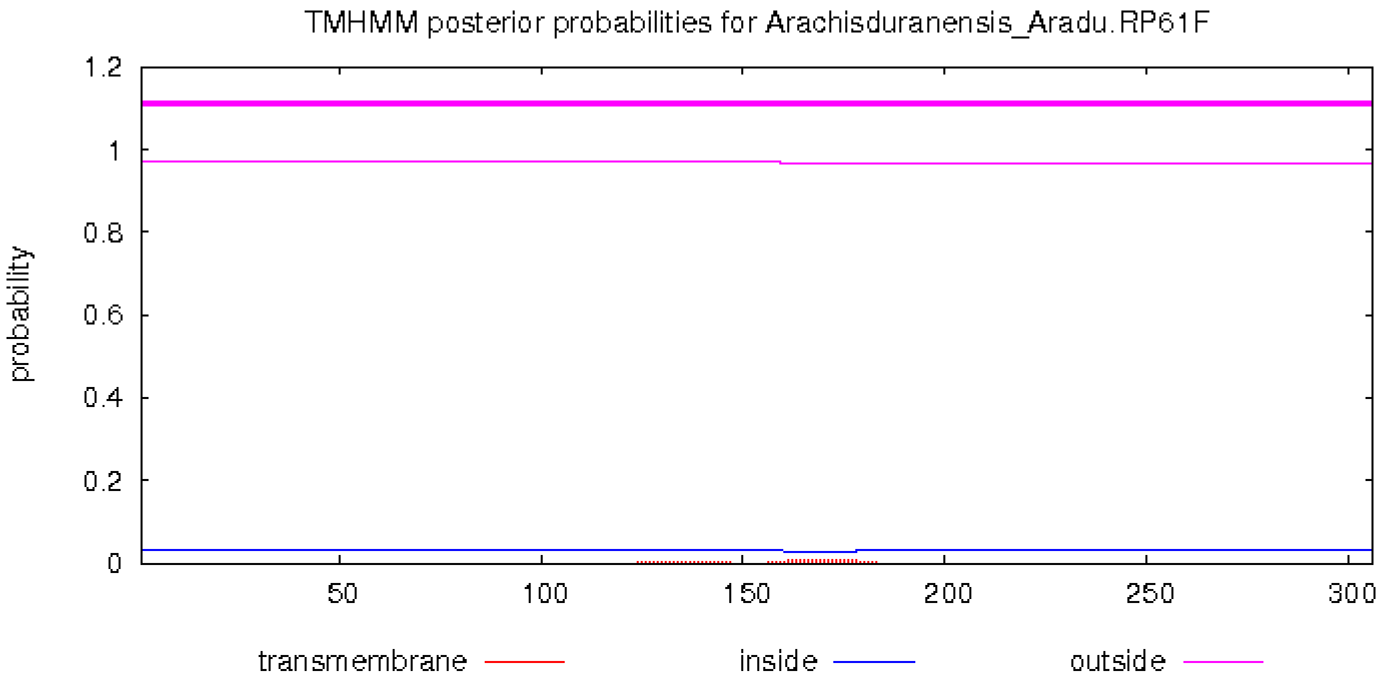

plot in postscript, script for making the plot in gnuplot, data for plot

---

```
# Arachisduranensis_Aradu.RRT20 Length: 394
# Arachisduranensis_Aradu.RRT20 Number of predicted TMHs:  0
# Arachisduranensis_Aradu.RRT20 Exp number of AAs in TMHs: 0.09104
# Arachisduranensis_Aradu.RRT20 Exp number, first 60 AAs:  0.0907000000000000001
# Arachisduranensis_Aradu.RRT20 Total prob of N-in:        0.00701
Arachisduranensis_Aradu.RRT20   TMHMM2.0        outside      1   394
```

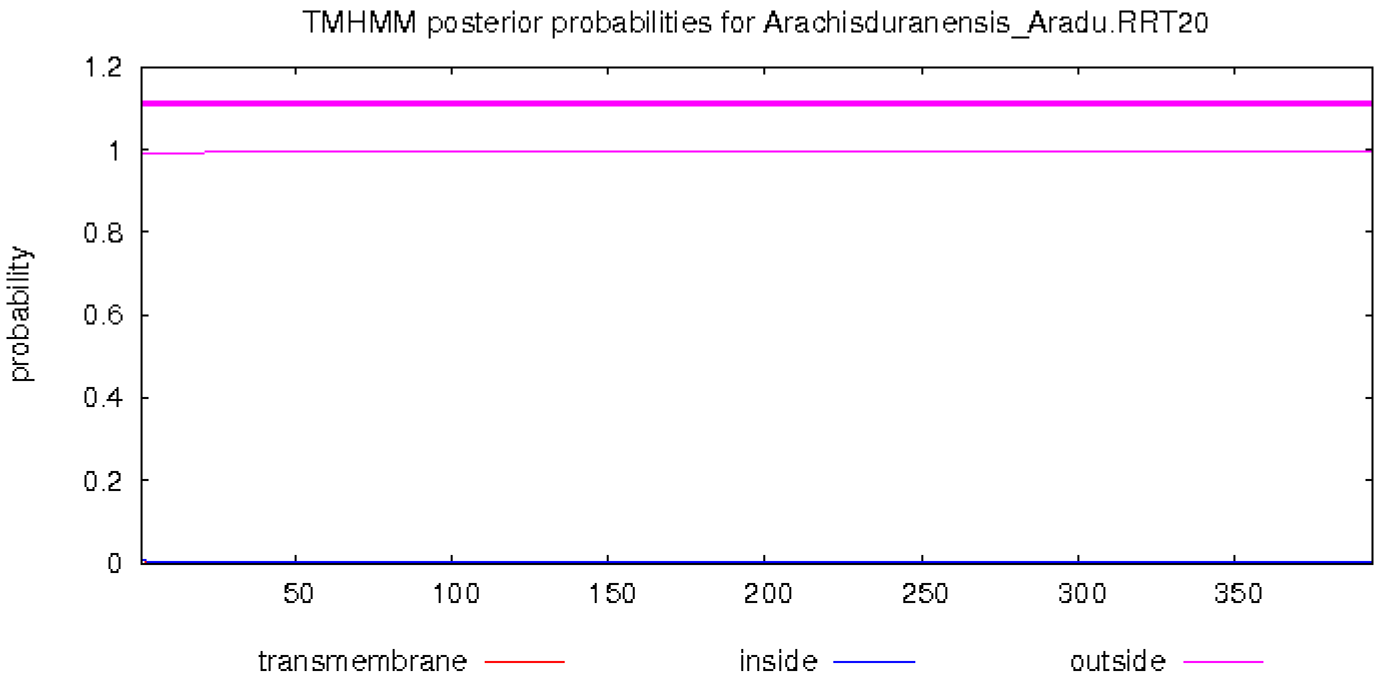

plot in postscript, script for making the plot in gnuplot, data for plot

---

```
# Arachisduranensis_Aradu.S13QQ Length: 344
# Arachisduranensis_Aradu.S13QQ Number of predicted TMHs:  0
# Arachisduranensis_Aradu.S13QQ Exp number of AAs in TMHs: 0.00624
# Arachisduranensis_Aradu.S13QQ Exp number, first 60 AAs:  0.00014
```

```
# Arachisduranensis_Aradu.S13QQ Total prob of N-in:        0.01923
Arachisduranensis_Aradu.S13QQ   TMHMM2.0        outside      1   344
```

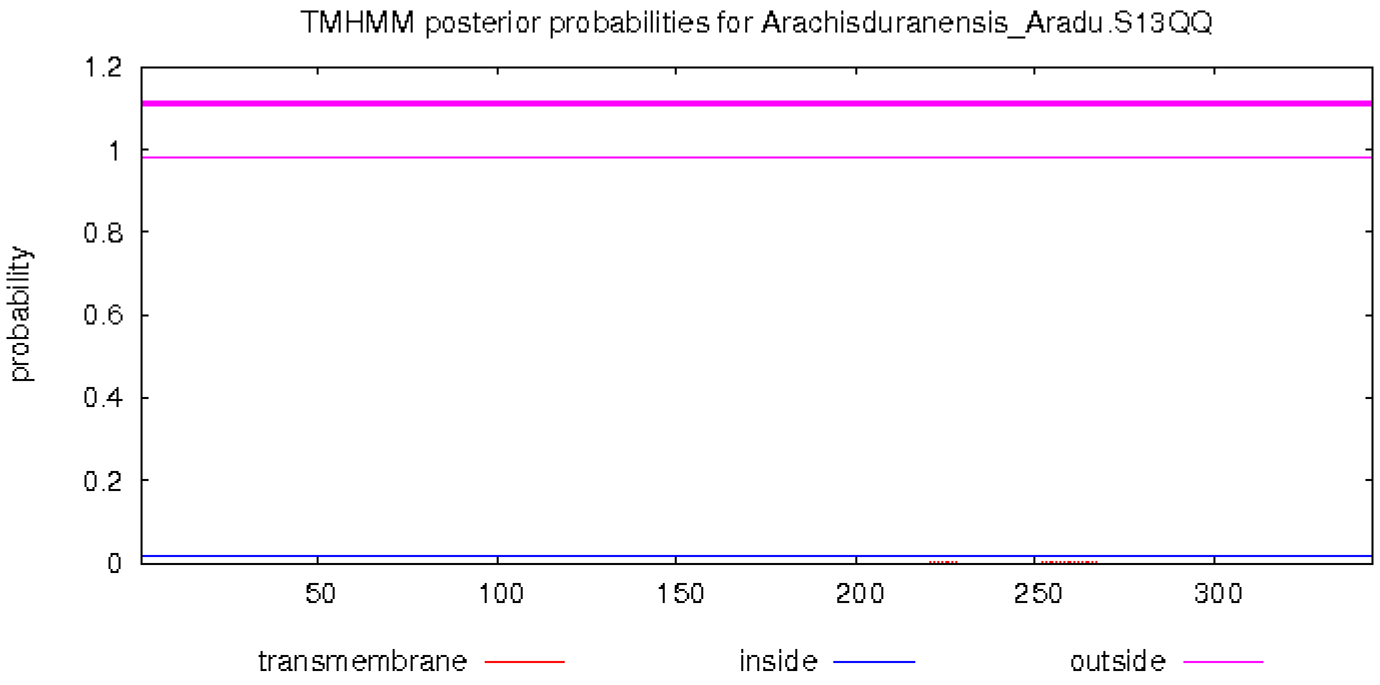

plot in postscript, script for making the plot in gnuplot, data for plot

---

```
# Arachisduranensis_Aradu.TGA11 Length: 315
# Arachisduranensis_Aradu.TGA11 Number of predicted TMHs:  0
# Arachisduranensis_Aradu.TGA11 Exp number of AAs in TMHs: 0.00094
# Arachisduranensis_Aradu.TGA11 Exp number, first 60 AAs:  0.00034
# Arachisduranensis_Aradu.TGA11 Total prob of N-in:        0.01161
Arachisduranensis_Aradu.TGA11   TMHMM2.0        outside      1   315
```

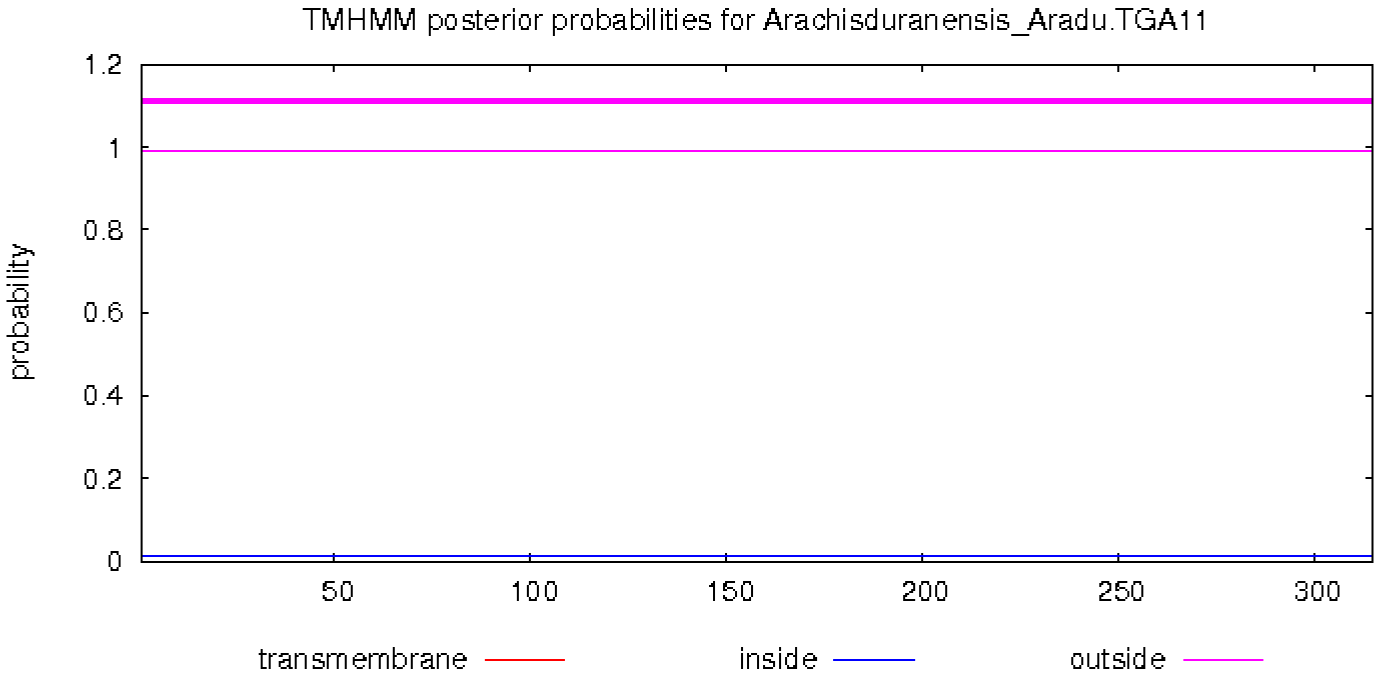

plot in postscript, script for making the plot in gnuplot, data for plot

---

```
# Arachisduranensis_Aradu.TI0Z7 Length: 322
# Arachisduranensis_Aradu.TI0Z7 Number of predicted TMHs:  0
# Arachisduranensis_Aradu.TI0Z7 Exp number of AAs in TMHs: 0.00055
# Arachisduranensis_Aradu.TI0Z7 Exp number, first 60 AAs:  0
```

```
# Arachisduranensis_Aradu.TI0Z7 Total prob of N-in:        0.02037
Arachisduranensis_Aradu.TI0Z7   TMHMM2.0        outside      1   322
```

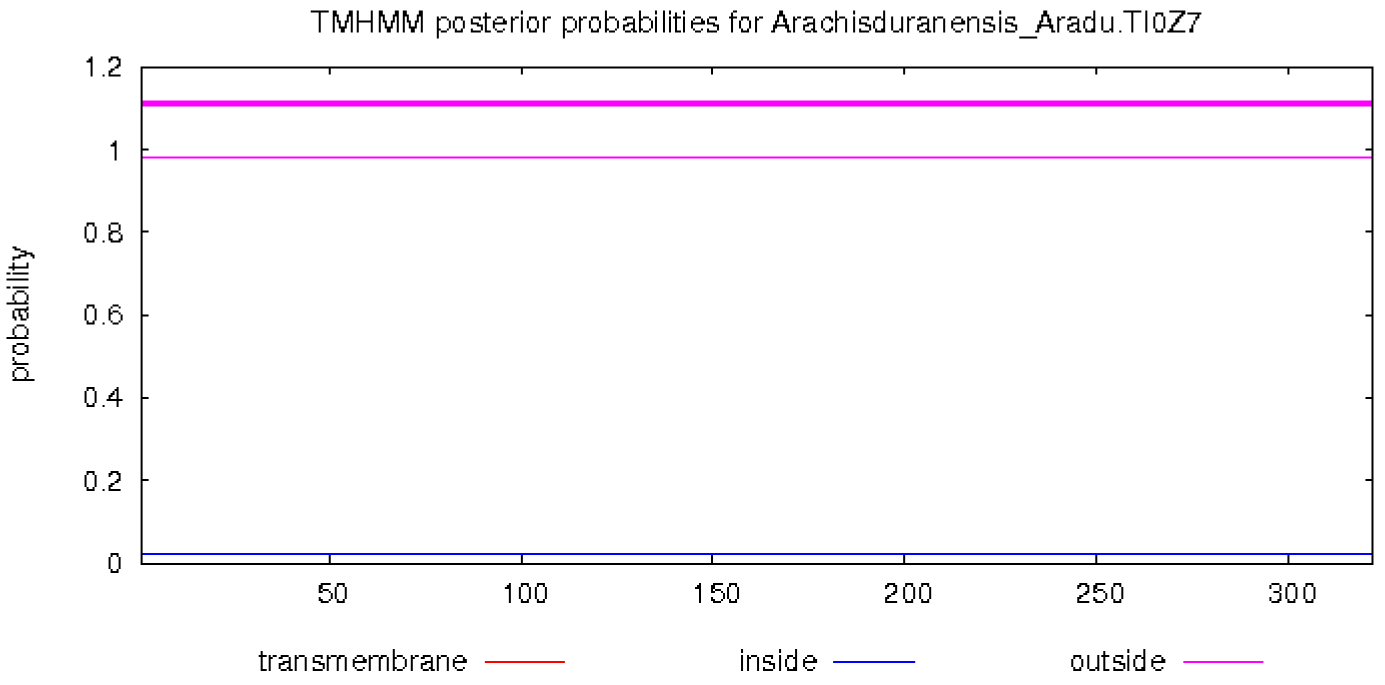

plot in postscript, script for making the plot in gnuplot, data for plot

---

```
# Arachisduranensis_Aradu.U974Q Length: 633
# Arachisduranensis_Aradu.U974Q Number of predicted TMHs:  2
# Arachisduranensis_Aradu.U974Q Exp number of AAs in TMHs: 40.88286
# Arachisduranensis_Aradu.U974Q Exp number, first 60 AAs:  0
# Arachisduranensis_Aradu.U974Q Total prob of N-in:        0.00005
Arachisduranensis_Aradu.U974Q   TMHMM2.0        outside      1   524
Arachisduranensis_Aradu.U974Q   TMHMM2.0        TMhelix    525   544
Arachisduranensis_Aradu.U974Q   TMHMM2.0        inside     545   611
Arachisduranensis_Aradu.U974Q   TMHMM2.0        TMhelix    612   631
Arachisduranensis_Aradu.U974Q   TMHMM2.0        outside    632   633
```

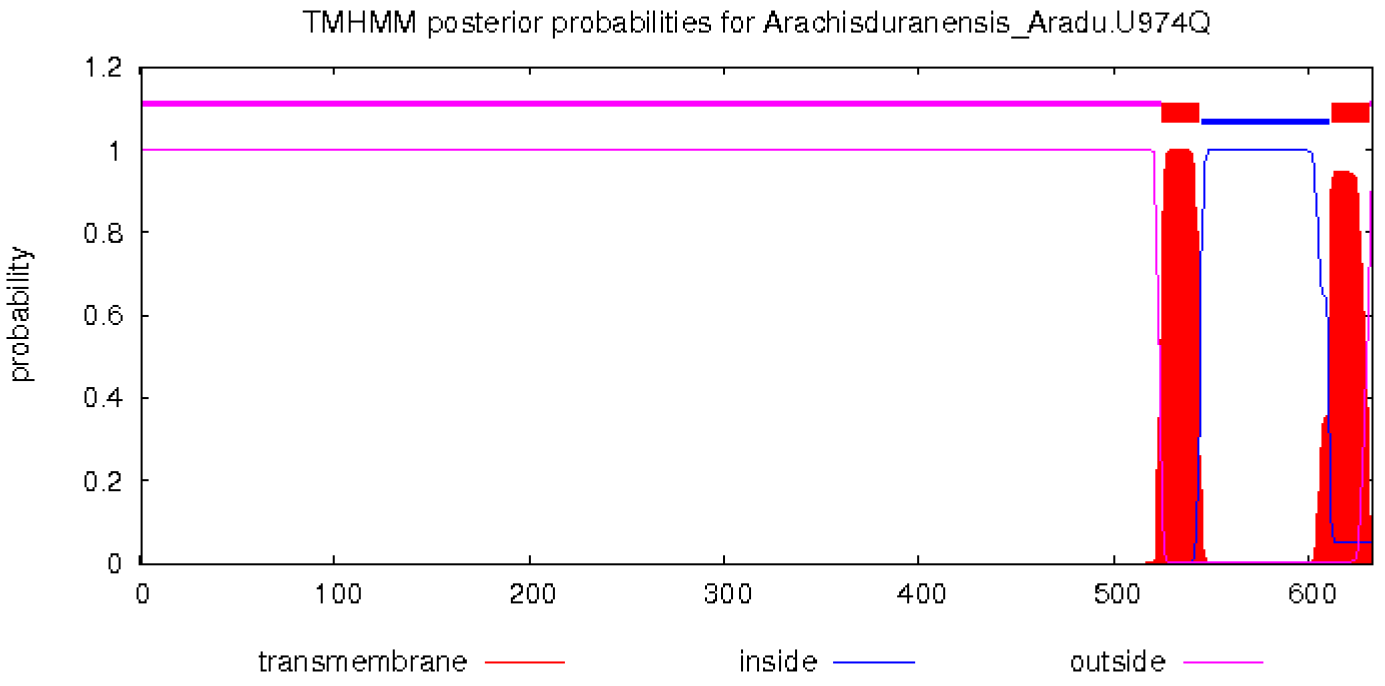

plot in postscript, script for making the plot in gnuplot, data for plot

---

```
# Arachisduranensis_Aradu.USH95 Length: 369
# Arachisduranensis_Aradu.USH95 Number of predicted TMHs:  0
# Arachisduranensis_Aradu.USH95 Exp number of AAs in TMHs: 0.00961
# Arachisduranensis_Aradu.USH95 Exp number, first 60 AAs:  0
# Arachisduranensis_Aradu.USH95 Total prob of N-in:        0.01618
Arachisduranensis_Aradu.USH95	TMHMM2.0	outside	     1   369
```

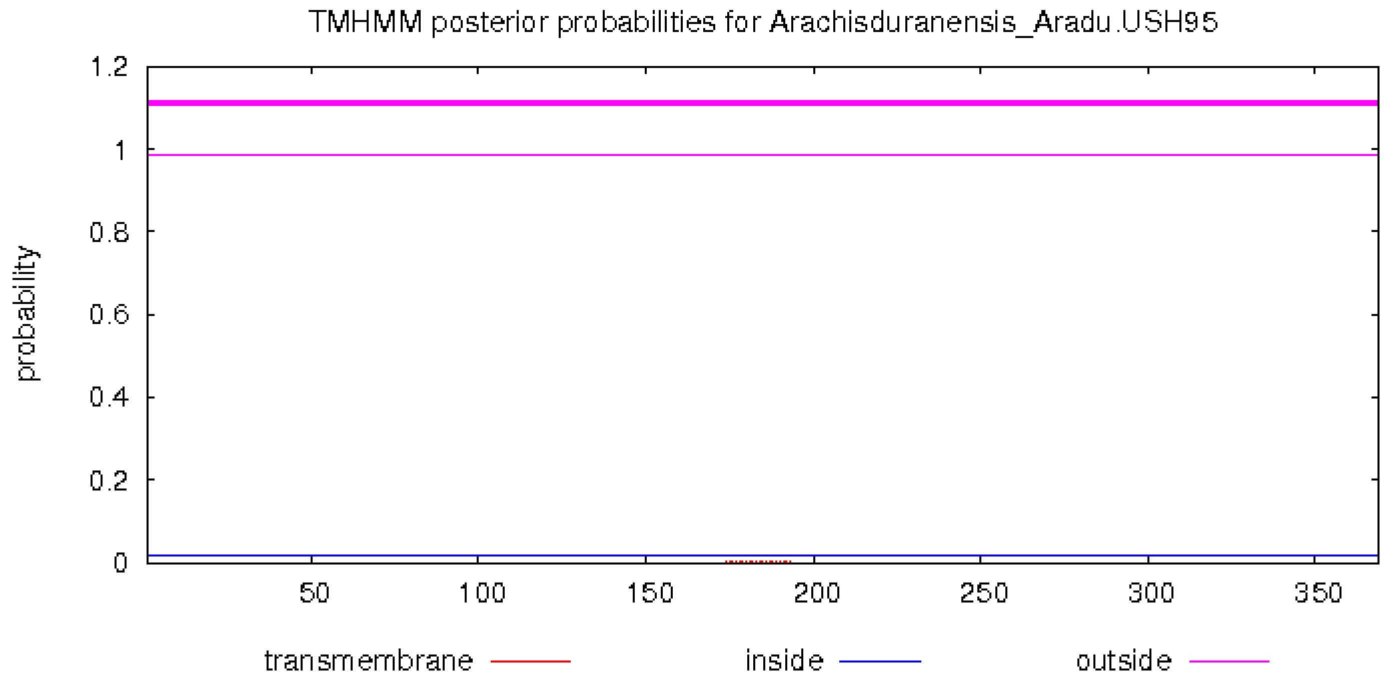

plot in postscript, script for making the plot in gnuplot, data for plot

---

```
# Arachisduranensis_Aradu.UXN6T Length: 304
# Arachisduranensis_Aradu.UXN6T Number of predicted TMHs:  0
# Arachisduranensis_Aradu.UXN6T Exp number of AAs in TMHs: 0
# Arachisduranensis_Aradu.UXN6T Exp number, first 60 AAs:  0
# Arachisduranensis_Aradu.UXN6T Total prob of N-in:        0.00144
Arachisduranensis_Aradu.UXN6T	TMHMM2.0	outside	     1   304
```

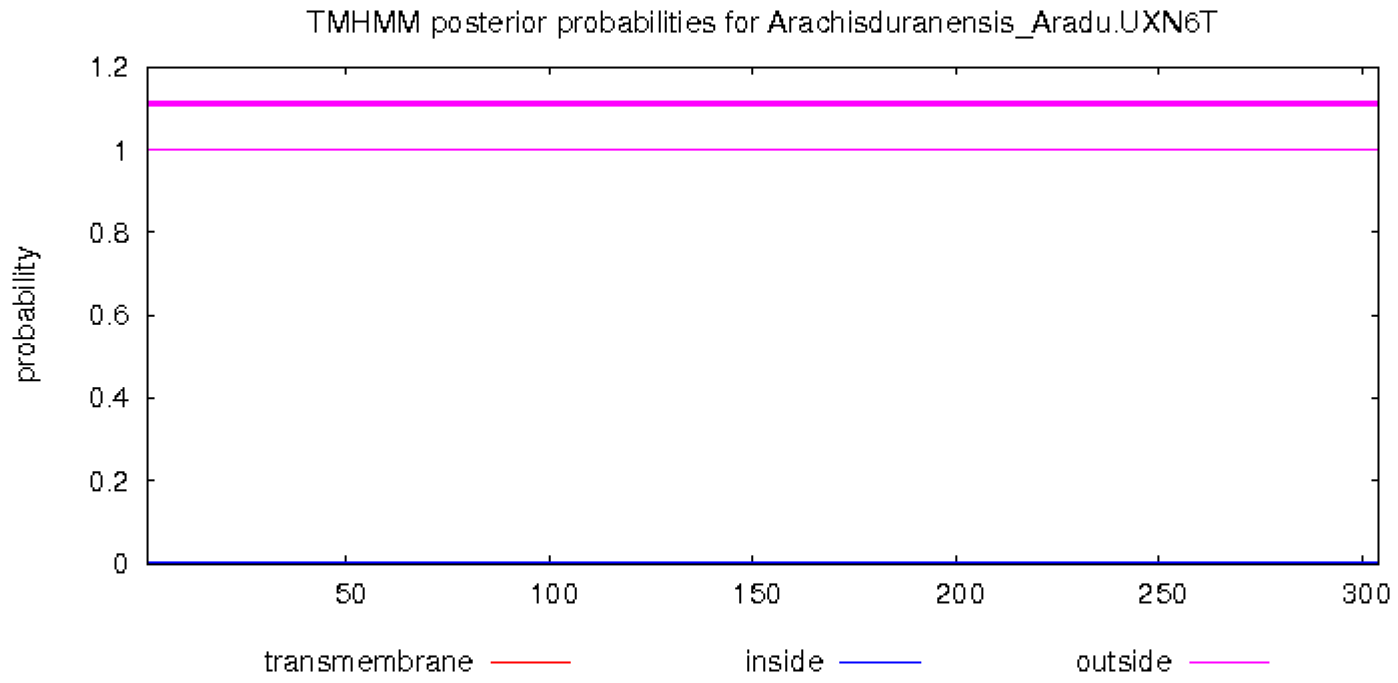

plot in postscript, script for making the plot in gnuplot, data for plot

---

```
# Arachisduranensis_Aradu.VUC67 Length: 321
# Arachisduranensis_Aradu.VUC67 Number of predicted TMHs:  0
# Arachisduranensis_Aradu.VUC67 Exp number of AAs in TMHs: 0
# Arachisduranensis_Aradu.VUC67 Exp number, first 60 AAs:  0
# Arachisduranensis_Aradu.VUC67 Total prob of N-in:        0.00614
Arachisduranensis_Aradu.VUC67   TMHMM2.0        outside      1   321
```

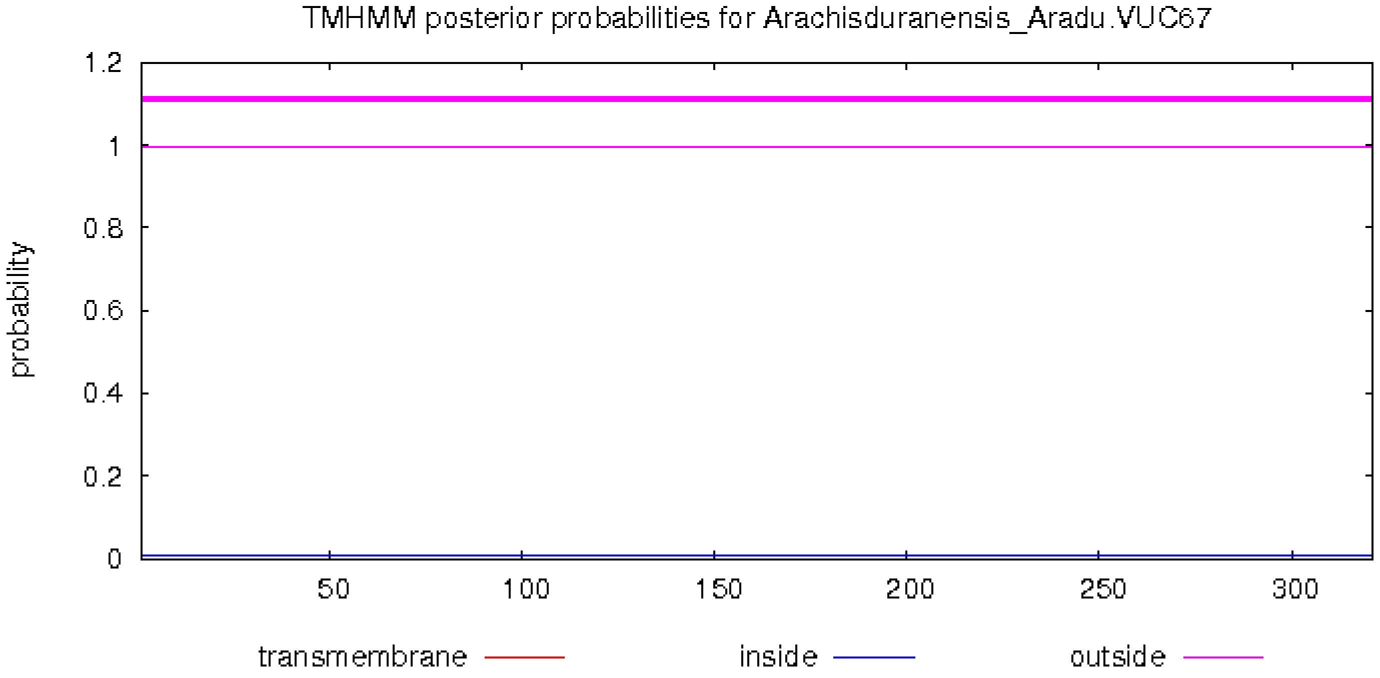

plot in postscript, script for making the plot in gnuplot, data for plot

---

```
# Arachisduranensis_Aradu.W3GLH Length: 679
# Arachisduranensis_Aradu.W3GLH Number of predicted TMHs:  2
# Arachisduranensis_Aradu.W3GLH Exp number of AAs in TMHs: 41.33532
# Arachisduranensis_Aradu.W3GLH Exp number, first 60 AAs:  0
# Arachisduranensis_Aradu.W3GLH Total prob of N-in:        0.00041
Arachisduranensis_Aradu.W3GLH   TMHMM2.0        outside      1   580
Arachisduranensis_Aradu.W3GLH   TMHMM2.0        TMhelix    581   603
Arachisduranensis_Aradu.W3GLH   TMHMM2.0        inside     604   656
Arachisduranensis_Aradu.W3GLH   TMHMM2.0        TMhelix    657   676
Arachisduranensis_Aradu.W3GLH   TMHMM2.0        outside    677   679
```

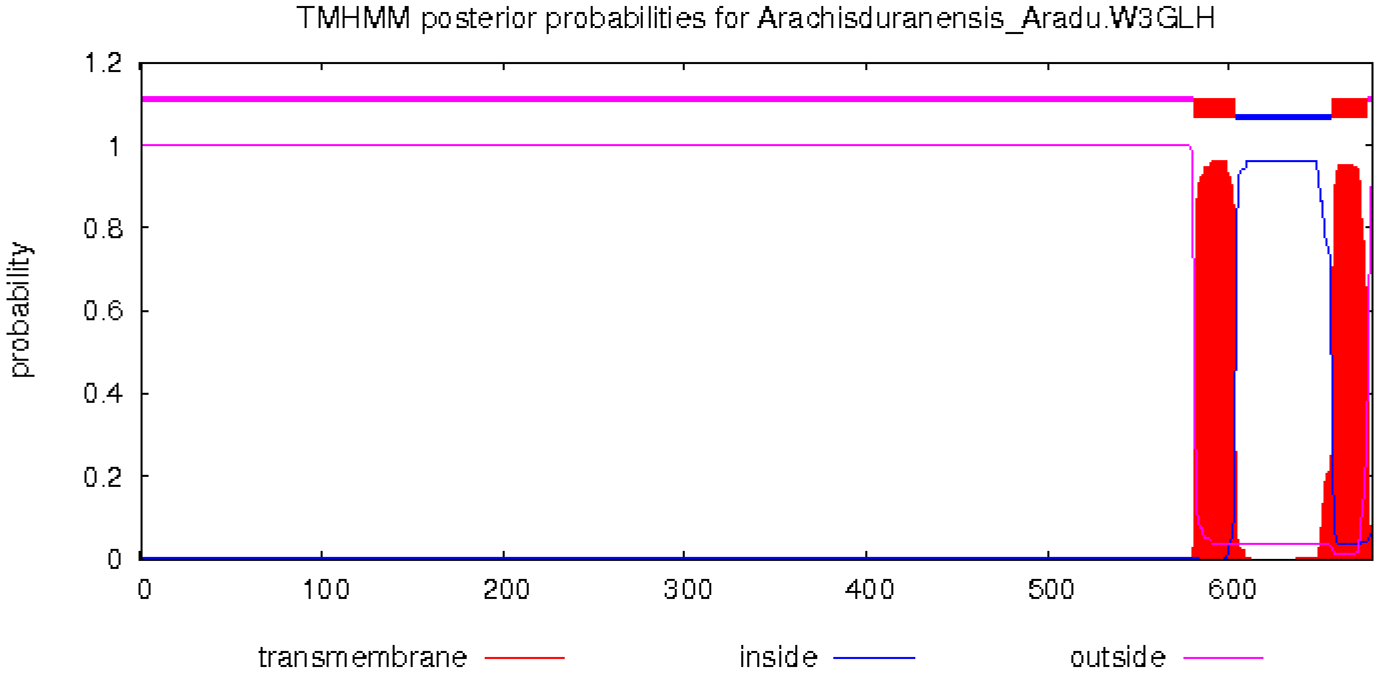

plot in postscript, script for making the plot in gnuplot, data for plot

```
# Arachisduranensis_Aradu.WIT0W Length: 346
# Arachisduranensis_Aradu.WIT0W Number of predicted TMHs:  0
# Arachisduranensis_Aradu.WIT0W Exp number of AAs in TMHs: 0
# Arachisduranensis_Aradu.WIT0W Exp number, first 60 AAs:  0
# Arachisduranensis_Aradu.WIT0W Total prob of N-in:        0.00082
Arachisduranensis_Aradu.WIT0W   TMHMM2.0        outside      1   346
```

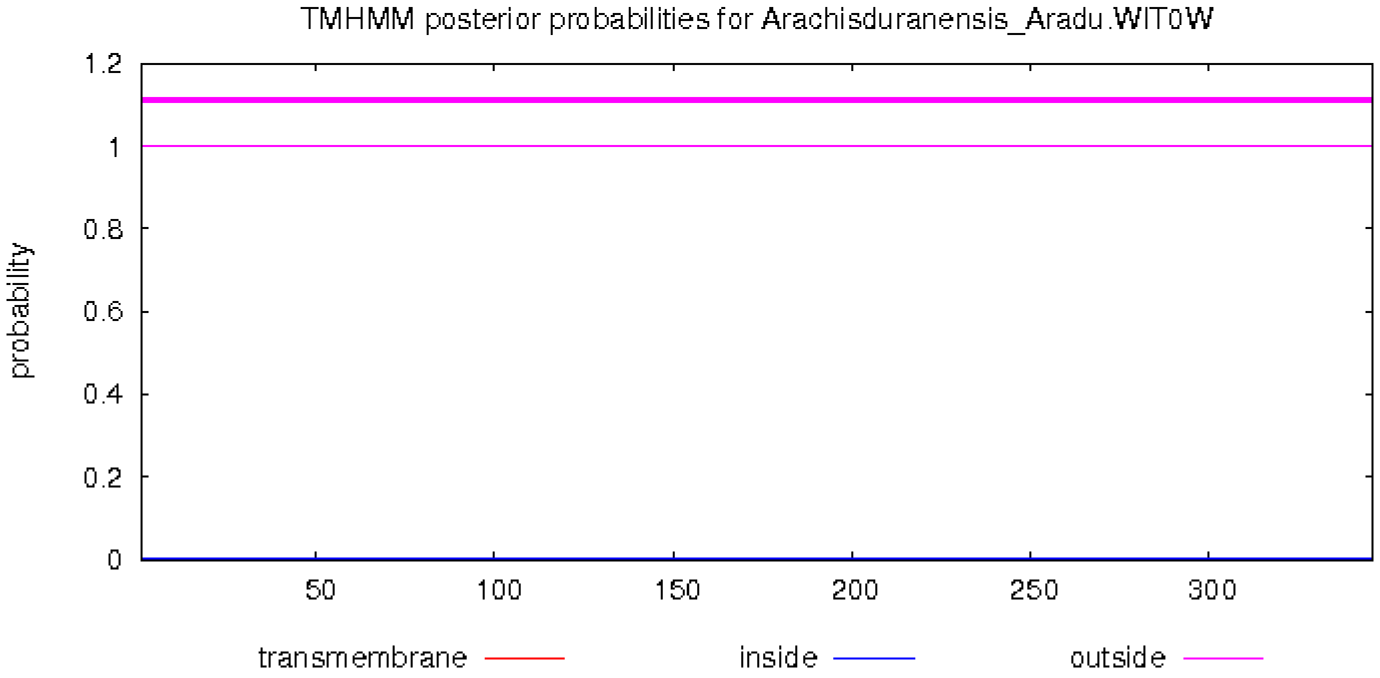

plot in postscript, script for making the plot in gnuplot, data for plot

```
# Arachisduranensis_Aradu.WS3DN Length: 213
# Arachisduranensis_Aradu.WS3DN Number of predicted TMHs:  0
# Arachisduranensis_Aradu.WS3DN Exp number of AAs in TMHs: 0
# Arachisduranensis_Aradu.WS3DN Exp number, first 60 AAs:  0
# Arachisduranensis_Aradu.WS3DN Total prob of N-in:        0.03081
Arachisduranensis_Aradu.WS3DN   TMHMM2.0        outside      1   213
```

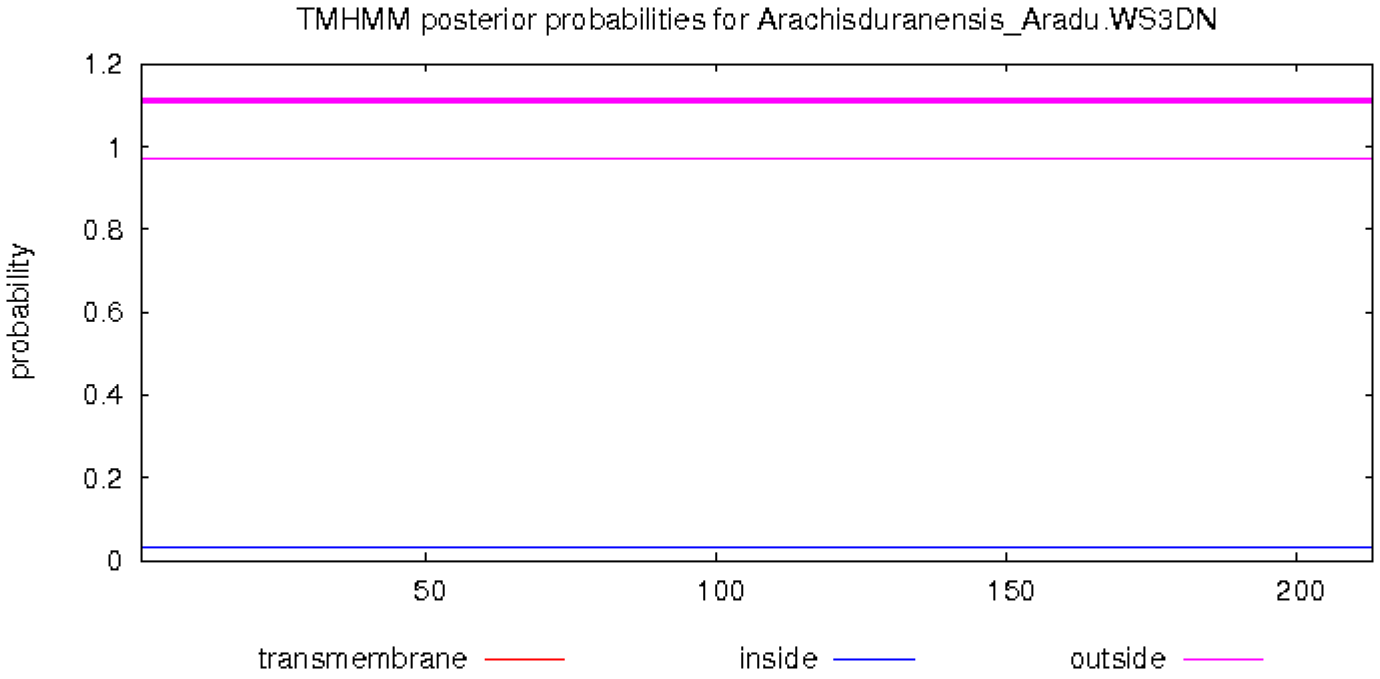

plot in postscript, script for making the plot in gnuplot, data for plot

---

```
# Arachisduranensis_Aradu.XE8WZ Length: 300
# Arachisduranensis_Aradu.XE8WZ Number of predicted TMHs:  0
# Arachisduranensis_Aradu.XE8WZ Exp number of AAs in TMHs: 0.690510000000000001
# Arachisduranensis_Aradu.XE8WZ Exp number, first 60 AAs:  0.69031
# Arachisduranensis_Aradu.XE8WZ Total prob of N-in:        0.20509
Arachisduranensis_Aradu.XE8WZ   TMHMM2.0        outside      1   300
```

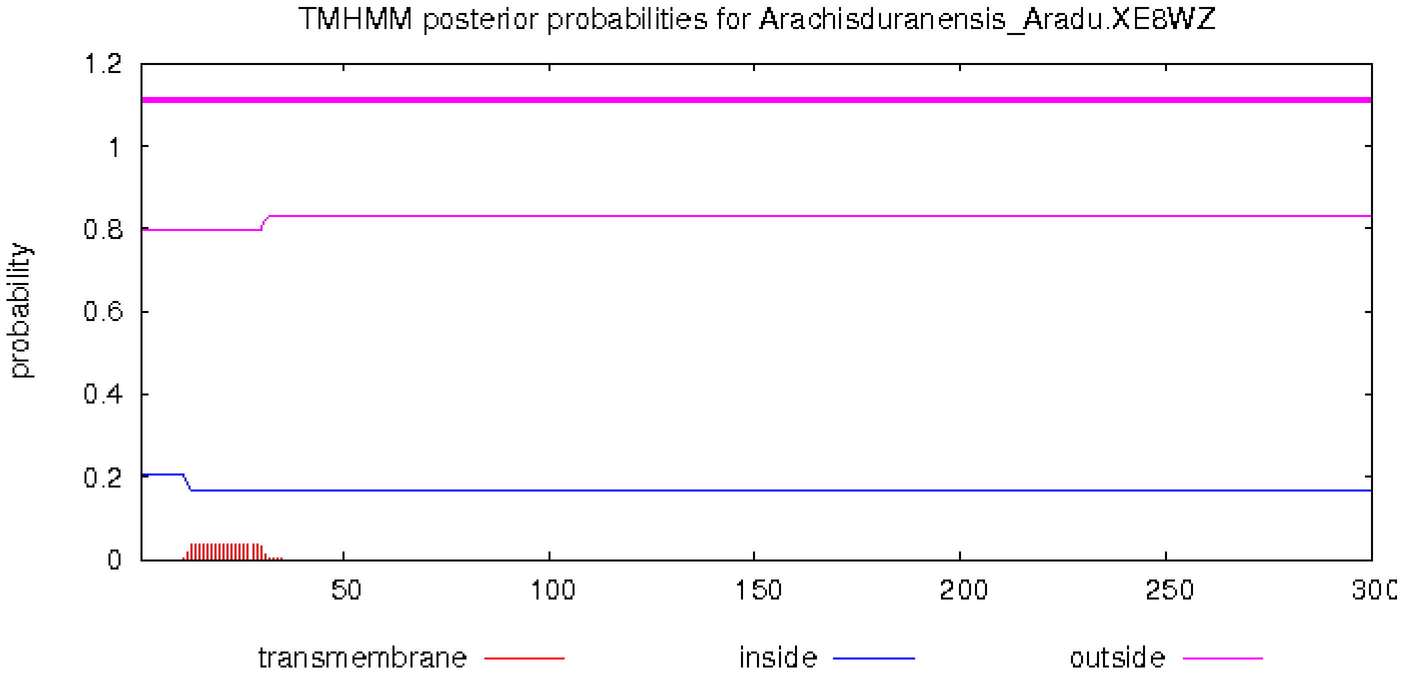

plot in postscript, script for making the plot in gnuplot, data for plot

---

```
# Arachisduranensis_Aradu.XJF09 Length: 396
# Arachisduranensis_Aradu.XJF09 Number of predicted TMHs:  0
# Arachisduranensis_Aradu.XJF09 Exp number of AAs in TMHs: 0
# Arachisduranensis_Aradu.XJF09 Exp number, first 60 AAs:  0
# Arachisduranensis_Aradu.XJF09 Total prob of N-in:        0.00880
Arachisduranensis_Aradu.XJF09   TMHMM2.0        outside      1   396
```

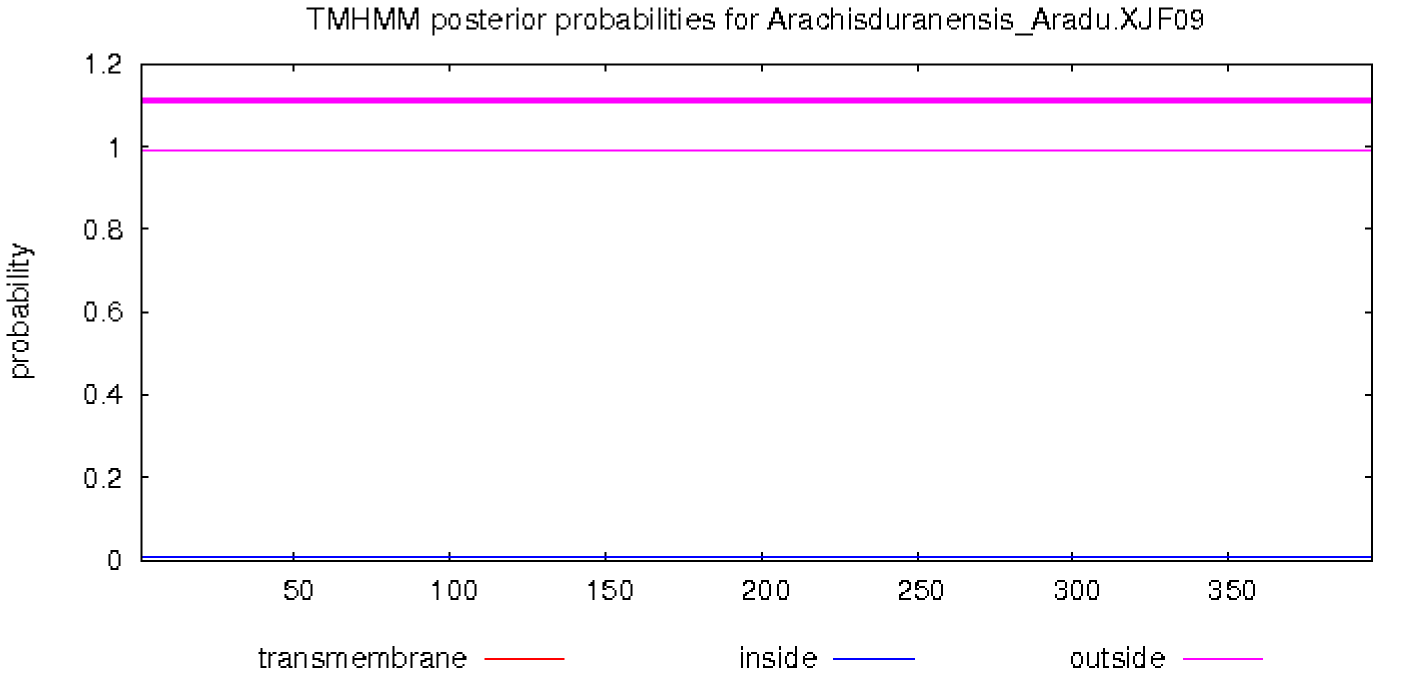

plot in postscript, script for making the plot in gnuplot, data for plot

```
# Arachisduranensis_Aradu.XQ4VP Length: 167
# Arachisduranensis_Aradu.XQ4VP Number of predicted TMHs:  0
# Arachisduranensis_Aradu.XQ4VP Exp number of AAs in TMHs: 0
# Arachisduranensis_Aradu.XQ4VP Exp number, first 60 AAs:  0
# Arachisduranensis_Aradu.XQ4VP Total prob of N-in:        0.00507
Arachisduranensis_Aradu.XQ4VP   TMHMM2.0        outside      1   167
```

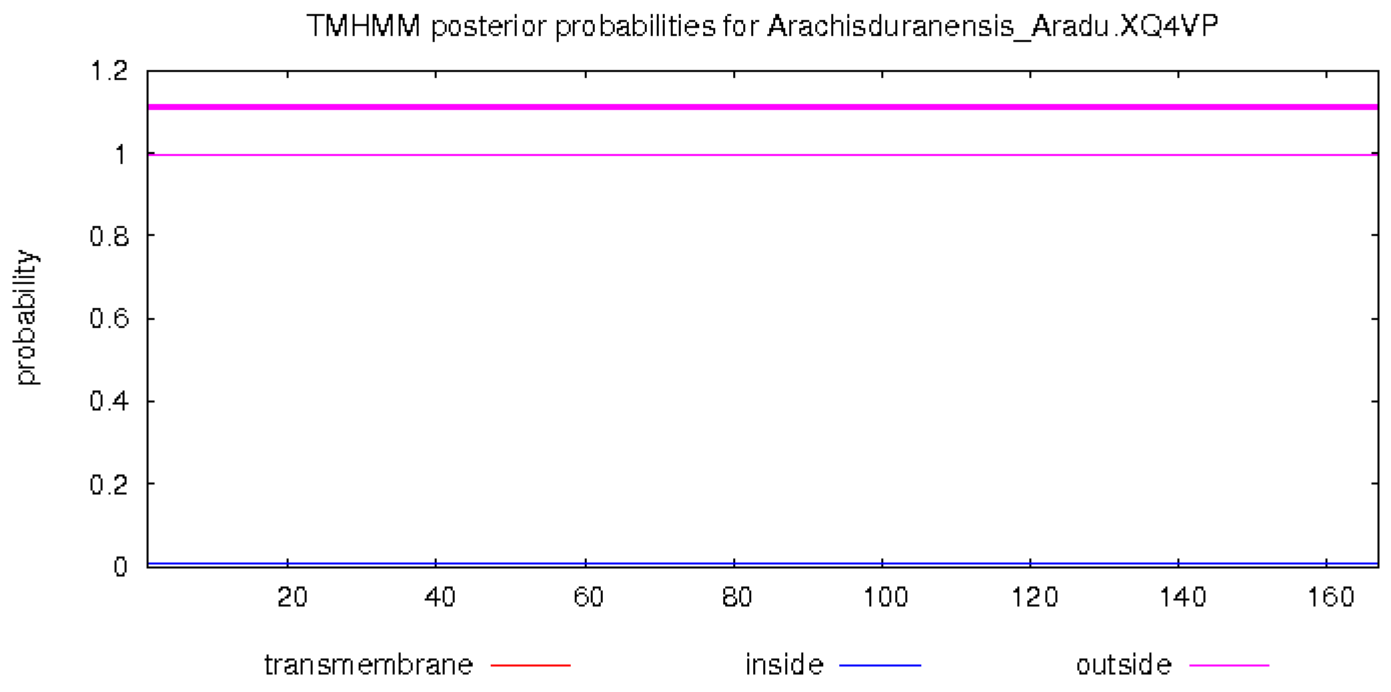

plot in postscript, script for making the plot in gnuplot, data for plot

```
# Arachisduranensis_Aradu.Y1DM8 Length: 396
# Arachisduranensis_Aradu.Y1DM8 Number of predicted TMHs:  0
# Arachisduranensis_Aradu.Y1DM8 Exp number of AAs in TMHs: 0.0002
# Arachisduranensis_Aradu.Y1DM8 Exp number, first 60 AAs:  0.0002
# Arachisduranensis_Aradu.Y1DM8 Total prob of N-in:        0.00575
Arachisduranensis_Aradu.Y1DM8   TMHMM2.0        outside      1   396
```

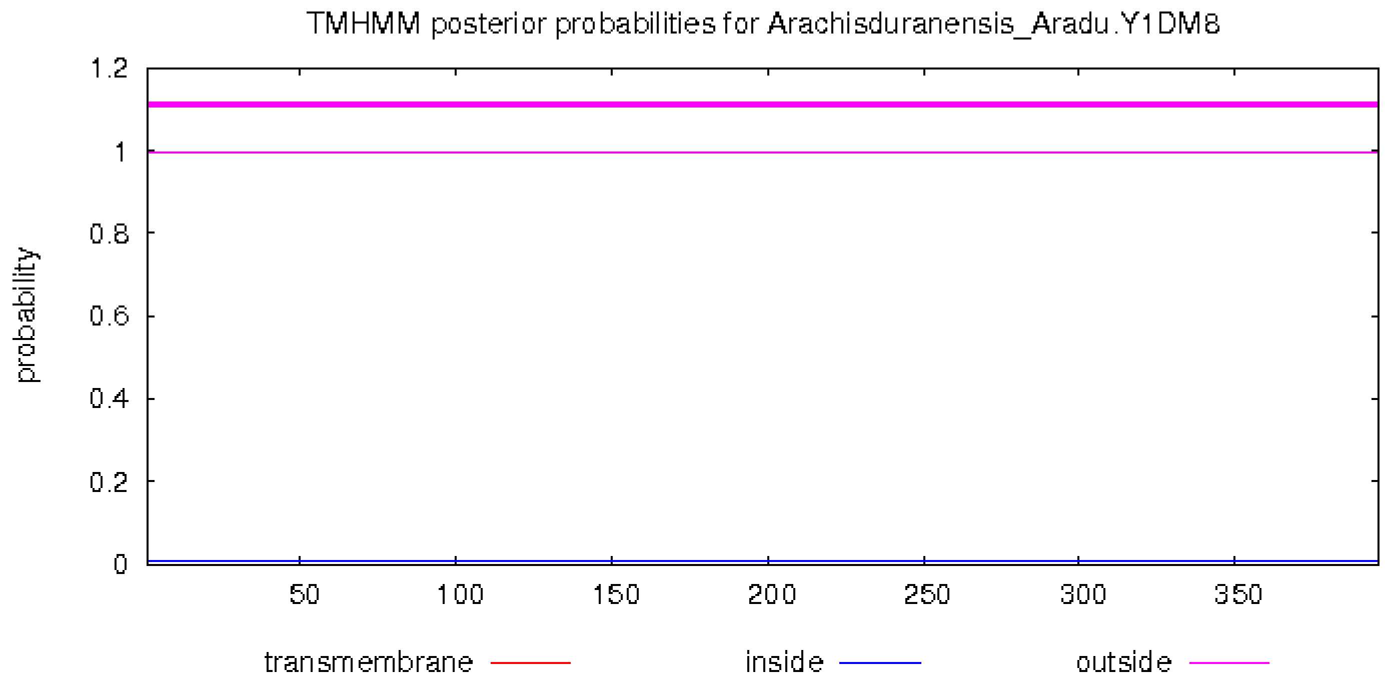

plot in postscript, script for making the plot in gnuplot, data for plot

---

```
# Arachisduranensis_Aradu.Y9JNS Length: 369
# Arachisduranensis_Aradu.Y9JNS Number of predicted TMHs:  0
# Arachisduranensis_Aradu.Y9JNS Exp number of AAs in TMHs: 0.01061
# Arachisduranensis_Aradu.Y9JNS Exp number, first 60 AAs:  0.00643
# Arachisduranensis_Aradu.Y9JNS Total prob of N-in:        0.00378
Arachisduranensis_Aradu.Y9JNS   TMHMM2.0        outside      1   369
```

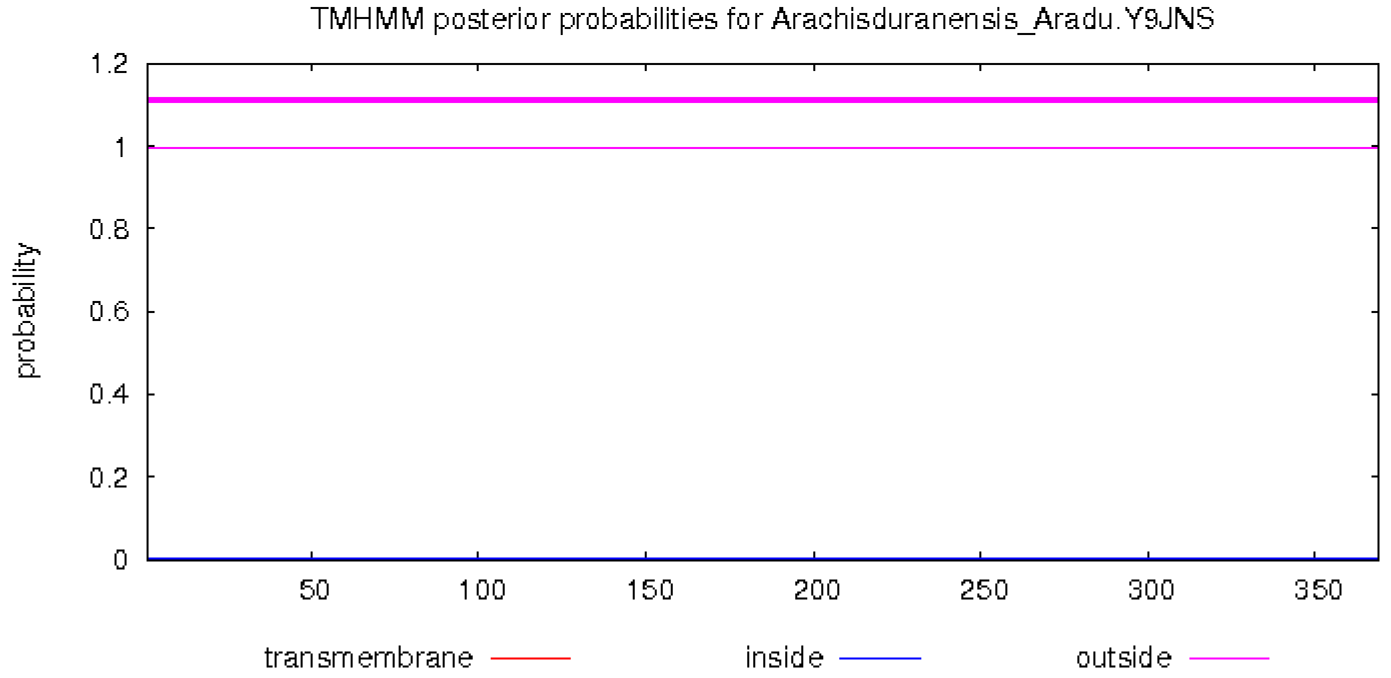

plot in postscript, script for making the plot in gnuplot, data for plot

---

```
# Arachisduranensis_Aradu.YFQ3P Length: 260
# Arachisduranensis_Aradu.YFQ3P Number of predicted TMHs:  0
# Arachisduranensis_Aradu.YFQ3P Exp number of AAs in TMHs: 0.0002
# Arachisduranensis_Aradu.YFQ3P Exp number, first 60 AAs:  0.0002
# Arachisduranensis_Aradu.YFQ3P Total prob of N-in:        0.00507
Arachisduranensis_Aradu.YFQ3P   TMHMM2.0        outside      1   260
```

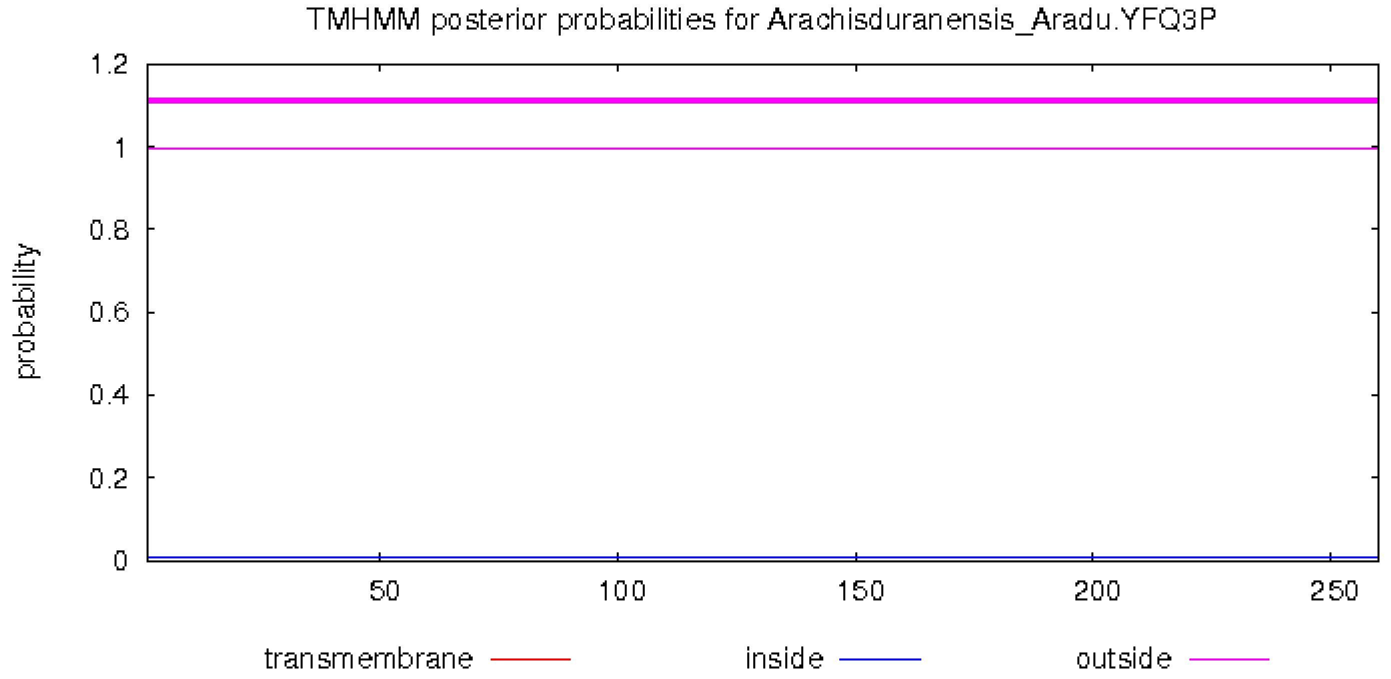

plot in postscript, script for making the plot in gnuplot, data for plot

```
# Arachisduranensis_Aradu.YIQ80 Length: 349
# Arachisduranensis_Aradu.YIQ80 Number of predicted TMHs:  0
# Arachisduranensis_Aradu.YIQ80 Exp number of AAs in TMHs: 0.0006
# Arachisduranensis_Aradu.YIQ80 Exp number, first 60 AAs:  0
# Arachisduranensis_Aradu.YIQ80 Total prob of N-in:        0.00698
Arachisduranensis_Aradu.YIQ80   TMHMM2.0        outside      1   349
```

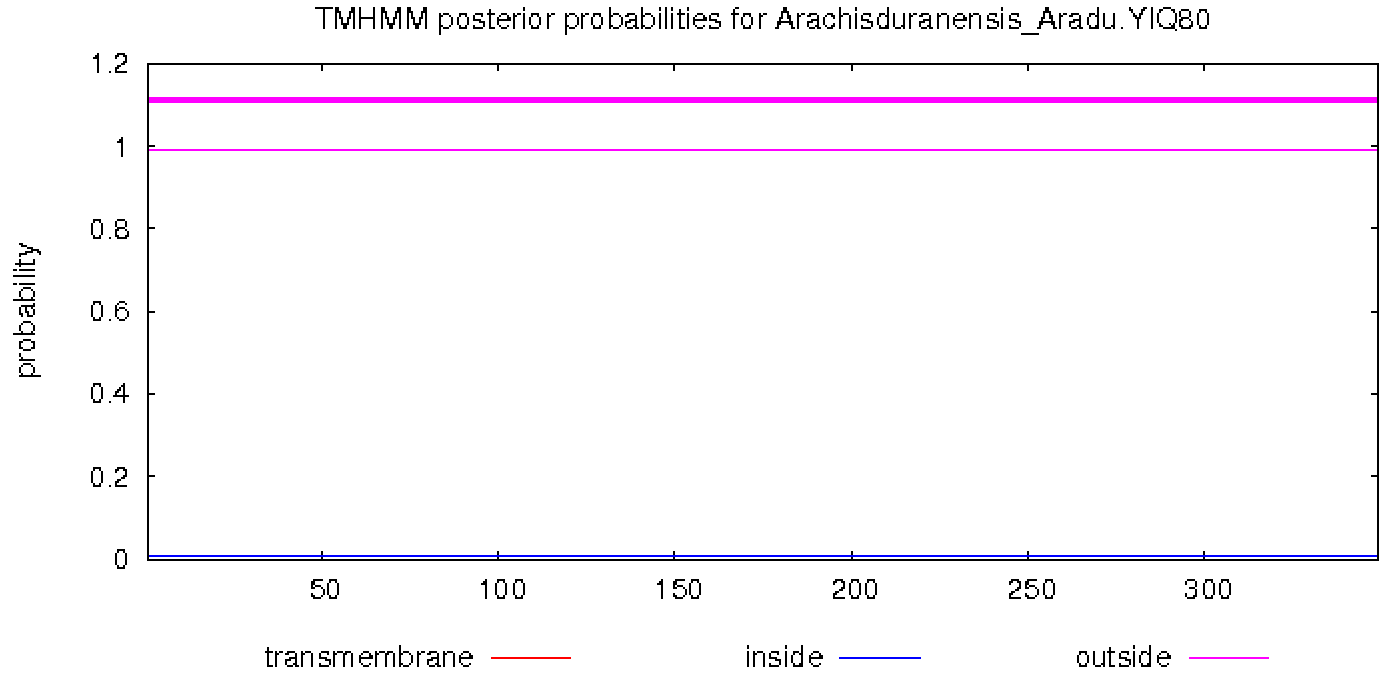

plot in postscript, script for making the plot in gnuplot, data for plot

```
# Arachisduranensis_Aradu.YXW0Z Length: 342
# Arachisduranensis_Aradu.YXW0Z Number of predicted TMHs:  0
# Arachisduranensis_Aradu.YXW0Z Exp number of AAs in TMHs: 0.00347
# Arachisduranensis_Aradu.YXW0Z Exp number, first 60 AAs:  0
# Arachisduranensis_Aradu.YXW0Z Total prob of N-in:        0.00898
Arachisduranensis_Aradu.YXW0Z   TMHMM2.0        outside      1   342
```

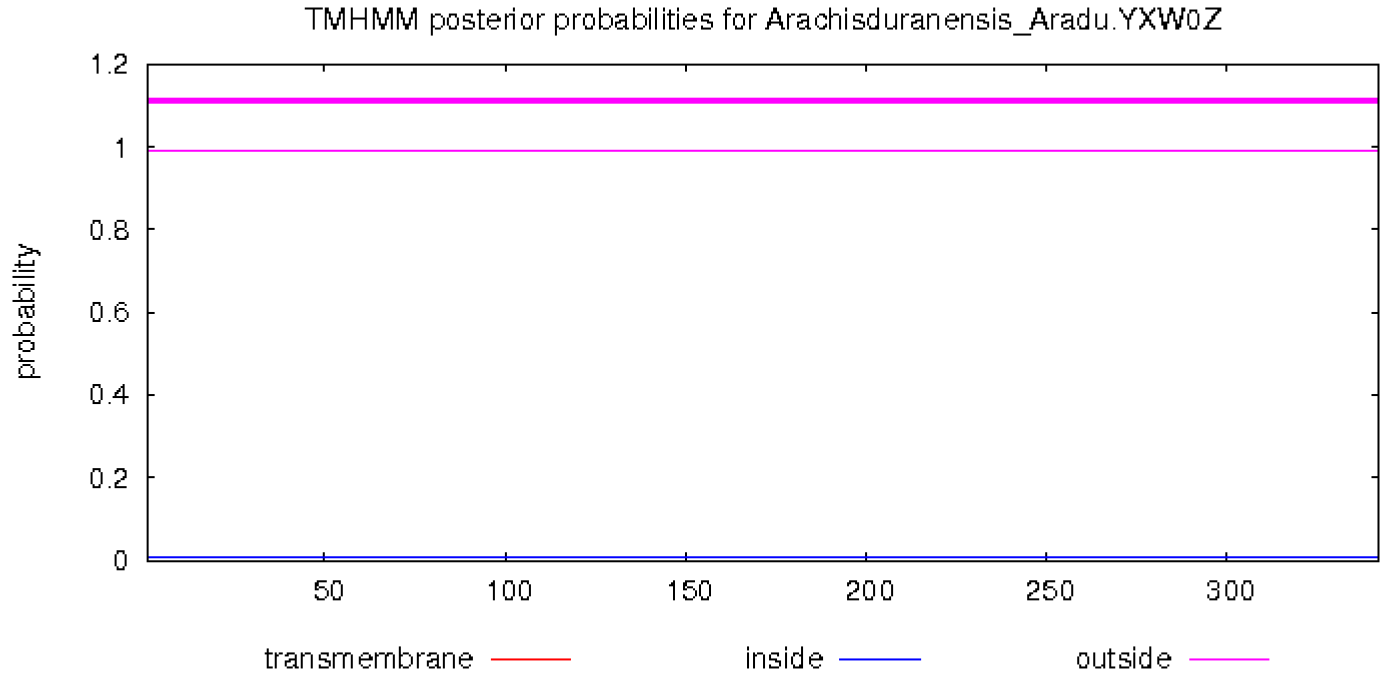

plot in postscript, script for making the plot in gnuplot, data for plot

```
# Arachisduranensis_Aradu.Z4K97 Length: 493
# Arachisduranensis_Aradu.Z4K97 Number of predicted TMHs:  1
# Arachisduranensis_Aradu.Z4K97 Exp number of AAs in TMHs: 22.83396
# Arachisduranensis_Aradu.Z4K97 Exp number, first 60 AAs:  0.08732
# Arachisduranensis_Aradu.Z4K97 Total prob of N-in:        0.24123
Arachisduranensis_Aradu.Z4K97   TMHMM2.0        outside      1    85
Arachisduranensis_Aradu.Z4K97   TMHMM2.0        TMhelix     86   108
Arachisduranensis_Aradu.Z4K97   TMHMM2.0        inside     109   493
```

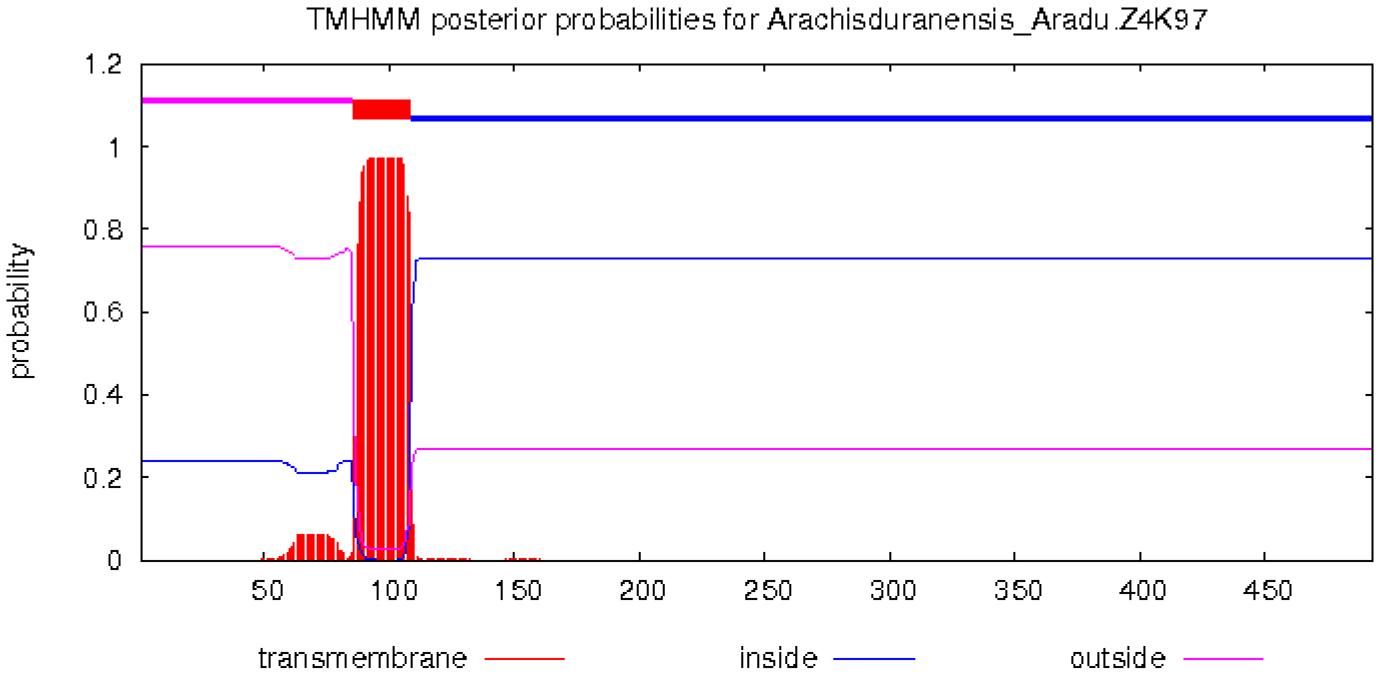

plot in postscript, script for making the plot in gnuplot, data for plot

```
# Arachisduranensis_Aradu.Z5H58 Length: 335
# Arachisduranensis_Aradu.Z5H58 Number of predicted TMHs:  0
# Arachisduranensis_Aradu.Z5H58 Exp number of AAs in TMHs: 0.00077000000000000001
# Arachisduranensis_Aradu.Z5H58 Exp number, first 60 AAs:  0.00056
# Arachisduranensis_Aradu.Z5H58 Total prob of N-in:        0.00396
Arachisduranensis_Aradu.Z5H58   TMHMM2.0        outside      1   335
```

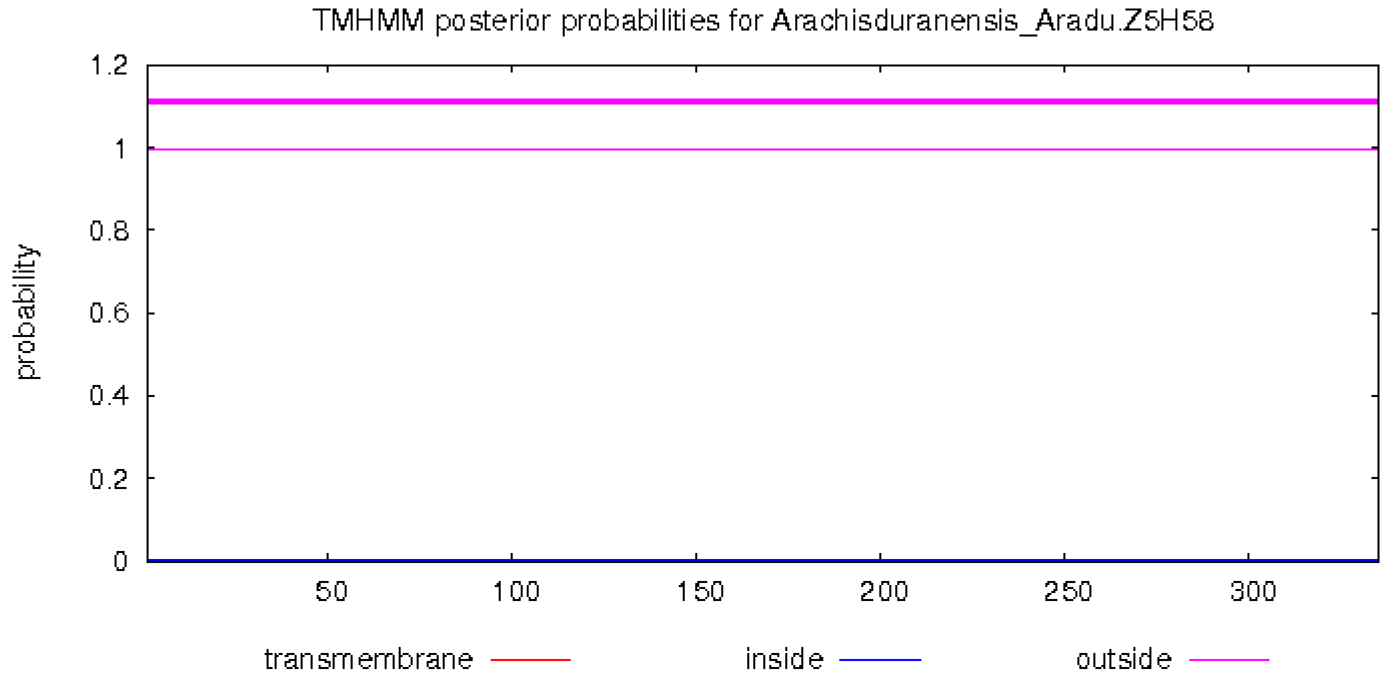

plot in postscript, script for making the plot in gnuplot, data for plot

---

```
# Arachisduranensis_Aradu.Z9Y3J Length: 330
# Arachisduranensis_Aradu.Z9Y3J Number of predicted TMHs:  0
# Arachisduranensis_Aradu.Z9Y3J Exp number of AAs in TMHs: 0.0009
# Arachisduranensis_Aradu.Z9Y3J Exp number, first 60 AAs:  0.0009
# Arachisduranensis_Aradu.Z9Y3J Total prob of N-in:        0.04170
Arachisduranensis_Aradu.Z9Y3J   TMHMM2.0        outside      1   330
```

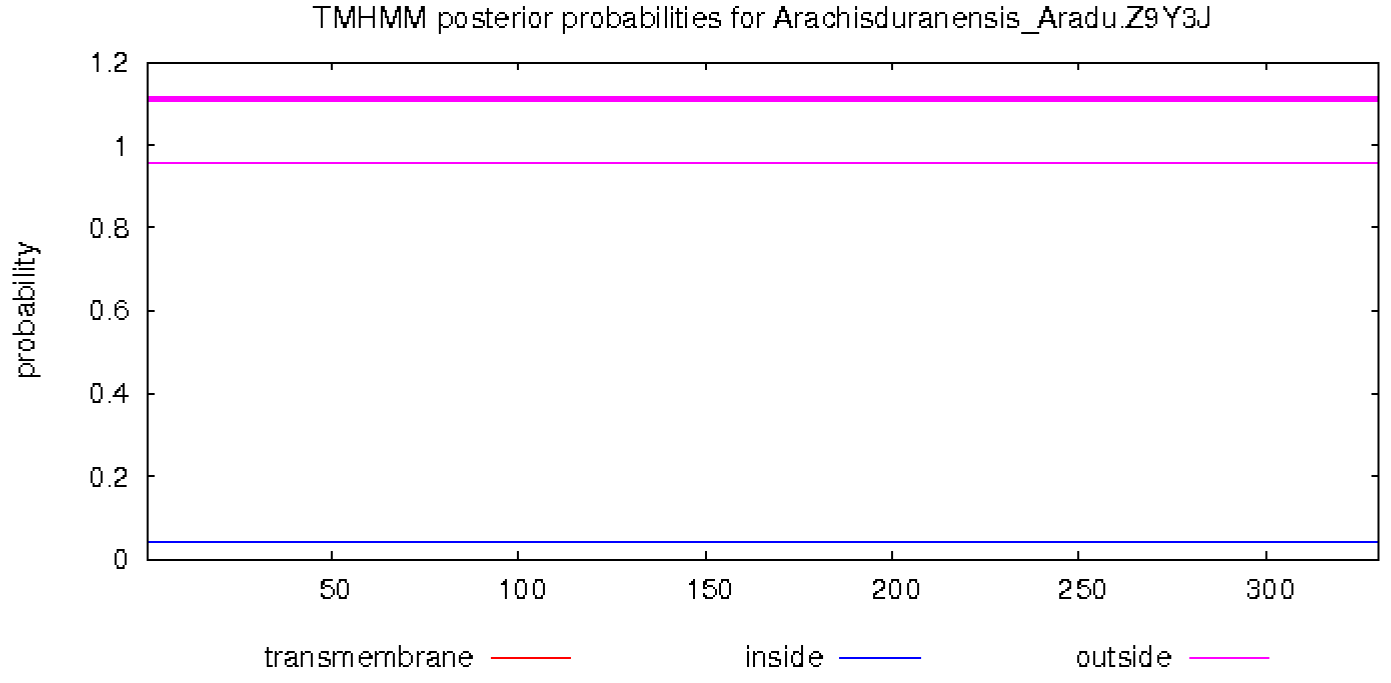

plot in postscript, script for making the plot in gnuplot, data for plot

---

```
# Arachisduranensis_Aradu.ZT2TE Length: 341
# Arachisduranensis_Aradu.ZT2TE Number of predicted TMHs:  0
# Arachisduranensis_Aradu.ZT2TE Exp number of AAs in TMHs: 0
# Arachisduranensis_Aradu.ZT2TE Exp number, first 60 AAs:  0
# Arachisduranensis_Aradu.ZT2TE Total prob of N-in:        0.00687
Arachisduranensis_Aradu.ZT2TE   TMHMM2.0        outside      1   341
```

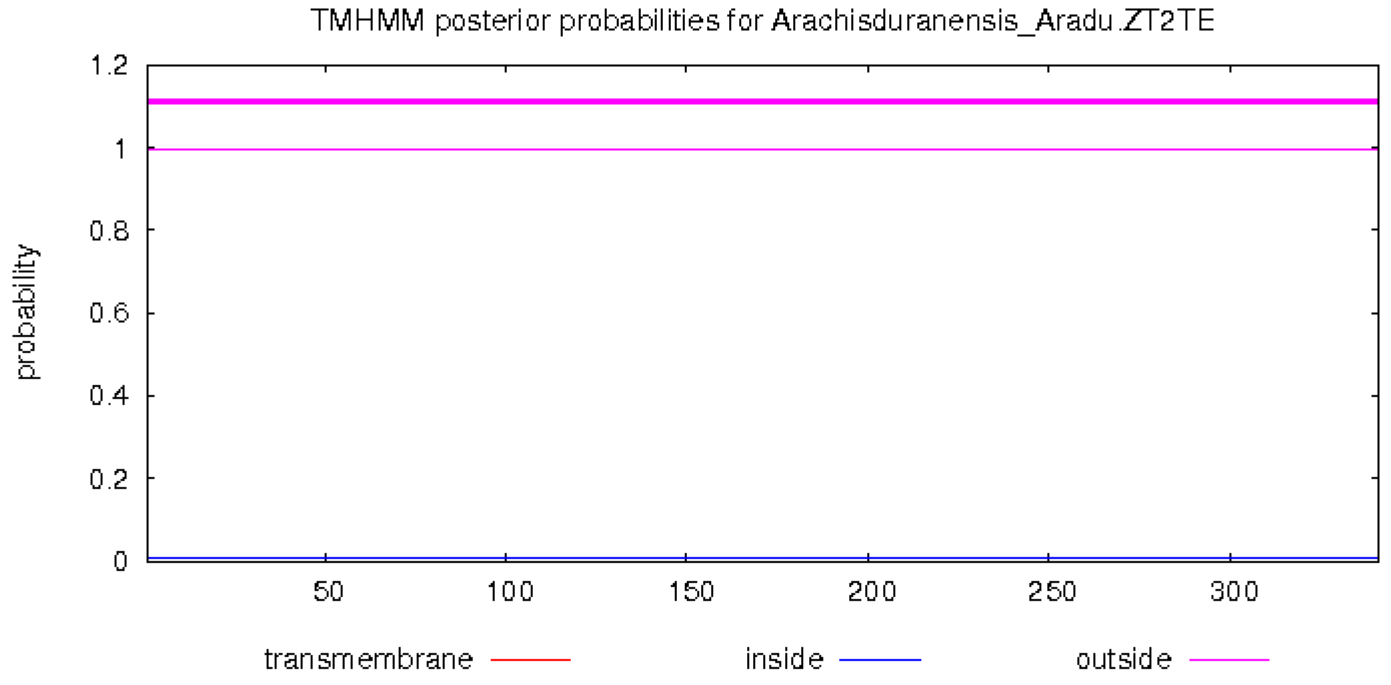

plot in postscript, script for making the plot in gnuplot, data for plot

```
# Arachishypogaea_Ahy003428 Length: 408
# Arachishypogaea_Ahy003428 Number of predicted TMHs:  0
# Arachishypogaea_Ahy003428 Exp number of AAs in TMHs: 0.00036
# Arachishypogaea_Ahy003428 Exp number, first 60 AAs:  0
# Arachishypogaea_Ahy003428 Total prob of N-in:        0.00076
Arachishypogaea_Ahy003428	TMHMM2.0	outside	     1   408
```

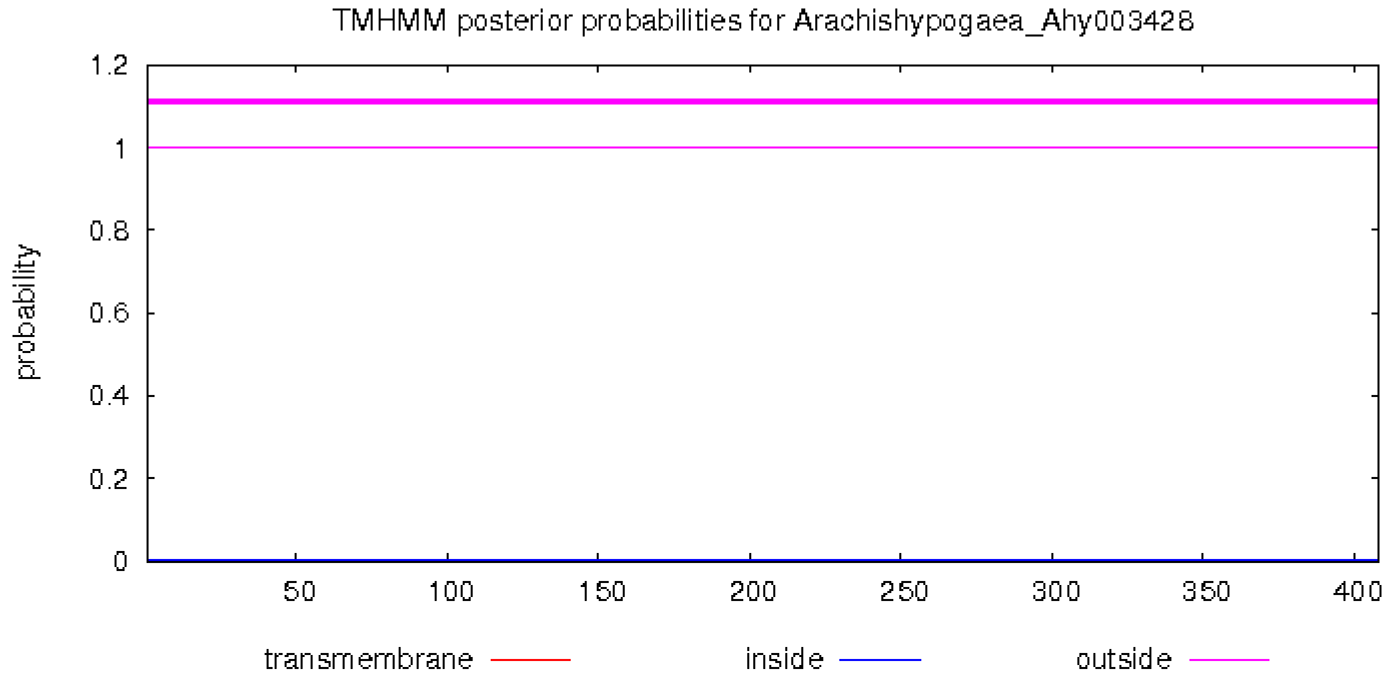

plot in postscript, script for making the plot in gnuplot, data for plot

```
# Arachishypogaea_Ahy004174 Length: 260
# Arachishypogaea_Ahy004174 Number of predicted TMHs:  0
# Arachishypogaea_Ahy004174 Exp number of AAs in TMHs: 0.0002
# Arachishypogaea_Ahy004174 Exp number, first 60 AAs:  0.0002
# Arachishypogaea_Ahy004174 Total prob of N-in:        0.00507
Arachishypogaea_Ahy004174	TMHMM2.0	outside	     1   260
```

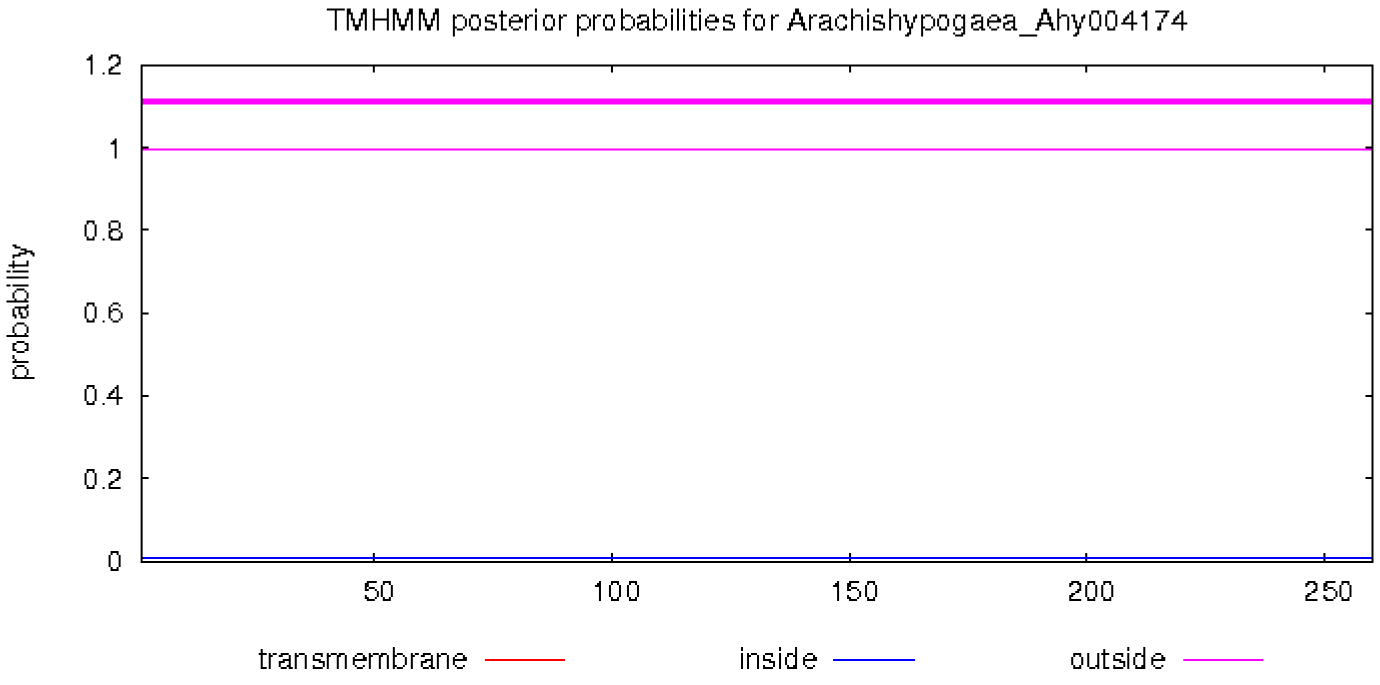

plot in postscript, script for making the plot in gnuplot, data for plot

---

```
# Arachishypogaea_Ahy007630 Length: 397
# Arachishypogaea_Ahy007630 Number of predicted TMHs:  0
# Arachishypogaea_Ahy007630 Exp number of AAs in TMHs: 0
# Arachishypogaea_Ahy007630 Exp number, first 60 AAs:  0
# Arachishypogaea_Ahy007630 Total prob of N-in:        0.00234
Arachishypogaea_Ahy007630       TMHMM2.0        outside      1   397
```

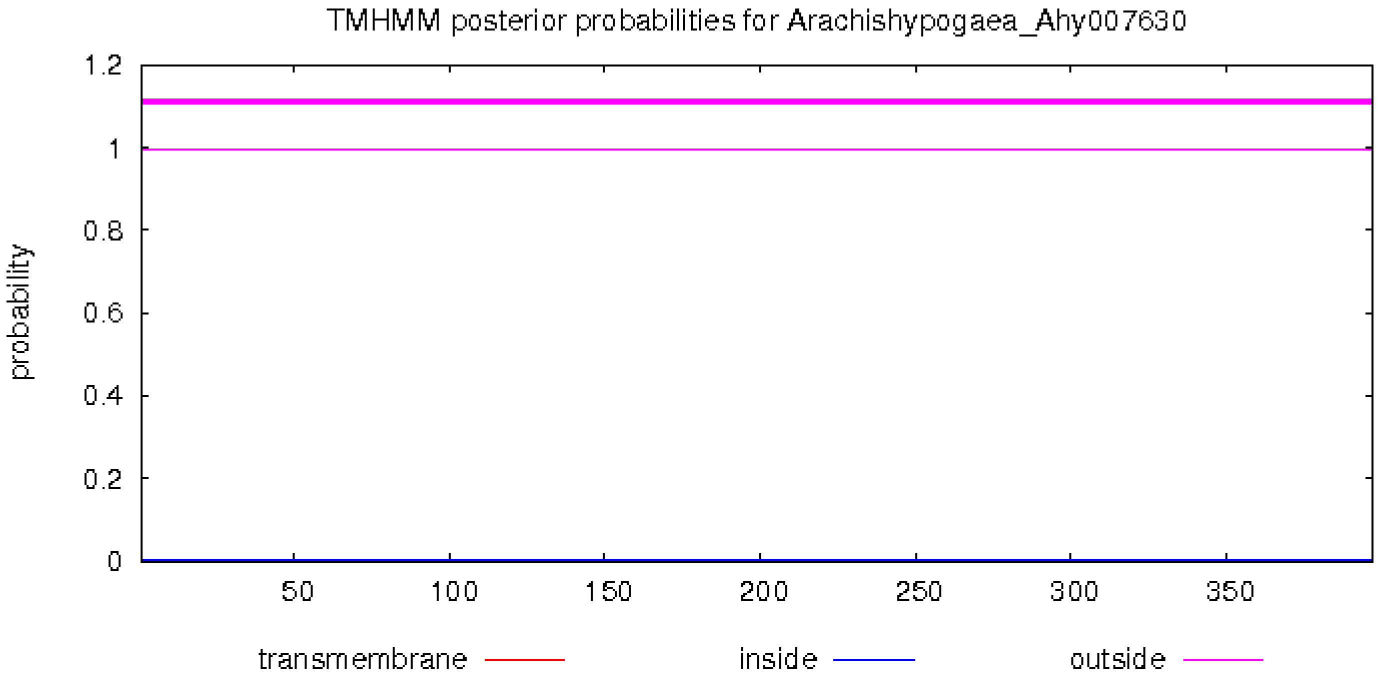

plot in postscript, script for making the plot in gnuplot, data for plot

---

```
# Arachishypogaea_Ahy008073 Length: 697
# Arachishypogaea_Ahy008073 Number of predicted TMHs:  1
# Arachishypogaea_Ahy008073 Exp number of AAs in TMHs: 22.60263
# Arachishypogaea_Ahy008073 Exp number, first 60 AAs:  0.01368
# Arachishypogaea_Ahy008073 Total prob of N-in:        0.00428
Arachishypogaea_Ahy008073       TMHMM2.0        outside      1   667
```

```
Arachishypogaea_Ahy008073       TMHMM2.0        TMhelix     668   690
Arachishypogaea_Ahy008073       TMHMM2.0        inside      691   697
```

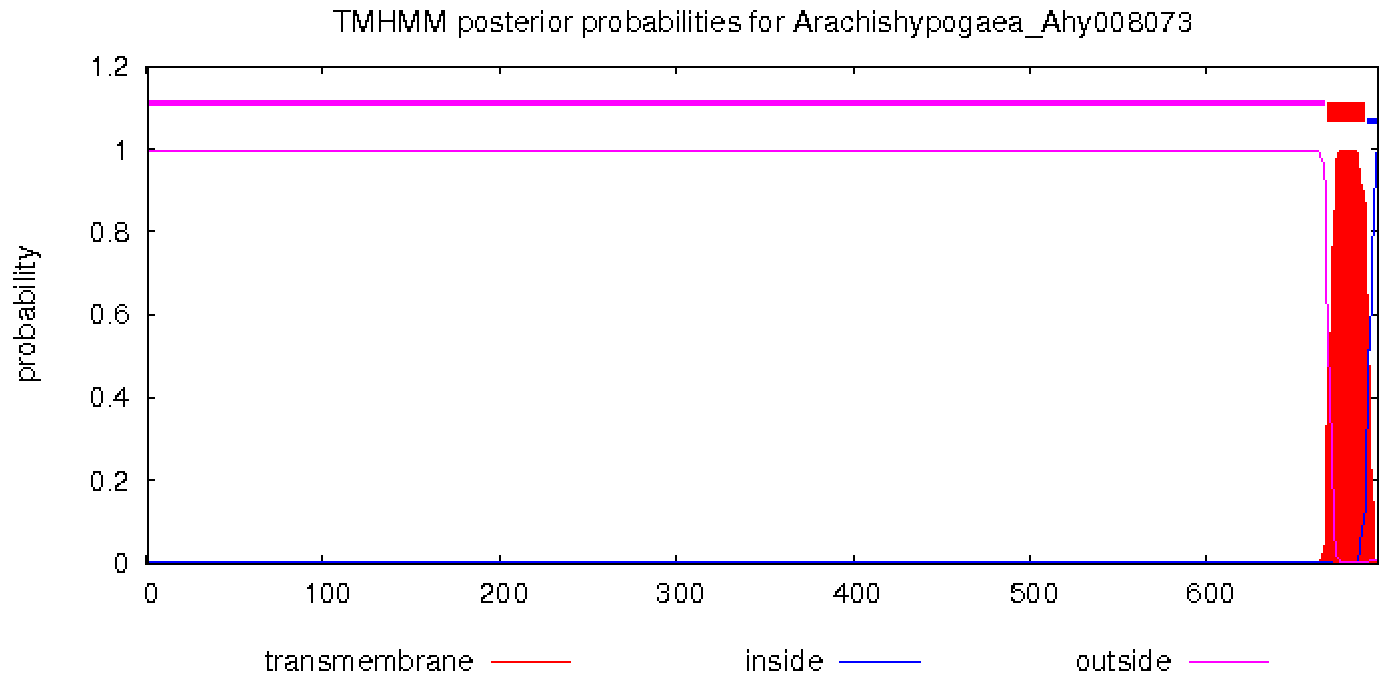

plot in postscript, script for making the plot in gnuplot, data for plot

---

```
# Arachishypogaea_Ahy008395 Length: 272
# Arachishypogaea_Ahy008395 Number of predicted TMHs:  0
# Arachishypogaea_Ahy008395 Exp number of AAs in TMHs: 0
# Arachishypogaea_Ahy008395 Exp number, first 60 AAs:  0
# Arachishypogaea_Ahy008395 Total prob of N-in:        0.00775
Arachishypogaea_Ahy008395       TMHMM2.0        outside      1   272
```

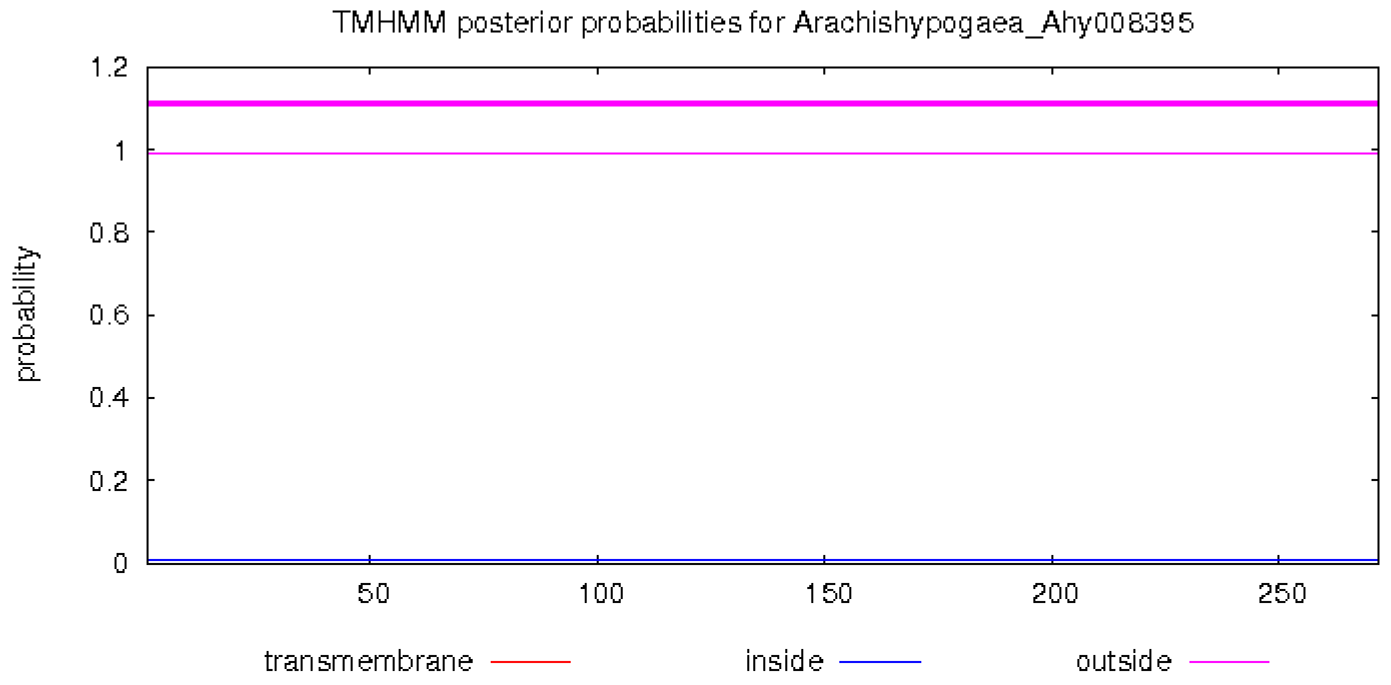

plot in postscript, script for making the plot in gnuplot, data for plot

---

```
# Arachishypogaea_Ahy008545 Length: 286
# Arachishypogaea_Ahy008545 Number of predicted TMHs:  0
# Arachishypogaea_Ahy008545 Exp number of AAs in TMHs: 0.01256
# Arachishypogaea_Ahy008545 Exp number, first 60 AAs:  0.0002
```

```
# Arachishypogaea_Ahy008545 Total prob of N-in:        0.02242
Arachishypogaea_Ahy008545       TMHMM2.0        outside      1   286
```

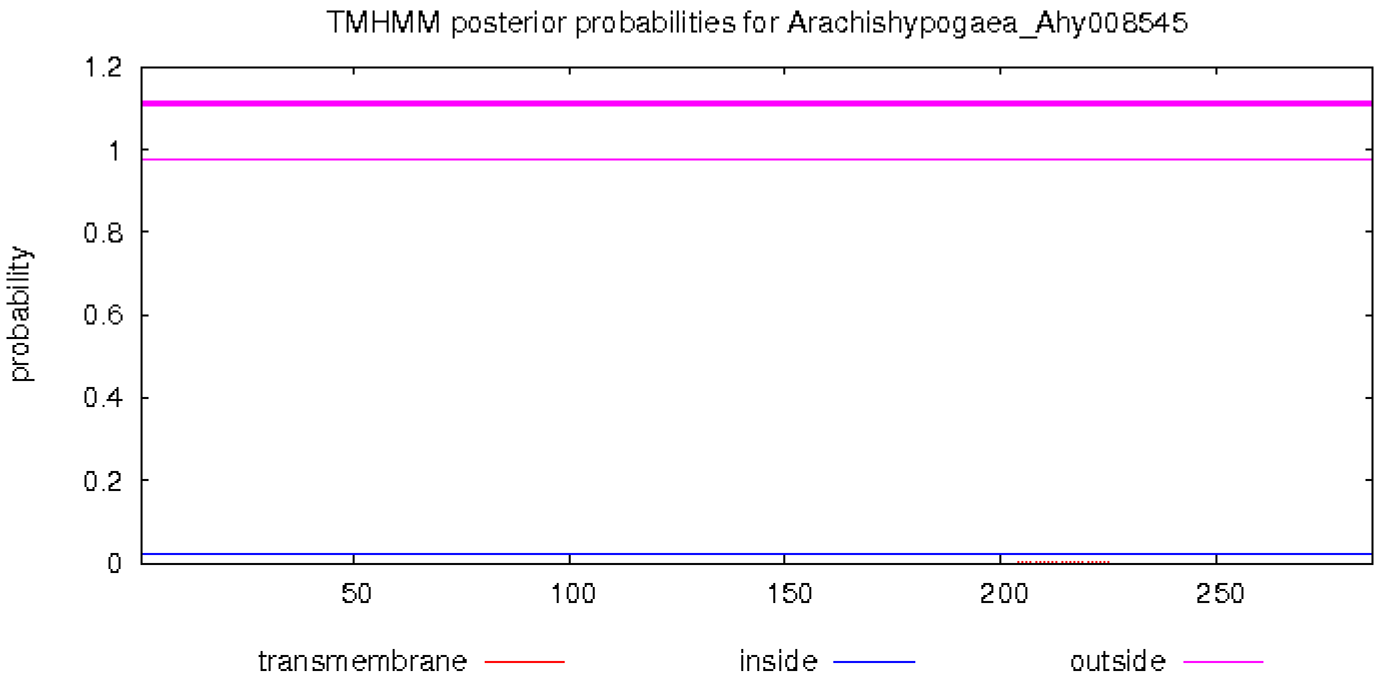

plot in postscript, script for making the plot in gnuplot, data for plot

---

```
# Arachishypogaea_Ahy008969 Length: 422
# Arachishypogaea_Ahy008969 Number of predicted TMHs:  0
# Arachishypogaea_Ahy008969 Exp number of AAs in TMHs: 0.05196
# Arachishypogaea_Ahy008969 Exp number, first 60 AAs:  0.00128
# Arachishypogaea_Ahy008969 Total prob of N-in:        0.00144
Arachishypogaea_Ahy008969       TMHMM2.0        outside      1   422
```

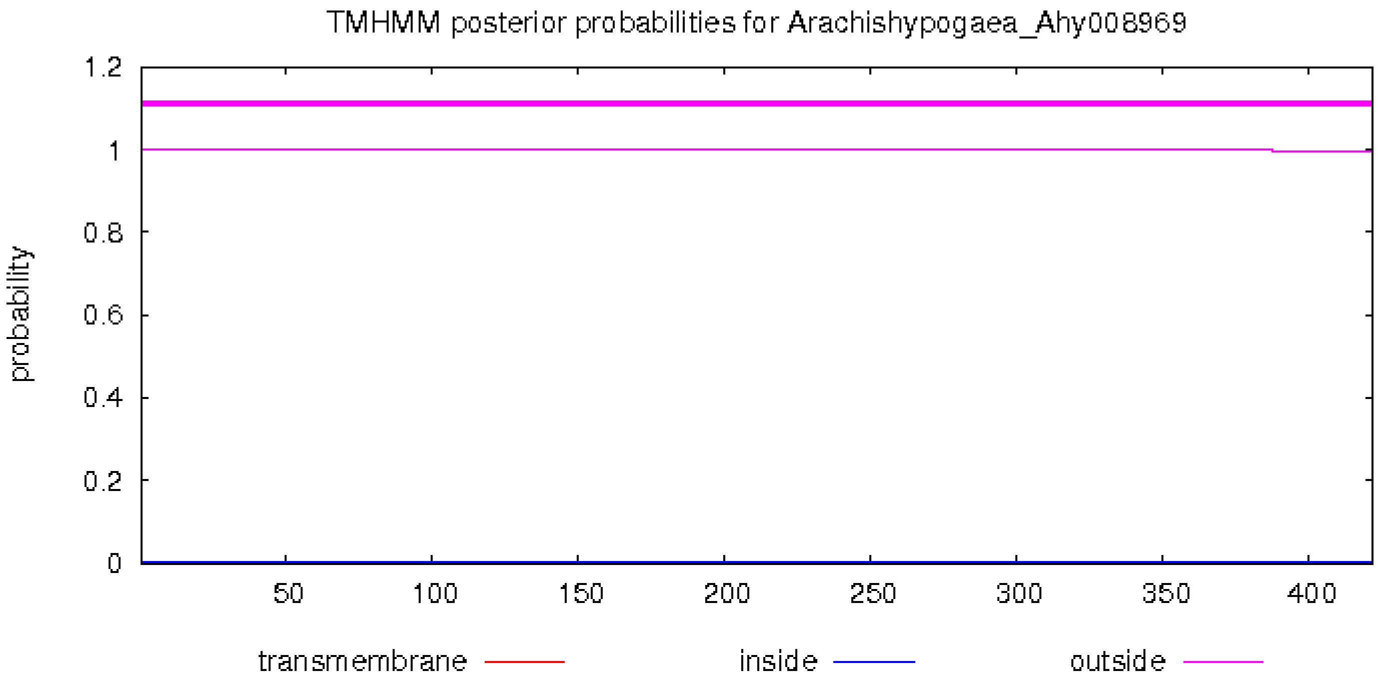

plot in postscript, script for making the plot in gnuplot, data for plot

---

```
# Arachishypogaea_Ahy009563 Length: 583
# Arachishypogaea_Ahy009563 Number of predicted TMHs:  2
# Arachishypogaea_Ahy009563 Exp number of AAs in TMHs: 39.26784
# Arachishypogaea_Ahy009563 Exp number, first 60 AAs:  0
```

```
# Arachishypogaea_Ahy009563 Total prob of N-in:        0.00356
Arachishypogaea_Ahy009563       TMHMM2.0        outside      1   530
Arachishypogaea_Ahy009563       TMHMM2.0        TMhelix    531   553
Arachishypogaea_Ahy009563       TMHMM2.0        inside     554   557
Arachishypogaea_Ahy009563       TMHMM2.0        TMhelix    558   580
Arachishypogaea_Ahy009563       TMHMM2.0        outside    581   583
```

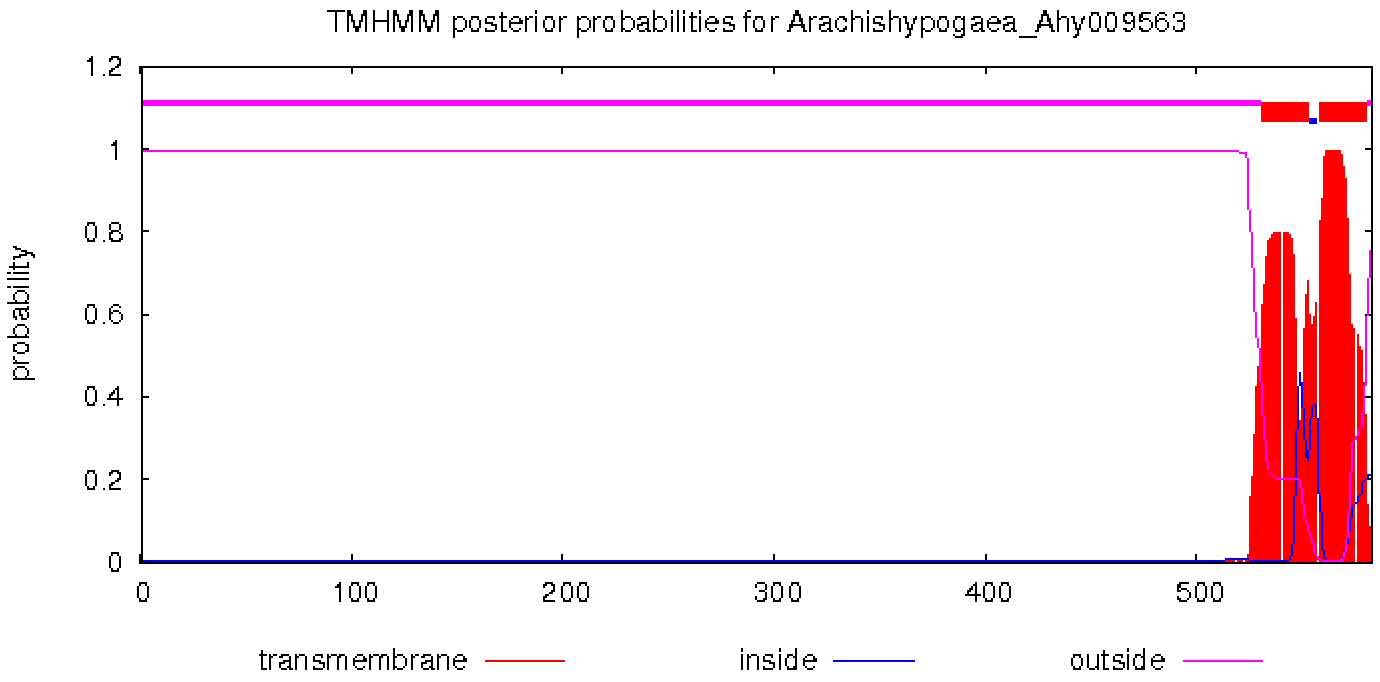

plot in postscript, script for making the plot in gnuplot, data for plot

---

```
# Arachishypogaea_Ahy009594 Length: 392
# Arachishypogaea_Ahy009594 Number of predicted TMHs:  0
# Arachishypogaea_Ahy009594 Exp number of AAs in TMHs: 0.00017
# Arachishypogaea_Ahy009594 Exp number, first 60 AAs:  0.00017
# Arachishypogaea_Ahy009594 Total prob of N-in:        0.03009
Arachishypogaea_Ahy009594       TMHMM2.0        outside      1   392
```

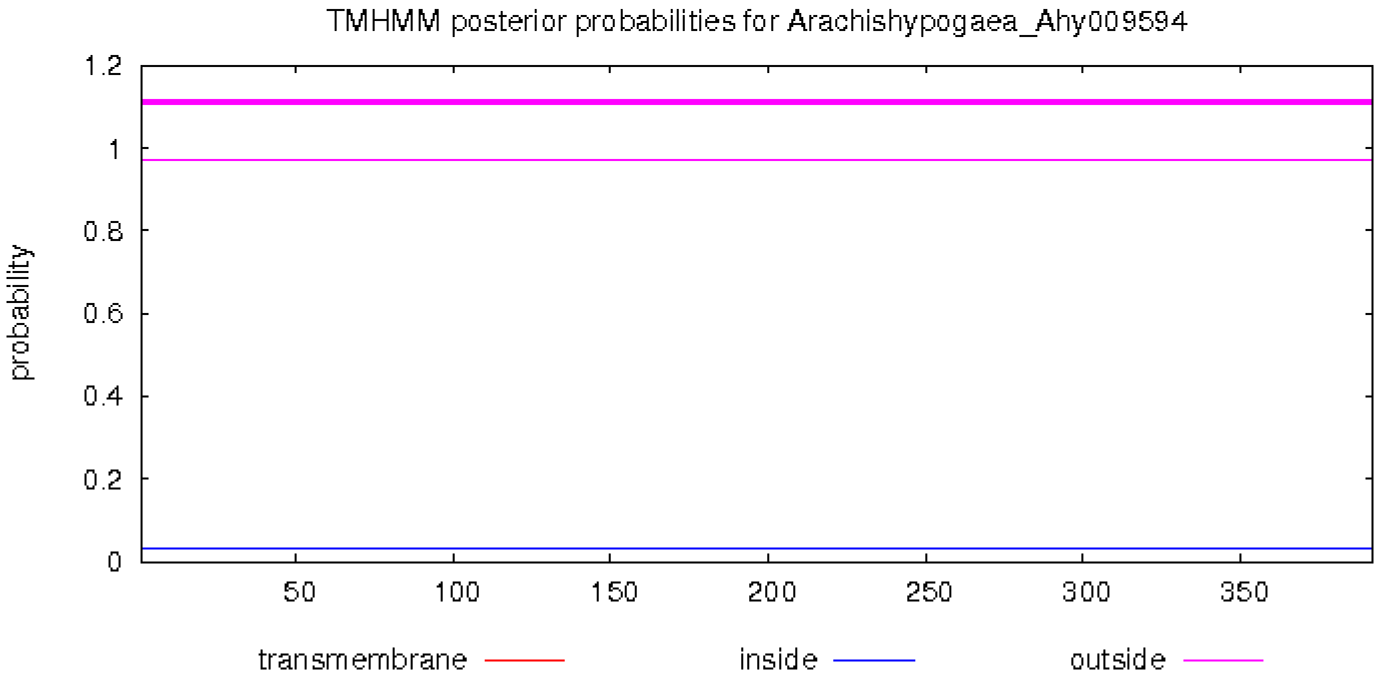

plot in postscript, script for making the plot in gnuplot, data for plot

---

```
# Arachishypogaea_Ahy009762 Length: 81
# Arachishypogaea_Ahy009762 Number of predicted TMHs:  0
# Arachishypogaea_Ahy009762 Exp number of AAs in TMHs: 0.00081
# Arachishypogaea_Ahy009762 Exp number, first 60 AAs:  0.00081
# Arachishypogaea_Ahy009762 Total prob of N-in:        0.34048
Arachishypogaea_Ahy009762       TMHMM2.0        outside      1    81
```

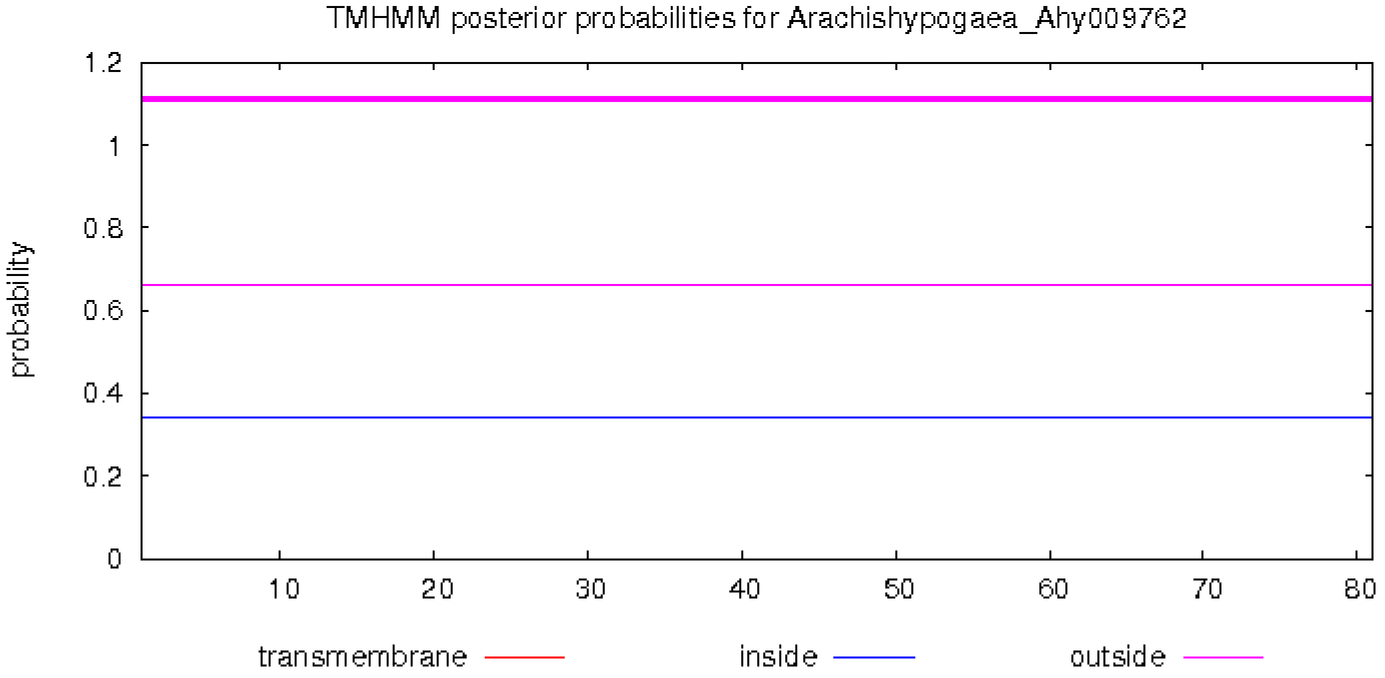

plot in postscript, script for making the plot in gnuplot, data for plot

---

```
# Arachishypogaea_Ahy010013 Length: 306
# Arachishypogaea_Ahy010013 Number of predicted TMHs:  0
# Arachishypogaea_Ahy010013 Exp number of AAs in TMHs: 0
# Arachishypogaea_Ahy010013 Exp number, first 60 AAs:  0
# Arachishypogaea_Ahy010013 Total prob of N-in:        0.00882
Arachishypogaea_Ahy010013       TMHMM2.0        outside      1   306
```

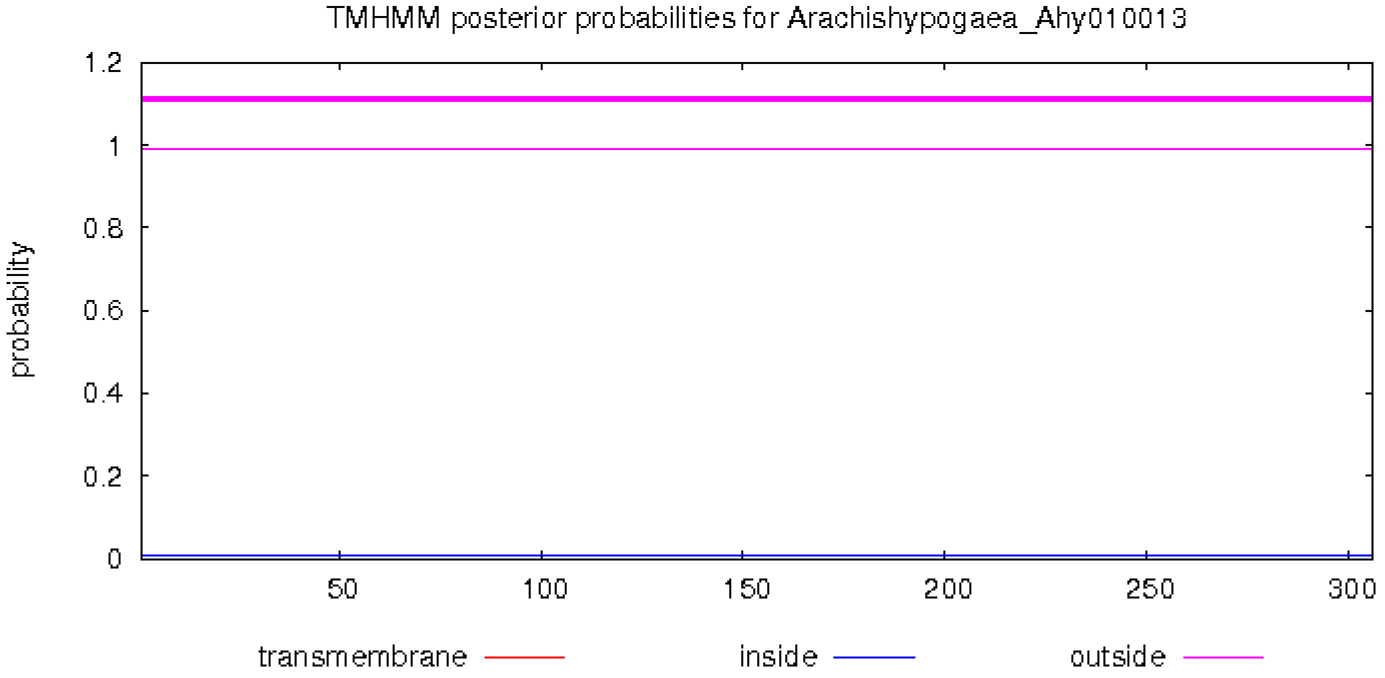

plot in postscript, script for making the plot in gnuplot, data for plot

---

```
# Arachishypogaea_Ahy010059 Length: 283
# Arachishypogaea_Ahy010059 Number of predicted TMHs:  0
# Arachishypogaea_Ahy010059 Exp number of AAs in TMHs: 0.0046
# Arachishypogaea_Ahy010059 Exp number, first 60 AAs:  0
# Arachishypogaea_Ahy010059 Total prob of N-in:        0.02422
Arachishypogaea_Ahy010059	TMHMM2.0	outside	     1   283
```

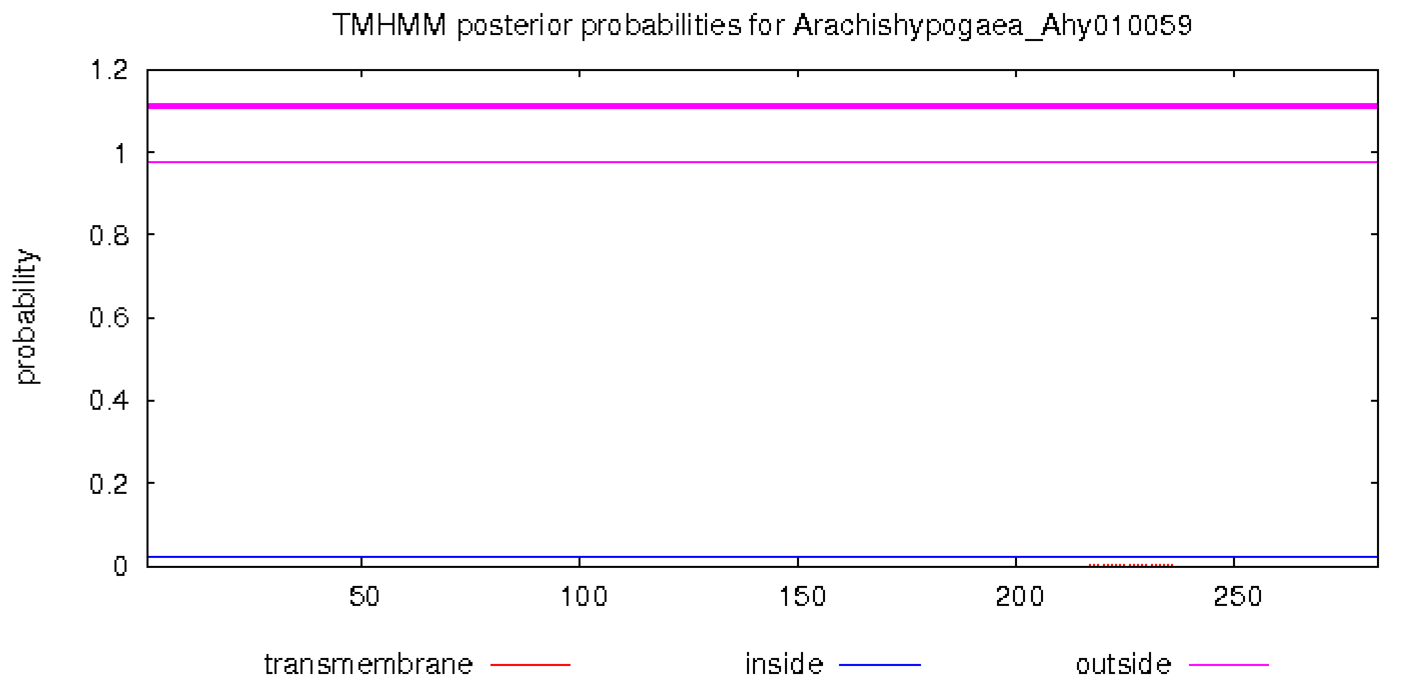

plot in postscript, script for making the plot in gnuplot, data for plot

```
# Arachishypogaea_Ahy010486 Length: 404
# Arachishypogaea_Ahy010486 Number of predicted TMHs:  0
# Arachishypogaea_Ahy010486 Exp number of AAs in TMHs: 0.05763
# Arachishypogaea_Ahy010486 Exp number, first 60 AAs:  0
# Arachishypogaea_Ahy010486 Total prob of N-in:        0.01004
Arachishypogaea_Ahy010486	TMHMM2.0	outside	     1   404
```

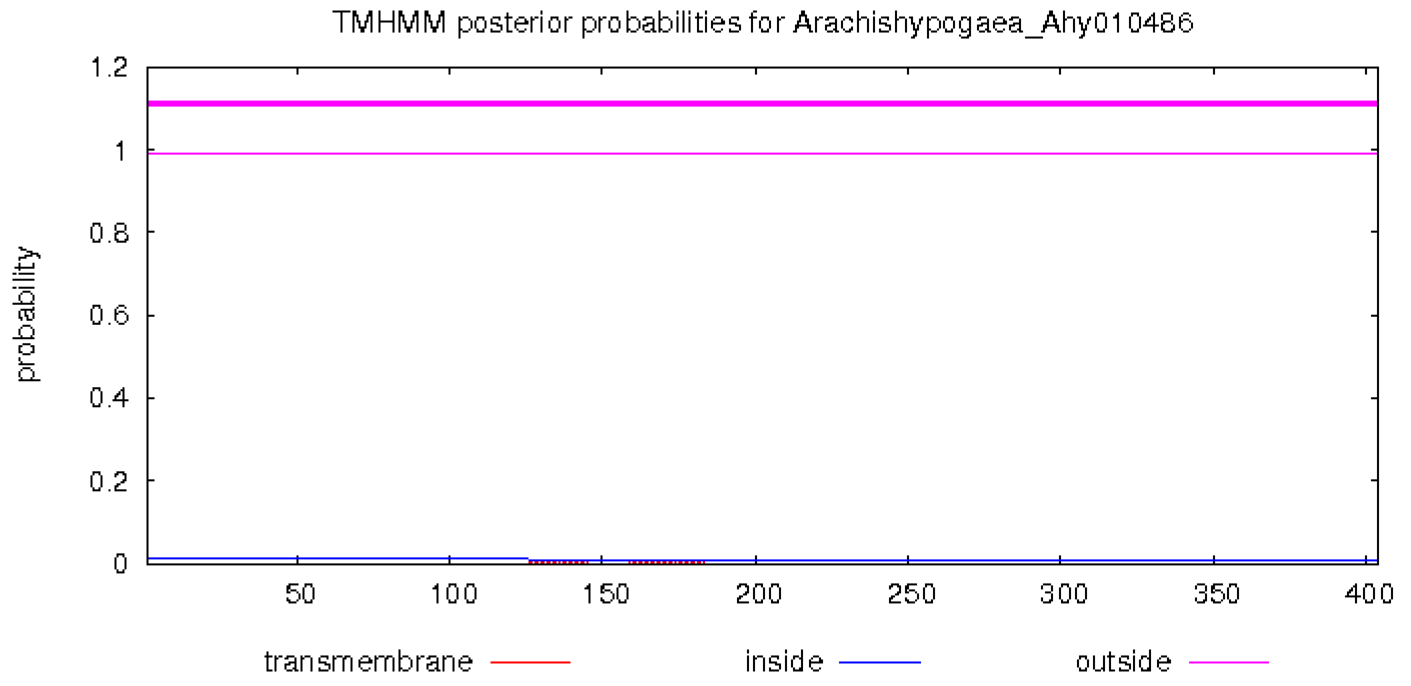

plot in postscript, script for making the plot in gnuplot, data for plot

```
# Arachishypogaea_Ahy011698 Length: 280
# Arachishypogaea_Ahy011698 Number of predicted TMHs:  0
# Arachishypogaea_Ahy011698 Exp number of AAs in TMHs: 0.00034
# Arachishypogaea_Ahy011698 Exp number, first 60 AAs:  0
# Arachishypogaea_Ahy011698 Total prob of N-in:        0.00840
Arachishypogaea_Ahy011698       TMHMM2.0        outside      1   280
```

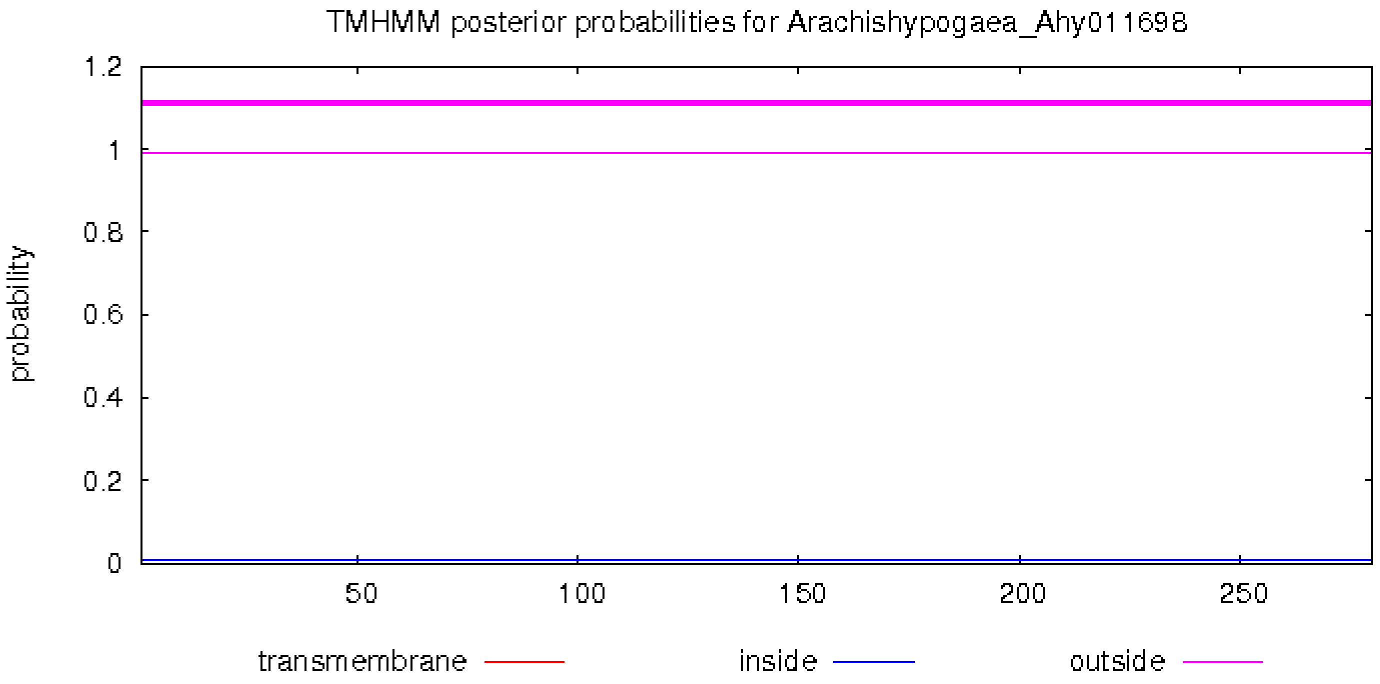

plot in postscript, script for making the plot in gnuplot, data for plot

---

```
# Arachishypogaea_Ahy012574 Length: 246
# Arachishypogaea_Ahy012574 Number of predicted TMHs:  0
# Arachishypogaea_Ahy012574 Exp number of AAs in TMHs: 0.0048
# Arachishypogaea_Ahy012574 Exp number, first 60 AAs:  0
# Arachishypogaea_Ahy012574 Total prob of N-in:        0.03579
Arachishypogaea_Ahy012574       TMHMM2.0        outside      1   246
```

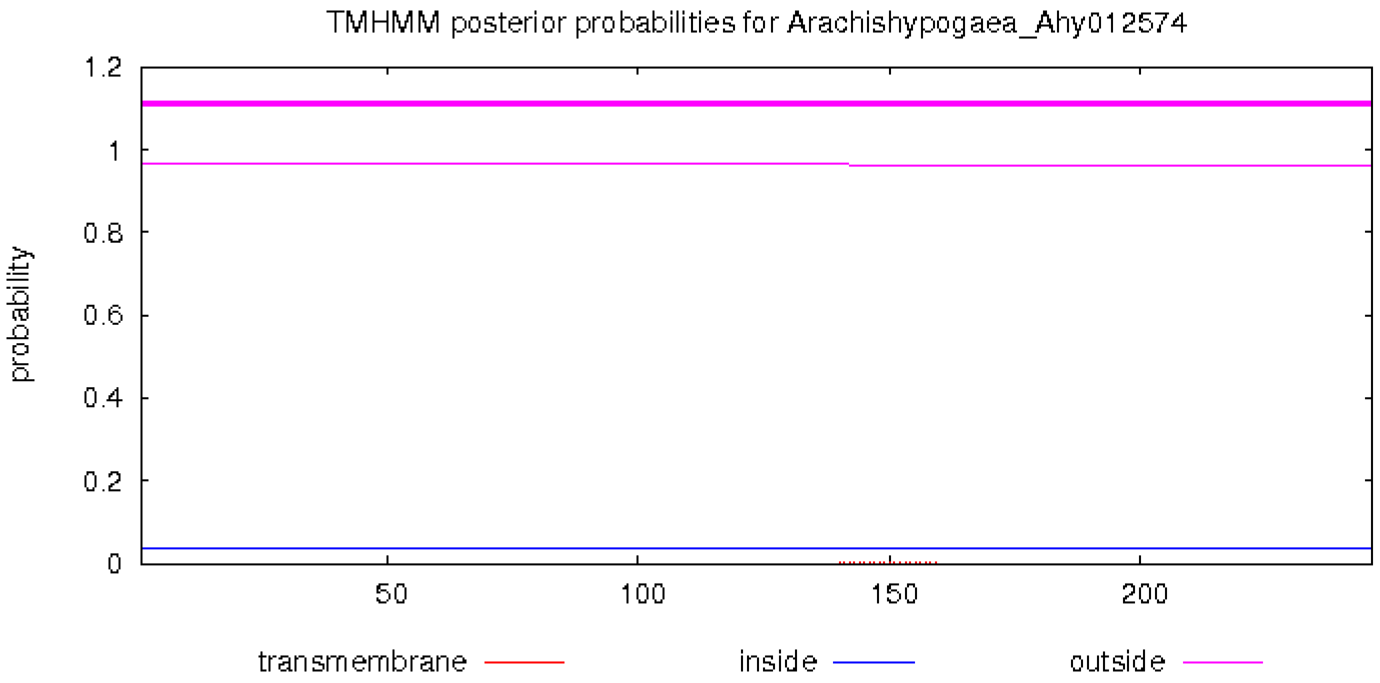

plot in postscript, script for making the plot in gnuplot, data for plot

---

```
# Arachishypogaea_Ahy012663 Length: 431
# Arachishypogaea_Ahy012663 Number of predicted TMHs:  0
# Arachishypogaea_Ahy012663 Exp number of AAs in TMHs: 0
# Arachishypogaea_Ahy012663 Exp number, first 60 AAs:  0
# Arachishypogaea_Ahy012663 Total prob of N-in:        0.00408
Arachishypogaea_Ahy012663       TMHMM2.0        outside      1   431
```

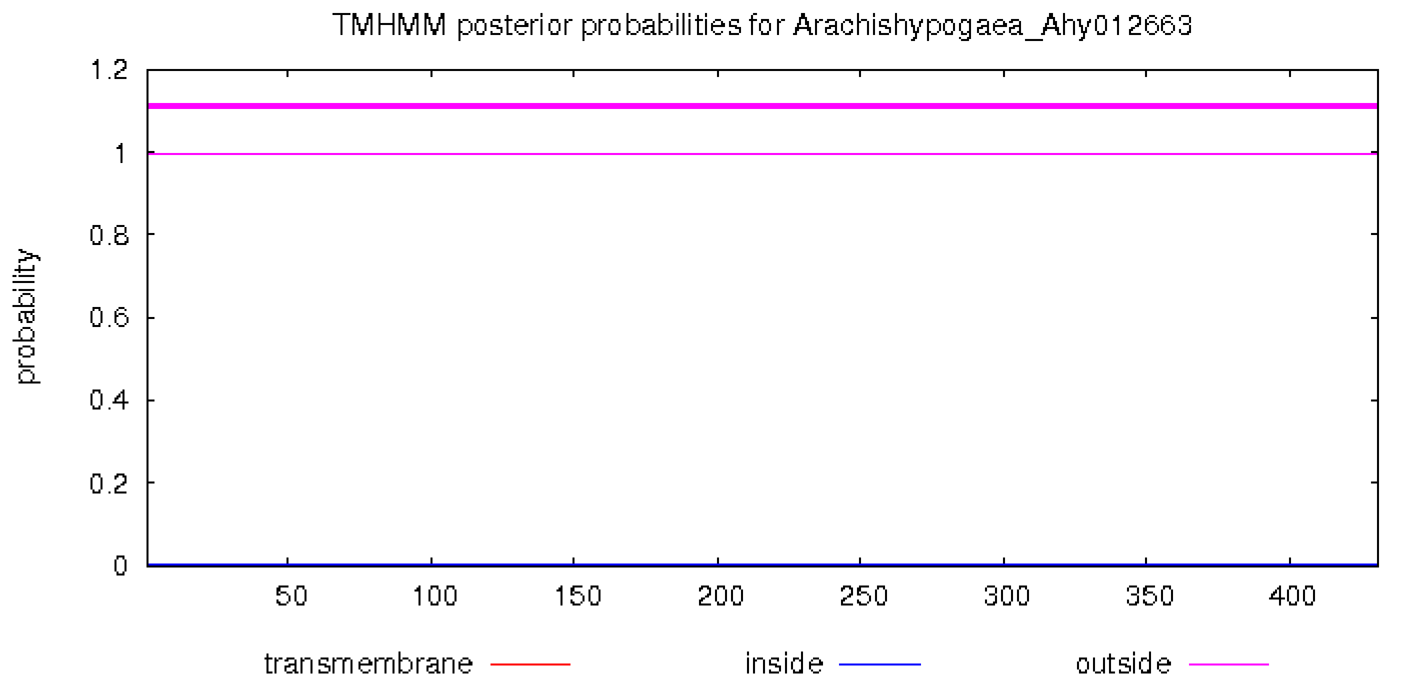

plot in postscript, script for making the plot in gnuplot, data for plot

---

```
# Arachishypogaea_Ahy012805 Length: 250
# Arachishypogaea_Ahy012805 Number of predicted TMHs:  0
# Arachishypogaea_Ahy012805 Exp number of AAs in TMHs: 0.00511
# Arachishypogaea_Ahy012805 Exp number, first 60 AAs:  0
# Arachishypogaea_Ahy012805 Total prob of N-in:        0.03250
Arachishypogaea_Ahy012805       TMHMM2.0        outside      1   250
```

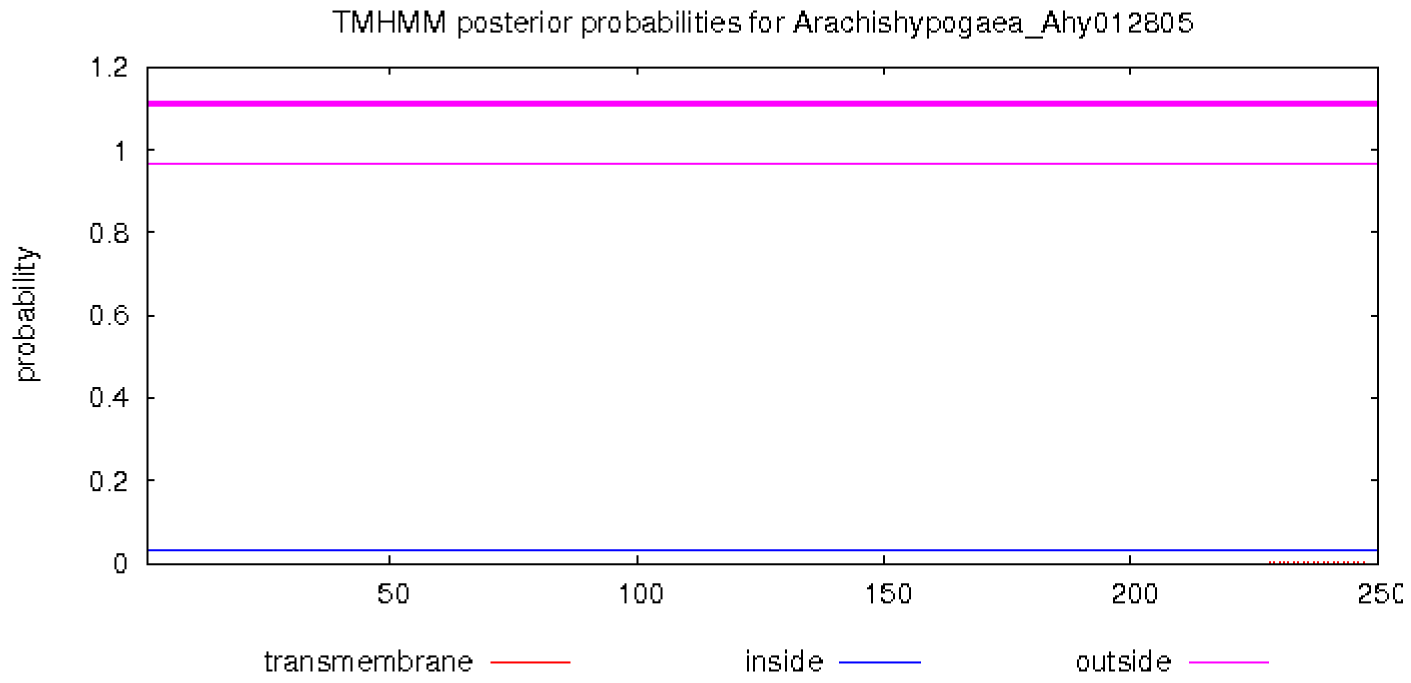

plot in postscript, script for making the plot in gnuplot, data for plot

---

```
# Arachishypogaea_Ahy014891 Length: 71
# Arachishypogaea_Ahy014891 Number of predicted TMHs:  0
# Arachishypogaea_Ahy014891 Exp number of AAs in TMHs: 0.0005
# Arachishypogaea_Ahy014891 Exp number, first 60 AAs:  0.0005
# Arachishypogaea_Ahy014891 Total prob of N-in:        0.20988
Arachishypogaea_Ahy014891	TMHMM2.0	outside	     1    71
```

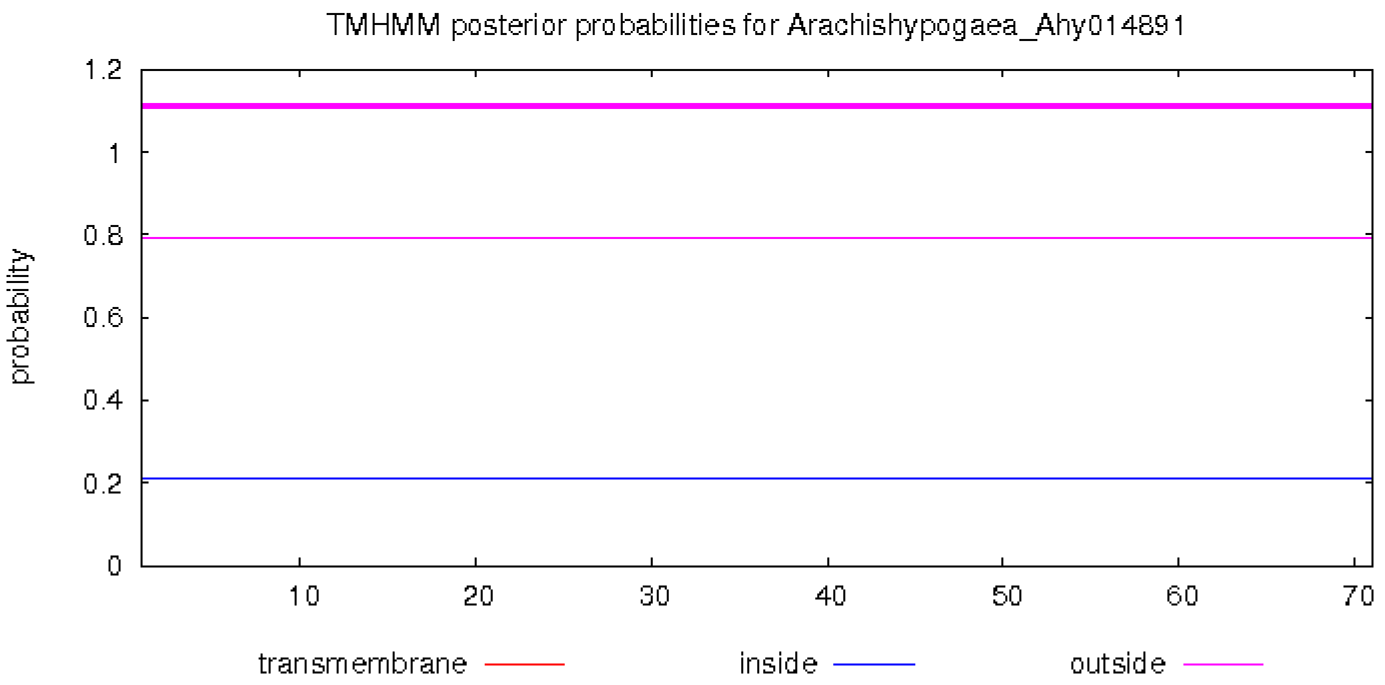

plot in postscript, script for making the plot in gnuplot, data for plot

---

```
# Arachishypogaea_Ahy016041 Length: 231
# Arachishypogaea_Ahy016041 Number of predicted TMHs:  0
# Arachishypogaea_Ahy016041 Exp number of AAs in TMHs: 0.00017
# Arachishypogaea_Ahy016041 Exp number, first 60 AAs:  0
# Arachishypogaea_Ahy016041 Total prob of N-in:        0.07252
Arachishypogaea_Ahy016041	TMHMM2.0	outside	     1   231
```

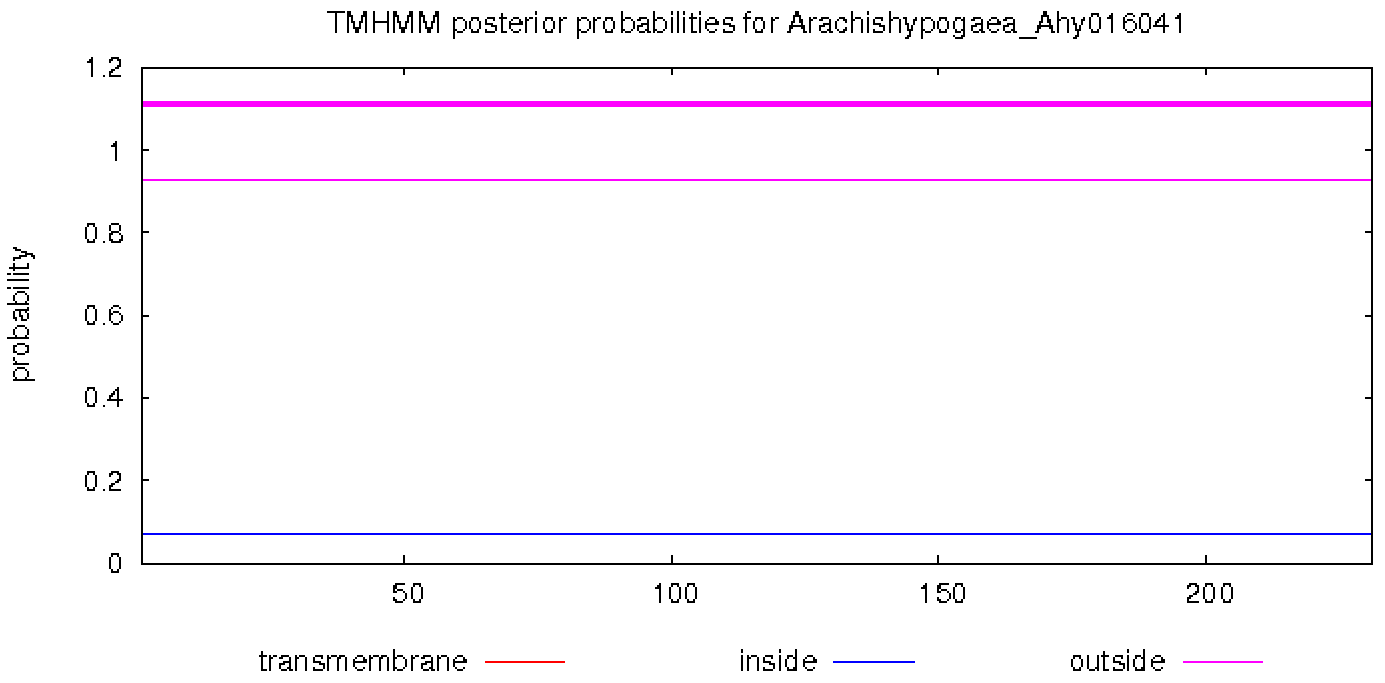

plot in postscript, script for making the plot in gnuplot, data for plot

---

```
# Arachishypogaea_Ahy016633 Length: 250
# Arachishypogaea_Ahy016633 Number of predicted TMHs:  0
# Arachishypogaea_Ahy016633 Exp number of AAs in TMHs: 0.0005
# Arachishypogaea_Ahy016633 Exp number, first 60 AAs:  0.00025
# Arachishypogaea_Ahy016633 Total prob of N-in:        0.04647
Arachishypogaea_Ahy016633	TMHMM2.0	outside	     1   250
```

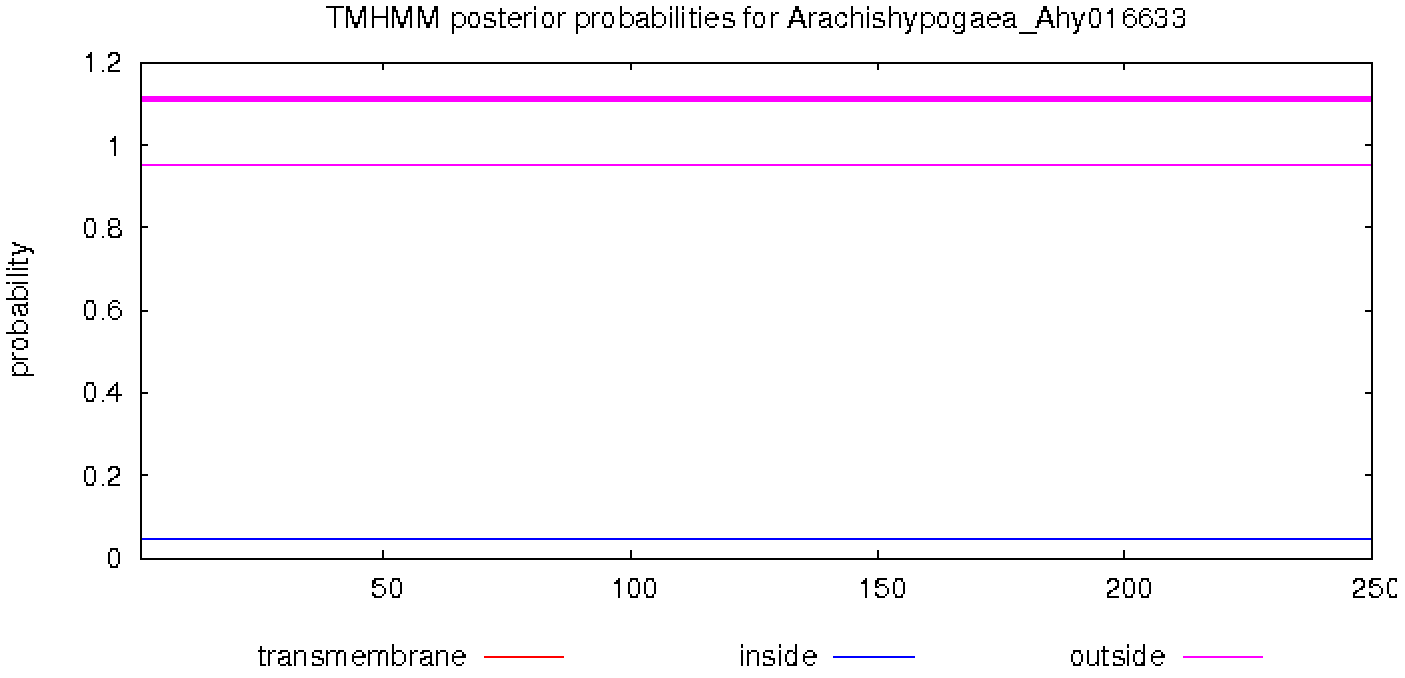

plot in postscript, script for making the plot in gnuplot, data for plot

---

```
# Arachishypogaea_Ahy016999 Length: 574
# Arachishypogaea_Ahy016999 Number of predicted TMHs:  1
# Arachishypogaea_Ahy016999 Exp number of AAs in TMHs: 22.15524
# Arachishypogaea_Ahy016999 Exp number, first 60 AAs:  0
# Arachishypogaea_Ahy016999 Total prob of N-in:        0.01756
Arachishypogaea_Ahy016999	TMHMM2.0	outside	     1   545
Arachishypogaea_Ahy016999	TMHMM2.0	TMhelix	   546   568
Arachishypogaea_Ahy016999	TMHMM2.0	inside	   569   574
```

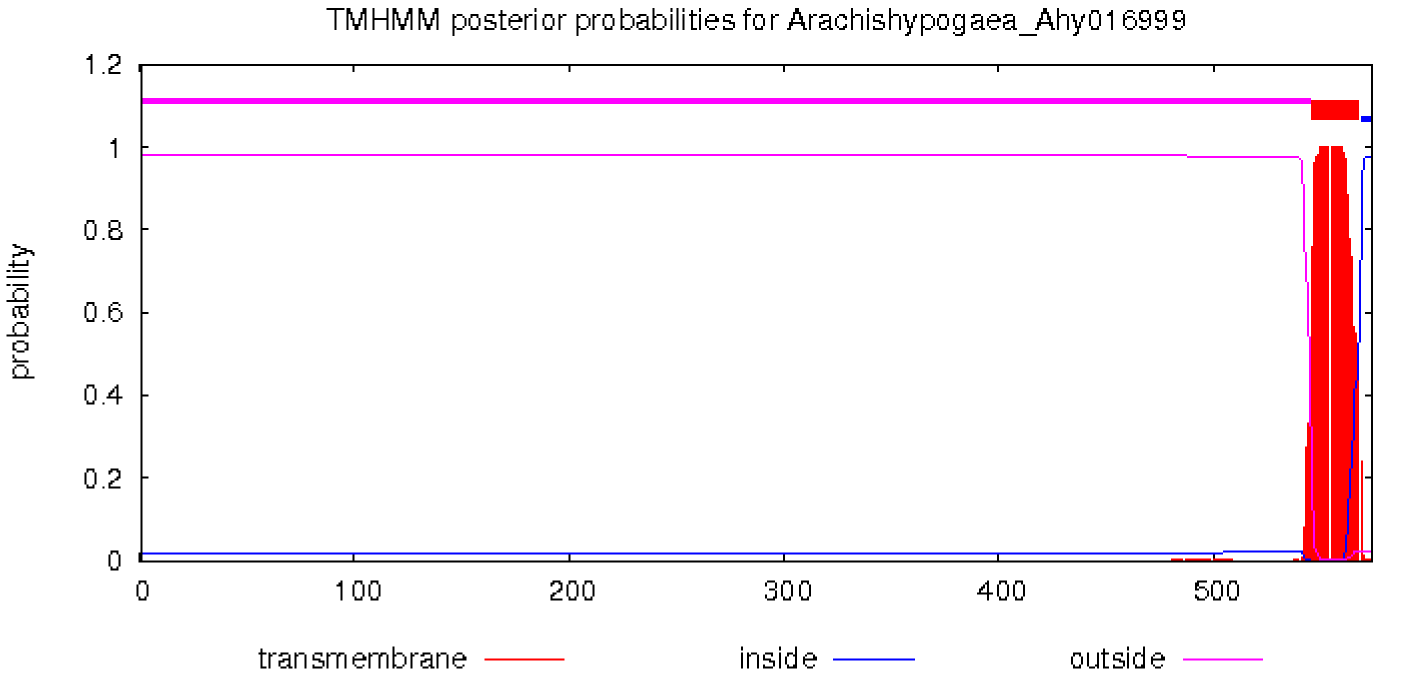

plot in postscript, script for making the plot in gnuplot, data for plot

```
# Arachishypogaea_Ahy018843 Length: 171
# Arachishypogaea_Ahy018843 Number of predicted TMHs:  0
# Arachishypogaea_Ahy018843 Exp number of AAs in TMHs: 0.00036
# Arachishypogaea_Ahy018843 Exp number, first 60 AAs:  0.00036
# Arachishypogaea_Ahy018843 Total prob of N-in:        0.03991
Arachishypogaea_Ahy018843       TMHMM2.0        outside      1   171
```

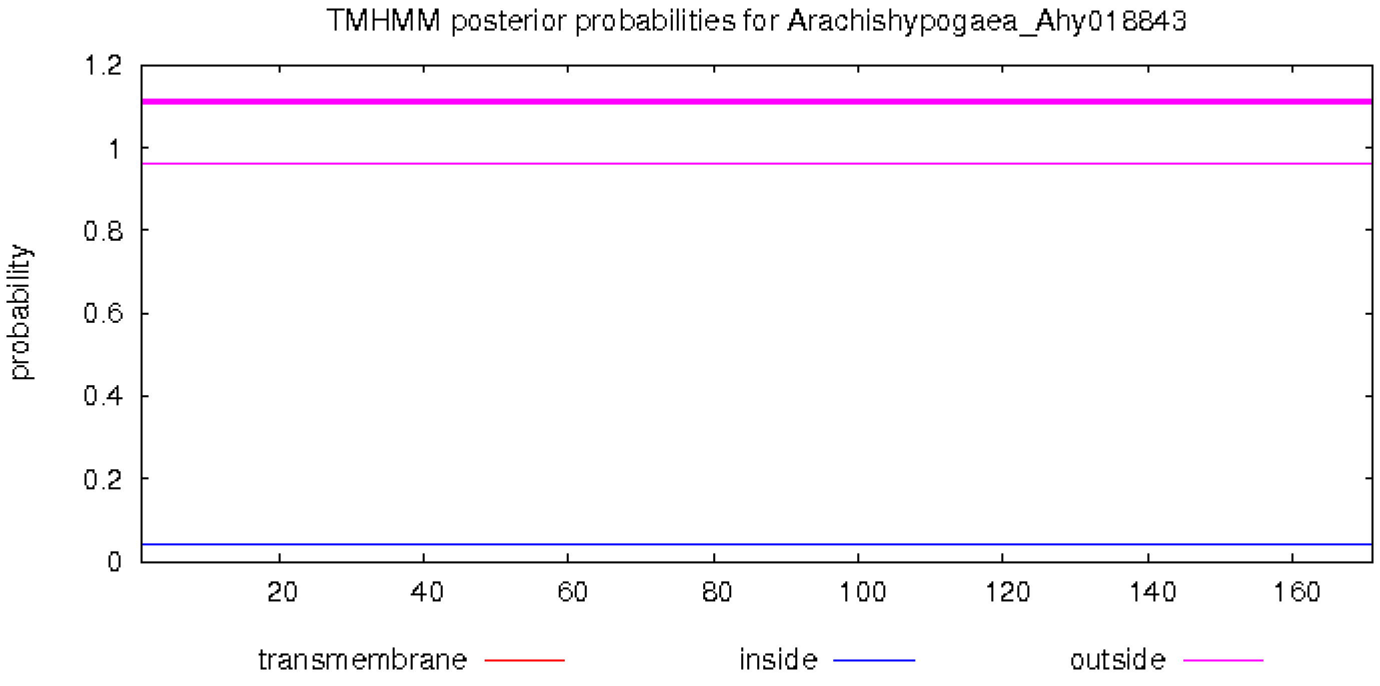

plot in postscript, script for making the plot in gnuplot, data for plot

```
# Arachishypogaea_Ahy020393 Length: 514
# Arachishypogaea_Ahy020393 Number of predicted TMHs:  1
# Arachishypogaea_Ahy020393 Exp number of AAs in TMHs: 22.55163
# Arachishypogaea_Ahy020393 Exp number, first 60 AAs:  0.00406
# Arachishypogaea_Ahy020393 Total prob of N-in:        0.03584
Arachishypogaea_Ahy020393       TMHMM2.0        outside      1   490
Arachishypogaea_Ahy020393       TMHMM2.0        TMhelix    491   513
Arachishypogaea_Ahy020393       TMHMM2.0        inside     514   514
```

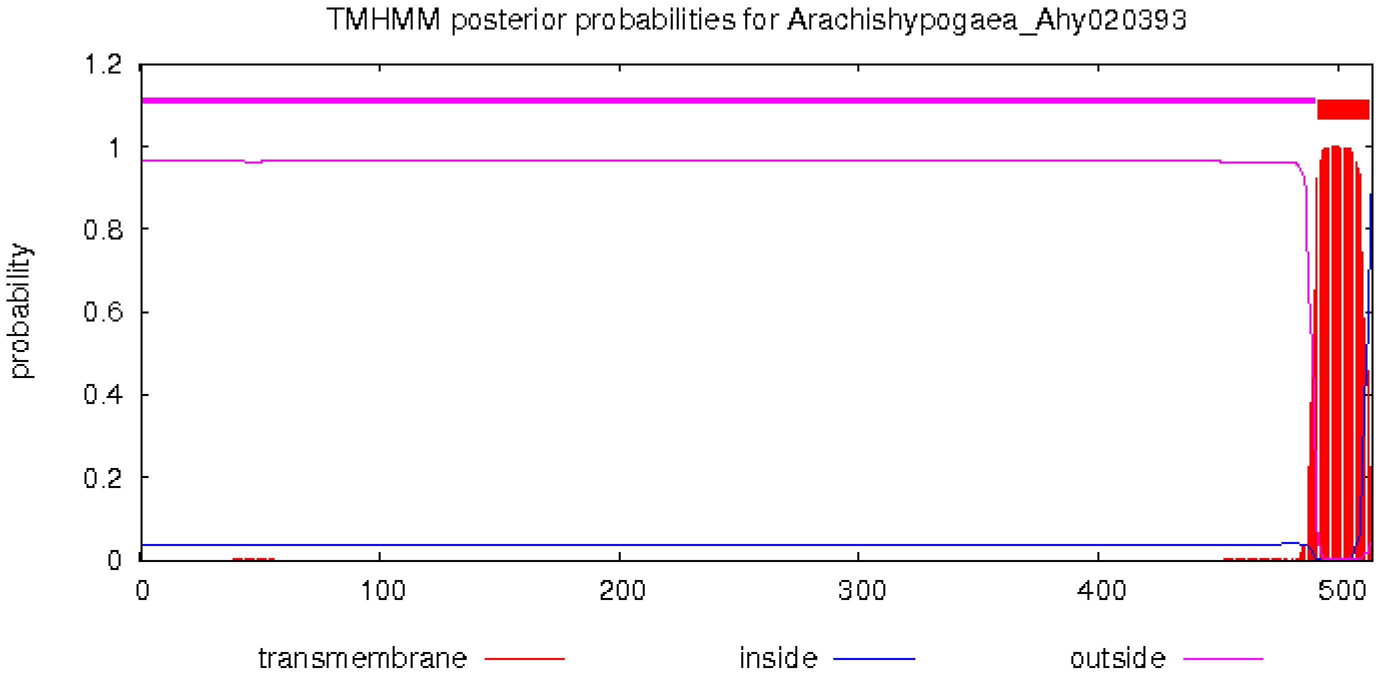

plot in postscript, script for making the plot in gnuplot, data for plot

---

```
# Arachishypogaea_Ahy020431 Length: 367
# Arachishypogaea_Ahy020431 Number of predicted TMHs:  0
# Arachishypogaea_Ahy020431 Exp number of AAs in TMHs: 0.00221
# Arachishypogaea_Ahy020431 Exp number, first 60 AAs:  0.00161
# Arachishypogaea_Ahy020431 Total prob of N-in:        0.01108
Arachishypogaea_Ahy020431       TMHMM2.0        outside      1   367
```

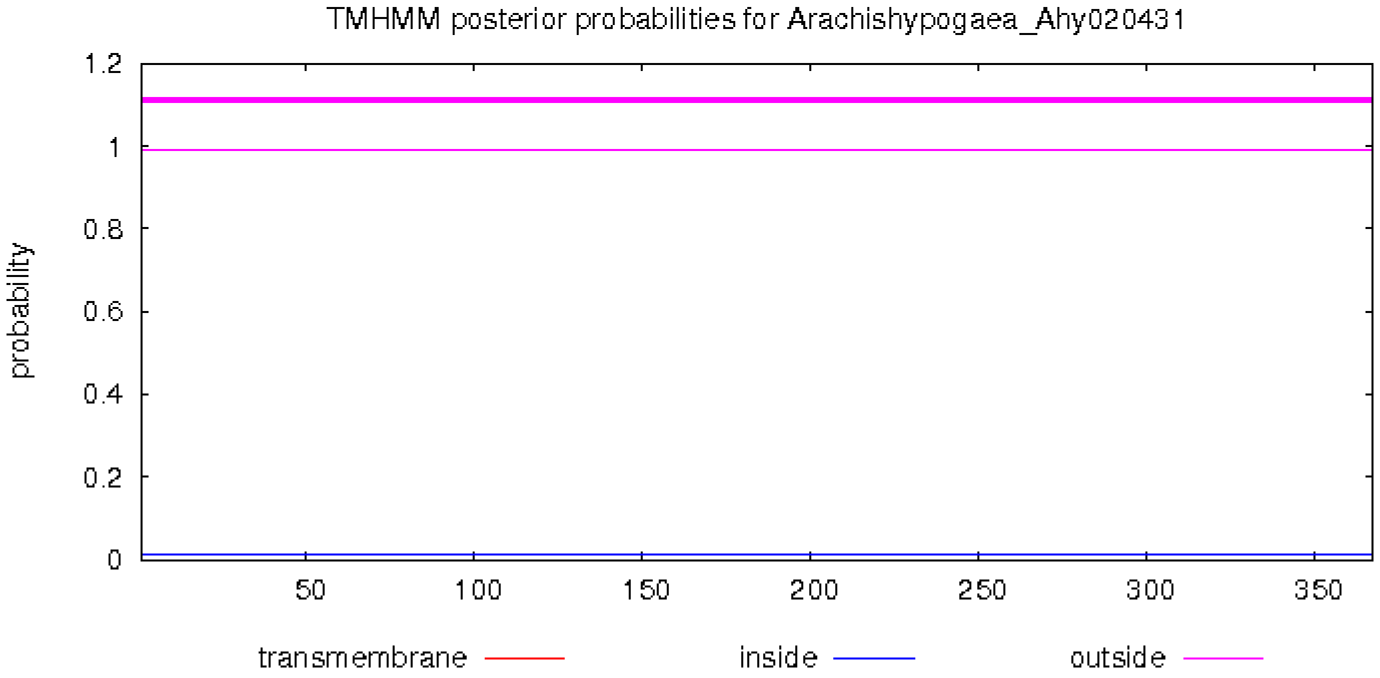

plot in postscript, script for making the plot in gnuplot, data for plot

---

```
# Arachishypogaea_Ahy020455 Length: 150
# Arachishypogaea_Ahy020455 Number of predicted TMHs:  0
# Arachishypogaea_Ahy020455 Exp number of AAs in TMHs: 0
# Arachishypogaea_Ahy020455 Exp number, first 60 AAs:  0
# Arachishypogaea_Ahy020455 Total prob of N-in:        0.01989
Arachishypogaea_Ahy020455       TMHMM2.0        outside      1   150
```

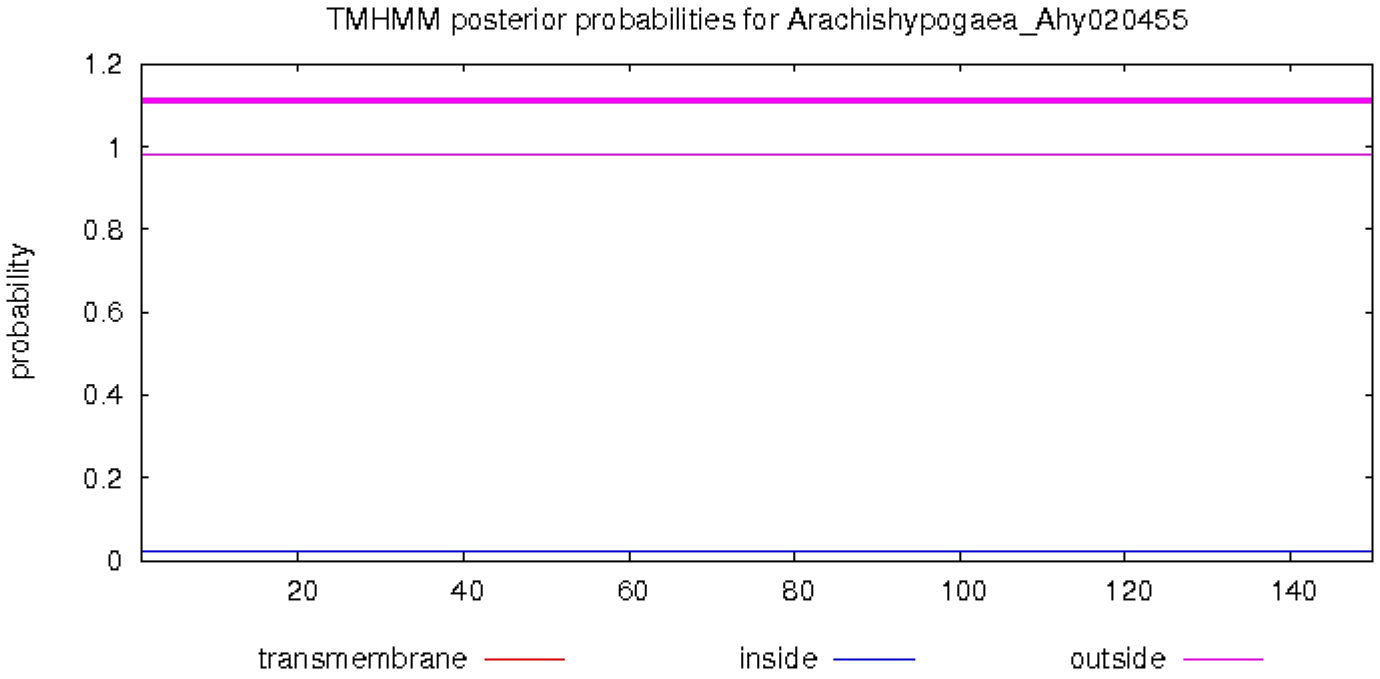

plot in postscript, script for making the plot in gnuplot, data for plot

---

```
# Arachishypogaea_Ahy020949 Length: 321
# Arachishypogaea_Ahy020949 Number of predicted TMHs:  0
# Arachishypogaea_Ahy020949 Exp number of AAs in TMHs: 0.00079
# Arachishypogaea_Ahy020949 Exp number, first 60 AAs:  0
# Arachishypogaea_Ahy020949 Total prob of N-in:        0.02063
Arachishypogaea_Ahy020949       TMHMM2.0        outside      1   321
```

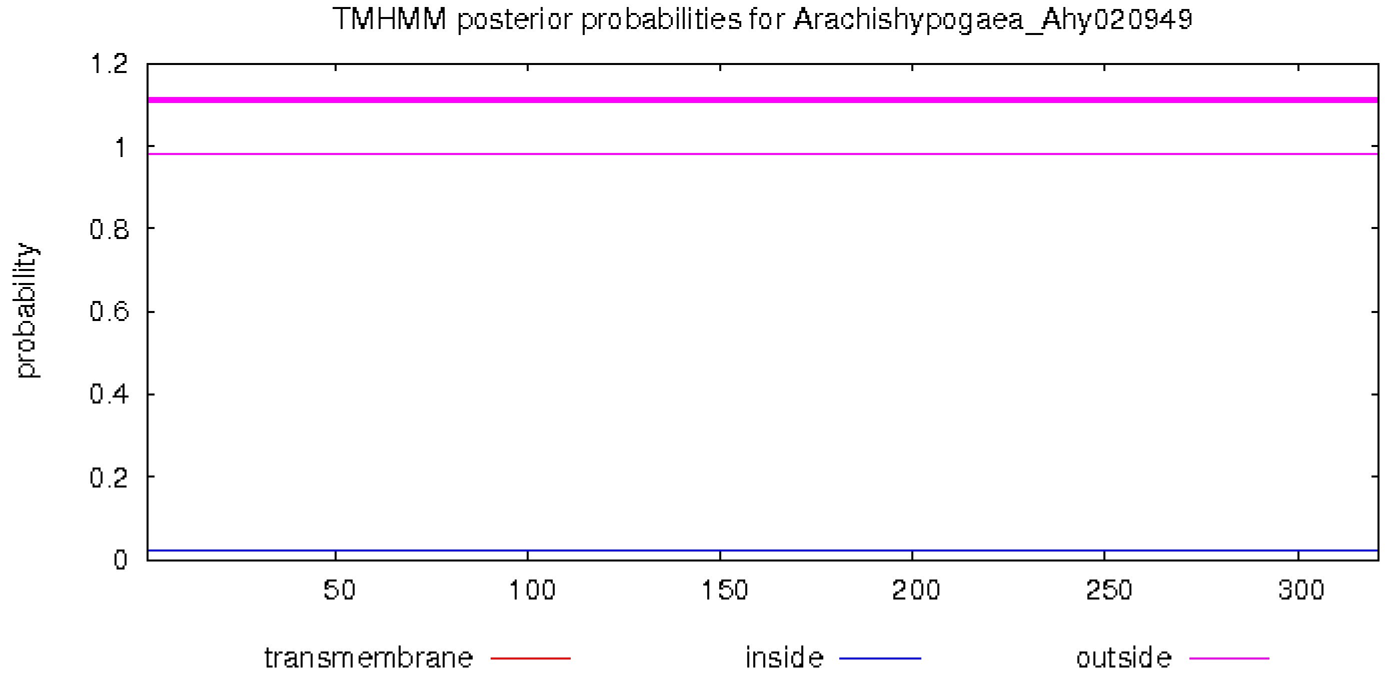

plot in postscript, script for making the plot in gnuplot, data for plot

---

```
# Arachishypogaea_Ahy021814 Length: 328
# Arachishypogaea_Ahy021814 Number of predicted TMHs:  0
# Arachishypogaea_Ahy021814 Exp number of AAs in TMHs: 0.00016
# Arachishypogaea_Ahy021814 Exp number, first 60 AAs:  0.00016
# Arachishypogaea_Ahy021814 Total prob of N-in:        0.02756
Arachishypogaea_Ahy021814       TMHMM2.0        outside      1   328
```

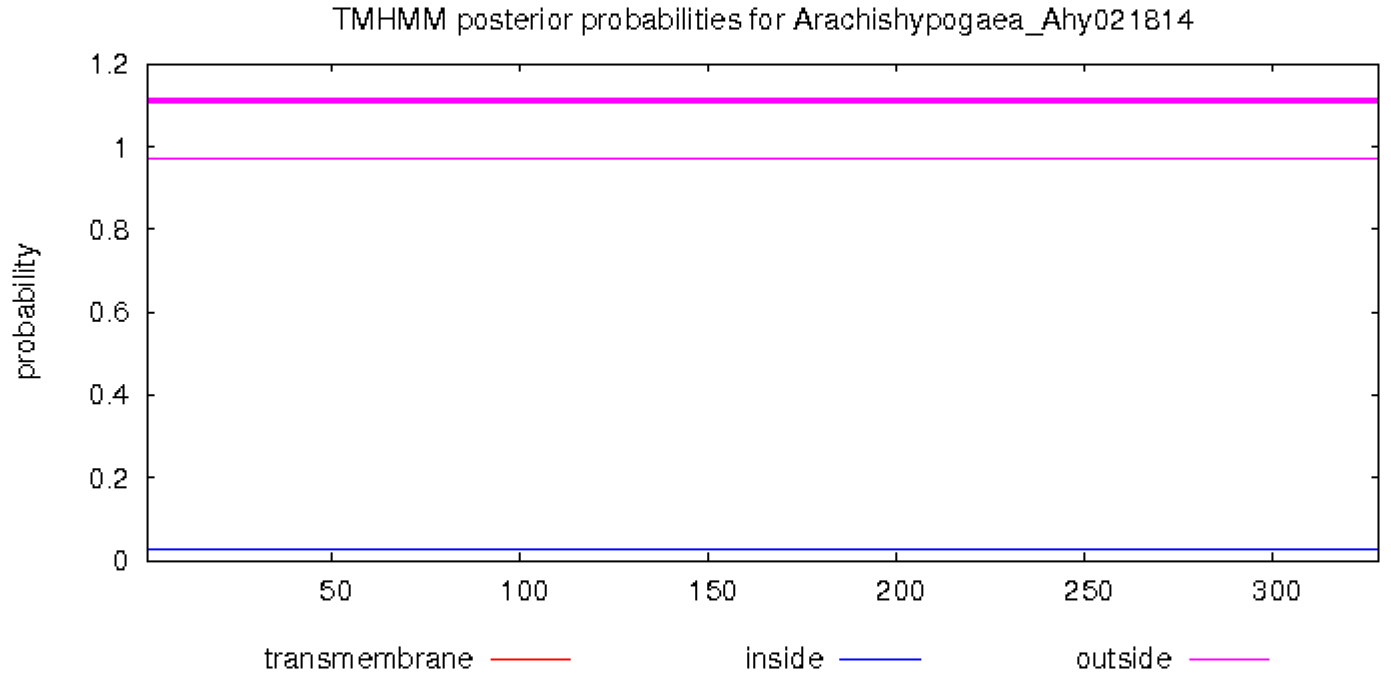

plot in postscript, script for making the plot in gnuplot, data for plot

```
# Arachishypogaea_Ahy021850 Length: 337
# Arachishypogaea_Ahy021850 Number of predicted TMHs:  0
# Arachishypogaea_Ahy021850 Exp number of AAs in TMHs: 0.00076
# Arachishypogaea_Ahy021850 Exp number, first 60 AAs:  0.00055
# Arachishypogaea_Ahy021850 Total prob of N-in:        0.00387
Arachishypogaea_Ahy021850	TMHMM2.0	outside	     1   337
```

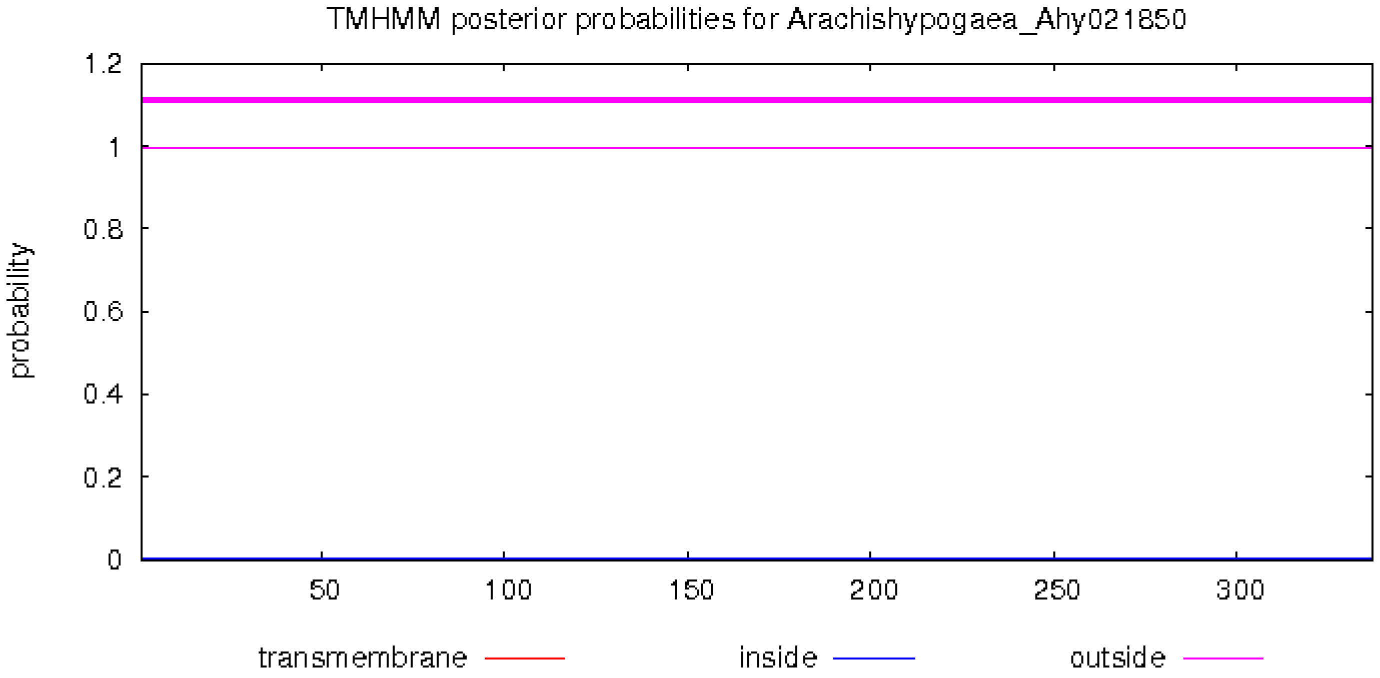

plot in postscript, script for making the plot in gnuplot, data for plot

```
# Arachishypogaea_Ahy022124 Length: 237
# Arachishypogaea_Ahy022124 Number of predicted TMHs:  0
# Arachishypogaea_Ahy022124 Exp number of AAs in TMHs: 0.00041
# Arachishypogaea_Ahy022124 Exp number, first 60 AAs:  0.00041
# Arachishypogaea_Ahy022124 Total prob of N-in:        0.00622
Arachishypogaea_Ahy022124	TMHMM2.0	outside	     1   237
```

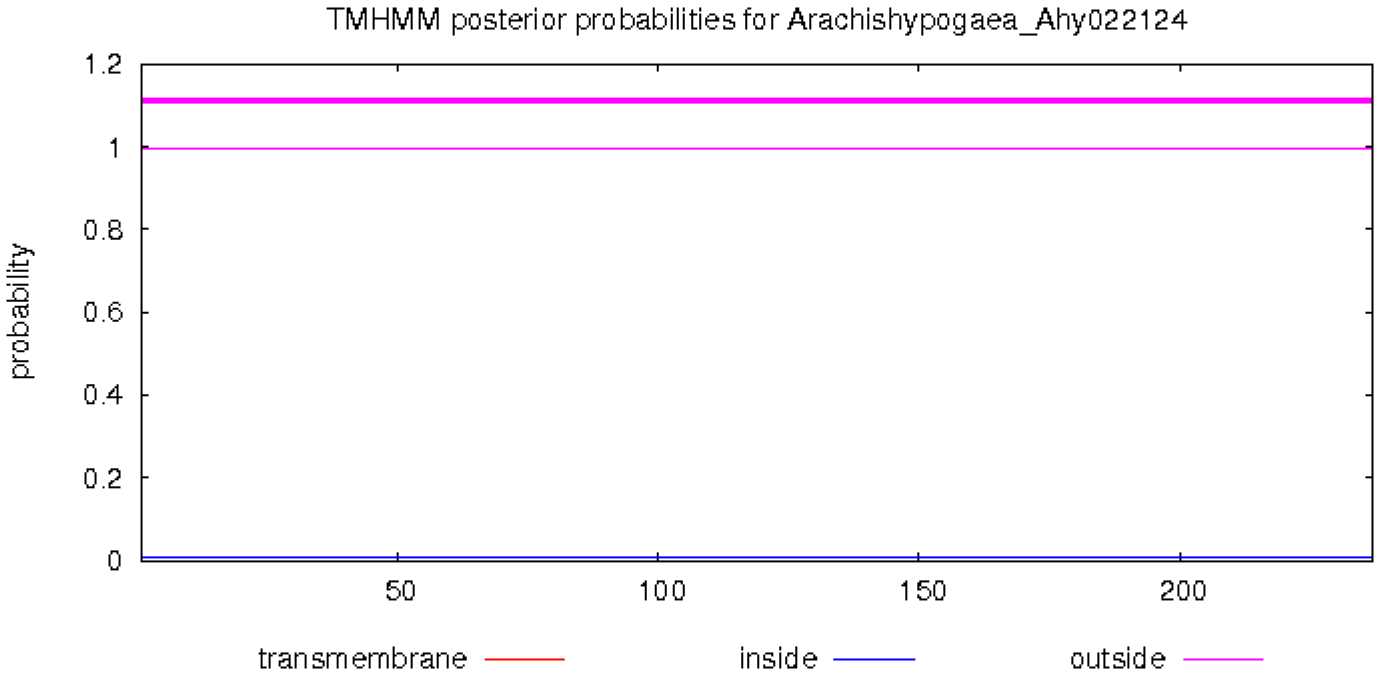

plot in postscript, script for making the plot in gnuplot, data for plot

---

```
# Arachishypogaea_Ahy022375 Length: 566
# Arachishypogaea_Ahy022375 Number of predicted TMHs:  0
# Arachishypogaea_Ahy022375 Exp number of AAs in TMHs: 0.11641
# Arachishypogaea_Ahy022375 Exp number, first 60 AAs:  0.03126
# Arachishypogaea_Ahy022375 Total prob of N-in:        0.00473
Arachishypogaea_Ahy022375	TMHMM2.0	outside	     1   566
```

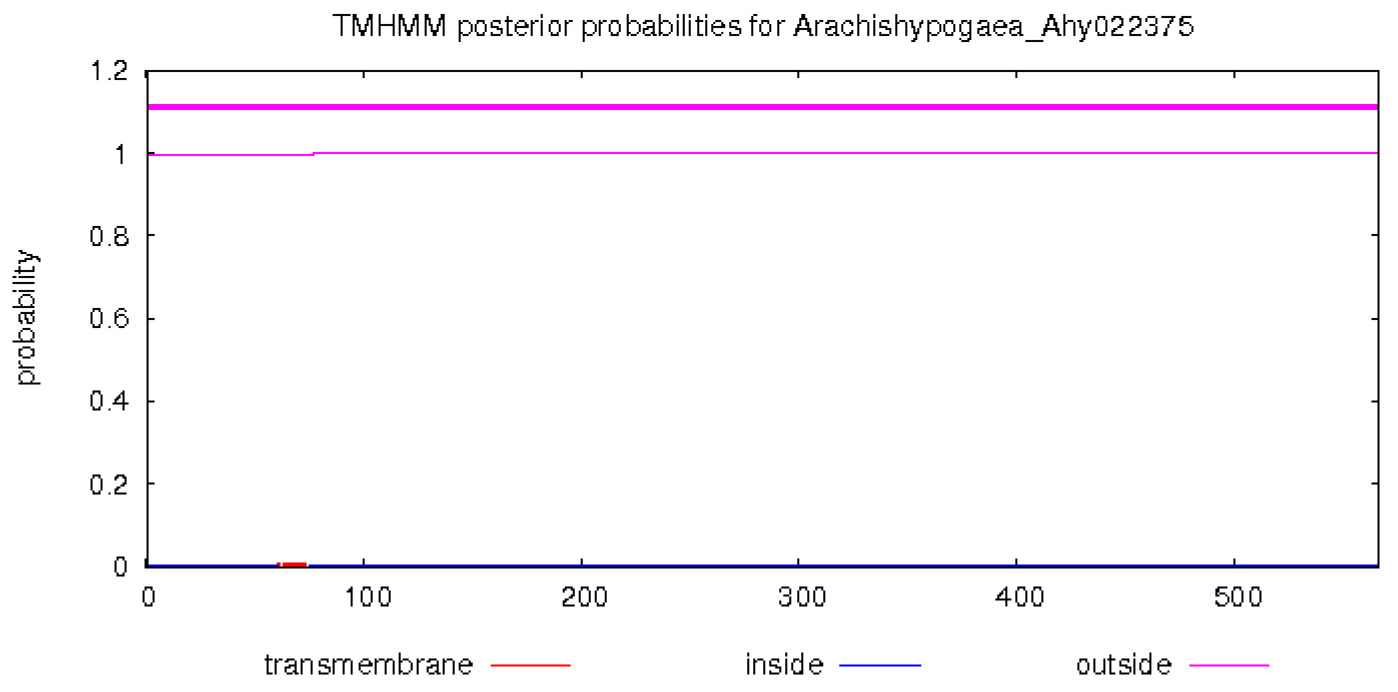

plot in postscript, script for making the plot in gnuplot, data for plot

---

```
# Arachishypogaea_Ahy022629 Length: 349
# Arachishypogaea_Ahy022629 Number of predicted TMHs:  0
# Arachishypogaea_Ahy022629 Exp number of AAs in TMHs: 0.00041
# Arachishypogaea_Ahy022629 Exp number, first 60 AAs:  0
# Arachishypogaea_Ahy022629 Total prob of N-in:        0.00698
Arachishypogaea_Ahy022629	TMHMM2.0	outside	     1   349
```

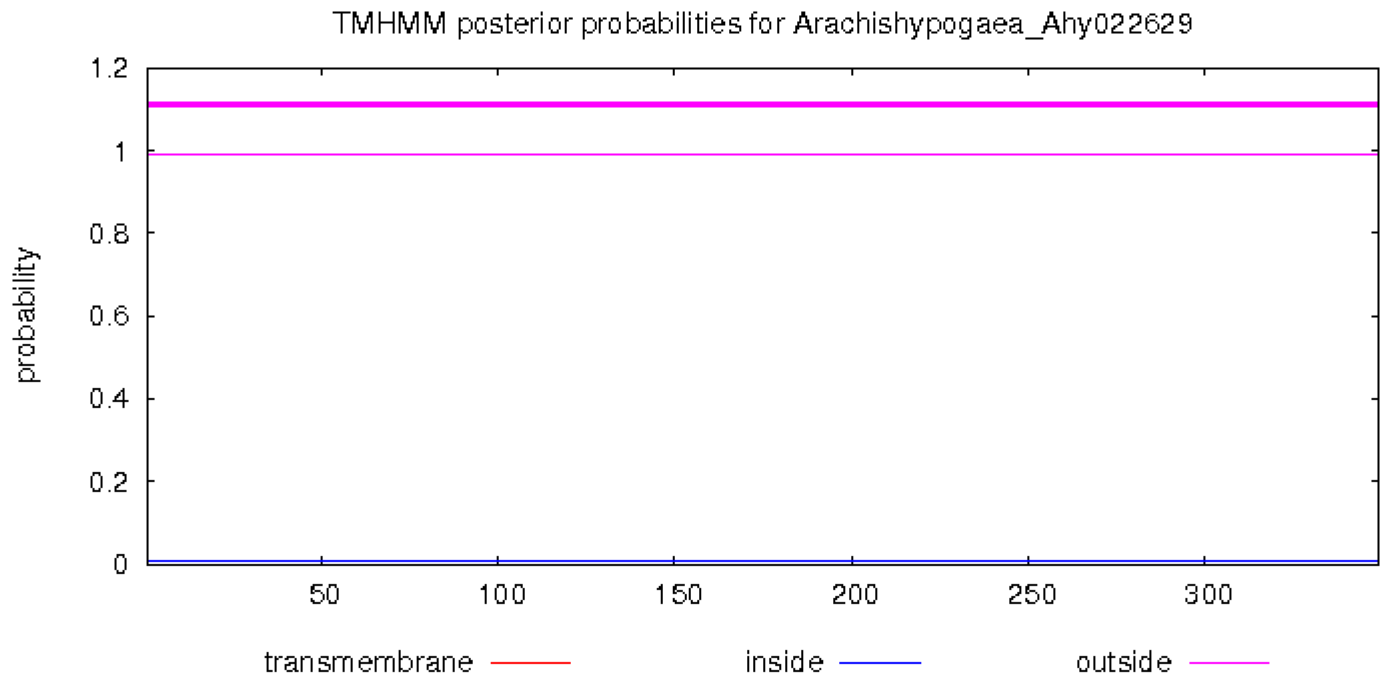

plot in postscript, script for making the plot in gnuplot, data for plot

```
# Arachishypogaea_Ahy023064 Length: 182
# Arachishypogaea_Ahy023064 Number of predicted TMHs:  0
# Arachishypogaea_Ahy023064 Exp number of AAs in TMHs: 0.00019
# Arachishypogaea_Ahy023064 Exp number, first 60 AAs:  0
# Arachishypogaea_Ahy023064 Total prob of N-in:        0.00677
Arachishypogaea_Ahy023064       TMHMM2.0        outside      1   182
```

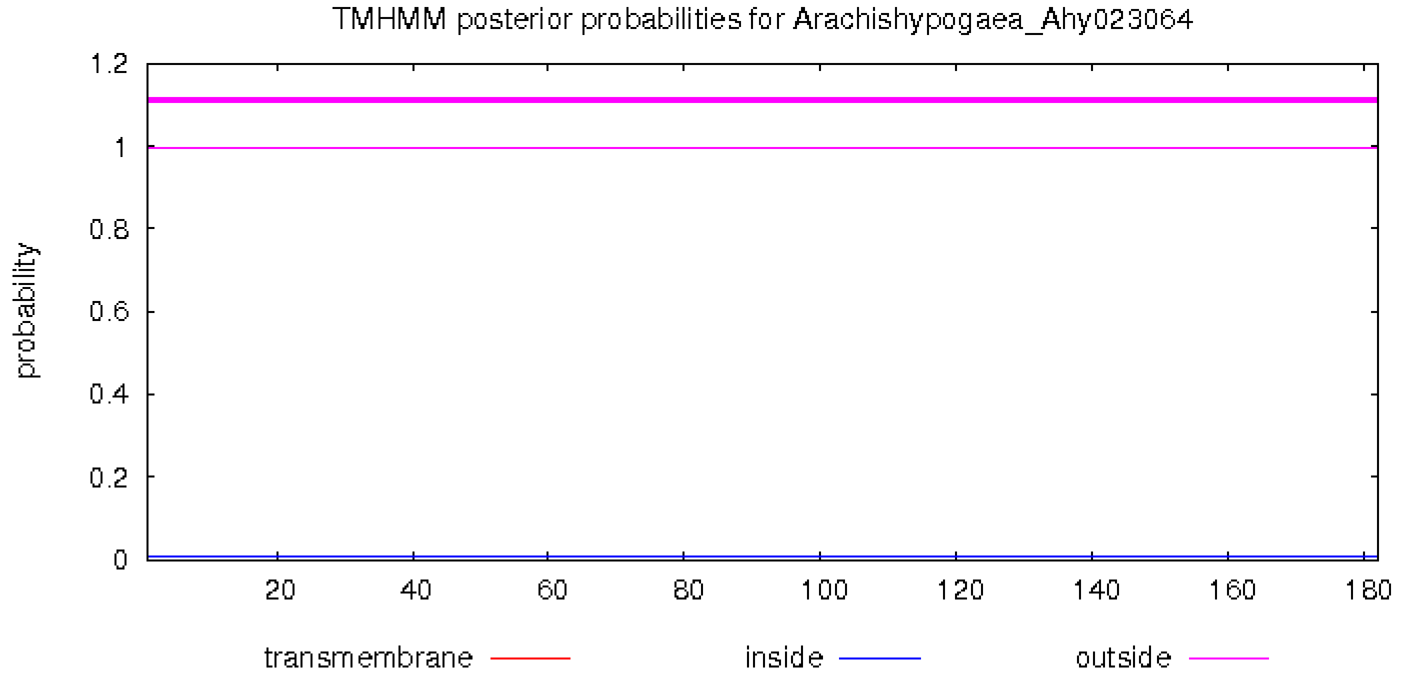

plot in postscript, script for making the plot in gnuplot, data for plot

```
# Arachisipaensis_Araip.0550R Length: 330
# Arachisipaensis_Araip.0550R Number of predicted TMHs:  0
# Arachisipaensis_Araip.0550R Exp number of AAs in TMHs: 0.00099
# Arachisipaensis_Araip.0550R Exp number, first 60 AAs:  0.00099
# Arachisipaensis_Araip.0550R Total prob of N-in:        0.00336
Arachisipaensis_Araip.0550R     TMHMM2.0        outside      1   330
```

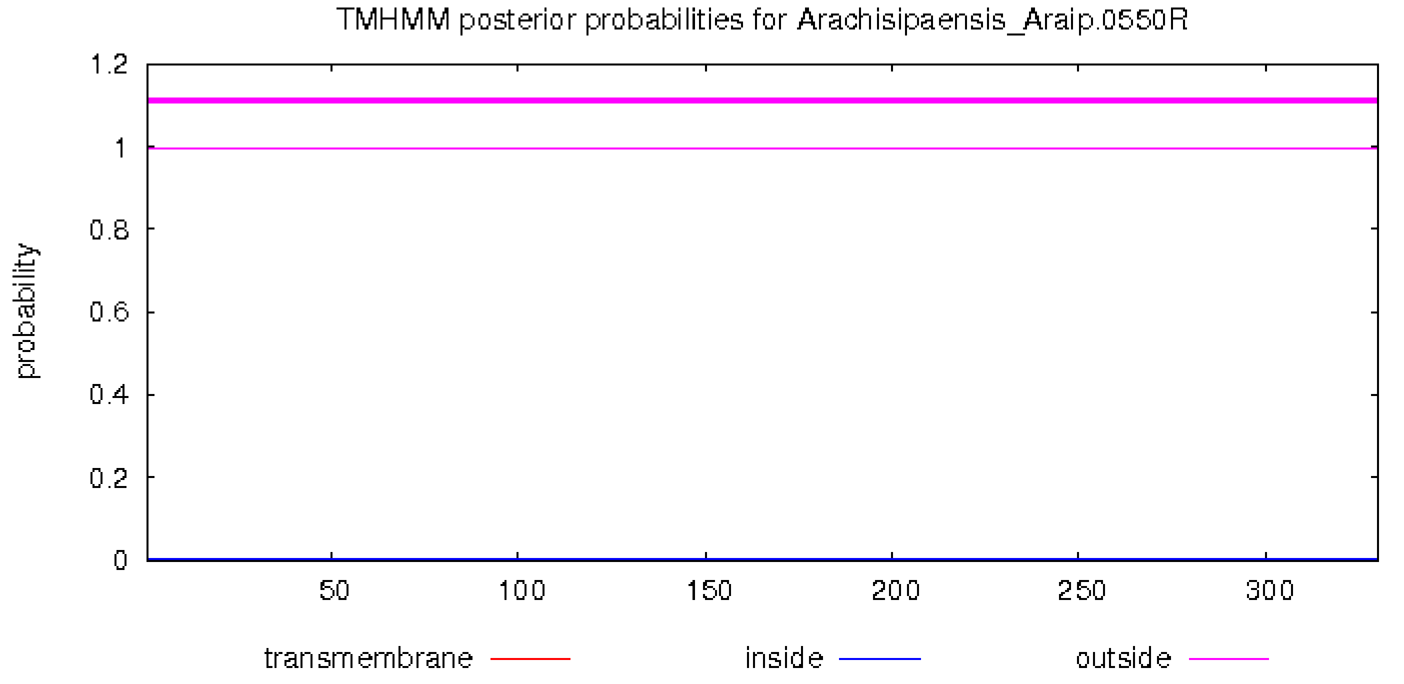

plot in postscript, script for making the plot in gnuplot, data for plot

```
# Arachisipaensis_Araip.0S3JI Length: 358
# Arachisipaensis_Araip.0S3JI Number of predicted TMHs:  0
# Arachisipaensis_Araip.0S3JI Exp number of AAs in TMHs: 0.00035
# Arachisipaensis_Araip.0S3JI Exp number, first 60 AAs:  0
# Arachisipaensis_Araip.0S3JI Total prob of N-in:        0.02892
Arachisipaensis_Araip.0S3JI     TMHMM2.0        outside      1   358
```

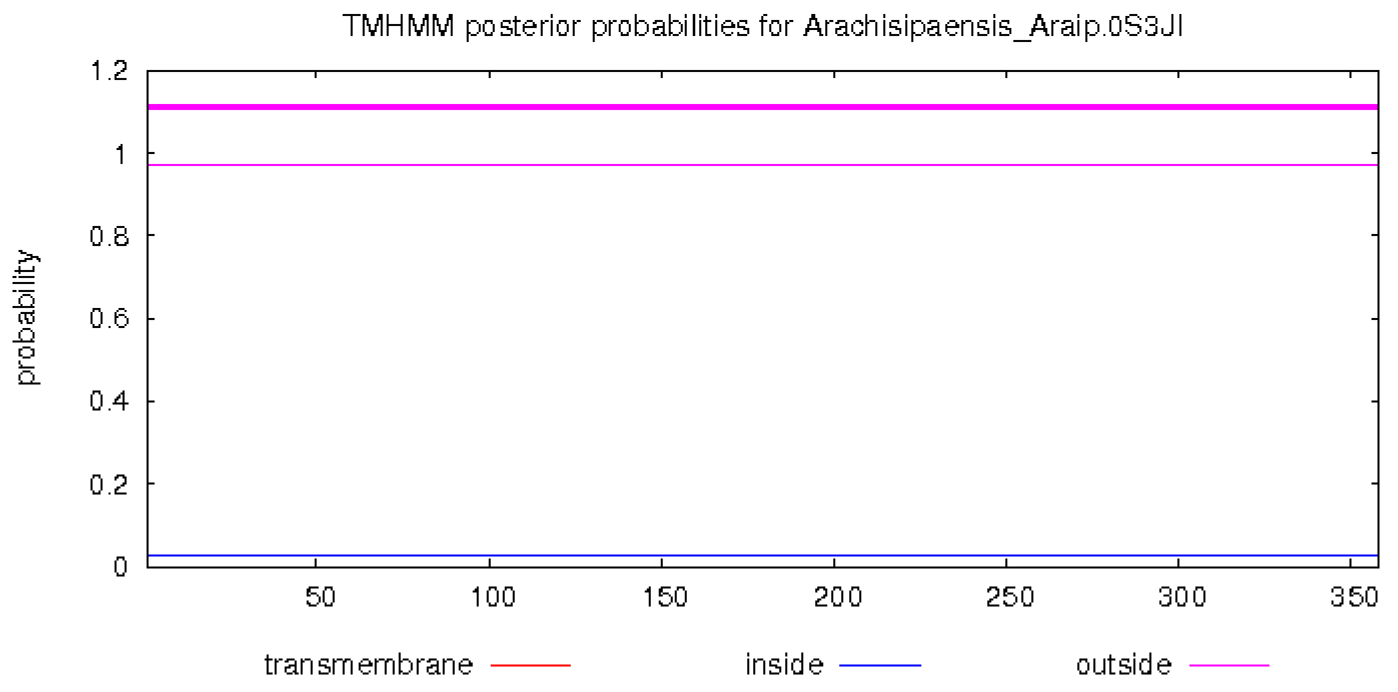

plot in postscript, script for making the plot in gnuplot, data for plot

```
# Arachisipaensis_Araip.1N7IP Length: 324
# Arachisipaensis_Araip.1N7IP Number of predicted TMHs:  0
# Arachisipaensis_Araip.1N7IP Exp number of AAs in TMHs: 0.07978
# Arachisipaensis_Araip.1N7IP Exp number, first 60 AAs:  0
# Arachisipaensis_Araip.1N7IP Total prob of N-in:        0.11283
Arachisipaensis_Araip.1N7IP     TMHMM2.0        outside      1   324
```

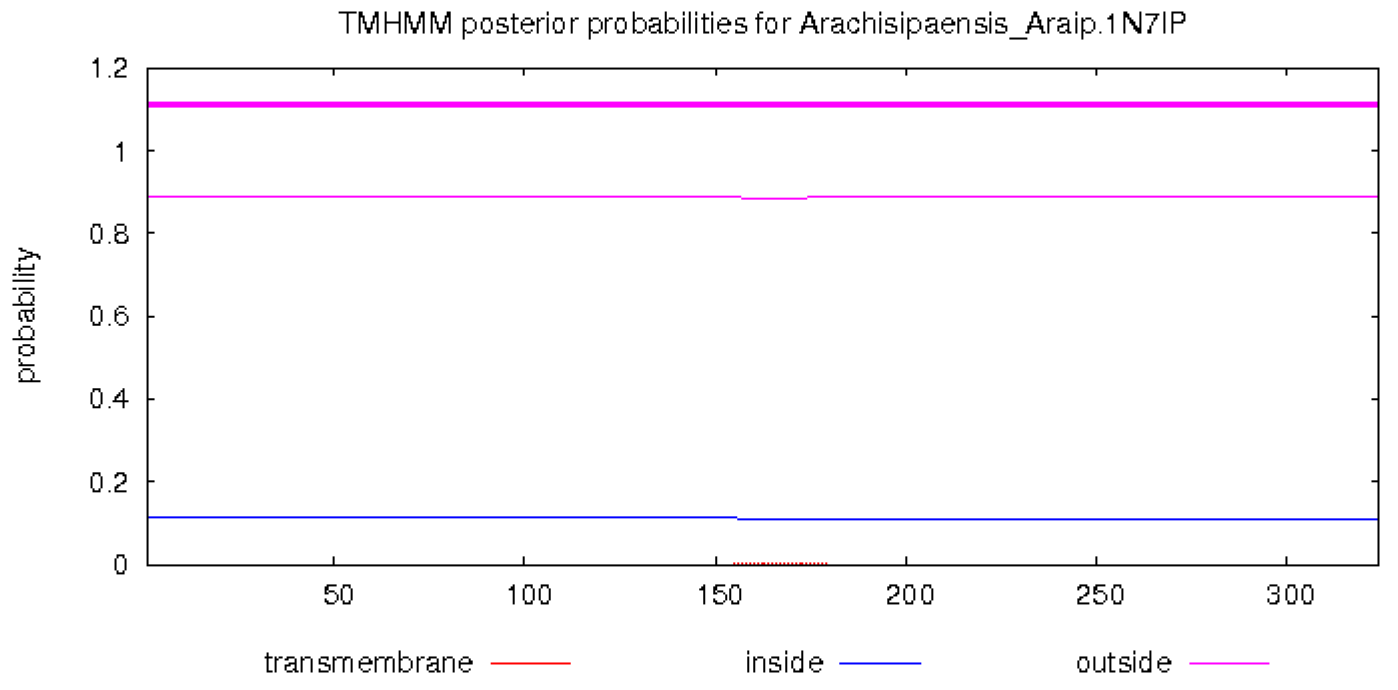

plot in postscript, script for making the plot in gnuplot, data for plot

---

```
# Arachisipaensis_Araip.1Z0SD Length: 381
# Arachisipaensis_Araip.1Z0SD Number of predicted TMHs:  0
# Arachisipaensis_Araip.1Z0SD Exp number of AAs in TMHs: 0
# Arachisipaensis_Araip.1Z0SD Exp number, first 60 AAs:  0
# Arachisipaensis_Araip.1Z0SD Total prob of N-in:        0.02113
Arachisipaensis_Araip.1Z0SD     TMHMM2.0        outside      1   381
```

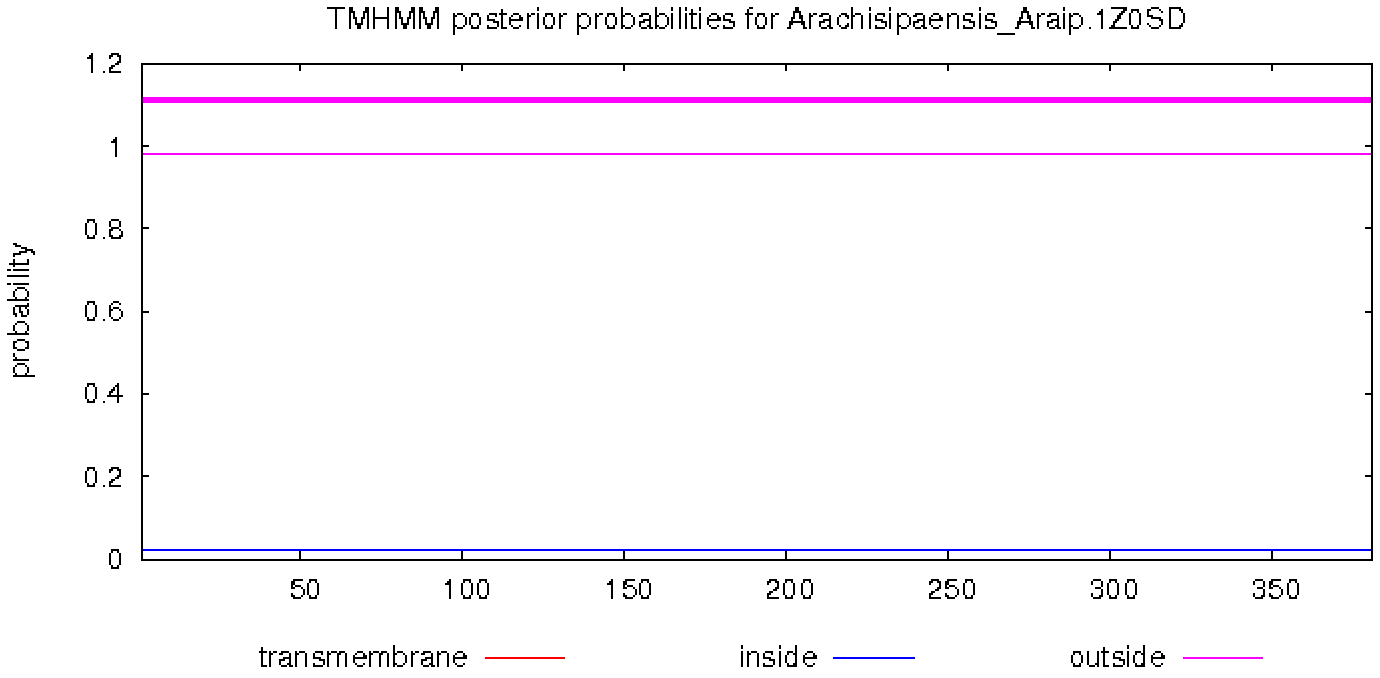

plot in postscript, script for making the plot in gnuplot, data for plot

---

```
# Arachisipaensis_Araip.2BL8E Length: 320
# Arachisipaensis_Araip.2BL8E Number of predicted TMHs:  1
# Arachisipaensis_Araip.2BL8E Exp number of AAs in TMHs: 22.53842
# Arachisipaensis_Araip.2BL8E Exp number, first 60 AAs:  0.01738
# Arachisipaensis_Araip.2BL8E Total prob of N-in:        0.13390
Arachisipaensis_Araip.2BL8E     TMHMM2.0        outside      1   281
Arachisipaensis_Araip.2BL8E     TMHMM2.0        TMhelix    282   304
Arachisipaensis_Araip.2BL8E     TMHMM2.0        inside     305   320
```

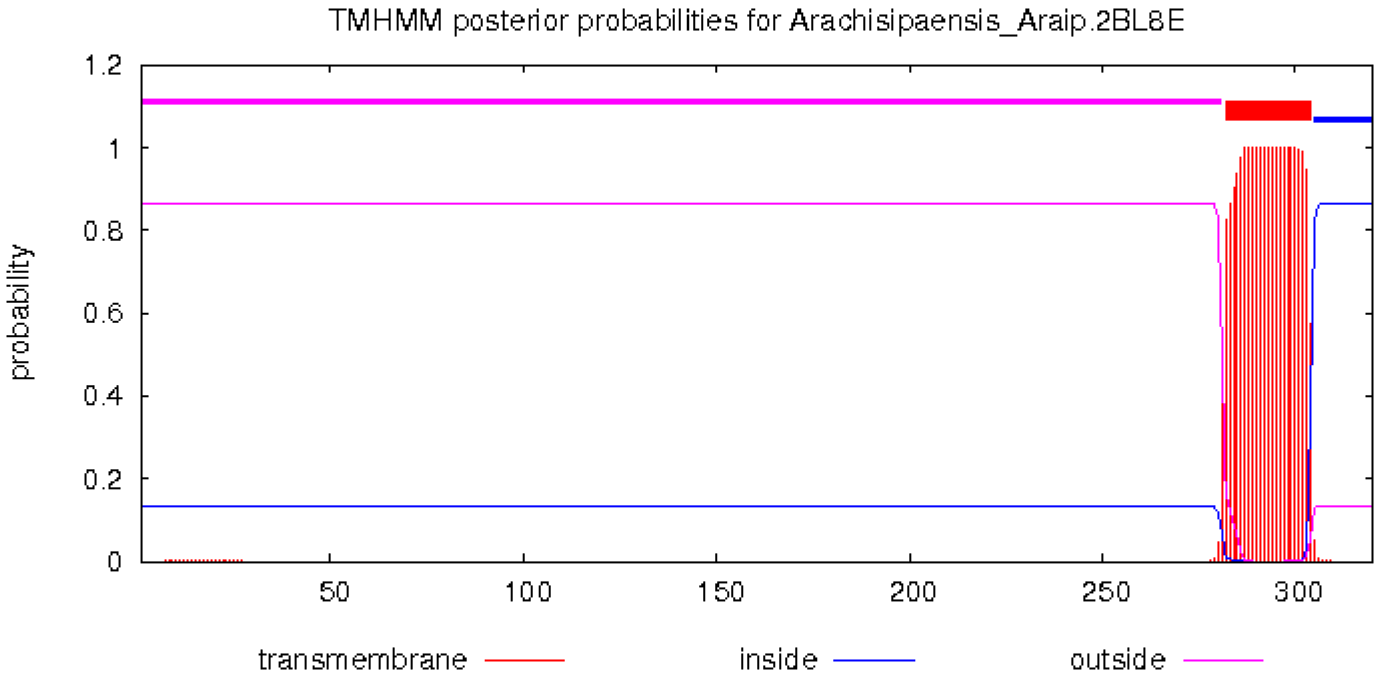

plot in postscript, script for making the plot in gnuplot, data for plot

---

```
# Arachisipaensis_Araip.2W5R5 Length: 592
# Arachisipaensis_Araip.2W5R5 Number of predicted TMHs:  1
# Arachisipaensis_Araip.2W5R5 Exp number of AAs in TMHs: 22.56847
# Arachisipaensis_Araip.2W5R5 Exp number, first 60 AAs:  0.01598
# Arachisipaensis_Araip.2W5R5 Total prob of N-in:        0.03114
Arachisipaensis_Araip.2W5R5     TMHMM2.0        outside      1   568
Arachisipaensis_Araip.2W5R5     TMHMM2.0        TMhelix    569   591
Arachisipaensis_Araip.2W5R5     TMHMM2.0        inside     592   592
```

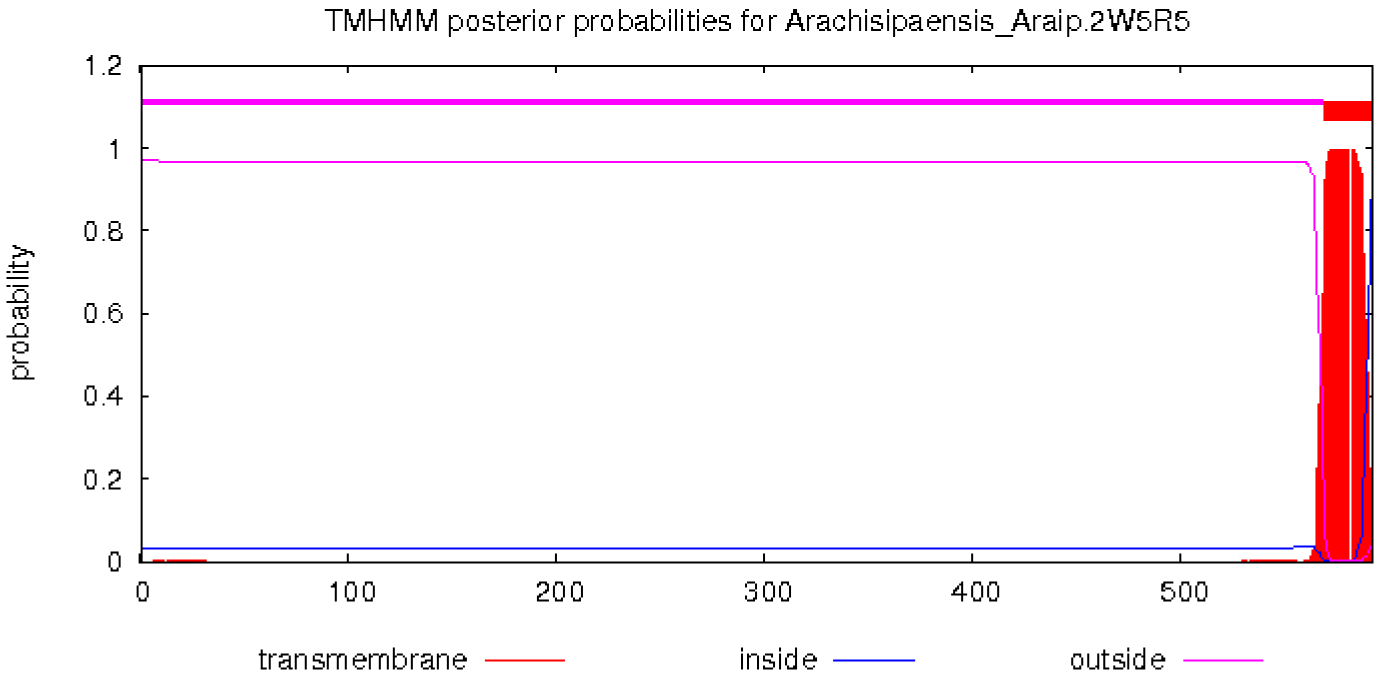

plot in postscript, script for making the plot in gnuplot, data for plot

---

```
# Arachisipaensis_Araip.310T2 Length: 301
# Arachisipaensis_Araip.310T2 Number of predicted TMHs:  0
# Arachisipaensis_Araip.310T2 Exp number of AAs in TMHs: 0.00098
# Arachisipaensis_Araip.310T2 Exp number, first 60 AAs:  0.00023
```

```
# Arachisipaensis_Araip.310T2 Total prob of N-in:        0.00283
Arachisipaensis_Araip.310T2     TMHMM2.0        outside      1   301
```

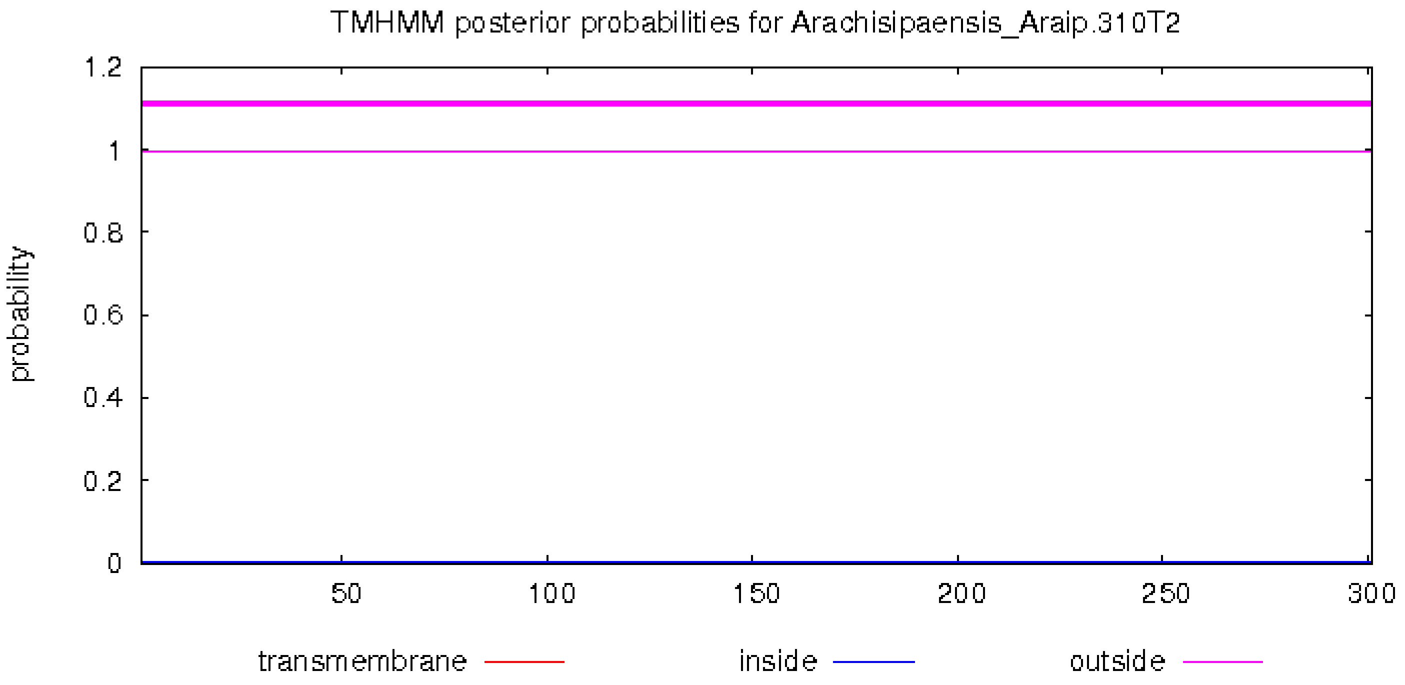

plot in postscript, script for making the plot in gnuplot, data for plot

---

```
# Arachisipaensis_Araip.31EFM Length: 410
# Arachisipaensis_Araip.31EFM Number of predicted TMHs:  0
# Arachisipaensis_Araip.31EFM Exp number of AAs in TMHs: 0
# Arachisipaensis_Araip.31EFM Exp number, first 60 AAs:  0
# Arachisipaensis_Araip.31EFM Total prob of N-in:        0.00308
Arachisipaensis_Araip.31EFM     TMHMM2.0        outside      1   410
```

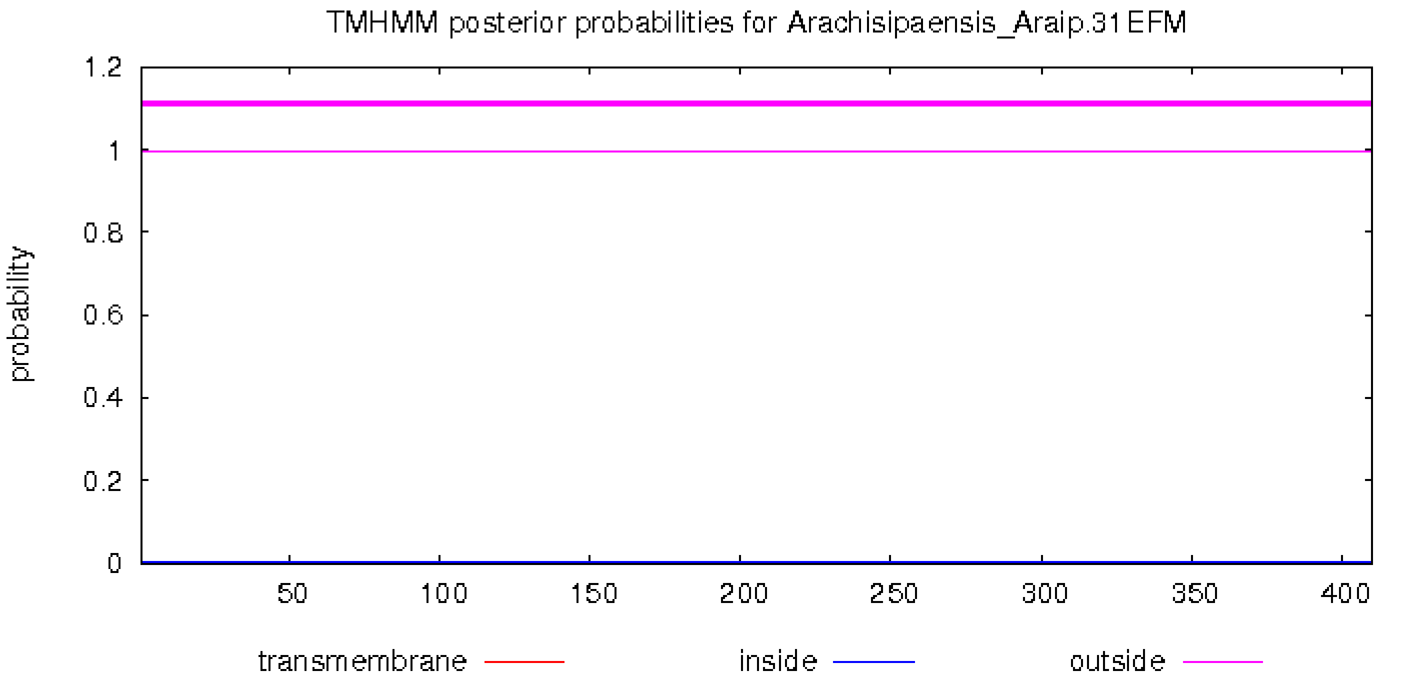

plot in postscript, script for making the plot in gnuplot, data for plot

---

```
# Arachisipaensis_Araip.333QY Length: 335
# Arachisipaensis_Araip.333QY Number of predicted TMHs:  0
# Arachisipaensis_Araip.333QY Exp number of AAs in TMHs: 0.000770000000000000001
# Arachisipaensis_Araip.333QY Exp number, first 60 AAs:  0.00056
```

```
# Arachisipaensis_Araip.333QY Total prob of N-in:        0.00396
Arachisipaensis_Araip.333QY     TMHMM2.0        outside      1   335
```

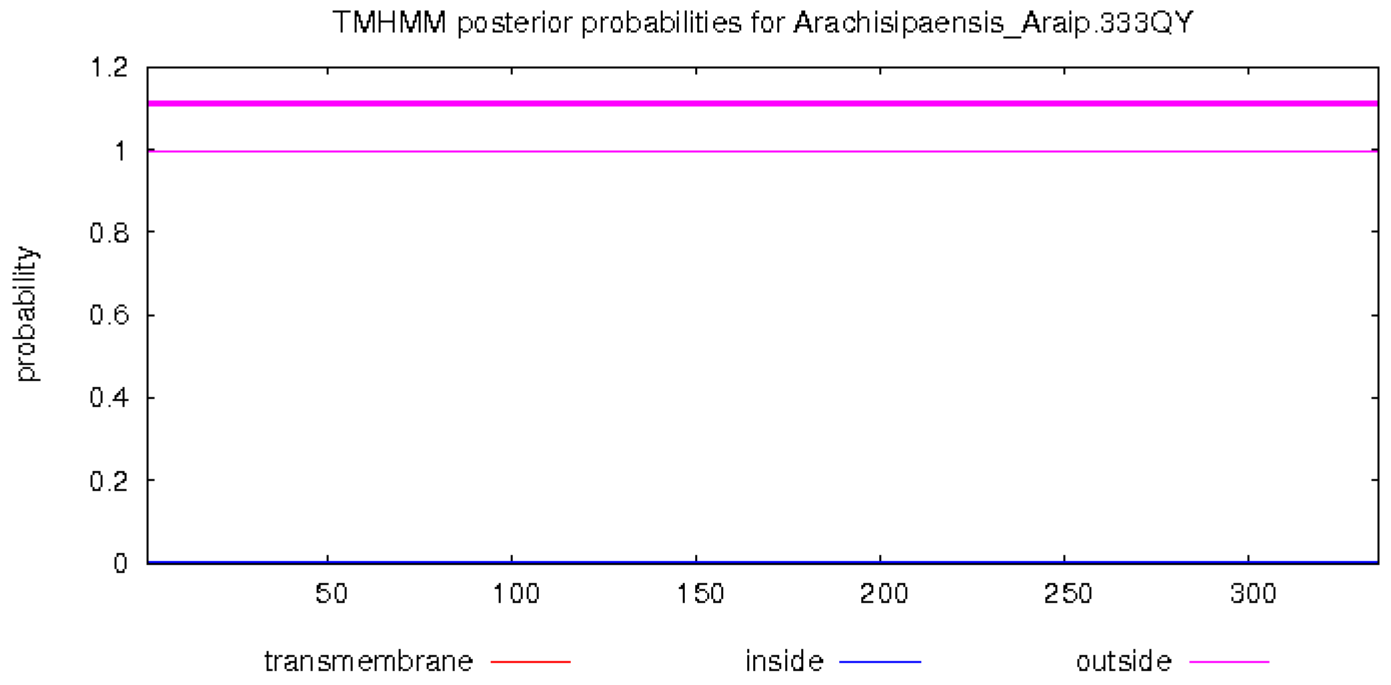

plot in postscript, script for making the plot in gnuplot, data for plot

---

```
# Arachisipaensis_Araip.4A49L Length: 285
# Arachisipaensis_Araip.4A49L Number of predicted TMHs:  0
# Arachisipaensis_Araip.4A49L Exp number of AAs in TMHs: 0.037
# Arachisipaensis_Araip.4A49L Exp number, first 60 AAs:  0.03554
# Arachisipaensis_Araip.4A49L Total prob of N-in:        0.05204
Arachisipaensis_Araip.4A49L     TMHMM2.0        outside      1   285
```

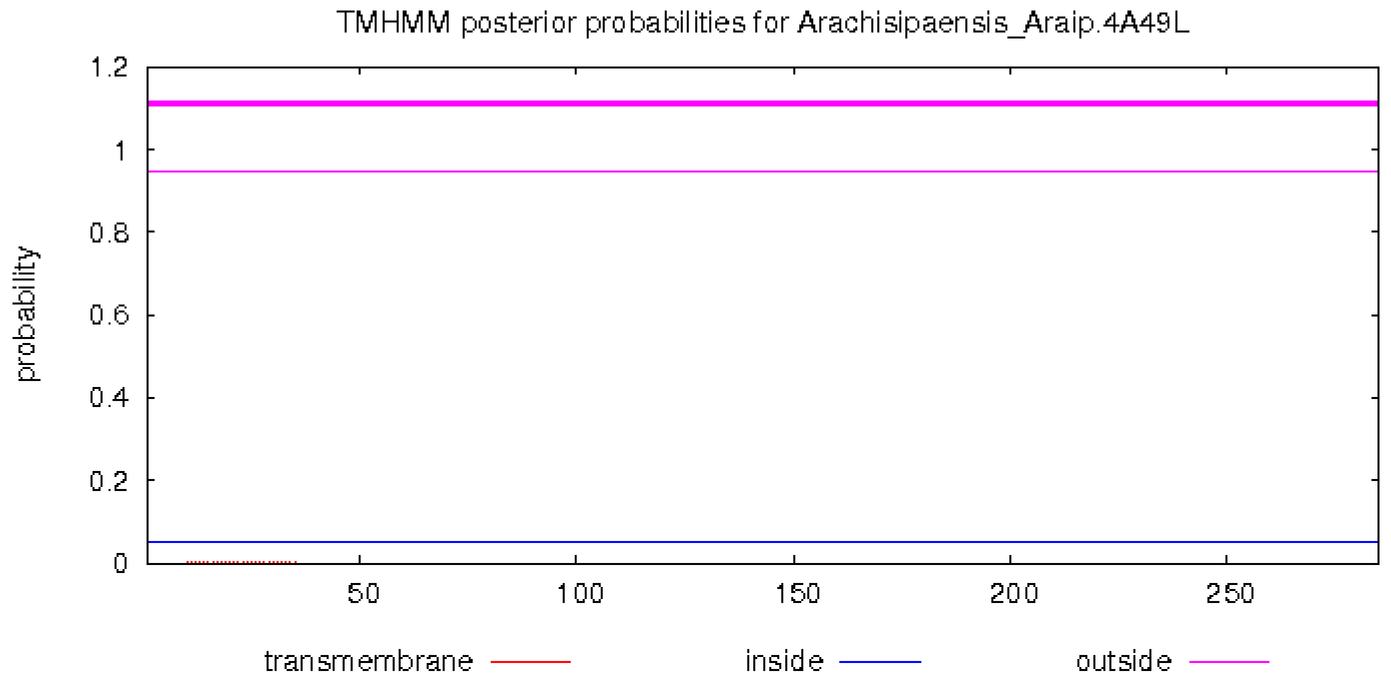

plot in postscript, script for making the plot in gnuplot, data for plot

---

```
# Arachisipaensis_Araip.4R4C1 Length: 69
# Arachisipaensis_Araip.4R4C1 Number of predicted TMHs:  0
# Arachisipaensis_Araip.4R4C1 Exp number of AAs in TMHs: 0.05959
# Arachisipaensis_Araip.4R4C1 Exp number, first 60 AAs:  0.05951
```

```
# Arachisipaensis_Araip.4R4C1 Total prob of N-in:        0.26959
Arachisipaensis_Araip.4R4C1     TMHMM2.0        outside      1    69
```

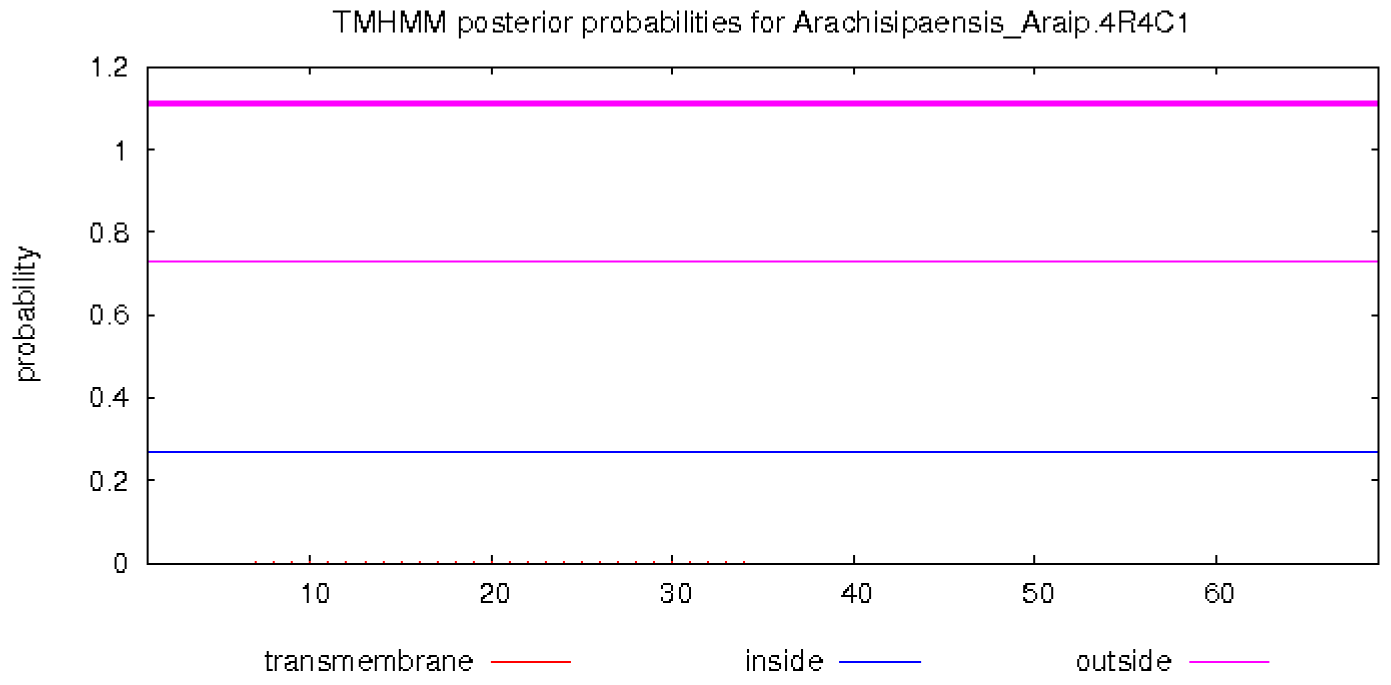

plot in postscript, script for making the plot in gnuplot, data for plot

---

```
# Arachisipaensis_Araip.609WS Length: 363
# Arachisipaensis_Araip.609WS Number of predicted TMHs:  0
# Arachisipaensis_Araip.609WS Exp number of AAs in TMHs: 0.00714
# Arachisipaensis_Araip.609WS Exp number, first 60 AAs:  0
# Arachisipaensis_Araip.609WS Total prob of N-in:        0.00206
Arachisipaensis_Araip.609WS     TMHMM2.0        outside      1   363
```

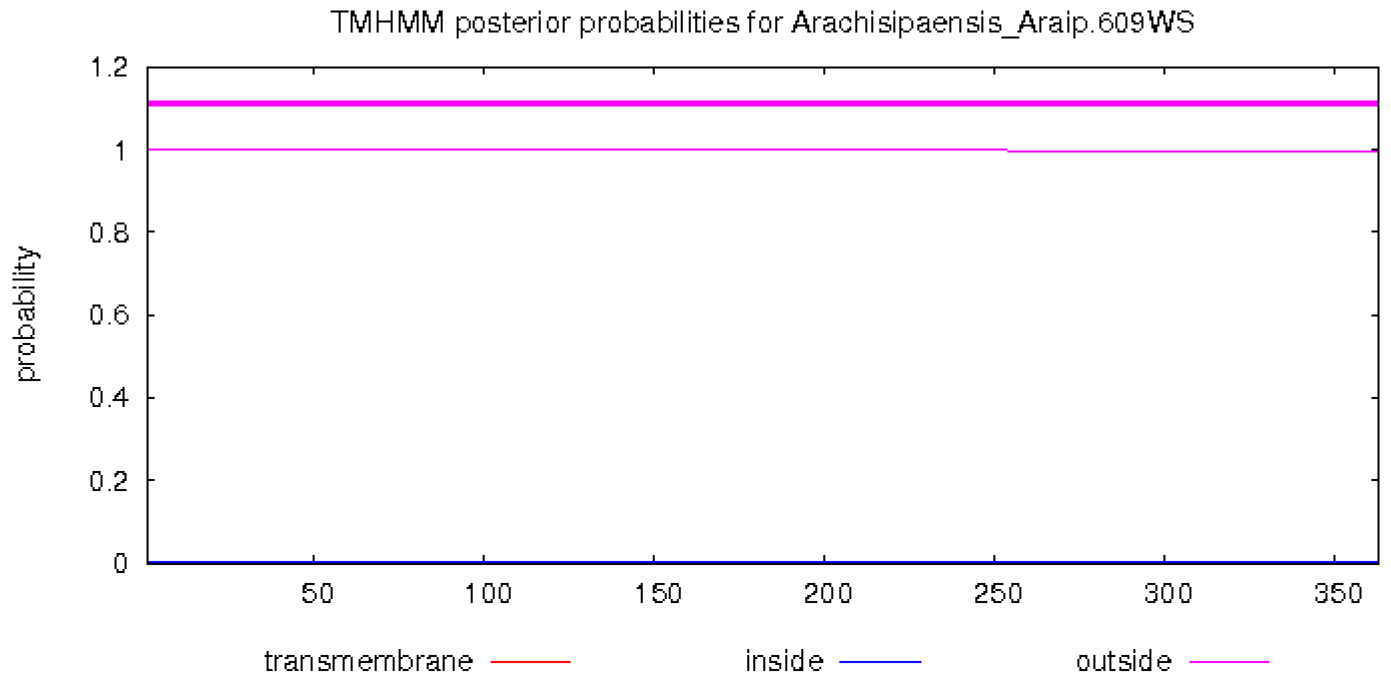

plot in postscript, script for making the plot in gnuplot, data for plot

---

```
# Arachisipaensis_Araip.64GCN Length: 308
# Arachisipaensis_Araip.64GCN Number of predicted TMHs:  0
# Arachisipaensis_Araip.64GCN Exp number of AAs in TMHs: 0.00118
# Arachisipaensis_Araip.64GCN Exp number, first 60 AAs:  0
```

```
# Arachisipaensis_Araip.64GCN Total prob of N-in:        0.04432
Arachisipaensis_Araip.64GCN     TMHMM2.0        outside      1   308
```

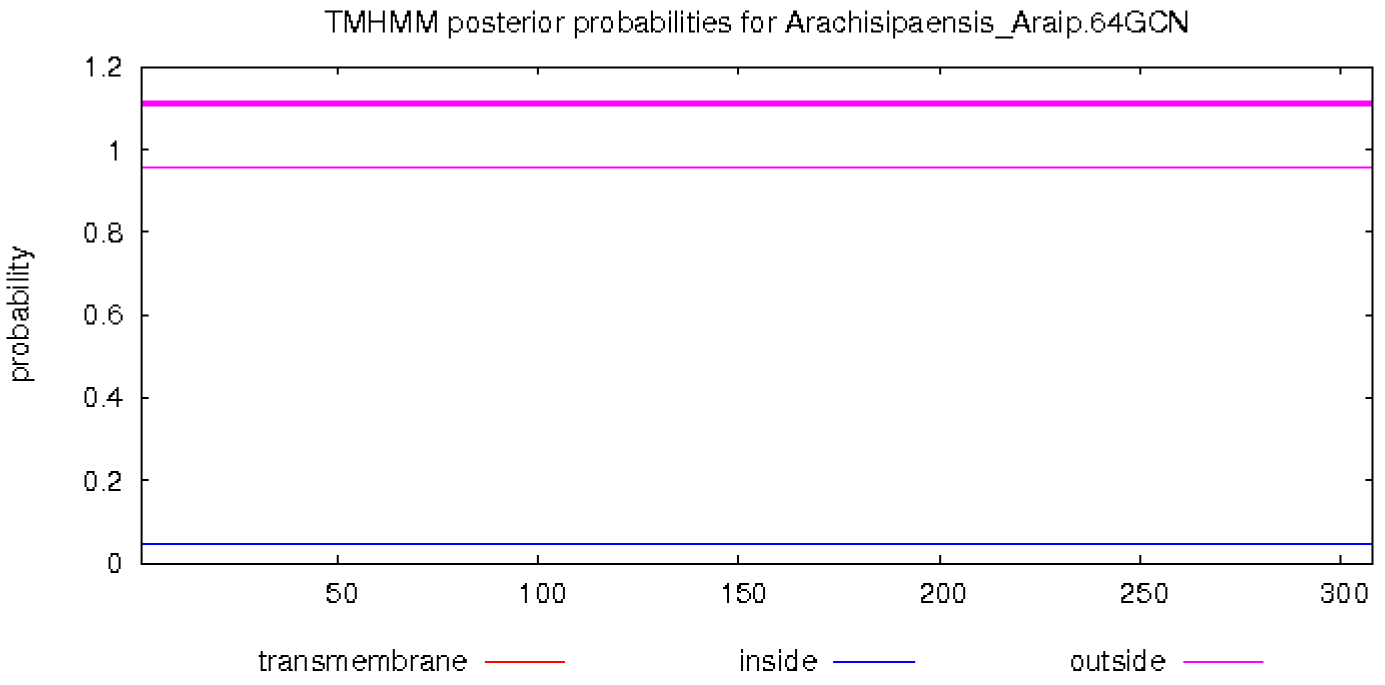

plot in postscript, script for making the plot in gnuplot, data for plot

---

```
# Arachisipaensis_Araip.67R8V Length: 471
# Arachisipaensis_Araip.67R8V Number of predicted TMHs:  0
# Arachisipaensis_Araip.67R8V Exp number of AAs in TMHs: 2.01569
# Arachisipaensis_Araip.67R8V Exp number, first 60 AAs:  2.01567
# Arachisipaensis_Araip.67R8V Total prob of N-in:        0.10091
Arachisipaensis_Araip.67R8V     TMHMM2.0        outside      1   471
```

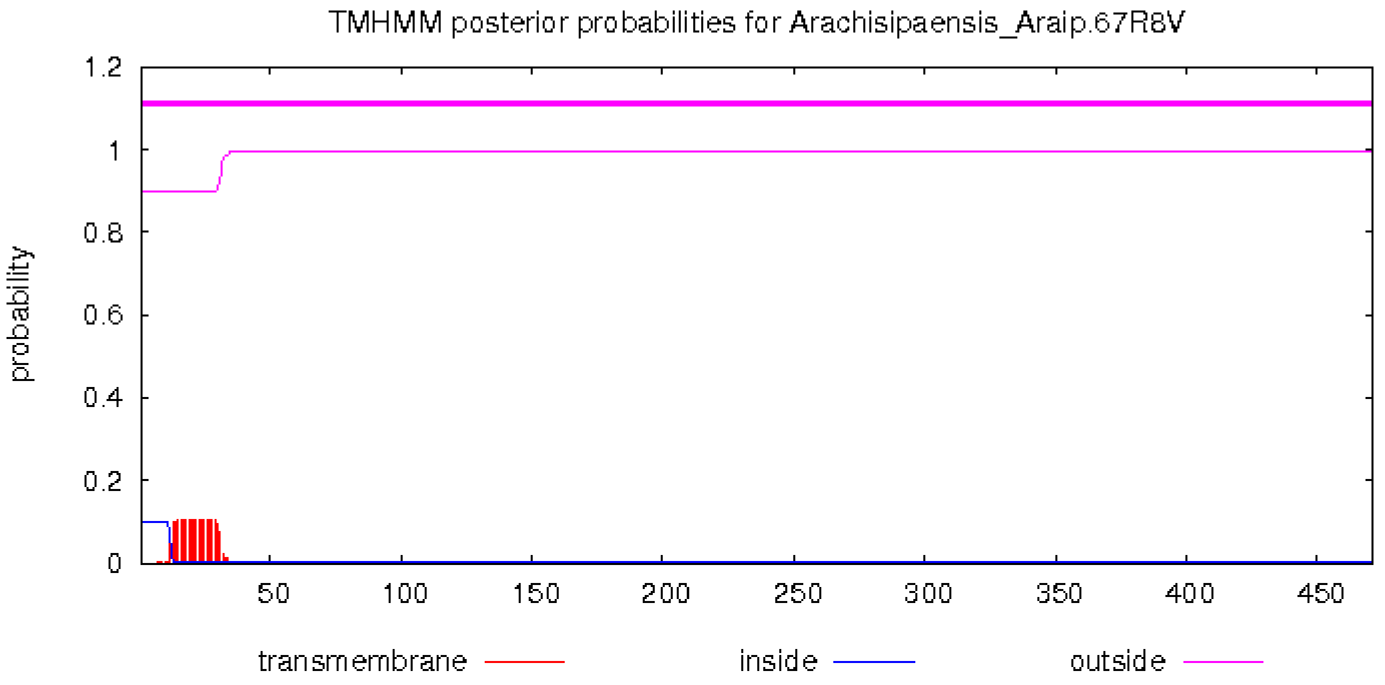

plot in postscript, script for making the plot in gnuplot, data for plot

---

```
# Arachisipaensis_Araip.6CI1F Length: 391
# Arachisipaensis_Araip.6CI1F Number of predicted TMHs:  1
# Arachisipaensis_Araip.6CI1F Exp number of AAs in TMHs: 26.94084
# Arachisipaensis_Araip.6CI1F Exp number, first 60 AAs:  3.93794
```

```
# Arachisipaensis_Araip.6CI1F Total prob of N-in:        0.78641
Arachisipaensis_Araip.6CI1F     TMHMM2.0        inside       1   105
Arachisipaensis_Araip.6CI1F     TMHMM2.0        TMhelix    106   128
Arachisipaensis_Araip.6CI1F     TMHMM2.0        outside    129   391
```

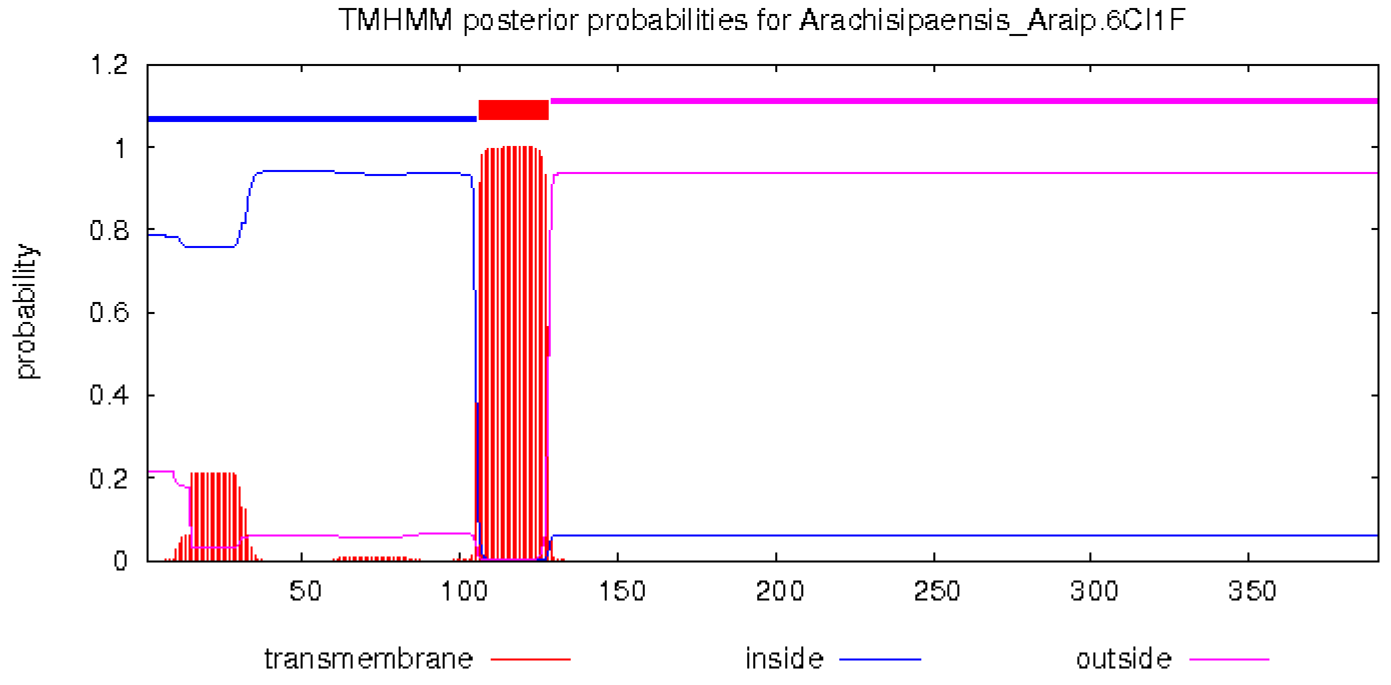

plot in postscript, script for making the plot in gnuplot, data for plot

---

```
# Arachisipaensis_Araip.6Y0GY Length: 280
# Arachisipaensis_Araip.6Y0GY Number of predicted TMHs:  0
# Arachisipaensis_Araip.6Y0GY Exp number of AAs in TMHs: 0.0002
# Arachisipaensis_Araip.6Y0GY Exp number, first 60 AAs:  0
# Arachisipaensis_Araip.6Y0GY Total prob of N-in:        0.07818
Arachisipaensis_Araip.6Y0GY     TMHMM2.0        outside      1   280
```

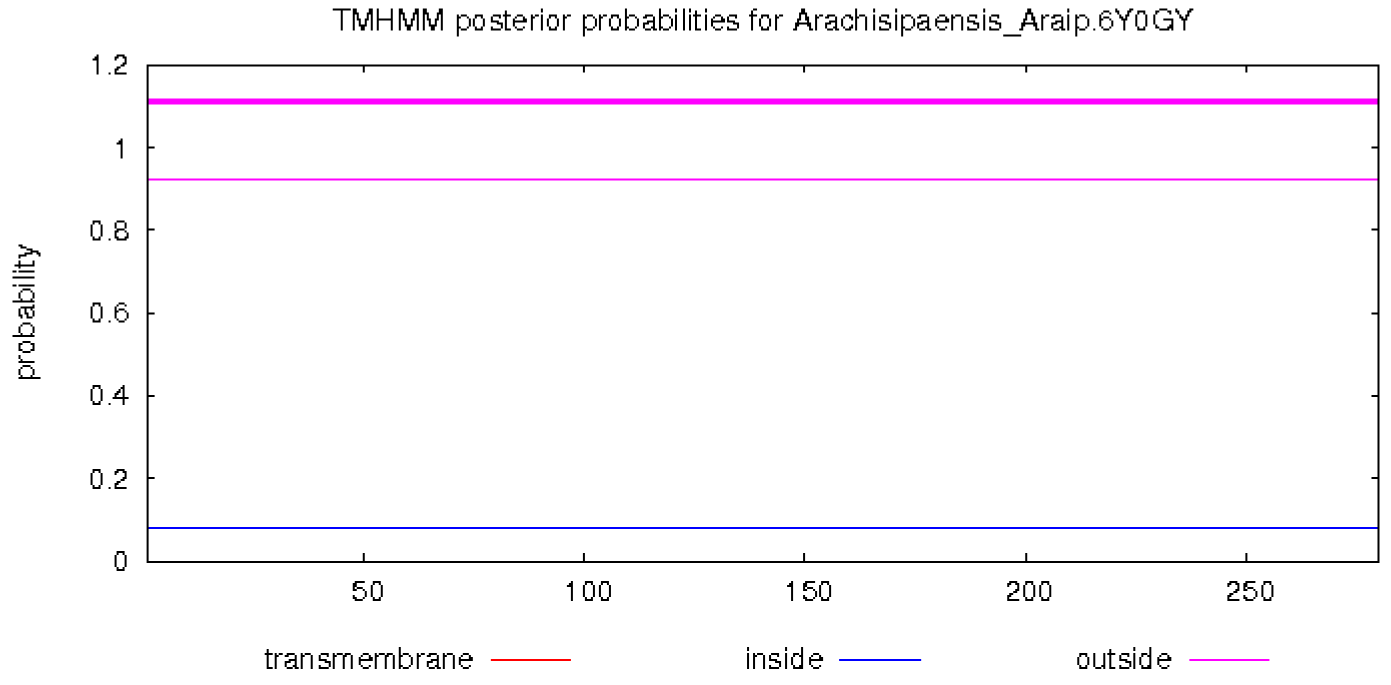

plot in postscript, script for making the plot in gnuplot, data for plot

---

```
# Arachisipaensis_Araip.714GL Length: 559
# Arachisipaensis_Araip.714GL Number of predicted TMHs:  1
```

```
# Arachisipaensis_Araip.714GL Exp number of AAs in TMHs: 22.14053
# Arachisipaensis_Araip.714GL Exp number, first 60 AAs:  0.00084
# Arachisipaensis_Araip.714GL Total prob of N-in:        0.06380
Arachisipaensis_Araip.714GL     TMHMM2.0        outside      1   530
Arachisipaensis_Araip.714GL     TMHMM2.0        TMhelix    531   553
Arachisipaensis_Araip.714GL     TMHMM2.0        inside     554   559
```

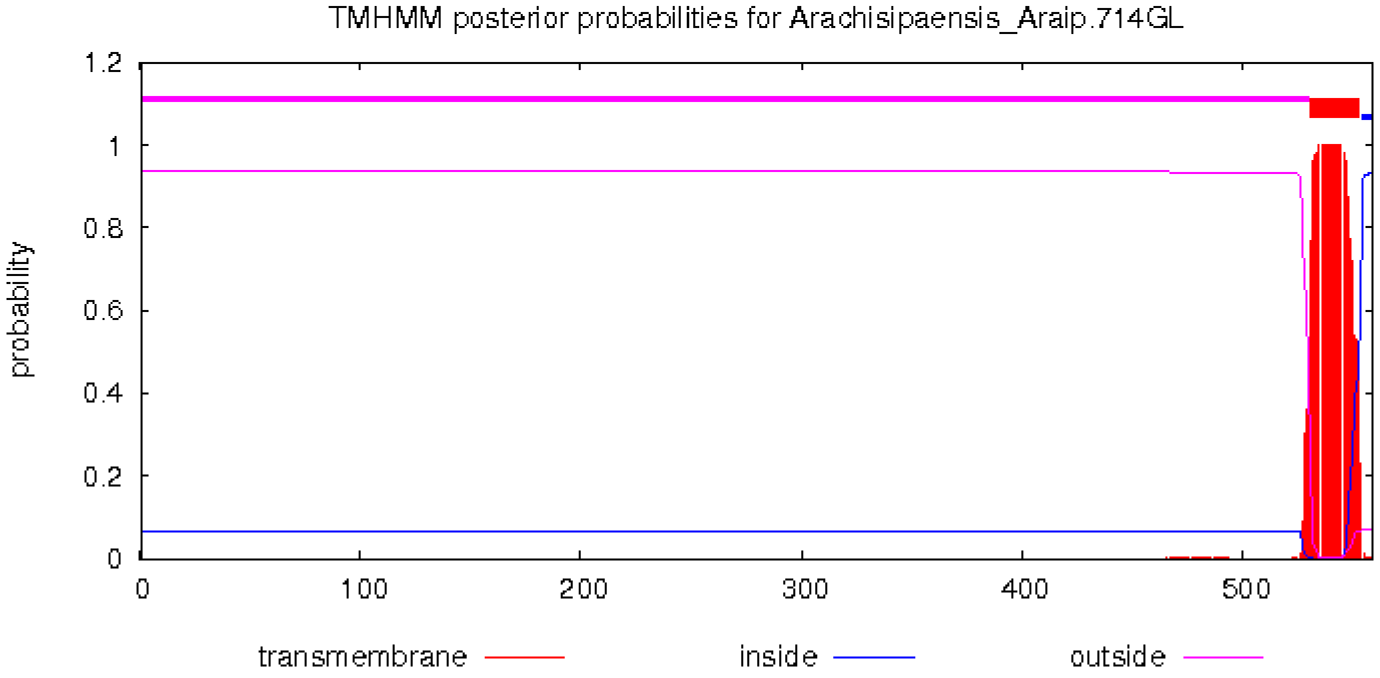

plot in postscript, script for making the plot in gnuplot, data for plot

---

```
# Arachisipaensis_Araip.71CS3 Length: 367
# Arachisipaensis_Araip.71CS3 Number of predicted TMHs:  0
# Arachisipaensis_Araip.71CS3 Exp number of AAs in TMHs: 0.00224
# Arachisipaensis_Araip.71CS3 Exp number, first 60 AAs:  0.00161
# Arachisipaensis_Araip.71CS3 Total prob of N-in:        0.01108
Arachisipaensis_Araip.71CS3     TMHMM2.0        outside      1   367
```

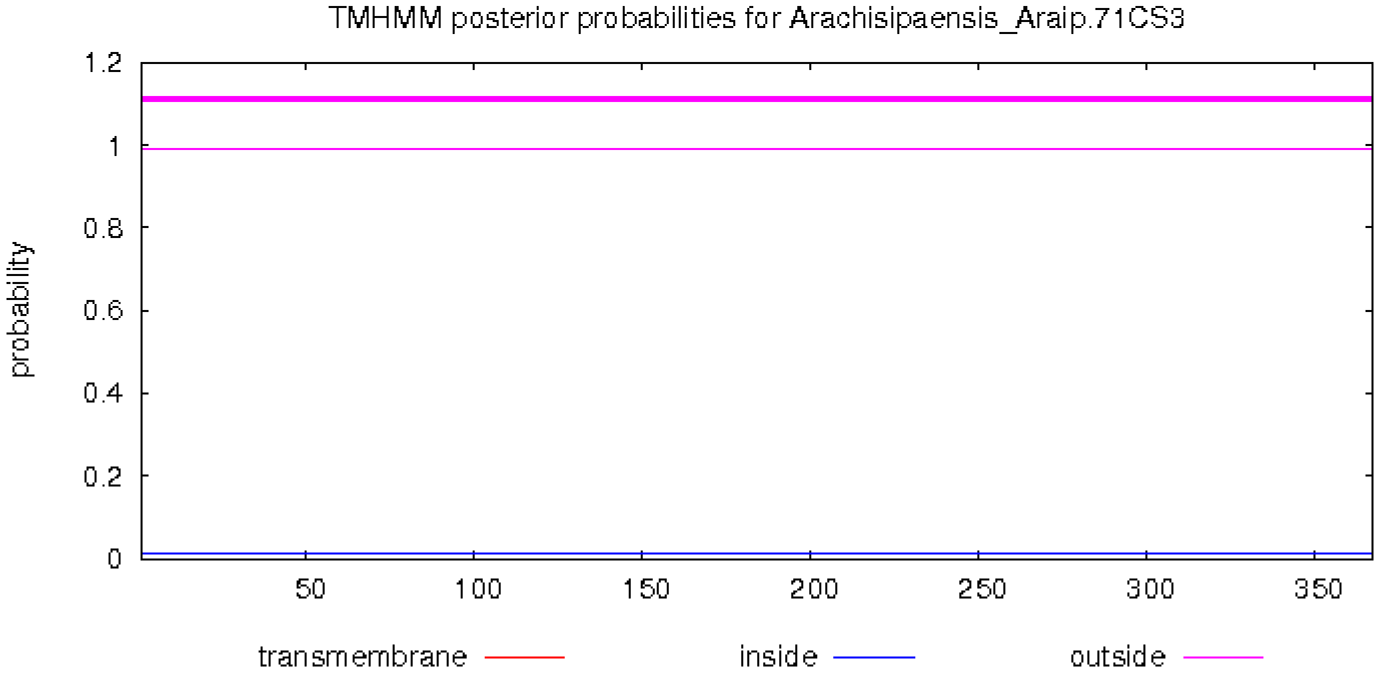

plot in postscript, script for making the plot in gnuplot, data for plot

---

```
# Arachisipaensis_Araip.77ISR Length: 257
# Arachisipaensis_Araip.77ISR Number of predicted TMHs:  0
# Arachisipaensis_Araip.77ISR Exp number of AAs in TMHs: 0.00075
# Arachisipaensis_Araip.77ISR Exp number, first 60 AAs:  0.00018
# Arachisipaensis_Araip.77ISR Total prob of N-in:        0.13980
Arachisipaensis_Araip.77ISR     TMHMM2.0        outside      1   257
```

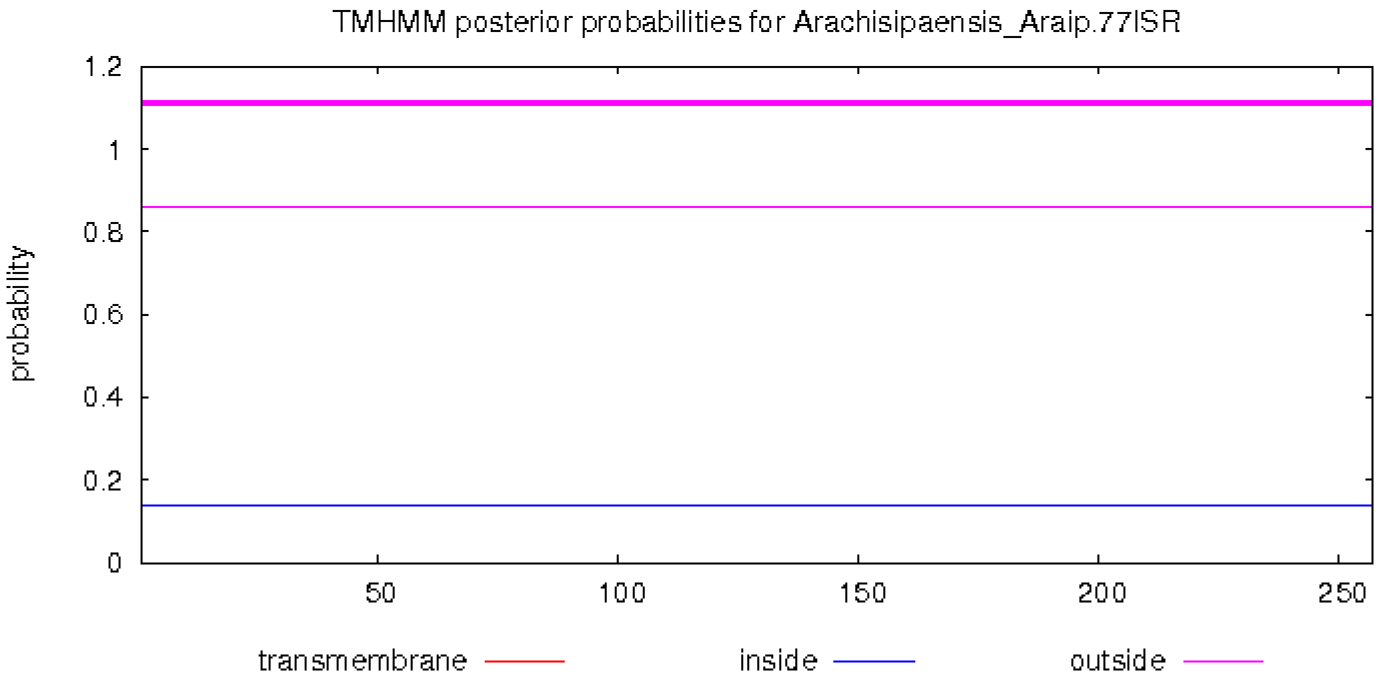

plot in postscript, script for making the plot in gnuplot, data for plot

---

```
# Arachisipaensis_Araip.78PTT Length: 366
# Arachisipaensis_Araip.78PTT Number of predicted TMHs:  0
# Arachisipaensis_Araip.78PTT Exp number of AAs in TMHs: 0.00565
# Arachisipaensis_Araip.78PTT Exp number, first 60 AAs:  0
# Arachisipaensis_Araip.78PTT Total prob of N-in:        0.01666
Arachisipaensis_Araip.78PTT     TMHMM2.0        outside      1   366
```

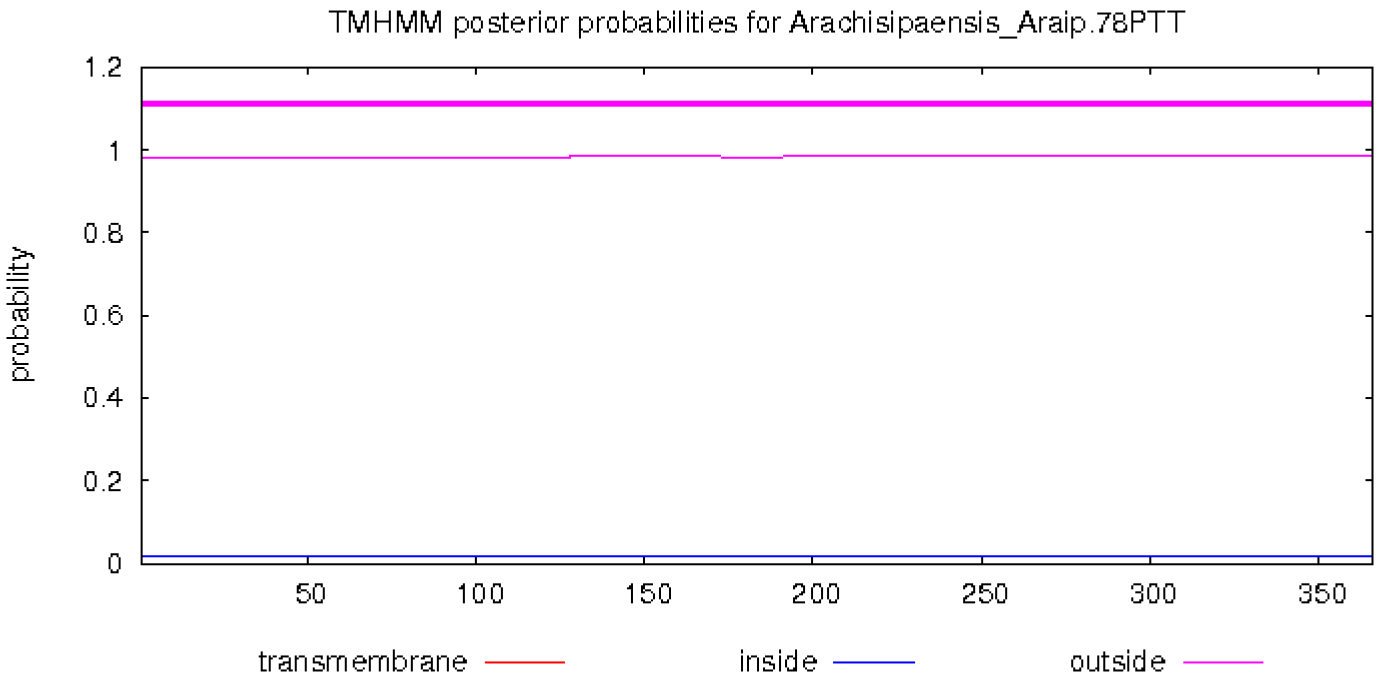

plot in postscript, script for making the plot in gnuplot, data for plot

---

```
# Arachisipaensis_Araip.79TDF Length: 678
# Arachisipaensis_Araip.79TDF Number of predicted TMHs:  2
# Arachisipaensis_Araip.79TDF Exp number of AAs in TMHs: 41.84687
# Arachisipaensis_Araip.79TDF Exp number, first 60 AAs:  0
# Arachisipaensis_Araip.79TDF Total prob of N-in:        0.00036
Arachisipaensis_Araip.79TDF     TMHMM2.0        outside      1   581
Arachisipaensis_Araip.79TDF     TMHMM2.0        TMhelix    582   604
Arachisipaensis_Araip.79TDF     TMHMM2.0        inside     605   655
Arachisipaensis_Araip.79TDF     TMHMM2.0        TMhelix    656   675
Arachisipaensis_Araip.79TDF     TMHMM2.0        outside    676   678
```

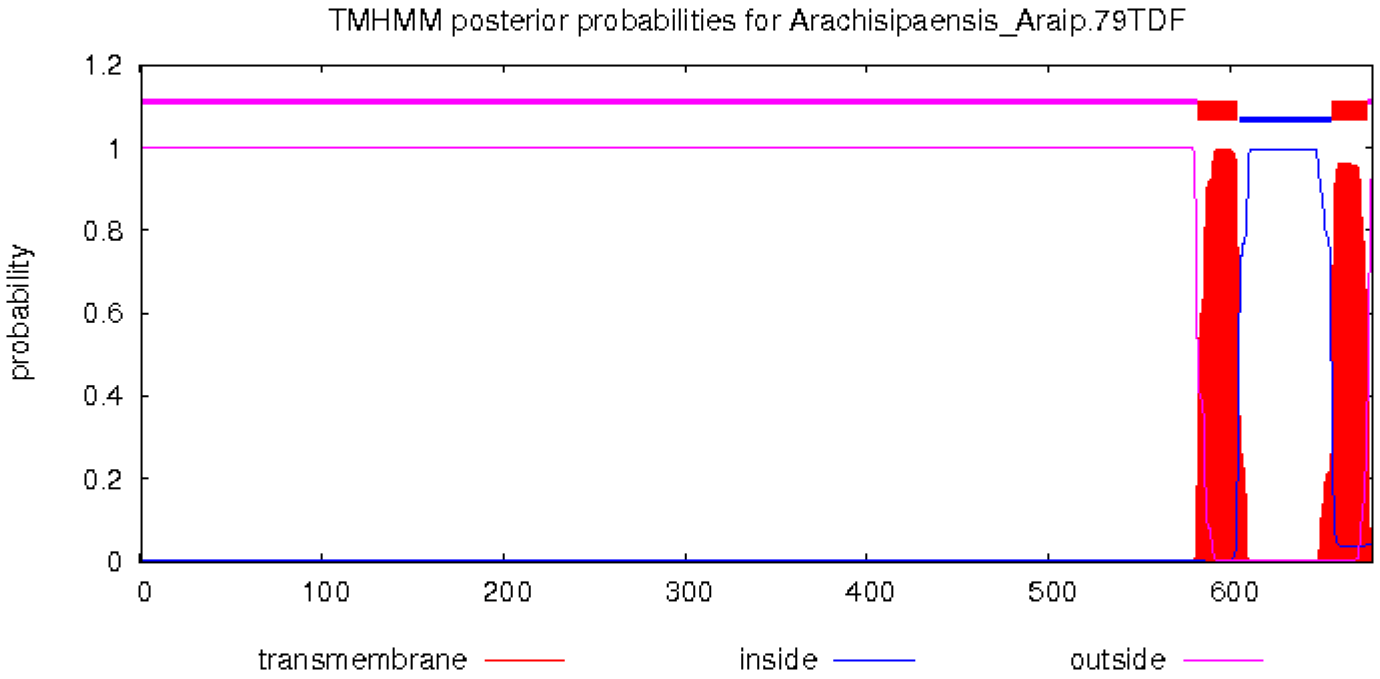

plot in postscript, script for making the plot in gnuplot, data for plot

---

```
# Arachisipaensis_Araip.7L9YW Length: 497
# Arachisipaensis_Araip.7L9YW Number of predicted TMHs:  1
# Arachisipaensis_Araip.7L9YW Exp number of AAs in TMHs: 22.06586
# Arachisipaensis_Araip.7L9YW Exp number, first 60 AAs:  22.06459
# Arachisipaensis_Araip.7L9YW Total prob of N-in:        0.97433
# Arachisipaensis_Araip.7L9YW POSSIBLE N-term signal sequence
Arachisipaensis_Araip.7L9YW     TMHMM2.0        inside       1     6
Arachisipaensis_Araip.7L9YW     TMHMM2.0        TMhelix      7    29
Arachisipaensis_Araip.7L9YW     TMHMM2.0        outside     30   497
```

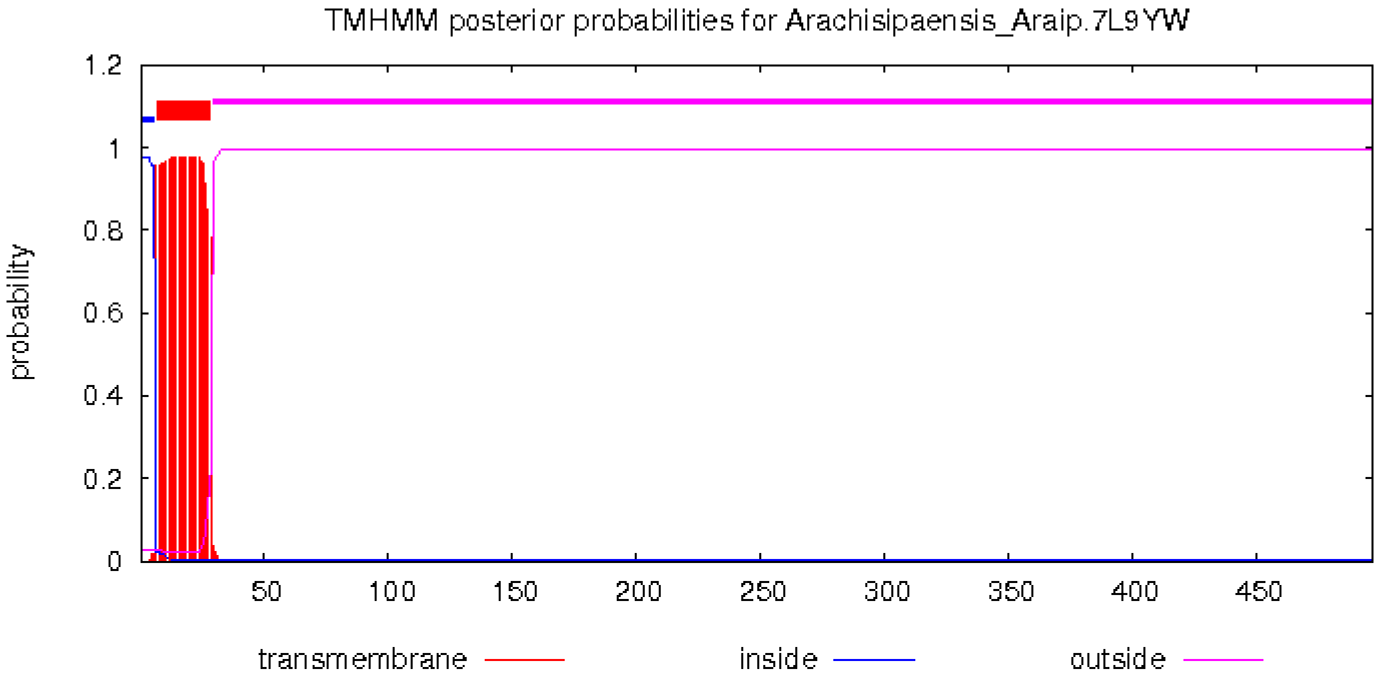

plot in postscript, script for making the plot in gnuplot, data for plot

---

```
# Arachisipaensis_Araip.8NR3H Length: 260
# Arachisipaensis_Araip.8NR3H Number of predicted TMHs:  0
# Arachisipaensis_Araip.8NR3H Exp number of AAs in TMHs: 0.0002
# Arachisipaensis_Araip.8NR3H Exp number, first 60 AAs:  0.0002
# Arachisipaensis_Araip.8NR3H Total prob of N-in:        0.00507
Arachisipaensis_Araip.8NR3H     TMHMM2.0        outside      1   260
```

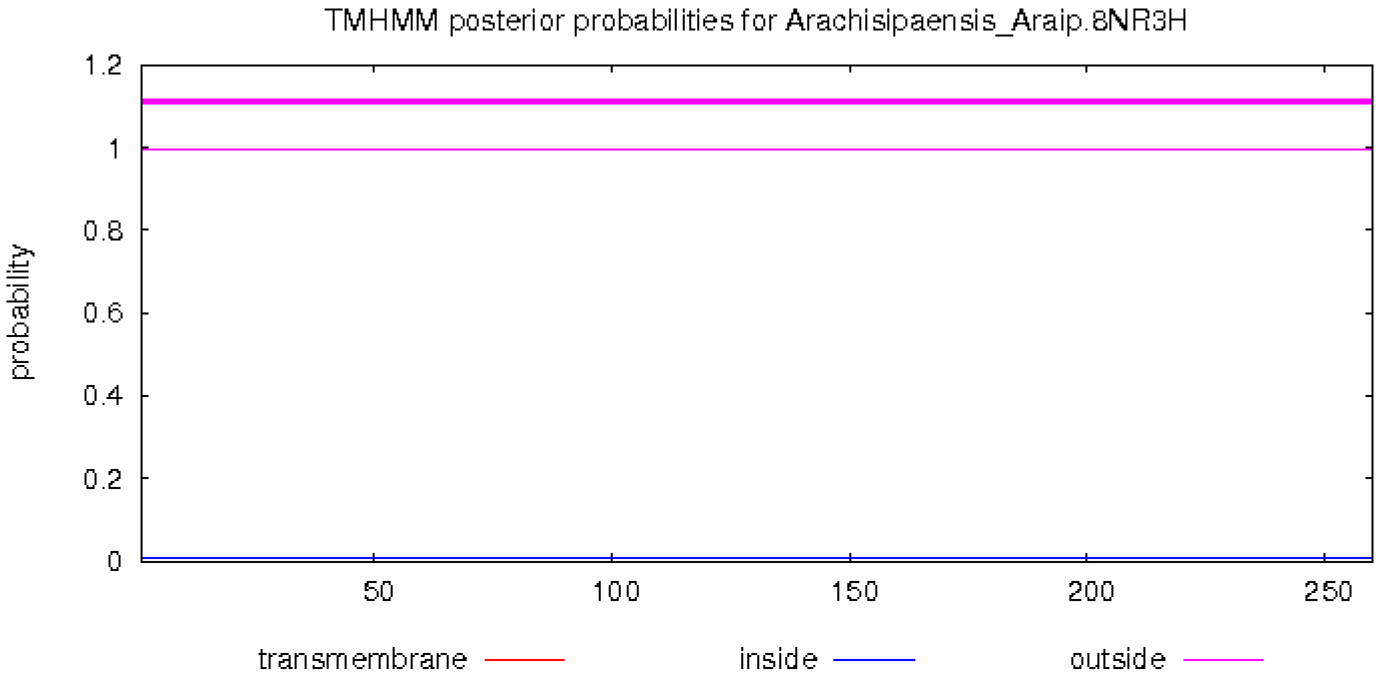

plot in postscript, script for making the plot in gnuplot, data for plot

---

```
# Arachisipaensis_Araip.92BTQ Length: 213
# Arachisipaensis_Araip.92BTQ Number of predicted TMHs:  0
# Arachisipaensis_Araip.92BTQ Exp number of AAs in TMHs: 0.00399
# Arachisipaensis_Araip.92BTQ Exp number, first 60 AAs:  0.0032
# Arachisipaensis_Araip.92BTQ Total prob of N-in:        0.24699
Arachisipaensis_Araip.92BTQ     TMHMM2.0        outside      1   213
```

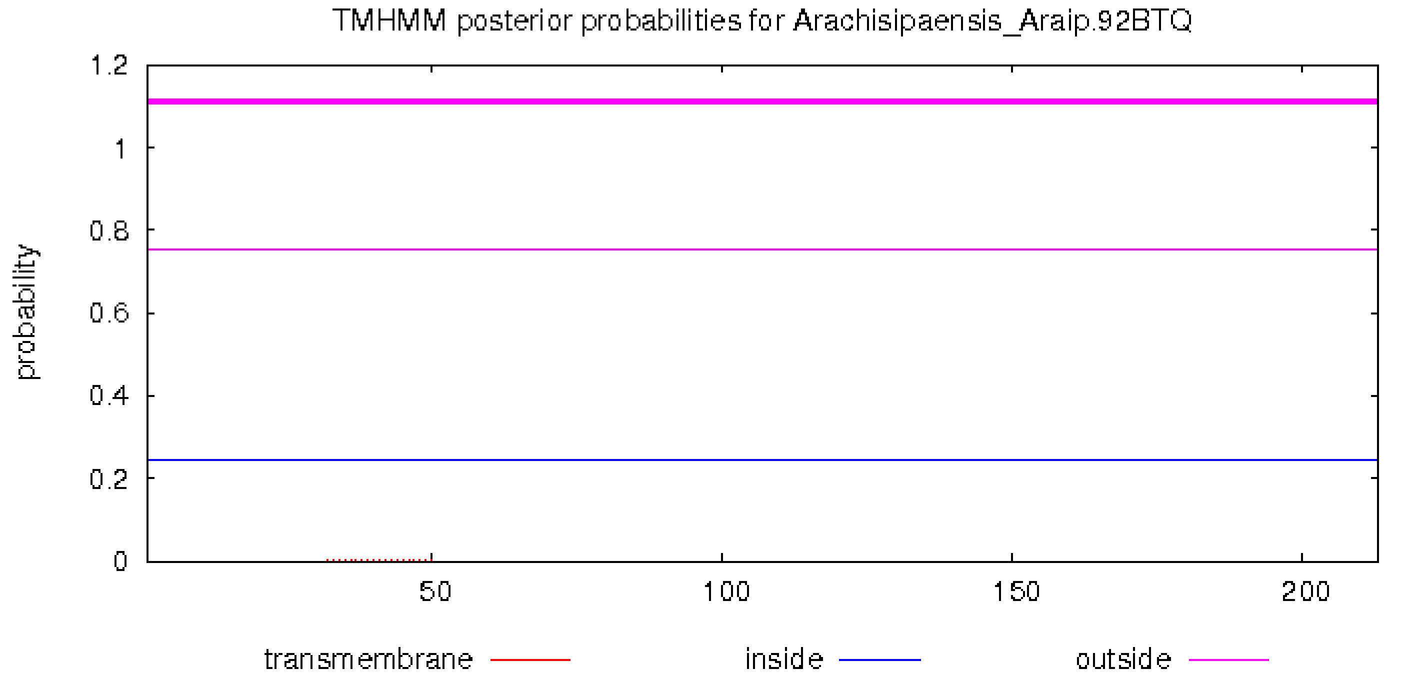

plot in postscript, script for making the plot in gnuplot, data for plot

---

```
# Arachisipaensis_Araip.9BR1Z Length: 202
# Arachisipaensis_Araip.9BR1Z Number of predicted TMHs:  0
# Arachisipaensis_Araip.9BR1Z Exp number of AAs in TMHs: 0.00061
# Arachisipaensis_Araip.9BR1Z Exp number, first 60 AAs:  0.00036
# Arachisipaensis_Araip.9BR1Z Total prob of N-in:        0.01072
Arachisipaensis_Araip.9BR1Z     TMHMM2.0        outside      1   202
```

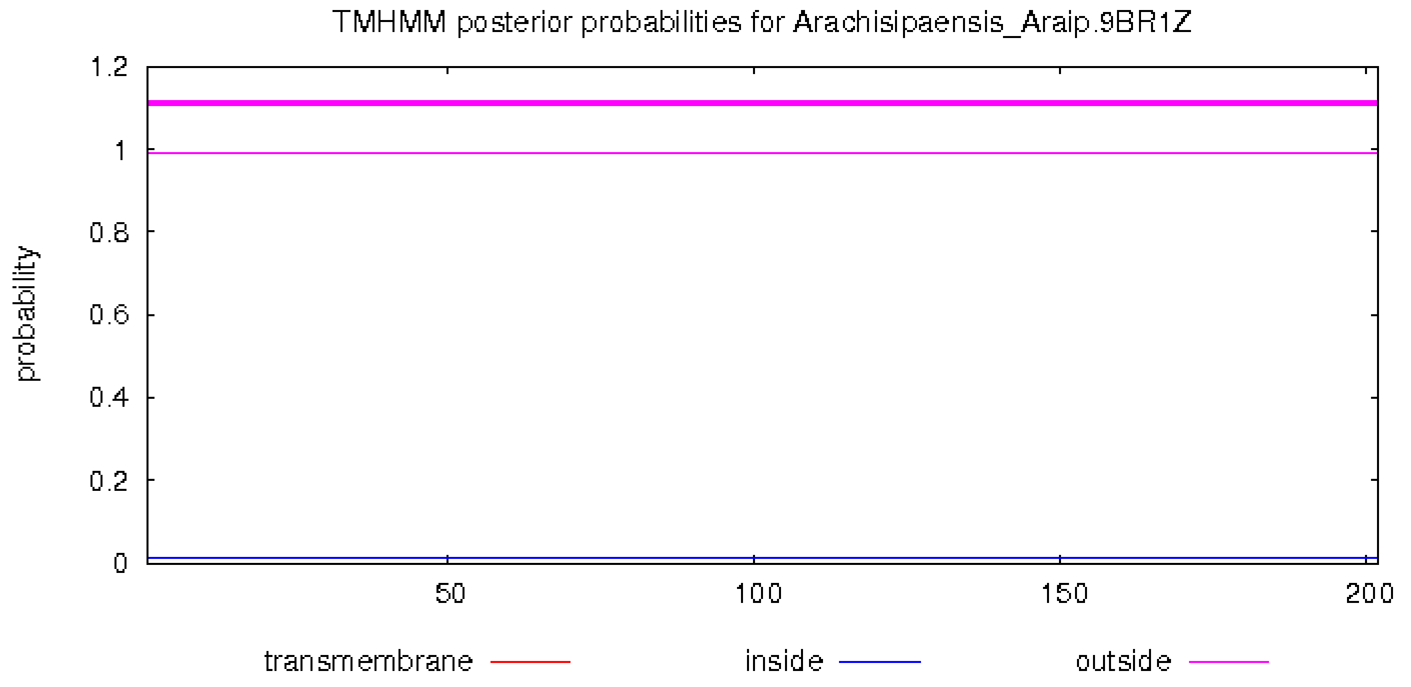

plot in postscript, script for making the plot in gnuplot, data for plot

---

```
# Arachisipaensis_Araip.9MG9F Length: 321
# Arachisipaensis_Araip.9MG9F Number of predicted TMHs:  0
# Arachisipaensis_Araip.9MG9F Exp number of AAs in TMHs: 0.00021
# Arachisipaensis_Araip.9MG9F Exp number, first 60 AAs:  0
# Arachisipaensis_Araip.9MG9F Total prob of N-in:        0.06769
Arachisipaensis_Araip.9MG9F     TMHMM2.0        outside      1   321
```

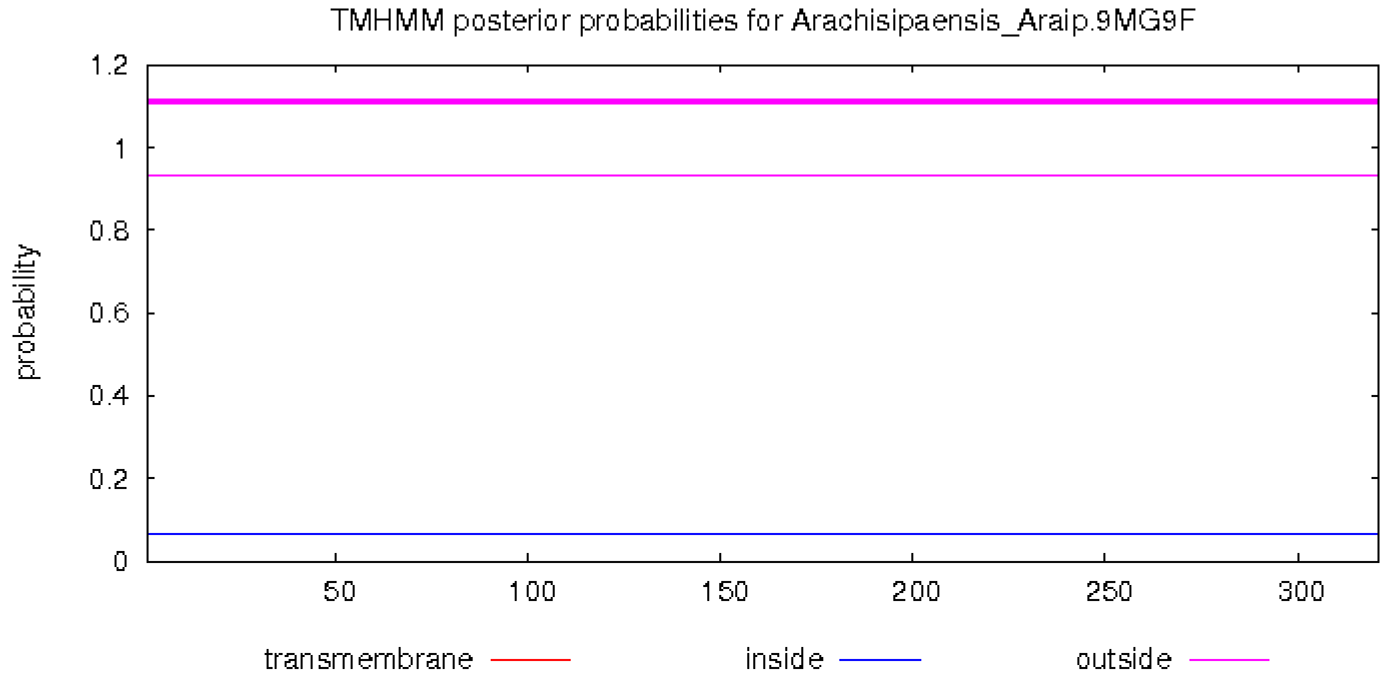

plot in postscript, script for making the plot in gnuplot, data for plot

---

```
# Arachisipaensis_Araip.9N5S4 Length: 338
# Arachisipaensis_Araip.9N5S4 Number of predicted TMHs:  0
# Arachisipaensis_Araip.9N5S4 Exp number of AAs in TMHs: 0
# Arachisipaensis_Araip.9N5S4 Exp number, first 60 AAs:  0
# Arachisipaensis_Araip.9N5S4 Total prob of N-in:        0.01996
Arachisipaensis_Araip.9N5S4     TMHMM2.0        outside      1   338
```

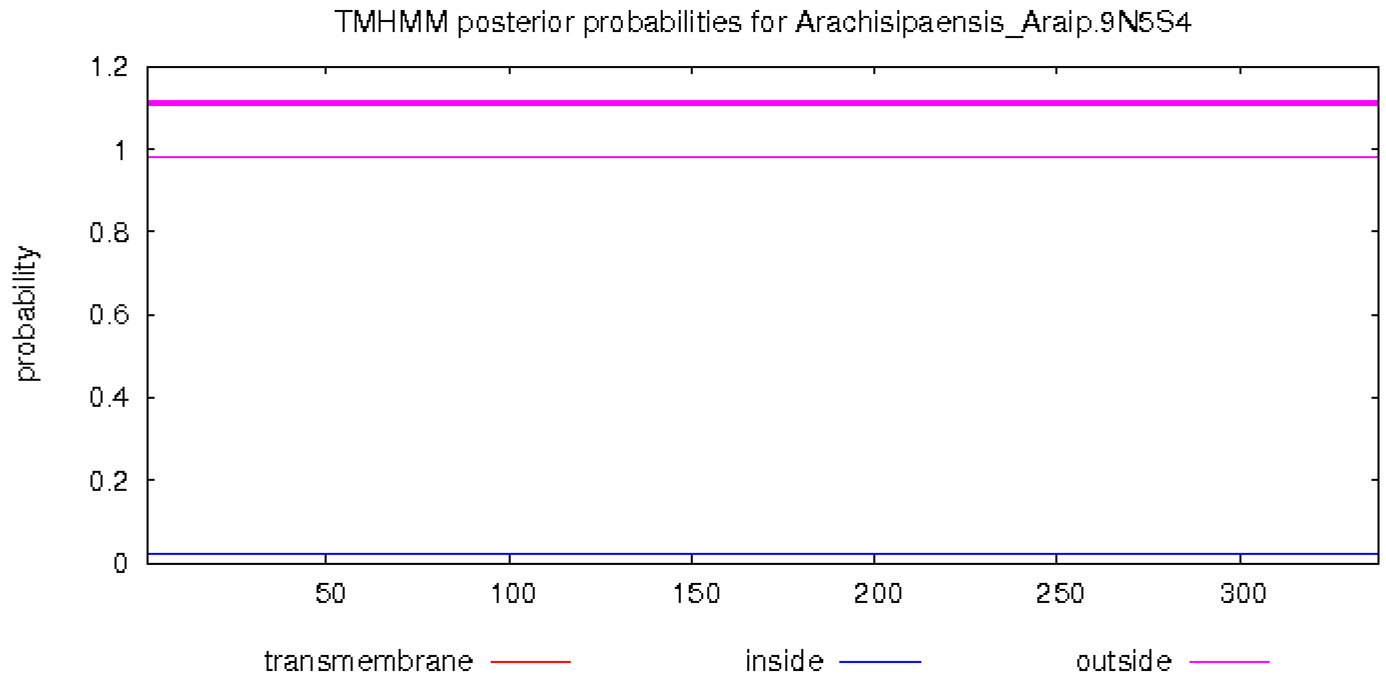

plot in postscript, script for making the plot in gnuplot, data for plot

---

```
# Arachisipaensis_Araip.9W6SR Length: 633
# Arachisipaensis_Araip.9W6SR Number of predicted TMHs:  2
# Arachisipaensis_Araip.9W6SR Exp number of AAs in TMHs: 40.88285
# Arachisipaensis_Araip.9W6SR Exp number, first 60 AAs:  0
# Arachisipaensis_Araip.9W6SR Total prob of N-in:        0.00005
Arachisipaensis_Araip.9W6SR     TMHMM2.0        outside      1   524
Arachisipaensis_Araip.9W6SR     TMHMM2.0        TMhelix    525   544
```

```
Arachisipaensis_Araip.9W6SR     TMHMM2.0        inside      545   611
Arachisipaensis_Araip.9W6SR     TMHMM2.0        TMhelix     612   631
Arachisipaensis_Araip.9W6SR     TMHMM2.0        outside     632   633
```

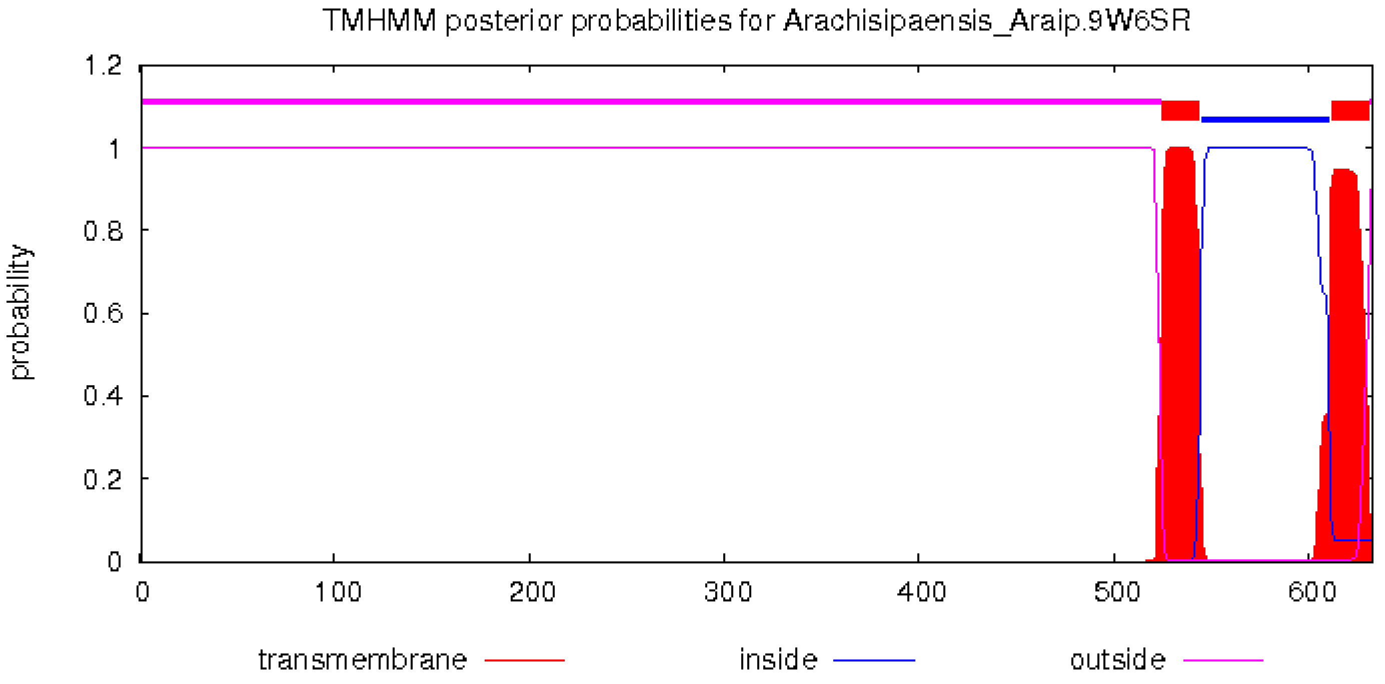

plot in postscript, script for making the plot in gnuplot, data for plot

---

```
# Arachisipaensis_Araip.A6QWC Length: 481
# Arachisipaensis_Araip.A6QWC Number of predicted TMHs:  1
# Arachisipaensis_Araip.A6QWC Exp number of AAs in TMHs: 19.08509
# Arachisipaensis_Araip.A6QWC Exp number, first 60 AAs:  0.00479
# Arachisipaensis_Araip.A6QWC Total prob of N-in:        0.04916
Arachisipaensis_Araip.A6QWC     TMHMM2.0        outside       1   457
Arachisipaensis_Araip.A6QWC     TMHMM2.0        TMhelix     458   480
Arachisipaensis_Araip.A6QWC     TMHMM2.0        inside      481   481
```

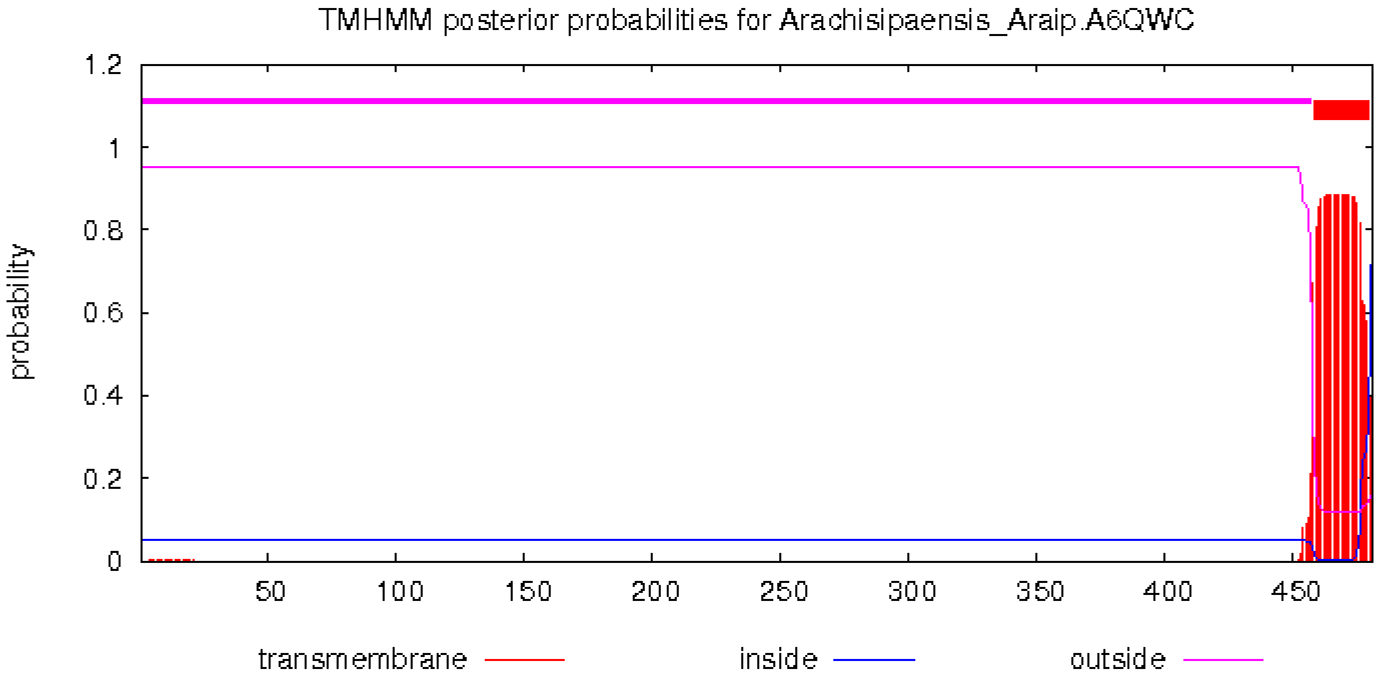

plot in postscript, script for making the plot in gnuplot, data for plot

---

```
# Arachisipaensis_Araip.AVV74 Length: 330
# Arachisipaensis_Araip.AVV74 Number of predicted TMHs:  0
# Arachisipaensis_Araip.AVV74 Exp number of AAs in TMHs: 0.0009
# Arachisipaensis_Araip.AVV74 Exp number, first 60 AAs:  0.0009
# Arachisipaensis_Araip.AVV74 Total prob of N-in:        0.04170
Arachisipaensis_Araip.AVV74     TMHMM2.0        outside      1   330
```

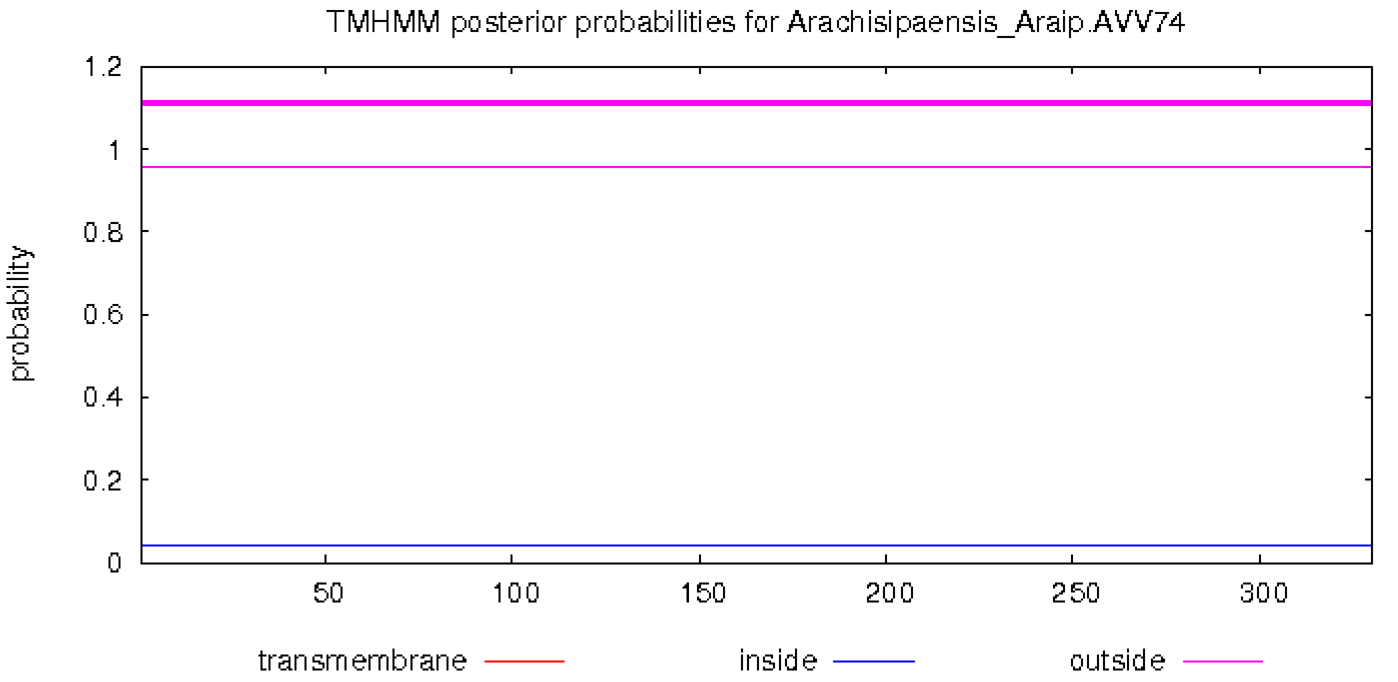

plot in postscript, script for making the plot in gnuplot, data for plot

---

```
# Arachisipaensis_Araip.AWF0A Length: 354
# Arachisipaensis_Araip.AWF0A Number of predicted TMHs:  0
# Arachisipaensis_Araip.AWF0A Exp number of AAs in TMHs: 0.00411
# Arachisipaensis_Araip.AWF0A Exp number, first 60 AAs:  0.00049
# Arachisipaensis_Araip.AWF0A Total prob of N-in:        0.00441
Arachisipaensis_Araip.AWF0A     TMHMM2.0        outside      1   354
```

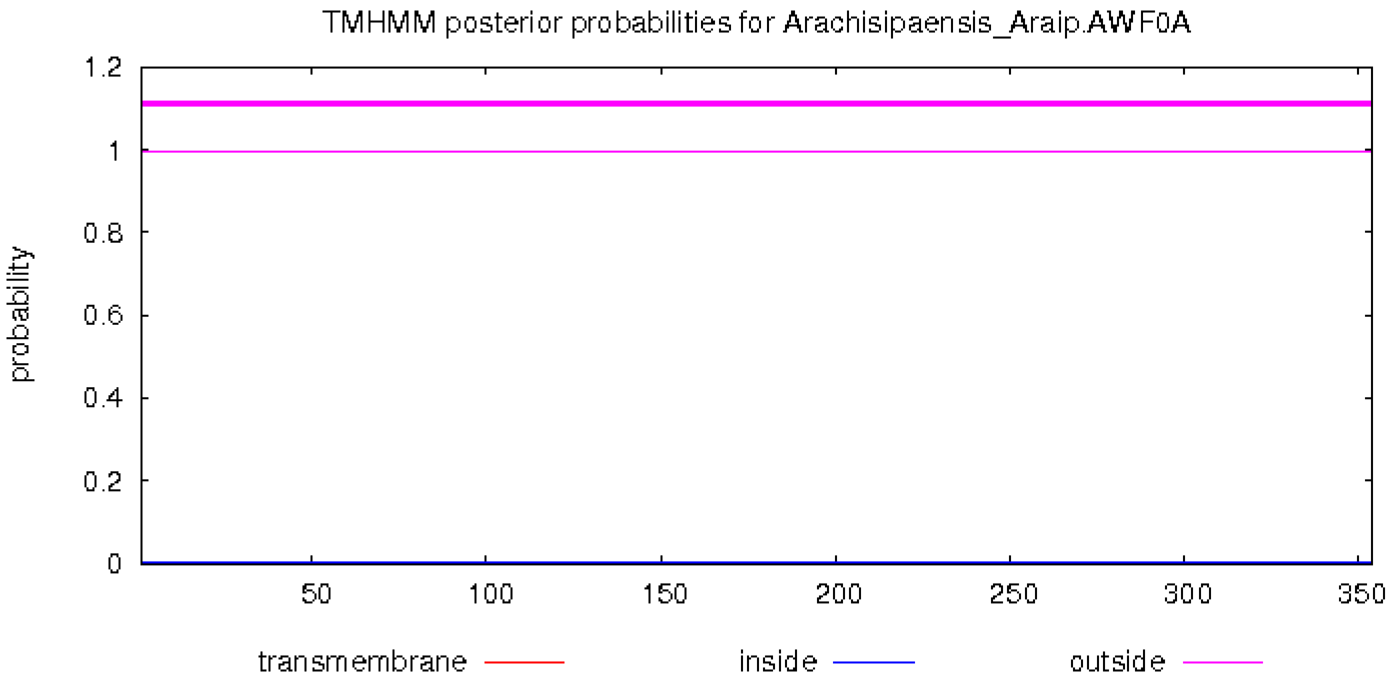

plot in postscript, script for making the plot in gnuplot, data for plot

---

```
# Arachisipaensis_Araip.CC7W1 Length: 461
# Arachisipaensis_Araip.CC7W1 Number of predicted TMHs:  0
# Arachisipaensis_Araip.CC7W1 Exp number of AAs in TMHs: 0.00118
# Arachisipaensis_Araip.CC7W1 Exp number, first 60 AAs:  0
# Arachisipaensis_Araip.CC7W1 Total prob of N-in:        0.00184
Arachisipaensis_Araip.CC7W1     TMHMM2.0        outside      1   461
```

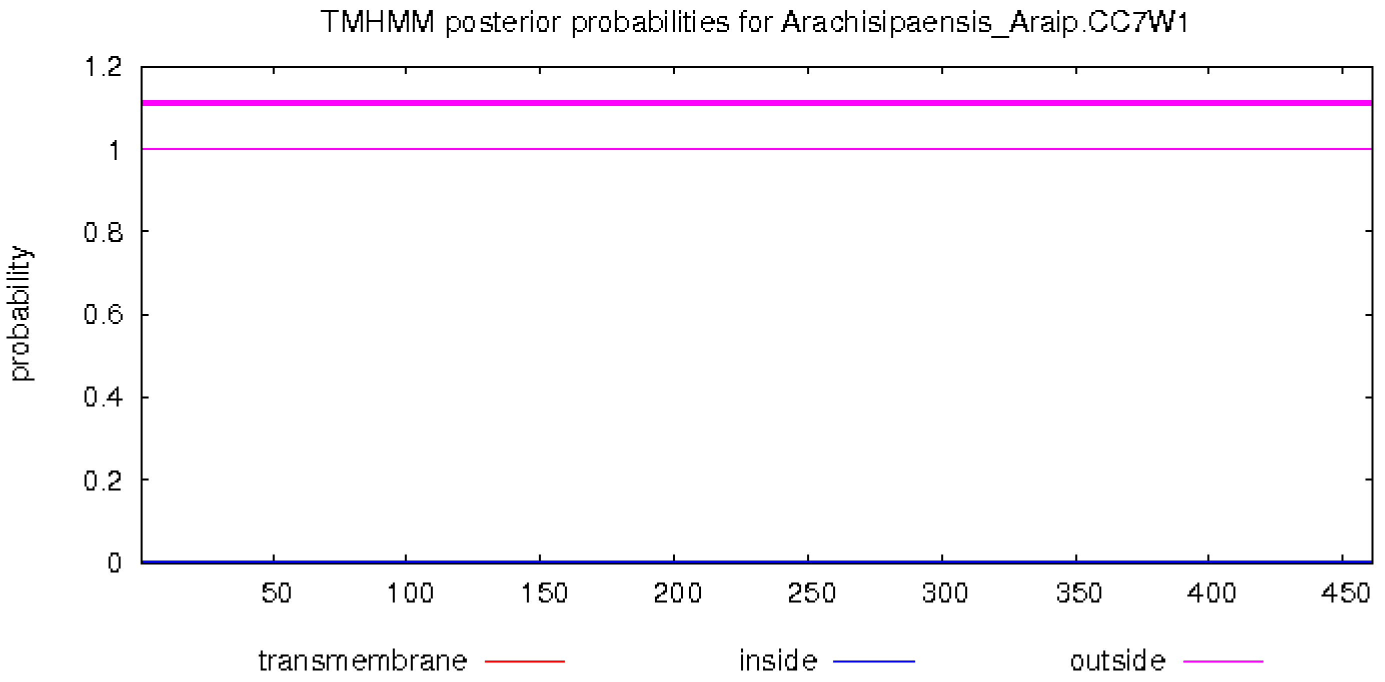

plot in postscript, script for making the plot in gnuplot, data for plot

---

```
# Arachisipaensis_Araip.D25HB Length: 390
# Arachisipaensis_Araip.D25HB Number of predicted TMHs:  0
# Arachisipaensis_Araip.D25HB Exp number of AAs in TMHs: 0.00197
# Arachisipaensis_Araip.D25HB Exp number, first 60 AAs:  0
# Arachisipaensis_Araip.D25HB Total prob of N-in:        0.00916
Arachisipaensis_Araip.D25HB     TMHMM2.0        outside      1   390
```

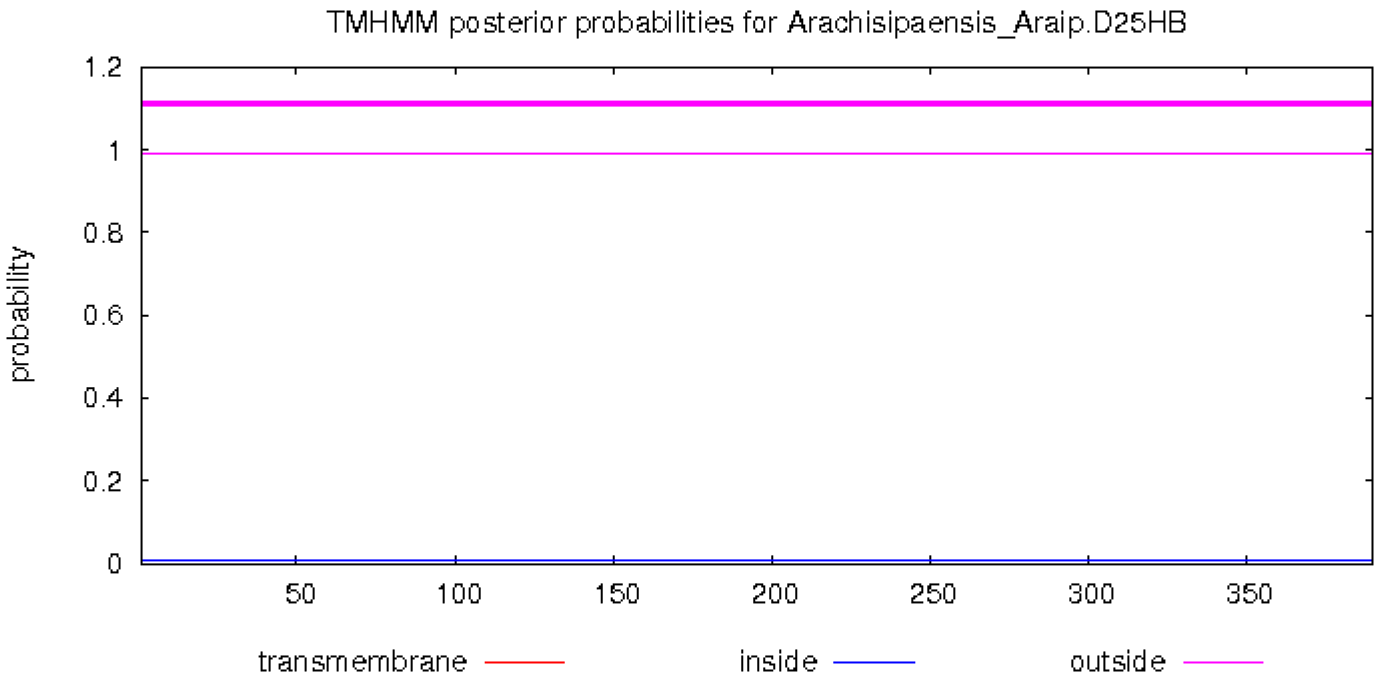

plot in postscript, script for making the plot in gnuplot, data for plot

---

```
# Arachisipaensis_Araip.D7N1Q Length: 142
# Arachisipaensis_Araip.D7N1Q Number of predicted TMHs:  0
# Arachisipaensis_Araip.D7N1Q Exp number of AAs in TMHs: 0.00348
# Arachisipaensis_Araip.D7N1Q Exp number, first 60 AAs:  0.00051
# Arachisipaensis_Araip.D7N1Q Total prob of N-in:        0.18562
Arachisipaensis_Araip.D7N1Q     TMHMM2.0        outside      1   142
```

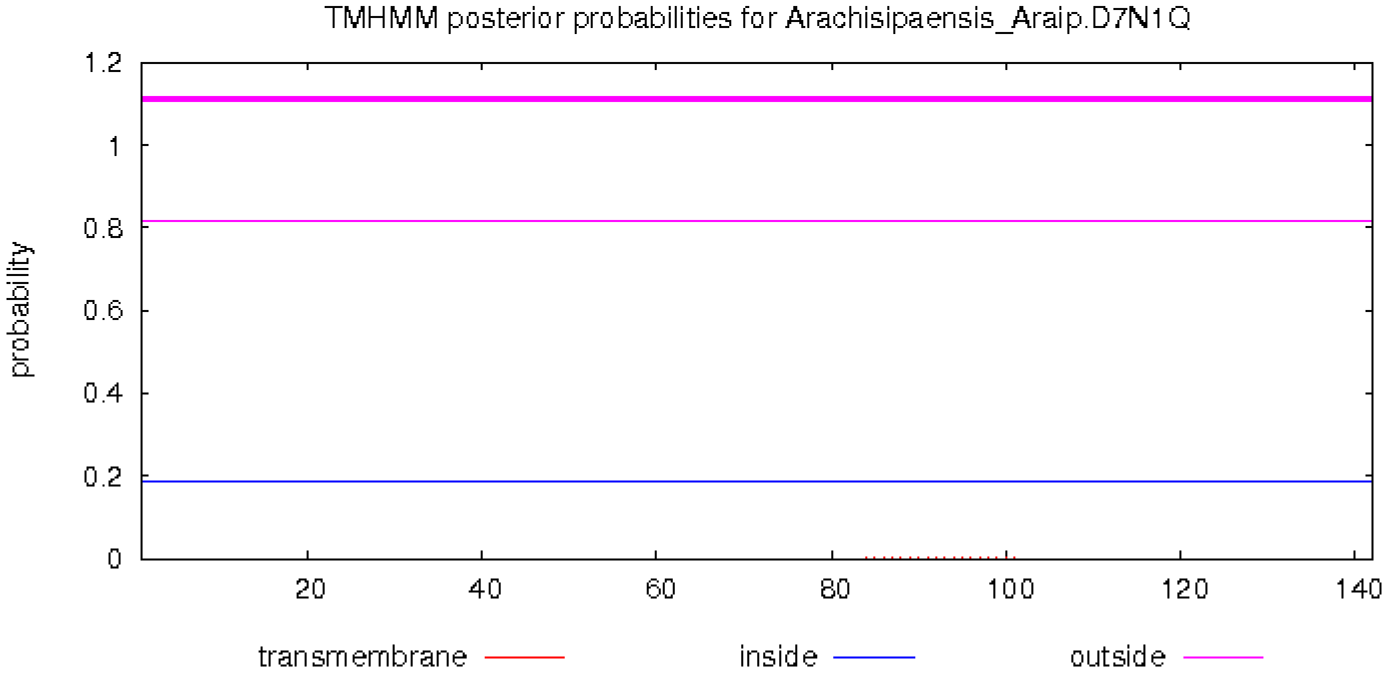

plot in postscript, script for making the plot in gnuplot, data for plot

```
# Arachisipaensis_Araip.DEH65 Length: 343
# Arachisipaensis_Araip.DEH65 Number of predicted TMHs:  0
# Arachisipaensis_Araip.DEH65 Exp number of AAs in TMHs: 0.02647
# Arachisipaensis_Araip.DEH65 Exp number, first 60 AAs:  0.02627
# Arachisipaensis_Araip.DEH65 Total prob of N-in:        0.00920
Arachisipaensis_Araip.DEH65     TMHMM2.0        outside      1   343
```

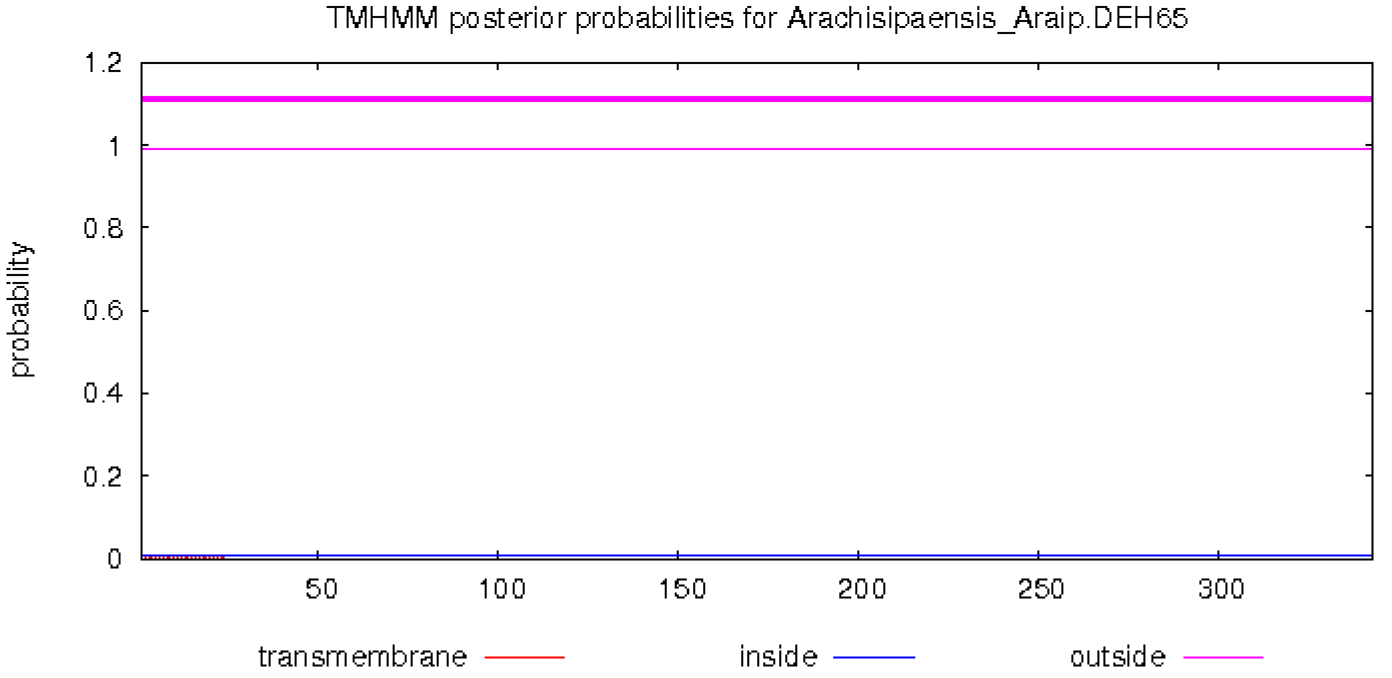

plot in postscript, script for making the plot in gnuplot, data for plot

```
# Arachisipaensis_Araip.DL86S Length: 349
# Arachisipaensis_Araip.DL86S Number of predicted TMHs:  0
# Arachisipaensis_Araip.DL86S Exp number of AAs in TMHs: 0.0006
# Arachisipaensis_Araip.DL86S Exp number, first 60 AAs:  0
# Arachisipaensis_Araip.DL86S Total prob of N-in:        0.00698
Arachisipaensis_Araip.DL86S     TMHMM2.0        outside      1   349
```

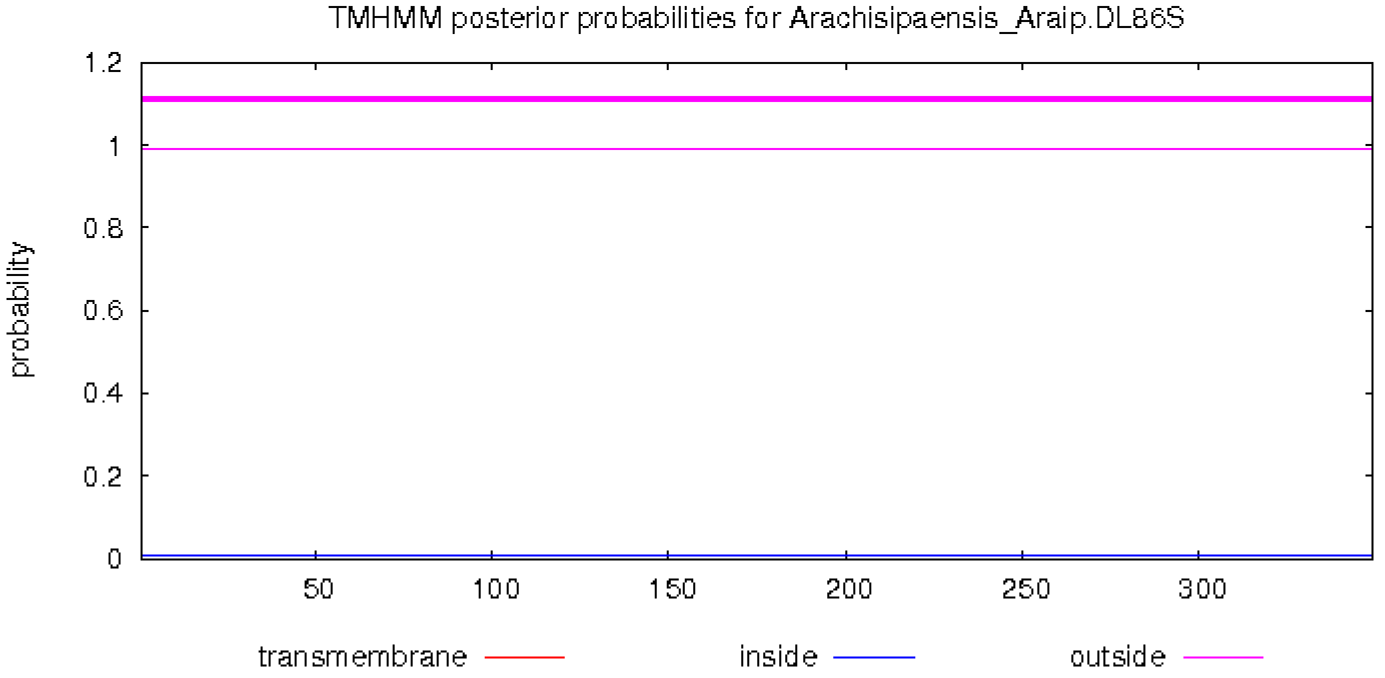

plot in postscript, script for making the plot in gnuplot, data for plot

---

```
# Arachisipaensis_Araip.DR280 Length: 304
# Arachisipaensis_Araip.DR280 Number of predicted TMHs:  0
# Arachisipaensis_Araip.DR280 Exp number of AAs in TMHs: 0.00023
# Arachisipaensis_Araip.DR280 Exp number, first 60 AAs:  0
# Arachisipaensis_Araip.DR280 Total prob of N-in:        0.00797
Arachisipaensis_Araip.DR280     TMHMM2.0        outside      1   304
```

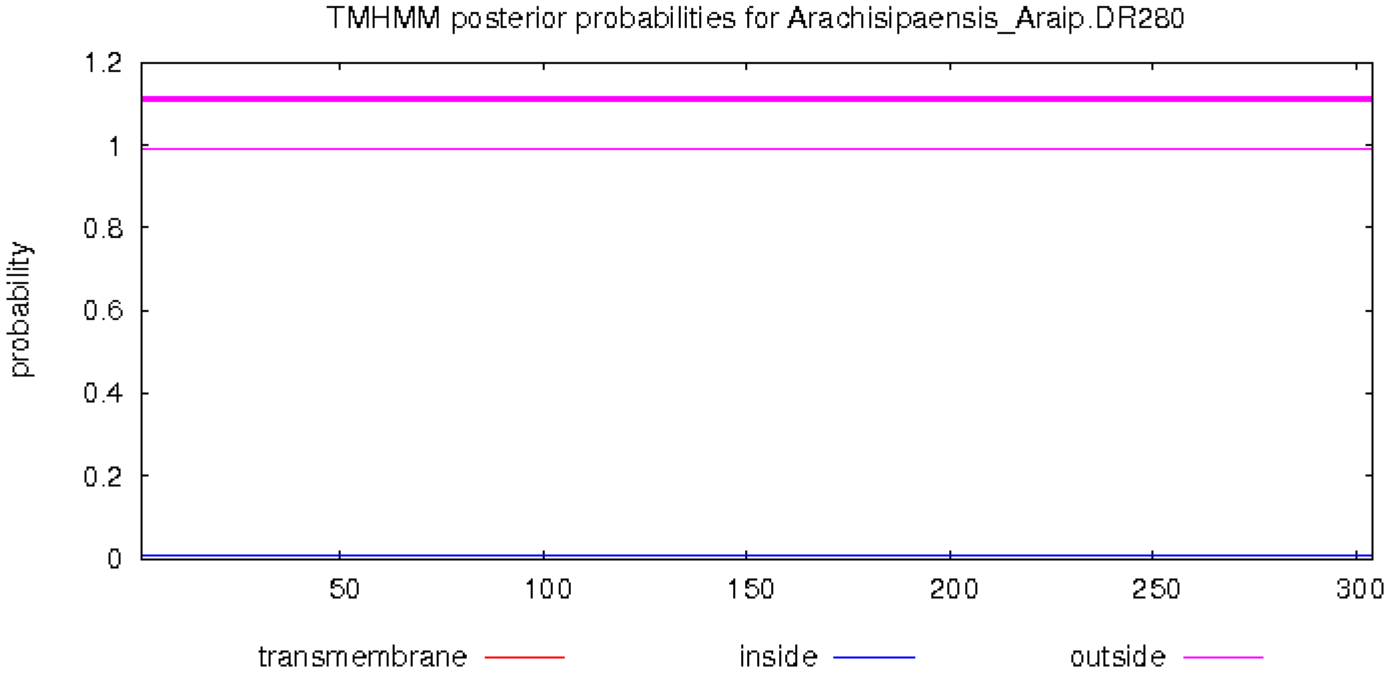

plot in postscript, script for making the plot in gnuplot, data for plot

---

```
# Arachisipaensis_Araip.E0NQ0 Length: 494
# Arachisipaensis_Araip.E0NQ0 Number of predicted TMHs:  1
# Arachisipaensis_Araip.E0NQ0 Exp number of AAs in TMHs: 22.35972
# Arachisipaensis_Araip.E0NQ0 Exp number, first 60 AAs:  0.07257
# Arachisipaensis_Araip.E0NQ0 Total prob of N-in:        0.23499
Arachisipaensis_Araip.E0NQ0     TMHMM2.0        outside      1    85
Arachisipaensis_Araip.E0NQ0     TMHMM2.0        TMhelix     86   108
Arachisipaensis_Araip.E0NQ0     TMHMM2.0        inside     109   494
```

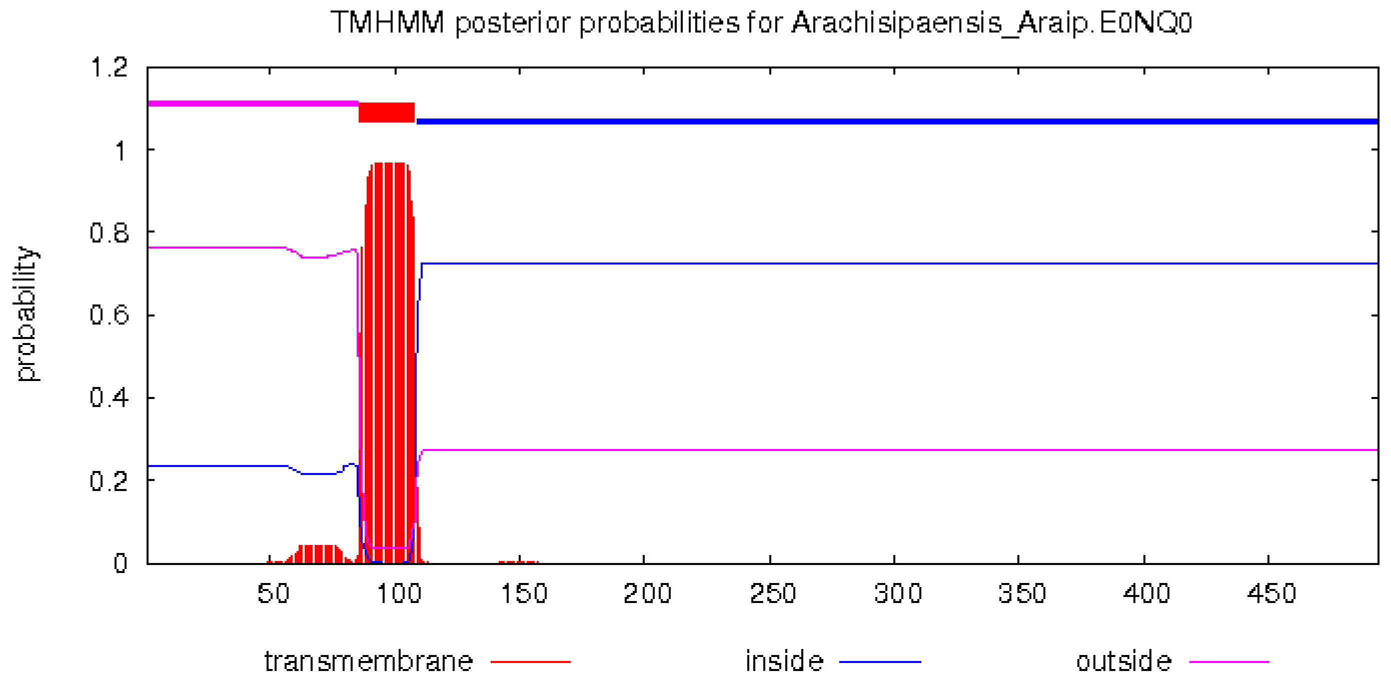

plot in postscript, script for making the plot in gnuplot, data for plot

---

```
# Arachisipaensis_Araip.F5AGL Length: 330
# Arachisipaensis_Araip.F5AGL Number of predicted TMHs:  0
# Arachisipaensis_Araip.F5AGL Exp number of AAs in TMHs: 0.00182
# Arachisipaensis_Araip.F5AGL Exp number, first 60 AAs:  0
# Arachisipaensis_Araip.F5AGL Total prob of N-in:        0.01303
Arachisipaensis_Araip.F5AGL     TMHMM2.0        outside      1   330
```

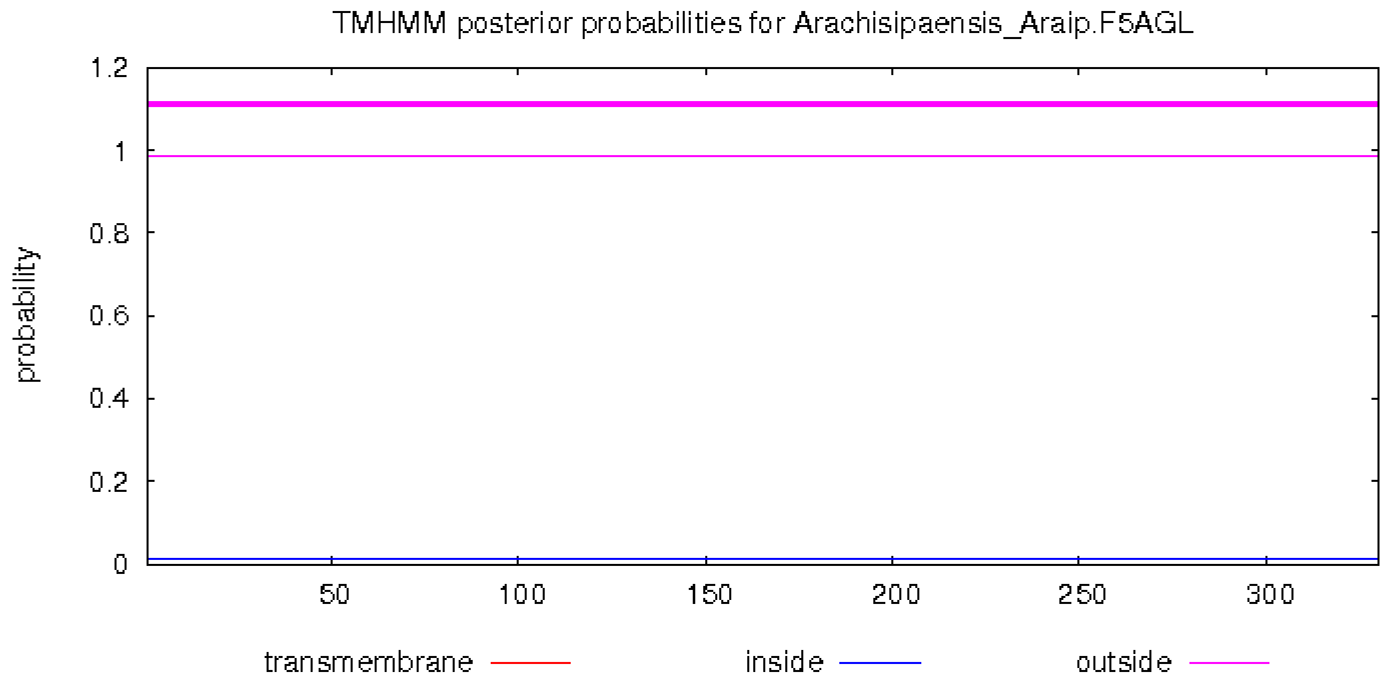

plot in postscript, script for making the plot in gnuplot, data for plot

```
# Arachisipaensis_Araip.F8I62 Length: 425
# Arachisipaensis_Araip.F8I62 Number of predicted TMHs:  0
# Arachisipaensis_Araip.F8I62 Exp number of AAs in TMHs: 0.0046
# Arachisipaensis_Araip.F8I62 Exp number, first 60 AAs:  0.00087
# Arachisipaensis_Araip.F8I62 Total prob of N-in:        0.00314
Arachisipaensis_Araip.F8I62     TMHMM2.0        outside      1   425
```

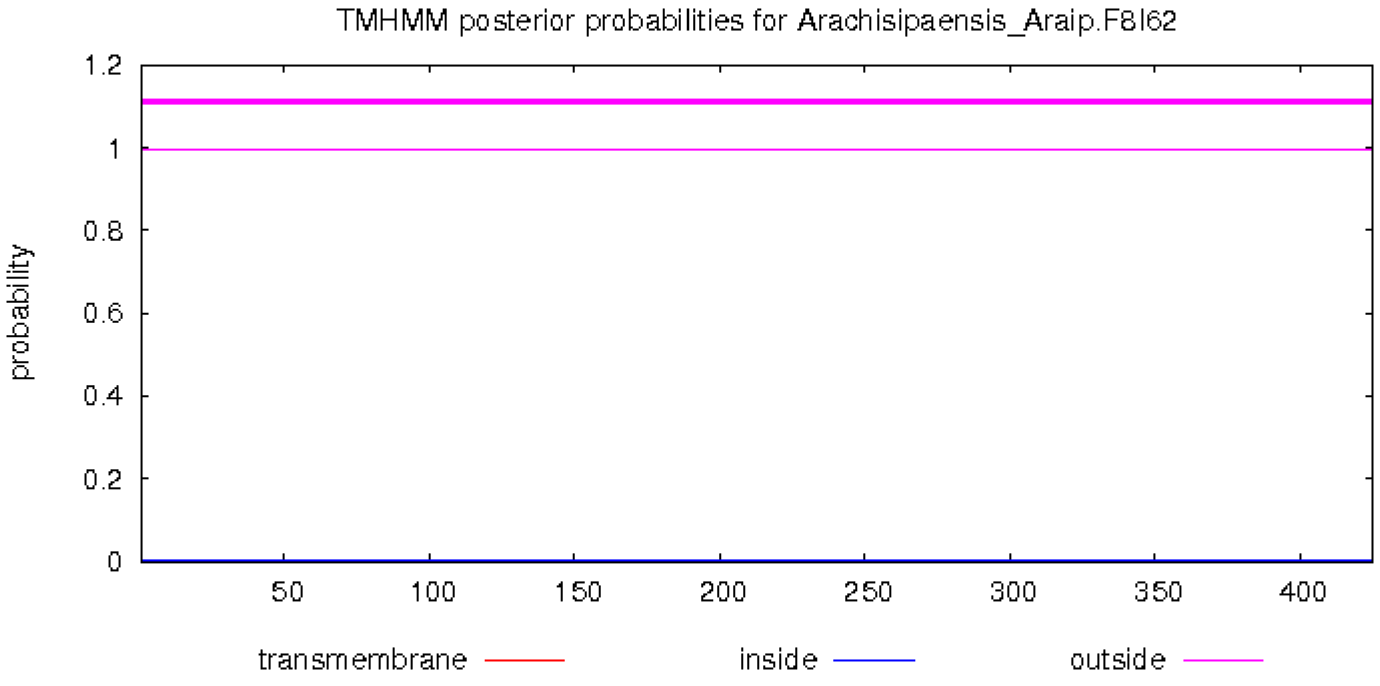

plot in postscript, script for making the plot in gnuplot, data for plot

```
# Arachisipaensis_Araip.FR0NA Length: 116
# Arachisipaensis_Araip.FR0NA Number of predicted TMHs:  0
# Arachisipaensis_Araip.FR0NA Exp number of AAs in TMHs: 0.02285
# Arachisipaensis_Araip.FR0NA Exp number, first 60 AAs:  0.00042
# Arachisipaensis_Araip.FR0NA Total prob of N-in:        0.53277
Arachisipaensis_Araip.FR0NA     TMHMM2.0        inside       1   116
```

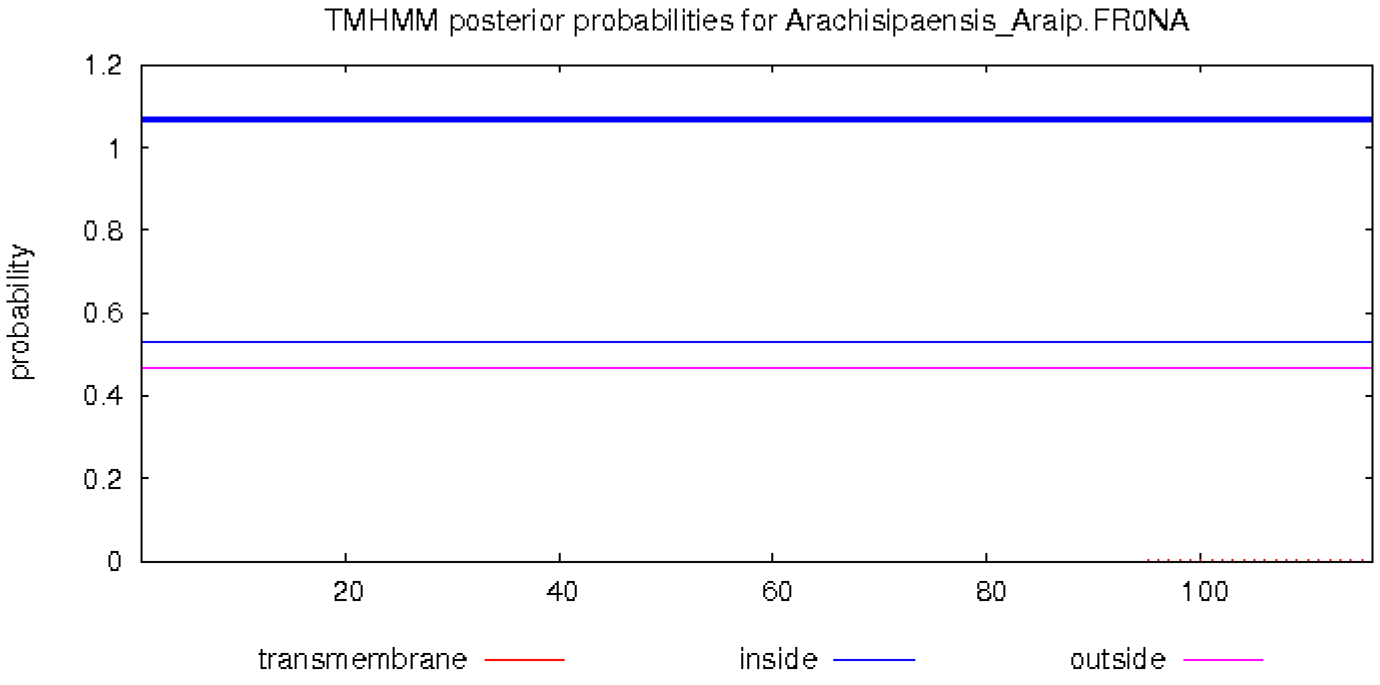

plot in postscript, script for making the plot in gnuplot, data for plot

```
# Arachisipaensis_Araip.FRS32 Length: 259
# Arachisipaensis_Araip.FRS32 Number of predicted TMHs:  0
# Arachisipaensis_Araip.FRS32 Exp number of AAs in TMHs: 0
# Arachisipaensis_Araip.FRS32 Exp number, first 60 AAs:  0
# Arachisipaensis_Araip.FRS32 Total prob of N-in:        0.01267
Arachisipaensis_Araip.FRS32     TMHMM2.0        outside      1   259
```

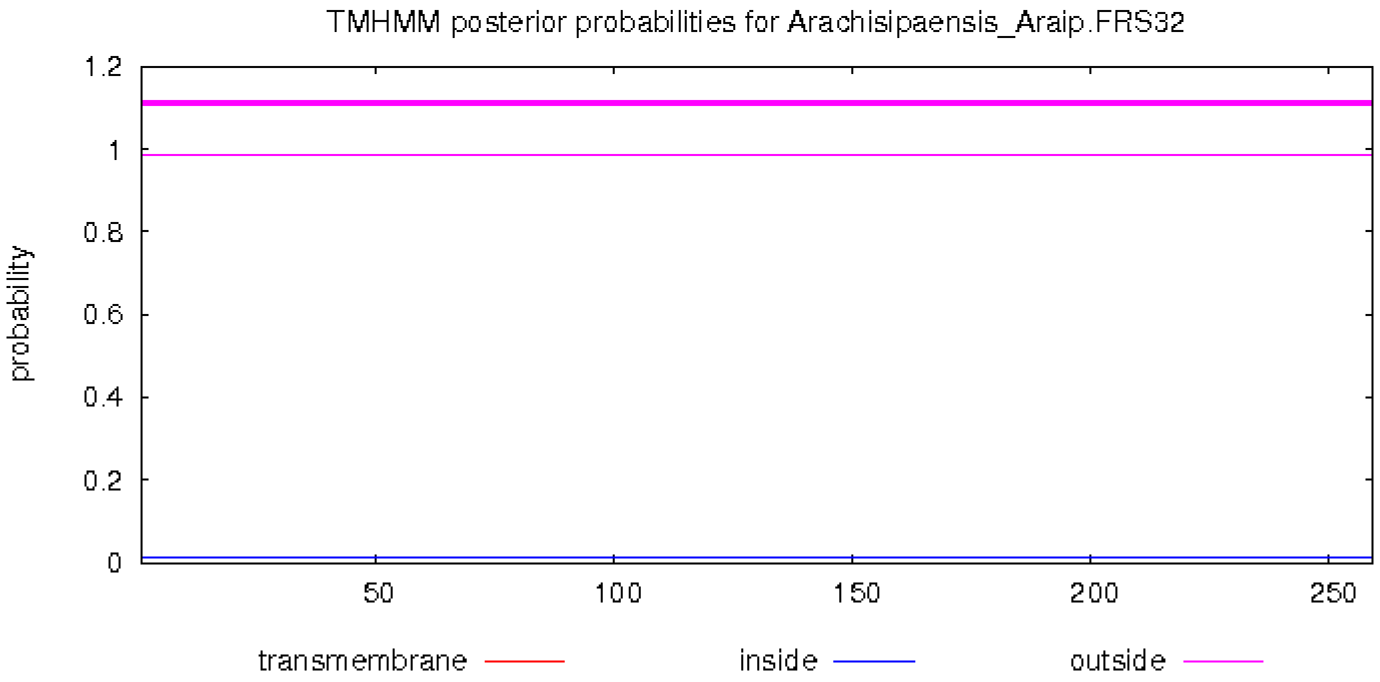

plot in postscript, script for making the plot in gnuplot, data for plot

---

```
# Arachisipaensis_Araip.G3ZLR Length: 321
# Arachisipaensis_Araip.G3ZLR Number of predicted TMHs:  0
# Arachisipaensis_Araip.G3ZLR Exp number of AAs in TMHs: 0.00107
# Arachisipaensis_Araip.G3ZLR Exp number, first 60 AAs:  0
# Arachisipaensis_Araip.G3ZLR Total prob of N-in:        0.00310
Arachisipaensis_Araip.G3ZLR     TMHMM2.0        outside      1   321
```

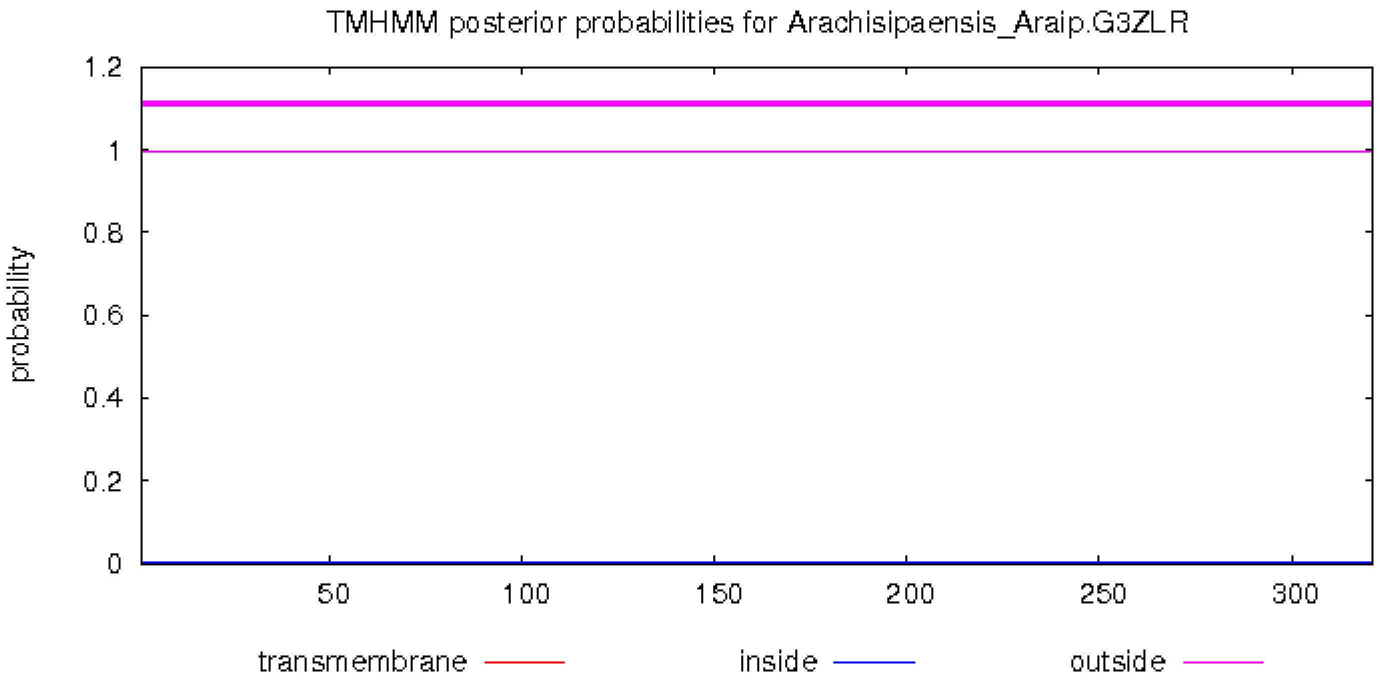

plot in postscript, script for making the plot in gnuplot, data for plot

---

```
# Arachisipaensis_Araip.G88UP Length: 330
# Arachisipaensis_Araip.G88UP Number of predicted TMHs:  0
# Arachisipaensis_Araip.G88UP Exp number of AAs in TMHs: 0.00717
# Arachisipaensis_Araip.G88UP Exp number, first 60 AAs:  0.00196
# Arachisipaensis_Araip.G88UP Total prob of N-in:        0.08475
Arachisipaensis_Araip.G88UP     TMHMM2.0        outside      1   330
```

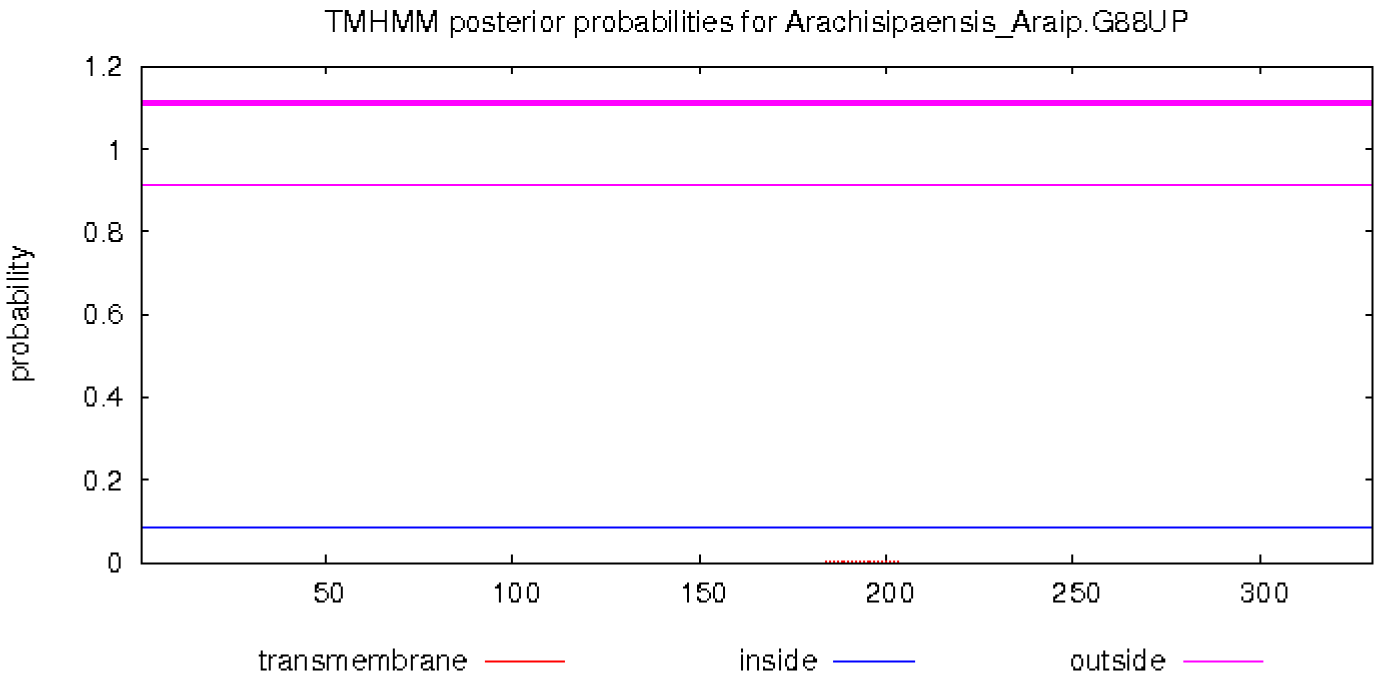

plot in postscript, script for making the plot in gnuplot, data for plot

---

```
# Arachisipaensis_Araip.HIJ9F Length: 418
# Arachisipaensis_Araip.HIJ9F Number of predicted TMHs:  0
# Arachisipaensis_Araip.HIJ9F Exp number of AAs in TMHs: 3.5087
# Arachisipaensis_Araip.HIJ9F Exp number, first 60 AAs:  2.15722
# Arachisipaensis_Araip.HIJ9F Total prob of N-in:        0.02353
Arachisipaensis_Araip.HIJ9F     TMHMM2.0        outside      1   418
```

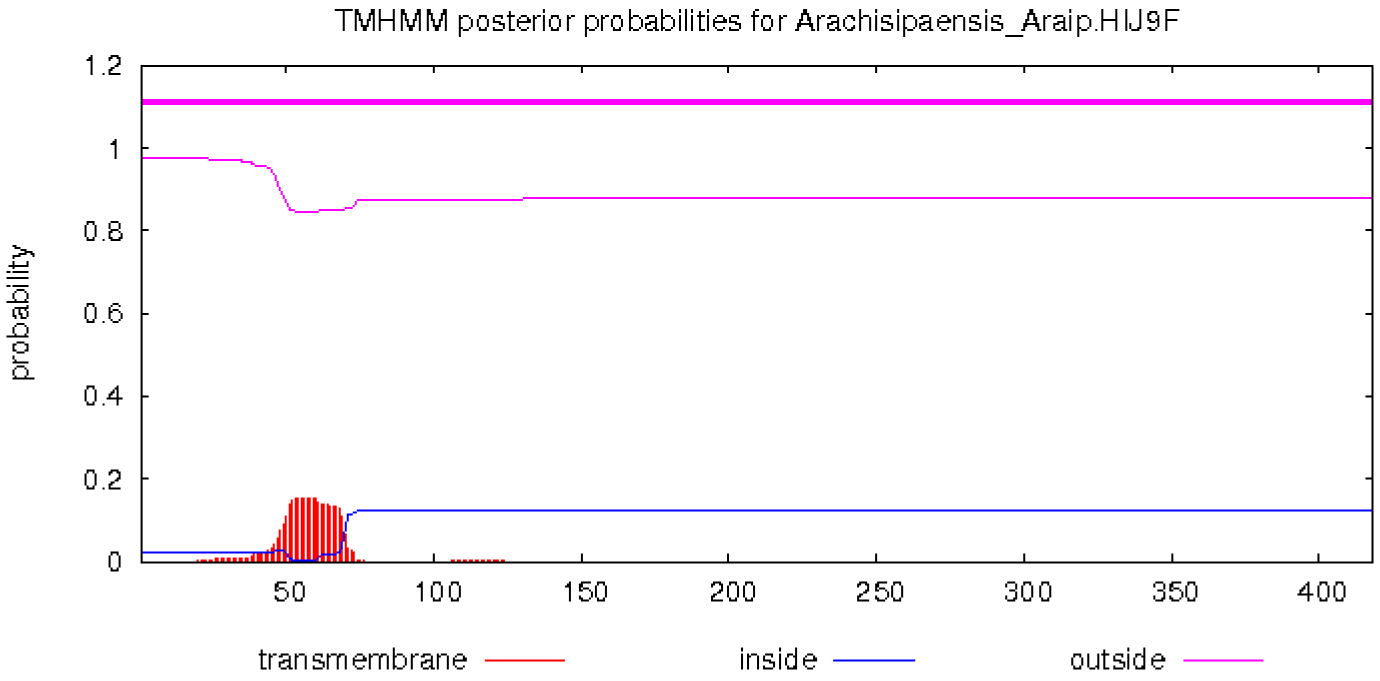

plot in postscript, script for making the plot in gnuplot, data for plot

---

```
# Arachisipaensis_Araip.HYM8C Length: 395
# Arachisipaensis_Araip.HYM8C Number of predicted TMHs:  0
# Arachisipaensis_Araip.HYM8C Exp number of AAs in TMHs: 0.0002
# Arachisipaensis_Araip.HYM8C Exp number, first 60 AAs:  0.0002
# Arachisipaensis_Araip.HYM8C Total prob of N-in:        0.00582
Arachisipaensis_Araip.HYM8C     TMHMM2.0        outside      1   395
```

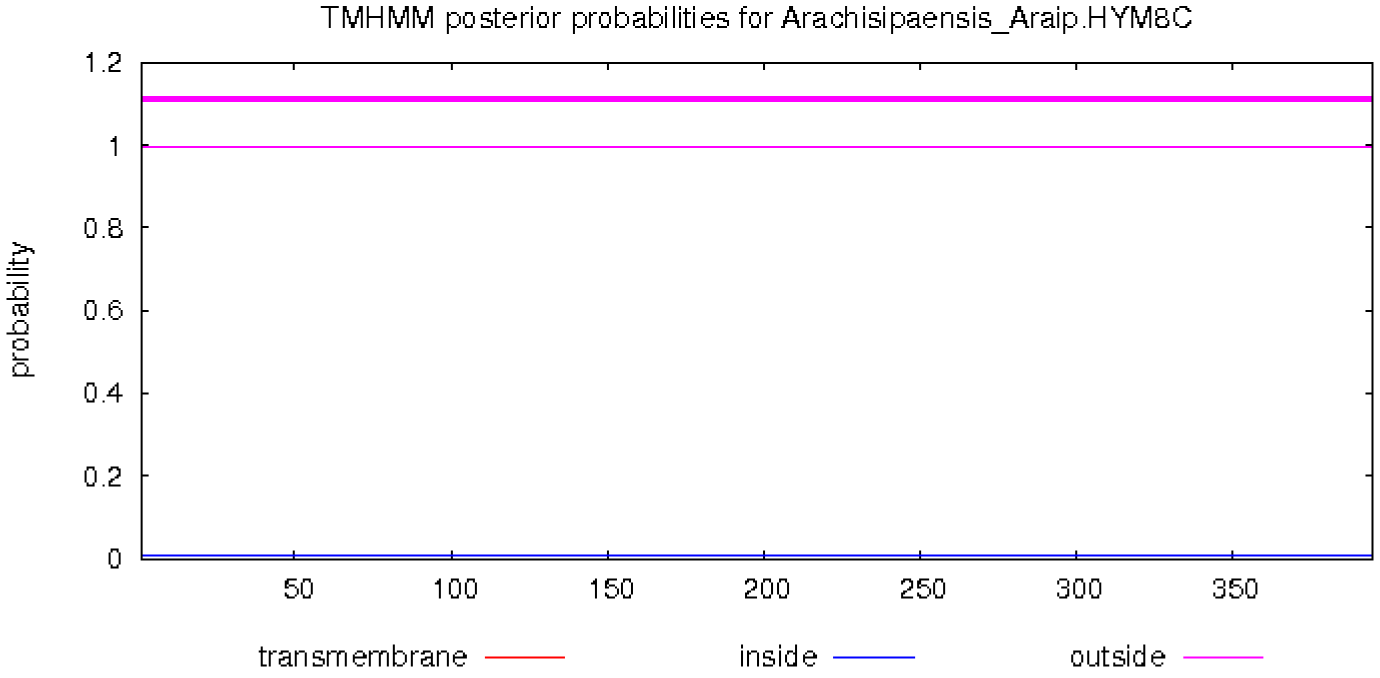

plot in postscript, script for making the plot in gnuplot, data for plot

---

```
# Arachisipaensis_Araip.I60BC Length: 234
# Arachisipaensis_Araip.I60BC Number of predicted TMHs:  0
# Arachisipaensis_Araip.I60BC Exp number of AAs in TMHs: 8e-05
# Arachisipaensis_Araip.I60BC Exp number, first 60 AAs:  0
# Arachisipaensis_Araip.I60BC Total prob of N-in:        0.01225
Arachisipaensis_Araip.I60BC     TMHMM2.0        outside      1   234
```

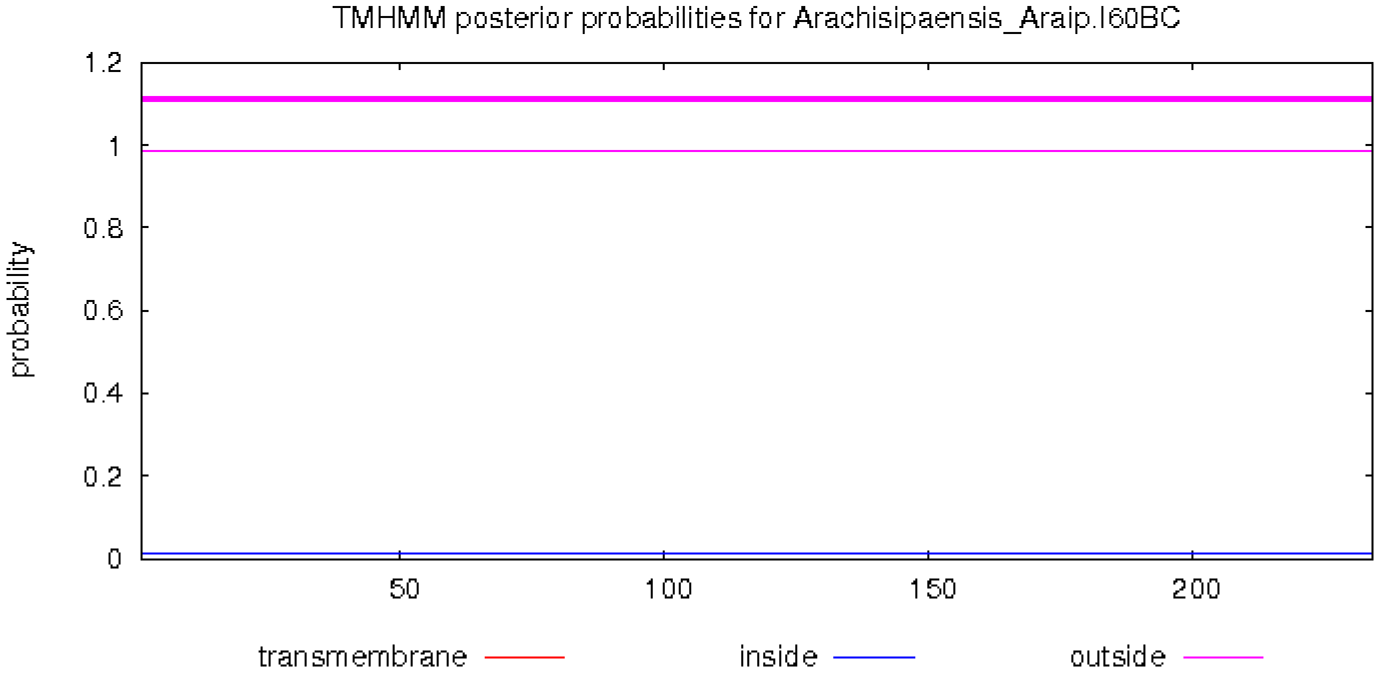

plot in postscript, script for making the plot in gnuplot, data for plot

---

```
# Arachisipaensis_Araip.I6LH9 Length: 318
# Arachisipaensis_Araip.I6LH9 Number of predicted TMHs:  0
# Arachisipaensis_Araip.I6LH9 Exp number of AAs in TMHs: 0.00019
# Arachisipaensis_Araip.I6LH9 Exp number, first 60 AAs:  0
# Arachisipaensis_Araip.I6LH9 Total prob of N-in:        0.02905
Arachisipaensis_Araip.I6LH9     TMHMM2.0        outside      1   318
```

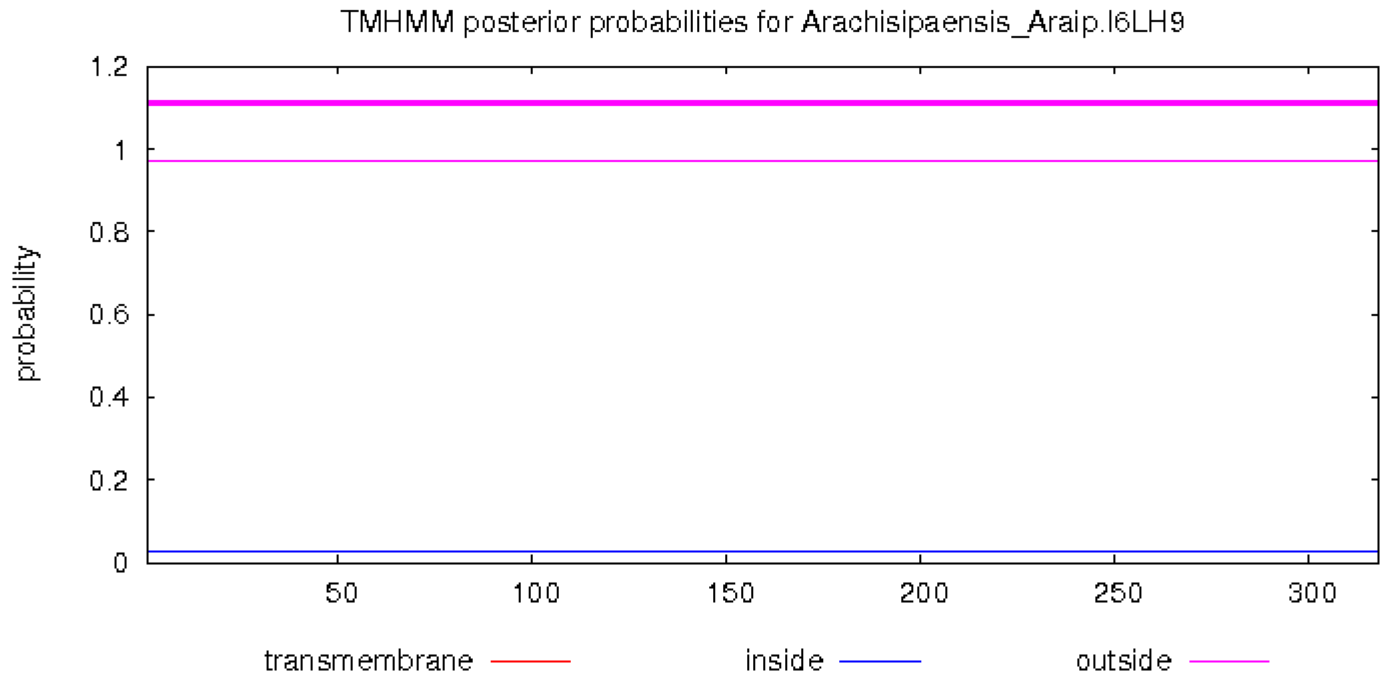

plot in postscript, script for making the plot in gnuplot, data for plot

```
# Arachisipaensis_Araip.J93FI Length: 294
# Arachisipaensis_Araip.J93FI Number of predicted TMHs:  0
# Arachisipaensis_Araip.J93FI Exp number of AAs in TMHs: 0.00074
# Arachisipaensis_Araip.J93FI Exp number, first 60 AAs:  0
# Arachisipaensis_Araip.J93FI Total prob of N-in:        0.08097
Arachisipaensis_Araip.J93FI     TMHMM2.0        outside      1   294
```

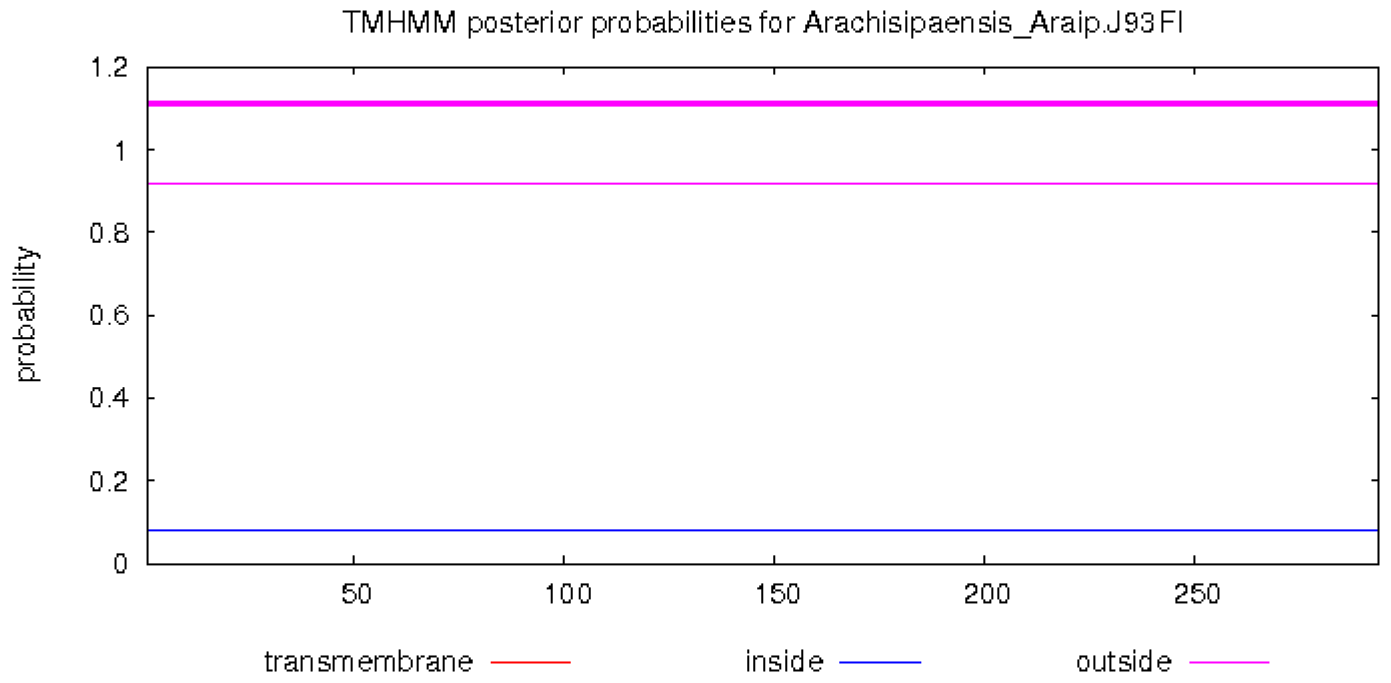

plot in postscript, script for making the plot in gnuplot, data for plot

```
# Arachisipaensis_Araip.J9WH5 Length: 375
# Arachisipaensis_Araip.J9WH5 Number of predicted TMHs:  0
# Arachisipaensis_Araip.J9WH5 Exp number of AAs in TMHs: 0
# Arachisipaensis_Araip.J9WH5 Exp number, first 60 AAs:  0
# Arachisipaensis_Araip.J9WH5 Total prob of N-in:        0.00416
Arachisipaensis_Araip.J9WH5     TMHMM2.0        outside      1   375
```

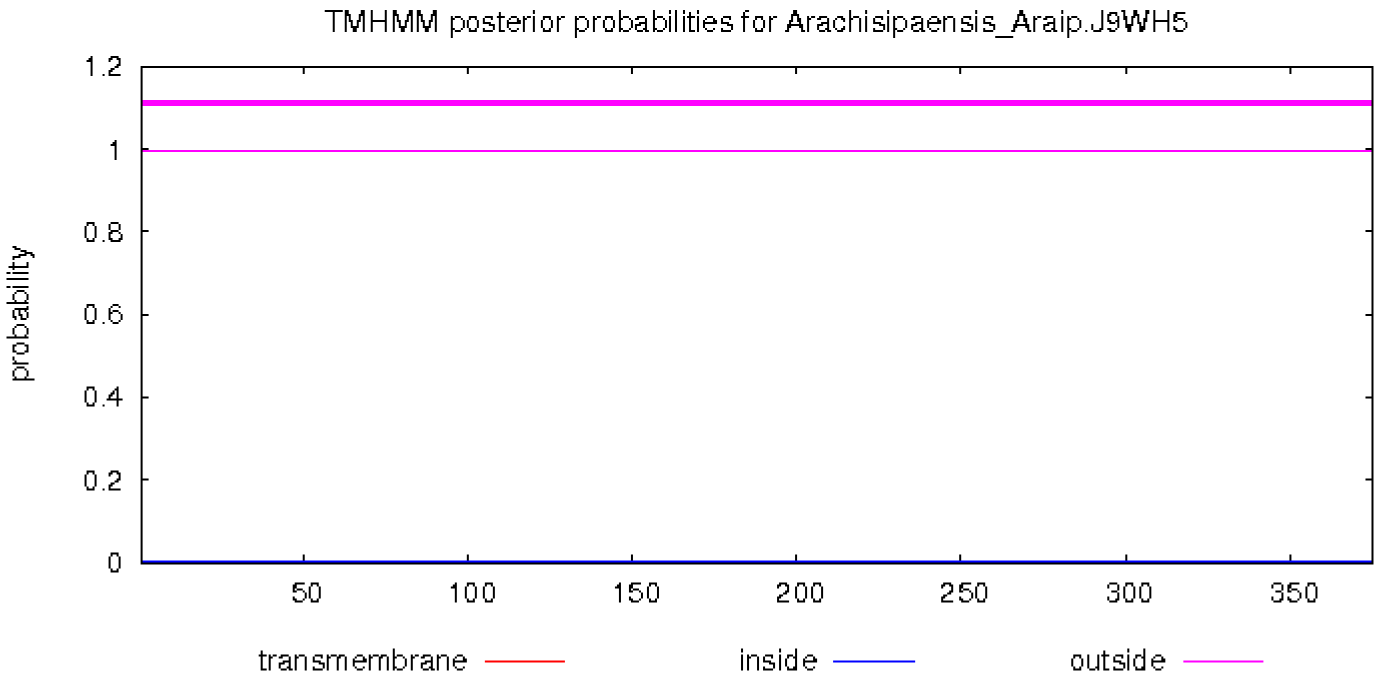

plot in postscript, script for making the plot in gnuplot, data for plot

```
# Arachisipaensis_Araip.KI83M Length: 306
# Arachisipaensis_Araip.KI83M Number of predicted TMHs:  0
# Arachisipaensis_Araip.KI83M Exp number of AAs in TMHs: 0.02257
# Arachisipaensis_Araip.KI83M Exp number, first 60 AAs:  0.02257
# Arachisipaensis_Araip.KI83M Total prob of N-in:        0.04036
Arachisipaensis_Araip.KI83M     TMHMM2.0        outside      1   306
```

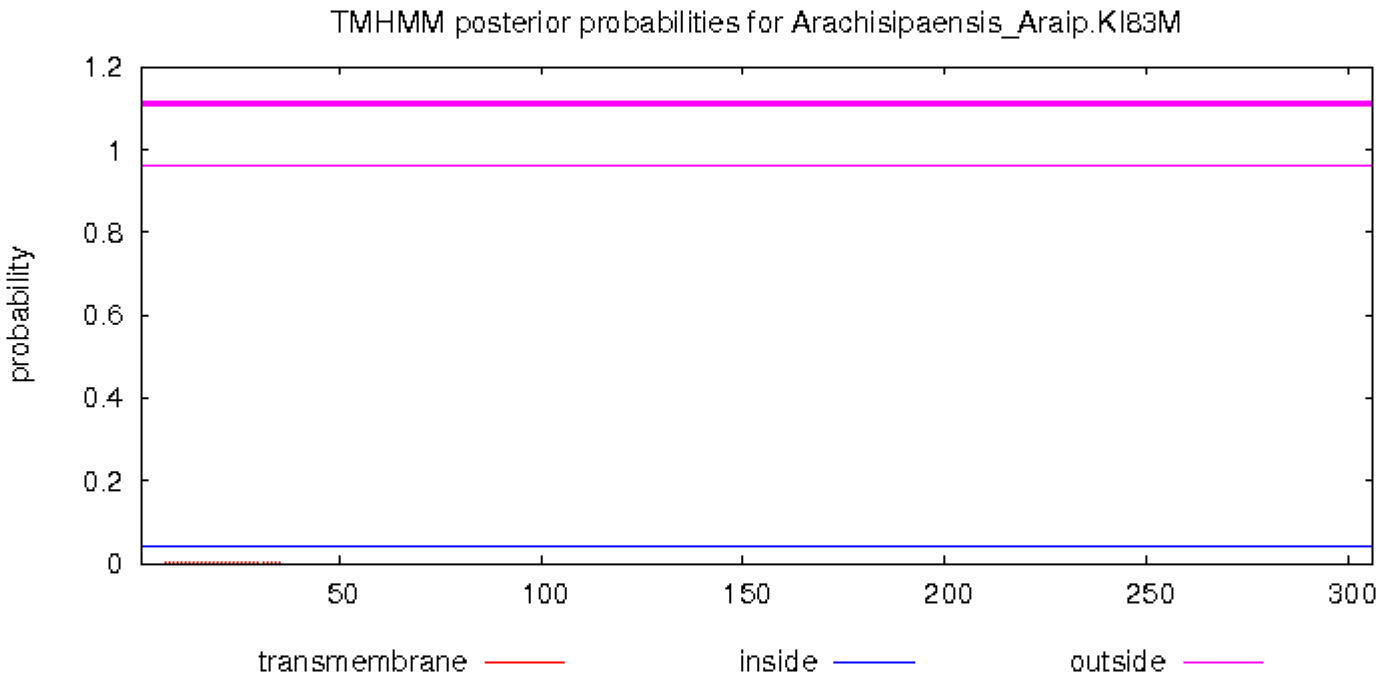

plot in postscript, script for making the plot in gnuplot, data for plot

```
# Arachisipaensis_Araip.KM0ZG Length: 143
# Arachisipaensis_Araip.KM0ZG Number of predicted TMHs:  0
# Arachisipaensis_Araip.KM0ZG Exp number of AAs in TMHs: 0.00151
# Arachisipaensis_Araip.KM0ZG Exp number, first 60 AAs:  0
# Arachisipaensis_Araip.KM0ZG Total prob of N-in:        0.06170
Arachisipaensis_Araip.KM0ZG     TMHMM2.0        outside      1   143
```

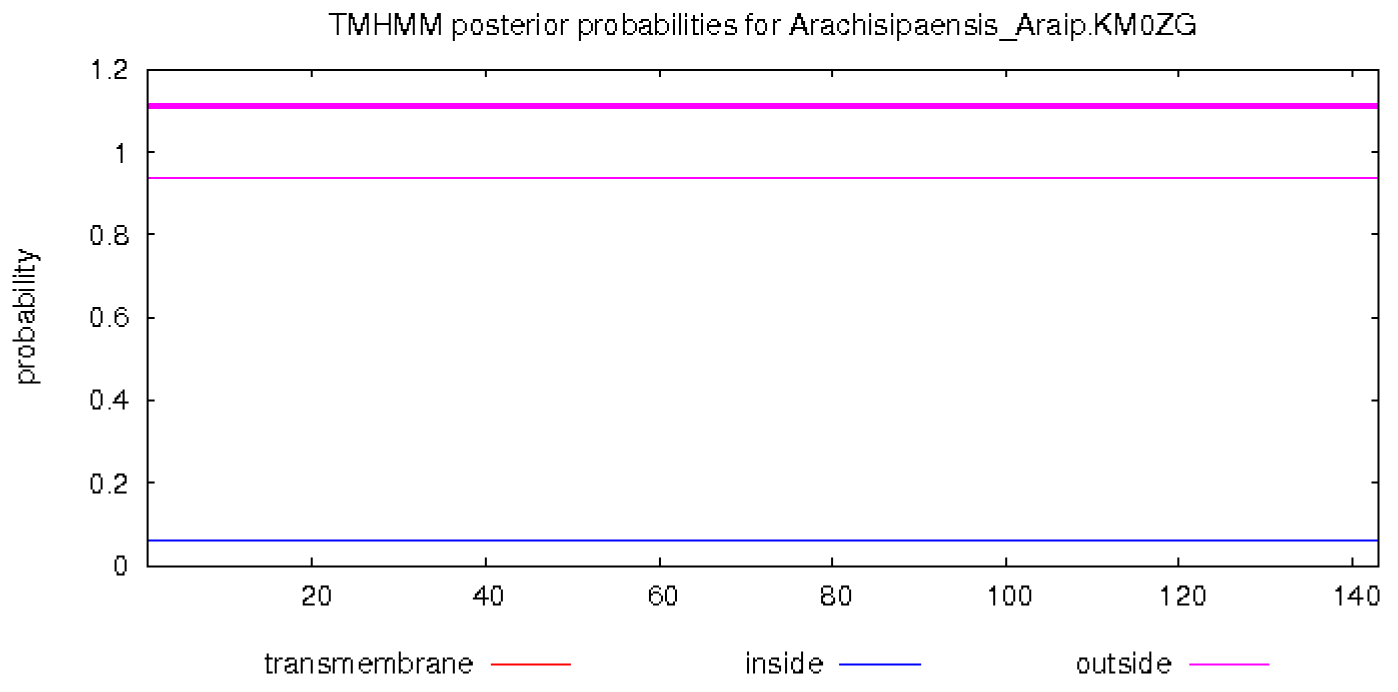

plot in postscript, script for making the plot in gnuplot, data for plot

---

```
# Arachisipaensis_Araip.KP5QZ Length: 362
# Arachisipaensis_Araip.KP5QZ Number of predicted TMHs:  0
# Arachisipaensis_Araip.KP5QZ Exp number of AAs in TMHs: 0.4738
# Arachisipaensis_Araip.KP5QZ Exp number, first 60 AAs:  0.00015
# Arachisipaensis_Araip.KP5QZ Total prob of N-in:        0.02531
Arachisipaensis_Araip.KP5QZ     TMHMM2.0        outside      1   362
```

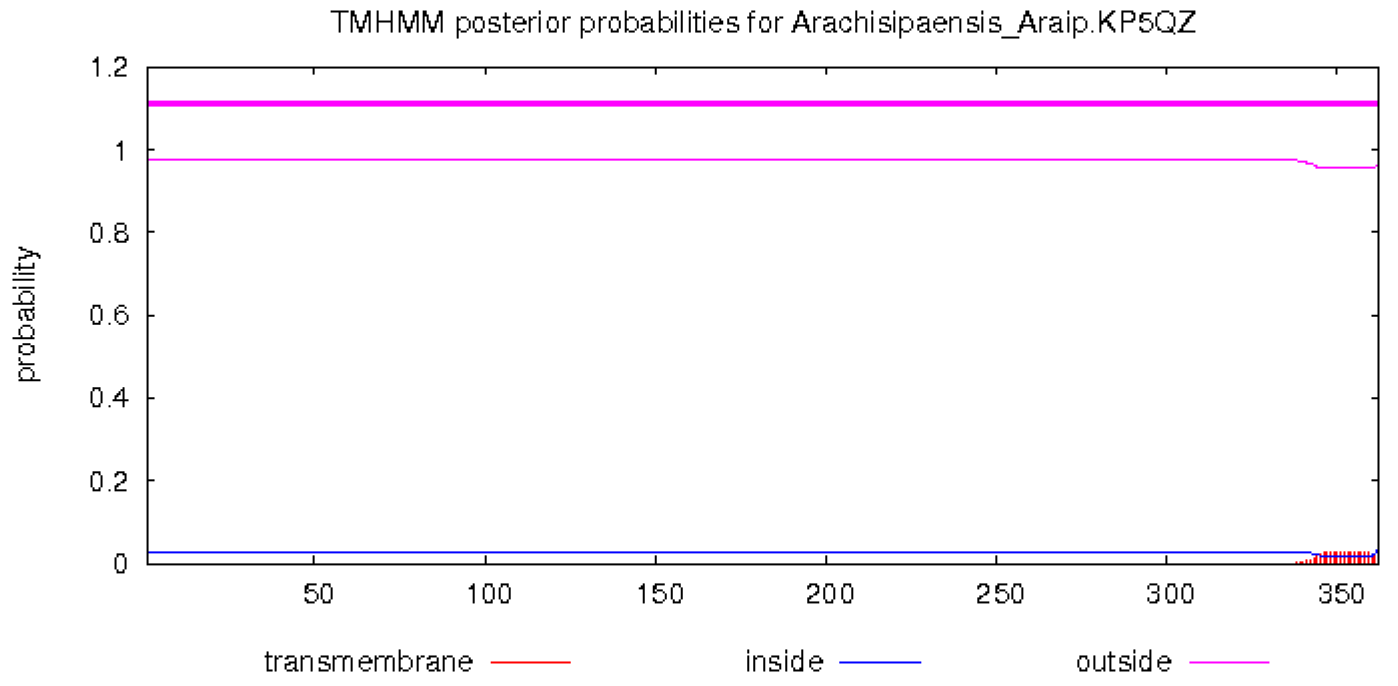

plot in postscript, script for making the plot in gnuplot, data for plot

---

```
# Arachisipaensis_Araip.KTL6D Length: 59
# Arachisipaensis_Araip.KTL6D Number of predicted TMHs:  0
# Arachisipaensis_Araip.KTL6D Exp number of AAs in TMHs: 0
# Arachisipaensis_Araip.KTL6D Exp number, first 60 AAs:  0
# Arachisipaensis_Araip.KTL6D Total prob of N-in:        0.13129
Arachisipaensis_Araip.KTL6D     TMHMM2.0        outside      1    59
```

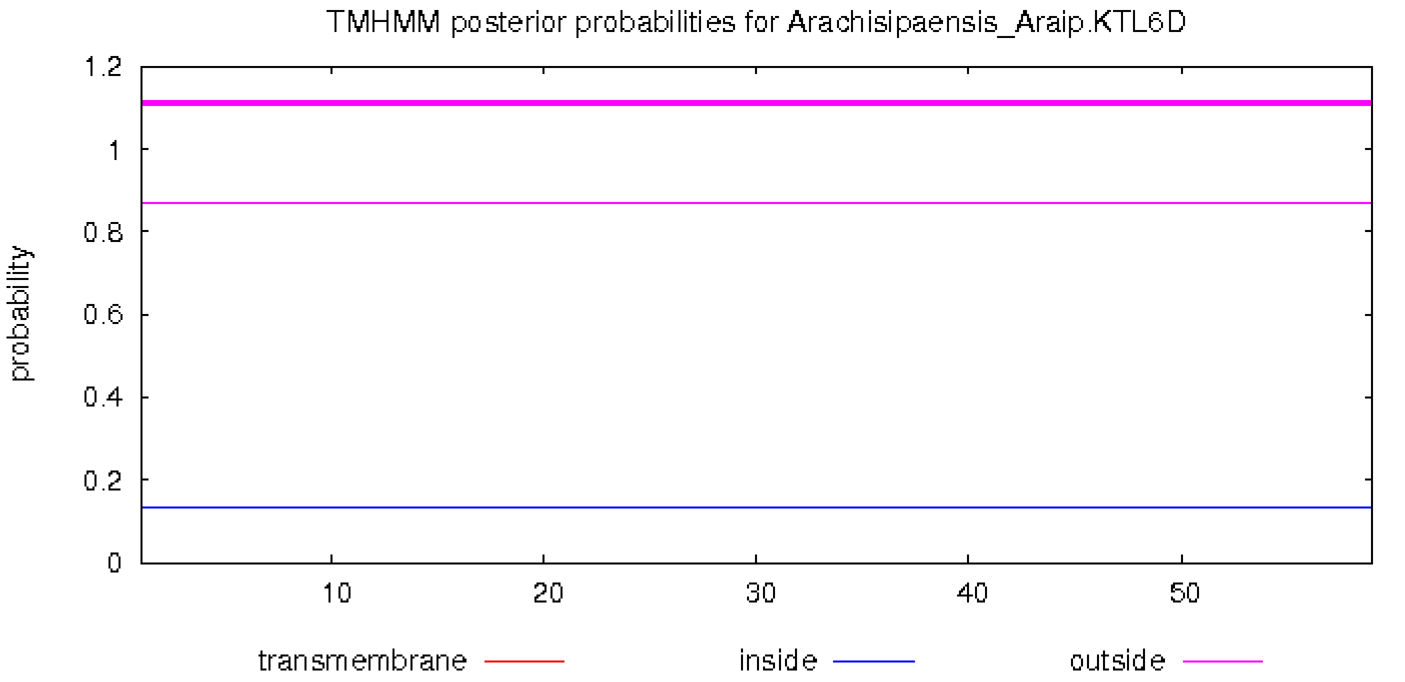

plot in postscript, script for making the plot in gnuplot, data for plot

---

```
# Arachisipaensis_Araip.KXD50|Arachis_ipaensis|NAC|Araip.KXD50 Length: 65
# Arachisipaensis_Araip.KXD50|Arachis_ipaensis|NAC|Araip.KXD50 Number of predicted TMHs:  0
# Arachisipaensis_Araip.KXD50|Arachis_ipaensis|NAC|Araip.KXD50 Exp number of AAs in TMHs: 0.07659
# Arachisipaensis_Araip.KXD50|Arachis_ipaensis|NAC|Araip.KXD50 Exp number, first 60 AAs:  0.0677
# Arachisipaensis_Araip.KXD50|Arachis_ipaensis|NAC|Araip.KXD50 Total prob of N-in:        0.65825
Arachisipaensis_Araip.KXD50|Arachis_ipaensis|NAC|Araip.KXD50    TMHMM2.0        inside       1    65
```

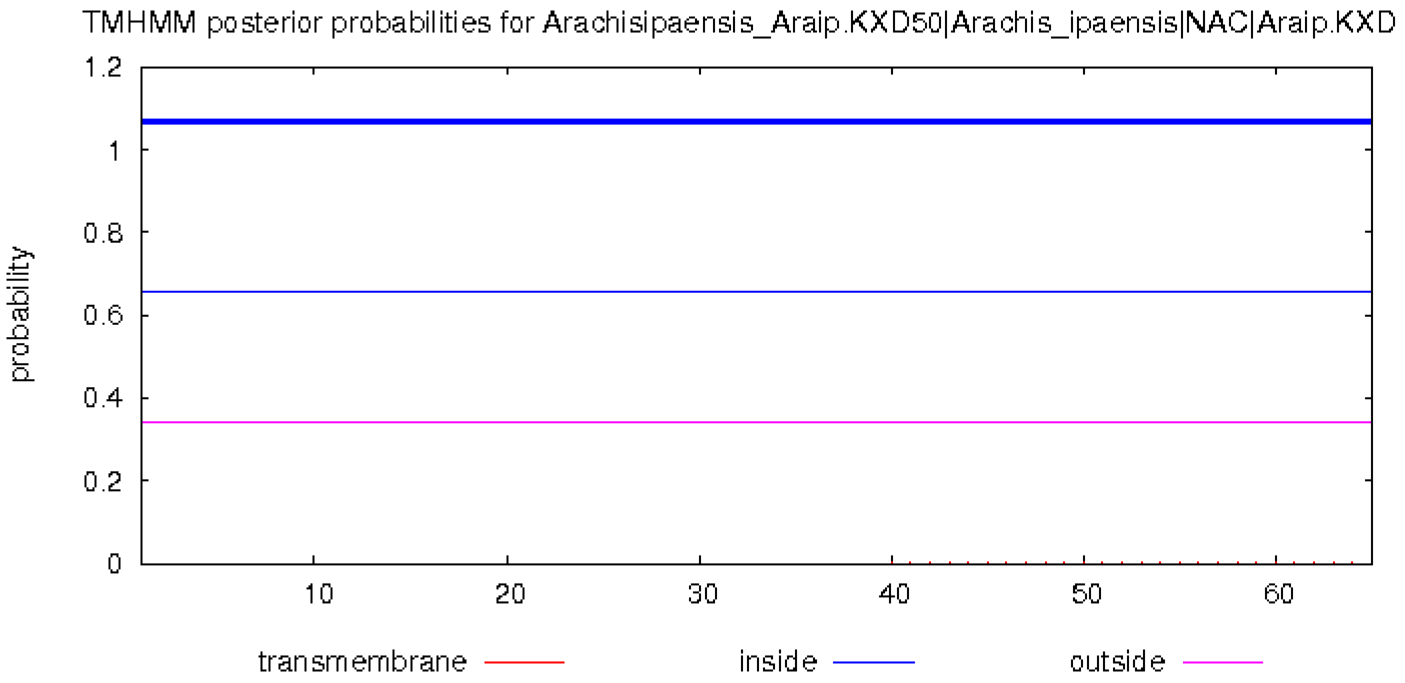

plot in postscript, script for making the plot in gnuplot, data for plot

---

```
# Arachisipaensis_Araip.L222I Length: 423
# Arachisipaensis_Araip.L222I Number of predicted TMHs:  0
# Arachisipaensis_Araip.L222I Exp number of AAs in TMHs: 0.00159
# Arachisipaensis_Araip.L222I Exp number, first 60 AAs:  0.00145
# Arachisipaensis_Araip.L222I Total prob of N-in:        0.00197
Arachisipaensis_Araip.L222I     TMHMM2.0        outside      1   423
```

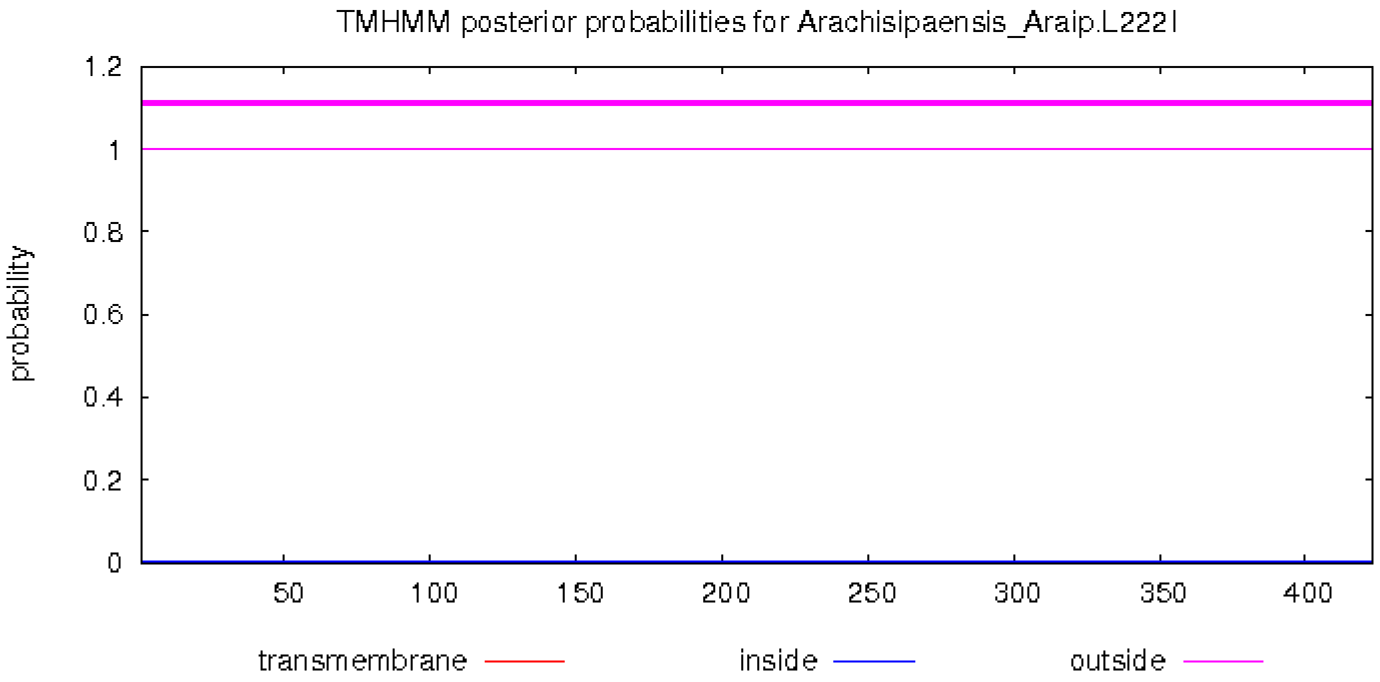

plot in postscript, script for making the plot in gnuplot, data for plot

---

```
# Arachisipaensis_Araip.MQD5S Length: 289
# Arachisipaensis_Araip.MQD5S Number of predicted TMHs:  0
# Arachisipaensis_Araip.MQD5S Exp number of AAs in TMHs: 0
# Arachisipaensis_Araip.MQD5S Exp number, first 60 AAs:  0
# Arachisipaensis_Araip.MQD5S Total prob of N-in:        0.01560
Arachisipaensis_Araip.MQD5S     TMHMM2.0        outside      1   289
```

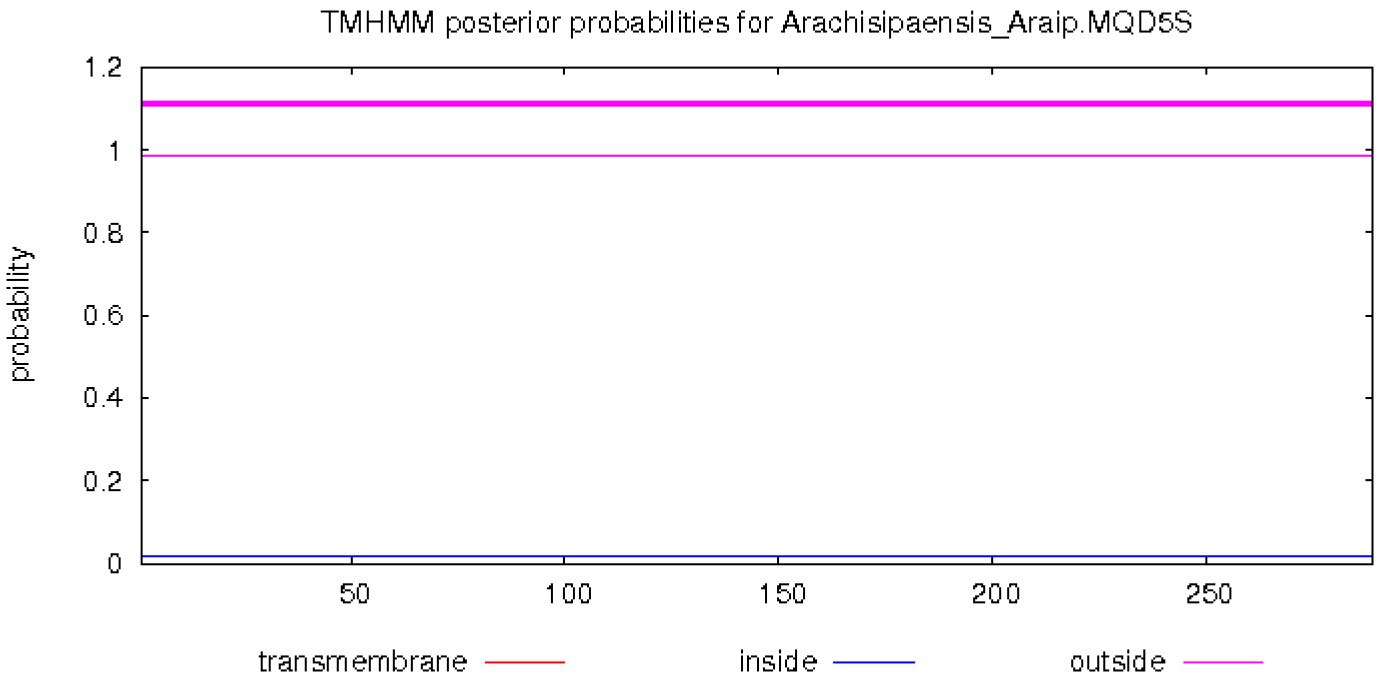

plot in postscript, script for making the plot in gnuplot, data for plot

---

```
# Arachisipaensis_Araip.NB7HU Length: 332
# Arachisipaensis_Araip.NB7HU Number of predicted TMHs:  0
# Arachisipaensis_Araip.NB7HU Exp number of AAs in TMHs: 0.00058
# Arachisipaensis_Araip.NB7HU Exp number, first 60 AAs:  0.00058
# Arachisipaensis_Araip.NB7HU Total prob of N-in:        0.01665
Arachisipaensis_Araip.NB7HU     TMHMM2.0        outside      1   332
```

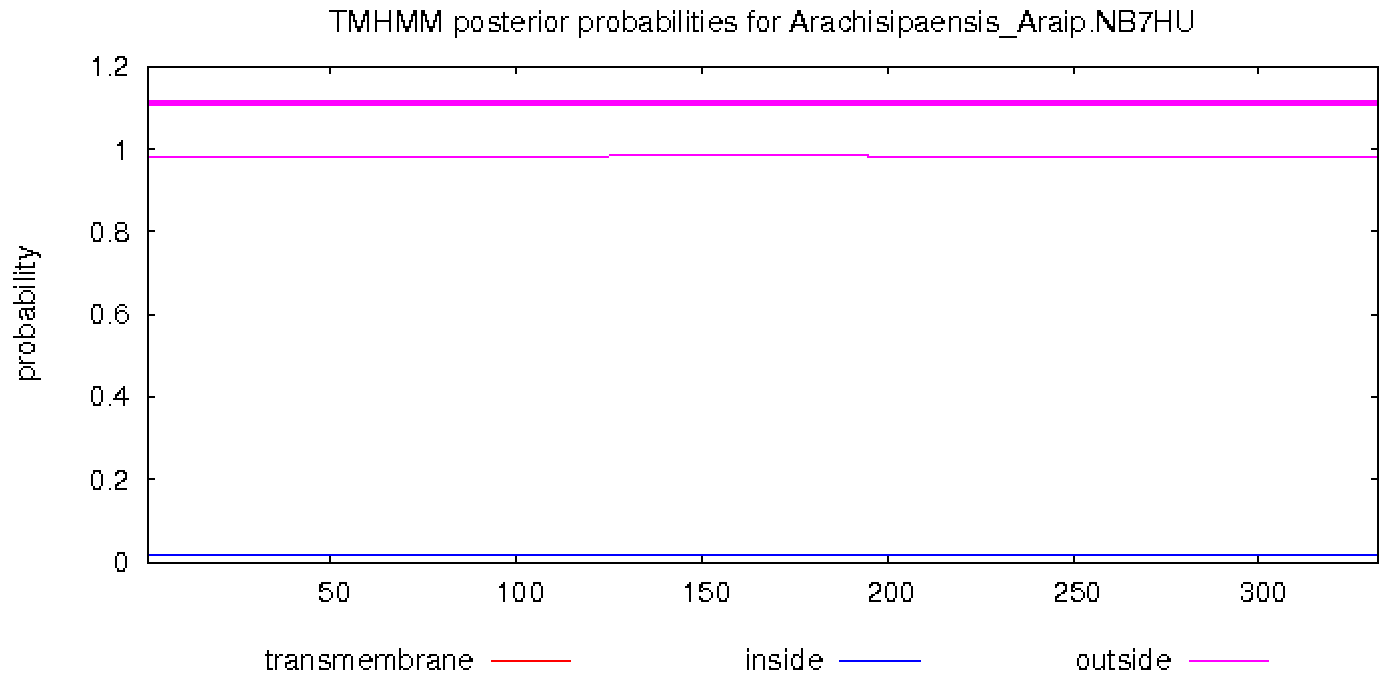

plot in postscript, script for making the plot in gnuplot, data for plot

---

```
# Arachisipaensis_Araip.NL359 Length: 255
# Arachisipaensis_Araip.NL359 Number of predicted TMHs:  0
# Arachisipaensis_Araip.NL359 Exp number of AAs in TMHs: 0
# Arachisipaensis_Araip.NL359 Exp number, first 60 AAs:  0
# Arachisipaensis_Araip.NL359 Total prob of N-in:        0.00173
Arachisipaensis_Araip.NL359     TMHMM2.0        outside      1   255
```

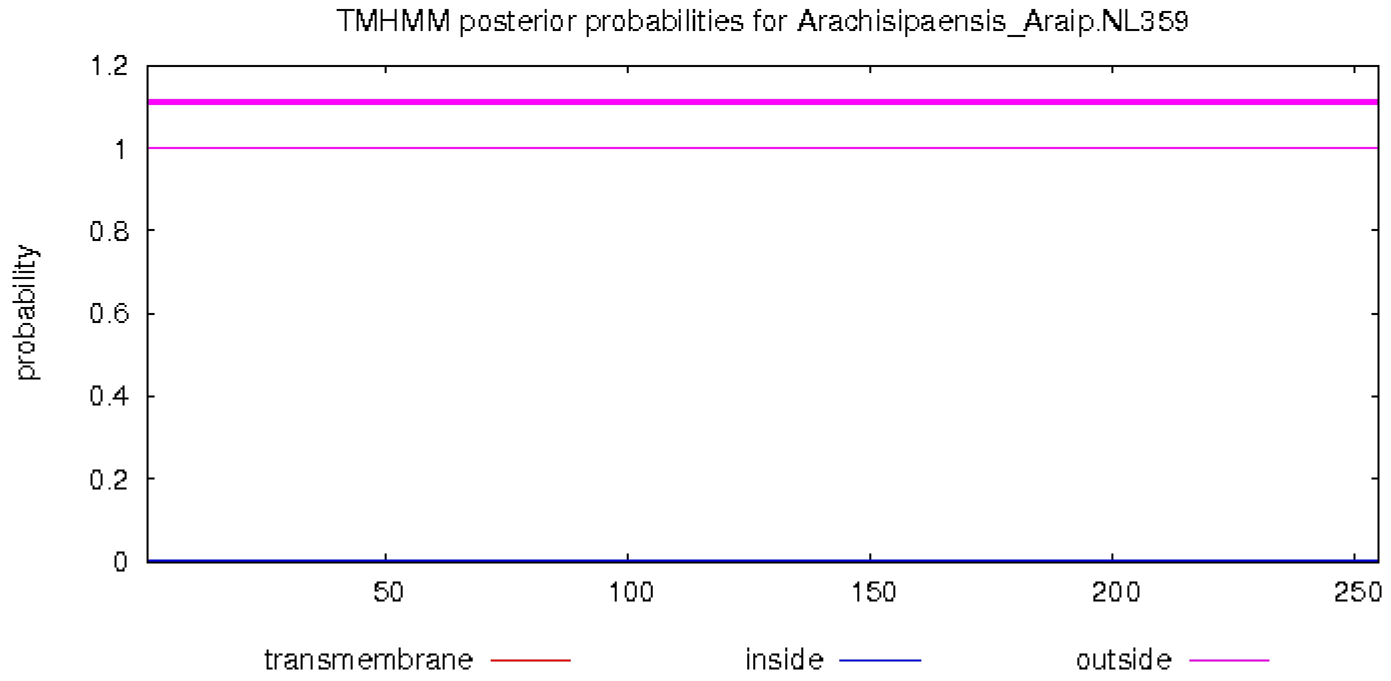

plot in postscript, script for making the plot in gnuplot, data for plot

---

```
# Arachisipaensis_Araip.PNX61 Length: 308
# Arachisipaensis_Araip.PNX61 Number of predicted TMHs:  0
# Arachisipaensis_Araip.PNX61 Exp number of AAs in TMHs: 0.0821
# Arachisipaensis_Araip.PNX61 Exp number, first 60 AAs:  0
# Arachisipaensis_Araip.PNX61 Total prob of N-in:        0.02919
Arachisipaensis_Araip.PNX61     TMHMM2.0        outside      1   308
```

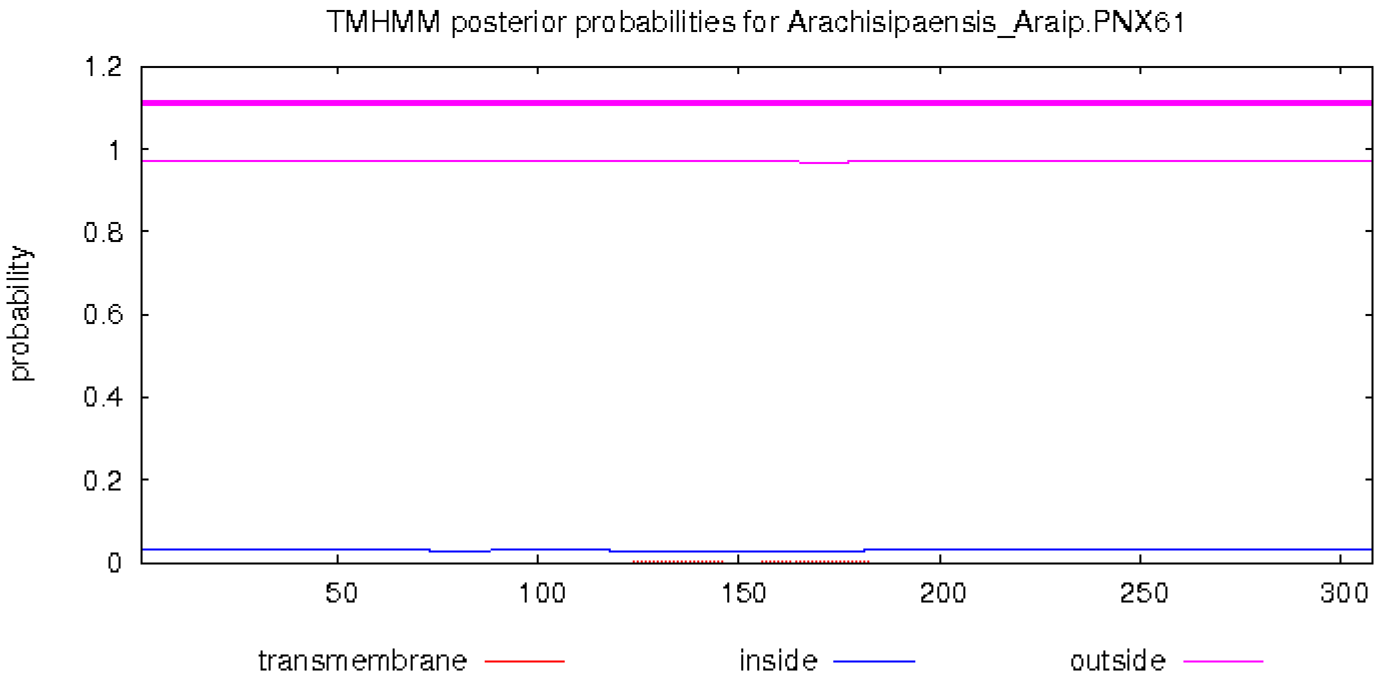

plot in postscript, script for making the plot in gnuplot, data for plot

---

```
# Arachisipaensis_Araip.PT231 Length: 343
# Arachisipaensis_Araip.PT231 Number of predicted TMHs:  0
# Arachisipaensis_Araip.PT231 Exp number of AAs in TMHs: 8e-05
# Arachisipaensis_Araip.PT231 Exp number, first 60 AAs:  8e-05
# Arachisipaensis_Araip.PT231 Total prob of N-in:        0.00312
Arachisipaensis_Araip.PT231     TMHMM2.0        outside      1   343
```

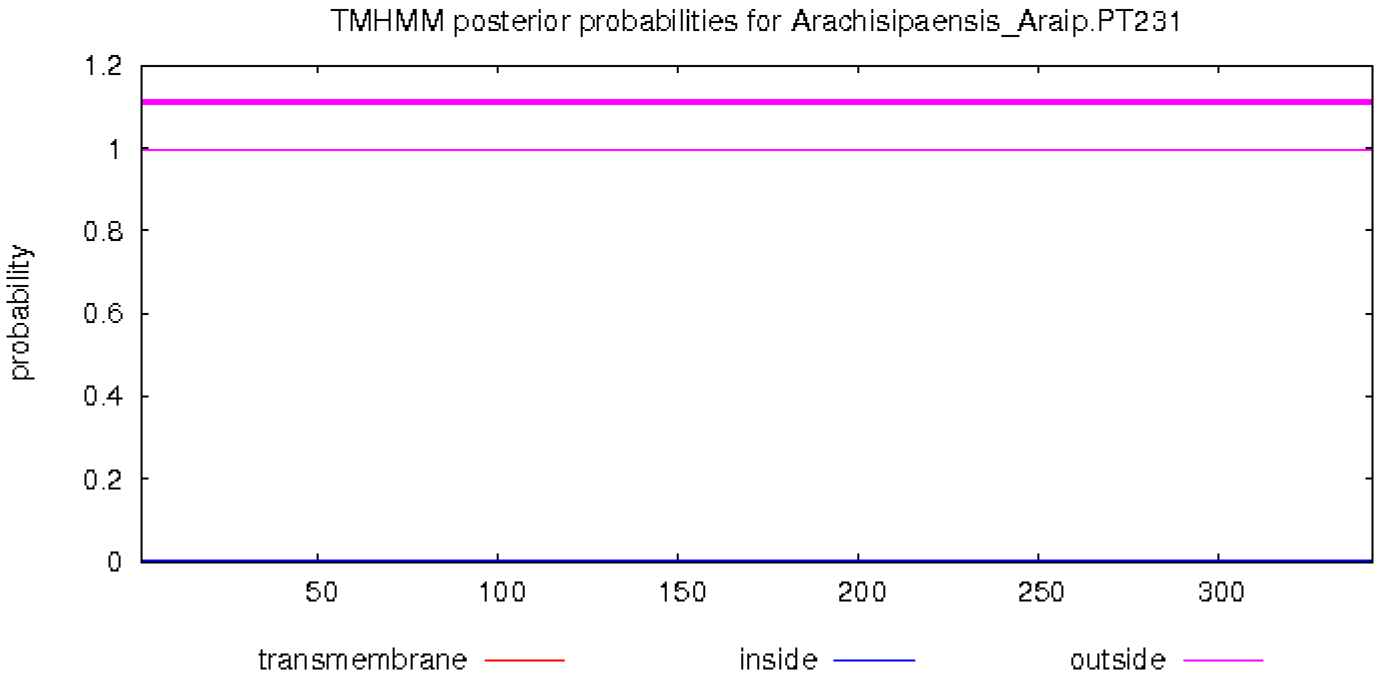

plot in postscript, script for making the plot in gnuplot, data for plot

---

```
# Arachisipaensis_Araip.PW8UQ Length: 256
# Arachisipaensis_Araip.PW8UQ Number of predicted TMHs:  0
# Arachisipaensis_Araip.PW8UQ Exp number of AAs in TMHs: 0.00078
# Arachisipaensis_Araip.PW8UQ Exp number, first 60 AAs:  0.00017
# Arachisipaensis_Araip.PW8UQ Total prob of N-in:        0.02392
Arachisipaensis_Araip.PW8UQ     TMHMM2.0        outside      1   256
```

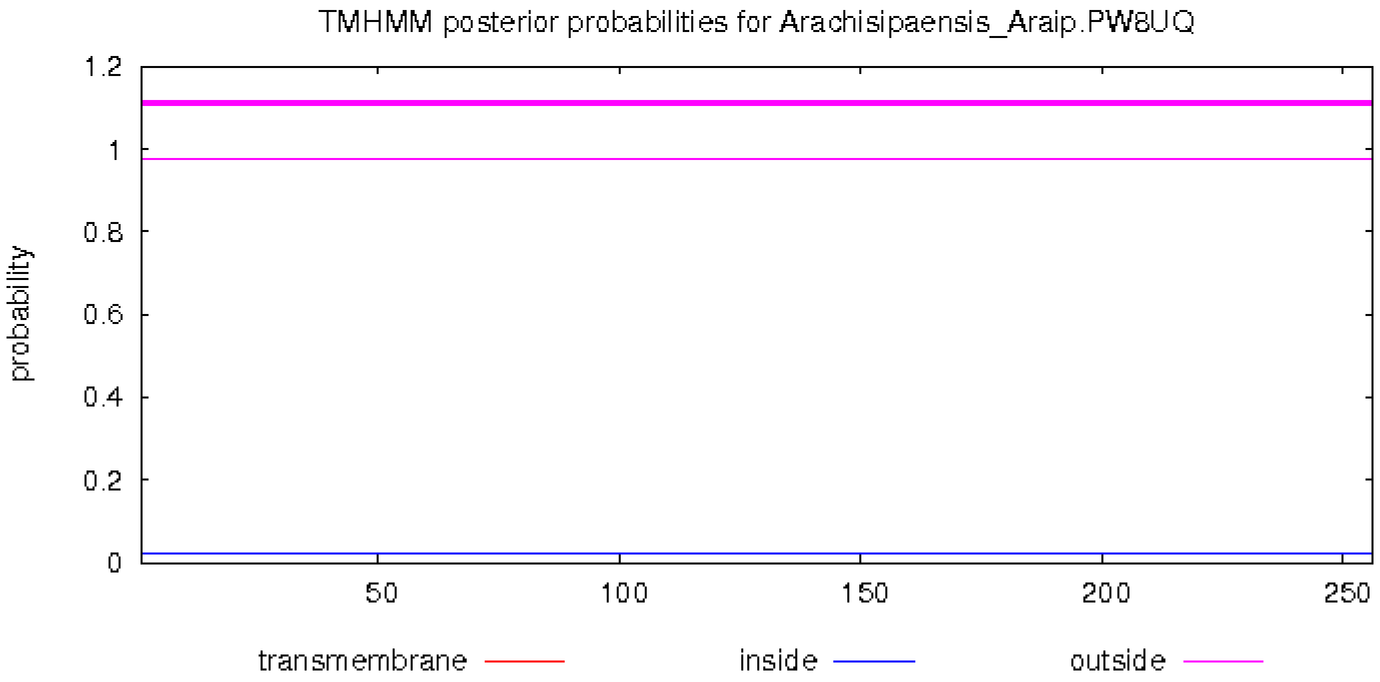

plot in postscript, script for making the plot in gnuplot, data for plot

---

```
# Arachisipaensis_Araip.PX0QP Length: 573
# Arachisipaensis_Araip.PX0QP Number of predicted TMHs:  1
# Arachisipaensis_Araip.PX0QP Exp number of AAs in TMHs: 22.54871
# Arachisipaensis_Araip.PX0QP Exp number, first 60 AAs:  0
# Arachisipaensis_Araip.PX0QP Total prob of N-in:        0.03747
Arachisipaensis_Araip.PX0QP     TMHMM2.0        outside      1   543
Arachisipaensis_Araip.PX0QP     TMHMM2.0        TMhelix    544   566
Arachisipaensis_Araip.PX0QP     TMHMM2.0        inside     567   573
```

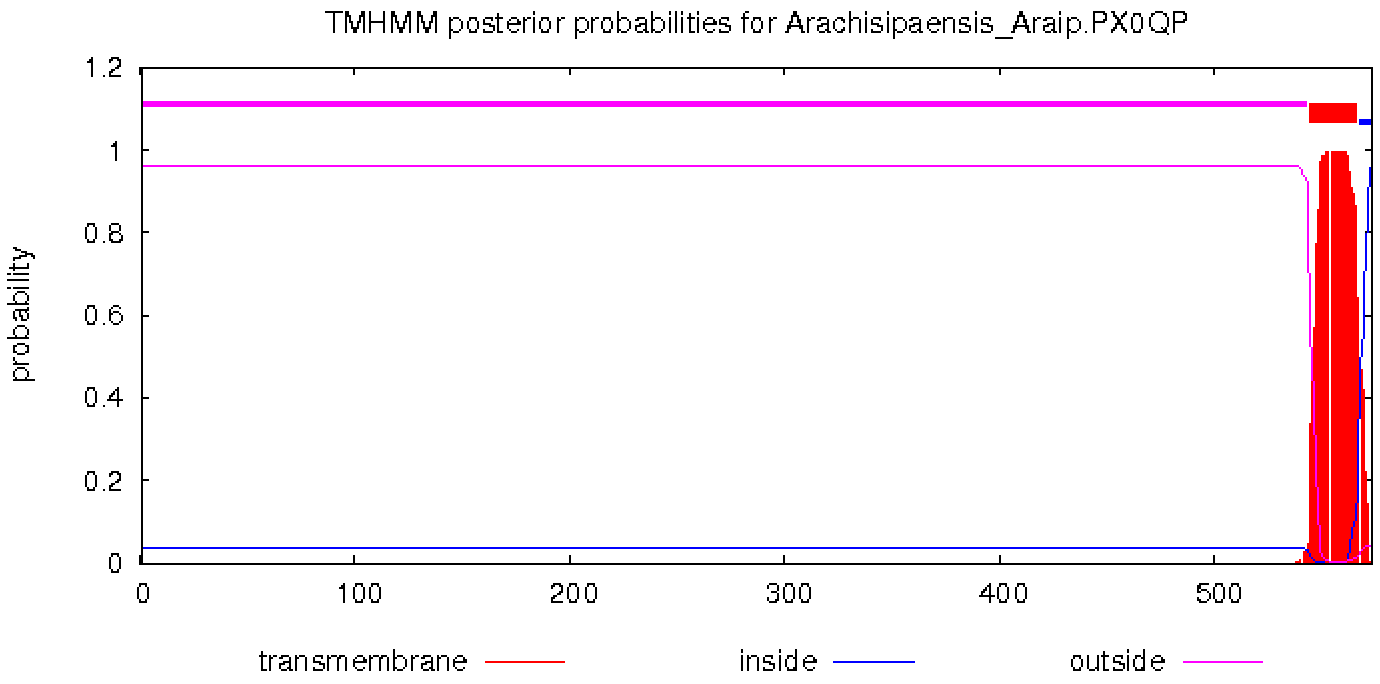

plot in postscript, script for making the plot in gnuplot, data for plot

```
# Arachisipaensis_Araip.Q1JTJ Length: 377
# Arachisipaensis_Araip.Q1JTJ Number of predicted TMHs:  0
# Arachisipaensis_Araip.Q1JTJ Exp number of AAs in TMHs: 0.00019
# Arachisipaensis_Araip.Q1JTJ Exp number, first 60 AAs:  0
# Arachisipaensis_Araip.Q1JTJ Total prob of N-in:        0.00286
Arachisipaensis_Araip.Q1JTJ     TMHMM2.0        outside      1   377
```

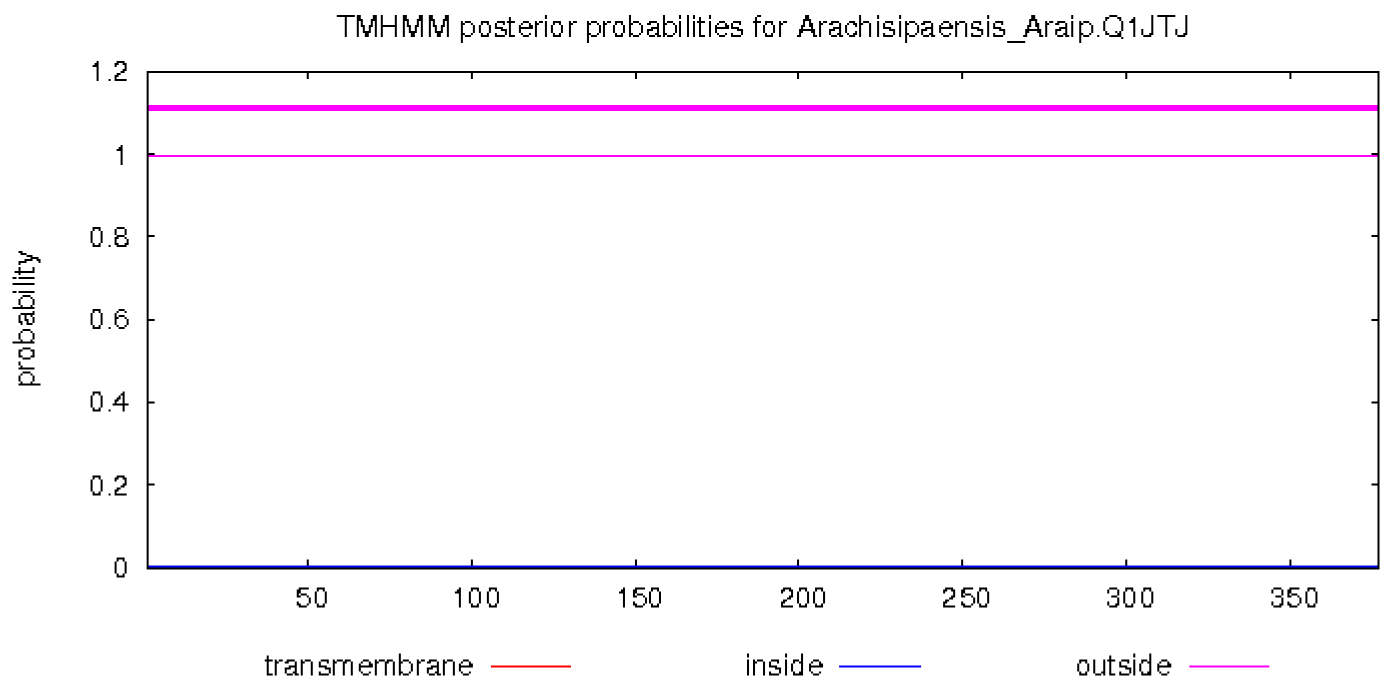

plot in postscript, script for making the plot in gnuplot, data for plot

```
# Arachisipaensis_Araip.Q3R6H Length: 360
# Arachisipaensis_Araip.Q3R6H Number of predicted TMHs:  0
# Arachisipaensis_Araip.Q3R6H Exp number of AAs in TMHs: 0.00123
# Arachisipaensis_Araip.Q3R6H Exp number, first 60 AAs:  0
# Arachisipaensis_Araip.Q3R6H Total prob of N-in:        0.00639
Arachisipaensis_Araip.Q3R6H     TMHMM2.0        outside      1   360
```

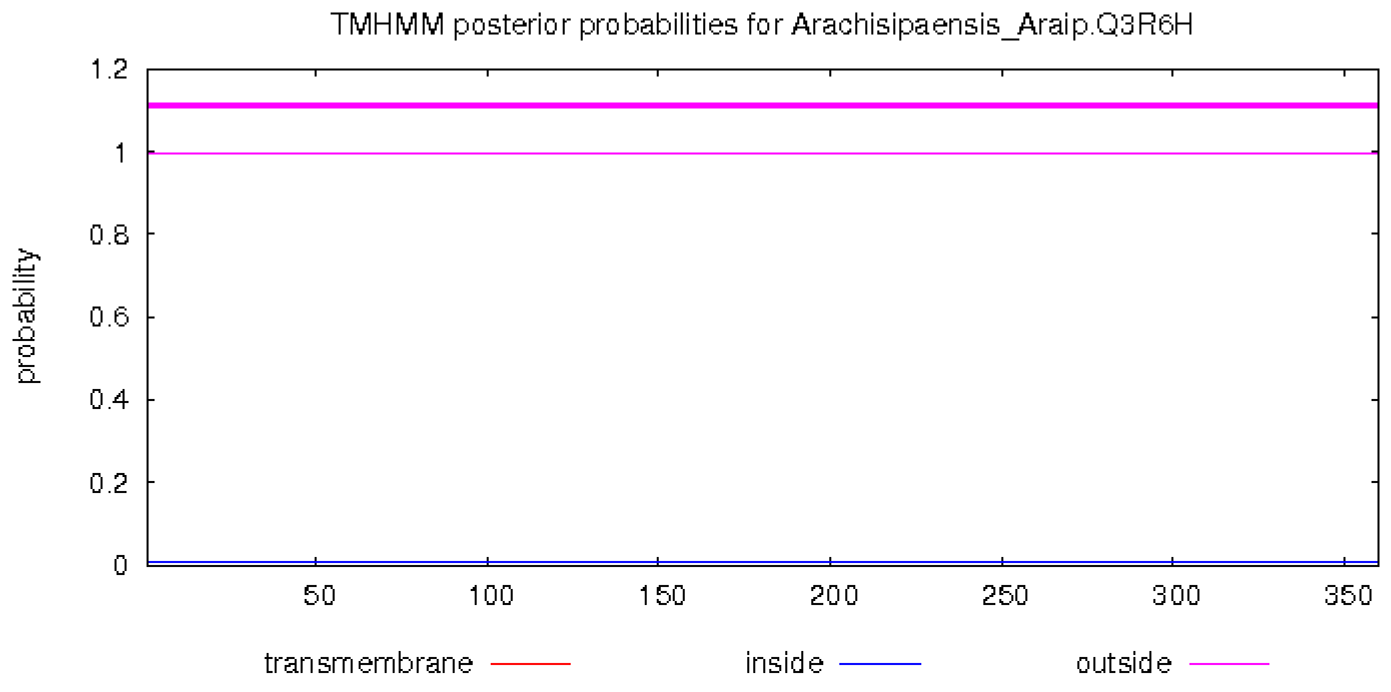

plot in postscript, script for making the plot in gnuplot, data for plot

```
# Arachisipaensis_Araip.QS7JY Length: 291
# Arachisipaensis_Araip.QS7JY Number of predicted TMHs:  0
# Arachisipaensis_Araip.QS7JY Exp number of AAs in TMHs: 0
# Arachisipaensis_Araip.QS7JY Exp number, first 60 AAs:  0
# Arachisipaensis_Araip.QS7JY Total prob of N-in:        0.00890
Arachisipaensis_Araip.QS7JY     TMHMM2.0        outside      1   291
```

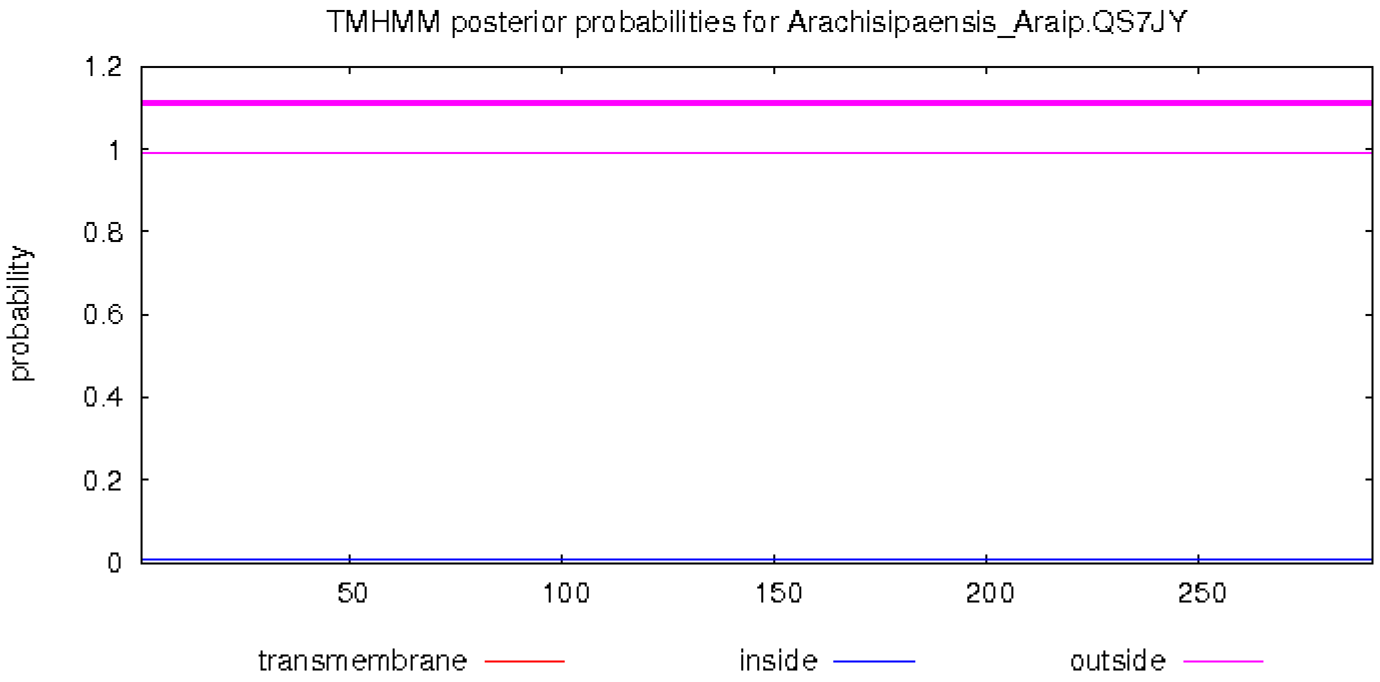

plot in postscript, script for making the plot in gnuplot, data for plot

---

```
# Arachisipaensis_Araip.R0657 Length: 211
# Arachisipaensis_Araip.R0657 Number of predicted TMHs:  0
# Arachisipaensis_Araip.R0657 Exp number of AAs in TMHs: 0.00224
# Arachisipaensis_Araip.R0657 Exp number, first 60 AAs:  0.00224
# Arachisipaensis_Araip.R0657 Total prob of N-in:        0.15080
Arachisipaensis_Araip.R0657     TMHMM2.0        outside      1   211
```

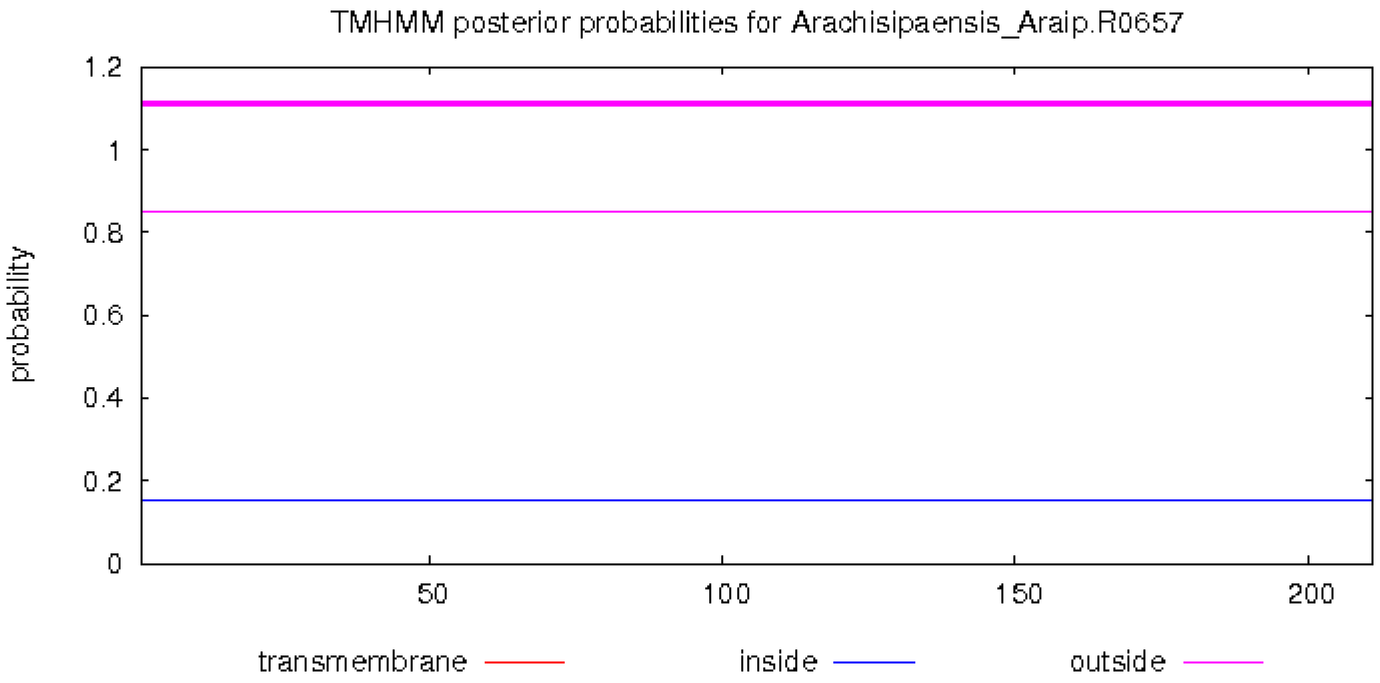

plot in postscript, script for making the plot in gnuplot, data for plot

---

```
# Arachisipaensis_Araip.T6ICI Length: 286
# Arachisipaensis_Araip.T6ICI Number of predicted TMHs:  0
# Arachisipaensis_Araip.T6ICI Exp number of AAs in TMHs: 0.01133
# Arachisipaensis_Araip.T6ICI Exp number, first 60 AAs:  0.0002
# Arachisipaensis_Araip.T6ICI Total prob of N-in:        0.02094
Arachisipaensis_Araip.T6ICI     TMHMM2.0        outside      1   286
```

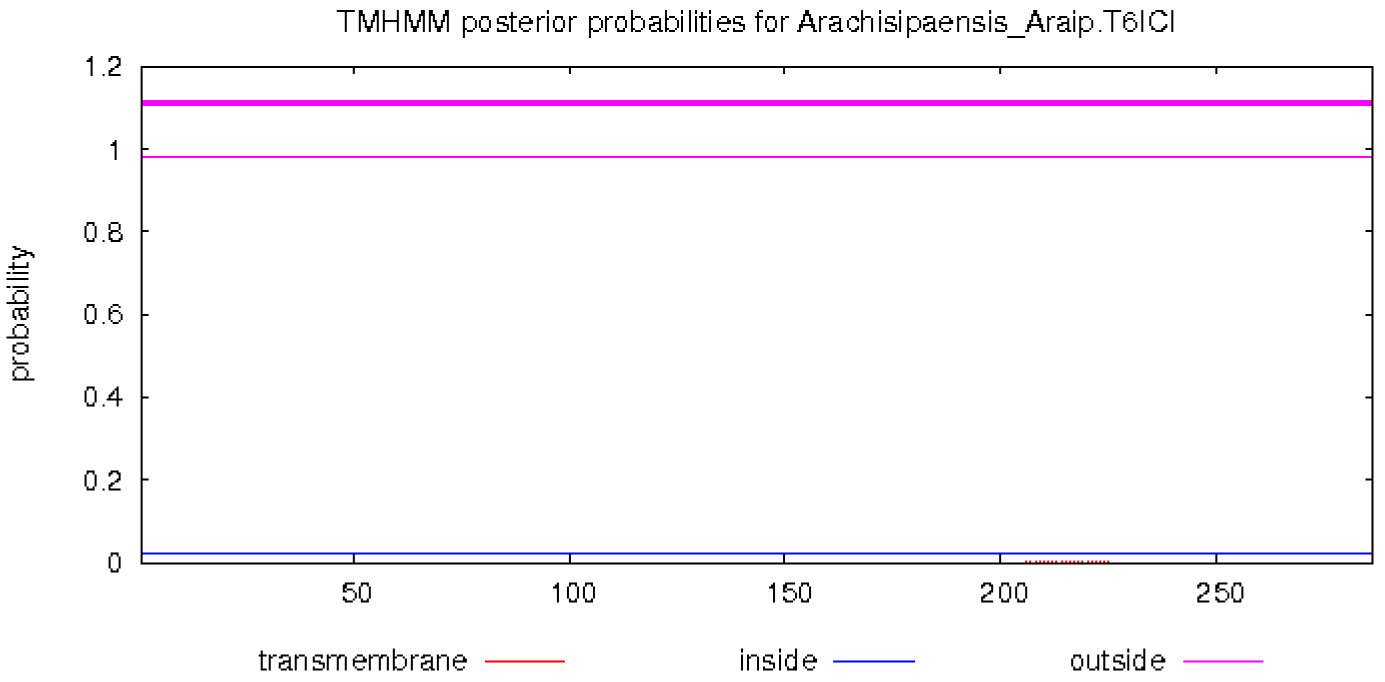

plot in postscript, script for making the plot in gnuplot, data for plot

---

```
# Arachisipaensis_Araip.TL0B5 Length: 234
# Arachisipaensis_Araip.TL0B5 Number of predicted TMHs:  0
# Arachisipaensis_Araip.TL0B5 Exp number of AAs in TMHs: 1.64942
# Arachisipaensis_Araip.TL0B5 Exp number, first 60 AAs:  1.64942
# Arachisipaensis_Araip.TL0B5 Total prob of N-in:        0.04961
Arachisipaensis_Araip.TL0B5     TMHMM2.0        outside      1   234
```

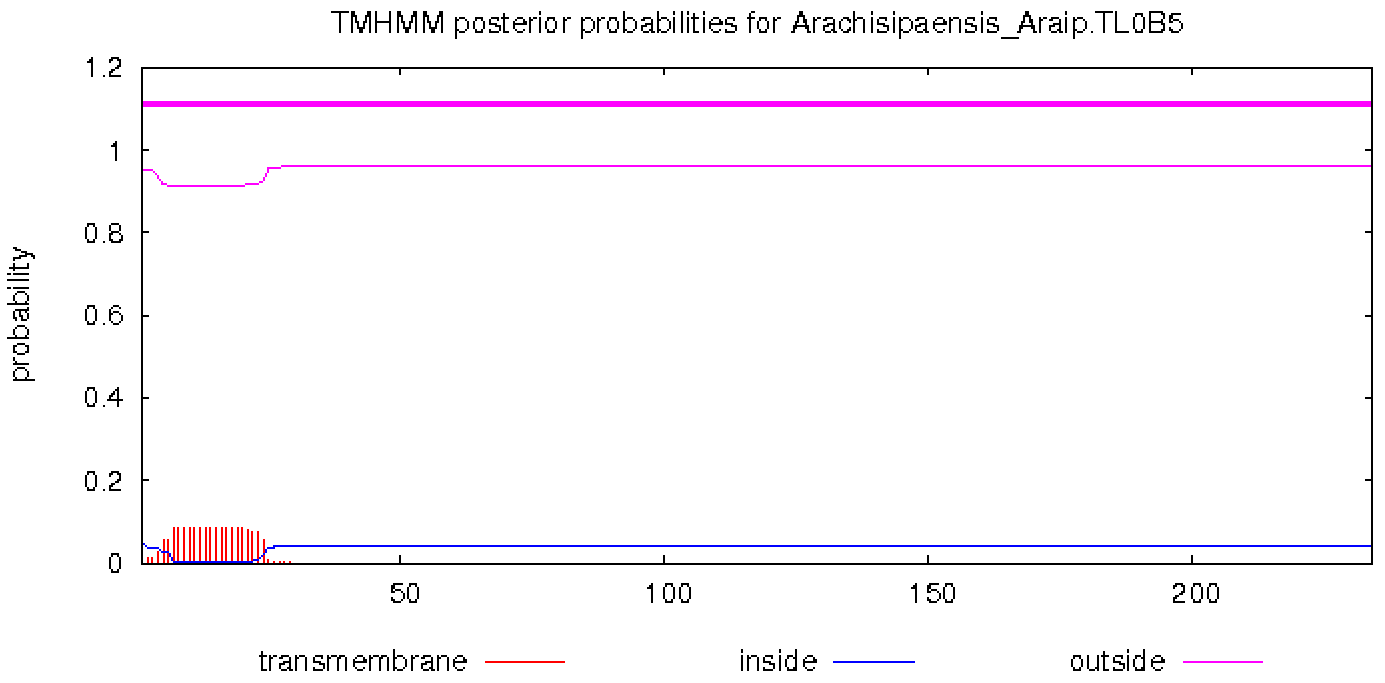

plot in postscript, script for making the plot in gnuplot, data for plot

---

```
# Arachisipaensis_Araip.U9RGH Length: 403
# Arachisipaensis_Araip.U9RGH Number of predicted TMHs:  0
# Arachisipaensis_Araip.U9RGH Exp number of AAs in TMHs: 0.00055
# Arachisipaensis_Araip.U9RGH Exp number, first 60 AAs:  0
# Arachisipaensis_Araip.U9RGH Total prob of N-in:        0.00518
Arachisipaensis_Araip.U9RGH     TMHMM2.0        outside      1   403
```

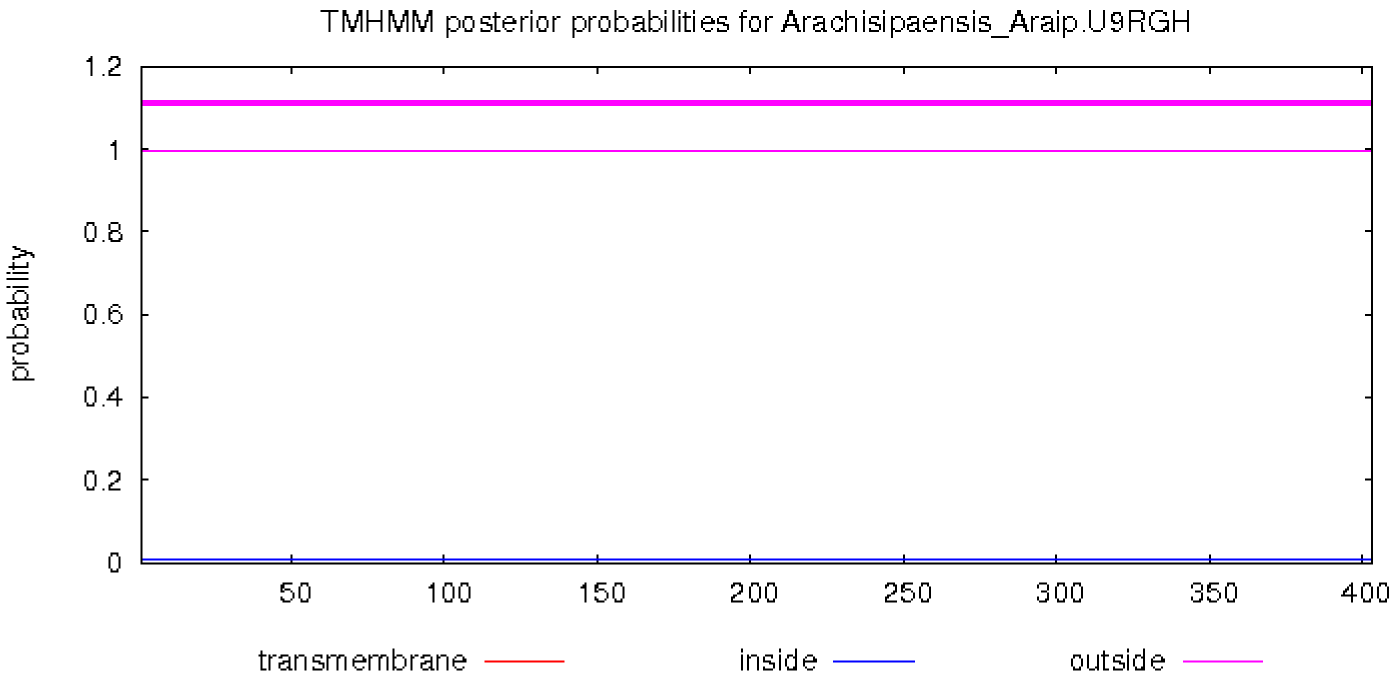

plot in postscript, script for making the plot in gnuplot, data for plot

---

```
# Arachisipaensis_Araip.UA0W9 Length: 277
# Arachisipaensis_Araip.UA0W9 Number of predicted TMHs:  0
# Arachisipaensis_Araip.UA0W9 Exp number of AAs in TMHs: 0.01194
# Arachisipaensis_Araip.UA0W9 Exp number, first 60 AAs:  0.01194
# Arachisipaensis_Araip.UA0W9 Total prob of N-in:        0.00425
Arachisipaensis_Araip.UA0W9     TMHMM2.0        outside      1   277
```

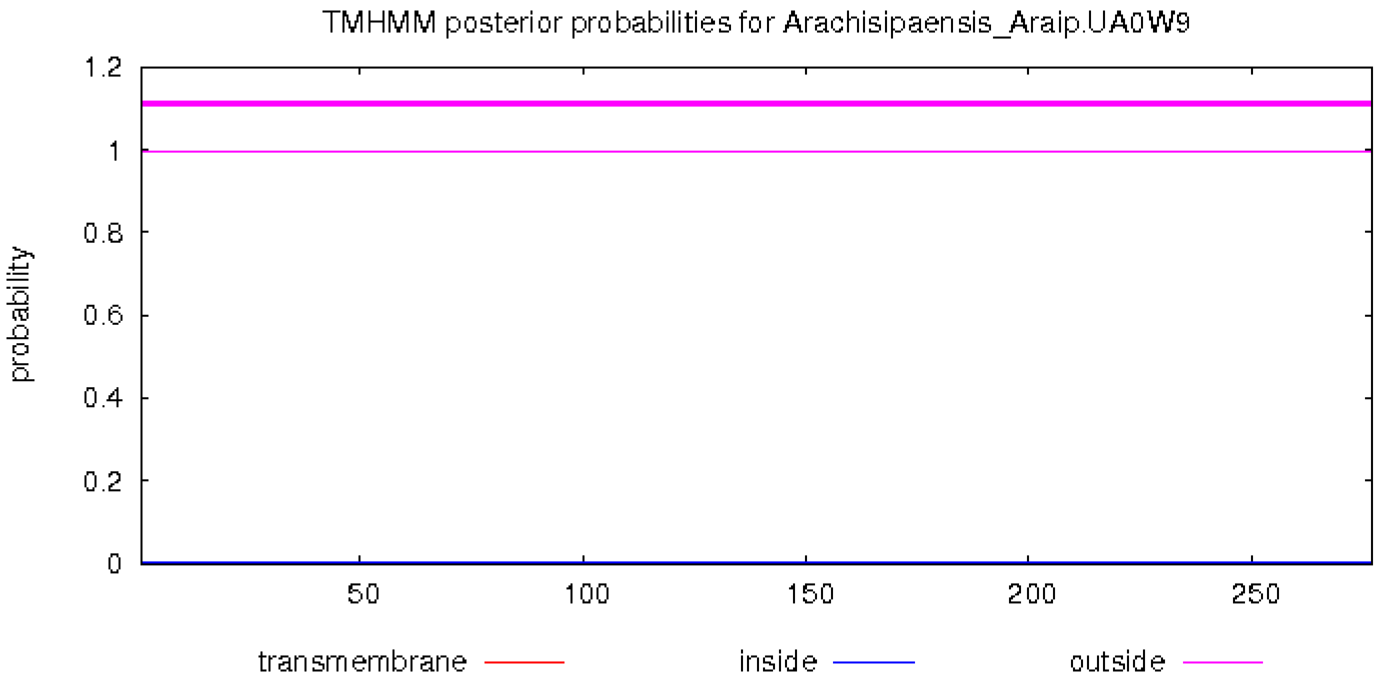

plot in postscript, script for making the plot in gnuplot, data for plot

---

```
# Arachisipaensis_Araip.WV14F Length: 342
# Arachisipaensis_Araip.WV14F Number of predicted TMHs:  0
# Arachisipaensis_Araip.WV14F Exp number of AAs in TMHs: 0
# Arachisipaensis_Araip.WV14F Exp number, first 60 AAs:  0
# Arachisipaensis_Araip.WV14F Total prob of N-in:        0.00273
Arachisipaensis_Araip.WV14F     TMHMM2.0        outside      1   342
```

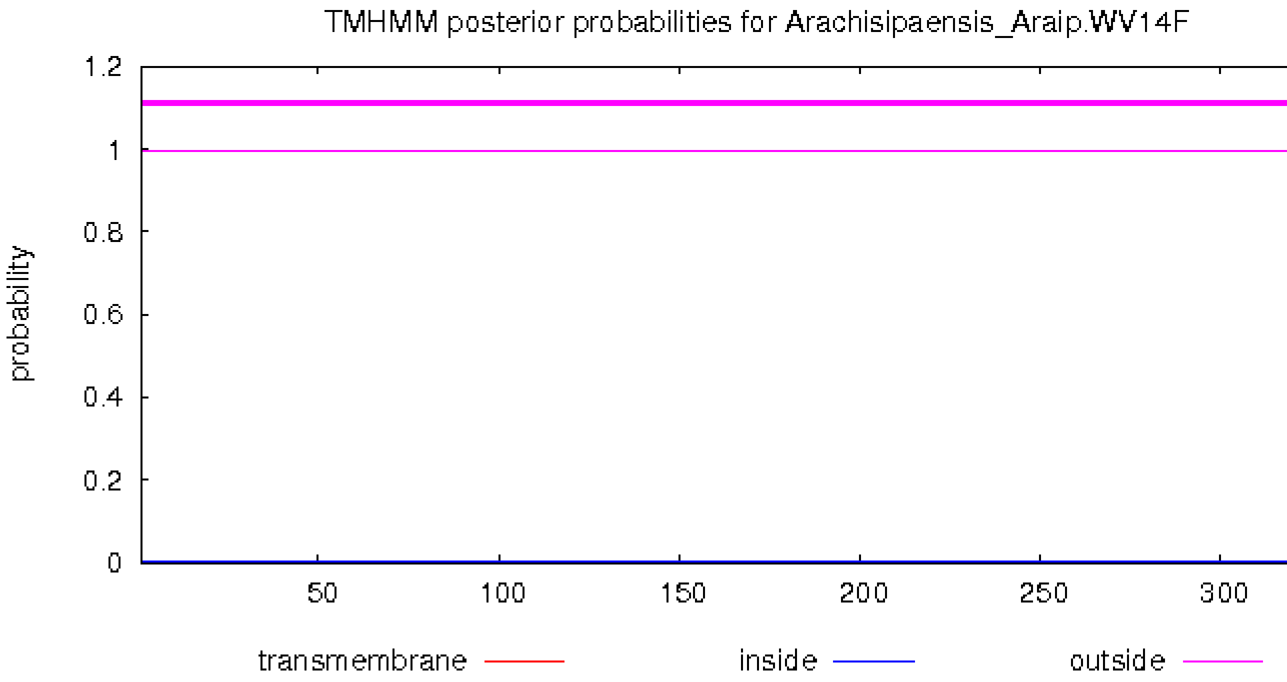

plot in postscript, script for making the plot in gnuplot, data for plot

---

```
# Arachisipaensis_Araip.X2KK1 Length: 352
# Arachisipaensis_Araip.X2KK1 Number of predicted TMHs:  0
# Arachisipaensis_Araip.X2KK1 Exp number of AAs in TMHs: 0
# Arachisipaensis_Araip.X2KK1 Exp number, first 60 AAs:  0
# Arachisipaensis_Araip.X2KK1 Total prob of N-in:        0.00078
Arachisipaensis_Araip.X2KK1     TMHMM2.0        outside      1   352
```

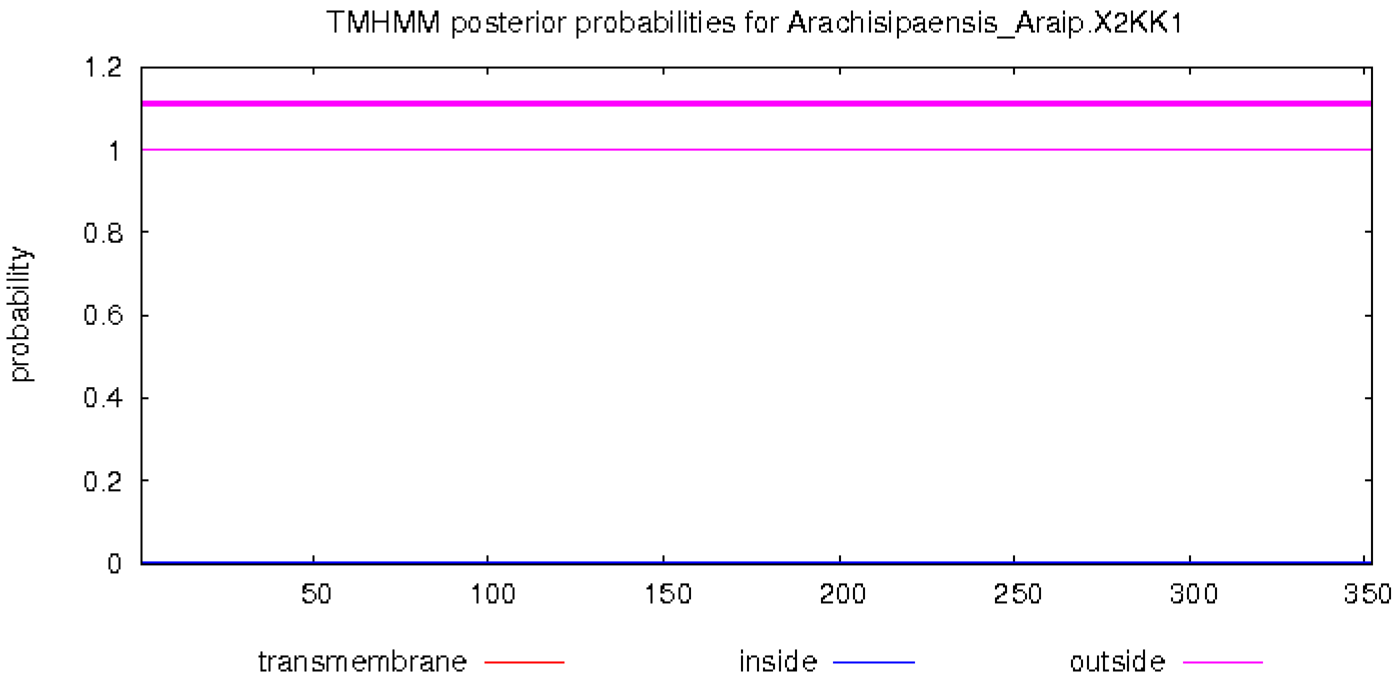

plot in postscript, script for making the plot in gnuplot, data for plot

---

```
# Arachisipaensis_Araip.XJ3T4 Length: 342
# Arachisipaensis_Araip.XJ3T4 Number of predicted TMHs:  0
# Arachisipaensis_Araip.XJ3T4 Exp number of AAs in TMHs: 0.17488
# Arachisipaensis_Araip.XJ3T4 Exp number, first 60 AAs:  0
# Arachisipaensis_Araip.XJ3T4 Total prob of N-in:        0.06087
Arachisipaensis_Araip.XJ3T4	TMHMM2.0	outside	     1   342
```

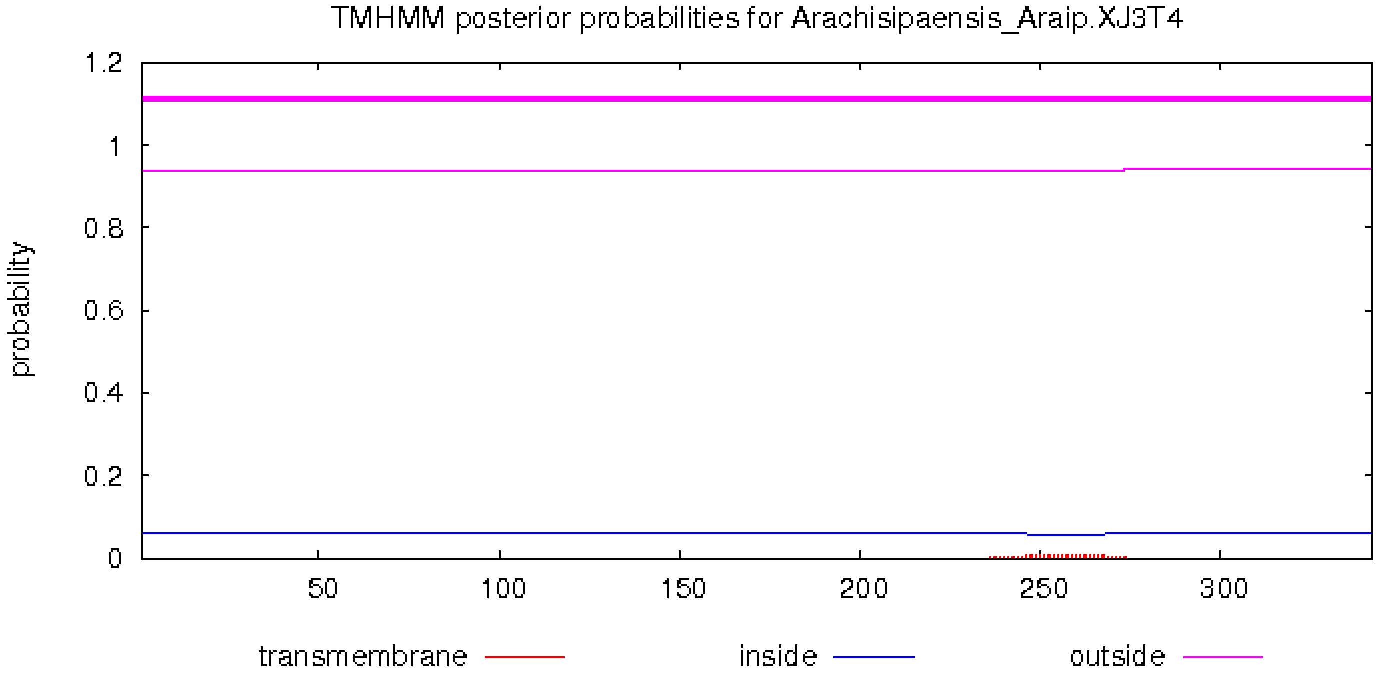

plot in postscript, script for making the plot in gnuplot, data for plot

---

```
# Arachisipaensis_Araip.XJX1I Length: 319
# Arachisipaensis_Araip.XJX1I Number of predicted TMHs:  0
# Arachisipaensis_Araip.XJX1I Exp number of AAs in TMHs: 0
# Arachisipaensis_Araip.XJX1I Exp number, first 60 AAs:  0
# Arachisipaensis_Araip.XJX1I Total prob of N-in:        0.00128
Arachisipaensis_Araip.XJX1I	TMHMM2.0	outside	     1   319
```

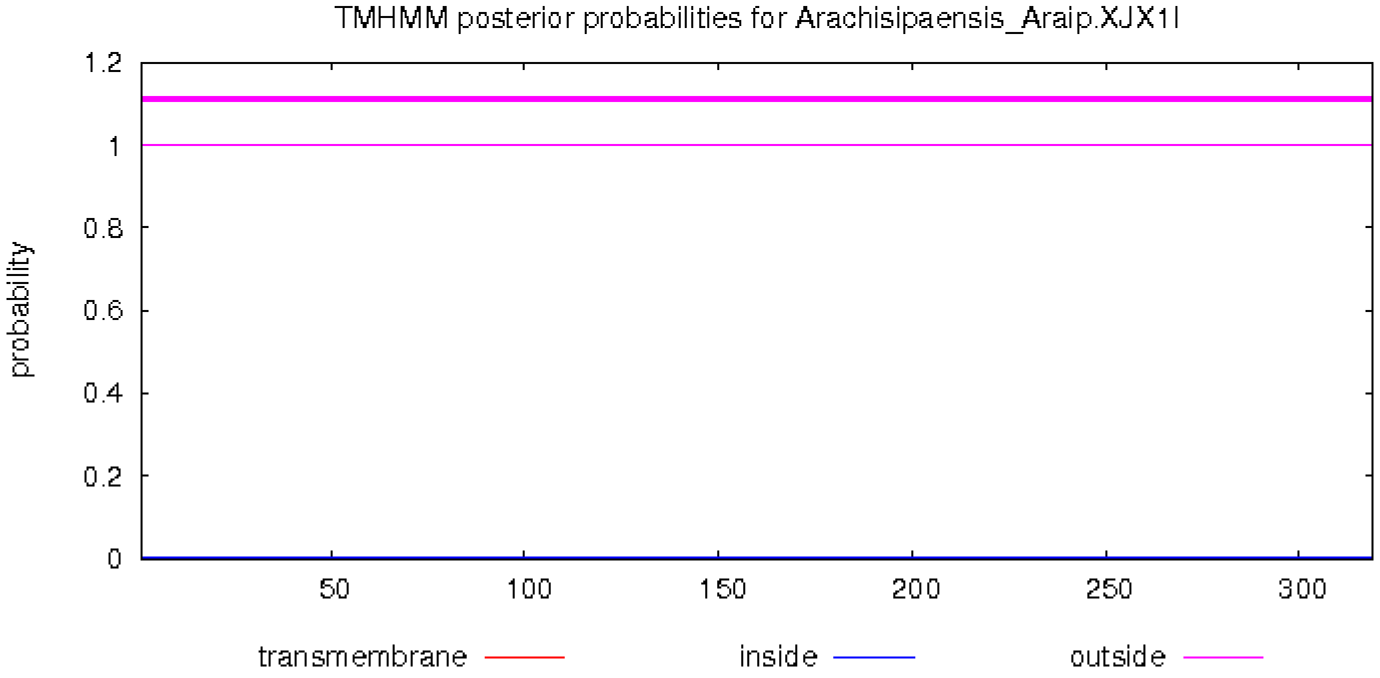

plot in postscript, script for making the plot in gnuplot, data for plot

---

```
# Arachisipaensis_Araip.XK9AB Length: 277
# Arachisipaensis_Araip.XK9AB Number of predicted TMHs:  0
# Arachisipaensis_Araip.XK9AB Exp number of AAs in TMHs: 0.00223
# Arachisipaensis_Araip.XK9AB Exp number, first 60 AAs:  0
# Arachisipaensis_Araip.XK9AB Total prob of N-in:        0.00171
Arachisipaensis_Araip.XK9AB     TMHMM2.0        outside      1   277
```

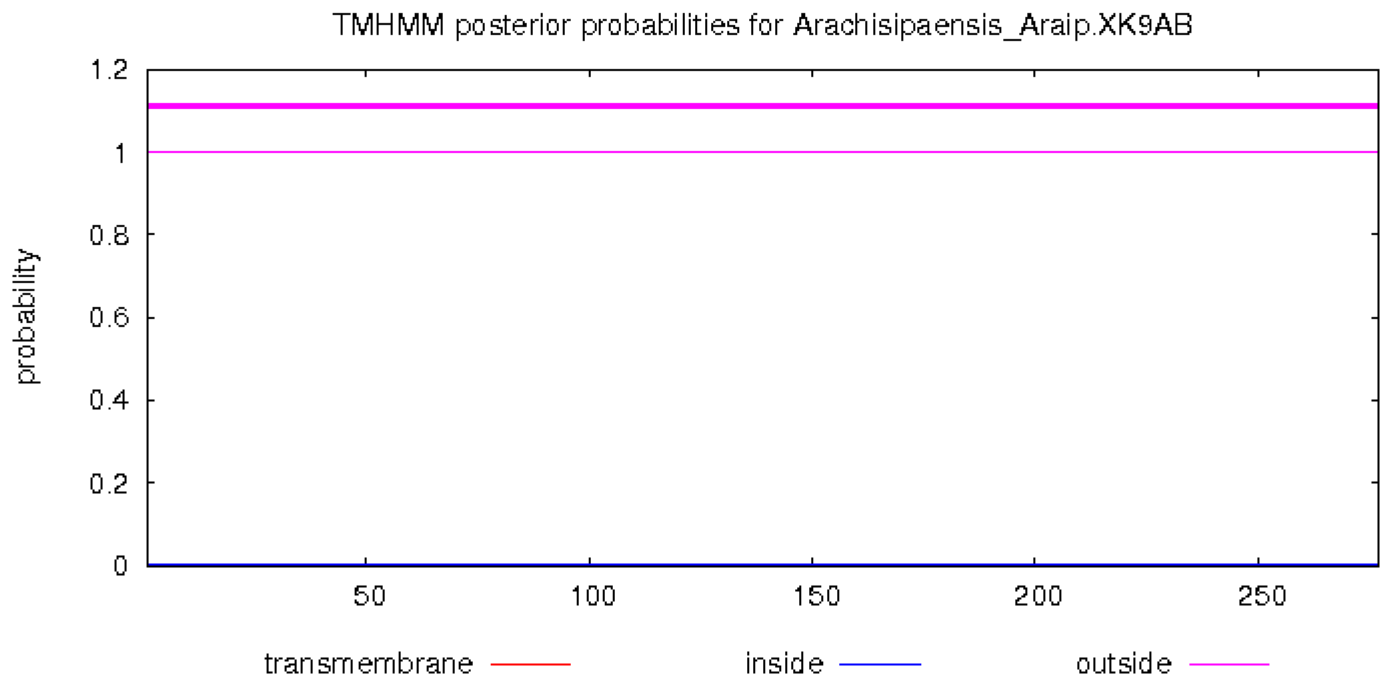

plot in postscript, script for making the plot in gnuplot, data for plot

---

```
# Arachisipaensis_Araip.XQA0A Length: 339
# Arachisipaensis_Araip.XQA0A Number of predicted TMHs:  0
# Arachisipaensis_Araip.XQA0A Exp number of AAs in TMHs: 0
# Arachisipaensis_Araip.XQA0A Exp number, first 60 AAs:  0
# Arachisipaensis_Araip.XQA0A Total prob of N-in:        0.00705
Arachisipaensis_Araip.XQA0A     TMHMM2.0        outside      1   339
```

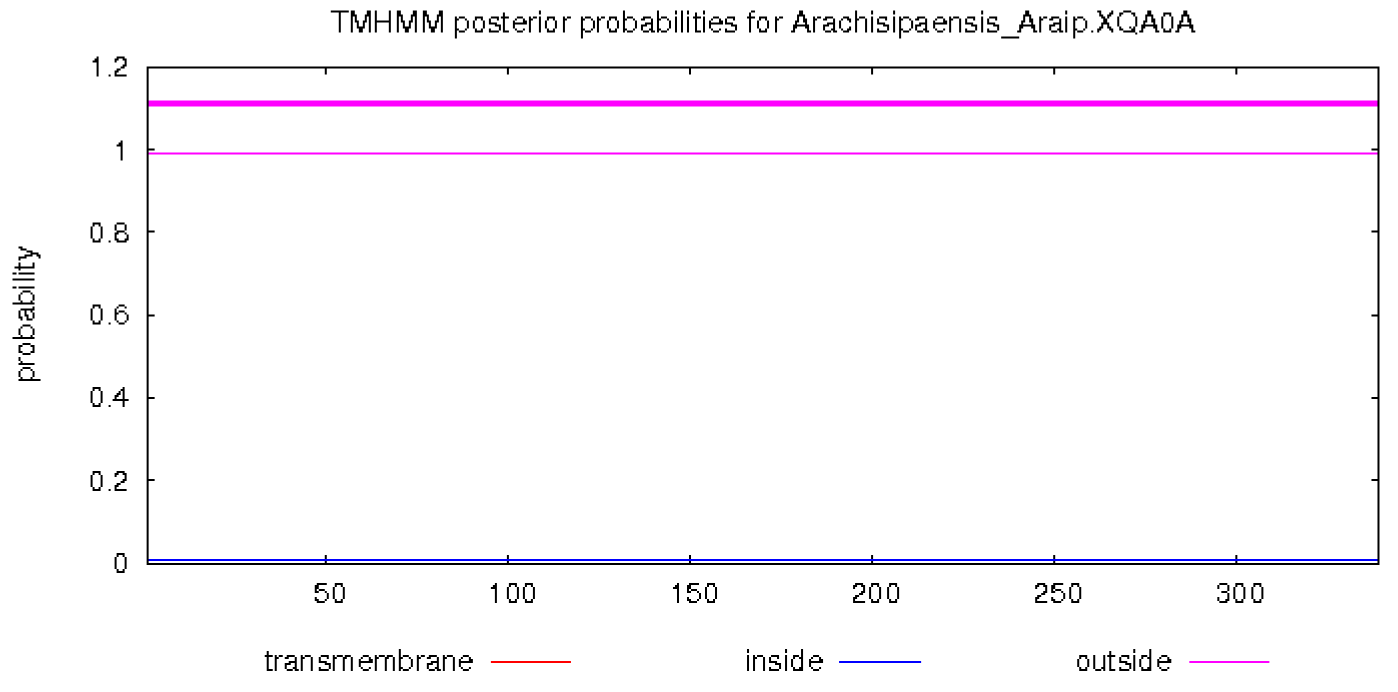

plot in postscript, script for making the plot in gnuplot, data for plot

---

```
# Arachisipaensis_Araip.XT8UZ Length: 365
# Arachisipaensis_Araip.XT8UZ Number of predicted TMHs:  0
# Arachisipaensis_Araip.XT8UZ Exp number of AAs in TMHs: 0.00043
# Arachisipaensis_Araip.XT8UZ Exp number, first 60 AAs:  0.00023
# Arachisipaensis_Araip.XT8UZ Total prob of N-in:        0.00360
Arachisipaensis_Araip.XT8UZ     TMHMM2.0        outside      1   365
```

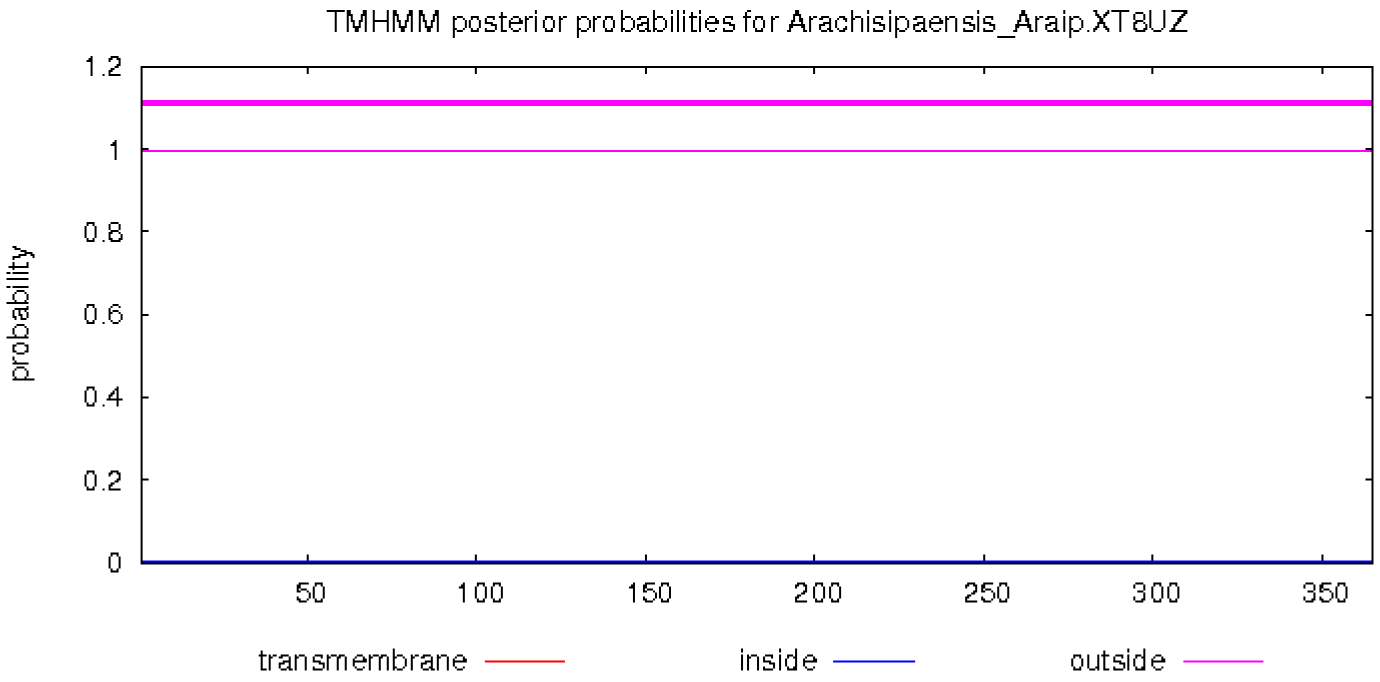

plot in postscript, script for making the plot in gnuplot, data for plot

---

```
# Arachisipaensis_Araip.YL288 Length: 282
# Arachisipaensis_Araip.YL288 Number of predicted TMHs:  0
# Arachisipaensis_Araip.YL288 Exp number of AAs in TMHs: 0.0095
# Arachisipaensis_Araip.YL288 Exp number, first 60 AAs:  0.00489
# Arachisipaensis_Araip.YL288 Total prob of N-in:        0.02796
Arachisipaensis_Araip.YL288     TMHMM2.0        outside      1   282
```

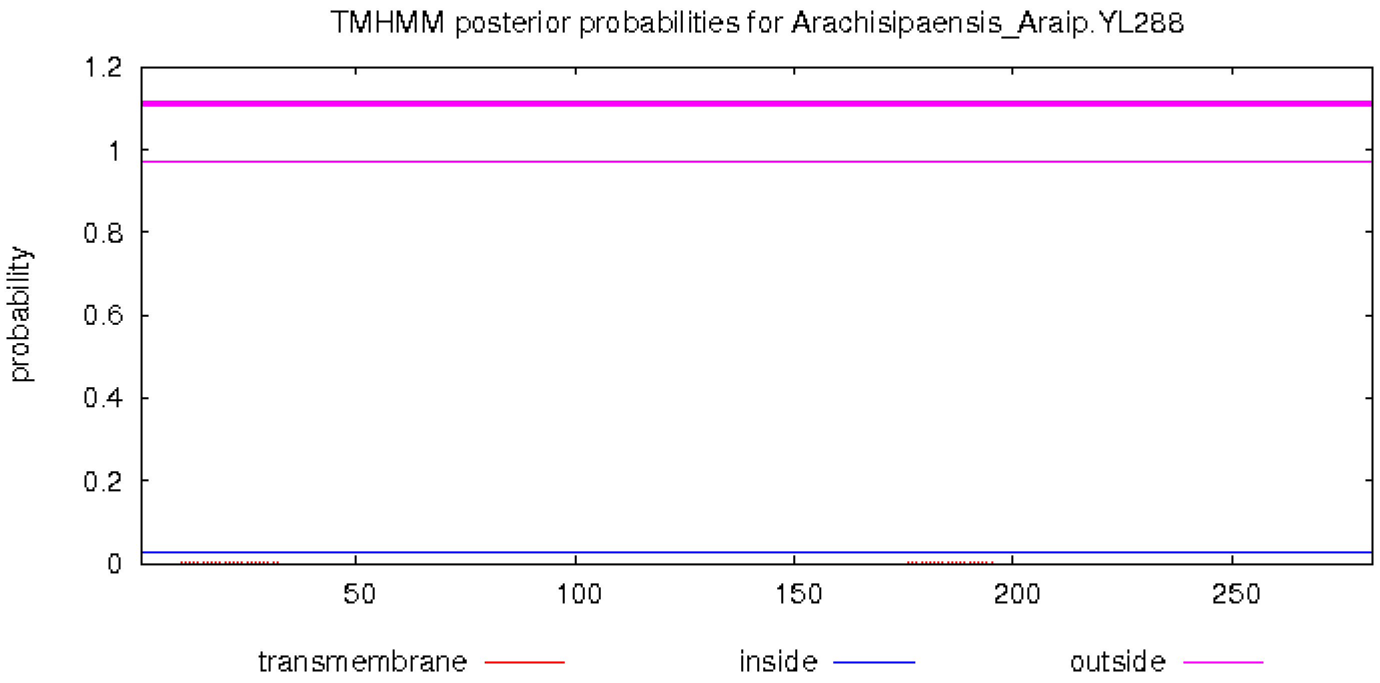

plot in postscript, script for making the plot in gnuplot, data for plot

---

```
# Arachisipaensis_Araip.YS3WM Length: 229
# Arachisipaensis_Araip.YS3WM Number of predicted TMHs:  0
# Arachisipaensis_Araip.YS3WM Exp number of AAs in TMHs: 2.34871
# Arachisipaensis_Araip.YS3WM Exp number, first 60 AAs:  2.34509
# Arachisipaensis_Araip.YS3WM Total prob of N-in:        0.21933
Arachisipaensis_Araip.YS3WM     TMHMM2.0        outside      1   229
```

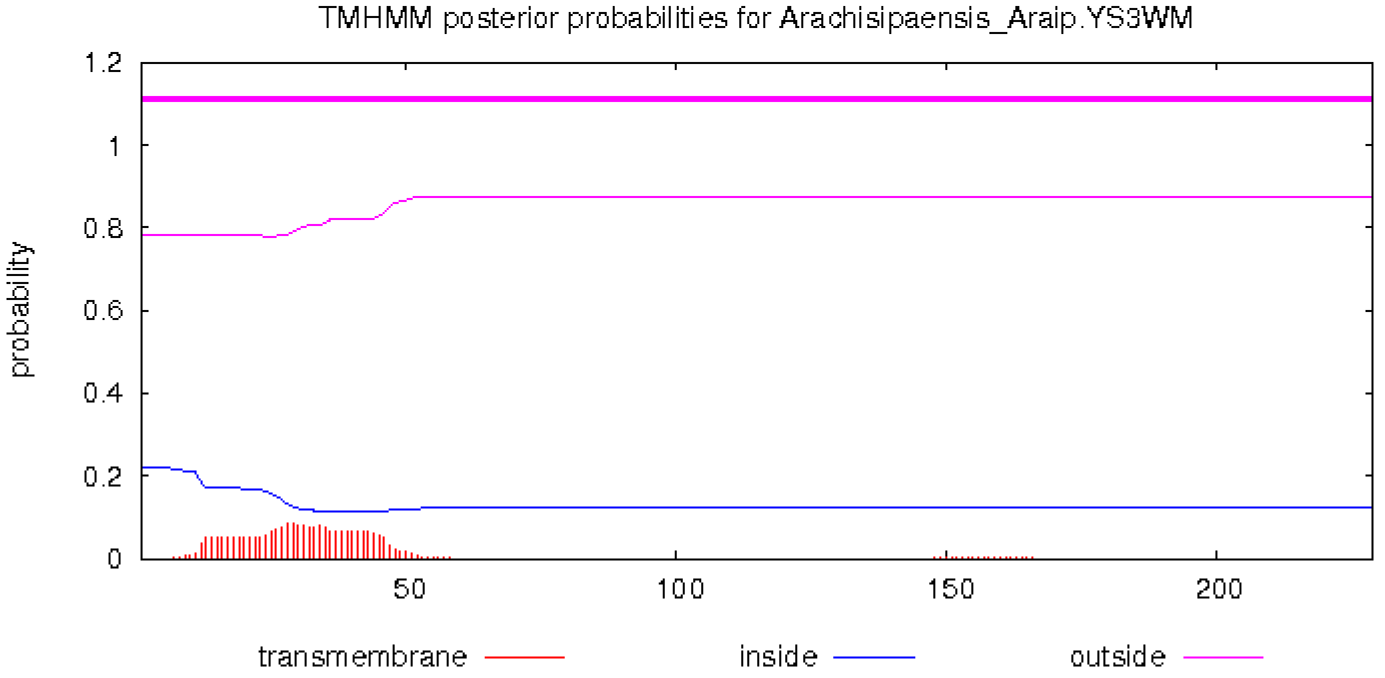

plot in postscript, script for making the plot in gnuplot, data for plot

---

```
# Arachisipaensis_Araip.Z57SD Length: 593
# Arachisipaensis_Araip.Z57SD Number of predicted TMHs:  2
# Arachisipaensis_Araip.Z57SD Exp number of AAs in TMHs: 50.98545
# Arachisipaensis_Araip.Z57SD Exp number, first 60 AAs:  0.00024
# Arachisipaensis_Araip.Z57SD Total prob of N-in:        0.32646
Arachisipaensis_Araip.Z57SD     TMHMM2.0        outside      1   540
Arachisipaensis_Araip.Z57SD     TMHMM2.0        TMhelix    541   563
Arachisipaensis_Araip.Z57SD     TMHMM2.0        inside     564   567
Arachisipaensis_Araip.Z57SD     TMHMM2.0        TMhelix    568   590
Arachisipaensis_Araip.Z57SD     TMHMM2.0        outside    591   593
```

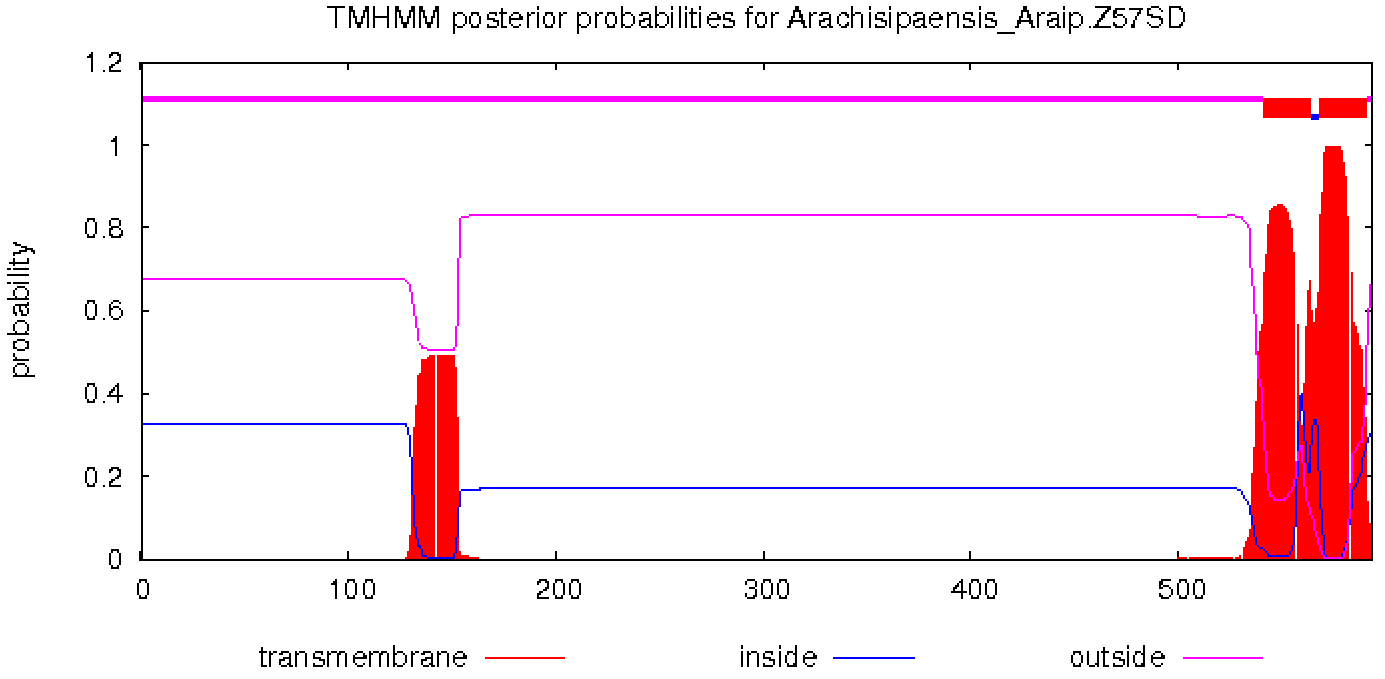

plot in postscript, script for making the plot in gnuplot, data for plot

---

```
# Arachisipaensis_Araip.ZUP60 Length: 350
# Arachisipaensis_Araip.ZUP60 Number of predicted TMHs:  0
# Arachisipaensis_Araip.ZUP60 Exp number of AAs in TMHs: 0
# Arachisipaensis_Araip.ZUP60 Exp number, first 60 AAs:  0
# Arachisipaensis_Araip.ZUP60 Total prob of N-in:        0.01024
Arachisipaensis_Araip.ZUP60     TMHMM2.0        outside      1   350
```

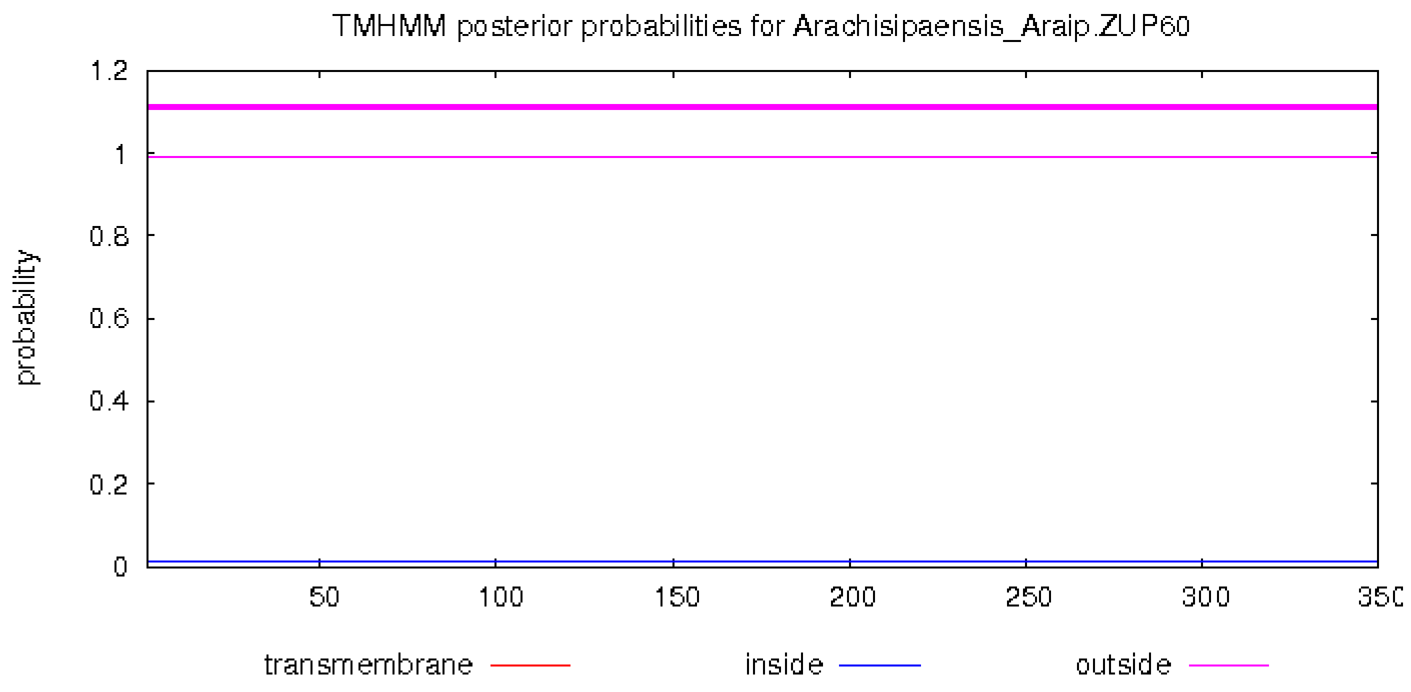

plot in postscript, script for making the plot in gnuplot, data for plot

---

```
# Arachisipaensis_Araip.ZX5IX Length: 259
# Arachisipaensis_Araip.ZX5IX Number of predicted TMHs:  0
# Arachisipaensis_Araip.ZX5IX Exp number of AAs in TMHs: 0
# Arachisipaensis_Araip.ZX5IX Exp number, first 60 AAs:  0
# Arachisipaensis_Araip.ZX5IX Total prob of N-in:        0.02575
Arachisipaensis_Araip.ZX5IX     TMHMM2.0        outside      1   259
```

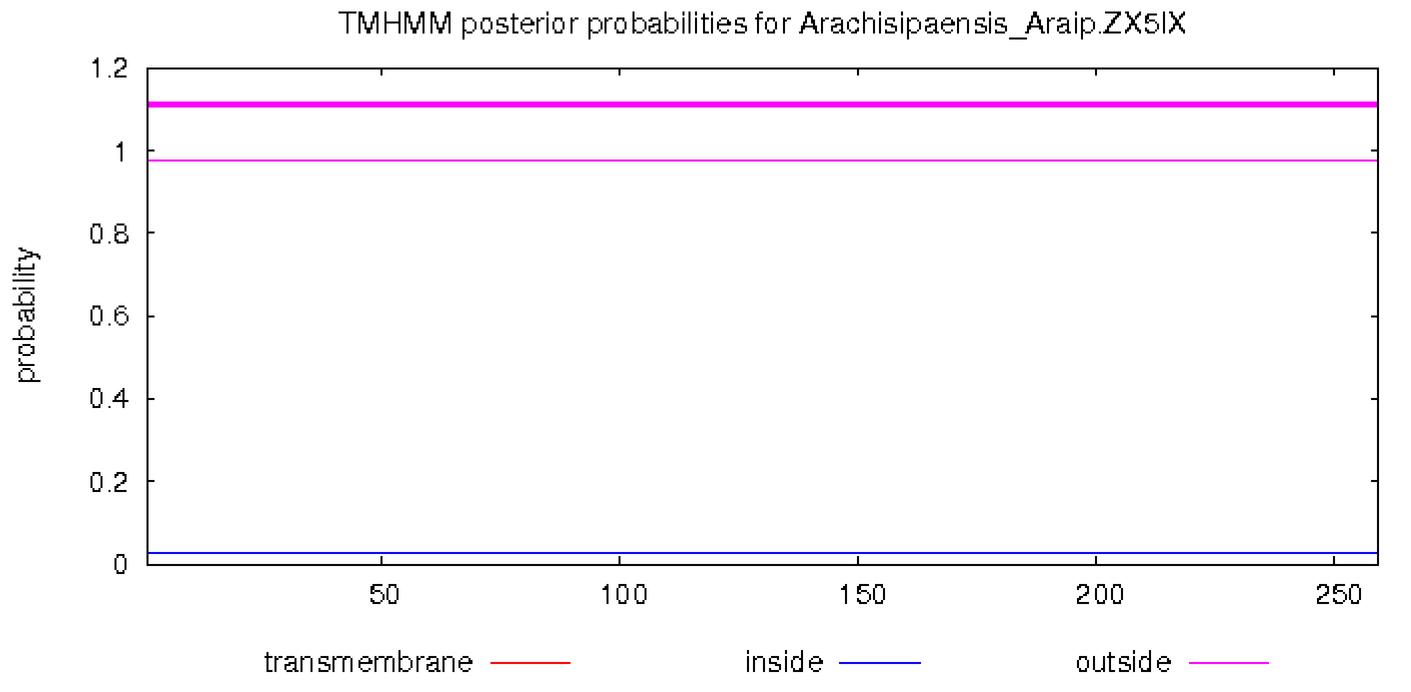

plot in postscript, script for making the plot in gnuplot, data for plot

```
# Artemisiaannua_Aan000926 Length: 305
# Artemisiaannua_Aan000926 Number of predicted TMHs:  0
# Artemisiaannua_Aan000926 Exp number of AAs in TMHs: 0.00702
# Artemisiaannua_Aan000926 Exp number, first 60 AAs:  0.00459
# Artemisiaannua_Aan000926 Total prob of N-in:        0.12158
Artemisiaannua_Aan000926        TMHMM2.0        outside      1   305
```

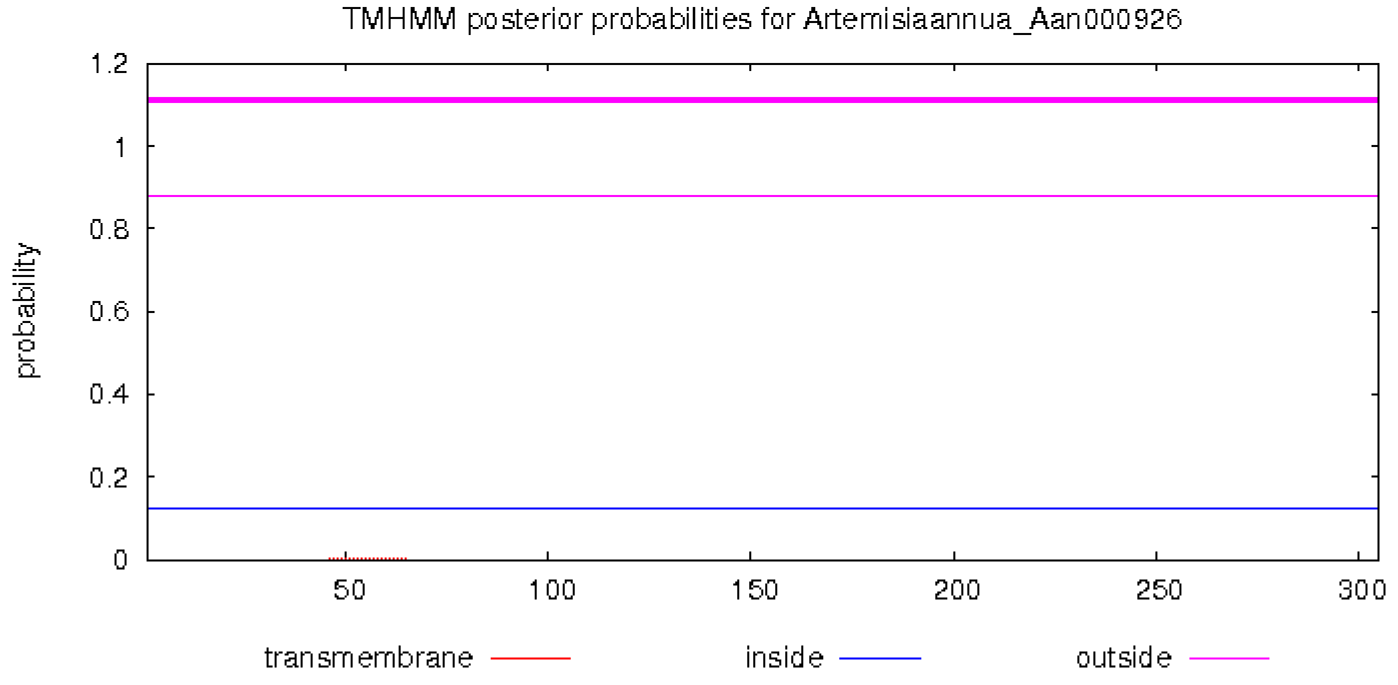

plot in postscript, script for making the plot in gnuplot, data for plot

```
# Artemisiaannua_Aan001843 Length: 394
# Artemisiaannua_Aan001843 Number of predicted TMHs:  0
# Artemisiaannua_Aan001843 Exp number of AAs in TMHs: 0
# Artemisiaannua_Aan001843 Exp number, first 60 AAs:  0
# Artemisiaannua_Aan001843 Total prob of N-in:        0.00239
Artemisiaannua_Aan001843        TMHMM2.0        outside      1   394
```

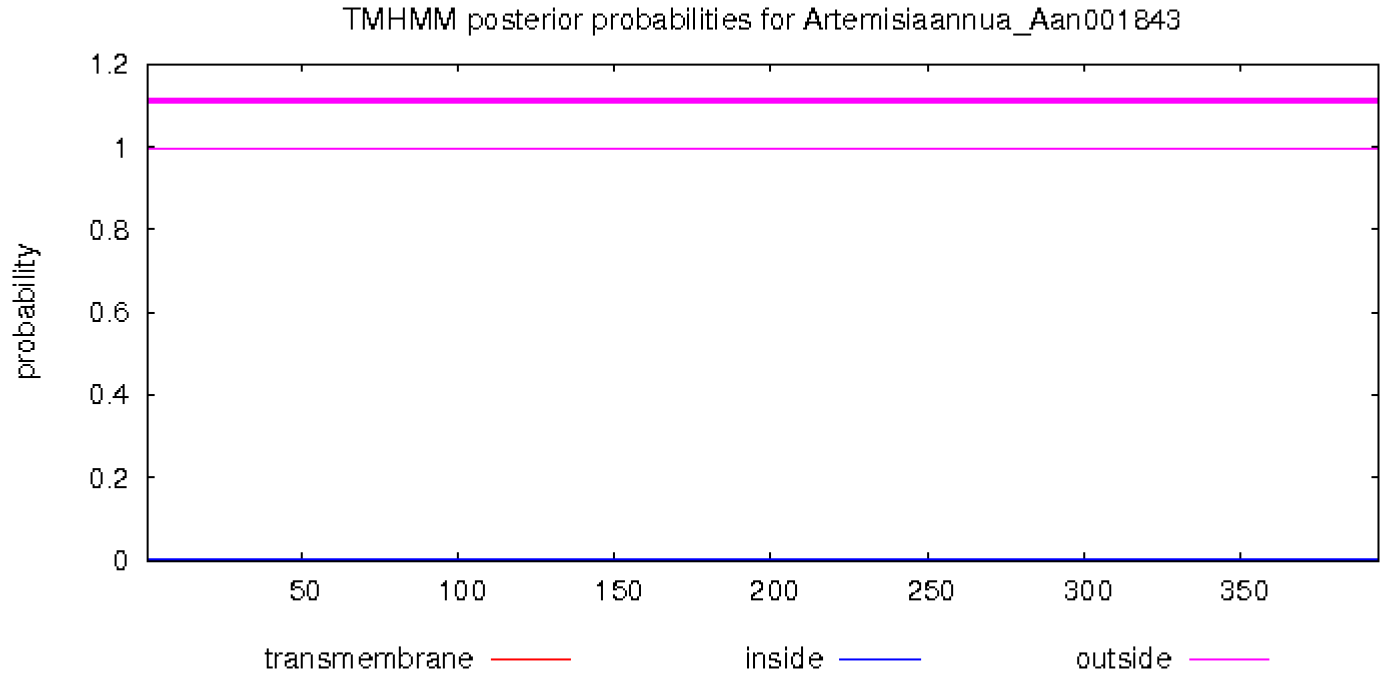

plot in postscript, script for making the plot in gnuplot, data for plot

```
# Artemisiaannua_Aan002468 Length: 335
# Artemisiaannua_Aan002468 Number of predicted TMHs:  0
# Artemisiaannua_Aan002468 Exp number of AAs in TMHs: 0
# Artemisiaannua_Aan002468 Exp number, first 60 AAs:  0
# Artemisiaannua_Aan002468 Total prob of N-in:        0.00633
Artemisiaannua_Aan002468        TMHMM2.0        outside      1   335
```

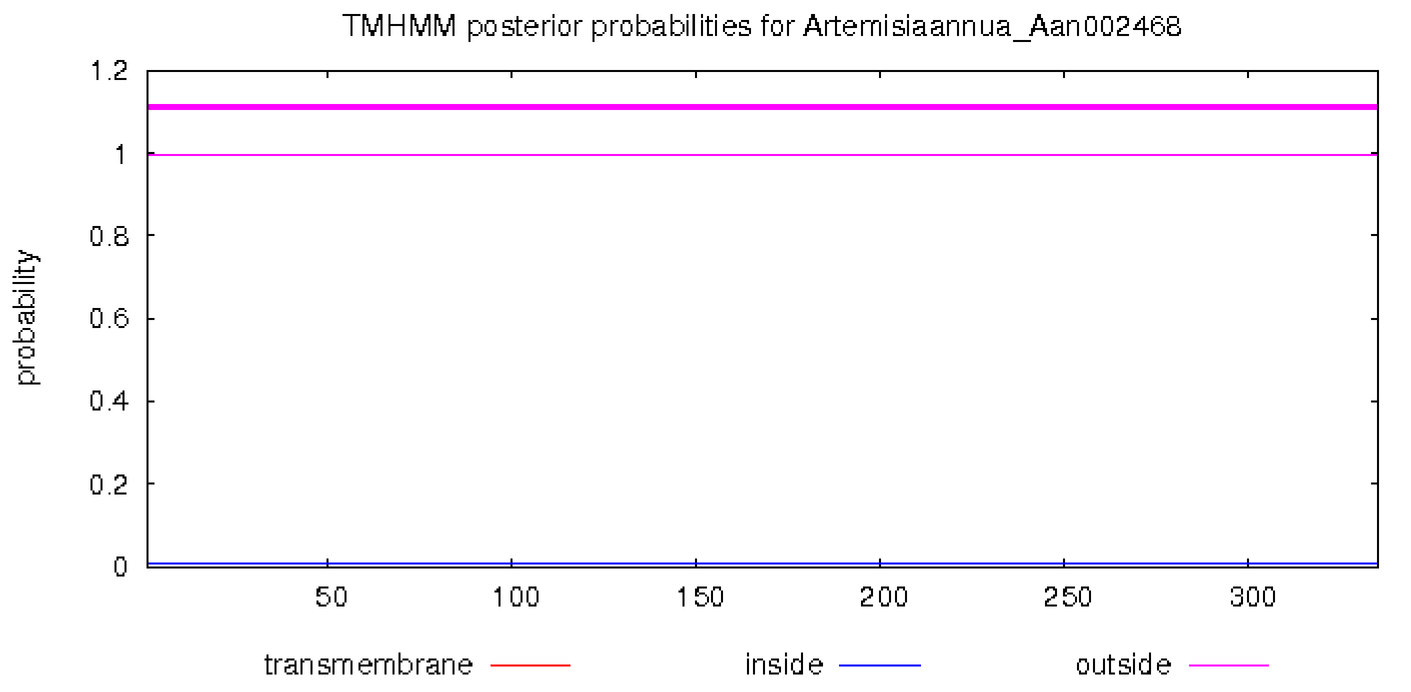

plot in postscript, script for making the plot in gnuplot, data for plot

```
# Artemisiaannua_Aan006201 Length: 302
# Artemisiaannua_Aan006201 Number of predicted TMHs:  0
# Artemisiaannua_Aan006201 Exp number of AAs in TMHs: 0.00103
# Artemisiaannua_Aan006201 Exp number, first 60 AAs:  0.00103
# Artemisiaannua_Aan006201 Total prob of N-in:        0.03950
Artemisiaannua_Aan006201        TMHMM2.0        outside      1   302
```

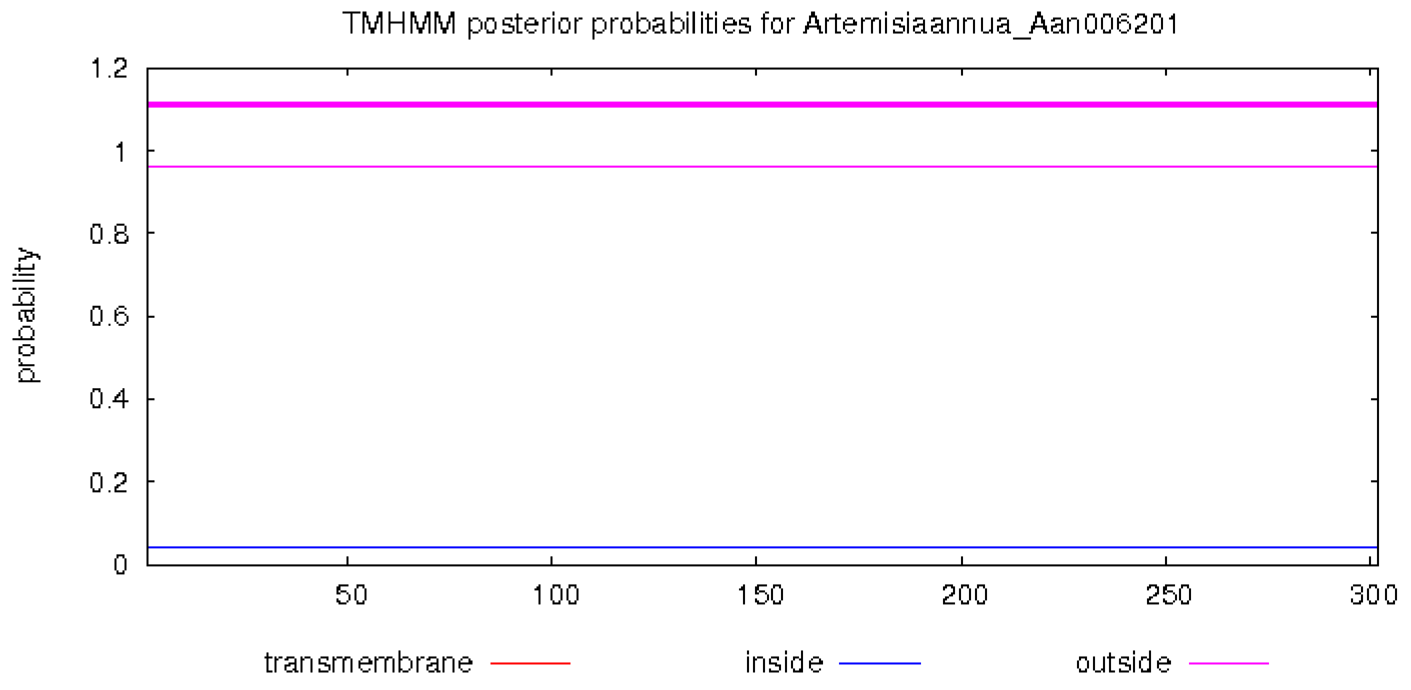

plot in postscript, script for making the plot in gnuplot, data for plot

```
# Artemisiaannua_Aan006361 Length: 290
# Artemisiaannua_Aan006361 Number of predicted TMHs:  0
# Artemisiaannua_Aan006361 Exp number of AAs in TMHs: 0.00399
# Artemisiaannua_Aan006361 Exp number, first 60 AAs:  0
# Artemisiaannua_Aan006361 Total prob of N-in:        0.05012
Artemisiaannua_Aan006361	TMHMM2.0	outside	     1   290
```

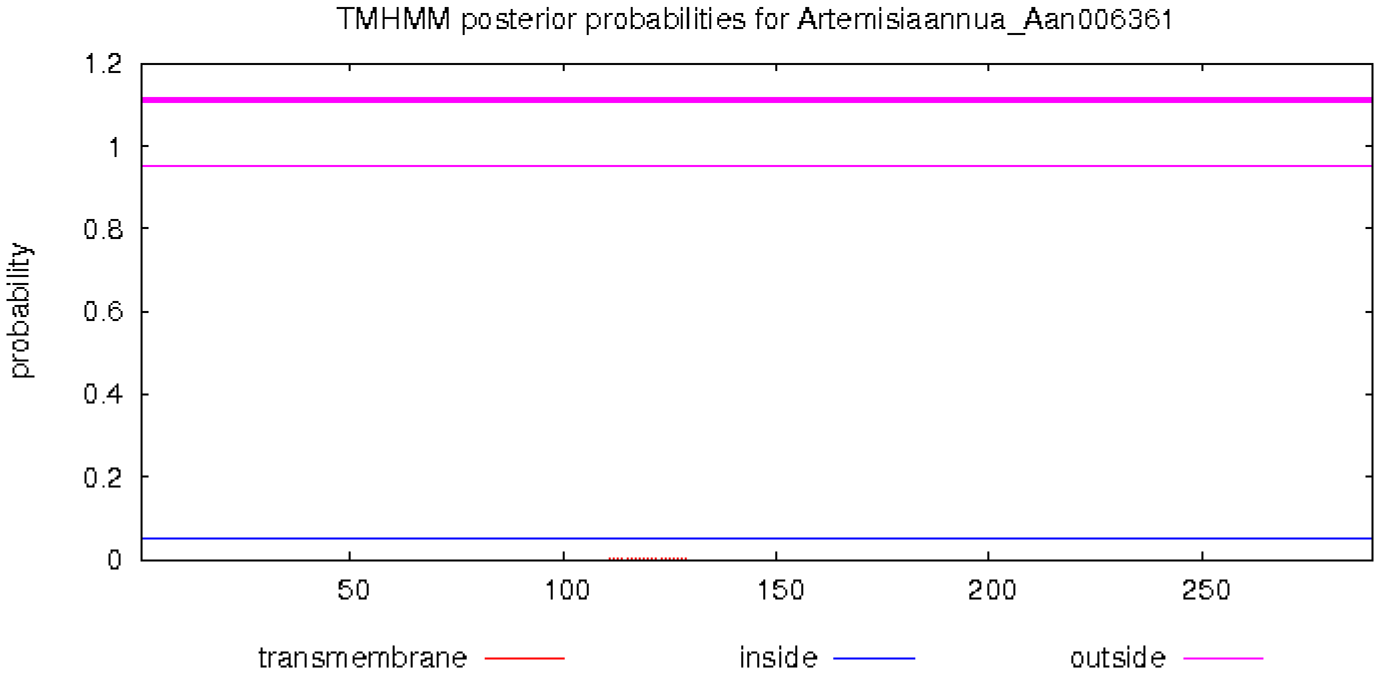

plot in postscript, script for making the plot in gnuplot, data for plot

```
# Artemisiaannua_Aan008548 Length: 214
# Artemisiaannua_Aan008548 Number of predicted TMHs:  0
# Artemisiaannua_Aan008548 Exp number of AAs in TMHs: 0
# Artemisiaannua_Aan008548 Exp number, first 60 AAs:  0
# Artemisiaannua_Aan008548 Total prob of N-in:        0.04959
Artemisiaannua_Aan008548	TMHMM2.0	outside	     1   214
```

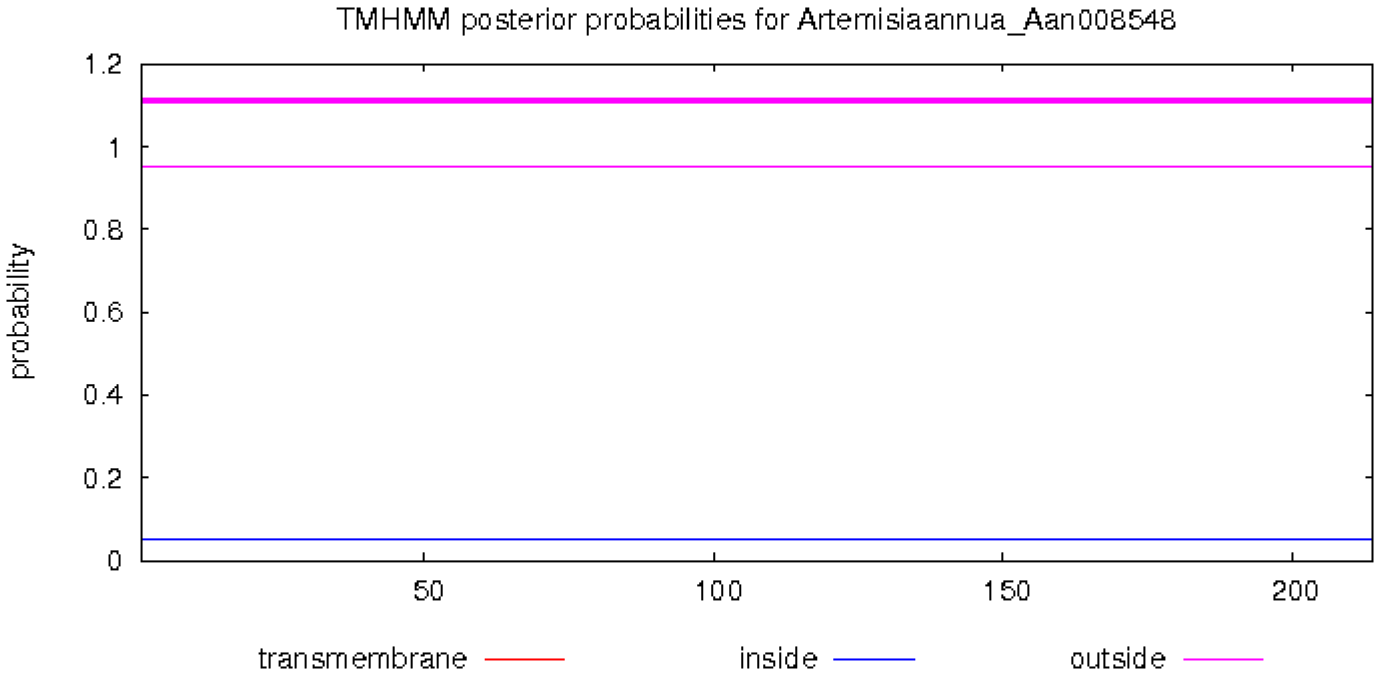

plot in postscript, script for making the plot in gnuplot, data for plot

```
# Artemisiaannua_Aan009721 Length: 179
# Artemisiaannua_Aan009721 Number of predicted TMHs:  0
# Artemisiaannua_Aan009721 Exp number of AAs in TMHs: 0.06591
# Artemisiaannua_Aan009721 Exp number, first 60 AAs:  0.02822
# Artemisiaannua_Aan009721 Total prob of N-in:        0.08526
Artemisiaannua_Aan009721	TMHMM2.0	outside	     1   179
```

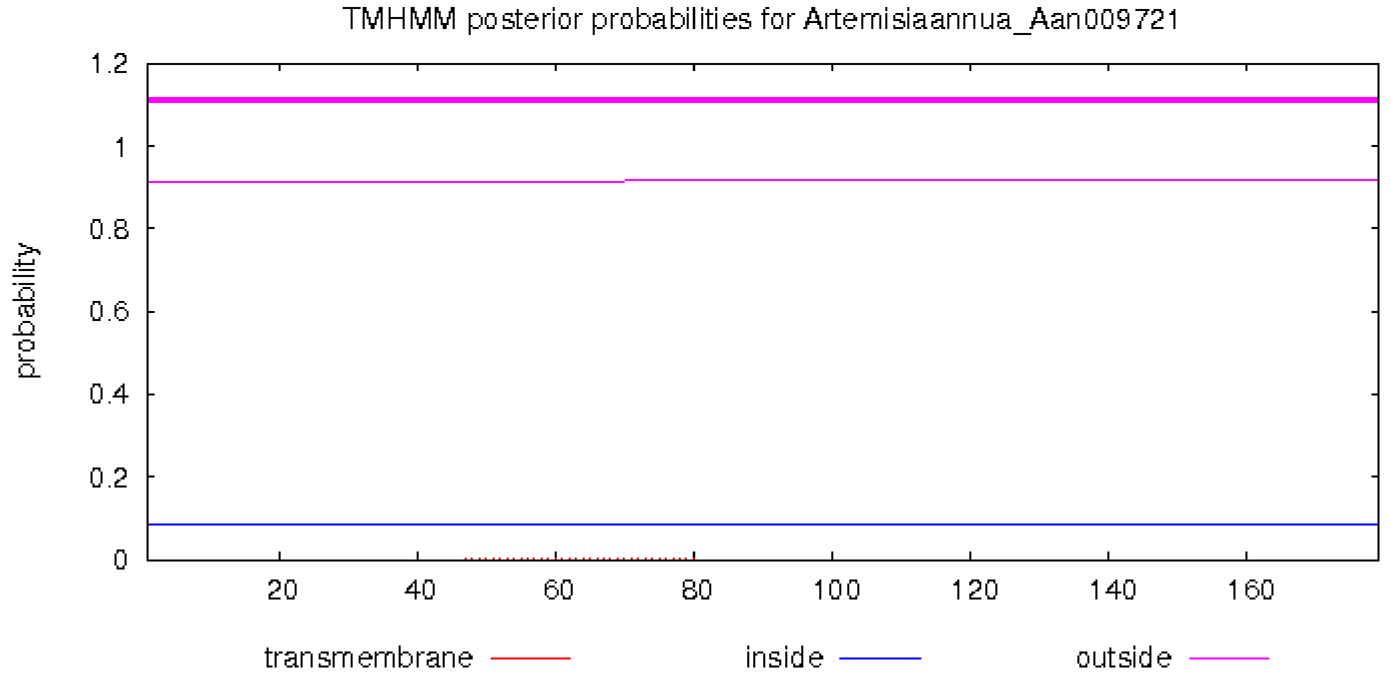

plot in postscript, script for making the plot in gnuplot, data for plot

```
# Artemisiaannua_Aan010235 Length: 165
# Artemisiaannua_Aan010235 Number of predicted TMHs:  0
# Artemisiaannua_Aan010235 Exp number of AAs in TMHs: 0.00099
# Artemisiaannua_Aan010235 Exp number, first 60 AAs:  0
# Artemisiaannua_Aan010235 Total prob of N-in:        0.17006
Artemisiaannua_Aan010235	TMHMM2.0	outside	     1   165
```

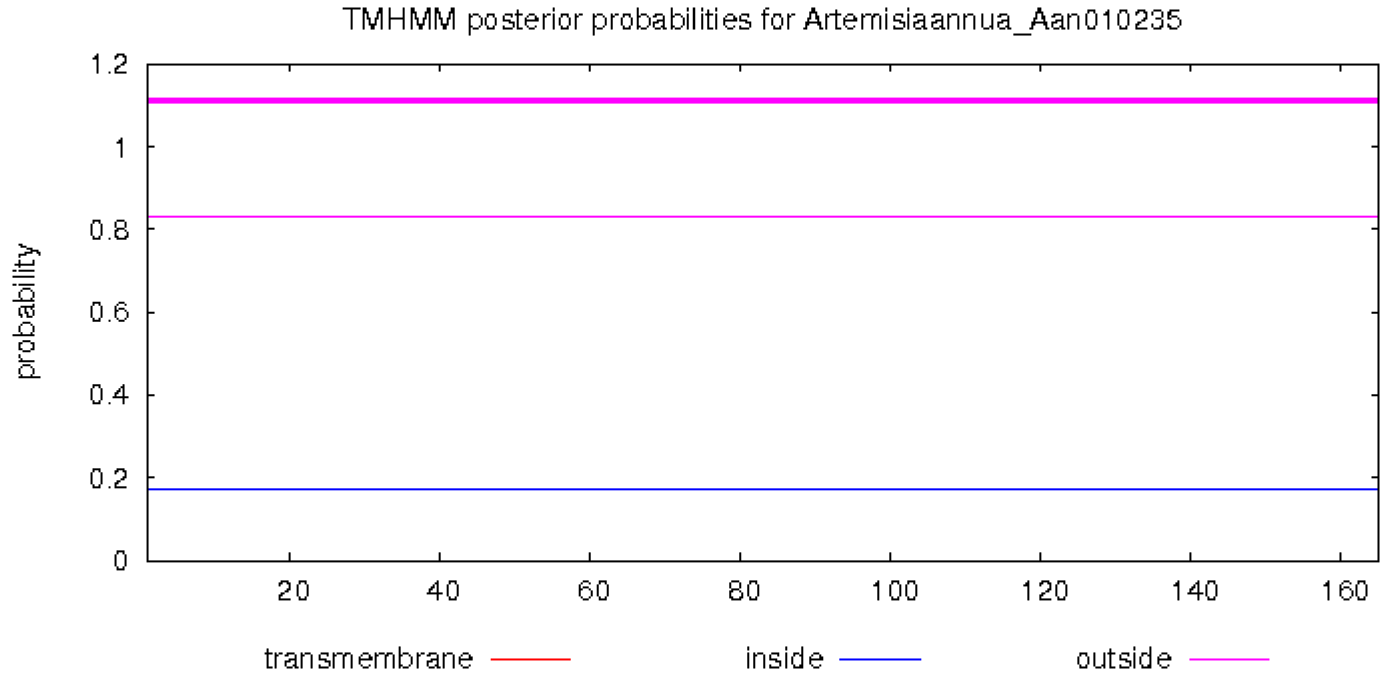

plot in postscript, script for making the plot in gnuplot, data for plot

```
# Artemisiaannua_Aan010386 Length: 160
# Artemisiaannua_Aan010386 Number of predicted TMHs:  0
# Artemisiaannua_Aan010386 Exp number of AAs in TMHs: 0.14157
# Artemisiaannua_Aan010386 Exp number, first 60 AAs:  0.14033
# Artemisiaannua_Aan010386 Total prob of N-in:        0.17172
Artemisiaannua_Aan010386        TMHMM2.0        outside      1   160
```

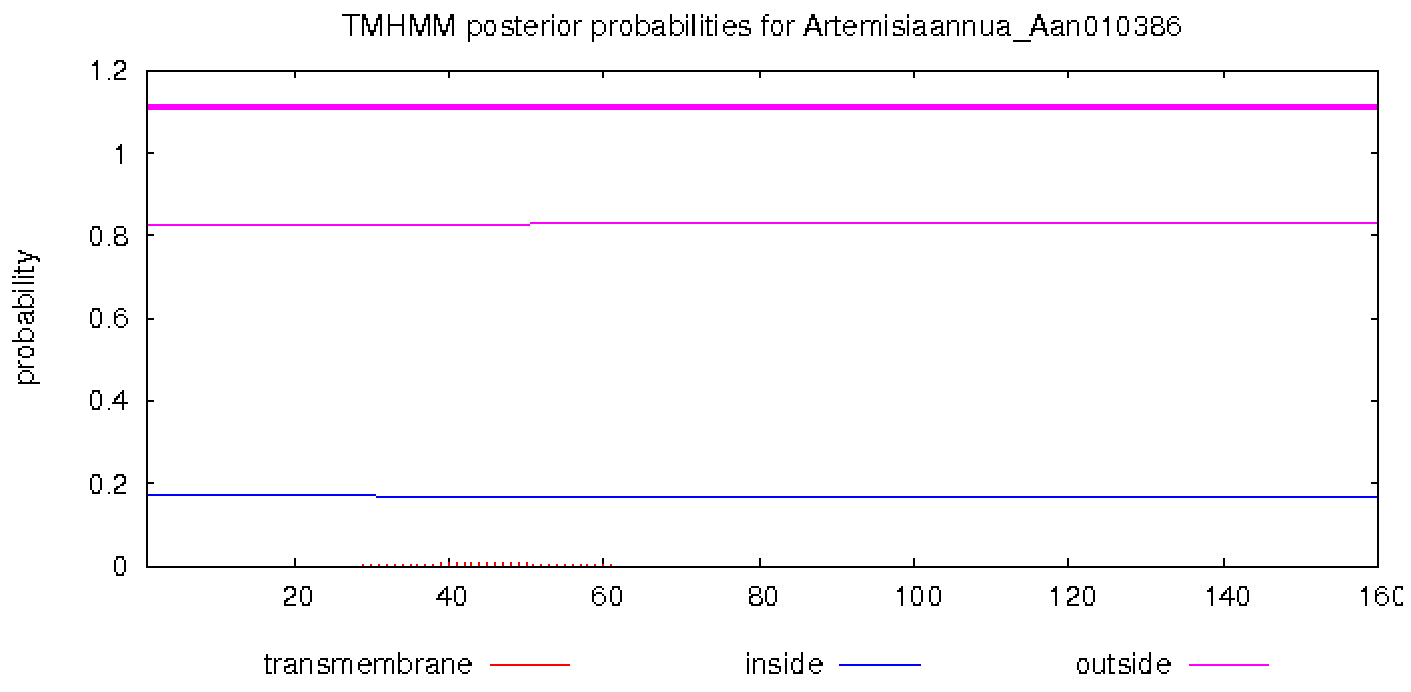

plot in postscript, script for making the plot in gnuplot, data for plot

```
# Artemisiaannua_Aan010878 Length: 146
# Artemisiaannua_Aan010878 Number of predicted TMHs:  0
# Artemisiaannua_Aan010878 Exp number of AAs in TMHs: 0.07177
# Artemisiaannua_Aan010878 Exp number, first 60 AAs:  0
# Artemisiaannua_Aan010878 Total prob of N-in:        0.06447
Artemisiaannua_Aan010878        TMHMM2.0        outside      1   146
```

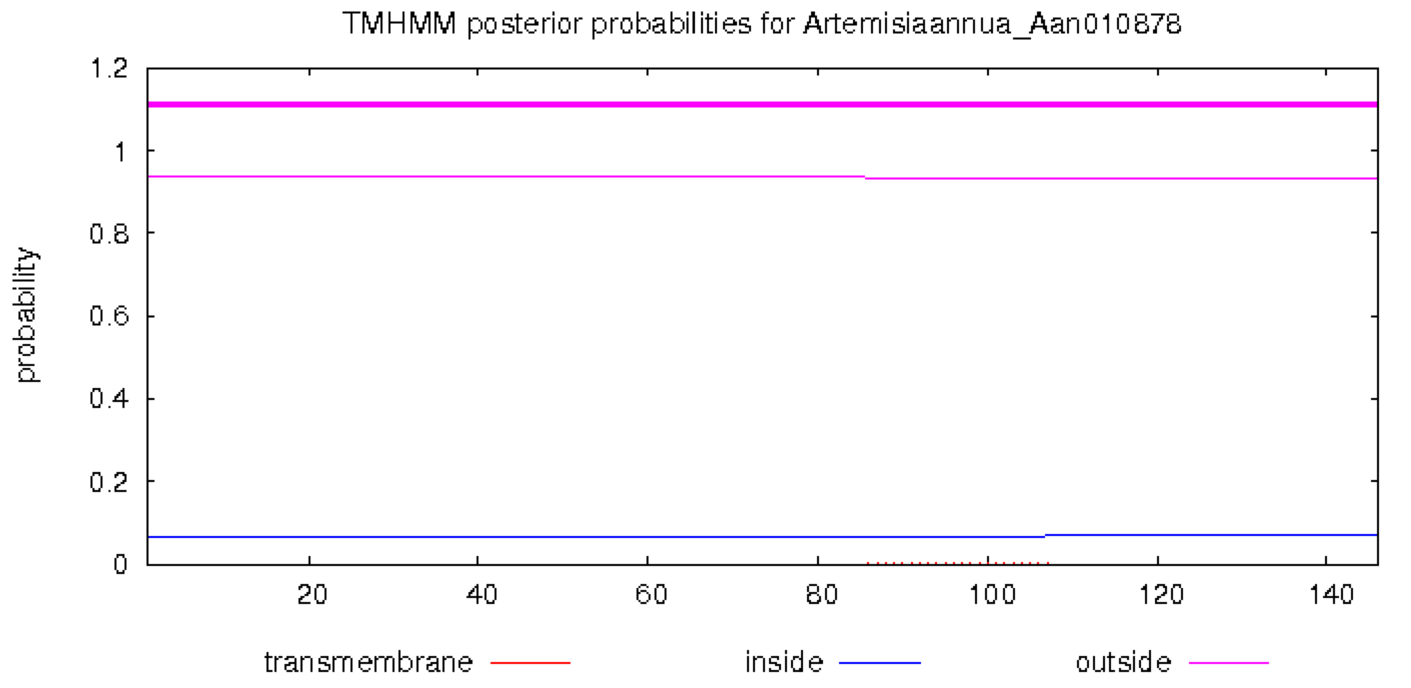

plot in postscript, script for making the plot in gnuplot, data for plot

```
# Artemisiaannua_Aan012884 Length: 69
# Artemisiaannua_Aan012884 Number of predicted TMHs:  0
# Artemisiaannua_Aan012884 Exp number of AAs in TMHs: 0.00021
# Artemisiaannua_Aan012884 Exp number, first 60 AAs:  0.00021
# Artemisiaannua_Aan012884 Total prob of N-in:        0.32453
Artemisiaannua_Aan012884        TMHMM2.0        outside      1    69
```

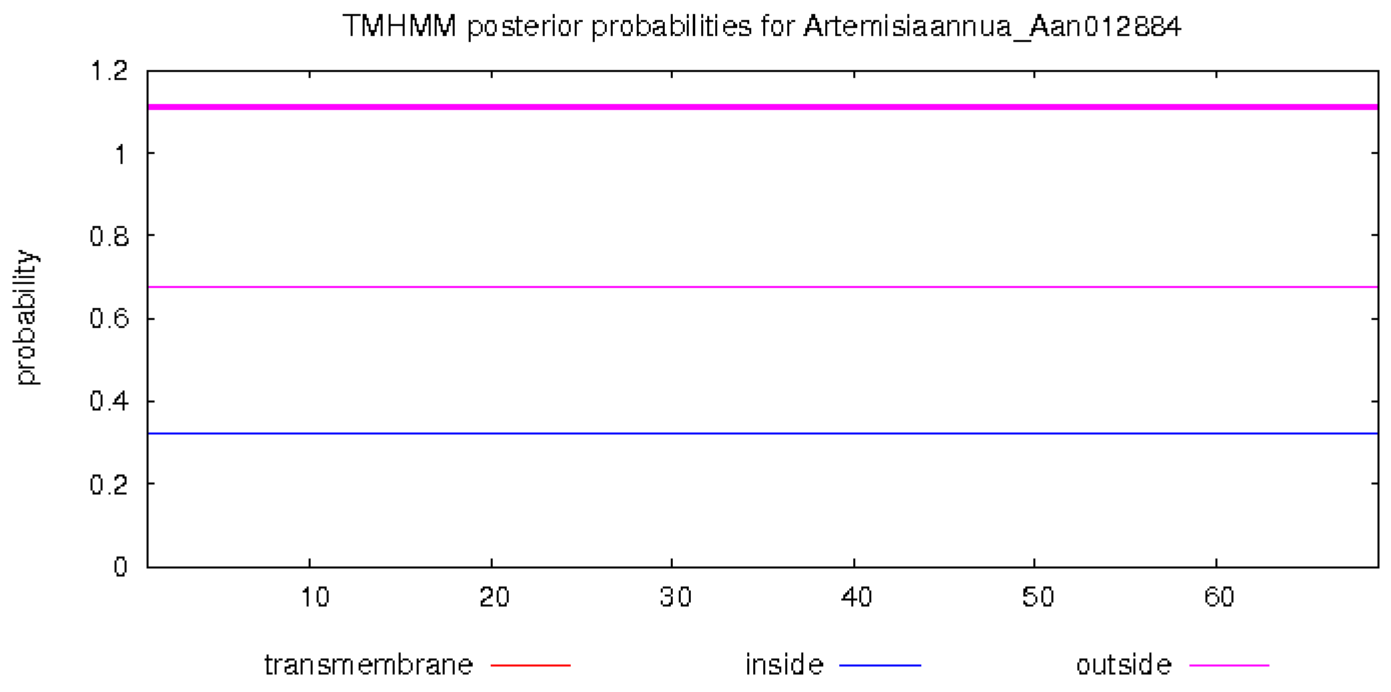

plot in postscript, script for making the plot in gnuplot, data for plot

```
# Artemisiaannua_Aan012975 Length: 63
# Artemisiaannua_Aan012975 Number of predicted TMHs:  0
# Artemisiaannua_Aan012975 Exp number of AAs in TMHs: 0.00202
# Artemisiaannua_Aan012975 Exp number, first 60 AAs:  0.00202
# Artemisiaannua_Aan012975 Total prob of N-in:        0.29614
Artemisiaannua_Aan012975        TMHMM2.0        outside      1    63
```

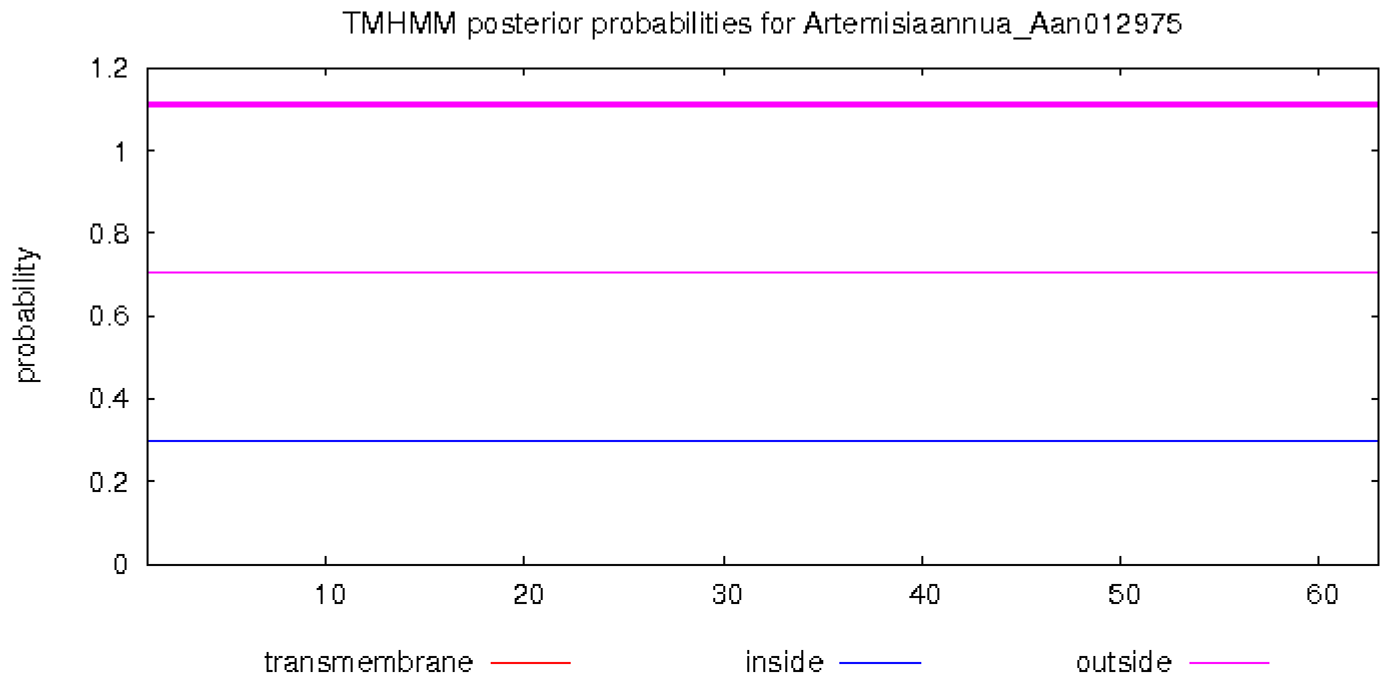

plot in postscript, script for making the plot in gnuplot, data for plot

---

```
# Artemisiaannua_Aan013239 Length: 79
# Artemisiaannua_Aan013239 Number of predicted TMHs:  0
# Artemisiaannua_Aan013239 Exp number of AAs in TMHs: 0.00019
# Artemisiaannua_Aan013239 Exp number, first 60 AAs:  0.00019
# Artemisiaannua_Aan013239 Total prob of N-in:        0.33781
Artemisiaannua_Aan013239        TMHMM2.0        outside      1    79
```

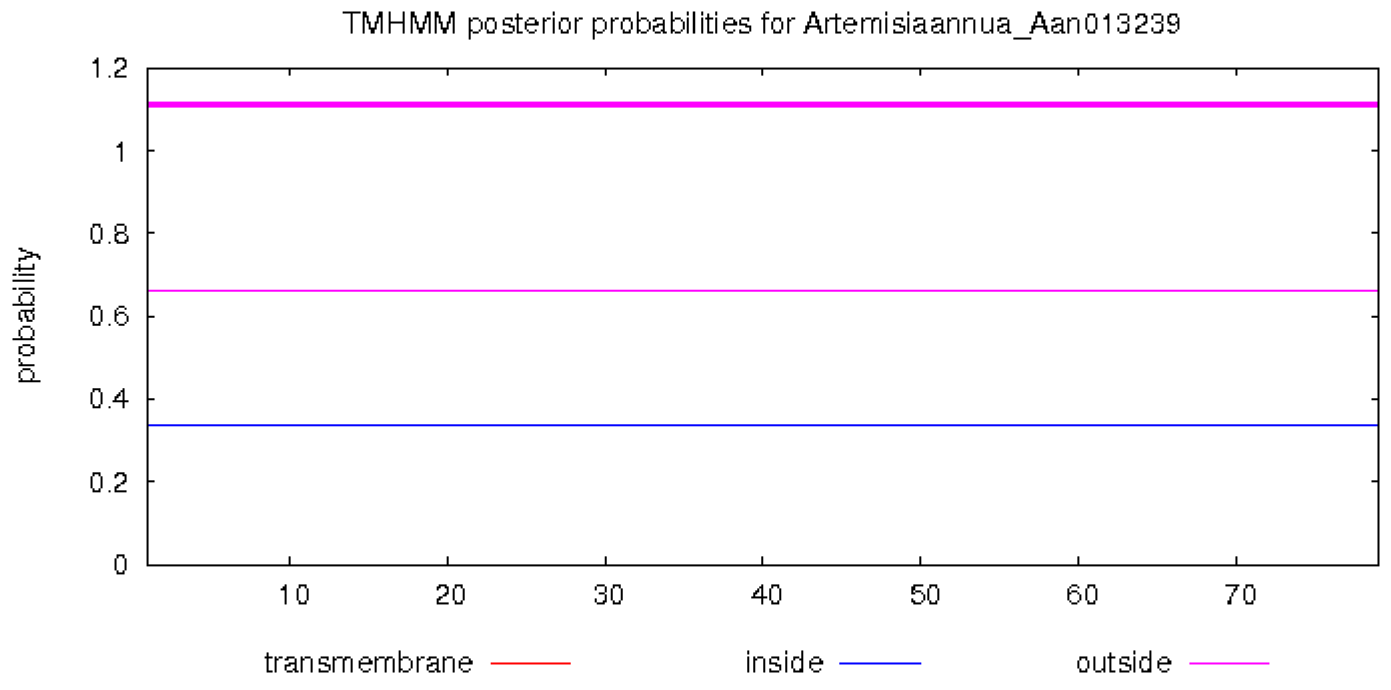

plot in postscript, script for making the plot in gnuplot, data for plot

---

```
# Artemisiaannua_Aan013749 Length: 430
# Artemisiaannua_Aan013749 Number of predicted TMHs:  0
# Artemisiaannua_Aan013749 Exp number of AAs in TMHs: 0.00018
# Artemisiaannua_Aan013749 Exp number, first 60 AAs:  0.00018
# Artemisiaannua_Aan013749 Total prob of N-in:        0.00465
Artemisiaannua_Aan013749        TMHMM2.0        outside      1   430
```

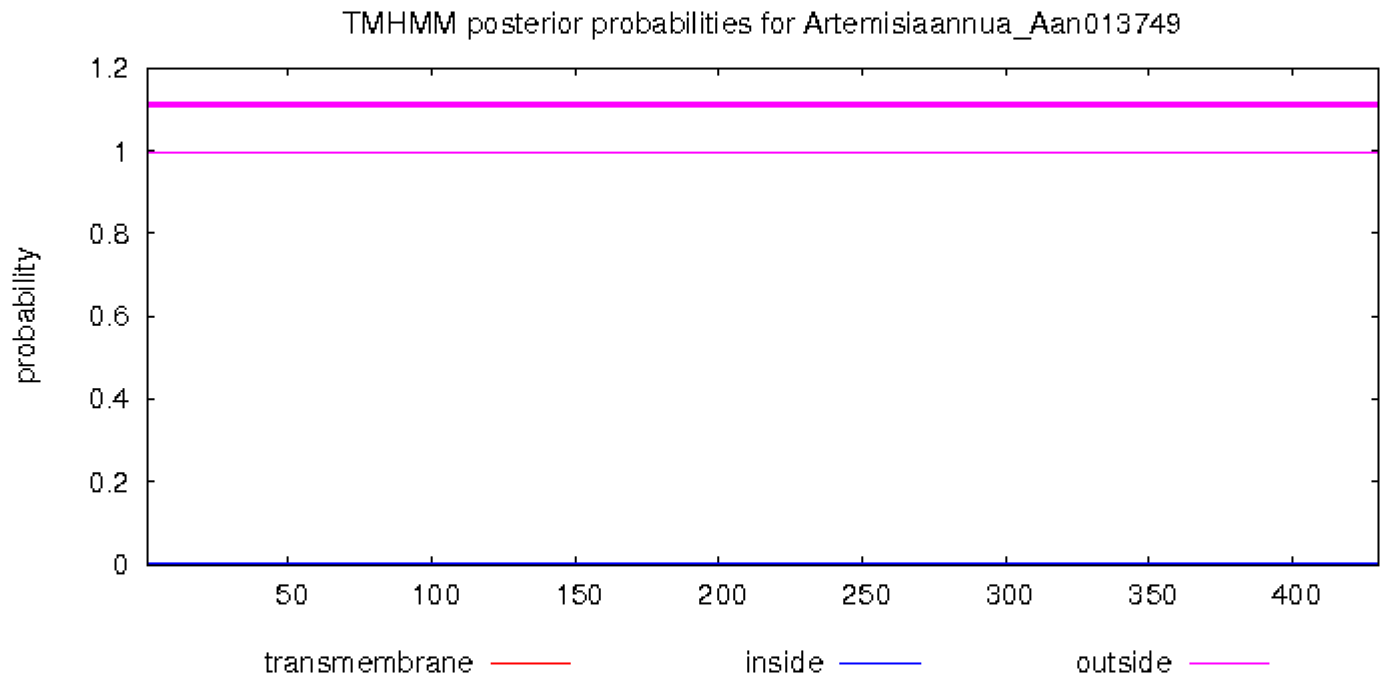

plot in postscript, script for making the plot in gnuplot, data for plot

---

```
# Artemisiaannua_Aan014755 Length: 262
# Artemisiaannua_Aan014755 Number of predicted TMHs:  0
# Artemisiaannua_Aan014755 Exp number of AAs in TMHs: 0.00041
# Artemisiaannua_Aan014755 Exp number, first 60 AAs:  0
# Artemisiaannua_Aan014755 Total prob of N-in:        0.07756
Artemisiaannua_Aan014755        TMHMM2.0        outside      1   262
```

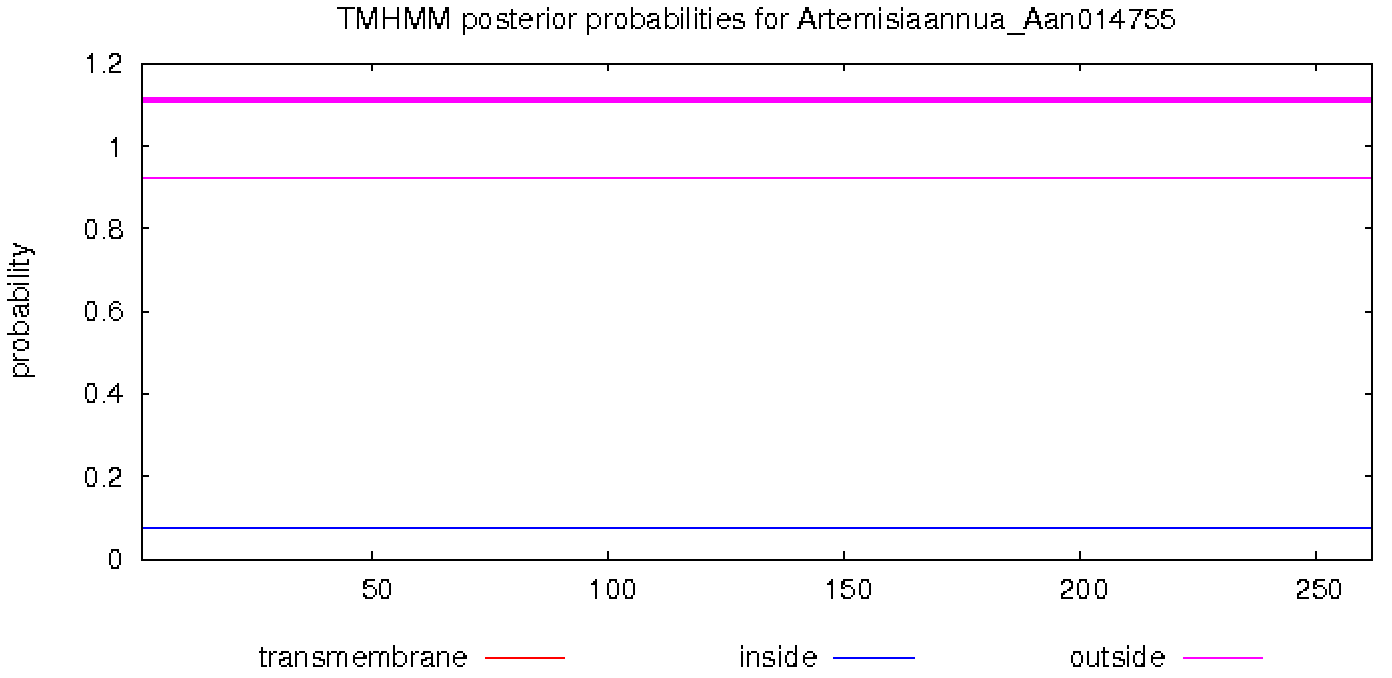

plot in postscript, script for making the plot in gnuplot, data for plot

---

```
# Artemisiaannua_Aan015209 Length: 203
# Artemisiaannua_Aan015209 Number of predicted TMHs:  0
# Artemisiaannua_Aan015209 Exp number of AAs in TMHs: 0.00028
# Artemisiaannua_Aan015209 Exp number, first 60 AAs:  0.00028
# Artemisiaannua_Aan015209 Total prob of N-in:        0.10655
Artemisiaannua_Aan015209        TMHMM2.0        outside      1   203
```

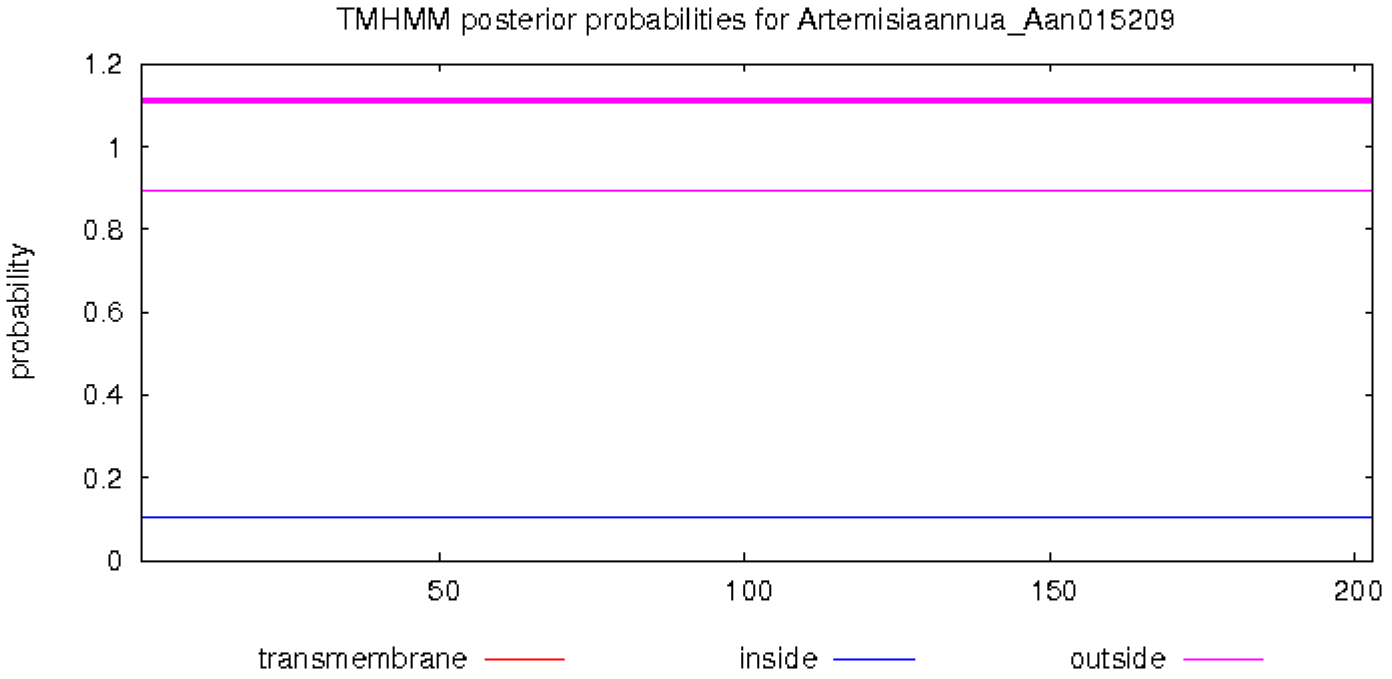

plot in postscript, script for making the plot in gnuplot, data for plot

```
# Artemisiaannua_Aan015985 Length: 217
# Artemisiaannua_Aan015985 Number of predicted TMHs:  0
# Artemisiaannua_Aan015985 Exp number of AAs in TMHs: 0.00431
# Artemisiaannua_Aan015985 Exp number, first 60 AAs:  0
# Artemisiaannua_Aan015985 Total prob of N-in:        0.06129
Artemisiaannua_Aan015985        TMHMM2.0        outside      1   217
```

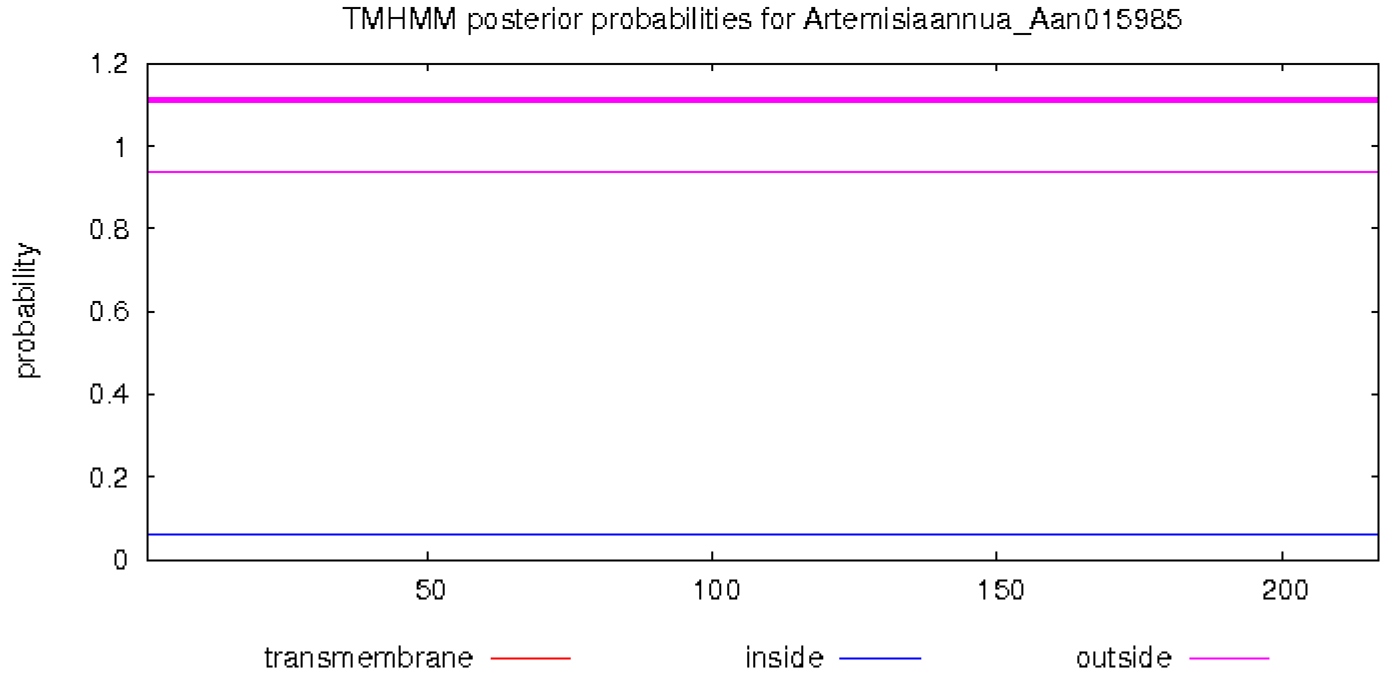

plot in postscript, script for making the plot in gnuplot, data for plot

```
# Artemisiaannua_Aan016410 Length: 320
# Artemisiaannua_Aan016410 Number of predicted TMHs:  0
# Artemisiaannua_Aan016410 Exp number of AAs in TMHs: 0.00031
# Artemisiaannua_Aan016410 Exp number, first 60 AAs:  0.00014
# Artemisiaannua_Aan016410 Total prob of N-in:        0.00571
Artemisiaannua_Aan016410        TMHMM2.0        outside      1   320
```

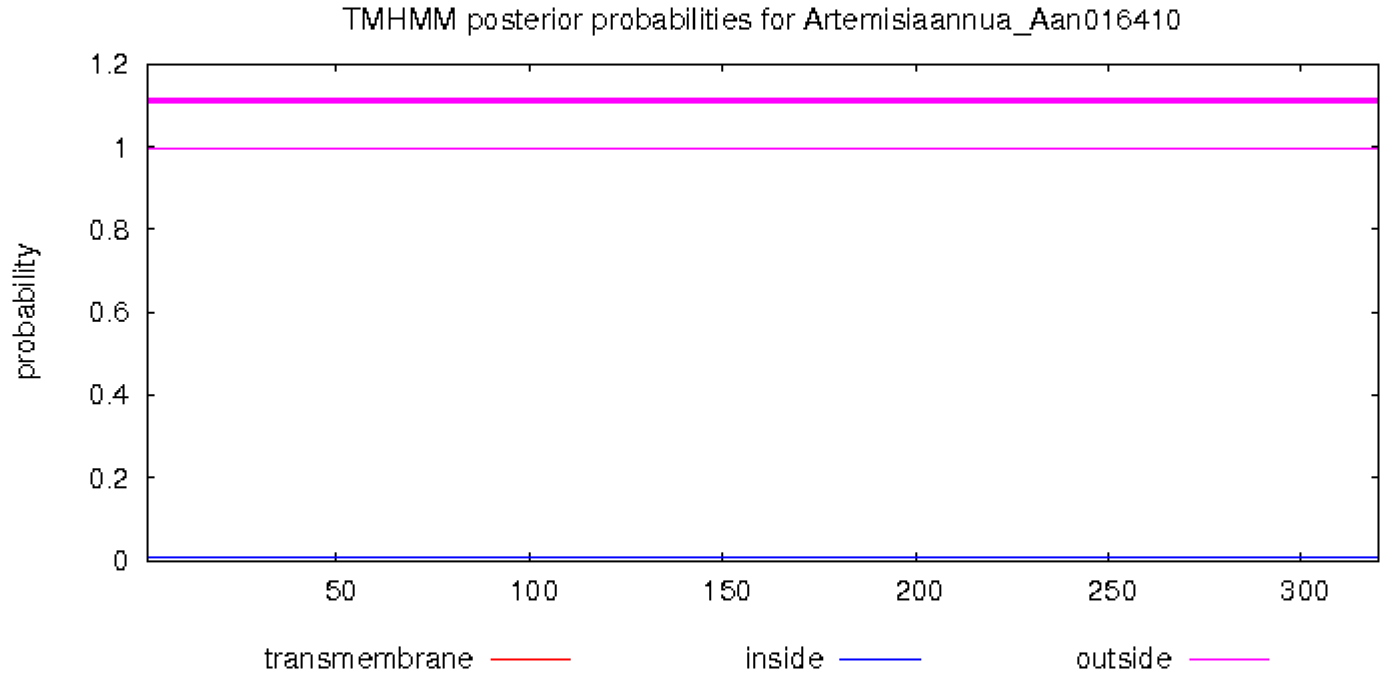

plot in postscript, script for making the plot in gnuplot, data for plot

```
# Artemisiaannua_Aan017655 Length: 333
# Artemisiaannua_Aan017655 Number of predicted TMHs:  0
# Artemisiaannua_Aan017655 Exp number of AAs in TMHs: 0
# Artemisiaannua_Aan017655 Exp number, first 60 AAs:  0
# Artemisiaannua_Aan017655 Total prob of N-in:        0.01349
Artemisiaannua_Aan017655        TMHMM2.0        outside      1   333
```

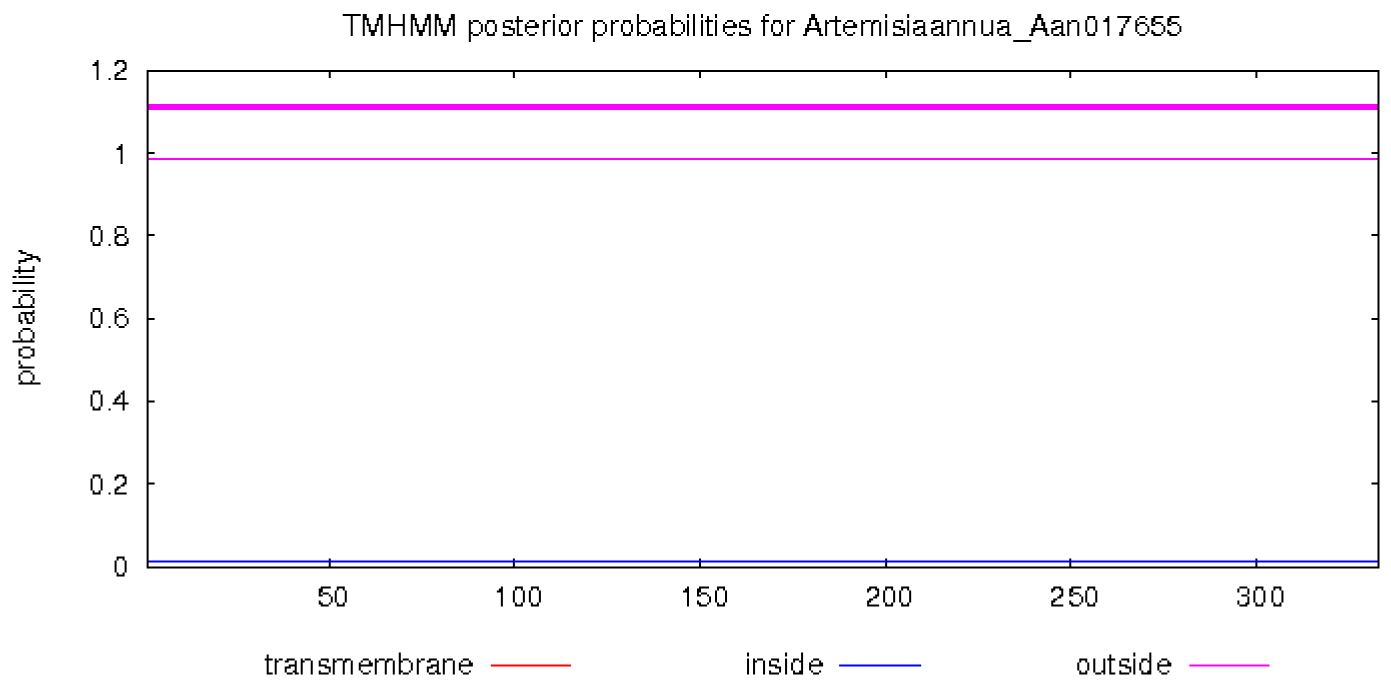

plot in postscript, script for making the plot in gnuplot, data for plot

```
# Artemisiaannua_Aan018369 Length: 333
# Artemisiaannua_Aan018369 Number of predicted TMHs:  0
# Artemisiaannua_Aan018369 Exp number of AAs in TMHs: 0
# Artemisiaannua_Aan018369 Exp number, first 60 AAs:  0
# Artemisiaannua_Aan018369 Total prob of N-in:        0.01017
Artemisiaannua_Aan018369        TMHMM2.0        outside      1   333
```

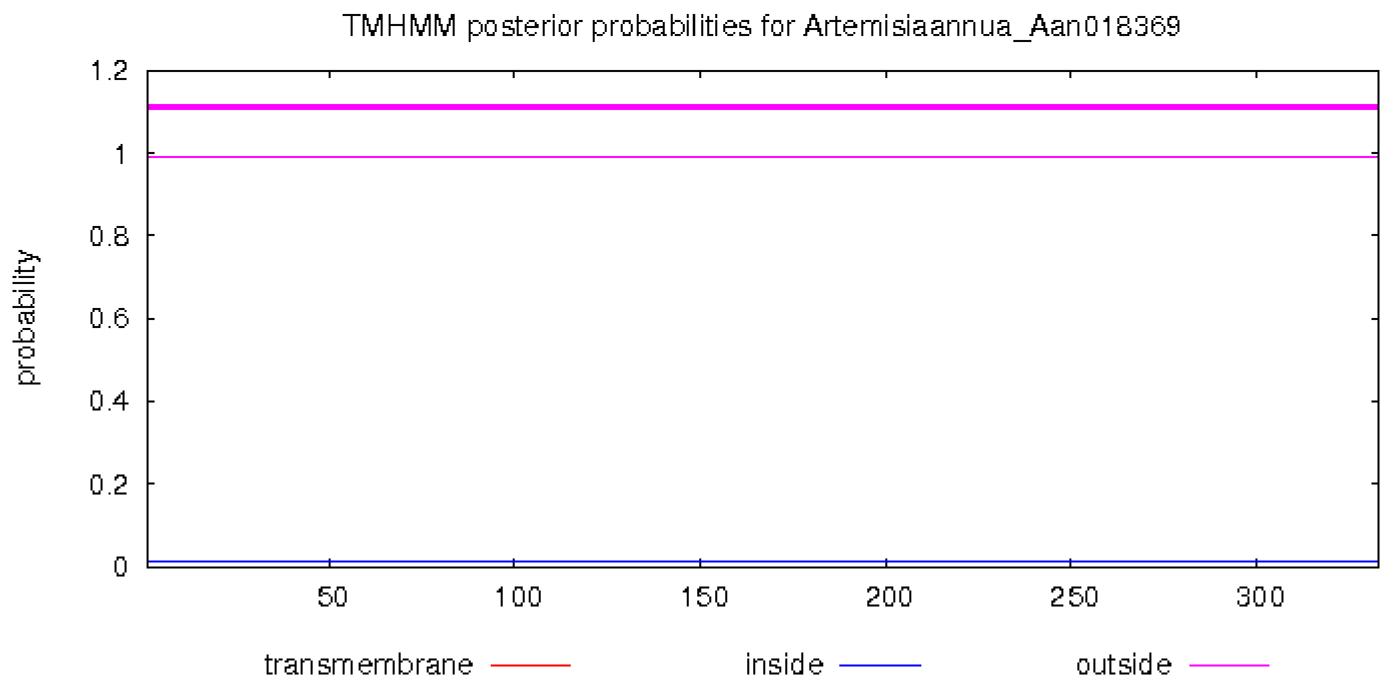

plot in postscript, script for making the plot in gnuplot, data for plot

---

```
# Artemisiaannua_Aan018435 Length: 416
# Artemisiaannua_Aan018435 Number of predicted TMHs:  0
# Artemisiaannua_Aan018435 Exp number of AAs in TMHs: 0.00248
# Artemisiaannua_Aan018435 Exp number, first 60 AAs:  0.00118
# Artemisiaannua_Aan018435 Total prob of N-in:        0.00130
Artemisiaannua_Aan018435        TMHMM2.0        outside      1   416
```

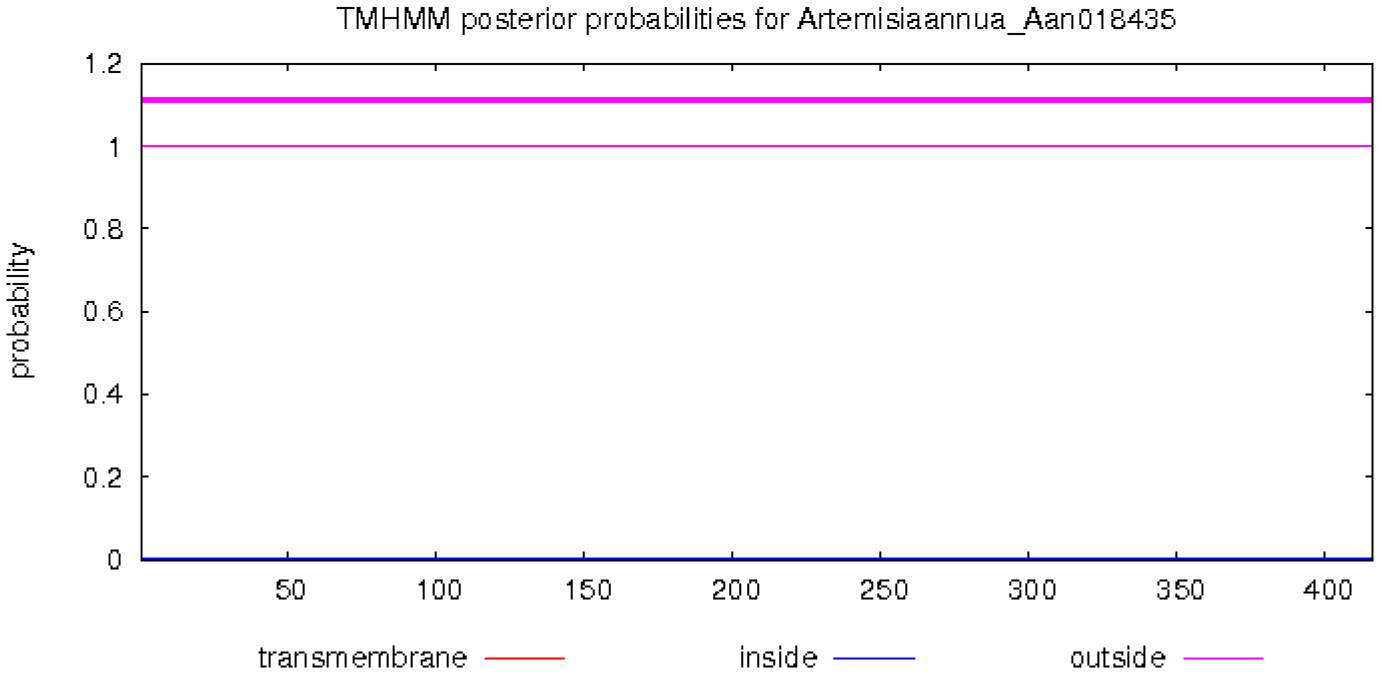

plot in postscript, script for making the plot in gnuplot, data for plot

---

```
# Artemisiaannua_Aan018568 Length: 152
# Artemisiaannua_Aan018568 Number of predicted TMHs:  0
# Artemisiaannua_Aan018568 Exp number of AAs in TMHs: 0.00038
# Artemisiaannua_Aan018568 Exp number, first 60 AAs:  0
# Artemisiaannua_Aan018568 Total prob of N-in:        0.13175
Artemisiaannua_Aan018568        TMHMM2.0        outside      1   152
```

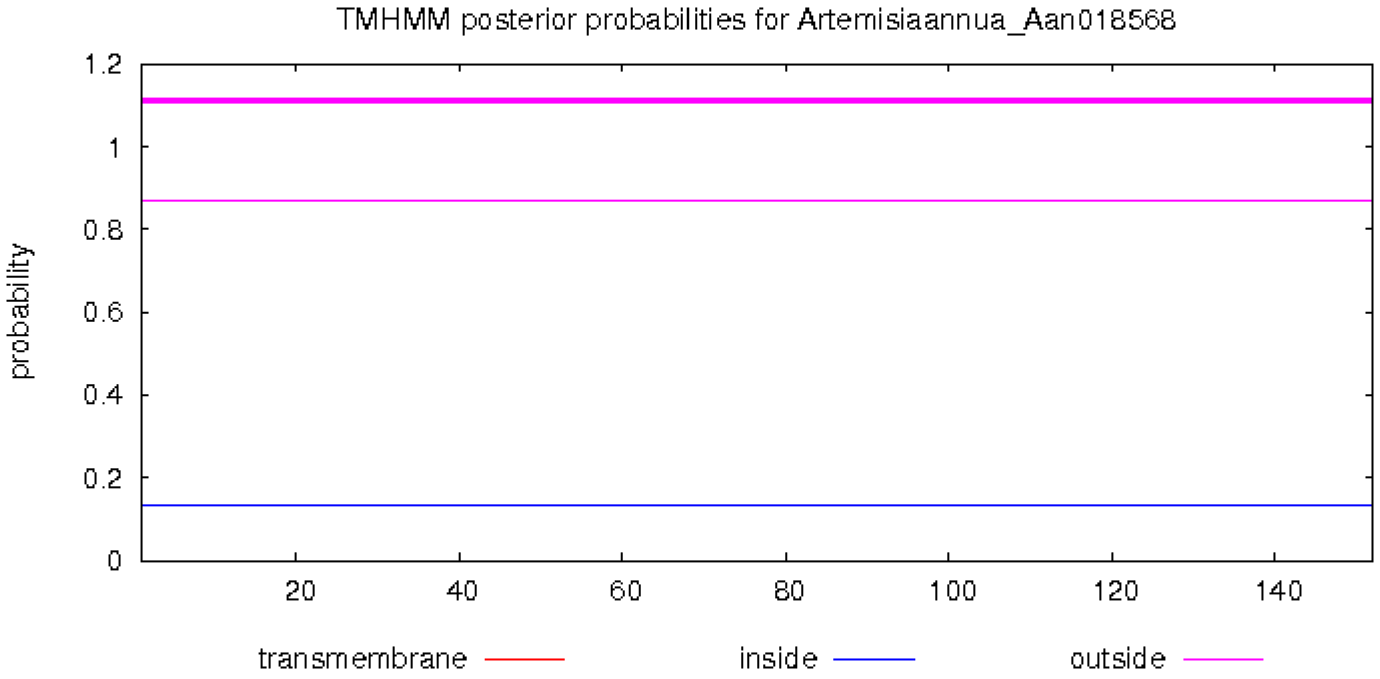

plot in postscript, script for making the plot in gnuplot, data for plot

---

```
# Artemisiaannua_Aan019479 Length: 250
# Artemisiaannua_Aan019479 Number of predicted TMHs:  0
# Artemisiaannua_Aan019479 Exp number of AAs in TMHs: 0
# Artemisiaannua_Aan019479 Exp number, first 60 AAs:  0
# Artemisiaannua_Aan019479 Total prob of N-in:        0.05368
Artemisiaannua_Aan019479        TMHMM2.0        outside      1   250
```

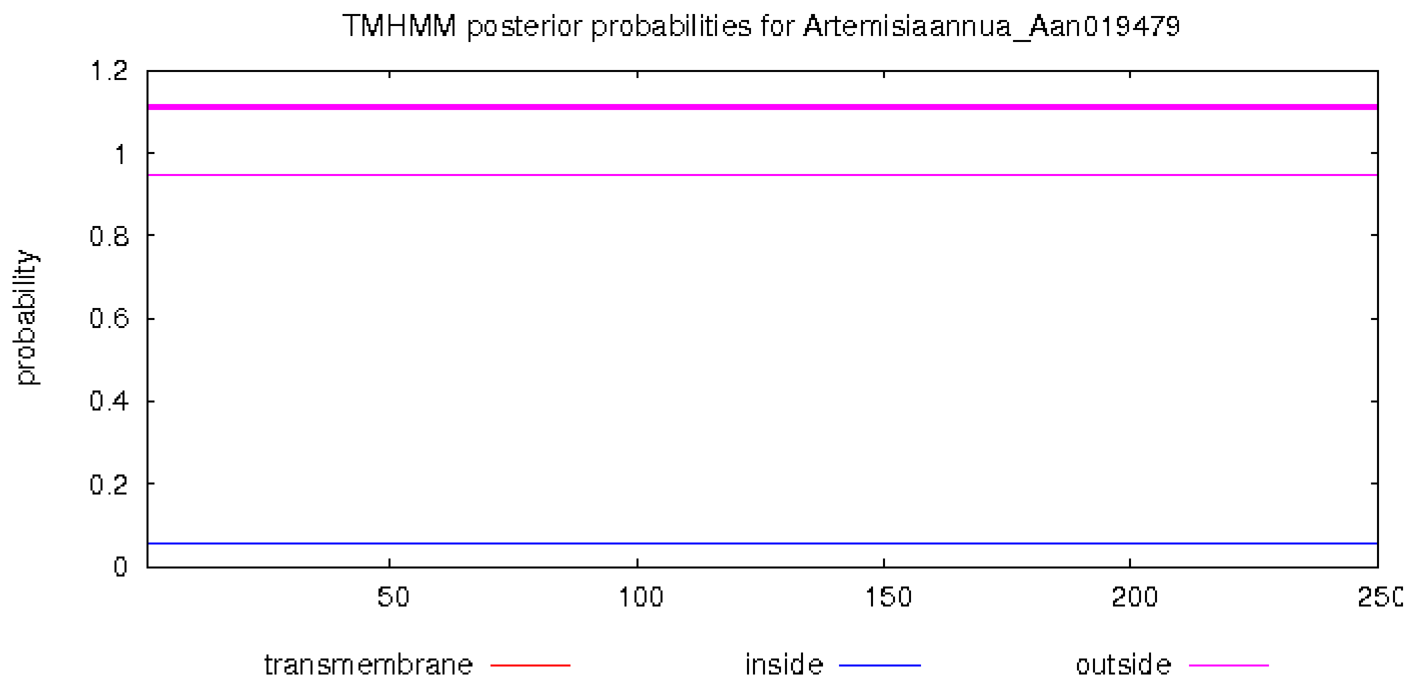

plot in postscript, script for making the plot in gnuplot, data for plot

---

```
# Artemisiaannua_Aan019671 Length: 314
# Artemisiaannua_Aan019671 Number of predicted TMHs:  0
# Artemisiaannua_Aan019671 Exp number of AAs in TMHs: 0.000880000000000000001
# Artemisiaannua_Aan019671 Exp number, first 60 AAs:  0.00026
# Artemisiaannua_Aan019671 Total prob of N-in:        0.03800
Artemisiaannua_Aan019671        TMHMM2.0        outside      1   314
```

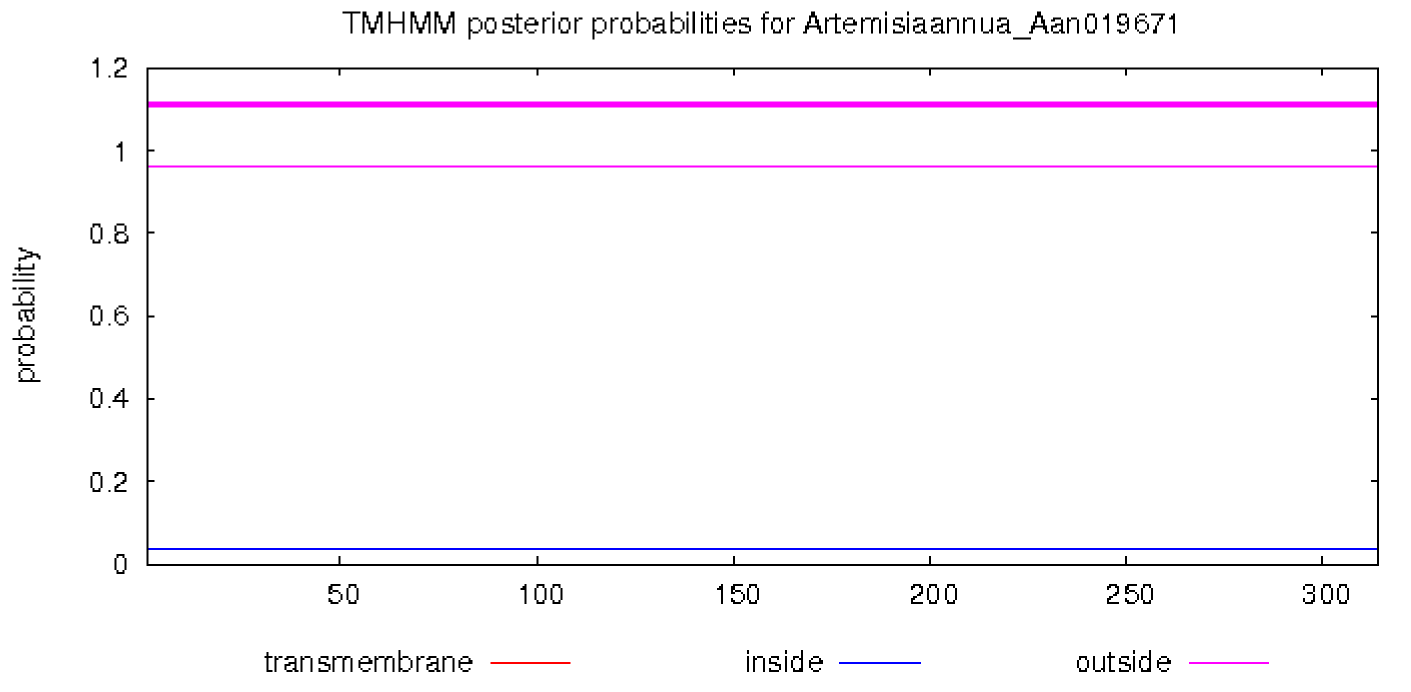

plot in postscript, script for making the plot in gnuplot, data for plot

---

```
# Artemisiaannua_Aan020678 Length: 271
# Artemisiaannua_Aan020678 Number of predicted TMHs:  0
# Artemisiaannua_Aan020678 Exp number of AAs in TMHs: 0.00124
# Artemisiaannua_Aan020678 Exp number, first 60 AAs:  0.00124
# Artemisiaannua_Aan020678 Total prob of N-in:        0.00582
Artemisiaannua_Aan020678	TMHMM2.0	outside	     1   271
```

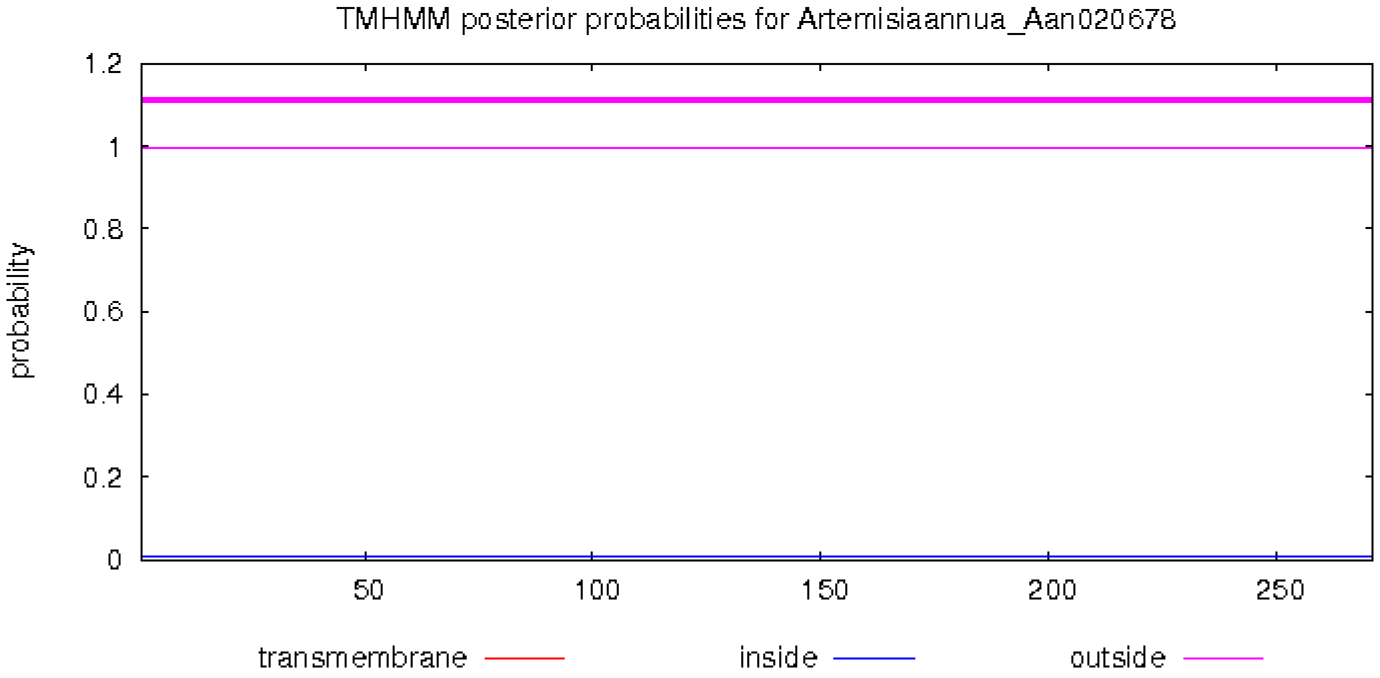

plot in postscript, script for making the plot in gnuplot, data for plot

---

```
# Artemisiaannua_Aan020942 Length: 467
# Artemisiaannua_Aan020942 Number of predicted TMHs:  0
# Artemisiaannua_Aan020942 Exp number of AAs in TMHs: 0.02529
# Artemisiaannua_Aan020942 Exp number, first 60 AAs:  0.0251
# Artemisiaannua_Aan020942 Total prob of N-in:        0.01259
Artemisiaannua_Aan020942	TMHMM2.0	outside	     1   467
```

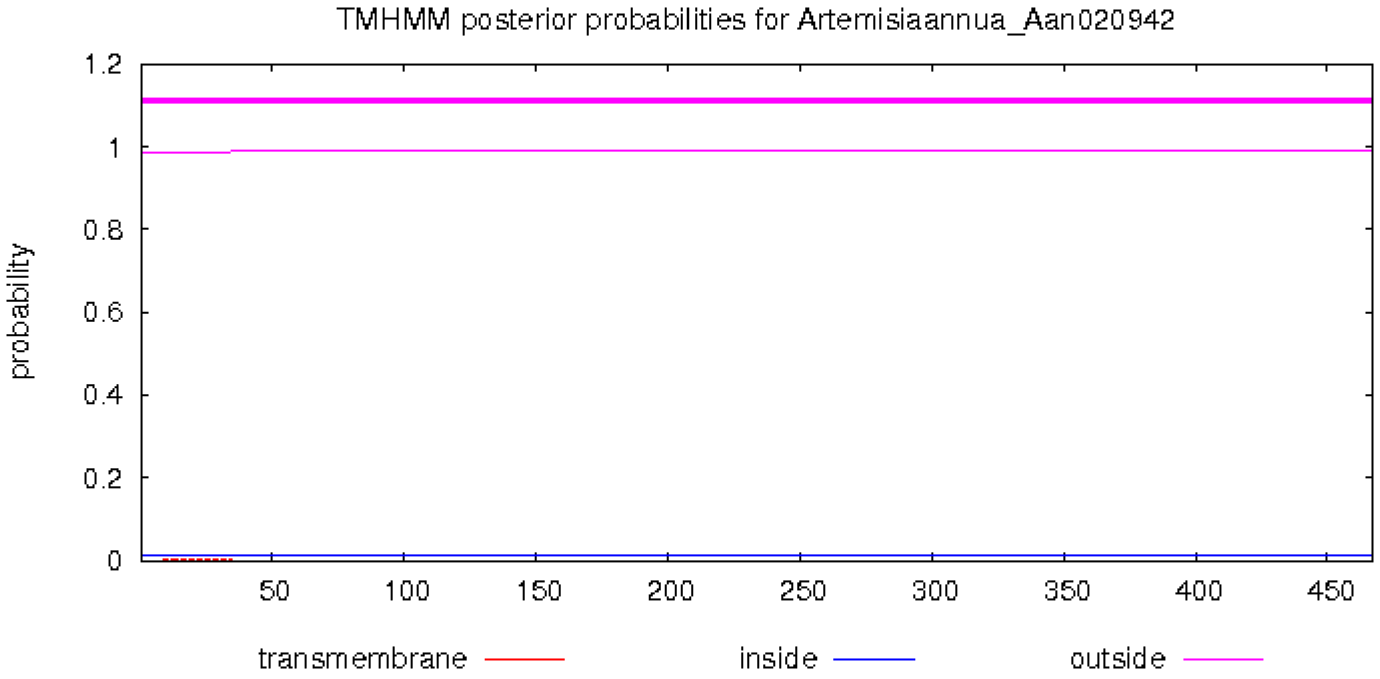

plot in postscript, script for making the plot in gnuplot, data for plot

```
# Artemisiaannua_Aan021211 Length: 423
# Artemisiaannua_Aan021211 Number of predicted TMHs:  0
# Artemisiaannua_Aan021211 Exp number of AAs in TMHs: 0
# Artemisiaannua_Aan021211 Exp number, first 60 AAs:  0
# Artemisiaannua_Aan021211 Total prob of N-in:        0.01169
Artemisiaannua_Aan021211        TMHMM2.0        outside      1   423
```

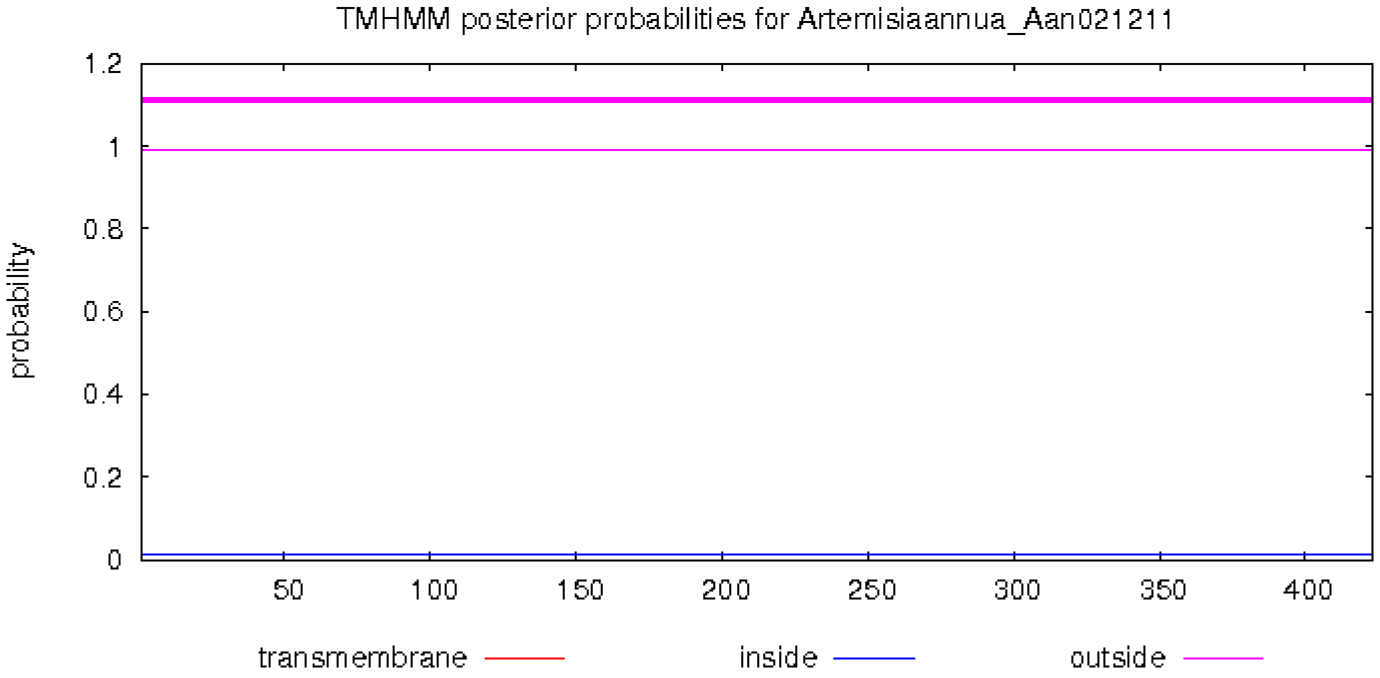

plot in postscript, script for making the plot in gnuplot, data for plot

```
# Artemisiaannua_Aan021242 Length: 370
# Artemisiaannua_Aan021242 Number of predicted TMHs:  0
# Artemisiaannua_Aan021242 Exp number of AAs in TMHs: 0.00241
# Artemisiaannua_Aan021242 Exp number, first 60 AAs:  0
# Artemisiaannua_Aan021242 Total prob of N-in:        0.00216
Artemisiaannua_Aan021242        TMHMM2.0        outside      1   370
```

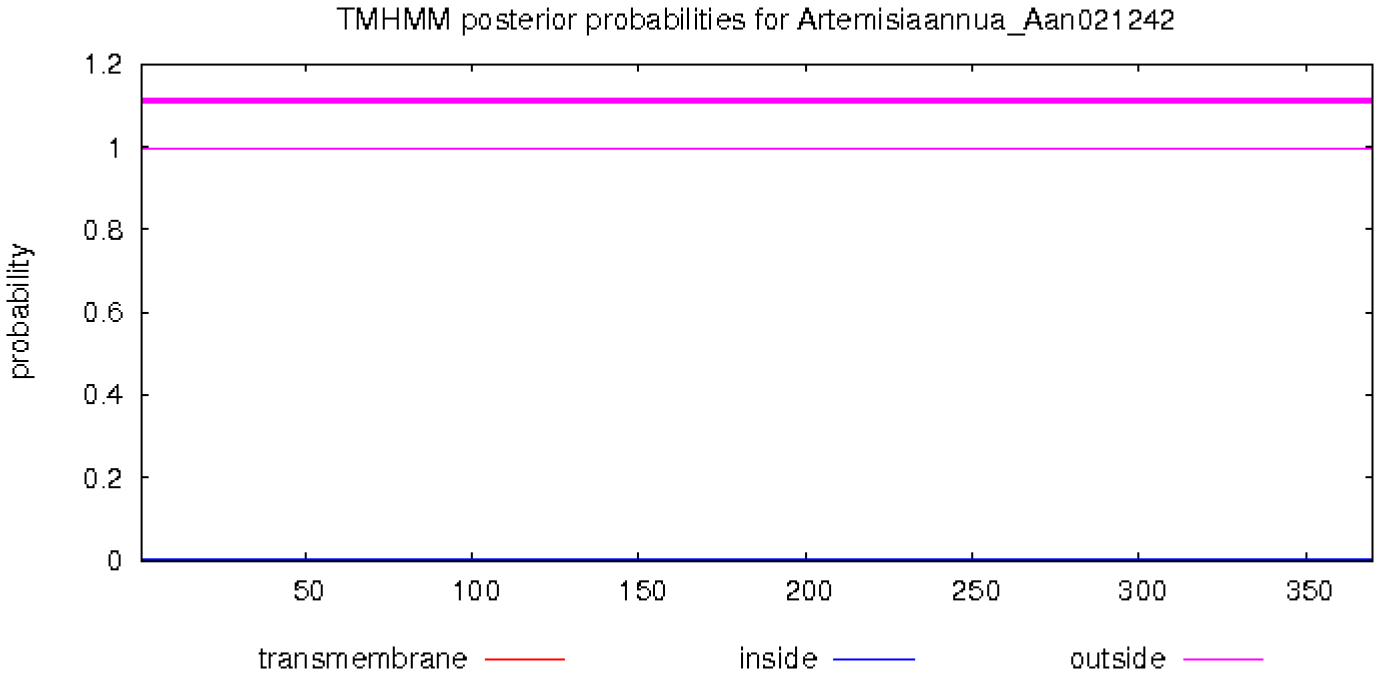

plot in postscript, script for making the plot in gnuplot, data for plot

---

```
# Azadirachtaindica_Neem_10055_f_1 Length: 382
# Azadirachtaindica_Neem_10055_f_1 Number of predicted TMHs:  0
# Azadirachtaindica_Neem_10055_f_1 Exp number of AAs in TMHs: 0.00033
# Azadirachtaindica_Neem_10055_f_1 Exp number, first 60 AAs:  0
# Azadirachtaindica_Neem_10055_f_1 Total prob of N-in:        0.01643
Azadirachtaindica_Neem_10055_f_1        TMHMM2.0        outside      1   382
```

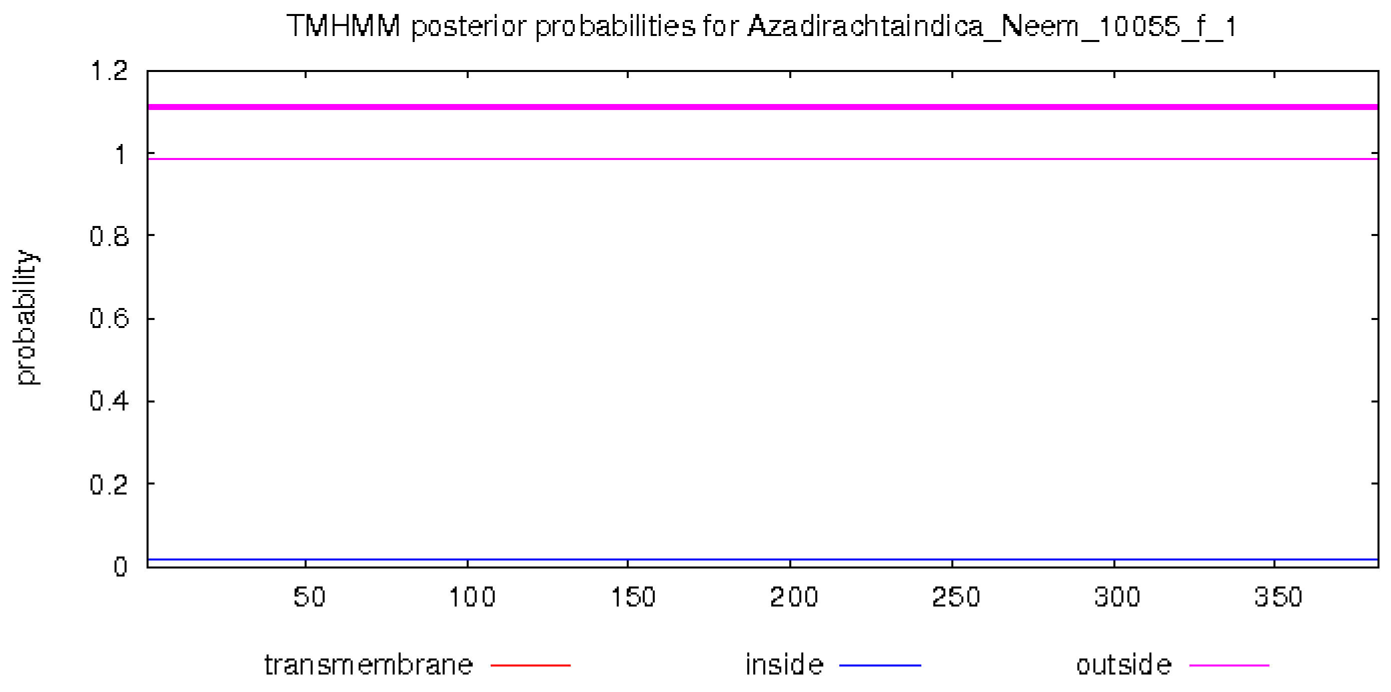

plot in postscript, script for making the plot in gnuplot, data for plot

---

```
# Azadirachtaindica_Neem_10068_f_1 Length: 231
# Azadirachtaindica_Neem_10068_f_1 Number of predicted TMHs:  0
# Azadirachtaindica_Neem_10068_f_1 Exp number of AAs in TMHs: 0.00023
# Azadirachtaindica_Neem_10068_f_1 Exp number, first 60 AAs:  6e-05
# Azadirachtaindica_Neem_10068_f_1 Total prob of N-in:        0.05375
Azadirachtaindica_Neem_10068_f_1        TMHMM2.0        outside      1   231
```

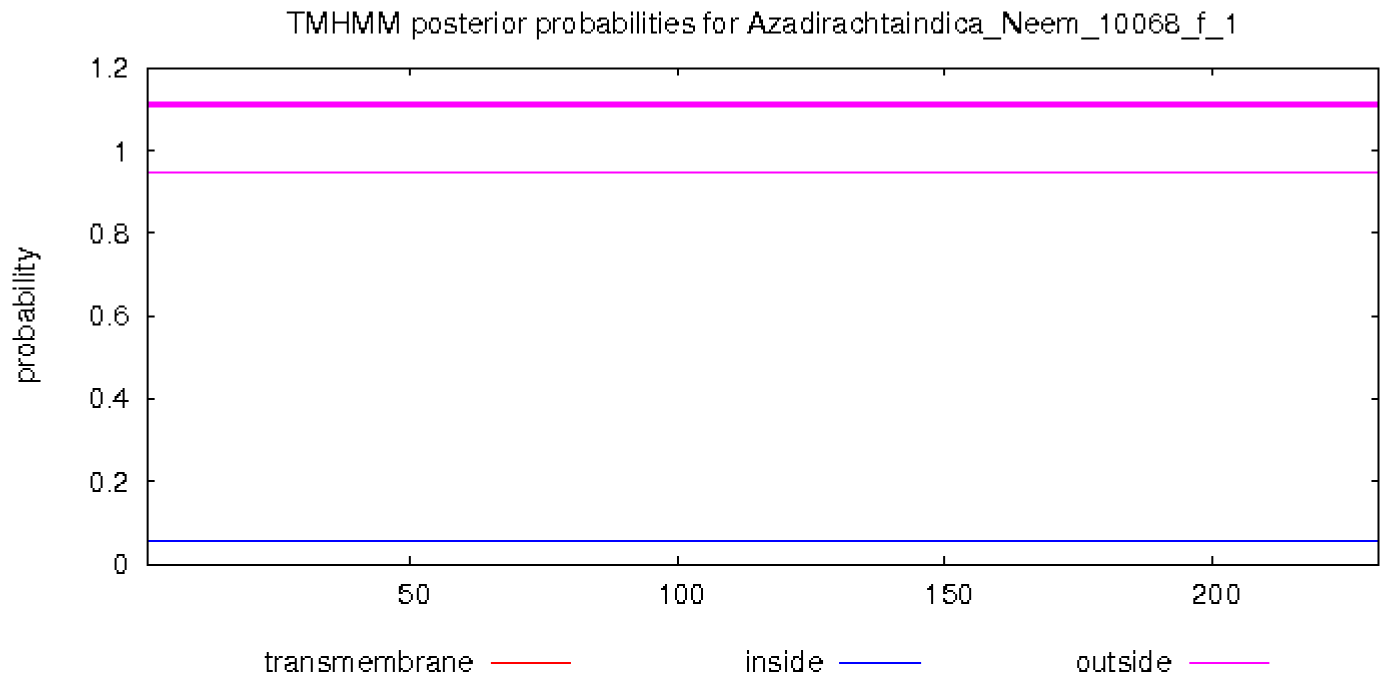

plot in postscript, script for making the plot in gnuplot, data for plot

---

```
# Azadirachtaindica_Neem_10068_f_2 Length: 219
# Azadirachtaindica_Neem_10068_f_2 Number of predicted TMHs:  0
# Azadirachtaindica_Neem_10068_f_2 Exp number of AAs in TMHs: 0.00286
# Azadirachtaindica_Neem_10068_f_2 Exp number, first 60 AAs:  0.00286
# Azadirachtaindica_Neem_10068_f_2 Total prob of N-in:        0.07831
Azadirachtaindica_Neem_10068_f_2        TMHMM2.0        outside      1   219
```

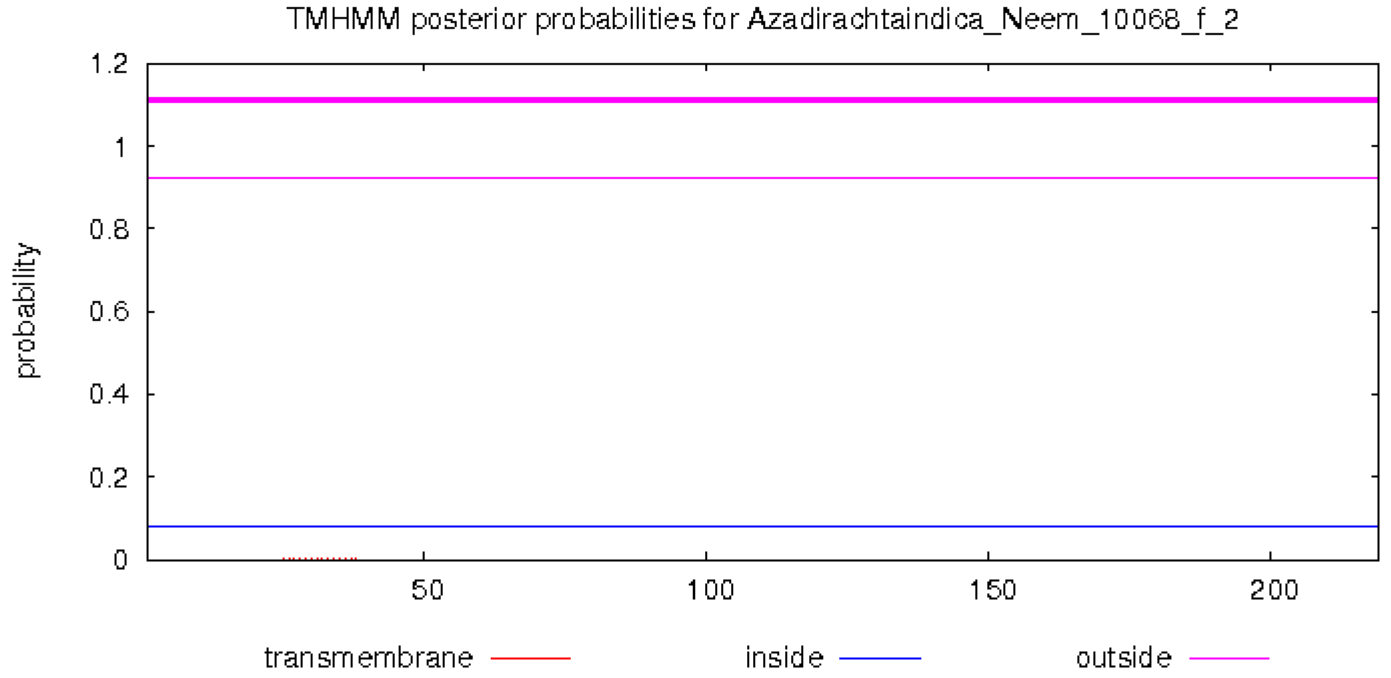

plot in postscript, script for making the plot in gnuplot, data for plot

---

```
# Azadirachtaindica_Neem_10194_f_1 Length: 264
# Azadirachtaindica_Neem_10194_f_1 Number of predicted TMHs:  0
# Azadirachtaindica_Neem_10194_f_1 Exp number of AAs in TMHs: 0.00951
# Azadirachtaindica_Neem_10194_f_1 Exp number, first 60 AAs:  0
# Azadirachtaindica_Neem_10194_f_1 Total prob of N-in:        0.00851
Azadirachtaindica_Neem_10194_f_1        TMHMM2.0        outside      1   264
```

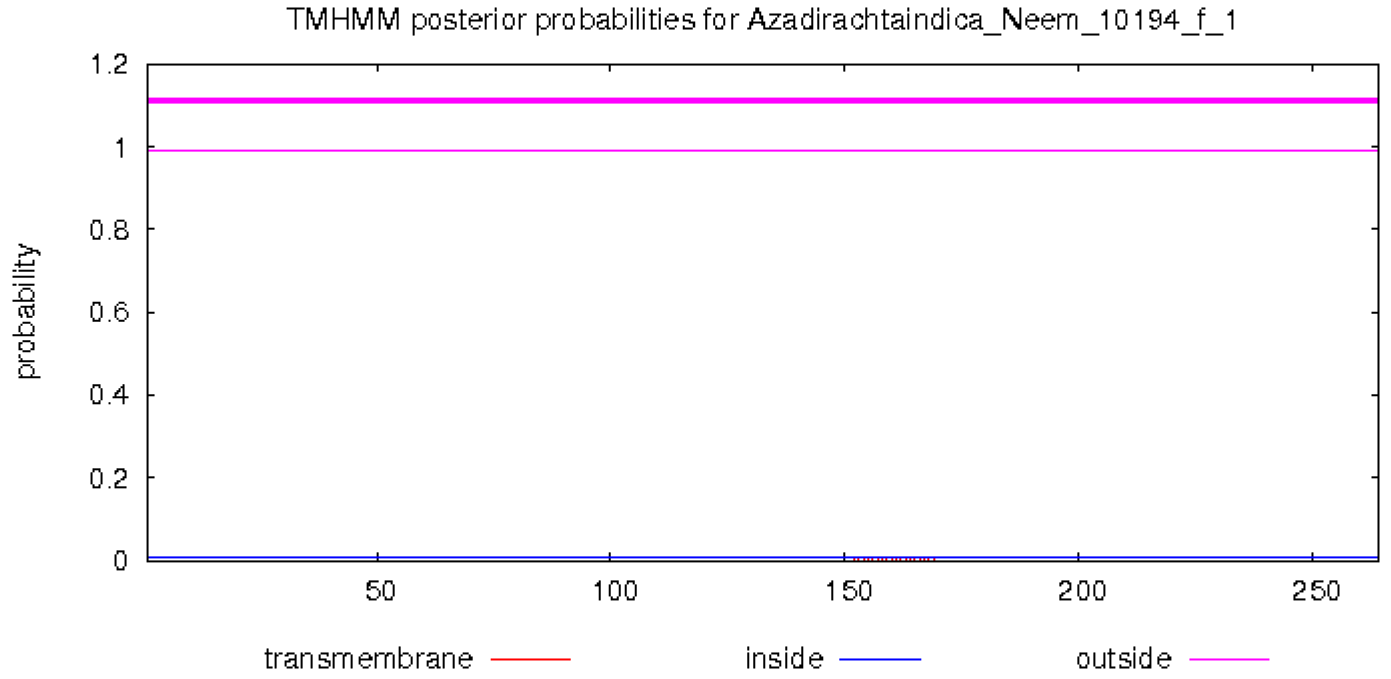

plot in postscript, script for making the plot in gnuplot, data for plot

```
# Azadirachtaindica_Neem_10248_f_2 Length: 244
# Azadirachtaindica_Neem_10248_f_2 Number of predicted TMHs:  0
# Azadirachtaindica_Neem_10248_f_2 Exp number of AAs in TMHs: 0.00039
# Azadirachtaindica_Neem_10248_f_2 Exp number, first 60 AAs:  0.00025
# Azadirachtaindica_Neem_10248_f_2 Total prob of N-in:        0.05327
Azadirachtaindica_Neem_10248_f_2        TMHMM2.0        outside      1   244
```

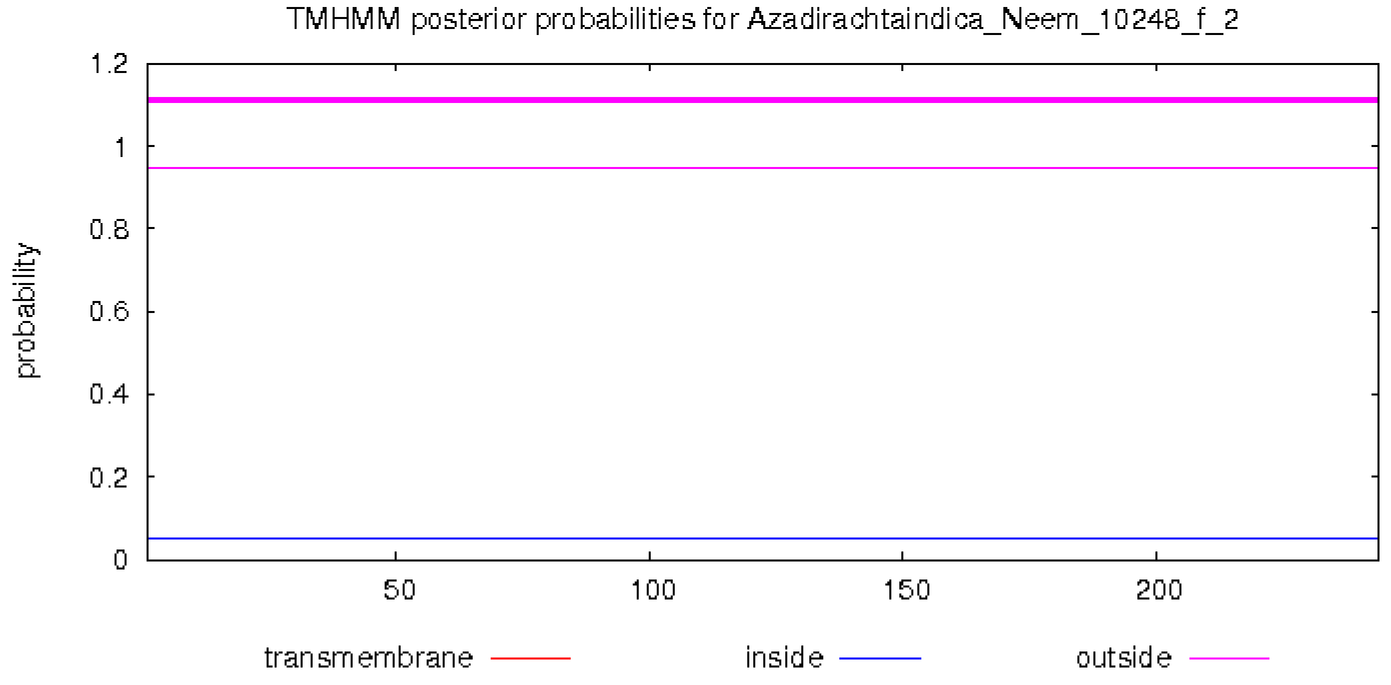

plot in postscript, script for making the plot in gnuplot, data for plot

```
# Azadirachtaindica_Neem_10408_f_4 Length: 286
# Azadirachtaindica_Neem_10408_f_4 Number of predicted TMHs:  0
# Azadirachtaindica_Neem_10408_f_4 Exp number of AAs in TMHs: 0.00015
# Azadirachtaindica_Neem_10408_f_4 Exp number, first 60 AAs:  0
# Azadirachtaindica_Neem_10408_f_4 Total prob of N-in:        0.01180
Azadirachtaindica_Neem_10408_f_4        TMHMM2.0        outside      1   286
```

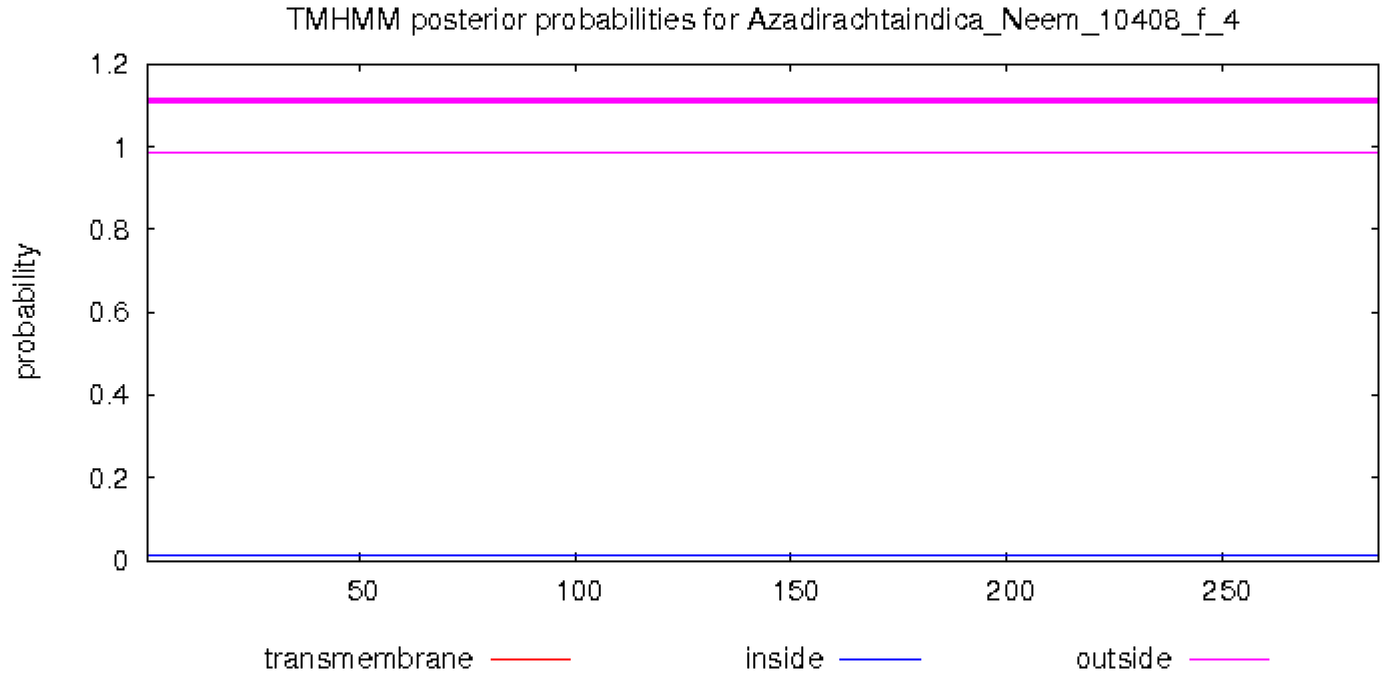

plot in postscript, script for making the plot in gnuplot, data for plot

---

```
# Azadirachtaindica_Neem_10484_f_1 Length: 301
# Azadirachtaindica_Neem_10484_f_1 Number of predicted TMHs:  0
# Azadirachtaindica_Neem_10484_f_1 Exp number of AAs in TMHs: 0.00017
# Azadirachtaindica_Neem_10484_f_1 Exp number, first 60 AAs:  0
# Azadirachtaindica_Neem_10484_f_1 Total prob of N-in:        0.02775
Azadirachtaindica_Neem_10484_f_1        TMHMM2.0        outside      1   301
```

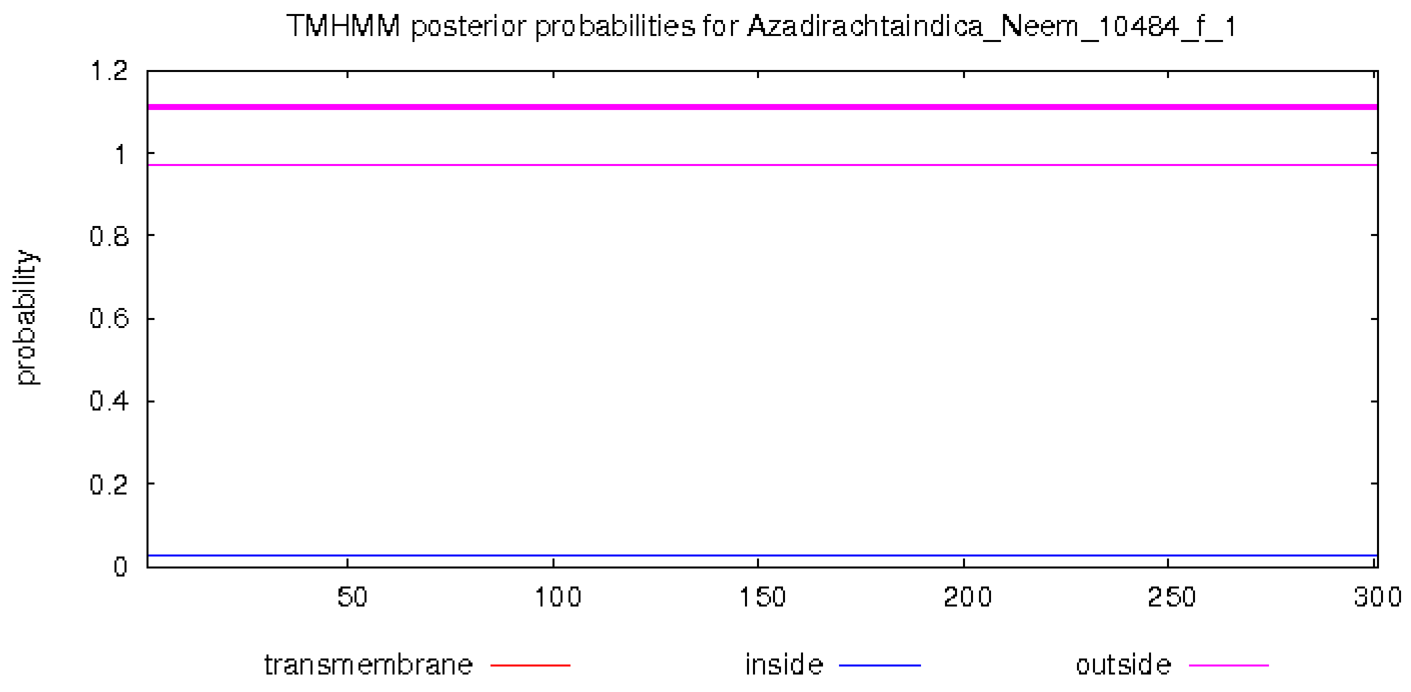

plot in postscript, script for making the plot in gnuplot, data for plot

---

```
# Azadirachtaindica_Neem_10791_f_1 Length: 249
# Azadirachtaindica_Neem_10791_f_1 Number of predicted TMHs:  0
# Azadirachtaindica_Neem_10791_f_1 Exp number of AAs in TMHs: 0
# Azadirachtaindica_Neem_10791_f_1 Exp number, first 60 AAs:  0
# Azadirachtaindica_Neem_10791_f_1 Total prob of N-in:        0.00214
Azadirachtaindica_Neem_10791_f_1        TMHMM2.0        outside      1   249
```

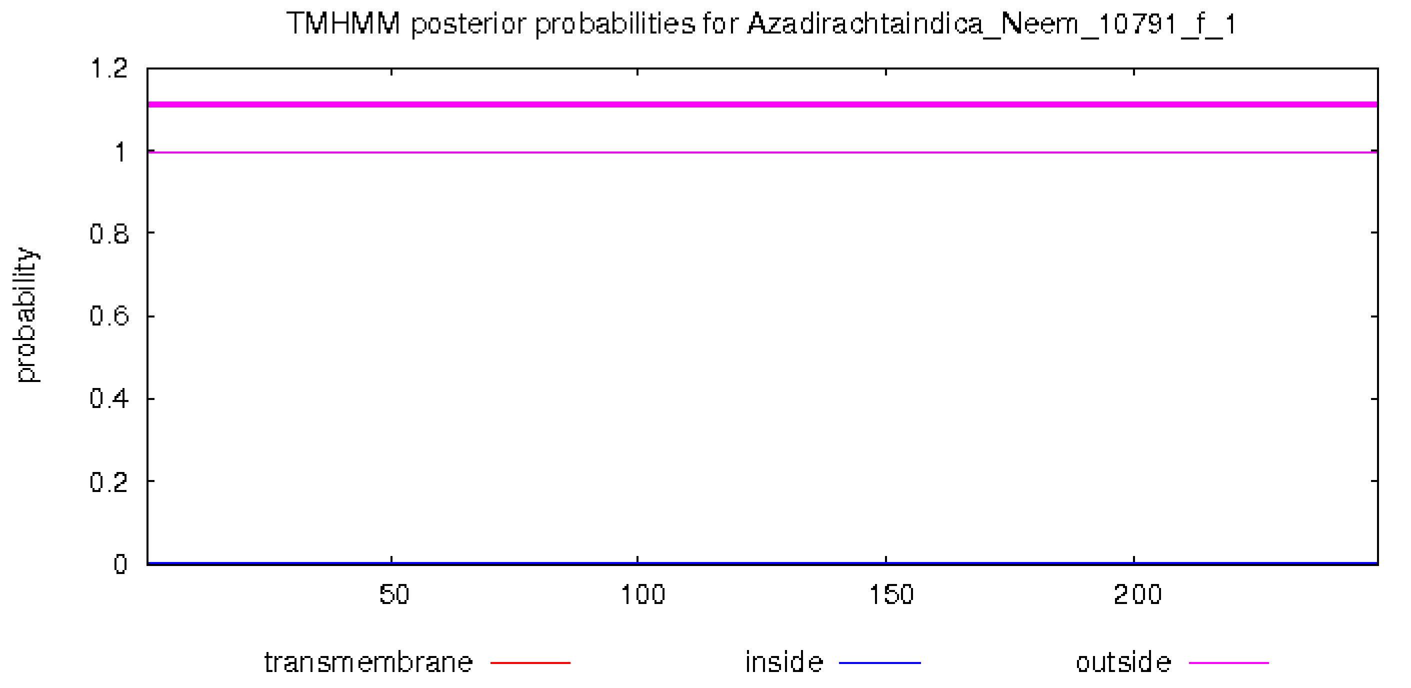

plot in postscript, script for making the plot in gnuplot, data for plot

```
# Azadirachtaindica_Neem_10795_f_2 Length: 379
# Azadirachtaindica_Neem_10795_f_2 Number of predicted TMHs:  0
# Azadirachtaindica_Neem_10795_f_2 Exp number of AAs in TMHs: 0.00295
# Azadirachtaindica_Neem_10795_f_2 Exp number, first 60 AAs:  0
# Azadirachtaindica_Neem_10795_f_2 Total prob of N-in:        0.01733
Azadirachtaindica_Neem_10795_f_2	TMHMM2.0	outside	     1   379
```

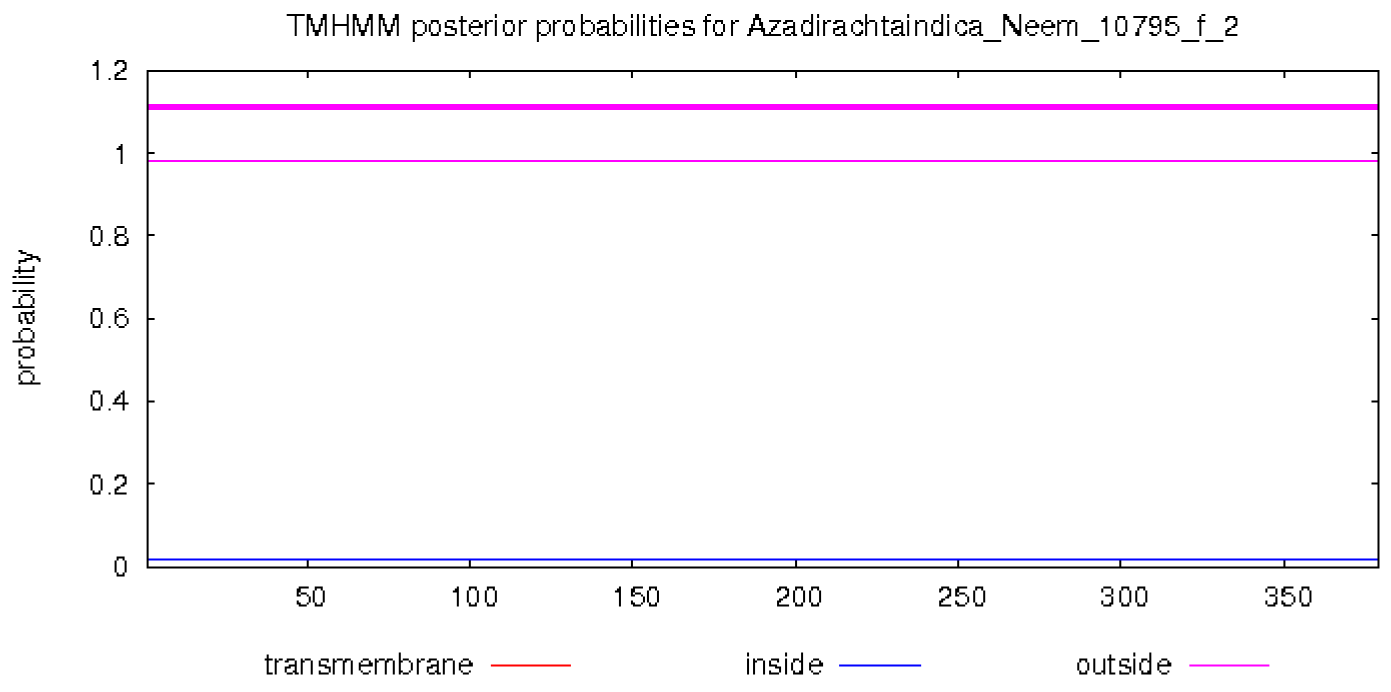

plot in postscript, script for making the plot in gnuplot, data for plot

```
# Azadirachtaindica_Neem_10879_f_1 Length: 268
# Azadirachtaindica_Neem_10879_f_1 Number of predicted TMHs:  0
# Azadirachtaindica_Neem_10879_f_1 Exp number of AAs in TMHs: 0.00507
# Azadirachtaindica_Neem_10879_f_1 Exp number, first 60 AAs:  9e-05
# Azadirachtaindica_Neem_10879_f_1 Total prob of N-in:        0.04752
Azadirachtaindica_Neem_10879_f_1	TMHMM2.0	outside	     1   268
```

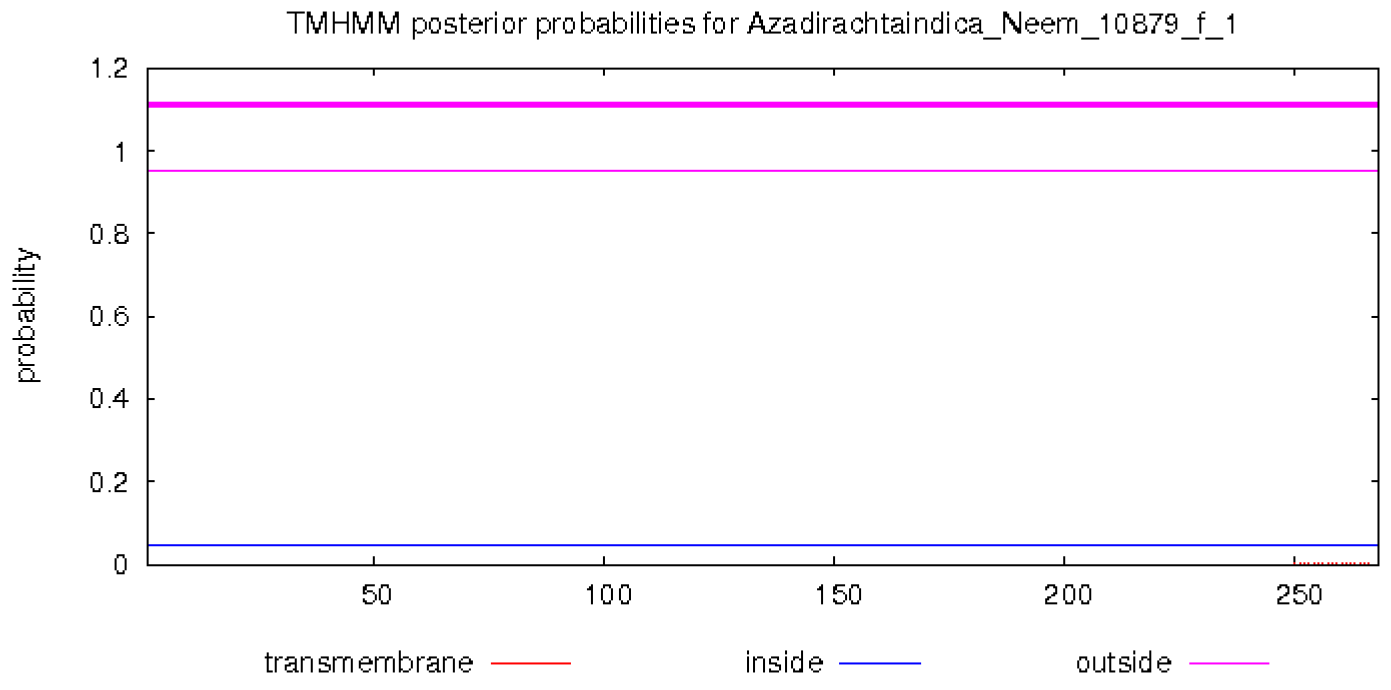

plot in postscript, script for making the plot in gnuplot, data for plot

---

```
# Azadirachtaindica_Neem_10938_f_1 Length: 365
# Azadirachtaindica_Neem_10938_f_1 Number of predicted TMHs:  0
# Azadirachtaindica_Neem_10938_f_1 Exp number of AAs in TMHs: 0.0254
# Azadirachtaindica_Neem_10938_f_1 Exp number, first 60 AAs:  0
# Azadirachtaindica_Neem_10938_f_1 Total prob of N-in:        0.00213
Azadirachtaindica_Neem_10938_f_1	TMHMM2.0	outside	     1   365
```

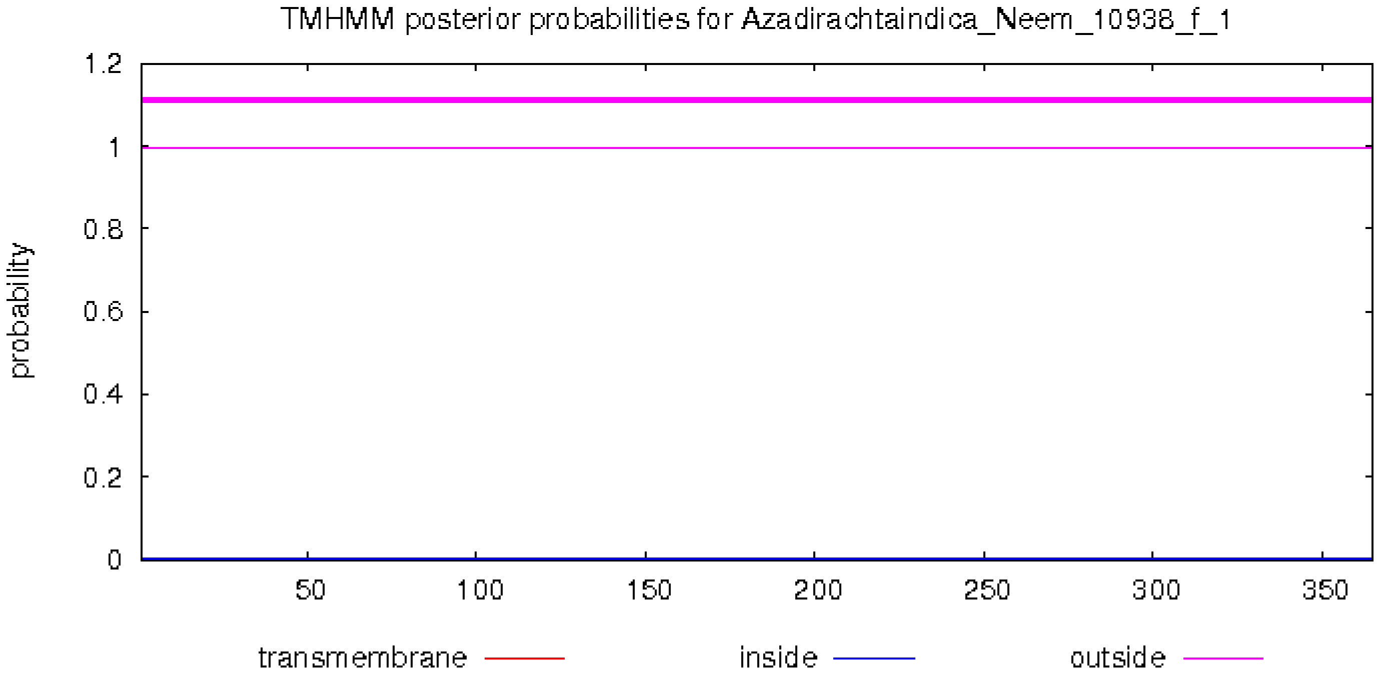

plot in postscript, script for making the plot in gnuplot, data for plot

---

```
# Azadirachtaindica_Neem_11412_f_1 Length: 271
# Azadirachtaindica_Neem_11412_f_1 Number of predicted TMHs:  0
# Azadirachtaindica_Neem_11412_f_1 Exp number of AAs in TMHs: 0
# Azadirachtaindica_Neem_11412_f_1 Exp number, first 60 AAs:  0
# Azadirachtaindica_Neem_11412_f_1 Total prob of N-in:        0.01228
Azadirachtaindica_Neem_11412_f_1	TMHMM2.0	outside	     1   271
```

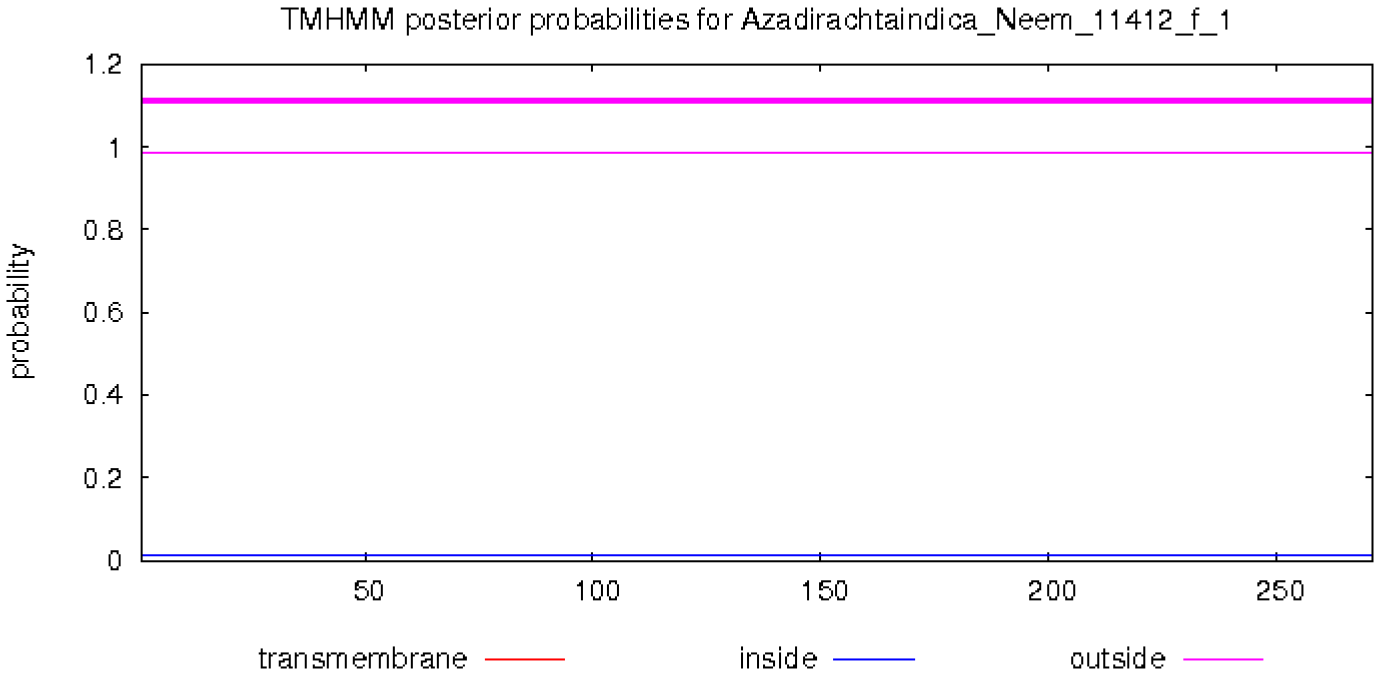

plot in postscript, script for making the plot in gnuplot, data for plot

---

```
# Azadirachtaindica_Neem_11486_f_1 Length: 221
# Azadirachtaindica_Neem_11486_f_1 Number of predicted TMHs:  0
# Azadirachtaindica_Neem_11486_f_1 Exp number of AAs in TMHs: 0.00173
# Azadirachtaindica_Neem_11486_f_1 Exp number, first 60 AAs:  0.00074
# Azadirachtaindica_Neem_11486_f_1 Total prob of N-in:        0.08692
Azadirachtaindica_Neem_11486_f_1	TMHMM2.0	outside	     1   221
```

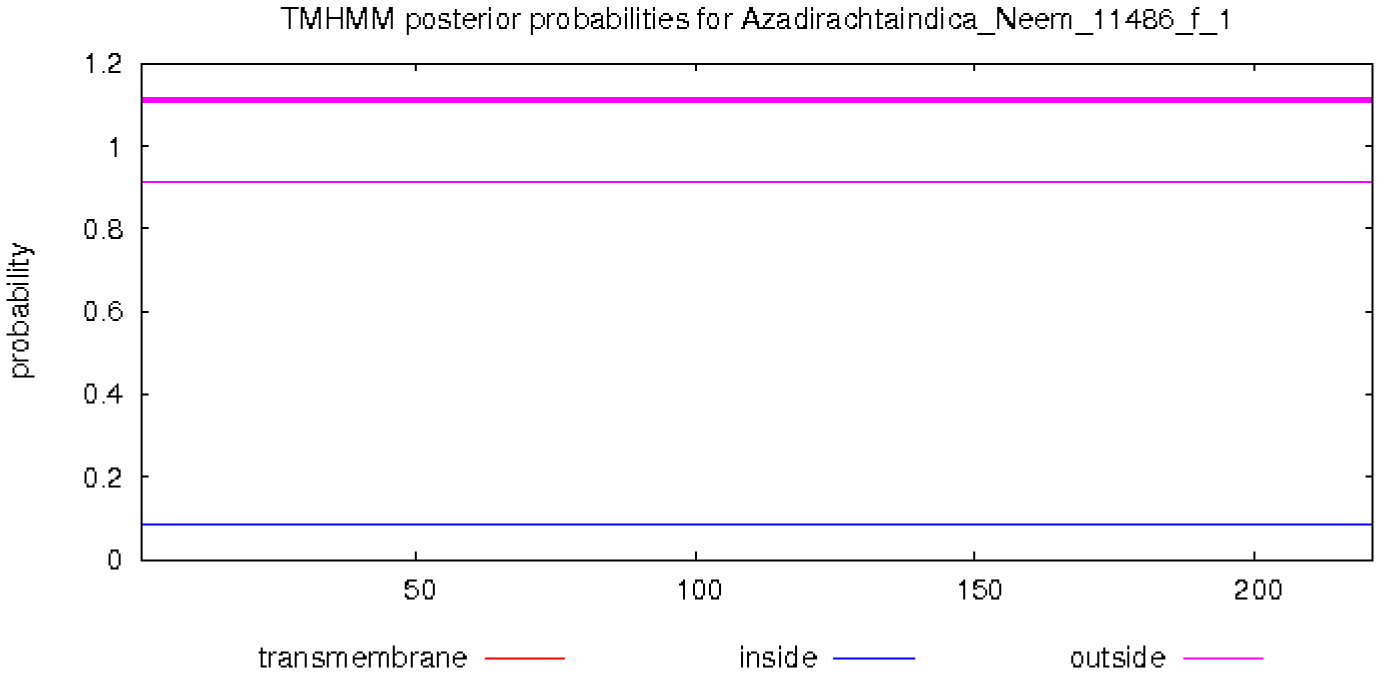

plot in postscript, script for making the plot in gnuplot, data for plot

---

```
# Azadirachtaindica_Neem_11567_f_1 Length: 431
# Azadirachtaindica_Neem_11567_f_1 Number of predicted TMHs:  0
# Azadirachtaindica_Neem_11567_f_1 Exp number of AAs in TMHs: 0.00047
# Azadirachtaindica_Neem_11567_f_1 Exp number, first 60 AAs:  0.00045
# Azadirachtaindica_Neem_11567_f_1 Total prob of N-in:        0.00111
Azadirachtaindica_Neem_11567_f_1	TMHMM2.0	outside	     1   431
```

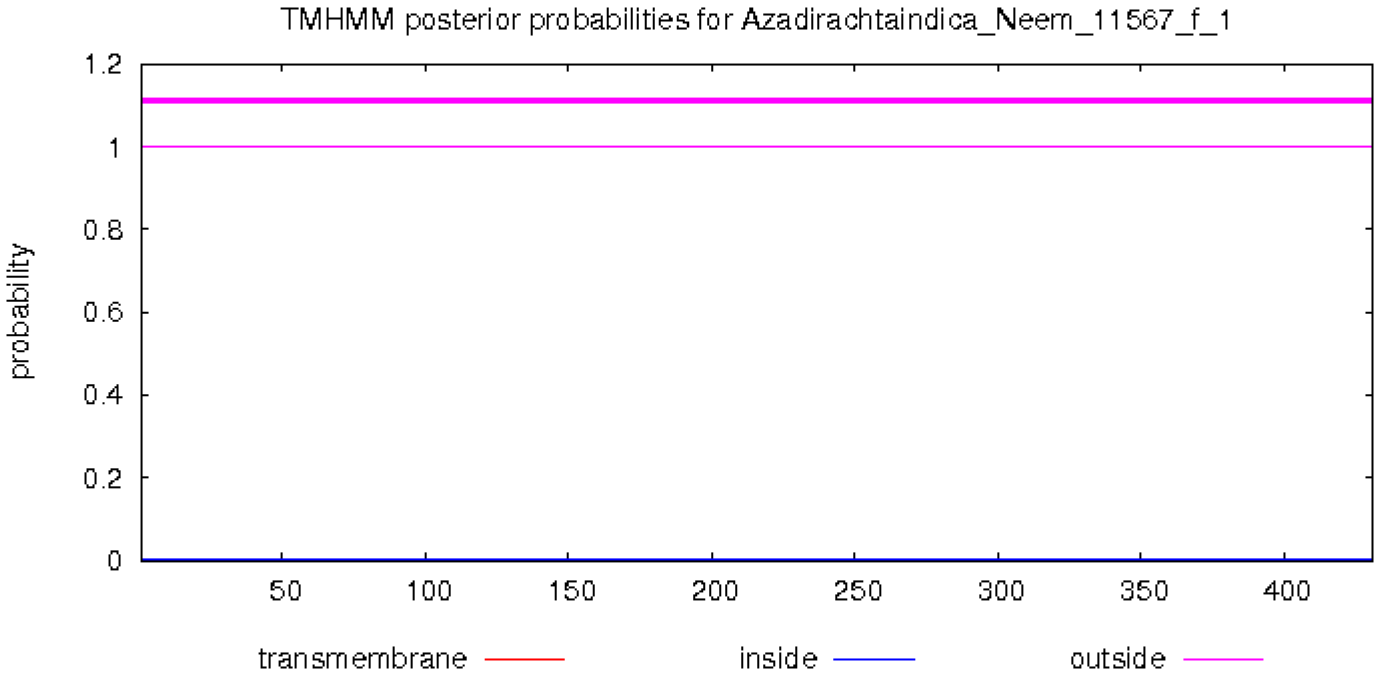

plot in postscript, script for making the plot in gnuplot, data for plot

---

```
# Azadirachtaindica_Neem_11643_f_3 Length: 298
# Azadirachtaindica_Neem_11643_f_3 Number of predicted TMHs:  0
# Azadirachtaindica_Neem_11643_f_3 Exp number of AAs in TMHs: 0.04441
# Azadirachtaindica_Neem_11643_f_3 Exp number, first 60 AAs:  0.00125
# Azadirachtaindica_Neem_11643_f_3 Total prob of N-in:        0.02866
Azadirachtaindica_Neem_11643_f_3        TMHMM2.0        outside      1   298
```

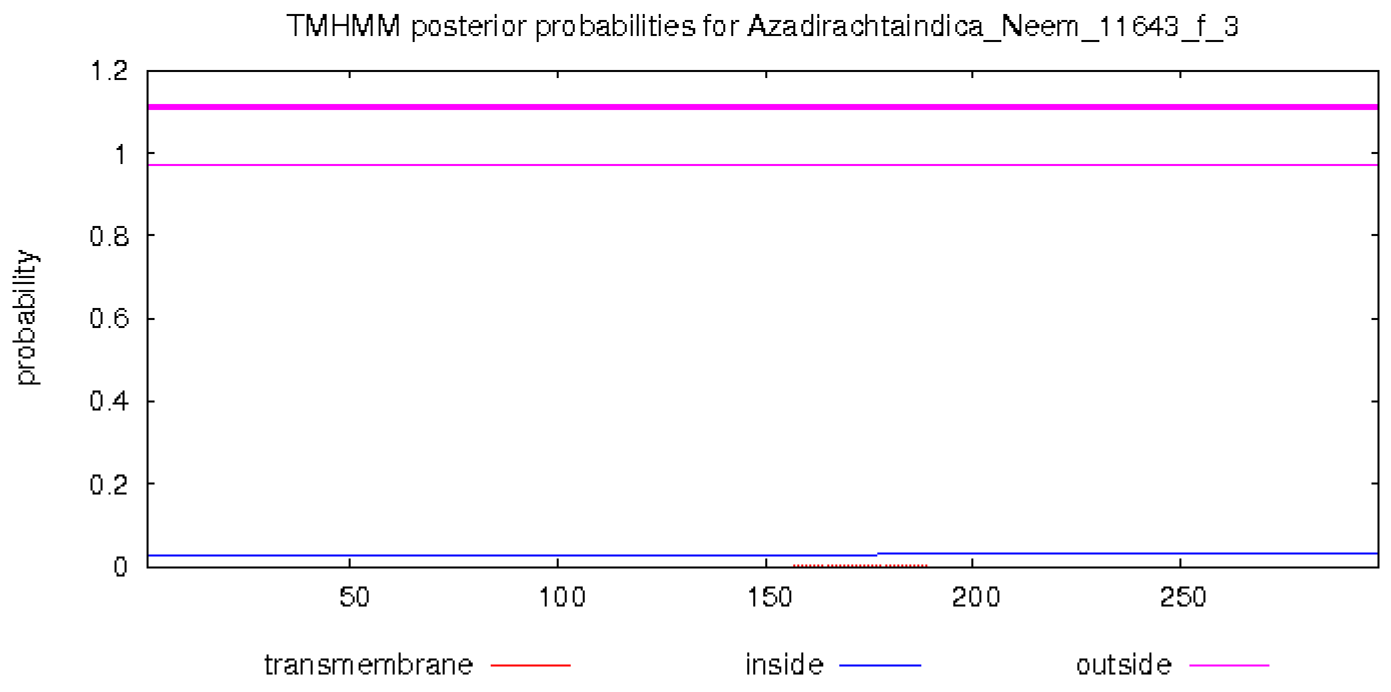

plot in postscript, script for making the plot in gnuplot, data for plot

---

```
# Azadirachtaindica_Neem_11660_f_5 Length: 384
# Azadirachtaindica_Neem_11660_f_5 Number of predicted TMHs:  0
# Azadirachtaindica_Neem_11660_f_5 Exp number of AAs in TMHs: 0.00082
# Azadirachtaindica_Neem_11660_f_5 Exp number, first 60 AAs:  0
# Azadirachtaindica_Neem_11660_f_5 Total prob of N-in:        0.02242
Azadirachtaindica_Neem_11660_f_5        TMHMM2.0        outside      1   384
```

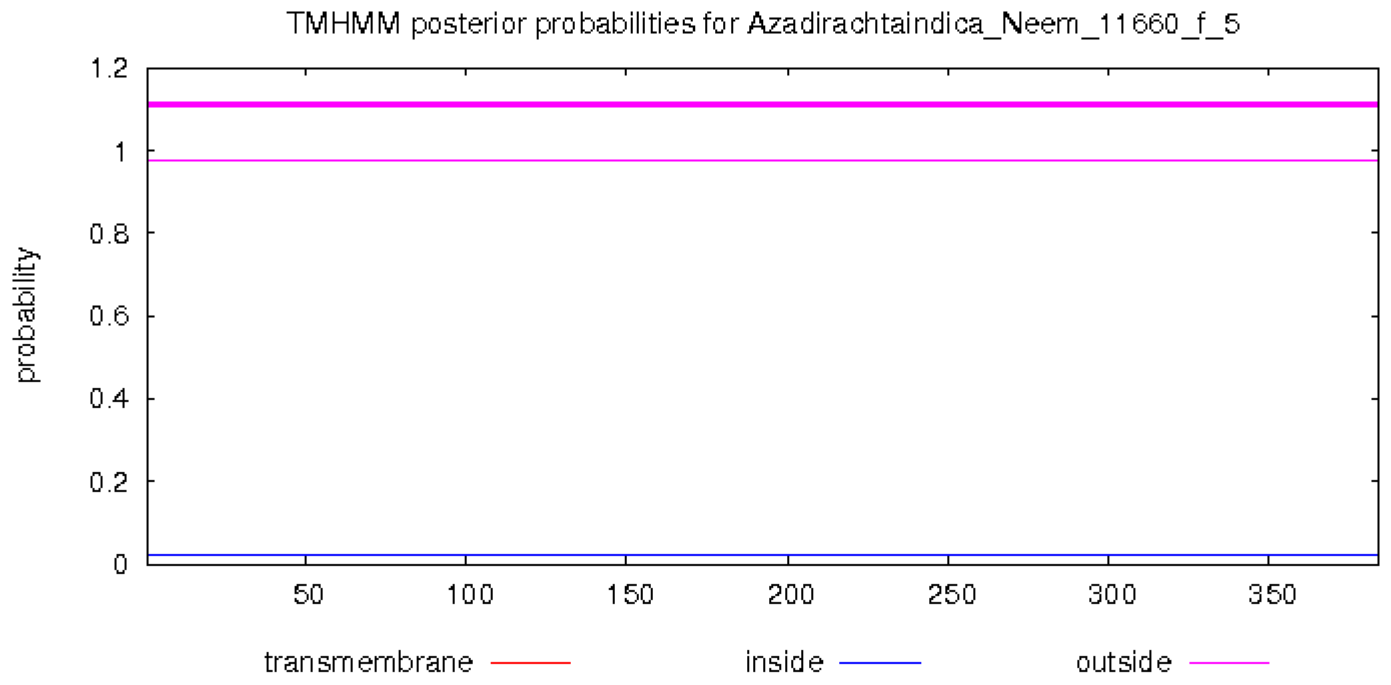

plot in postscript, script for making the plot in gnuplot, data for plot

---

```
# Azadirachtaindica_Neem_11713_f_2 Length: 604
# Azadirachtaindica_Neem_11713_f_2 Number of predicted TMHs:  1
# Azadirachtaindica_Neem_11713_f_2 Exp number of AAs in TMHs: 21.17329
# Azadirachtaindica_Neem_11713_f_2 Exp number, first 60 AAs:  0
# Azadirachtaindica_Neem_11713_f_2 Total prob of N-in:        0.00066
Azadirachtaindica_Neem_11713_f_2        TMHMM2.0        outside      1   574
Azadirachtaindica_Neem_11713_f_2        TMHMM2.0        TMhelix    575   597
Azadirachtaindica_Neem_11713_f_2        TMHMM2.0        inside     598   604
```

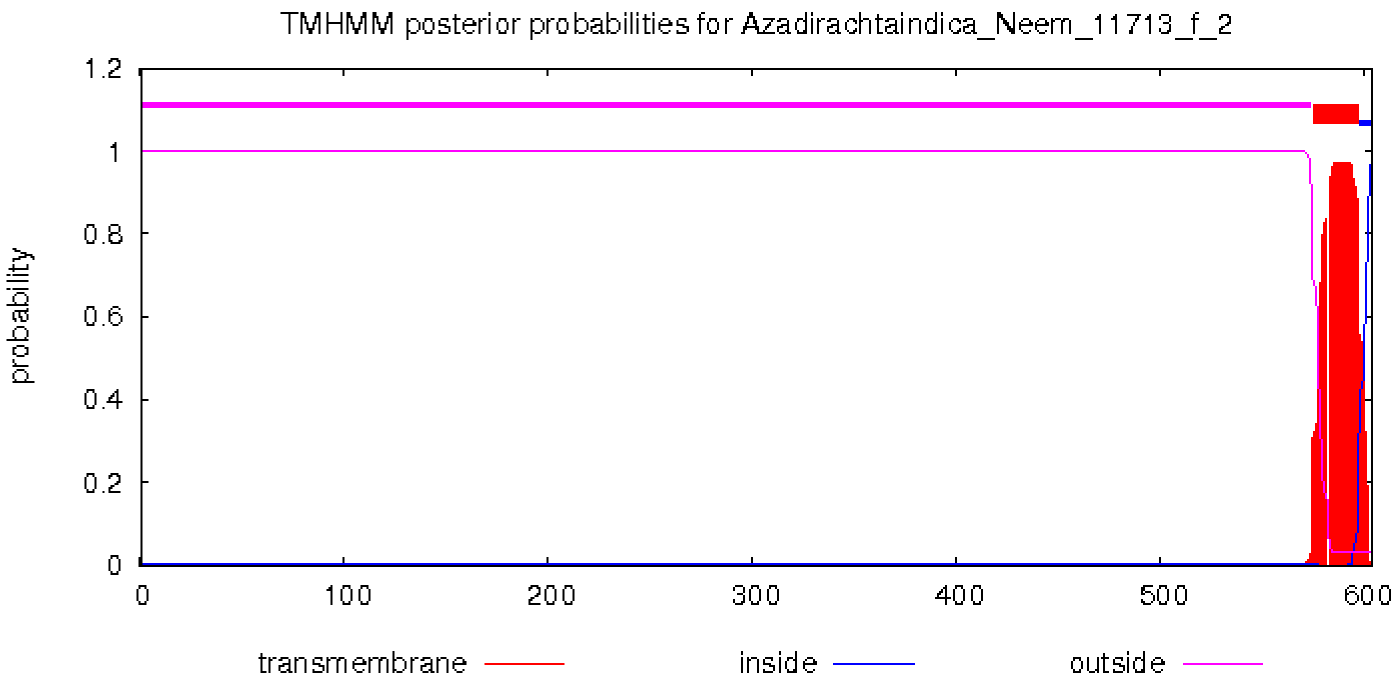

plot in postscript, script for making the plot in gnuplot, data for plot

---

```
# Azadirachtaindica_Neem_11719_f_1 Length: 164
# Azadirachtaindica_Neem_11719_f_1 Number of predicted TMHs:  0
# Azadirachtaindica_Neem_11719_f_1 Exp number of AAs in TMHs: 0.00054
# Azadirachtaindica_Neem_11719_f_1 Exp number, first 60 AAs:  0.00032
# Azadirachtaindica_Neem_11719_f_1 Total prob of N-in:        0.24484
Azadirachtaindica_Neem_11719_f_1        TMHMM2.0        outside      1   164
```

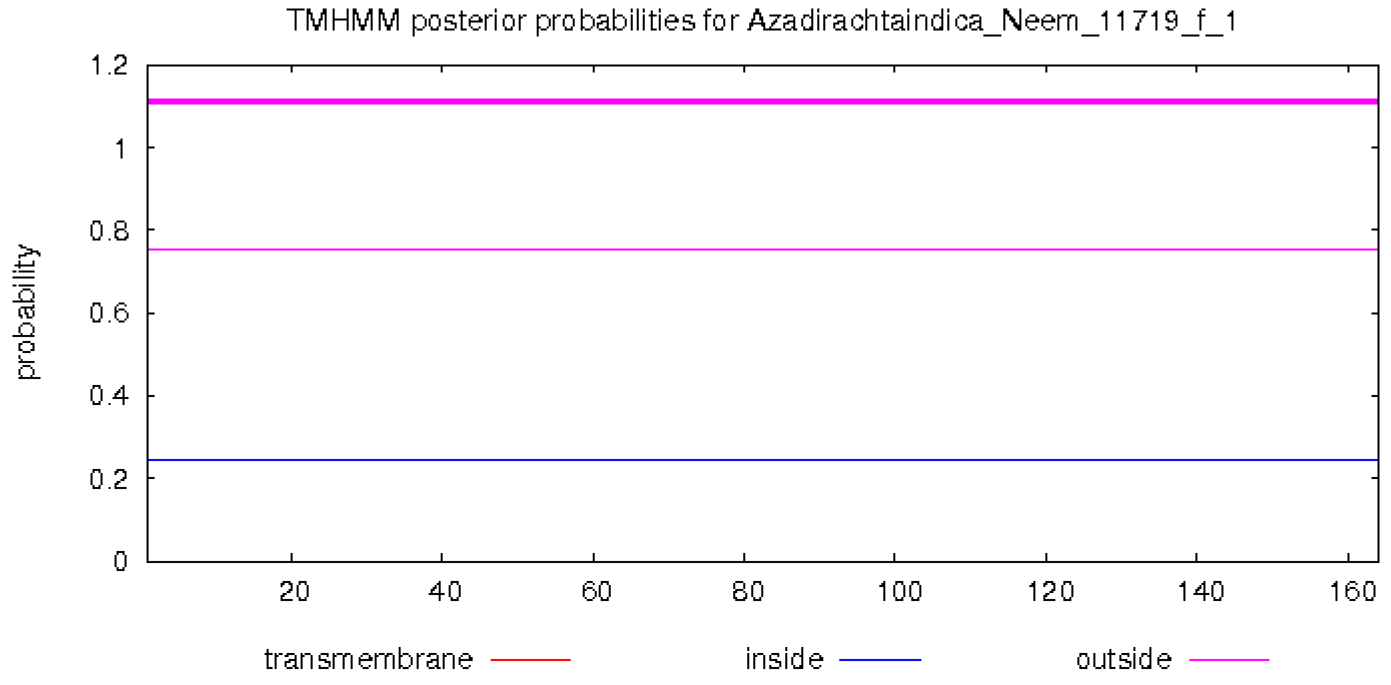

plot in postscript, script for making the plot in gnuplot, data for plot

---

```
# Azadirachtaindica_Neem_12089_f_2 Length: 297
# Azadirachtaindica_Neem_12089_f_2 Number of predicted TMHs:  0
# Azadirachtaindica_Neem_12089_f_2 Exp number of AAs in TMHs: 0.0004
# Azadirachtaindica_Neem_12089_f_2 Exp number, first 60 AAs:  0
# Azadirachtaindica_Neem_12089_f_2 Total prob of N-in:        0.08326
Azadirachtaindica_Neem_12089_f_2        TMHMM2.0        outside      1   297
```

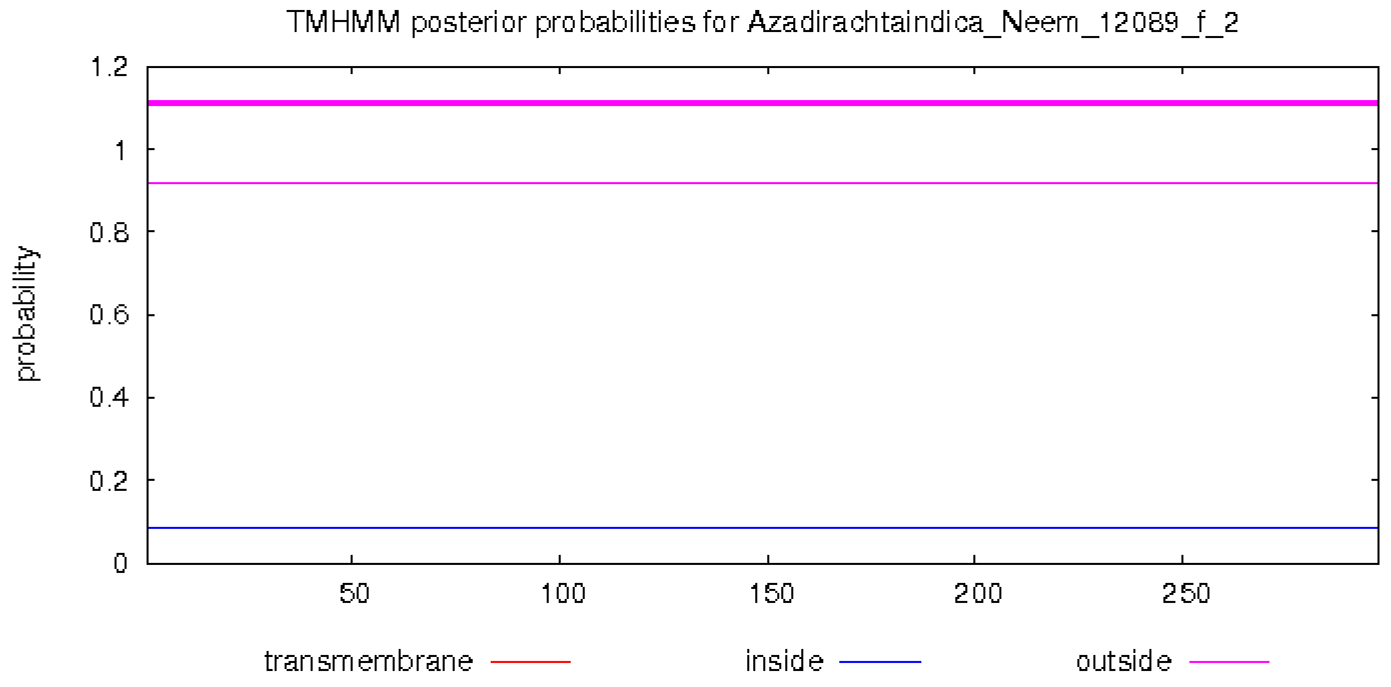

plot in postscript, script for making the plot in gnuplot, data for plot

---

```
# Azadirachtaindica_Neem_12411_f_2 Length: 328
# Azadirachtaindica_Neem_12411_f_2 Number of predicted TMHs:  0
# Azadirachtaindica_Neem_12411_f_2 Exp number of AAs in TMHs: 0
# Azadirachtaindica_Neem_12411_f_2 Exp number, first 60 AAs:  0
# Azadirachtaindica_Neem_12411_f_2 Total prob of N-in:        0.01993
Azadirachtaindica_Neem_12411_f_2        TMHMM2.0        outside      1   328
```

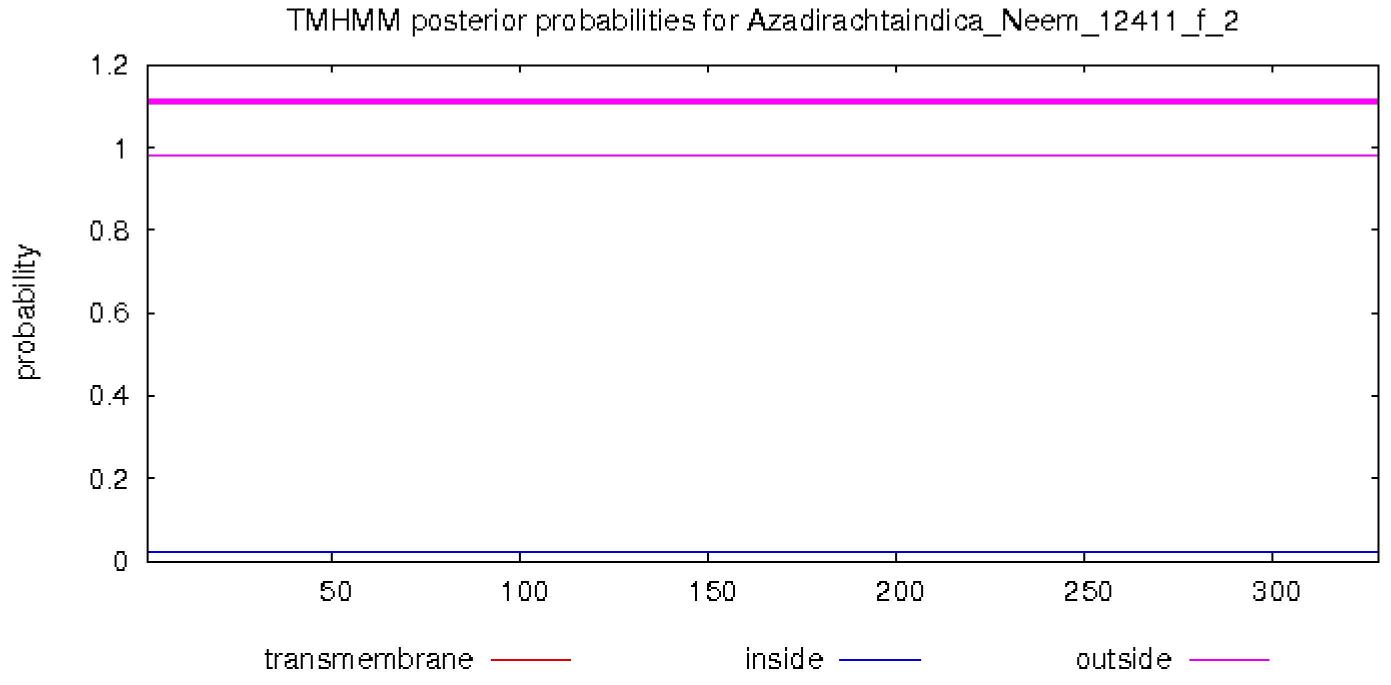

plot in postscript, script for making the plot in gnuplot, data for plot

---

```
# Azadirachtaindica_Neem_12411_f_3 Length: 283
# Azadirachtaindica_Neem_12411_f_3 Number of predicted TMHs:  0
# Azadirachtaindica_Neem_12411_f_3 Exp number of AAs in TMHs: 0
# Azadirachtaindica_Neem_12411_f_3 Exp number, first 60 AAs:  0
# Azadirachtaindica_Neem_12411_f_3 Total prob of N-in:        0.03959
Azadirachtaindica_Neem_12411_f_3        TMHMM2.0        outside      1   283
```

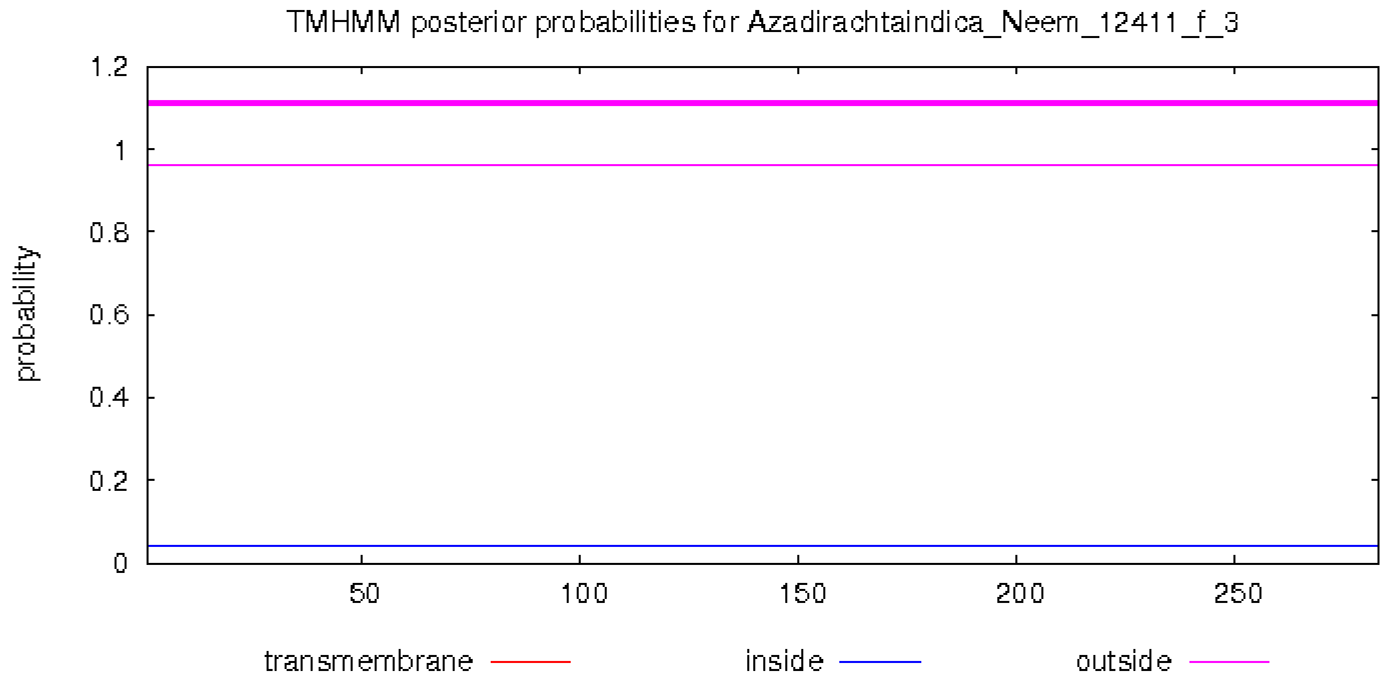

plot in postscript, script for making the plot in gnuplot, data for plot

---

```
# Azadirachtaindica_Neem_12571_f_1 Length: 301
# Azadirachtaindica_Neem_12571_f_1 Number of predicted TMHs:  0
# Azadirachtaindica_Neem_12571_f_1 Exp number of AAs in TMHs: 0.00017
# Azadirachtaindica_Neem_12571_f_1 Exp number, first 60 AAs:  0
# Azadirachtaindica_Neem_12571_f_1 Total prob of N-in:        0.02775
Azadirachtaindica_Neem_12571_f_1        TMHMM2.0        outside      1   301
```

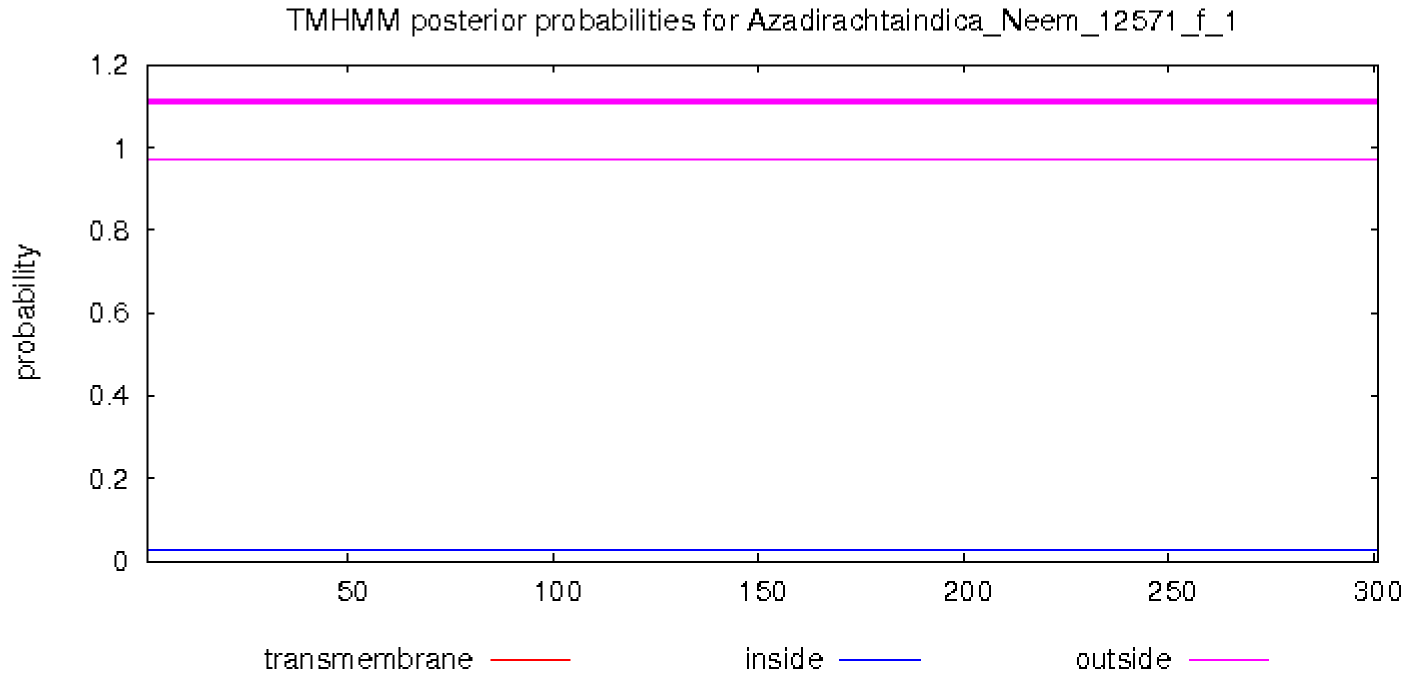

plot in postscript, script for making the plot in gnuplot, data for plot

---

```
# Azadirachtaindica_Neem_12632_f_1 Length: 156
# Azadirachtaindica_Neem_12632_f_1 Number of predicted TMHs:  0
# Azadirachtaindica_Neem_12632_f_1 Exp number of AAs in TMHs: 0
# Azadirachtaindica_Neem_12632_f_1 Exp number, first 60 AAs:  0
# Azadirachtaindica_Neem_12632_f_1 Total prob of N-in:        0.01471
Azadirachtaindica_Neem_12632_f_1        TMHMM2.0        outside      1   156
```

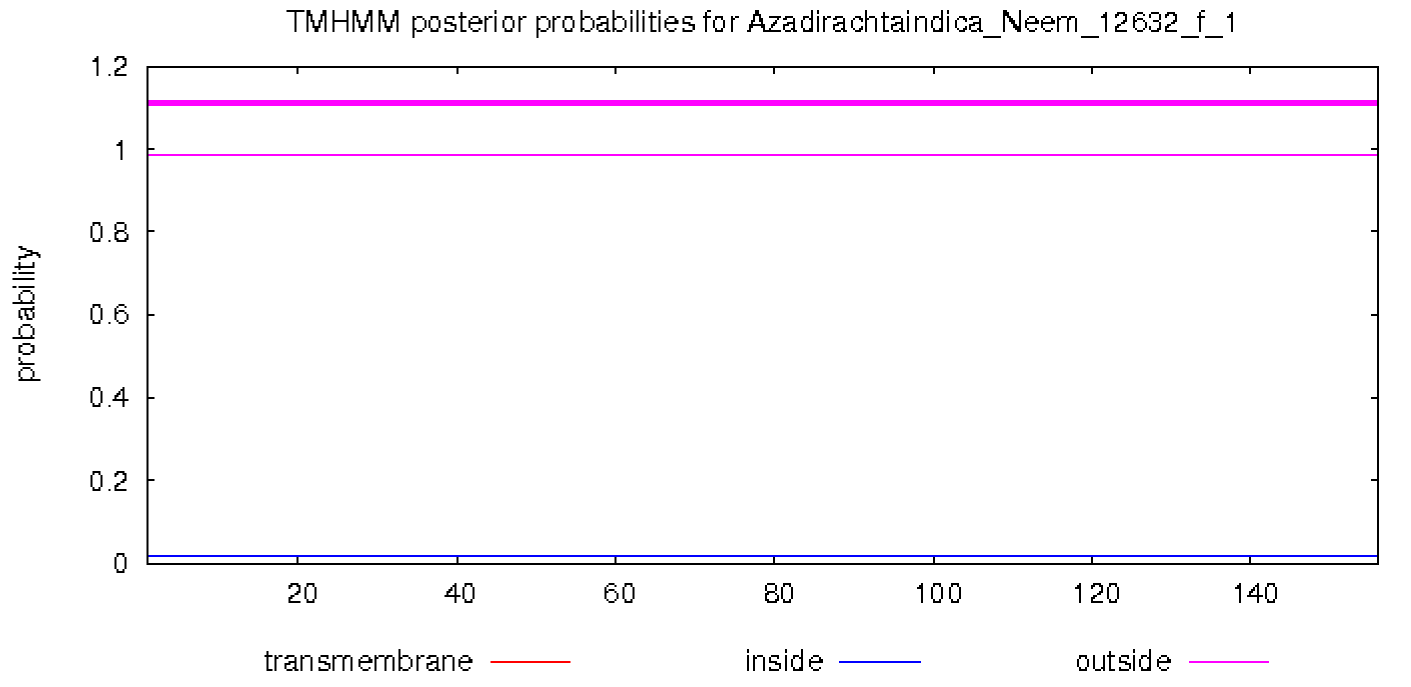

plot in postscript, script for making the plot in gnuplot, data for plot

---

```
# Azadirachtaindica_Neem_12730_f_1 Length: 341
# Azadirachtaindica_Neem_12730_f_1 Number of predicted TMHs:  0
# Azadirachtaindica_Neem_12730_f_1 Exp number of AAs in TMHs: 0
# Azadirachtaindica_Neem_12730_f_1 Exp number, first 60 AAs:  0
# Azadirachtaindica_Neem_12730_f_1 Total prob of N-in:        0.00074
Azadirachtaindica_Neem_12730_f_1        TMHMM2.0        outside      1   341
```

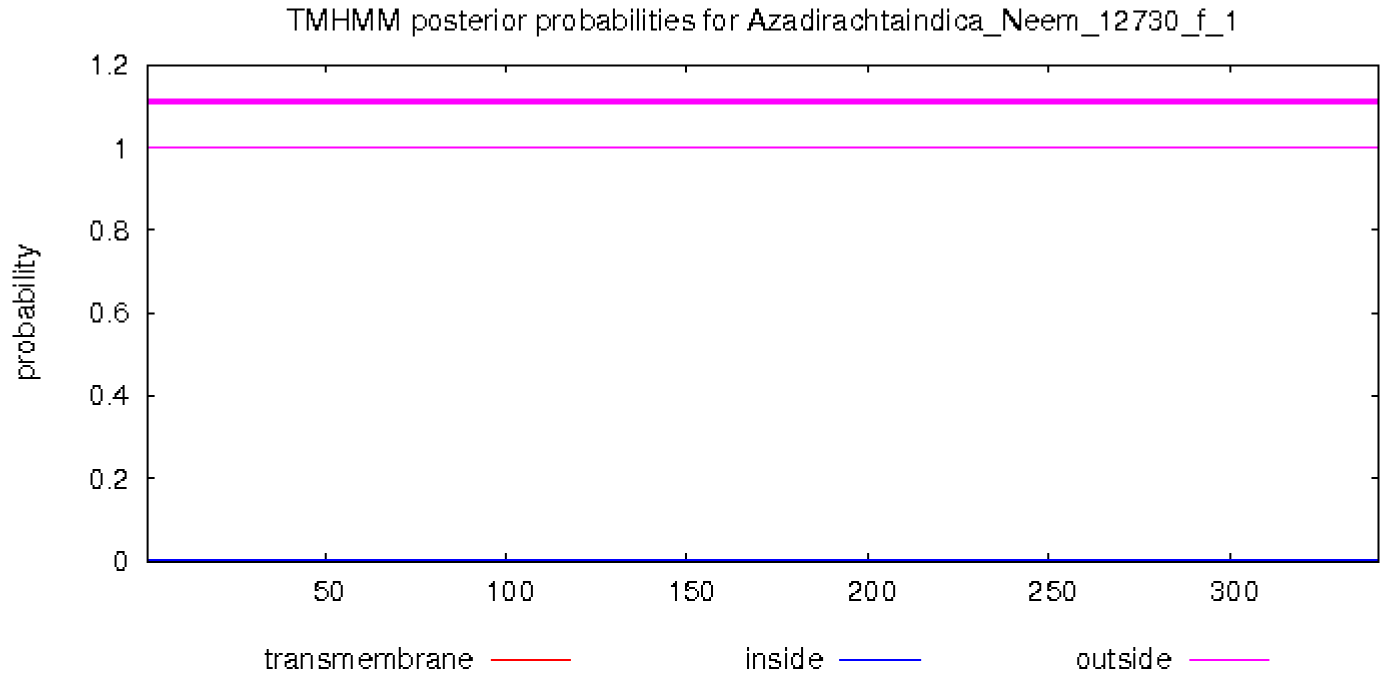

plot in postscript, script for making the plot in gnuplot, data for plot

---

```
# Azadirachtaindica_Neem_12856_f_1 Length: 355
# Azadirachtaindica_Neem_12856_f_1 Number of predicted TMHs:  0
# Azadirachtaindica_Neem_12856_f_1 Exp number of AAs in TMHs: 0.00021
# Azadirachtaindica_Neem_12856_f_1 Exp number, first 60 AAs:  0
# Azadirachtaindica_Neem_12856_f_1 Total prob of N-in:        0.01512
Azadirachtaindica_Neem_12856_f_1        TMHMM2.0        outside      1   355
```

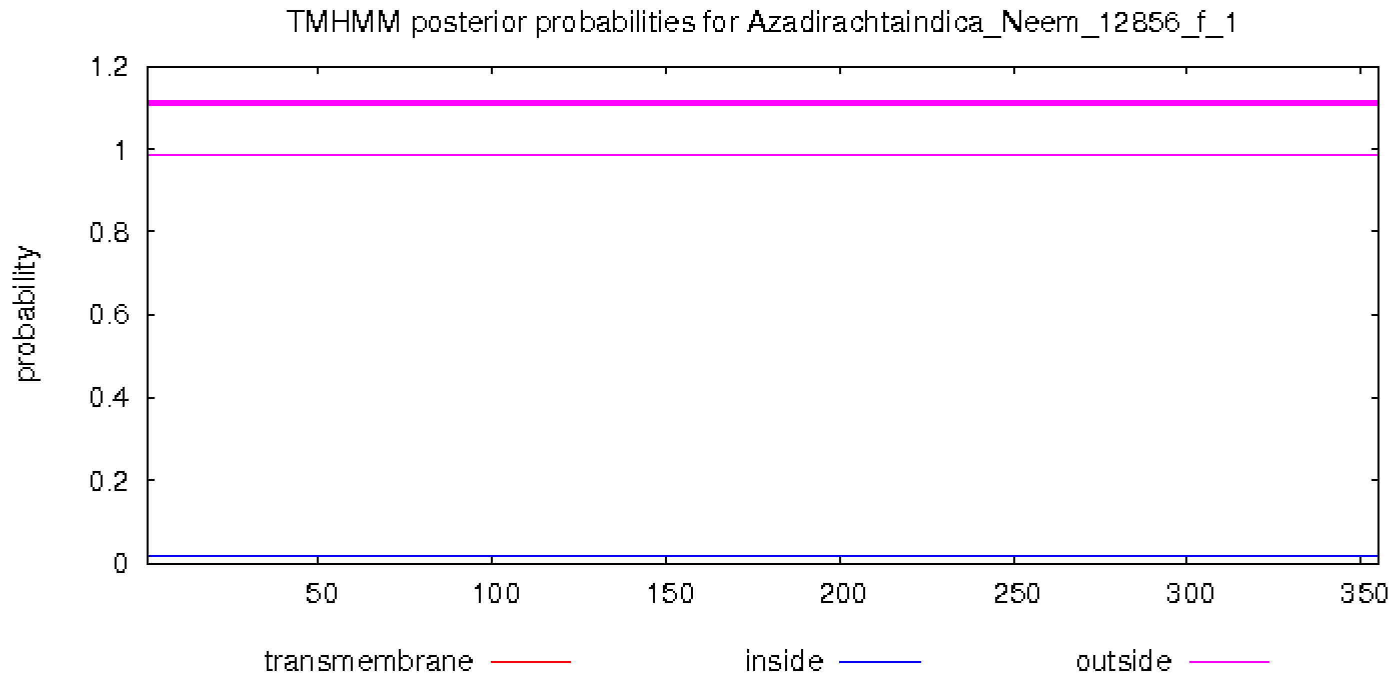

plot in postscript, script for making the plot in gnuplot, data for plot

---

```
# Azadirachtaindica_Neem_1311_f_4 Length: 363
# Azadirachtaindica_Neem_1311_f_4 Number of predicted TMHs:  0
# Azadirachtaindica_Neem_1311_f_4 Exp number of AAs in TMHs: 0.02425
# Azadirachtaindica_Neem_1311_f_4 Exp number, first 60 AAs:  0.00041
# Azadirachtaindica_Neem_1311_f_4 Total prob of N-in:        0.01797
Azadirachtaindica_Neem_1311_f_4 TMHMM2.0        outside      1   363
```

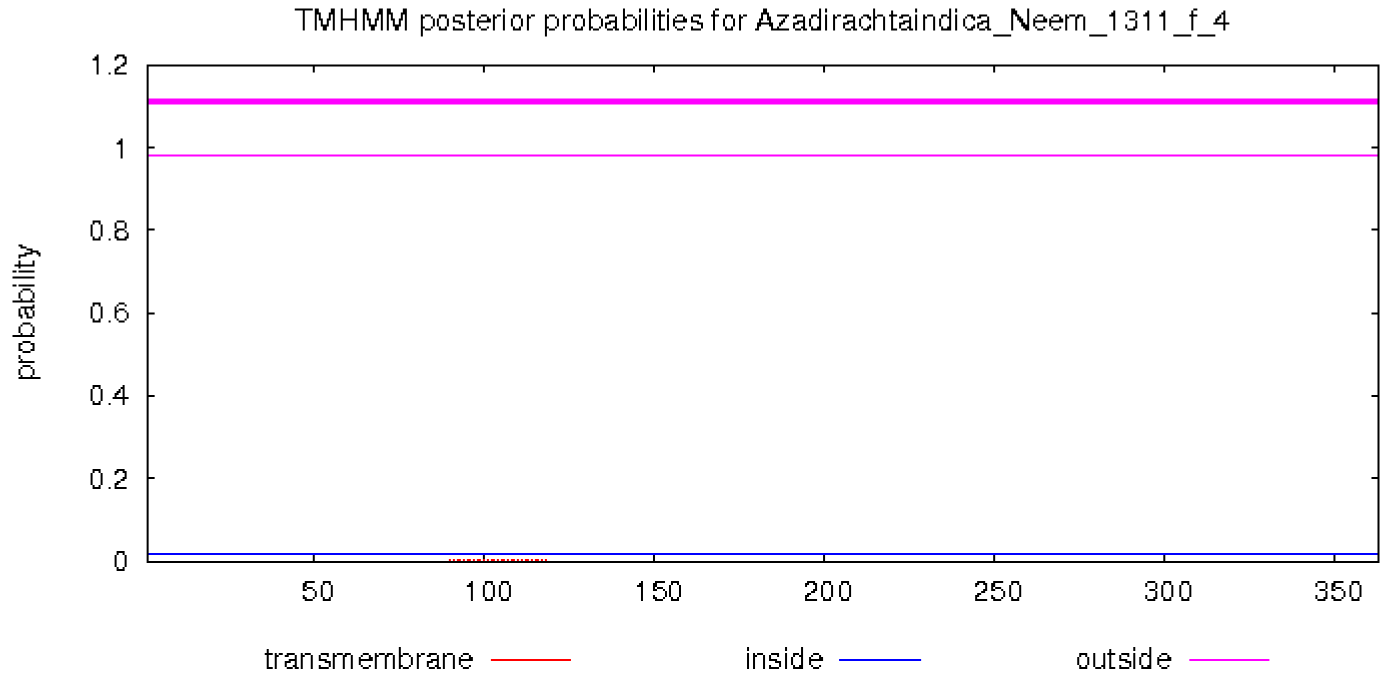

plot in postscript, script for making the plot in gnuplot, data for plot

---

```
# Azadirachtaindica_Neem_1368_f_4 Length: 295
# Azadirachtaindica_Neem_1368_f_4 Number of predicted TMHs:  0
# Azadirachtaindica_Neem_1368_f_4 Exp number of AAs in TMHs: 0
# Azadirachtaindica_Neem_1368_f_4 Exp number, first 60 AAs:  0
# Azadirachtaindica_Neem_1368_f_4 Total prob of N-in:        0.00468
Azadirachtaindica_Neem_1368_f_4 TMHMM2.0        outside      1   295
```

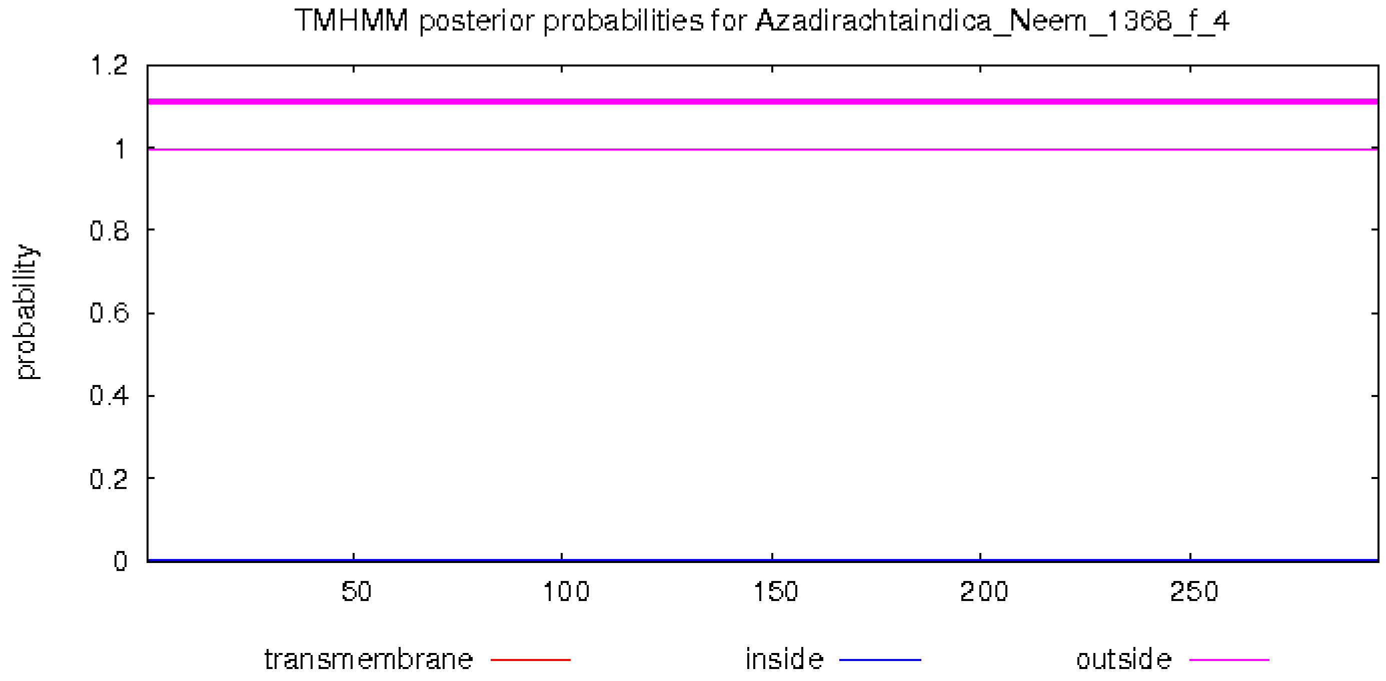

plot in postscript, script for making the plot in gnuplot, data for plot

---

```
# Azadirachtaindica_Neem_1375_f_2 Length: 201
# Azadirachtaindica_Neem_1375_f_2 Number of predicted TMHs:  0
# Azadirachtaindica_Neem_1375_f_2 Exp number of AAs in TMHs: 0
# Azadirachtaindica_Neem_1375_f_2 Exp number, first 60 AAs:  0
# Azadirachtaindica_Neem_1375_f_2 Total prob of N-in:        0.01731
Azadirachtaindica_Neem_1375_f_2 TMHMM2.0        outside      1   201
```

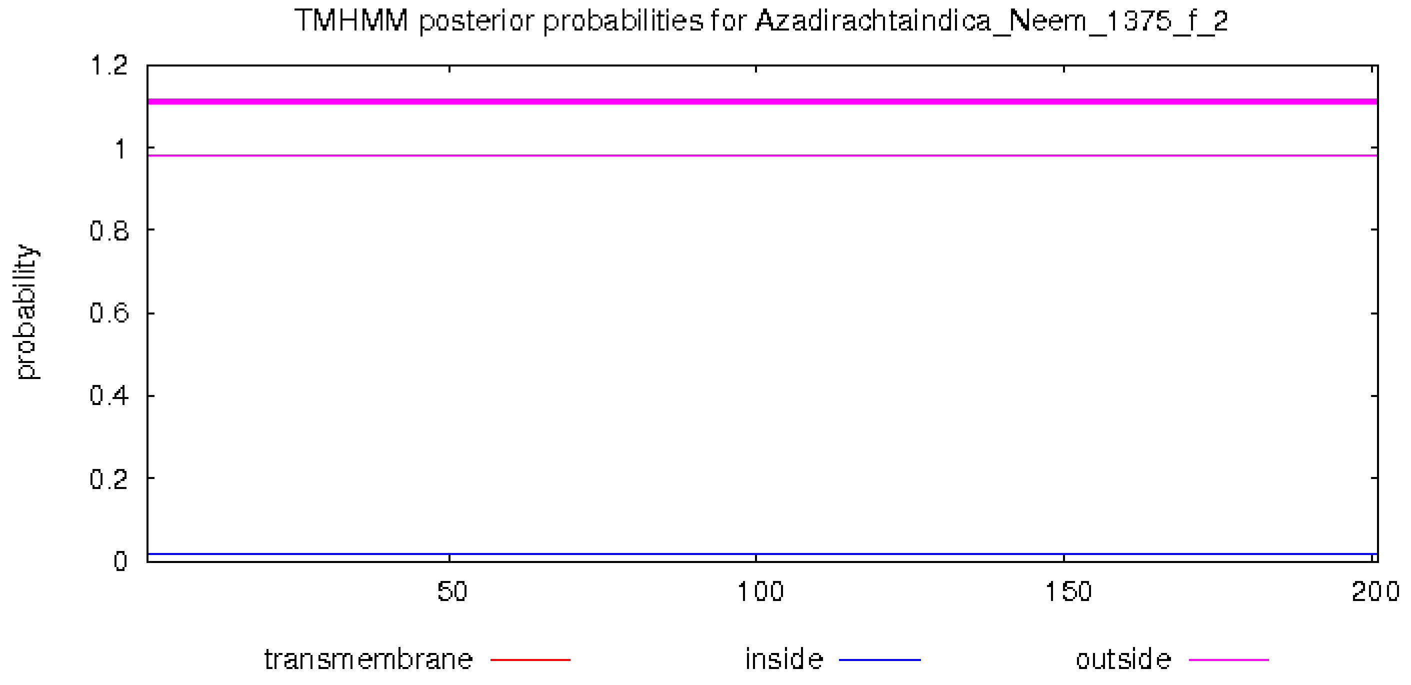

plot in postscript, script for making the plot in gnuplot, data for plot

---

```
# Azadirachtaindica_Neem_13837_f_1 Length: 123
# Azadirachtaindica_Neem_13837_f_1 Number of predicted TMHs:  0
# Azadirachtaindica_Neem_13837_f_1 Exp number of AAs in TMHs: 0
# Azadirachtaindica_Neem_13837_f_1 Exp number, first 60 AAs:  0
# Azadirachtaindica_Neem_13837_f_1 Total prob of N-in:        0.44292
Azadirachtaindica_Neem_13837_f_1        TMHMM2.0        outside      1   123
```

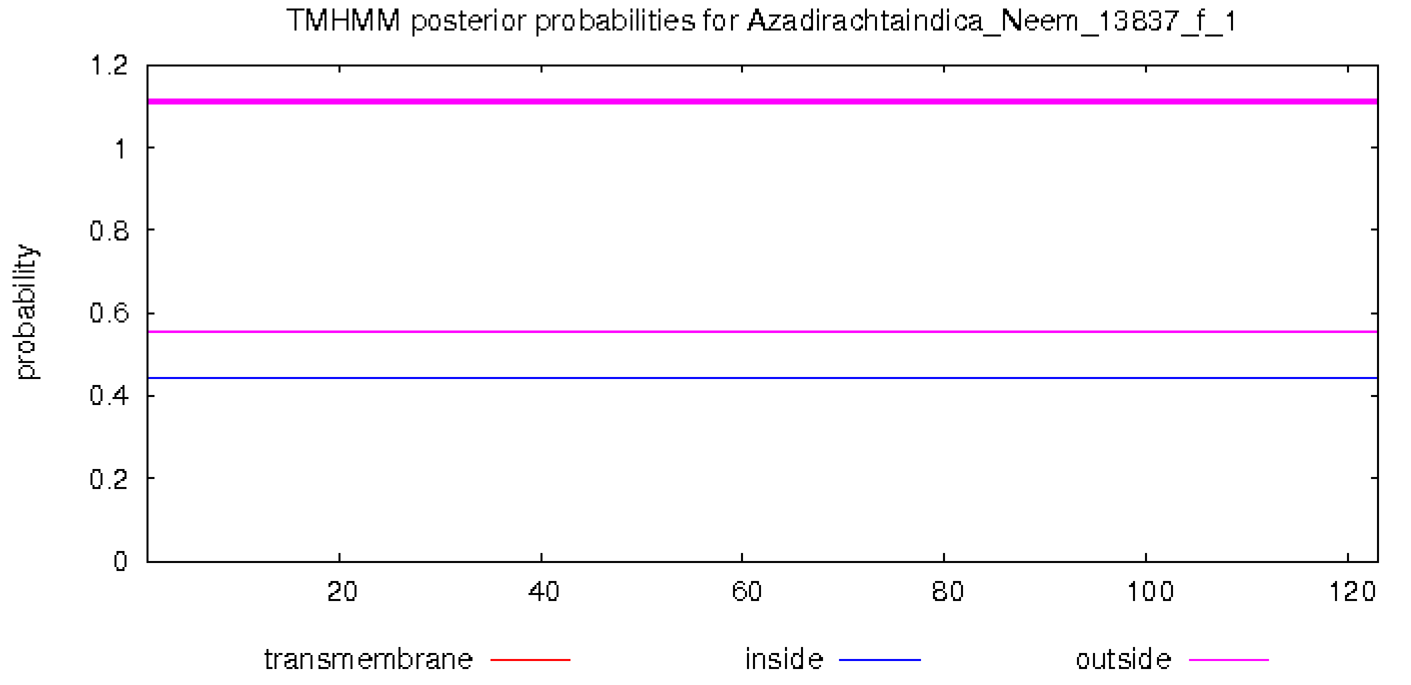

plot in postscript, script for making the plot in gnuplot, data for plot

---

```
# Azadirachtaindica_Neem_13860_f_1 Length: 340
# Azadirachtaindica_Neem_13860_f_1 Number of predicted TMHs:  0
# Azadirachtaindica_Neem_13860_f_1 Exp number of AAs in TMHs: 0.44028
# Azadirachtaindica_Neem_13860_f_1 Exp number, first 60 AAs:  0.00718
# Azadirachtaindica_Neem_13860_f_1 Total prob of N-in:        0.01680
Azadirachtaindica_Neem_13860_f_1        TMHMM2.0        outside      1   340
```

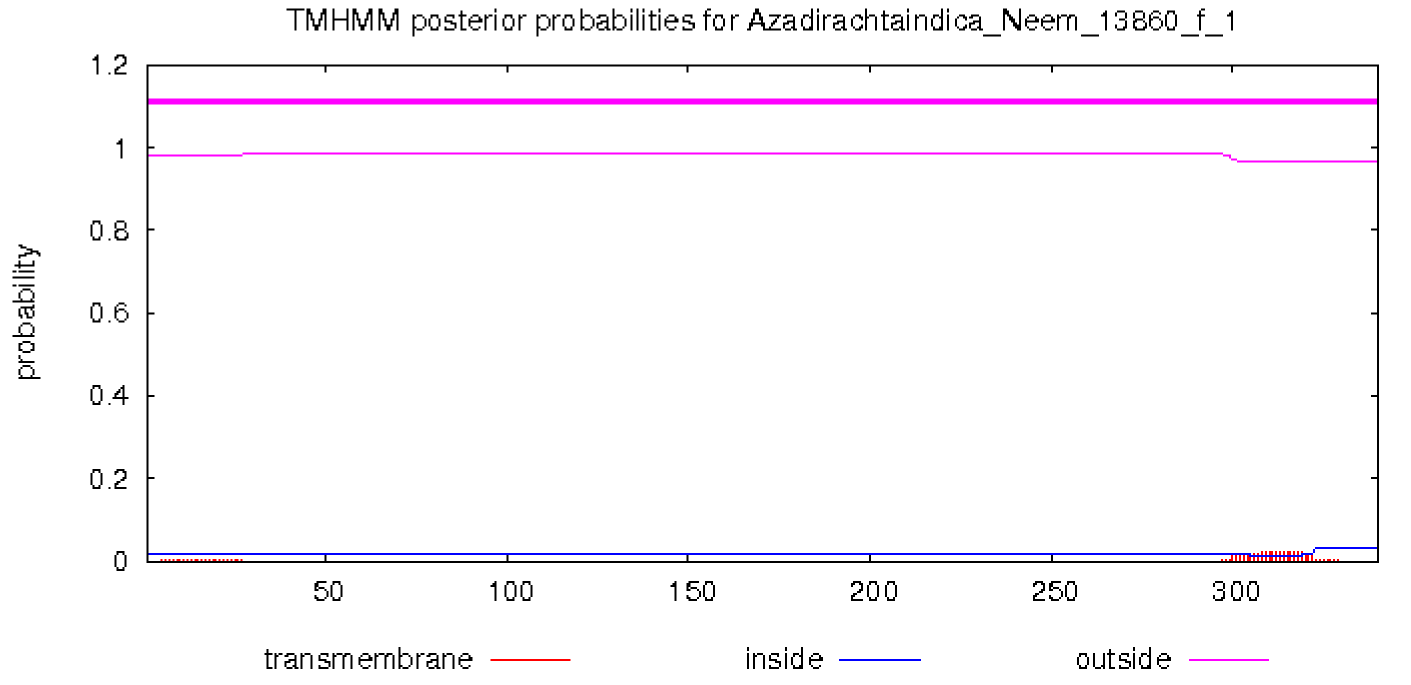

plot in postscript, script for making the plot in gnuplot, data for plot

---

```
# Azadirachtaindica_Neem_13873_f_2 Length: 303
# Azadirachtaindica_Neem_13873_f_2 Number of predicted TMHs:  0
# Azadirachtaindica_Neem_13873_f_2 Exp number of AAs in TMHs: 0.00137
# Azadirachtaindica_Neem_13873_f_2 Exp number, first 60 AAs:  0
# Azadirachtaindica_Neem_13873_f_2 Total prob of N-in:        0.02974
Azadirachtaindica_Neem_13873_f_2        TMHMM2.0        outside      1   303
```

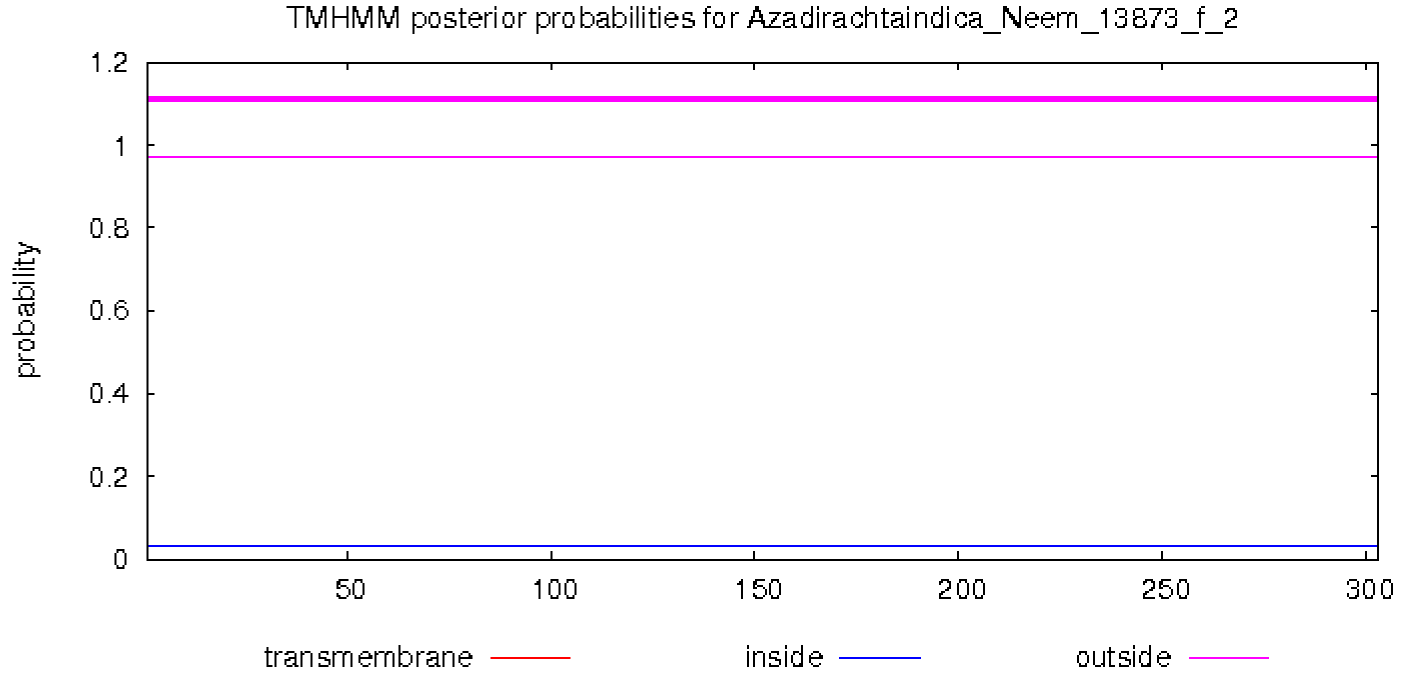

plot in postscript, script for making the plot in gnuplot, data for plot

---

```
# Azadirachtaindica_Neem_14406_f_1 Length: 323
# Azadirachtaindica_Neem_14406_f_1 Number of predicted TMHs:  0
# Azadirachtaindica_Neem_14406_f_1 Exp number of AAs in TMHs: 0
# Azadirachtaindica_Neem_14406_f_1 Exp number, first 60 AAs:  0
# Azadirachtaindica_Neem_14406_f_1 Total prob of N-in:        0.00431
Azadirachtaindica_Neem_14406_f_1        TMHMM2.0        outside      1   323
```

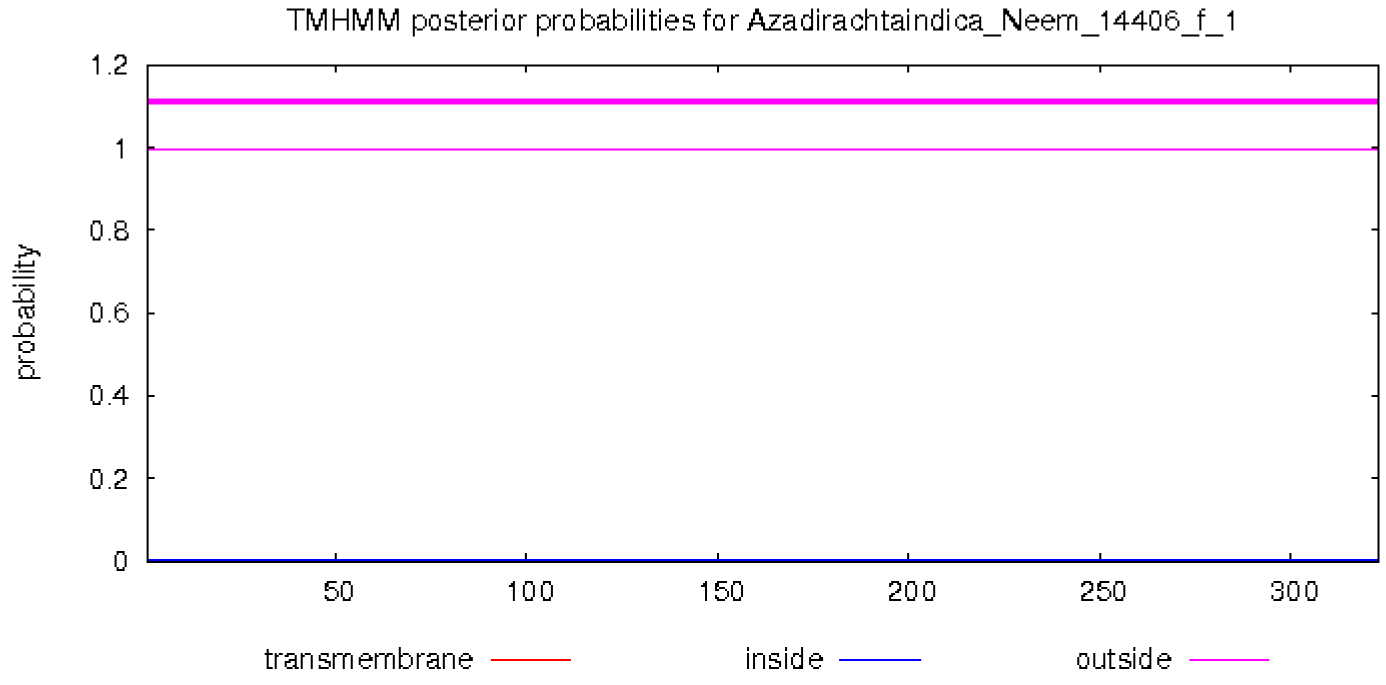

plot in postscript, script for making the plot in gnuplot, data for plot

---

```
# Azadirachtaindica_Neem_14495_f_1 Length: 391
# Azadirachtaindica_Neem_14495_f_1 Number of predicted TMHs:  0
# Azadirachtaindica_Neem_14495_f_1 Exp number of AAs in TMHs: 0
# Azadirachtaindica_Neem_14495_f_1 Exp number, first 60 AAs:  0
# Azadirachtaindica_Neem_14495_f_1 Total prob of N-in:        0.00752
Azadirachtaindica_Neem_14495_f_1        TMHMM2.0        outside      1   391
```

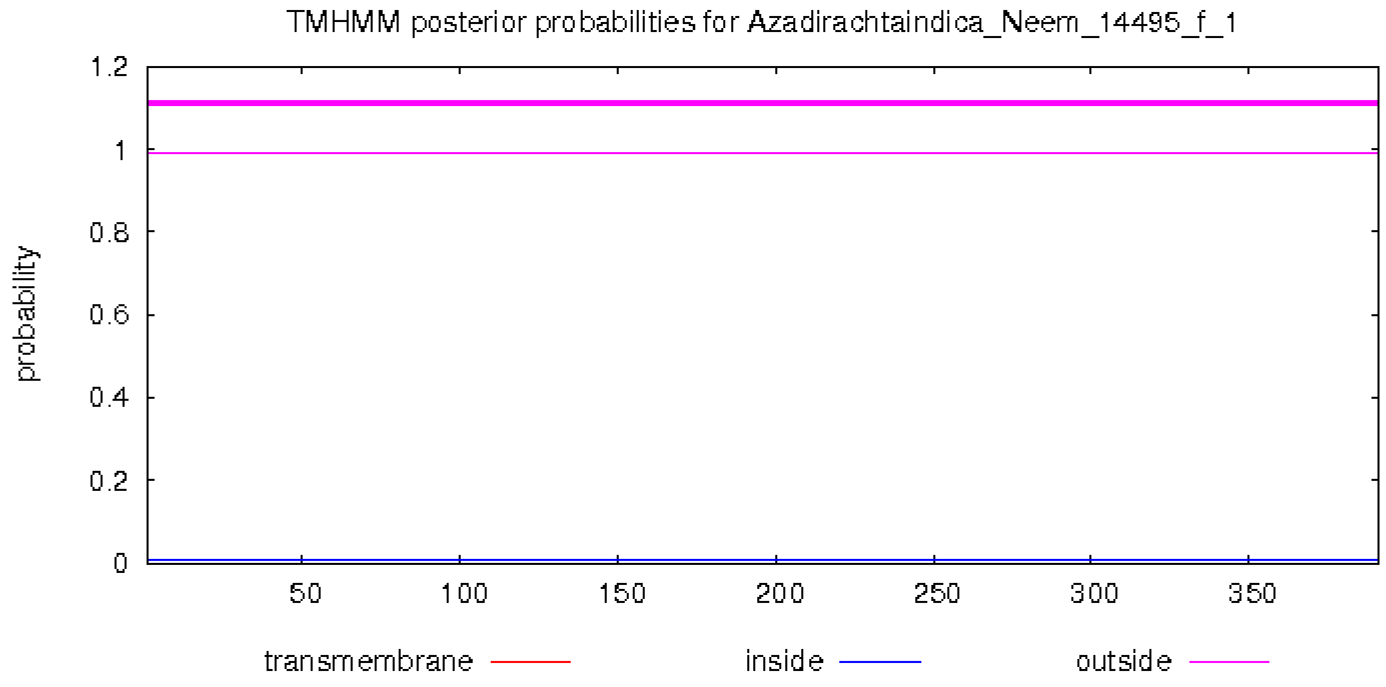

plot in postscript, script for making the plot in gnuplot, data for plot

---

```
# Azadirachtaindica_Neem_14534_f_2 Length: 297
# Azadirachtaindica_Neem_14534_f_2 Number of predicted TMHs:  0
# Azadirachtaindica_Neem_14534_f_2 Exp number of AAs in TMHs: 0.0004
# Azadirachtaindica_Neem_14534_f_2 Exp number, first 60 AAs:  0
# Azadirachtaindica_Neem_14534_f_2 Total prob of N-in:        0.08326
Azadirachtaindica_Neem_14534_f_2        TMHMM2.0        outside      1   297
```

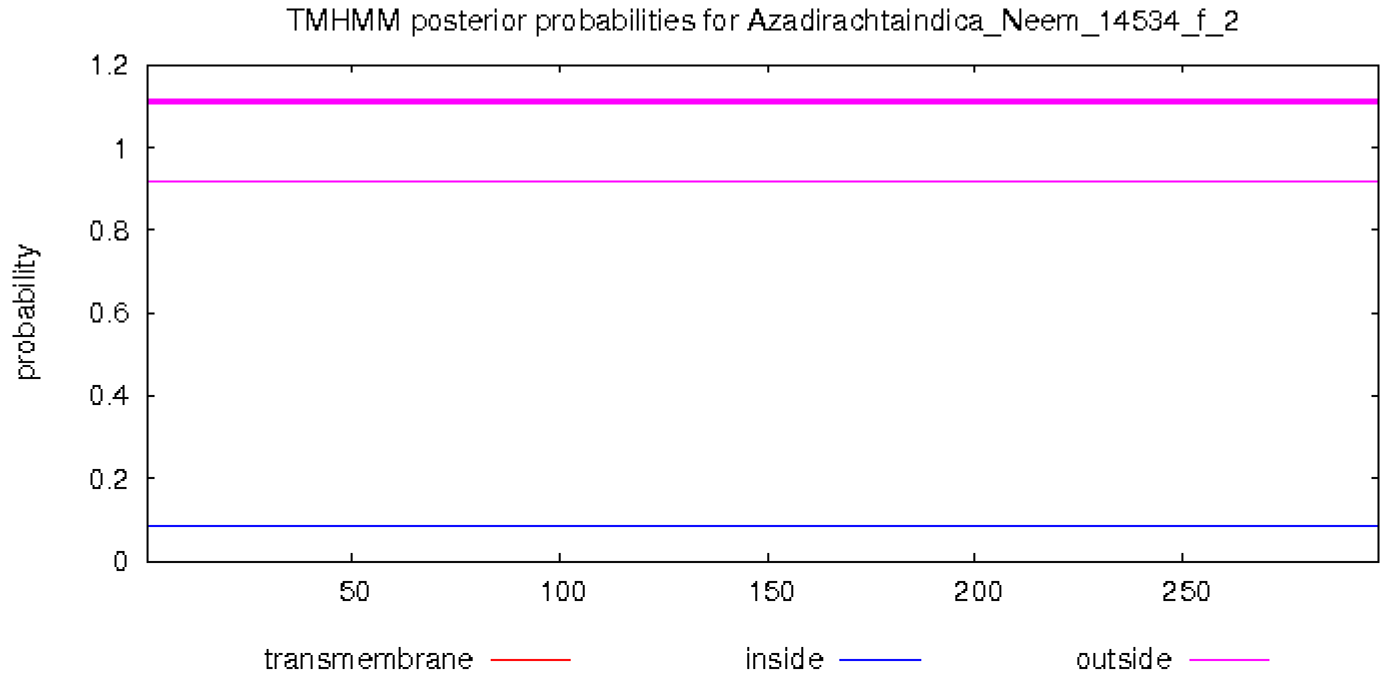

plot in postscript, script for making the plot in gnuplot, data for plot

---

```
# Azadirachtaindica_Neem_14749_f_1 Length: 172
# Azadirachtaindica_Neem_14749_f_1 Number of predicted TMHs:  0
# Azadirachtaindica_Neem_14749_f_1 Exp number of AAs in TMHs: 0.00114
# Azadirachtaindica_Neem_14749_f_1 Exp number, first 60 AAs:  0.00085
# Azadirachtaindica_Neem_14749_f_1 Total prob of N-in:        0.18429
Azadirachtaindica_Neem_14749_f_1        TMHMM2.0        outside      1   172
```

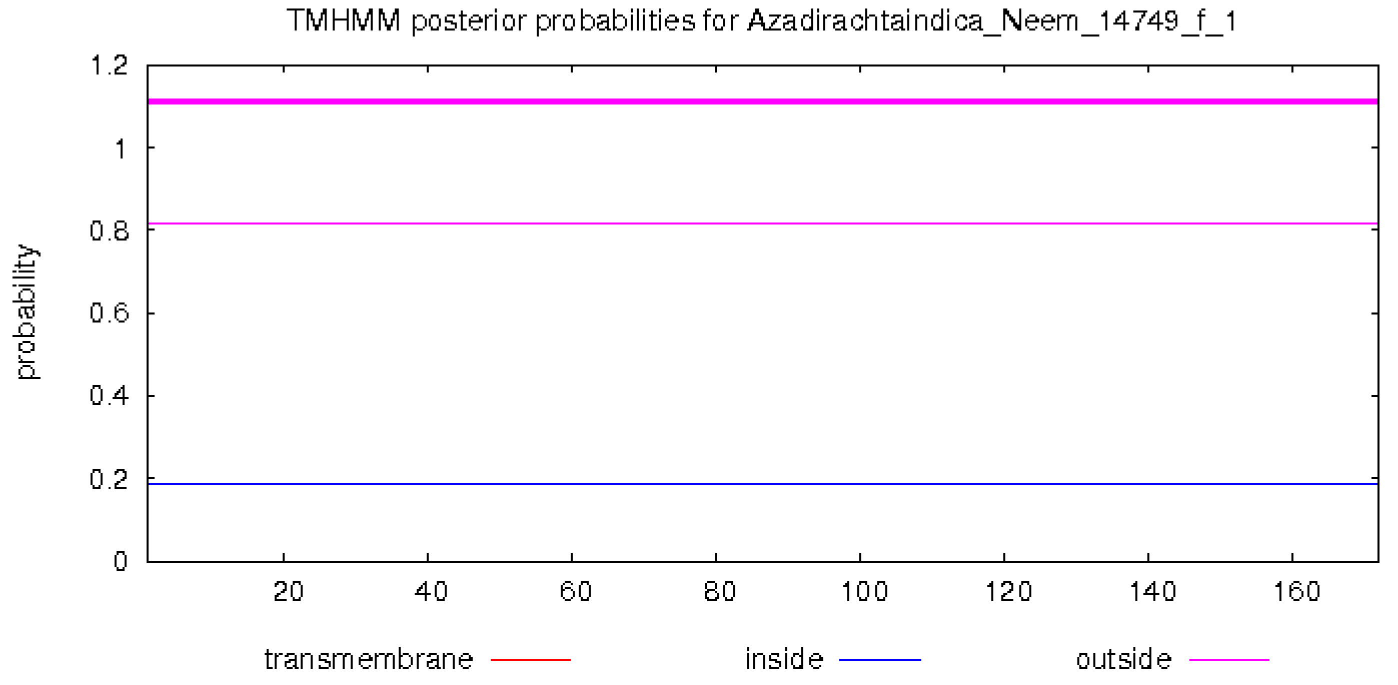

plot in postscript, script for making the plot in gnuplot, data for plot

---

```
# Azadirachtaindica_Neem_15575_f_1 Length: 519
# Azadirachtaindica_Neem_15575_f_1 Number of predicted TMHs:  0
# Azadirachtaindica_Neem_15575_f_1 Exp number of AAs in TMHs: 0.00937
# Azadirachtaindica_Neem_15575_f_1 Exp number, first 60 AAs:  0
# Azadirachtaindica_Neem_15575_f_1 Total prob of N-in:        0.00278
Azadirachtaindica_Neem_15575_f_1        TMHMM2.0        outside      1   519
```

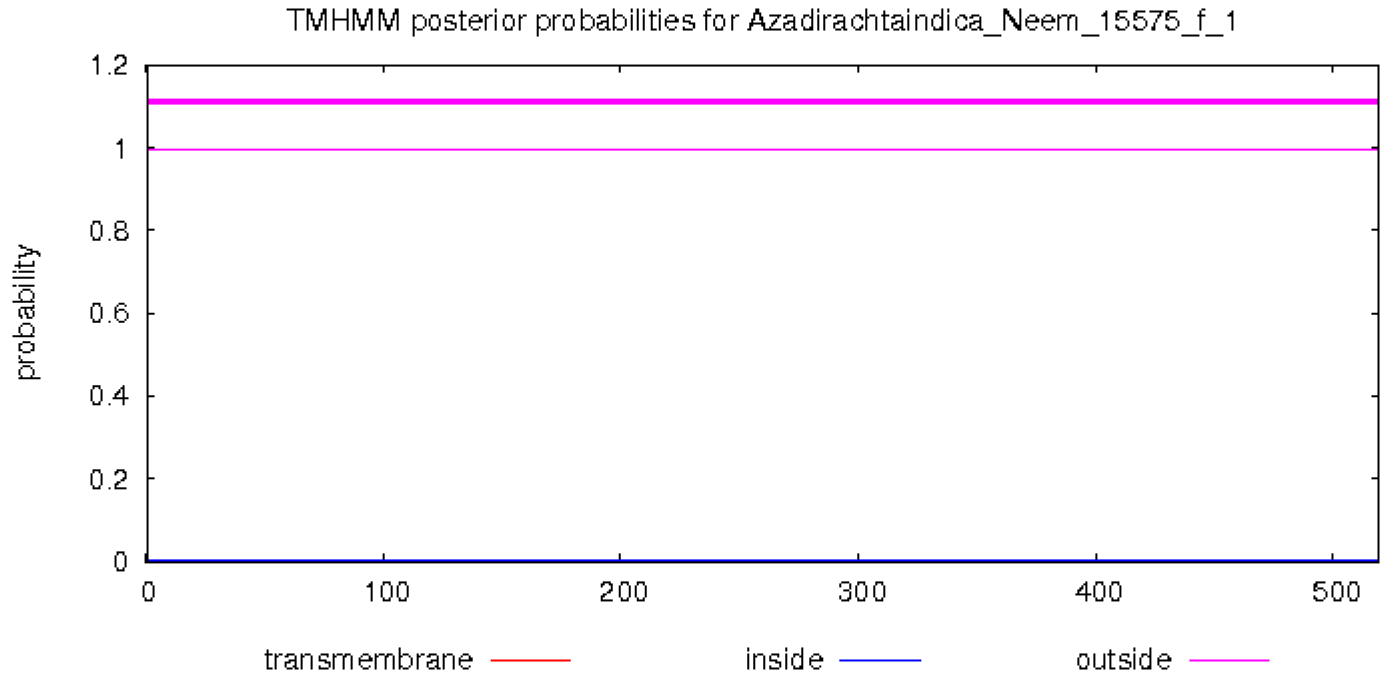

plot in postscript, script for making the plot in gnuplot, data for plot

---

```
# Azadirachtaindica_Neem_15803_f_1 Length: 328
# Azadirachtaindica_Neem_15803_f_1 Number of predicted TMHs:  0
# Azadirachtaindica_Neem_15803_f_1 Exp number of AAs in TMHs: 0
# Azadirachtaindica_Neem_15803_f_1 Exp number, first 60 AAs:  0
# Azadirachtaindica_Neem_15803_f_1 Total prob of N-in:        0.01993
Azadirachtaindica_Neem_15803_f_1        TMHMM2.0        outside      1   328
```

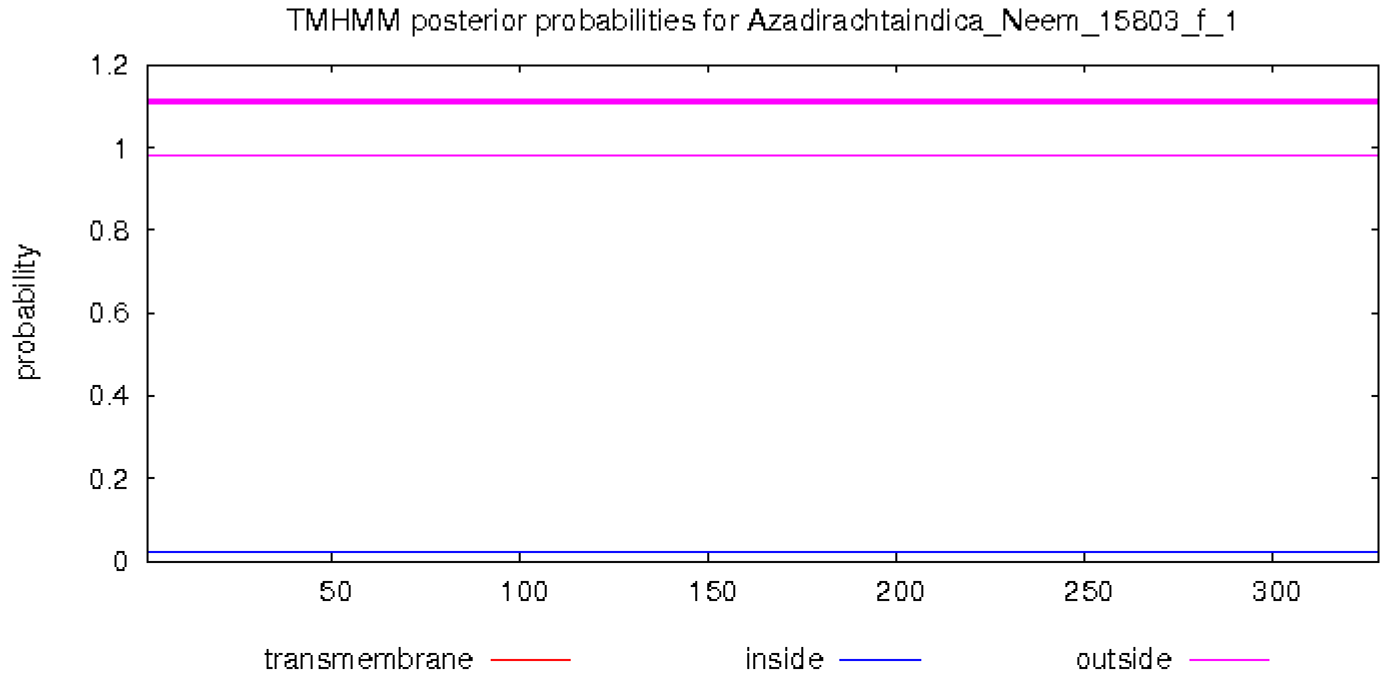

plot in postscript, script for making the plot in gnuplot, data for plot

---

```
# Azadirachtaindica_Neem_16015_f_1 Length: 72
# Azadirachtaindica_Neem_16015_f_1 Number of predicted TMHs:  0
# Azadirachtaindica_Neem_16015_f_1 Exp number of AAs in TMHs: 0.11766
# Azadirachtaindica_Neem_16015_f_1 Exp number, first 60 AAs:  0.11766
# Azadirachtaindica_Neem_16015_f_1 Total prob of N-in:        0.27130
Azadirachtaindica_Neem_16015_f_1        TMHMM2.0        outside      1    72
```

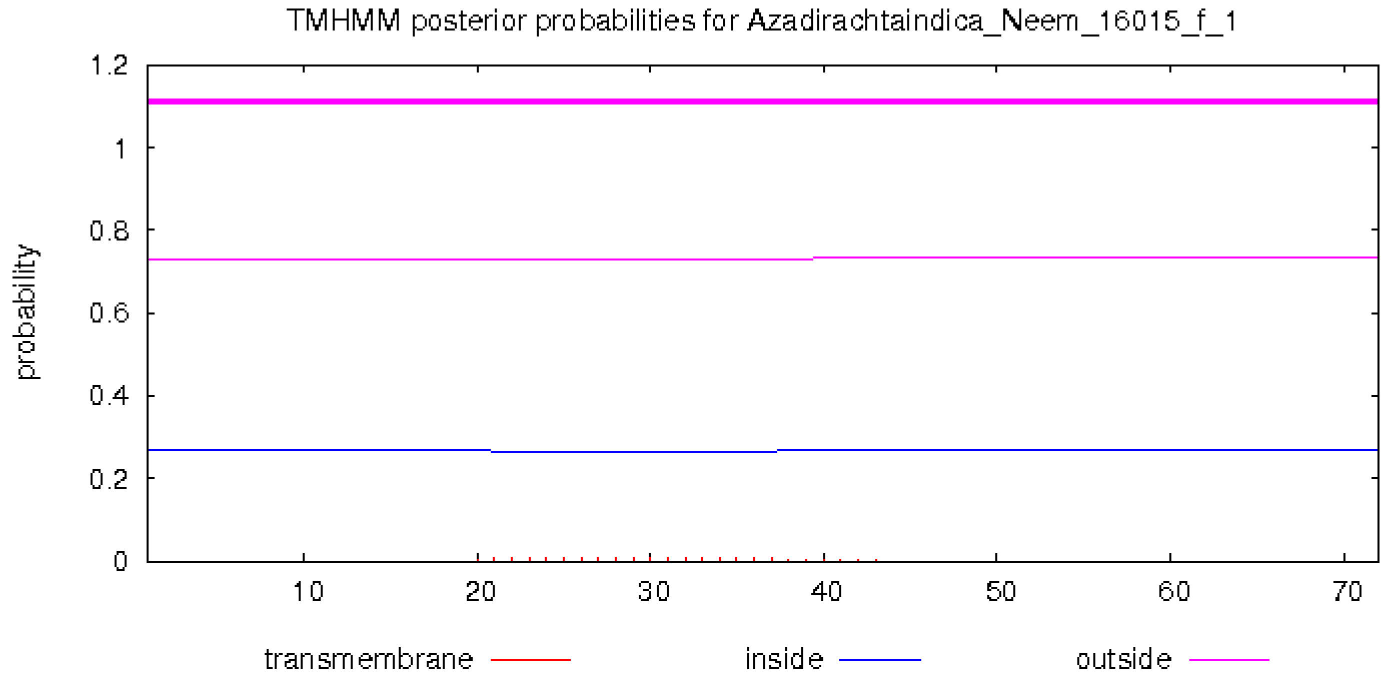

plot in postscript, script for making the plot in gnuplot, data for plot

```
# Azadirachtaindica_Neem_16434_f_1 Length: 355
# Azadirachtaindica_Neem_16434_f_1 Number of predicted TMHs:  0
# Azadirachtaindica_Neem_16434_f_1 Exp number of AAs in TMHs: 0.30959
# Azadirachtaindica_Neem_16434_f_1 Exp number, first 60 AAs:  0.08442
# Azadirachtaindica_Neem_16434_f_1 Total prob of N-in:        0.01208
Azadirachtaindica_Neem_16434_f_1	TMHMM2.0	outside	     1   355
```

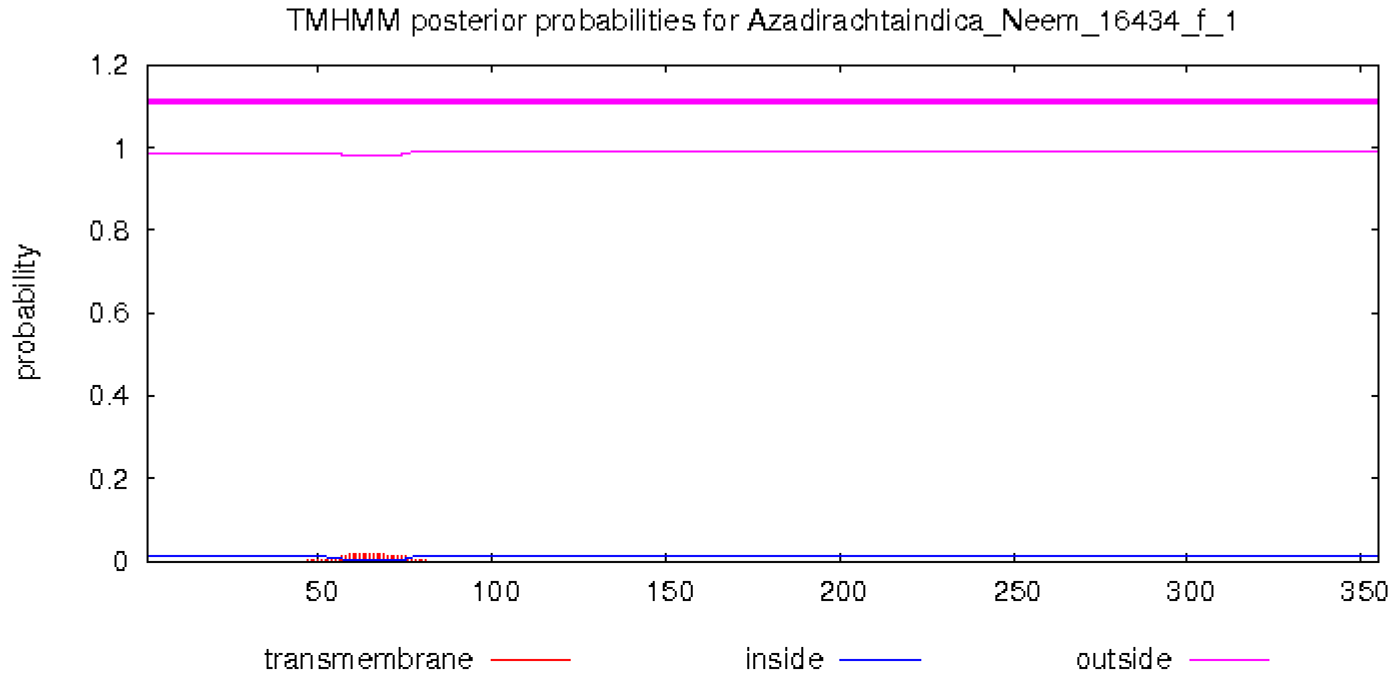

plot in postscript, script for making the plot in gnuplot, data for plot

```
# Azadirachtaindica_Neem_16489_f_1 Length: 135
# Azadirachtaindica_Neem_16489_f_1 Number of predicted TMHs:  0
# Azadirachtaindica_Neem_16489_f_1 Exp number of AAs in TMHs: 0.02208
# Azadirachtaindica_Neem_16489_f_1 Exp number, first 60 AAs:  0.0219
# Azadirachtaindica_Neem_16489_f_1 Total prob of N-in:        0.26434
Azadirachtaindica_Neem_16489_f_1	TMHMM2.0	outside	     1   135
```

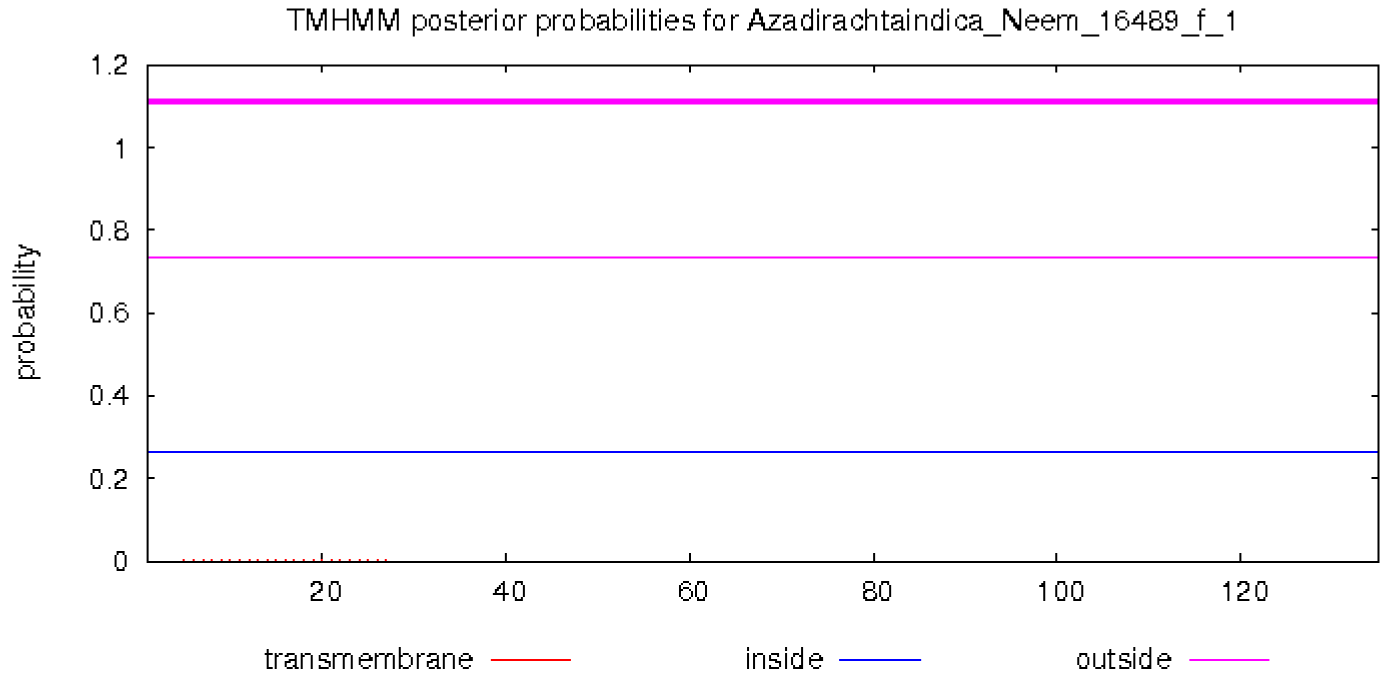

plot in postscript, script for making the plot in gnuplot, data for plot

---

```
# Azadirachtaindica_Neem_1648_f_2 Length: 155
# Azadirachtaindica_Neem_1648_f_2 Number of predicted TMHs:  0
# Azadirachtaindica_Neem_1648_f_2 Exp number of AAs in TMHs: 0.00604
# Azadirachtaindica_Neem_1648_f_2 Exp number, first 60 AAs:  0.00018
# Azadirachtaindica_Neem_1648_f_2 Total prob of N-in:        0.08386
Azadirachtaindica_Neem_1648_f_2 TMHMM2.0        outside      1   155
```

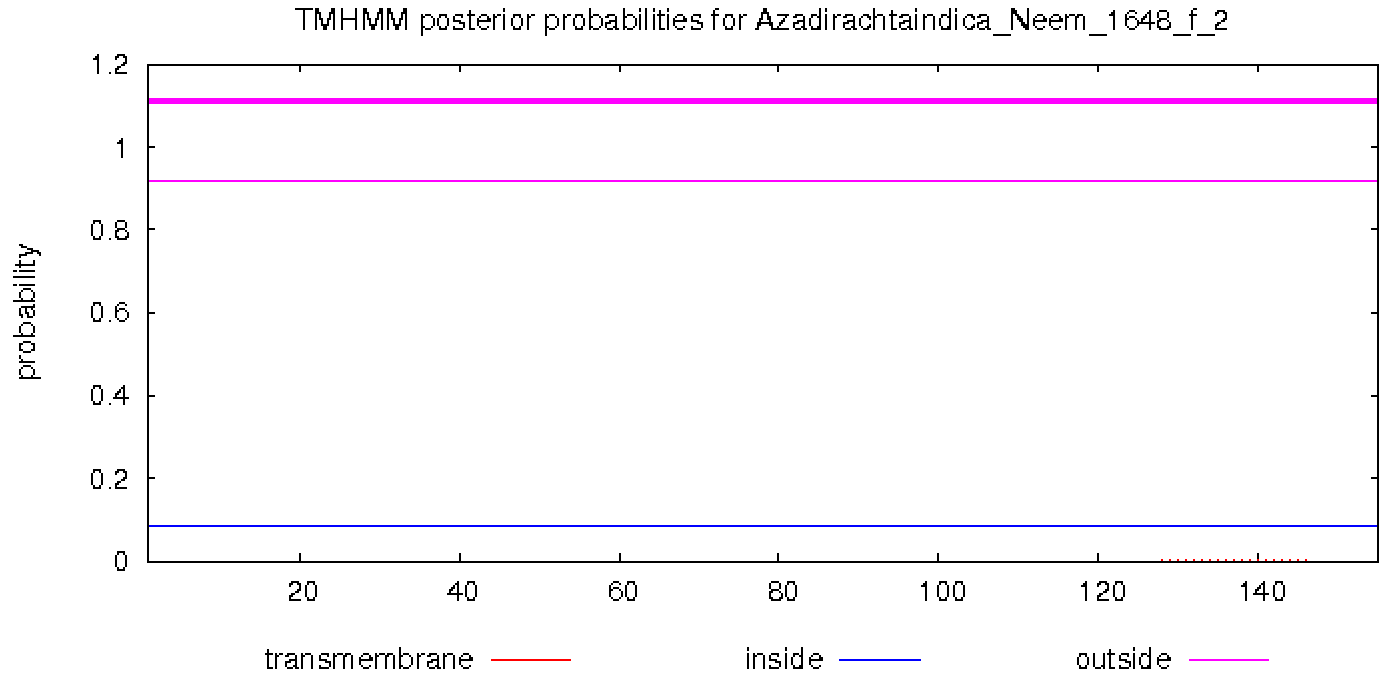

plot in postscript, script for making the plot in gnuplot, data for plot

---

```
# Azadirachtaindica_Neem_16822_f_1 Length: 327
# Azadirachtaindica_Neem_16822_f_1 Number of predicted TMHs:  0
# Azadirachtaindica_Neem_16822_f_1 Exp number of AAs in TMHs: 0
# Azadirachtaindica_Neem_16822_f_1 Exp number, first 60 AAs:  0
# Azadirachtaindica_Neem_16822_f_1 Total prob of N-in:        0.01574
Azadirachtaindica_Neem_16822_f_1        TMHMM2.0        outside      1   327
```

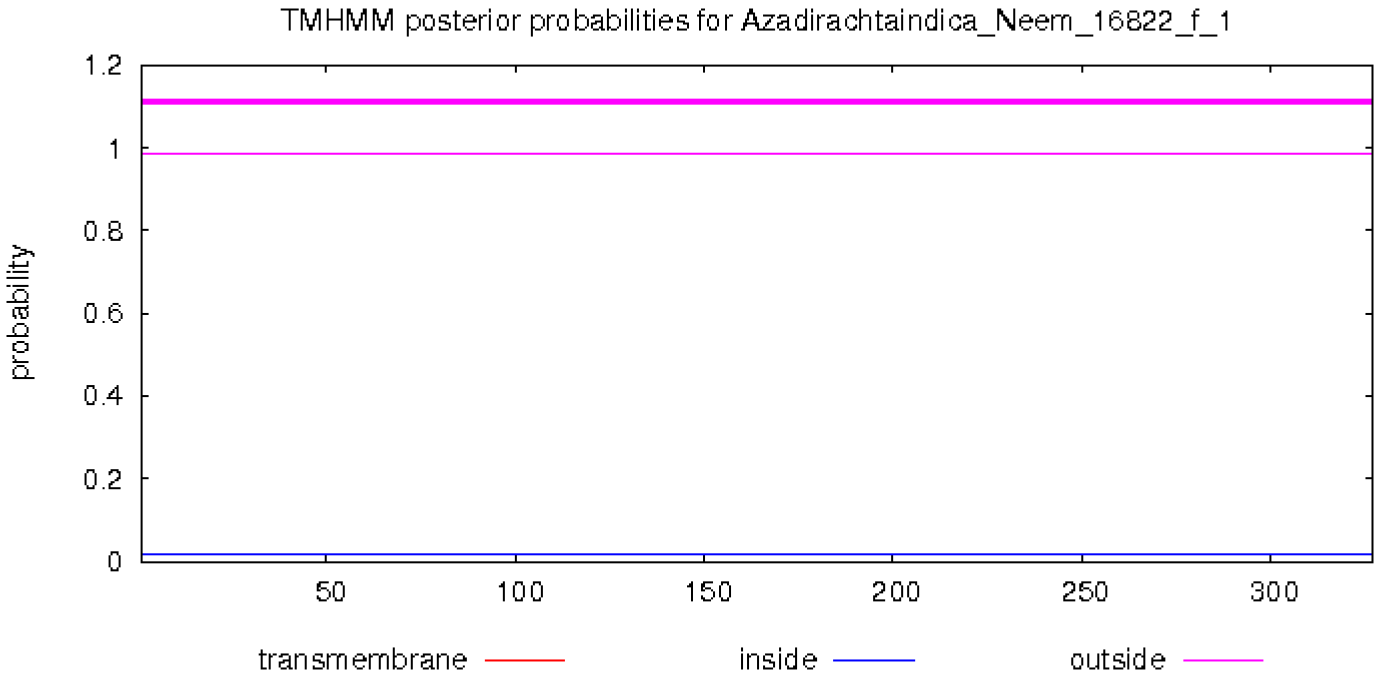

plot in postscript, script for making the plot in gnuplot, data for plot

---

```
# Azadirachtaindica_Neem_17060_f_1 Length: 155
# Azadirachtaindica_Neem_17060_f_1 Number of predicted TMHs:  0
# Azadirachtaindica_Neem_17060_f_1 Exp number of AAs in TMHs: 0.09609
# Azadirachtaindica_Neem_17060_f_1 Exp number, first 60 AAs:  0
# Azadirachtaindica_Neem_17060_f_1 Total prob of N-in:        0.04302
Azadirachtaindica_Neem_17060_f_1        TMHMM2.0        outside      1   155
```

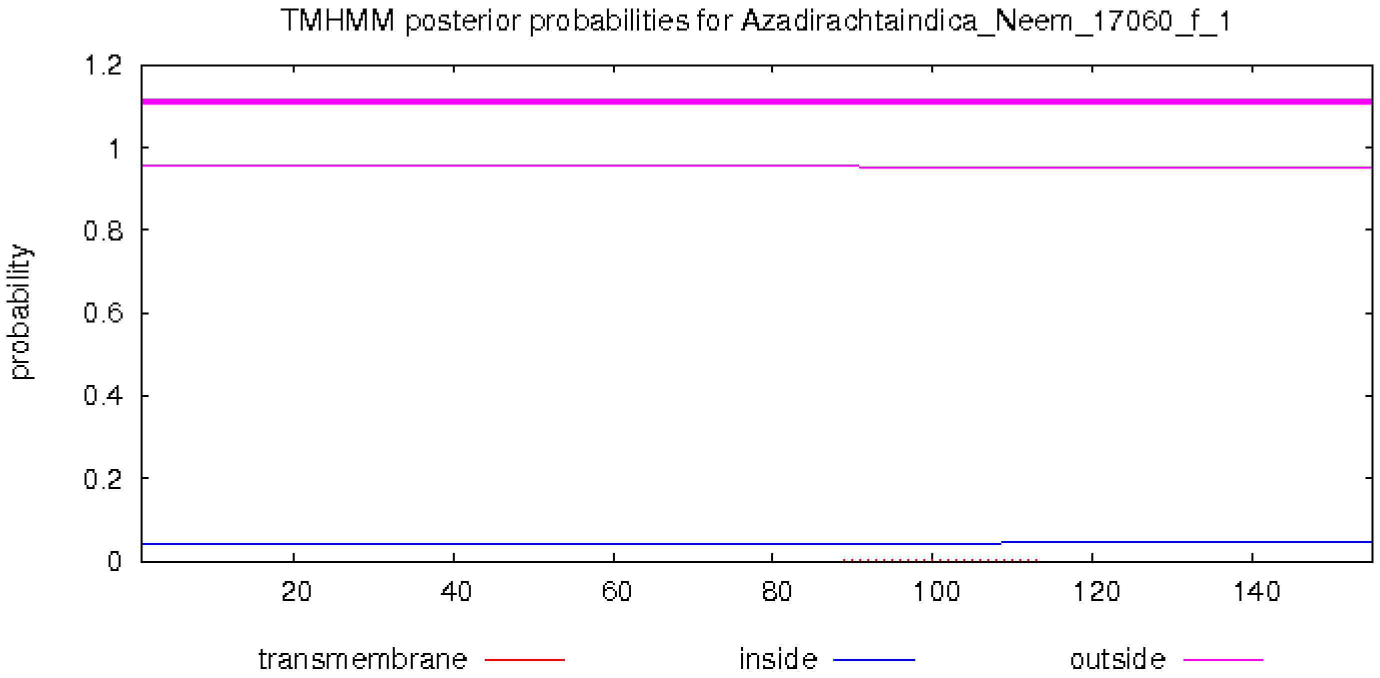

plot in postscript, script for making the plot in gnuplot, data for plot

---

```
# Azadirachtaindica_Neem_17097_f_1 Length: 265
# Azadirachtaindica_Neem_17097_f_1 Number of predicted TMHs:  0
# Azadirachtaindica_Neem_17097_f_1 Exp number of AAs in TMHs: 0.00034
# Azadirachtaindica_Neem_17097_f_1 Exp number, first 60 AAs:  0
# Azadirachtaindica_Neem_17097_f_1 Total prob of N-in:        0.55200
Azadirachtaindica_Neem_17097_f_1        TMHMM2.0        inside       1   265
```

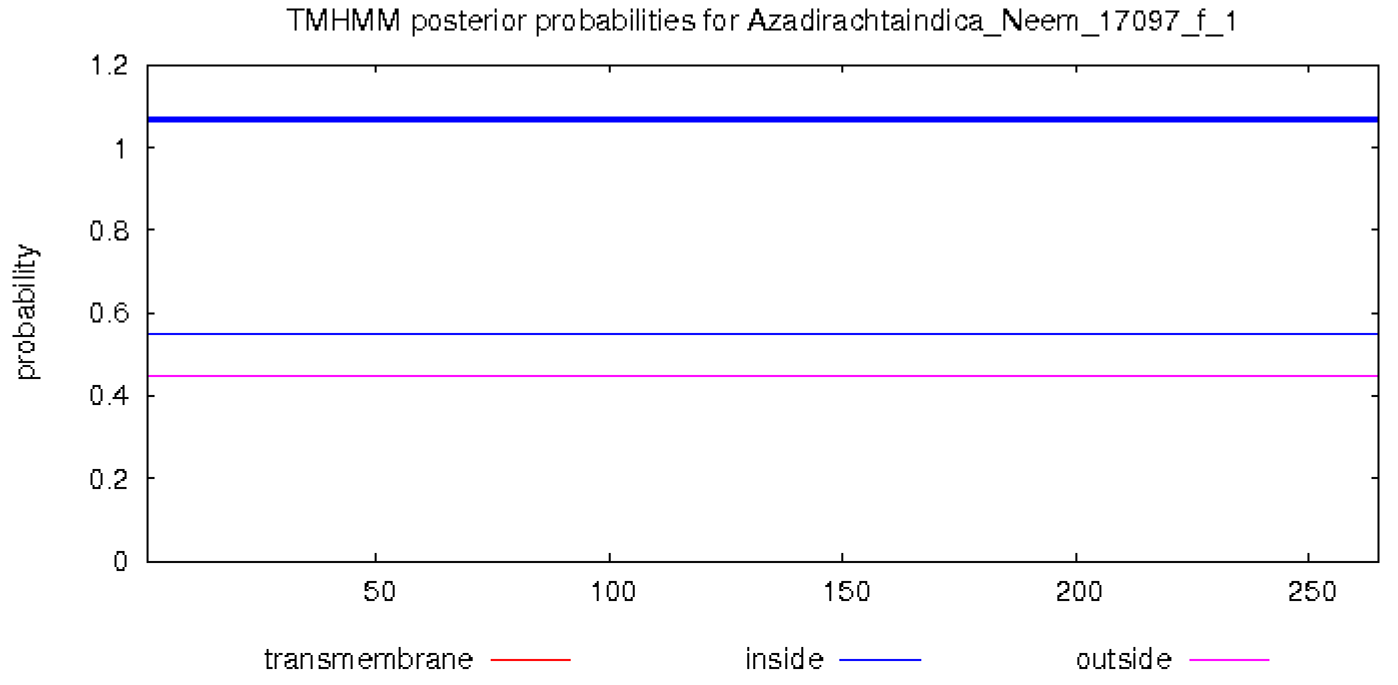

plot in postscript, script for making the plot in gnuplot, data for plot

---

```
# Azadirachtaindica_Neem_17126_f_1 Length: 254
# Azadirachtaindica_Neem_17126_f_1 Number of predicted TMHs:  0
# Azadirachtaindica_Neem_17126_f_1 Exp number of AAs in TMHs: 0.00033
# Azadirachtaindica_Neem_17126_f_1 Exp number, first 60 AAs:  0.00016
# Azadirachtaindica_Neem_17126_f_1 Total prob of N-in:        0.07591
Azadirachtaindica_Neem_17126_f_1	TMHMM2.0	outside	     1   254
```

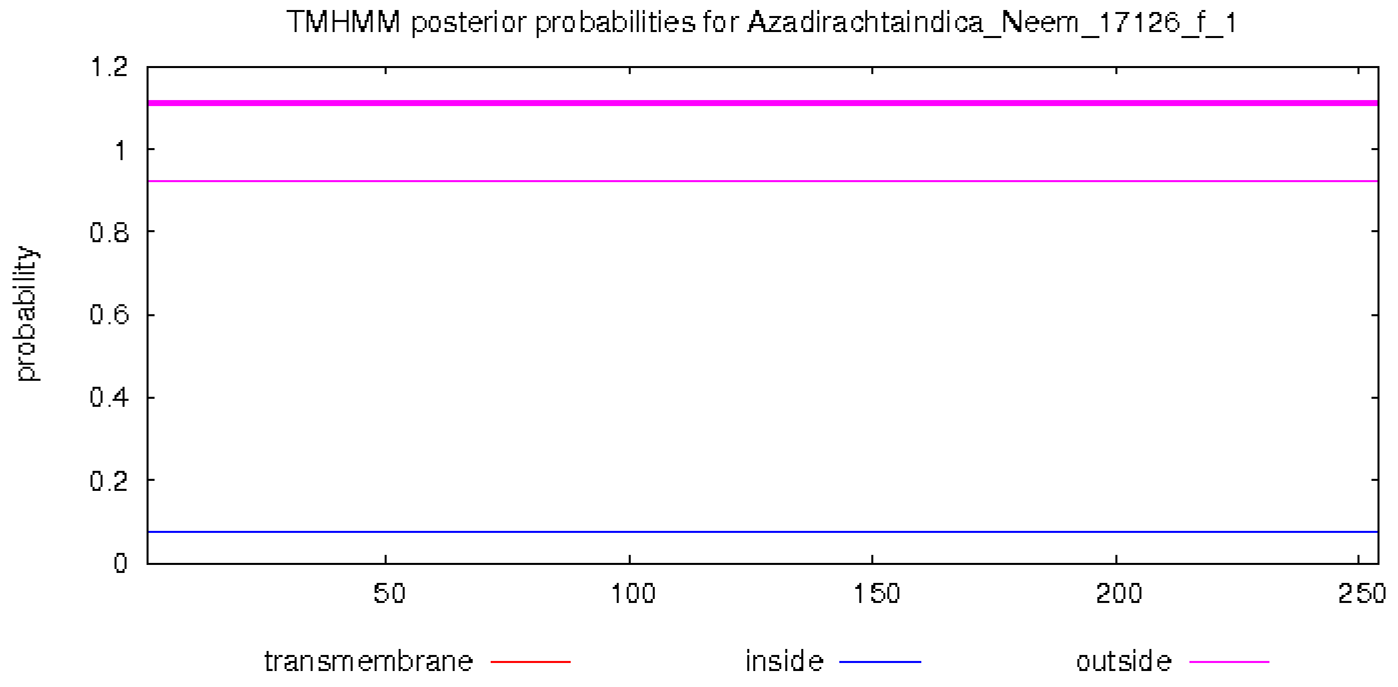

plot in postscript, script for making the plot in gnuplot, data for plot

---

```
# Azadirachtaindica_Neem_17557_f_3 Length: 184
# Azadirachtaindica_Neem_17557_f_3 Number of predicted TMHs:  0
# Azadirachtaindica_Neem_17557_f_3 Exp number of AAs in TMHs: 0.28646
# Azadirachtaindica_Neem_17557_f_3 Exp number, first 60 AAs:  0
# Azadirachtaindica_Neem_17557_f_3 Total prob of N-in:        0.03797
Azadirachtaindica_Neem_17557_f_3	TMHMM2.0	outside	     1   184
```

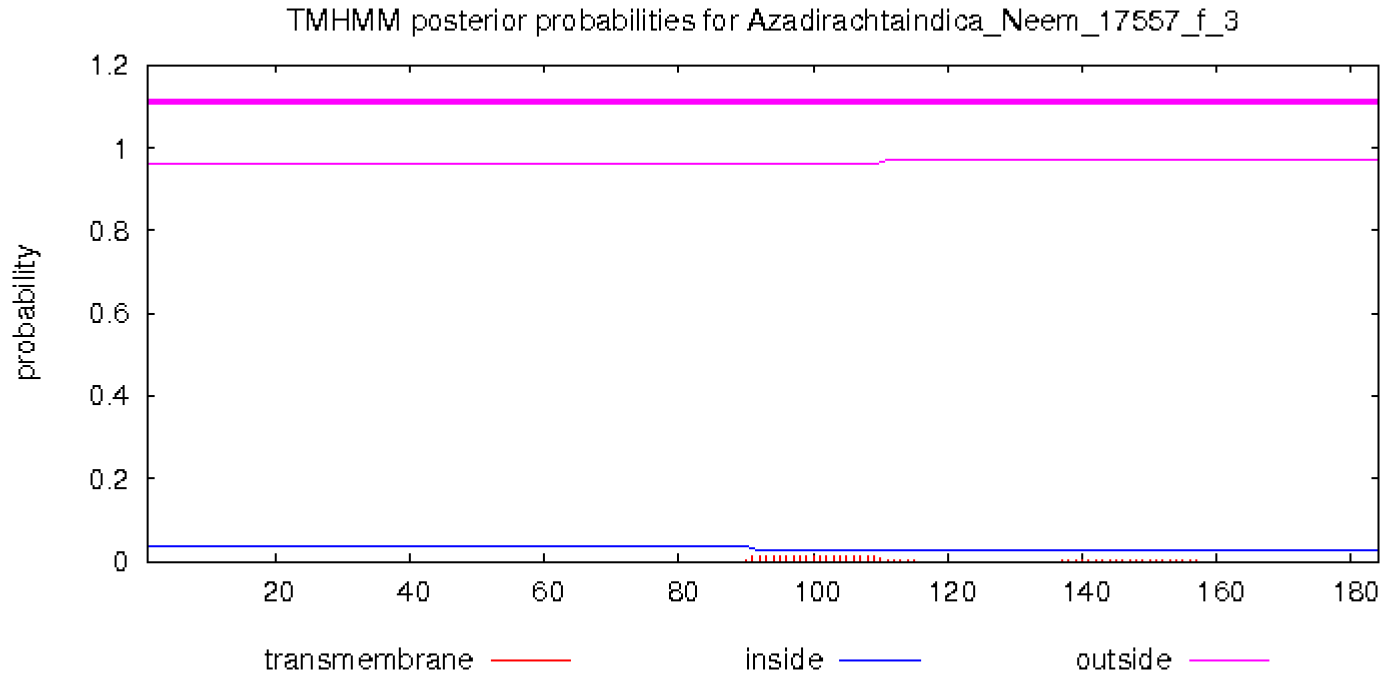

plot in postscript, script for making the plot in gnuplot, data for plot

---

```
# Azadirachtaindica_Neem_17601_f_1 Length: 378
# Azadirachtaindica_Neem_17601_f_1 Number of predicted TMHs:  0
# Azadirachtaindica_Neem_17601_f_1 Exp number of AAs in TMHs: 0.0451
# Azadirachtaindica_Neem_17601_f_1 Exp number, first 60 AAs:  0.00831
# Azadirachtaindica_Neem_17601_f_1 Total prob of N-in:        0.01098
Azadirachtaindica_Neem_17601_f_1        TMHMM2.0        outside      1   378
```

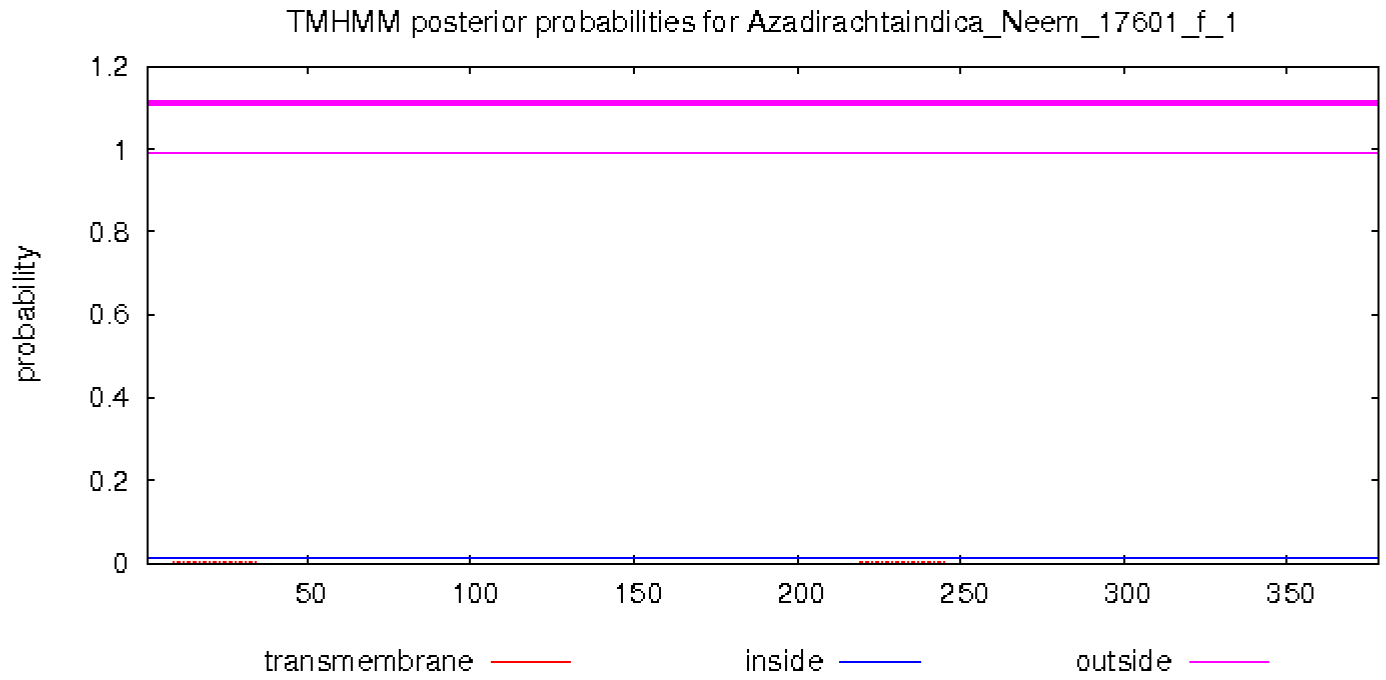

plot in postscript, script for making the plot in gnuplot, data for plot

---

```
# Azadirachtaindica_Neem_17750_f_1 Length: 253
# Azadirachtaindica_Neem_17750_f_1 Number of predicted TMHs:  0
# Azadirachtaindica_Neem_17750_f_1 Exp number of AAs in TMHs: 0.00865
# Azadirachtaindica_Neem_17750_f_1 Exp number, first 60 AAs:  0
# Azadirachtaindica_Neem_17750_f_1 Total prob of N-in:        0.13536
Azadirachtaindica_Neem_17750_f_1        TMHMM2.0        outside      1   253
```

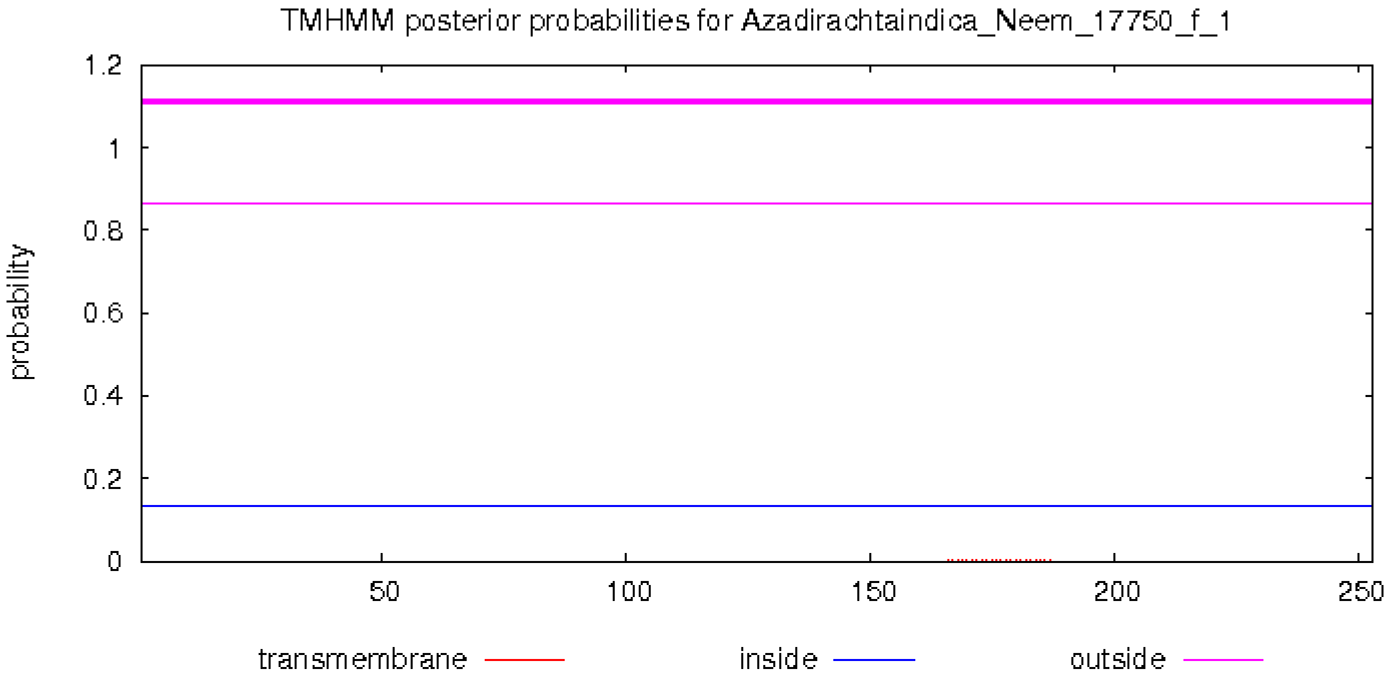

plot in postscript, script for making the plot in gnuplot, data for plot

---

```
# Azadirachtaindica_Neem_17887_f_1 Length: 233
# Azadirachtaindica_Neem_17887_f_1 Number of predicted TMHs:  0
# Azadirachtaindica_Neem_17887_f_1 Exp number of AAs in TMHs: 0
# Azadirachtaindica_Neem_17887_f_1 Exp number, first 60 AAs:  0
# Azadirachtaindica_Neem_17887_f_1 Total prob of N-in:        0.01681
Azadirachtaindica_Neem_17887_f_1        TMHMM2.0        outside      1   233
```

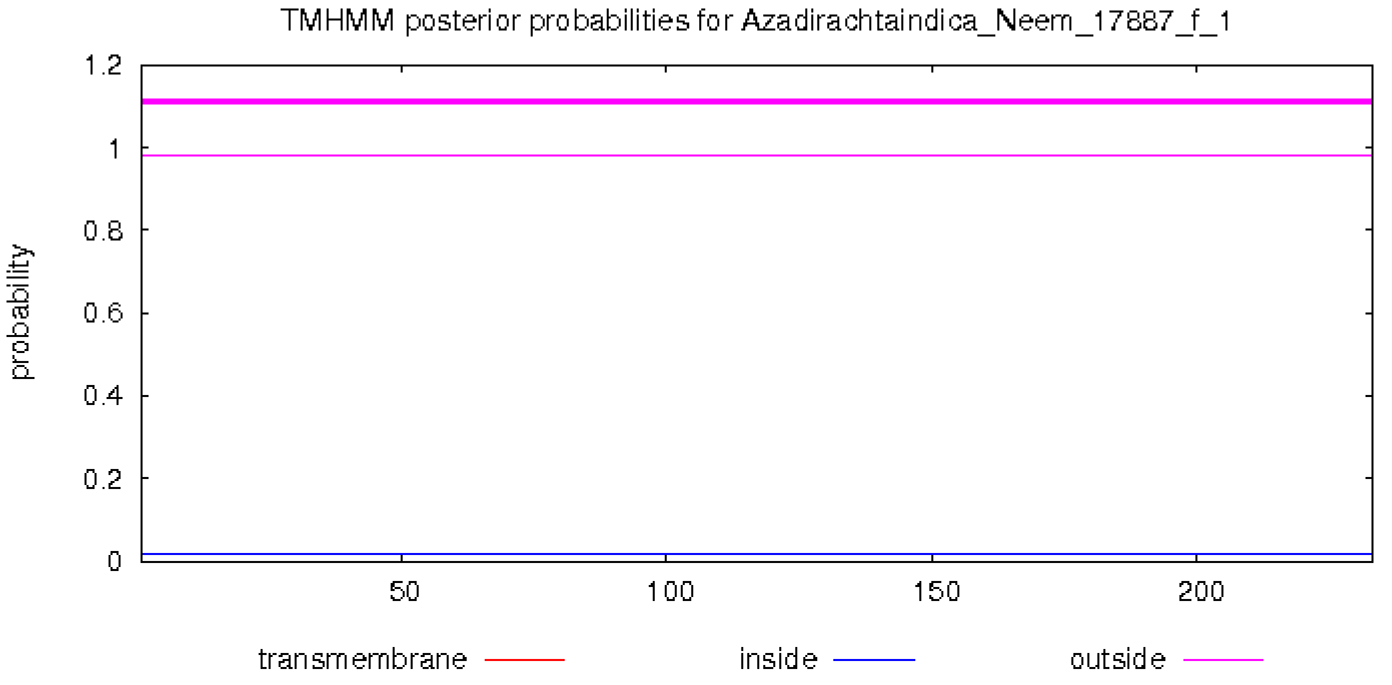

plot in postscript, script for making the plot in gnuplot, data for plot

---

```
# Azadirachtaindica_Neem_17921_f_1 Length: 340
# Azadirachtaindica_Neem_17921_f_1 Number of predicted TMHs:  0
# Azadirachtaindica_Neem_17921_f_1 Exp number of AAs in TMHs: 0.44028
# Azadirachtaindica_Neem_17921_f_1 Exp number, first 60 AAs:  0.00718
# Azadirachtaindica_Neem_17921_f_1 Total prob of N-in:        0.01680
Azadirachtaindica_Neem_17921_f_1        TMHMM2.0        outside      1   340
```

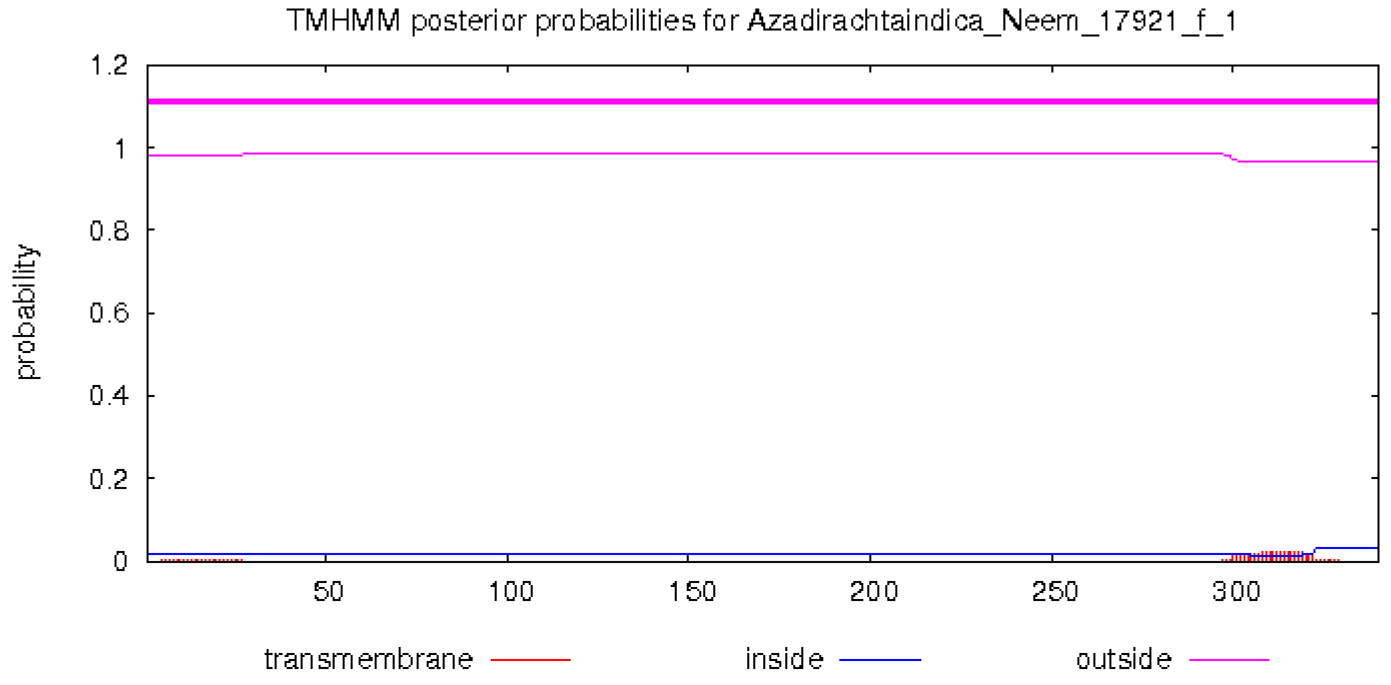

plot in postscript, script for making the plot in gnuplot, data for plot

---

```
# Azadirachtaindica_Neem_17921_f_2 Length: 625
# Azadirachtaindica_Neem_17921_f_2 Number of predicted TMHs:  1
# Azadirachtaindica_Neem_17921_f_2 Exp number of AAs in TMHs: 21.66529
# Azadirachtaindica_Neem_17921_f_2 Exp number, first 60 AAs:  0
# Azadirachtaindica_Neem_17921_f_2 Total prob of N-in:        0.00189
Azadirachtaindica_Neem_17921_f_2        TMHMM2.0        outside      1   562
Azadirachtaindica_Neem_17921_f_2        TMHMM2.0        TMhelix    563   585
Azadirachtaindica_Neem_17921_f_2        TMHMM2.0        inside     586   625
```

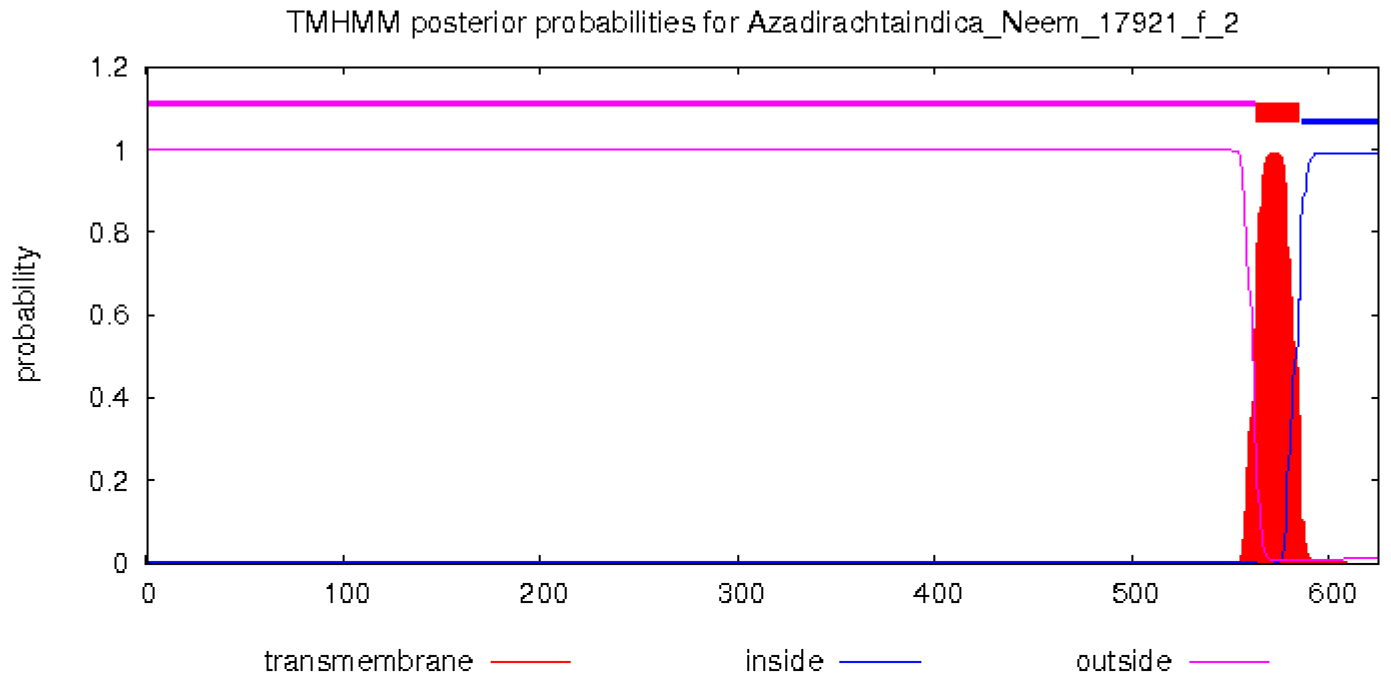

plot in postscript, script for making the plot in gnuplot, data for plot

---

```
# Azadirachtaindica_Neem_18453_f_1 Length: 305
# Azadirachtaindica_Neem_18453_f_1 Number of predicted TMHs:  0
# Azadirachtaindica_Neem_18453_f_1 Exp number of AAs in TMHs: 0.00128
# Azadirachtaindica_Neem_18453_f_1 Exp number, first 60 AAs:  0.00088
```

```
# Azadirachtaindica_Neem_18453_f_1 Total prob of N-in:        0.00444
Azadirachtaindica_Neem_18453_f_1        TMHMM2.0        outside      1   305
```

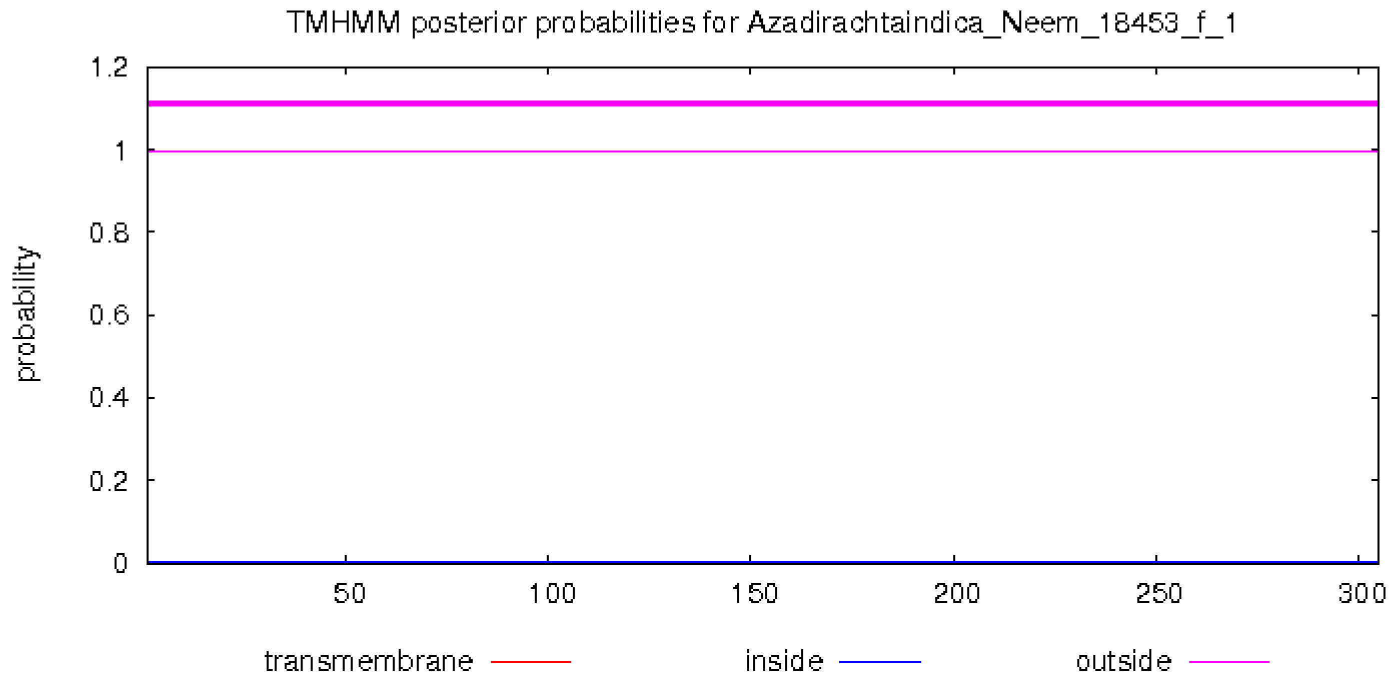

plot in postscript, script for making the plot in gnuplot, data for plot

---

```
# Azadirachtaindica_Neem_18705_f_2 Length: 361
# Azadirachtaindica_Neem_18705_f_2 Number of predicted TMHs:  0
# Azadirachtaindica_Neem_18705_f_2 Exp number of AAs in TMHs: 0.00096
# Azadirachtaindica_Neem_18705_f_2 Exp number, first 60 AAs:  0
# Azadirachtaindica_Neem_18705_f_2 Total prob of N-in:        0.00967
Azadirachtaindica_Neem_18705_f_2        TMHMM2.0        outside      1   361
```

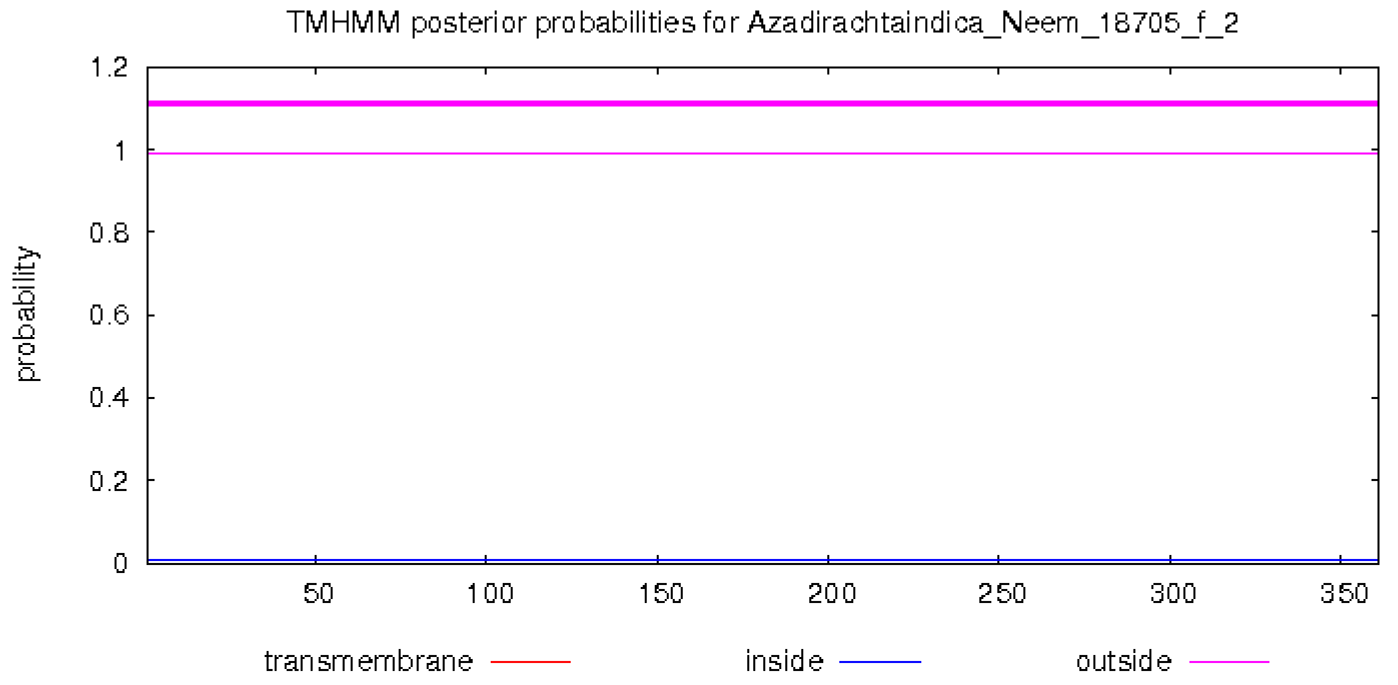

plot in postscript, script for making the plot in gnuplot, data for plot

---

```
# Azadirachtaindica_Neem_19267_f_1 Length: 275
# Azadirachtaindica_Neem_19267_f_1 Number of predicted TMHs:  0
# Azadirachtaindica_Neem_19267_f_1 Exp number of AAs in TMHs: 0.00018
# Azadirachtaindica_Neem_19267_f_1 Exp number, first 60 AAs:  0
```

```
# Azadirachtaindica_Neem_19267_f_1 Total prob of N-in:        0.05152
Azadirachtaindica_Neem_19267_f_1	TMHMM2.0	outside	     1   275
```

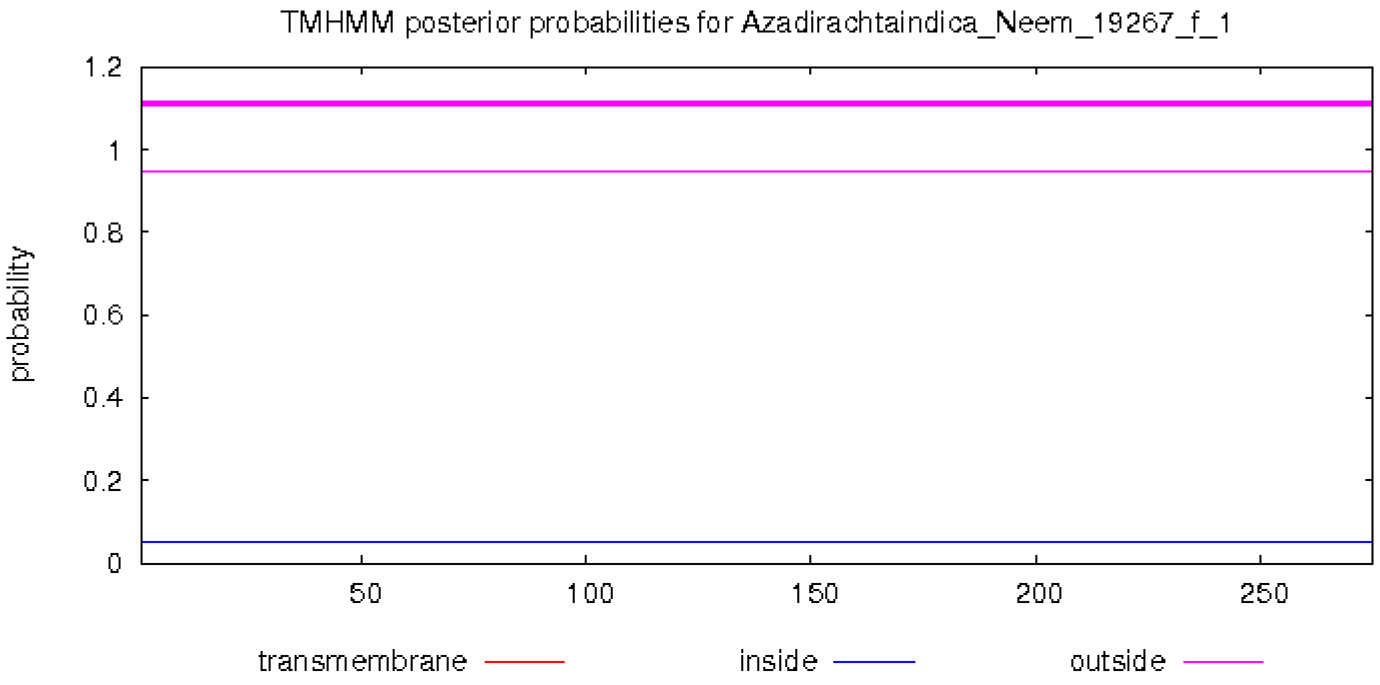

plot in postscript, script for making the plot in gnuplot, data for plot

---

```
# Azadirachtaindica_Neem_20502_f_1 Length: 308
# Azadirachtaindica_Neem_20502_f_1 Number of predicted TMHs:  0
# Azadirachtaindica_Neem_20502_f_1 Exp number of AAs in TMHs: 0.00328
# Azadirachtaindica_Neem_20502_f_1 Exp number, first 60 AAs:  0
# Azadirachtaindica_Neem_20502_f_1 Total prob of N-in:        0.03073
Azadirachtaindica_Neem_20502_f_1	TMHMM2.0	outside	     1   308
```

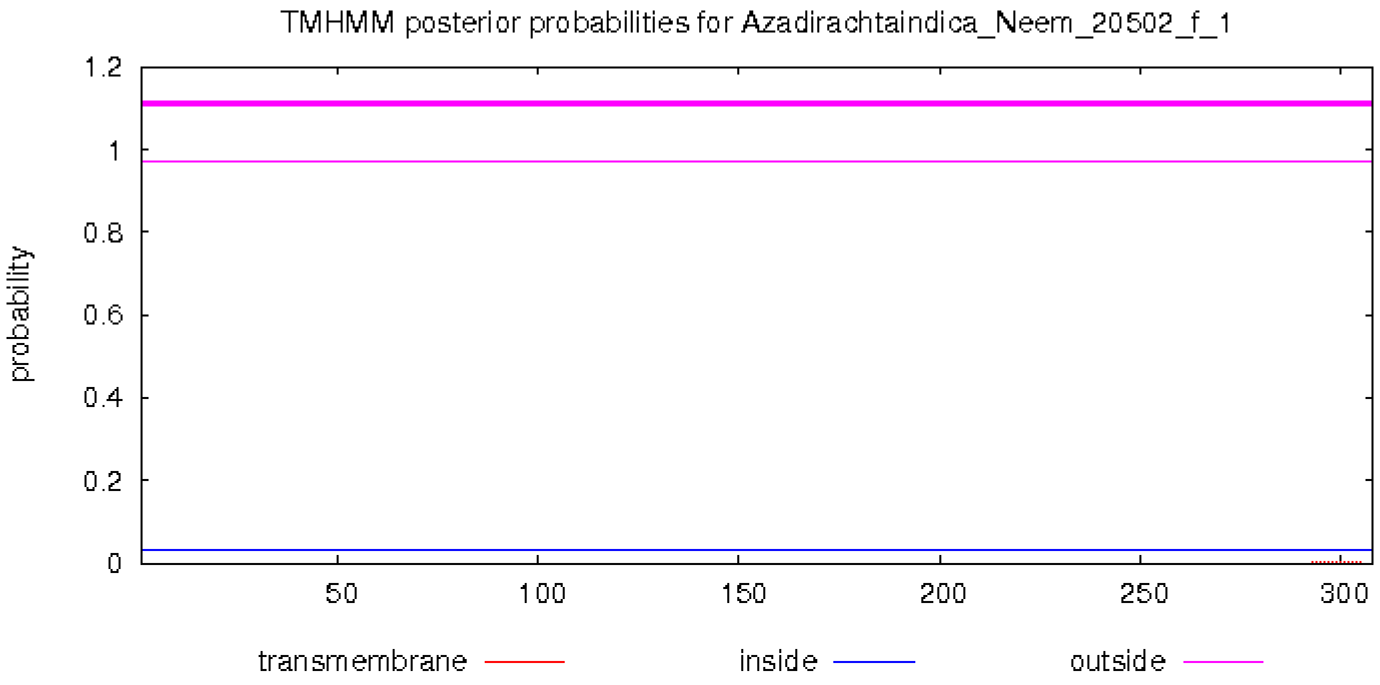

plot in postscript, script for making the plot in gnuplot, data for plot

---

```
# Azadirachtaindica_Neem_20955_f_1 Length: 321
# Azadirachtaindica_Neem_20955_f_1 Number of predicted TMHs:  0
# Azadirachtaindica_Neem_20955_f_1 Exp number of AAs in TMHs: 0.09055
# Azadirachtaindica_Neem_20955_f_1 Exp number, first 60 AAs:  0
```

```
# Azadirachtaindica_Neem_20955_f_1 Total prob of N-in:        0.02504
Azadirachtaindica_Neem_20955_f_1        TMHMM2.0        outside      1   321
```

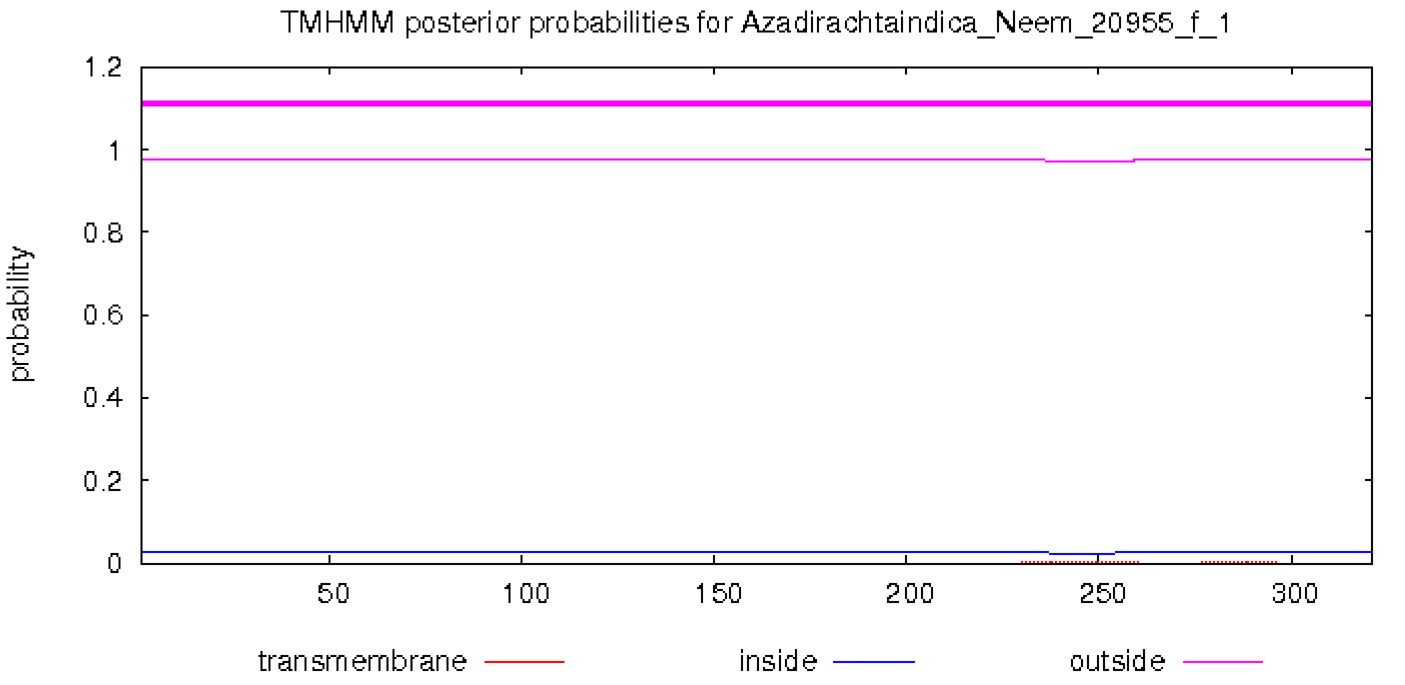

plot in postscript, script for making the plot in gnuplot, data for plot

```
# Azadirachtaindica_Neem_21394_f_3 Length: 604
# Azadirachtaindica_Neem_21394_f_3 Number of predicted TMHs:  1
# Azadirachtaindica_Neem_21394_f_3 Exp number of AAs in TMHs: 21.17329
# Azadirachtaindica_Neem_21394_f_3 Exp number, first 60 AAs:  0
# Azadirachtaindica_Neem_21394_f_3 Total prob of N-in:        0.00066
Azadirachtaindica_Neem_21394_f_3        TMHMM2.0        outside      1   574
Azadirachtaindica_Neem_21394_f_3        TMHMM2.0        TMhelix    575   597
Azadirachtaindica_Neem_21394_f_3        TMHMM2.0        inside     598   604
```

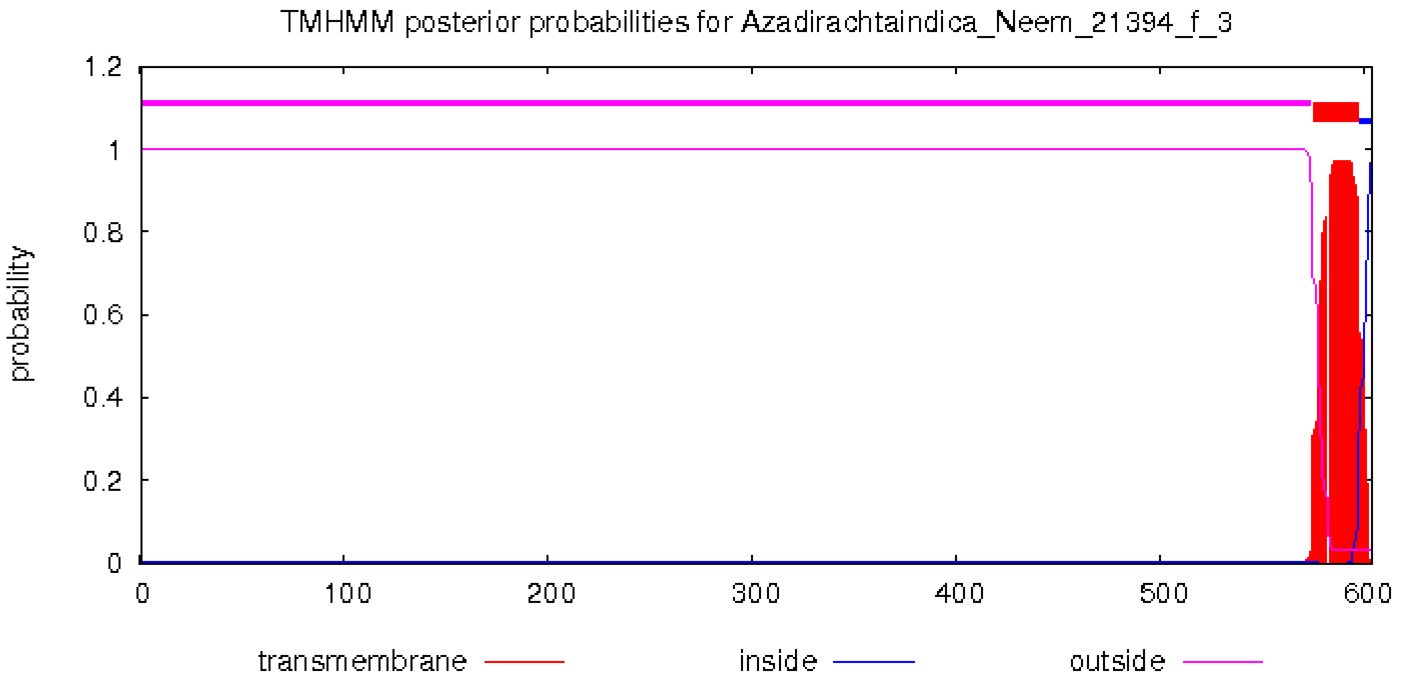

plot in postscript, script for making the plot in gnuplot, data for plot

```
# Azadirachtaindica_Neem_2174_f_6 Length: 247
# Azadirachtaindica_Neem_2174_f_6 Number of predicted TMHs:  0
```

```
# Azadirachtaindica_Neem_2174_f_6 Exp number of AAs in TMHs: 0.00024
# Azadirachtaindica_Neem_2174_f_6 Exp number, first 60 AAs:  0
# Azadirachtaindica_Neem_2174_f_6 Total prob of N-in:        0.05208
Azadirachtaindica_Neem_2174_f_6 TMHMM2.0        outside      1   247
```

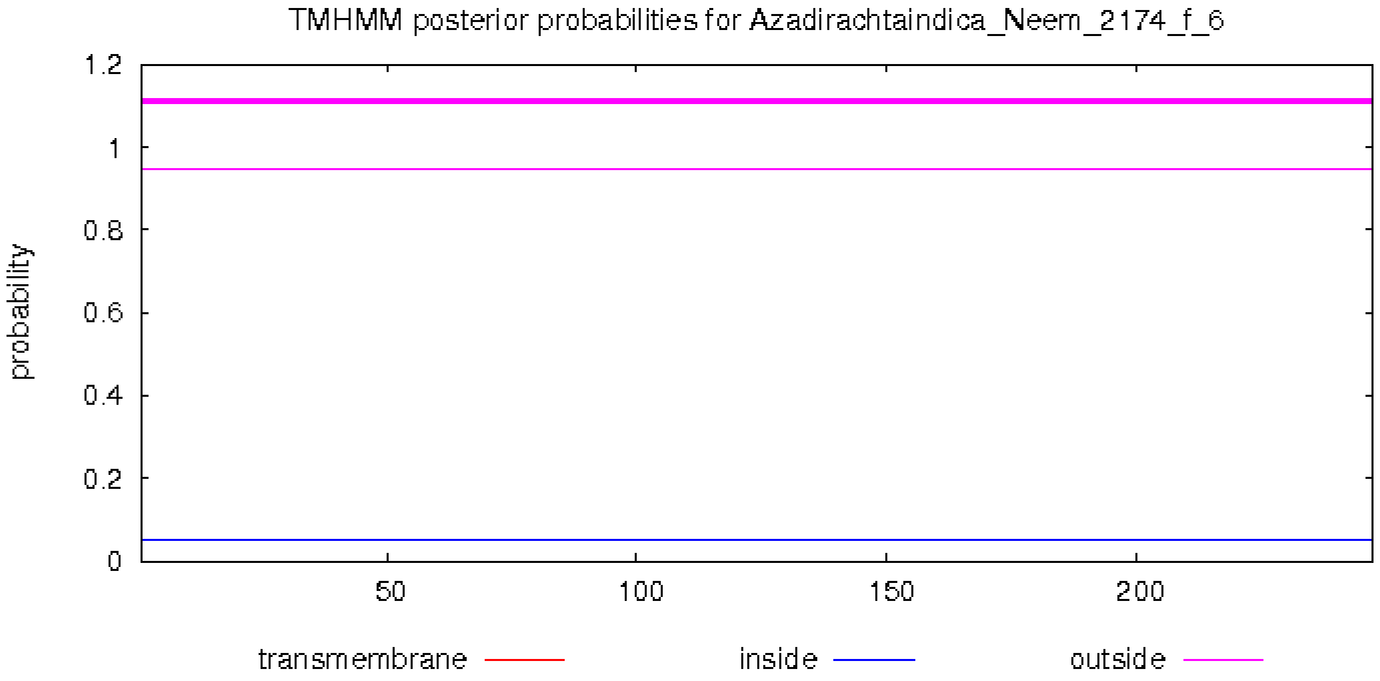

plot in postscript, script for making the plot in gnuplot, data for plot

---

```
# Azadirachtaindica_Neem_21789_f_1 Length: 281
# Azadirachtaindica_Neem_21789_f_1 Number of predicted TMHs:  0
# Azadirachtaindica_Neem_21789_f_1 Exp number of AAs in TMHs: 0.00082
# Azadirachtaindica_Neem_21789_f_1 Exp number, first 60 AAs:  0.00063
# Azadirachtaindica_Neem_21789_f_1 Total prob of N-in:        0.00234
Azadirachtaindica_Neem_21789_f_1        TMHMM2.0        outside      1   281
```

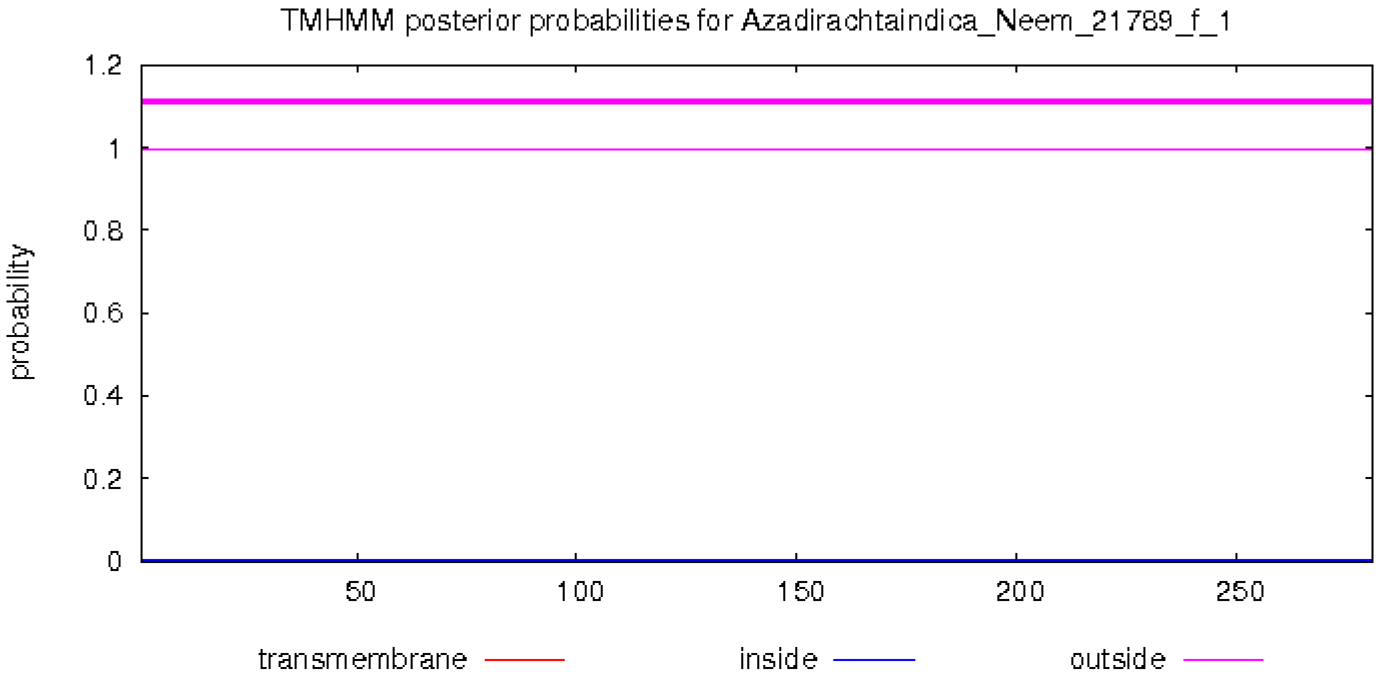

plot in postscript, script for making the plot in gnuplot, data for plot

---

```
# Azadirachtaindica_Neem_22363_f_2 Length: 249
# Azadirachtaindica_Neem_22363_f_2 Number of predicted TMHs:  0
```

```
# Azadirachtaindica_Neem_22363_f_2 Exp number of AAs in TMHs: 0.02152
# Azadirachtaindica_Neem_22363_f_2 Exp number, first 60 AAs:  0.00257
# Azadirachtaindica_Neem_22363_f_2 Total prob of N-in:        0.07158
Azadirachtaindica_Neem_22363_f_2        TMHMM2.0        outside      1   249
```

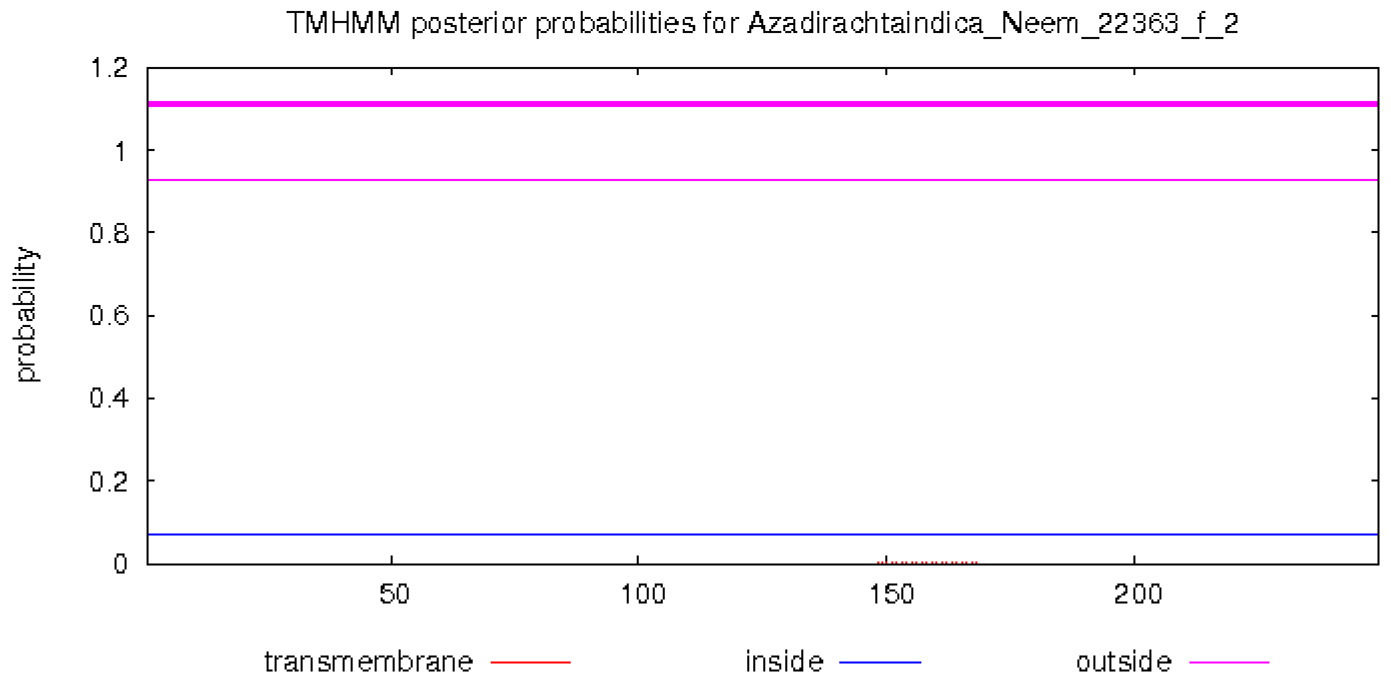

plot in postscript, script for making the plot in gnuplot, data for plot

---

```
# Azadirachtaindica_Neem_22839_f_1 Length: 249
# Azadirachtaindica_Neem_22839_f_1 Number of predicted TMHs:  0
# Azadirachtaindica_Neem_22839_f_1 Exp number of AAs in TMHs: 0
# Azadirachtaindica_Neem_22839_f_1 Exp number, first 60 AAs:  0
# Azadirachtaindica_Neem_22839_f_1 Total prob of N-in:        0.00214
Azadirachtaindica_Neem_22839_f_1        TMHMM2.0        outside      1   249
```

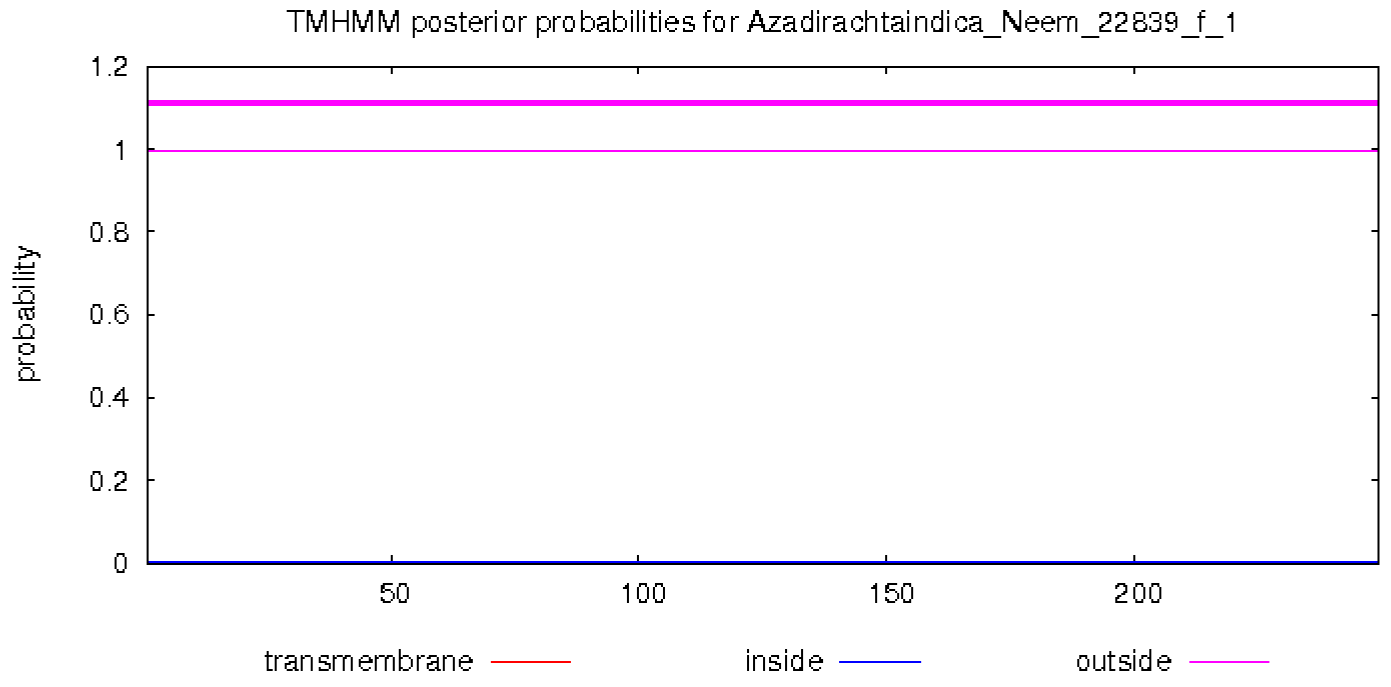

plot in postscript, script for making the plot in gnuplot, data for plot

---

```
# Azadirachtaindica_Neem_23091_f_1 Length: 166
# Azadirachtaindica_Neem_23091_f_1 Number of predicted TMHs:  0
```

```
# Azadirachtaindica_Neem_23091_f_1 Exp number of AAs in TMHs: 0
# Azadirachtaindica_Neem_23091_f_1 Exp number, first 60 AAs:  0
# Azadirachtaindica_Neem_23091_f_1 Total prob of N-in:        0.09528
Azadirachtaindica_Neem_23091_f_1        TMHMM2.0        outside      1   166
```

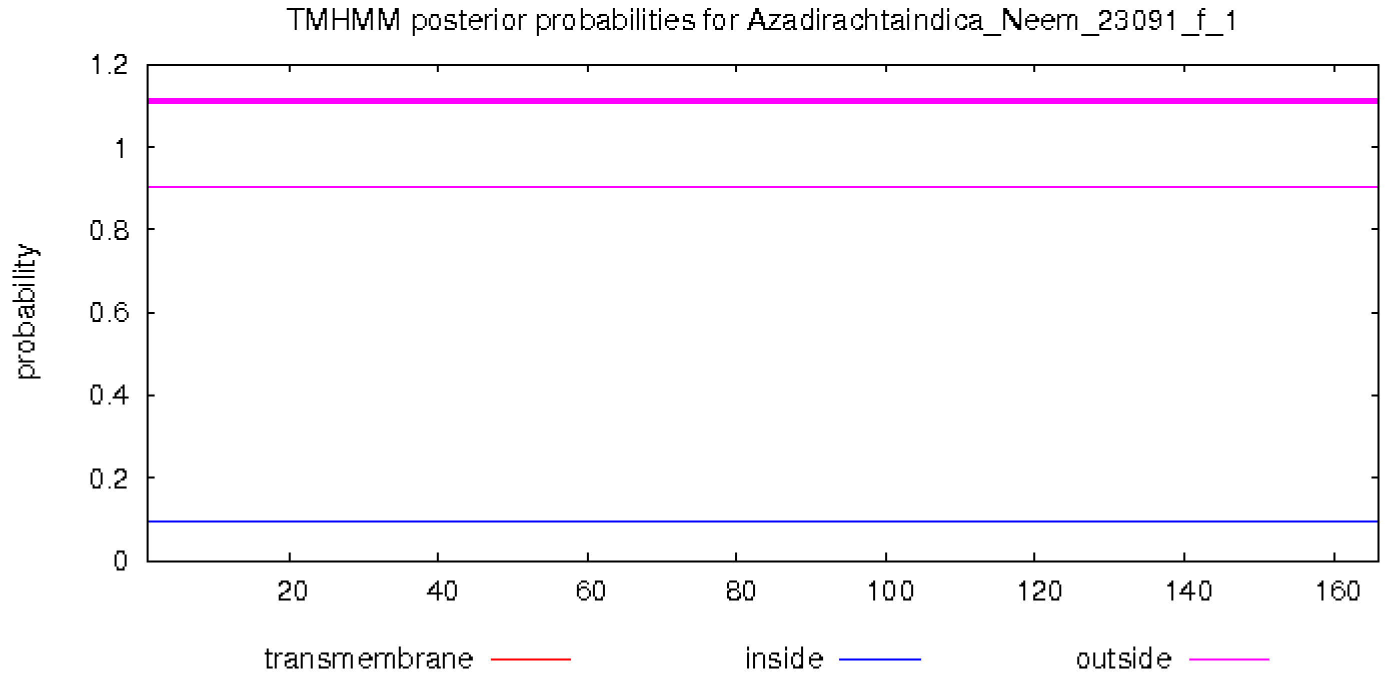

plot in postscript, script for making the plot in gnuplot, data for plot

---

```
# Azadirachtaindica_Neem_2314_f_2 Length: 335
# Azadirachtaindica_Neem_2314_f_2 Number of predicted TMHs:  0
# Azadirachtaindica_Neem_2314_f_2 Exp number of AAs in TMHs: 0.00211
# Azadirachtaindica_Neem_2314_f_2 Exp number, first 60 AAs:  0
# Azadirachtaindica_Neem_2314_f_2 Total prob of N-in:        0.09865
Azadirachtaindica_Neem_2314_f_2 TMHMM2.0        outside      1   335
```

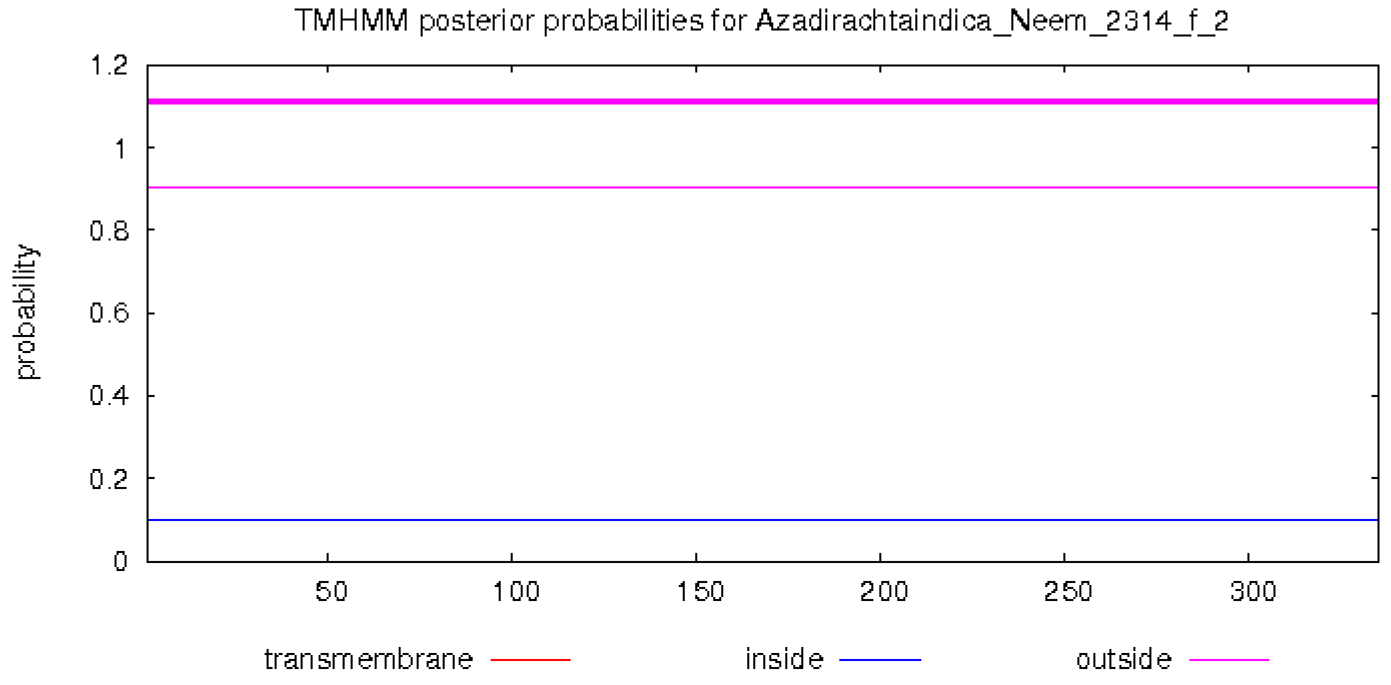

plot in postscript, script for making the plot in gnuplot, data for plot

---

```
# Azadirachtaindica_Neem_23158_f_1 Length: 182
# Azadirachtaindica_Neem_23158_f_1 Number of predicted TMHs:  0
```

```
# Azadirachtaindica_Neem_23158_f_1 Exp number of AAs in TMHs: 0.00142
# Azadirachtaindica_Neem_23158_f_1 Exp number, first 60 AAs:  0.00031
# Azadirachtaindica_Neem_23158_f_1 Total prob of N-in:        0.08846
Azadirachtaindica_Neem_23158_f_1        TMHMM2.0        outside      1   182
```

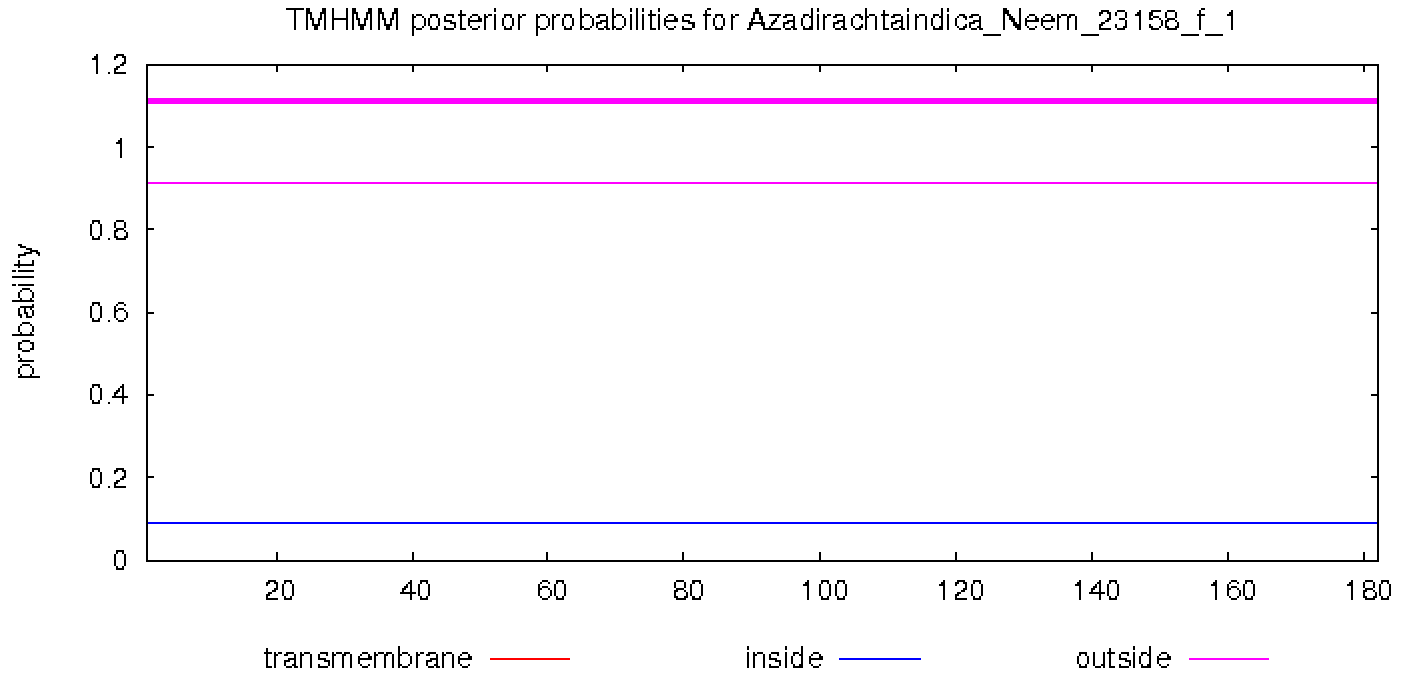

plot in postscript, script for making the plot in gnuplot, data for plot

---

```
# Azadirachtaindica_Neem_2328_f_1 Length: 269
# Azadirachtaindica_Neem_2328_f_1 Number of predicted TMHs:  0
# Azadirachtaindica_Neem_2328_f_1 Exp number of AAs in TMHs: 0.00664
# Azadirachtaindica_Neem_2328_f_1 Exp number, first 60 AAs:  0.00015
# Azadirachtaindica_Neem_2328_f_1 Total prob of N-in:        0.01157
Azadirachtaindica_Neem_2328_f_1 TMHMM2.0        outside      1   269
```

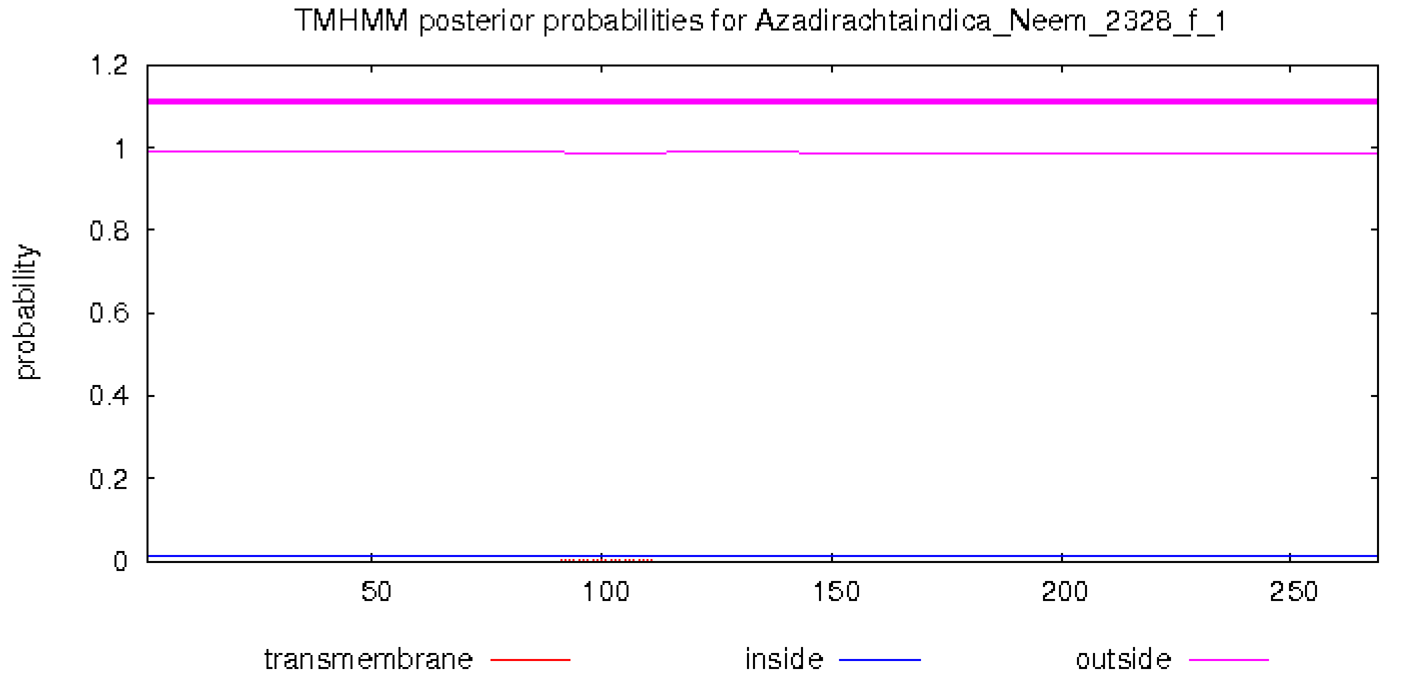

plot in postscript, script for making the plot in gnuplot, data for plot

---

```
# Azadirachtaindica_Neem_23413_f_1 Length: 244
# Azadirachtaindica_Neem_23413_f_1 Number of predicted TMHs:  0
```

```
# Azadirachtaindica_Neem_23413_f_1 Exp number of AAs in TMHs: 0.00039
# Azadirachtaindica_Neem_23413_f_1 Exp number, first 60 AAs:  0.00025
# Azadirachtaindica_Neem_23413_f_1 Total prob of N-in:        0.05327
Azadirachtaindica_Neem_23413_f_1        TMHMM2.0        outside      1   244
```

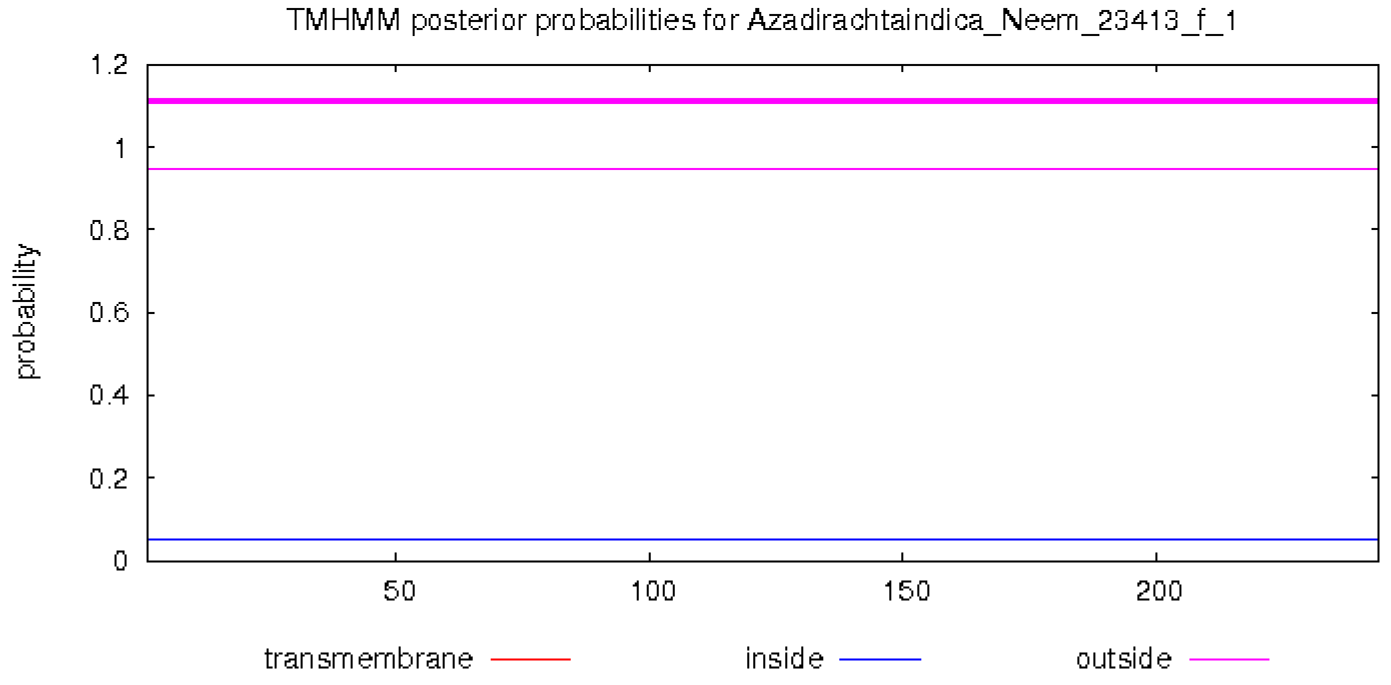

plot in postscript, script for making the plot in gnuplot, data for plot

```
# Azadirachtaindica_Neem_23449_f_2 Length: 112
# Azadirachtaindica_Neem_23449_f_2 Number of predicted TMHs:  0
# Azadirachtaindica_Neem_23449_f_2 Exp number of AAs in TMHs: 0.63738
# Azadirachtaindica_Neem_23449_f_2 Exp number, first 60 AAs:  0.01201
# Azadirachtaindica_Neem_23449_f_2 Total prob of N-in:        0.17724
Azadirachtaindica_Neem_23449_f_2        TMHMM2.0        outside      1   112
```

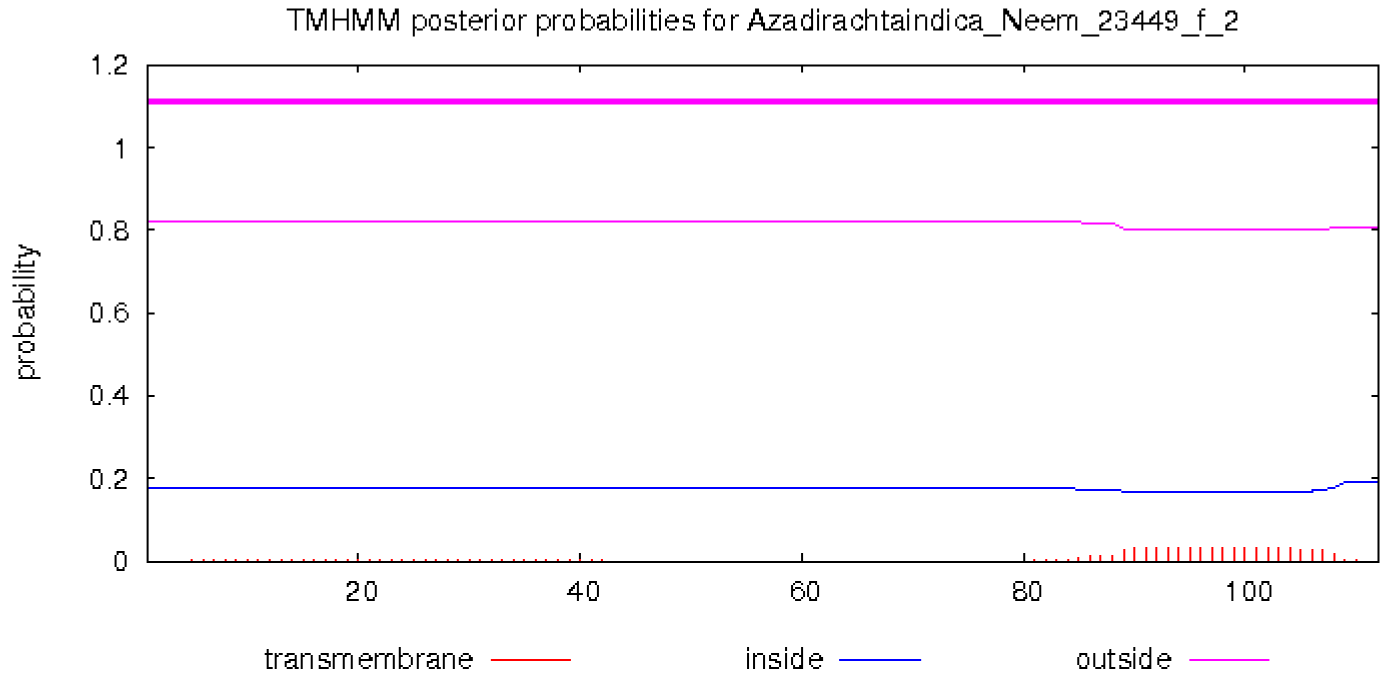

plot in postscript, script for making the plot in gnuplot, data for plot

```
# Azadirachtaindica_Neem_2345_f_3 Length: 661
# Azadirachtaindica_Neem_2345_f_3 Number of predicted TMHs:  0
```

```
# Azadirachtaindica_Neem_2345_f_3 Exp number of AAs in TMHs: 23.40141
# Azadirachtaindica_Neem_2345_f_3 Exp number, first 60 AAs:  0
# Azadirachtaindica_Neem_2345_f_3 Total prob of N-in:        0.00015
Azadirachtaindica_Neem_2345_f_3 TMHMM2.0        outside      1   661
```

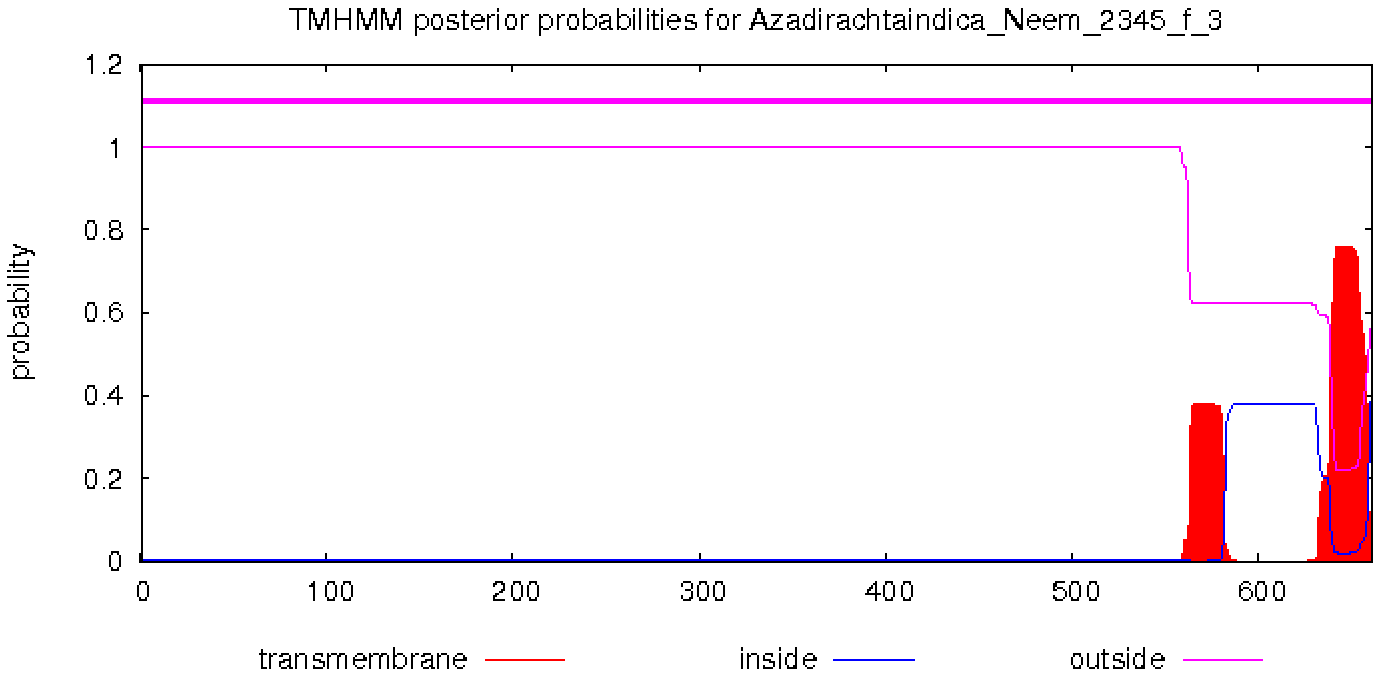

plot in postscript, script for making the plot in gnuplot, data for plot

---

```
# Azadirachtaindica_Neem_23729_f_1 Length: 182
# Azadirachtaindica_Neem_23729_f_1 Number of predicted TMHs:  0
# Azadirachtaindica_Neem_23729_f_1 Exp number of AAs in TMHs: 0.05727
# Azadirachtaindica_Neem_23729_f_1 Exp number, first 60 AAs:  0.00017
# Azadirachtaindica_Neem_23729_f_1 Total prob of N-in:        0.16034
Azadirachtaindica_Neem_23729_f_1        TMHMM2.0        outside      1   182
```

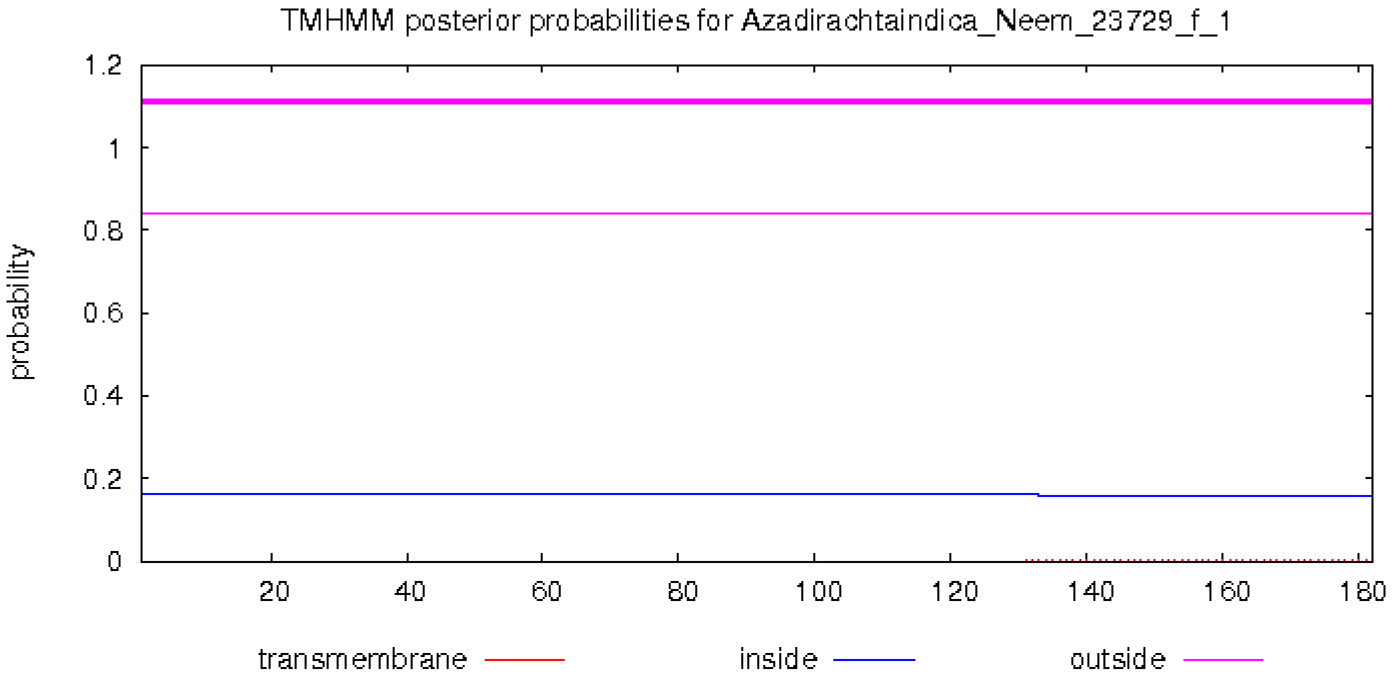

plot in postscript, script for making the plot in gnuplot, data for plot

---

```
# Azadirachtaindica_Neem_2377_f_1 Length: 327
# Azadirachtaindica_Neem_2377_f_1 Number of predicted TMHs:  0
```

```
# Azadirachtaindica_Neem_2377_f_1 Exp number of AAs in TMHs: 0
# Azadirachtaindica_Neem_2377_f_1 Exp number, first 60 AAs:  0
# Azadirachtaindica_Neem_2377_f_1 Total prob of N-in:        0.02737
Azadirachtaindica_Neem_2377_f_1 TMHMM2.0        outside      1   327
```

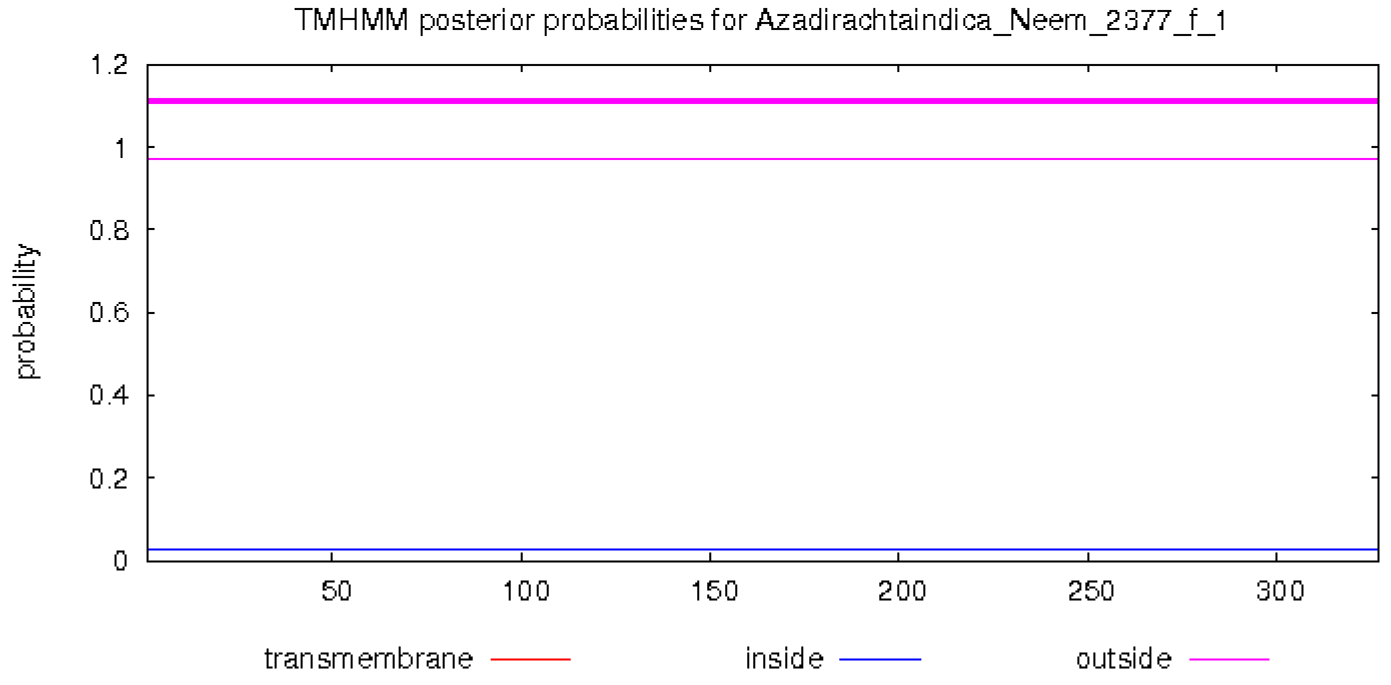

plot in postscript, script for making the plot in gnuplot, data for plot

---

```
# Azadirachtaindica_Neem_24521_f_3 Length: 294
# Azadirachtaindica_Neem_24521_f_3 Number of predicted TMHs:  0
# Azadirachtaindica_Neem_24521_f_3 Exp number of AAs in TMHs: 0.00035
# Azadirachtaindica_Neem_24521_f_3 Exp number, first 60 AAs:  0.00035
# Azadirachtaindica_Neem_24521_f_3 Total prob of N-in:        0.03611
Azadirachtaindica_Neem_24521_f_3        TMHMM2.0        outside      1   294
```

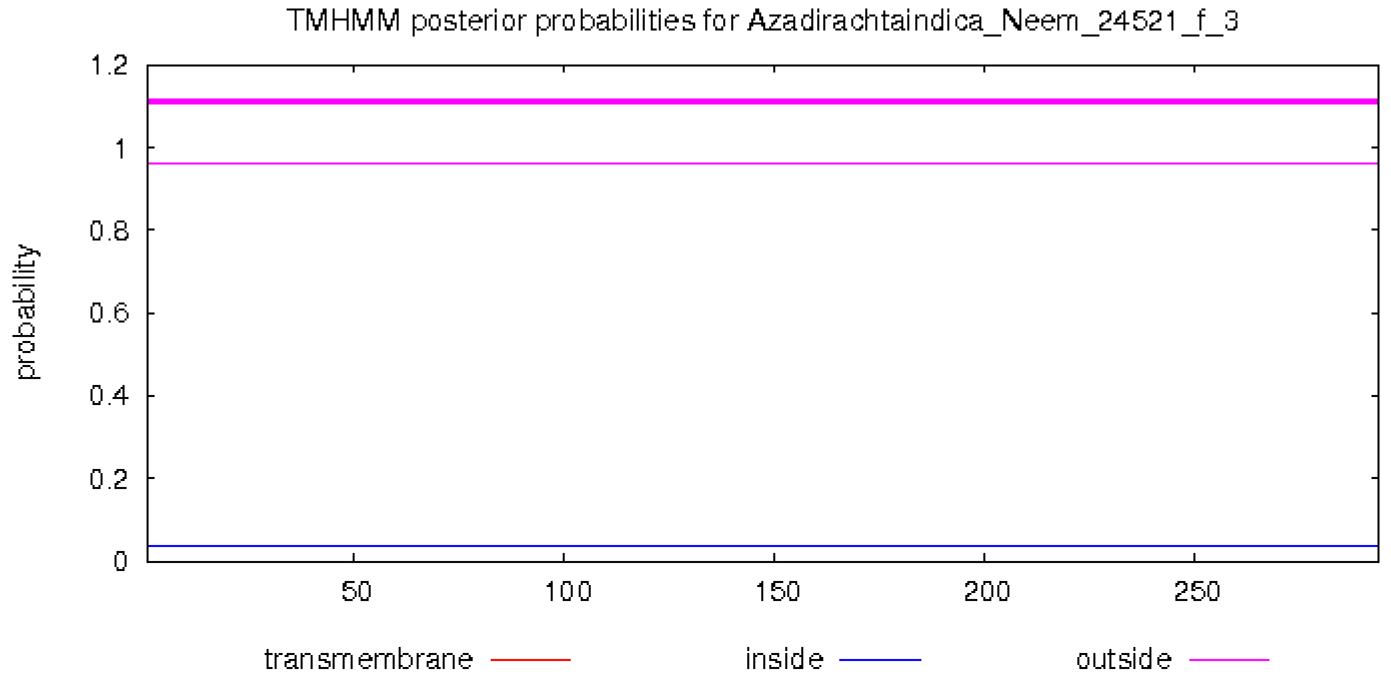

plot in postscript, script for making the plot in gnuplot, data for plot

---

```
# Azadirachtaindica_Neem_2455_f_1 Length: 271
# Azadirachtaindica_Neem_2455_f_1 Number of predicted TMHs:  0
```

```
# Azadirachtaindica_Neem_2455_f_1 Exp number of AAs in TMHs: 0
# Azadirachtaindica_Neem_2455_f_1 Exp number, first 60 AAs:  0
# Azadirachtaindica_Neem_2455_f_1 Total prob of N-in:        0.03241
Azadirachtaindica_Neem_2455_f_1 TMHMM2.0        outside      1   271
```

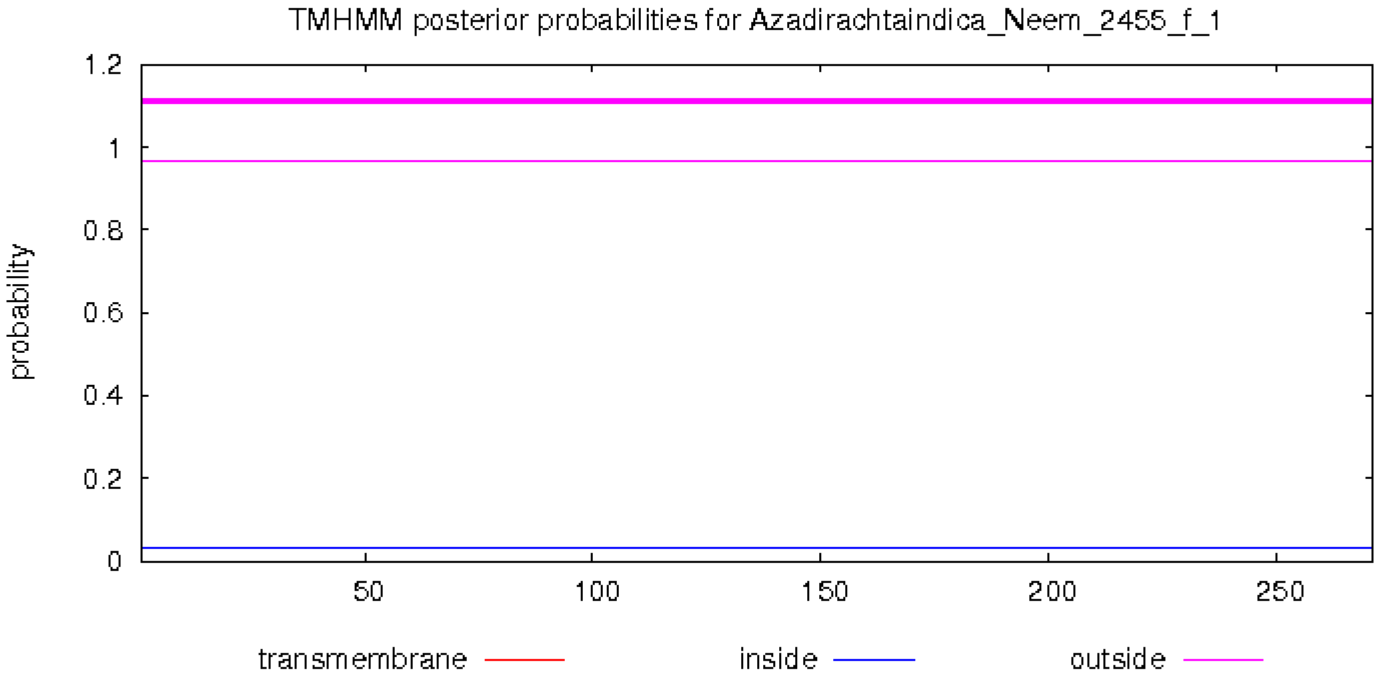

plot in postscript, script for making the plot in gnuplot, data for plot

---

```
# Azadirachtaindica_Neem_2493_f_1 Length: 230
# Azadirachtaindica_Neem_2493_f_1 Number of predicted TMHs:  0
# Azadirachtaindica_Neem_2493_f_1 Exp number of AAs in TMHs: 0.00036
# Azadirachtaindica_Neem_2493_f_1 Exp number, first 60 AAs:  0.00036
# Azadirachtaindica_Neem_2493_f_1 Total prob of N-in:        0.05828
Azadirachtaindica_Neem_2493_f_1 TMHMM2.0        outside      1   230
```

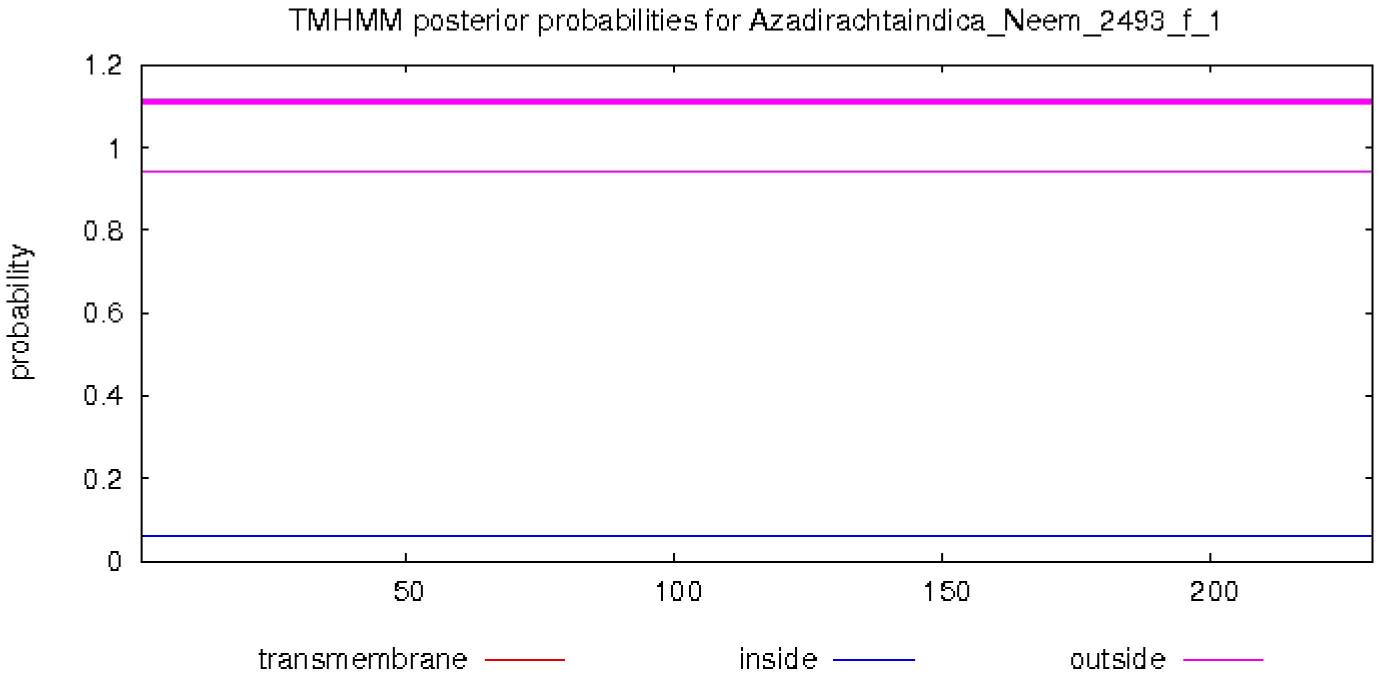

plot in postscript, script for making the plot in gnuplot, data for plot

---

```
# Azadirachtaindica_Neem_24960_f_1 Length: 334
# Azadirachtaindica_Neem_24960_f_1 Number of predicted TMHs:  0
```

```
# Azadirachtaindica_Neem_24960_f_1 Exp number of AAs in TMHs: 0
# Azadirachtaindica_Neem_24960_f_1 Exp number, first 60 AAs:  0
# Azadirachtaindica_Neem_24960_f_1 Total prob of N-in:        0.00288
Azadirachtaindica_Neem_24960_f_1        TMHMM2.0        outside      1   334
```

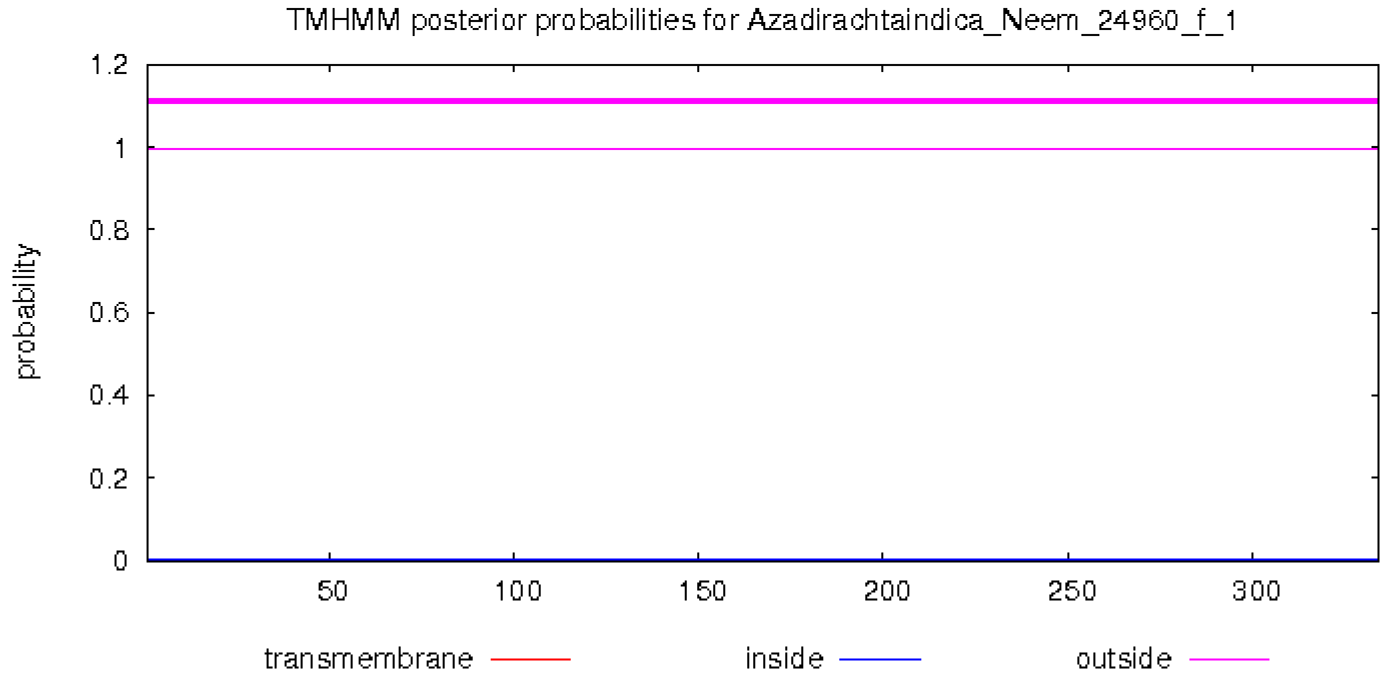

plot in postscript, script for making the plot in gnuplot, data for plot

---

```
# Azadirachtaindica_Neem_2507_f_1 Length: 223
# Azadirachtaindica_Neem_2507_f_1 Number of predicted TMHs:  0
# Azadirachtaindica_Neem_2507_f_1 Exp number of AAs in TMHs: 0.97691
# Azadirachtaindica_Neem_2507_f_1 Exp number, first 60 AAs:  0.97478
# Azadirachtaindica_Neem_2507_f_1 Total prob of N-in:        0.23103
Azadirachtaindica_Neem_2507_f_1 TMHMM2.0        outside      1   223
```

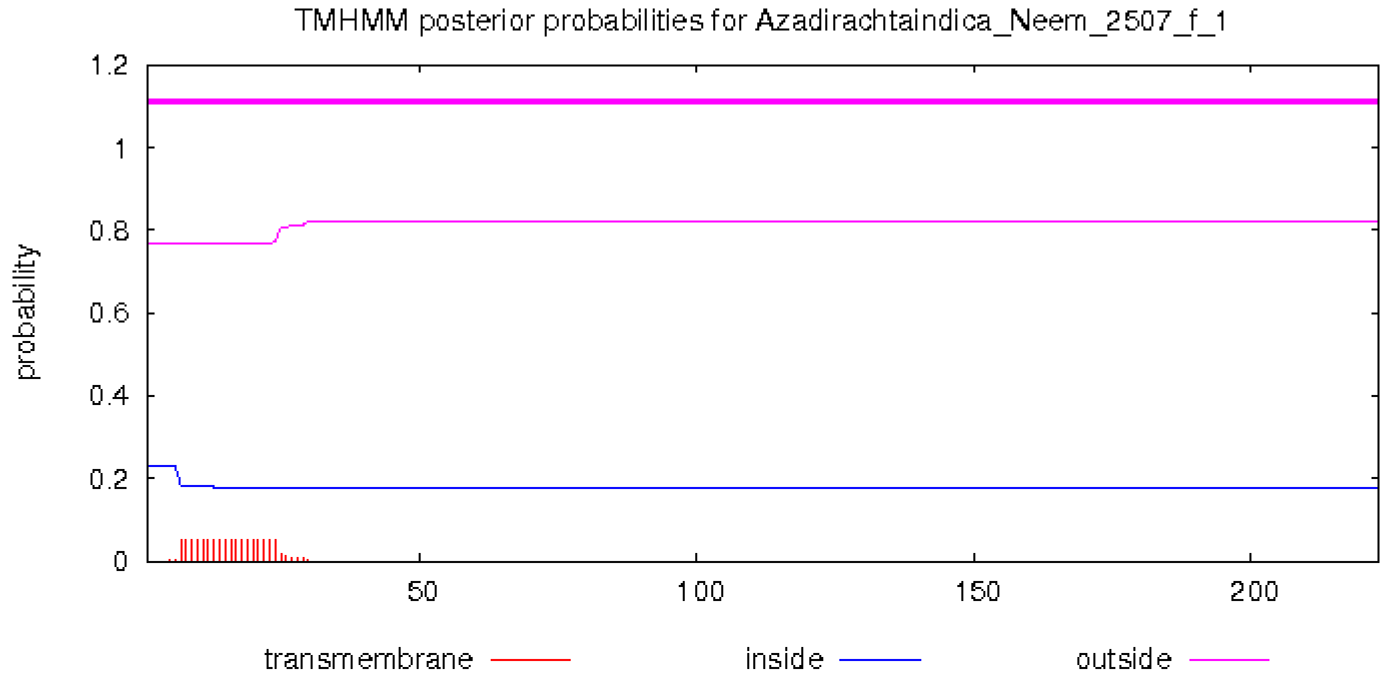

plot in postscript, script for making the plot in gnuplot, data for plot

---

```
# Azadirachtaindica_Neem_25879_f_1 Length: 212
# Azadirachtaindica_Neem_25879_f_1 Number of predicted TMHs:  0
```

```
# Azadirachtaindica_Neem_25879_f_1 Exp number of AAs in TMHs: 0.00293
# Azadirachtaindica_Neem_25879_f_1 Exp number, first 60 AAs:  0.00158
# Azadirachtaindica_Neem_25879_f_1 Total prob of N-in:        0.06808
Azadirachtaindica_Neem_25879_f_1        TMHMM2.0        outside      1   212
```

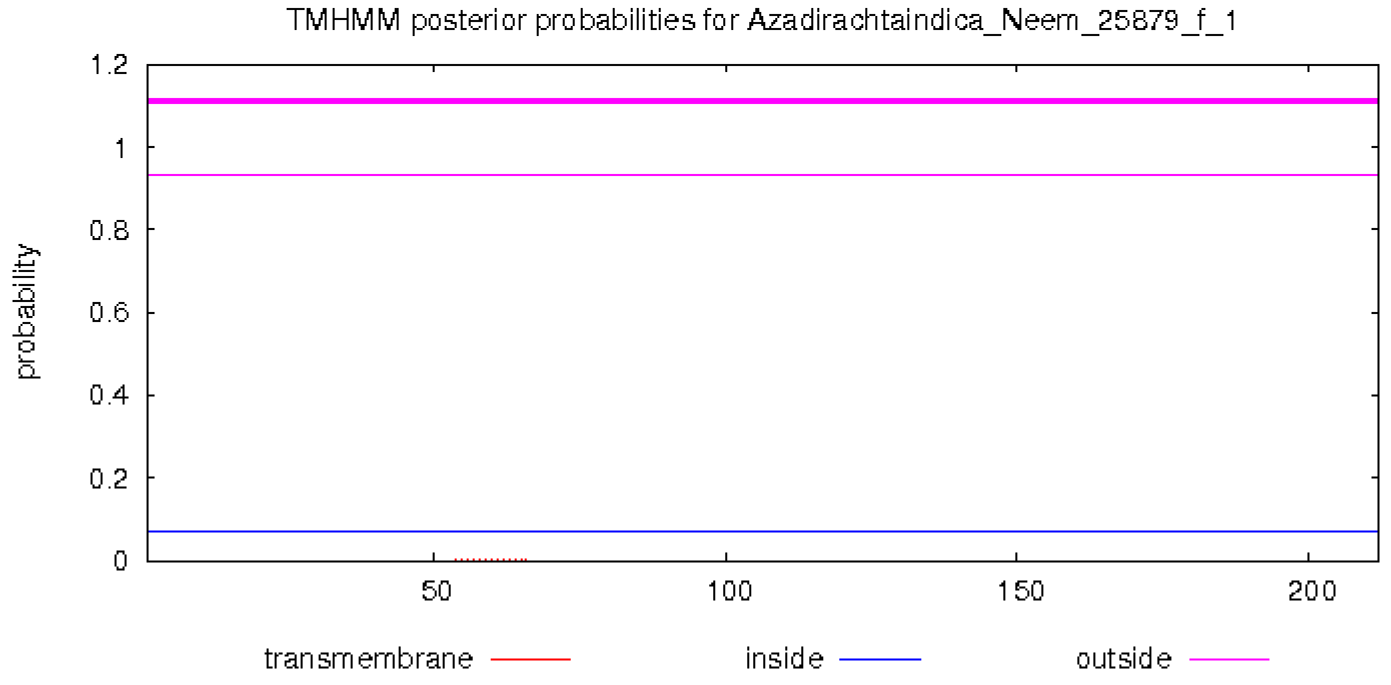

plot in postscript, script for making the plot in gnuplot, data for plot

---

```
# Azadirachtaindica_Neem_2659_f_2 Length: 314
# Azadirachtaindica_Neem_2659_f_2 Number of predicted TMHs:  0
# Azadirachtaindica_Neem_2659_f_2 Exp number of AAs in TMHs: 0.0002
# Azadirachtaindica_Neem_2659_f_2 Exp number, first 60 AAs:  0.0001
# Azadirachtaindica_Neem_2659_f_2 Total prob of N-in:        0.02387
Azadirachtaindica_Neem_2659_f_2 TMHMM2.0        outside      1   314
```

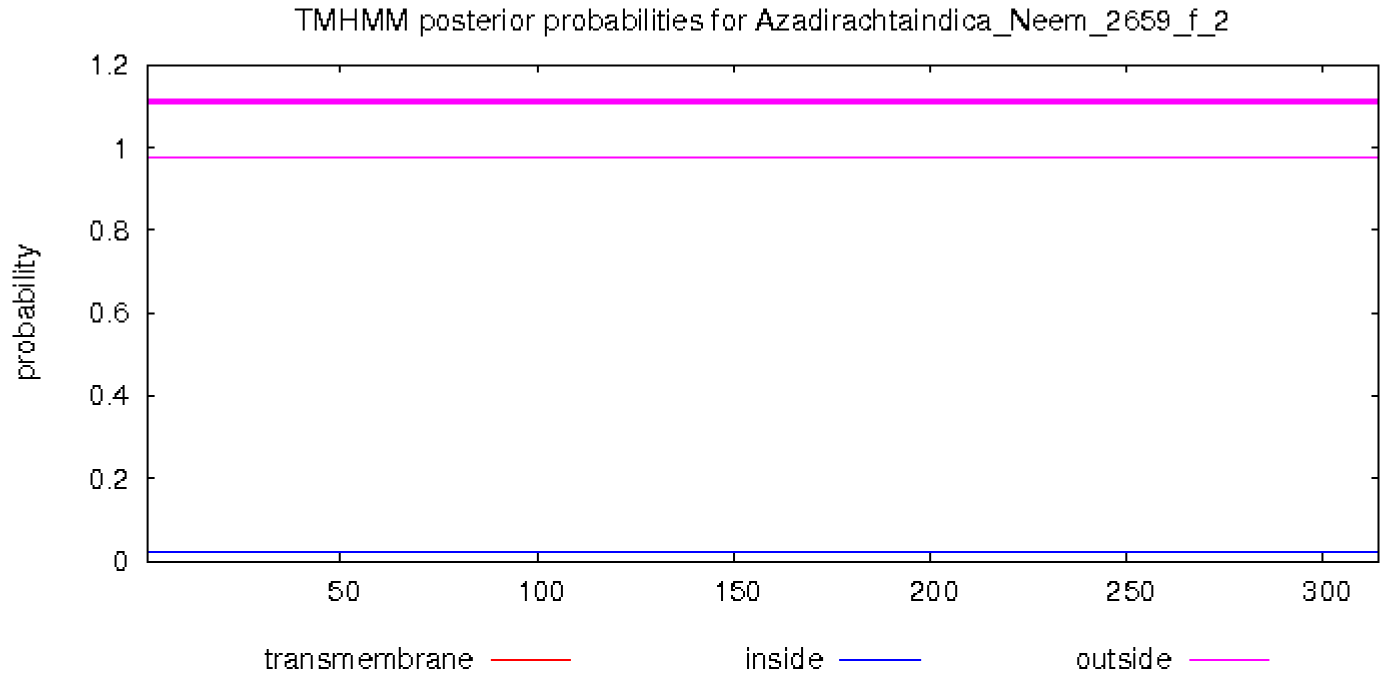

plot in postscript, script for making the plot in gnuplot, data for plot

---

```
# Azadirachtaindica_Neem_26821_f_2 Length: 354
# Azadirachtaindica_Neem_26821_f_2 Number of predicted TMHs:  0
```

```
# Azadirachtaindica_Neem_26821_f_2 Exp number of AAs in TMHs: 0.00062
# Azadirachtaindica_Neem_26821_f_2 Exp number, first 60 AAs:  0.00042
# Azadirachtaindica_Neem_26821_f_2 Total prob of N-in:        0.01630
Azadirachtaindica_Neem_26821_f_2        TMHMM2.0        outside      1   354
```

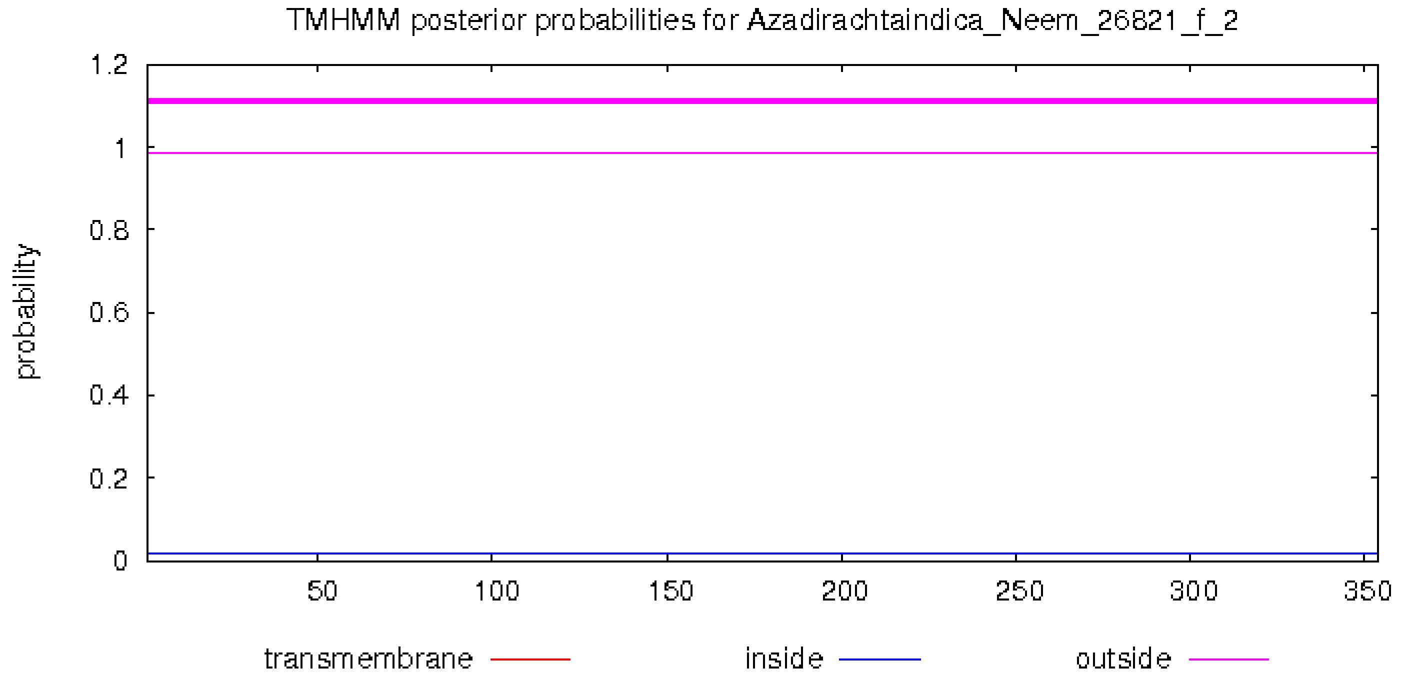

plot in postscript, script for making the plot in gnuplot, data for plot

---

```
# Azadirachtaindica_Neem_27088_f_1 Length: 309
# Azadirachtaindica_Neem_27088_f_1 Number of predicted TMHs:  0
# Azadirachtaindica_Neem_27088_f_1 Exp number of AAs in TMHs: 0.00127
# Azadirachtaindica_Neem_27088_f_1 Exp number, first 60 AAs:  0
# Azadirachtaindica_Neem_27088_f_1 Total prob of N-in:        0.02126
Azadirachtaindica_Neem_27088_f_1        TMHMM2.0        outside      1   309
```

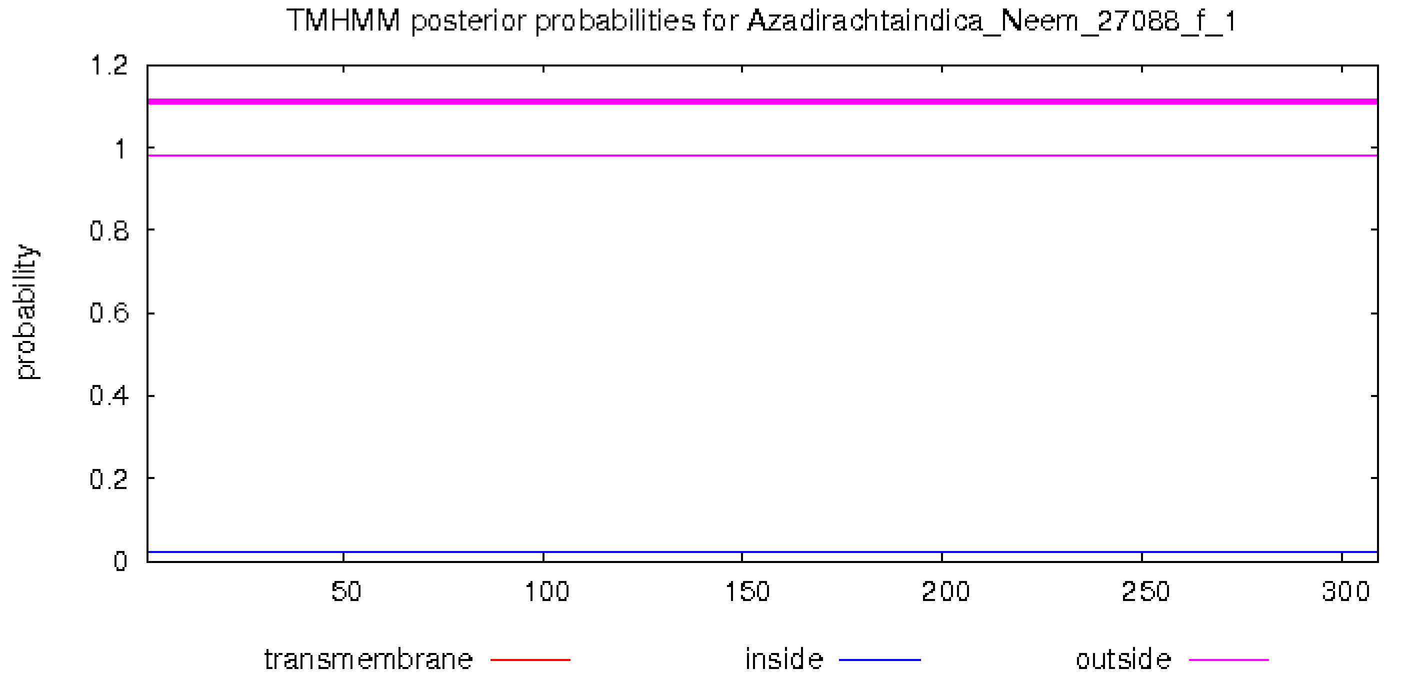

plot in postscript, script for making the plot in gnuplot, data for plot

---

```
# Azadirachtaindica_Neem_27137_f_3 Length: 384
# Azadirachtaindica_Neem_27137_f_3 Number of predicted TMHs:  0
```

```
# Azadirachtaindica_Neem_27137_f_3 Exp number of AAs in TMHs: 0.00082
# Azadirachtaindica_Neem_27137_f_3 Exp number, first 60 AAs:  0
# Azadirachtaindica_Neem_27137_f_3 Total prob of N-in:        0.02242
Azadirachtaindica_Neem_27137_f_3        TMHMM2.0        outside      1   384
```

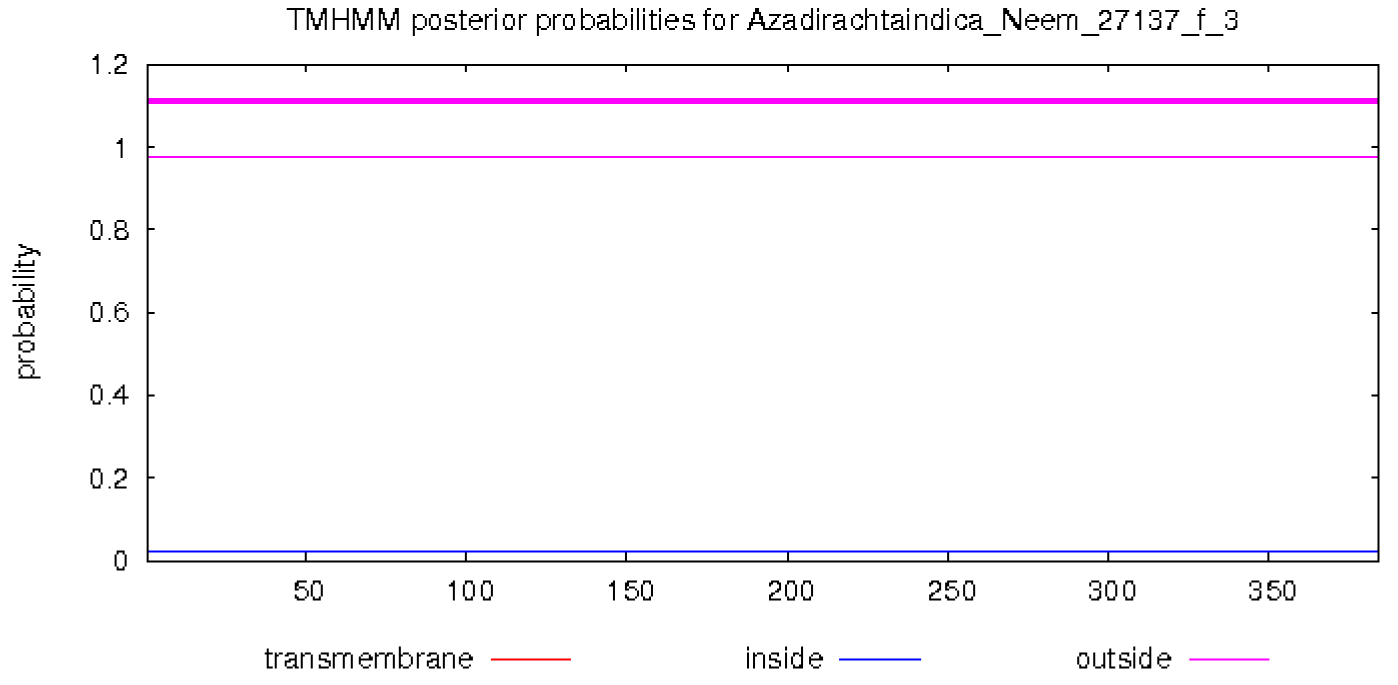

plot in postscript, script for making the plot in gnuplot, data for plot

---

```
# Azadirachtaindica_Neem_2718_f_2 Length: 253
# Azadirachtaindica_Neem_2718_f_2 Number of predicted TMHs:  0
# Azadirachtaindica_Neem_2718_f_2 Exp number of AAs in TMHs: 0.0066
# Azadirachtaindica_Neem_2718_f_2 Exp number, first 60 AAs:  0.00188
# Azadirachtaindica_Neem_2718_f_2 Total prob of N-in:        0.07946
Azadirachtaindica_Neem_2718_f_2 TMHMM2.0        outside      1   253
```

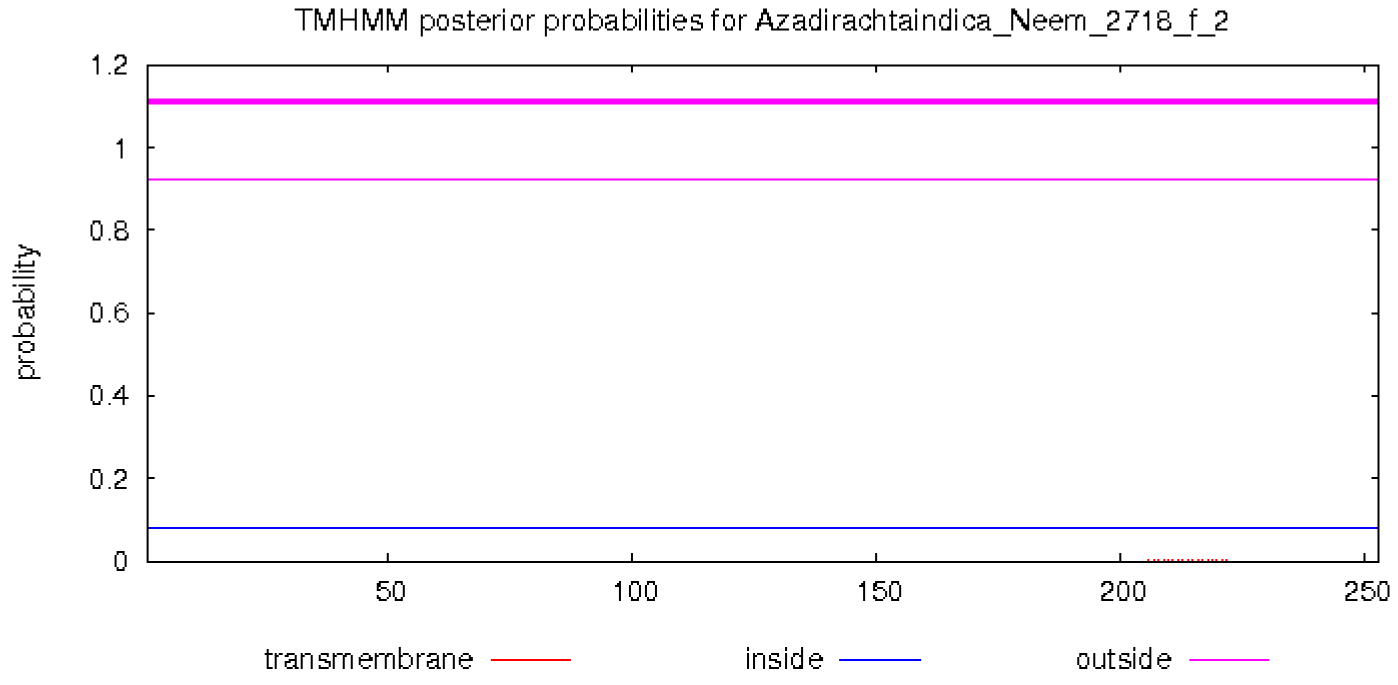

plot in postscript, script for making the plot in gnuplot, data for plot

---

```
# Azadirachtaindica_Neem_27369_f_1 Length: 361
# Azadirachtaindica_Neem_27369_f_1 Number of predicted TMHs:  0
```

```
# Azadirachtaindica_Neem_27369_f_1 Exp number of AAs in TMHs: 0.00096
# Azadirachtaindica_Neem_27369_f_1 Exp number, first 60 AAs:  0
# Azadirachtaindica_Neem_27369_f_1 Total prob of N-in:        0.00967
Azadirachtaindica_Neem_27369_f_1        TMHMM2.0        outside      1   361
```

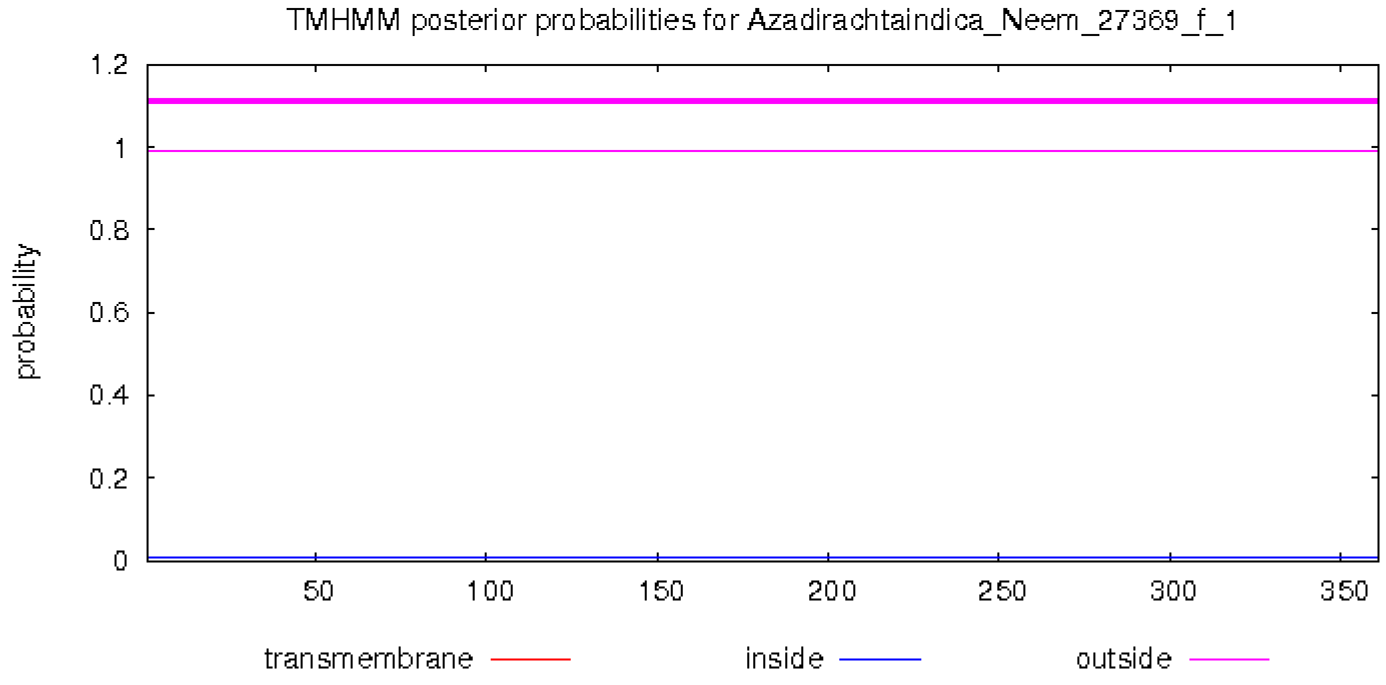

plot in postscript, script for making the plot in gnuplot, data for plot

---

```
# Azadirachtaindica_Neem_27894_f_1 Length: 191
# Azadirachtaindica_Neem_27894_f_1 Number of predicted TMHs:  0
# Azadirachtaindica_Neem_27894_f_1 Exp number of AAs in TMHs: 0
# Azadirachtaindica_Neem_27894_f_1 Exp number, first 60 AAs:  0
# Azadirachtaindica_Neem_27894_f_1 Total prob of N-in:        0.03624
Azadirachtaindica_Neem_27894_f_1        TMHMM2.0        outside      1   191
```

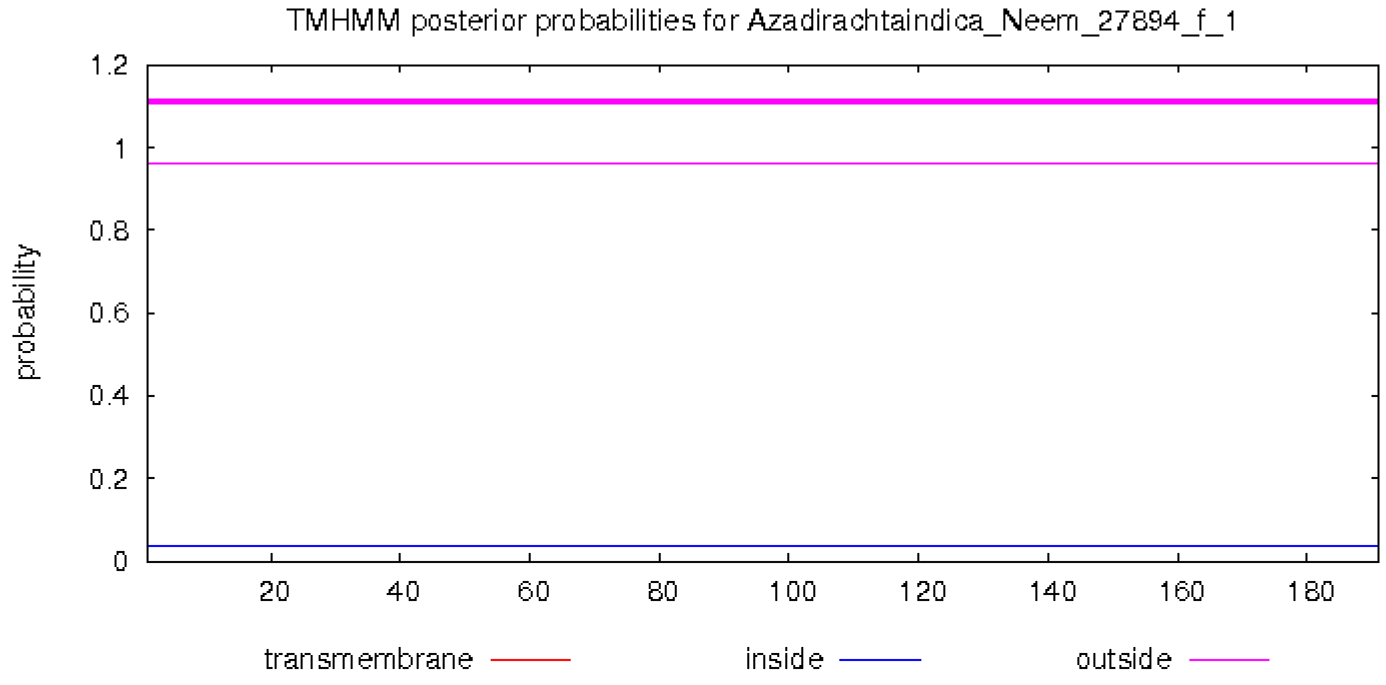

plot in postscript, script for making the plot in gnuplot, data for plot

---

```
# Azadirachtaindica_Neem_28050_f_1 Length: 298
# Azadirachtaindica_Neem_28050_f_1 Number of predicted TMHs:  0
```

```
# Azadirachtaindica_Neem_28050_f_1 Exp number of AAs in TMHs: 0.04441
# Azadirachtaindica_Neem_28050_f_1 Exp number, first 60 AAs:  0.00125
# Azadirachtaindica_Neem_28050_f_1 Total prob of N-in:        0.02866
Azadirachtaindica_Neem_28050_f_1        TMHMM2.0        outside      1   298
```

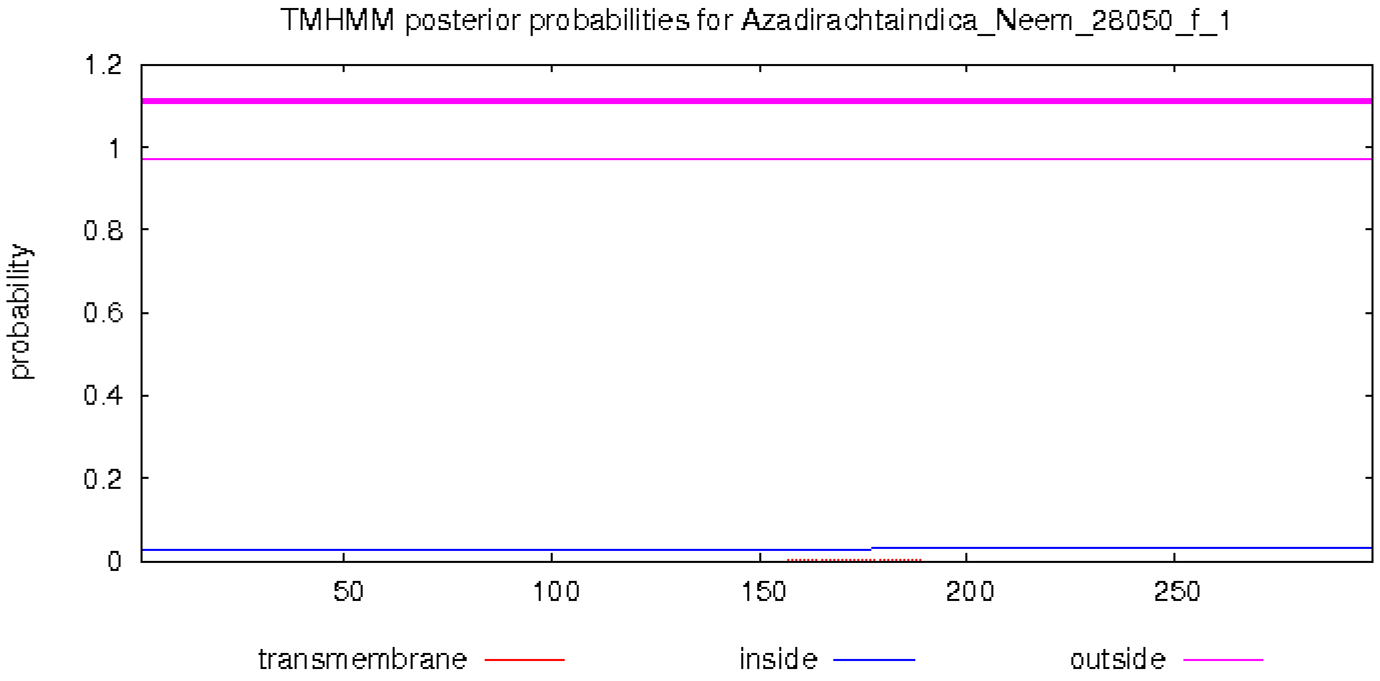

plot in postscript, script for making the plot in gnuplot, data for plot

---

```
# Azadirachtaindica_Neem_28263_f_1 Length: 155
# Azadirachtaindica_Neem_28263_f_1 Number of predicted TMHs:  0
# Azadirachtaindica_Neem_28263_f_1 Exp number of AAs in TMHs: 0.09609
# Azadirachtaindica_Neem_28263_f_1 Exp number, first 60 AAs:  0
# Azadirachtaindica_Neem_28263_f_1 Total prob of N-in:        0.04302
Azadirachtaindica_Neem_28263_f_1        TMHMM2.0        outside      1   155
```

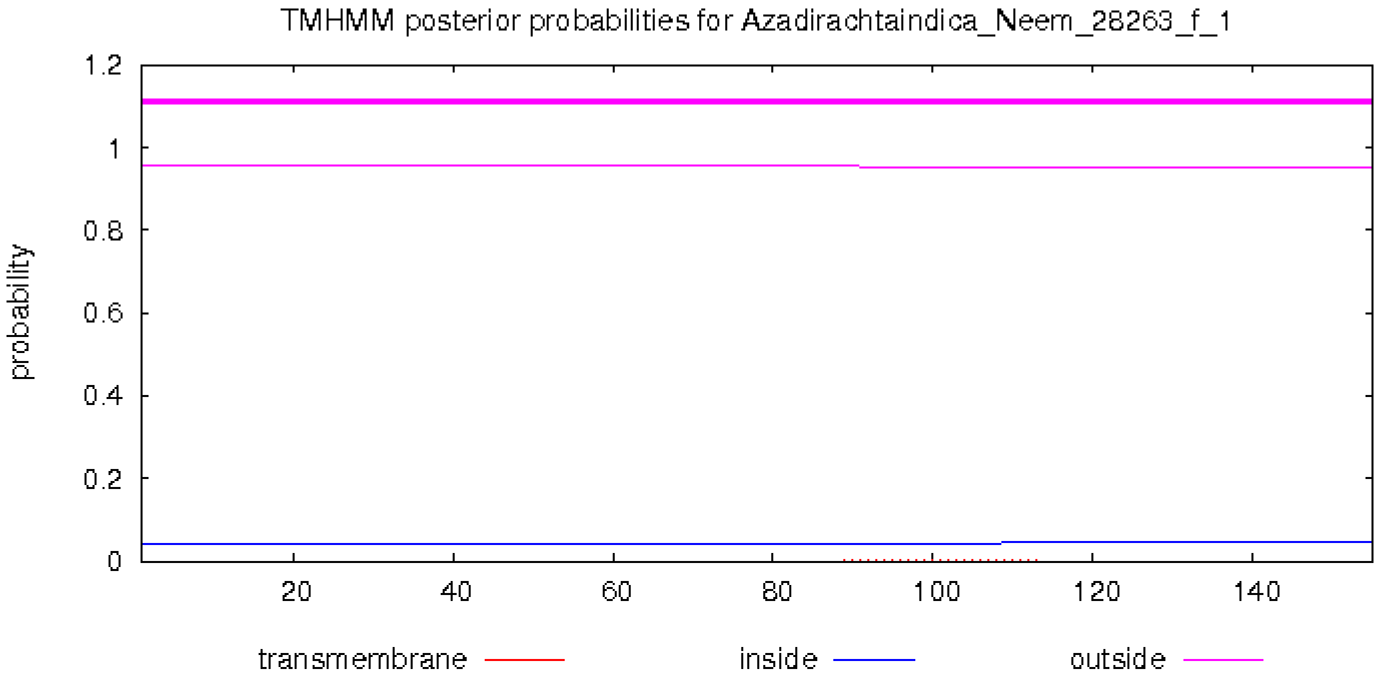

plot in postscript, script for making the plot in gnuplot, data for plot

---

```
# Azadirachtaindica_Neem_28678_f_1 Length: 417
# Azadirachtaindica_Neem_28678_f_1 Number of predicted TMHs:  0
```

```
# Azadirachtaindica_Neem_28678_f_1 Exp number of AAs in TMHs: 0
# Azadirachtaindica_Neem_28678_f_1 Exp number, first 60 AAs:  0
# Azadirachtaindica_Neem_28678_f_1 Total prob of N-in:        0.00382
Azadirachtaindica_Neem_28678_f_1        TMHMM2.0        outside      1   417
```

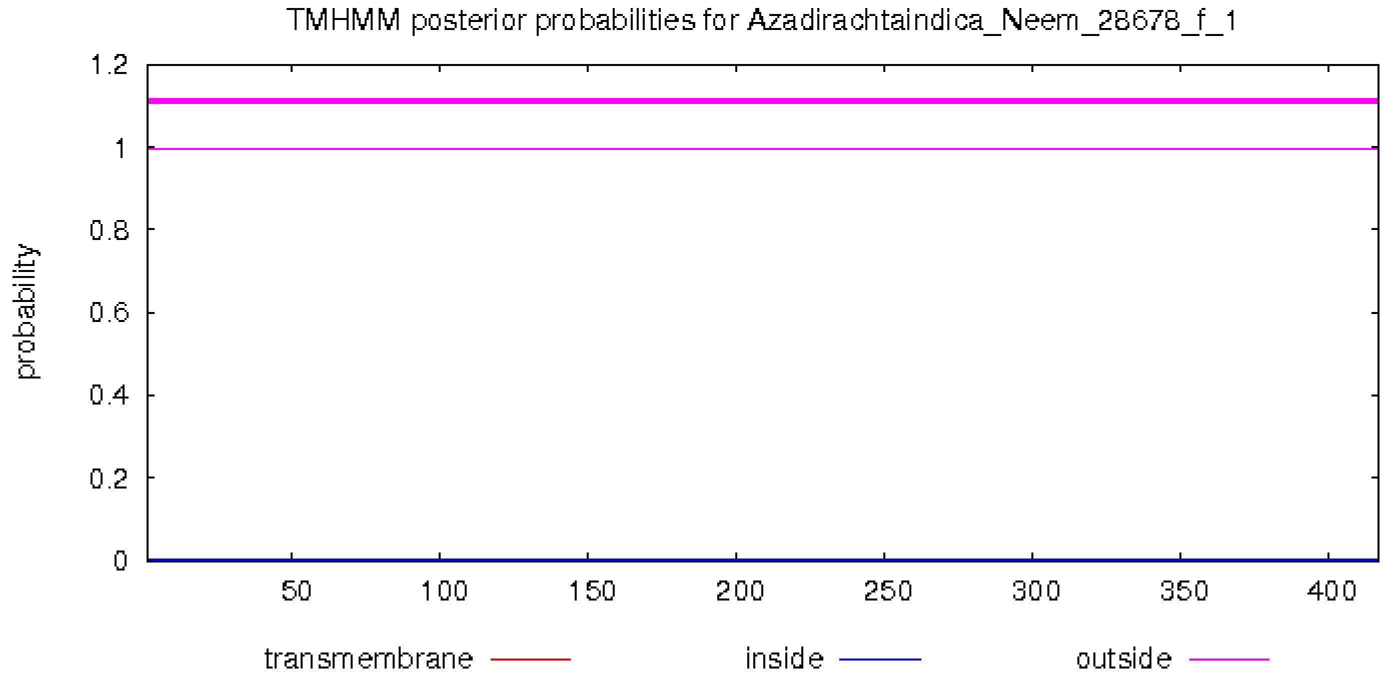

plot in postscript, script for making the plot in gnuplot, data for plot

---

```
# Azadirachtaindica_Neem_2867_f_1 Length: 305
# Azadirachtaindica_Neem_2867_f_1 Number of predicted TMHs:  0
# Azadirachtaindica_Neem_2867_f_1 Exp number of AAs in TMHs: 0
# Azadirachtaindica_Neem_2867_f_1 Exp number, first 60 AAs:  0
# Azadirachtaindica_Neem_2867_f_1 Total prob of N-in:        0.03608
Azadirachtaindica_Neem_2867_f_1 TMHMM2.0        outside      1   305
```

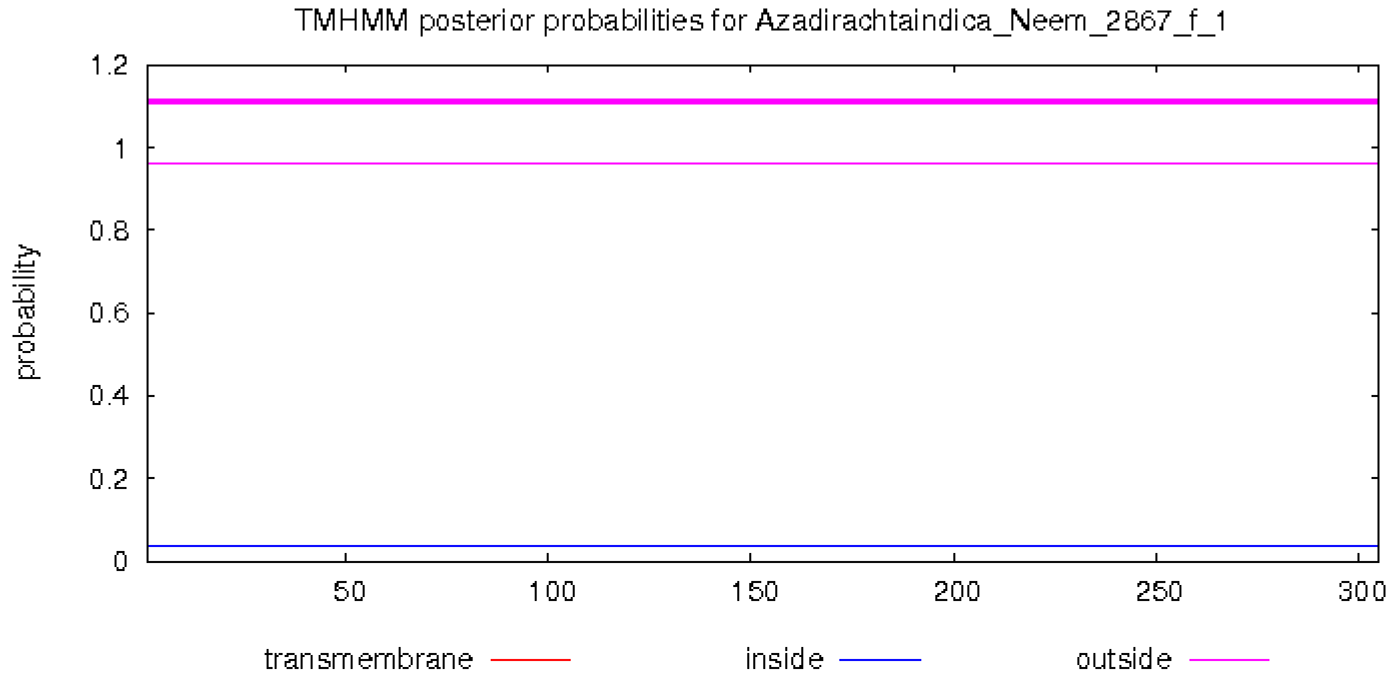

plot in postscript, script for making the plot in gnuplot, data for plot

---

```
# Azadirachtaindica_Neem_28837_f_1 Length: 333
# Azadirachtaindica_Neem_28837_f_1 Number of predicted TMHs:  0
```

```
# Azadirachtaindica_Neem_28837_f_1 Exp number of AAs in TMHs: 0.00034
# Azadirachtaindica_Neem_28837_f_1 Exp number, first 60 AAs:  0
# Azadirachtaindica_Neem_28837_f_1 Total prob of N-in:        0.00501
Azadirachtaindica_Neem_28837_f_1        TMHMM2.0        outside      1   333
```

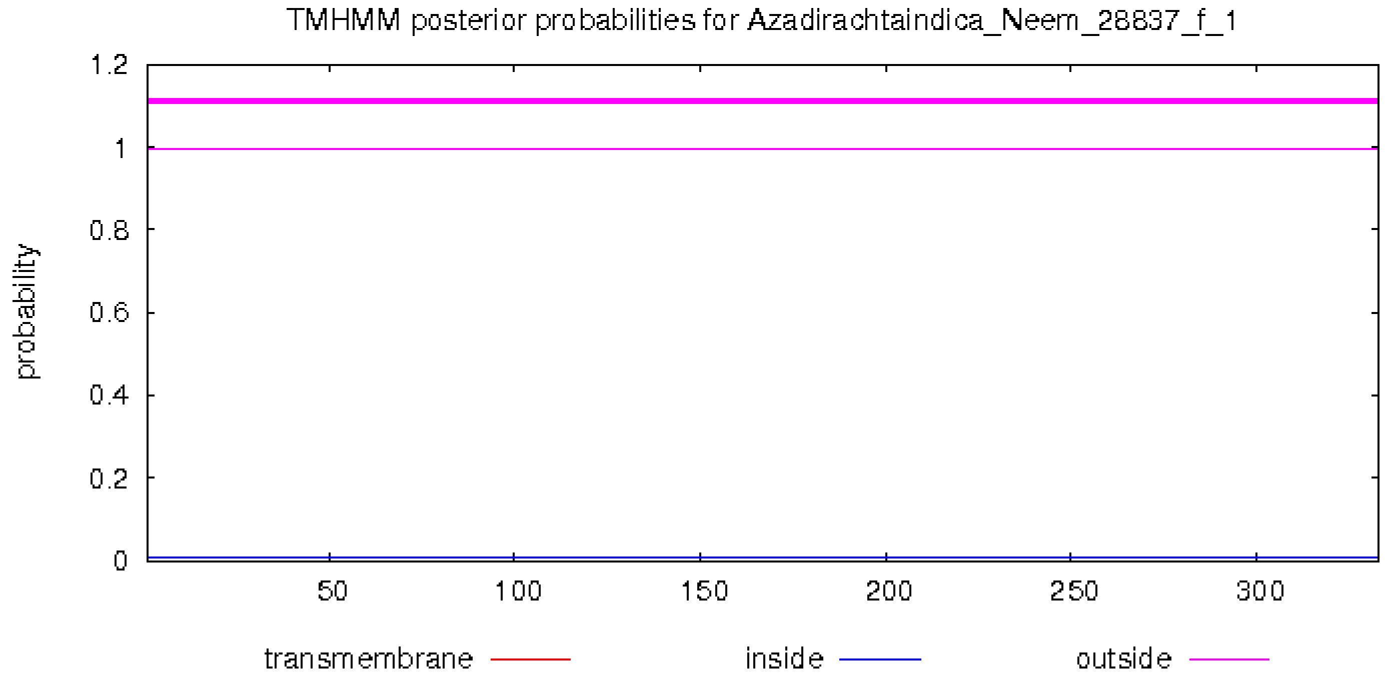

plot in postscript, script for making the plot in gnuplot, data for plot

---

```
# Azadirachtaindica_Neem_29032_f_1 Length: 209
# Azadirachtaindica_Neem_29032_f_1 Number of predicted TMHs:  0
# Azadirachtaindica_Neem_29032_f_1 Exp number of AAs in TMHs: 0.00056
# Azadirachtaindica_Neem_29032_f_1 Exp number, first 60 AAs:  0
# Azadirachtaindica_Neem_29032_f_1 Total prob of N-in:        0.06700
Azadirachtaindica_Neem_29032_f_1        TMHMM2.0        outside      1   209
```

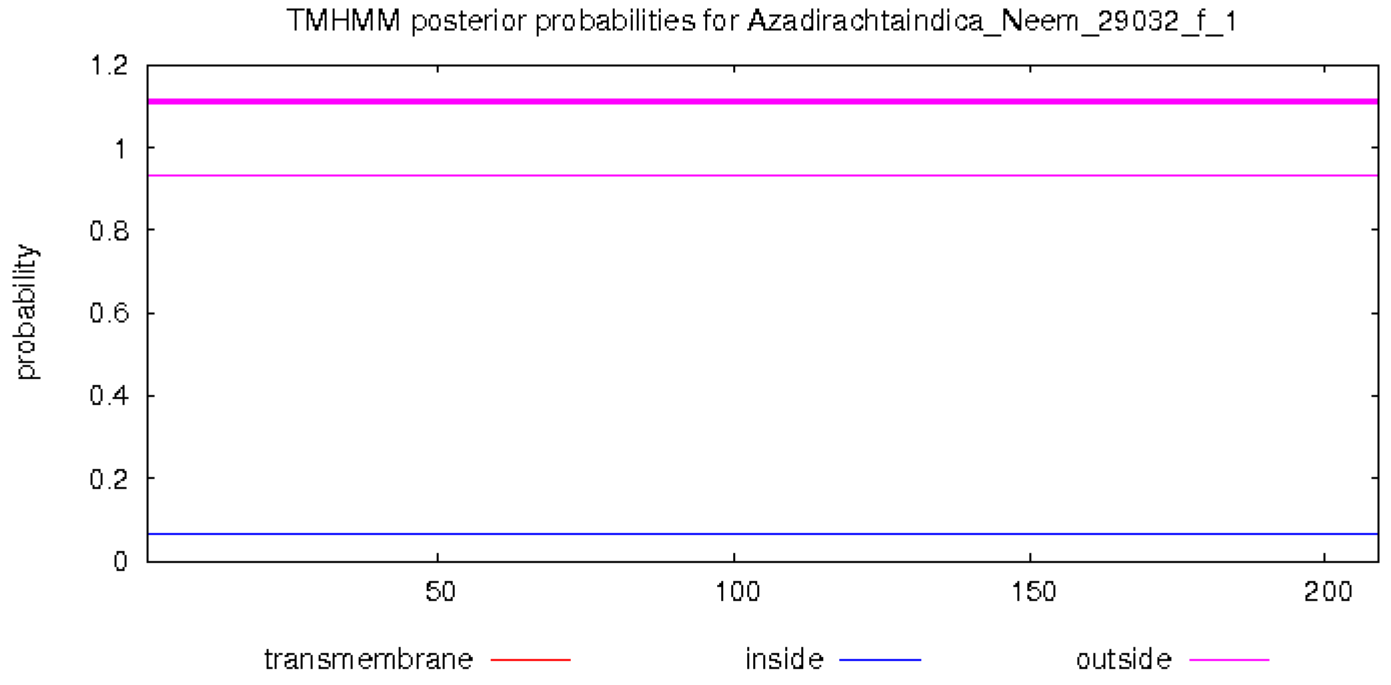

plot in postscript, script for making the plot in gnuplot, data for plot

---

```
# Azadirachtaindica_Neem_29819_f_1 Length: 313
# Azadirachtaindica_Neem_29819_f_1 Number of predicted TMHs:  0
```

```
# Azadirachtaindica_Neem_29819_f_1 Exp number of AAs in TMHs: 0.01643
# Azadirachtaindica_Neem_29819_f_1 Exp number, first 60 AAs:  0.0004
# Azadirachtaindica_Neem_29819_f_1 Total prob of N-in:        0.00873
Azadirachtaindica_Neem_29819_f_1        TMHMM2.0        outside      1   313
```

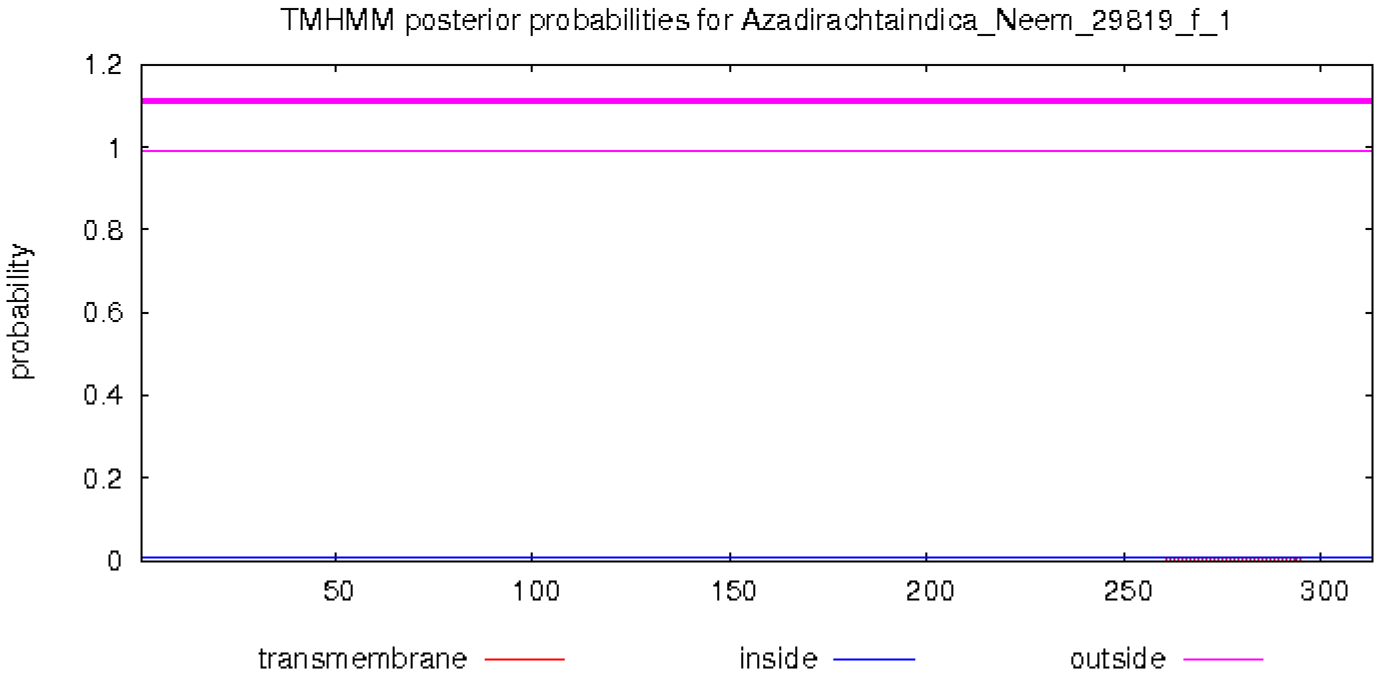

plot in postscript, script for making the plot in gnuplot, data for plot

---

```
# Azadirachtaindica_Neem_2986_a_1 Length: 262
# Azadirachtaindica_Neem_2986_a_1 Number of predicted TMHs:  0
# Azadirachtaindica_Neem_2986_a_1 Exp number of AAs in TMHs: 0.572460000000000001
# Azadirachtaindica_Neem_2986_a_1 Exp number, first 60 AAs:  0.1161
# Azadirachtaindica_Neem_2986_a_1 Total prob of N-in:        0.03628
Azadirachtaindica_Neem_2986_a_1 TMHMM2.0        outside      1   262
```

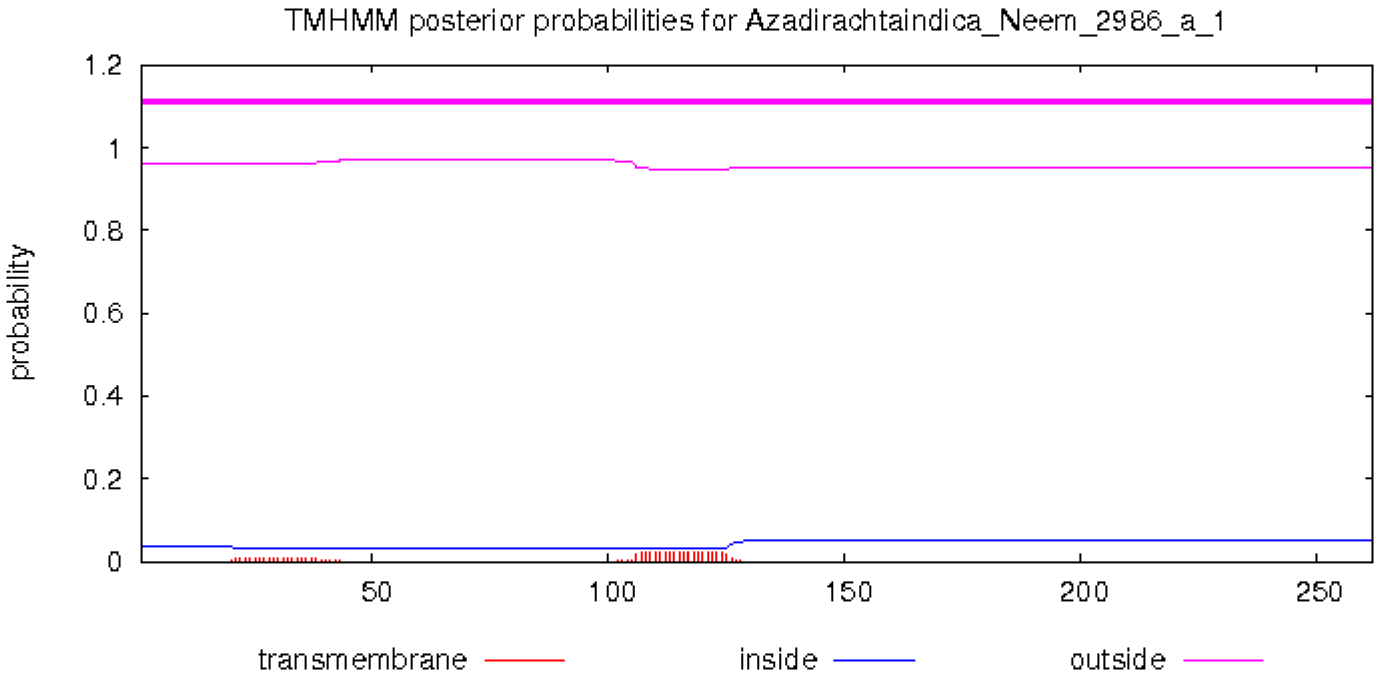

plot in postscript, script for making the plot in gnuplot, data for plot

---

```
# Azadirachtaindica_Neem_29920_f_1 Length: 262
# Azadirachtaindica_Neem_29920_f_1 Number of predicted TMHs:  0
```

```
# Azadirachtaindica_Neem_29920_f_1 Exp number of AAs in TMHs: 0.00107
# Azadirachtaindica_Neem_29920_f_1 Exp number, first 60 AAs:  0
# Azadirachtaindica_Neem_29920_f_1 Total prob of N-in:        0.04726
Azadirachtaindica_Neem_29920_f_1        TMHMM2.0        outside      1   262
```

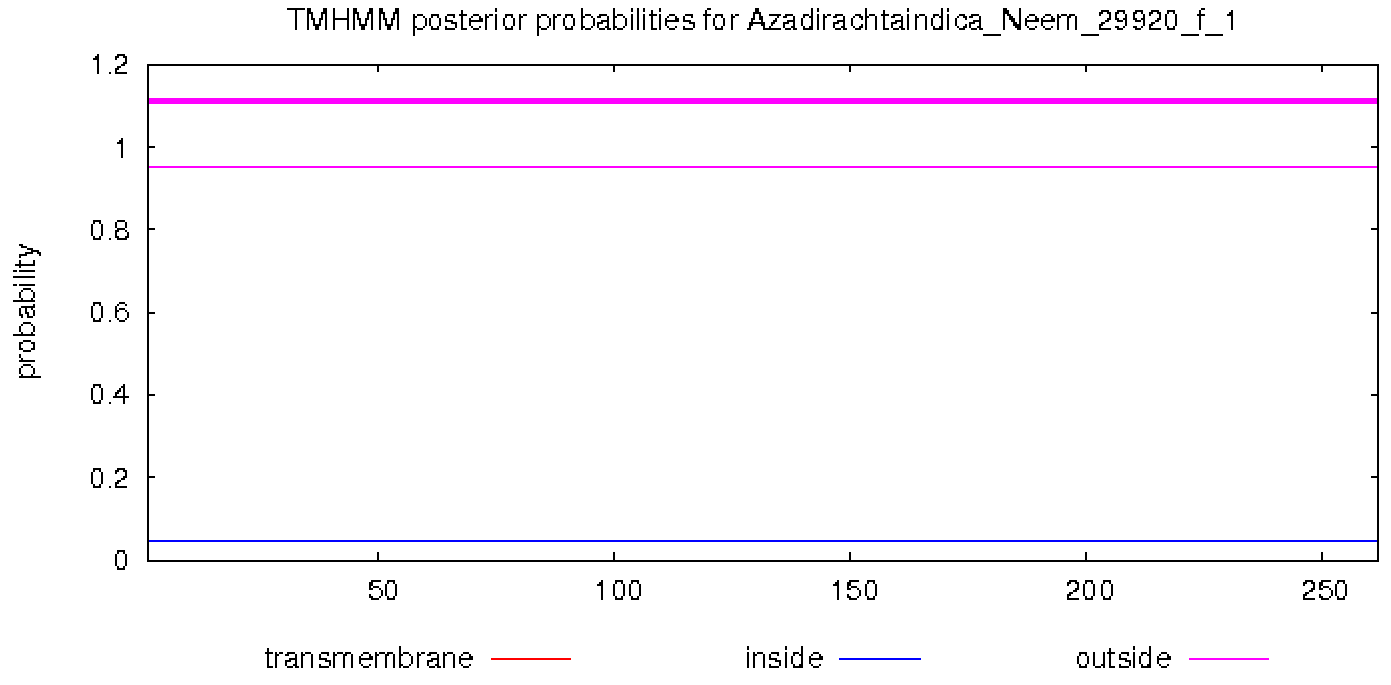

plot in postscript, script for making the plot in gnuplot, data for plot

---

```
# Azadirachtaindica_Neem_30138_f_1 Length: 153
# Azadirachtaindica_Neem_30138_f_1 Number of predicted TMHs:  0
# Azadirachtaindica_Neem_30138_f_1 Exp number of AAs in TMHs: 0.01888
# Azadirachtaindica_Neem_30138_f_1 Exp number, first 60 AAs:  0
# Azadirachtaindica_Neem_30138_f_1 Total prob of N-in:        0.04351
Azadirachtaindica_Neem_30138_f_1        TMHMM2.0        outside      1   153
```

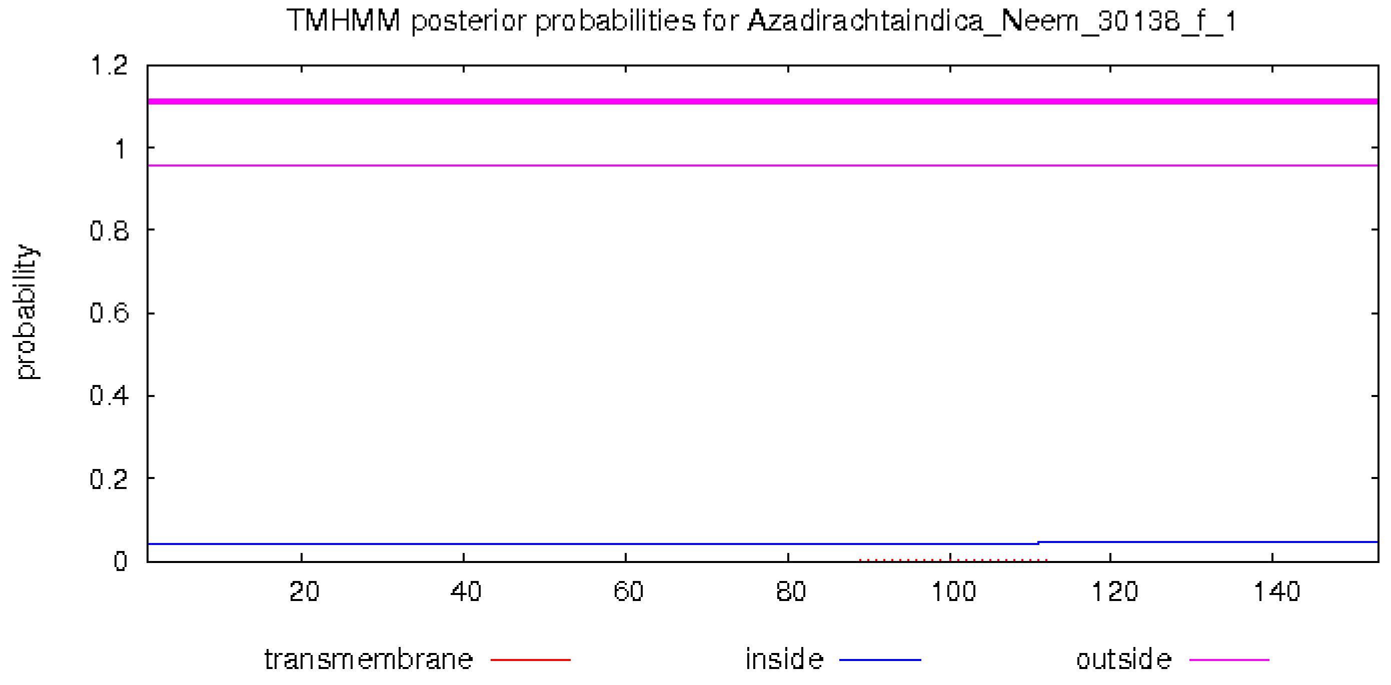

plot in postscript, script for making the plot in gnuplot, data for plot

---

```
# Azadirachtaindica_Neem_30361_f_1 Length: 150
# Azadirachtaindica_Neem_30361_f_1 Number of predicted TMHs:  0
```

```
# Azadirachtaindica_Neem_30361_f_1 Exp number of AAs in TMHs: 0.00052
# Azadirachtaindica_Neem_30361_f_1 Exp number, first 60 AAs:  0.00015
# Azadirachtaindica_Neem_30361_f_1 Total prob of N-in:        0.03163
Azadirachtaindica_Neem_30361_f_1        TMHMM2.0        outside      1   150
```

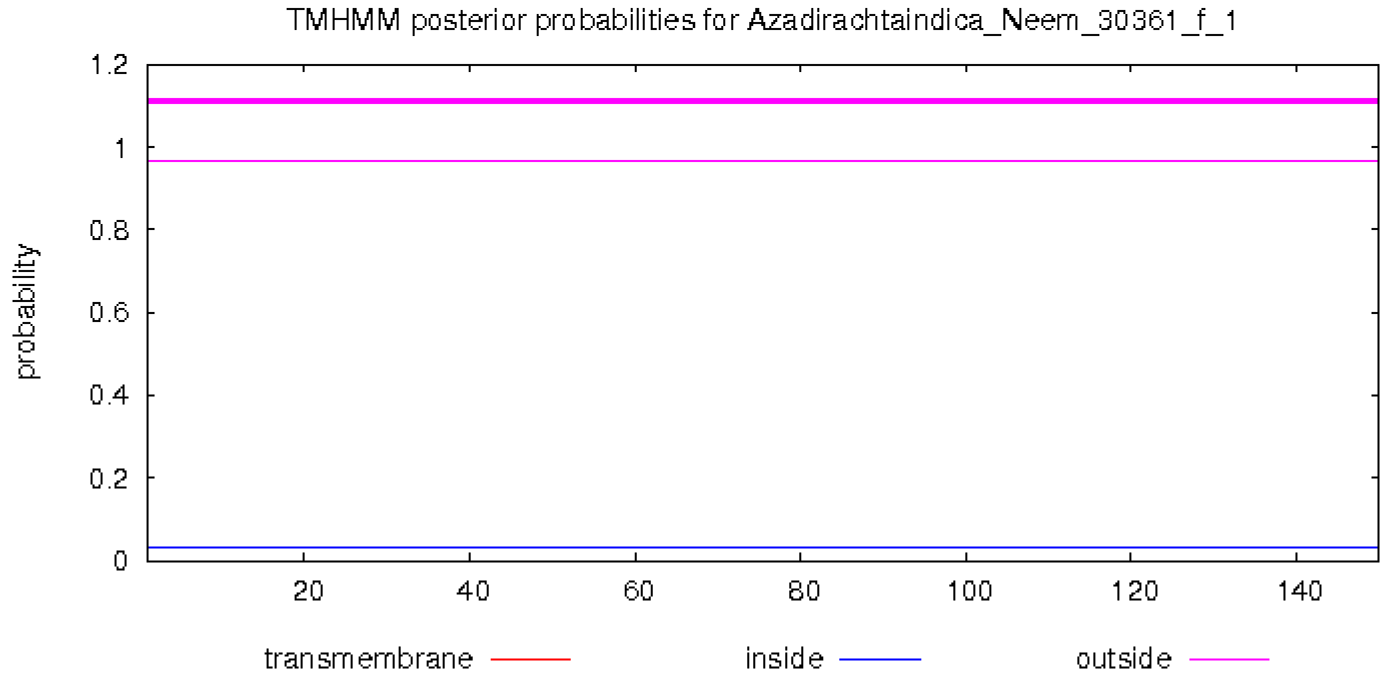

plot in postscript, script for making the plot in gnuplot, data for plot

---

```
# Azadirachtaindica_Neem_3043_f_3 Length: 362
# Azadirachtaindica_Neem_3043_f_3 Number of predicted TMHs:  0
# Azadirachtaindica_Neem_3043_f_3 Exp number of AAs in TMHs: 0
# Azadirachtaindica_Neem_3043_f_3 Exp number, first 60 AAs:  0
# Azadirachtaindica_Neem_3043_f_3 Total prob of N-in:        0.12080
Azadirachtaindica_Neem_3043_f_3 TMHMM2.0        outside      1   362
```

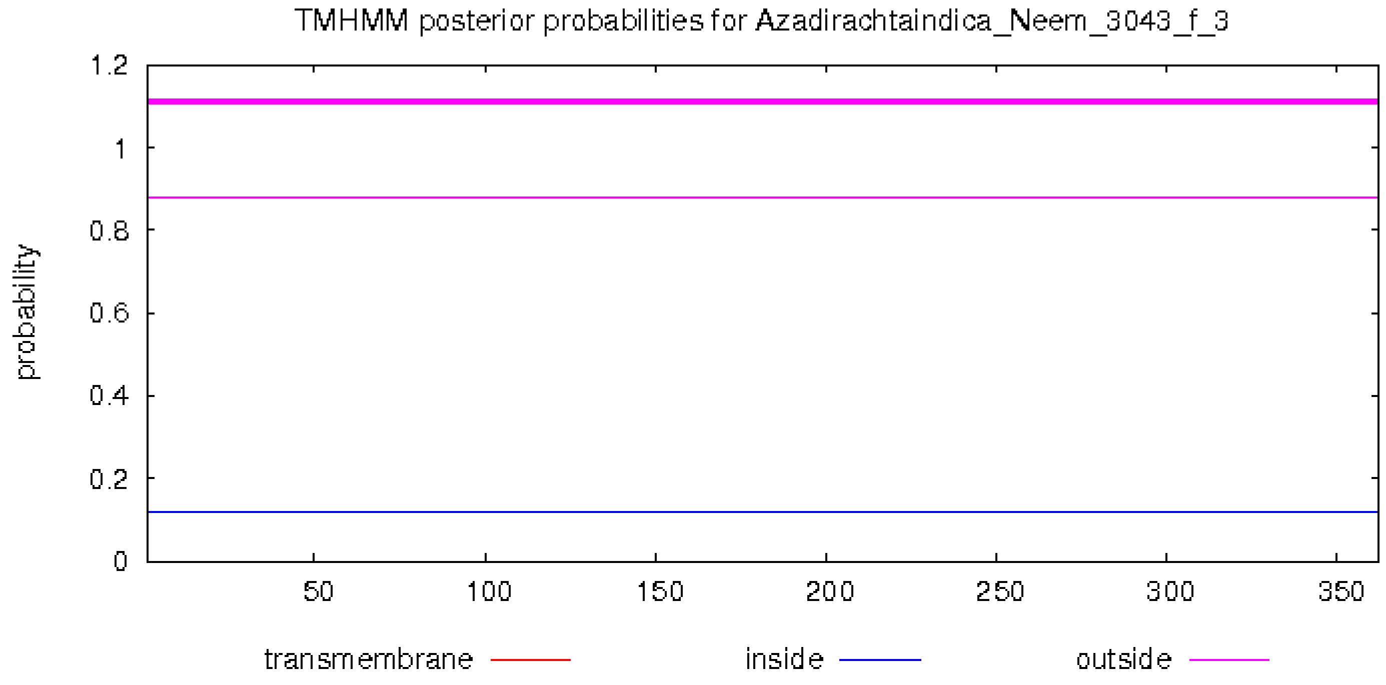

plot in postscript, script for making the plot in gnuplot, data for plot

---

```
# Azadirachtaindica_Neem_30599_f_1 Length: 115
# Azadirachtaindica_Neem_30599_f_1 Number of predicted TMHs:  0
```

```
# Azadirachtaindica_Neem_30599_f_1 Exp number of AAs in TMHs: 0.00185
# Azadirachtaindica_Neem_30599_f_1 Exp number, first 60 AAs:  0.00185
# Azadirachtaindica_Neem_30599_f_1 Total prob of N-in:        0.15468
Azadirachtaindica_Neem_30599_f_1        TMHMM2.0        outside      1   115
```

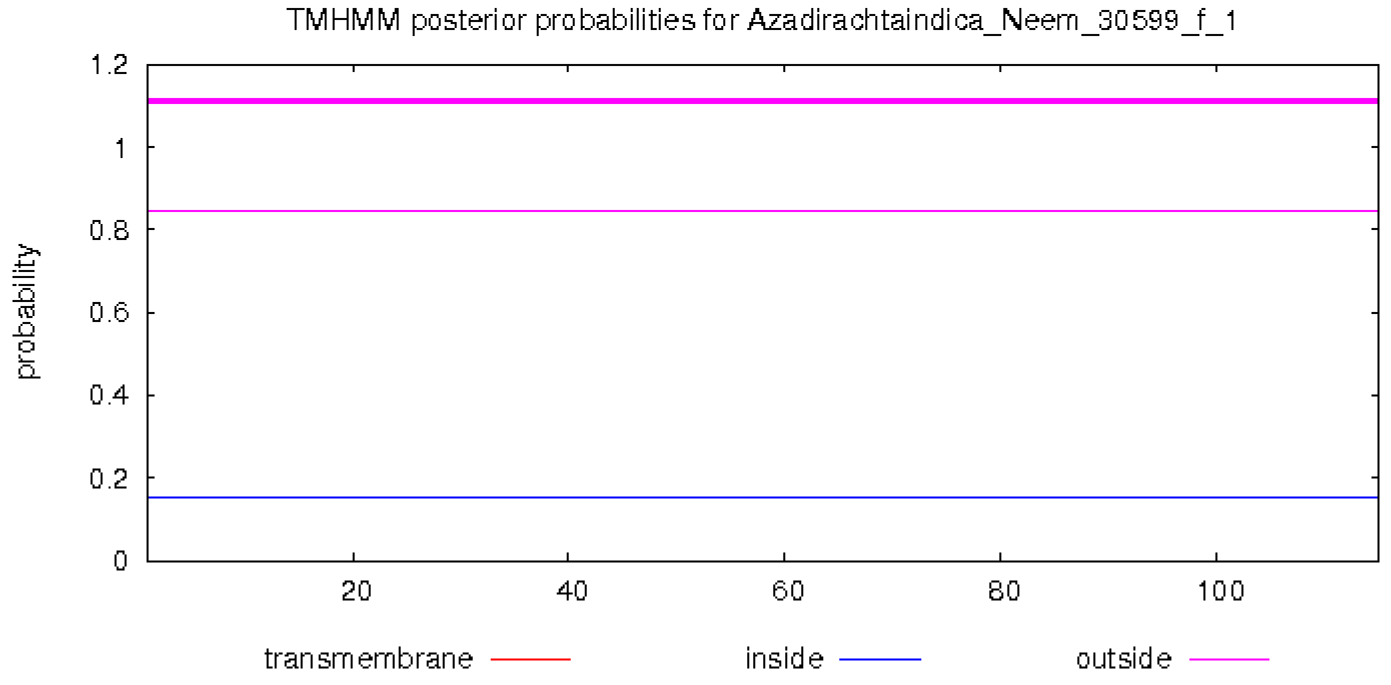

plot in postscript, script for making the plot in gnuplot, data for plot

---

```
# Azadirachtaindica_Neem_30788_f_1 Length: 363
# Azadirachtaindica_Neem_30788_f_1 Number of predicted TMHs:  0
# Azadirachtaindica_Neem_30788_f_1 Exp number of AAs in TMHs: 0.02425
# Azadirachtaindica_Neem_30788_f_1 Exp number, first 60 AAs:  0.00041
# Azadirachtaindica_Neem_30788_f_1 Total prob of N-in:        0.01797
Azadirachtaindica_Neem_30788_f_1        TMHMM2.0        outside      1   363
```

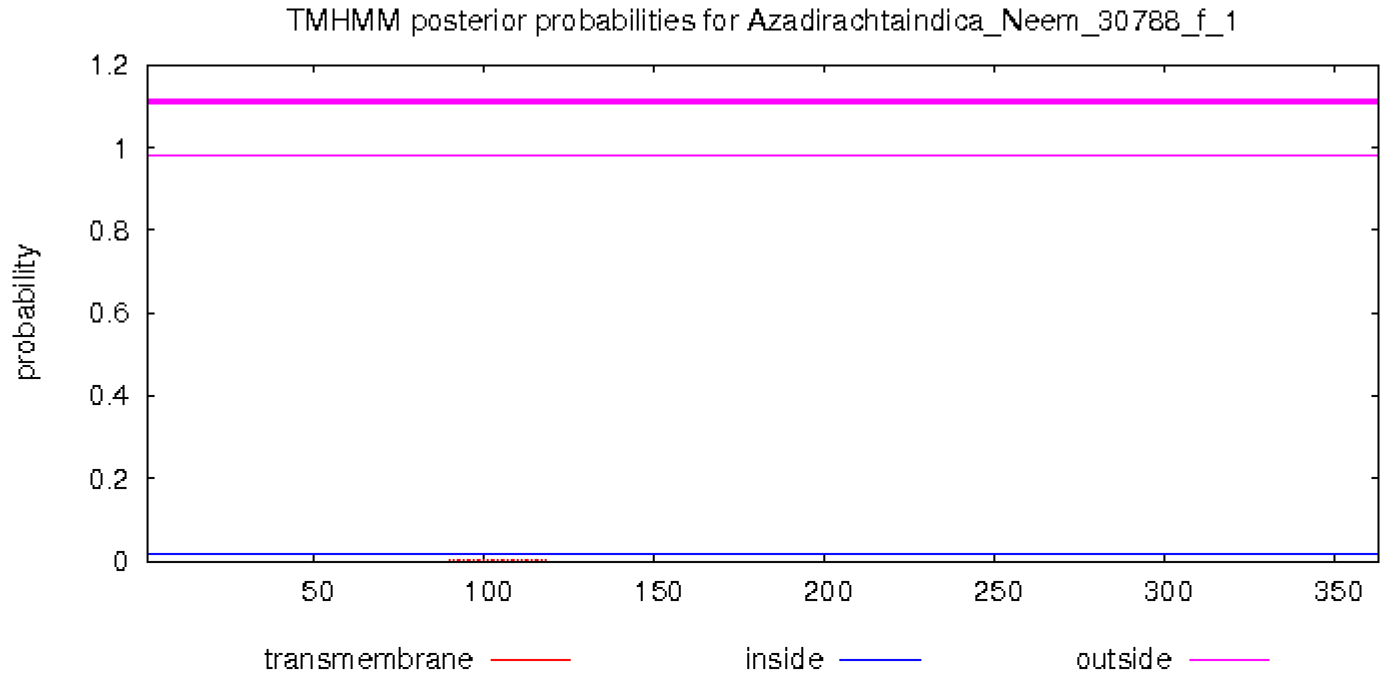

plot in postscript, script for making the plot in gnuplot, data for plot

---

```
# Azadirachtaindica_Neem_30861_f_1 Length: 217
# Azadirachtaindica_Neem_30861_f_1 Number of predicted TMHs:  0
```

```
# Azadirachtaindica_Neem_30861_f_1 Exp number of AAs in TMHs: 0.0029
# Azadirachtaindica_Neem_30861_f_1 Exp number, first 60 AAs:  0
# Azadirachtaindica_Neem_30861_f_1 Total prob of N-in:        0.01709
Azadirachtaindica_Neem_30861_f_1        TMHMM2.0        outside      1   217
```

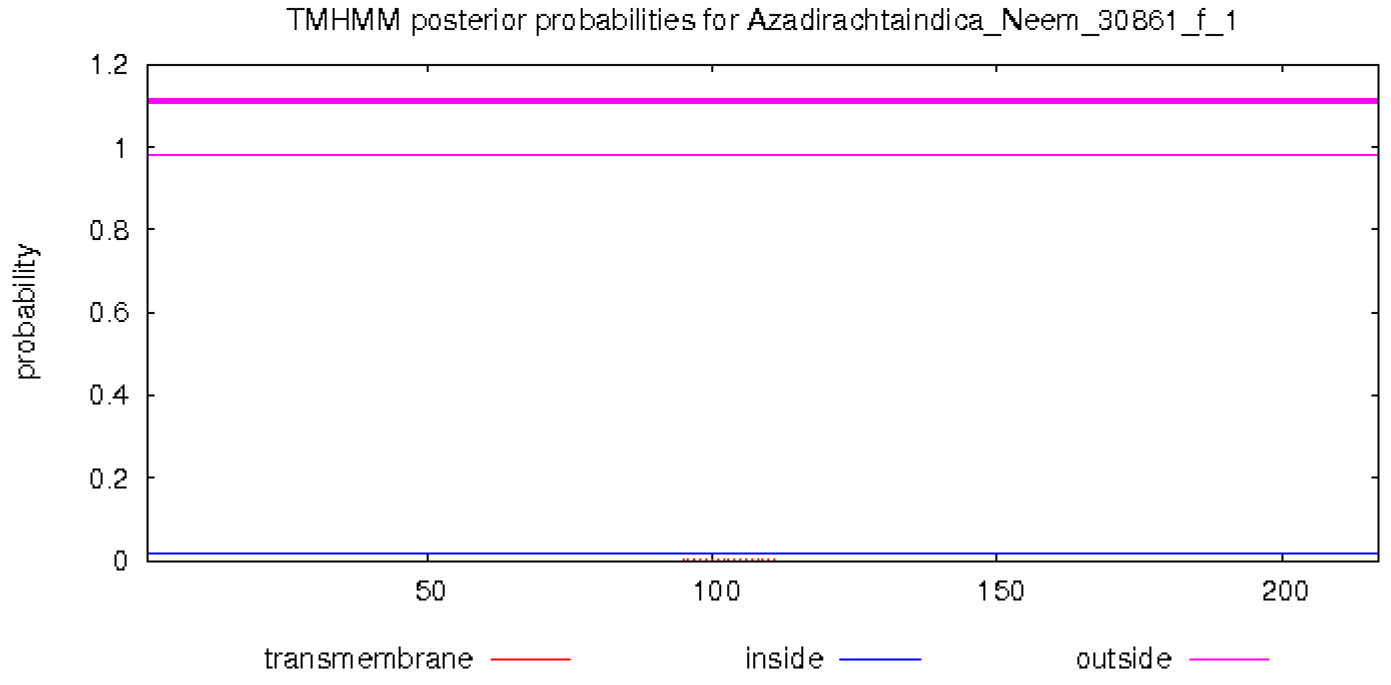

plot in postscript, script for making the plot in gnuplot, data for plot

---

```
# Azadirachtaindica_Neem_30988_f_2 Length: 172
# Azadirachtaindica_Neem_30988_f_2 Number of predicted TMHs:  0
# Azadirachtaindica_Neem_30988_f_2 Exp number of AAs in TMHs: 0.00114
# Azadirachtaindica_Neem_30988_f_2 Exp number, first 60 AAs:  0.00085
# Azadirachtaindica_Neem_30988_f_2 Total prob of N-in:        0.18429
Azadirachtaindica_Neem_30988_f_2        TMHMM2.0        outside      1   172
```

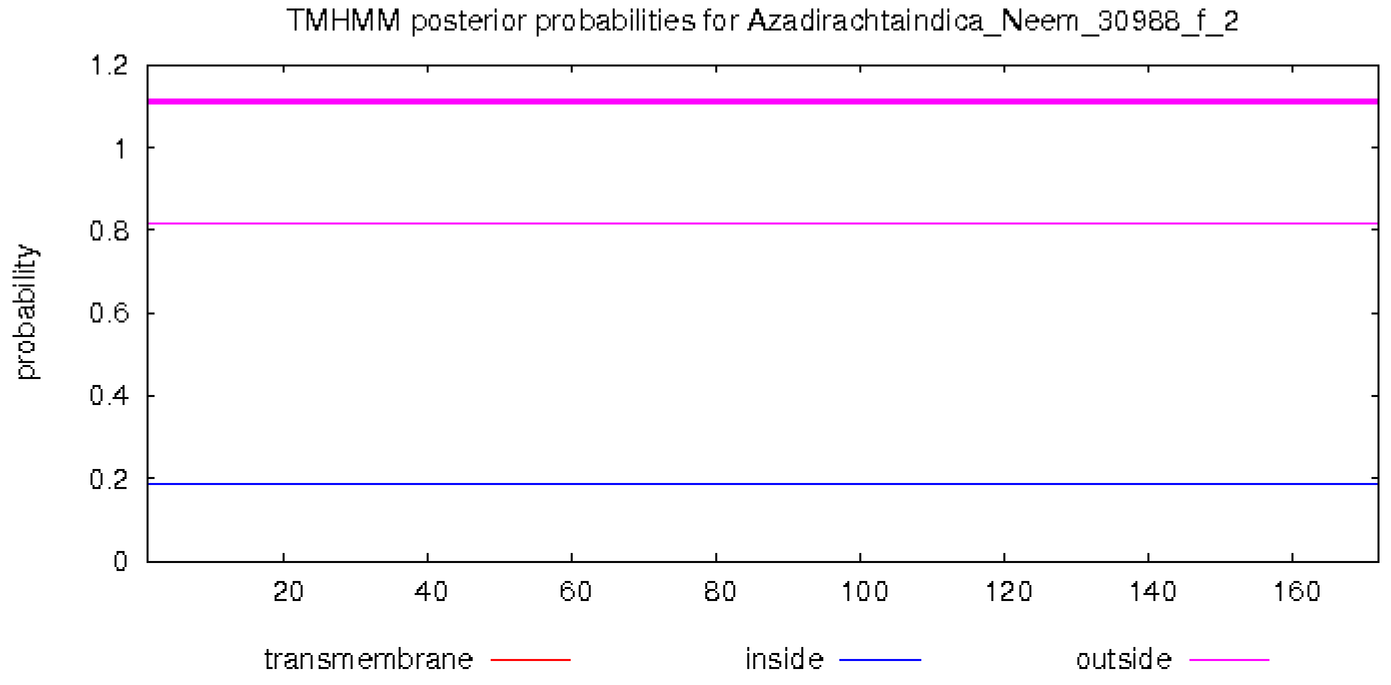

plot in postscript, script for making the plot in gnuplot, data for plot

---

```
# Azadirachtaindica_Neem_3103_f_14 Length: 347
# Azadirachtaindica_Neem_3103_f_14 Number of predicted TMHs:  0
```

```
# Azadirachtaindica_Neem_3103_f_14 Exp number of AAs in TMHs: 0.00036
# Azadirachtaindica_Neem_3103_f_14 Exp number, first 60 AAs:  0
# Azadirachtaindica_Neem_3103_f_14 Total prob of N-in:        0.03038
Azadirachtaindica_Neem_3103_f_14        TMHMM2.0        outside      1   347
```

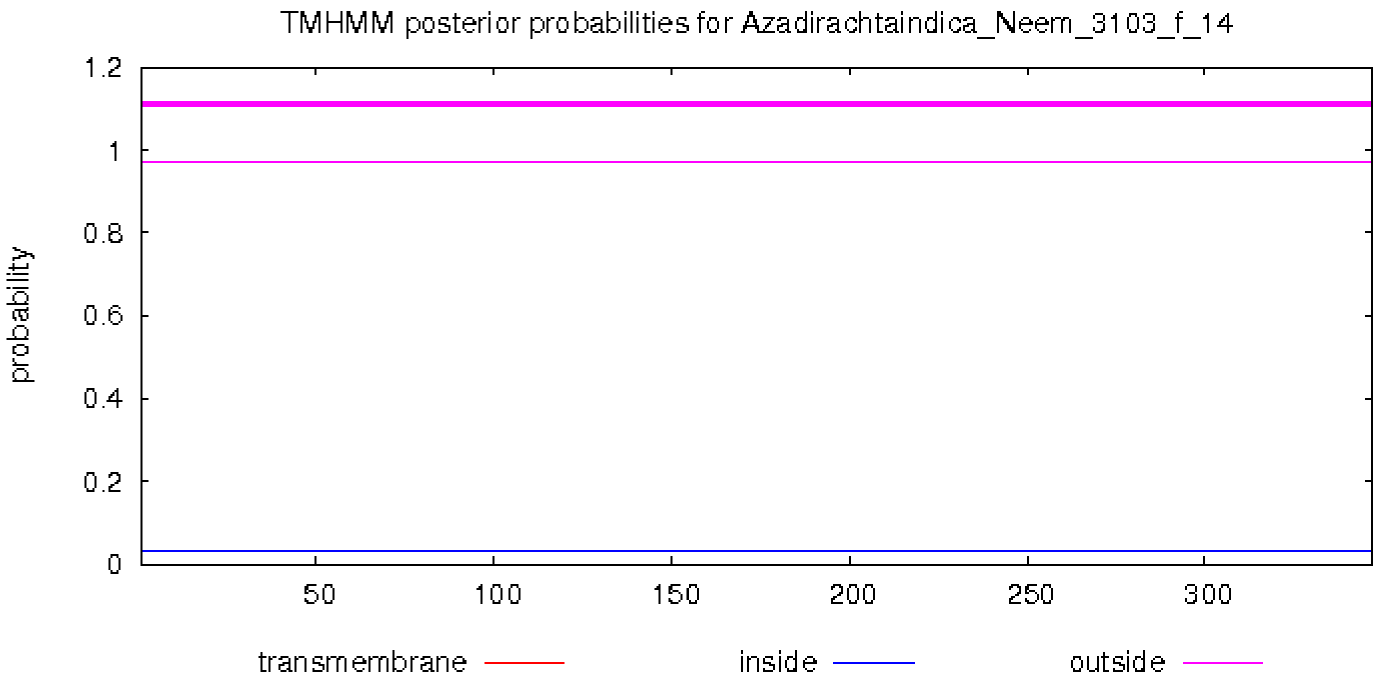

plot in postscript, script for making the plot in gnuplot, data for plot

---

```
# Azadirachtaindica_Neem_31069_f_2 Length: 299
# Azadirachtaindica_Neem_31069_f_2 Number of predicted TMHs:  0
# Azadirachtaindica_Neem_31069_f_2 Exp number of AAs in TMHs: 0.00057
# Azadirachtaindica_Neem_31069_f_2 Exp number, first 60 AAs:  0
# Azadirachtaindica_Neem_31069_f_2 Total prob of N-in:        0.09036
Azadirachtaindica_Neem_31069_f_2        TMHMM2.0        outside      1   299
```

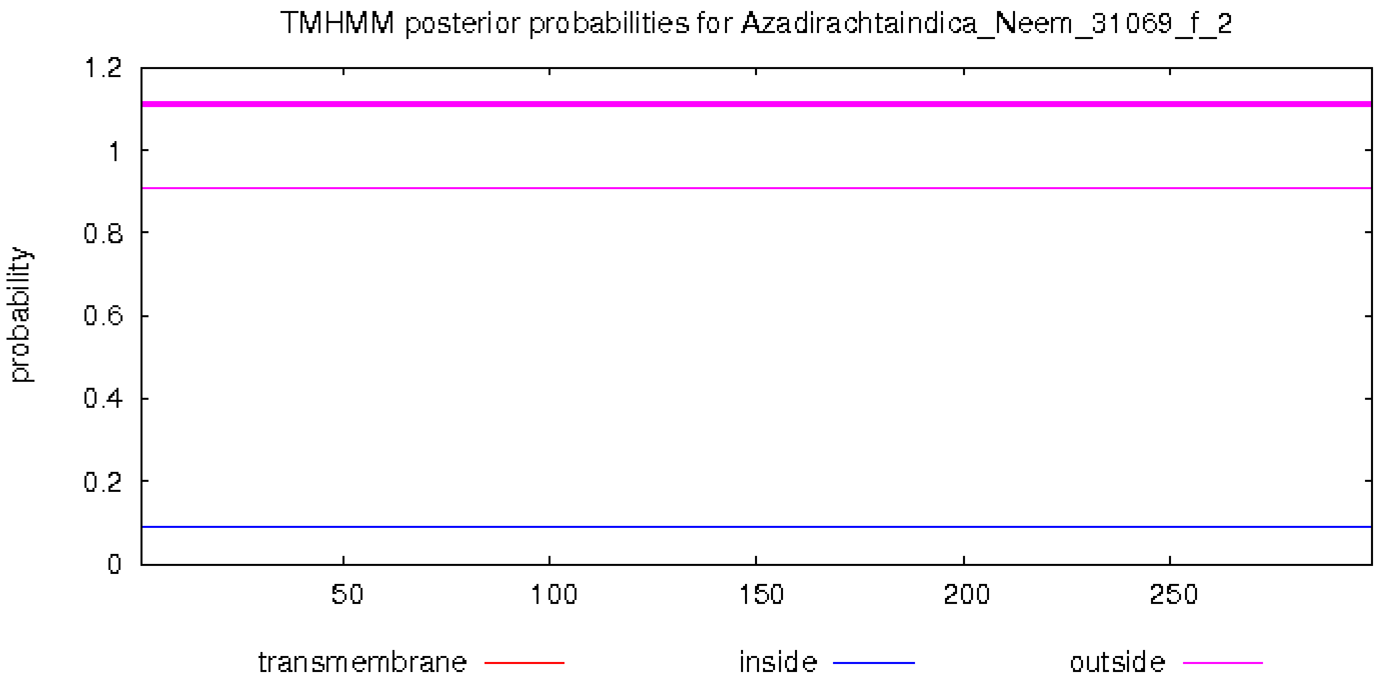

plot in postscript, script for making the plot in gnuplot, data for plot

---

```
# Azadirachtaindica_Neem_31746_f_1 Length: 136
# Azadirachtaindica_Neem_31746_f_1 Number of predicted TMHs:  0
```

```
# Azadirachtaindica_Neem_31746_f_1 Exp number of AAs in TMHs: 0
# Azadirachtaindica_Neem_31746_f_1 Exp number, first 60 AAs:  0
# Azadirachtaindica_Neem_31746_f_1 Total prob of N-in:        0.05867
Azadirachtaindica_Neem_31746_f_1        TMHMM2.0        outside      1   136
```

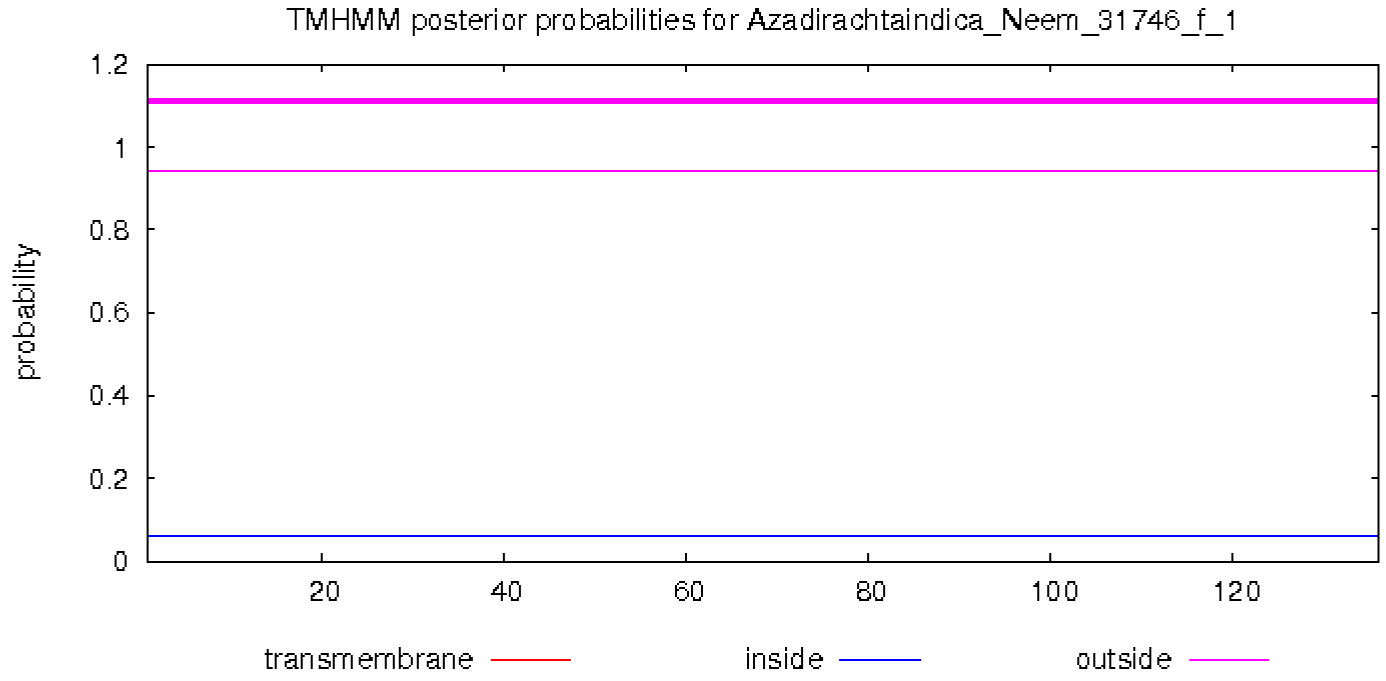

plot in postscript, script for making the plot in gnuplot, data for plot

---

```
# Azadirachtaindica_Neem_31990_f_1 Length: 157
# Azadirachtaindica_Neem_31990_f_1 Number of predicted TMHs:  0
# Azadirachtaindica_Neem_31990_f_1 Exp number of AAs in TMHs: 0.00035
# Azadirachtaindica_Neem_31990_f_1 Exp number, first 60 AAs:  3e-05
# Azadirachtaindica_Neem_31990_f_1 Total prob of N-in:        0.10268
Azadirachtaindica_Neem_31990_f_1        TMHMM2.0        outside      1   157
```

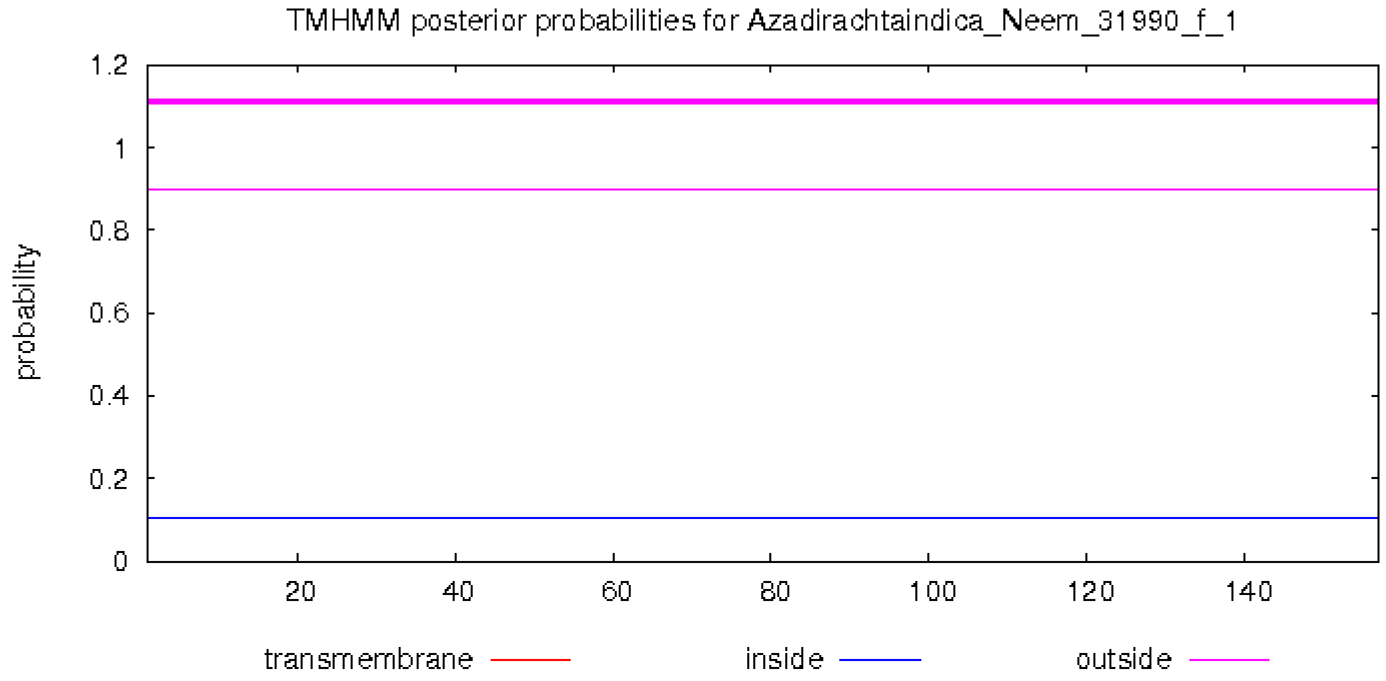

plot in postscript, script for making the plot in gnuplot, data for plot

---

```
# Azadirachtaindica_Neem_32411_f_1 Length: 88
# Azadirachtaindica_Neem_32411_f_1 Number of predicted TMHs:  0
```

```
# Azadirachtaindica_Neem_32411_f_1 Exp number of AAs in TMHs: 0.01356
# Azadirachtaindica_Neem_32411_f_1 Exp number, first 60 AAs:  0.00639
# Azadirachtaindica_Neem_32411_f_1 Total prob of N-in:        0.35844
Azadirachtaindica_Neem_32411_f_1        TMHMM2.0        outside      1    88
```

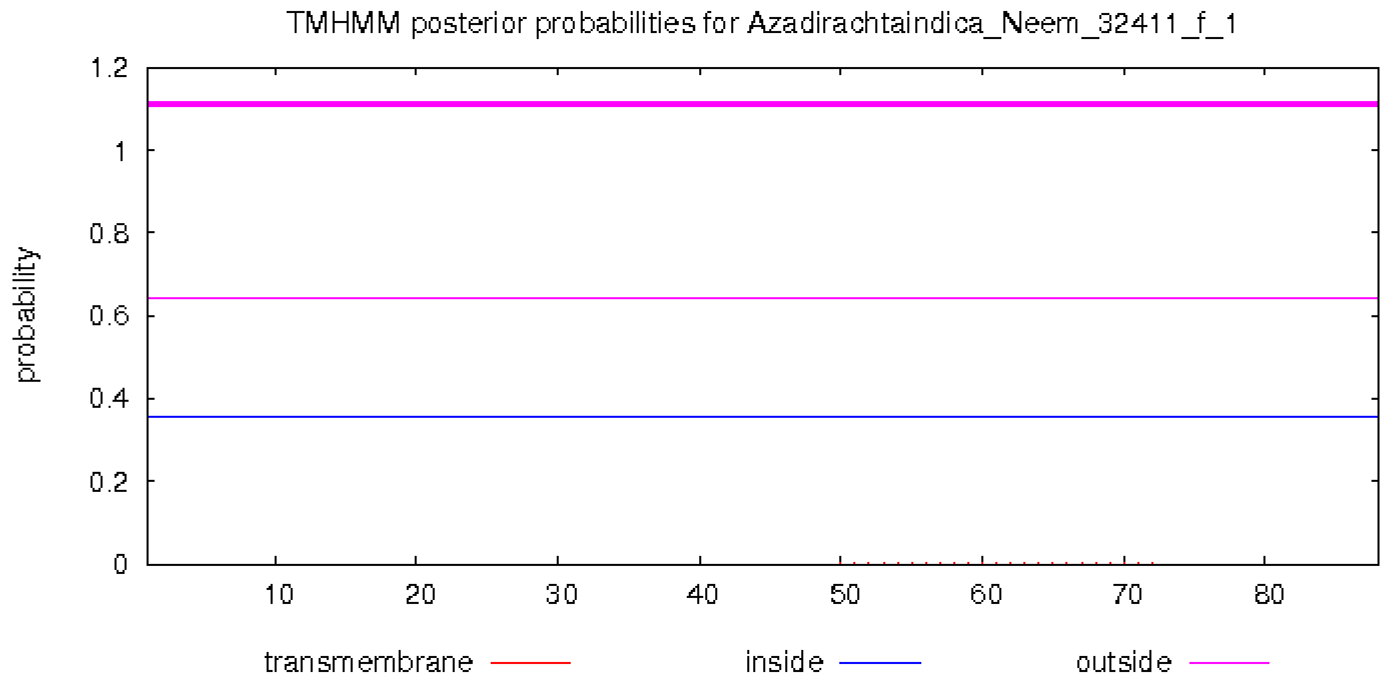

plot in postscript, script for making the plot in gnuplot, data for plot

---

```
# Azadirachtaindica_Neem_32518_f_2 Length: 266
# Azadirachtaindica_Neem_32518_f_2 Number of predicted TMHs:  0
# Azadirachtaindica_Neem_32518_f_2 Exp number of AAs in TMHs: 0.00023
# Azadirachtaindica_Neem_32518_f_2 Exp number, first 60 AAs:  1e-05
# Azadirachtaindica_Neem_32518_f_2 Total prob of N-in:        0.00659
Azadirachtaindica_Neem_32518_f_2        TMHMM2.0        outside      1   266
```

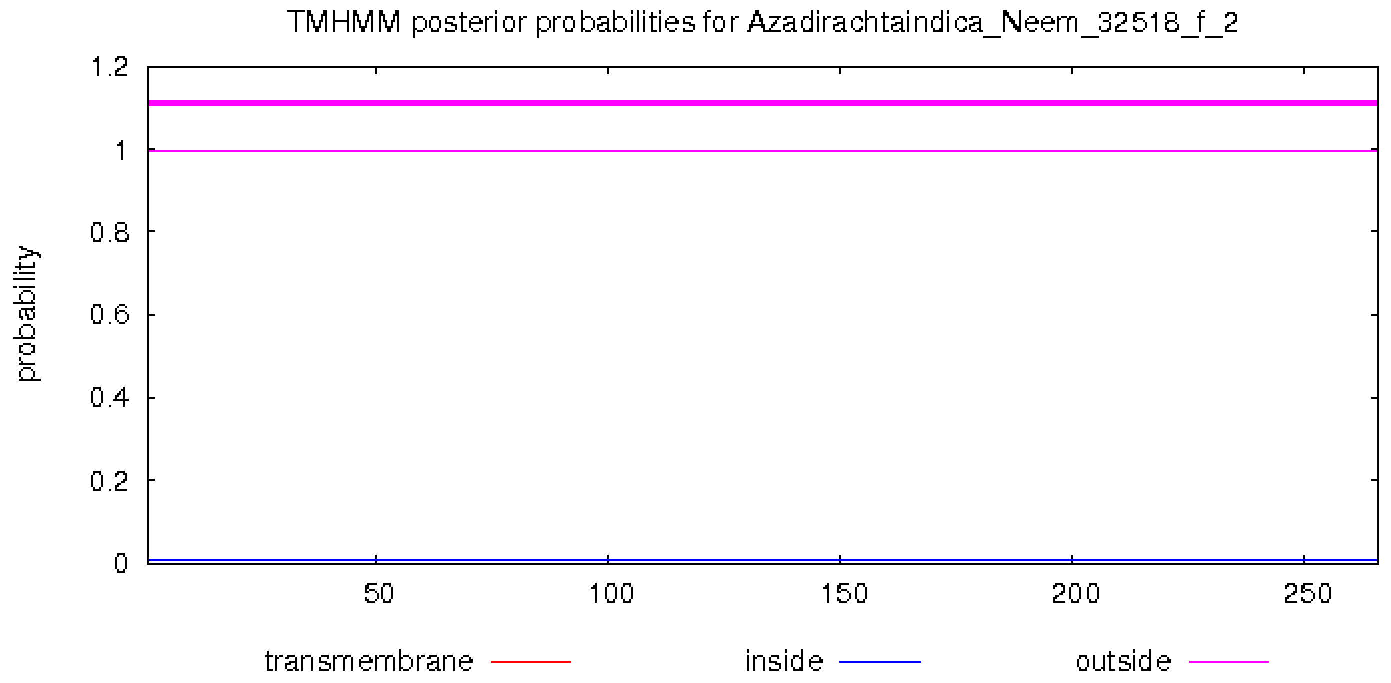

plot in postscript, script for making the plot in gnuplot, data for plot

---

```
# Azadirachtaindica_Neem_32626_f_1 Length: 261
# Azadirachtaindica_Neem_32626_f_1 Number of predicted TMHs:  0
```

```
# Azadirachtaindica_Neem_32626_f_1 Exp number of AAs in TMHs: 0.0072
# Azadirachtaindica_Neem_32626_f_1 Exp number, first 60 AAs:  0
# Azadirachtaindica_Neem_32626_f_1 Total prob of N-in:        0.01276
Azadirachtaindica_Neem_32626_f_1        TMHMM2.0        outside      1   261
```

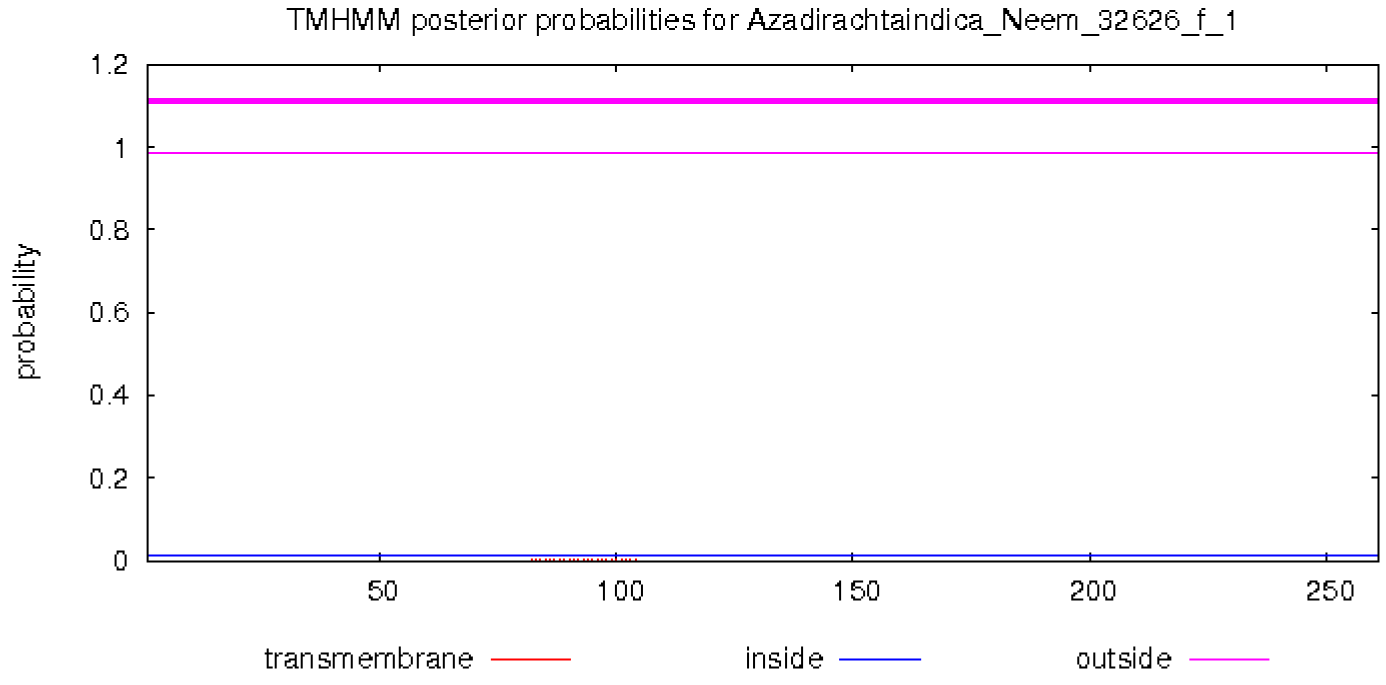

plot in postscript, script for making the plot in gnuplot, data for plot

---

```
# Azadirachtaindica_Neem_32792_f_2 Length: 345
# Azadirachtaindica_Neem_32792_f_2 Number of predicted TMHs:  0
# Azadirachtaindica_Neem_32792_f_2 Exp number of AAs in TMHs: 0.00041
# Azadirachtaindica_Neem_32792_f_2 Exp number, first 60 AAs:  0
# Azadirachtaindica_Neem_32792_f_2 Total prob of N-in:        0.00090
Azadirachtaindica_Neem_32792_f_2        TMHMM2.0        outside      1   345
```

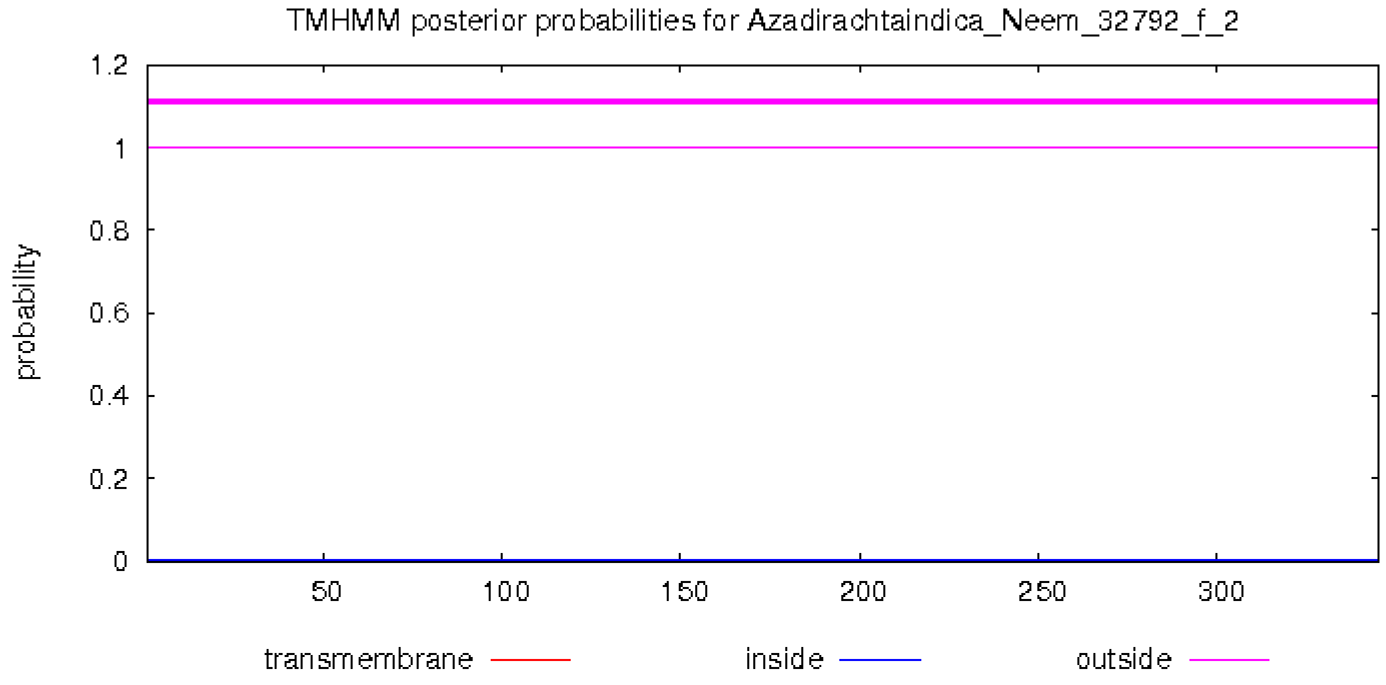

plot in postscript, script for making the plot in gnuplot, data for plot

---

```
# Azadirachtaindica_Neem_327_f_1 Length: 362
# Azadirachtaindica_Neem_327_f_1 Number of predicted TMHs:  0
```

```
# Azadirachtaindica_Neem_327_f_1 Exp number of AAs in TMHs: 0.00055
# Azadirachtaindica_Neem_327_f_1 Exp number, first 60 AAs:  0
# Azadirachtaindica_Neem_327_f_1 Total prob of N-in:        0.01562
Azadirachtaindica_Neem_327_f_1  TMHMM2.0        outside      1   362
```

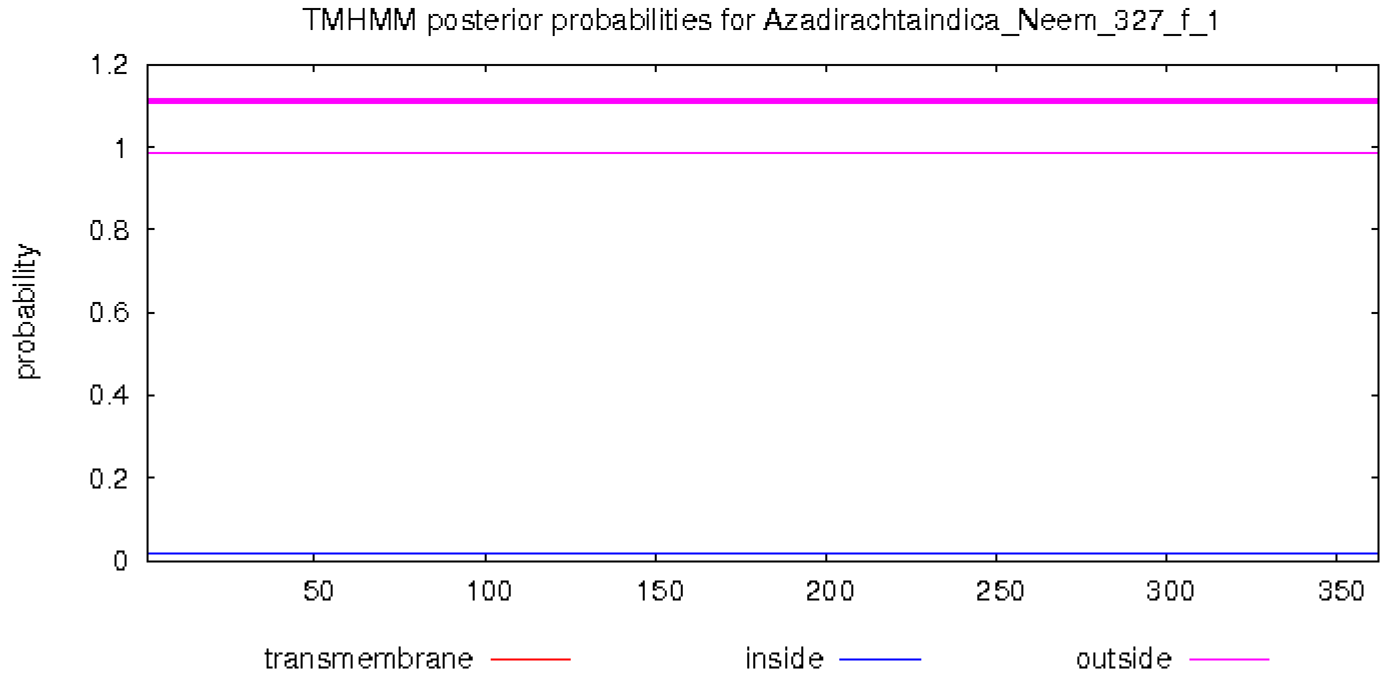

plot in postscript, script for making the plot in gnuplot, data for plot

---

```
# Azadirachtaindica_Neem_3287_a_1 Length: 172
# Azadirachtaindica_Neem_3287_a_1 Number of predicted TMHs:  0
# Azadirachtaindica_Neem_3287_a_1 Exp number of AAs in TMHs: 0.00036
# Azadirachtaindica_Neem_3287_a_1 Exp number, first 60 AAs:  0
# Azadirachtaindica_Neem_3287_a_1 Total prob of N-in:        0.13089
Azadirachtaindica_Neem_3287_a_1 TMHMM2.0        outside      1   172
```

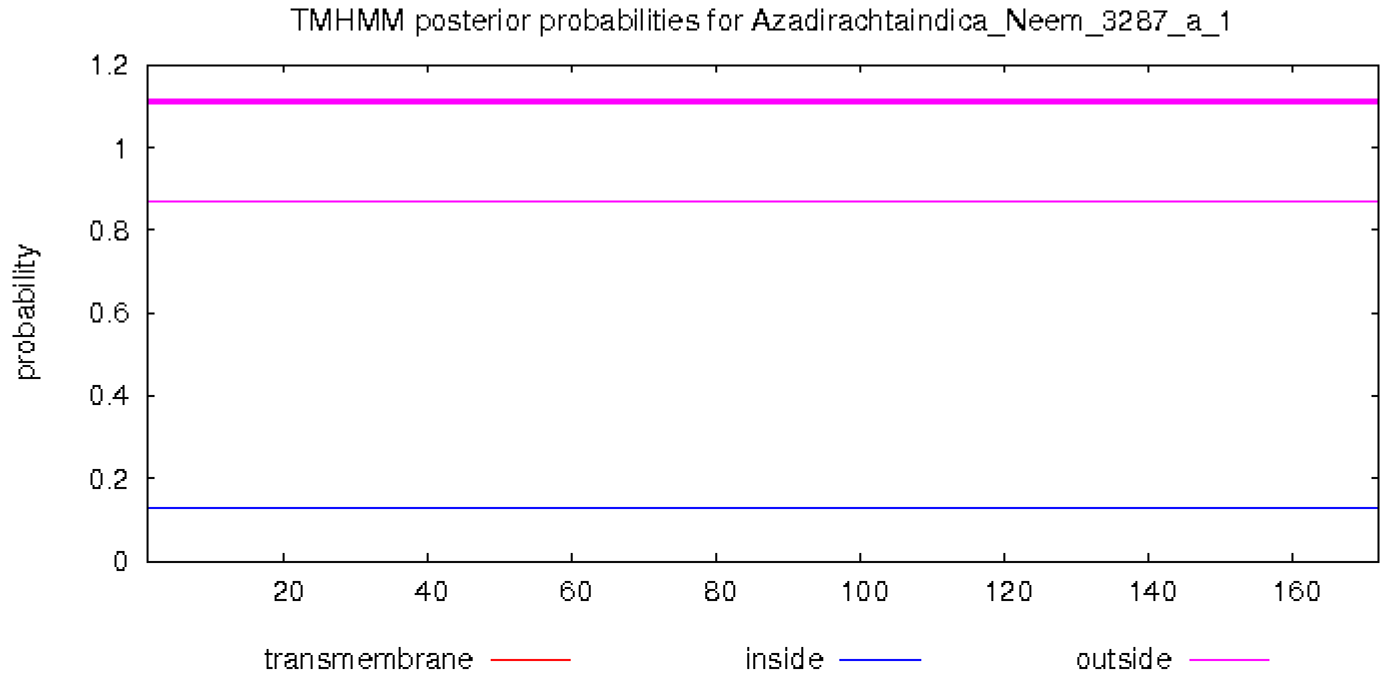

plot in postscript, script for making the plot in gnuplot, data for plot

---

```
# Azadirachtaindica_Neem_32940_f_1 Length: 135
# Azadirachtaindica_Neem_32940_f_1 Number of predicted TMHs:  0
```

```
# Azadirachtaindica_Neem_32940_f_1 Exp number of AAs in TMHs: 0
# Azadirachtaindica_Neem_32940_f_1 Exp number, first 60 AAs:  0
# Azadirachtaindica_Neem_32940_f_1 Total prob of N-in:        0.16903
Azadirachtaindica_Neem_32940_f_1        TMHMM2.0        outside      1   135
```

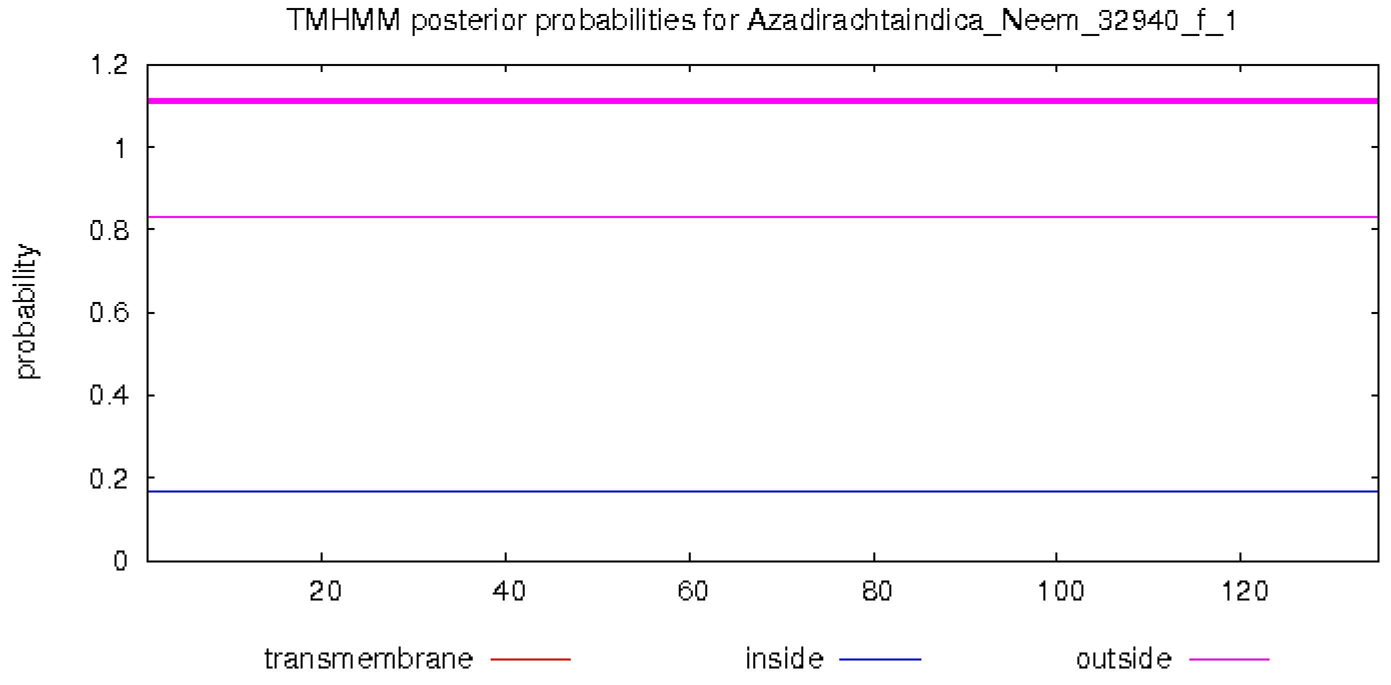

plot in postscript, script for making the plot in gnuplot, data for plot

```
# Azadirachtaindica_Neem_33457_f_1 Length: 204
# Azadirachtaindica_Neem_33457_f_1 Number of predicted TMHs:  0
# Azadirachtaindica_Neem_33457_f_1 Exp number of AAs in TMHs: 9.2075
# Azadirachtaindica_Neem_33457_f_1 Exp number, first 60 AAs:  0
# Azadirachtaindica_Neem_33457_f_1 Total prob of N-in:        0.25601
Azadirachtaindica_Neem_33457_f_1        TMHMM2.0        outside      1   204
```

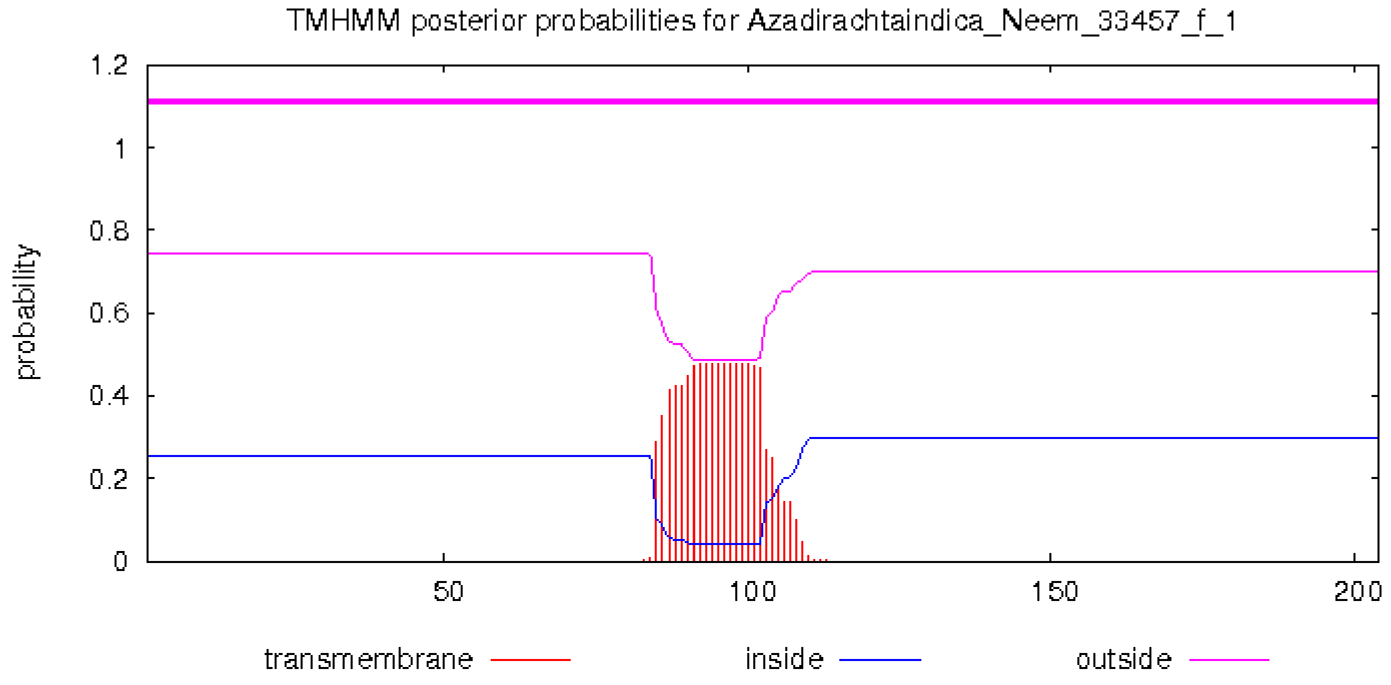

plot in postscript, script for making the plot in gnuplot, data for plot

```
# Azadirachtaindica_Neem_33765_f_1 Length: 116
# Azadirachtaindica_Neem_33765_f_1 Number of predicted TMHs:  0
```

```
# Azadirachtaindica_Neem_33765_f_1 Exp number of AAs in TMHs: 0.00035
# Azadirachtaindica_Neem_33765_f_1 Exp number, first 60 AAs:  0
# Azadirachtaindica_Neem_33765_f_1 Total prob of N-in:        0.29826
Azadirachtaindica_Neem_33765_f_1        TMHMM2.0        outside      1   116
```

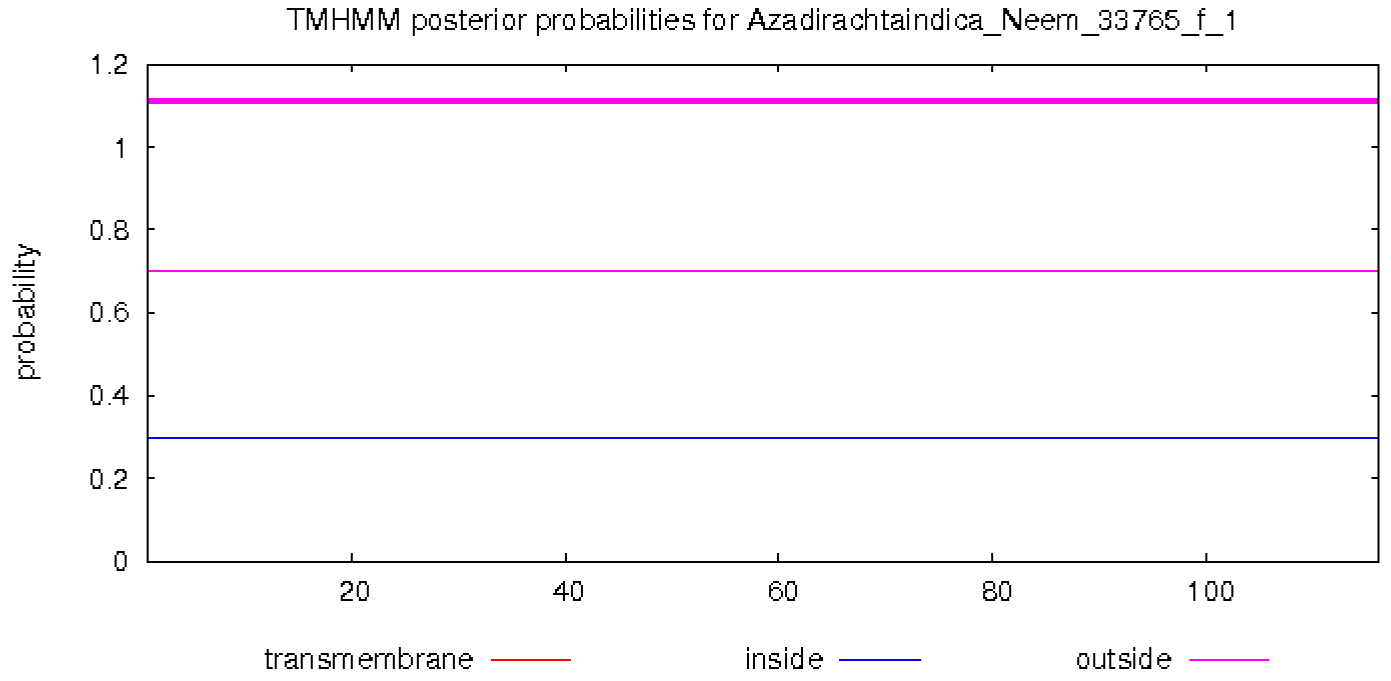

plot in postscript, script for making the plot in gnuplot, data for plot

---

```
# Azadirachtaindica_Neem_34010_f_1 Length: 168
# Azadirachtaindica_Neem_34010_f_1 Number of predicted TMHs:  0
# Azadirachtaindica_Neem_34010_f_1 Exp number of AAs in TMHs: 0.66365
# Azadirachtaindica_Neem_34010_f_1 Exp number, first 60 AAs:  0.00895
# Azadirachtaindica_Neem_34010_f_1 Total prob of N-in:        0.03720
Azadirachtaindica_Neem_34010_f_1        TMHMM2.0        outside      1   168
```

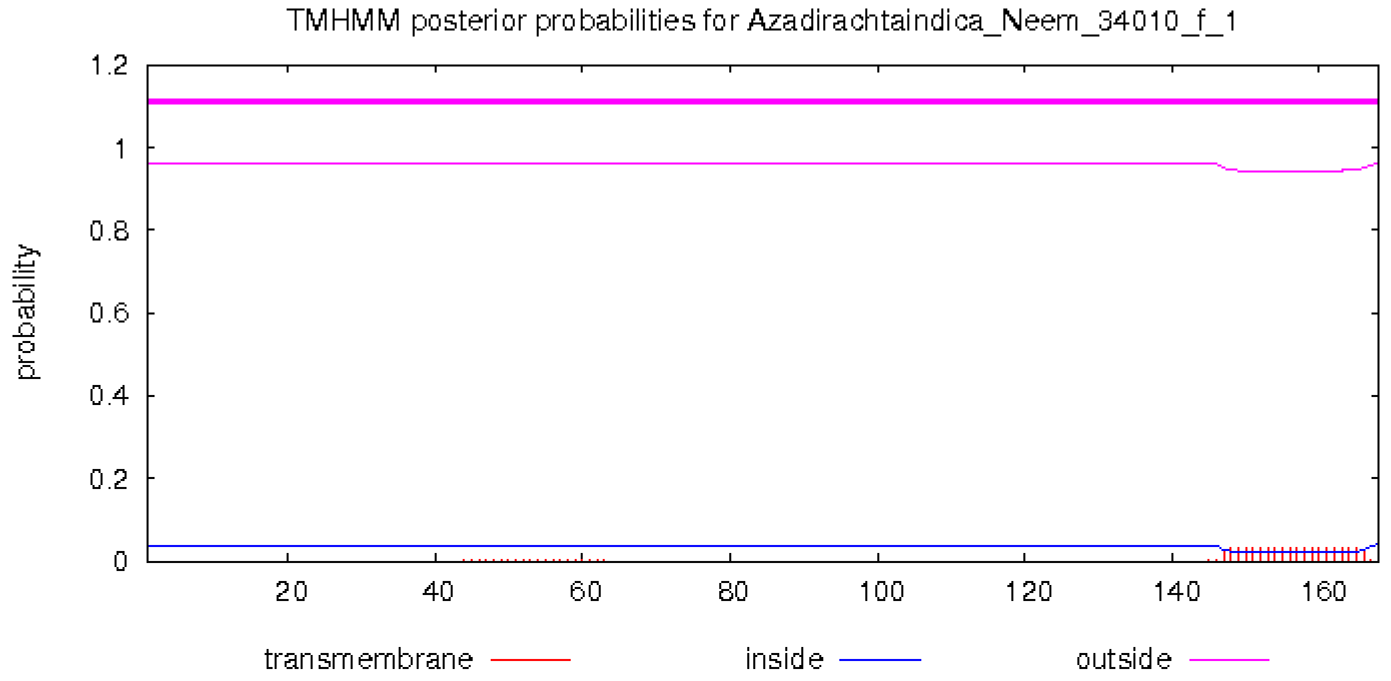

plot in postscript, script for making the plot in gnuplot, data for plot

---

```
# Azadirachtaindica_Neem_34120_f_1 Length: 235
# Azadirachtaindica_Neem_34120_f_1 Number of predicted TMHs:  0
```

```
# Azadirachtaindica_Neem_34120_f_1 Exp number of AAs in TMHs: 0.00023
# Azadirachtaindica_Neem_34120_f_1 Exp number, first 60 AAs:  2e-05
# Azadirachtaindica_Neem_34120_f_1 Total prob of N-in:        0.05127
Azadirachtaindica_Neem_34120_f_1        TMHMM2.0        outside      1   235
```

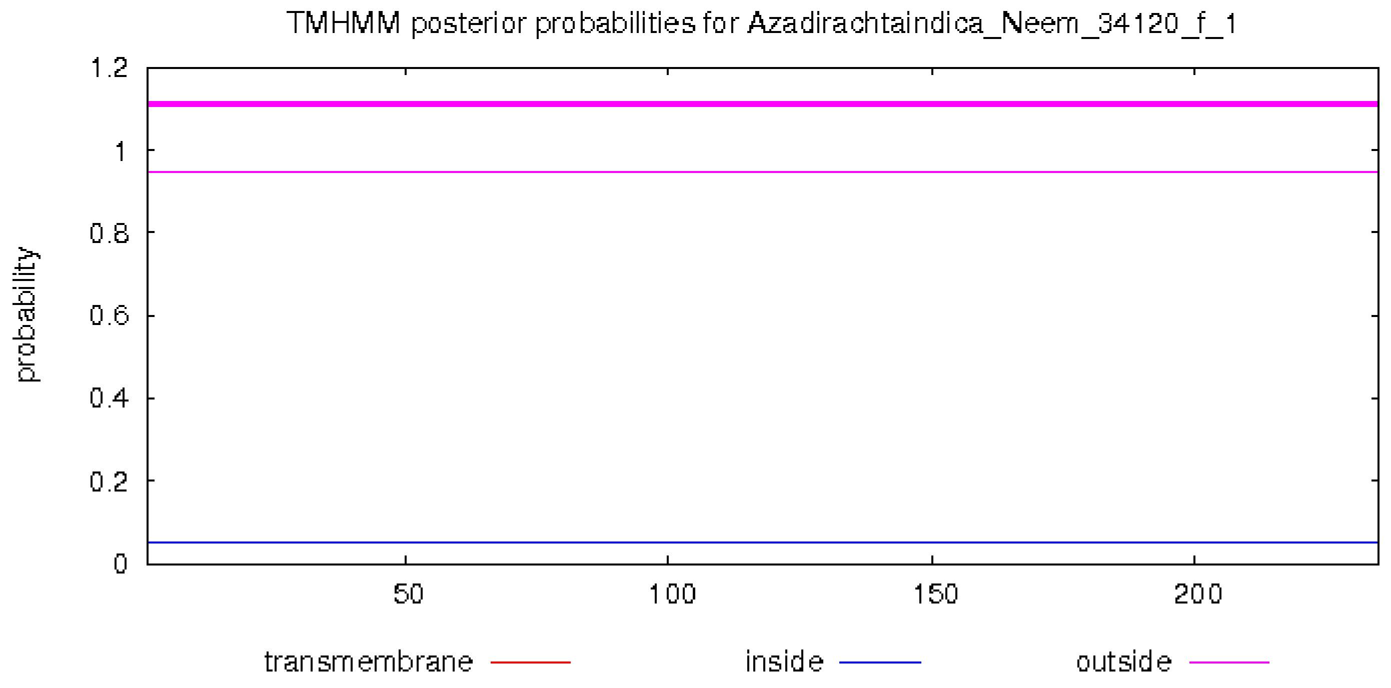

plot in postscript, script for making the plot in gnuplot, data for plot

---

```
# Azadirachtaindica_Neem_34330_f_2 Length: 148
# Azadirachtaindica_Neem_34330_f_2 Number of predicted TMHs:  0
# Azadirachtaindica_Neem_34330_f_2 Exp number of AAs in TMHs: 0
# Azadirachtaindica_Neem_34330_f_2 Exp number, first 60 AAs:  0
# Azadirachtaindica_Neem_34330_f_2 Total prob of N-in:        0.14342
Azadirachtaindica_Neem_34330_f_2        TMHMM2.0        outside      1   148
```

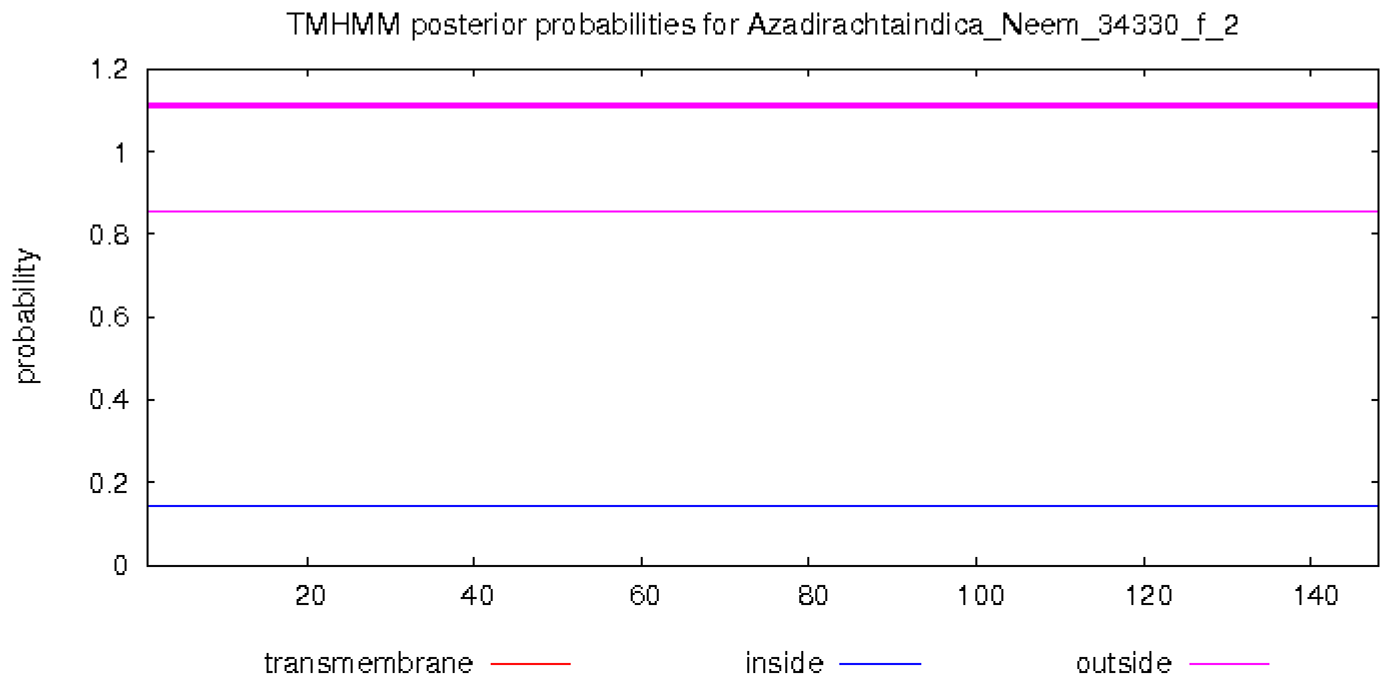

plot in postscript, script for making the plot in gnuplot, data for plot

---

```
# Azadirachtaindica_Neem_34778_f_1 Length: 335
# Azadirachtaindica_Neem_34778_f_1 Number of predicted TMHs:  0
```

```
# Azadirachtaindica_Neem_34778_f_1 Exp number of AAs in TMHs: 0
# Azadirachtaindica_Neem_34778_f_1 Exp number, first 60 AAs:  0
# Azadirachtaindica_Neem_34778_f_1 Total prob of N-in:        0.01931
Azadirachtaindica_Neem_34778_f_1        TMHMM2.0        outside      1   335
```

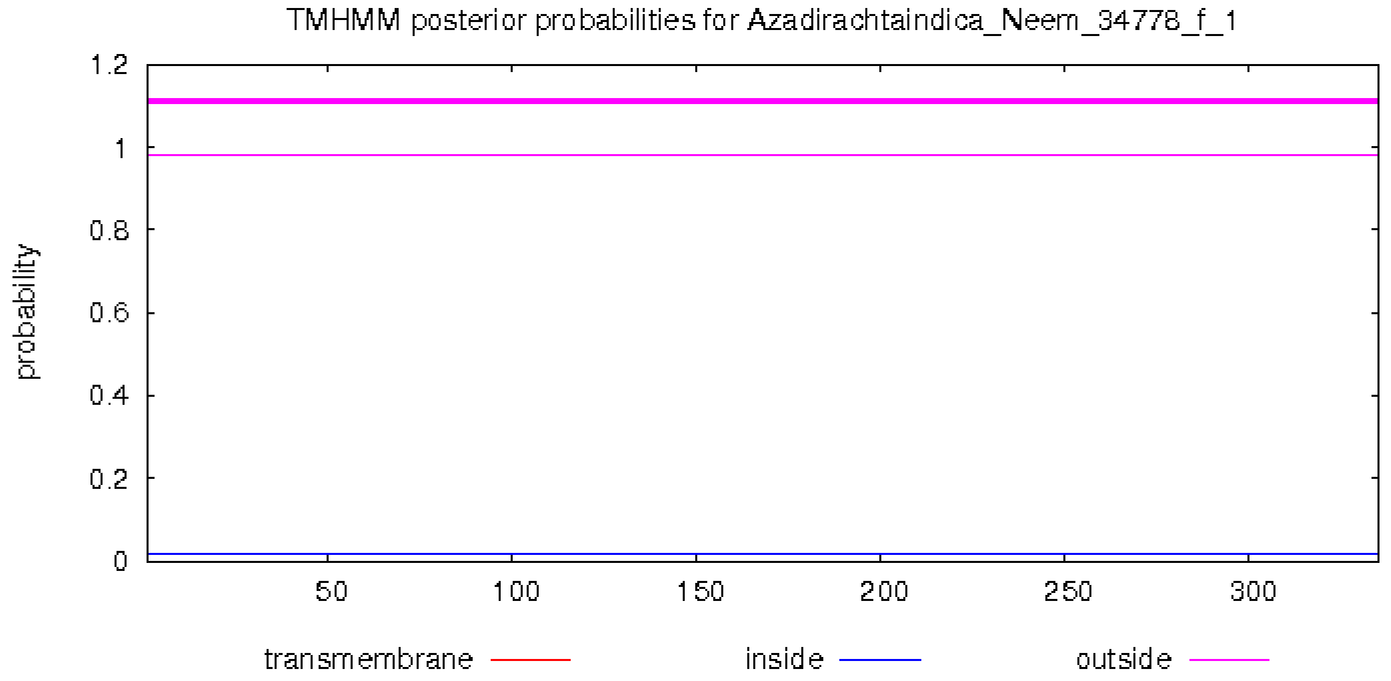

plot in postscript, script for making the plot in gnuplot, data for plot

---

```
# Azadirachtaindica_Neem_34841_f_1 Length: 208
# Azadirachtaindica_Neem_34841_f_1 Number of predicted TMHs:  0
# Azadirachtaindica_Neem_34841_f_1 Exp number of AAs in TMHs: 0.17635
# Azadirachtaindica_Neem_34841_f_1 Exp number, first 60 AAs:  0.00025
# Azadirachtaindica_Neem_34841_f_1 Total prob of N-in:        0.12956
Azadirachtaindica_Neem_34841_f_1        TMHMM2.0        outside      1   208
```

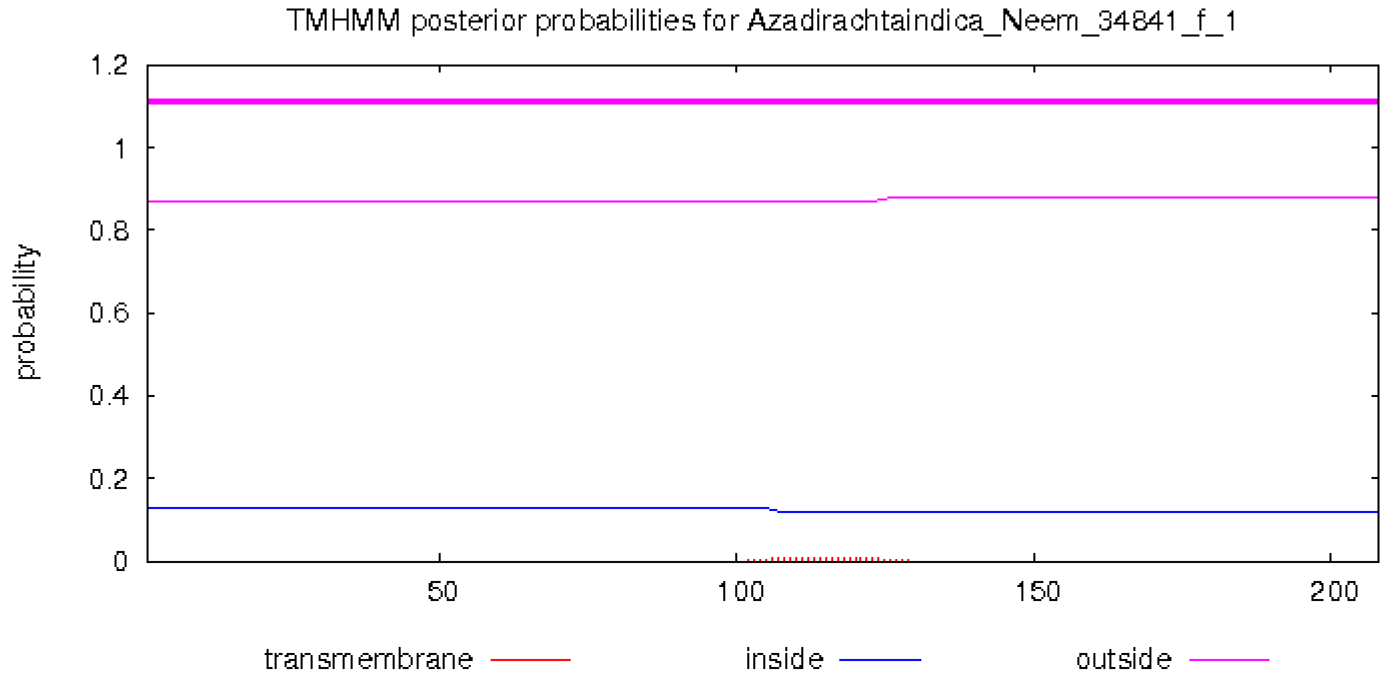

plot in postscript, script for making the plot in gnuplot, data for plot

---

```
# Azadirachtaindica_Neem_34950_f_1 Length: 335
# Azadirachtaindica_Neem_34950_f_1 Number of predicted TMHs:  0
```

```
# Azadirachtaindica_Neem_34950_f_1 Exp number of AAs in TMHs: 0
# Azadirachtaindica_Neem_34950_f_1 Exp number, first 60 AAs:  0
# Azadirachtaindica_Neem_34950_f_1 Total prob of N-in:        0.00343
Azadirachtaindica_Neem_34950_f_1	TMHMM2.0	outside	     1   335
```

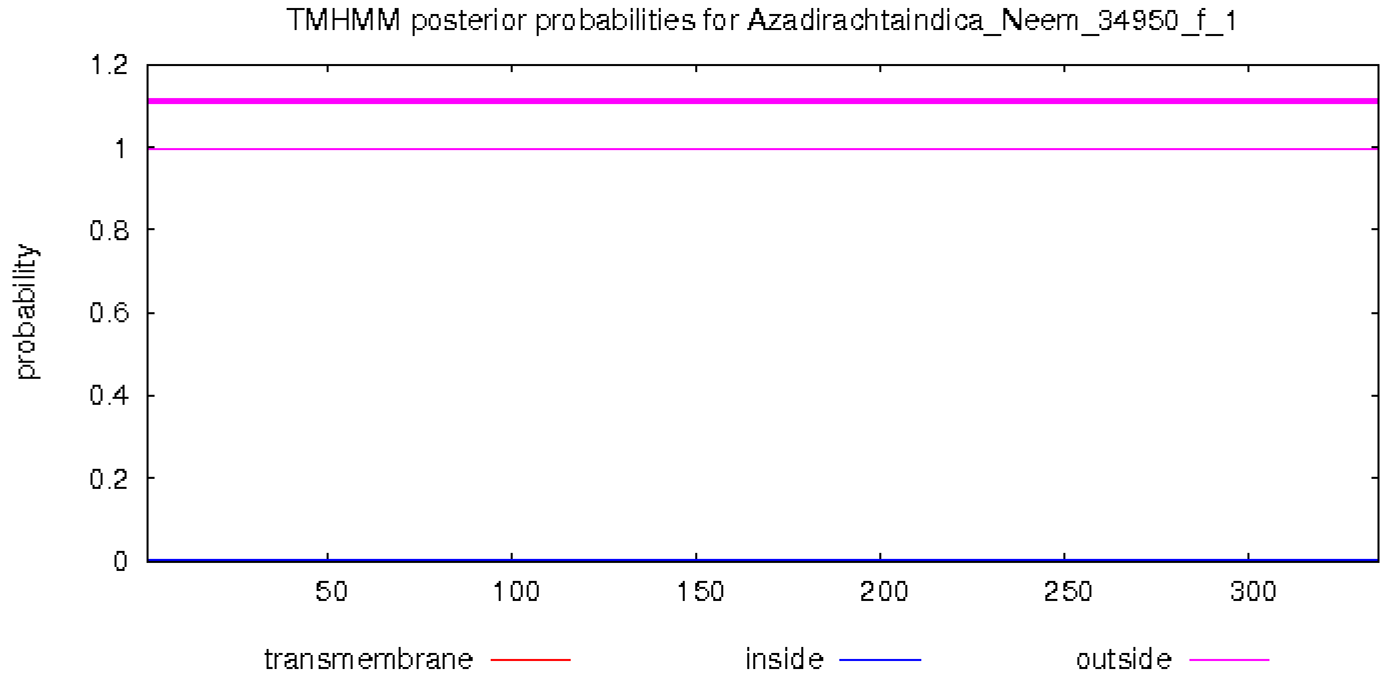

plot in postscript, script for making the plot in gnuplot, data for plot

---

```
# Azadirachtaindica_Neem_35390_f_1 Length: 162
# Azadirachtaindica_Neem_35390_f_1 Number of predicted TMHs:  0
# Azadirachtaindica_Neem_35390_f_1 Exp number of AAs in TMHs: 0.00571
# Azadirachtaindica_Neem_35390_f_1 Exp number, first 60 AAs:  0.00018
# Azadirachtaindica_Neem_35390_f_1 Total prob of N-in:        0.03981
Azadirachtaindica_Neem_35390_f_1	TMHMM2.0	outside	     1   162
```

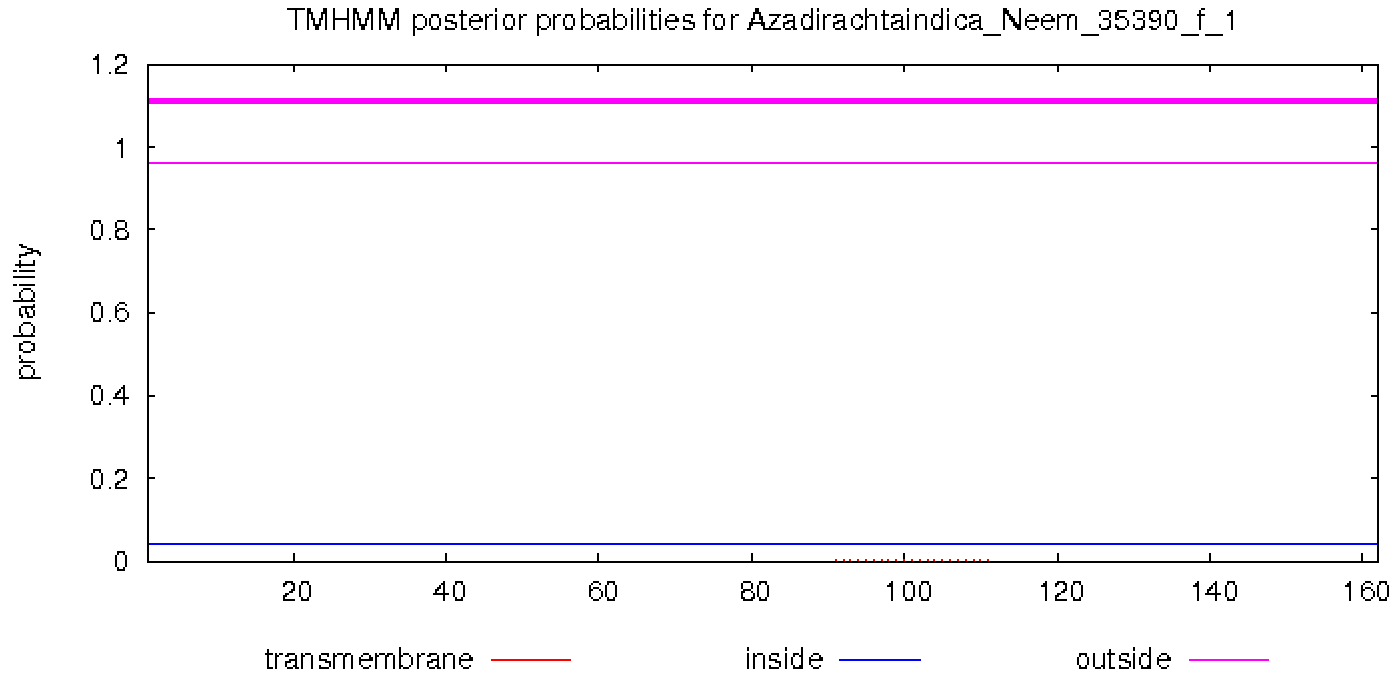

plot in postscript, script for making the plot in gnuplot, data for plot

---

```
# Azadirachtaindica_Neem_35472_f_1 Length: 347
# Azadirachtaindica_Neem_35472_f_1 Number of predicted TMHs:  0
```

```
# Azadirachtaindica_Neem_35472_f_1 Exp number of AAs in TMHs: 0.00036
# Azadirachtaindica_Neem_35472_f_1 Exp number, first 60 AAs:  0
# Azadirachtaindica_Neem_35472_f_1 Total prob of N-in:        0.03038
Azadirachtaindica_Neem_35472_f_1        TMHMM2.0        outside      1   347
```

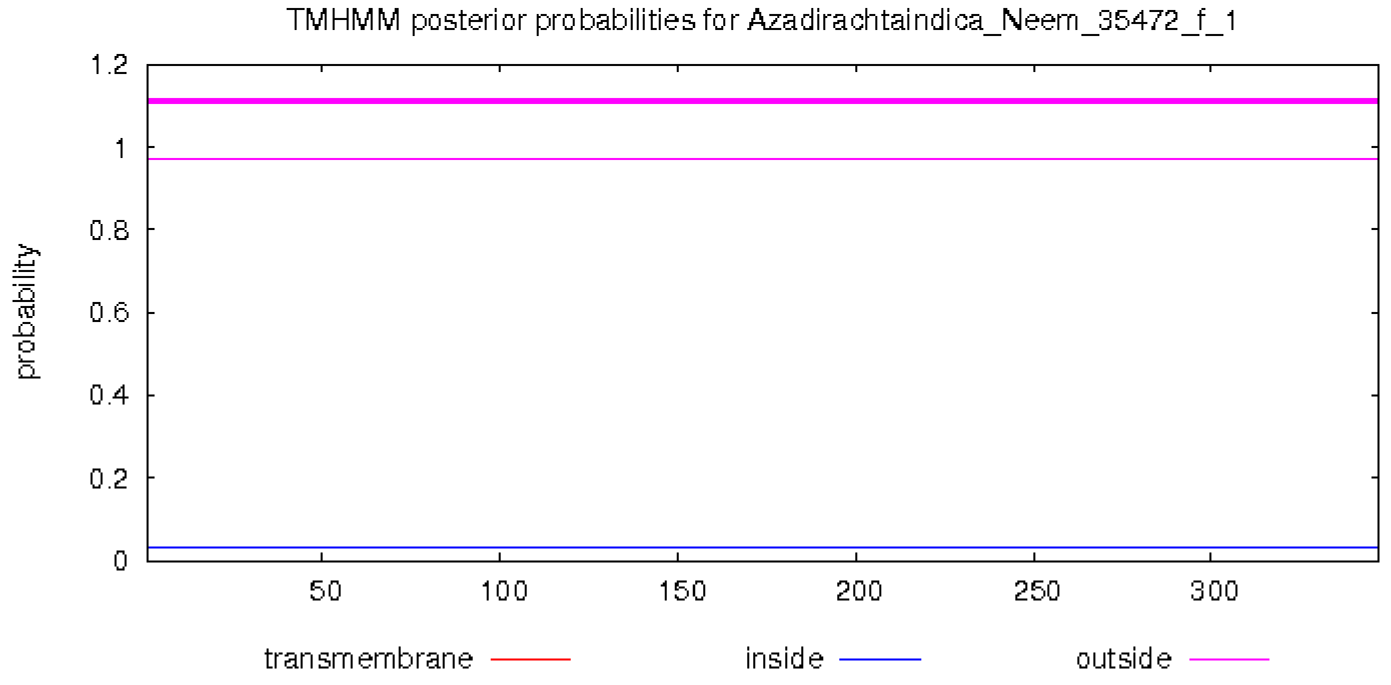

plot in postscript, script for making the plot in gnuplot, data for plot

---

```
# Azadirachtaindica_Neem_35720_f_1 Length: 286
# Azadirachtaindica_Neem_35720_f_1 Number of predicted TMHs:  0
# Azadirachtaindica_Neem_35720_f_1 Exp number of AAs in TMHs: 0.00015
# Azadirachtaindica_Neem_35720_f_1 Exp number, first 60 AAs:  0
# Azadirachtaindica_Neem_35720_f_1 Total prob of N-in:        0.01180
Azadirachtaindica_Neem_35720_f_1        TMHMM2.0        outside      1   286
```

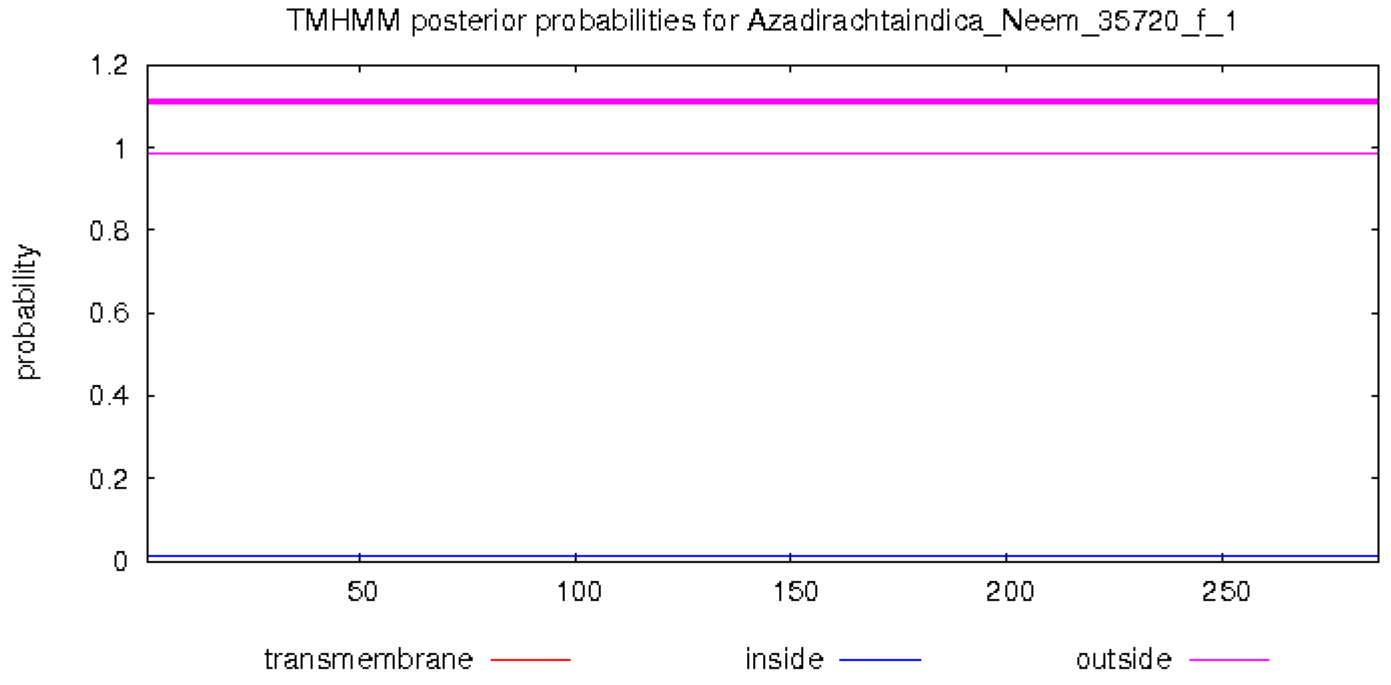

plot in postscript, script for making the plot in gnuplot, data for plot

---

```
# Azadirachtaindica_Neem_3589_f_8 Length: 291
# Azadirachtaindica_Neem_3589_f_8 Number of predicted TMHs:  0
```

```
# Azadirachtaindica_Neem_3589_f_8 Exp number of AAs in TMHs: 0.0006
# Azadirachtaindica_Neem_3589_f_8 Exp number, first 60 AAs:  0
# Azadirachtaindica_Neem_3589_f_8 Total prob of N-in:        0.01431
Azadirachtaindica_Neem_3589_f_8 TMHMM2.0        outside      1   291
```

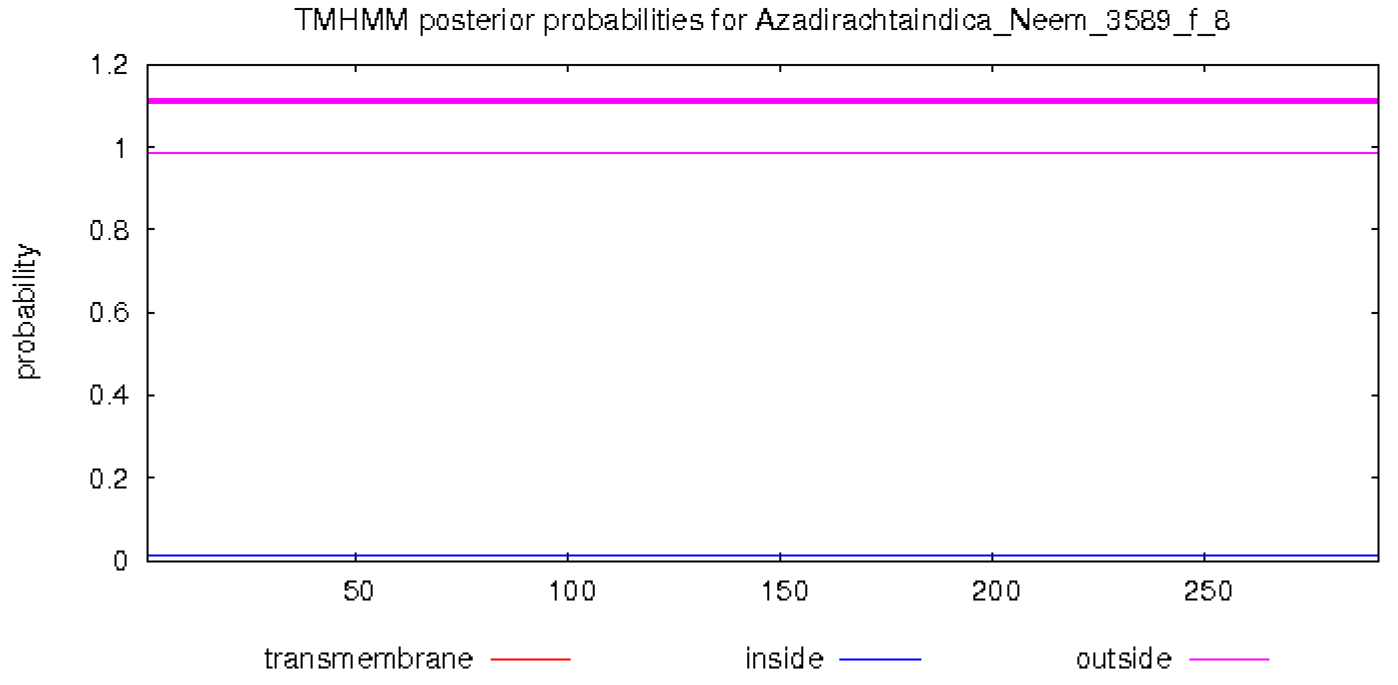

plot in postscript, script for making the plot in gnuplot, data for plot

---

```
# Azadirachtaindica_Neem_36116_a_1 Length: 80
# Azadirachtaindica_Neem_36116_a_1 Number of predicted TMHs:  0
# Azadirachtaindica_Neem_36116_a_1 Exp number of AAs in TMHs: 12.31768
# Azadirachtaindica_Neem_36116_a_1 Exp number, first 60 AAs:  1.85817
# Azadirachtaindica_Neem_36116_a_1 Total prob of N-in:        0.47076
Azadirachtaindica_Neem_36116_a_1        TMHMM2.0        outside      1    80
```

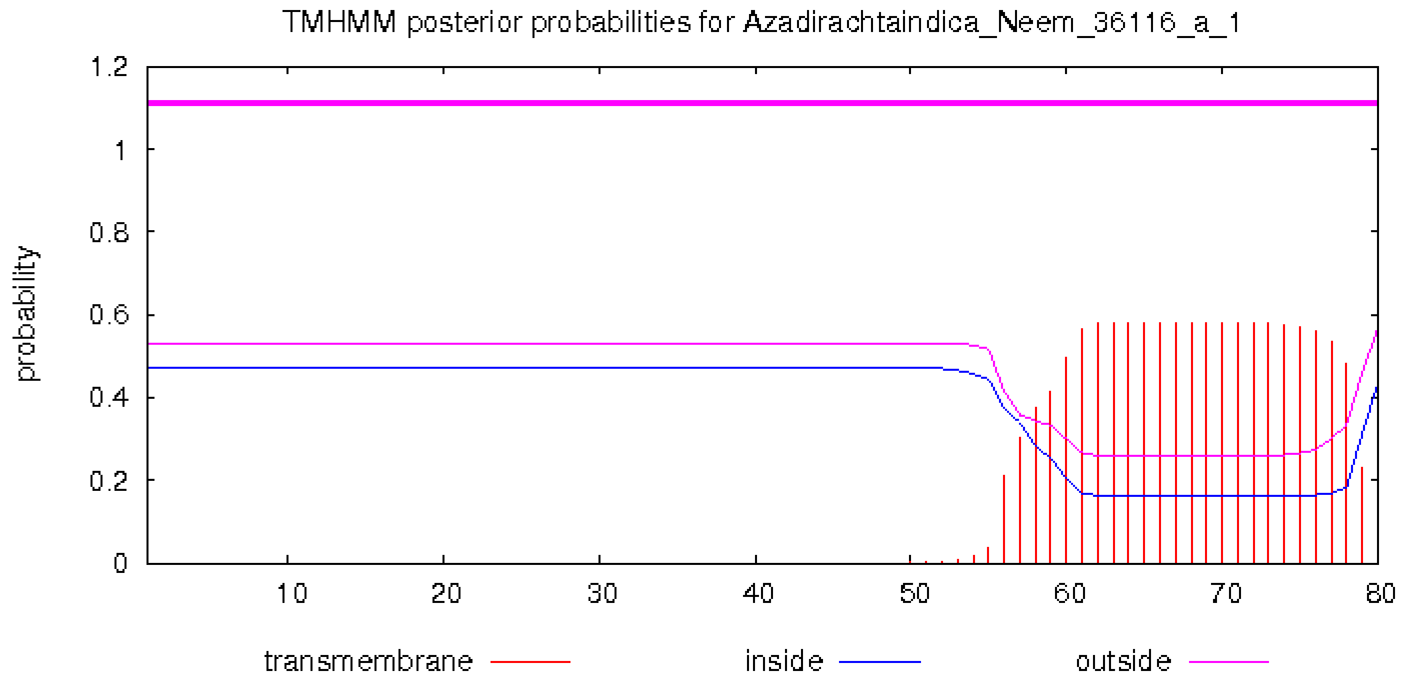

plot in postscript, script for making the plot in gnuplot, data for plot

---

```
# Azadirachtaindica_Neem_3629_f_1 Length: 277
# Azadirachtaindica_Neem_3629_f_1 Number of predicted TMHs:  0
```

```
# Azadirachtaindica_Neem_3629_f_1 Exp number of AAs in TMHs: 0.00022
# Azadirachtaindica_Neem_3629_f_1 Exp number, first 60 AAs:  0
# Azadirachtaindica_Neem_3629_f_1 Total prob of N-in:        0.01521
Azadirachtaindica_Neem_3629_f_1 TMHMM2.0        outside      1   277
```

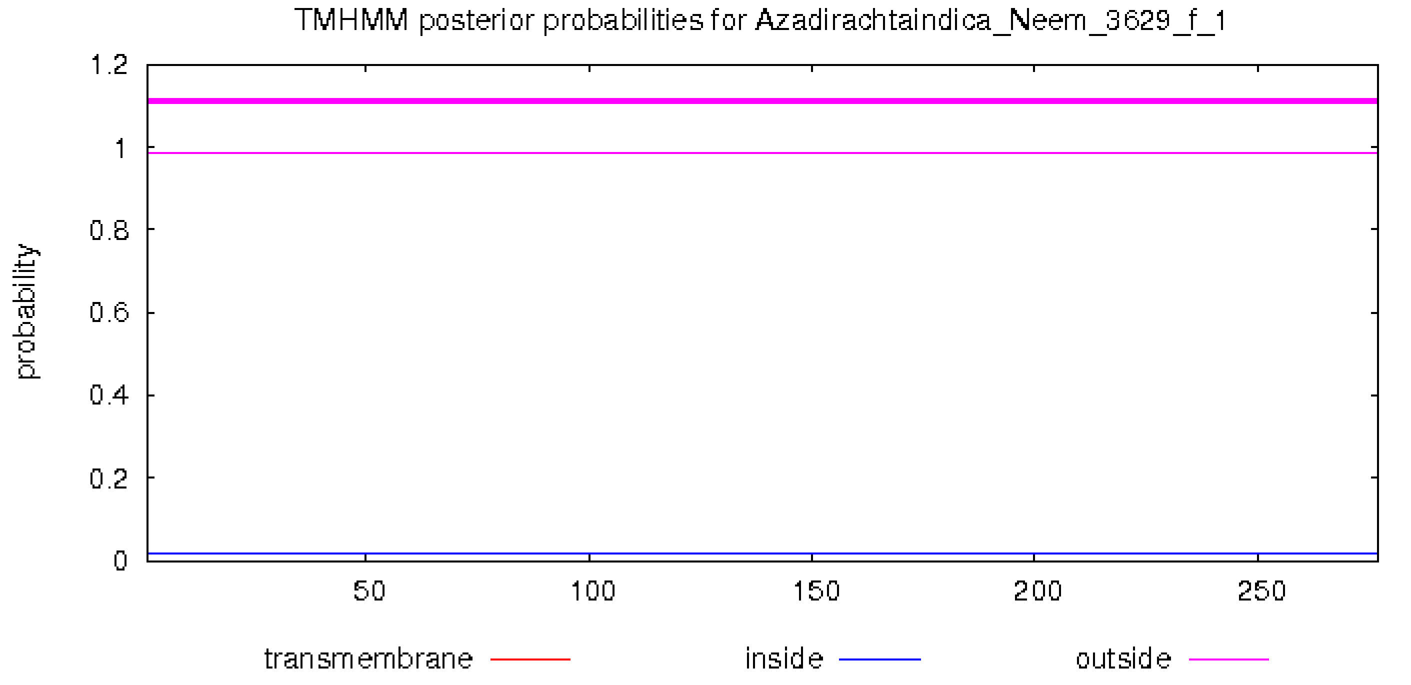

plot in postscript, script for making the plot in gnuplot, data for plot

---

```
# Azadirachtaindica_Neem_36363_f_1 Length: 180
# Azadirachtaindica_Neem_36363_f_1 Number of predicted TMHs:  0
# Azadirachtaindica_Neem_36363_f_1 Exp number of AAs in TMHs: 0.00657
# Azadirachtaindica_Neem_36363_f_1 Exp number, first 60 AAs:  0.00012
# Azadirachtaindica_Neem_36363_f_1 Total prob of N-in:        0.23906
Azadirachtaindica_Neem_36363_f_1        TMHMM2.0        outside      1   180
```

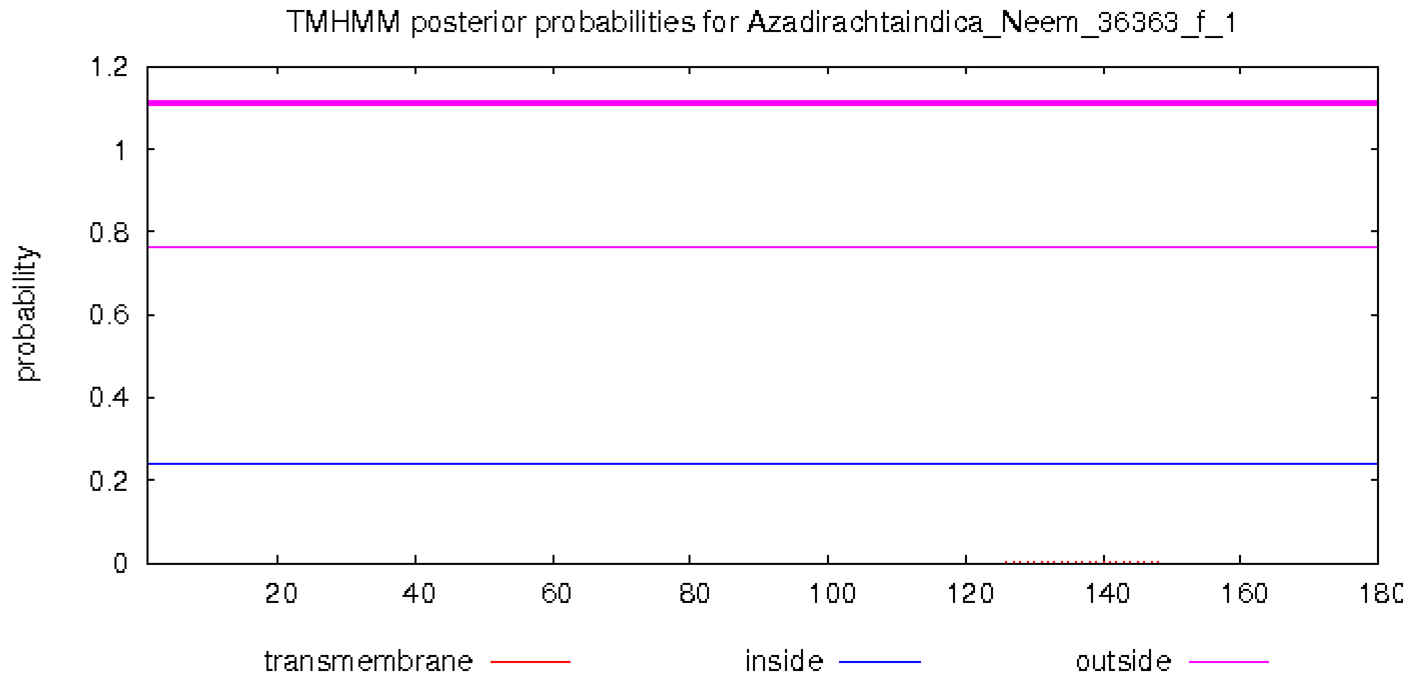

plot in postscript, script for making the plot in gnuplot, data for plot

---

```
# Azadirachtaindica_Neem_37635_f_3 Length: 184
# Azadirachtaindica_Neem_37635_f_3 Number of predicted TMHs:  0
```

```
# Azadirachtaindica_Neem_37635_f_3 Exp number of AAs in TMHs: 0.28646
# Azadirachtaindica_Neem_37635_f_3 Exp number, first 60 AAs:  0
# Azadirachtaindica_Neem_37635_f_3 Total prob of N-in:        0.03797
Azadirachtaindica_Neem_37635_f_3        TMHMM2.0        outside      1   184
```

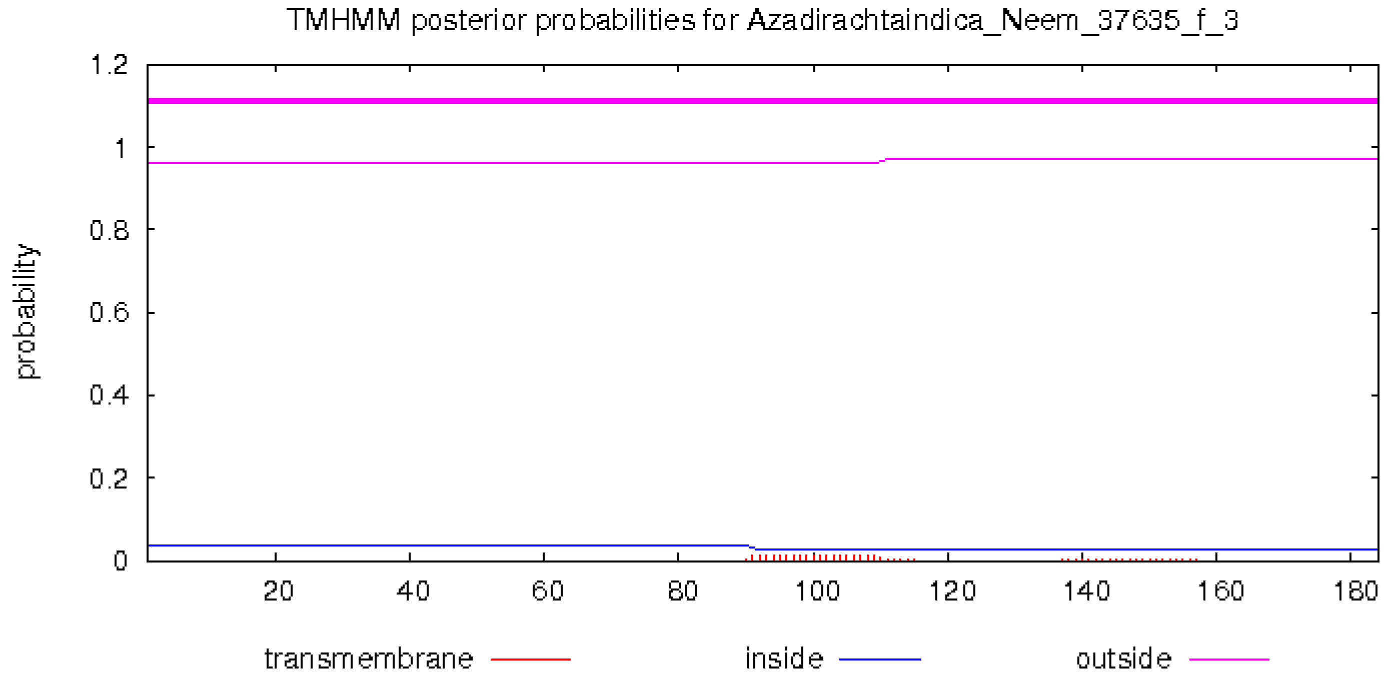

plot in postscript, script for making the plot in gnuplot, data for plot

---

```
# Azadirachtaindica_Neem_38603_f_1 Length: 317
# Azadirachtaindica_Neem_38603_f_1 Number of predicted TMHs:  0
# Azadirachtaindica_Neem_38603_f_1 Exp number of AAs in TMHs: 0.05282
# Azadirachtaindica_Neem_38603_f_1 Exp number, first 60 AAs:  0.04167
# Azadirachtaindica_Neem_38603_f_1 Total prob of N-in:        0.01408
Azadirachtaindica_Neem_38603_f_1        TMHMM2.0        outside      1   317
```

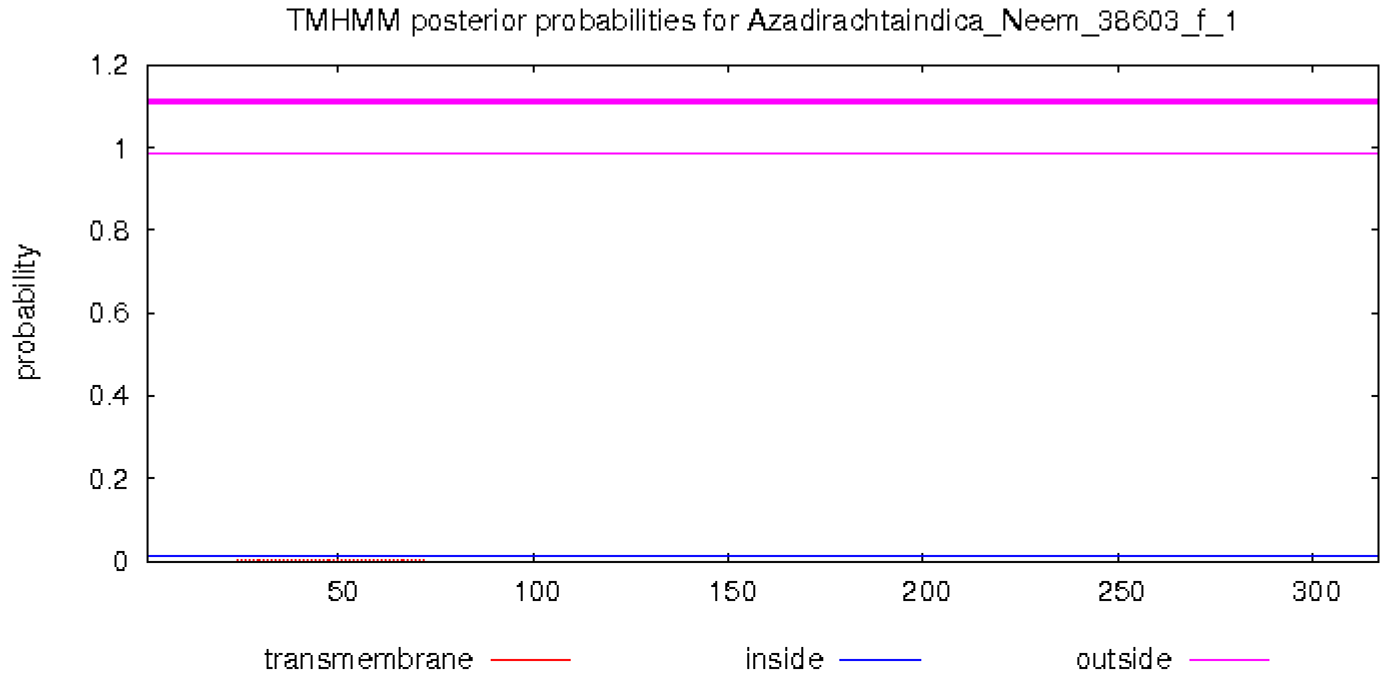

plot in postscript, script for making the plot in gnuplot, data for plot

---

```
# Azadirachtaindica_Neem_39437_f_1 Length: 155
# Azadirachtaindica_Neem_39437_f_1 Number of predicted TMHs:  0
```

```
# Azadirachtaindica_Neem_39437_f_1 Exp number of AAs in TMHs: 0.01328
# Azadirachtaindica_Neem_39437_f_1 Exp number, first 60 AAs:  0
# Azadirachtaindica_Neem_39437_f_1 Total prob of N-in:        0.04312
Azadirachtaindica_Neem_39437_f_1        TMHMM2.0        outside      1   155
```

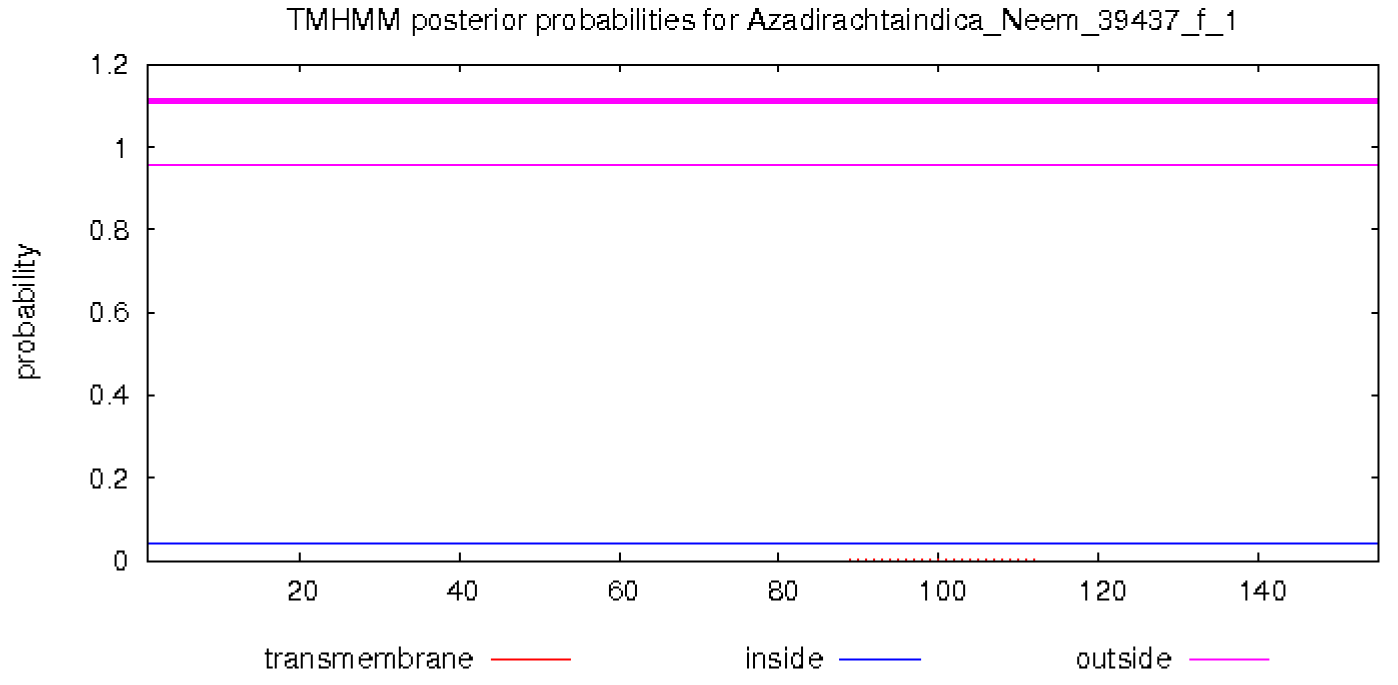

plot in postscript, script for making the plot in gnuplot, data for plot

---

```
# Azadirachtaindica_Neem_39493_f_2 Length: 268
# Azadirachtaindica_Neem_39493_f_2 Number of predicted TMHs:  0
# Azadirachtaindica_Neem_39493_f_2 Exp number of AAs in TMHs: 0.00455
# Azadirachtaindica_Neem_39493_f_2 Exp number, first 60 AAs:  4e-05
# Azadirachtaindica_Neem_39493_f_2 Total prob of N-in:        0.05272
Azadirachtaindica_Neem_39493_f_2        TMHMM2.0        outside      1   268
```

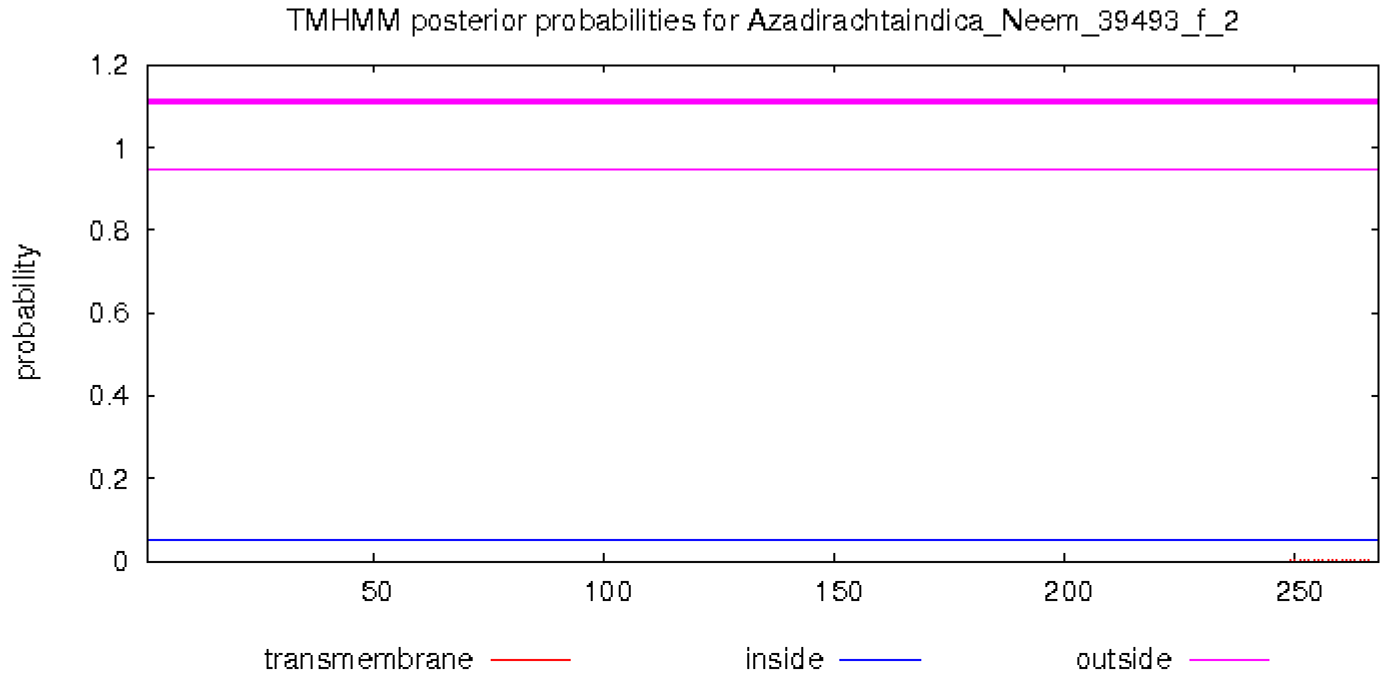

plot in postscript, script for making the plot in gnuplot, data for plot

---

```
# Azadirachtaindica_Neem_3950_f_1 Length: 312
# Azadirachtaindica_Neem_3950_f_1 Number of predicted TMHs:  0
```

```
# Azadirachtaindica_Neem_3950_f_1 Exp number of AAs in TMHs: 0.00018
# Azadirachtaindica_Neem_3950_f_1 Exp number, first 60 AAs:  0
# Azadirachtaindica_Neem_3950_f_1 Total prob of N-in:        0.00805
Azadirachtaindica_Neem_3950_f_1 TMHMM2.0        outside      1   312
```

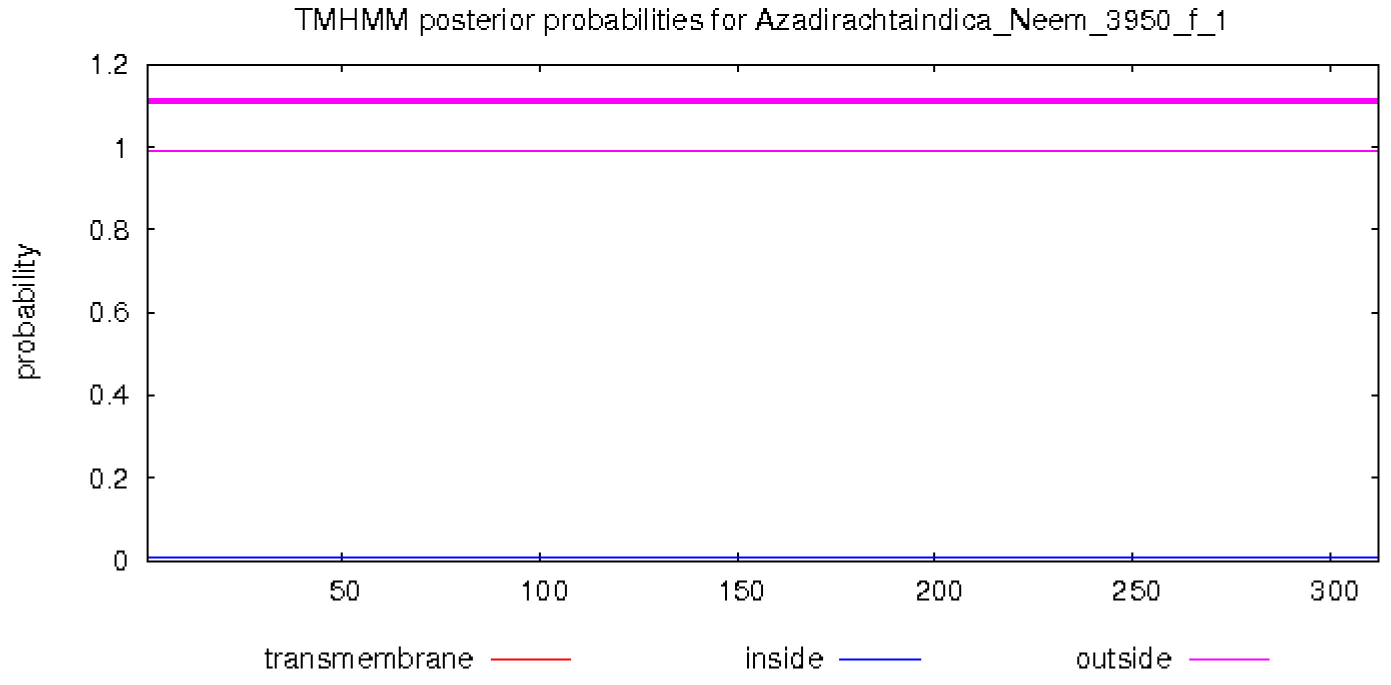

plot in postscript, script for making the plot in gnuplot, data for plot

---

```
# Azadirachtaindica_Neem_39610_f_1 Length: 223
# Azadirachtaindica_Neem_39610_f_1 Number of predicted TMHs:  0
# Azadirachtaindica_Neem_39610_f_1 Exp number of AAs in TMHs: 9e-05
# Azadirachtaindica_Neem_39610_f_1 Exp number, first 60 AAs:  6e-05
# Azadirachtaindica_Neem_39610_f_1 Total prob of N-in:        0.02131
Azadirachtaindica_Neem_39610_f_1        TMHMM2.0        outside      1   223
```

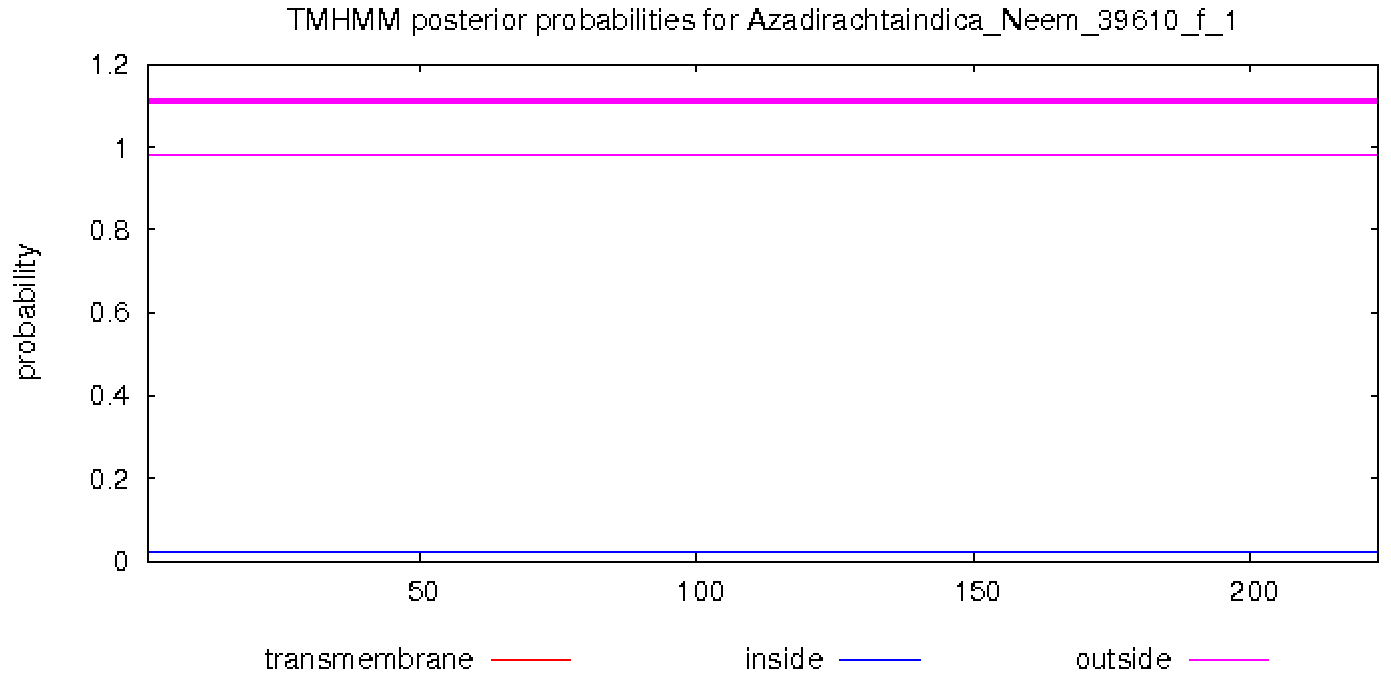

plot in postscript, script for making the plot in gnuplot, data for plot

---

```
# Azadirachtaindica_Neem_39628_f_1 Length: 416
# Azadirachtaindica_Neem_39628_f_1 Number of predicted TMHs:  0
```

```
# Azadirachtaindica_Neem_39628_f_1 Exp number of AAs in TMHs: 0.0002
# Azadirachtaindica_Neem_39628_f_1 Exp number, first 60 AAs:  0
# Azadirachtaindica_Neem_39628_f_1 Total prob of N-in:        0.01171
Azadirachtaindica_Neem_39628_f_1        TMHMM2.0        outside      1   416
```

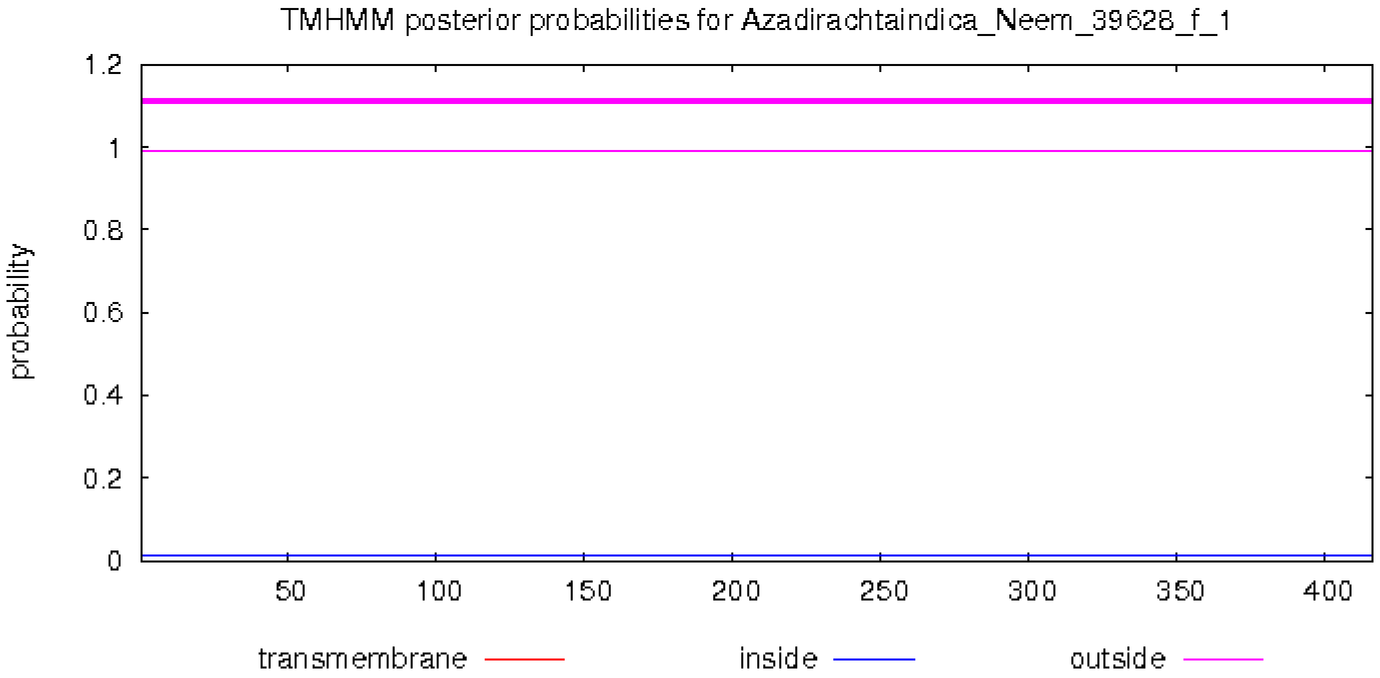

plot in postscript, script for making the plot in gnuplot, data for plot

---

```
# Azadirachtaindica_Neem_39666_f_1 Length: 441
# Azadirachtaindica_Neem_39666_f_1 Number of predicted TMHs:  0
# Azadirachtaindica_Neem_39666_f_1 Exp number of AAs in TMHs: 28.06097
# Azadirachtaindica_Neem_39666_f_1 Exp number, first 60 AAs:  0.00062
# Azadirachtaindica_Neem_39666_f_1 Total prob of N-in:        0.51707
Azadirachtaindica_Neem_39666_f_1        TMHMM2.0        outside      1   441
```

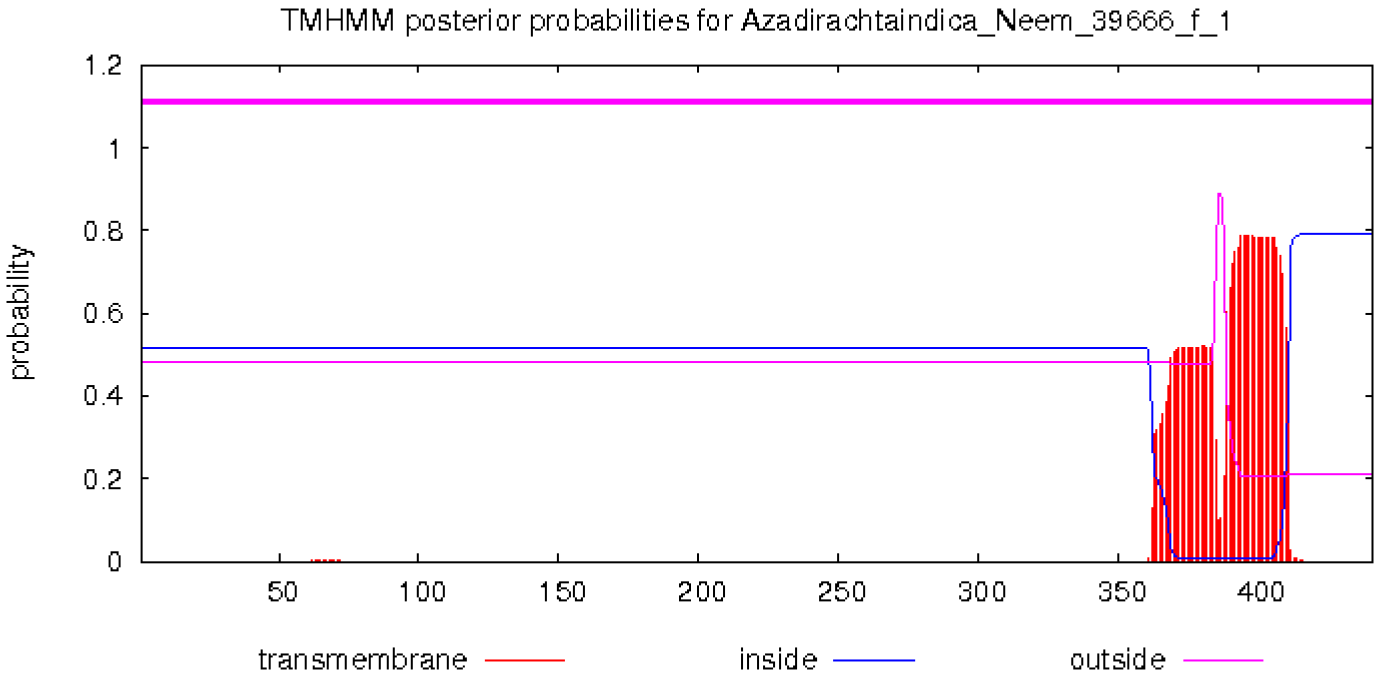

plot in postscript, script for making the plot in gnuplot, data for plot

---

```
# Azadirachtaindica_Neem_409_f_2 Length: 321
# Azadirachtaindica_Neem_409_f_2 Number of predicted TMHs:  0
```

```
# Azadirachtaindica_Neem_409_f_2 Exp number of AAs in TMHs: 0.09055
# Azadirachtaindica_Neem_409_f_2 Exp number, first 60 AAs:  0
# Azadirachtaindica_Neem_409_f_2 Total prob of N-in:        0.02504
Azadirachtaindica_Neem_409_f_2  TMHMM2.0        outside      1   321
```

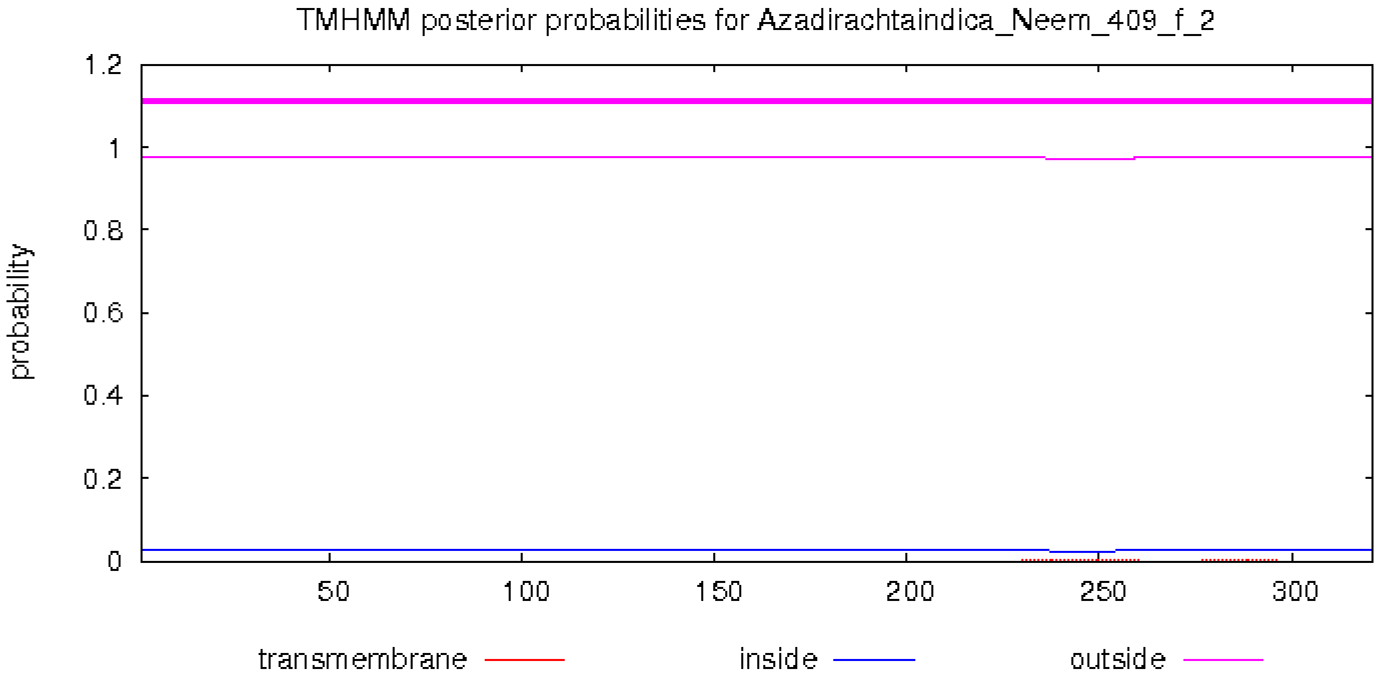

plot in postscript, script for making the plot in gnuplot, data for plot

---

```
# Azadirachtaindica_Neem_4377_a_2 Length: 175
# Azadirachtaindica_Neem_4377_a_2 Number of predicted TMHs:  0
# Azadirachtaindica_Neem_4377_a_2 Exp number of AAs in TMHs: 0.00044
# Azadirachtaindica_Neem_4377_a_2 Exp number, first 60 AAs:  0.00044
# Azadirachtaindica_Neem_4377_a_2 Total prob of N-in:        0.04380
Azadirachtaindica_Neem_4377_a_2 TMHMM2.0        outside      1   175
```

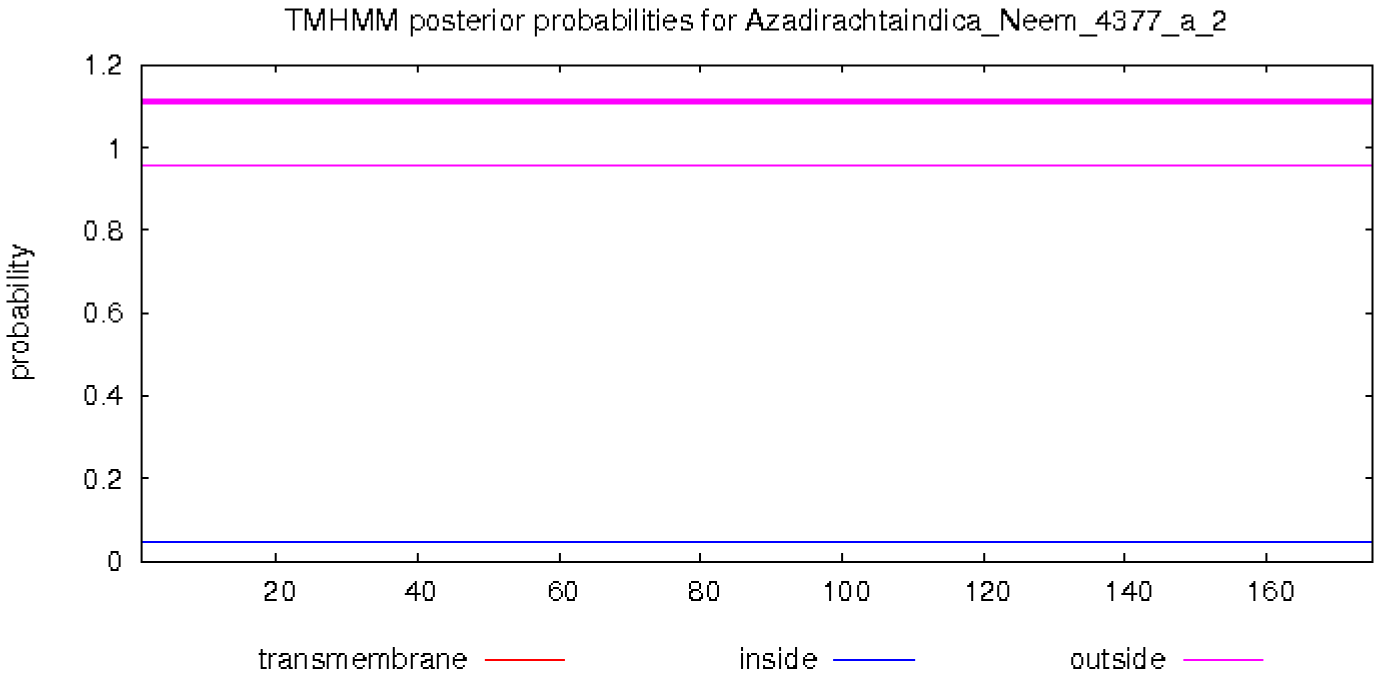

plot in postscript, script for making the plot in gnuplot, data for plot

---

```
# Azadirachtaindica_Neem_4530_f_1 Length: 577
# Azadirachtaindica_Neem_4530_f_1 Number of predicted TMHs:  0
```

```
# Azadirachtaindica_Neem_4530_f_1 Exp number of AAs in TMHs: 0.00199
# Azadirachtaindica_Neem_4530_f_1 Exp number, first 60 AAs:  0.00021
# Azadirachtaindica_Neem_4530_f_1 Total prob of N-in:        0.00269
Azadirachtaindica_Neem_4530_f_1 TMHMM2.0        outside      1   577
```

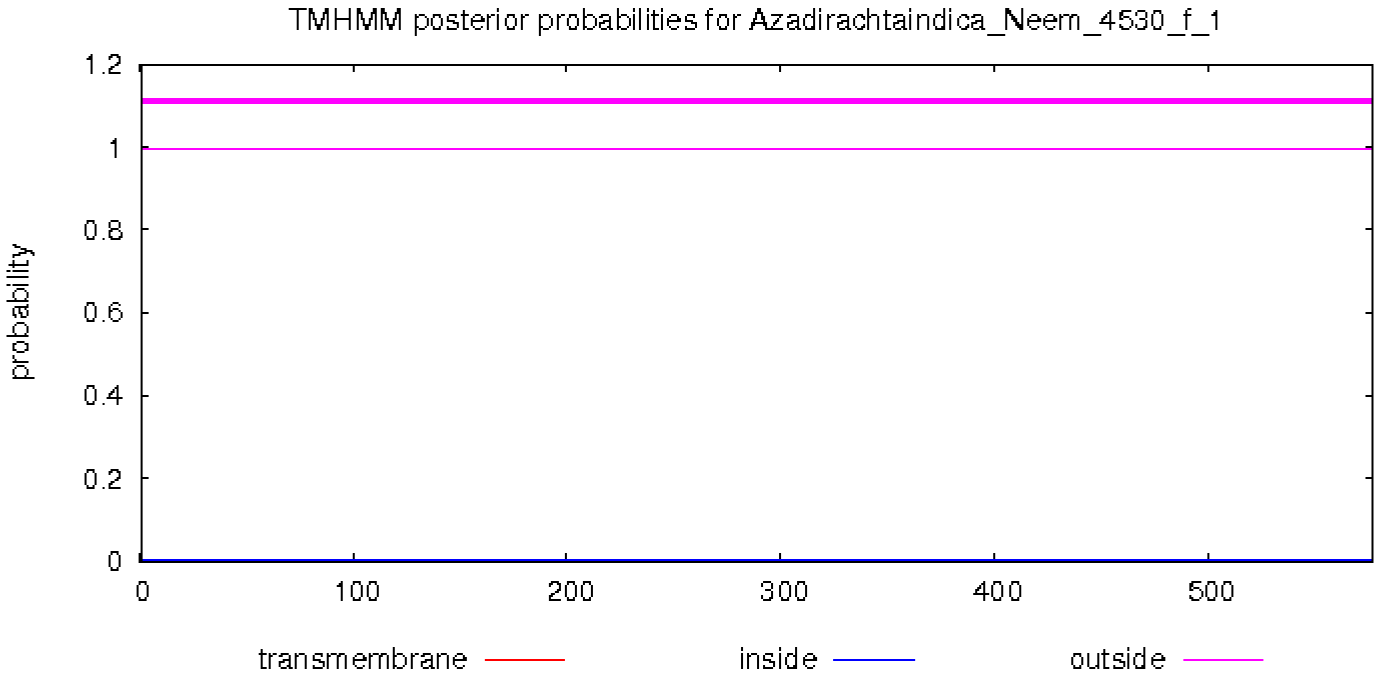

plot in postscript, script for making the plot in gnuplot, data for plot

---

```
# Azadirachtaindica_Neem_4815_f_1 Length: 347
# Azadirachtaindica_Neem_4815_f_1 Number of predicted TMHs:  0
# Azadirachtaindica_Neem_4815_f_1 Exp number of AAs in TMHs: 0.00019
# Azadirachtaindica_Neem_4815_f_1 Exp number, first 60 AAs:  0.00019
# Azadirachtaindica_Neem_4815_f_1 Total prob of N-in:        0.01488
Azadirachtaindica_Neem_4815_f_1 TMHMM2.0        outside      1   347
```

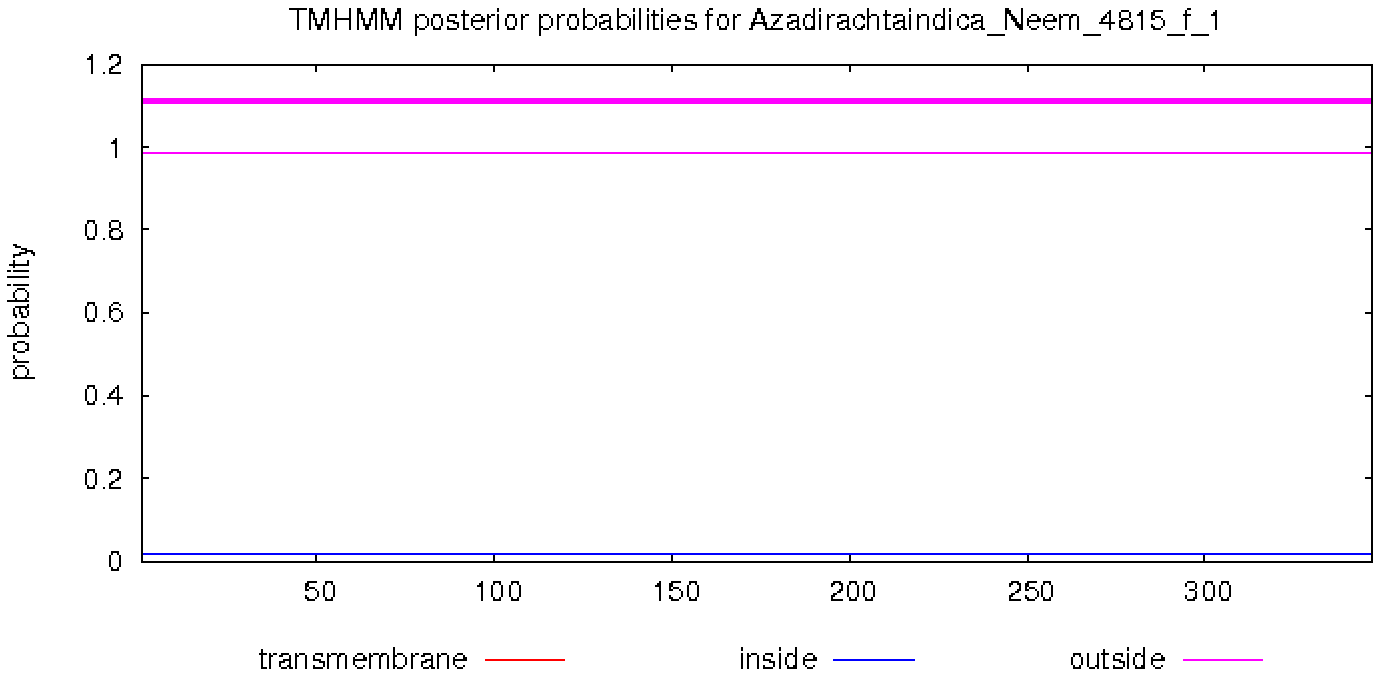

plot in postscript, script for making the plot in gnuplot, data for plot

---

```
# Azadirachtaindica_Neem_4983_f_1 Length: 399
# Azadirachtaindica_Neem_4983_f_1 Number of predicted TMHs:  0
```

```
# Azadirachtaindica_Neem_4983_f_1 Exp number of AAs in TMHs: 0.07764
# Azadirachtaindica_Neem_4983_f_1 Exp number, first 60 AAs:  0.00404
# Azadirachtaindica_Neem_4983_f_1 Total prob of N-in:        0.02007
Azadirachtaindica_Neem_4983_f_1 TMHMM2.0        outside      1   399
```

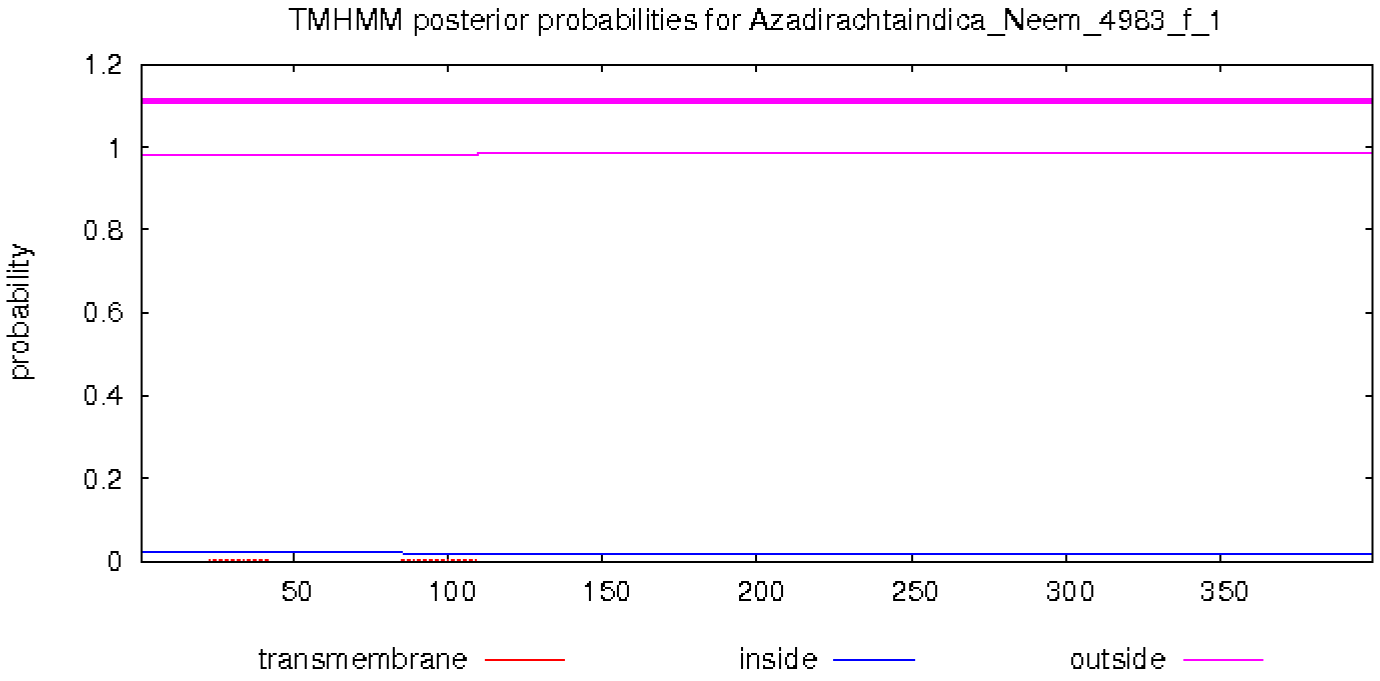

plot in postscript, script for making the plot in gnuplot, data for plot

---

```
# Azadirachtaindica_Neem_517_f_8 Length: 249
# Azadirachtaindica_Neem_517_f_8 Number of predicted TMHs:  0
# Azadirachtaindica_Neem_517_f_8 Exp number of AAs in TMHs: 0.02152
# Azadirachtaindica_Neem_517_f_8 Exp number, first 60 AAs:  0.00257
# Azadirachtaindica_Neem_517_f_8 Total prob of N-in:        0.07158
Azadirachtaindica_Neem_517_f_8  TMHMM2.0        outside      1   249
```

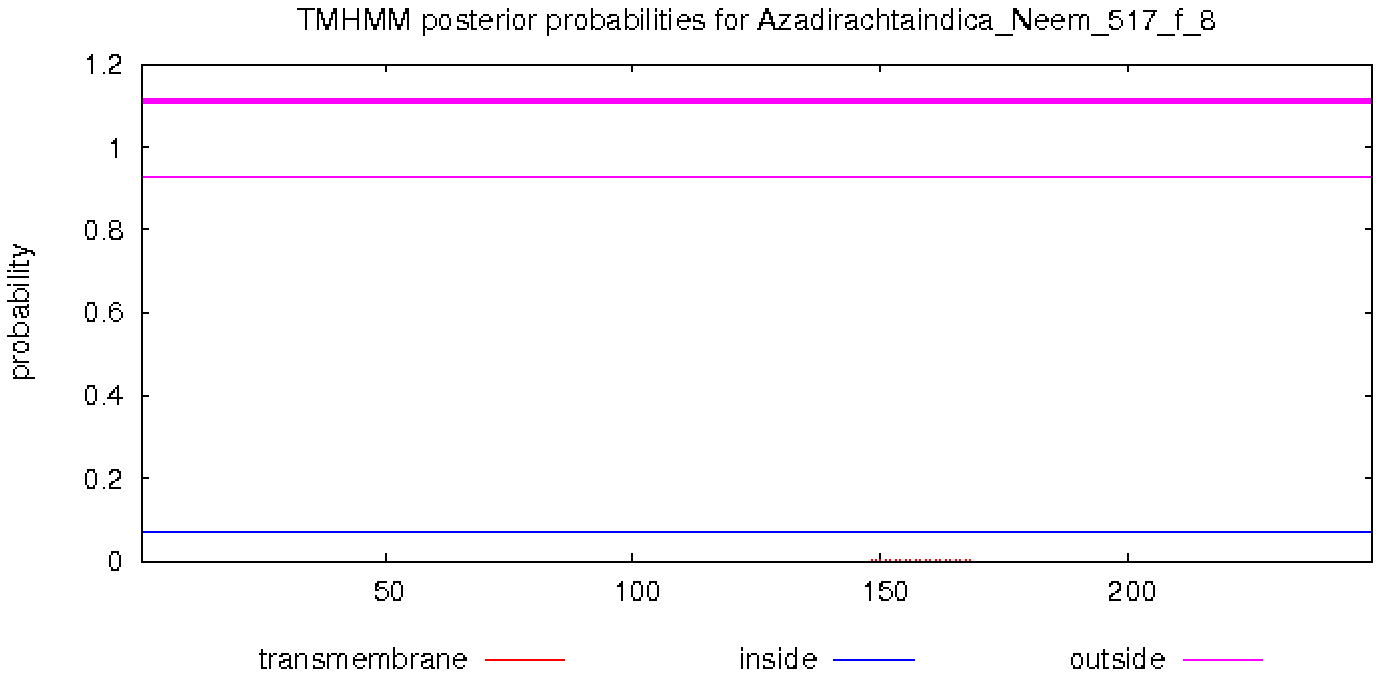

plot in postscript, script for making the plot in gnuplot, data for plot

---

```
# Azadirachtaindica_Neem_5254_f_1 Length: 264
# Azadirachtaindica_Neem_5254_f_1 Number of predicted TMHs:  0
```

```
# Azadirachtaindica_Neem_5254_f_1 Exp number of AAs in TMHs: 0
# Azadirachtaindica_Neem_5254_f_1 Exp number, first 60 AAs:  0
# Azadirachtaindica_Neem_5254_f_1 Total prob of N-in:        0.01855
Azadirachtaindica_Neem_5254_f_1 TMHMM2.0        outside      1   264
```

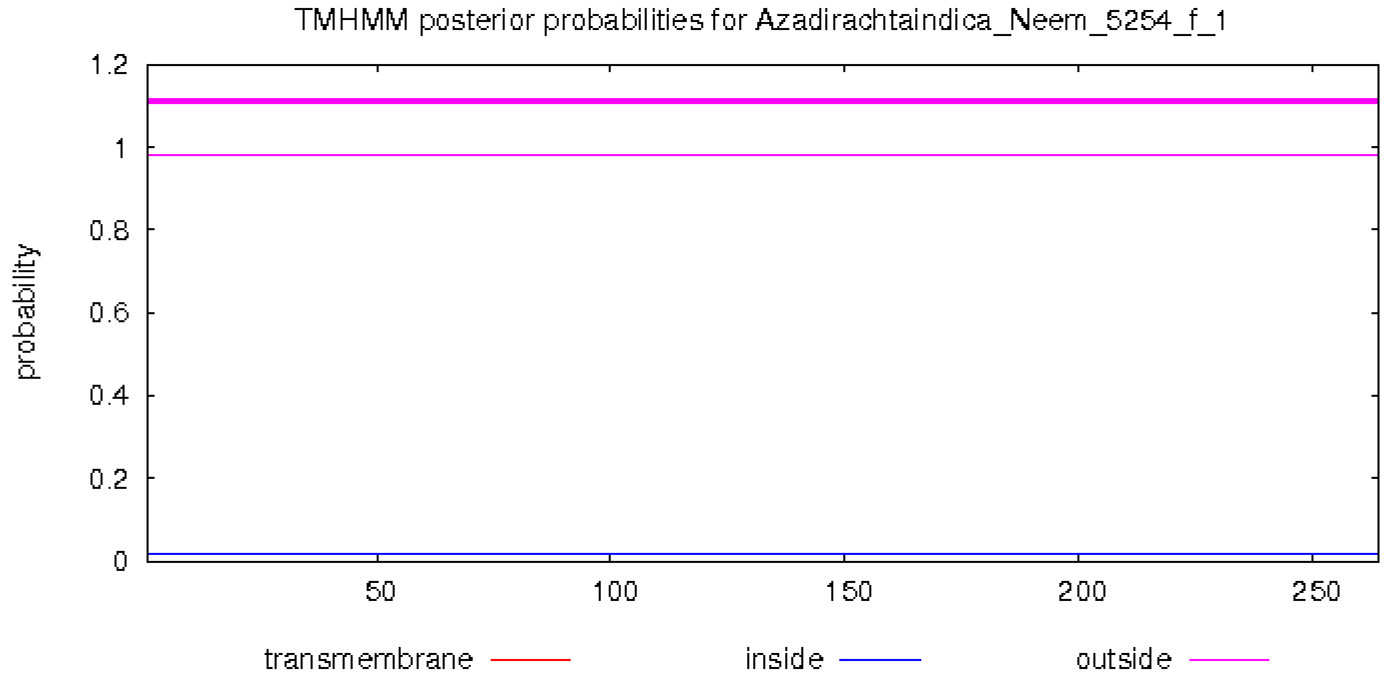

plot in postscript, script for making the plot in gnuplot, data for plot

---

```
# Azadirachtaindica_Neem_530_f_5 Length: 407
# Azadirachtaindica_Neem_530_f_5 Number of predicted TMHs:  0
# Azadirachtaindica_Neem_530_f_5 Exp number of AAs in TMHs: 0.0002
# Azadirachtaindica_Neem_530_f_5 Exp number, first 60 AAs:  0.0002
# Azadirachtaindica_Neem_530_f_5 Total prob of N-in:        0.00417
Azadirachtaindica_Neem_530_f_5  TMHMM2.0        outside      1   407
```

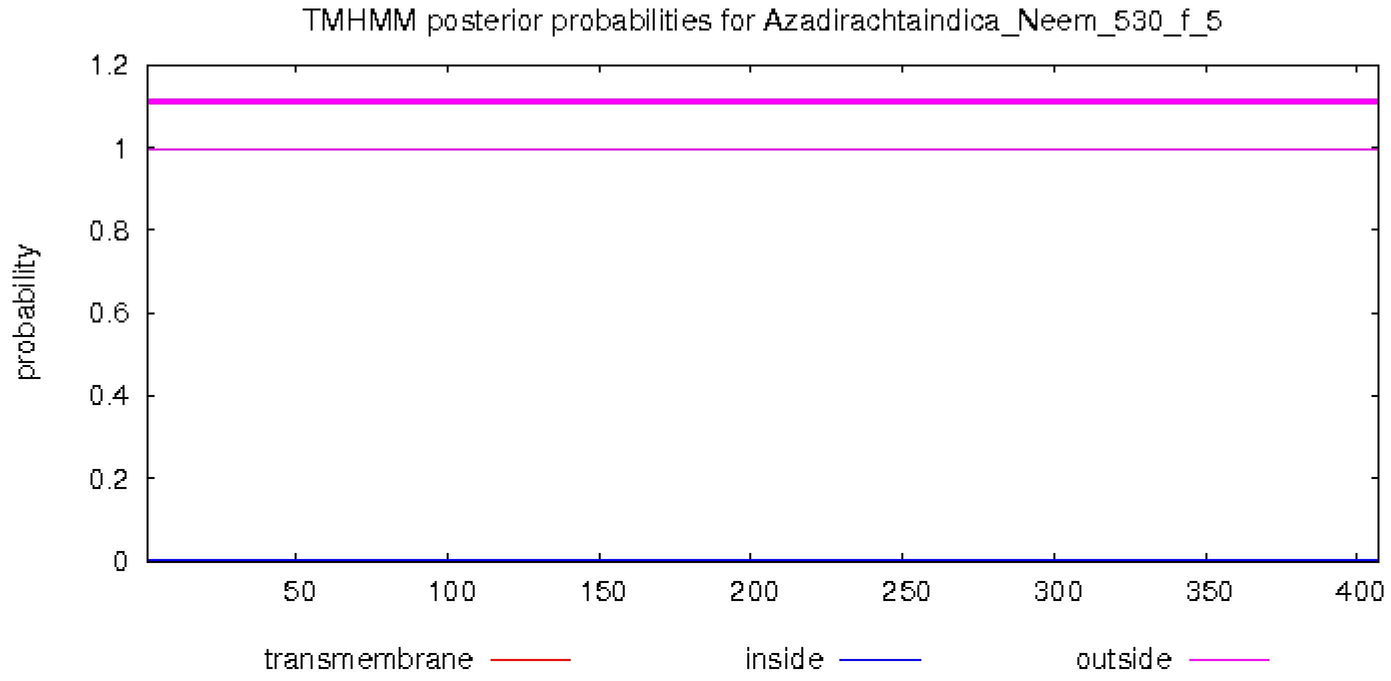

plot in postscript, script for making the plot in gnuplot, data for plot

---

```
# Azadirachtaindica_Neem_5527_a_2 Length: 171
# Azadirachtaindica_Neem_5527_a_2 Number of predicted TMHs:  0
```

```
# Azadirachtaindica_Neem_5527_a_2 Exp number of AAs in TMHs: 0.01217
# Azadirachtaindica_Neem_5527_a_2 Exp number, first 60 AAs:  0
# Azadirachtaindica_Neem_5527_a_2 Total prob of N-in:        0.02891
Azadirachtaindica_Neem_5527_a_2 TMHMM2.0        outside      1   171
```

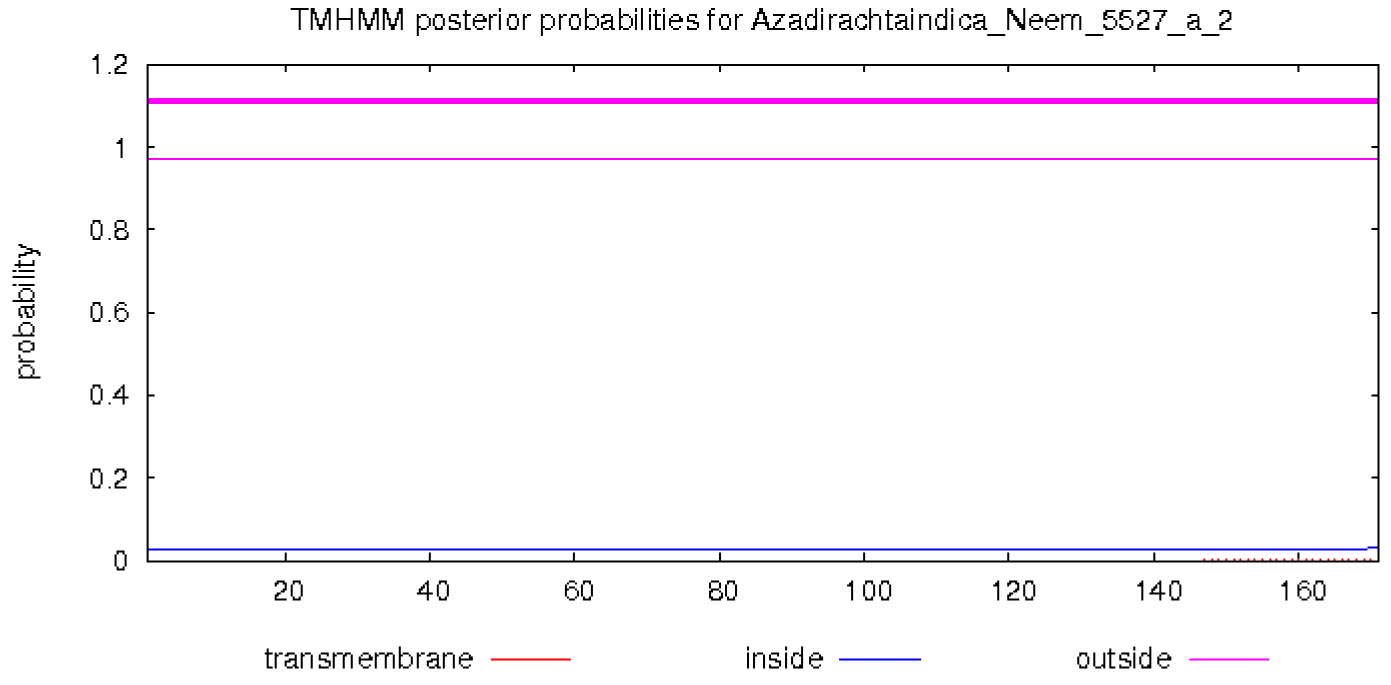

plot in postscript, script for making the plot in gnuplot, data for plot

```
# Azadirachtaindica_Neem_5527_f_1 Length: 363
# Azadirachtaindica_Neem_5527_f_1 Number of predicted TMHs:  0
# Azadirachtaindica_Neem_5527_f_1 Exp number of AAs in TMHs: 0.00053
# Azadirachtaindica_Neem_5527_f_1 Exp number, first 60 AAs:  0
# Azadirachtaindica_Neem_5527_f_1 Total prob of N-in:        0.00283
Azadirachtaindica_Neem_5527_f_1 TMHMM2.0        outside      1   363
```

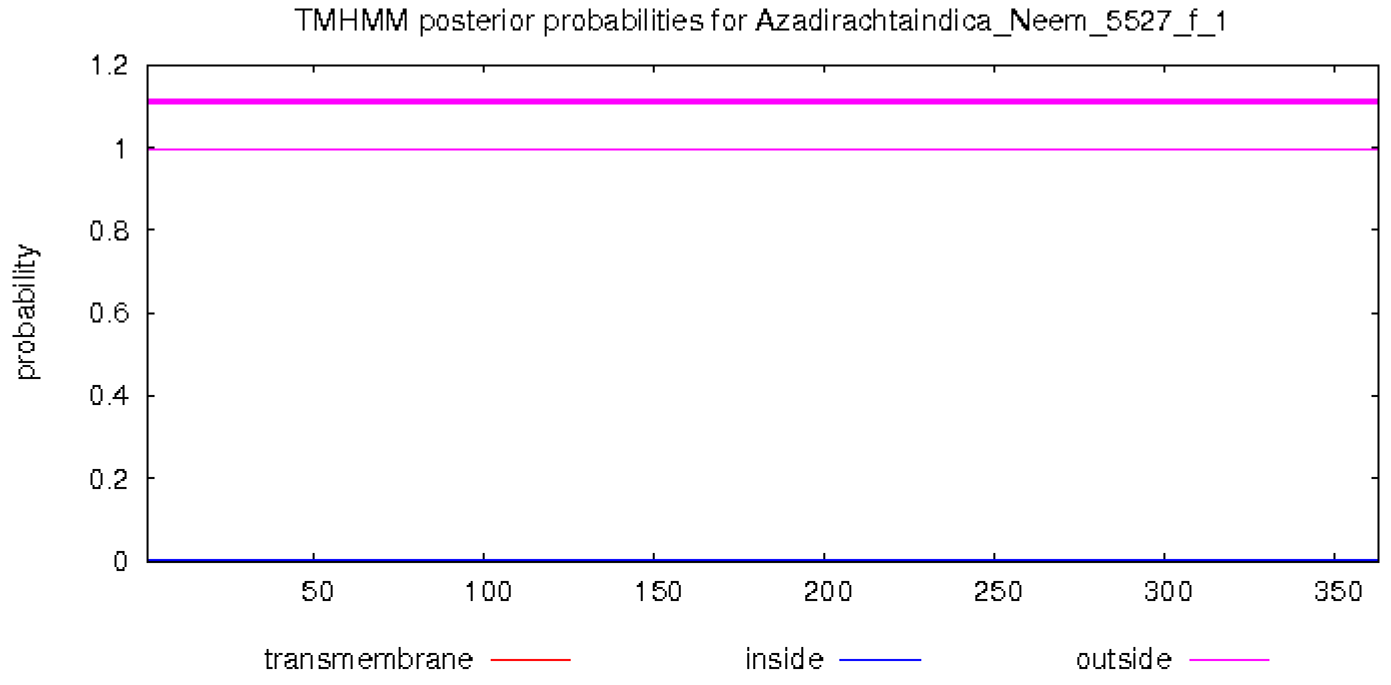

plot in postscript, script for making the plot in gnuplot, data for plot

```
# Azadirachtaindica_Neem_5630_f_1 Length: 335
# Azadirachtaindica_Neem_5630_f_1 Number of predicted TMHs:  0
```

```
# Azadirachtaindica_Neem_5630_f_1 Exp number of AAs in TMHs: 0
# Azadirachtaindica_Neem_5630_f_1 Exp number, first 60 AAs:  0
# Azadirachtaindica_Neem_5630_f_1 Total prob of N-in:        0.00343
Azadirachtaindica_Neem_5630_f_1 TMHMM2.0        outside      1   335
```

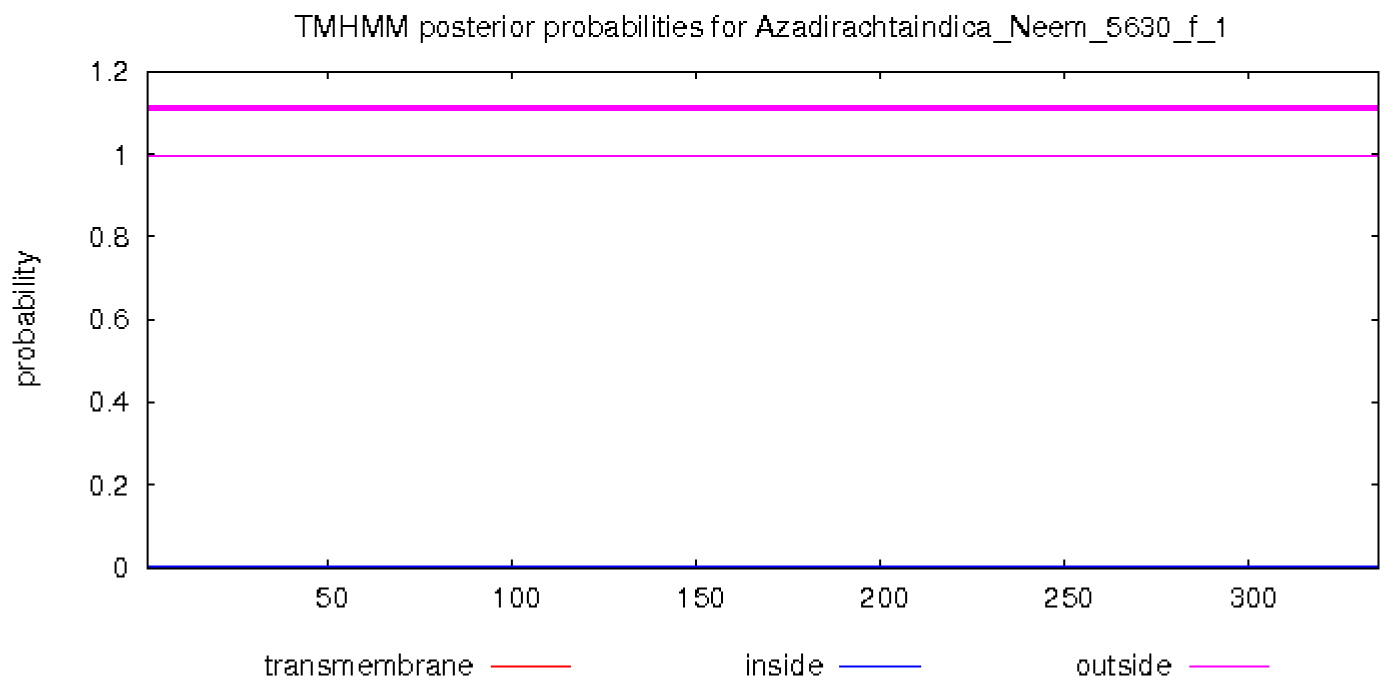

plot in postscript, script for making the plot in gnuplot, data for plot

---

```
# Azadirachtaindica_Neem_5679_f_1 Length: 166
# Azadirachtaindica_Neem_5679_f_1 Number of predicted TMHs:  0
# Azadirachtaindica_Neem_5679_f_1 Exp number of AAs in TMHs: 0
# Azadirachtaindica_Neem_5679_f_1 Exp number, first 60 AAs:  0
# Azadirachtaindica_Neem_5679_f_1 Total prob of N-in:        0.09528
Azadirachtaindica_Neem_5679_f_1 TMHMM2.0        outside      1   166
```

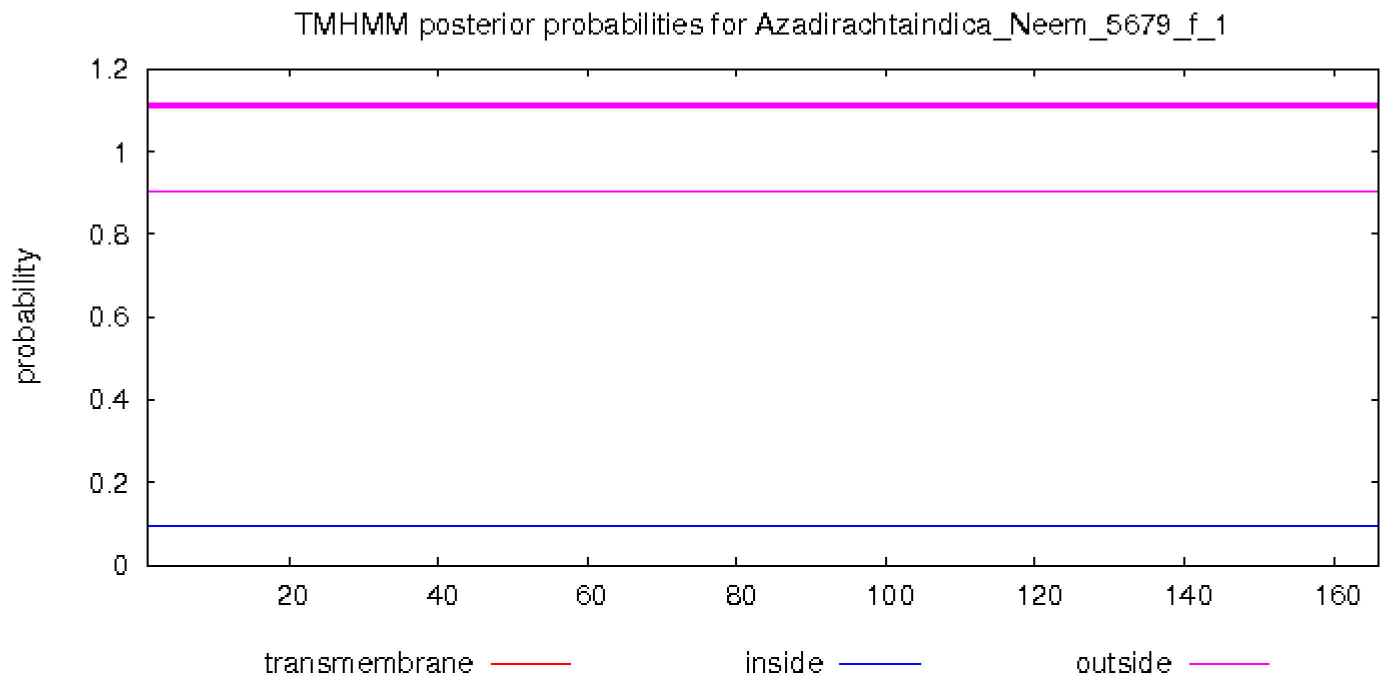

plot in postscript, script for making the plot in gnuplot, data for plot

---

```
# Azadirachtaindica_Neem_5801_f_1 Length: 333
# Azadirachtaindica_Neem_5801_f_1 Number of predicted TMHs:  0
```

```
# Azadirachtaindica_Neem_5801_f_1 Exp number of AAs in TMHs: 0.00021
# Azadirachtaindica_Neem_5801_f_1 Exp number, first 60 AAs:  0
# Azadirachtaindica_Neem_5801_f_1 Total prob of N-in:        0.00490
Azadirachtaindica_Neem_5801_f_1 TMHMM2.0        outside      1   333
```

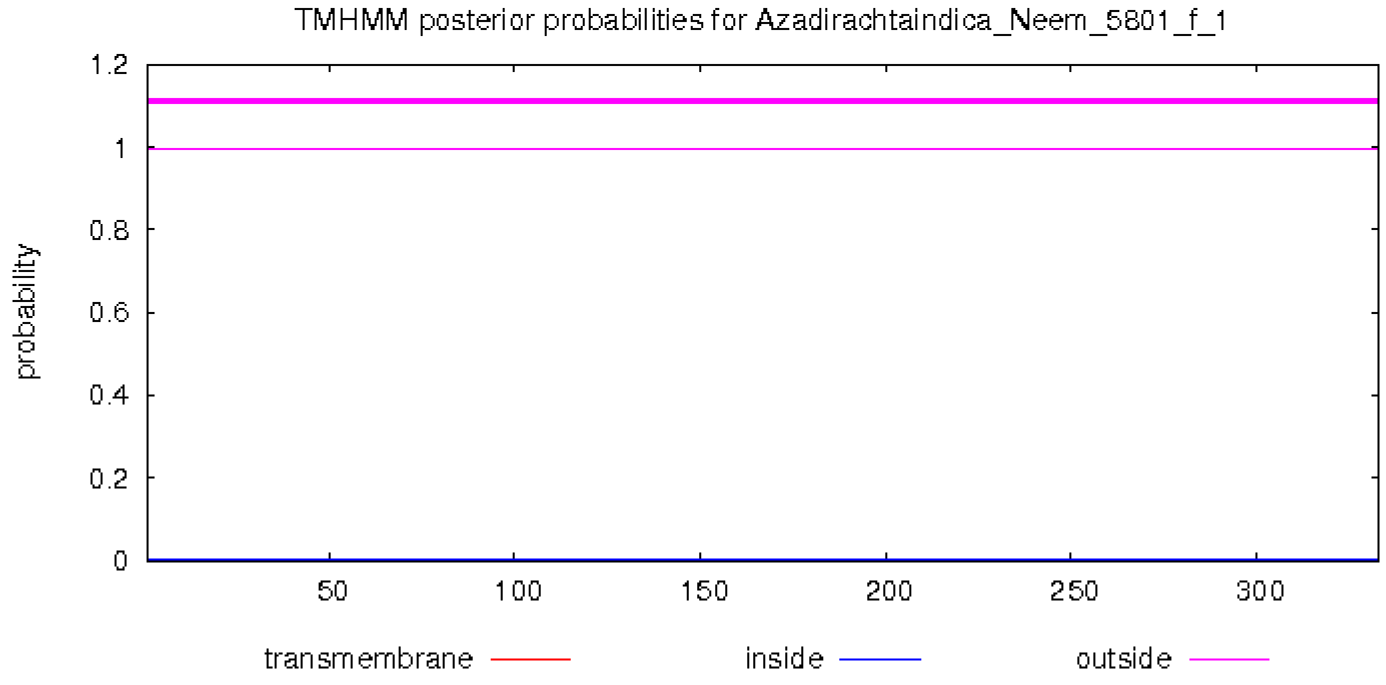

plot in postscript, script for making the plot in gnuplot, data for plot

---

```
# Azadirachtaindica_Neem_585_f_6 Length: 299
# Azadirachtaindica_Neem_585_f_6 Number of predicted TMHs:  0
# Azadirachtaindica_Neem_585_f_6 Exp number of AAs in TMHs: 0.00057
# Azadirachtaindica_Neem_585_f_6 Exp number, first 60 AAs:  0
# Azadirachtaindica_Neem_585_f_6 Total prob of N-in:        0.09036
Azadirachtaindica_Neem_585_f_6  TMHMM2.0        outside      1   299
```

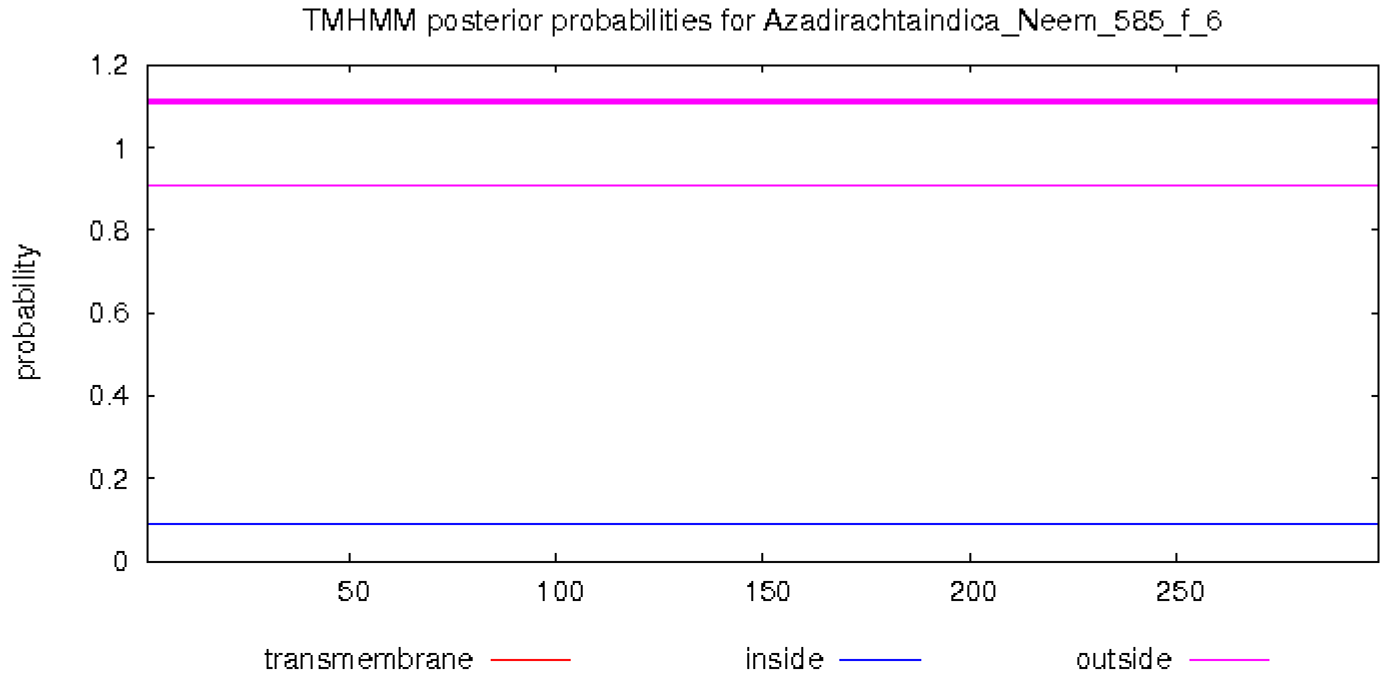

plot in postscript, script for making the plot in gnuplot, data for plot

---

```
# Azadirachtaindica_Neem_5959_f_1 Length: 157
# Azadirachtaindica_Neem_5959_f_1 Number of predicted TMHs:  0
```

```
# Azadirachtaindica_Neem_5959_f_1 Exp number of AAs in TMHs: 0.00035
# Azadirachtaindica_Neem_5959_f_1 Exp number, first 60 AAs:  3e-05
# Azadirachtaindica_Neem_5959_f_1 Total prob of N-in:        0.10268
Azadirachtaindica_Neem_5959_f_1 TMHMM2.0        outside      1   157
```

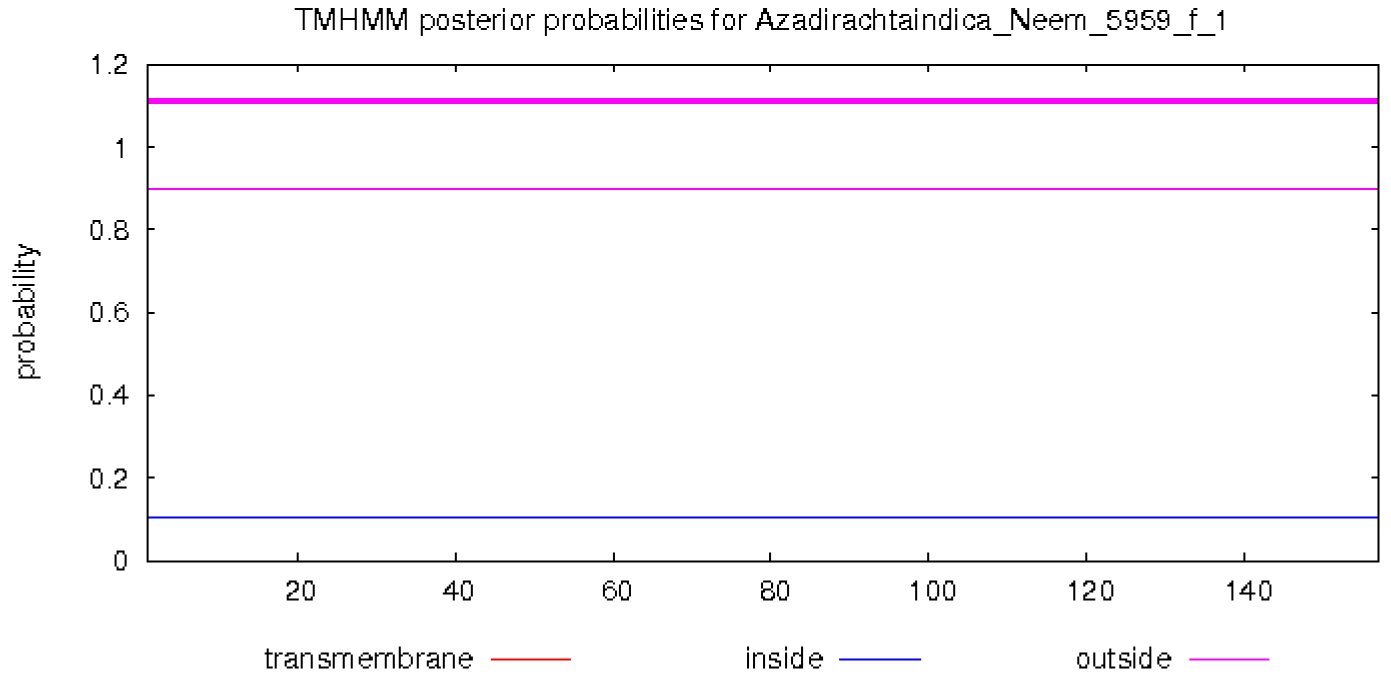

plot in postscript, script for making the plot in gnuplot, data for plot

---

```
# Azadirachtaindica_Neem_6031_f_1 Length: 317
# Azadirachtaindica_Neem_6031_f_1 Number of predicted TMHs:  0
# Azadirachtaindica_Neem_6031_f_1 Exp number of AAs in TMHs: 0.05282
# Azadirachtaindica_Neem_6031_f_1 Exp number, first 60 AAs:  0.04167
# Azadirachtaindica_Neem_6031_f_1 Total prob of N-in:        0.01408
Azadirachtaindica_Neem_6031_f_1 TMHMM2.0        outside      1   317
```

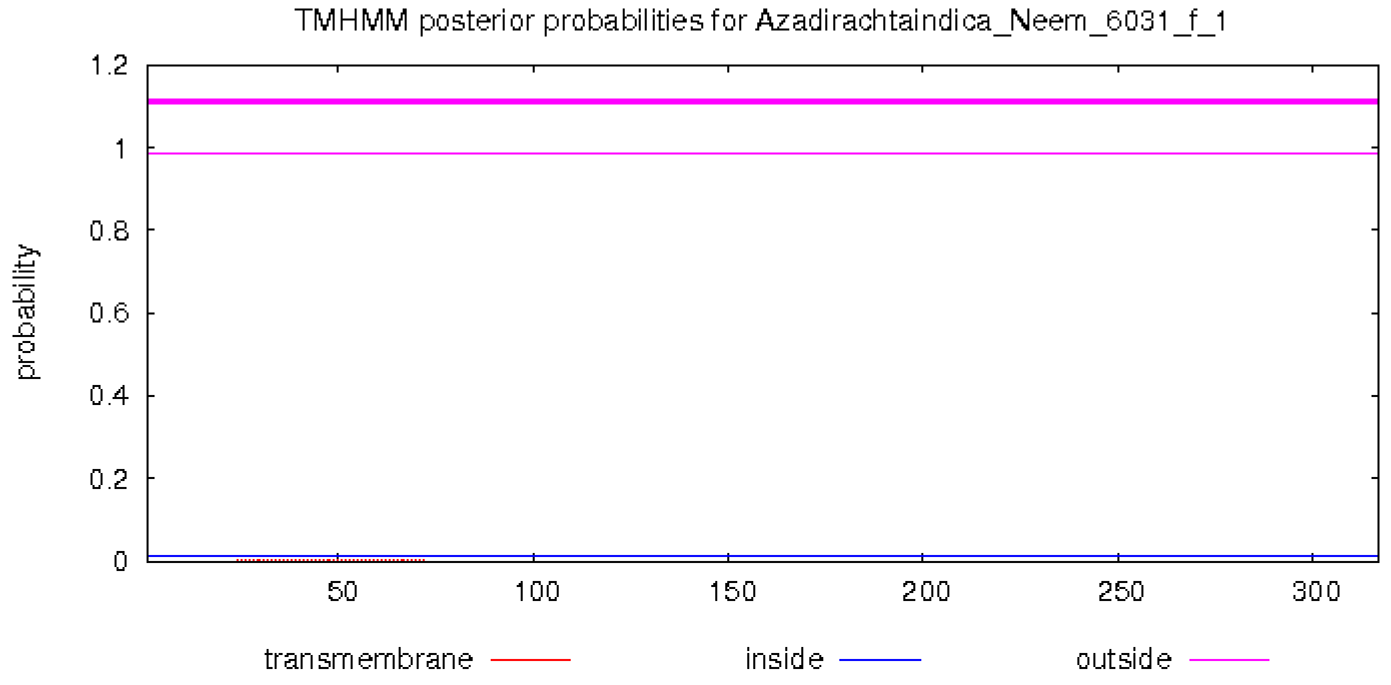

plot in postscript, script for making the plot in gnuplot, data for plot

---

```
# Azadirachtaindica_Neem_6040_a_4 Length: 118
# Azadirachtaindica_Neem_6040_a_4 Number of predicted TMHs:  0
```

```
# Azadirachtaindica_Neem_6040_a_4 Exp number of AAs in TMHs: 0.00978
# Azadirachtaindica_Neem_6040_a_4 Exp number, first 60 AAs:  0.00207
# Azadirachtaindica_Neem_6040_a_4 Total prob of N-in:        0.58232
Azadirachtaindica_Neem_6040_a_4 TMHMM2.0        inside       1   118
```

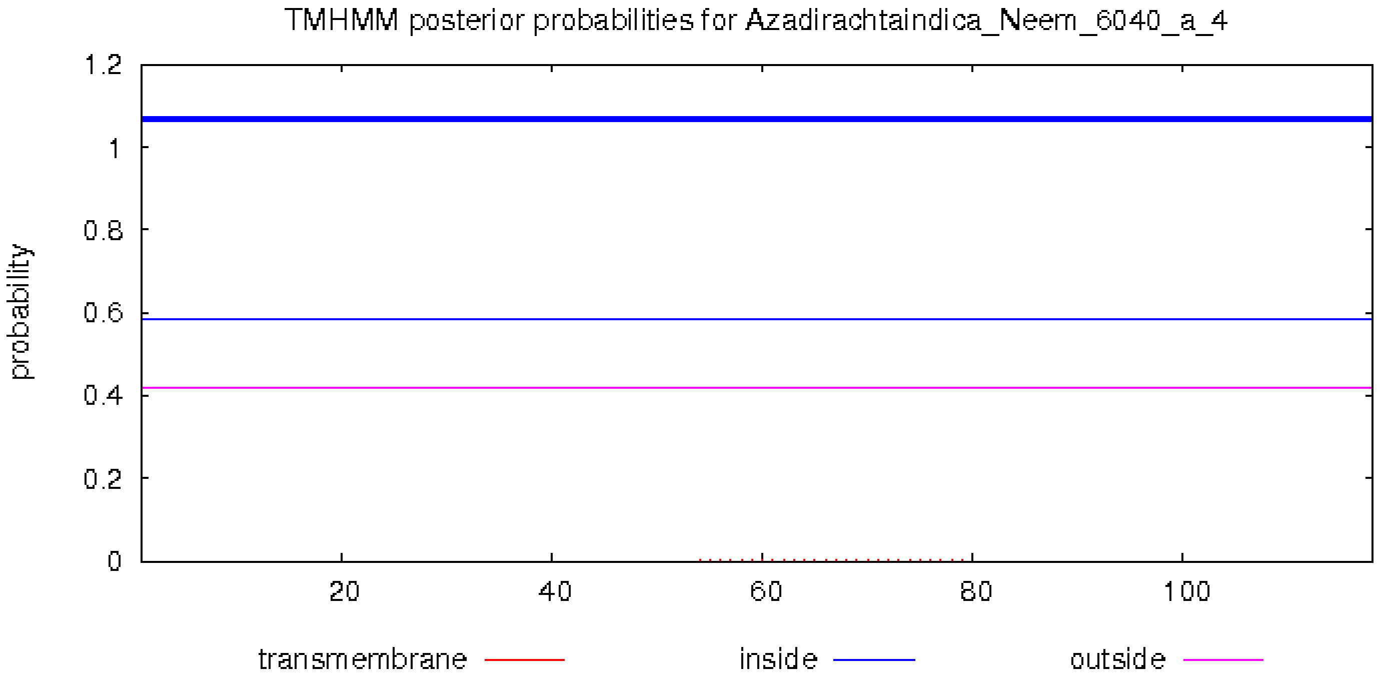

plot in postscript, script for making the plot in gnuplot, data for plot

---

```
# Azadirachtaindica_Neem_6403_f_1 Length: 294
# Azadirachtaindica_Neem_6403_f_1 Number of predicted TMHs:  0
# Azadirachtaindica_Neem_6403_f_1 Exp number of AAs in TMHs: 0.00035
# Azadirachtaindica_Neem_6403_f_1 Exp number, first 60 AAs:  0.00035
# Azadirachtaindica_Neem_6403_f_1 Total prob of N-in:        0.03611
Azadirachtaindica_Neem_6403_f_1 TMHMM2.0        outside      1   294
```

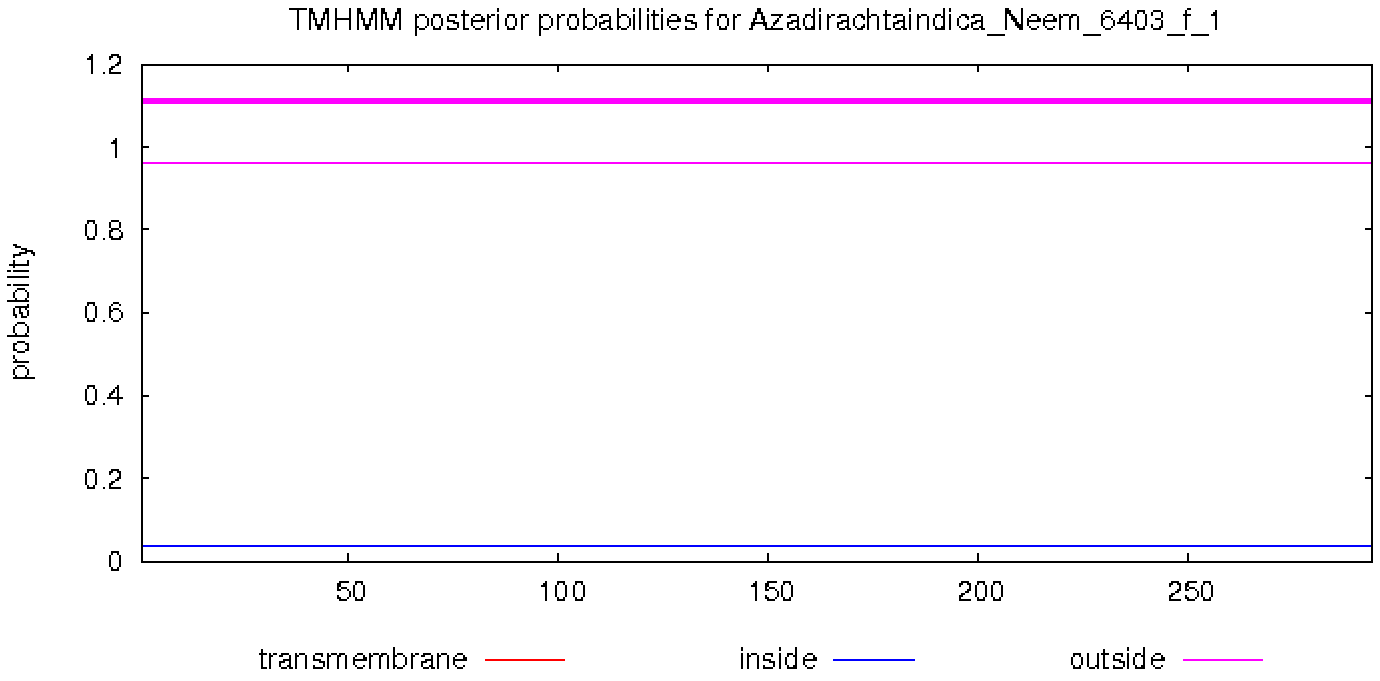

plot in postscript, script for making the plot in gnuplot, data for plot

---

```
# Azadirachtaindica_Neem_6492_f_1 Length: 376
# Azadirachtaindica_Neem_6492_f_1 Number of predicted TMHs:  0
```

```
# Azadirachtaindica_Neem_6492_f_1 Exp number of AAs in TMHs: 1.59581
# Azadirachtaindica_Neem_6492_f_1 Exp number, first 60 AAs:  0.00094
# Azadirachtaindica_Neem_6492_f_1 Total prob of N-in:        0.00373
Azadirachtaindica_Neem_6492_f_1 TMHMM2.0        outside      1   376
```

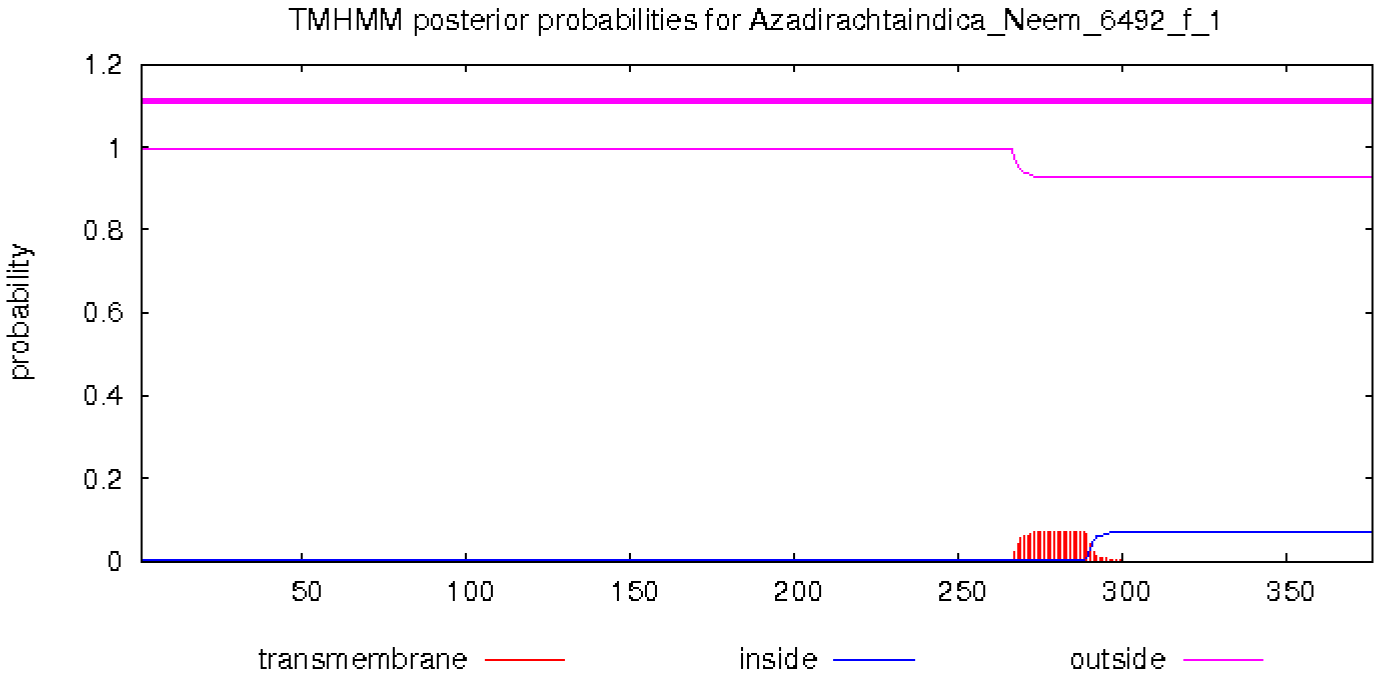

plot in postscript, script for making the plot in gnuplot, data for plot

---

```
# Azadirachtaindica_Neem_6716_f_1 Length: 281
# Azadirachtaindica_Neem_6716_f_1 Number of predicted TMHs:  0
# Azadirachtaindica_Neem_6716_f_1 Exp number of AAs in TMHs: 0.00082
# Azadirachtaindica_Neem_6716_f_1 Exp number, first 60 AAs:  0.00063
# Azadirachtaindica_Neem_6716_f_1 Total prob of N-in:        0.00234
Azadirachtaindica_Neem_6716_f_1 TMHMM2.0        outside      1   281
```

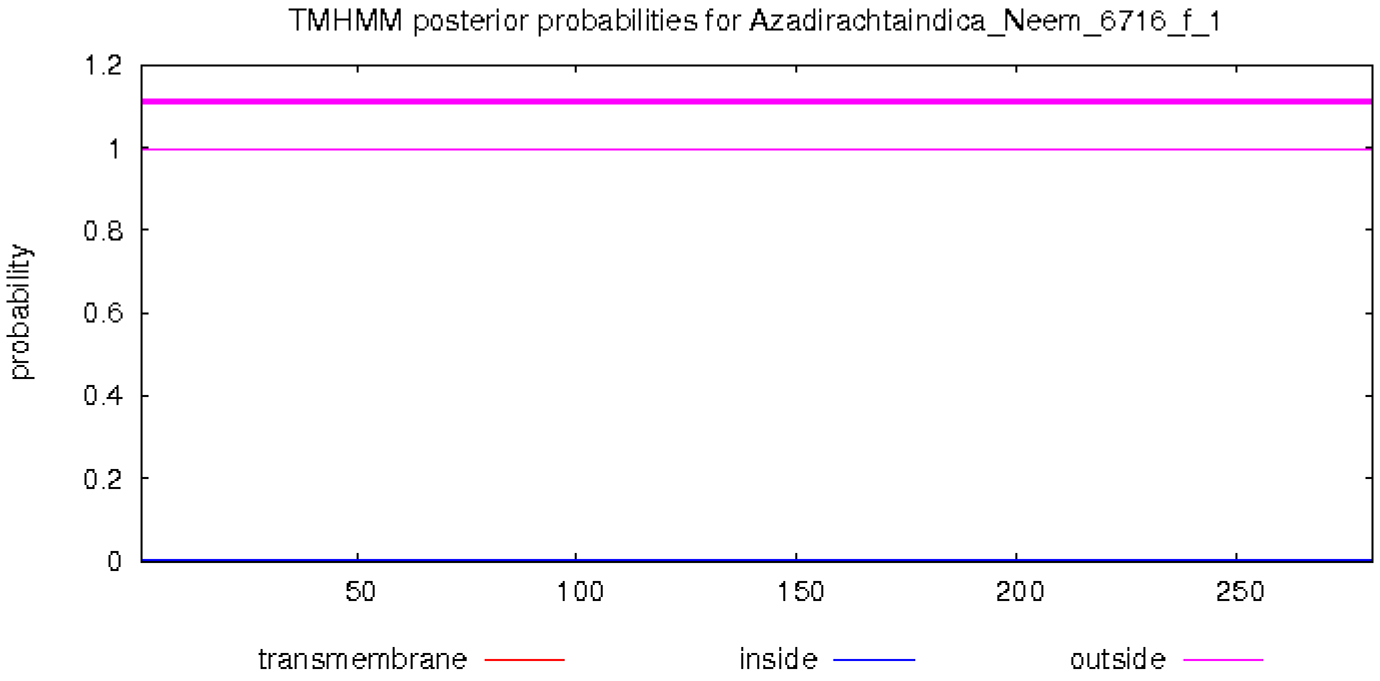

plot in postscript, script for making the plot in gnuplot, data for plot

---

```
# Azadirachtaindica_Neem_6790_f_1 Length: 209
# Azadirachtaindica_Neem_6790_f_1 Number of predicted TMHs:  0
```

```
# Azadirachtaindica_Neem_6790_f_1 Exp number of AAs in TMHs: 2.7306
# Azadirachtaindica_Neem_6790_f_1 Exp number, first 60 AAs:  0
# Azadirachtaindica_Neem_6790_f_1 Total prob of N-in:        0.00770
Azadirachtaindica_Neem_6790_f_1 TMHMM2.0        outside      1   209
```

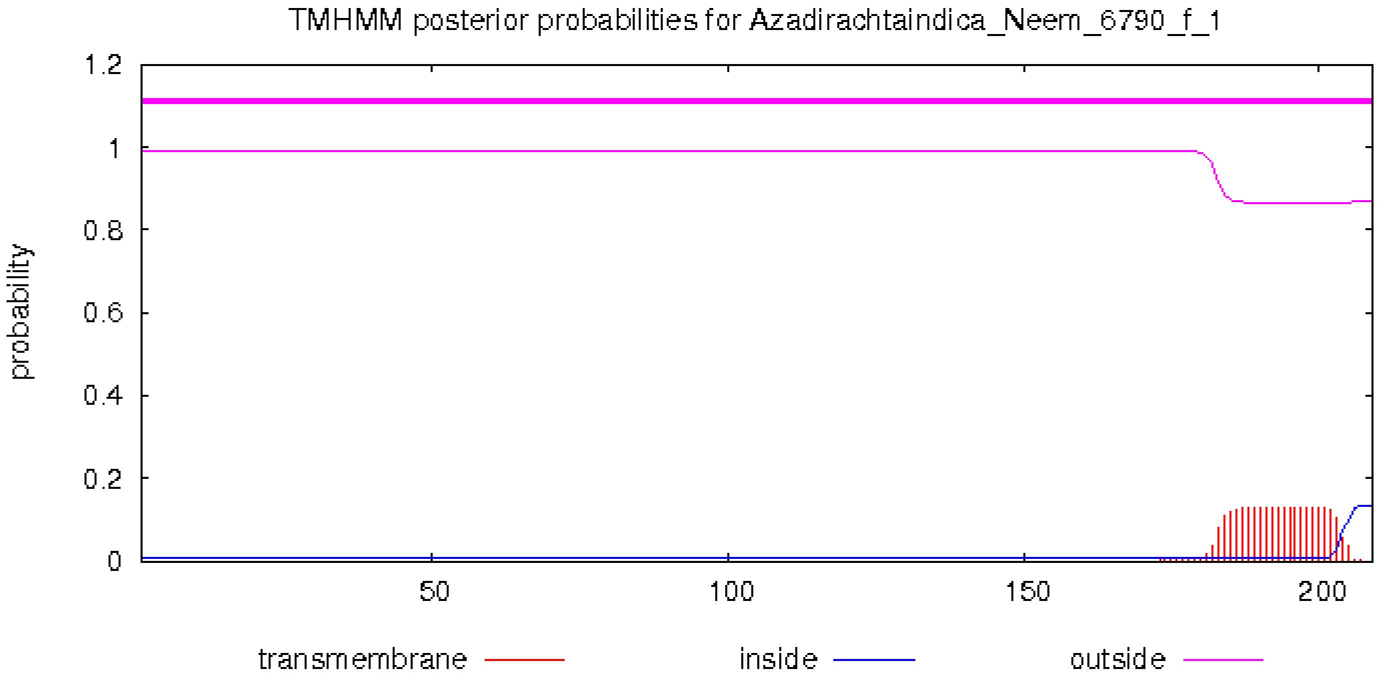

plot in postscript, script for making the plot in gnuplot, data for plot

---

```
# Azadirachtaindica_Neem_7002_f_4 Length: 313
# Azadirachtaindica_Neem_7002_f_4 Number of predicted TMHs:  0
# Azadirachtaindica_Neem_7002_f_4 Exp number of AAs in TMHs: 0.01643
# Azadirachtaindica_Neem_7002_f_4 Exp number, first 60 AAs:  0.0004
# Azadirachtaindica_Neem_7002_f_4 Total prob of N-in:        0.00873
Azadirachtaindica_Neem_7002_f_4 TMHMM2.0        outside      1   313
```

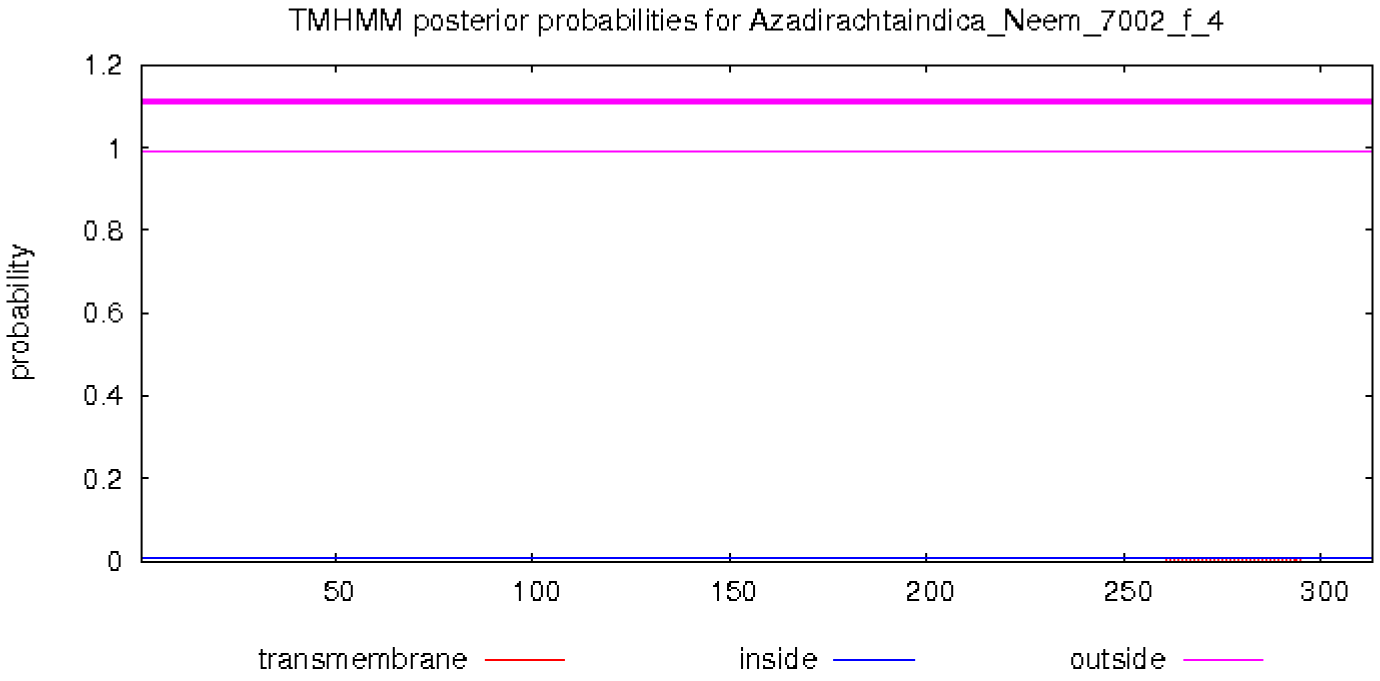

plot in postscript, script for making the plot in gnuplot, data for plot

---

```
# Azadirachtaindica_Neem_7195_f_2 Length: 417
# Azadirachtaindica_Neem_7195_f_2 Number of predicted TMHs:  0
```

```
# Azadirachtaindica_Neem_7195_f_2 Exp number of AAs in TMHs: 0
# Azadirachtaindica_Neem_7195_f_2 Exp number, first 60 AAs:  0
# Azadirachtaindica_Neem_7195_f_2 Total prob of N-in:        0.00382
Azadirachtaindica_Neem_7195_f_2 TMHMM2.0        outside      1   417
```

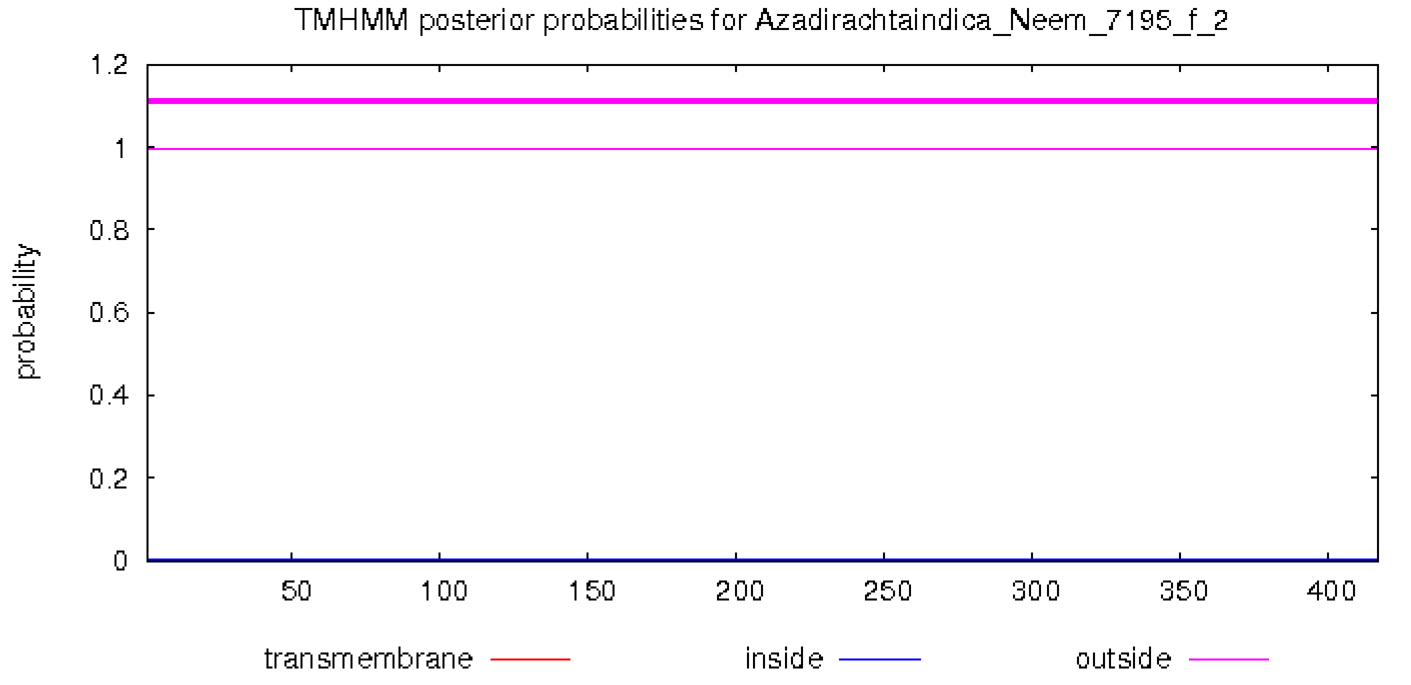

plot in postscript, script for making the plot in gnuplot, data for plot

---

```
# Azadirachtaindica_Neem_7280_f_1 Length: 250
# Azadirachtaindica_Neem_7280_f_1 Number of predicted TMHs:  0
# Azadirachtaindica_Neem_7280_f_1 Exp number of AAs in TMHs: 0.00038
# Azadirachtaindica_Neem_7280_f_1 Exp number, first 60 AAs:  0
# Azadirachtaindica_Neem_7280_f_1 Total prob of N-in:        0.08449
Azadirachtaindica_Neem_7280_f_1 TMHMM2.0        outside      1   250
```

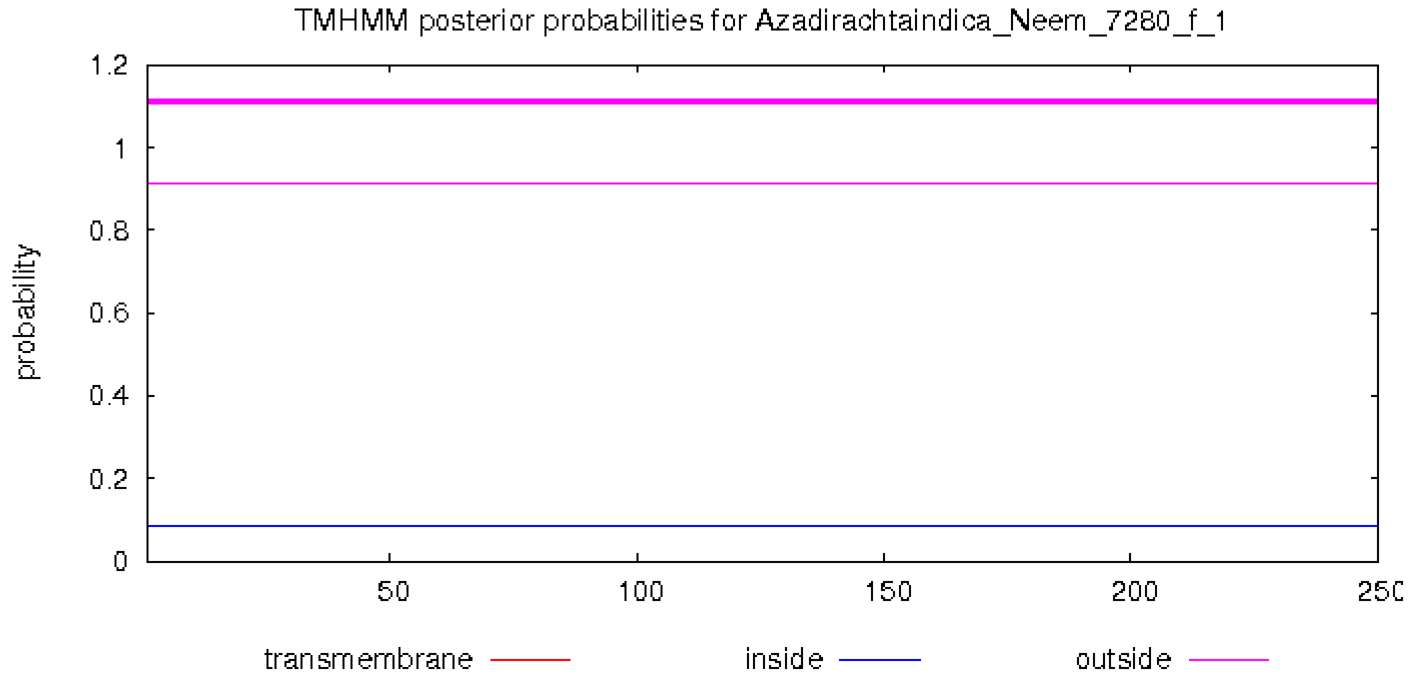

plot in postscript, script for making the plot in gnuplot, data for plot

---

```
# Azadirachtaindica_Neem_7328_f_1 Length: 361
# Azadirachtaindica_Neem_7328_f_1 Number of predicted TMHs:  0
```

```
# Azadirachtaindica_Neem_7328_f_1 Exp number of AAs in TMHs: 0.01004
# Azadirachtaindica_Neem_7328_f_1 Exp number, first 60 AAs:  0.00649
# Azadirachtaindica_Neem_7328_f_1 Total prob of N-in:        0.01657
Azadirachtaindica_Neem_7328_f_1 TMHMM2.0        outside      1   361
```

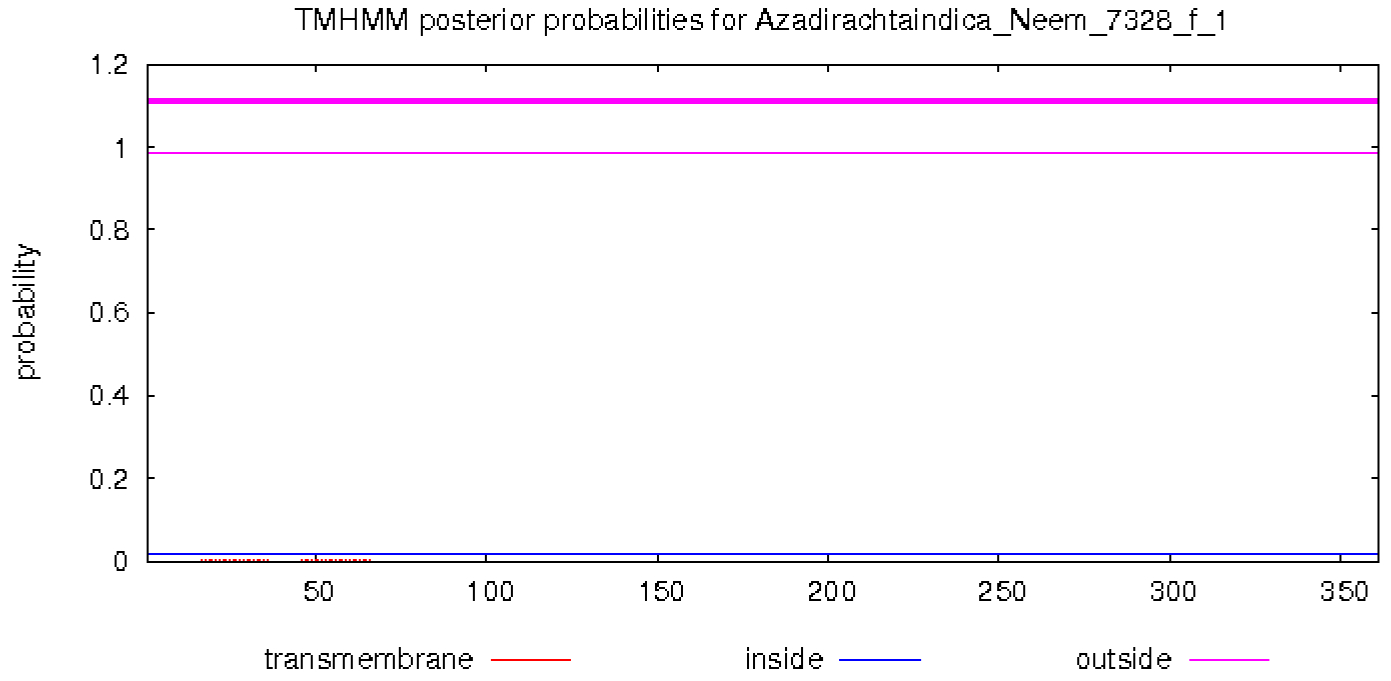

plot in postscript, script for making the plot in gnuplot, data for plot

---

```
# Azadirachtaindica_Neem_7400_f_1 Length: 222
# Azadirachtaindica_Neem_7400_f_1 Number of predicted TMHs:  0
# Azadirachtaindica_Neem_7400_f_1 Exp number of AAs in TMHs: 0.00303
# Azadirachtaindica_Neem_7400_f_1 Exp number, first 60 AAs:  0
# Azadirachtaindica_Neem_7400_f_1 Total prob of N-in:        0.02387
Azadirachtaindica_Neem_7400_f_1 TMHMM2.0        outside      1   222
```

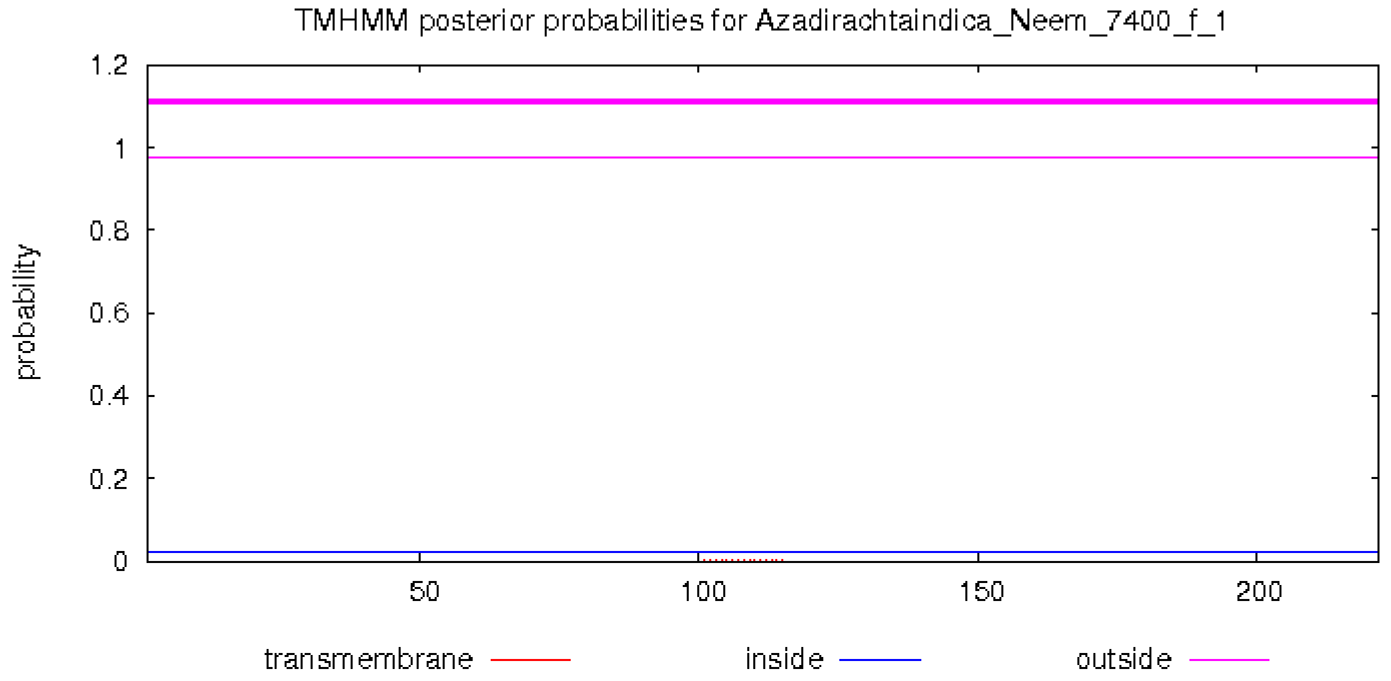

plot in postscript, script for making the plot in gnuplot, data for plot

---

```
# Azadirachtaindica_Neem_7585_f_1 Length: 262
# Azadirachtaindica_Neem_7585_f_1 Number of predicted TMHs:  0
```

```
# Azadirachtaindica_Neem_7585_f_1 Exp number of AAs in TMHs: 0.00861
# Azadirachtaindica_Neem_7585_f_1 Exp number, first 60 AAs:  0
# Azadirachtaindica_Neem_7585_f_1 Total prob of N-in:        0.01328
Azadirachtaindica_Neem_7585_f_1 TMHMM2.0        outside      1   262
```

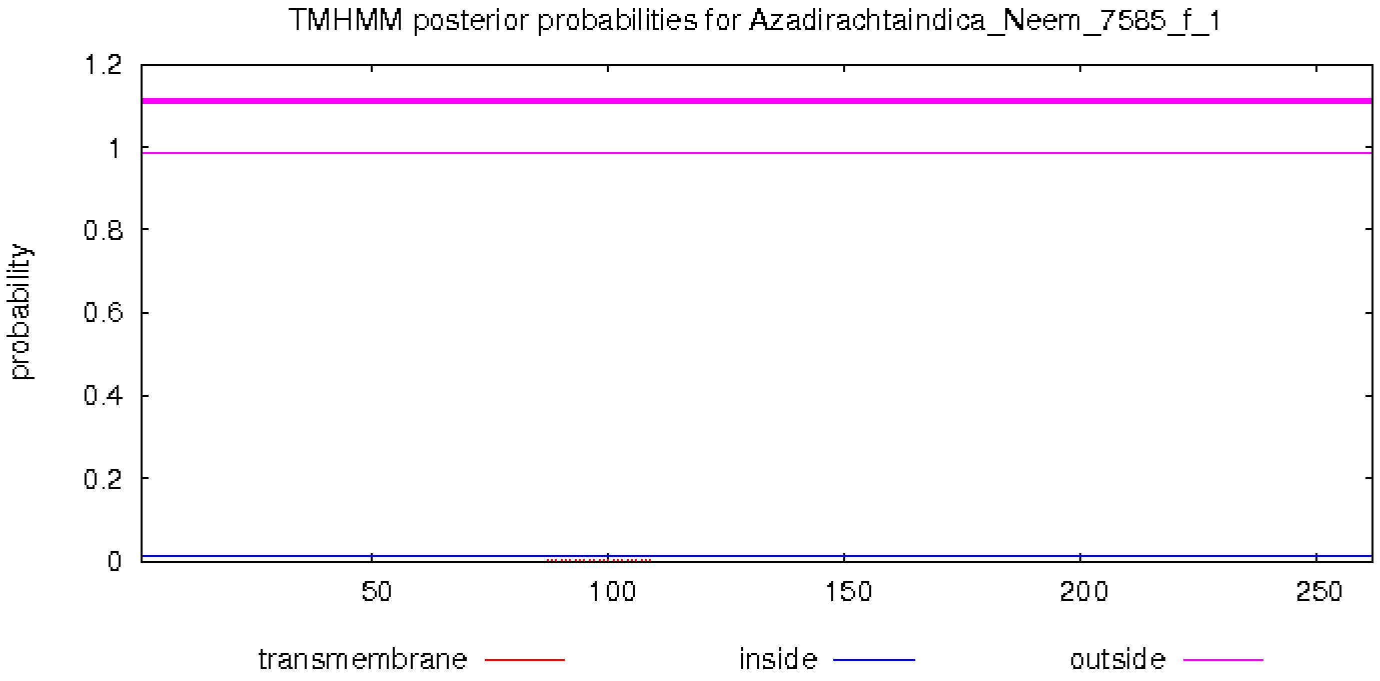

plot in postscript, script for making the plot in gnuplot, data for plot

---

```
# Azadirachtaindica_Neem_7585_f_2 Length: 213
# Azadirachtaindica_Neem_7585_f_2 Number of predicted TMHs:  0
# Azadirachtaindica_Neem_7585_f_2 Exp number of AAs in TMHs: 0.00321
# Azadirachtaindica_Neem_7585_f_2 Exp number, first 60 AAs:  0.00014
# Azadirachtaindica_Neem_7585_f_2 Total prob of N-in:        0.02142
Azadirachtaindica_Neem_7585_f_2 TMHMM2.0        outside      1   213
```

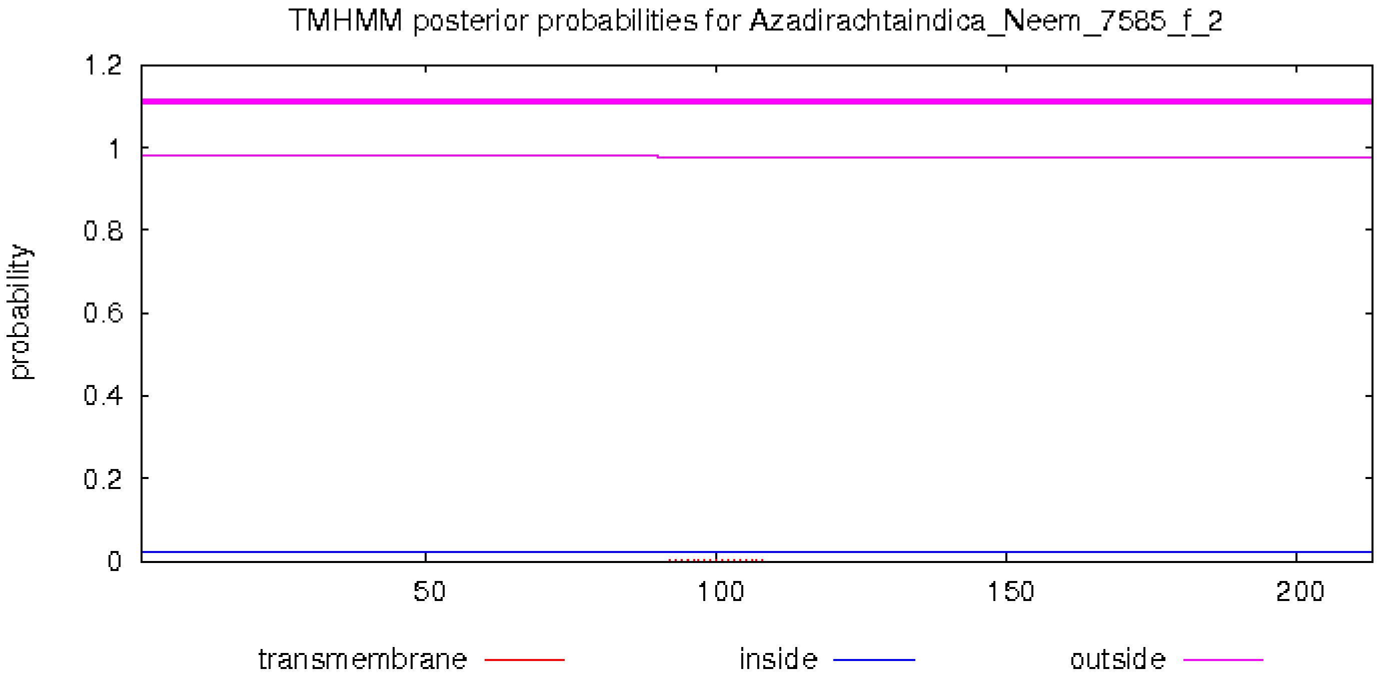

plot in postscript, script for making the plot in gnuplot, data for plot

---

```
# Azadirachtaindica_Neem_7585_f_4 Length: 154
# Azadirachtaindica_Neem_7585_f_4 Number of predicted TMHs:  0
```

```
# Azadirachtaindica_Neem_7585_f_4 Exp number of AAs in TMHs: 0.01865
# Azadirachtaindica_Neem_7585_f_4 Exp number, first 60 AAs:  0
# Azadirachtaindica_Neem_7585_f_4 Total prob of N-in:        0.04265
Azadirachtaindica_Neem_7585_f_4 TMHMM2.0        outside      1   154
```

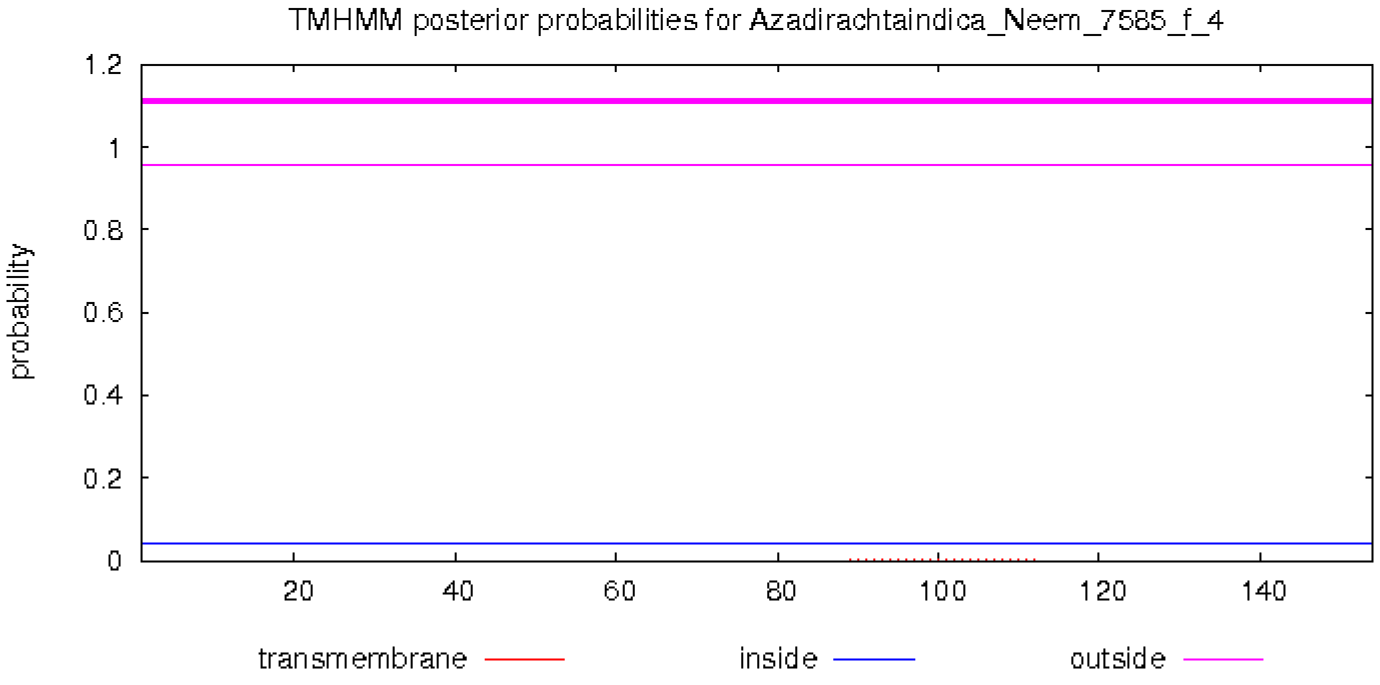

plot in postscript, script for making the plot in gnuplot, data for plot

---

```
# Azadirachtaindica_Neem_7585_f_7 Length: 272
# Azadirachtaindica_Neem_7585_f_7 Number of predicted TMHs:  0
# Azadirachtaindica_Neem_7585_f_7 Exp number of AAs in TMHs: 0.0037
# Azadirachtaindica_Neem_7585_f_7 Exp number, first 60 AAs:  0
# Azadirachtaindica_Neem_7585_f_7 Total prob of N-in:        0.01031
Azadirachtaindica_Neem_7585_f_7 TMHMM2.0        outside      1   272
```

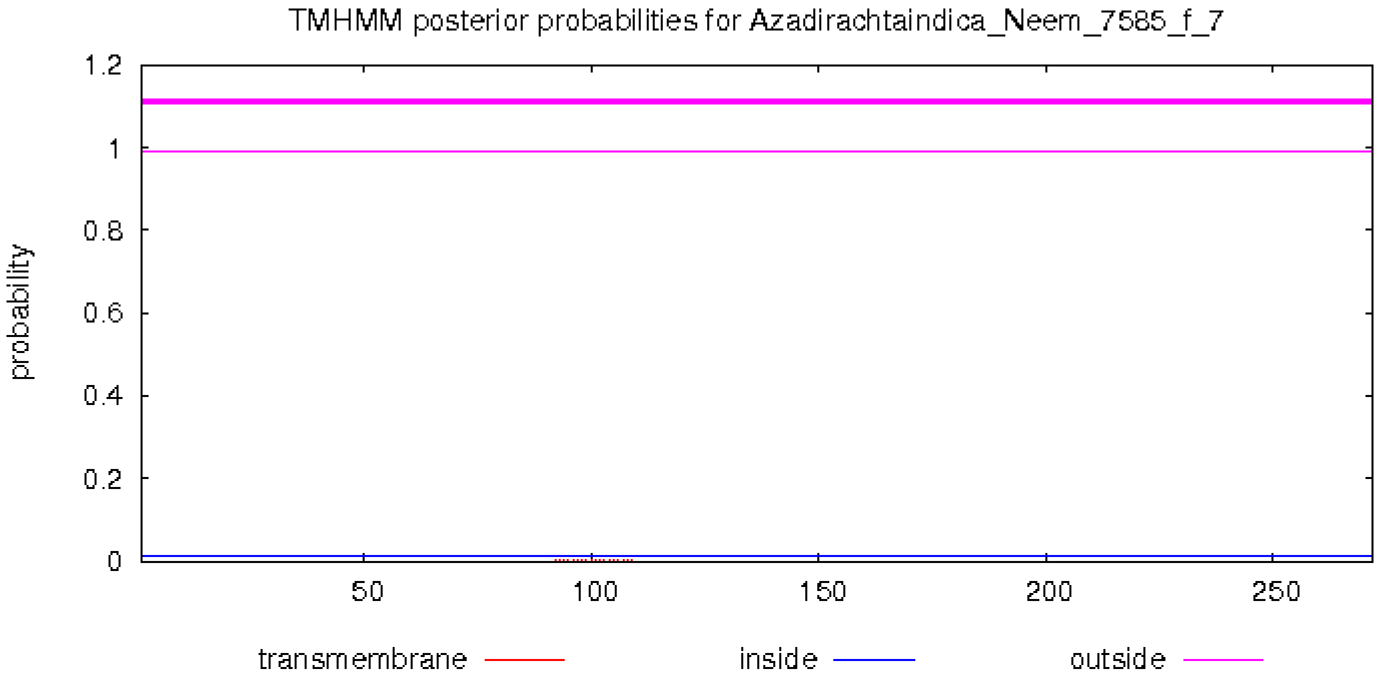

plot in postscript, script for making the plot in gnuplot, data for plot

---

```
# Azadirachtaindica_Neem_7680_f_1 Length: 113
# Azadirachtaindica_Neem_7680_f_1 Number of predicted TMHs:  0
```

```
# Azadirachtaindica_Neem_7680_f_1 Exp number of AAs in TMHs: 0.00151
# Azadirachtaindica_Neem_7680_f_1 Exp number, first 60 AAs:  0.0005
# Azadirachtaindica_Neem_7680_f_1 Total prob of N-in:        0.10280
Azadirachtaindica_Neem_7680_f_1 TMHMM2.0        outside      1   113
```

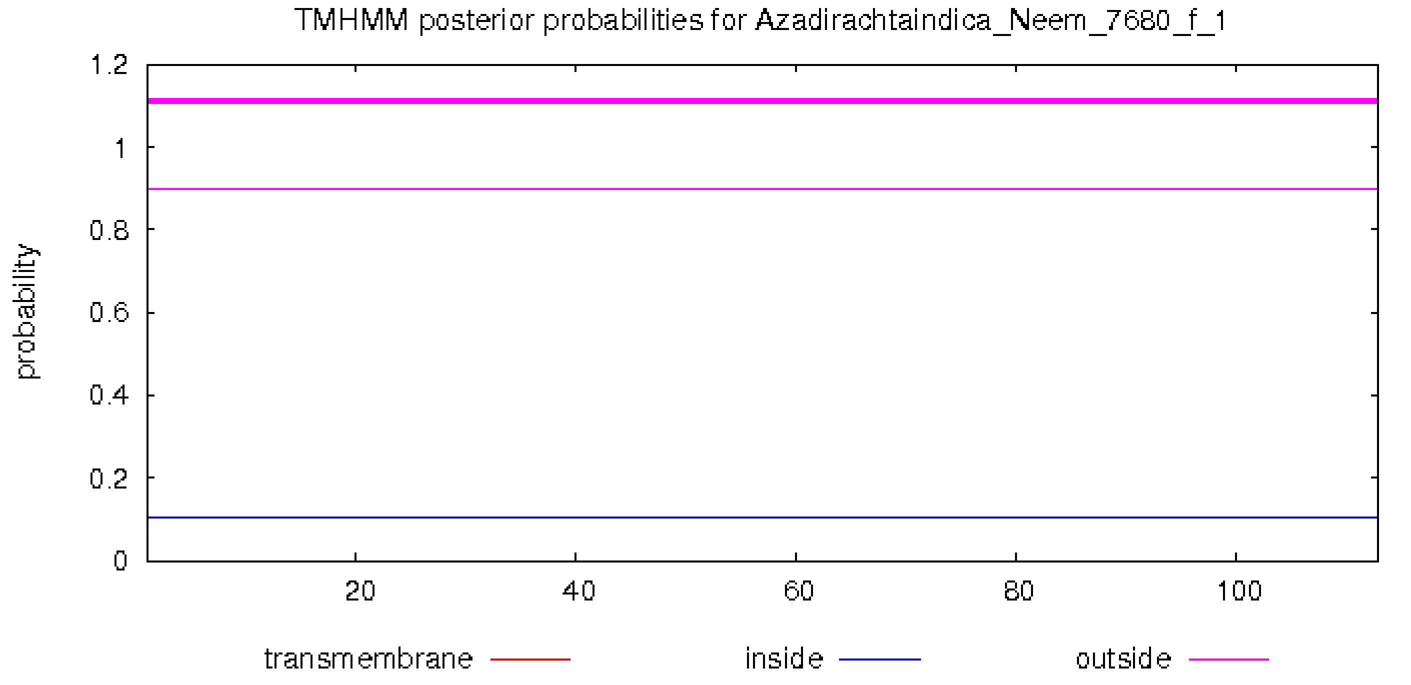

plot in postscript, script for making the plot in gnuplot, data for plot

---

```
# Azadirachtaindica_Neem_7766_f_1 Length: 450
# Azadirachtaindica_Neem_7766_f_1 Number of predicted TMHs:  0
# Azadirachtaindica_Neem_7766_f_1 Exp number of AAs in TMHs: 0
# Azadirachtaindica_Neem_7766_f_1 Exp number, first 60 AAs:  0
# Azadirachtaindica_Neem_7766_f_1 Total prob of N-in:        0.00370
Azadirachtaindica_Neem_7766_f_1 TMHMM2.0        outside      1   450
```

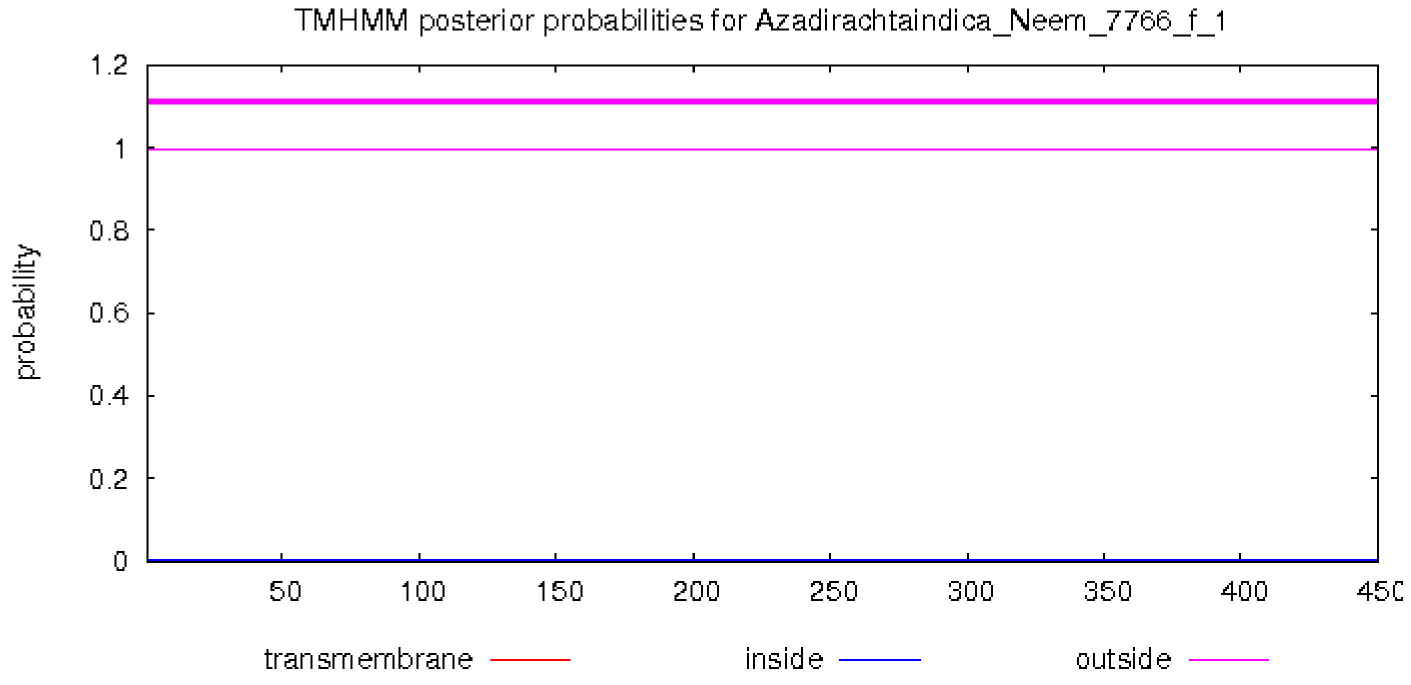

plot in postscript, script for making the plot in gnuplot, data for plot

---

```
# Azadirachtaindica_Neem_8133_f_4 Length: 394
# Azadirachtaindica_Neem_8133_f_4 Number of predicted TMHs:  0
```

```
# Azadirachtaindica_Neem_8133_f_4 Exp number of AAs in TMHs: 0.00021
# Azadirachtaindica_Neem_8133_f_4 Exp number, first 60 AAs:  0
# Azadirachtaindica_Neem_8133_f_4 Total prob of N-in:        0.00818
Azadirachtaindica_Neem_8133_f_4 TMHMM2.0        outside      1   394
```

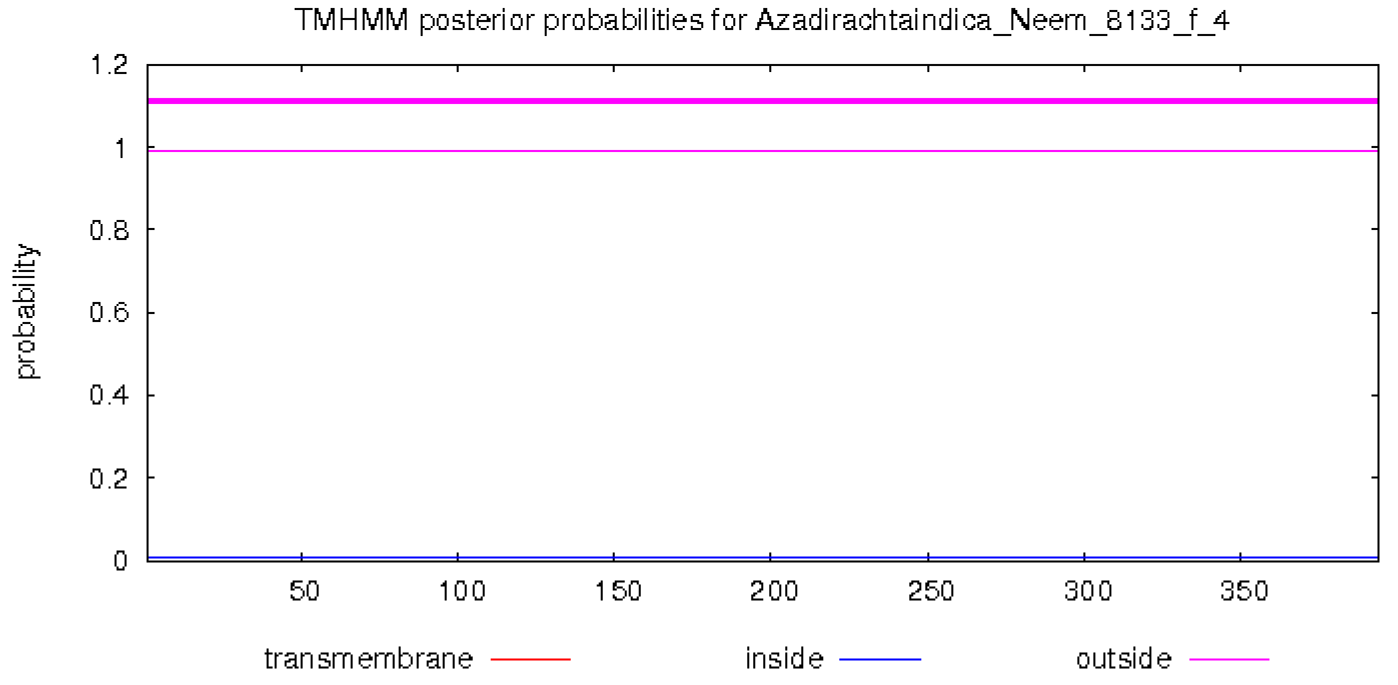

plot in postscript, script for making the plot in gnuplot, data for plot

---

```
# Azadirachtaindica_Neem_8272_f_2 Length: 354
# Azadirachtaindica_Neem_8272_f_2 Number of predicted TMHs:  0
# Azadirachtaindica_Neem_8272_f_2 Exp number of AAs in TMHs: 0.00062
# Azadirachtaindica_Neem_8272_f_2 Exp number, first 60 AAs:  0.00042
# Azadirachtaindica_Neem_8272_f_2 Total prob of N-in:        0.01630
Azadirachtaindica_Neem_8272_f_2 TMHMM2.0        outside      1   354
```

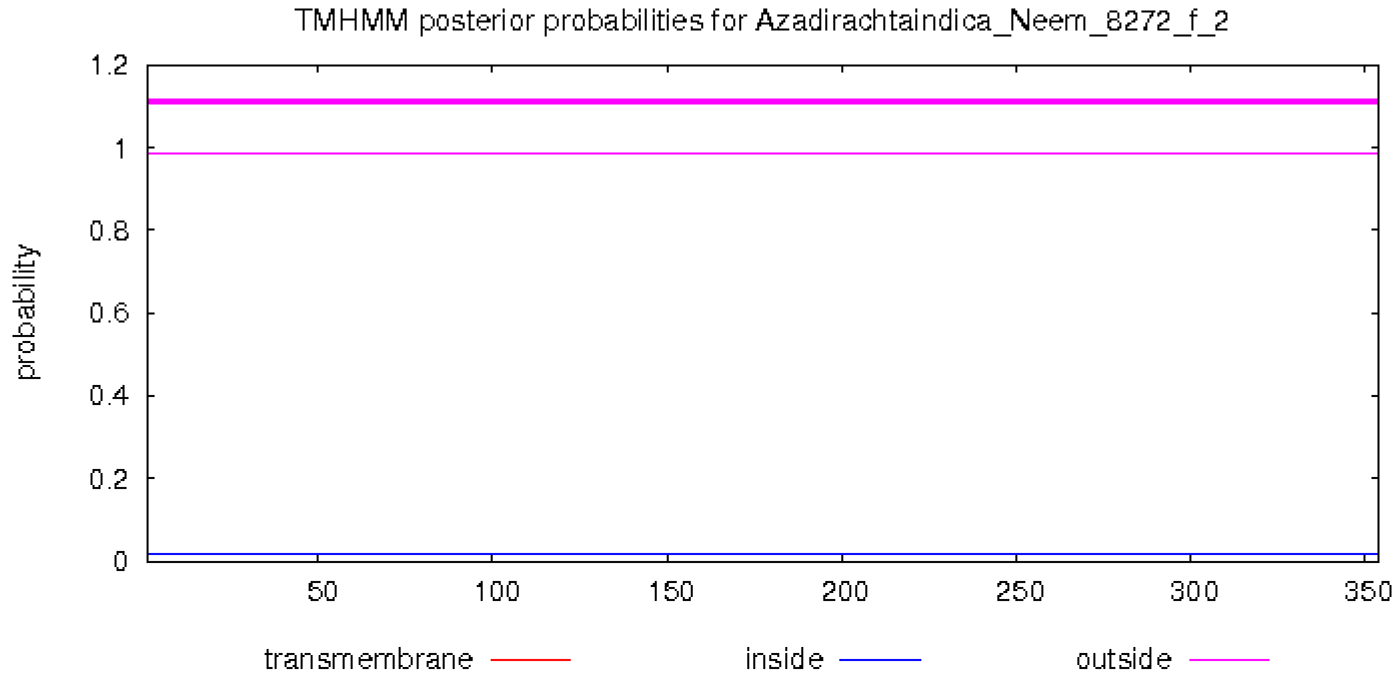

plot in postscript, script for making the plot in gnuplot, data for plot

---

```
# Azadirachtaindica_Neem_8368_f_2 Length: 345
# Azadirachtaindica_Neem_8368_f_2 Number of predicted TMHs:  0
```

```
# Azadirachtaindica_Neem_8368_f_2 Exp number of AAs in TMHs: 0.00041
# Azadirachtaindica_Neem_8368_f_2 Exp number, first 60 AAs:  0
# Azadirachtaindica_Neem_8368_f_2 Total prob of N-in:        0.00090
Azadirachtaindica_Neem_8368_f_2 TMHMM2.0        outside      1   345
```

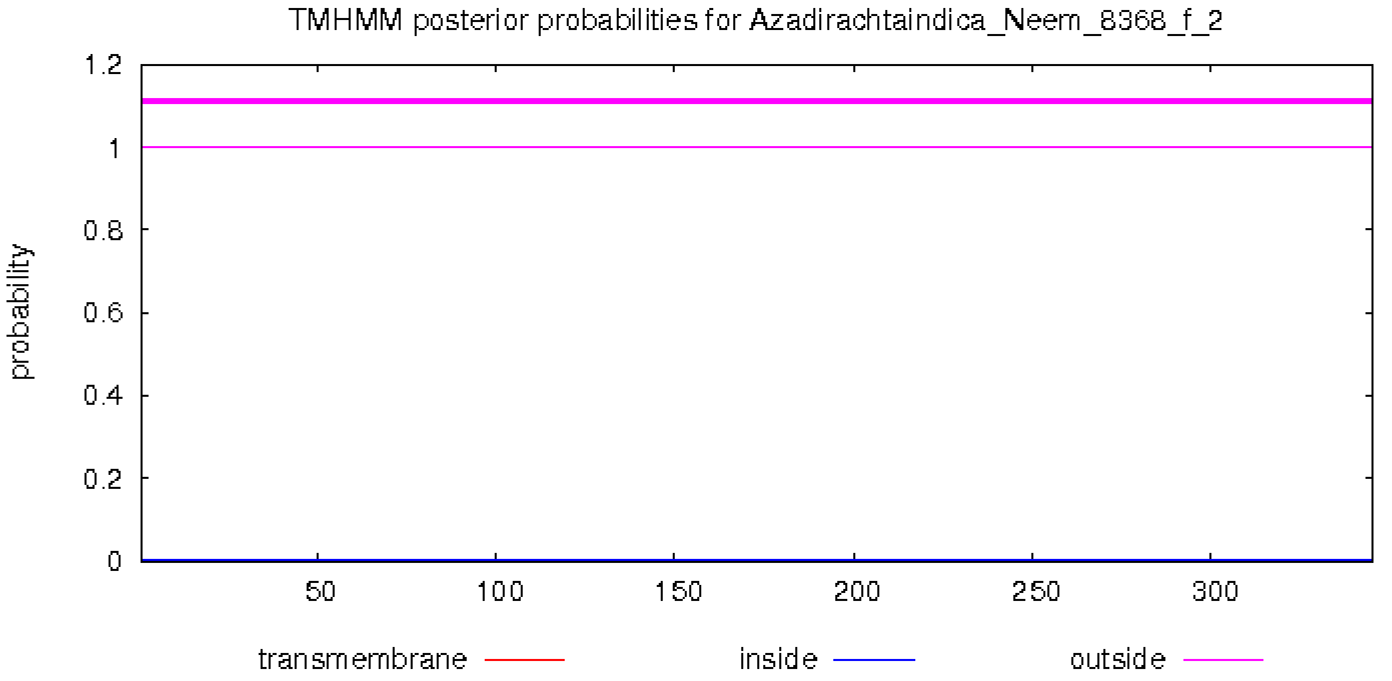

plot in postscript, script for making the plot in gnuplot, data for plot

---

```
# Azadirachtaindica_Neem_8411_f_2 Length: 182
# Azadirachtaindica_Neem_8411_f_2 Number of predicted TMHs:  0
# Azadirachtaindica_Neem_8411_f_2 Exp number of AAs in TMHs: 0.00142
# Azadirachtaindica_Neem_8411_f_2 Exp number, first 60 AAs:  0.00031
# Azadirachtaindica_Neem_8411_f_2 Total prob of N-in:        0.08846
Azadirachtaindica_Neem_8411_f_2 TMHMM2.0        outside      1   182
```

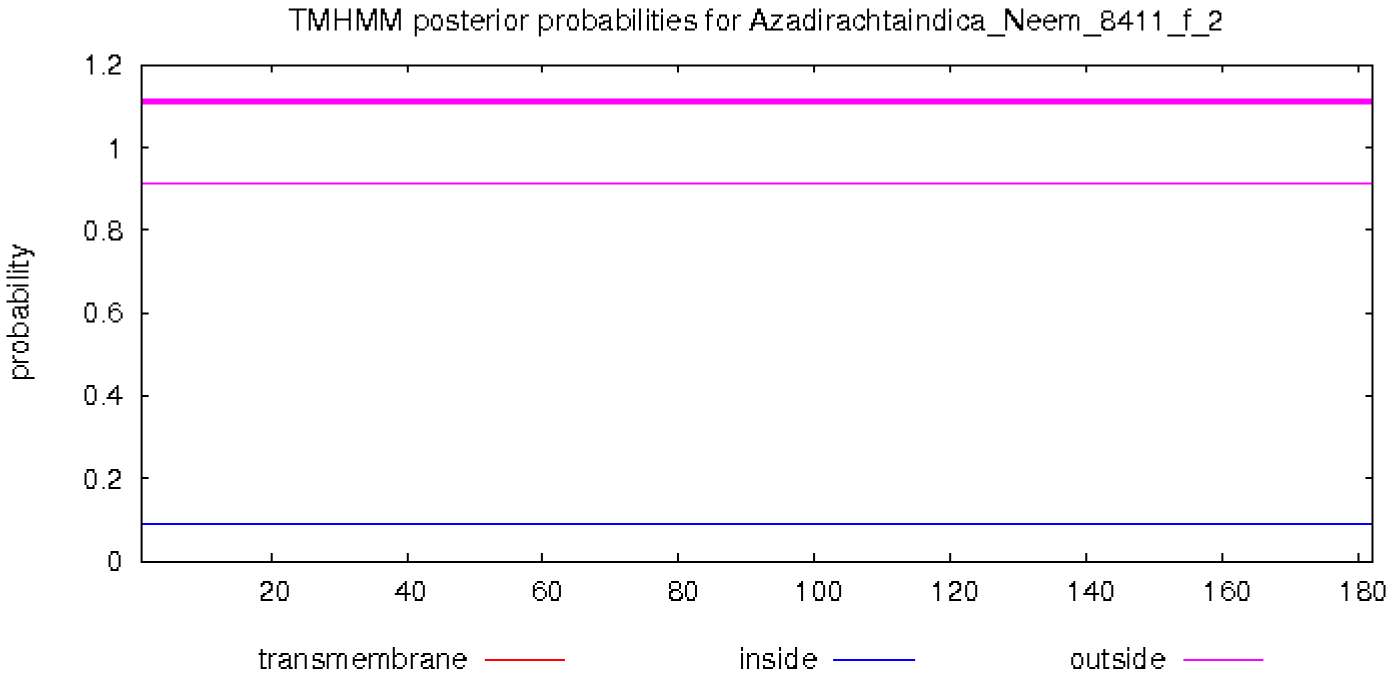

plot in postscript, script for making the plot in gnuplot, data for plot

---

```
# Azadirachtaindica_Neem_8434_f_2 Length: 334
# Azadirachtaindica_Neem_8434_f_2 Number of predicted TMHs:  0
```

```
# Azadirachtaindica_Neem_8434_f_2 Exp number of AAs in TMHs: 0.00428
# Azadirachtaindica_Neem_8434_f_2 Exp number, first 60 AAs:  0.00328
# Azadirachtaindica_Neem_8434_f_2 Total prob of N-in:        0.12766
Azadirachtaindica_Neem_8434_f_2 TMHMM2.0        outside      1   334
```

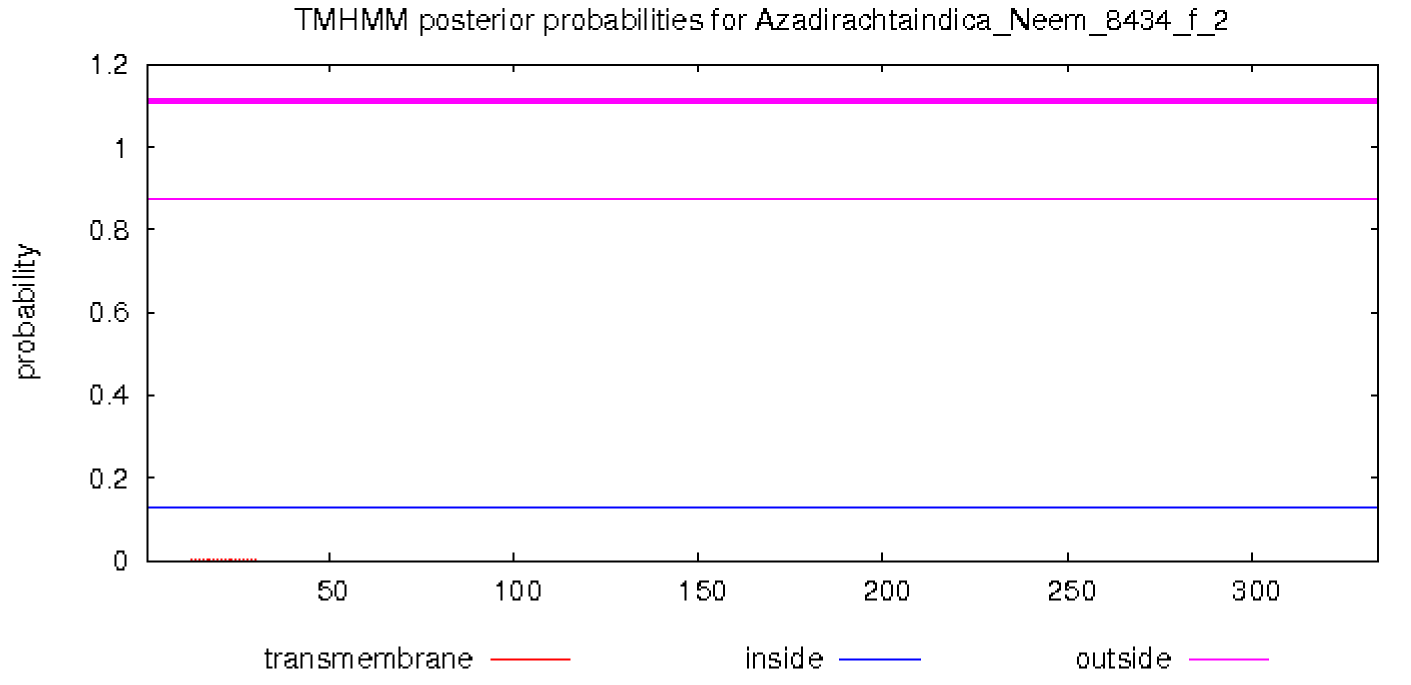

plot in postscript, script for making the plot in gnuplot, data for plot

---

```
# Azadirachtaindica_Neem_8476_f_1 Length: 60
# Azadirachtaindica_Neem_8476_f_1 Number of predicted TMHs:  0
# Azadirachtaindica_Neem_8476_f_1 Exp number of AAs in TMHs: 0.00019
# Azadirachtaindica_Neem_8476_f_1 Exp number, first 60 AAs:  0.00019
# Azadirachtaindica_Neem_8476_f_1 Total prob of N-in:        0.17410
Azadirachtaindica_Neem_8476_f_1 TMHMM2.0        outside      1    60
```

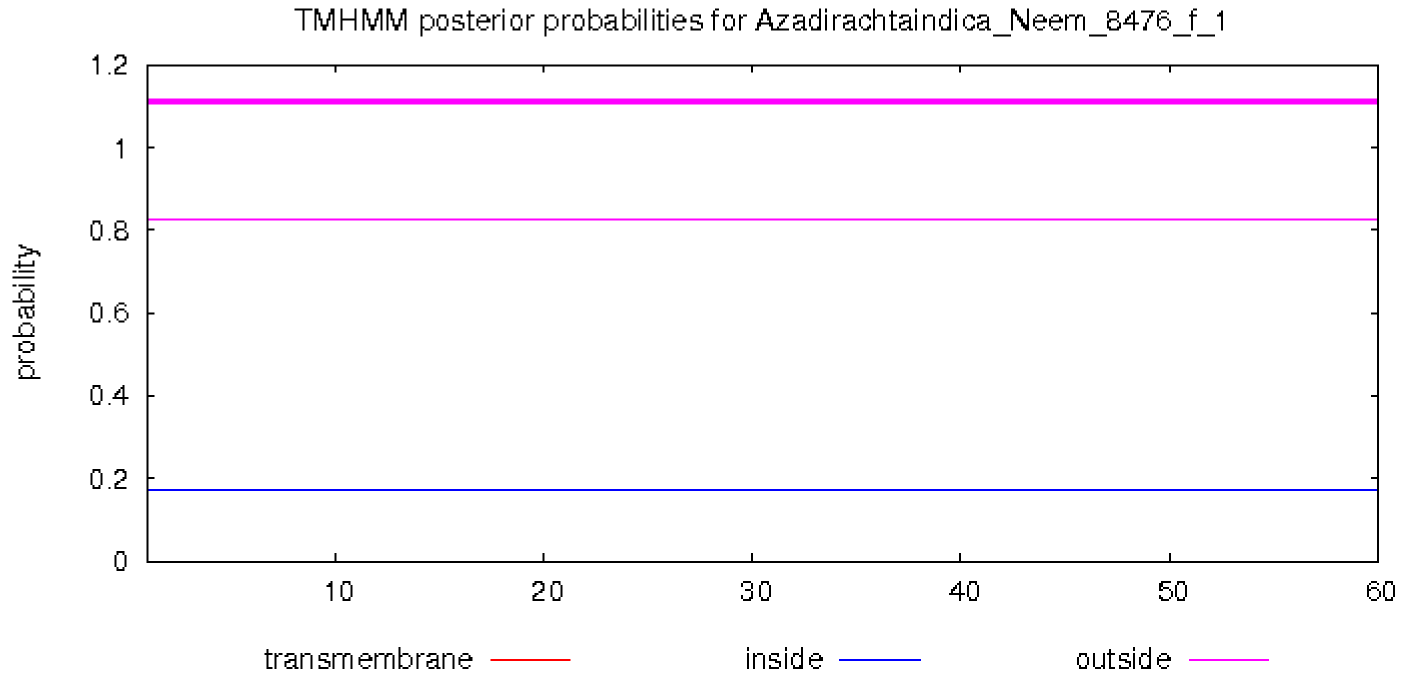

plot in postscript, script for making the plot in gnuplot, data for plot

---

```
# Azadirachtaindica_Neem_8476_f_2 Length: 391
# Azadirachtaindica_Neem_8476_f_2 Number of predicted TMHs:  0
```

```
# Azadirachtaindica_Neem_8476_f_2 Exp number of AAs in TMHs: 0.00055
# Azadirachtaindica_Neem_8476_f_2 Exp number, first 60 AAs:  0.00019
# Azadirachtaindica_Neem_8476_f_2 Total prob of N-in:        0.00728
Azadirachtaindica_Neem_8476_f_2 TMHMM2.0        outside      1   391
```

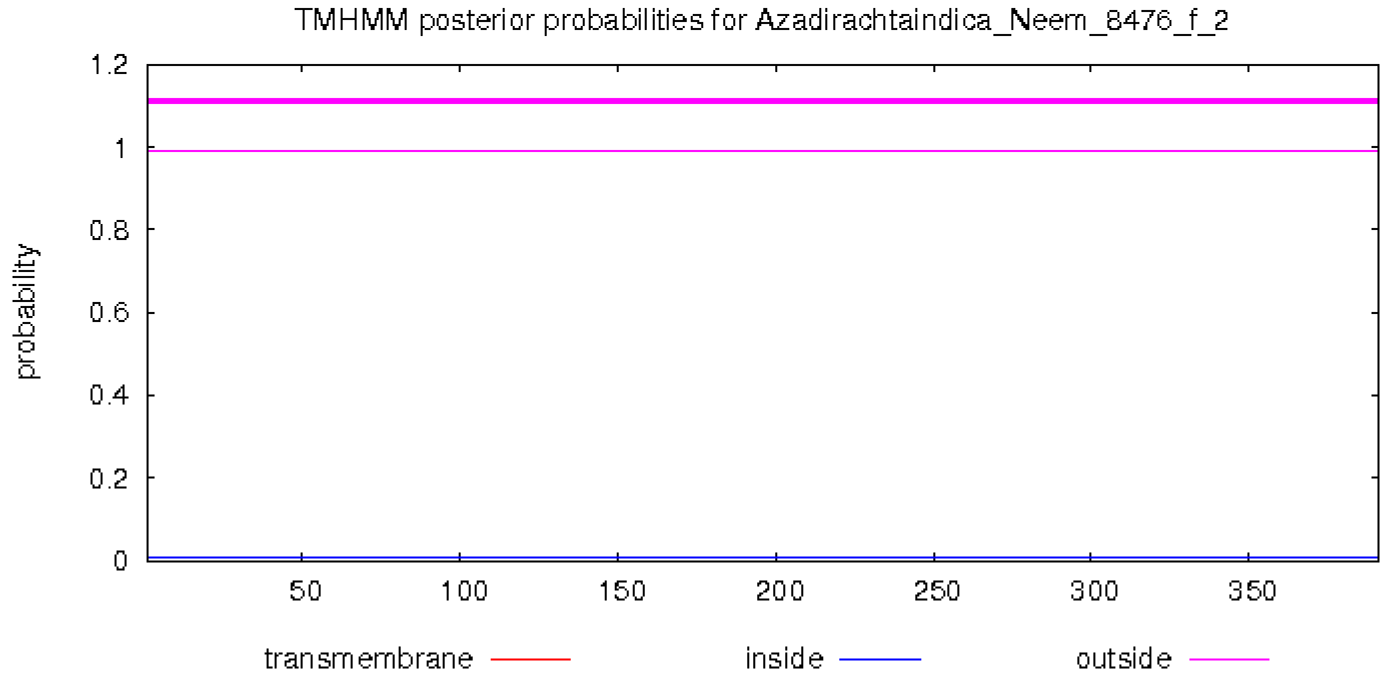

plot in postscript, script for making the plot in gnuplot, data for plot

---

```
# Azadirachtaindica_Neem_9030_f_1 Length: 208
# Azadirachtaindica_Neem_9030_f_1 Number of predicted TMHs:  0
# Azadirachtaindica_Neem_9030_f_1 Exp number of AAs in TMHs: 0.17635
# Azadirachtaindica_Neem_9030_f_1 Exp number, first 60 AAs:  0.00025
# Azadirachtaindica_Neem_9030_f_1 Total prob of N-in:        0.12956
Azadirachtaindica_Neem_9030_f_1 TMHMM2.0        outside      1   208
```

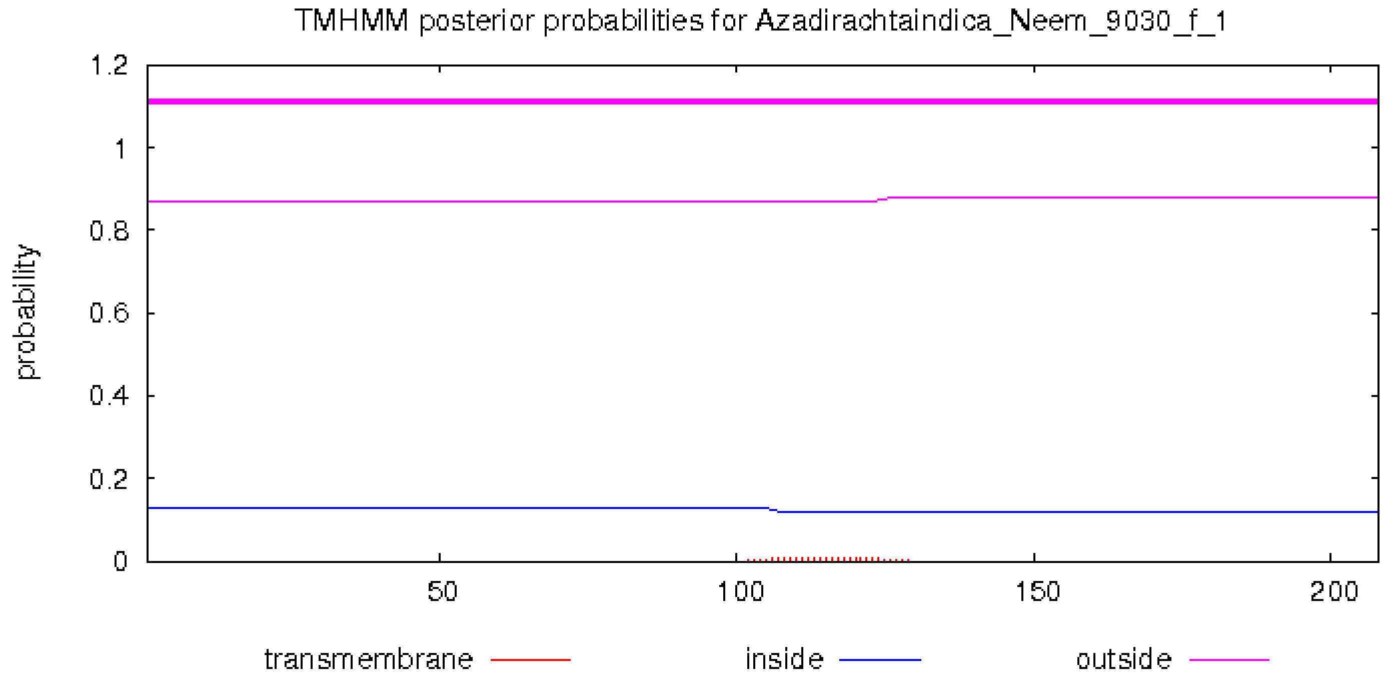

plot in postscript, script for making the plot in gnuplot, data for plot

---

```
# Azadirachtaindica_Neem_9151_f_6 Length: 309
# Azadirachtaindica_Neem_9151_f_6 Number of predicted TMHs:  0
```

```
# Azadirachtaindica_Neem_9151_f_6 Exp number of AAs in TMHs: 0.04452
# Azadirachtaindica_Neem_9151_f_6 Exp number, first 60 AAs:  0
# Azadirachtaindica_Neem_9151_f_6 Total prob of N-in:        0.06631
Azadirachtaindica_Neem_9151_f_6 TMHMM2.0        outside      1   309
```

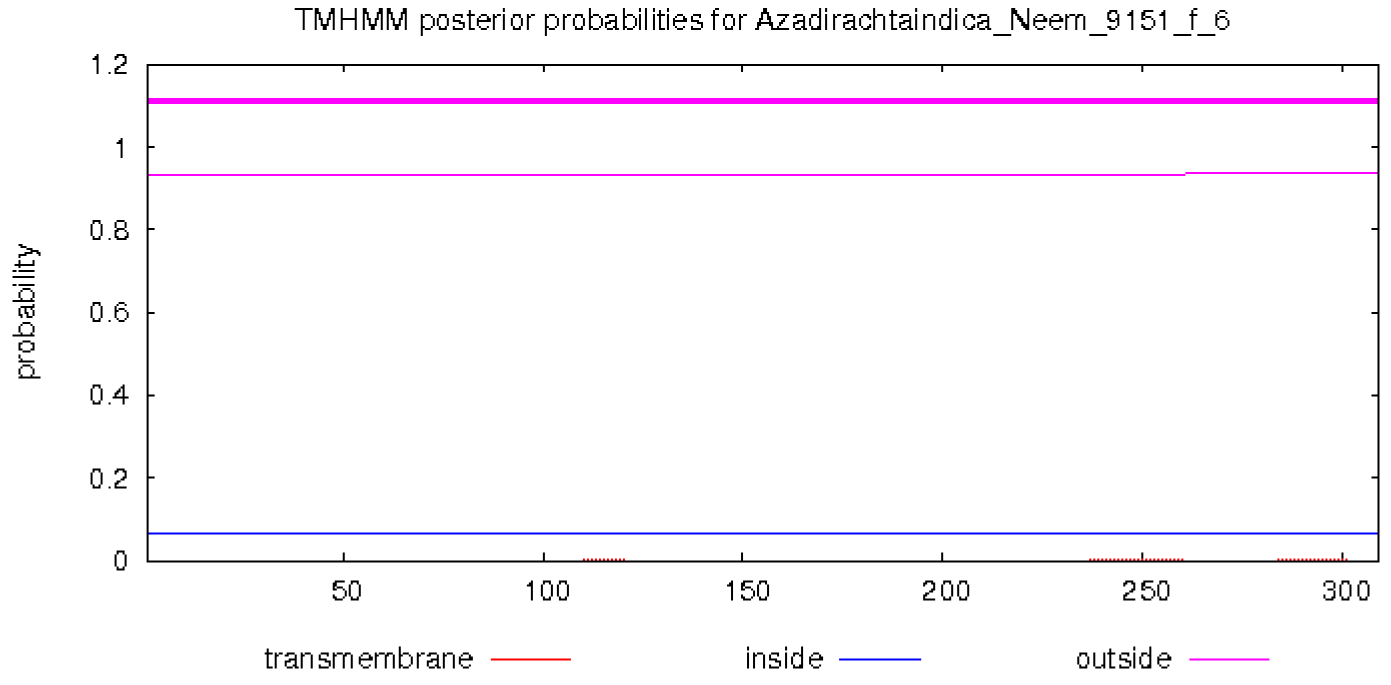

plot in postscript, script for making the plot in gnuplot, data for plot

---

```
# Azadirachtaindica_Neem_923_f_1 Length: 283
# Azadirachtaindica_Neem_923_f_1 Number of predicted TMHs:  0
# Azadirachtaindica_Neem_923_f_1 Exp number of AAs in TMHs: 0.00153
# Azadirachtaindica_Neem_923_f_1 Exp number, first 60 AAs:  0
# Azadirachtaindica_Neem_923_f_1 Total prob of N-in:        0.02917
Azadirachtaindica_Neem_923_f_1  TMHMM2.0        outside      1   283
```

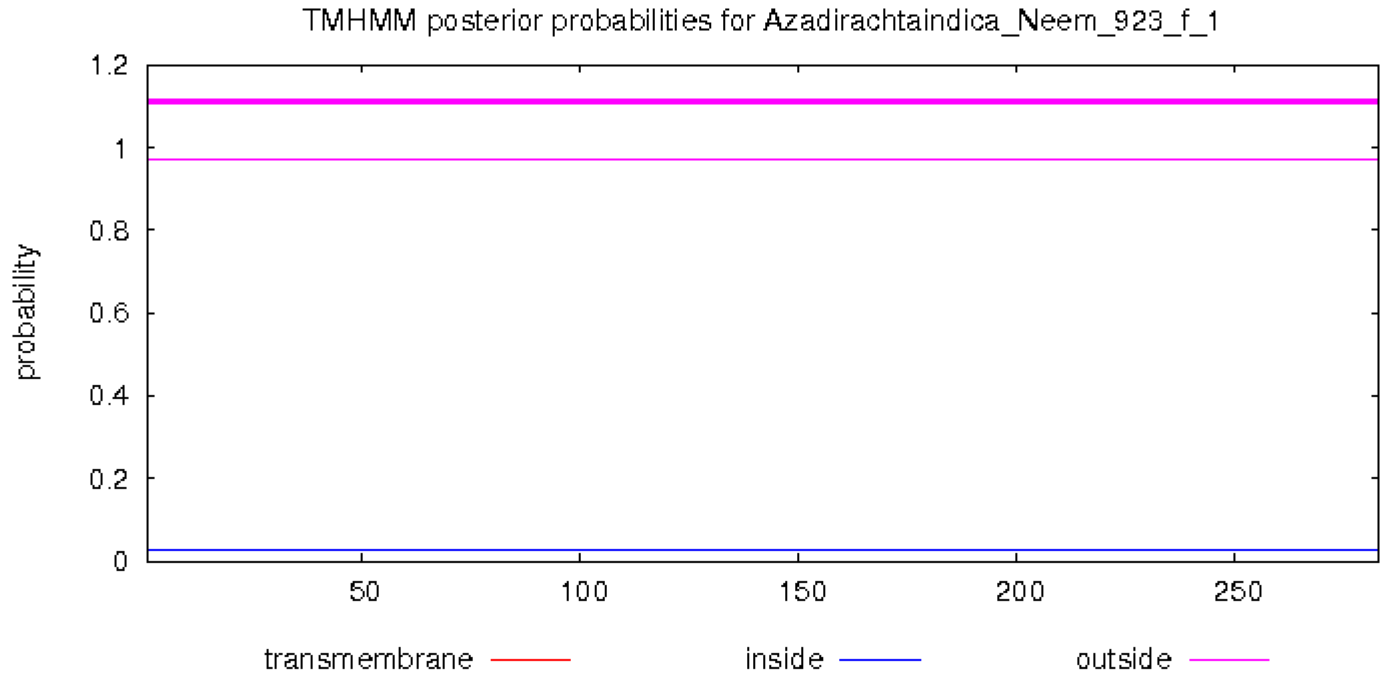

plot in postscript, script for making the plot in gnuplot, data for plot

---

```
# Azadirachtaindica_Neem_9263_f_10 Length: 137
# Azadirachtaindica_Neem_9263_f_10 Number of predicted TMHs:  0
```

```
# Azadirachtaindica_Neem_9263_f_10 Exp number of AAs in TMHs: 11.03483
# Azadirachtaindica_Neem_9263_f_10 Exp number, first 60 AAs:  0
# Azadirachtaindica_Neem_9263_f_10 Total prob of N-in:        0.45536
Azadirachtaindica_Neem_9263_f_10        TMHMM2.0        outside      1   137
```

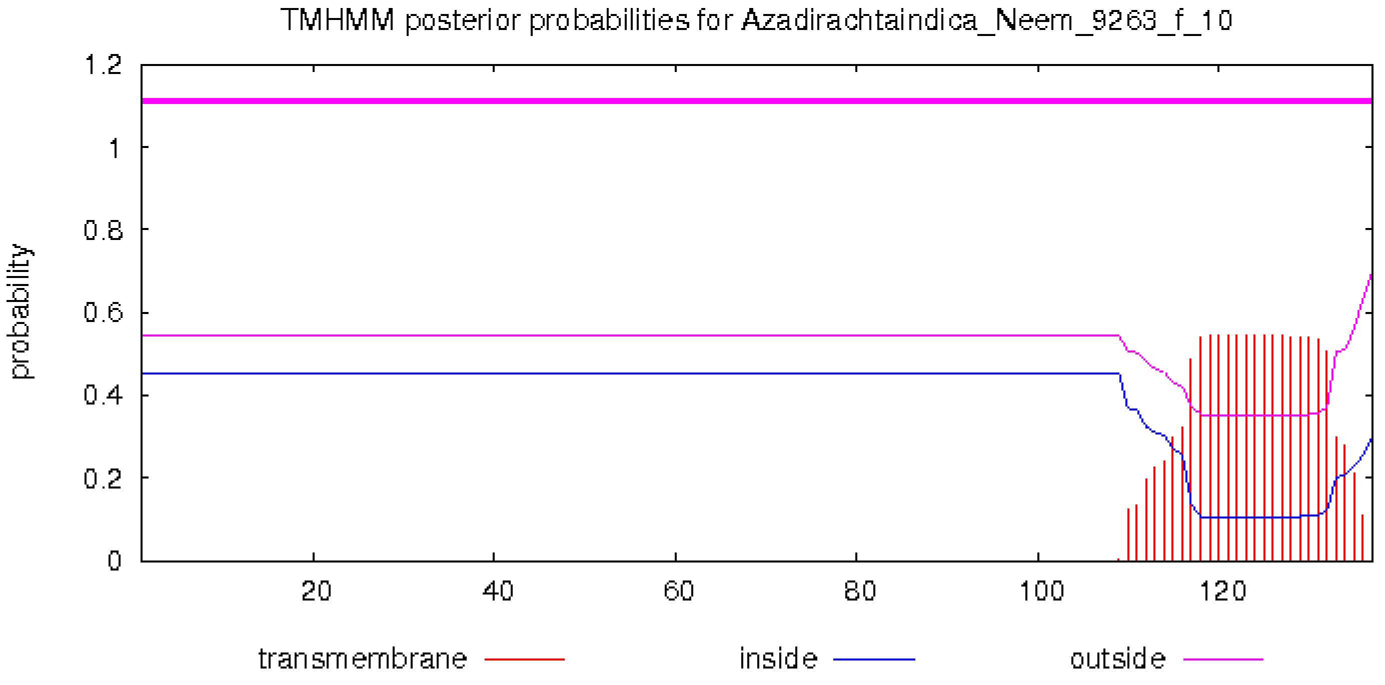

plot in postscript, script for making the plot in gnuplot, data for plot

---

```
# Azadirachtaindica_Neem_9263_f_12 Length: 484
# Azadirachtaindica_Neem_9263_f_12 Number of predicted TMHs:  1
# Azadirachtaindica_Neem_9263_f_12 Exp number of AAs in TMHs: 19.60918
# Azadirachtaindica_Neem_9263_f_12 Exp number, first 60 AAs:  0
# Azadirachtaindica_Neem_9263_f_12 Total prob of N-in:        0.01261
Azadirachtaindica_Neem_9263_f_12        TMHMM2.0        outside      1   459
Azadirachtaindica_Neem_9263_f_12        TMHMM2.0        TMhelix    460   477
Azadirachtaindica_Neem_9263_f_12        TMHMM2.0        inside     478   484
```

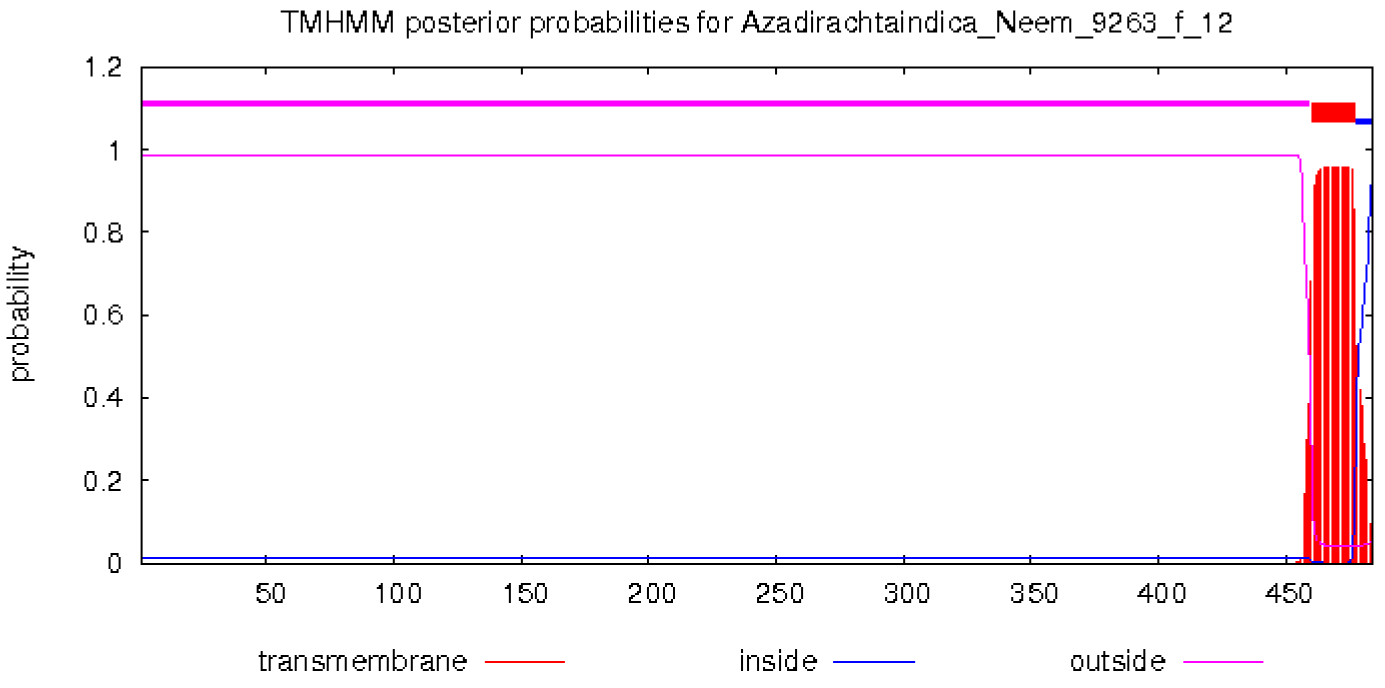

plot in postscript, script for making the plot in gnuplot, data for plot

---

```
# Azadirachtaindica_Neem_9263_f_8 Length: 153
# Azadirachtaindica_Neem_9263_f_8 Number of predicted TMHs:  0
# Azadirachtaindica_Neem_9263_f_8 Exp number of AAs in TMHs: 0.00714
# Azadirachtaindica_Neem_9263_f_8 Exp number, first 60 AAs:  0.00017
# Azadirachtaindica_Neem_9263_f_8 Total prob of N-in:        0.21253
Azadirachtaindica_Neem_9263_f_8 TMHMM2.0        outside      1   153
```

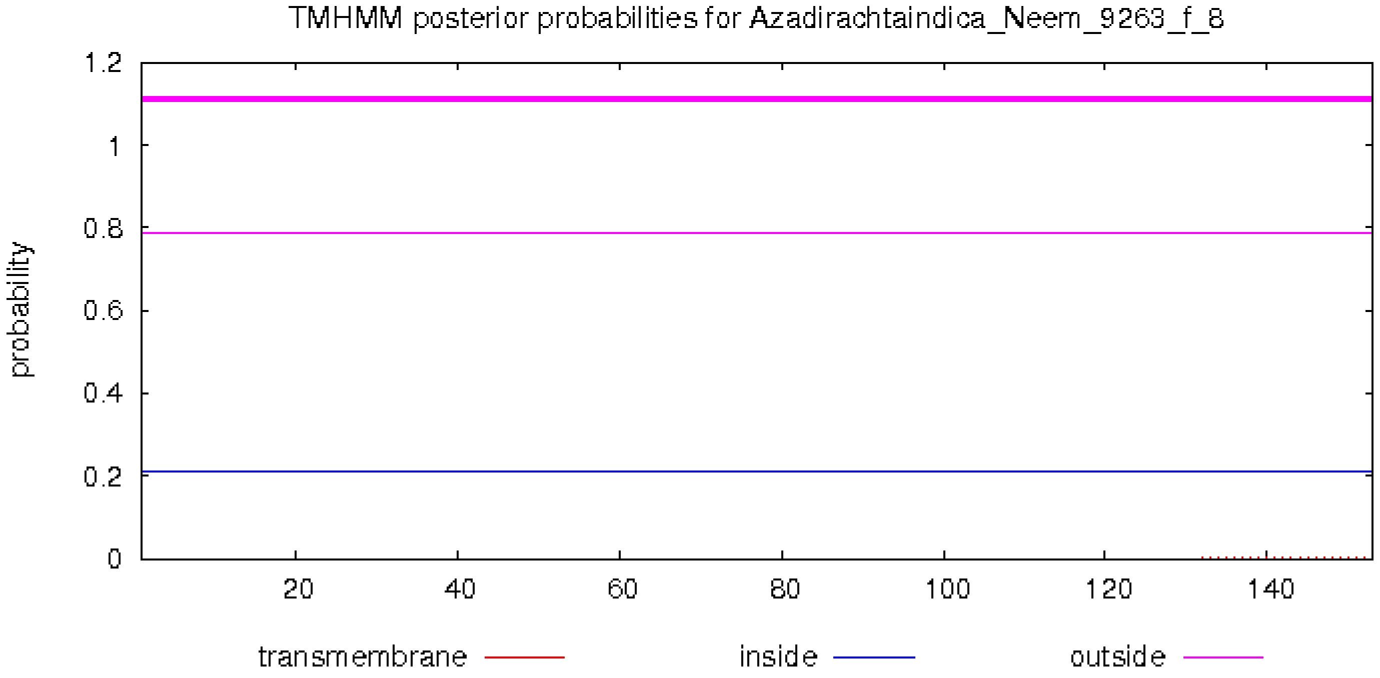

plot in postscript, script for making the plot in gnuplot, data for plot

---

```
# Azadirachtaindica_Neem_9263_f_9 Length: 159
# Azadirachtaindica_Neem_9263_f_9 Number of predicted TMHs:  0
# Azadirachtaindica_Neem_9263_f_9 Exp number of AAs in TMHs: 0
# Azadirachtaindica_Neem_9263_f_9 Exp number, first 60 AAs:  0
# Azadirachtaindica_Neem_9263_f_9 Total prob of N-in:        0.14447
Azadirachtaindica_Neem_9263_f_9 TMHMM2.0        outside      1   159
```

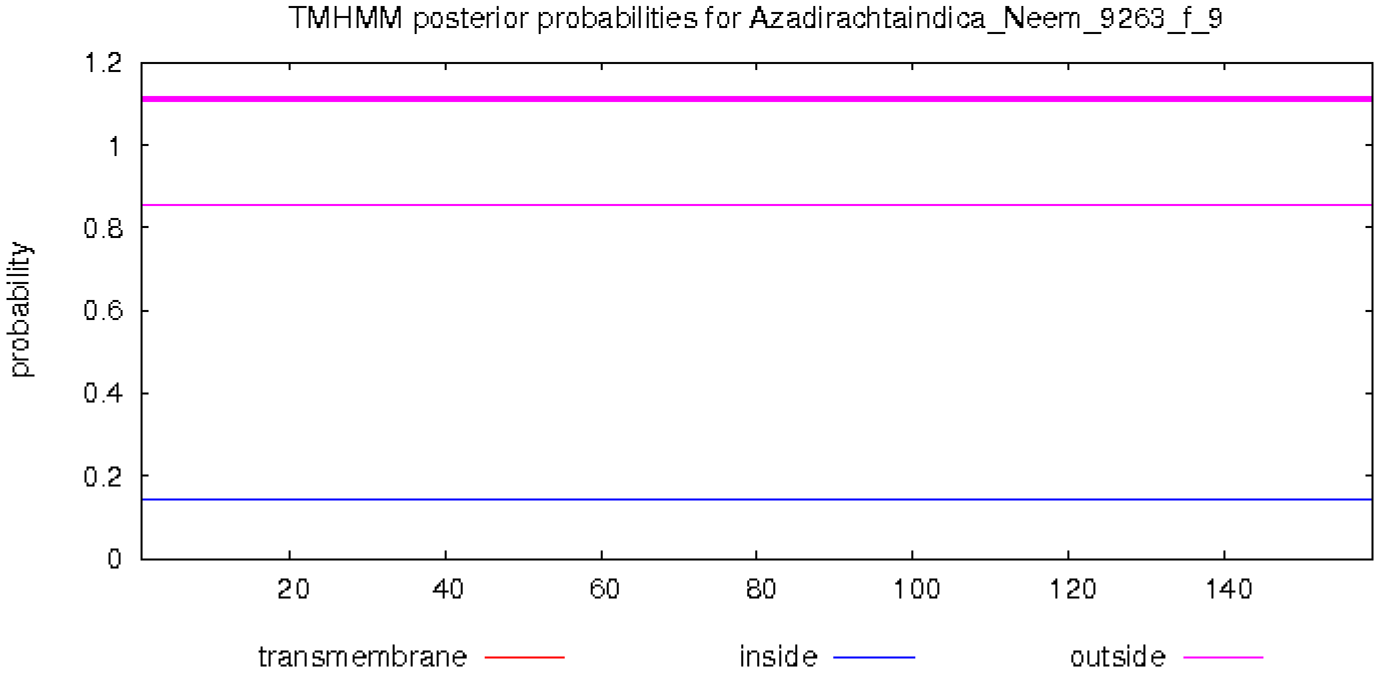

plot in postscript, script for making the plot in gnuplot, data for plot

---

```
# Azadirachtaindica_Neem_9411_f_1 Length: 277
# Azadirachtaindica_Neem_9411_f_1 Number of predicted TMHs:  0
# Azadirachtaindica_Neem_9411_f_1 Exp number of AAs in TMHs: 0.00022
# Azadirachtaindica_Neem_9411_f_1 Exp number, first 60 AAs:  0
# Azadirachtaindica_Neem_9411_f_1 Total prob of N-in:        0.01521
Azadirachtaindica_Neem_9411_f_1 TMHMM2.0        outside      1   277
```

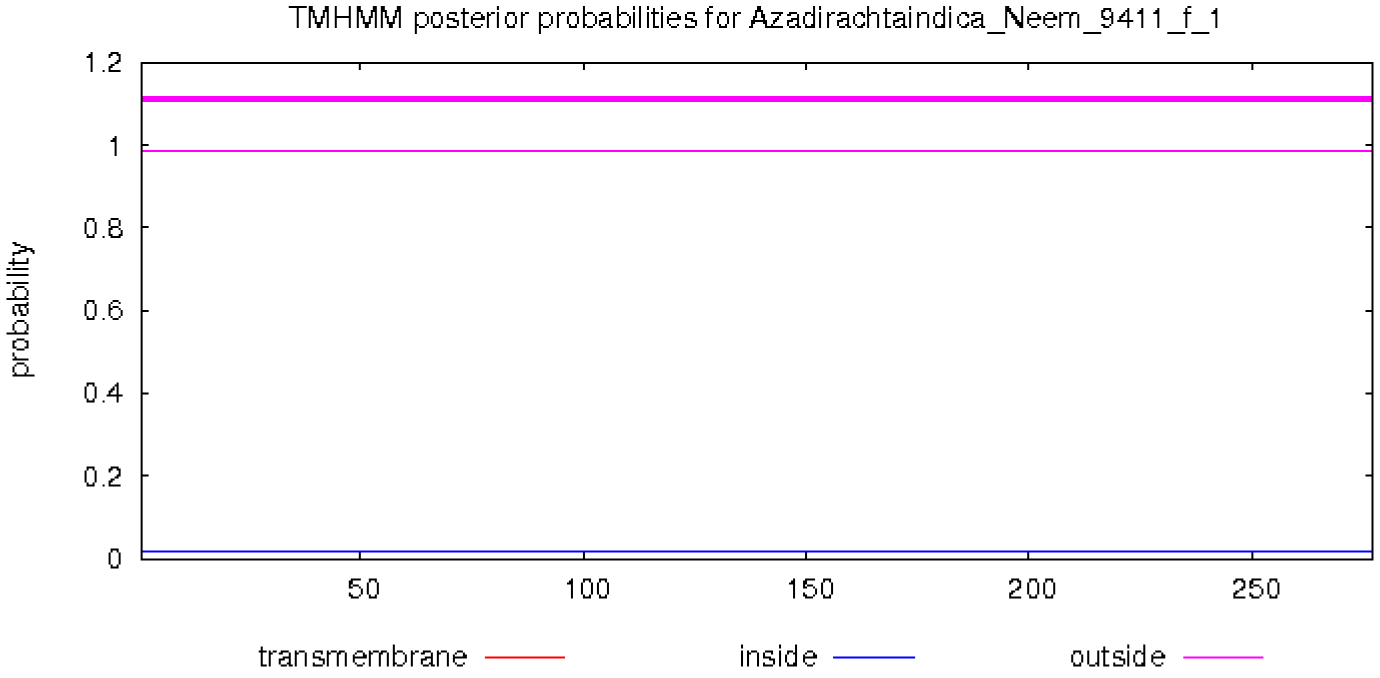

plot in postscript, script for making the plot in gnuplot, data for plot

---

```
# Azadirachtaindica_Neem_9517_f_1 Length: 262
# Azadirachtaindica_Neem_9517_f_1 Number of predicted TMHs:  0
# Azadirachtaindica_Neem_9517_f_1 Exp number of AAs in TMHs: 0.00107
# Azadirachtaindica_Neem_9517_f_1 Exp number, first 60 AAs:  0
# Azadirachtaindica_Neem_9517_f_1 Total prob of N-in:        0.04726
Azadirachtaindica_Neem_9517_f_1 TMHMM2.0        outside      1   262
```

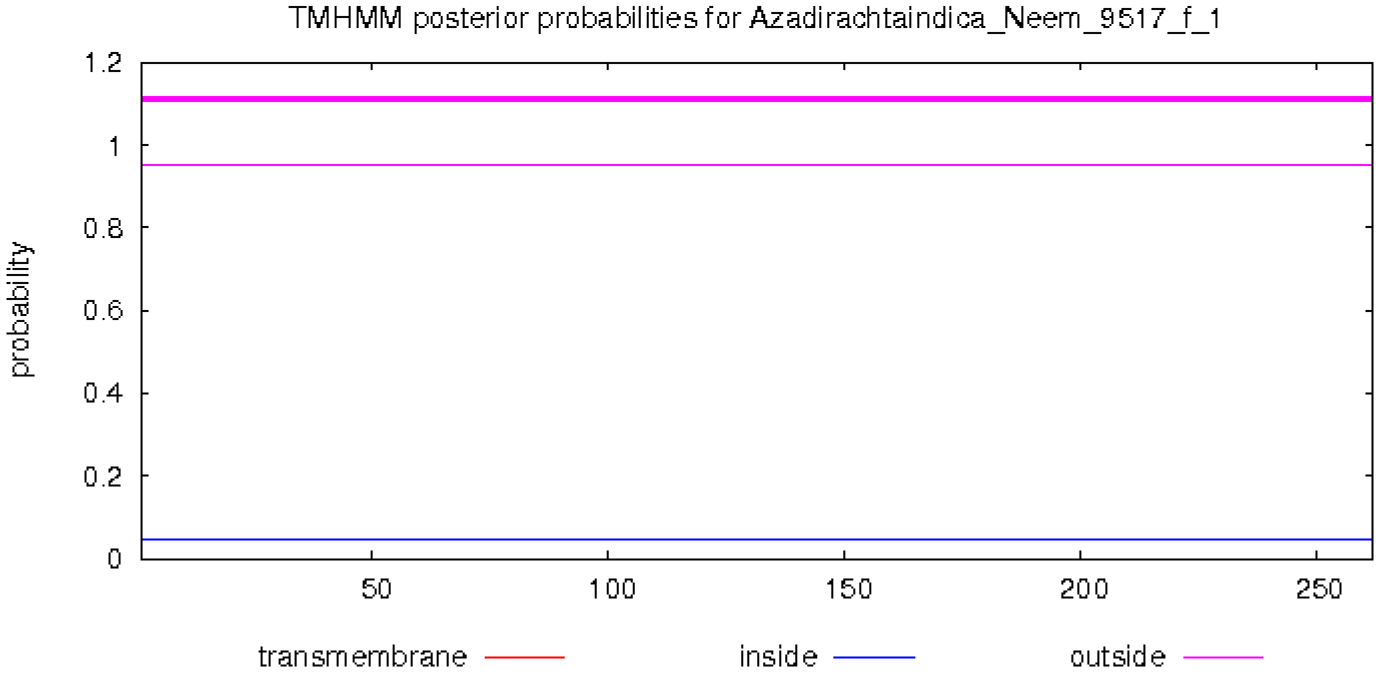

plot in postscript, script for making the plot in gnuplot, data for plot

---

```
# Azadirachtaindica_Neem_9779_f_3 Length: 341
# Azadirachtaindica_Neem_9779_f_3 Number of predicted TMHs:  0
# Azadirachtaindica_Neem_9779_f_3 Exp number of AAs in TMHs: 0
# Azadirachtaindica_Neem_9779_f_3 Exp number, first 60 AAs:  0
# Azadirachtaindica_Neem_9779_f_3 Total prob of N-in:        0.00074
Azadirachtaindica_Neem_9779_f_3 TMHMM2.0        outside      1   341
```

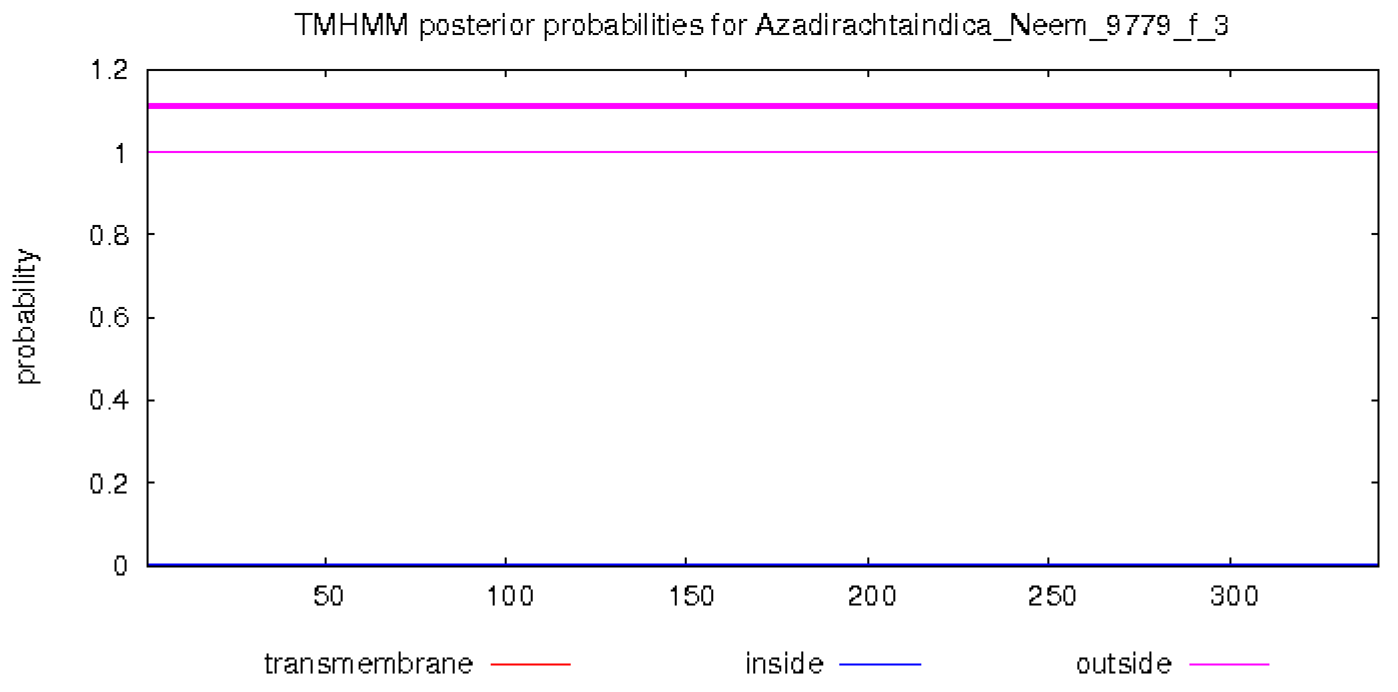

plot in postscript, script for making the plot in gnuplot, data for plot

---

```
# Azadirachtaindica_Neem_9982_f_1 Length: 359
# Azadirachtaindica_Neem_9982_f_1 Number of predicted TMHs:  0
# Azadirachtaindica_Neem_9982_f_1 Exp number of AAs in TMHs: 0.00036
# Azadirachtaindica_Neem_9982_f_1 Exp number, first 60 AAs:  0.00036
# Azadirachtaindica_Neem_9982_f_1 Total prob of N-in:        0.01529
Azadirachtaindica_Neem_9982_f_1 TMHMM2.0        outside      1   359
```

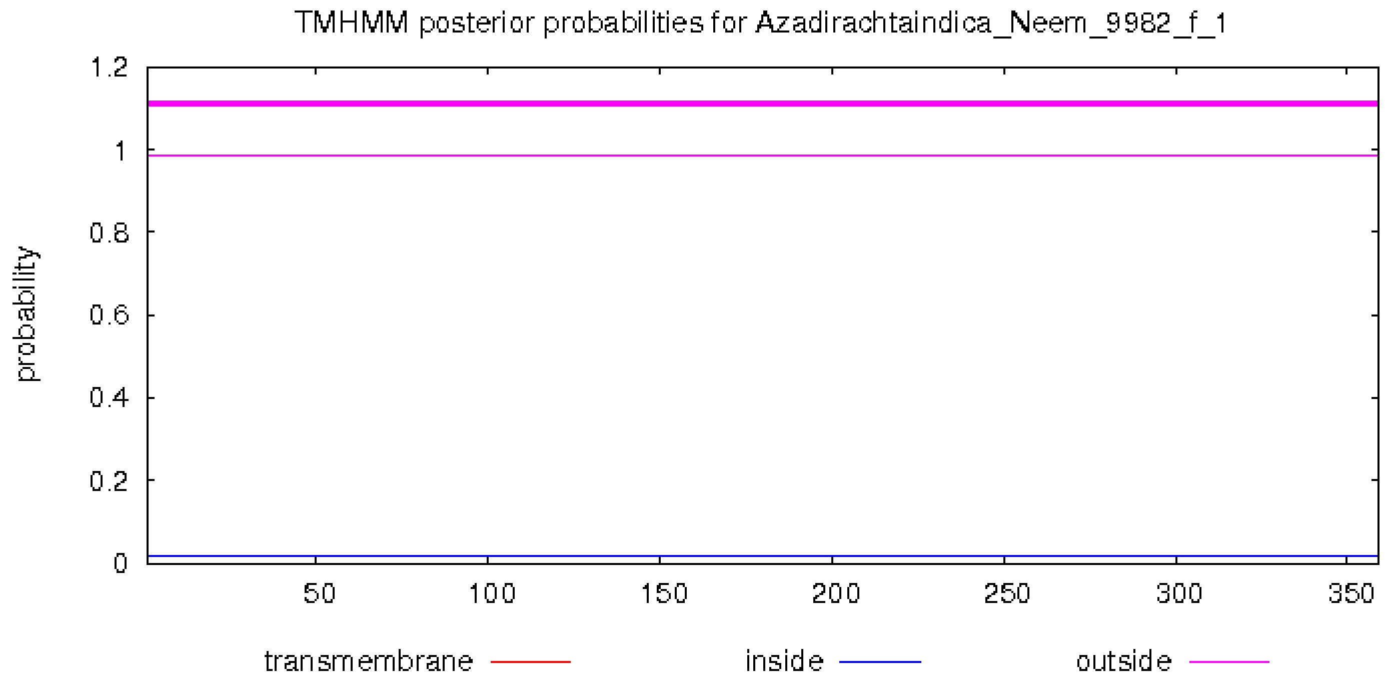

plot in postscript, script for making the plot in gnuplot, data for plot

---

```
# Betavulgaris_Bv_000470_dyzk.t1 Length: 320
# Betavulgaris_Bv_000470_dyzk.t1 Number of predicted TMHs:  0
# Betavulgaris_Bv_000470_dyzk.t1 Exp number of AAs in TMHs: 0.0002
# Betavulgaris_Bv_000470_dyzk.t1 Exp number, first 60 AAs:  0
# Betavulgaris_Bv_000470_dyzk.t1 Total prob of N-in:        0.02208
Betavulgaris_Bv_000470_dyzk.t1  TMHMM2.0        outside      1   320
```

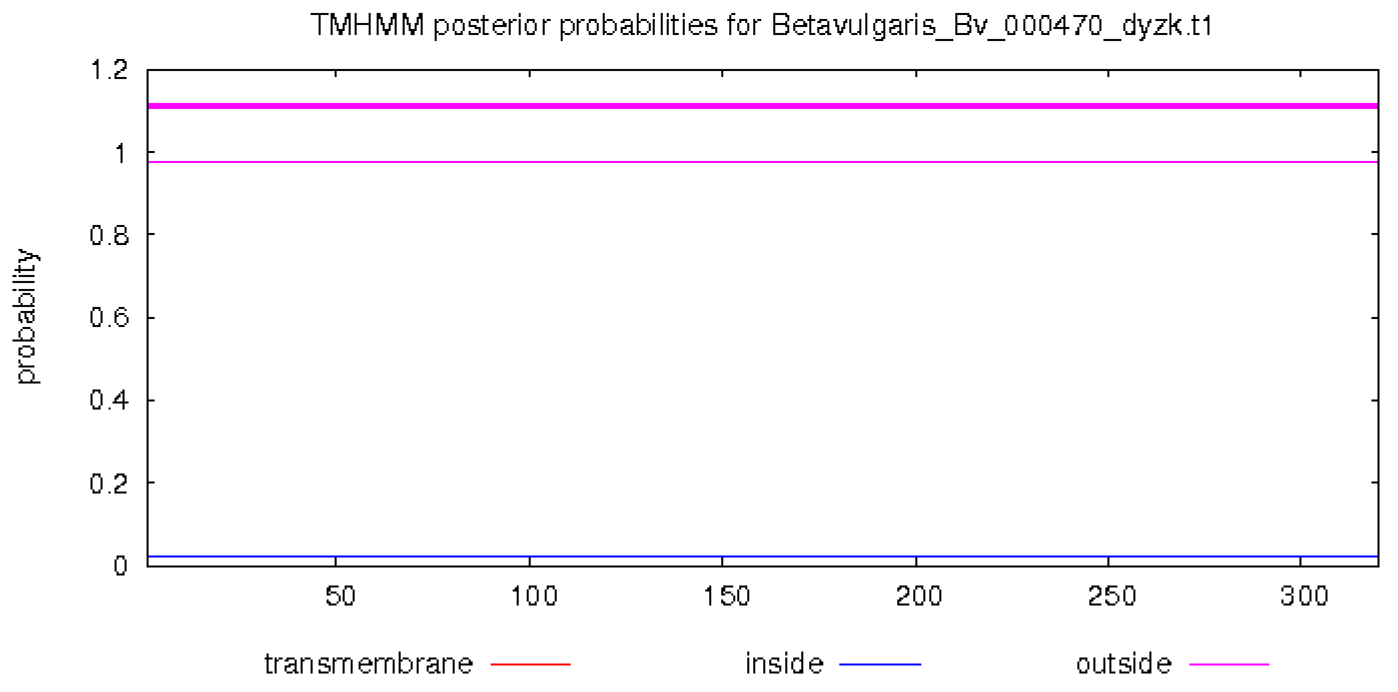

plot in postscript, script for making the plot in gnuplot, data for plot

```
# Betavulgaris_Bv1_001030_strk.t1 Length: 349
# Betavulgaris_Bv1_001030_strk.t1 Number of predicted TMHs:  0
# Betavulgaris_Bv1_001030_strk.t1 Exp number of AAs in TMHs: 0
# Betavulgaris_Bv1_001030_strk.t1 Exp number, first 60 AAs:  0
# Betavulgaris_Bv1_001030_strk.t1 Total prob of N-in:        0.00862
Betavulgaris_Bv1_001030_strk.t1 TMHMM2.0        outside      1   349
```

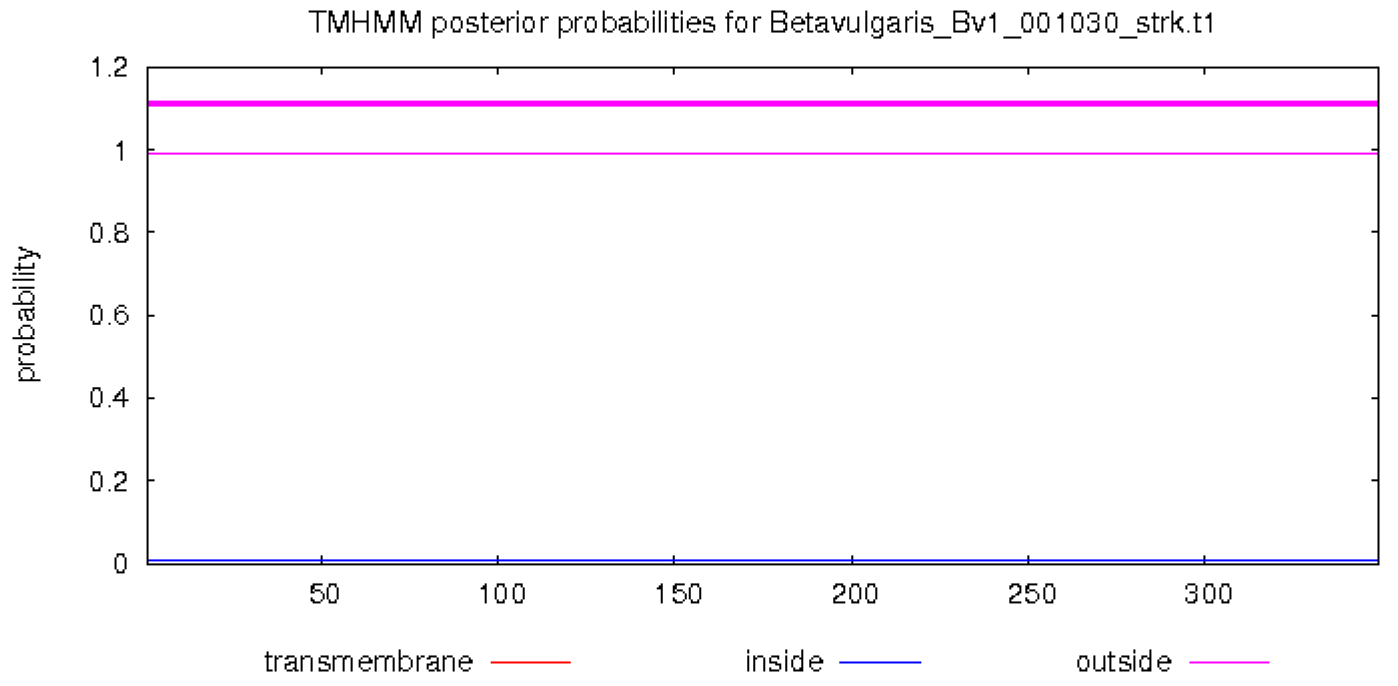

plot in postscript, script for making the plot in gnuplot, data for plot

```
# Betavulgaris_Bv1_001950_ekgh.t1 Length: 388
# Betavulgaris_Bv1_001950_ekgh.t1 Number of predicted TMHs:  0
# Betavulgaris_Bv1_001950_ekgh.t1 Exp number of AAs in TMHs: 0
# Betavulgaris_Bv1_001950_ekgh.t1 Exp number, first 60 AAs:  0
# Betavulgaris_Bv1_001950_ekgh.t1 Total prob of N-in:        0.02012
Betavulgaris_Bv1_001950_ekgh.t1 TMHMM2.0        outside      1   388
```

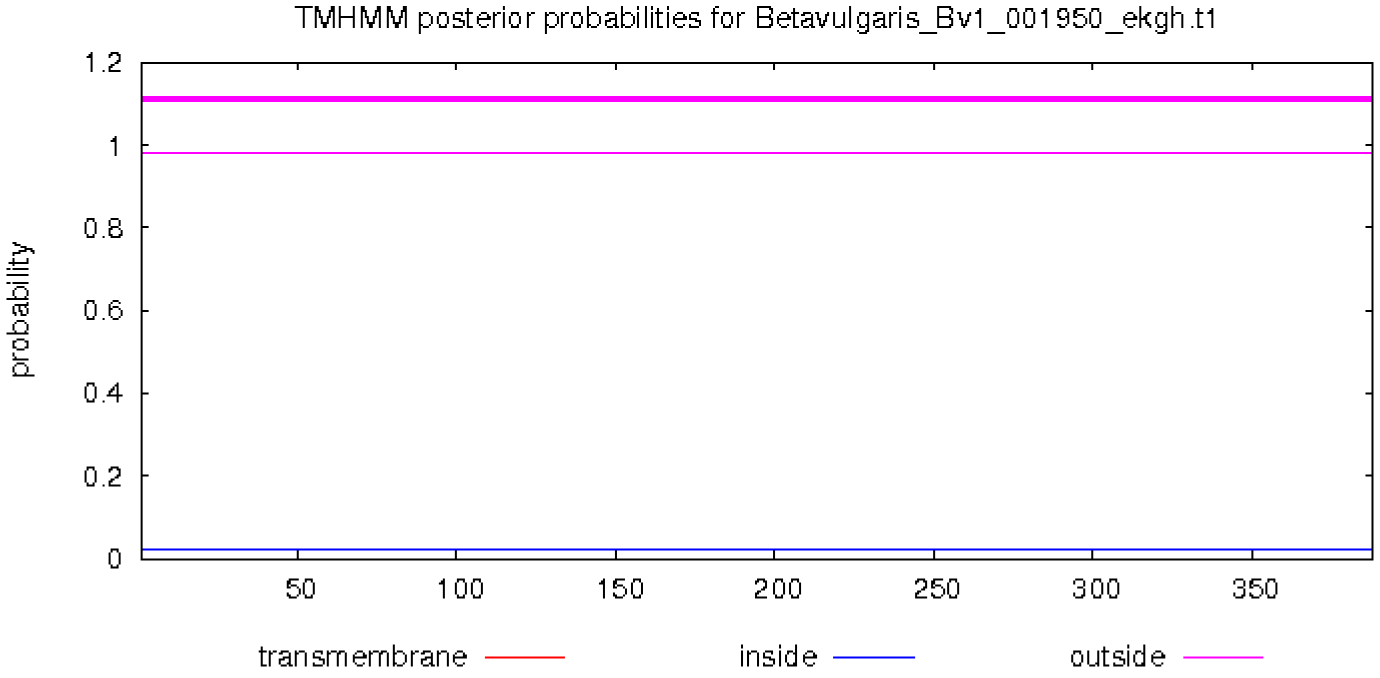

plot in postscript, script for making the plot in gnuplot, data for plot

---

```
# Betavulgaris_Bv1_002240_rapp.t1 Length: 292
# Betavulgaris_Bv1_002240_rapp.t1 Number of predicted TMHs:  0
# Betavulgaris_Bv1_002240_rapp.t1 Exp number of AAs in TMHs: 0.00017
# Betavulgaris_Bv1_002240_rapp.t1 Exp number, first 60 AAs:  0
# Betavulgaris_Bv1_002240_rapp.t1 Total prob of N-in:        0.06031
Betavulgaris_Bv1_002240_rapp.t1 TMHMM2.0        outside      1   292
```

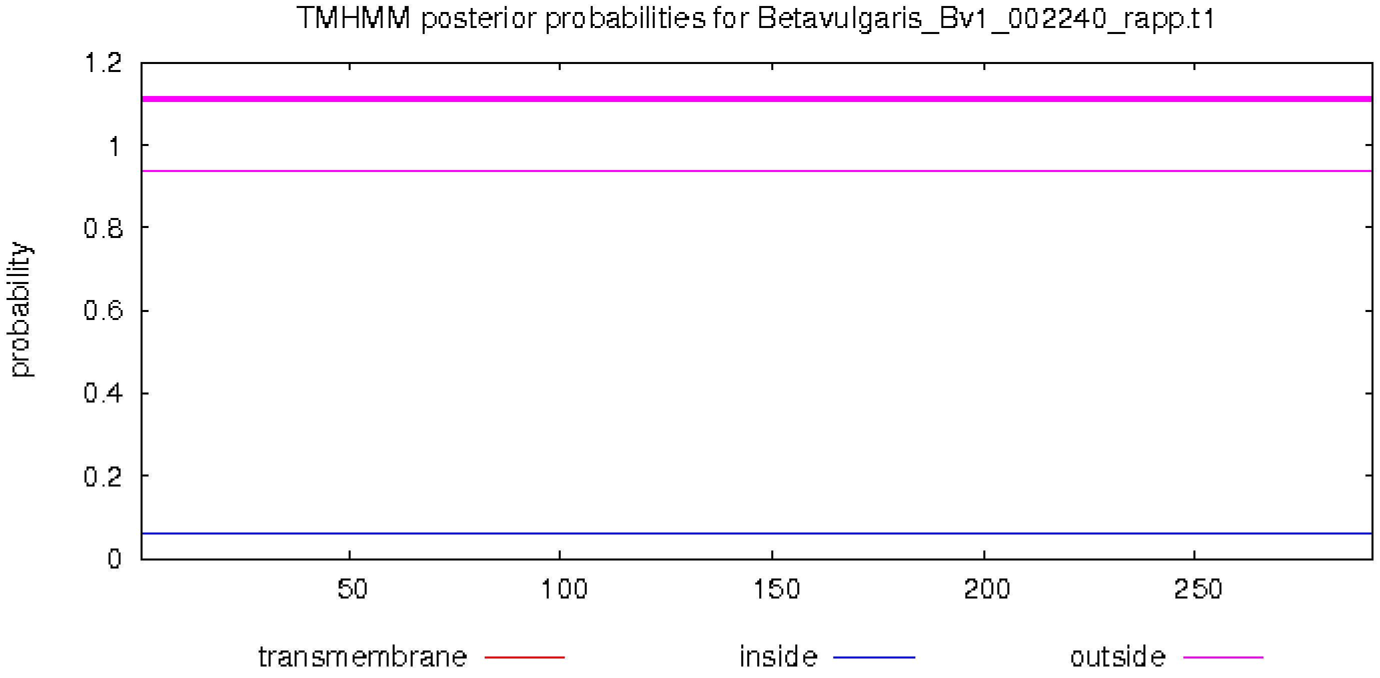

plot in postscript, script for making the plot in gnuplot, data for plot

---

```
# Betavulgaris_Bv1_011210_zihs.t1 Length: 358
# Betavulgaris_Bv1_011210_zihs.t1 Number of predicted TMHs:  0
# Betavulgaris_Bv1_011210_zihs.t1 Exp number of AAs in TMHs: 0.00769
# Betavulgaris_Bv1_011210_zihs.t1 Exp number, first 60 AAs:  0
# Betavulgaris_Bv1_011210_zihs.t1 Total prob of N-in:        0.00763
Betavulgaris_Bv1_011210_zihs.t1 TMHMM2.0        outside      1   358
```

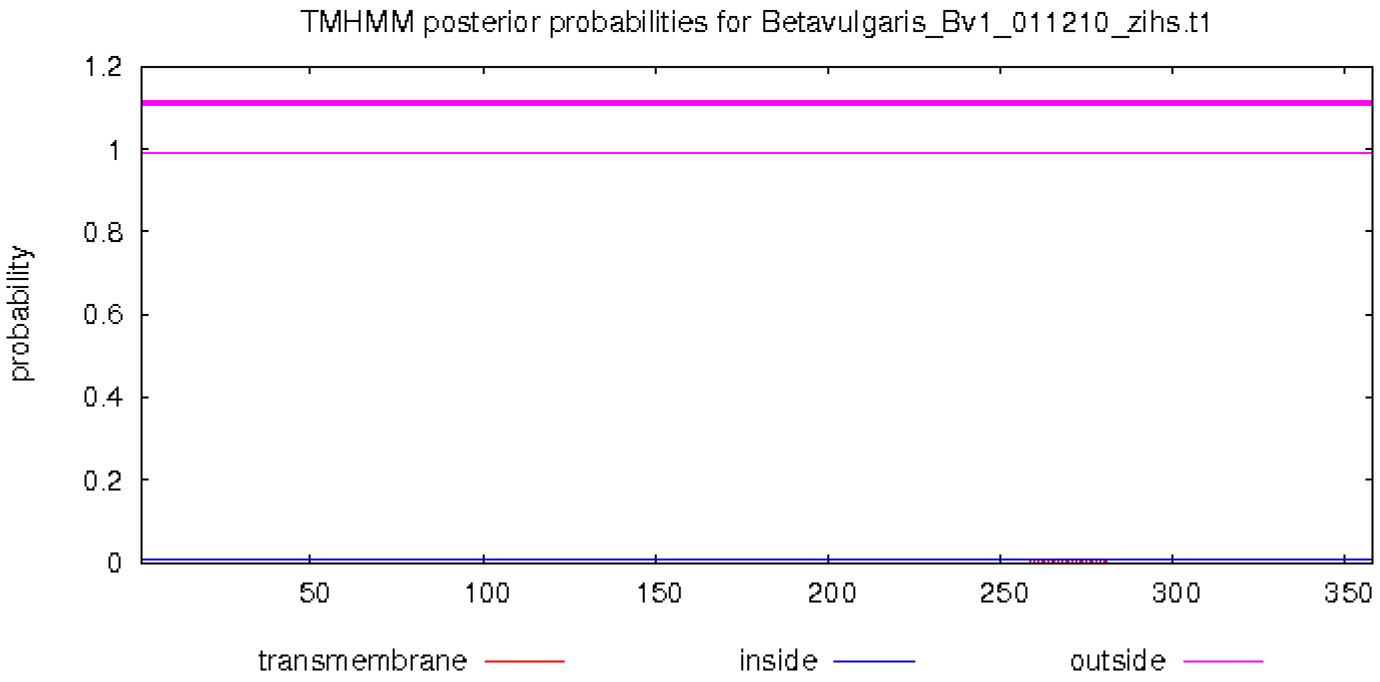

plot in postscript, script for making the plot in gnuplot, data for plot

---

```
# Betavulgaris_Bv1_011220_zfhk.t1 Length: 361
# Betavulgaris_Bv1_011220_zfhk.t1 Number of predicted TMHs:  0
# Betavulgaris_Bv1_011220_zfhk.t1 Exp number of AAs in TMHs: 0.00041
# Betavulgaris_Bv1_011220_zfhk.t1 Exp number, first 60 AAs:  0
# Betavulgaris_Bv1_011220_zfhk.t1 Total prob of N-in:        0.00417
Betavulgaris_Bv1_011220_zfhk.t1 TMHMM2.0        outside      1   361
```

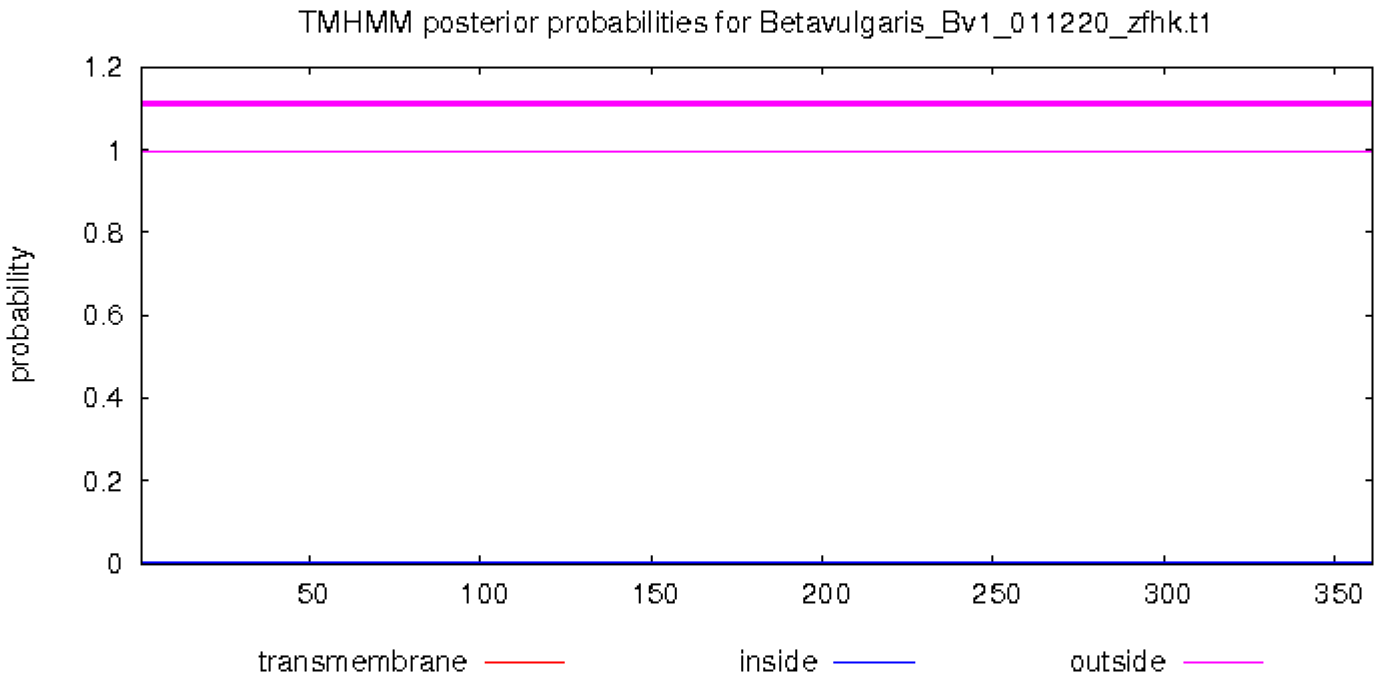

plot in postscript, script for making the plot in gnuplot, data for plot

---

```
# Betavulgaris_Bv1_014170_yzgr.t1 Length: 444
# Betavulgaris_Bv1_014170_yzgr.t1 Number of predicted TMHs:  0
# Betavulgaris_Bv1_014170_yzgr.t1 Exp number of AAs in TMHs: 0.73144
# Betavulgaris_Bv1_014170_yzgr.t1 Exp number, first 60 AAs:  0.0281
# Betavulgaris_Bv1_014170_yzgr.t1 Total prob of N-in:        0.04405
Betavulgaris_Bv1_014170_yzgr.t1 TMHMM2.0        outside      1   444
```

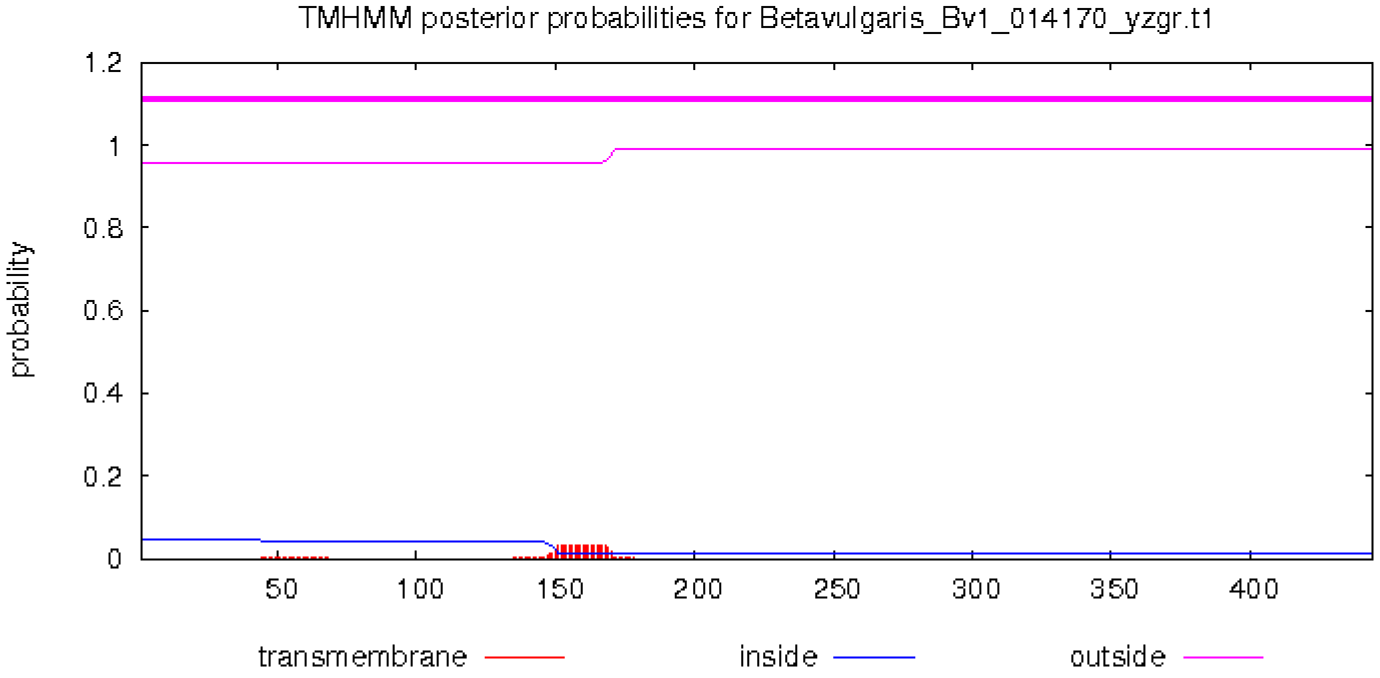

plot in postscript, script for making the plot in gnuplot, data for plot

---

```
# Betavulgaris_Bv1_022030_ueac.t1 Length: 387
# Betavulgaris_Bv1_022030_ueac.t1 Number of predicted TMHs:  1
# Betavulgaris_Bv1_022030_ueac.t1 Exp number of AAs in TMHs: 22.39182
# Betavulgaris_Bv1_022030_ueac.t1 Exp number, first 60 AAs:  0.00799
# Betavulgaris_Bv1_022030_ueac.t1 Total prob of N-in:        0.84654
Betavulgaris_Bv1_022030_ueac.t1 TMHMM2.0        inside       1   362
Betavulgaris_Bv1_022030_ueac.t1 TMHMM2.0        TMhelix    363   385
Betavulgaris_Bv1_022030_ueac.t1 TMHMM2.0        outside    386   387
```

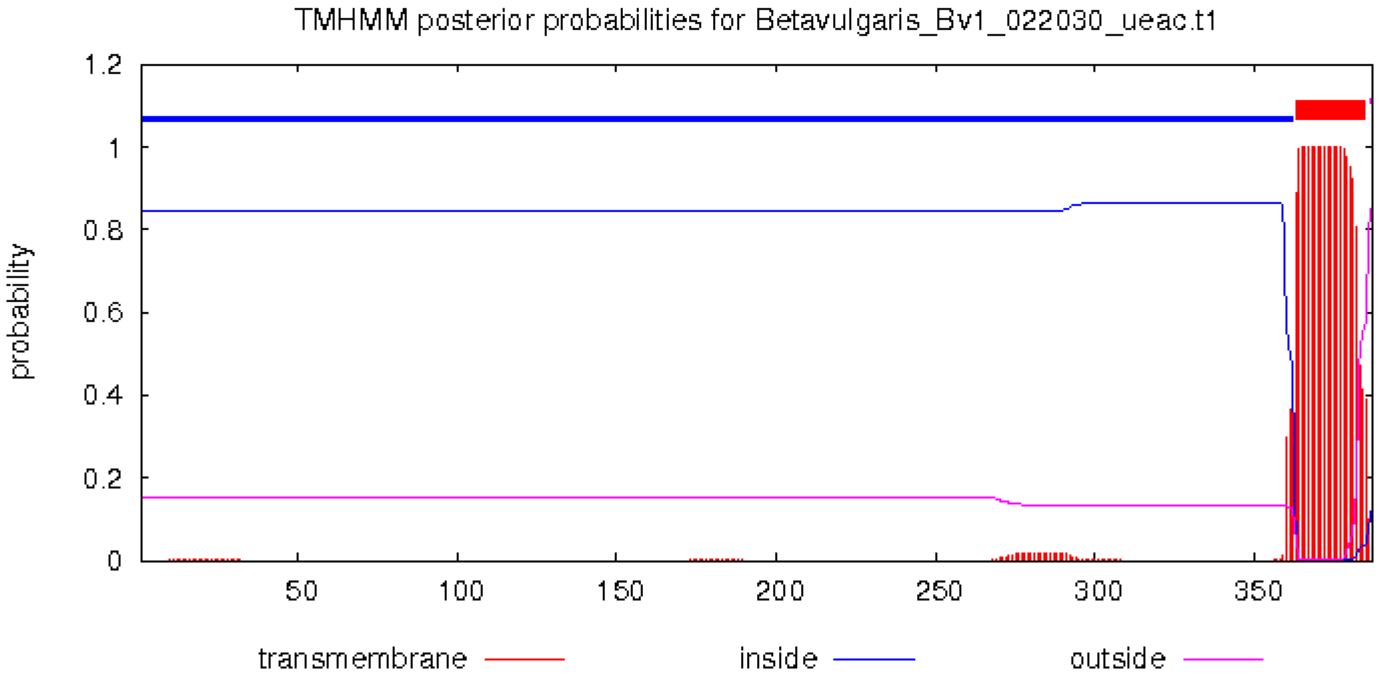

plot in postscript, script for making the plot in gnuplot, data for plot

```
# Betavulgaris_Bv2_032870_nige.t1 Length: 393
# Betavulgaris_Bv2_032870_nige.t1 Number of predicted TMHs:  1
# Betavulgaris_Bv2_032870_nige.t1 Exp number of AAs in TMHs: 21.7821
# Betavulgaris_Bv2_032870_nige.t1 Exp number, first 60 AAs:  0.00668
# Betavulgaris_Bv2_032870_nige.t1 Total prob of N-in:        0.48226
Betavulgaris_Bv2_032870_nige.t1 TMHMM2.0        inside       1   365
Betavulgaris_Bv2_032870_nige.t1 TMHMM2.0        TMhelix    366   388
Betavulgaris_Bv2_032870_nige.t1 TMHMM2.0        outside    389   393
```

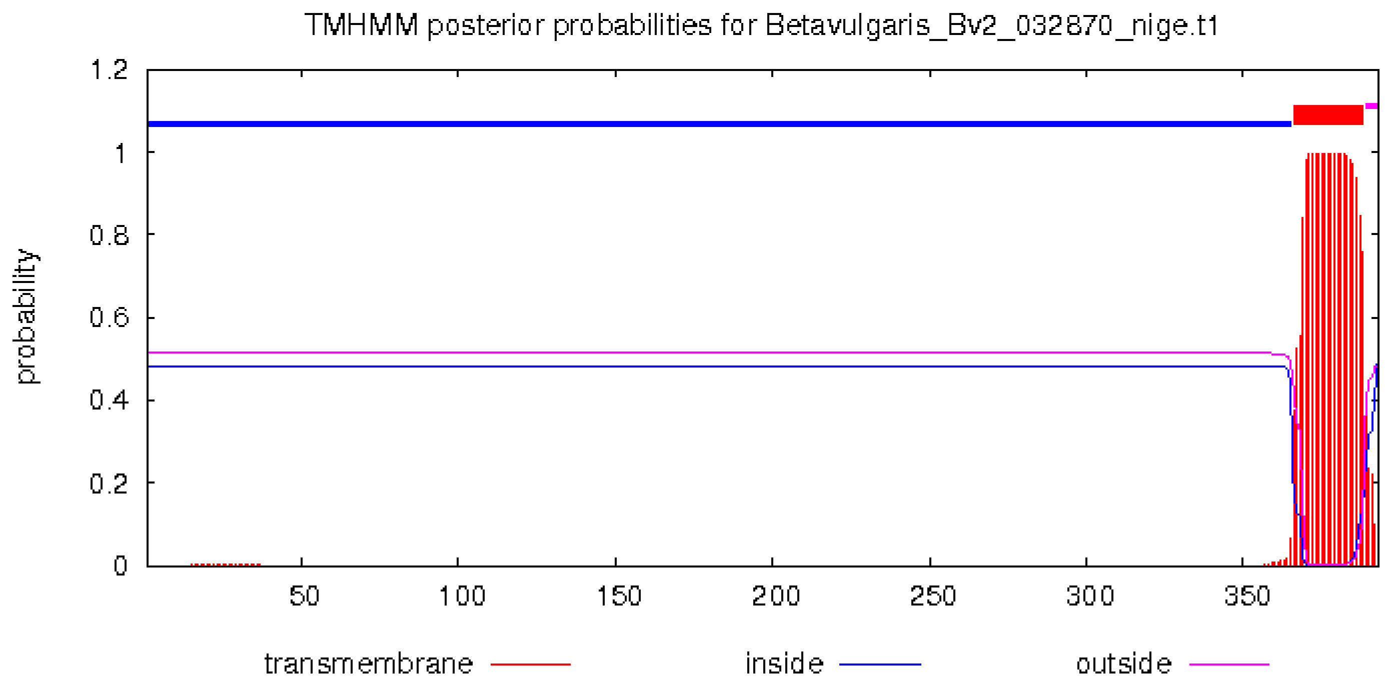

plot in postscript, script for making the plot in gnuplot, data for plot

```
# Betavulgaris_Bv2_035010_gcaa.t1 Length: 335
# Betavulgaris_Bv2_035010_gcaa.t1 Number of predicted TMHs:  0
# Betavulgaris_Bv2_035010_gcaa.t1 Exp number of AAs in TMHs: 0.00861
# Betavulgaris_Bv2_035010_gcaa.t1 Exp number, first 60 AAs:  0
# Betavulgaris_Bv2_035010_gcaa.t1 Total prob of N-in:        0.01421
Betavulgaris_Bv2_035010_gcaa.t1 TMHMM2.0        outside      1   335
```

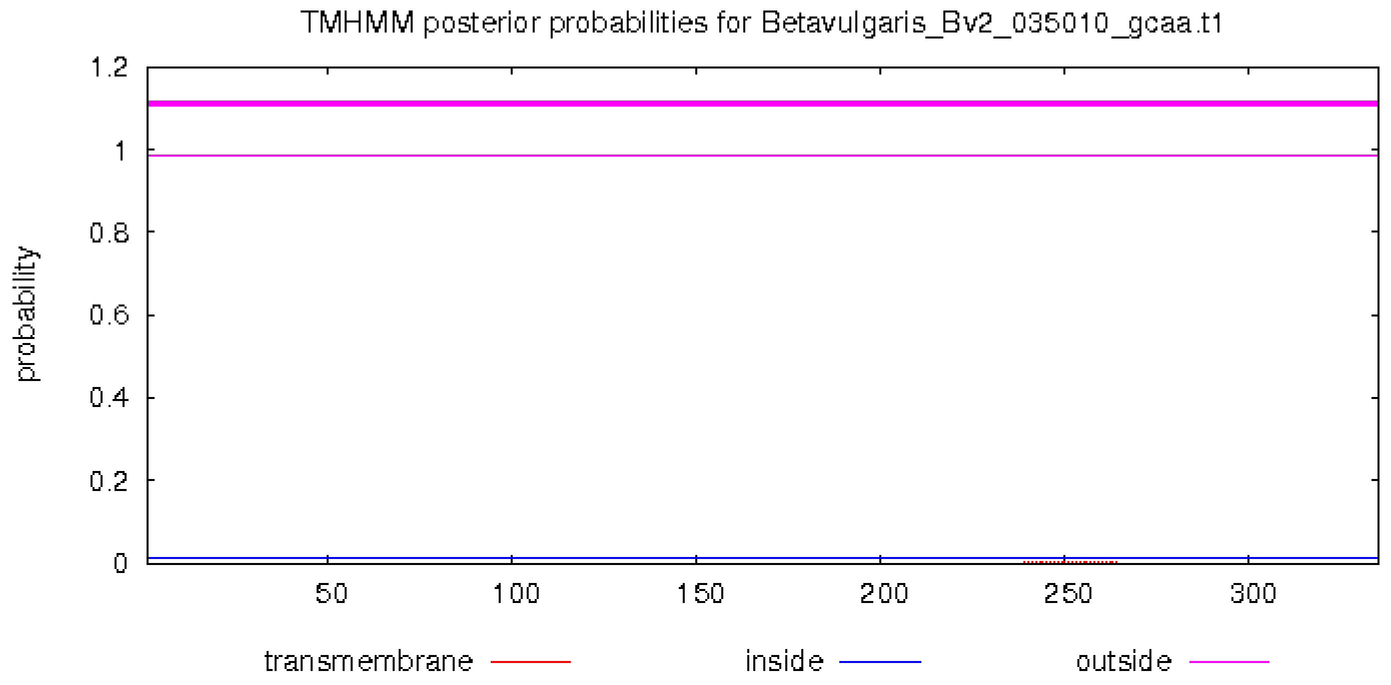

plot in postscript, script for making the plot in gnuplot, data for plot

```
# Betavulgaris_Bv2_040580_mnyx.t1 Length: 209
# Betavulgaris_Bv2_040580_mnyx.t1 Number of predicted TMHs:  0
# Betavulgaris_Bv2_040580_mnyx.t1 Exp number of AAs in TMHs: 0
# Betavulgaris_Bv2_040580_mnyx.t1 Exp number, first 60 AAs:  0
# Betavulgaris_Bv2_040580_mnyx.t1 Total prob of N-in:        0.06821
Betavulgaris_Bv2_040580_mnyx.t1 TMHMM2.0        outside      1   209
```

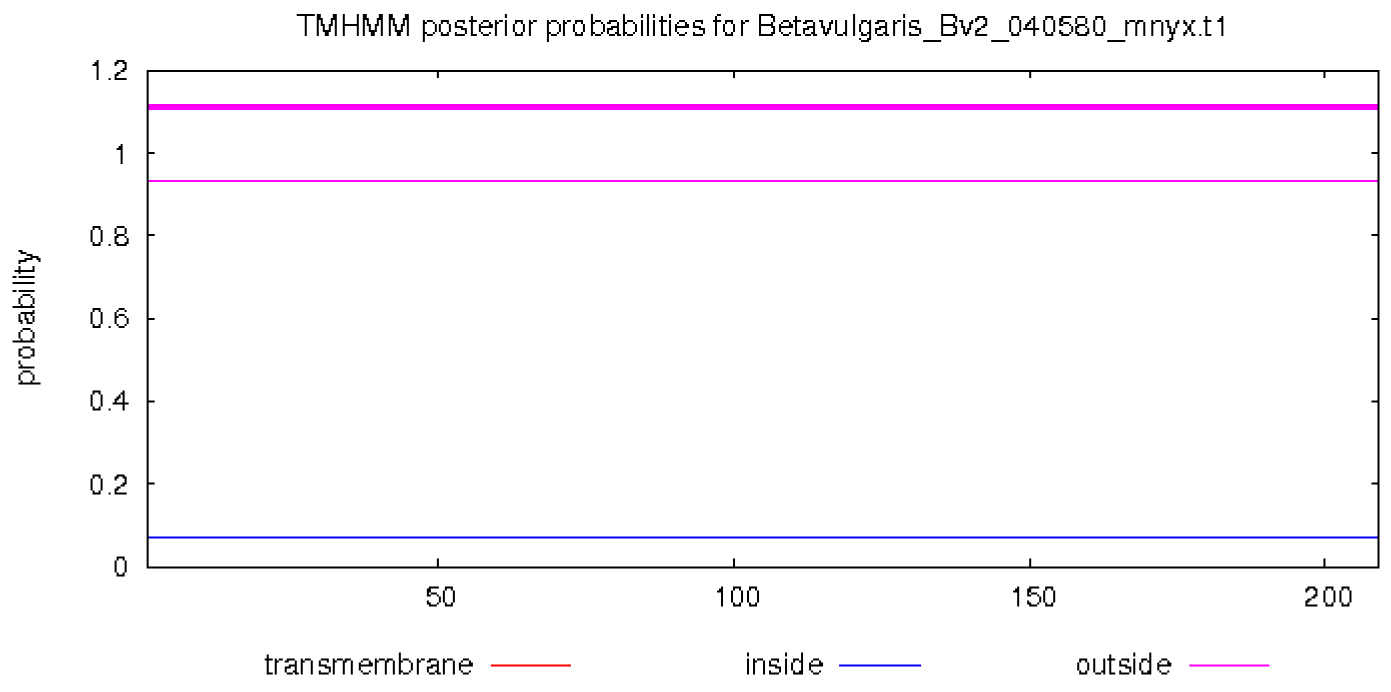

plot in postscript, script for making the plot in gnuplot, data for plot

```
# Betavulgaris_Bv2_046230_sgnn.t1 Length: 336
# Betavulgaris_Bv2_046230_sgnn.t1 Number of predicted TMHs:  0
# Betavulgaris_Bv2_046230_sgnn.t1 Exp number of AAs in TMHs: 0.00016
# Betavulgaris_Bv2_046230_sgnn.t1 Exp number, first 60 AAs:  0
# Betavulgaris_Bv2_046230_sgnn.t1 Total prob of N-in:        0.08638
Betavulgaris_Bv2_046230_sgnn.t1 TMHMM2.0        outside      1   336
```

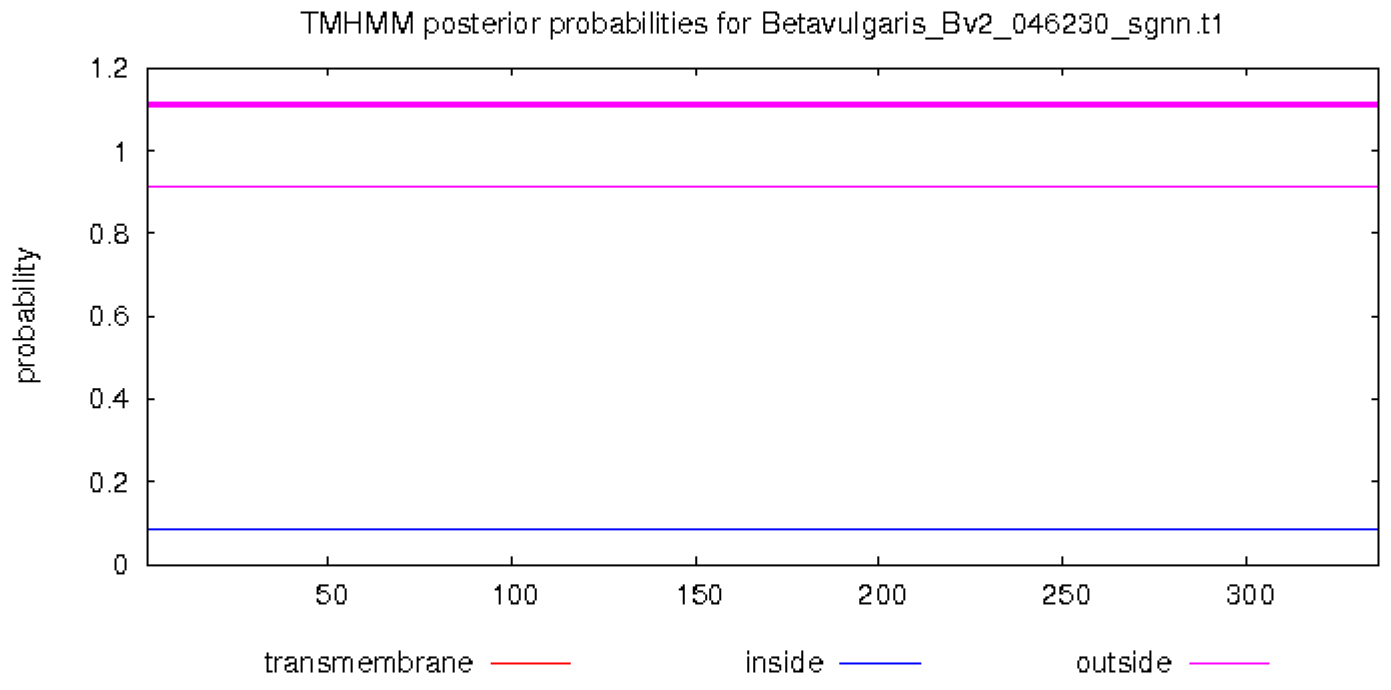

plot in postscript, script for making the plot in gnuplot, data for plot

```
# Betavulgaris_Bv2_046650_xzdt.t1 Length: 387
# Betavulgaris_Bv2_046650_xzdt.t1 Number of predicted TMHs:  0
# Betavulgaris_Bv2_046650_xzdt.t1 Exp number of AAs in TMHs: 0.01208
# Betavulgaris_Bv2_046650_xzdt.t1 Exp number, first 60 AAs:  0
# Betavulgaris_Bv2_046650_xzdt.t1 Total prob of N-in:        0.04530
Betavulgaris_Bv2_046650_xzdt.t1 TMHMM2.0        outside      1   387
```

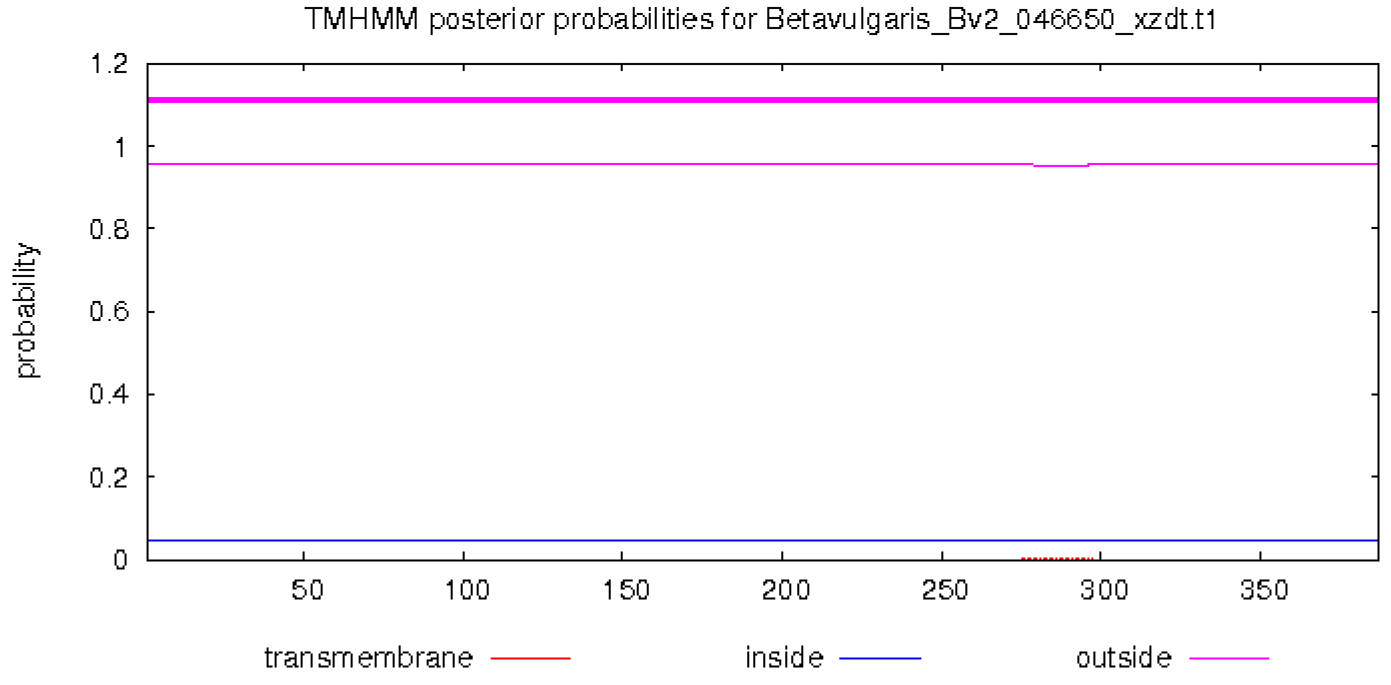

plot in postscript, script for making the plot in gnuplot, data for plot

```
# Betavulgaris_Bv2_046950_hasi.t1 Length: 324
# Betavulgaris_Bv2_046950_hasi.t1 Number of predicted TMHs:  0
# Betavulgaris_Bv2_046950_hasi.t1 Exp number of AAs in TMHs: 0.0006
# Betavulgaris_Bv2_046950_hasi.t1 Exp number, first 60 AAs:  0
# Betavulgaris_Bv2_046950_hasi.t1 Total prob of N-in:        0.02409
Betavulgaris_Bv2_046950_hasi.t1 TMHMM2.0        outside      1   324
```

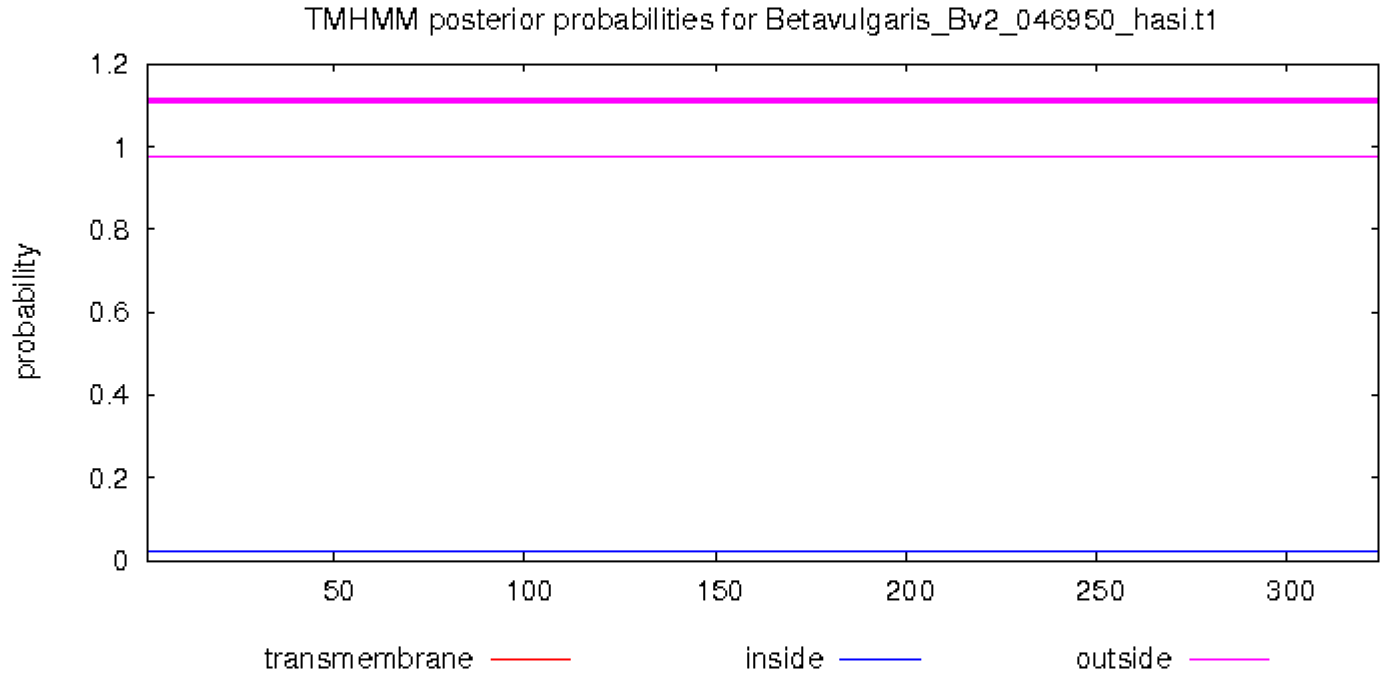

plot in postscript, script for making the plot in gnuplot, data for plot

---

```
# Betavulgaris_Bv2_047470_qmgy.t1 Length: 228
# Betavulgaris_Bv2_047470_qmgy.t1 Number of predicted TMHs:  0
# Betavulgaris_Bv2_047470_qmgy.t1 Exp number of AAs in TMHs: 0.00202
# Betavulgaris_Bv2_047470_qmgy.t1 Exp number, first 60 AAs:  0.00072
# Betavulgaris_Bv2_047470_qmgy.t1 Total prob of N-in:        0.05373
Betavulgaris_Bv2_047470_qmgy.t1 TMHMM2.0        outside      1   228
```

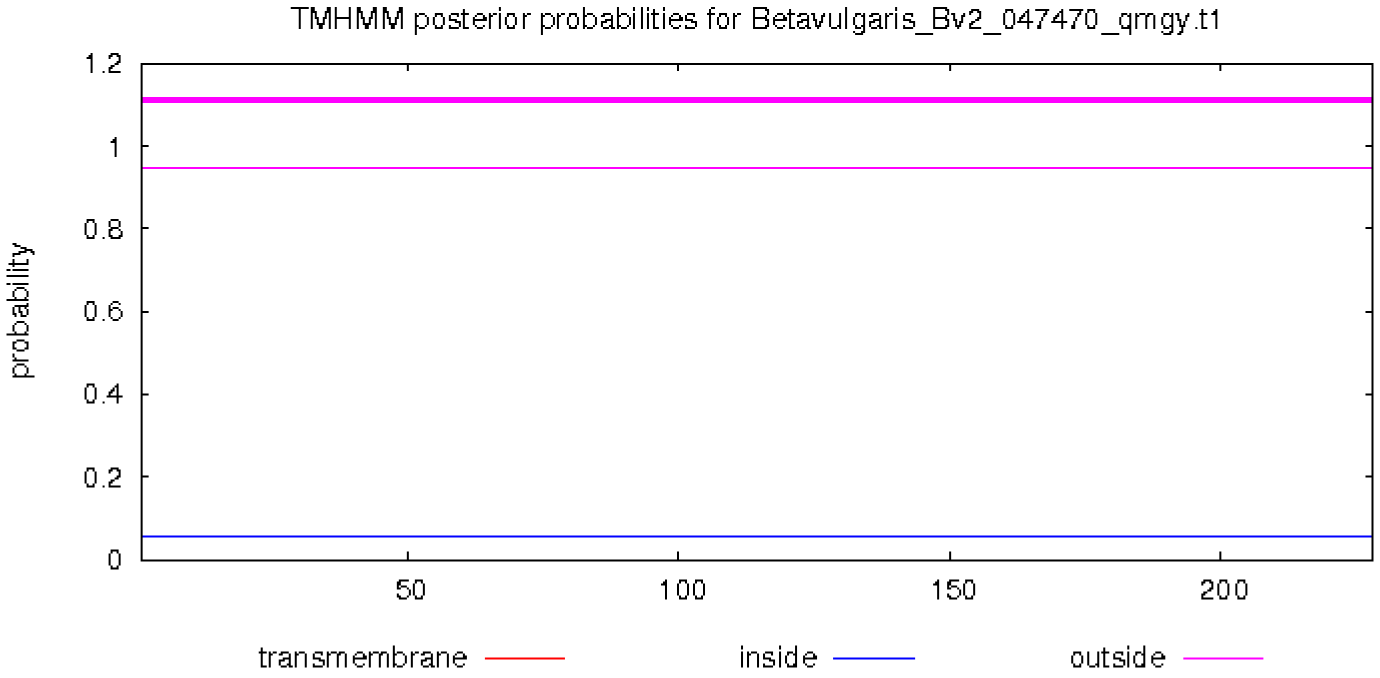

plot in postscript, script for making the plot in gnuplot, data for plot

---

```
# Betavulgaris_Bv3_050780_usim.t1 Length: 637
# Betavulgaris_Bv3_050780_usim.t1 Number of predicted TMHs:  1
# Betavulgaris_Bv3_050780_usim.t1 Exp number of AAs in TMHs: 22.35623
# Betavulgaris_Bv3_050780_usim.t1 Exp number, first 60 AAs:  0
# Betavulgaris_Bv3_050780_usim.t1 Total prob of N-in:        0.00718
Betavulgaris_Bv3_050780_usim.t1 TMHMM2.0        outside      1   606
Betavulgaris_Bv3_050780_usim.t1 TMHMM2.0        TMhelix    607   629
Betavulgaris_Bv3_050780_usim.t1 TMHMM2.0        inside     630   637
```

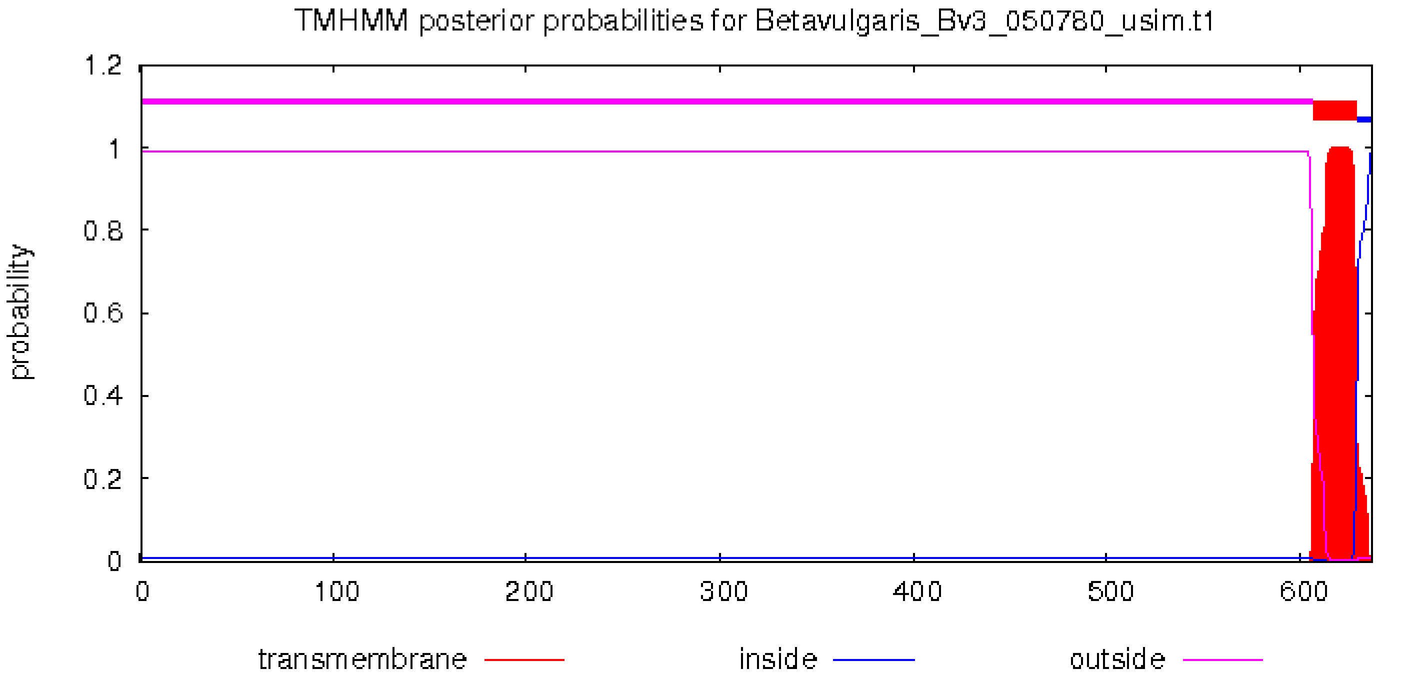

plot in postscript, script for making the plot in gnuplot, data for plot

```
# Betavulgaris_Bv3_056020_uozo.t1 Length: 401
# Betavulgaris_Bv3_056020_uozo.t1 Number of predicted TMHs:  0
# Betavulgaris_Bv3_056020_uozo.t1 Exp number of AAs in TMHs: 4e-05
# Betavulgaris_Bv3_056020_uozo.t1 Exp number, first 60 AAs:  0
# Betavulgaris_Bv3_056020_uozo.t1 Total prob of N-in:        0.00484
Betavulgaris_Bv3_056020_uozo.t1 TMHMM2.0        outside      1   401
```

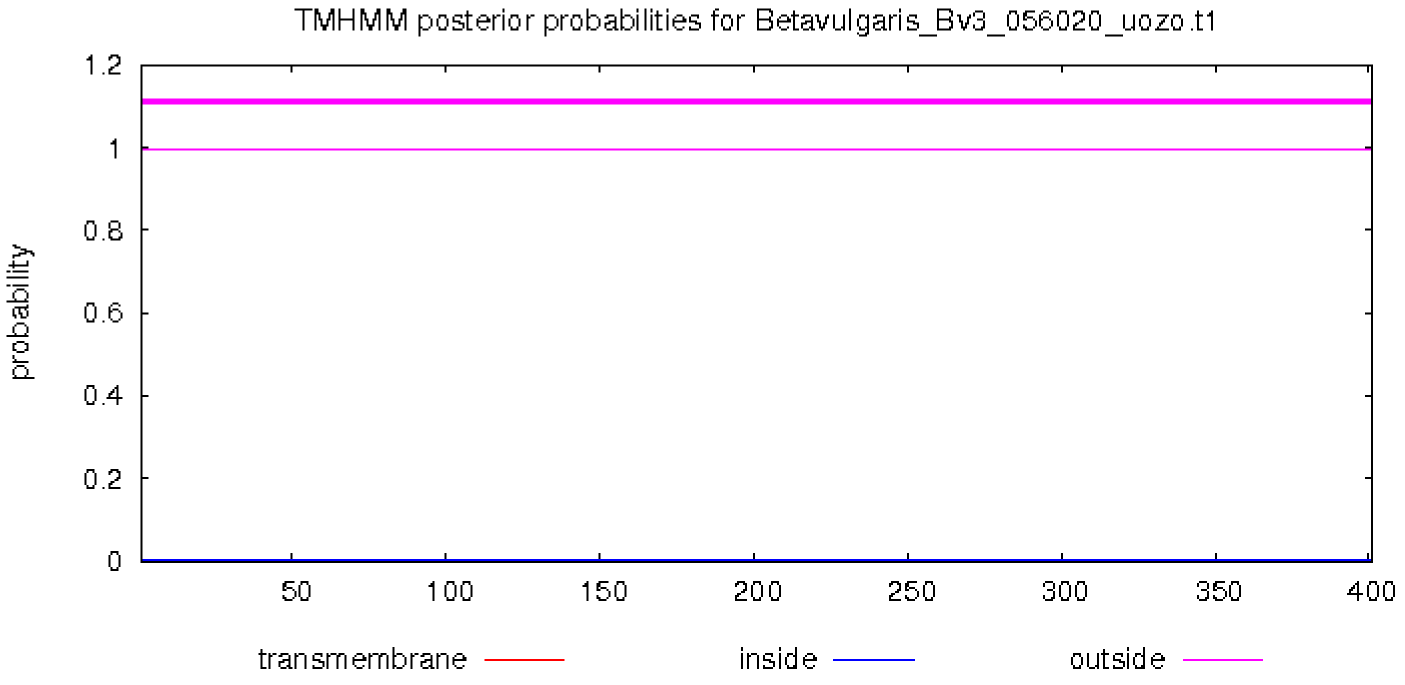

plot in postscript, script for making the plot in gnuplot, data for plot

```
# Betavulgaris_Bv4_078190_hzcx.t1 Length: 303
# Betavulgaris_Bv4_078190_hzcx.t1 Number of predicted TMHs:  0
# Betavulgaris_Bv4_078190_hzcx.t1 Exp number of AAs in TMHs: 0
# Betavulgaris_Bv4_078190_hzcx.t1 Exp number, first 60 AAs:  0
# Betavulgaris_Bv4_078190_hzcx.t1 Total prob of N-in:        0.00275
Betavulgaris_Bv4_078190_hzcx.t1 TMHMM2.0        outside      1   303
```

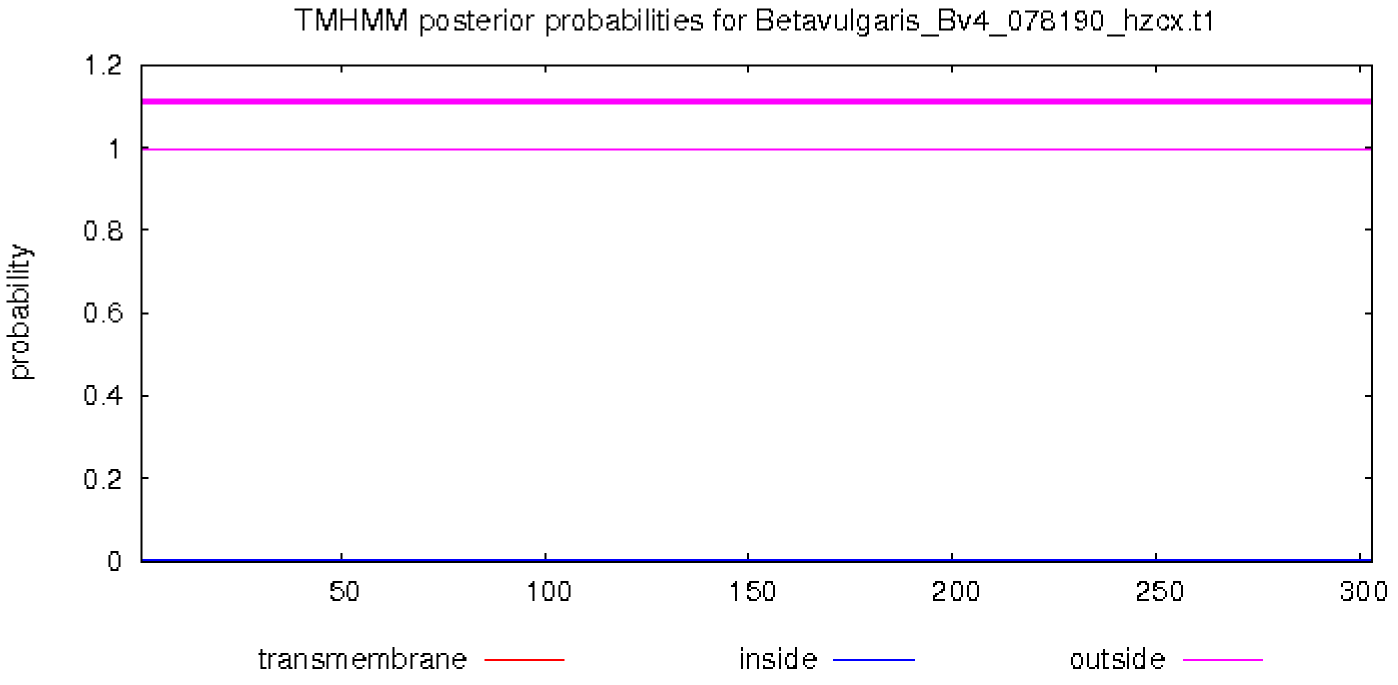

plot in postscript, script for making the plot in gnuplot, data for plot

---

```
# Betavulgaris_Bv4_079110_tgus.t1 Length: 347
# Betavulgaris_Bv4_079110_tgus.t1 Number of predicted TMHs:  0
# Betavulgaris_Bv4_079110_tgus.t1 Exp number of AAs in TMHs: 0.01784
# Betavulgaris_Bv4_079110_tgus.t1 Exp number, first 60 AAs:  0
# Betavulgaris_Bv4_079110_tgus.t1 Total prob of N-in:        0.00204
Betavulgaris_Bv4_079110_tgus.t1 TMHMM2.0        outside      1   347
```

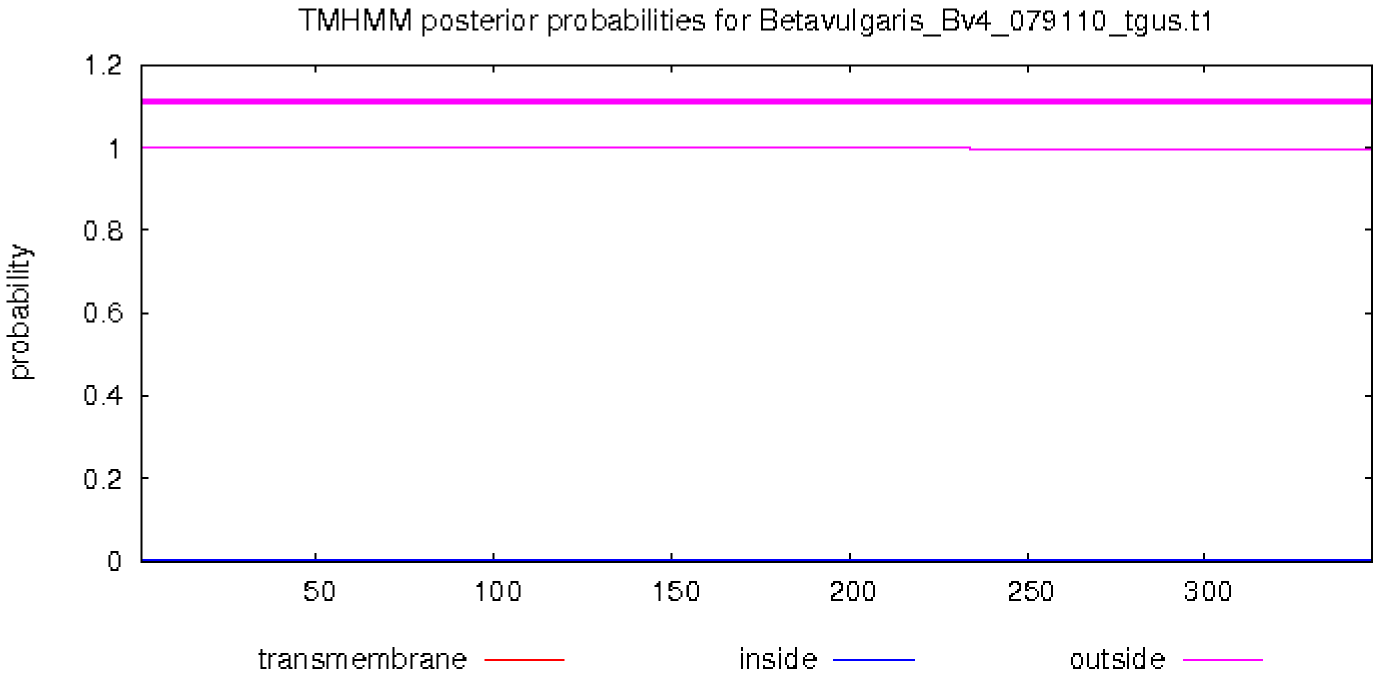

plot in postscript, script for making the plot in gnuplot, data for plot

---

```
# Betavulgaris_Bv4_086610_cugw.t1 Length: 404
# Betavulgaris_Bv4_086610_cugw.t1 Number of predicted TMHs:  0
# Betavulgaris_Bv4_086610_cugw.t1 Exp number of AAs in TMHs: 0.00916
# Betavulgaris_Bv4_086610_cugw.t1 Exp number, first 60 AAs:  0
# Betavulgaris_Bv4_086610_cugw.t1 Total prob of N-in:        0.00092
Betavulgaris_Bv4_086610_cugw.t1 TMHMM2.0        outside      1   404
```

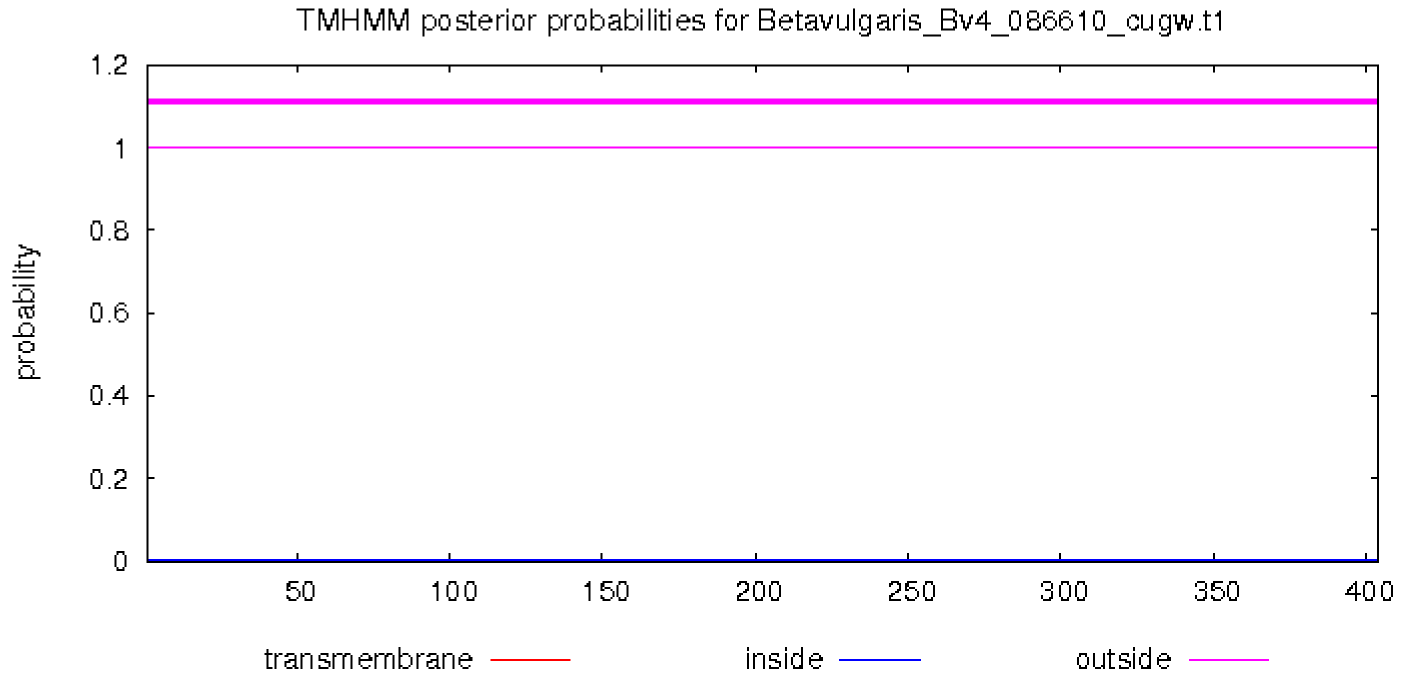

plot in postscript, script for making the plot in gnuplot, data for plot

---

```
# Betavulgaris_Bv4_087410_qejj.t1 Length: 410
# Betavulgaris_Bv4_087410_qejj.t1 Number of predicted TMHs:  0
# Betavulgaris_Bv4_087410_qejj.t1 Exp number of AAs in TMHs: 0.00126
# Betavulgaris_Bv4_087410_qejj.t1 Exp number, first 60 AAs:  0
# Betavulgaris_Bv4_087410_qejj.t1 Total prob of N-in:        0.00852
Betavulgaris_Bv4_087410_qejj.t1 TMHMM2.0        outside      1   410
```

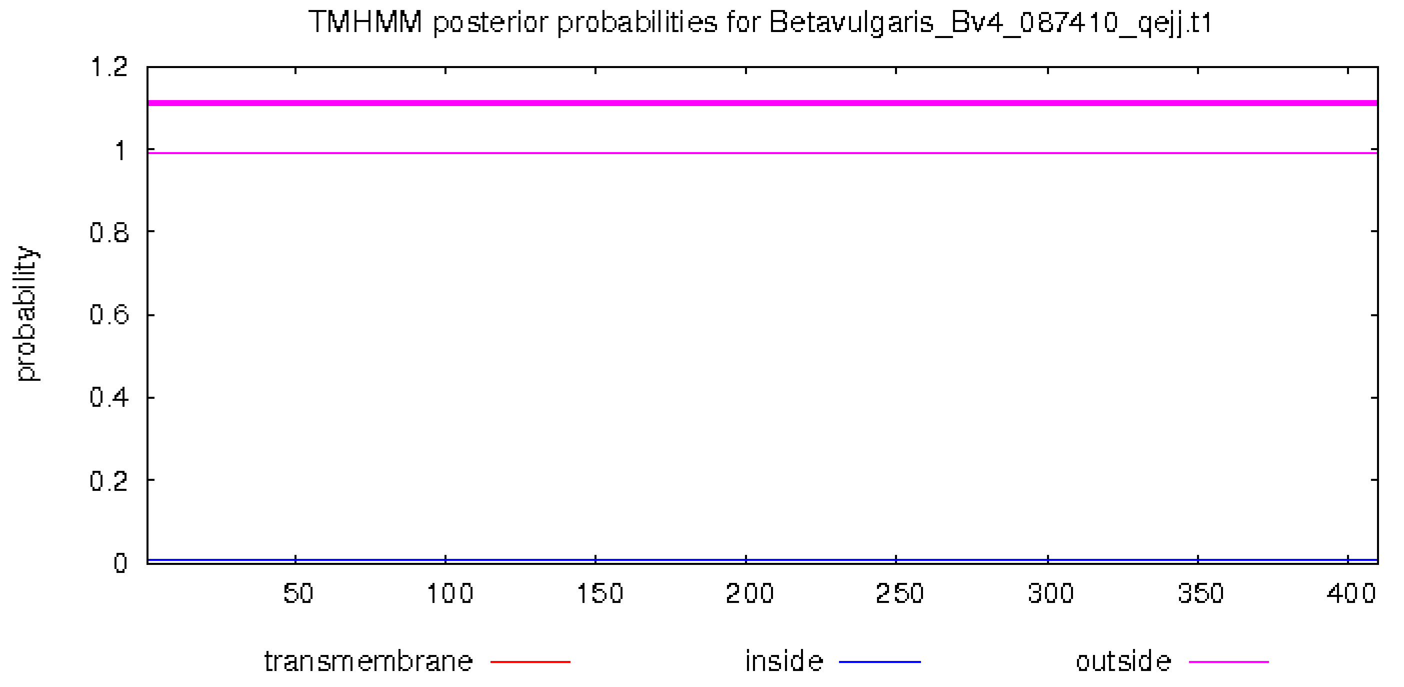

plot in postscript, script for making the plot in gnuplot, data for plot

---

```
# Betavulgaris_Bv4_087440_twas.t1 Length: 364
# Betavulgaris_Bv4_087440_twas.t1 Number of predicted TMHs:  0
# Betavulgaris_Bv4_087440_twas.t1 Exp number of AAs in TMHs: 0.00458
# Betavulgaris_Bv4_087440_twas.t1 Exp number, first 60 AAs:  0.00022
# Betavulgaris_Bv4_087440_twas.t1 Total prob of N-in:        0.00901
Betavulgaris_Bv4_087440_twas.t1 TMHMM2.0        outside      1   364
```

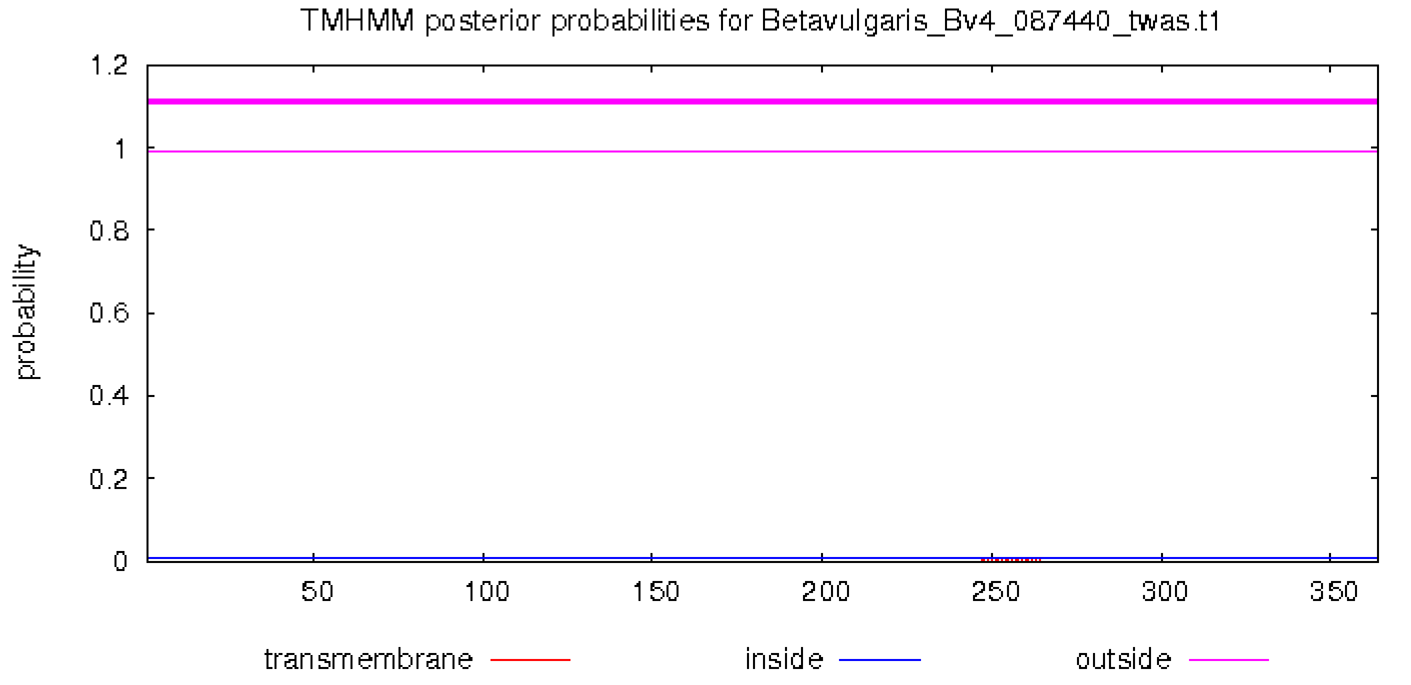

plot in postscript, script for making the plot in gnuplot, data for plot

---

```
# Betavulgaris_Bv4_093610_xiqu.t1 Length: 401
# Betavulgaris_Bv4_093610_xiqu.t1 Number of predicted TMHs:  0
# Betavulgaris_Bv4_093610_xiqu.t1 Exp number of AAs in TMHs: 0.00053
# Betavulgaris_Bv4_093610_xiqu.t1 Exp number, first 60 AAs:  0.00053
# Betavulgaris_Bv4_093610_xiqu.t1 Total prob of N-in:        0.00125
Betavulgaris_Bv4_093610_xiqu.t1 TMHMM2.0        outside      1   401
```

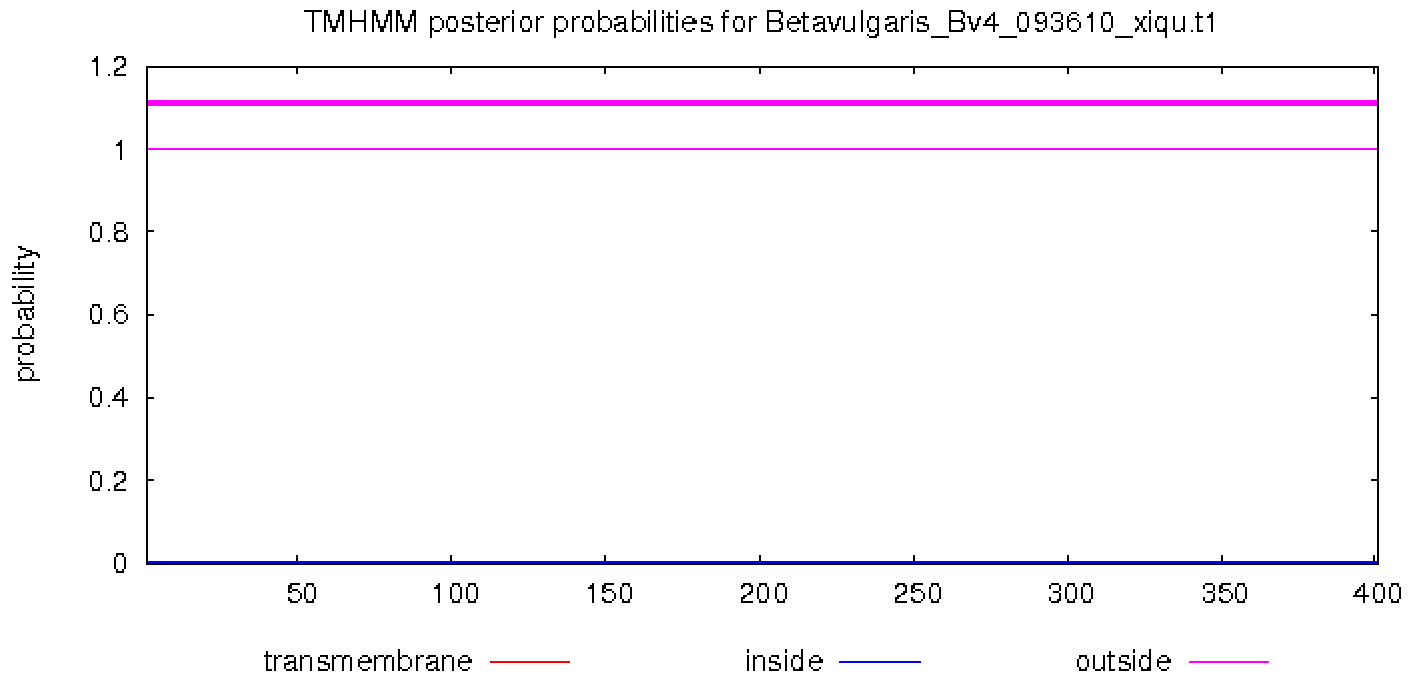

plot in postscript, script for making the plot in gnuplot, data for plot

---

```
# Betavulgaris_Bv4_093720_guiw.t1 Length: 540
# Betavulgaris_Bv4_093720_guiw.t1 Number of predicted TMHs:  1
# Betavulgaris_Bv4_093720_guiw.t1 Exp number of AAs in TMHs: 21.56868
# Betavulgaris_Bv4_093720_guiw.t1 Exp number, first 60 AAs:  0
# Betavulgaris_Bv4_093720_guiw.t1 Total prob of N-in:        0.02645
Betavulgaris_Bv4_093720_guiw.t1 TMHMM2.0        outside      1   516
```

```
Betavulgaris_Bv4_093720_guiw.t1 TMHMM2.0        TMhelix    517   539
Betavulgaris_Bv4_093720_guiw.t1 TMHMM2.0        inside     540   540
```

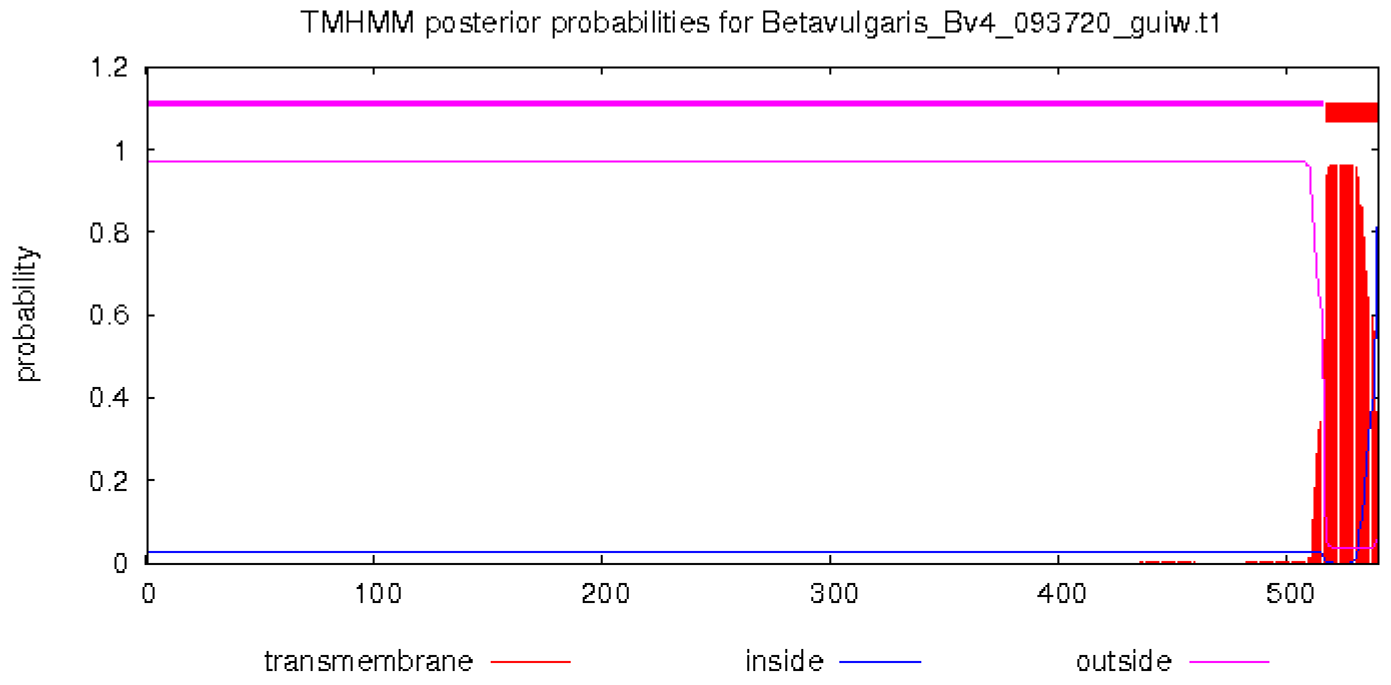

plot in postscript, script for making the plot in gnuplot, data for plot

---

```
# Betavulgaris_Bv4_095900_twpc.t1 Length: 264
# Betavulgaris_Bv4_095900_twpc.t1 Number of predicted TMHs:  0
# Betavulgaris_Bv4_095900_twpc.t1 Exp number of AAs in TMHs: 0
# Betavulgaris_Bv4_095900_twpc.t1 Exp number, first 60 AAs:  0
# Betavulgaris_Bv4_095900_twpc.t1 Total prob of N-in:        0.19523
Betavulgaris_Bv4_095900_twpc.t1 TMHMM2.0        outside      1   264
```

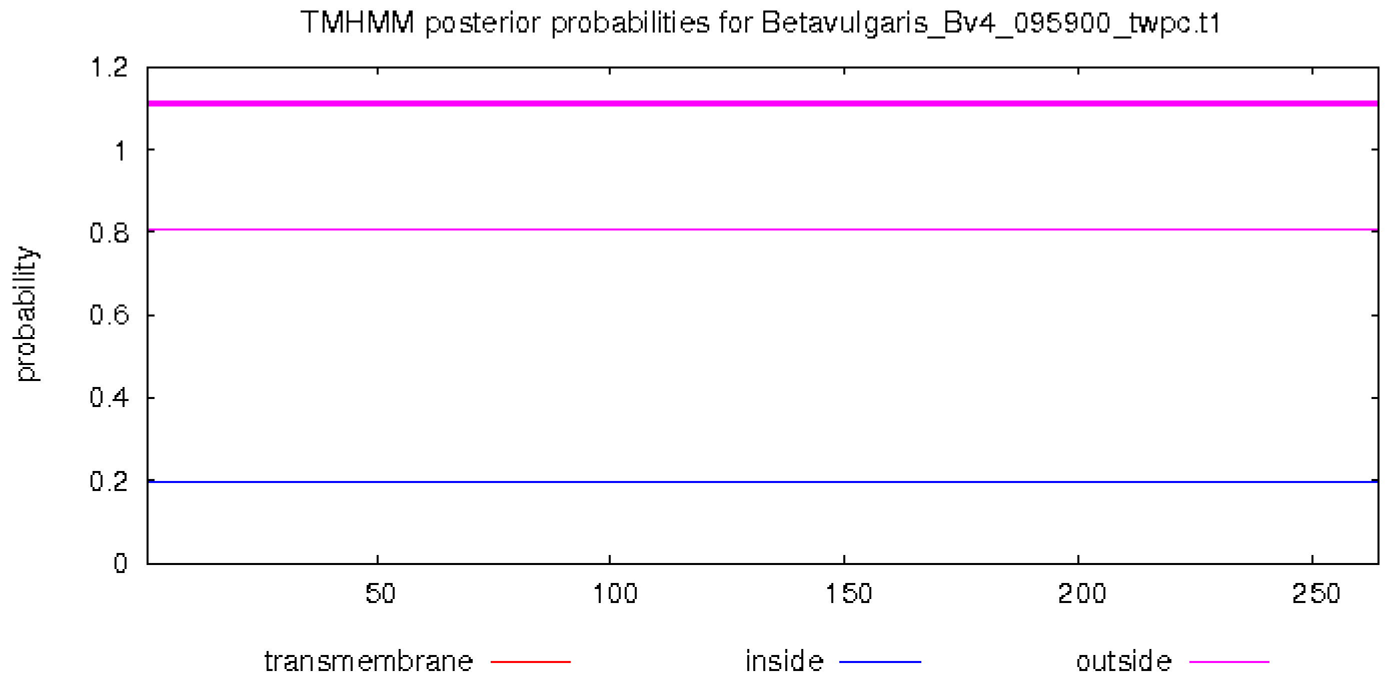

plot in postscript, script for making the plot in gnuplot, data for plot

---

```
# Betavulgaris_Bv4_096620_mhnx.t1 Length: 496
# Betavulgaris_Bv4_096620_mhnx.t1 Number of predicted TMHs:  0
# Betavulgaris_Bv4_096620_mhnx.t1 Exp number of AAs in TMHs: 5.15643
# Betavulgaris_Bv4_096620_mhnx.t1 Exp number, first 60 AAs:  0
```

```
# Betavulgaris_Bv4_096620_mhnx.t1 Total prob of N-in:        0.17044
Betavulgaris_Bv4_096620_mhnx.t1 TMHMM2.0        outside      1   496
```

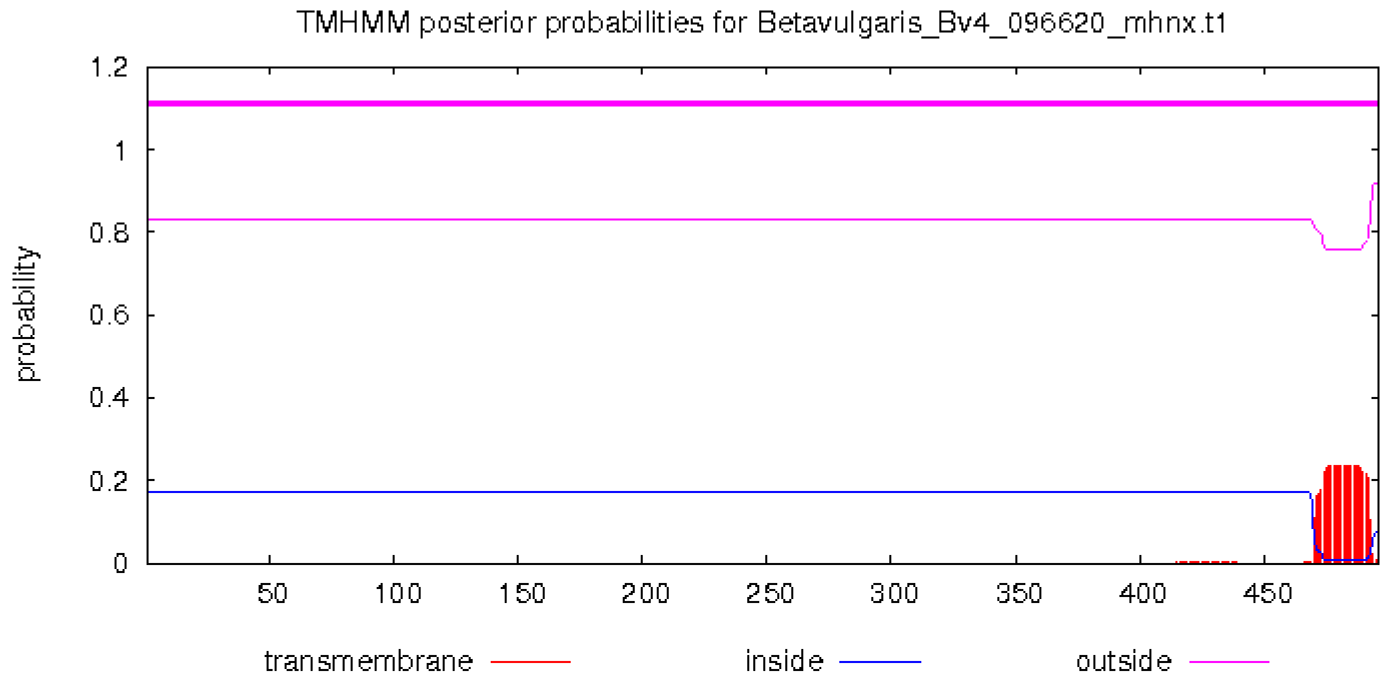

plot in postscript, script for making the plot in gnuplot, data for plot

---

```
# Betavulgaris_Bv4_096650_oxms.t1 Length: 241
# Betavulgaris_Bv4_096650_oxms.t1 Number of predicted TMHs:  0
# Betavulgaris_Bv4_096650_oxms.t1 Exp number of AAs in TMHs: 0
# Betavulgaris_Bv4_096650_oxms.t1 Exp number, first 60 AAs:  0
# Betavulgaris_Bv4_096650_oxms.t1 Total prob of N-in:        0.00327
Betavulgaris_Bv4_096650_oxms.t1 TMHMM2.0        outside      1   241
```

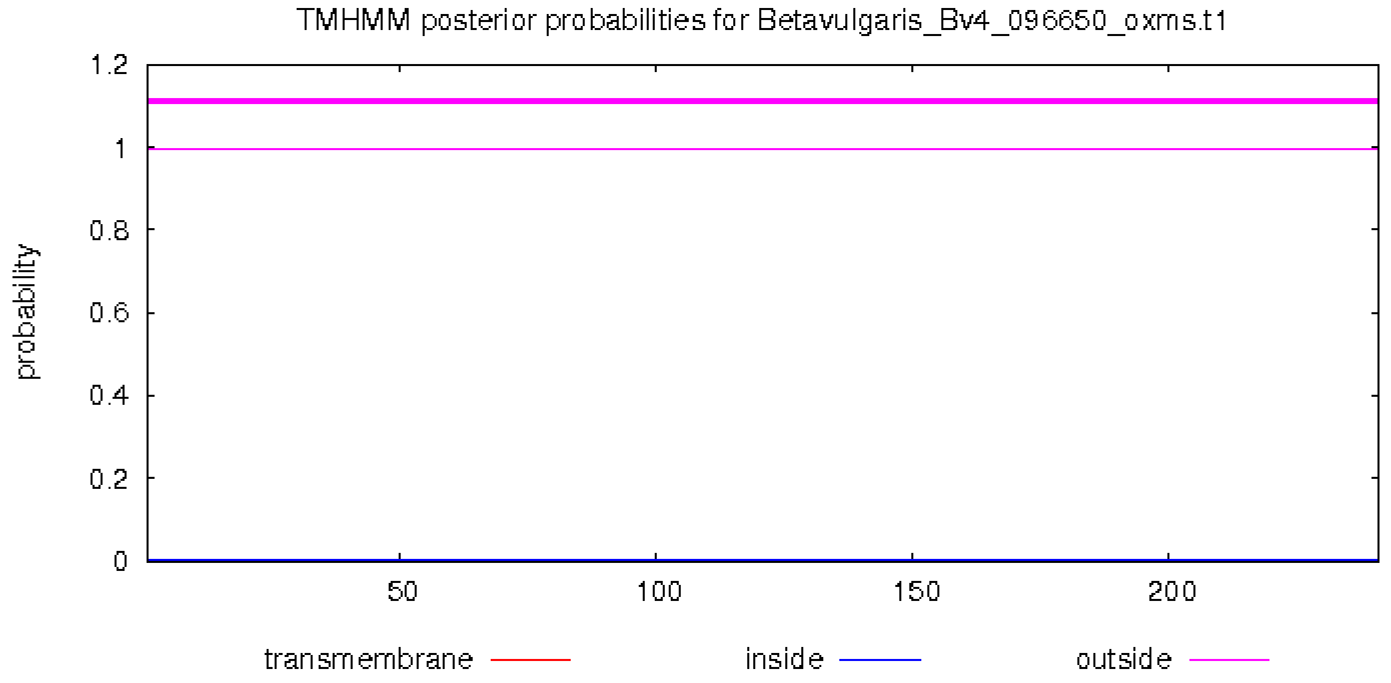

plot in postscript, script for making the plot in gnuplot, data for plot

---

```
# Betavulgaris_Bv5_097990_fpfn.t1 Length: 365
# Betavulgaris_Bv5_097990_fpfn.t1 Number of predicted TMHs:  0
# Betavulgaris_Bv5_097990_fpfn.t1 Exp number of AAs in TMHs: 0
# Betavulgaris_Bv5_097990_fpfn.t1 Exp number, first 60 AAs:  0
```

```
# Betavulgaris_Bv5_097990_fpfn.t1 Total prob of N-in:        0.00378
Betavulgaris_Bv5_097990_fpfn.t1 TMHMM2.0        outside      1   365
```

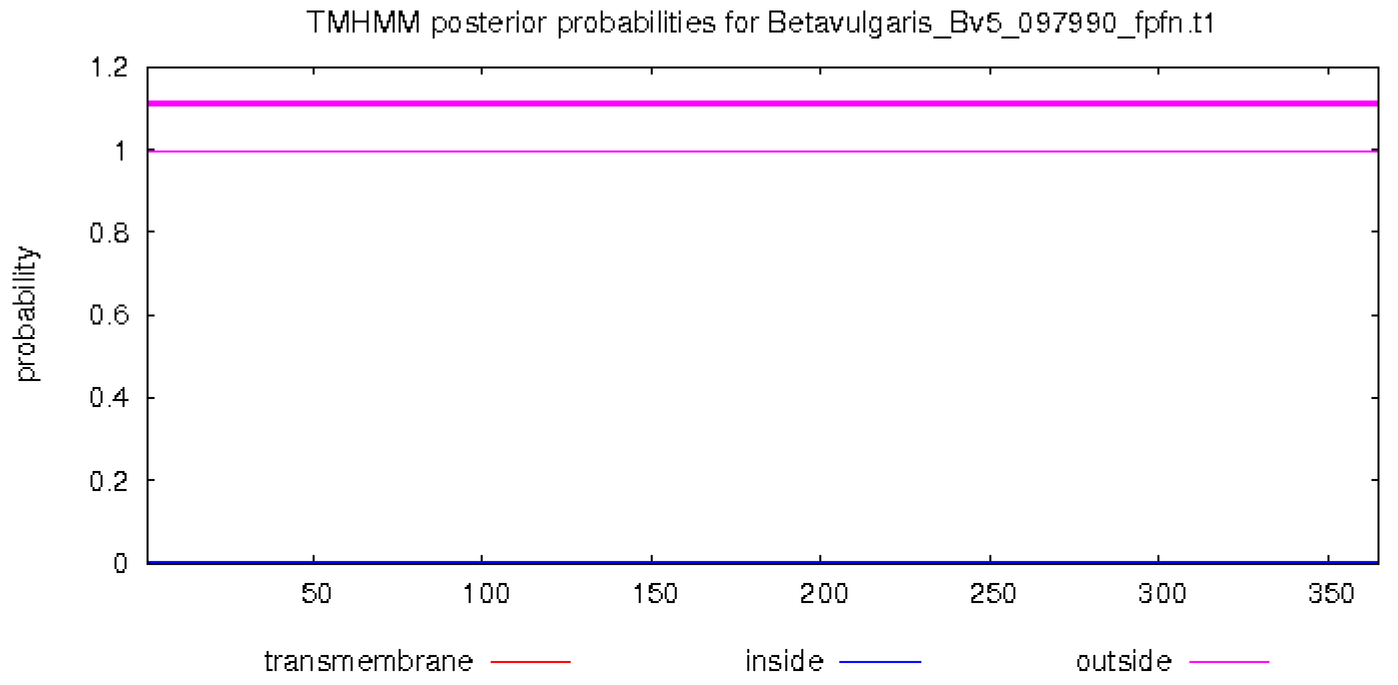

plot in postscript, script for making the plot in gnuplot, data for plot

---

```
# Betavulgaris_Bv5_104170_xrgj.t1 Length: 355
# Betavulgaris_Bv5_104170_xrgj.t1 Number of predicted TMHs:  0
# Betavulgaris_Bv5_104170_xrgj.t1 Exp number of AAs in TMHs: 0
# Betavulgaris_Bv5_104170_xrgj.t1 Exp number, first 60 AAs:  0
# Betavulgaris_Bv5_104170_xrgj.t1 Total prob of N-in:        0.01524
Betavulgaris_Bv5_104170_xrgj.t1 TMHMM2.0        outside      1   355
```

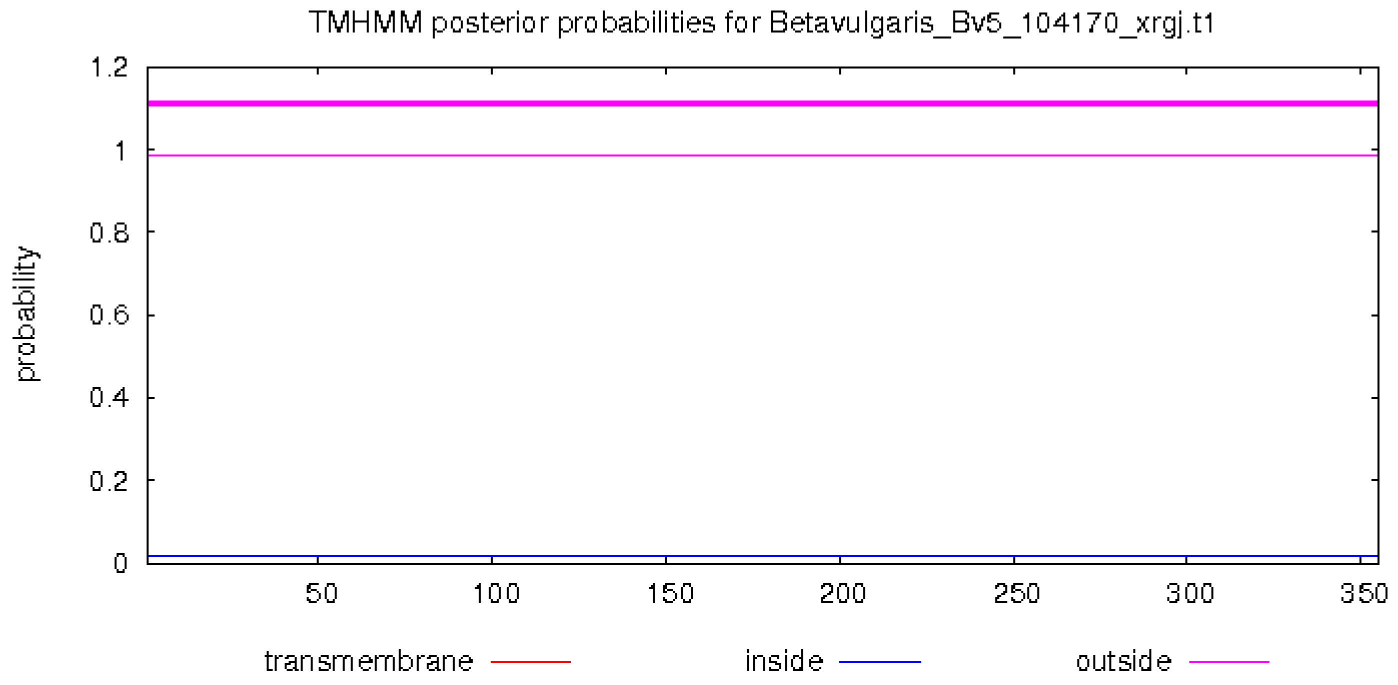

plot in postscript, script for making the plot in gnuplot, data for plot

---

```
# Betavulgaris_Bv5_106480_gzwx.t1 Length: 329
# Betavulgaris_Bv5_106480_gzwx.t1 Number of predicted TMHs:  0
# Betavulgaris_Bv5_106480_gzwx.t1 Exp number of AAs in TMHs: 0.00042
# Betavulgaris_Bv5_106480_gzwx.t1 Exp number, first 60 AAs:  0
```

```
# Betavulgaris_Bv5_106480_gzwx.t1 Total prob of N-in:        0.00228
Betavulgaris_Bv5_106480_gzwx.t1 TMHMM2.0        outside      1   329
```

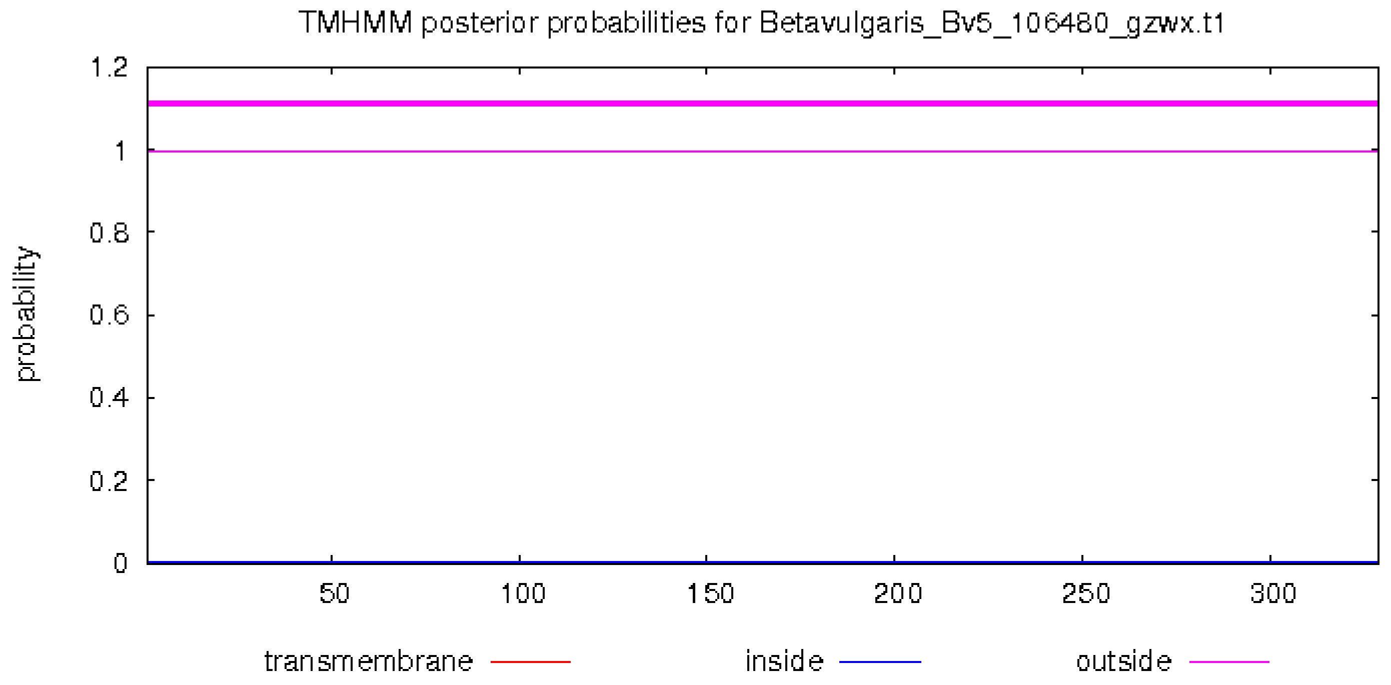

plot in postscript, script for making the plot in gnuplot, data for plot

---

```
# Betavulgaris_Bv5_114130_gzxk.t1 Length: 338
# Betavulgaris_Bv5_114130_gzxk.t1 Number of predicted TMHs:  0
# Betavulgaris_Bv5_114130_gzxk.t1 Exp number of AAs in TMHs: 0.0015
# Betavulgaris_Bv5_114130_gzxk.t1 Exp number, first 60 AAs:  0
# Betavulgaris_Bv5_114130_gzxk.t1 Total prob of N-in:        0.00304
Betavulgaris_Bv5_114130_gzxk.t1 TMHMM2.0        outside      1   338
```

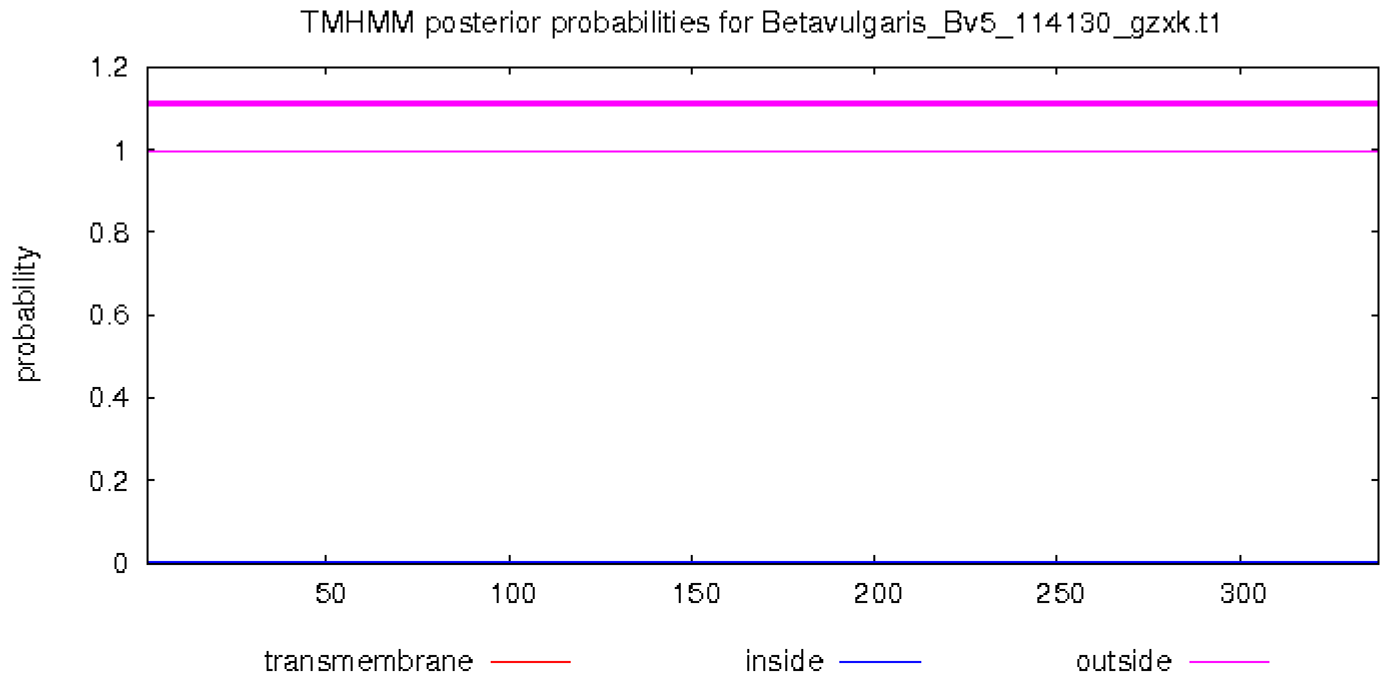

plot in postscript, script for making the plot in gnuplot, data for plot

---

```
# Betavulgaris_Bv5_114390_pjnp.t1 Length: 361
# Betavulgaris_Bv5_114390_pjnp.t1 Number of predicted TMHs:  0
# Betavulgaris_Bv5_114390_pjnp.t1 Exp number of AAs in TMHs: 0.00018
# Betavulgaris_Bv5_114390_pjnp.t1 Exp number, first 60 AAs:  0
```

```
# Betavulgaris_Bv5_114390_pjnp.t1 Total prob of N-in:        0.01313
Betavulgaris_Bv5_114390_pjnp.t1 TMHMM2.0        outside      1   361
```

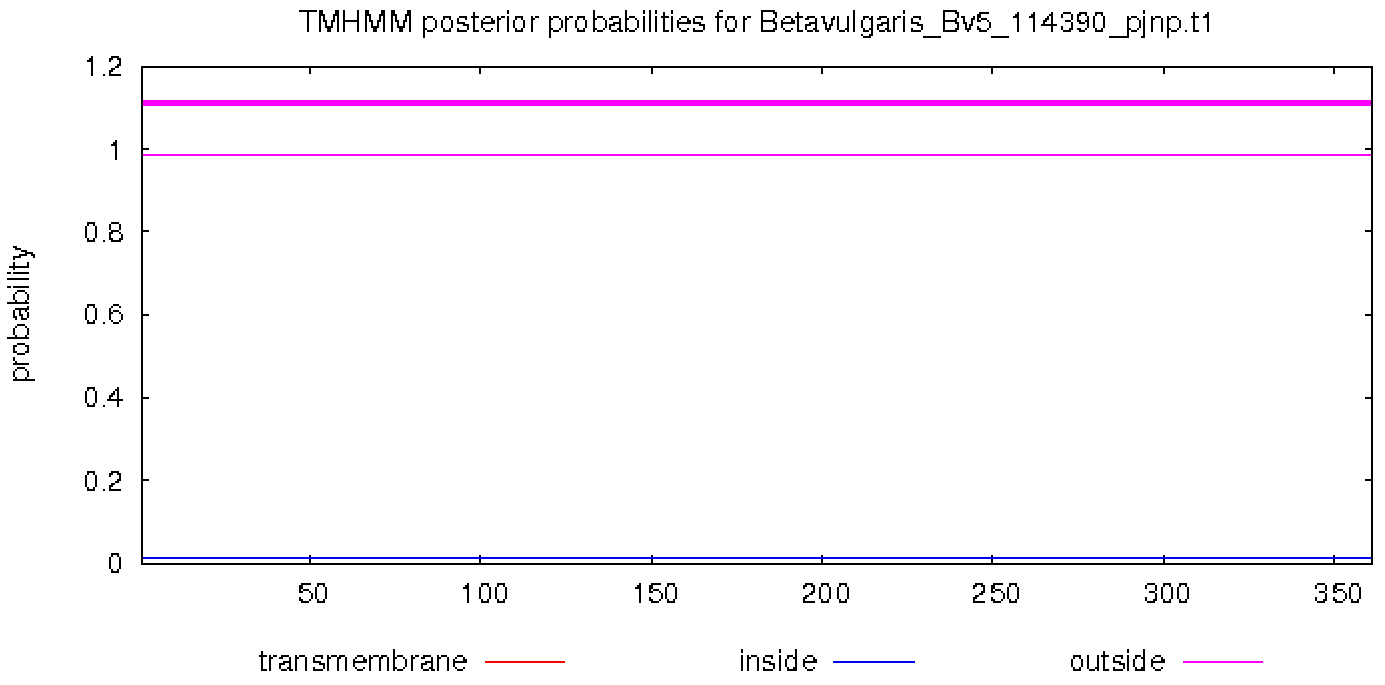

plot in postscript, script for making the plot in gnuplot, data for plot

```
# Betavulgaris_Bv5_121330_zrdc.t1 Length: 304
# Betavulgaris_Bv5_121330_zrdc.t1 Number of predicted TMHs:  0
# Betavulgaris_Bv5_121330_zrdc.t1 Exp number of AAs in TMHs: 0
# Betavulgaris_Bv5_121330_zrdc.t1 Exp number, first 60 AAs:  0
# Betavulgaris_Bv5_121330_zrdc.t1 Total prob of N-in:        0.00734
Betavulgaris_Bv5_121330_zrdc.t1 TMHMM2.0        outside      1   304
```

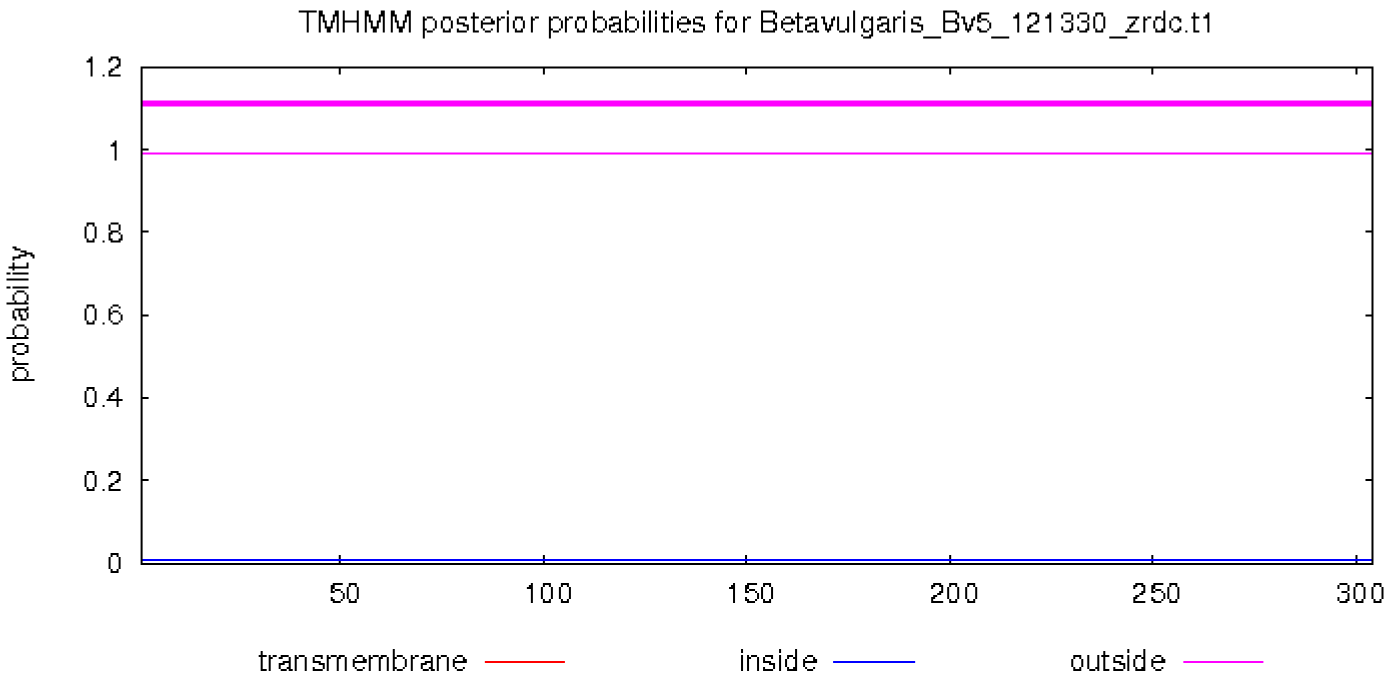

plot in postscript, script for making the plot in gnuplot, data for plot

```
# Betavulgaris_Bv6_129910_cmip.t1 Length: 401
# Betavulgaris_Bv6_129910_cmip.t1 Number of predicted TMHs:  0
# Betavulgaris_Bv6_129910_cmip.t1 Exp number of AAs in TMHs: 0.0002
# Betavulgaris_Bv6_129910_cmip.t1 Exp number, first 60 AAs:  0
```

```
# Betavulgaris_Bv6_129910_cmip.t1 Total prob of N-in:        0.00326
Betavulgaris_Bv6_129910_cmip.t1 TMHMM2.0        outside      1   401
```

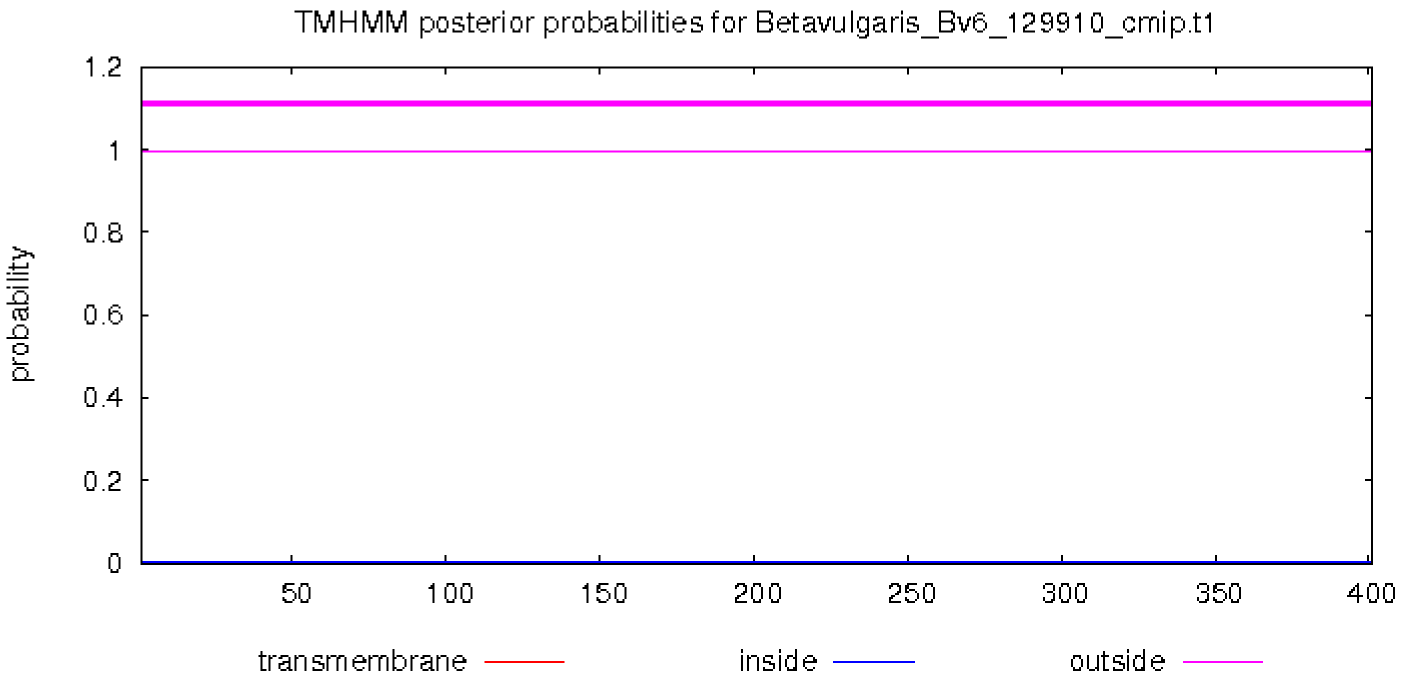

plot in postscript, script for making the plot in gnuplot, data for plot

---

```
# Betavulgaris_Bv6_132760_wkcm.t2 Length: 222
# Betavulgaris_Bv6_132760_wkcm.t2 Number of predicted TMHs:  0
# Betavulgaris_Bv6_132760_wkcm.t2 Exp number of AAs in TMHs: 0.00022
# Betavulgaris_Bv6_132760_wkcm.t2 Exp number, first 60 AAs:  1e-05
# Betavulgaris_Bv6_132760_wkcm.t2 Total prob of N-in:        0.17369
Betavulgaris_Bv6_132760_wkcm.t2 TMHMM2.0        outside      1   222
```

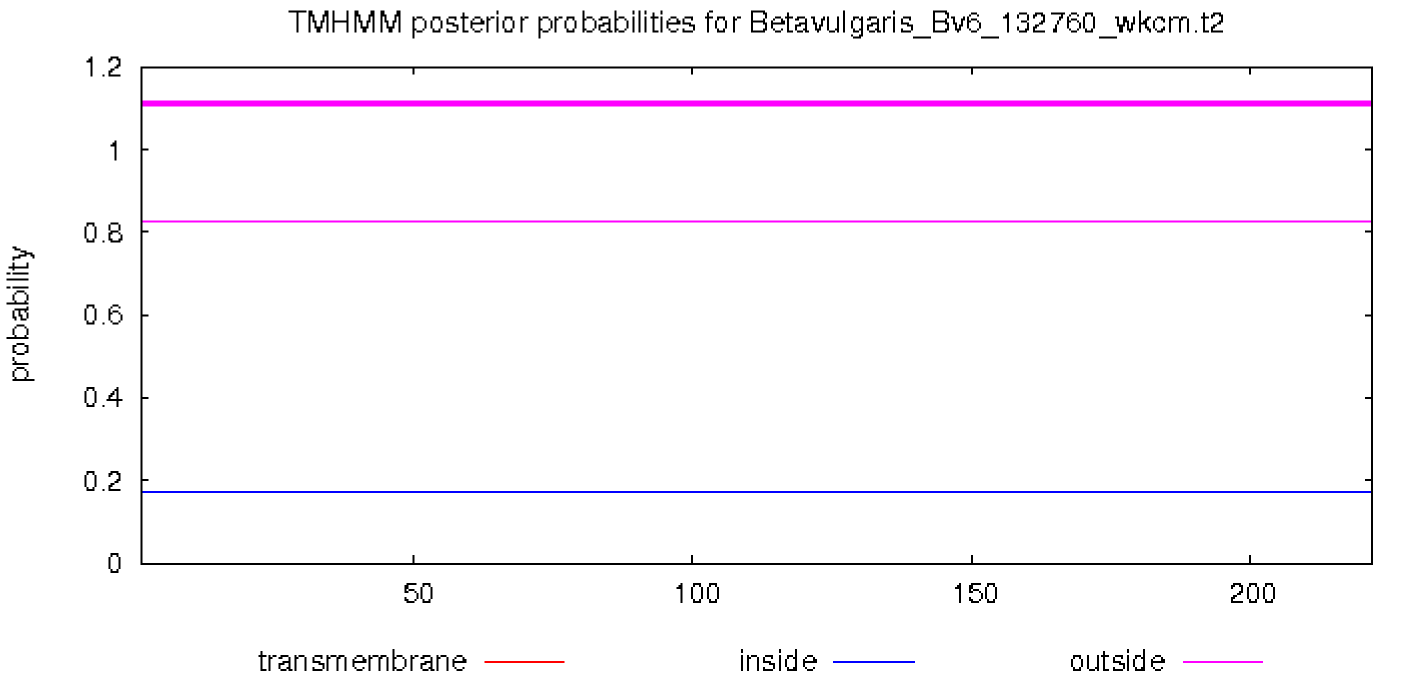

plot in postscript, script for making the plot in gnuplot, data for plot

---

```
# Betavulgaris_Bv6_132770_wshi.t1 Length: 208
# Betavulgaris_Bv6_132770_wshi.t1 Number of predicted TMHs:  0
# Betavulgaris_Bv6_132770_wshi.t1 Exp number of AAs in TMHs: 0
# Betavulgaris_Bv6_132770_wshi.t1 Exp number, first 60 AAs:  0
```

```
# Betavulgaris_Bv6_132770_wshi.t1 Total prob of N-in:        0.03152
Betavulgaris_Bv6_132770_wshi.t1 TMHMM2.0        outside      1   208
```

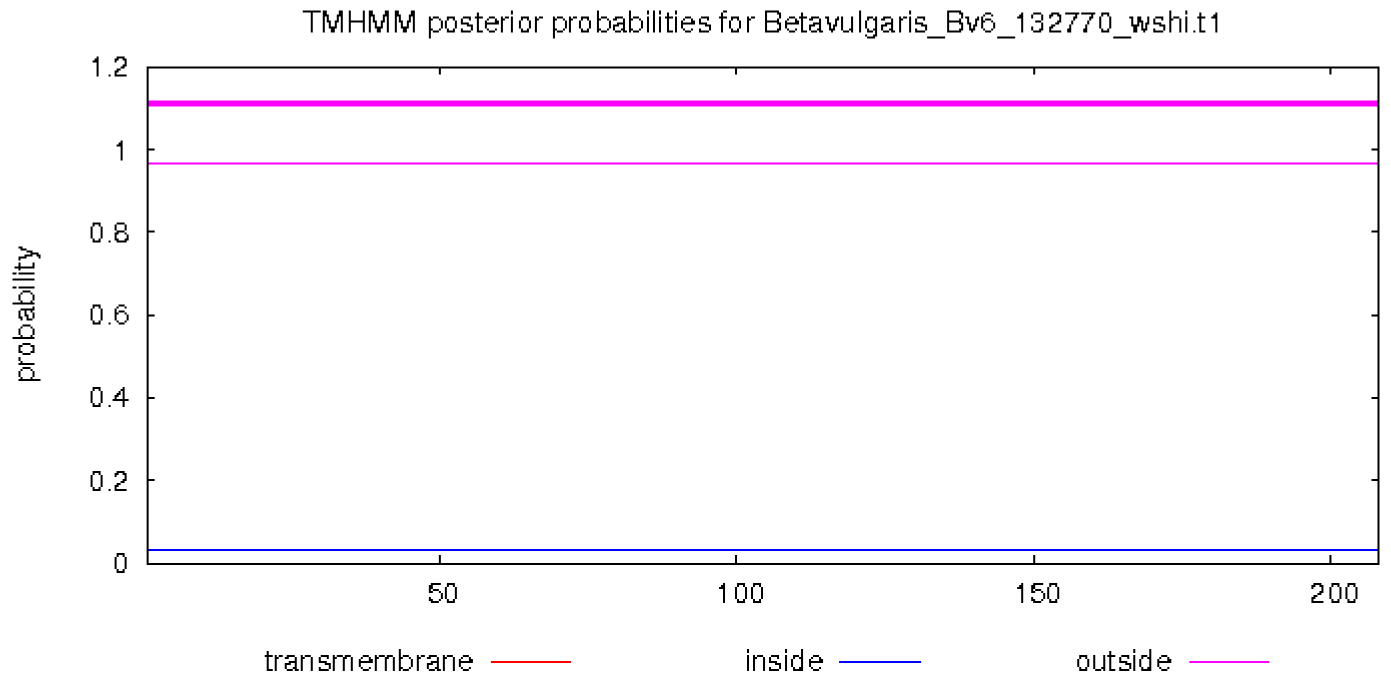

plot in postscript, script for making the plot in gnuplot, data for plot

---

```
# Betavulgaris_Bv6_136600_detz.t1 Length: 282
# Betavulgaris_Bv6_136600_detz.t1 Number of predicted TMHs:  0
# Betavulgaris_Bv6_136600_detz.t1 Exp number of AAs in TMHs: 0.05864
# Betavulgaris_Bv6_136600_detz.t1 Exp number, first 60 AAs:  0.05503
# Betavulgaris_Bv6_136600_detz.t1 Total prob of N-in:        0.04242
Betavulgaris_Bv6_136600_detz.t1 TMHMM2.0        outside      1   282
```

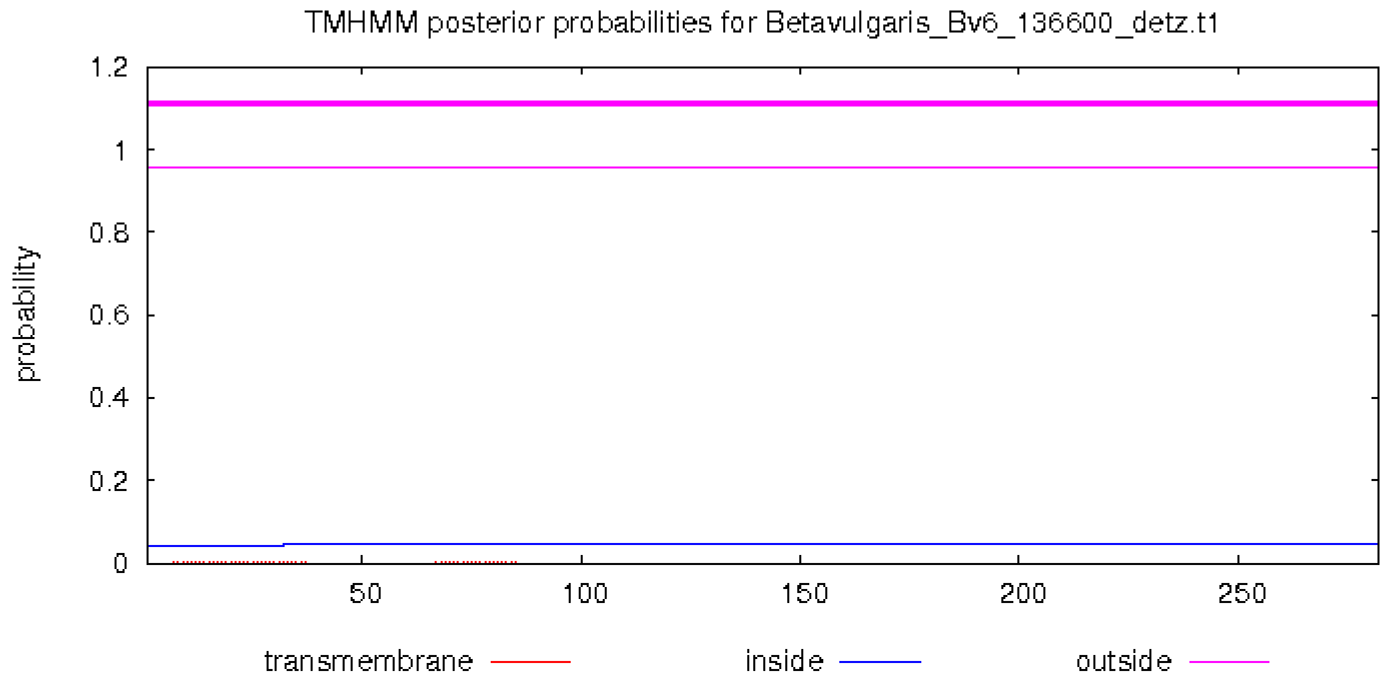

plot in postscript, script for making the plot in gnuplot, data for plot

---

```
# Betavulgaris_Bv6_136620_pzjj.t1 Length: 234
# Betavulgaris_Bv6_136620_pzjj.t1 Number of predicted TMHs:  0
# Betavulgaris_Bv6_136620_pzjj.t1 Exp number of AAs in TMHs: 0.00145
# Betavulgaris_Bv6_136620_pzjj.t1 Exp number, first 60 AAs:  0.00145
```

```
# Betavulgaris_Bv6_136620_pzjj.t1 Total prob of N-in:        0.08750
Betavulgaris_Bv6_136620_pzjj.t1 TMHMM2.0        outside      1   234
```

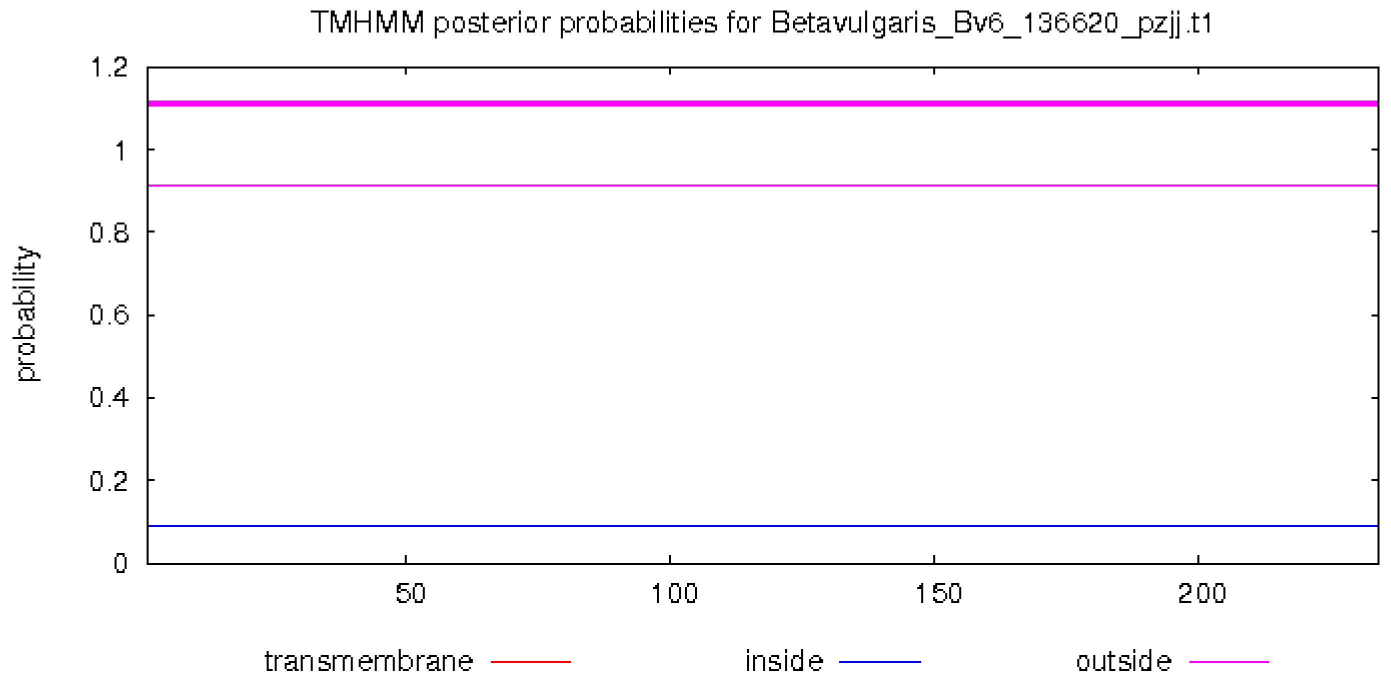

plot in postscript, script for making the plot in gnuplot, data for plot

---

```
# Betavulgaris_Bv6_145550_jkxz.t1 Length: 518
# Betavulgaris_Bv6_145550_jkxz.t1 Number of predicted TMHs:  0
# Betavulgaris_Bv6_145550_jkxz.t1 Exp number of AAs in TMHs: 0.00832
# Betavulgaris_Bv6_145550_jkxz.t1 Exp number, first 60 AAs:  0
# Betavulgaris_Bv6_145550_jkxz.t1 Total prob of N-in:        0.00388
Betavulgaris_Bv6_145550_jkxz.t1 TMHMM2.0        outside      1   518
```

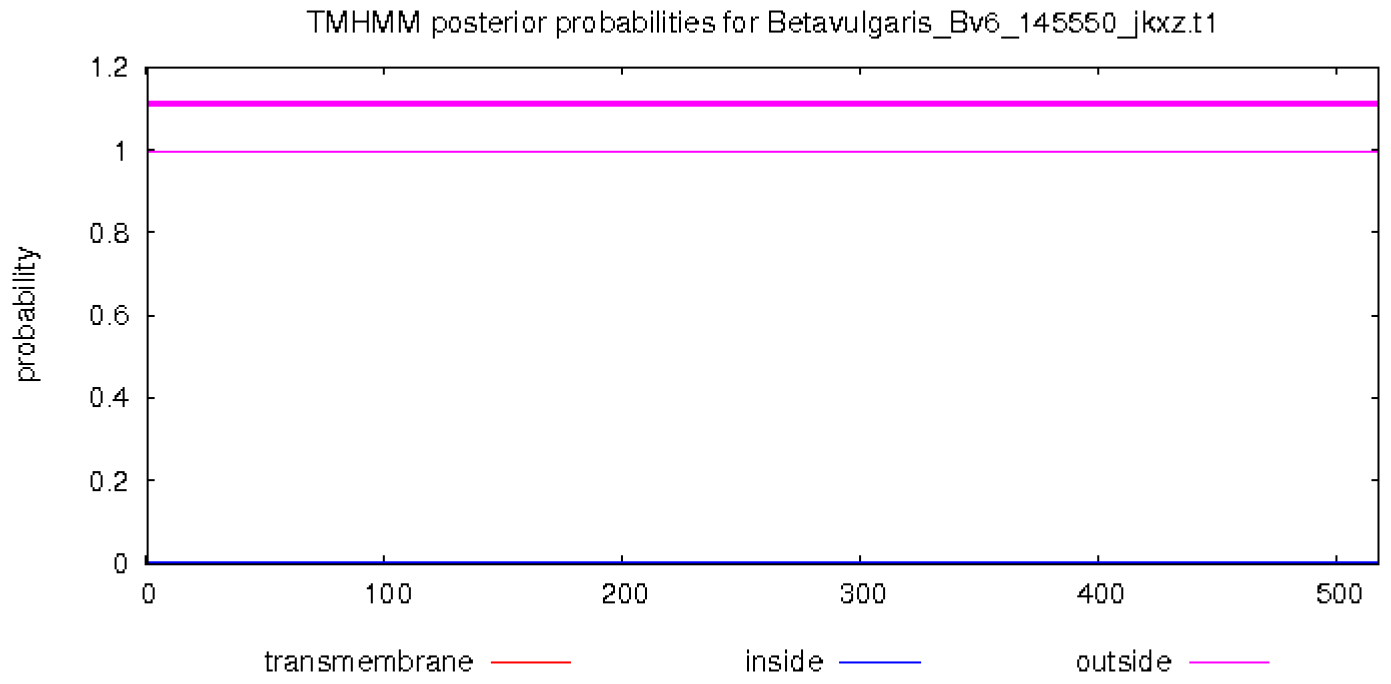

plot in postscript, script for making the plot in gnuplot, data for plot

---

```
# Betavulgaris_Bv6_150040_hdfh.t1 Length: 303
# Betavulgaris_Bv6_150040_hdfh.t1 Number of predicted TMHs:  0
# Betavulgaris_Bv6_150040_hdfh.t1 Exp number of AAs in TMHs: 0.00033
# Betavulgaris_Bv6_150040_hdfh.t1 Exp number, first 60 AAs:  0
```

```
# Betavulgaris_Bv6_150040_hdfh.t1 Total prob of N-in:        0.05164
Betavulgaris_Bv6_150040_hdfh.t1 TMHMM2.0        outside      1   303
```

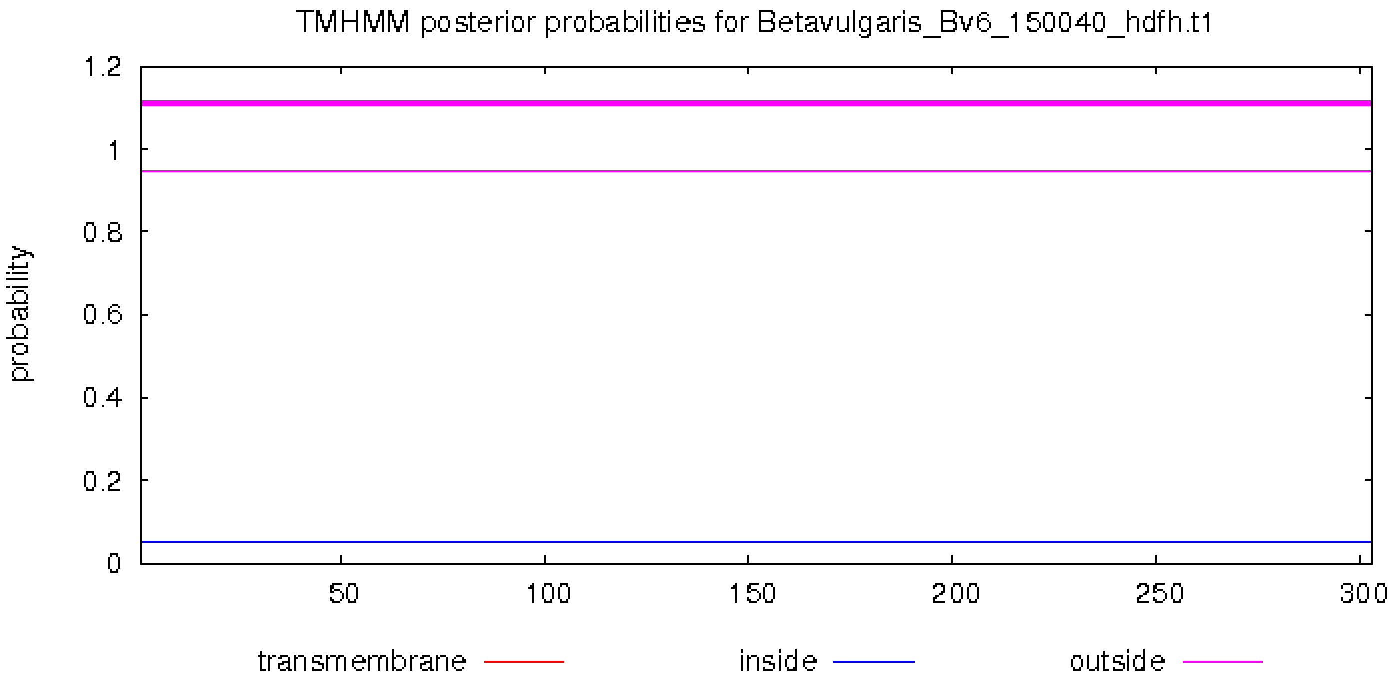

plot in postscript, script for making the plot in gnuplot, data for plot

---

```
# Betavulgaris_Bv6_153540_qoyx.t1 Length: 274
# Betavulgaris_Bv6_153540_qoyx.t1 Number of predicted TMHs:  0
# Betavulgaris_Bv6_153540_qoyx.t1 Exp number of AAs in TMHs: 0.02036
# Betavulgaris_Bv6_153540_qoyx.t1 Exp number, first 60 AAs:  0
# Betavulgaris_Bv6_153540_qoyx.t1 Total prob of N-in:        0.21332
Betavulgaris_Bv6_153540_qoyx.t1 TMHMM2.0        outside      1   274
```

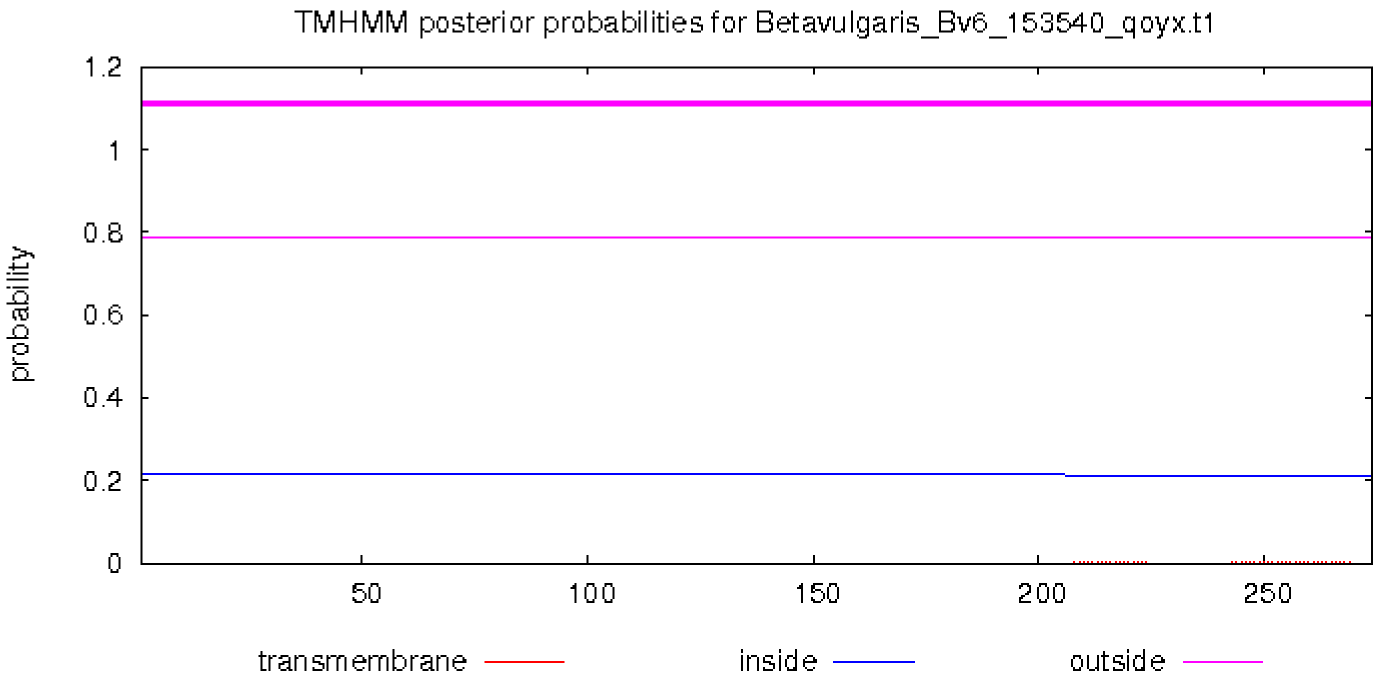

plot in postscript, script for making the plot in gnuplot, data for plot

---

```
# Betavulgaris_Bv6_155570_rpmu.t1 Length: 429
# Betavulgaris_Bv6_155570_rpmu.t1 Number of predicted TMHs:  0
# Betavulgaris_Bv6_155570_rpmu.t1 Exp number of AAs in TMHs: 0.00019
# Betavulgaris_Bv6_155570_rpmu.t1 Exp number, first 60 AAs:  0
```

```
# Betavulgaris_Bv6_155570_rpmu.t1 Total prob of N-in:        0.00312
Betavulgaris_Bv6_155570_rpmu.t1 TMHMM2.0        outside      1   429
```

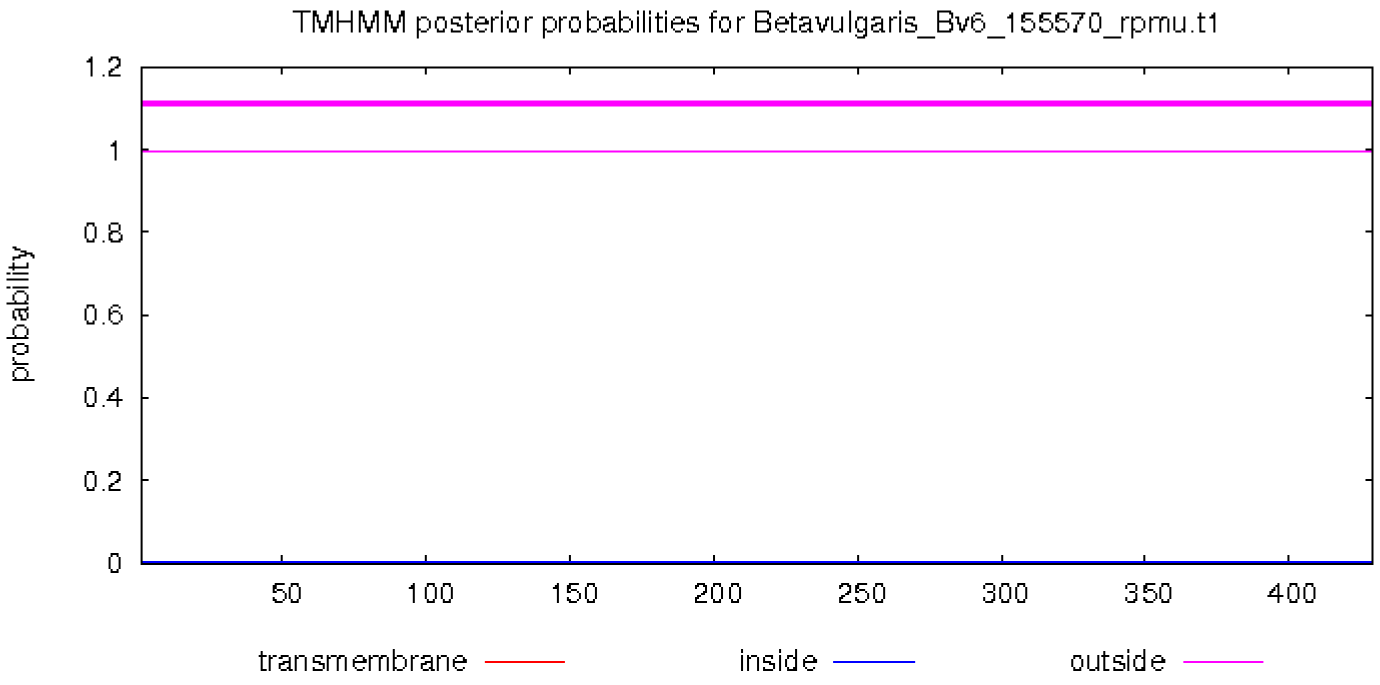

plot in postscript, script for making the plot in gnuplot, data for plot

---

```
# Betavulgaris_Bv7_159010_tzwj.t1 Length: 282
# Betavulgaris_Bv7_159010_tzwj.t1 Number of predicted TMHs:  0
# Betavulgaris_Bv7_159010_tzwj.t1 Exp number of AAs in TMHs: 0.00054
# Betavulgaris_Bv7_159010_tzwj.t1 Exp number, first 60 AAs:  0
# Betavulgaris_Bv7_159010_tzwj.t1 Total prob of N-in:        0.01131
Betavulgaris_Bv7_159010_tzwj.t1 TMHMM2.0        outside      1   282
```

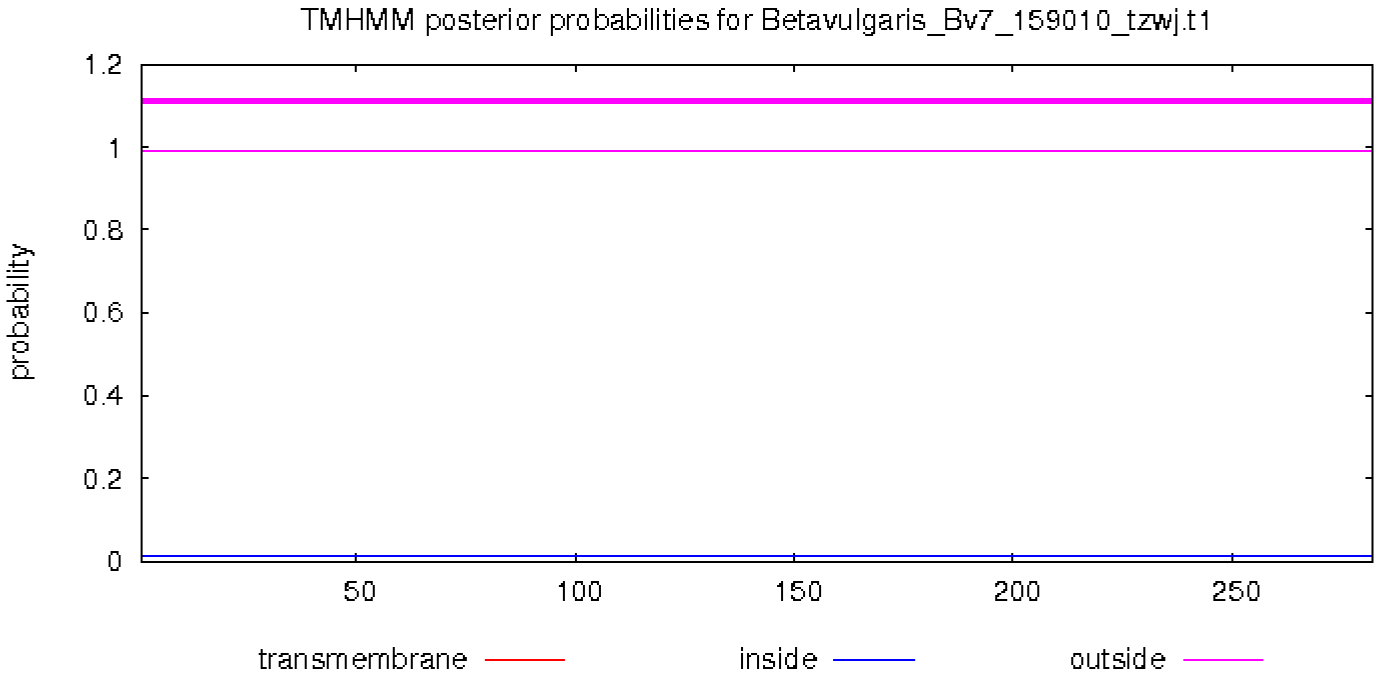

plot in postscript, script for making the plot in gnuplot, data for plot

---

```
# Betavulgaris_Bv7_179460_yqtj.t1 Length: 254
# Betavulgaris_Bv7_179460_yqtj.t1 Number of predicted TMHs:  0
# Betavulgaris_Bv7_179460_yqtj.t1 Exp number of AAs in TMHs: 0.00014
# Betavulgaris_Bv7_179460_yqtj.t1 Exp number, first 60 AAs:  0
```

```
# Betavulgaris_Bv7_179460_yqtj.t1 Total prob of N-in:        0.07930
Betavulgaris_Bv7_179460_yqtj.t1 TMHMM2.0        outside      1   254
```

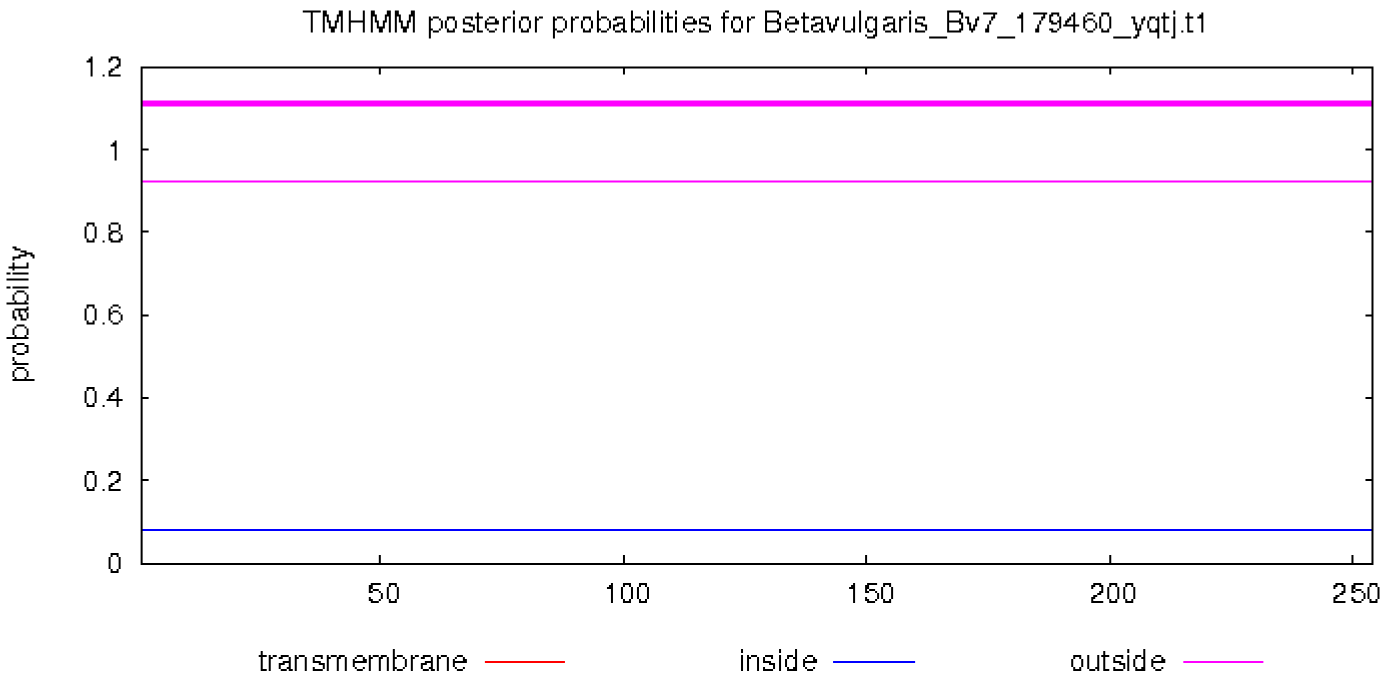

plot in postscript, script for making the plot in gnuplot, data for plot

---

```
# Betavulgaris_Bv8_182450_hisu.t1 Length: 315
# Betavulgaris_Bv8_182450_hisu.t1 Number of predicted TMHs:  0
# Betavulgaris_Bv8_182450_hisu.t1 Exp number of AAs in TMHs: 0.00759
# Betavulgaris_Bv8_182450_hisu.t1 Exp number, first 60 AAs:  0
# Betavulgaris_Bv8_182450_hisu.t1 Total prob of N-in:        0.05090
Betavulgaris_Bv8_182450_hisu.t1 TMHMM2.0        outside      1   315
```

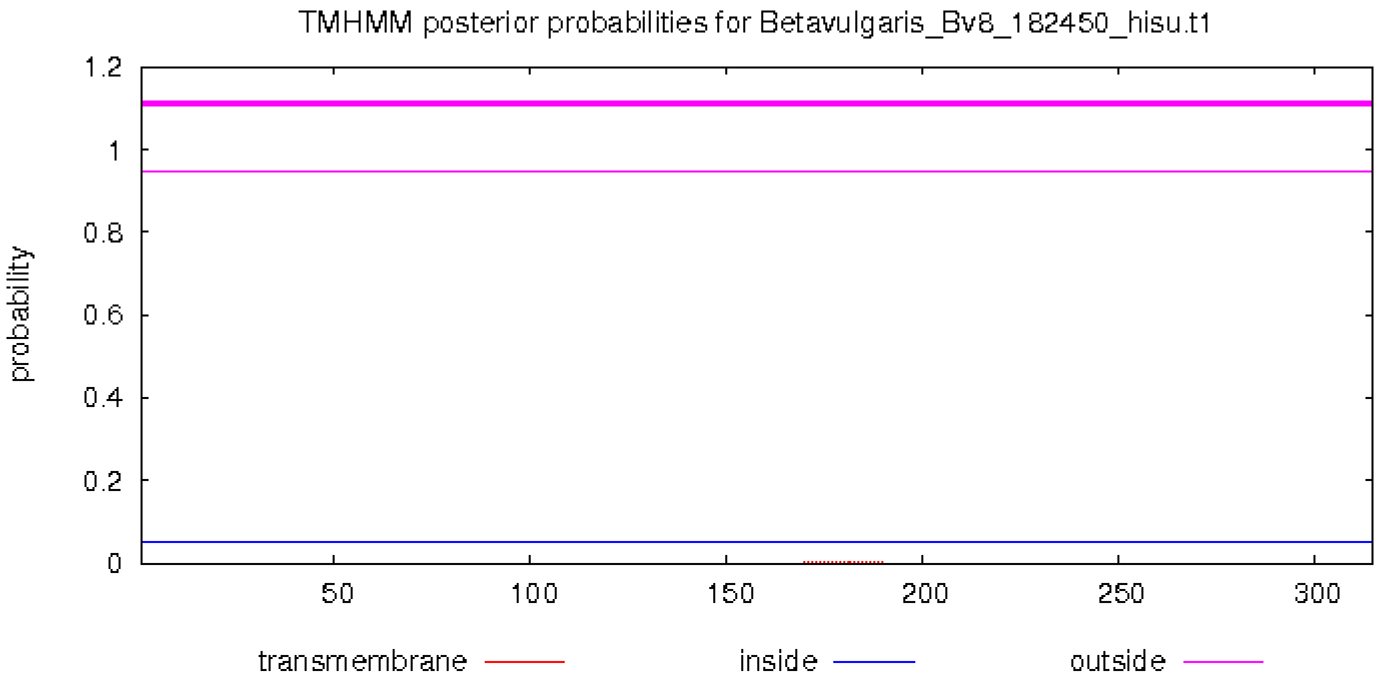

plot in postscript, script for making the plot in gnuplot, data for plot

---

```
# Betavulgaris_Bv8_184020_pfso.t1 Length: 341
# Betavulgaris_Bv8_184020_pfso.t1 Number of predicted TMHs:  0
# Betavulgaris_Bv8_184020_pfso.t1 Exp number of AAs in TMHs: 0.00745
# Betavulgaris_Bv8_184020_pfso.t1 Exp number, first 60 AAs:  0
```

```
# Betavulgaris_Bv8_184020_pfso.t1 Total prob of N-in:        0.02653
Betavulgaris_Bv8_184020_pfso.t1 TMHMM2.0        outside      1   341
```

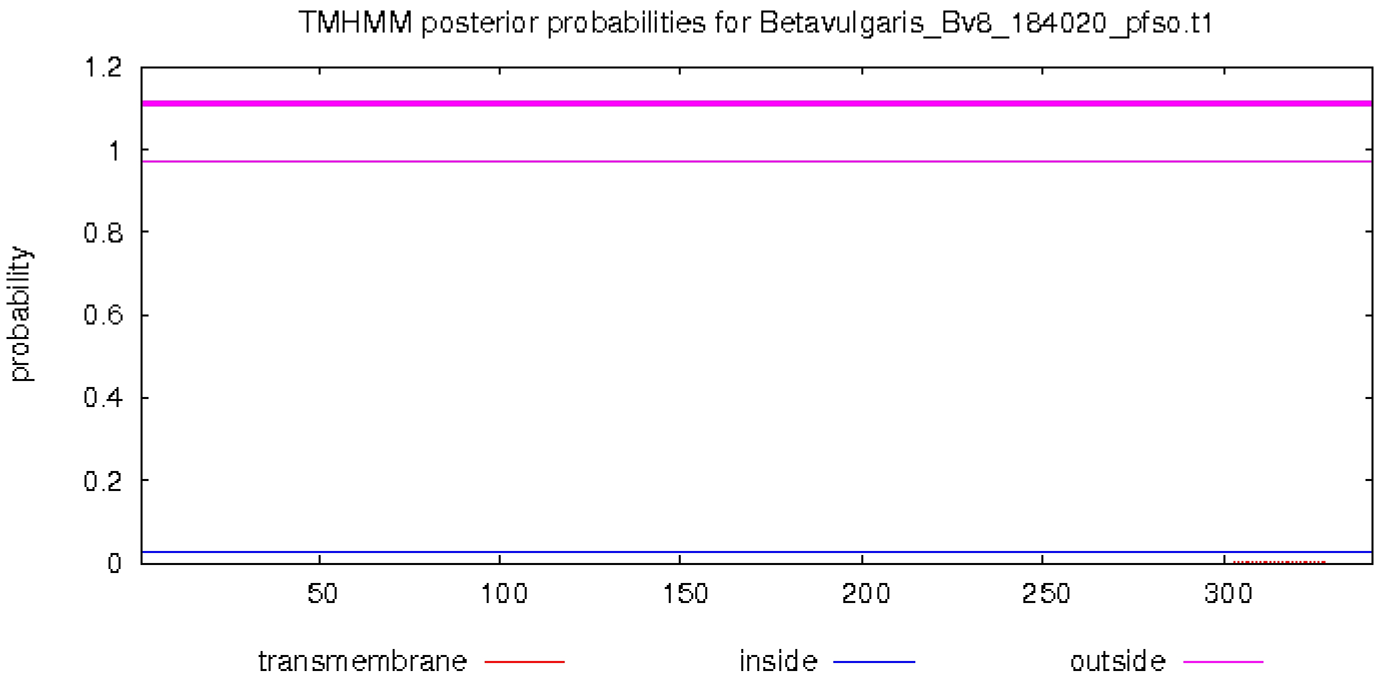

plot in postscript, script for making the plot in gnuplot, data for plot

---

```
# Betavulgaris_Bv8_190180_ktgn.t1 Length: 191
# Betavulgaris_Bv8_190180_ktgn.t1 Number of predicted TMHs:  0
# Betavulgaris_Bv8_190180_ktgn.t1 Exp number of AAs in TMHs: 0.02218
# Betavulgaris_Bv8_190180_ktgn.t1 Exp number, first 60 AAs:  0
# Betavulgaris_Bv8_190180_ktgn.t1 Total prob of N-in:        0.01131
Betavulgaris_Bv8_190180_ktgn.t1 TMHMM2.0        outside      1   191
```

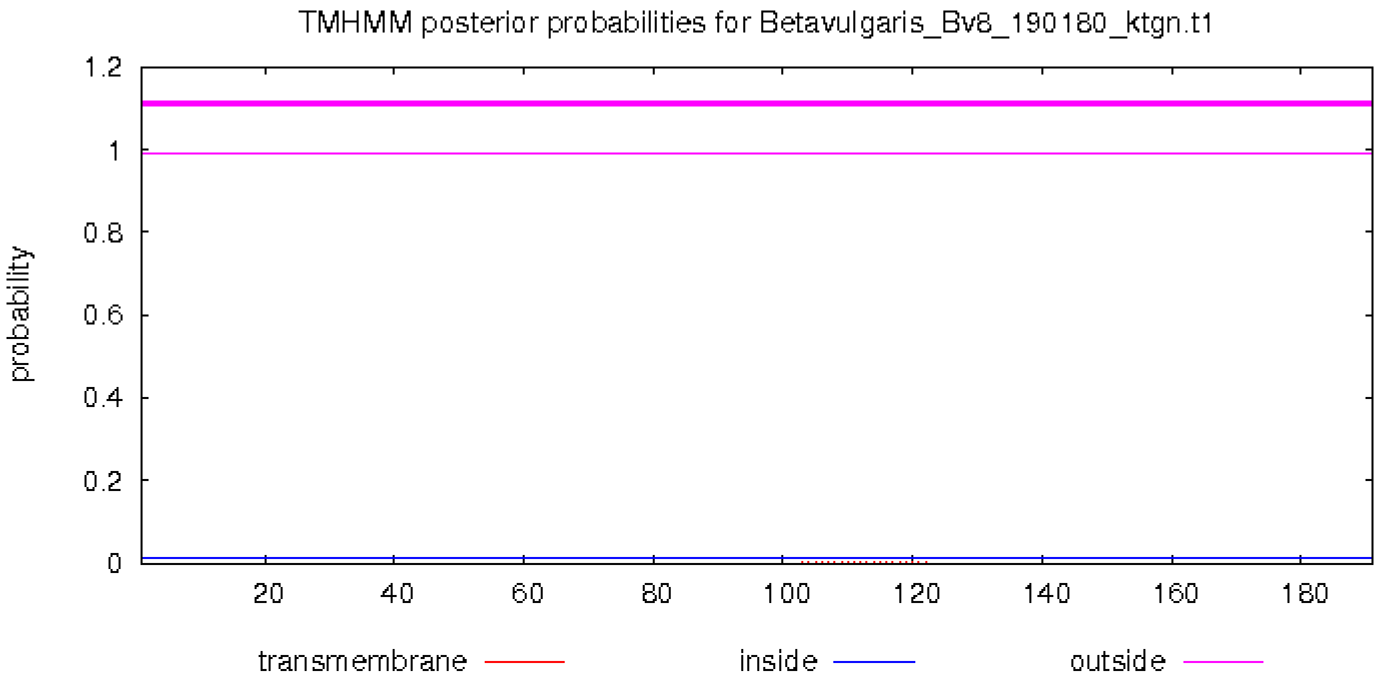

plot in postscript, script for making the plot in gnuplot, data for plot

---

```
# Betavulgaris_Bv8_197850_tdfd.t1 Length: 519
# Betavulgaris_Bv8_197850_tdfd.t1 Number of predicted TMHs:  0
# Betavulgaris_Bv8_197850_tdfd.t1 Exp number of AAs in TMHs: 0.01463
# Betavulgaris_Bv8_197850_tdfd.t1 Exp number, first 60 AAs:  0
```

```
# Betavulgaris_Bv8_197850_tdfd.t1 Total prob of N-in:        0.00881
Betavulgaris_Bv8_197850_tdfd.t1 TMHMM2.0        outside      1   519
```

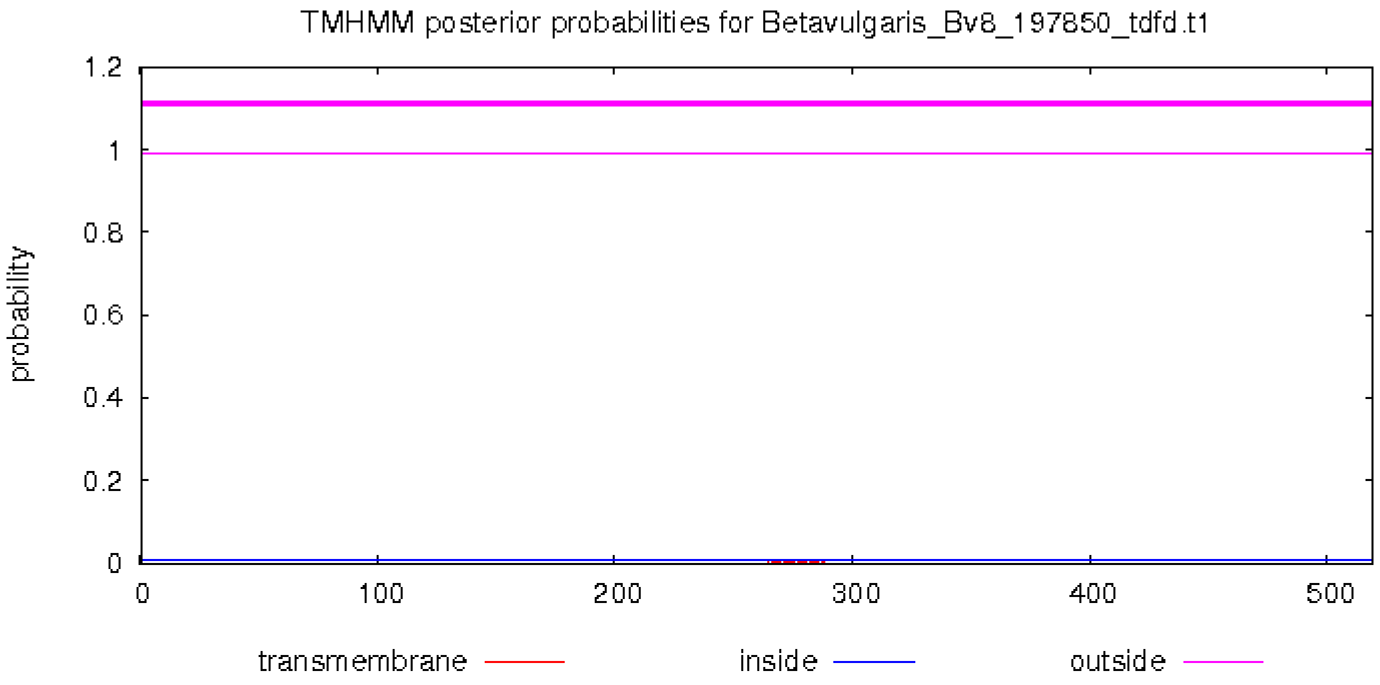

plot in postscript, script for making the plot in gnuplot, data for plot

---

```
# Betavulgaris_Bv8_199060_znkf.t1 Length: 518
# Betavulgaris_Bv8_199060_znkf.t1 Number of predicted TMHs:  1
# Betavulgaris_Bv8_199060_znkf.t1 Exp number of AAs in TMHs: 22.71723
# Betavulgaris_Bv8_199060_znkf.t1 Exp number, first 60 AAs:  0
# Betavulgaris_Bv8_199060_znkf.t1 Total prob of N-in:        0.01086
Betavulgaris_Bv8_199060_znkf.t1 TMHMM2.0        outside      1   486
Betavulgaris_Bv8_199060_znkf.t1 TMHMM2.0        TMhelix    487   509
Betavulgaris_Bv8_199060_znkf.t1 TMHMM2.0        inside     510   518
```

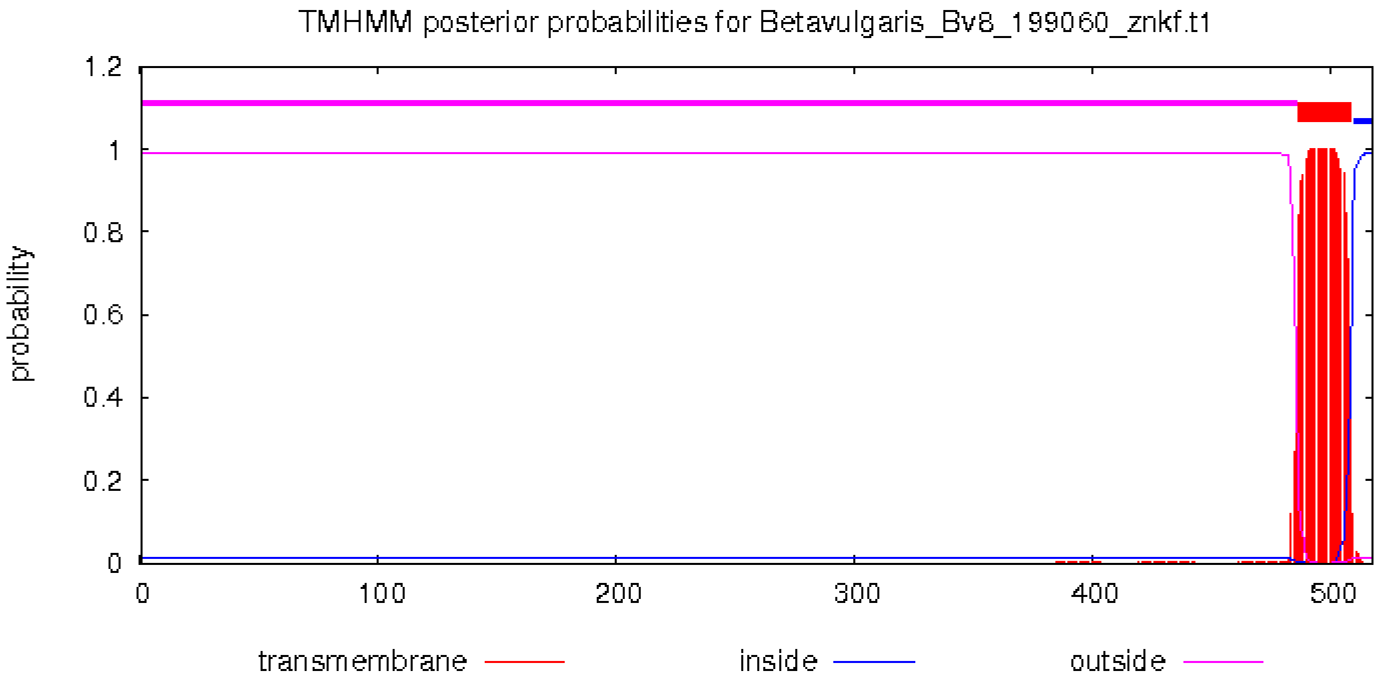

plot in postscript, script for making the plot in gnuplot, data for plot

---

```
# Betavulgaris_Bv9_203240_qoku.t1 Length: 346
# Betavulgaris_Bv9_203240_qoku.t1 Number of predicted TMHs:  0
```

```
# Betavulgaris_Bv9_203240_qoku.t1 Exp number of AAs in TMHs: 0
# Betavulgaris_Bv9_203240_qoku.t1 Exp number, first 60 AAs:  0
# Betavulgaris_Bv9_203240_qoku.t1 Total prob of N-in:        0.00421
Betavulgaris_Bv9_203240_qoku.t1 TMHMM2.0        outside      1   346
```

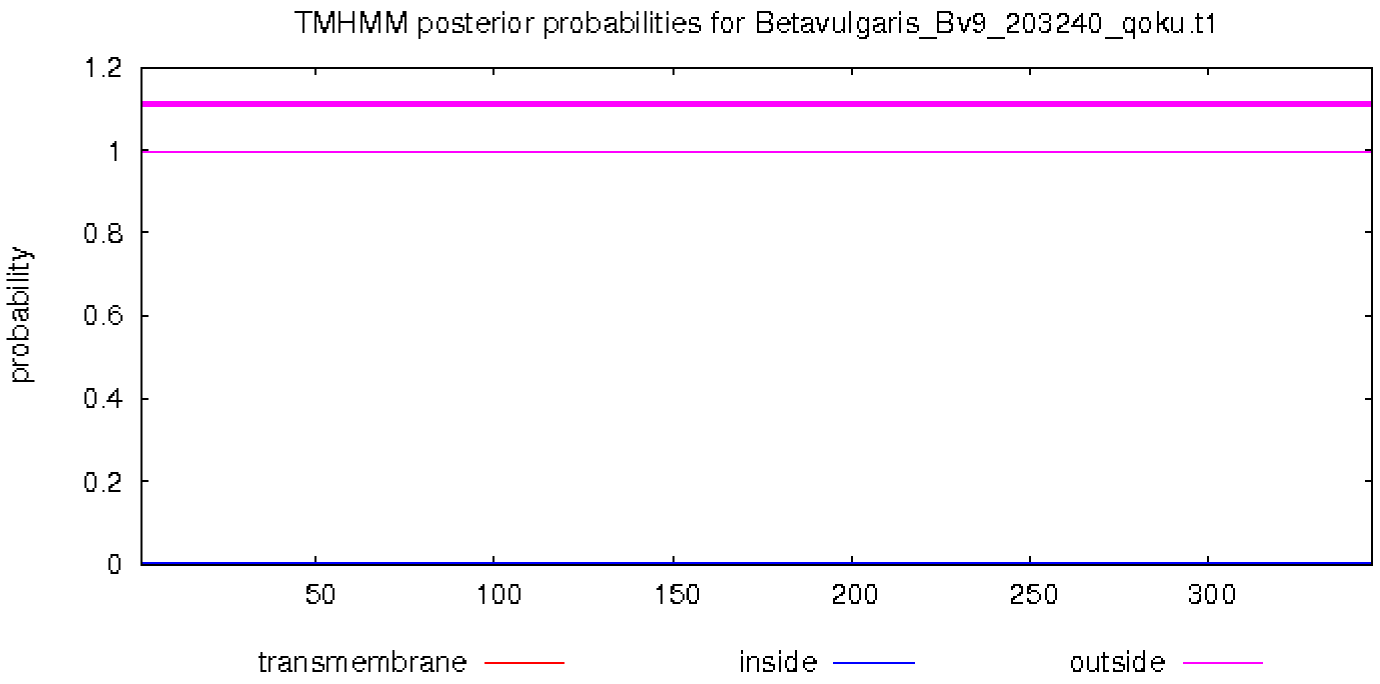

plot in postscript, script for making the plot in gnuplot, data for plot

---

```
# Betavulgaris_Bv9_209530_aktr.t1 Length: 272
# Betavulgaris_Bv9_209530_aktr.t1 Number of predicted TMHs:  0
# Betavulgaris_Bv9_209530_aktr.t1 Exp number of AAs in TMHs: 0
# Betavulgaris_Bv9_209530_aktr.t1 Exp number, first 60 AAs:  0
# Betavulgaris_Bv9_209530_aktr.t1 Total prob of N-in:        0.10013
Betavulgaris_Bv9_209530_aktr.t1 TMHMM2.0        outside      1   272
```

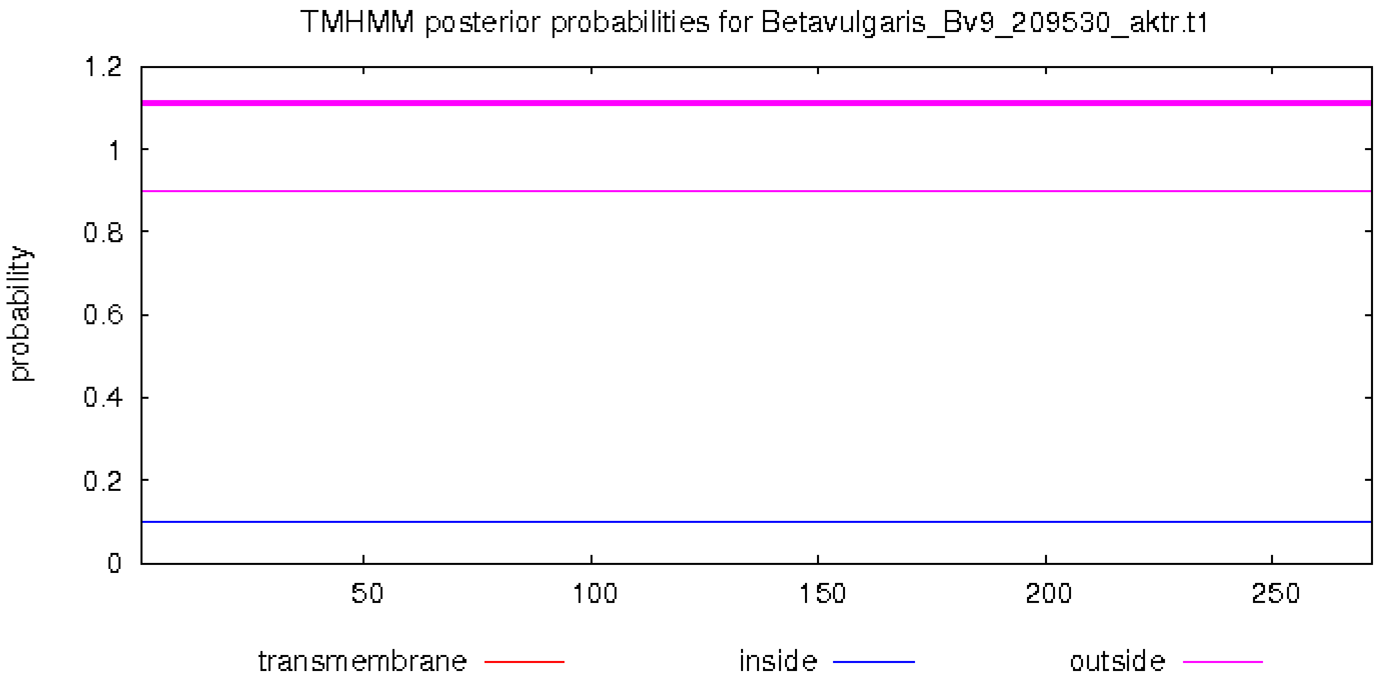

plot in postscript, script for making the plot in gnuplot, data for plot

---

```
# Betavulgaris_Bv9_212910_kjzy.t1 Length: 298
# Betavulgaris_Bv9_212910_kjzy.t1 Number of predicted TMHs:  0
```

```
# Betavulgaris_Bv9_212910_kjzy.t1 Exp number of AAs in TMHs: 0
# Betavulgaris_Bv9_212910_kjzy.t1 Exp number, first 60 AAs:  0
# Betavulgaris_Bv9_212910_kjzy.t1 Total prob of N-in:        0.00709
Betavulgaris_Bv9_212910_kjzy.t1 TMHMM2.0        outside      1   298
```

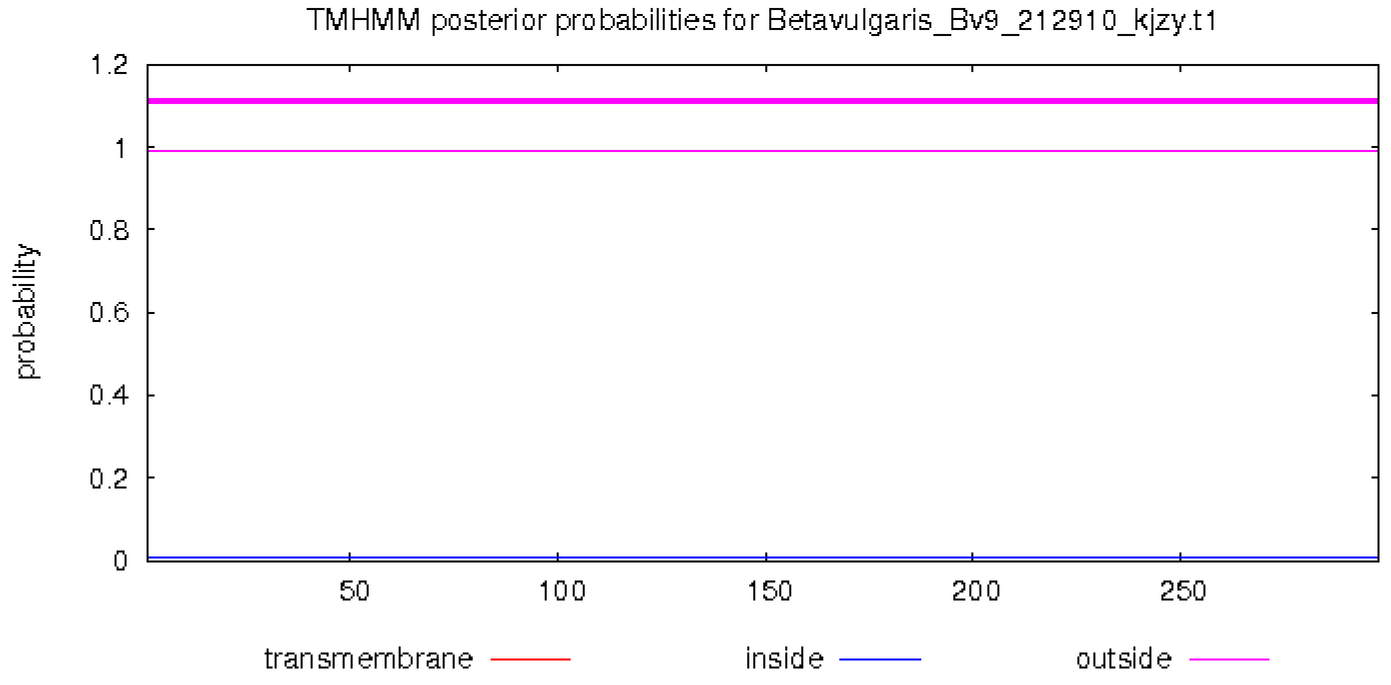

plot in postscript, script for making the plot in gnuplot, data for plot

---

```
# Betavulgaris_Bv9_214490_ofji.t1 Length: 351
# Betavulgaris_Bv9_214490_ofji.t1 Number of predicted TMHs:  0
# Betavulgaris_Bv9_214490_ofji.t1 Exp number of AAs in TMHs: 0.00015
# Betavulgaris_Bv9_214490_ofji.t1 Exp number, first 60 AAs:  0
# Betavulgaris_Bv9_214490_ofji.t1 Total prob of N-in:        0.00072
Betavulgaris_Bv9_214490_ofji.t1 TMHMM2.0        outside      1   351
```

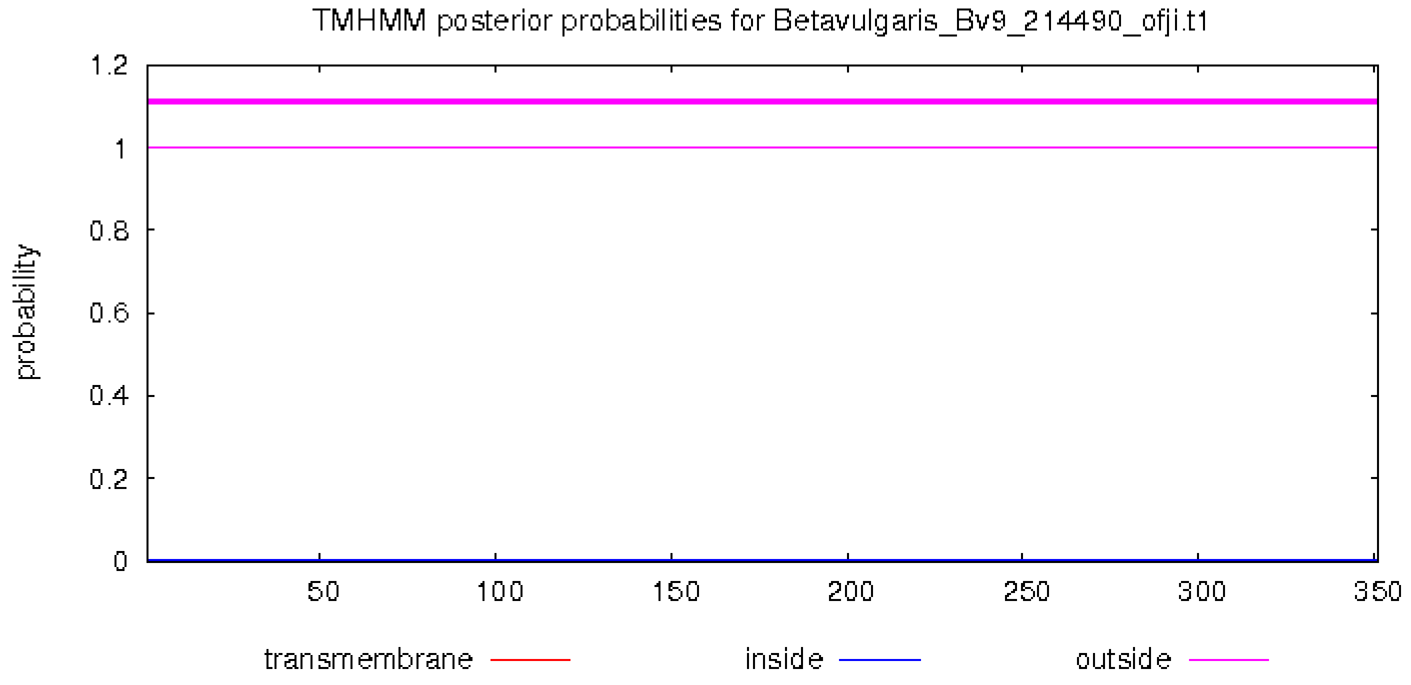

plot in postscript, script for making the plot in gnuplot, data for plot

---

```
# Boecherastricta_Bostr.0124s0081.1.p Length: 318
# Boecherastricta_Bostr.0124s0081.1.p Number of predicted TMHs:  0
```

```
# Boecherastricta_Bostr.0124s0081.1.p Exp number of AAs in TMHs: 0.00101
# Boecherastricta_Bostr.0124s0081.1.p Exp number, first 60 AAs:  0.0001
# Boecherastricta_Bostr.0124s0081.1.p Total prob of N-in:        0.01362
Boecherastricta_Bostr.0124s0081.1.p     TMHMM2.0        outside      1   318
```

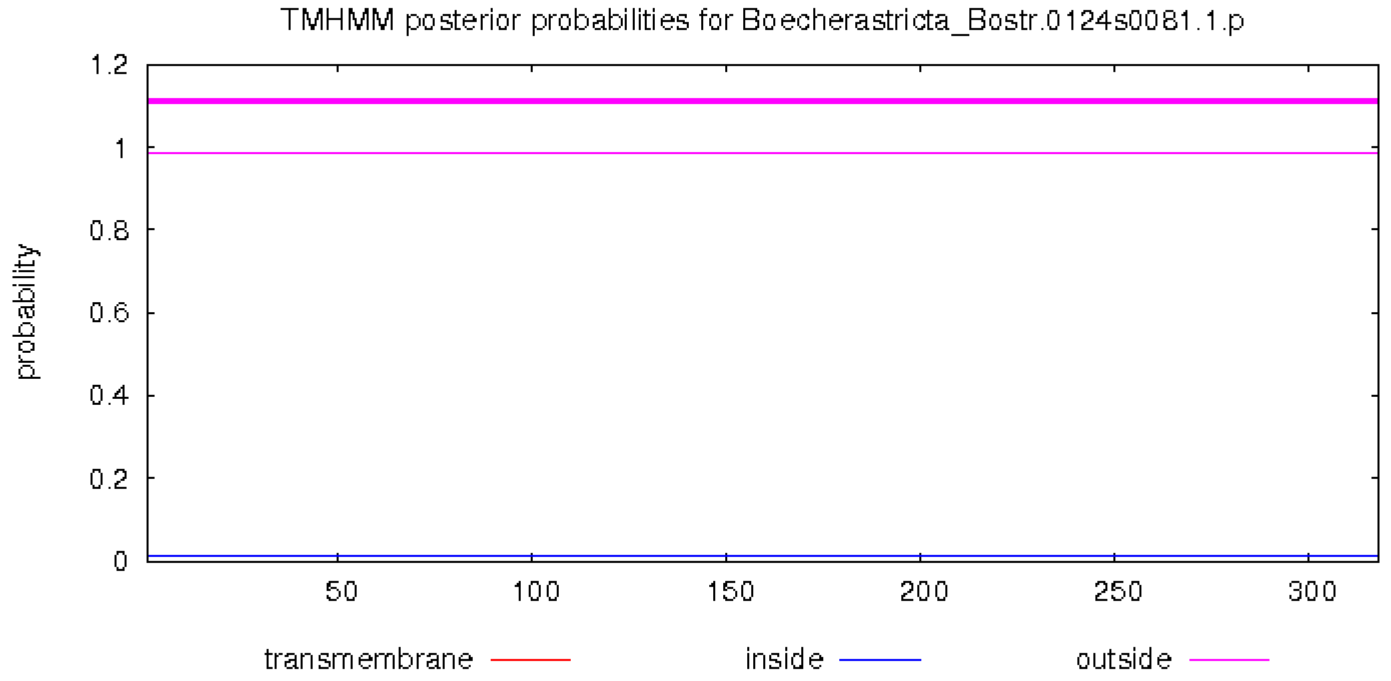

plot in postscript, script for making the plot in gnuplot, data for plot

```
# Boecherastricta_Bostr.0124s0082.1.p Length: 318
# Boecherastricta_Bostr.0124s0082.1.p Number of predicted TMHs:  0
# Boecherastricta_Bostr.0124s0082.1.p Exp number of AAs in TMHs: 0.03629
# Boecherastricta_Bostr.0124s0082.1.p Exp number, first 60 AAs:  0.00022
# Boecherastricta_Bostr.0124s0082.1.p Total prob of N-in:        0.00958
Boecherastricta_Bostr.0124s0082.1.p     TMHMM2.0        outside      1   318
```

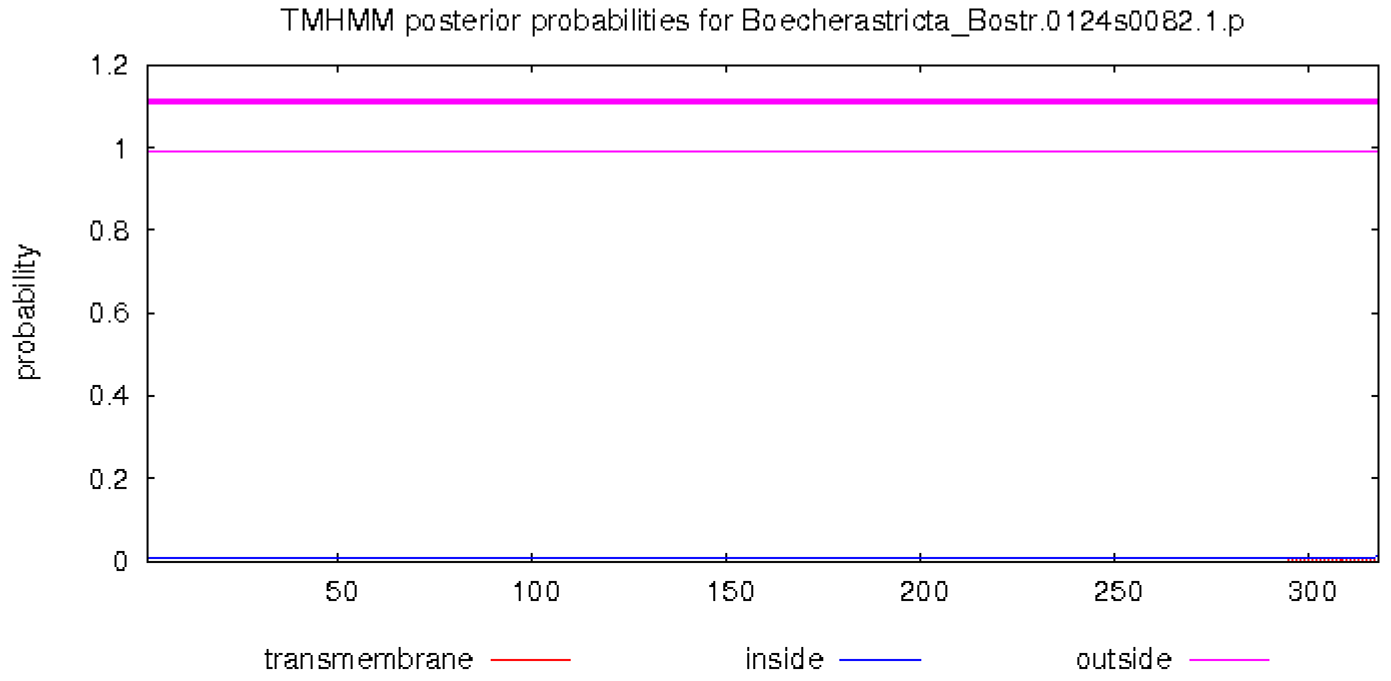

plot in postscript, script for making the plot in gnuplot, data for plot

```
# Boecherastricta_Bostr.0556s0069.1.p Length: 415
# Boecherastricta_Bostr.0556s0069.1.p Number of predicted TMHs:  0
```

```
# Boecherastricta_Bostr.0556s0069.1.p Exp number of AAs in TMHs: 0.03387
# Boecherastricta_Bostr.0556s0069.1.p Exp number, first 60 AAs:  0
# Boecherastricta_Bostr.0556s0069.1.p Total prob of N-in:        0.00962
Boecherastricta_Bostr.0556s0069.1.p     TMHMM2.0        outside      1   415
```

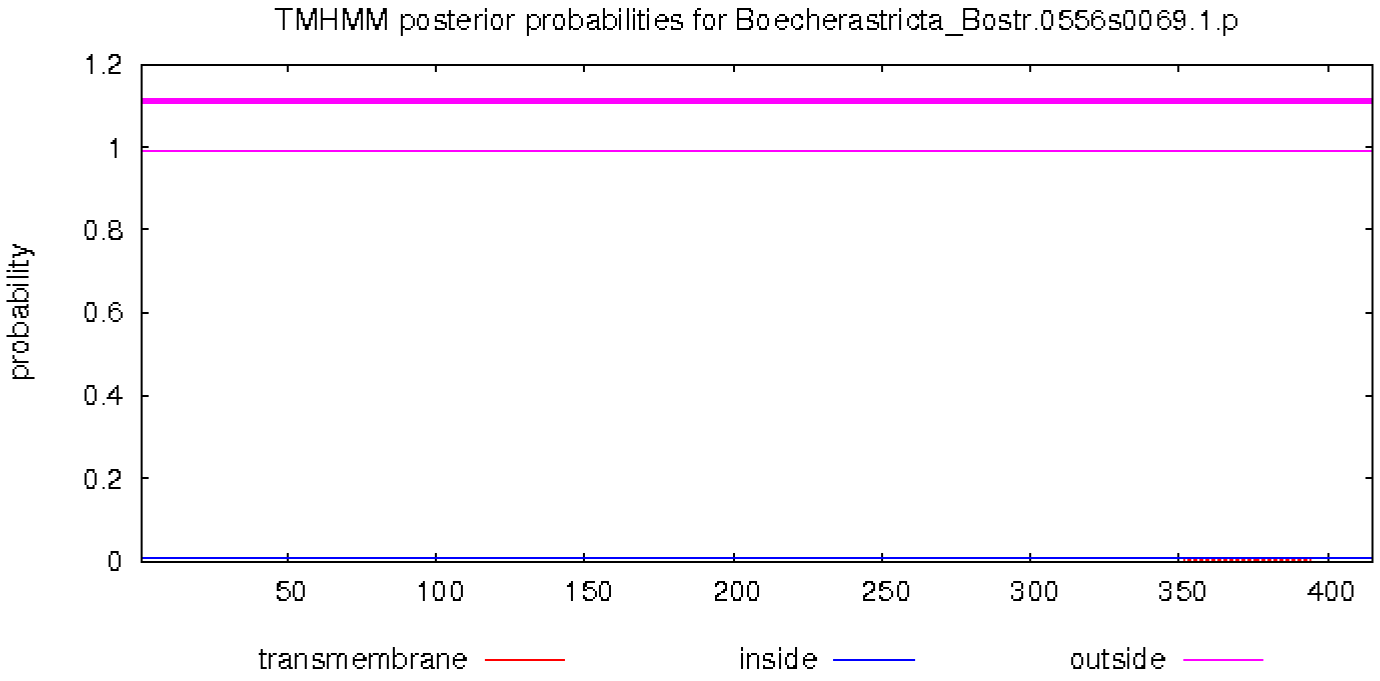

plot in postscript, script for making the plot in gnuplot, data for plot

---

```
# Boecherastricta_Bostr.0556s0555.1.p Length: 325
# Boecherastricta_Bostr.0556s0555.1.p Number of predicted TMHs:  0
# Boecherastricta_Bostr.0556s0555.1.p Exp number of AAs in TMHs: 0.0077
# Boecherastricta_Bostr.0556s0555.1.p Exp number, first 60 AAs:  0.00049
# Boecherastricta_Bostr.0556s0555.1.p Total prob of N-in:        0.00942
Boecherastricta_Bostr.0556s0555.1.p     TMHMM2.0        outside      1   325
```

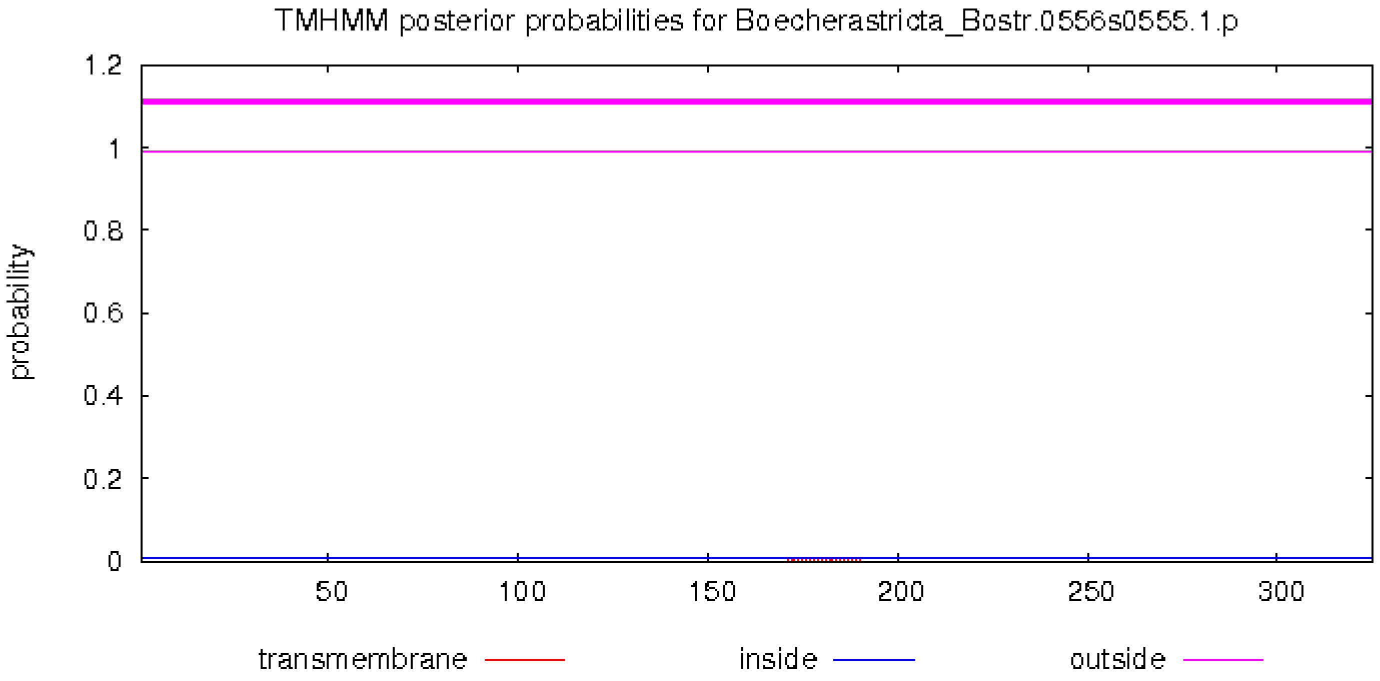

plot in postscript, script for making the plot in gnuplot, data for plot

---

```
# Boecherastricta_Bostr.0556s0726.1.p Length: 335
# Boecherastricta_Bostr.0556s0726.1.p Number of predicted TMHs:  1
```

```
# Boecherastricta_Bostr.0556s0726.1.p Exp number of AAs in TMHs: 15.95705
# Boecherastricta_Bostr.0556s0726.1.p Exp number, first 60 AAs:  0.06619
# Boecherastricta_Bostr.0556s0726.1.p Total prob of N-in:        0.12924
Boecherastricta_Bostr.0556s0726.1.p     TMHMM2.0        outside      1   316
Boecherastricta_Bostr.0556s0726.1.p     TMHMM2.0        TMhelix    317   334
Boecherastricta_Bostr.0556s0726.1.p     TMHMM2.0        inside     335   335
```

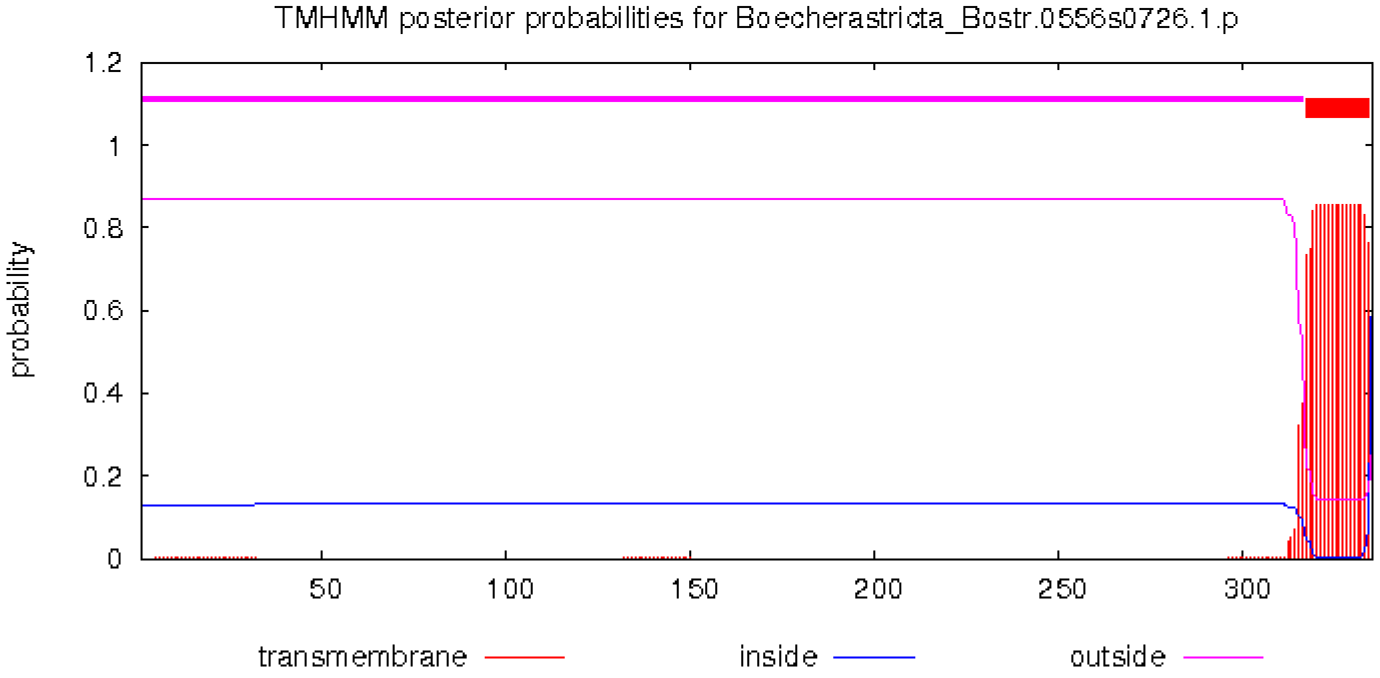

plot in postscript, script for making the plot in gnuplot, data for plot

---

```
# Boecherastricta_Bostr.0556s0732.1.p Length: 245
# Boecherastricta_Bostr.0556s0732.1.p Number of predicted TMHs:  0
# Boecherastricta_Bostr.0556s0732.1.p Exp number of AAs in TMHs: 0.01339
# Boecherastricta_Bostr.0556s0732.1.p Exp number, first 60 AAs:  0.01339
# Boecherastricta_Bostr.0556s0732.1.p Total prob of N-in:        0.00847
Boecherastricta_Bostr.0556s0732.1.p     TMHMM2.0        outside      1   245
```

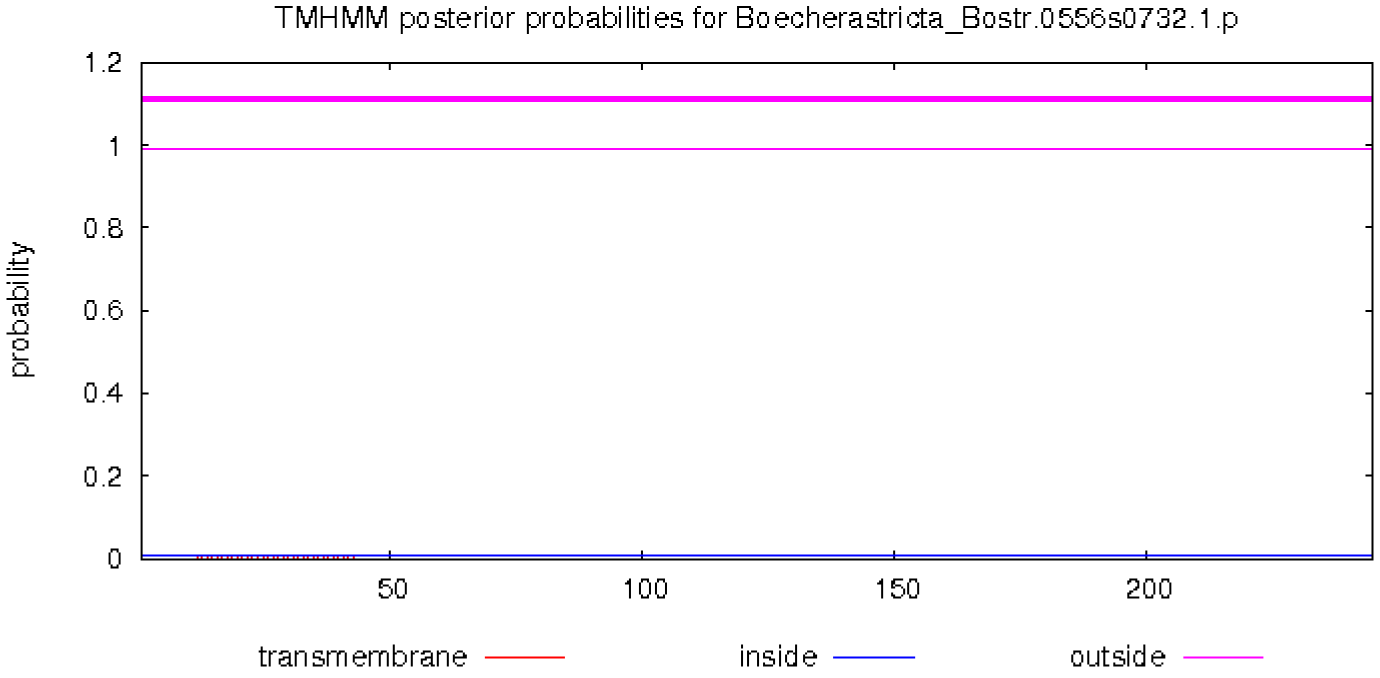

plot in postscript, script for making the plot in gnuplot, data for plot

---

```
# Boecherastricta_Bostr.0556s0784.1.p Length: 121
# Boecherastricta_Bostr.0556s0784.1.p Number of predicted TMHs:  0
# Boecherastricta_Bostr.0556s0784.1.p Exp number of AAs in TMHs: 0.00817
# Boecherastricta_Bostr.0556s0784.1.p Exp number, first 60 AAs:  0.00019
# Boecherastricta_Bostr.0556s0784.1.p Total prob of N-in:        0.06723
Boecherastricta_Bostr.0556s0784.1.p     TMHMM2.0        outside      1   121
```

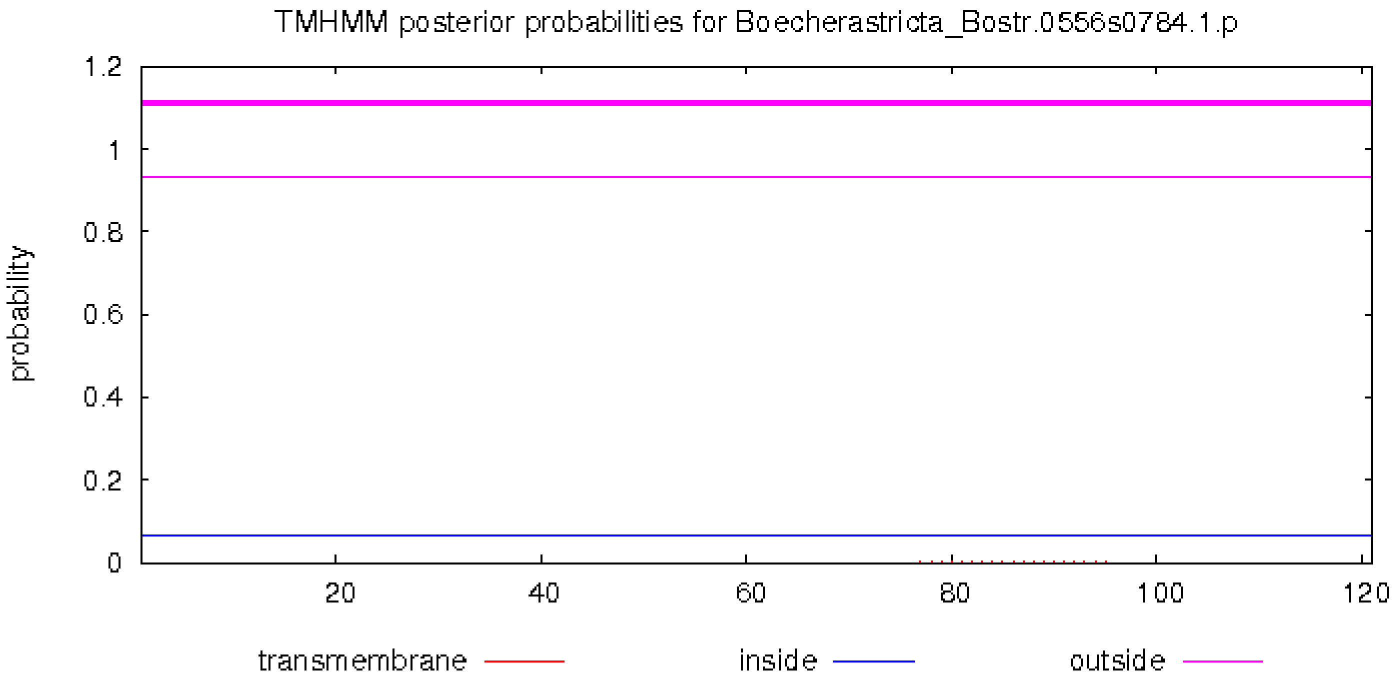

plot in postscript, script for making the plot in gnuplot, data for plot

---

```
# Boecherastricta_Bostr.0568s0042.1.p Length: 290
# Boecherastricta_Bostr.0568s0042.1.p Number of predicted TMHs:  0
# Boecherastricta_Bostr.0568s0042.1.p Exp number of AAs in TMHs: 0.00038
# Boecherastricta_Bostr.0568s0042.1.p Exp number, first 60 AAs:  0
# Boecherastricta_Bostr.0568s0042.1.p Total prob of N-in:        0.04005
Boecherastricta_Bostr.0568s0042.1.p     TMHMM2.0        outside      1   290
```

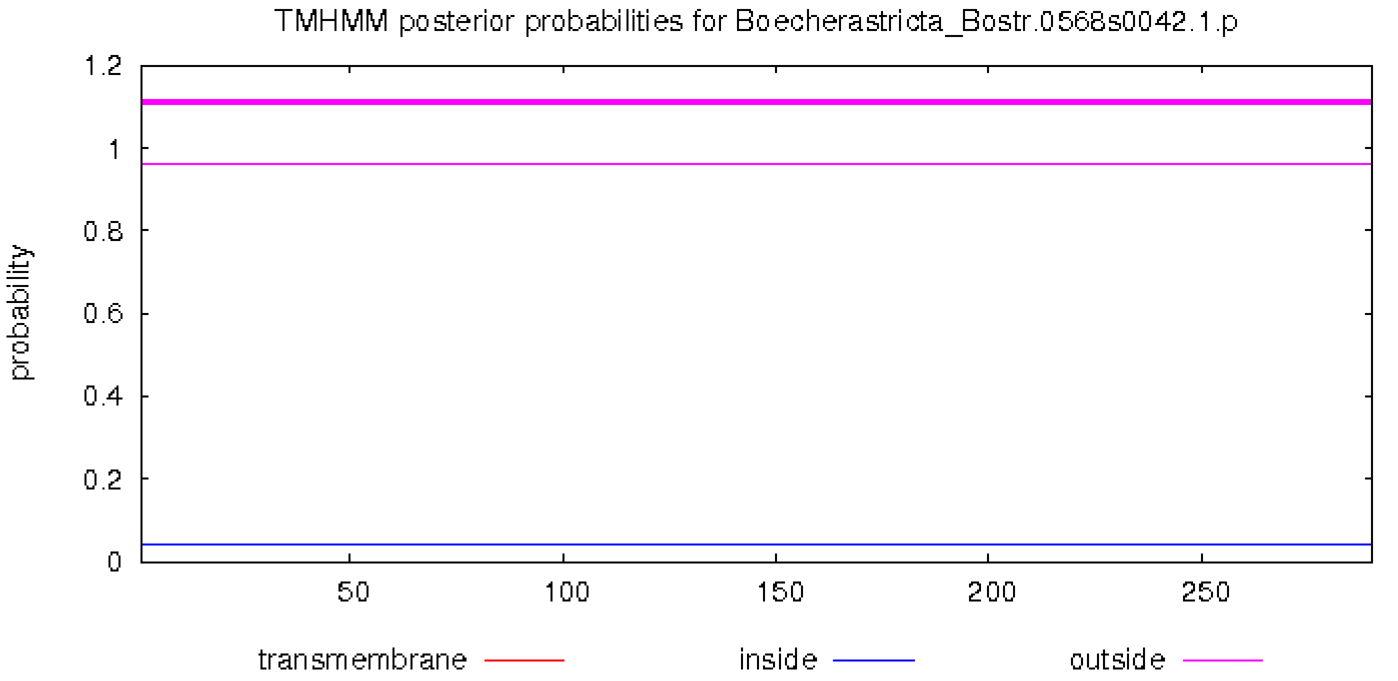

plot in postscript, script for making the plot in gnuplot, data for plot

---

```
# Boecherastricta_Bostr.0568s0345.1.p Length: 188
# Boecherastricta_Bostr.0568s0345.1.p Number of predicted TMHs:  0
# Boecherastricta_Bostr.0568s0345.1.p Exp number of AAs in TMHs: 0
# Boecherastricta_Bostr.0568s0345.1.p Exp number, first 60 AAs:  0
# Boecherastricta_Bostr.0568s0345.1.p Total prob of N-in:        0.00724
Boecherastricta_Bostr.0568s0345.1.p     TMHMM2.0        outside      1   188
```

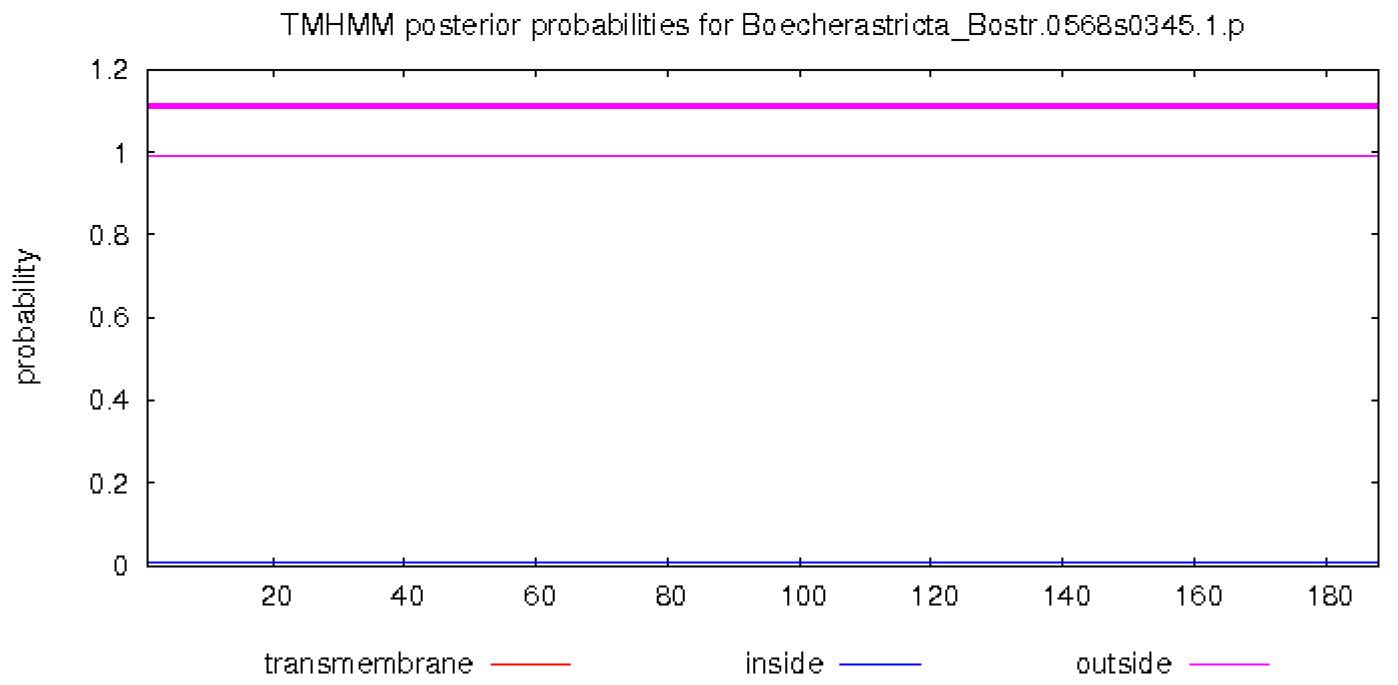

plot in postscript, script for making the plot in gnuplot, data for plot

---

```
# Boecherastricta_Bostr.0568s0398.1.p Length: 357
# Boecherastricta_Bostr.0568s0398.1.p Number of predicted TMHs:  0
# Boecherastricta_Bostr.0568s0398.1.p Exp number of AAs in TMHs: 0.00038
# Boecherastricta_Bostr.0568s0398.1.p Exp number, first 60 AAs:  0.00018
# Boecherastricta_Bostr.0568s0398.1.p Total prob of N-in:        0.00469
Boecherastricta_Bostr.0568s0398.1.p     TMHMM2.0        outside      1   357
```

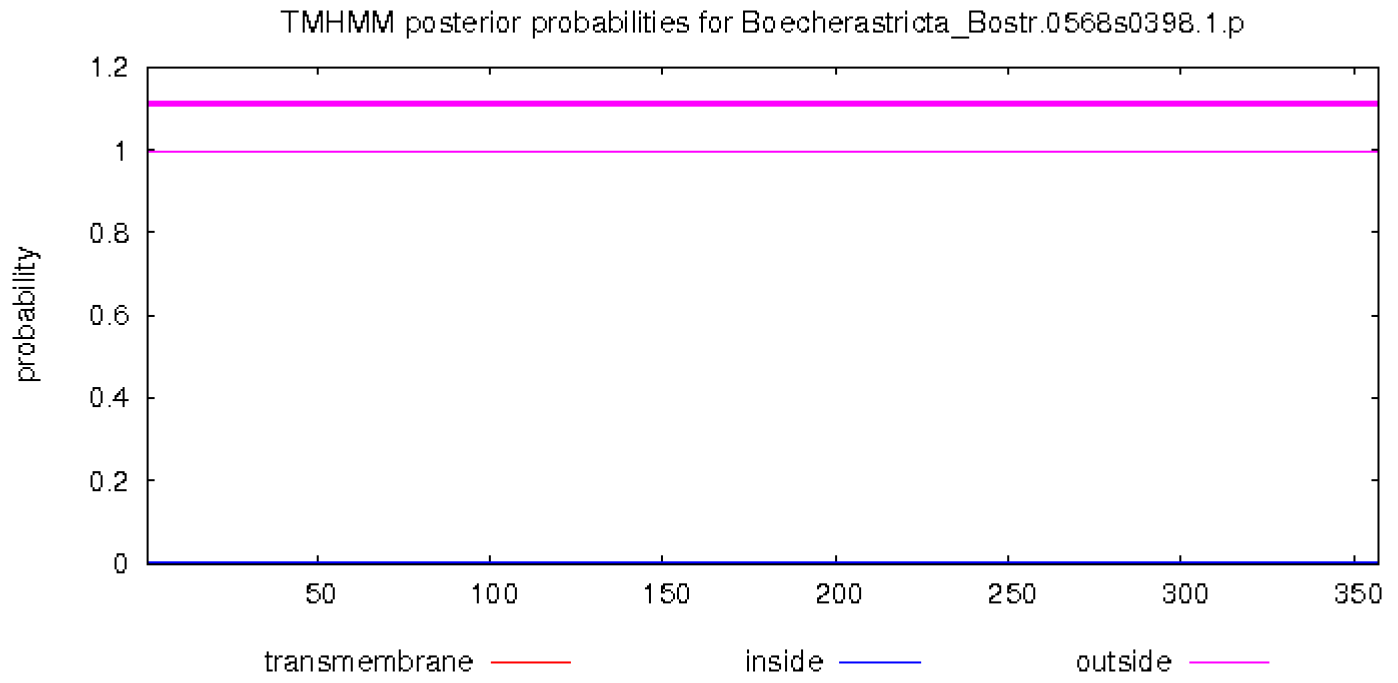

plot in postscript, script for making the plot in gnuplot, data for plot

---

```
# Boecherastricta_Bostr.0568s0429.1.p Length: 270
# Boecherastricta_Bostr.0568s0429.1.p Number of predicted TMHs:  0
# Boecherastricta_Bostr.0568s0429.1.p Exp number of AAs in TMHs: 0.00019
# Boecherastricta_Bostr.0568s0429.1.p Exp number, first 60 AAs:  0
# Boecherastricta_Bostr.0568s0429.1.p Total prob of N-in:        0.01222
Boecherastricta_Bostr.0568s0429.1.p	TMHMM2.0	outside	     1   270
```

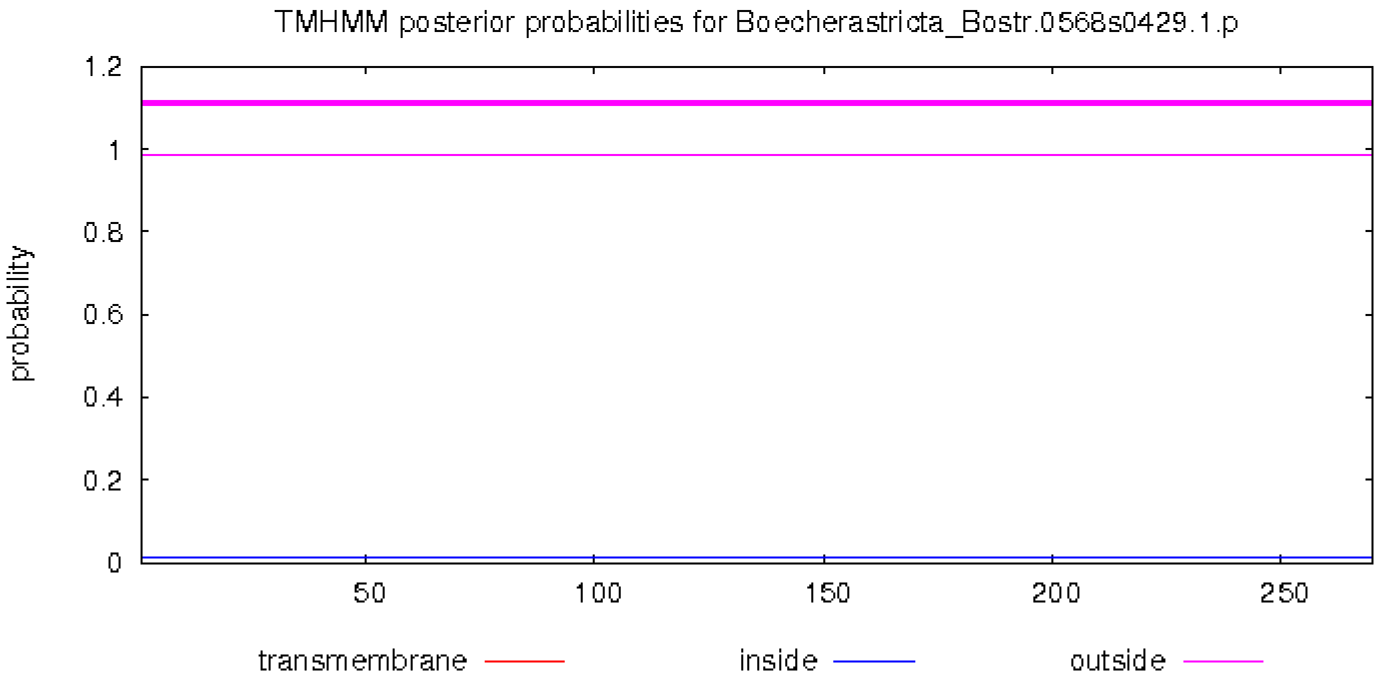

plot in postscript, script for making the plot in gnuplot, data for plot

---

```
# Boecherastricta_Bostr.0568s0569.1.p Length: 347
# Boecherastricta_Bostr.0568s0569.1.p Number of predicted TMHs:  0
# Boecherastricta_Bostr.0568s0569.1.p Exp number of AAs in TMHs: 0.00059
# Boecherastricta_Bostr.0568s0569.1.p Exp number, first 60 AAs:  0
# Boecherastricta_Bostr.0568s0569.1.p Total prob of N-in:        0.00194
Boecherastricta_Bostr.0568s0569.1.p	TMHMM2.0	outside	     1   347
```

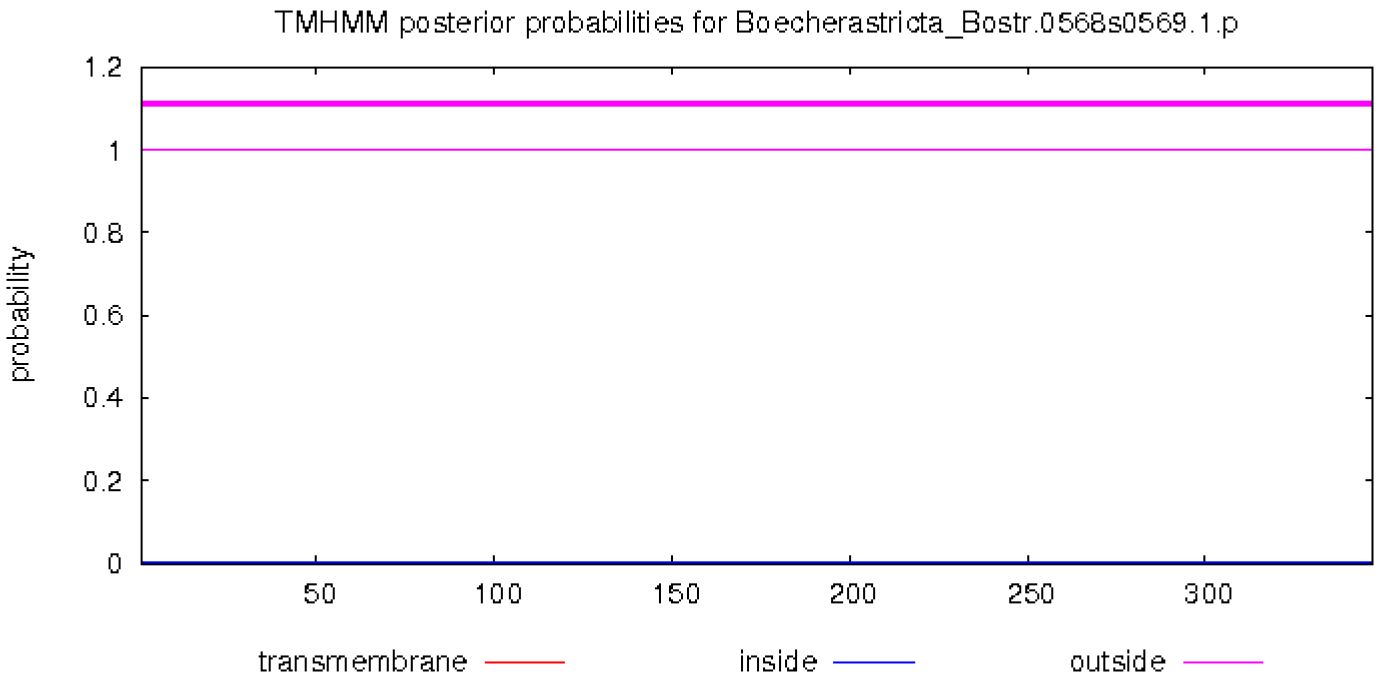

plot in postscript, script for making the plot in gnuplot, data for plot

---

```
# Boecherastricta_Bostr.10040s0089.1.p Length: 321
# Boecherastricta_Bostr.10040s0089.1.p Number of predicted TMHs:  0
# Boecherastricta_Bostr.10040s0089.1.p Exp number of AAs in TMHs: 0.0004
# Boecherastricta_Bostr.10040s0089.1.p Exp number, first 60 AAs:  0.00019
# Boecherastricta_Bostr.10040s0089.1.p Total prob of N-in:        0.01170
Boecherastricta_Bostr.10040s0089.1.p	TMHMM2.0	outside	     1   321
```

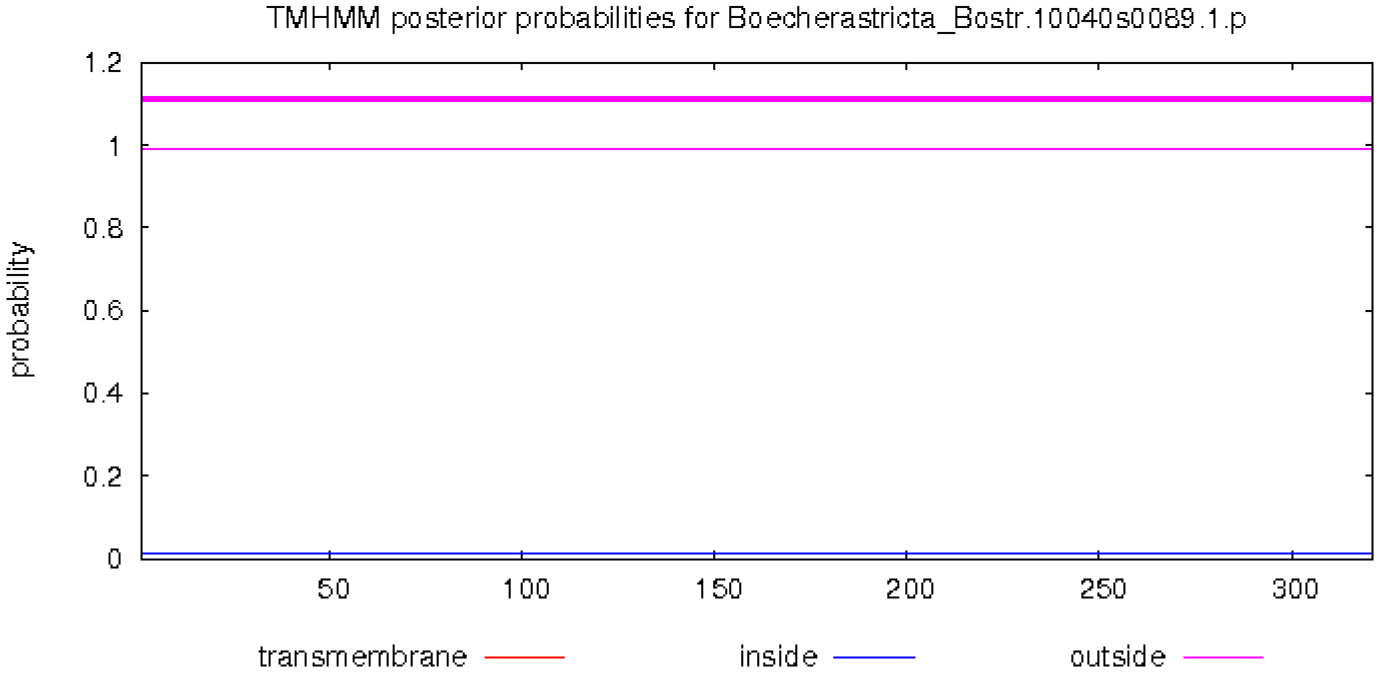

plot in postscript, script for making the plot in gnuplot, data for plot

---

```
# Boecherastricta_Bostr.10040s0187.1.p Length: 276
# Boecherastricta_Bostr.10040s0187.1.p Number of predicted TMHs:  0
# Boecherastricta_Bostr.10040s0187.1.p Exp number of AAs in TMHs: 0.0048
# Boecherastricta_Bostr.10040s0187.1.p Exp number, first 60 AAs:  0.00016
# Boecherastricta_Bostr.10040s0187.1.p Total prob of N-in:        0.02803
Boecherastricta_Bostr.10040s0187.1.p	TMHMM2.0	outside	     1   276
```

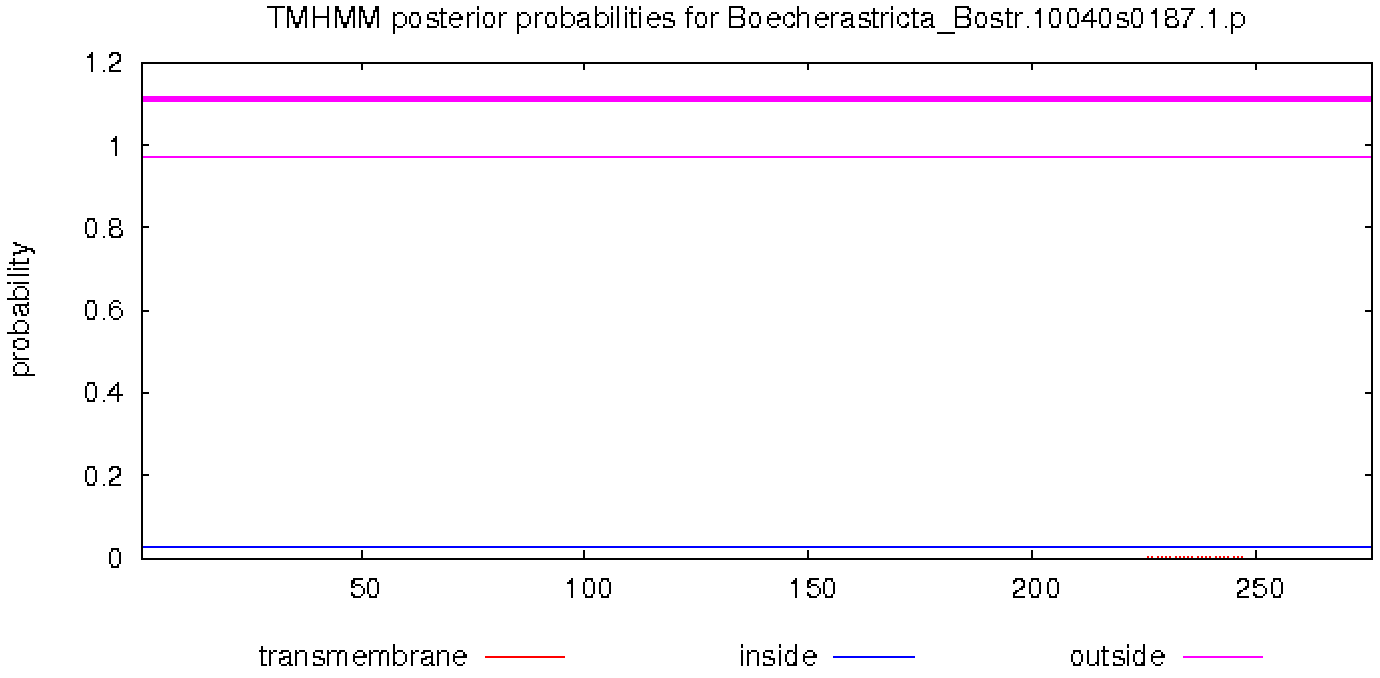

plot in postscript, script for making the plot in gnuplot, data for plot

---

```
# Boecherastricta_Bostr.10040s0190.1.p Length: 312
# Boecherastricta_Bostr.10040s0190.1.p Number of predicted TMHs:  0
# Boecherastricta_Bostr.10040s0190.1.p Exp number of AAs in TMHs: 0.00071
# Boecherastricta_Bostr.10040s0190.1.p Exp number, first 60 AAs:  0.00071
# Boecherastricta_Bostr.10040s0190.1.p Total prob of N-in:        0.01191
Boecherastricta_Bostr.10040s0190.1.p    TMHMM2.0        outside      1   312
```

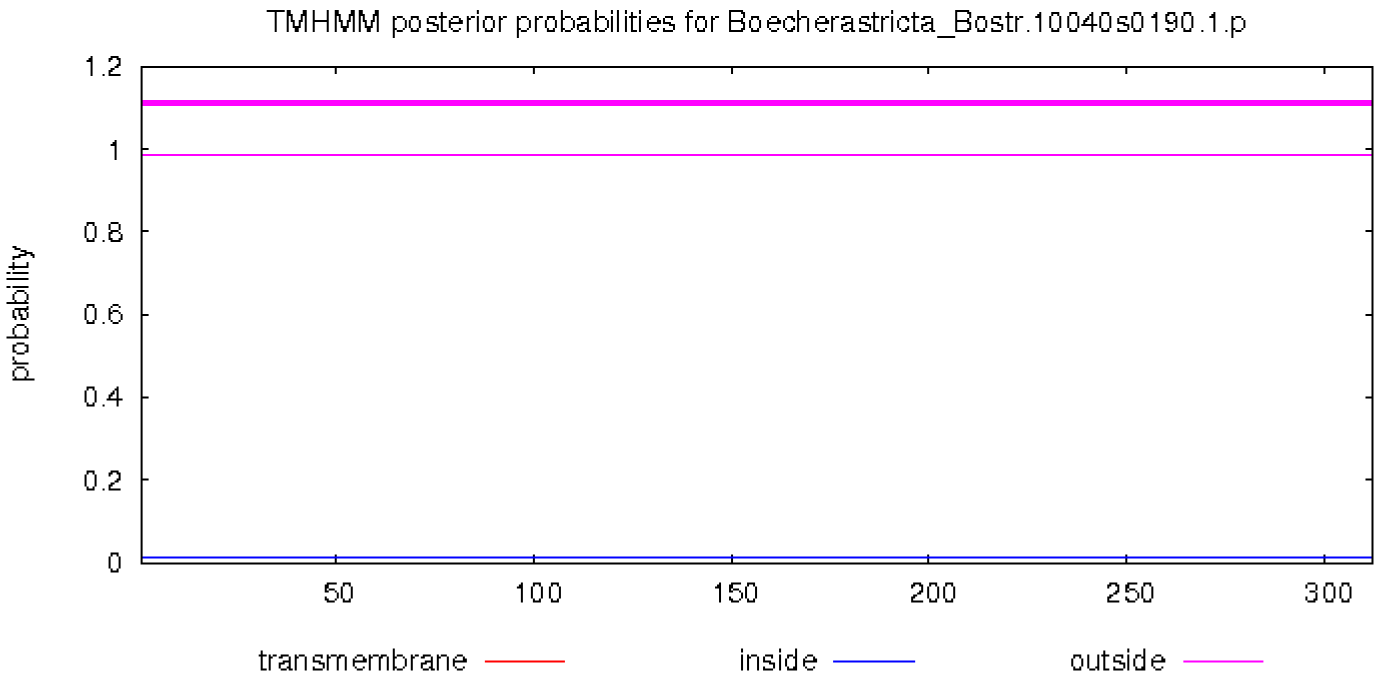

plot in postscript, script for making the plot in gnuplot, data for plot

---

```
# Boecherastricta_Bostr.10040s0194.1.p Length: 184
# Boecherastricta_Bostr.10040s0194.1.p Number of predicted TMHs:  0
# Boecherastricta_Bostr.10040s0194.1.p Exp number of AAs in TMHs: 0
# Boecherastricta_Bostr.10040s0194.1.p Exp number, first 60 AAs:  0
# Boecherastricta_Bostr.10040s0194.1.p Total prob of N-in:        0.10719
Boecherastricta_Bostr.10040s0194.1.p    TMHMM2.0        outside      1   184
```

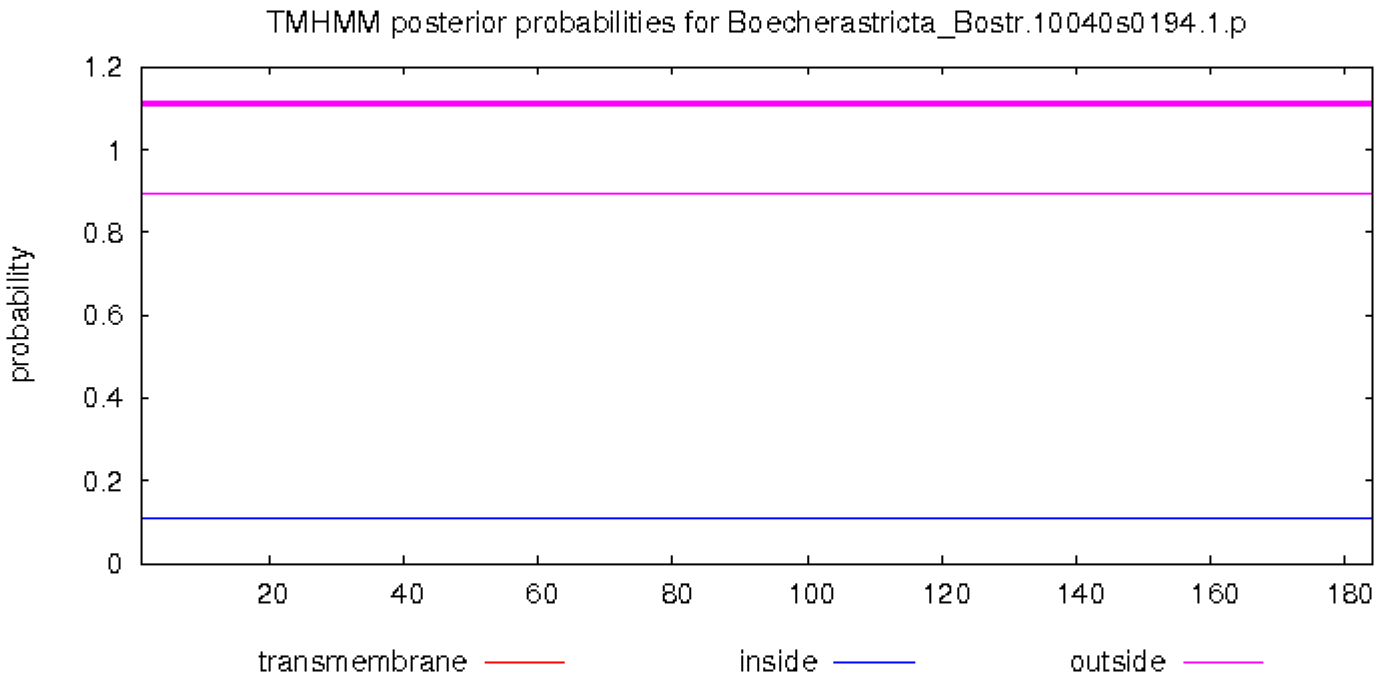

plot in postscript, script for making the plot in gnuplot, data for plot

---

```
# Boecherastricta_Bostr.10040s0202.1.p Length: 290
# Boecherastricta_Bostr.10040s0202.1.p Number of predicted TMHs:  0
# Boecherastricta_Bostr.10040s0202.1.p Exp number of AAs in TMHs: 0.00045
# Boecherastricta_Bostr.10040s0202.1.p Exp number, first 60 AAs:  0
# Boecherastricta_Bostr.10040s0202.1.p Total prob of N-in:        0.01247
Boecherastricta_Bostr.10040s0202.1.p    TMHMM2.0        outside      1   290
```

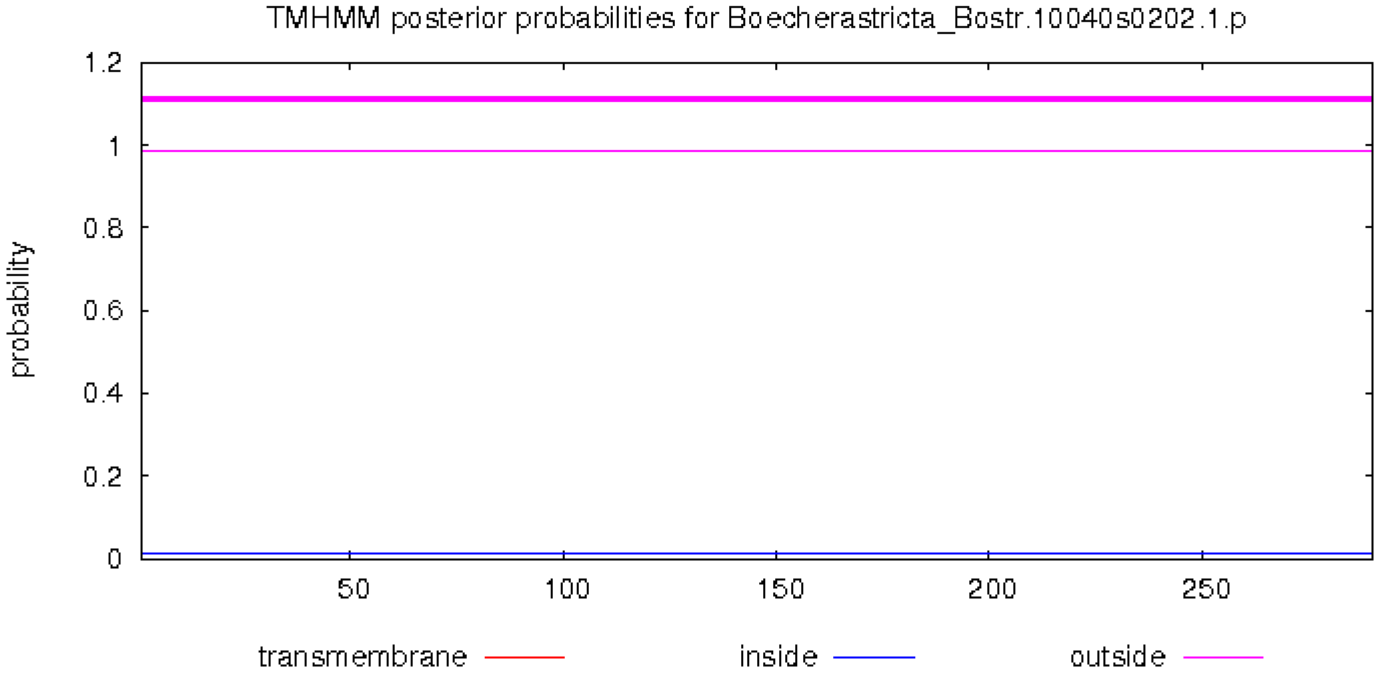

plot in postscript, script for making the plot in gnuplot, data for plot

---

```
# Boecherastricta_Bostr.10058s0013.1.p Length: 341
# Boecherastricta_Bostr.10058s0013.1.p Number of predicted TMHs:  0
# Boecherastricta_Bostr.10058s0013.1.p Exp number of AAs in TMHs: 0.00059
# Boecherastricta_Bostr.10058s0013.1.p Exp number, first 60 AAs:  0
# Boecherastricta_Bostr.10058s0013.1.p Total prob of N-in:        0.00940
Boecherastricta_Bostr.10058s0013.1.p    TMHMM2.0        outside      1   341
```

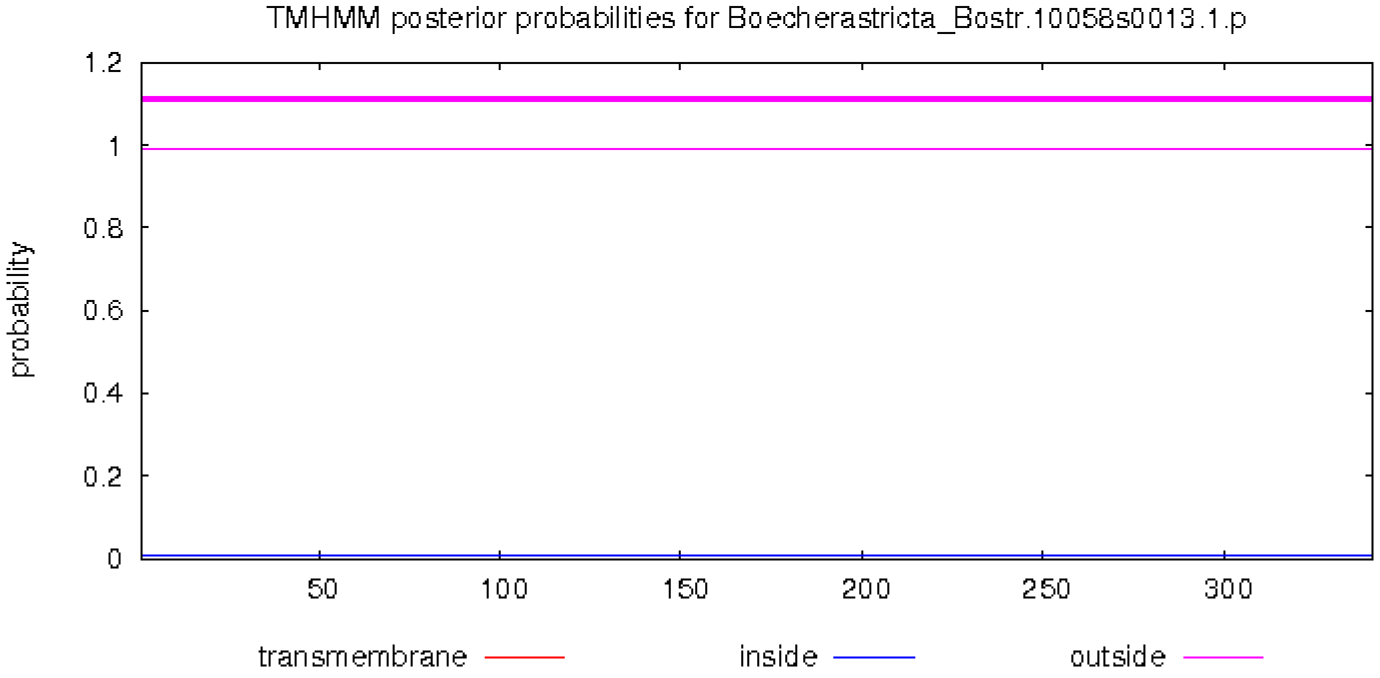

plot in postscript, script for making the plot in gnuplot, data for plot

---

```
# Boecherastricta_Bostr.10058s0039.1.p Length: 289
# Boecherastricta_Bostr.10058s0039.1.p Number of predicted TMHs:  0
# Boecherastricta_Bostr.10058s0039.1.p Exp number of AAs in TMHs: 0.00141
# Boecherastricta_Bostr.10058s0039.1.p Exp number, first 60 AAs:  0.00123
# Boecherastricta_Bostr.10058s0039.1.p Total prob of N-in:        0.10761
Boecherastricta_Bostr.10058s0039.1.p	TMHMM2.0	outside	     1   289
```

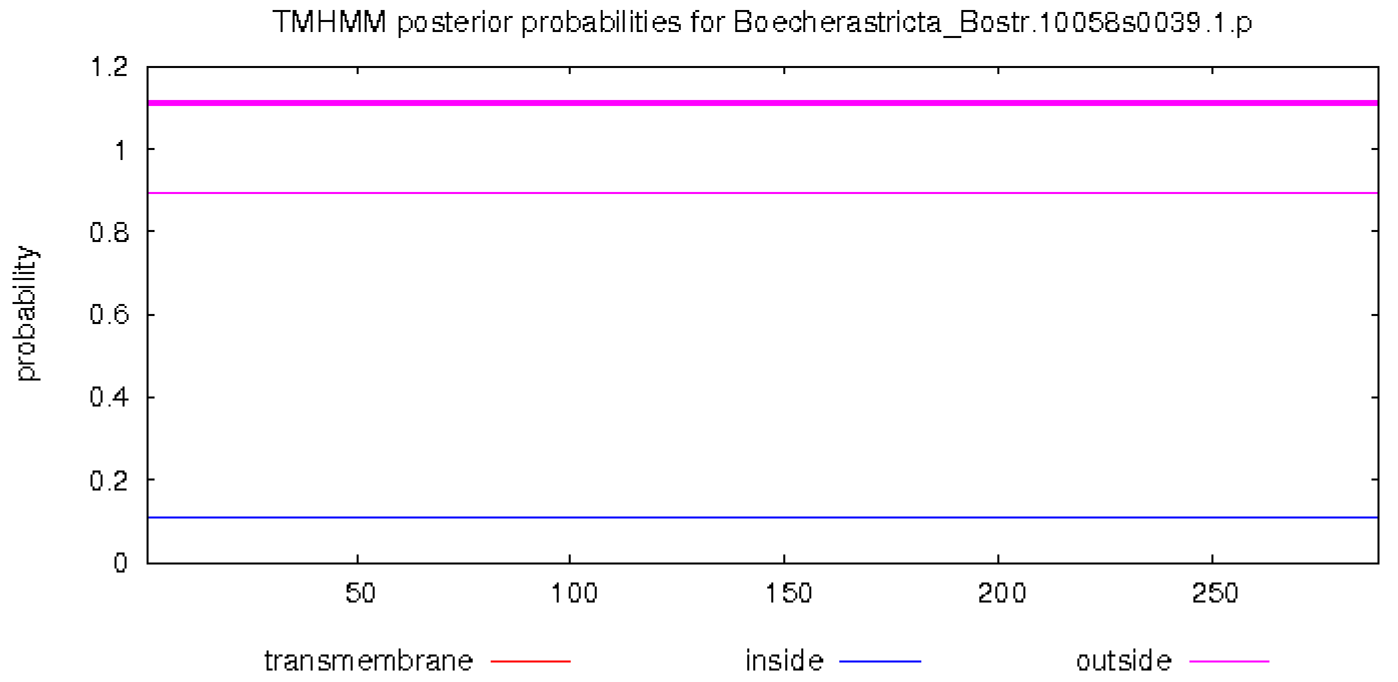

plot in postscript, script for making the plot in gnuplot, data for plot

```
# Boecherastricta_Bostr.10064s0010.1.p Length: 157
# Boecherastricta_Bostr.10064s0010.1.p Number of predicted TMHs:  0
# Boecherastricta_Bostr.10064s0010.1.p Exp number of AAs in TMHs: 0.00107
# Boecherastricta_Bostr.10064s0010.1.p Exp number, first 60 AAs:  0.00077
# Boecherastricta_Bostr.10064s0010.1.p Total prob of N-in:        0.04402
Boecherastricta_Bostr.10064s0010.1.p	TMHMM2.0	outside	     1   157
```

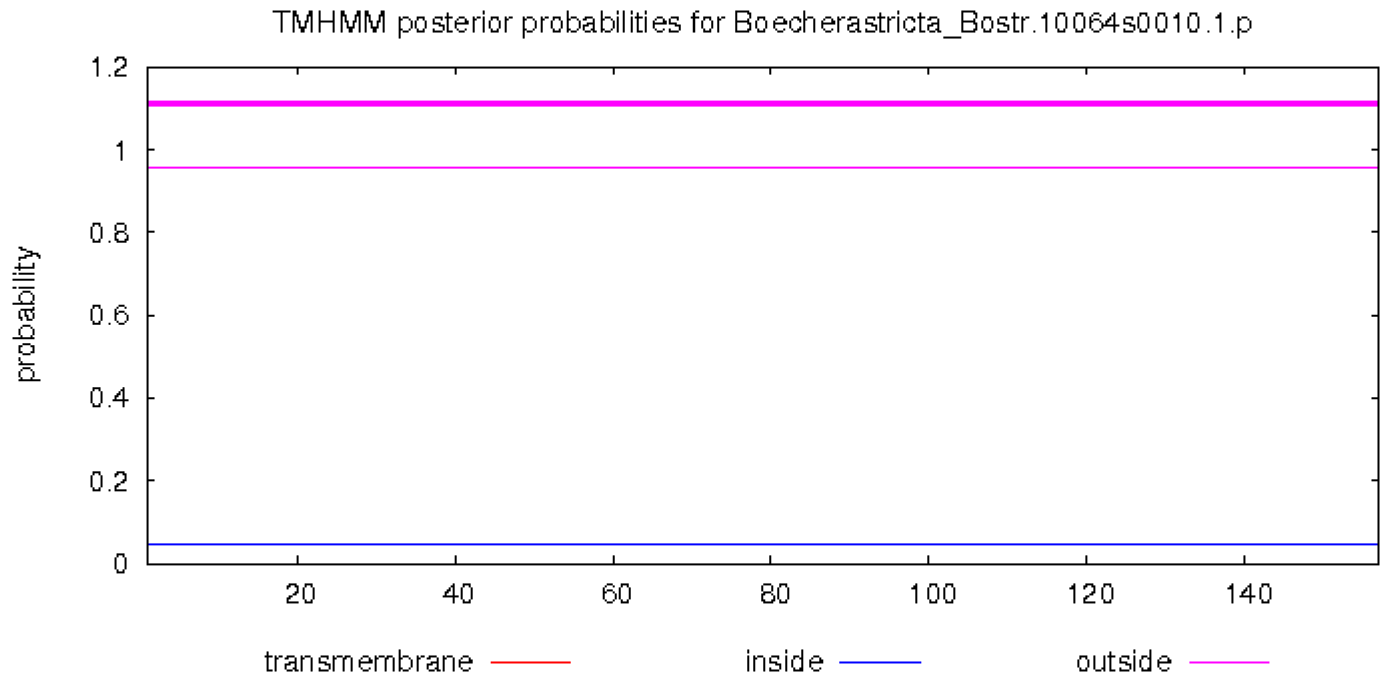

plot in postscript, script for making the plot in gnuplot, data for plot

```
# Boecherastricta_Bostr.10273s0160.1.p Length: 326
# Boecherastricta_Bostr.10273s0160.1.p Number of predicted TMHs:  0
# Boecherastricta_Bostr.10273s0160.1.p Exp number of AAs in TMHs: 0
# Boecherastricta_Bostr.10273s0160.1.p Exp number, first 60 AAs:  0
# Boecherastricta_Bostr.10273s0160.1.p Total prob of N-in:        0.01060
Boecherastricta_Bostr.10273s0160.1.p    TMHMM2.0        outside      1   326
```

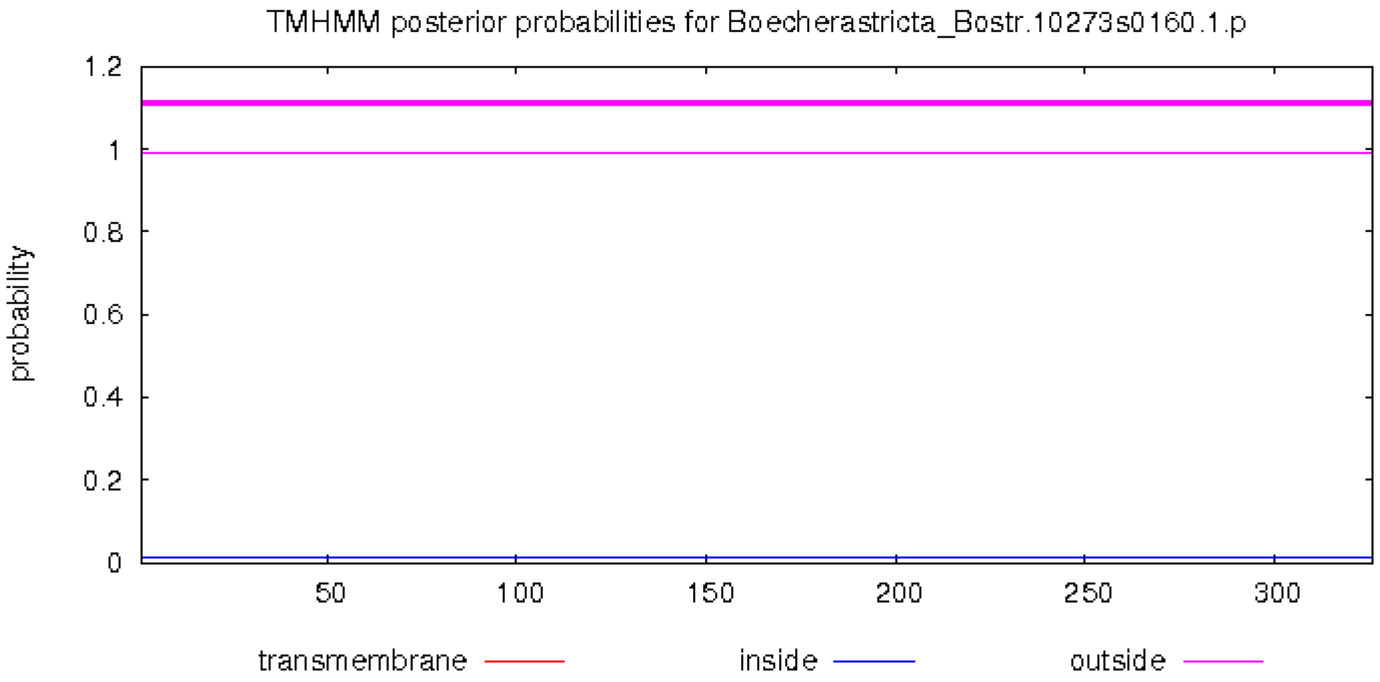

plot in postscript, script for making the plot in gnuplot, data for plot

---

```
# Boecherastricta_Bostr.10273s0400.1.p Length: 374
# Boecherastricta_Bostr.10273s0400.1.p Number of predicted TMHs:  0
# Boecherastricta_Bostr.10273s0400.1.p Exp number of AAs in TMHs: 0.00018
# Boecherastricta_Bostr.10273s0400.1.p Exp number, first 60 AAs:  0
# Boecherastricta_Bostr.10273s0400.1.p Total prob of N-in:        0.01876
Boecherastricta_Bostr.10273s0400.1.p    TMHMM2.0        outside      1   374
```

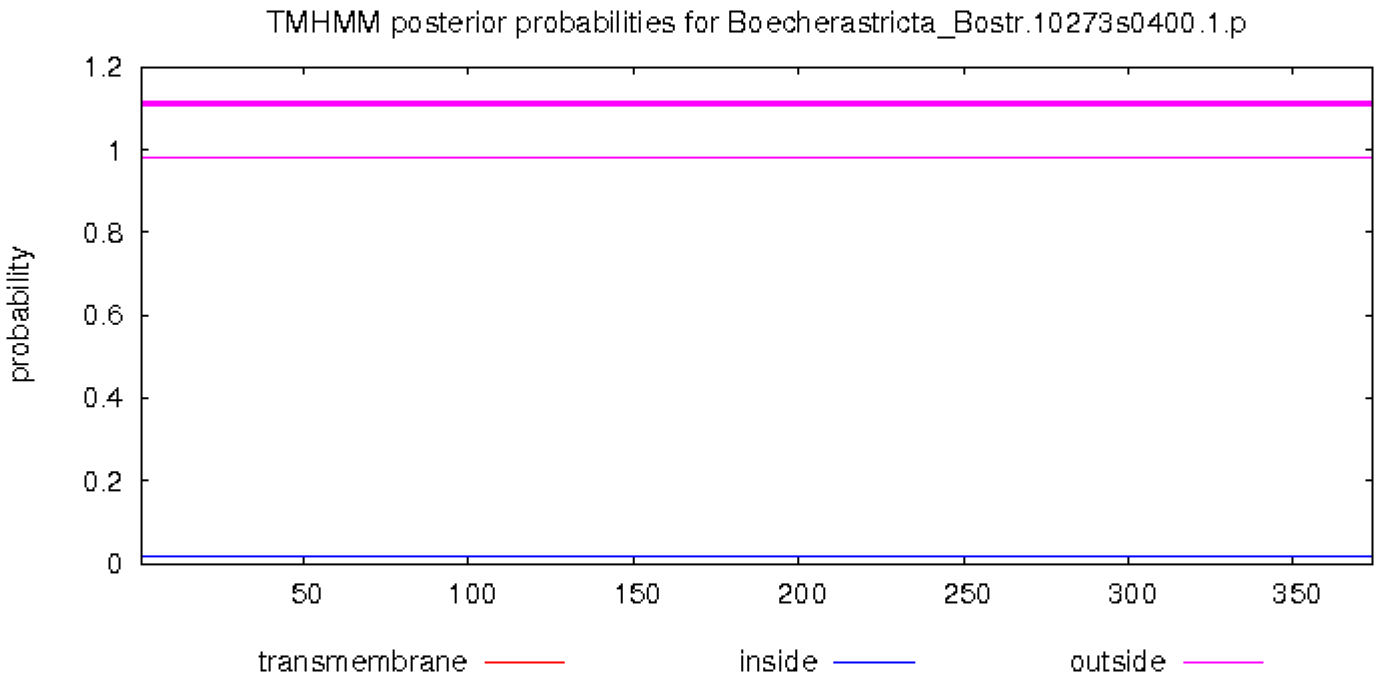

plot in postscript, script for making the plot in gnuplot, data for plot

---

```
# Boecherastricta_Bostr.10273s0418.1.p Length: 269
# Boecherastricta_Bostr.10273s0418.1.p Number of predicted TMHs:  0
# Boecherastricta_Bostr.10273s0418.1.p Exp number of AAs in TMHs: 0.00078
# Boecherastricta_Bostr.10273s0418.1.p Exp number, first 60 AAs:  0
# Boecherastricta_Bostr.10273s0418.1.p Total prob of N-in:        0.03792
Boecherastricta_Bostr.10273s0418.1.p	TMHMM2.0	outside	     1   269
```

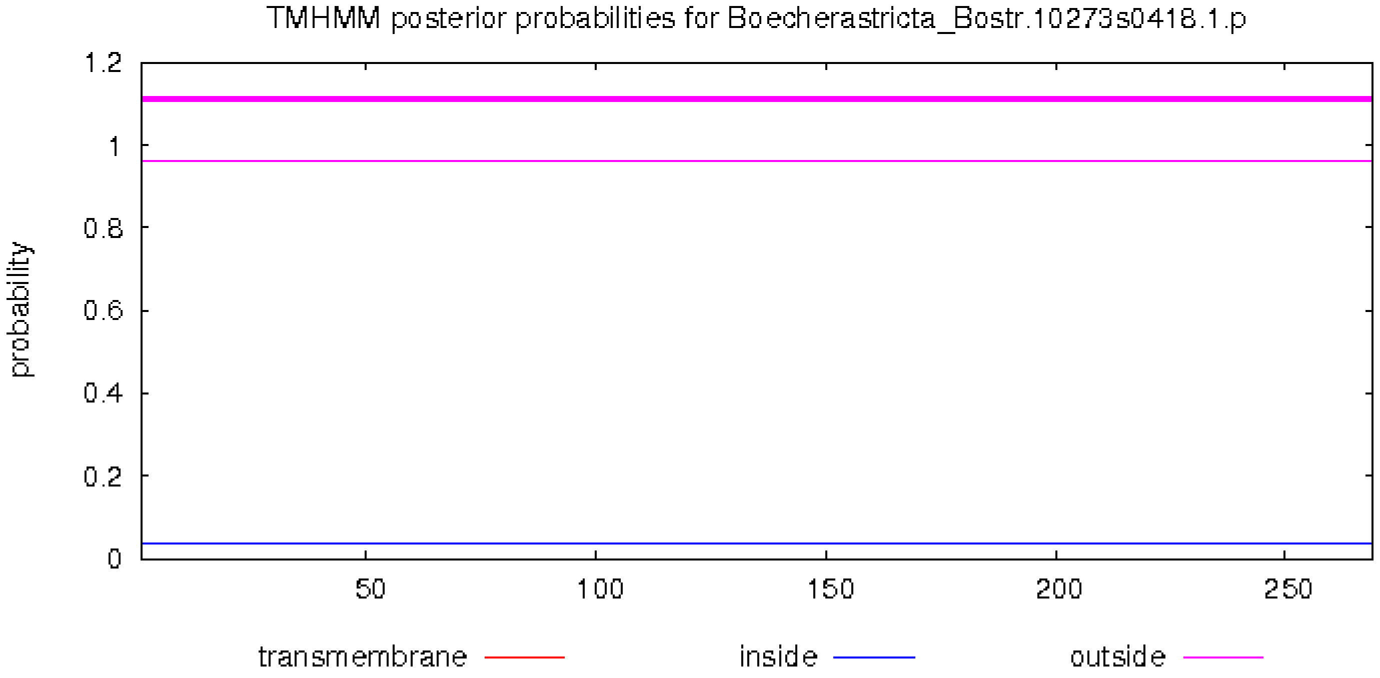

plot in postscript, script for making the plot in gnuplot, data for plot

---

```
# Boecherastricta_Bostr.1040s0050.1.p Length: 559
# Boecherastricta_Bostr.1040s0050.1.p Number of predicted TMHs:  1
# Boecherastricta_Bostr.1040s0050.1.p Exp number of AAs in TMHs: 21.43976
# Boecherastricta_Bostr.1040s0050.1.p Exp number, first 60 AAs:  0.00613
# Boecherastricta_Bostr.1040s0050.1.p Total prob of N-in:        0.08150
Boecherastricta_Bostr.1040s0050.1.p	TMHMM2.0	outside	     1   529
Boecherastricta_Bostr.1040s0050.1.p	TMHMM2.0	TMhelix	   530   552
Boecherastricta_Bostr.1040s0050.1.p	TMHMM2.0	inside	   553   559
```

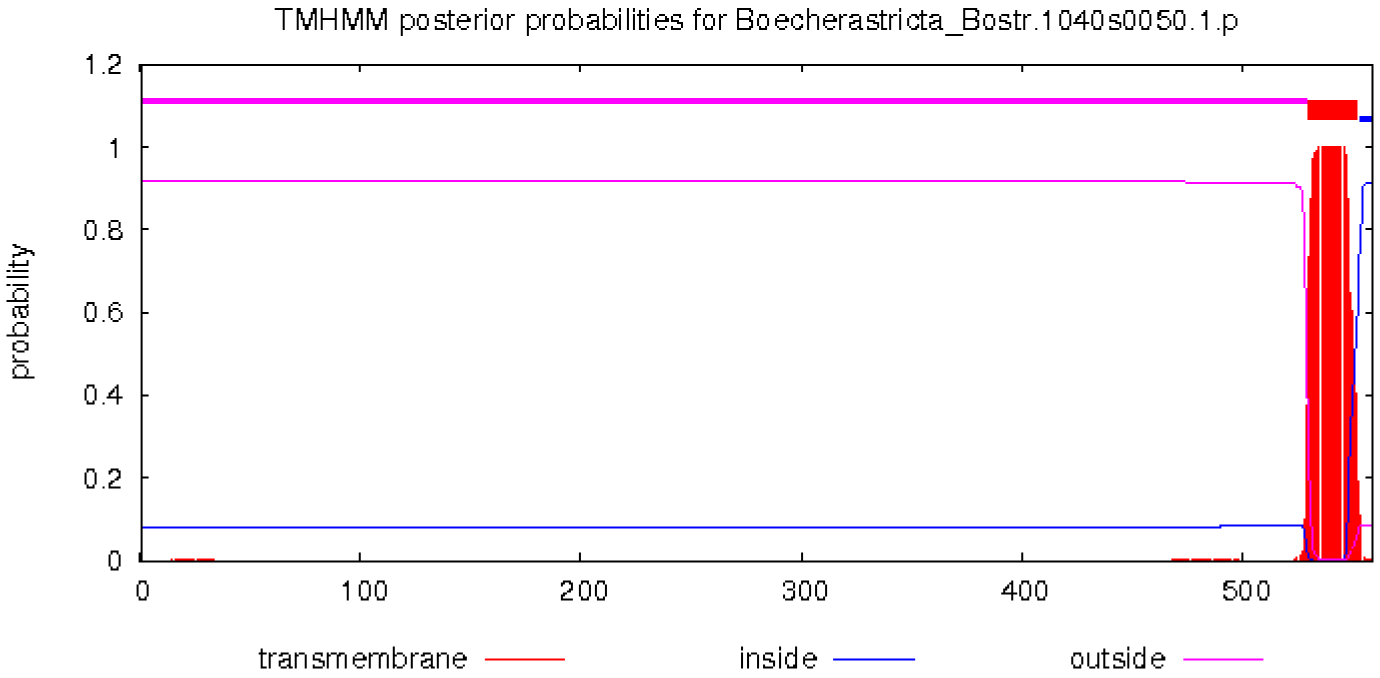

plot in postscript, script for making the plot in gnuplot, data for plot

```
# Boecherastricta_Bostr.1040s0051.1.p Length: 573
# Boecherastricta_Bostr.1040s0051.1.p Number of predicted TMHs:  1
# Boecherastricta_Bostr.1040s0051.1.p Exp number of AAs in TMHs: 23.02934
# Boecherastricta_Bostr.1040s0051.1.p Exp number, first 60 AAs:  0.00563
# Boecherastricta_Bostr.1040s0051.1.p Total prob of N-in:        0.11701
Boecherastricta_Bostr.1040s0051.1.p     TMHMM2.0        outside      1   544
Boecherastricta_Bostr.1040s0051.1.p     TMHMM2.0        TMhelix    545   567
Boecherastricta_Bostr.1040s0051.1.p     TMHMM2.0        inside     568   573
```

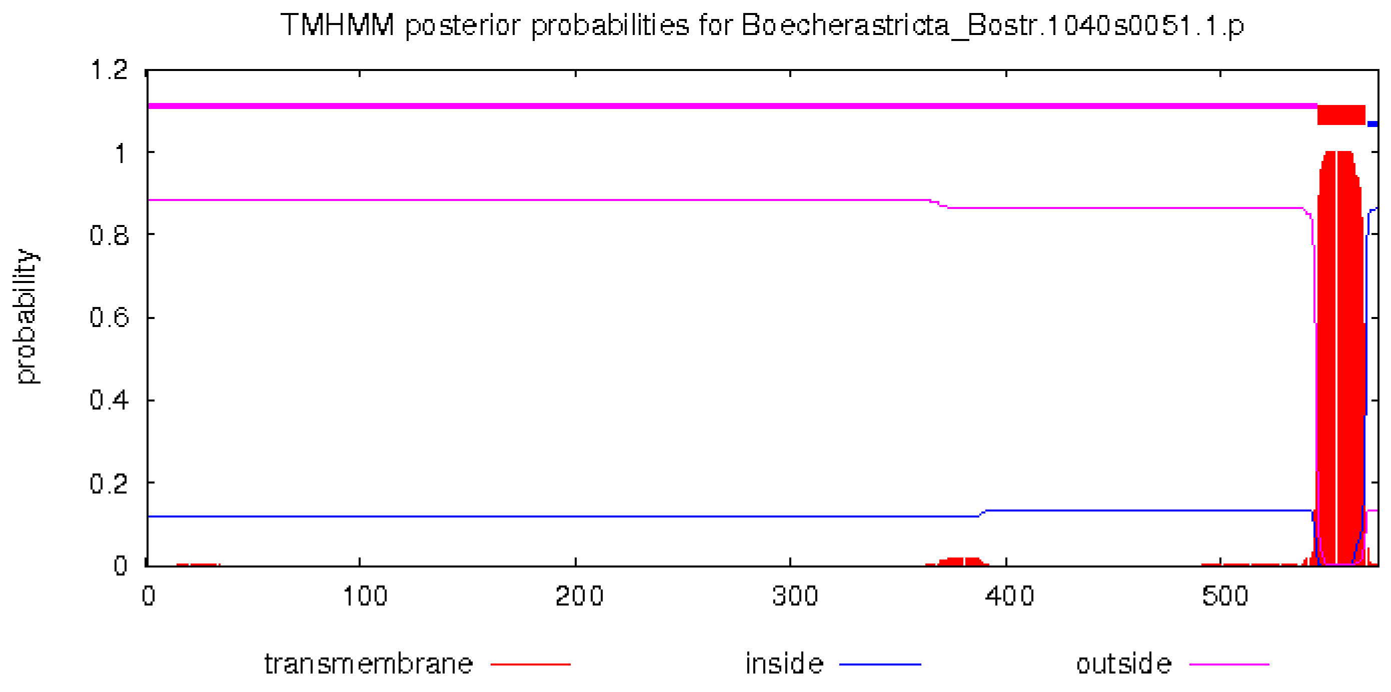

plot in postscript, script for making the plot in gnuplot, data for plot

```
# Boecherastricta_Bostr.12659s0312.1.p Length: 417
# Boecherastricta_Bostr.12659s0312.1.p Number of predicted TMHs:  0
# Boecherastricta_Bostr.12659s0312.1.p Exp number of AAs in TMHs: 0.00157
# Boecherastricta_Bostr.12659s0312.1.p Exp number, first 60 AAs:  0
# Boecherastricta_Bostr.12659s0312.1.p Total prob of N-in:        0.01165
Boecherastricta_Bostr.12659s0312.1.p    TMHMM2.0        outside      1   417
```

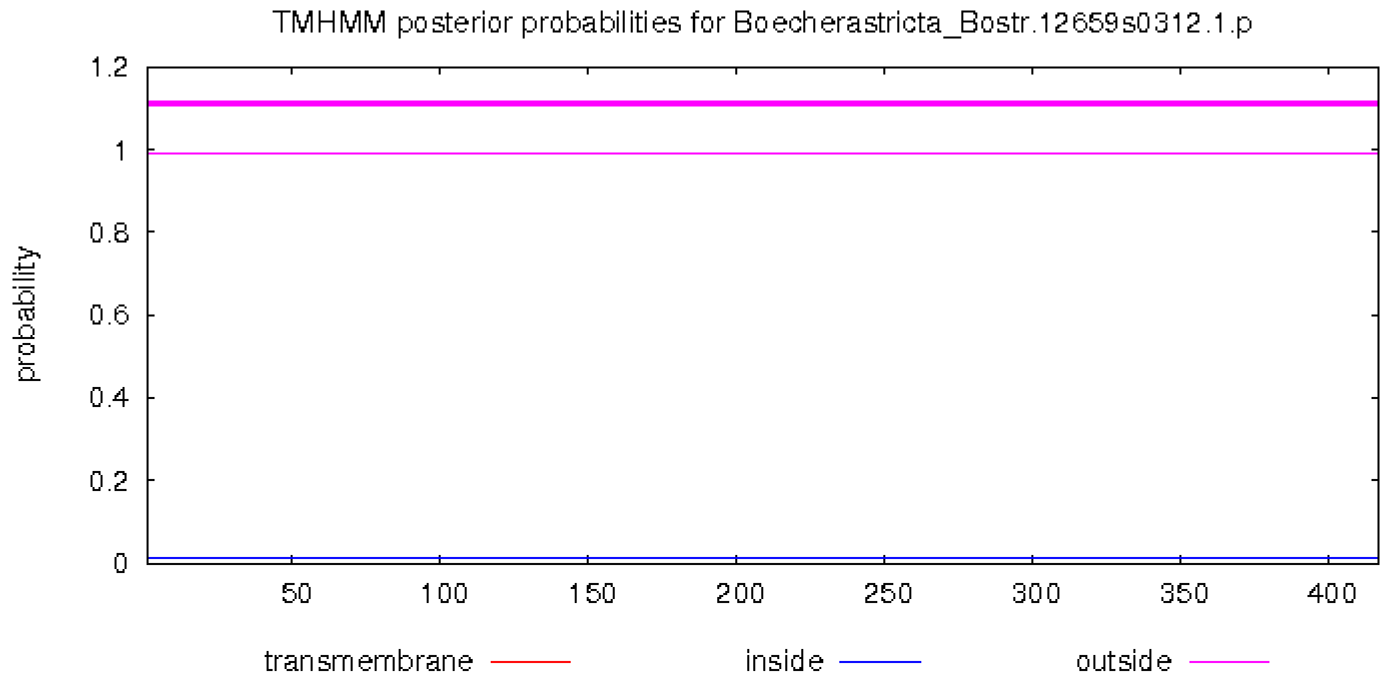

plot in postscript, script for making the plot in gnuplot, data for plot

---

```
# Boecherastricta_Bostr.12659s0396.1.p Length: 455
# Boecherastricta_Bostr.12659s0396.1.p Number of predicted TMHs:  0
# Boecherastricta_Bostr.12659s0396.1.p Exp number of AAs in TMHs: 0.00075
# Boecherastricta_Bostr.12659s0396.1.p Exp number, first 60 AAs:  0
# Boecherastricta_Bostr.12659s0396.1.p Total prob of N-in:        0.00281
Boecherastricta_Bostr.12659s0396.1.p    TMHMM2.0        outside      1   455
```

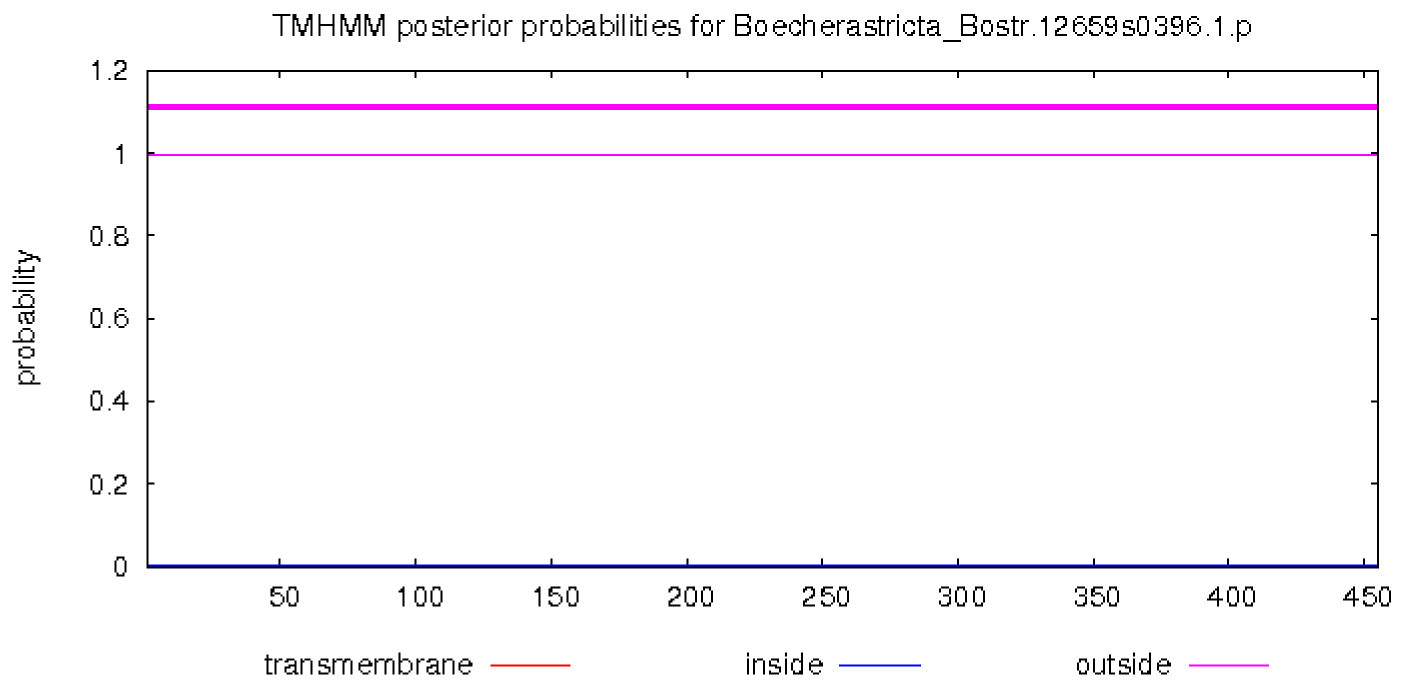

plot in postscript, script for making the plot in gnuplot, data for plot

---

```
# Boecherastricta_Bostr.13129s0062.1.p Length: 388
# Boecherastricta_Bostr.13129s0062.1.p Number of predicted TMHs:  0
# Boecherastricta_Bostr.13129s0062.1.p Exp number of AAs in TMHs: 0.00035
# Boecherastricta_Bostr.13129s0062.1.p Exp number, first 60 AAs:  0.00035
# Boecherastricta_Bostr.13129s0062.1.p Total prob of N-in:        0.00316
Boecherastricta_Bostr.13129s0062.1.p    TMHMM2.0        outside      1   388
```

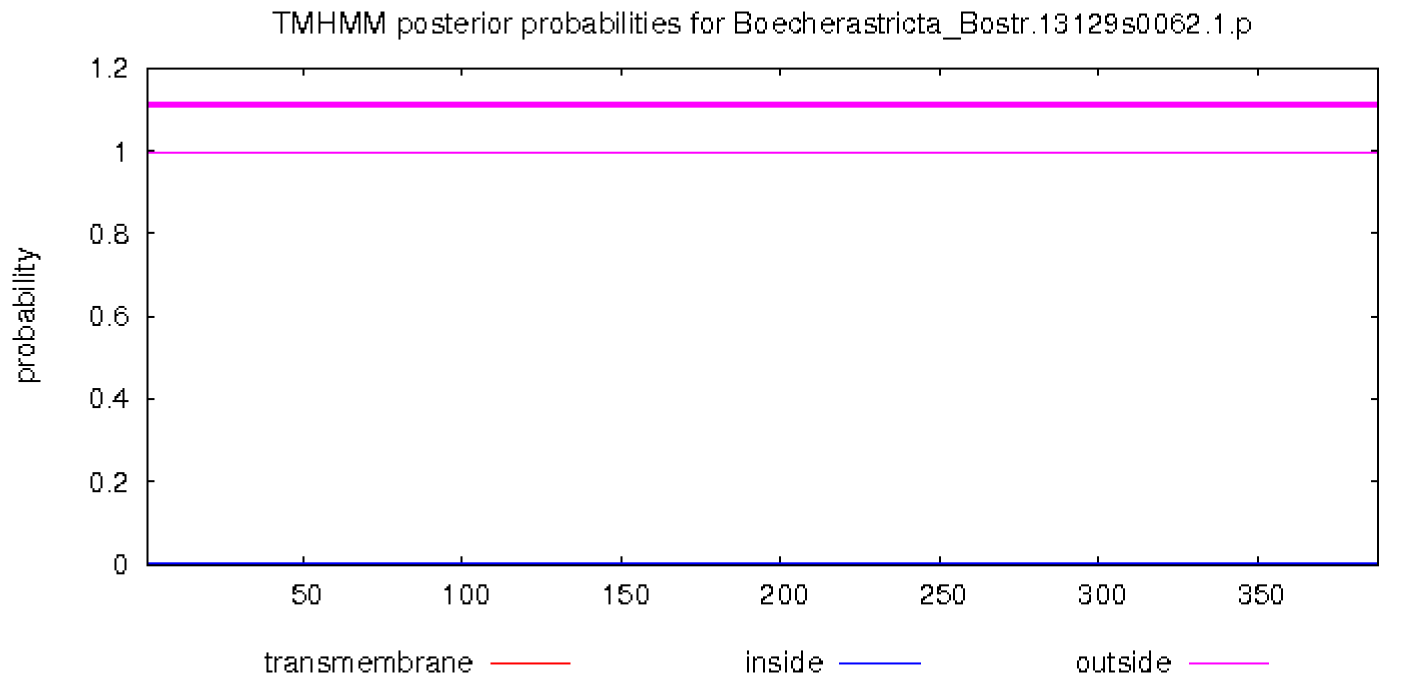

plot in postscript, script for making the plot in gnuplot, data for plot

---

```
# Boecherastricta_Bostr.13129s0073.1.p Length: 283
# Boecherastricta_Bostr.13129s0073.1.p Number of predicted TMHs:  0
# Boecherastricta_Bostr.13129s0073.1.p Exp number of AAs in TMHs: 0.00019
# Boecherastricta_Bostr.13129s0073.1.p Exp number, first 60 AAs:  0
# Boecherastricta_Bostr.13129s0073.1.p Total prob of N-in:        0.01031
Boecherastricta_Bostr.13129s0073.1.p	TMHMM2.0	outside	     1   283
```

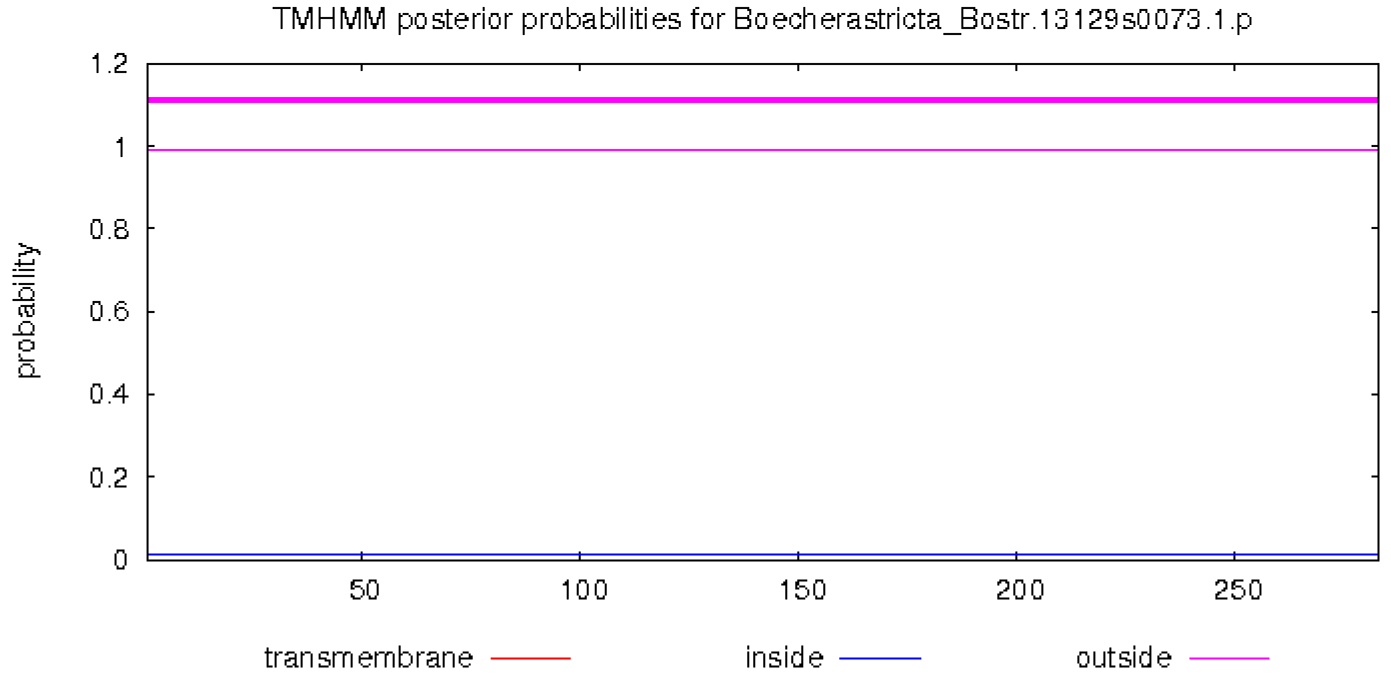

plot in postscript, script for making the plot in gnuplot, data for plot

---

```
# Boecherastricta_Bostr.13129s0198.1.p Length: 341
# Boecherastricta_Bostr.13129s0198.1.p Number of predicted TMHs:  0
# Boecherastricta_Bostr.13129s0198.1.p Exp number of AAs in TMHs: 0.00057
# Boecherastricta_Bostr.13129s0198.1.p Exp number, first 60 AAs:  0
# Boecherastricta_Bostr.13129s0198.1.p Total prob of N-in:        0.00563
Boecherastricta_Bostr.13129s0198.1.p	TMHMM2.0	outside	     1   341
```

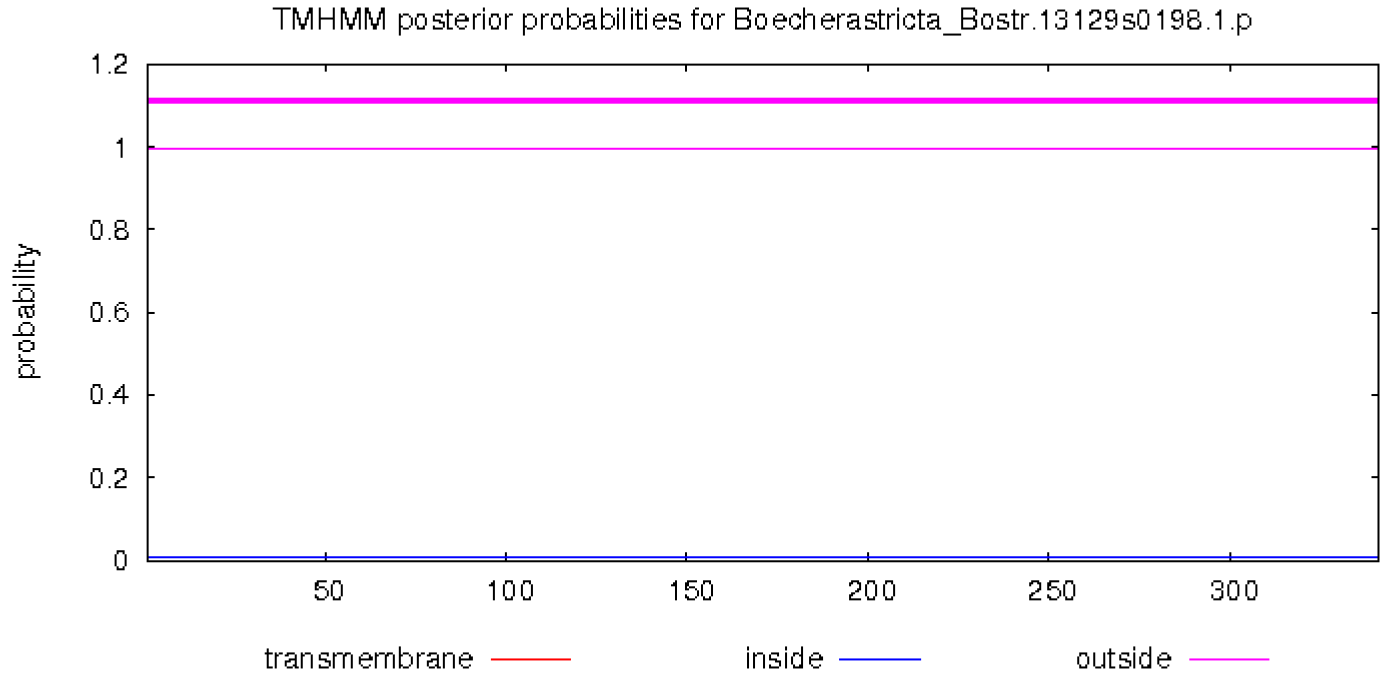

plot in postscript, script for making the plot in gnuplot, data for plot

---

```
# Boecherastricta_Bostr.13158s0196.1.p Length: 339
# Boecherastricta_Bostr.13158s0196.1.p Number of predicted TMHs:  0
# Boecherastricta_Bostr.13158s0196.1.p Exp number of AAs in TMHs: 0
# Boecherastricta_Bostr.13158s0196.1.p Exp number, first 60 AAs:  0
# Boecherastricta_Bostr.13158s0196.1.p Total prob of N-in:        0.02603
Boecherastricta_Bostr.13158s0196.1.p	TMHMM2.0	outside	     1   339
```

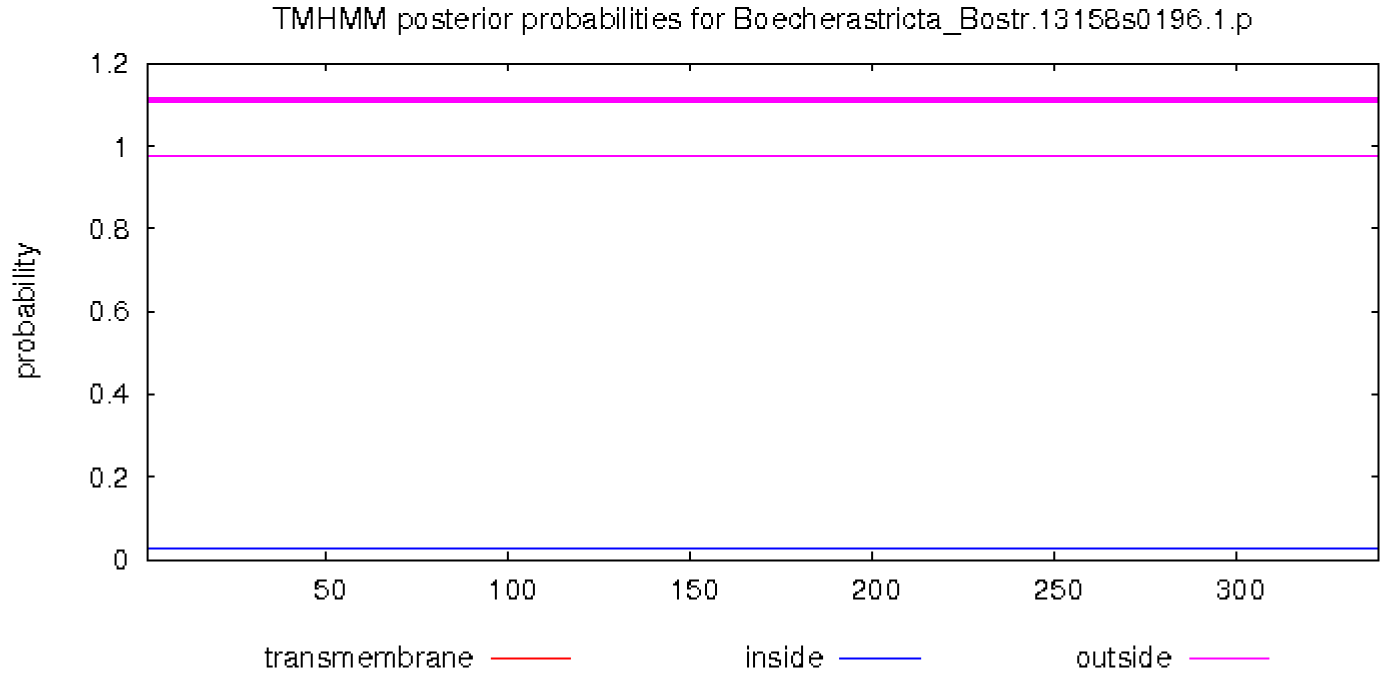

plot in postscript, script for making the plot in gnuplot, data for plot

---

```
# Boecherastricta_Bostr.13671s0233.1.p Length: 342
# Boecherastricta_Bostr.13671s0233.1.p Number of predicted TMHs:  0
# Boecherastricta_Bostr.13671s0233.1.p Exp number of AAs in TMHs: 0.00018
# Boecherastricta_Bostr.13671s0233.1.p Exp number, first 60 AAs:  0
# Boecherastricta_Bostr.13671s0233.1.p Total prob of N-in:        0.00322
Boecherastricta_Bostr.13671s0233.1.p	TMHMM2.0	outside	     1   342
```

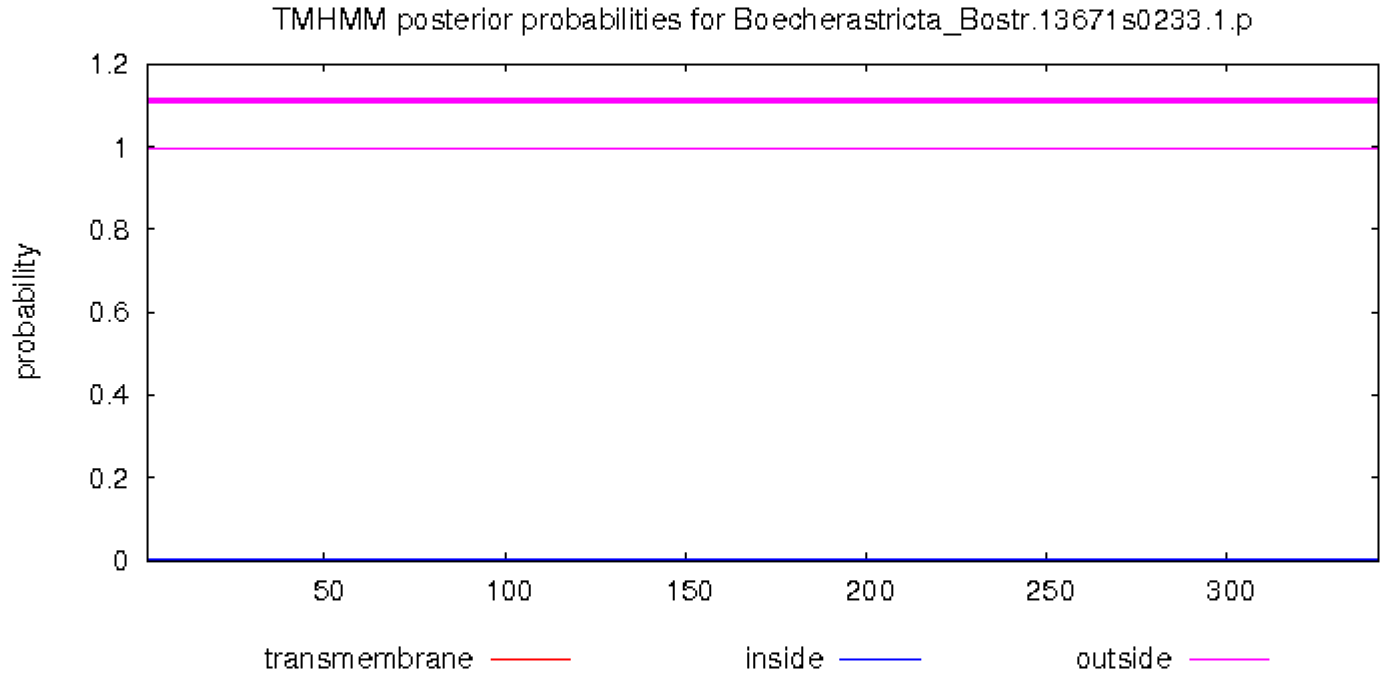

plot in postscript, script for making the plot in gnuplot, data for plot

```
# Boecherastricta_Bostr.1460s0050.1.p Length: 365
# Boecherastricta_Bostr.1460s0050.1.p Number of predicted TMHs:  0
# Boecherastricta_Bostr.1460s0050.1.p Exp number of AAs in TMHs: 0.00018
# Boecherastricta_Bostr.1460s0050.1.p Exp number, first 60 AAs:  0
# Boecherastricta_Bostr.1460s0050.1.p Total prob of N-in:        0.02764
Boecherastricta_Bostr.1460s0050.1.p     TMHMM2.0        outside      1   365
```

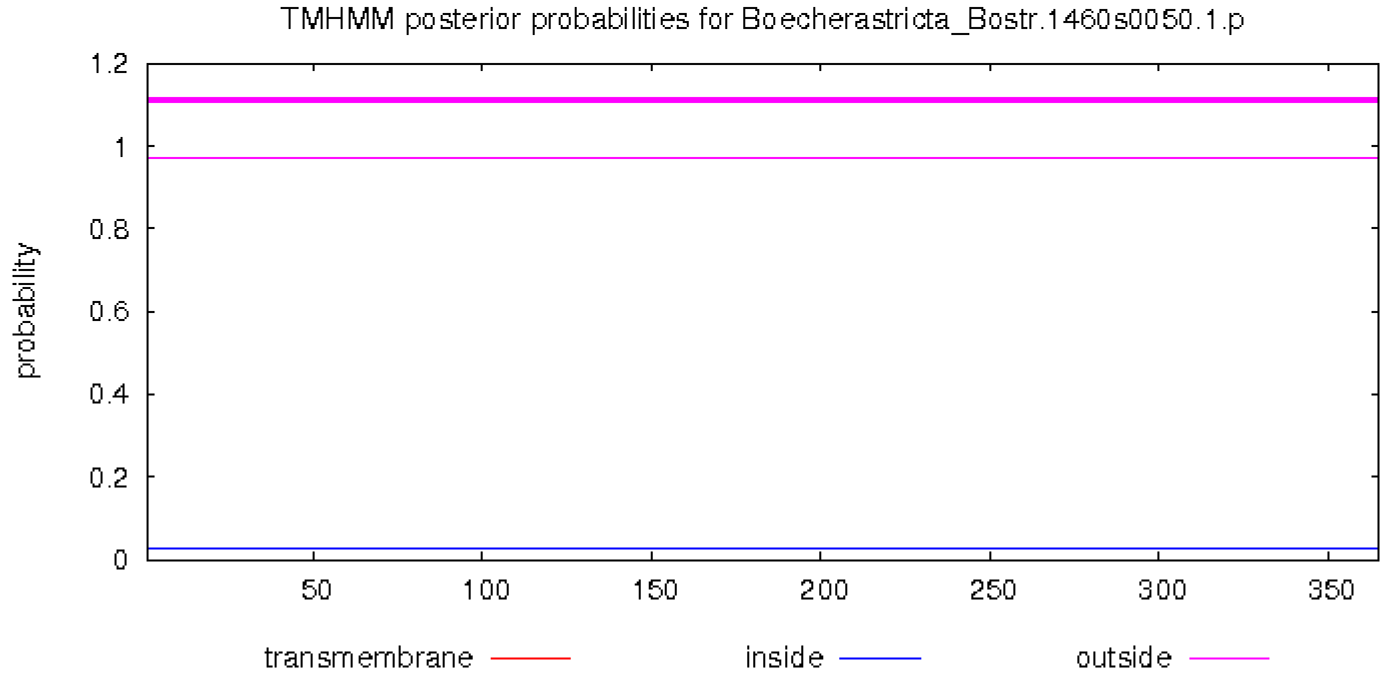

plot in postscript, script for making the plot in gnuplot, data for plot

```
# Boecherastricta_Bostr.15697s0168.1.p Length: 316
# Boecherastricta_Bostr.15697s0168.1.p Number of predicted TMHs:  0
# Boecherastricta_Bostr.15697s0168.1.p Exp number of AAs in TMHs: 0.0002
# Boecherastricta_Bostr.15697s0168.1.p Exp number, first 60 AAs:  0
# Boecherastricta_Bostr.15697s0168.1.p Total prob of N-in:        0.02878
Boecherastricta_Bostr.15697s0168.1.p    TMHMM2.0        outside      1   316
```

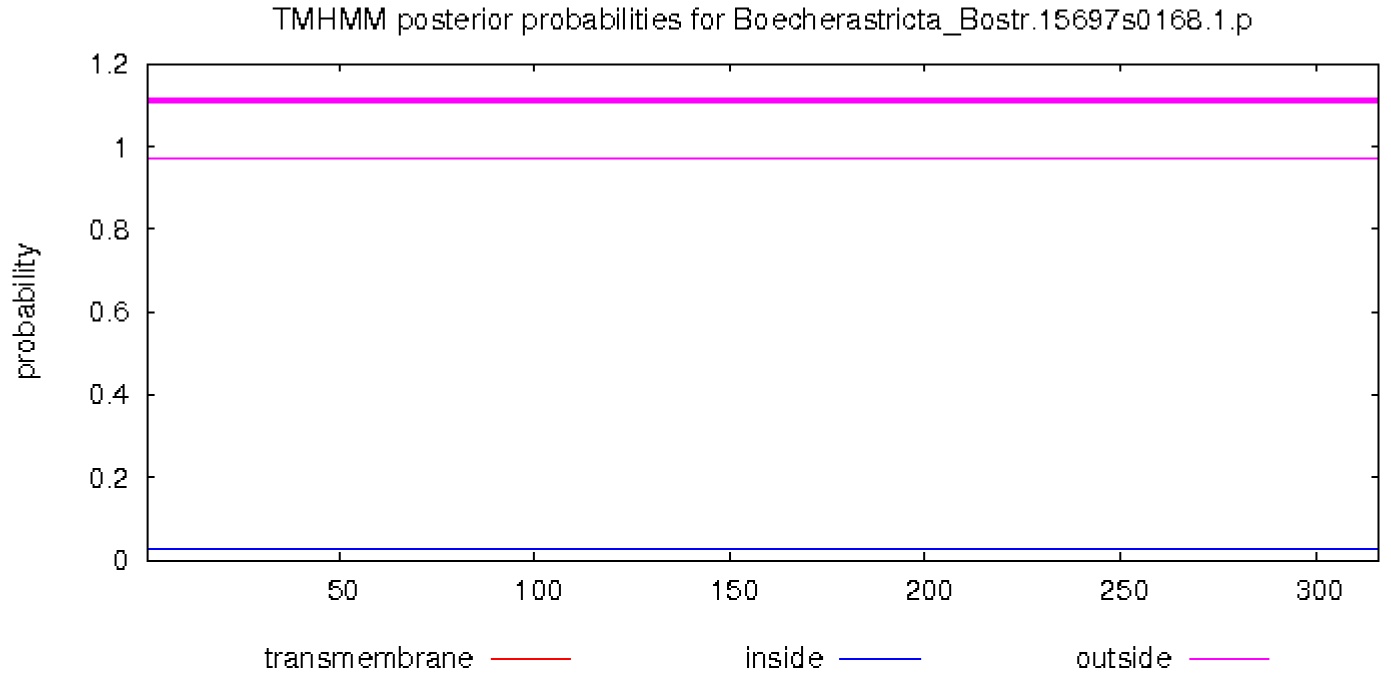

plot in postscript, script for making the plot in gnuplot, data for plot

---

```
# Boecherastricta_Bostr.15697s0466.1.p Length: 259
# Boecherastricta_Bostr.15697s0466.1.p Number of predicted TMHs:  0
# Boecherastricta_Bostr.15697s0466.1.p Exp number of AAs in TMHs: 3.04775
# Boecherastricta_Bostr.15697s0466.1.p Exp number, first 60 AAs:  0.00114
# Boecherastricta_Bostr.15697s0466.1.p Total prob of N-in:        0.01511
Boecherastricta_Bostr.15697s0466.1.p	TMHMM2.0	outside	     1   259
```

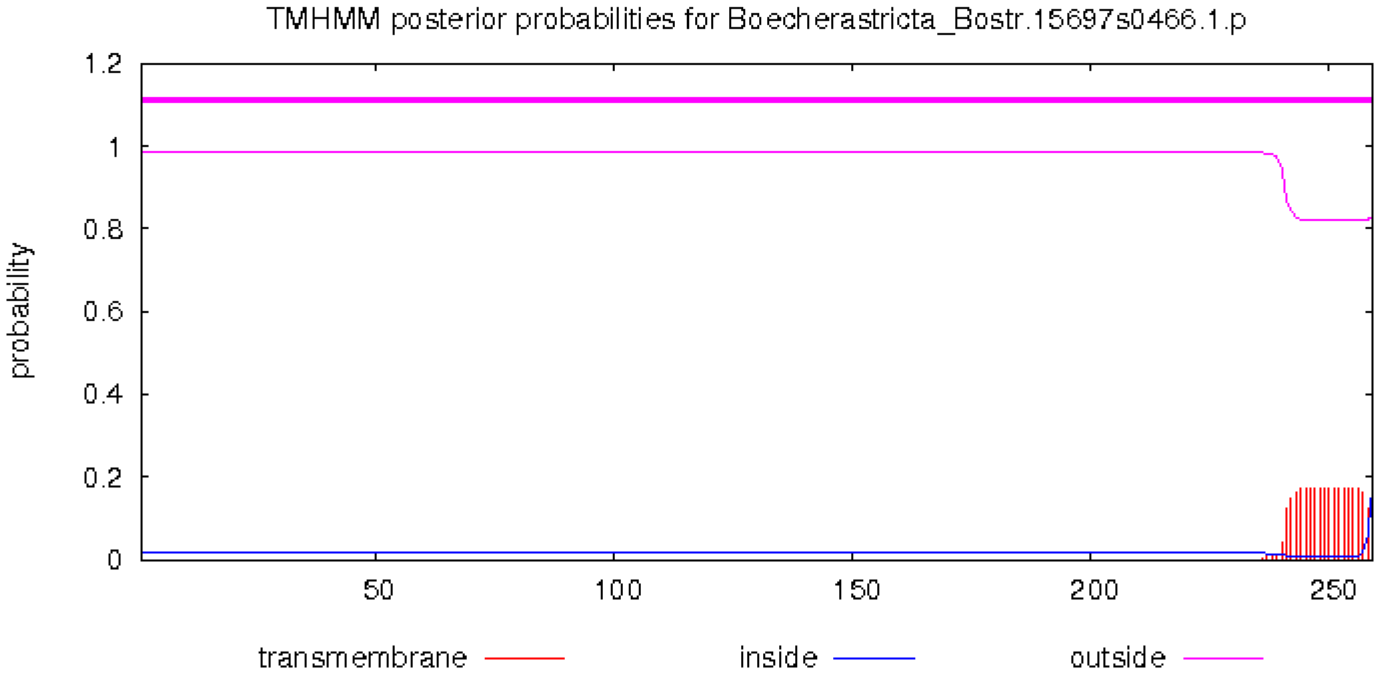

plot in postscript, script for making the plot in gnuplot, data for plot

---

```
# Boecherastricta_Bostr.15774s0177.1.p Length: 186
# Boecherastricta_Bostr.15774s0177.1.p Number of predicted TMHs:  0
# Boecherastricta_Bostr.15774s0177.1.p Exp number of AAs in TMHs: 0.00129
# Boecherastricta_Bostr.15774s0177.1.p Exp number, first 60 AAs:  0.00016
# Boecherastricta_Bostr.15774s0177.1.p Total prob of N-in:        0.08884
Boecherastricta_Bostr.15774s0177.1.p	TMHMM2.0	outside	     1   186
```

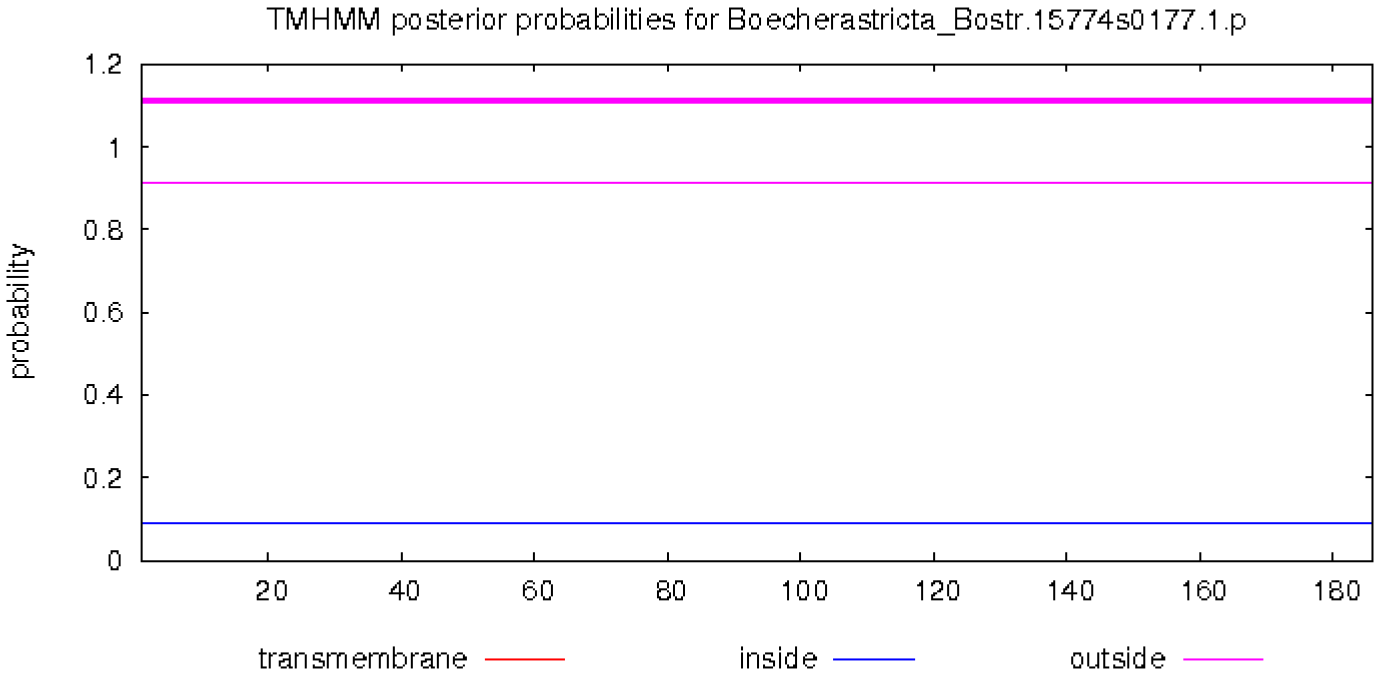

plot in postscript, script for making the plot in gnuplot, data for plot

```
# Boecherastricta_Bostr.15774s0178.1.p Length: 234
# Boecherastricta_Bostr.15774s0178.1.p Number of predicted TMHs:  0
# Boecherastricta_Bostr.15774s0178.1.p Exp number of AAs in TMHs: 0.01104
# Boecherastricta_Bostr.15774s0178.1.p Exp number, first 60 AAs:  0.00031
# Boecherastricta_Bostr.15774s0178.1.p Total prob of N-in:        0.03581
Boecherastricta_Bostr.15774s0178.1.p	TMHMM2.0	outside	     1   234
```

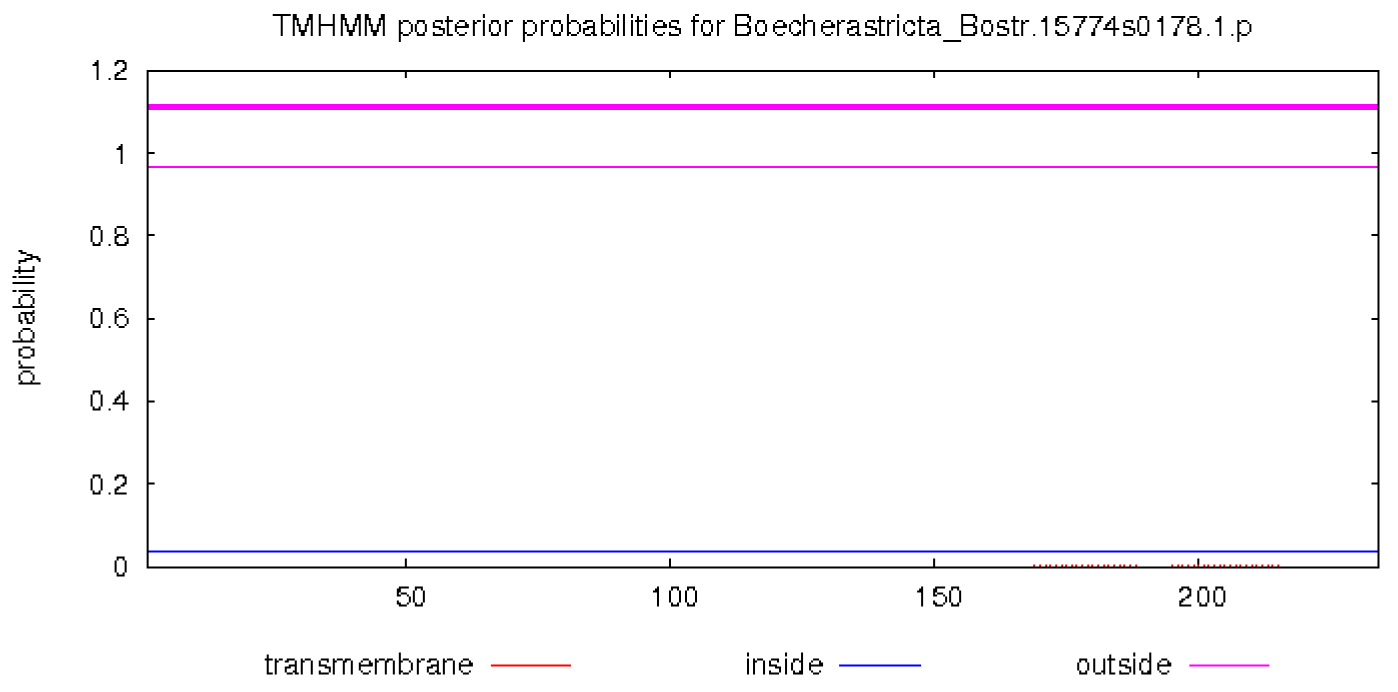

plot in postscript, script for making the plot in gnuplot, data for plot

```
# Boecherastricta_Bostr.15774s0222.1.p Length: 193
# Boecherastricta_Bostr.15774s0222.1.p Number of predicted TMHs:  0
# Boecherastricta_Bostr.15774s0222.1.p Exp number of AAs in TMHs: 0.00058
# Boecherastricta_Bostr.15774s0222.1.p Exp number, first 60 AAs:  0
# Boecherastricta_Bostr.15774s0222.1.p Total prob of N-in:        0.12748
Boecherastricta_Bostr.15774s0222.1.p	TMHMM2.0	outside	     1   193
```

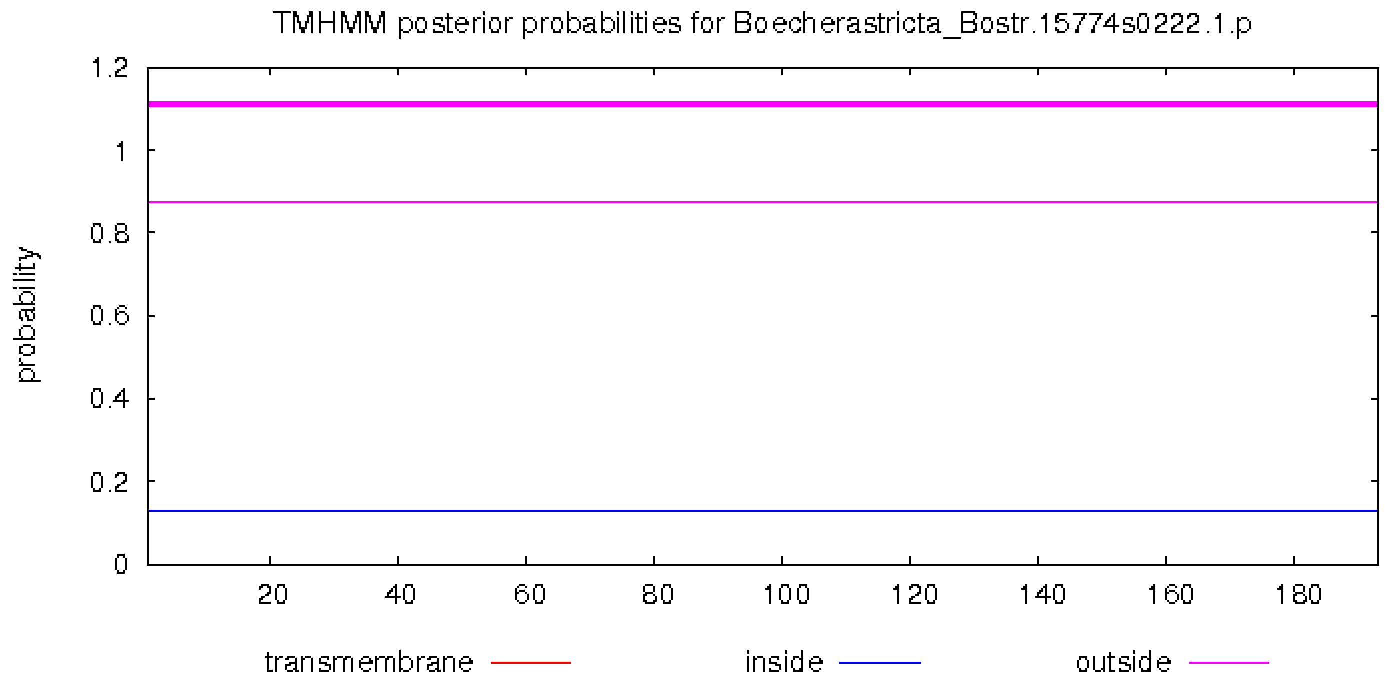

plot in postscript, script for making the plot in gnuplot, data for plot

```
# Boecherastricta_Bostr.18351s0021.1.p Length: 480
# Boecherastricta_Bostr.18351s0021.1.p Number of predicted TMHs:  0
# Boecherastricta_Bostr.18351s0021.1.p Exp number of AAs in TMHs: 0.00079
# Boecherastricta_Bostr.18351s0021.1.p Exp number, first 60 AAs:  0.0002
# Boecherastricta_Bostr.18351s0021.1.p Total prob of N-in:        0.01451
Boecherastricta_Bostr.18351s0021.1.p    TMHMM2.0        outside      1   480
```

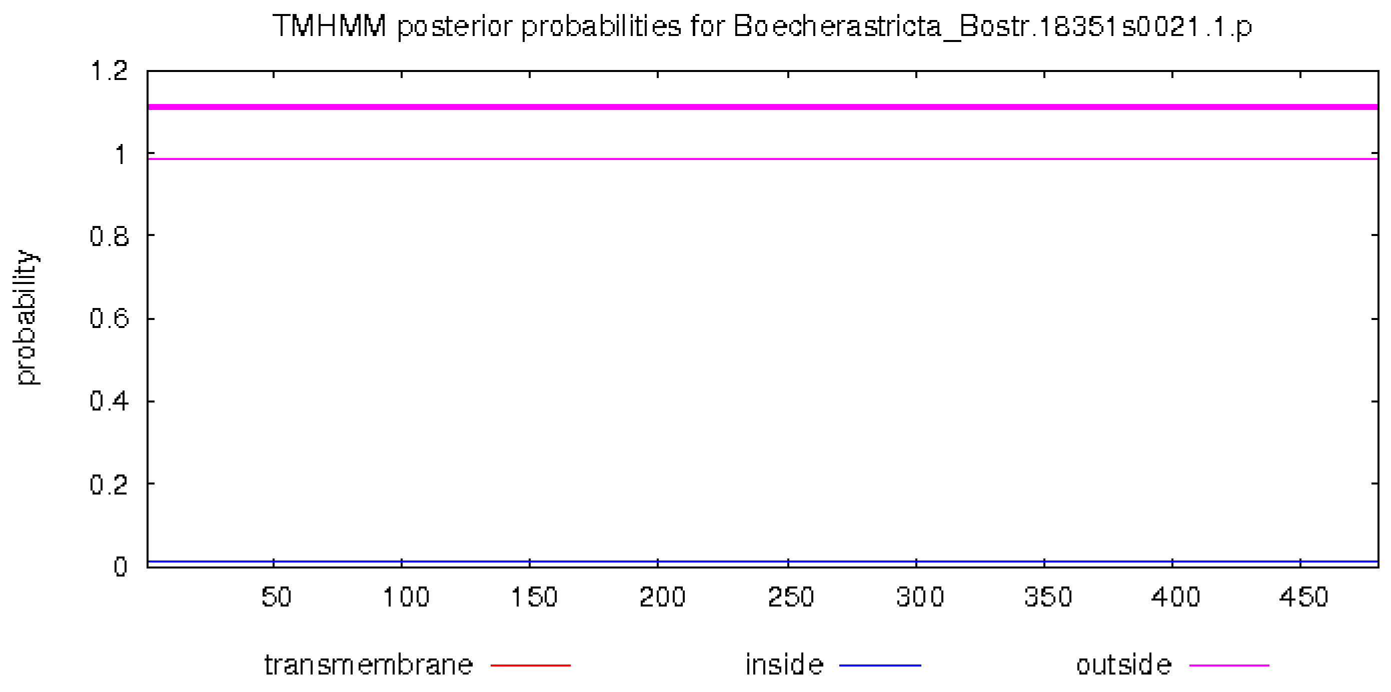

plot in postscript, script for making the plot in gnuplot, data for plot

```
# Boecherastricta_Bostr.18351s0165.1.p Length: 280
# Boecherastricta_Bostr.18351s0165.1.p Number of predicted TMHs:  0
# Boecherastricta_Bostr.18351s0165.1.p Exp number of AAs in TMHs: 0.00081
# Boecherastricta_Bostr.18351s0165.1.p Exp number, first 60 AAs:  0
# Boecherastricta_Bostr.18351s0165.1.p Total prob of N-in:        0.00650
Boecherastricta_Bostr.18351s0165.1.p    TMHMM2.0        outside      1   280
```

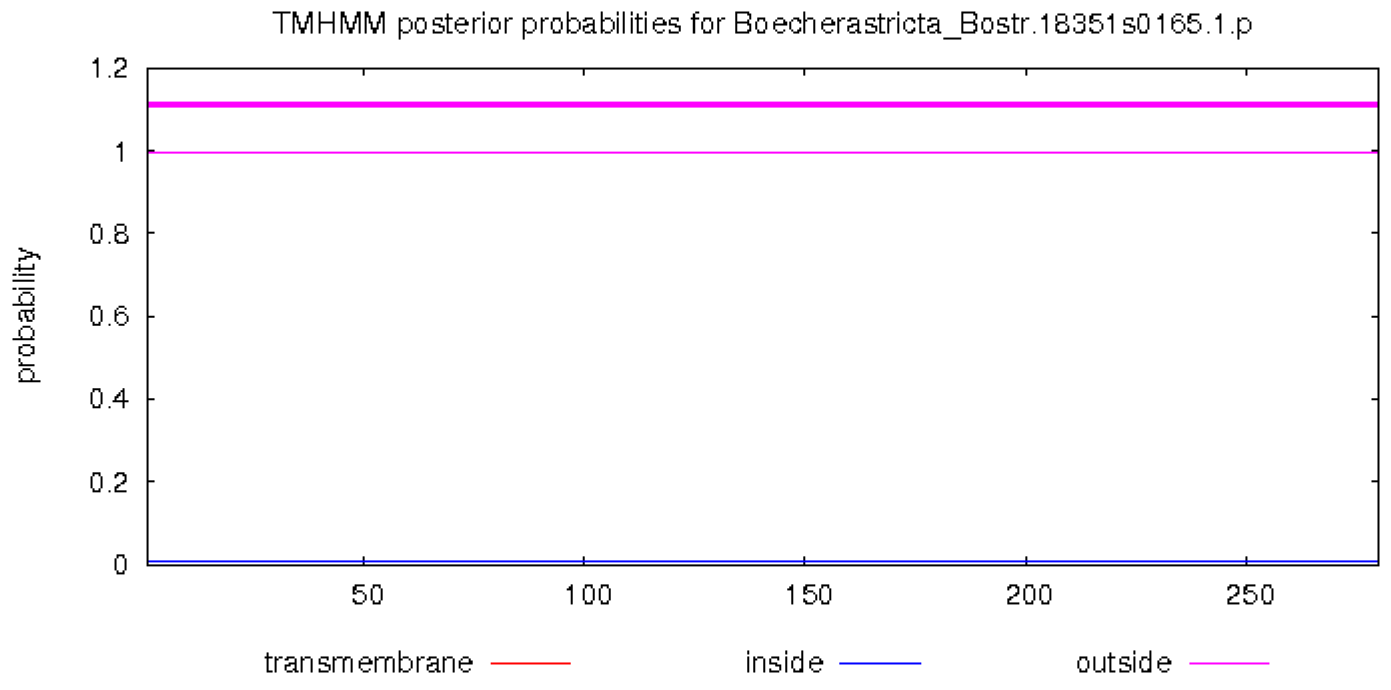

plot in postscript, script for making the plot in gnuplot, data for plot

```
# Boecherastricta_Bostr.18351s0273.1.p Length: 365
# Boecherastricta_Bostr.18351s0273.1.p Number of predicted TMHs:  0
# Boecherastricta_Bostr.18351s0273.1.p Exp number of AAs in TMHs: 0.00019
# Boecherastricta_Bostr.18351s0273.1.p Exp number, first 60 AAs:  0
# Boecherastricta_Bostr.18351s0273.1.p Total prob of N-in:        0.00601
Boecherastricta_Bostr.18351s0273.1.p	TMHMM2.0	outside	     1   365
```

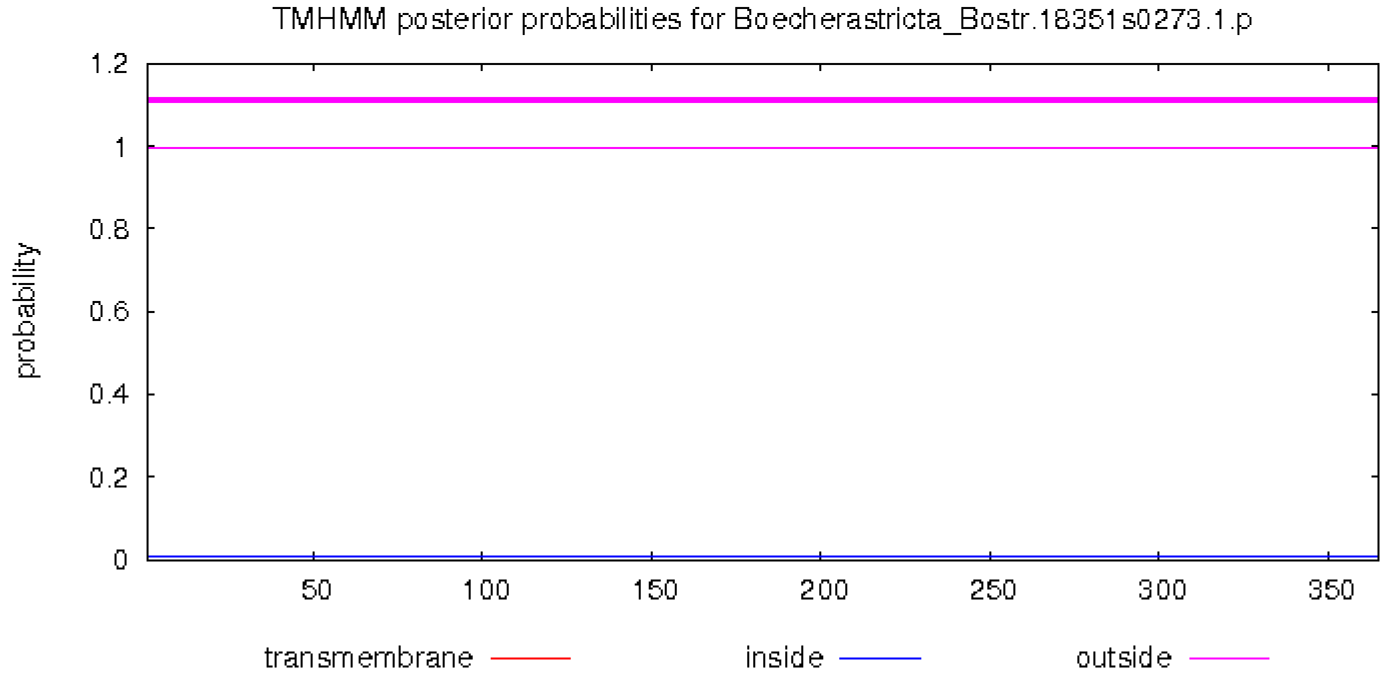

plot in postscript, script for making the plot in gnuplot, data for plot

```
# Boecherastricta_Bostr.19046s0031.1.p Length: 479
# Boecherastricta_Bostr.19046s0031.1.p Number of predicted TMHs:  0
# Boecherastricta_Bostr.19046s0031.1.p Exp number of AAs in TMHs: 0.00182
# Boecherastricta_Bostr.19046s0031.1.p Exp number, first 60 AAs:  0
# Boecherastricta_Bostr.19046s0031.1.p Total prob of N-in:        0.00016
Boecherastricta_Bostr.19046s0031.1.p	TMHMM2.0	outside	     1   479
```

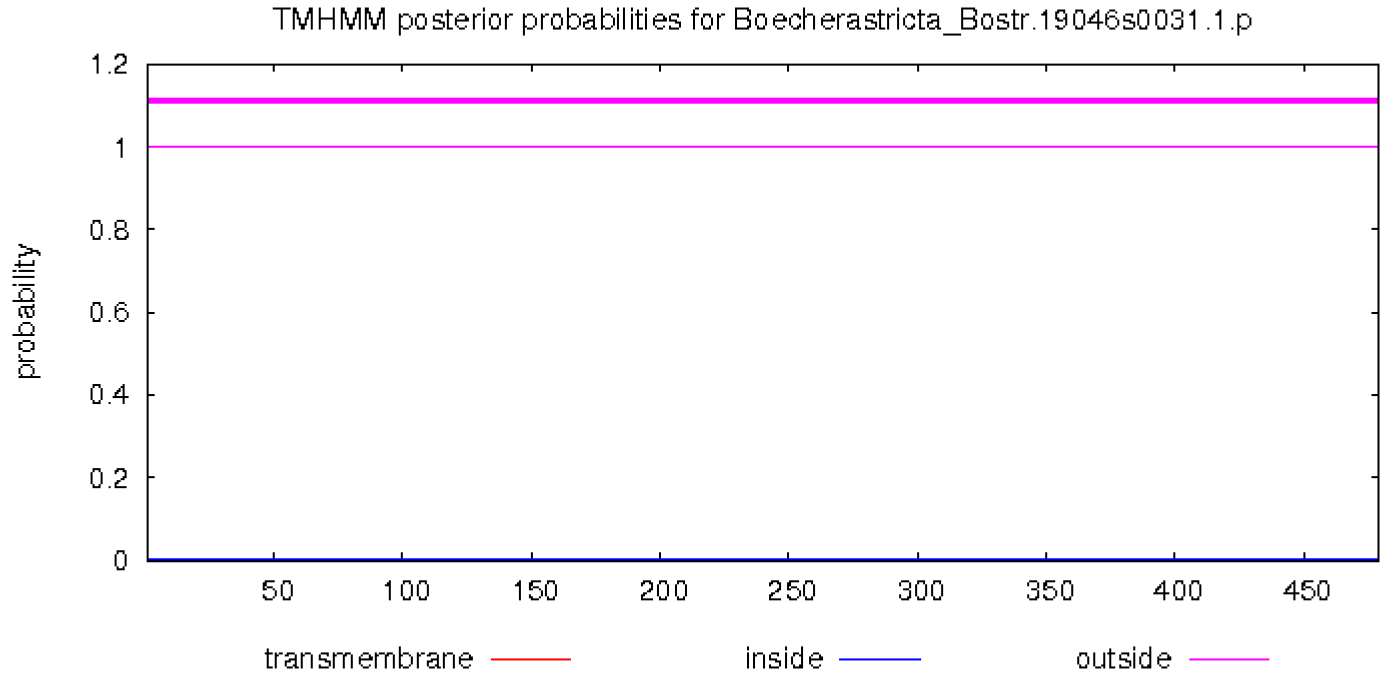

plot in postscript, script for making the plot in gnuplot, data for plot

---

```
# Boecherastricta_Bostr.19424s0822.1.p Length: 311
# Boecherastricta_Bostr.19424s0822.1.p Number of predicted TMHs:  0
# Boecherastricta_Bostr.19424s0822.1.p Exp number of AAs in TMHs: 0.07732
# Boecherastricta_Bostr.19424s0822.1.p Exp number, first 60 AAs:  0
# Boecherastricta_Bostr.19424s0822.1.p Total prob of N-in:        0.00513
Boecherastricta_Bostr.19424s0822.1.p	TMHMM2.0	outside	     1   311
```

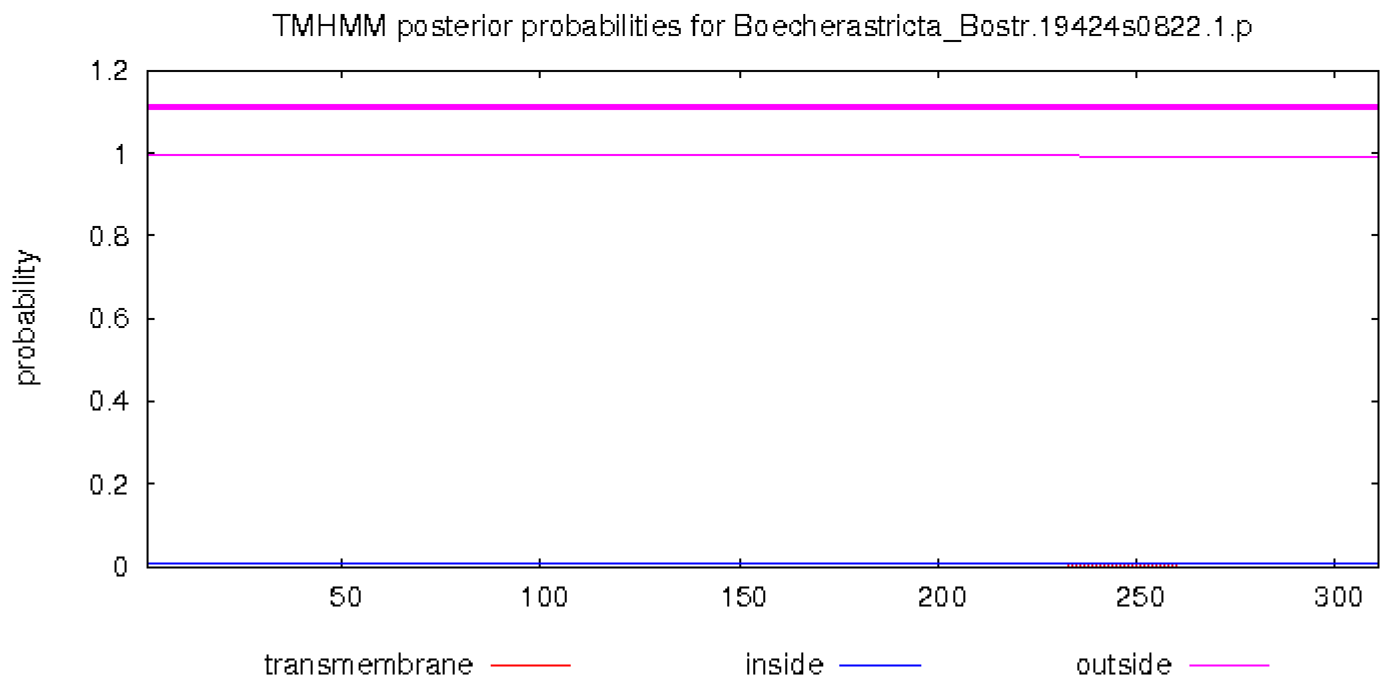

plot in postscript, script for making the plot in gnuplot, data for plot

---

```
# Boecherastricta_Bostr.19424s0897.1.p Length: 244
# Boecherastricta_Bostr.19424s0897.1.p Number of predicted TMHs:  0
# Boecherastricta_Bostr.19424s0897.1.p Exp number of AAs in TMHs: 0
# Boecherastricta_Bostr.19424s0897.1.p Exp number, first 60 AAs:  0
# Boecherastricta_Bostr.19424s0897.1.p Total prob of N-in:        0.00228
Boecherastricta_Bostr.19424s0897.1.p	TMHMM2.0	outside	     1   244
```

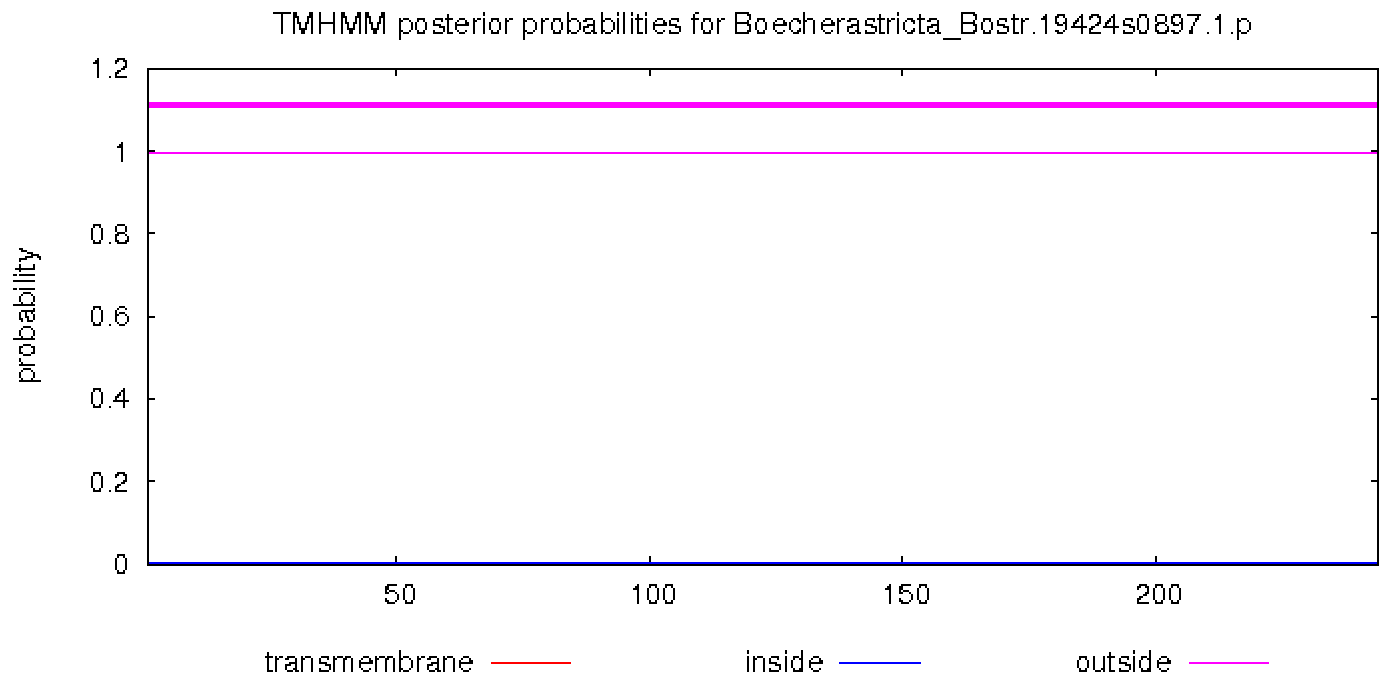

plot in postscript, script for making the plot in gnuplot, data for plot

---

```
# Boecherastricta_Bostr.20129s0079.1.p Length: 341
# Boecherastricta_Bostr.20129s0079.1.p Number of predicted TMHs:  0
# Boecherastricta_Bostr.20129s0079.1.p Exp number of AAs in TMHs: 0.01079
# Boecherastricta_Bostr.20129s0079.1.p Exp number, first 60 AAs:  0.0049
# Boecherastricta_Bostr.20129s0079.1.p Total prob of N-in:        0.00472
Boecherastricta_Bostr.20129s0079.1.p	TMHMM2.0	outside	     1   341
```

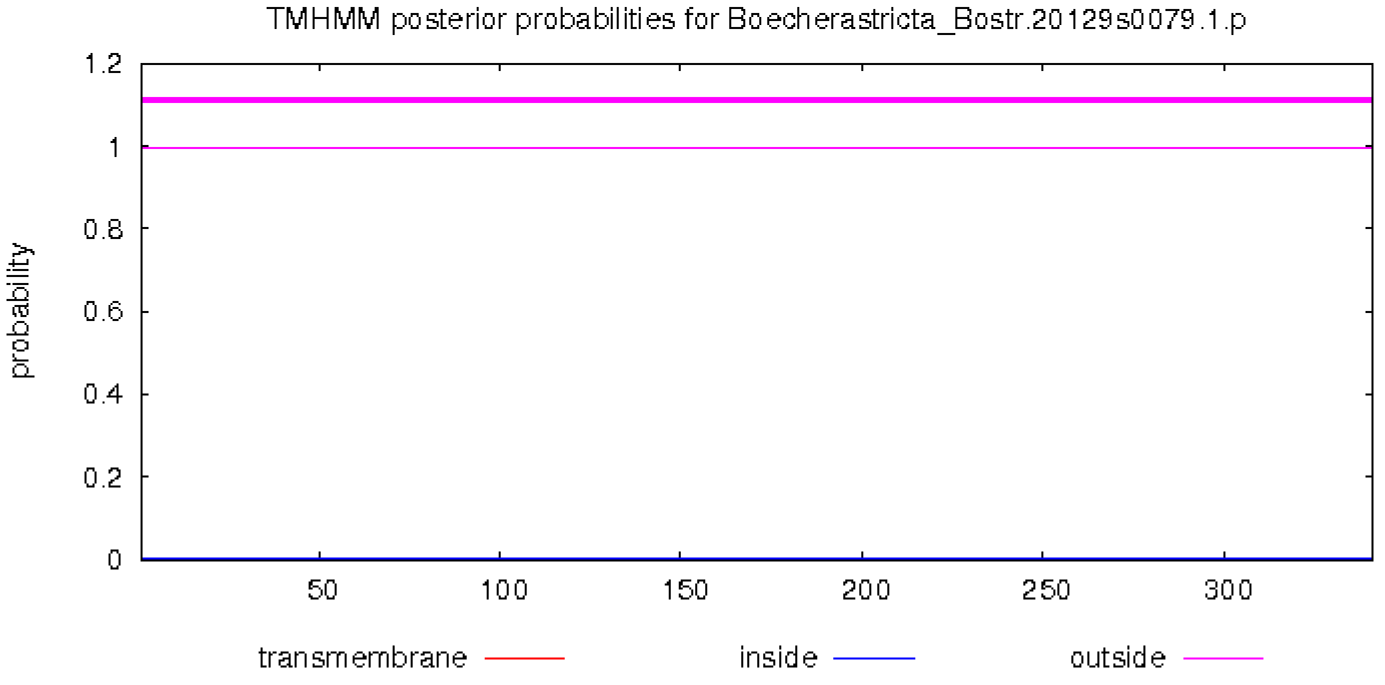

plot in postscript, script for making the plot in gnuplot, data for plot

---

```
# Boecherastricta_Bostr.20129s0180.1.p Length: 255
# Boecherastricta_Bostr.20129s0180.1.p Number of predicted TMHs:  0
# Boecherastricta_Bostr.20129s0180.1.p Exp number of AAs in TMHs: 0.00019
# Boecherastricta_Bostr.20129s0180.1.p Exp number, first 60 AAs:  0
# Boecherastricta_Bostr.20129s0180.1.p Total prob of N-in:        0.03267
Boecherastricta_Bostr.20129s0180.1.p	TMHMM2.0	outside	     1   255
```

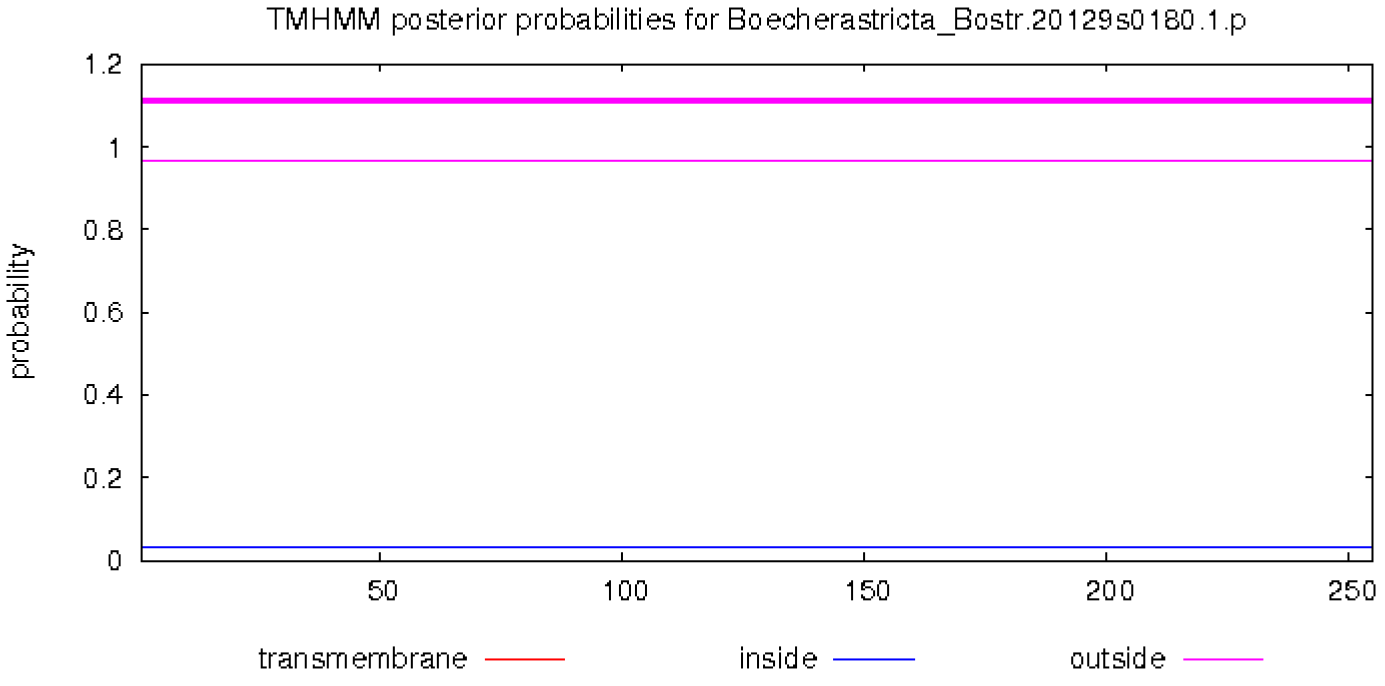

plot in postscript, script for making the plot in gnuplot, data for plot

---

```
# Boecherastricta_Bostr.20129s0396.1.p Length: 376
# Boecherastricta_Bostr.20129s0396.1.p Number of predicted TMHs:  0
# Boecherastricta_Bostr.20129s0396.1.p Exp number of AAs in TMHs: 0.00765
# Boecherastricta_Bostr.20129s0396.1.p Exp number, first 60 AAs:  0.00096
# Boecherastricta_Bostr.20129s0396.1.p Total prob of N-in:        0.00721
Boecherastricta_Bostr.20129s0396.1.p	TMHMM2.0	outside	     1   376
```

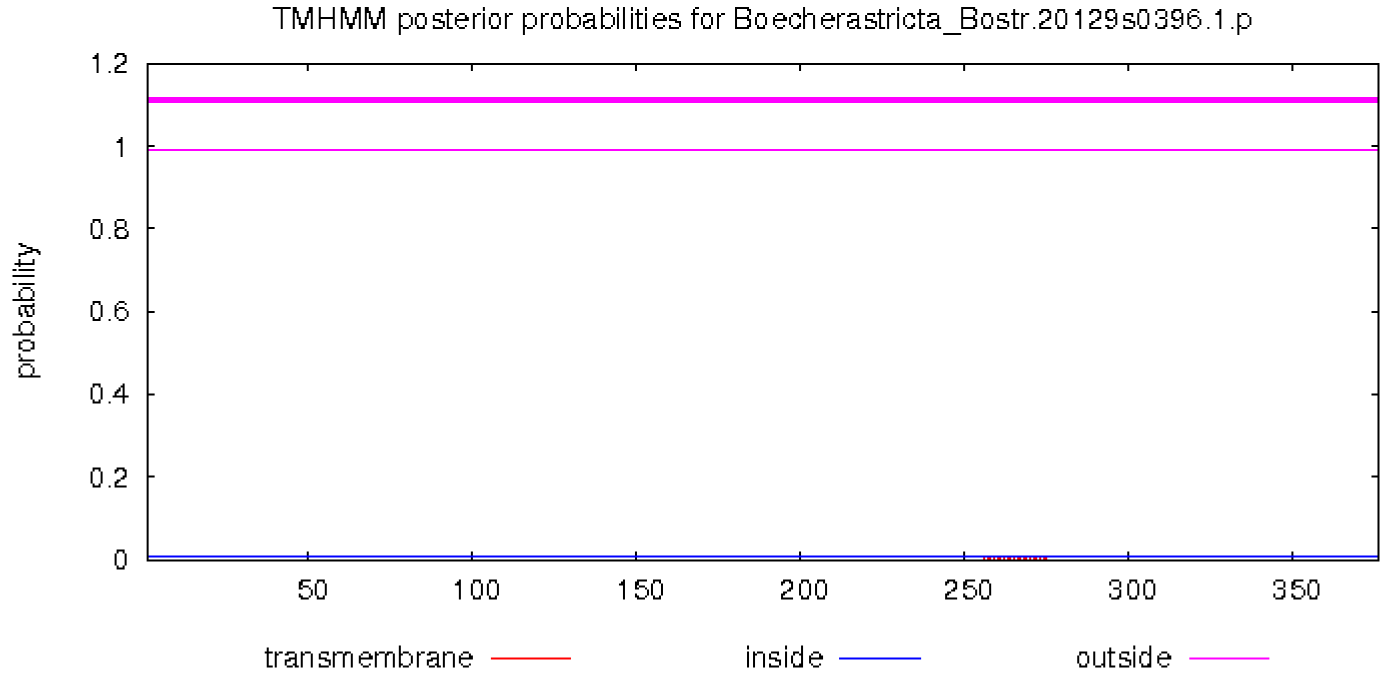

plot in postscript, script for making the plot in gnuplot, data for plot

---

```
# Boecherastricta_Bostr.20505s0101.1.p Length: 299
# Boecherastricta_Bostr.20505s0101.1.p Number of predicted TMHs:  0
# Boecherastricta_Bostr.20505s0101.1.p Exp number of AAs in TMHs: 0.00019
# Boecherastricta_Bostr.20505s0101.1.p Exp number, first 60 AAs:  0
# Boecherastricta_Bostr.20505s0101.1.p Total prob of N-in:        0.08381
Boecherastricta_Bostr.20505s0101.1.p	TMHMM2.0	outside	     1   299
```

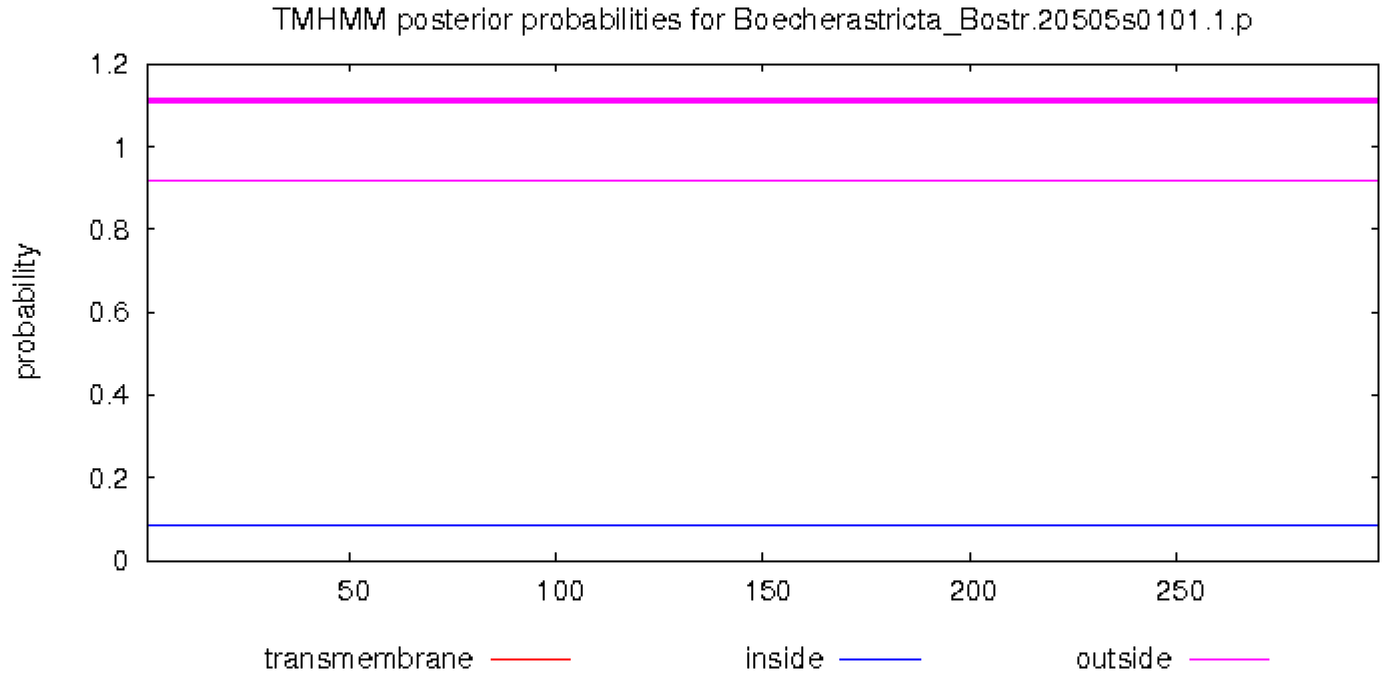

plot in postscript, script for making the plot in gnuplot, data for plot

---

```
# Boecherastricta_Bostr.20505s0102.1.p Length: 168
# Boecherastricta_Bostr.20505s0102.1.p Number of predicted TMHs:  0
# Boecherastricta_Bostr.20505s0102.1.p Exp number of AAs in TMHs: 0.00016
# Boecherastricta_Bostr.20505s0102.1.p Exp number, first 60 AAs:  0
# Boecherastricta_Bostr.20505s0102.1.p Total prob of N-in:        0.04978
Boecherastricta_Bostr.20505s0102.1.p	TMHMM2.0	outside	     1   168
```

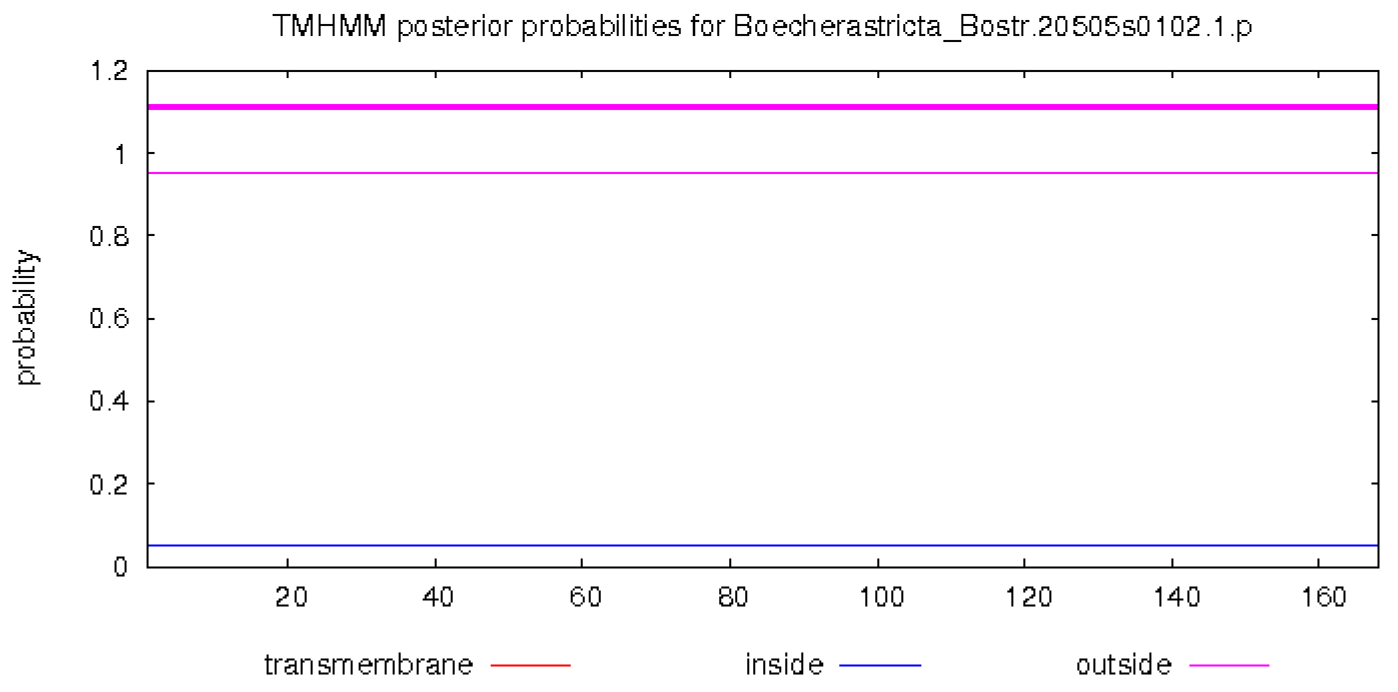

plot in postscript, script for making the plot in gnuplot, data for plot

---

```
# Boecherastricta_Bostr.21132s0013.1.p Length: 301
# Boecherastricta_Bostr.21132s0013.1.p Number of predicted TMHs:  0
# Boecherastricta_Bostr.21132s0013.1.p Exp number of AAs in TMHs: 0
# Boecherastricta_Bostr.21132s0013.1.p Exp number, first 60 AAs:  0
# Boecherastricta_Bostr.21132s0013.1.p Total prob of N-in:        0.05563
Boecherastricta_Bostr.21132s0013.1.p	TMHMM2.0	outside	     1   301
```

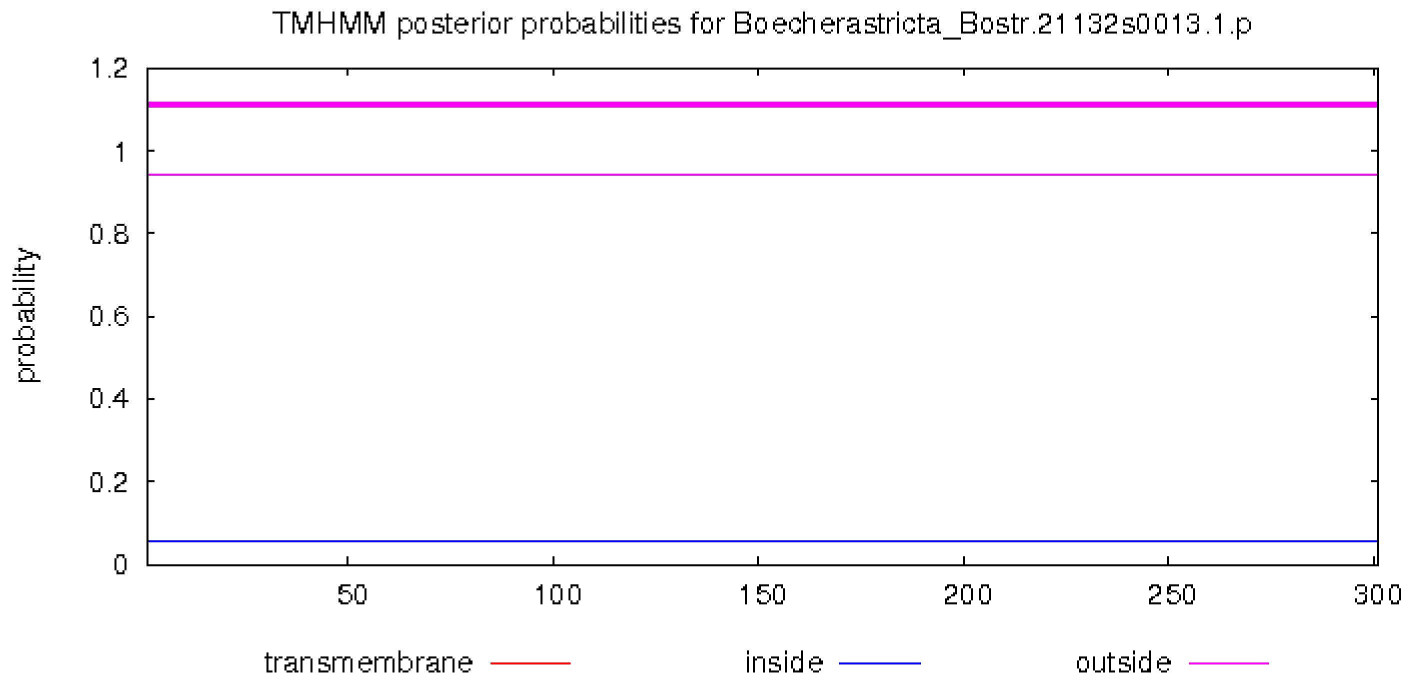

plot in postscript, script for making the plot in gnuplot, data for plot

---

```
# Boecherastricta_Bostr.2128s0007.1.p Length: 563
# Boecherastricta_Bostr.2128s0007.1.p Number of predicted TMHs:  1
# Boecherastricta_Bostr.2128s0007.1.p Exp number of AAs in TMHs: 20.8559
# Boecherastricta_Bostr.2128s0007.1.p Exp number, first 60 AAs:  0
# Boecherastricta_Bostr.2128s0007.1.p Total prob of N-in:        0.01862
Boecherastricta_Bostr.2128s0007.1.p     TMHMM2.0        outside      1   537
Boecherastricta_Bostr.2128s0007.1.p     TMHMM2.0        TMhelix    538   560
Boecherastricta_Bostr.2128s0007.1.p     TMHMM2.0        inside     561   563
```

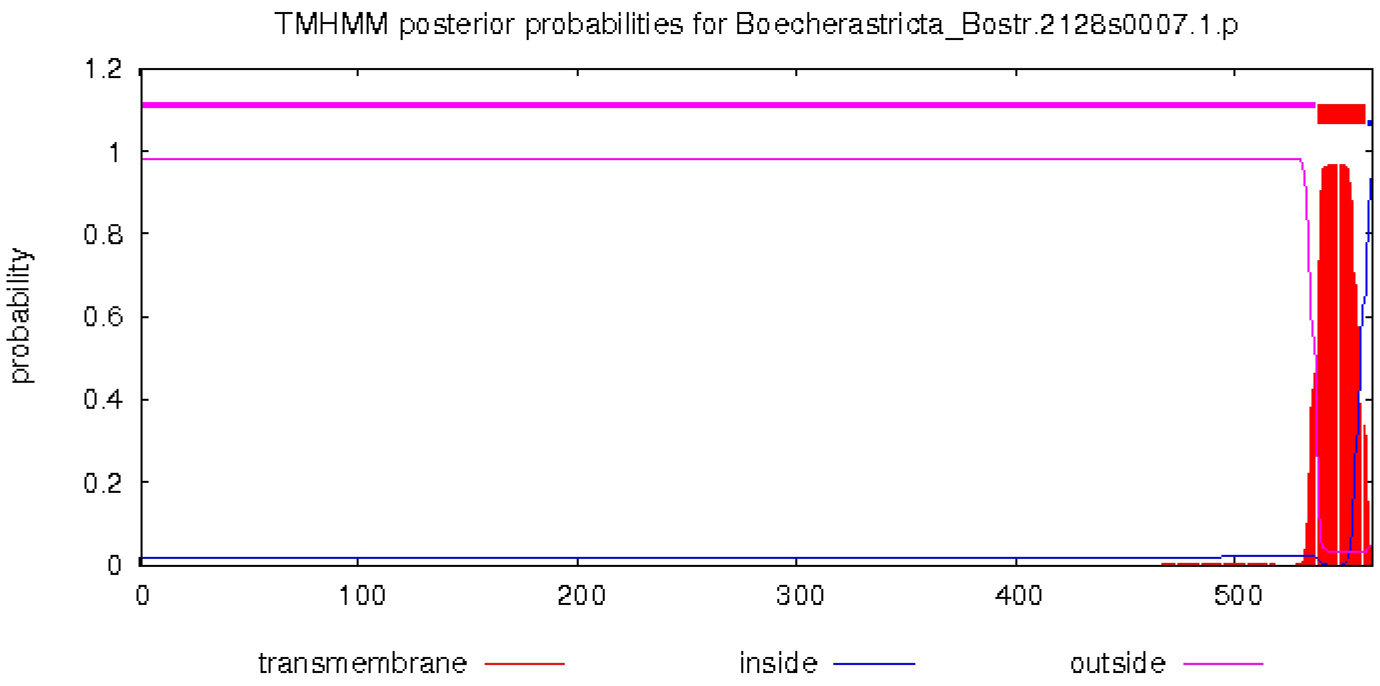

plot in postscript, script for making the plot in gnuplot, data for plot

---

```
# Boecherastricta_Bostr.2128s0008.1.p Length: 405
# Boecherastricta_Bostr.2128s0008.1.p Number of predicted TMHs:  0
# Boecherastricta_Bostr.2128s0008.1.p Exp number of AAs in TMHs: 0.826740000000000001
# Boecherastricta_Bostr.2128s0008.1.p Exp number, first 60 AAs:  0.0043
# Boecherastricta_Bostr.2128s0008.1.p Total prob of N-in:        0.05920
Boecherastricta_Bostr.2128s0008.1.p     TMHMM2.0        outside      1   405
```

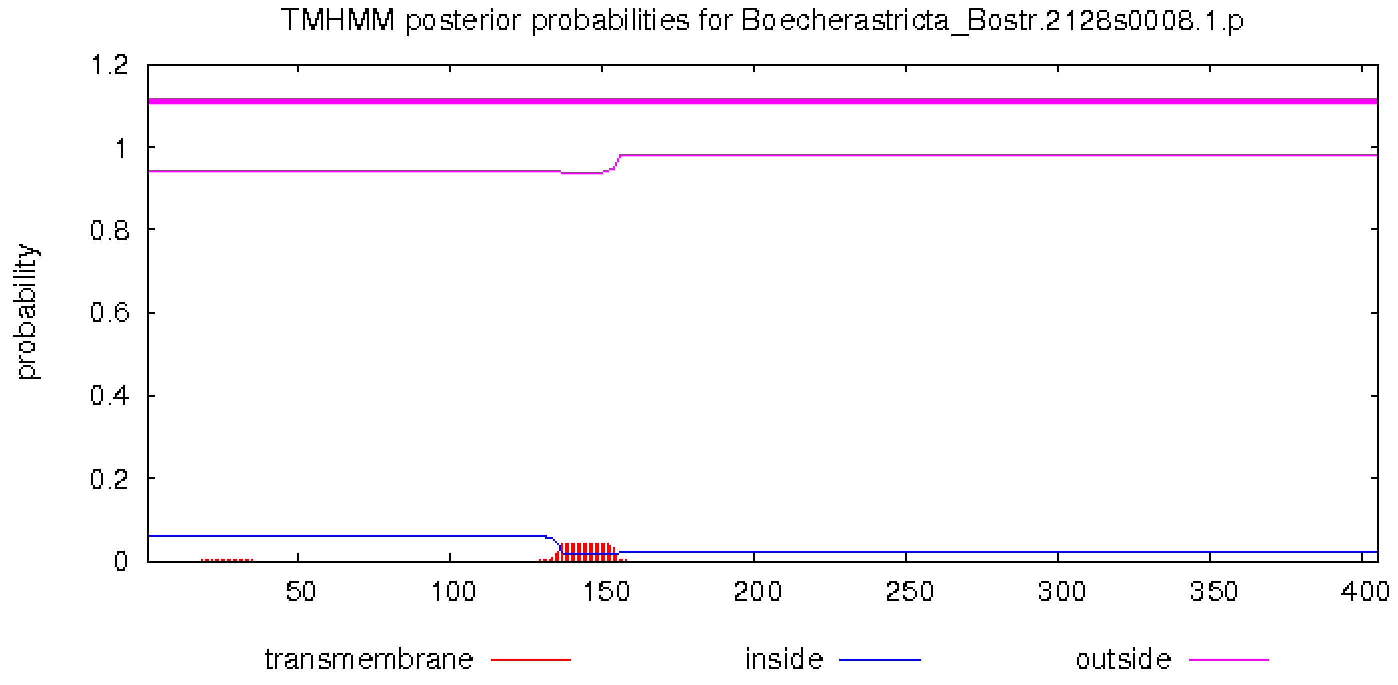

plot in postscript, script for making the plot in gnuplot, data for plot

---

```
# Boecherastricta_Bostr.22157s0066.1.p Length: 409
# Boecherastricta_Bostr.22157s0066.1.p Number of predicted TMHs:  0
# Boecherastricta_Bostr.22157s0066.1.p Exp number of AAs in TMHs: 0.00018
# Boecherastricta_Bostr.22157s0066.1.p Exp number, first 60 AAs:  0
# Boecherastricta_Bostr.22157s0066.1.p Total prob of N-in:        0.00169
Boecherastricta_Bostr.22157s0066.1.p    TMHMM2.0        outside      1   409
```

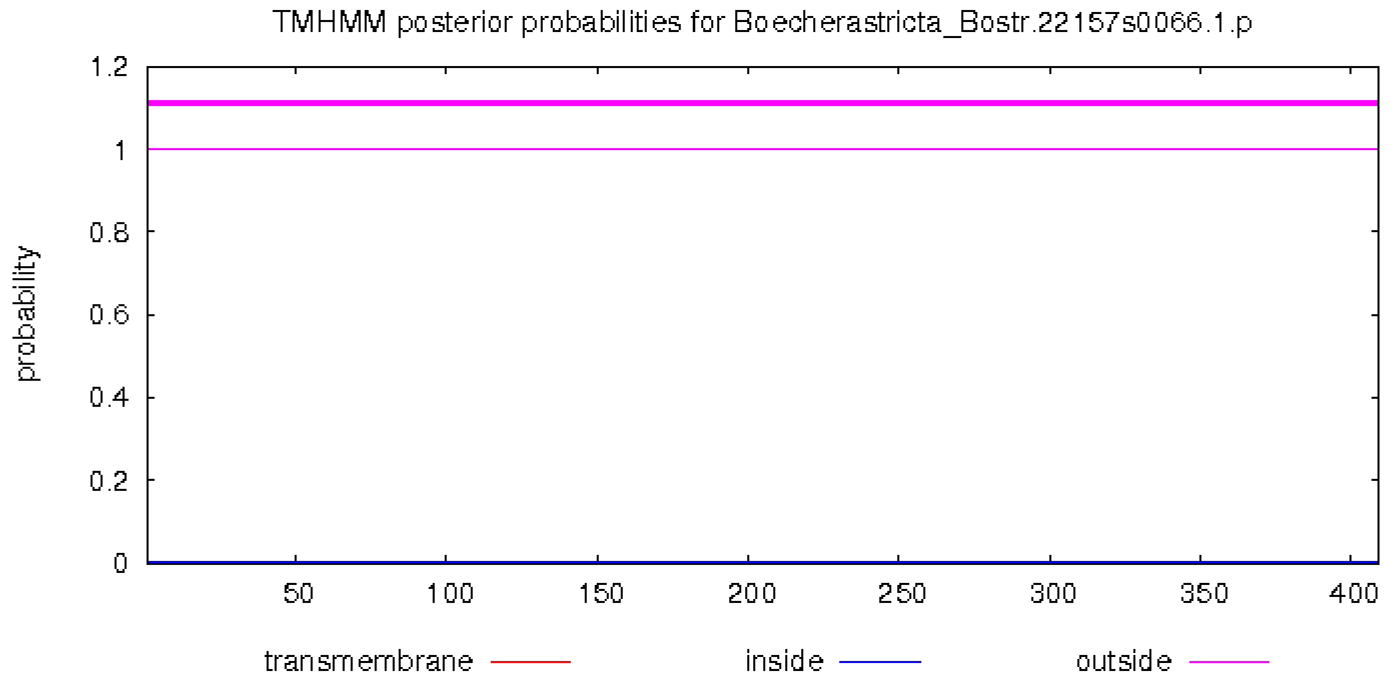

plot in postscript, script for making the plot in gnuplot, data for plot

---

```
# Boecherastricta_Bostr.22252s0010.1.p Length: 558
# Boecherastricta_Bostr.22252s0010.1.p Number of predicted TMHs:  1
# Boecherastricta_Bostr.22252s0010.1.p Exp number of AAs in TMHs: 21.62134
# Boecherastricta_Bostr.22252s0010.1.p Exp number, first 60 AAs:  0
# Boecherastricta_Bostr.22252s0010.1.p Total prob of N-in:        0.00725
Boecherastricta_Bostr.22252s0010.1.p    TMHMM2.0        outside      1   533
```

```
Boecherastricta_Bostr.22252s0010.1.p    TMHMM2.0        TMhelix    534   556
Boecherastricta_Bostr.22252s0010.1.p    TMHMM2.0        inside     557   558
```

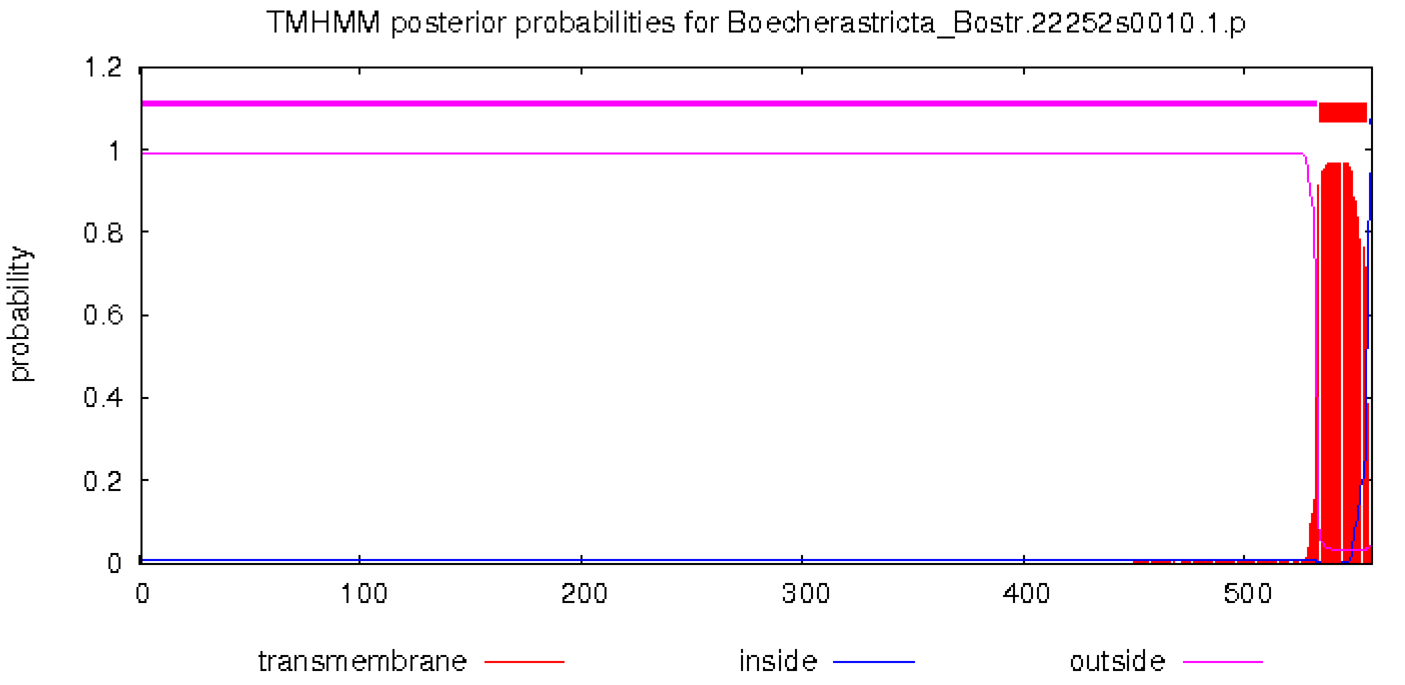

plot in postscript, script for making the plot in gnuplot, data for plot

---

```
# Boecherastricta_Bostr.22252s0011.1.p Length: 450
# Boecherastricta_Bostr.22252s0011.1.p Number of predicted TMHs:  0
# Boecherastricta_Bostr.22252s0011.1.p Exp number of AAs in TMHs: 0.43797
# Boecherastricta_Bostr.22252s0011.1.p Exp number, first 60 AAs:  0.42454
# Boecherastricta_Bostr.22252s0011.1.p Total prob of N-in:        0.02562
Boecherastricta_Bostr.22252s0011.1.p    TMHMM2.0        outside      1   450
```

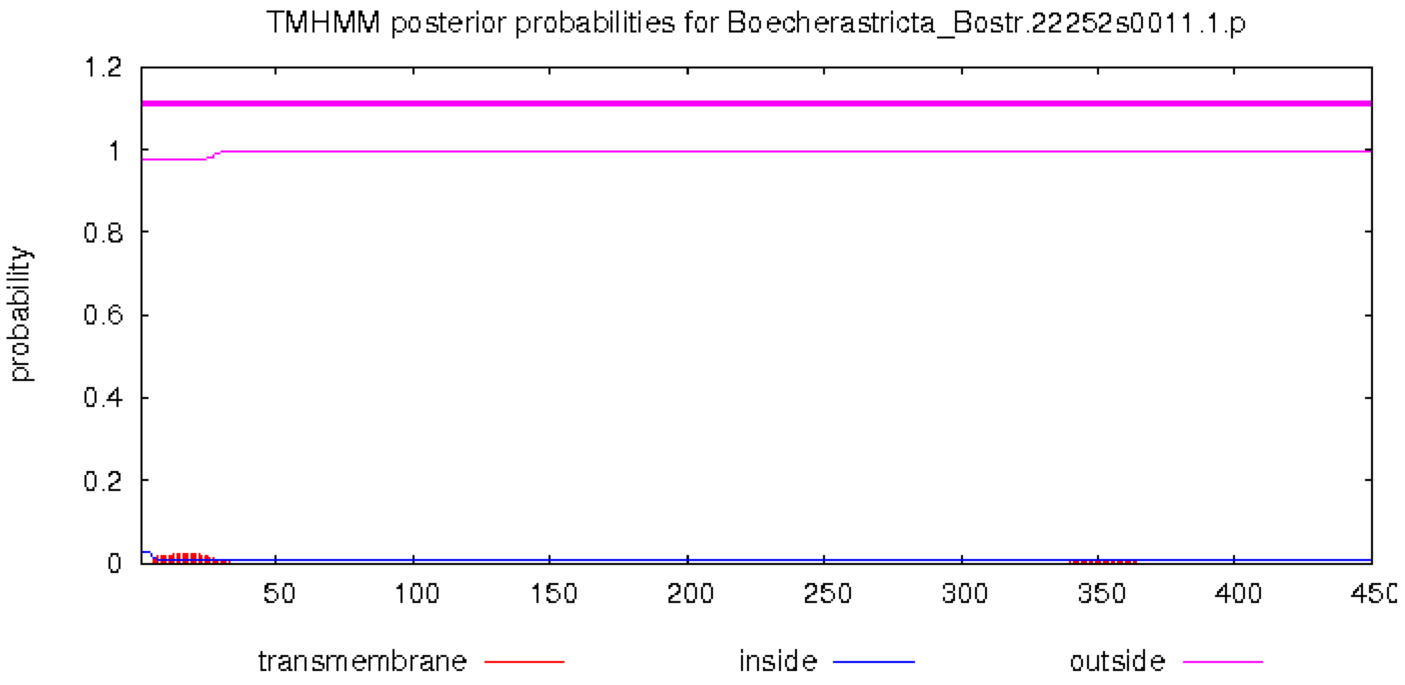

plot in postscript, script for making the plot in gnuplot, data for plot

---

```
# Boecherastricta_Bostr.22252s0011.2.p Length: 447
# Boecherastricta_Bostr.22252s0011.2.p Number of predicted TMHs:  0
# Boecherastricta_Bostr.22252s0011.2.p Exp number of AAs in TMHs: 0.43807
# Boecherastricta_Bostr.22252s0011.2.p Exp number, first 60 AAs:  0.42449
```

```
# Boecherastricta_Bostr.22252s0011.2.p Total prob of N-in:        0.02584
Boecherastricta_Bostr.22252s0011.2.p    TMHMM2.0        outside      1   447
```

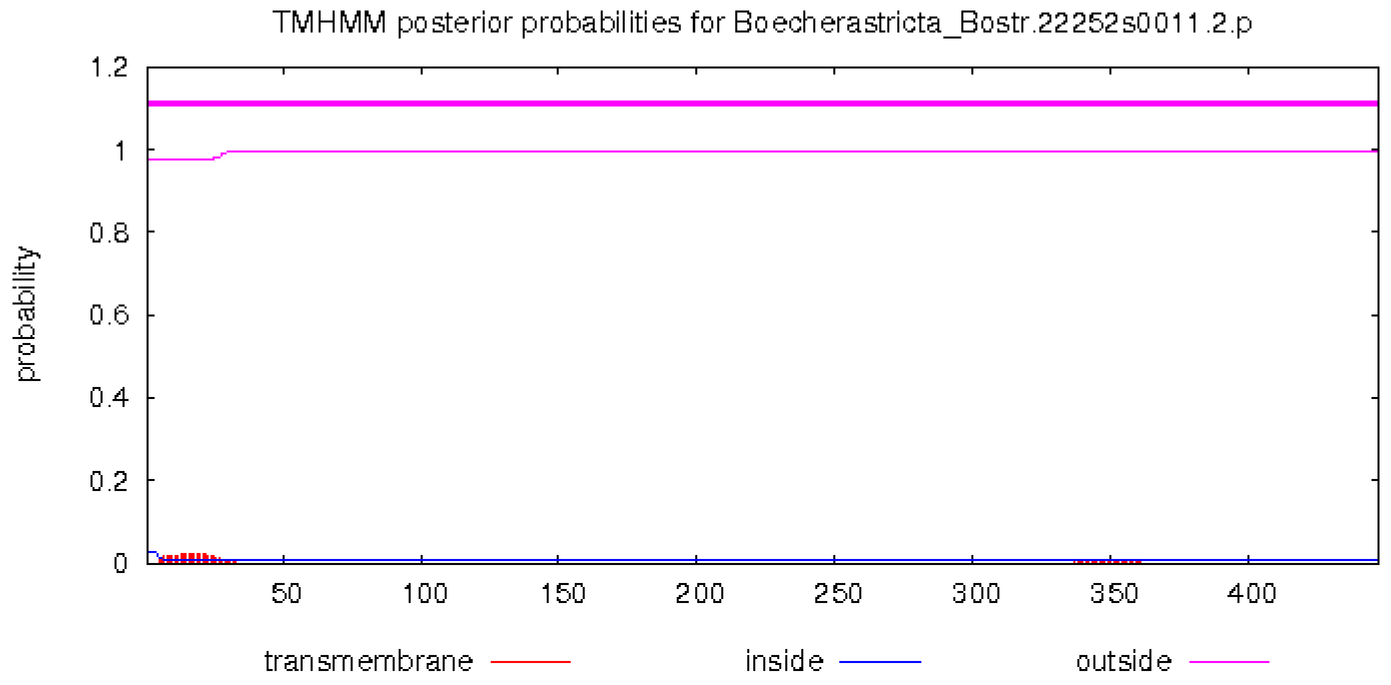

plot in postscript, script for making the plot in gnuplot, data for plot

---

```
# Boecherastricta_Bostr.22252s0012.1.p Length: 449
# Boecherastricta_Bostr.22252s0012.1.p Number of predicted TMHs:  0
# Boecherastricta_Bostr.22252s0012.1.p Exp number of AAs in TMHs: 0.11848
# Boecherastricta_Bostr.22252s0012.1.p Exp number, first 60 AAs:  0.10159
# Boecherastricta_Bostr.22252s0012.1.p Total prob of N-in:        0.00828
Boecherastricta_Bostr.22252s0012.1.p    TMHMM2.0        outside      1   449
```

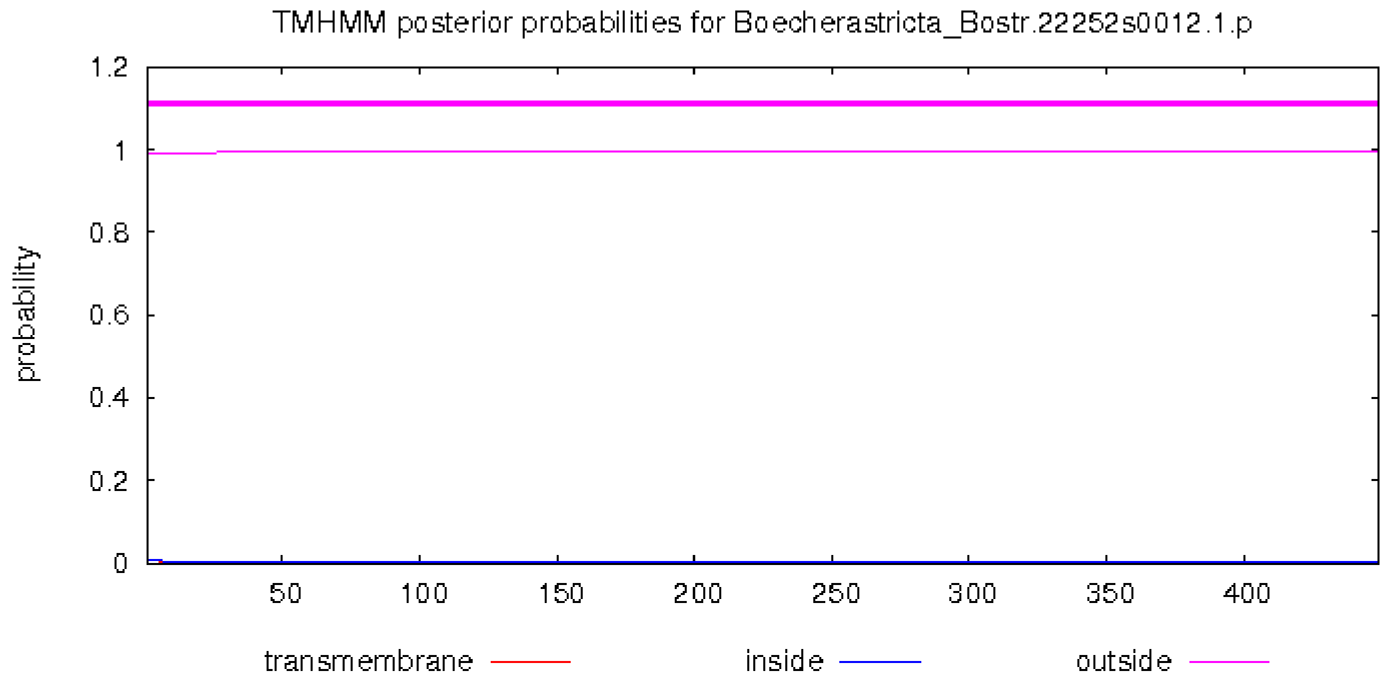

plot in postscript, script for making the plot in gnuplot, data for plot

---

```
# Boecherastricta_Bostr.23794s0753.1.p Length: 268
# Boecherastricta_Bostr.23794s0753.1.p Number of predicted TMHs:  0
# Boecherastricta_Bostr.23794s0753.1.p Exp number of AAs in TMHs: 0.02121
# Boecherastricta_Bostr.23794s0753.1.p Exp number, first 60 AAs:  0
```

```
# Boecherastricta_Bostr.23794s0753.1.p Total prob of N-in:        0.09661
Boecherastricta_Bostr.23794s0753.1.p	TMHMM2.0	outside	     1   268
```

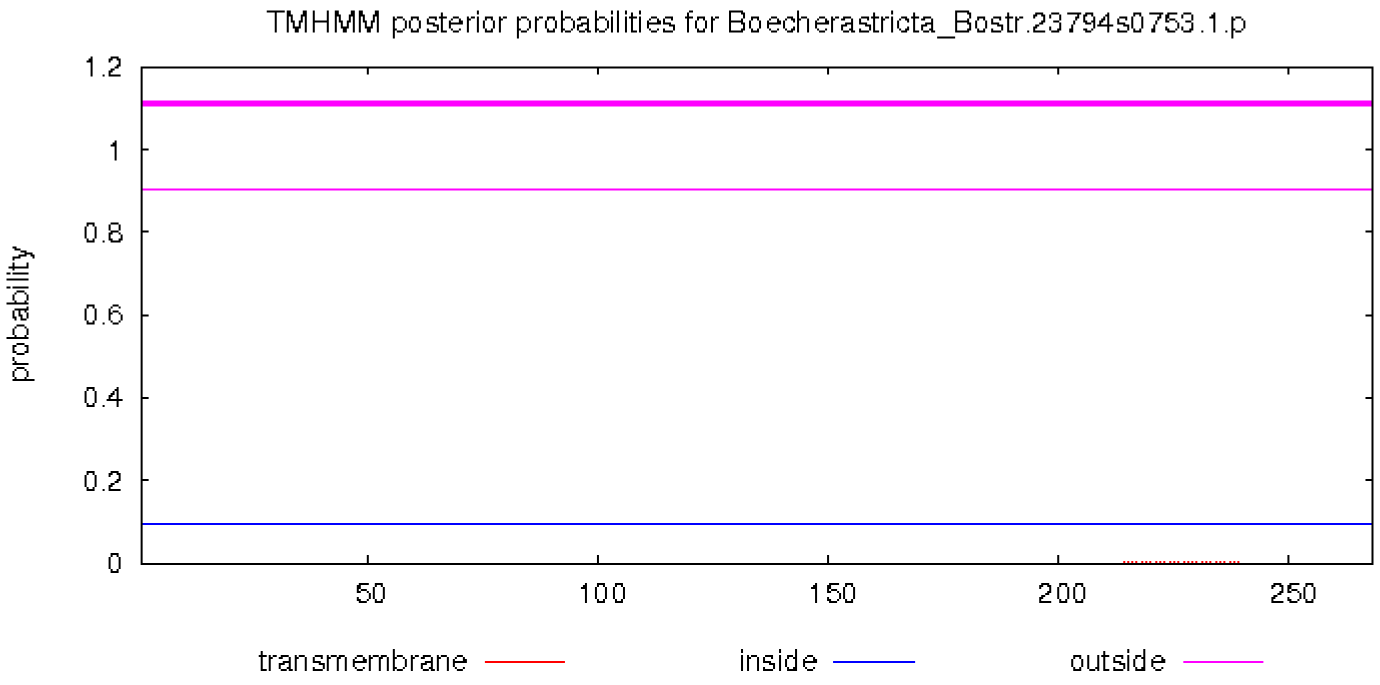

plot in postscript, script for making the plot in gnuplot, data for plot

---

```
# Boecherastricta_Bostr.25375s0002.1.p Length: 335
# Boecherastricta_Bostr.25375s0002.1.p Number of predicted TMHs:  0
# Boecherastricta_Bostr.25375s0002.1.p Exp number of AAs in TMHs: 0.00023
# Boecherastricta_Bostr.25375s0002.1.p Exp number, first 60 AAs:  0.00023
# Boecherastricta_Bostr.25375s0002.1.p Total prob of N-in:        0.00756
Boecherastricta_Bostr.25375s0002.1.p	TMHMM2.0	outside	     1   335
```

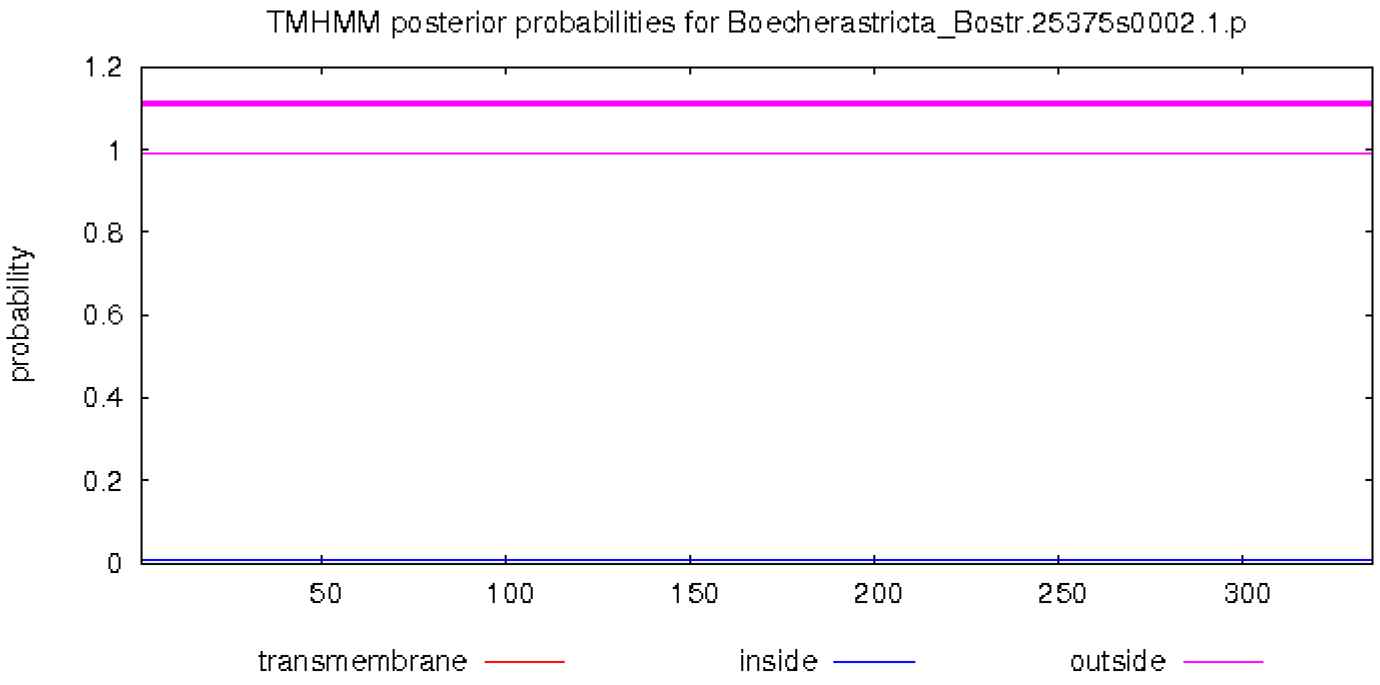

plot in postscript, script for making the plot in gnuplot, data for plot

---

```
# Boecherastricta_Bostr.25463s0283.1.p Length: 341
# Boecherastricta_Bostr.25463s0283.1.p Number of predicted TMHs:  0
# Boecherastricta_Bostr.25463s0283.1.p Exp number of AAs in TMHs: 0
# Boecherastricta_Bostr.25463s0283.1.p Exp number, first 60 AAs:  0
```

```
# Boecherastricta_Bostr.25463s0283.1.p Total prob of N-in:        0.00436
Boecherastricta_Bostr.25463s0283.1.p    TMHMM2.0        outside      1   341
```

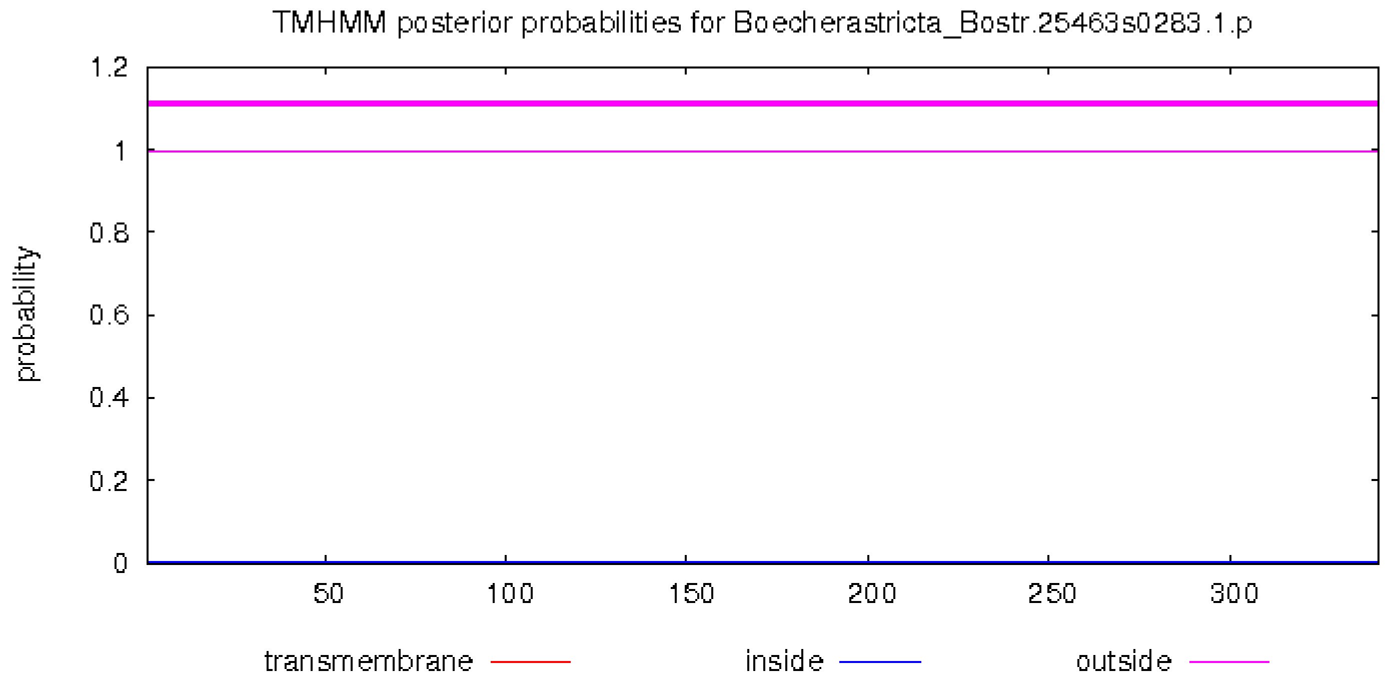

plot in postscript, script for making the plot in gnuplot, data for plot

---

```
# Boecherastricta_Bostr.25463s0411.1.p Length: 421
# Boecherastricta_Bostr.25463s0411.1.p Number of predicted TMHs:  0
# Boecherastricta_Bostr.25463s0411.1.p Exp number of AAs in TMHs: 0.00072
# Boecherastricta_Bostr.25463s0411.1.p Exp number, first 60 AAs:  0
# Boecherastricta_Bostr.25463s0411.1.p Total prob of N-in:        0.00724
Boecherastricta_Bostr.25463s0411.1.p    TMHMM2.0        outside      1   421
```

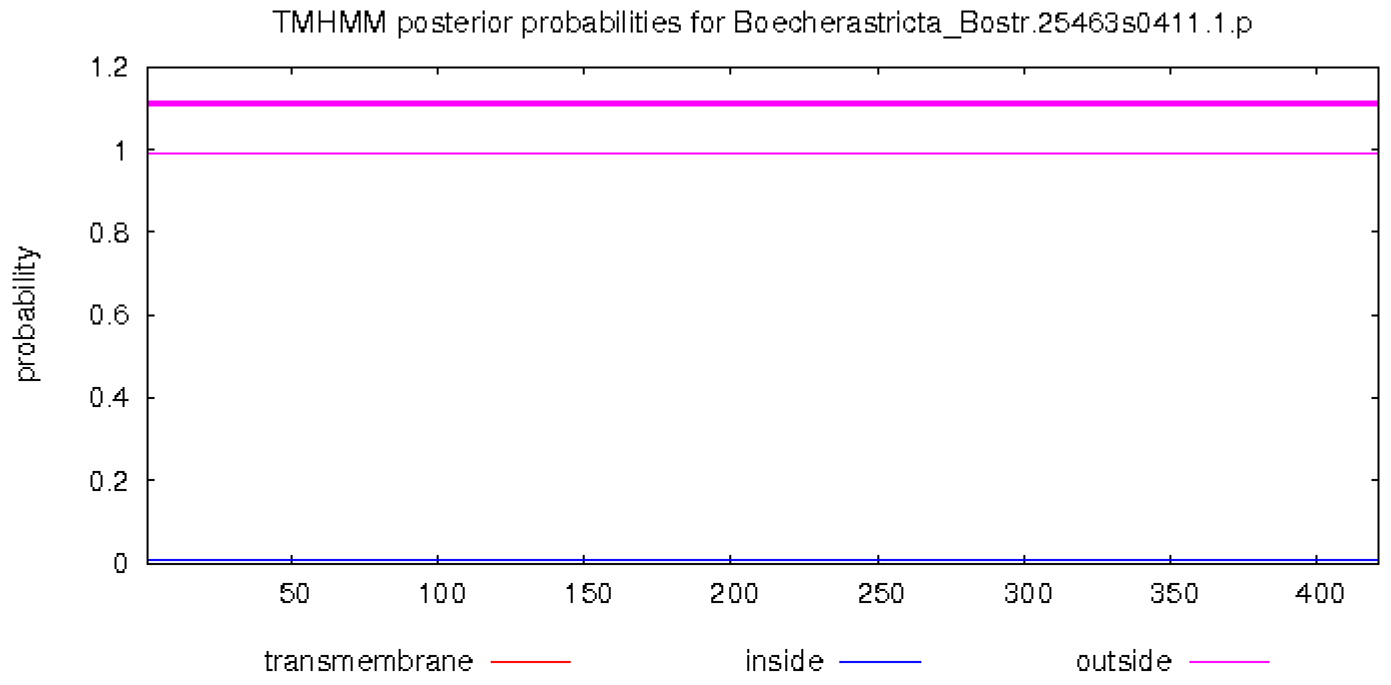

plot in postscript, script for making the plot in gnuplot, data for plot

---

```
# Boecherastricta_Bostr.2570s0301.1.p Length: 349
# Boecherastricta_Bostr.2570s0301.1.p Number of predicted TMHs:  0
# Boecherastricta_Bostr.2570s0301.1.p Exp number of AAs in TMHs: 0
# Boecherastricta_Bostr.2570s0301.1.p Exp number, first 60 AAs:  0
```

```
# Boecherastricta_Bostr.2570s0301.1.p Total prob of N-in:        0.01308
Boecherastricta_Bostr.2570s0301.1.p     TMHMM2.0        outside      1   349
```

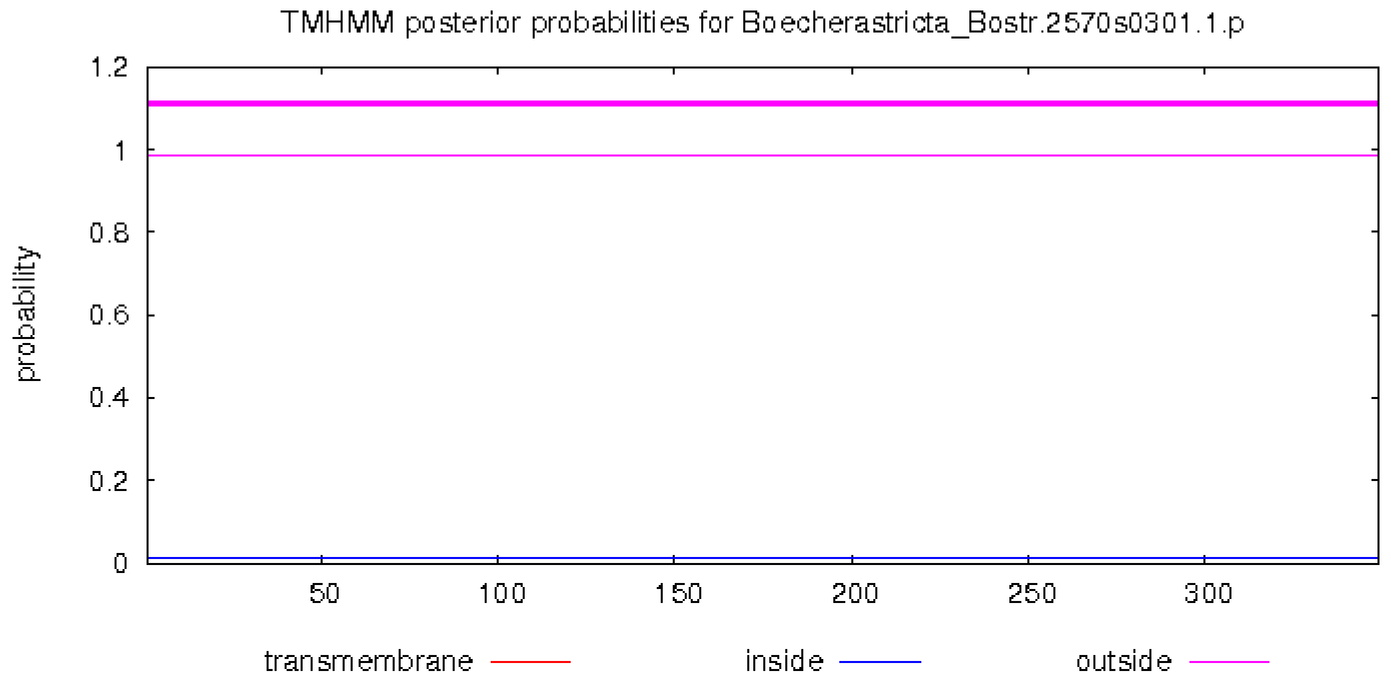

plot in postscript, script for making the plot in gnuplot, data for plot

---

```
# Boecherastricta_Bostr.2570s0302.1.p Length: 339
# Boecherastricta_Bostr.2570s0302.1.p Number of predicted TMHs:  0
# Boecherastricta_Bostr.2570s0302.1.p Exp number of AAs in TMHs: 0.00018
# Boecherastricta_Bostr.2570s0302.1.p Exp number, first 60 AAs:  0
# Boecherastricta_Bostr.2570s0302.1.p Total prob of N-in:        0.00755
Boecherastricta_Bostr.2570s0302.1.p     TMHMM2.0        outside      1   339
```

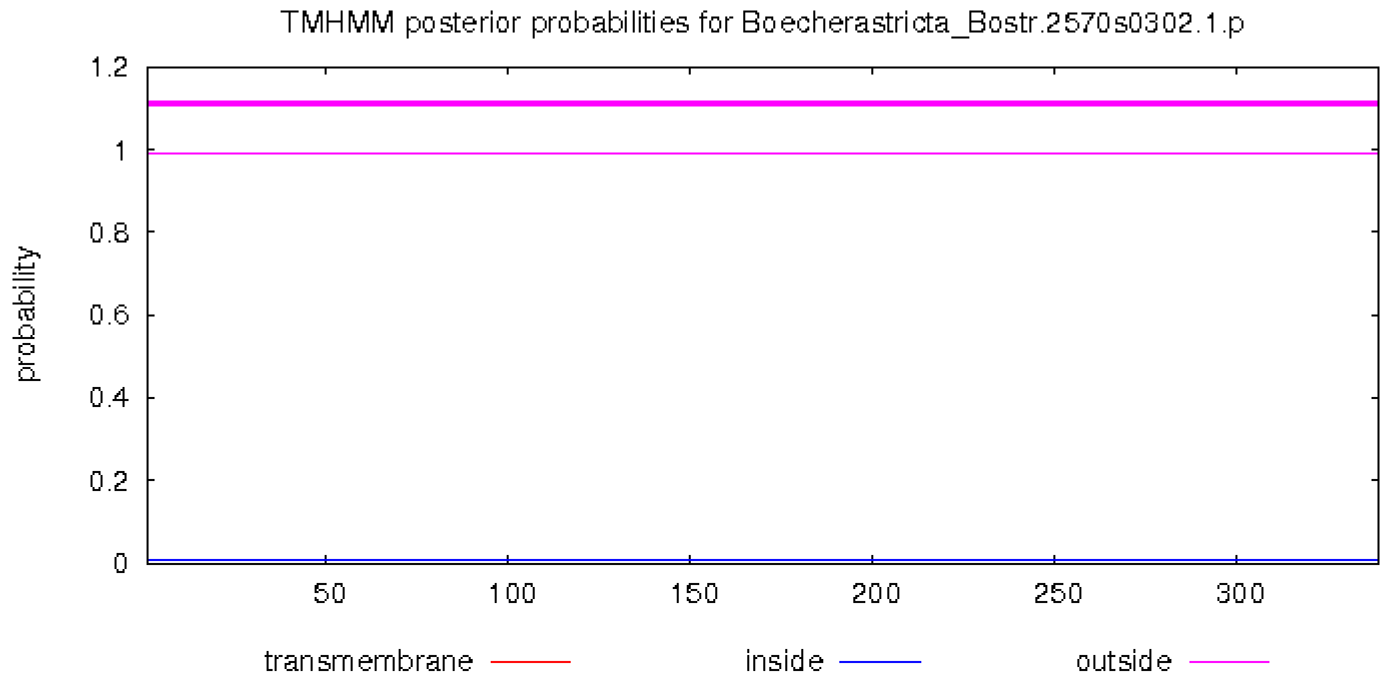

plot in postscript, script for making the plot in gnuplot, data for plot

---

```
# Boecherastricta_Bostr.25993s0090.1.p Length: 358
# Boecherastricta_Bostr.25993s0090.1.p Number of predicted TMHs:  0
# Boecherastricta_Bostr.25993s0090.1.p Exp number of AAs in TMHs: 0.00017
# Boecherastricta_Bostr.25993s0090.1.p Exp number, first 60 AAs:  0
```

```
# Boecherastricta_Bostr.25993s0090.1.p Total prob of N-in:        0.01348
Boecherastricta_Bostr.25993s0090.1.p    TMHMM2.0        outside      1   358
```

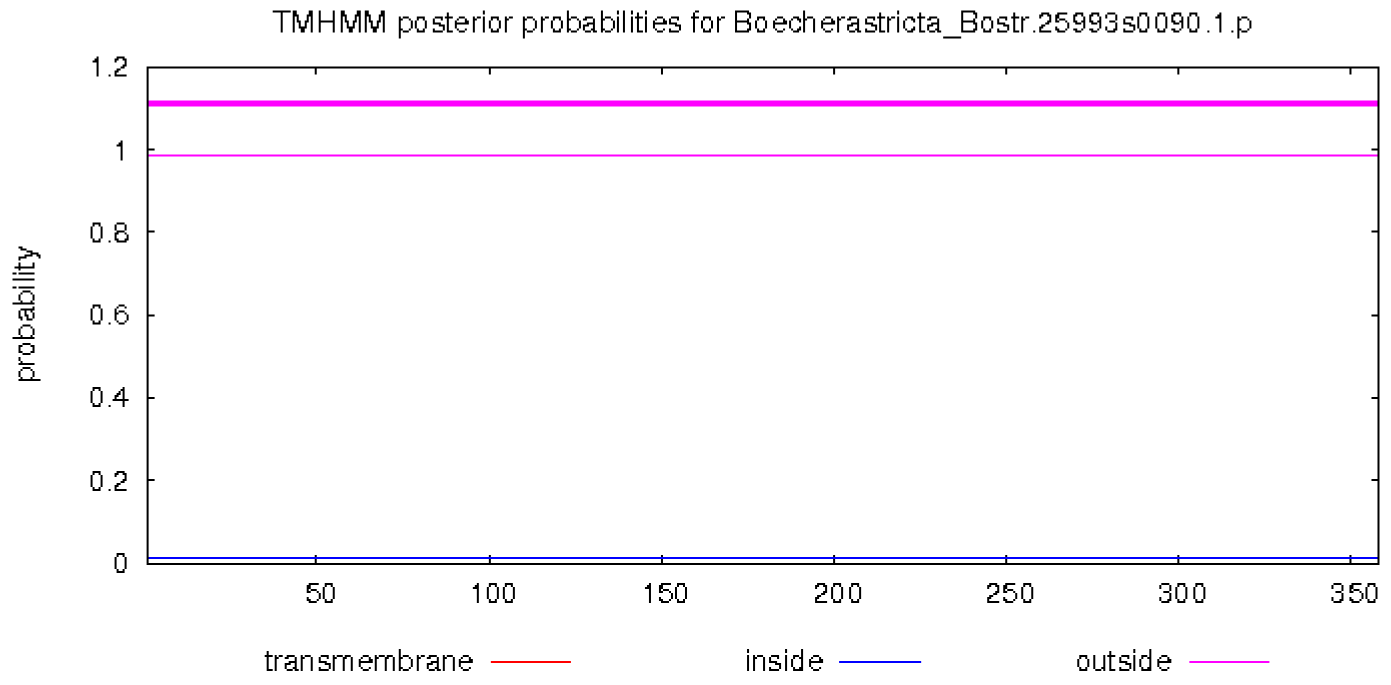

plot in postscript, script for making the plot in gnuplot, data for plot

---

```
# Boecherastricta_Bostr.25993s0479.1.p Length: 275
# Boecherastricta_Bostr.25993s0479.1.p Number of predicted TMHs:  0
# Boecherastricta_Bostr.25993s0479.1.p Exp number of AAs in TMHs: 0.00049
# Boecherastricta_Bostr.25993s0479.1.p Exp number, first 60 AAs:  0
# Boecherastricta_Bostr.25993s0479.1.p Total prob of N-in:        0.01185
Boecherastricta_Bostr.25993s0479.1.p    TMHMM2.0        outside      1   275
```

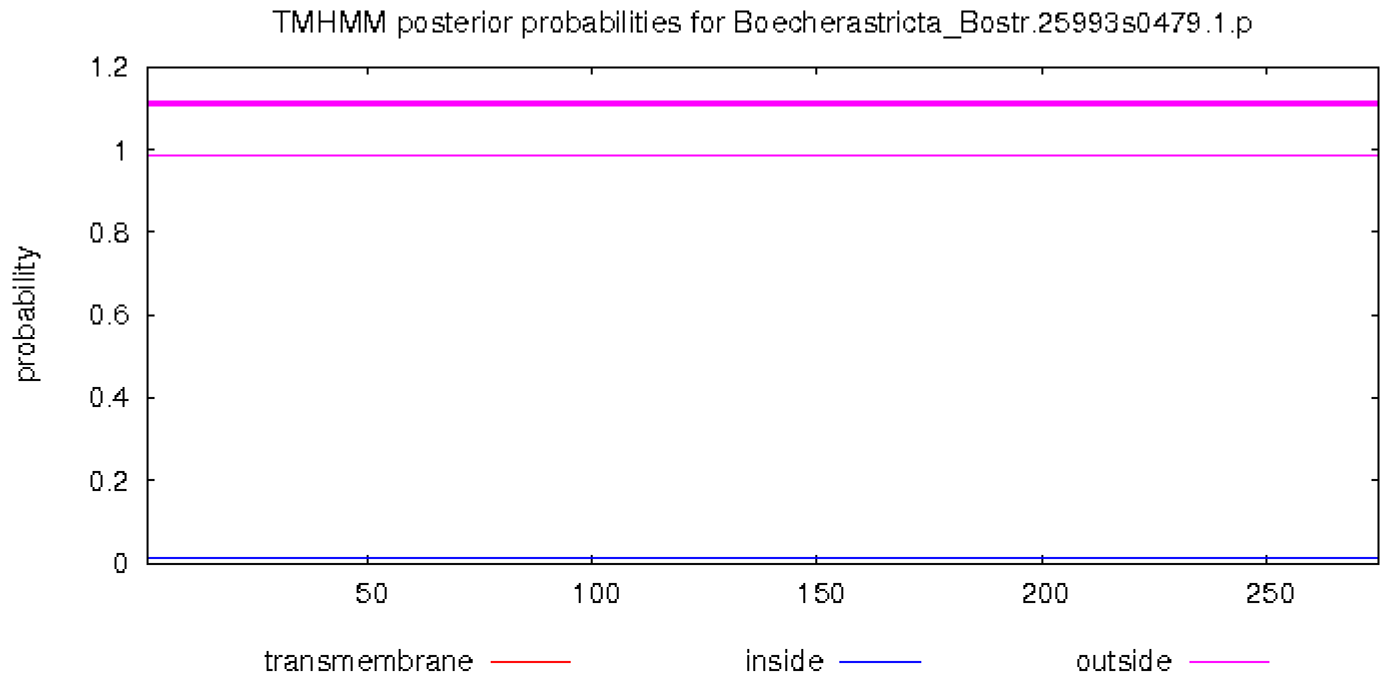

plot in postscript, script for making the plot in gnuplot, data for plot

---

```
# Boecherastricta_Bostr.2618s0061.1.p Length: 466
# Boecherastricta_Bostr.2618s0061.1.p Number of predicted TMHs:  0
# Boecherastricta_Bostr.2618s0061.1.p Exp number of AAs in TMHs: 0.00037
# Boecherastricta_Bostr.2618s0061.1.p Exp number, first 60 AAs:  0
```

```
# Boecherastricta_Bostr.2618s0061.1.p Total prob of N-in:        0.00020
Boecherastricta_Bostr.2618s0061.1.p     TMHMM2.0        outside      1   466
```

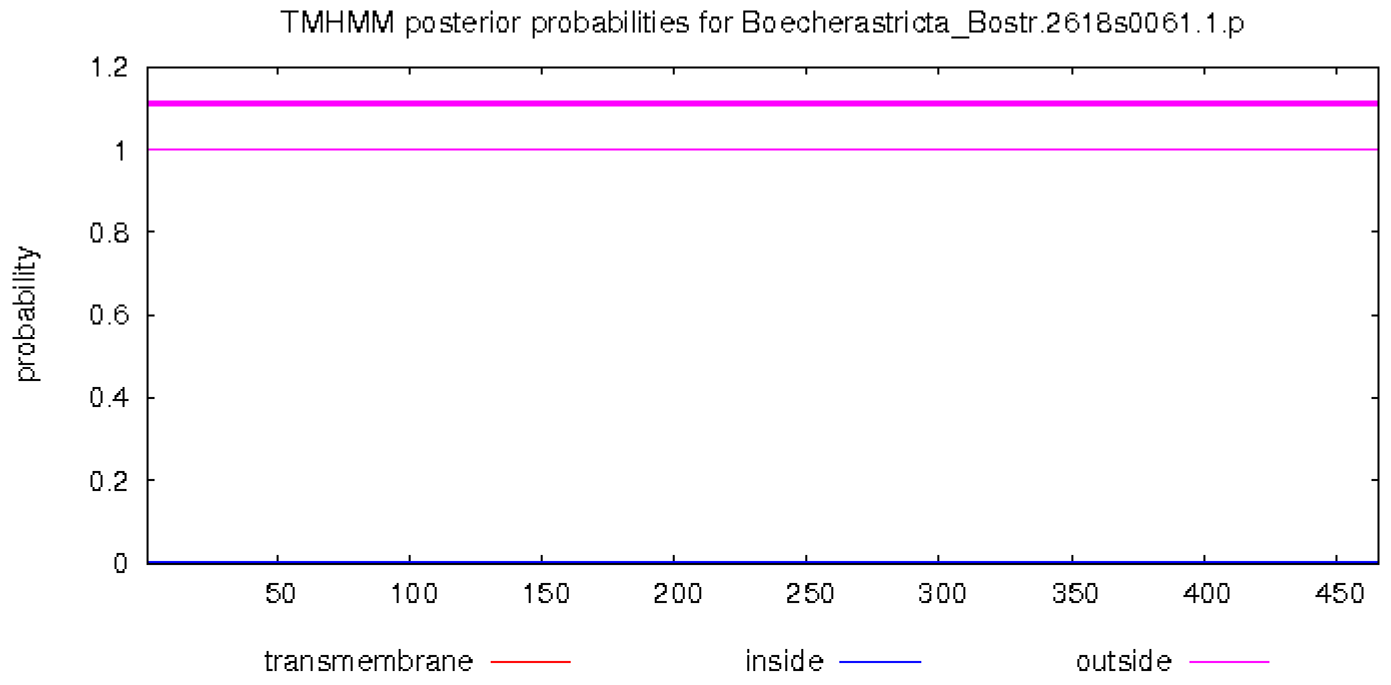

plot in postscript, script for making the plot in gnuplot, data for plot

---

```
# Boecherastricta_Bostr.26527s0039.1.p Length: 236
# Boecherastricta_Bostr.26527s0039.1.p Number of predicted TMHs:  0
# Boecherastricta_Bostr.26527s0039.1.p Exp number of AAs in TMHs: 0
# Boecherastricta_Bostr.26527s0039.1.p Exp number, first 60 AAs:  0
# Boecherastricta_Bostr.26527s0039.1.p Total prob of N-in:        0.04762
Boecherastricta_Bostr.26527s0039.1.p    TMHMM2.0        outside      1   236
```

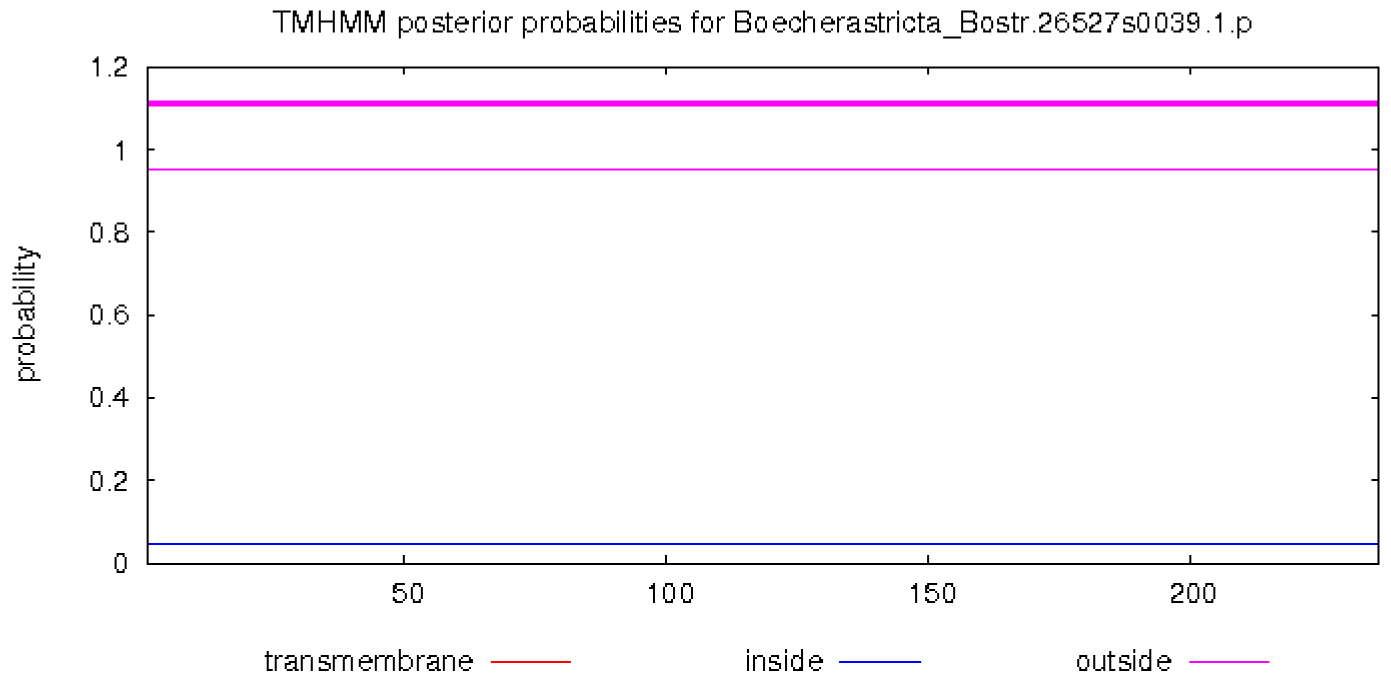

plot in postscript, script for making the plot in gnuplot, data for plot

---

```
# Boecherastricta_Bostr.26527s0049.1.p Length: 341
# Boecherastricta_Bostr.26527s0049.1.p Number of predicted TMHs:  0
# Boecherastricta_Bostr.26527s0049.1.p Exp number of AAs in TMHs: 7.42701
# Boecherastricta_Bostr.26527s0049.1.p Exp number, first 60 AAs:  0.01159
```

```
# Boecherastricta_Bostr.26527s0049.1.p Total prob of N-in:        0.19757
Boecherastricta_Bostr.26527s0049.1.p    TMHMM2.0        outside      1   341
```

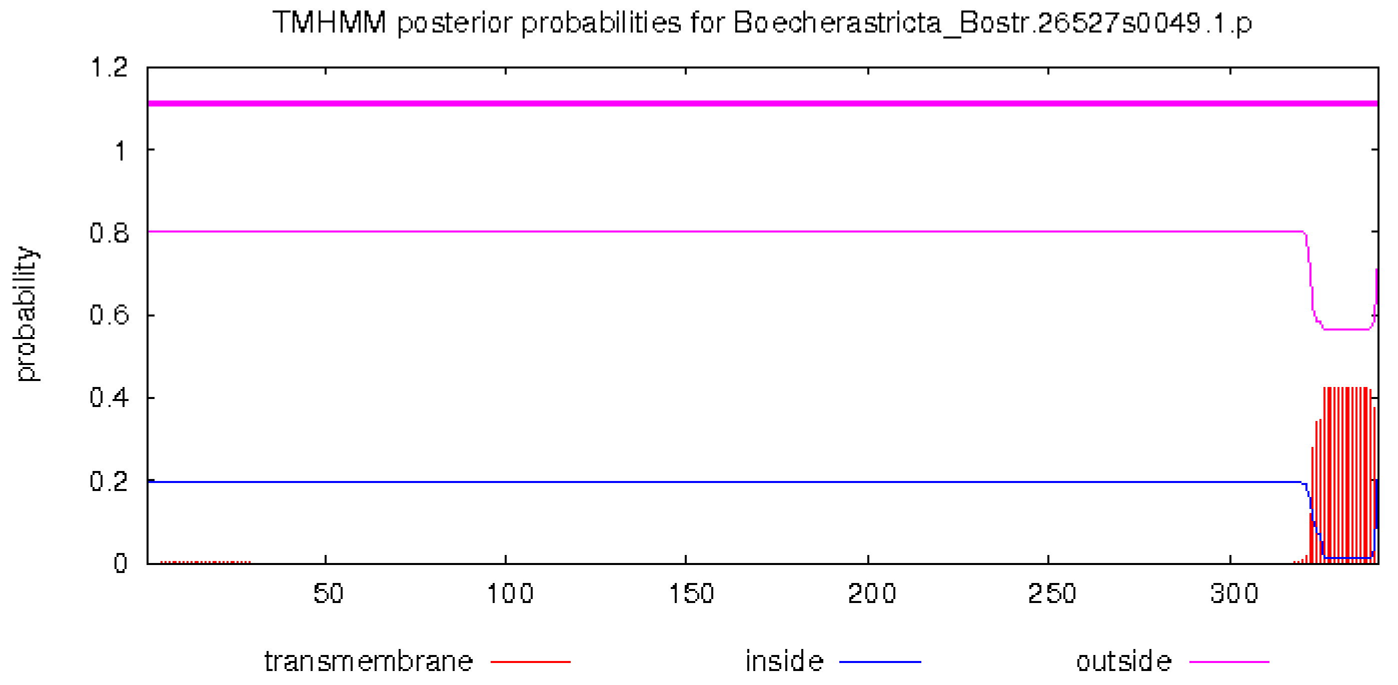

plot in postscript, script for making the plot in gnuplot, data for plot

---

```
# Boecherastricta_Bostr.26527s0378.1.p Length: 114
# Boecherastricta_Bostr.26527s0378.1.p Number of predicted TMHs:  0
# Boecherastricta_Bostr.26527s0378.1.p Exp number of AAs in TMHs: 0.01493
# Boecherastricta_Bostr.26527s0378.1.p Exp number, first 60 AAs:  0.01493
# Boecherastricta_Bostr.26527s0378.1.p Total prob of N-in:        0.10123
Boecherastricta_Bostr.26527s0378.1.p    TMHMM2.0        outside      1   114
```

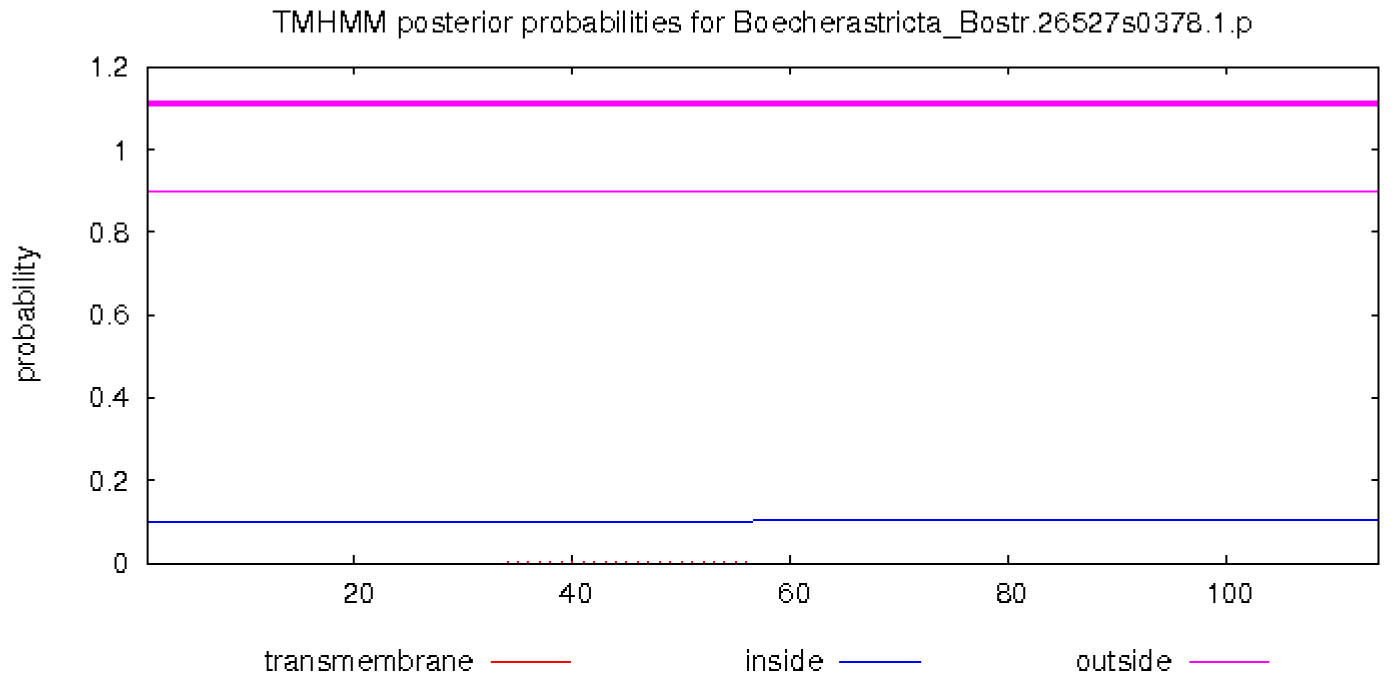

plot in postscript, script for making the plot in gnuplot, data for plot

---

```
# Boecherastricta_Bostr.26527s0381.1.p Length: 336
# Boecherastricta_Bostr.26527s0381.1.p Number of predicted TMHs:  0
# Boecherastricta_Bostr.26527s0381.1.p Exp number of AAs in TMHs: 0.00031
# Boecherastricta_Bostr.26527s0381.1.p Exp number, first 60 AAs:  0
```

```
# Boecherastricta_Bostr.26527s0381.1.p Total prob of N-in:        0.00862
Boecherastricta_Bostr.26527s0381.1.p	TMHMM2.0	outside	     1   336
```

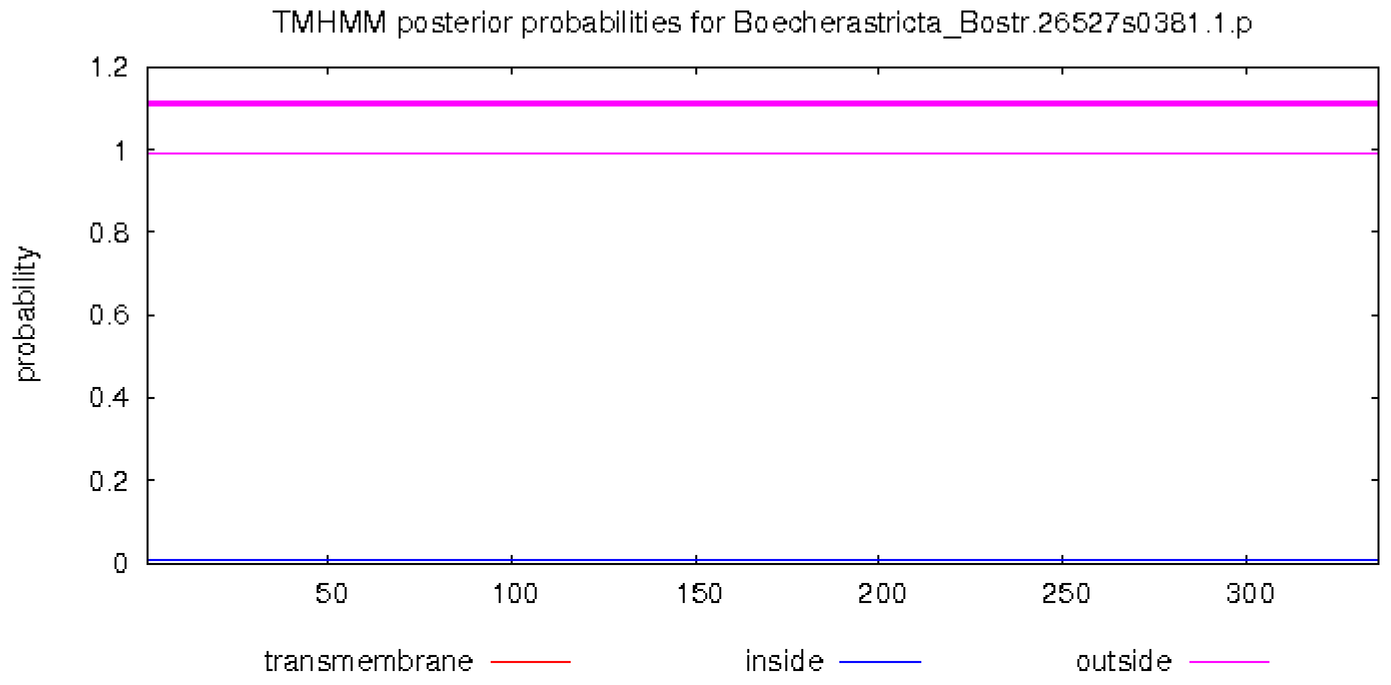

plot in postscript, script for making the plot in gnuplot, data for plot

---

```
# Boecherastricta_Bostr.26527s0404.1.p Length: 266
# Boecherastricta_Bostr.26527s0404.1.p Number of predicted TMHs:  0
# Boecherastricta_Bostr.26527s0404.1.p Exp number of AAs in TMHs: 0
# Boecherastricta_Bostr.26527s0404.1.p Exp number, first 60 AAs:  0
# Boecherastricta_Bostr.26527s0404.1.p Total prob of N-in:        0.14977
Boecherastricta_Bostr.26527s0404.1.p	TMHMM2.0	outside	     1   266
```

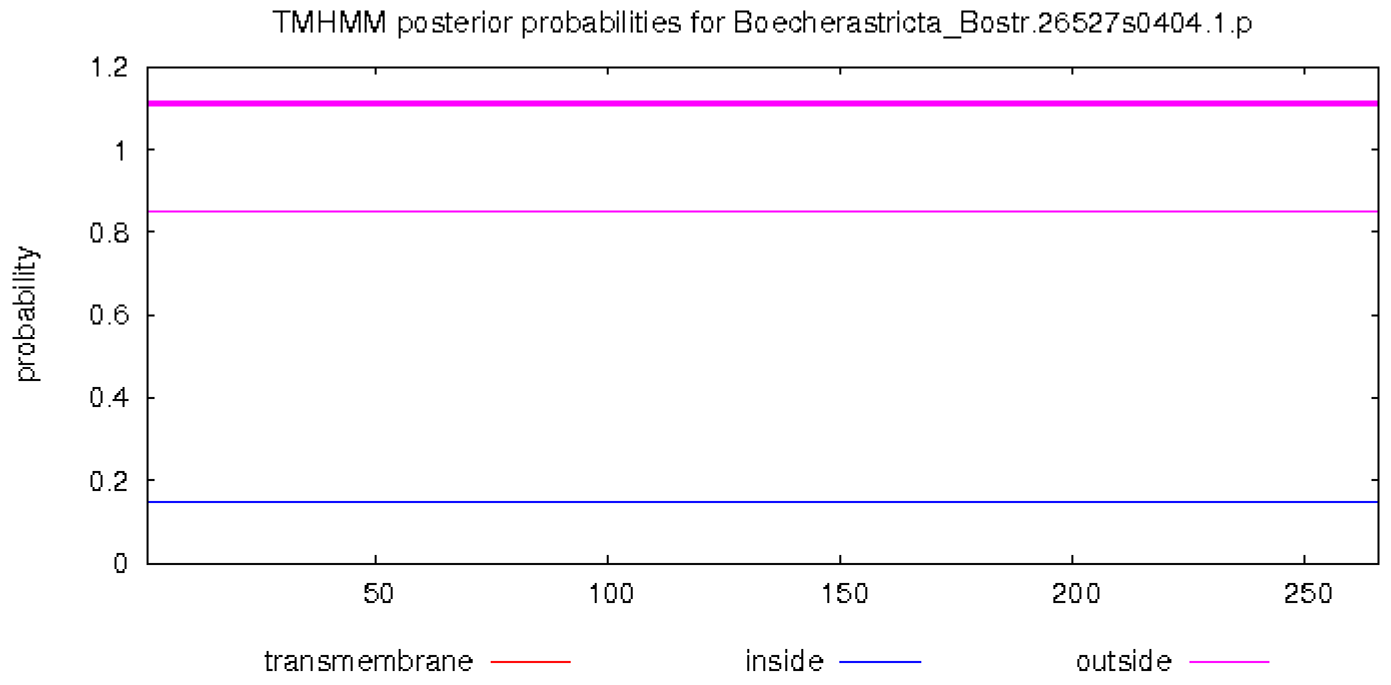

plot in postscript, script for making the plot in gnuplot, data for plot

---

```
# Boecherastricta_Bostr.26675s0262.1.p Length: 273
# Boecherastricta_Bostr.26675s0262.1.p Number of predicted TMHs:  0
# Boecherastricta_Bostr.26675s0262.1.p Exp number of AAs in TMHs: 0.00984
# Boecherastricta_Bostr.26675s0262.1.p Exp number, first 60 AAs:  0
```

```
# Boecherastricta_Bostr.26675s0262.1.p Total prob of N-in:        0.00902
Boecherastricta_Bostr.26675s0262.1.p    TMHMM2.0        outside      1   273
```

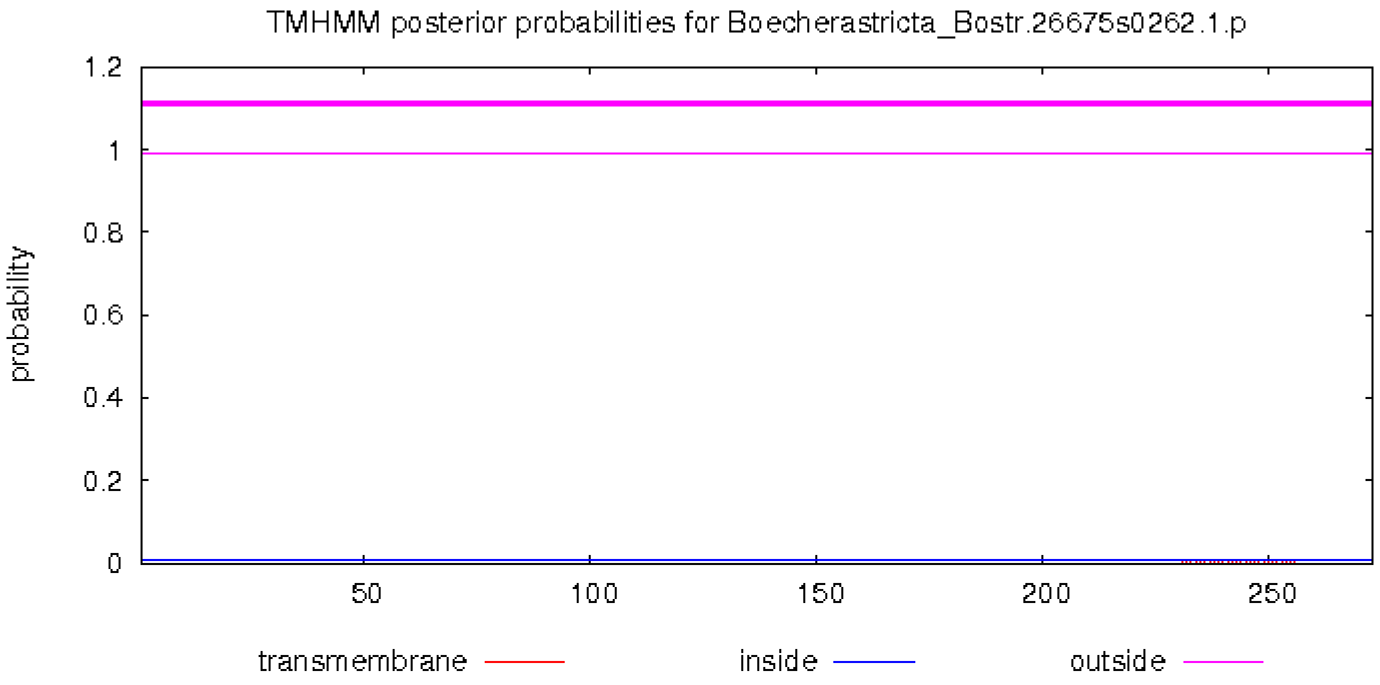

plot in postscript, script for making the plot in gnuplot, data for plot

---

```
# Boecherastricta_Bostr.26833s0095.1.p Length: 336
# Boecherastricta_Bostr.26833s0095.1.p Number of predicted TMHs:  0
# Boecherastricta_Bostr.26833s0095.1.p Exp number of AAs in TMHs: 0
# Boecherastricta_Bostr.26833s0095.1.p Exp number, first 60 AAs:  0
# Boecherastricta_Bostr.26833s0095.1.p Total prob of N-in:        0.00854
Boecherastricta_Bostr.26833s0095.1.p    TMHMM2.0        outside      1   336
```

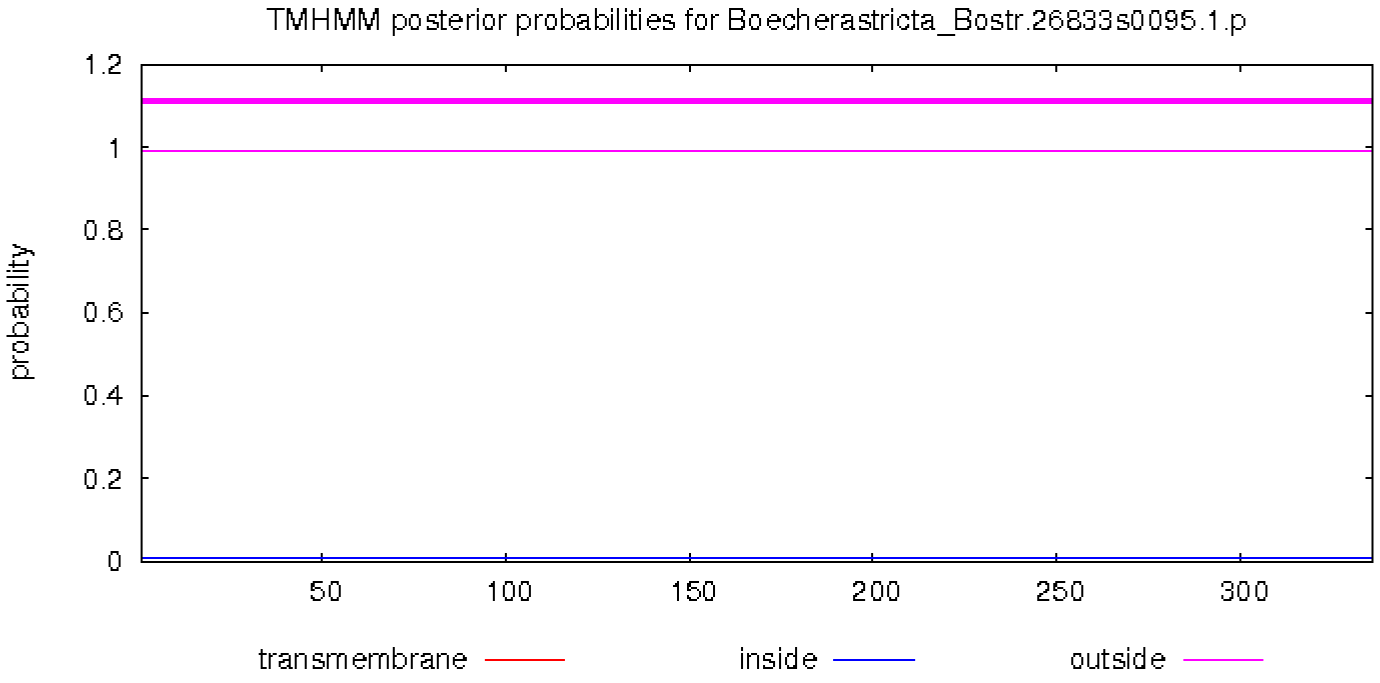

plot in postscript, script for making the plot in gnuplot, data for plot

---

```
# Boecherastricta_Bostr.26833s0275.1.p Length: 352
# Boecherastricta_Bostr.26833s0275.1.p Number of predicted TMHs:  0
# Boecherastricta_Bostr.26833s0275.1.p Exp number of AAs in TMHs: 0.00018
# Boecherastricta_Bostr.26833s0275.1.p Exp number, first 60 AAs:  0
```

```
# Boecherastricta_Bostr.26833s0275.1.p Total prob of N-in:        0.01504
Boecherastricta_Bostr.26833s0275.1.p    TMHMM2.0        outside      1   352
```

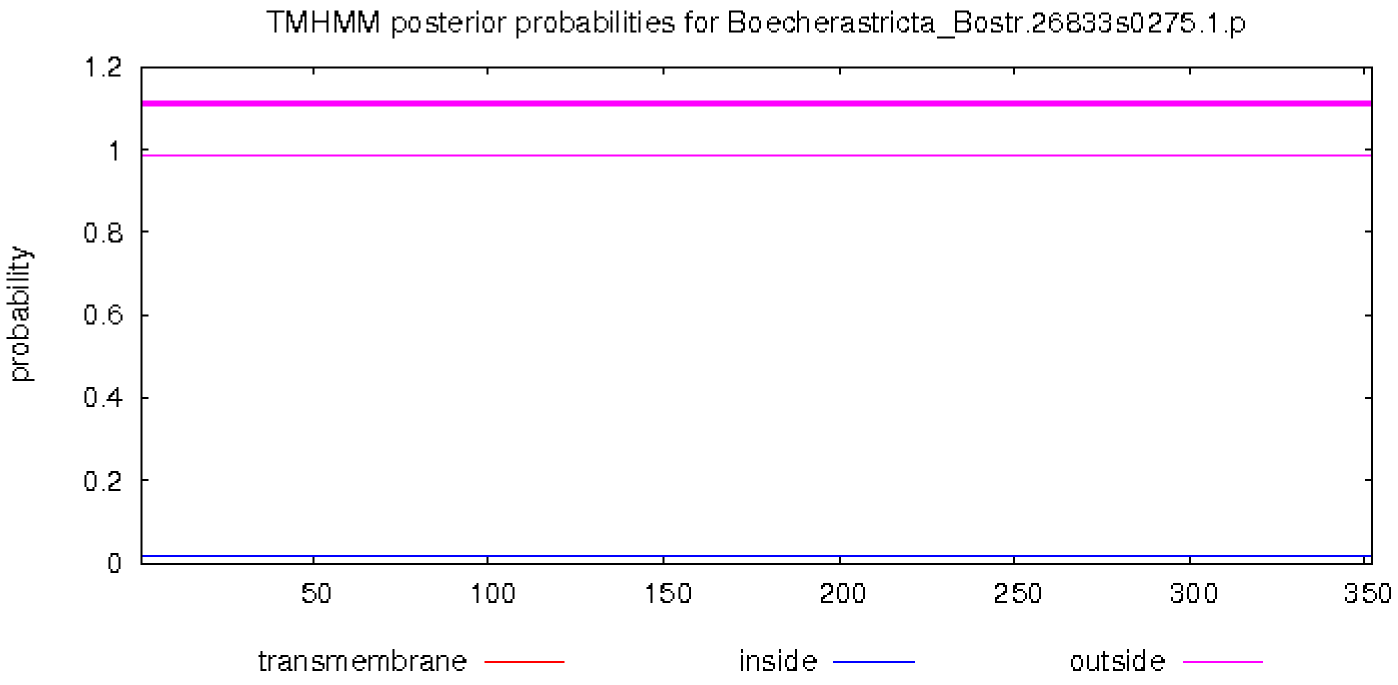

plot in postscript, script for making the plot in gnuplot, data for plot

---

```
# Boecherastricta_Bostr.26833s0491.1.p Length: 183
# Boecherastricta_Bostr.26833s0491.1.p Number of predicted TMHs:  0
# Boecherastricta_Bostr.26833s0491.1.p Exp number of AAs in TMHs: 0.10533
# Boecherastricta_Bostr.26833s0491.1.p Exp number, first 60 AAs:  0.00964
# Boecherastricta_Bostr.26833s0491.1.p Total prob of N-in:        0.21596
Boecherastricta_Bostr.26833s0491.1.p    TMHMM2.0        outside      1   183
```

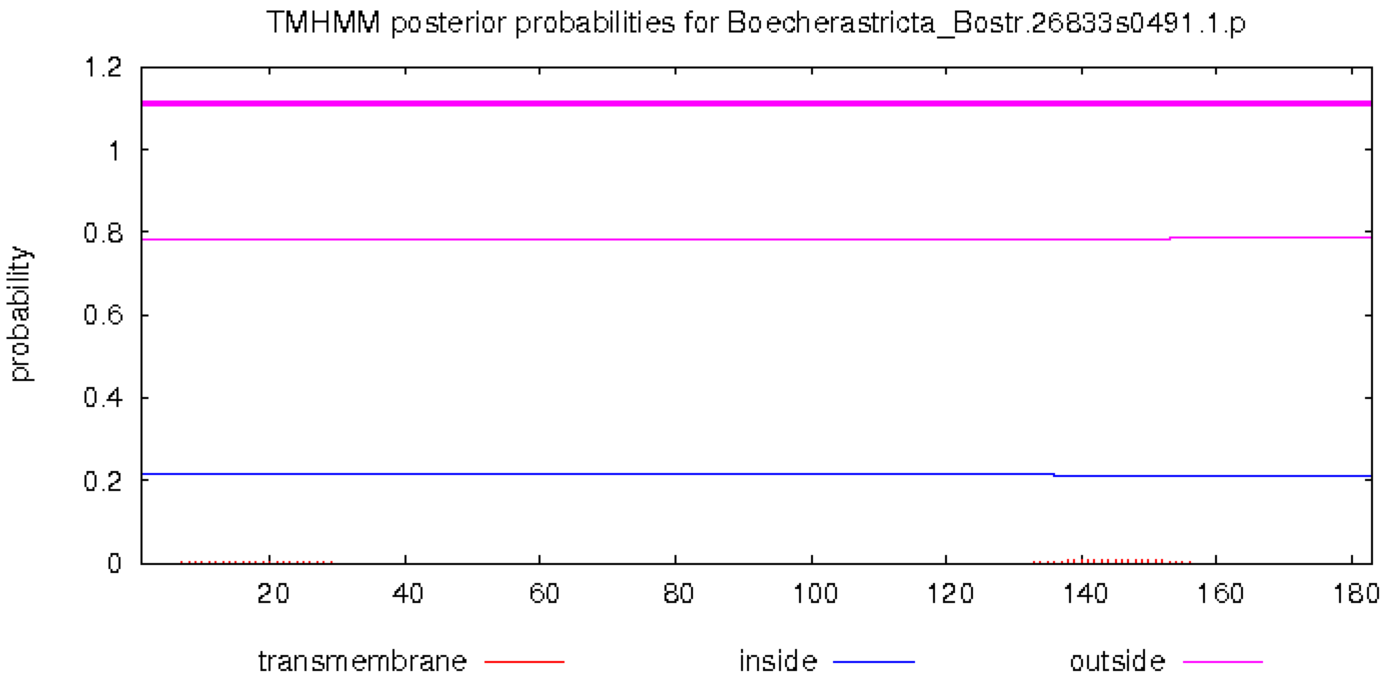

plot in postscript, script for making the plot in gnuplot, data for plot

---

```
# Boecherastricta_Bostr.26833s0596.1.p Length: 411
# Boecherastricta_Bostr.26833s0596.1.p Number of predicted TMHs:  0
# Boecherastricta_Bostr.26833s0596.1.p Exp number of AAs in TMHs: 0.00078
# Boecherastricta_Bostr.26833s0596.1.p Exp number, first 60 AAs:  0
```

```
# Boecherastricta_Bostr.26833s0596.1.p Total prob of N-in:        0.02337
Boecherastricta_Bostr.26833s0596.1.p	TMHMM2.0	outside	     1   411
```

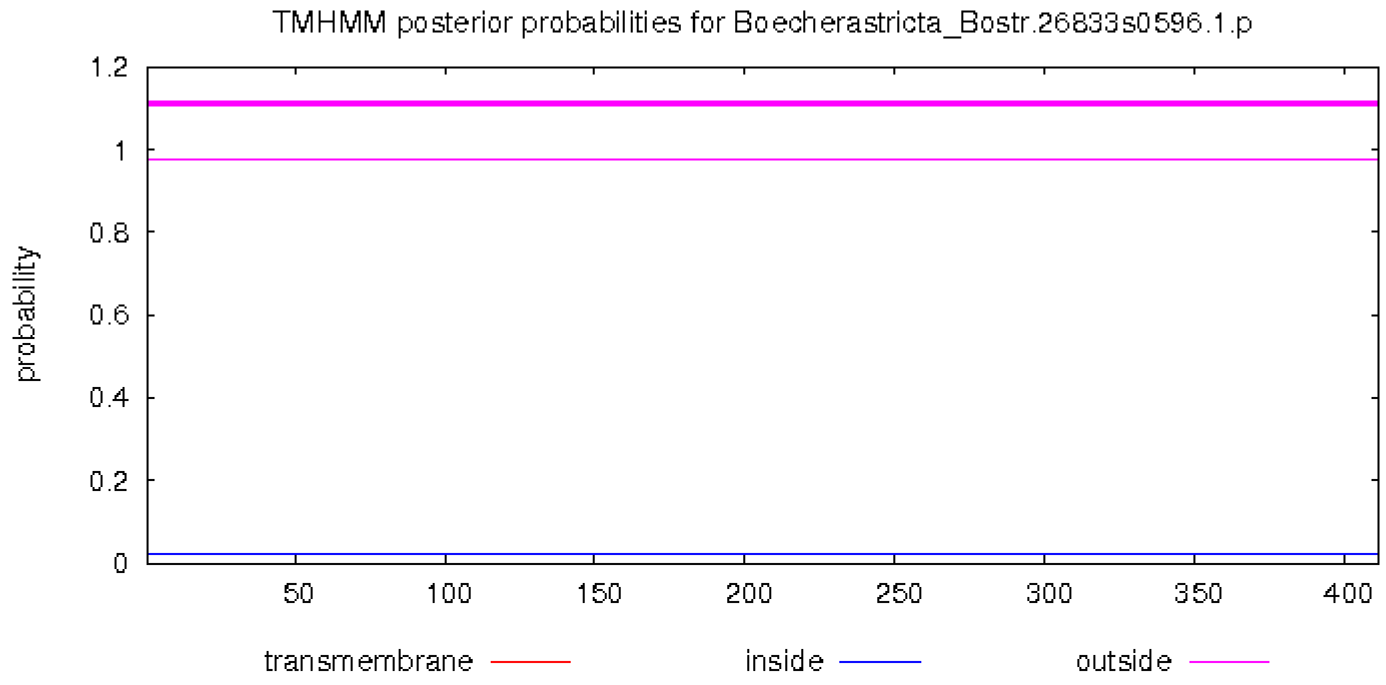

plot in postscript, script for making the plot in gnuplot, data for plot

---

```
# Boecherastricta_Bostr.26833s0880.1.p Length: 370
# Boecherastricta_Bostr.26833s0880.1.p Number of predicted TMHs:  0
# Boecherastricta_Bostr.26833s0880.1.p Exp number of AAs in TMHs: 0.01095
# Boecherastricta_Bostr.26833s0880.1.p Exp number, first 60 AAs:  0
# Boecherastricta_Bostr.26833s0880.1.p Total prob of N-in:        0.02261
Boecherastricta_Bostr.26833s0880.1.p	TMHMM2.0	outside	     1   370
```

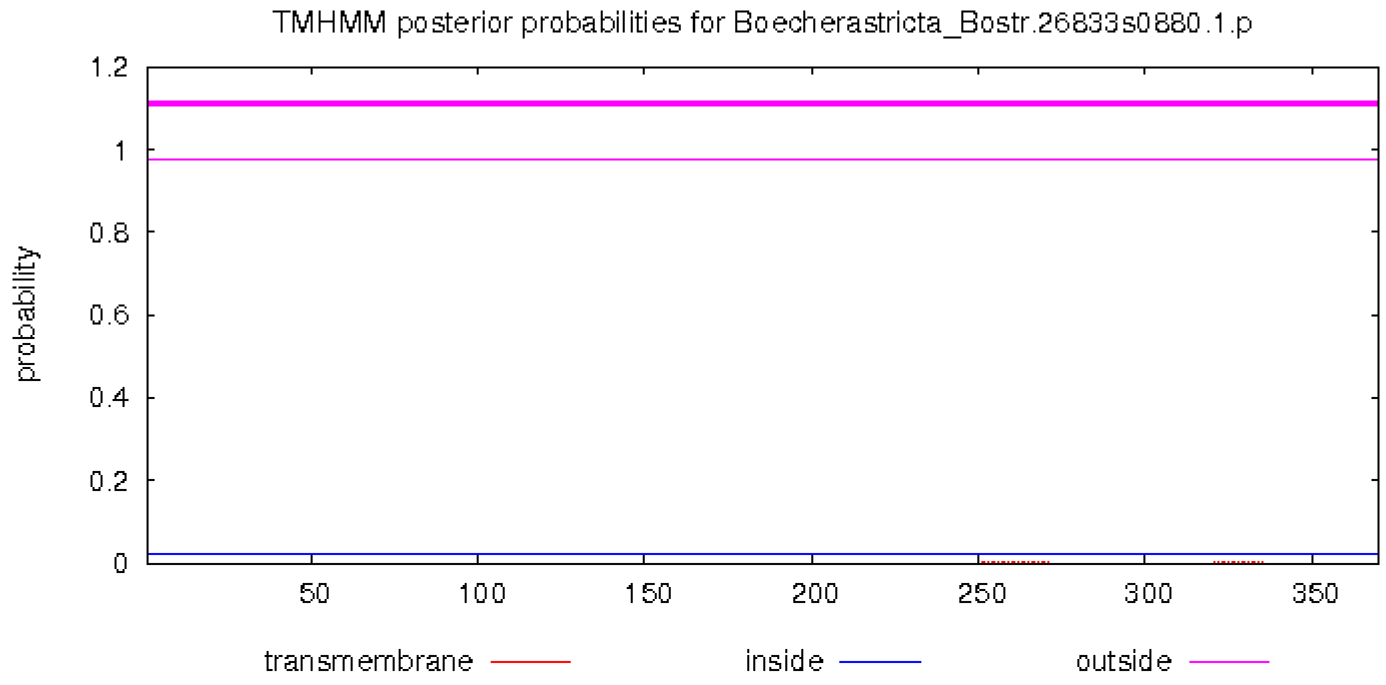

plot in postscript, script for making the plot in gnuplot, data for plot

---

```
# Boecherastricta_Bostr.26959s0323.1.p Length: 630
# Boecherastricta_Bostr.26959s0323.1.p Number of predicted TMHs:  0
# Boecherastricta_Bostr.26959s0323.1.p Exp number of AAs in TMHs: 14.62184
# Boecherastricta_Bostr.26959s0323.1.p Exp number, first 60 AAs:  0
```

```
# Boecherastricta_Bostr.26959s0323.1.p Total prob of N-in:        0.00022
Boecherastricta_Bostr.26959s0323.1.p    TMHMM2.0        outside      1   630
```

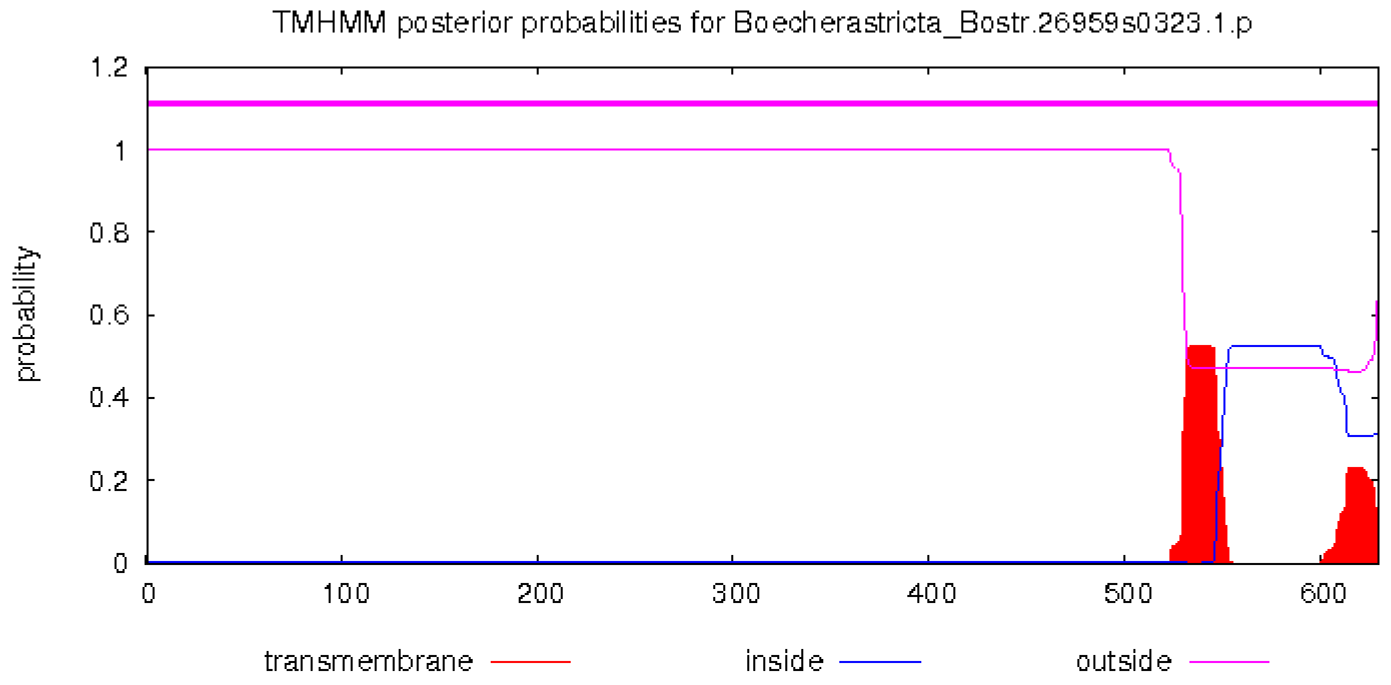

plot in postscript, script for making the plot in gnuplot, data for plot

---

```
# Boecherastricta_Bostr.27895s0039.1.p Length: 314
# Boecherastricta_Bostr.27895s0039.1.p Number of predicted TMHs:  0
# Boecherastricta_Bostr.27895s0039.1.p Exp number of AAs in TMHs: 0.02331
# Boecherastricta_Bostr.27895s0039.1.p Exp number, first 60 AAs:  0
# Boecherastricta_Bostr.27895s0039.1.p Total prob of N-in:        0.07602
Boecherastricta_Bostr.27895s0039.1.p    TMHMM2.0        outside      1   314
```

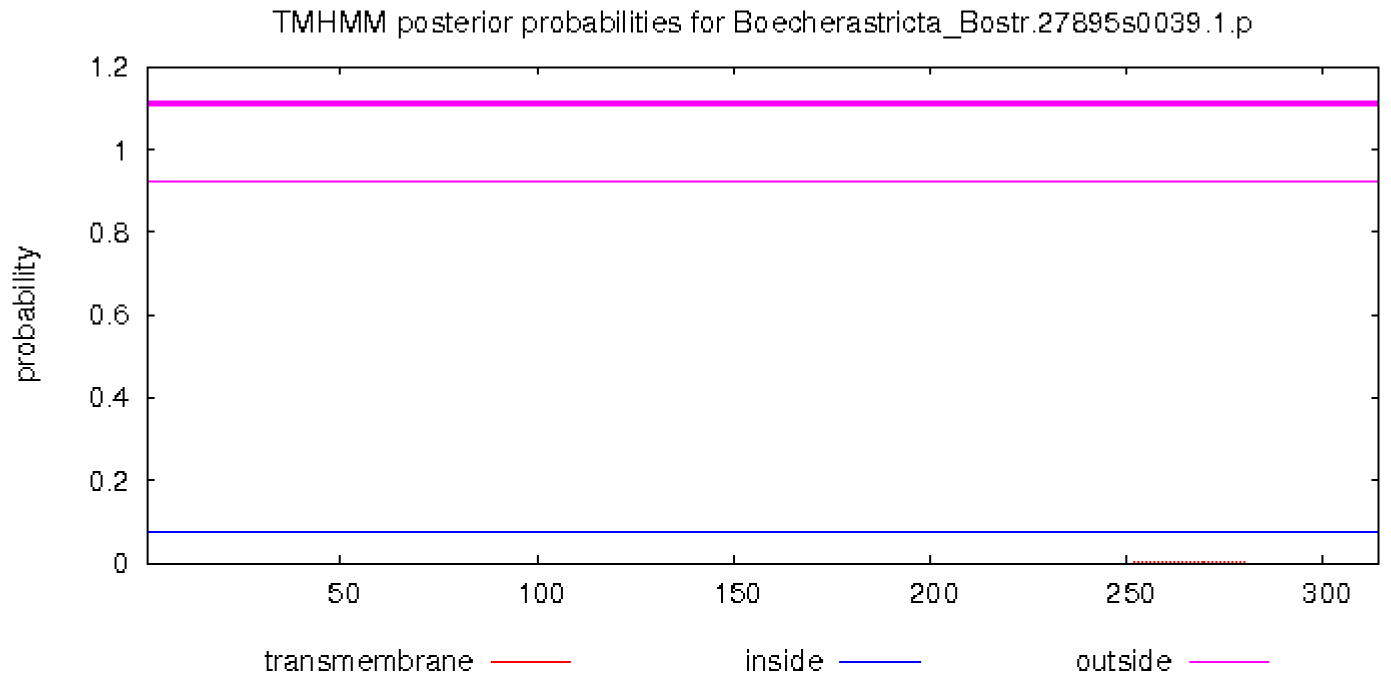

plot in postscript, script for making the plot in gnuplot, data for plot

---

```
# Boecherastricta_Bostr.28625s0148.1.p Length: 364
# Boecherastricta_Bostr.28625s0148.1.p Number of predicted TMHs:  0
# Boecherastricta_Bostr.28625s0148.1.p Exp number of AAs in TMHs: 0.00239
# Boecherastricta_Bostr.28625s0148.1.p Exp number, first 60 AAs:  0.00017
```

```
# Boecherastricta_Bostr.28625s0148.1.p Total prob of N-in:        0.00414
Boecherastricta_Bostr.28625s0148.1.p	TMHMM2.0	outside	     1   364
```

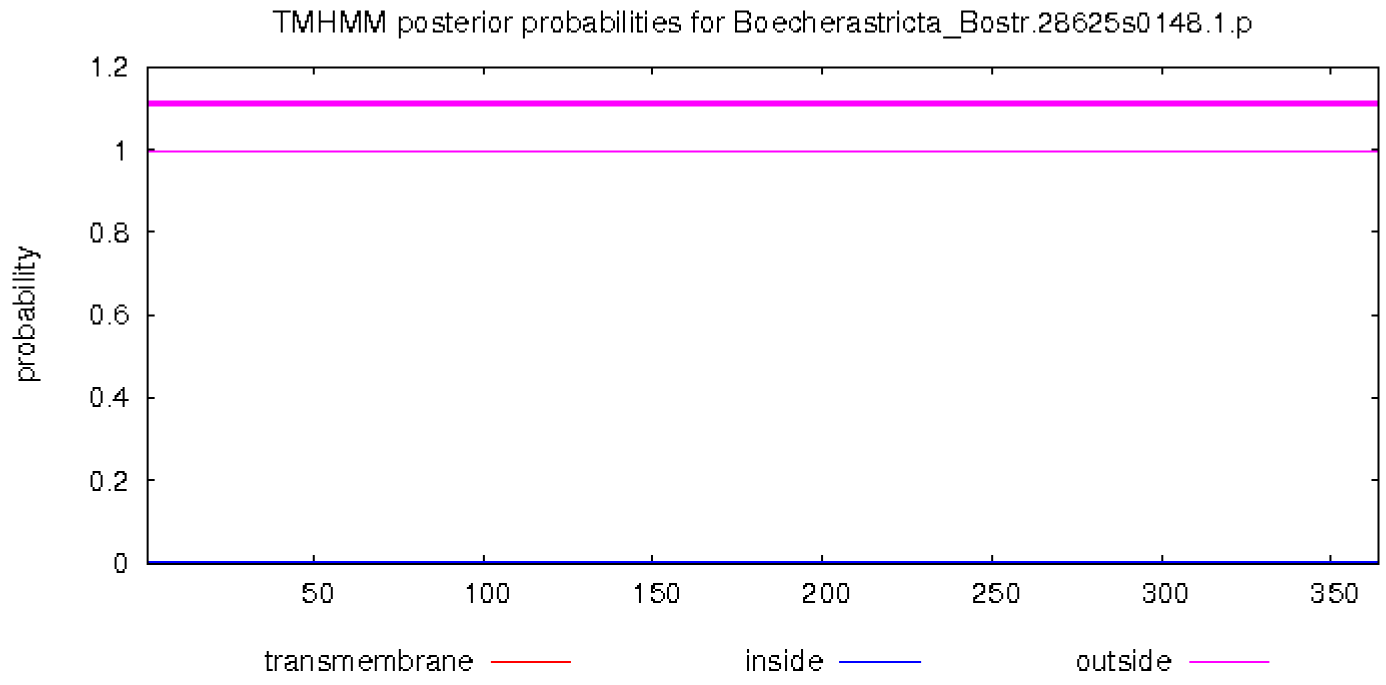

plot in postscript, script for making the plot in gnuplot, data for plot

---

```
# Boecherastricta_Bostr.28625s0149.1.p Length: 313
# Boecherastricta_Bostr.28625s0149.1.p Number of predicted TMHs:  0
# Boecherastricta_Bostr.28625s0149.1.p Exp number of AAs in TMHs: 0
# Boecherastricta_Bostr.28625s0149.1.p Exp number, first 60 AAs:  0
# Boecherastricta_Bostr.28625s0149.1.p Total prob of N-in:        0.00250
Boecherastricta_Bostr.28625s0149.1.p	TMHMM2.0	outside	     1   313
```

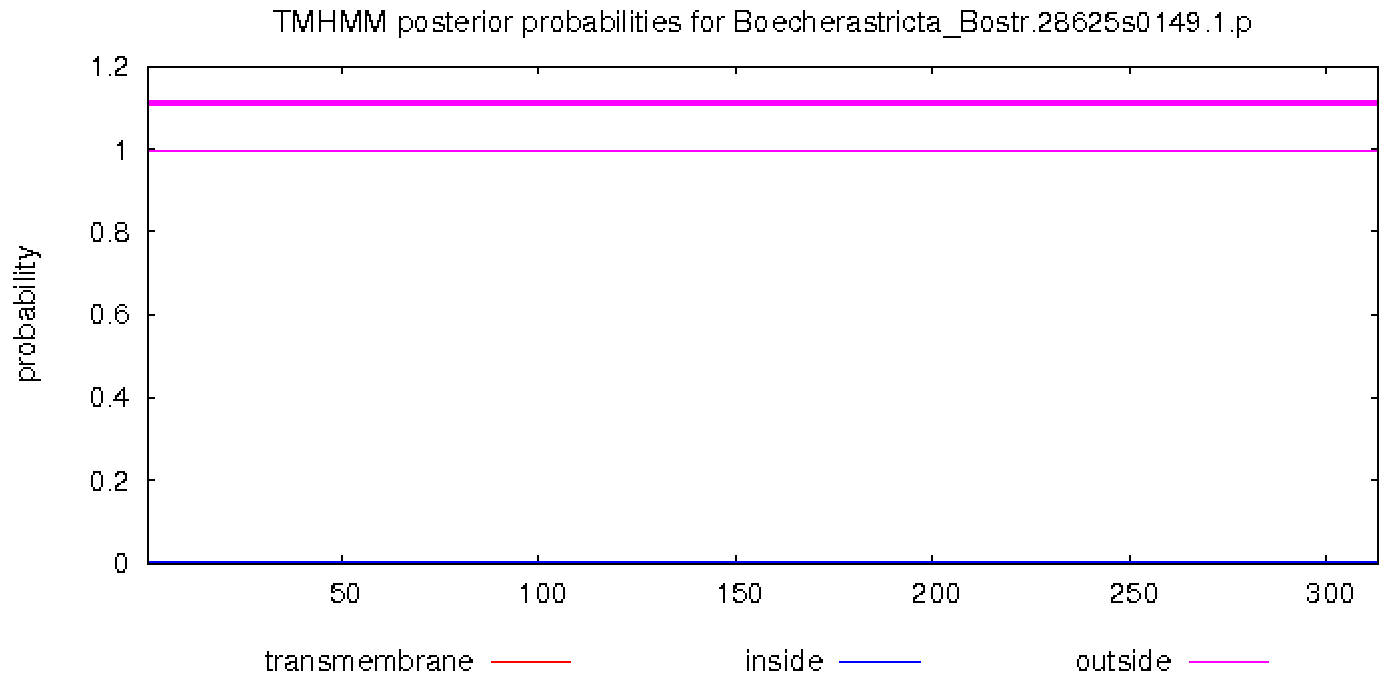

plot in postscript, script for making the plot in gnuplot, data for plot

---

```
# Boecherastricta_Bostr.28625s0180.1.p Length: 307
# Boecherastricta_Bostr.28625s0180.1.p Number of predicted TMHs:  0
# Boecherastricta_Bostr.28625s0180.1.p Exp number of AAs in TMHs: 0.12772
# Boecherastricta_Bostr.28625s0180.1.p Exp number, first 60 AAs:  0
```

```
# Boecherastricta_Bostr.28625s0180.1.p Total prob of N-in:        0.00905
Boecherastricta_Bostr.28625s0180.1.p	TMHMM2.0	outside	     1   307
```

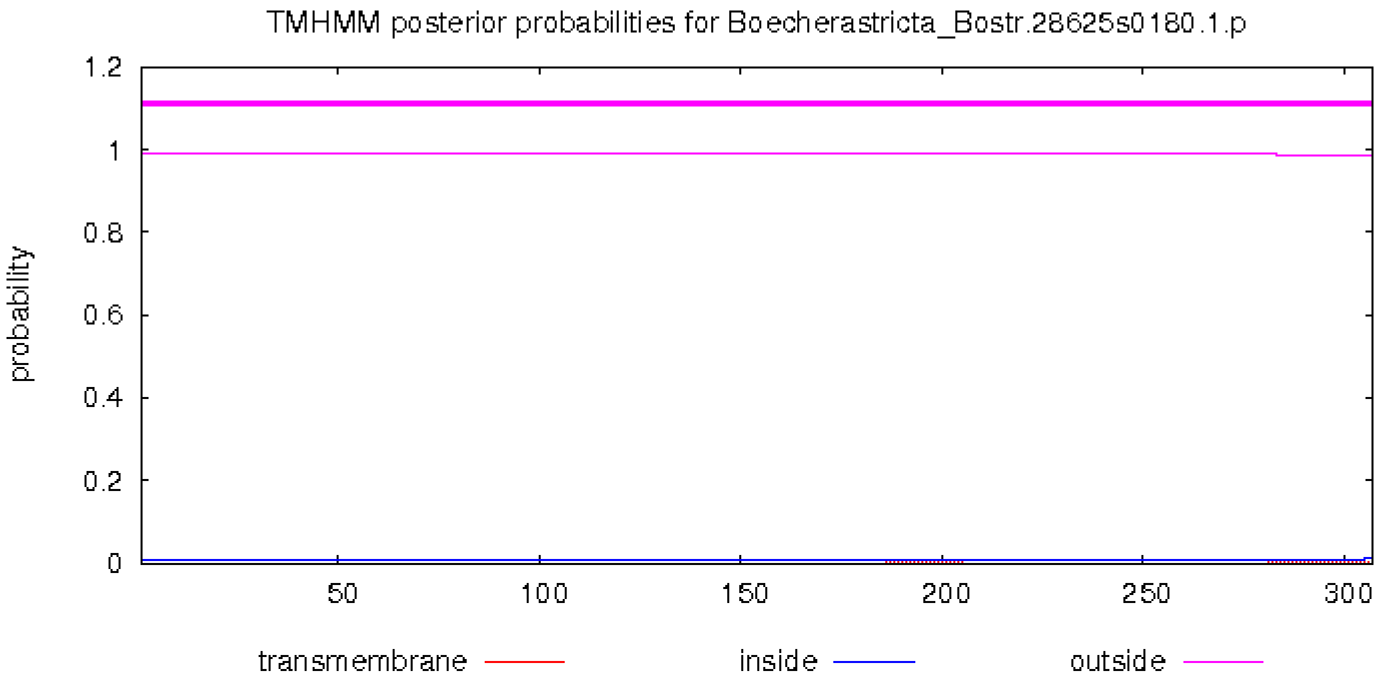

plot in postscript, script for making the plot in gnuplot, data for plot

---

```
# Boecherastricta_Bostr.28794s0029.1.p Length: 340
# Boecherastricta_Bostr.28794s0029.1.p Number of predicted TMHs:  1
# Boecherastricta_Bostr.28794s0029.1.p Exp number of AAs in TMHs: 21.08689
# Boecherastricta_Bostr.28794s0029.1.p Exp number, first 60 AAs:  0.08029
# Boecherastricta_Bostr.28794s0029.1.p Total prob of N-in:        0.50127
Boecherastricta_Bostr.28794s0029.1.p	TMHMM2.0	outside	     1   315
Boecherastricta_Bostr.28794s0029.1.p	TMHMM2.0	TMhelix	   316   335
Boecherastricta_Bostr.28794s0029.1.p	TMHMM2.0	inside	   336   340
```

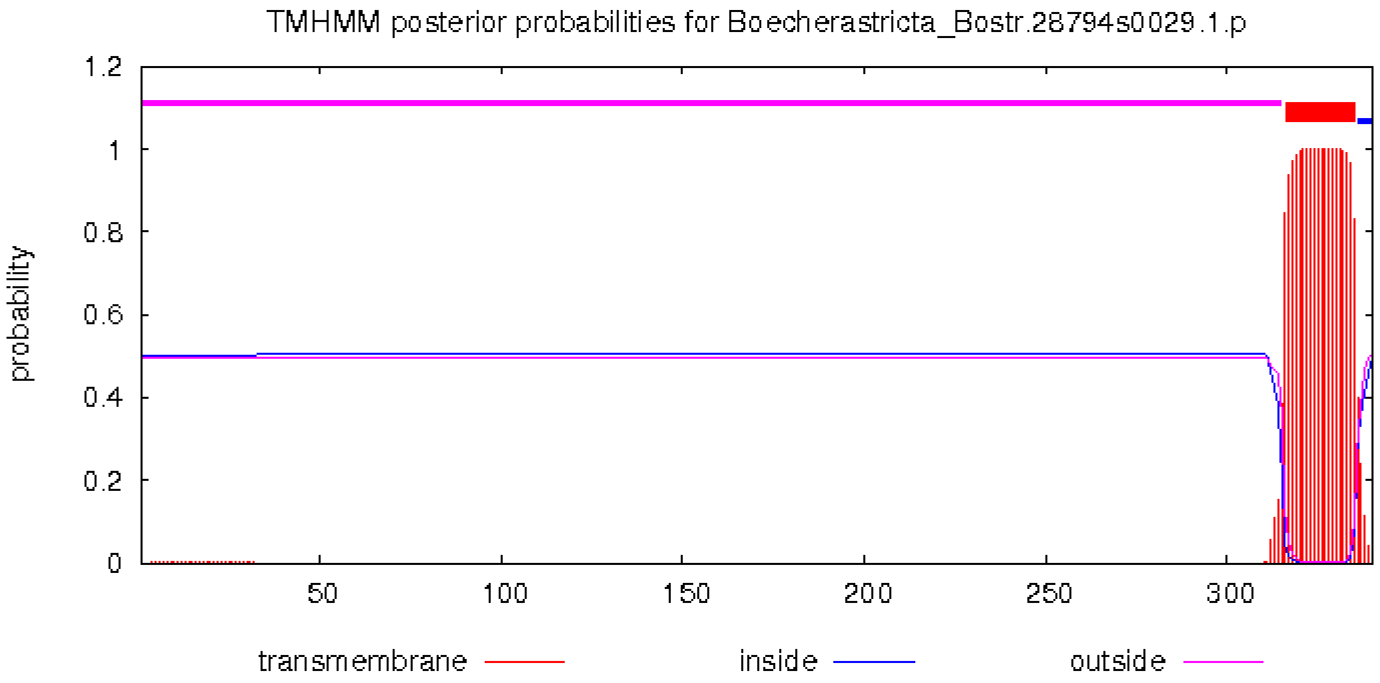

plot in postscript, script for making the plot in gnuplot, data for plot

---

```
# Boecherastricta_Bostr.2902s0053.1.p Length: 254
# Boecherastricta_Bostr.2902s0053.1.p Number of predicted TMHs:  0
```

```
# Boecherastricta_Bostr.2902s0053.1.p Exp number of AAs in TMHs: 0.00053
# Boecherastricta_Bostr.2902s0053.1.p Exp number, first 60 AAs:  0
# Boecherastricta_Bostr.2902s0053.1.p Total prob of N-in:        0.10866
Boecherastricta_Bostr.2902s0053.1.p     TMHMM2.0        outside      1   254
```

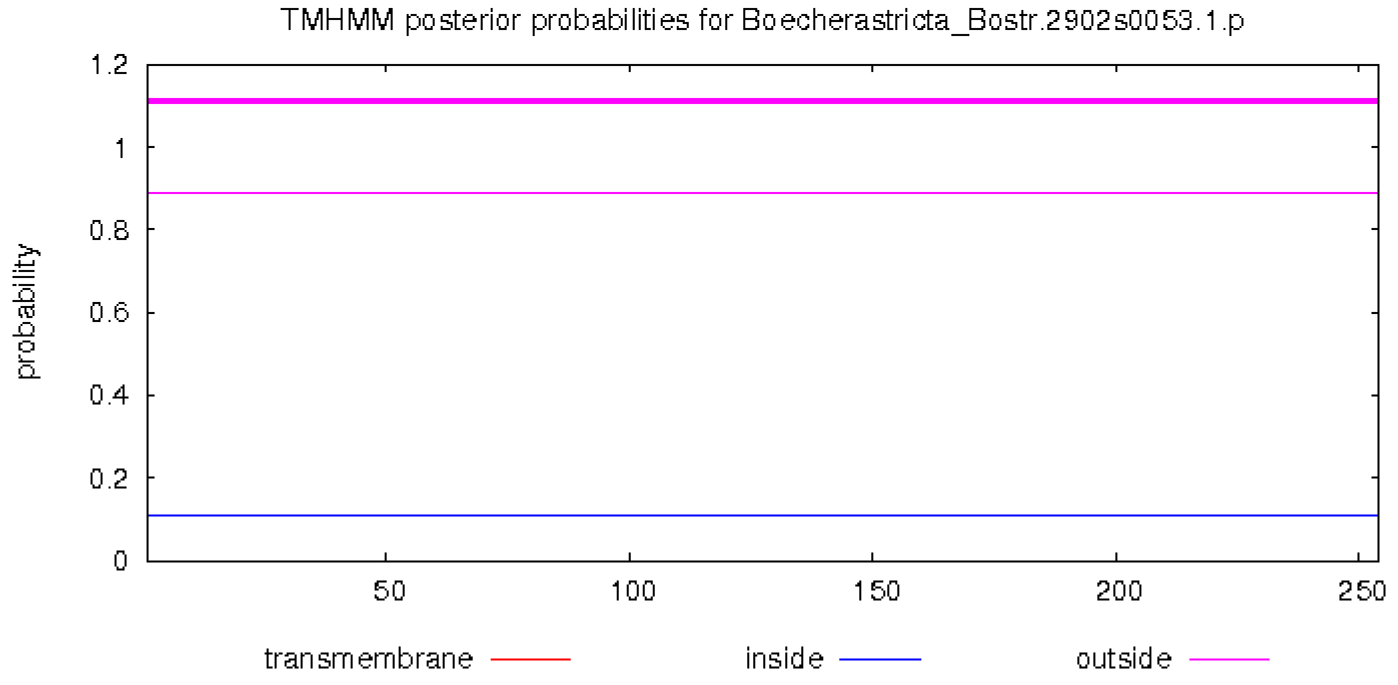

plot in postscript, script for making the plot in gnuplot, data for plot

---

```
# Boecherastricta_Bostr.2902s0136.1.p Length: 207
# Boecherastricta_Bostr.2902s0136.1.p Number of predicted TMHs:  0
# Boecherastricta_Bostr.2902s0136.1.p Exp number of AAs in TMHs: 1.40732
# Boecherastricta_Bostr.2902s0136.1.p Exp number, first 60 AAs:  1.10968
# Boecherastricta_Bostr.2902s0136.1.p Total prob of N-in:        0.20886
Boecherastricta_Bostr.2902s0136.1.p     TMHMM2.0        outside      1   207
```

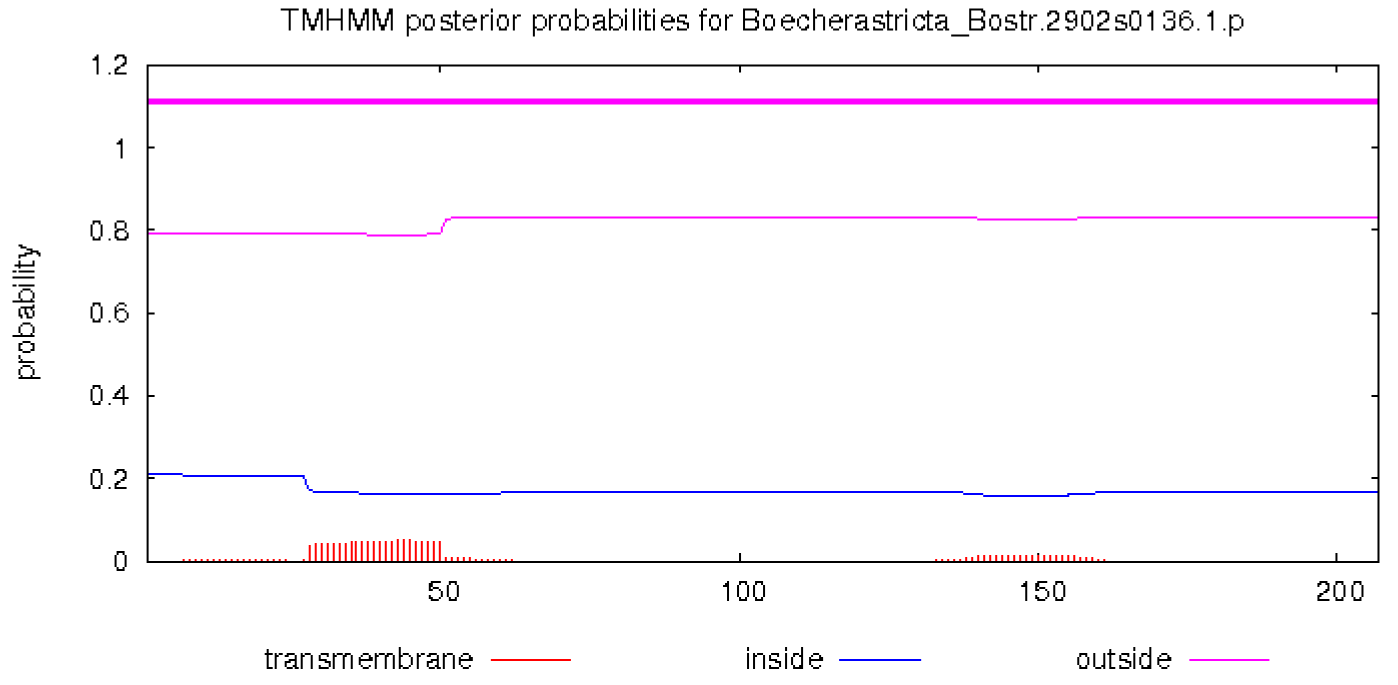

plot in postscript, script for making the plot in gnuplot, data for plot

---

```
# Boecherastricta_Bostr.2902s0186.1.p Length: 365
# Boecherastricta_Bostr.2902s0186.1.p Number of predicted TMHs:  0
```

```
# Boecherastricta_Bostr.2902s0186.1.p Exp number of AAs in TMHs: 9e-05
# Boecherastricta_Bostr.2902s0186.1.p Exp number, first 60 AAs:  9e-05
# Boecherastricta_Bostr.2902s0186.1.p Total prob of N-in:        0.04276
Boecherastricta_Bostr.2902s0186.1.p     TMHMM2.0        outside      1   365
```

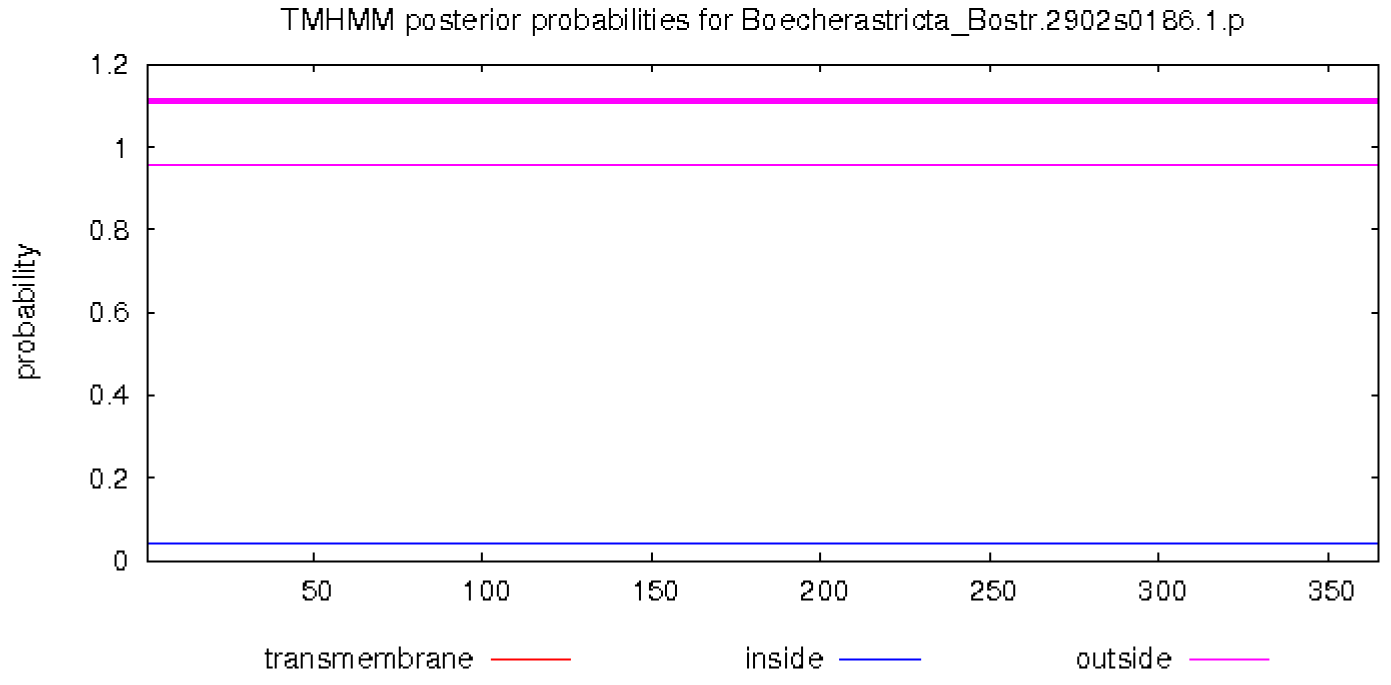

plot in postscript, script for making the plot in gnuplot, data for plot

---

```
# Boecherastricta_Bostr.30275s0267.1.p Length: 292
# Boecherastricta_Bostr.30275s0267.1.p Number of predicted TMHs:  0
# Boecherastricta_Bostr.30275s0267.1.p Exp number of AAs in TMHs: 0
# Boecherastricta_Bostr.30275s0267.1.p Exp number, first 60 AAs:  0
# Boecherastricta_Bostr.30275s0267.1.p Total prob of N-in:        0.00699
Boecherastricta_Bostr.30275s0267.1.p    TMHMM2.0        outside      1   292
```

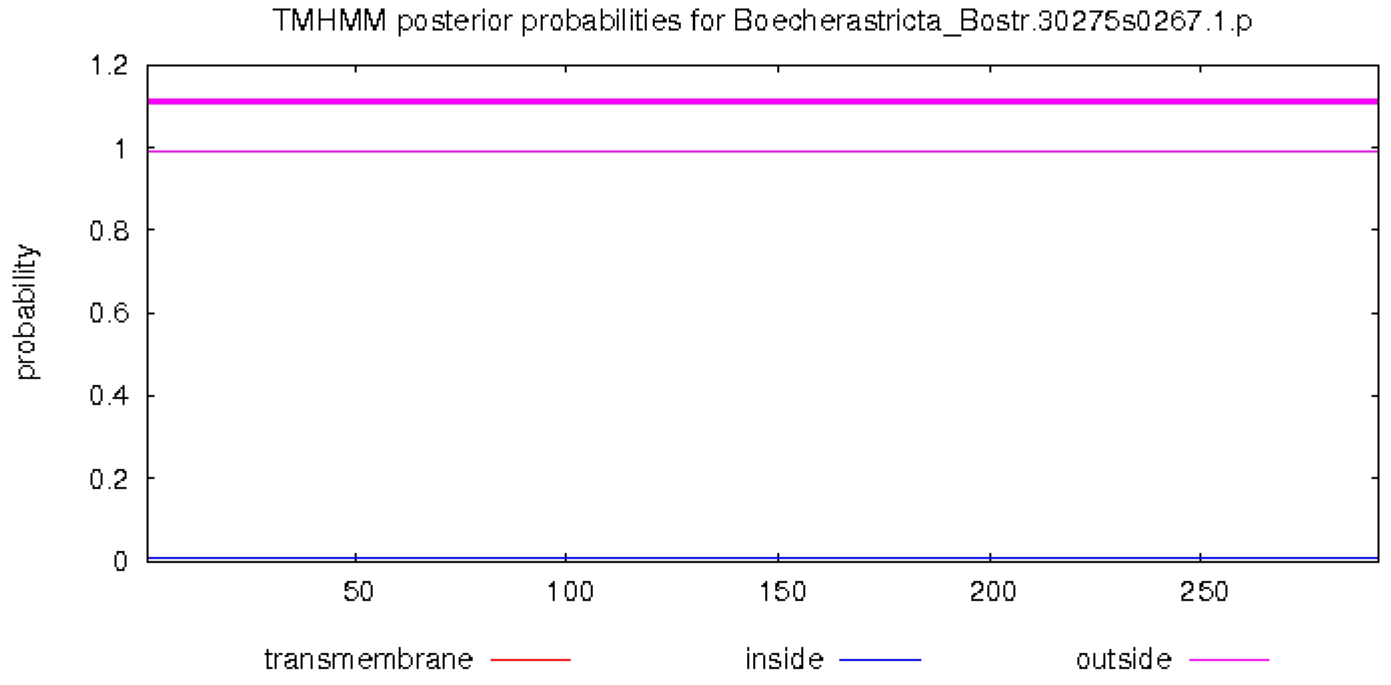

plot in postscript, script for making the plot in gnuplot, data for plot

---

```
# Boecherastricta_Bostr.3359s0042.1.p Length: 305
# Boecherastricta_Bostr.3359s0042.1.p Number of predicted TMHs:  0
```

```
# Boecherastricta_Bostr.3359s0042.1.p Exp number of AAs in TMHs: 0
# Boecherastricta_Bostr.3359s0042.1.p Exp number, first 60 AAs:  0
# Boecherastricta_Bostr.3359s0042.1.p Total prob of N-in:        0.00497
Boecherastricta_Bostr.3359s0042.1.p     TMHMM2.0        outside      1   305
```

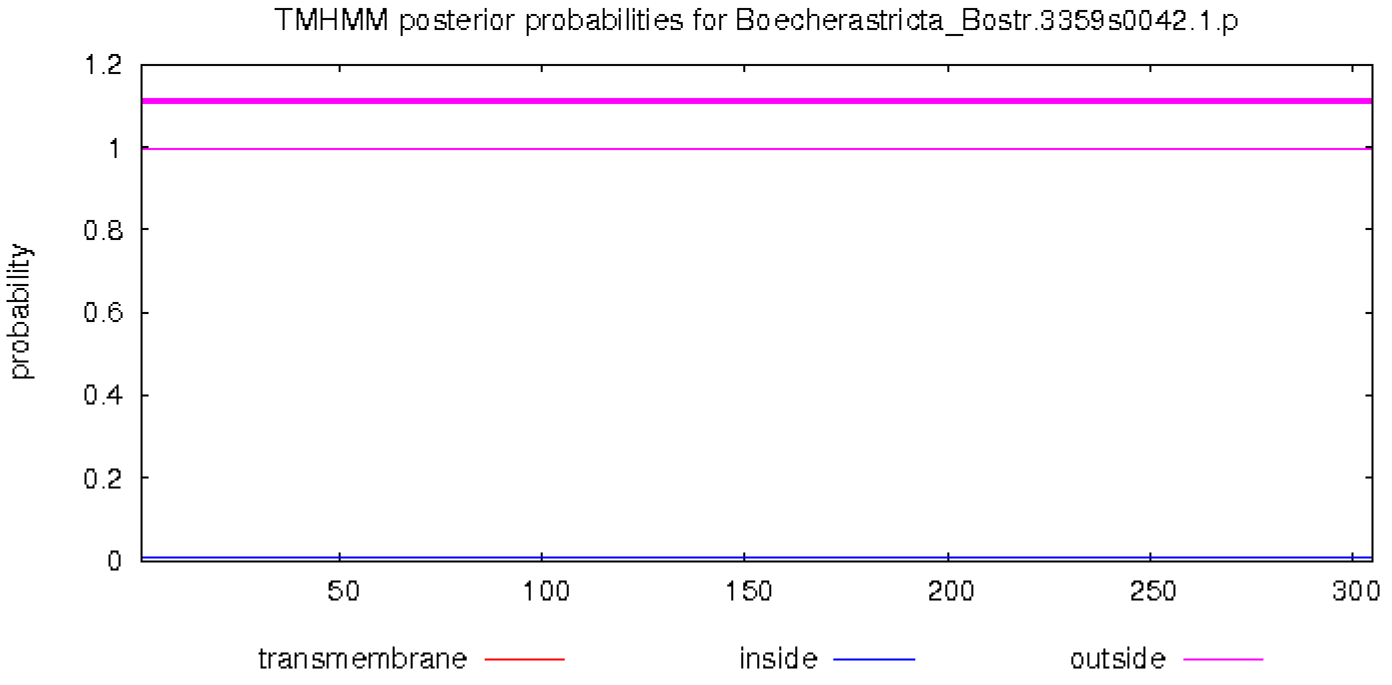

plot in postscript, script for making the plot in gnuplot, data for plot

---

```
# Boecherastricta_Bostr.3359s0059.1.p Length: 674
# Boecherastricta_Bostr.3359s0059.1.p Number of predicted TMHs:  1
# Boecherastricta_Bostr.3359s0059.1.p Exp number of AAs in TMHs: 20.13146
# Boecherastricta_Bostr.3359s0059.1.p Exp number, first 60 AAs:  0.00203
# Boecherastricta_Bostr.3359s0059.1.p Total prob of N-in:        0.23243
Boecherastricta_Bostr.3359s0059.1.p     TMHMM2.0        outside      1   631
Boecherastricta_Bostr.3359s0059.1.p     TMHMM2.0        TMhelix    632   651
Boecherastricta_Bostr.3359s0059.1.p     TMHMM2.0        inside     652   674
```

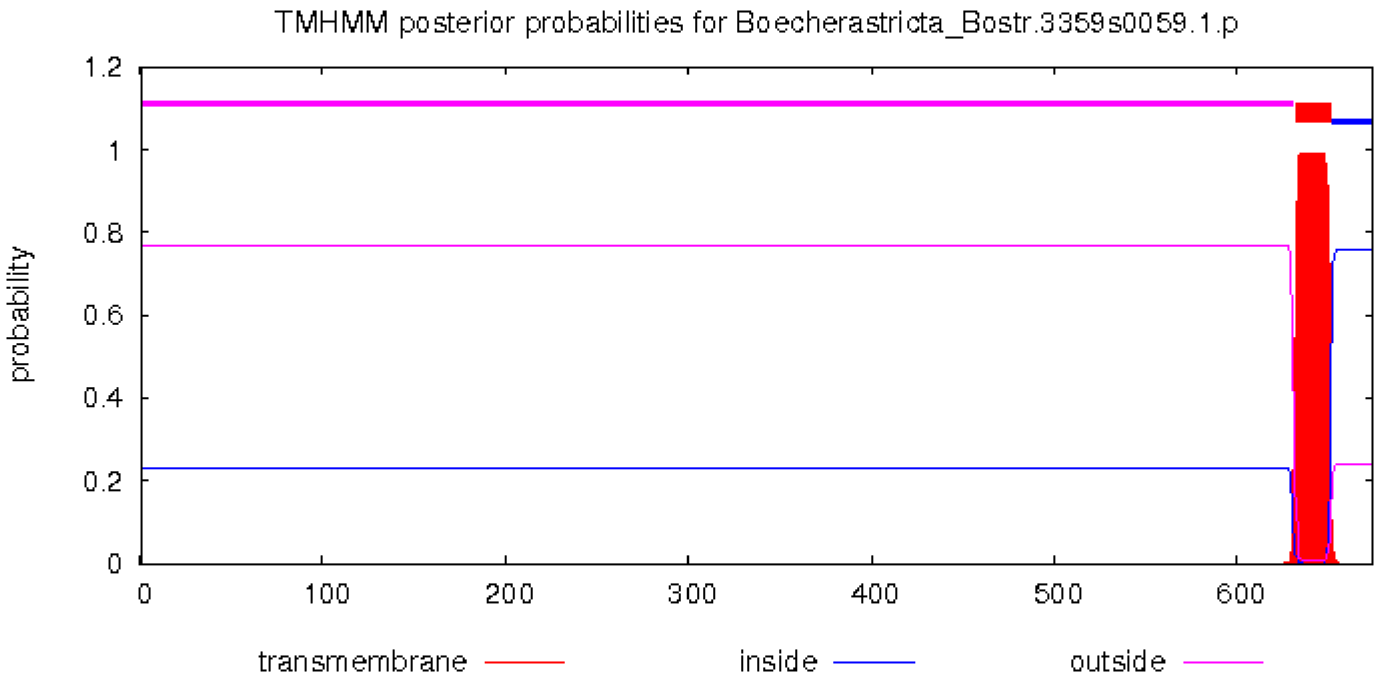

plot in postscript, script for making the plot in gnuplot, data for plot

---

```
# Boecherastricta_Bostr.3359s0074.1.p Length: 533
# Boecherastricta_Bostr.3359s0074.1.p Number of predicted TMHs:  1
# Boecherastricta_Bostr.3359s0074.1.p Exp number of AAs in TMHs: 22.87044
# Boecherastricta_Bostr.3359s0074.1.p Exp number, first 60 AAs:  0.02606
# Boecherastricta_Bostr.3359s0074.1.p Total prob of N-in:        0.72323
Boecherastricta_Bostr.3359s0074.1.p     TMHMM2.0        inside       1   503
Boecherastricta_Bostr.3359s0074.1.p     TMHMM2.0        TMhelix    504   526
Boecherastricta_Bostr.3359s0074.1.p     TMHMM2.0        outside    527   533
```

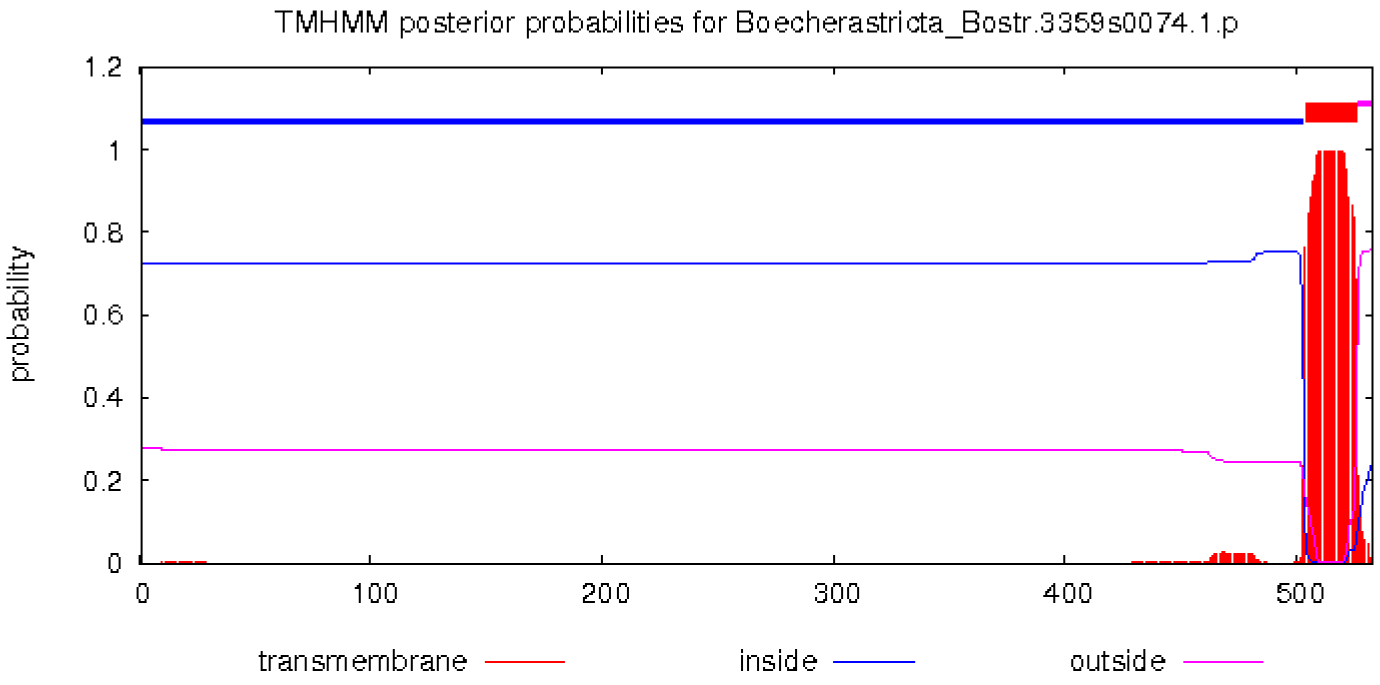

plot in postscript, script for making the plot in gnuplot, data for plot

---

```
# Boecherastricta_Bostr.3359s0081.1.p Length: 362
# Boecherastricta_Bostr.3359s0081.1.p Number of predicted TMHs:  0
# Boecherastricta_Bostr.3359s0081.1.p Exp number of AAs in TMHs: 0.00042
# Boecherastricta_Bostr.3359s0081.1.p Exp number, first 60 AAs:  0
# Boecherastricta_Bostr.3359s0081.1.p Total prob of N-in:        0.03140
Boecherastricta_Bostr.3359s0081.1.p     TMHMM2.0        outside      1   362
```

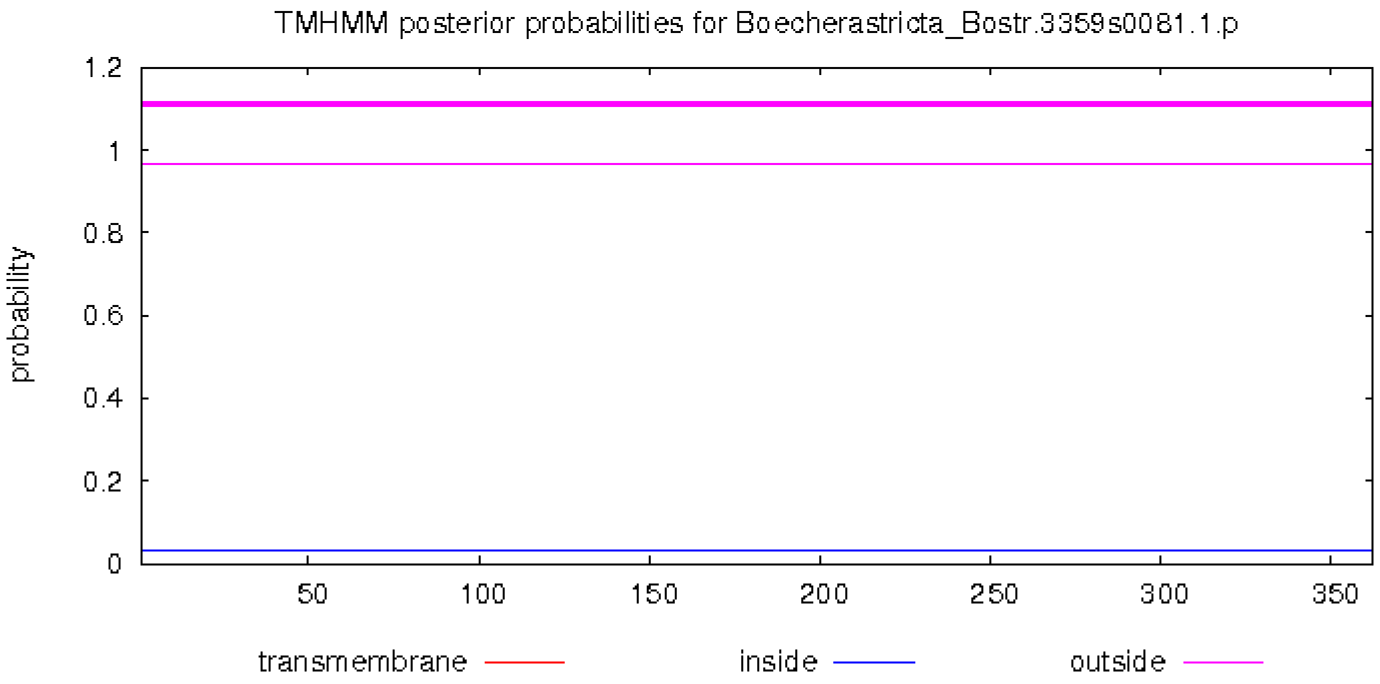

plot in postscript, script for making the plot in gnuplot, data for plot

```
# Boecherastricta_Bostr.3359s0109.1.p Length: 283
# Boecherastricta_Bostr.3359s0109.1.p Number of predicted TMHs:  0
# Boecherastricta_Bostr.3359s0109.1.p Exp number of AAs in TMHs: 0
# Boecherastricta_Bostr.3359s0109.1.p Exp number, first 60 AAs:  0
# Boecherastricta_Bostr.3359s0109.1.p Total prob of N-in:        0.00231
Boecherastricta_Bostr.3359s0109.1.p     TMHMM2.0        outside      1   283
```

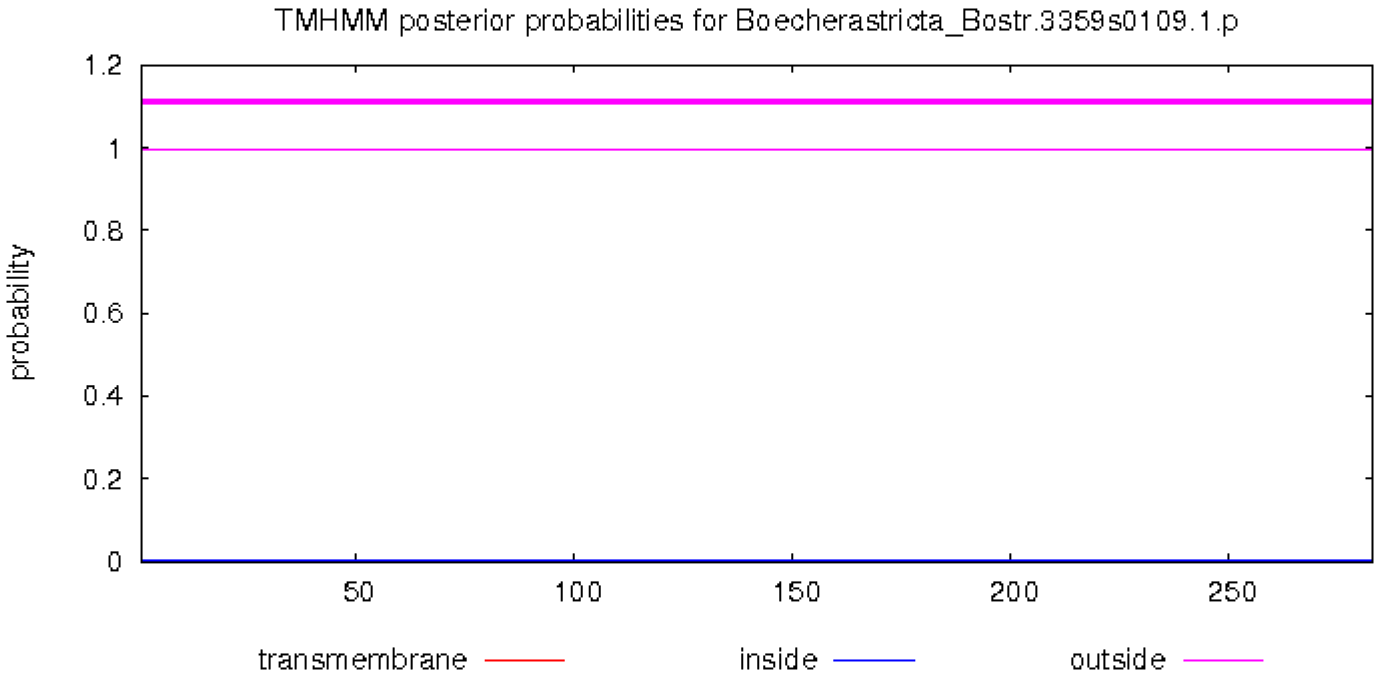

plot in postscript, script for making the plot in gnuplot, data for plot

```
# Boecherastricta_Bostr.3640s0064.1.p Length: 300
# Boecherastricta_Bostr.3640s0064.1.p Number of predicted TMHs:  0
# Boecherastricta_Bostr.3640s0064.1.p Exp number of AAs in TMHs: 0.00034
# Boecherastricta_Bostr.3640s0064.1.p Exp number, first 60 AAs:  0
# Boecherastricta_Bostr.3640s0064.1.p Total prob of N-in:        0.01699
Boecherastricta_Bostr.3640s0064.1.p     TMHMM2.0        outside      1   300
```

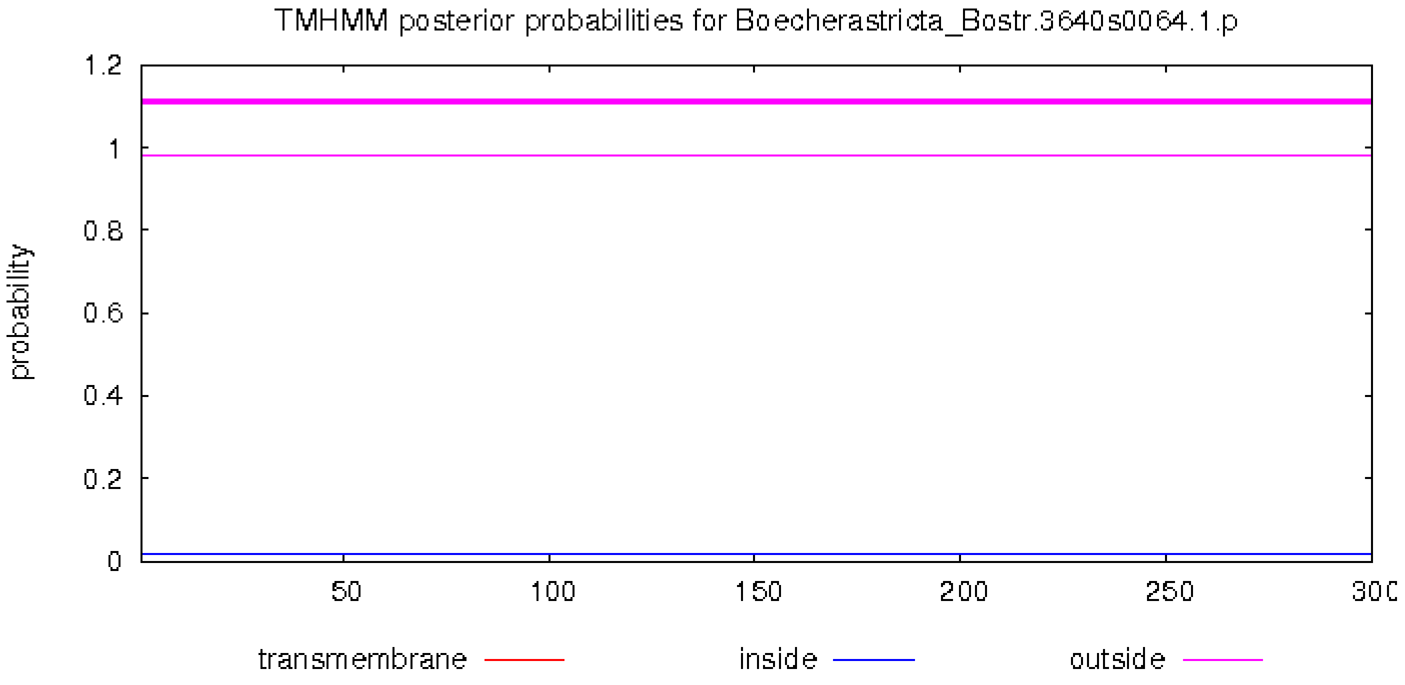

plot in postscript, script for making the plot in gnuplot, data for plot

```
# Boecherastricta_Bostr.3640s0073.1.p Length: 278
# Boecherastricta_Bostr.3640s0073.1.p Number of predicted TMHs:  0
# Boecherastricta_Bostr.3640s0073.1.p Exp number of AAs in TMHs: 0.00039
# Boecherastricta_Bostr.3640s0073.1.p Exp number, first 60 AAs:  0
# Boecherastricta_Bostr.3640s0073.1.p Total prob of N-in:        0.09463
Boecherastricta_Bostr.3640s0073.1.p     TMHMM2.0        outside      1   278
```

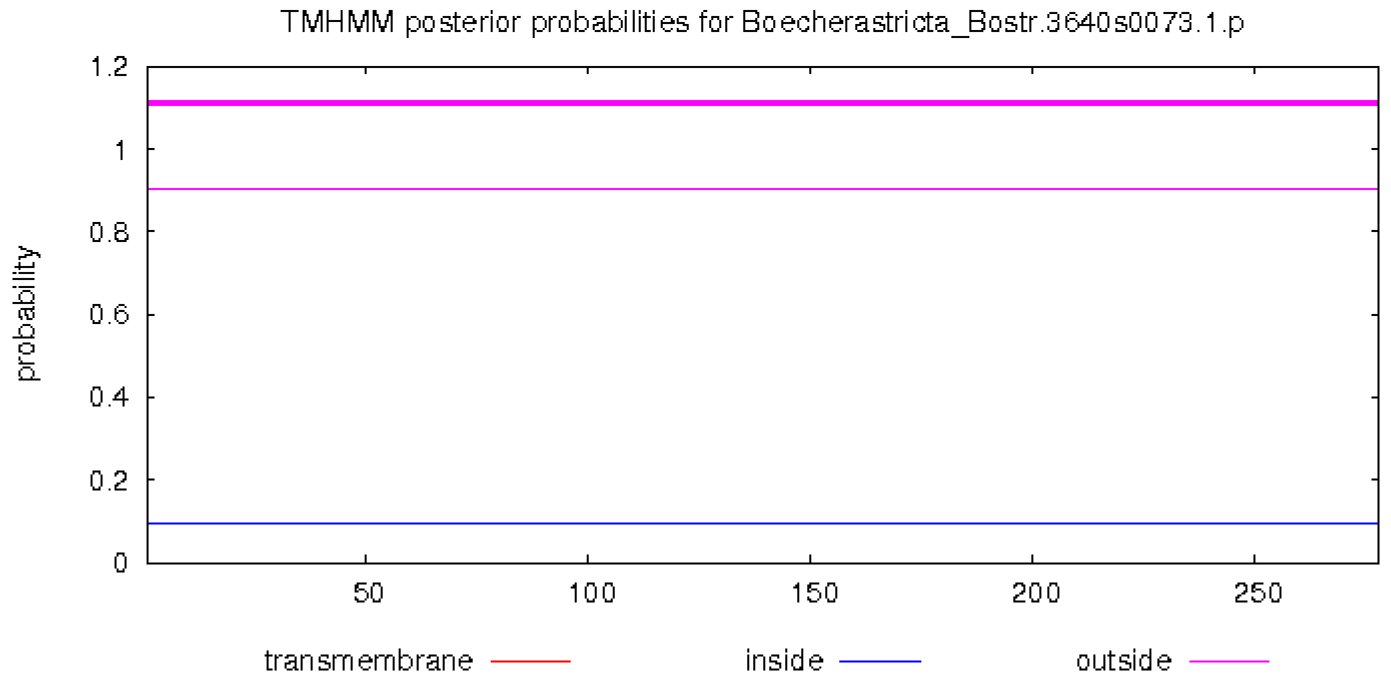

plot in postscript, script for making the plot in gnuplot, data for plot

---

```
# Boecherastricta_Bostr.4485s0006.1.p Length: 284
# Boecherastricta_Bostr.4485s0006.1.p Number of predicted TMHs:  0
# Boecherastricta_Bostr.4485s0006.1.p Exp number of AAs in TMHs: 0.00017
# Boecherastricta_Bostr.4485s0006.1.p Exp number, first 60 AAs:  0
# Boecherastricta_Bostr.4485s0006.1.p Total prob of N-in:        0.00242
Boecherastricta_Bostr.4485s0006.1.p     TMHMM2.0        outside      1   284
```

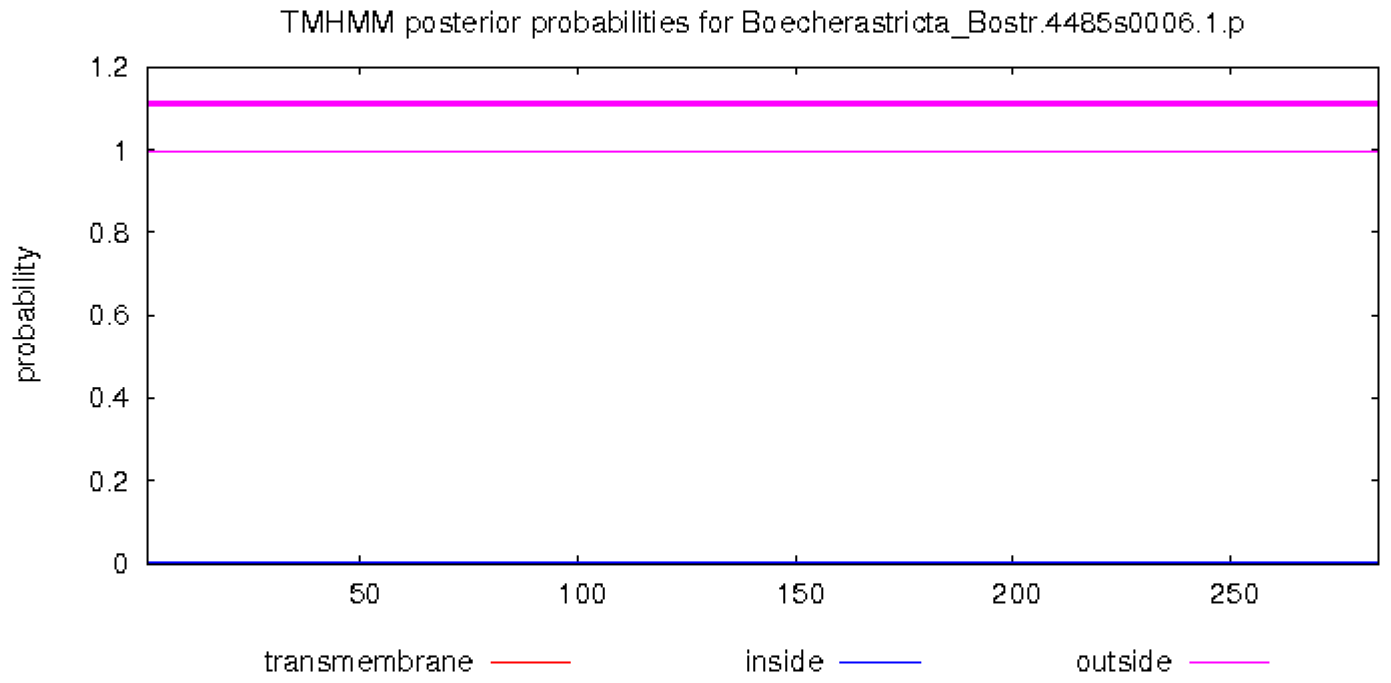

plot in postscript, script for making the plot in gnuplot, data for plot

---

```
# Boecherastricta_Bostr.4825s0021.1.p Length: 636
# Boecherastricta_Bostr.4825s0021.1.p Number of predicted TMHs:  1
# Boecherastricta_Bostr.4825s0021.1.p Exp number of AAs in TMHs: 21.8901
# Boecherastricta_Bostr.4825s0021.1.p Exp number, first 60 AAs:  0
# Boecherastricta_Bostr.4825s0021.1.p Total prob of N-in:        0.00421
Boecherastricta_Bostr.4825s0021.1.p     TMHMM2.0        outside      1   609
Boecherastricta_Bostr.4825s0021.1.p     TMHMM2.0        TMhelix    610   632
Boecherastricta_Bostr.4825s0021.1.p     TMHMM2.0        inside     633   636
```

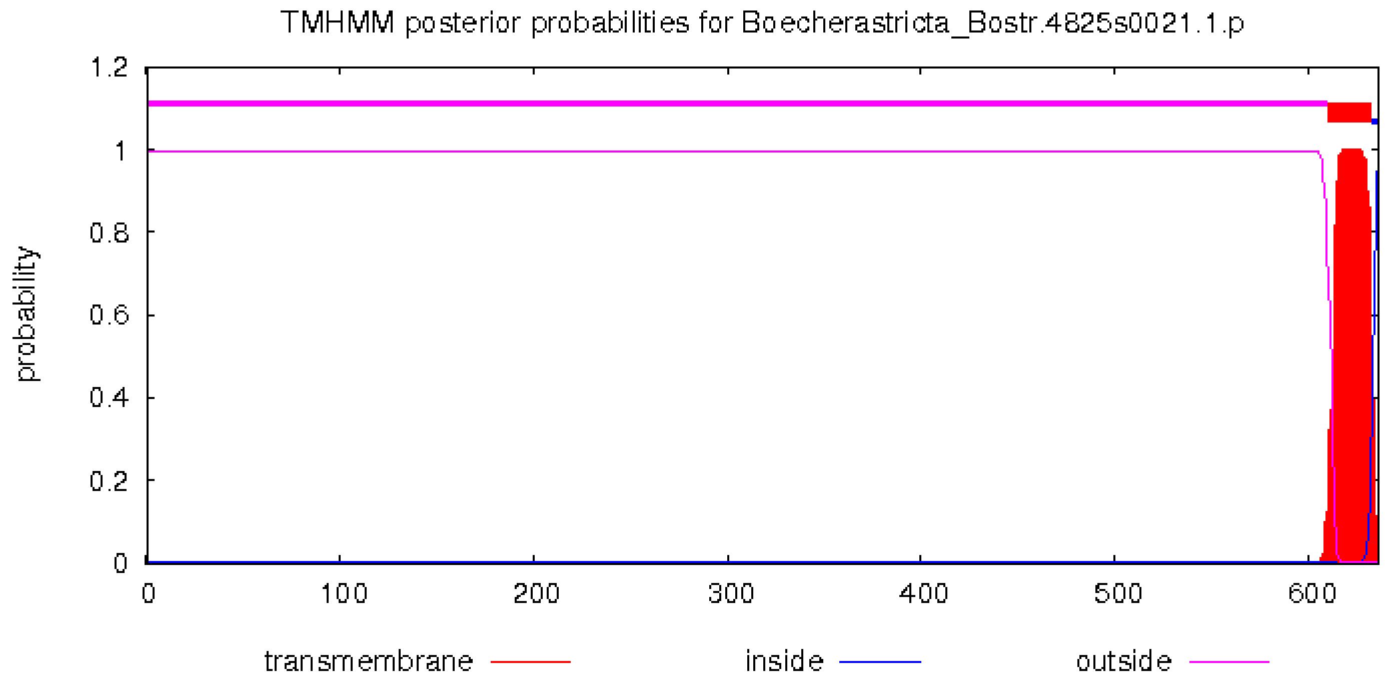

plot in postscript, script for making the plot in gnuplot, data for plot

---

```
# Boecherastricta_Bostr.4825s0023.1.p Length: 445
# Boecherastricta_Bostr.4825s0023.1.p Number of predicted TMHs:  1
# Boecherastricta_Bostr.4825s0023.1.p Exp number of AAs in TMHs: 21.92784
# Boecherastricta_Bostr.4825s0023.1.p Exp number, first 60 AAs:  0.00074
# Boecherastricta_Bostr.4825s0023.1.p Total prob of N-in:        0.06628
Boecherastricta_Bostr.4825s0023.1.p     TMHMM2.0        outside      1   418
Boecherastricta_Bostr.4825s0023.1.p     TMHMM2.0        TMhelix    419   441
Boecherastricta_Bostr.4825s0023.1.p     TMHMM2.0        inside     442   445
```

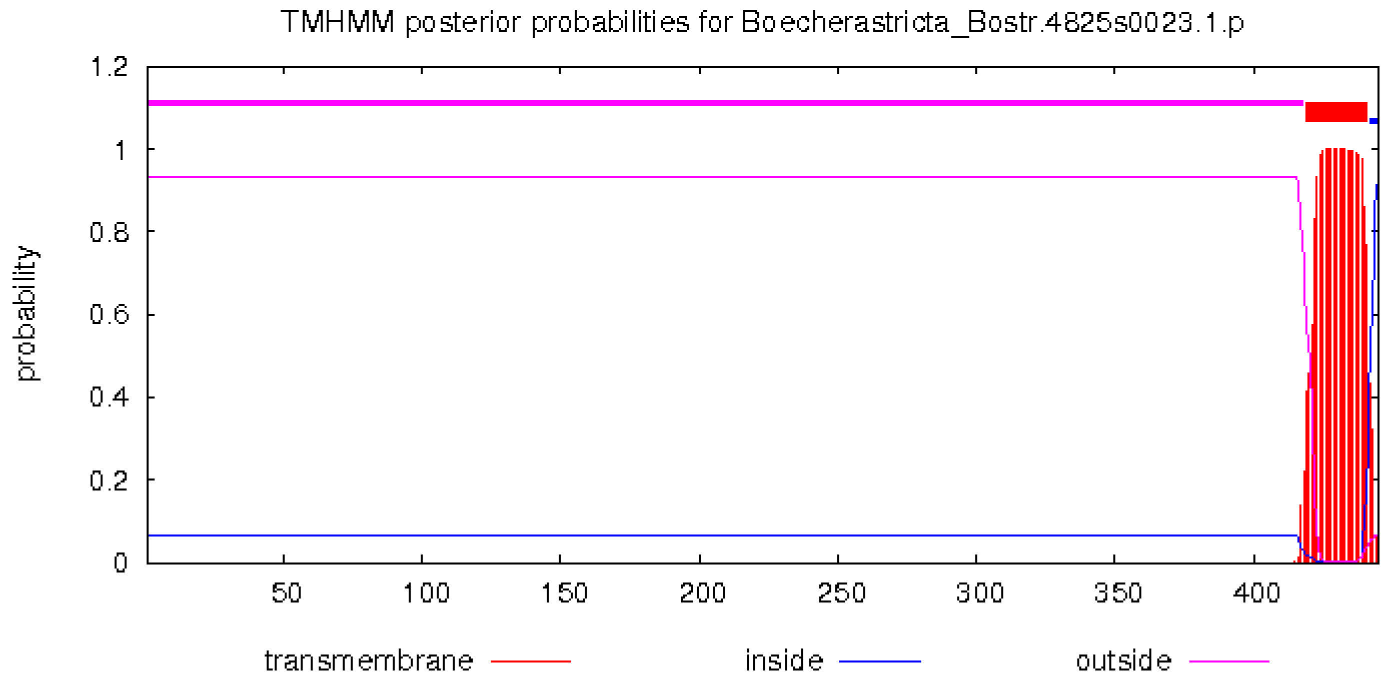

plot in postscript, script for making the plot in gnuplot, data for plot

```
# Boecherastricta_Bostr.4825s0024.1.p Length: 437
# Boecherastricta_Bostr.4825s0024.1.p Number of predicted TMHs:  1
# Boecherastricta_Bostr.4825s0024.1.p Exp number of AAs in TMHs: 18.14077
# Boecherastricta_Bostr.4825s0024.1.p Exp number, first 60 AAs:  0.00018
# Boecherastricta_Bostr.4825s0024.1.p Total prob of N-in:        0.00561
Boecherastricta_Bostr.4825s0024.1.p     TMHMM2.0        outside      1   416
Boecherastricta_Bostr.4825s0024.1.p     TMHMM2.0        TMhelix    417   436
Boecherastricta_Bostr.4825s0024.1.p     TMHMM2.0        inside     437   437
```

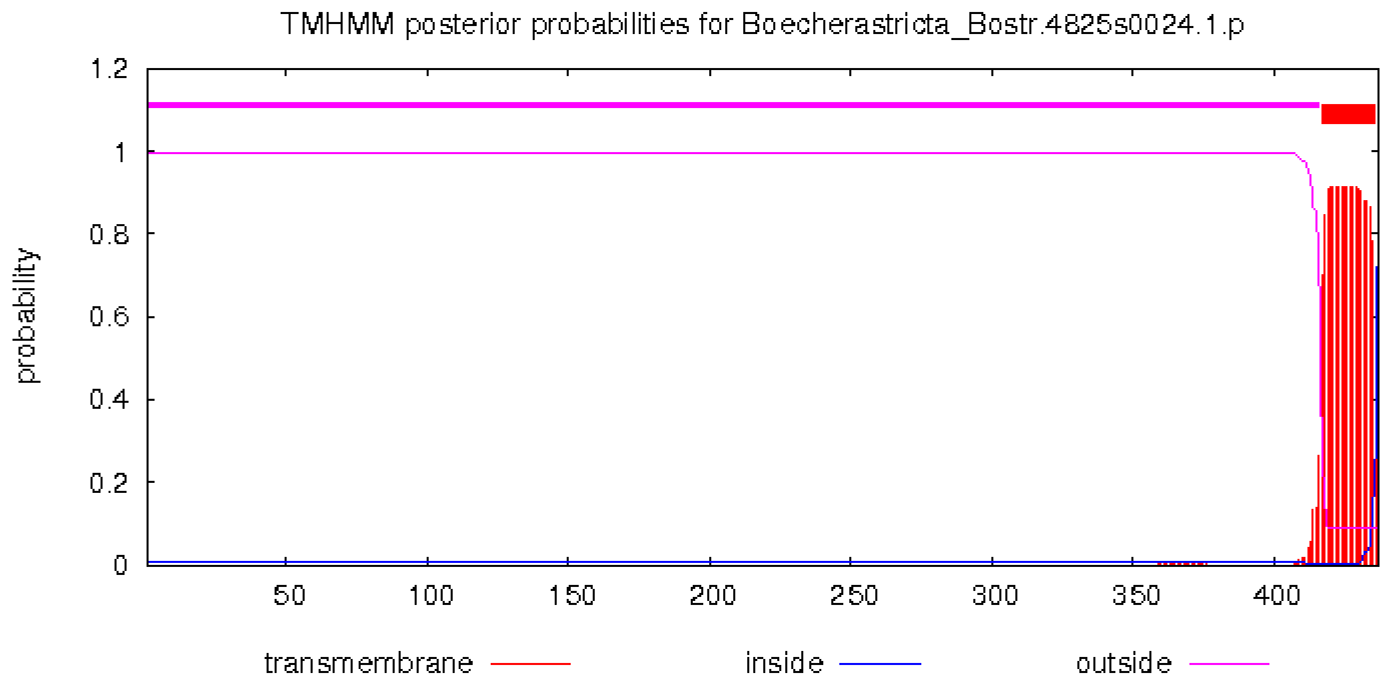

plot in postscript, script for making the plot in gnuplot, data for plot

```
# Boecherastricta_Bostr.5325s0006.1.p Length: 272
# Boecherastricta_Bostr.5325s0006.1.p Number of predicted TMHs:  0
# Boecherastricta_Bostr.5325s0006.1.p Exp number of AAs in TMHs: 0
# Boecherastricta_Bostr.5325s0006.1.p Exp number, first 60 AAs:  0
# Boecherastricta_Bostr.5325s0006.1.p Total prob of N-in:        0.02301
Boecherastricta_Bostr.5325s0006.1.p     TMHMM2.0        outside      1   272
```

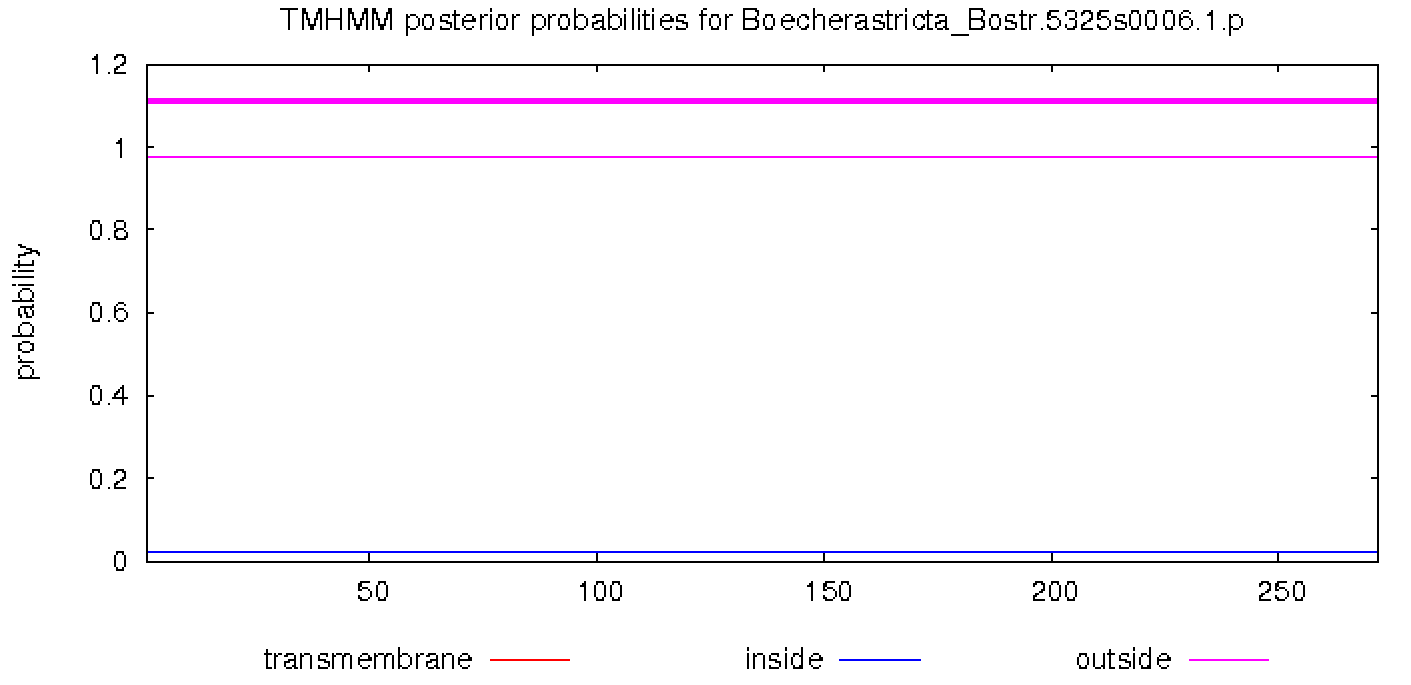

plot in postscript, script for making the plot in gnuplot, data for plot

---

```
# Boecherastricta_Bostr.5325s0007.1.p Length: 140
# Boecherastricta_Bostr.5325s0007.1.p Number of predicted TMHs:  0
# Boecherastricta_Bostr.5325s0007.1.p Exp number of AAs in TMHs: 0.00573
# Boecherastricta_Bostr.5325s0007.1.p Exp number, first 60 AAs:  0.00162
# Boecherastricta_Bostr.5325s0007.1.p Total prob of N-in:        0.06647
Boecherastricta_Bostr.5325s0007.1.p	TMHMM2.0	outside	     1   140
```

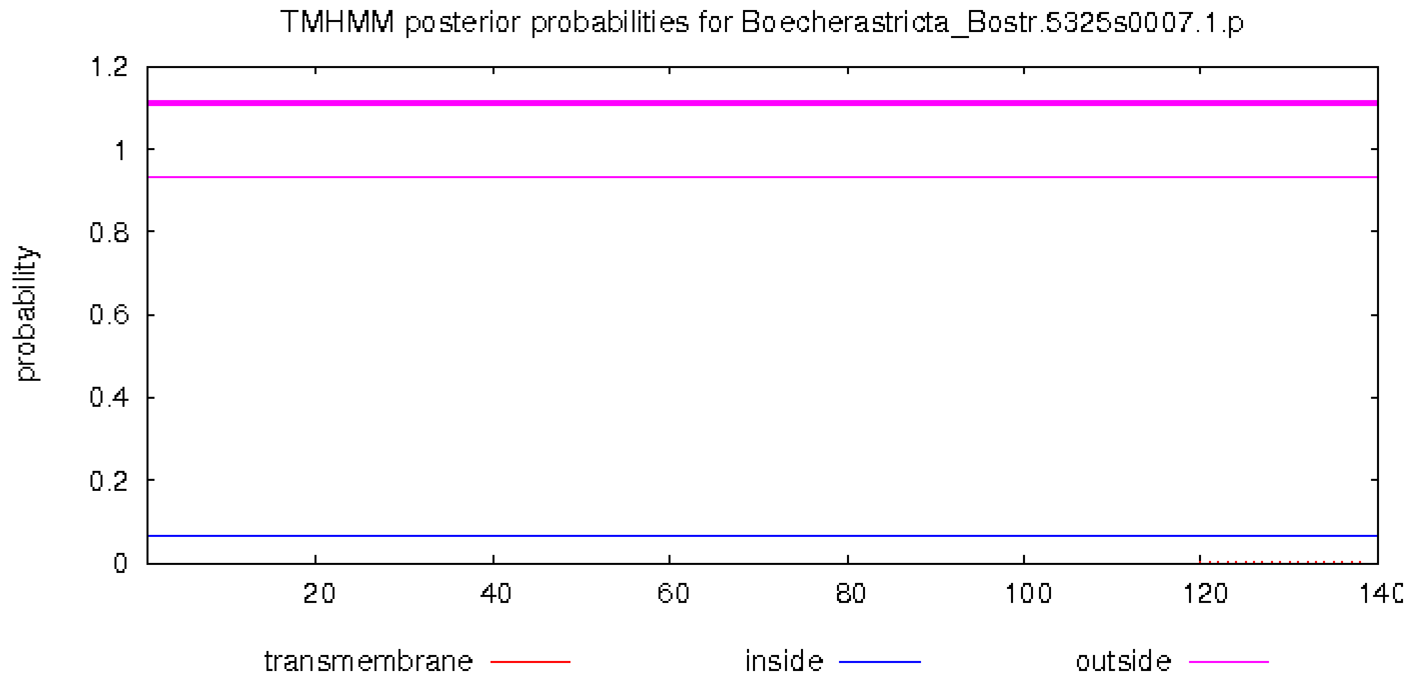

plot in postscript, script for making the plot in gnuplot, data for plot

---

```
# Boecherastricta_Bostr.5325s0008.1.p Length: 226
# Boecherastricta_Bostr.5325s0008.1.p Number of predicted TMHs:  0
# Boecherastricta_Bostr.5325s0008.1.p Exp number of AAs in TMHs: 0.00013
# Boecherastricta_Bostr.5325s0008.1.p Exp number, first 60 AAs:  5e-05
# Boecherastricta_Bostr.5325s0008.1.p Total prob of N-in:        0.04736
Boecherastricta_Bostr.5325s0008.1.p	TMHMM2.0	outside	     1   226
```

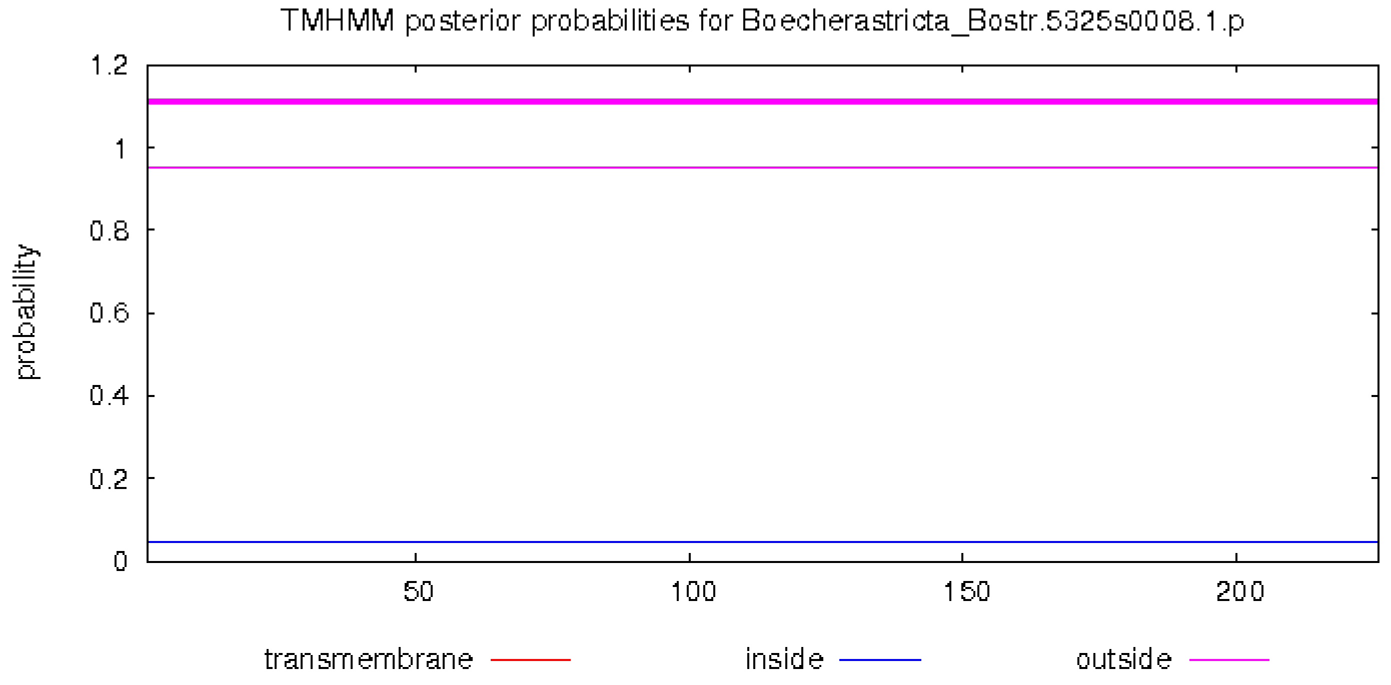

plot in postscript, script for making the plot in gnuplot, data for plot

---

```
# Boecherastricta_Bostr.5325s0009.1.p Length: 423
# Boecherastricta_Bostr.5325s0009.1.p Number of predicted TMHs:  1
# Boecherastricta_Bostr.5325s0009.1.p Exp number of AAs in TMHs: 21.07839
# Boecherastricta_Bostr.5325s0009.1.p Exp number, first 60 AAs:  0.00039
# Boecherastricta_Bostr.5325s0009.1.p Total prob of N-in:        0.63944
Boecherastricta_Bostr.5325s0009.1.p     TMHMM2.0        inside       1   399
Boecherastricta_Bostr.5325s0009.1.p     TMHMM2.0        TMhelix    400   422
Boecherastricta_Bostr.5325s0009.1.p     TMHMM2.0        outside    423   423
```

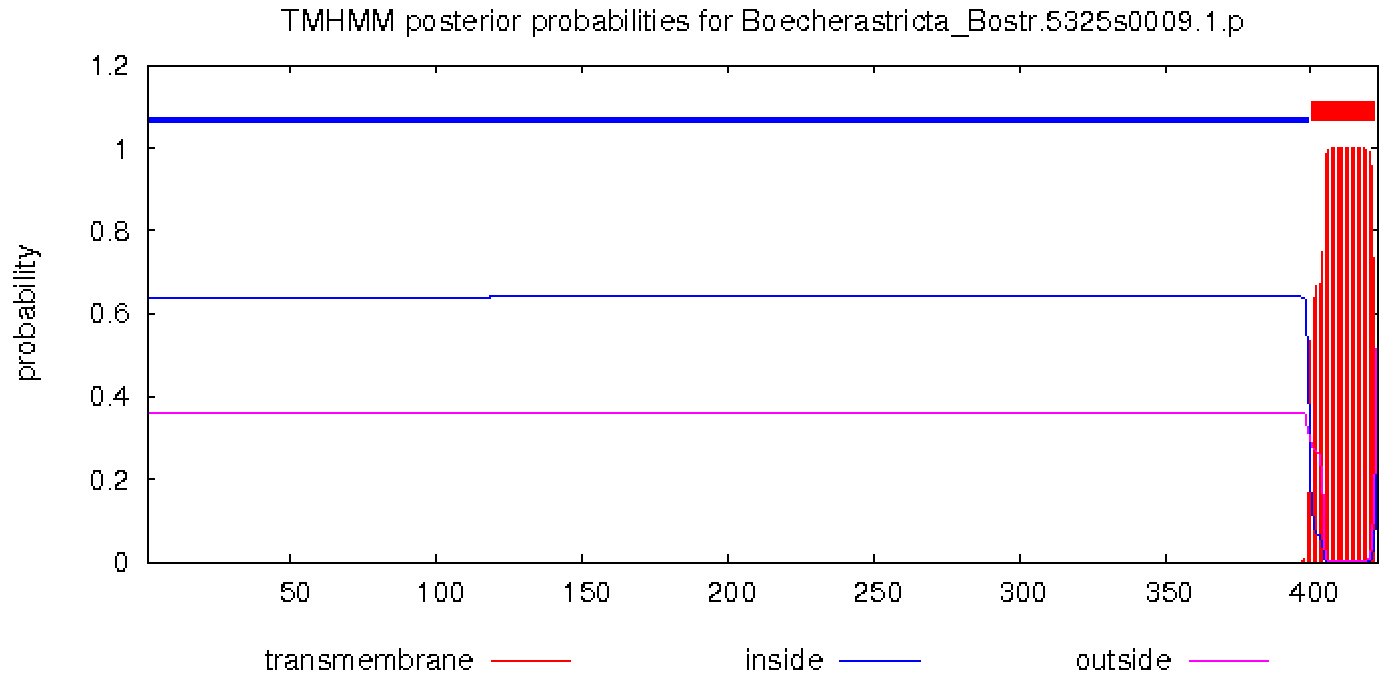

plot in postscript, script for making the plot in gnuplot, data for plot

---

```
# Boecherastricta_Bostr.5325s0010.1.p Length: 361
# Boecherastricta_Bostr.5325s0010.1.p Number of predicted TMHs:  0
# Boecherastricta_Bostr.5325s0010.1.p Exp number of AAs in TMHs: 0.00447
# Boecherastricta_Bostr.5325s0010.1.p Exp number, first 60 AAs:  0.00018
```

```
# Boecherastricta_Bostr.5325s0010.1.p Total prob of N-in:        0.00489
Boecherastricta_Bostr.5325s0010.1.p     TMHMM2.0        outside      1   361
```

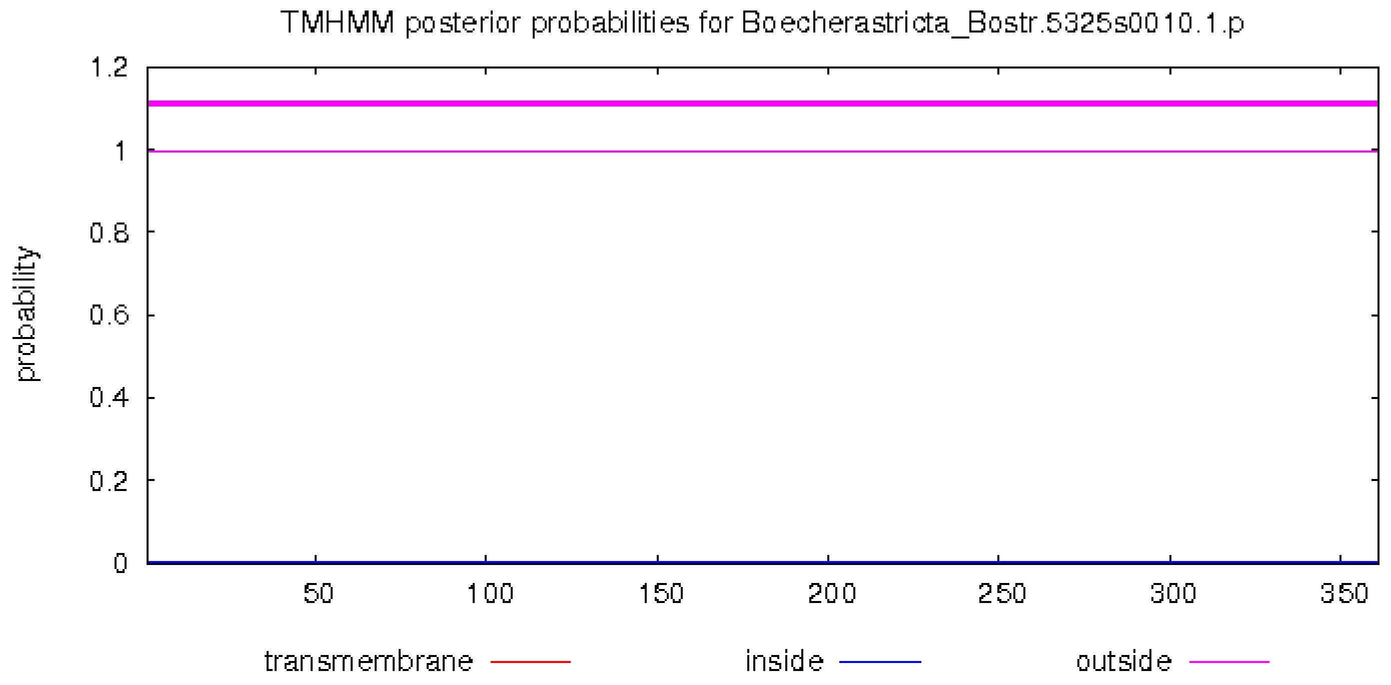

plot in postscript, script for making the plot in gnuplot, data for plot

---

```
# Boecherastricta_Bostr.5325s0011.1.p Length: 348
# Boecherastricta_Bostr.5325s0011.1.p Number of predicted TMHs:  1
# Boecherastricta_Bostr.5325s0011.1.p Exp number of AAs in TMHs: 22.48746
# Boecherastricta_Bostr.5325s0011.1.p Exp number, first 60 AAs:  0.00081
# Boecherastricta_Bostr.5325s0011.1.p Total prob of N-in:        0.75648
Boecherastricta_Bostr.5325s0011.1.p     TMHMM2.0        inside       1   324
Boecherastricta_Bostr.5325s0011.1.p     TMHMM2.0        TMhelix    325   347
Boecherastricta_Bostr.5325s0011.1.p     TMHMM2.0        outside    348   348
```

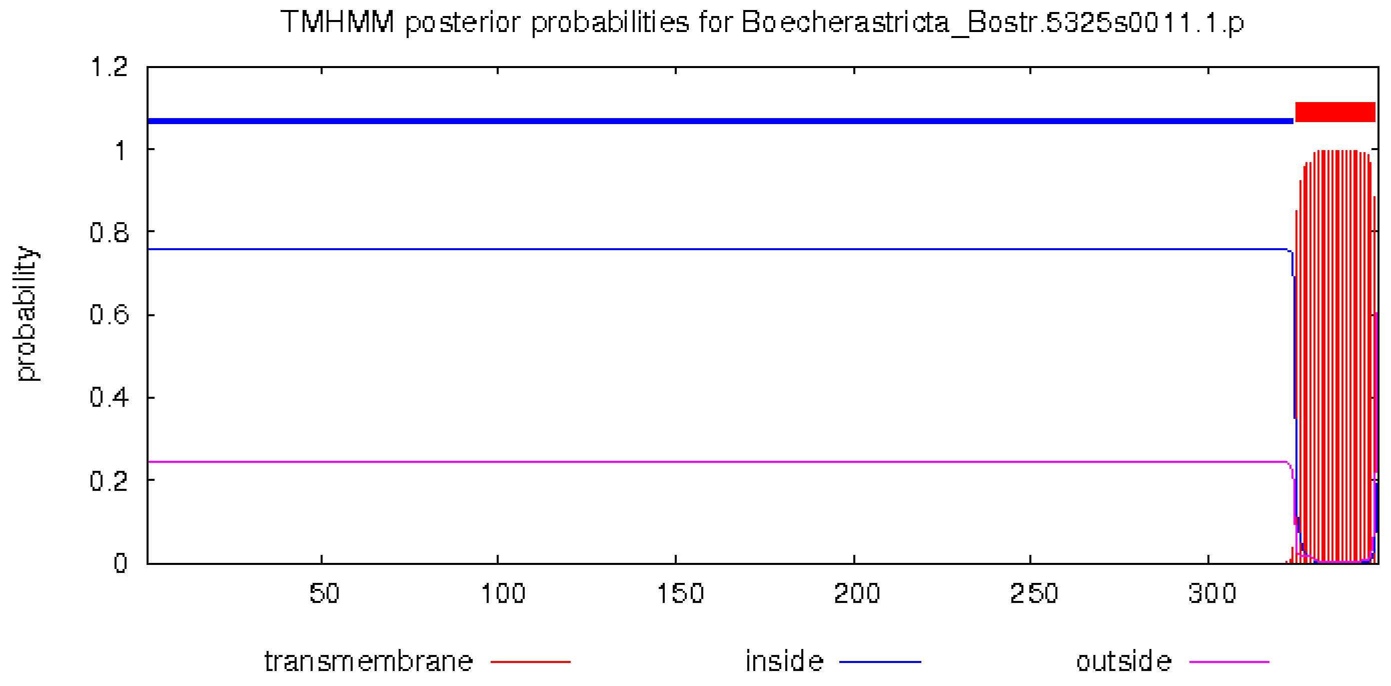

plot in postscript, script for making the plot in gnuplot, data for plot

---

```
# Boecherastricta_Bostr.5325s0013.1.p Length: 73
# Boecherastricta_Bostr.5325s0013.1.p Number of predicted TMHs:  0
```

```
# Boecherastricta_Bostr.5325s0013.1.p Exp number of AAs in TMHs: 0
# Boecherastricta_Bostr.5325s0013.1.p Exp number, first 60 AAs:  0
# Boecherastricta_Bostr.5325s0013.1.p Total prob of N-in:        0.38333
Boecherastricta_Bostr.5325s0013.1.p     TMHMM2.0        outside      1    73
```

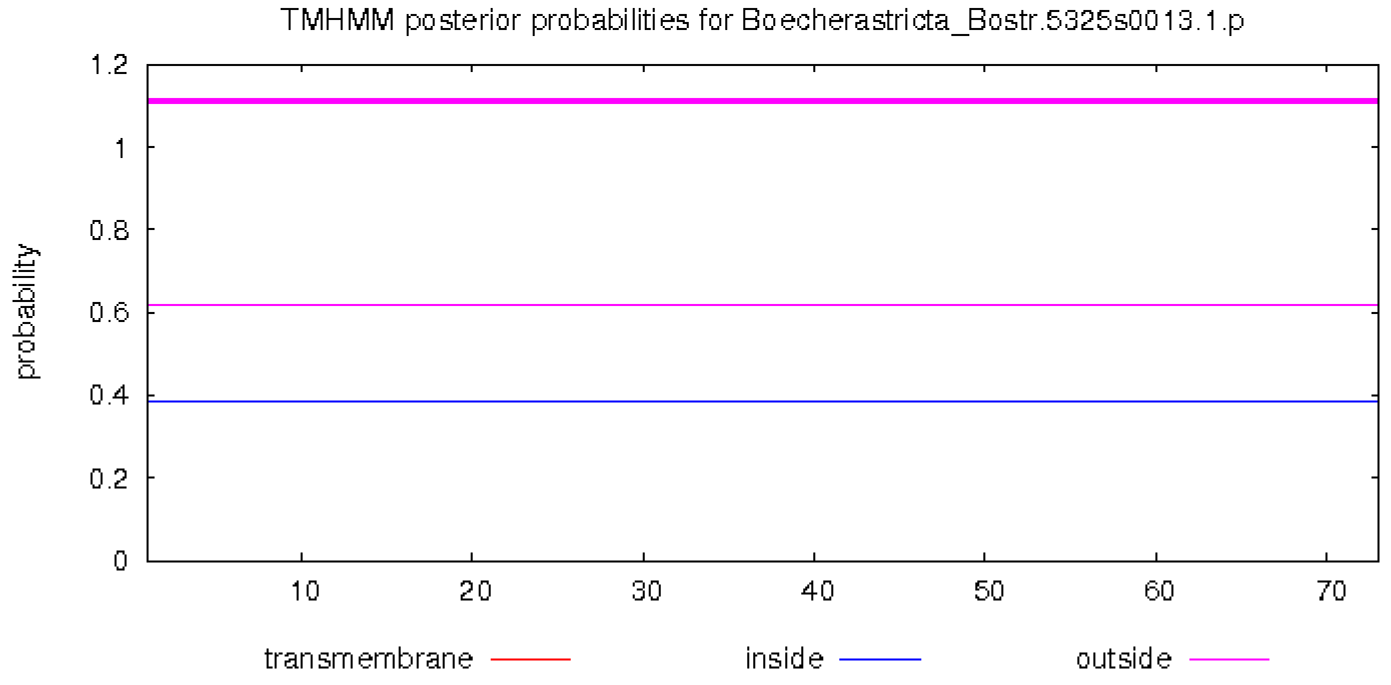

plot in postscript, script for making the plot in gnuplot, data for plot

---

```
# Boecherastricta_Bostr.5325s0014.1.p Length: 170
# Boecherastricta_Bostr.5325s0014.1.p Number of predicted TMHs:  0
# Boecherastricta_Bostr.5325s0014.1.p Exp number of AAs in TMHs: 0.00017
# Boecherastricta_Bostr.5325s0014.1.p Exp number, first 60 AAs:  6e-05
# Boecherastricta_Bostr.5325s0014.1.p Total prob of N-in:        0.09874
Boecherastricta_Bostr.5325s0014.1.p     TMHMM2.0        outside      1   170
```

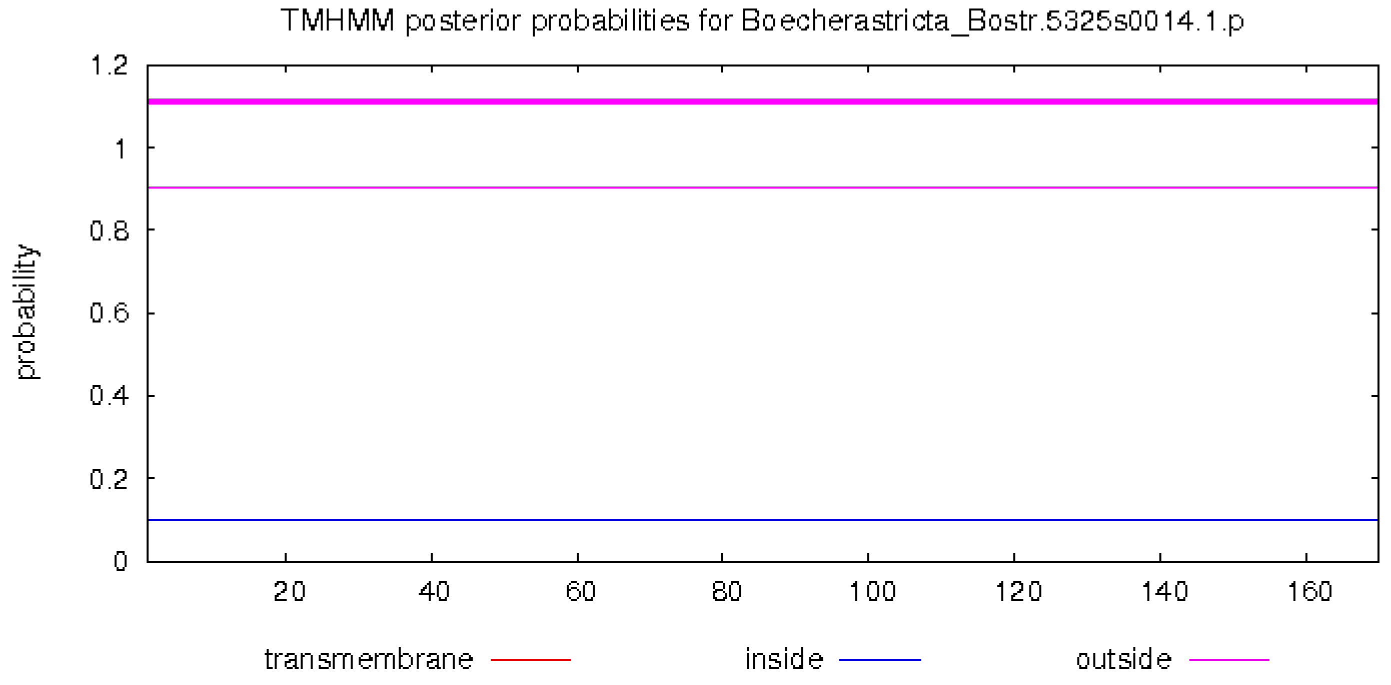

plot in postscript, script for making the plot in gnuplot, data for plot

---

```
# Boecherastricta_Bostr.5325s0016.1.p Length: 350
# Boecherastricta_Bostr.5325s0016.1.p Number of predicted TMHs:  0
```

```
# Boecherastricta_Bostr.5325s0016.1.p Exp number of AAs in TMHs: 0
# Boecherastricta_Bostr.5325s0016.1.p Exp number, first 60 AAs:  0
# Boecherastricta_Bostr.5325s0016.1.p Total prob of N-in:        0.01111
Boecherastricta_Bostr.5325s0016.1.p     TMHMM2.0        outside      1   350
```

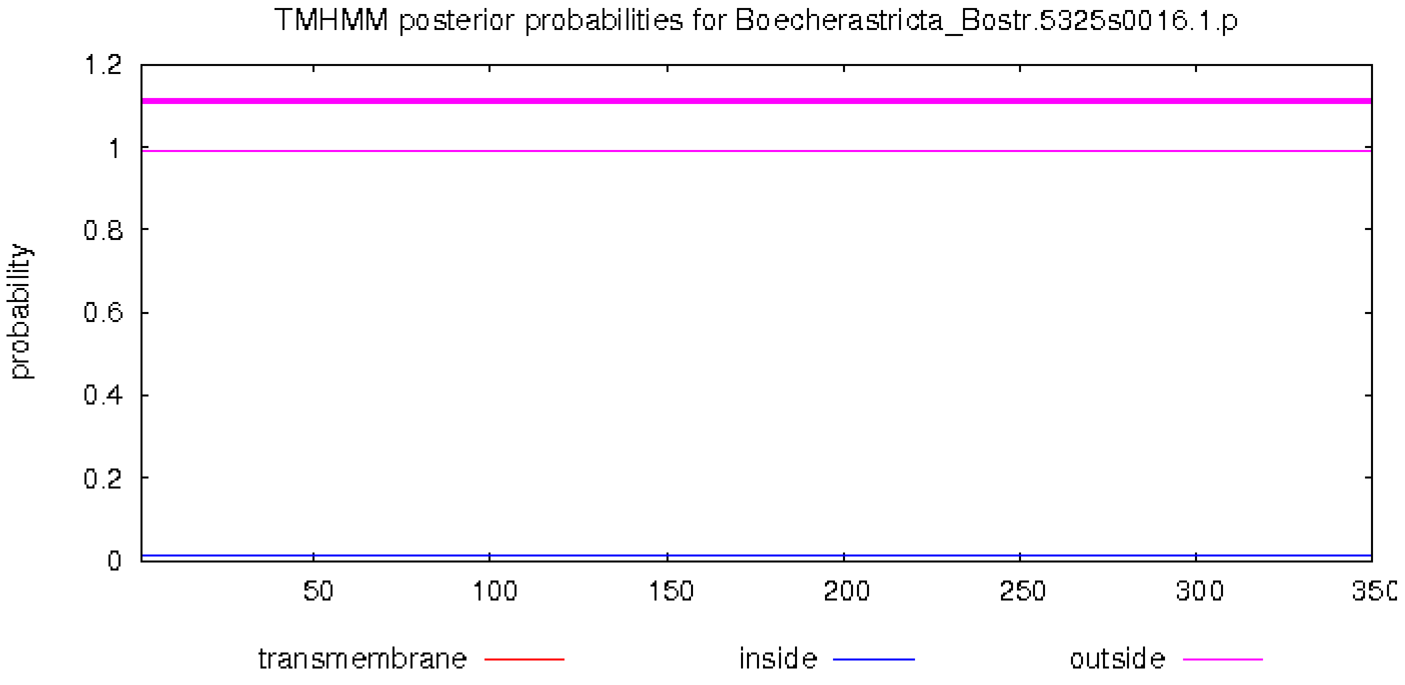

plot in postscript, script for making the plot in gnuplot, data for plot

---

```
# Boecherastricta_Bostr.5325s0017.1.p Length: 216
# Boecherastricta_Bostr.5325s0017.1.p Number of predicted TMHs:  0
# Boecherastricta_Bostr.5325s0017.1.p Exp number of AAs in TMHs: 1.26565
# Boecherastricta_Bostr.5325s0017.1.p Exp number, first 60 AAs:  0.71531
# Boecherastricta_Bostr.5325s0017.1.p Total prob of N-in:        0.33163
Boecherastricta_Bostr.5325s0017.1.p     TMHMM2.0        outside      1   216
```

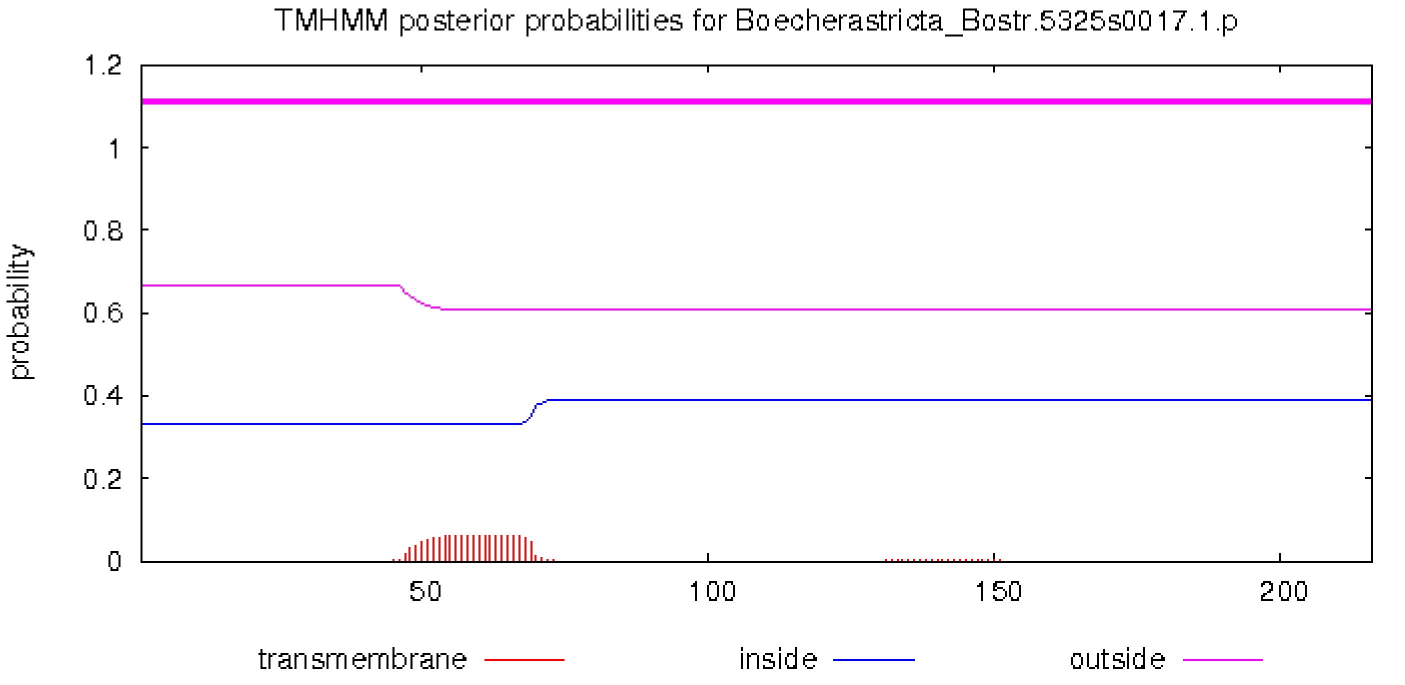

plot in postscript, script for making the plot in gnuplot, data for plot

---

```
# Boecherastricta_Bostr.5325s0018.1.p Length: 234
# Boecherastricta_Bostr.5325s0018.1.p Number of predicted TMHs:  0
```

```
# Boecherastricta_Bostr.5325s0018.1.p Exp number of AAs in TMHs: 0.00014
# Boecherastricta_Bostr.5325s0018.1.p Exp number, first 60 AAs:  6e-05
# Boecherastricta_Bostr.5325s0018.1.p Total prob of N-in:        0.04412
Boecherastricta_Bostr.5325s0018.1.p     TMHMM2.0        outside      1   234
```

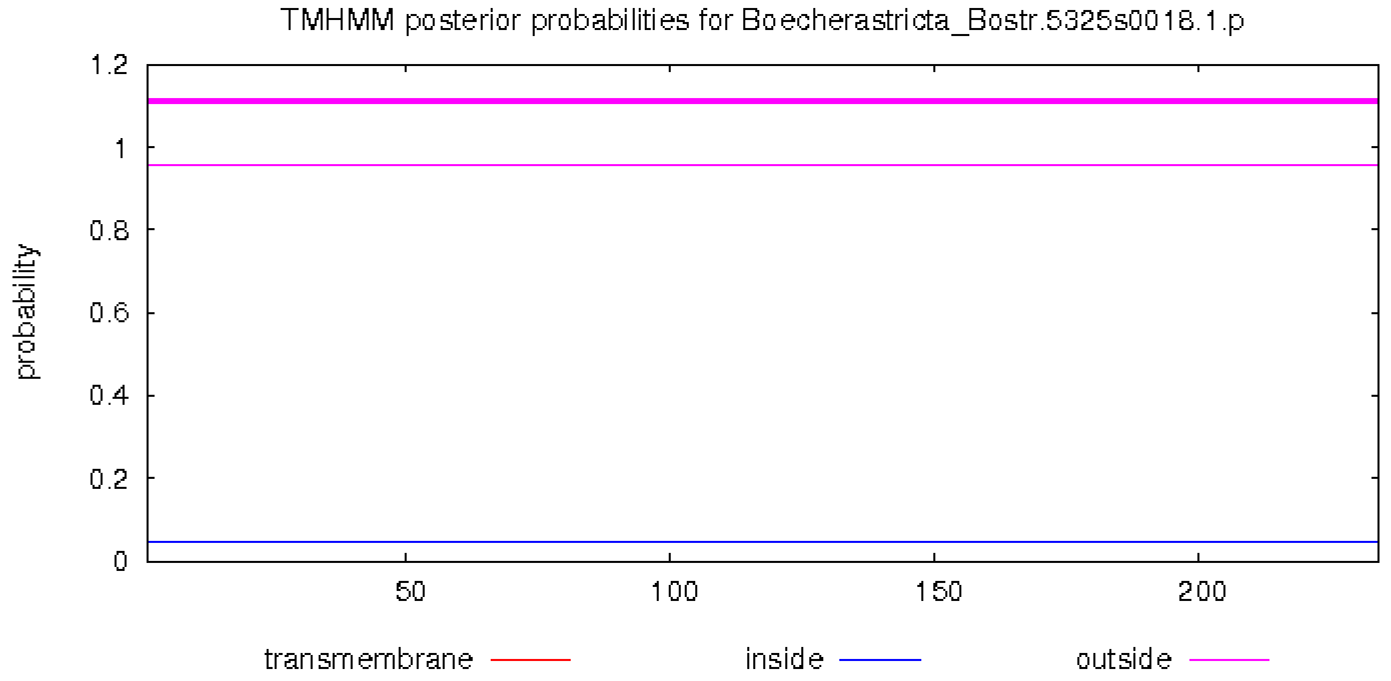

plot in postscript, script for making the plot in gnuplot, data for plot

```
# Boecherastricta_Bostr.5325s0019.1.p Length: 467
# Boecherastricta_Bostr.5325s0019.1.p Number of predicted TMHs:  1
# Boecherastricta_Bostr.5325s0019.1.p Exp number of AAs in TMHs: 20.77591
# Boecherastricta_Bostr.5325s0019.1.p Exp number, first 60 AAs:  0.00077
# Boecherastricta_Bostr.5325s0019.1.p Total prob of N-in:        0.53704
Boecherastricta_Bostr.5325s0019.1.p     TMHMM2.0        inside       1   443
Boecherastricta_Bostr.5325s0019.1.p     TMHMM2.0        TMhelix    444   466
Boecherastricta_Bostr.5325s0019.1.p     TMHMM2.0        outside    467   467
```

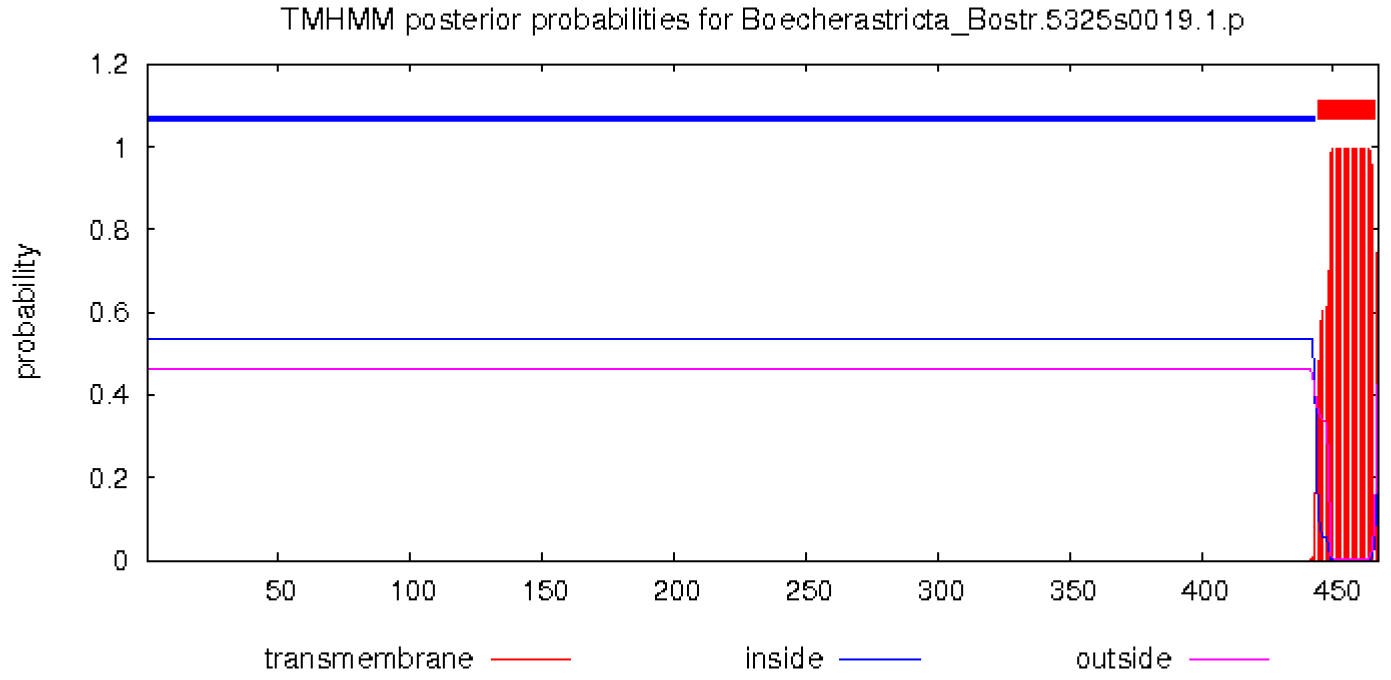

plot in postscript, script for making the plot in gnuplot, data for plot

```
# Boecherastricta_Bostr.5325s0094.1.p Length: 287
# Boecherastricta_Bostr.5325s0094.1.p Number of predicted TMHs:  0
# Boecherastricta_Bostr.5325s0094.1.p Exp number of AAs in TMHs: 0.00123
# Boecherastricta_Bostr.5325s0094.1.p Exp number, first 60 AAs:  0.0002
# Boecherastricta_Bostr.5325s0094.1.p Total prob of N-in:        0.00337
Boecherastricta_Bostr.5325s0094.1.p     TMHMM2.0        outside      1   287
```

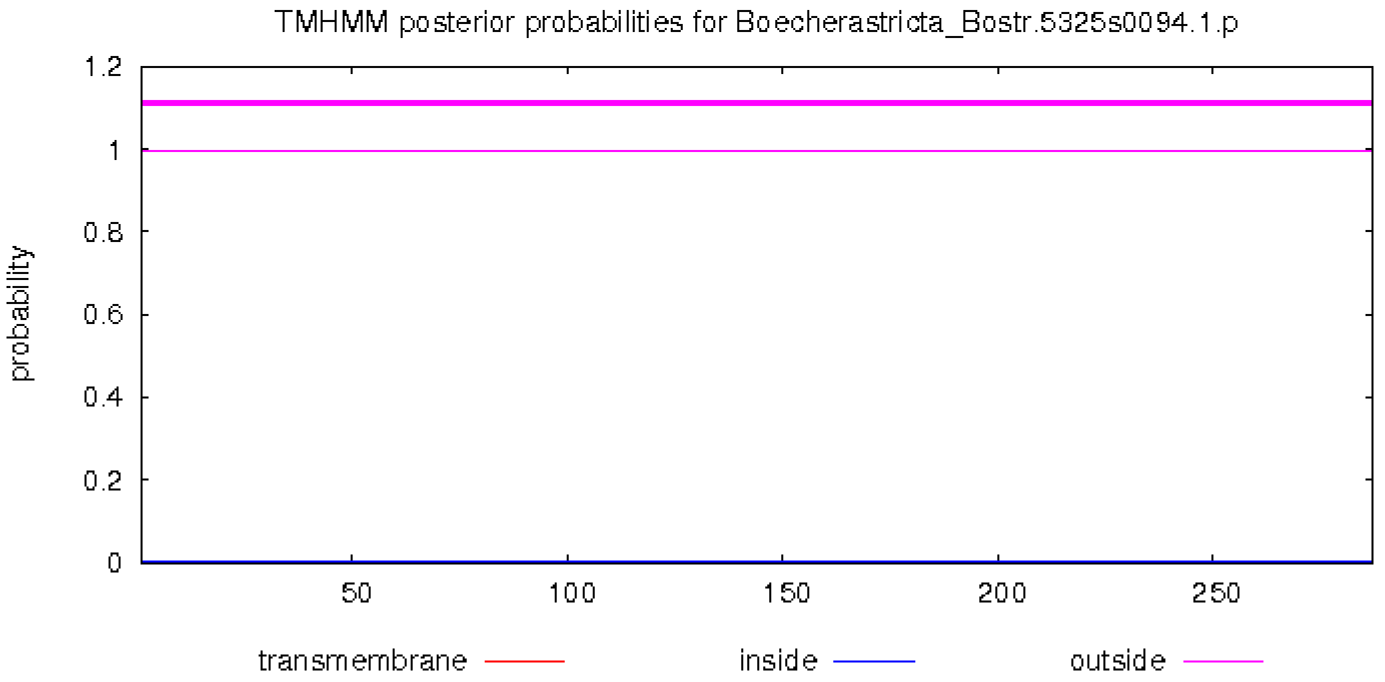

plot in postscript, script for making the plot in gnuplot, data for plot

---

```
# Boecherastricta_Bostr.5763s0059.1.p Length: 468
# Boecherastricta_Bostr.5763s0059.1.p Number of predicted TMHs:  1
# Boecherastricta_Bostr.5763s0059.1.p Exp number of AAs in TMHs: 13.60559
# Boecherastricta_Bostr.5763s0059.1.p Exp number, first 60 AAs:  0.00446
# Boecherastricta_Bostr.5763s0059.1.p Total prob of N-in:        0.01846
Boecherastricta_Bostr.5763s0059.1.p     TMHMM2.0        outside      1   447
Boecherastricta_Bostr.5763s0059.1.p     TMHMM2.0        TMhelix    448   467
Boecherastricta_Bostr.5763s0059.1.p     TMHMM2.0        inside     468   468
```

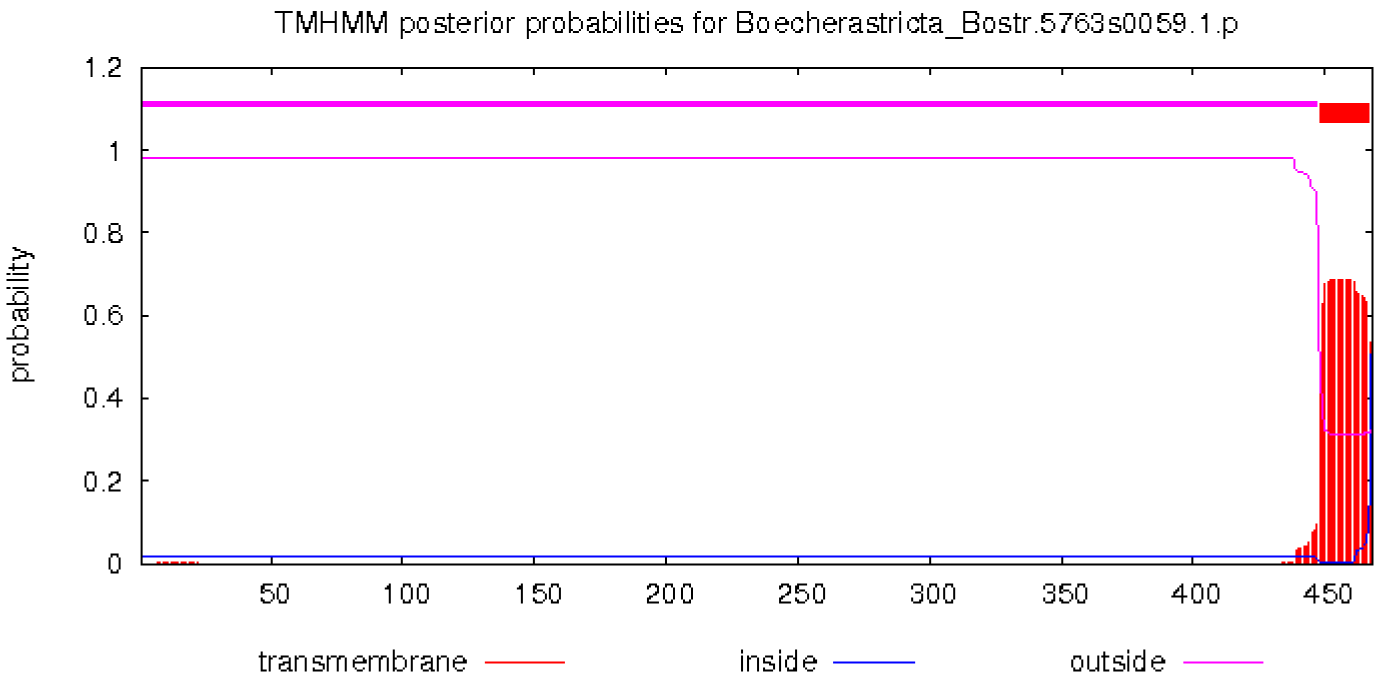

plot in postscript, script for making the plot in gnuplot, data for plot

```
# Boecherastricta_Bostr.6758s0055.1.p Length: 255
# Boecherastricta_Bostr.6758s0055.1.p Number of predicted TMHs:  0
# Boecherastricta_Bostr.6758s0055.1.p Exp number of AAs in TMHs: 0.00127
# Boecherastricta_Bostr.6758s0055.1.p Exp number, first 60 AAs:  0.00079
# Boecherastricta_Bostr.6758s0055.1.p Total prob of N-in:        0.07201
Boecherastricta_Bostr.6758s0055.1.p     TMHMM2.0        outside      1   255
```

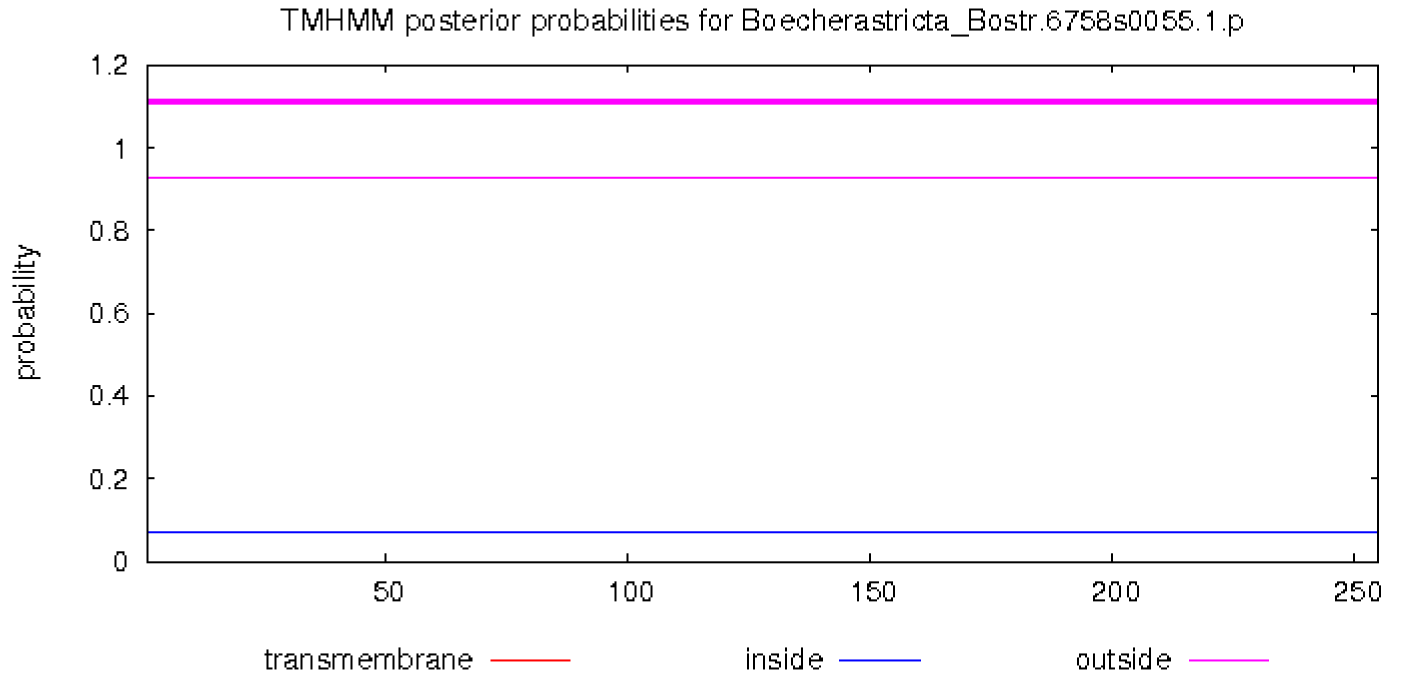

plot in postscript, script for making the plot in gnuplot, data for plot

```
# Boecherastricta_Bostr.6758s0078.1.p Length: 300
# Boecherastricta_Bostr.6758s0078.1.p Number of predicted TMHs:  0
# Boecherastricta_Bostr.6758s0078.1.p Exp number of AAs in TMHs: 0
# Boecherastricta_Bostr.6758s0078.1.p Exp number, first 60 AAs:  0
# Boecherastricta_Bostr.6758s0078.1.p Total prob of N-in:        0.00144
Boecherastricta_Bostr.6758s0078.1.p     TMHMM2.0        outside      1   300
```

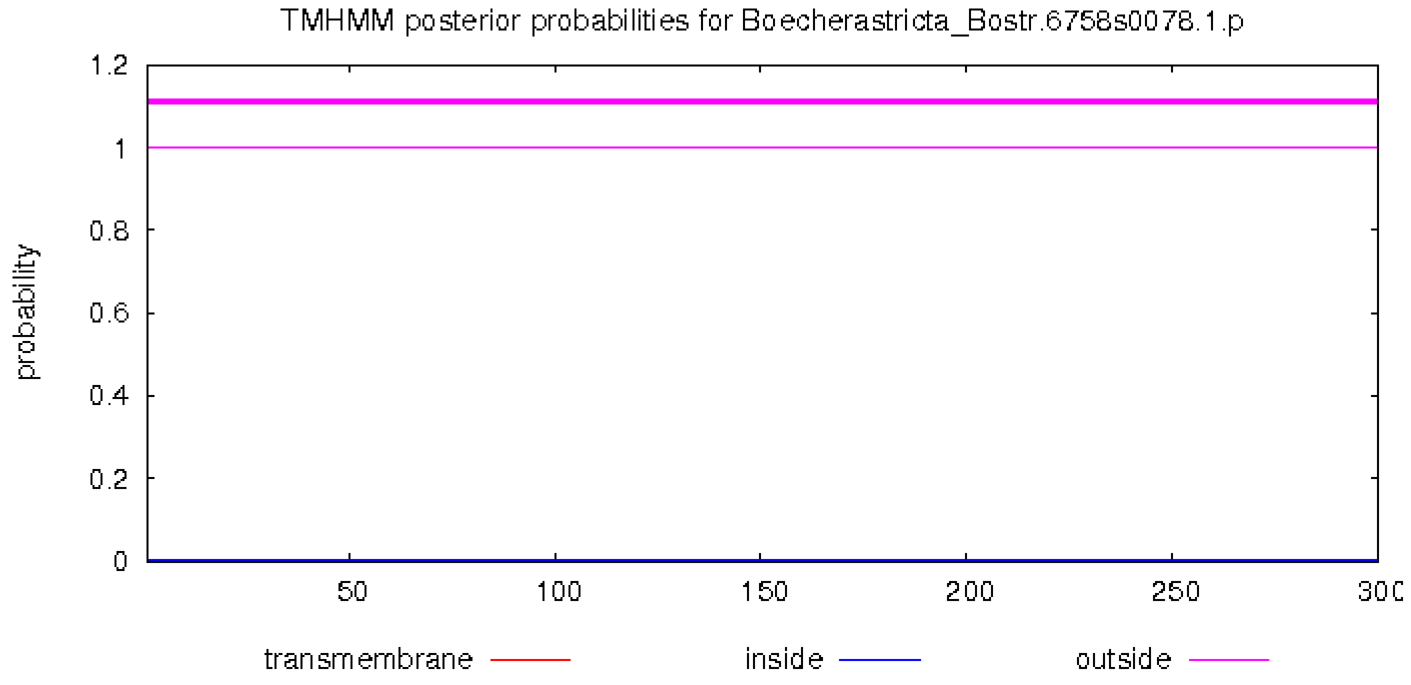

plot in postscript, script for making the plot in gnuplot, data for plot

```
# Boecherastricta_Bostr.6864s0295.1.p Length: 463
# Boecherastricta_Bostr.6864s0295.1.p Number of predicted TMHs:  1
# Boecherastricta_Bostr.6864s0295.1.p Exp number of AAs in TMHs: 21.18524
# Boecherastricta_Bostr.6864s0295.1.p Exp number, first 60 AAs:  0
# Boecherastricta_Bostr.6864s0295.1.p Total prob of N-in:        0.06770
Boecherastricta_Bostr.6864s0295.1.p     TMHMM2.0        outside      1   432
Boecherastricta_Bostr.6864s0295.1.p     TMHMM2.0        TMhelix    433   455
Boecherastricta_Bostr.6864s0295.1.p     TMHMM2.0        inside     456   463
```

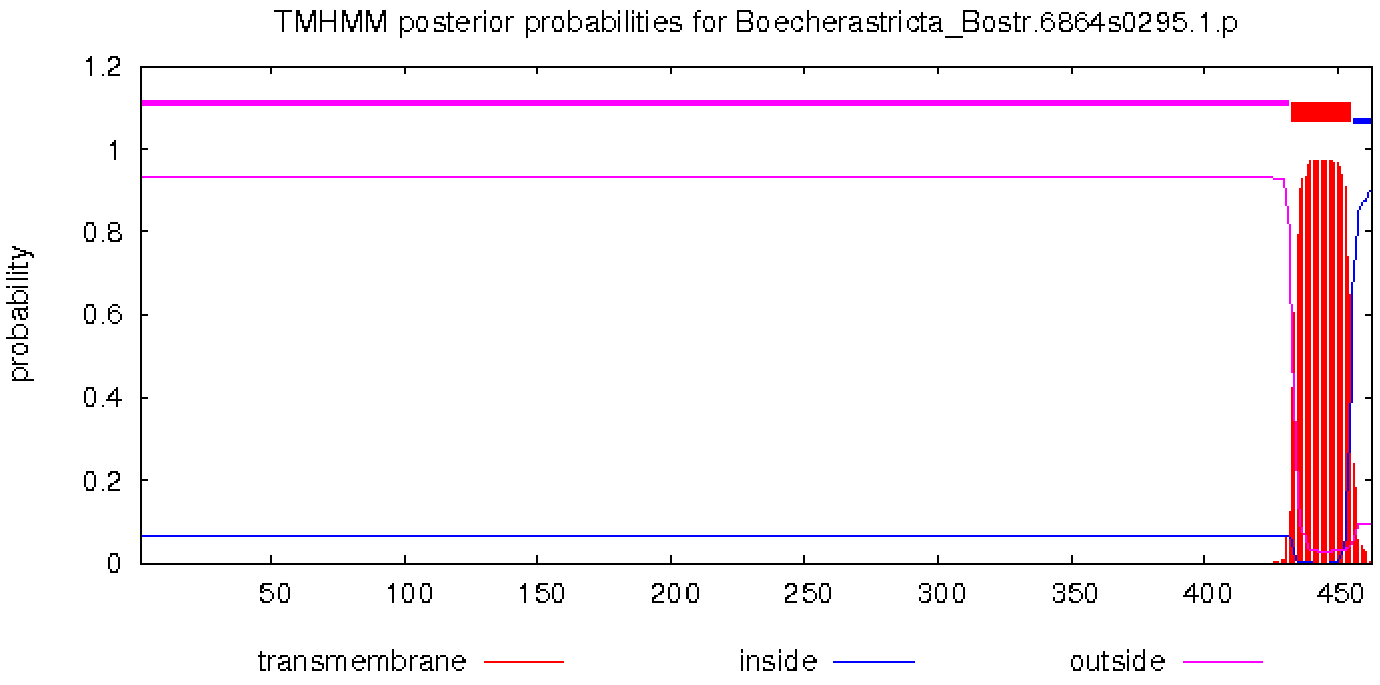

plot in postscript, script for making the plot in gnuplot, data for plot

---

```
# Boecherastricta_Bostr.7200s0128.1.p Length: 214
# Boecherastricta_Bostr.7200s0128.1.p Number of predicted TMHs:  0
# Boecherastricta_Bostr.7200s0128.1.p Exp number of AAs in TMHs: 0.01018
# Boecherastricta_Bostr.7200s0128.1.p Exp number, first 60 AAs:  0.00999
# Boecherastricta_Bostr.7200s0128.1.p Total prob of N-in:        0.03279
Boecherastricta_Bostr.7200s0128.1.p     TMHMM2.0        outside      1   214
```

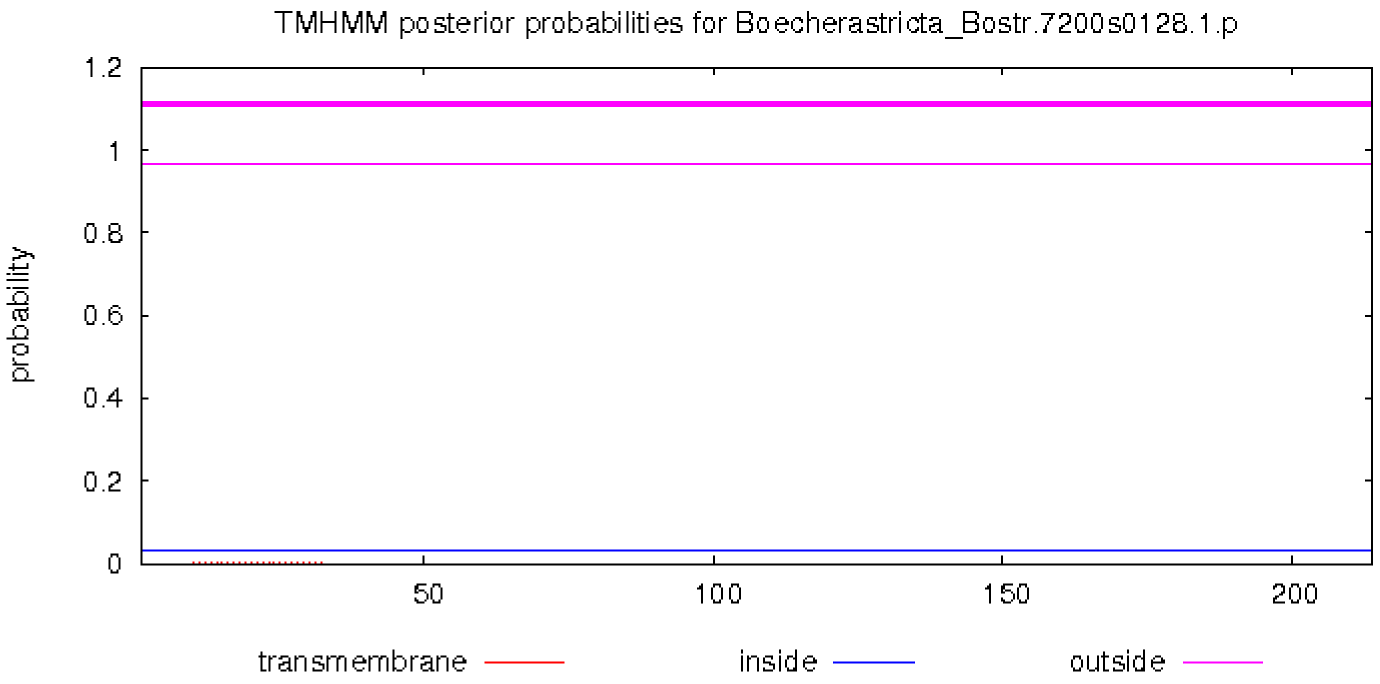

plot in postscript, script for making the plot in gnuplot, data for plot

```
# Boecherastricta_Bostr.7867s0562.1.p Length: 301
# Boecherastricta_Bostr.7867s0562.1.p Number of predicted TMHs:  0
# Boecherastricta_Bostr.7867s0562.1.p Exp number of AAs in TMHs: 0.00224
# Boecherastricta_Bostr.7867s0562.1.p Exp number, first 60 AAs:  0.00039
# Boecherastricta_Bostr.7867s0562.1.p Total prob of N-in:        0.02095
Boecherastricta_Bostr.7867s0562.1.p     TMHMM2.0        outside      1   301
```

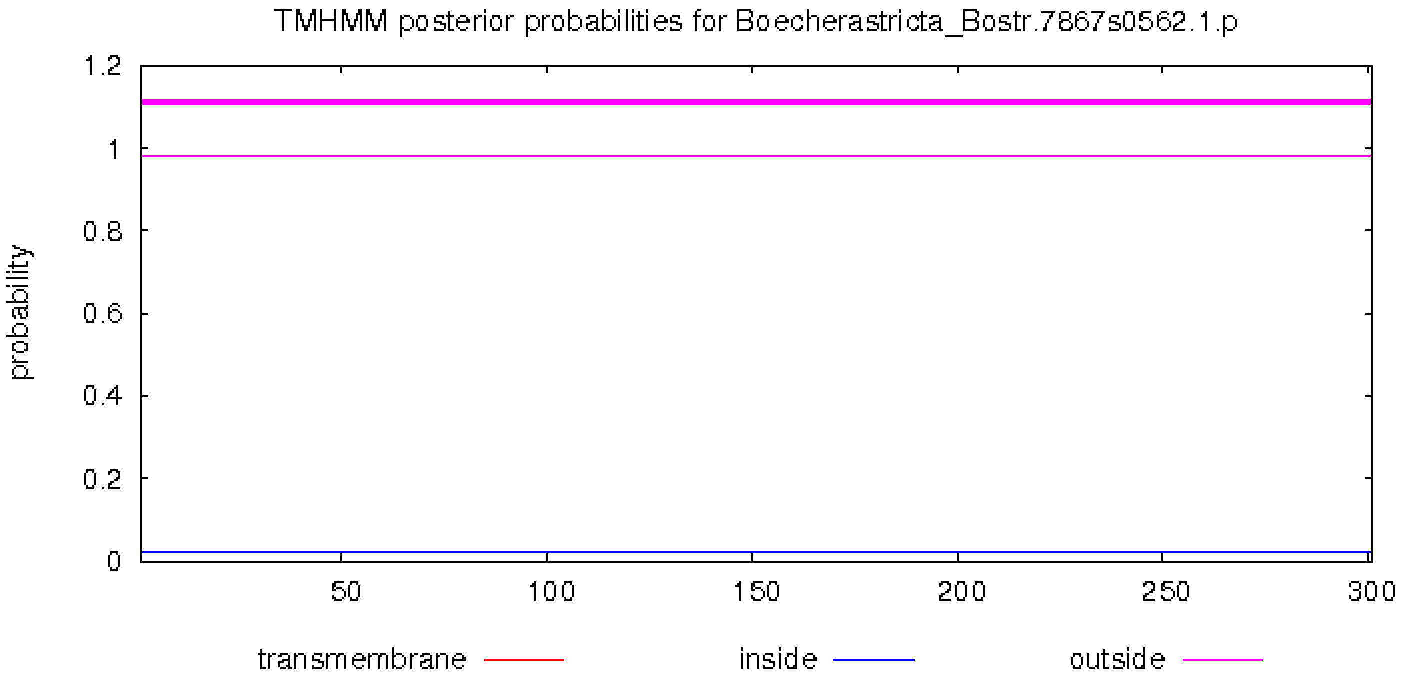

plot in postscript, script for making the plot in gnuplot, data for plot

```
# Boecherastricta_Bostr.7867s0691.1.p Length: 308
# Boecherastricta_Bostr.7867s0691.1.p Number of predicted TMHs:  0
# Boecherastricta_Bostr.7867s0691.1.p Exp number of AAs in TMHs: 0.00175
# Boecherastricta_Bostr.7867s0691.1.p Exp number, first 60 AAs:  0
# Boecherastricta_Bostr.7867s0691.1.p Total prob of N-in:        0.09541
Boecherastricta_Bostr.7867s0691.1.p     TMHMM2.0        outside      1   308
```

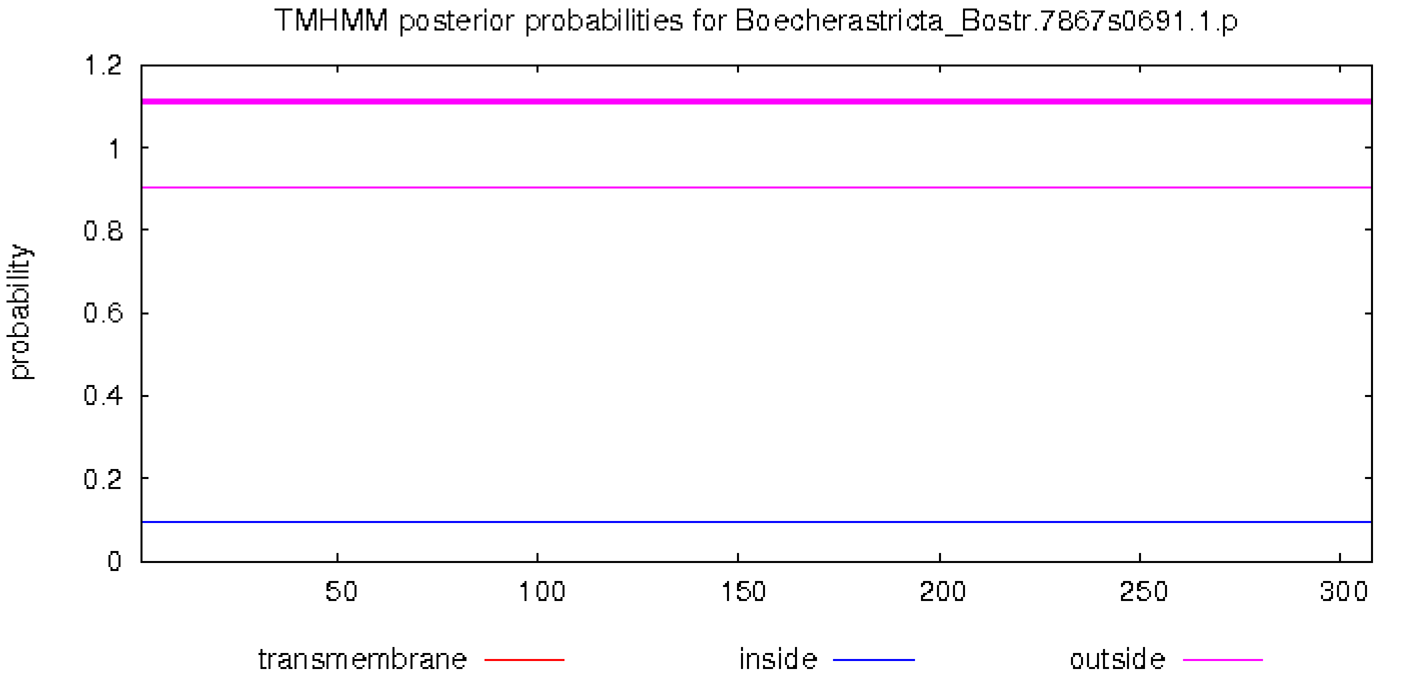

plot in postscript, script for making the plot in gnuplot, data for plot

```
# Boecherastricta_Bostr.7867s0694.1.p Length: 350
# Boecherastricta_Bostr.7867s0694.1.p Number of predicted TMHs:  0
# Boecherastricta_Bostr.7867s0694.1.p Exp number of AAs in TMHs: 0
# Boecherastricta_Bostr.7867s0694.1.p Exp number, first 60 AAs:  0
# Boecherastricta_Bostr.7867s0694.1.p Total prob of N-in:        0.00578
Boecherastricta_Bostr.7867s0694.1.p     TMHMM2.0        outside      1   350
```

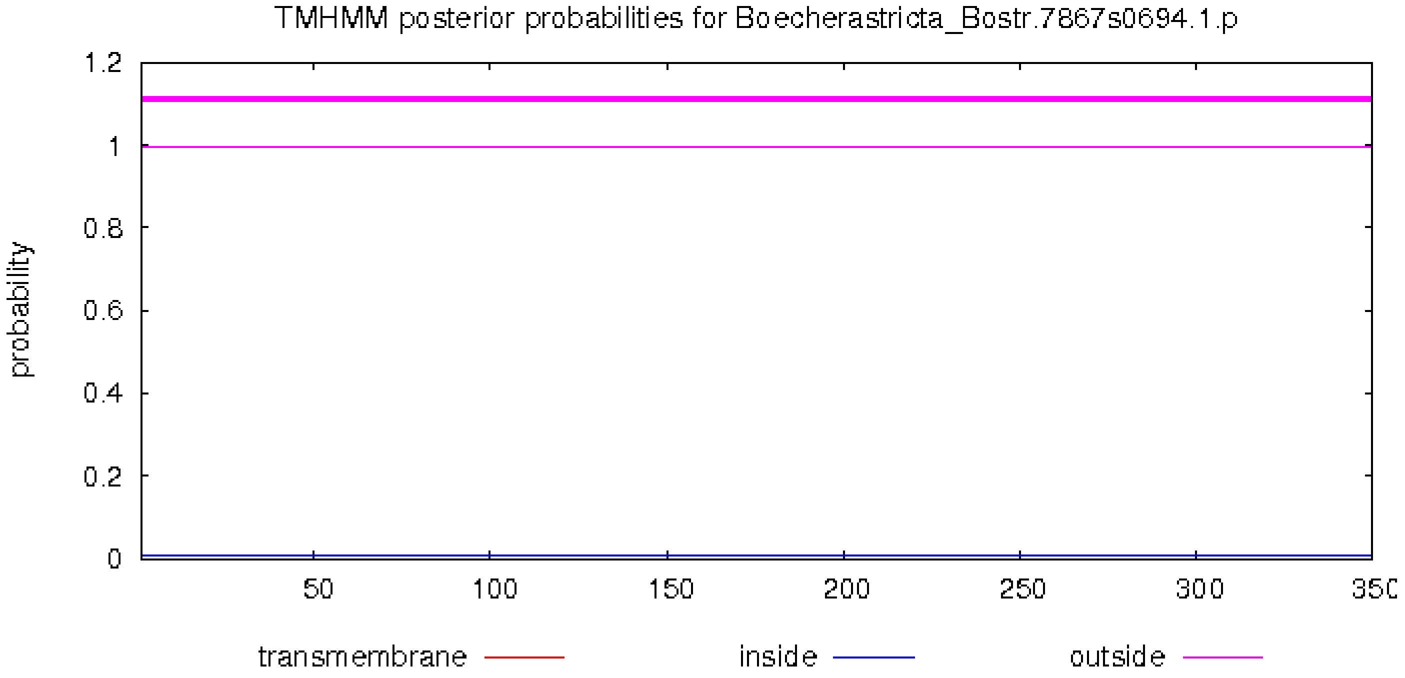

plot in postscript, script for making the plot in gnuplot, data for plot

---

```
# Boecherastricta_Bostr.7867s0767.1.p Length: 497
# Boecherastricta_Bostr.7867s0767.1.p Number of predicted TMHs:  0
# Boecherastricta_Bostr.7867s0767.1.p Exp number of AAs in TMHs: 3.01675
# Boecherastricta_Bostr.7867s0767.1.p Exp number, first 60 AAs:  0
# Boecherastricta_Bostr.7867s0767.1.p Total prob of N-in:        0.12133
Boecherastricta_Bostr.7867s0767.1.p     TMHMM2.0        outside      1   497
```

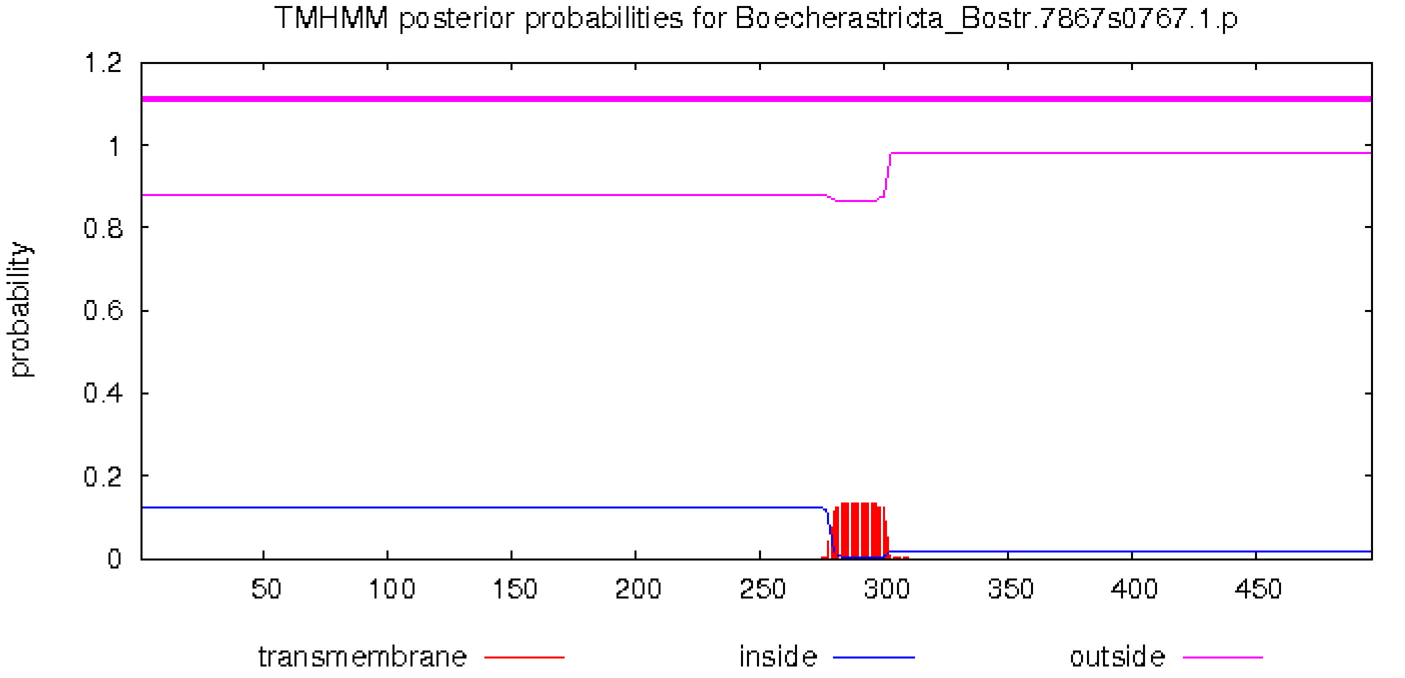

plot in postscript, script for making the plot in gnuplot, data for plot

---

```
# Boecherastricta_Bostr.7867s1416.1.p Length: 523
# Boecherastricta_Bostr.7867s1416.1.p Number of predicted TMHs:  1
# Boecherastricta_Bostr.7867s1416.1.p Exp number of AAs in TMHs: 19.89632
# Boecherastricta_Bostr.7867s1416.1.p Exp number, first 60 AAs:  0
# Boecherastricta_Bostr.7867s1416.1.p Total prob of N-in:        0.05962
Boecherastricta_Bostr.7867s1416.1.p     TMHMM2.0        outside      1   499
Boecherastricta_Bostr.7867s1416.1.p     TMHMM2.0        TMhelix    500   522
Boecherastricta_Bostr.7867s1416.1.p     TMHMM2.0        inside     523   523
```

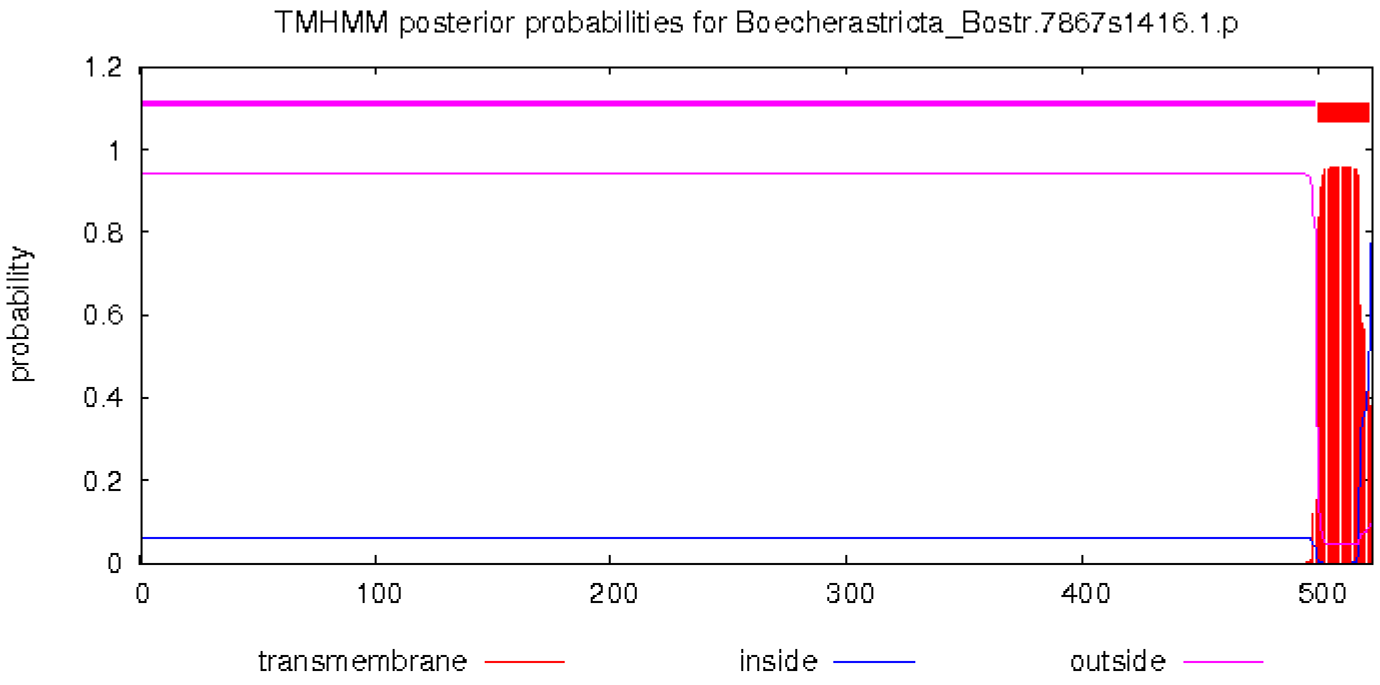

plot in postscript, script for making the plot in gnuplot, data for plot

---

```
# Boecherastricta_Bostr.7867s1483.1.p Length: 376
# Boecherastricta_Bostr.7867s1483.1.p Number of predicted TMHs:  0
# Boecherastricta_Bostr.7867s1483.1.p Exp number of AAs in TMHs: 0.00018
# Boecherastricta_Bostr.7867s1483.1.p Exp number, first 60 AAs:  0
# Boecherastricta_Bostr.7867s1483.1.p Total prob of N-in:        0.01269
Boecherastricta_Bostr.7867s1483.1.p     TMHMM2.0        outside      1   376
```

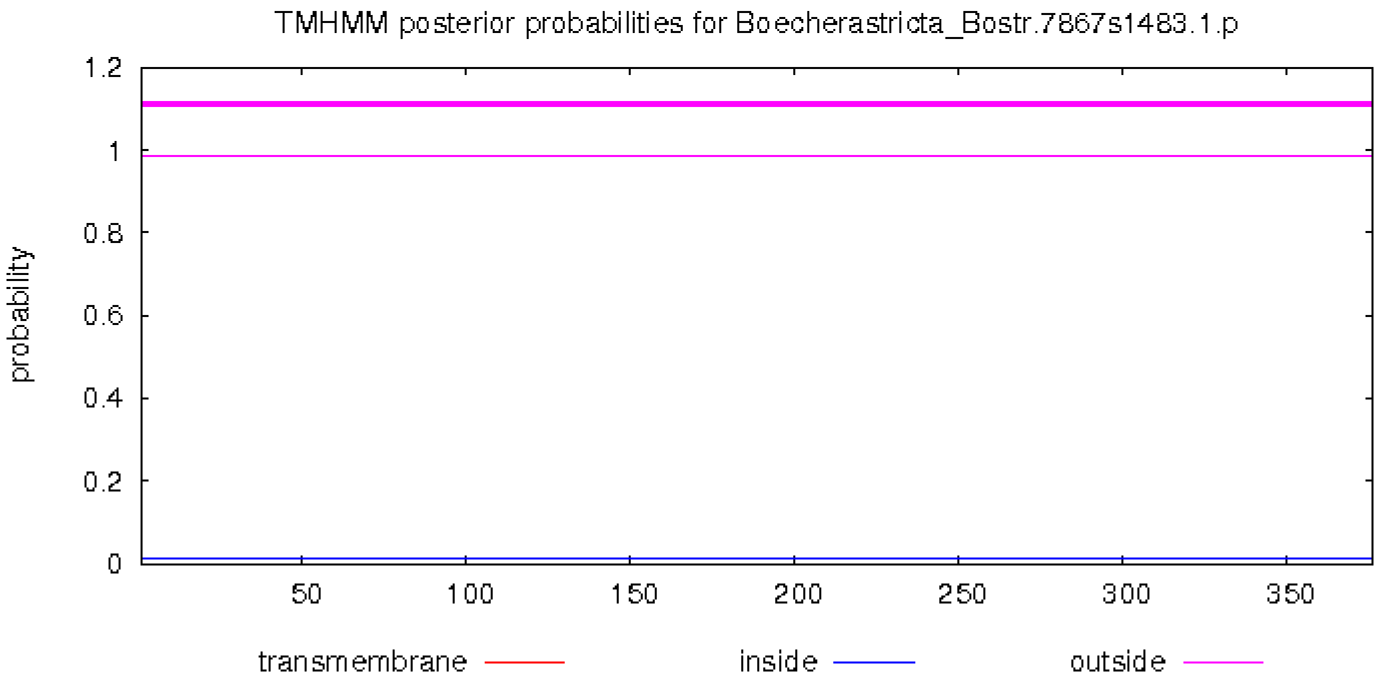

plot in postscript, script for making the plot in gnuplot, data for plot

```
# Boecherastricta_Bostr.8819s0187.1.p Length: 334
# Boecherastricta_Bostr.8819s0187.1.p Number of predicted TMHs:  0
# Boecherastricta_Bostr.8819s0187.1.p Exp number of AAs in TMHs: 0.00374
# Boecherastricta_Bostr.8819s0187.1.p Exp number, first 60 AAs:  0
# Boecherastricta_Bostr.8819s0187.1.p Total prob of N-in:        0.01146
Boecherastricta_Bostr.8819s0187.1.p     TMHMM2.0        outside      1   334
```

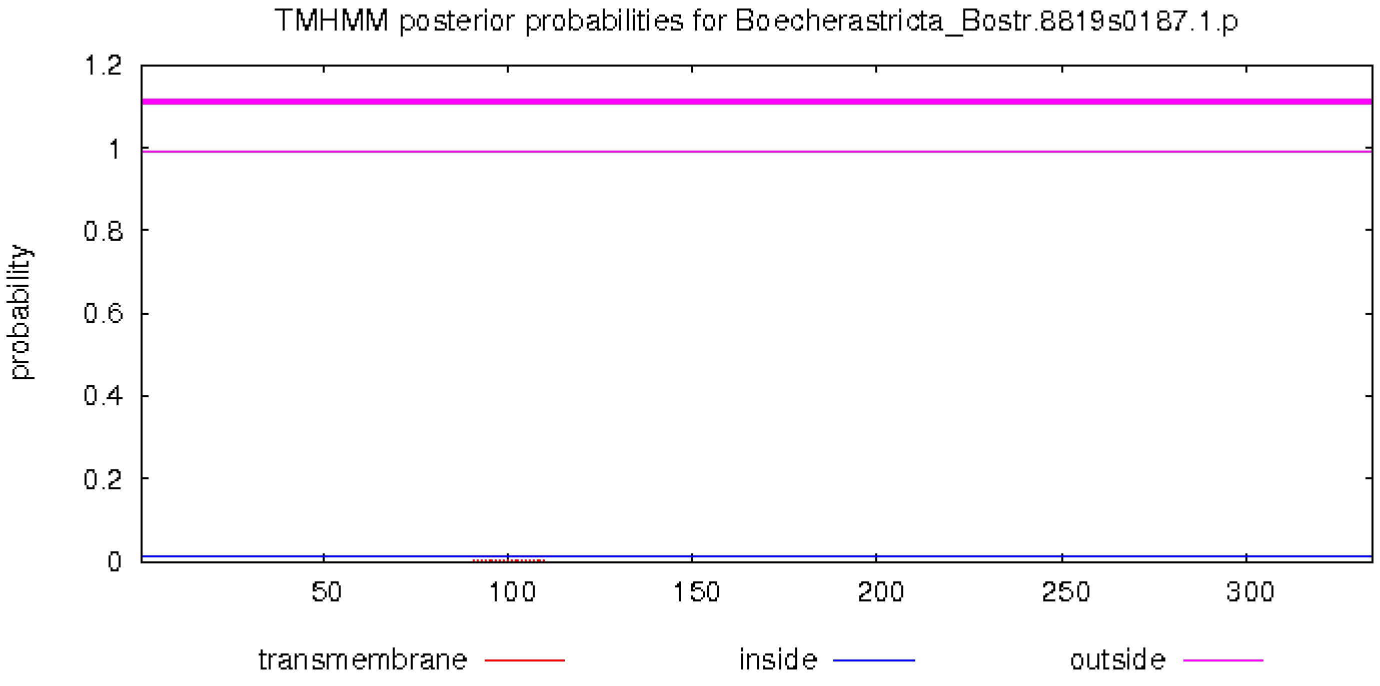

plot in postscript, script for making the plot in gnuplot, data for plot

```
# Brachypodiumdistachyon_Bradi1g01640.2.p Length: 655
# Brachypodiumdistachyon_Bradi1g01640.2.p Number of predicted TMHs:  0
# Brachypodiumdistachyon_Bradi1g01640.2.p Exp number of AAs in TMHs: 0.00944
# Brachypodiumdistachyon_Bradi1g01640.2.p Exp number, first 60 AAs:  0
# Brachypodiumdistachyon_Bradi1g01640.2.p Total prob of N-in:        0.00023
Brachypodiumdistachyon_Bradi1g01640.2.p TMHMM2.0        outside      1   655
```

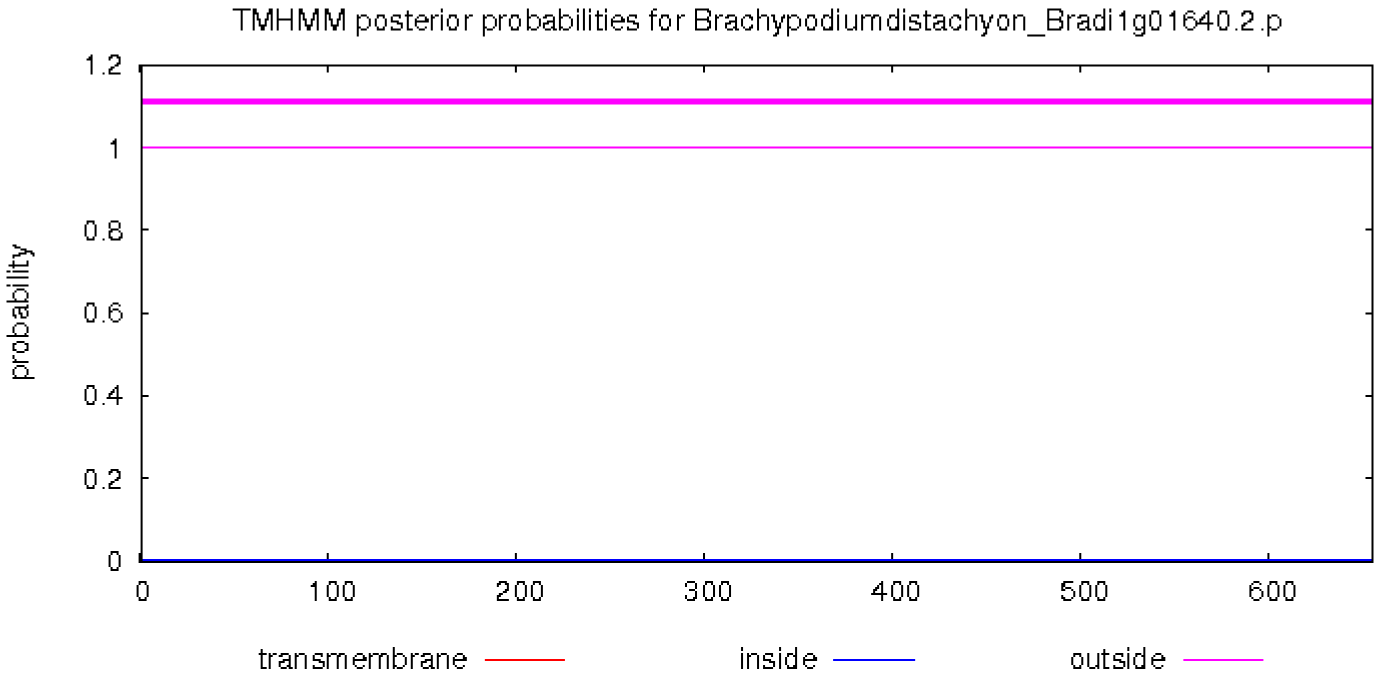

plot in postscript, script for making the plot in gnuplot, data for plot

```
# Brachypodiumdistachyon_Bradi1g01650.1.p Length: 368
# Brachypodiumdistachyon_Bradi1g01650.1.p Number of predicted TMHs:  0
# Brachypodiumdistachyon_Bradi1g01650.1.p Exp number of AAs in TMHs: 0.01908
# Brachypodiumdistachyon_Bradi1g01650.1.p Exp number, first 60 AAs:  0
# Brachypodiumdistachyon_Bradi1g01650.1.p Total prob of N-in:        0.02047
Brachypodiumdistachyon_Bradi1g01650.1.p TMHMM2.0        outside      1   368
```

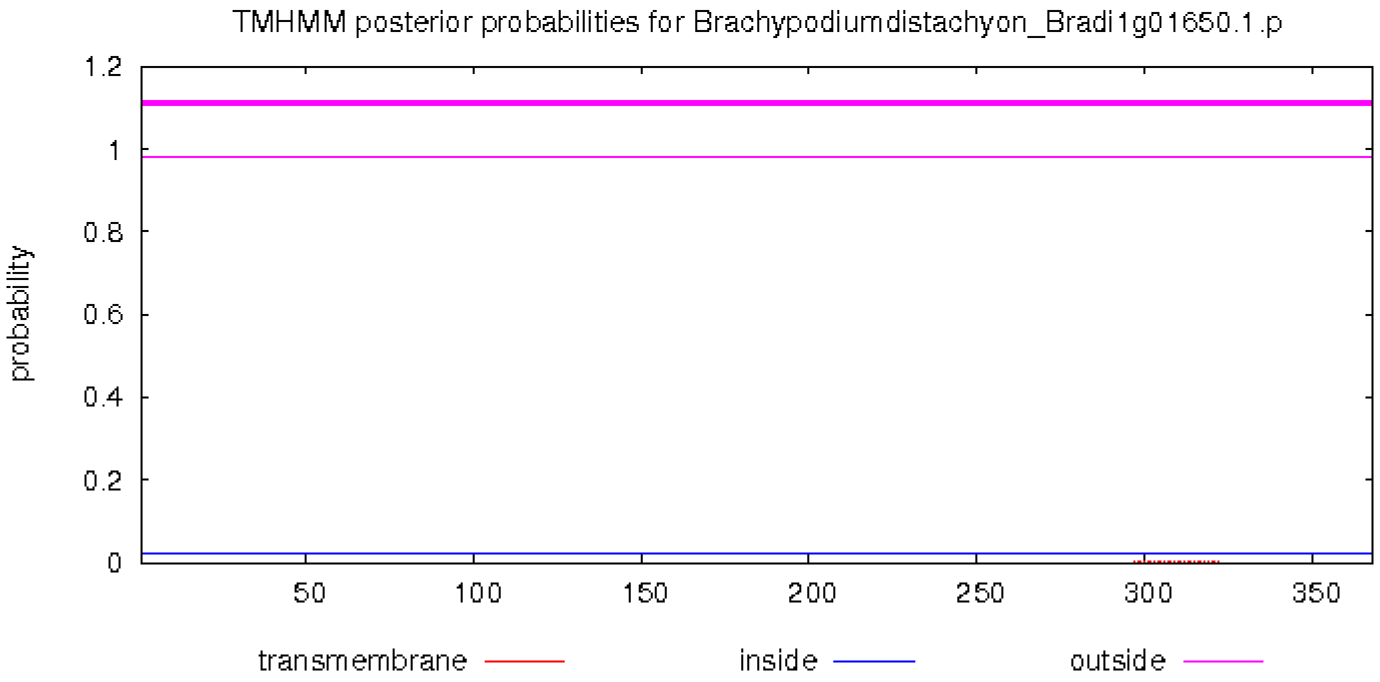

plot in postscript, script for making the plot in gnuplot, data for plot

---

```
# Brachypodiumdistachyon_Bradi1g01683.1.p Length: 360
# Brachypodiumdistachyon_Bradi1g01683.1.p Number of predicted TMHs:  0
# Brachypodiumdistachyon_Bradi1g01683.1.p Exp number of AAs in TMHs: 8e-05
# Brachypodiumdistachyon_Bradi1g01683.1.p Exp number, first 60 AAs:  0
# Brachypodiumdistachyon_Bradi1g01683.1.p Total prob of N-in:        0.00371
Brachypodiumdistachyon_Bradi1g01683.1.p TMHMM2.0        outside      1   360
```

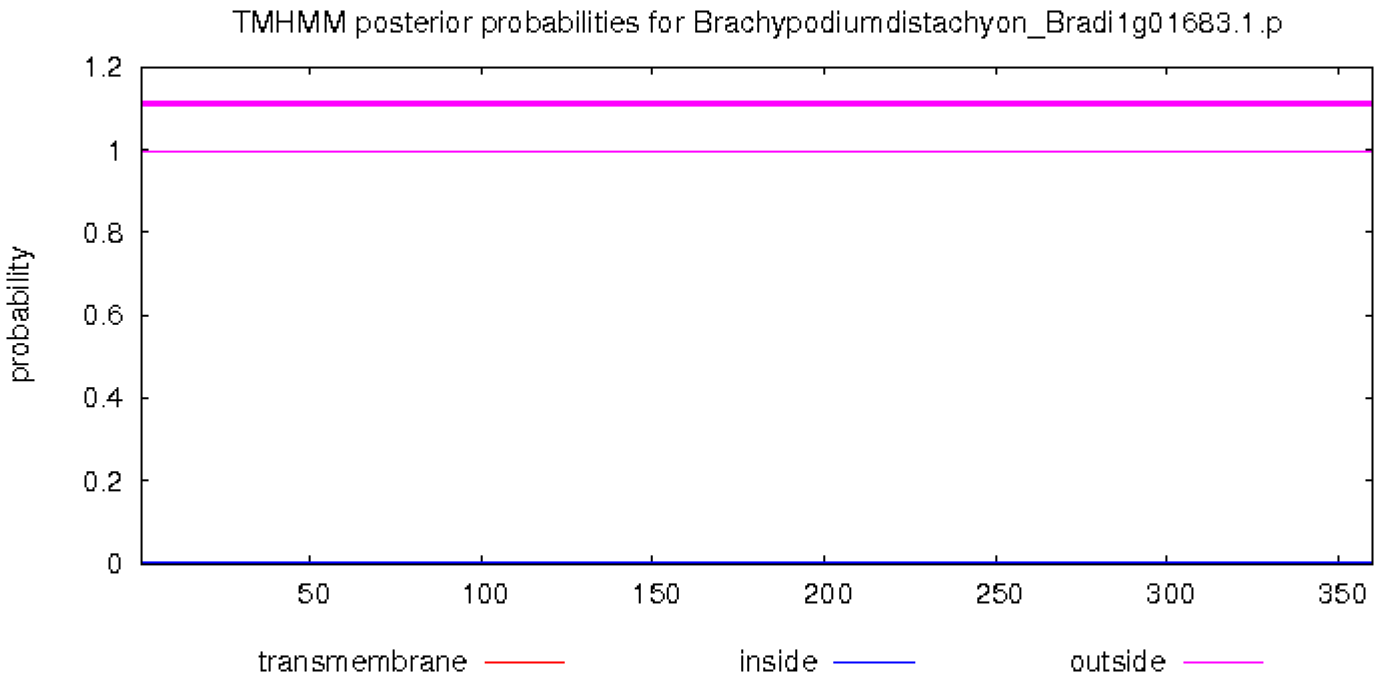

plot in postscript, script for making the plot in gnuplot, data for plot

---

```
# Brachypodiumdistachyon_Bradi1g01695.1.p Length: 267
# Brachypodiumdistachyon_Bradi1g01695.1.p Number of predicted TMHs:  0
# Brachypodiumdistachyon_Bradi1g01695.1.p Exp number of AAs in TMHs: 0.00019
# Brachypodiumdistachyon_Bradi1g01695.1.p Exp number, first 60 AAs:  0
# Brachypodiumdistachyon_Bradi1g01695.1.p Total prob of N-in:        0.01177
Brachypodiumdistachyon_Bradi1g01695.1.p TMHMM2.0        outside      1   267
```

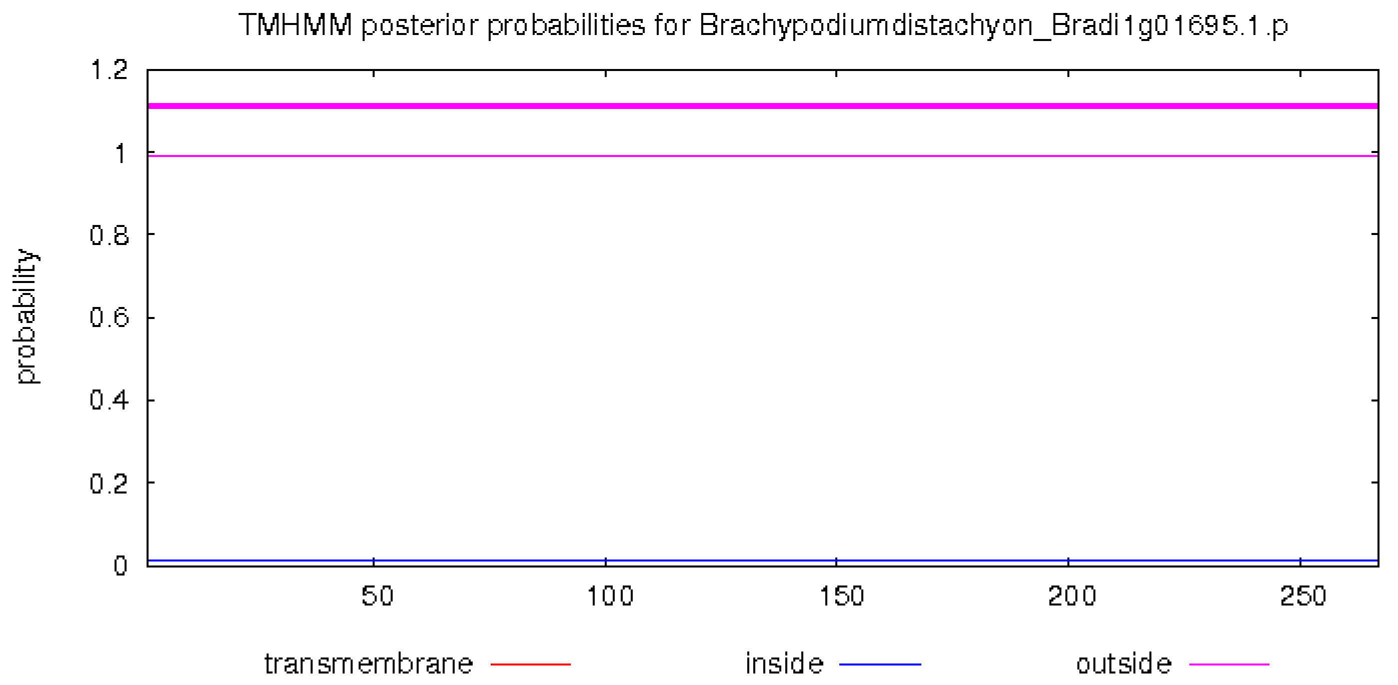

plot in postscript, script for making the plot in gnuplot, data for plot

```
# Brachypodiumdistachyon_Bradi1g01801.1.p Length: 259
# Brachypodiumdistachyon_Bradi1g01801.1.p Number of predicted TMHs:  0
# Brachypodiumdistachyon_Bradi1g01801.1.p Exp number of AAs in TMHs: 0.00037
# Brachypodiumdistachyon_Bradi1g01801.1.p Exp number, first 60 AAs:  0.00021
# Brachypodiumdistachyon_Bradi1g01801.1.p Total prob of N-in:        0.01389
Brachypodiumdistachyon_Bradi1g01801.1.p TMHMM2.0        outside      1   259
```

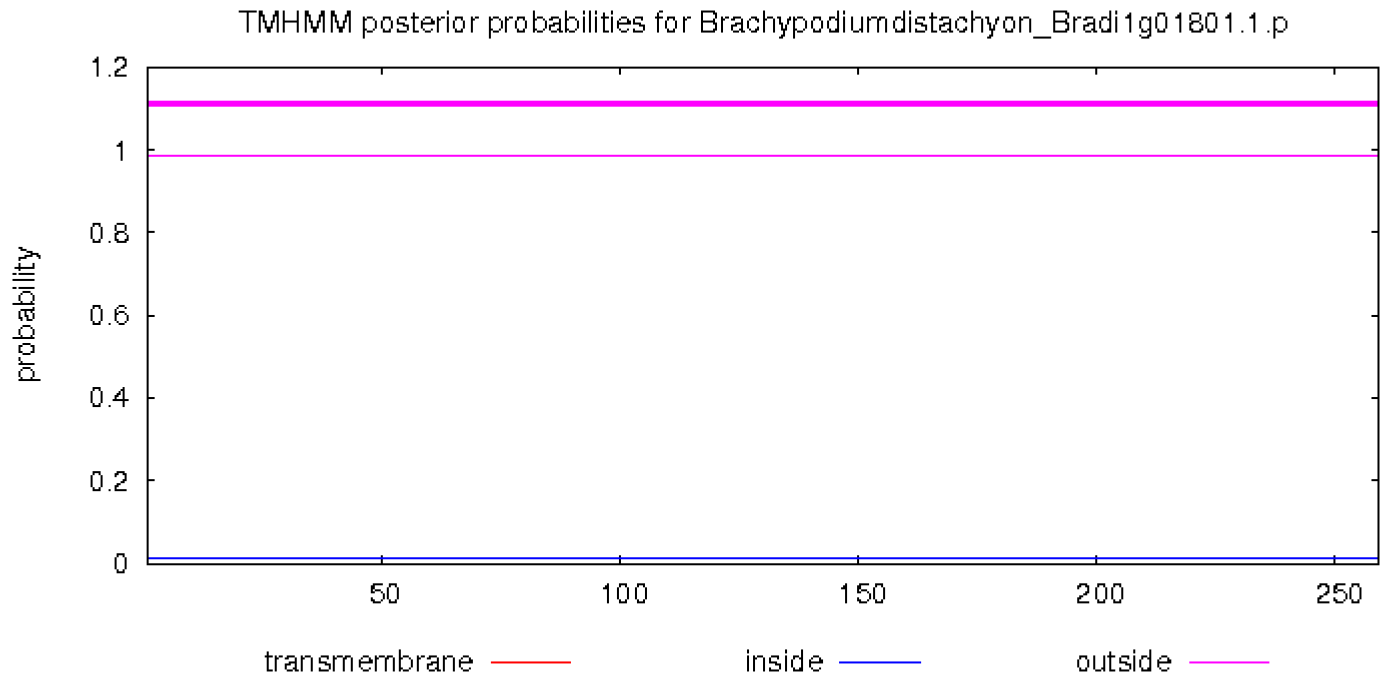

plot in postscript, script for making the plot in gnuplot, data for plot

```
# Brachypodiumdistachyon_Bradi1g01807.1.p Length: 356
# Brachypodiumdistachyon_Bradi1g01807.1.p Number of predicted TMHs:  0
# Brachypodiumdistachyon_Bradi1g01807.1.p Exp number of AAs in TMHs: 0
# Brachypodiumdistachyon_Bradi1g01807.1.p Exp number, first 60 AAs:  0
# Brachypodiumdistachyon_Bradi1g01807.1.p Total prob of N-in:        0.00288
Brachypodiumdistachyon_Bradi1g01807.1.p TMHMM2.0        outside      1   356
```

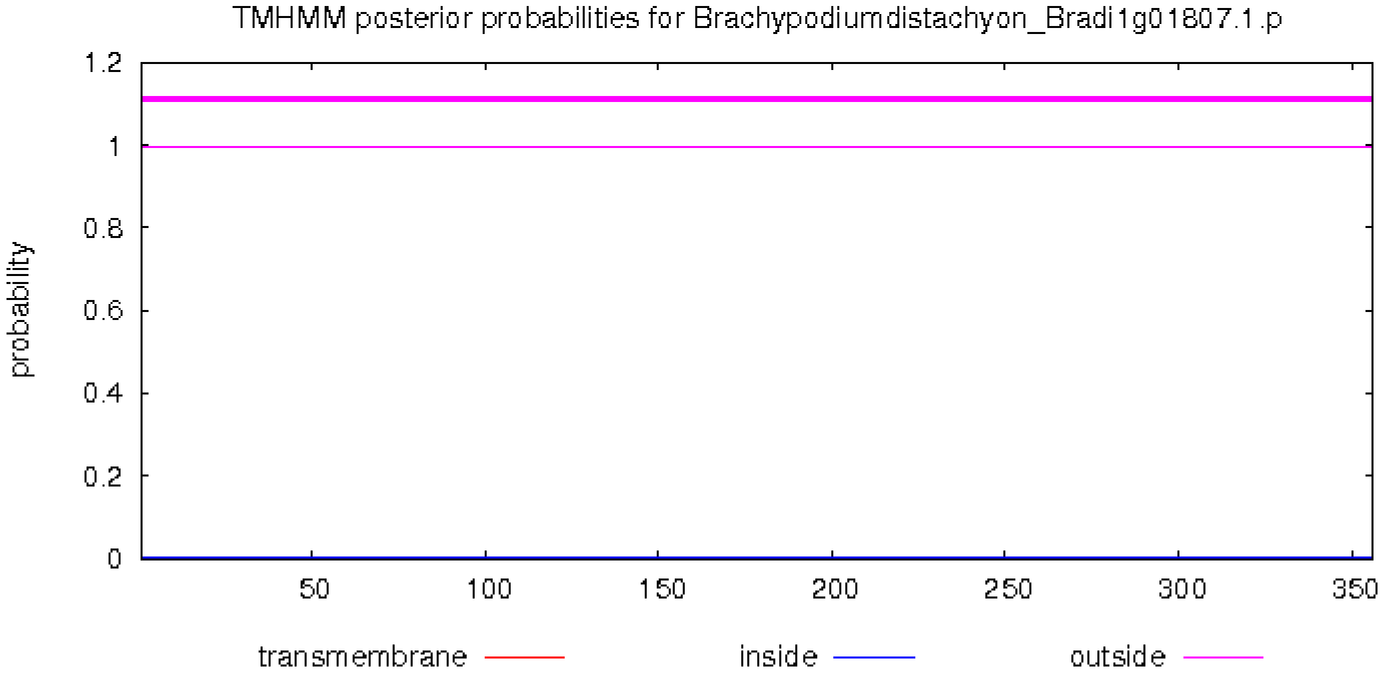

plot in postscript, script for making the plot in gnuplot, data for plot

---

```
# Brachypodiumdistachyon_Bradi1g03146.1.p Length: 216
# Brachypodiumdistachyon_Bradi1g03146.1.p Number of predicted TMHs:  0
# Brachypodiumdistachyon_Bradi1g03146.1.p Exp number of AAs in TMHs: 0.01691
# Brachypodiumdistachyon_Bradi1g03146.1.p Exp number, first 60 AAs:  0.00232
# Brachypodiumdistachyon_Bradi1g03146.1.p Total prob of N-in:        0.12143
Brachypodiumdistachyon_Bradi1g03146.1.p TMHMM2.0        outside      1   216
```

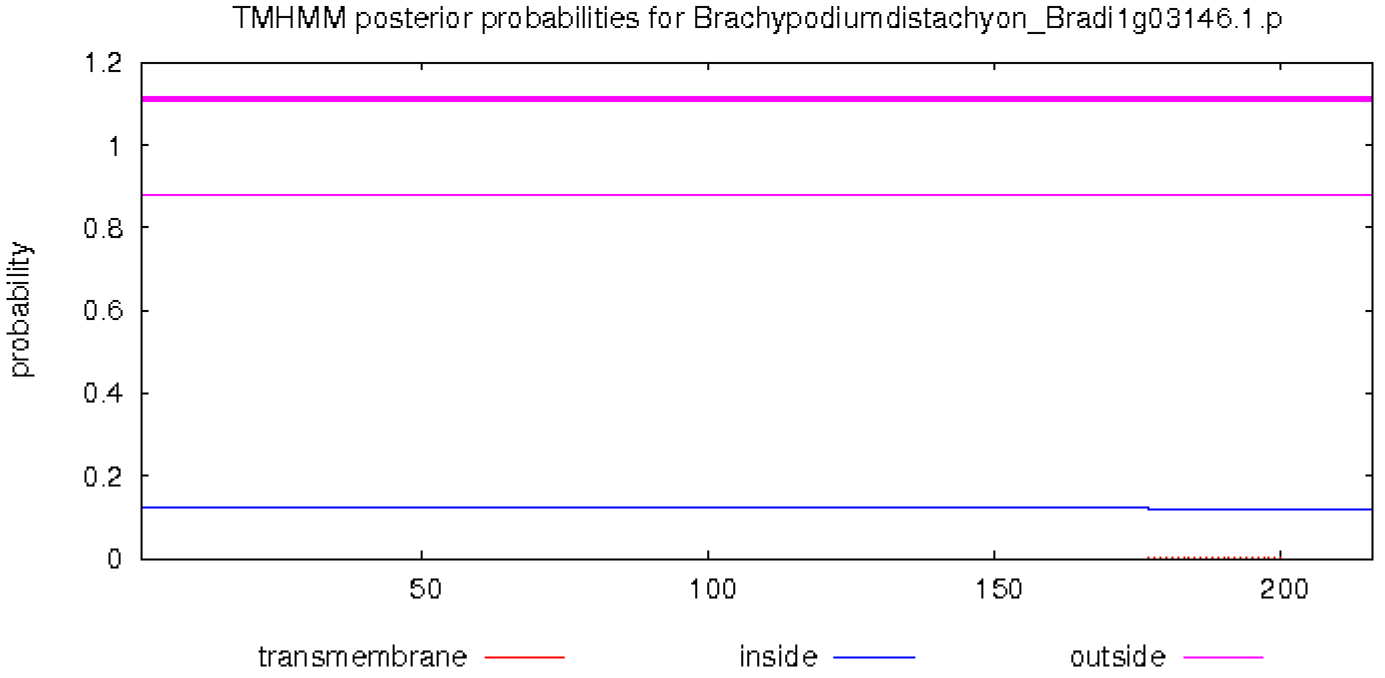

plot in postscript, script for making the plot in gnuplot, data for plot

---

```
# Brachypodiumdistachyon_Bradi1g03245.1.p Length: 331
# Brachypodiumdistachyon_Bradi1g03245.1.p Number of predicted TMHs:  0
# Brachypodiumdistachyon_Bradi1g03245.1.p Exp number of AAs in TMHs: 0.00078
# Brachypodiumdistachyon_Bradi1g03245.1.p Exp number, first 60 AAs:  0.0006
# Brachypodiumdistachyon_Bradi1g03245.1.p Total prob of N-in:        0.01108
Brachypodiumdistachyon_Bradi1g03245.1.p TMHMM2.0        outside      1   331
```

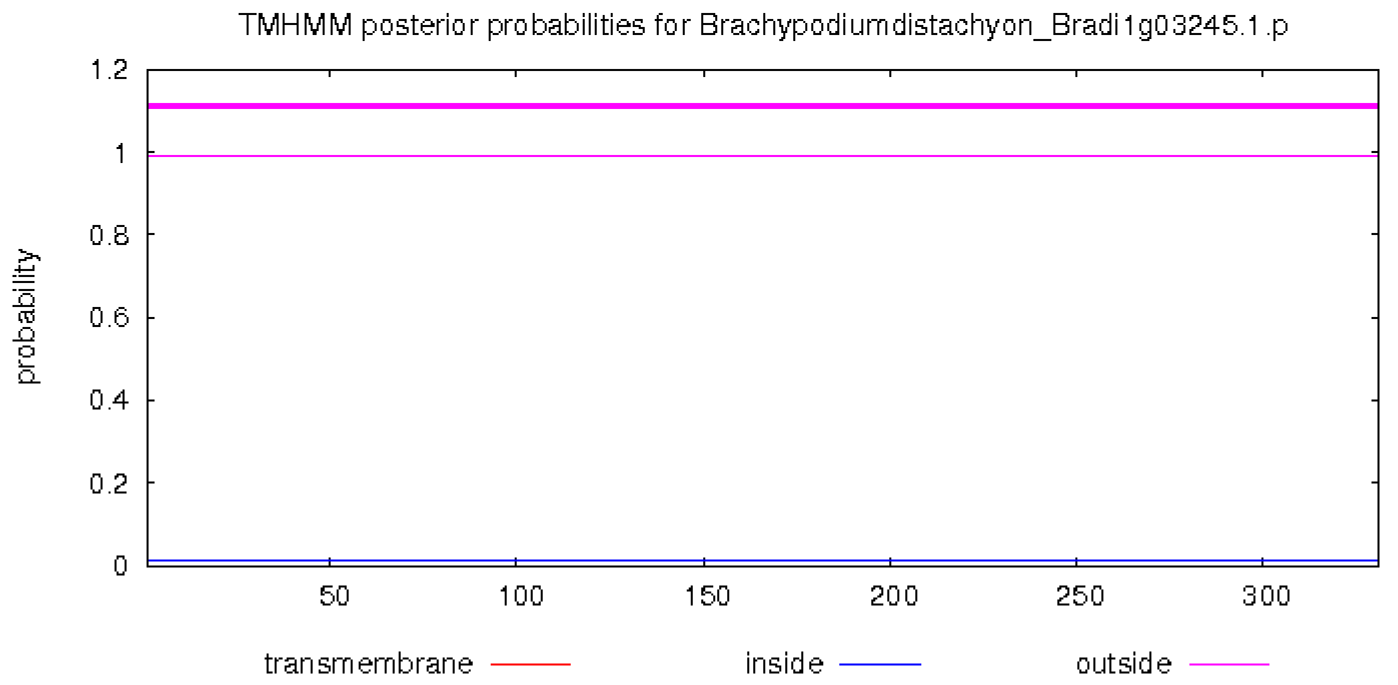

plot in postscript, script for making the plot in gnuplot, data for plot

---

```
# Brachypodiumdistachyon_Bradi1g03313.1.p Length: 244
# Brachypodiumdistachyon_Bradi1g03313.1.p Number of predicted TMHs:  0
# Brachypodiumdistachyon_Bradi1g03313.1.p Exp number of AAs in TMHs: 0.00058
# Brachypodiumdistachyon_Bradi1g03313.1.p Exp number, first 60 AAs:  0
# Brachypodiumdistachyon_Bradi1g03313.1.p Total prob of N-in:        0.08957
Brachypodiumdistachyon_Bradi1g03313.1.p TMHMM2.0        outside      1   244
```

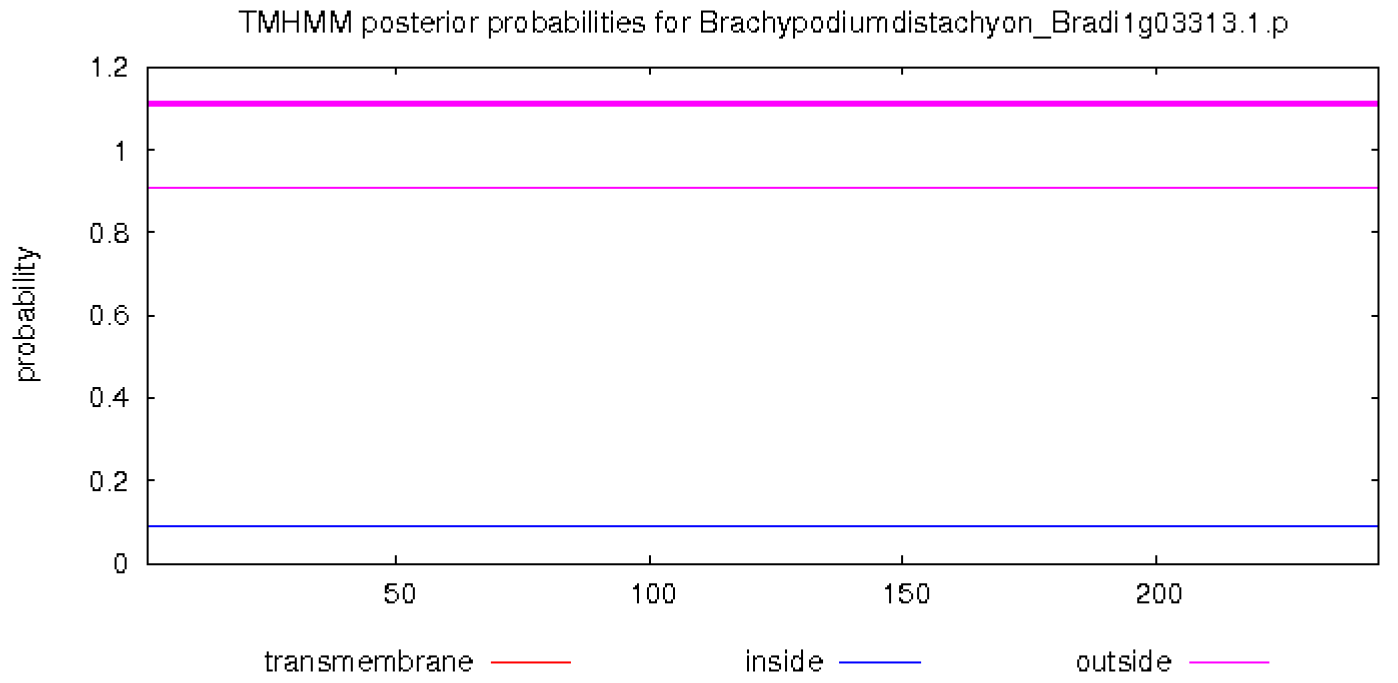

plot in postscript, script for making the plot in gnuplot, data for plot

---

```
# Brachypodiumdistachyon_Bradi1g04150.1.p Length: 325
# Brachypodiumdistachyon_Bradi1g04150.1.p Number of predicted TMHs:  0
# Brachypodiumdistachyon_Bradi1g04150.1.p Exp number of AAs in TMHs: 0.00208
# Brachypodiumdistachyon_Bradi1g04150.1.p Exp number, first 60 AAs:  0.00129
# Brachypodiumdistachyon_Bradi1g04150.1.p Total prob of N-in:        0.13207
Brachypodiumdistachyon_Bradi1g04150.1.p TMHMM2.0        outside      1   325
```

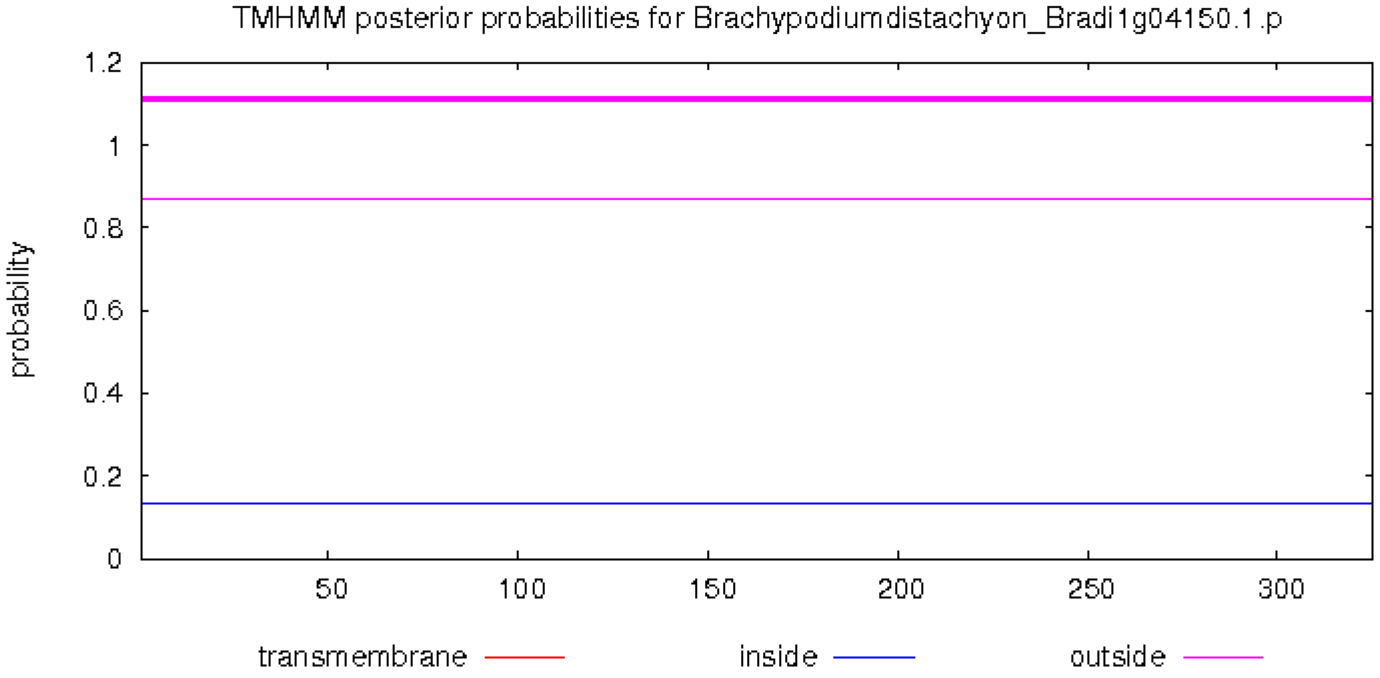

plot in postscript, script for making the plot in gnuplot, data for plot

---

```
# Brachypodiumdistachyon_Bradi1g04229.1.p Length: 365
# Brachypodiumdistachyon_Bradi1g04229.1.p Number of predicted TMHs:  0
# Brachypodiumdistachyon_Bradi1g04229.1.p Exp number of AAs in TMHs: 0.00146
# Brachypodiumdistachyon_Bradi1g04229.1.p Exp number, first 60 AAs:  0.00016
# Brachypodiumdistachyon_Bradi1g04229.1.p Total prob of N-in:        0.00191
Brachypodiumdistachyon_Bradi1g04229.1.p TMHMM2.0        outside      1   365
```

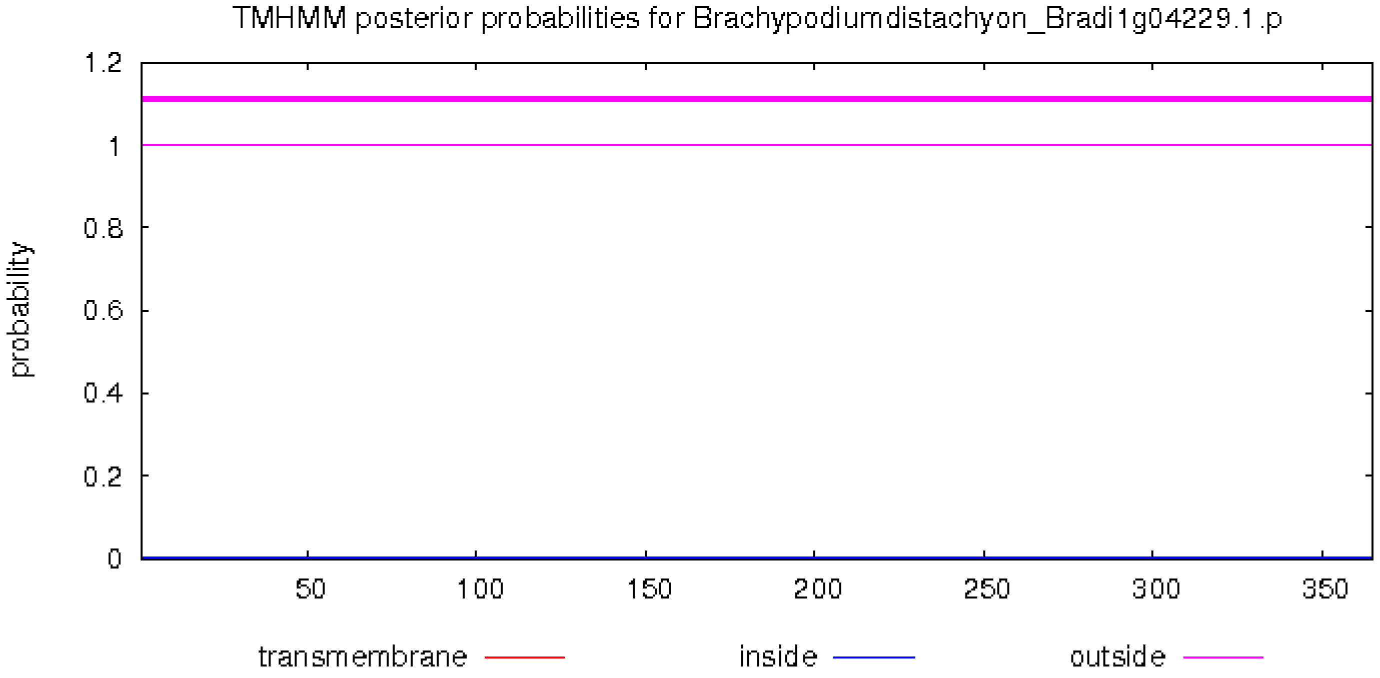

plot in postscript, script for making the plot in gnuplot, data for plot

---

```
# Brachypodiumdistachyon_Bradi1g06970.1.p Length: 344
# Brachypodiumdistachyon_Bradi1g06970.1.p Number of predicted TMHs:  0
# Brachypodiumdistachyon_Bradi1g06970.1.p Exp number of AAs in TMHs: 0.00285
# Brachypodiumdistachyon_Bradi1g06970.1.p Exp number, first 60 AAs:  0
# Brachypodiumdistachyon_Bradi1g06970.1.p Total prob of N-in:        0.09529
Brachypodiumdistachyon_Bradi1g06970.1.p TMHMM2.0        outside      1   344
```

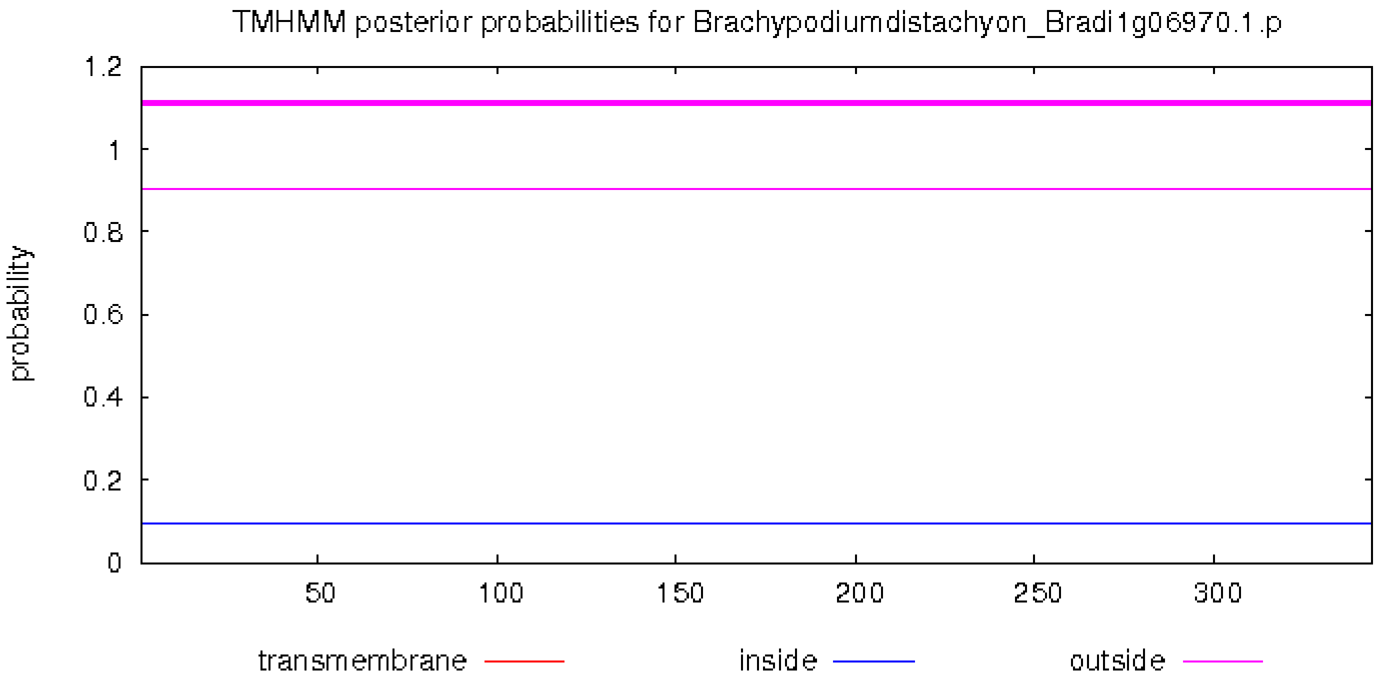

plot in postscript, script for making the plot in gnuplot, data for plot

```
# Brachypodiumdistachyon_Bradi1g14467.1.p Length: 302
# Brachypodiumdistachyon_Bradi1g14467.1.p Number of predicted TMHs:  0
# Brachypodiumdistachyon_Bradi1g14467.1.p Exp number of AAs in TMHs: 0.91323
# Brachypodiumdistachyon_Bradi1g14467.1.p Exp number, first 60 AAs:  0
# Brachypodiumdistachyon_Bradi1g14467.1.p Total prob of N-in:        0.00587
Brachypodiumdistachyon_Bradi1g14467.1.p TMHMM2.0        outside      1   302
```

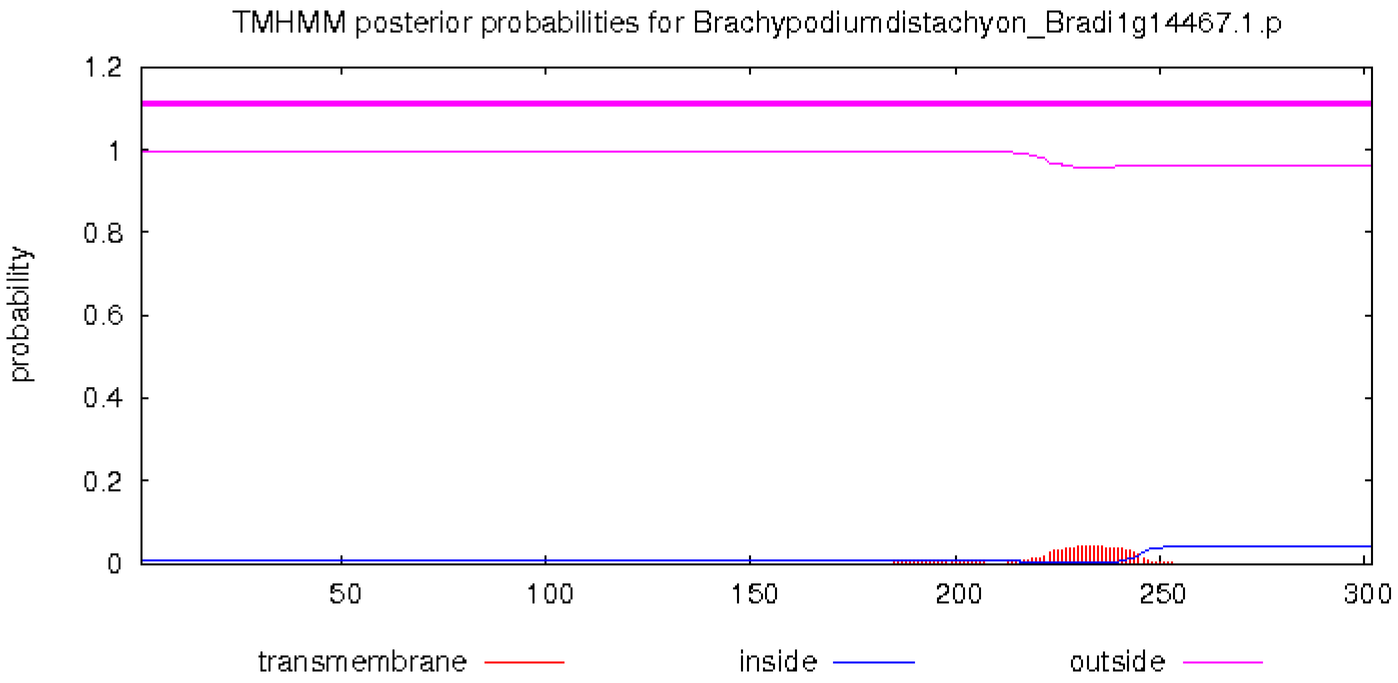

plot in postscript, script for making the plot in gnuplot, data for plot

```
# Brachypodiumdistachyon_Bradi1g17440.1.p Length: 302
# Brachypodiumdistachyon_Bradi1g17440.1.p Number of predicted TMHs:  0
# Brachypodiumdistachyon_Bradi1g17440.1.p Exp number of AAs in TMHs: 0.003
# Brachypodiumdistachyon_Bradi1g17440.1.p Exp number, first 60 AAs:  0
# Brachypodiumdistachyon_Bradi1g17440.1.p Total prob of N-in:        0.02344
Brachypodiumdistachyon_Bradi1g17440.1.p TMHMM2.0        outside      1   302
```

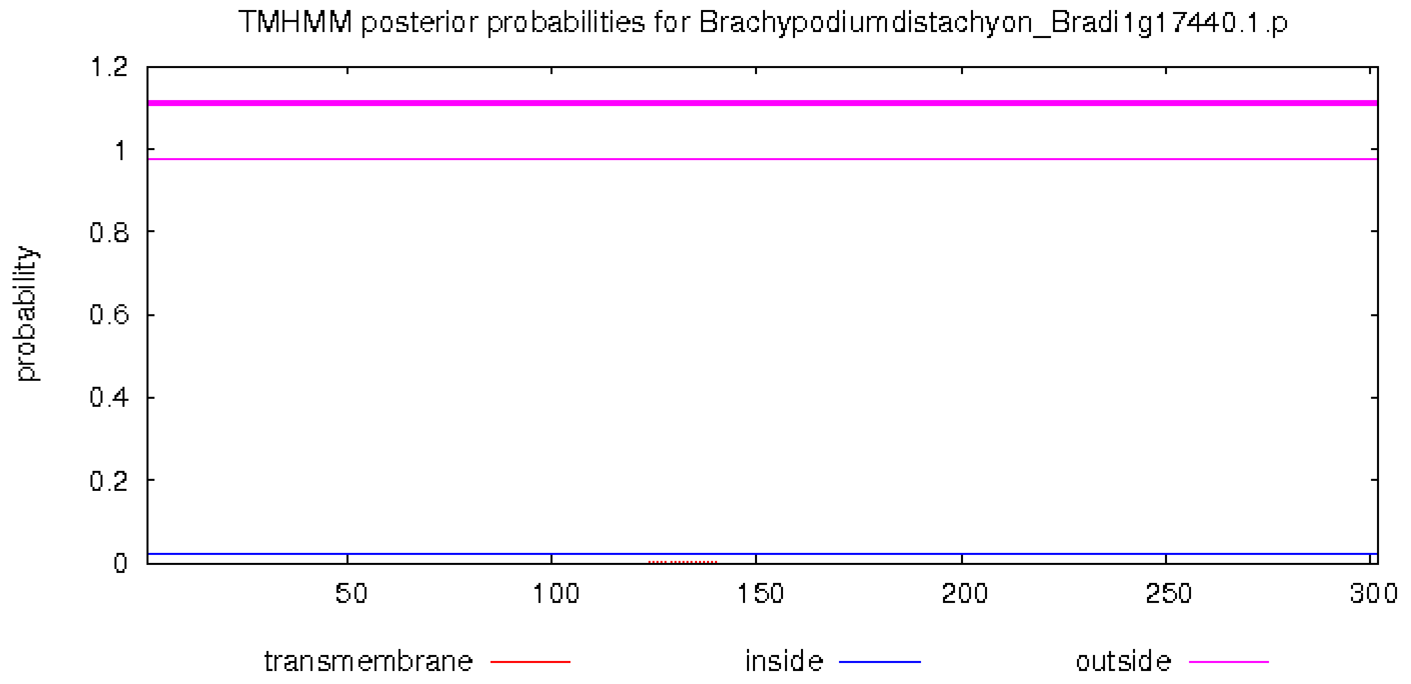

plot in postscript, script for making the plot in gnuplot, data for plot

---

```
# Brachypodiumdistachyon_Bradi1g17480.1.p Length: 375
# Brachypodiumdistachyon_Bradi1g17480.1.p Number of predicted TMHs:  0
# Brachypodiumdistachyon_Bradi1g17480.1.p Exp number of AAs in TMHs: 0.00794
# Brachypodiumdistachyon_Bradi1g17480.1.p Exp number, first 60 AAs:  0.00016
# Brachypodiumdistachyon_Bradi1g17480.1.p Total prob of N-in:        0.00459
Brachypodiumdistachyon_Bradi1g17480.1.p TMHMM2.0        outside      1   375
```

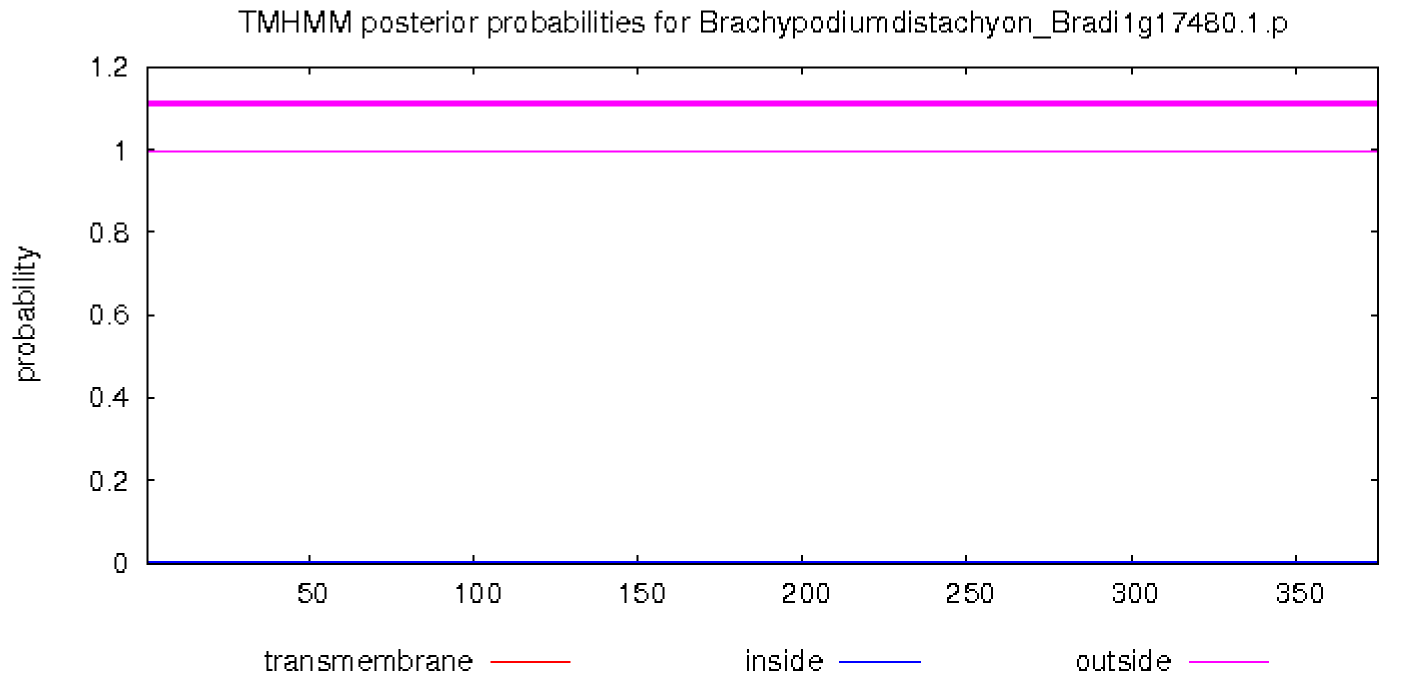

plot in postscript, script for making the plot in gnuplot, data for plot

---

```
# Brachypodiumdistachyon_Bradi1g20063.2.p Length: 378
# Brachypodiumdistachyon_Bradi1g20063.2.p Number of predicted TMHs:  0
# Brachypodiumdistachyon_Bradi1g20063.2.p Exp number of AAs in TMHs: 0.00371
# Brachypodiumdistachyon_Bradi1g20063.2.p Exp number, first 60 AAs:  0
# Brachypodiumdistachyon_Bradi1g20063.2.p Total prob of N-in:        0.03695
Brachypodiumdistachyon_Bradi1g20063.2.p TMHMM2.0        outside      1   378
```

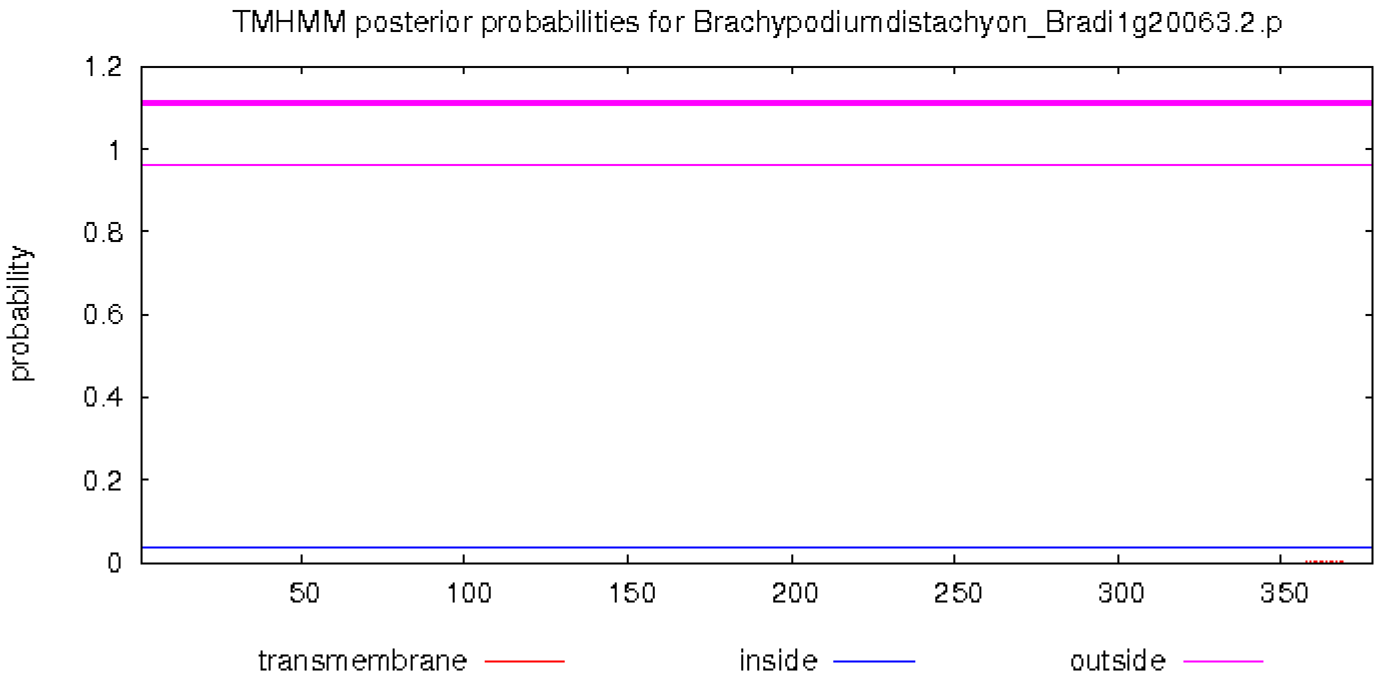

plot in postscript, script for making the plot in gnuplot, data for plot

---

```
# Brachypodiumdistachyon_Bradi1g20801.1.p Length: 297
# Brachypodiumdistachyon_Bradi1g20801.1.p Number of predicted TMHs:  0
# Brachypodiumdistachyon_Bradi1g20801.1.p Exp number of AAs in TMHs: 0.00365
# Brachypodiumdistachyon_Bradi1g20801.1.p Exp number, first 60 AAs:  0
# Brachypodiumdistachyon_Bradi1g20801.1.p Total prob of N-in:        0.02113
Brachypodiumdistachyon_Bradi1g20801.1.p TMHMM2.0        outside      1   297
```

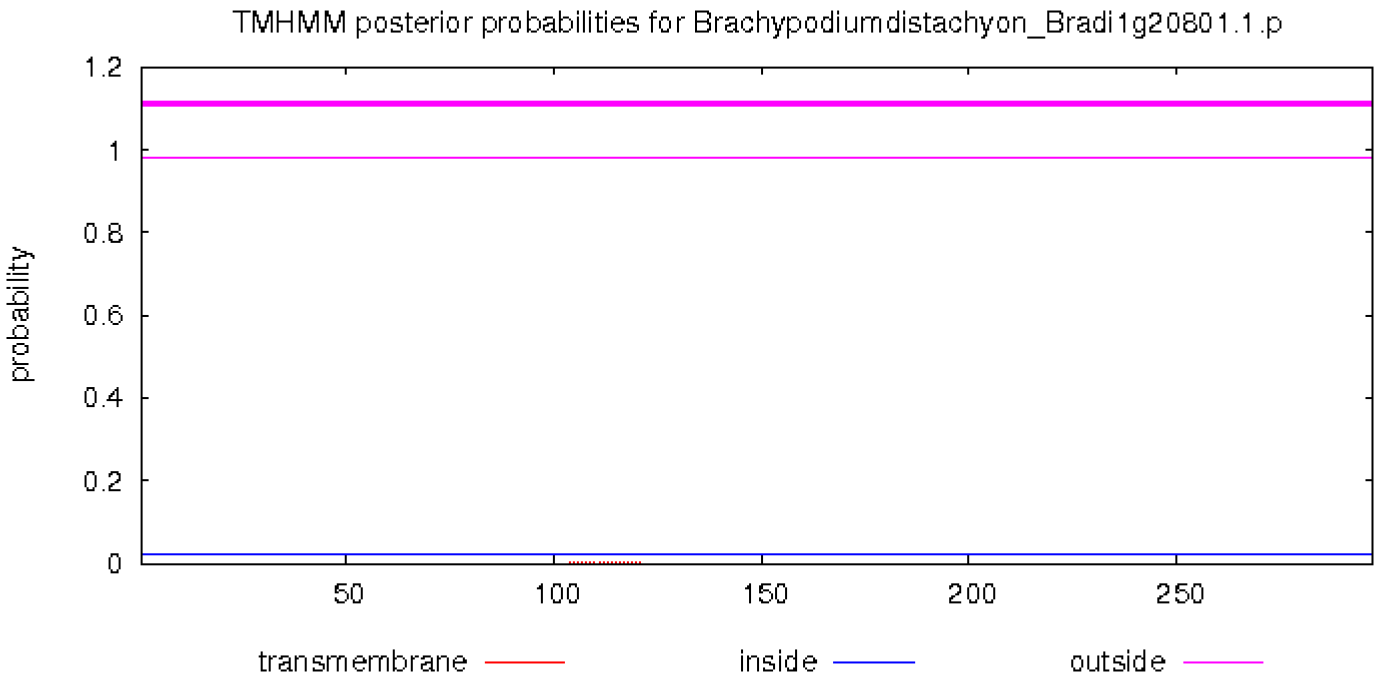

plot in postscript, script for making the plot in gnuplot, data for plot

---

```
# Brachypodiumdistachyon_Bradi1g20805.1.p Length: 316
# Brachypodiumdistachyon_Bradi1g20805.1.p Number of predicted TMHs:  0
# Brachypodiumdistachyon_Bradi1g20805.1.p Exp number of AAs in TMHs: 0.04319
# Brachypodiumdistachyon_Bradi1g20805.1.p Exp number, first 60 AAs:  0
# Brachypodiumdistachyon_Bradi1g20805.1.p Total prob of N-in:        0.00958
Brachypodiumdistachyon_Bradi1g20805.1.p TMHMM2.0        outside      1   316
```

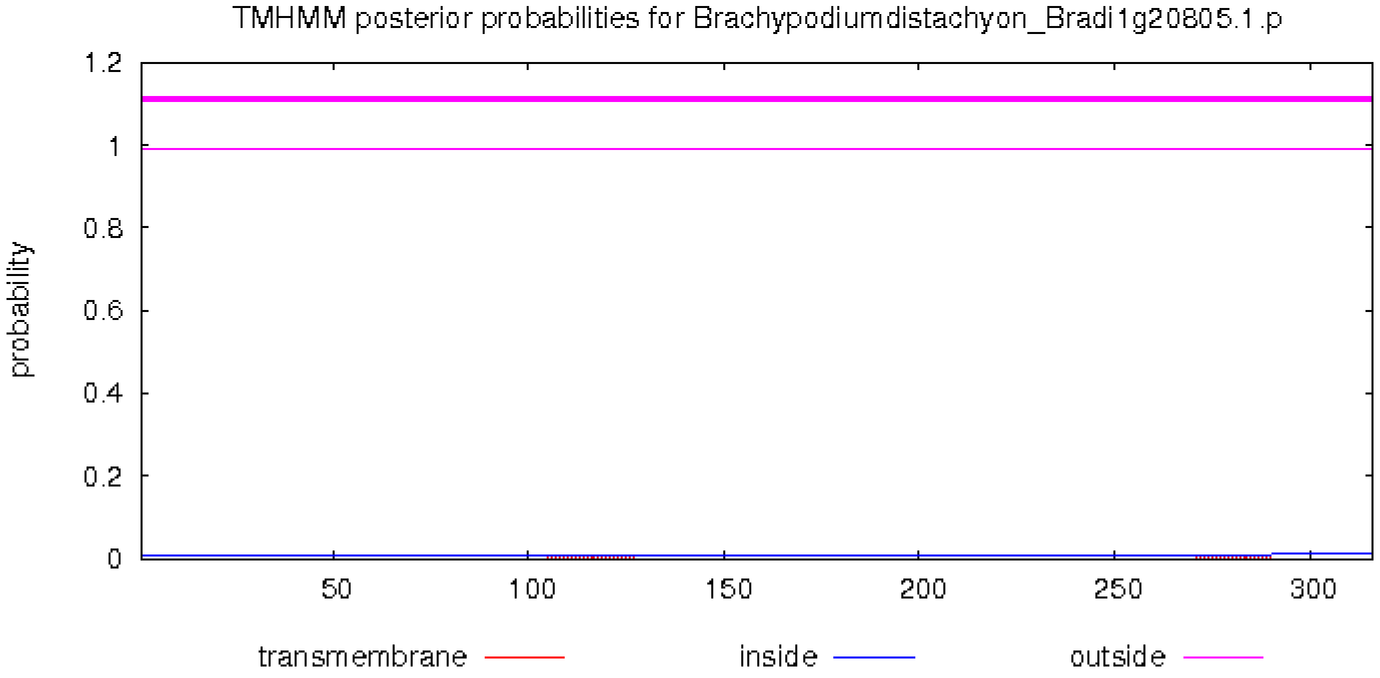

plot in postscript, script for making the plot in gnuplot, data for plot

---

```
# Brachypodiumdistachyon_Bradi1g24390.1.p Length: 435
# Brachypodiumdistachyon_Bradi1g24390.1.p Number of predicted TMHs:  0
# Brachypodiumdistachyon_Bradi1g24390.1.p Exp number of AAs in TMHs: 0.00163
# Brachypodiumdistachyon_Bradi1g24390.1.p Exp number, first 60 AAs:  0
# Brachypodiumdistachyon_Bradi1g24390.1.p Total prob of N-in:        0.00309
Brachypodiumdistachyon_Bradi1g24390.1.p TMHMM2.0        outside      1   435
```

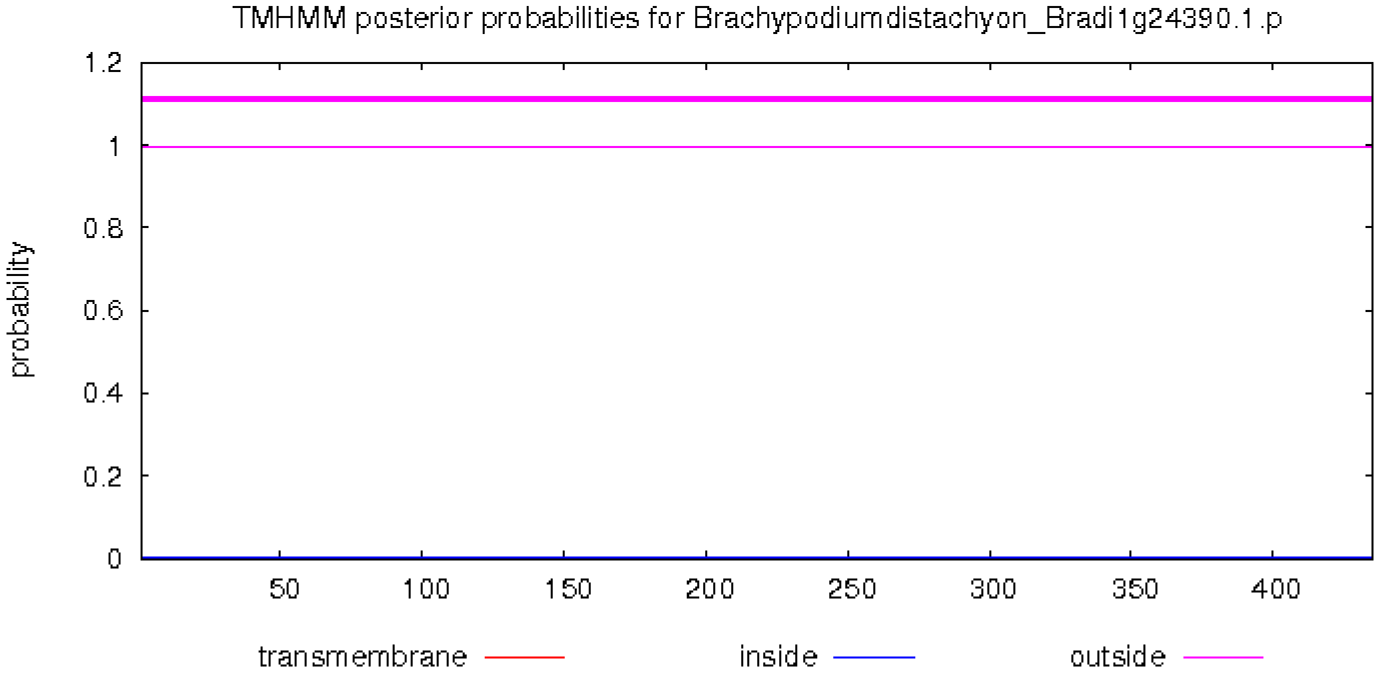

plot in postscript, script for making the plot in gnuplot, data for plot

---

```
# Brachypodiumdistachyon_Bradi1g29857.1.p Length: 304
# Brachypodiumdistachyon_Bradi1g29857.1.p Number of predicted TMHs:  0
# Brachypodiumdistachyon_Bradi1g29857.1.p Exp number of AAs in TMHs: 0.00056
# Brachypodiumdistachyon_Bradi1g29857.1.p Exp number, first 60 AAs:  0
# Brachypodiumdistachyon_Bradi1g29857.1.p Total prob of N-in:        0.00174
Brachypodiumdistachyon_Bradi1g29857.1.p TMHMM2.0        outside      1   304
```

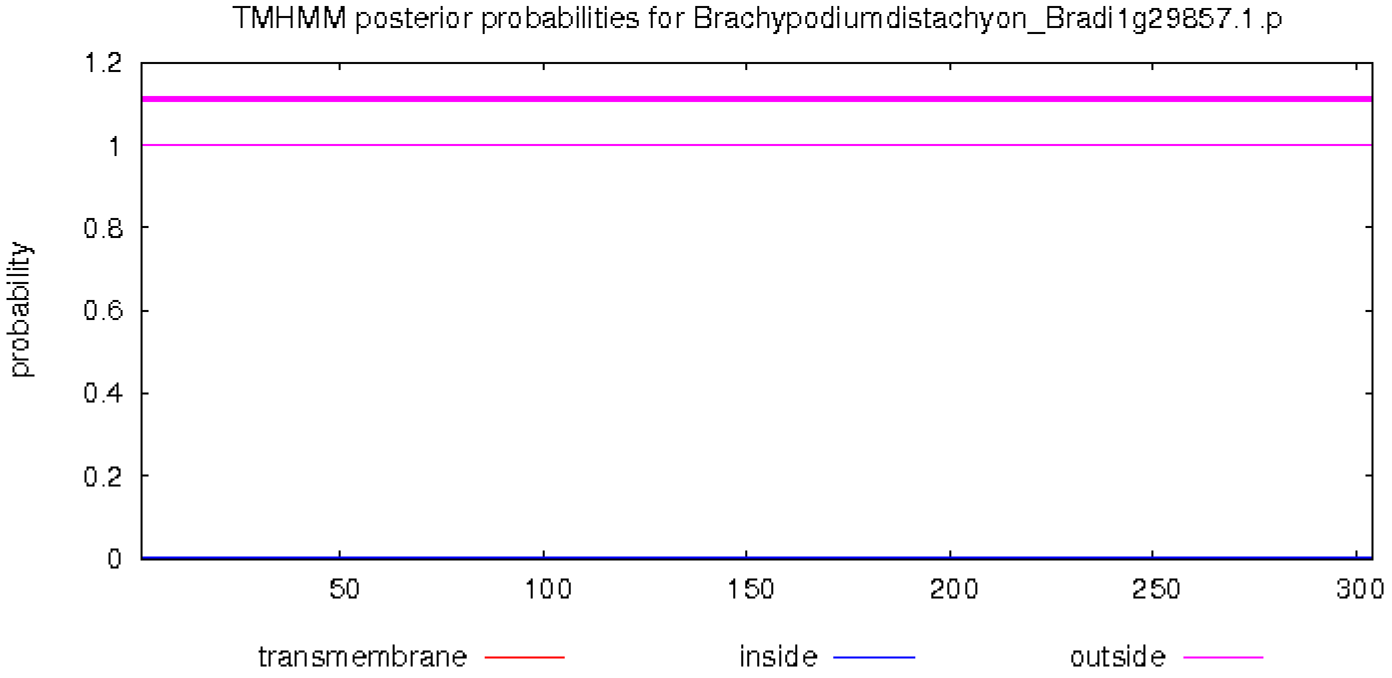

plot in postscript, script for making the plot in gnuplot, data for plot

---

```
# Brachypodiumdistachyon_Bradi1g32660.1.p Length: 303
# Brachypodiumdistachyon_Bradi1g32660.1.p Number of predicted TMHs:  0
# Brachypodiumdistachyon_Bradi1g32660.1.p Exp number of AAs in TMHs: 0.02032
# Brachypodiumdistachyon_Bradi1g32660.1.p Exp number, first 60 AAs:  0.01578
# Brachypodiumdistachyon_Bradi1g32660.1.p Total prob of N-in:        0.02871
Brachypodiumdistachyon_Bradi1g32660.1.p TMHMM2.0        outside      1   303
```

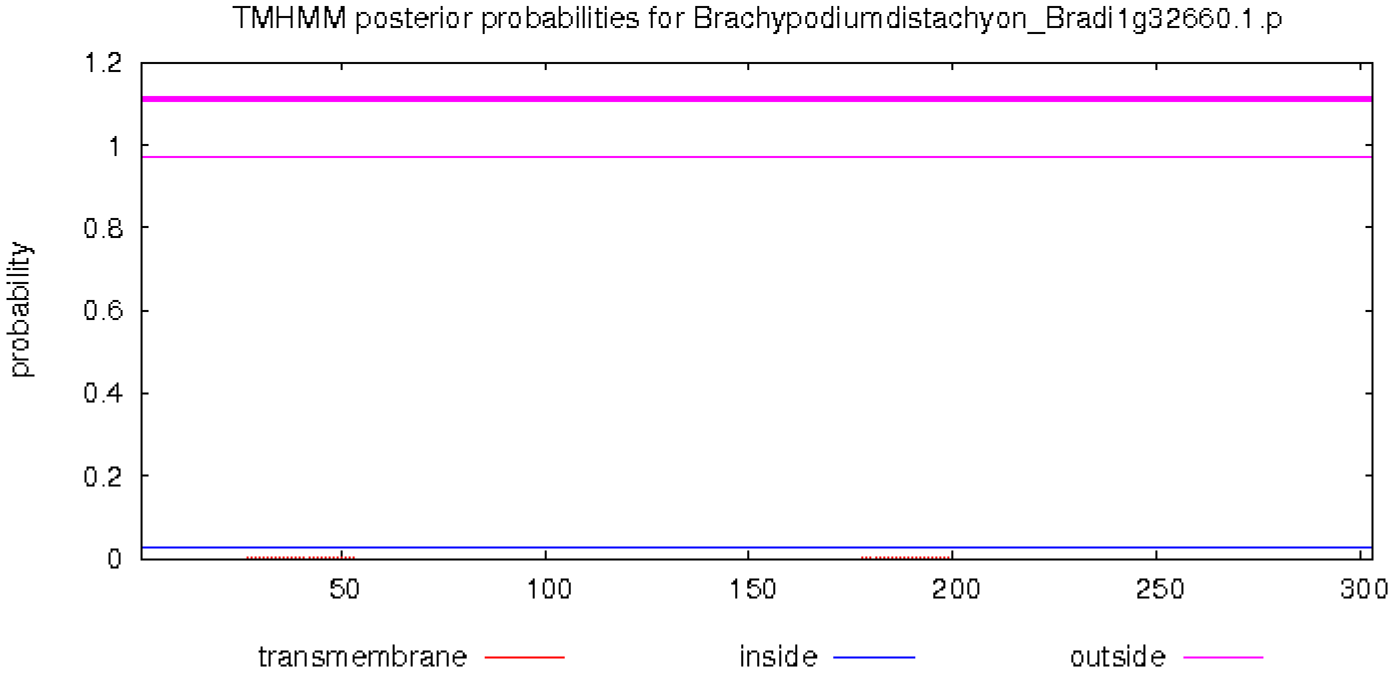

plot in postscript, script for making the plot in gnuplot, data for plot

---

```
# Brachypodiumdistachyon_Bradi1g36200.1.p Length: 324
# Brachypodiumdistachyon_Bradi1g36200.1.p Number of predicted TMHs:  0
# Brachypodiumdistachyon_Bradi1g36200.1.p Exp number of AAs in TMHs: 0.03242
# Brachypodiumdistachyon_Bradi1g36200.1.p Exp number, first 60 AAs:  0
# Brachypodiumdistachyon_Bradi1g36200.1.p Total prob of N-in:        0.01492
Brachypodiumdistachyon_Bradi1g36200.1.p TMHMM2.0        outside      1   324
```

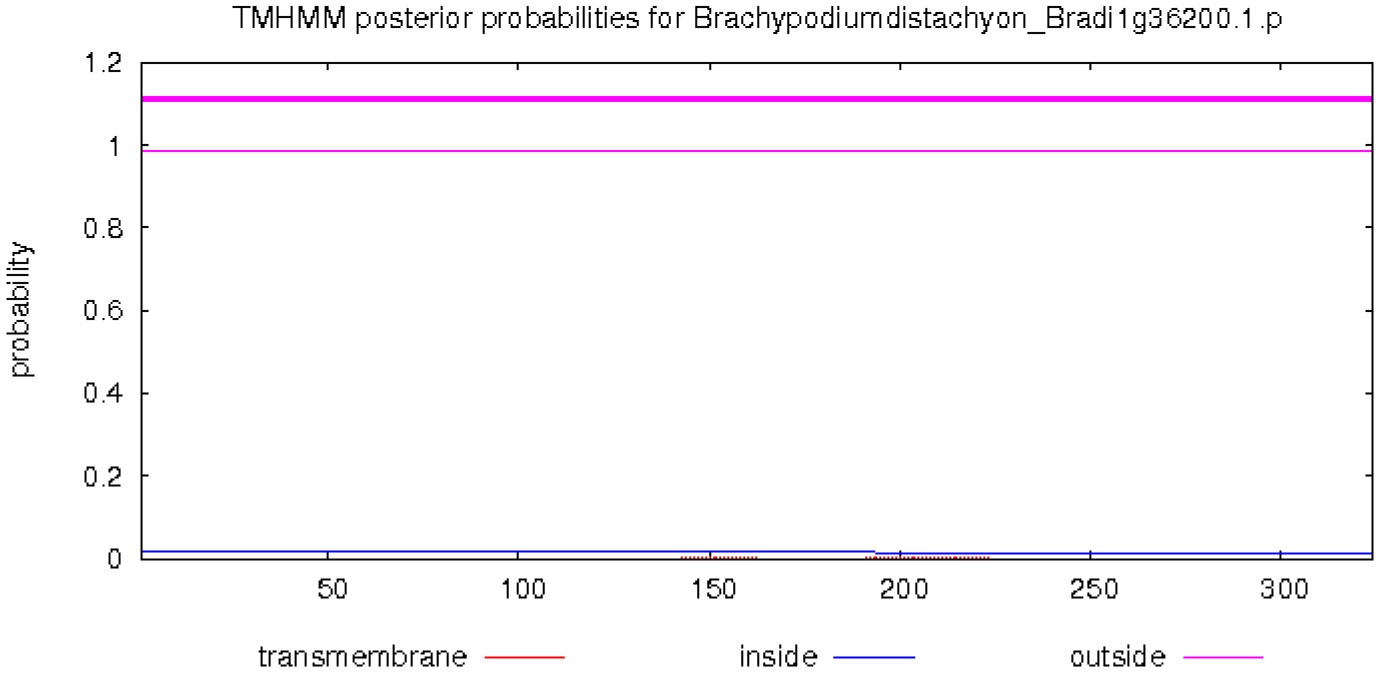

plot in postscript, script for making the plot in gnuplot, data for plot

---

```
# Brachypodiumdistachyon_Bradi1g37898.1.p Length: 486
# Brachypodiumdistachyon_Bradi1g37898.1.p Number of predicted TMHs:  0
# Brachypodiumdistachyon_Bradi1g37898.1.p Exp number of AAs in TMHs: 0.00418
# Brachypodiumdistachyon_Bradi1g37898.1.p Exp number, first 60 AAs:  0.00018
# Brachypodiumdistachyon_Bradi1g37898.1.p Total prob of N-in:        0.00254
Brachypodiumdistachyon_Bradi1g37898.1.p TMHMM2.0        outside      1   486
```

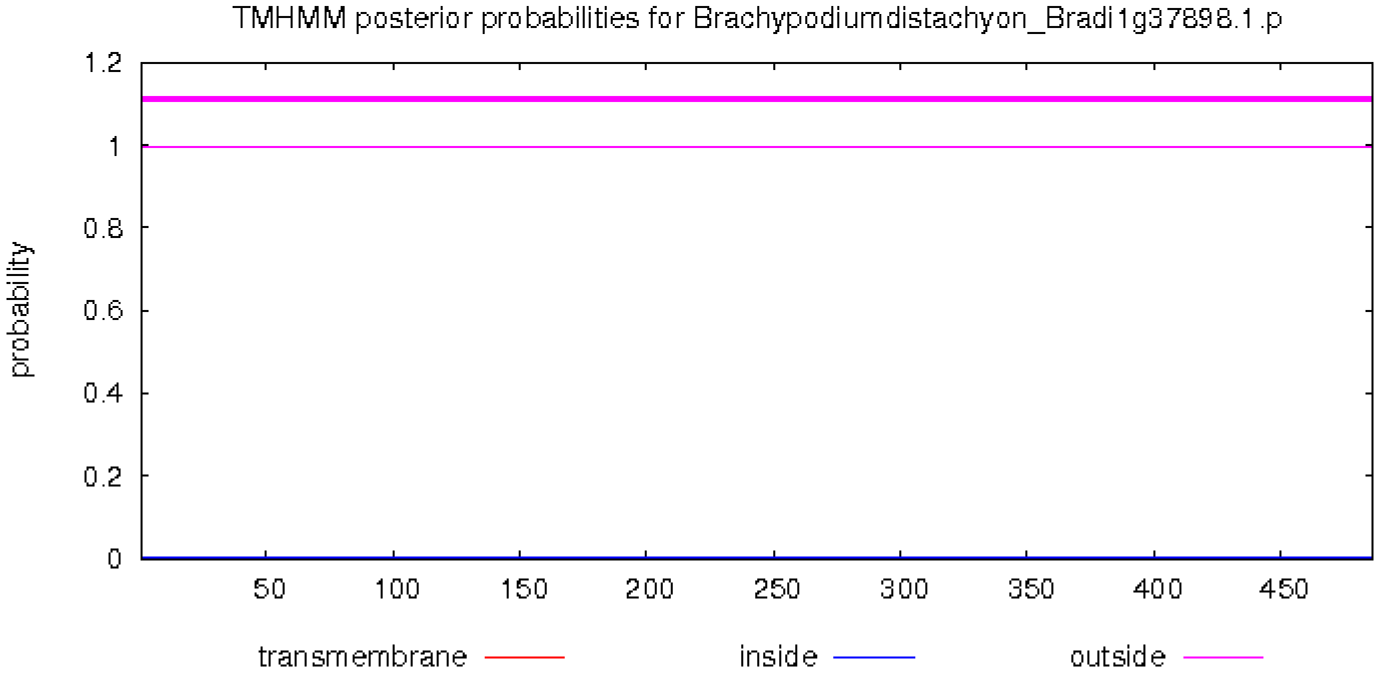

plot in postscript, script for making the plot in gnuplot, data for plot

---

```
# Brachypodiumdistachyon_Bradi1g38730.1.p Length: 364
# Brachypodiumdistachyon_Bradi1g38730.1.p Number of predicted TMHs:  0
# Brachypodiumdistachyon_Bradi1g38730.1.p Exp number of AAs in TMHs: 0.01117
# Brachypodiumdistachyon_Bradi1g38730.1.p Exp number, first 60 AAs:  0
# Brachypodiumdistachyon_Bradi1g38730.1.p Total prob of N-in:        0.00519
Brachypodiumdistachyon_Bradi1g38730.1.p TMHMM2.0        outside      1   364
```

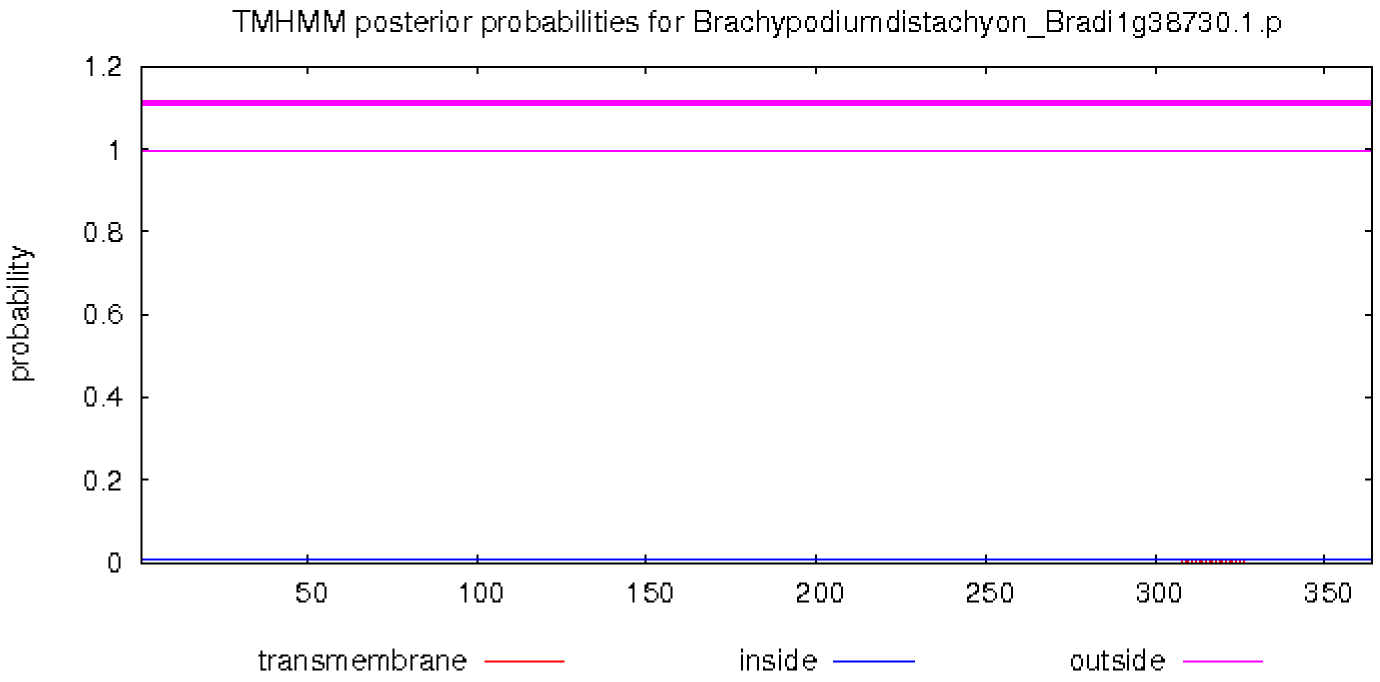

plot in postscript, script for making the plot in gnuplot, data for plot

---

```
# Brachypodiumdistachyon_Bradi1g41712.2.p Length: 346
# Brachypodiumdistachyon_Bradi1g41712.2.p Number of predicted TMHs:  0
# Brachypodiumdistachyon_Bradi1g41712.2.p Exp number of AAs in TMHs: 0.36153
# Brachypodiumdistachyon_Bradi1g41712.2.p Exp number, first 60 AAs:  0
# Brachypodiumdistachyon_Bradi1g41712.2.p Total prob of N-in:        0.02338
Brachypodiumdistachyon_Bradi1g41712.2.p TMHMM2.0        outside      1   346
```

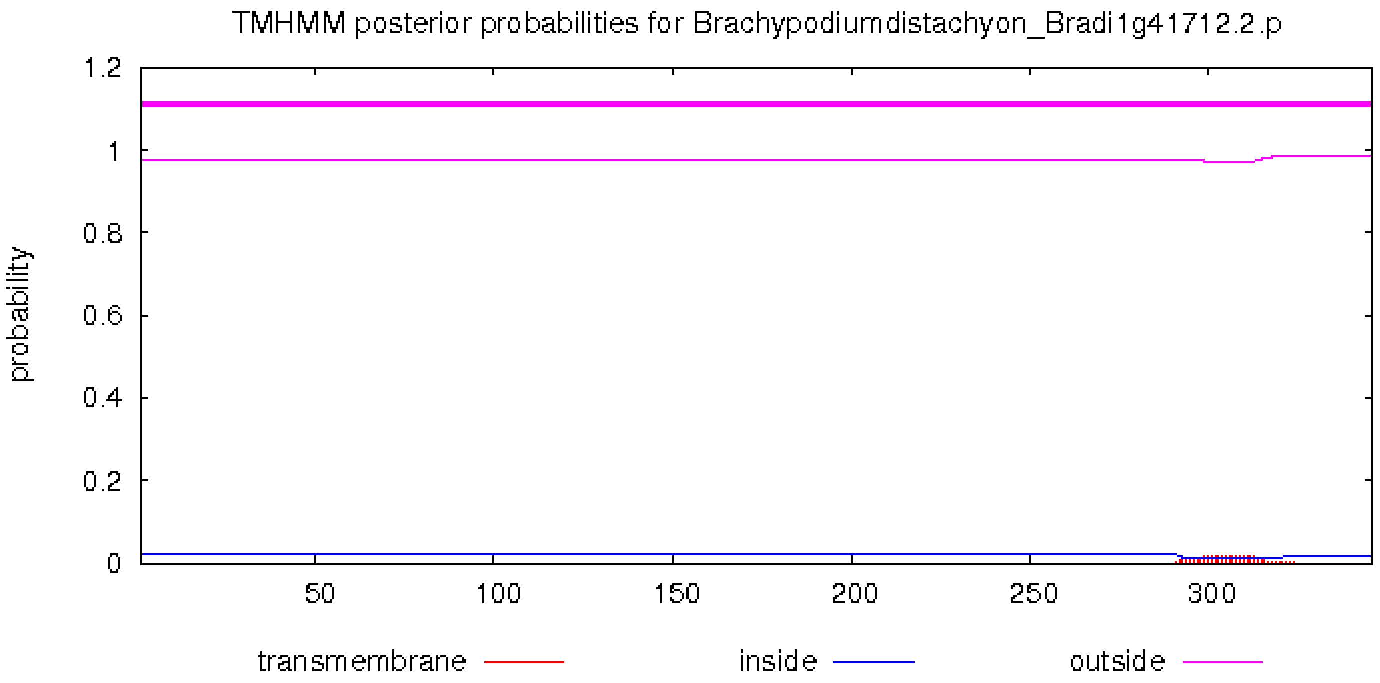

plot in postscript, script for making the plot in gnuplot, data for plot

---

```
# Brachypodiumdistachyon_Bradi1g43840.1.p Length: 481
# Brachypodiumdistachyon_Bradi1g43840.1.p Number of predicted TMHs:  0
# Brachypodiumdistachyon_Bradi1g43840.1.p Exp number of AAs in TMHs: 0
# Brachypodiumdistachyon_Bradi1g43840.1.p Exp number, first 60 AAs:  0
# Brachypodiumdistachyon_Bradi1g43840.1.p Total prob of N-in:        0.00195
Brachypodiumdistachyon_Bradi1g43840.1.p TMHMM2.0        outside      1   481
```

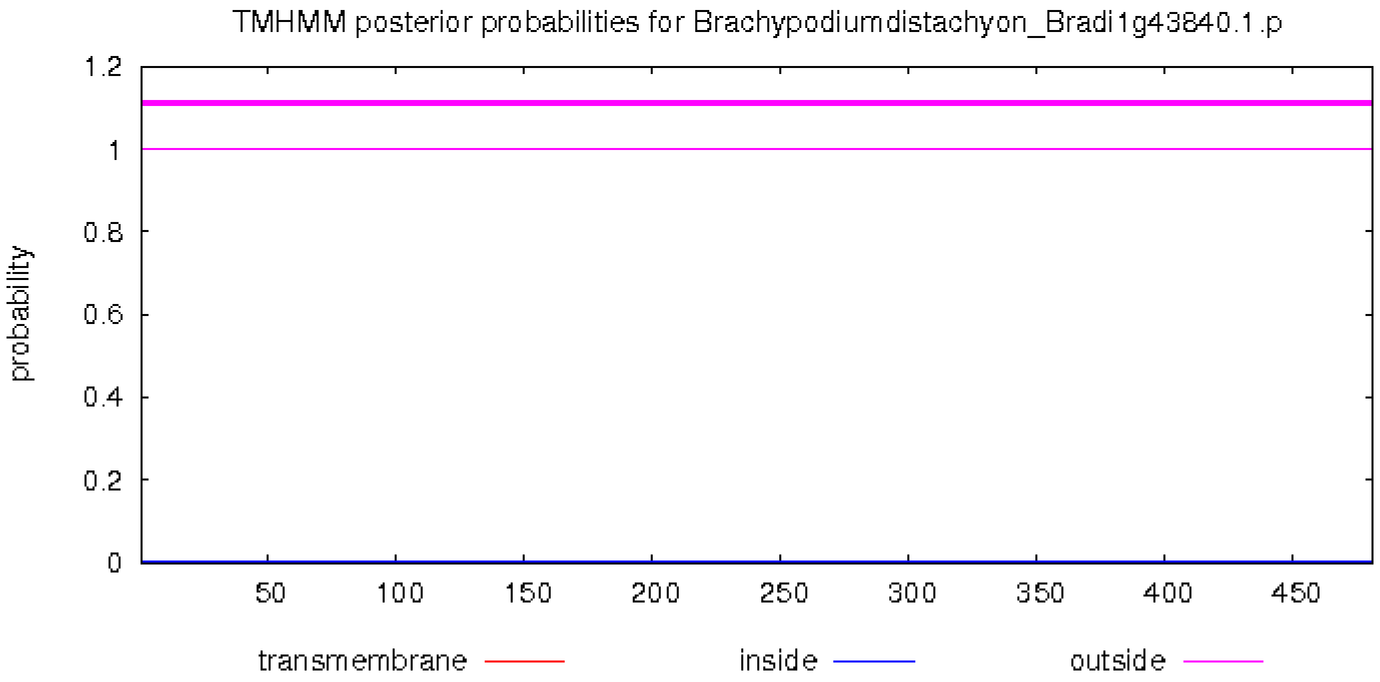

plot in postscript, script for making the plot in gnuplot, data for plot

---

```
# Brachypodiumdistachyon_Bradi1g50057.1.p Length: 415
# Brachypodiumdistachyon_Bradi1g50057.1.p Number of predicted TMHs:  0
# Brachypodiumdistachyon_Bradi1g50057.1.p Exp number of AAs in TMHs: 0
# Brachypodiumdistachyon_Bradi1g50057.1.p Exp number, first 60 AAs:  0
# Brachypodiumdistachyon_Bradi1g50057.1.p Total prob of N-in:        0.00573
Brachypodiumdistachyon_Bradi1g50057.1.p TMHMM2.0        outside      1   415
```

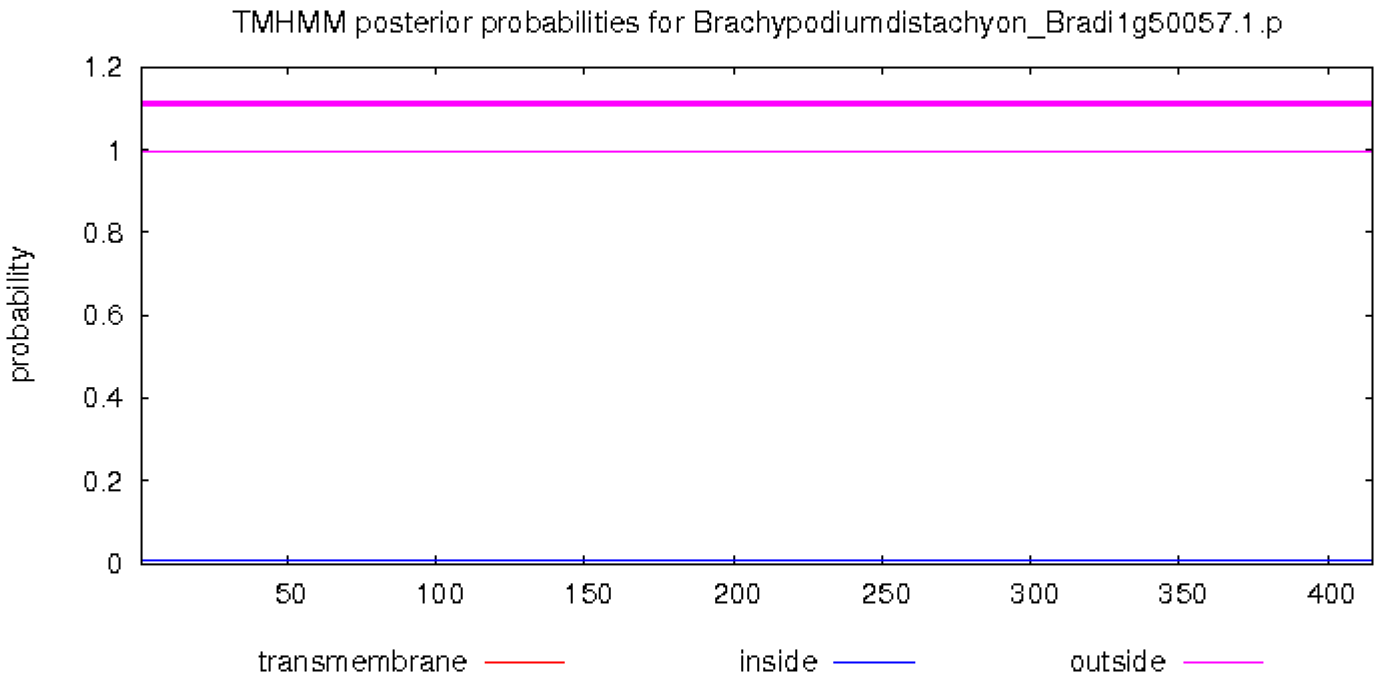

plot in postscript, script for making the plot in gnuplot, data for plot

---

```
# Brachypodiumdistachyon_Bradi1g52187.2.p Length: 336
# Brachypodiumdistachyon_Bradi1g52187.2.p Number of predicted TMHs:  0
# Brachypodiumdistachyon_Bradi1g52187.2.p Exp number of AAs in TMHs: 0.00016
# Brachypodiumdistachyon_Bradi1g52187.2.p Exp number, first 60 AAs:  0
# Brachypodiumdistachyon_Bradi1g52187.2.p Total prob of N-in:        0.07016
Brachypodiumdistachyon_Bradi1g52187.2.p TMHMM2.0        outside      1   336
```

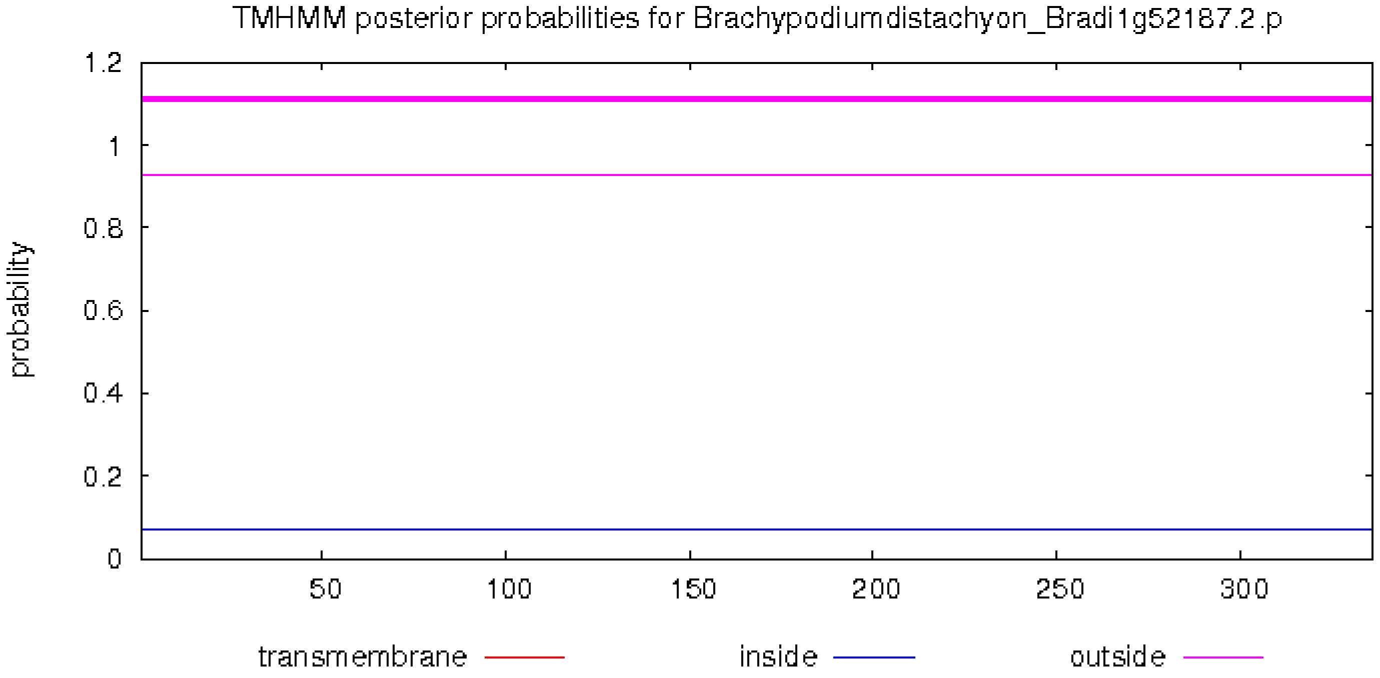

plot in postscript, script for making the plot in gnuplot, data for plot

```
# Brachypodiumdistachyon_Bradi1g52480.2.p Length: 638
# Brachypodiumdistachyon_Bradi1g52480.2.p Number of predicted TMHs:  1
# Brachypodiumdistachyon_Bradi1g52480.2.p Exp number of AAs in TMHs: 22.35513
# Brachypodiumdistachyon_Bradi1g52480.2.p Exp number, first 60 AAs:  0.00128
# Brachypodiumdistachyon_Bradi1g52480.2.p Total prob of N-in:        0.01157
Brachypodiumdistachyon_Bradi1g52480.2.p TMHMM2.0        outside      1   611
Brachypodiumdistachyon_Bradi1g52480.2.p TMHMM2.0        TMhelix    612   634
Brachypodiumdistachyon_Bradi1g52480.2.p TMHMM2.0        inside     635   638
```

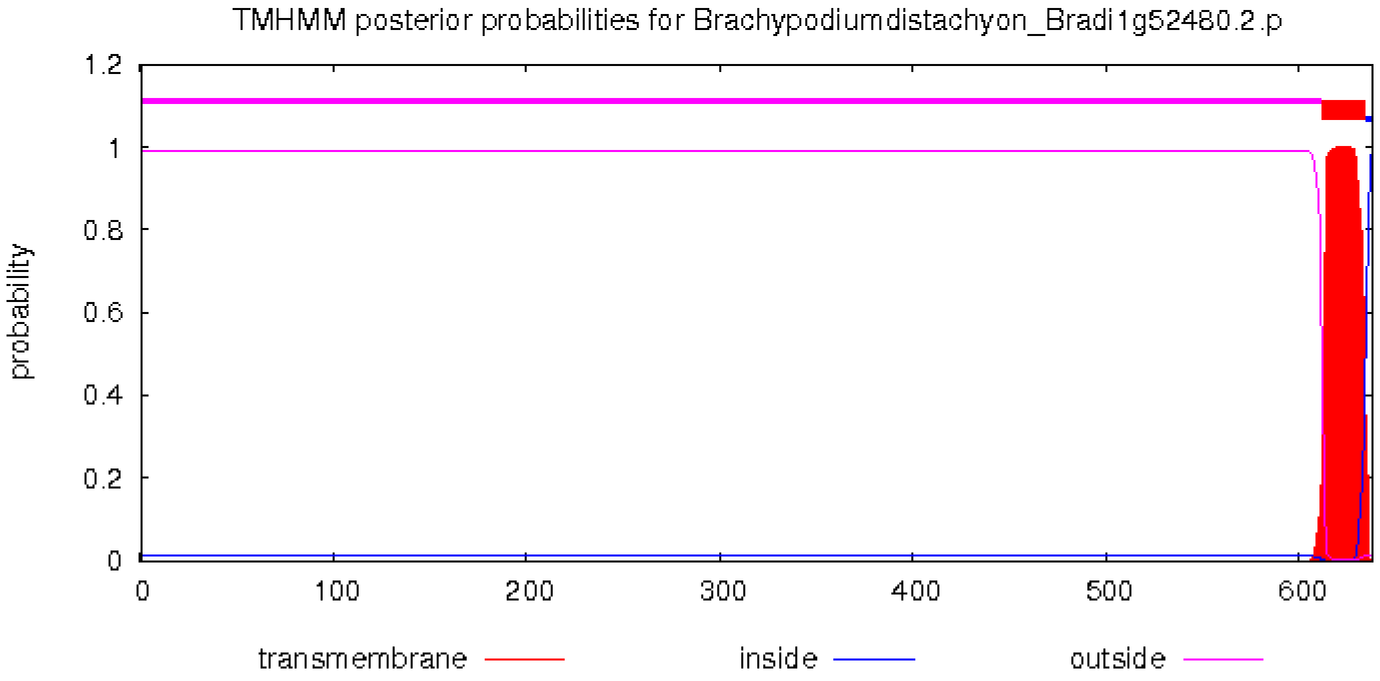

plot in postscript, script for making the plot in gnuplot, data for plot

```
# Brachypodiumdistachyon_Bradi1g53656.1.p Length: 102
# Brachypodiumdistachyon_Bradi1g53656.1.p Number of predicted TMHs:  0
# Brachypodiumdistachyon_Bradi1g53656.1.p Exp number of AAs in TMHs: 0.01912
# Brachypodiumdistachyon_Bradi1g53656.1.p Exp number, first 60 AAs:  0.01912
# Brachypodiumdistachyon_Bradi1g53656.1.p Total prob of N-in:        0.08237
Brachypodiumdistachyon_Bradi1g53656.1.p TMHMM2.0        outside      1   102
```

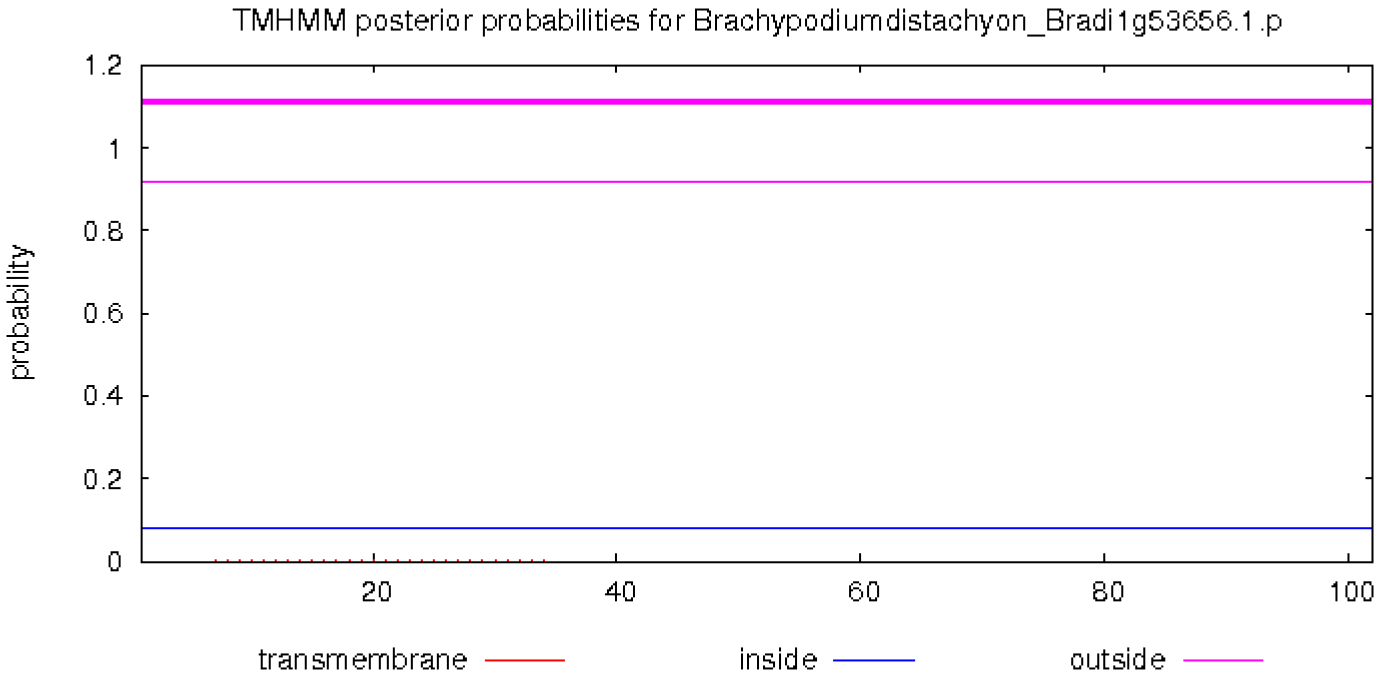

plot in postscript, script for making the plot in gnuplot, data for plot

```
# Brachypodiumdistachyon_Bradi1g53770.1.p Length: 279
# Brachypodiumdistachyon_Bradi1g53770.1.p Number of predicted TMHs:  0
# Brachypodiumdistachyon_Bradi1g53770.1.p Exp number of AAs in TMHs: 0.0004
# Brachypodiumdistachyon_Bradi1g53770.1.p Exp number, first 60 AAs:  0.00024
# Brachypodiumdistachyon_Bradi1g53770.1.p Total prob of N-in:        0.04705
Brachypodiumdistachyon_Bradi1g53770.1.p TMHMM2.0        outside      1   279
```

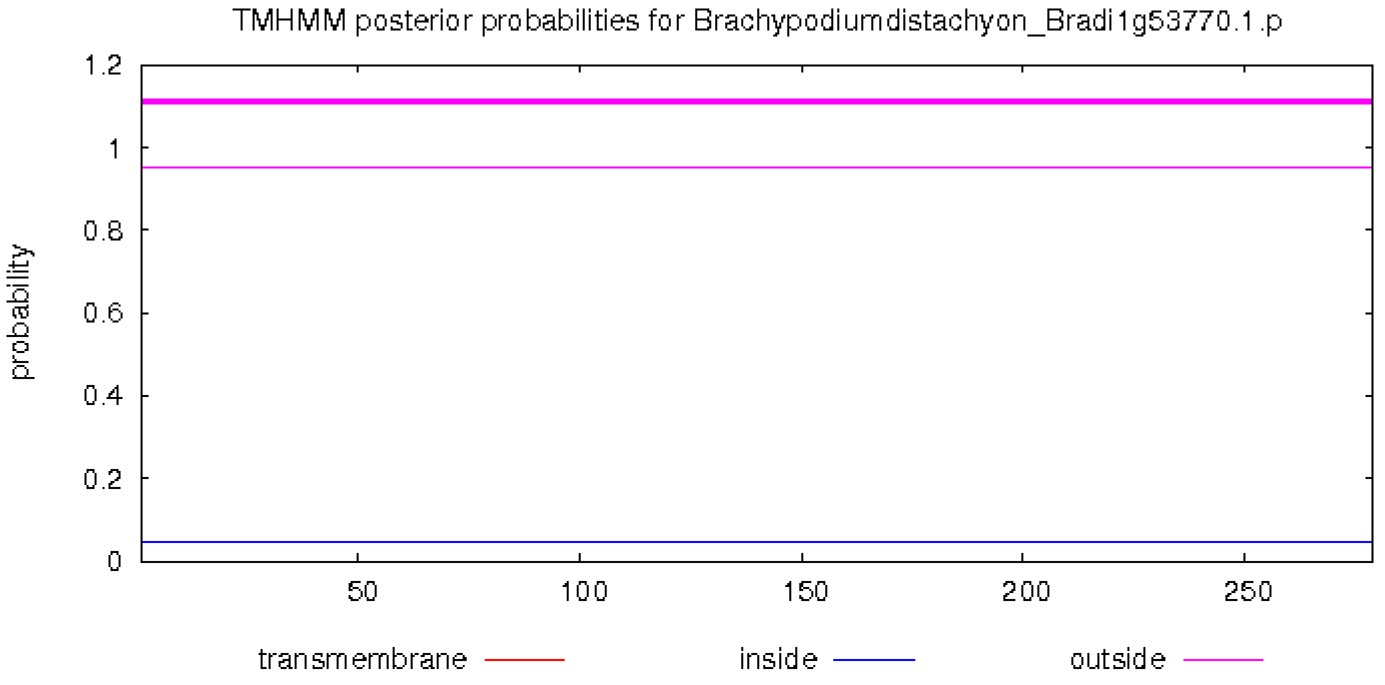

plot in postscript, script for making the plot in gnuplot, data for plot

```
# Brachypodiumdistachyon_Bradi1g58057.1.p Length: 328
# Brachypodiumdistachyon_Bradi1g58057.1.p Number of predicted TMHs:  0
# Brachypodiumdistachyon_Bradi1g58057.1.p Exp number of AAs in TMHs: 0.00132
# Brachypodiumdistachyon_Bradi1g58057.1.p Exp number, first 60 AAs:  0
# Brachypodiumdistachyon_Bradi1g58057.1.p Total prob of N-in:        0.01359
Brachypodiumdistachyon_Bradi1g58057.1.p TMHMM2.0        outside      1   328
```

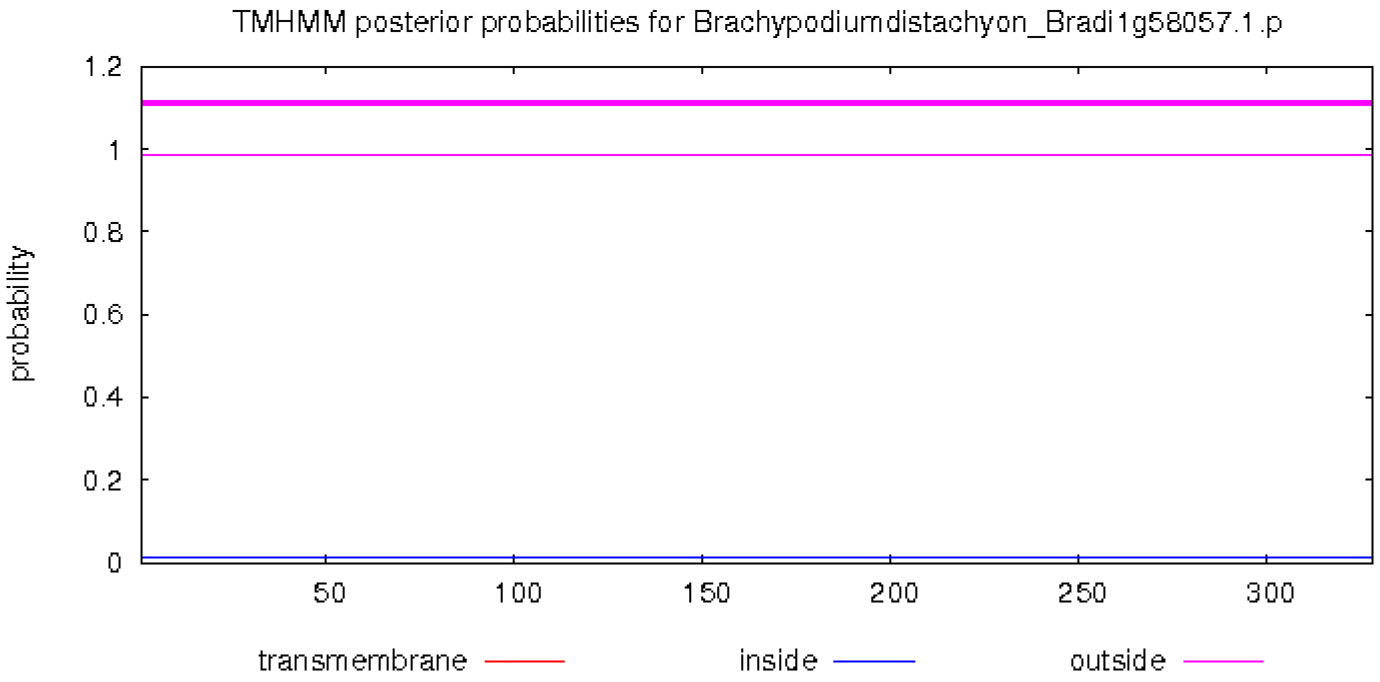

plot in postscript, script for making the plot in gnuplot, data for plot

---

```
# Brachypodiumdistachyon_Bradi1g61525.1.p Length: 205
# Brachypodiumdistachyon_Bradi1g61525.1.p Number of predicted TMHs:  0
# Brachypodiumdistachyon_Bradi1g61525.1.p Exp number of AAs in TMHs: 0.0015
# Brachypodiumdistachyon_Bradi1g61525.1.p Exp number, first 60 AAs:  0.0015
# Brachypodiumdistachyon_Bradi1g61525.1.p Total prob of N-in:        0.06800
Brachypodiumdistachyon_Bradi1g61525.1.p TMHMM2.0        outside      1   205
```

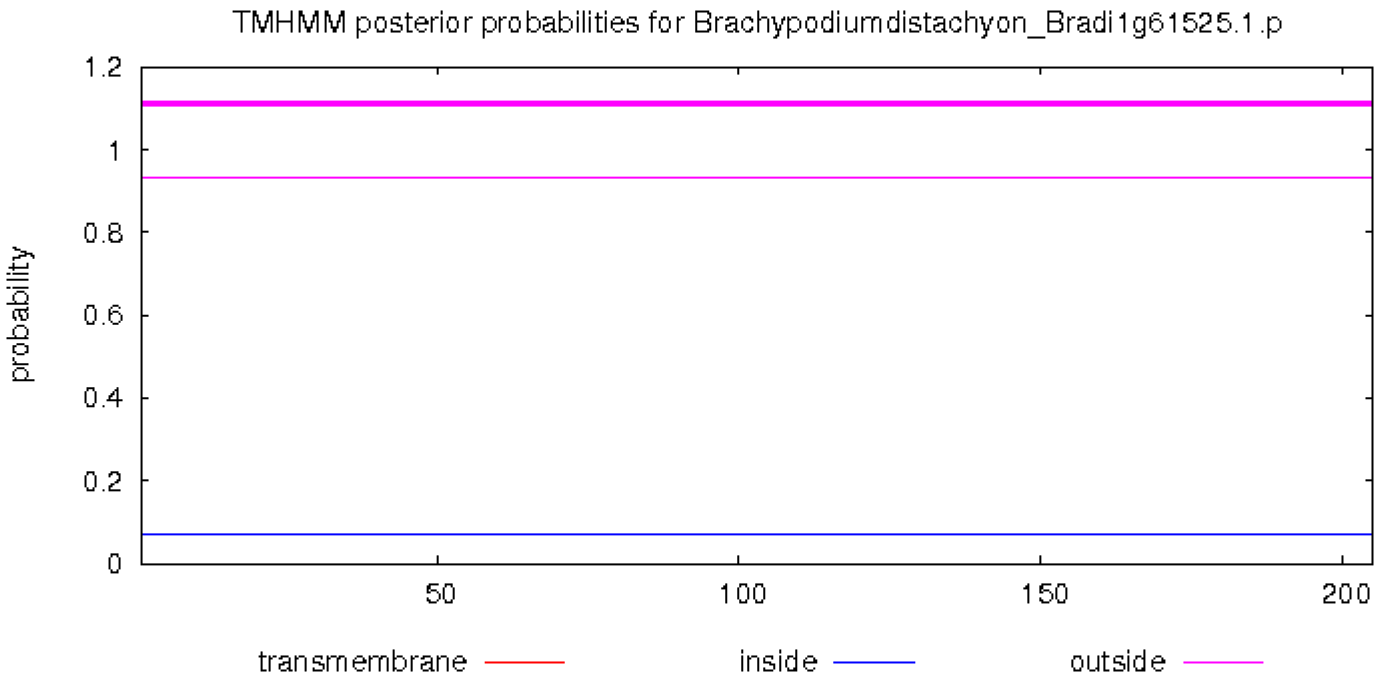

plot in postscript, script for making the plot in gnuplot, data for plot

---

```
# Brachypodiumdistachyon_Bradi1g63600.1.p Length: 347
# Brachypodiumdistachyon_Bradi1g63600.1.p Number of predicted TMHs:  0
# Brachypodiumdistachyon_Bradi1g63600.1.p Exp number of AAs in TMHs: 0.00231
# Brachypodiumdistachyon_Bradi1g63600.1.p Exp number, first 60 AAs:  0
# Brachypodiumdistachyon_Bradi1g63600.1.p Total prob of N-in:        0.00329
Brachypodiumdistachyon_Bradi1g63600.1.p TMHMM2.0        outside      1   347
```

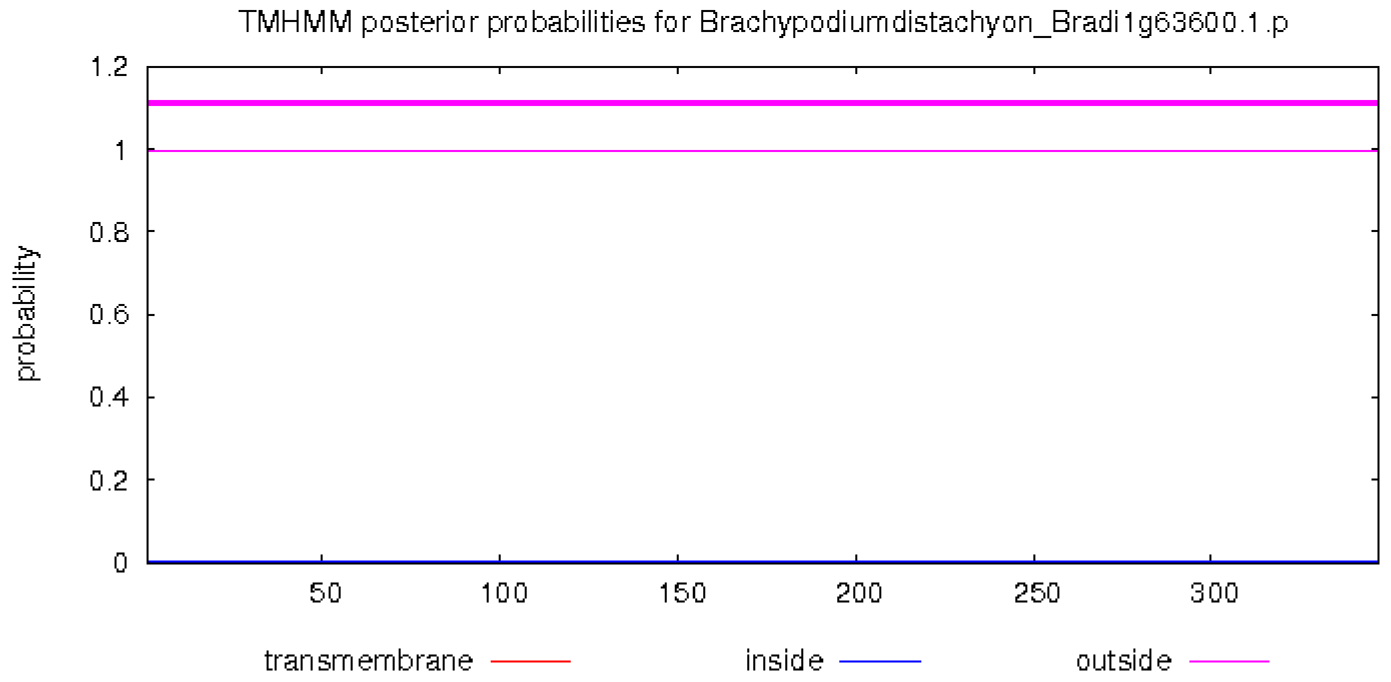

plot in postscript, script for making the plot in gnuplot, data for plot

---

```
# Brachypodiumdistachyon_Bradi1g63630.1.p Length: 358
# Brachypodiumdistachyon_Bradi1g63630.1.p Number of predicted TMHs:  0
# Brachypodiumdistachyon_Bradi1g63630.1.p Exp number of AAs in TMHs: 0.00469
# Brachypodiumdistachyon_Bradi1g63630.1.p Exp number, first 60 AAs:  0
# Brachypodiumdistachyon_Bradi1g63630.1.p Total prob of N-in:        0.01736
Brachypodiumdistachyon_Bradi1g63630.1.p TMHMM2.0        outside      1   358
```

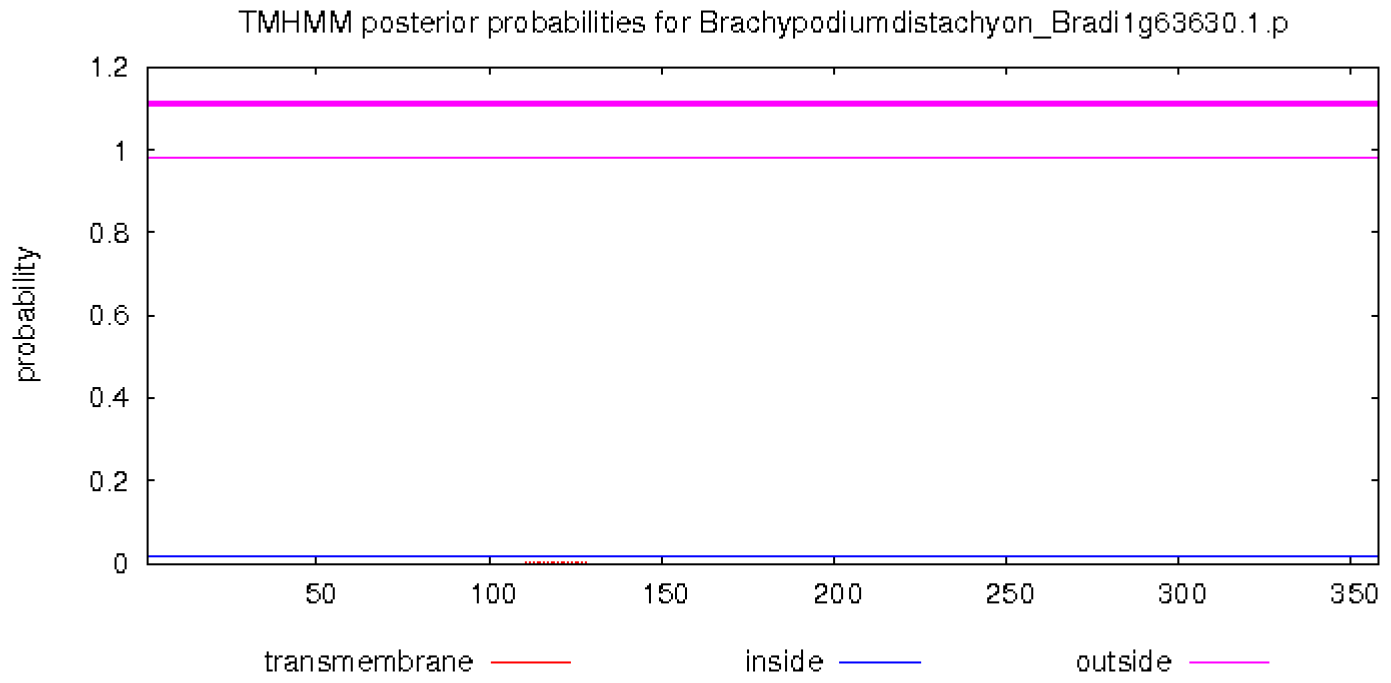

plot in postscript, script for making the plot in gnuplot, data for plot

---

```
# Brachypodiumdistachyon_Bradi1g69617.2.p Length: 846
# Brachypodiumdistachyon_Bradi1g69617.2.p Number of predicted TMHs:  0
# Brachypodiumdistachyon_Bradi1g69617.2.p Exp number of AAs in TMHs: 0.00402
# Brachypodiumdistachyon_Bradi1g69617.2.p Exp number, first 60 AAs:  0
# Brachypodiumdistachyon_Bradi1g69617.2.p Total prob of N-in:        0.00010
Brachypodiumdistachyon_Bradi1g69617.2.p TMHMM2.0        outside      1   846
```

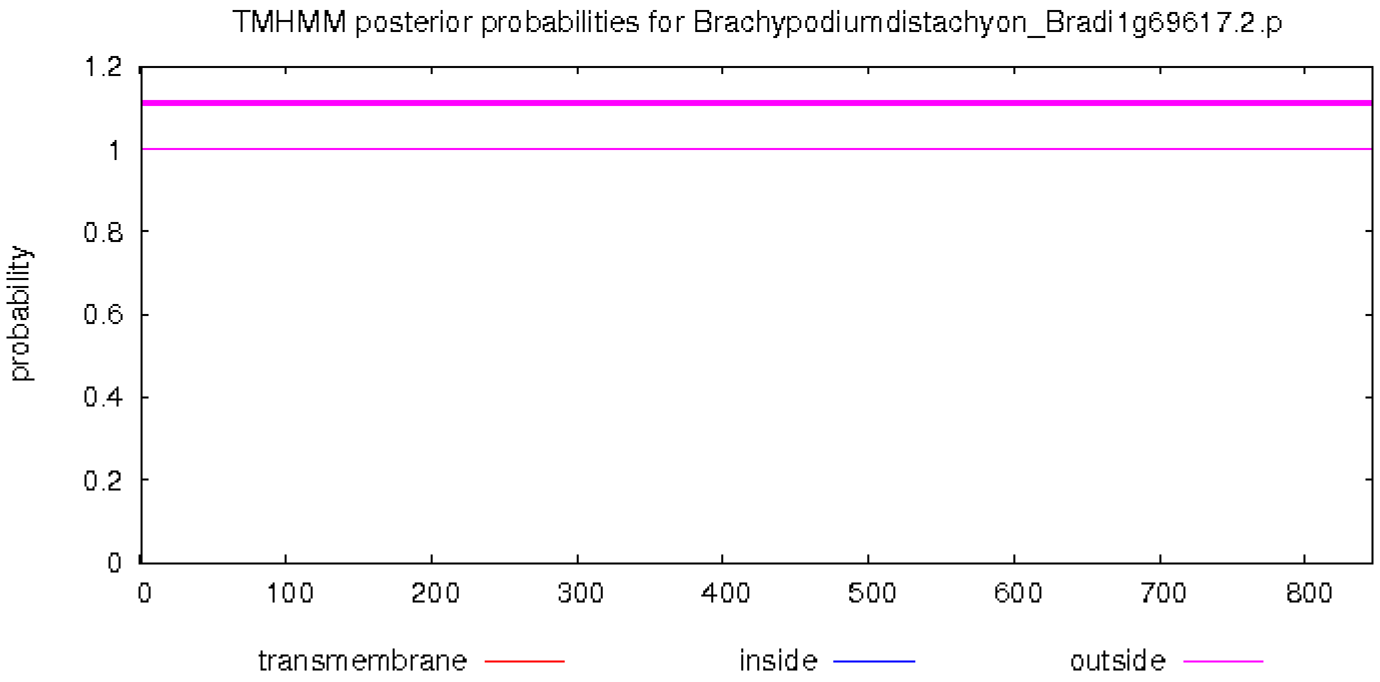

plot in postscript, script for making the plot in gnuplot, data for plot

---

```
# Brachypodiumdistachyon_Bradi1g76207.2.p Length: 363
# Brachypodiumdistachyon_Bradi1g76207.2.p Number of predicted TMHs:  0
# Brachypodiumdistachyon_Bradi1g76207.2.p Exp number of AAs in TMHs: 0.0004
# Brachypodiumdistachyon_Bradi1g76207.2.p Exp number, first 60 AAs:  0.00023
# Brachypodiumdistachyon_Bradi1g76207.2.p Total prob of N-in:        0.01011
Brachypodiumdistachyon_Bradi1g76207.2.p TMHMM2.0        outside      1   363
```

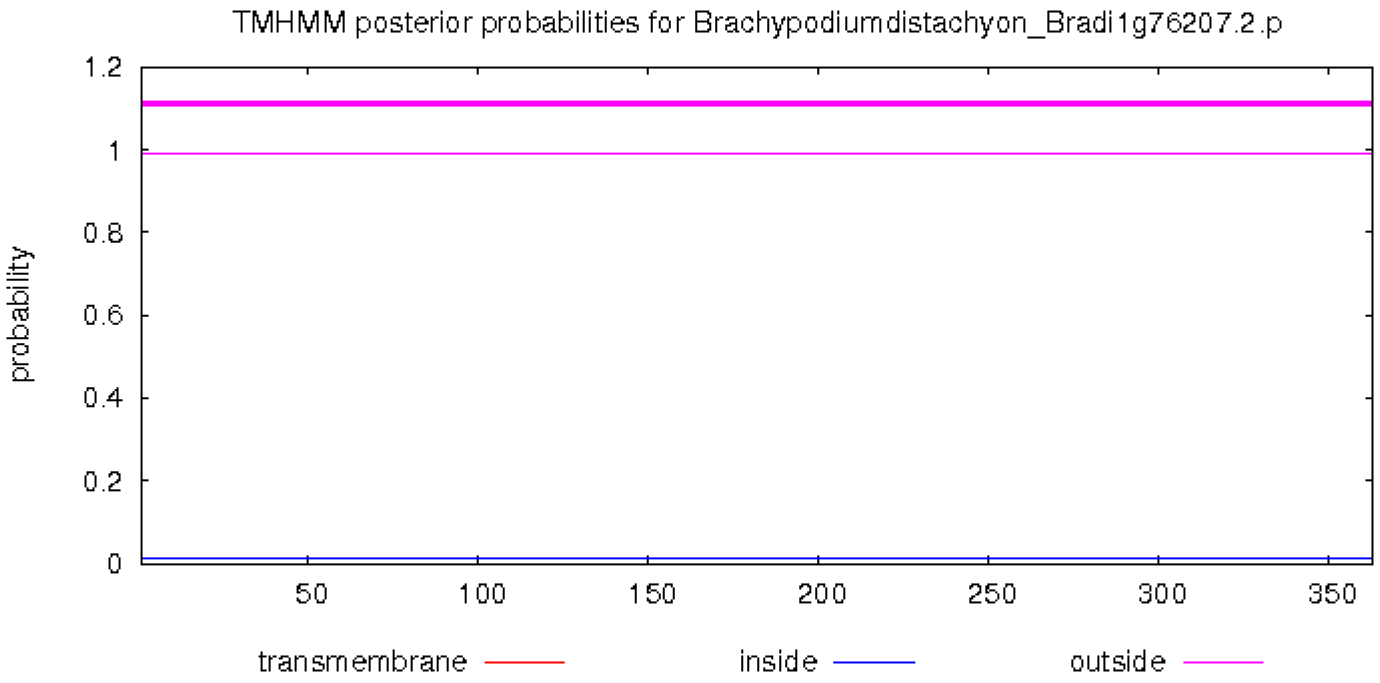

plot in postscript, script for making the plot in gnuplot, data for plot

---

```
# Brachypodiumdistachyon_Bradi1g76732.1.p Length: 364
# Brachypodiumdistachyon_Bradi1g76732.1.p Number of predicted TMHs:  0
# Brachypodiumdistachyon_Bradi1g76732.1.p Exp number of AAs in TMHs: 0.00936
# Brachypodiumdistachyon_Bradi1g76732.1.p Exp number, first 60 AAs:  0
# Brachypodiumdistachyon_Bradi1g76732.1.p Total prob of N-in:        0.00503
Brachypodiumdistachyon_Bradi1g76732.1.p TMHMM2.0        outside      1   364
```

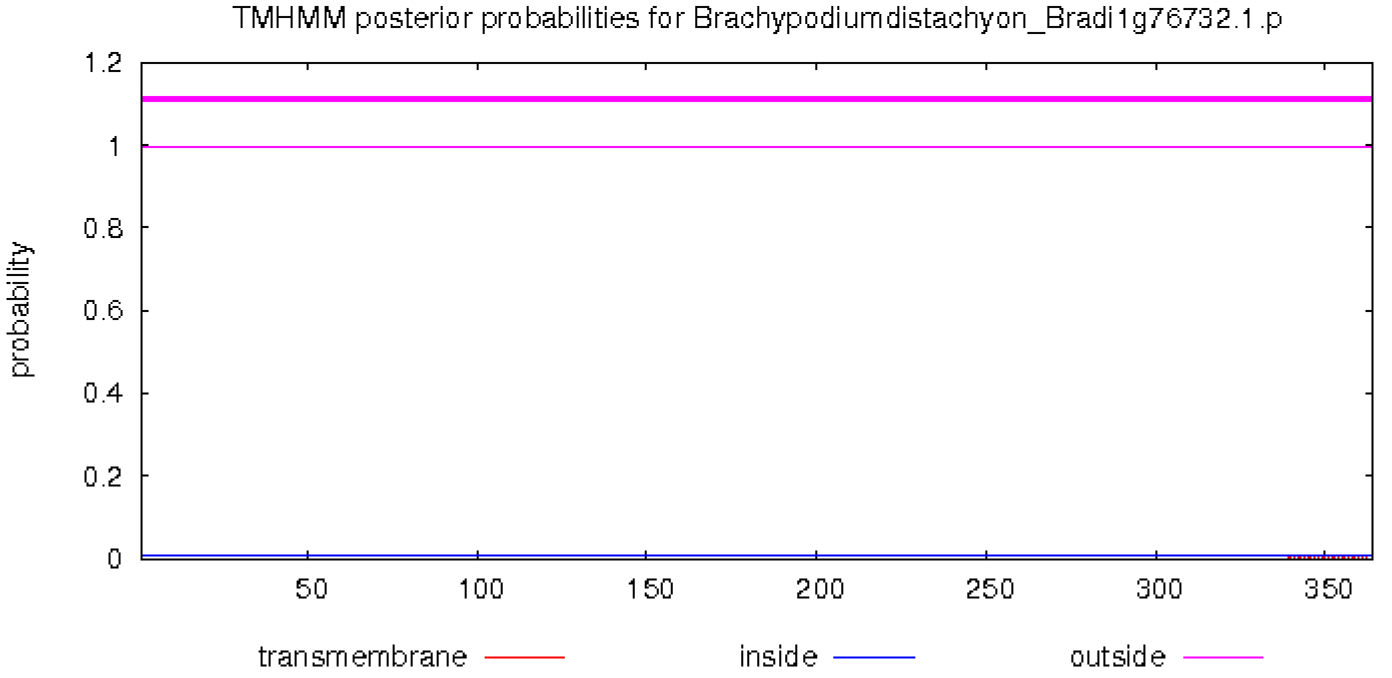

plot in postscript, script for making the plot in gnuplot, data for plot

```
# Brachypodiumdistachyon_Bradi1g77217.1.p Length: 710
# Brachypodiumdistachyon_Bradi1g77217.1.p Number of predicted TMHs:  0
# Brachypodiumdistachyon_Bradi1g77217.1.p Exp number of AAs in TMHs: 0.60461
# Brachypodiumdistachyon_Bradi1g77217.1.p Exp number, first 60 AAs:  0
# Brachypodiumdistachyon_Bradi1g77217.1.p Total prob of N-in:        0.00002
Brachypodiumdistachyon_Bradi1g77217.1.p TMHMM2.0        outside      1   710
```

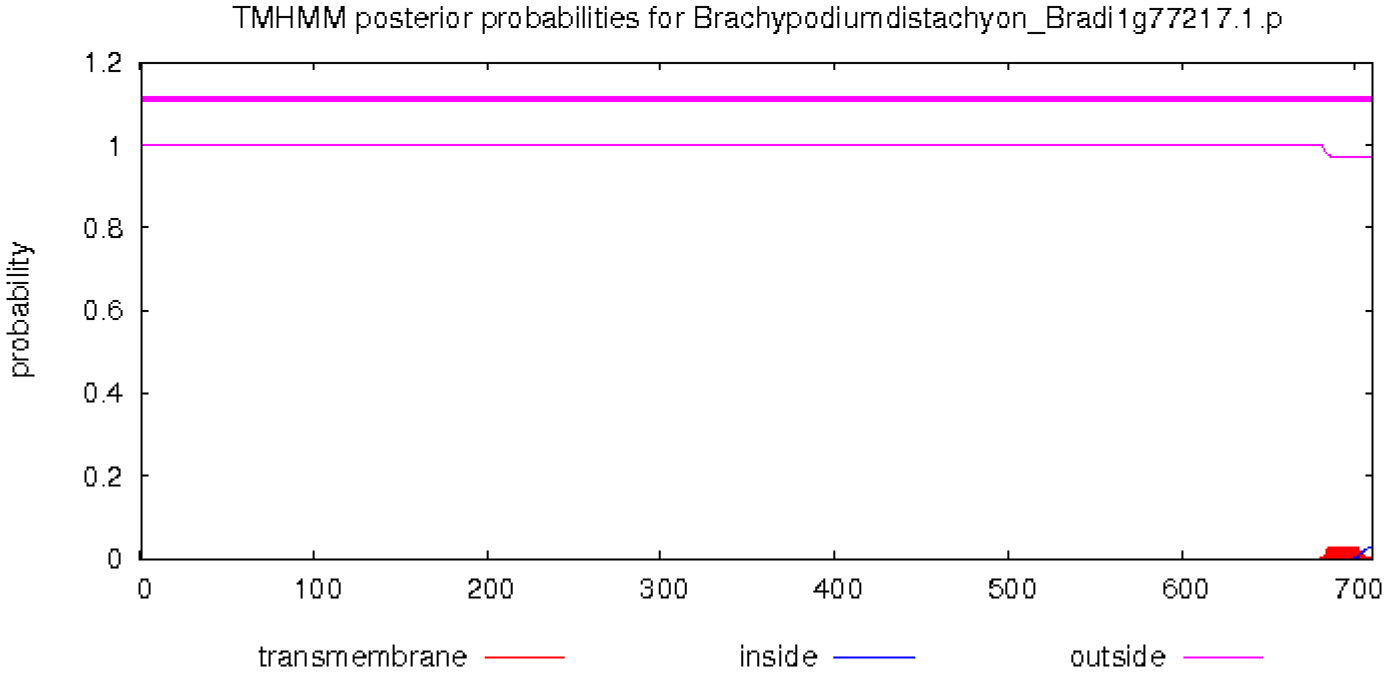

plot in postscript, script for making the plot in gnuplot, data for plot

```
# Brachypodiumdistachyon_Bradi2g03448.1.p Length: 350
# Brachypodiumdistachyon_Bradi2g03448.1.p Number of predicted TMHs:  0
# Brachypodiumdistachyon_Bradi2g03448.1.p Exp number of AAs in TMHs: 0.0058
# Brachypodiumdistachyon_Bradi2g03448.1.p Exp number, first 60 AAs:  0
# Brachypodiumdistachyon_Bradi2g03448.1.p Total prob of N-in:        0.03552
Brachypodiumdistachyon_Bradi2g03448.1.p TMHMM2.0        outside      1   350
```

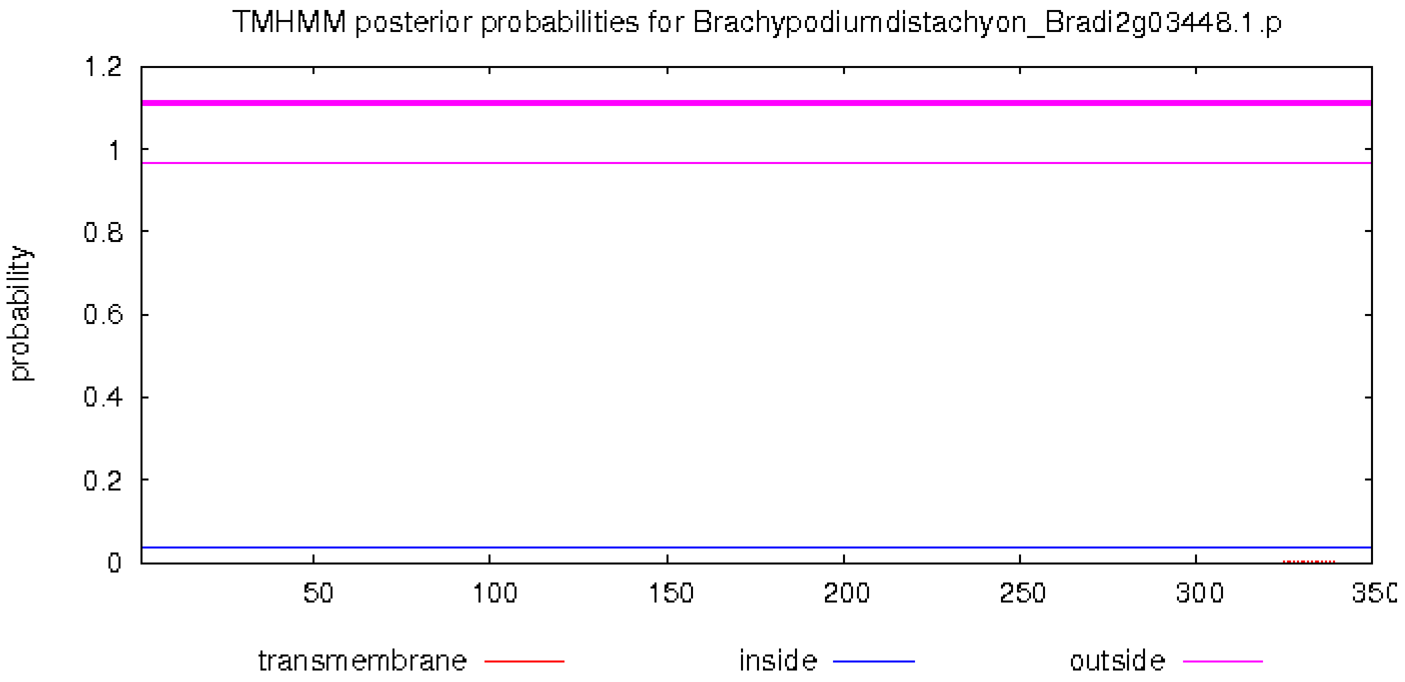

plot in postscript, script for making the plot in gnuplot, data for plot

---

```
# Brachypodiumdistachyon_Bradi2g03467.1.p Length: 337
# Brachypodiumdistachyon_Bradi2g03467.1.p Number of predicted TMHs:  0
# Brachypodiumdistachyon_Bradi2g03467.1.p Exp number of AAs in TMHs: 0.00133
# Brachypodiumdistachyon_Bradi2g03467.1.p Exp number, first 60 AAs:  0.00055
# Brachypodiumdistachyon_Bradi2g03467.1.p Total prob of N-in:        0.14936
Brachypodiumdistachyon_Bradi2g03467.1.p TMHMM2.0        outside      1   337
```

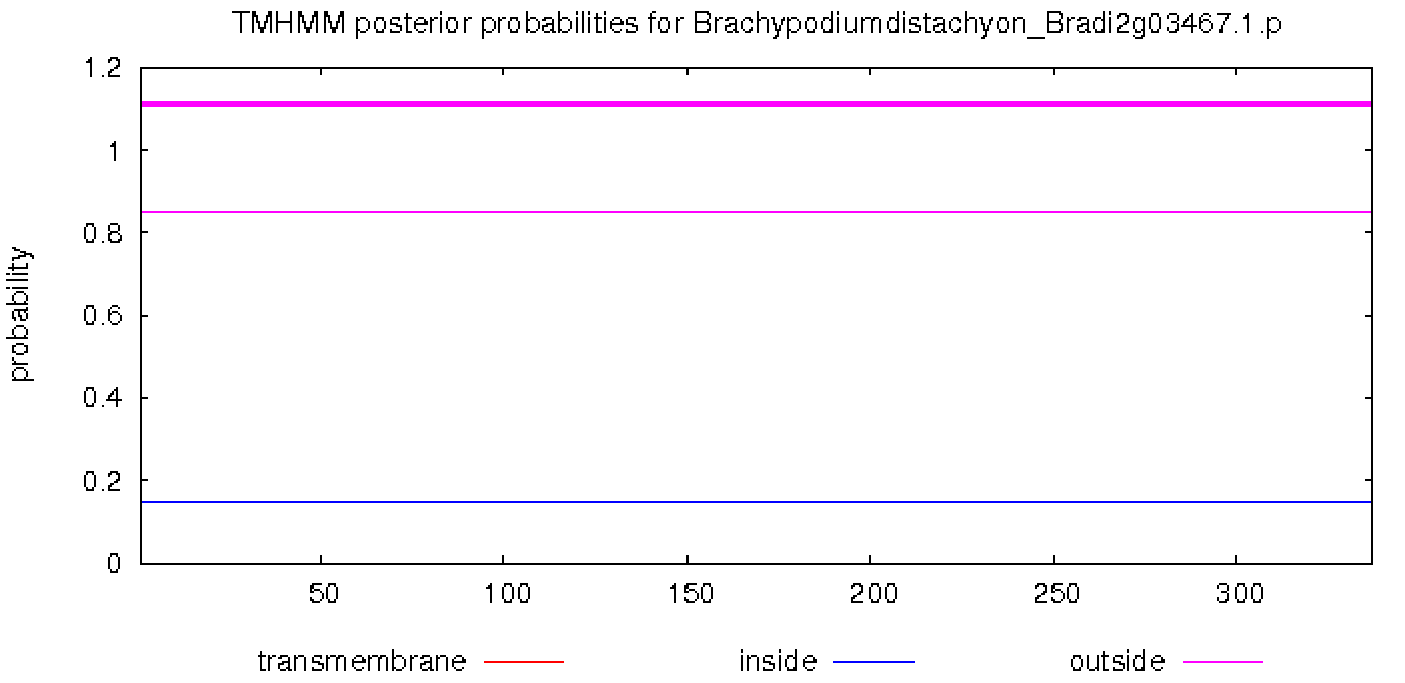

plot in postscript, script for making the plot in gnuplot, data for plot

---

```
# Brachypodiumdistachyon_Bradi2g05700.2.p Length: 439
# Brachypodiumdistachyon_Bradi2g05700.2.p Number of predicted TMHs:  0
# Brachypodiumdistachyon_Bradi2g05700.2.p Exp number of AAs in TMHs: 0.0134
# Brachypodiumdistachyon_Bradi2g05700.2.p Exp number, first 60 AAs:  0
# Brachypodiumdistachyon_Bradi2g05700.2.p Total prob of N-in:        0.00430
Brachypodiumdistachyon_Bradi2g05700.2.p TMHMM2.0        outside      1   439
```

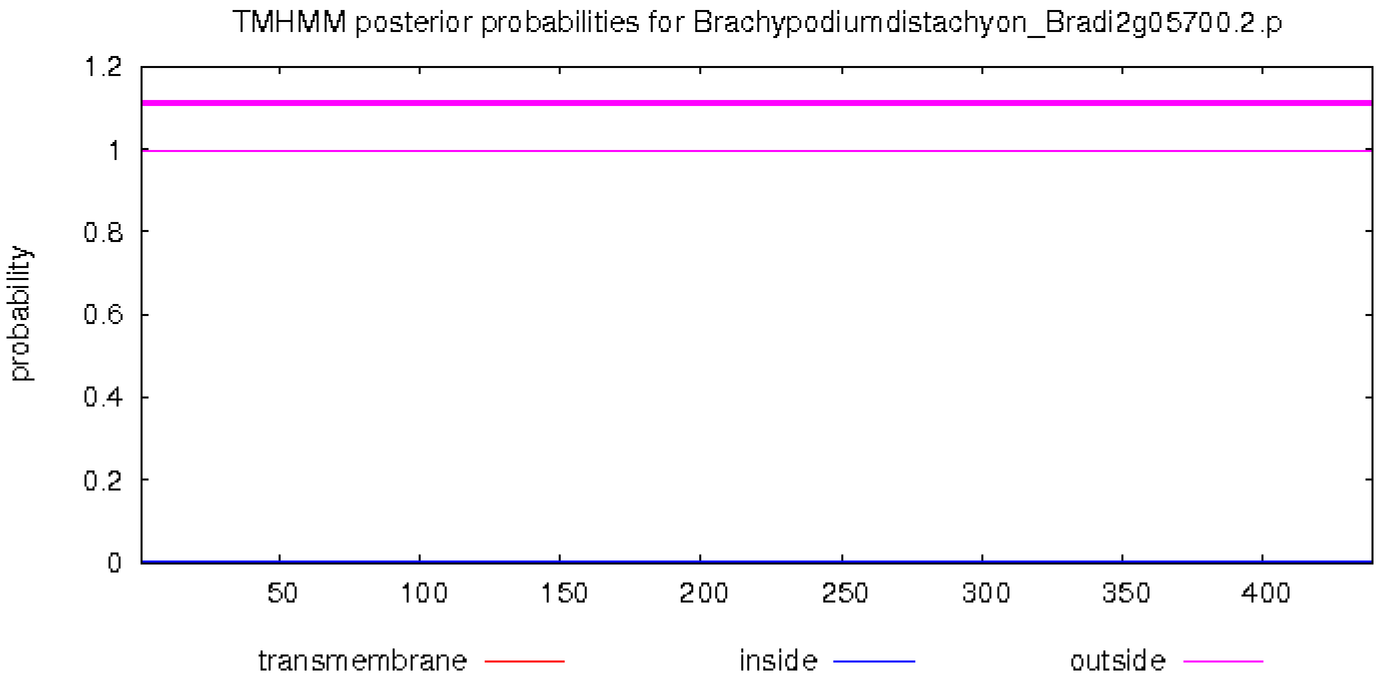

plot in postscript, script for making the plot in gnuplot, data for plot

---

```
# Brachypodiumdistachyon_Bradi2g08366.1.p Length: 287
# Brachypodiumdistachyon_Bradi2g08366.1.p Number of predicted TMHs:  0
# Brachypodiumdistachyon_Bradi2g08366.1.p Exp number of AAs in TMHs: 0.00149
# Brachypodiumdistachyon_Bradi2g08366.1.p Exp number, first 60 AAs:  0.00098
# Brachypodiumdistachyon_Bradi2g08366.1.p Total prob of N-in:        0.00788
Brachypodiumdistachyon_Bradi2g08366.1.p TMHMM2.0        outside      1   287
```

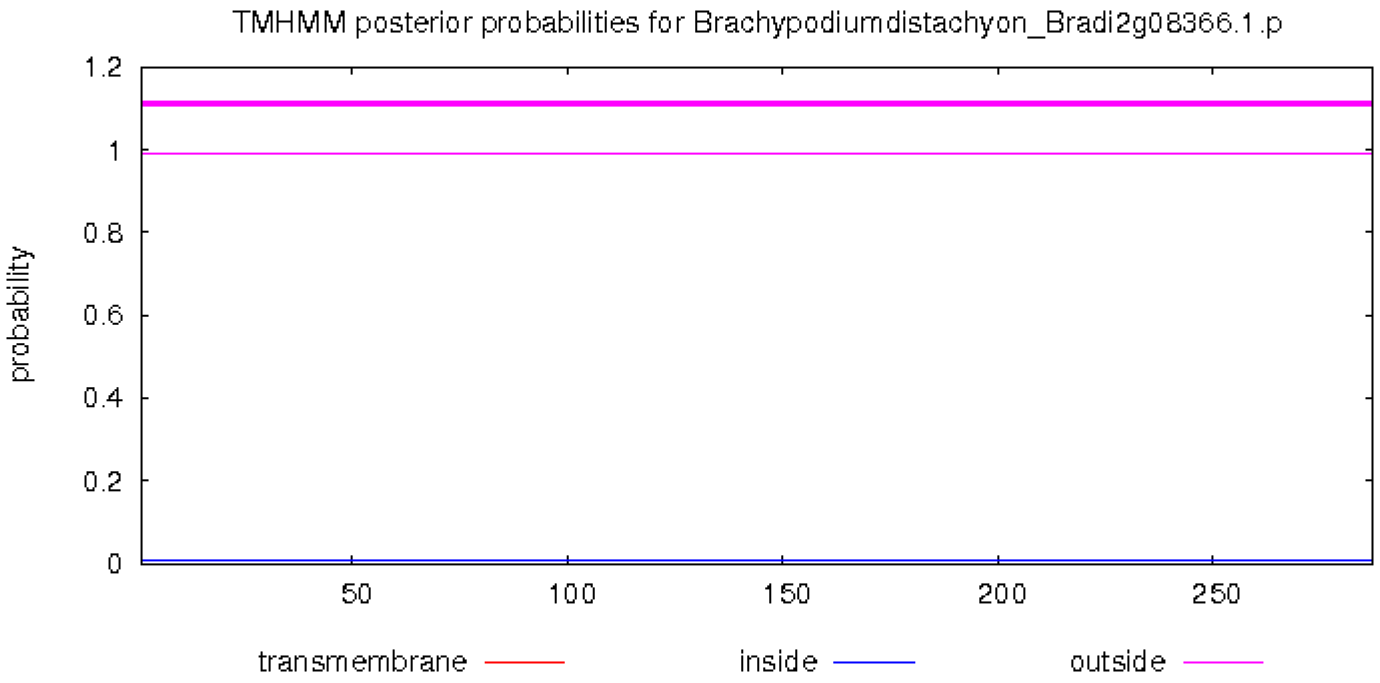

plot in postscript, script for making the plot in gnuplot, data for plot

---

```
# Brachypodiumdistachyon_Bradi2g09530.1.p Length: 495
# Brachypodiumdistachyon_Bradi2g09530.1.p Number of predicted TMHs:  1
# Brachypodiumdistachyon_Bradi2g09530.1.p Exp number of AAs in TMHs: 21.72776
# Brachypodiumdistachyon_Bradi2g09530.1.p Exp number, first 60 AAs:  0
# Brachypodiumdistachyon_Bradi2g09530.1.p Total prob of N-in:        0.08402
Brachypodiumdistachyon_Bradi2g09530.1.p TMHMM2.0        outside      1   460
Brachypodiumdistachyon_Bradi2g09530.1.p TMHMM2.0        TMhelix    461   483
Brachypodiumdistachyon_Bradi2g09530.1.p TMHMM2.0        inside     484   495
```

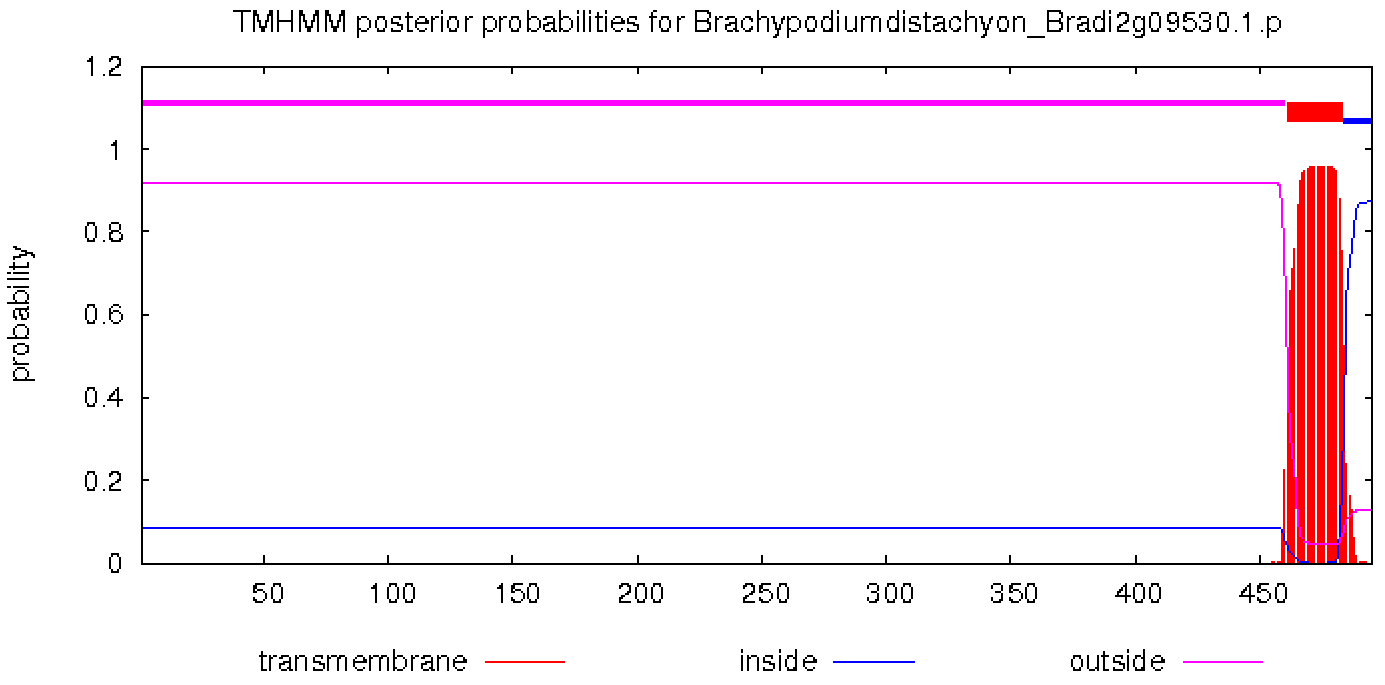

plot in postscript, script for making the plot in gnuplot, data for plot

---

```
# Brachypodiumdistachyon_Bradi2g20590.1.p Length: 340
# Brachypodiumdistachyon_Bradi2g20590.1.p Number of predicted TMHs:  0
# Brachypodiumdistachyon_Bradi2g20590.1.p Exp number of AAs in TMHs: 0.0002
# Brachypodiumdistachyon_Bradi2g20590.1.p Exp number, first 60 AAs:  1e-05
# Brachypodiumdistachyon_Bradi2g20590.1.p Total prob of N-in:        0.00346
Brachypodiumdistachyon_Bradi2g20590.1.p TMHMM2.0        outside      1   340
```

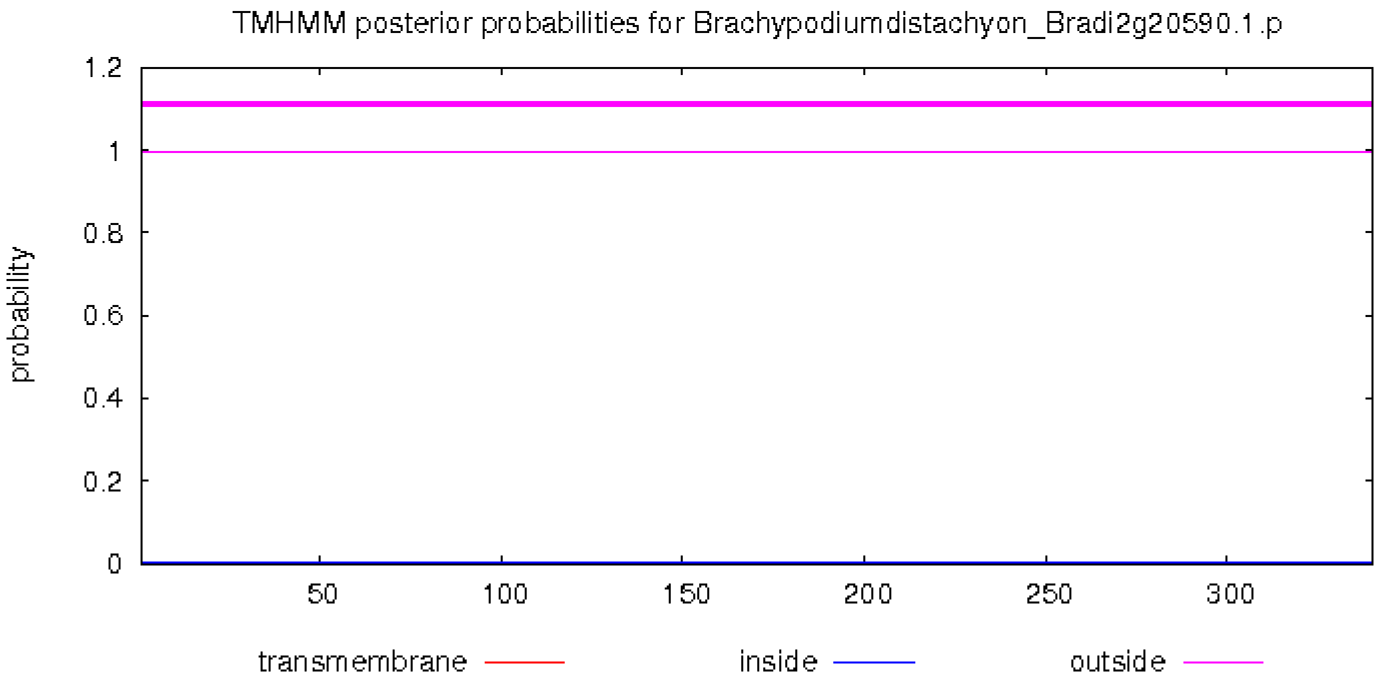

plot in postscript, script for making the plot in gnuplot, data for plot

```
# Brachypodiumdistachyon_Bradi2g23967.1.p Length: 264
# Brachypodiumdistachyon_Bradi2g23967.1.p Number of predicted TMHs:  0
# Brachypodiumdistachyon_Bradi2g23967.1.p Exp number of AAs in TMHs: 0.00283
# Brachypodiumdistachyon_Bradi2g23967.1.p Exp number, first 60 AAs:  0
# Brachypodiumdistachyon_Bradi2g23967.1.p Total prob of N-in:        0.00541
Brachypodiumdistachyon_Bradi2g23967.1.p TMHMM2.0        outside      1   264
```

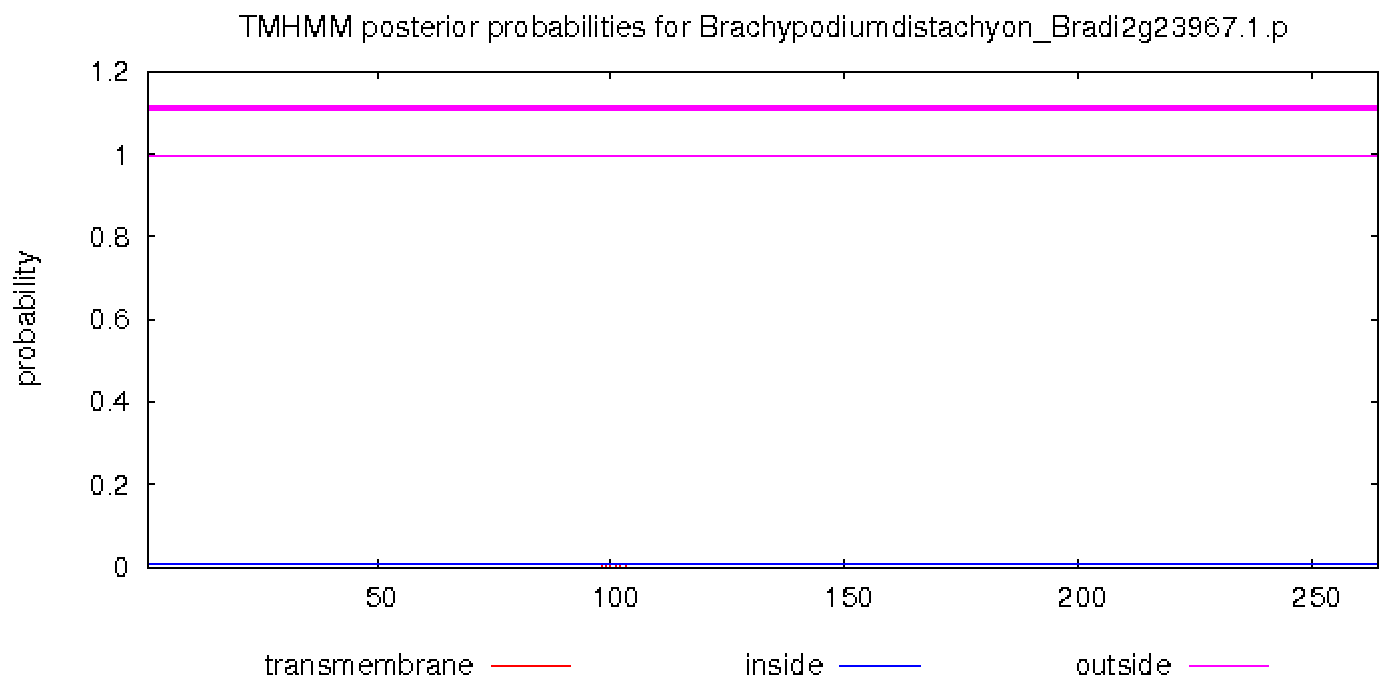

plot in postscript, script for making the plot in gnuplot, data for plot

```
# Brachypodiumdistachyon_Bradi2g24790.1.p Length: 464
# Brachypodiumdistachyon_Bradi2g24790.1.p Number of predicted TMHs:  0
# Brachypodiumdistachyon_Bradi2g24790.1.p Exp number of AAs in TMHs: 0.01072
# Brachypodiumdistachyon_Bradi2g24790.1.p Exp number, first 60 AAs:  0.00033
# Brachypodiumdistachyon_Bradi2g24790.1.p Total prob of N-in:        0.00082
Brachypodiumdistachyon_Bradi2g24790.1.p TMHMM2.0        outside      1   464
```

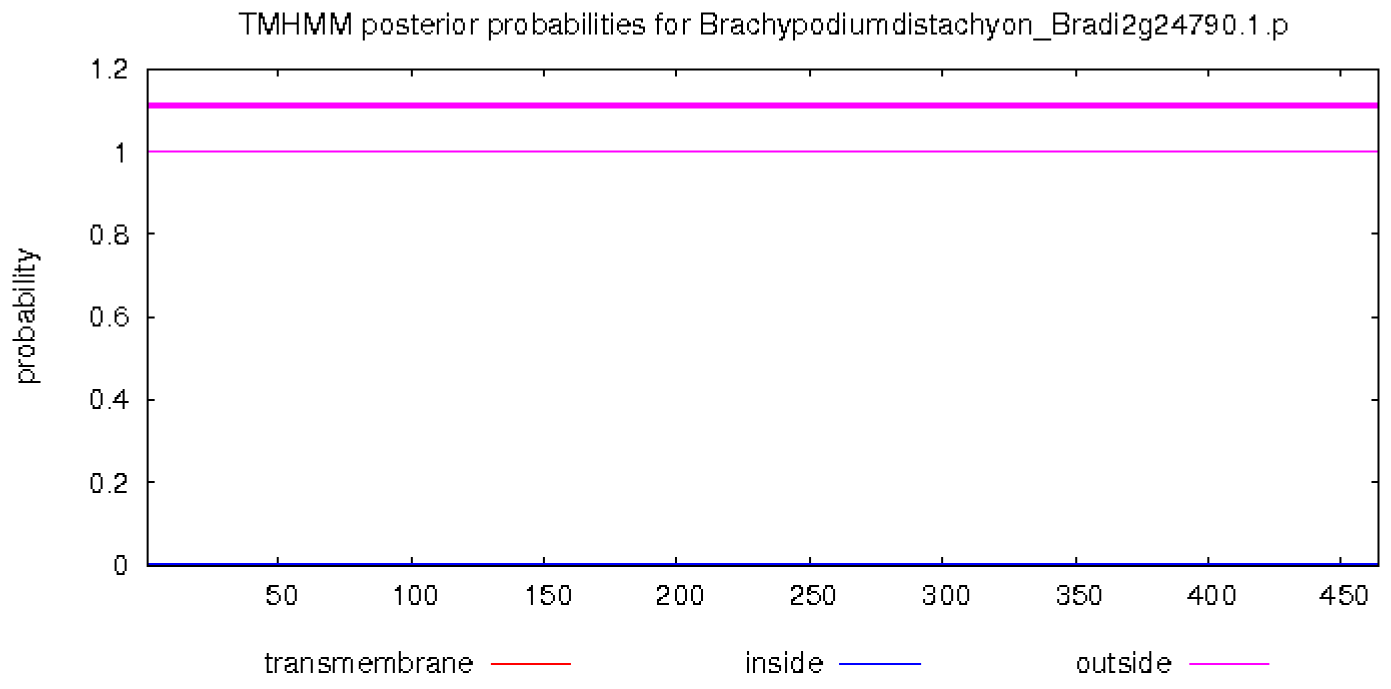

plot in postscript, script for making the plot in gnuplot, data for plot

```
# Brachypodiumdistachyon_Bradi2g24987.1.p Length: 308
# Brachypodiumdistachyon_Bradi2g24987.1.p Number of predicted TMHs:  0
# Brachypodiumdistachyon_Bradi2g24987.1.p Exp number of AAs in TMHs: 0.00295
# Brachypodiumdistachyon_Bradi2g24987.1.p Exp number, first 60 AAs:  0.00155
# Brachypodiumdistachyon_Bradi2g24987.1.p Total prob of N-in:        0.00506
Brachypodiumdistachyon_Bradi2g24987.1.p TMHMM2.0        outside      1   308
```

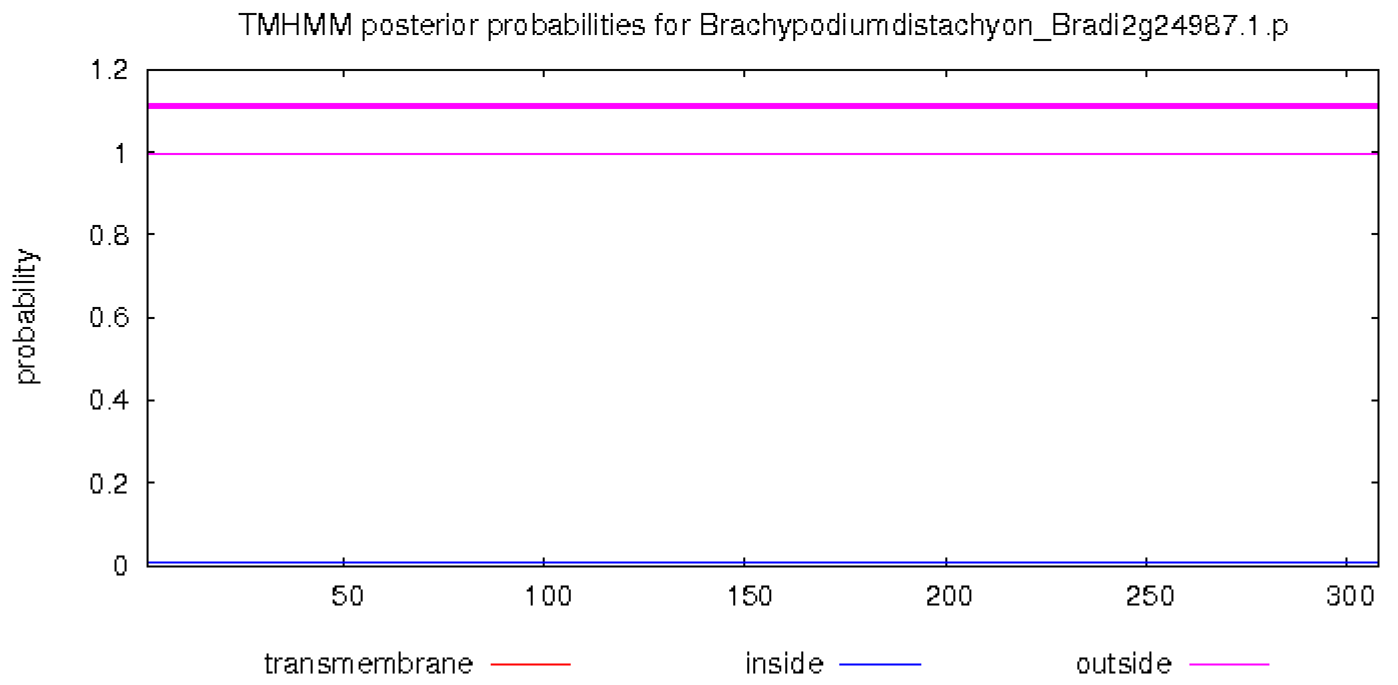

plot in postscript, script for making the plot in gnuplot, data for plot

---

```
# Brachypodiumdistachyon_Bradi2g25150.1.p Length: 368
# Brachypodiumdistachyon_Bradi2g25150.1.p Number of predicted TMHs:  0
# Brachypodiumdistachyon_Bradi2g25150.1.p Exp number of AAs in TMHs: 0.00207
# Brachypodiumdistachyon_Bradi2g25150.1.p Exp number, first 60 AAs:  0
# Brachypodiumdistachyon_Bradi2g25150.1.p Total prob of N-in:        0.01253
Brachypodiumdistachyon_Bradi2g25150.1.p TMHMM2.0        outside      1   368
```

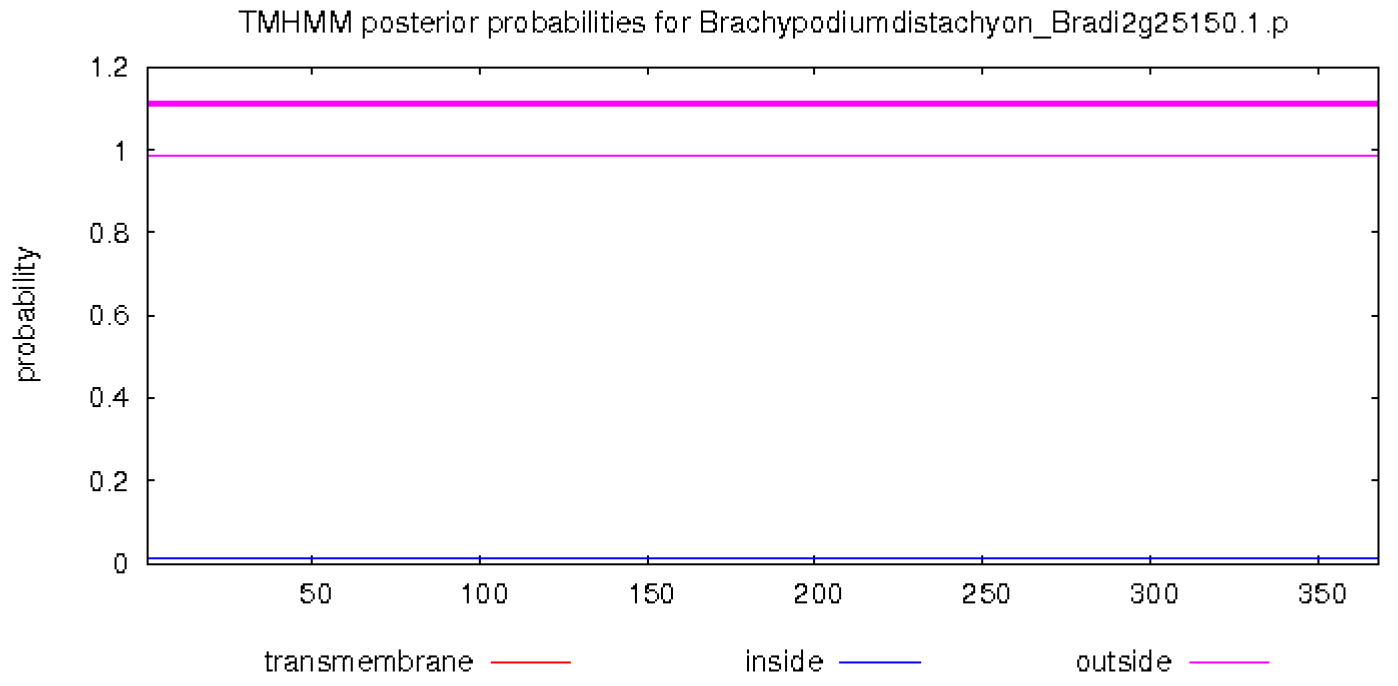

plot in postscript, script for making the plot in gnuplot, data for plot

---

```
# Brachypodiumdistachyon_Bradi2g28020.1.p Length: 338
# Brachypodiumdistachyon_Bradi2g28020.1.p Number of predicted TMHs:  0
# Brachypodiumdistachyon_Bradi2g28020.1.p Exp number of AAs in TMHs: 0.00116
# Brachypodiumdistachyon_Bradi2g28020.1.p Exp number, first 60 AAs:  0.00096
# Brachypodiumdistachyon_Bradi2g28020.1.p Total prob of N-in:        0.00478
Brachypodiumdistachyon_Bradi2g28020.1.p TMHMM2.0        outside      1   338
```

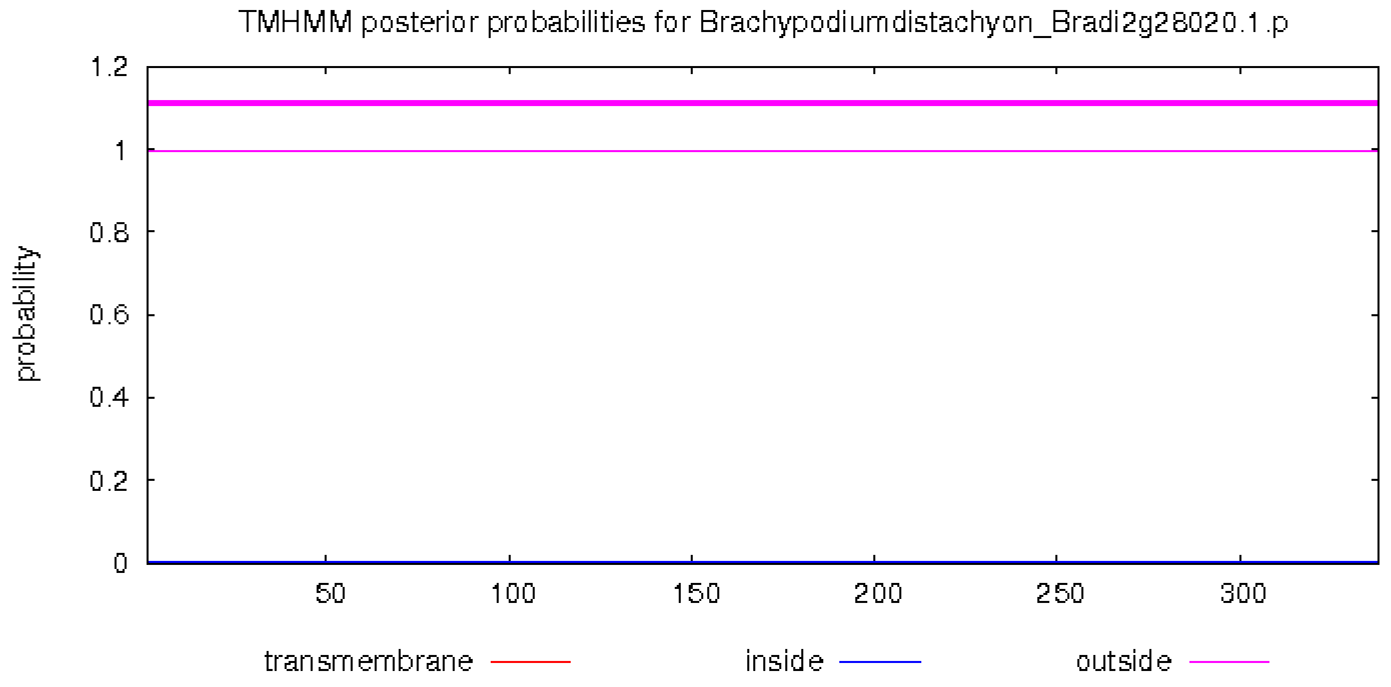

plot in postscript, script for making the plot in gnuplot, data for plot

---

```
# Brachypodiumdistachyon_Bradi2g33307.2.p Length: 262
# Brachypodiumdistachyon_Bradi2g33307.2.p Number of predicted TMHs:  0
# Brachypodiumdistachyon_Bradi2g33307.2.p Exp number of AAs in TMHs: 2.55809
# Brachypodiumdistachyon_Bradi2g33307.2.p Exp number, first 60 AAs:  0
# Brachypodiumdistachyon_Bradi2g33307.2.p Total prob of N-in:        0.15374
Brachypodiumdistachyon_Bradi2g33307.2.p TMHMM2.0        outside      1   262
```

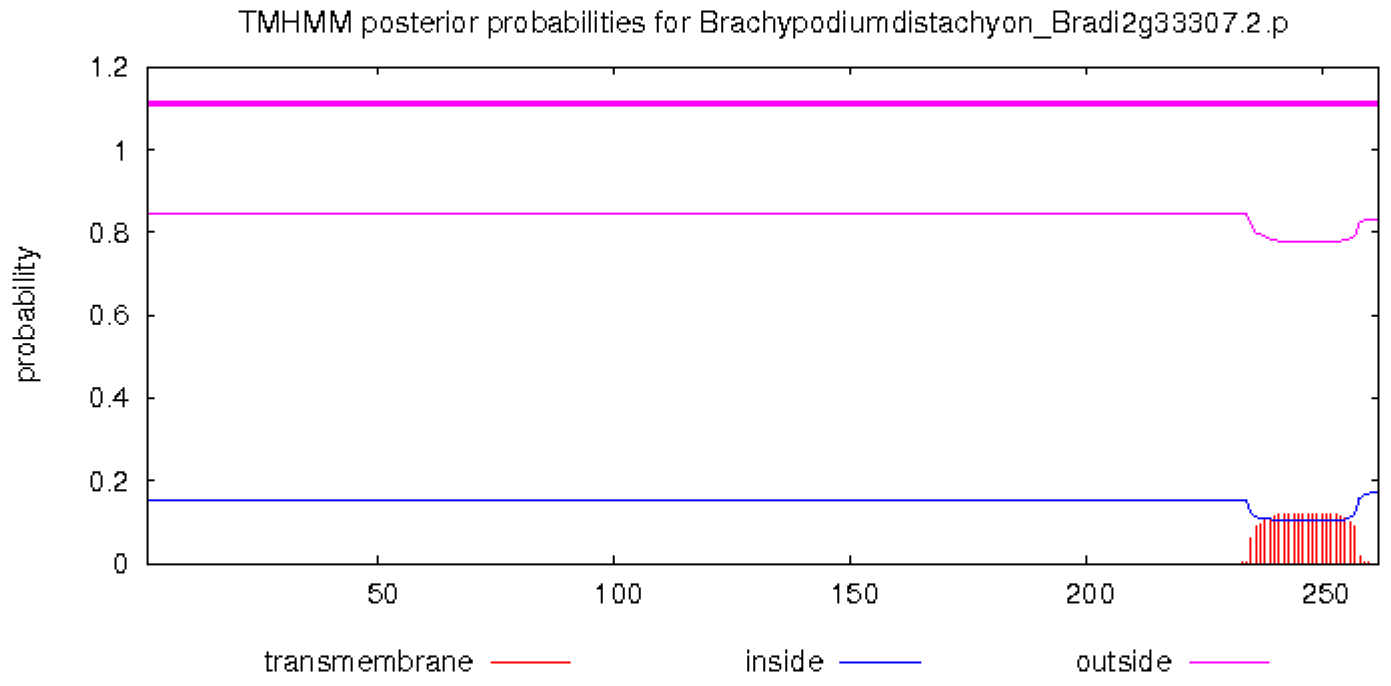

plot in postscript, script for making the plot in gnuplot, data for plot

---

```
# Brachypodiumdistachyon_Bradi2g40026.1.p Length: 354
# Brachypodiumdistachyon_Bradi2g40026.1.p Number of predicted TMHs:  0
# Brachypodiumdistachyon_Bradi2g40026.1.p Exp number of AAs in TMHs: 0.11354
# Brachypodiumdistachyon_Bradi2g40026.1.p Exp number, first 60 AAs:  0.00558
# Brachypodiumdistachyon_Bradi2g40026.1.p Total prob of N-in:        0.01003
Brachypodiumdistachyon_Bradi2g40026.1.p TMHMM2.0        outside      1   354
```

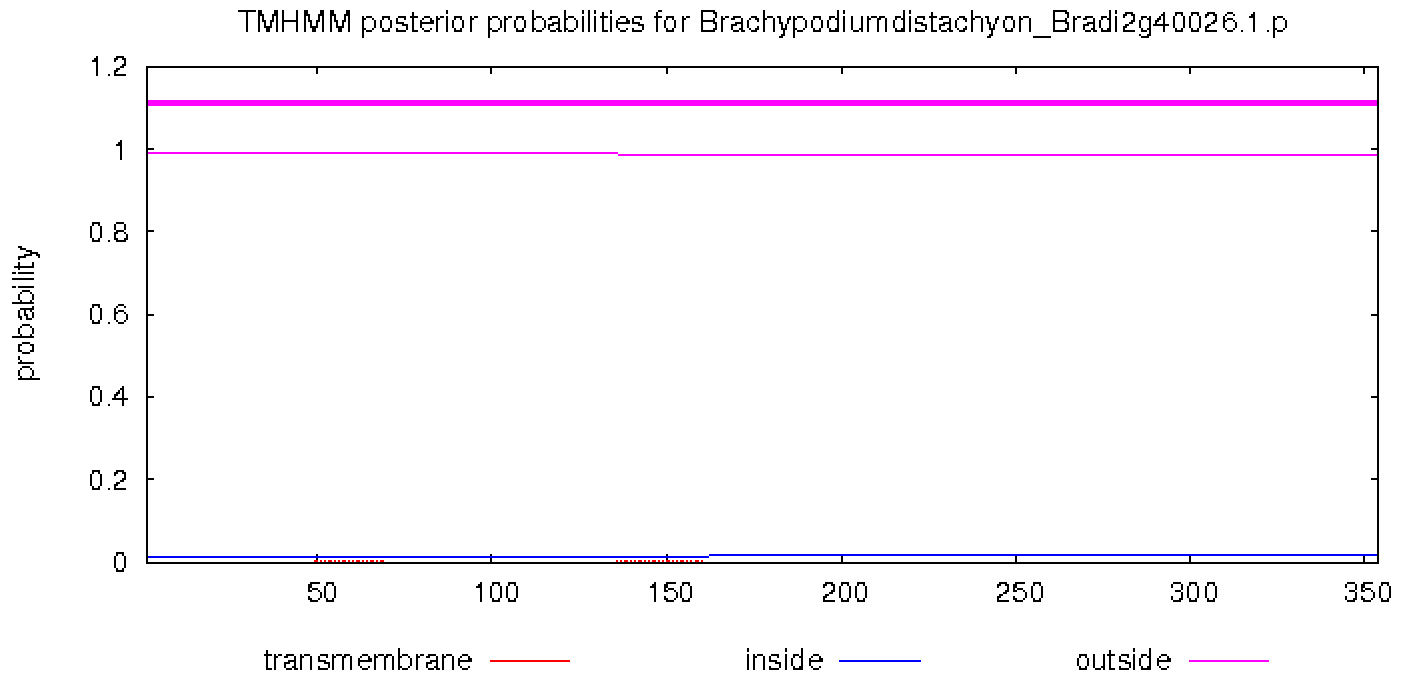

plot in postscript, script for making the plot in gnuplot, data for plot

---

```
# Brachypodiumdistachyon_Bradi2g46020.1.p Length: 350
# Brachypodiumdistachyon_Bradi2g46020.1.p Number of predicted TMHs:  0
# Brachypodiumdistachyon_Bradi2g46020.1.p Exp number of AAs in TMHs: 0.00019
# Brachypodiumdistachyon_Bradi2g46020.1.p Exp number, first 60 AAs:  0
# Brachypodiumdistachyon_Bradi2g46020.1.p Total prob of N-in:        0.02359
Brachypodiumdistachyon_Bradi2g46020.1.p TMHMM2.0        outside      1   350
```

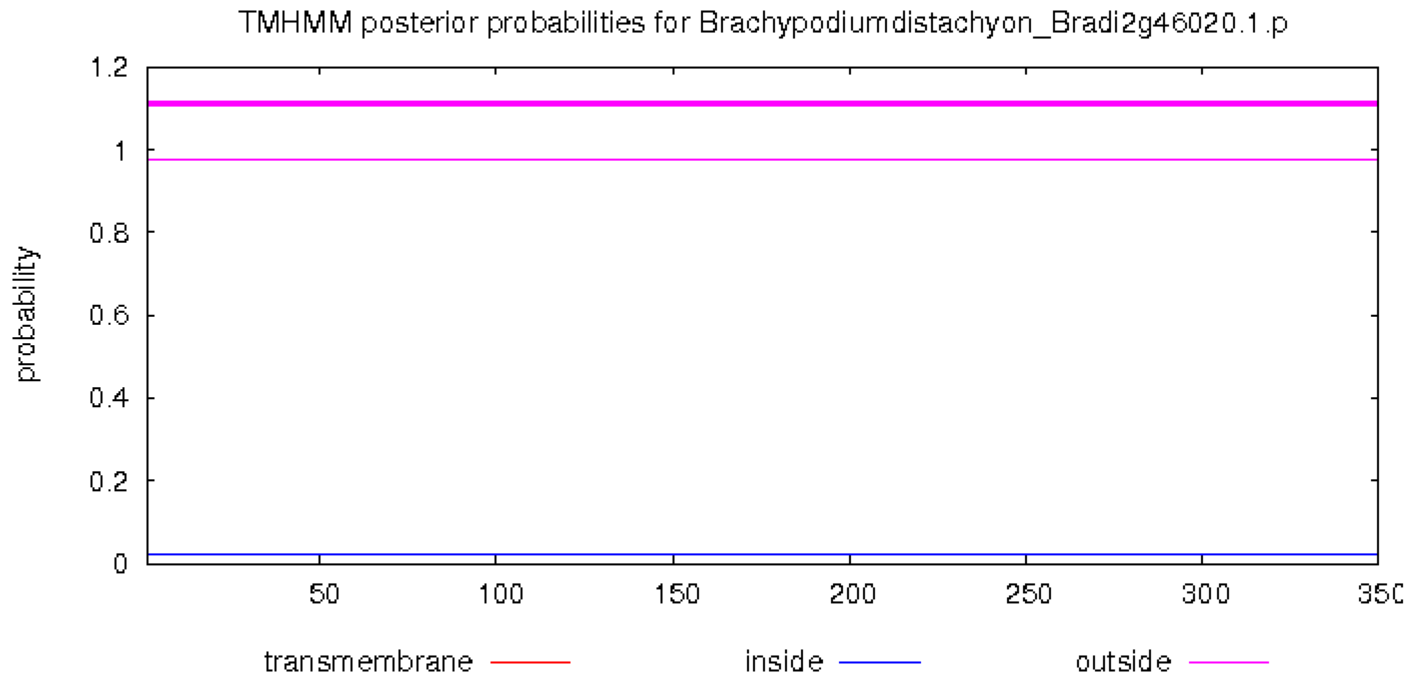

plot in postscript, script for making the plot in gnuplot, data for plot

---

```
# Brachypodiumdistachyon_Bradi2g46197.2.p Length: 336
# Brachypodiumdistachyon_Bradi2g46197.2.p Number of predicted TMHs:  0
# Brachypodiumdistachyon_Bradi2g46197.2.p Exp number of AAs in TMHs: 0.02437
# Brachypodiumdistachyon_Bradi2g46197.2.p Exp number, first 60 AAs:  0.00602
# Brachypodiumdistachyon_Bradi2g46197.2.p Total prob of N-in:        0.03035
Brachypodiumdistachyon_Bradi2g46197.2.p TMHMM2.0        outside      1   336
```

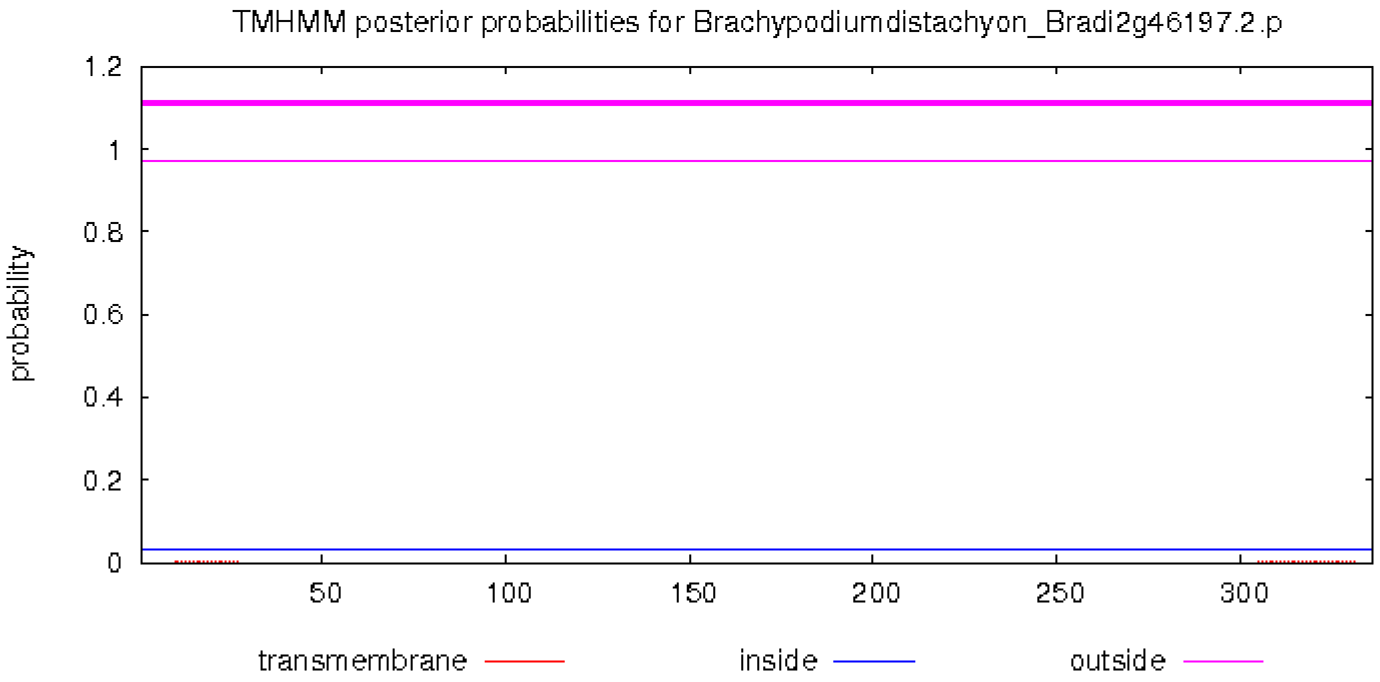

plot in postscript, script for making the plot in gnuplot, data for plot

---

```
# Brachypodiumdistachyon_Bradi2g46427.1.p Length: 556
# Brachypodiumdistachyon_Bradi2g46427.1.p Number of predicted TMHs:  0
# Brachypodiumdistachyon_Bradi2g46427.1.p Exp number of AAs in TMHs: 0.00018
# Brachypodiumdistachyon_Bradi2g46427.1.p Exp number, first 60 AAs:  0
# Brachypodiumdistachyon_Bradi2g46427.1.p Total prob of N-in:        0.00174
Brachypodiumdistachyon_Bradi2g46427.1.p TMHMM2.0        outside      1   556
```

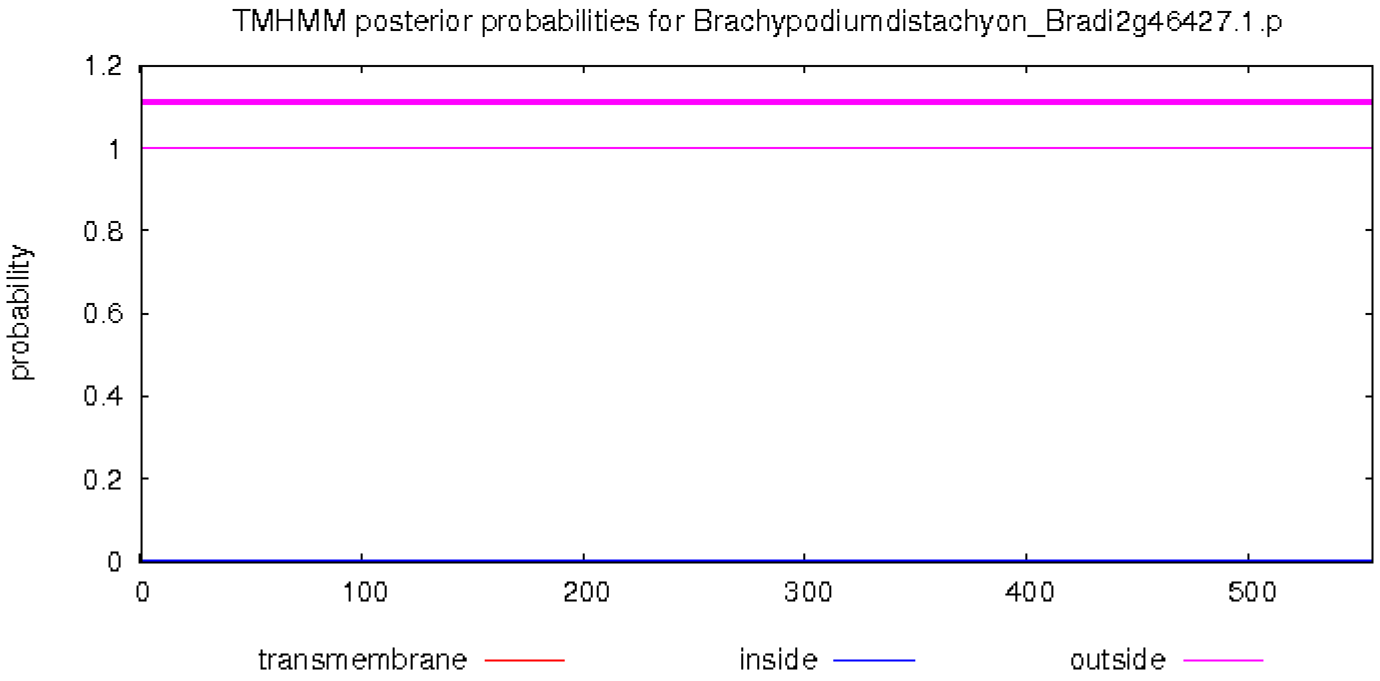

plot in postscript, script for making the plot in gnuplot, data for plot

---

```
# Brachypodiumdistachyon_Bradi2g46483.1.p Length: 273
# Brachypodiumdistachyon_Bradi2g46483.1.p Number of predicted TMHs:  0
# Brachypodiumdistachyon_Bradi2g46483.1.p Exp number of AAs in TMHs: 0
# Brachypodiumdistachyon_Bradi2g46483.1.p Exp number, first 60 AAs:  0
# Brachypodiumdistachyon_Bradi2g46483.1.p Total prob of N-in:        0.01693
Brachypodiumdistachyon_Bradi2g46483.1.p TMHMM2.0        outside      1   273
```

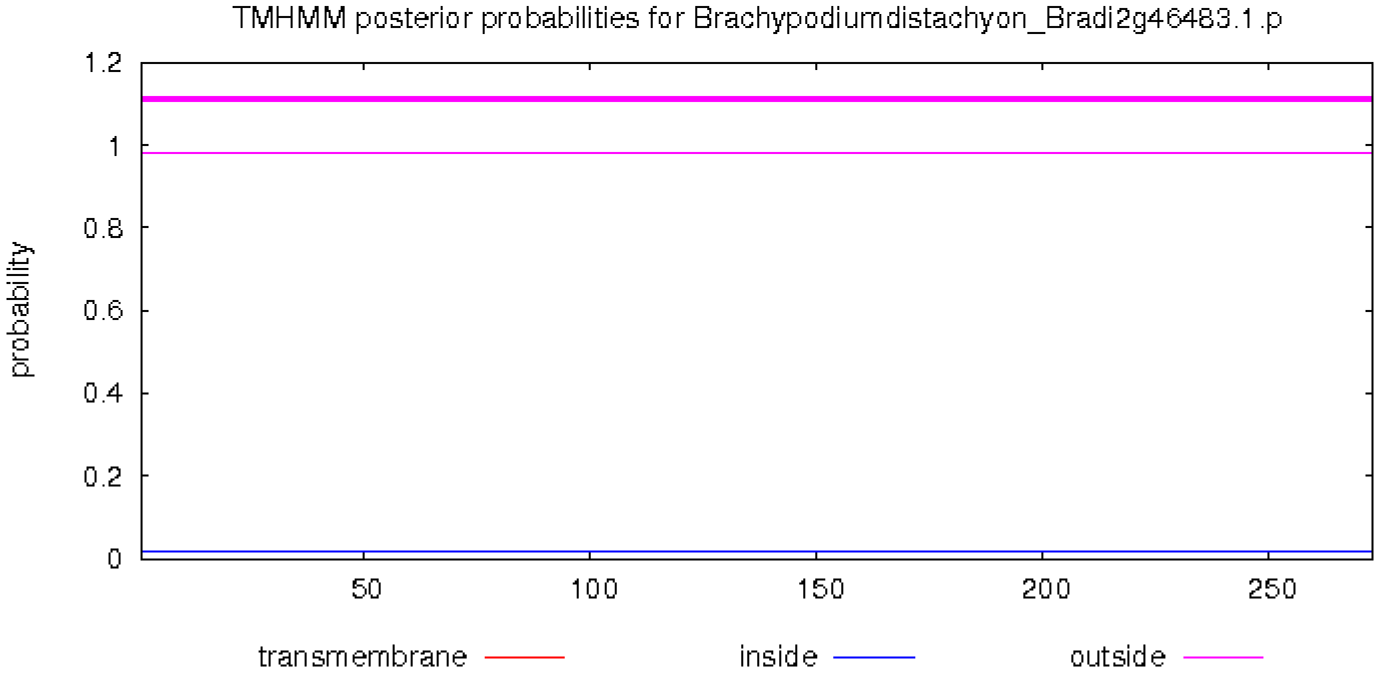

plot in postscript, script for making the plot in gnuplot, data for plot

```
# Brachypodiumdistachyon_Bradi2g46980.1.p Length: 388
# Brachypodiumdistachyon_Bradi2g46980.1.p Number of predicted TMHs:  0
# Brachypodiumdistachyon_Bradi2g46980.1.p Exp number of AAs in TMHs: 0.04463
# Brachypodiumdistachyon_Bradi2g46980.1.p Exp number, first 60 AAs:  0.04452
# Brachypodiumdistachyon_Bradi2g46980.1.p Total prob of N-in:        0.00447
Brachypodiumdistachyon_Bradi2g46980.1.p TMHMM2.0        outside      1   388
```

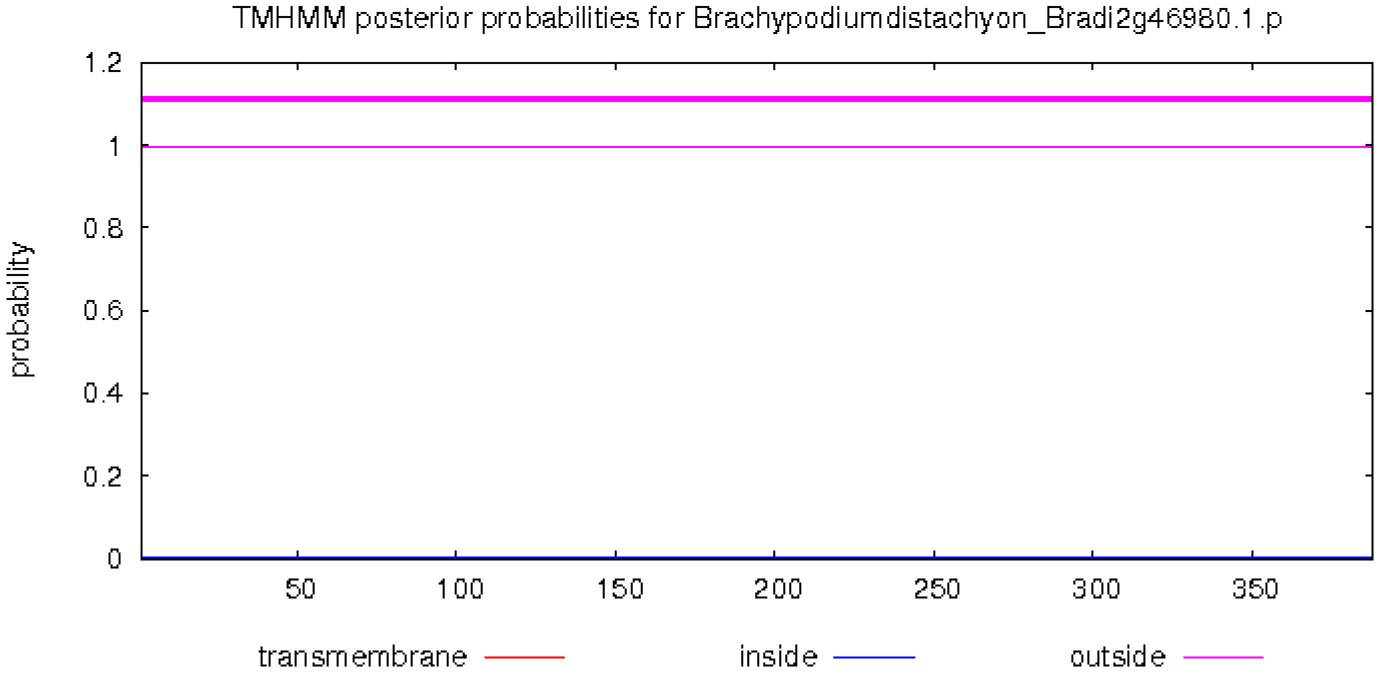

plot in postscript, script for making the plot in gnuplot, data for plot

```
# Brachypodiumdistachyon_Bradi2g53260.1.p Length: 310
# Brachypodiumdistachyon_Bradi2g53260.1.p Number of predicted TMHs:  0
# Brachypodiumdistachyon_Bradi2g53260.1.p Exp number of AAs in TMHs: 0.00661
# Brachypodiumdistachyon_Bradi2g53260.1.p Exp number, first 60 AAs:  0
# Brachypodiumdistachyon_Bradi2g53260.1.p Total prob of N-in:        0.04163
Brachypodiumdistachyon_Bradi2g53260.1.p TMHMM2.0        outside      1   310
```

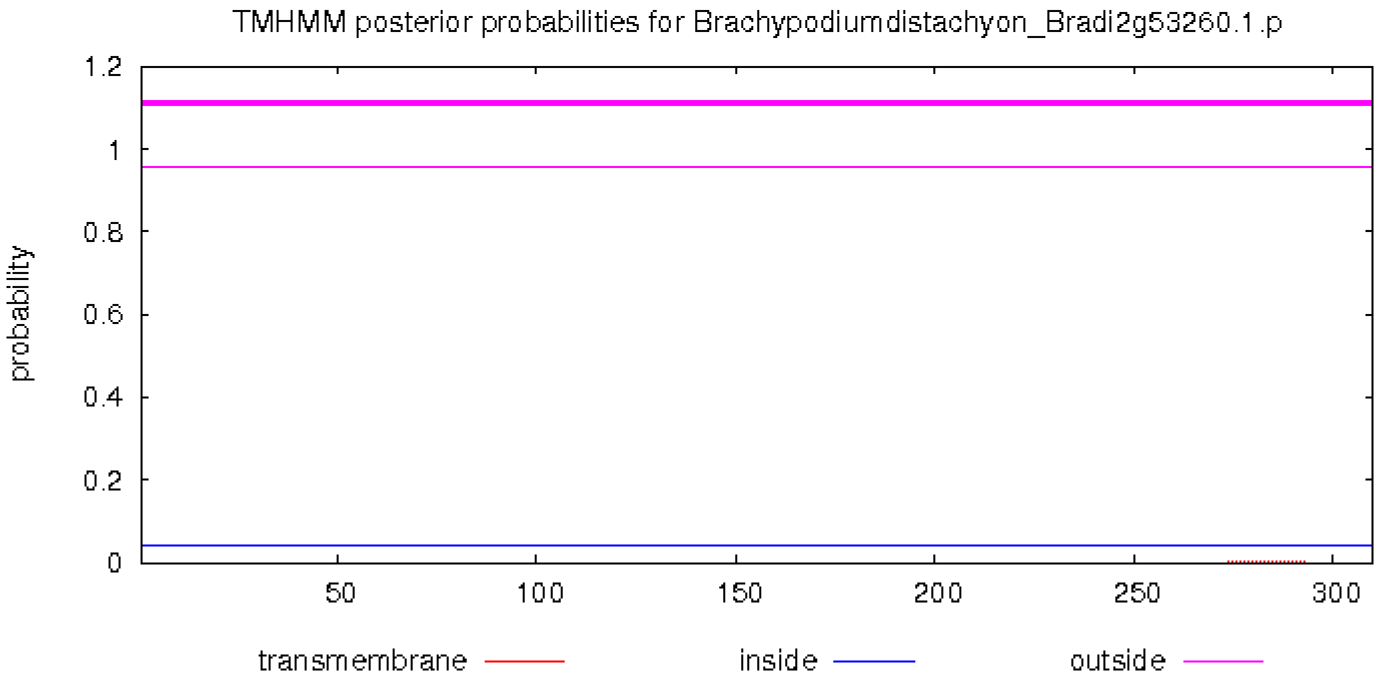

plot in postscript, script for making the plot in gnuplot, data for plot

---

```
# Brachypodiumdistachyon_Bradi2g55780.1.p Length: 266
# Brachypodiumdistachyon_Bradi2g55780.1.p Number of predicted TMHs:  0
# Brachypodiumdistachyon_Bradi2g55780.1.p Exp number of AAs in TMHs: 0.00914
# Brachypodiumdistachyon_Bradi2g55780.1.p Exp number, first 60 AAs:  0
# Brachypodiumdistachyon_Bradi2g55780.1.p Total prob of N-in:        0.00665
Brachypodiumdistachyon_Bradi2g55780.1.p TMHMM2.0        outside      1   266
```

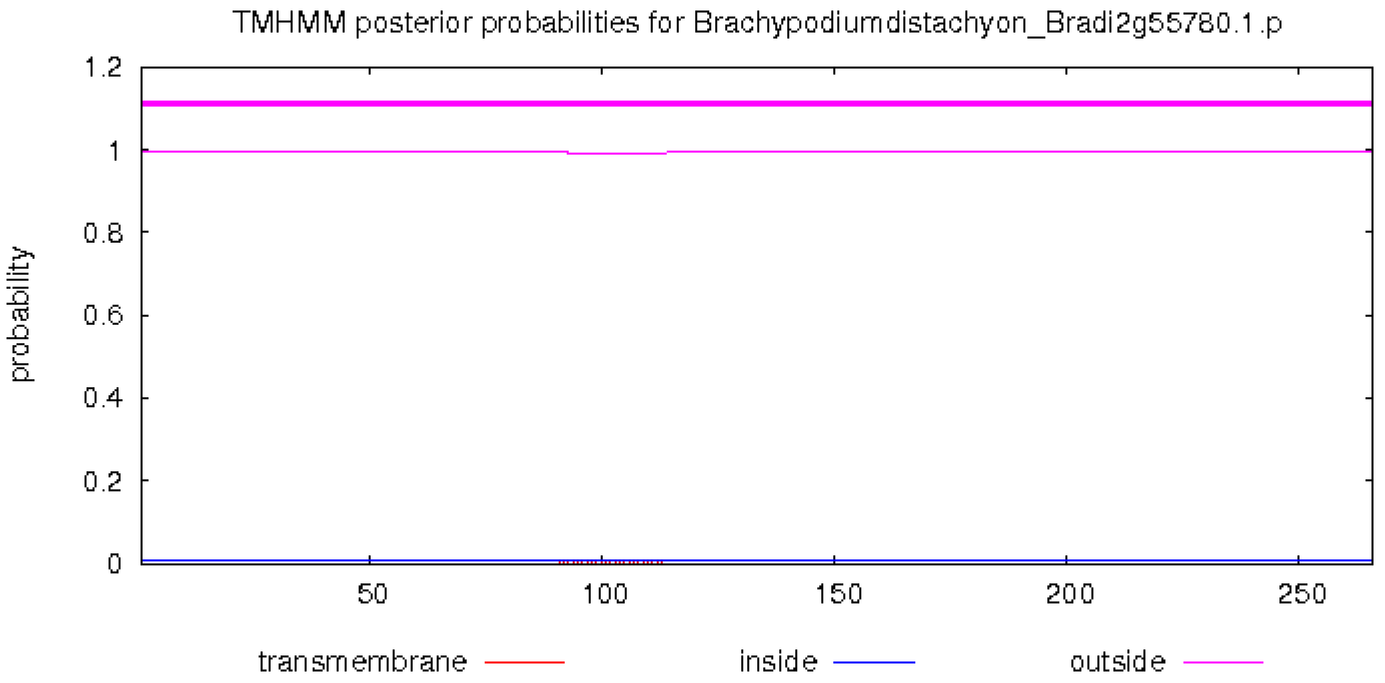

plot in postscript, script for making the plot in gnuplot, data for plot

---

```
# Brachypodiumdistachyon_Bradi2g57100.1.p Length: 292
# Brachypodiumdistachyon_Bradi2g57100.1.p Number of predicted TMHs:  0
# Brachypodiumdistachyon_Bradi2g57100.1.p Exp number of AAs in TMHs: 0.00642
# Brachypodiumdistachyon_Bradi2g57100.1.p Exp number, first 60 AAs:  0.00424
# Brachypodiumdistachyon_Bradi2g57100.1.p Total prob of N-in:        0.00584
Brachypodiumdistachyon_Bradi2g57100.1.p TMHMM2.0        outside      1   292
```

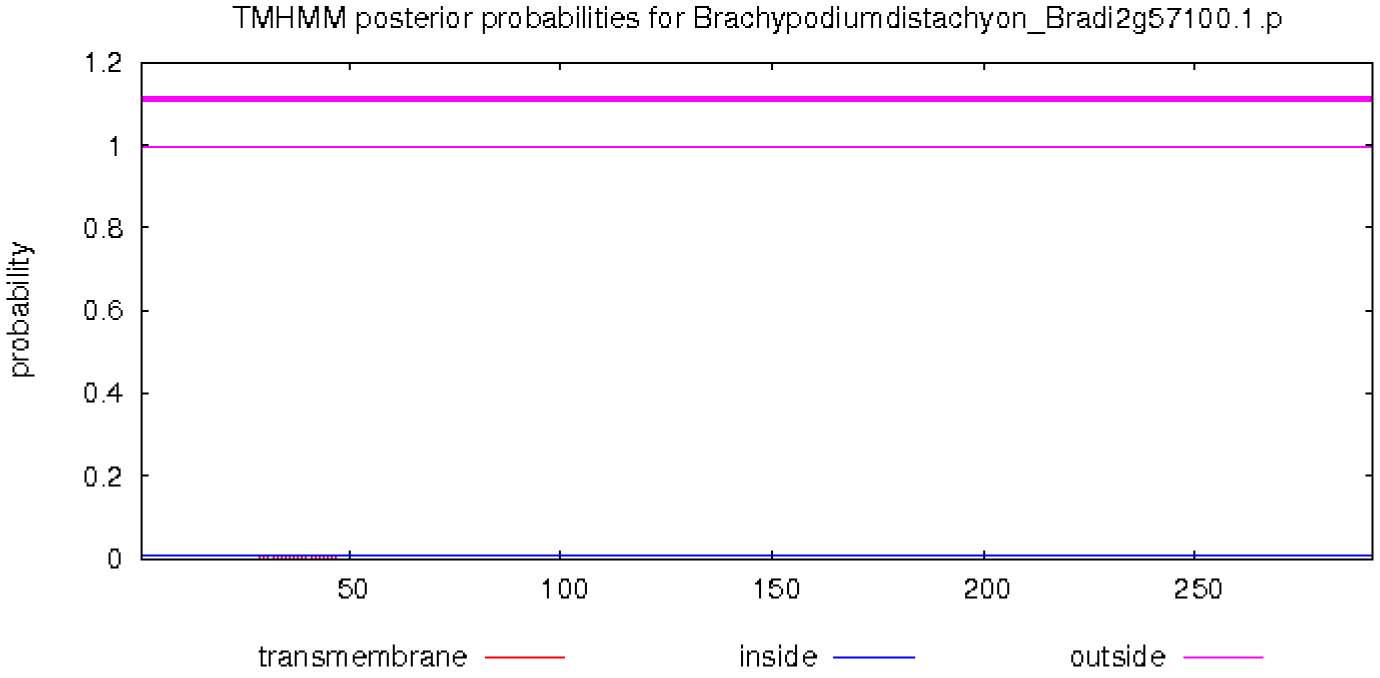

plot in postscript, script for making the plot in gnuplot, data for plot

---

```
# Brachypodiumdistachyon_Bradi2g57297.1.p Length: 413
# Brachypodiumdistachyon_Bradi2g57297.1.p Number of predicted TMHs:  0
# Brachypodiumdistachyon_Bradi2g57297.1.p Exp number of AAs in TMHs: 0.1332
# Brachypodiumdistachyon_Bradi2g57297.1.p Exp number, first 60 AAs:  0.00018
# Brachypodiumdistachyon_Bradi2g57297.1.p Total prob of N-in:        0.00599
Brachypodiumdistachyon_Bradi2g57297.1.p TMHMM2.0        outside      1   413
```

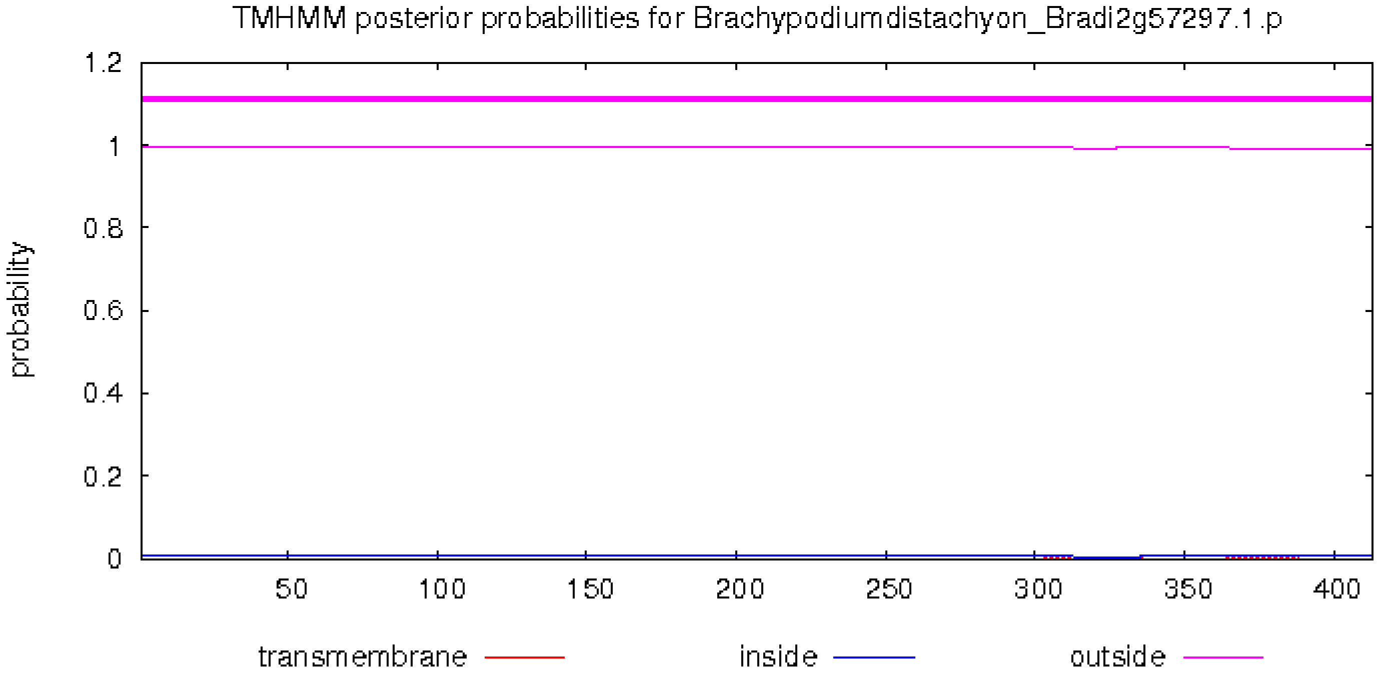

plot in postscript, script for making the plot in gnuplot, data for plot

---

```
# Brachypodiumdistachyon_Bradi2g60620.1.p Length: 219
# Brachypodiumdistachyon_Bradi2g60620.1.p Number of predicted TMHs:  0
# Brachypodiumdistachyon_Bradi2g60620.1.p Exp number of AAs in TMHs: 0.00059
# Brachypodiumdistachyon_Bradi2g60620.1.p Exp number, first 60 AAs:  0
# Brachypodiumdistachyon_Bradi2g60620.1.p Total prob of N-in:        0.02066
Brachypodiumdistachyon_Bradi2g60620.1.p TMHMM2.0        outside      1   219
```

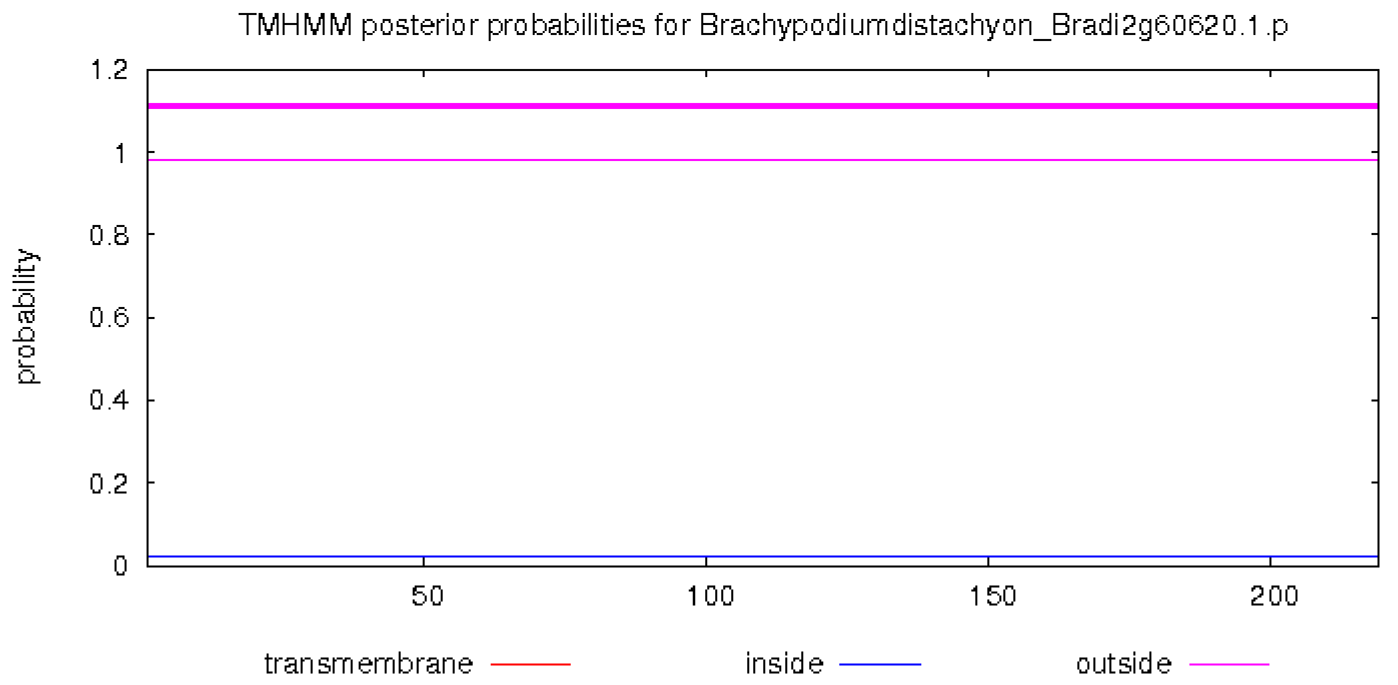

plot in postscript, script for making the plot in gnuplot, data for plot

---

```
# Brachypodiumdistachyon_Bradi3g09520.2.p Length: 378
# Brachypodiumdistachyon_Bradi3g09520.2.p Number of predicted TMHs:  0
# Brachypodiumdistachyon_Bradi3g09520.2.p Exp number of AAs in TMHs: 0.00176
# Brachypodiumdistachyon_Bradi3g09520.2.p Exp number, first 60 AAs:  0
# Brachypodiumdistachyon_Bradi3g09520.2.p Total prob of N-in:        0.00495
Brachypodiumdistachyon_Bradi3g09520.2.p TMHMM2.0        outside      1   378
```

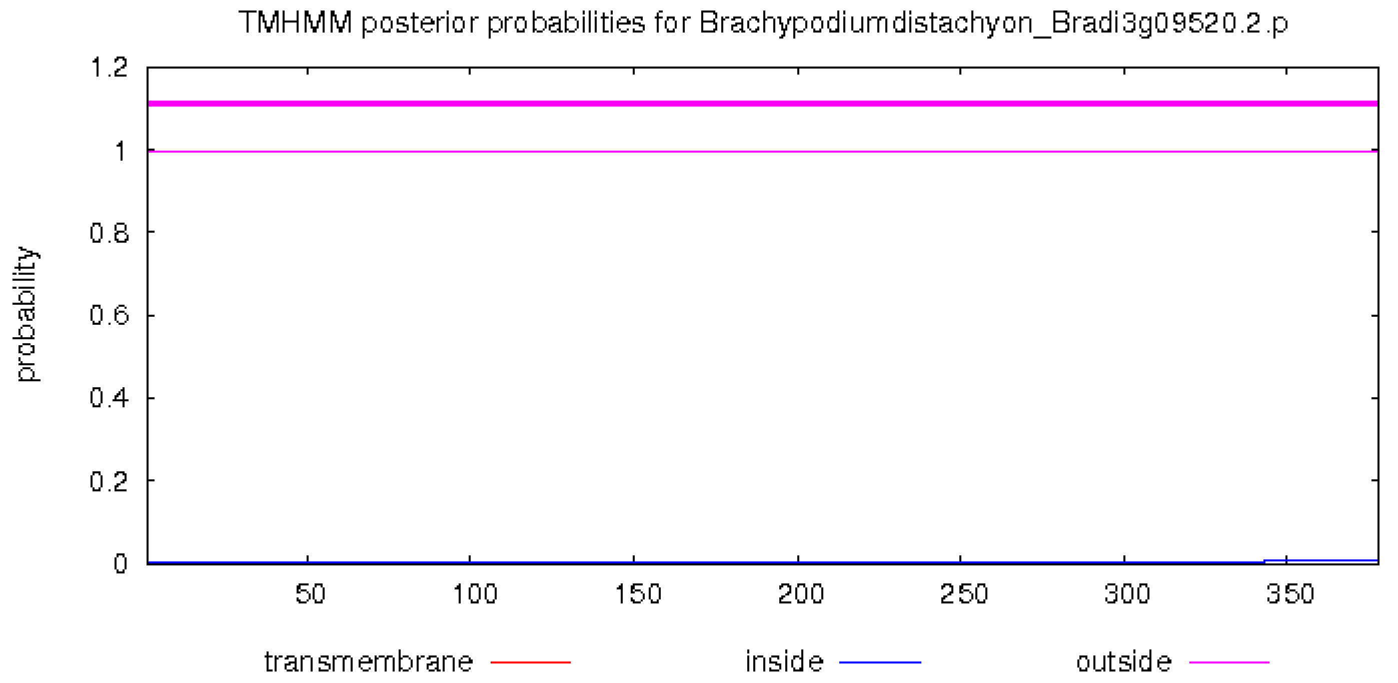

plot in postscript, script for making the plot in gnuplot, data for plot

---

```
# Brachypodiumdistachyon_Bradi3g10414.1.p Length: 365
# Brachypodiumdistachyon_Bradi3g10414.1.p Number of predicted TMHs:  0
# Brachypodiumdistachyon_Bradi3g10414.1.p Exp number of AAs in TMHs: 0.00516
# Brachypodiumdistachyon_Bradi3g10414.1.p Exp number, first 60 AAs:  0
# Brachypodiumdistachyon_Bradi3g10414.1.p Total prob of N-in:        0.03574
Brachypodiumdistachyon_Bradi3g10414.1.p TMHMM2.0        outside      1   365
```

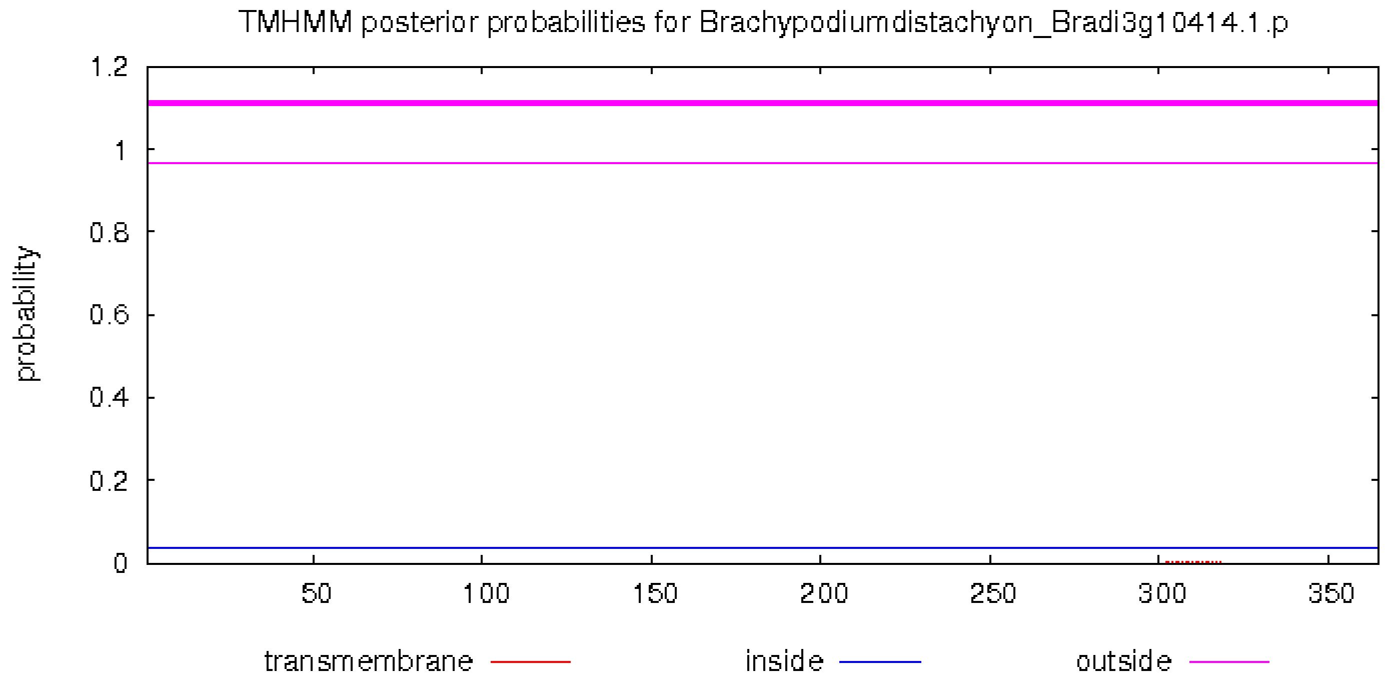

plot in postscript, script for making the plot in gnuplot, data for plot

---

```
# Brachypodiumdistachyon_Bradi3g10540.2.p Length: 342
# Brachypodiumdistachyon_Bradi3g10540.2.p Number of predicted TMHs:  0
# Brachypodiumdistachyon_Bradi3g10540.2.p Exp number of AAs in TMHs: 0.00168
# Brachypodiumdistachyon_Bradi3g10540.2.p Exp number, first 60 AAs:  0
# Brachypodiumdistachyon_Bradi3g10540.2.p Total prob of N-in:        0.00641
Brachypodiumdistachyon_Bradi3g10540.2.p TMHMM2.0        outside      1   342
```

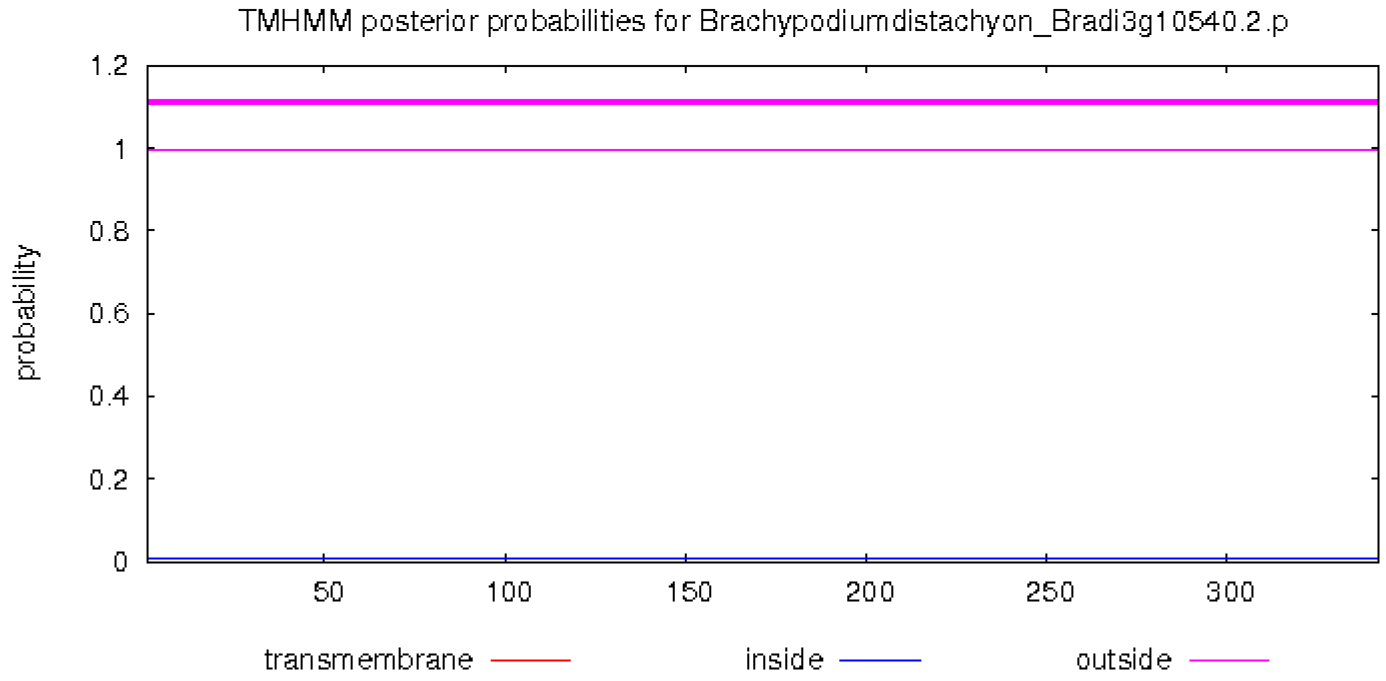

plot in postscript, script for making the plot in gnuplot, data for plot

---

```
# Brachypodiumdistachyon_Bradi3g12470.1.p Length: 653
# Brachypodiumdistachyon_Bradi3g12470.1.p Number of predicted TMHs:  1
# Brachypodiumdistachyon_Bradi3g12470.1.p Exp number of AAs in TMHs: 20.4793
# Brachypodiumdistachyon_Bradi3g12470.1.p Exp number, first 60 AAs:  0.00015
# Brachypodiumdistachyon_Bradi3g12470.1.p Total prob of N-in:        0.00126
Brachypodiumdistachyon_Bradi3g12470.1.p TMHMM2.0        outside      1   629
Brachypodiumdistachyon_Bradi3g12470.1.p TMHMM2.0        TMhelix    630   652
Brachypodiumdistachyon_Bradi3g12470.1.p TMHMM2.0        inside     653   653
```

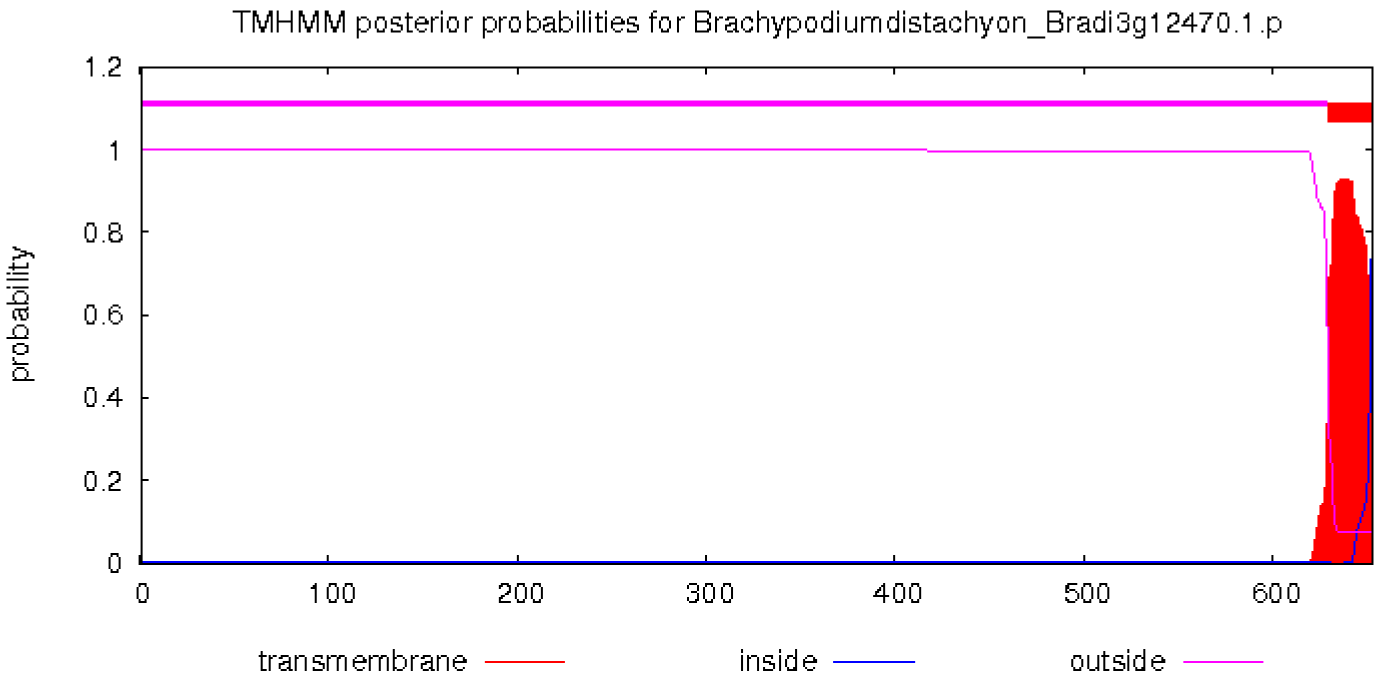

plot in postscript, script for making the plot in gnuplot, data for plot

---

```
# Brachypodiumdistachyon_Bradi3g13117.1.p Length: 371
# Brachypodiumdistachyon_Bradi3g13117.1.p Number of predicted TMHs:  0
# Brachypodiumdistachyon_Bradi3g13117.1.p Exp number of AAs in TMHs: 0.04309
# Brachypodiumdistachyon_Bradi3g13117.1.p Exp number, first 60 AAs:  0
# Brachypodiumdistachyon_Bradi3g13117.1.p Total prob of N-in:        0.00085
Brachypodiumdistachyon_Bradi3g13117.1.p TMHMM2.0        outside      1   371
```

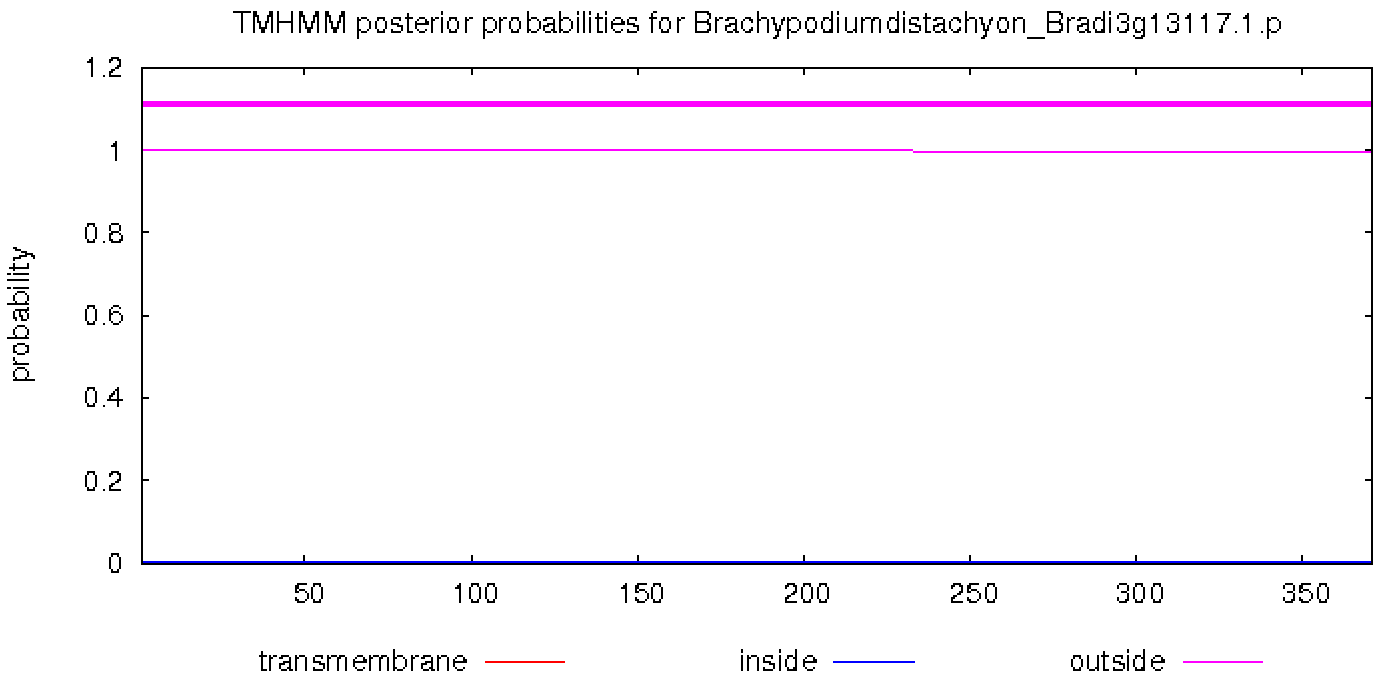

plot in postscript, script for making the plot in gnuplot, data for plot

```
# Brachypodiumdistachyon_Bradi3g13630.1.p Length: 396
# Brachypodiumdistachyon_Bradi3g13630.1.p Number of predicted TMHs:  0
# Brachypodiumdistachyon_Bradi3g13630.1.p Exp number of AAs in TMHs: 0.01298
# Brachypodiumdistachyon_Bradi3g13630.1.p Exp number, first 60 AAs:  0.00513
# Brachypodiumdistachyon_Bradi3g13630.1.p Total prob of N-in:        0.01541
Brachypodiumdistachyon_Bradi3g13630.1.p TMHMM2.0        outside      1   396
```

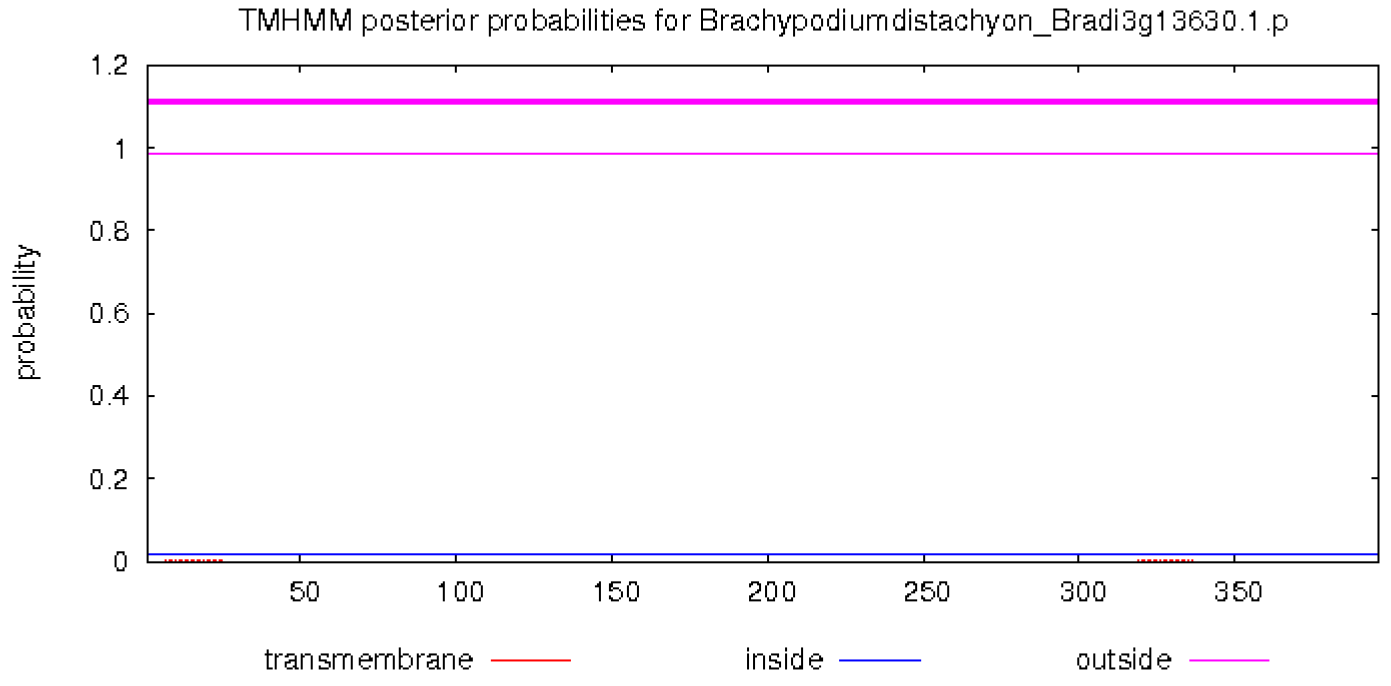

plot in postscript, script for making the plot in gnuplot, data for plot

```
# Brachypodiumdistachyon_Bradi3g13727.1.p Length: 390
# Brachypodiumdistachyon_Bradi3g13727.1.p Number of predicted TMHs:  0
# Brachypodiumdistachyon_Bradi3g13727.1.p Exp number of AAs in TMHs: 0.00547
# Brachypodiumdistachyon_Bradi3g13727.1.p Exp number, first 60 AAs:  0
# Brachypodiumdistachyon_Bradi3g13727.1.p Total prob of N-in:        0.00999
Brachypodiumdistachyon_Bradi3g13727.1.p TMHMM2.0        outside      1   390
```

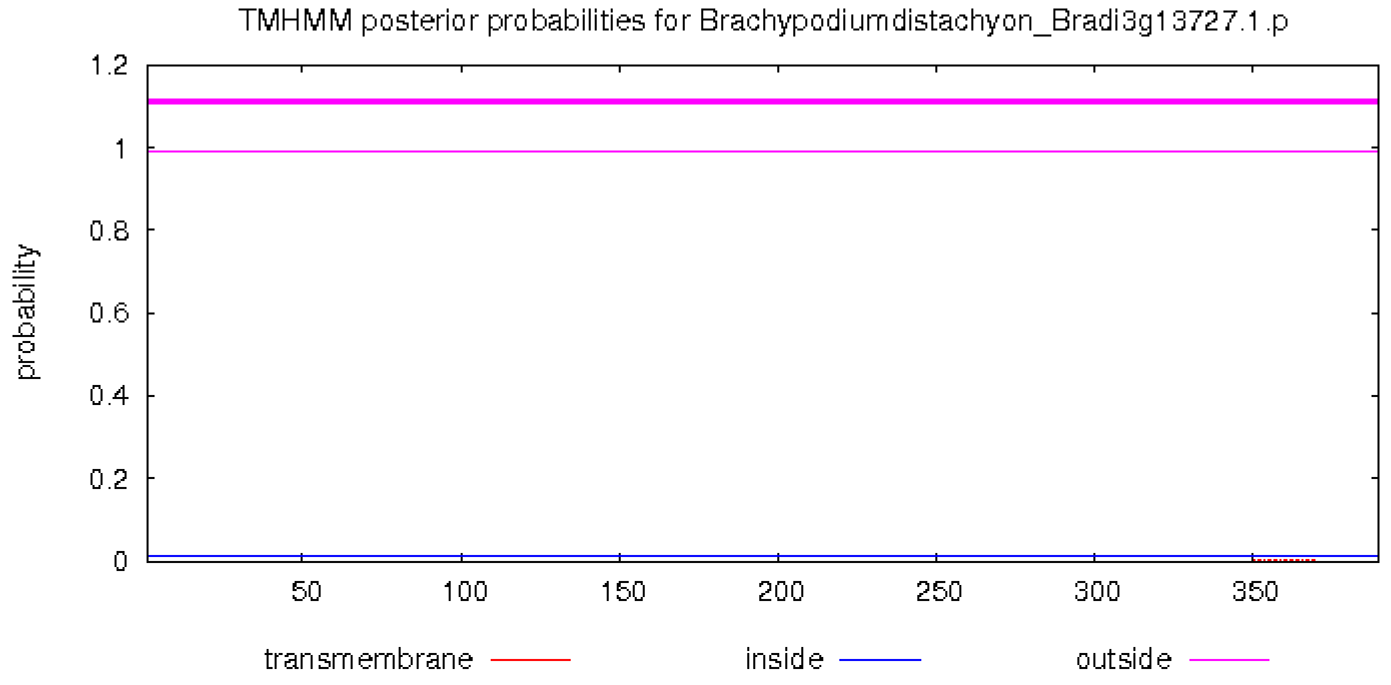

plot in postscript, script for making the plot in gnuplot, data for plot

```
# Brachypodiumdistachyon_Bradi3g16480.1.p Length: 800
# Brachypodiumdistachyon_Bradi3g16480.1.p Number of predicted TMHs:  1
# Brachypodiumdistachyon_Bradi3g16480.1.p Exp number of AAs in TMHs: 22.58566
# Brachypodiumdistachyon_Bradi3g16480.1.p Exp number, first 60 AAs:  0
# Brachypodiumdistachyon_Bradi3g16480.1.p Total prob of N-in:        0.00016
Brachypodiumdistachyon_Bradi3g16480.1.p TMHMM2.0        outside      1   776
Brachypodiumdistachyon_Bradi3g16480.1.p TMHMM2.0        TMhelix    777   799
Brachypodiumdistachyon_Bradi3g16480.1.p TMHMM2.0        inside     800   800
```

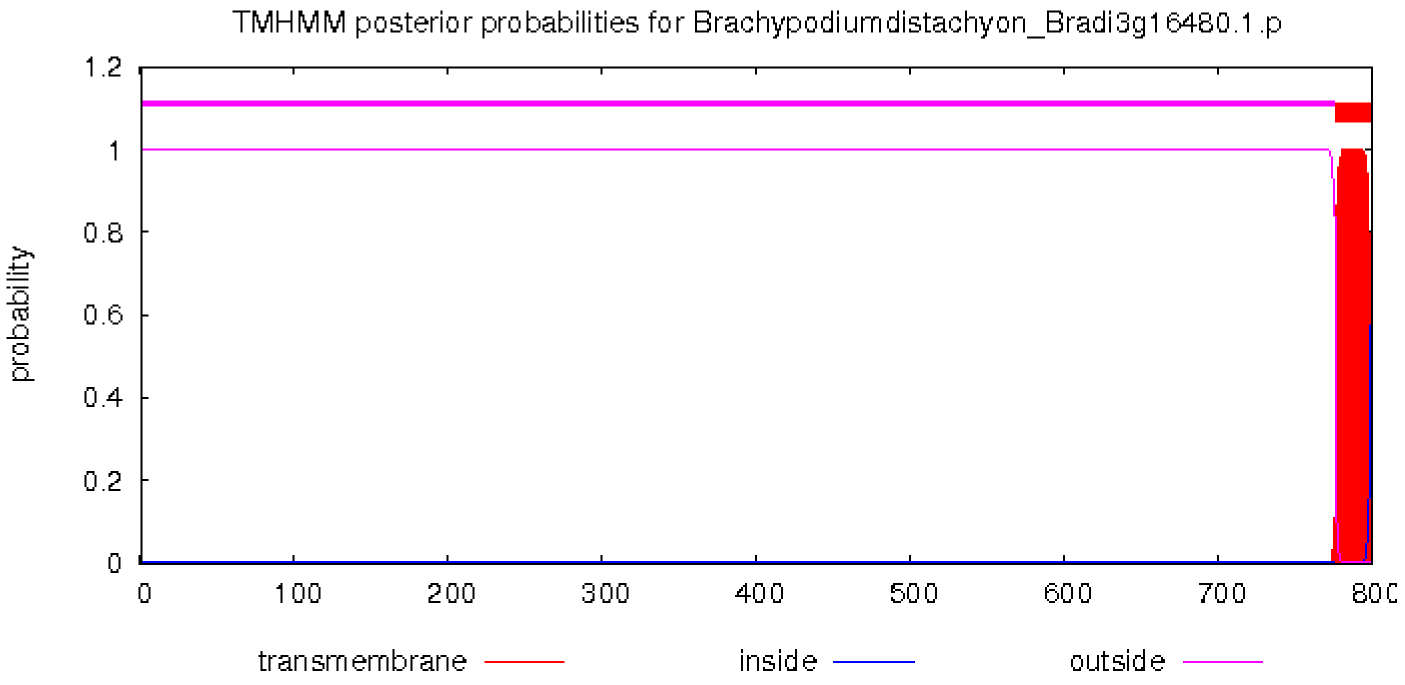

plot in postscript, script for making the plot in gnuplot, data for plot

---

```
# Brachypodiumdistachyon_Bradi3g17287.1.p Length: 285
# Brachypodiumdistachyon_Bradi3g17287.1.p Number of predicted TMHs:  0
# Brachypodiumdistachyon_Bradi3g17287.1.p Exp number of AAs in TMHs: 0.00586
# Brachypodiumdistachyon_Bradi3g17287.1.p Exp number, first 60 AAs:  0.00125
# Brachypodiumdistachyon_Bradi3g17287.1.p Total prob of N-in:        0.04038
Brachypodiumdistachyon_Bradi3g17287.1.p TMHMM2.0        outside      1   285
```

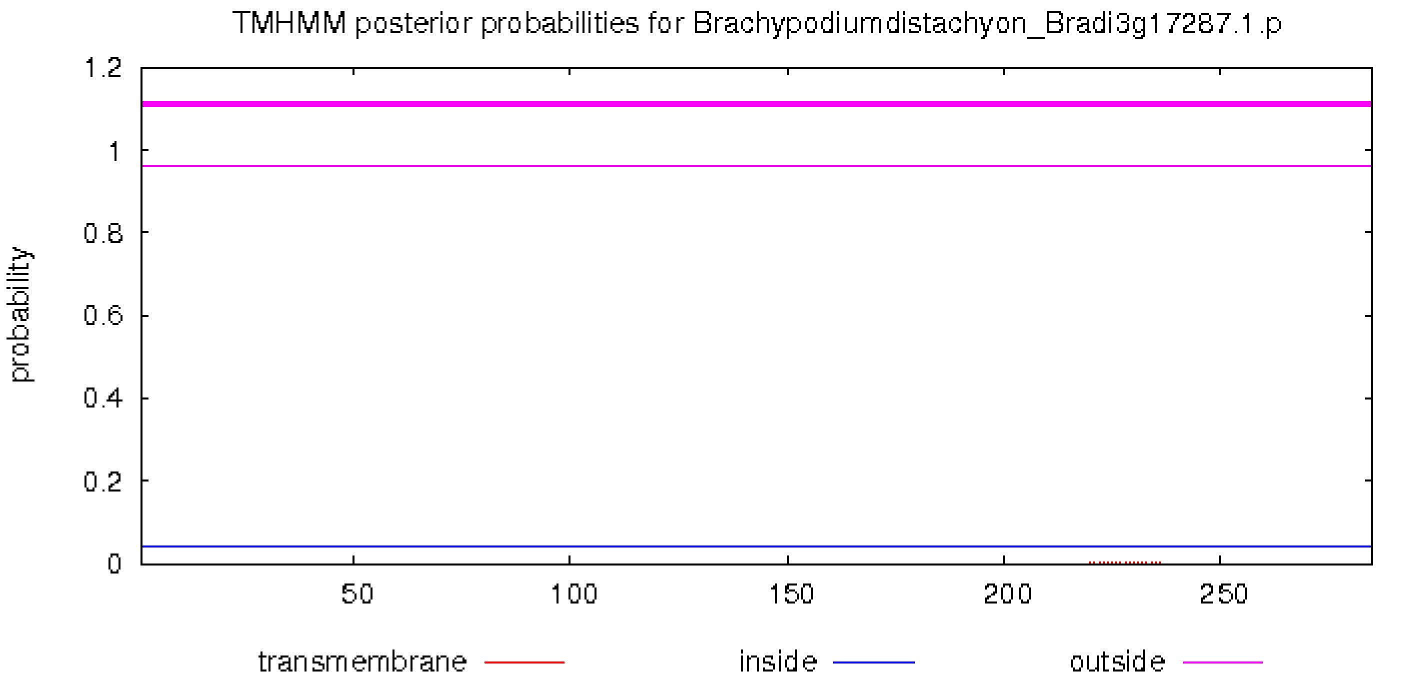

plot in postscript, script for making the plot in gnuplot, data for plot

```
# Brachypodiumdistachyon_Bradi3g17573.1.p Length: 197
# Brachypodiumdistachyon_Bradi3g17573.1.p Number of predicted TMHs:  0
# Brachypodiumdistachyon_Bradi3g17573.1.p Exp number of AAs in TMHs: 0.00913
# Brachypodiumdistachyon_Bradi3g17573.1.p Exp number, first 60 AAs:  0.00444
# Brachypodiumdistachyon_Bradi3g17573.1.p Total prob of N-in:        0.12899
Brachypodiumdistachyon_Bradi3g17573.1.p TMHMM2.0        outside      1   197
```

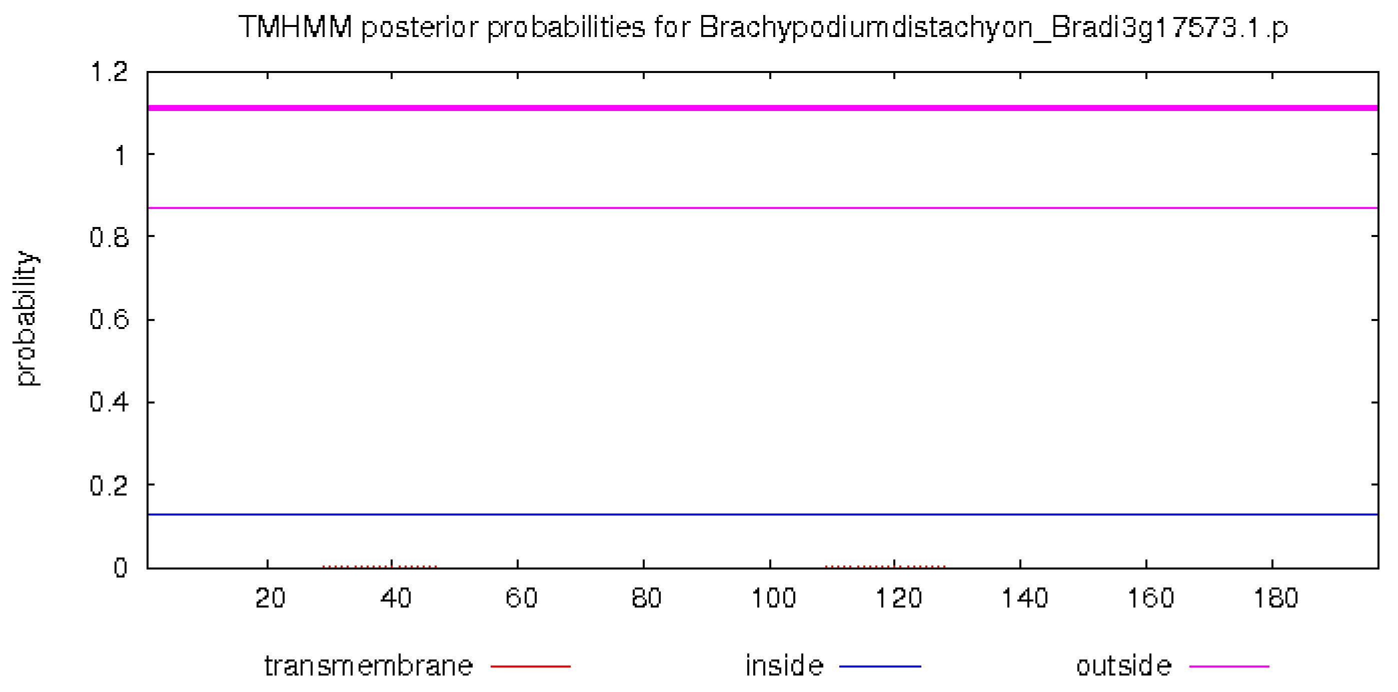

plot in postscript, script for making the plot in gnuplot, data for plot

```
# Brachypodiumdistachyon_Bradi3g26665.1.p Length: 437
# Brachypodiumdistachyon_Bradi3g26665.1.p Number of predicted TMHs:  0
# Brachypodiumdistachyon_Bradi3g26665.1.p Exp number of AAs in TMHs: 0.0015
# Brachypodiumdistachyon_Bradi3g26665.1.p Exp number, first 60 AAs:  0
# Brachypodiumdistachyon_Bradi3g26665.1.p Total prob of N-in:        0.01195
Brachypodiumdistachyon_Bradi3g26665.1.p TMHMM2.0        outside      1   437
```

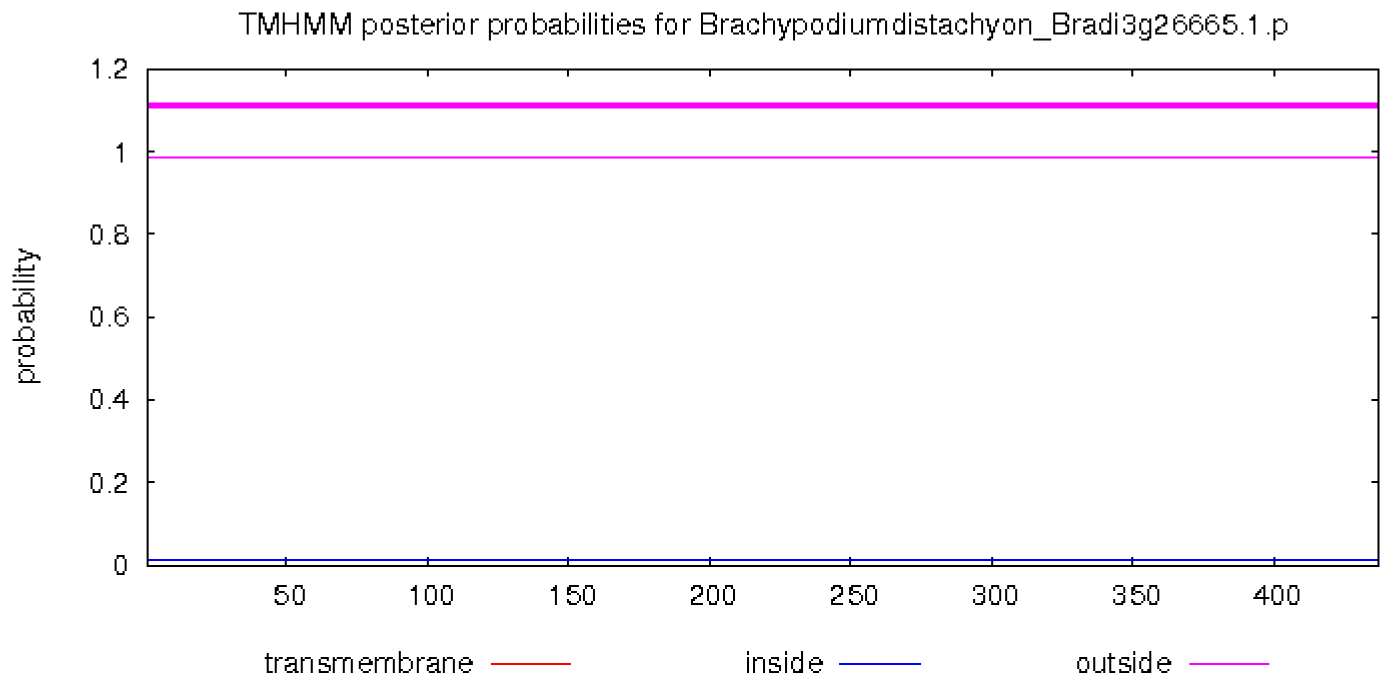

plot in postscript, script for making the plot in gnuplot, data for plot

```
# Brachypodiumdistachyon_Bradi3g33320.3.p Length: 358
# Brachypodiumdistachyon_Bradi3g33320.3.p Number of predicted TMHs:  0
# Brachypodiumdistachyon_Bradi3g33320.3.p Exp number of AAs in TMHs: 0.017690000000000001
# Brachypodiumdistachyon_Bradi3g33320.3.p Exp number, first 60 AAs:  0.01708
# Brachypodiumdistachyon_Bradi3g33320.3.p Total prob of N-in:        0.20309
Brachypodiumdistachyon_Bradi3g33320.3.p TMHMM2.0        outside      1   358
```

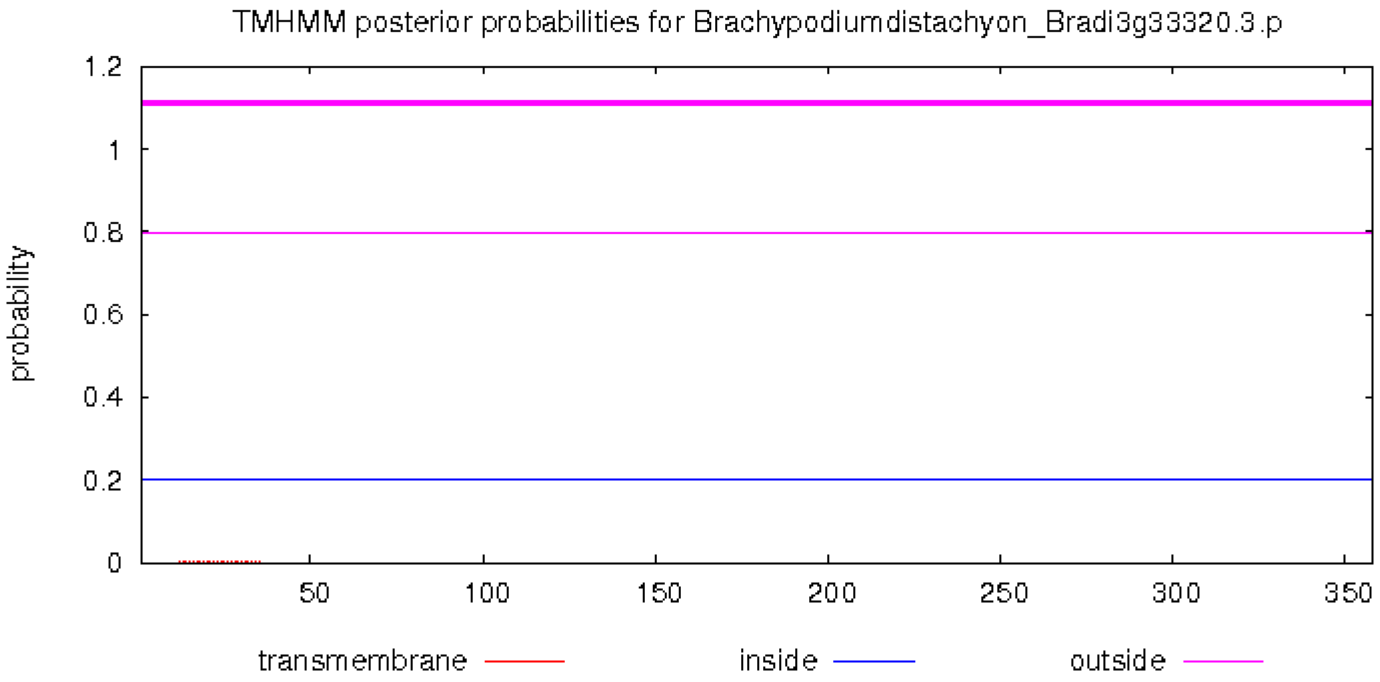

plot in postscript, script for making the plot in gnuplot, data for plot

```
# Brachypodiumdistachyon_Bradi3g33680.2.p Length: 226
# Brachypodiumdistachyon_Bradi3g33680.2.p Number of predicted TMHs:  0
# Brachypodiumdistachyon_Bradi3g33680.2.p Exp number of AAs in TMHs: 0.0761
# Brachypodiumdistachyon_Bradi3g33680.2.p Exp number, first 60 AAs:  0
# Brachypodiumdistachyon_Bradi3g33680.2.p Total prob of N-in:        0.01181
Brachypodiumdistachyon_Bradi3g33680.2.p TMHMM2.0        outside      1   226
```

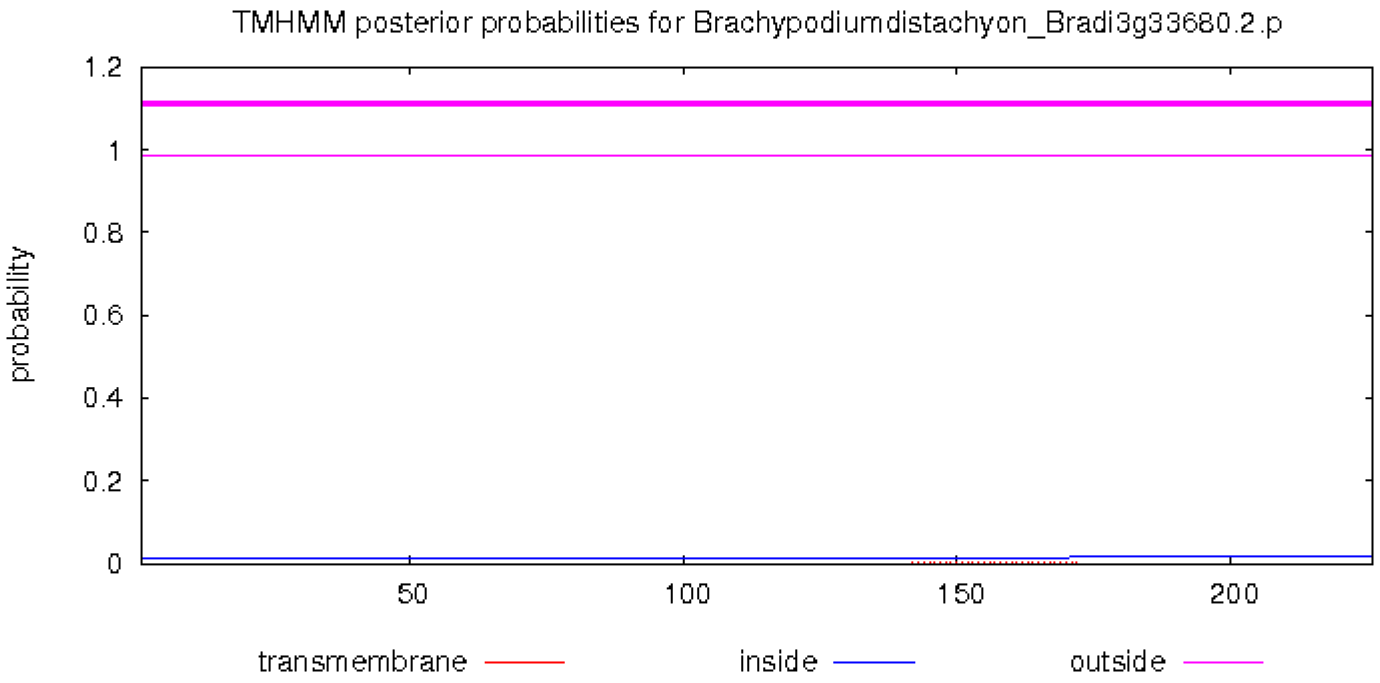

plot in postscript, script for making the plot in gnuplot, data for plot

```
# Brachypodiumdistachyon_Bradi3g33957.2.p Length: 350
# Brachypodiumdistachyon_Bradi3g33957.2.p Number of predicted TMHs:  0
# Brachypodiumdistachyon_Bradi3g33957.2.p Exp number of AAs in TMHs: 0.01423
# Brachypodiumdistachyon_Bradi3g33957.2.p Exp number, first 60 AAs:  0
# Brachypodiumdistachyon_Bradi3g33957.2.p Total prob of N-in:        0.00189
Brachypodiumdistachyon_Bradi3g33957.2.p TMHMM2.0        outside      1   350
```

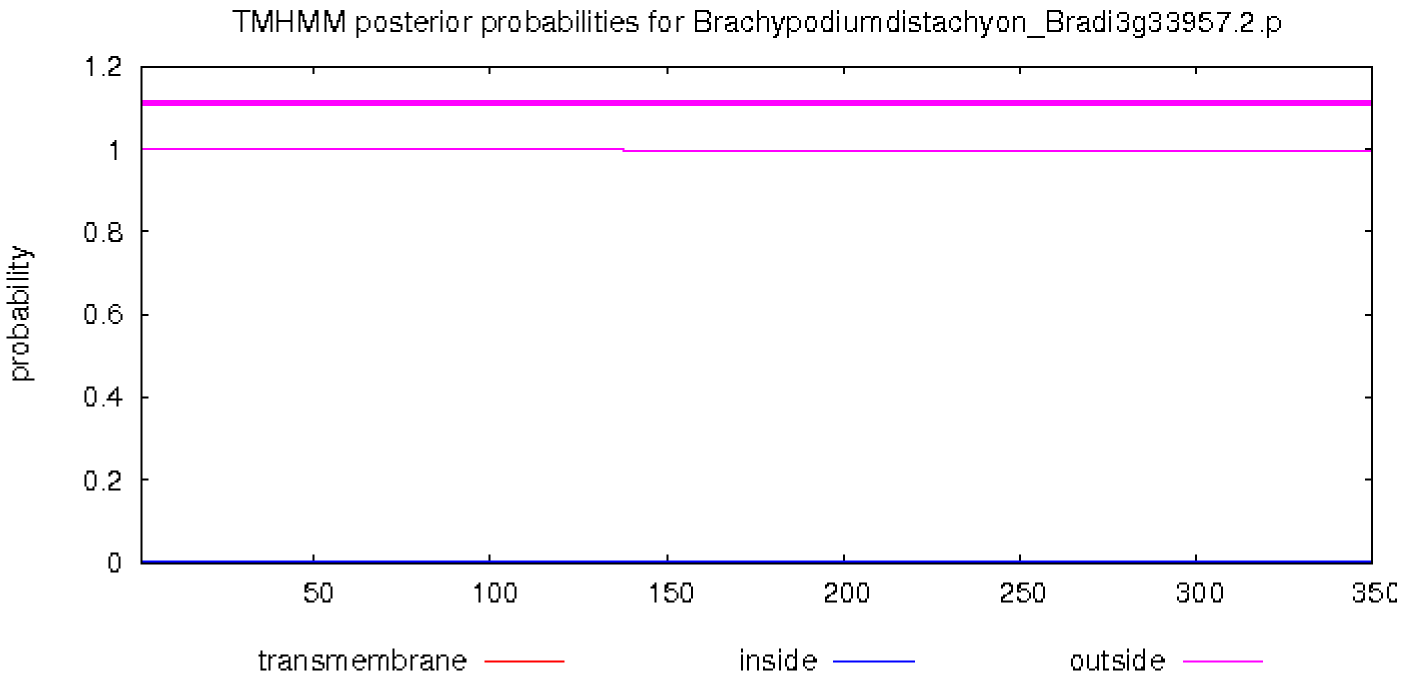

plot in postscript, script for making the plot in gnuplot, data for plot

---

```
# Brachypodiumdistachyon_Bradi3g36670.1.p Length: 281
# Brachypodiumdistachyon_Bradi3g36670.1.p Number of predicted TMHs:  0
# Brachypodiumdistachyon_Bradi3g36670.1.p Exp number of AAs in TMHs: 0
# Brachypodiumdistachyon_Bradi3g36670.1.p Exp number, first 60 AAs:  0
# Brachypodiumdistachyon_Bradi3g36670.1.p Total prob of N-in:        0.02139
Brachypodiumdistachyon_Bradi3g36670.1.p TMHMM2.0        outside      1   281
```

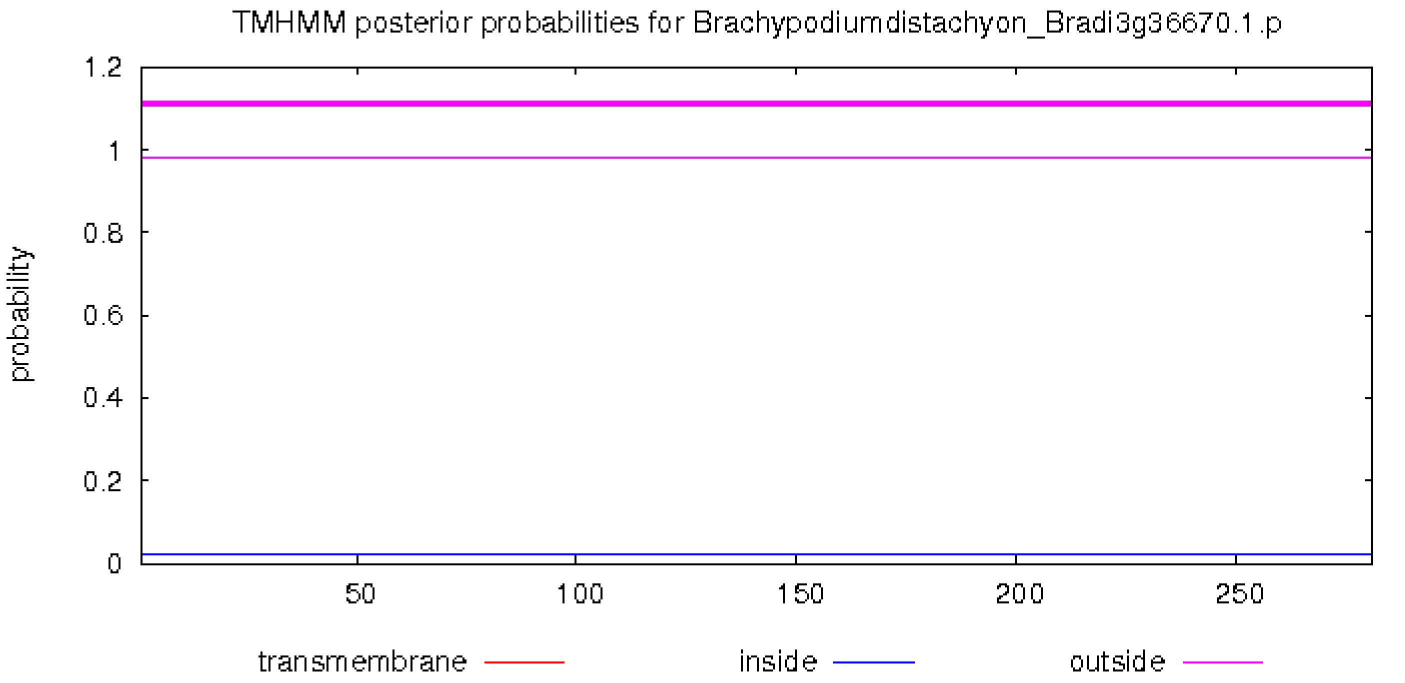

plot in postscript, script for making the plot in gnuplot, data for plot

---

```
# Brachypodiumdistachyon_Bradi3g37067.1.p Length: 381
# Brachypodiumdistachyon_Bradi3g37067.1.p Number of predicted TMHs:  0
# Brachypodiumdistachyon_Bradi3g37067.1.p Exp number of AAs in TMHs: 0
# Brachypodiumdistachyon_Bradi3g37067.1.p Exp number, first 60 AAs:  0
# Brachypodiumdistachyon_Bradi3g37067.1.p Total prob of N-in:        0.01503
Brachypodiumdistachyon_Bradi3g37067.1.p TMHMM2.0        outside      1   381
```

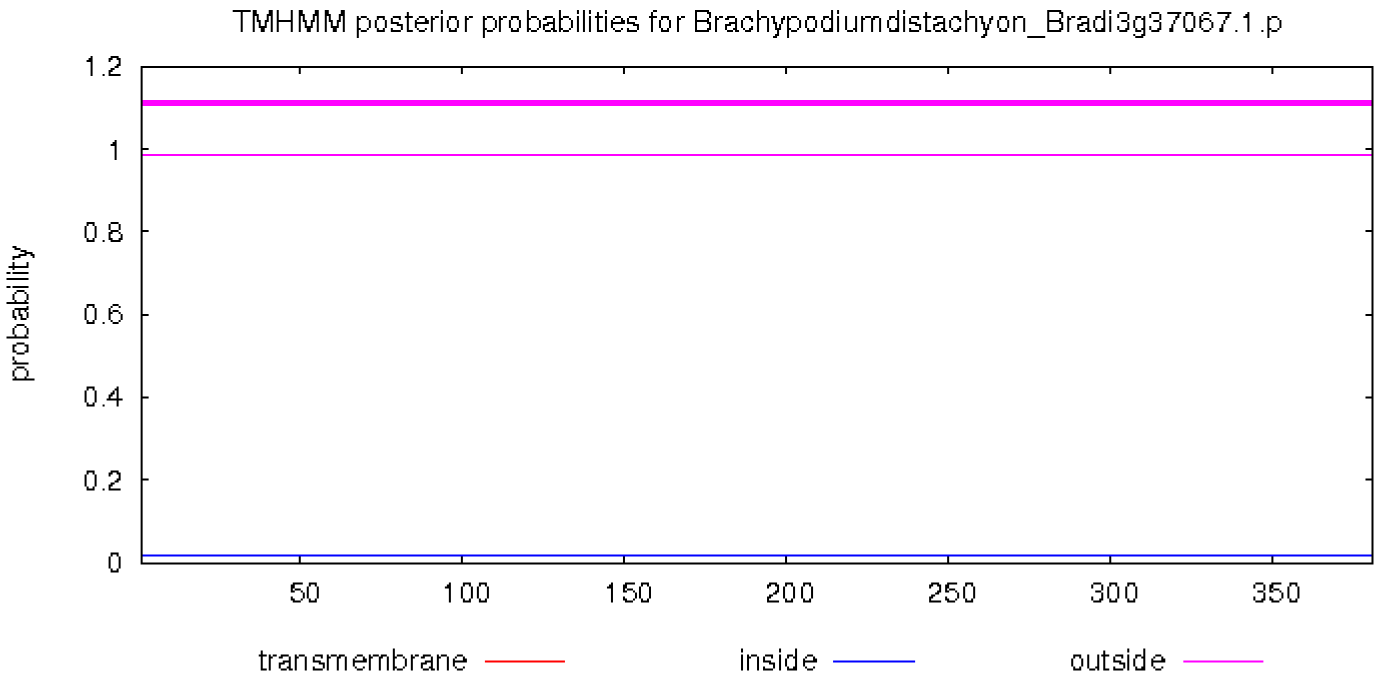

plot in postscript, script for making the plot in gnuplot, data for plot

---

```
# Brachypodiumdistachyon_Bradi3g40085.1.p Length: 372
# Brachypodiumdistachyon_Bradi3g40085.1.p Number of predicted TMHs:  0
# Brachypodiumdistachyon_Bradi3g40085.1.p Exp number of AAs in TMHs: 0.03334
# Brachypodiumdistachyon_Bradi3g40085.1.p Exp number, first 60 AAs:  0.00234
# Brachypodiumdistachyon_Bradi3g40085.1.p Total prob of N-in:        0.01854
Brachypodiumdistachyon_Bradi3g40085.1.p TMHMM2.0        outside      1   372
```

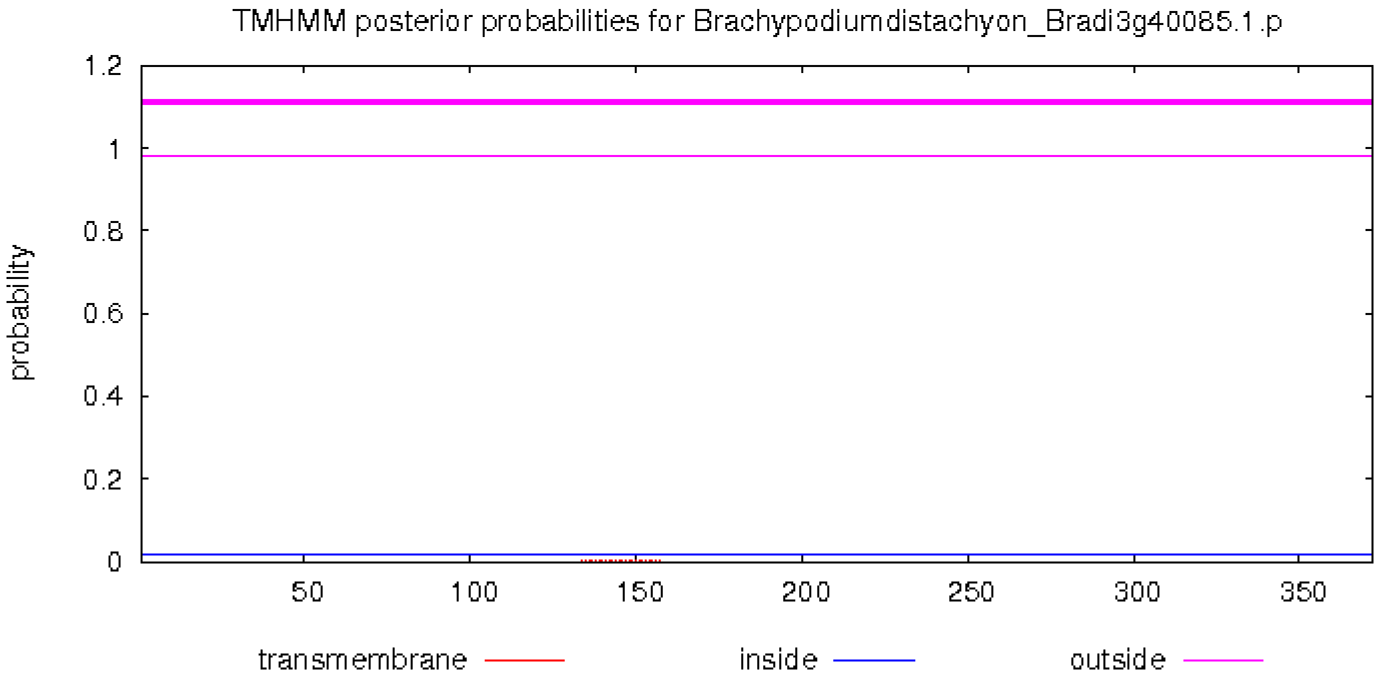

plot in postscript, script for making the plot in gnuplot, data for plot

---

```
# Brachypodiumdistachyon_Bradi3g41470.1.p Length: 251
# Brachypodiumdistachyon_Bradi3g41470.1.p Number of predicted TMHs:  0
# Brachypodiumdistachyon_Bradi3g41470.1.p Exp number of AAs in TMHs: 0.00052
# Brachypodiumdistachyon_Bradi3g41470.1.p Exp number, first 60 AAs:  0.00015
# Brachypodiumdistachyon_Bradi3g41470.1.p Total prob of N-in:        0.01941
Brachypodiumdistachyon_Bradi3g41470.1.p TMHMM2.0        outside      1   251
```

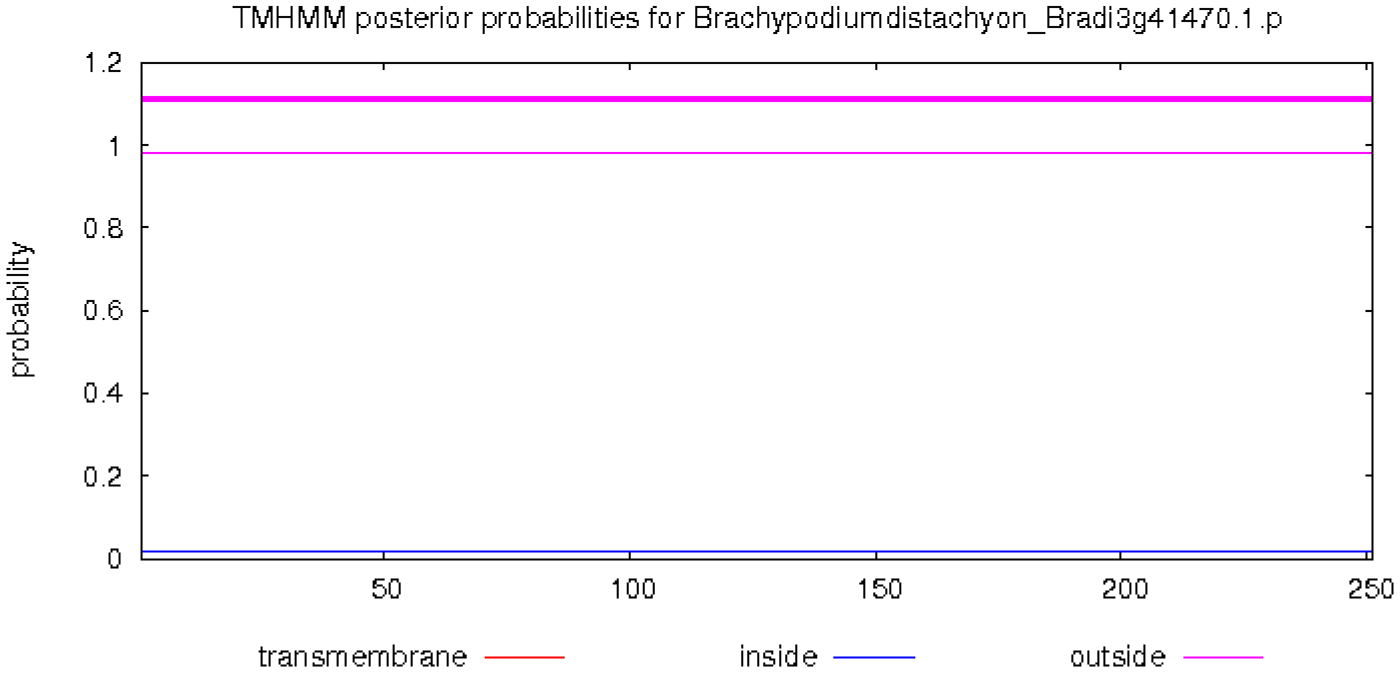

plot in postscript, script for making the plot in gnuplot, data for plot

---

```
# Brachypodiumdistachyon_Bradi3g46900.1.p Length: 344
# Brachypodiumdistachyon_Bradi3g46900.1.p Number of predicted TMHs:  0
# Brachypodiumdistachyon_Bradi3g46900.1.p Exp number of AAs in TMHs: 0.00874999999999999999
# Brachypodiumdistachyon_Bradi3g46900.1.p Exp number, first 60 AAs:  0
# Brachypodiumdistachyon_Bradi3g46900.1.p Total prob of N-in:        0.02148
Brachypodiumdistachyon_Bradi3g46900.1.p TMHMM2.0        outside      1   344
```

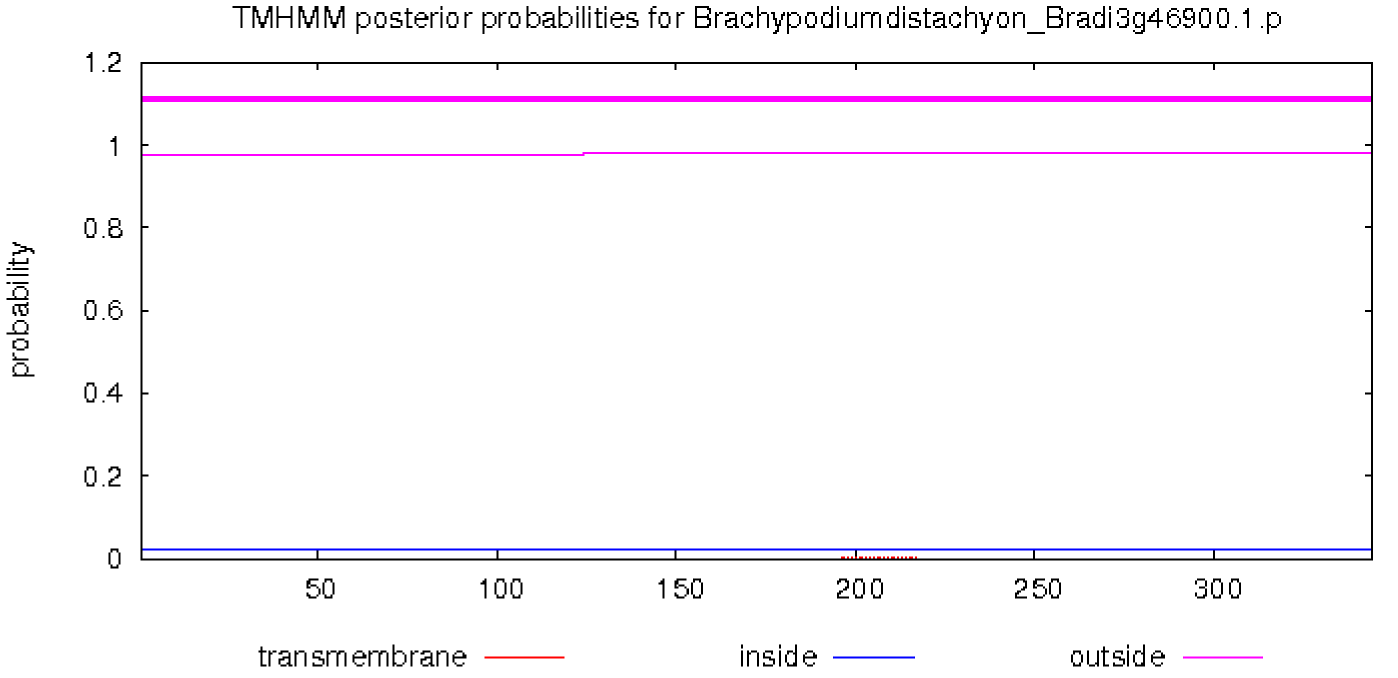

plot in postscript, script for making the plot in gnuplot, data for plot

---

```
# Brachypodiumdistachyon_Bradi3g47627.1.p Length: 382
# Brachypodiumdistachyon_Bradi3g47627.1.p Number of predicted TMHs:  0
# Brachypodiumdistachyon_Bradi3g47627.1.p Exp number of AAs in TMHs: 0.00043
# Brachypodiumdistachyon_Bradi3g47627.1.p Exp number, first 60 AAs:  0
# Brachypodiumdistachyon_Bradi3g47627.1.p Total prob of N-in:        0.02582
Brachypodiumdistachyon_Bradi3g47627.1.p TMHMM2.0        outside      1   382
```

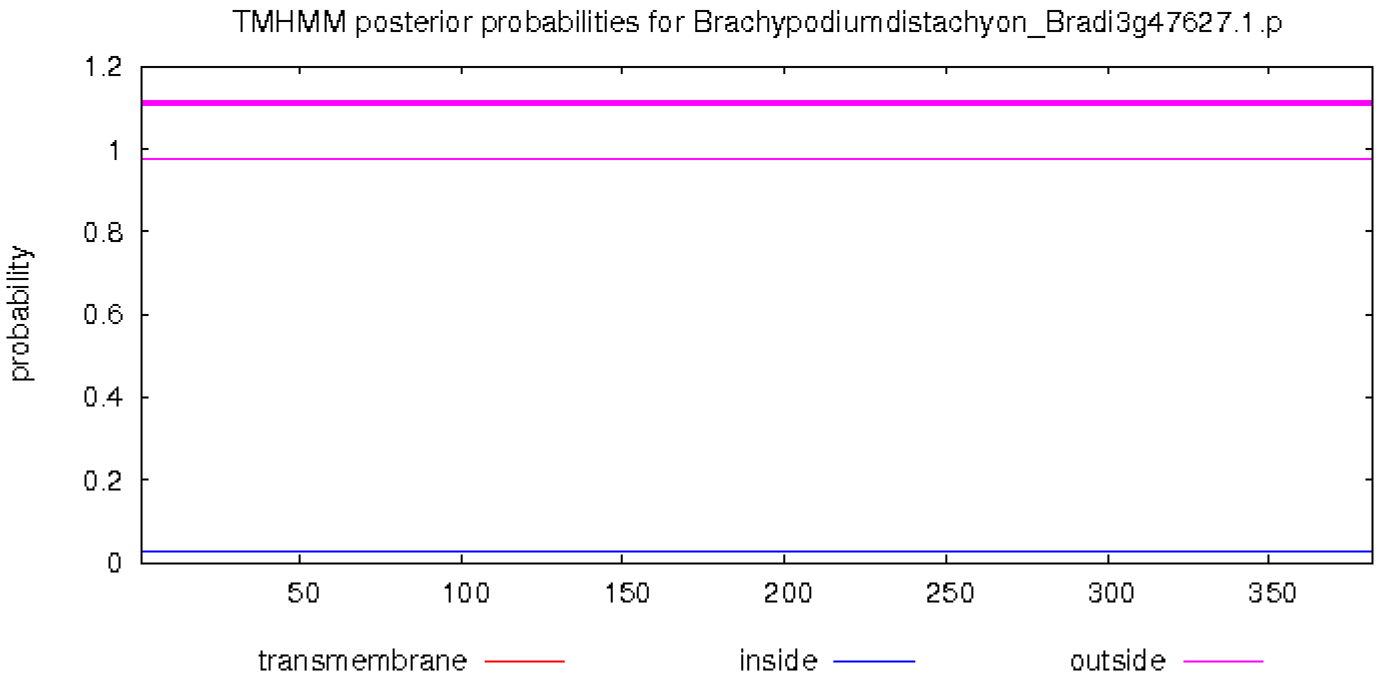

plot in postscript, script for making the plot in gnuplot, data for plot

---

```
# Brachypodiumdistachyon_Bradi3g50067.1.p Length: 344
# Brachypodiumdistachyon_Bradi3g50067.1.p Number of predicted TMHs:  0
# Brachypodiumdistachyon_Bradi3g50067.1.p Exp number of AAs in TMHs: 0.00019
# Brachypodiumdistachyon_Bradi3g50067.1.p Exp number, first 60 AAs:  0
# Brachypodiumdistachyon_Bradi3g50067.1.p Total prob of N-in:        0.00381
Brachypodiumdistachyon_Bradi3g50067.1.p TMHMM2.0        outside      1   344
```

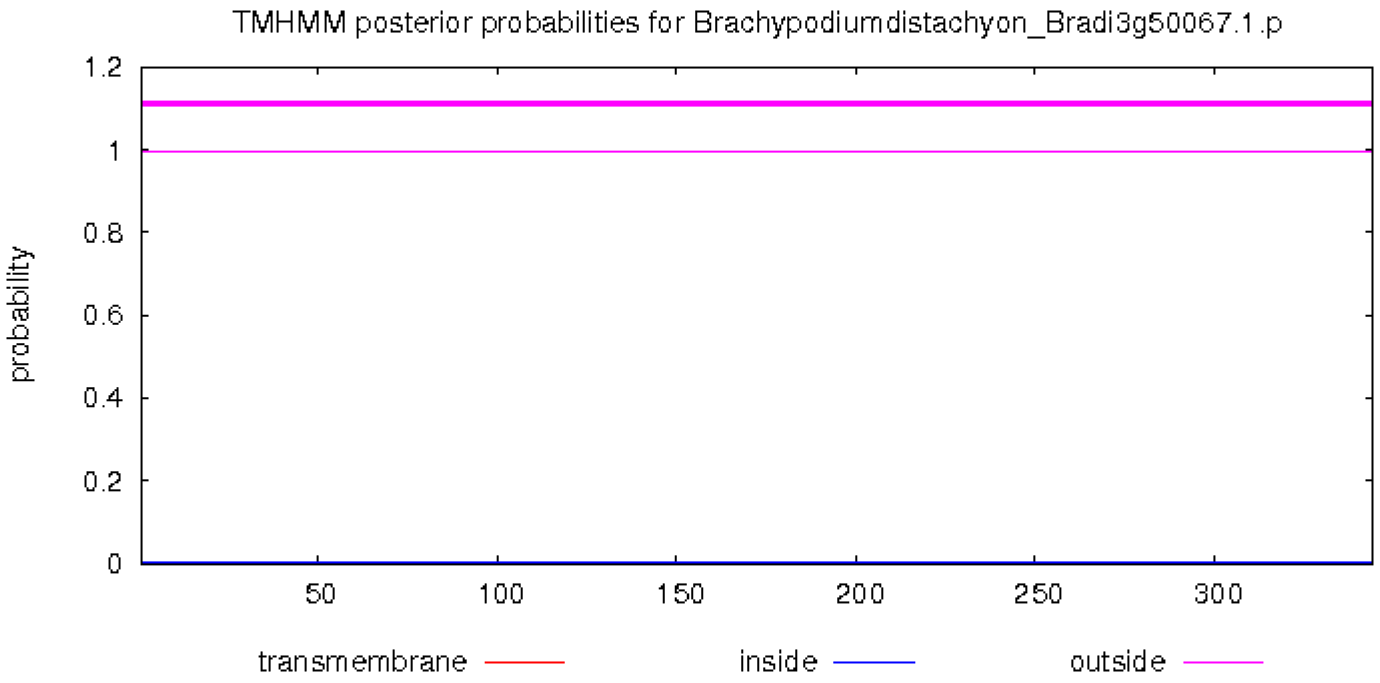

plot in postscript, script for making the plot in gnuplot, data for plot

---

```
# Brachypodiumdistachyon_Bradi3g54817.1.p Length: 301
# Brachypodiumdistachyon_Bradi3g54817.1.p Number of predicted TMHs:  0
# Brachypodiumdistachyon_Bradi3g54817.1.p Exp number of AAs in TMHs: 0.00164
# Brachypodiumdistachyon_Bradi3g54817.1.p Exp number, first 60 AAs:  0
# Brachypodiumdistachyon_Bradi3g54817.1.p Total prob of N-in:        0.01117
Brachypodiumdistachyon_Bradi3g54817.1.p TMHMM2.0        outside      1   301
```

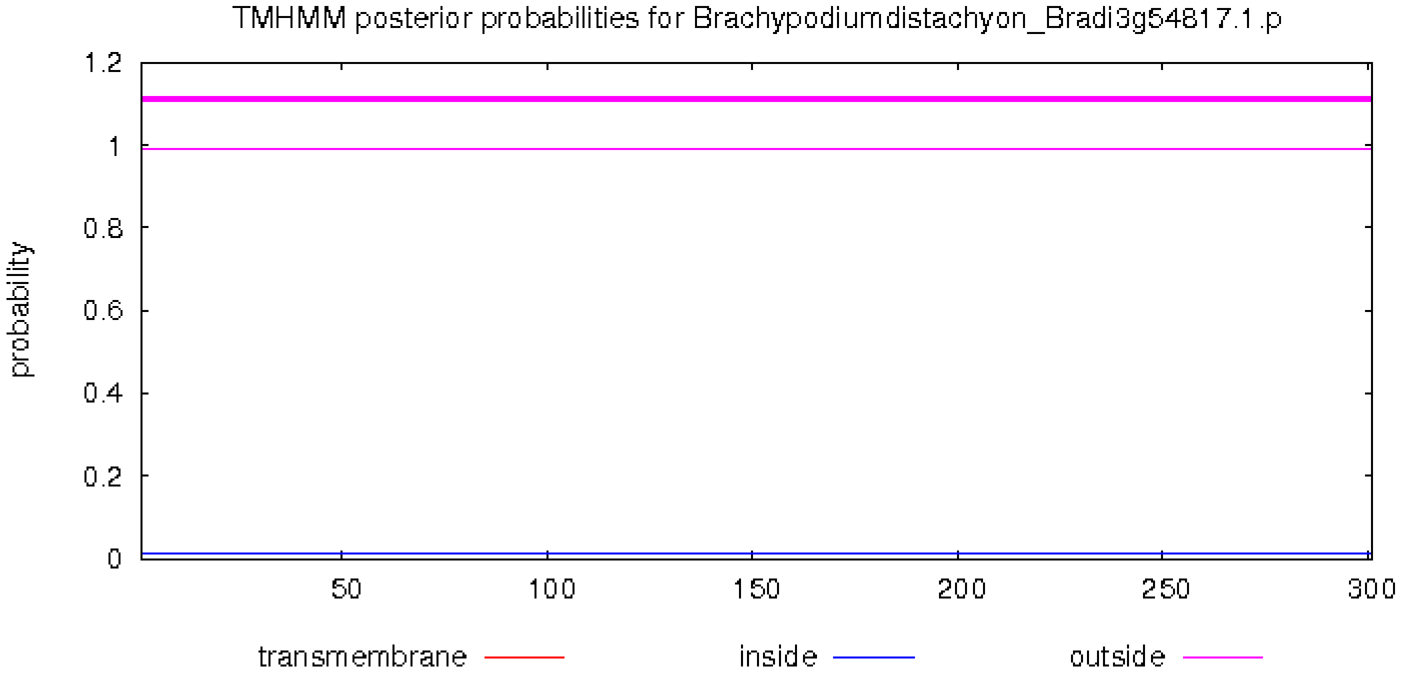

plot in postscript, script for making the plot in gnuplot, data for plot

---

```
# Brachypodiumdistachyon_Bradi3g56080.1.p Length: 648
# Brachypodiumdistachyon_Bradi3g56080.1.p Number of predicted TMHs:  1
# Brachypodiumdistachyon_Bradi3g56080.1.p Exp number of AAs in TMHs: 21.6645
# Brachypodiumdistachyon_Bradi3g56080.1.p Exp number, first 60 AAs:  0
# Brachypodiumdistachyon_Bradi3g56080.1.p Total prob of N-in:        0.00009
Brachypodiumdistachyon_Bradi3g56080.1.p TMHMM2.0        outside      1   623
Brachypodiumdistachyon_Bradi3g56080.1.p TMHMM2.0        TMhelix    624   646
Brachypodiumdistachyon_Bradi3g56080.1.p TMHMM2.0        inside     647   648
```

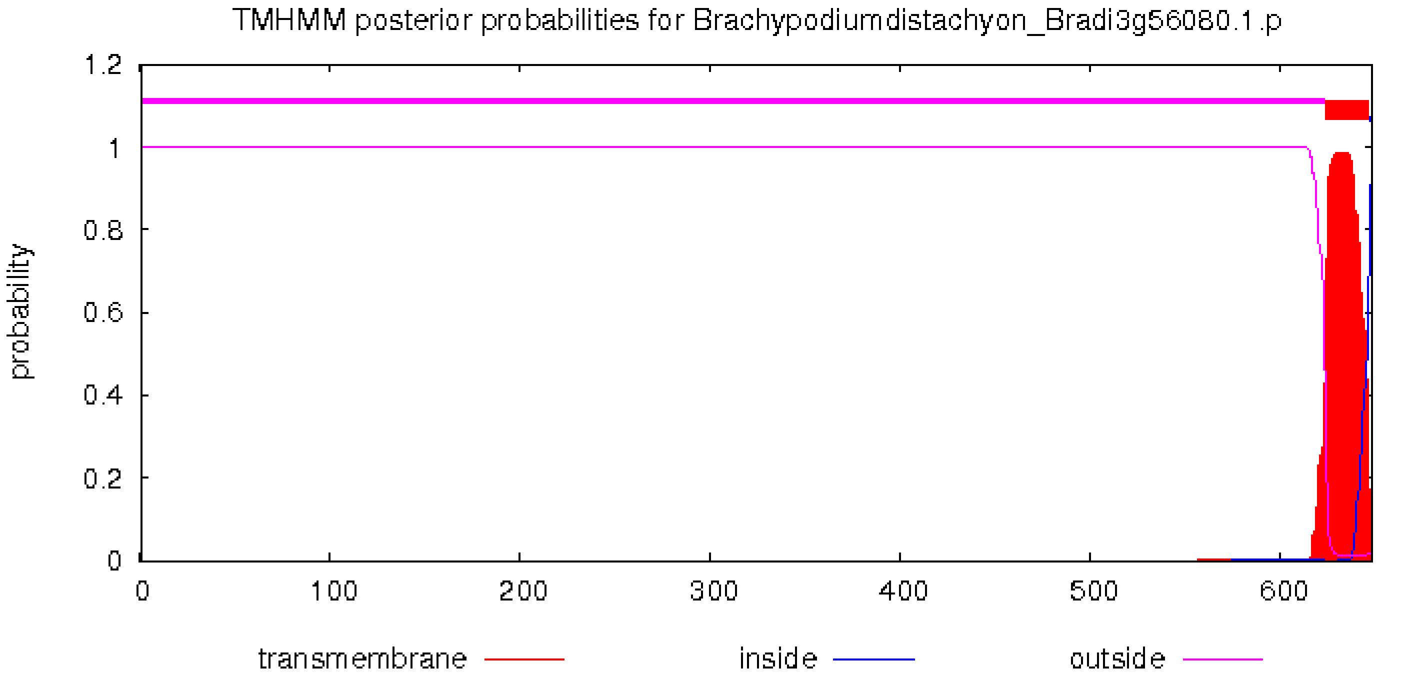

plot in postscript, script for making the plot in gnuplot, data for plot

```
# Brachypodiumdistachyon_Bradi3g59380.1.p Length: 387
# Brachypodiumdistachyon_Bradi3g59380.1.p Number of predicted TMHs:  0
# Brachypodiumdistachyon_Bradi3g59380.1.p Exp number of AAs in TMHs: 0.00224
# Brachypodiumdistachyon_Bradi3g59380.1.p Exp number, first 60 AAs:  0
# Brachypodiumdistachyon_Bradi3g59380.1.p Total prob of N-in:        0.01497
Brachypodiumdistachyon_Bradi3g59380.1.p TMHMM2.0        outside      1   387
```

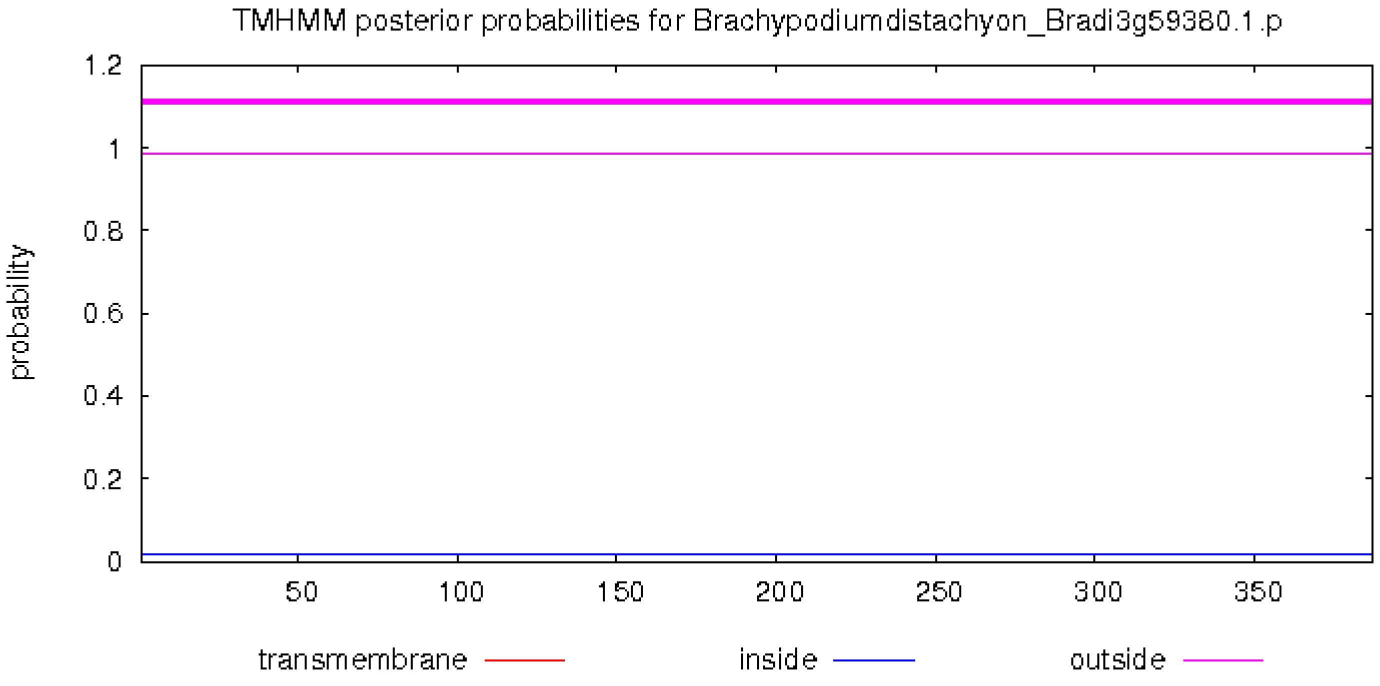

plot in postscript, script for making the plot in gnuplot, data for plot

```
# Brachypodiumdistachyon_Bradi3g59866.1.p Length: 591
# Brachypodiumdistachyon_Bradi3g59866.1.p Number of predicted TMHs:  0
# Brachypodiumdistachyon_Bradi3g59866.1.p Exp number of AAs in TMHs: 0.00085
# Brachypodiumdistachyon_Bradi3g59866.1.p Exp number, first 60 AAs:  0.00018
# Brachypodiumdistachyon_Bradi3g59866.1.p Total prob of N-in:        0.00197
Brachypodiumdistachyon_Bradi3g59866.1.p TMHMM2.0        outside      1   591
```

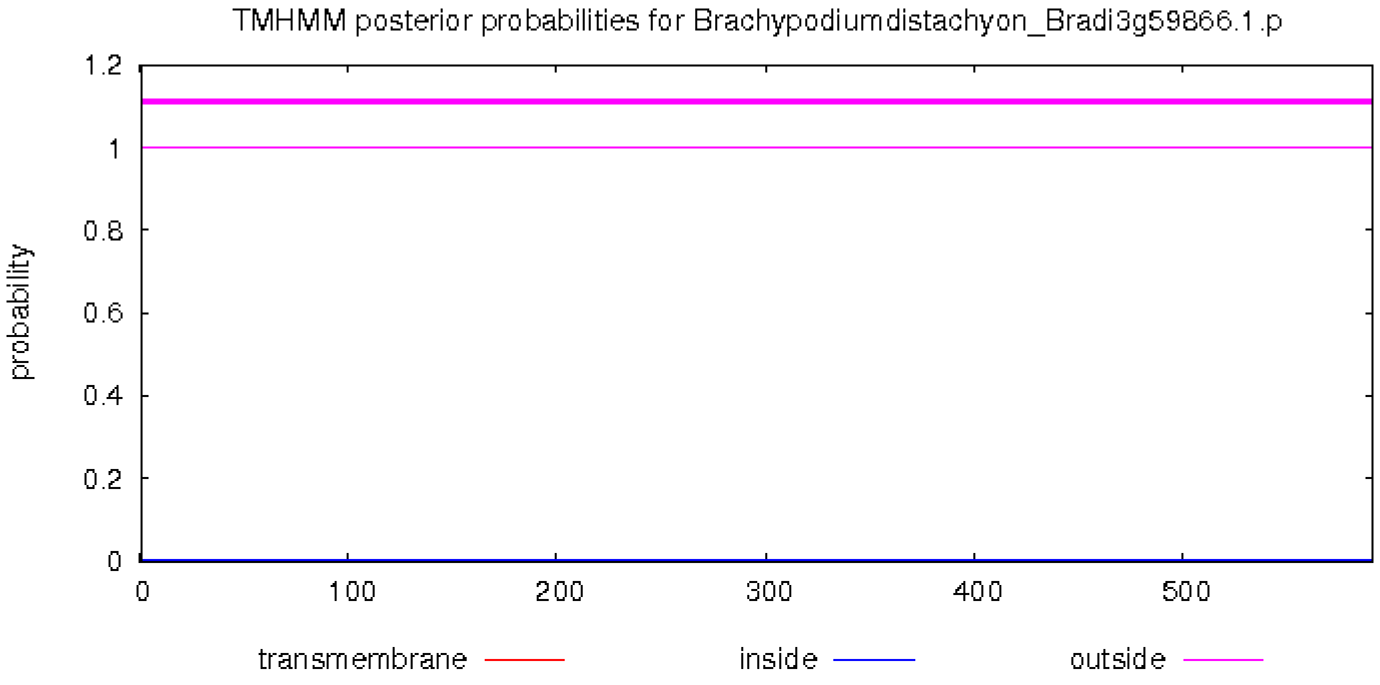

plot in postscript, script for making the plot in gnuplot, data for plot

```
# Brachypodiumdistachyon_Bradi4g02060.2.p Length: 313
# Brachypodiumdistachyon_Bradi4g02060.2.p Number of predicted TMHs:  0
# Brachypodiumdistachyon_Bradi4g02060.2.p Exp number of AAs in TMHs: 0.005
# Brachypodiumdistachyon_Bradi4g02060.2.p Exp number, first 60 AAs:  0.00016
# Brachypodiumdistachyon_Bradi4g02060.2.p Total prob of N-in:        0.00567
Brachypodiumdistachyon_Bradi4g02060.2.p TMHMM2.0        outside      1   313
```

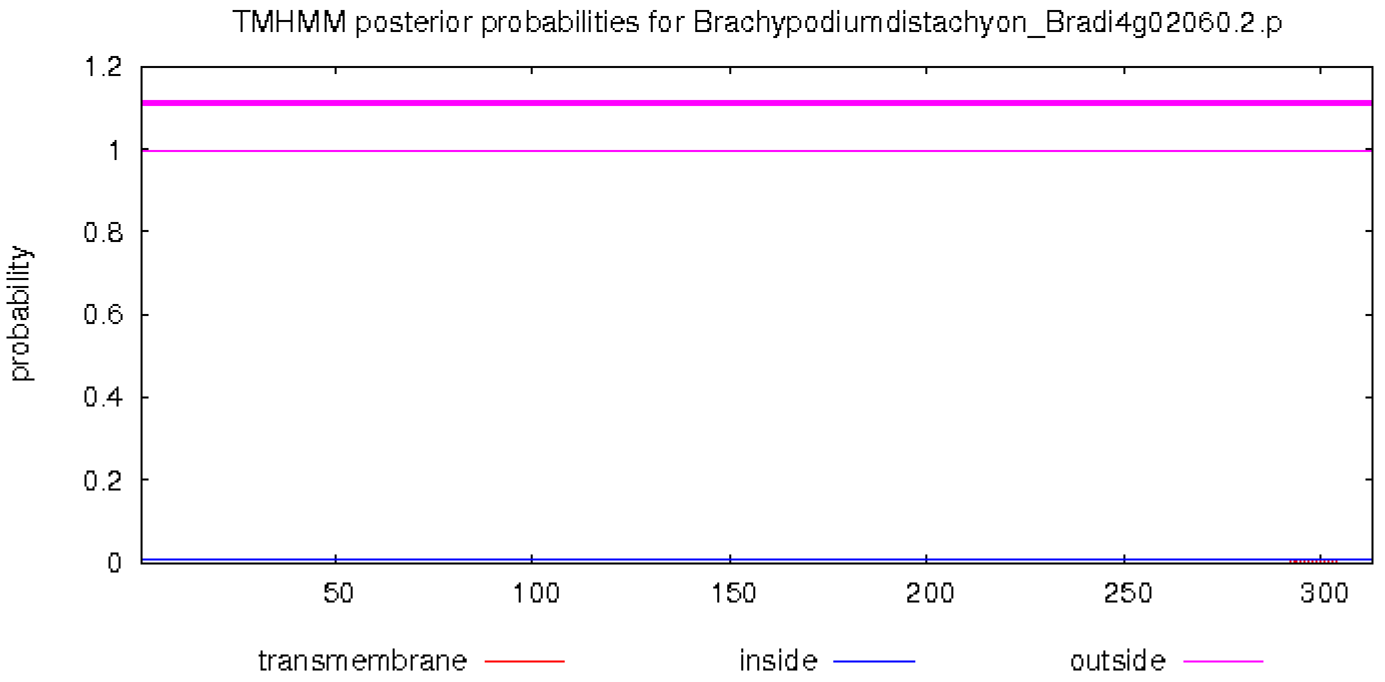

plot in postscript, script for making the plot in gnuplot, data for plot

---

```
# Brachypodiumdistachyon_Bradi4g03620.1.p Length: 576
# Brachypodiumdistachyon_Bradi4g03620.1.p Number of predicted TMHs:  0
# Brachypodiumdistachyon_Bradi4g03620.1.p Exp number of AAs in TMHs: 0.00252
# Brachypodiumdistachyon_Bradi4g03620.1.p Exp number, first 60 AAs:  0.00022
# Brachypodiumdistachyon_Bradi4g03620.1.p Total prob of N-in:        0.00021
Brachypodiumdistachyon_Bradi4g03620.1.p TMHMM2.0        outside      1   576
```

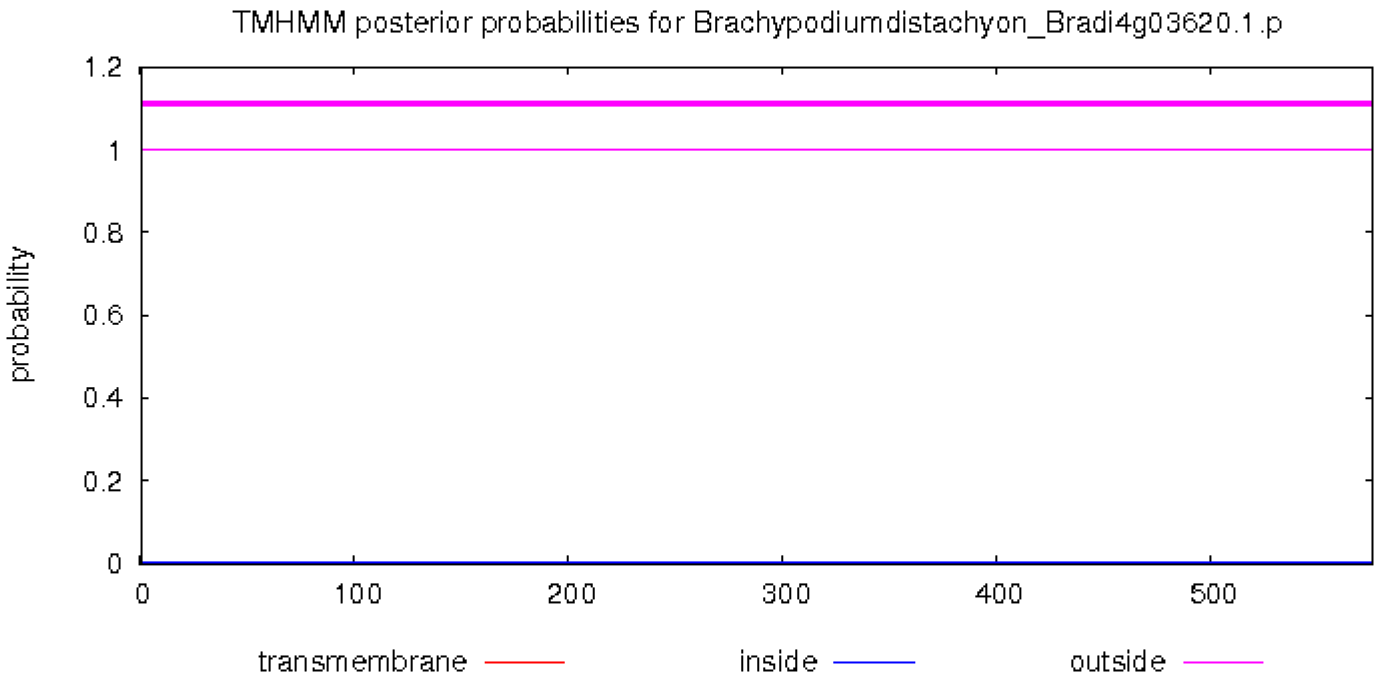

plot in postscript, script for making the plot in gnuplot, data for plot

---

```
# Brachypodiumdistachyon_Bradi4g07527.1.p Length: 252
# Brachypodiumdistachyon_Bradi4g07527.1.p Number of predicted TMHs:  0
# Brachypodiumdistachyon_Bradi4g07527.1.p Exp number of AAs in TMHs: 0.12377
# Brachypodiumdistachyon_Bradi4g07527.1.p Exp number, first 60 AAs:  0.00014
# Brachypodiumdistachyon_Bradi4g07527.1.p Total prob of N-in:        0.11121
Brachypodiumdistachyon_Bradi4g07527.1.p TMHMM2.0        outside      1   252
```

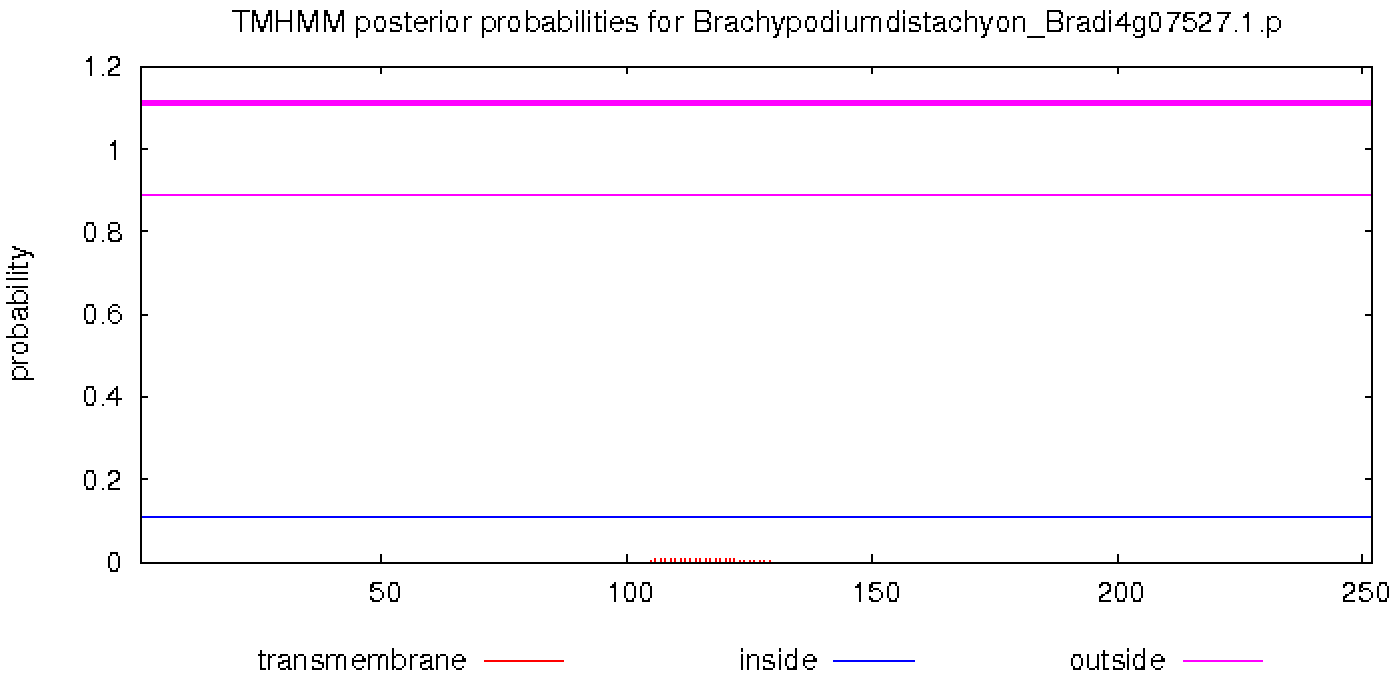

plot in postscript, script for making the plot in gnuplot, data for plot

---

```
# Brachypodiumdistachyon_Bradi4g12065.1.p Length: 294
# Brachypodiumdistachyon_Bradi4g12065.1.p Number of predicted TMHs:  0
# Brachypodiumdistachyon_Bradi4g12065.1.p Exp number of AAs in TMHs: 0.00044
# Brachypodiumdistachyon_Bradi4g12065.1.p Exp number, first 60 AAs:  0
# Brachypodiumdistachyon_Bradi4g12065.1.p Total prob of N-in:        0.00316
Brachypodiumdistachyon_Bradi4g12065.1.p TMHMM2.0        outside      1   294
```

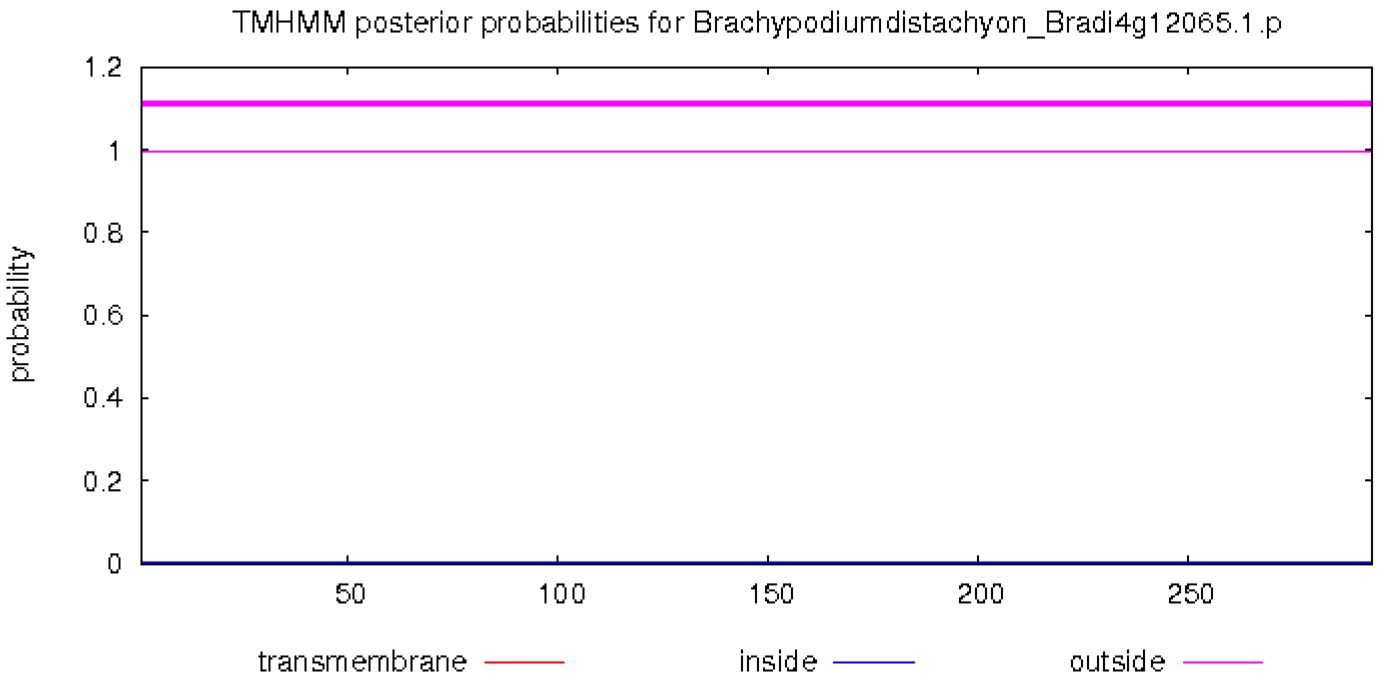

plot in postscript, script for making the plot in gnuplot, data for plot

---

```
# Brachypodiumdistachyon_Bradi4g13562.1.p Length: 106
# Brachypodiumdistachyon_Bradi4g13562.1.p Number of predicted TMHs:  0
# Brachypodiumdistachyon_Bradi4g13562.1.p Exp number of AAs in TMHs: 0.01214
# Brachypodiumdistachyon_Bradi4g13562.1.p Exp number, first 60 AAs:  0.01214
# Brachypodiumdistachyon_Bradi4g13562.1.p Total prob of N-in:        0.31581
Brachypodiumdistachyon_Bradi4g13562.1.p TMHMM2.0        outside      1   106
```

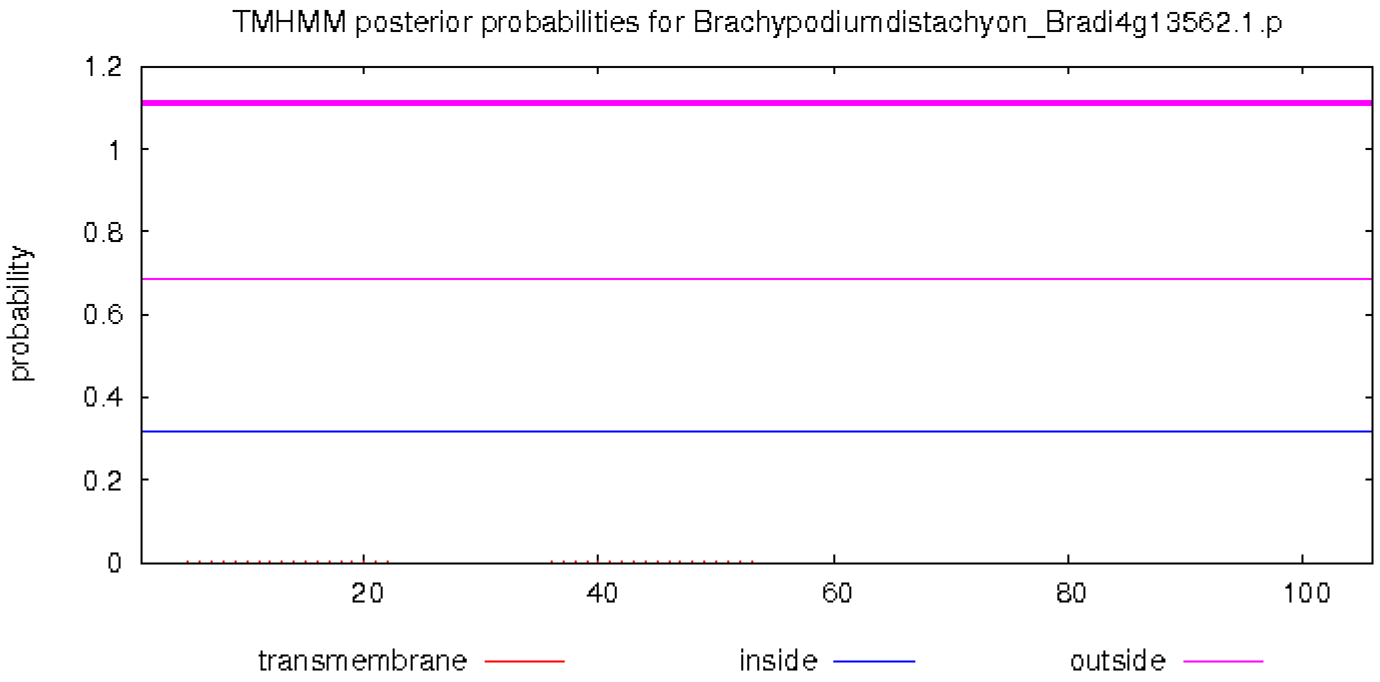

plot in postscript, script for making the plot in gnuplot, data for plot

---

```
# Brachypodiumdistachyon_Bradi4g13570.1.p Length: 75
# Brachypodiumdistachyon_Bradi4g13570.1.p Number of predicted TMHs:  0
# Brachypodiumdistachyon_Bradi4g13570.1.p Exp number of AAs in TMHs: 1.39734
# Brachypodiumdistachyon_Bradi4g13570.1.p Exp number, first 60 AAs:  1.39734
# Brachypodiumdistachyon_Bradi4g13570.1.p Total prob of N-in:        0.15696
Brachypodiumdistachyon_Bradi4g13570.1.p TMHMM2.0        outside      1    75
```

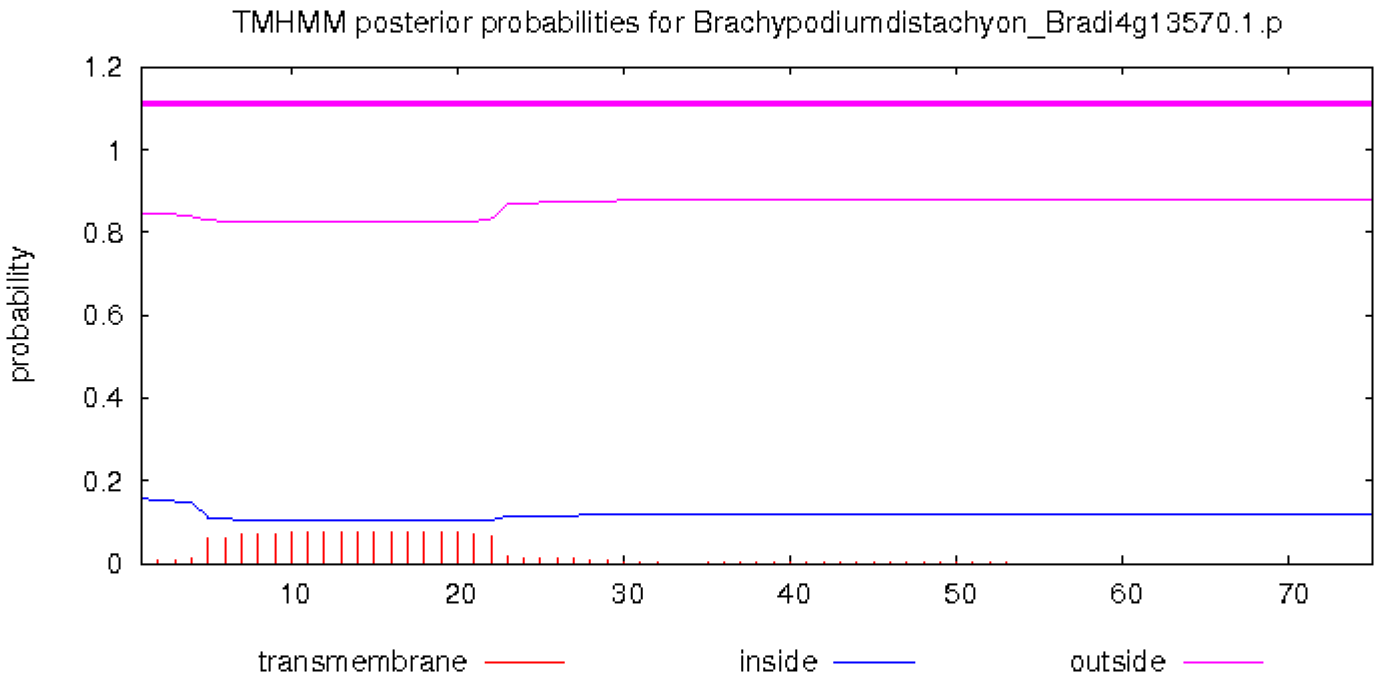

plot in postscript, script for making the plot in gnuplot, data for plot

---

```
# Brachypodiumdistachyon_Bradi4g13575.1.p Length: 138
# Brachypodiumdistachyon_Bradi4g13575.1.p Number of predicted TMHs:  0
# Brachypodiumdistachyon_Bradi4g13575.1.p Exp number of AAs in TMHs: 0.00203
# Brachypodiumdistachyon_Bradi4g13575.1.p Exp number, first 60 AAs:  0.00185
# Brachypodiumdistachyon_Bradi4g13575.1.p Total prob of N-in:        0.10508
Brachypodiumdistachyon_Bradi4g13575.1.p TMHMM2.0        outside      1   138
```

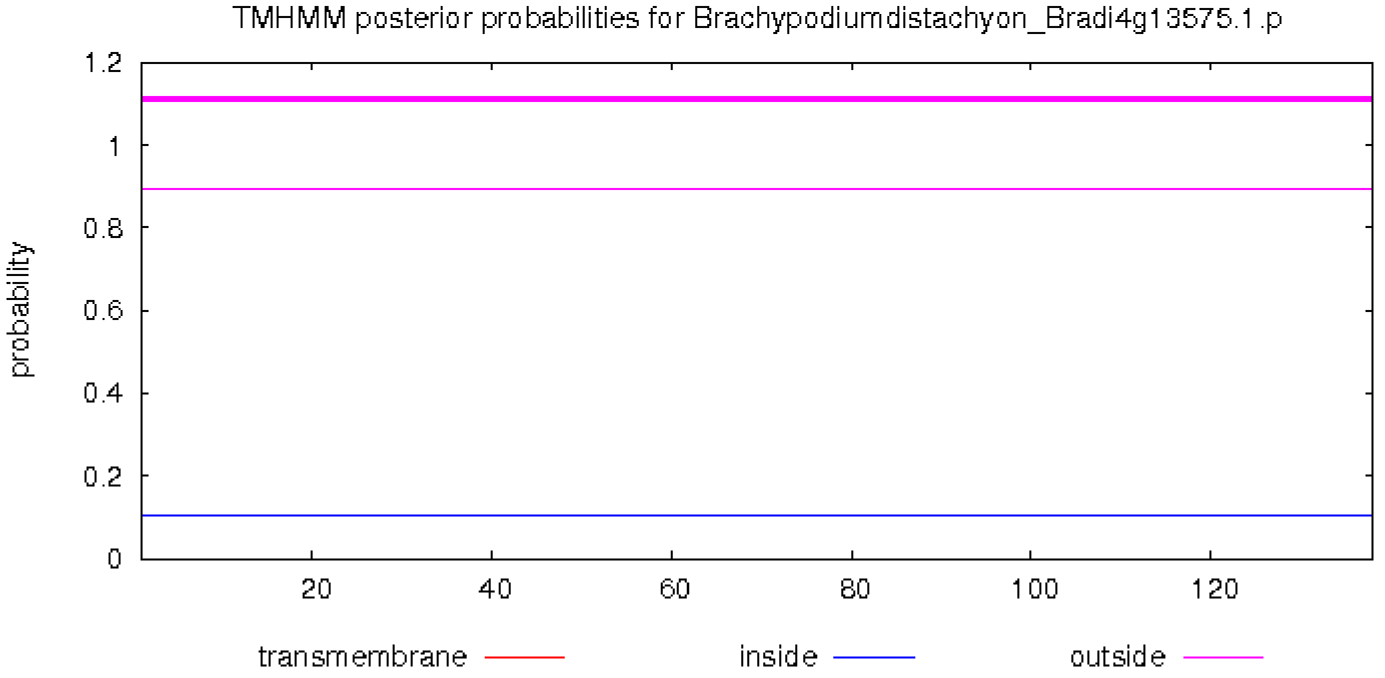

plot in postscript, script for making the plot in gnuplot, data for plot

---

```
# Brachypodiumdistachyon_Bradi4g13586.1.p Length: 204
# Brachypodiumdistachyon_Bradi4g13586.1.p Number of predicted TMHs:  1
# Brachypodiumdistachyon_Bradi4g13586.1.p Exp number of AAs in TMHs: 18.68062
# Brachypodiumdistachyon_Bradi4g13586.1.p Exp number, first 60 AAs:  18.65908
# Brachypodiumdistachyon_Bradi4g13586.1.p Total prob of N-in:        0.88865
# Brachypodiumdistachyon_Bradi4g13586.1.p POSSIBLE N-term signal sequence
Brachypodiumdistachyon_Bradi4g13586.1.p TMHMM2.0        inside       1     4
Brachypodiumdistachyon_Bradi4g13586.1.p TMHMM2.0        TMhelix      5    27
Brachypodiumdistachyon_Bradi4g13586.1.p TMHMM2.0        outside     28   204
```

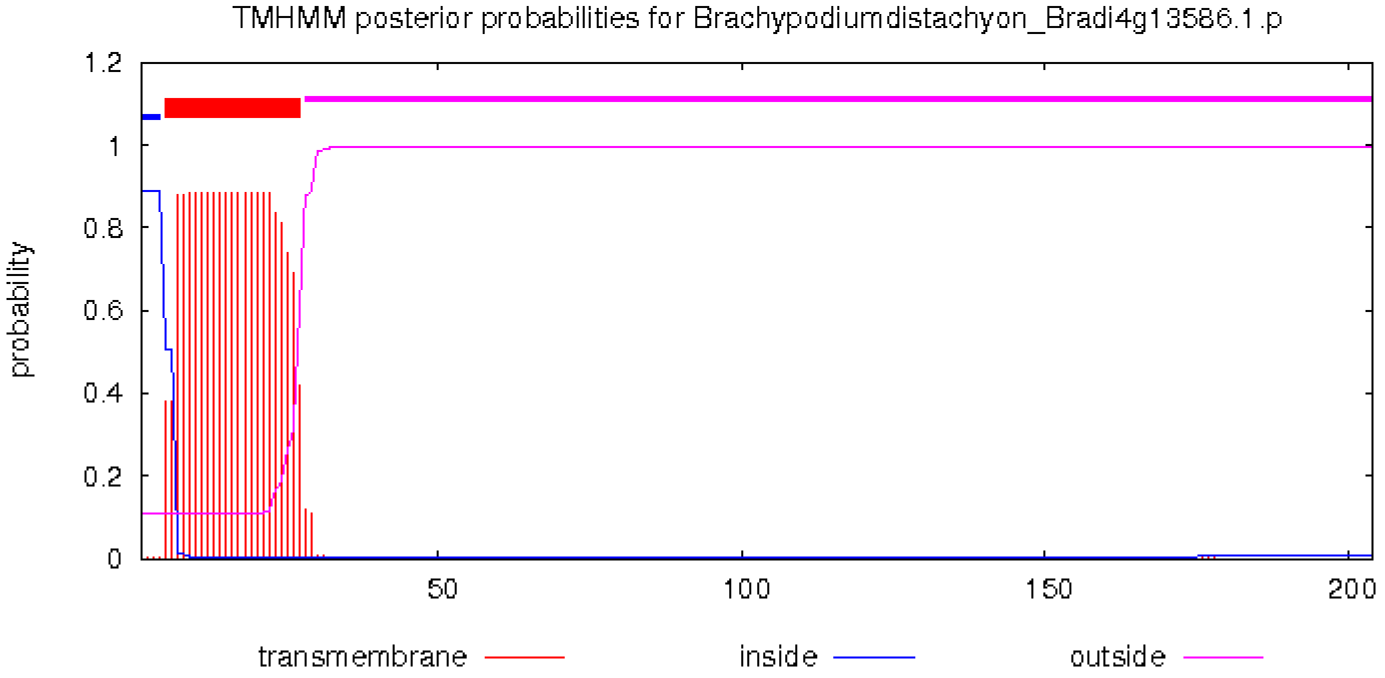

plot in postscript, script for making the plot in gnuplot, data for plot

```
# Brachypodiumdistachyon_Bradi4g22195.1.p Length: 179
# Brachypodiumdistachyon_Bradi4g22195.1.p Number of predicted TMHs:  0
# Brachypodiumdistachyon_Bradi4g22195.1.p Exp number of AAs in TMHs: 0.04839
# Brachypodiumdistachyon_Bradi4g22195.1.p Exp number, first 60 AAs:  0.01028
# Brachypodiumdistachyon_Bradi4g22195.1.p Total prob of N-in:        0.04264
Brachypodiumdistachyon_Bradi4g22195.1.p TMHMM2.0        outside      1   179
```

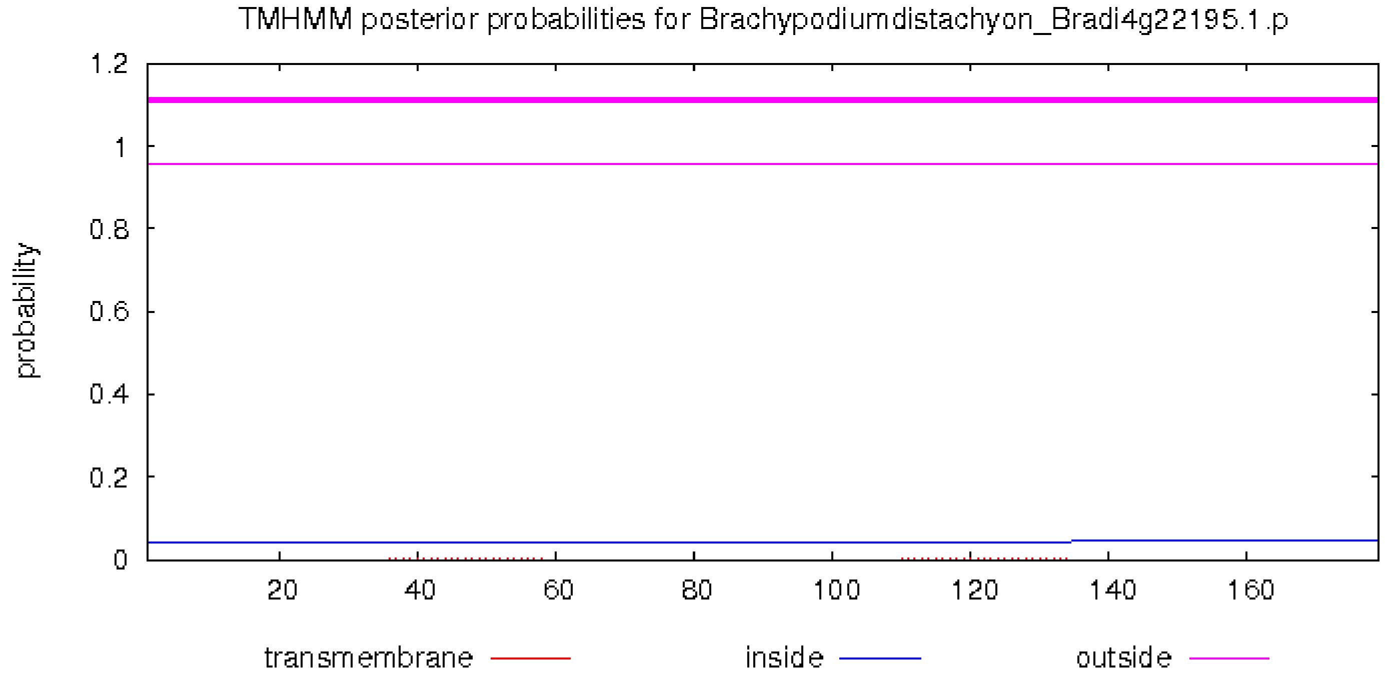

plot in postscript, script for making the plot in gnuplot, data for plot

```
# Brachypodiumdistachyon_Bradi4g22216.2.p Length: 172
# Brachypodiumdistachyon_Bradi4g22216.2.p Number of predicted TMHs:  0
# Brachypodiumdistachyon_Bradi4g22216.2.p Exp number of AAs in TMHs: 1.08766
# Brachypodiumdistachyon_Bradi4g22216.2.p Exp number, first 60 AAs:  0.00053
# Brachypodiumdistachyon_Bradi4g22216.2.p Total prob of N-in:        0.06909
Brachypodiumdistachyon_Bradi4g22216.2.p TMHMM2.0        outside      1   172
```

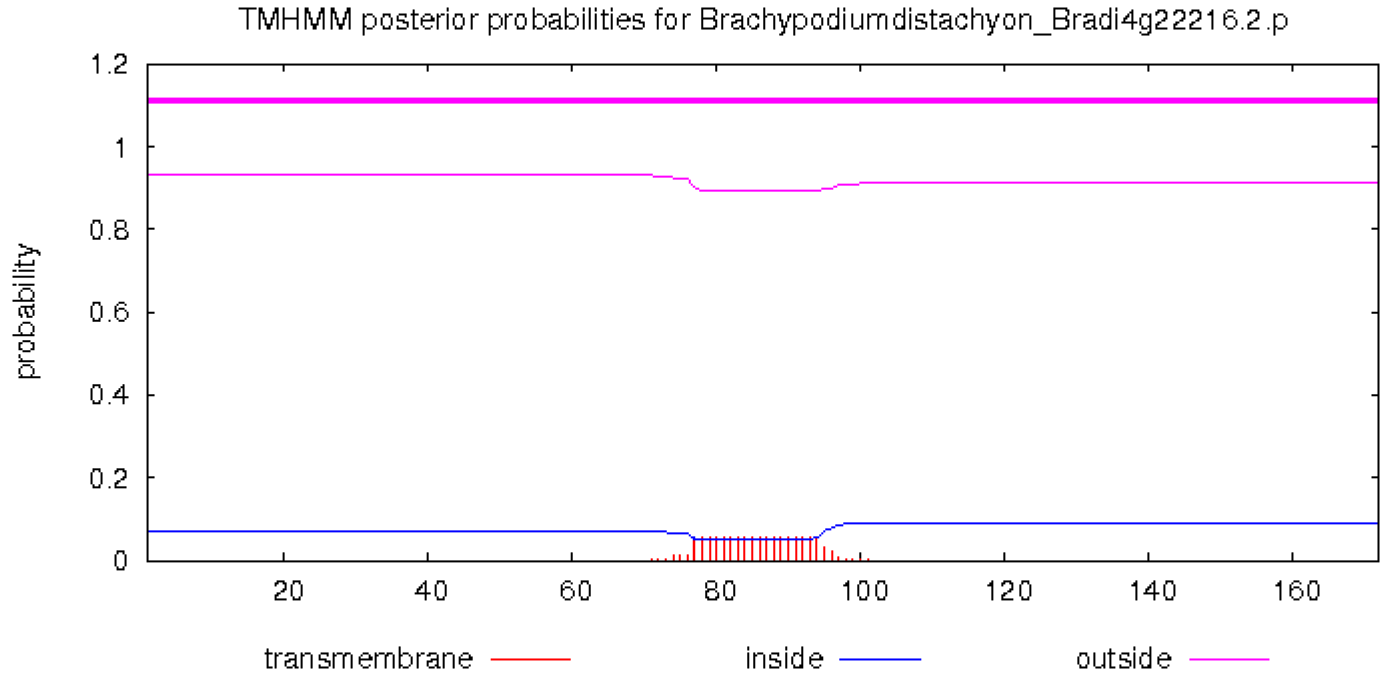

plot in postscript, script for making the plot in gnuplot, data for plot

---

```
# Brachypodiumdistachyon_Bradi4g22681.1.p Length: 191
# Brachypodiumdistachyon_Bradi4g22681.1.p Number of predicted TMHs:  0
# Brachypodiumdistachyon_Bradi4g22681.1.p Exp number of AAs in TMHs: 0.10042
# Brachypodiumdistachyon_Bradi4g22681.1.p Exp number, first 60 AAs:  0.00392
# Brachypodiumdistachyon_Bradi4g22681.1.p Total prob of N-in:        0.05705
Brachypodiumdistachyon_Bradi4g22681.1.p TMHMM2.0        outside      1   191
```

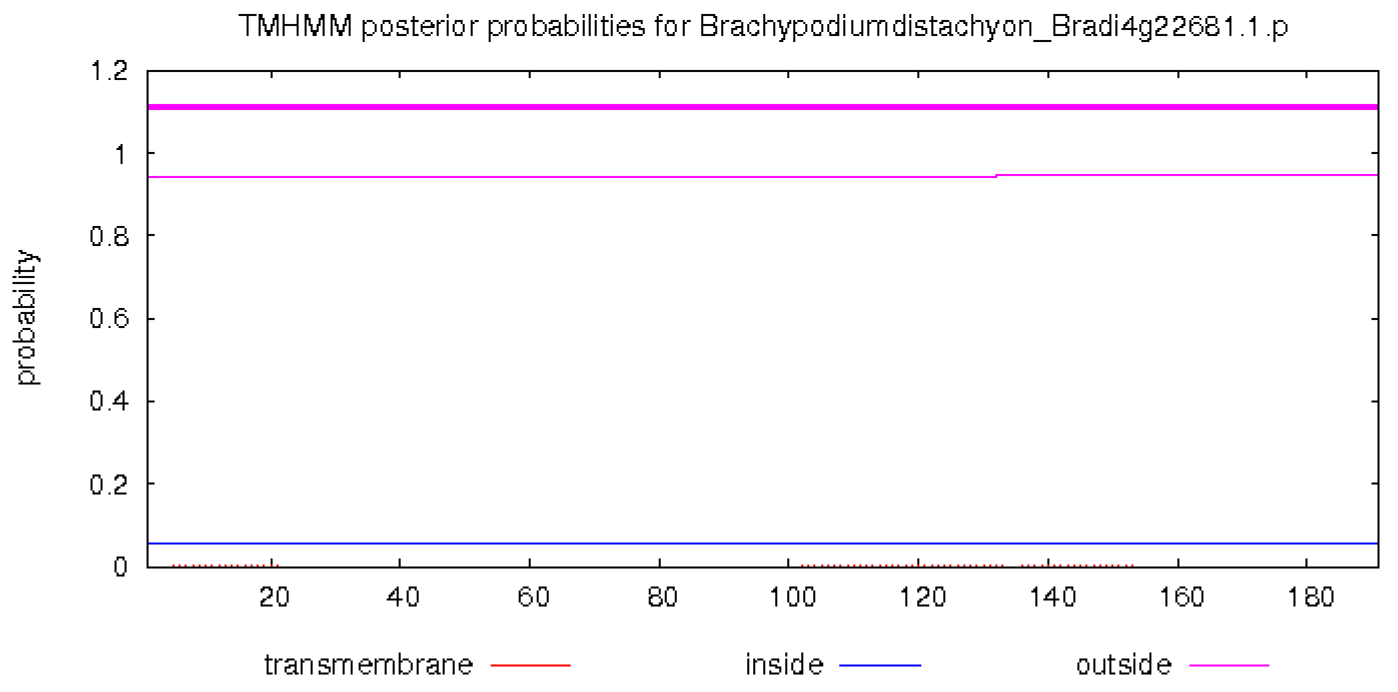

plot in postscript, script for making the plot in gnuplot, data for plot

---

```
# Brachypodiumdistachyon_Bradi4g23640.1.p Length: 366
# Brachypodiumdistachyon_Bradi4g23640.1.p Number of predicted TMHs:  0
# Brachypodiumdistachyon_Bradi4g23640.1.p Exp number of AAs in TMHs: 0.00457
# Brachypodiumdistachyon_Bradi4g23640.1.p Exp number, first 60 AAs:  0.00394
# Brachypodiumdistachyon_Bradi4g23640.1.p Total prob of N-in:        0.00979
Brachypodiumdistachyon_Bradi4g23640.1.p TMHMM2.0        outside      1   366
```

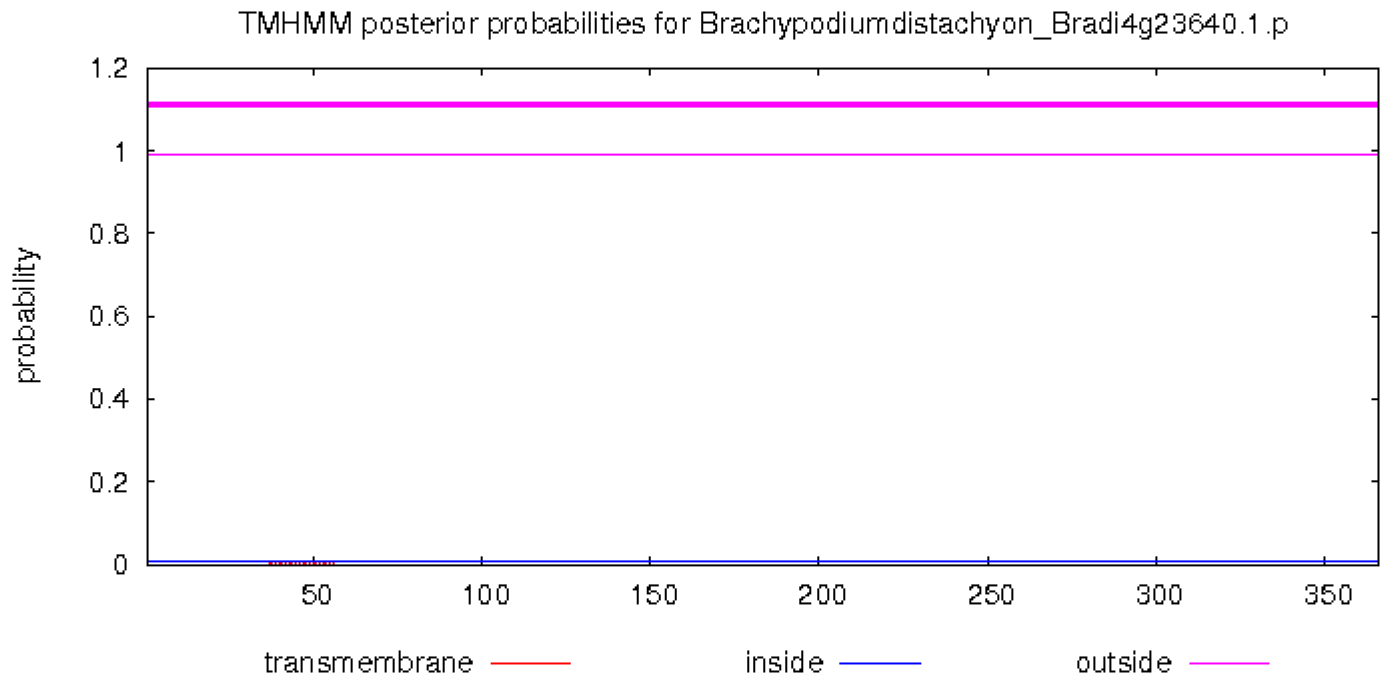

plot in postscript, script for making the plot in gnuplot, data for plot

```
# Brachypodiumdistachyon_Bradi4g24060.1.p Length: 304
# Brachypodiumdistachyon_Bradi4g24060.1.p Number of predicted TMHs:  0
# Brachypodiumdistachyon_Bradi4g24060.1.p Exp number of AAs in TMHs: 0.00754
# Brachypodiumdistachyon_Bradi4g24060.1.p Exp number, first 60 AAs:  0.00754
# Brachypodiumdistachyon_Bradi4g24060.1.p Total prob of N-in:        0.03164
Brachypodiumdistachyon_Bradi4g24060.1.p TMHMM2.0        outside      1   304
```

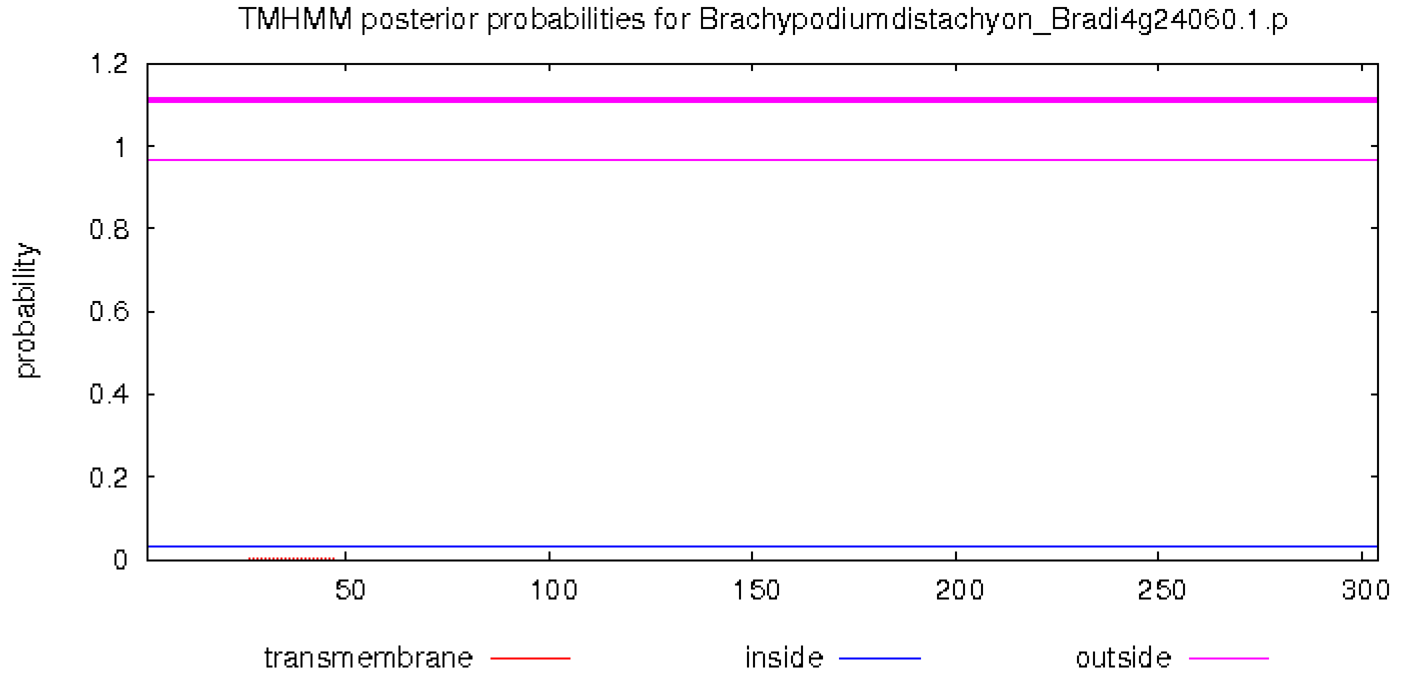

plot in postscript, script for making the plot in gnuplot, data for plot

```
# Brachypodiumdistachyon_Bradi4g24100.1.p Length: 353
# Brachypodiumdistachyon_Bradi4g24100.1.p Number of predicted TMHs:  0
# Brachypodiumdistachyon_Bradi4g24100.1.p Exp number of AAs in TMHs: 0.0566
# Brachypodiumdistachyon_Bradi4g24100.1.p Exp number, first 60 AAs:  0
# Brachypodiumdistachyon_Bradi4g24100.1.p Total prob of N-in:        0.01292
Brachypodiumdistachyon_Bradi4g24100.1.p TMHMM2.0        outside      1   353
```

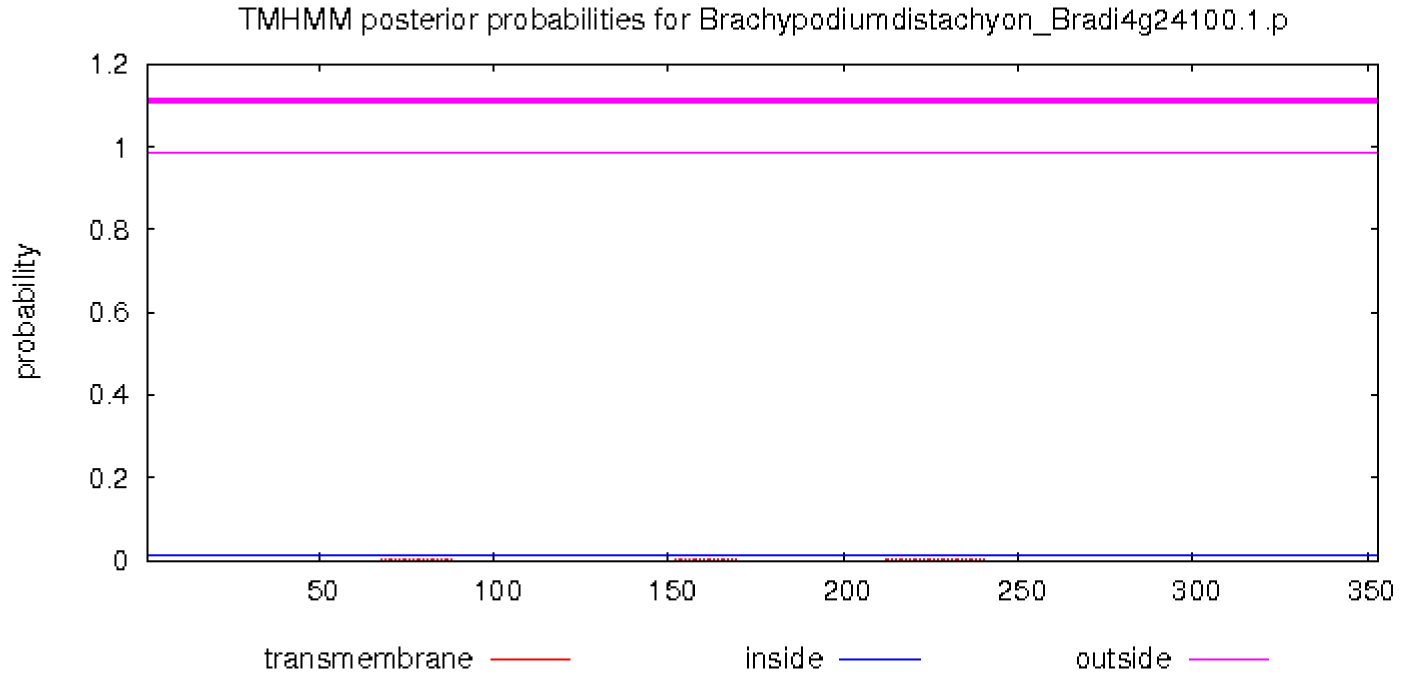

plot in postscript, script for making the plot in gnuplot, data for plot

---

```
# Brachypodiumdistachyon_Bradi4g26275.1.p Length: 438
# Brachypodiumdistachyon_Bradi4g26275.1.p Number of predicted TMHs:  0
# Brachypodiumdistachyon_Bradi4g26275.1.p Exp number of AAs in TMHs: 0.00055
# Brachypodiumdistachyon_Bradi4g26275.1.p Exp number, first 60 AAs:  0.00055
# Brachypodiumdistachyon_Bradi4g26275.1.p Total prob of N-in:        0.00148
Brachypodiumdistachyon_Bradi4g26275.1.p TMHMM2.0        outside      1   438
```

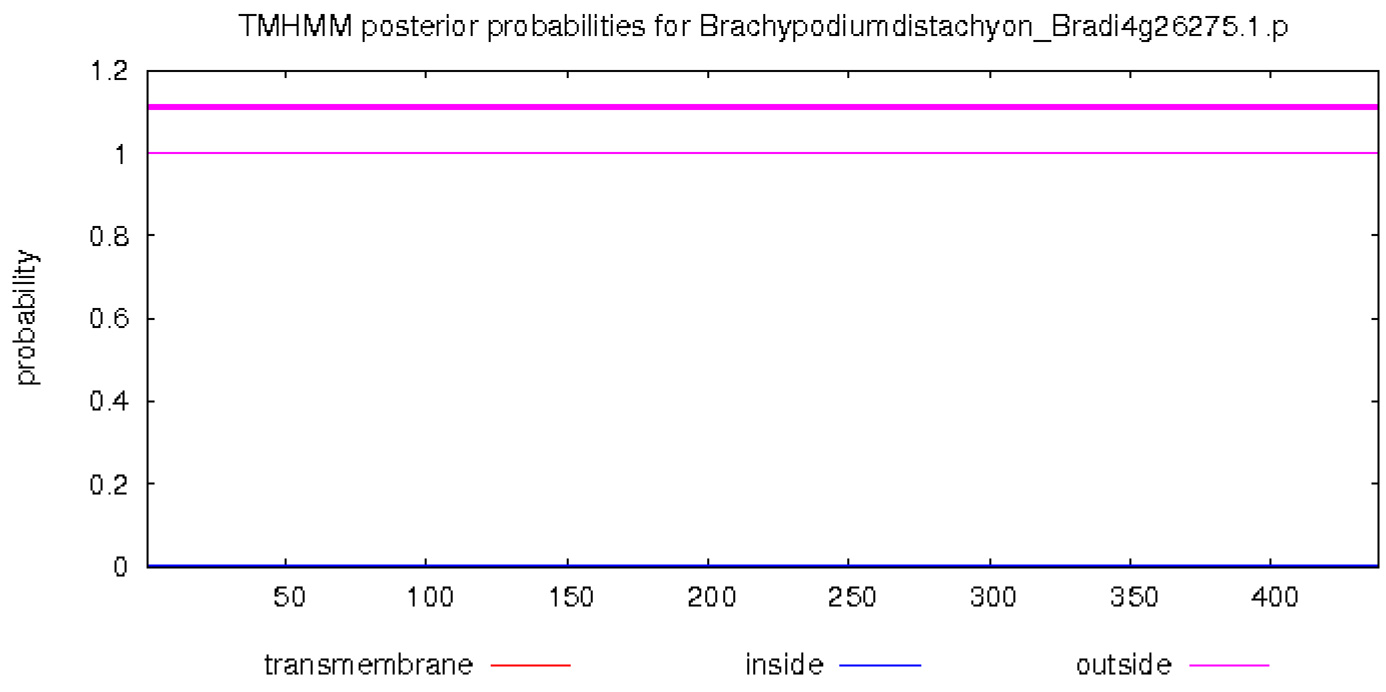

plot in postscript, script for making the plot in gnuplot, data for plot

---

```
# Brachypodiumdistachyon_Bradi4g26470.2.p Length: 425
# Brachypodiumdistachyon_Bradi4g26470.2.p Number of predicted TMHs:  0
# Brachypodiumdistachyon_Bradi4g26470.2.p Exp number of AAs in TMHs: 0.00117
# Brachypodiumdistachyon_Bradi4g26470.2.p Exp number, first 60 AAs:  0.00059
# Brachypodiumdistachyon_Bradi4g26470.2.p Total prob of N-in:        0.01608
Brachypodiumdistachyon_Bradi4g26470.2.p TMHMM2.0        outside      1   425
```

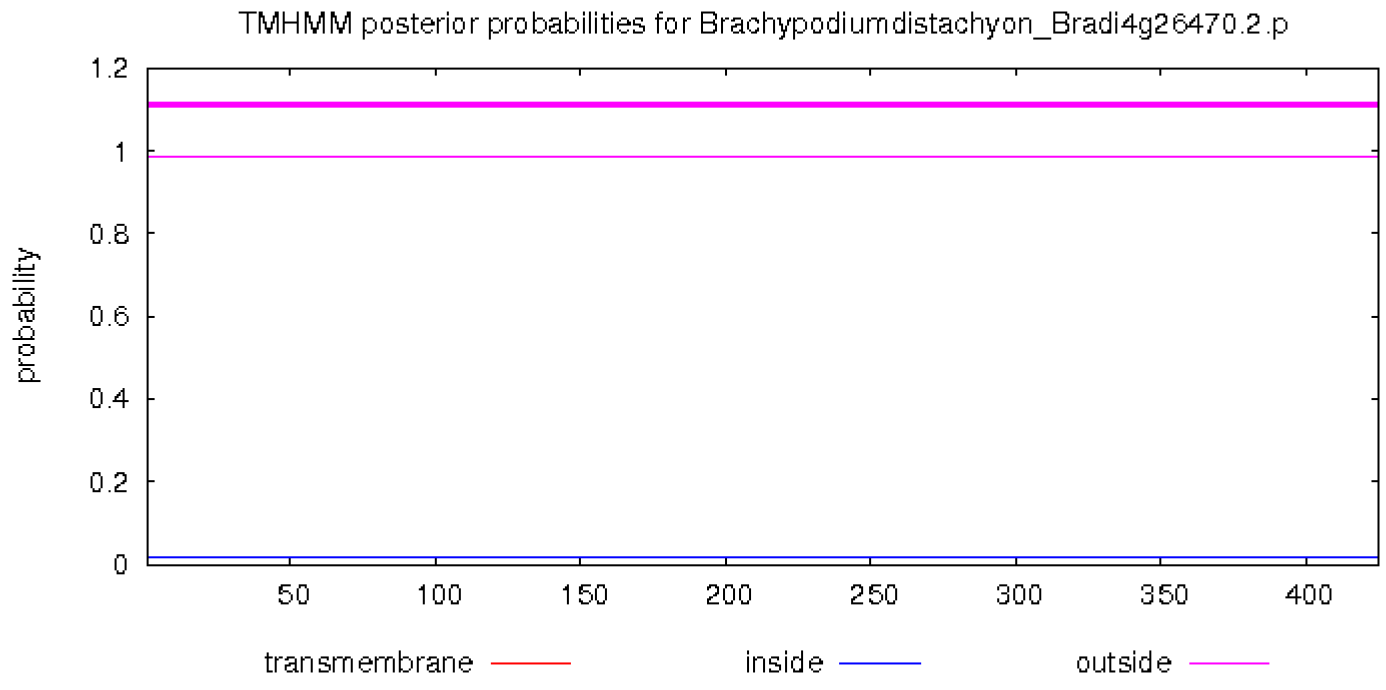

plot in postscript, script for making the plot in gnuplot, data for plot

---

```
# Brachypodiumdistachyon_Bradi4g26600.1.p Length: 523
# Brachypodiumdistachyon_Bradi4g26600.1.p Number of predicted TMHs:  0
# Brachypodiumdistachyon_Bradi4g26600.1.p Exp number of AAs in TMHs: 0.00018
# Brachypodiumdistachyon_Bradi4g26600.1.p Exp number, first 60 AAs:  0
# Brachypodiumdistachyon_Bradi4g26600.1.p Total prob of N-in:        0.00094
Brachypodiumdistachyon_Bradi4g26600.1.p TMHMM2.0        outside      1   523
```

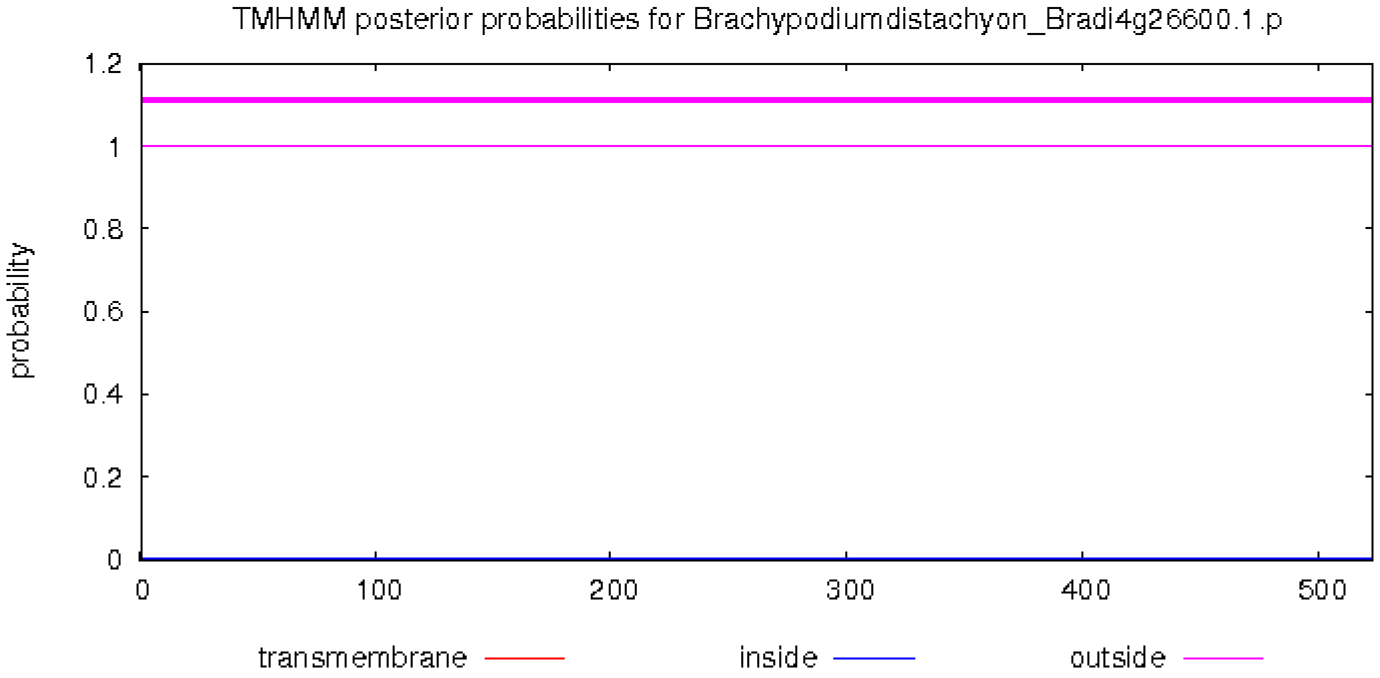

plot in postscript, script for making the plot in gnuplot, data for plot

---

```
# Brachypodiumdistachyon_Bradi4g26605.1.p Length: 489
# Brachypodiumdistachyon_Bradi4g26605.1.p Number of predicted TMHs:  0
# Brachypodiumdistachyon_Bradi4g26605.1.p Exp number of AAs in TMHs: 0.00021
# Brachypodiumdistachyon_Bradi4g26605.1.p Exp number, first 60 AAs:  0
# Brachypodiumdistachyon_Bradi4g26605.1.p Total prob of N-in:        0.00156
Brachypodiumdistachyon_Bradi4g26605.1.p TMHMM2.0        outside      1   489
```

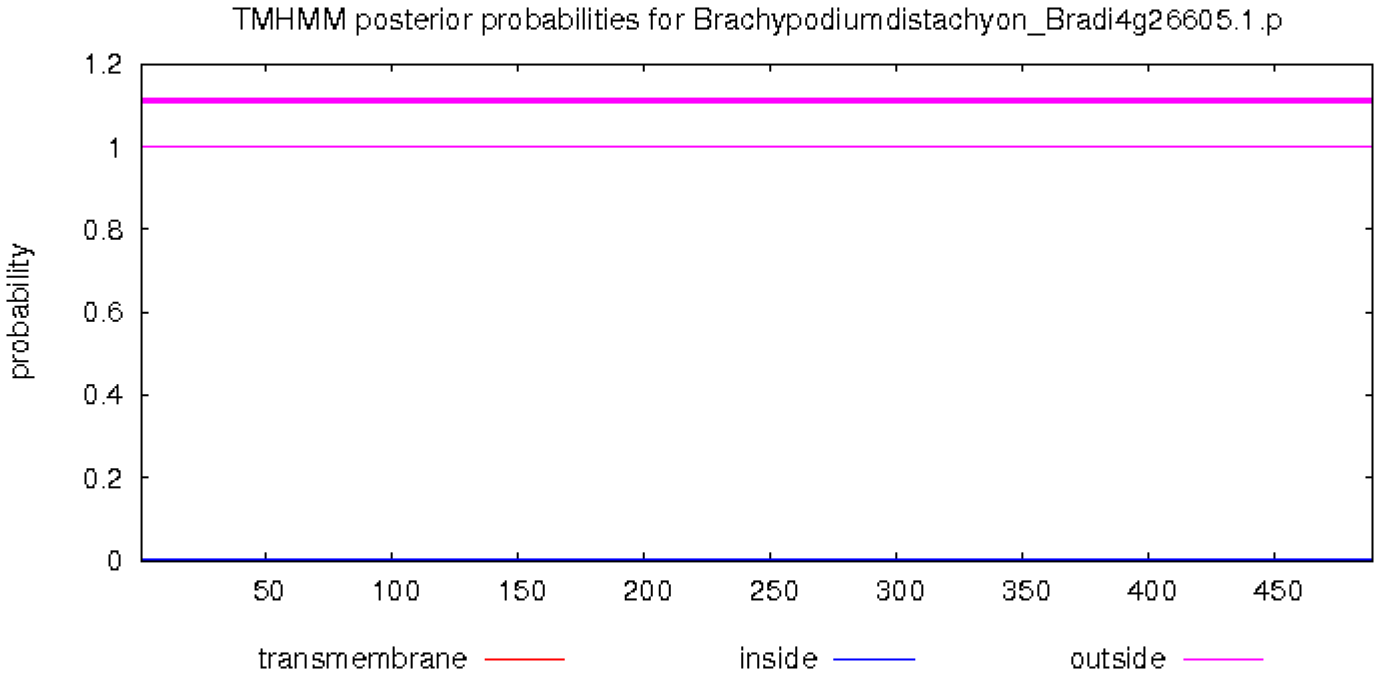

[plot](#) in postscript, [script](#) for making the plot in gnuplot, [data](#) for plot

---

```
# Brachypodiumdistachyon_Bradi4g30177.1.p Length: 405
# Brachypodiumdistachyon_Bradi4g30177.1.p Number of predicted TMHs:  0
# Brachypodiumdistachyon_Bradi4g30177.1.p Exp number of AAs in TMHs: 0.00053
# Brachypodiumdistachyon_Bradi4g30177.1.p Exp number, first 60 AAs:  0
# Brachypodiumdistachyon_Bradi4g30177.1.p Total prob of N-in:        0.03261
Brachypodiumdistachyon_Bradi4g30177.1.p TMHMM2.0        outside      1   405
```

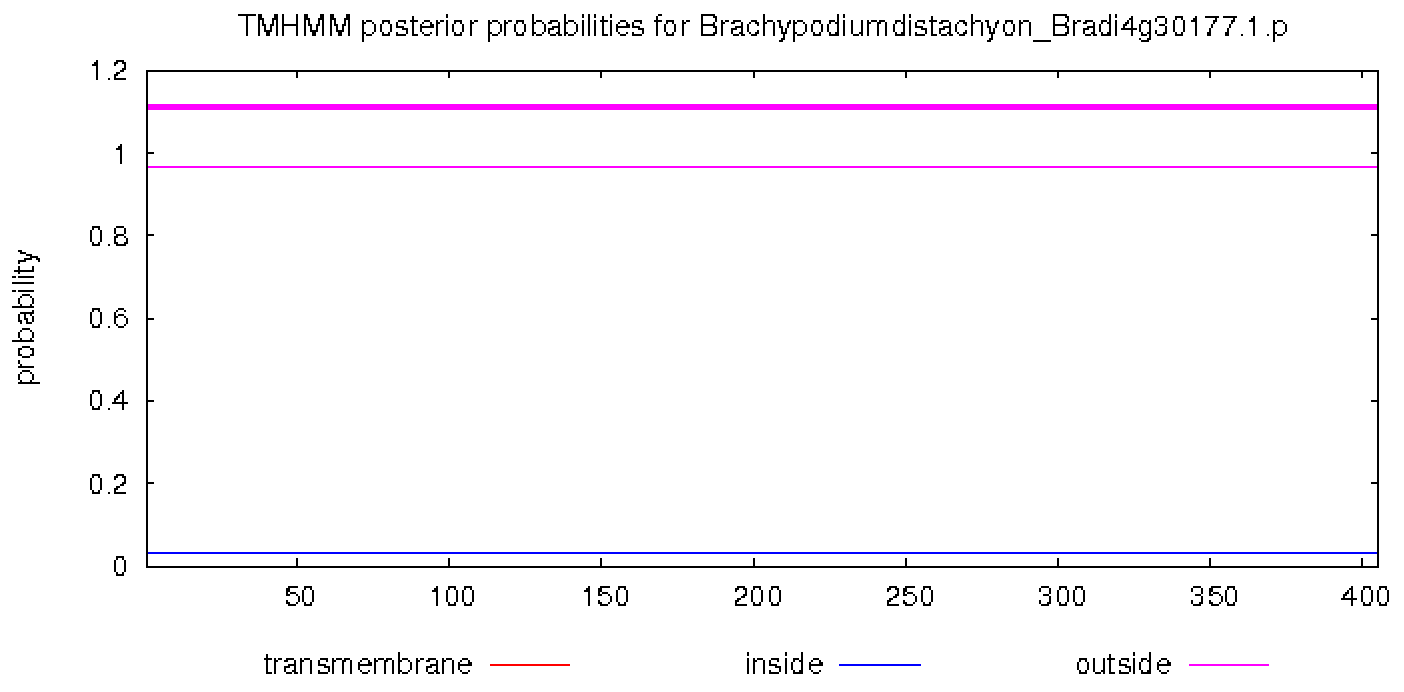

[plot](#) in postscript, [script](#) for making the plot in gnuplot, [data](#) for plot

---

```
# Brachypodiumdistachyon_Bradi4g34022.1.p Length: 689
# Brachypodiumdistachyon_Bradi4g34022.1.p Number of predicted TMHs:  1
# Brachypodiumdistachyon_Bradi4g34022.1.p Exp number of AAs in TMHs: 21.27879
# Brachypodiumdistachyon_Bradi4g34022.1.p Exp number, first 60 AAs:  0
# Brachypodiumdistachyon_Bradi4g34022.1.p Total prob of N-in:        0.00189
Brachypodiumdistachyon_Bradi4g34022.1.p TMHMM2.0        outside      1   651
Brachypodiumdistachyon_Bradi4g34022.1.p TMHMM2.0        TMhelix    652   674
Brachypodiumdistachyon_Bradi4g34022.1.p TMHMM2.0        inside     675   689
```

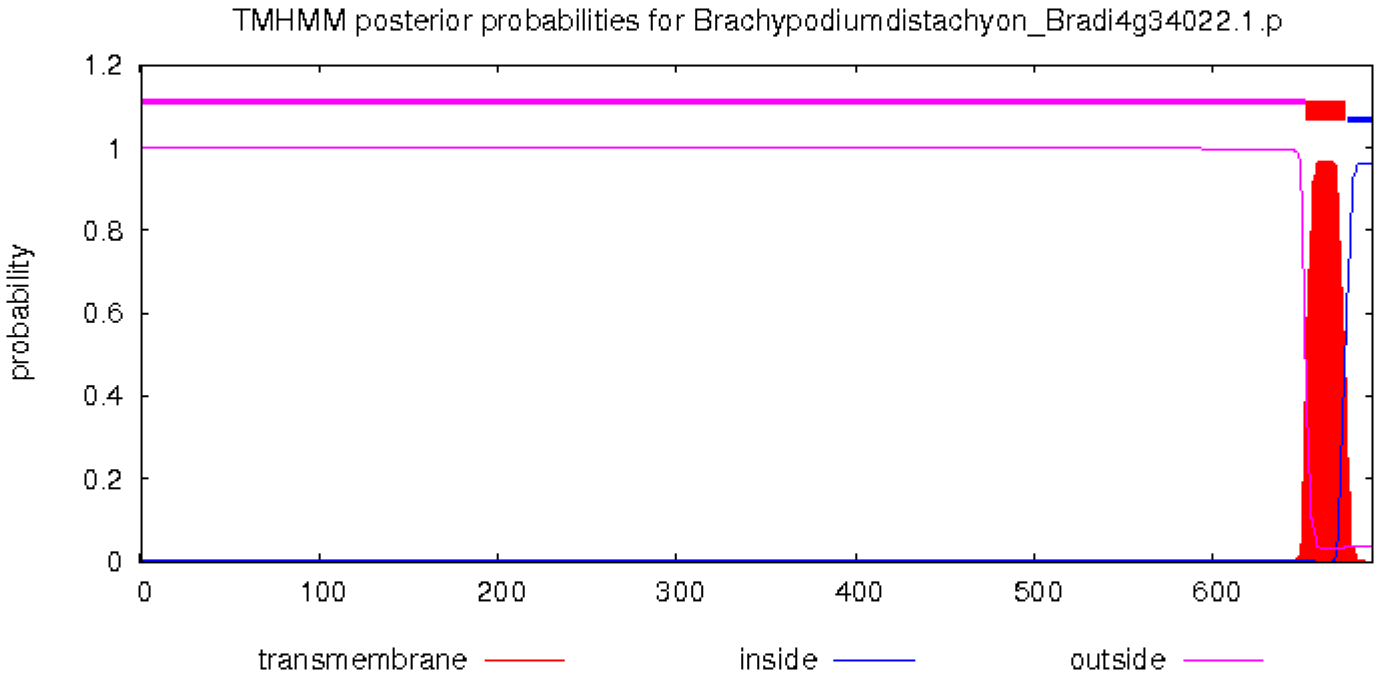

plot in postscript, script for making the plot in gnuplot, data for plot

---

```
# Brachypodiumdistachyon_Bradi4g34157.1.p Length: 354
# Brachypodiumdistachyon_Bradi4g34157.1.p Number of predicted TMHs:  0
# Brachypodiumdistachyon_Bradi4g34157.1.p Exp number of AAs in TMHs: 0.00159
# Brachypodiumdistachyon_Bradi4g34157.1.p Exp number, first 60 AAs:  0
# Brachypodiumdistachyon_Bradi4g34157.1.p Total prob of N-in:        0.00126
Brachypodiumdistachyon_Bradi4g34157.1.p TMHMM2.0        outside      1   354
```

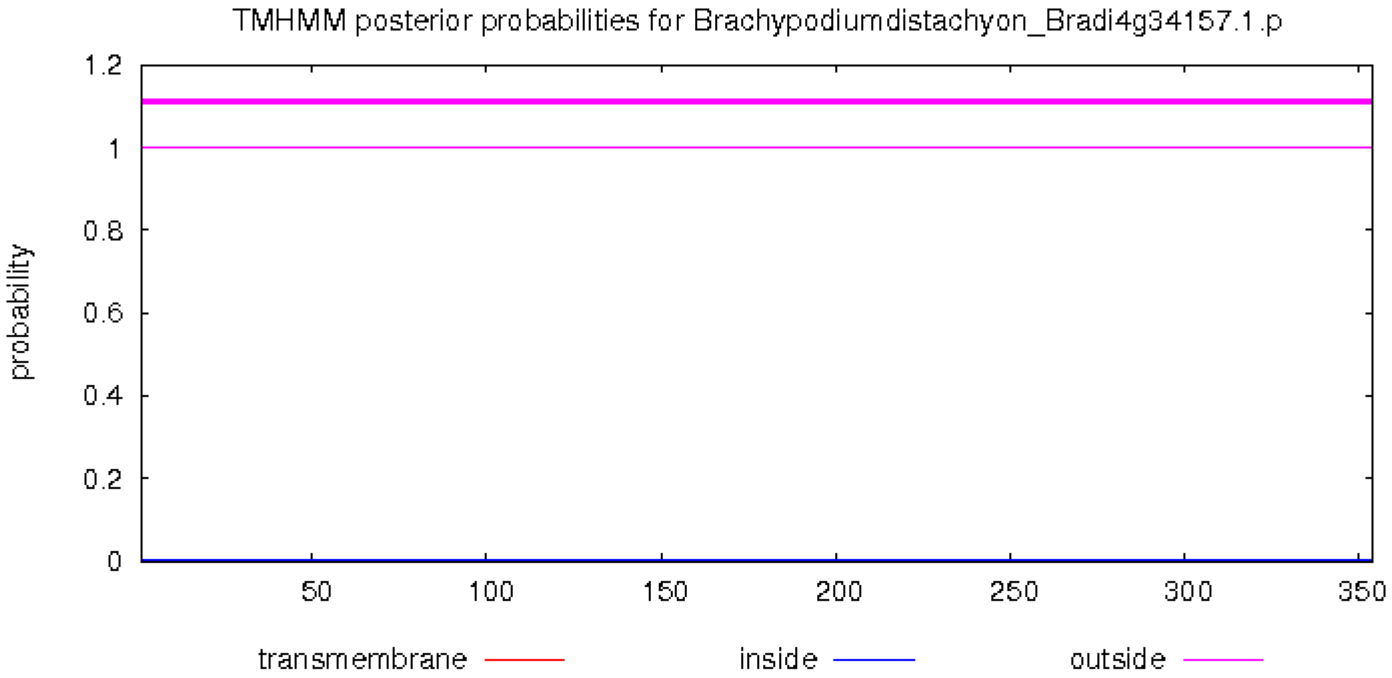

plot in postscript, script for making the plot in gnuplot, data for plot

---

```
# Brachypodiumdistachyon_Bradi4g34157.2.p Length: 302
# Brachypodiumdistachyon_Bradi4g34157.2.p Number of predicted TMHs:  0
# Brachypodiumdistachyon_Bradi4g34157.2.p Exp number of AAs in TMHs: 0.00188
# Brachypodiumdistachyon_Bradi4g34157.2.p Exp number, first 60 AAs:  0
# Brachypodiumdistachyon_Bradi4g34157.2.p Total prob of N-in:        0.01048
Brachypodiumdistachyon_Bradi4g34157.2.p TMHMM2.0        outside      1   302
```

TMHMM posterior probabilities for Brachypodiumdistachyon_Bradi4g34157.2.p

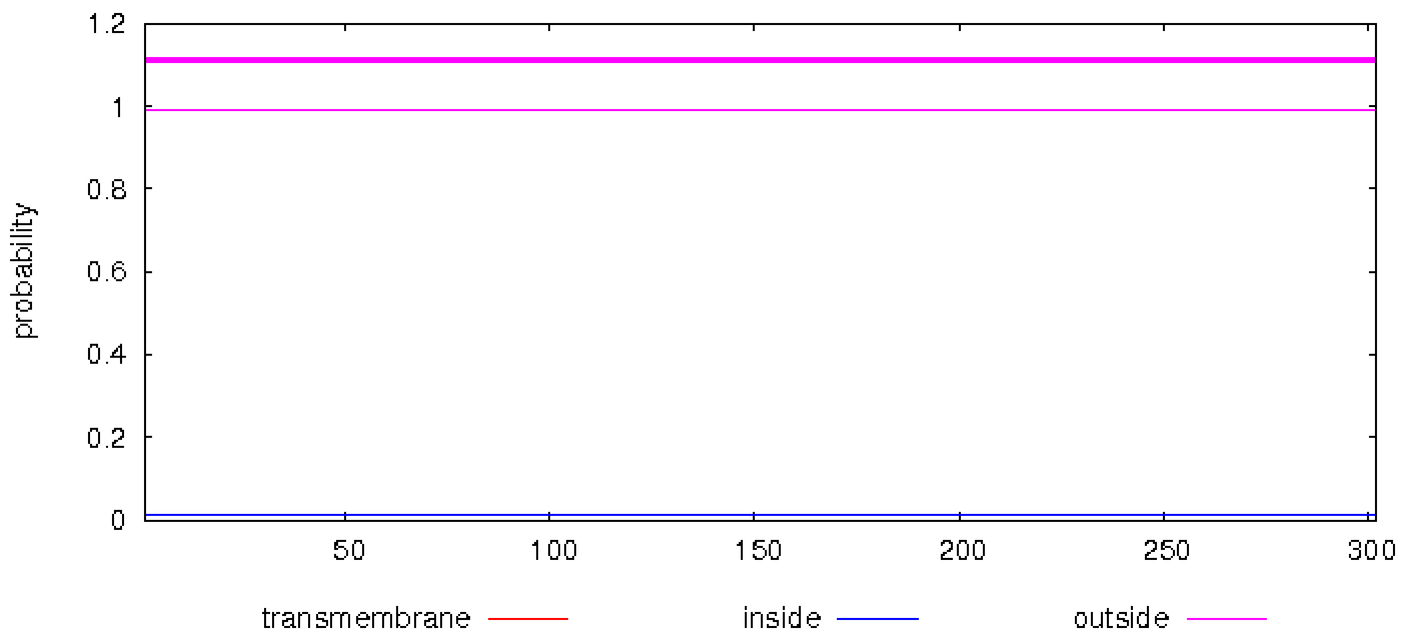

plot in postscript, script for making the plot in gnuplot, data for plot

---

```
# Brachypodiumdistachyon_Bradi4g34800.1.p Length: 230
# Brachypodiumdistachyon_Bradi4g34800.1.p Number of predicted TMHs:  0
# Brachypodiumdistachyon_Bradi4g34800.1.p Exp number of AAs in TMHs: 0.07212
# Brachypodiumdistachyon_Bradi4g34800.1.p Exp number, first 60 AAs:  0.06832
# Brachypodiumdistachyon_Bradi4g34800.1.p Total prob of N-in:        0.08495
Brachypodiumdistachyon_Bradi4g34800.1.p TMHMM2.0        outside      1   230
```

TMHMM posterior probabilities for Brachypodiumdistachyon_Bradi4g34800.1.p

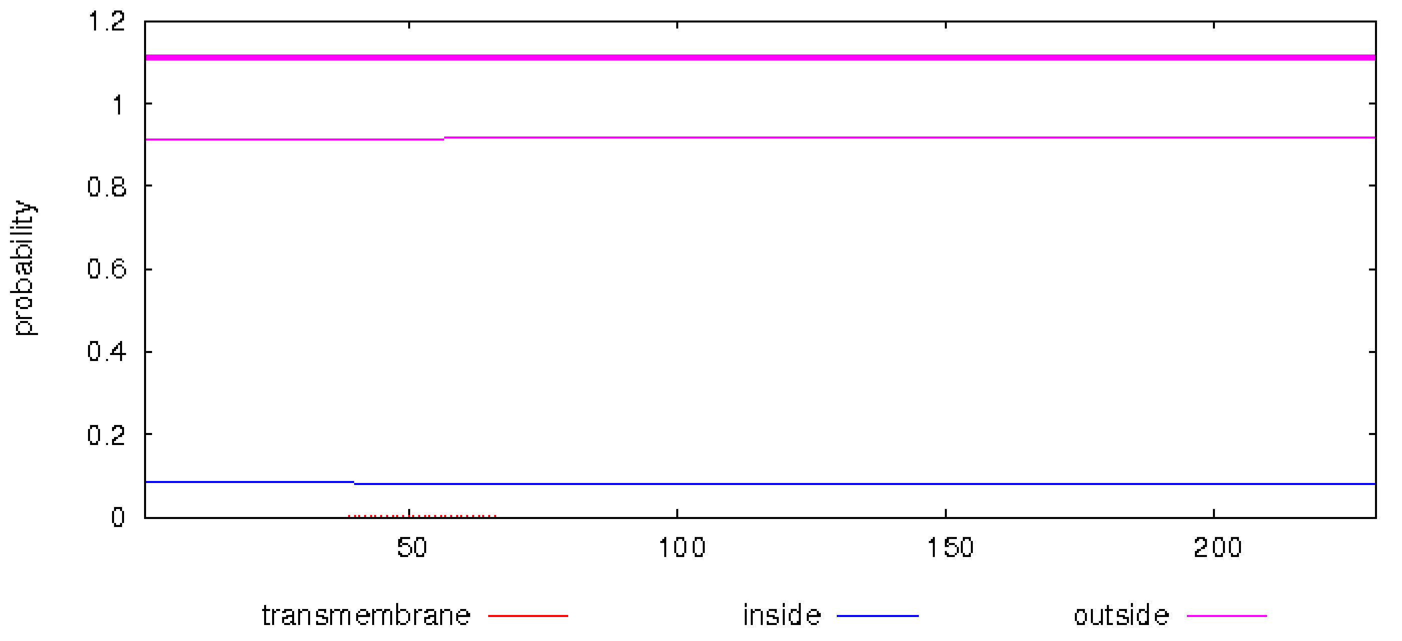

plot in postscript, script for making the plot in gnuplot, data for plot

---

```
# Brachypodiumdistachyon_Bradi4g37318.1.p Length: 255
# Brachypodiumdistachyon_Bradi4g37318.1.p Number of predicted TMHs:  0
# Brachypodiumdistachyon_Bradi4g37318.1.p Exp number of AAs in TMHs: 0.00329
# Brachypodiumdistachyon_Bradi4g37318.1.p Exp number, first 60 AAs:  0
# Brachypodiumdistachyon_Bradi4g37318.1.p Total prob of N-in:        0.00190
Brachypodiumdistachyon_Bradi4g37318.1.p TMHMM2.0        outside      1   255
```

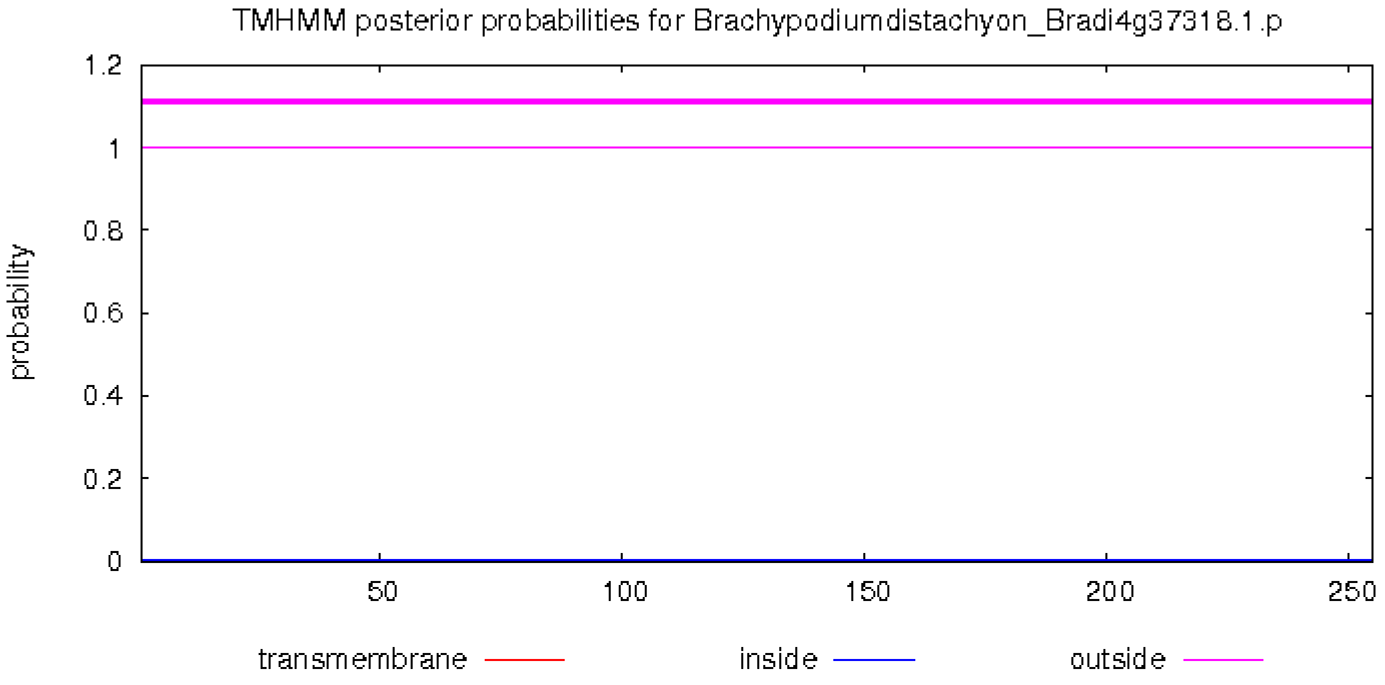

plot in postscript, script for making the plot in gnuplot, data for plot

```
# Brachypodiumdistachyon_Bradi4g37327.1.p Length: 217
# Brachypodiumdistachyon_Bradi4g37327.1.p Number of predicted TMHs:  0
# Brachypodiumdistachyon_Bradi4g37327.1.p Exp number of AAs in TMHs: 0
# Brachypodiumdistachyon_Bradi4g37327.1.p Exp number, first 60 AAs:  0
# Brachypodiumdistachyon_Bradi4g37327.1.p Total prob of N-in:        0.00428
Brachypodiumdistachyon_Bradi4g37327.1.p TMHMM2.0        outside      1   217
```

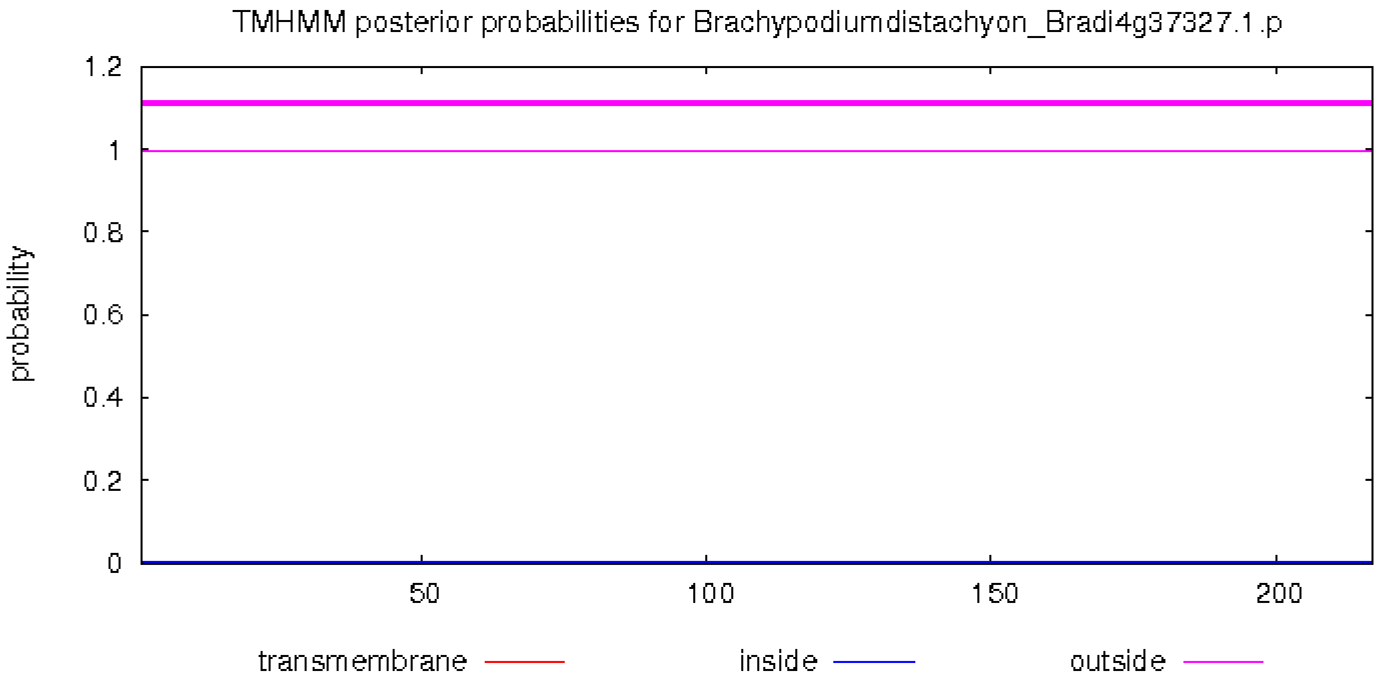

plot in postscript, script for making the plot in gnuplot, data for plot

```
# Brachypodiumdistachyon_Bradi4g40880.1.p Length: 346
# Brachypodiumdistachyon_Bradi4g40880.1.p Number of predicted TMHs:  0
# Brachypodiumdistachyon_Bradi4g40880.1.p Exp number of AAs in TMHs: 0.00023
# Brachypodiumdistachyon_Bradi4g40880.1.p Exp number, first 60 AAs:  5e-05
# Brachypodiumdistachyon_Bradi4g40880.1.p Total prob of N-in:        0.01503
Brachypodiumdistachyon_Bradi4g40880.1.p TMHMM2.0        outside      1   346
```

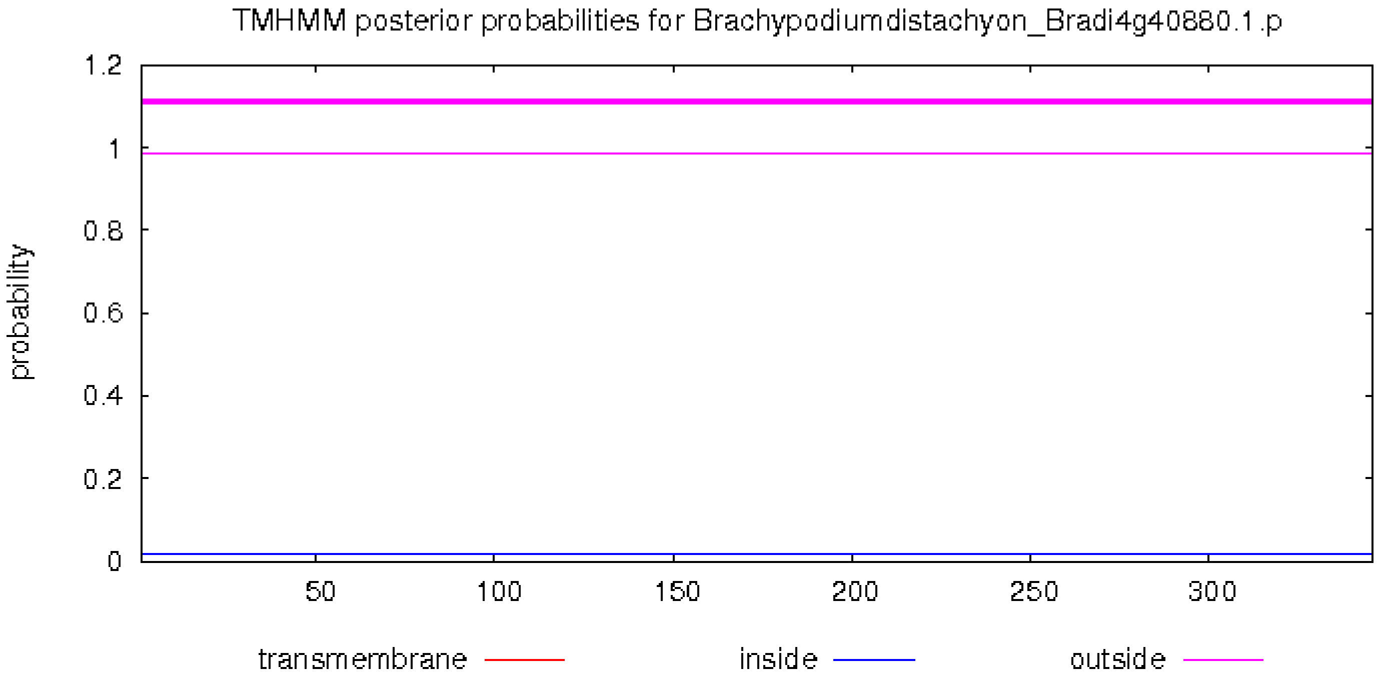

plot in postscript, script for making the plot in gnuplot, data for plot

```
# Brachypodiumdistachyon_Bradi4g41200.1.p Length: 455
# Brachypodiumdistachyon_Bradi4g41200.1.p Number of predicted TMHs:  0
# Brachypodiumdistachyon_Bradi4g41200.1.p Exp number of AAs in TMHs: 0.58857
# Brachypodiumdistachyon_Bradi4g41200.1.p Exp number, first 60 AAs:  0.50834
# Brachypodiumdistachyon_Bradi4g41200.1.p Total prob of N-in:        0.10680
Brachypodiumdistachyon_Bradi4g41200.1.p TMHMM2.0        outside      1   455
```

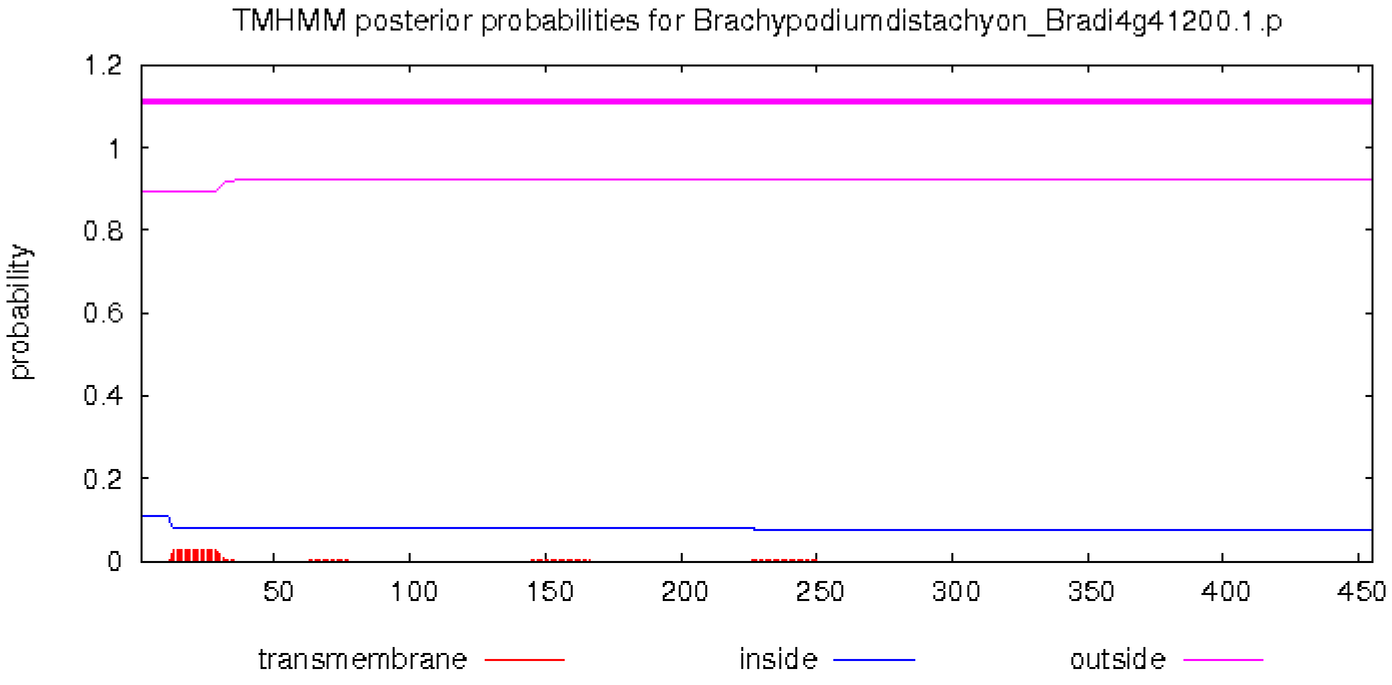

plot in postscript, script for making the plot in gnuplot, data for plot

```
# Brachypodiumdistachyon_Bradi4g42180.1.p Length: 263
# Brachypodiumdistachyon_Bradi4g42180.1.p Number of predicted TMHs:  0
# Brachypodiumdistachyon_Bradi4g42180.1.p Exp number of AAs in TMHs: 0.02342
# Brachypodiumdistachyon_Bradi4g42180.1.p Exp number, first 60 AAs:  8e-05
# Brachypodiumdistachyon_Bradi4g42180.1.p Total prob of N-in:        0.11122
Brachypodiumdistachyon_Bradi4g42180.1.p TMHMM2.0        outside      1   263
```

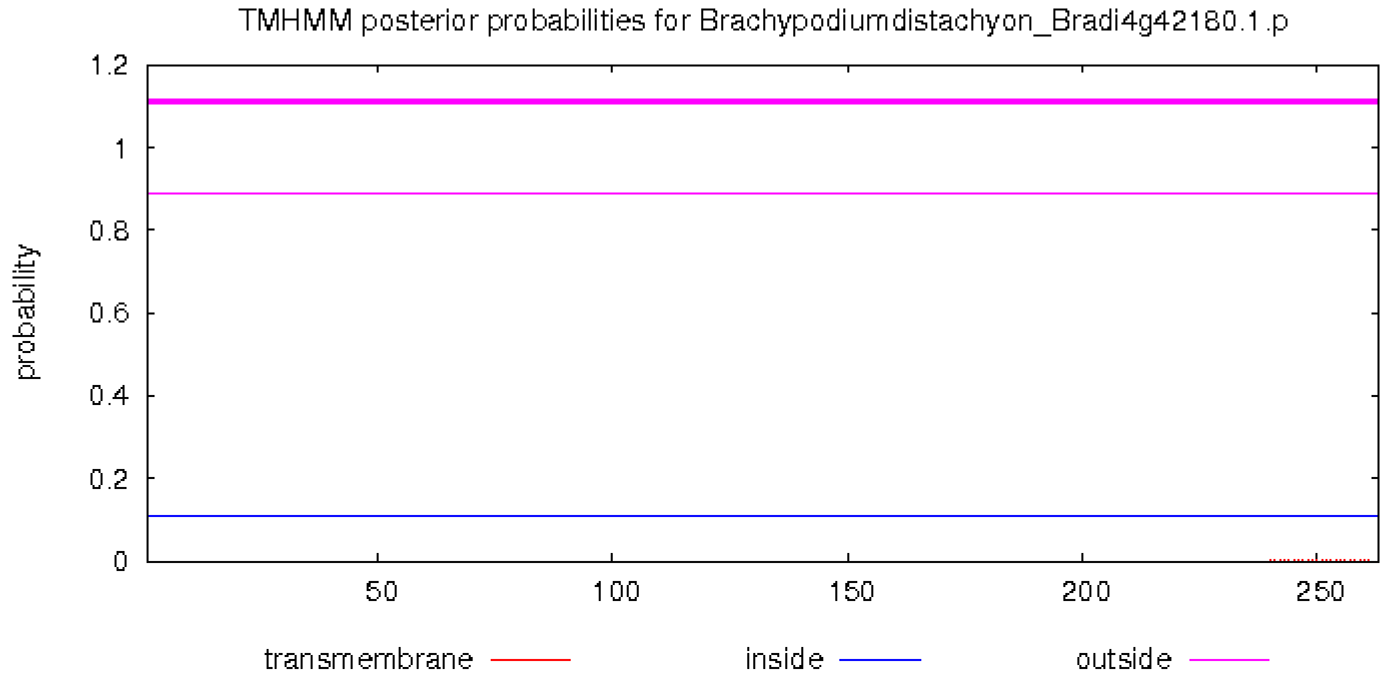

plot in postscript, script for making the plot in gnuplot, data for plot

---

```
# Brachypodiumdistachyon_Bradi4g43010.1.p Length: 337
# Brachypodiumdistachyon_Bradi4g43010.1.p Number of predicted TMHs:  0
# Brachypodiumdistachyon_Bradi4g43010.1.p Exp number of AAs in TMHs: 0.00243
# Brachypodiumdistachyon_Bradi4g43010.1.p Exp number, first 60 AAs:  0.00243
# Brachypodiumdistachyon_Bradi4g43010.1.p Total prob of N-in:        0.00634
Brachypodiumdistachyon_Bradi4g43010.1.p TMHMM2.0        outside      1   337
```

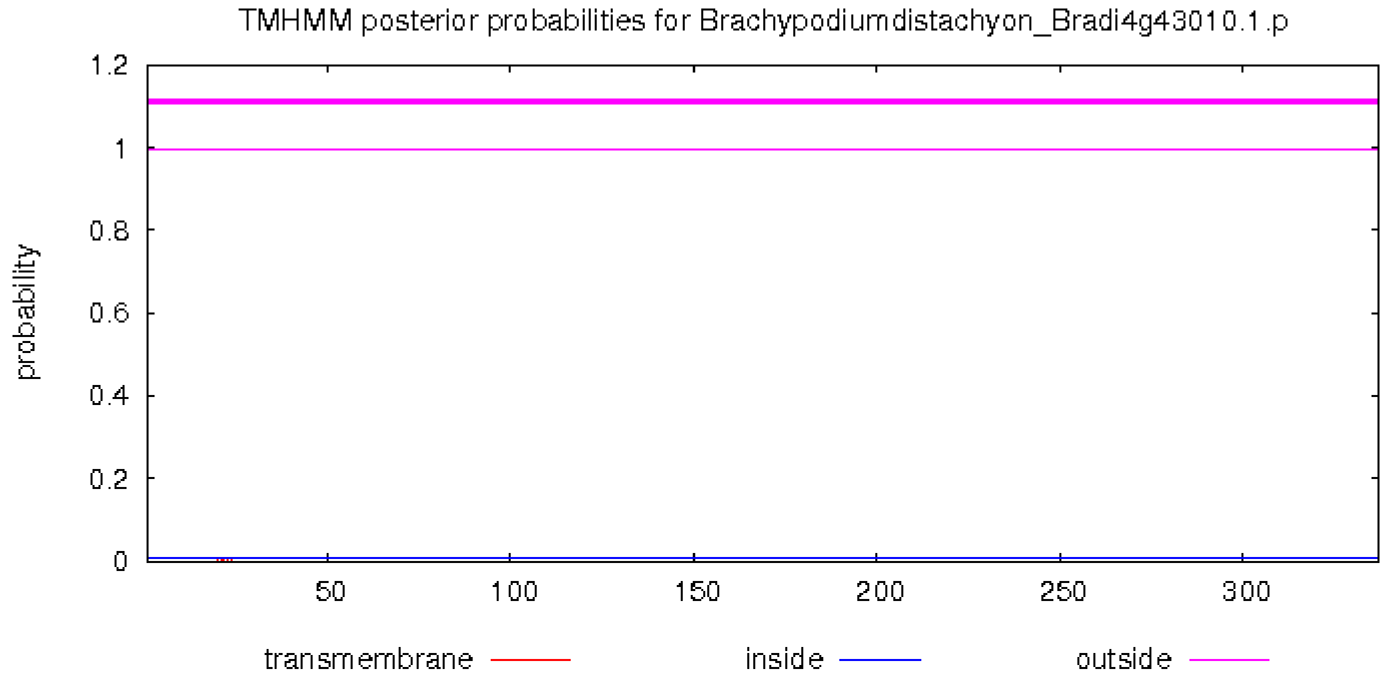

plot in postscript, script for making the plot in gnuplot, data for plot

---

```
# Brachypodiumdistachyon_Bradi4g43020.1.p Length: 360
# Brachypodiumdistachyon_Bradi4g43020.1.p Number of predicted TMHs:  0
# Brachypodiumdistachyon_Bradi4g43020.1.p Exp number of AAs in TMHs: 0.00055
# Brachypodiumdistachyon_Bradi4g43020.1.p Exp number, first 60 AAs:  0.00055
# Brachypodiumdistachyon_Bradi4g43020.1.p Total prob of N-in:        0.02502
Brachypodiumdistachyon_Bradi4g43020.1.p TMHMM2.0        outside      1   360
```

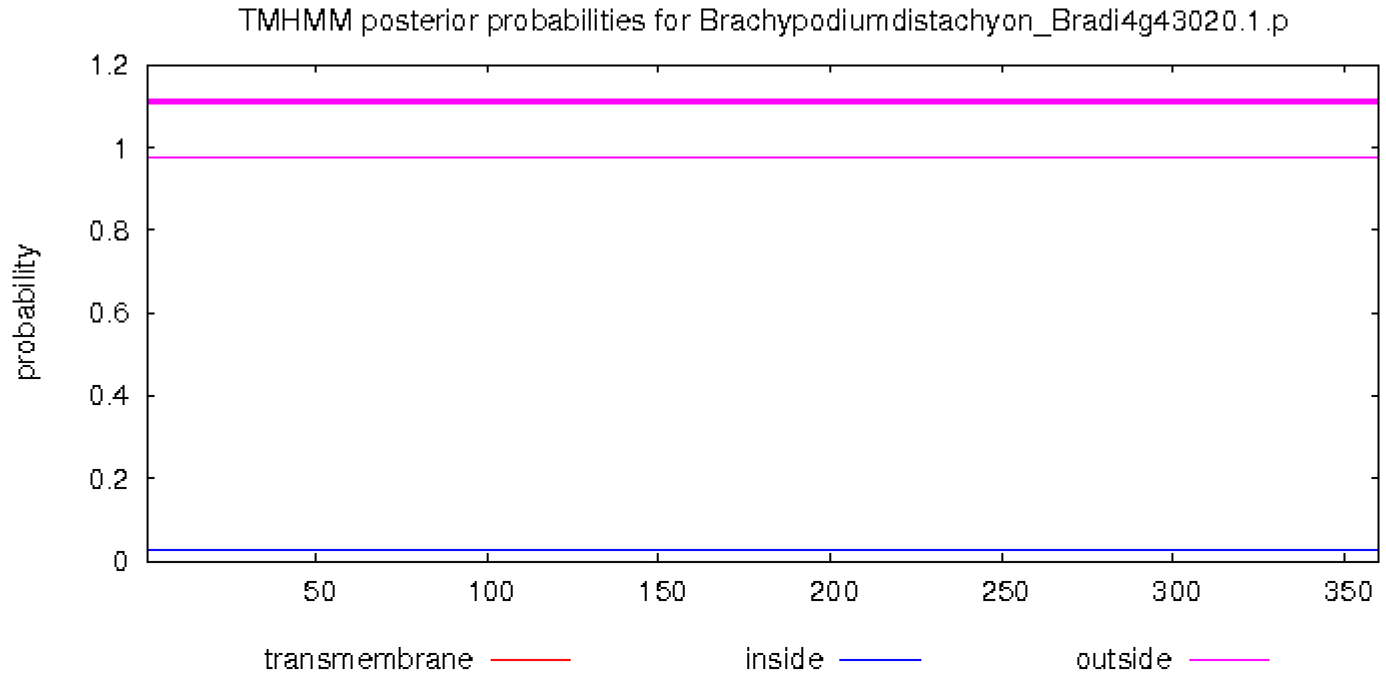

plot in postscript, script for making the plot in gnuplot, data for plot

---

```
# Brachypodiumdistachyon_Bradi4g43330.1.p Length: 359
# Brachypodiumdistachyon_Bradi4g43330.1.p Number of predicted TMHs:  0
# Brachypodiumdistachyon_Bradi4g43330.1.p Exp number of AAs in TMHs: 0.00035
# Brachypodiumdistachyon_Bradi4g43330.1.p Exp number, first 60 AAs:  0.00012
# Brachypodiumdistachyon_Bradi4g43330.1.p Total prob of N-in:        0.00351
Brachypodiumdistachyon_Bradi4g43330.1.p TMHMM2.0        outside      1   359
```

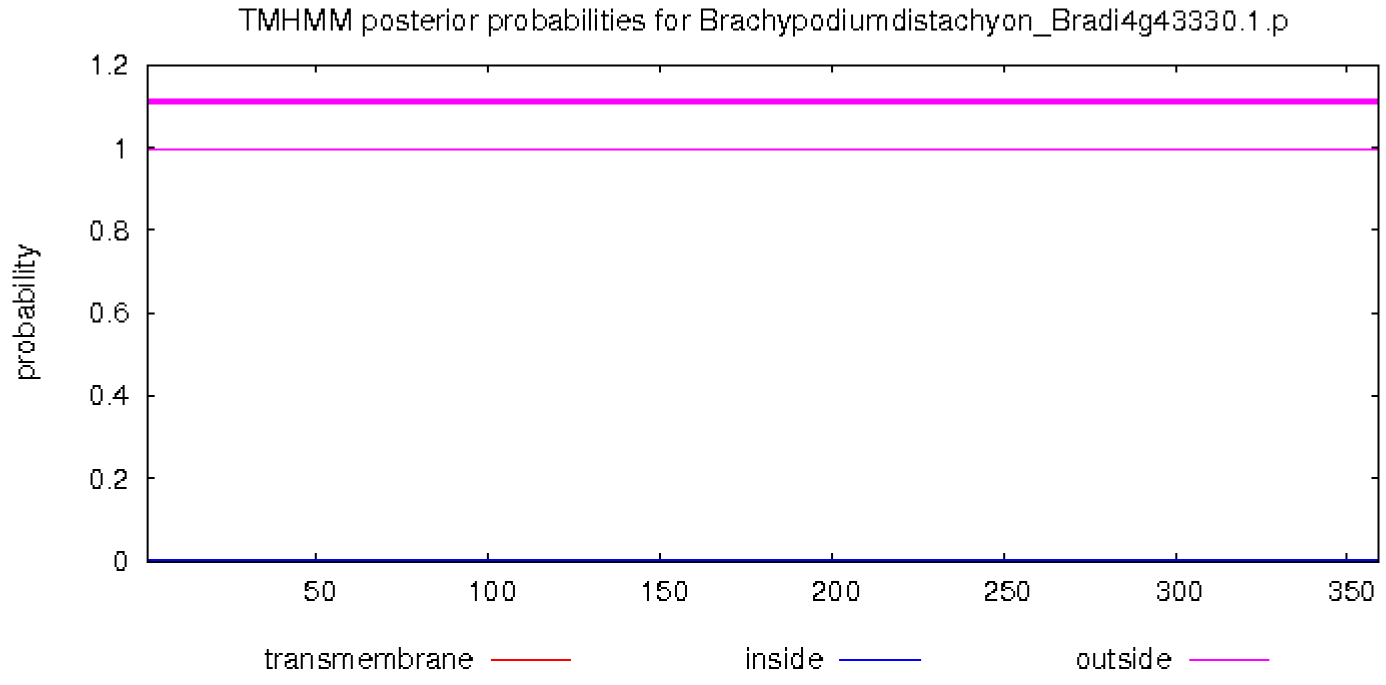

plot in postscript, script for making the plot in gnuplot, data for plot

---

```
# Brachypodiumdistachyon_Bradi4g43346.1.p Length: 335
# Brachypodiumdistachyon_Bradi4g43346.1.p Number of predicted TMHs:  0
# Brachypodiumdistachyon_Bradi4g43346.1.p Exp number of AAs in TMHs: 0.00064
# Brachypodiumdistachyon_Bradi4g43346.1.p Exp number, first 60 AAs:  0.00046
# Brachypodiumdistachyon_Bradi4g43346.1.p Total prob of N-in:        0.00348
Brachypodiumdistachyon_Bradi4g43346.1.p TMHMM2.0        outside      1   335
```

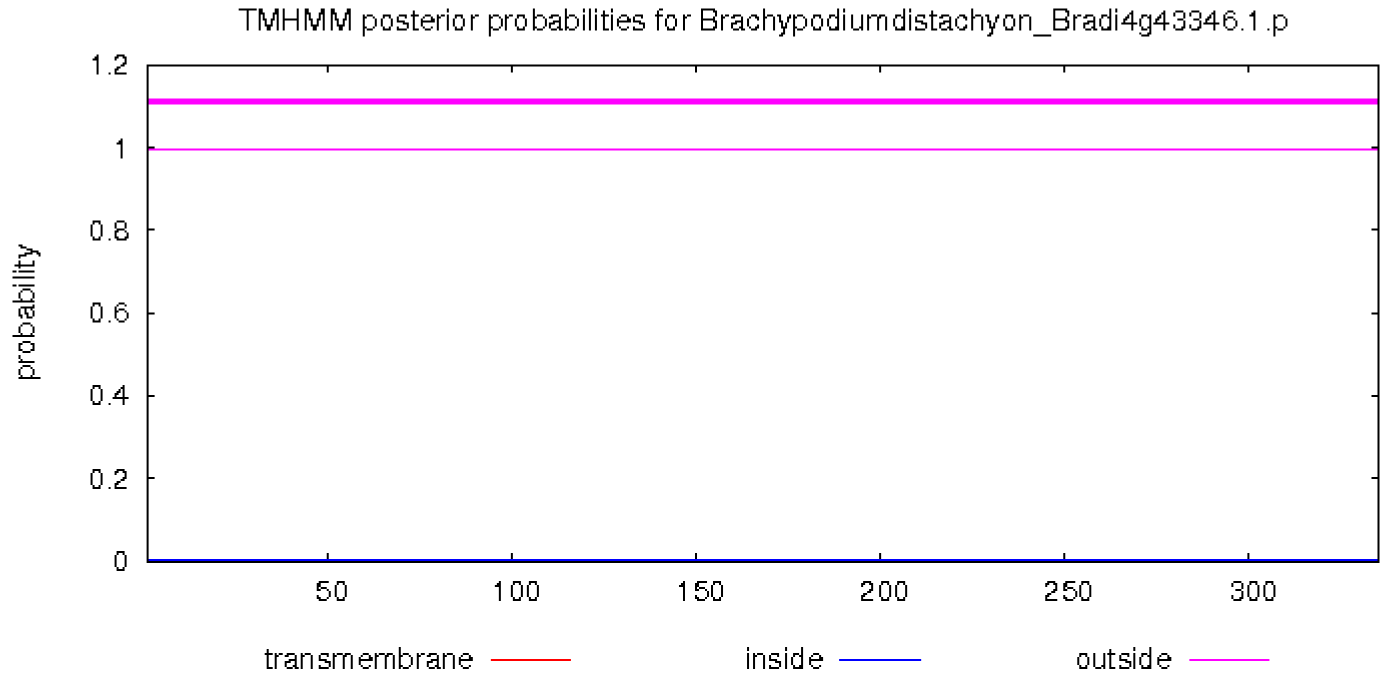

plot in postscript, script for making the plot in gnuplot, data for plot

---

```
# Brachypodiumdistachyon_Bradi4g43995.1.p Length: 433
# Brachypodiumdistachyon_Bradi4g43995.1.p Number of predicted TMHs:  0
# Brachypodiumdistachyon_Bradi4g43995.1.p Exp number of AAs in TMHs: 0.05092
# Brachypodiumdistachyon_Bradi4g43995.1.p Exp number, first 60 AAs:  0.02107
# Brachypodiumdistachyon_Bradi4g43995.1.p Total prob of N-in:        0.00444
Brachypodiumdistachyon_Bradi4g43995.1.p TMHMM2.0        outside      1   433
```

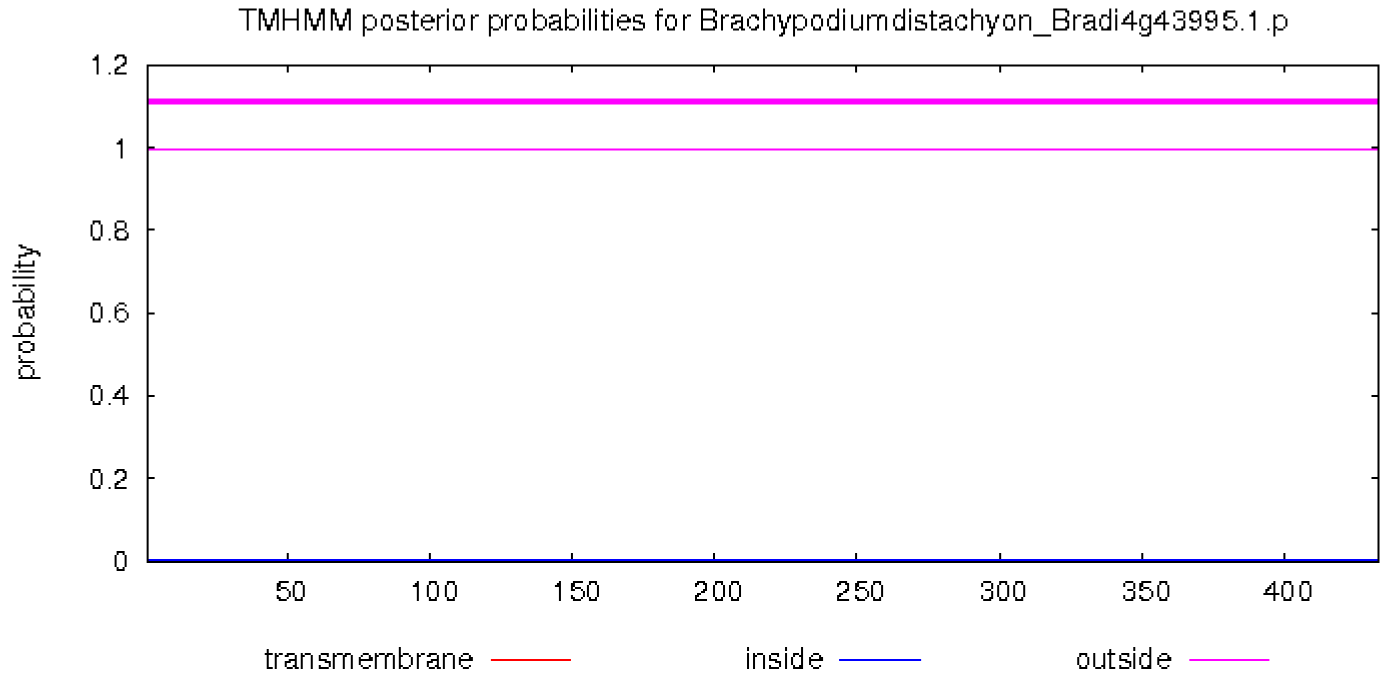

plot in postscript, script for making the plot in gnuplot, data for plot

---

```
# Brachypodiumdistachyon_Bradi4g44000.1.p Length: 400
# Brachypodiumdistachyon_Bradi4g44000.1.p Number of predicted TMHs:  0
# Brachypodiumdistachyon_Bradi4g44000.1.p Exp number of AAs in TMHs: 0.00676
# Brachypodiumdistachyon_Bradi4g44000.1.p Exp number, first 60 AAs:  0.00017
# Brachypodiumdistachyon_Bradi4g44000.1.p Total prob of N-in:        0.00290
Brachypodiumdistachyon_Bradi4g44000.1.p TMHMM2.0        outside      1   400
```

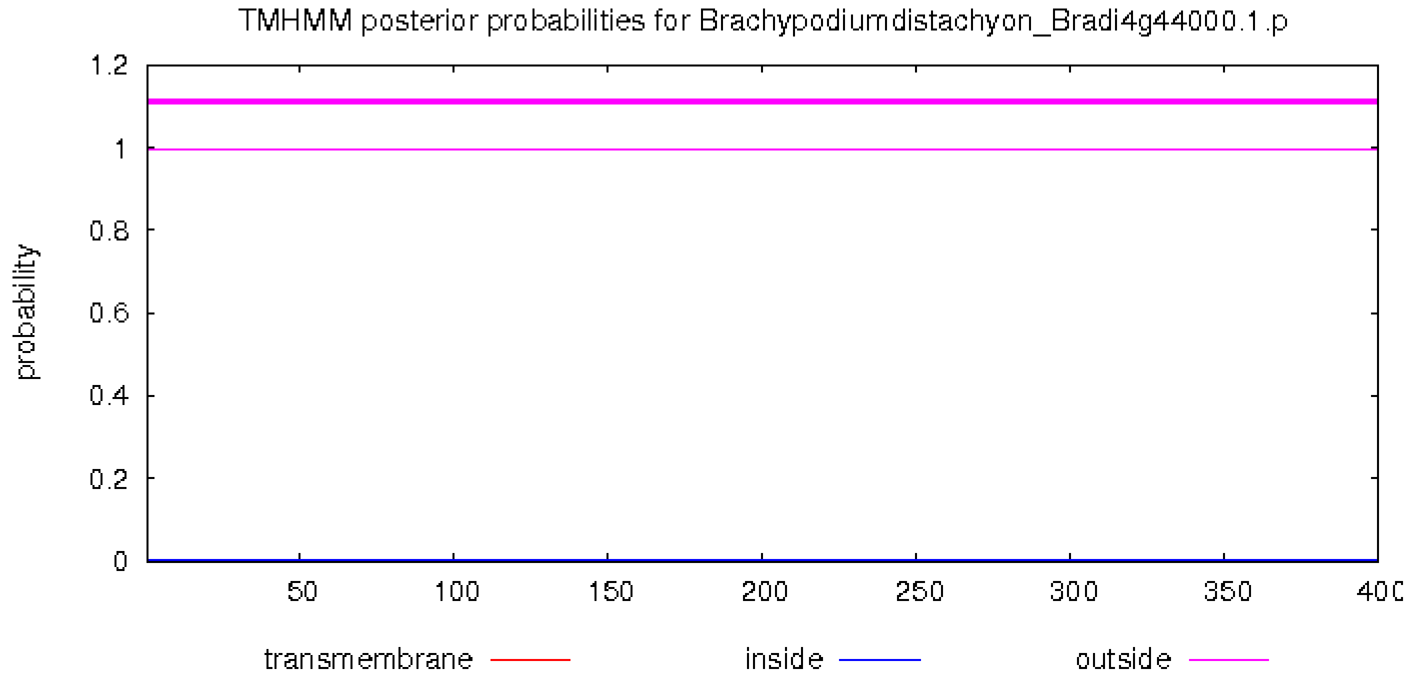

plot in postscript, script for making the plot in gnuplot, data for plot

---

```
# Brachypodiumdistachyon_Bradi5g10640.1.p Length: 199
# Brachypodiumdistachyon_Bradi5g10640.1.p Number of predicted TMHs:  0
# Brachypodiumdistachyon_Bradi5g10640.1.p Exp number of AAs in TMHs: 0.00014
# Brachypodiumdistachyon_Bradi5g10640.1.p Exp number, first 60 AAs:  0.00014
# Brachypodiumdistachyon_Bradi5g10640.1.p Total prob of N-in:        0.01063
Brachypodiumdistachyon_Bradi5g10640.1.p TMHMM2.0        outside      1   199
```

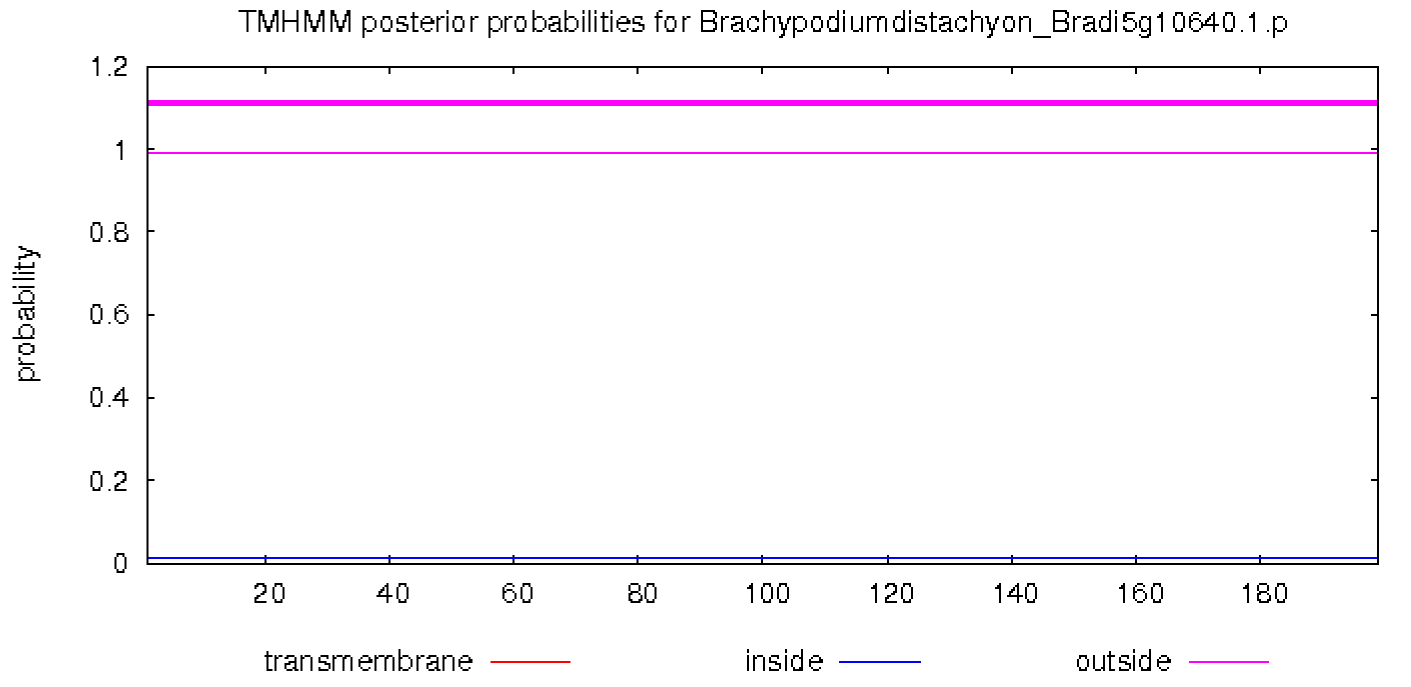

plot in postscript, script for making the plot in gnuplot, data for plot

---

```
# Brachypodiumdistachyon_Bradi5g11247.1.p Length: 345
# Brachypodiumdistachyon_Bradi5g11247.1.p Number of predicted TMHs:  0
# Brachypodiumdistachyon_Bradi5g11247.1.p Exp number of AAs in TMHs: 0.00058
# Brachypodiumdistachyon_Bradi5g11247.1.p Exp number, first 60 AAs:  0
# Brachypodiumdistachyon_Bradi5g11247.1.p Total prob of N-in:        0.00823
Brachypodiumdistachyon_Bradi5g11247.1.p TMHMM2.0        outside      1   345
```

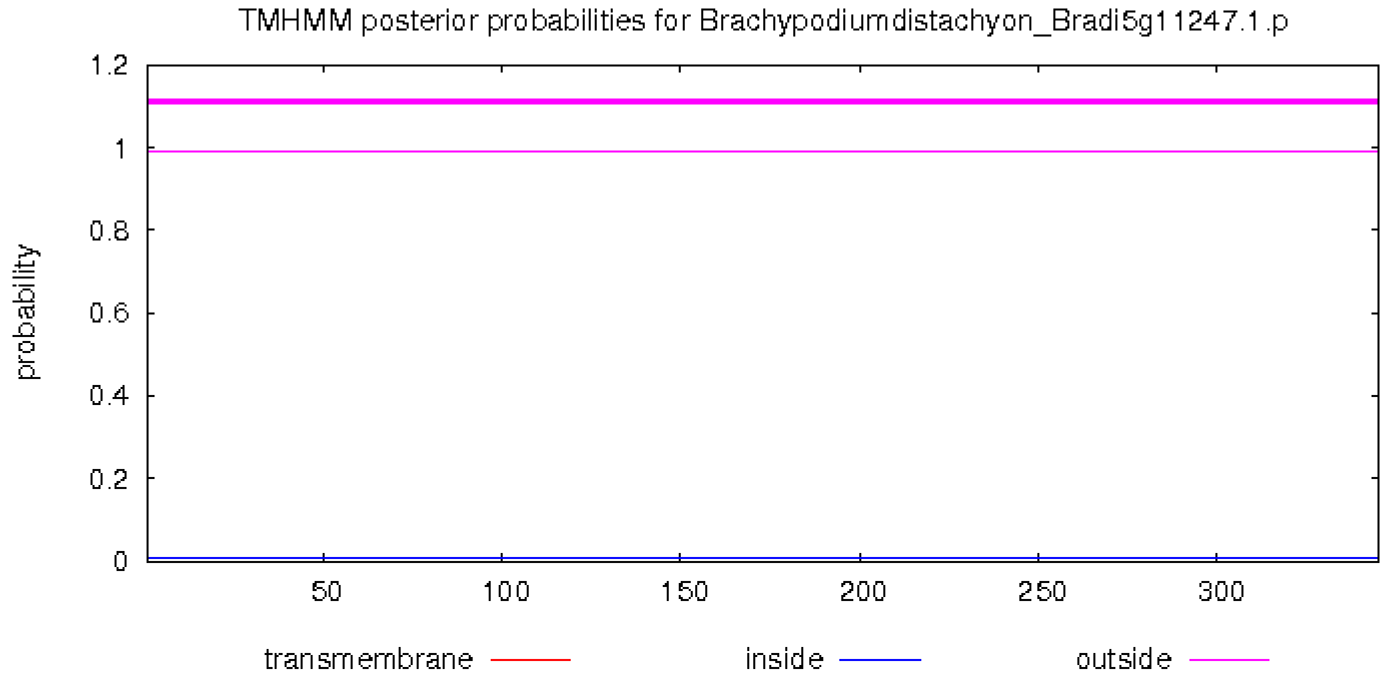

[plot](#) in postscript, [script](#) for making the plot in gnuplot, [data](#) for plot

---

```
# Brachypodiumdistachyon_Bradi5g12407.4.p Length: 340
# Brachypodiumdistachyon_Bradi5g12407.4.p Number of predicted TMHs:  0
# Brachypodiumdistachyon_Bradi5g12407.4.p Exp number of AAs in TMHs: 0.02064
# Brachypodiumdistachyon_Bradi5g12407.4.p Exp number, first 60 AAs:  0
# Brachypodiumdistachyon_Bradi5g12407.4.p Total prob of N-in:        0.02513
Brachypodiumdistachyon_Bradi5g12407.4.p TMHMM2.0        outside      1   340
```

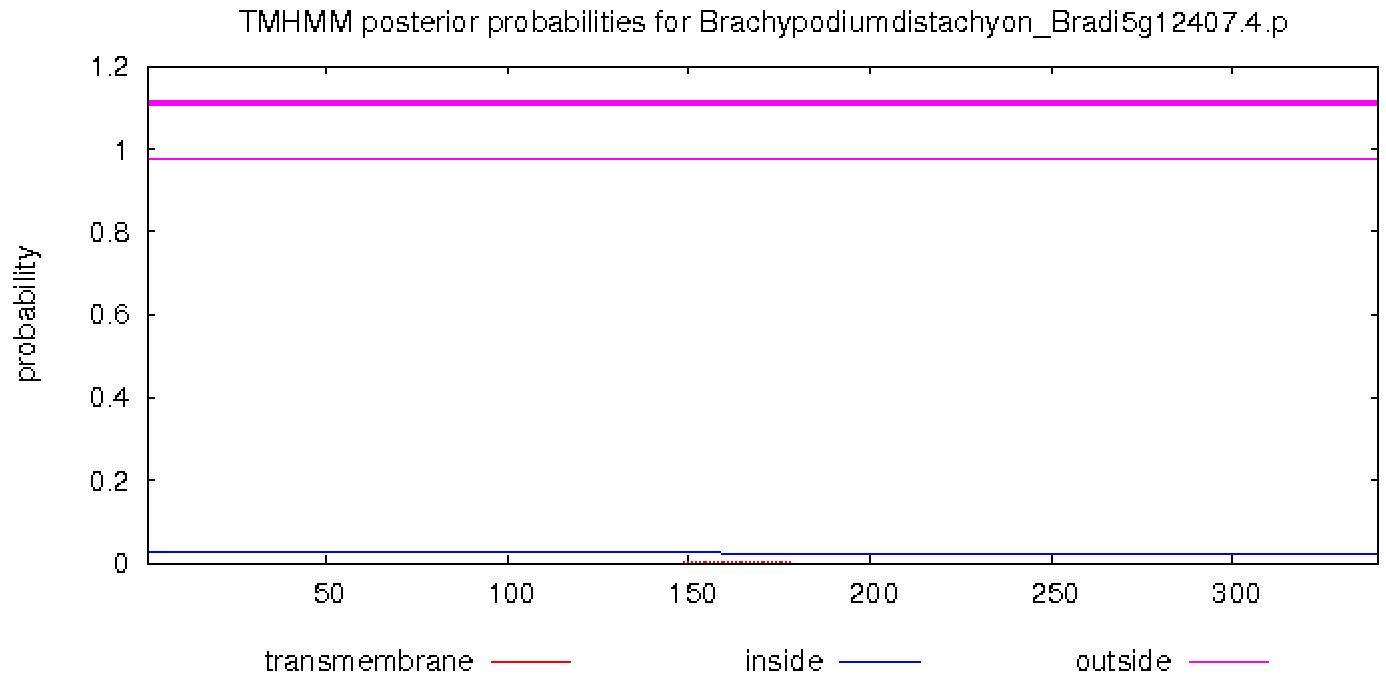

[plot](#) in postscript, [script](#) for making the plot in gnuplot, [data](#) for plot

---

```
# Brachypodiumdistachyon_Bradi5g13300.1.p Length: 222
# Brachypodiumdistachyon_Bradi5g13300.1.p Number of predicted TMHs:  0
# Brachypodiumdistachyon_Bradi5g13300.1.p Exp number of AAs in TMHs: 0.01993
# Brachypodiumdistachyon_Bradi5g13300.1.p Exp number, first 60 AAs:  0.00135
# Brachypodiumdistachyon_Bradi5g13300.1.p Total prob of N-in:        0.13314
Brachypodiumdistachyon_Bradi5g13300.1.p TMHMM2.0        outside      1   222
```

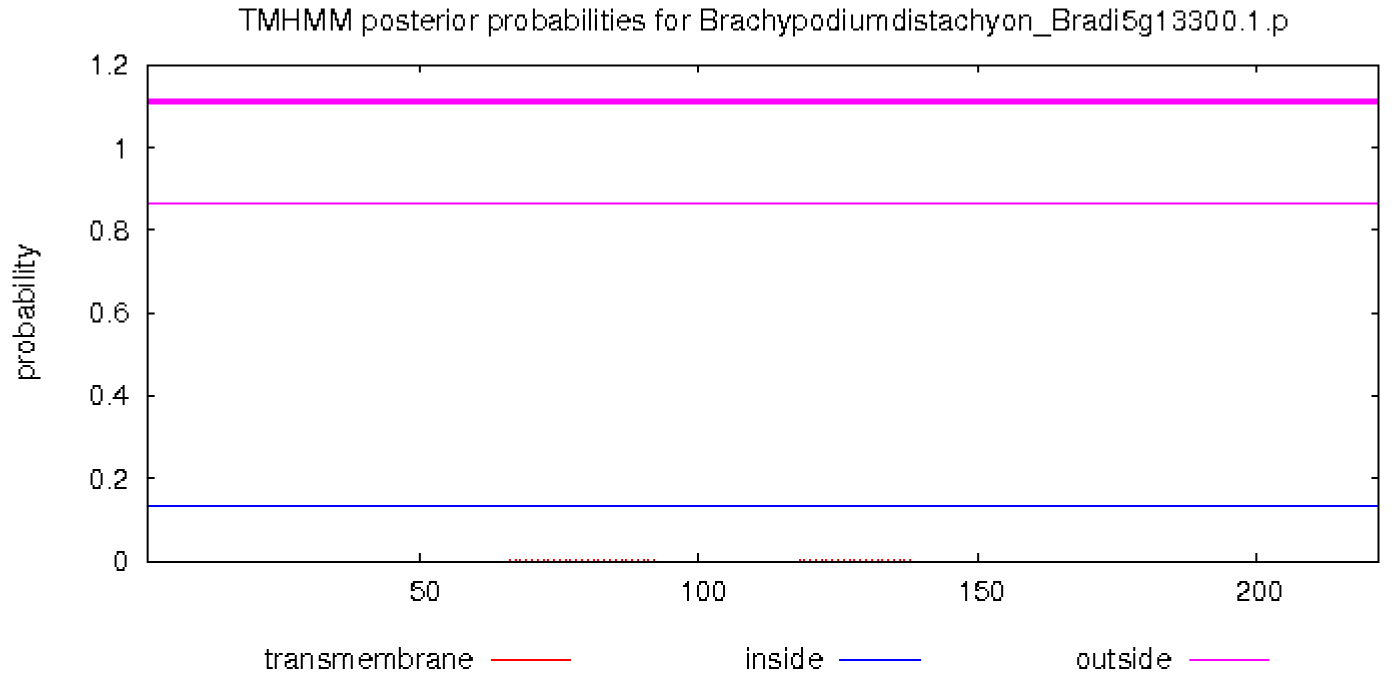

plot in postscript, script for making the plot in gnuplot, data for plot

---

```
# Brachypodiumdistachyon_Bradi5g13450.1.p Length: 356
# Brachypodiumdistachyon_Bradi5g13450.1.p Number of predicted TMHs:  0
# Brachypodiumdistachyon_Bradi5g13450.1.p Exp number of AAs in TMHs: 5e-05
# Brachypodiumdistachyon_Bradi5g13450.1.p Exp number, first 60 AAs:  0
# Brachypodiumdistachyon_Bradi5g13450.1.p Total prob of N-in:        0.01081
Brachypodiumdistachyon_Bradi5g13450.1.p TMHMM2.0        outside      1   356
```

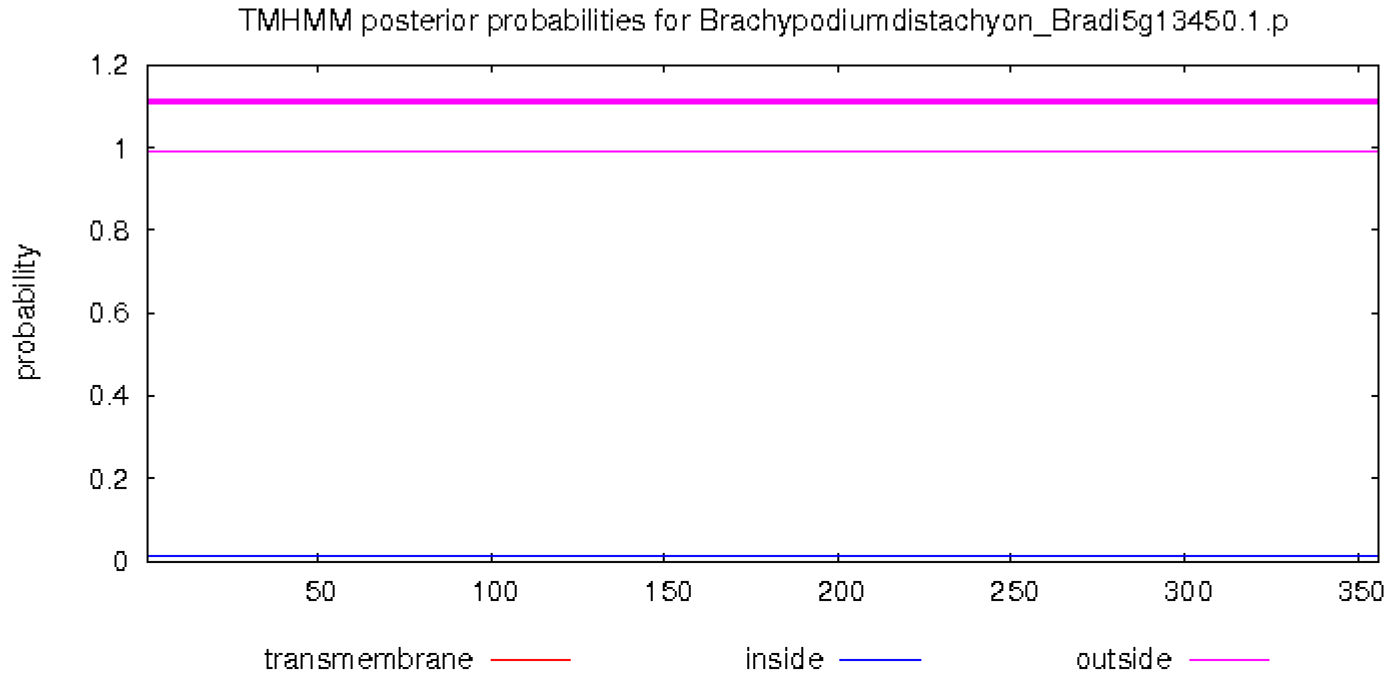

plot in postscript, script for making the plot in gnuplot, data for plot

---

```
# Brachypodiumdistachyon_Bradi5g13816.1.p Length: 309
# Brachypodiumdistachyon_Bradi5g13816.1.p Number of predicted TMHs:  0
# Brachypodiumdistachyon_Bradi5g13816.1.p Exp number of AAs in TMHs: 0.00592
# Brachypodiumdistachyon_Bradi5g13816.1.p Exp number, first 60 AAs:  0
# Brachypodiumdistachyon_Bradi5g13816.1.p Total prob of N-in:        0.03145
Brachypodiumdistachyon_Bradi5g13816.1.p TMHMM2.0        outside      1   309
```

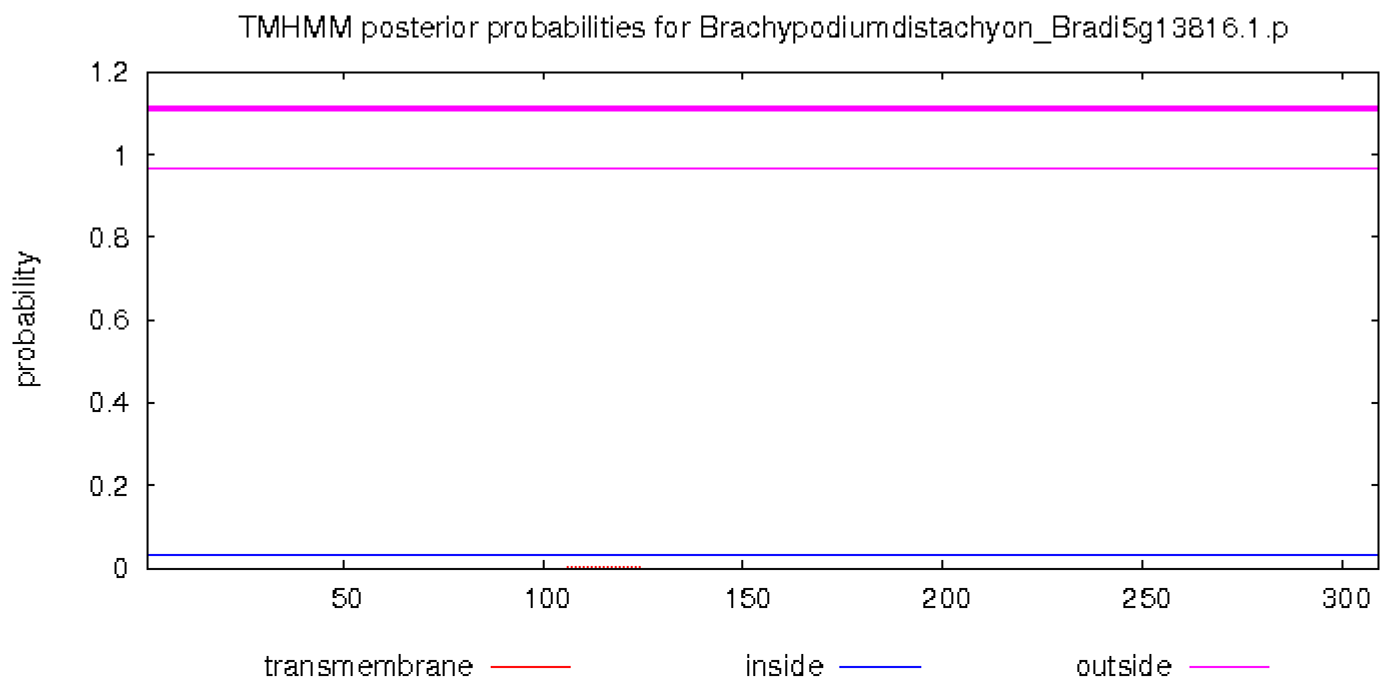

plot in postscript, script for making the plot in gnuplot, data for plot

---

```
# Brachypodiumdistachyon_Bradi5g14736.1.p Length: 242
# Brachypodiumdistachyon_Bradi5g14736.1.p Number of predicted TMHs:  0
# Brachypodiumdistachyon_Bradi5g14736.1.p Exp number of AAs in TMHs: 0.00702
# Brachypodiumdistachyon_Bradi5g14736.1.p Exp number, first 60 AAs:  0.00078
# Brachypodiumdistachyon_Bradi5g14736.1.p Total prob of N-in:        0.15834
Brachypodiumdistachyon_Bradi5g14736.1.p TMHMM2.0        outside      1   242
```

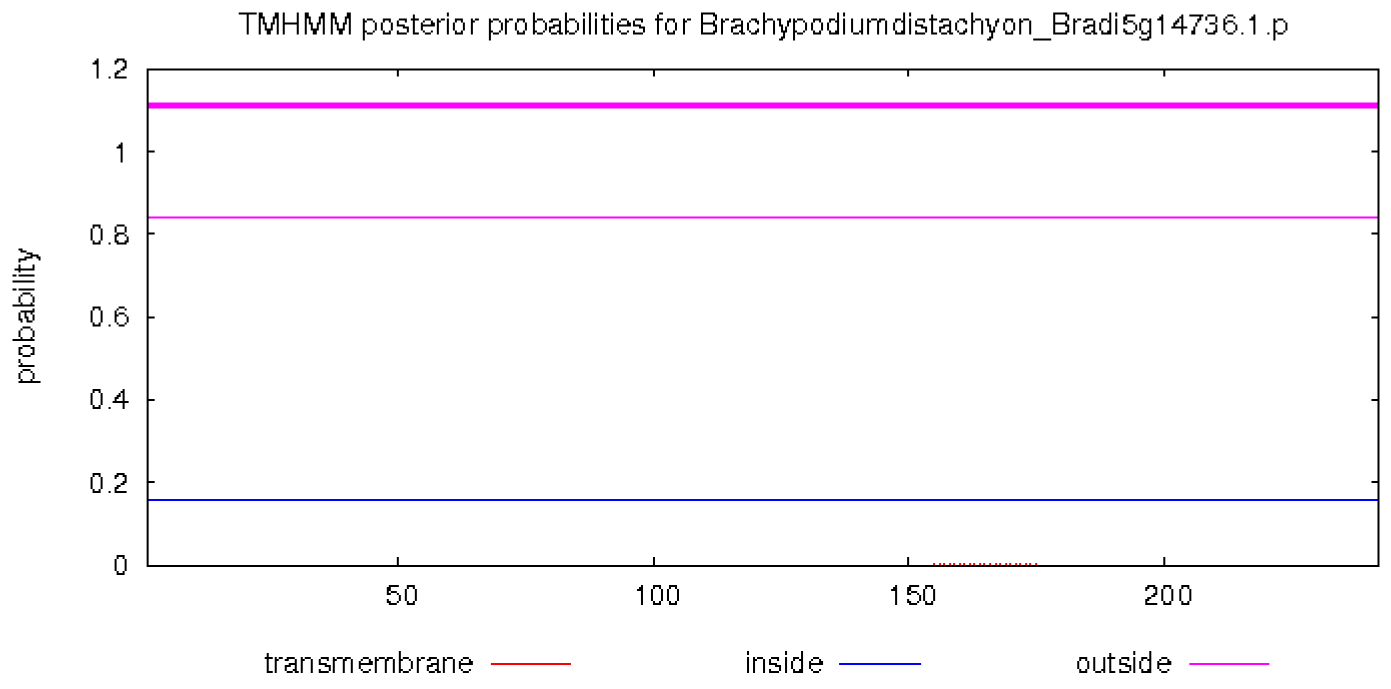

plot in postscript, script for making the plot in gnuplot, data for plot

---

```
# Brachypodiumdistachyon_Bradi5g14952.1.p Length: 184
# Brachypodiumdistachyon_Bradi5g14952.1.p Number of predicted TMHs:  0
# Brachypodiumdistachyon_Bradi5g14952.1.p Exp number of AAs in TMHs: 0.00072
# Brachypodiumdistachyon_Bradi5g14952.1.p Exp number, first 60 AAs:  0.00036
# Brachypodiumdistachyon_Bradi5g14952.1.p Total prob of N-in:        0.27636
Brachypodiumdistachyon_Bradi5g14952.1.p TMHMM2.0        outside      1   184
```

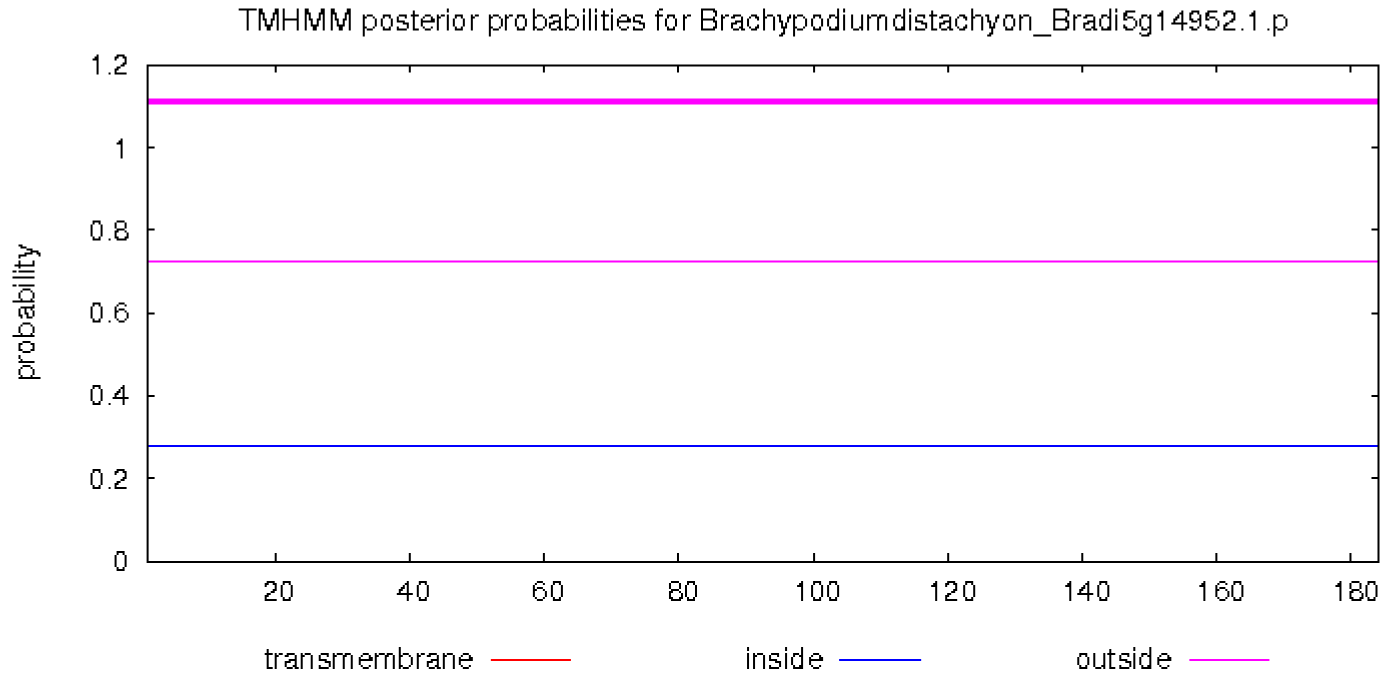

plot in postscript, script for making the plot in gnuplot, data for plot

---

```
# Brachypodiumdistachyon_Bradi5g15230.1.p Length: 393
# Brachypodiumdistachyon_Bradi5g15230.1.p Number of predicted TMHs:  0
# Brachypodiumdistachyon_Bradi5g15230.1.p Exp number of AAs in TMHs: 0.13035
# Brachypodiumdistachyon_Bradi5g15230.1.p Exp number, first 60 AAs:  0
# Brachypodiumdistachyon_Bradi5g15230.1.p Total prob of N-in:        0.01832
Brachypodiumdistachyon_Bradi5g15230.1.p TMHMM2.0        outside      1   393
```

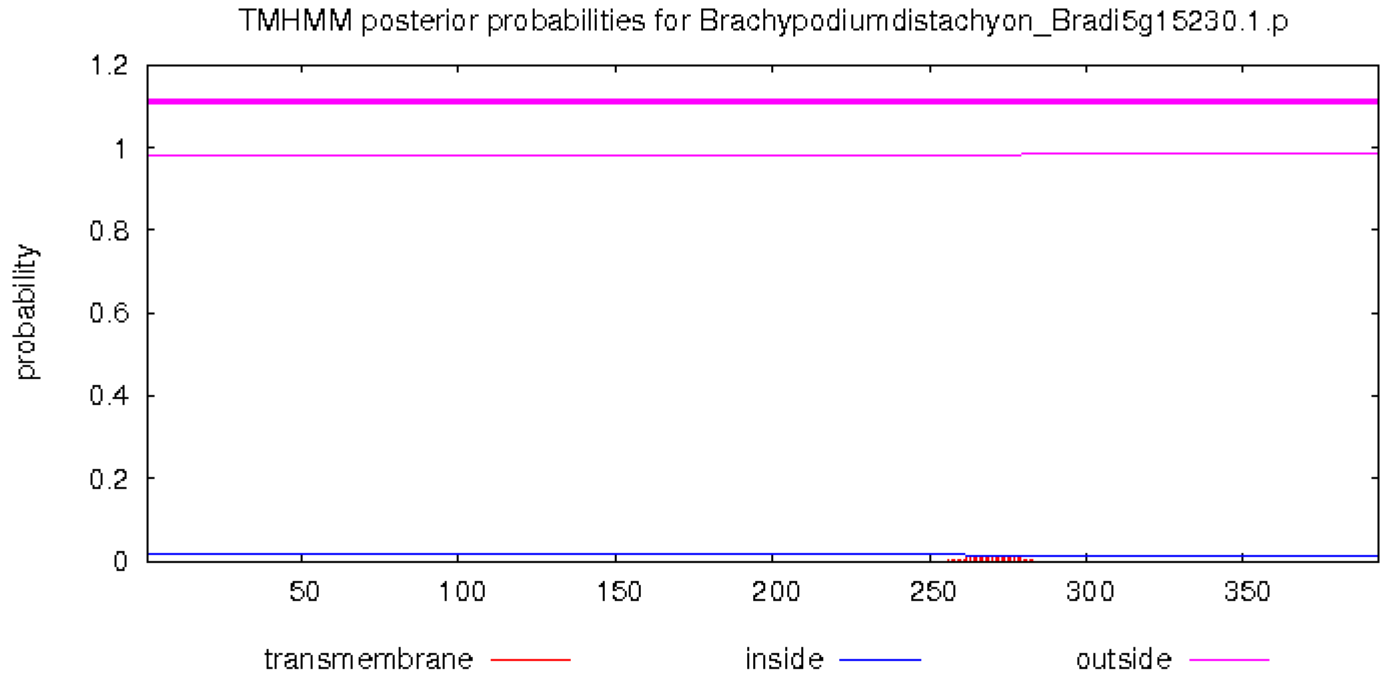

plot in postscript, script for making the plot in gnuplot, data for plot

---

```
# Brachypodiumdistachyon_Bradi5g15587.1.p Length: 288
# Brachypodiumdistachyon_Bradi5g15587.1.p Number of predicted TMHs:  0
# Brachypodiumdistachyon_Bradi5g15587.1.p Exp number of AAs in TMHs: 0.00019
# Brachypodiumdistachyon_Bradi5g15587.1.p Exp number, first 60 AAs:  0
# Brachypodiumdistachyon_Bradi5g15587.1.p Total prob of N-in:        0.01178
Brachypodiumdistachyon_Bradi5g15587.1.p TMHMM2.0        outside      1   288
```

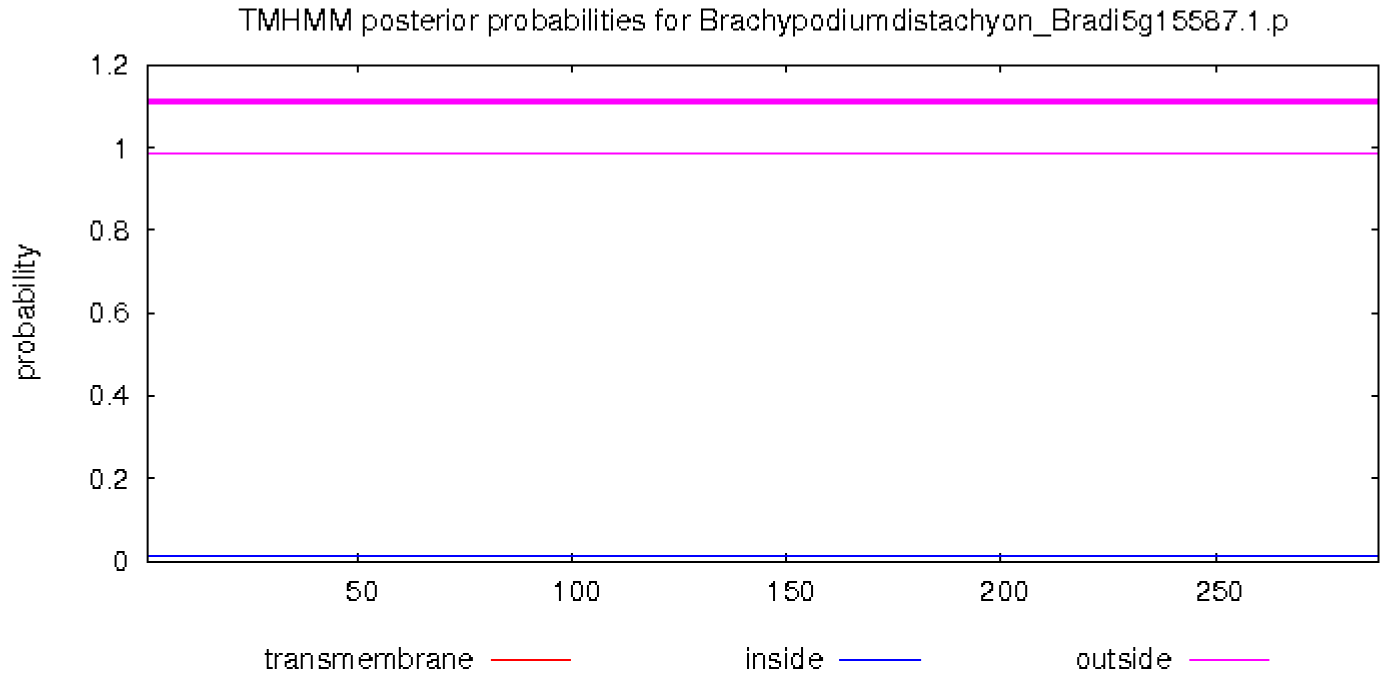

plot in postscript, script for making the plot in gnuplot, data for plot

---

```
# Brachypodiumdistachyon_Bradi5g16917.1.p Length: 334
# Brachypodiumdistachyon_Bradi5g16917.1.p Number of predicted TMHs:  0
# Brachypodiumdistachyon_Bradi5g16917.1.p Exp number of AAs in TMHs: 0
# Brachypodiumdistachyon_Bradi5g16917.1.p Exp number, first 60 AAs:  0
# Brachypodiumdistachyon_Bradi5g16917.1.p Total prob of N-in:        0.00396
Brachypodiumdistachyon_Bradi5g16917.1.p TMHMM2.0        outside      1   334
```

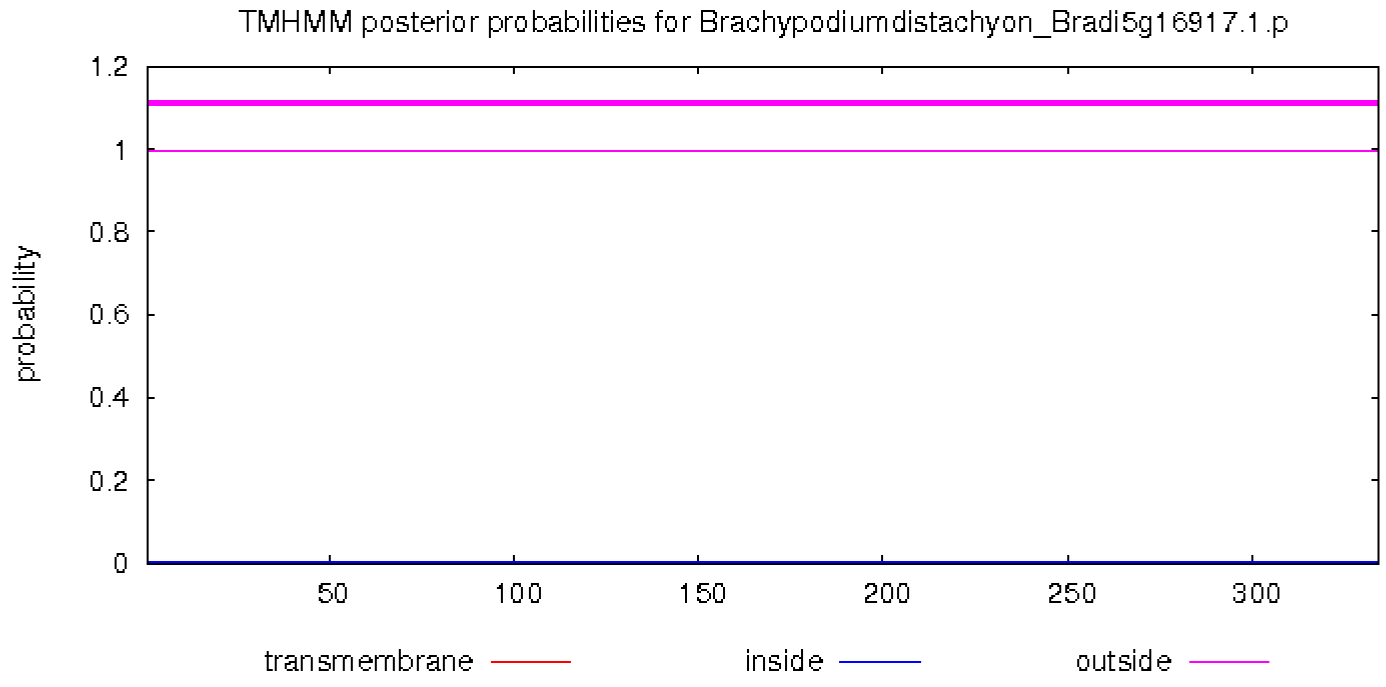

plot in postscript, script for making the plot in gnuplot, data for plot

---

```
# Brachypodiumdistachyon_Bradi5g18044.2.p Length: 595
# Brachypodiumdistachyon_Bradi5g18044.2.p Number of predicted TMHs:  0
# Brachypodiumdistachyon_Bradi5g18044.2.p Exp number of AAs in TMHs: 0.00193
# Brachypodiumdistachyon_Bradi5g18044.2.p Exp number, first 60 AAs:  0
# Brachypodiumdistachyon_Bradi5g18044.2.p Total prob of N-in:        0.00153
Brachypodiumdistachyon_Bradi5g18044.2.p TMHMM2.0        outside      1   595
```

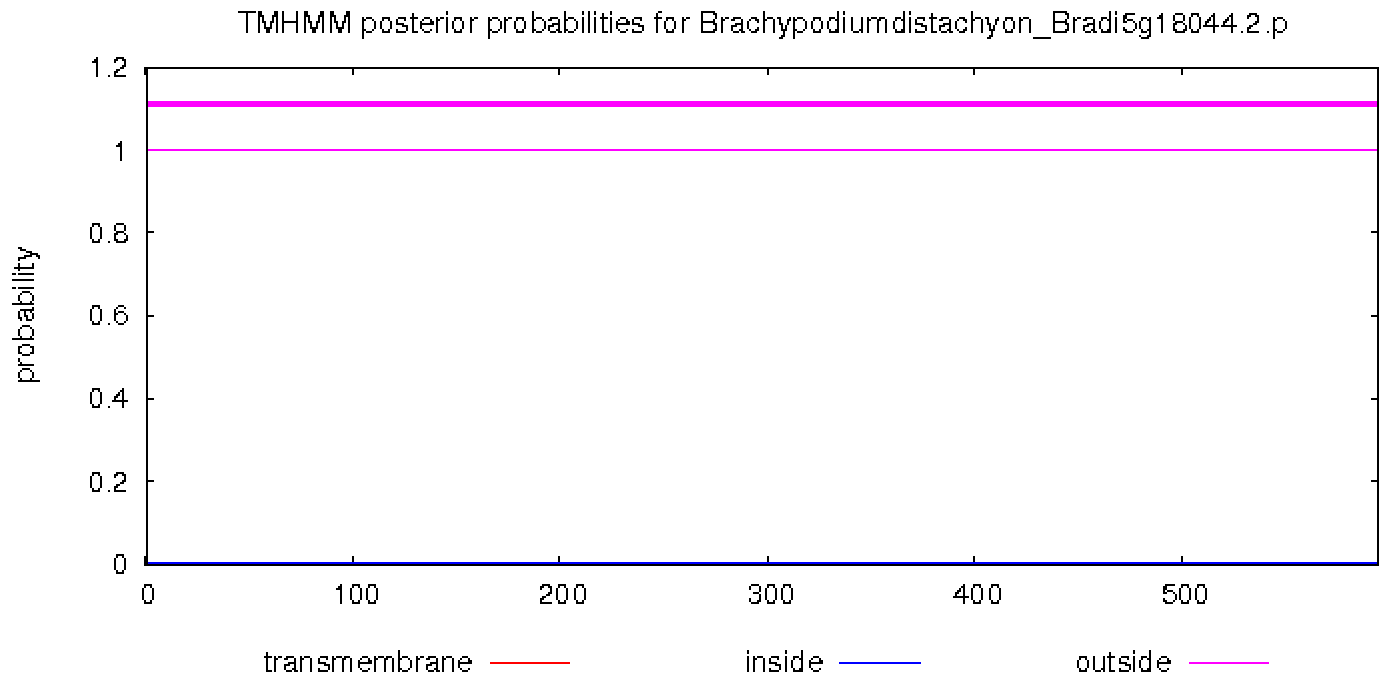

[plot](#) in postscript, [script](#) for making the plot in gnuplot, [data](#) for plot

---

```
# Brachypodiumdistachyon_Bradi5g21010.1.p Length: 390
# Brachypodiumdistachyon_Bradi5g21010.1.p Number of predicted TMHs:  0
# Brachypodiumdistachyon_Bradi5g21010.1.p Exp number of AAs in TMHs: 0
# Brachypodiumdistachyon_Bradi5g21010.1.p Exp number, first 60 AAs:  0
# Brachypodiumdistachyon_Bradi5g21010.1.p Total prob of N-in:        0.00378
Brachypodiumdistachyon_Bradi5g21010.1.p TMHMM2.0        outside      1   390
```

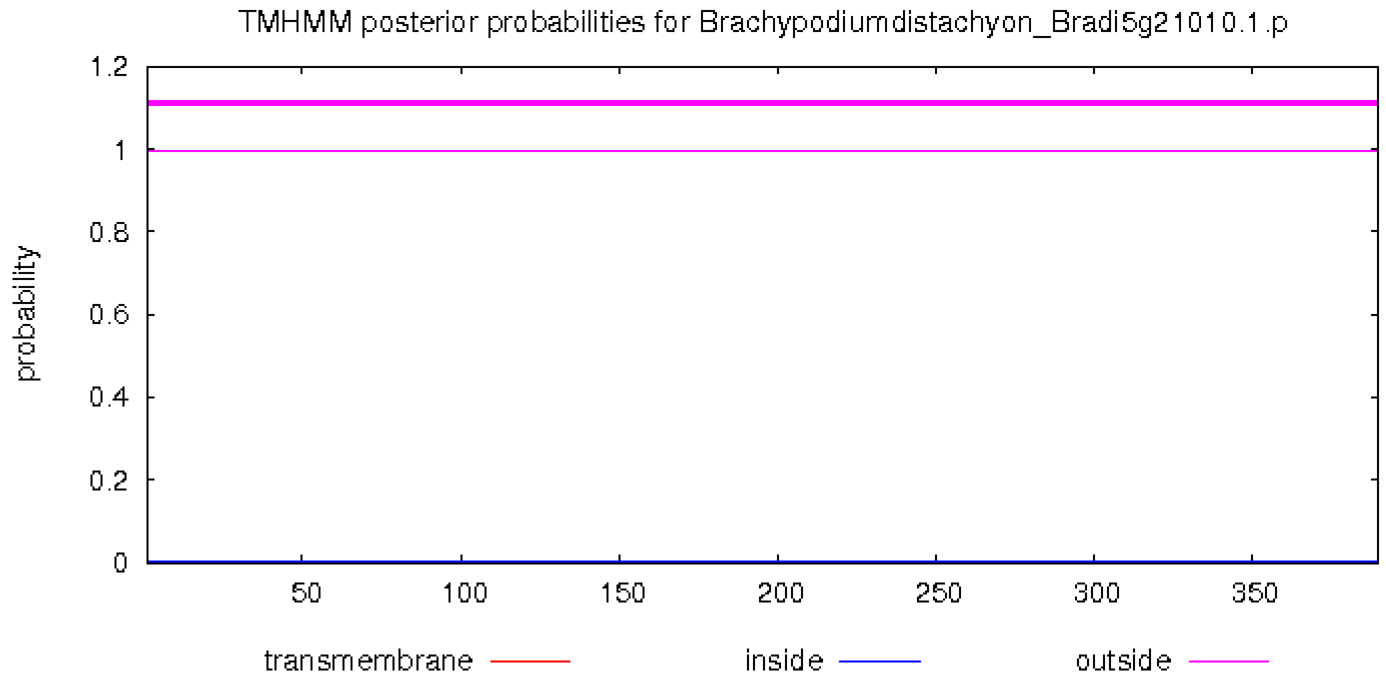

[plot](#) in postscript, [script](#) for making the plot in gnuplot, [data](#) for plot

---

```
# Brachypodiumdistachyon_Bradi5g21983.1.p Length: 283
# Brachypodiumdistachyon_Bradi5g21983.1.p Number of predicted TMHs:  0
# Brachypodiumdistachyon_Bradi5g21983.1.p Exp number of AAs in TMHs: 0.08418
# Brachypodiumdistachyon_Bradi5g21983.1.p Exp number, first 60 AAs:  0.00291
# Brachypodiumdistachyon_Bradi5g21983.1.p Total prob of N-in:        0.07637
Brachypodiumdistachyon_Bradi5g21983.1.p TMHMM2.0        outside      1   283
```

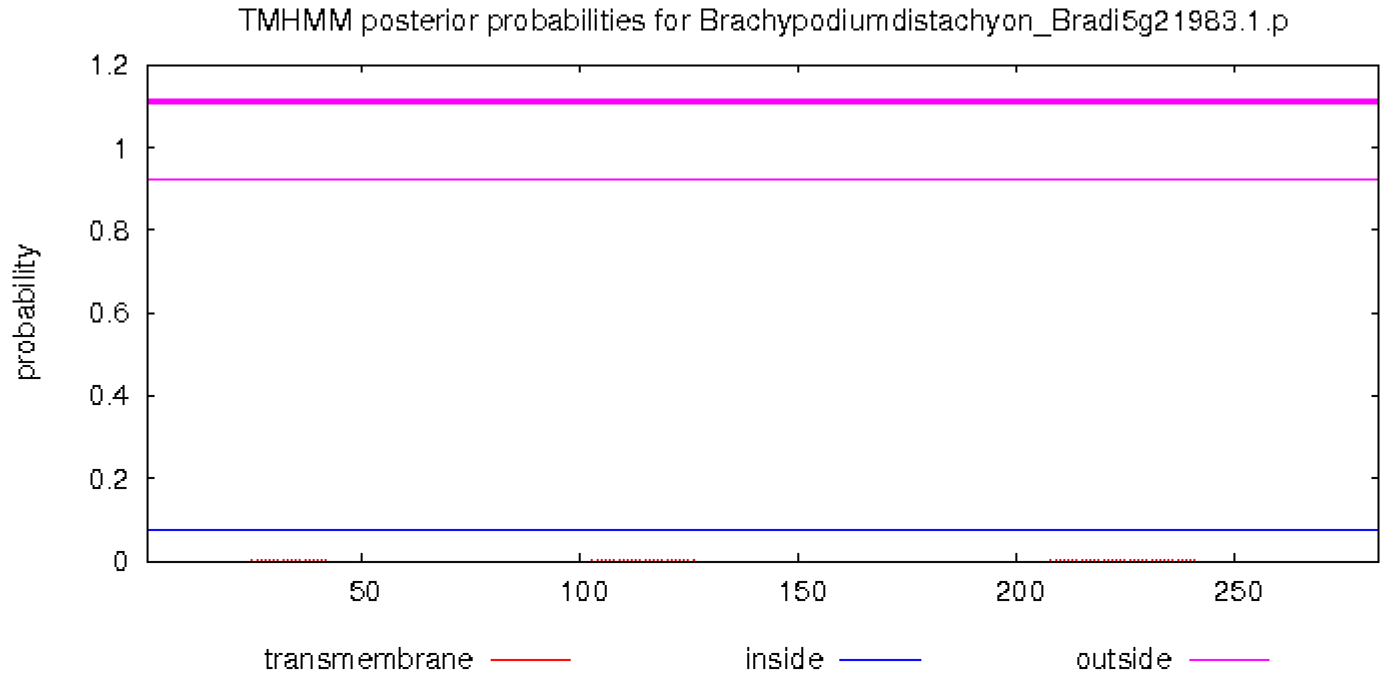

plot in postscript, script for making the plot in gnuplot, data for plot

---

```
# Brachypodiumdistachyon_Bradi5g27467.2.p Length: 449
# Brachypodiumdistachyon_Bradi5g27467.2.p Number of predicted TMHs:  0
# Brachypodiumdistachyon_Bradi5g27467.2.p Exp number of AAs in TMHs: 0.12135
# Brachypodiumdistachyon_Bradi5g27467.2.p Exp number, first 60 AAs:  0.10963
# Brachypodiumdistachyon_Bradi5g27467.2.p Total prob of N-in:        0.00430
Brachypodiumdistachyon_Bradi5g27467.2.p TMHMM2.0        outside      1   449
```

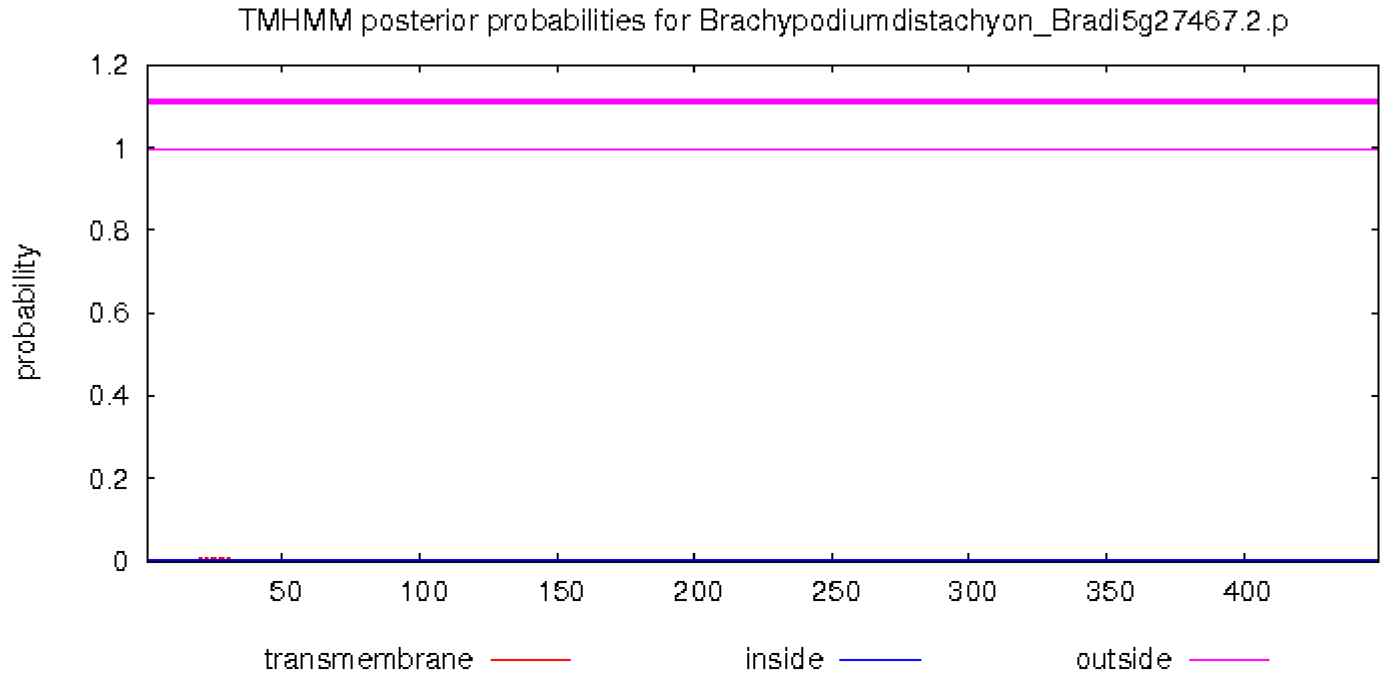

plot in postscript, script for making the plot in gnuplot, data for plot

---

```
# Brachypodiumstacei_Brast01G023500.1.p Length: 211
# Brachypodiumstacei_Brast01G023500.1.p Number of predicted TMHs:  0
# Brachypodiumstacei_Brast01G023500.1.p Exp number of AAs in TMHs: 0.01411
# Brachypodiumstacei_Brast01G023500.1.p Exp number, first 60 AAs:  0
# Brachypodiumstacei_Brast01G023500.1.p Total prob of N-in:        0.05063
Brachypodiumstacei_Brast01G023500.1.p   TMHMM2.0        outside      1   211
```

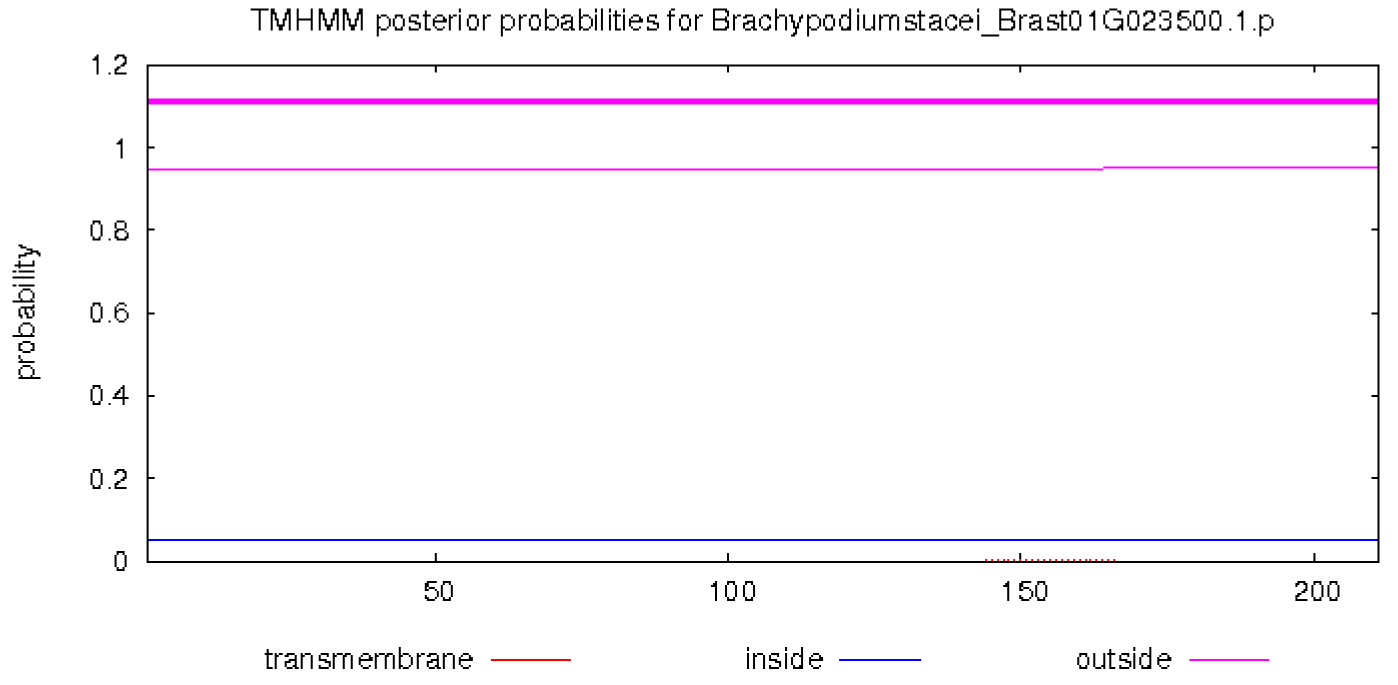

plot in postscript, script for making the plot in gnuplot, data for plot

---

```
# Brachypodiumstacei_Brast01G038400.1.p Length: 202
# Brachypodiumstacei_Brast01G038400.1.p Number of predicted TMHs:  0
# Brachypodiumstacei_Brast01G038400.1.p Exp number of AAs in TMHs: 0.0017
# Brachypodiumstacei_Brast01G038400.1.p Exp number, first 60 AAs:  0.00115
# Brachypodiumstacei_Brast01G038400.1.p Total prob of N-in:        0.02203
Brachypodiumstacei_Brast01G038400.1.p   TMHMM2.0        outside      1   202
```

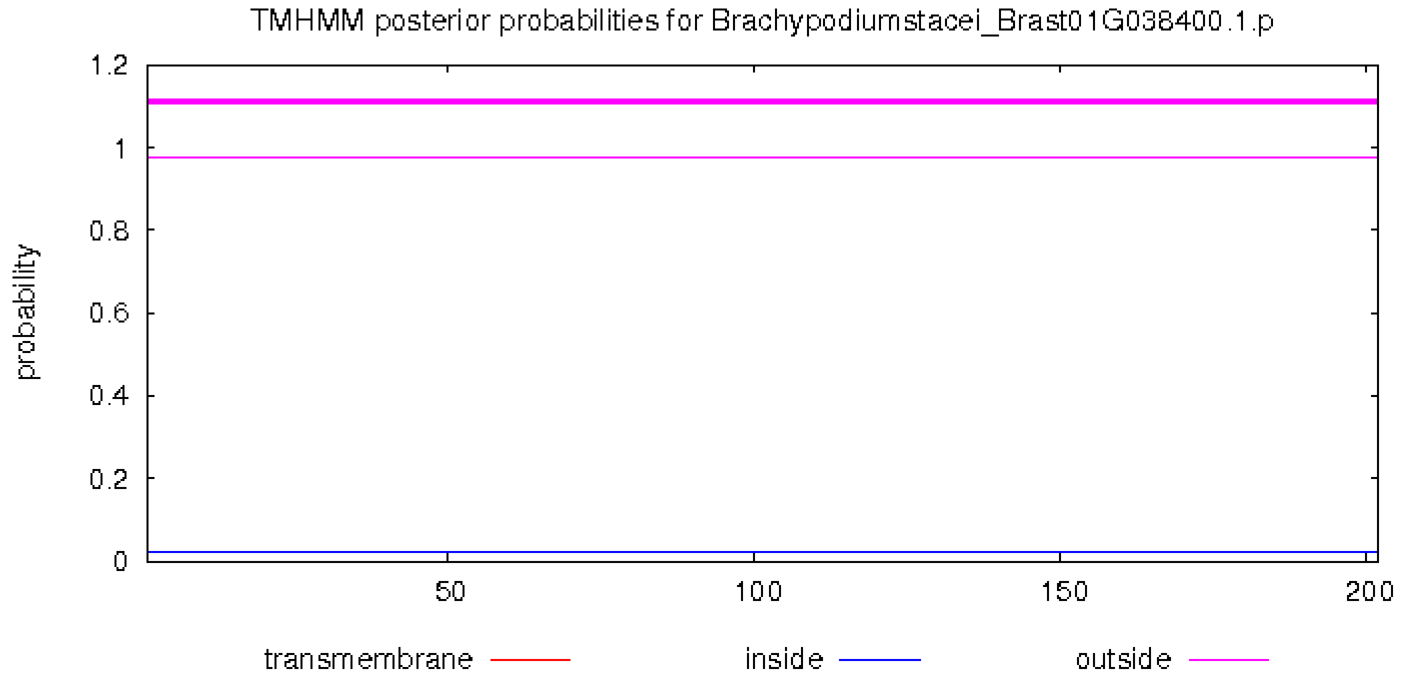

plot in postscript, script for making the plot in gnuplot, data for plot

---

```
# Brachypodiumstacei_Brast01G038600.1.p Length: 203
# Brachypodiumstacei_Brast01G038600.1.p Number of predicted TMHs:  0
# Brachypodiumstacei_Brast01G038600.1.p Exp number of AAs in TMHs: 0.0056
# Brachypodiumstacei_Brast01G038600.1.p Exp number, first 60 AAs:  0.00419
# Brachypodiumstacei_Brast01G038600.1.p Total prob of N-in:        0.02174
Brachypodiumstacei_Brast01G038600.1.p   TMHMM2.0        outside      1   203
```

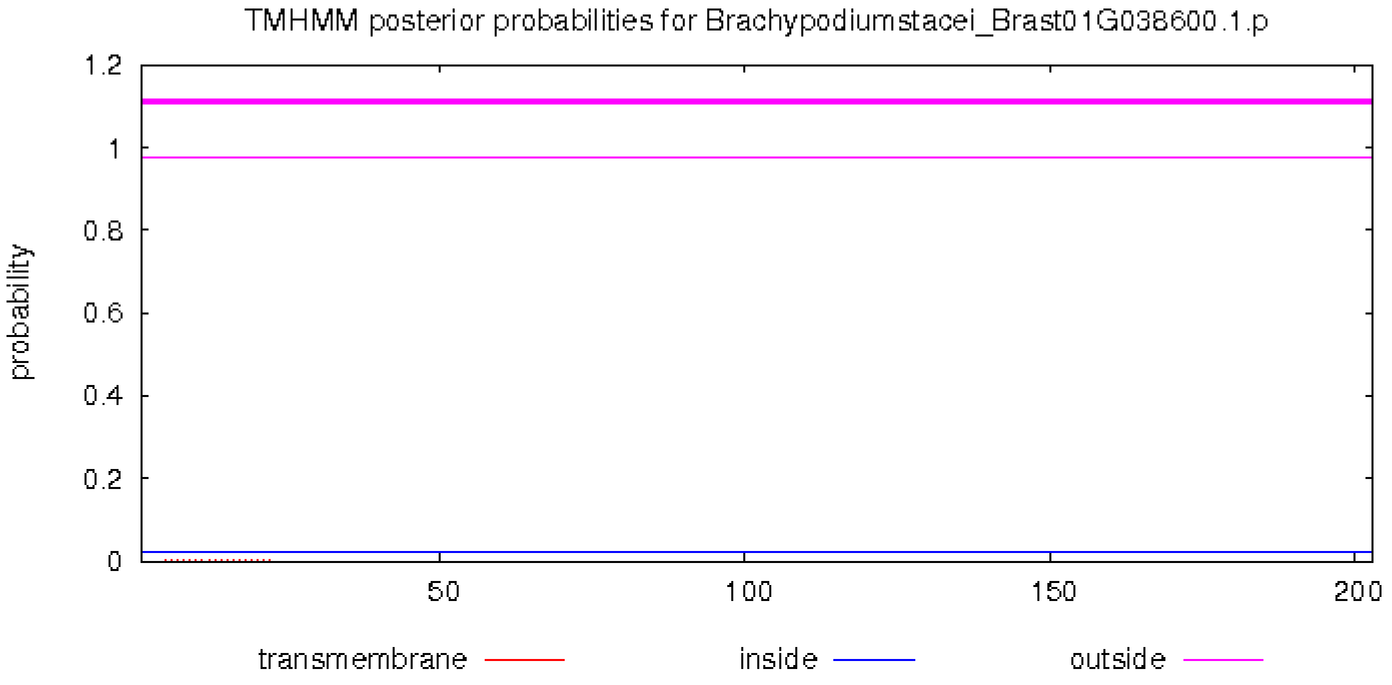

plot in postscript, script for making the plot in gnuplot, data for plot

---

```
# Brachypodiumstacei_Brast01G063400.1.p Length: 395
# Brachypodiumstacei_Brast01G063400.1.p Number of predicted TMHs:  0
# Brachypodiumstacei_Brast01G063400.1.p Exp number of AAs in TMHs: 0.08757
# Brachypodiumstacei_Brast01G063400.1.p Exp number, first 60 AAs:  0.00018
# Brachypodiumstacei_Brast01G063400.1.p Total prob of N-in:        0.00696
Brachypodiumstacei_Brast01G063400.1.p   TMHMM2.0        outside      1   395
```

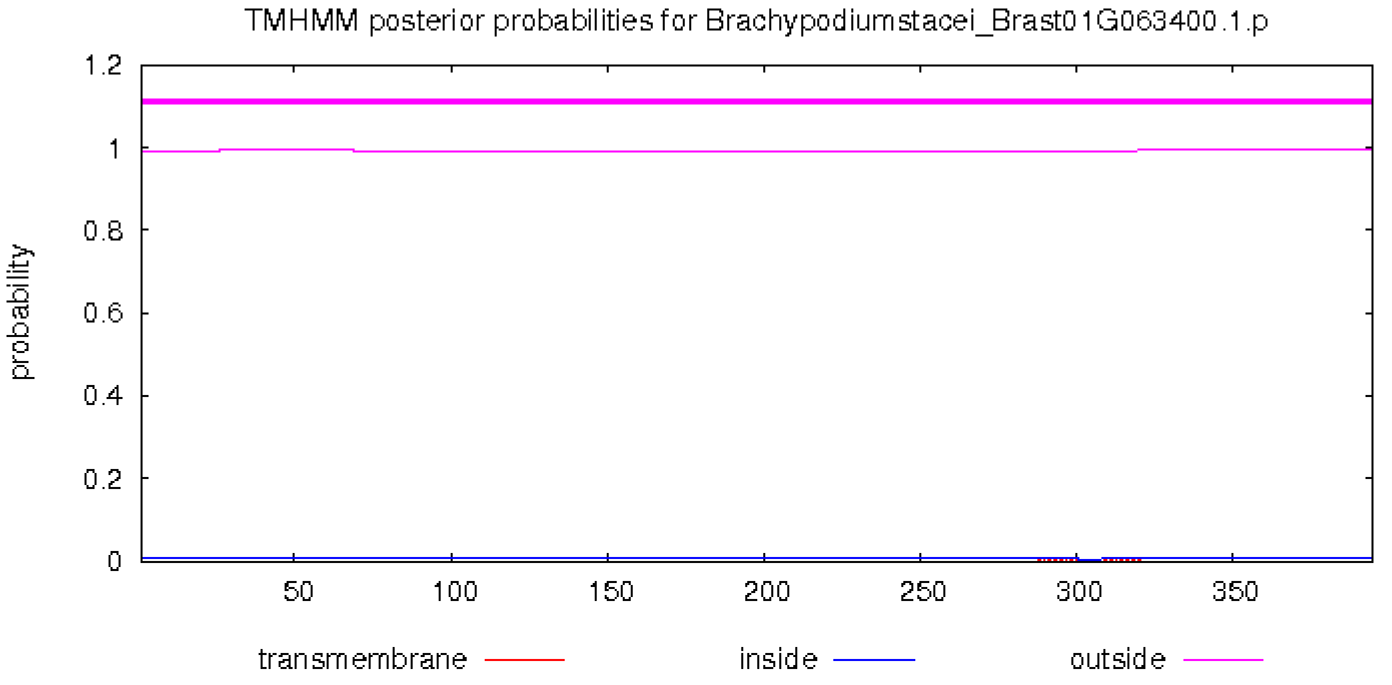

plot in postscript, script for making the plot in gnuplot, data for plot

---

```
# Brachypodiumstacei_Brast01G065900.1.p Length: 295
# Brachypodiumstacei_Brast01G065900.1.p Number of predicted TMHs:  0
# Brachypodiumstacei_Brast01G065900.1.p Exp number of AAs in TMHs: 0.00642
# Brachypodiumstacei_Brast01G065900.1.p Exp number, first 60 AAs:  0.00423
# Brachypodiumstacei_Brast01G065900.1.p Total prob of N-in:        0.00562
Brachypodiumstacei_Brast01G065900.1.p   TMHMM2.0        outside      1   295
```

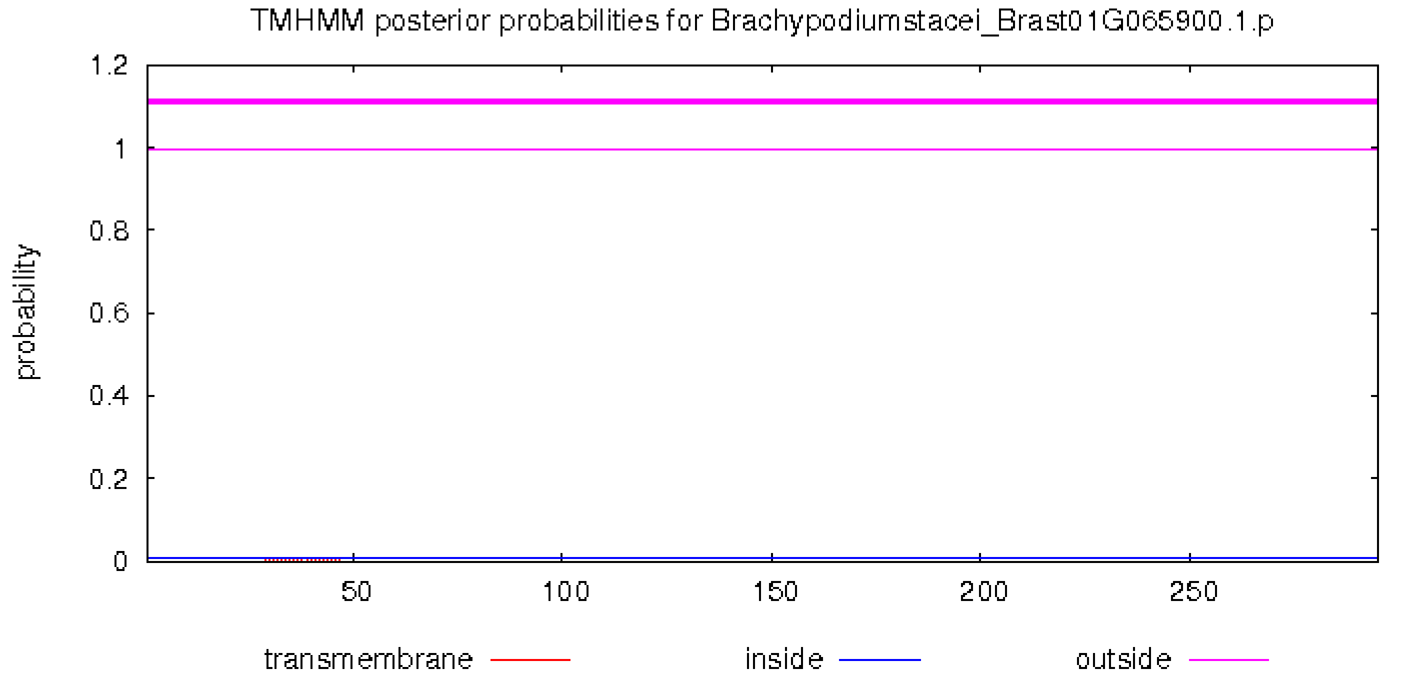

plot in postscript, script for making the plot in gnuplot, data for plot

```
# Brachypodiumstacei_Brast01G080400.1.p Length: 251
# Brachypodiumstacei_Brast01G080400.1.p Number of predicted TMHs:  0
# Brachypodiumstacei_Brast01G080400.1.p Exp number of AAs in TMHs: 0.00997
# Brachypodiumstacei_Brast01G080400.1.p Exp number, first 60 AAs:  0
# Brachypodiumstacei_Brast01G080400.1.p Total prob of N-in:        0.01091
Brachypodiumstacei_Brast01G080400.1.p	TMHMM2.0	outside	     1   251
```

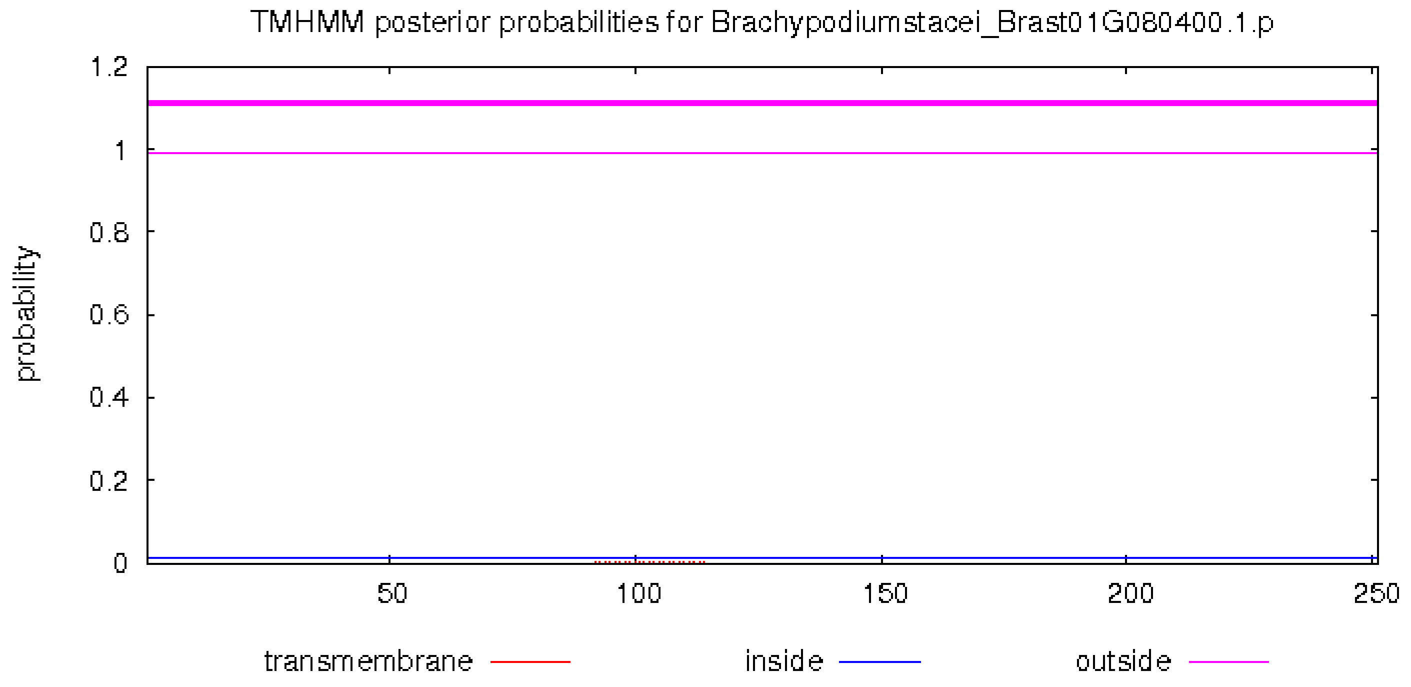

plot in postscript, script for making the plot in gnuplot, data for plot

```
# Brachypodiumstacei_Brast01G108000.1.p Length: 310
# Brachypodiumstacei_Brast01G108000.1.p Number of predicted TMHs:  0
# Brachypodiumstacei_Brast01G108000.1.p Exp number of AAs in TMHs: 0.01682
# Brachypodiumstacei_Brast01G108000.1.p Exp number, first 60 AAs:  0
# Brachypodiumstacei_Brast01G108000.1.p Total prob of N-in:        0.02822
Brachypodiumstacei_Brast01G108000.1.p	TMHMM2.0	outside	     1   310
```

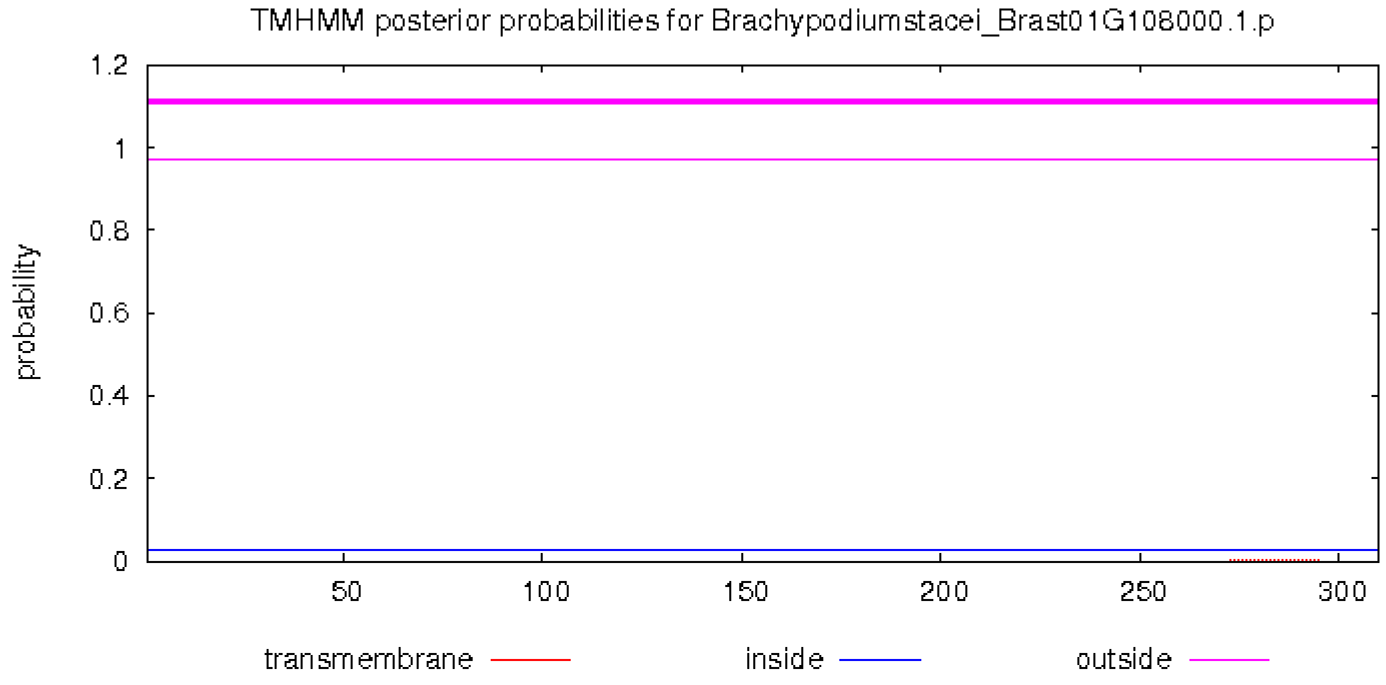

plot in postscript, script for making the plot in gnuplot, data for plot

---

```
# Brachypodiumstacei_Brast01G111300.1.p Length: 179
# Brachypodiumstacei_Brast01G111300.1.p Number of predicted TMHs:  0
# Brachypodiumstacei_Brast01G111300.1.p Exp number of AAs in TMHs: 0
# Brachypodiumstacei_Brast01G111300.1.p Exp number, first 60 AAs:  0
# Brachypodiumstacei_Brast01G111300.1.p Total prob of N-in:        0.07686
Brachypodiumstacei_Brast01G111300.1.p	TMHMM2.0	outside	     1   179
```

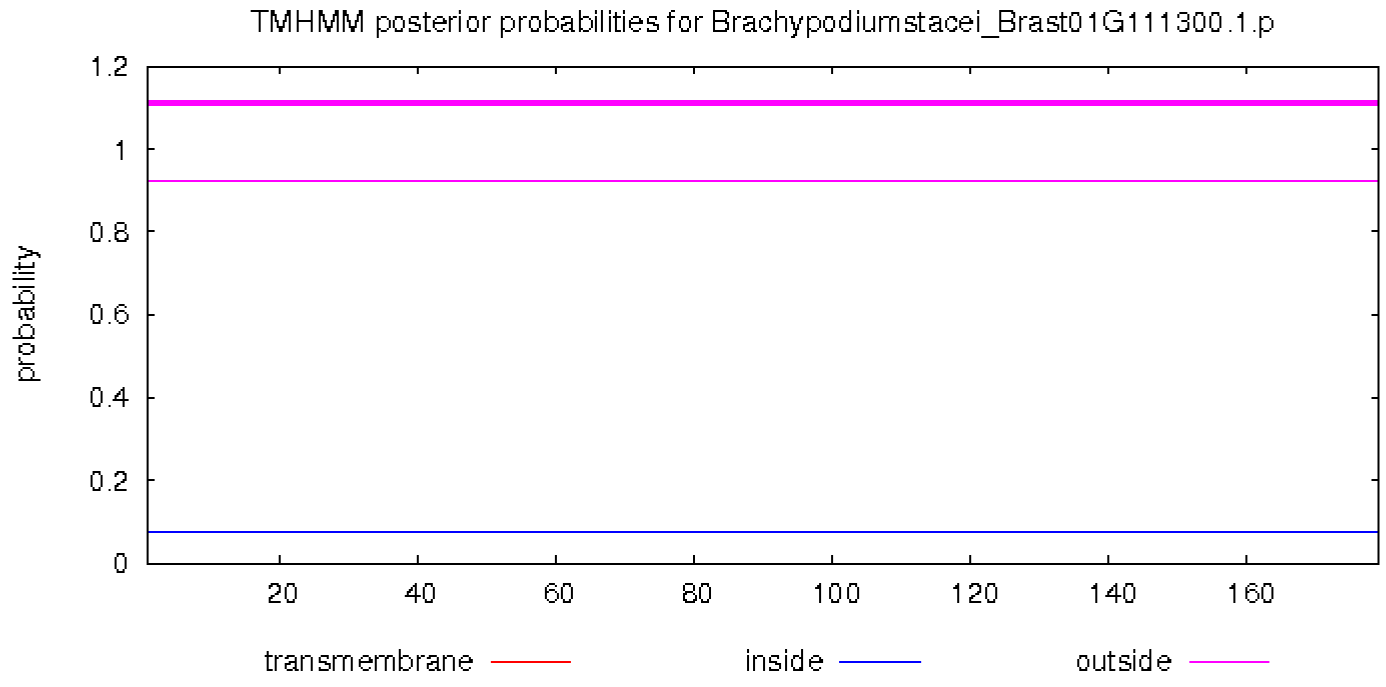

plot in postscript, script for making the plot in gnuplot, data for plot

---

```
# Brachypodiumstacei_Brast01G184700.1.p Length: 291
# Brachypodiumstacei_Brast01G184700.1.p Number of predicted TMHs:  0
# Brachypodiumstacei_Brast01G184700.1.p Exp number of AAs in TMHs: 0.00223
# Brachypodiumstacei_Brast01G184700.1.p Exp number, first 60 AAs:  0.00017
# Brachypodiumstacei_Brast01G184700.1.p Total prob of N-in:        0.01045
Brachypodiumstacei_Brast01G184700.1.p	TMHMM2.0	outside	     1   291
```

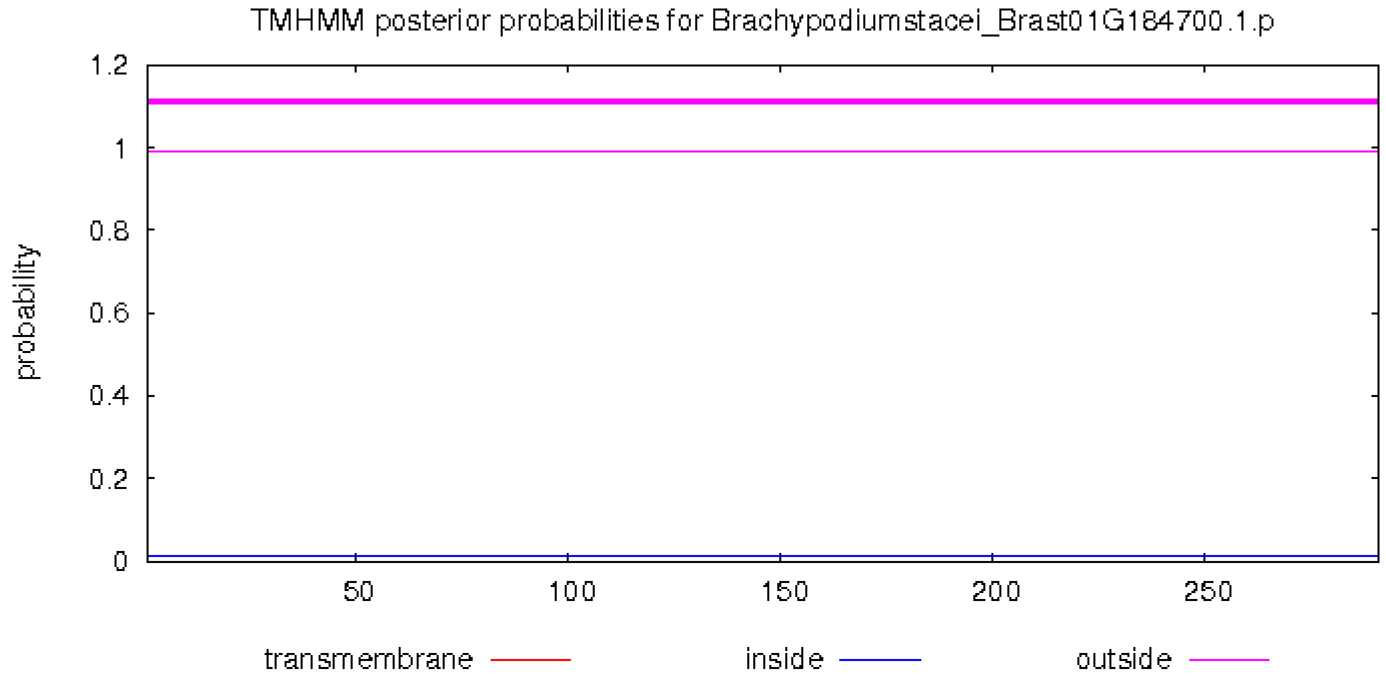

plot in postscript, script for making the plot in gnuplot, data for plot

---

```
# Brachypodiumstacei_Brast01G185400.1.p Length: 588
# Brachypodiumstacei_Brast01G185400.1.p Number of predicted TMHs:  0
# Brachypodiumstacei_Brast01G185400.1.p Exp number of AAs in TMHs: 0.00039
# Brachypodiumstacei_Brast01G185400.1.p Exp number, first 60 AAs:  0
# Brachypodiumstacei_Brast01G185400.1.p Total prob of N-in:        0.00146
Brachypodiumstacei_Brast01G185400.1.p	TMHMM2.0	outside	     1   588
```

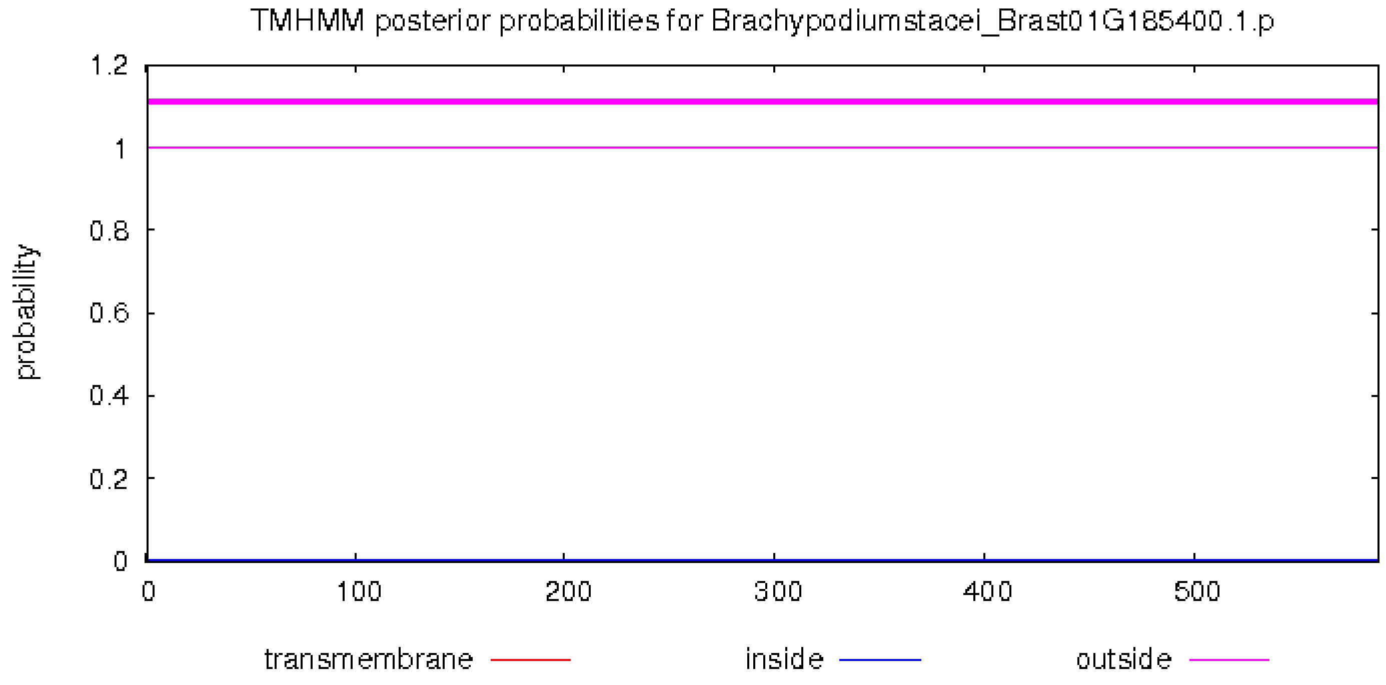

plot in postscript, script for making the plot in gnuplot, data for plot

---

```
# Brachypodiumstacei_Brast01G188200.1.p Length: 331
# Brachypodiumstacei_Brast01G188200.1.p Number of predicted TMHs:  0
# Brachypodiumstacei_Brast01G188200.1.p Exp number of AAs in TMHs: 0.01925
# Brachypodiumstacei_Brast01G188200.1.p Exp number, first 60 AAs:  0.0062
# Brachypodiumstacei_Brast01G188200.1.p Total prob of N-in:        0.03188
Brachypodiumstacei_Brast01G188200.1.p	TMHMM2.0	outside	     1   331
```

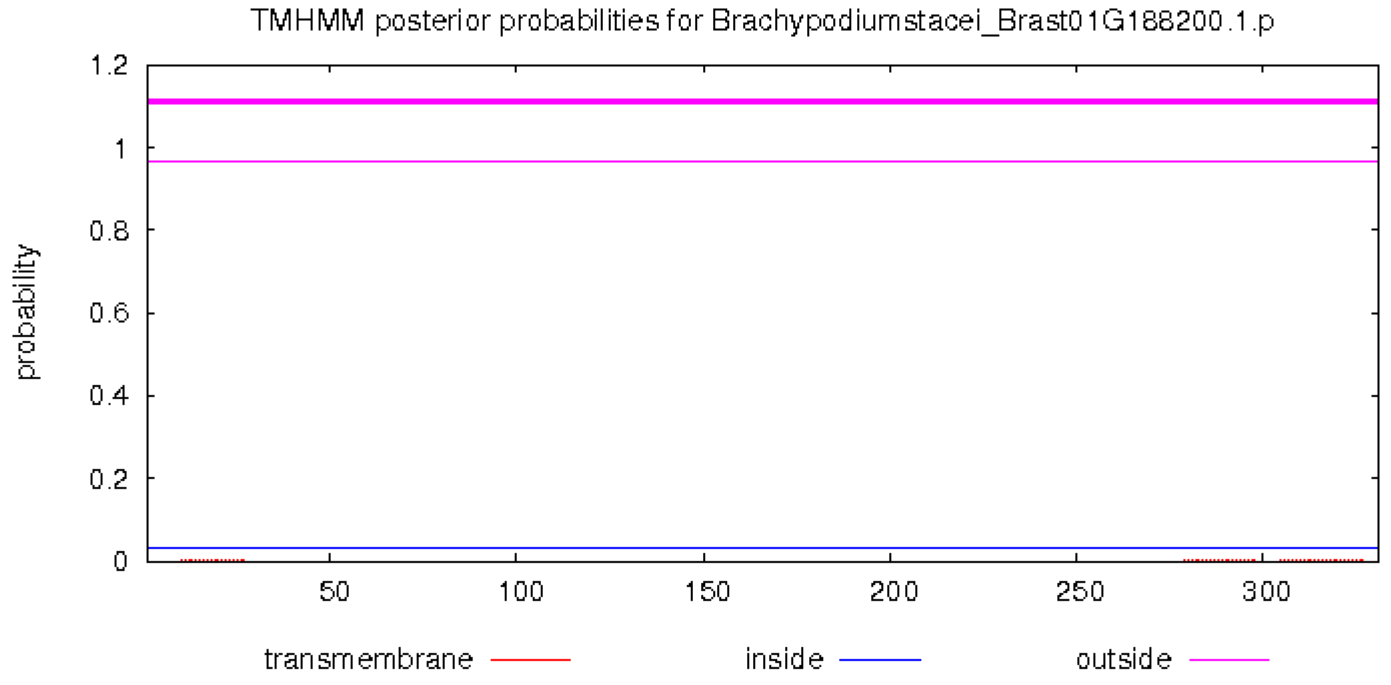

plot in postscript, script for making the plot in gnuplot, data for plot

---

```
# Brachypodiumstacei_Brast01G190200.1.p Length: 369
# Brachypodiumstacei_Brast01G190200.1.p Number of predicted TMHs:  0
# Brachypodiumstacei_Brast01G190200.1.p Exp number of AAs in TMHs: 0.0016
# Brachypodiumstacei_Brast01G190200.1.p Exp number, first 60 AAs:  0.00011
# Brachypodiumstacei_Brast01G190200.1.p Total prob of N-in:        0.01360
Brachypodiumstacei_Brast01G190200.1.p   TMHMM2.0        outside      1   369
```

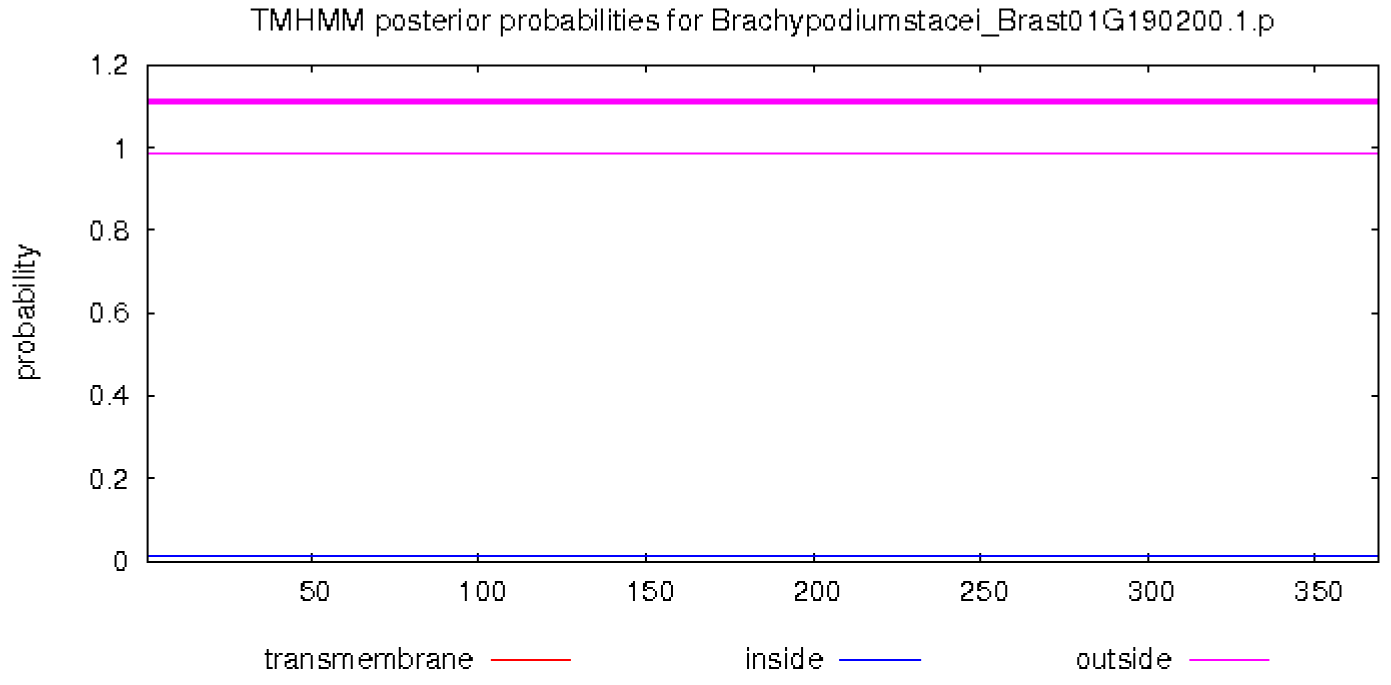

plot in postscript, script for making the plot in gnuplot, data for plot

---

```
# Brachypodiumstacei_Brast01G304500.1.p Length: 94
# Brachypodiumstacei_Brast01G304500.1.p Number of predicted TMHs:  0
# Brachypodiumstacei_Brast01G304500.1.p Exp number of AAs in TMHs: 0.00286
# Brachypodiumstacei_Brast01G304500.1.p Exp number, first 60 AAs:  0.00286
# Brachypodiumstacei_Brast01G304500.1.p Total prob of N-in:        0.07226
Brachypodiumstacei_Brast01G304500.1.p   TMHMM2.0        outside      1    94
```

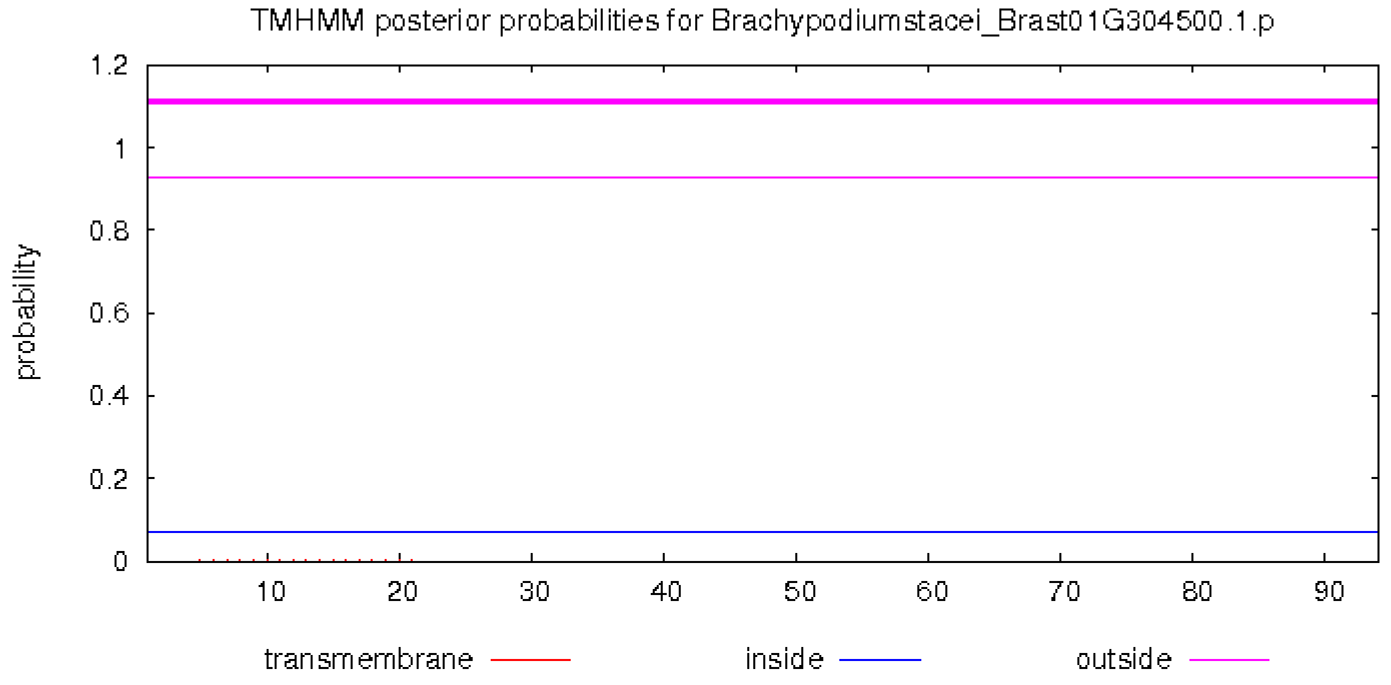

plot in postscript, script for making the plot in gnuplot, data for plot

---

```
# Brachypodiumstacei_Brast01G307800.1.p Length: 492
# Brachypodiumstacei_Brast01G307800.1.p Number of predicted TMHs:  1
# Brachypodiumstacei_Brast01G307800.1.p Exp number of AAs in TMHs: 22.30262
# Brachypodiumstacei_Brast01G307800.1.p Exp number, first 60 AAs:  0
# Brachypodiumstacei_Brast01G307800.1.p Total prob of N-in:        0.05598
Brachypodiumstacei_Brast01G307800.1.p   TMHMM2.0        outside      1   457
Brachypodiumstacei_Brast01G307800.1.p   TMHMM2.0        TMhelix    458   480
Brachypodiumstacei_Brast01G307800.1.p   TMHMM2.0        inside     481   492
```

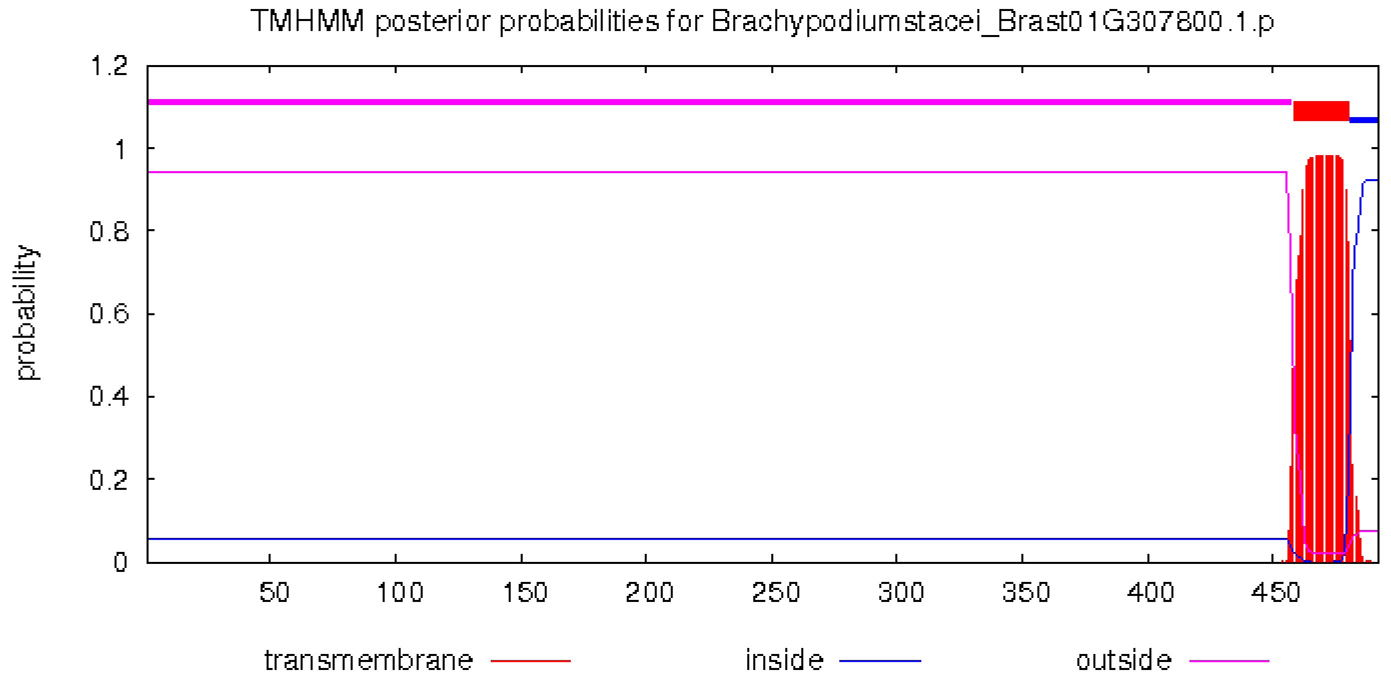

plot in postscript, script for making the plot in gnuplot, data for plot

---

```
# Brachypodiumstacei_Brast01G322200.1.p Length: 292
# Brachypodiumstacei_Brast01G322200.1.p Number of predicted TMHs:  0
# Brachypodiumstacei_Brast01G322200.1.p Exp number of AAs in TMHs: 0.000730000000000000001
# Brachypodiumstacei_Brast01G322200.1.p Exp number, first 60 AAs:  0.0003
```

```
# Brachypodiumstacei_Brast01G322200.1.p Total prob of N-in:        0.00744
Brachypodiumstacei_Brast01G322200.1.p	TMHMM2.0	outside	     1   292
```

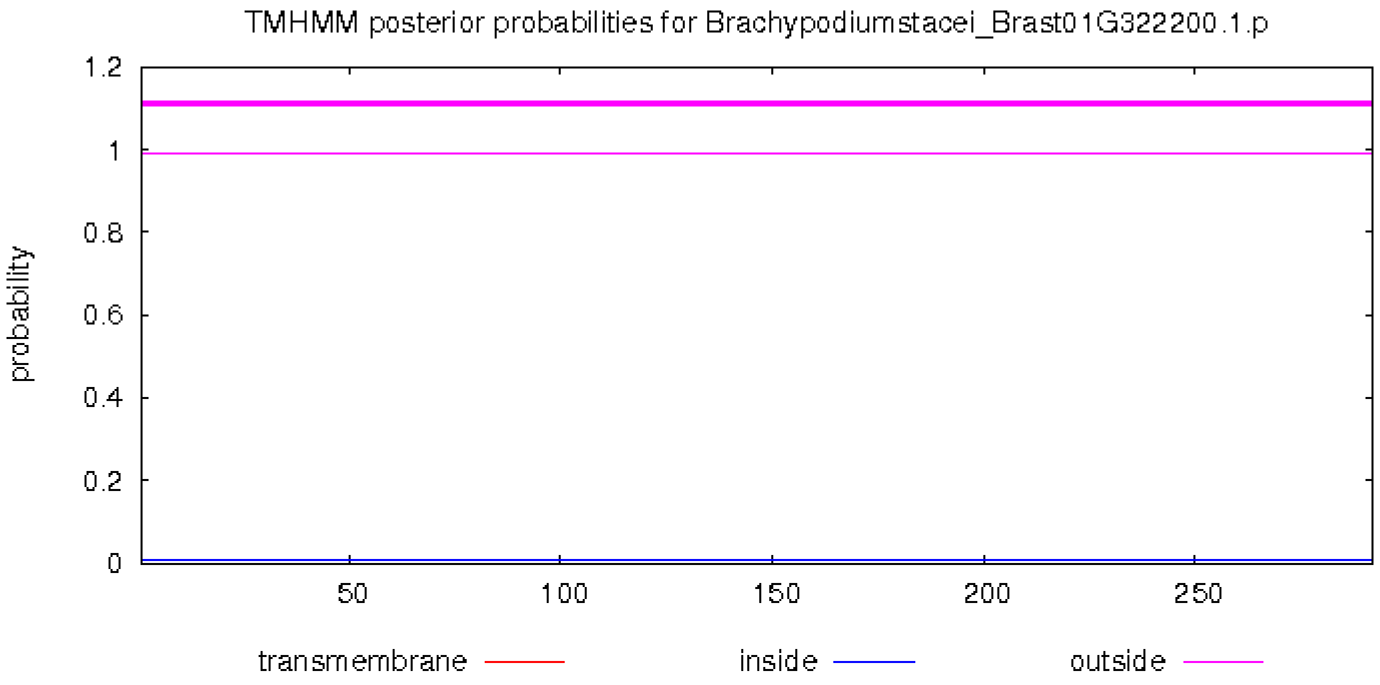

plot in postscript, script for making the plot in gnuplot, data for plot

---

```
# Brachypodiumstacei_Brast01G345300.1.p Length: 344
# Brachypodiumstacei_Brast01G345300.1.p Number of predicted TMHs:  0
# Brachypodiumstacei_Brast01G345300.1.p Exp number of AAs in TMHs: 0.00589
# Brachypodiumstacei_Brast01G345300.1.p Exp number, first 60 AAs:  0.00556
# Brachypodiumstacei_Brast01G345300.1.p Total prob of N-in:        0.00111
Brachypodiumstacei_Brast01G345300.1.p	TMHMM2.0	outside	     1   344
```

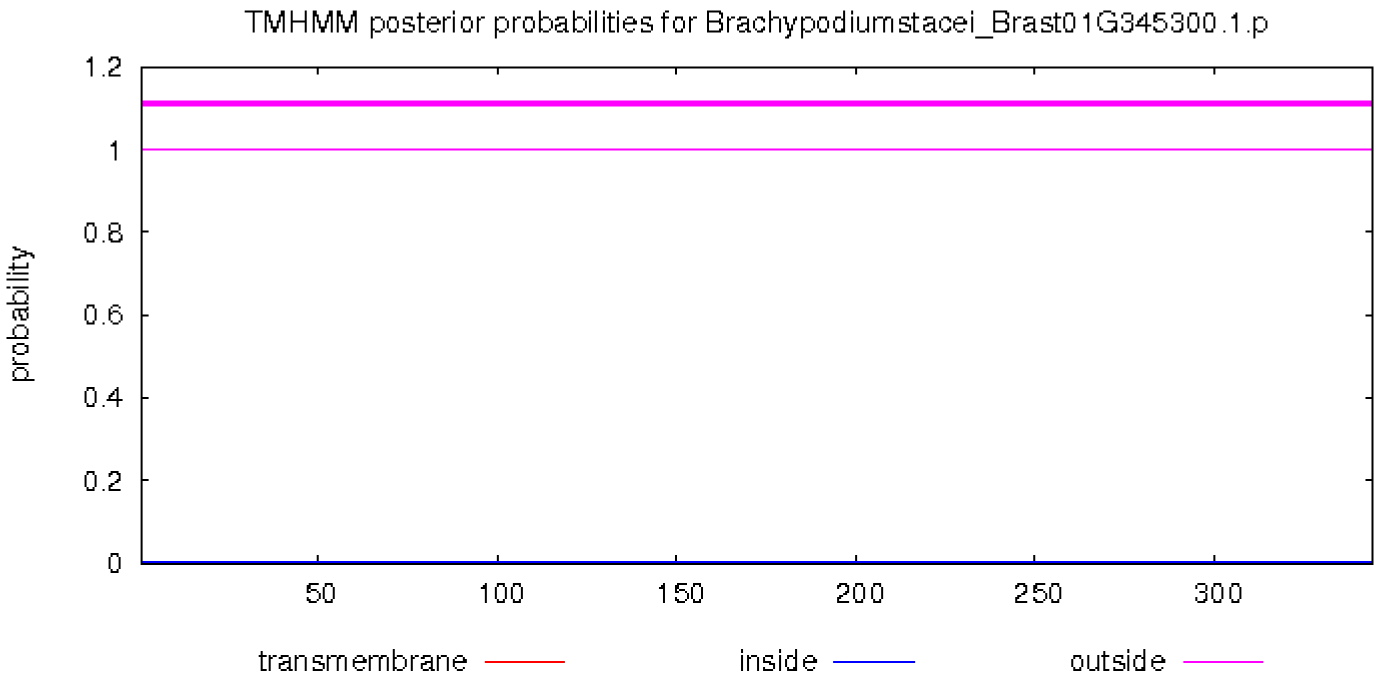

plot in postscript, script for making the plot in gnuplot, data for plot

---

```
# Brachypodiumstacei_Brast01G355100.1.p Length: 442
# Brachypodiumstacei_Brast01G355100.1.p Number of predicted TMHs:  0
# Brachypodiumstacei_Brast01G355100.1.p Exp number of AAs in TMHs: 0.01028
# Brachypodiumstacei_Brast01G355100.1.p Exp number, first 60 AAs:  0
```

```
# Brachypodiumstacei_Brast01G355100.1.p Total prob of N-in:        0.00589
Brachypodiumstacei_Brast01G355100.1.p   TMHMM2.0        outside      1   442
```

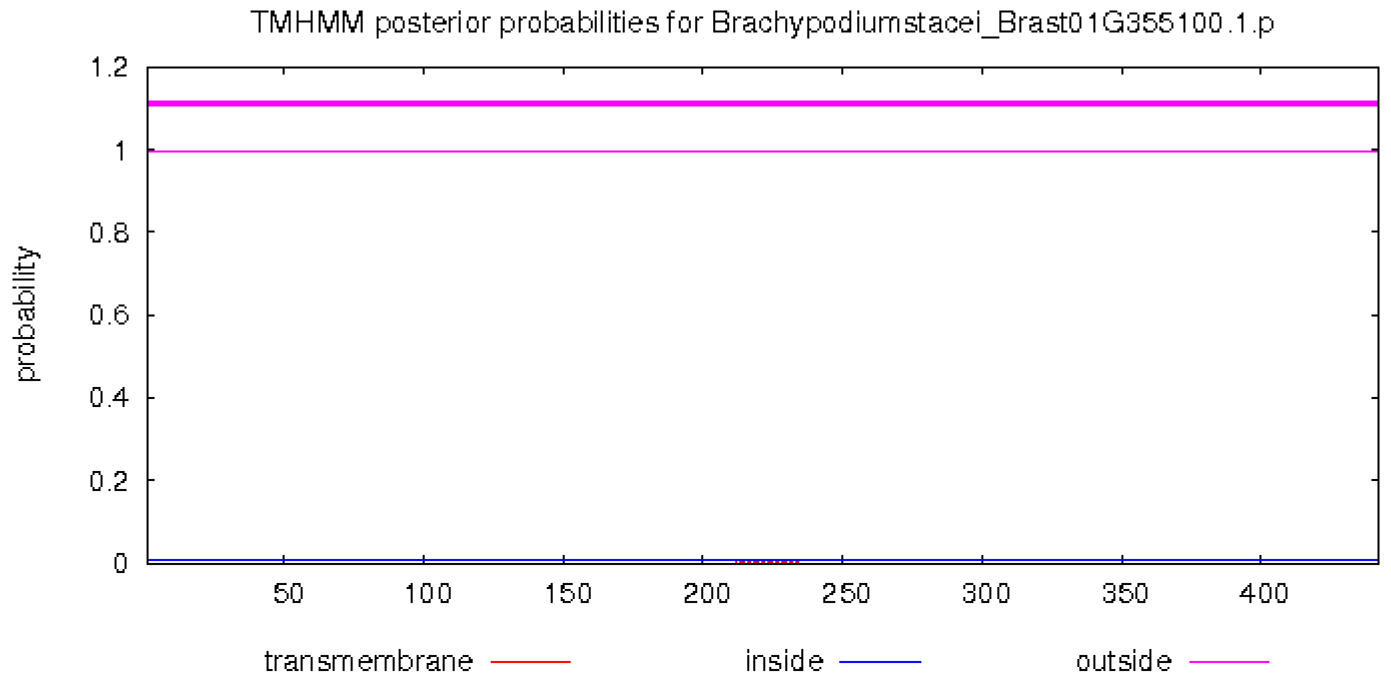

plot in postscript, script for making the plot in gnuplot, data for plot

```
# Brachypodiumstacei_Brast01G380000.1.p Length: 725
# Brachypodiumstacei_Brast01G380000.1.p Number of predicted TMHs:  0
# Brachypodiumstacei_Brast01G380000.1.p Exp number of AAs in TMHs: 0.00391
# Brachypodiumstacei_Brast01G380000.1.p Exp number, first 60 AAs:  0.0002
# Brachypodiumstacei_Brast01G380000.1.p Total prob of N-in:        0.00079
Brachypodiumstacei_Brast01G380000.1.p   TMHMM2.0        outside      1   725
```

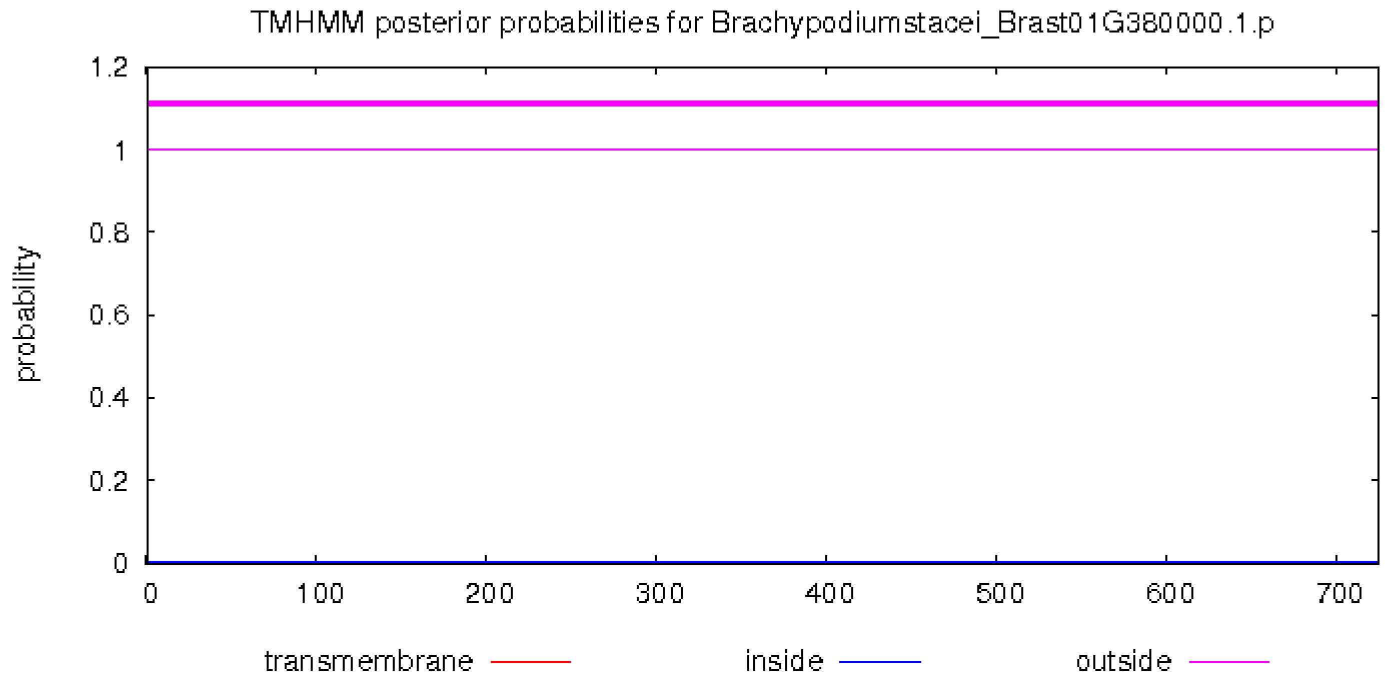

plot in postscript, script for making the plot in gnuplot, data for plot

```
# Brachypodiumstacei_Brast01G380100.1.p Length: 359
# Brachypodiumstacei_Brast01G380100.1.p Number of predicted TMHs:  0
# Brachypodiumstacei_Brast01G380100.1.p Exp number of AAs in TMHs: 0.00938
# Brachypodiumstacei_Brast01G380100.1.p Exp number, first 60 AAs:  0
```

```
# Brachypodiumstacei_Brast01G380100.1.p Total prob of N-in:        0.04984
Brachypodiumstacei_Brast01G380100.1.p	TMHMM2.0	outside	     1   359
```

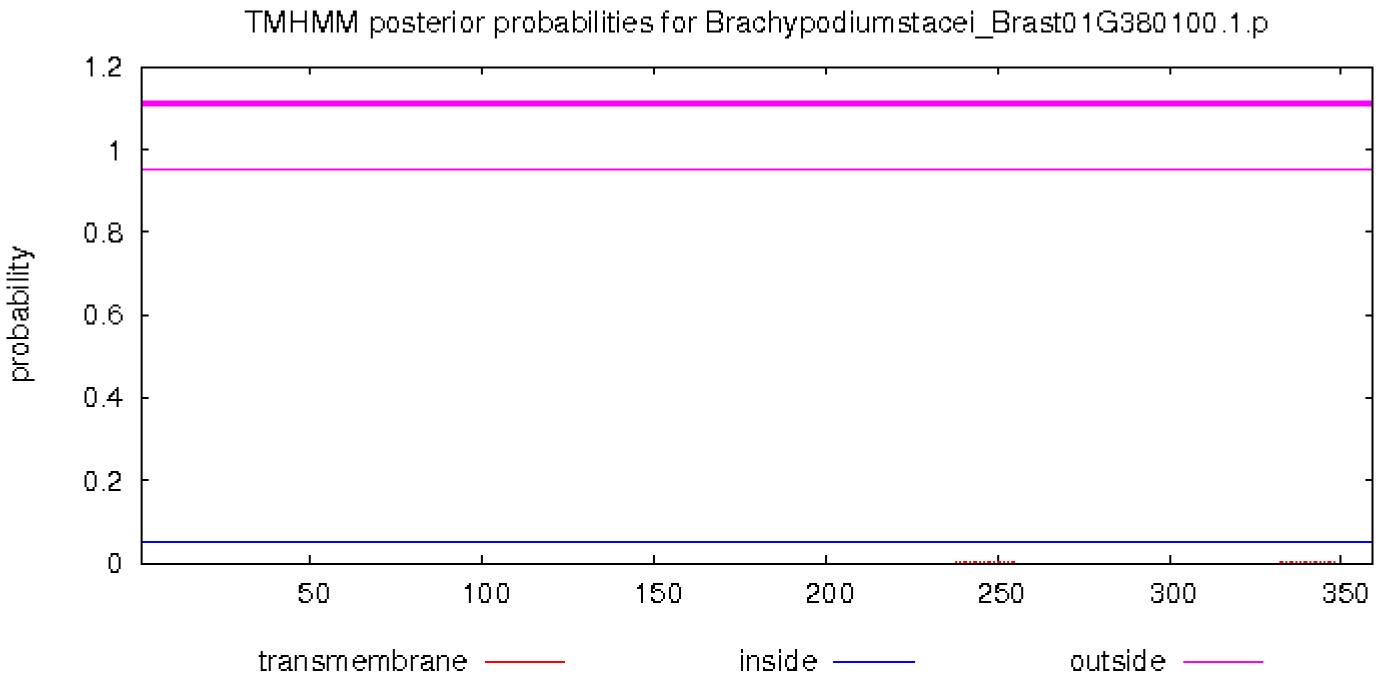

plot in postscript, script for making the plot in gnuplot, data for plot

---

```
# Brachypodiumstacei_Brast02G009100.1.p Length: 414
# Brachypodiumstacei_Brast02G009100.1.p Number of predicted TMHs:  0
# Brachypodiumstacei_Brast02G009100.1.p Exp number of AAs in TMHs: 0.00557
# Brachypodiumstacei_Brast02G009100.1.p Exp number, first 60 AAs:  0.00206
# Brachypodiumstacei_Brast02G009100.1.p Total prob of N-in:        0.00185
Brachypodiumstacei_Brast02G009100.1.p	TMHMM2.0	outside	     1   414
```

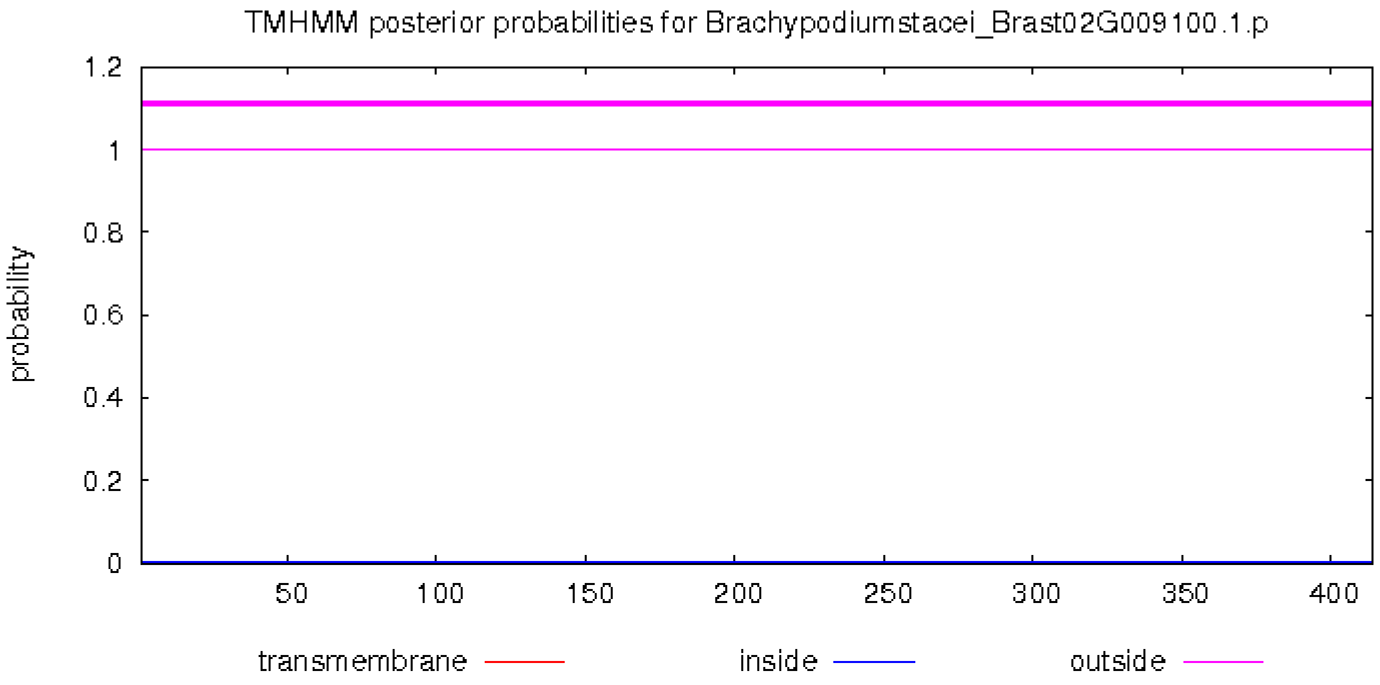

plot in postscript, script for making the plot in gnuplot, data for plot

---

```
# Brachypodiumstacei_Brast02G018500.1.p Length: 702
# Brachypodiumstacei_Brast02G018500.1.p Number of predicted TMHs:  0
# Brachypodiumstacei_Brast02G018500.1.p Exp number of AAs in TMHs: 0.82554
# Brachypodiumstacei_Brast02G018500.1.p Exp number, first 60 AAs:  0
```

```
# Brachypodiumstacei_Brast02G018500.1.p Total prob of N-in:        0.00003
Brachypodiumstacei_Brast02G018500.1.p   TMHMM2.0        outside      1   702
```

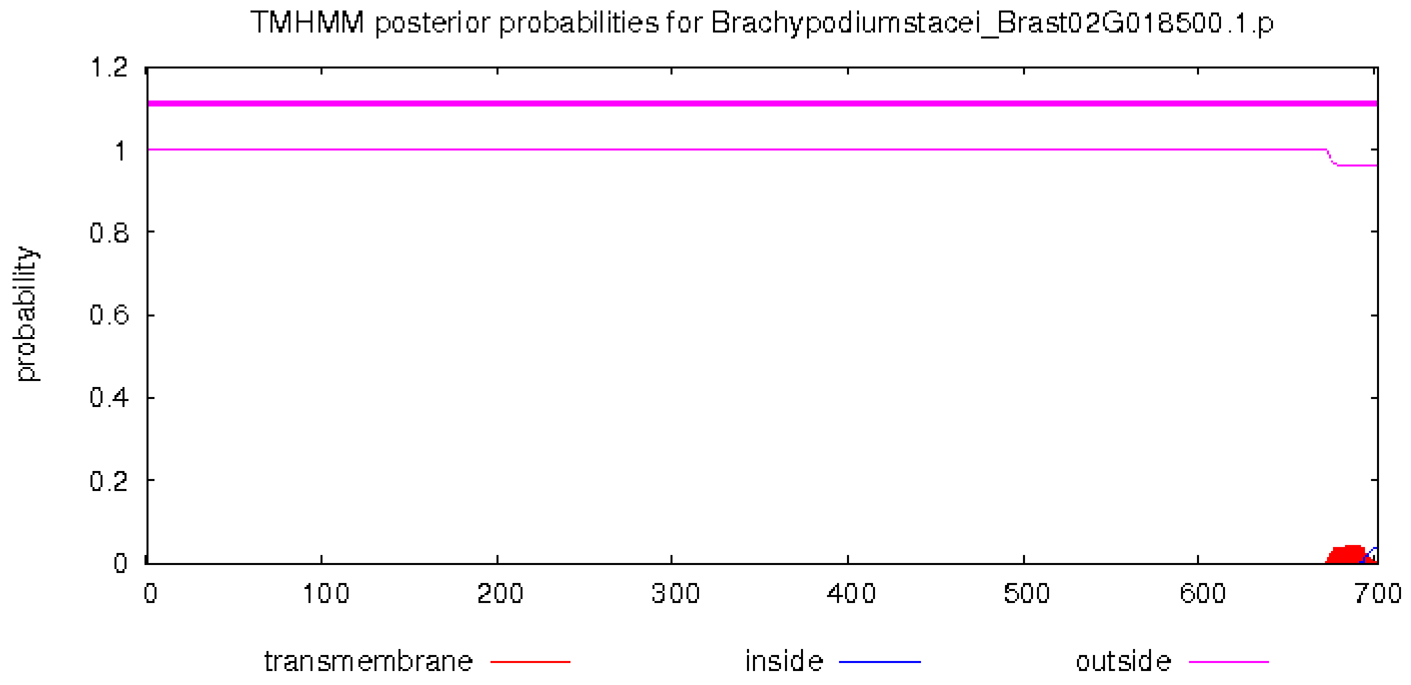

plot in postscript, script for making the plot in gnuplot, data for plot

```
# Brachypodiumstacei_Brast02G024100.1.p Length: 364
# Brachypodiumstacei_Brast02G024100.1.p Number of predicted TMHs:  0
# Brachypodiumstacei_Brast02G024100.1.p Exp number of AAs in TMHs: 0.01018
# Brachypodiumstacei_Brast02G024100.1.p Exp number, first 60 AAs:  0
# Brachypodiumstacei_Brast02G024100.1.p Total prob of N-in:        0.00503
Brachypodiumstacei_Brast02G024100.1.p   TMHMM2.0        outside      1   364
```

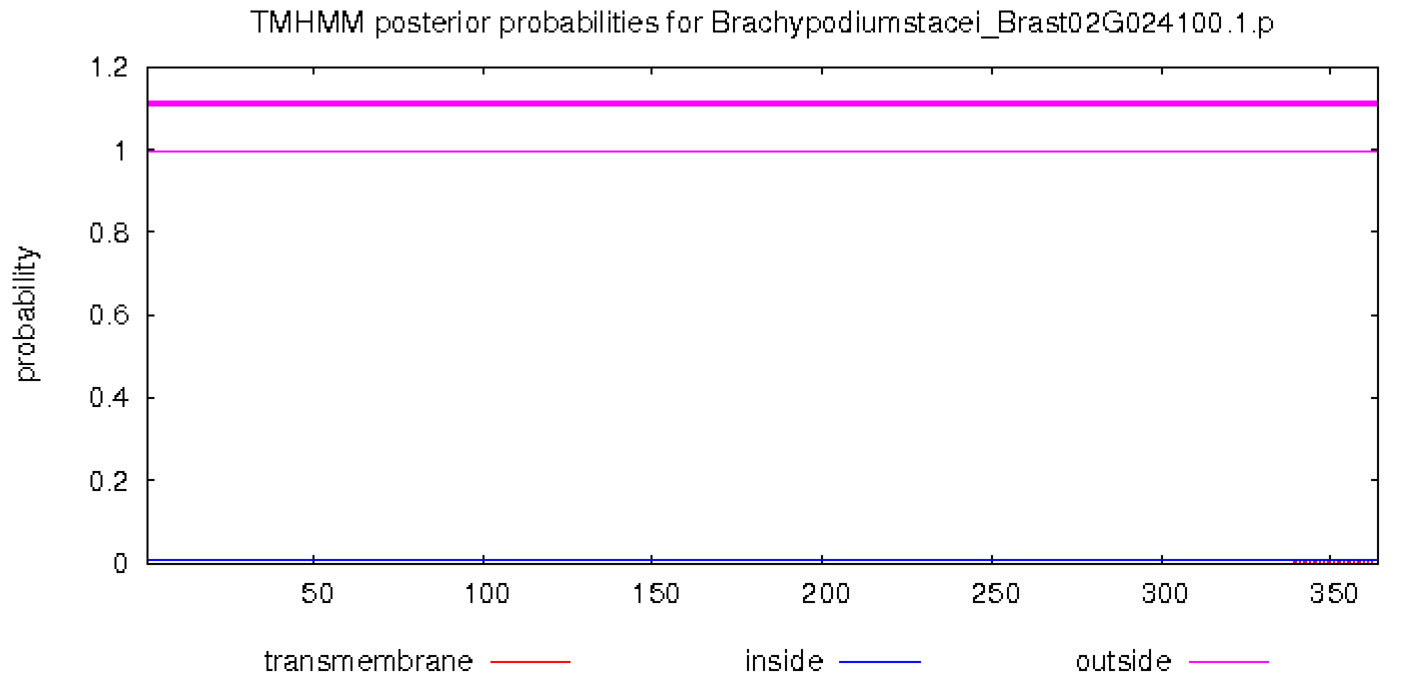

plot in postscript, script for making the plot in gnuplot, data for plot

```
# Brachypodiumstacei_Brast02G030400.1.p Length: 374
# Brachypodiumstacei_Brast02G030400.1.p Number of predicted TMHs:  0
# Brachypodiumstacei_Brast02G030400.1.p Exp number of AAs in TMHs: 0.0006
# Brachypodiumstacei_Brast02G030400.1.p Exp number, first 60 AAs:  0.0006
```

```
# Brachypodiumstacei_Brast02G030400.1.p Total prob of N-in:        0.01978
Brachypodiumstacei_Brast02G030400.1.p   TMHMM2.0        outside      1   374
```

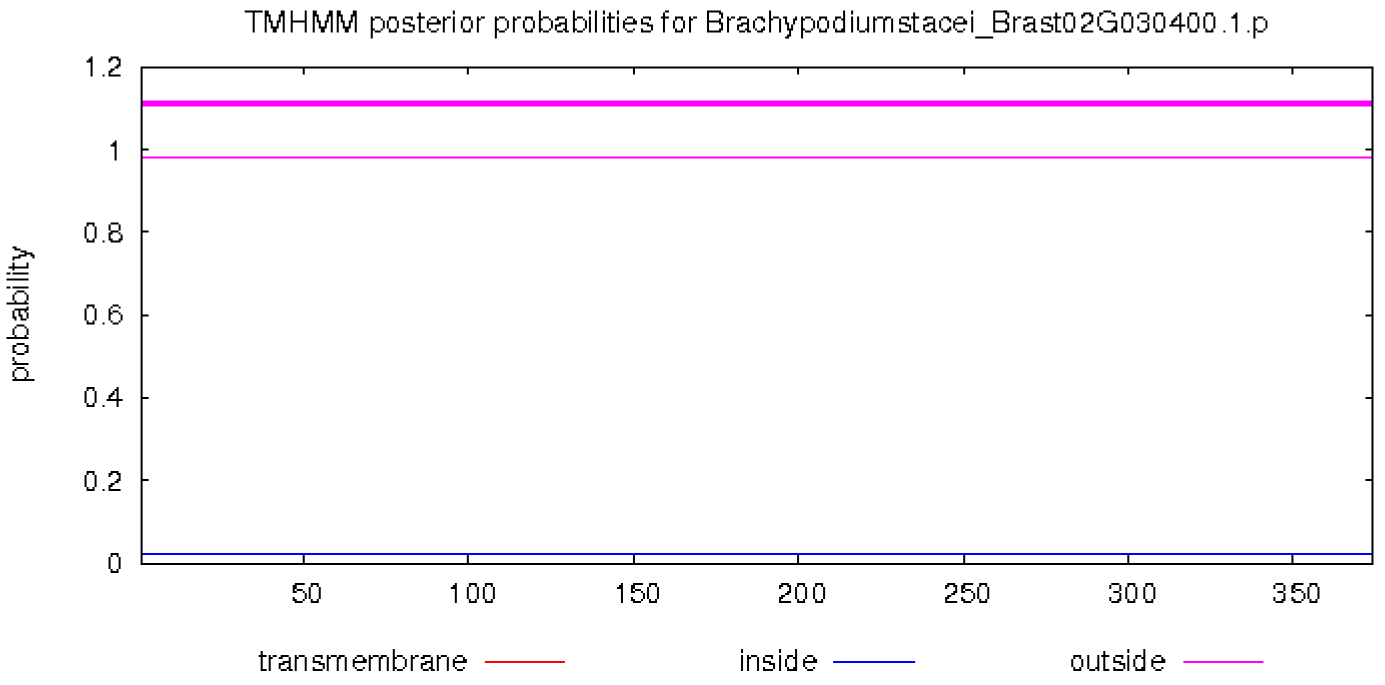

[plot](#) in postscript, [script](#) for making the plot in gnuplot, [data](#) for plot

---

```
# Brachypodiumstacei_Brast02G031500.1.p Length: 227
# Brachypodiumstacei_Brast02G031500.1.p Number of predicted TMHs:  0
# Brachypodiumstacei_Brast02G031500.1.p Exp number of AAs in TMHs: 0.00015
# Brachypodiumstacei_Brast02G031500.1.p Exp number, first 60 AAs:  0
# Brachypodiumstacei_Brast02G031500.1.p Total prob of N-in:        0.04580
Brachypodiumstacei_Brast02G031500.1.p   TMHMM2.0        outside      1   227
```

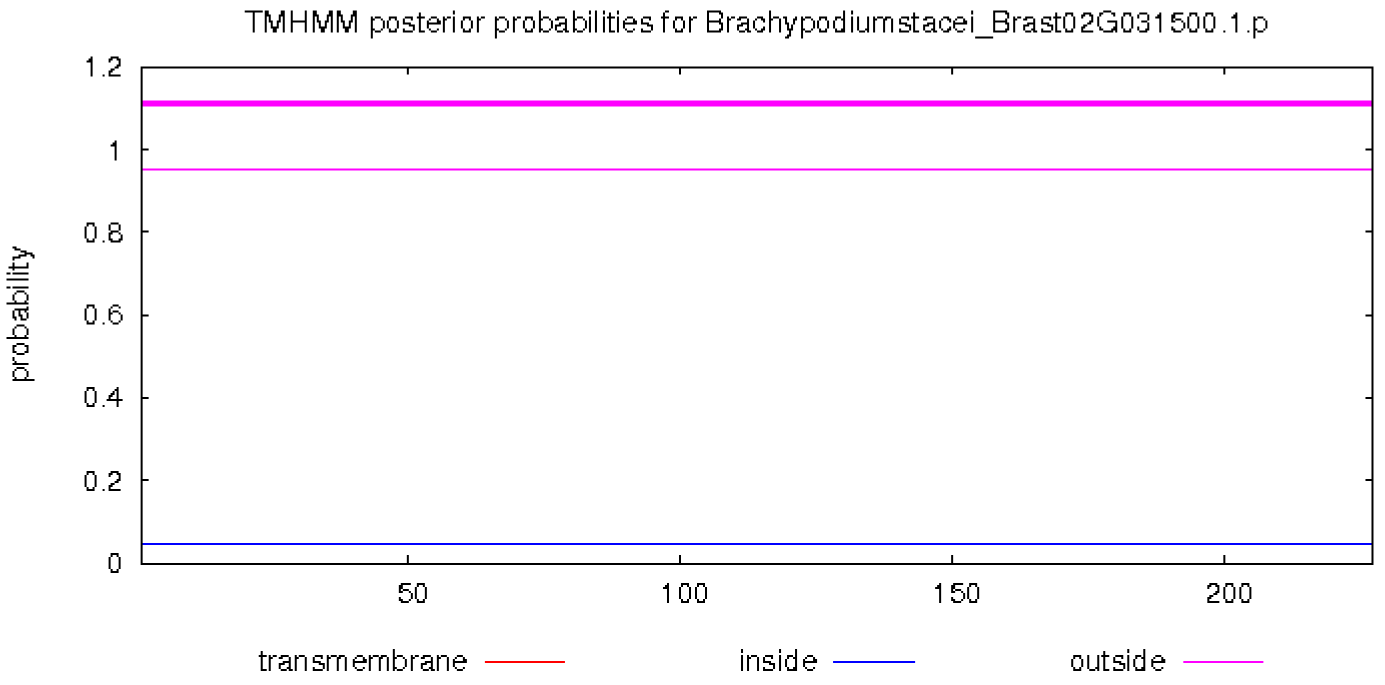

[plot](#) in postscript, [script](#) for making the plot in gnuplot, [data](#) for plot

---

```
# Brachypodiumstacei_Brast02G098700.1.p Length: 846
# Brachypodiumstacei_Brast02G098700.1.p Number of predicted TMHs:  0
# Brachypodiumstacei_Brast02G098700.1.p Exp number of AAs in TMHs: 0.00327
# Brachypodiumstacei_Brast02G098700.1.p Exp number, first 60 AAs:  0
```

```
# Brachypodiumstacei_Brast02G098700.1.p Total prob of N-in:        0.00010
Brachypodiumstacei_Brast02G098700.1.p	TMHMM2.0	outside	     1   846
```

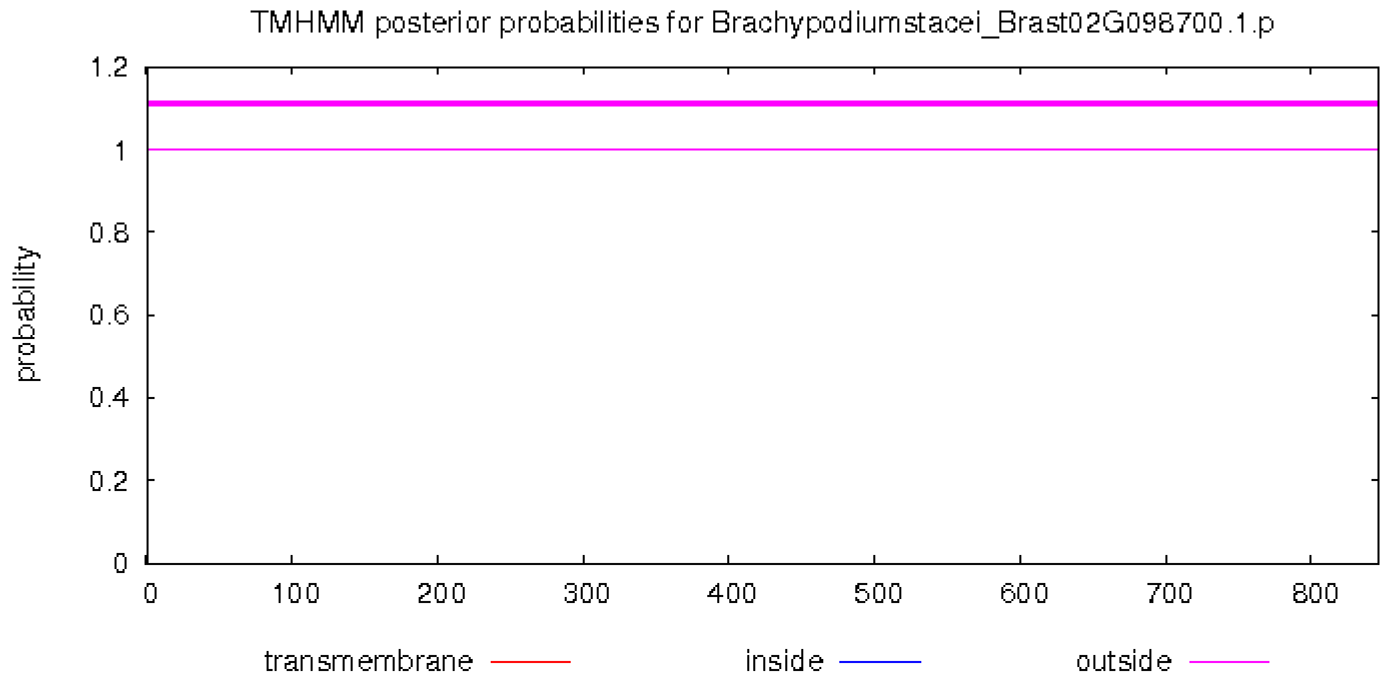

plot in postscript, script for making the plot in gnuplot, data for plot

---

```
# Brachypodiumstacei_Brast02G110400.1.p Length: 427
# Brachypodiumstacei_Brast02G110400.1.p Number of predicted TMHs:  0
# Brachypodiumstacei_Brast02G110400.1.p Exp number of AAs in TMHs: 0.01867
# Brachypodiumstacei_Brast02G110400.1.p Exp number, first 60 AAs:  0.00854
# Brachypodiumstacei_Brast02G110400.1.p Total prob of N-in:        0.00428
Brachypodiumstacei_Brast02G110400.1.p	TMHMM2.0	outside	     1   427
```

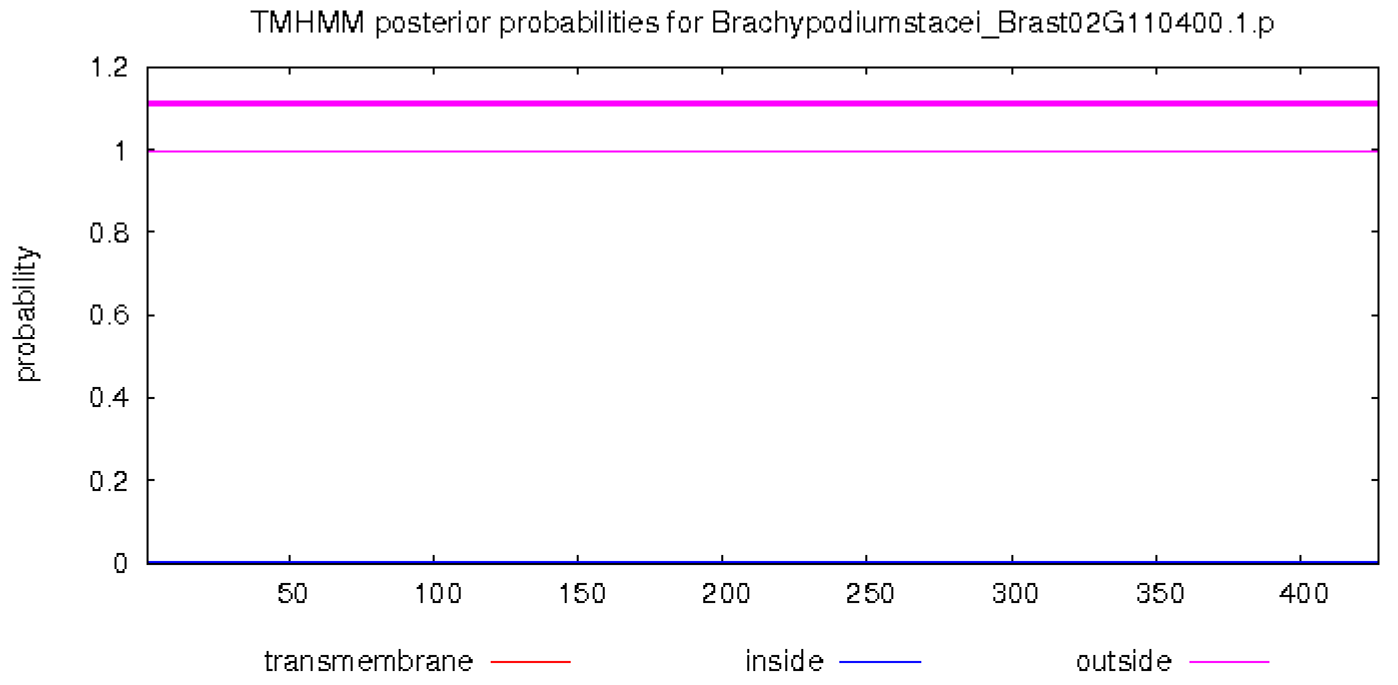

plot in postscript, script for making the plot in gnuplot, data for plot

---

```
# Brachypodiumstacei_Brast02G166800.1.p Length: 355
# Brachypodiumstacei_Brast02G166800.1.p Number of predicted TMHs:  0
# Brachypodiumstacei_Brast02G166800.1.p Exp number of AAs in TMHs: 0.00473
# Brachypodiumstacei_Brast02G166800.1.p Exp number, first 60 AAs:  0
```

```
# Brachypodiumstacei_Brast02G166800.1.p Total prob of N-in:        0.01800
Brachypodiumstacei_Brast02G166800.1.p	TMHMM2.0	outside	     1   355
```

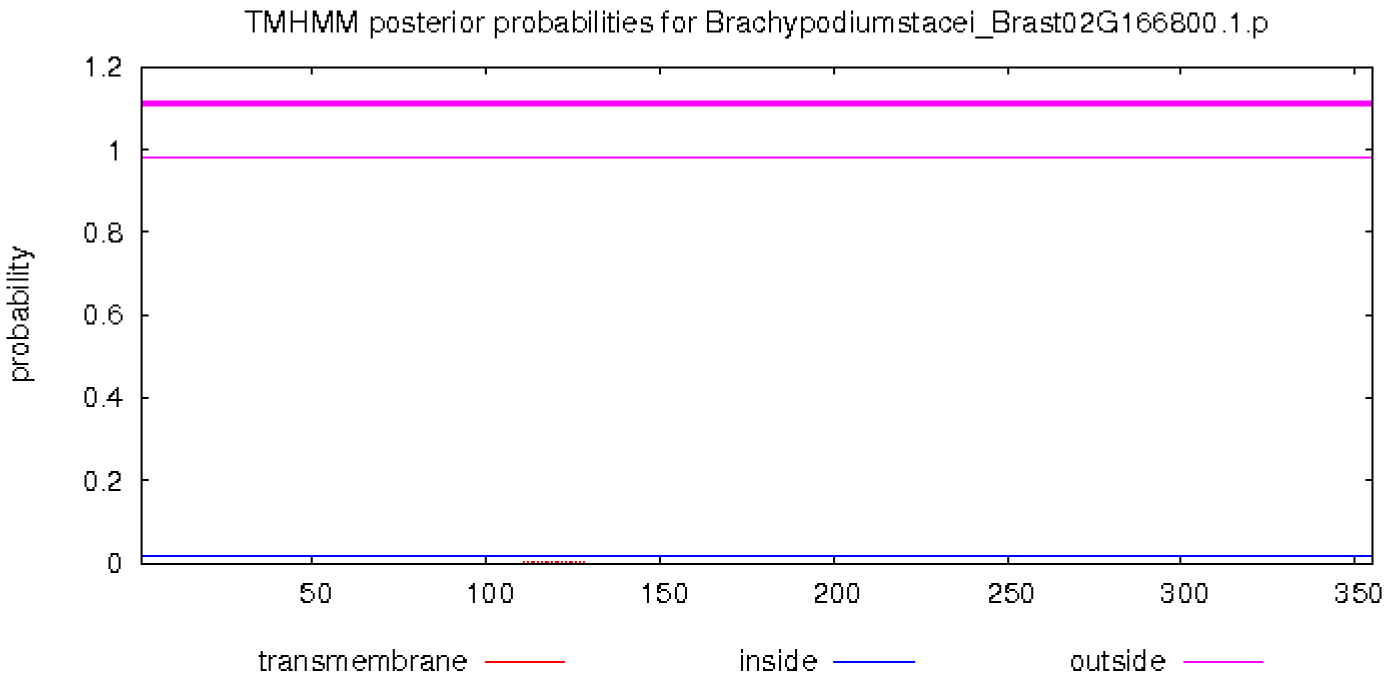

plot in postscript, script for making the plot in gnuplot, data for plot

---

```
# Brachypodiumstacei_Brast02G167100.1.p Length: 345
# Brachypodiumstacei_Brast02G167100.1.p Number of predicted TMHs:  0
# Brachypodiumstacei_Brast02G167100.1.p Exp number of AAs in TMHs: 0.00357
# Brachypodiumstacei_Brast02G167100.1.p Exp number, first 60 AAs:  0
# Brachypodiumstacei_Brast02G167100.1.p Total prob of N-in:        0.00339
Brachypodiumstacei_Brast02G167100.1.p	TMHMM2.0	outside	     1   345
```

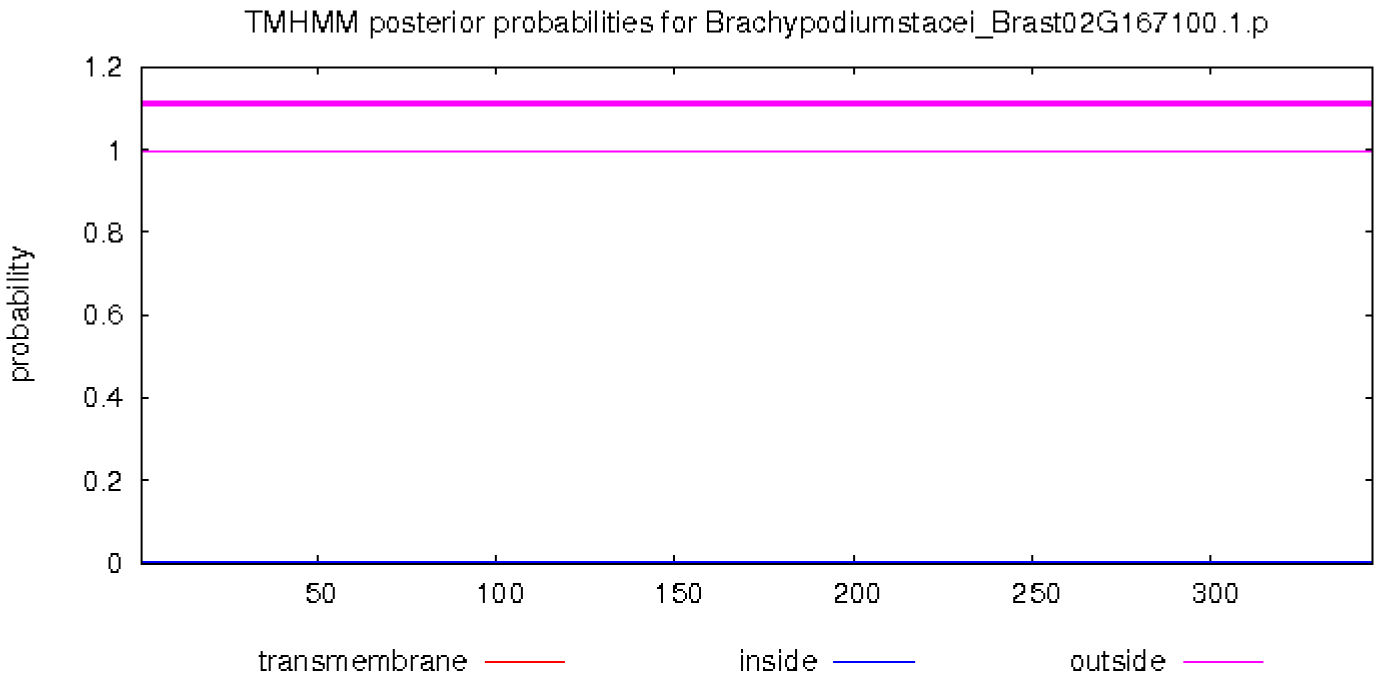

plot in postscript, script for making the plot in gnuplot, data for plot

---

```
# Brachypodiumstacei_Brast02G192700.1.p Length: 456
# Brachypodiumstacei_Brast02G192700.1.p Number of predicted TMHs:  0
# Brachypodiumstacei_Brast02G192700.1.p Exp number of AAs in TMHs: 0.01742
# Brachypodiumstacei_Brast02G192700.1.p Exp number, first 60 AAs:  0.00243
```

```
# Brachypodiumstacei_Brast02G192700.1.p Total prob of N-in:        0.00192
Brachypodiumstacei_Brast02G192700.1.p   TMHMM2.0        outside      1   456
```

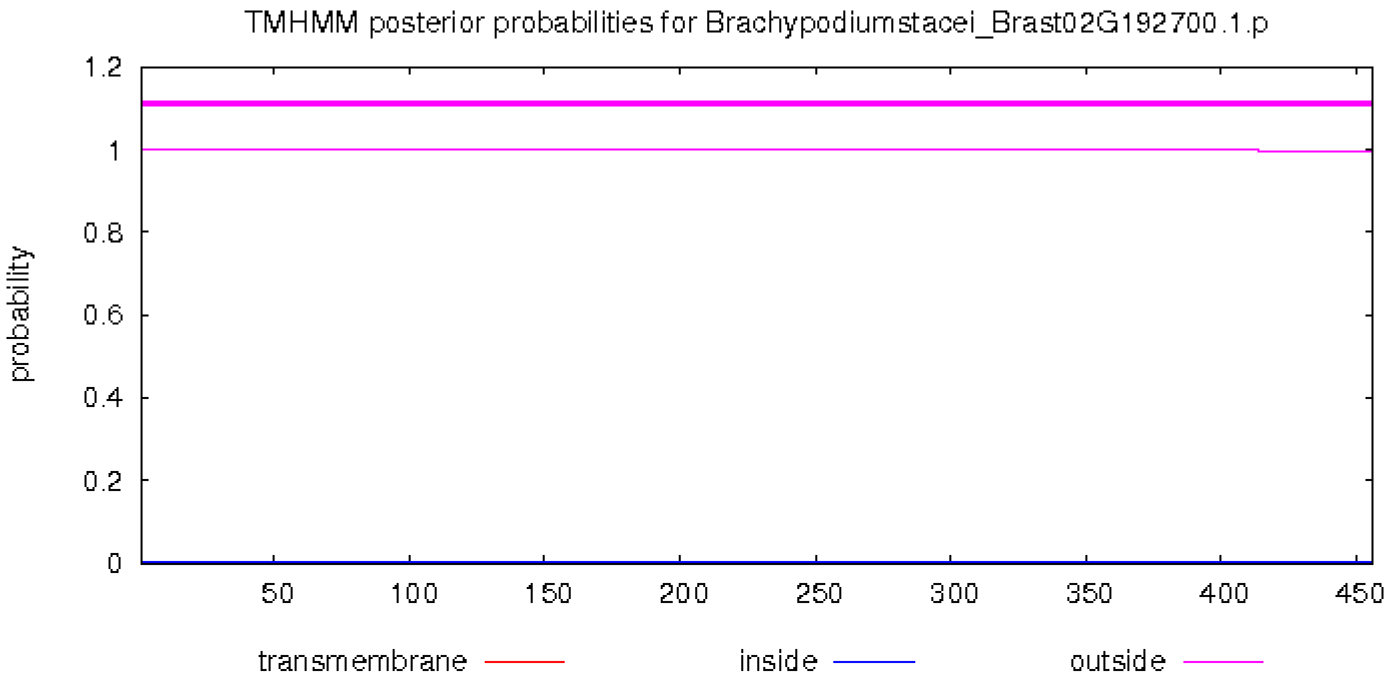

plot in postscript, script for making the plot in gnuplot, data for plot

---

```
# Brachypodiumstacei_Brast02G236200.1.p Length: 304
# Brachypodiumstacei_Brast02G236200.1.p Number of predicted TMHs:  0
# Brachypodiumstacei_Brast02G236200.1.p Exp number of AAs in TMHs: 0.62340000000000001
# Brachypodiumstacei_Brast02G236200.1.p Exp number, first 60 AAs:  0
# Brachypodiumstacei_Brast02G236200.1.p Total prob of N-in:        0.00778
Brachypodiumstacei_Brast02G236200.1.p   TMHMM2.0        outside      1   304
```

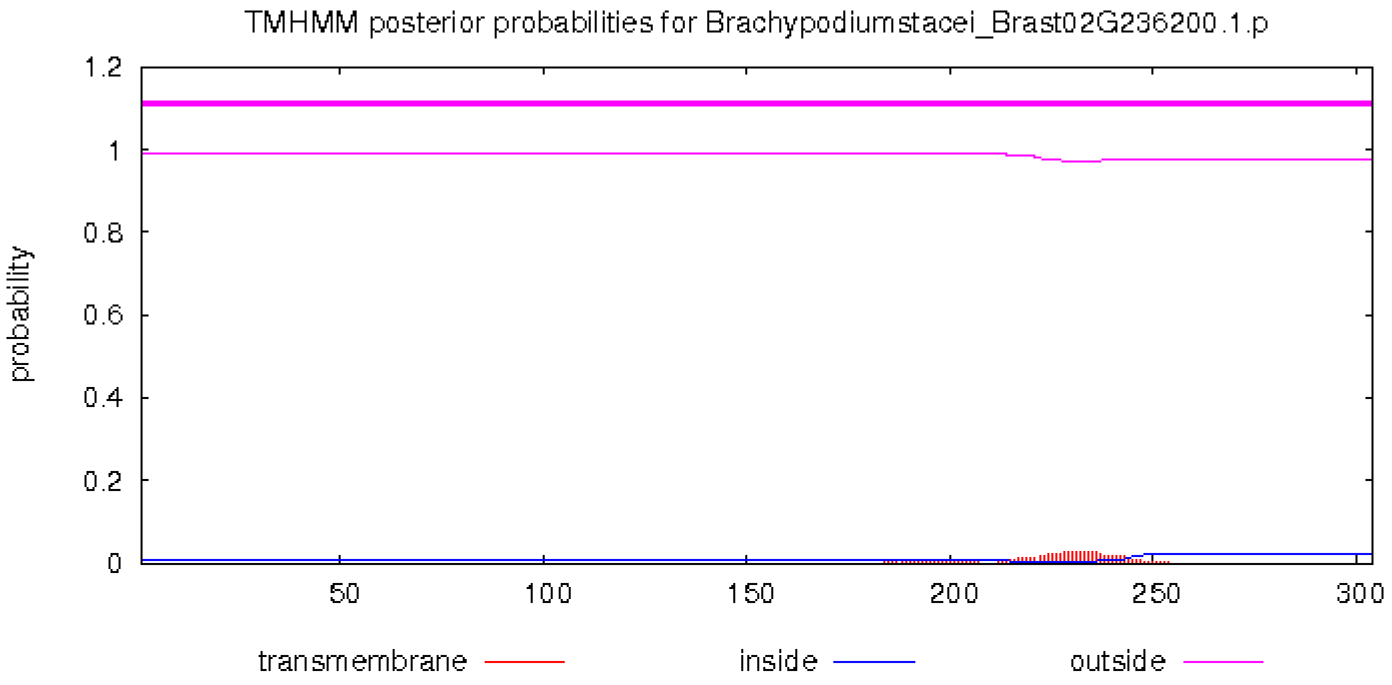

plot in postscript, script for making the plot in gnuplot, data for plot

---

```
# Brachypodiumstacei_Brast02G325400.1.p Length: 335
# Brachypodiumstacei_Brast02G325400.1.p Number of predicted TMHs:  0
# Brachypodiumstacei_Brast02G325400.1.p Exp number of AAs in TMHs: 0.00213
# Brachypodiumstacei_Brast02G325400.1.p Exp number, first 60 AAs:  0
```

```
# Brachypodiumstacei_Brast02G325400.1.p Total prob of N-in:        0.05382
Brachypodiumstacei_Brast02G325400.1.p	TMHMM2.0	outside	     1   335
```

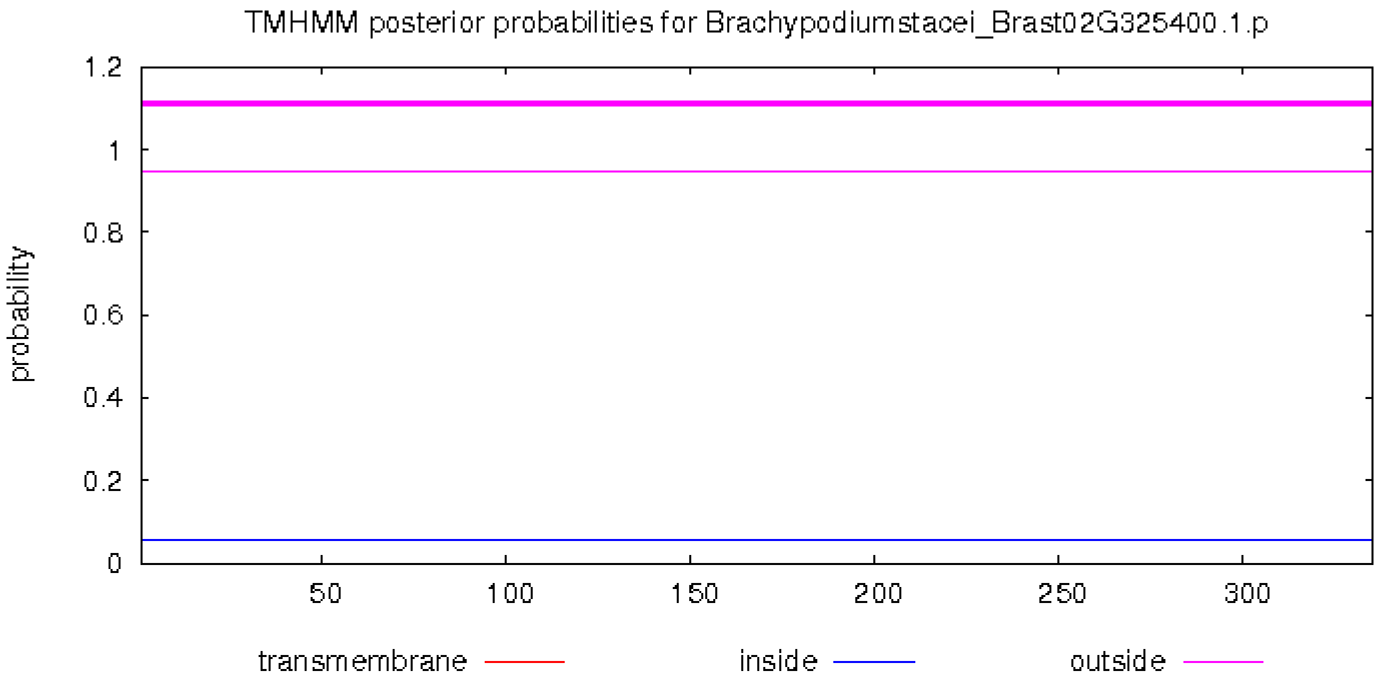

plot in postscript, script for making the plot in gnuplot, data for plot

---

```
# Brachypodiumstacei_Brast02G355100.1.p Length: 574
# Brachypodiumstacei_Brast02G355100.1.p Number of predicted TMHs:  0
# Brachypodiumstacei_Brast02G355100.1.p Exp number of AAs in TMHs: 0
# Brachypodiumstacei_Brast02G355100.1.p Exp number, first 60 AAs:  0
# Brachypodiumstacei_Brast02G355100.1.p Total prob of N-in:        0.00200
Brachypodiumstacei_Brast02G355100.1.p	TMHMM2.0	outside	     1   574
```

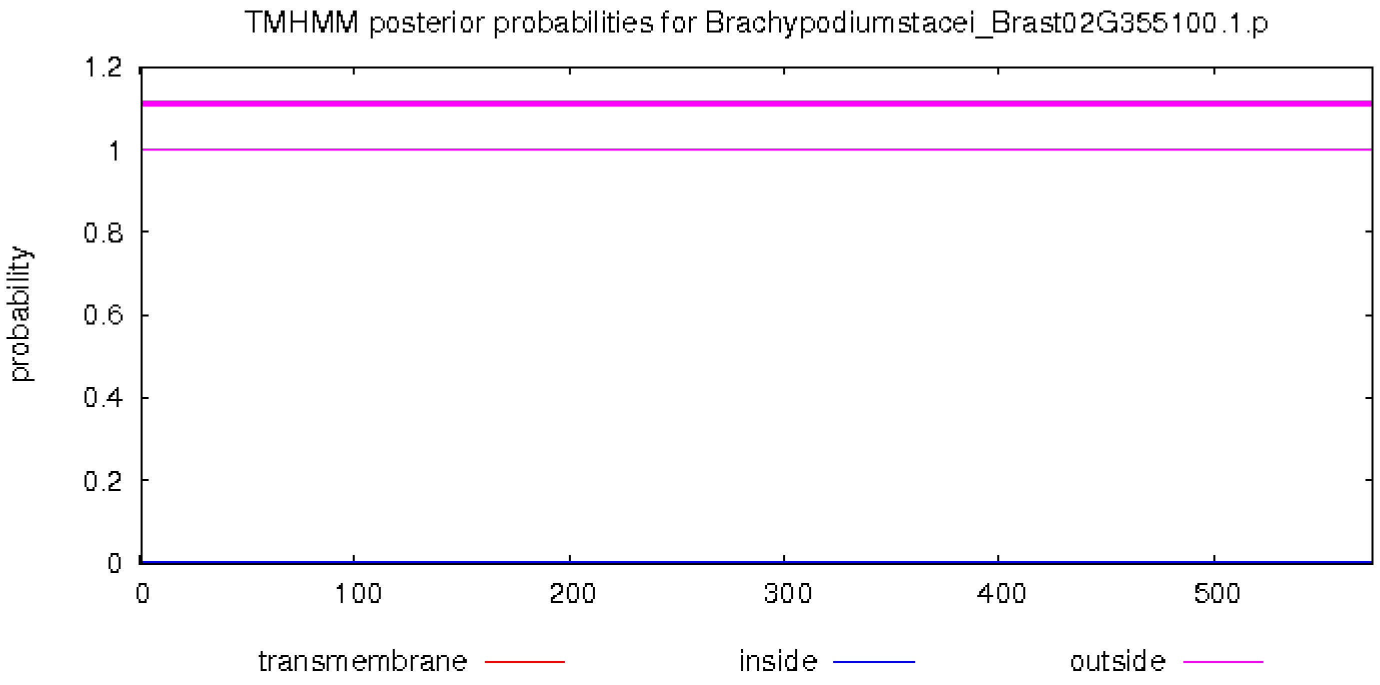

plot in postscript, script for making the plot in gnuplot, data for plot

---

```
# Brachypodiumstacei_Brast02G356100.1.p Length: 323
# Brachypodiumstacei_Brast02G356100.1.p Number of predicted TMHs:  0
# Brachypodiumstacei_Brast02G356100.1.p Exp number of AAs in TMHs: 0.00147
# Brachypodiumstacei_Brast02G356100.1.p Exp number, first 60 AAs:  0.00097
```

```
# Brachypodiumstacei_Brast02G356100.1.p Total prob of N-in:        0.10532
Brachypodiumstacei_Brast02G356100.1.p	TMHMM2.0	outside	     1   323
```

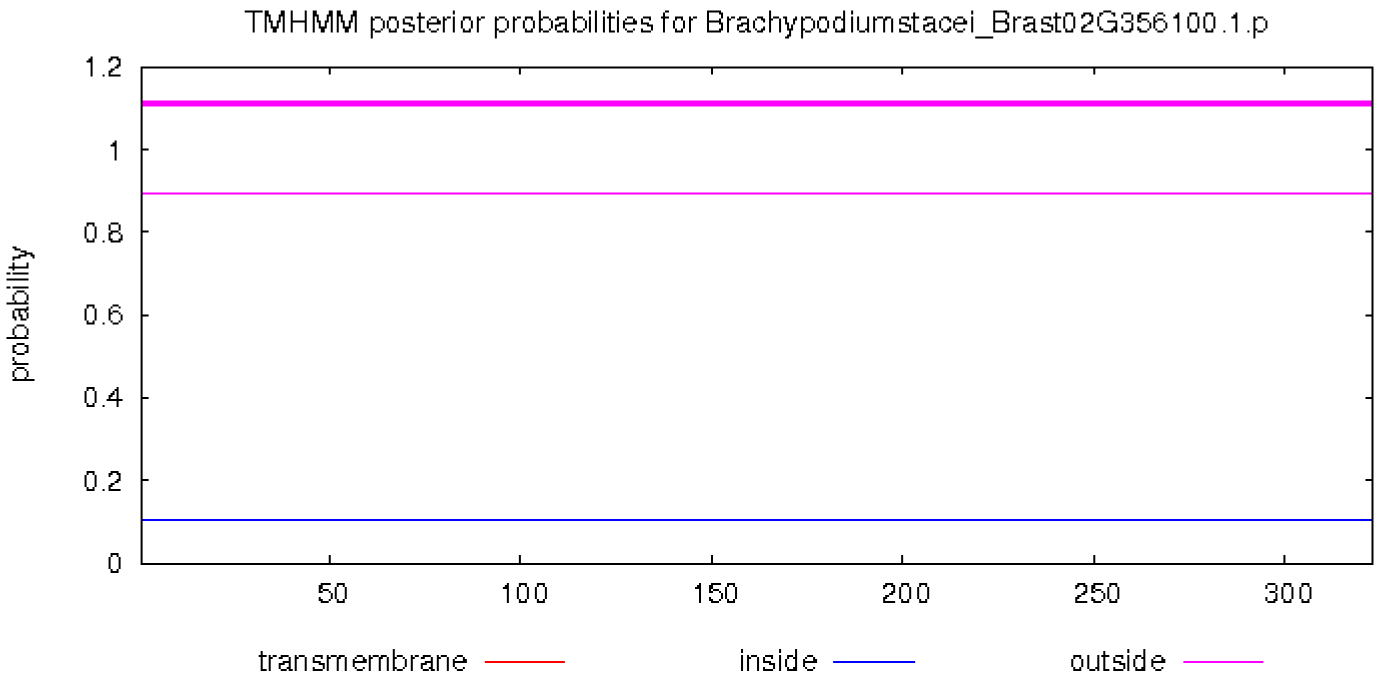

plot in postscript, script for making the plot in gnuplot, data for plot

---

```
# Brachypodiumstacei_Brast02G382000.1.p Length: 747
# Brachypodiumstacei_Brast02G382000.1.p Number of predicted TMHs:  0
# Brachypodiumstacei_Brast02G382000.1.p Exp number of AAs in TMHs: 0.01356
# Brachypodiumstacei_Brast02G382000.1.p Exp number, first 60 AAs:  0
# Brachypodiumstacei_Brast02G382000.1.p Total prob of N-in:        0.00015
Brachypodiumstacei_Brast02G382000.1.p	TMHMM2.0	outside	     1   747
```

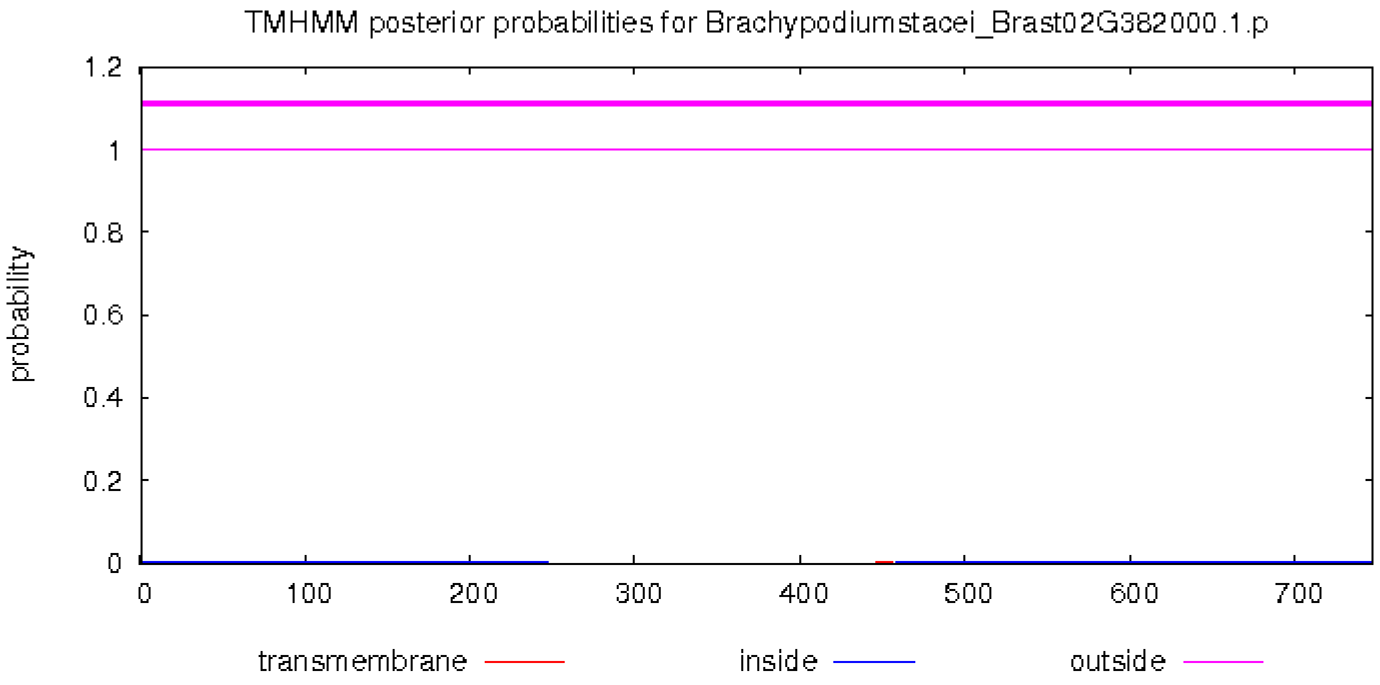

plot in postscript, script for making the plot in gnuplot, data for plot

---

```
# Brachypodiumstacei_Brast02G383500.1.p Length: 328
# Brachypodiumstacei_Brast02G383500.1.p Number of predicted TMHs:  0
# Brachypodiumstacei_Brast02G383500.1.p Exp number of AAs in TMHs: 0.00018
# Brachypodiumstacei_Brast02G383500.1.p Exp number, first 60 AAs:  0
```

```
# Brachypodiumstacei_Brast02G383500.1.p Total prob of N-in:        0.01845
Brachypodiumstacei_Brast02G383500.1.p   TMHMM2.0        outside      1   328
```

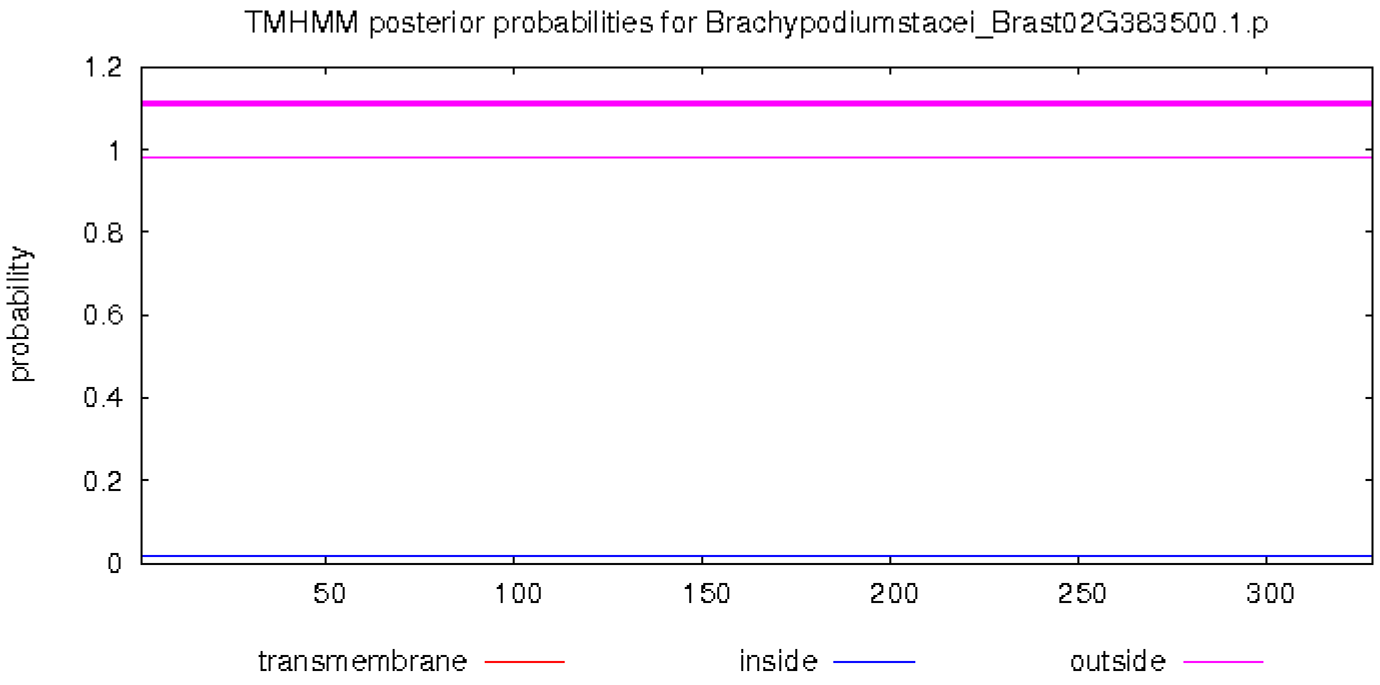

plot in postscript, script for making the plot in gnuplot, data for plot

---

```
# Brachypodiumstacei_Brast02G383700.1.p Length: 445
# Brachypodiumstacei_Brast02G383700.1.p Number of predicted TMHs:  0
# Brachypodiumstacei_Brast02G383700.1.p Exp number of AAs in TMHs: 0.02156
# Brachypodiumstacei_Brast02G383700.1.p Exp number, first 60 AAs:  0.00018
# Brachypodiumstacei_Brast02G383700.1.p Total prob of N-in:        0.00035
Brachypodiumstacei_Brast02G383700.1.p   TMHMM2.0        outside      1   445
```

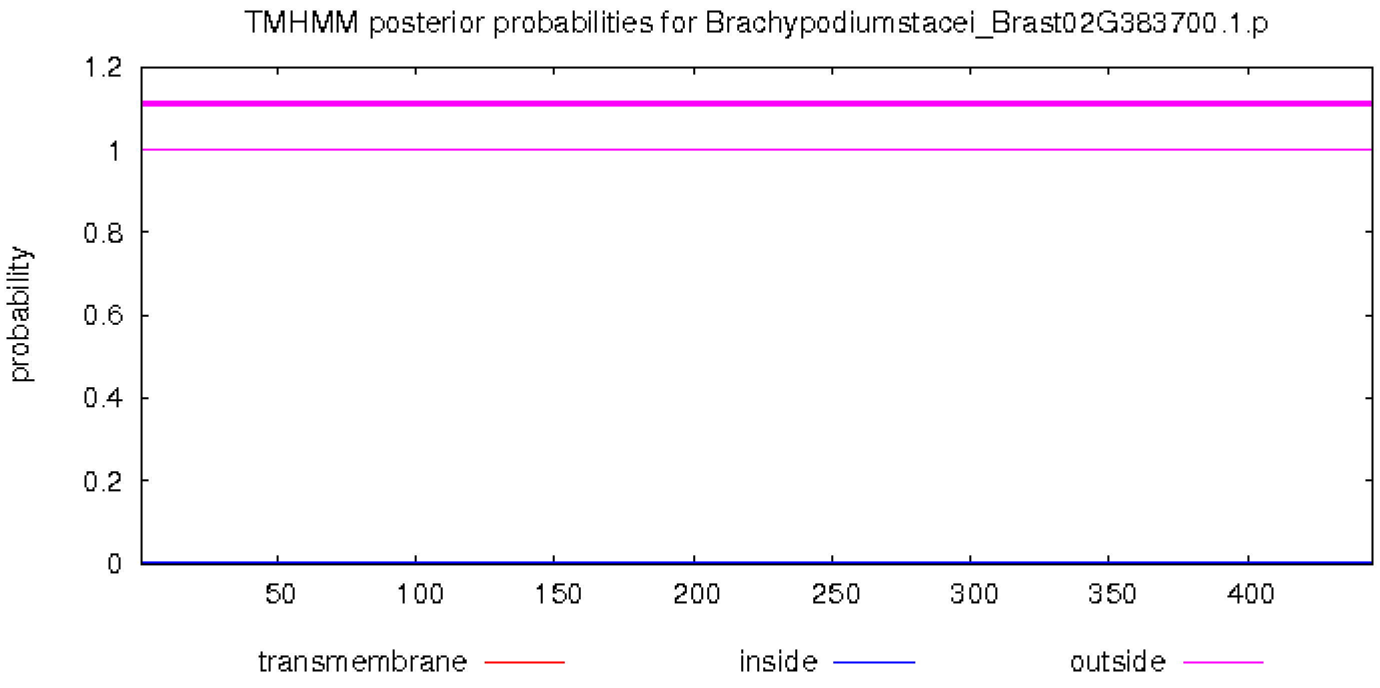

plot in postscript, script for making the plot in gnuplot, data for plot

---

```
# Brachypodiumstacei_Brast03G008200.1.p Length: 186
# Brachypodiumstacei_Brast03G008200.1.p Number of predicted TMHs:  0
# Brachypodiumstacei_Brast03G008200.1.p Exp number of AAs in TMHs: 0.26916
# Brachypodiumstacei_Brast03G008200.1.p Exp number, first 60 AAs:  0.25245
```

```
# Brachypodiumstacei_Brast03G008200.1.p Total prob of N-in:        0.21936
Brachypodiumstacei_Brast03G008200.1.p   TMHMM2.0        outside      1   186
```

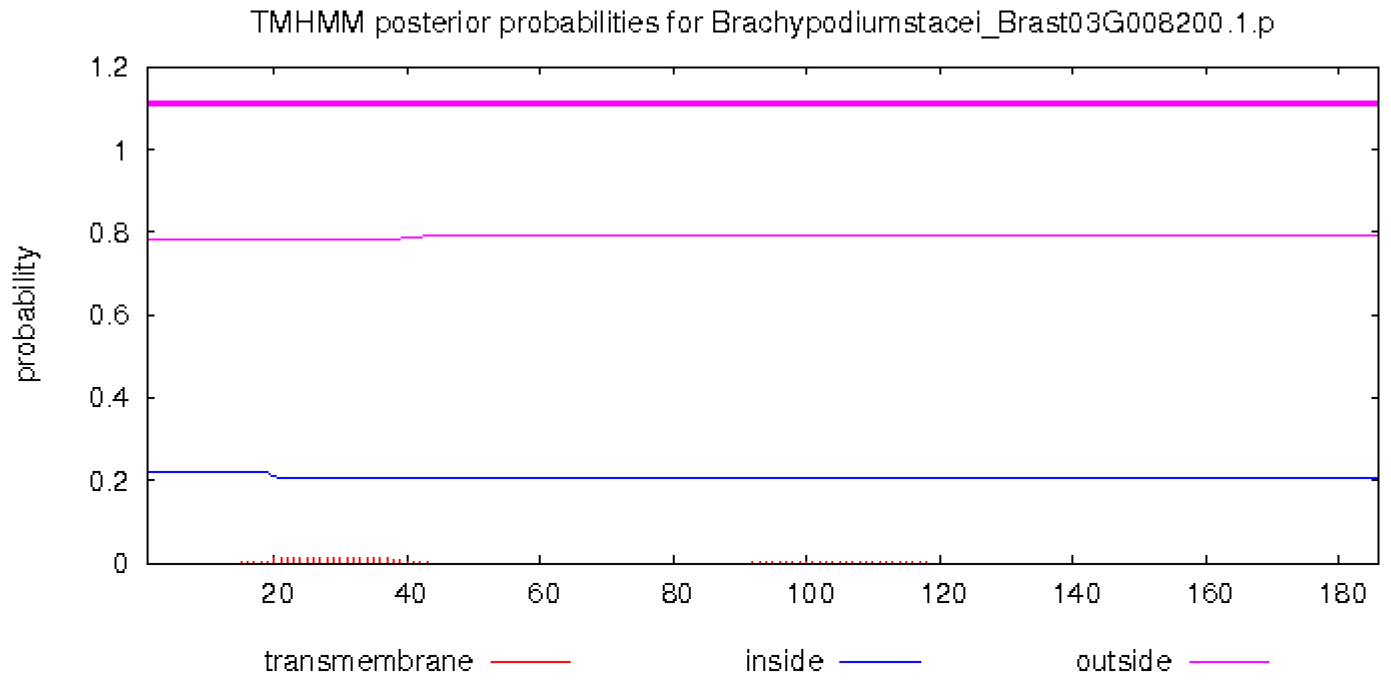

plot in postscript, script for making the plot in gnuplot, data for plot

---

```
# Brachypodiumstacei_Brast03G010700.1.p Length: 287
# Brachypodiumstacei_Brast03G010700.1.p Number of predicted TMHs:  0
# Brachypodiumstacei_Brast03G010700.1.p Exp number of AAs in TMHs: 0.00354
# Brachypodiumstacei_Brast03G010700.1.p Exp number, first 60 AAs:  0.00128
# Brachypodiumstacei_Brast03G010700.1.p Total prob of N-in:        0.03934
Brachypodiumstacei_Brast03G010700.1.p   TMHMM2.0        outside      1   287
```

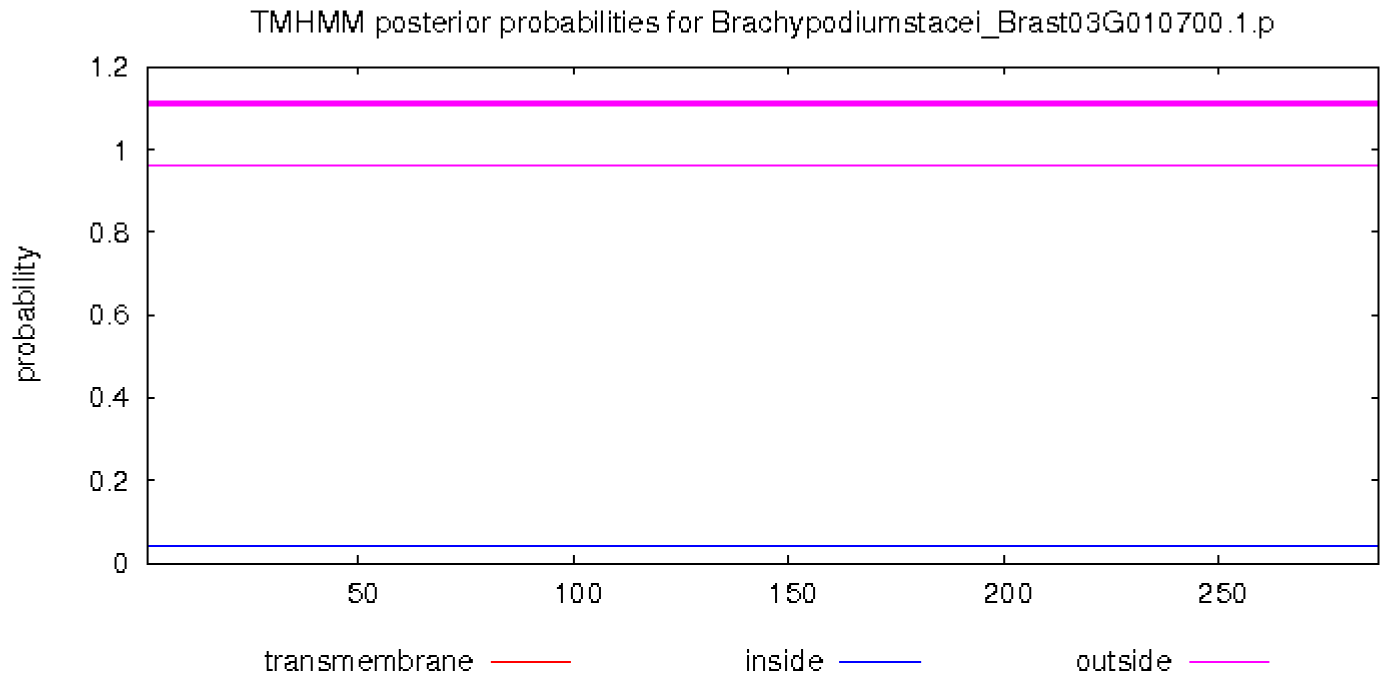

plot in postscript, script for making the plot in gnuplot, data for plot

---

```
# Brachypodiumstacei_Brast03G018400.1.p Length: 799
# Brachypodiumstacei_Brast03G018400.1.p Number of predicted TMHs:  1
# Brachypodiumstacei_Brast03G018400.1.p Exp number of AAs in TMHs: 22.66616
# Brachypodiumstacei_Brast03G018400.1.p Exp number, first 60 AAs:  0
```

```
# Brachypodiumstacei_Brast03G018400.1.p Total prob of N-in:        0.00029
Brachypodiumstacei_Brast03G018400.1.p   TMHMM2.0        outside      1   775
Brachypodiumstacei_Brast03G018400.1.p   TMHMM2.0        TMhelix    776   798
Brachypodiumstacei_Brast03G018400.1.p   TMHMM2.0        inside     799   799
```

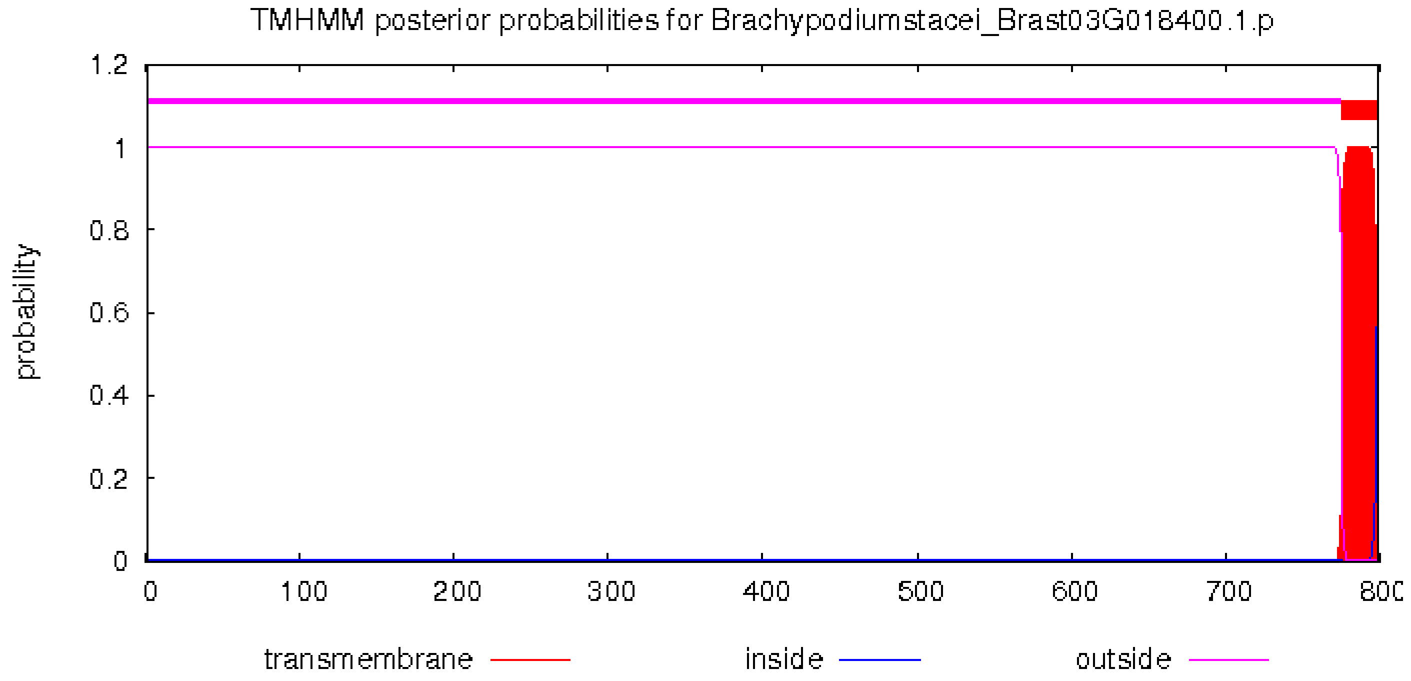

plot in postscript, script for making the plot in gnuplot, data for plot

---

```
# Brachypodiumstacei_Brast03G048700.1.p Length: 381
# Brachypodiumstacei_Brast03G048700.1.p Number of predicted TMHs:  0
# Brachypodiumstacei_Brast03G048700.1.p Exp number of AAs in TMHs: 0.00129
# Brachypodiumstacei_Brast03G048700.1.p Exp number, first 60 AAs:  0
# Brachypodiumstacei_Brast03G048700.1.p Total prob of N-in:        0.01104
Brachypodiumstacei_Brast03G048700.1.p   TMHMM2.0        outside      1   381
```

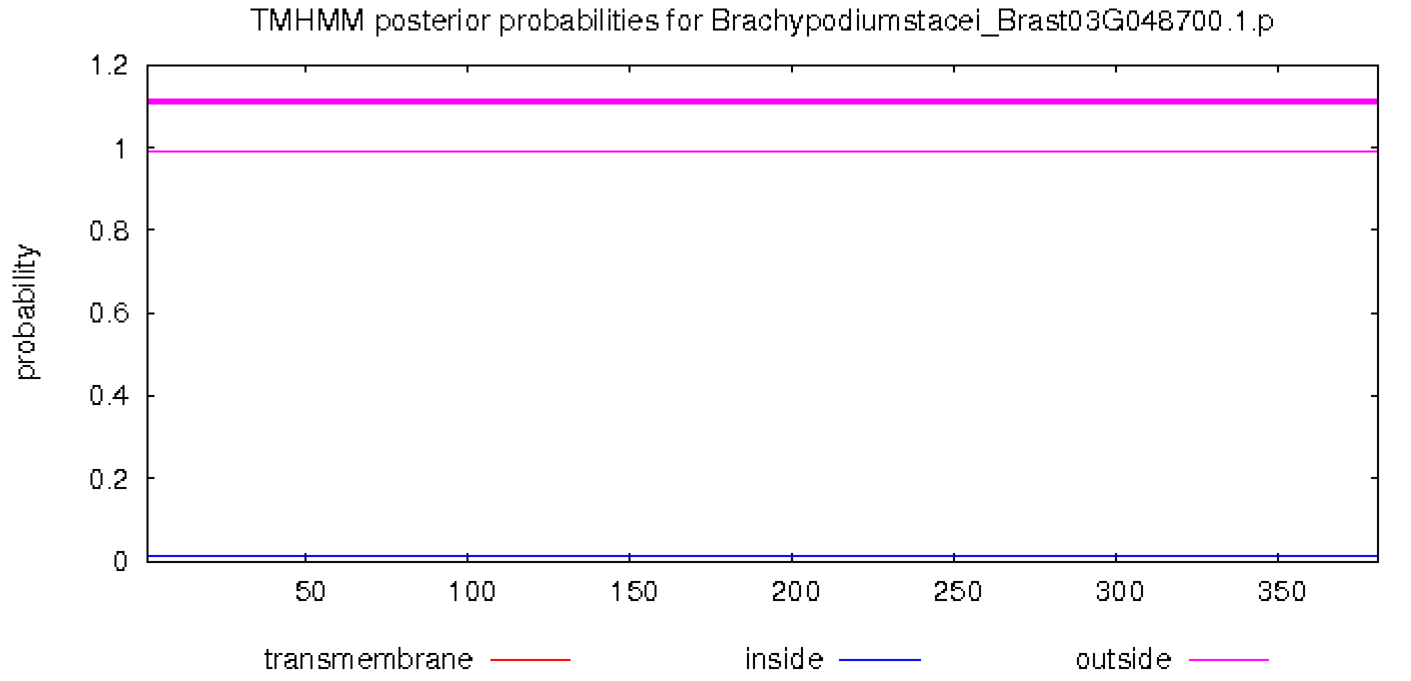

plot in postscript, script for making the plot in gnuplot, data for plot

---

```
# Brachypodiumstacei_Brast03G050200.1.p Length: 404
# Brachypodiumstacei_Brast03G050200.1.p Number of predicted TMHs:  0
```

```
# Brachypodiumstacei_Brast03G050200.1.p Exp number of AAs in TMHs: 0.00669
# Brachypodiumstacei_Brast03G050200.1.p Exp number, first 60 AAs:  0.00037
# Brachypodiumstacei_Brast03G050200.1.p Total prob of N-in:        0.01368
Brachypodiumstacei_Brast03G050200.1.p	TMHMM2.0	outside	     1   404
```

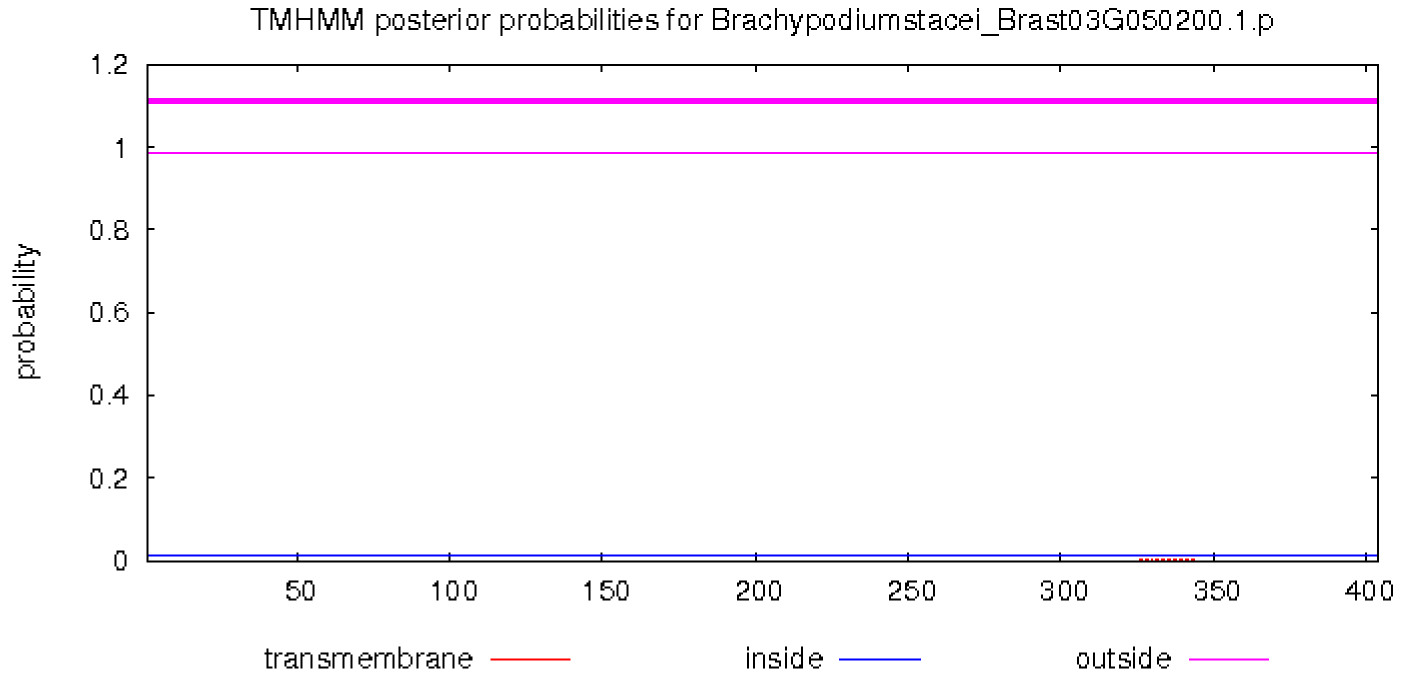

plot in postscript, script for making the plot in gnuplot, data for plot

---

```
# Brachypodiumstacei_Brast03G055500.1.p Length: 333
# Brachypodiumstacei_Brast03G055500.1.p Number of predicted TMHs:  0
# Brachypodiumstacei_Brast03G055500.1.p Exp number of AAs in TMHs: 0.11076
# Brachypodiumstacei_Brast03G055500.1.p Exp number, first 60 AAs:  0.00056
# Brachypodiumstacei_Brast03G055500.1.p Total prob of N-in:        0.01408
Brachypodiumstacei_Brast03G055500.1.p	TMHMM2.0	outside	     1   333
```

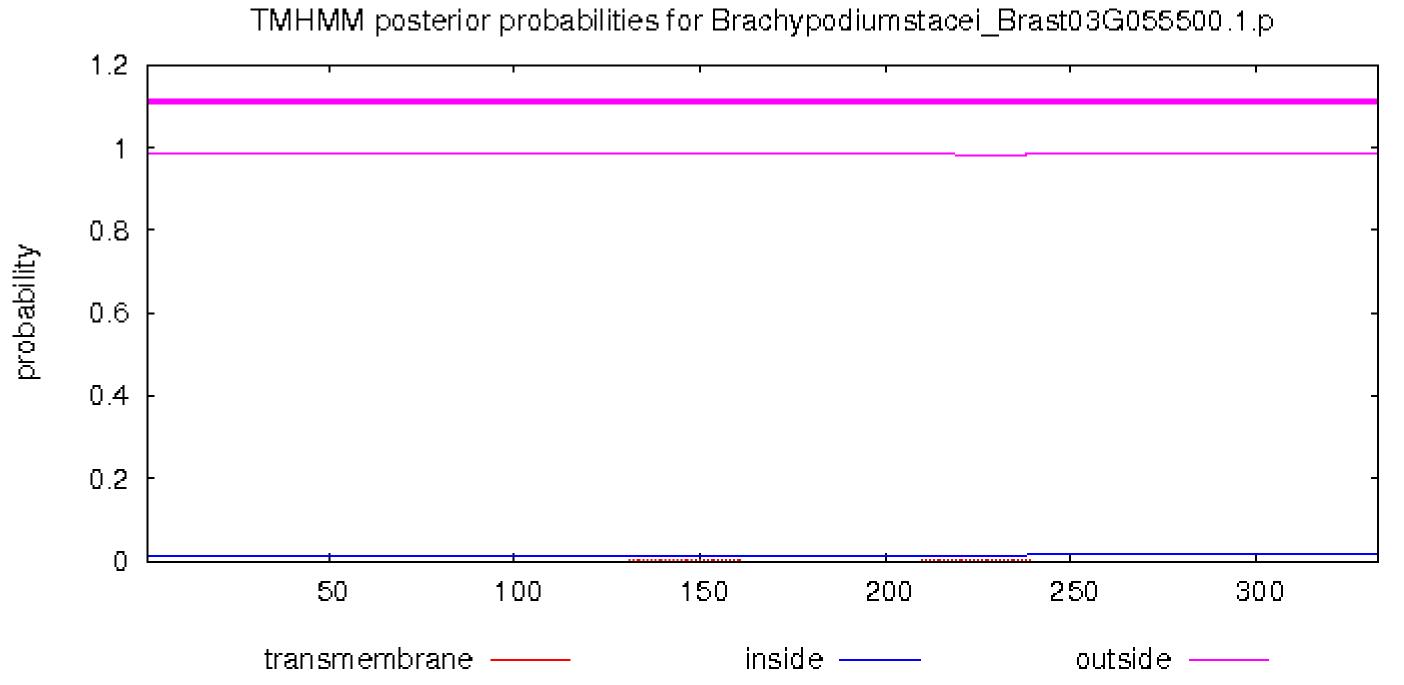

plot in postscript, script for making the plot in gnuplot, data for plot

---

```
# Brachypodiumstacei_Brast03G114000.1.p Length: 359
# Brachypodiumstacei_Brast03G114000.1.p Number of predicted TMHs:  0
```

```
# Brachypodiumstacei_Brast03G114000.1.p Exp number of AAs in TMHs: 0.00047
# Brachypodiumstacei_Brast03G114000.1.p Exp number, first 60 AAs:  0
# Brachypodiumstacei_Brast03G114000.1.p Total prob of N-in:        0.02907
Brachypodiumstacei_Brast03G114000.1.p	TMHMM2.0	outside	     1   359
```

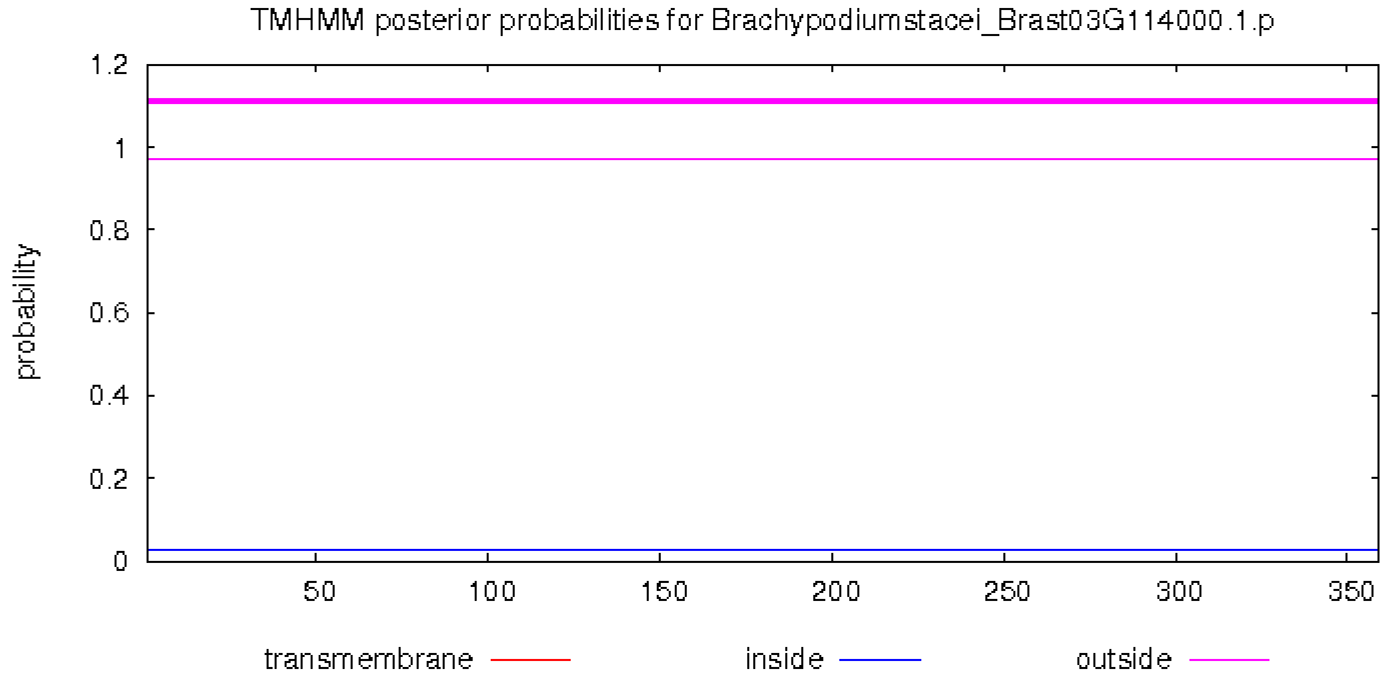

plot in postscript, script for making the plot in gnuplot, data for plot

---

```
# Brachypodiumstacei_Brast03G115600.1.p Length: 96
# Brachypodiumstacei_Brast03G115600.1.p Number of predicted TMHs:  0
# Brachypodiumstacei_Brast03G115600.1.p Exp number of AAs in TMHs: 0.01176
# Brachypodiumstacei_Brast03G115600.1.p Exp number, first 60 AAs:  0.00014
# Brachypodiumstacei_Brast03G115600.1.p Total prob of N-in:        0.24282
Brachypodiumstacei_Brast03G115600.1.p	TMHMM2.0	outside	     1    96
```

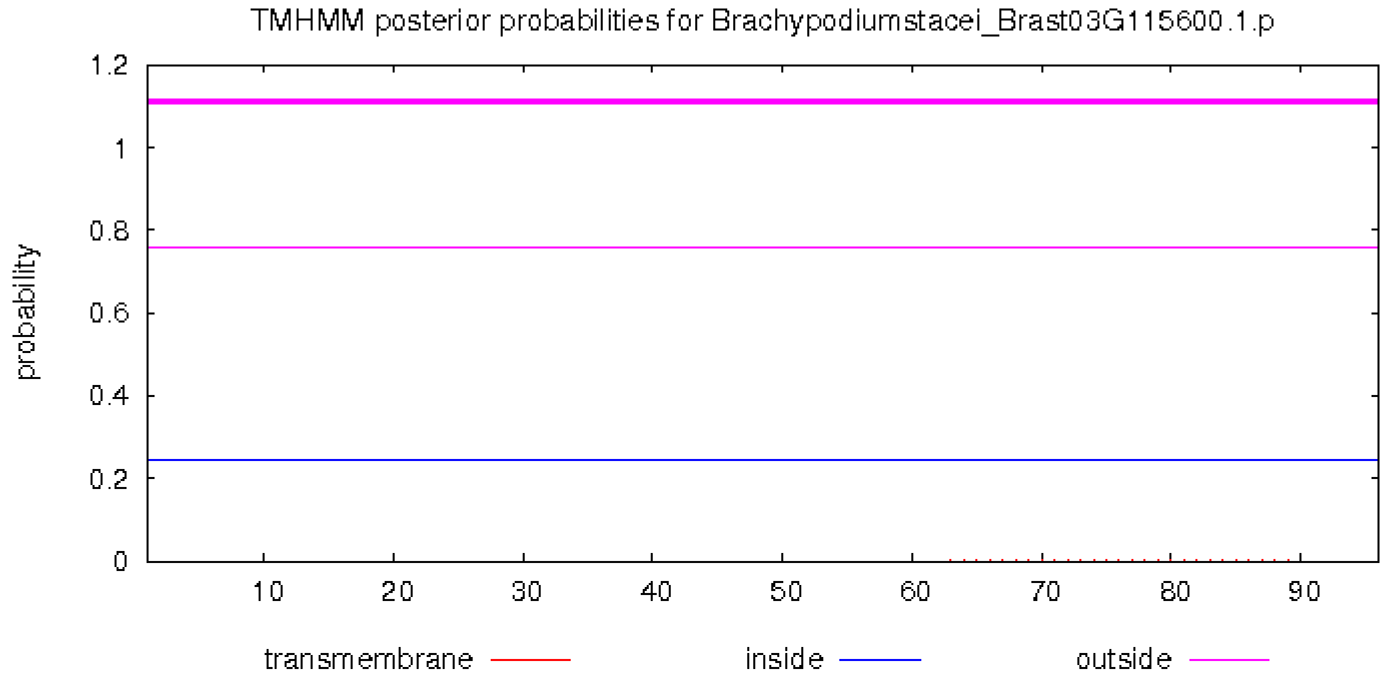

plot in postscript, script for making the plot in gnuplot, data for plot

---

```
# Brachypodiumstacei_Brast03G197400.1.p Length: 353
# Brachypodiumstacei_Brast03G197400.1.p Number of predicted TMHs:  0
```

```
# Brachypodiumstacei_Brast03G197400.1.p Exp number of AAs in TMHs: 0.01218
# Brachypodiumstacei_Brast03G197400.1.p Exp number, first 60 AAs:  0.00878
# Brachypodiumstacei_Brast03G197400.1.p Total prob of N-in:        0.13755
Brachypodiumstacei_Brast03G197400.1.p	TMHMM2.0	outside	     1   353
```

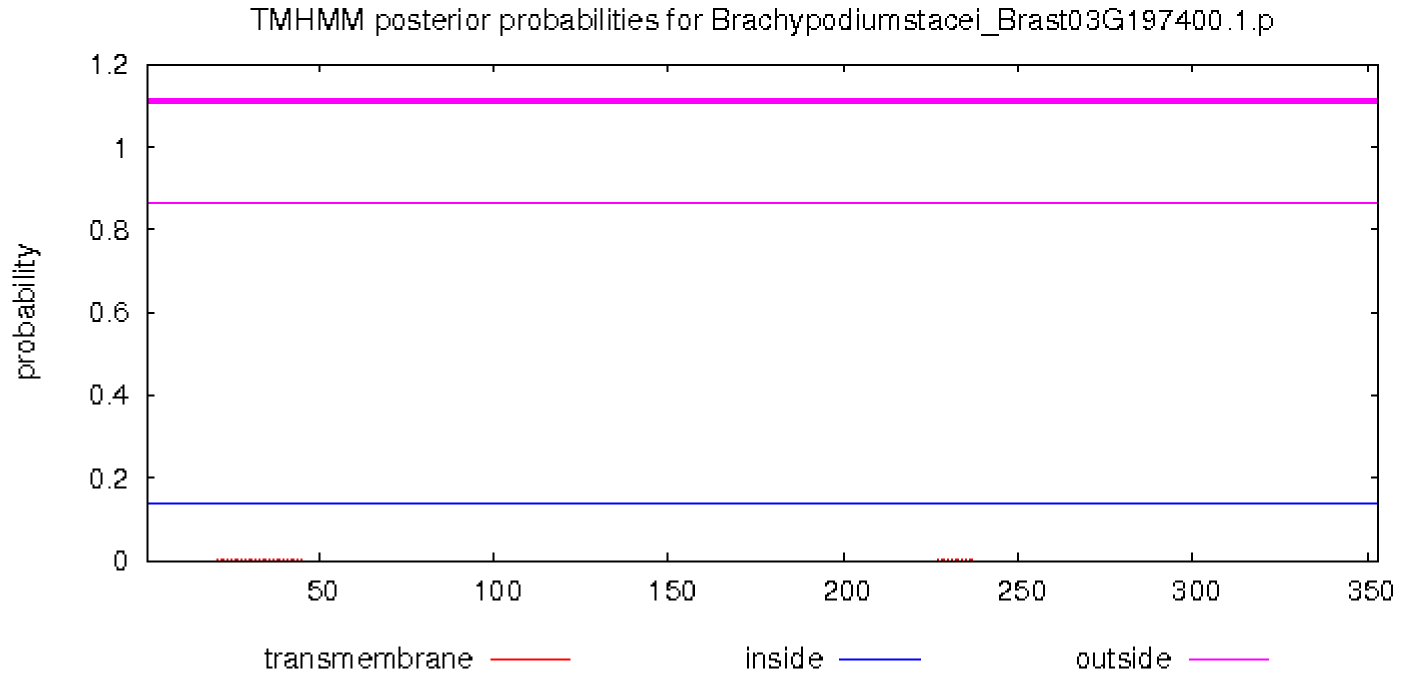

plot in postscript, script for making the plot in gnuplot, data for plot

---

```
# Brachypodiumstacei_Brast03G197500.1.p Length: 261
# Brachypodiumstacei_Brast03G197500.1.p Number of predicted TMHs:  0
# Brachypodiumstacei_Brast03G197500.1.p Exp number of AAs in TMHs: 0.03425
# Brachypodiumstacei_Brast03G197500.1.p Exp number, first 60 AAs:  3e-05
# Brachypodiumstacei_Brast03G197500.1.p Total prob of N-in:        0.01086
Brachypodiumstacei_Brast03G197500.1.p	TMHMM2.0	outside	     1   261
```

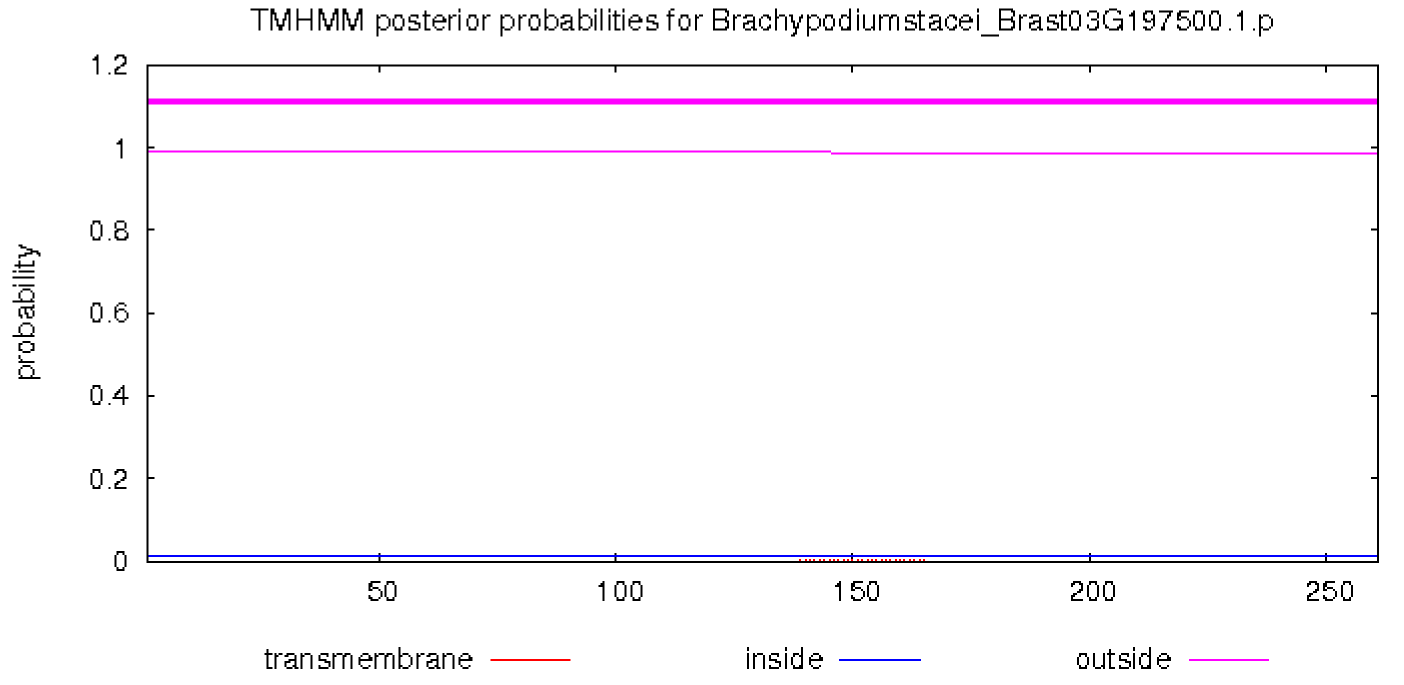

plot in postscript, script for making the plot in gnuplot, data for plot

---

```
# Brachypodiumstacei_Brast03G202100.1.p Length: 266
# Brachypodiumstacei_Brast03G202100.1.p Number of predicted TMHs:  0
```

```
# Brachypodiumstacei_Brast03G202100.1.p Exp number of AAs in TMHs: 0.02686
# Brachypodiumstacei_Brast03G202100.1.p Exp number, first 60 AAs:  3e-05
# Brachypodiumstacei_Brast03G202100.1.p Total prob of N-in:        0.00898
Brachypodiumstacei_Brast03G202100.1.p	TMHMM2.0	outside	     1   266
```

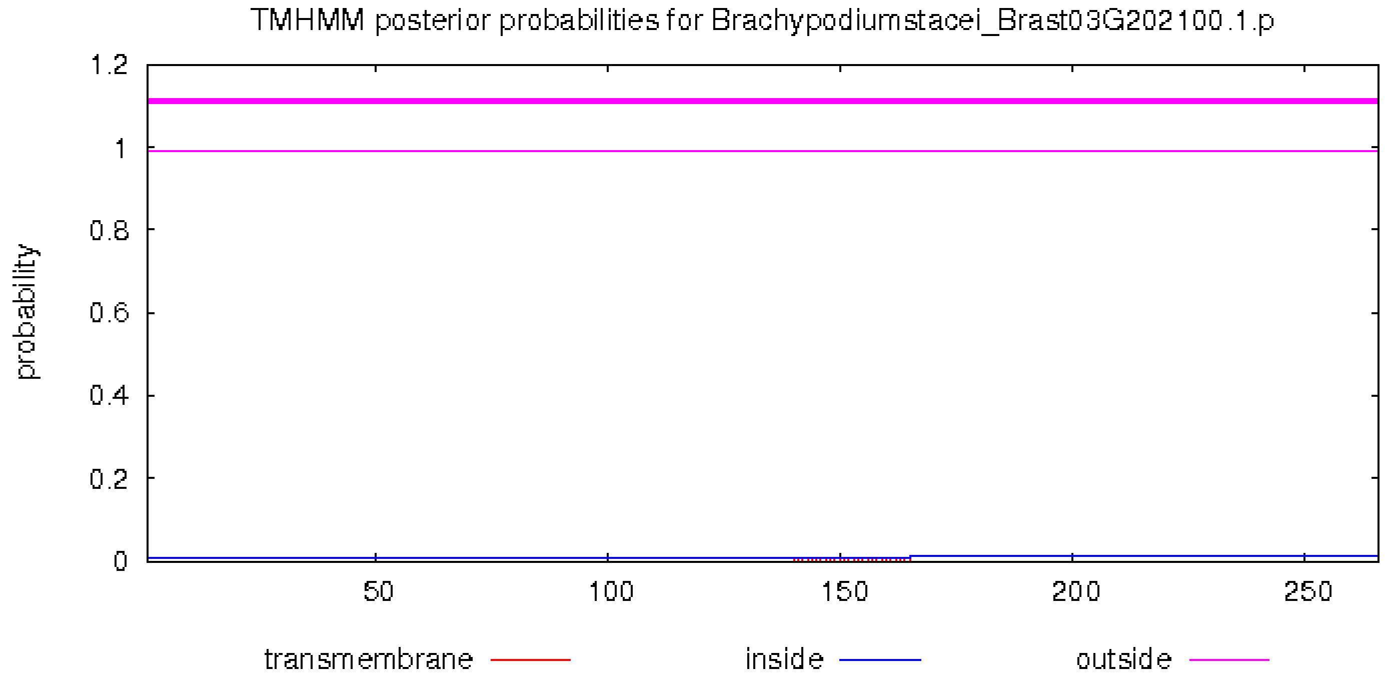

plot in postscript, script for making the plot in gnuplot, data for plot

---

```
# Brachypodiumstacei_Brast03G205400.1.p Length: 348
# Brachypodiumstacei_Brast03G205400.1.p Number of predicted TMHs:  0
# Brachypodiumstacei_Brast03G205400.1.p Exp number of AAs in TMHs: 0.01444
# Brachypodiumstacei_Brast03G205400.1.p Exp number, first 60 AAs:  0
# Brachypodiumstacei_Brast03G205400.1.p Total prob of N-in:        0.00194
Brachypodiumstacei_Brast03G205400.1.p	TMHMM2.0	outside	     1   348
```

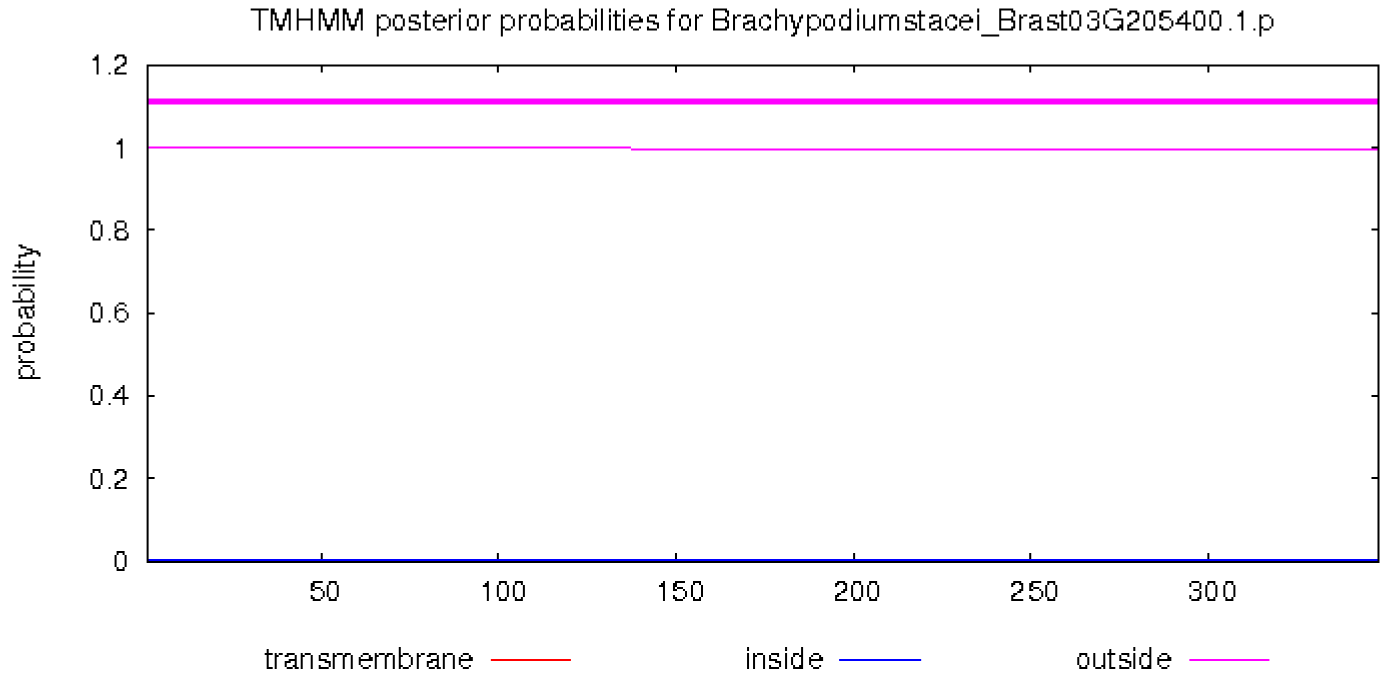

plot in postscript, script for making the plot in gnuplot, data for plot

---

```
# Brachypodiumstacei_Brast03G237200.1.p Length: 325
# Brachypodiumstacei_Brast03G237200.1.p Number of predicted TMHs:  0
```

```
# Brachypodiumstacei_Brast03G237200.1.p Exp number of AAs in TMHs: 0.00085
# Brachypodiumstacei_Brast03G237200.1.p Exp number, first 60 AAs:  0.00068
# Brachypodiumstacei_Brast03G237200.1.p Total prob of N-in:        0.01056
Brachypodiumstacei_Brast03G237200.1.p   TMHMM2.0        outside      1   325
```

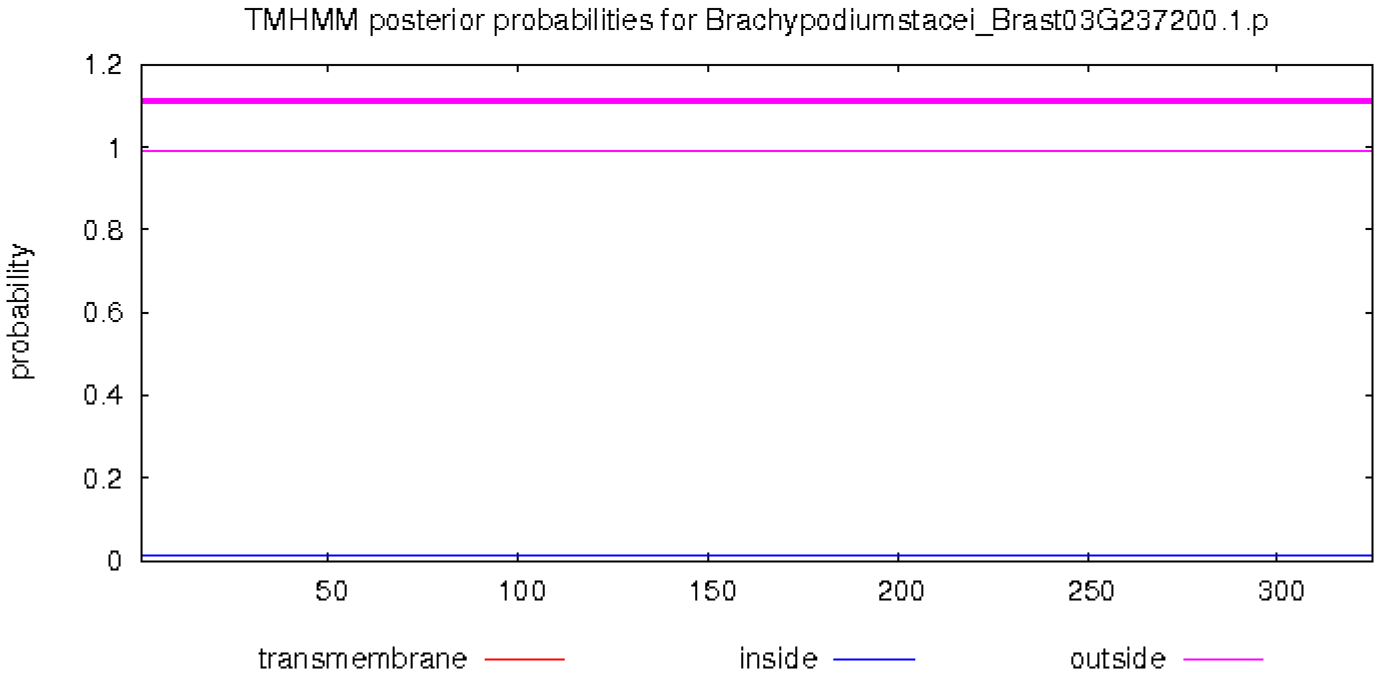

plot in postscript, script for making the plot in gnuplot, data for plot

```
# Brachypodiumstacei_Brast03G241500.1.p Length: 381
# Brachypodiumstacei_Brast03G241500.1.p Number of predicted TMHs:  0
# Brachypodiumstacei_Brast03G241500.1.p Exp number of AAs in TMHs: 0
# Brachypodiumstacei_Brast03G241500.1.p Exp number, first 60 AAs:  0
# Brachypodiumstacei_Brast03G241500.1.p Total prob of N-in:        0.01503
Brachypodiumstacei_Brast03G241500.1.p   TMHMM2.0        outside      1   381
```

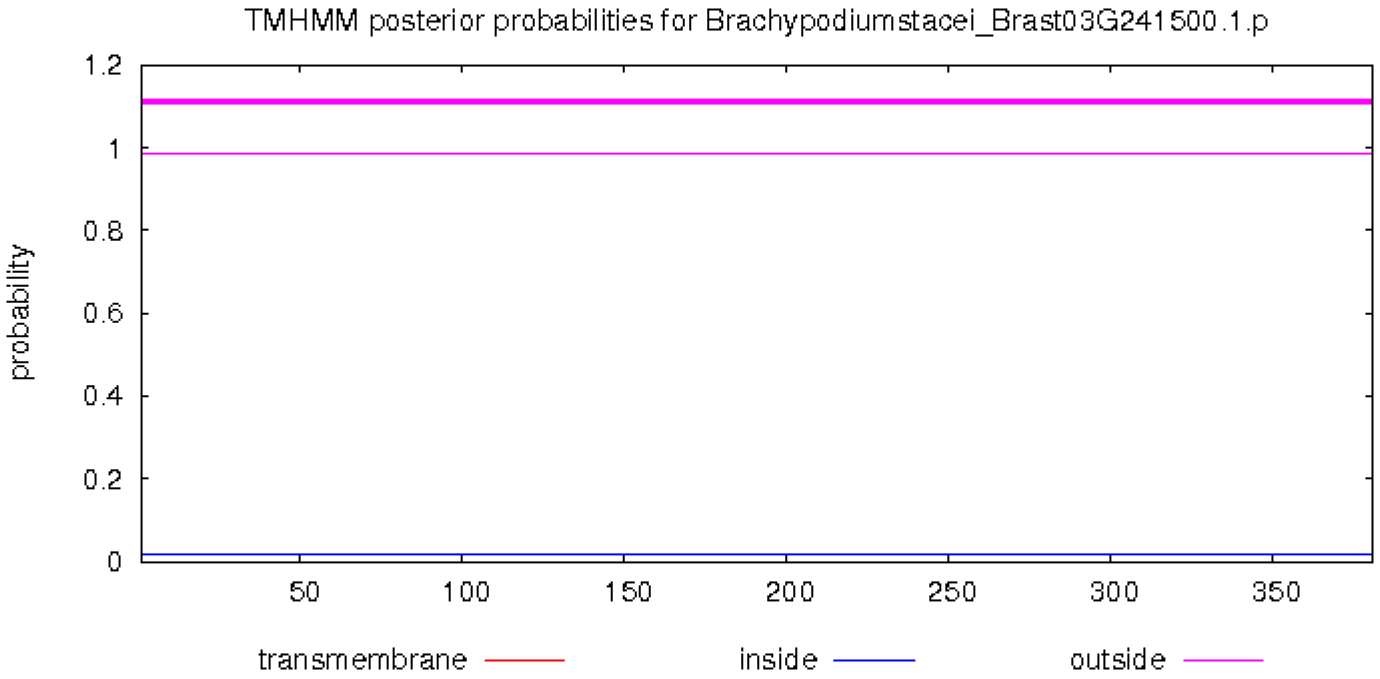

plot in postscript, script for making the plot in gnuplot, data for plot

```
# Brachypodiumstacei_Brast03G275500.1.p Length: 381
# Brachypodiumstacei_Brast03G275500.1.p Number of predicted TMHs:  0
```

```
# Brachypodiumstacei_Brast03G275500.1.p Exp number of AAs in TMHs: 0.02751
# Brachypodiumstacei_Brast03G275500.1.p Exp number, first 60 AAs:  0.00277
# Brachypodiumstacei_Brast03G275500.1.p Total prob of N-in:        0.01034
Brachypodiumstacei_Brast03G275500.1.p	TMHMM2.0	outside	     1   381
```

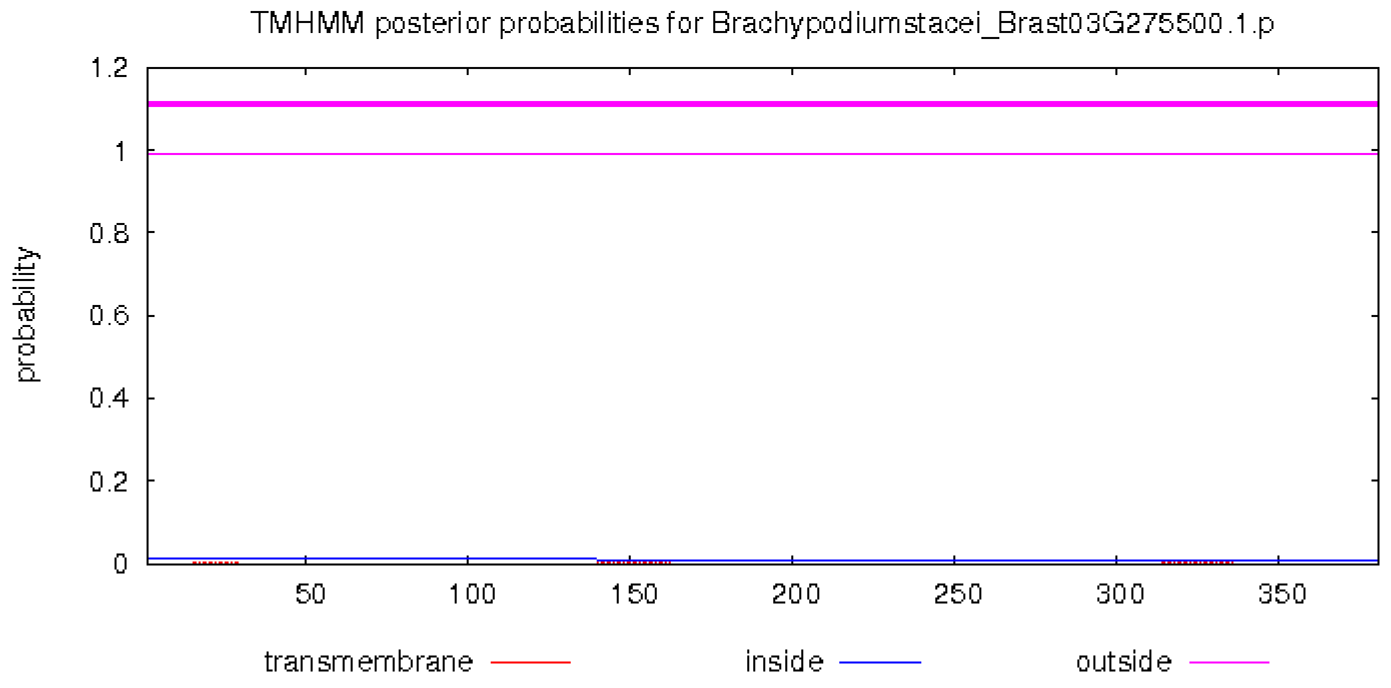

plot in postscript, script for making the plot in gnuplot, data for plot

---

```
# Brachypodiumstacei_Brast03G291700.1.p Length: 257
# Brachypodiumstacei_Brast03G291700.1.p Number of predicted TMHs:  0
# Brachypodiumstacei_Brast03G291700.1.p Exp number of AAs in TMHs: 0.00053
# Brachypodiumstacei_Brast03G291700.1.p Exp number, first 60 AAs:  0
# Brachypodiumstacei_Brast03G291700.1.p Total prob of N-in:        0.01982
Brachypodiumstacei_Brast03G291700.1.p	TMHMM2.0	outside	     1   257
```

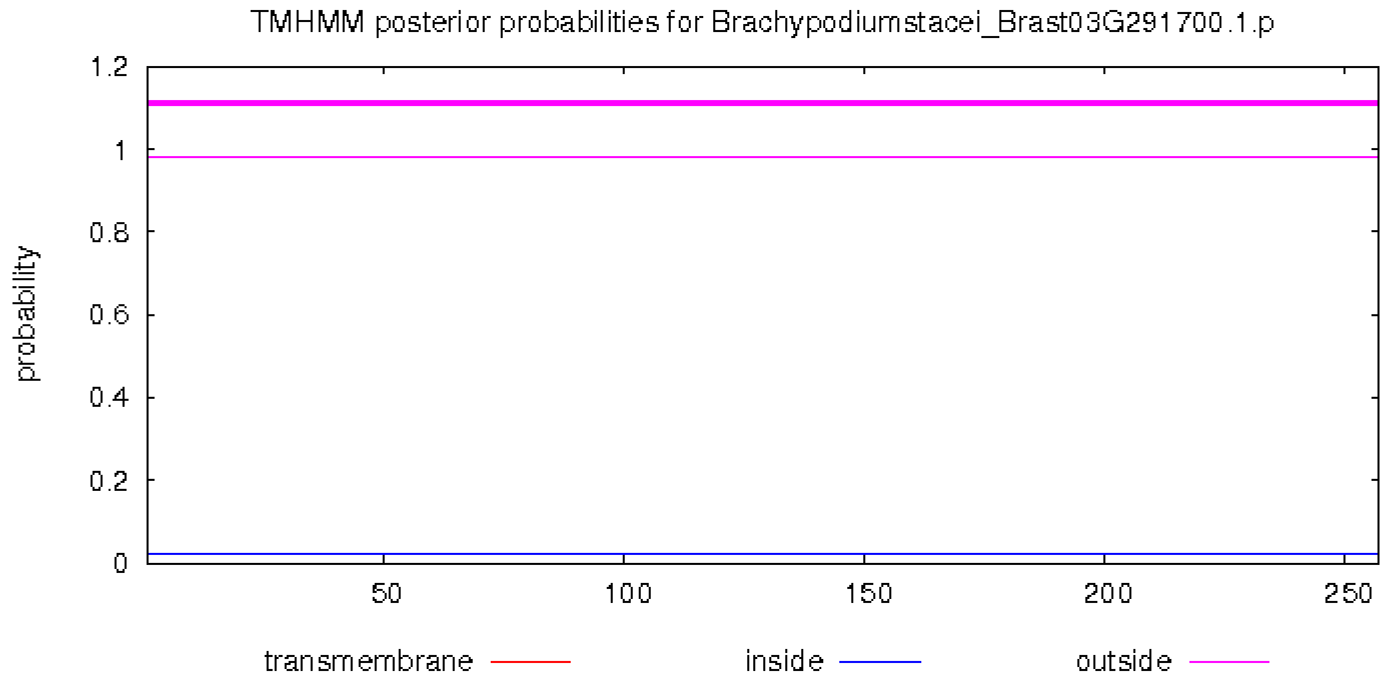

plot in postscript, script for making the plot in gnuplot, data for plot

---

```
# Brachypodiumstacei_Brast03G309200.1.p Length: 655
# Brachypodiumstacei_Brast03G309200.1.p Number of predicted TMHs:  1
```

```
# Brachypodiumstacei_Brast03G309200.1.p Exp number of AAs in TMHs: 20.17241
# Brachypodiumstacei_Brast03G309200.1.p Exp number, first 60 AAs:  0
# Brachypodiumstacei_Brast03G309200.1.p Total prob of N-in:        0.00383
Brachypodiumstacei_Brast03G309200.1.p   TMHMM2.0        outside      1   631
Brachypodiumstacei_Brast03G309200.1.p   TMHMM2.0        TMhelix    632   654
Brachypodiumstacei_Brast03G309200.1.p   TMHMM2.0        inside     655   655
```

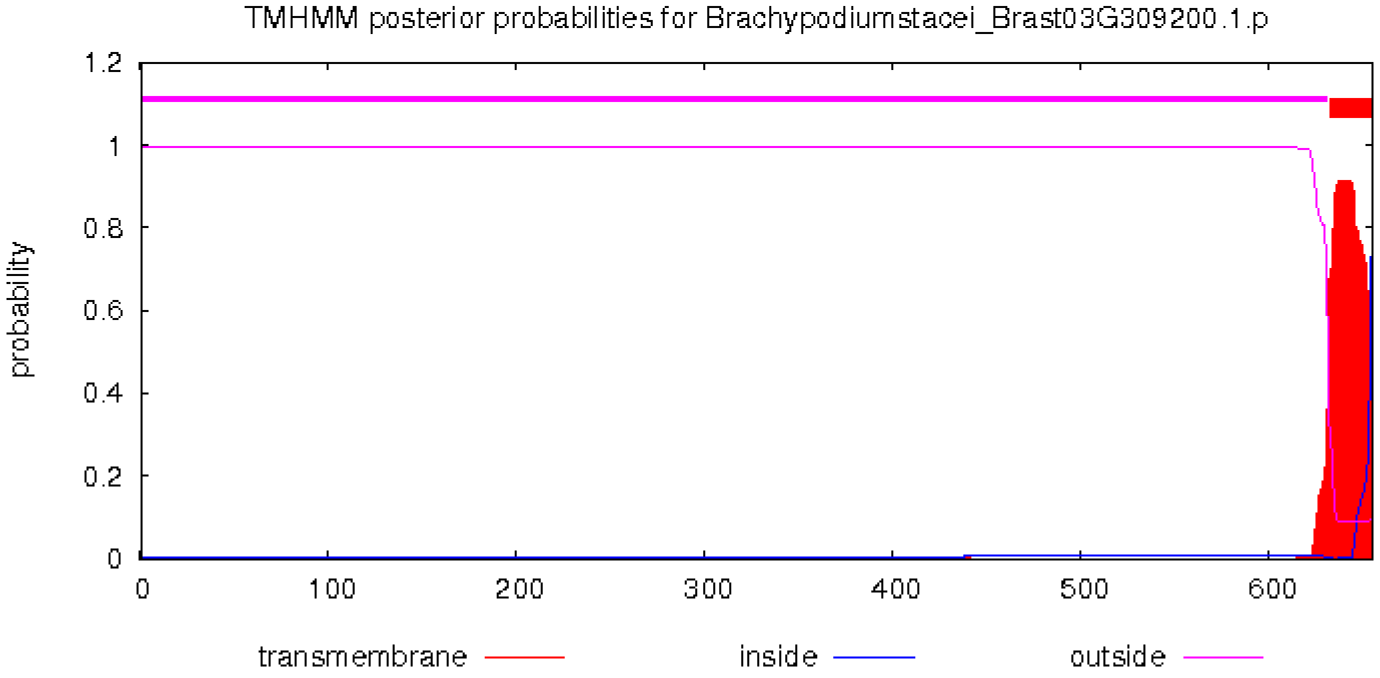

plot in postscript, script for making the plot in gnuplot, data for plot

---

```
# Brachypodiumstacei_Brast04G010800.1.p Length: 597
# Brachypodiumstacei_Brast04G010800.1.p Number of predicted TMHs:  0
# Brachypodiumstacei_Brast04G010800.1.p Exp number of AAs in TMHs: 0.00082
# Brachypodiumstacei_Brast04G010800.1.p Exp number, first 60 AAs:  0
# Brachypodiumstacei_Brast04G010800.1.p Total prob of N-in:        0.00062
Brachypodiumstacei_Brast04G010800.1.p   TMHMM2.0        outside      1   597
```

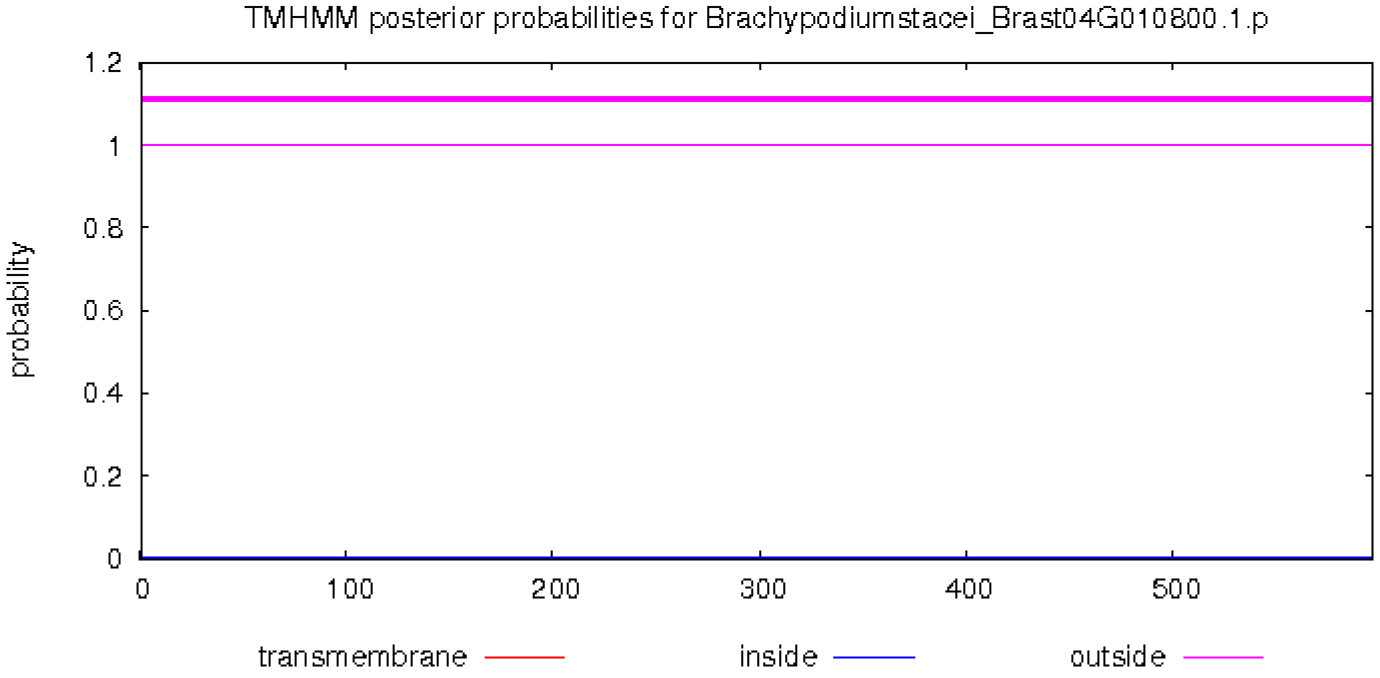

plot in postscript, script for making the plot in gnuplot, data for plot

---

```
# Brachypodiumstacei_Brast04G016800.1.p Length: 387
# Brachypodiumstacei_Brast04G016800.1.p Number of predicted TMHs:  0
# Brachypodiumstacei_Brast04G016800.1.p Exp number of AAs in TMHs: 0.0043
# Brachypodiumstacei_Brast04G016800.1.p Exp number, first 60 AAs:  0
# Brachypodiumstacei_Brast04G016800.1.p Total prob of N-in:        0.01245
Brachypodiumstacei_Brast04G016800.1.p   TMHMM2.0        outside      1   387
```

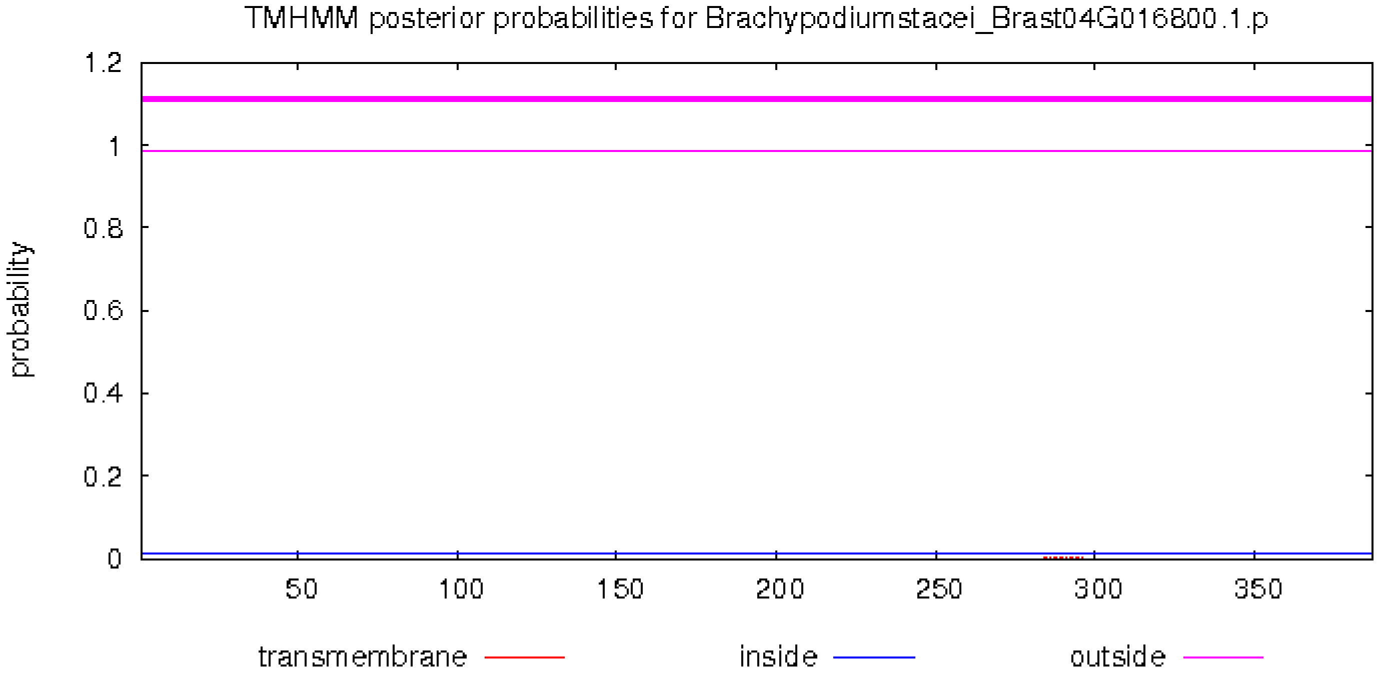

plot in postscript, script for making the plot in gnuplot, data for plot

```
# Brachypodiumstacei_Brast04G052200.1.p Length: 670
# Brachypodiumstacei_Brast04G052200.1.p Number of predicted TMHs:  1
# Brachypodiumstacei_Brast04G052200.1.p Exp number of AAs in TMHs: 21.59348
# Brachypodiumstacei_Brast04G052200.1.p Exp number, first 60 AAs:  0
# Brachypodiumstacei_Brast04G052200.1.p Total prob of N-in:        0.00007
Brachypodiumstacei_Brast04G052200.1.p   TMHMM2.0        outside      1   645
Brachypodiumstacei_Brast04G052200.1.p   TMHMM2.0        TMhelix    646   668
Brachypodiumstacei_Brast04G052200.1.p   TMHMM2.0        inside     669   670
```

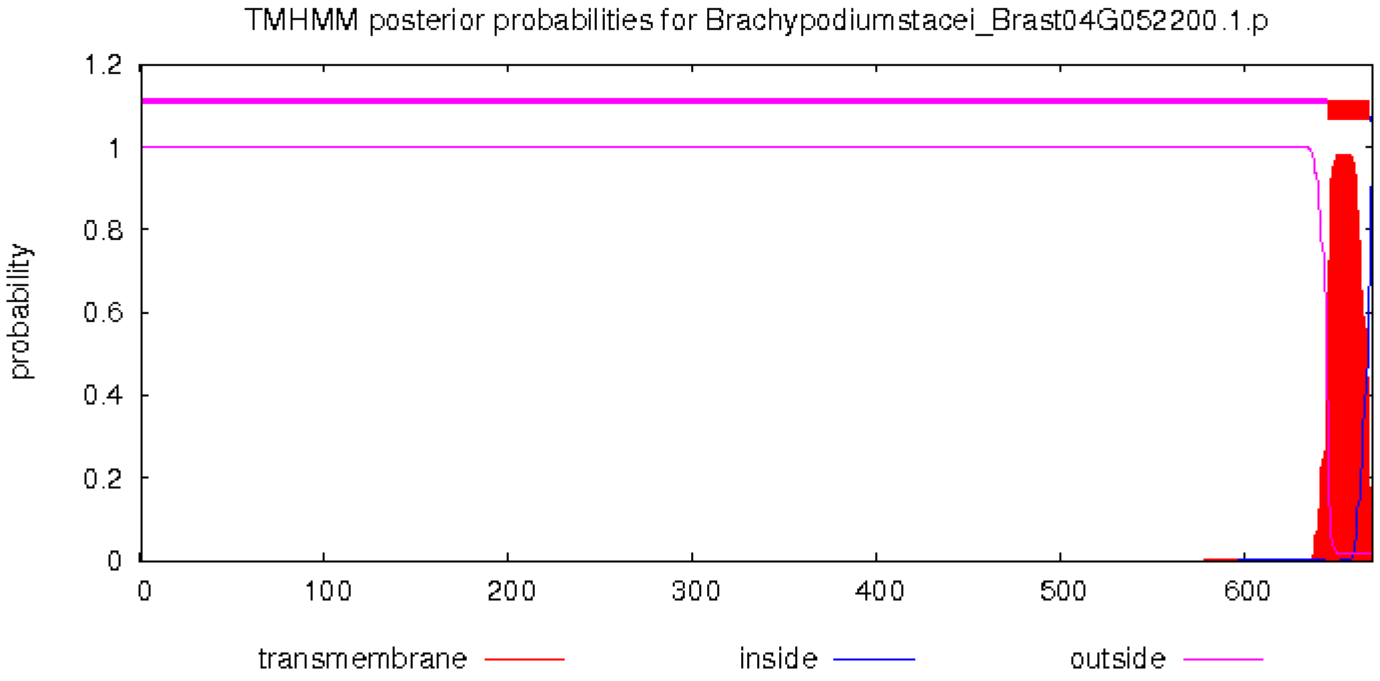

plot in postscript, script for making the plot in gnuplot, data for plot

```
# Brachypodiumstacei_Brast04G066800.1.p Length: 301
# Brachypodiumstacei_Brast04G066800.1.p Number of predicted TMHs:  0
# Brachypodiumstacei_Brast04G066800.1.p Exp number of AAs in TMHs: 0.00284
# Brachypodiumstacei_Brast04G066800.1.p Exp number, first 60 AAs:  0
# Brachypodiumstacei_Brast04G066800.1.p Total prob of N-in:        0.01106
Brachypodiumstacei_Brast04G066800.1.p	TMHMM2.0	outside	     1   301
```

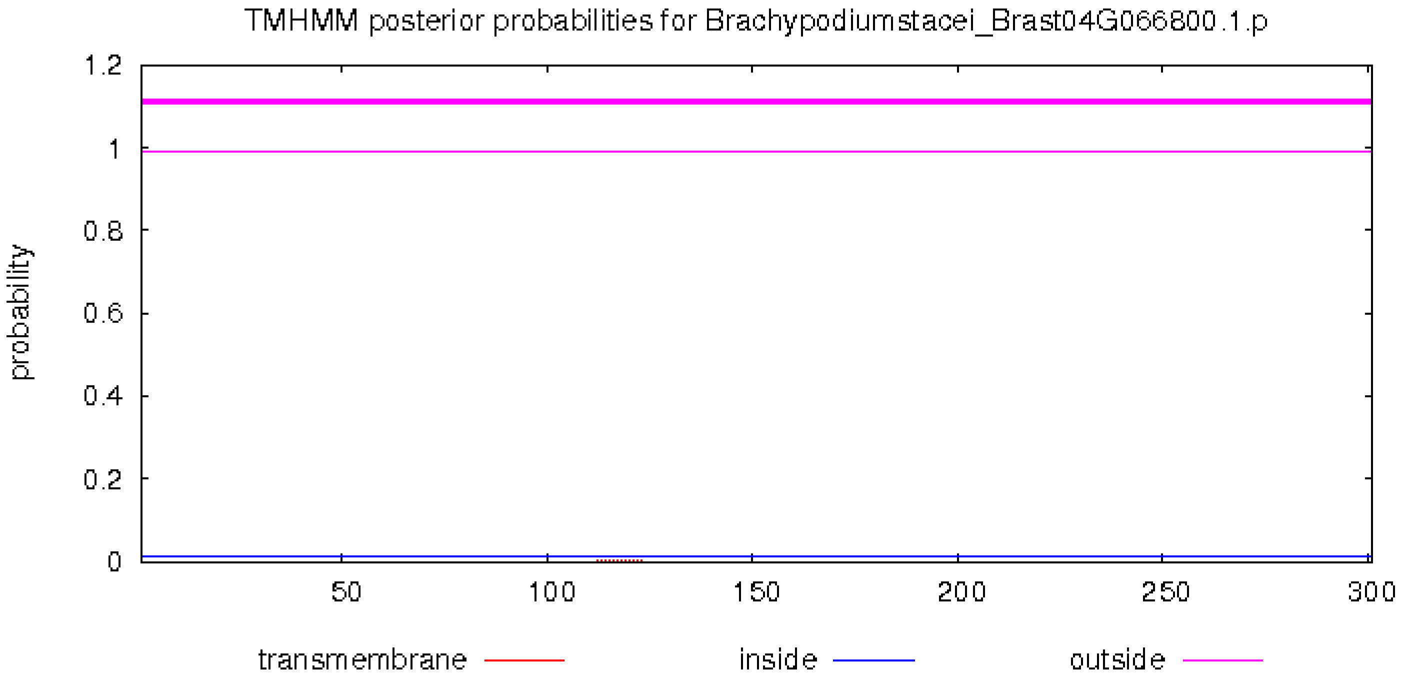

plot in postscript, script for making the plot in gnuplot, data for plot

```
# Brachypodiumstacei_Brast04G123400.1.p Length: 351
# Brachypodiumstacei_Brast04G123400.1.p Number of predicted TMHs:  0
# Brachypodiumstacei_Brast04G123400.1.p Exp number of AAs in TMHs: 0.00019
# Brachypodiumstacei_Brast04G123400.1.p Exp number, first 60 AAs:  0
# Brachypodiumstacei_Brast04G123400.1.p Total prob of N-in:        0.00349
Brachypodiumstacei_Brast04G123400.1.p	TMHMM2.0	outside	     1   351
```

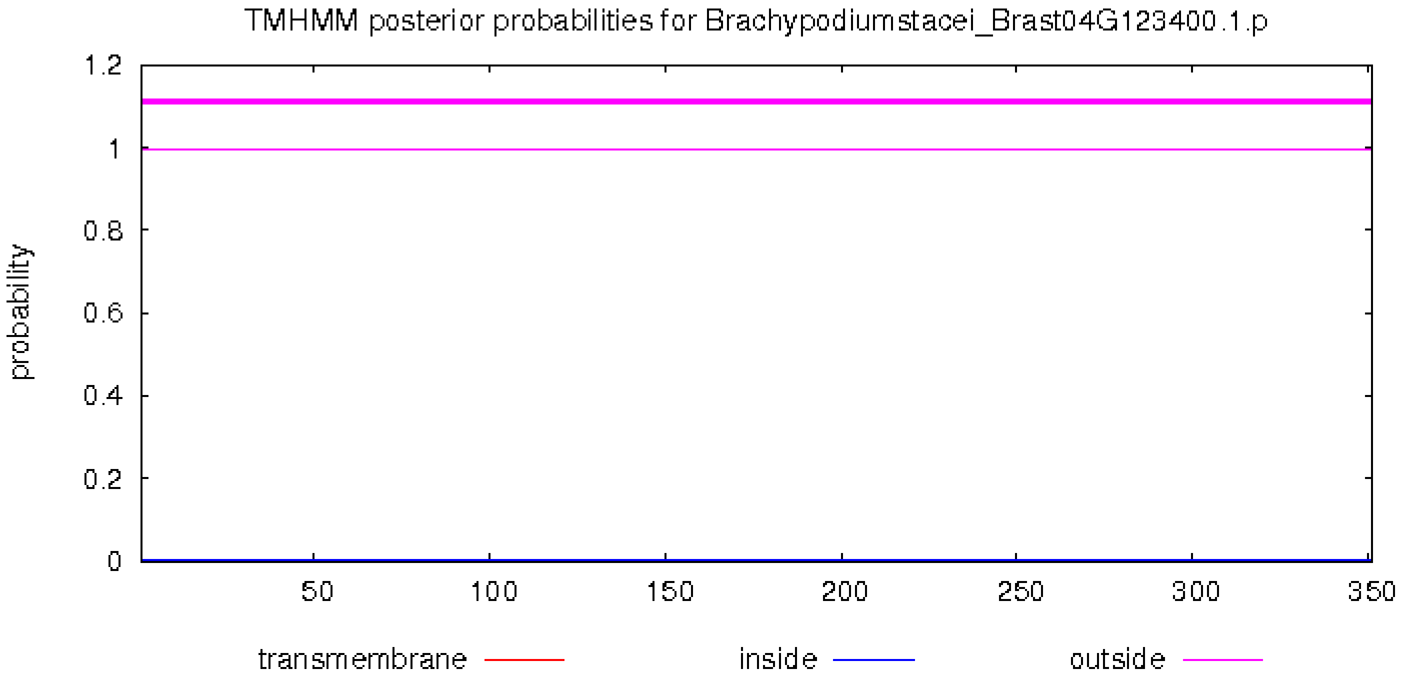

plot in postscript, script for making the plot in gnuplot, data for plot

```
# Brachypodiumstacei_Brast04G151000.1.p Length: 384
# Brachypodiumstacei_Brast04G151000.1.p Number of predicted TMHs:  0
# Brachypodiumstacei_Brast04G151000.1.p Exp number of AAs in TMHs: 0.00042
# Brachypodiumstacei_Brast04G151000.1.p Exp number, first 60 AAs:  0
# Brachypodiumstacei_Brast04G151000.1.p Total prob of N-in:        0.02520
Brachypodiumstacei_Brast04G151000.1.p	TMHMM2.0	outside	     1   384
```

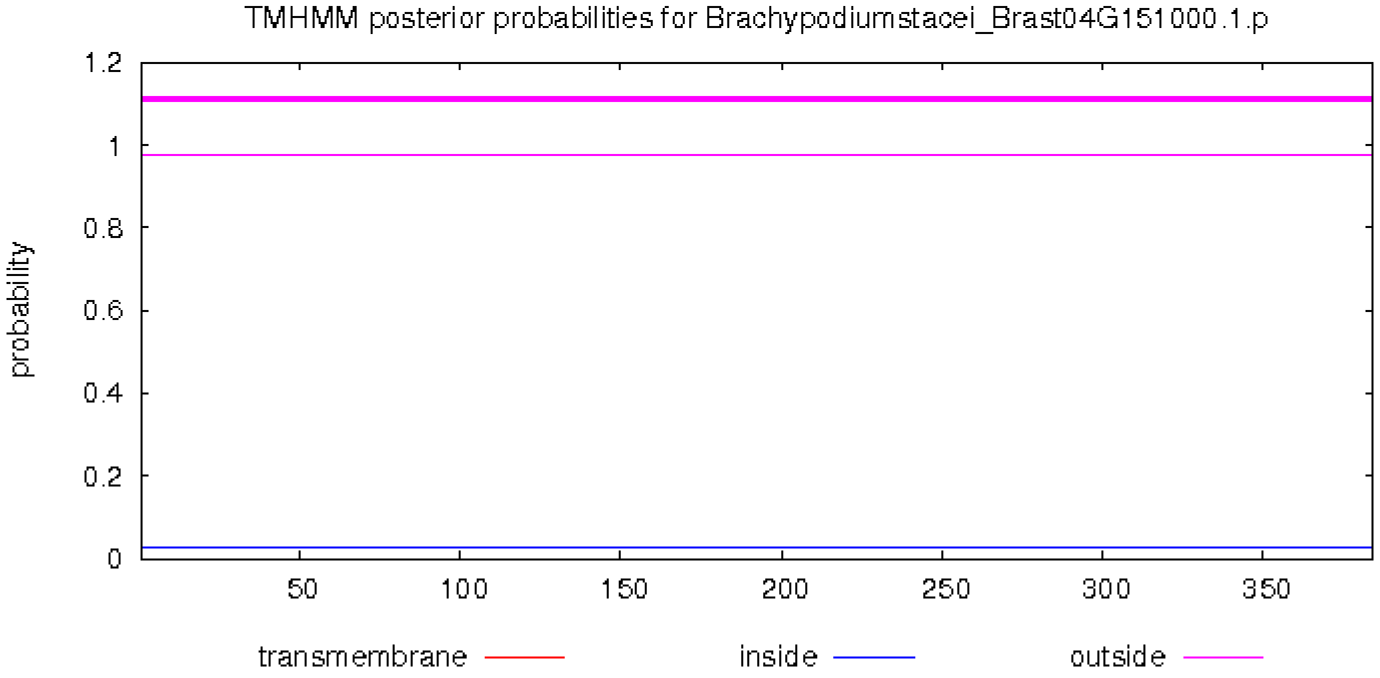

plot in postscript, script for making the plot in gnuplot, data for plot

```
# Brachypodiumstacei_Brast04G158600.1.p Length: 333
# Brachypodiumstacei_Brast04G158600.1.p Number of predicted TMHs:  0
# Brachypodiumstacei_Brast04G158600.1.p Exp number of AAs in TMHs: 0.01113
# Brachypodiumstacei_Brast04G158600.1.p Exp number, first 60 AAs:  0
# Brachypodiumstacei_Brast04G158600.1.p Total prob of N-in:        0.01817
Brachypodiumstacei_Brast04G158600.1.p	TMHMM2.0	outside	     1   333
```

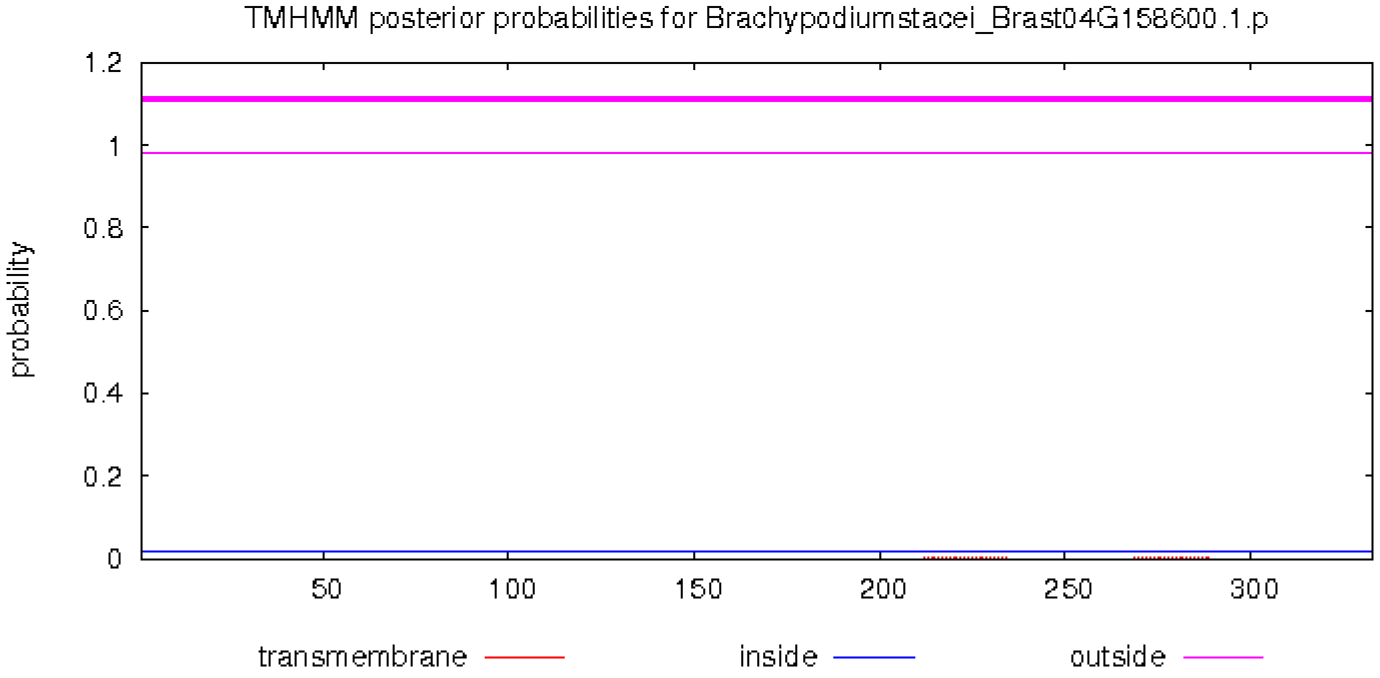

plot in postscript, script for making the plot in gnuplot, data for plot

```
# Brachypodiumstacei_Brast04G217600.1.p Length: 372
# Brachypodiumstacei_Brast04G217600.1.p Number of predicted TMHs:  0
# Brachypodiumstacei_Brast04G217600.1.p Exp number of AAs in TMHs: 0.00043
# Brachypodiumstacei_Brast04G217600.1.p Exp number, first 60 AAs:  0
# Brachypodiumstacei_Brast04G217600.1.p Total prob of N-in:        0.00588
Brachypodiumstacei_Brast04G217600.1.p	TMHMM2.0	outside	     1   372
```

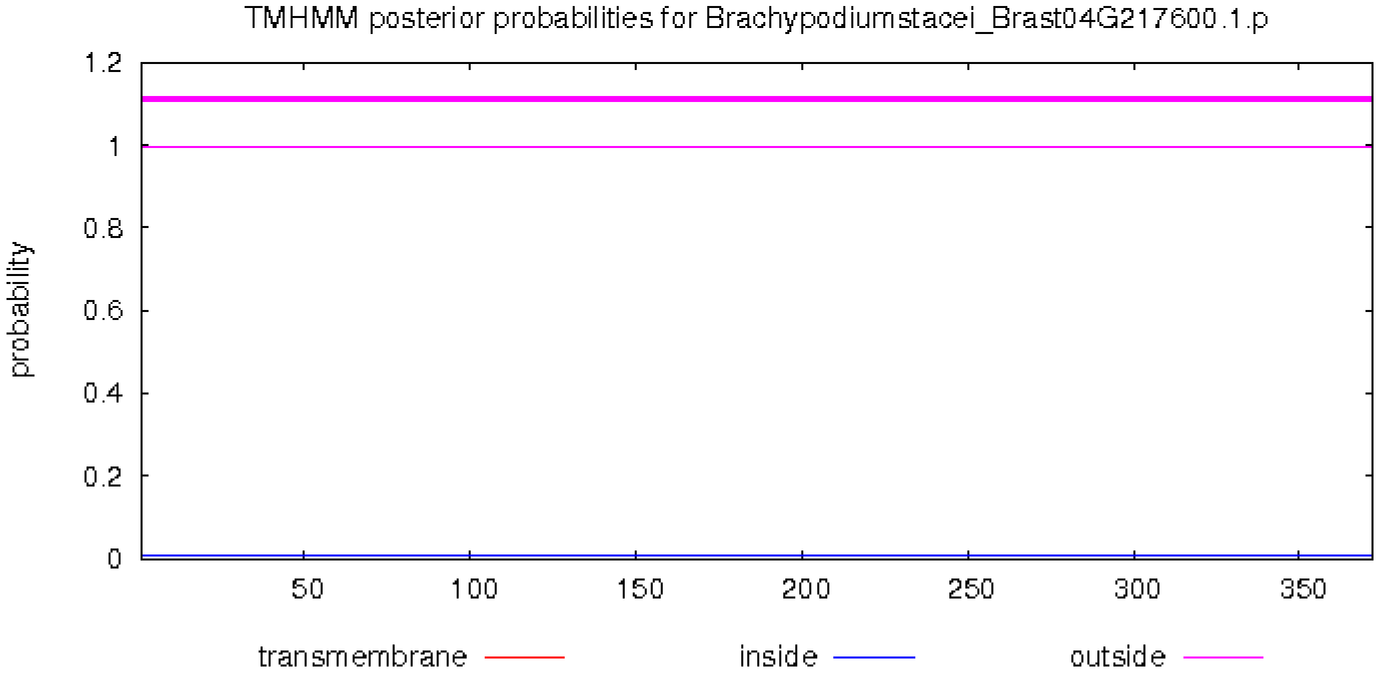

plot in postscript, script for making the plot in gnuplot, data for plot

```
# Brachypodiumstacei_Brast04G233200.1.p Length: 381
# Brachypodiumstacei_Brast04G233200.1.p Number of predicted TMHs:  0
# Brachypodiumstacei_Brast04G233200.1.p Exp number of AAs in TMHs: 0.00157
# Brachypodiumstacei_Brast04G233200.1.p Exp number, first 60 AAs:  0
# Brachypodiumstacei_Brast04G233200.1.p Total prob of N-in:        0.00428
Brachypodiumstacei_Brast04G233200.1.p	TMHMM2.0	outside	     1   381
```

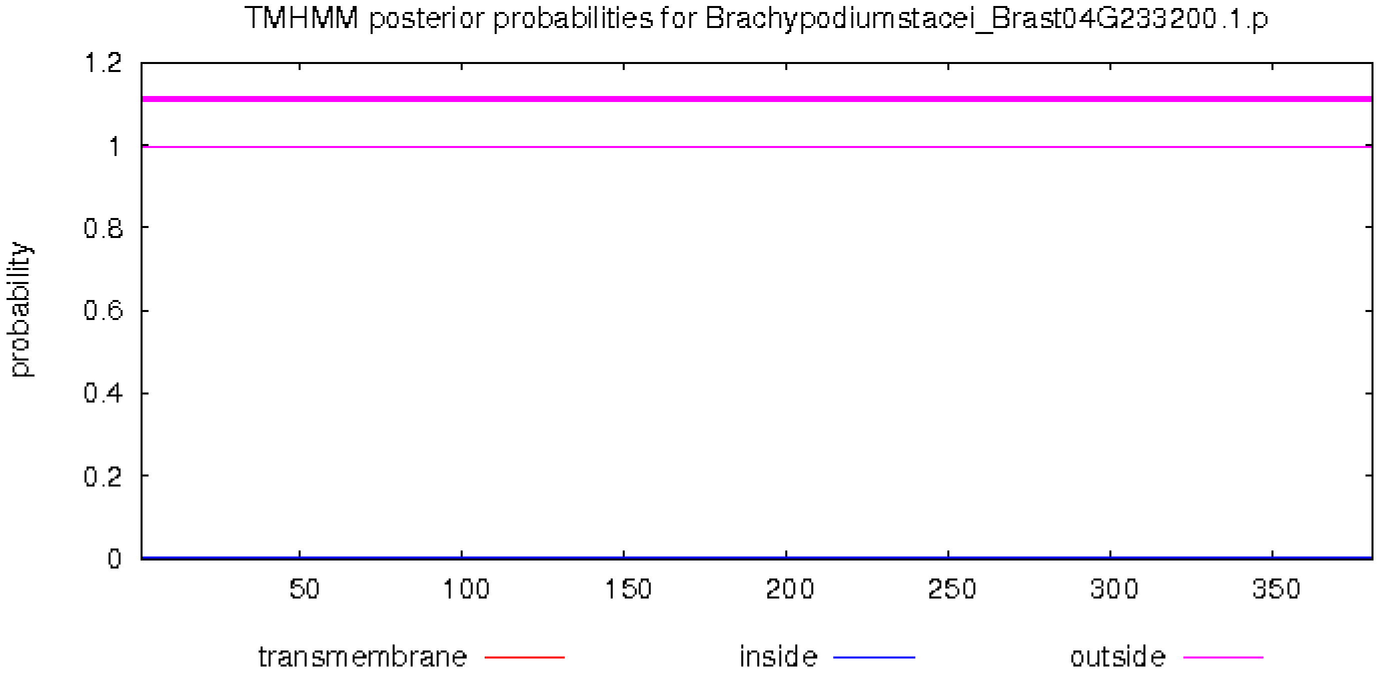

plot in postscript, script for making the plot in gnuplot, data for plot

```
# Brachypodiumstacei_Brast05G004300.1.p Length: 532
# Brachypodiumstacei_Brast05G004300.1.p Number of predicted TMHs:  0
# Brachypodiumstacei_Brast05G004300.1.p Exp number of AAs in TMHs: 0.00855
# Brachypodiumstacei_Brast05G004300.1.p Exp number, first 60 AAs:  0
# Brachypodiumstacei_Brast05G004300.1.p Total prob of N-in:        0.00057
Brachypodiumstacei_Brast05G004300.1.p	TMHMM2.0	outside	     1   532
```

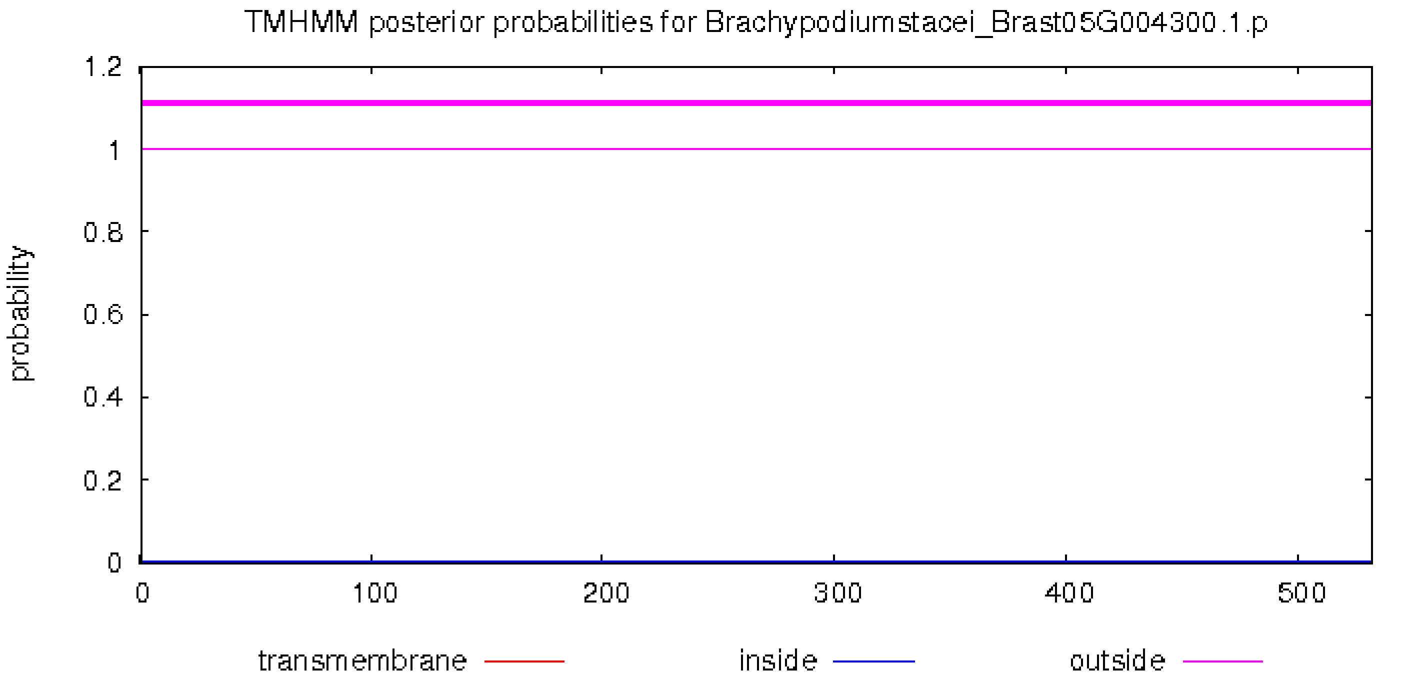

plot in postscript, script for making the plot in gnuplot, data for plot

---

```
# Brachypodiumstacei_Brast05G004400.1.p Length: 452
# Brachypodiumstacei_Brast05G004400.1.p Number of predicted TMHs:  0
# Brachypodiumstacei_Brast05G004400.1.p Exp number of AAs in TMHs: 0.11007
# Brachypodiumstacei_Brast05G004400.1.p Exp number, first 60 AAs:  0.00551
# Brachypodiumstacei_Brast05G004400.1.p Total prob of N-in:        0.03491
Brachypodiumstacei_Brast05G004400.1.p	TMHMM2.0	outside	     1   452
```

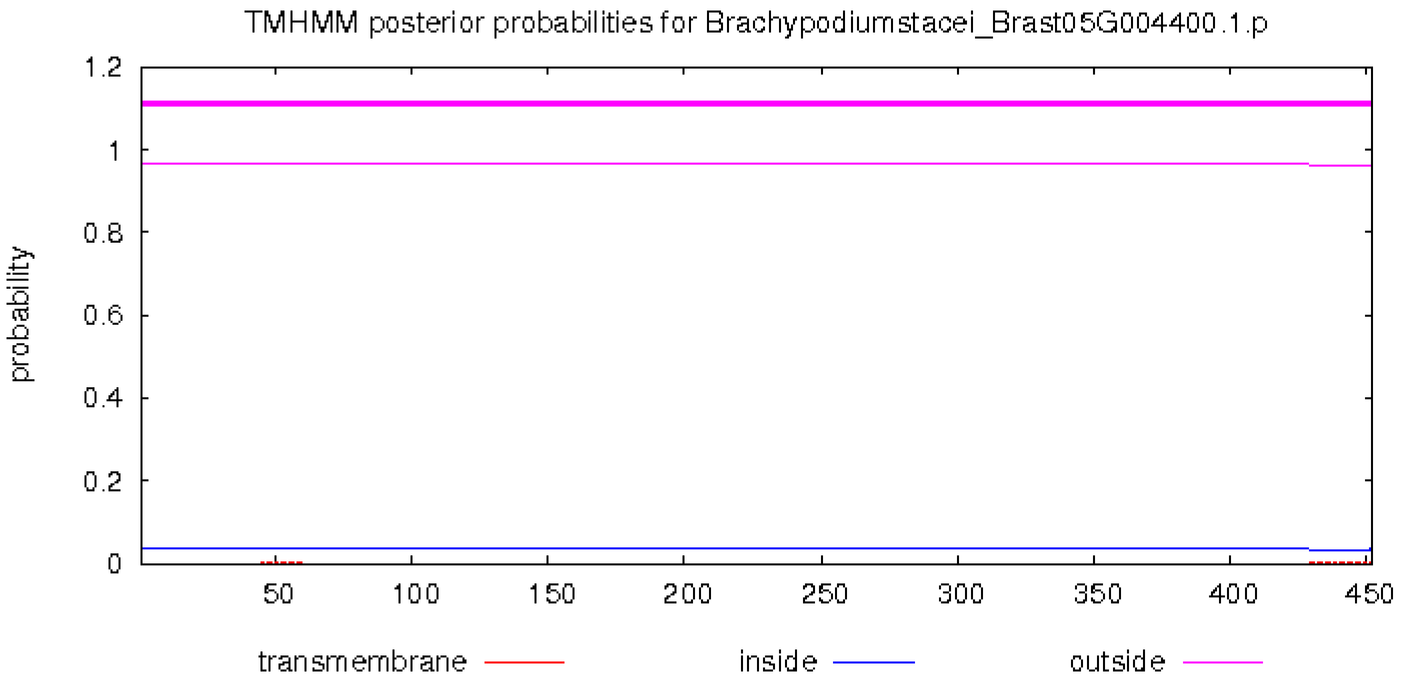

plot in postscript, script for making the plot in gnuplot, data for plot

---

```
# Brachypodiumstacei_Brast05G005600.1.p Length: 404
# Brachypodiumstacei_Brast05G005600.1.p Number of predicted TMHs:  0
# Brachypodiumstacei_Brast05G005600.1.p Exp number of AAs in TMHs: 0.03728
# Brachypodiumstacei_Brast05G005600.1.p Exp number, first 60 AAs:  0.03728
# Brachypodiumstacei_Brast05G005600.1.p Total prob of N-in:        0.00260
Brachypodiumstacei_Brast05G005600.1.p	TMHMM2.0	outside	     1   404
```

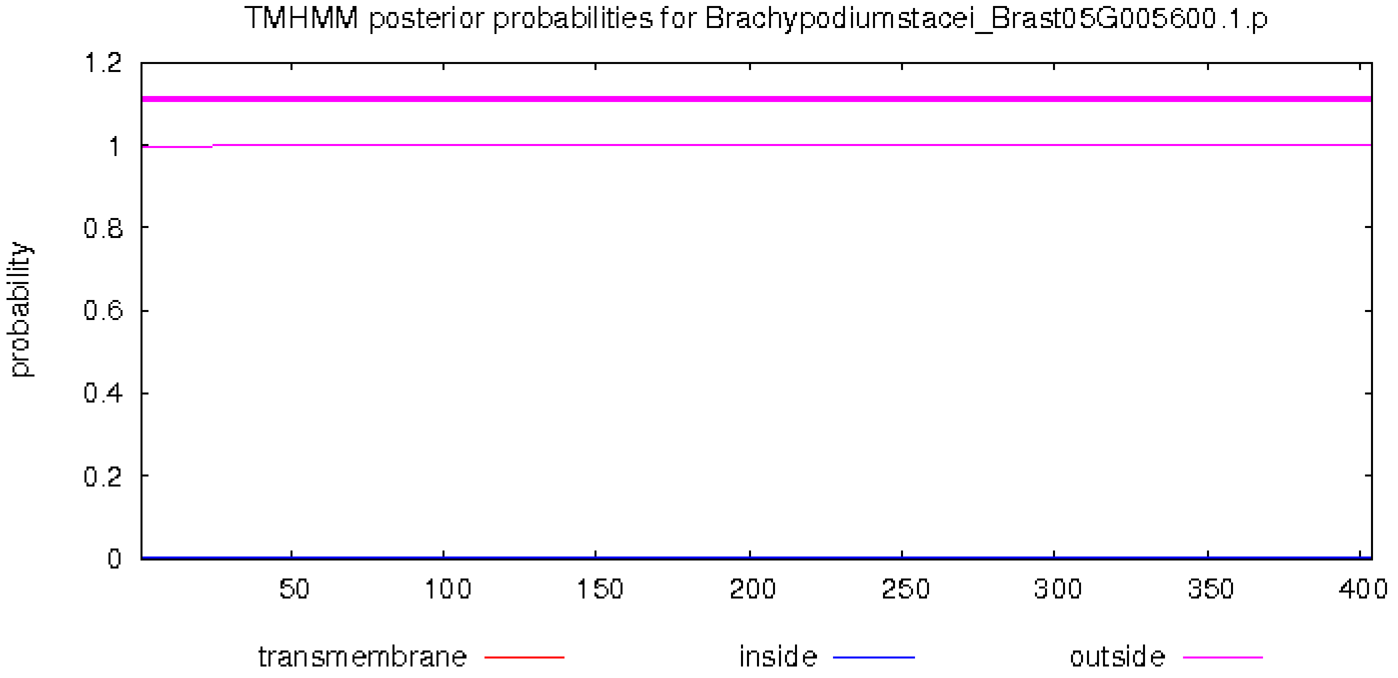

plot in postscript, script for making the plot in gnuplot, data for plot

```
# Brachypodiumstacei_Brast05G007900.1.p Length: 375
# Brachypodiumstacei_Brast05G007900.1.p Number of predicted TMHs:  0
# Brachypodiumstacei_Brast05G007900.1.p Exp number of AAs in TMHs: 0.03814
# Brachypodiumstacei_Brast05G007900.1.p Exp number, first 60 AAs:  0.03616
# Brachypodiumstacei_Brast05G007900.1.p Total prob of N-in:        0.00338
Brachypodiumstacei_Brast05G007900.1.p	TMHMM2.0	outside	     1   375
```

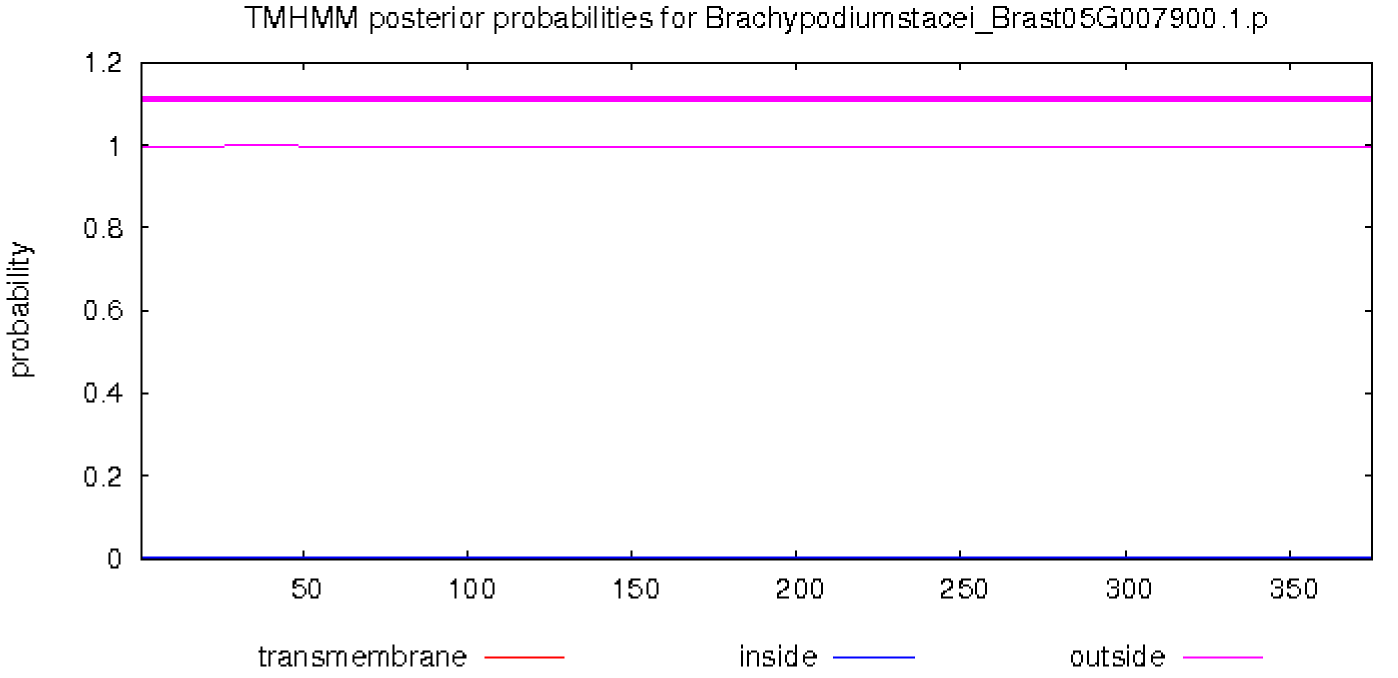

plot in postscript, script for making the plot in gnuplot, data for plot

```
# Brachypodiumstacei_Brast05G029300.1.p Length: 343
# Brachypodiumstacei_Brast05G029300.1.p Number of predicted TMHs:  0
# Brachypodiumstacei_Brast05G029300.1.p Exp number of AAs in TMHs: 0.00902
# Brachypodiumstacei_Brast05G029300.1.p Exp number, first 60 AAs:  0
# Brachypodiumstacei_Brast05G029300.1.p Total prob of N-in:        0.00828
Brachypodiumstacei_Brast05G029300.1.p	TMHMM2.0	outside	     1   343
```

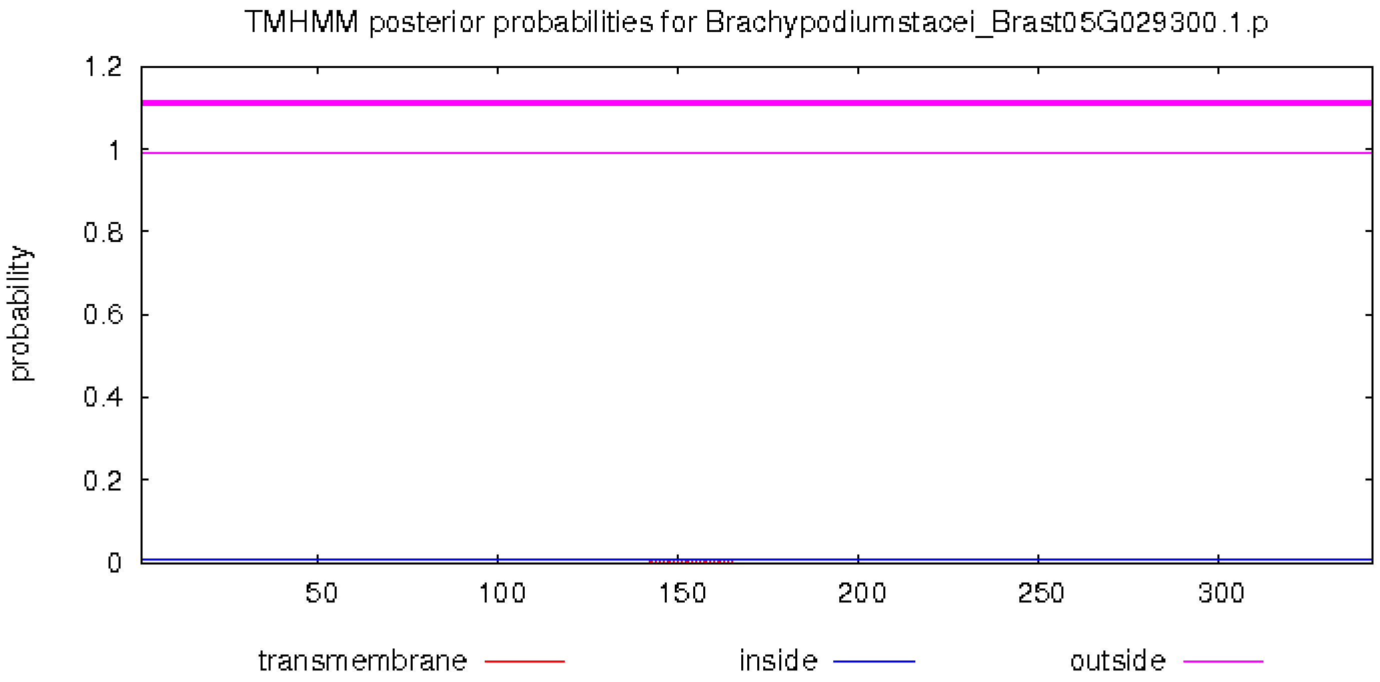

plot in postscript, script for making the plot in gnuplot, data for plot

---

```
# Brachypodiumstacei_Brast05G029400.1.p Length: 281
# Brachypodiumstacei_Brast05G029400.1.p Number of predicted TMHs:  0
# Brachypodiumstacei_Brast05G029400.1.p Exp number of AAs in TMHs: 0
# Brachypodiumstacei_Brast05G029400.1.p Exp number, first 60 AAs:  0
# Brachypodiumstacei_Brast05G029400.1.p Total prob of N-in:        0.00893
Brachypodiumstacei_Brast05G029400.1.p	TMHMM2.0	outside	     1   281
```

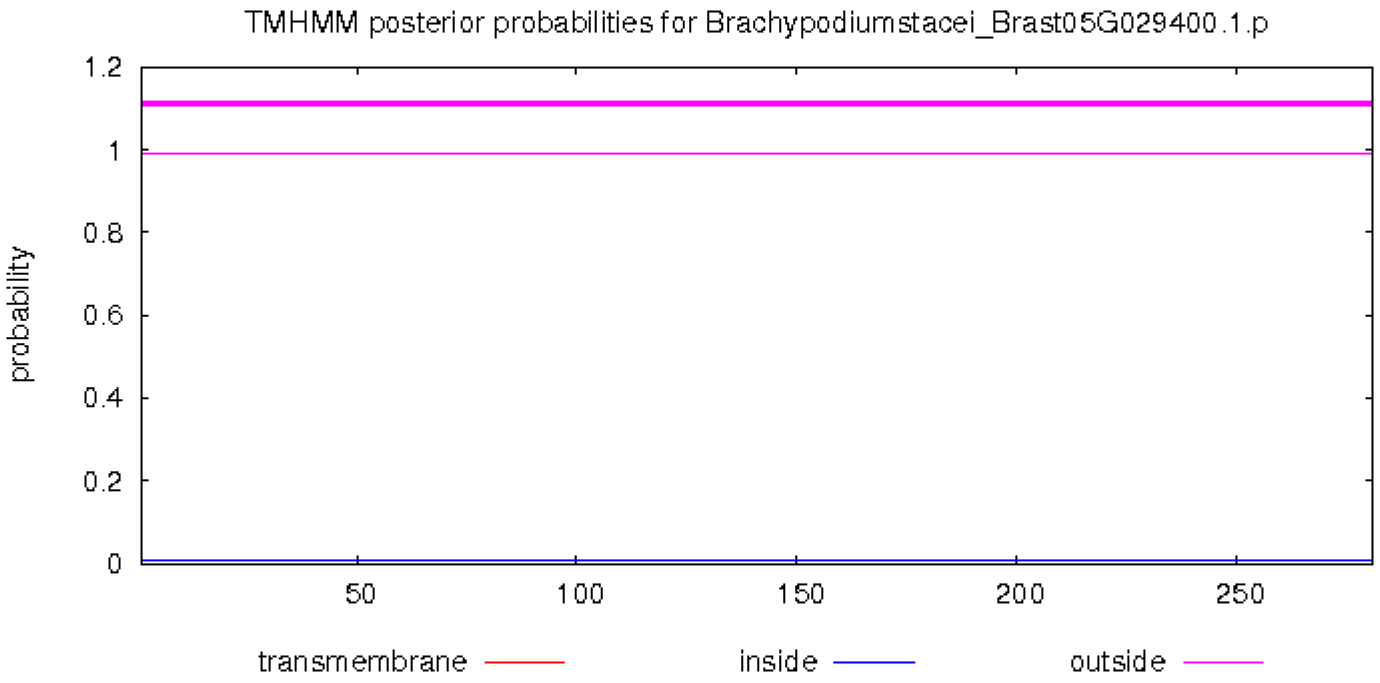

plot in postscript, script for making the plot in gnuplot, data for plot

---

```
# Brachypodiumstacei_Brast05G030000.1.p Length: 343
# Brachypodiumstacei_Brast05G030000.1.p Number of predicted TMHs:  0
# Brachypodiumstacei_Brast05G030000.1.p Exp number of AAs in TMHs: 0.00902
# Brachypodiumstacei_Brast05G030000.1.p Exp number, first 60 AAs:  0
# Brachypodiumstacei_Brast05G030000.1.p Total prob of N-in:        0.00828
Brachypodiumstacei_Brast05G030000.1.p	TMHMM2.0	outside	     1   343
```

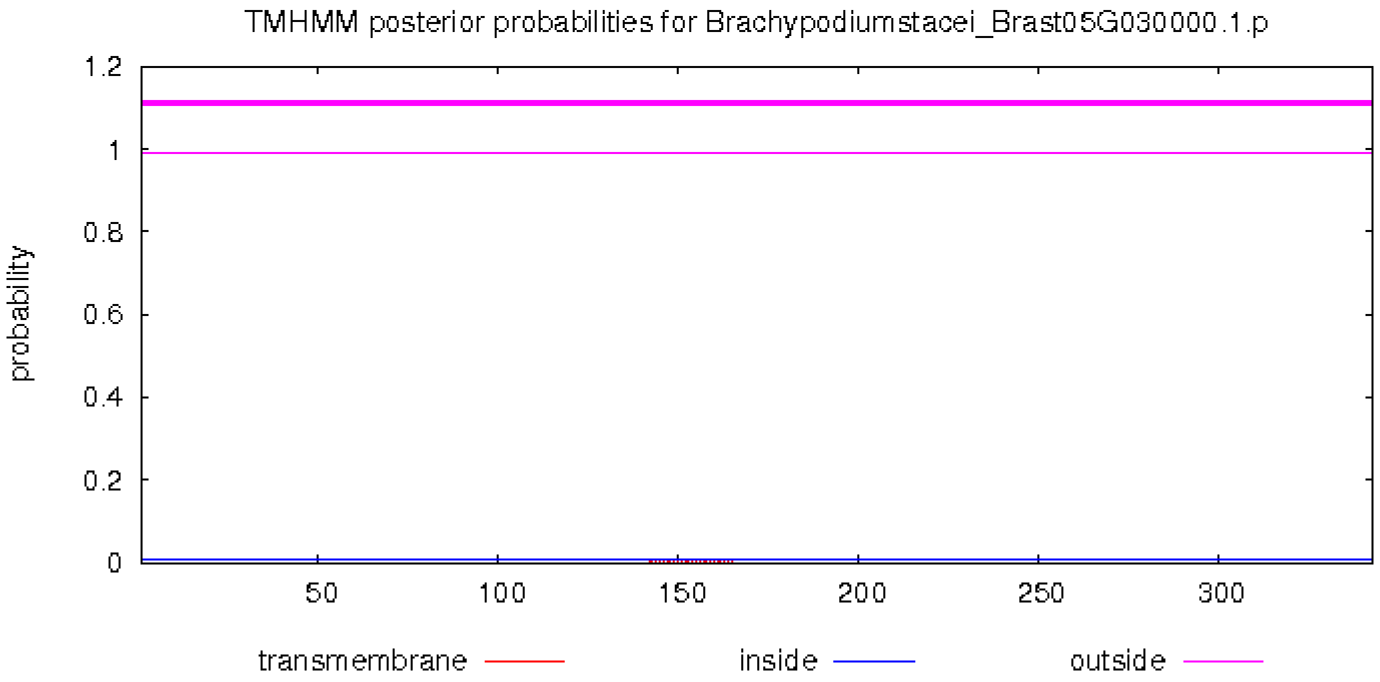

plot in postscript, script for making the plot in gnuplot, data for plot

---

```
# Brachypodiumstacei_Brast05G035500.1.p Length: 358
# Brachypodiumstacei_Brast05G035500.1.p Number of predicted TMHs:  0
# Brachypodiumstacei_Brast05G035500.1.p Exp number of AAs in TMHs: 0.00423
# Brachypodiumstacei_Brast05G035500.1.p Exp number, first 60 AAs:  0.0035
# Brachypodiumstacei_Brast05G035500.1.p Total prob of N-in:        0.01014
Brachypodiumstacei_Brast05G035500.1.p	TMHMM2.0	outside	     1   358
```

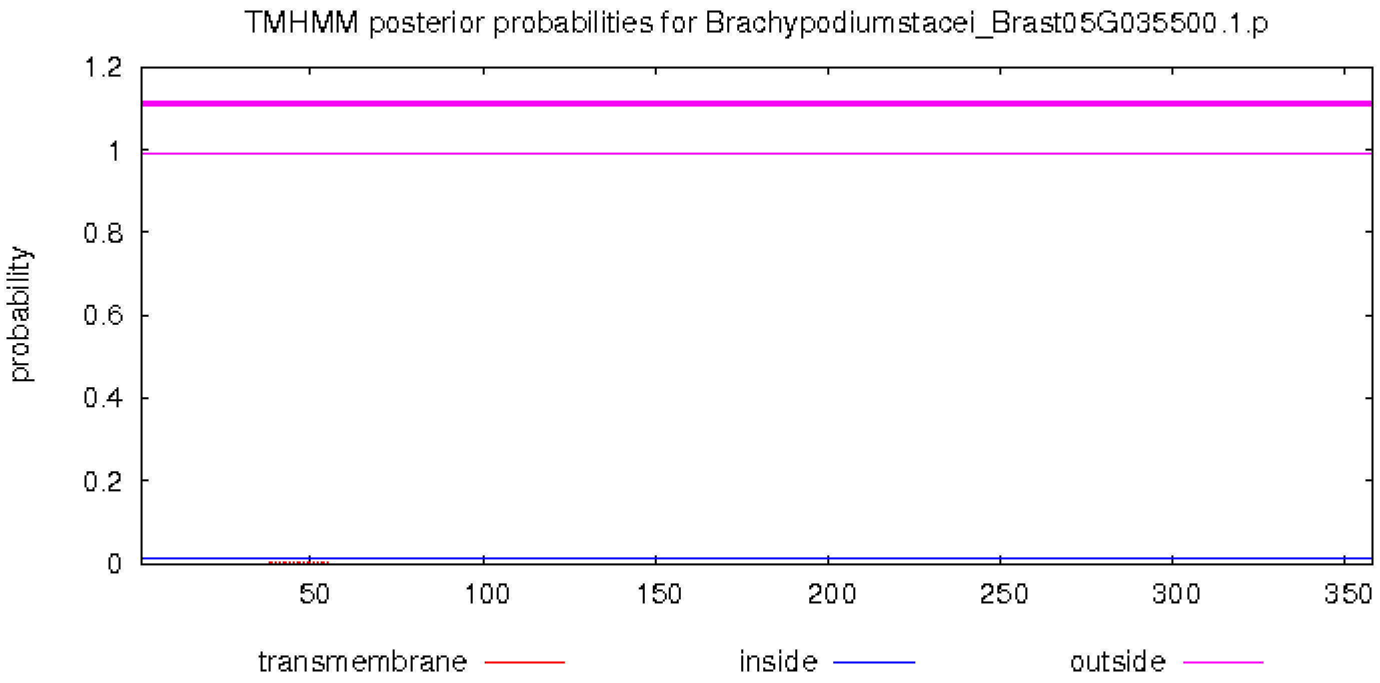

plot in postscript, script for making the plot in gnuplot, data for plot

---

```
# Brachypodiumstacei_Brast05G067100.1.p Length: 292
# Brachypodiumstacei_Brast05G067100.1.p Number of predicted TMHs:  0
# Brachypodiumstacei_Brast05G067100.1.p Exp number of AAs in TMHs: 0.0158
# Brachypodiumstacei_Brast05G067100.1.p Exp number, first 60 AAs:  0.00705
# Brachypodiumstacei_Brast05G067100.1.p Total prob of N-in:        0.00220
Brachypodiumstacei_Brast05G067100.1.p   TMHMM2.0        outside      1   292
```

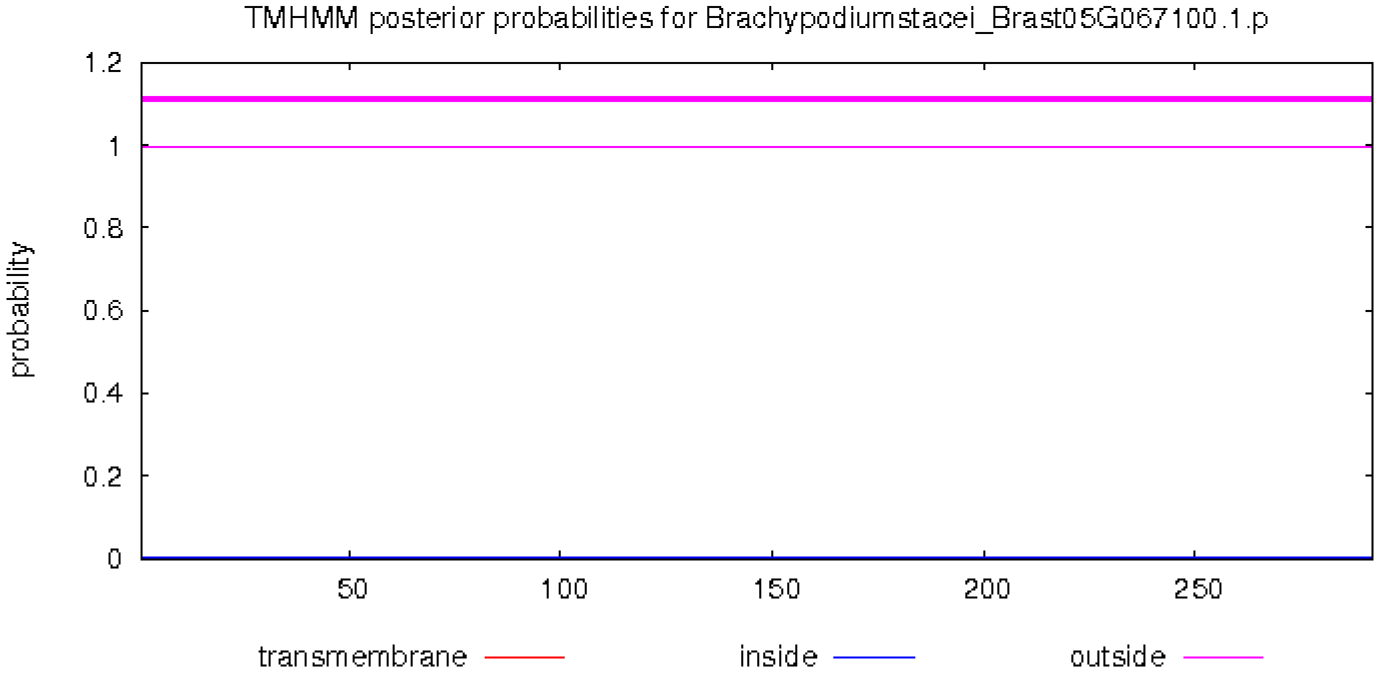

plot in postscript, script for making the plot in gnuplot, data for plot

```
# Brachypodiumstacei_Brast05G124400.1.p Length: 381
# Brachypodiumstacei_Brast05G124400.1.p Number of predicted TMHs:  0
# Brachypodiumstacei_Brast05G124400.1.p Exp number of AAs in TMHs: 0.00055
# Brachypodiumstacei_Brast05G124400.1.p Exp number, first 60 AAs:  0
# Brachypodiumstacei_Brast05G124400.1.p Total prob of N-in:        0.03552
Brachypodiumstacei_Brast05G124400.1.p   TMHMM2.0        outside      1   381
```

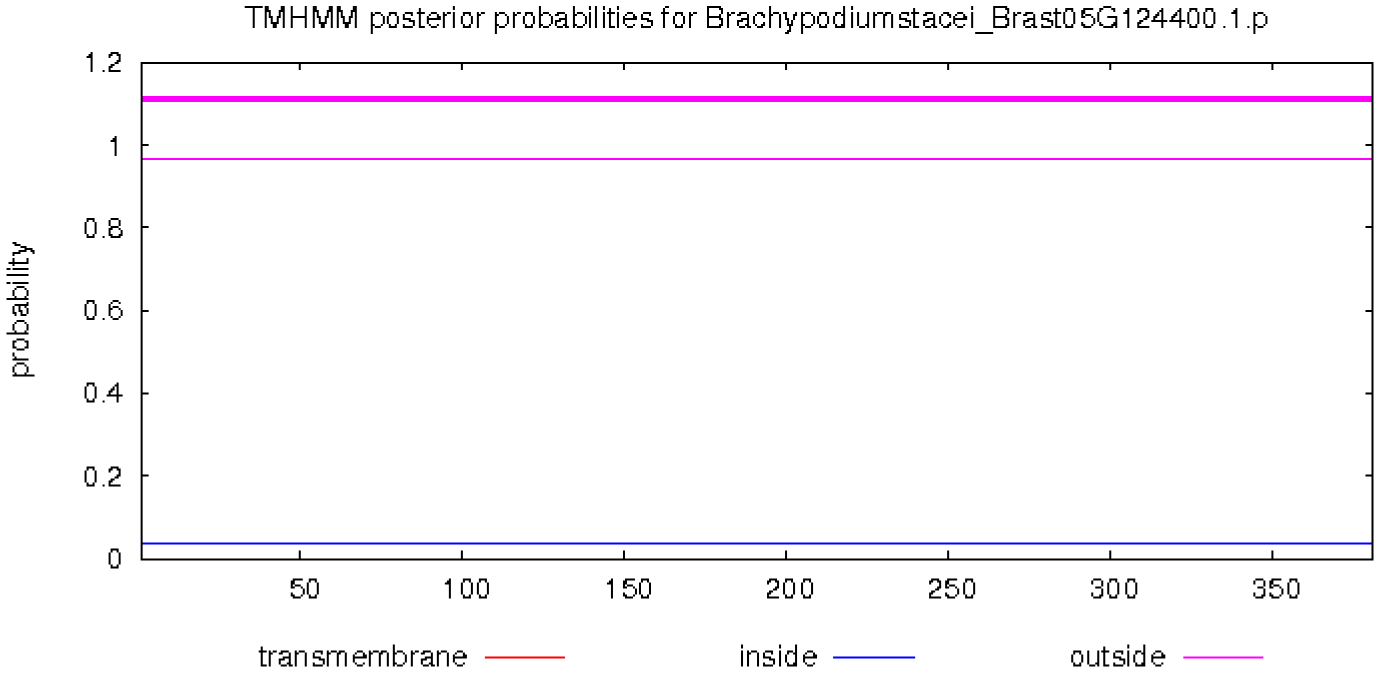

plot in postscript, script for making the plot in gnuplot, data for plot

```
# Brachypodiumstacei_Brast05G168300.1.p Length: 688
# Brachypodiumstacei_Brast05G168300.1.p Number of predicted TMHs:  1
# Brachypodiumstacei_Brast05G168300.1.p Exp number of AAs in TMHs: 21.2799
# Brachypodiumstacei_Brast05G168300.1.p Exp number, first 60 AAs:  0
# Brachypodiumstacei_Brast05G168300.1.p Total prob of N-in:        0.00192
Brachypodiumstacei_Brast05G168300.1.p   TMHMM2.0        outside      1   650
Brachypodiumstacei_Brast05G168300.1.p   TMHMM2.0        TMhelix    651   673
Brachypodiumstacei_Brast05G168300.1.p   TMHMM2.0        inside     674   688
```

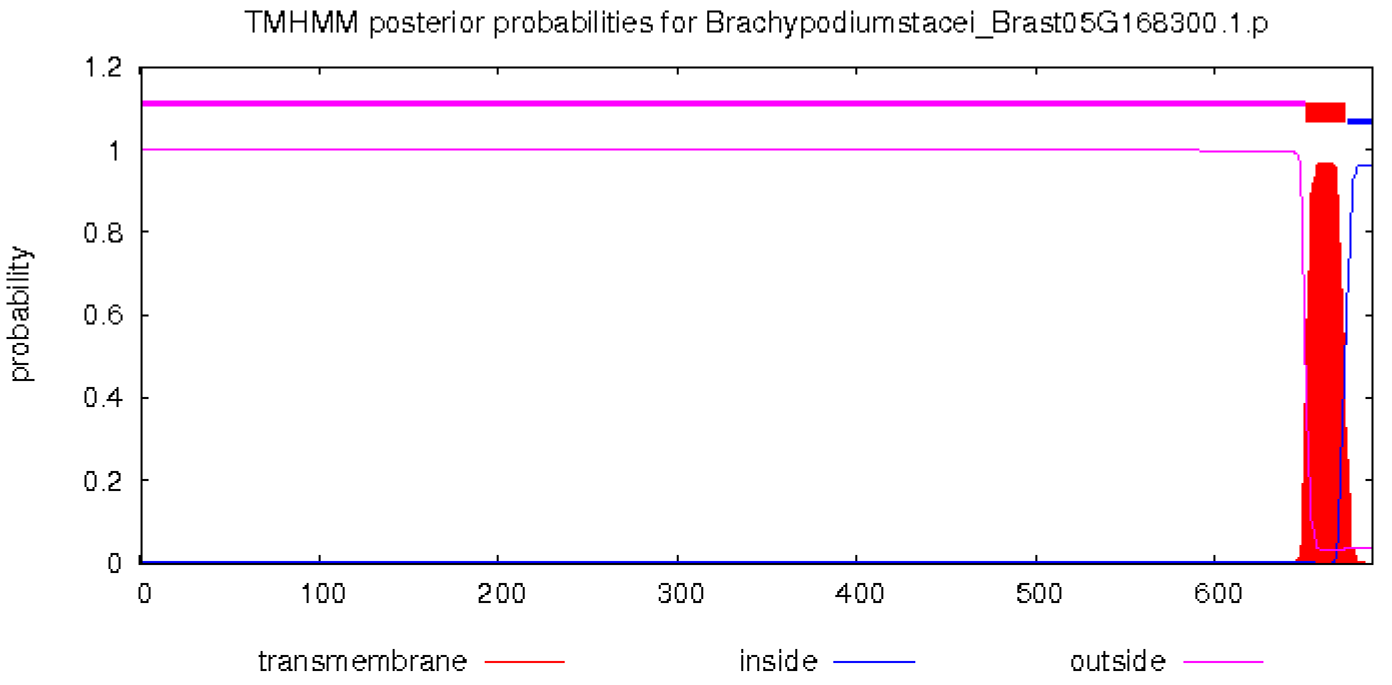

plot in postscript, script for making the plot in gnuplot, data for plot

---

```
# Brachypodiumstacei_Brast05G169900.1.p Length: 354
# Brachypodiumstacei_Brast05G169900.1.p Number of predicted TMHs:  0
# Brachypodiumstacei_Brast05G169900.1.p Exp number of AAs in TMHs: 0.00342
# Brachypodiumstacei_Brast05G169900.1.p Exp number, first 60 AAs:  0
# Brachypodiumstacei_Brast05G169900.1.p Total prob of N-in:        0.00217
Brachypodiumstacei_Brast05G169900.1.p   TMHMM2.0        outside      1   354
```

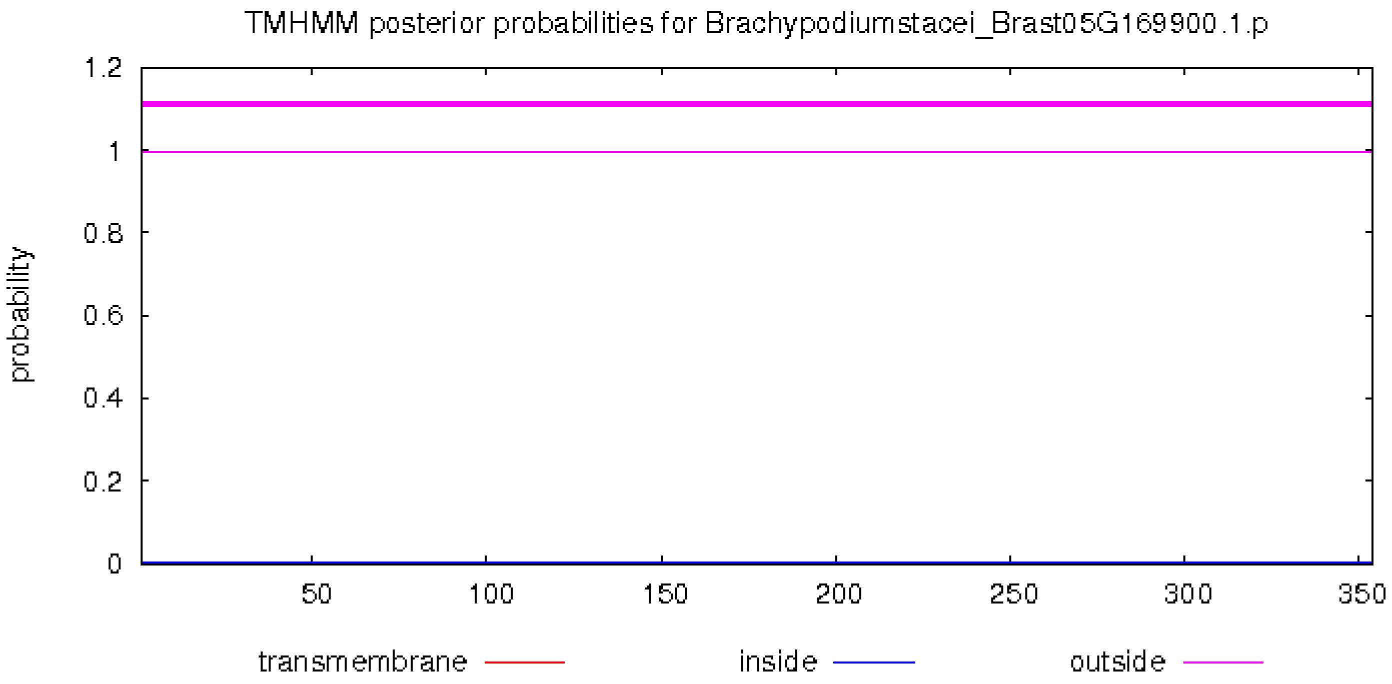

plot in postscript, script for making the plot in gnuplot, data for plot

```
# Brachypodiumstacei_Brast05G177000.1.p Length: 229
# Brachypodiumstacei_Brast05G177000.1.p Number of predicted TMHs:  0
# Brachypodiumstacei_Brast05G177000.1.p Exp number of AAs in TMHs: 0.06733
# Brachypodiumstacei_Brast05G177000.1.p Exp number, first 60 AAs:  0.06206
# Brachypodiumstacei_Brast05G177000.1.p Total prob of N-in:        0.07895
Brachypodiumstacei_Brast05G177000.1.p	TMHMM2.0	outside	     1   229
```

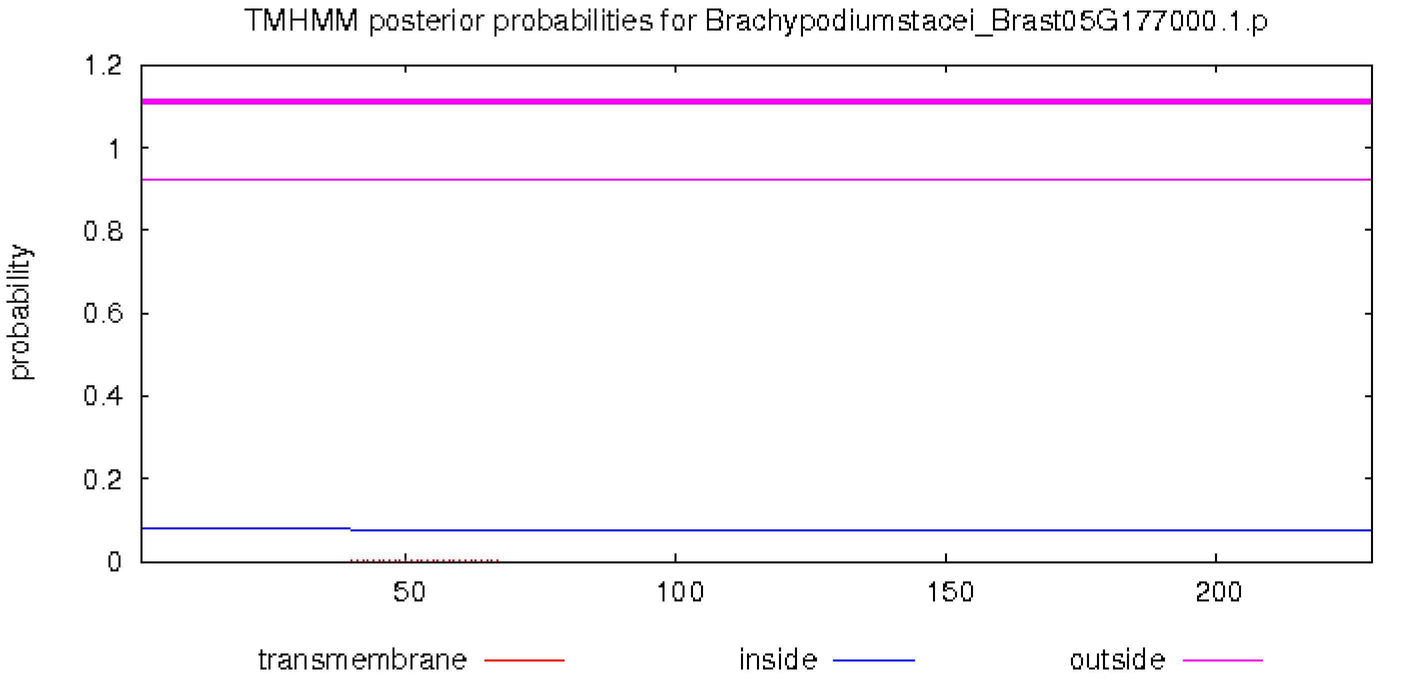

plot in postscript, script for making the plot in gnuplot, data for plot

```
# Brachypodiumstacei_Brast05G207900.1.p Length: 251
# Brachypodiumstacei_Brast05G207900.1.p Number of predicted TMHs:  0
# Brachypodiumstacei_Brast05G207900.1.p Exp number of AAs in TMHs: 0.00015
# Brachypodiumstacei_Brast05G207900.1.p Exp number, first 60 AAs:  0
# Brachypodiumstacei_Brast05G207900.1.p Total prob of N-in:        0.00199
Brachypodiumstacei_Brast05G207900.1.p	TMHMM2.0	outside	     1   251
```

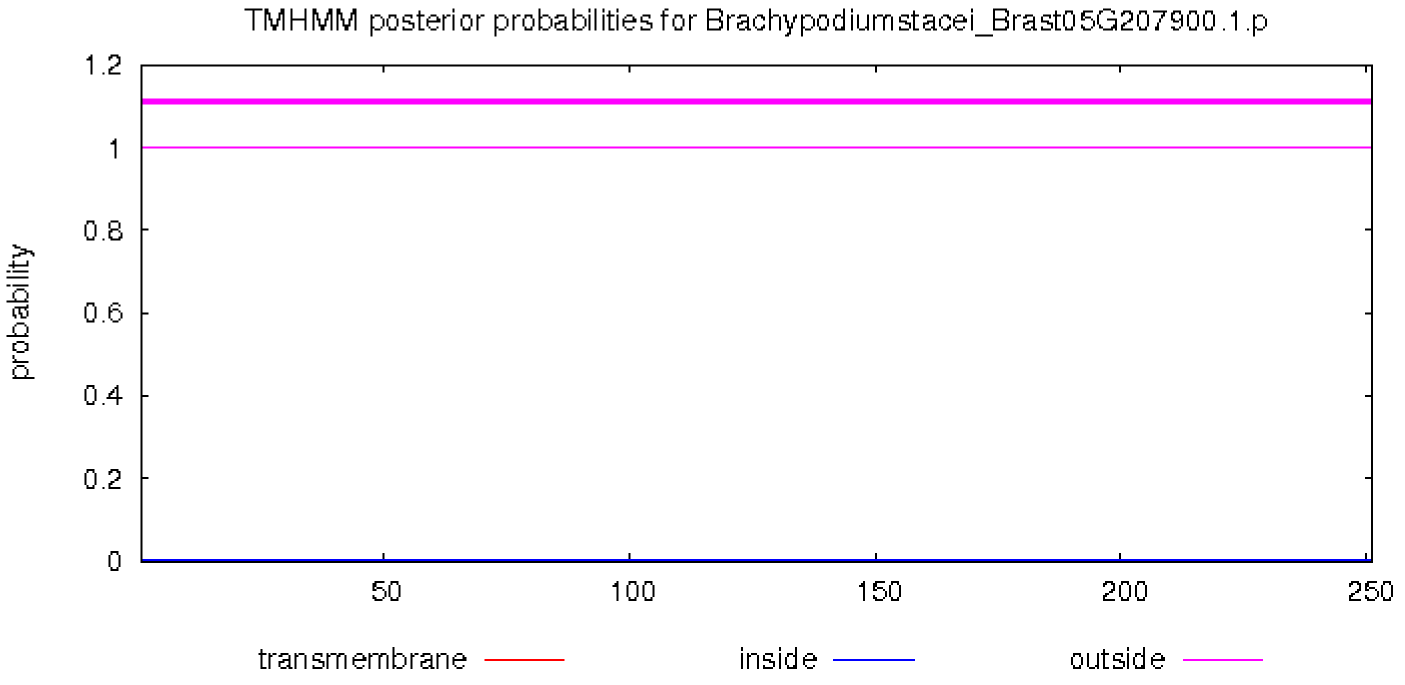

plot in postscript, script for making the plot in gnuplot, data for plot

```
# Brachypodiumstacei_Brast05G208100.1.p Length: 217
# Brachypodiumstacei_Brast05G208100.1.p Number of predicted TMHs:  0
# Brachypodiumstacei_Brast05G208100.1.p Exp number of AAs in TMHs: 0
# Brachypodiumstacei_Brast05G208100.1.p Exp number, first 60 AAs:  0
# Brachypodiumstacei_Brast05G208100.1.p Total prob of N-in:        0.00428
Brachypodiumstacei_Brast05G208100.1.p	TMHMM2.0	outside	     1   217
```

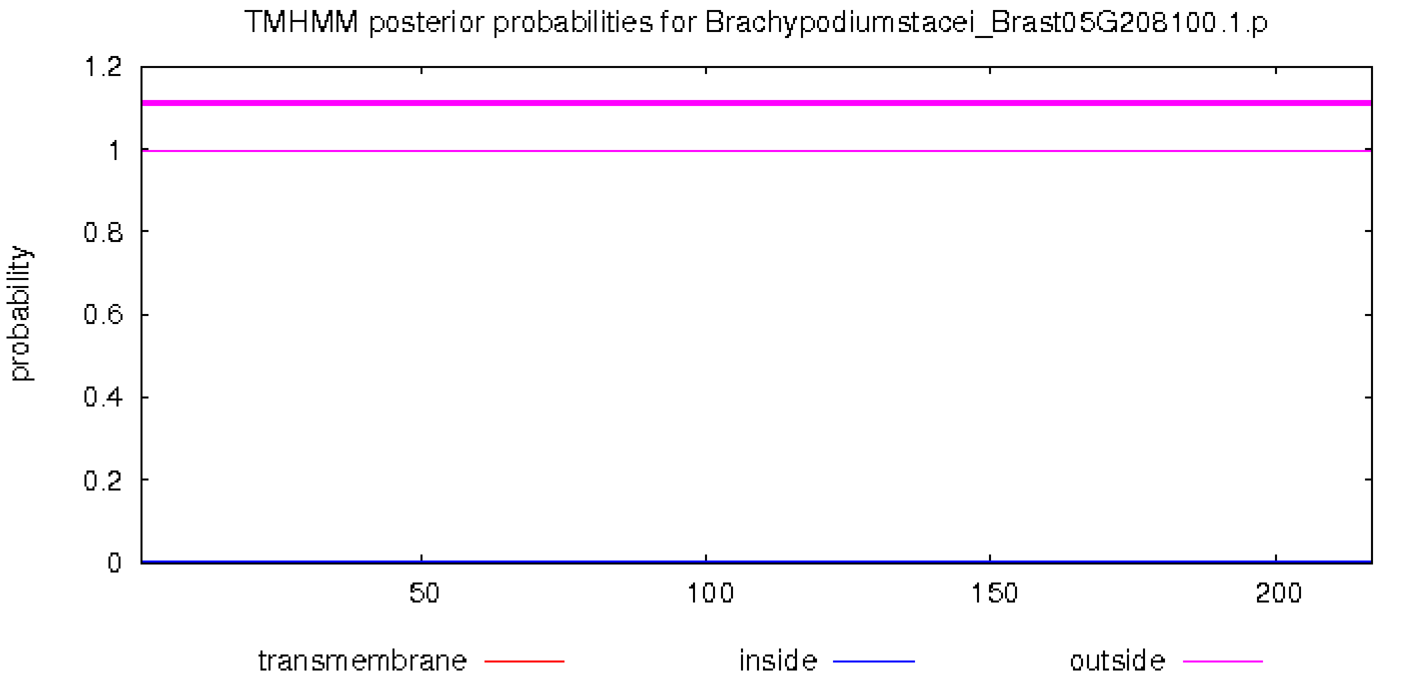

plot in postscript, script for making the plot in gnuplot, data for plot

---

```
# Brachypodiumstacei_Brast05G253000.1.p Length: 452
# Brachypodiumstacei_Brast05G253000.1.p Number of predicted TMHs:  0
# Brachypodiumstacei_Brast05G253000.1.p Exp number of AAs in TMHs: 2.16523
# Brachypodiumstacei_Brast05G253000.1.p Exp number, first 60 AAs:  2.144
# Brachypodiumstacei_Brast05G253000.1.p Total prob of N-in:        0.17727
Brachypodiumstacei_Brast05G253000.1.p	TMHMM2.0	outside	     1   452
```

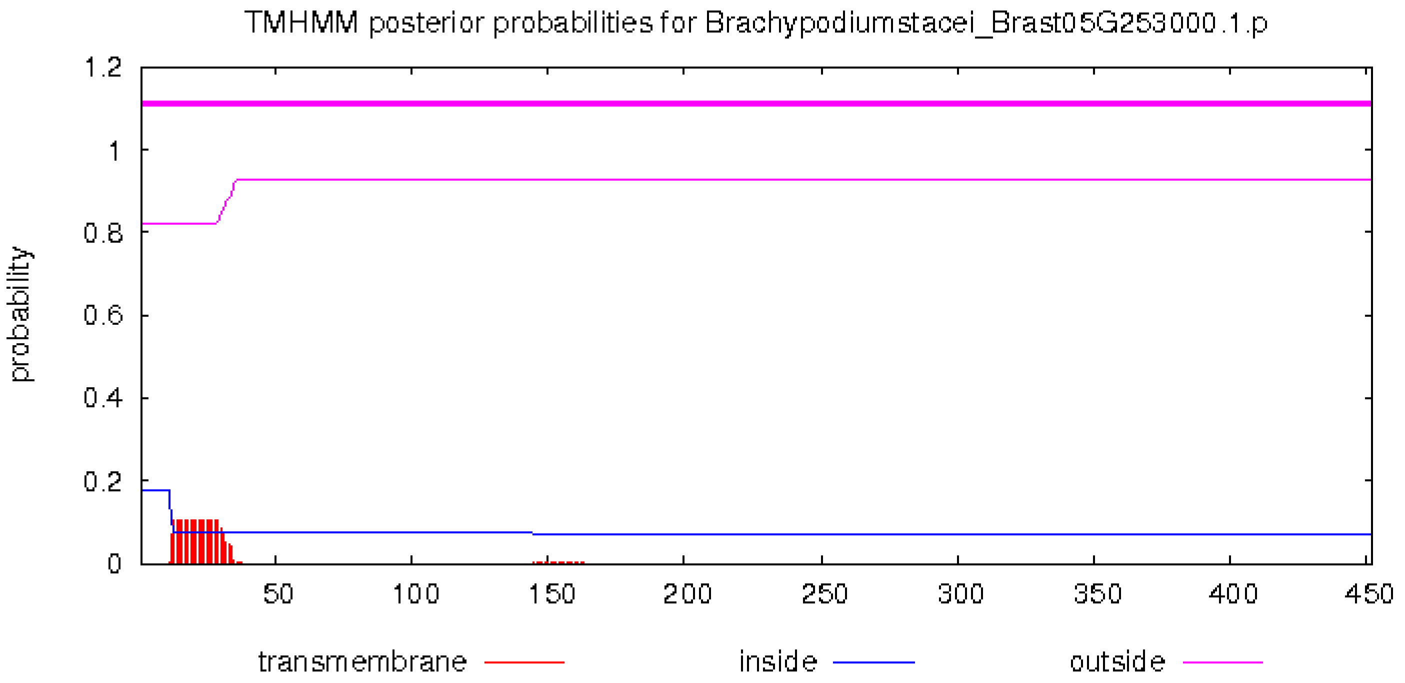

plot in postscript, script for making the plot in gnuplot, data for plot

---

```
# Brachypodiumstacei_Brast05G265600.1.p Length: 253
# Brachypodiumstacei_Brast05G265600.1.p Number of predicted TMHs:  0
# Brachypodiumstacei_Brast05G265600.1.p Exp number of AAs in TMHs: 0.00192
# Brachypodiumstacei_Brast05G265600.1.p Exp number, first 60 AAs:  0.00021
# Brachypodiumstacei_Brast05G265600.1.p Total prob of N-in:        0.11625
Brachypodiumstacei_Brast05G265600.1.p	TMHMM2.0	outside	     1   253
```

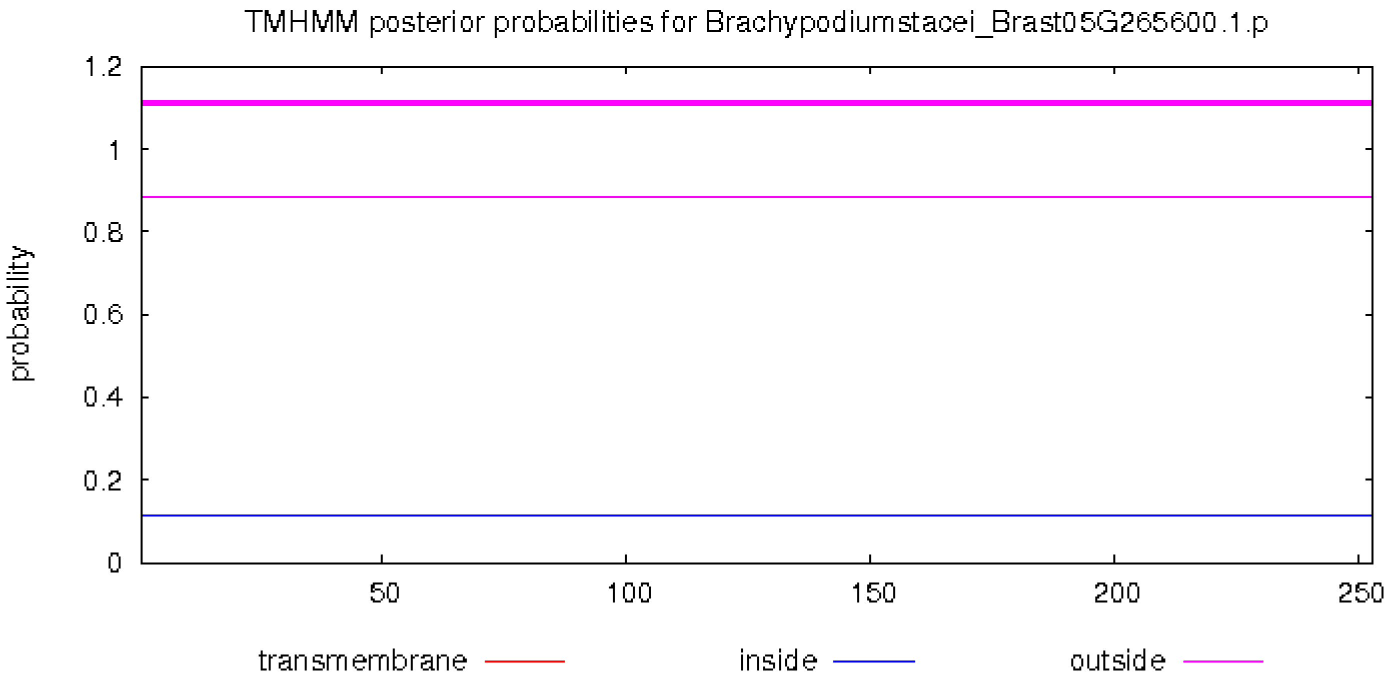

plot in postscript, script for making the plot in gnuplot, data for plot

```
# Brachypodiumstacei_Brast05G275400.1.p Length: 395
# Brachypodiumstacei_Brast05G275400.1.p Number of predicted TMHs:  0
# Brachypodiumstacei_Brast05G275400.1.p Exp number of AAs in TMHs: 0.01249
# Brachypodiumstacei_Brast05G275400.1.p Exp number, first 60 AAs:  0.01249
# Brachypodiumstacei_Brast05G275400.1.p Total prob of N-in:        0.00191
Brachypodiumstacei_Brast05G275400.1.p	TMHMM2.0	outside	     1   395
```

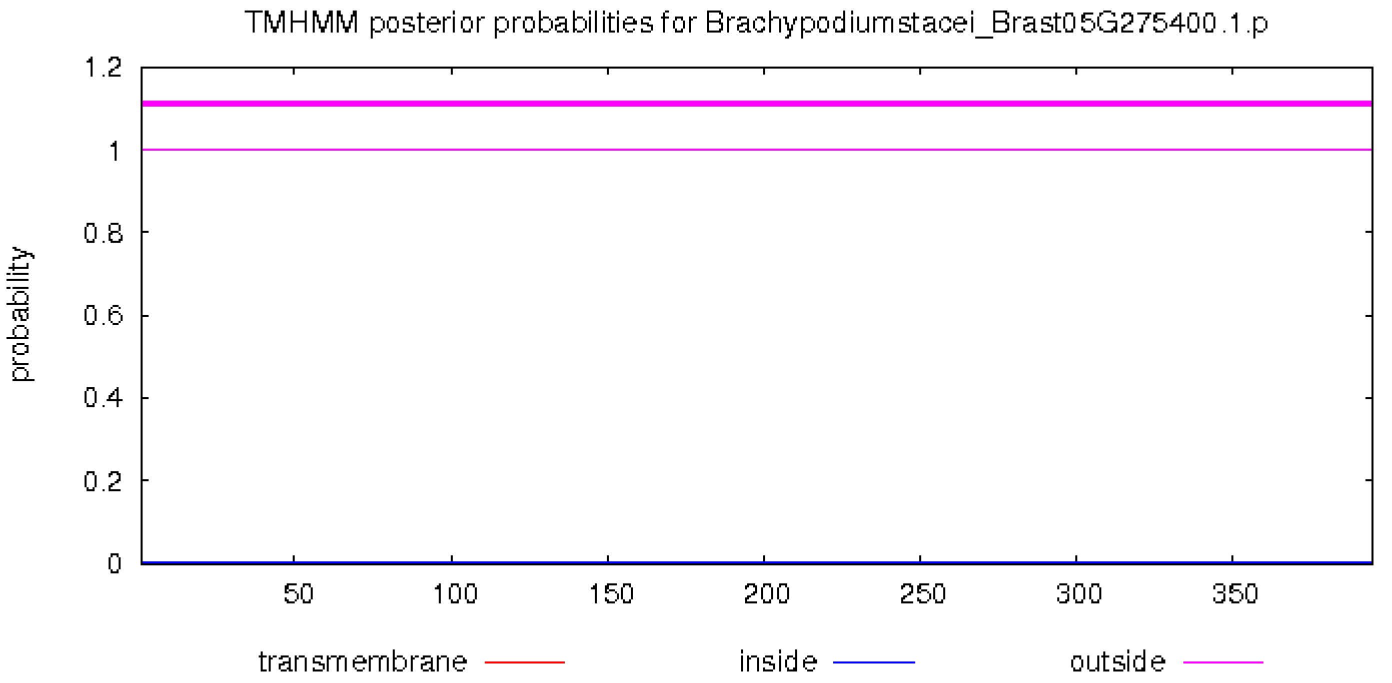

plot in postscript, script for making the plot in gnuplot, data for plot

```
# Brachypodiumstacei_Brast05G276000.1.p Length: 365
# Brachypodiumstacei_Brast05G276000.1.p Number of predicted TMHs:  0
# Brachypodiumstacei_Brast05G276000.1.p Exp number of AAs in TMHs: 0.01267
# Brachypodiumstacei_Brast05G276000.1.p Exp number, first 60 AAs:  0.01267
# Brachypodiumstacei_Brast05G276000.1.p Total prob of N-in:        0.00257
Brachypodiumstacei_Brast05G276000.1.p	TMHMM2.0	outside	     1   365
```

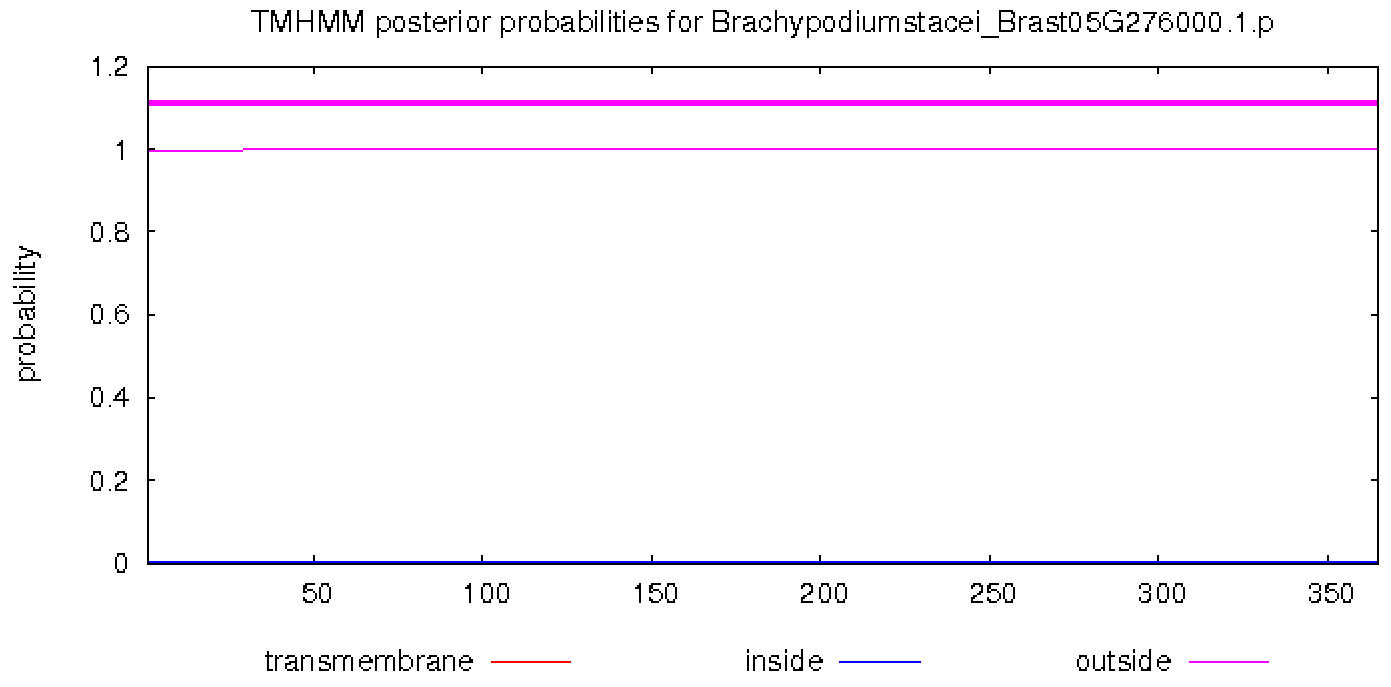

plot in postscript, script for making the plot in gnuplot, data for plot

---

```
# Brachypodiumstacei_Brast05G276100.1.p Length: 368
# Brachypodiumstacei_Brast05G276100.1.p Number of predicted TMHs:  0
# Brachypodiumstacei_Brast05G276100.1.p Exp number of AAs in TMHs: 0.00312
# Brachypodiumstacei_Brast05G276100.1.p Exp number, first 60 AAs:  0.00312
# Brachypodiumstacei_Brast05G276100.1.p Total prob of N-in:        0.00471
Brachypodiumstacei_Brast05G276100.1.p	TMHMM2.0	outside	     1   368
```

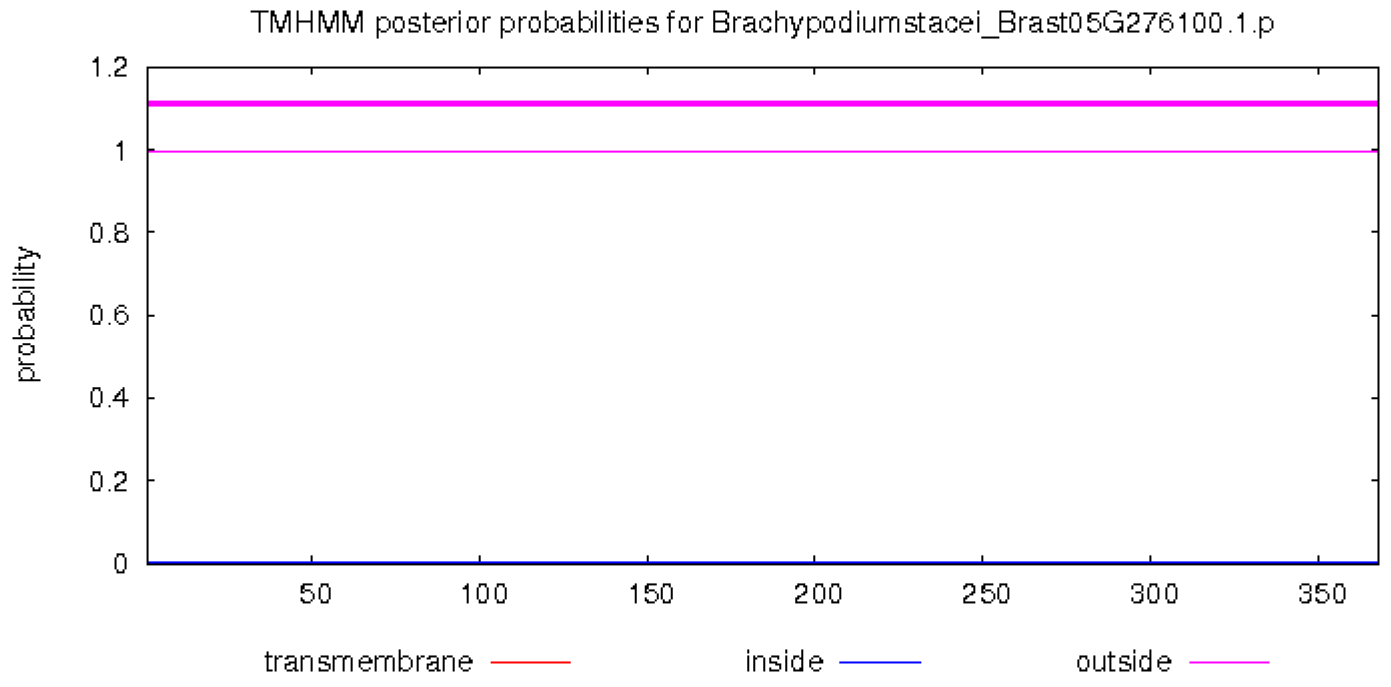

plot in postscript, script for making the plot in gnuplot, data for plot

---

```
# Brachypodiumstacei_Brast05G280400.1.p Length: 386
# Brachypodiumstacei_Brast05G280400.1.p Number of predicted TMHs:  0
# Brachypodiumstacei_Brast05G280400.1.p Exp number of AAs in TMHs: 0.01254
# Brachypodiumstacei_Brast05G280400.1.p Exp number, first 60 AAs:  0.01254
# Brachypodiumstacei_Brast05G280400.1.p Total prob of N-in:        0.00208
Brachypodiumstacei_Brast05G280400.1.p   TMHMM2.0        outside      1   386
```

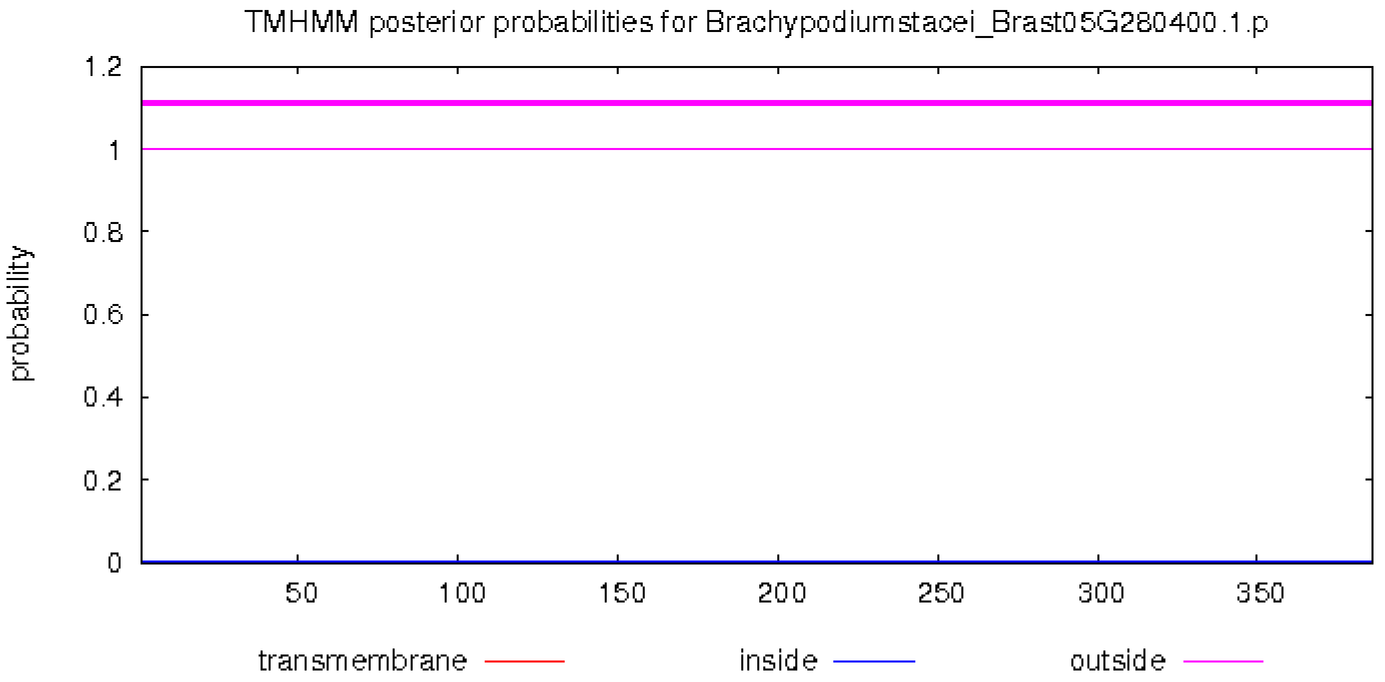

plot in postscript, script for making the plot in gnuplot, data for plot

---

```
# Brachypodiumstacei_Brast05G287500.1.p Length: 377
# Brachypodiumstacei_Brast05G287500.1.p Number of predicted TMHs:  0
# Brachypodiumstacei_Brast05G287500.1.p Exp number of AAs in TMHs: 0.15387
# Brachypodiumstacei_Brast05G287500.1.p Exp number, first 60 AAs:  0.01783
# Brachypodiumstacei_Brast05G287500.1.p Total prob of N-in:        0.01208
Brachypodiumstacei_Brast05G287500.1.p   TMHMM2.0        outside      1   377
```

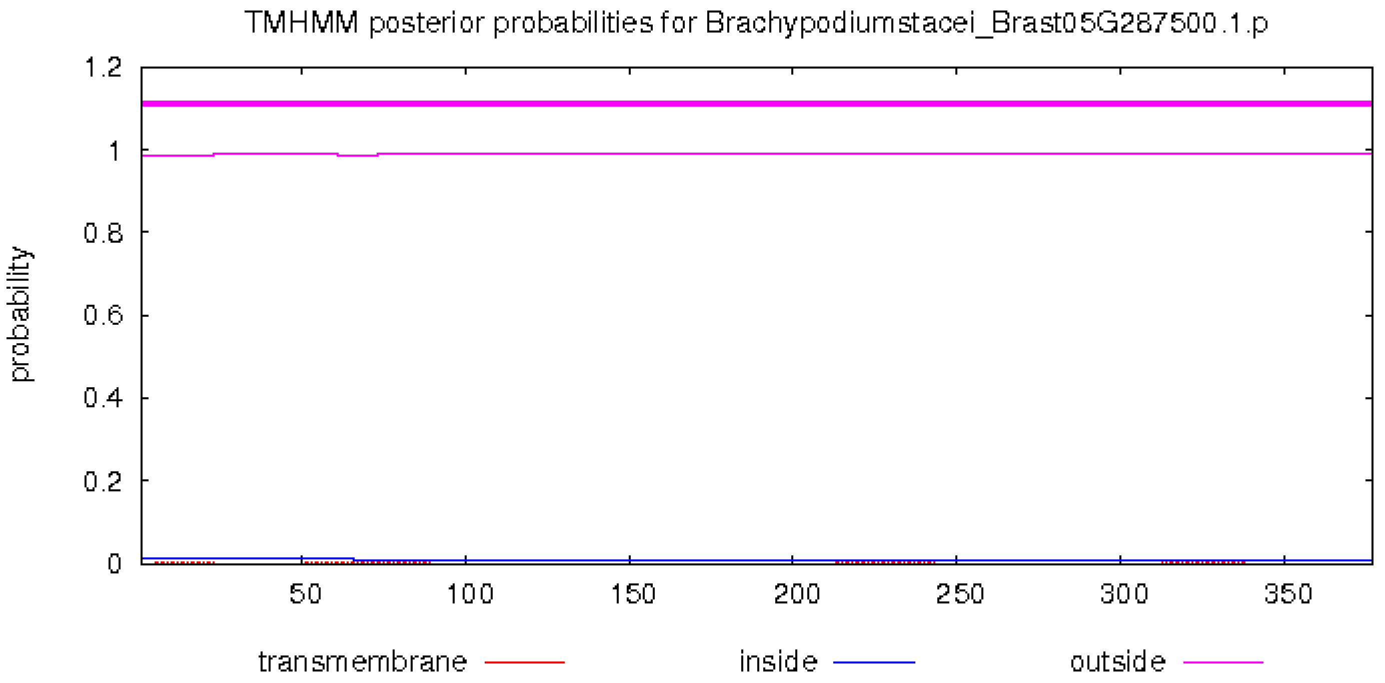

plot in postscript, script for making the plot in gnuplot, data for plot

---

```
# Brachypodiumstacei_Brast05G287600.1.p Length: 396
# Brachypodiumstacei_Brast05G287600.1.p Number of predicted TMHs:  0
# Brachypodiumstacei_Brast05G287600.1.p Exp number of AAs in TMHs: 0.001
# Brachypodiumstacei_Brast05G287600.1.p Exp number, first 60 AAs:  0
# Brachypodiumstacei_Brast05G287600.1.p Total prob of N-in:        0.00525
Brachypodiumstacei_Brast05G287600.1.p   TMHMM2.0        outside      1   396
```

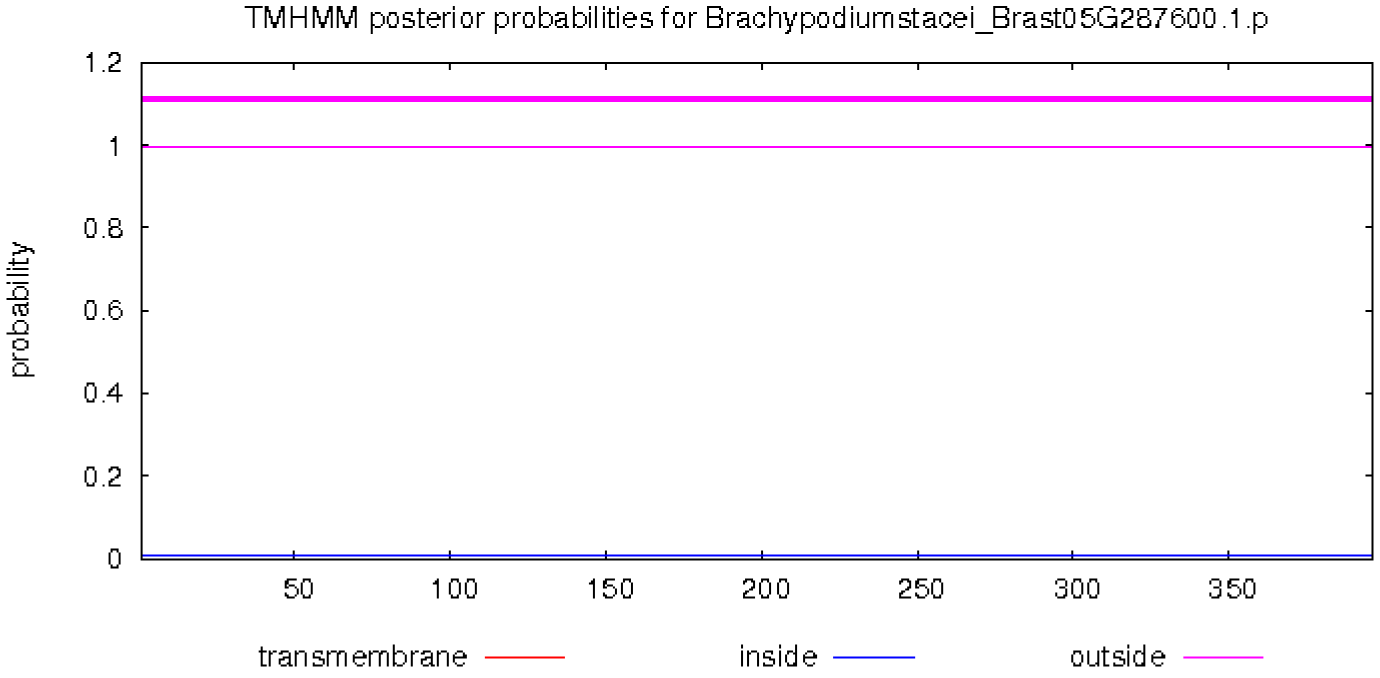

plot in postscript, script for making the plot in gnuplot, data for plot

```
# Brachypodiumstacei_Brast06G009100.1.p Length: 293
# Brachypodiumstacei_Brast06G009100.1.p Number of predicted TMHs:  0
# Brachypodiumstacei_Brast06G009100.1.p Exp number of AAs in TMHs: 0.0035
# Brachypodiumstacei_Brast06G009100.1.p Exp number, first 60 AAs:  0
# Brachypodiumstacei_Brast06G009100.1.p Total prob of N-in:        0.02918
Brachypodiumstacei_Brast06G009100.1.p   TMHMM2.0        outside      1   293
```

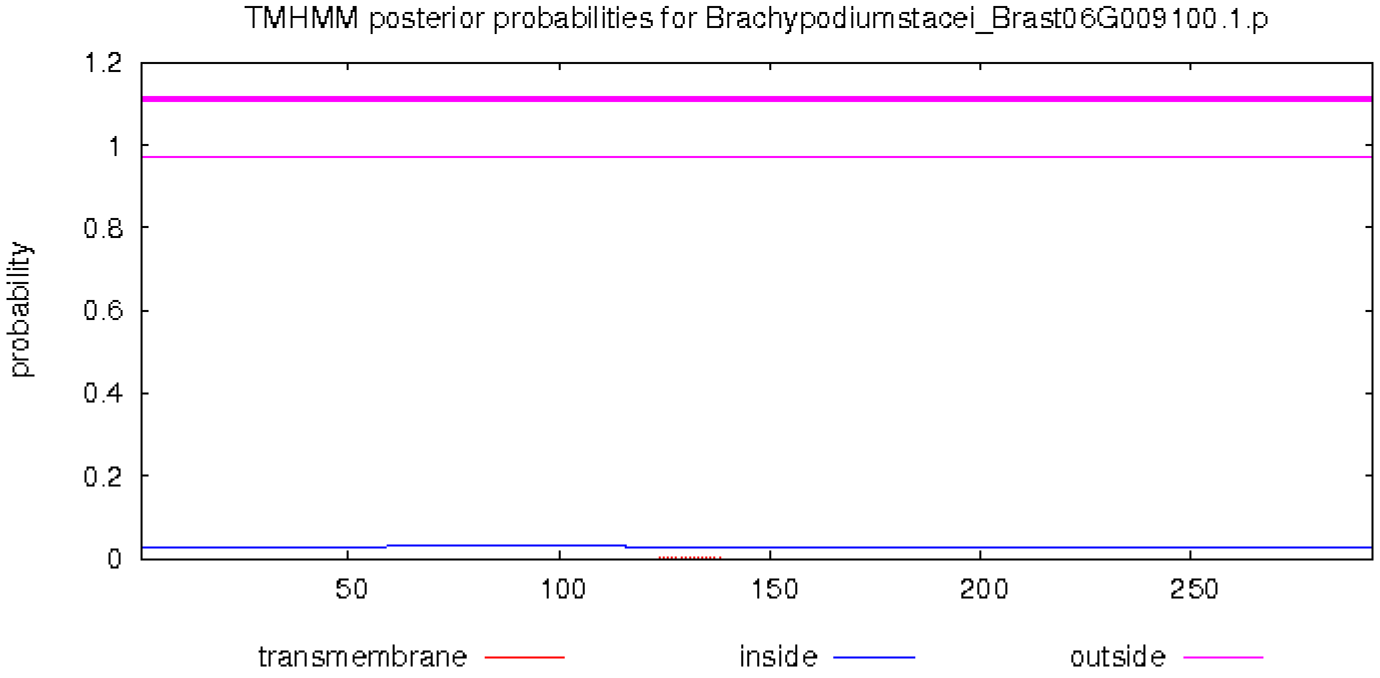

plot in postscript, script for making the plot in gnuplot, data for plot

```
# Brachypodiumstacei_Brast06G009400.1.p Length: 365
# Brachypodiumstacei_Brast06G009400.1.p Number of predicted TMHs:  0
# Brachypodiumstacei_Brast06G009400.1.p Exp number of AAs in TMHs: 0.00092
# Brachypodiumstacei_Brast06G009400.1.p Exp number, first 60 AAs:  0
# Brachypodiumstacei_Brast06G009400.1.p Total prob of N-in:        0.00282
Brachypodiumstacei_Brast06G009400.1.p	TMHMM2.0	outside	     1   365
```

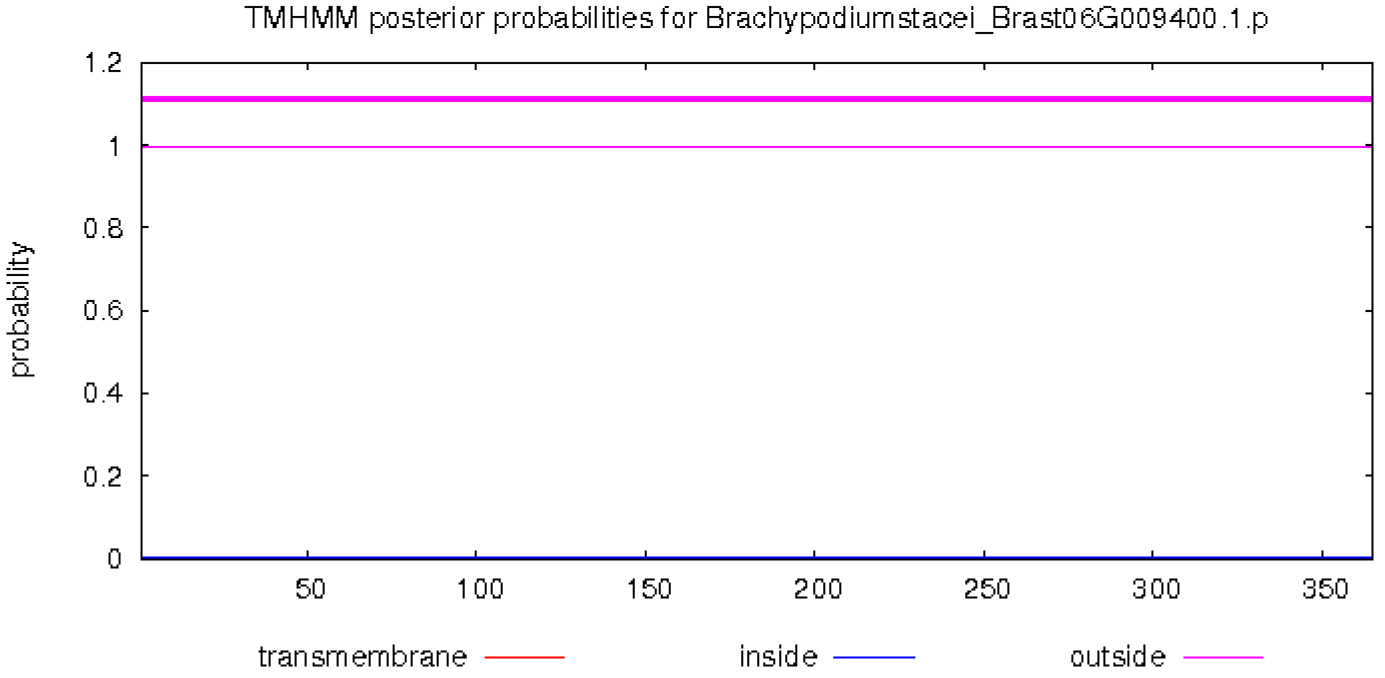

plot in postscript, script for making the plot in gnuplot, data for plot

---

```
# Brachypodiumstacei_Brast06G038200.1.p Length: 345
# Brachypodiumstacei_Brast06G038200.1.p Number of predicted TMHs:  0
# Brachypodiumstacei_Brast06G038200.1.p Exp number of AAs in TMHs: 0.00106
# Brachypodiumstacei_Brast06G038200.1.p Exp number, first 60 AAs:  0.00088
# Brachypodiumstacei_Brast06G038200.1.p Total prob of N-in:        0.02416
Brachypodiumstacei_Brast06G038200.1.p	TMHMM2.0	outside	     1   345
```

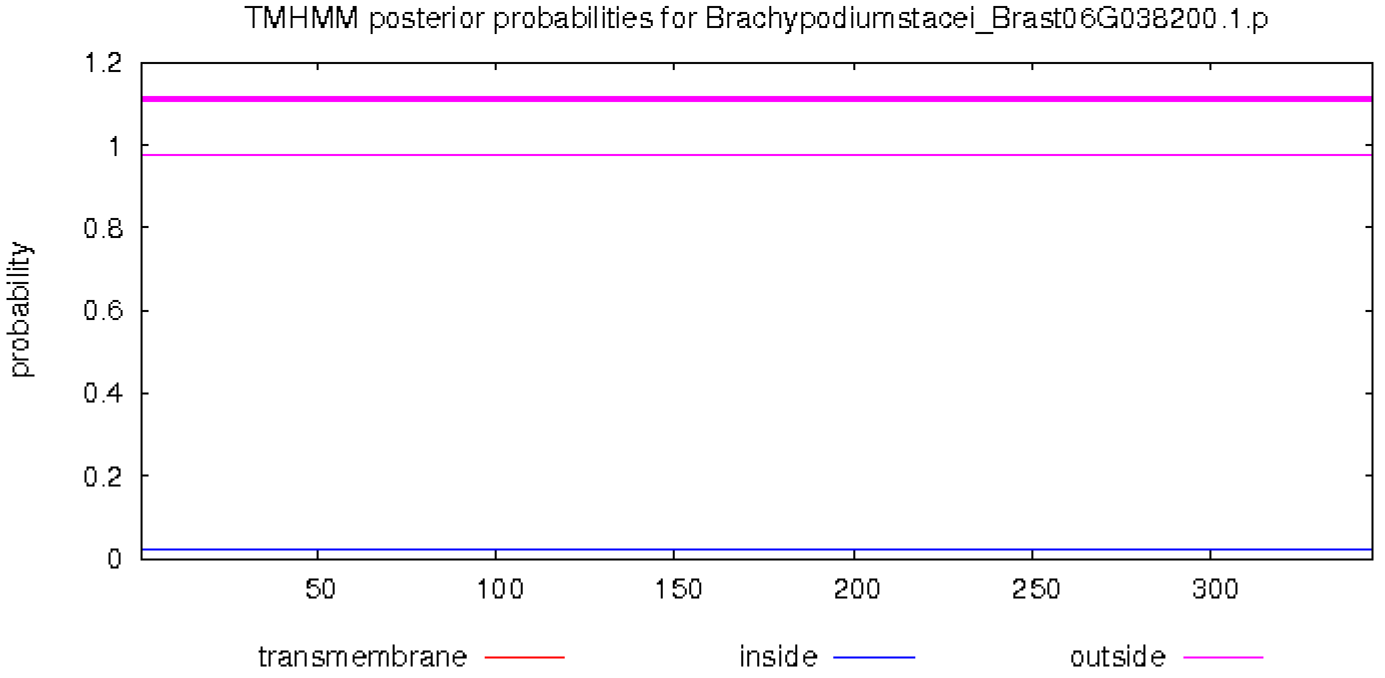

plot in postscript, script for making the plot in gnuplot, data for plot

---

```
# Brachypodiumstacei_Brast06G046700.1.p Length: 314
# Brachypodiumstacei_Brast06G046700.1.p Number of predicted TMHs:  0
# Brachypodiumstacei_Brast06G046700.1.p Exp number of AAs in TMHs: 0.02635
# Brachypodiumstacei_Brast06G046700.1.p Exp number, first 60 AAs:  0.00023
# Brachypodiumstacei_Brast06G046700.1.p Total prob of N-in:        0.04895
Brachypodiumstacei_Brast06G046700.1.p   TMHMM2.0        outside      1   314
```

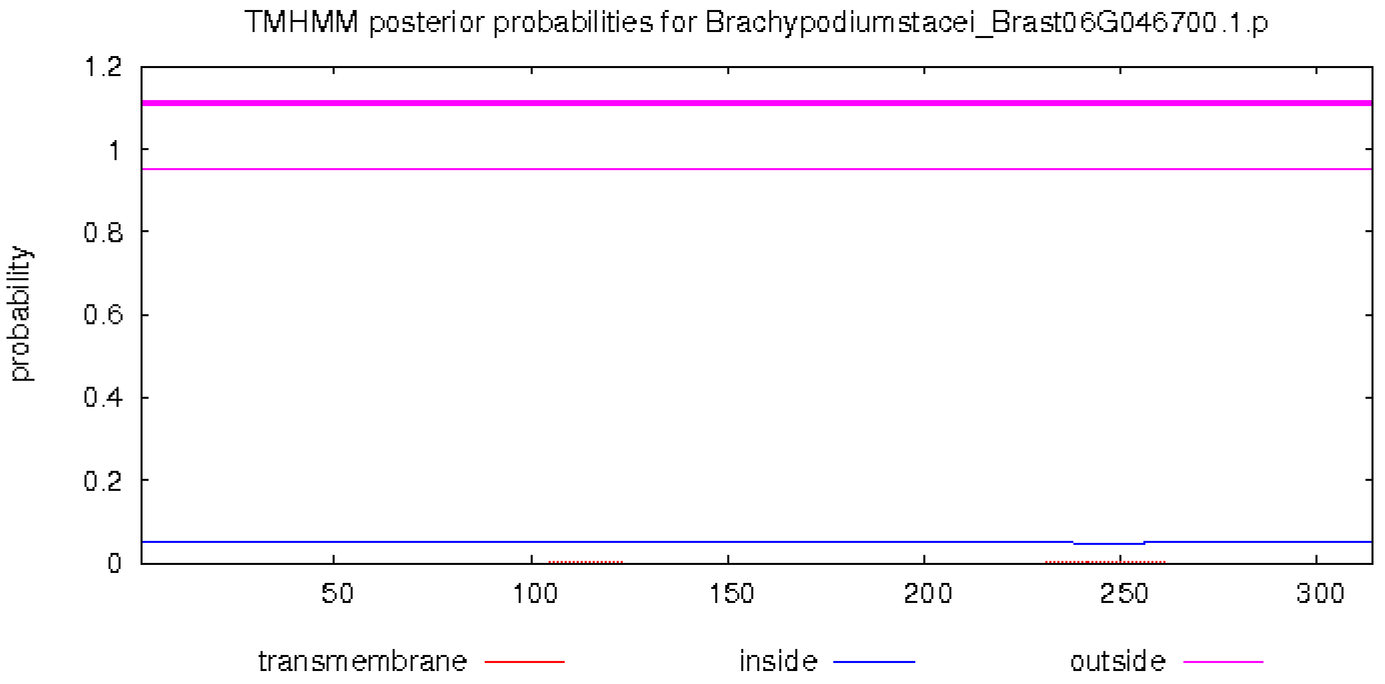

plot in postscript, script for making the plot in gnuplot, data for plot

---

```
# Brachypodiumstacei_Brast06G046900.1.p Length: 316
# Brachypodiumstacei_Brast06G046900.1.p Number of predicted TMHs:  0
# Brachypodiumstacei_Brast06G046900.1.p Exp number of AAs in TMHs: 0.10383
# Brachypodiumstacei_Brast06G046900.1.p Exp number, first 60 AAs:  0
# Brachypodiumstacei_Brast06G046900.1.p Total prob of N-in:        0.05694
Brachypodiumstacei_Brast06G046900.1.p   TMHMM2.0        outside      1   316
```

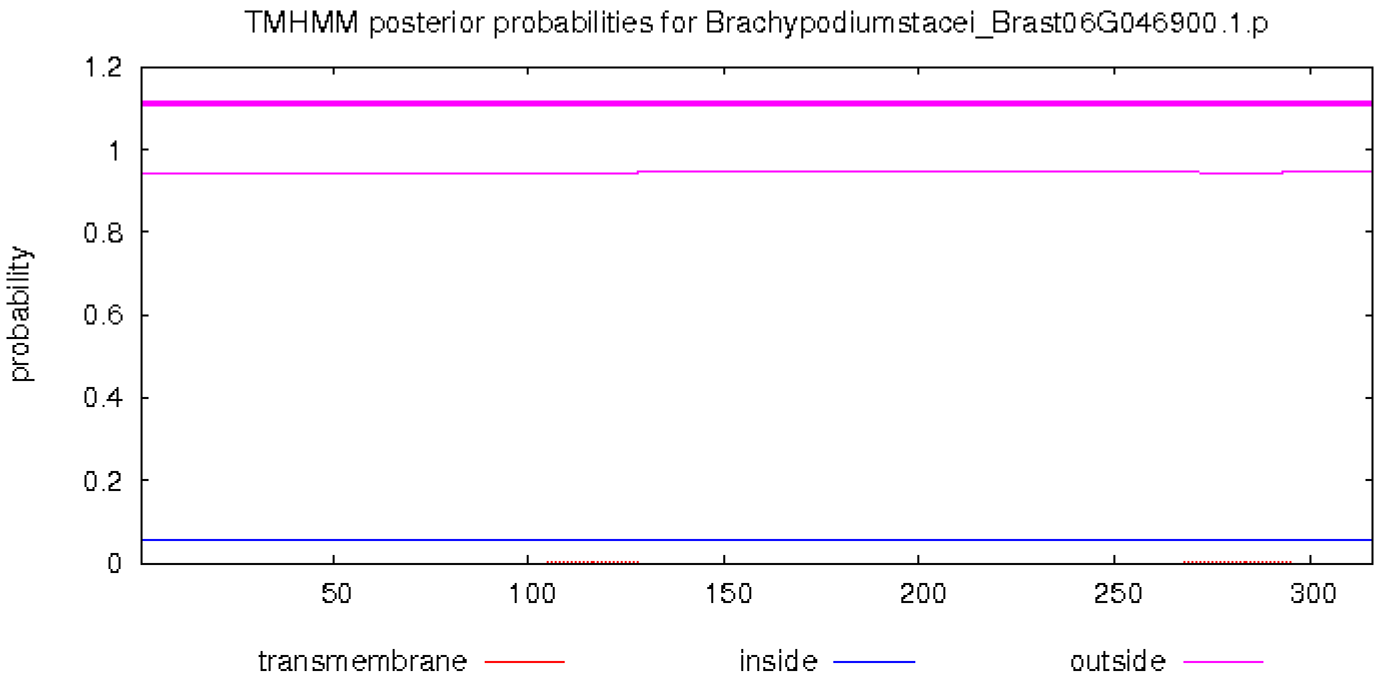

plot in postscript, script for making the plot in gnuplot, data for plot

---

```
# Brachypodiumstacei_Brast06G088900.1.p Length: 432
# Brachypodiumstacei_Brast06G088900.1.p Number of predicted TMHs:  0
# Brachypodiumstacei_Brast06G088900.1.p Exp number of AAs in TMHs: 0.00501
# Brachypodiumstacei_Brast06G088900.1.p Exp number, first 60 AAs:  0
# Brachypodiumstacei_Brast06G088900.1.p Total prob of N-in:        0.00326
Brachypodiumstacei_Brast06G088900.1.p   TMHMM2.0        outside      1   432
```

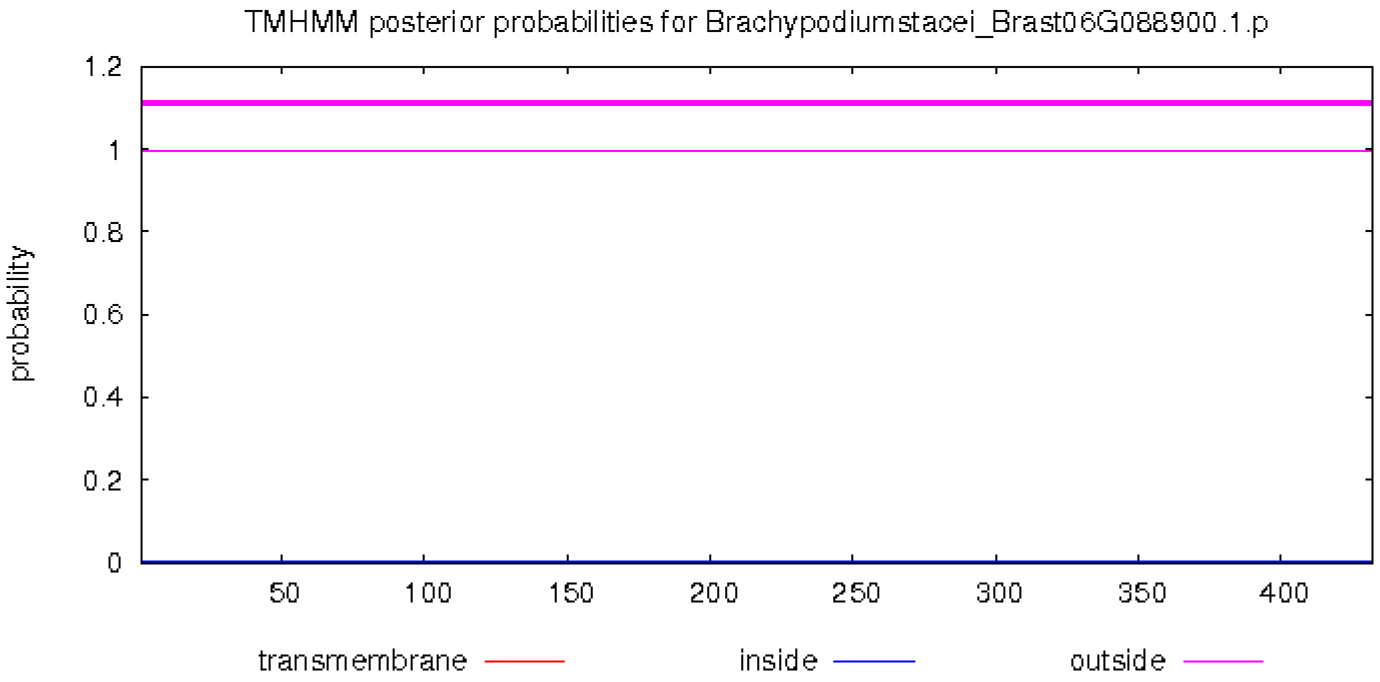

plot in postscript, script for making the plot in gnuplot, data for plot

---

```
# Brachypodiumstacei_Brast06G167100.1.p Length: 285
# Brachypodiumstacei_Brast06G167100.1.p Number of predicted TMHs:  0
# Brachypodiumstacei_Brast06G167100.1.p Exp number of AAs in TMHs: 0.12242
# Brachypodiumstacei_Brast06G167100.1.p Exp number, first 60 AAs:  0.12116
# Brachypodiumstacei_Brast06G167100.1.p Total prob of N-in:        0.13720
Brachypodiumstacei_Brast06G167100.1.p   TMHMM2.0        outside      1   285
```

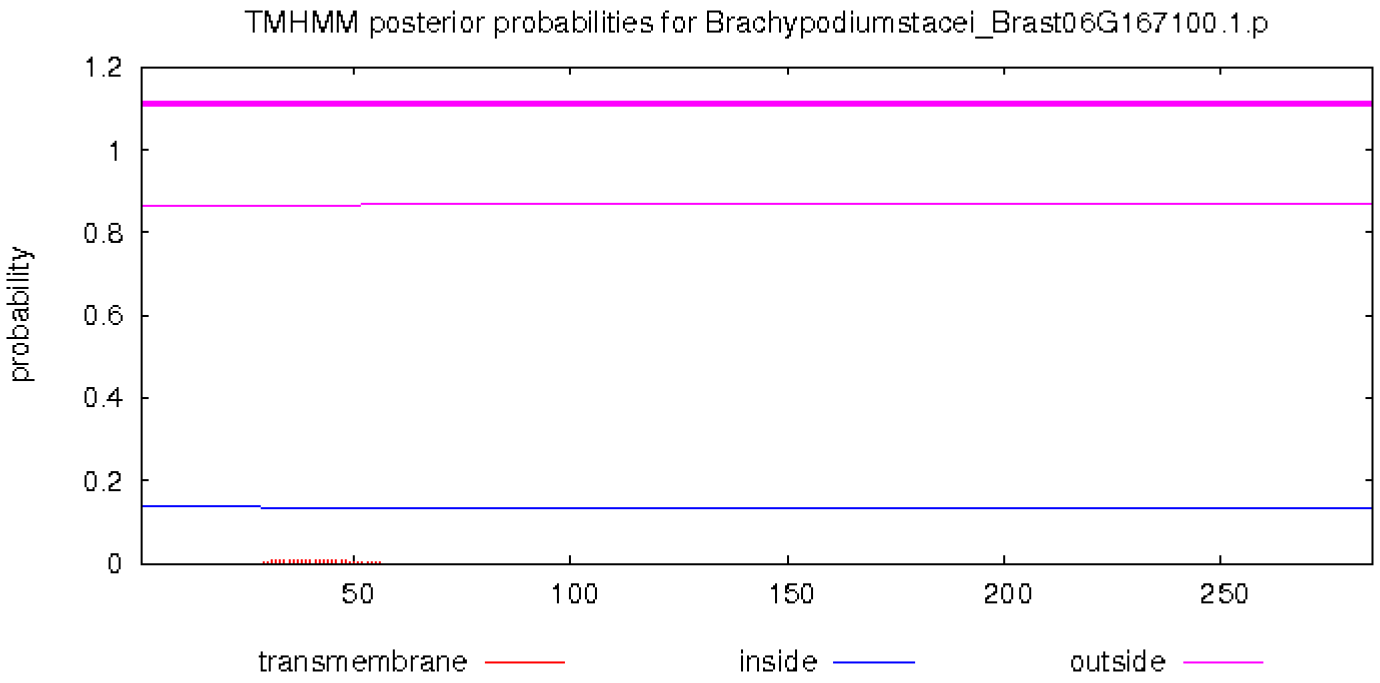

plot in postscript, script for making the plot in gnuplot, data for plot

---

```
# Brachypodiumstacei_Brast06G224900.1.p Length: 332
# Brachypodiumstacei_Brast06G224900.1.p Number of predicted TMHs:  0
# Brachypodiumstacei_Brast06G224900.1.p Exp number of AAs in TMHs: 0.00175
# Brachypodiumstacei_Brast06G224900.1.p Exp number, first 60 AAs:  0
# Brachypodiumstacei_Brast06G224900.1.p Total prob of N-in:        0.01437
Brachypodiumstacei_Brast06G224900.1.p   TMHMM2.0        outside      1   332
```

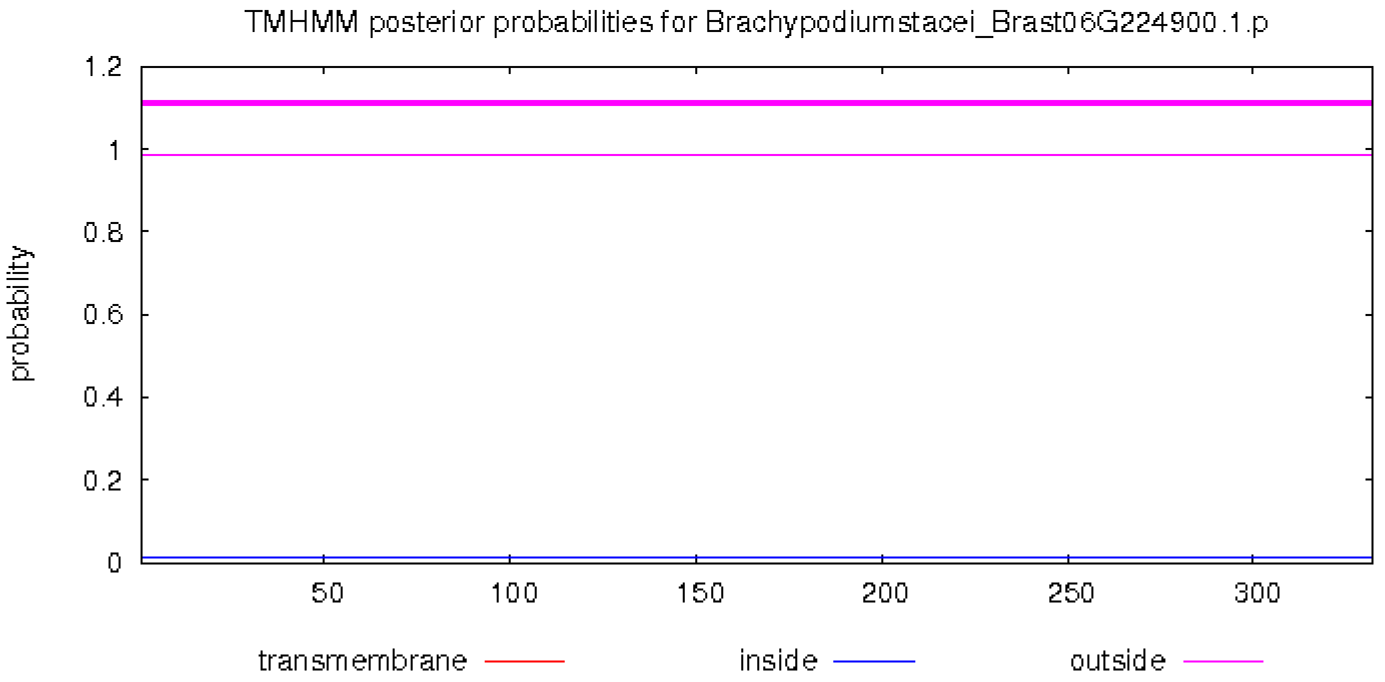

plot in postscript, script for making the plot in gnuplot, data for plot

---

```
# Brachypodiumstacei_Brast07G003500.1.p Length: 338
# Brachypodiumstacei_Brast07G003500.1.p Number of predicted TMHs:  0
# Brachypodiumstacei_Brast07G003500.1.p Exp number of AAs in TMHs: 0.00017
# Brachypodiumstacei_Brast07G003500.1.p Exp number, first 60 AAs:  0
# Brachypodiumstacei_Brast07G003500.1.p Total prob of N-in:        0.09103
Brachypodiumstacei_Brast07G003500.1.p   TMHMM2.0        outside      1   338
```

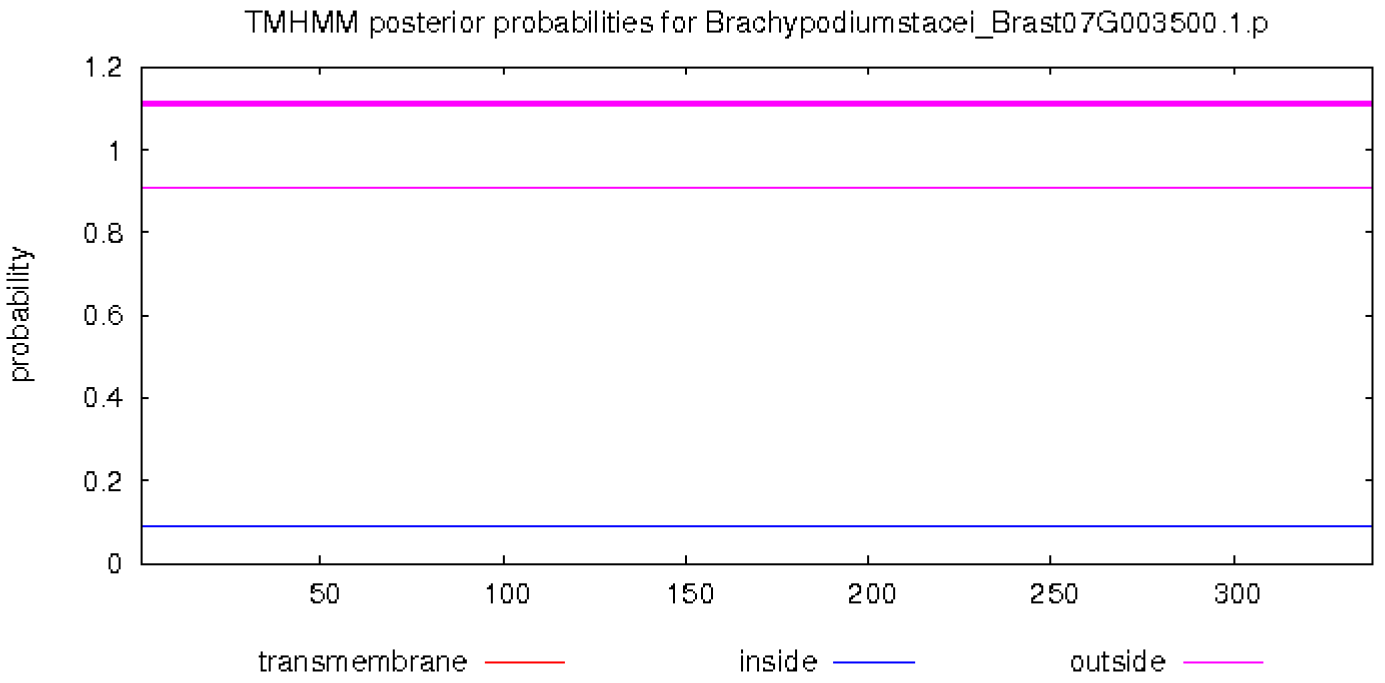

plot in postscript, script for making the plot in gnuplot, data for plot

---

```
# Brachypodiumstacei_Brast07G026300.1.p Length: 400
# Brachypodiumstacei_Brast07G026300.1.p Number of predicted TMHs:  0
# Brachypodiumstacei_Brast07G026300.1.p Exp number of AAs in TMHs: 0.00248
# Brachypodiumstacei_Brast07G026300.1.p Exp number, first 60 AAs:  0
# Brachypodiumstacei_Brast07G026300.1.p Total prob of N-in:        0.00741
Brachypodiumstacei_Brast07G026300.1.p	TMHMM2.0	outside	     1   400
```

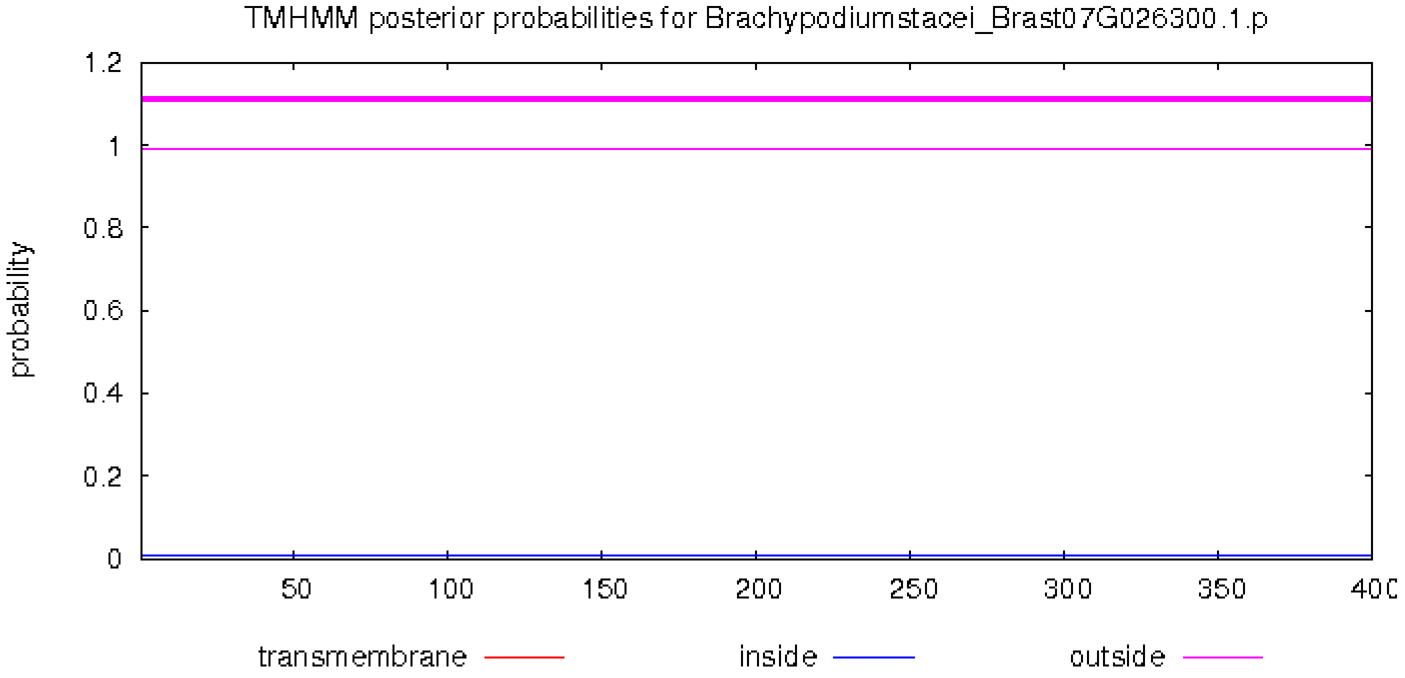

plot in postscript, script for making the plot in gnuplot, data for plot

```
# Brachypodiumstacei_Brast07G098400.1.p Length: 483
# Brachypodiumstacei_Brast07G098400.1.p Number of predicted TMHs:  0
# Brachypodiumstacei_Brast07G098400.1.p Exp number of AAs in TMHs: 0
# Brachypodiumstacei_Brast07G098400.1.p Exp number, first 60 AAs:  0
# Brachypodiumstacei_Brast07G098400.1.p Total prob of N-in:        0.00300
Brachypodiumstacei_Brast07G098400.1.p	TMHMM2.0	outside	     1   483
```

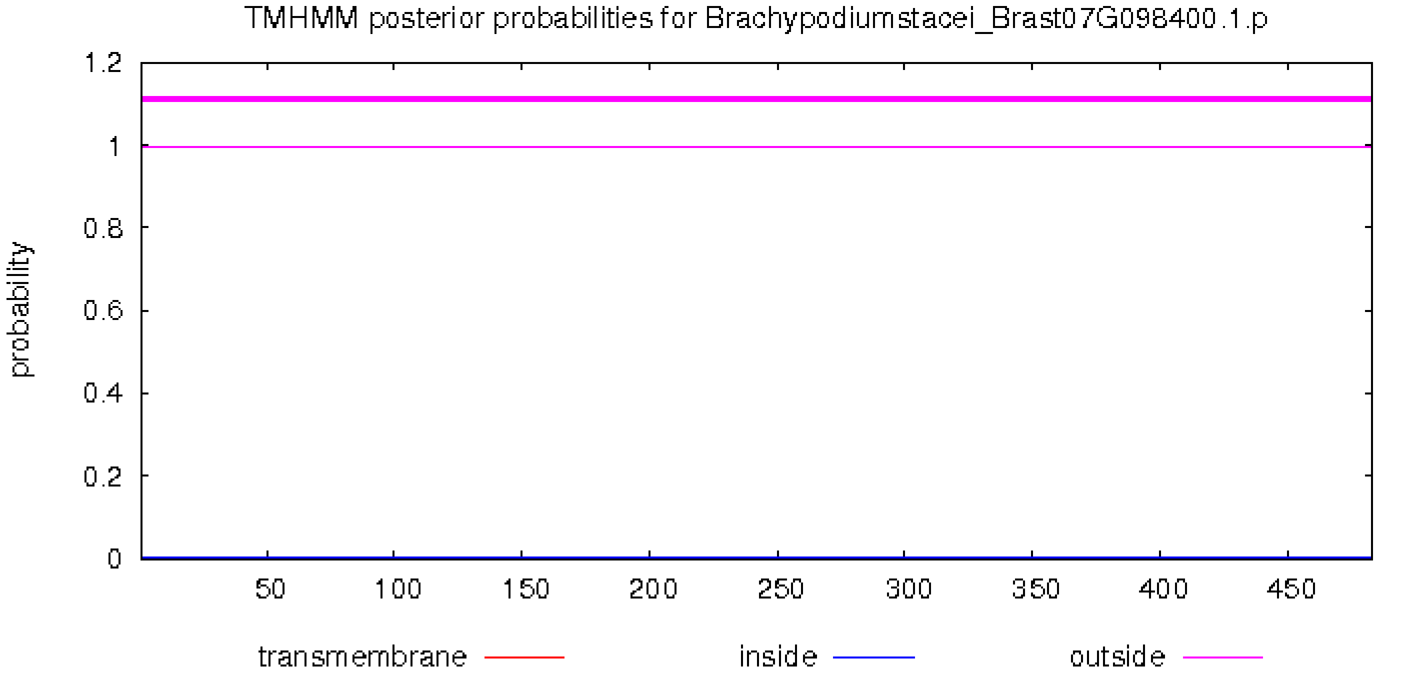

plot in postscript, script for making the plot in gnuplot, data for plot

```
# Brachypodiumstacei_Brast07G127300.1.p Length: 330
# Brachypodiumstacei_Brast07G127300.1.p Number of predicted TMHs:  0
# Brachypodiumstacei_Brast07G127300.1.p Exp number of AAs in TMHs: 0.40889
# Brachypodiumstacei_Brast07G127300.1.p Exp number, first 60 AAs:  0.00036
# Brachypodiumstacei_Brast07G127300.1.p Total prob of N-in:        0.08320
Brachypodiumstacei_Brast07G127300.1.p   TMHMM2.0        outside      1   330
```

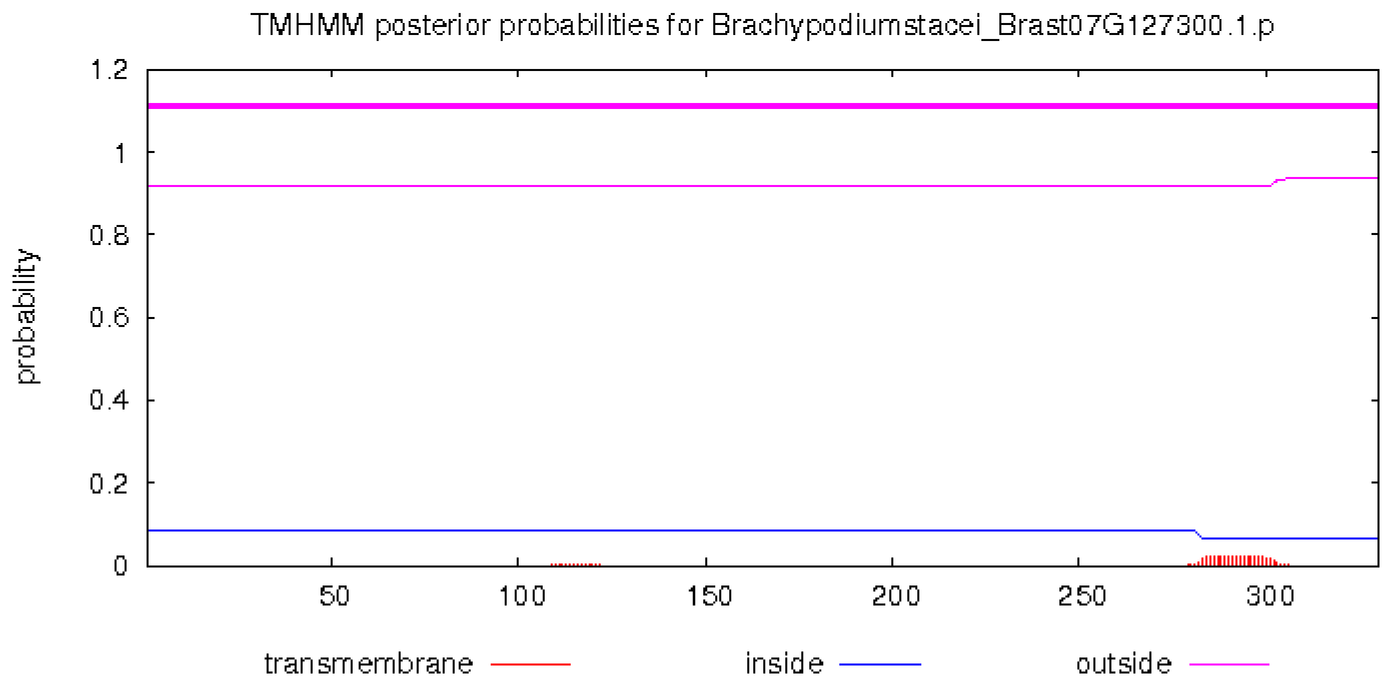

plot in postscript, script for making the plot in gnuplot, data for plot

---

```
# Brachypodiumstacei_Brast07G134300.1.p Length: 361
# Brachypodiumstacei_Brast07G134300.1.p Number of predicted TMHs:  0
# Brachypodiumstacei_Brast07G134300.1.p Exp number of AAs in TMHs: 0.00036
# Brachypodiumstacei_Brast07G134300.1.p Exp number, first 60 AAs:  0
# Brachypodiumstacei_Brast07G134300.1.p Total prob of N-in:        0.00537
Brachypodiumstacei_Brast07G134300.1.p   TMHMM2.0        outside      1   361
```

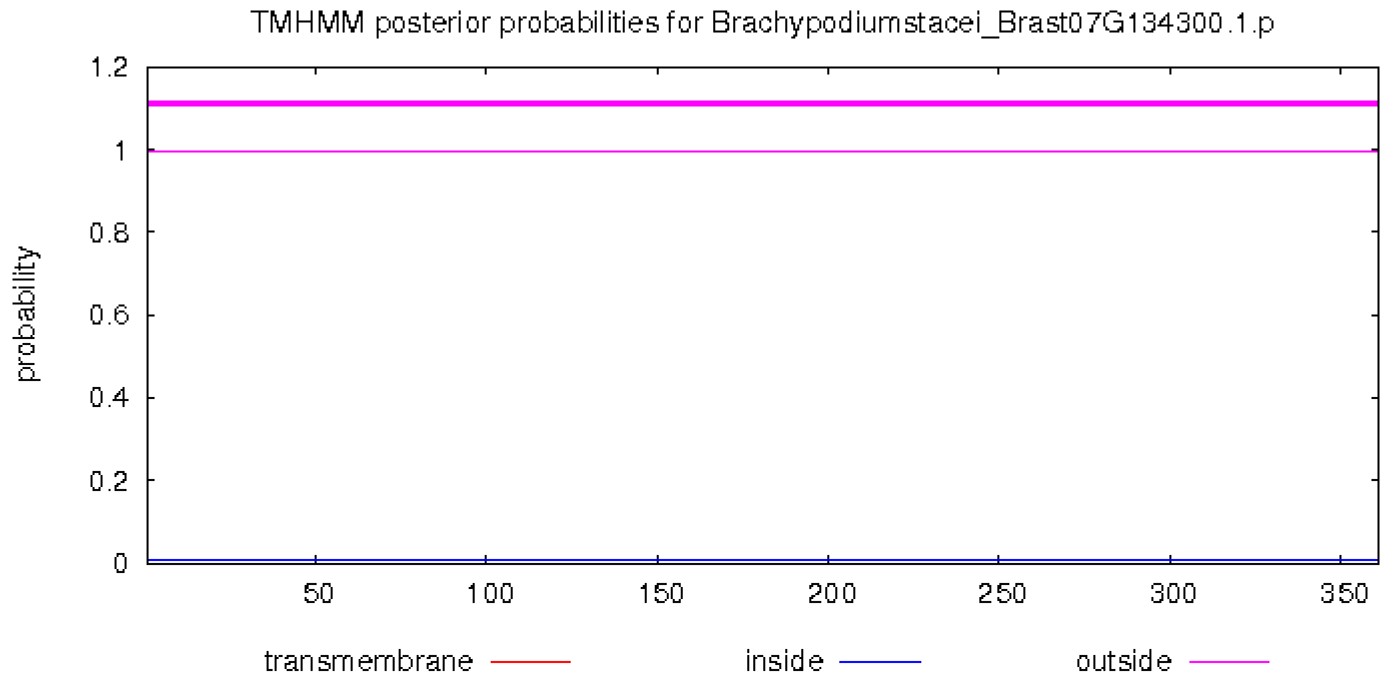

plot in postscript, script for making the plot in gnuplot, data for plot

---

```
# Brachypodiumstacei_Brast07G145000.1.p Length: 479
# Brachypodiumstacei_Brast07G145000.1.p Number of predicted TMHs:  0
# Brachypodiumstacei_Brast07G145000.1.p Exp number of AAs in TMHs: 0.00321
# Brachypodiumstacei_Brast07G145000.1.p Exp number, first 60 AAs:  0
# Brachypodiumstacei_Brast07G145000.1.p Total prob of N-in:        0.00778
Brachypodiumstacei_Brast07G145000.1.p   TMHMM2.0        outside      1   479
```

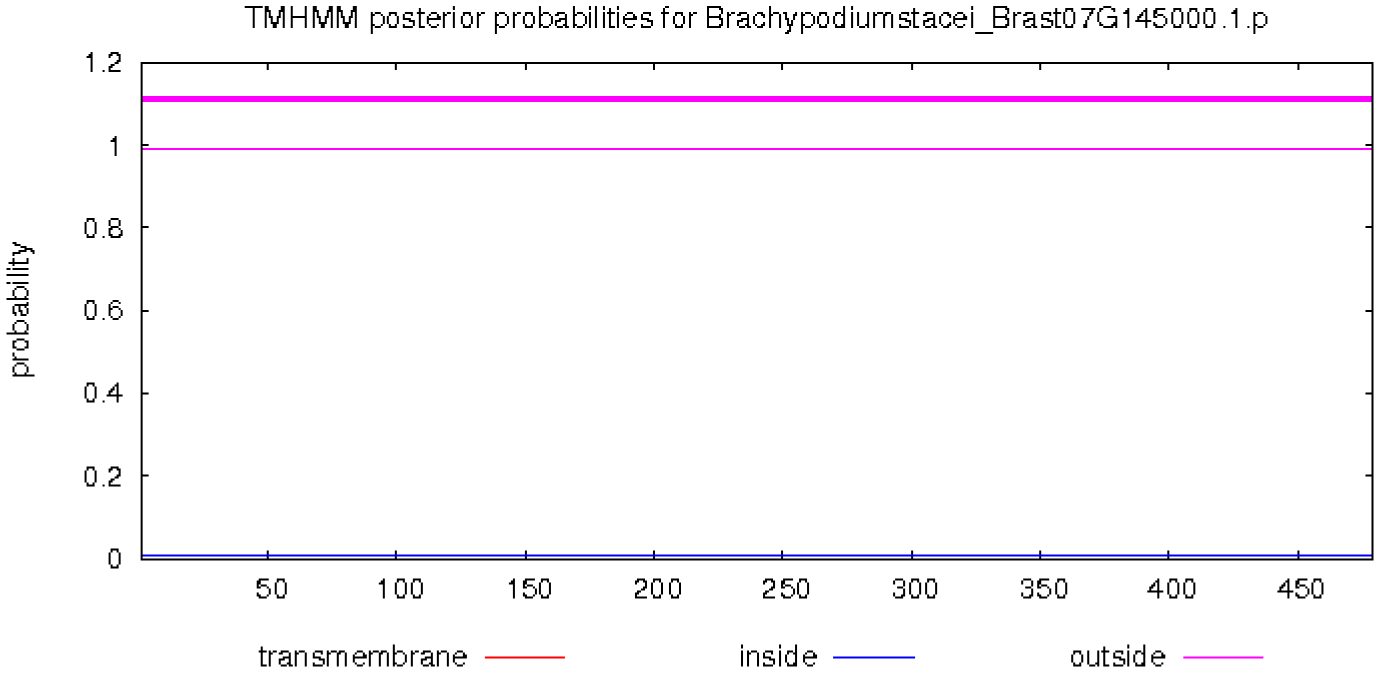

plot in postscript, script for making the plot in gnuplot, data for plot

---

```
# Brachypodiumstacei_Brast07G209700.1.p Length: 303
# Brachypodiumstacei_Brast07G209700.1.p Number of predicted TMHs:  0
# Brachypodiumstacei_Brast07G209700.1.p Exp number of AAs in TMHs: 0.01849
# Brachypodiumstacei_Brast07G209700.1.p Exp number, first 60 AAs:  0.01321
# Brachypodiumstacei_Brast07G209700.1.p Total prob of N-in:        0.02242
Brachypodiumstacei_Brast07G209700.1.p   TMHMM2.0        outside      1   303
```

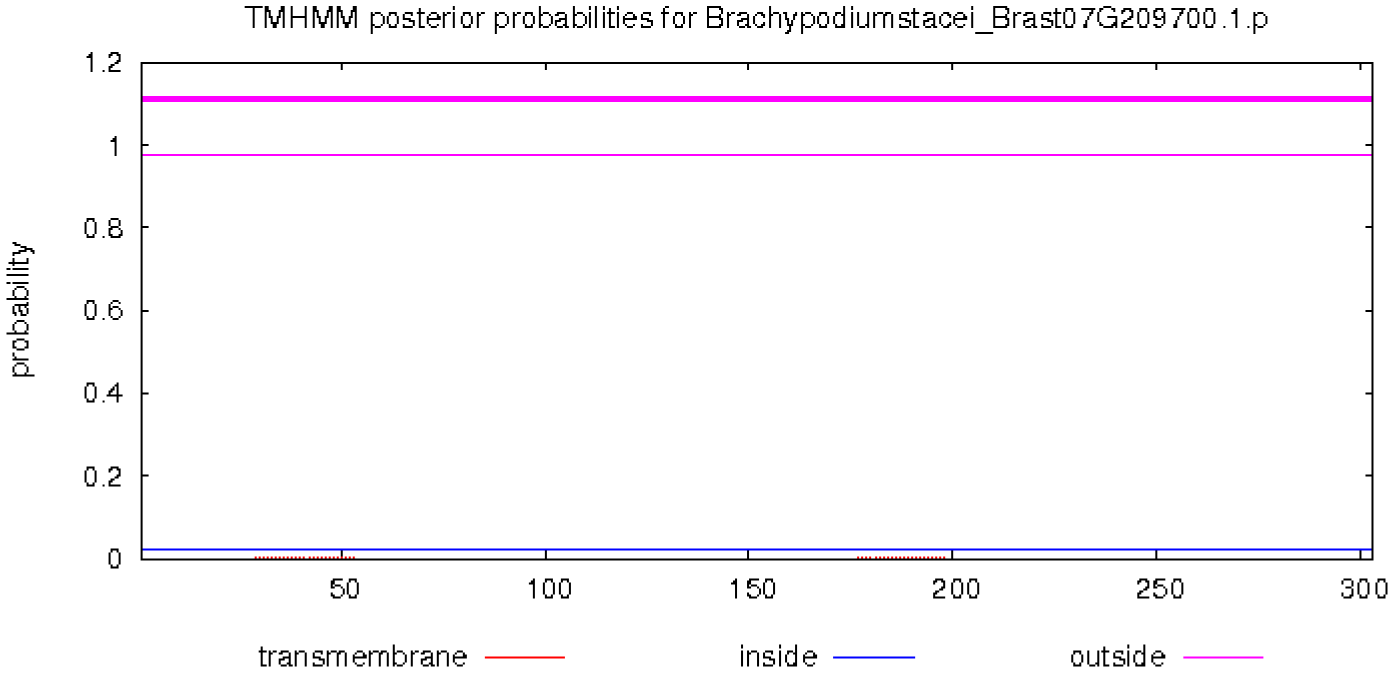

plot in postscript, script for making the plot in gnuplot, data for plot

---

```
# Brachypodiumstacei_Brast07G238700.1.p Length: 300
# Brachypodiumstacei_Brast07G238700.1.p Number of predicted TMHs:  0
# Brachypodiumstacei_Brast07G238700.1.p Exp number of AAs in TMHs: 0.0011
# Brachypodiumstacei_Brast07G238700.1.p Exp number, first 60 AAs:  0
# Brachypodiumstacei_Brast07G238700.1.p Total prob of N-in:        0.00182
Brachypodiumstacei_Brast07G238700.1.p	TMHMM2.0	outside	     1   300
```

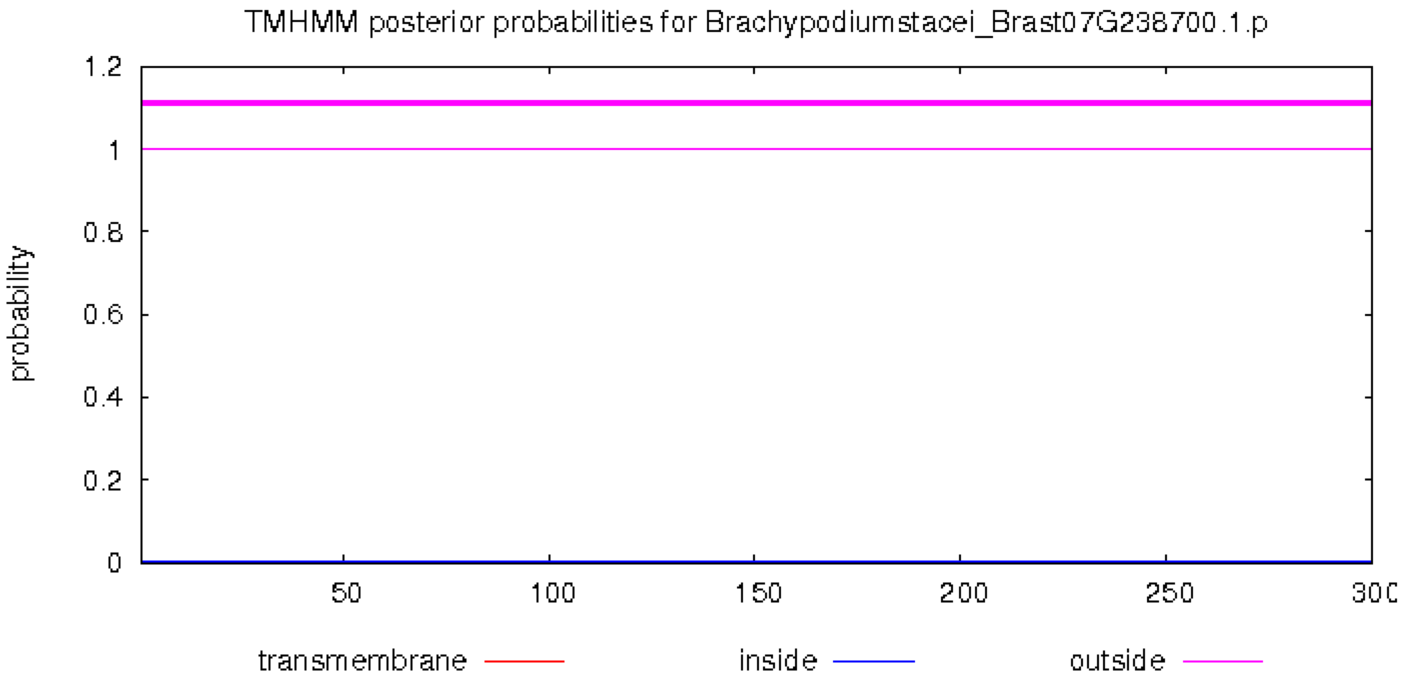

plot in postscript, script for making the plot in gnuplot, data for plot

---

```
# Brachypodiumstacei_Brast08G069400.1.p Length: 431
# Brachypodiumstacei_Brast08G069400.1.p Number of predicted TMHs:  0
# Brachypodiumstacei_Brast08G069400.1.p Exp number of AAs in TMHs: 0.00307
# Brachypodiumstacei_Brast08G069400.1.p Exp number, first 60 AAs:  0.00012
# Brachypodiumstacei_Brast08G069400.1.p Total prob of N-in:        0.00082
Brachypodiumstacei_Brast08G069400.1.p	TMHMM2.0	outside	     1   431
```

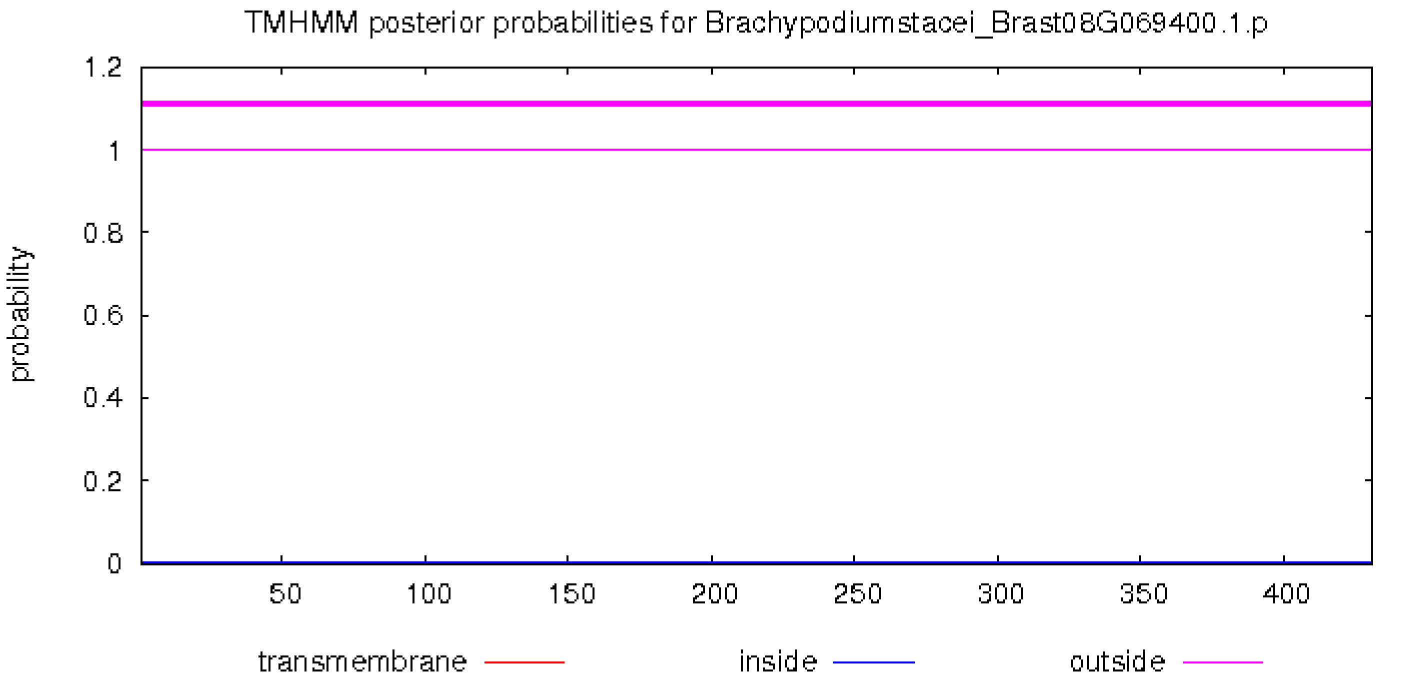

plot in postscript, script for making the plot in gnuplot, data for plot

---

```
# Brachypodiumstacei_Brast08G105300.1.p Length: 255
# Brachypodiumstacei_Brast08G105300.1.p Number of predicted TMHs:  0
# Brachypodiumstacei_Brast08G105300.1.p Exp number of AAs in TMHs: 0
# Brachypodiumstacei_Brast08G105300.1.p Exp number, first 60 AAs:  0
# Brachypodiumstacei_Brast08G105300.1.p Total prob of N-in:        0.00599
Brachypodiumstacei_Brast08G105300.1.p   TMHMM2.0        outside      1   255
```

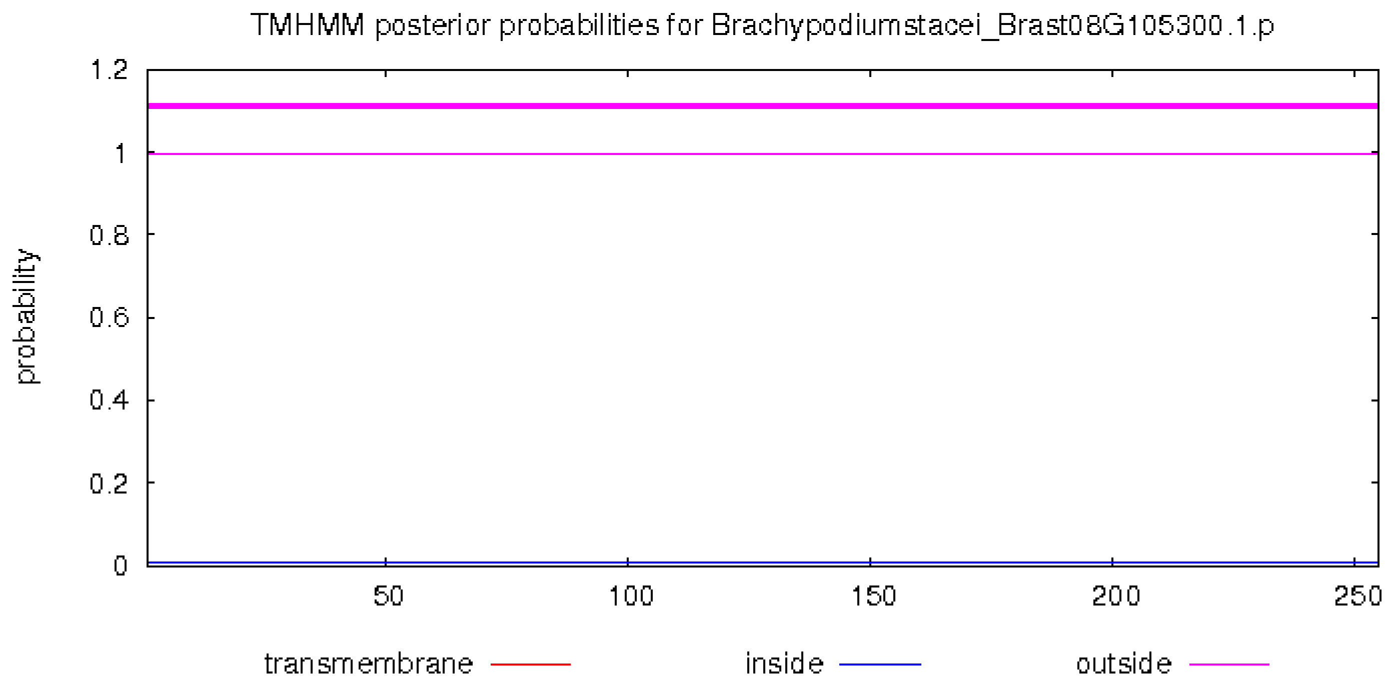

plot in postscript, script for making the plot in gnuplot, data for plot

---

```
# Brachypodiumstacei_Brast08G113200.1.p Length: 462
# Brachypodiumstacei_Brast08G113200.1.p Number of predicted TMHs:  0
# Brachypodiumstacei_Brast08G113200.1.p Exp number of AAs in TMHs: 0.00266
# Brachypodiumstacei_Brast08G113200.1.p Exp number, first 60 AAs:  0.00033
# Brachypodiumstacei_Brast08G113200.1.p Total prob of N-in:        0.00081
Brachypodiumstacei_Brast08G113200.1.p   TMHMM2.0        outside      1   462
```

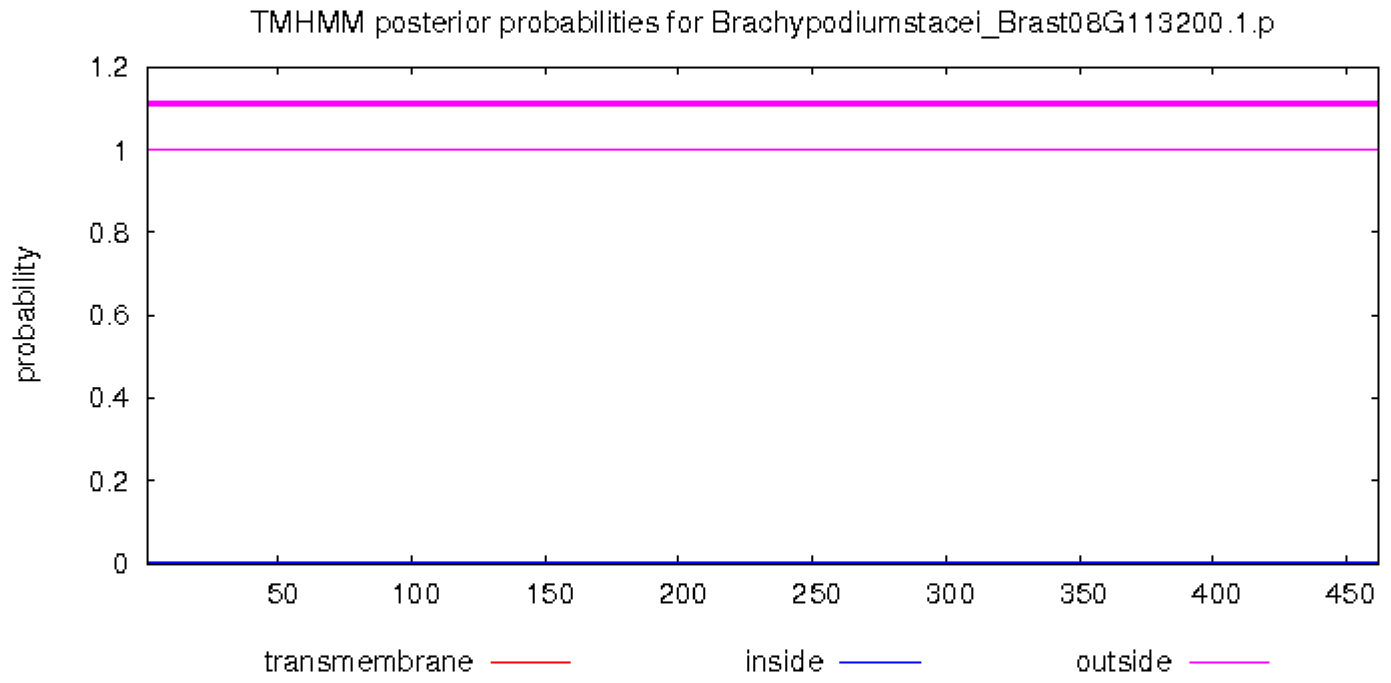

plot in postscript, script for making the plot in gnuplot, data for plot

---

```
# Brachypodiumstacei_Brast08G114900.1.p Length: 309
# Brachypodiumstacei_Brast08G114900.1.p Number of predicted TMHs:  0
# Brachypodiumstacei_Brast08G114900.1.p Exp number of AAs in TMHs: 0.00394
# Brachypodiumstacei_Brast08G114900.1.p Exp number, first 60 AAs:  0.0033
# Brachypodiumstacei_Brast08G114900.1.p Total prob of N-in:        0.01420
Brachypodiumstacei_Brast08G114900.1.p   TMHMM2.0        outside      1   309
```

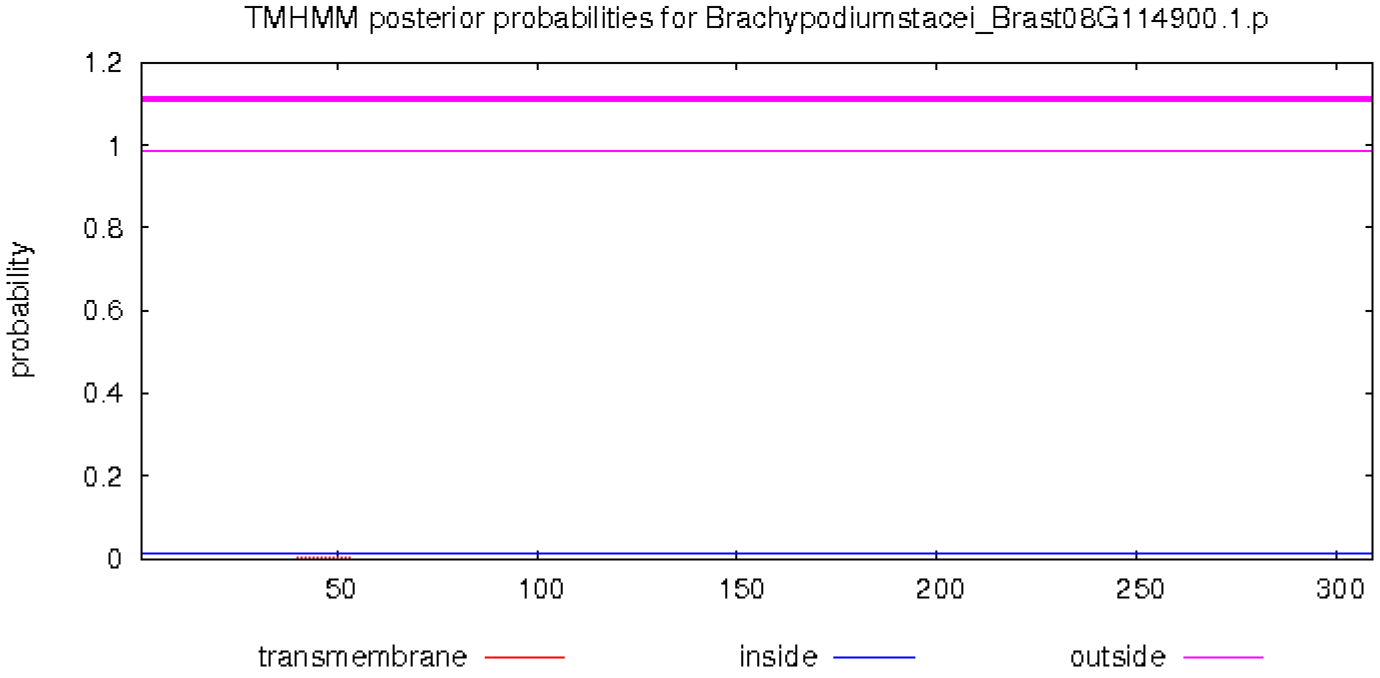

plot in postscript, script for making the plot in gnuplot, data for plot

---

```
# Brachypodiumstacei_Brast08G116600.1.p Length: 358
# Brachypodiumstacei_Brast08G116600.1.p Number of predicted TMHs:  0
# Brachypodiumstacei_Brast08G116600.1.p Exp number of AAs in TMHs: 0.00231
# Brachypodiumstacei_Brast08G116600.1.p Exp number, first 60 AAs:  0
# Brachypodiumstacei_Brast08G116600.1.p Total prob of N-in:        0.01431
Brachypodiumstacei_Brast08G116600.1.p   TMHMM2.0        outside      1   358
```

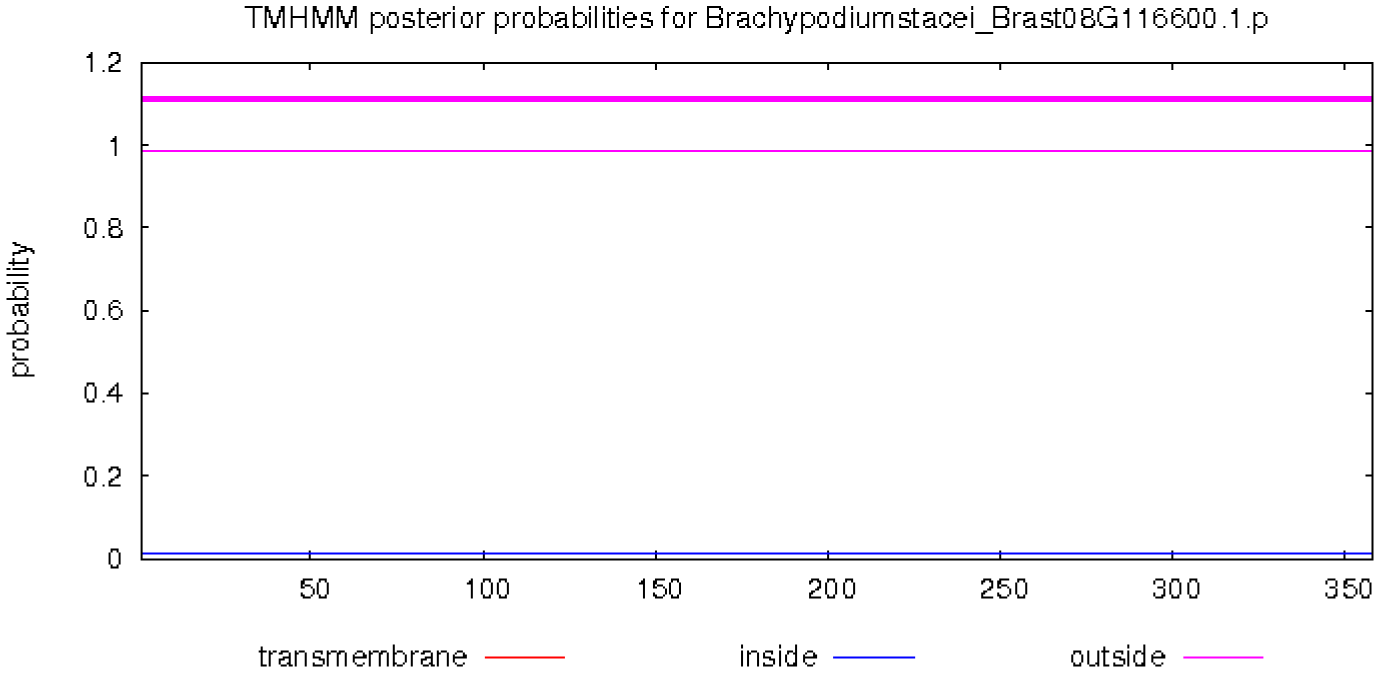

plot in postscript, script for making the plot in gnuplot, data for plot

---

```
# Brachypodiumstacei_Brast08G178700.1.p Length: 181
# Brachypodiumstacei_Brast08G178700.1.p Number of predicted TMHs:  0
# Brachypodiumstacei_Brast08G178700.1.p Exp number of AAs in TMHs: 0
# Brachypodiumstacei_Brast08G178700.1.p Exp number, first 60 AAs:  0
# Brachypodiumstacei_Brast08G178700.1.p Total prob of N-in:        0.14116
Brachypodiumstacei_Brast08G178700.1.p	TMHMM2.0	outside	     1   181
```

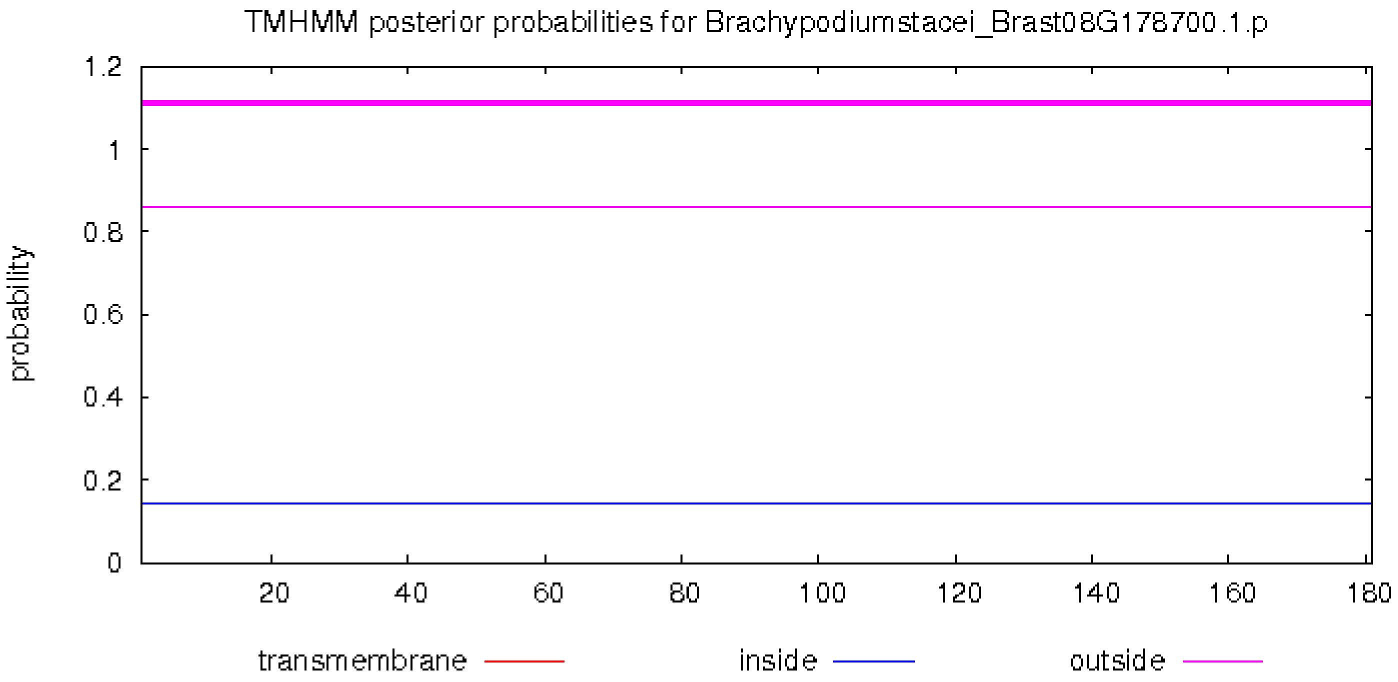

plot in postscript, script for making the plot in gnuplot, data for plot

---

```
# Brachypodiumstacei_Brast08G249300.1.p Length: 310
# Brachypodiumstacei_Brast08G249300.1.p Number of predicted TMHs:  0
# Brachypodiumstacei_Brast08G249300.1.p Exp number of AAs in TMHs: 0.0722899999999999999
# Brachypodiumstacei_Brast08G249300.1.p Exp number, first 60 AAs:  0.00036
# Brachypodiumstacei_Brast08G249300.1.p Total prob of N-in:        0.00546
Brachypodiumstacei_Brast08G249300.1.p	TMHMM2.0	outside	     1   310
```

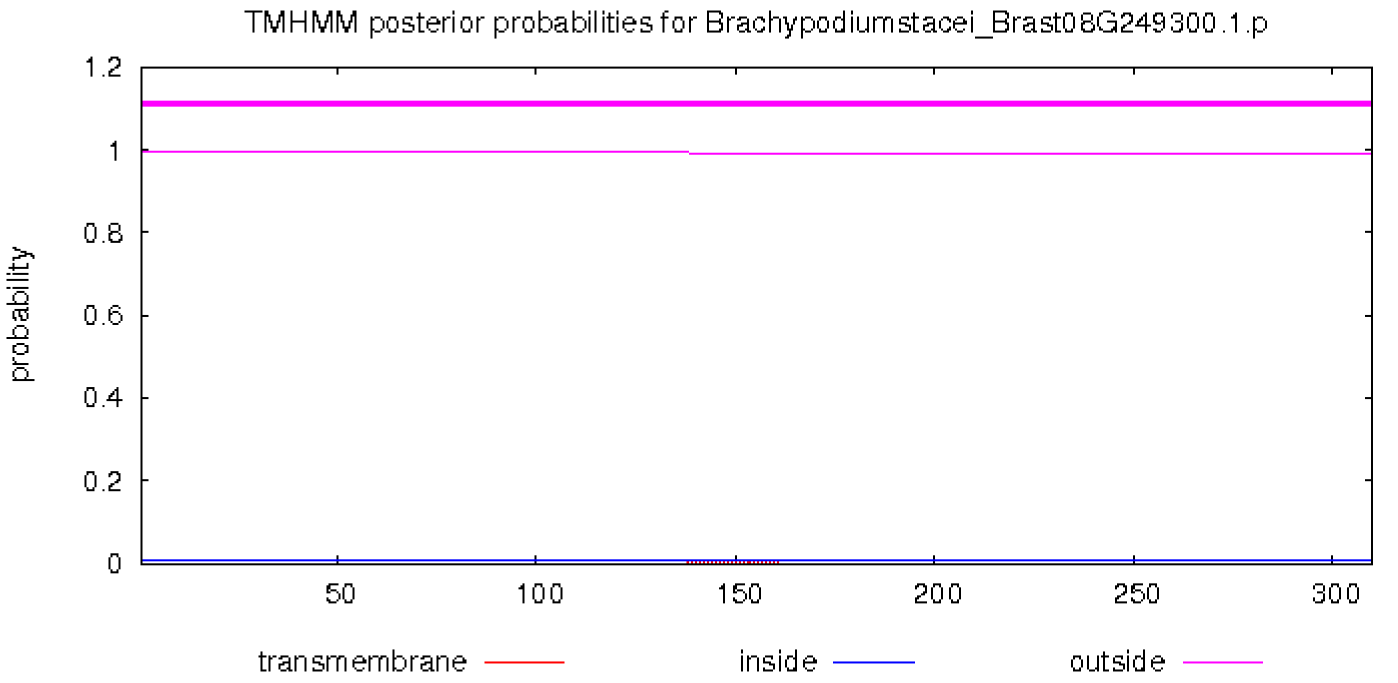

plot in postscript, script for making the plot in gnuplot, data for plot

---

```
# Brachypodiumstacei_Brast09G087300.1.p Length: 199
# Brachypodiumstacei_Brast09G087300.1.p Number of predicted TMHs:  0
# Brachypodiumstacei_Brast09G087300.1.p Exp number of AAs in TMHs: 0
# Brachypodiumstacei_Brast09G087300.1.p Exp number, first 60 AAs:  0
# Brachypodiumstacei_Brast09G087300.1.p Total prob of N-in:        0.01063
Brachypodiumstacei_Brast09G087300.1.p   TMHMM2.0        outside      1   199
```

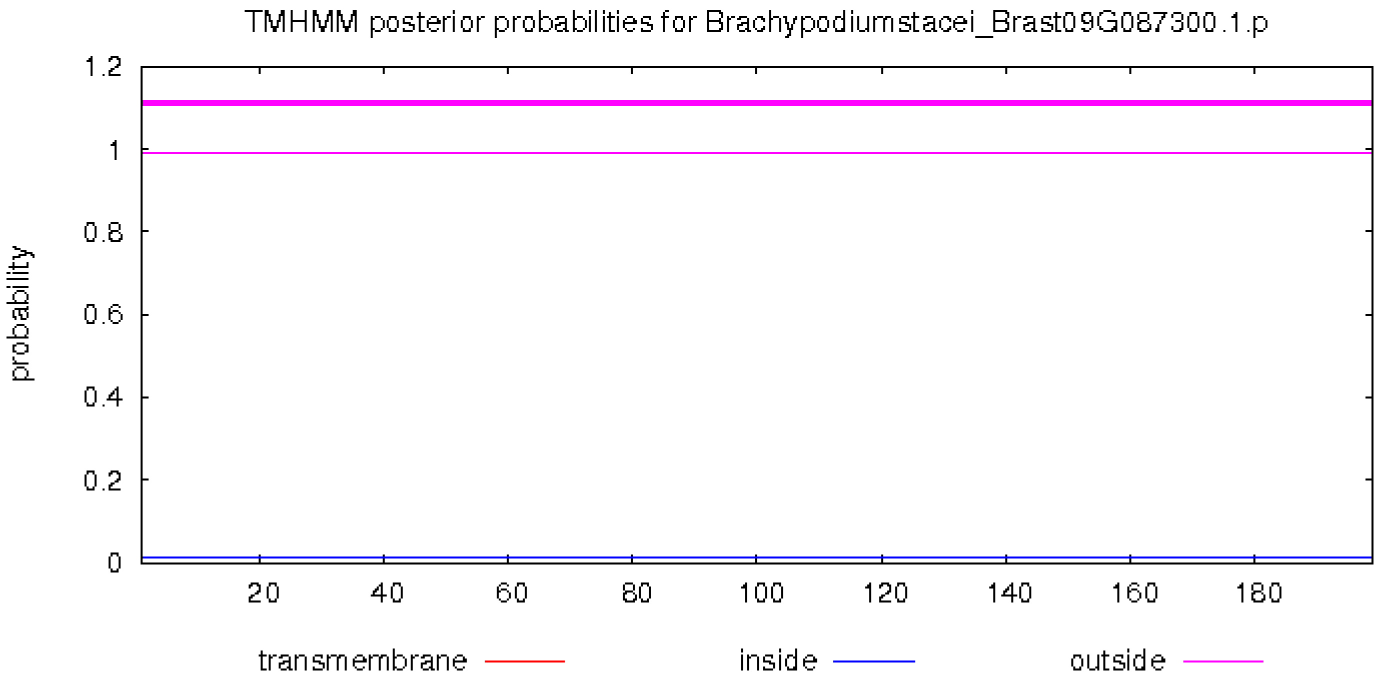

plot in postscript, script for making the plot in gnuplot, data for plot

```
# Brachypodiumstacei_Brast09G093500.1.p Length: 336
# Brachypodiumstacei_Brast09G093500.1.p Number of predicted TMHs:  0
# Brachypodiumstacei_Brast09G093500.1.p Exp number of AAs in TMHs: 0.00021
# Brachypodiumstacei_Brast09G093500.1.p Exp number, first 60 AAs:  0
# Brachypodiumstacei_Brast09G093500.1.p Total prob of N-in:        0.00918
Brachypodiumstacei_Brast09G093500.1.p   TMHMM2.0        outside      1   336
```

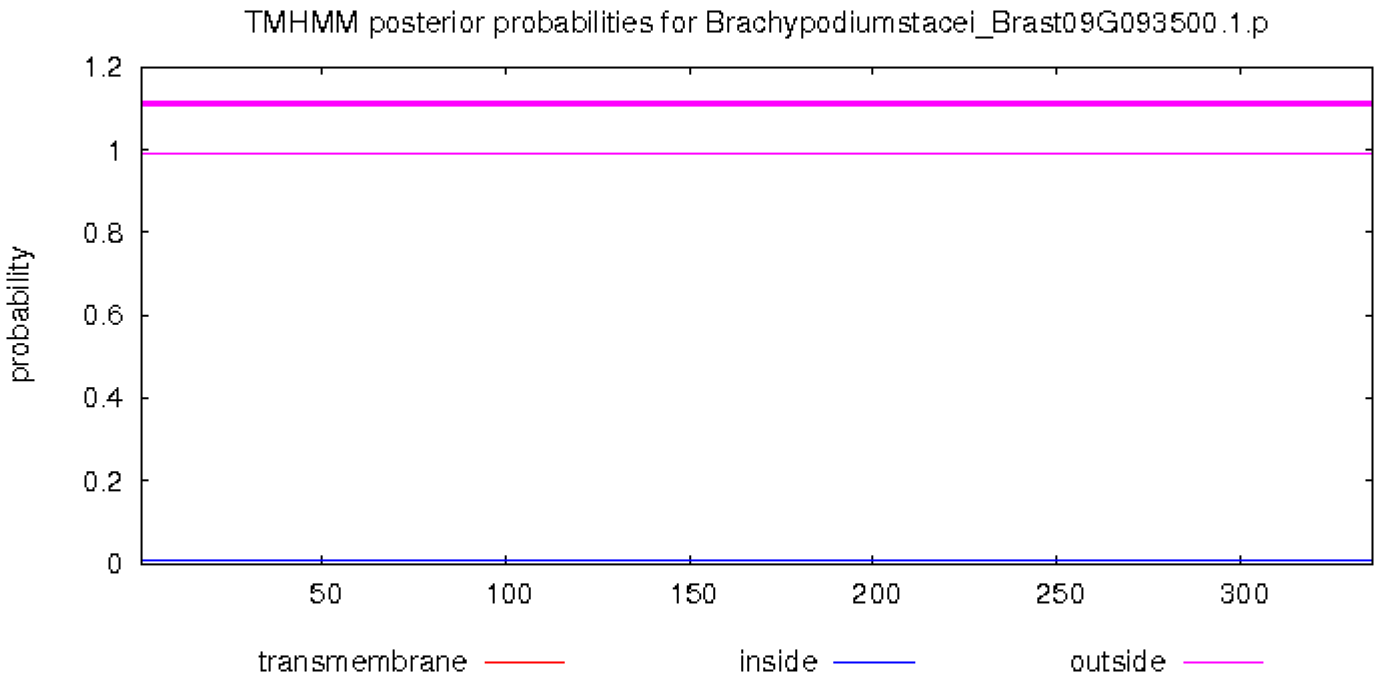

plot in postscript, script for making the plot in gnuplot, data for plot

```
# Brachypodiumstacei_Brast09G106400.1.p Length: 316
# Brachypodiumstacei_Brast09G106400.1.p Number of predicted TMHs:  0
# Brachypodiumstacei_Brast09G106400.1.p Exp number of AAs in TMHs: 0.01443
# Brachypodiumstacei_Brast09G106400.1.p Exp number, first 60 AAs:  0
# Brachypodiumstacei_Brast09G106400.1.p Total prob of N-in:        0.03301
Brachypodiumstacei_Brast09G106400.1.p	TMHMM2.0	outside	     1   316
```

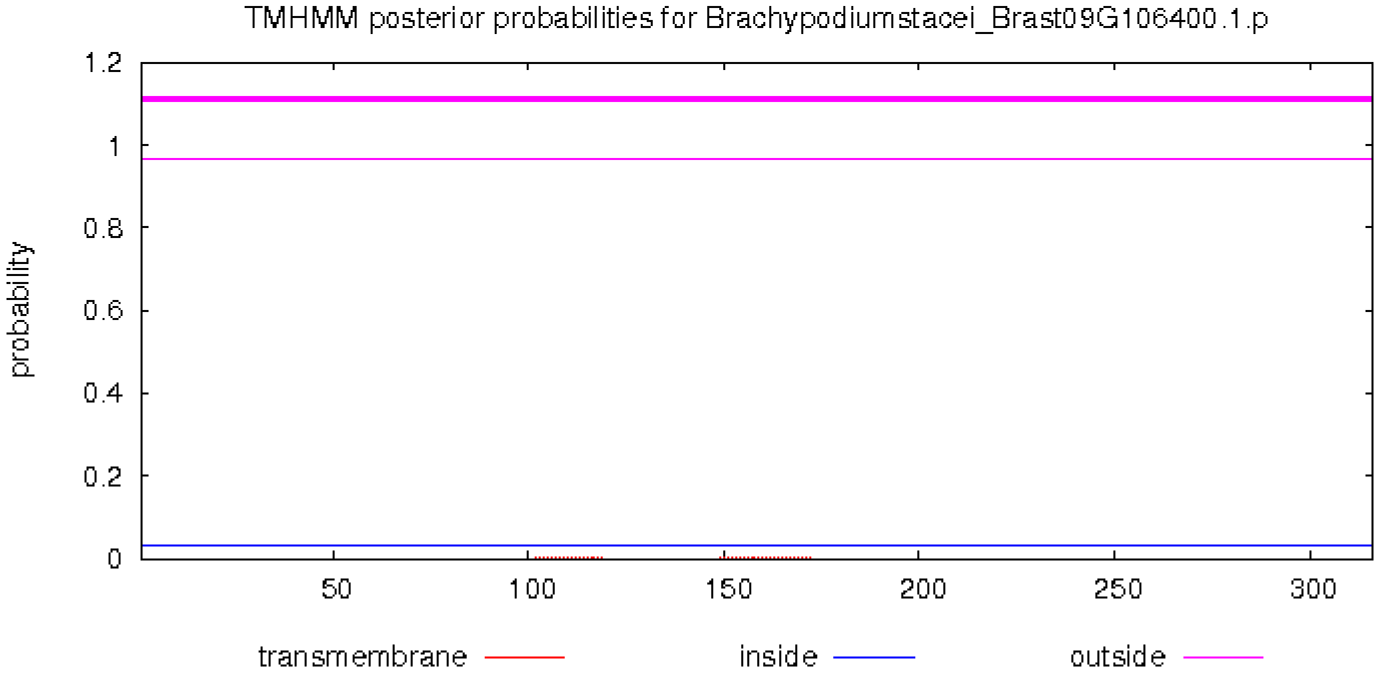

plot in postscript, script for making the plot in gnuplot, data for plot

---

```
# Brachypodiumstacei_Brast09G117500.1.p Length: 218
# Brachypodiumstacei_Brast09G117500.1.p Number of predicted TMHs:  0
# Brachypodiumstacei_Brast09G117500.1.p Exp number of AAs in TMHs: 0.02476
# Brachypodiumstacei_Brast09G117500.1.p Exp number, first 60 AAs:  0.00245
# Brachypodiumstacei_Brast09G117500.1.p Total prob of N-in:        0.26565
Brachypodiumstacei_Brast09G117500.1.p	TMHMM2.0	outside	     1   218
```

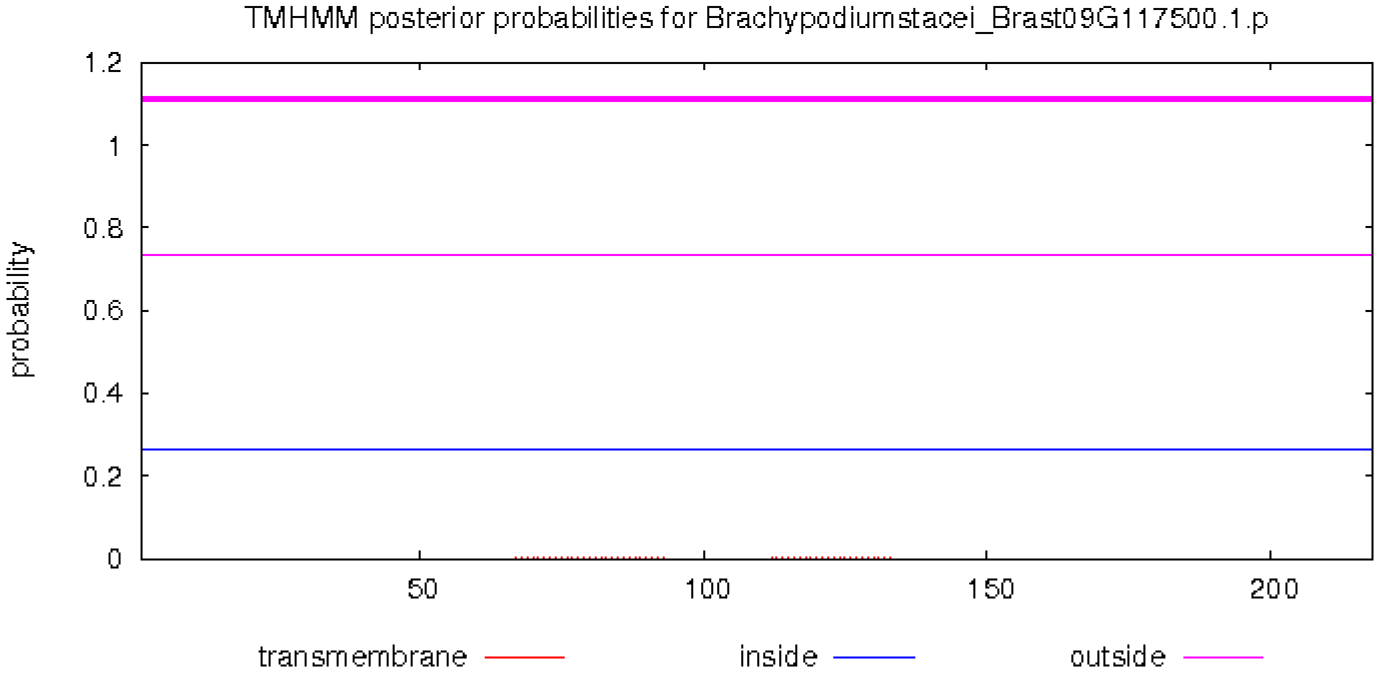

plot in postscript, script for making the plot in gnuplot, data for plot

---

```
# Brachypodiumstacei_Brast09G117600.1.p Length: 862
# Brachypodiumstacei_Brast09G117600.1.p Number of predicted TMHs:  0
# Brachypodiumstacei_Brast09G117600.1.p Exp number of AAs in TMHs: 0.01865
# Brachypodiumstacei_Brast09G117600.1.p Exp number, first 60 AAs:  0.00301
# Brachypodiumstacei_Brast09G117600.1.p Total prob of N-in:        0.00016
Brachypodiumstacei_Brast09G117600.1.p   TMHMM2.0        outside      1   862
```

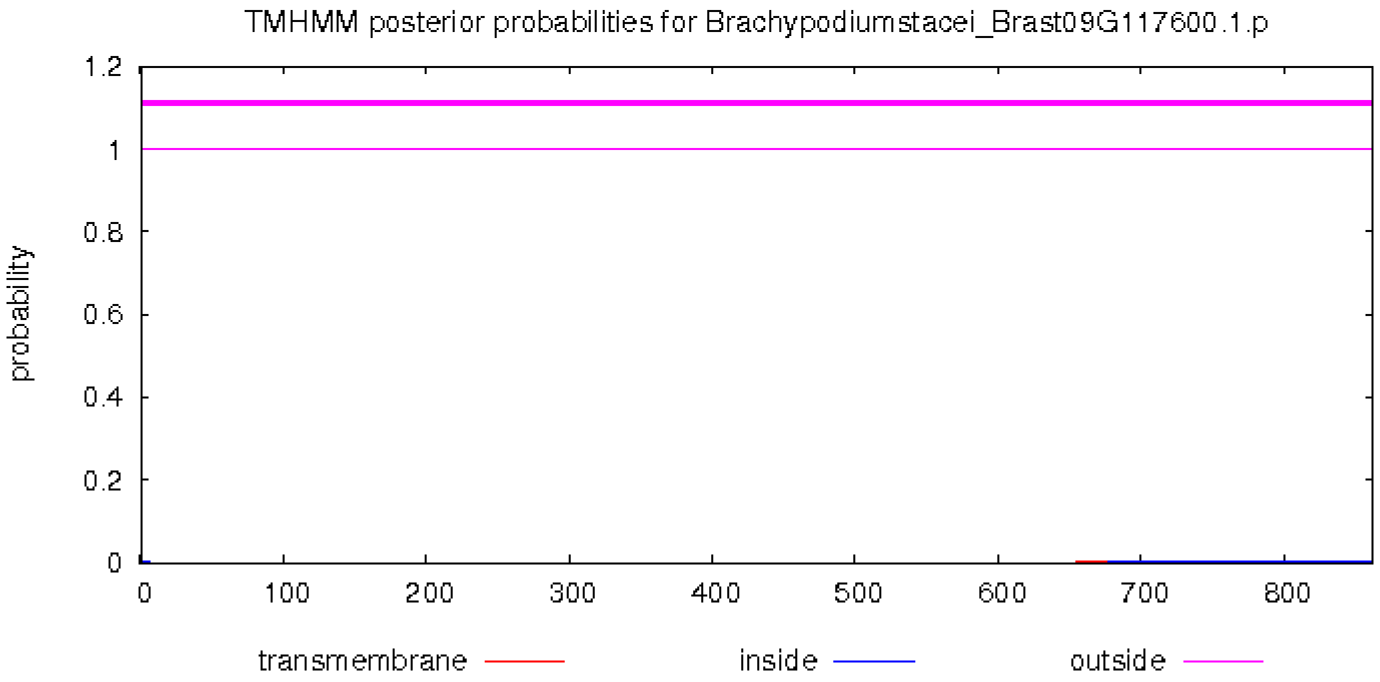

plot in postscript, script for making the plot in gnuplot, data for plot

---

```
# Brachypodiumstacei_Brast09G119200.1.p Length: 383
# Brachypodiumstacei_Brast09G119200.1.p Number of predicted TMHs:  0
# Brachypodiumstacei_Brast09G119200.1.p Exp number of AAs in TMHs: 0.00645
# Brachypodiumstacei_Brast09G119200.1.p Exp number, first 60 AAs:  0.00645
# Brachypodiumstacei_Brast09G119200.1.p Total prob of N-in:        0.00949
Brachypodiumstacei_Brast09G119200.1.p   TMHMM2.0        outside      1   383
```

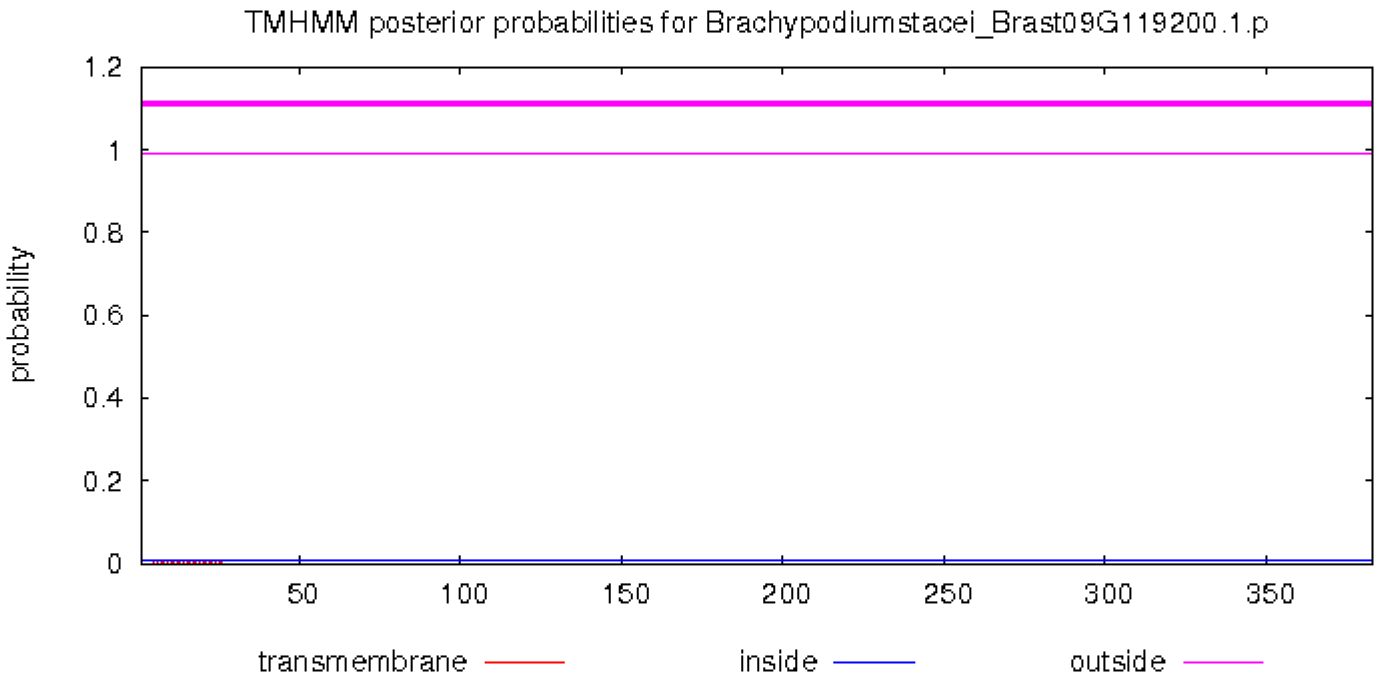

plot in postscript, script for making the plot in gnuplot, data for plot

---

```
# Brachypodiumstacei_Brast09G119300.1.p Length: 357
# Brachypodiumstacei_Brast09G119300.1.p Number of predicted TMHs:  0
# Brachypodiumstacei_Brast09G119300.1.p Exp number of AAs in TMHs: 0.00207
# Brachypodiumstacei_Brast09G119300.1.p Exp number, first 60 AAs:  0.00151
# Brachypodiumstacei_Brast09G119300.1.p Total prob of N-in:        0.00720
Brachypodiumstacei_Brast09G119300.1.p   TMHMM2.0        outside      1   357
```

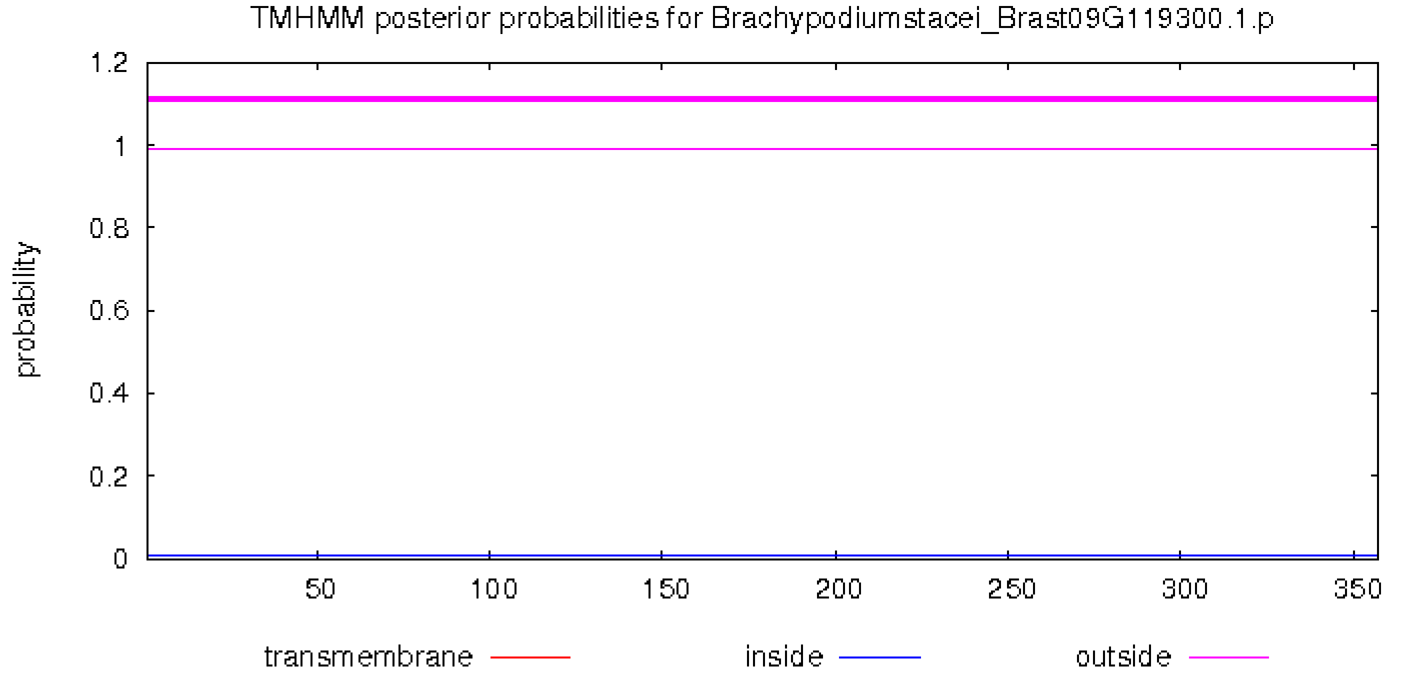

plot in postscript, script for making the plot in gnuplot, data for plot

---

```
# Brachypodiumstacei_Brast09G123100.1.p Length: 303
# Brachypodiumstacei_Brast09G123100.1.p Number of predicted TMHs:  0
# Brachypodiumstacei_Brast09G123100.1.p Exp number of AAs in TMHs: 0.08388
# Brachypodiumstacei_Brast09G123100.1.p Exp number, first 60 AAs:  0
# Brachypodiumstacei_Brast09G123100.1.p Total prob of N-in:        0.03050
Brachypodiumstacei_Brast09G123100.1.p   TMHMM2.0        outside      1   303
```

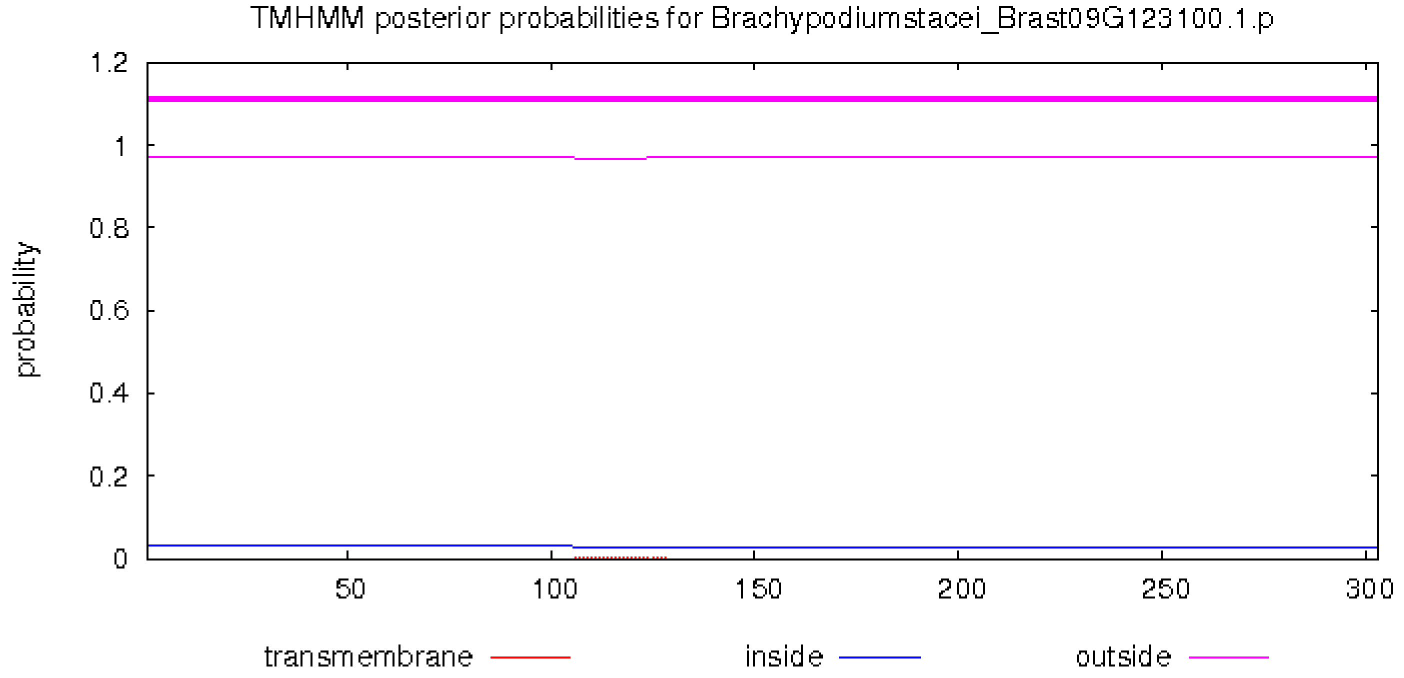

plot in postscript, script for making the plot in gnuplot, data for plot

---

```
# Brachypodiumstacei_Brast09G133100.1.p Length: 239
# Brachypodiumstacei_Brast09G133100.1.p Number of predicted TMHs:  0
# Brachypodiumstacei_Brast09G133100.1.p Exp number of AAs in TMHs: 0.0618
# Brachypodiumstacei_Brast09G133100.1.p Exp number, first 60 AAs:  0.00178
# Brachypodiumstacei_Brast09G133100.1.p Total prob of N-in:        0.20725
Brachypodiumstacei_Brast09G133100.1.p   TMHMM2.0        outside      1   239
```

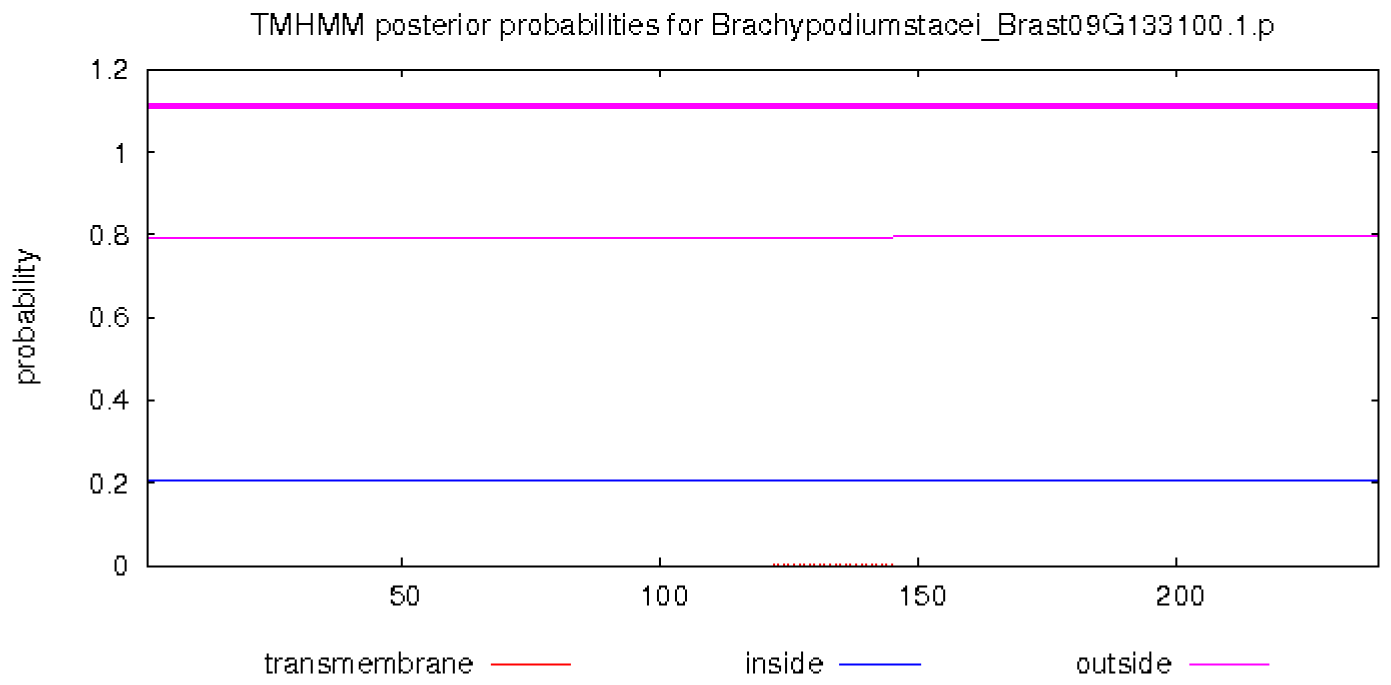

plot in postscript, script for making the plot in gnuplot, data for plot

```
# Brachypodiumstacei_Brast09G135700.1.p Length: 549
# Brachypodiumstacei_Brast09G135700.1.p Number of predicted TMHs:  0
# Brachypodiumstacei_Brast09G135700.1.p Exp number of AAs in TMHs: 2.20419
# Brachypodiumstacei_Brast09G135700.1.p Exp number, first 60 AAs:  2.20419
# Brachypodiumstacei_Brast09G135700.1.p Total prob of N-in:        0.09012
Brachypodiumstacei_Brast09G135700.1.p   TMHMM2.0        outside      1   549
```

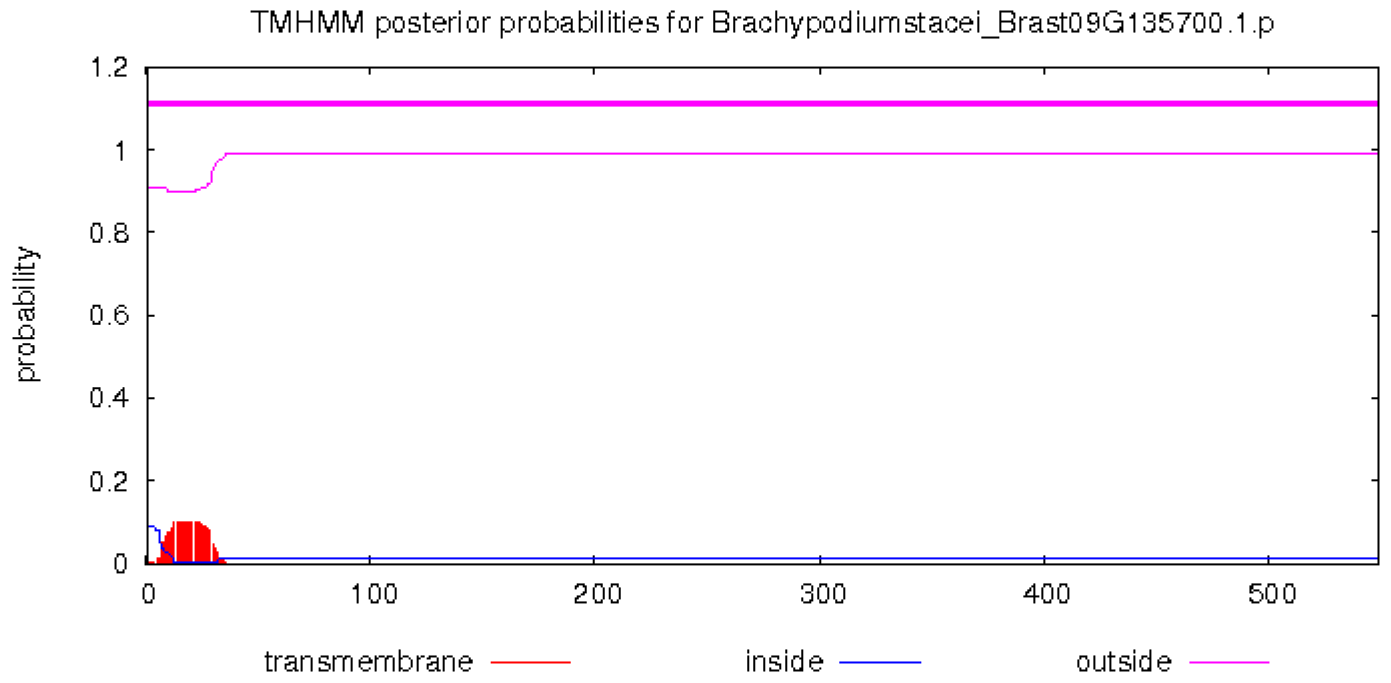

plot in postscript, script for making the plot in gnuplot, data for plot

```
# Brachypodiumstacei_Brast09G138900.1.p Length: 378
# Brachypodiumstacei_Brast09G138900.1.p Number of predicted TMHs:  0
# Brachypodiumstacei_Brast09G138900.1.p Exp number of AAs in TMHs: 0.10189
# Brachypodiumstacei_Brast09G138900.1.p Exp number, first 60 AAs:  0.00074
# Brachypodiumstacei_Brast09G138900.1.p Total prob of N-in:        0.01700
Brachypodiumstacei_Brast09G138900.1.p	TMHMM2.0	outside	     1   378
```

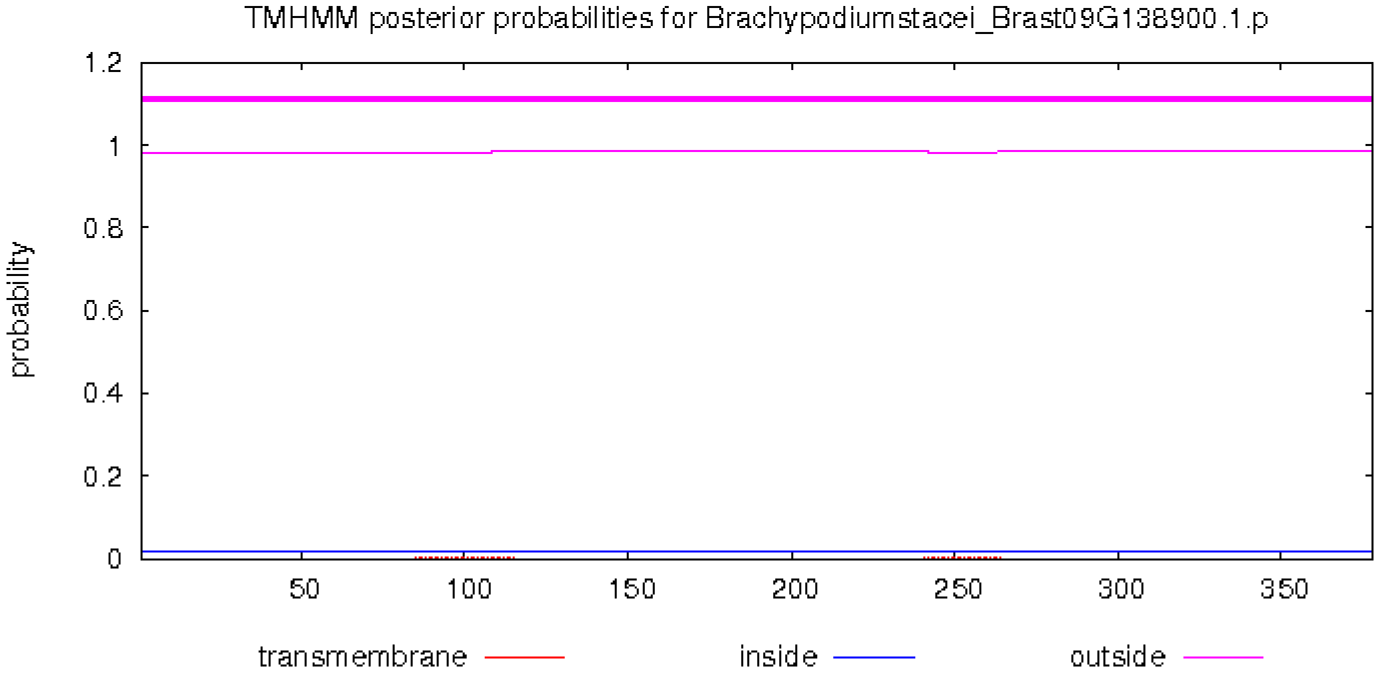

plot in postscript, script for making the plot in gnuplot, data for plot

---

```
# Brachypodiumstacei_Brast09G142600.1.p Length: 286
# Brachypodiumstacei_Brast09G142600.1.p Number of predicted TMHs:  0
# Brachypodiumstacei_Brast09G142600.1.p Exp number of AAs in TMHs: 0.00018
# Brachypodiumstacei_Brast09G142600.1.p Exp number, first 60 AAs:  0
# Brachypodiumstacei_Brast09G142600.1.p Total prob of N-in:        0.01208
Brachypodiumstacei_Brast09G142600.1.p	TMHMM2.0	outside	     1   286
```

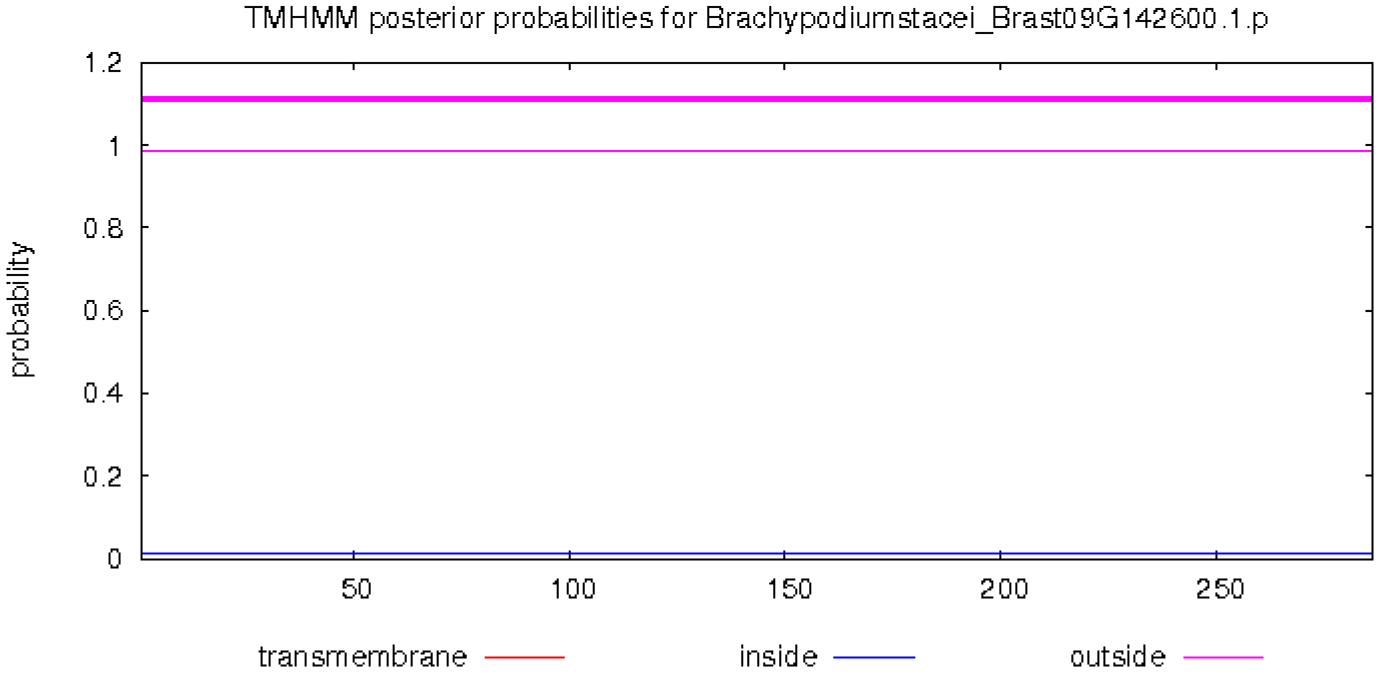

plot in postscript, script for making the plot in gnuplot, data for plot

---

```
# Brachypodiumstacei_Brast09G156400.1.p Length: 340
# Brachypodiumstacei_Brast09G156400.1.p Number of predicted TMHs:  0
# Brachypodiumstacei_Brast09G156400.1.p Exp number of AAs in TMHs: 0
# Brachypodiumstacei_Brast09G156400.1.p Exp number, first 60 AAs:  0
# Brachypodiumstacei_Brast09G156400.1.p Total prob of N-in:        0.00363
Brachypodiumstacei_Brast09G156400.1.p   TMHMM2.0        outside      1   340
```

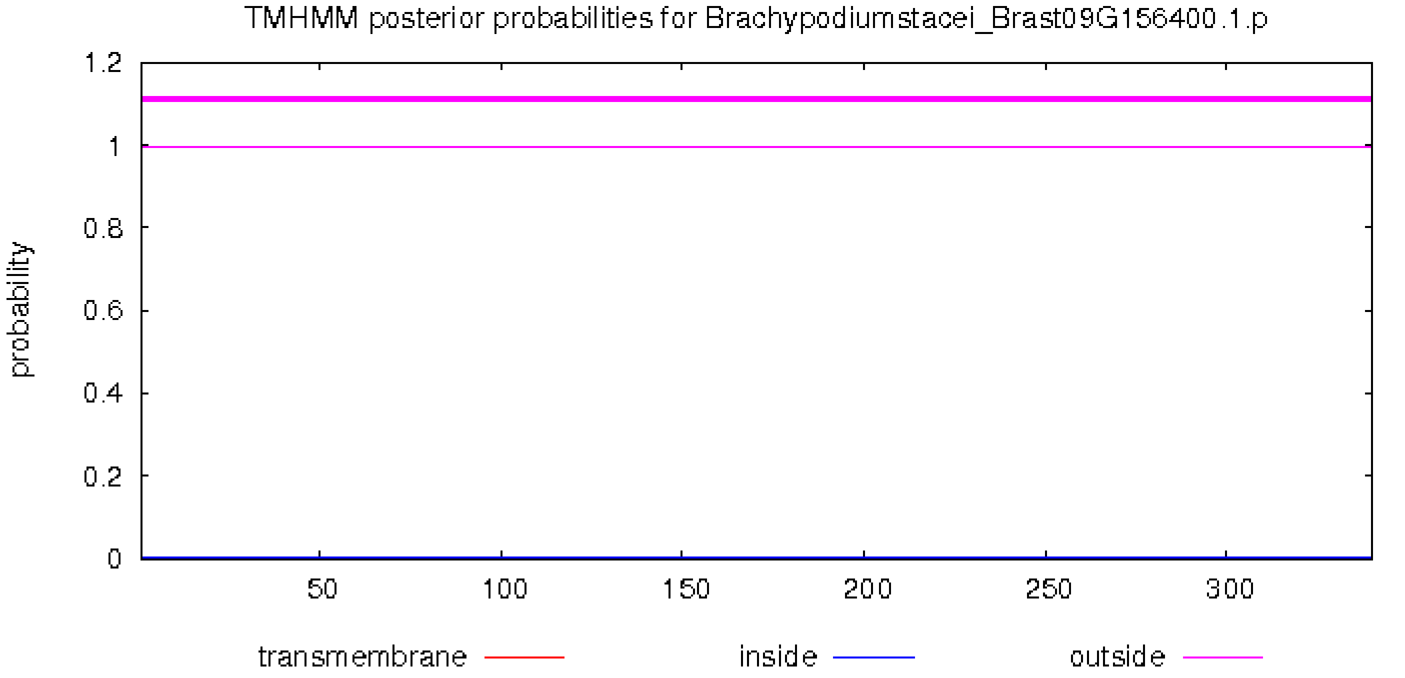

plot in postscript, script for making the plot in gnuplot, data for plot

---

```
# Brachypodiumstacei_Brast09G168600.1.p Length: 600
# Brachypodiumstacei_Brast09G168600.1.p Number of predicted TMHs:  0
# Brachypodiumstacei_Brast09G168600.1.p Exp number of AAs in TMHs: 0.00041
# Brachypodiumstacei_Brast09G168600.1.p Exp number, first 60 AAs:  0
# Brachypodiumstacei_Brast09G168600.1.p Total prob of N-in:        0.00141
Brachypodiumstacei_Brast09G168600.1.p   TMHMM2.0        outside      1   600
```

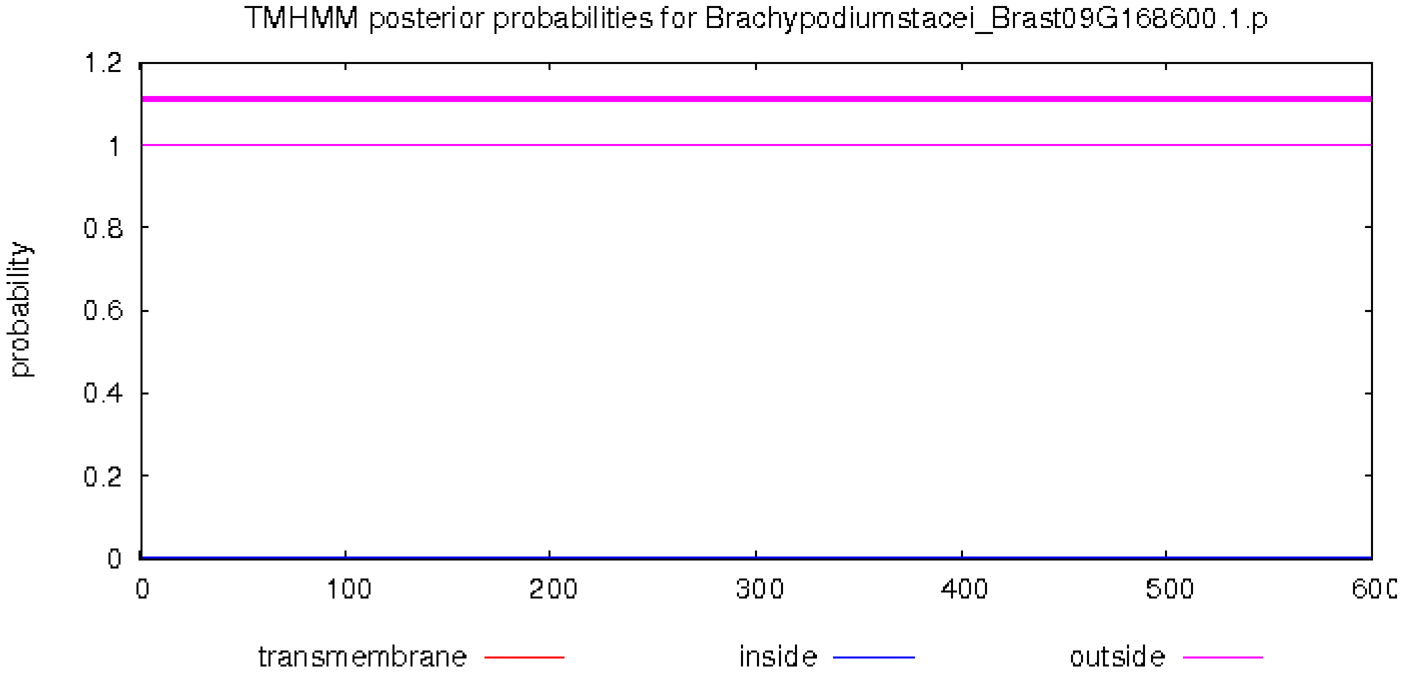

plot in postscript, script for making the plot in gnuplot, data for plot

---

```
# Brachypodiumstacei_Brast09G200100.1.p Length: 391
# Brachypodiumstacei_Brast09G200100.1.p Number of predicted TMHs:  0
# Brachypodiumstacei_Brast09G200100.1.p Exp number of AAs in TMHs: 0.0002
# Brachypodiumstacei_Brast09G200100.1.p Exp number, first 60 AAs:  0
# Brachypodiumstacei_Brast09G200100.1.p Total prob of N-in:        0.00376
Brachypodiumstacei_Brast09G200100.1.p   TMHMM2.0        outside      1   391
```

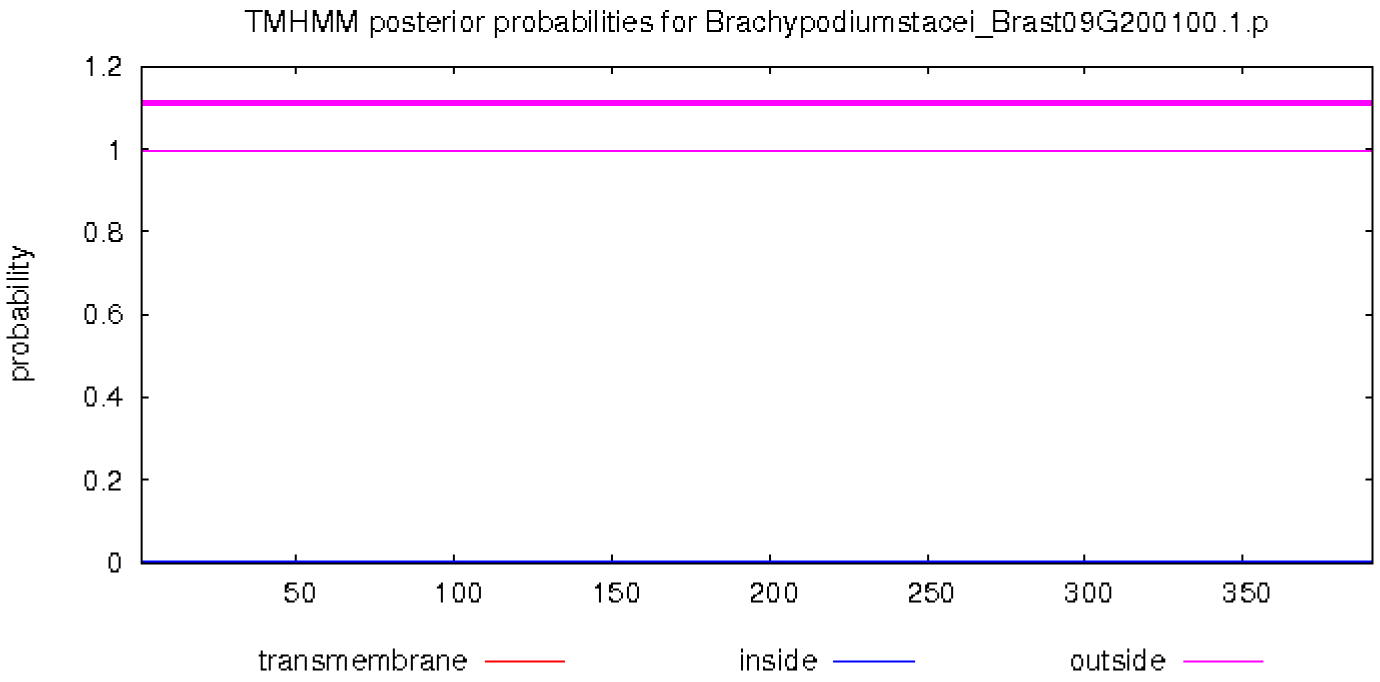

plot in postscript, script for making the plot in gnuplot, data for plot

---

```
# Brachypodiumstacei_Brast09G210800.1.p Length: 279
# Brachypodiumstacei_Brast09G210800.1.p Number of predicted TMHs:  0
# Brachypodiumstacei_Brast09G210800.1.p Exp number of AAs in TMHs: 0.35911
# Brachypodiumstacei_Brast09G210800.1.p Exp number, first 60 AAs:  0.00036
# Brachypodiumstacei_Brast09G210800.1.p Total prob of N-in:        0.09225
Brachypodiumstacei_Brast09G210800.1.p   TMHMM2.0        outside      1   279
```

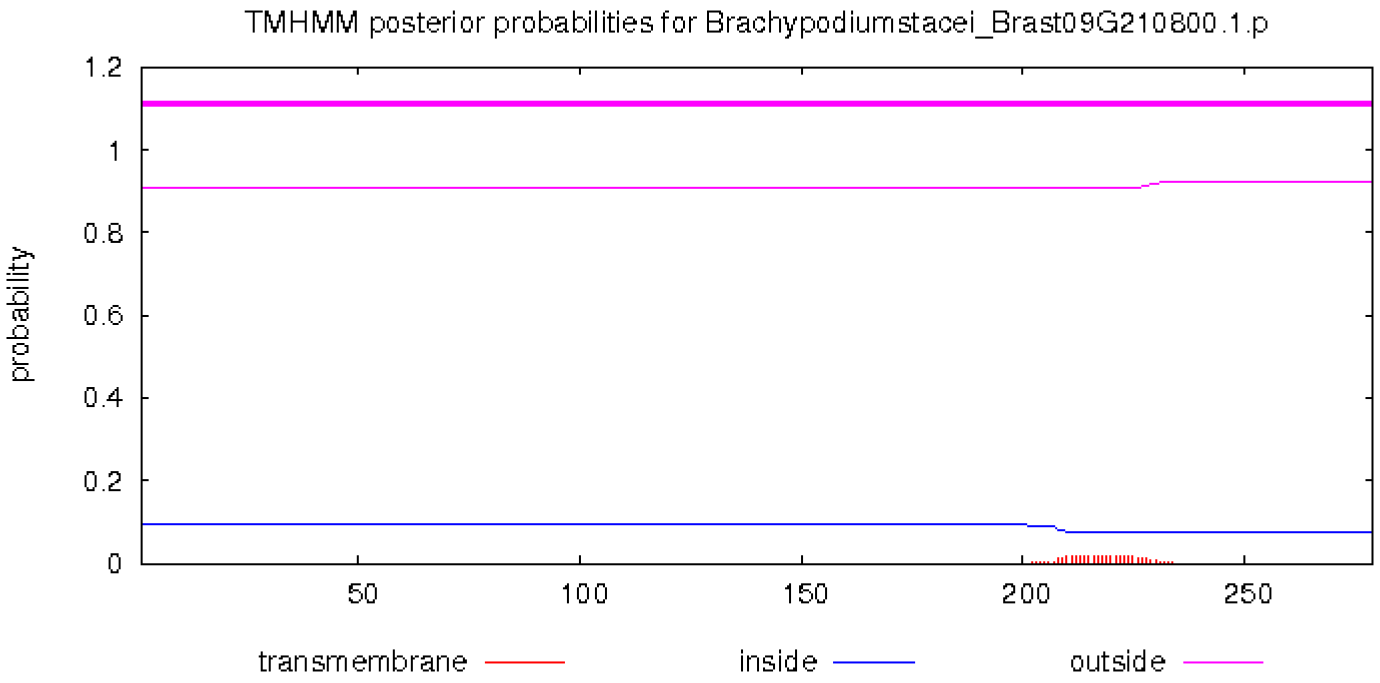

plot in postscript, script for making the plot in gnuplot, data for plot

---

```
# Brachypodiumstacei_Brast09G268800.1.p Length: 337
# Brachypodiumstacei_Brast09G268800.1.p Number of predicted TMHs:  0
# Brachypodiumstacei_Brast09G268800.1.p Exp number of AAs in TMHs: 0.06167
# Brachypodiumstacei_Brast09G268800.1.p Exp number, first 60 AAs:  0
# Brachypodiumstacei_Brast09G268800.1.p Total prob of N-in:        0.03814
Brachypodiumstacei_Brast09G268800.1.p   TMHMM2.0        outside      1   337
```

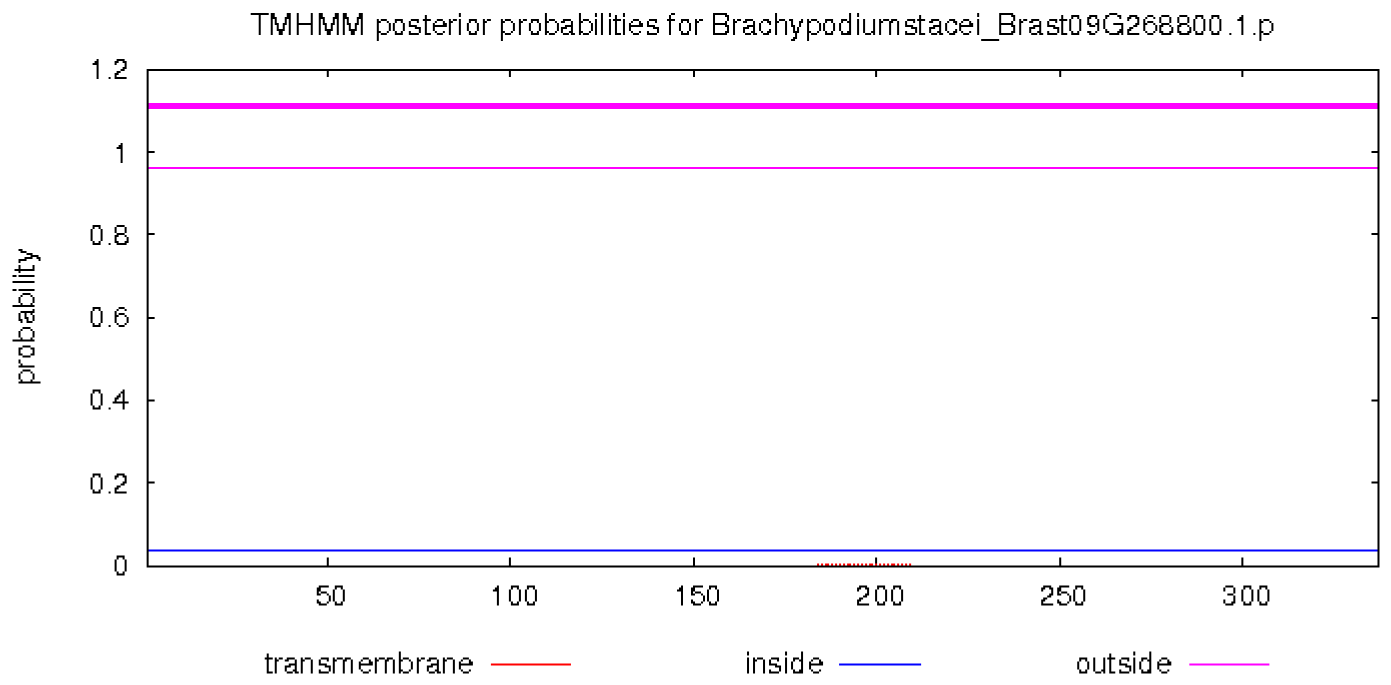

plot in postscript, script for making the plot in gnuplot, data for plot

---

```
# Brachypodiumstacei_Brast10G019700.1.p Length: 319
# Brachypodiumstacei_Brast10G019700.1.p Number of predicted TMHs:  0
# Brachypodiumstacei_Brast10G019700.1.p Exp number of AAs in TMHs: 0.00344
# Brachypodiumstacei_Brast10G019700.1.p Exp number, first 60 AAs:  0.00016
# Brachypodiumstacei_Brast10G019700.1.p Total prob of N-in:        0.00526
Brachypodiumstacei_Brast10G019700.1.p   TMHMM2.0        outside      1   319
```

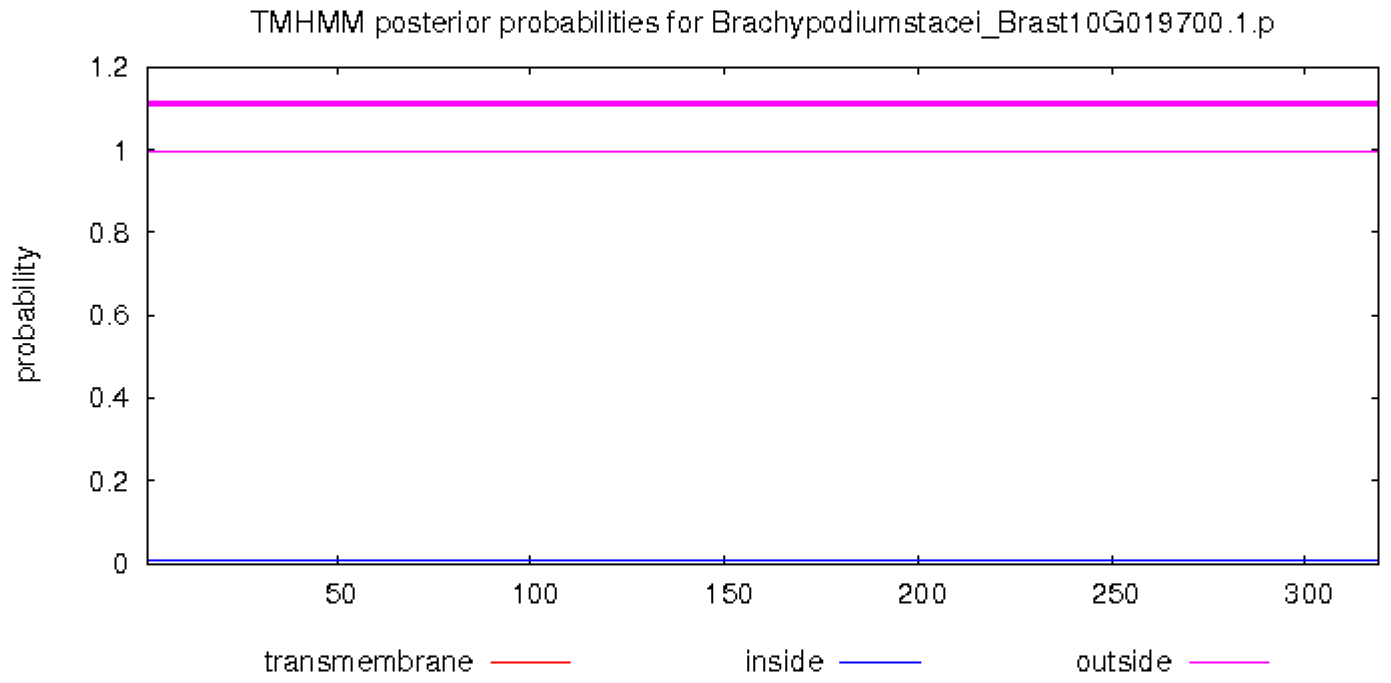

plot in postscript, script for making the plot in gnuplot, data for plot

---

```
# Brachypodiumstacei_Brast10G036500.1.p Length: 223
# Brachypodiumstacei_Brast10G036500.1.p Number of predicted TMHs:  0
# Brachypodiumstacei_Brast10G036500.1.p Exp number of AAs in TMHs: 0.00263
# Brachypodiumstacei_Brast10G036500.1.p Exp number, first 60 AAs:  0
# Brachypodiumstacei_Brast10G036500.1.p Total prob of N-in:        0.01197
Brachypodiumstacei_Brast10G036500.1.p	TMHMM2.0	outside	     1   223
```

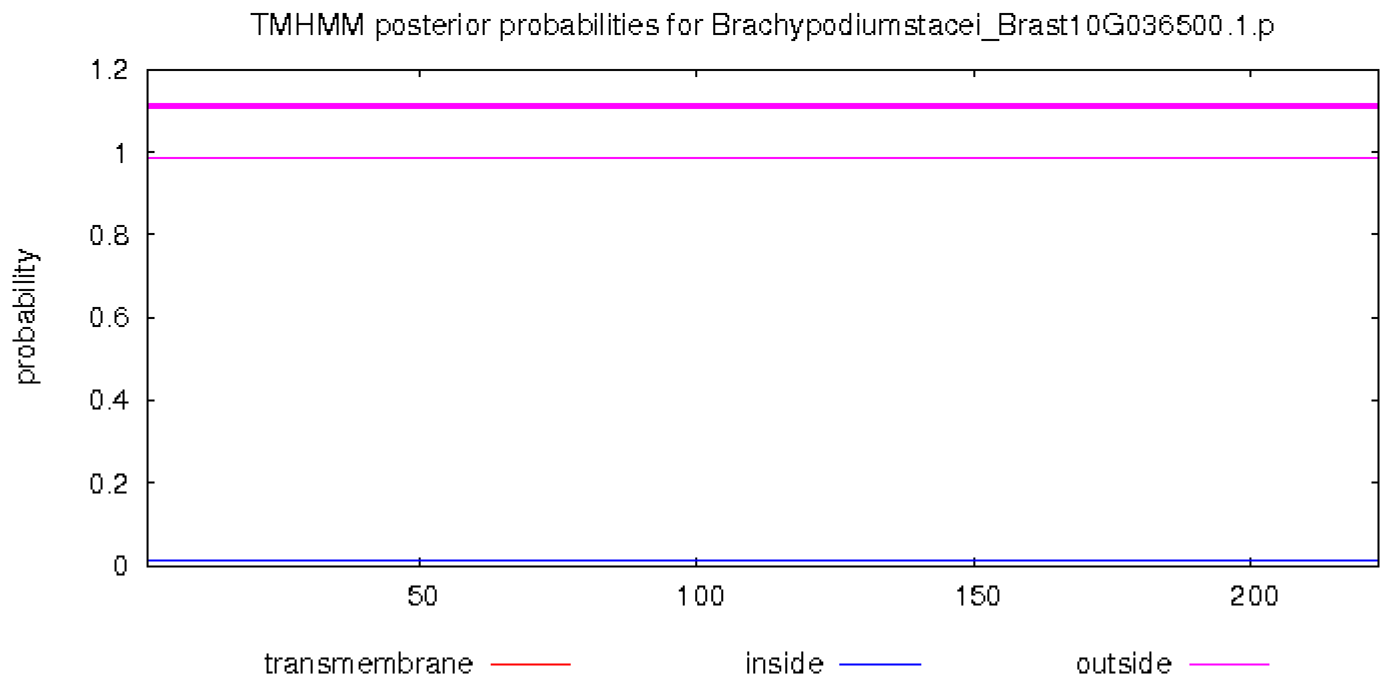

plot in postscript, script for making the plot in gnuplot, data for plot

---

```
# Brachypodiumstacei_Brast10G085400.1.p Length: 249
# Brachypodiumstacei_Brast10G085400.1.p Number of predicted TMHs:  0
# Brachypodiumstacei_Brast10G085400.1.p Exp number of AAs in TMHs: 0.08305
# Brachypodiumstacei_Brast10G085400.1.p Exp number, first 60 AAs:  0
# Brachypodiumstacei_Brast10G085400.1.p Total prob of N-in:        0.10610
Brachypodiumstacei_Brast10G085400.1.p	TMHMM2.0	outside	     1   249
```

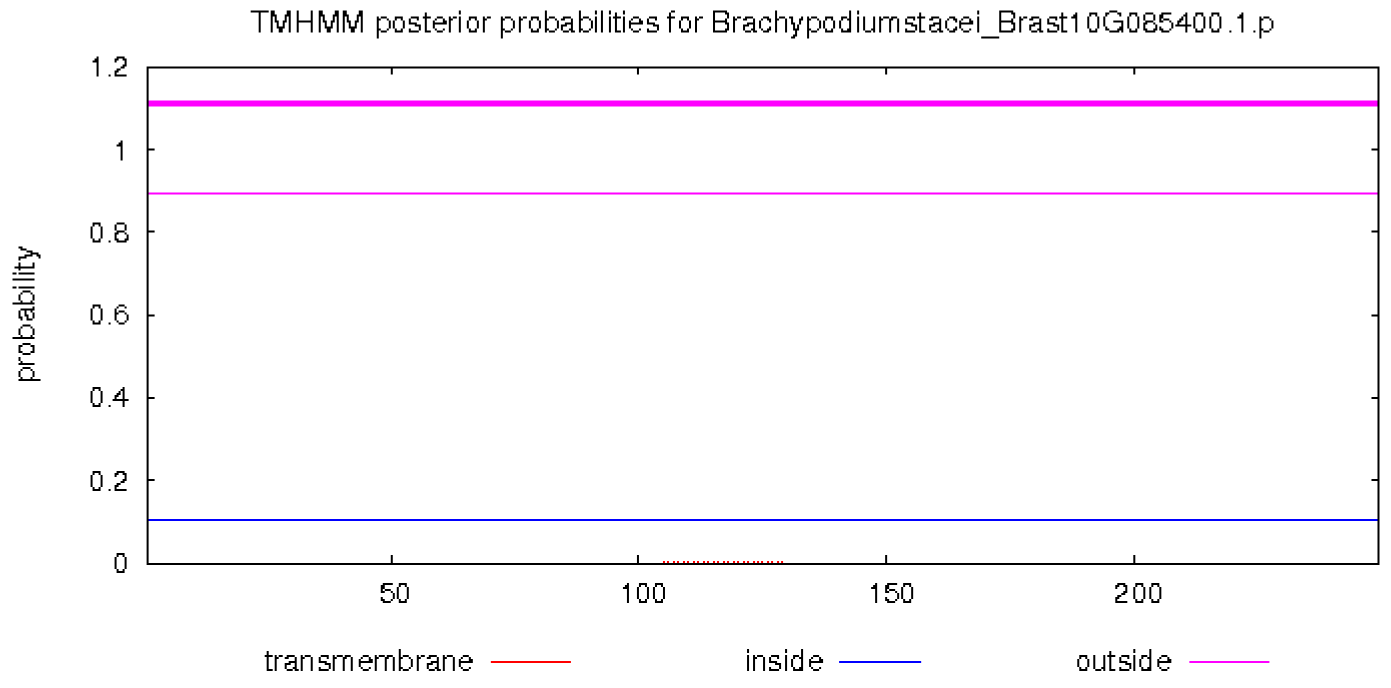

plot in postscript, script for making the plot in gnuplot, data for plot

---

```
# Brassicanapus_GSBRNA2T00000394001 Length: 455
# Brassicanapus_GSBRNA2T00000394001 Number of predicted TMHs:  0
# Brassicanapus_GSBRNA2T00000394001 Exp number of AAs in TMHs: 0.11818
# Brassicanapus_GSBRNA2T00000394001 Exp number, first 60 AAs:  0.08842
# Brassicanapus_GSBRNA2T00000394001 Total prob of N-in:        0.00764
Brassicanapus_GSBRNA2T00000394001       TMHMM2.0        outside      1   455
```

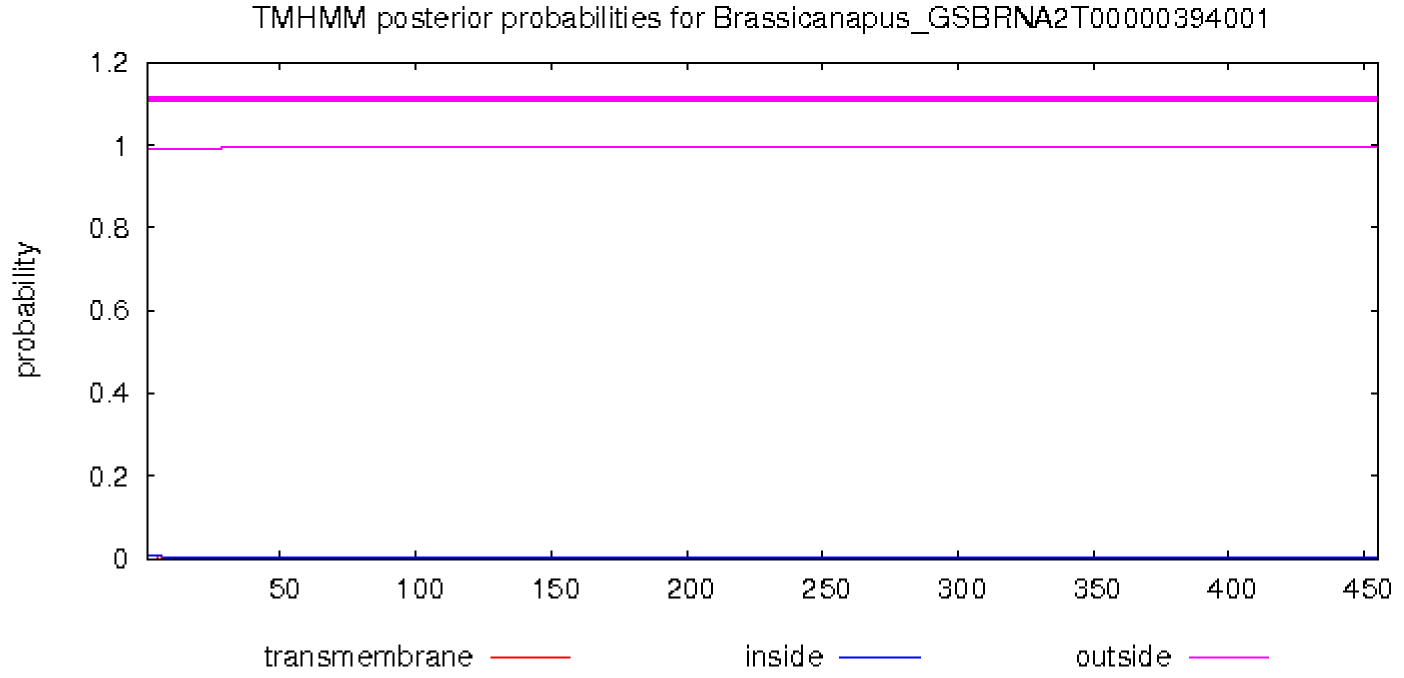

plot in postscript, script for making the plot in gnuplot, data for plot

---

```
# Brassicanapus_GSBRNA2T00000580001 Length: 180
# Brassicanapus_GSBRNA2T00000580001 Number of predicted TMHs:  0
# Brassicanapus_GSBRNA2T00000580001 Exp number of AAs in TMHs: 1.5762
# Brassicanapus_GSBRNA2T00000580001 Exp number, first 60 AAs:  0.02258
# Brassicanapus_GSBRNA2T00000580001 Total prob of N-in:        0.08226
Brassicanapus_GSBRNA2T00000580001       TMHMM2.0        outside      1   180
```

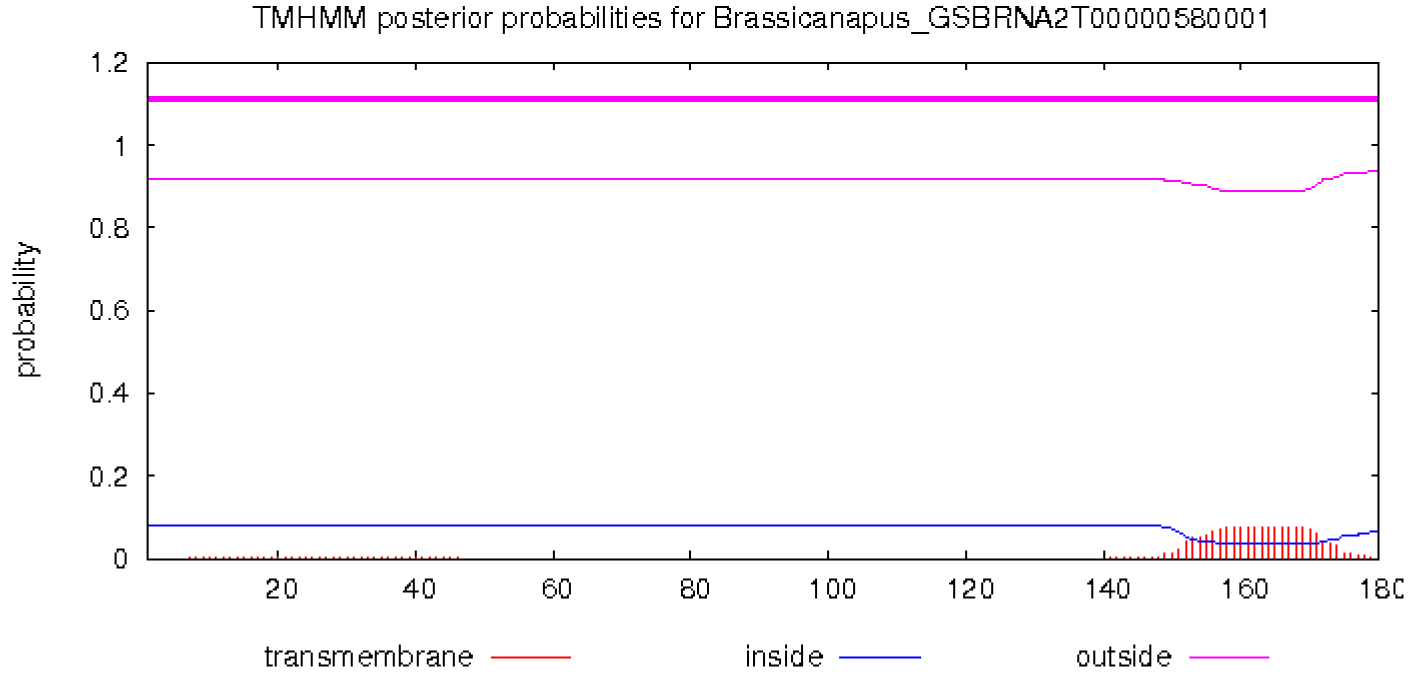

plot in postscript, script for making the plot in gnuplot, data for plot

---

```
# Brassicanapus_GSBRNA2T00001356001 Length: 343
# Brassicanapus_GSBRNA2T00001356001 Number of predicted TMHs:  0
# Brassicanapus_GSBRNA2T00001356001 Exp number of AAs in TMHs: 10.14168
# Brassicanapus_GSBRNA2T00001356001 Exp number, first 60 AAs:  0.00982
# Brassicanapus_GSBRNA2T00001356001 Total prob of N-in:        0.05612
Brassicanapus_GSBRNA2T00001356001	TMHMM2.0	outside	     1   343
```

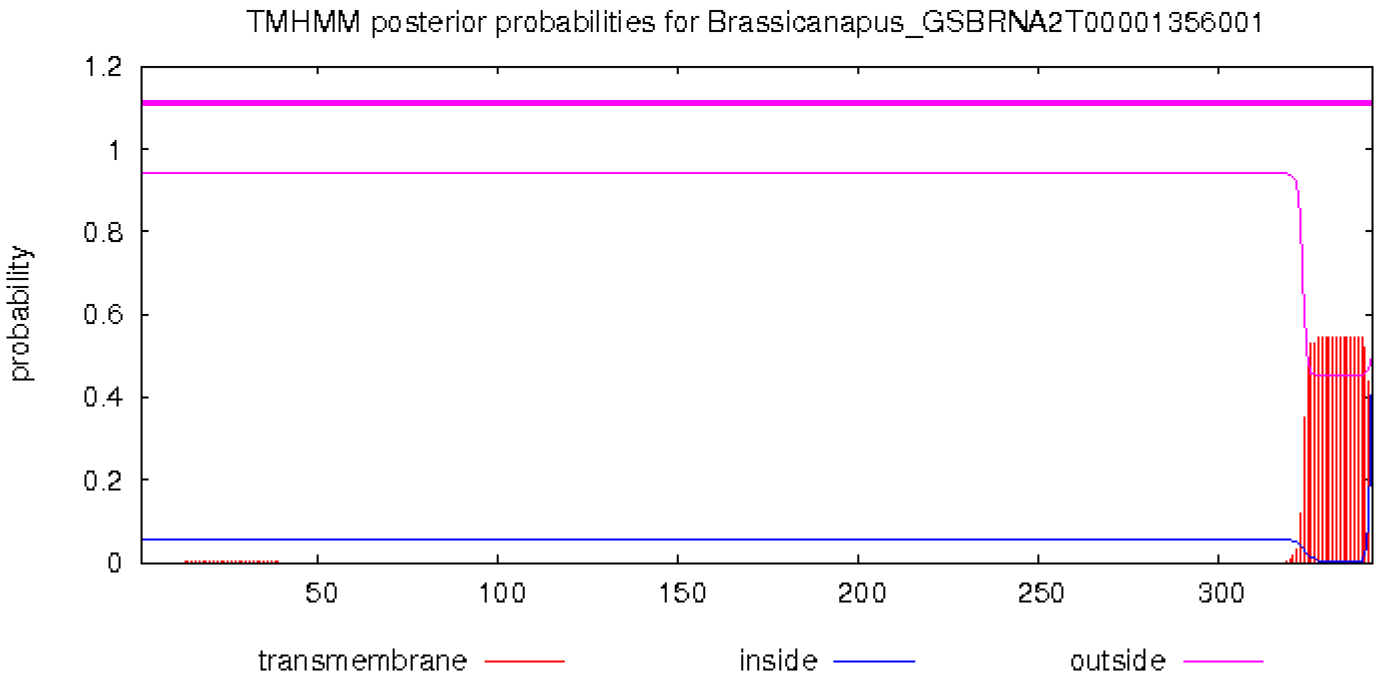

plot in postscript, script for making the plot in gnuplot, data for plot

---

```
# Brassicanapus_GSBRNA2T00001362001 Length: 235
# Brassicanapus_GSBRNA2T00001362001 Number of predicted TMHs:  0
# Brassicanapus_GSBRNA2T00001362001 Exp number of AAs in TMHs: 0
# Brassicanapus_GSBRNA2T00001362001 Exp number, first 60 AAs:  0
# Brassicanapus_GSBRNA2T00001362001 Total prob of N-in:        0.02517
Brassicanapus_GSBRNA2T00001362001	TMHMM2.0	outside	     1   235
```

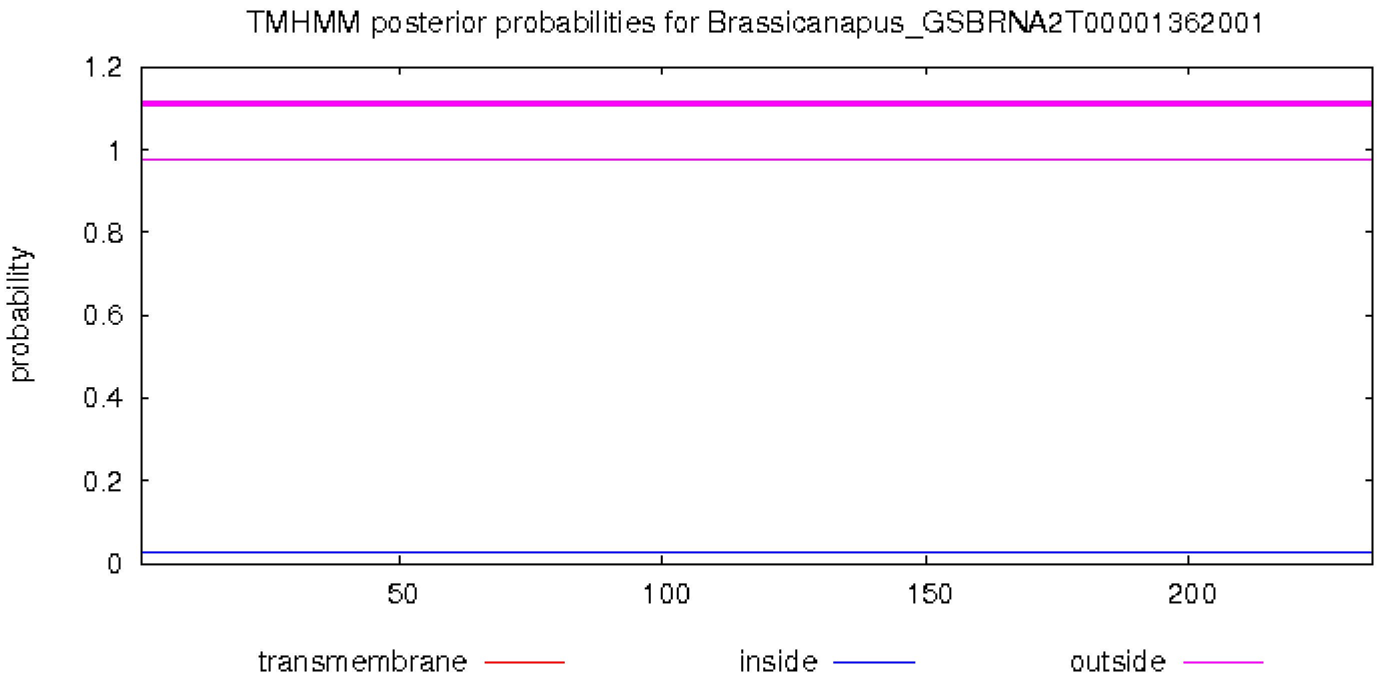

plot in postscript, script for making the plot in gnuplot, data for plot

---

```
# Brassicanapus_GSBRNA2T00001799001 Length: 417
# Brassicanapus_GSBRNA2T00001799001 Number of predicted TMHs:  0
# Brassicanapus_GSBRNA2T00001799001 Exp number of AAs in TMHs: 0.03475
# Brassicanapus_GSBRNA2T00001799001 Exp number, first 60 AAs:  0
# Brassicanapus_GSBRNA2T00001799001 Total prob of N-in:        0.00878
Brassicanapus_GSBRNA2T00001799001       TMHMM2.0        outside      1   417
```

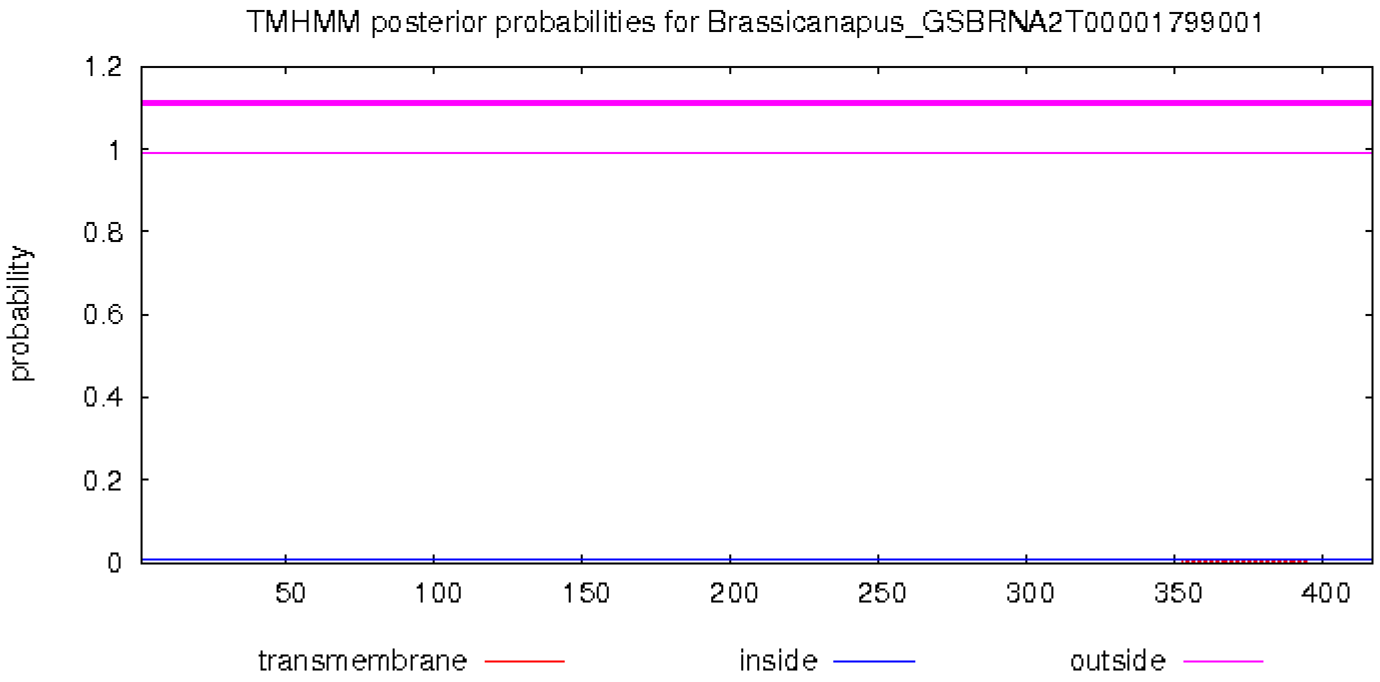

plot in postscript, script for making the plot in gnuplot, data for plot

---

```
# Brassicanapus_GSBRNA2T00002998001 Length: 337
# Brassicanapus_GSBRNA2T00002998001 Number of predicted TMHs:  0
# Brassicanapus_GSBRNA2T00002998001 Exp number of AAs in TMHs: 0.00059
# Brassicanapus_GSBRNA2T00002998001 Exp number, first 60 AAs:  0
# Brassicanapus_GSBRNA2T00002998001 Total prob of N-in:        0.00891
Brassicanapus_GSBRNA2T00002998001       TMHMM2.0        outside      1   337
```

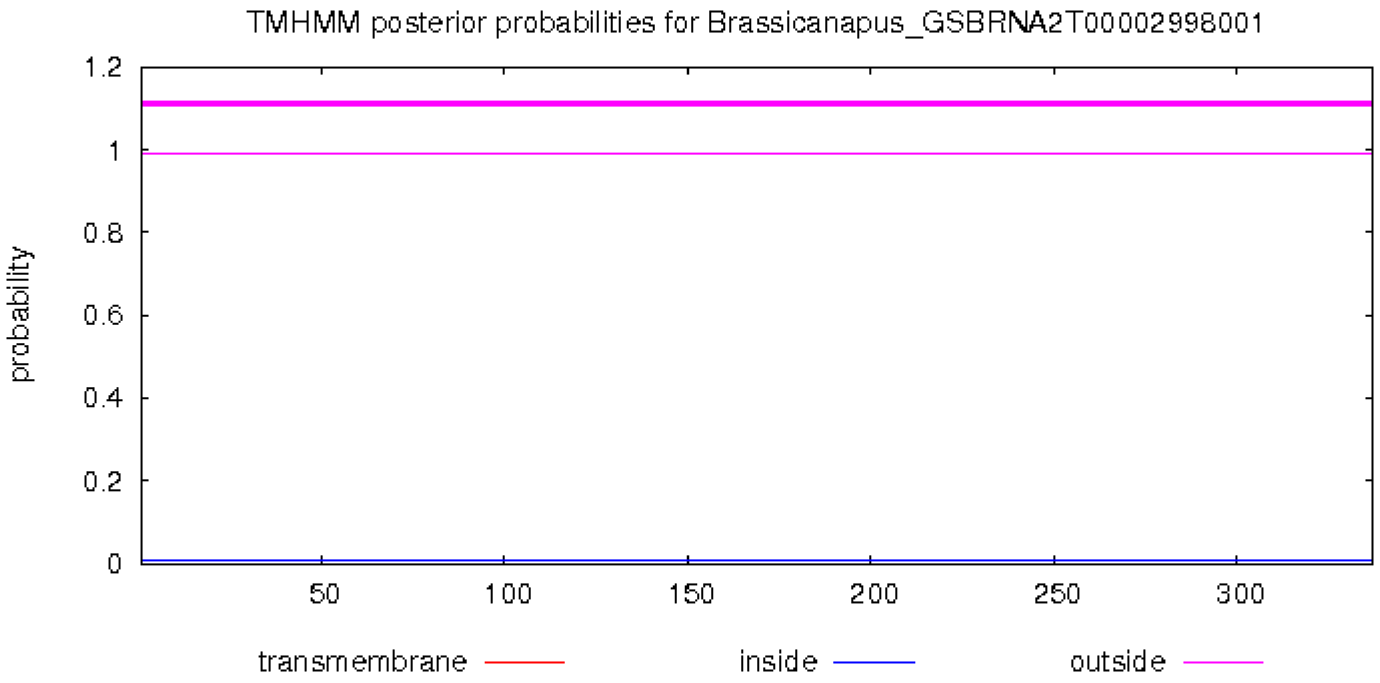

plot in postscript, script for making the plot in gnuplot, data for plot

---

```
# Brassicanapus_GSBRNA2T00003061001 Length: 492
# Brassicanapus_GSBRNA2T00003061001 Number of predicted TMHs:  0
# Brassicanapus_GSBRNA2T00003061001 Exp number of AAs in TMHs: 0
# Brassicanapus_GSBRNA2T00003061001 Exp number, first 60 AAs:  0
# Brassicanapus_GSBRNA2T00003061001 Total prob of N-in:        0.00705
Brassicanapus_GSBRNA2T00003061001       TMHMM2.0        outside      1   492
```

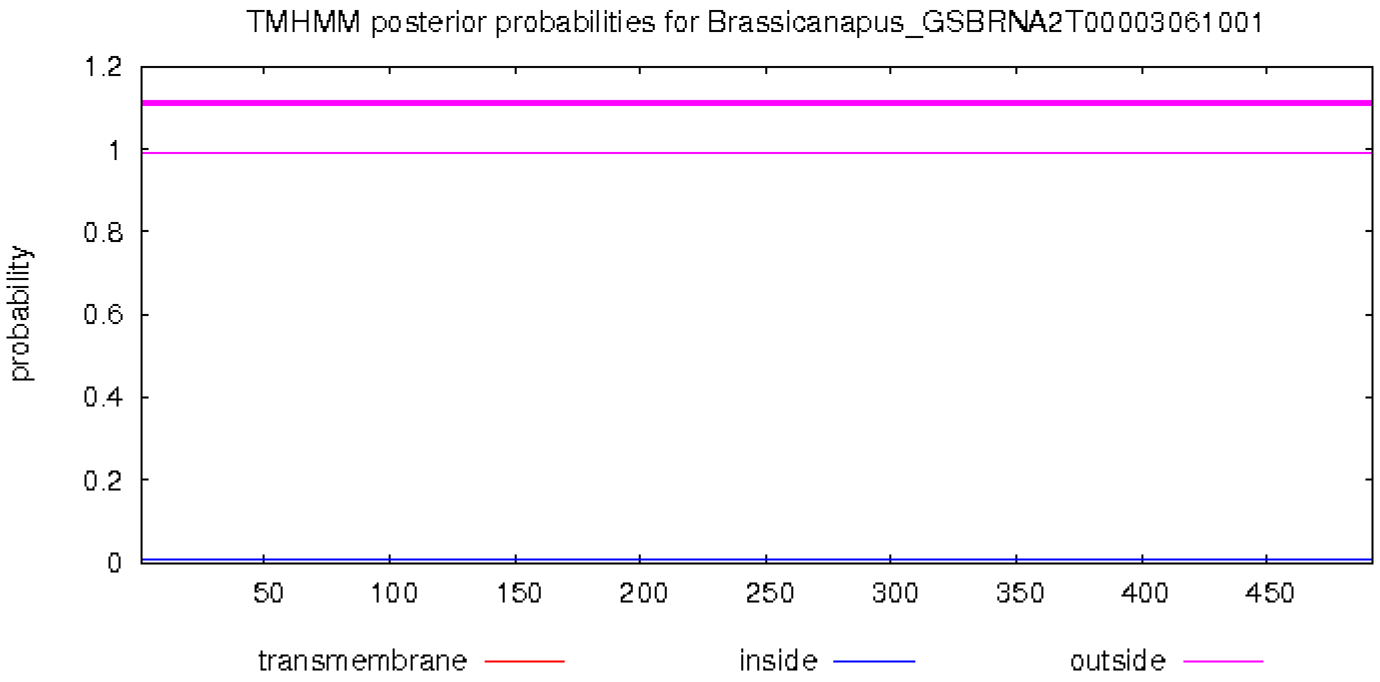

plot in postscript, script for making the plot in gnuplot, data for plot

---

```
# Brassicanapus_GSBRNA2T00003098001 Length: 188
# Brassicanapus_GSBRNA2T00003098001 Number of predicted TMHs:  0
# Brassicanapus_GSBRNA2T00003098001 Exp number of AAs in TMHs: 0
# Brassicanapus_GSBRNA2T00003098001 Exp number, first 60 AAs:  0
# Brassicanapus_GSBRNA2T00003098001 Total prob of N-in:        0.00724
Brassicanapus_GSBRNA2T00003098001       TMHMM2.0        outside      1   188
```

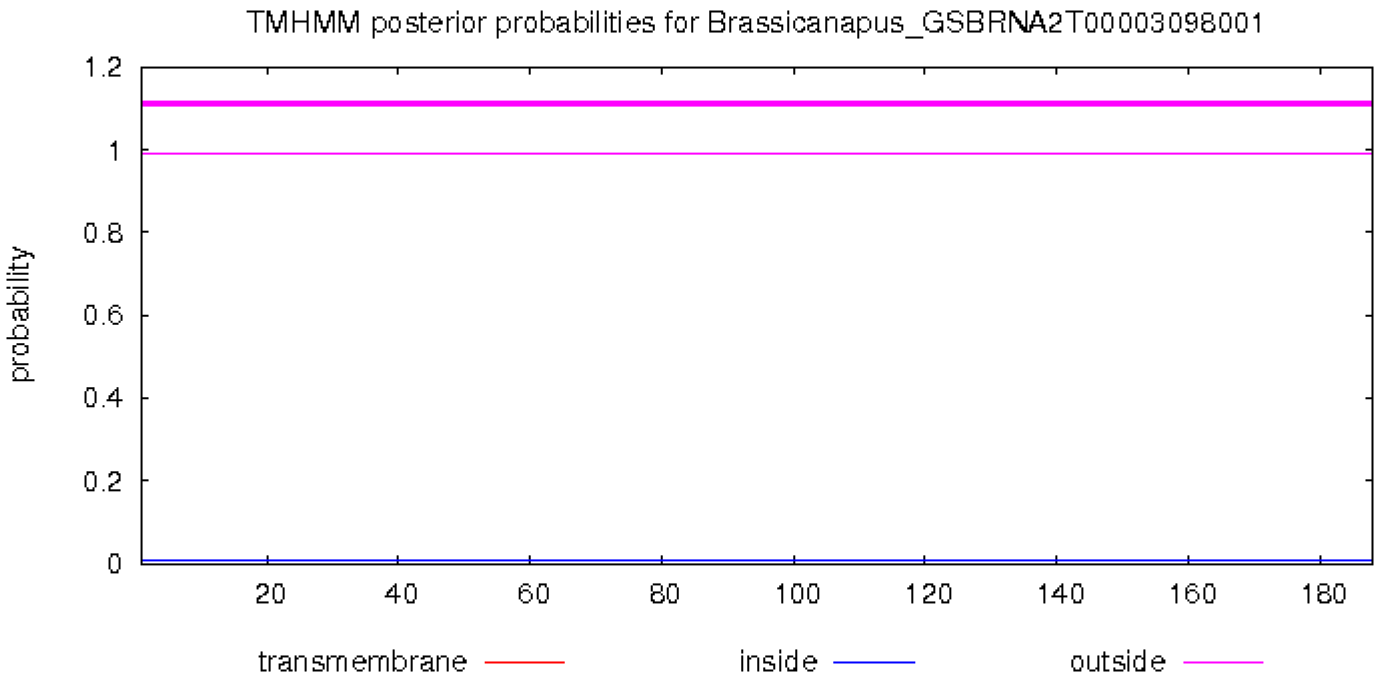

plot in postscript, script for making the plot in gnuplot, data for plot

---

```
# Brassicanapus_GSBRNA2T00003406001 Length: 253
# Brassicanapus_GSBRNA2T00003406001 Number of predicted TMHs:  0
# Brassicanapus_GSBRNA2T00003406001 Exp number of AAs in TMHs: 0.0004
# Brassicanapus_GSBRNA2T00003406001 Exp number, first 60 AAs:  0
# Brassicanapus_GSBRNA2T00003406001 Total prob of N-in:        0.09859
Brassicanapus_GSBRNA2T00003406001       TMHMM2.0        outside      1   253
```

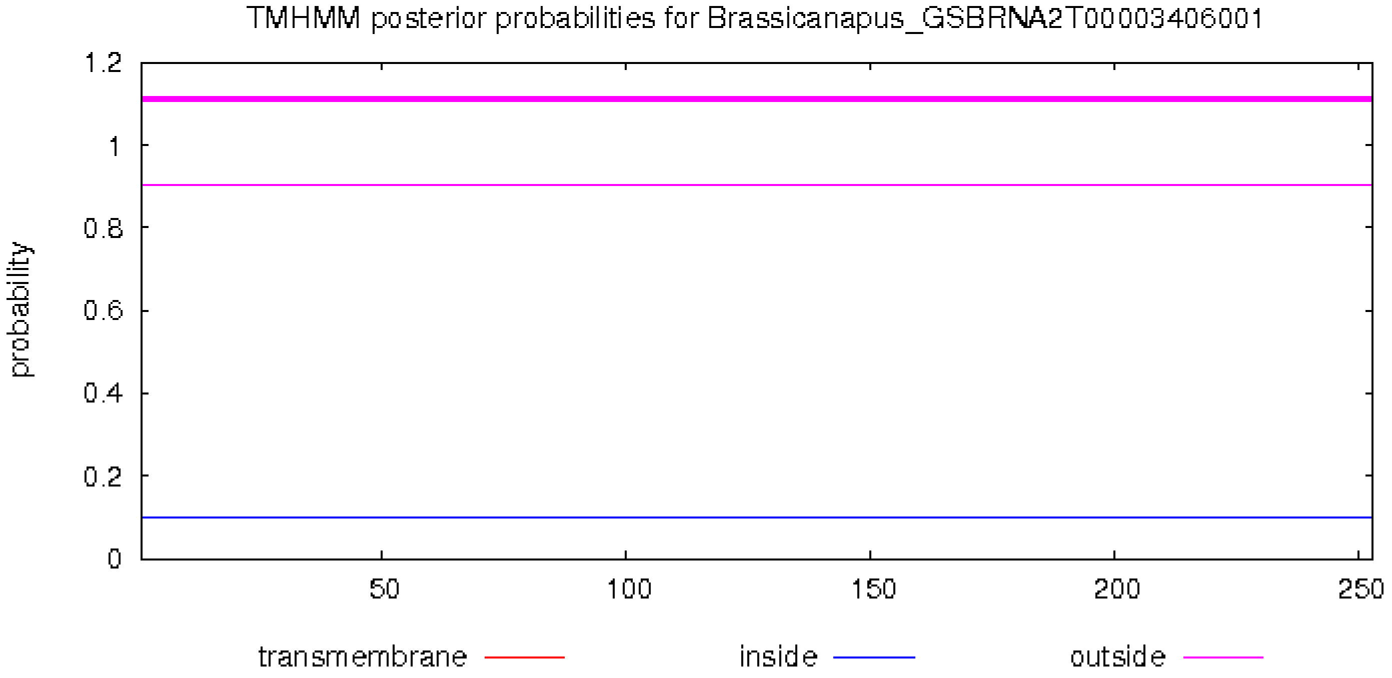

plot in postscript, script for making the plot in gnuplot, data for plot

```
# Brassicanapus_GSBRNA2T00003593001 Length: 272
# Brassicanapus_GSBRNA2T00003593001 Number of predicted TMHs:  0
# Brassicanapus_GSBRNA2T00003593001 Exp number of AAs in TMHs: 0.00803
# Brassicanapus_GSBRNA2T00003593001 Exp number, first 60 AAs:  0
# Brassicanapus_GSBRNA2T00003593001 Total prob of N-in:        0.05785
Brassicanapus_GSBRNA2T00003593001       TMHMM2.0        outside      1   272
```

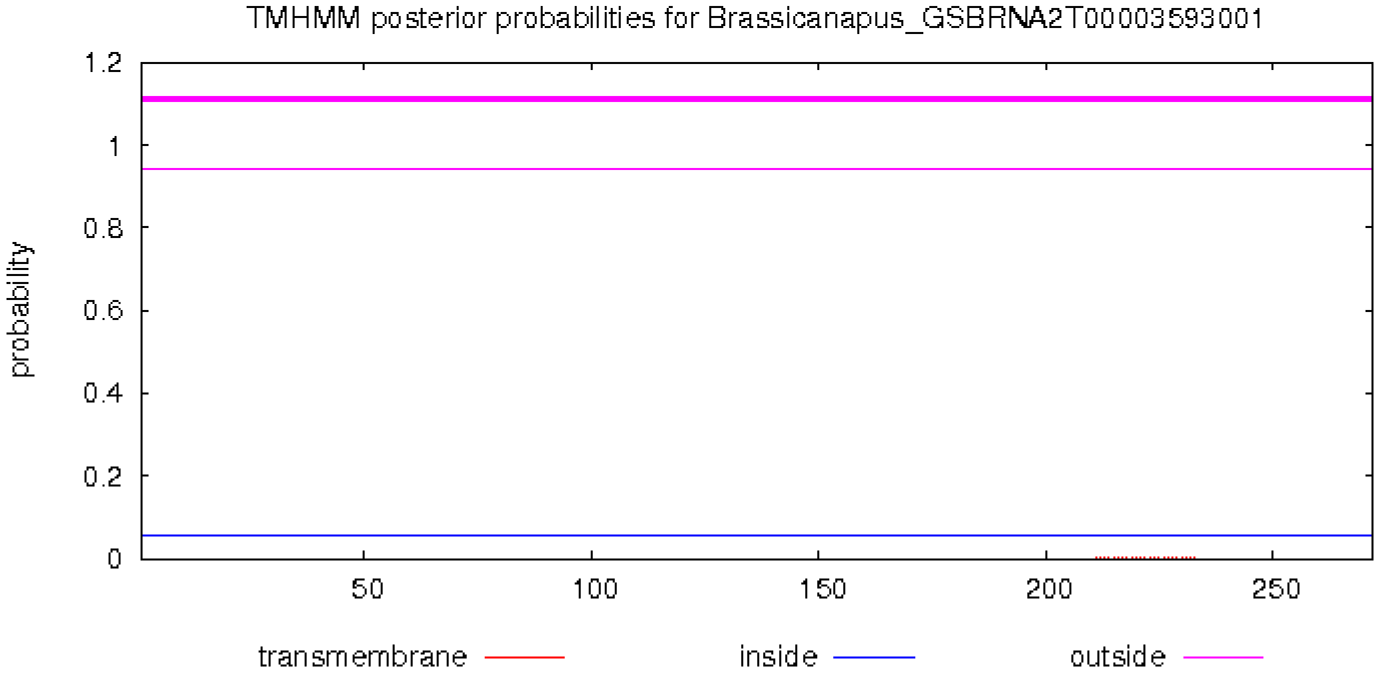

plot in postscript, script for making the plot in gnuplot, data for plot

```
# Brassicanapus_GSBRNA2T00004056001 Length: 158
# Brassicanapus_GSBRNA2T00004056001 Number of predicted TMHs:  0
# Brassicanapus_GSBRNA2T00004056001 Exp number of AAs in TMHs: 0.00018
# Brassicanapus_GSBRNA2T00004056001 Exp number, first 60 AAs:  0
# Brassicanapus_GSBRNA2T00004056001 Total prob of N-in:        0.07300
Brassicanapus_GSBRNA2T00004056001	TMHMM2.0	outside	     1   158
```

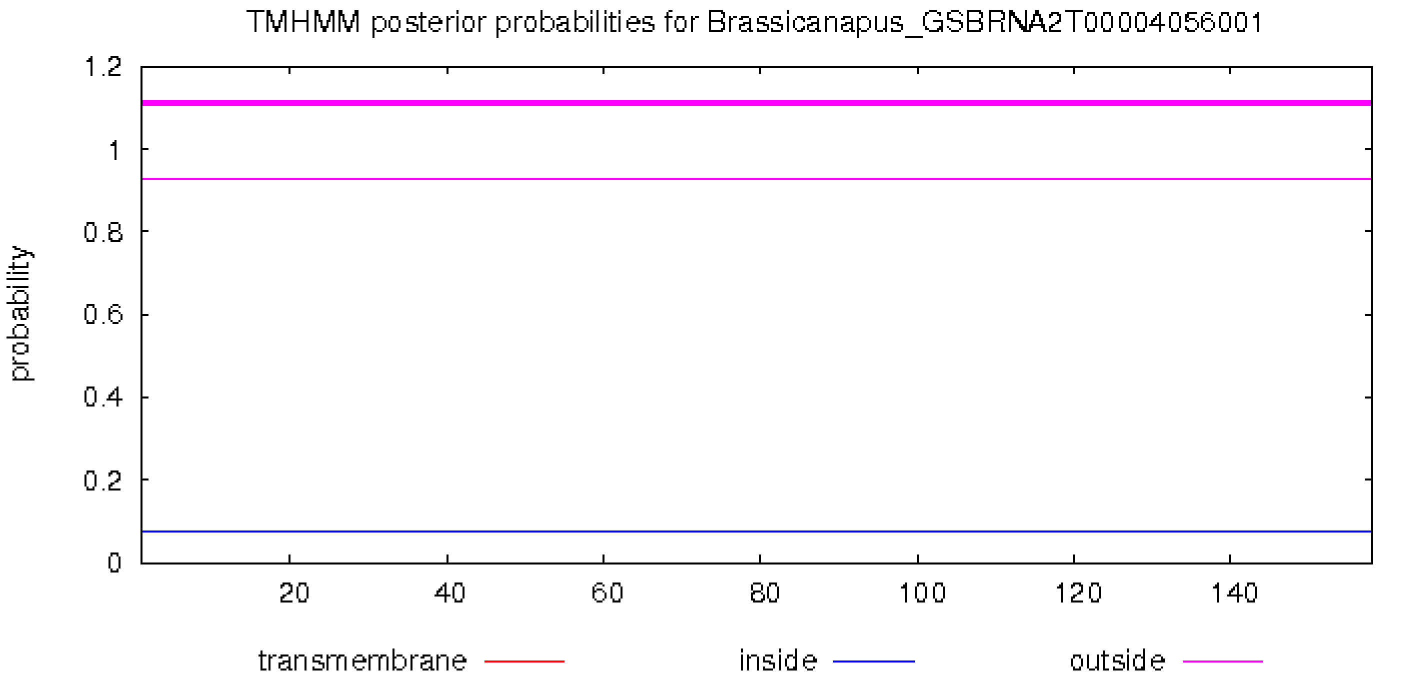

plot in postscript, script for making the plot in gnuplot, data for plot

```
# Brassicanapus_GSBRNA2T00004489001 Length: 250
# Brassicanapus_GSBRNA2T00004489001 Number of predicted TMHs:  0
# Brassicanapus_GSBRNA2T00004489001 Exp number of AAs in TMHs: 0.00017
# Brassicanapus_GSBRNA2T00004489001 Exp number, first 60 AAs:  0
# Brassicanapus_GSBRNA2T00004489001 Total prob of N-in:        0.08527
Brassicanapus_GSBRNA2T00004489001	TMHMM2.0	outside	     1   250
```

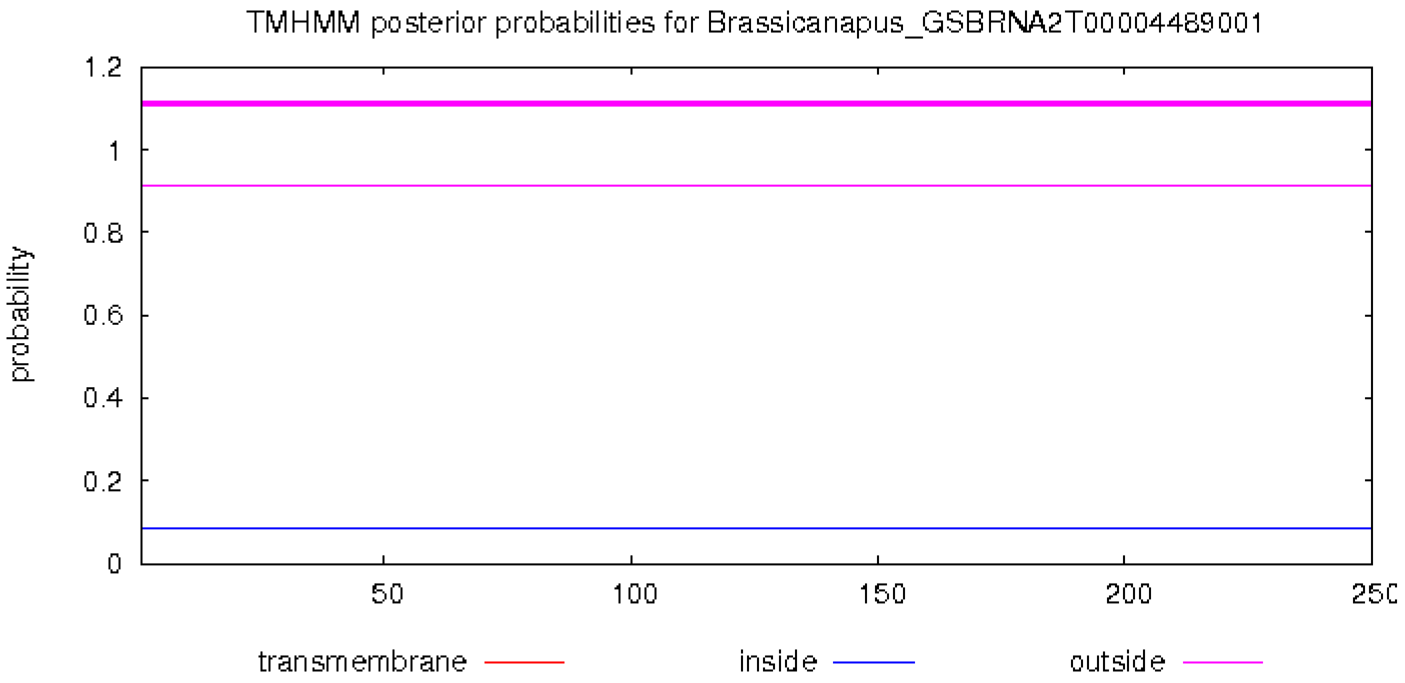

plot in postscript, script for making the plot in gnuplot, data for plot

```
# Brassicanapus_GSBRNA2T00004530001 Length: 299
# Brassicanapus_GSBRNA2T00004530001 Number of predicted TMHs:  0
# Brassicanapus_GSBRNA2T00004530001 Exp number of AAs in TMHs: 0.00407
# Brassicanapus_GSBRNA2T00004530001 Exp number, first 60 AAs:  0
# Brassicanapus_GSBRNA2T00004530001 Total prob of N-in:        0.00484
Brassicanapus_GSBRNA2T00004530001	TMHMM2.0	outside	     1   299
```

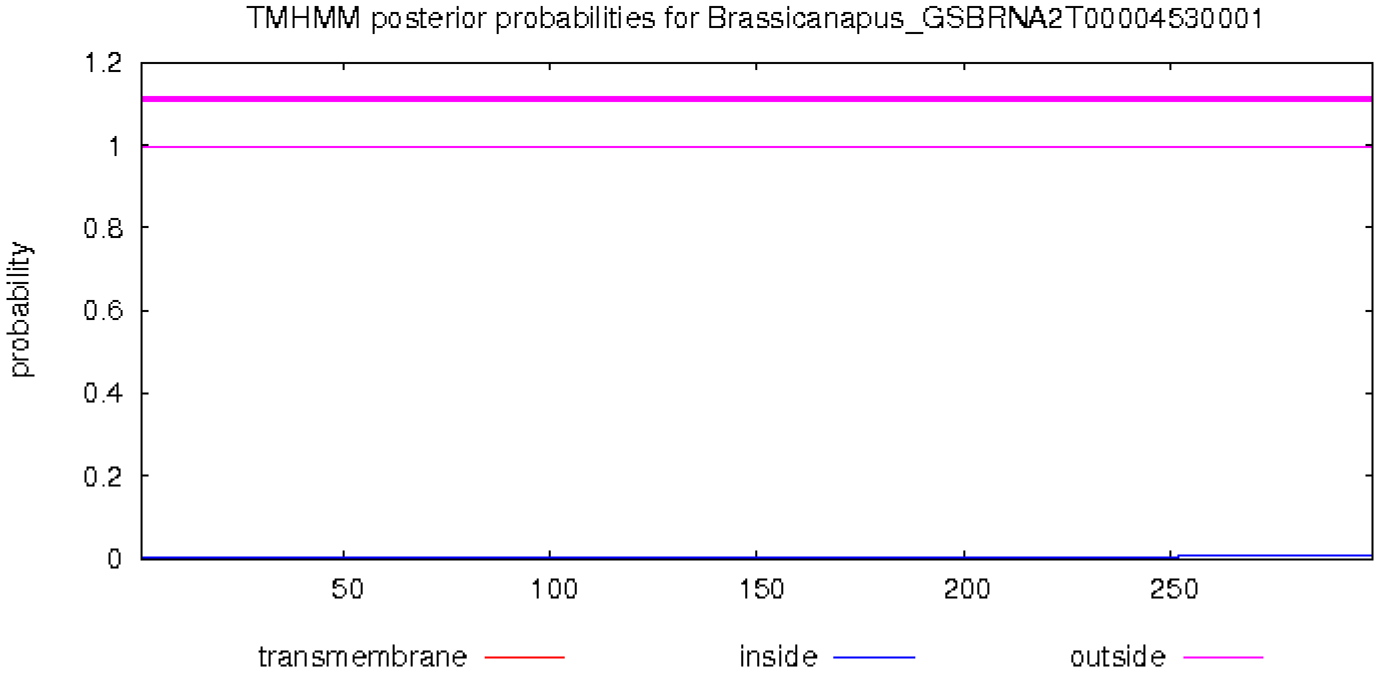

plot in postscript, script for making the plot in gnuplot, data for plot

---

```
# Brassicanapus_GSBRNA2T00004862001 Length: 165
# Brassicanapus_GSBRNA2T00004862001 Number of predicted TMHs:  0
# Brassicanapus_GSBRNA2T00004862001 Exp number of AAs in TMHs: 0
# Brassicanapus_GSBRNA2T00004862001 Exp number, first 60 AAs:  0
# Brassicanapus_GSBRNA2T00004862001 Total prob of N-in:        0.14552
Brassicanapus_GSBRNA2T00004862001	TMHMM2.0	outside	     1   165
```

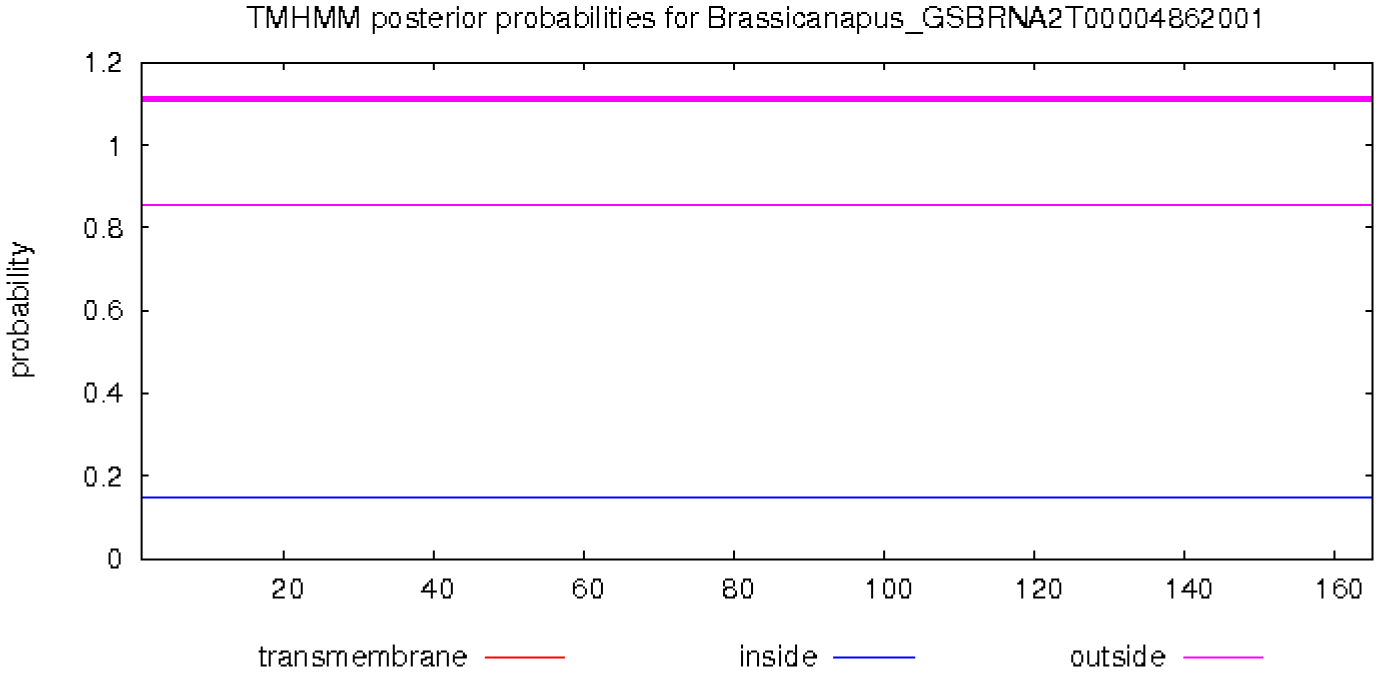

plot in postscript, script for making the plot in gnuplot, data for plot

---

```
# Brassicanapus_GSBRNA2T00004863001 Length: 396
# Brassicanapus_GSBRNA2T00004863001 Number of predicted TMHs:  1
# Brassicanapus_GSBRNA2T00004863001 Exp number of AAs in TMHs: 19.54567
# Brassicanapus_GSBRNA2T00004863001 Exp number, first 60 AAs:  19.00262
# Brassicanapus_GSBRNA2T00004863001 Total prob of N-in:        0.16233
# Brassicanapus_GSBRNA2T00004863001 POSSIBLE N-term signal sequence
Brassicanapus_GSBRNA2T00004863001       TMHMM2.0        outside      1    14
Brassicanapus_GSBRNA2T00004863001       TMHMM2.0        TMhelix     15    34
Brassicanapus_GSBRNA2T00004863001       TMHMM2.0        inside      35   396
```

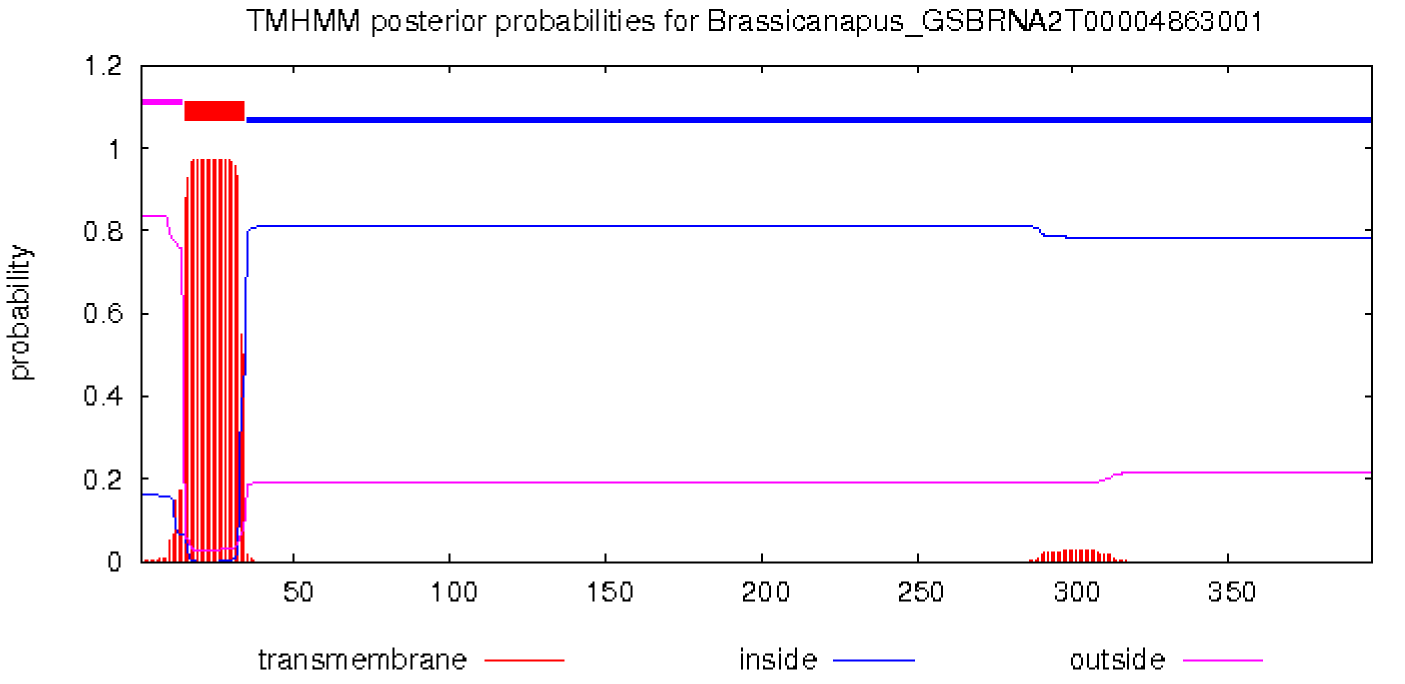

plot in postscript, script for making the plot in gnuplot, data for plot

---

```
# Brassicanapus_GSBRNA2T00005539001 Length: 302
# Brassicanapus_GSBRNA2T00005539001 Number of predicted TMHs:  0
# Brassicanapus_GSBRNA2T00005539001 Exp number of AAs in TMHs: 0.4622
# Brassicanapus_GSBRNA2T00005539001 Exp number, first 60 AAs:  0
# Brassicanapus_GSBRNA2T00005539001 Total prob of N-in:        0.00531
Brassicanapus_GSBRNA2T00005539001       TMHMM2.0        outside      1   302
```

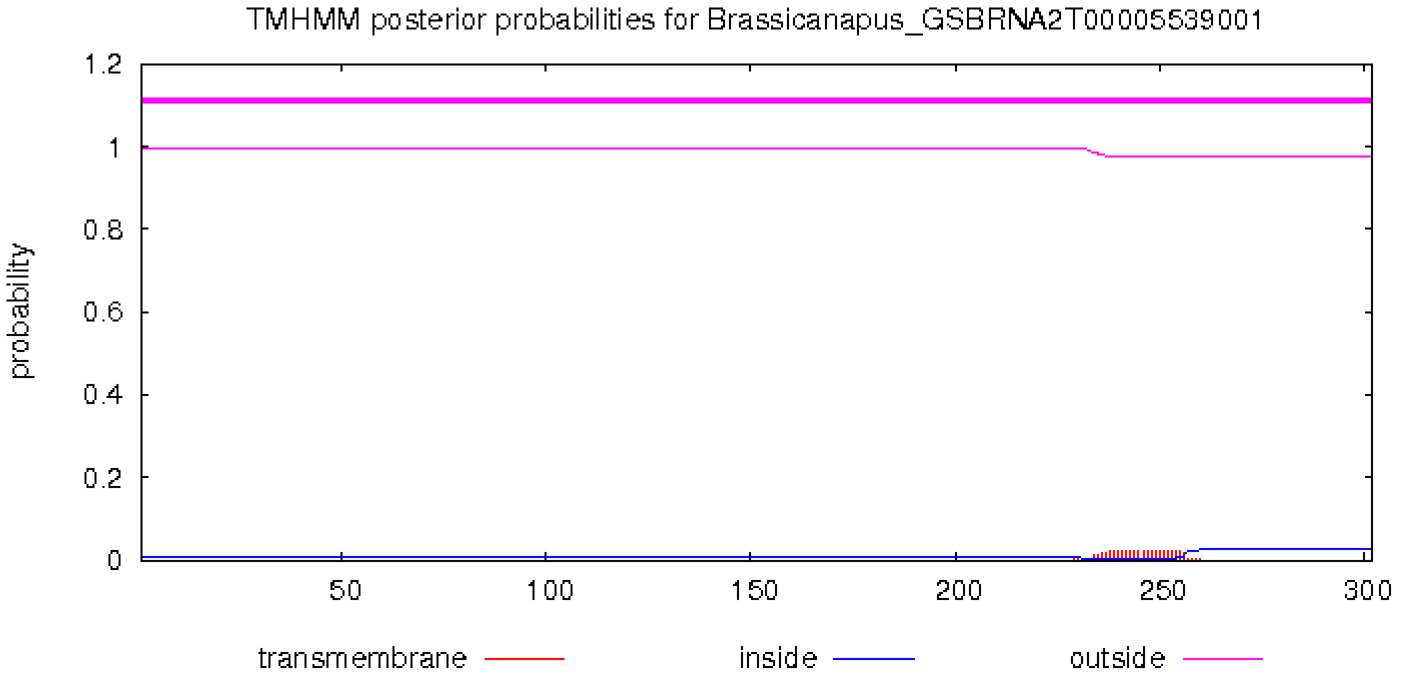

plot in postscript, script for making the plot in gnuplot, data for plot

```
# Brassicanapus_GSBRNA2T00005792001 Length: 188
# Brassicanapus_GSBRNA2T00005792001 Number of predicted TMHs:  0
# Brassicanapus_GSBRNA2T00005792001 Exp number of AAs in TMHs: 0
# Brassicanapus_GSBRNA2T00005792001 Exp number, first 60 AAs:  0
# Brassicanapus_GSBRNA2T00005792001 Total prob of N-in:        0.00724
Brassicanapus_GSBRNA2T00005792001       TMHMM2.0        outside      1   188
```

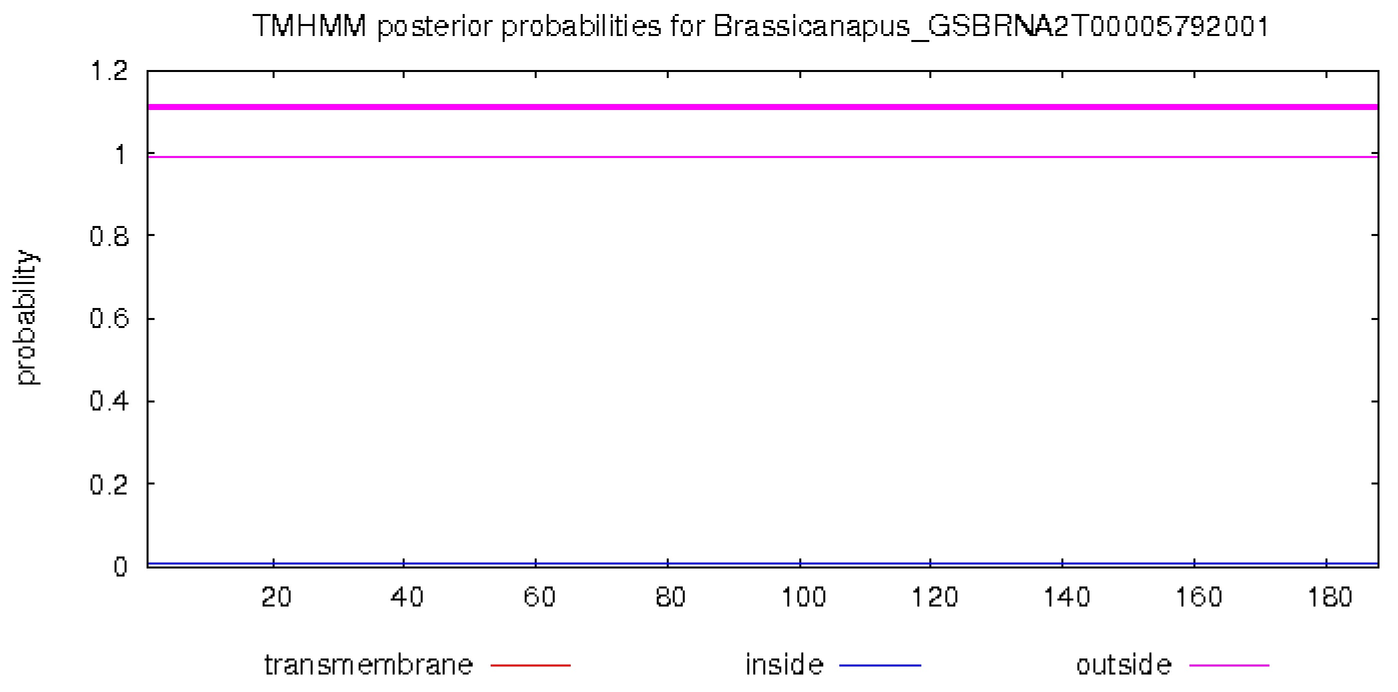

plot in postscript, script for making the plot in gnuplot, data for plot

```
# Brassicanapus_GSBRNA2T00007082001 Length: 179
# Brassicanapus_GSBRNA2T00007082001 Number of predicted TMHs:  0
# Brassicanapus_GSBRNA2T00007082001 Exp number of AAs in TMHs: 0.00099
# Brassicanapus_GSBRNA2T00007082001 Exp number, first 60 AAs:  0.00099
# Brassicanapus_GSBRNA2T00007082001 Total prob of N-in:        0.07126
Brassicanapus_GSBRNA2T00007082001       TMHMM2.0        outside      1   179
```

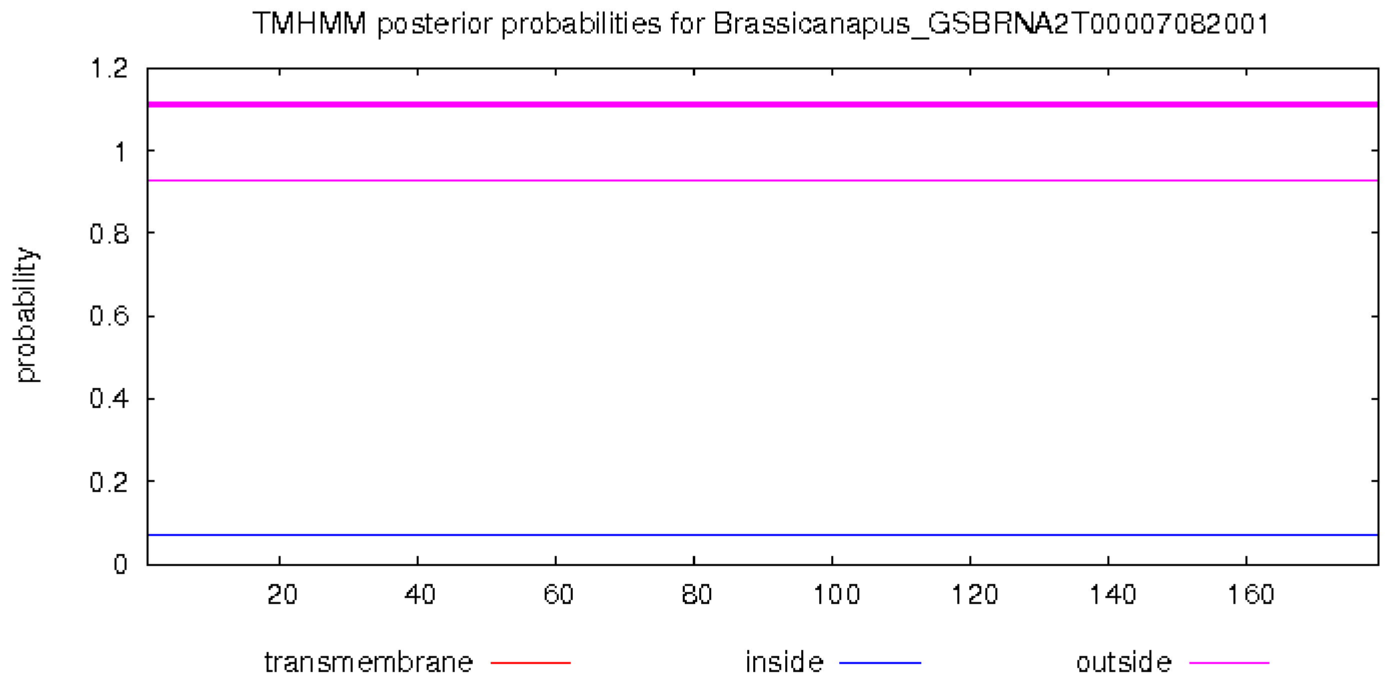

plot in postscript, script for making the plot in gnuplot, data for plot

---

```
# Brassicanapus_GSBRNA2T00009098001 Length: 252
# Brassicanapus_GSBRNA2T00009098001 Number of predicted TMHs:  0
# Brassicanapus_GSBRNA2T00009098001 Exp number of AAs in TMHs: 0.00527
# Brassicanapus_GSBRNA2T00009098001 Exp number, first 60 AAs:  0.00459
# Brassicanapus_GSBRNA2T00009098001 Total prob of N-in:        0.15475
Brassicanapus_GSBRNA2T00009098001	TMHMM2.0	outside	     1   252
```

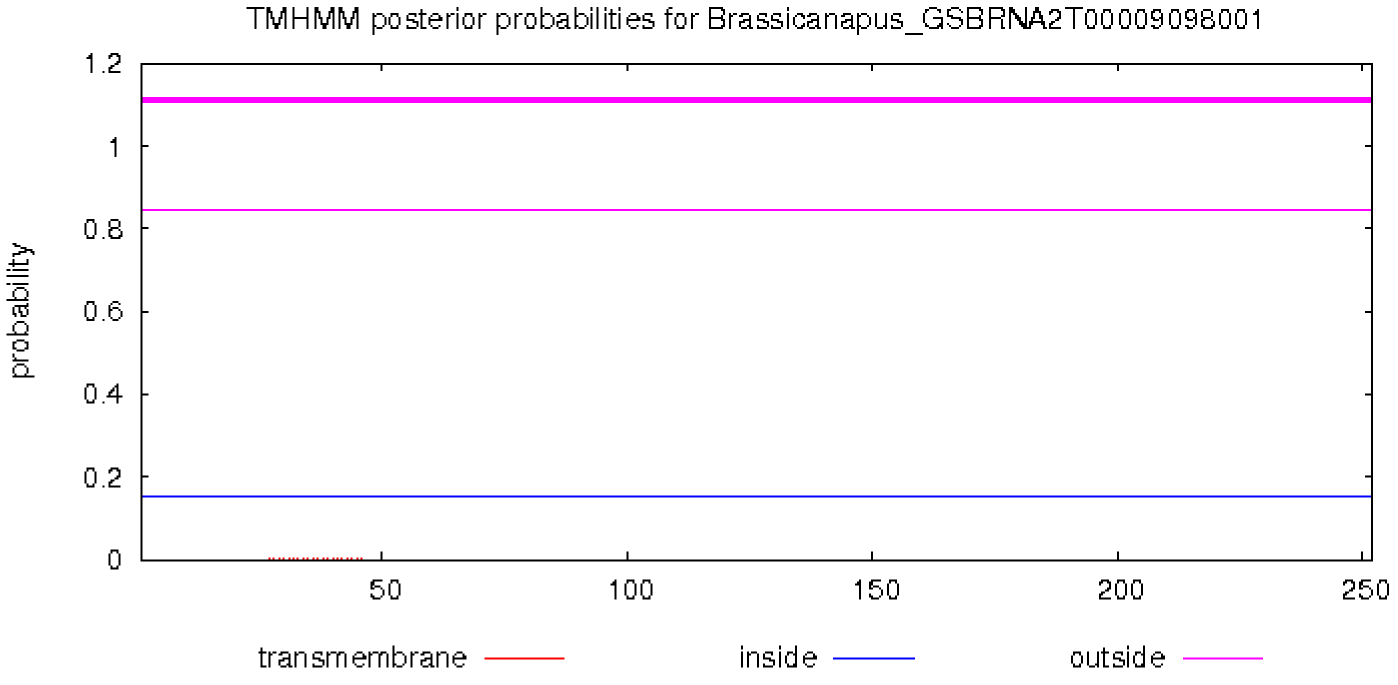

plot in postscript, script for making the plot in gnuplot, data for plot

---

```
# Brassicanapus_GSBRNA2T00009100001 Length: 251
# Brassicanapus_GSBRNA2T00009100001 Number of predicted TMHs:  0
# Brassicanapus_GSBRNA2T00009100001 Exp number of AAs in TMHs: 0.00616999999999999
# Brassicanapus_GSBRNA2T00009100001 Exp number, first 60 AAs:  0.00459
# Brassicanapus_GSBRNA2T00009100001 Total prob of N-in:        0.15684
Brassicanapus_GSBRNA2T00009100001	TMHMM2.0	outside	     1   251
```

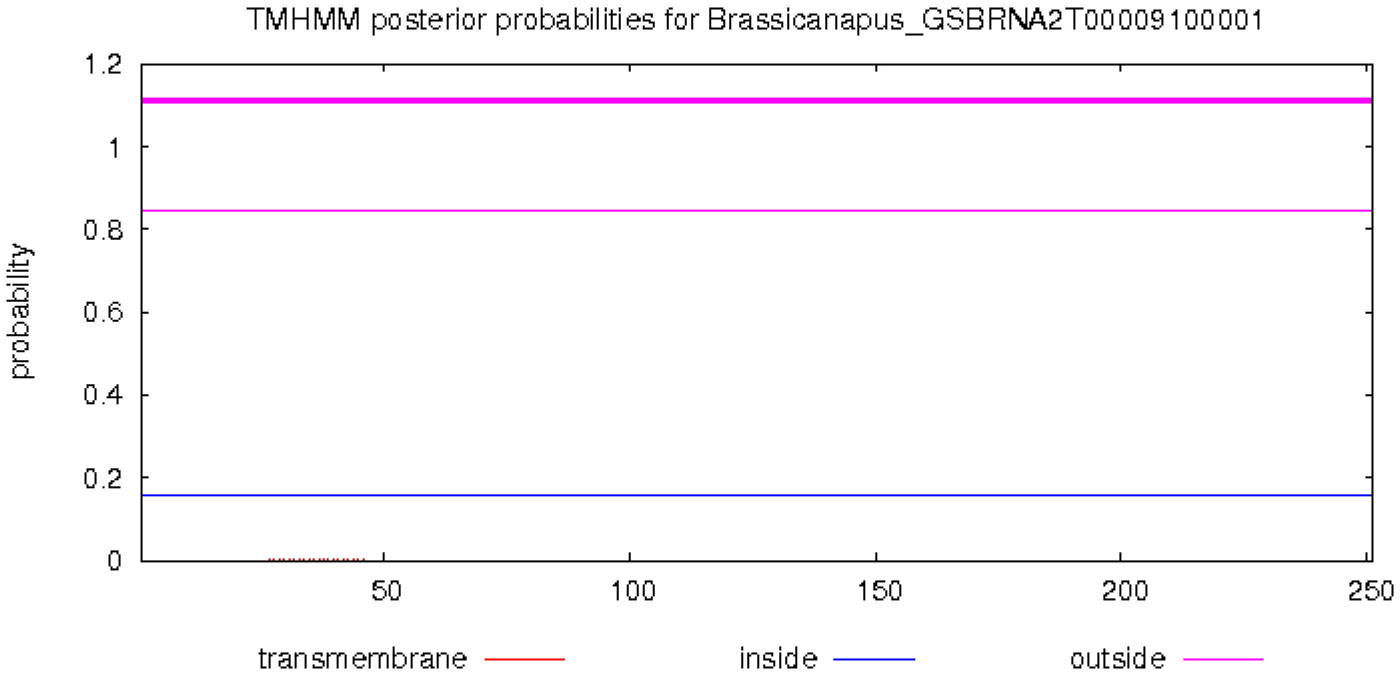

plot in postscript, script for making the plot in gnuplot, data for plot

```
# Brassicanapus_GSBRNA2T00009589001 Length: 445
# Brassicanapus_GSBRNA2T00009589001 Number of predicted TMHs:  0
# Brassicanapus_GSBRNA2T00009589001 Exp number of AAs in TMHs: 10.82447
# Brassicanapus_GSBRNA2T00009589001 Exp number, first 60 AAs:  0
# Brassicanapus_GSBRNA2T00009589001 Total prob of N-in:        0.00959
Brassicanapus_GSBRNA2T00009589001	TMHMM2.0	outside	     1   445
```

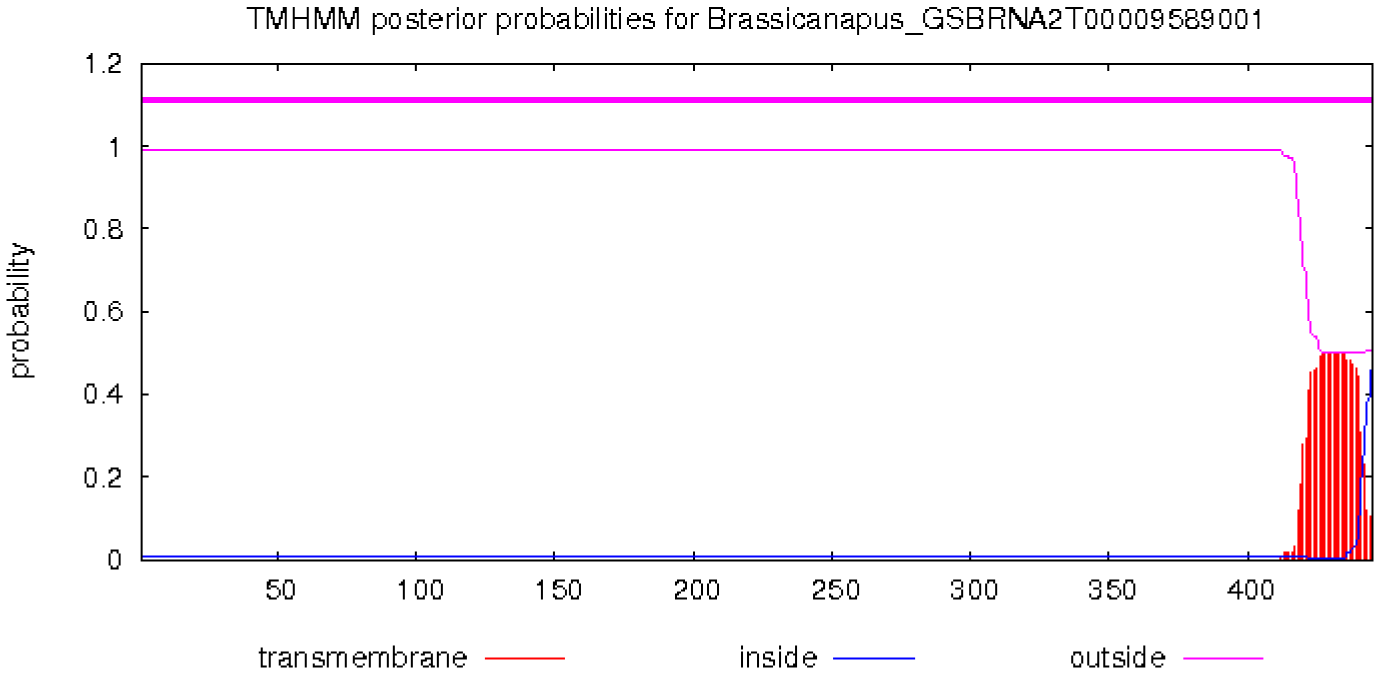

plot in postscript, script for making the plot in gnuplot, data for plot

```
# Brassicanapus_GSBRNA2T00009829001 Length: 372
# Brassicanapus_GSBRNA2T00009829001 Number of predicted TMHs:  0
# Brassicanapus_GSBRNA2T00009829001 Exp number of AAs in TMHs: 0.00899
# Brassicanapus_GSBRNA2T00009829001 Exp number, first 60 AAs:  0.00489
# Brassicanapus_GSBRNA2T00009829001 Total prob of N-in:        0.02395
Brassicanapus_GSBRNA2T00009829001	TMHMM2.0	outside	     1   372
```

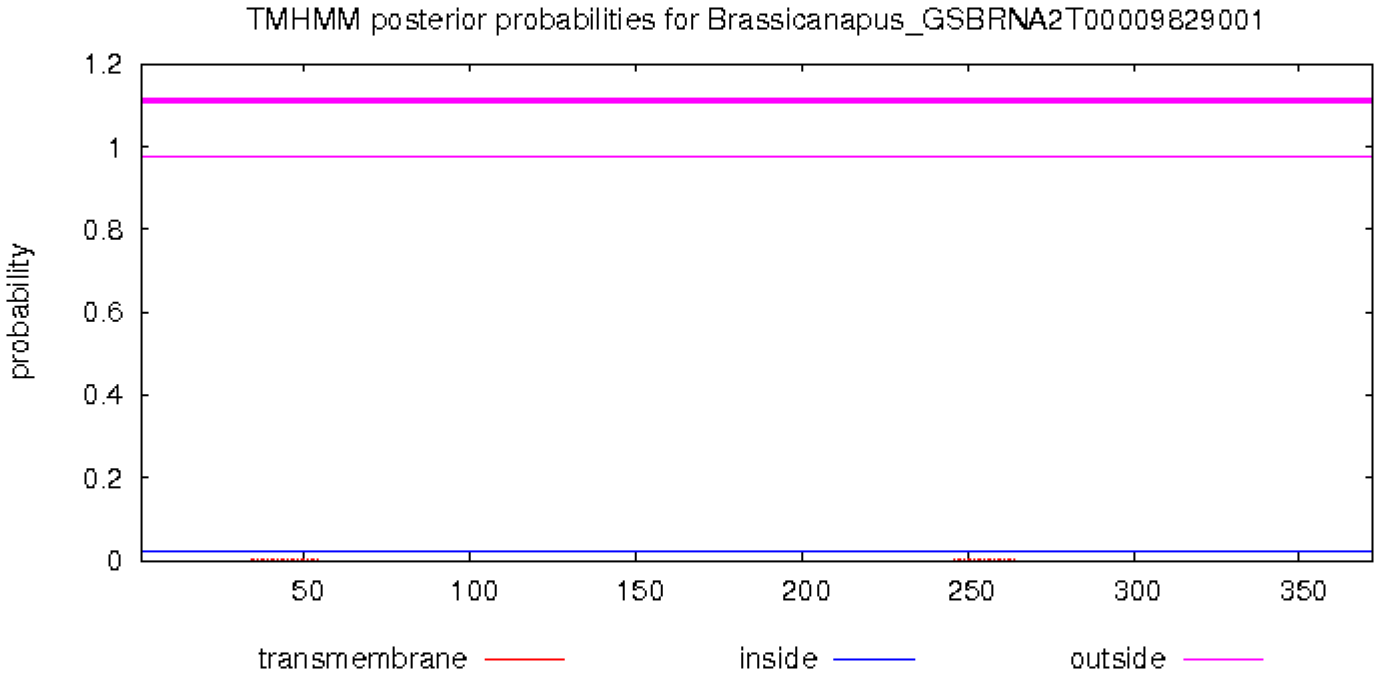

plot in postscript, script for making the plot in gnuplot, data for plot

---

```
# Brassicanapus_GSBRNA2T00009956001 Length: 303
# Brassicanapus_GSBRNA2T00009956001 Number of predicted TMHs:  0
# Brassicanapus_GSBRNA2T00009956001 Exp number of AAs in TMHs: 0.00153
# Brassicanapus_GSBRNA2T00009956001 Exp number, first 60 AAs:  0.00042
# Brassicanapus_GSBRNA2T00009956001 Total prob of N-in:        0.01052
Brassicanapus_GSBRNA2T00009956001       TMHMM2.0        outside      1   303
```

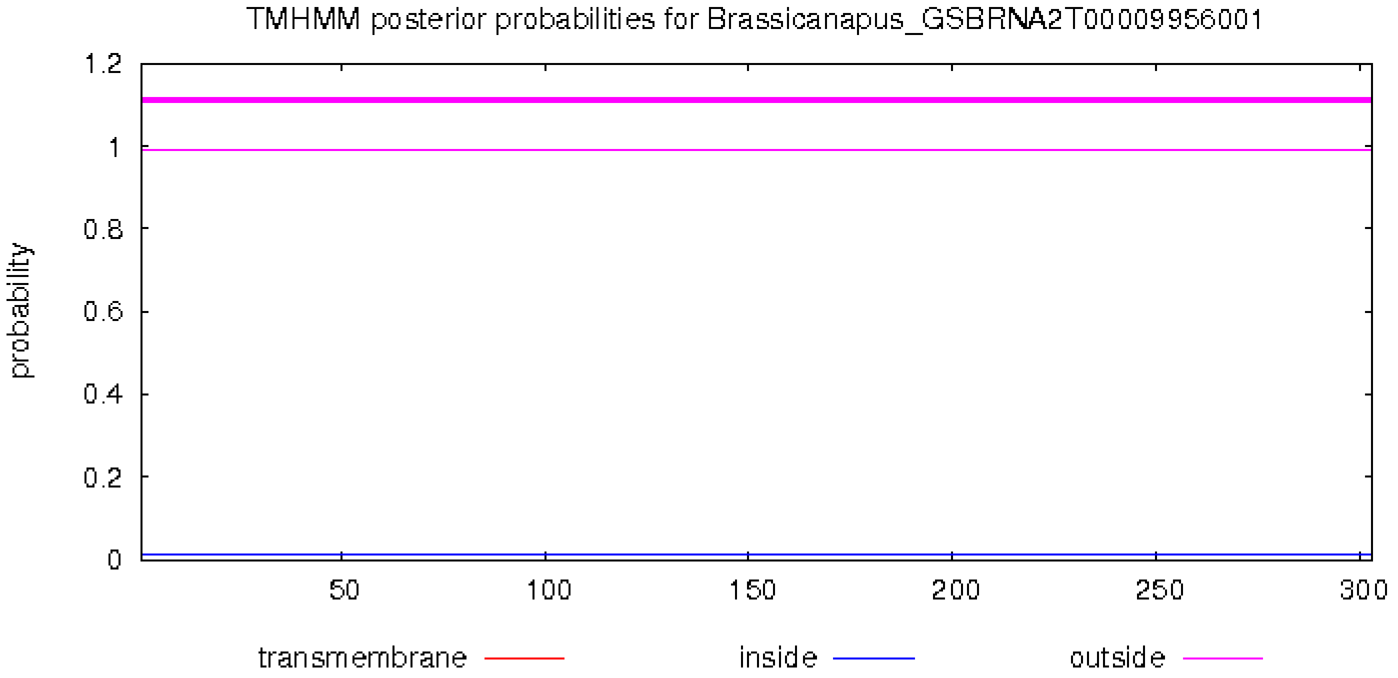

plot in postscript, script for making the plot in gnuplot, data for plot

---

```
# Brassicanapus_GSBRNA2T00009965001 Length: 317
# Brassicanapus_GSBRNA2T00009965001 Number of predicted TMHs:  0
# Brassicanapus_GSBRNA2T00009965001 Exp number of AAs in TMHs: 0
# Brassicanapus_GSBRNA2T00009965001 Exp number, first 60 AAs:  0
# Brassicanapus_GSBRNA2T00009965001 Total prob of N-in:        0.00384
Brassicanapus_GSBRNA2T00009965001       TMHMM2.0        outside      1   317
```

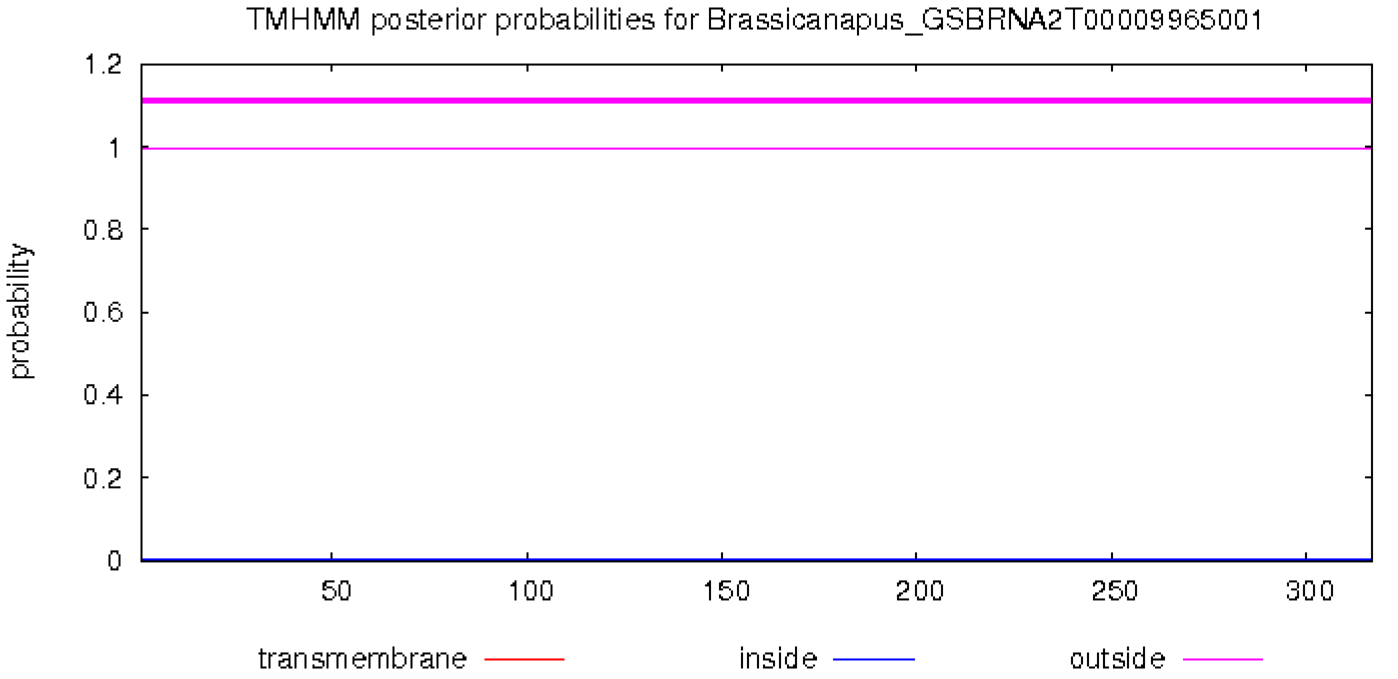

plot in postscript, script for making the plot in gnuplot, data for plot

```
# Brassicanapus_GSBRNA2T00009990001 Length: 350
# Brassicanapus_GSBRNA2T00009990001 Number of predicted TMHs:  0
# Brassicanapus_GSBRNA2T00009990001 Exp number of AAs in TMHs: 0.000810000000000000001
# Brassicanapus_GSBRNA2T00009990001 Exp number, first 60 AAs:  0
# Brassicanapus_GSBRNA2T00009990001 Total prob of N-in:        0.00488
Brassicanapus_GSBRNA2T00009990001       TMHMM2.0        outside      1   350
```

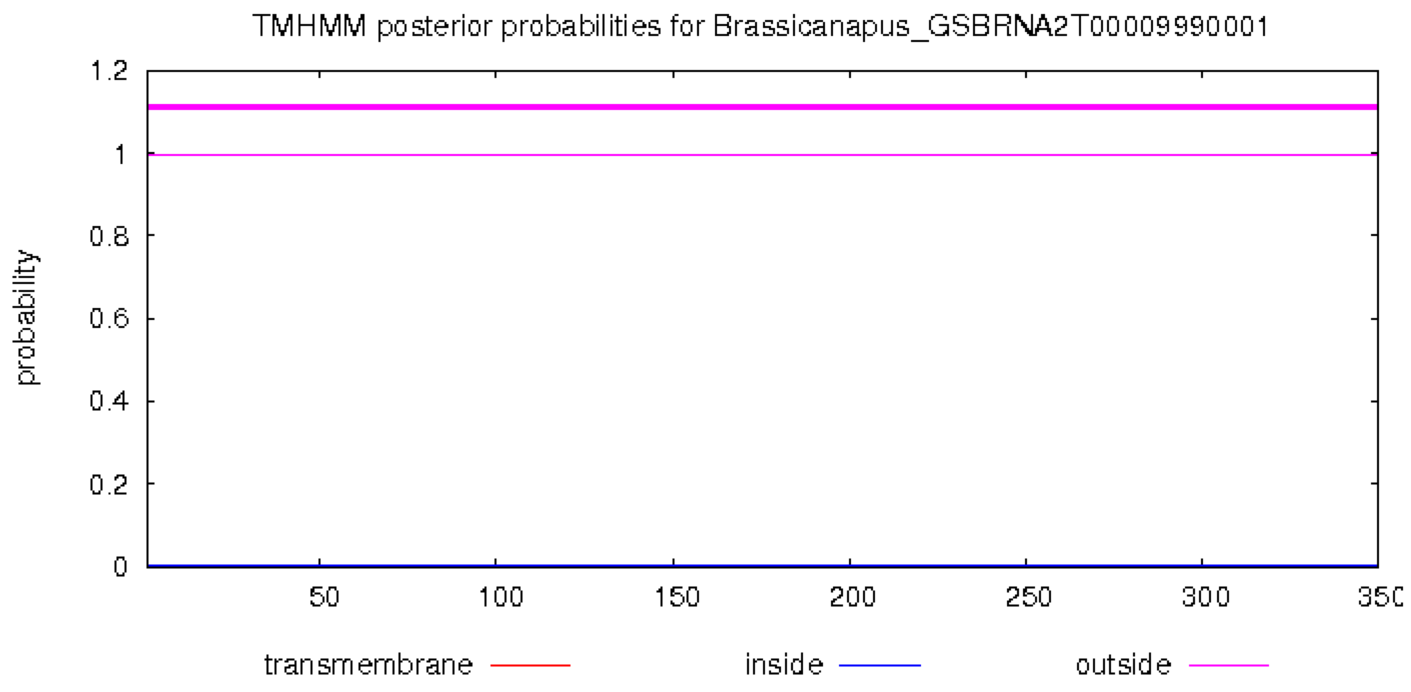

plot in postscript, script for making the plot in gnuplot, data for plot

```
# Brassicanapus_GSBRNA2T00010274001 Length: 336
# Brassicanapus_GSBRNA2T00010274001 Number of predicted TMHs:  0
# Brassicanapus_GSBRNA2T00010274001 Exp number of AAs in TMHs: 0.00017
# Brassicanapus_GSBRNA2T00010274001 Exp number, first 60 AAs:  0
# Brassicanapus_GSBRNA2T00010274001 Total prob of N-in:        0.00866
Brassicanapus_GSBRNA2T00010274001       TMHMM2.0        outside      1   336
```

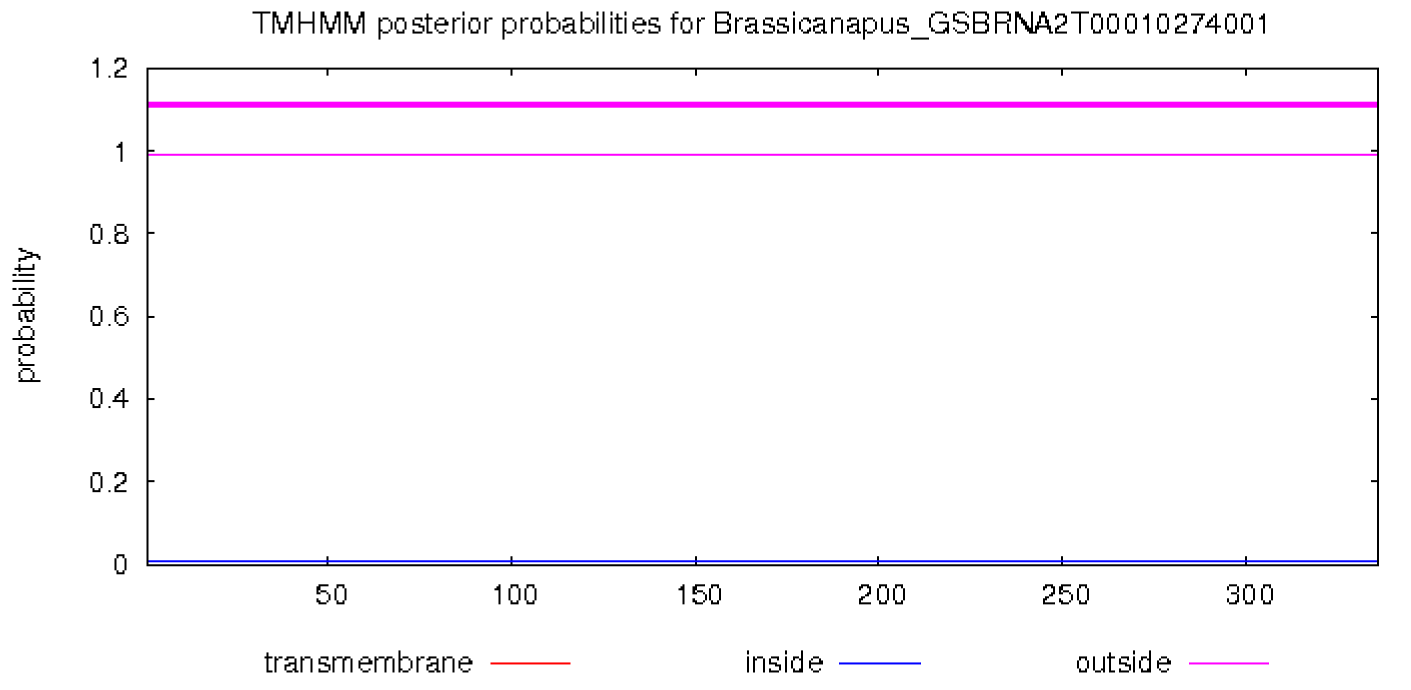

plot in postscript, script for making the plot in gnuplot, data for plot

```
# Brassicanapus_GSBRNA2T00010843001 Length: 140
# Brassicanapus_GSBRNA2T00010843001 Number of predicted TMHs:  0
# Brassicanapus_GSBRNA2T00010843001 Exp number of AAs in TMHs: 0.00244
# Brassicanapus_GSBRNA2T00010843001 Exp number, first 60 AAs:  0
# Brassicanapus_GSBRNA2T00010843001 Total prob of N-in:        0.06198
Brassicanapus_GSBRNA2T00010843001       TMHMM2.0        outside      1   140
```

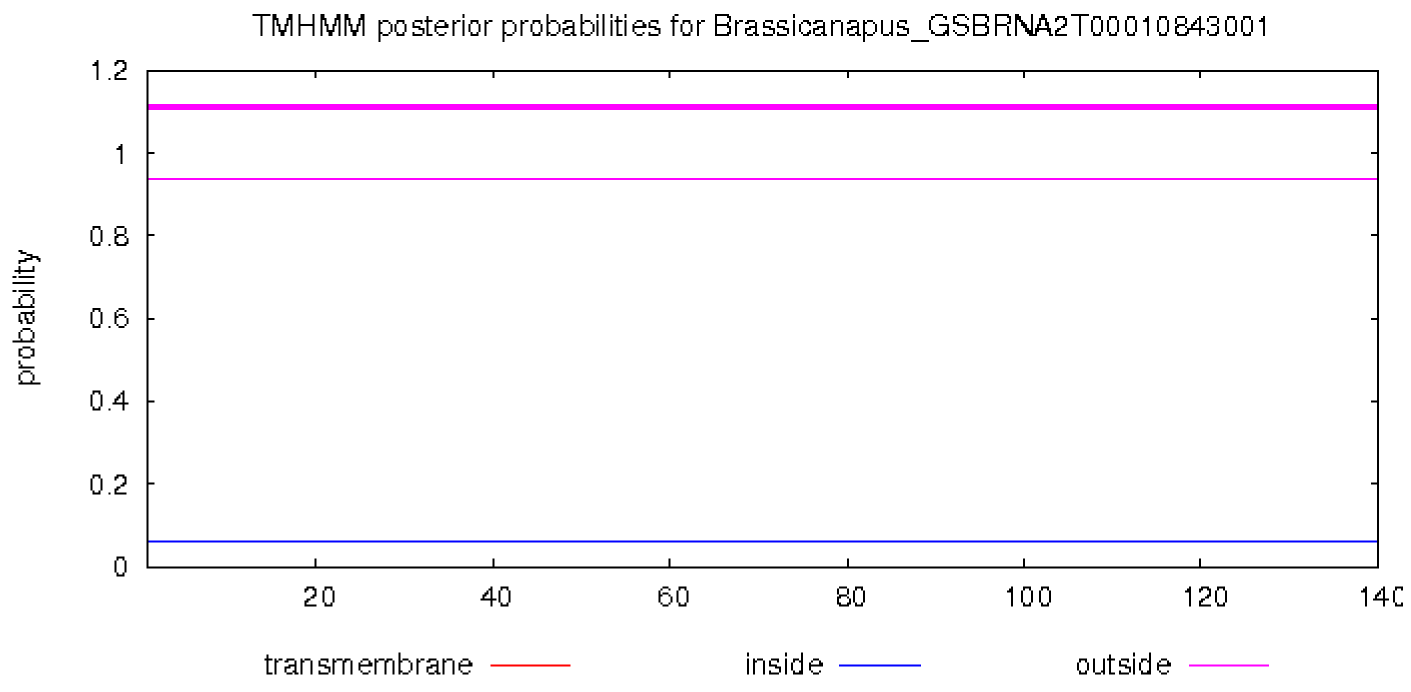

plot in postscript, script for making the plot in gnuplot, data for plot

```
# Brassicanapus_GSBRNA2T00011094001 Length: 248
# Brassicanapus_GSBRNA2T00011094001 Number of predicted TMHs:  0
# Brassicanapus_GSBRNA2T00011094001 Exp number of AAs in TMHs: 0.00125
# Brassicanapus_GSBRNA2T00011094001 Exp number, first 60 AAs:  0
# Brassicanapus_GSBRNA2T00011094001 Total prob of N-in:        0.16191
Brassicanapus_GSBRNA2T00011094001       TMHMM2.0        outside      1   248
```

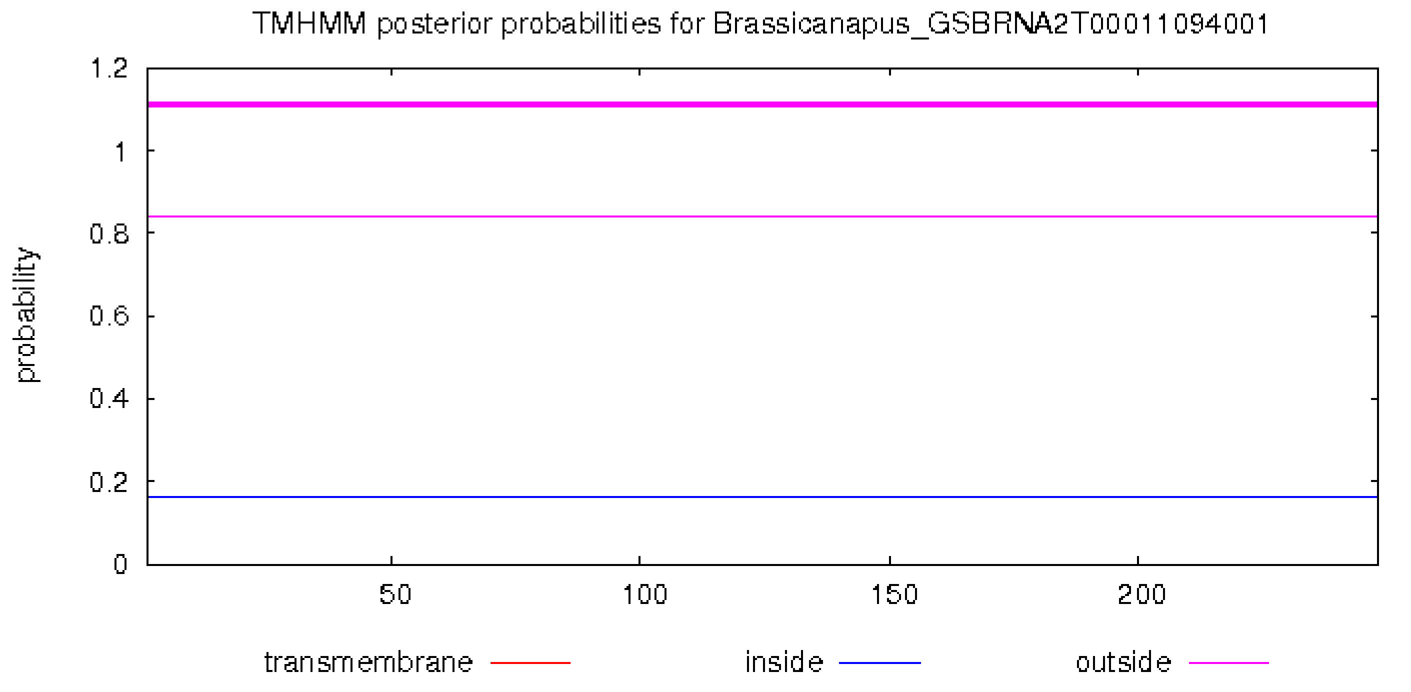

[plot](#) in postscript, [script](#) for making the plot in gnuplot, [data](#) for plot

---

```
# Brassicanapus_GSBRNA2T00011138001 Length: 279
# Brassicanapus_GSBRNA2T00011138001 Number of predicted TMHs:  0
# Brassicanapus_GSBRNA2T00011138001 Exp number of AAs in TMHs: 0.00103
# Brassicanapus_GSBRNA2T00011138001 Exp number, first 60 AAs:  0
# Brassicanapus_GSBRNA2T00011138001 Total prob of N-in:        0.04916
Brassicanapus_GSBRNA2T00011138001	TMHMM2.0	outside	     1   279
```

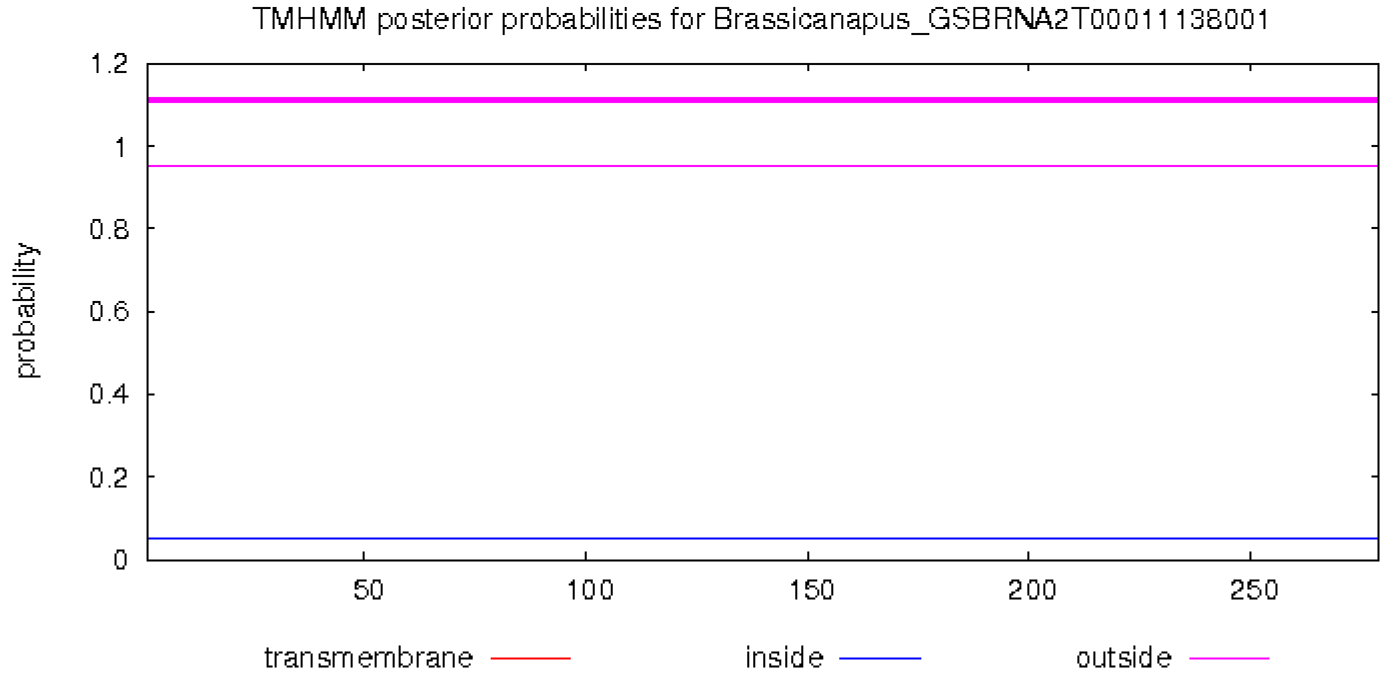

[plot](#) in postscript, [script](#) for making the plot in gnuplot, [data](#) for plot

---

```
# Brassicanapus_GSBRNA2T00011481001 Length: 318
# Brassicanapus_GSBRNA2T00011481001 Number of predicted TMHs:  0
# Brassicanapus_GSBRNA2T00011481001 Exp number of AAs in TMHs: 0.00059
# Brassicanapus_GSBRNA2T00011481001 Exp number, first 60 AAs:  0
# Brassicanapus_GSBRNA2T00011481001 Total prob of N-in:        0.03940
Brassicanapus_GSBRNA2T00011481001	TMHMM2.0	outside	     1   318
```

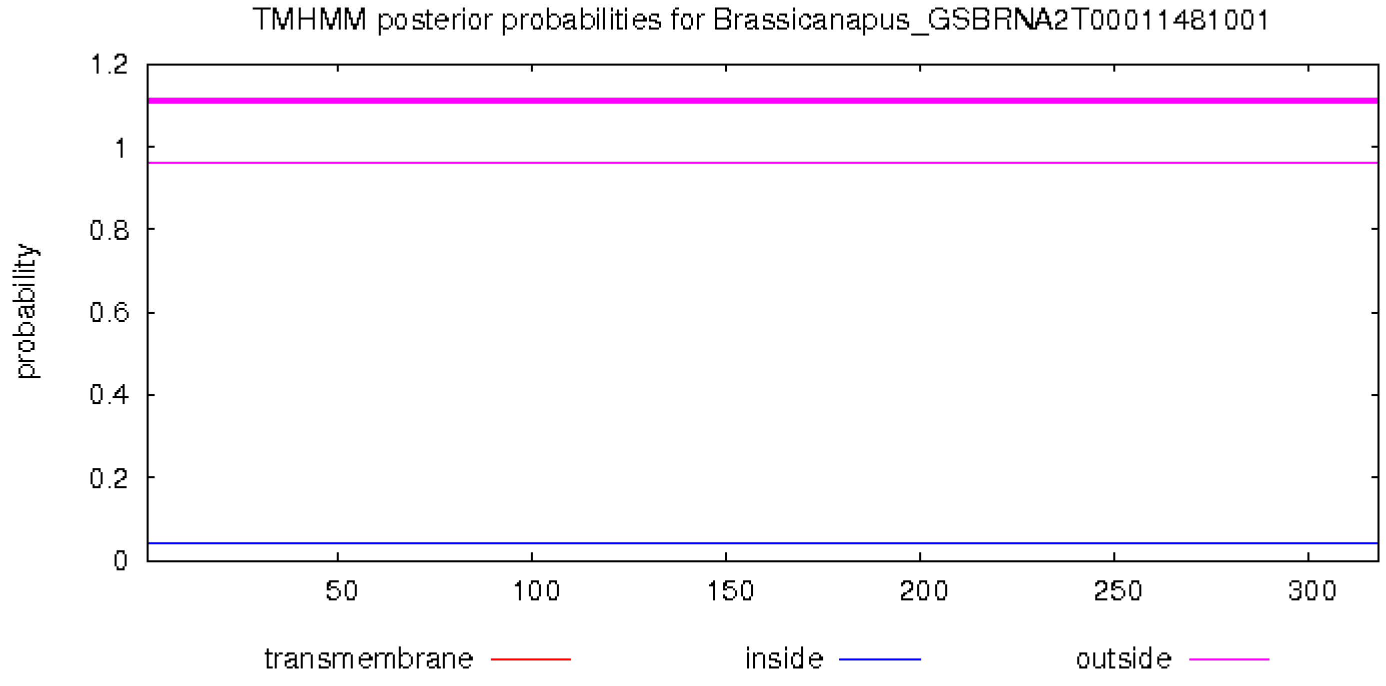

plot in postscript, script for making the plot in gnuplot, data for plot

---

```
# Brassicanapus_GSBRNA2T00011918001 Length: 308
# Brassicanapus_GSBRNA2T00011918001 Number of predicted TMHs:  0
# Brassicanapus_GSBRNA2T00011918001 Exp number of AAs in TMHs: 0.00893
# Brassicanapus_GSBRNA2T00011918001 Exp number, first 60 AAs:  0.00893
# Brassicanapus_GSBRNA2T00011918001 Total prob of N-in:        0.00696
Brassicanapus_GSBRNA2T00011918001	TMHMM2.0	outside	     1   308
```

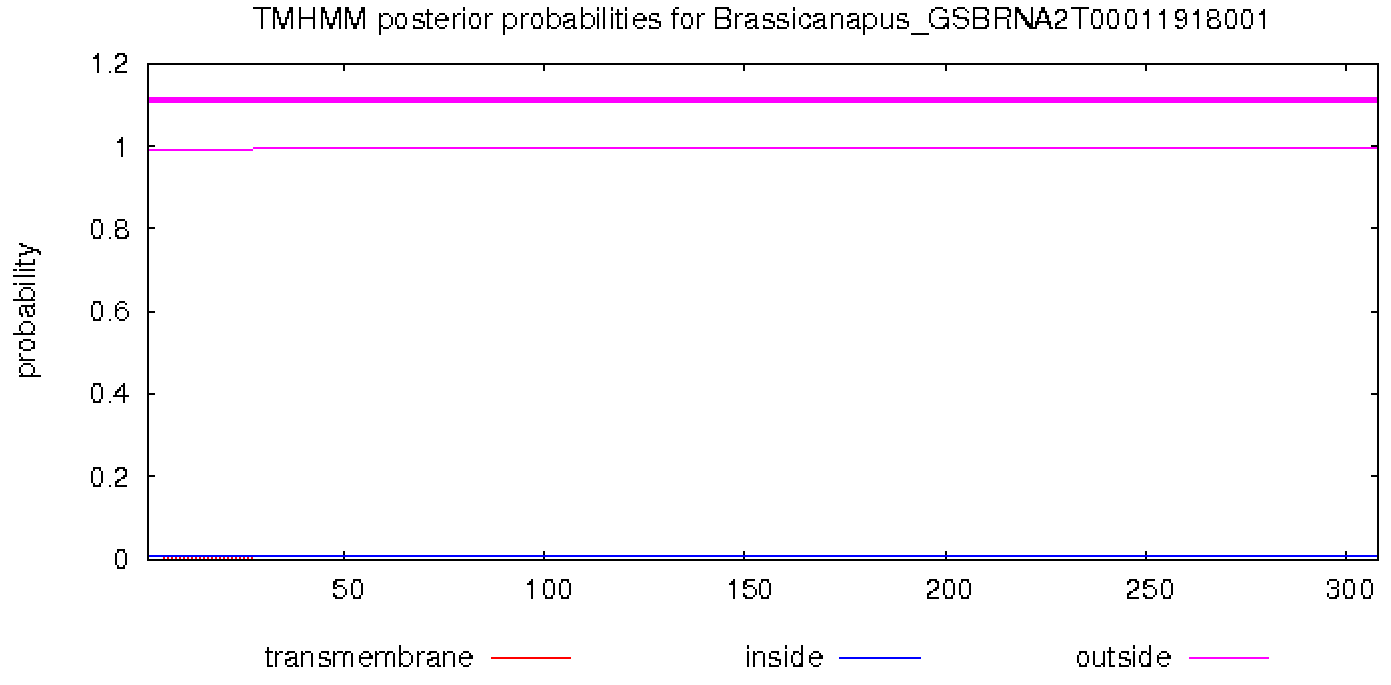

plot in postscript, script for making the plot in gnuplot, data for plot

---

```
# Brassicanapus_GSBRNA2T00012247001 Length: 317
# Brassicanapus_GSBRNA2T00012247001 Number of predicted TMHs:  0
# Brassicanapus_GSBRNA2T00012247001 Exp number of AAs in TMHs: 0.01844
# Brassicanapus_GSBRNA2T00012247001 Exp number, first 60 AAs:  0
# Brassicanapus_GSBRNA2T00012247001 Total prob of N-in:        0.07327
Brassicanapus_GSBRNA2T00012247001	TMHMM2.0	outside	     1   317
```

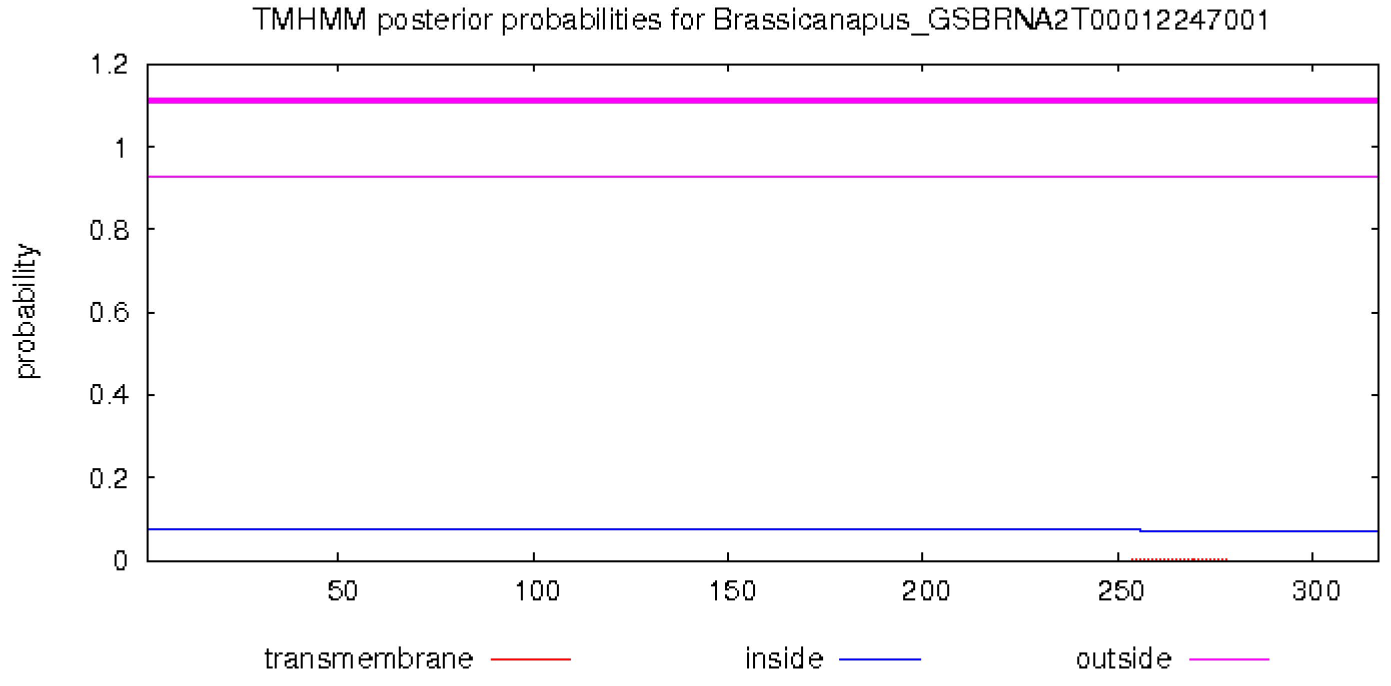

plot in postscript, script for making the plot in gnuplot, data for plot

```
# Brassicanapus_GSBRNA2T00012336001 Length: 339
# Brassicanapus_GSBRNA2T00012336001 Number of predicted TMHs:  1
# Brassicanapus_GSBRNA2T00012336001 Exp number of AAs in TMHs: 18.02341
# Brassicanapus_GSBRNA2T00012336001 Exp number, first 60 AAs:  0.19608
# Brassicanapus_GSBRNA2T00012336001 Total prob of N-in:        0.30510
Brassicanapus_GSBRNA2T00012336001       TMHMM2.0        outside      1   320
Brassicanapus_GSBRNA2T00012336001       TMHMM2.0        TMhelix    321   338
Brassicanapus_GSBRNA2T00012336001       TMHMM2.0        inside     339   339
```

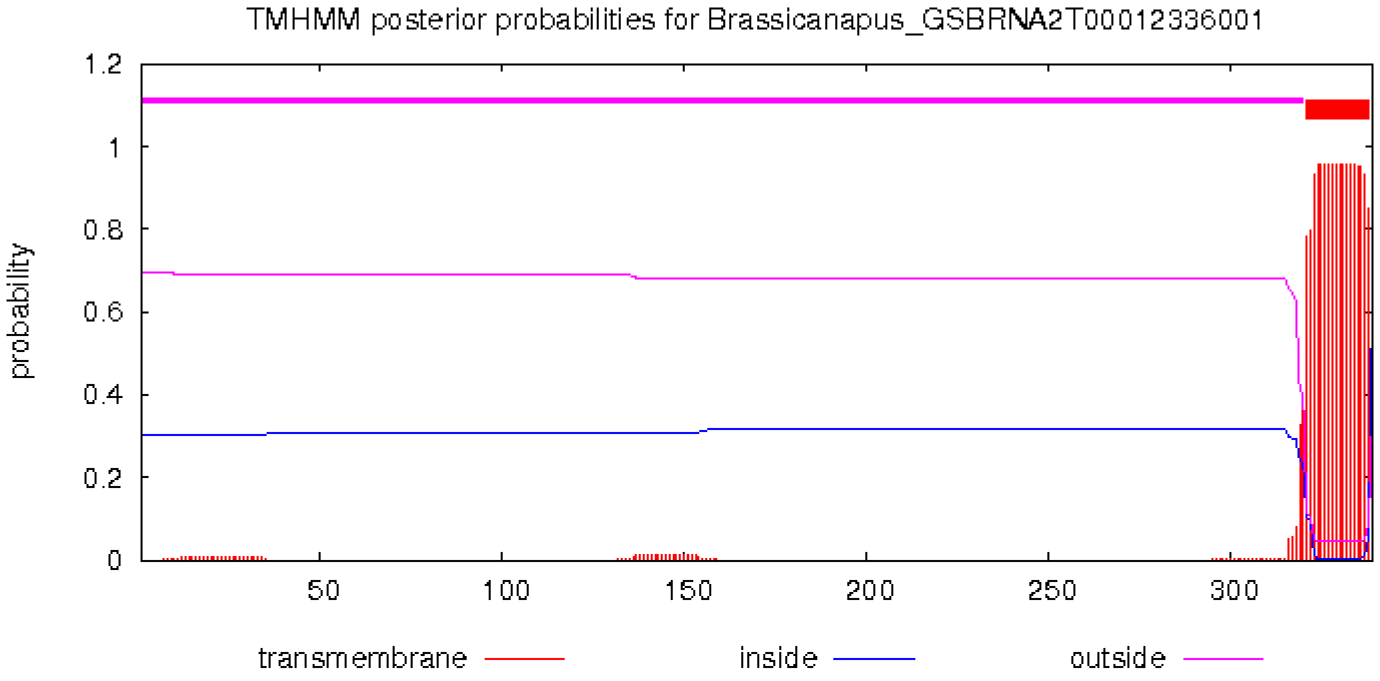

plot in postscript, script for making the plot in gnuplot, data for plot

```
# Brassicanapus_GSBRNA2T00012468001 Length: 321
# Brassicanapus_GSBRNA2T00012468001 Number of predicted TMHs:  0
# Brassicanapus_GSBRNA2T00012468001 Exp number of AAs in TMHs: 0.03603
# Brassicanapus_GSBRNA2T00012468001 Exp number, first 60 AAs:  0.02206
# Brassicanapus_GSBRNA2T00012468001 Total prob of N-in:        0.00466
Brassicanapus_GSBRNA2T00012468001       TMHMM2.0        outside      1   321
```

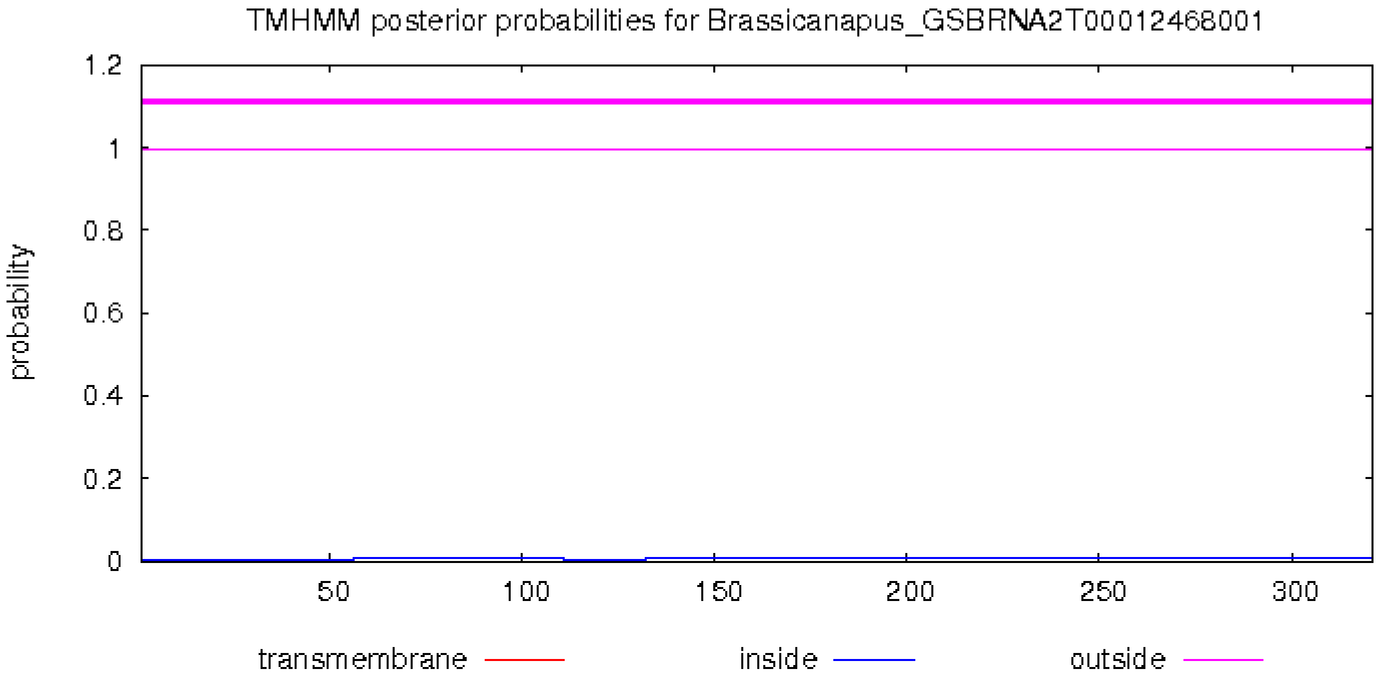

plot in postscript, script for making the plot in gnuplot, data for plot

---

```
# Brassicanapus_GSBRNA2T00012643001 Length: 461
# Brassicanapus_GSBRNA2T00012643001 Number of predicted TMHs:  0
# Brassicanapus_GSBRNA2T00012643001 Exp number of AAs in TMHs: 0.00112
# Brassicanapus_GSBRNA2T00012643001 Exp number, first 60 AAs:  0
# Brassicanapus_GSBRNA2T00012643001 Total prob of N-in:        0.00547
Brassicanapus_GSBRNA2T00012643001       TMHMM2.0        outside      1   461
```

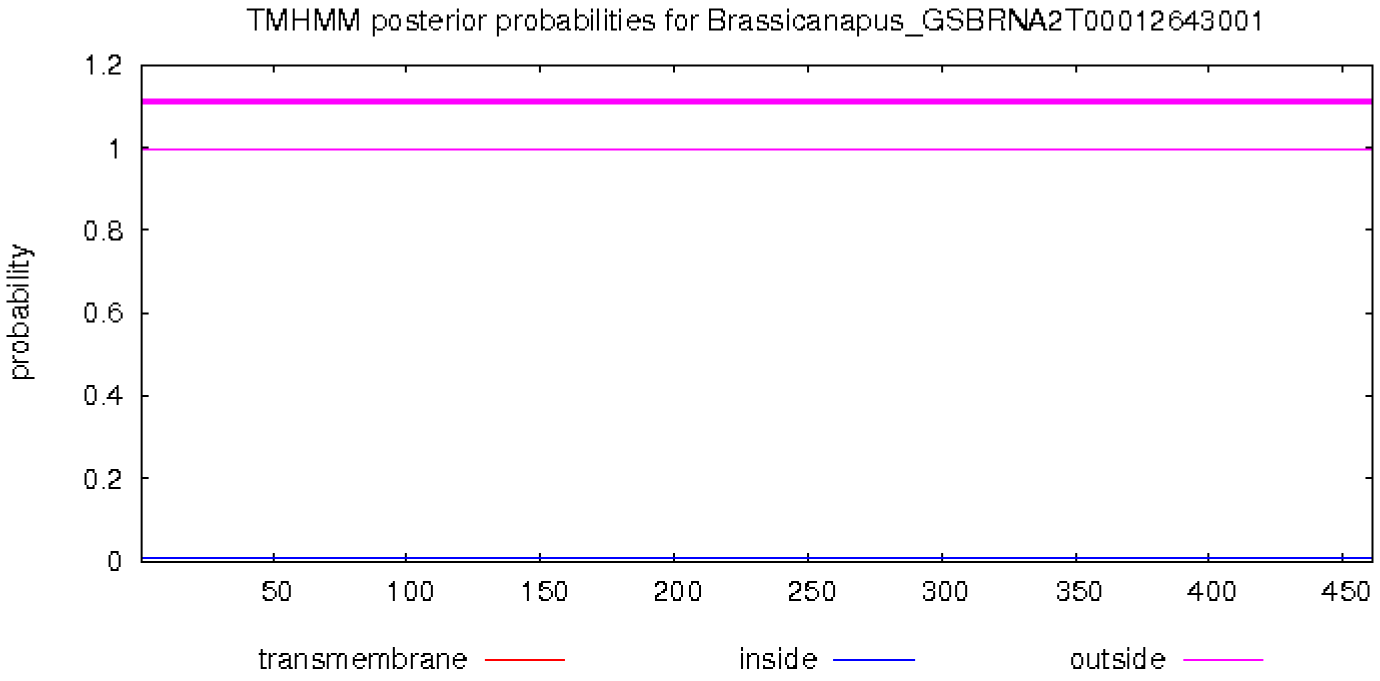

plot in postscript, script for making the plot in gnuplot, data for plot

---

```
# Brassicanapus_GSBRNA2T00013424001 Length: 255
# Brassicanapus_GSBRNA2T00013424001 Number of predicted TMHs:  0
# Brassicanapus_GSBRNA2T00013424001 Exp number of AAs in TMHs: 0.00043
# Brassicanapus_GSBRNA2T00013424001 Exp number, first 60 AAs:  0
# Brassicanapus_GSBRNA2T00013424001 Total prob of N-in:        0.22983
Brassicanapus_GSBRNA2T00013424001       TMHMM2.0        outside      1   255
```

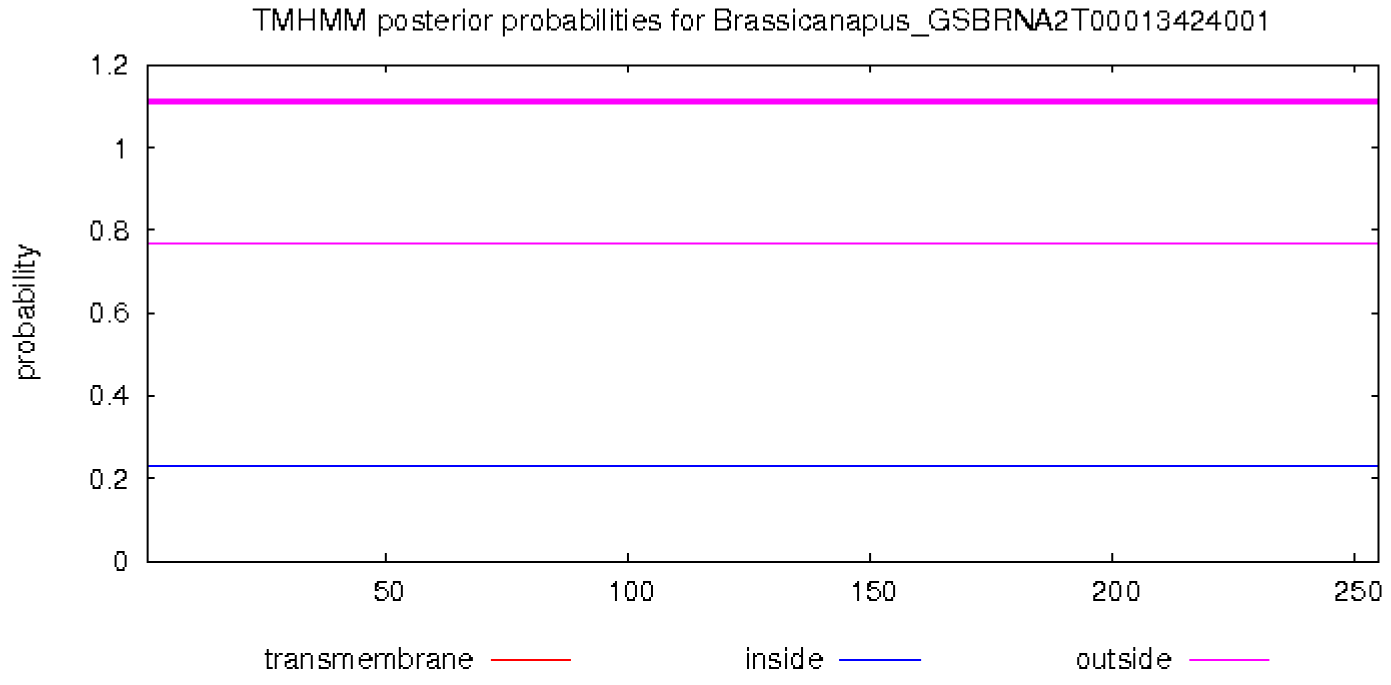

plot in postscript, script for making the plot in gnuplot, data for plot

---

```
# Brassicanapus_GSBRNA2T00013675001 Length: 178
# Brassicanapus_GSBRNA2T00013675001 Number of predicted TMHs:  0
# Brassicanapus_GSBRNA2T00013675001 Exp number of AAs in TMHs: 0.03273
# Brassicanapus_GSBRNA2T00013675001 Exp number, first 60 AAs:  0
# Brassicanapus_GSBRNA2T00013675001 Total prob of N-in:        0.00938
Brassicanapus_GSBRNA2T00013675001	TMHMM2.0	outside	     1   178
```

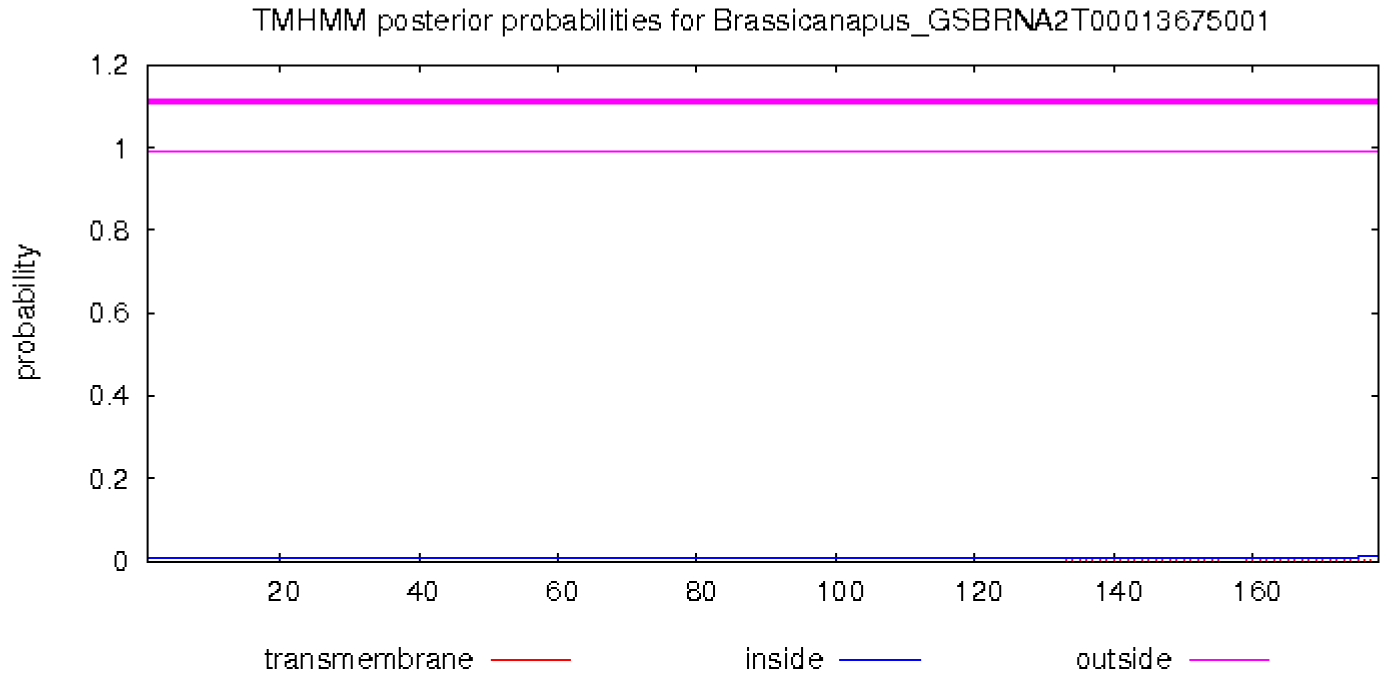

plot in postscript, script for making the plot in gnuplot, data for plot

---

```
# Brassicanapus_GSBRNA2T00014031001 Length: 127
# Brassicanapus_GSBRNA2T00014031001 Number of predicted TMHs:  0
# Brassicanapus_GSBRNA2T00014031001 Exp number of AAs in TMHs: 0.0003
# Brassicanapus_GSBRNA2T00014031001 Exp number, first 60 AAs:  7e-05
# Brassicanapus_GSBRNA2T00014031001 Total prob of N-in:        0.21701
Brassicanapus_GSBRNA2T00014031001	TMHMM2.0	outside	     1   127
```

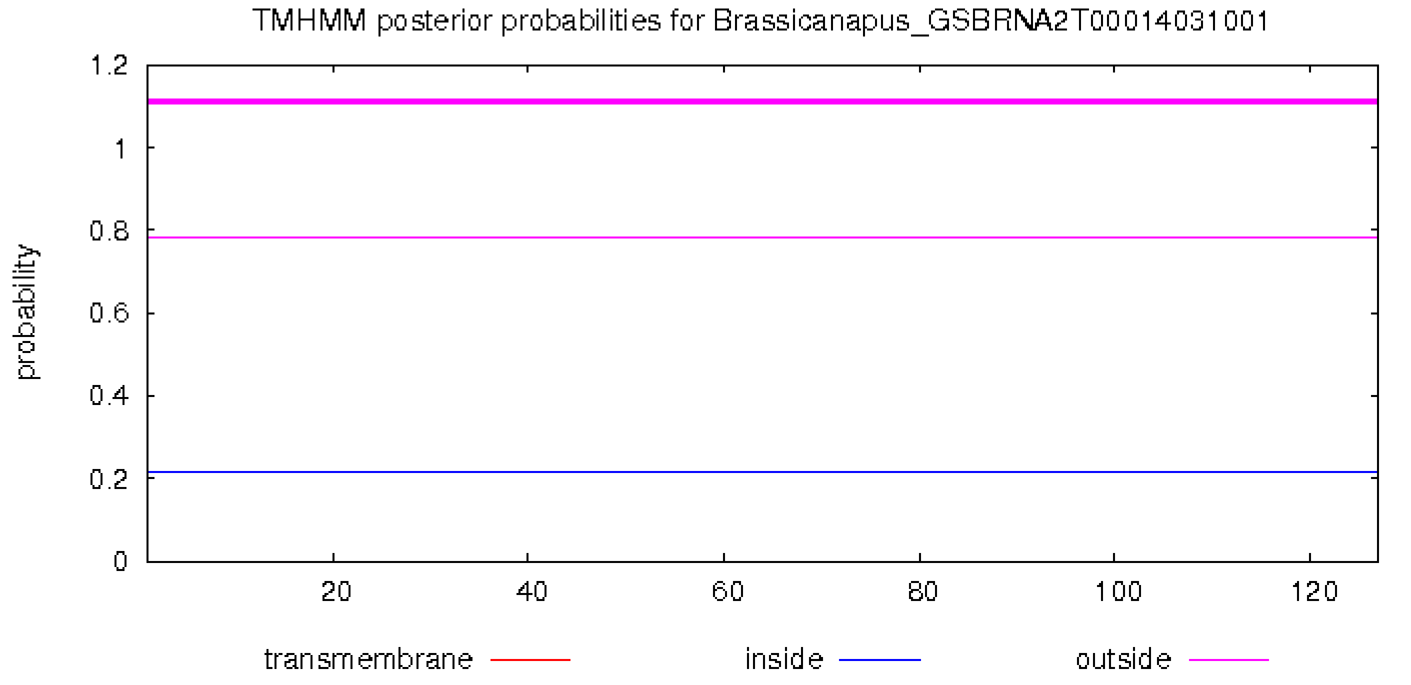

plot in postscript, script for making the plot in gnuplot, data for plot

---

```
# Brassicanapus_GSBRNA2T00015204001 Length: 353
# Brassicanapus_GSBRNA2T00015204001 Number of predicted TMHs:  0
# Brassicanapus_GSBRNA2T00015204001 Exp number of AAs in TMHs: 0.00018
# Brassicanapus_GSBRNA2T00015204001 Exp number, first 60 AAs:  0
# Brassicanapus_GSBRNA2T00015204001 Total prob of N-in:        0.00281
Brassicanapus_GSBRNA2T00015204001       TMHMM2.0        outside      1   353
```

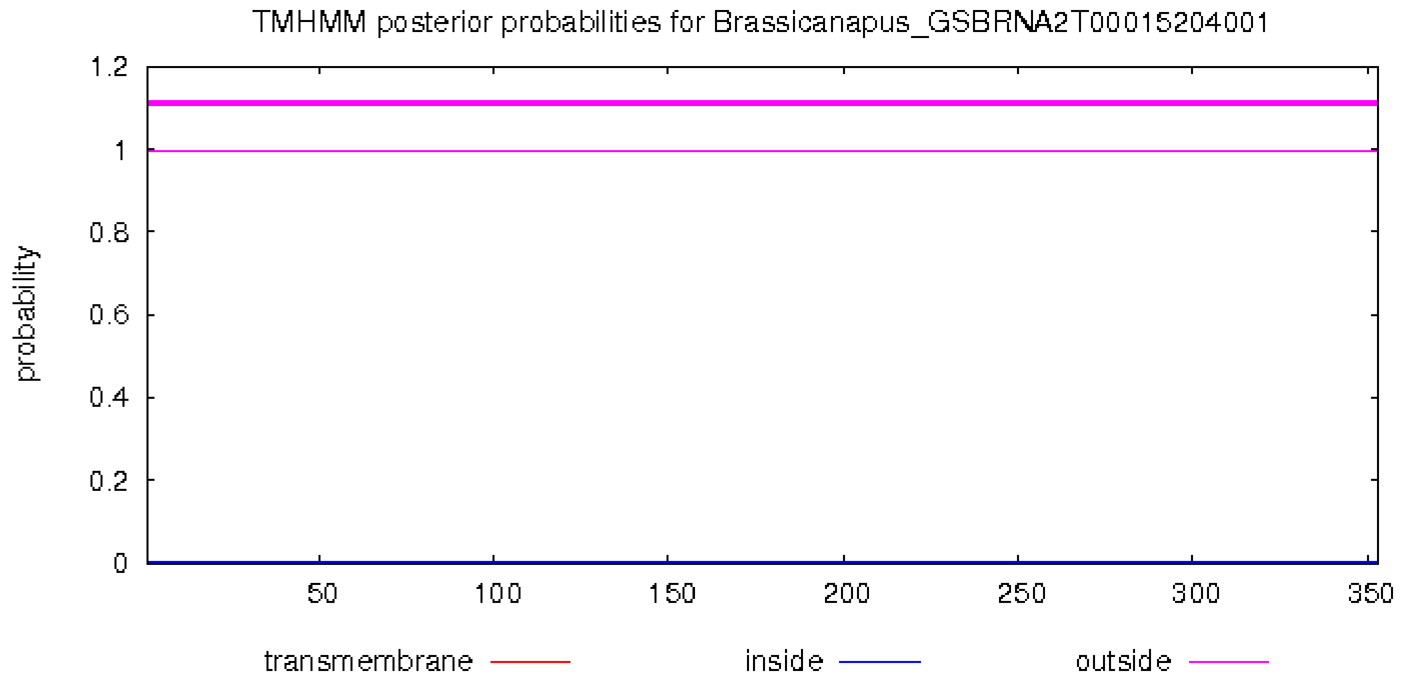

plot in postscript, script for making the plot in gnuplot, data for plot

---

```
# Brassicanapus_GSBRNA2T00015428001 Length: 430
# Brassicanapus_GSBRNA2T00015428001 Number of predicted TMHs:  0
# Brassicanapus_GSBRNA2T00015428001 Exp number of AAs in TMHs: 0.00629
# Brassicanapus_GSBRNA2T00015428001 Exp number, first 60 AAs:  0
# Brassicanapus_GSBRNA2T00015428001 Total prob of N-in:        0.00210
Brassicanapus_GSBRNA2T00015428001       TMHMM2.0        outside      1   430
```

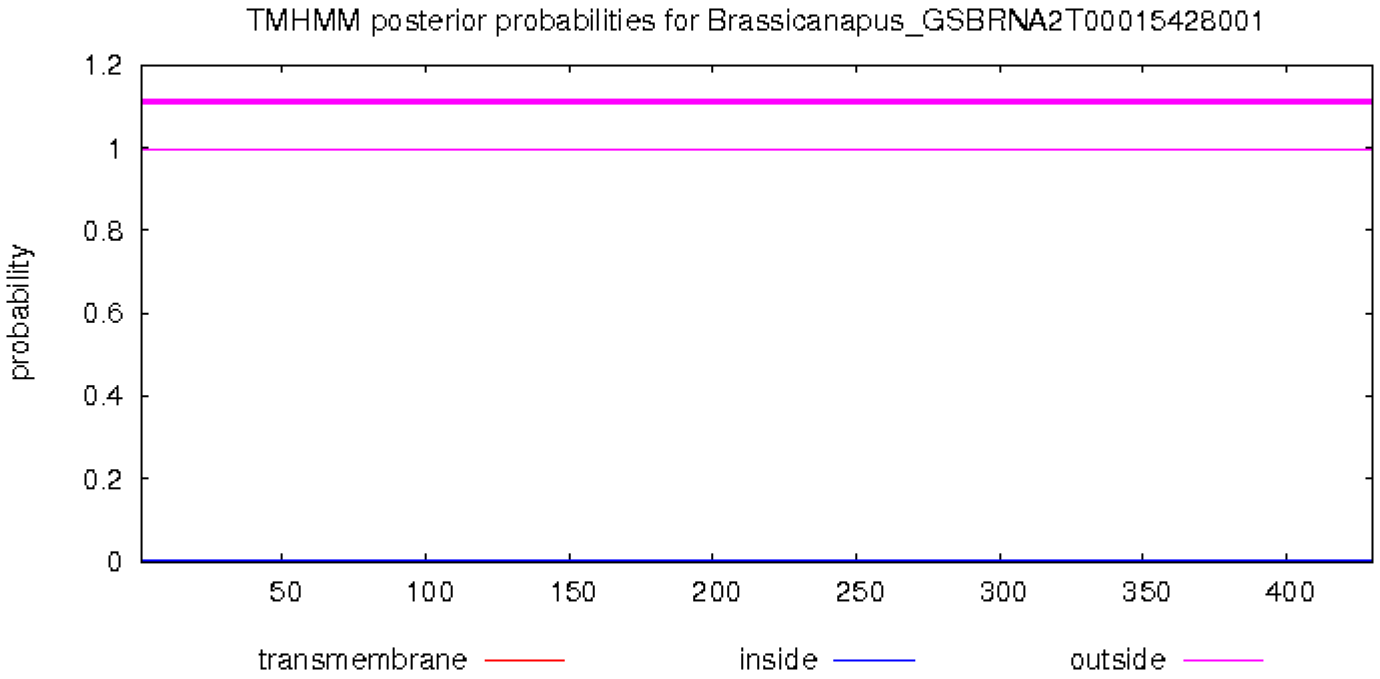

plot in postscript, script for making the plot in gnuplot, data for plot

---

```
# Brassicanapus_GSBRNA2T00015476001 Length: 270
# Brassicanapus_GSBRNA2T00015476001 Number of predicted TMHs:  0
# Brassicanapus_GSBRNA2T00015476001 Exp number of AAs in TMHs: 0.04384
# Brassicanapus_GSBRNA2T00015476001 Exp number, first 60 AAs:  0.00736
# Brassicanapus_GSBRNA2T00015476001 Total prob of N-in:        0.00283
Brassicanapus_GSBRNA2T00015476001       TMHMM2.0        outside      1   270
```

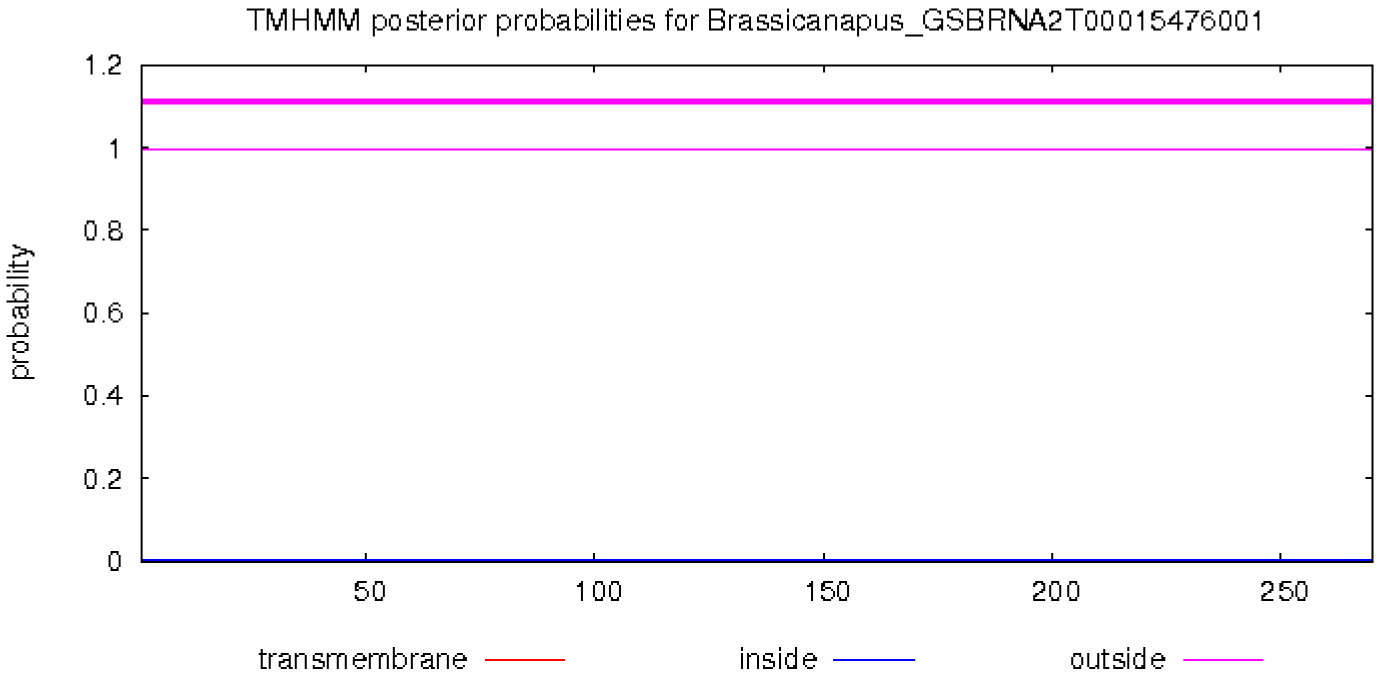

plot in postscript, script for making the plot in gnuplot, data for plot

---

```
# Brassicanapus_GSBRNA2T00016028001 Length: 278
# Brassicanapus_GSBRNA2T00016028001 Number of predicted TMHs:  0
# Brassicanapus_GSBRNA2T00016028001 Exp number of AAs in TMHs: 0.00019
# Brassicanapus_GSBRNA2T00016028001 Exp number, first 60 AAs:  0
# Brassicanapus_GSBRNA2T00016028001 Total prob of N-in:        0.01083
Brassicanapus_GSBRNA2T00016028001       TMHMM2.0        outside      1   278
```

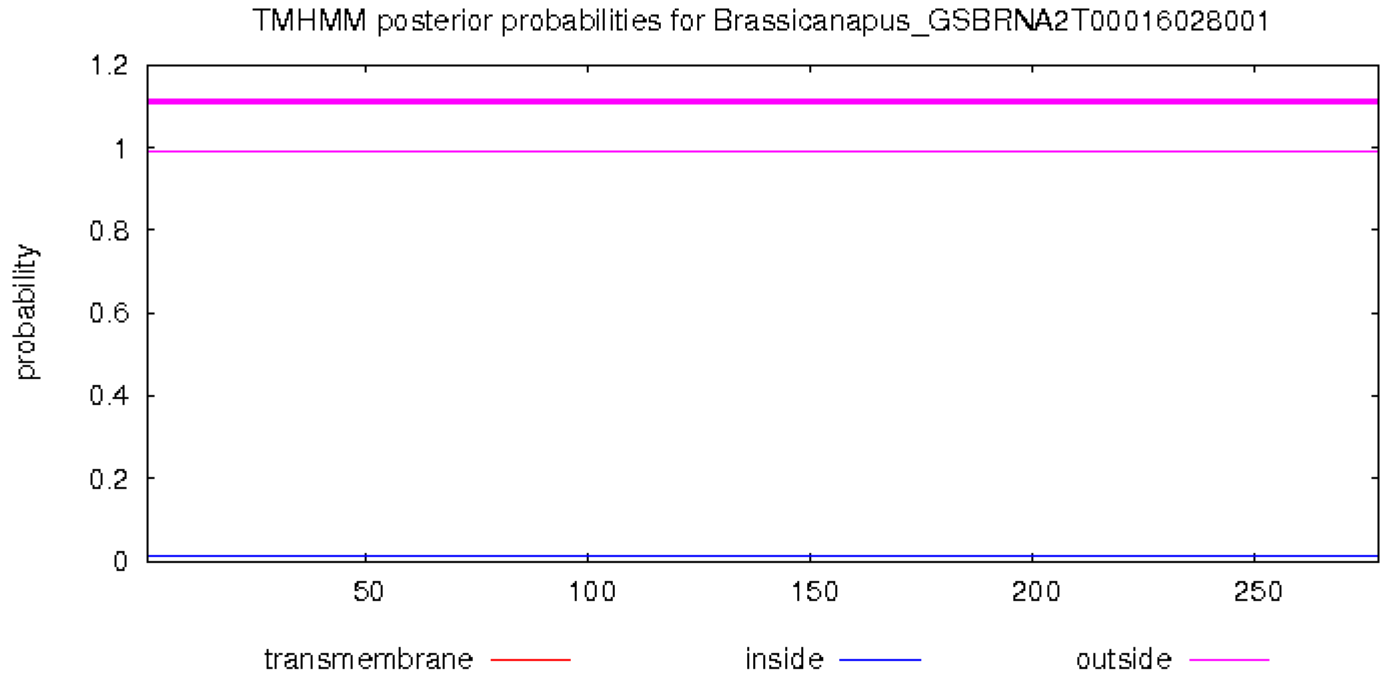

plot in postscript, script for making the plot in gnuplot, data for plot

---

```
# Brassicanapus_GSBRNA2T00016038001 Length: 434
# Brassicanapus_GSBRNA2T00016038001 Number of predicted TMHs:  0
# Brassicanapus_GSBRNA2T00016038001 Exp number of AAs in TMHs: 0
# Brassicanapus_GSBRNA2T00016038001 Exp number, first 60 AAs:  0
# Brassicanapus_GSBRNA2T00016038001 Total prob of N-in:        0.00134
Brassicanapus_GSBRNA2T00016038001       TMHMM2.0        outside      1   434
```

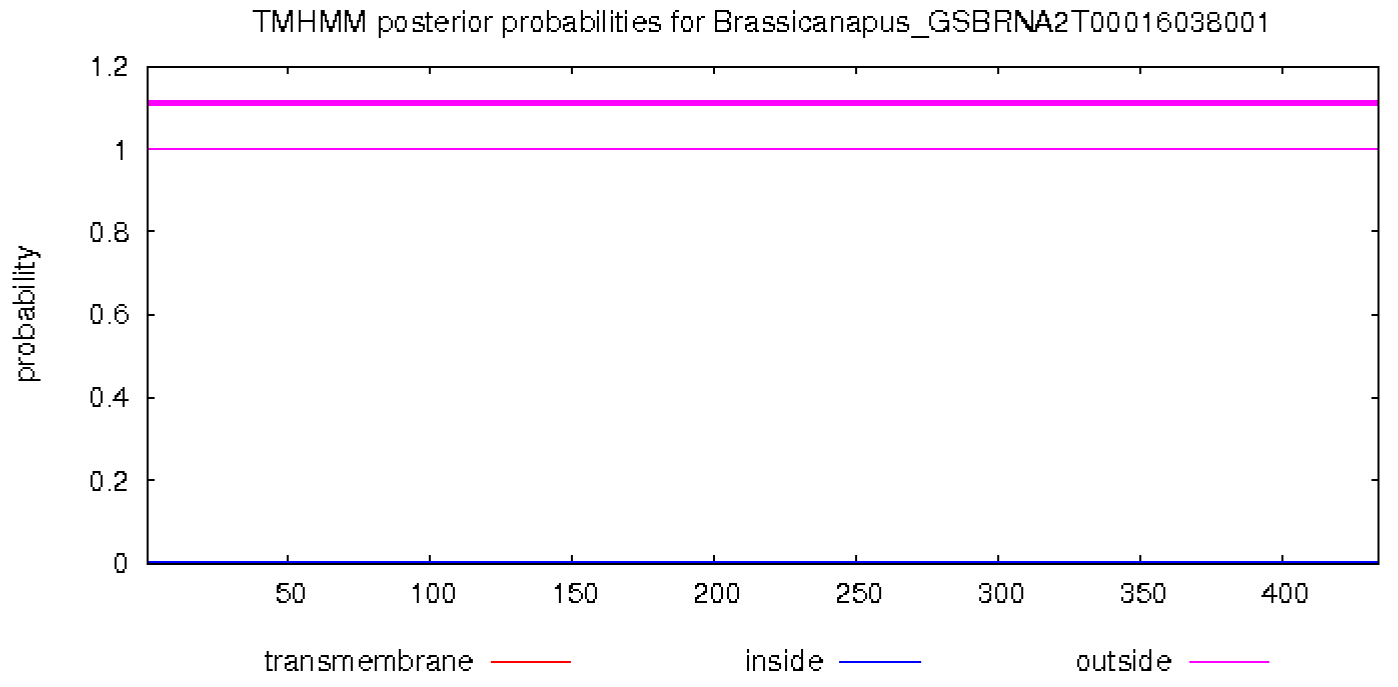

plot in postscript, script for making the plot in gnuplot, data for plot

---

```
# Brassicanapus_GSBRNA2T00016676001 Length: 254
# Brassicanapus_GSBRNA2T00016676001 Number of predicted TMHs:  0
# Brassicanapus_GSBRNA2T00016676001 Exp number of AAs in TMHs: 0
# Brassicanapus_GSBRNA2T00016676001 Exp number, first 60 AAs:  0
# Brassicanapus_GSBRNA2T00016676001 Total prob of N-in:        0.02451
Brassicanapus_GSBRNA2T00016676001       TMHMM2.0        outside      1   254
```

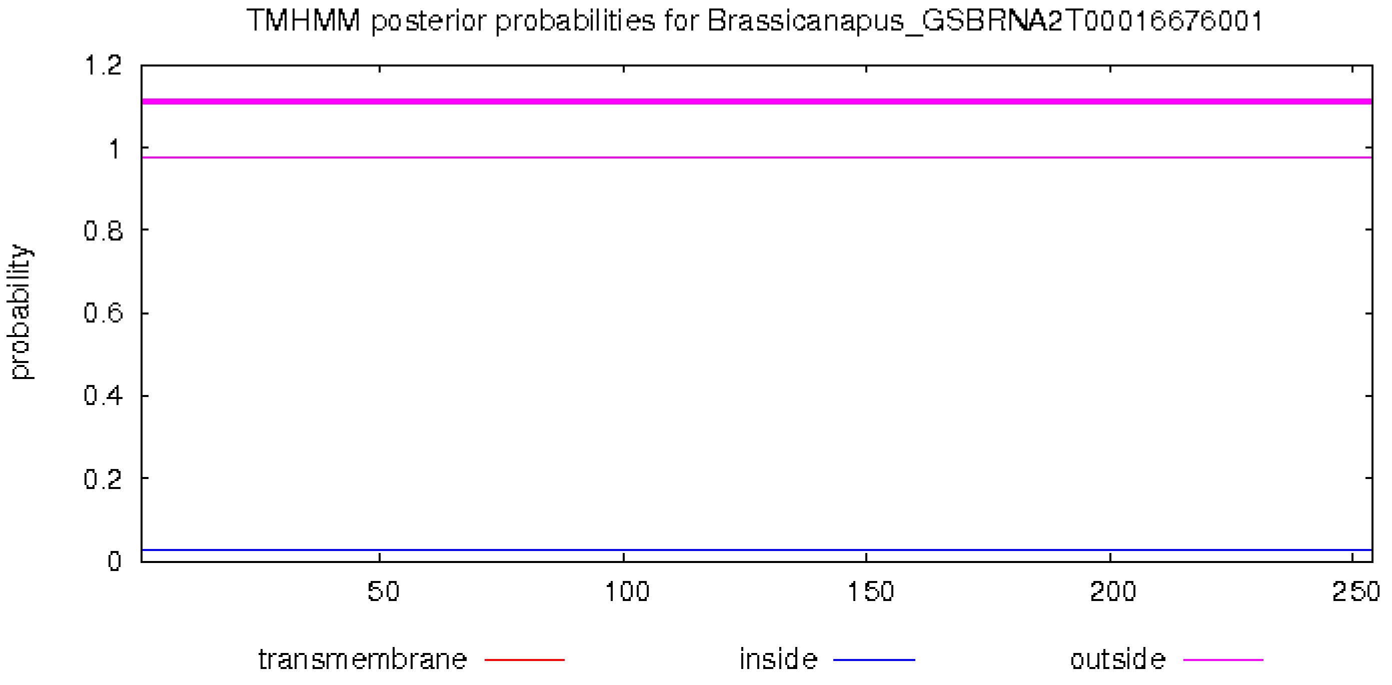

plot in postscript, script for making the plot in gnuplot, data for plot

---

```
# Brassicanapus_GSBRNA2T00016677001 Length: 242
# Brassicanapus_GSBRNA2T00016677001 Number of predicted TMHs:  0
# Brassicanapus_GSBRNA2T00016677001 Exp number of AAs in TMHs: 0
# Brassicanapus_GSBRNA2T00016677001 Exp number, first 60 AAs:  0
# Brassicanapus_GSBRNA2T00016677001 Total prob of N-in:        0.01384
Brassicanapus_GSBRNA2T00016677001       TMHMM2.0        outside      1   242
```

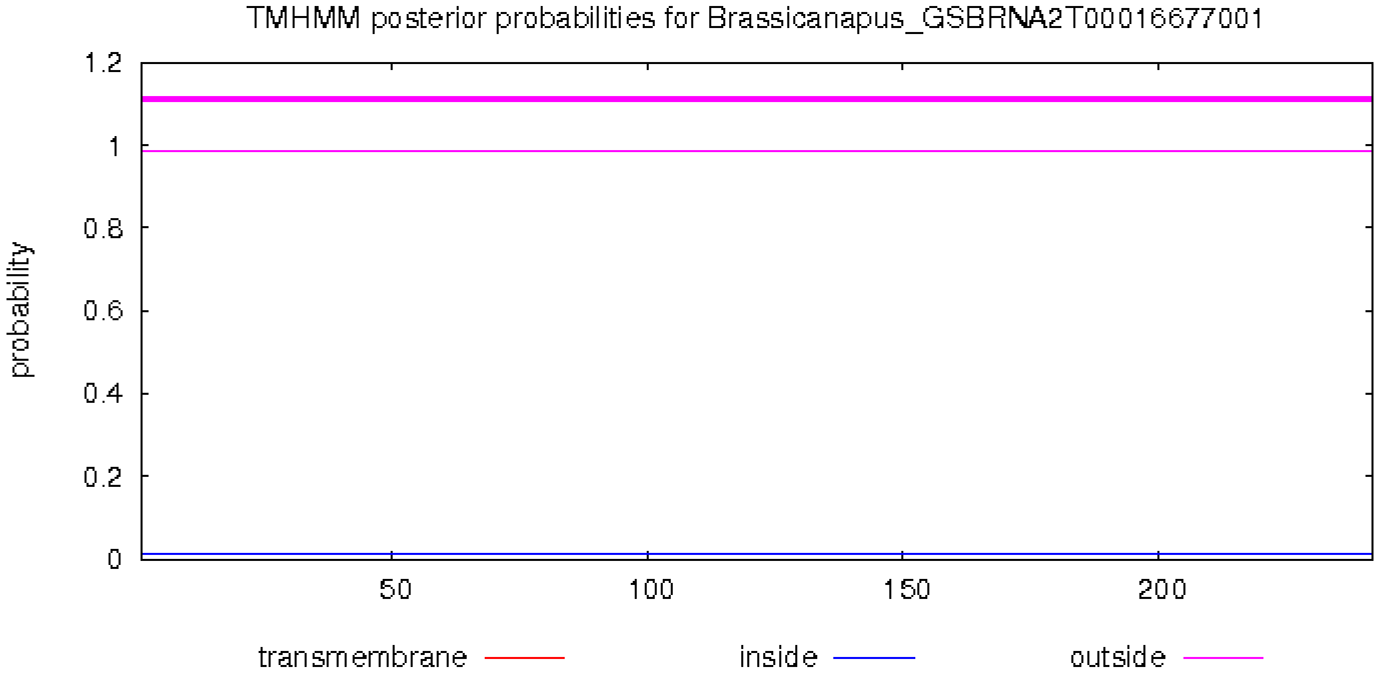

plot in postscript, script for making the plot in gnuplot, data for plot

---

```
# Brassicanapus_GSBRNA2T00016973001 Length: 279
# Brassicanapus_GSBRNA2T00016973001 Number of predicted TMHs:  0
# Brassicanapus_GSBRNA2T00016973001 Exp number of AAs in TMHs: 0.00124
# Brassicanapus_GSBRNA2T00016973001 Exp number, first 60 AAs:  0
# Brassicanapus_GSBRNA2T00016973001 Total prob of N-in:        0.03794
Brassicanapus_GSBRNA2T00016973001       TMHMM2.0        outside      1   279
```

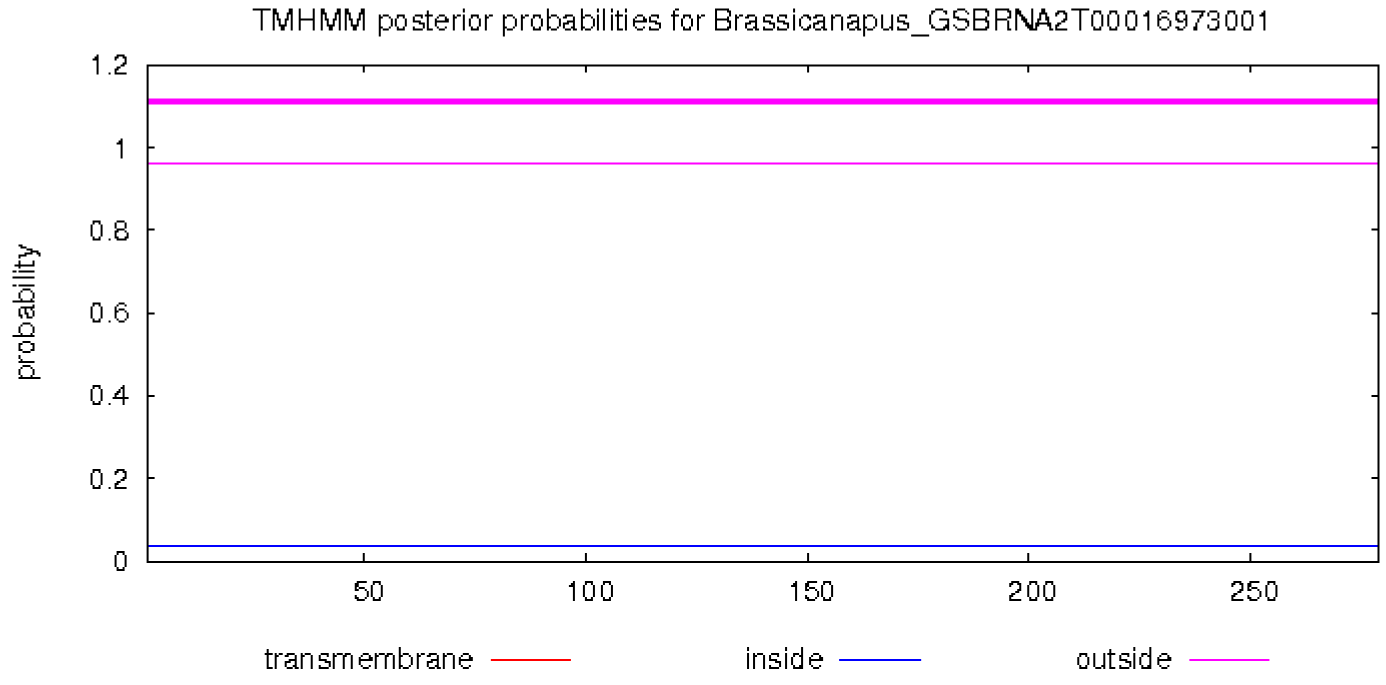

plot in postscript, script for making the plot in gnuplot, data for plot

---

```
# Brassicanapus_GSBRNA2T00018140001 Length: 317
# Brassicanapus_GSBRNA2T00018140001 Number of predicted TMHs:  0
# Brassicanapus_GSBRNA2T00018140001 Exp number of AAs in TMHs: 0
# Brassicanapus_GSBRNA2T00018140001 Exp number, first 60 AAs:  0
# Brassicanapus_GSBRNA2T00018140001 Total prob of N-in:        0.00384
Brassicanapus_GSBRNA2T00018140001       TMHMM2.0        outside      1   317
```

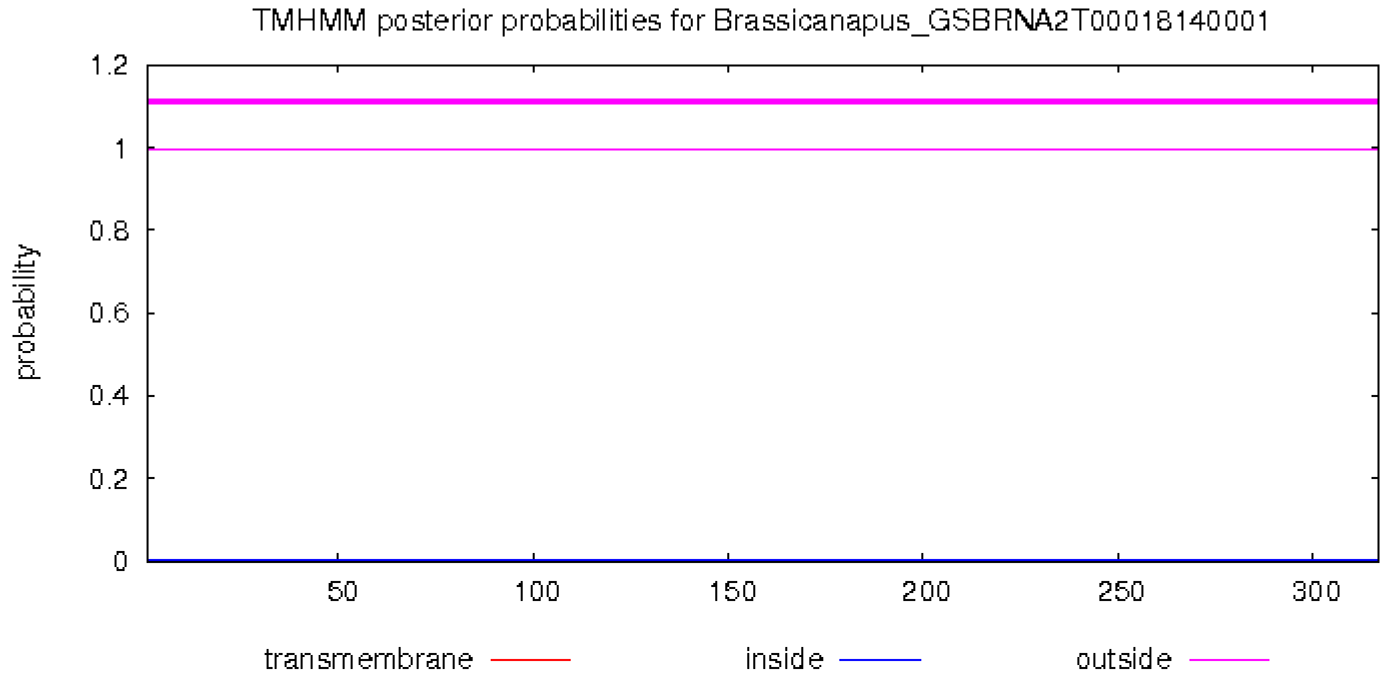

plot in postscript, script for making the plot in gnuplot, data for plot

---

```
# Brassicanapus_GSBRNA2T00018364001 Length: 708
# Brassicanapus_GSBRNA2T00018364001 Number of predicted TMHs:  0
# Brassicanapus_GSBRNA2T00018364001 Exp number of AAs in TMHs: 0.00036
# Brassicanapus_GSBRNA2T00018364001 Exp number, first 60 AAs:  0
# Brassicanapus_GSBRNA2T00018364001 Total prob of N-in:        0.00019
Brassicanapus_GSBRNA2T00018364001       TMHMM2.0        outside      1   708
```

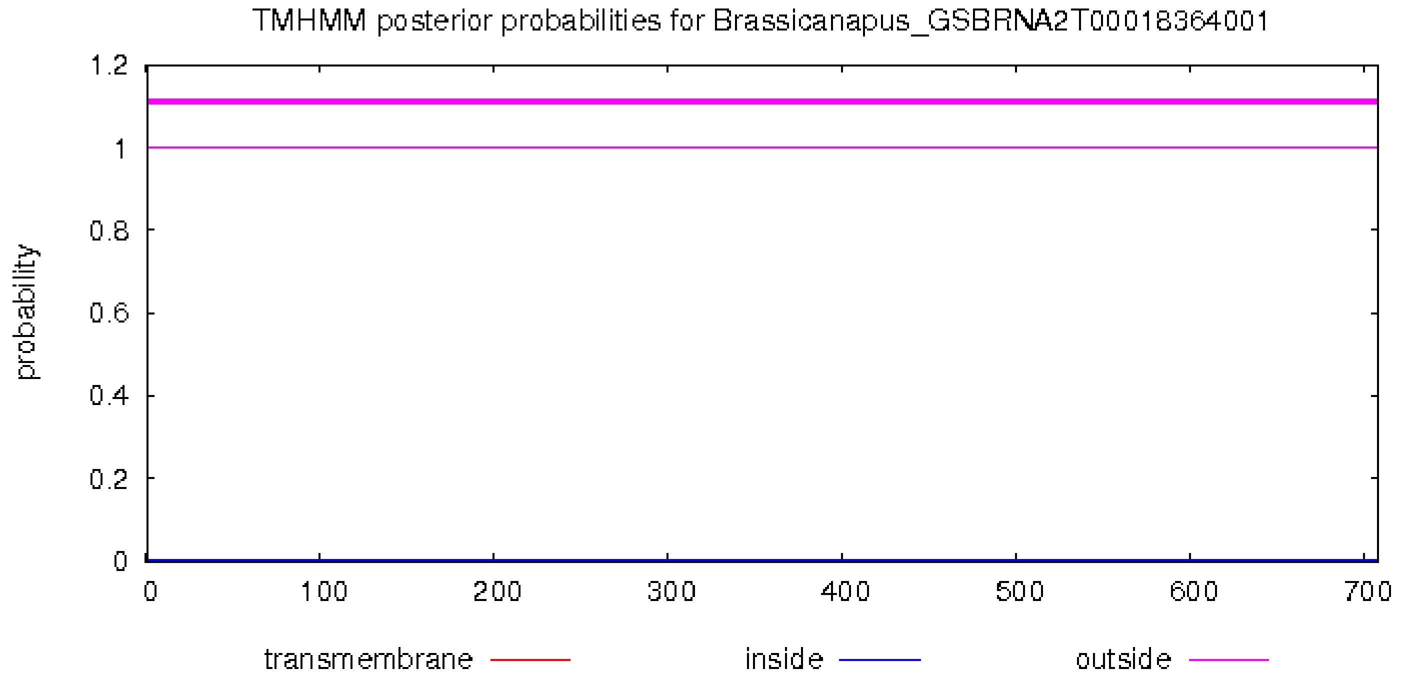

plot in postscript, script for making the plot in gnuplot, data for plot

---

```
# Brassicanapus_GSBRNA2T00018373001 Length: 411
# Brassicanapus_GSBRNA2T00018373001 Number of predicted TMHs:  0
# Brassicanapus_GSBRNA2T00018373001 Exp number of AAs in TMHs: 0.00117
# Brassicanapus_GSBRNA2T00018373001 Exp number, first 60 AAs:  0
# Brassicanapus_GSBRNA2T00018373001 Total prob of N-in:        0.00775
Brassicanapus_GSBRNA2T00018373001       TMHMM2.0        outside      1   411
```

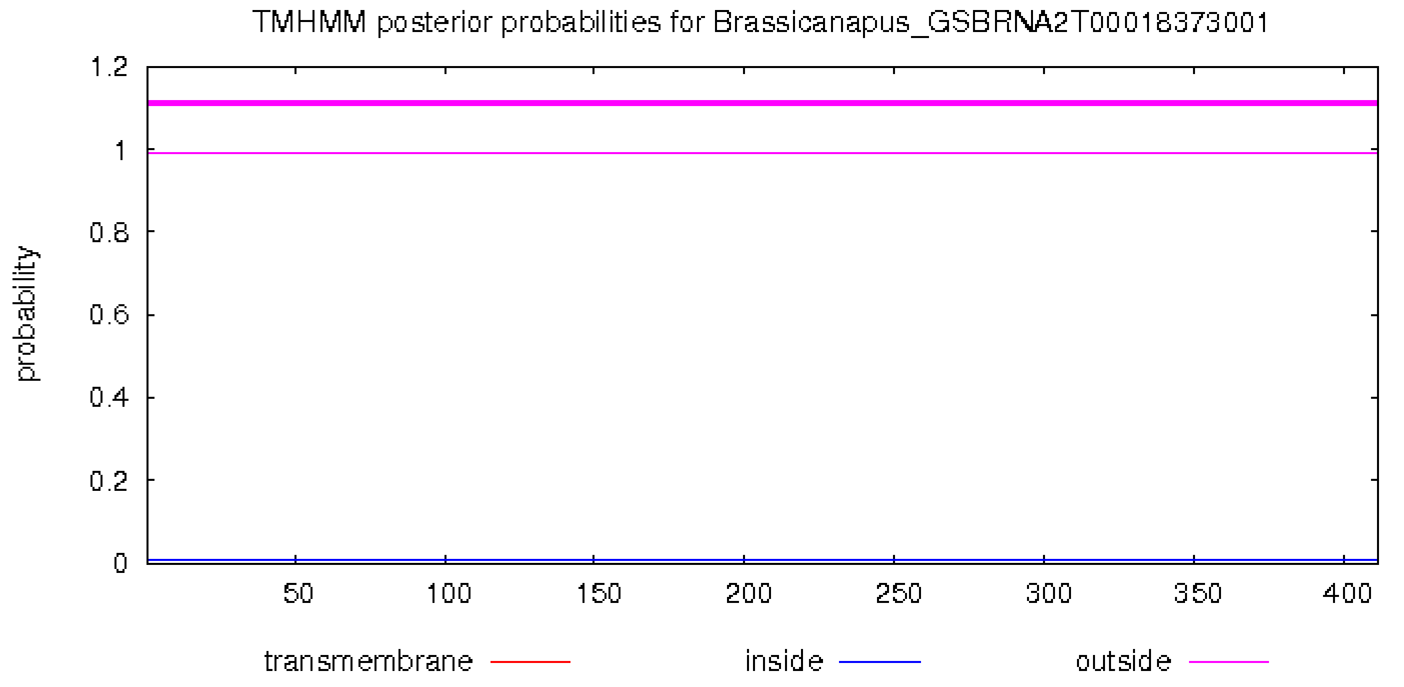

plot in postscript, script for making the plot in gnuplot, data for plot

---

```
# Brassicanapus_GSBRNA2T00018374001 Length: 552
# Brassicanapus_GSBRNA2T00018374001 Number of predicted TMHs:  0
# Brassicanapus_GSBRNA2T00018374001 Exp number of AAs in TMHs: 0.00122
# Brassicanapus_GSBRNA2T00018374001 Exp number, first 60 AAs:  0.00088
# Brassicanapus_GSBRNA2T00018374001 Total prob of N-in:        0.00514
Brassicanapus_GSBRNA2T00018374001       TMHMM2.0        outside      1   552
```

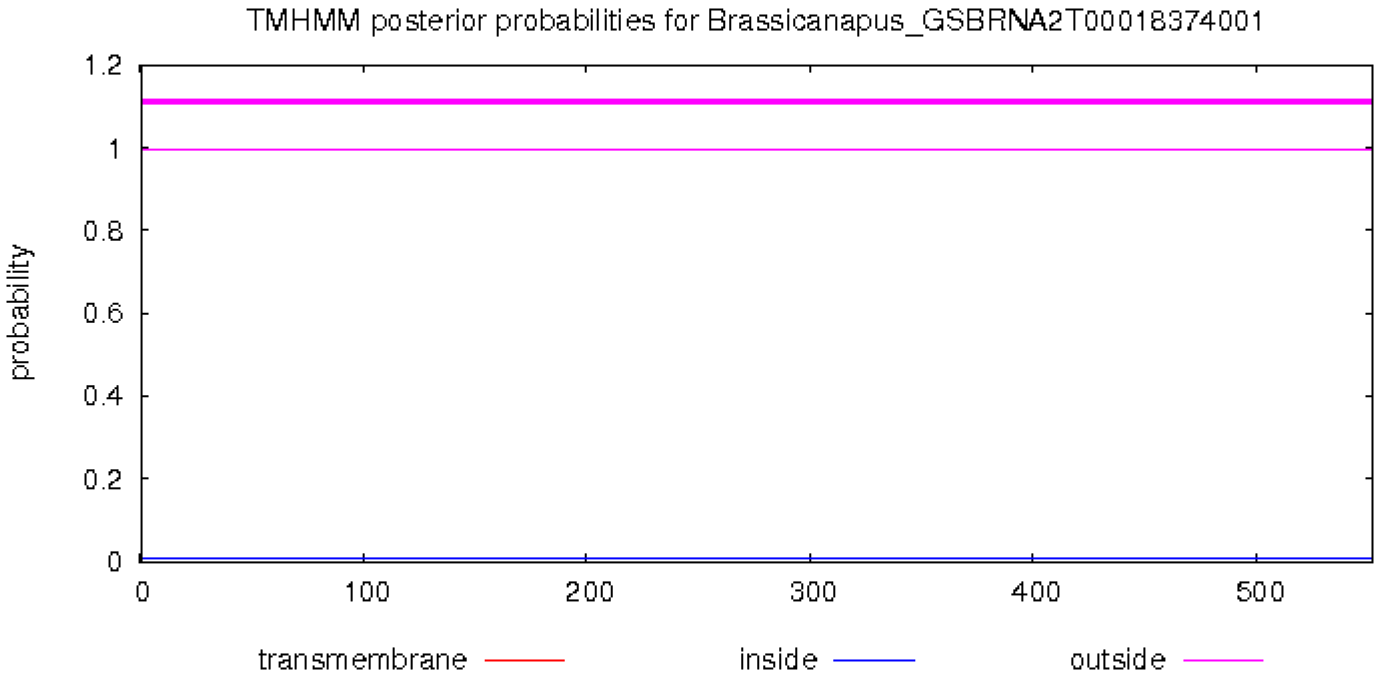

plot in postscript, script for making the plot in gnuplot, data for plot

---

```
# Brassicanapus_GSBRNA2T00018746001 Length: 347
# Brassicanapus_GSBRNA2T00018746001 Number of predicted TMHs:  0
# Brassicanapus_GSBRNA2T00018746001 Exp number of AAs in TMHs: 0.0002
# Brassicanapus_GSBRNA2T00018746001 Exp number, first 60 AAs:  0.0002
# Brassicanapus_GSBRNA2T00018746001 Total prob of N-in:        0.01369
Brassicanapus_GSBRNA2T00018746001       TMHMM2.0        outside      1   347
```

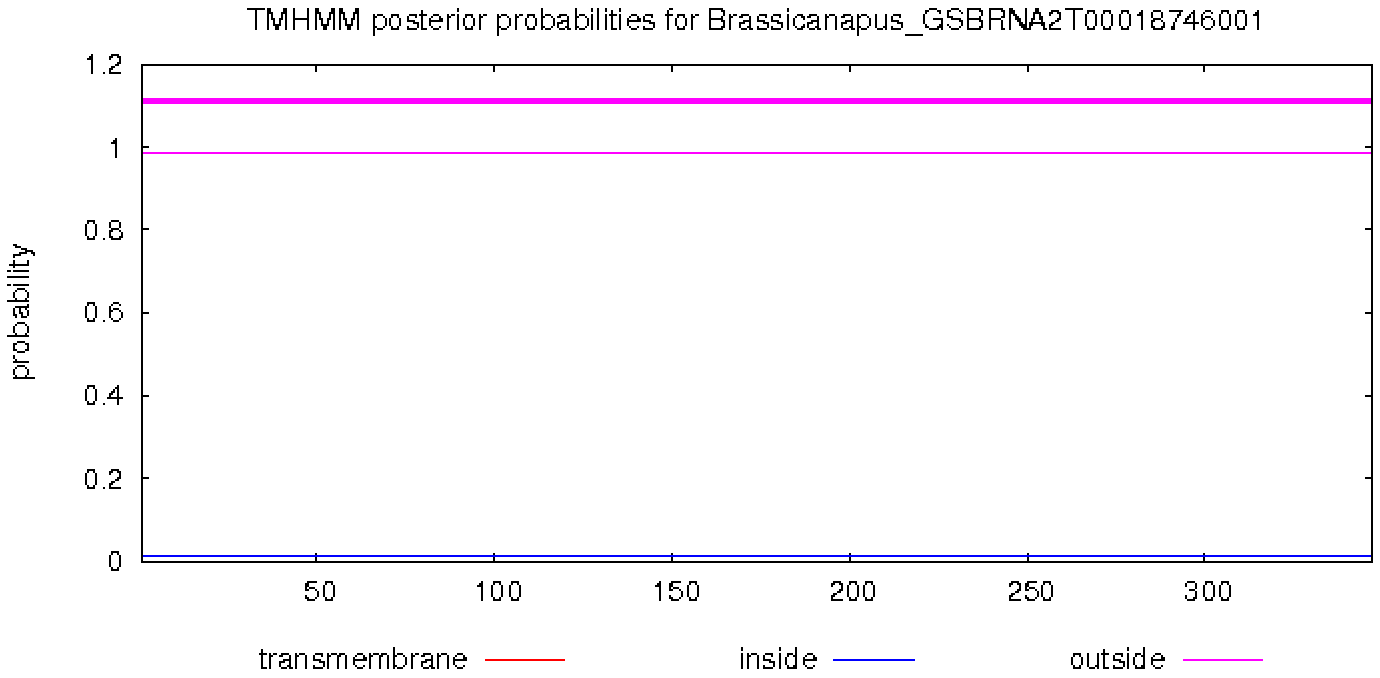

plot in postscript, script for making the plot in gnuplot, data for plot

---

```
# Brassicanapus_GSBRNA2T00019258001 Length: 135
# Brassicanapus_GSBRNA2T00019258001 Number of predicted TMHs:  0
# Brassicanapus_GSBRNA2T00019258001 Exp number of AAs in TMHs: 0.06957
# Brassicanapus_GSBRNA2T00019258001 Exp number, first 60 AAs:  0.00188
# Brassicanapus_GSBRNA2T00019258001 Total prob of N-in:        0.64795
Brassicanapus_GSBRNA2T00019258001       TMHMM2.0        inside       1   135
```

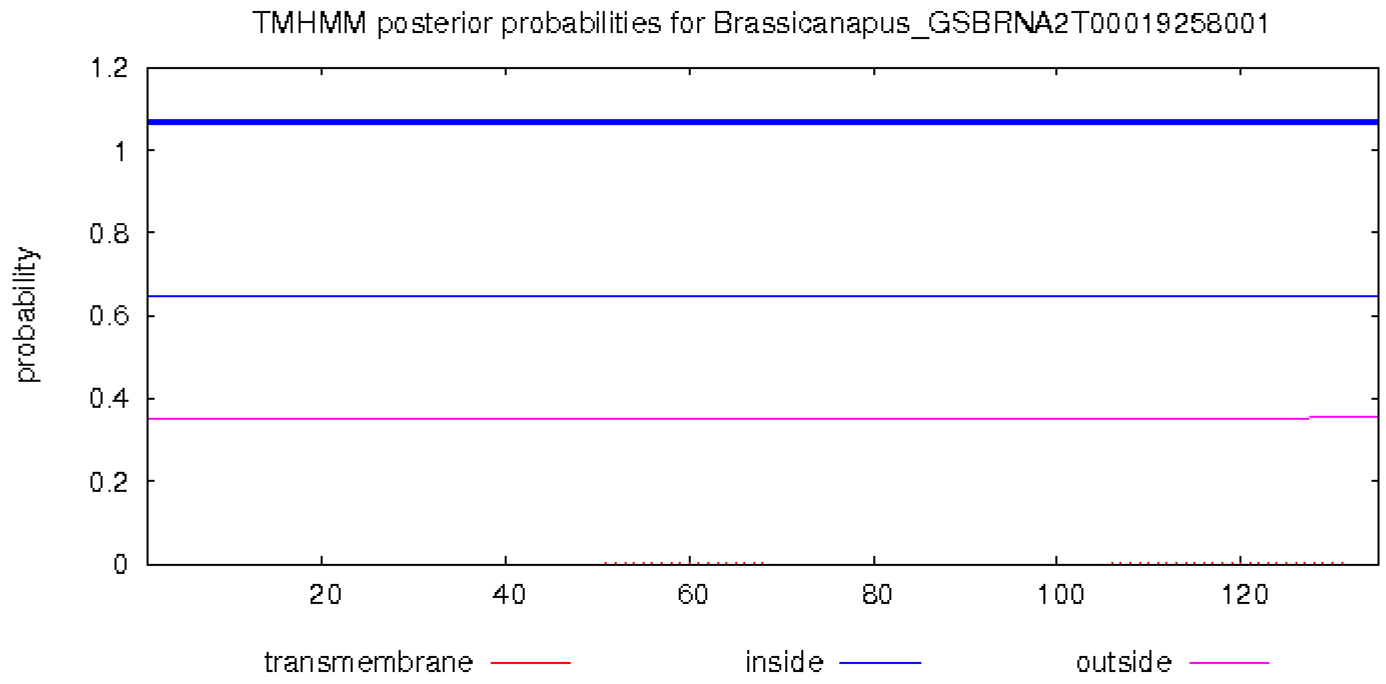

plot in postscript, script for making the plot in gnuplot, data for plot

---

```
# Brassicanapus_GSBRNA2T00019342001 Length: 199
# Brassicanapus_GSBRNA2T00019342001 Number of predicted TMHs:  0
# Brassicanapus_GSBRNA2T00019342001 Exp number of AAs in TMHs: 0.0023
# Brassicanapus_GSBRNA2T00019342001 Exp number, first 60 AAs:  0.0023
# Brassicanapus_GSBRNA2T00019342001 Total prob of N-in:        0.03336
Brassicanapus_GSBRNA2T00019342001       TMHMM2.0        outside      1   199
```

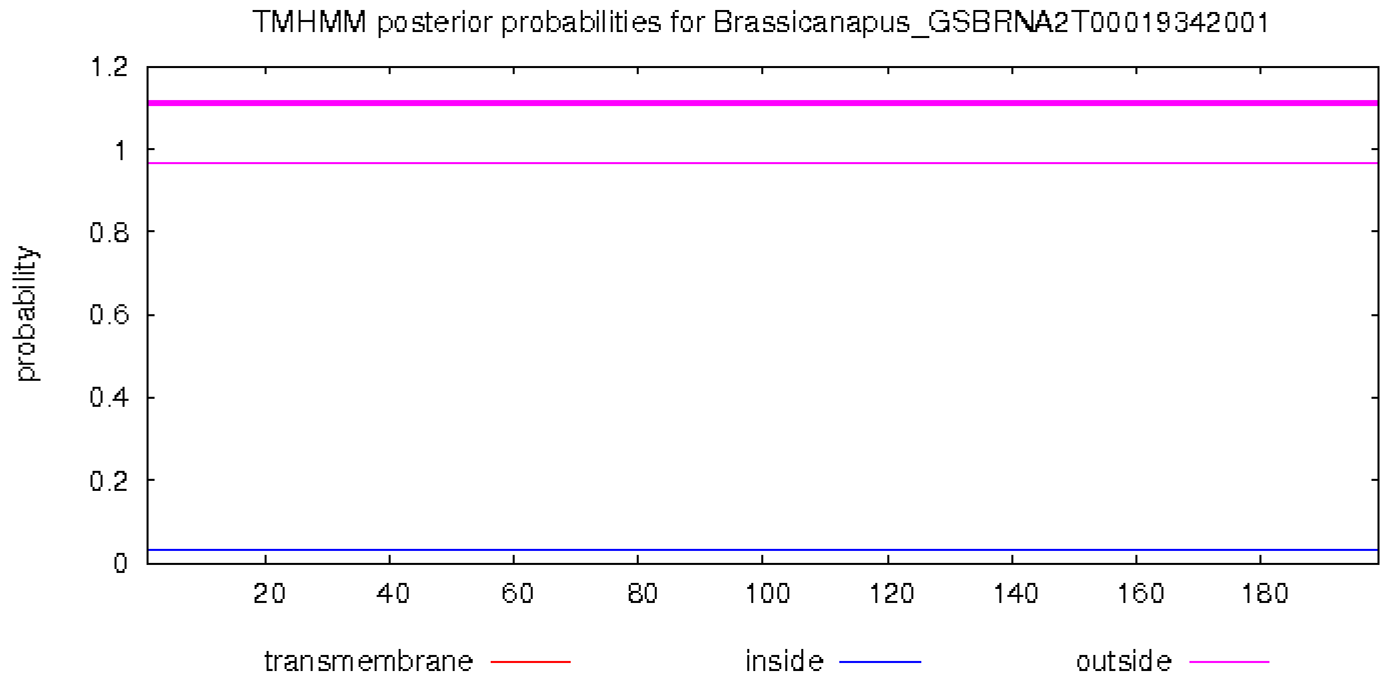

plot in postscript, script for making the plot in gnuplot, data for plot

---

```
# Brassicanapus_GSBRNA2T00019578001 Length: 327
# Brassicanapus_GSBRNA2T00019578001 Number of predicted TMHs:  0
# Brassicanapus_GSBRNA2T00019578001 Exp number of AAs in TMHs: 0.00024
# Brassicanapus_GSBRNA2T00019578001 Exp number, first 60 AAs:  0
# Brassicanapus_GSBRNA2T00019578001 Total prob of N-in:        0.00647
Brassicanapus_GSBRNA2T00019578001       TMHMM2.0        outside      1   327
```

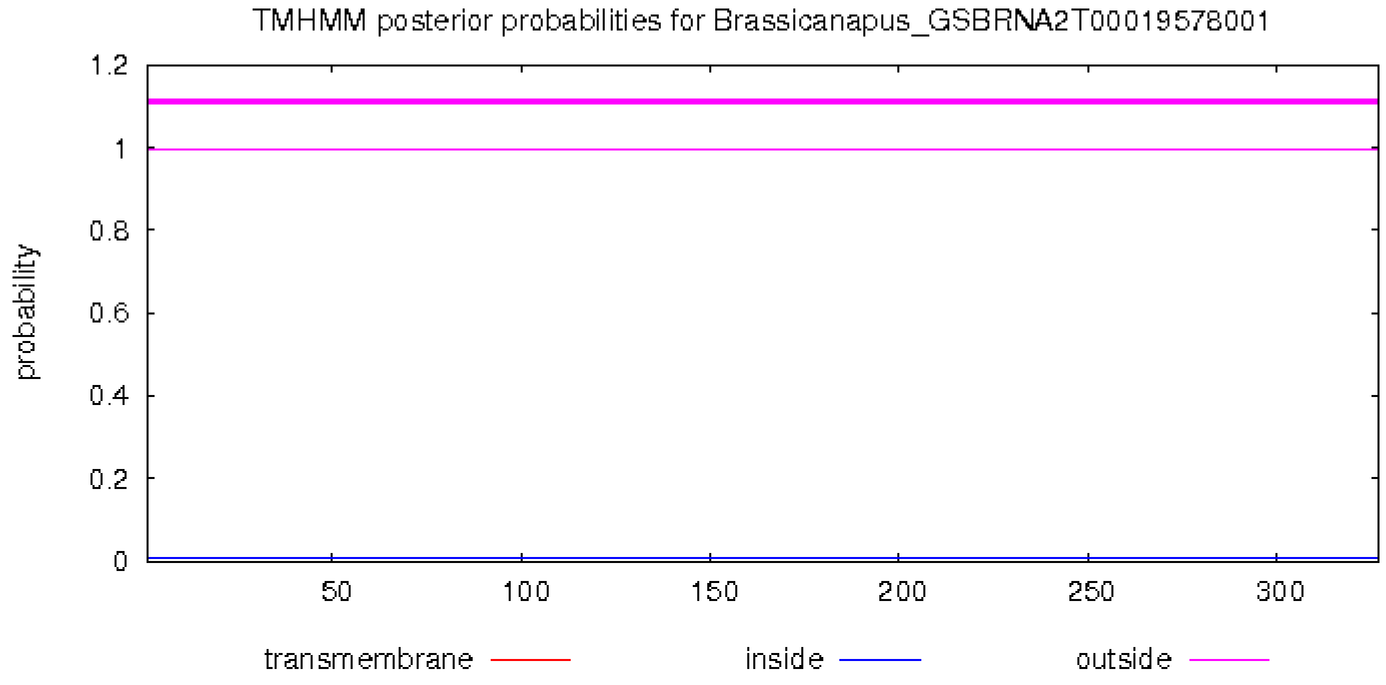

plot in postscript, script for making the plot in gnuplot, data for plot

```
# Brassicanapus_GSBRNA2T00019640001 Length: 188
# Brassicanapus_GSBRNA2T00019640001 Number of predicted TMHs:  0
# Brassicanapus_GSBRNA2T00019640001 Exp number of AAs in TMHs: 0
# Brassicanapus_GSBRNA2T00019640001 Exp number, first 60 AAs:  0
# Brassicanapus_GSBRNA2T00019640001 Total prob of N-in:        0.00724
Brassicanapus_GSBRNA2T00019640001       TMHMM2.0        outside      1   188
```

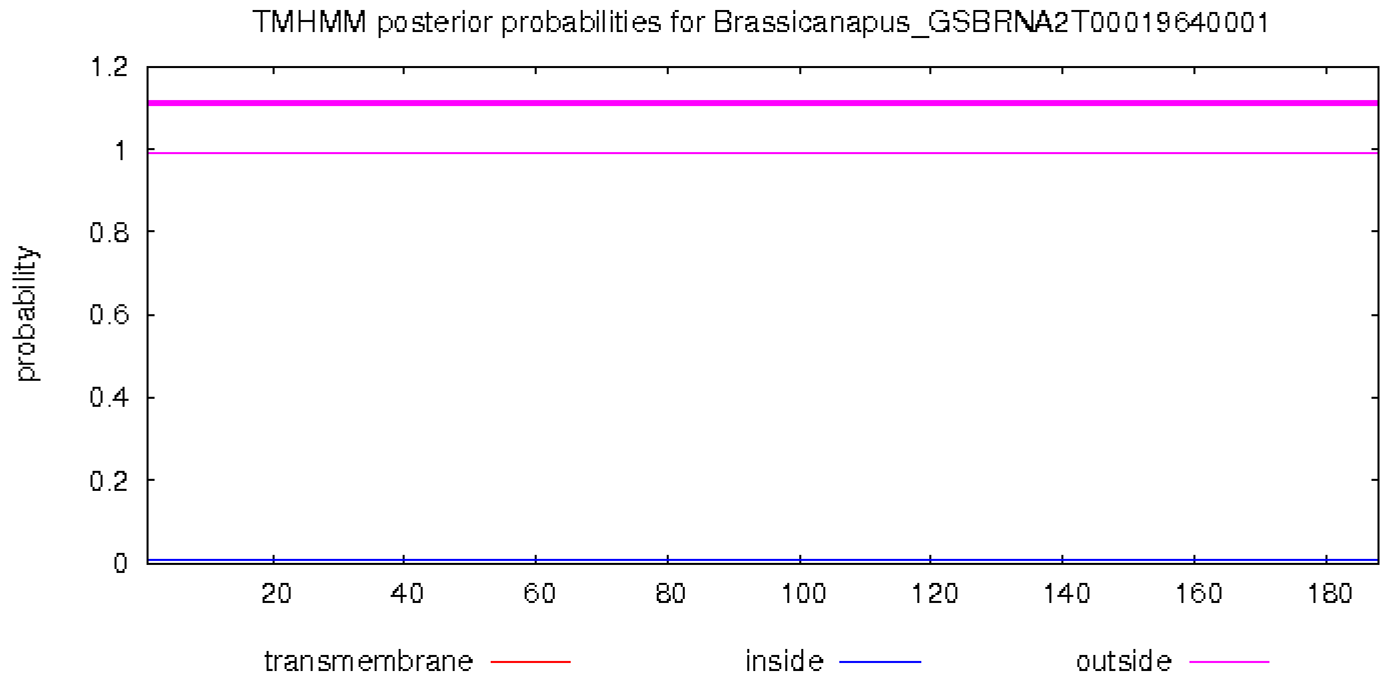

plot in postscript, script for making the plot in gnuplot, data for plot

```
# Brassicanapus_GSBRNA2T00020292001 Length: 327
# Brassicanapus_GSBRNA2T00020292001 Number of predicted TMHs:  0
# Brassicanapus_GSBRNA2T00020292001 Exp number of AAs in TMHs: 0.00033
# Brassicanapus_GSBRNA2T00020292001 Exp number, first 60 AAs:  0
# Brassicanapus_GSBRNA2T00020292001 Total prob of N-in:        0.01774
Brassicanapus_GSBRNA2T00020292001       TMHMM2.0        outside      1   327
```

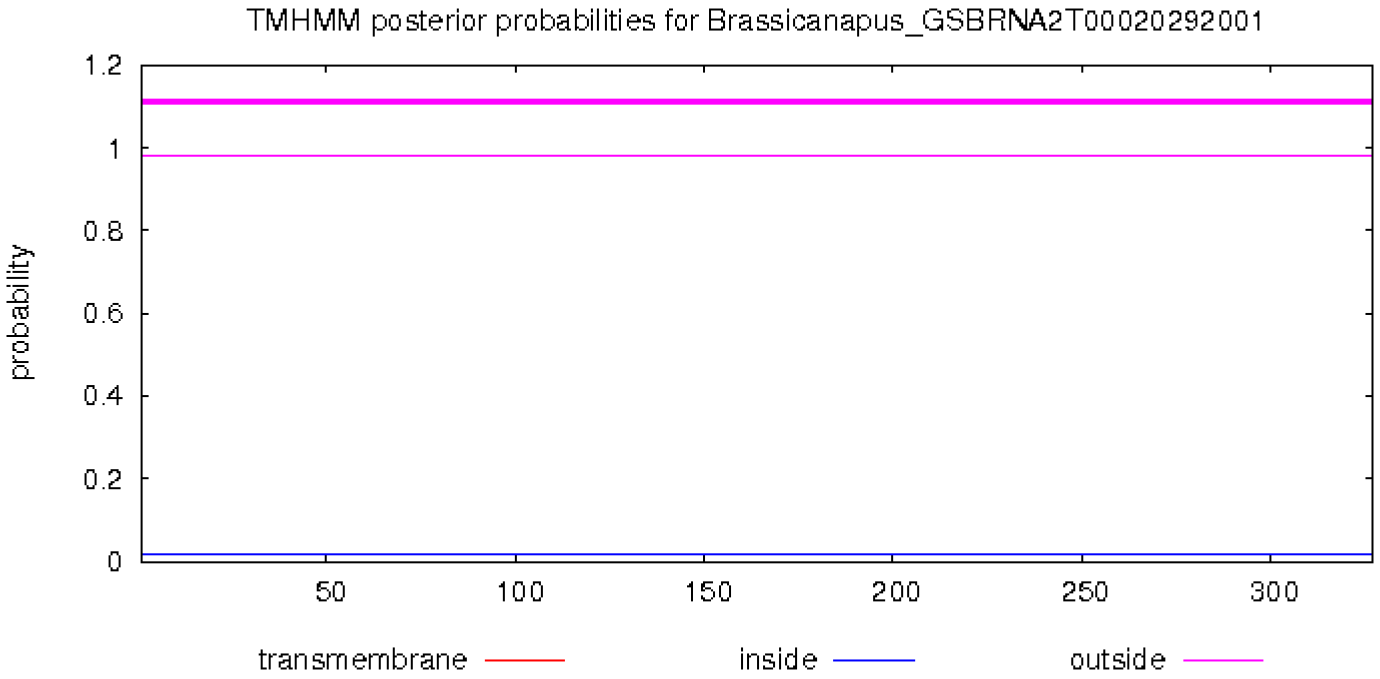

plot in postscript, script for making the plot in gnuplot, data for plot

---

```
# Brassicanapus_GSBRNA2T00020965001 Length: 325
# Brassicanapus_GSBRNA2T00020965001 Number of predicted TMHs:  0
# Brassicanapus_GSBRNA2T00020965001 Exp number of AAs in TMHs: 0.00114
# Brassicanapus_GSBRNA2T00020965001 Exp number, first 60 AAs:  0.00039
# Brassicanapus_GSBRNA2T00020965001 Total prob of N-in:        0.01326
Brassicanapus_GSBRNA2T00020965001       TMHMM2.0        outside      1   325
```

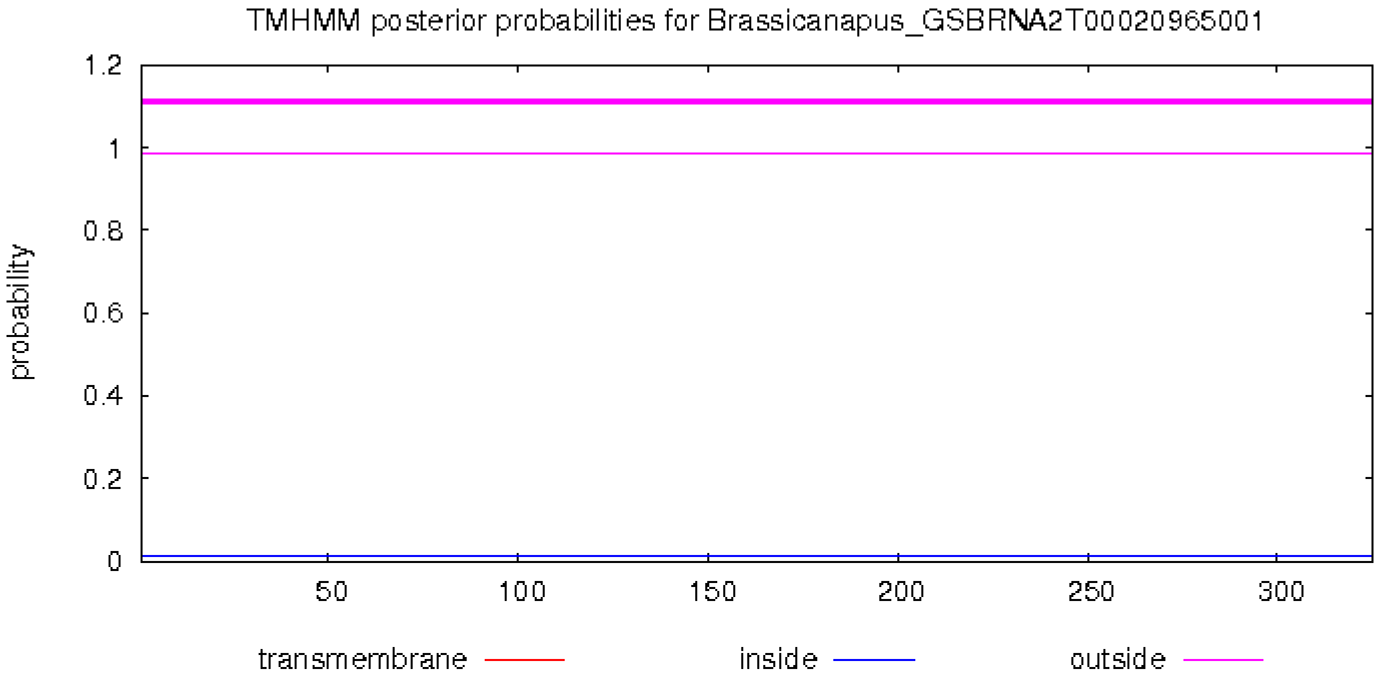

plot in postscript, script for making the plot in gnuplot, data for plot

---

```
# Brassicanapus_GSBRNA2T00021663001 Length: 2245
# Brassicanapus_GSBRNA2T00021663001 Number of predicted TMHs:  0
# Brassicanapus_GSBRNA2T00021663001 Exp number of AAs in TMHs: 0.0253500000000000001
# Brassicanapus_GSBRNA2T00021663001 Exp number, first 60 AAs:  0.00095
# Brassicanapus_GSBRNA2T00021663001 Total prob of N-in:        0.00126
Brassicanapus_GSBRNA2T00021663001       TMHMM2.0        outside      1  2245
```

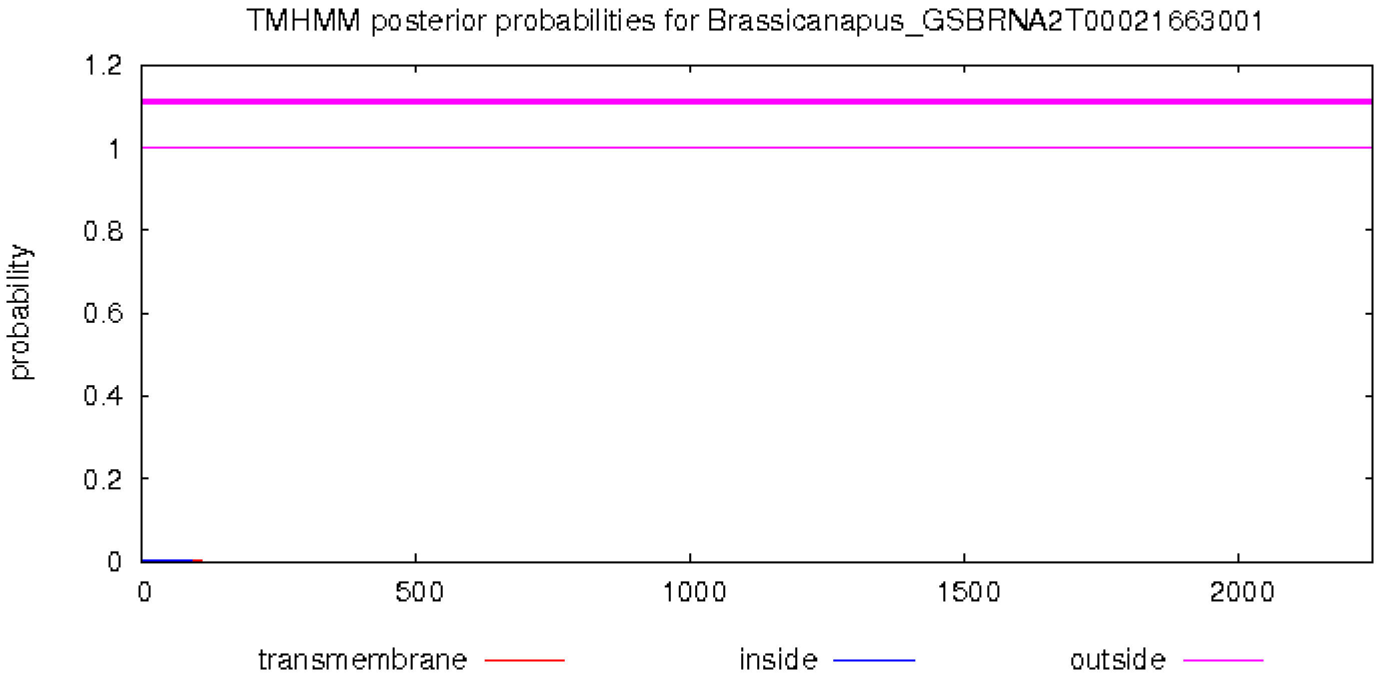

plot in postscript, script for making the plot in gnuplot, data for plot

---

```
# Brassicanapus_GSBRNA2T00022937001 Length: 348
# Brassicanapus_GSBRNA2T00022937001 Number of predicted TMHs:  0
# Brassicanapus_GSBRNA2T00022937001 Exp number of AAs in TMHs: 0.00019
# Brassicanapus_GSBRNA2T00022937001 Exp number, first 60 AAs:  0
# Brassicanapus_GSBRNA2T00022937001 Total prob of N-in:        0.00281
Brassicanapus_GSBRNA2T00022937001       TMHMM2.0        outside      1   348
```

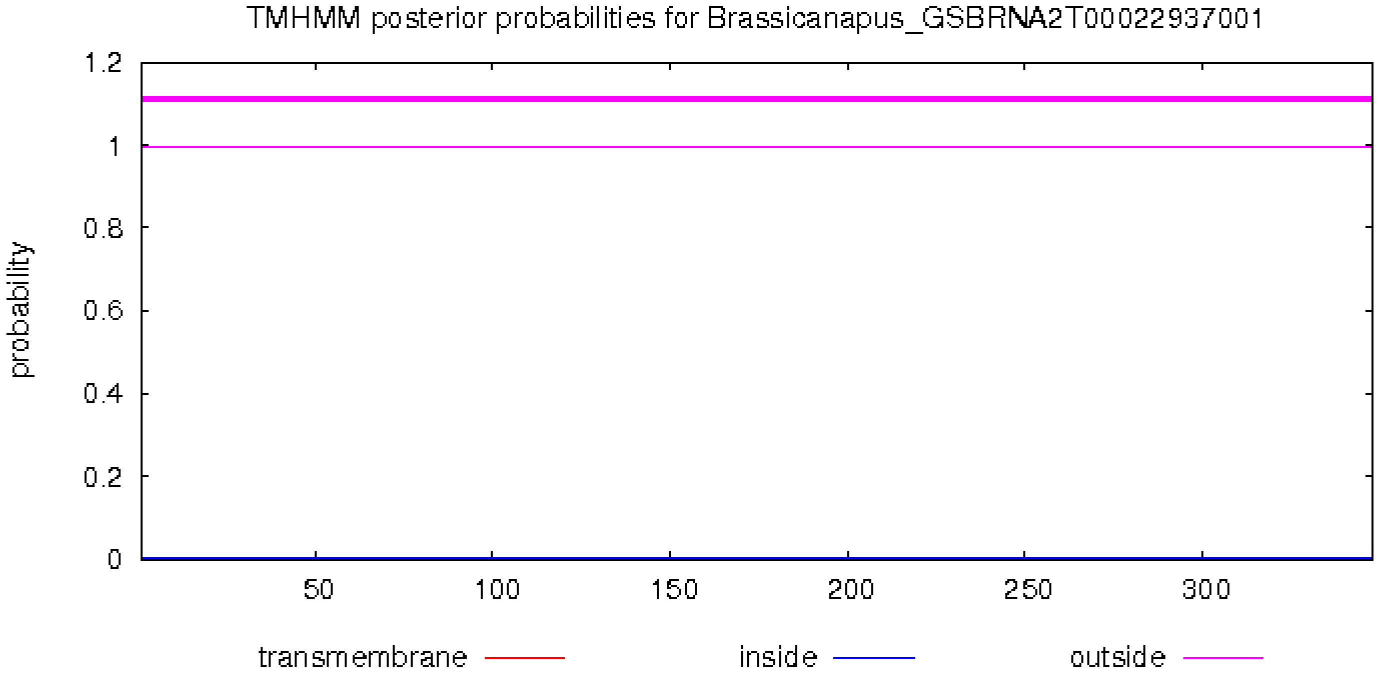

plot in postscript, script for making the plot in gnuplot, data for plot

---

```
# Brassicanapus_GSBRNA2T00022943001 Length: 349
# Brassicanapus_GSBRNA2T00022943001 Number of predicted TMHs:  0
# Brassicanapus_GSBRNA2T00022943001 Exp number of AAs in TMHs: 0.00096
# Brassicanapus_GSBRNA2T00022943001 Exp number, first 60 AAs:  0.00096
# Brassicanapus_GSBRNA2T00022943001 Total prob of N-in:        0.01005
Brassicanapus_GSBRNA2T00022943001       TMHMM2.0        outside      1   349
```

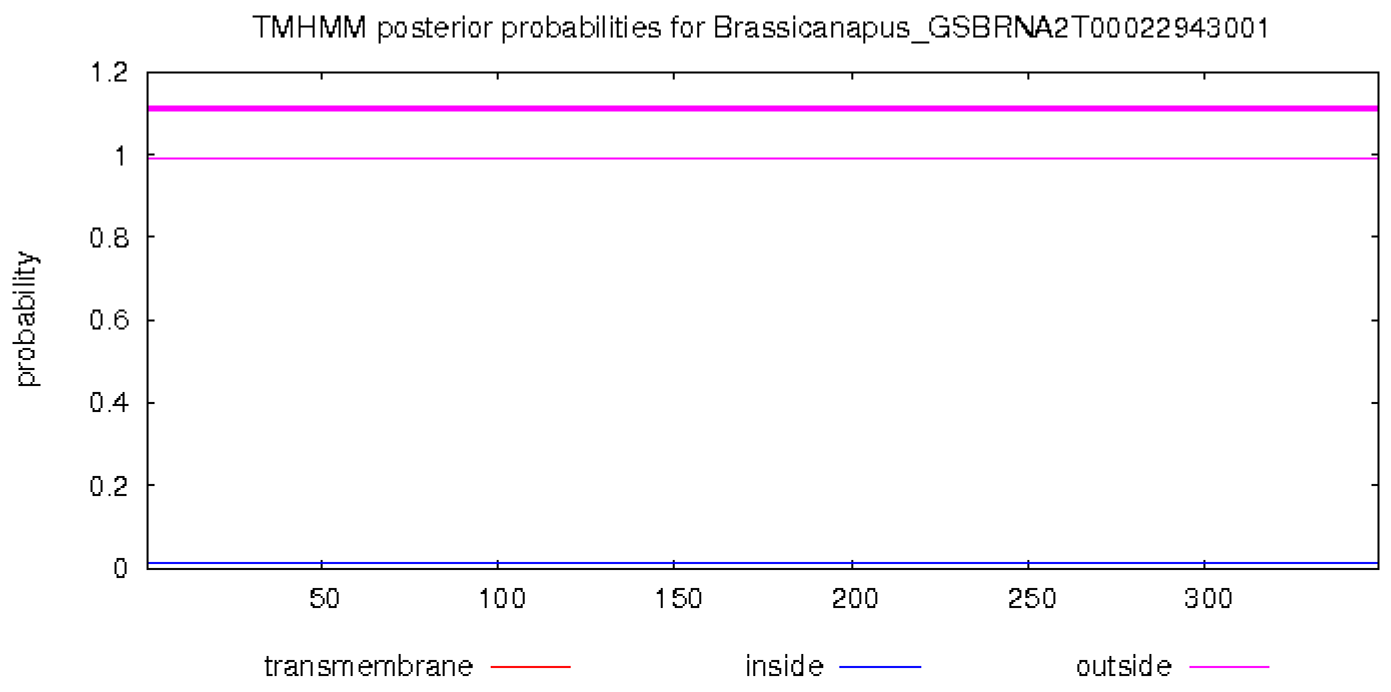

plot in postscript, script for making the plot in gnuplot, data for plot

---

```
# Brassicanapus_GSBRNA2T00023614001 Length: 556
# Brassicanapus_GSBRNA2T00023614001 Number of predicted TMHs:  1
# Brassicanapus_GSBRNA2T00023614001 Exp number of AAs in TMHs: 21.24627
# Brassicanapus_GSBRNA2T00023614001 Exp number, first 60 AAs:  0
# Brassicanapus_GSBRNA2T00023614001 Total prob of N-in:        0.00757
Brassicanapus_GSBRNA2T00023614001	TMHMM2.0	outside	     1   530
Brassicanapus_GSBRNA2T00023614001	TMHMM2.0	TMhelix	   531   553
Brassicanapus_GSBRNA2T00023614001	TMHMM2.0	inside	   554   556
```

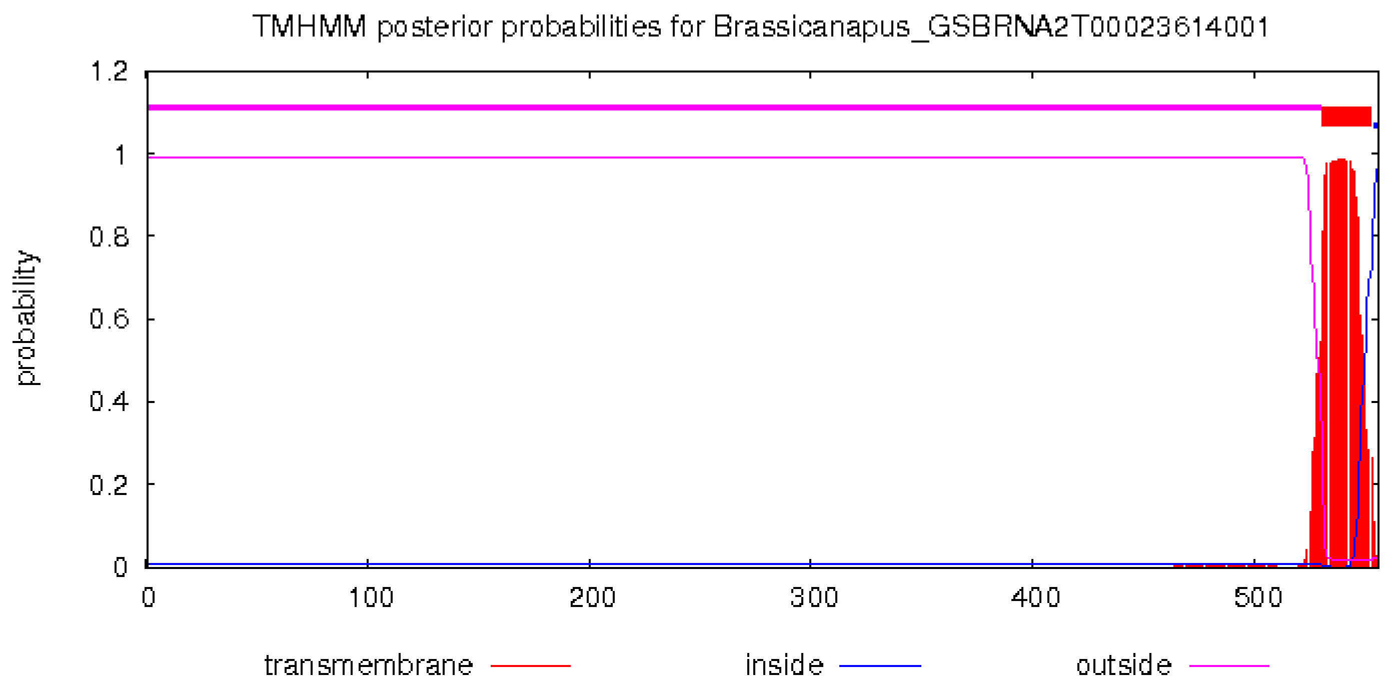

plot in postscript, script for making the plot in gnuplot, data for plot

---

```
# Brassicanapus_GSBRNA2T00023738001 Length: 375
# Brassicanapus_GSBRNA2T00023738001 Number of predicted TMHs:  0
# Brassicanapus_GSBRNA2T00023738001 Exp number of AAs in TMHs: 0.00069
# Brassicanapus_GSBRNA2T00023738001 Exp number, first 60 AAs:  0
```

```
# Brassicanapus_GSBRNA2T00023738001 Total prob of N-in:        0.01077
Brassicanapus_GSBRNA2T00023738001	TMHMM2.0	outside	     1   375
```

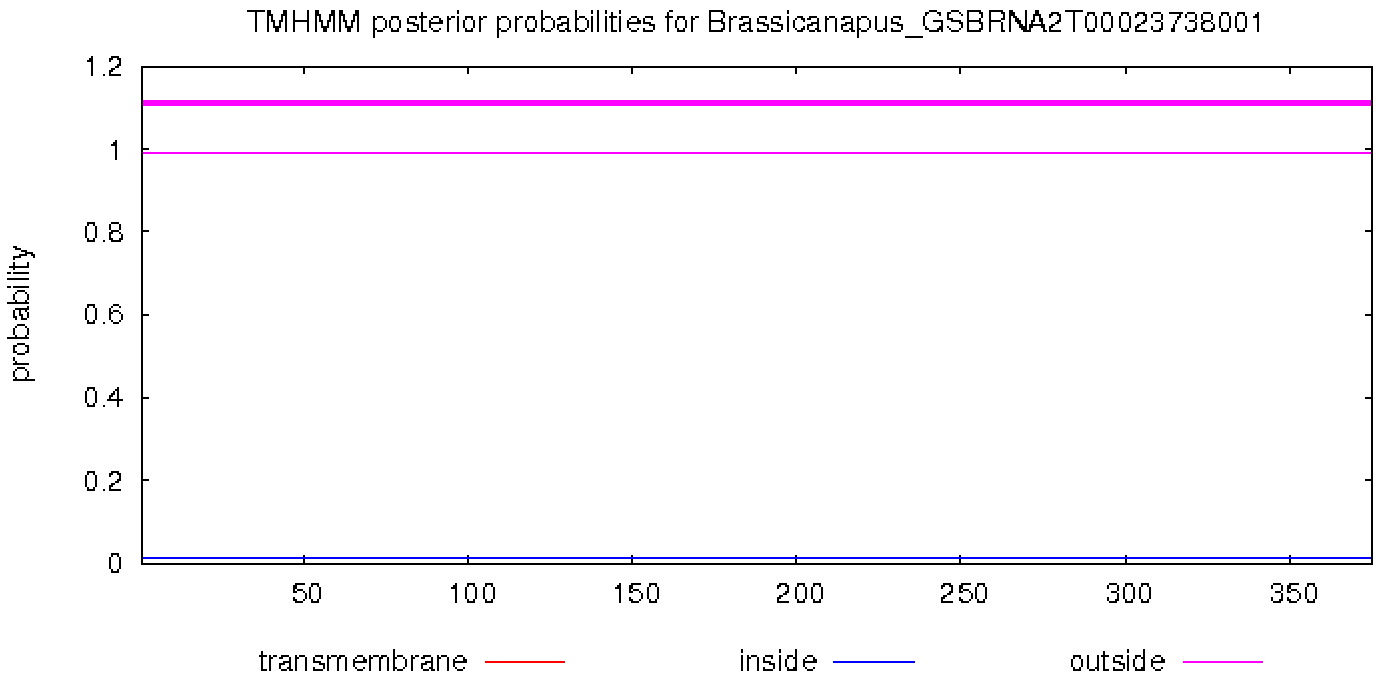

plot in postscript, script for making the plot in gnuplot, data for plot

---

```
# Brassicanapus_GSBRNA2T00023746001 Length: 274
# Brassicanapus_GSBRNA2T00023746001 Number of predicted TMHs:  0
# Brassicanapus_GSBRNA2T00023746001 Exp number of AAs in TMHs: 0.00151
# Brassicanapus_GSBRNA2T00023746001 Exp number, first 60 AAs:  0
# Brassicanapus_GSBRNA2T00023746001 Total prob of N-in:        0.04002
Brassicanapus_GSBRNA2T00023746001	TMHMM2.0	outside	     1   274
```

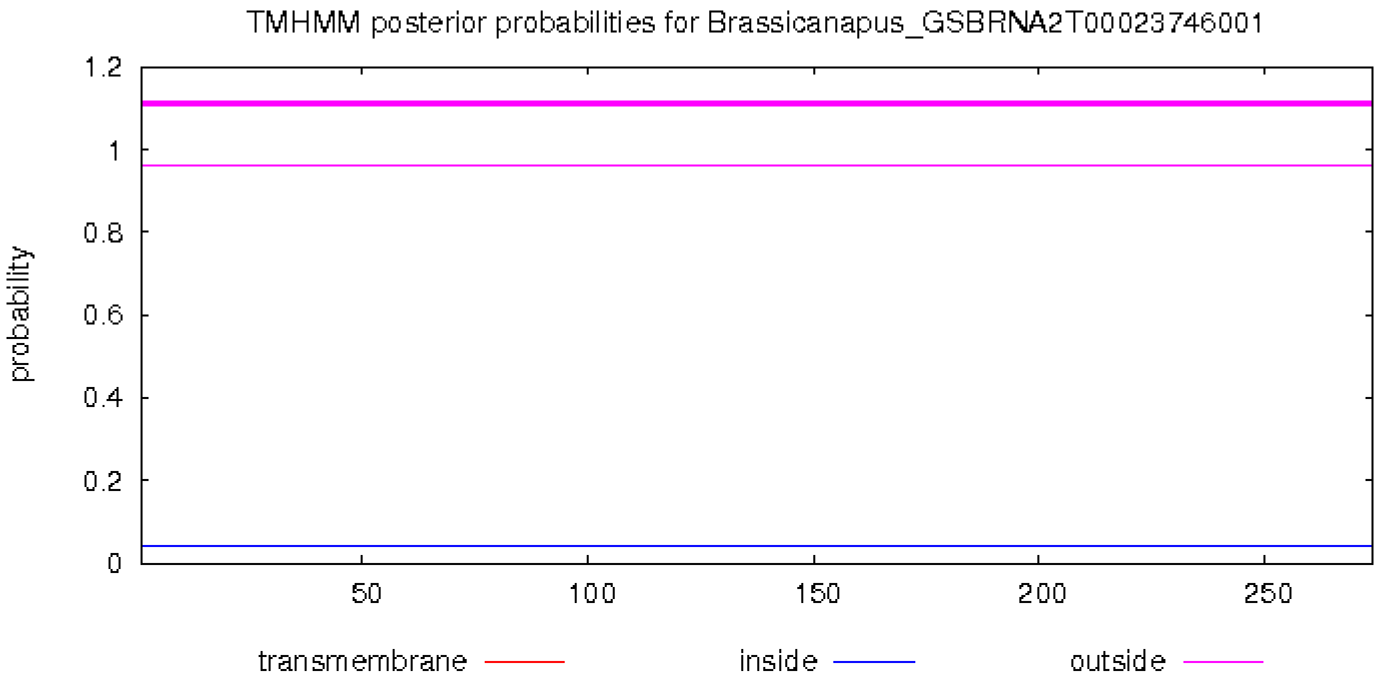

plot in postscript, script for making the plot in gnuplot, data for plot

---

```
# Brassicanapus_GSBRNA2T00024057001 Length: 255
# Brassicanapus_GSBRNA2T00024057001 Number of predicted TMHs:  0
# Brassicanapus_GSBRNA2T00024057001 Exp number of AAs in TMHs: 0.02487
# Brassicanapus_GSBRNA2T00024057001 Exp number, first 60 AAs:  0.0002
```

```
# Brassicanapus_GSBRNA2T00024057001 Total prob of N-in:        0.12587
Brassicanapus_GSBRNA2T00024057001       TMHMM2.0        outside      1   255
```

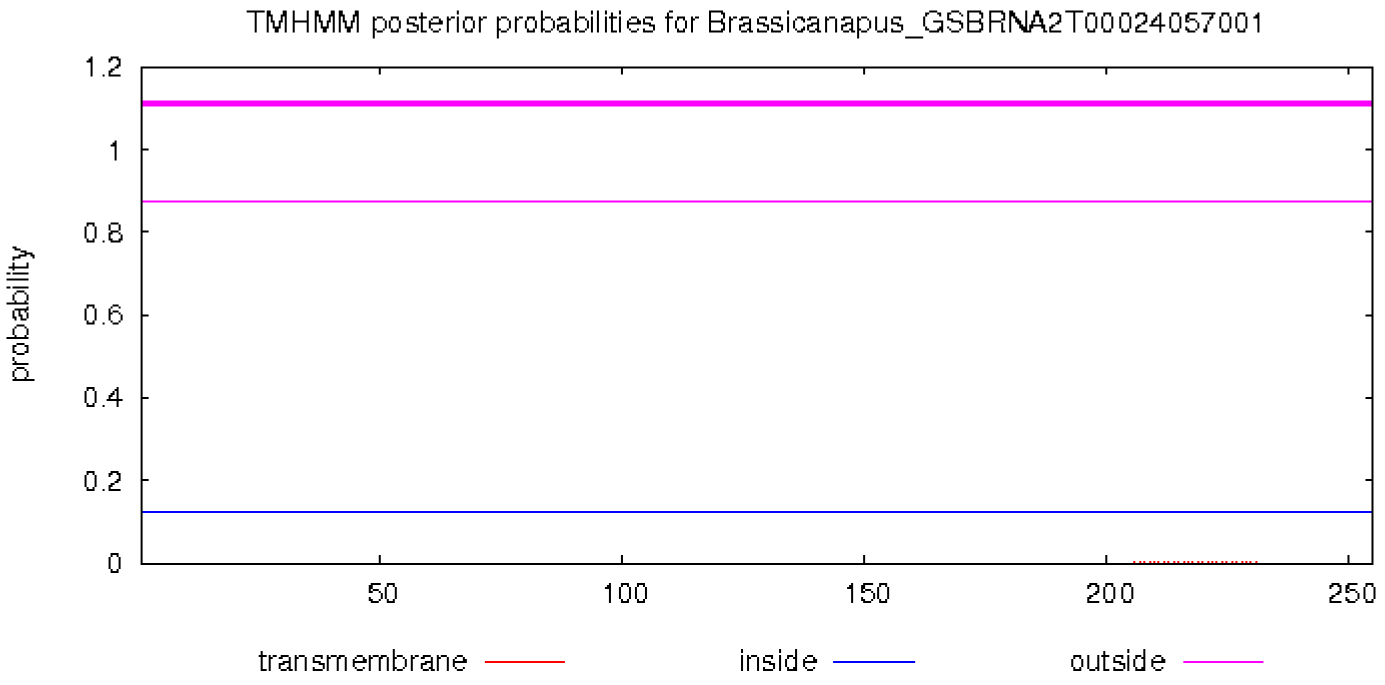

plot in postscript, script for making the plot in gnuplot, data for plot

---

```
# Brassicanapus_GSBRNA2T00024330001 Length: 243
# Brassicanapus_GSBRNA2T00024330001 Number of predicted TMHs:  0
# Brassicanapus_GSBRNA2T00024330001 Exp number of AAs in TMHs: 0.0002
# Brassicanapus_GSBRNA2T00024330001 Exp number, first 60 AAs:  0
# Brassicanapus_GSBRNA2T00024330001 Total prob of N-in:        0.22579
Brassicanapus_GSBRNA2T00024330001       TMHMM2.0        outside      1   243
```

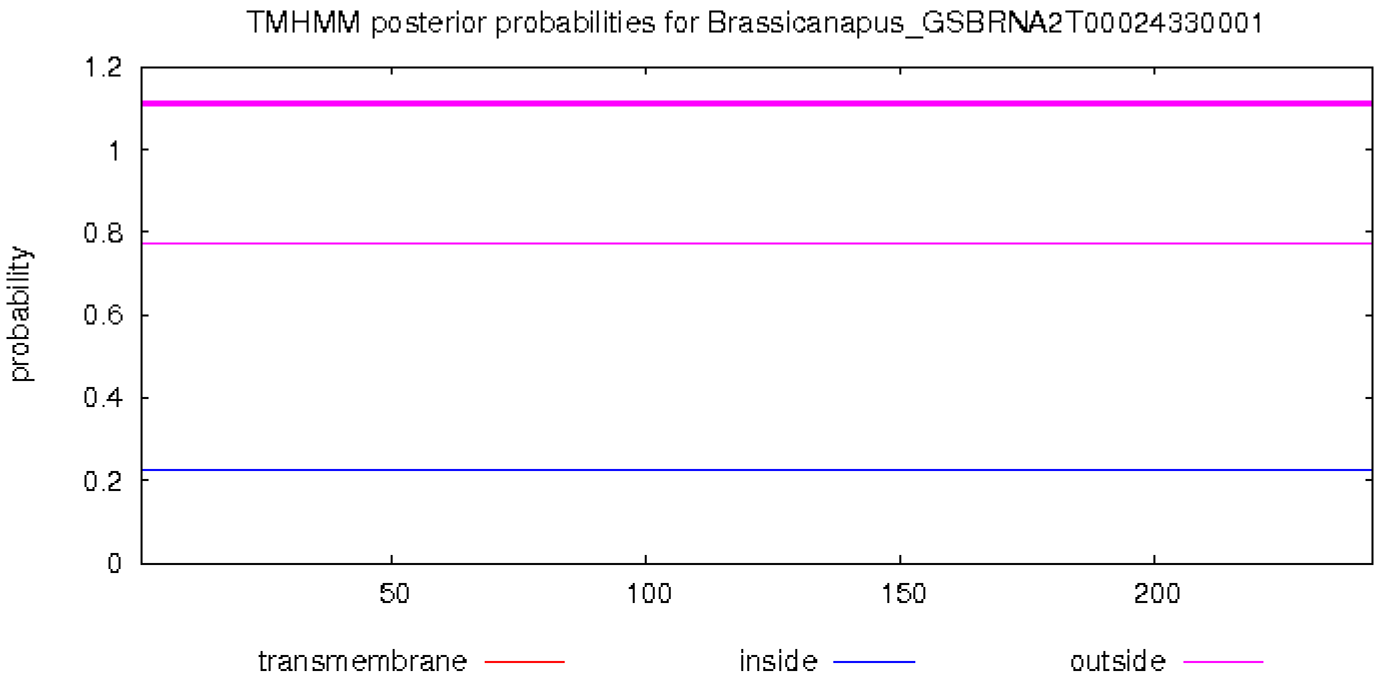

plot in postscript, script for making the plot in gnuplot, data for plot

---

```
# Brassicanapus_GSBRNA2T00024336001 Length: 368
# Brassicanapus_GSBRNA2T00024336001 Number of predicted TMHs:  0
# Brassicanapus_GSBRNA2T00024336001 Exp number of AAs in TMHs: 0.00332
# Brassicanapus_GSBRNA2T00024336001 Exp number, first 60 AAs:  0.00332
```

```
# Brassicanapus_GSBRNA2T00024336001 Total prob of N-in:        0.04305
Brassicanapus_GSBRNA2T00024336001       TMHMM2.0        outside      1   368
```

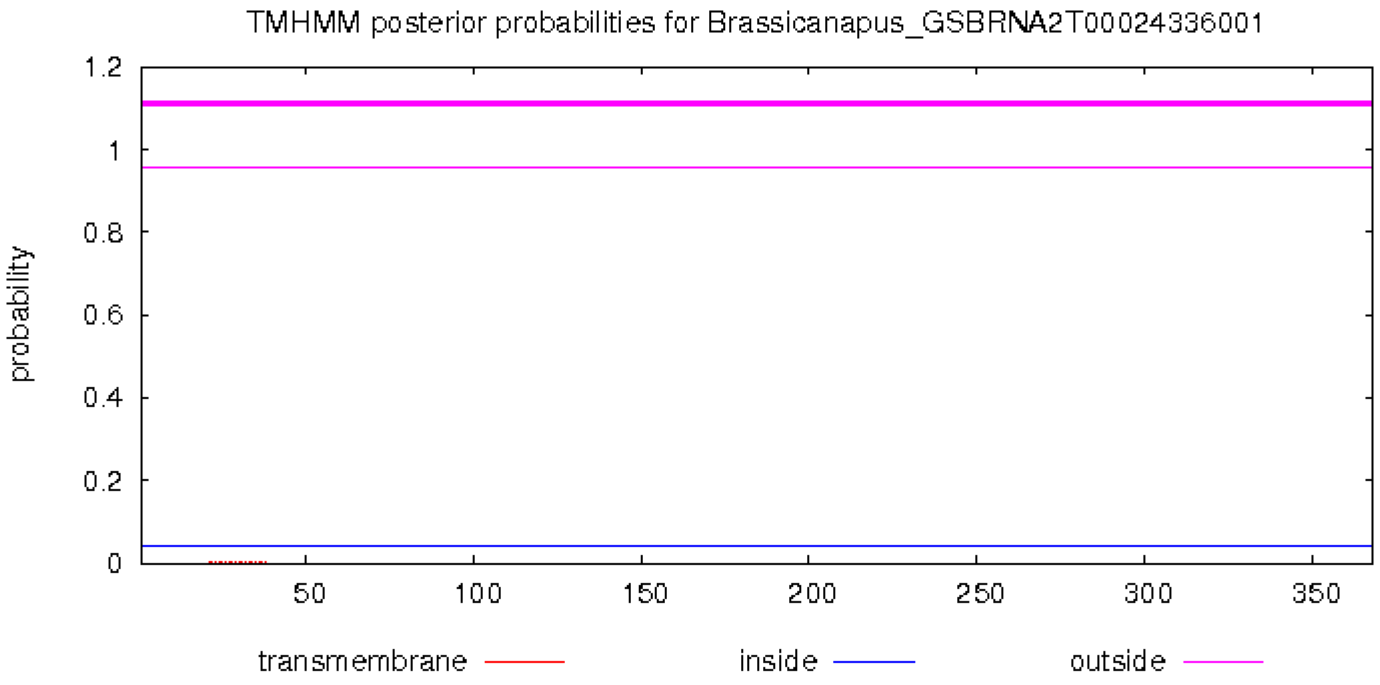

plot in postscript, script for making the plot in gnuplot, data for plot

---

```
# Brassicanapus_GSBRNA2T00026353001 Length: 307
# Brassicanapus_GSBRNA2T00026353001 Number of predicted TMHs:  0
# Brassicanapus_GSBRNA2T00026353001 Exp number of AAs in TMHs: 0.00114
# Brassicanapus_GSBRNA2T00026353001 Exp number, first 60 AAs:  0
# Brassicanapus_GSBRNA2T00026353001 Total prob of N-in:        0.02084
Brassicanapus_GSBRNA2T00026353001       TMHMM2.0        outside      1   307
```

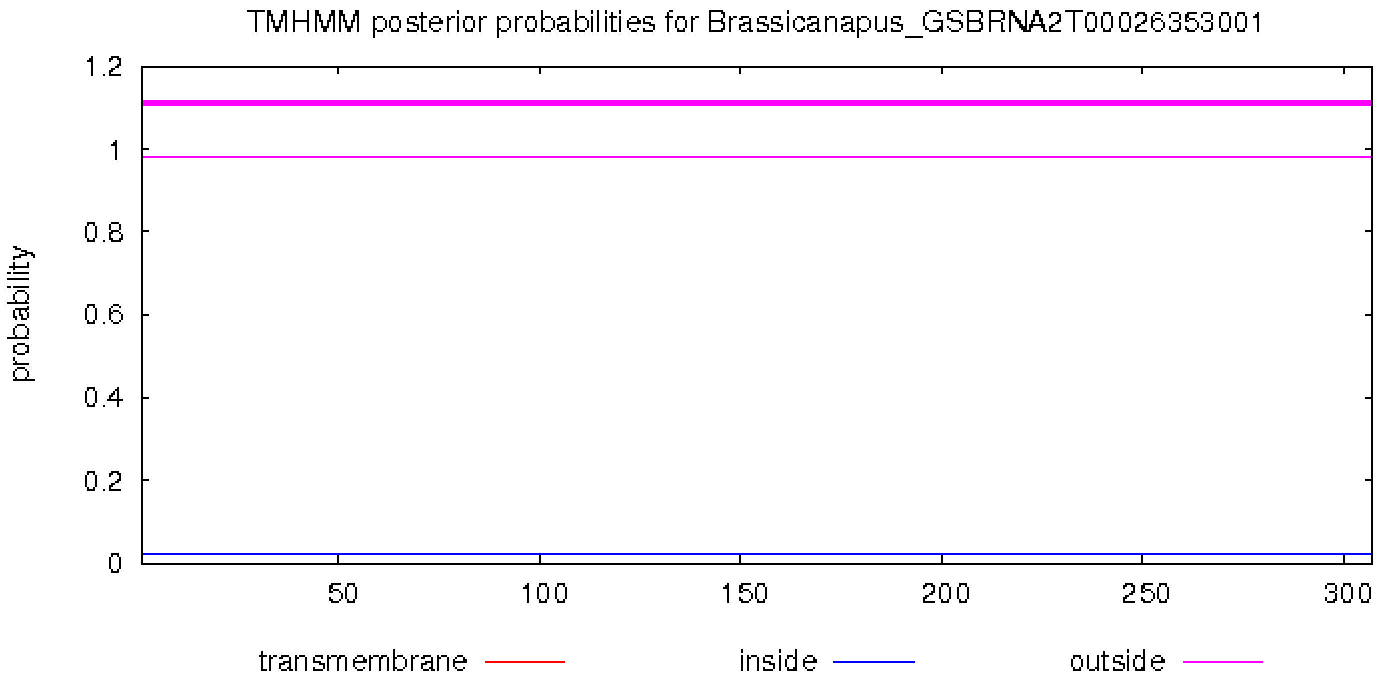

plot in postscript, script for making the plot in gnuplot, data for plot

---

```
# Brassicanapus_GSBRNA2T00026805001 Length: 272
# Brassicanapus_GSBRNA2T00026805001 Number of predicted TMHs:  0
# Brassicanapus_GSBRNA2T00026805001 Exp number of AAs in TMHs: 0.00172
# Brassicanapus_GSBRNA2T00026805001 Exp number, first 60 AAs:  0
```

```
# Brassicanapus_GSBRNA2T00026805001 Total prob of N-in:        0.12548
Brassicanapus_GSBRNA2T00026805001	TMHMM2.0	outside	     1   272
```

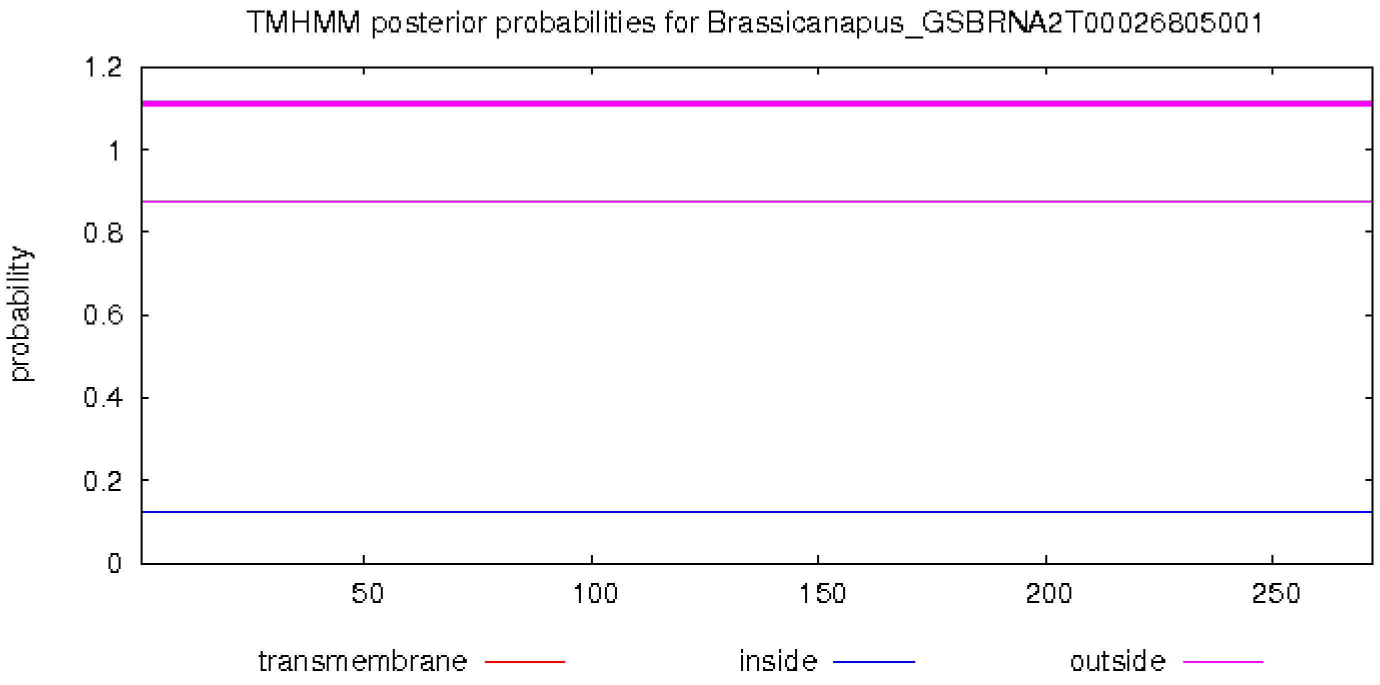

plot in postscript, script for making the plot in gnuplot, data for plot

---

```
# Brassicanapus_GSBRNA2T00026833001 Length: 326
# Brassicanapus_GSBRNA2T00026833001 Number of predicted TMHs:  0
# Brassicanapus_GSBRNA2T00026833001 Exp number of AAs in TMHs: 0.00104
# Brassicanapus_GSBRNA2T00026833001 Exp number, first 60 AAs:  0
# Brassicanapus_GSBRNA2T00026833001 Total prob of N-in:        0.06817
Brassicanapus_GSBRNA2T00026833001	TMHMM2.0	outside	     1   326
```

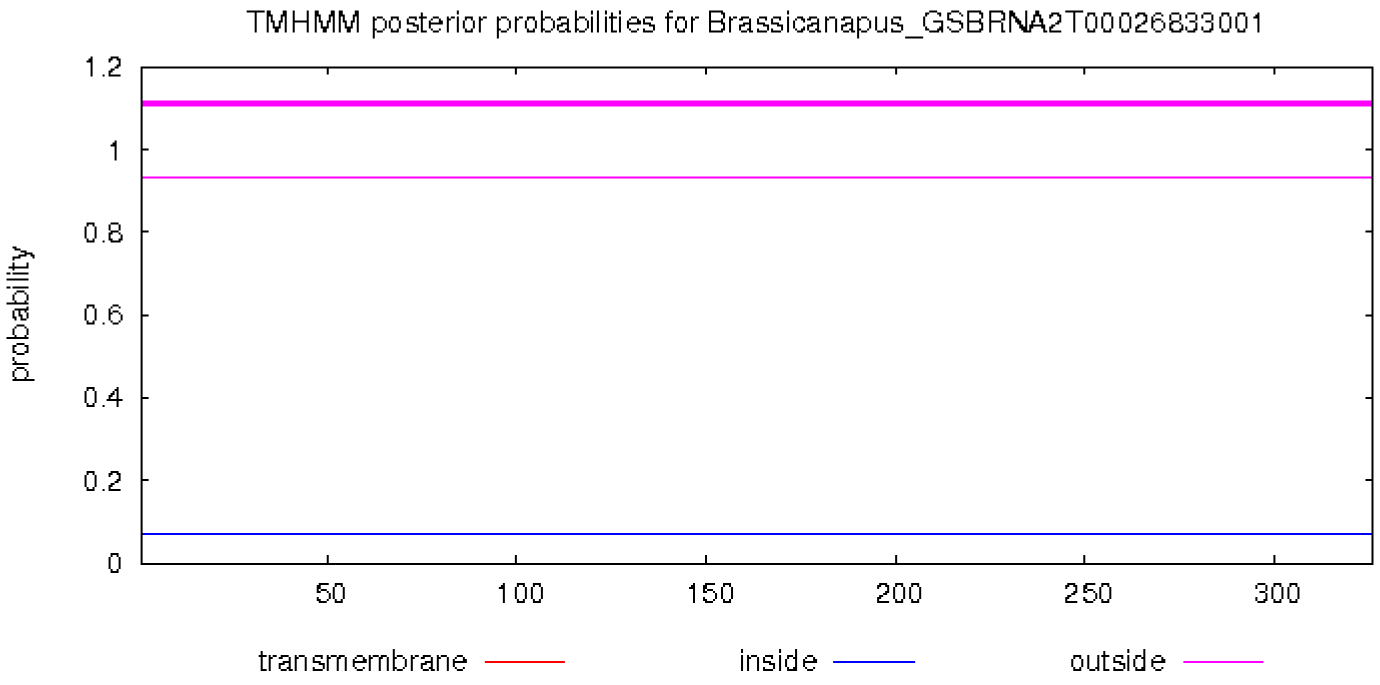

plot in postscript, script for making the plot in gnuplot, data for plot

---

```
# Brassicanapus_GSBRNA2T00027379001 Length: 365
# Brassicanapus_GSBRNA2T00027379001 Number of predicted TMHs:  0
# Brassicanapus_GSBRNA2T00027379001 Exp number of AAs in TMHs: 0
# Brassicanapus_GSBRNA2T00027379001 Exp number, first 60 AAs:  0
```

```
# Brassicanapus_GSBRNA2T00027379001 Total prob of N-in:        0.00850
Brassicanapus_GSBRNA2T00027379001       TMHMM2.0        outside      1   365
```

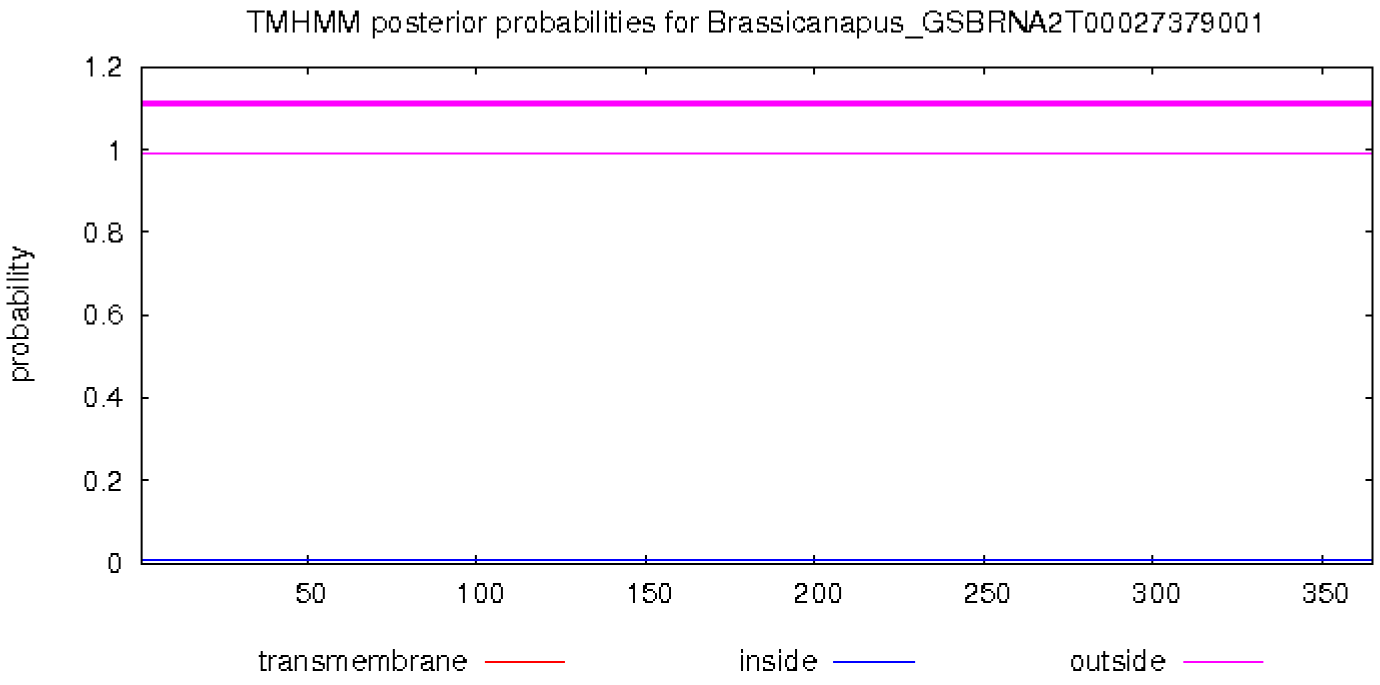

plot in postscript, script for making the plot in gnuplot, data for plot

---

```
# Brassicanapus_GSBRNA2T00027399001 Length: 294
# Brassicanapus_GSBRNA2T00027399001 Number of predicted TMHs:  0
# Brassicanapus_GSBRNA2T00027399001 Exp number of AAs in TMHs: 0
# Brassicanapus_GSBRNA2T00027399001 Exp number, first 60 AAs:  0
# Brassicanapus_GSBRNA2T00027399001 Total prob of N-in:        0.00206
Brassicanapus_GSBRNA2T00027399001       TMHMM2.0        outside      1   294
```

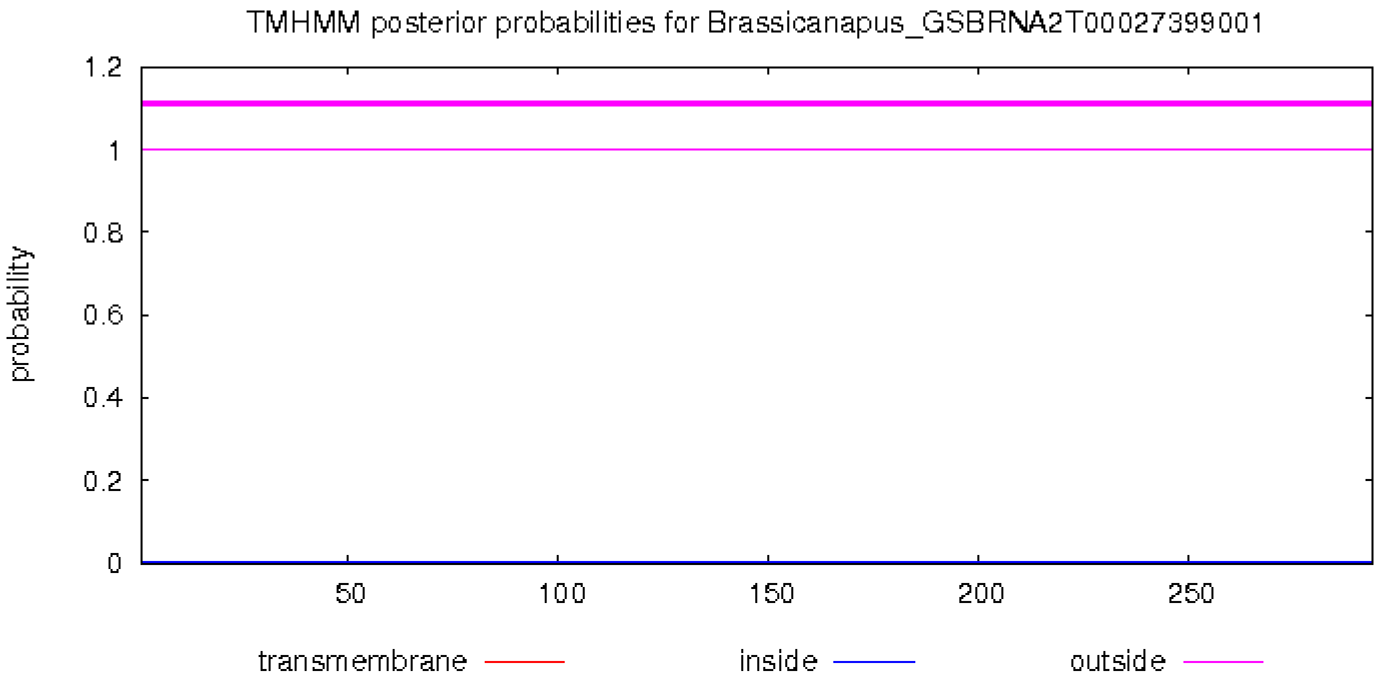

plot in postscript, script for making the plot in gnuplot, data for plot

---

```
# Brassicanapus_GSBRNA2T00027750001 Length: 451
# Brassicanapus_GSBRNA2T00027750001 Number of predicted TMHs:  0
# Brassicanapus_GSBRNA2T00027750001 Exp number of AAs in TMHs: 0.00933999999999999999
# Brassicanapus_GSBRNA2T00027750001 Exp number, first 60 AAs:  0.00143
```

```
# Brassicanapus_GSBRNA2T00027750001 Total prob of N-in:        0.01507
Brassicanapus_GSBRNA2T00027750001       TMHMM2.0        outside      1   451
```

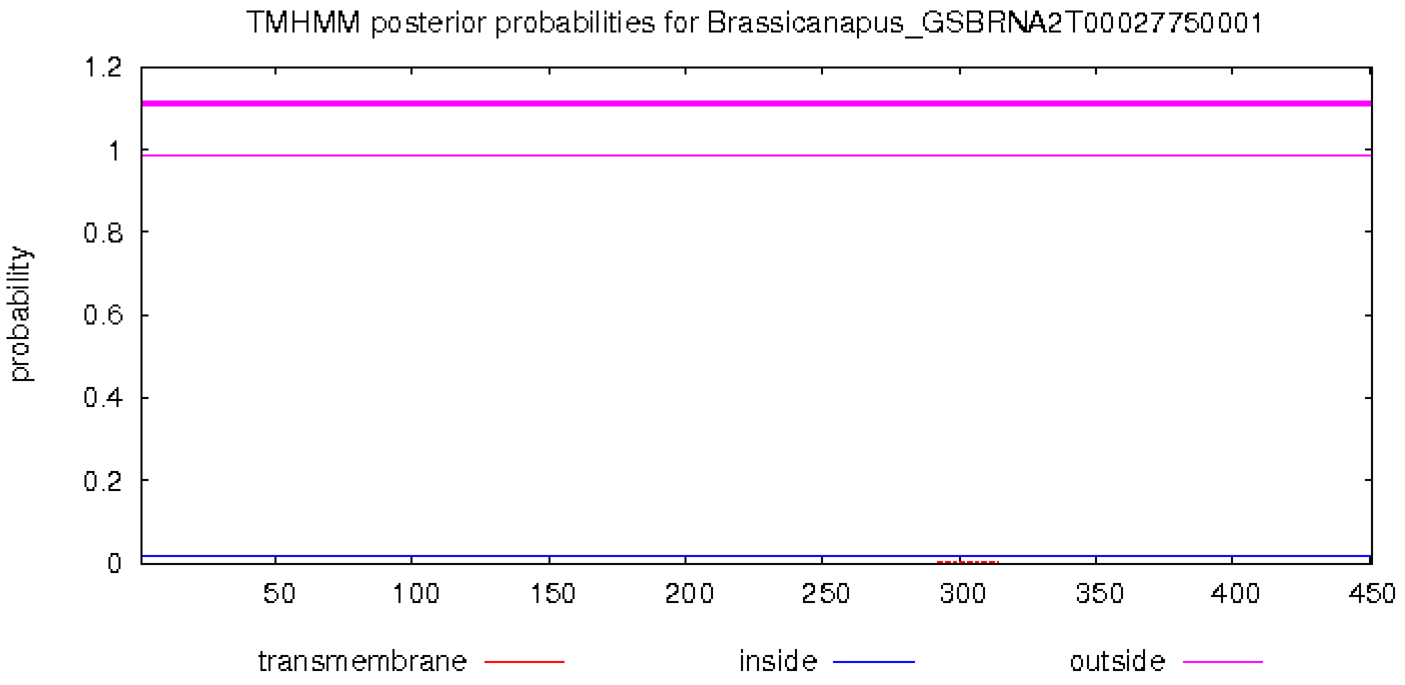

plot in postscript, script for making the plot in gnuplot, data for plot

---

```
# Brassicanapus_GSBRNA2T00027751001 Length: 425
# Brassicanapus_GSBRNA2T00027751001 Number of predicted TMHs:  1
# Brassicanapus_GSBRNA2T00027751001 Exp number of AAs in TMHs: 22.4619
# Brassicanapus_GSBRNA2T00027751001 Exp number, first 60 AAs:  0.0009
# Brassicanapus_GSBRNA2T00027751001 Total prob of N-in:        0.71591
Brassicanapus_GSBRNA2T00027751001       TMHMM2.0        inside       1   396
Brassicanapus_GSBRNA2T00027751001       TMHMM2.0        TMhelix    397   419
Brassicanapus_GSBRNA2T00027751001       TMHMM2.0        outside    420   425
```

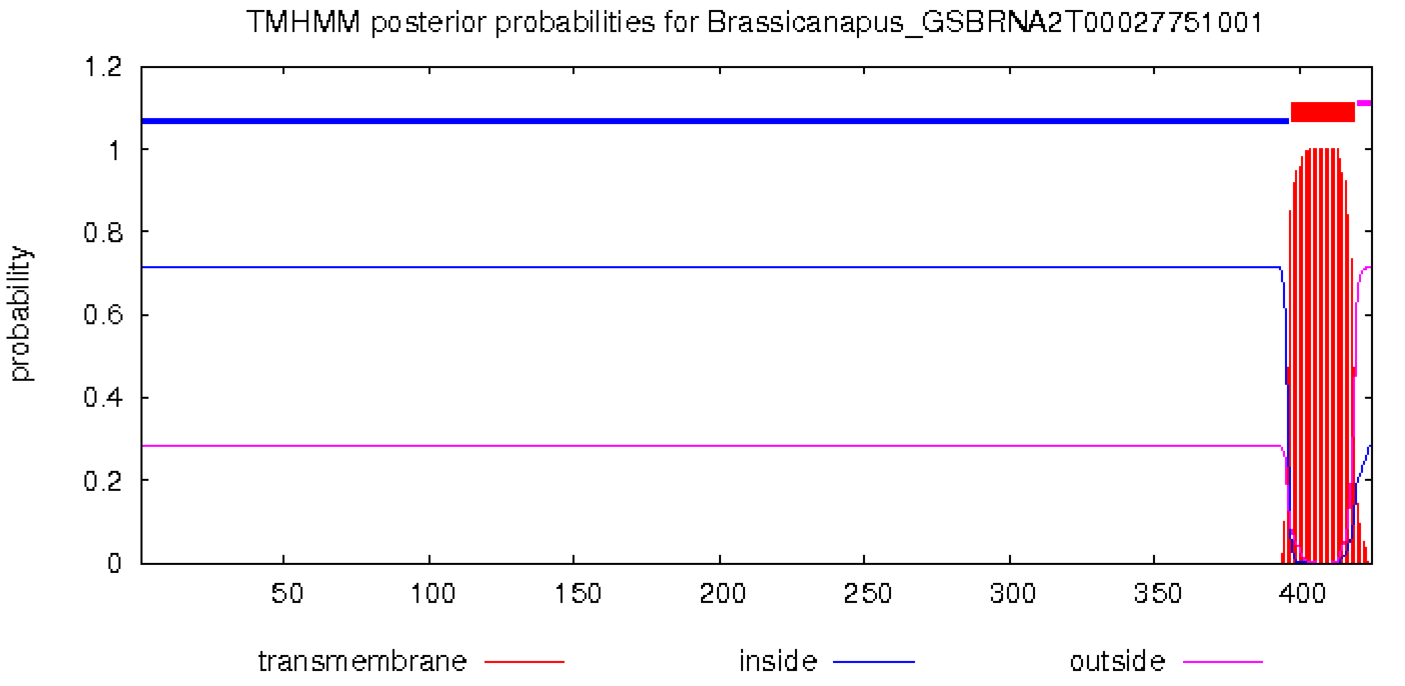

plot in postscript, script for making the plot in gnuplot, data for plot

---

```
# Brassicanapus_GSBRNA2T00027754001 Length: 267
# Brassicanapus_GSBRNA2T00027754001 Number of predicted TMHs:  0
```

```
# Brassicanapus_GSBRNA2T00027754001 Exp number of AAs in TMHs: 0.00023
# Brassicanapus_GSBRNA2T00027754001 Exp number, first 60 AAs:  0.00017
# Brassicanapus_GSBRNA2T00027754001 Total prob of N-in:        0.02430
Brassicanapus_GSBRNA2T00027754001       TMHMM2.0        outside      1   267
```

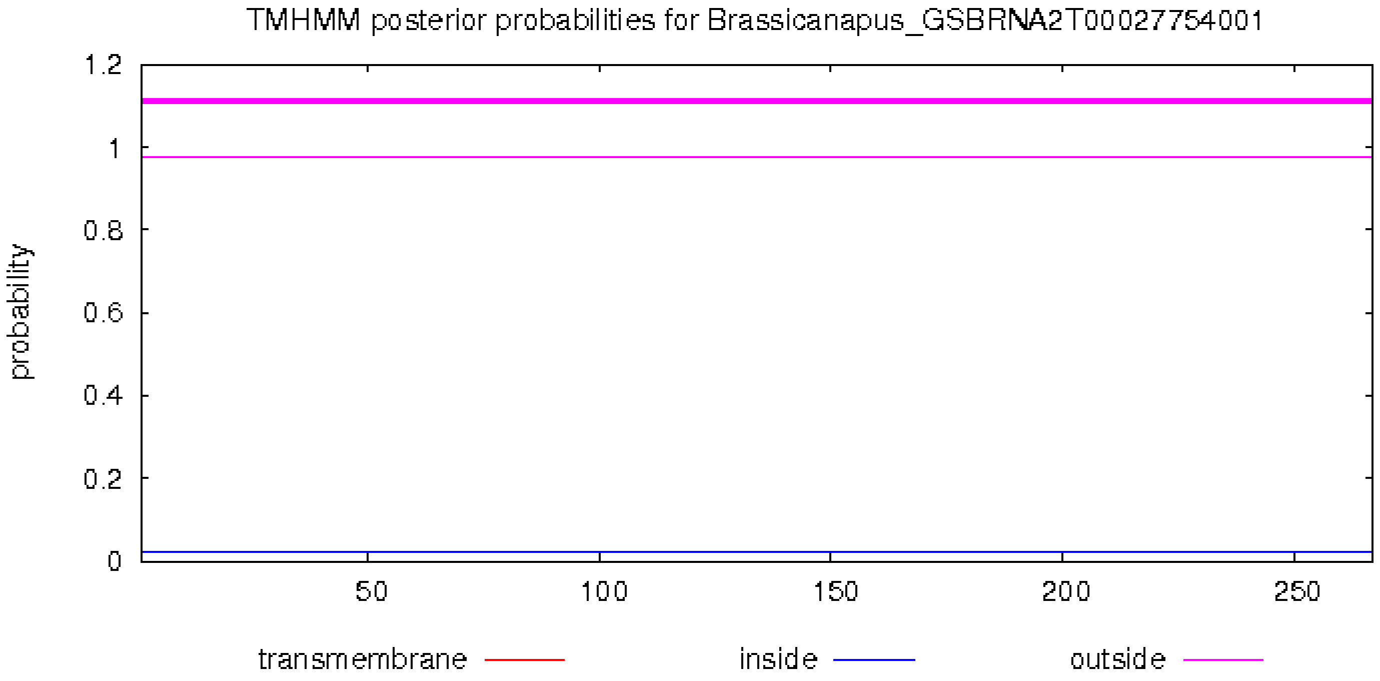

plot in postscript, script for making the plot in gnuplot, data for plot

---

```
# Brassicanapus_GSBRNA2T00028210001 Length: 279
# Brassicanapus_GSBRNA2T00028210001 Number of predicted TMHs:  0
# Brassicanapus_GSBRNA2T00028210001 Exp number of AAs in TMHs: 0.00017
# Brassicanapus_GSBRNA2T00028210001 Exp number, first 60 AAs:  0
# Brassicanapus_GSBRNA2T00028210001 Total prob of N-in:        0.04086
Brassicanapus_GSBRNA2T00028210001       TMHMM2.0        outside      1   279
```

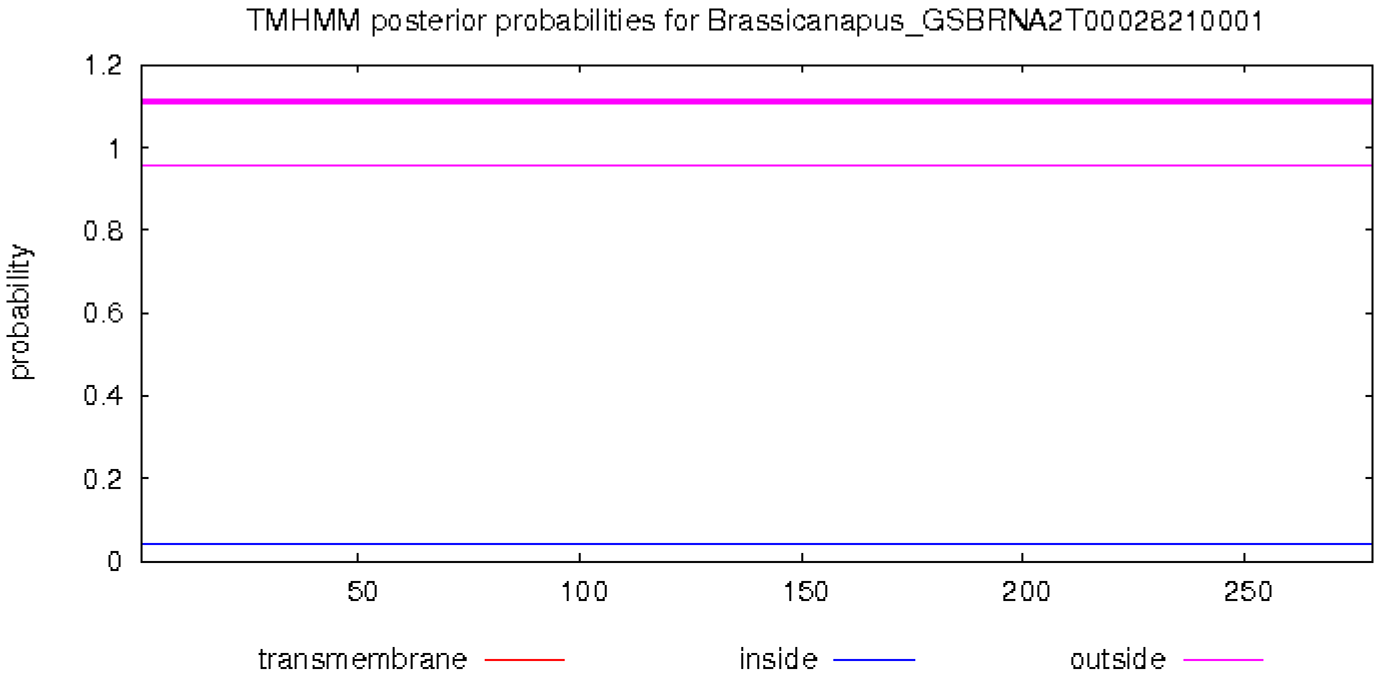

plot in postscript, script for making the plot in gnuplot, data for plot

---

```
# Brassicanapus_GSBRNA2T00028217001 Length: 327
# Brassicanapus_GSBRNA2T00028217001 Number of predicted TMHs:  0
```

```
# Brassicanapus_GSBRNA2T00028217001 Exp number of AAs in TMHs: 0.00147
# Brassicanapus_GSBRNA2T00028217001 Exp number, first 60 AAs:  0
# Brassicanapus_GSBRNA2T00028217001 Total prob of N-in:        0.01940
Brassicanapus_GSBRNA2T00028217001	TMHMM2.0	outside	     1   327
```

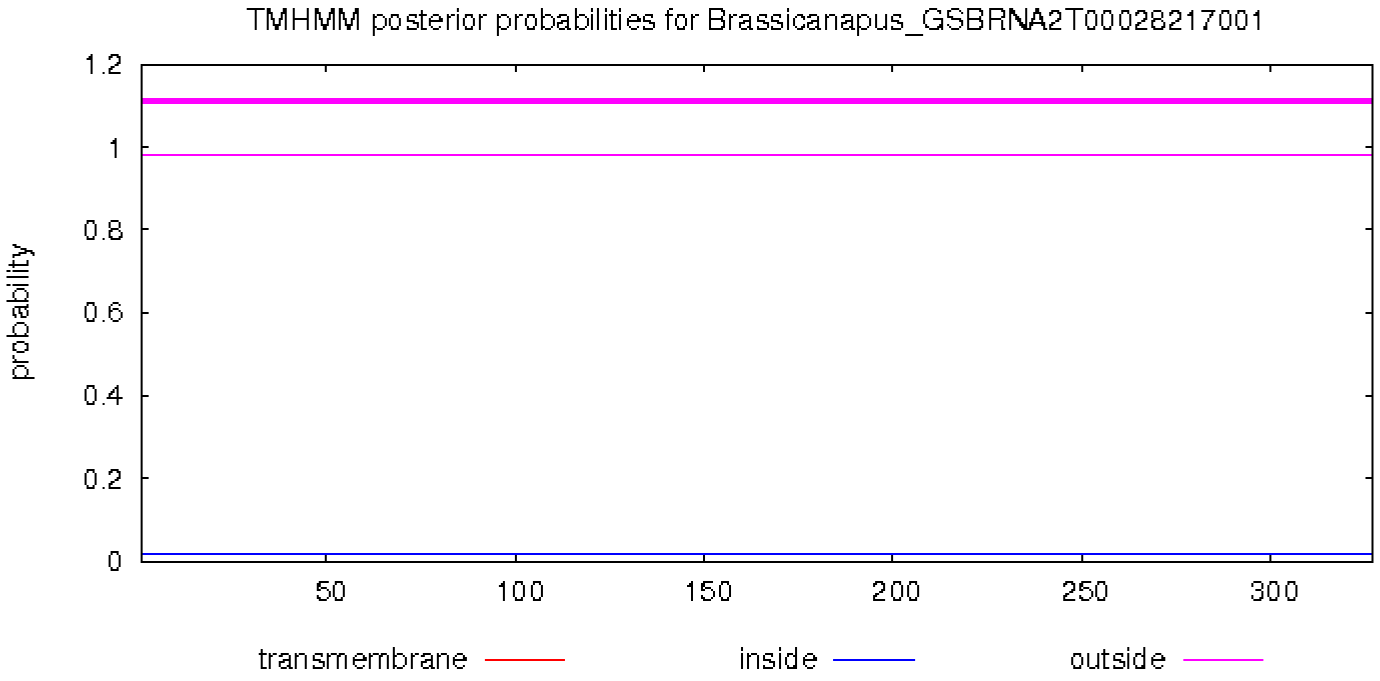

plot in postscript, script for making the plot in gnuplot, data for plot

---

```
# Brassicanapus_GSBRNA2T00028681001 Length: 62
# Brassicanapus_GSBRNA2T00028681001 Number of predicted TMHs:  0
# Brassicanapus_GSBRNA2T00028681001 Exp number of AAs in TMHs: 9.16488
# Brassicanapus_GSBRNA2T00028681001 Exp number, first 60 AAs:  9.16488
# Brassicanapus_GSBRNA2T00028681001 Total prob of N-in:        0.43331
Brassicanapus_GSBRNA2T00028681001	TMHMM2.0	inside	     1    62
```

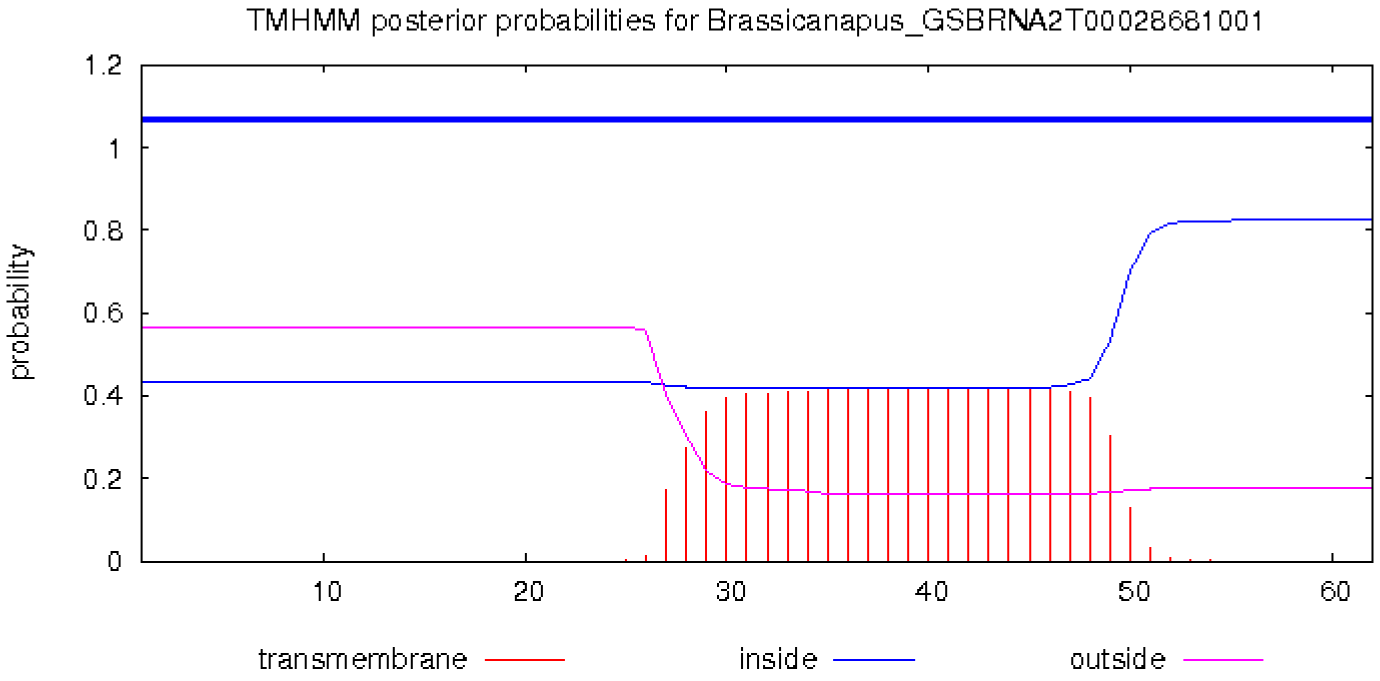

plot in postscript, script for making the plot in gnuplot, data for plot

---

```
# Brassicanapus_GSBRNA2T00028935001 Length: 326
# Brassicanapus_GSBRNA2T00028935001 Number of predicted TMHs:  0
```

```
# Brassicanapus_GSBRNA2T00028935001 Exp number of AAs in TMHs: 0
# Brassicanapus_GSBRNA2T00028935001 Exp number, first 60 AAs:  0
# Brassicanapus_GSBRNA2T00028935001 Total prob of N-in:        0.03326
Brassicanapus_GSBRNA2T00028935001       TMHMM2.0        outside      1   326
```

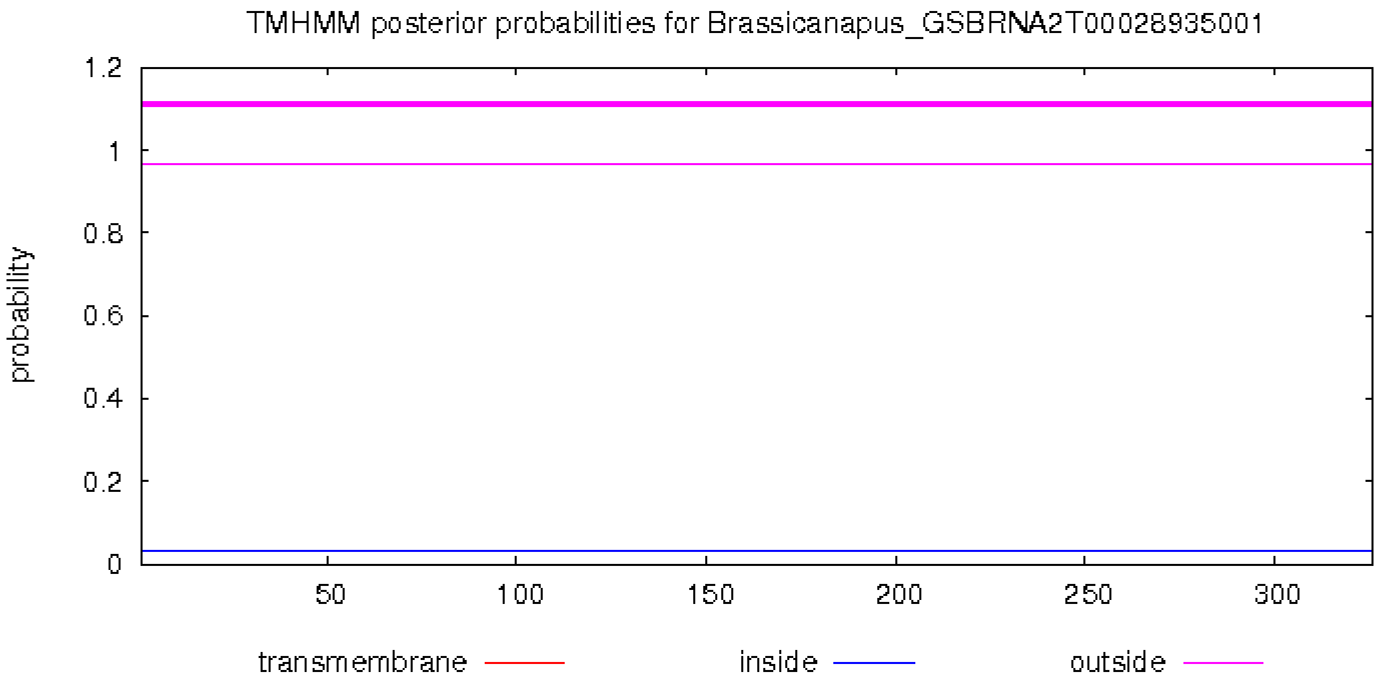

plot in postscript, script for making the plot in gnuplot, data for plot

---

```
# Brassicanapus_GSBRNA2T00030157001 Length: 577
# Brassicanapus_GSBRNA2T00030157001 Number of predicted TMHs:  0
# Brassicanapus_GSBRNA2T00030157001 Exp number of AAs in TMHs: 0.00308
# Brassicanapus_GSBRNA2T00030157001 Exp number, first 60 AAs:  0
# Brassicanapus_GSBRNA2T00030157001 Total prob of N-in:        0.00008
Brassicanapus_GSBRNA2T00030157001       TMHMM2.0        outside      1   577
```

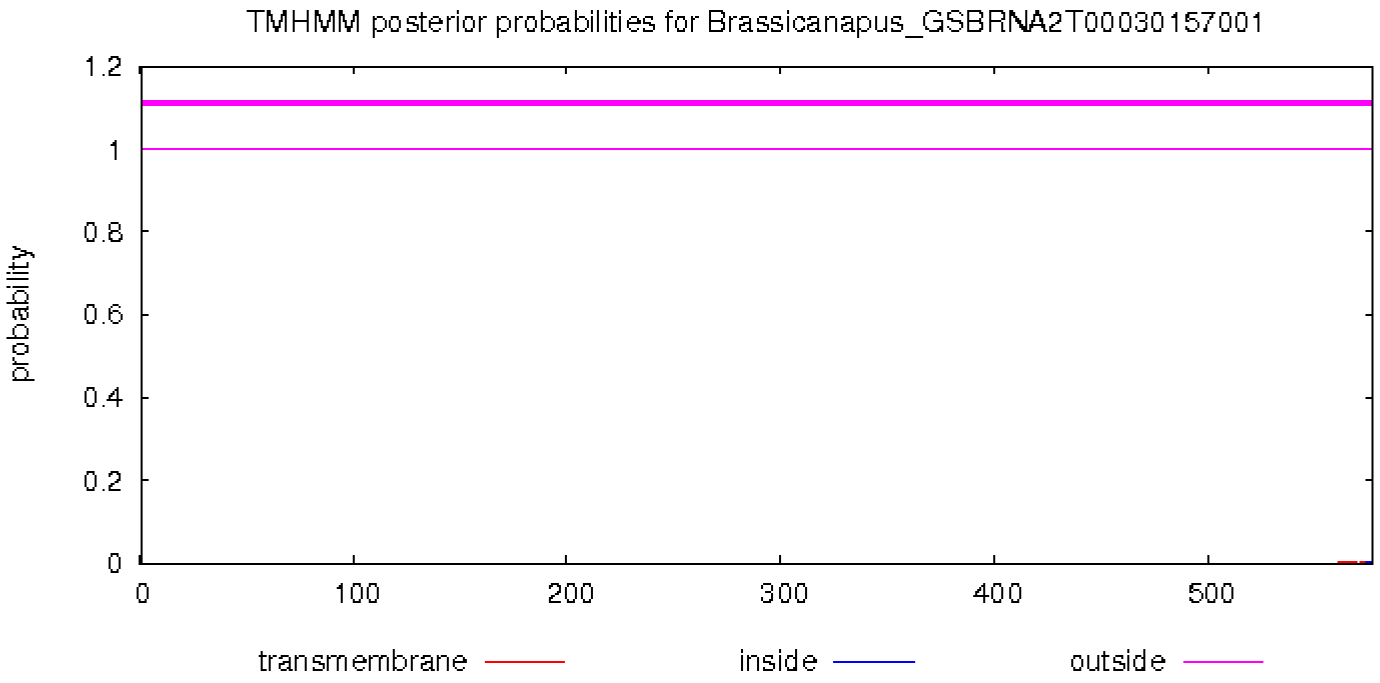

plot in postscript, script for making the plot in gnuplot, data for plot

---

```
# Brassicanapus_GSBRNA2T00030633001 Length: 298
# Brassicanapus_GSBRNA2T00030633001 Number of predicted TMHs:  0
```

```
# Brassicanapus_GSBRNA2T00030633001 Exp number of AAs in TMHs: 0.00018
# Brassicanapus_GSBRNA2T00030633001 Exp number, first 60 AAs:  0
# Brassicanapus_GSBRNA2T00030633001 Total prob of N-in:        0.07930
Brassicanapus_GSBRNA2T00030633001       TMHMM2.0        outside      1   298
```

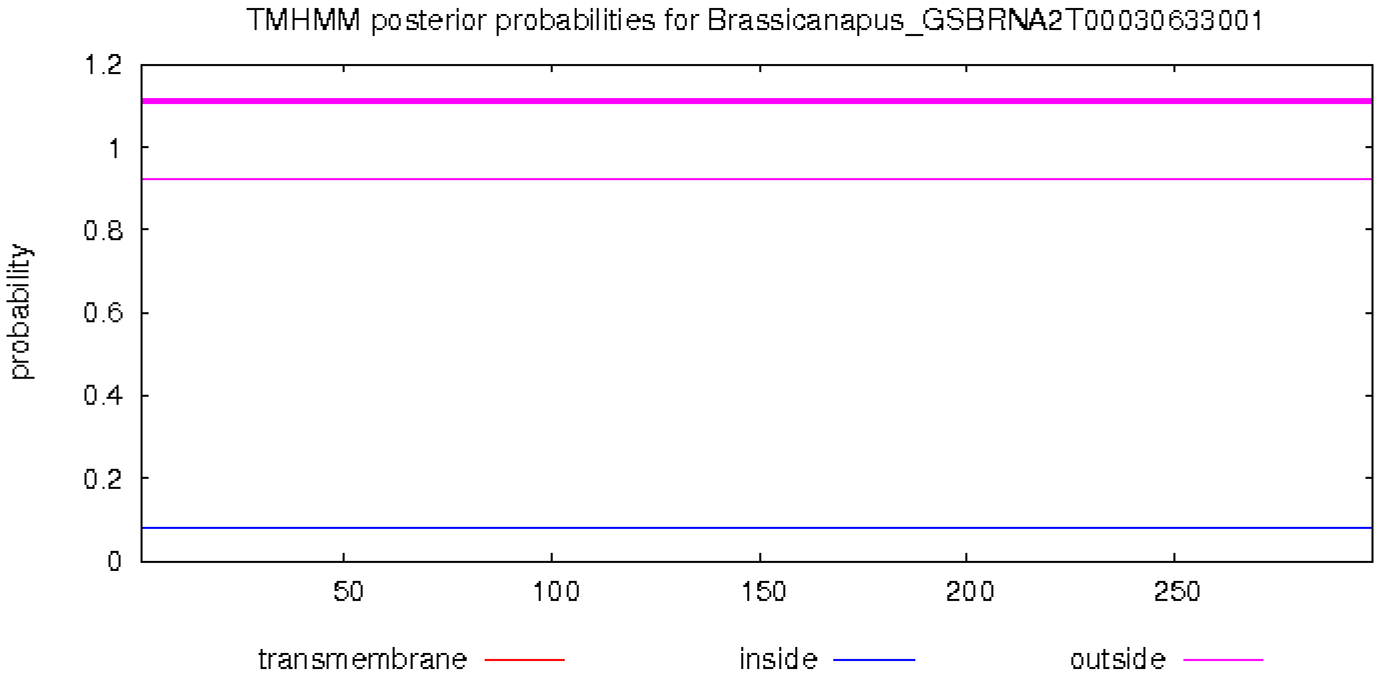

plot in postscript, script for making the plot in gnuplot, data for plot

---

```
# Brassicanapus_GSBRNA2T00030840001 Length: 406
# Brassicanapus_GSBRNA2T00030840001 Number of predicted TMHs:  0
# Brassicanapus_GSBRNA2T00030840001 Exp number of AAs in TMHs: 0.00264
# Brassicanapus_GSBRNA2T00030840001 Exp number, first 60 AAs:  0
# Brassicanapus_GSBRNA2T00030840001 Total prob of N-in:        0.00707
Brassicanapus_GSBRNA2T00030840001       TMHMM2.0        outside      1   406
```

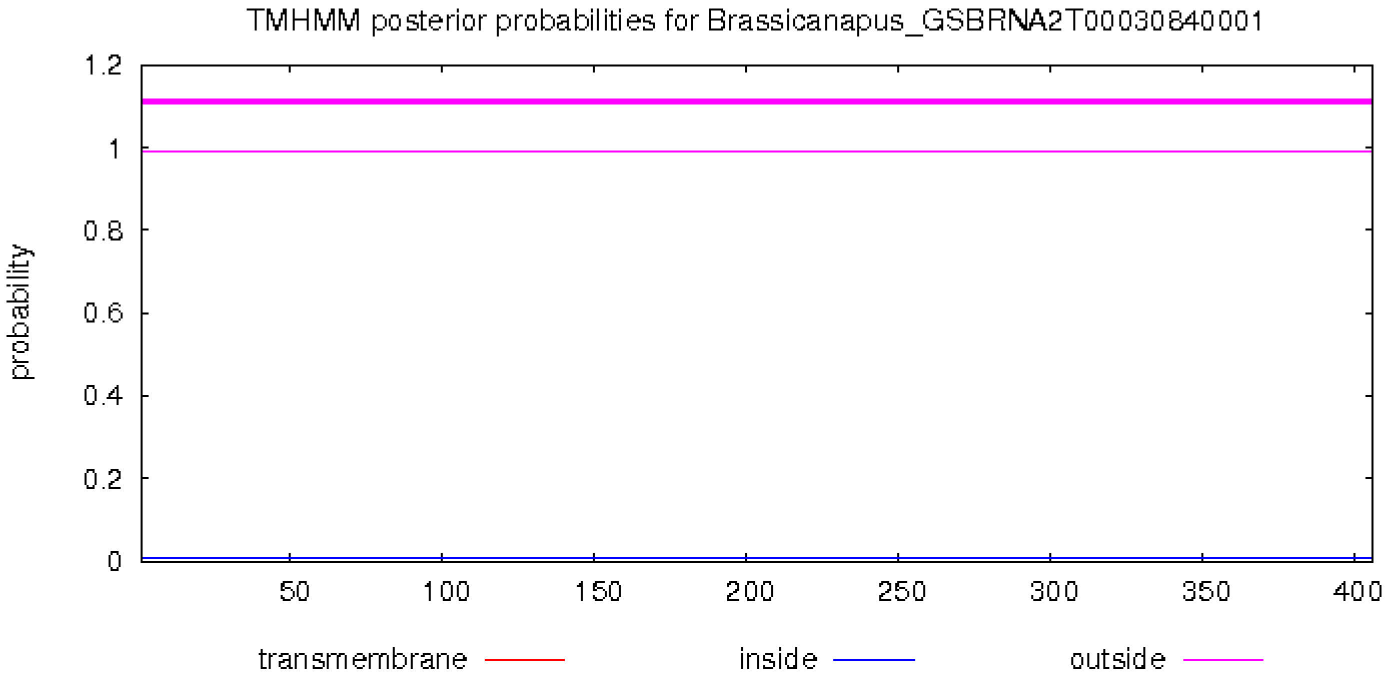

plot in postscript, script for making the plot in gnuplot, data for plot

---

```
# Brassicanapus_GSBRNA2T00031473001 Length: 276
# Brassicanapus_GSBRNA2T00031473001 Number of predicted TMHs:  0
```

```
# Brassicanapus_GSBRNA2T00031473001 Exp number of AAs in TMHs: 0.06065
# Brassicanapus_GSBRNA2T00031473001 Exp number, first 60 AAs:  0.05986
# Brassicanapus_GSBRNA2T00031473001 Total prob of N-in:        0.03530
Brassicanapus_GSBRNA2T00031473001       TMHMM2.0        outside      1   276
```

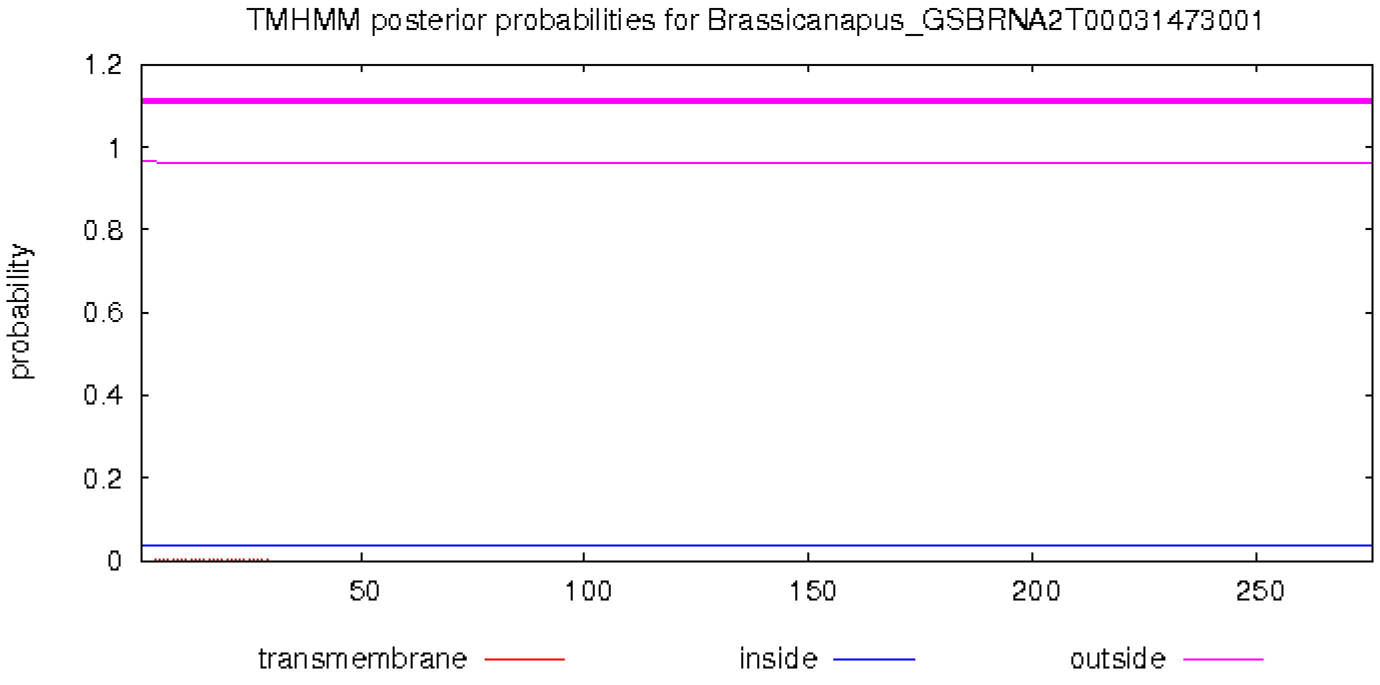

plot in postscript, script for making the plot in gnuplot, data for plot

```
# Brassicanapus_GSBRNA2T00031577001 Length: 199
# Brassicanapus_GSBRNA2T00031577001 Number of predicted TMHs:  0
# Brassicanapus_GSBRNA2T00031577001 Exp number of AAs in TMHs: 2.61399
# Brassicanapus_GSBRNA2T00031577001 Exp number, first 60 AAs:  2.59015
# Brassicanapus_GSBRNA2T00031577001 Total prob of N-in:        0.11503
Brassicanapus_GSBRNA2T00031577001       TMHMM2.0        outside      1   199
```

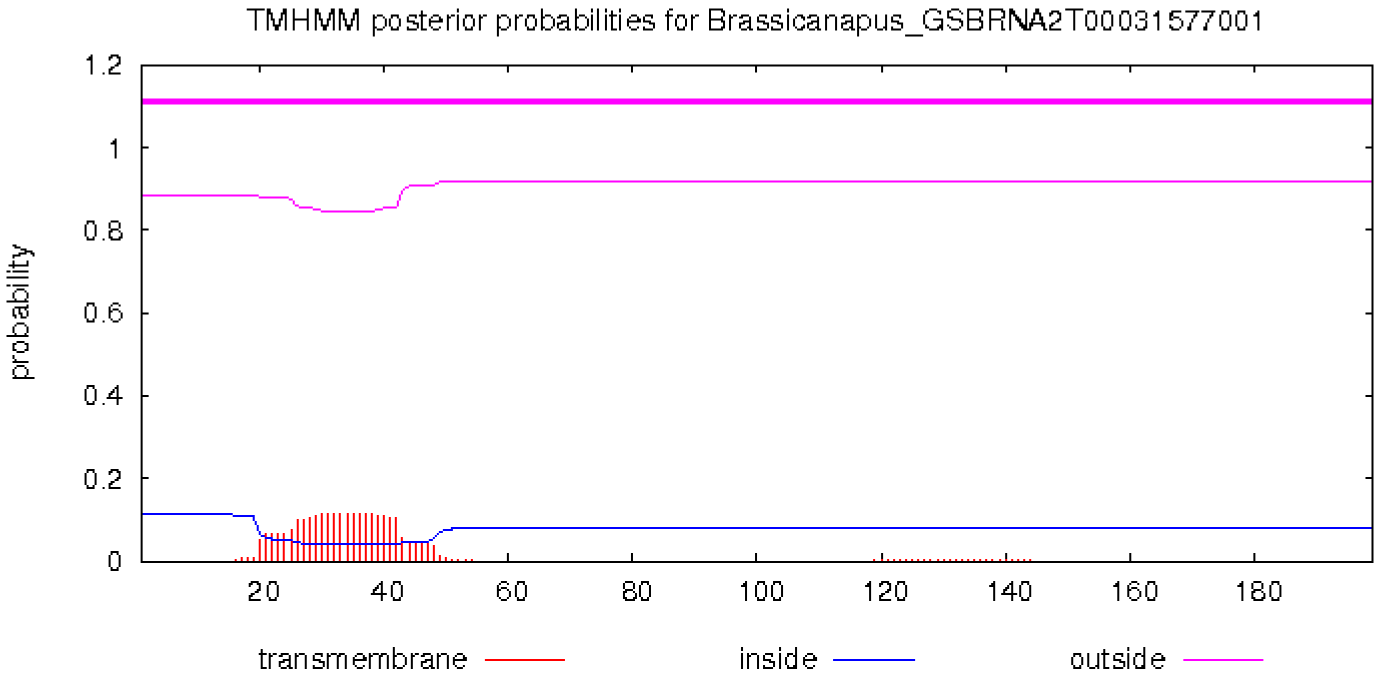

plot in postscript, script for making the plot in gnuplot, data for plot

```
# Brassicanapus_GSBRNA2T00032449001 Length: 307
# Brassicanapus_GSBRNA2T00032449001 Number of predicted TMHs:  0
```

```
# Brassicanapus_GSBRNA2T00032449001 Exp number of AAs in TMHs: 0
# Brassicanapus_GSBRNA2T00032449001 Exp number, first 60 AAs:  0
# Brassicanapus_GSBRNA2T00032449001 Total prob of N-in:        0.00130
Brassicanapus_GSBRNA2T00032449001       TMHMM2.0        outside      1   307
```

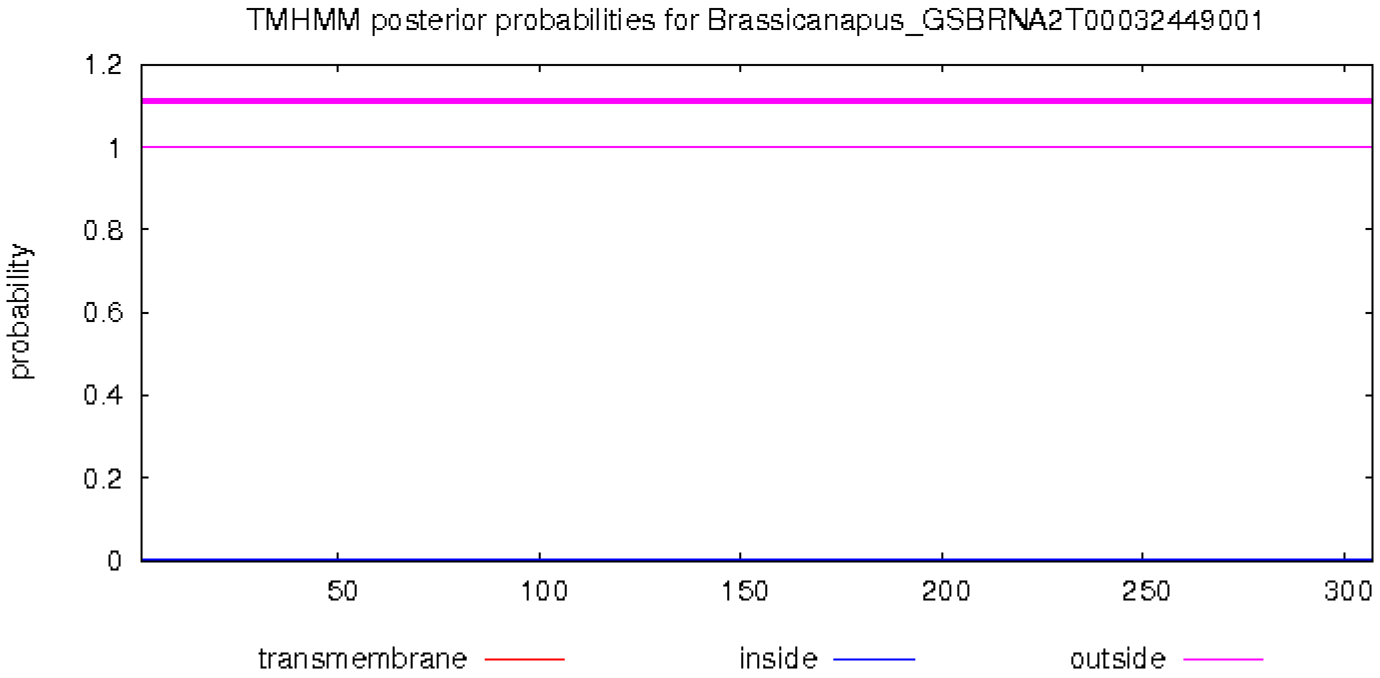

plot in postscript, script for making the plot in gnuplot, data for plot

---

```
# Brassicanapus_GSBRNA2T00032467001 Length: 322
# Brassicanapus_GSBRNA2T00032467001 Number of predicted TMHs:  0
# Brassicanapus_GSBRNA2T00032467001 Exp number of AAs in TMHs: 0.00041
# Brassicanapus_GSBRNA2T00032467001 Exp number, first 60 AAs:  0
# Brassicanapus_GSBRNA2T00032467001 Total prob of N-in:        0.01797
Brassicanapus_GSBRNA2T00032467001       TMHMM2.0        outside      1   322
```

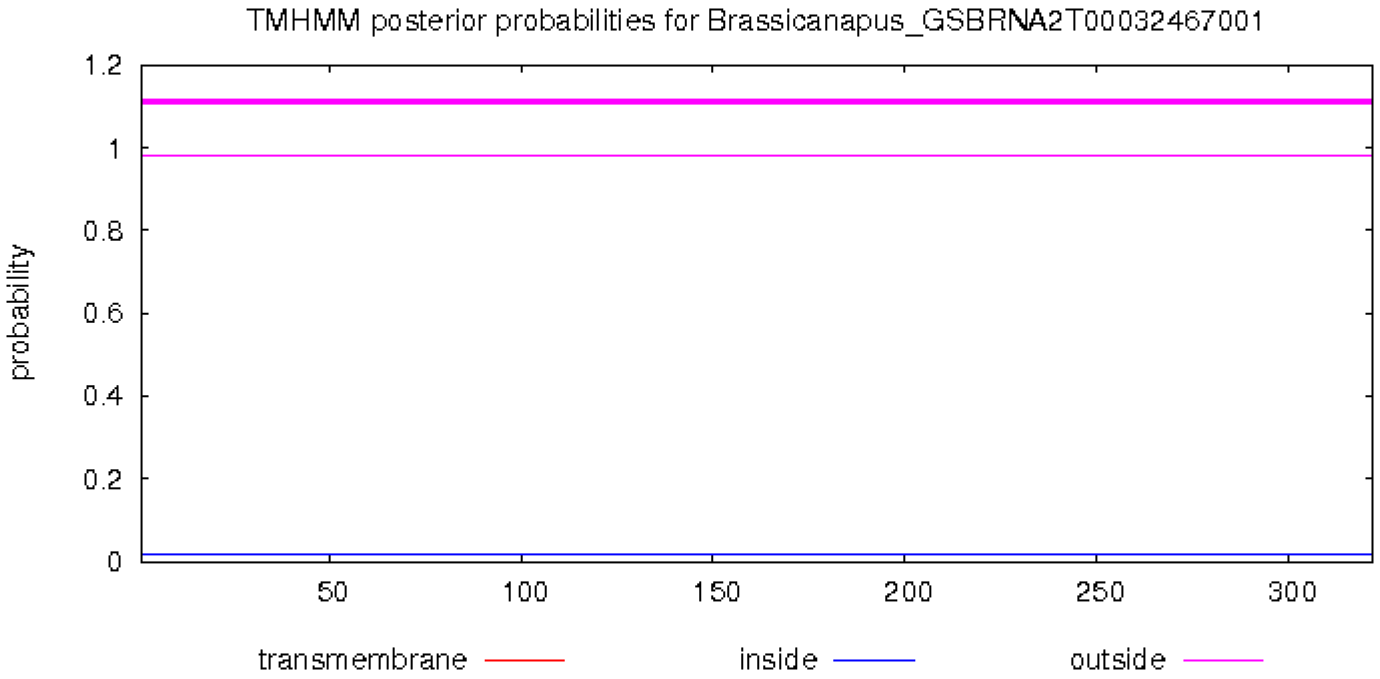

plot in postscript, script for making the plot in gnuplot, data for plot

---

```
# Brassicanapus_GSBRNA2T00033138001 Length: 509
# Brassicanapus_GSBRNA2T00033138001 Number of predicted TMHs:  1
```

```
# Brassicanapus_GSBRNA2T00033138001 Exp number of AAs in TMHs: 20.68
# Brassicanapus_GSBRNA2T00033138001 Exp number, first 60 AAs:  0.00512
# Brassicanapus_GSBRNA2T00033138001 Total prob of N-in:        0.91048
Brassicanapus_GSBRNA2T00033138001       TMHMM2.0        inside       1   481
Brassicanapus_GSBRNA2T00033138001       TMHMM2.0        TMhelix    482   504
Brassicanapus_GSBRNA2T00033138001       TMHMM2.0        outside    505   509
```

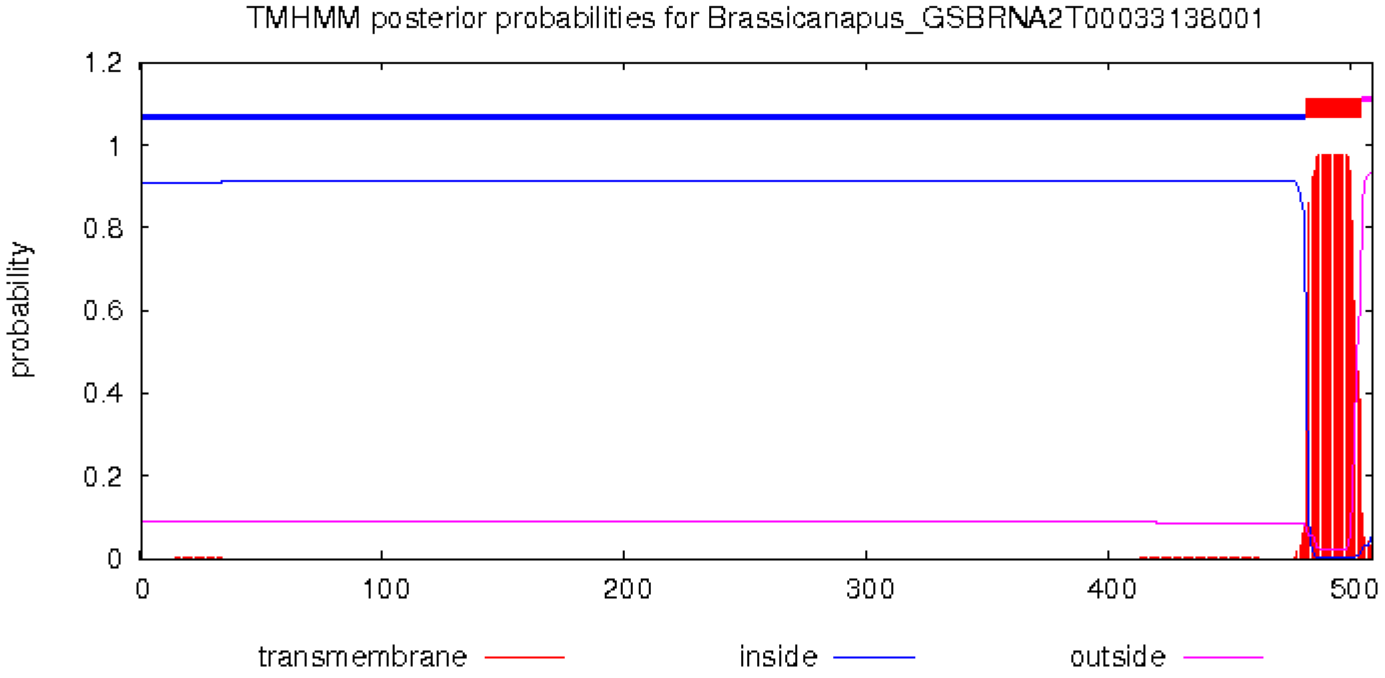

plot in postscript, script for making the plot in gnuplot, data for plot

---

```
# Brassicanapus_GSBRNA2T00033572001 Length: 151
# Brassicanapus_GSBRNA2T00033572001 Number of predicted TMHs:  0
# Brassicanapus_GSBRNA2T00033572001 Exp number of AAs in TMHs: 0.00092
# Brassicanapus_GSBRNA2T00033572001 Exp number, first 60 AAs:  0.00035
# Brassicanapus_GSBRNA2T00033572001 Total prob of N-in:        0.06985
Brassicanapus_GSBRNA2T00033572001       TMHMM2.0        outside      1   151
```

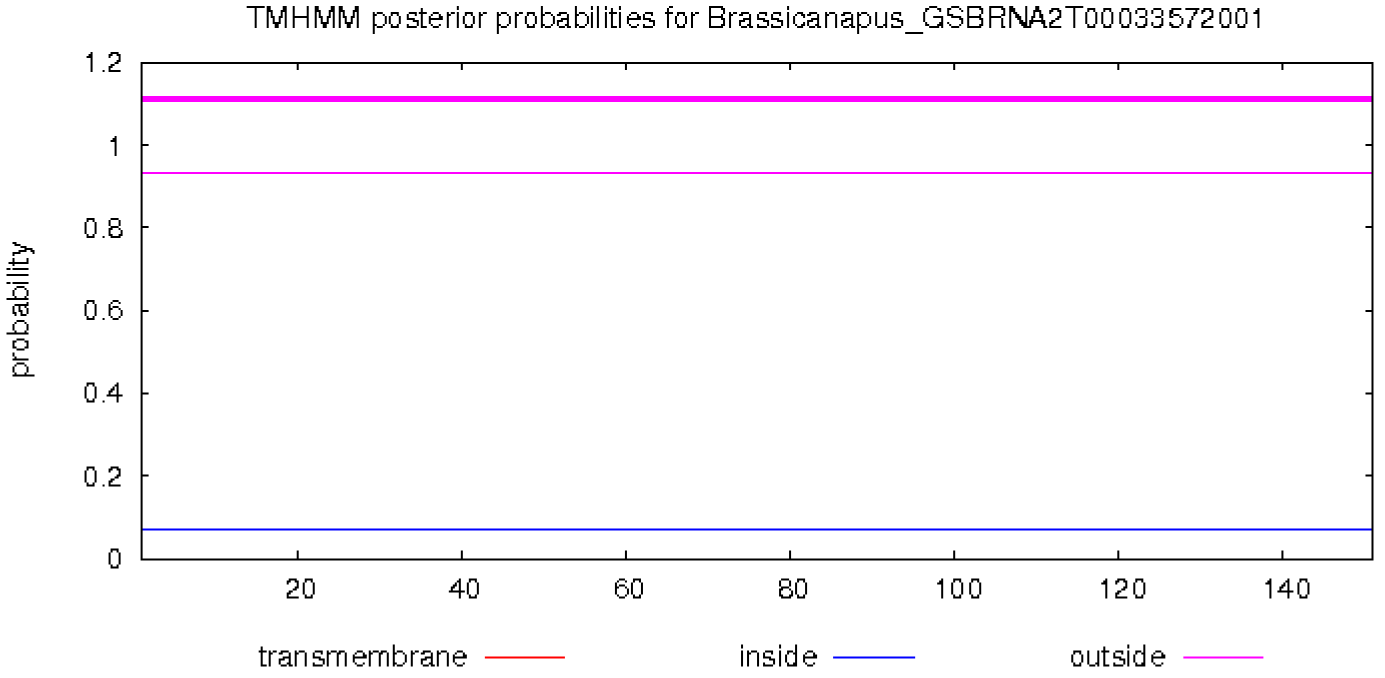

plot in postscript, script for making the plot in gnuplot, data for plot

---

```
# Brassicanapus_GSBRNA2T00033573001 Length: 161
# Brassicanapus_GSBRNA2T00033573001 Number of predicted TMHs:  0
# Brassicanapus_GSBRNA2T00033573001 Exp number of AAs in TMHs: 0.00071
# Brassicanapus_GSBRNA2T00033573001 Exp number, first 60 AAs:  0.00032
# Brassicanapus_GSBRNA2T00033573001 Total prob of N-in:        0.07092
Brassicanapus_GSBRNA2T00033573001       TMHMM2.0        outside      1   161
```

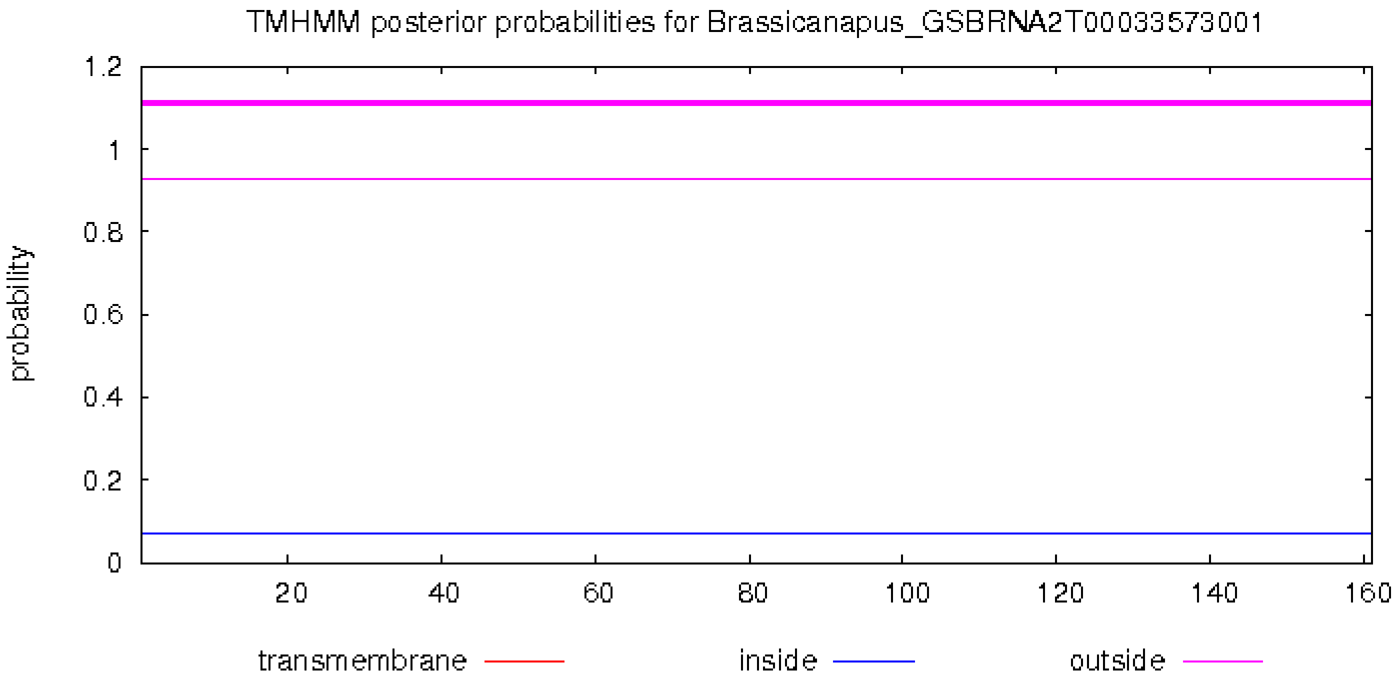

plot in postscript, script for making the plot in gnuplot, data for plot

---

```
# Brassicanapus_GSBRNA2T00034156001 Length: 292
# Brassicanapus_GSBRNA2T00034156001 Number of predicted TMHs:  0
# Brassicanapus_GSBRNA2T00034156001 Exp number of AAs in TMHs: 0.00088
# Brassicanapus_GSBRNA2T00034156001 Exp number, first 60 AAs:  0.00088
# Brassicanapus_GSBRNA2T00034156001 Total prob of N-in:        0.06866
Brassicanapus_GSBRNA2T00034156001       TMHMM2.0        outside      1   292
```

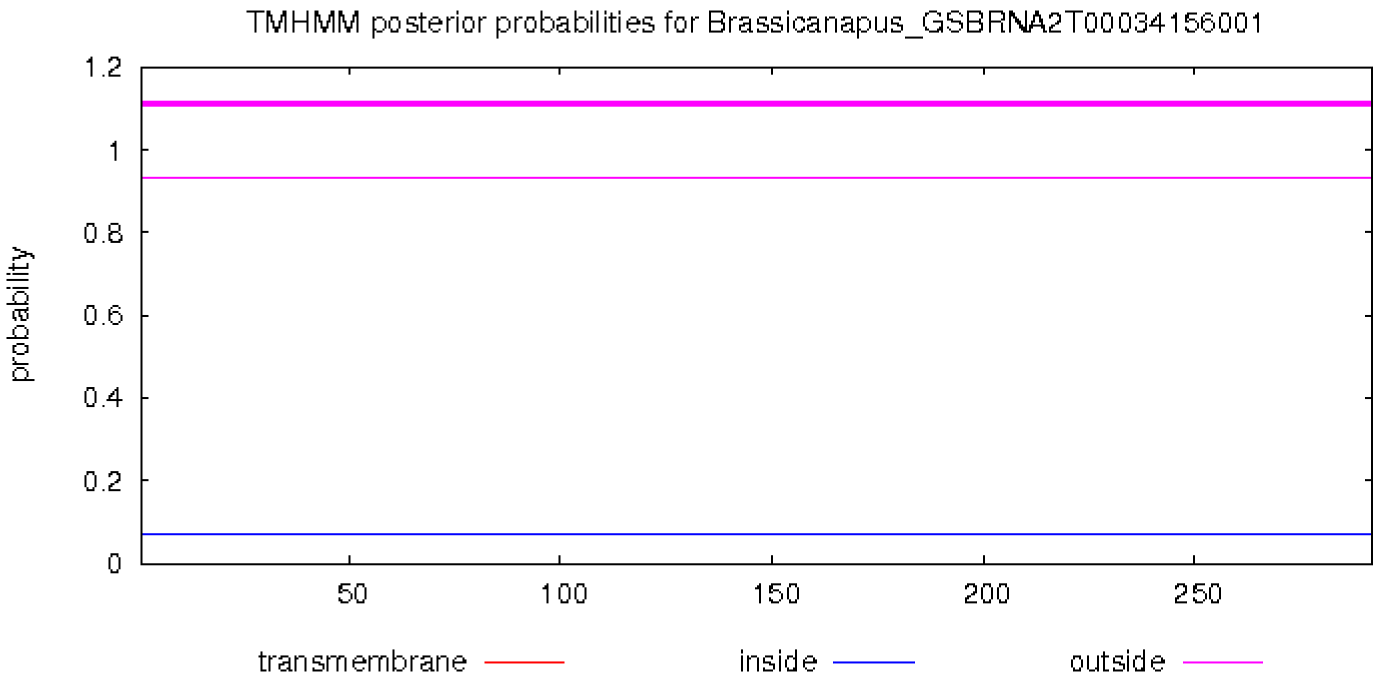

plot in postscript, script for making the plot in gnuplot, data for plot

---

```
# Brassicanapus_GSBRNA2T00034316001 Length: 163
# Brassicanapus_GSBRNA2T00034316001 Number of predicted TMHs:  0
# Brassicanapus_GSBRNA2T00034316001 Exp number of AAs in TMHs: 0
# Brassicanapus_GSBRNA2T00034316001 Exp number, first 60 AAs:  0
# Brassicanapus_GSBRNA2T00034316001 Total prob of N-in:        0.07879
Brassicanapus_GSBRNA2T00034316001       TMHMM2.0        outside      1   163
```

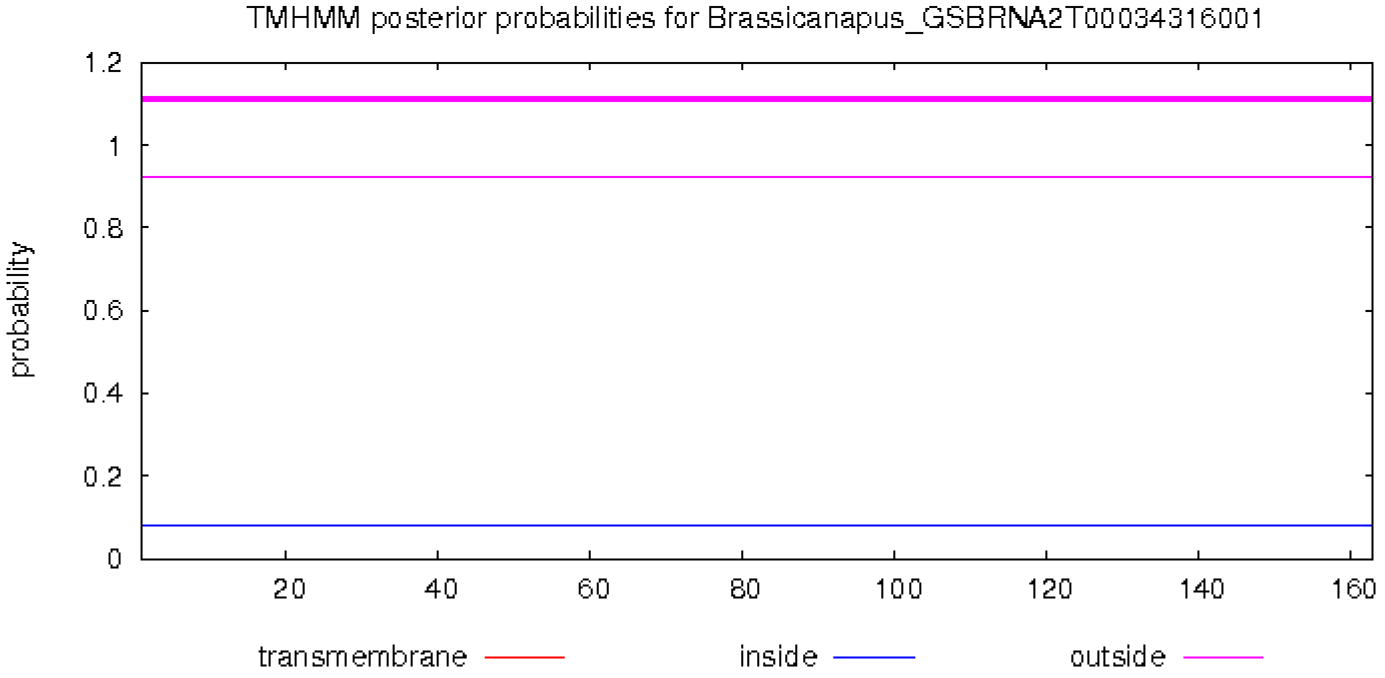

plot in postscript, script for making the plot in gnuplot, data for plot

---

```
# Brassicanapus_GSBRNA2T00034345001 Length: 519
# Brassicanapus_GSBRNA2T00034345001 Number of predicted TMHs:  1
# Brassicanapus_GSBRNA2T00034345001 Exp number of AAs in TMHs: 22.10728
# Brassicanapus_GSBRNA2T00034345001 Exp number, first 60 AAs:  0
# Brassicanapus_GSBRNA2T00034345001 Total prob of N-in:        0.01272
Brassicanapus_GSBRNA2T00034345001       TMHMM2.0        outside      1   493
Brassicanapus_GSBRNA2T00034345001       TMHMM2.0        TMhelix    494   516
Brassicanapus_GSBRNA2T00034345001       TMHMM2.0        inside     517   519
```

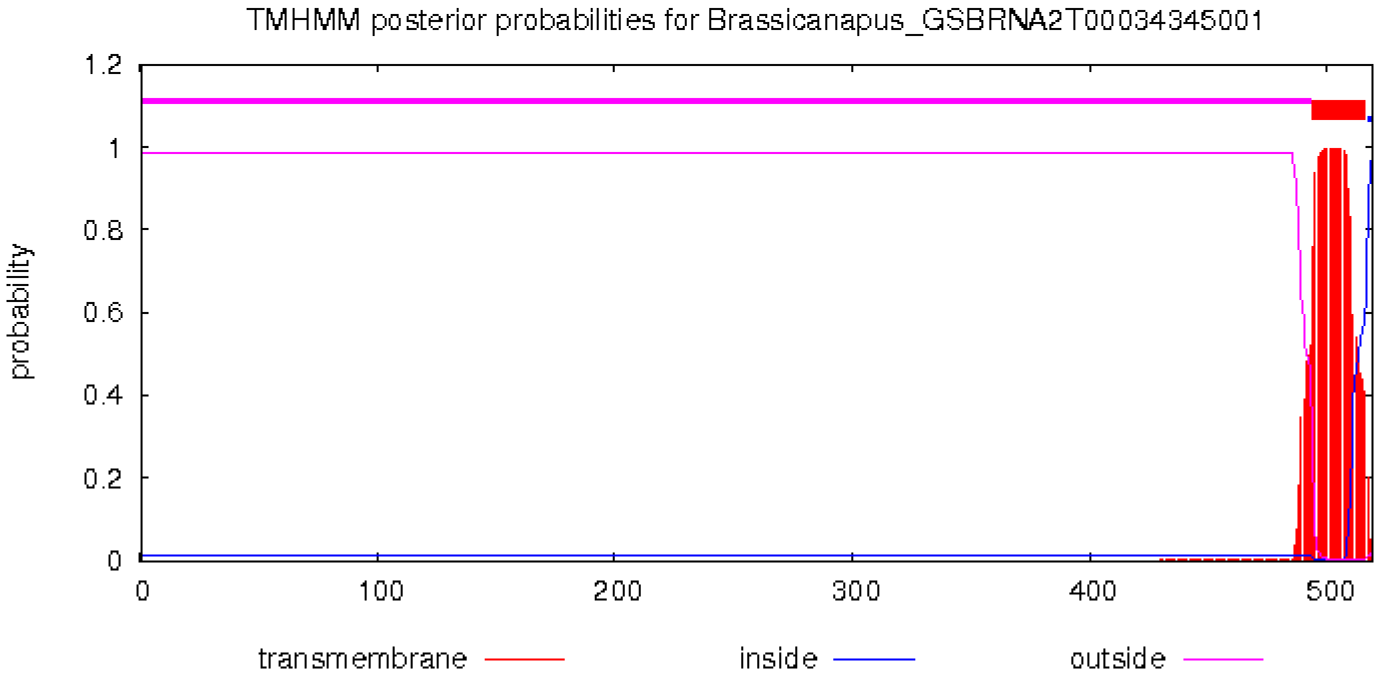

plot in postscript, script for making the plot in gnuplot, data for plot

```
# Brassicanapus_GSBRNA2T00035413001 Length: 340
# Brassicanapus_GSBRNA2T00035413001 Number of predicted TMHs:  0
# Brassicanapus_GSBRNA2T00035413001 Exp number of AAs in TMHs: 0.00215
# Brassicanapus_GSBRNA2T00035413001 Exp number, first 60 AAs:  0
# Brassicanapus_GSBRNA2T00035413001 Total prob of N-in:        0.00337
Brassicanapus_GSBRNA2T00035413001       TMHMM2.0        outside      1   340
```

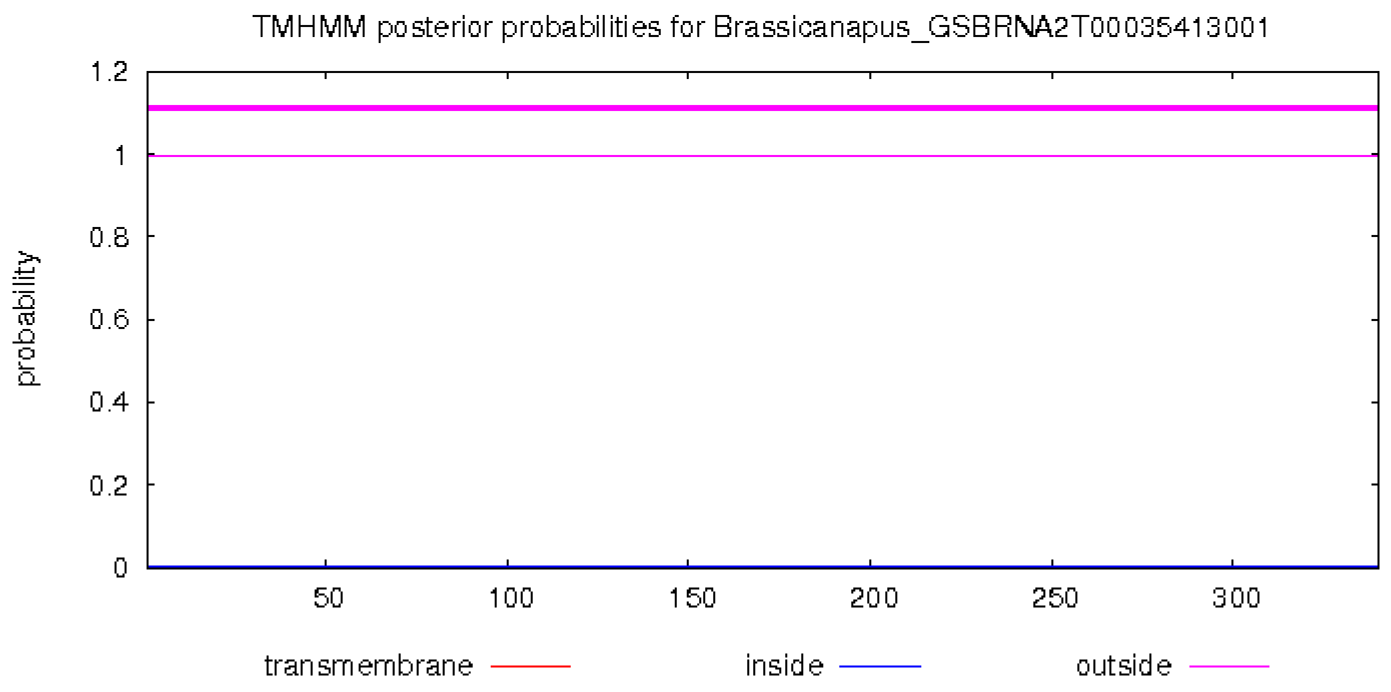

plot in postscript, script for making the plot in gnuplot, data for plot

```
# Brassicanapus_GSBRNA2T00035731001 Length: 279
# Brassicanapus_GSBRNA2T00035731001 Number of predicted TMHs:  0
# Brassicanapus_GSBRNA2T00035731001 Exp number of AAs in TMHs: 0.00228
# Brassicanapus_GSBRNA2T00035731001 Exp number, first 60 AAs:  0.00036
# Brassicanapus_GSBRNA2T00035731001 Total prob of N-in:        0.00561
Brassicanapus_GSBRNA2T00035731001       TMHMM2.0        outside      1   279
```

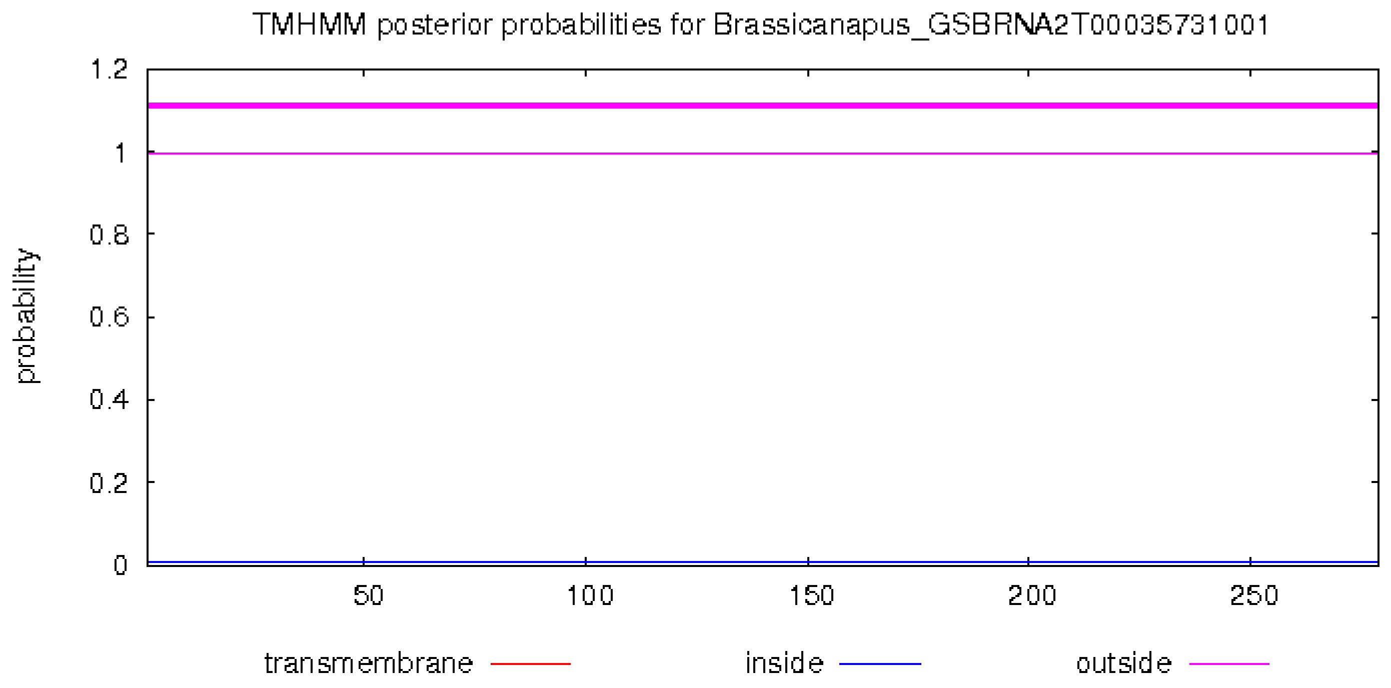

plot in postscript, script for making the plot in gnuplot, data for plot

```
# Brassicanapus_GSBRNA2T00036630001 Length: 308
# Brassicanapus_GSBRNA2T00036630001 Number of predicted TMHs:  0
# Brassicanapus_GSBRNA2T00036630001 Exp number of AAs in TMHs: 0.00017
# Brassicanapus_GSBRNA2T00036630001 Exp number, first 60 AAs:  0
# Brassicanapus_GSBRNA2T00036630001 Total prob of N-in:        0.02893
Brassicanapus_GSBRNA2T00036630001	TMHMM2.0	outside	     1   308
```

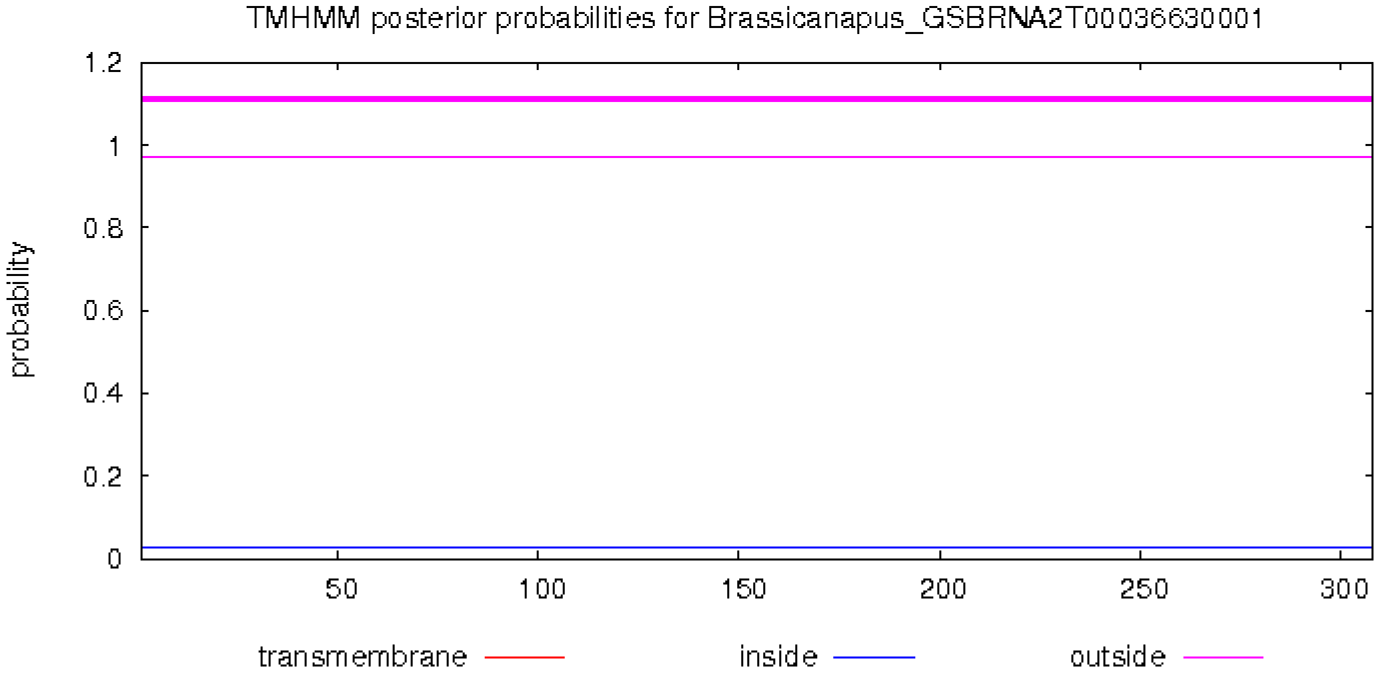

plot in postscript, script for making the plot in gnuplot, data for plot

```
# Brassicanapus_GSBRNA2T00036642001 Length: 636
# Brassicanapus_GSBRNA2T00036642001 Number of predicted TMHs:  1
# Brassicanapus_GSBRNA2T00036642001 Exp number of AAs in TMHs: 17.67923
# Brassicanapus_GSBRNA2T00036642001 Exp number, first 60 AAs:  0
# Brassicanapus_GSBRNA2T00036642001 Total prob of N-in:        0.19341
Brassicanapus_GSBRNA2T00036642001	TMHMM2.0	outside	     1   595
Brassicanapus_GSBRNA2T00036642001	TMHMM2.0	TMhelix	   596   613
Brassicanapus_GSBRNA2T00036642001	TMHMM2.0	inside	   614   636
```

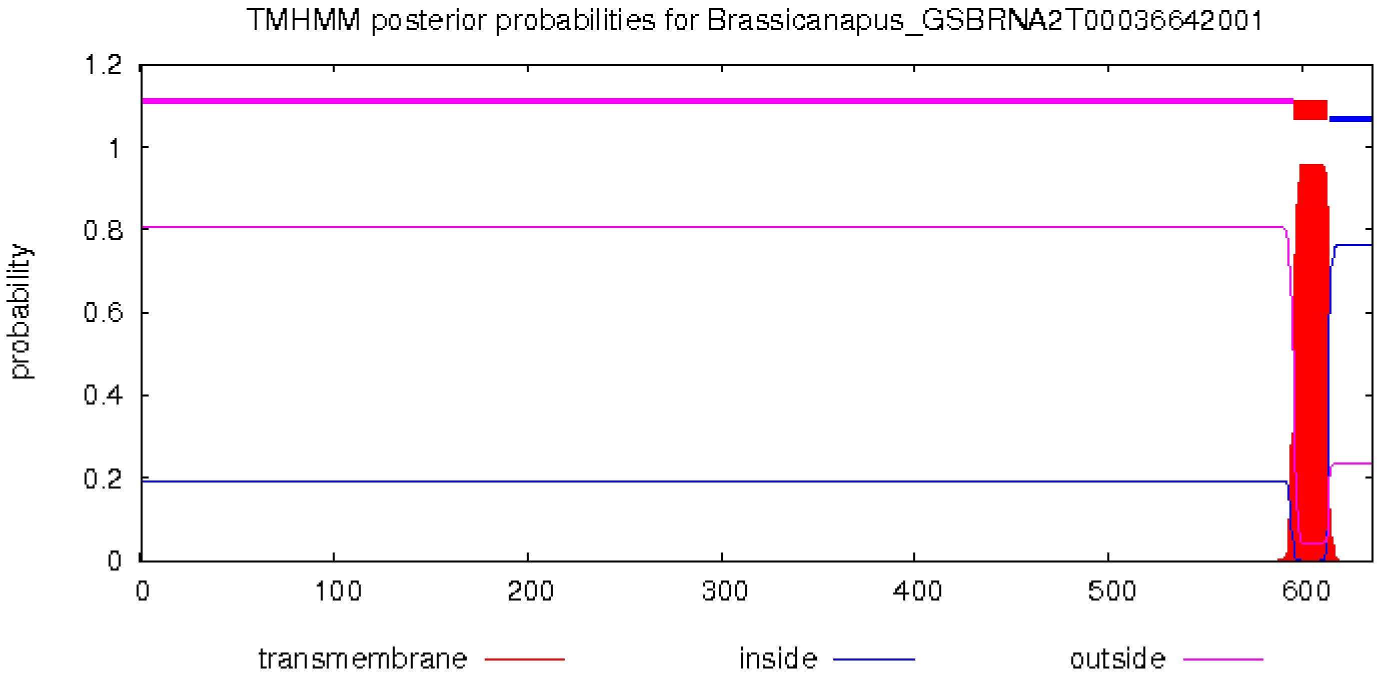

plot in postscript, script for making the plot in gnuplot, data for plot

```
# Brassicanapus_GSBRNA2T00037075001 Length: 330
# Brassicanapus_GSBRNA2T00037075001 Number of predicted TMHs:  0
# Brassicanapus_GSBRNA2T00037075001 Exp number of AAs in TMHs: 0.00032
# Brassicanapus_GSBRNA2T00037075001 Exp number, first 60 AAs:  0
# Brassicanapus_GSBRNA2T00037075001 Total prob of N-in:        0.02852
Brassicanapus_GSBRNA2T00037075001	TMHMM2.0	outside	     1   330
```

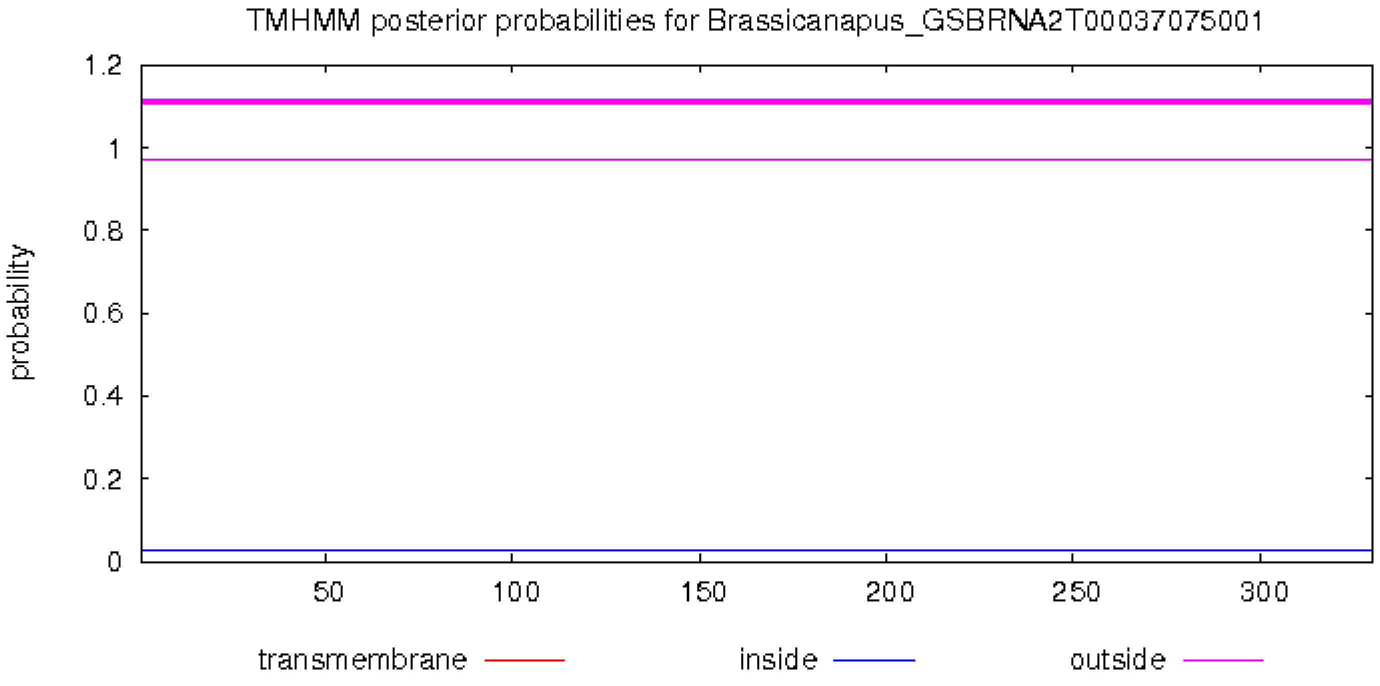

plot in postscript, script for making the plot in gnuplot, data for plot

```
# Brassicanapus_GSBRNA2T00037593001 Length: 158
# Brassicanapus_GSBRNA2T00037593001 Number of predicted TMHs:  0
# Brassicanapus_GSBRNA2T00037593001 Exp number of AAs in TMHs: 0.00018
# Brassicanapus_GSBRNA2T00037593001 Exp number, first 60 AAs:  0.00018
# Brassicanapus_GSBRNA2T00037593001 Total prob of N-in:        0.05996
Brassicanapus_GSBRNA2T00037593001	TMHMM2.0	outside	     1   158
```

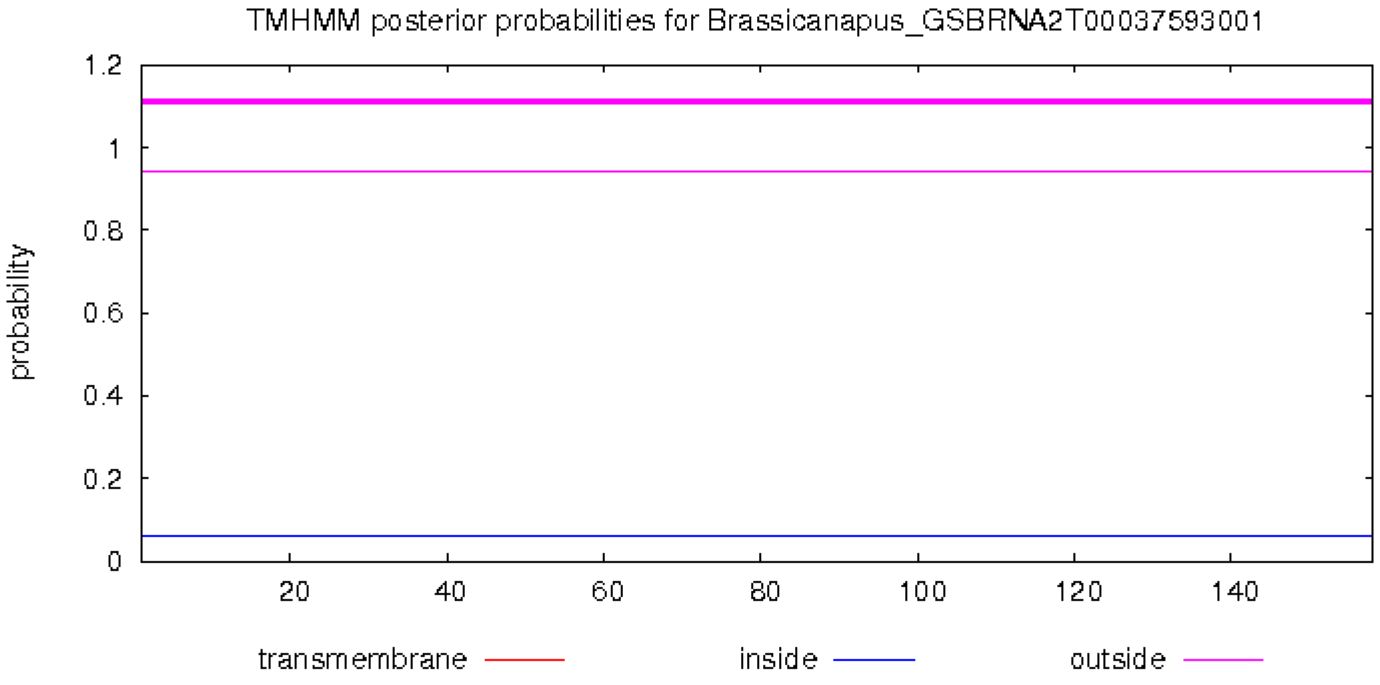

plot in postscript, script for making the plot in gnuplot, data for plot

```
# Brassicanapus_GSBRNA2T00037877001 Length: 290
# Brassicanapus_GSBRNA2T00037877001 Number of predicted TMHs:  0
# Brassicanapus_GSBRNA2T00037877001 Exp number of AAs in TMHs: 0
# Brassicanapus_GSBRNA2T00037877001 Exp number, first 60 AAs:  0
# Brassicanapus_GSBRNA2T00037877001 Total prob of N-in:        0.00159
Brassicanapus_GSBRNA2T00037877001       TMHMM2.0        outside      1   290
```

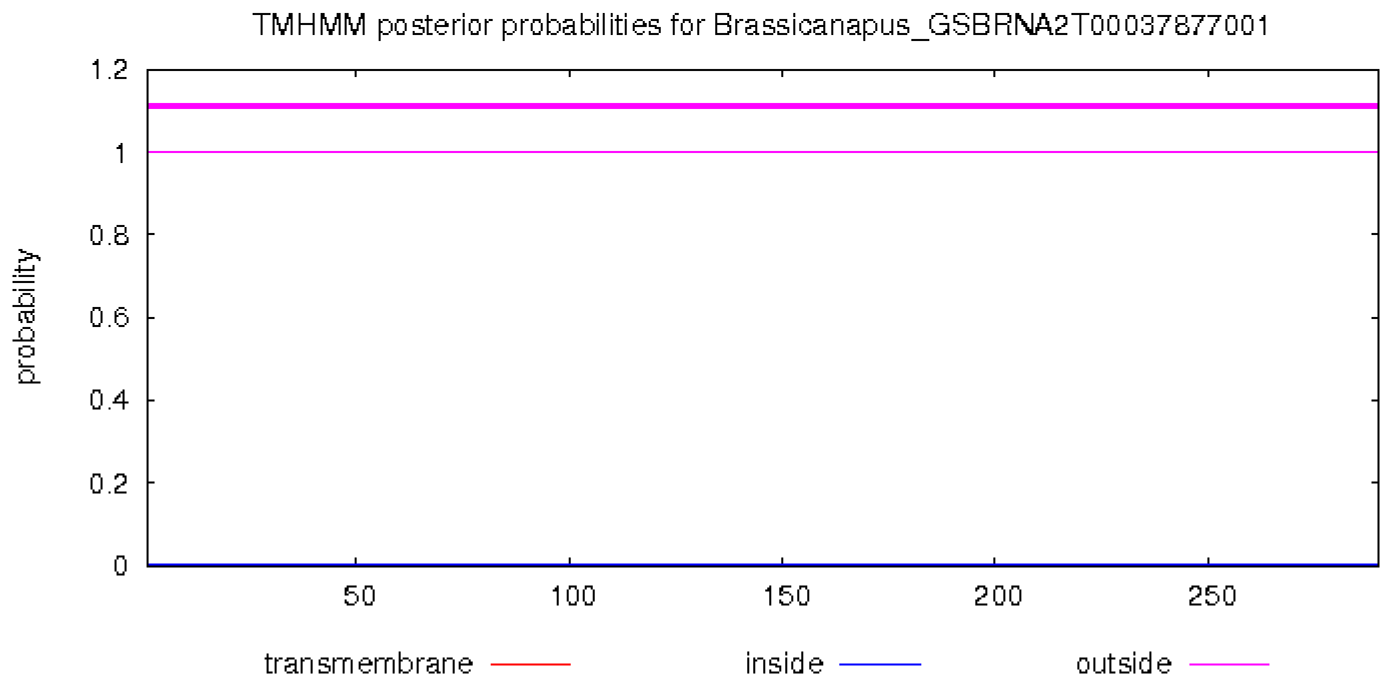

plot in postscript, script for making the plot in gnuplot, data for plot

---

```
# Brassicanapus_GSBRNA2T00038201001 Length: 364
# Brassicanapus_GSBRNA2T00038201001 Number of predicted TMHs:  0
# Brassicanapus_GSBRNA2T00038201001 Exp number of AAs in TMHs: 0.00039
# Brassicanapus_GSBRNA2T00038201001 Exp number, first 60 AAs:  0
# Brassicanapus_GSBRNA2T00038201001 Total prob of N-in:        0.00680
Brassicanapus_GSBRNA2T00038201001       TMHMM2.0        outside      1   364
```

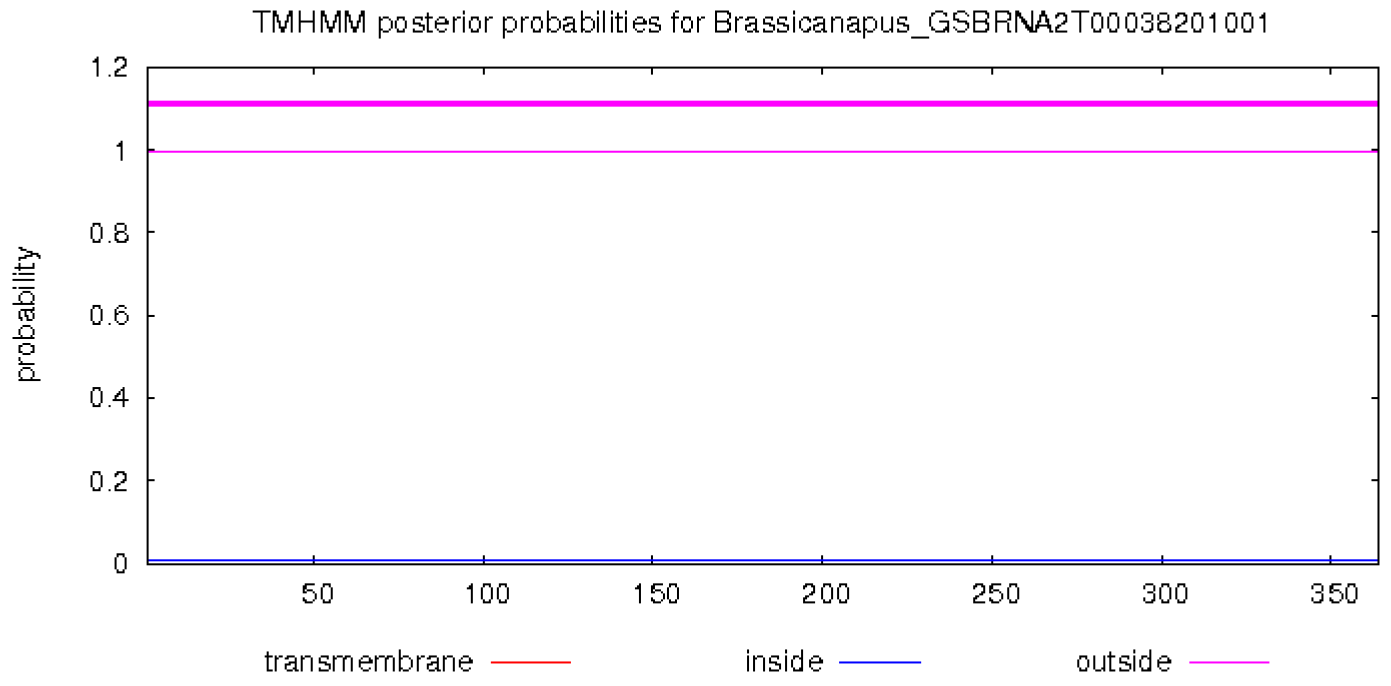

plot in postscript, script for making the plot in gnuplot, data for plot

---

```
# Brassicanapus_GSBRNA2T00038789001 Length: 510
# Brassicanapus_GSBRNA2T00038789001 Number of predicted TMHs:  2
# Brassicanapus_GSBRNA2T00038789001 Exp number of AAs in TMHs: 48.3965999999999999
# Brassicanapus_GSBRNA2T00038789001 Exp number, first 60 AAs:  1.84436
# Brassicanapus_GSBRNA2T00038789001 Total prob of N-in:        0.08140
Brassicanapus_GSBRNA2T00038789001	TMHMM2.0	outside	     1    79
Brassicanapus_GSBRNA2T00038789001	TMHMM2.0	TMhelix	    80   102
Brassicanapus_GSBRNA2T00038789001	TMHMM2.0	inside	   103   137
Brassicanapus_GSBRNA2T00038789001	TMHMM2.0	TMhelix	   138   160
Brassicanapus_GSBRNA2T00038789001	TMHMM2.0	outside	   161   510
```

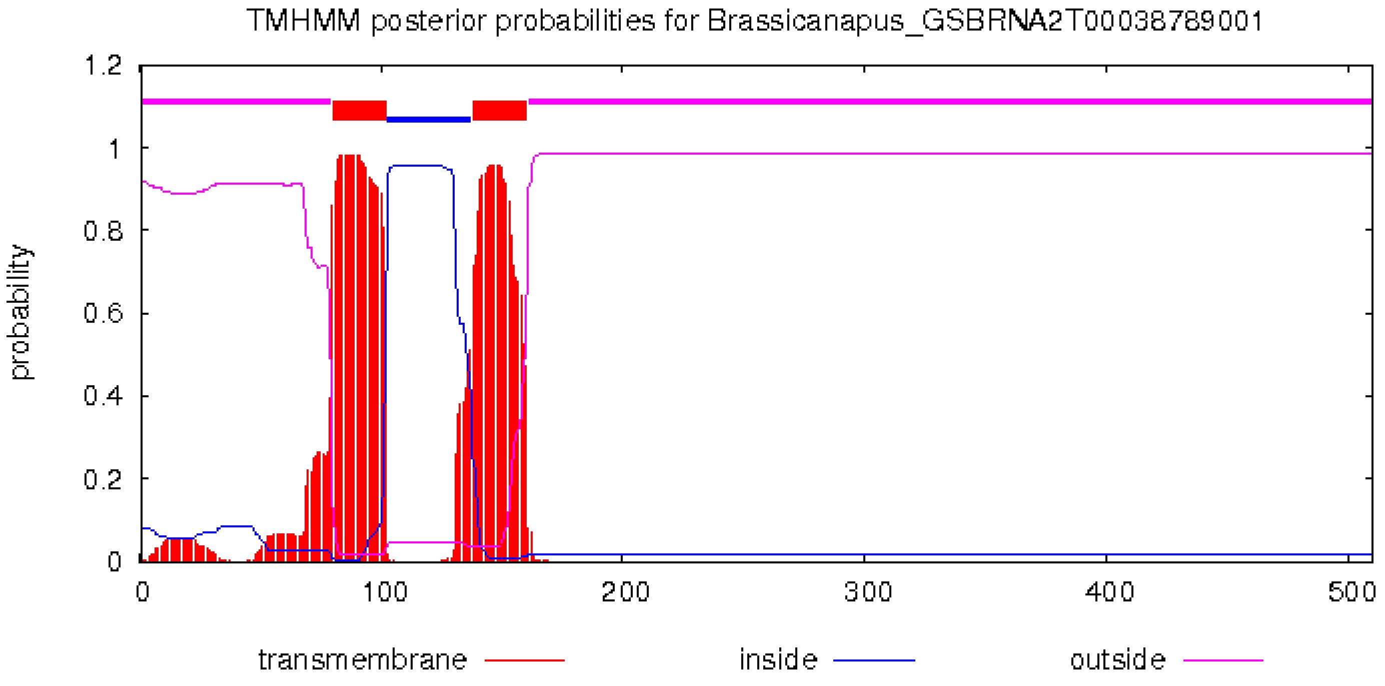

plot in postscript, script for making the plot in gnuplot, data for plot

---

```
# Brassicanapus_GSBRNA2T00040227001 Length: 578
# Brassicanapus_GSBRNA2T00040227001 Number of predicted TMHs:  1
# Brassicanapus_GSBRNA2T00040227001 Exp number of AAs in TMHs: 22.57695
# Brassicanapus_GSBRNA2T00040227001 Exp number, first 60 AAs:  0.0022
# Brassicanapus_GSBRNA2T00040227001 Total prob of N-in:        0.14245
Brassicanapus_GSBRNA2T00040227001	TMHMM2.0	outside	     1   545
Brassicanapus_GSBRNA2T00040227001	TMHMM2.0	TMhelix	   546   568
Brassicanapus_GSBRNA2T00040227001	TMHMM2.0	inside	   569   578
```

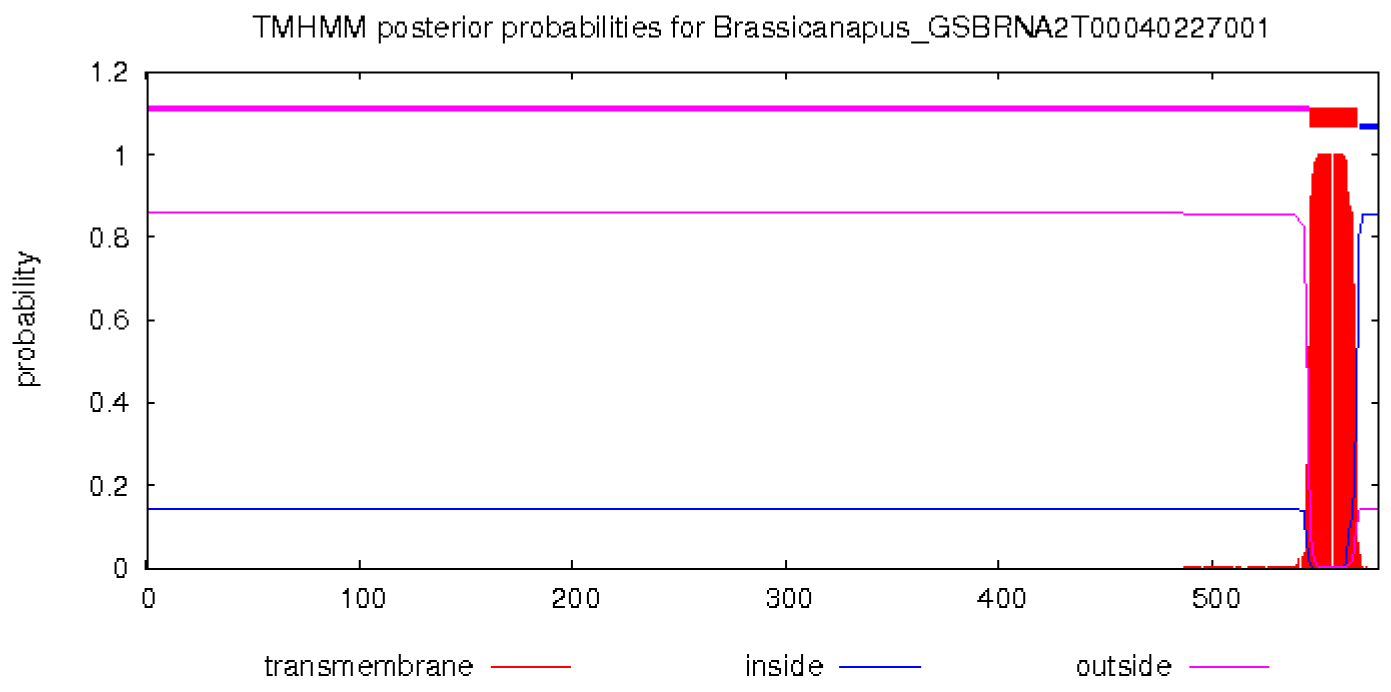

plot in postscript, script for making the plot in gnuplot, data for plot

---

```
# Brassicanapus_GSBRNA2T00040233001 Length: 546
# Brassicanapus_GSBRNA2T00040233001 Number of predicted TMHs:  1
# Brassicanapus_GSBRNA2T00040233001 Exp number of AAs in TMHs: 22.18742
# Brassicanapus_GSBRNA2T00040233001 Exp number, first 60 AAs:  0.03959
# Brassicanapus_GSBRNA2T00040233001 Total prob of N-in:        0.63825
Brassicanapus_GSBRNA2T00040233001       TMHMM2.0        inside       1   516
Brassicanapus_GSBRNA2T00040233001       TMHMM2.0        TMhelix    517   539
Brassicanapus_GSBRNA2T00040233001       TMHMM2.0        outside    540   546
```

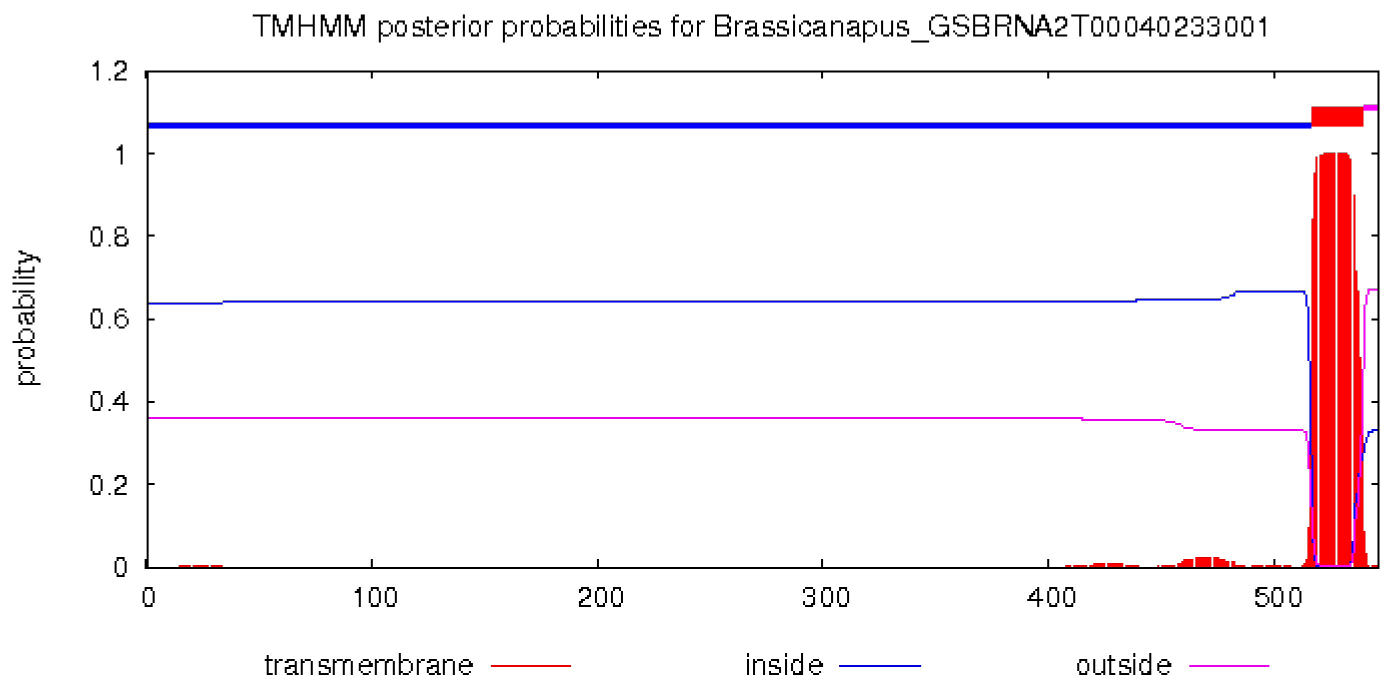

plot in postscript, script for making the plot in gnuplot, data for plot

---

```
# Brassicanapus_GSBRNA2T00040675001 Length: 325
# Brassicanapus_GSBRNA2T00040675001 Number of predicted TMHs:  0
# Brassicanapus_GSBRNA2T00040675001 Exp number of AAs in TMHs: 0.00354
# Brassicanapus_GSBRNA2T00040675001 Exp number, first 60 AAs:  0
```

```
# Brassicanapus_GSBRNA2T00040675001 Total prob of N-in:        0.02400
Brassicanapus_GSBRNA2T00040675001       TMHMM2.0        outside      1   325
```

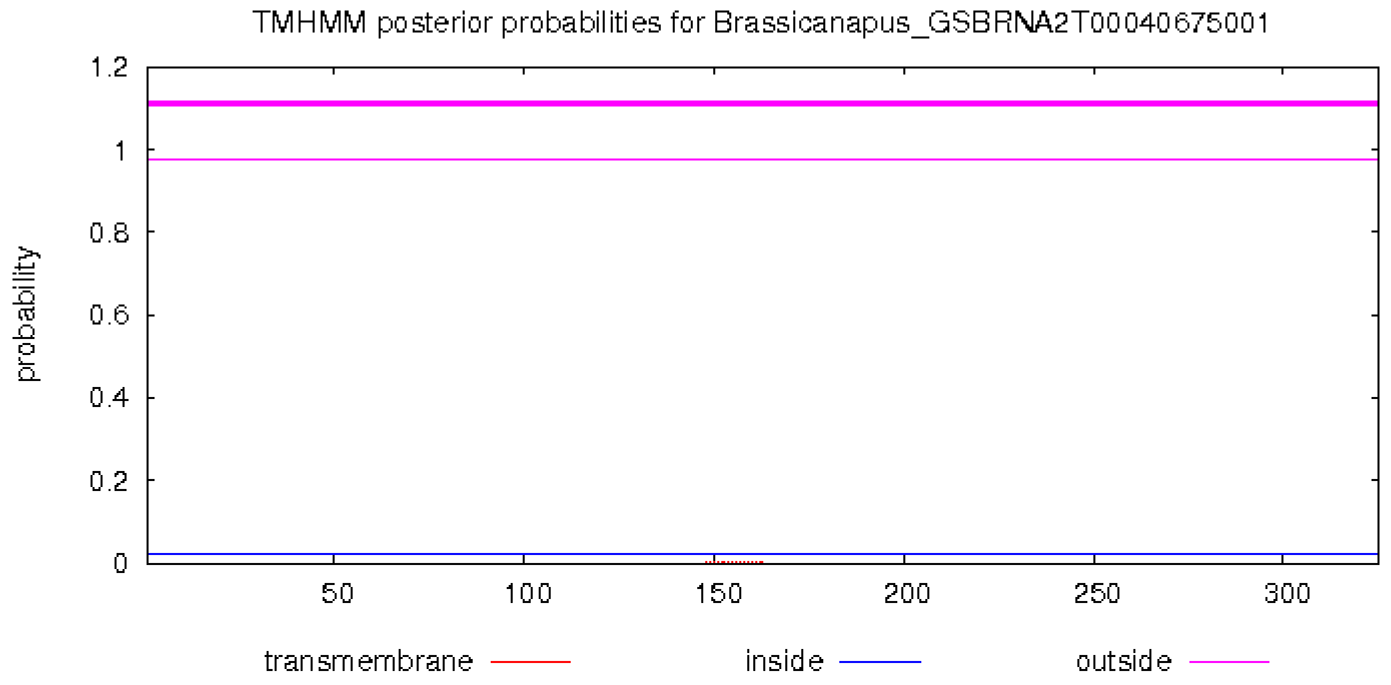

plot in postscript, script for making the plot in gnuplot, data for plot

---

```
# Brassicanapus_GSBRNA2T00040908001 Length: 489
# Brassicanapus_GSBRNA2T00040908001 Number of predicted TMHs:  0
# Brassicanapus_GSBRNA2T00040908001 Exp number of AAs in TMHs: 0.00379
# Brassicanapus_GSBRNA2T00040908001 Exp number, first 60 AAs:  0.00343
# Brassicanapus_GSBRNA2T00040908001 Total prob of N-in:        0.00550
Brassicanapus_GSBRNA2T00040908001       TMHMM2.0        outside      1   489
```

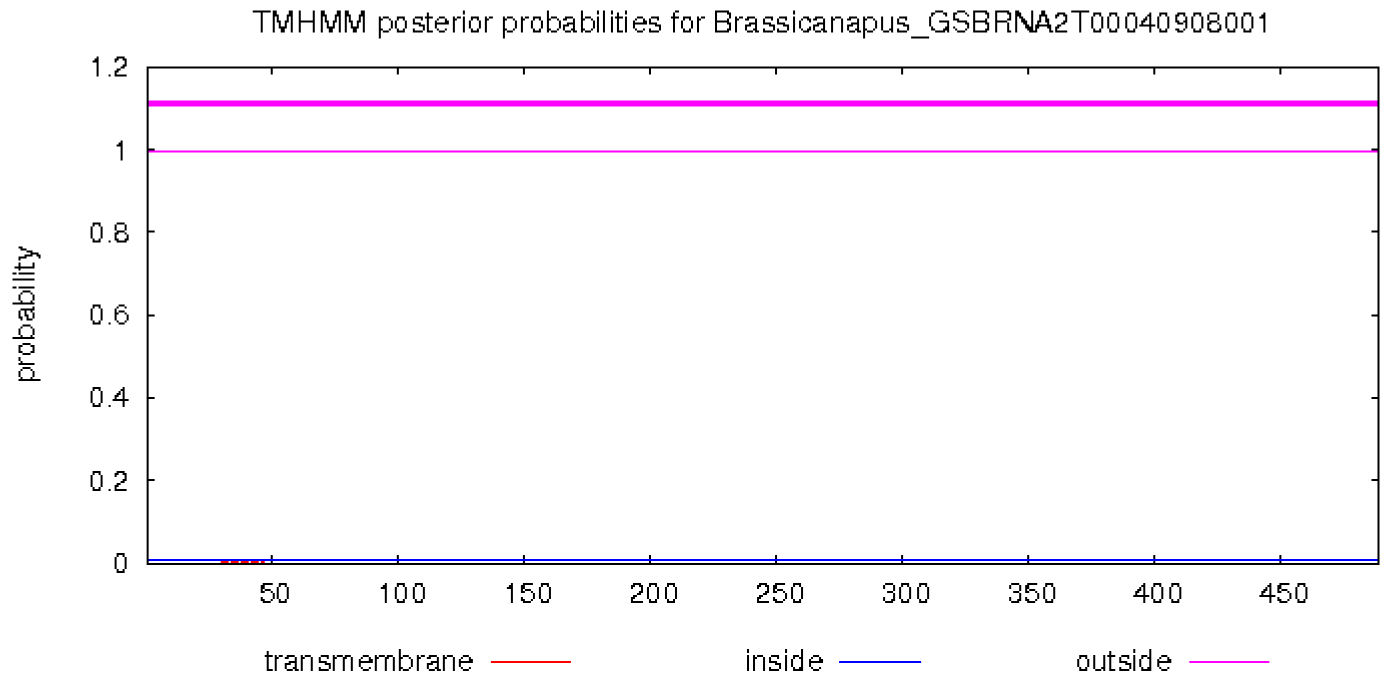

plot in postscript, script for making the plot in gnuplot, data for plot

---

```
# Brassicanapus_GSBRNA2T00041385001 Length: 478
# Brassicanapus_GSBRNA2T00041385001 Number of predicted TMHs:  0
# Brassicanapus_GSBRNA2T00041385001 Exp number of AAs in TMHs: 0.00091
# Brassicanapus_GSBRNA2T00041385001 Exp number, first 60 AAs:  0
```

```
# Brassicanapus_GSBRNA2T00041385001 Total prob of N-in:        0.00018
Brassicanapus_GSBRNA2T00041385001       TMHMM2.0        outside      1   478
```

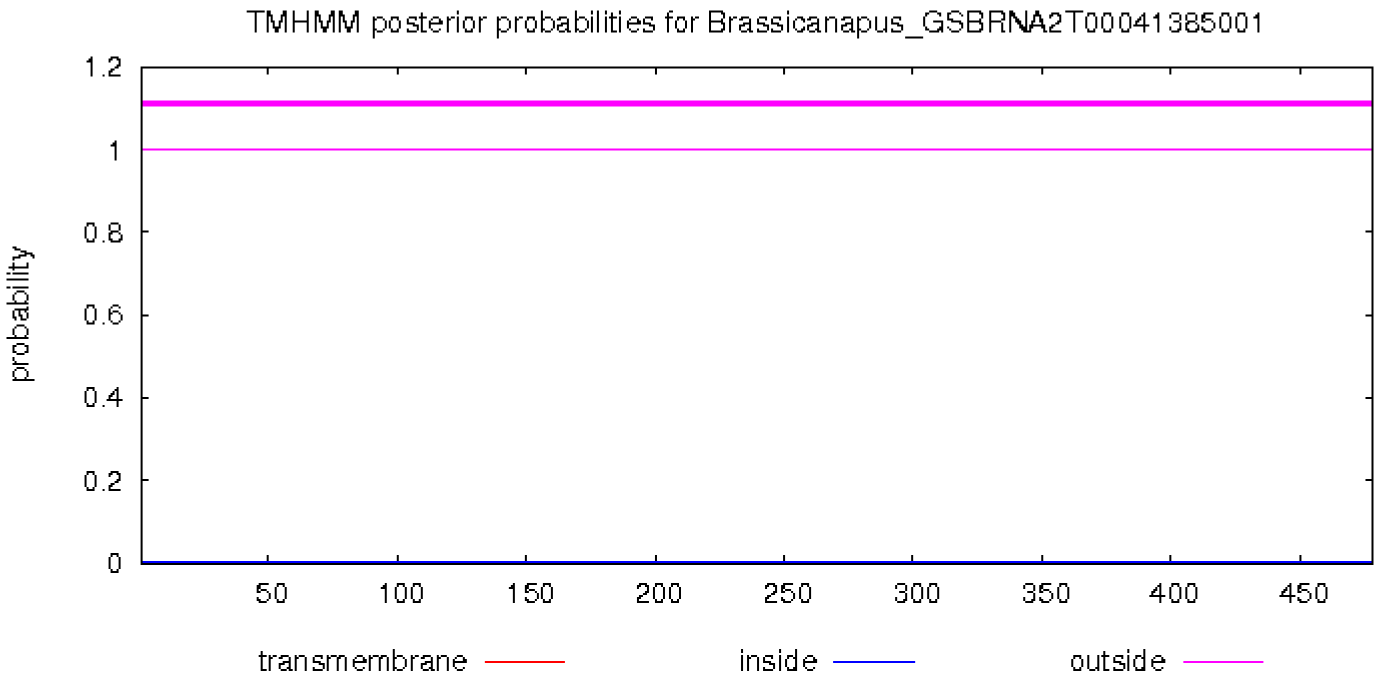

plot in postscript, script for making the plot in gnuplot, data for plot

---

```
# Brassicanapus_GSBRNA2T00041459001 Length: 367
# Brassicanapus_GSBRNA2T00041459001 Number of predicted TMHs:  0
# Brassicanapus_GSBRNA2T00041459001 Exp number of AAs in TMHs: 0.00349
# Brassicanapus_GSBRNA2T00041459001 Exp number, first 60 AAs:  0
# Brassicanapus_GSBRNA2T00041459001 Total prob of N-in:        0.01798
Brassicanapus_GSBRNA2T00041459001       TMHMM2.0        outside      1   367
```

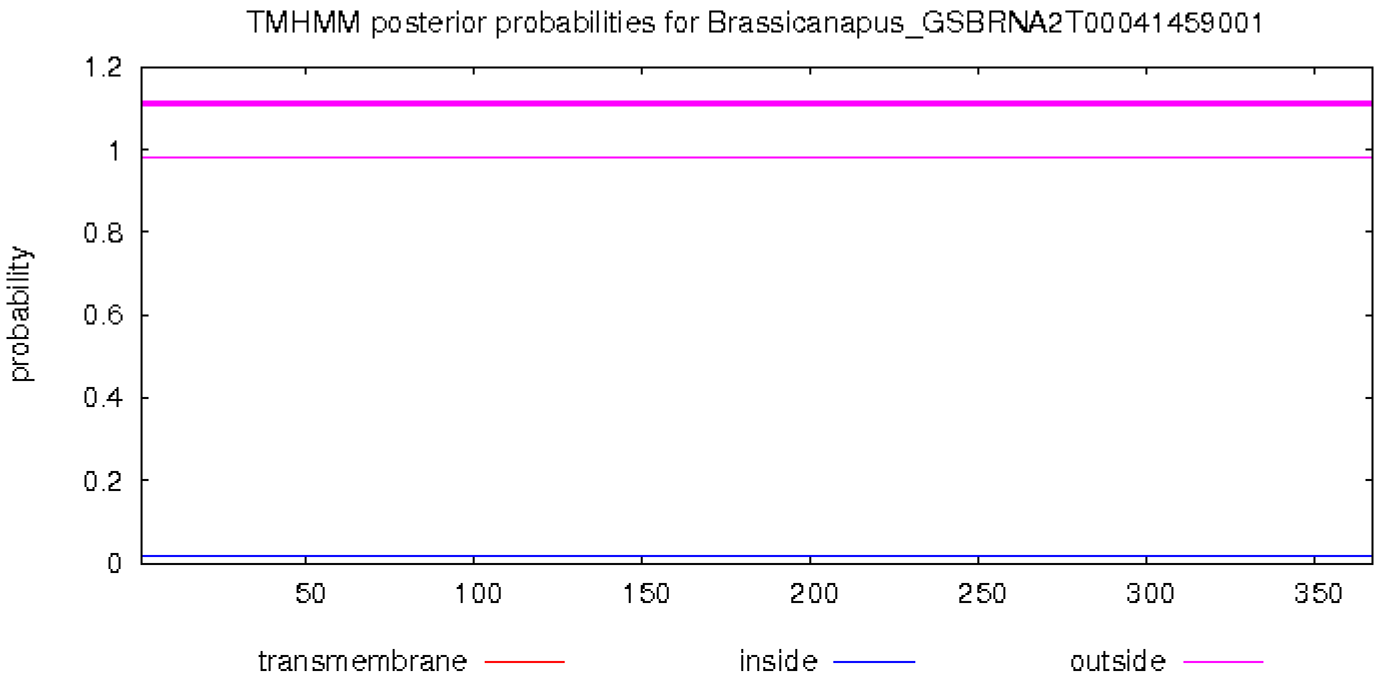

plot in postscript, script for making the plot in gnuplot, data for plot

---

```
# Brassicanapus_GSBRNA2T00042092001 Length: 323
# Brassicanapus_GSBRNA2T00042092001 Number of predicted TMHs:  0
# Brassicanapus_GSBRNA2T00042092001 Exp number of AAs in TMHs: 0.00475
# Brassicanapus_GSBRNA2T00042092001 Exp number, first 60 AAs:  0
```

```
# Brassicanapus_GSBRNA2T00042092001 Total prob of N-in:        0.03445
Brassicanapus_GSBRNA2T00042092001	TMHMM2.0	outside	     1   323
```

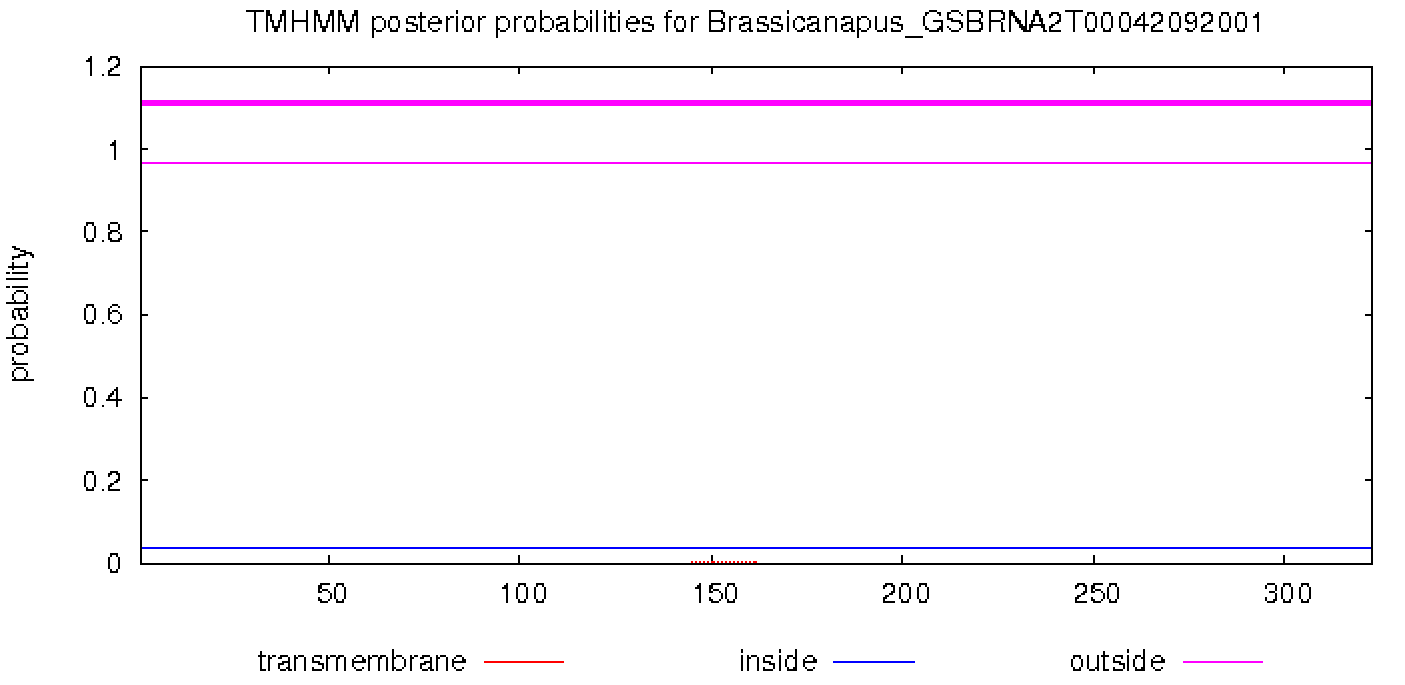

plot in postscript, script for making the plot in gnuplot, data for plot

---

```
# Brassicanapus_GSBRNA2T00042260001 Length: 300
# Brassicanapus_GSBRNA2T00042260001 Number of predicted TMHs:  0
# Brassicanapus_GSBRNA2T00042260001 Exp number of AAs in TMHs: 0.00339
# Brassicanapus_GSBRNA2T00042260001 Exp number, first 60 AAs:  0.00062
# Brassicanapus_GSBRNA2T00042260001 Total prob of N-in:        0.02422
Brassicanapus_GSBRNA2T00042260001	TMHMM2.0	outside	     1   300
```

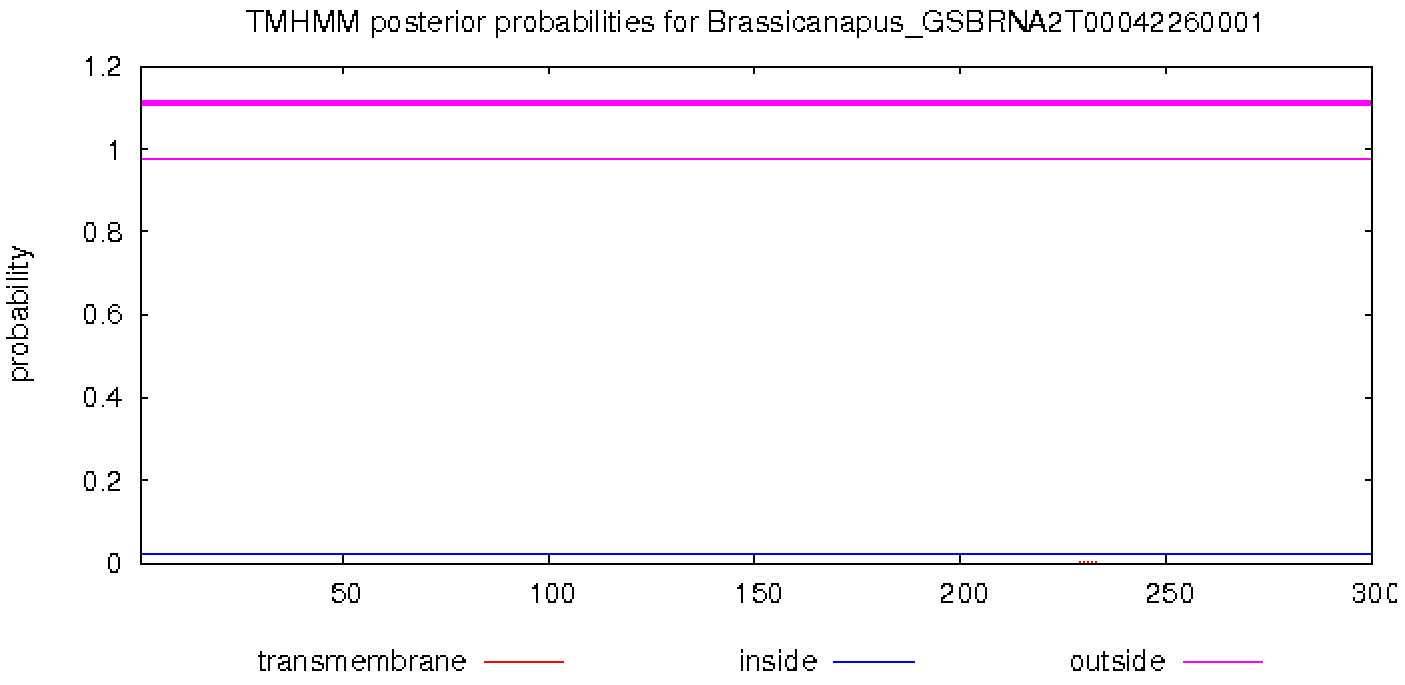

plot in postscript, script for making the plot in gnuplot, data for plot

---

```
# Brassicanapus_GSBRNA2T00042350001 Length: 294
# Brassicanapus_GSBRNA2T00042350001 Number of predicted TMHs:  0
# Brassicanapus_GSBRNA2T00042350001 Exp number of AAs in TMHs: 0.00131
# Brassicanapus_GSBRNA2T00042350001 Exp number, first 60 AAs:  0
```

```
# Brassicanapus_GSBRNA2T00042350001 Total prob of N-in:        0.08626
Brassicanapus_GSBRNA2T00042350001       TMHMM2.0        outside      1   294
```

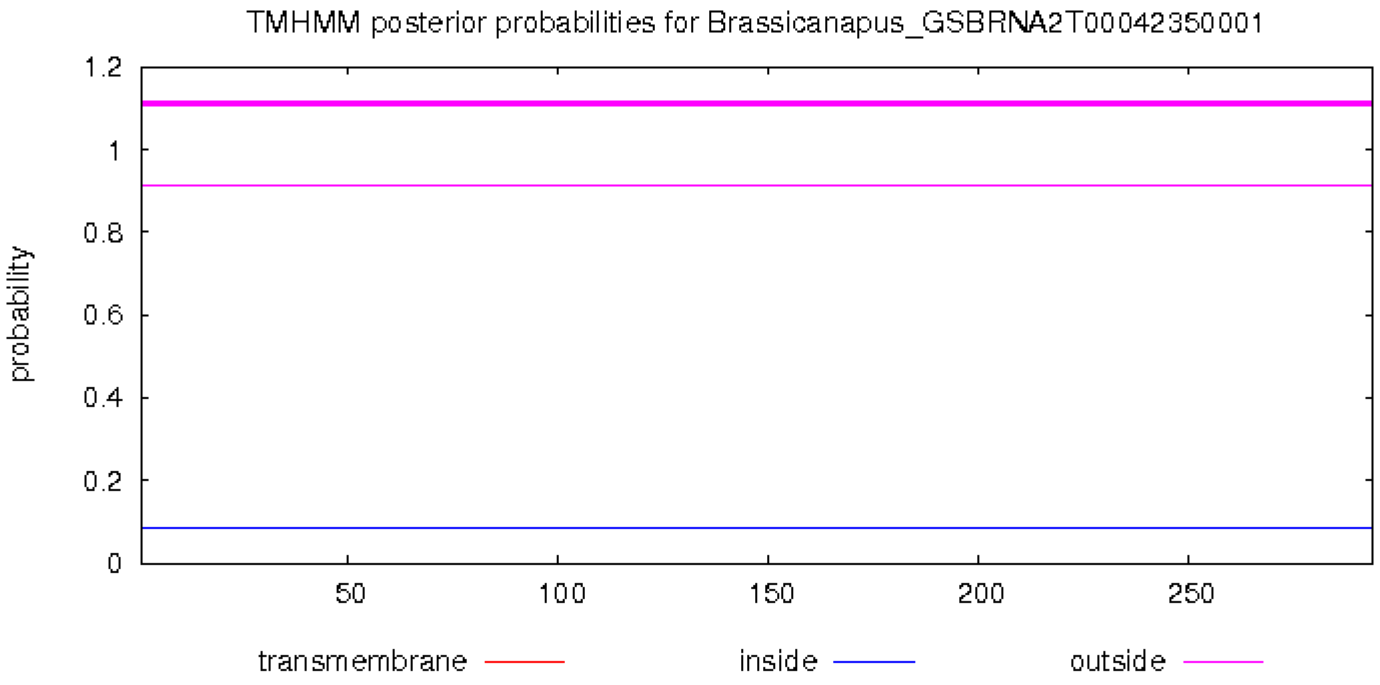

plot in postscript, script for making the plot in gnuplot, data for plot

---

```
# Brassicanapus_GSBRNA2T00042526001 Length: 283
# Brassicanapus_GSBRNA2T00042526001 Number of predicted TMHs:  0
# Brassicanapus_GSBRNA2T00042526001 Exp number of AAs in TMHs: 0.0034
# Brassicanapus_GSBRNA2T00042526001 Exp number, first 60 AAs:  0
# Brassicanapus_GSBRNA2T00042526001 Total prob of N-in:        0.01731
Brassicanapus_GSBRNA2T00042526001       TMHMM2.0        outside      1   283
```

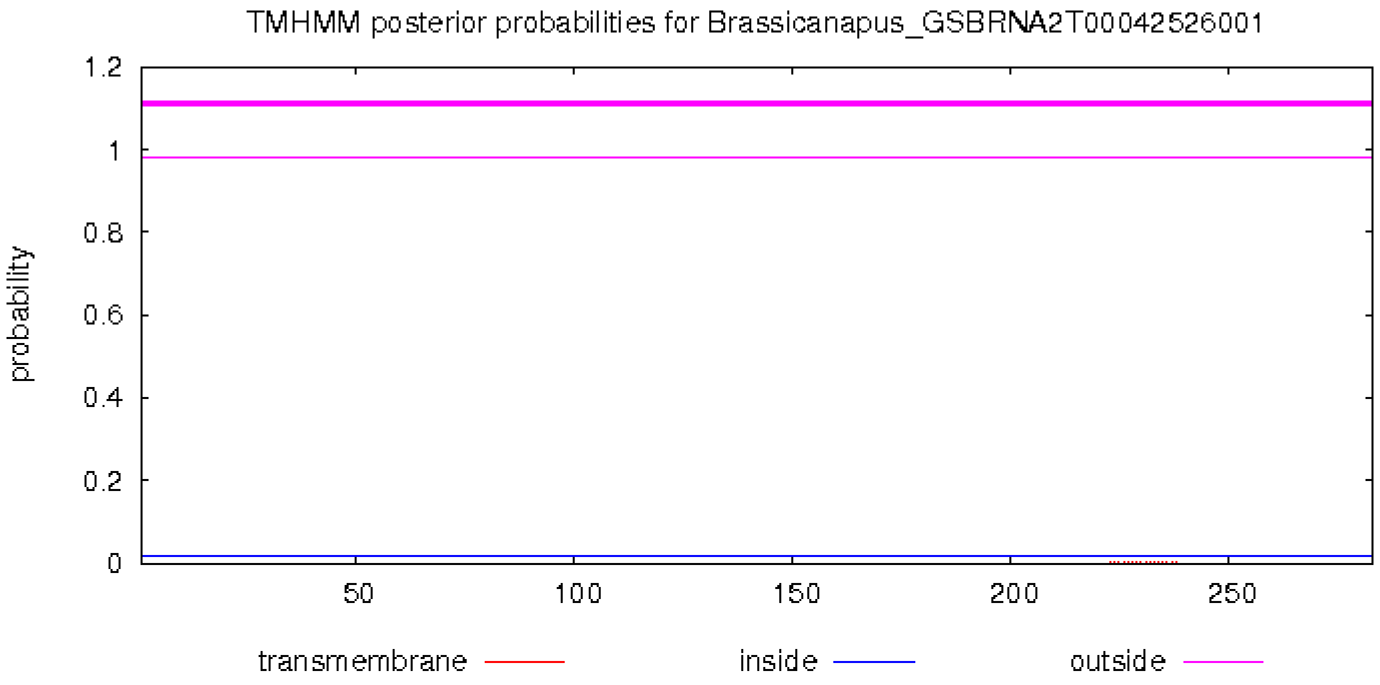

plot in postscript, script for making the plot in gnuplot, data for plot

---

```
# Brassicanapus_GSBRNA2T00043949001 Length: 400
# Brassicanapus_GSBRNA2T00043949001 Number of predicted TMHs:  0
# Brassicanapus_GSBRNA2T00043949001 Exp number of AAs in TMHs: 0.00028
# Brassicanapus_GSBRNA2T00043949001 Exp number, first 60 AAs:  0
```

```
# Brassicanapus_GSBRNA2T00043949001 Total prob of N-in:        0.02074
Brassicanapus_GSBRNA2T00043949001       TMHMM2.0        outside      1   400
```

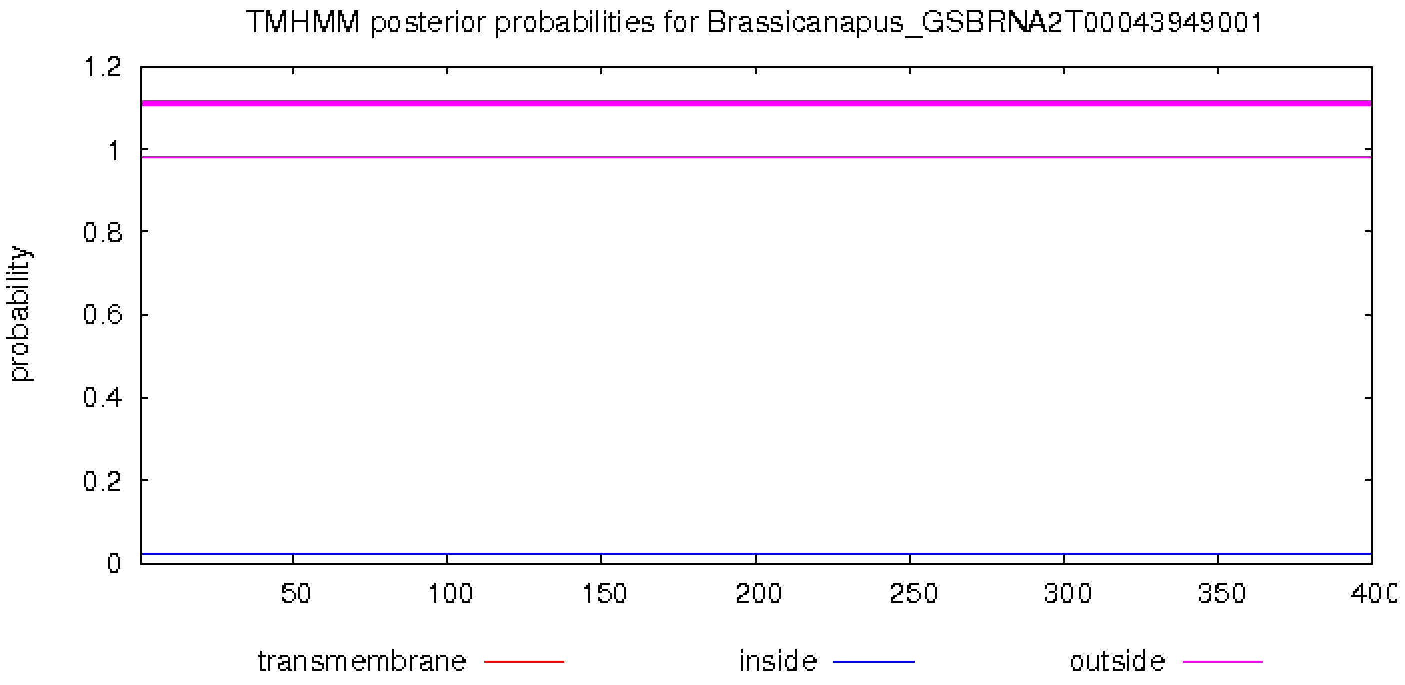

plot in postscript, script for making the plot in gnuplot, data for plot

---

```
# Brassicanapus_GSBRNA2T00044271001 Length: 300
# Brassicanapus_GSBRNA2T00044271001 Number of predicted TMHs:  0
# Brassicanapus_GSBRNA2T00044271001 Exp number of AAs in TMHs: 0.00219
# Brassicanapus_GSBRNA2T00044271001 Exp number, first 60 AAs:  0.00032
# Brassicanapus_GSBRNA2T00044271001 Total prob of N-in:        0.01333
Brassicanapus_GSBRNA2T00044271001       TMHMM2.0        outside      1   300
```

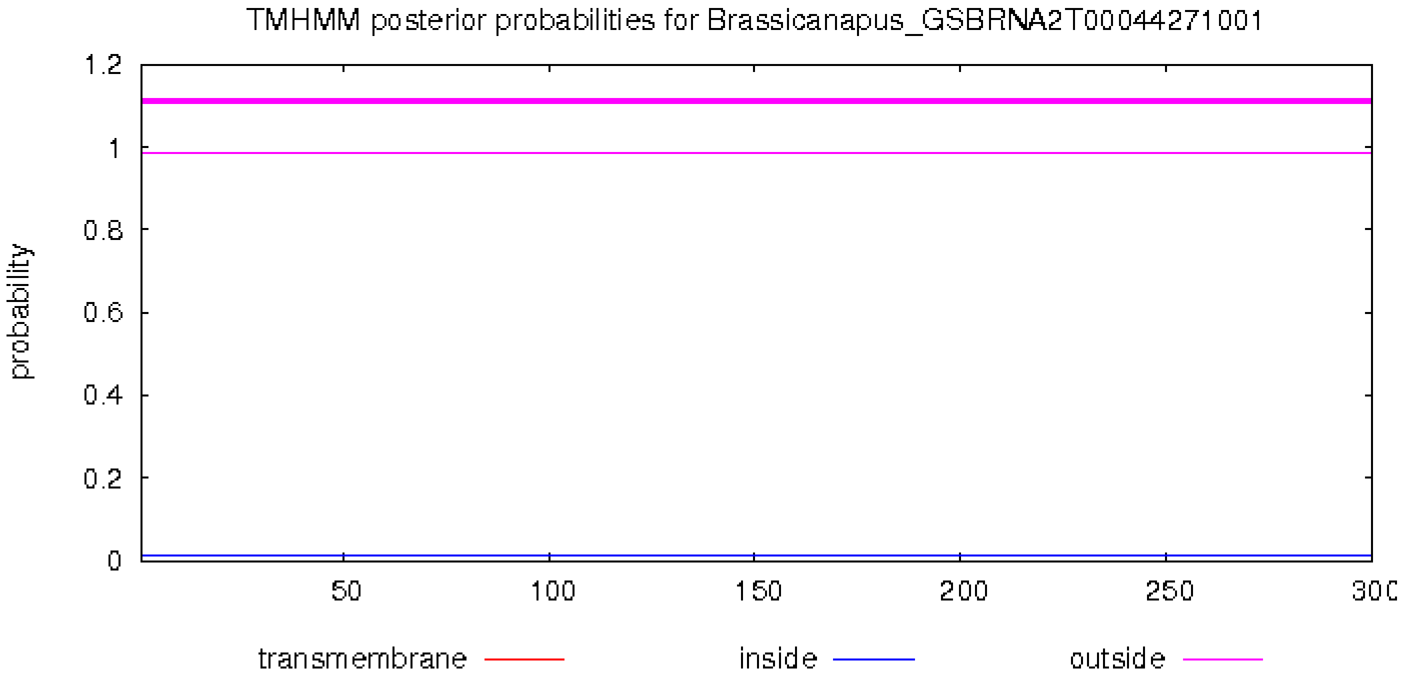

plot in postscript, script for making the plot in gnuplot, data for plot

---

```
# Brassicanapus_GSBRNA2T00044371001 Length: 305
# Brassicanapus_GSBRNA2T00044371001 Number of predicted TMHs:  0
# Brassicanapus_GSBRNA2T00044371001 Exp number of AAs in TMHs: 0.0009
# Brassicanapus_GSBRNA2T00044371001 Exp number, first 60 AAs:  0
```

```
# Brassicanapus_GSBRNA2T00044371001 Total prob of N-in:        0.05537
Brassicanapus_GSBRNA2T00044371001       TMHMM2.0        outside      1   305
```

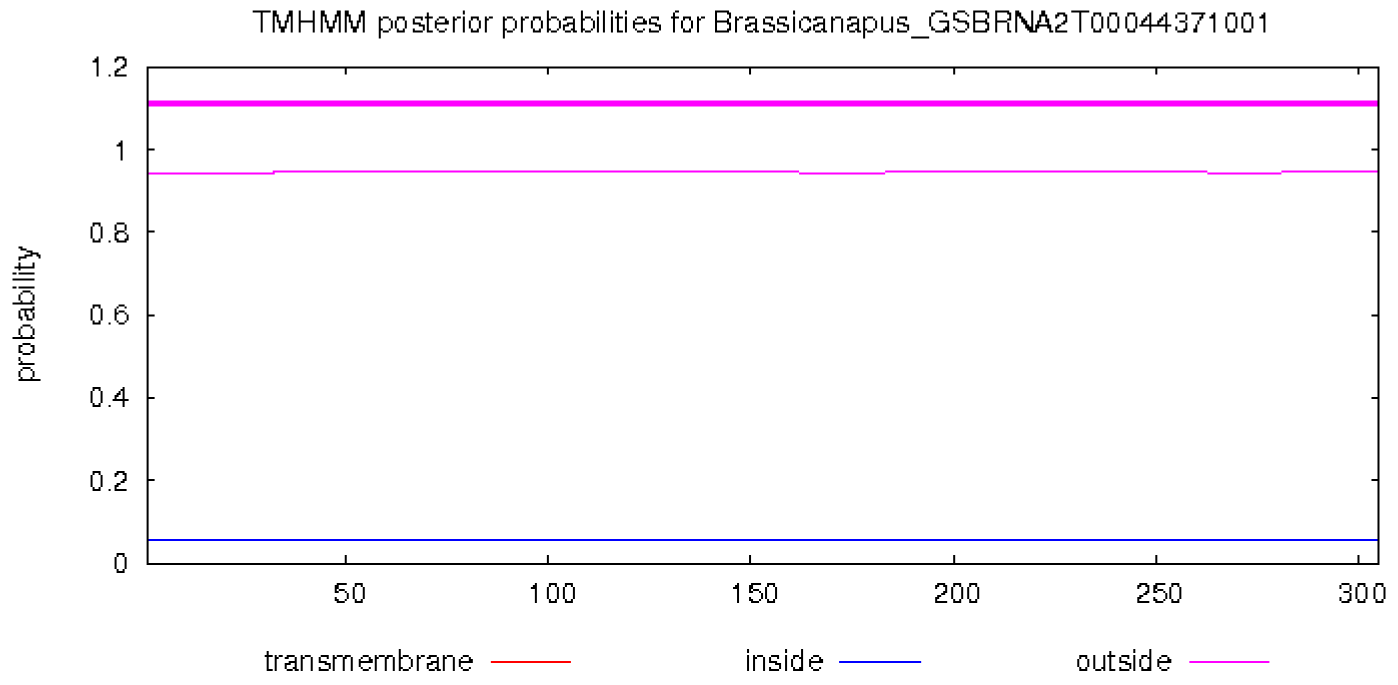

plot in postscript, script for making the plot in gnuplot, data for plot

---

```
# Brassicanapus_GSBRNA2T00044503001 Length: 172
# Brassicanapus_GSBRNA2T00044503001 Number of predicted TMHs:  0
# Brassicanapus_GSBRNA2T00044503001 Exp number of AAs in TMHs: 0.00157
# Brassicanapus_GSBRNA2T00044503001 Exp number, first 60 AAs:  0.00011
# Brassicanapus_GSBRNA2T00044503001 Total prob of N-in:        0.03602
Brassicanapus_GSBRNA2T00044503001       TMHMM2.0        outside      1   172
```

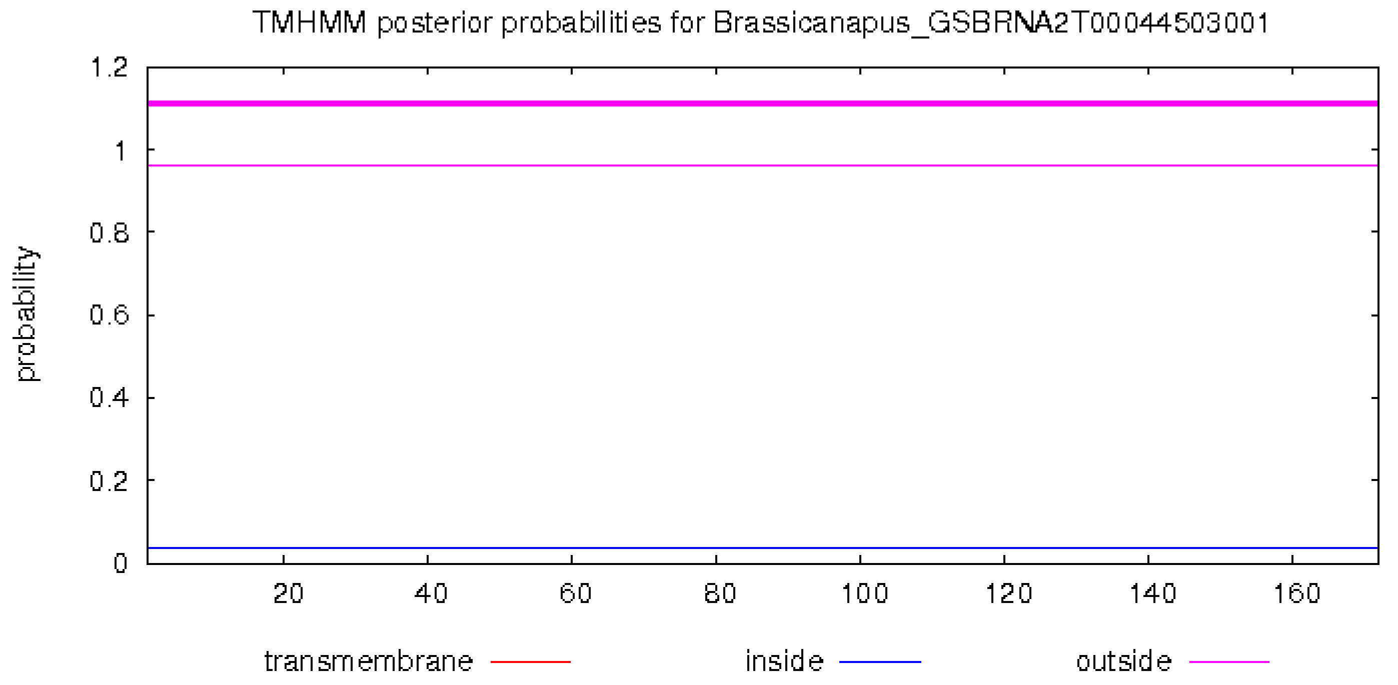

plot in postscript, script for making the plot in gnuplot, data for plot

---

```
# Brassicanapus_GSBRNA2T00044505001 Length: 355
# Brassicanapus_GSBRNA2T00044505001 Number of predicted TMHs:  0
# Brassicanapus_GSBRNA2T00044505001 Exp number of AAs in TMHs: 0.00036
# Brassicanapus_GSBRNA2T00044505001 Exp number, first 60 AAs:  0.00036
```

```
# Brassicanapus_GSBRNA2T00044505001 Total prob of N-in:        0.01043
Brassicanapus_GSBRNA2T00044505001       TMHMM2.0        outside      1   355
```

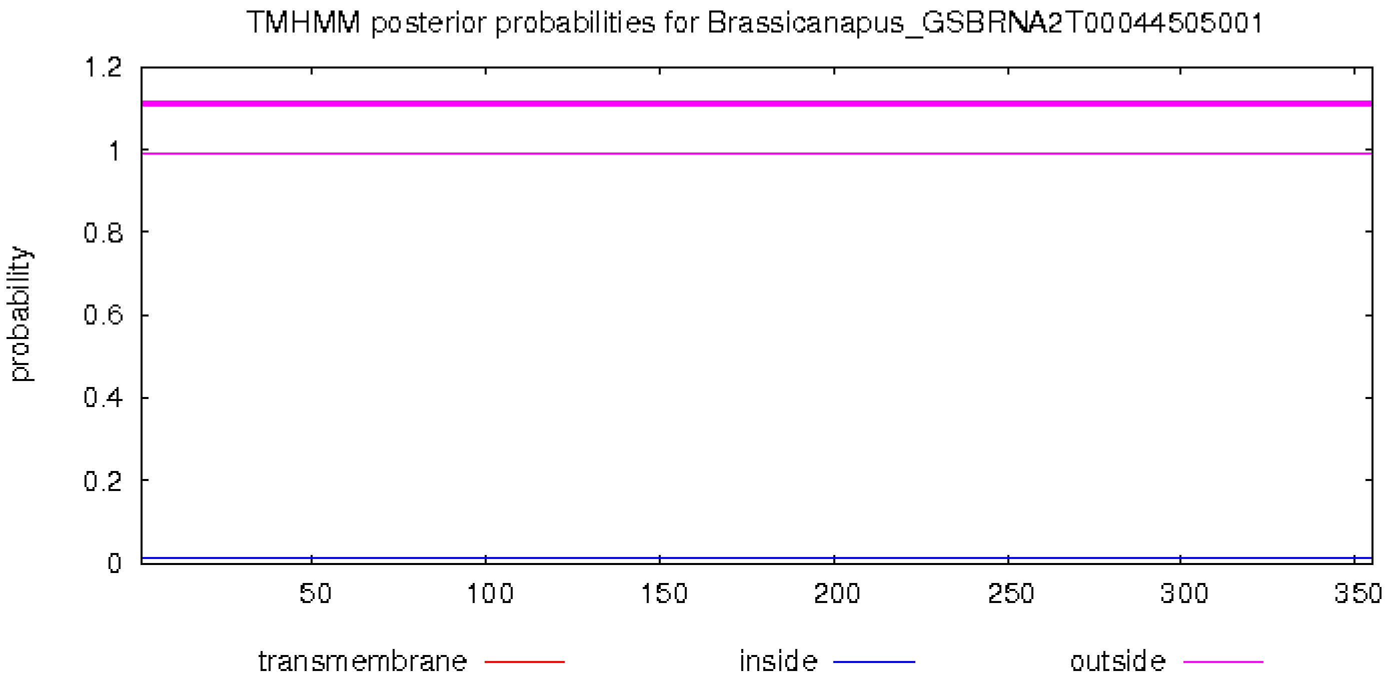

plot in postscript, script for making the plot in gnuplot, data for plot

```
# Brassicanapus_GSBRNA2T00044605001 Length: 394
# Brassicanapus_GSBRNA2T00044605001 Number of predicted TMHs:  0
# Brassicanapus_GSBRNA2T00044605001 Exp number of AAs in TMHs: 7e-05
# Brassicanapus_GSBRNA2T00044605001 Exp number, first 60 AAs:  0
# Brassicanapus_GSBRNA2T00044605001 Total prob of N-in:        0.00089
Brassicanapus_GSBRNA2T00044605001       TMHMM2.0        outside      1   394
```

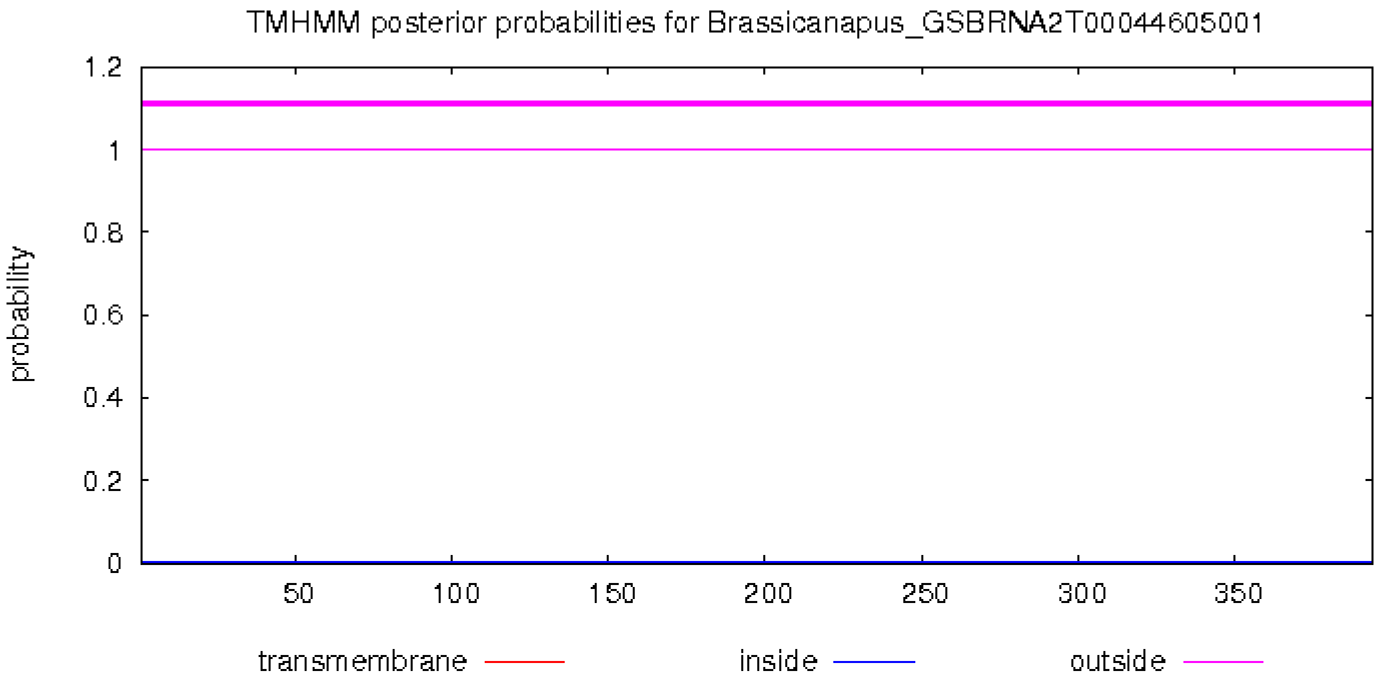

plot in postscript, script for making the plot in gnuplot, data for plot

```
# Brassicanapus_GSBRNA2T00044843001 Length: 484
# Brassicanapus_GSBRNA2T00044843001 Number of predicted TMHs:  0
# Brassicanapus_GSBRNA2T00044843001 Exp number of AAs in TMHs: 0.01629
# Brassicanapus_GSBRNA2T00044843001 Exp number, first 60 AAs:  0
```

```
# Brassicanapus_GSBRNA2T00044843001 Total prob of N-in:        0.00434
Brassicanapus_GSBRNA2T00044843001       TMHMM2.0        outside      1   484
```

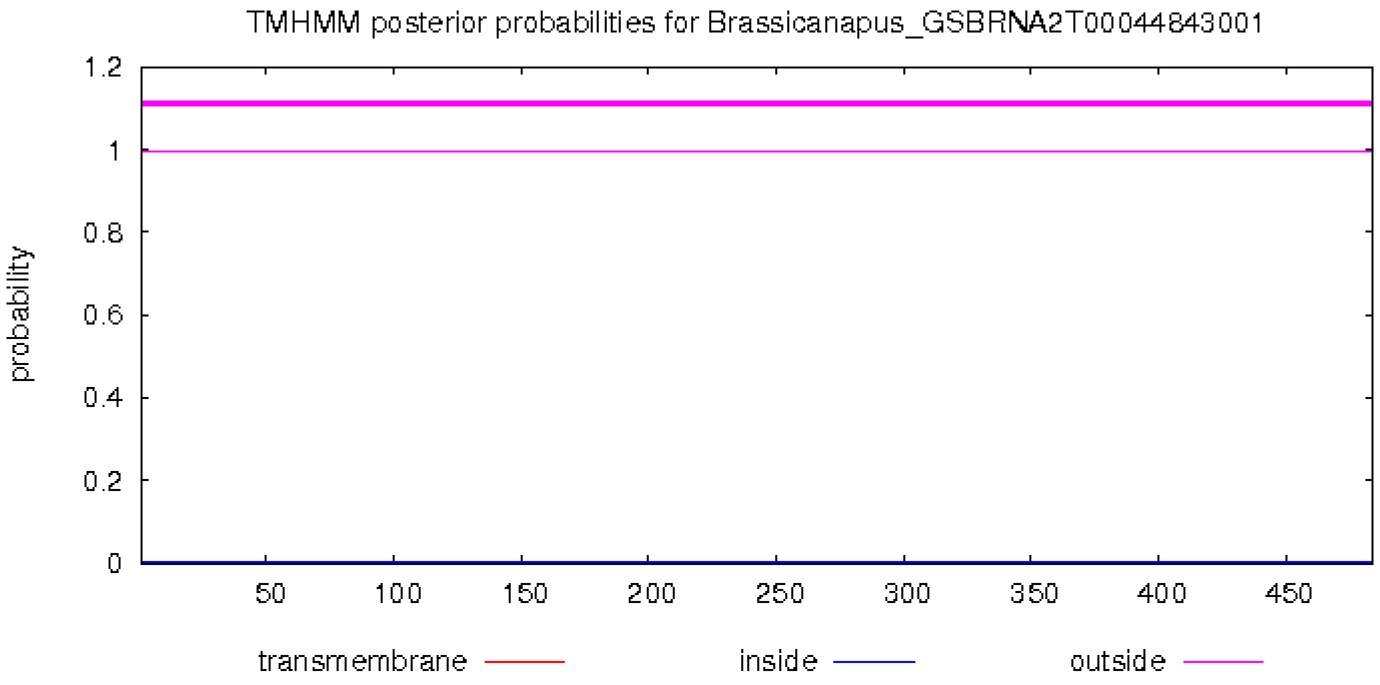

plot in postscript, script for making the plot in gnuplot, data for plot

---

```
# Brassicanapus_GSBRNA2T00044895001 Length: 289
# Brassicanapus_GSBRNA2T00044895001 Number of predicted TMHs:  0
# Brassicanapus_GSBRNA2T00044895001 Exp number of AAs in TMHs: 0.00072
# Brassicanapus_GSBRNA2T00044895001 Exp number, first 60 AAs:  0.00071
# Brassicanapus_GSBRNA2T00044895001 Total prob of N-in:        0.05495
Brassicanapus_GSBRNA2T00044895001       TMHMM2.0        outside      1   289
```

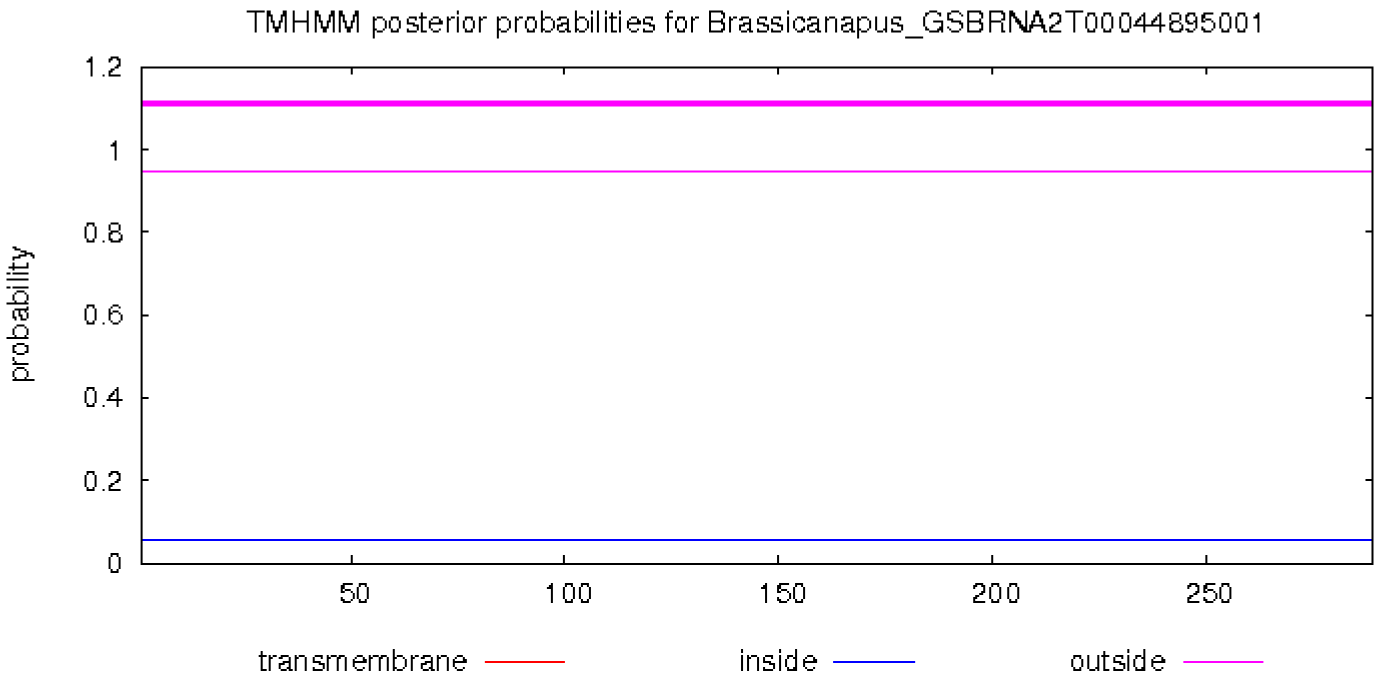

plot in postscript, script for making the plot in gnuplot, data for plot

---

```
# Brassicanapus_GSBRNA2T00045062001 Length: 277
# Brassicanapus_GSBRNA2T00045062001 Number of predicted TMHs:  0
# Brassicanapus_GSBRNA2T00045062001 Exp number of AAs in TMHs: 0.00108
# Brassicanapus_GSBRNA2T00045062001 Exp number, first 60 AAs:  0
```

```
# Brassicanapus_GSBRNA2T00045062001 Total prob of N-in:        0.02400
Brassicanapus_GSBRNA2T00045062001       TMHMM2.0        outside      1   277
```

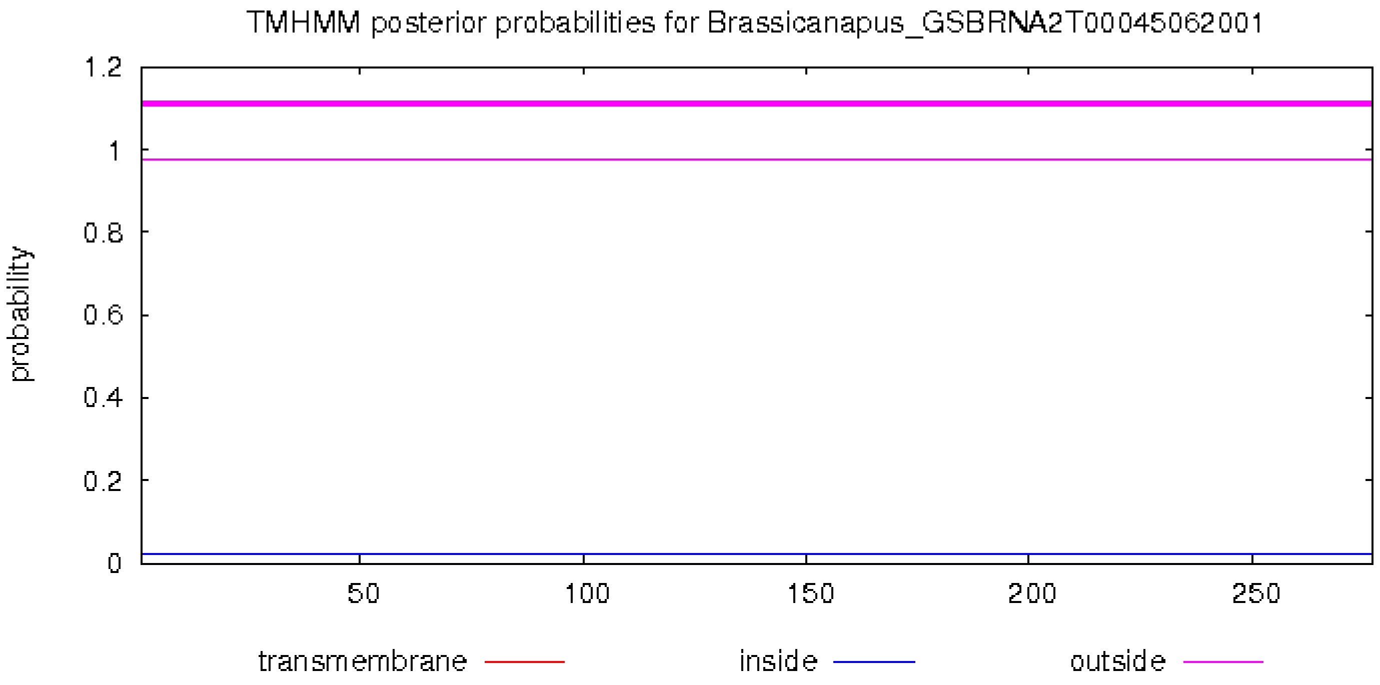

plot in postscript, script for making the plot in gnuplot, data for plot

---

```
# Brassicanapus_GSBRNA2T00045496001 Length: 395
# Brassicanapus_GSBRNA2T00045496001 Number of predicted TMHs:  0
# Brassicanapus_GSBRNA2T00045496001 Exp number of AAs in TMHs: 0
# Brassicanapus_GSBRNA2T00045496001 Exp number, first 60 AAs:  0
# Brassicanapus_GSBRNA2T00045496001 Total prob of N-in:        0.02352
Brassicanapus_GSBRNA2T00045496001       TMHMM2.0        outside      1   395
```

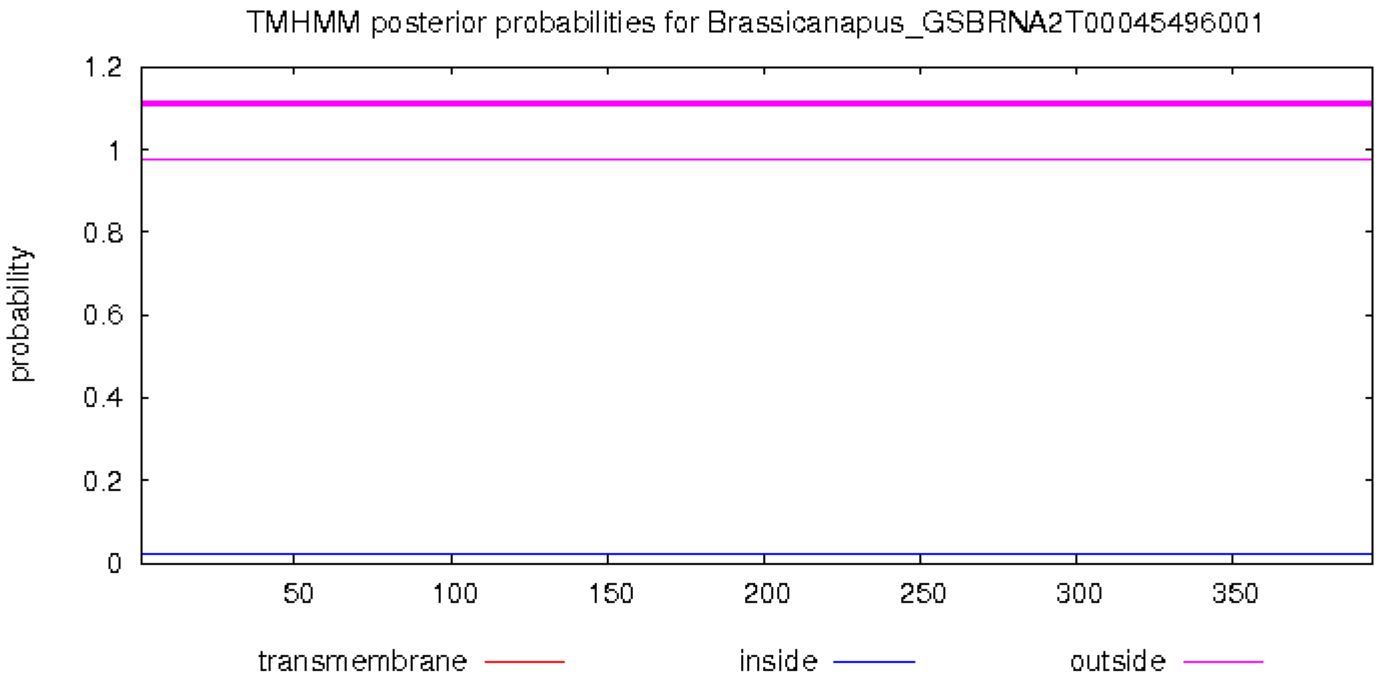

plot in postscript, script for making the plot in gnuplot, data for plot

---

```
# Brassicanapus_GSBRNA2T00046056001 Length: 361
# Brassicanapus_GSBRNA2T00046056001 Number of predicted TMHs:  0
# Brassicanapus_GSBRNA2T00046056001 Exp number of AAs in TMHs: 0.00057
# Brassicanapus_GSBRNA2T00046056001 Exp number, first 60 AAs:  0
```

```
# Brassicanapus_GSBRNA2T00046056001 Total prob of N-in:        0.00234
Brassicanapus_GSBRNA2T00046056001       TMHMM2.0        outside      1   361
```

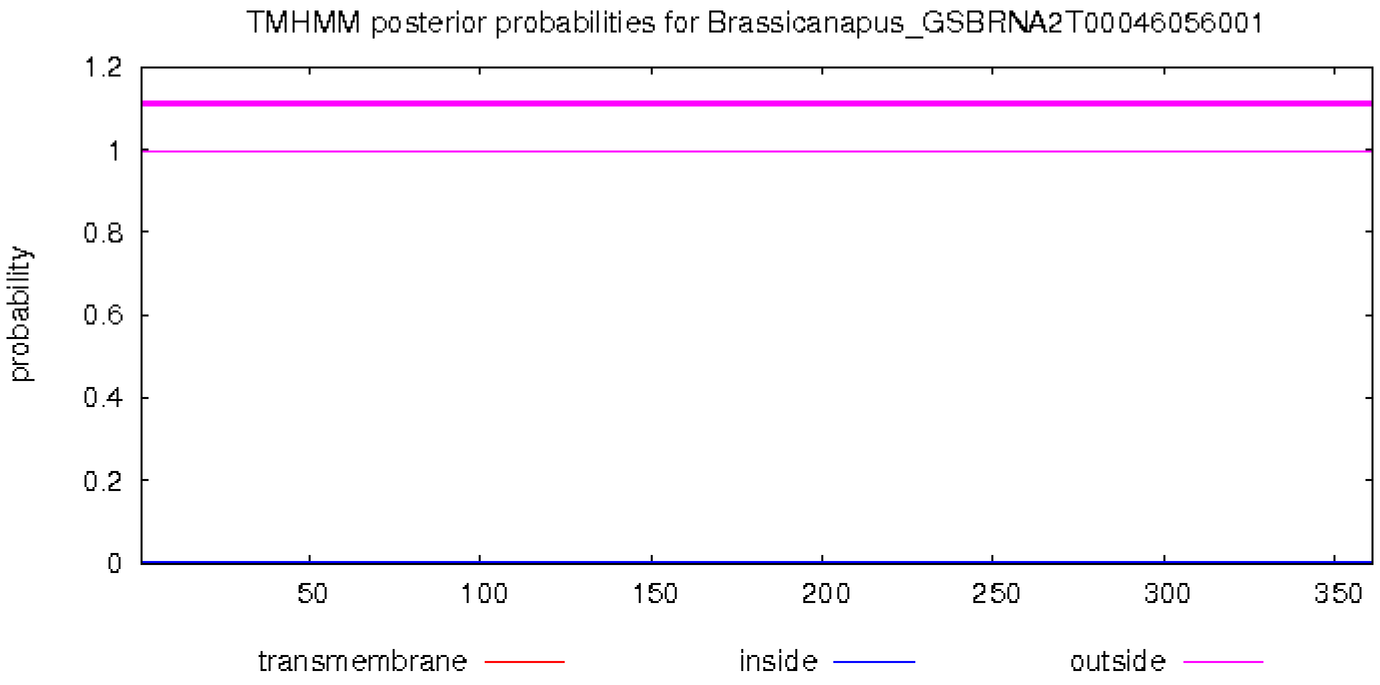

plot in postscript, script for making the plot in gnuplot, data for plot

---

```
# Brassicanapus_GSBRNA2T00046205001 Length: 295
# Brassicanapus_GSBRNA2T00046205001 Number of predicted TMHs:  0
# Brassicanapus_GSBRNA2T00046205001 Exp number of AAs in TMHs: 0.00018
# Brassicanapus_GSBRNA2T00046205001 Exp number, first 60 AAs:  0
# Brassicanapus_GSBRNA2T00046205001 Total prob of N-in:        0.01305
Brassicanapus_GSBRNA2T00046205001       TMHMM2.0        outside      1   295
```

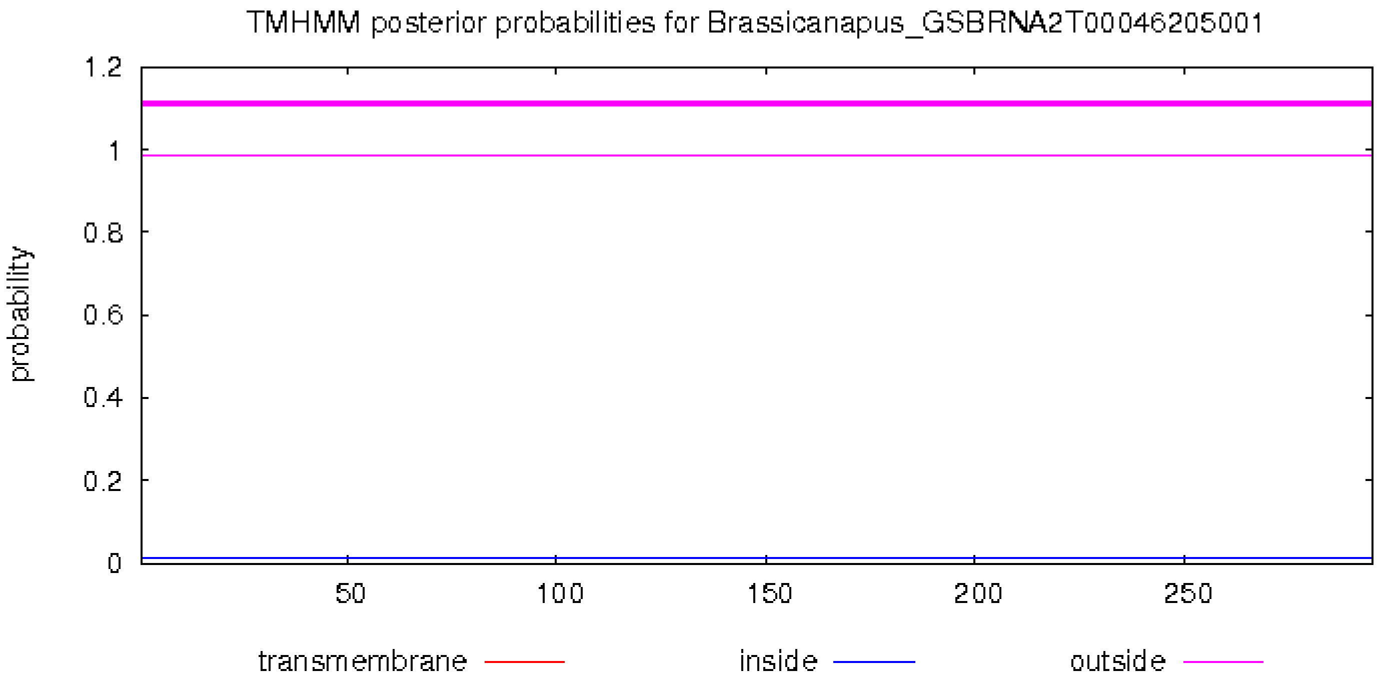

plot in postscript, script for making the plot in gnuplot, data for plot

---

```
# Brassicanapus_GSBRNA2T00046259001 Length: 312
# Brassicanapus_GSBRNA2T00046259001 Number of predicted TMHs:  0
# Brassicanapus_GSBRNA2T00046259001 Exp number of AAs in TMHs: 0.0002
# Brassicanapus_GSBRNA2T00046259001 Exp number, first 60 AAs:  0
```

```
# Brassicanapus_GSBRNA2T00046259001 Total prob of N-in:        0.02072
Brassicanapus_GSBRNA2T00046259001	TMHMM2.0	outside	     1   312
```

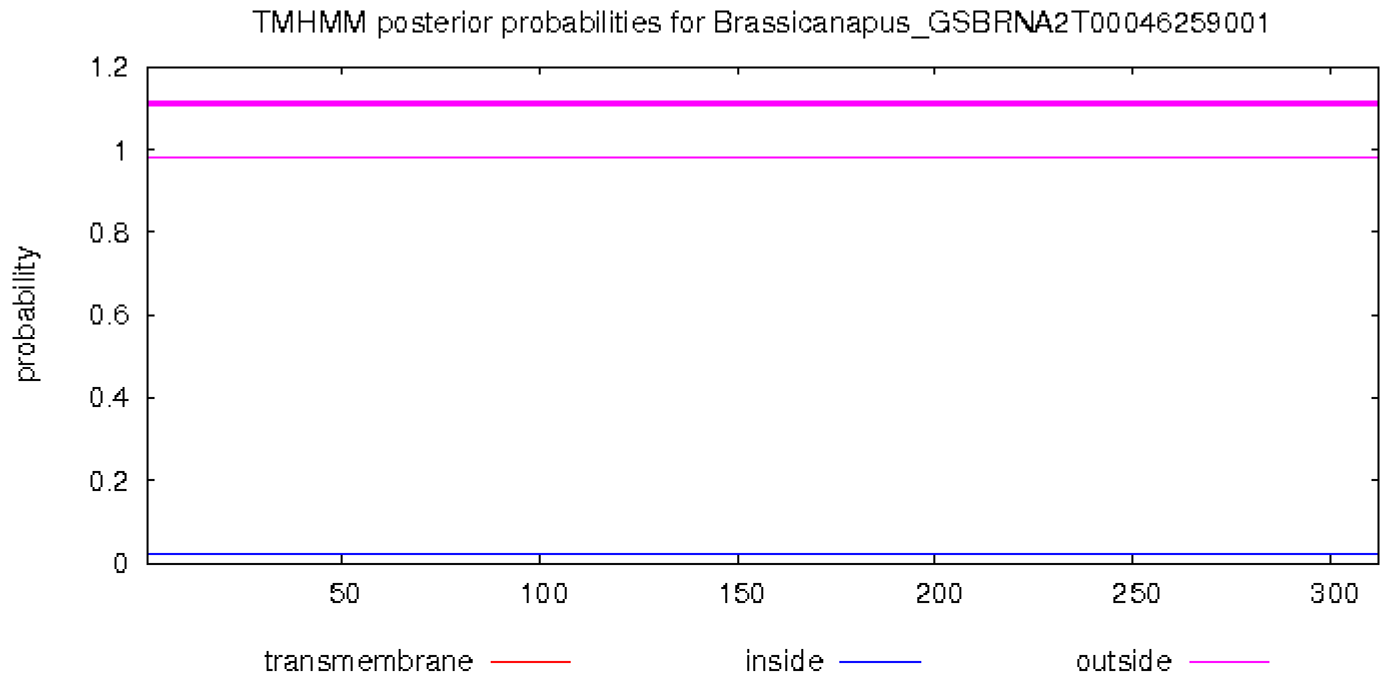

plot in postscript, script for making the plot in gnuplot, data for plot

---

```
# Brassicanapus_GSBRNA2T00046501001 Length: 243
# Brassicanapus_GSBRNA2T00046501001 Number of predicted TMHs:  0
# Brassicanapus_GSBRNA2T00046501001 Exp number of AAs in TMHs: 0.00947
# Brassicanapus_GSBRNA2T00046501001 Exp number, first 60 AAs:  0.00931
# Brassicanapus_GSBRNA2T00046501001 Total prob of N-in:        0.00385
Brassicanapus_GSBRNA2T00046501001	TMHMM2.0	outside	     1   243
```

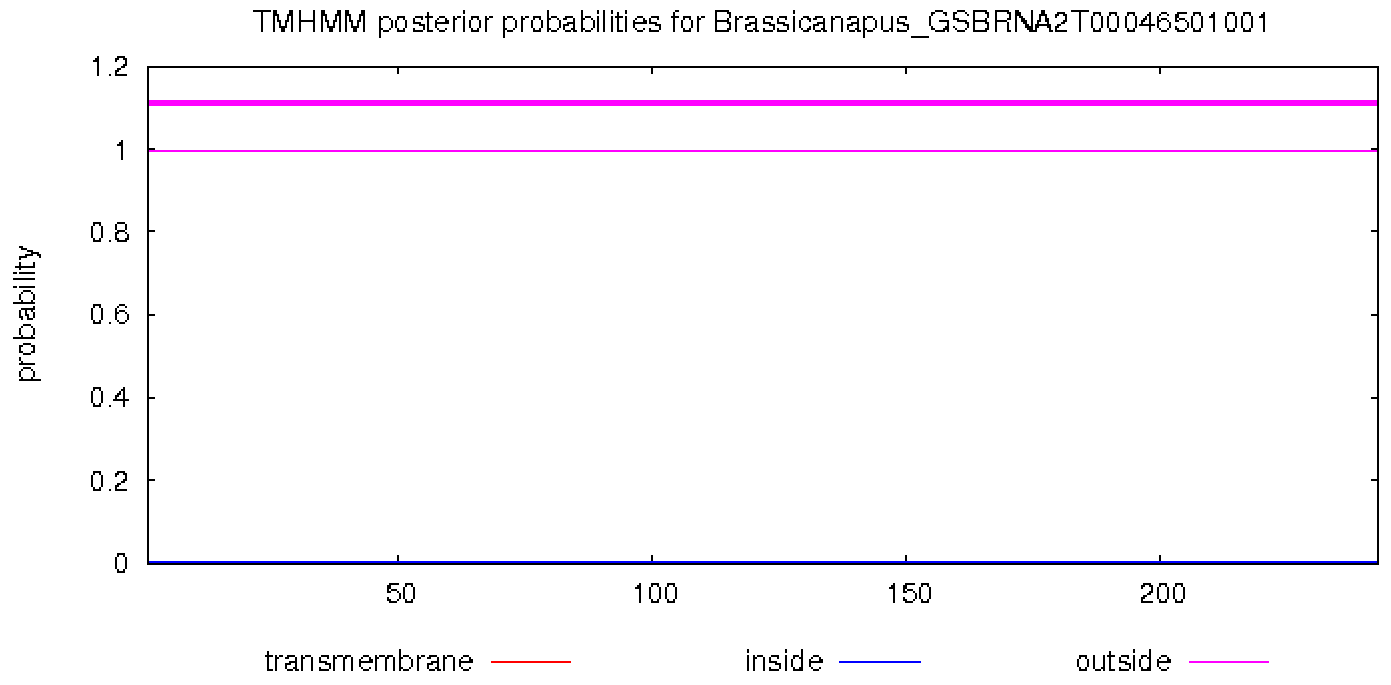

plot in postscript, script for making the plot in gnuplot, data for plot

---

```
# Brassicanapus_GSBRNA2T00047395001 Length: 365
# Brassicanapus_GSBRNA2T00047395001 Number of predicted TMHs:  0
# Brassicanapus_GSBRNA2T00047395001 Exp number of AAs in TMHs: 0.00018
# Brassicanapus_GSBRNA2T00047395001 Exp number, first 60 AAs:  0
```

```
# Brassicanapus_GSBRNA2T00047395001 Total prob of N-in:        0.00242
Brassicanapus_GSBRNA2T00047395001       TMHMM2.0        outside      1   365
```

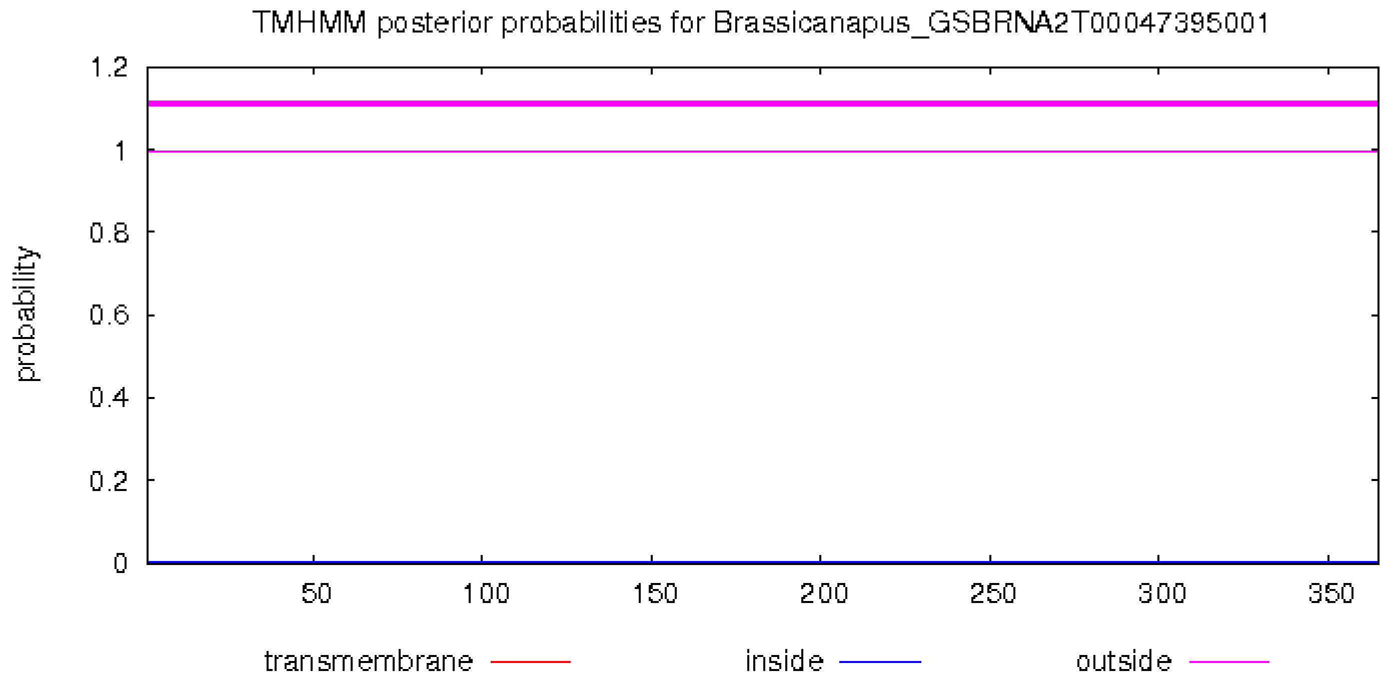

plot in postscript, script for making the plot in gnuplot, data for plot

---

```
# Brassicanapus_GSBRNA2T00047488001 Length: 185
# Brassicanapus_GSBRNA2T00047488001 Number of predicted TMHs:  0
# Brassicanapus_GSBRNA2T00047488001 Exp number of AAs in TMHs: 0
# Brassicanapus_GSBRNA2T00047488001 Exp number, first 60 AAs:  0
# Brassicanapus_GSBRNA2T00047488001 Total prob of N-in:        0.00752
Brassicanapus_GSBRNA2T00047488001       TMHMM2.0        outside      1   185
```

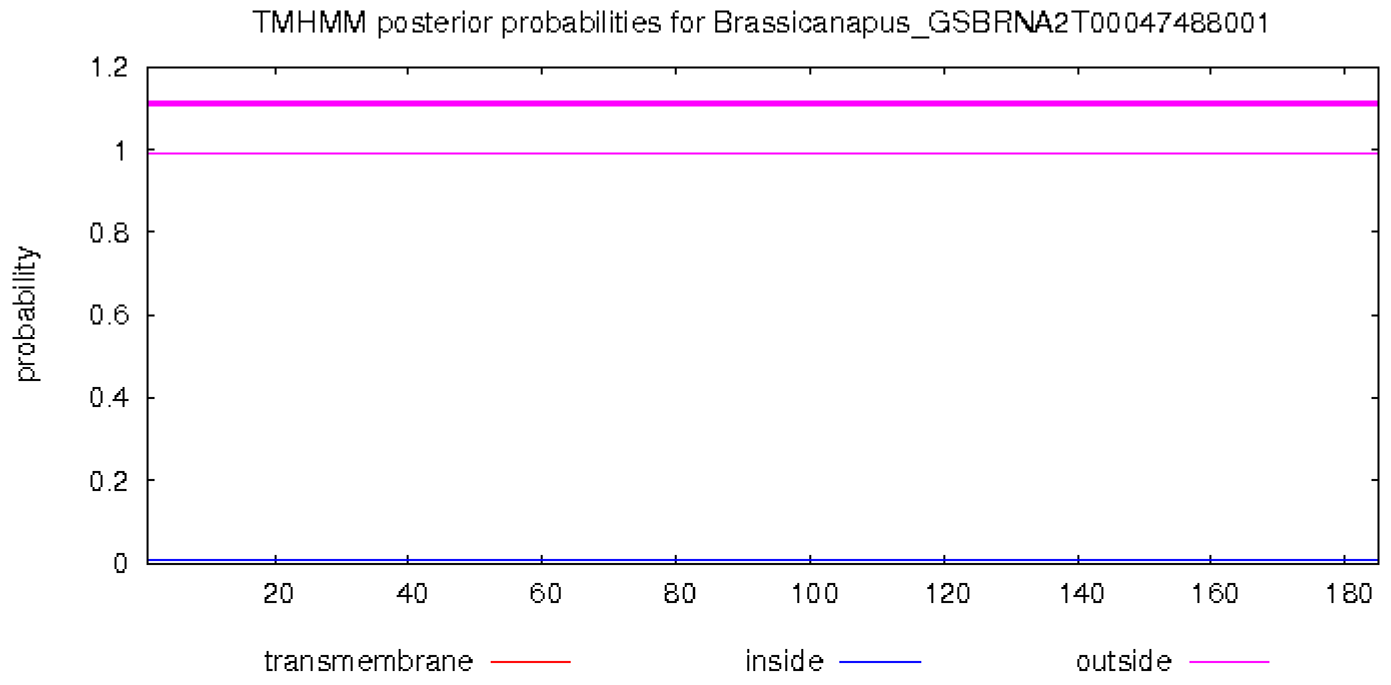

plot in postscript, script for making the plot in gnuplot, data for plot

---

```
# Brassicanapus_GSBRNA2T00047541001 Length: 265
# Brassicanapus_GSBRNA2T00047541001 Number of predicted TMHs:  0
# Brassicanapus_GSBRNA2T00047541001 Exp number of AAs in TMHs: 0
# Brassicanapus_GSBRNA2T00047541001 Exp number, first 60 AAs:  0
```

```
# Brassicanapus_GSBRNA2T00047541001 Total prob of N-in:        0.01285
Brassicanapus_GSBRNA2T00047541001	TMHMM2.0	outside	     1   265
```

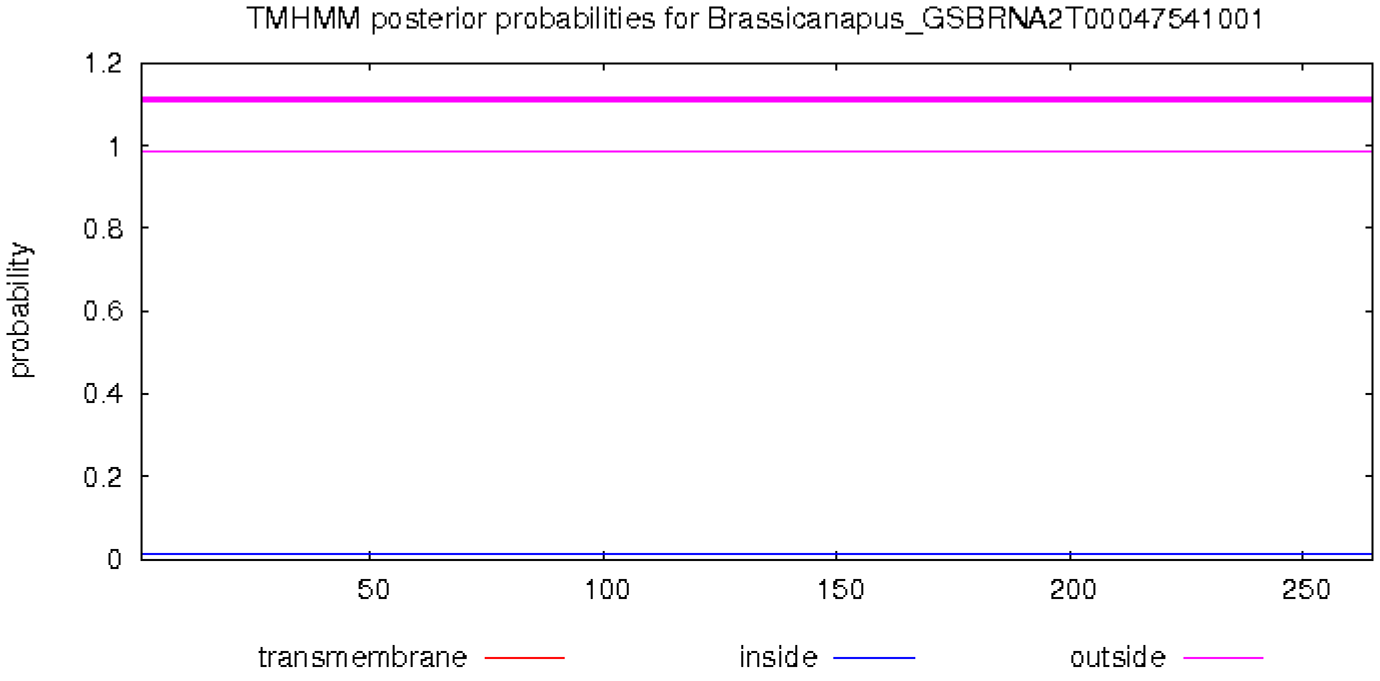

plot in postscript, script for making the plot in gnuplot, data for plot

---

```
# Brassicanapus_GSBRNA2T00047591001 Length: 319
# Brassicanapus_GSBRNA2T00047591001 Number of predicted TMHs:  0
# Brassicanapus_GSBRNA2T00047591001 Exp number of AAs in TMHs: 0.01372
# Brassicanapus_GSBRNA2T00047591001 Exp number, first 60 AAs:  0.00138
# Brassicanapus_GSBRNA2T00047591001 Total prob of N-in:        0.00256
Brassicanapus_GSBRNA2T00047591001	TMHMM2.0	outside	     1   319
```

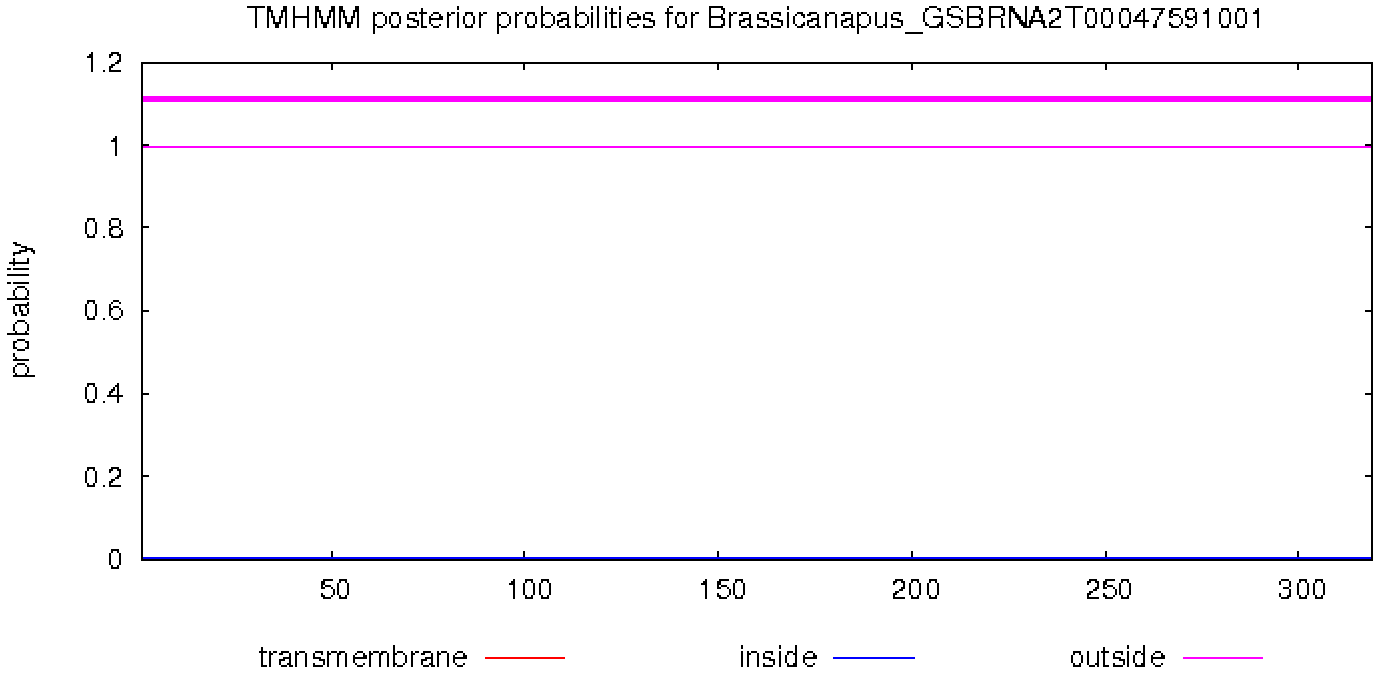

plot in postscript, script for making the plot in gnuplot, data for plot

---

```
# Brassicanapus_GSBRNA2T00049322001 Length: 566
# Brassicanapus_GSBRNA2T00049322001 Number of predicted TMHs:  1
# Brassicanapus_GSBRNA2T00049322001 Exp number of AAs in TMHs: 21.26993
# Brassicanapus_GSBRNA2T00049322001 Exp number, first 60 AAs:  0
```

```
# Brassicanapus_GSBRNA2T00049322001 Total prob of N-in:        0.00773
Brassicanapus_GSBRNA2T00049322001       TMHMM2.0        outside      1   540
Brassicanapus_GSBRNA2T00049322001       TMHMM2.0        TMhelix    541   563
Brassicanapus_GSBRNA2T00049322001       TMHMM2.0        inside     564   566
```

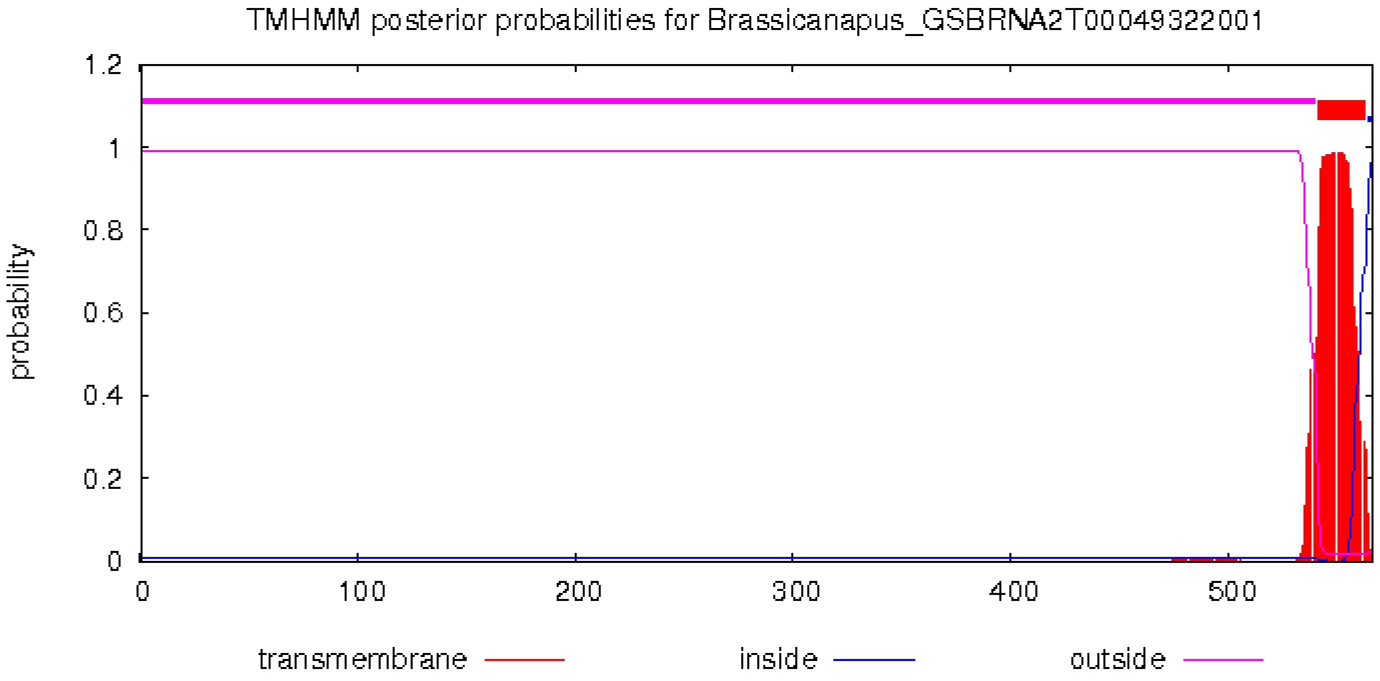

plot in postscript, script for making the plot in gnuplot, data for plot

---

```
# Brassicanapus_GSBRNA2T00049441001 Length: 322
# Brassicanapus_GSBRNA2T00049441001 Number of predicted TMHs:  0
# Brassicanapus_GSBRNA2T00049441001 Exp number of AAs in TMHs: 0
# Brassicanapus_GSBRNA2T00049441001 Exp number, first 60 AAs:  0
# Brassicanapus_GSBRNA2T00049441001 Total prob of N-in:        0.03347
Brassicanapus_GSBRNA2T00049441001       TMHMM2.0        outside      1   322
```

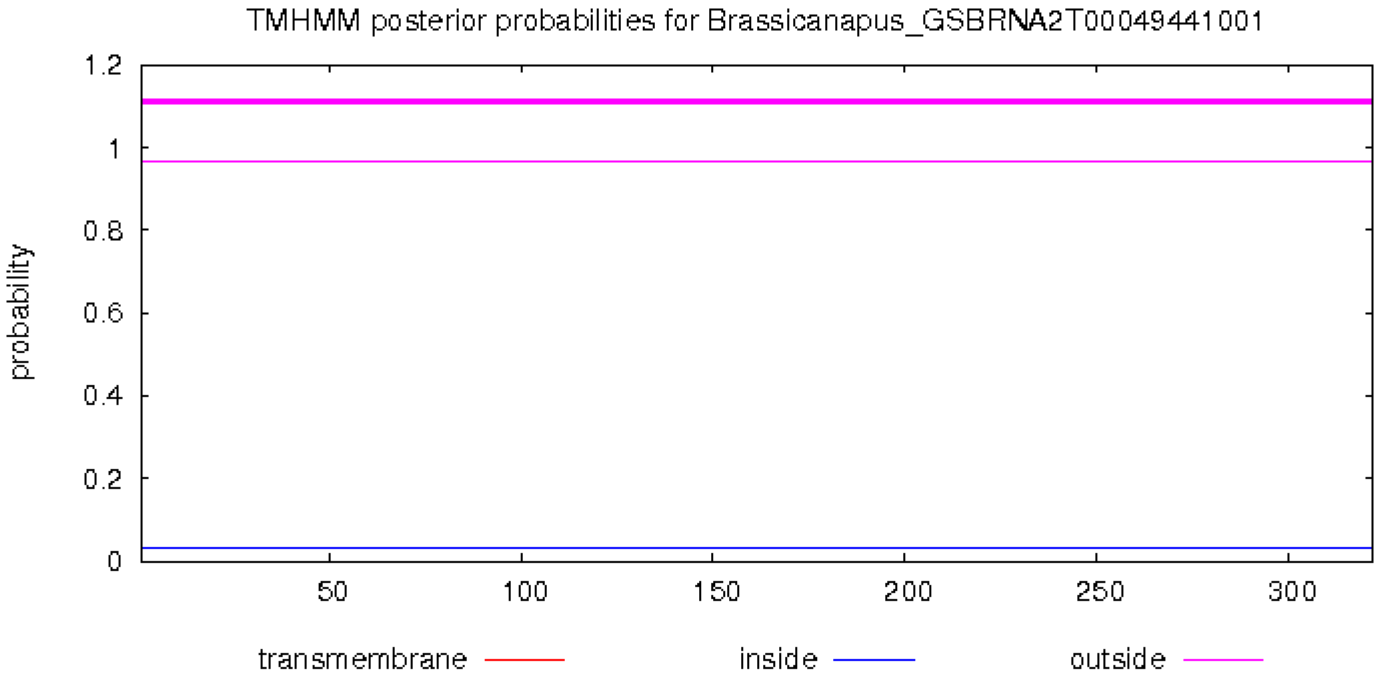

plot in postscript, script for making the plot in gnuplot, data for plot

---

```
# Brassicanapus_GSBRNA2T00049808001 Length: 335
# Brassicanapus_GSBRNA2T00049808001 Number of predicted TMHs:  0
```

```
# Brassicanapus_GSBRNA2T00049808001 Exp number of AAs in TMHs: 0.00621
# Brassicanapus_GSBRNA2T00049808001 Exp number, first 60 AAs:  0
# Brassicanapus_GSBRNA2T00049808001 Total prob of N-in:        0.02926
Brassicanapus_GSBRNA2T00049808001       TMHMM2.0        outside      1   335
```

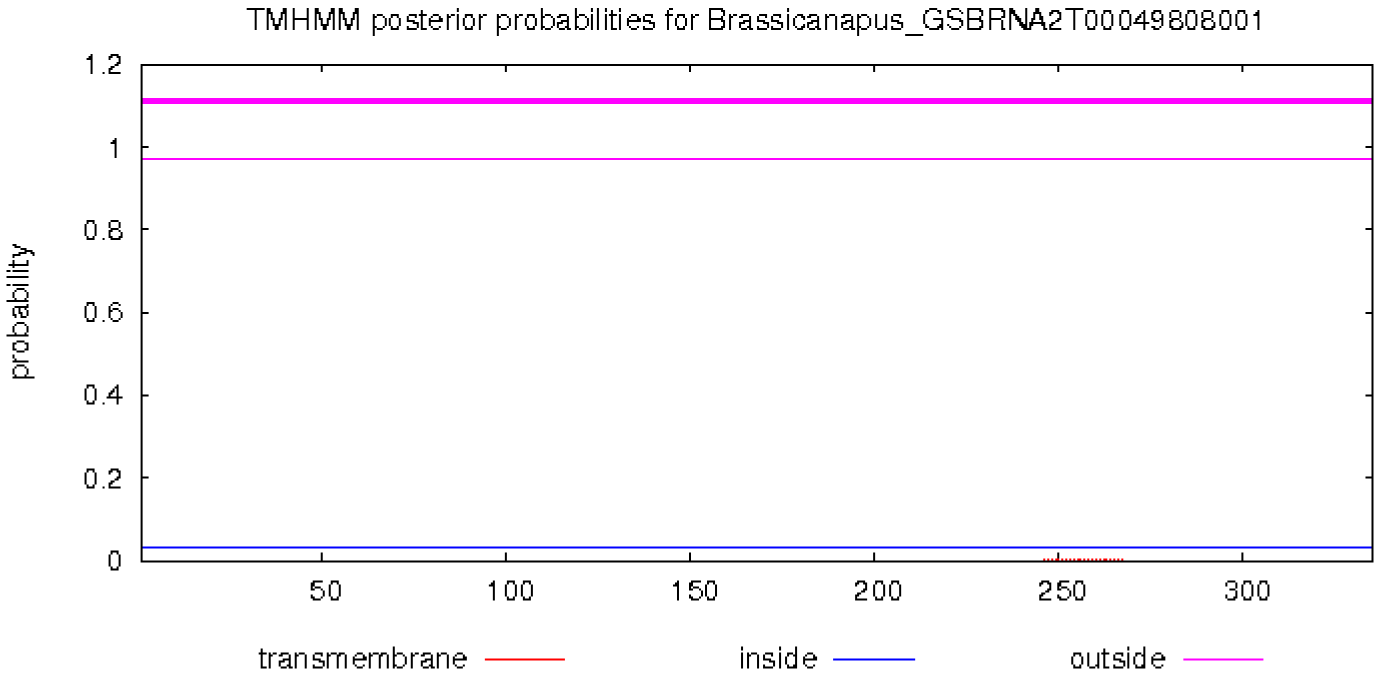

plot in postscript, script for making the plot in gnuplot, data for plot

---

```
# Brassicanapus_GSBRNA2T00049948001 Length: 198
# Brassicanapus_GSBRNA2T00049948001 Number of predicted TMHs:  0
# Brassicanapus_GSBRNA2T00049948001 Exp number of AAs in TMHs: 0
# Brassicanapus_GSBRNA2T00049948001 Exp number, first 60 AAs:  0
# Brassicanapus_GSBRNA2T00049948001 Total prob of N-in:        0.04816
Brassicanapus_GSBRNA2T00049948001       TMHMM2.0        outside      1   198
```

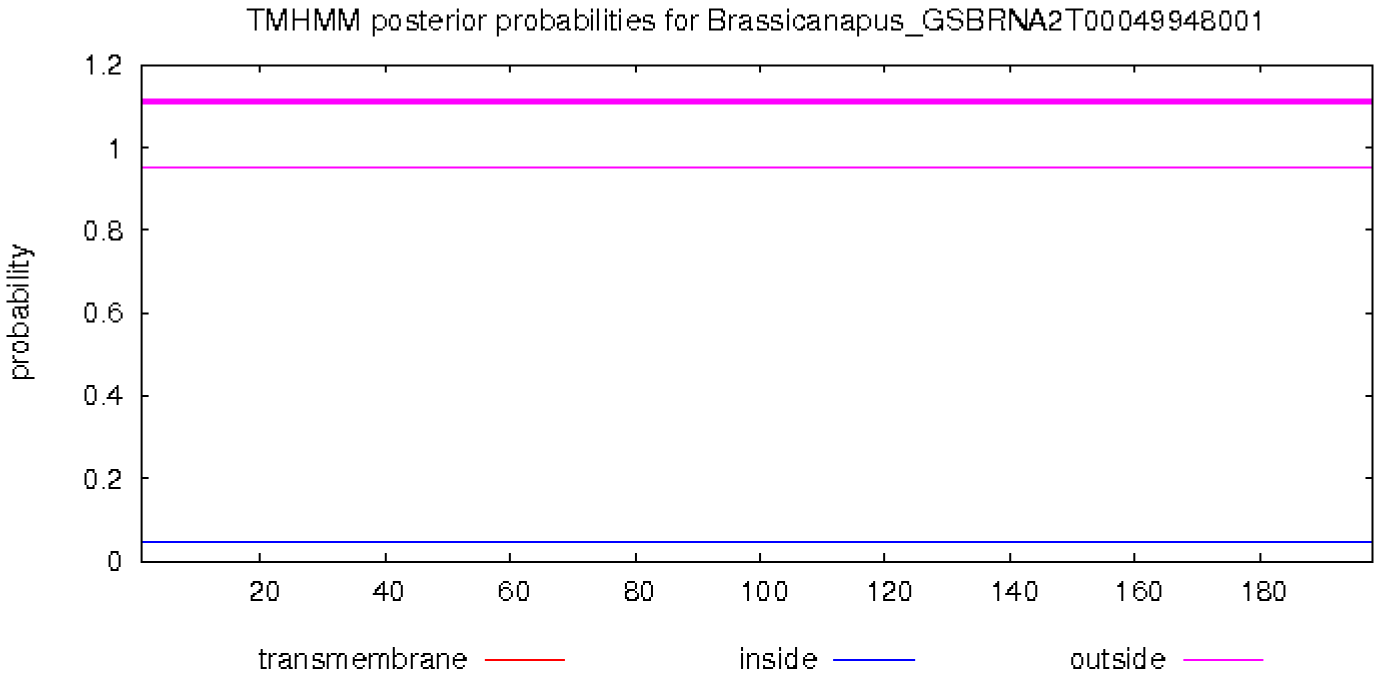

plot in postscript, script for making the plot in gnuplot, data for plot

---

```
# Brassicanapus_GSBRNA2T00049955001 Length: 139
# Brassicanapus_GSBRNA2T00049955001 Number of predicted TMHs:  0
```

```
# Brassicanapus_GSBRNA2T00049955001 Exp number of AAs in TMHs: 0.00393
# Brassicanapus_GSBRNA2T00049955001 Exp number, first 60 AAs:  0.00011
# Brassicanapus_GSBRNA2T00049955001 Total prob of N-in:        0.36224
Brassicanapus_GSBRNA2T00049955001       TMHMM2.0        outside      1   139
```

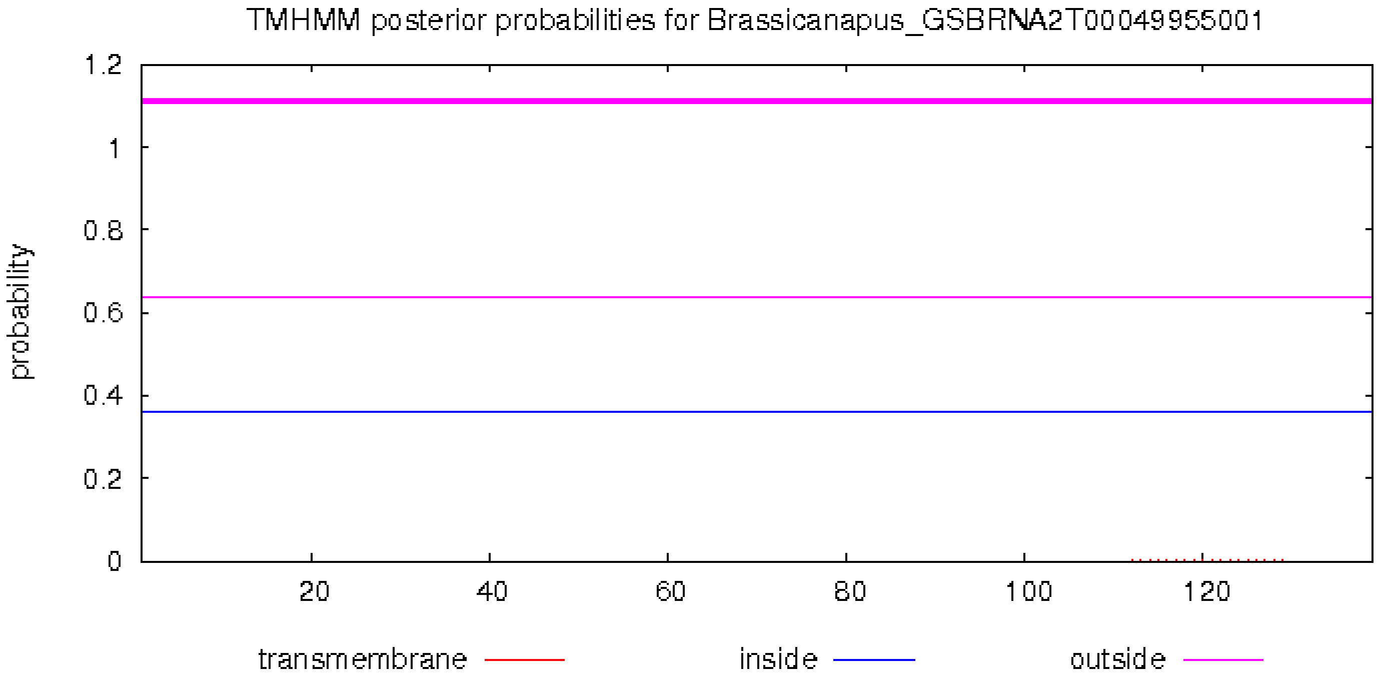

plot in postscript, script for making the plot in gnuplot, data for plot

---

```
# Brassicanapus_GSBRNA2T00050536001 Length: 361
# Brassicanapus_GSBRNA2T00050536001 Number of predicted TMHs:  0
# Brassicanapus_GSBRNA2T00050536001 Exp number of AAs in TMHs: 0.00018
# Brassicanapus_GSBRNA2T00050536001 Exp number, first 60 AAs:  0
# Brassicanapus_GSBRNA2T00050536001 Total prob of N-in:        0.00273
Brassicanapus_GSBRNA2T00050536001       TMHMM2.0        outside      1   361
```

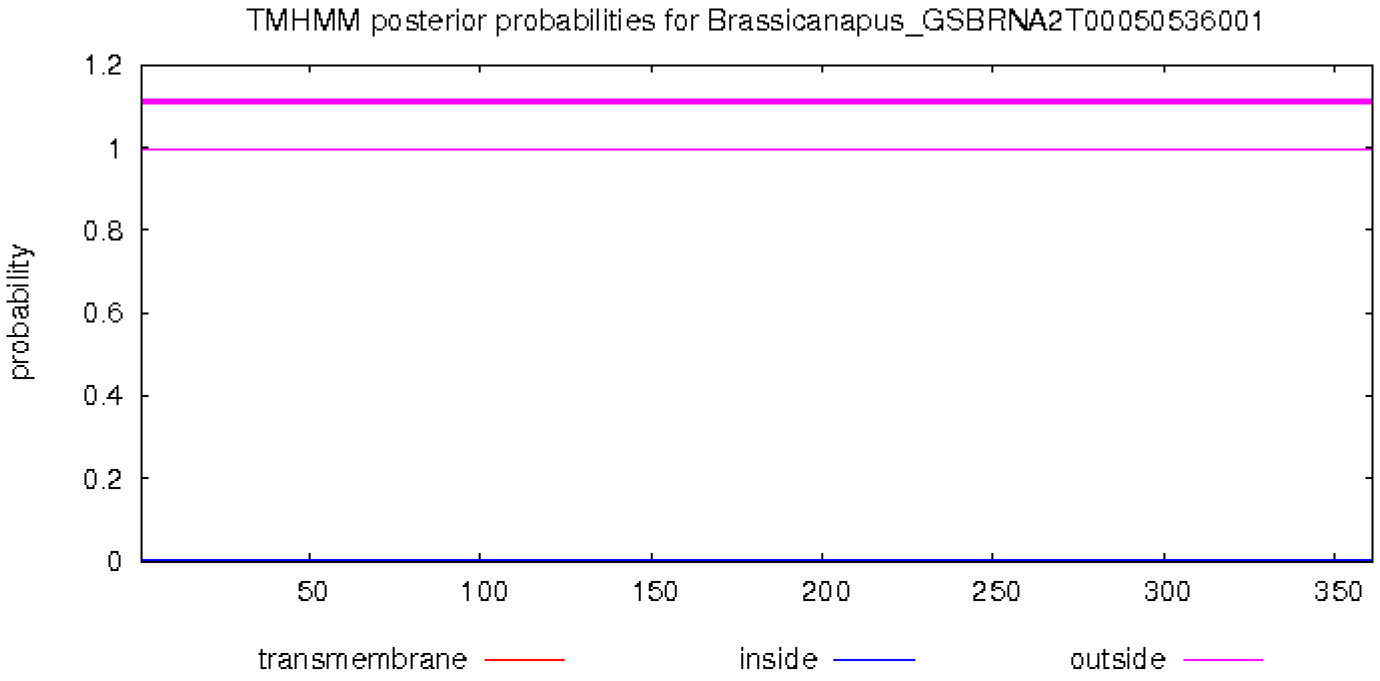

plot in postscript, script for making the plot in gnuplot, data for plot

---

```
# Brassicanapus_GSBRNA2T00050859001 Length: 354
# Brassicanapus_GSBRNA2T00050859001 Number of predicted TMHs:  0
```

```
# Brassicanapus_GSBRNA2T00050859001 Exp number of AAs in TMHs: 0.01244
# Brassicanapus_GSBRNA2T00050859001 Exp number, first 60 AAs:  0.00013
# Brassicanapus_GSBRNA2T00050859001 Total prob of N-in:        0.02691
Brassicanapus_GSBRNA2T00050859001	TMHMM2.0	outside	     1   354
```

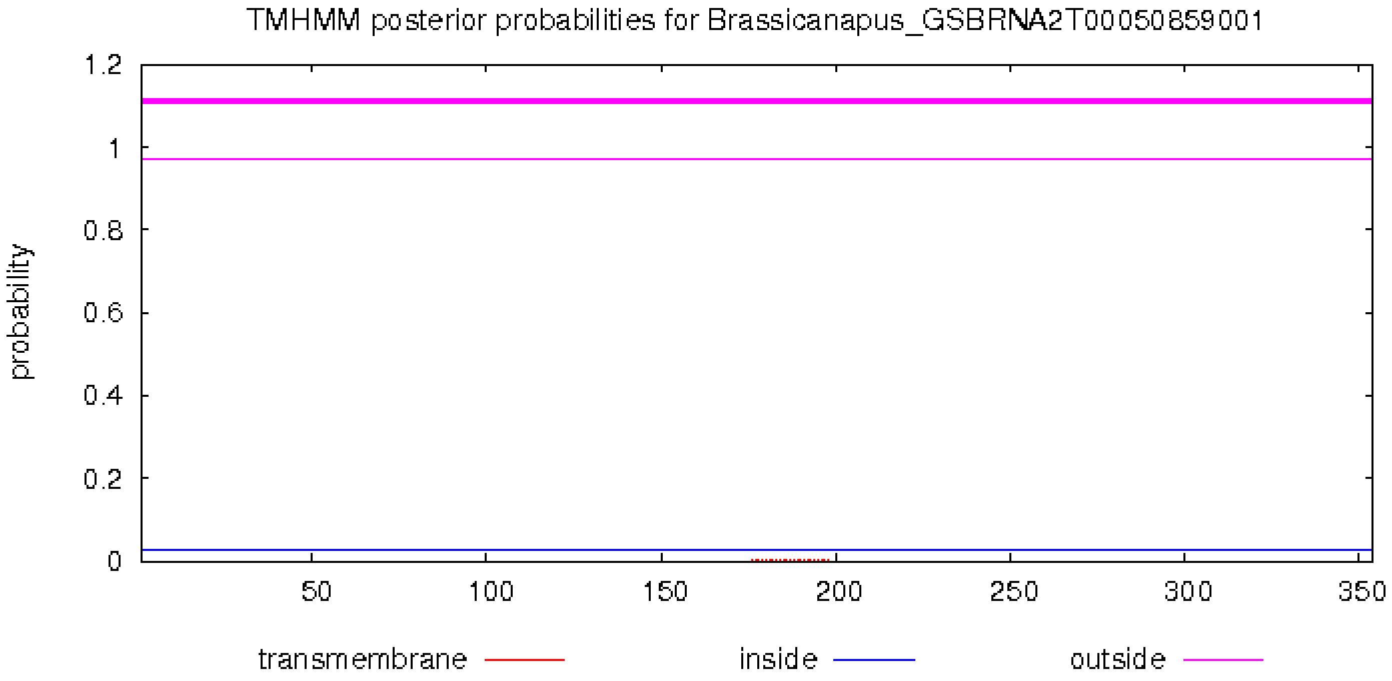

plot in postscript, script for making the plot in gnuplot, data for plot

---

```
# Brassicanapus_GSBRNA2T00050907001 Length: 376
# Brassicanapus_GSBRNA2T00050907001 Number of predicted TMHs:  0
# Brassicanapus_GSBRNA2T00050907001 Exp number of AAs in TMHs: 0.00066
# Brassicanapus_GSBRNA2T00050907001 Exp number, first 60 AAs:  0
# Brassicanapus_GSBRNA2T00050907001 Total prob of N-in:        0.00463
Brassicanapus_GSBRNA2T00050907001	TMHMM2.0	outside	     1   376
```

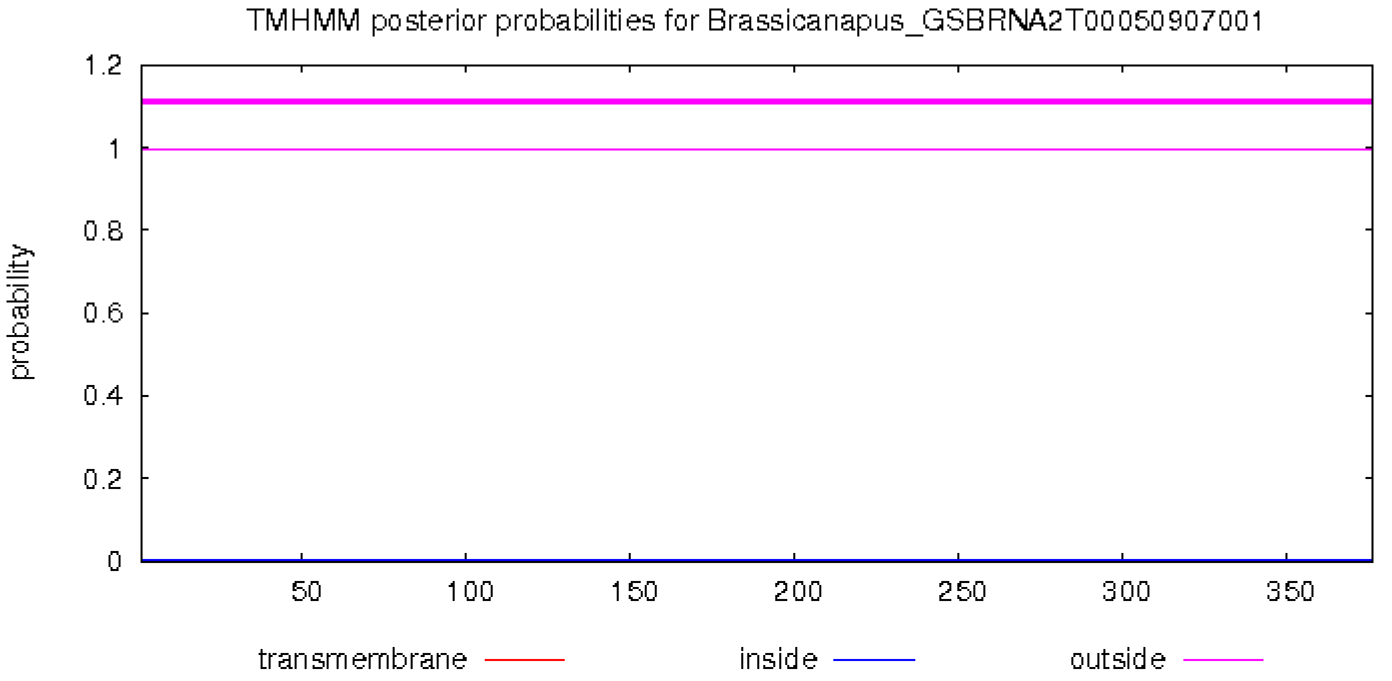

plot in postscript, script for making the plot in gnuplot, data for plot

---

```
# Brassicanapus_GSBRNA2T00051194001 Length: 259
# Brassicanapus_GSBRNA2T00051194001 Number of predicted TMHs:  0
```

```
# Brassicanapus_GSBRNA2T00051194001 Exp number of AAs in TMHs: 0.00839
# Brassicanapus_GSBRNA2T00051194001 Exp number, first 60 AAs:  0.00515
# Brassicanapus_GSBRNA2T00051194001 Total prob of N-in:        0.07826
Brassicanapus_GSBRNA2T00051194001       TMHMM2.0        outside      1   259
```

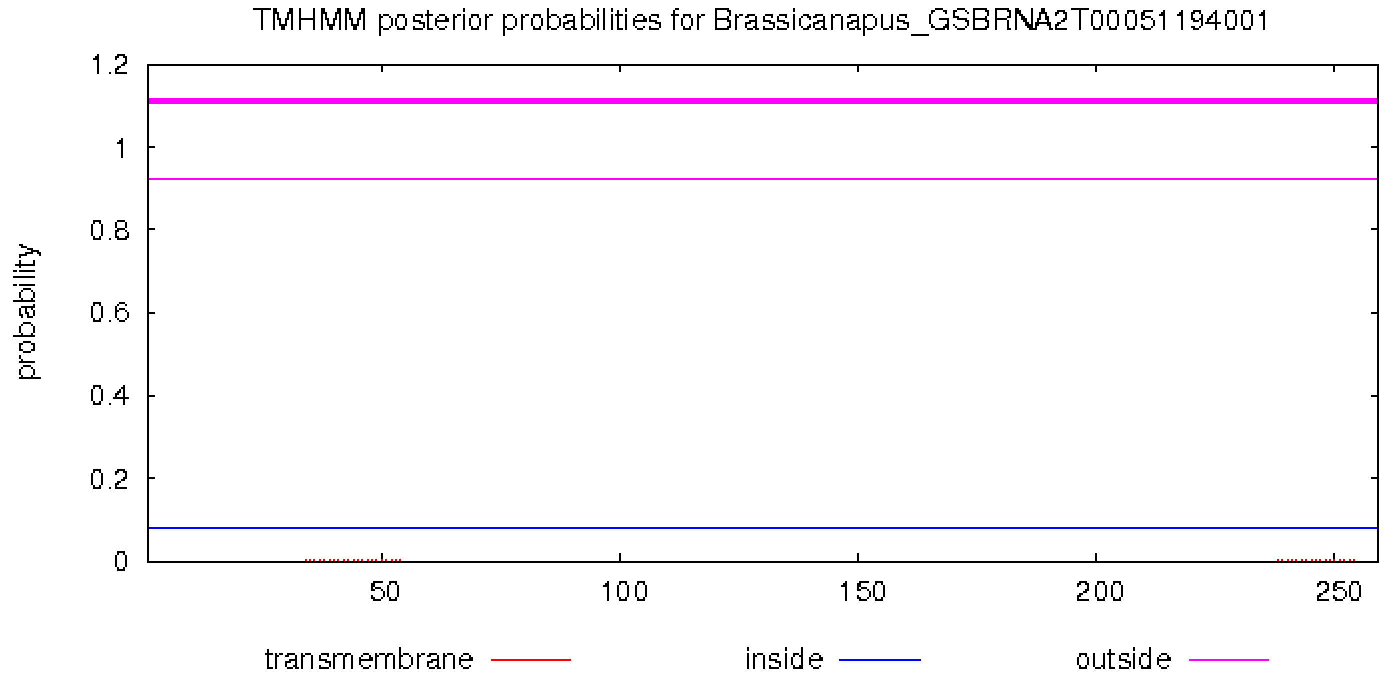

plot in postscript, script for making the plot in gnuplot, data for plot

---

```
# Brassicanapus_GSBRNA2T00051253001 Length: 286
# Brassicanapus_GSBRNA2T00051253001 Number of predicted TMHs:  0
# Brassicanapus_GSBRNA2T00051253001 Exp number of AAs in TMHs: 0.00029
# Brassicanapus_GSBRNA2T00051253001 Exp number, first 60 AAs:  0
# Brassicanapus_GSBRNA2T00051253001 Total prob of N-in:        0.00682
Brassicanapus_GSBRNA2T00051253001       TMHMM2.0        outside      1   286
```

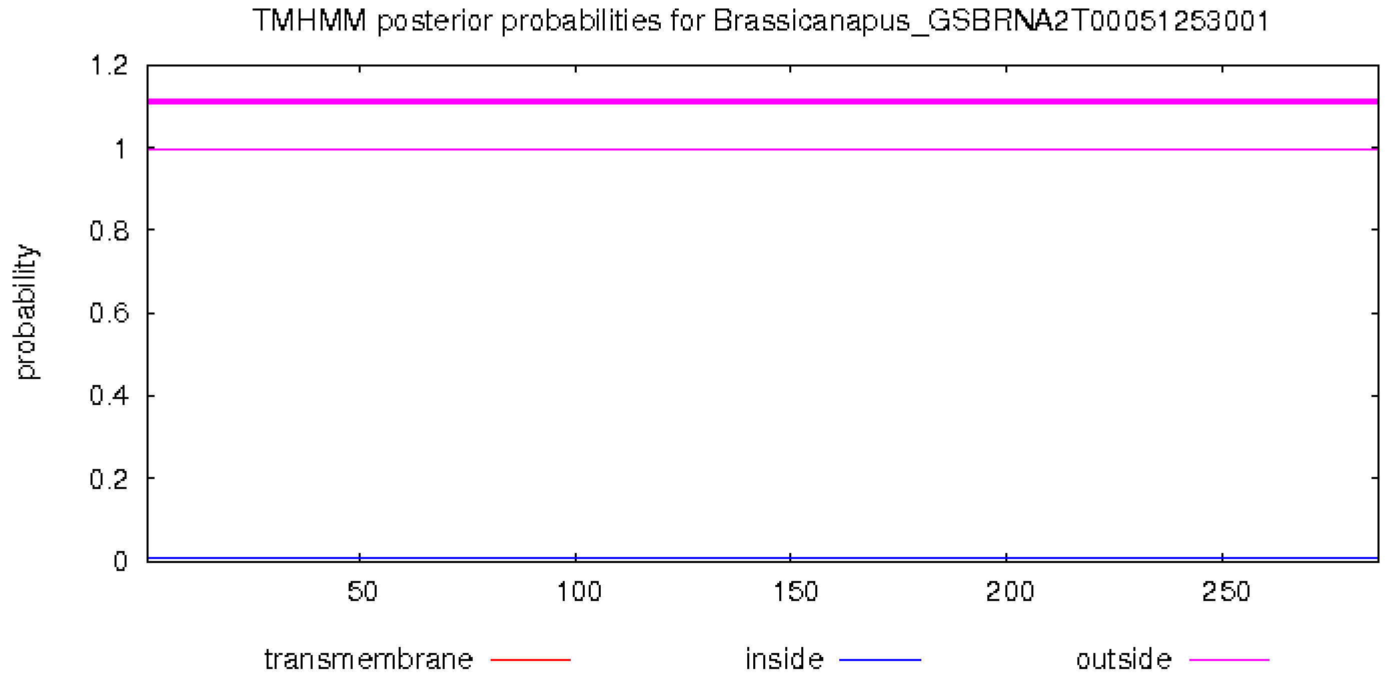

plot in postscript, script for making the plot in gnuplot, data for plot

---

```
# Brassicanapus_GSBRNA2T00051813001 Length: 307
# Brassicanapus_GSBRNA2T00051813001 Number of predicted TMHs:  0
```

```
# Brassicanapus_GSBRNA2T00051813001 Exp number of AAs in TMHs: 0.00192
# Brassicanapus_GSBRNA2T00051813001 Exp number, first 60 AAs:  0.00041
# Brassicanapus_GSBRNA2T00051813001 Total prob of N-in:        0.01003
Brassicanapus_GSBRNA2T00051813001       TMHMM2.0        outside      1   307
```

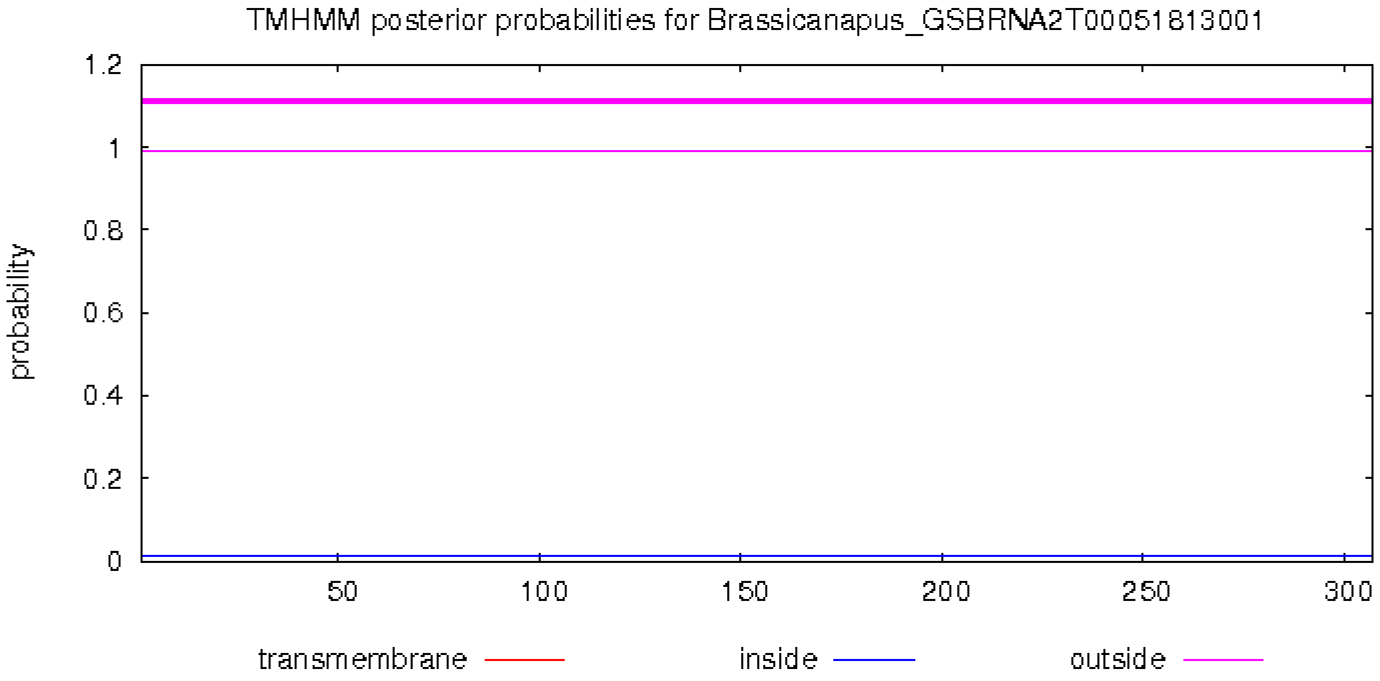

plot in postscript, script for making the plot in gnuplot, data for plot

---

```
# Brassicanapus_GSBRNA2T00052195001 Length: 635
# Brassicanapus_GSBRNA2T00052195001 Number of predicted TMHs:  1
# Brassicanapus_GSBRNA2T00052195001 Exp number of AAs in TMHs: 17.50902
# Brassicanapus_GSBRNA2T00052195001 Exp number, first 60 AAs:  0
# Brassicanapus_GSBRNA2T00052195001 Total prob of N-in:        0.10749
Brassicanapus_GSBRNA2T00052195001       TMHMM2.0        outside      1   594
Brassicanapus_GSBRNA2T00052195001       TMHMM2.0        TMhelix    595   612
Brassicanapus_GSBRNA2T00052195001       TMHMM2.0        inside     613   635
```

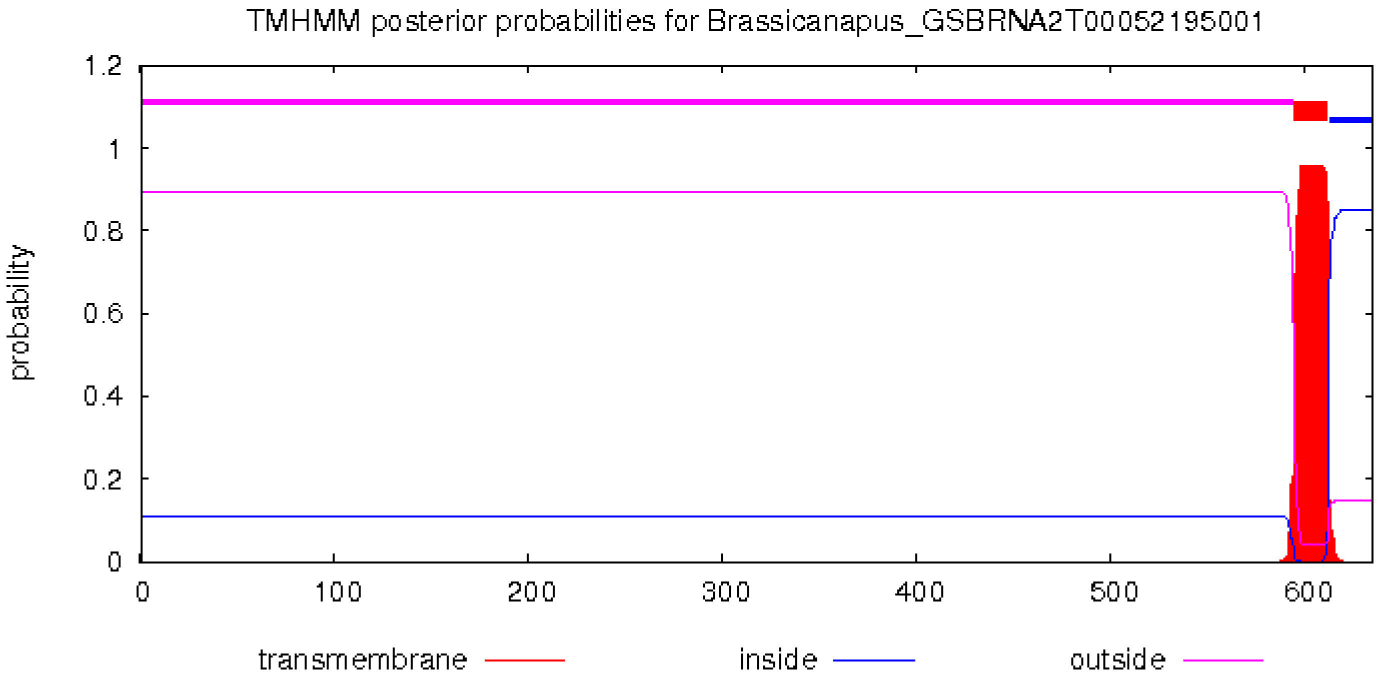

plot in postscript, script for making the plot in gnuplot, data for plot

---

```
# Brassicanapus_GSBRNA2T00052418001 Length: 224
# Brassicanapus_GSBRNA2T00052418001 Number of predicted TMHs:  0
# Brassicanapus_GSBRNA2T00052418001 Exp number of AAs in TMHs: 0
# Brassicanapus_GSBRNA2T00052418001 Exp number, first 60 AAs:  0
# Brassicanapus_GSBRNA2T00052418001 Total prob of N-in:        0.03474
Brassicanapus_GSBRNA2T00052418001       TMHMM2.0        outside      1   224
```

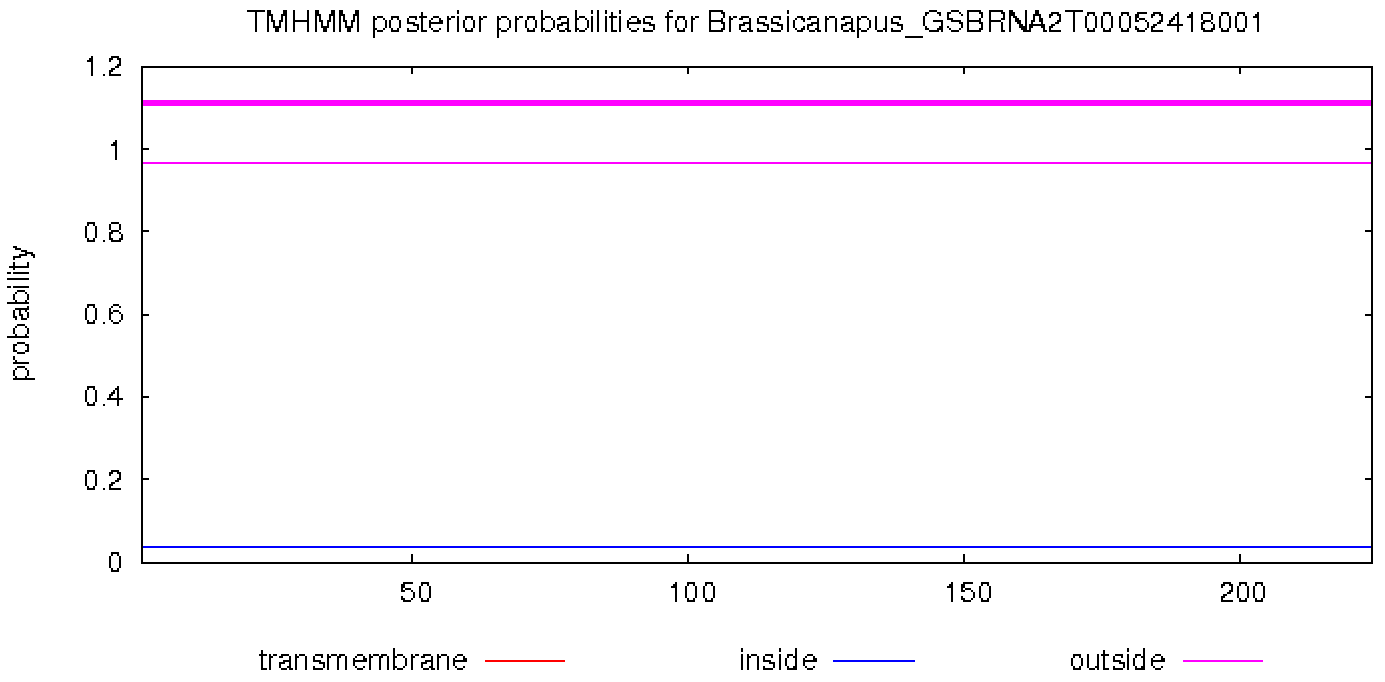

plot in postscript, script for making the plot in gnuplot, data for plot

---

```
# Brassicanapus_GSBRNA2T00052419001 Length: 224
# Brassicanapus_GSBRNA2T00052419001 Number of predicted TMHs:  0
# Brassicanapus_GSBRNA2T00052419001 Exp number of AAs in TMHs: 0
# Brassicanapus_GSBRNA2T00052419001 Exp number, first 60 AAs:  0
# Brassicanapus_GSBRNA2T00052419001 Total prob of N-in:        0.03474
Brassicanapus_GSBRNA2T00052419001       TMHMM2.0        outside      1   224
```

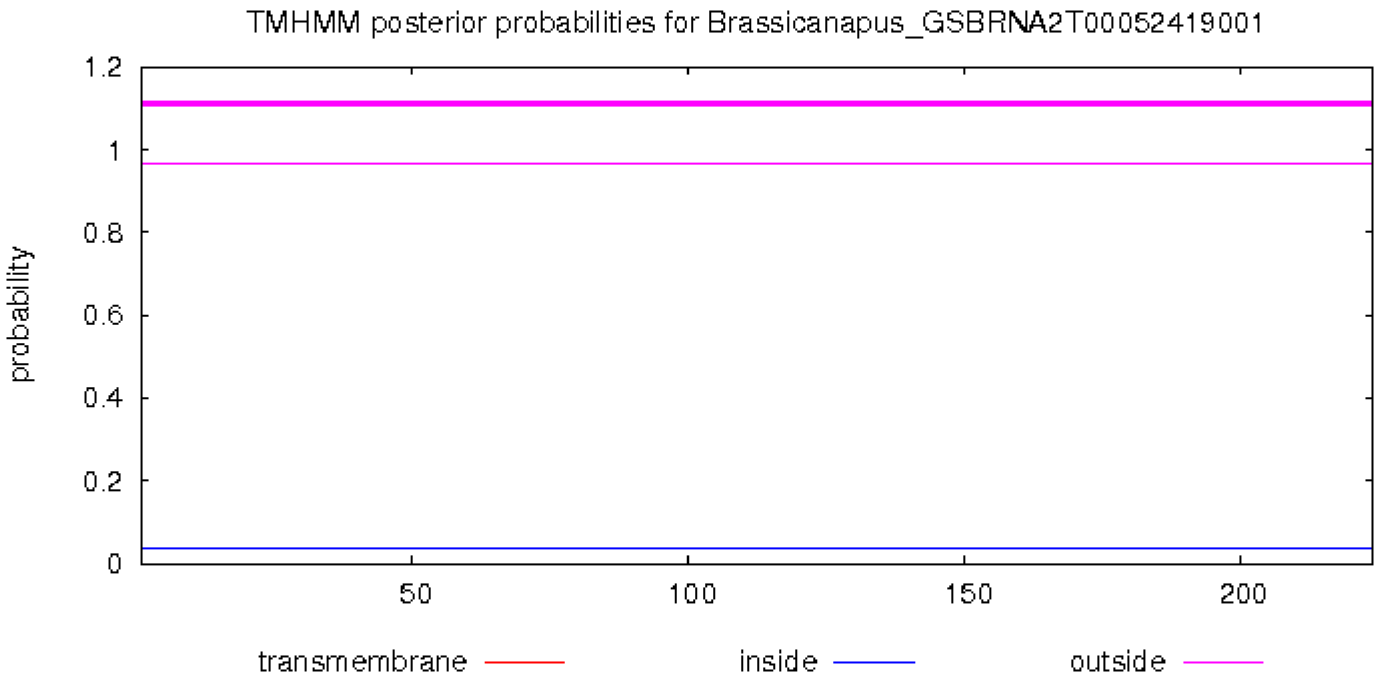

plot in postscript, script for making the plot in gnuplot, data for plot

---

```
# Brassicanapus_GSBRNA2T00053001001 Length: 401
# Brassicanapus_GSBRNA2T00053001001 Number of predicted TMHs:  0
# Brassicanapus_GSBRNA2T00053001001 Exp number of AAs in TMHs: 0.00088
# Brassicanapus_GSBRNA2T00053001001 Exp number, first 60 AAs:  0
# Brassicanapus_GSBRNA2T00053001001 Total prob of N-in:        0.00926
Brassicanapus_GSBRNA2T00053001001       TMHMM2.0        outside      1   401
```

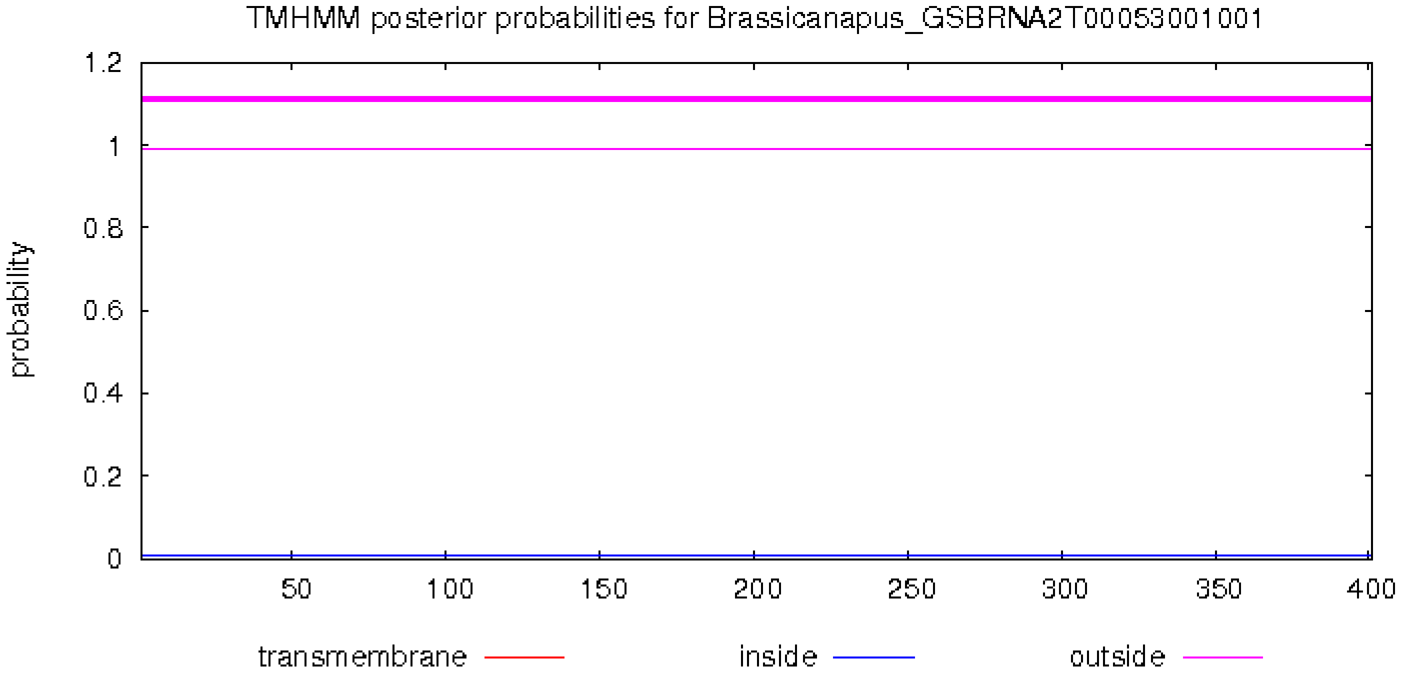

plot in postscript, script for making the plot in gnuplot, data for plot

---

```
# Brassicanapus_GSBRNA2T00056083001 Length: 175
# Brassicanapus_GSBRNA2T00056083001 Number of predicted TMHs:  0
# Brassicanapus_GSBRNA2T00056083001 Exp number of AAs in TMHs: 0.00013
# Brassicanapus_GSBRNA2T00056083001 Exp number, first 60 AAs:  0
# Brassicanapus_GSBRNA2T00056083001 Total prob of N-in:        0.06576
Brassicanapus_GSBRNA2T00056083001       TMHMM2.0        outside      1   175
```

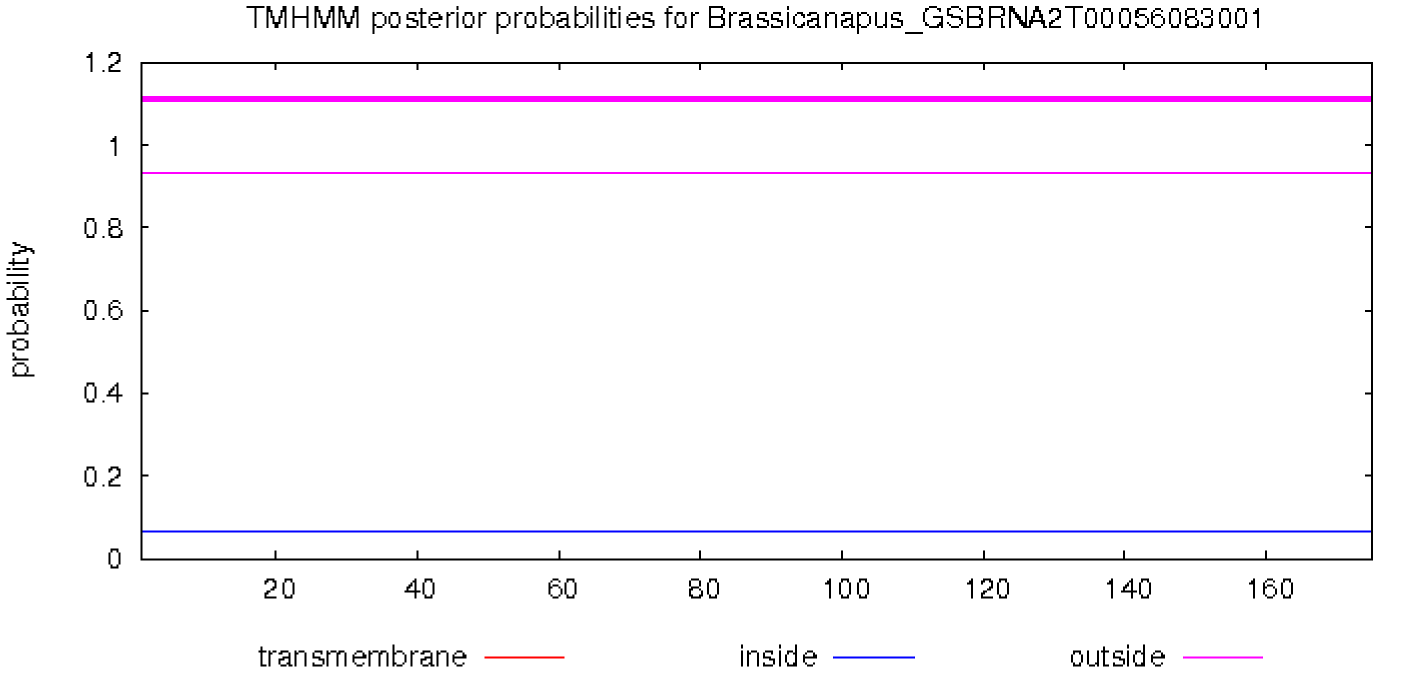

plot in postscript, script for making the plot in gnuplot, data for plot

---

```
# Brassicanapus_GSBRNA2T00057235001 Length: 338
# Brassicanapus_GSBRNA2T00057235001 Number of predicted TMHs:  1
# Brassicanapus_GSBRNA2T00057235001 Exp number of AAs in TMHs: 18.16979
# Brassicanapus_GSBRNA2T00057235001 Exp number, first 60 AAs:  0.26313
# Brassicanapus_GSBRNA2T00057235001 Total prob of N-in:        0.32749
Brassicanapus_GSBRNA2T00057235001       TMHMM2.0        outside      1   319
Brassicanapus_GSBRNA2T00057235001       TMHMM2.0        TMhelix    320   337
Brassicanapus_GSBRNA2T00057235001       TMHMM2.0        inside     338   338
```

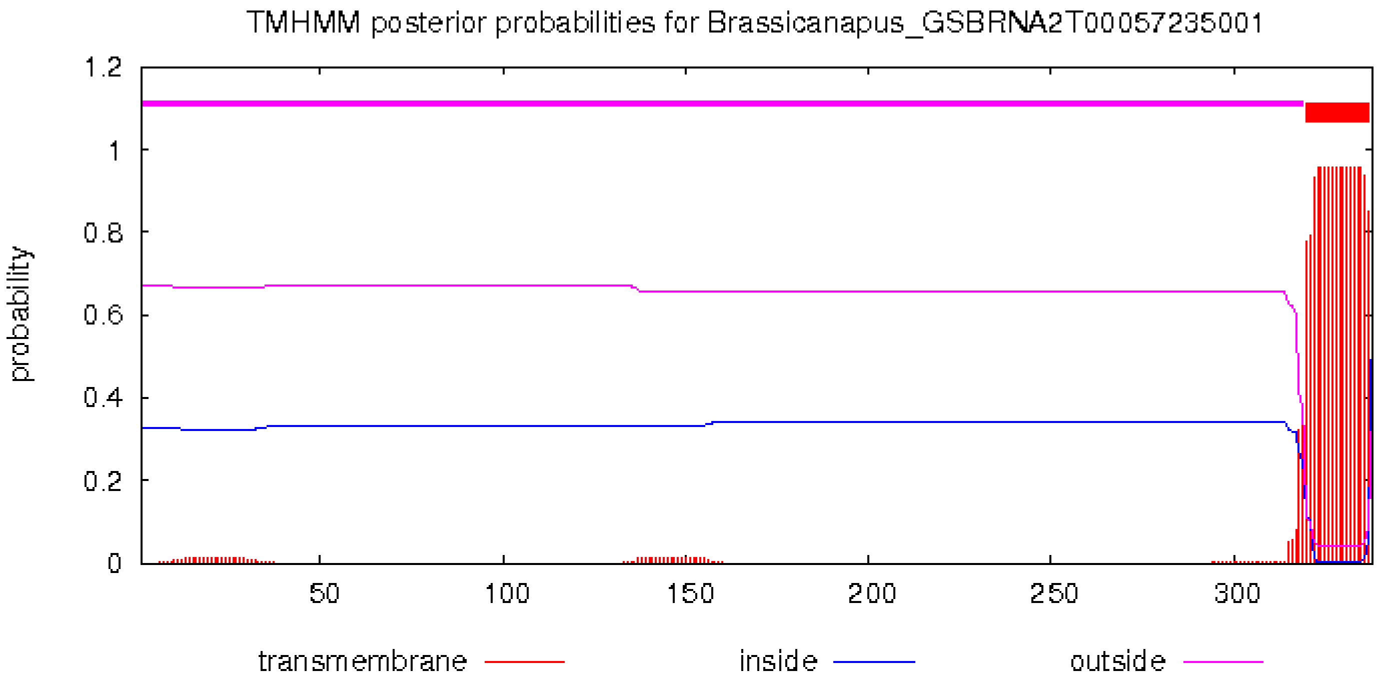

plot in postscript, script for making the plot in gnuplot, data for plot

---

```
# Brassicanapus_GSBRNA2T00057237001 Length: 294
# Brassicanapus_GSBRNA2T00057237001 Number of predicted TMHs:  1
# Brassicanapus_GSBRNA2T00057237001 Exp number of AAs in TMHs: 18.00772
# Brassicanapus_GSBRNA2T00057237001 Exp number, first 60 AAs:  0
# Brassicanapus_GSBRNA2T00057237001 Total prob of N-in:        0.56000
Brassicanapus_GSBRNA2T00057237001       TMHMM2.0        inside       1   275
Brassicanapus_GSBRNA2T00057237001       TMHMM2.0        TMhelix    276   293
Brassicanapus_GSBRNA2T00057237001       TMHMM2.0        outside    294   294
```

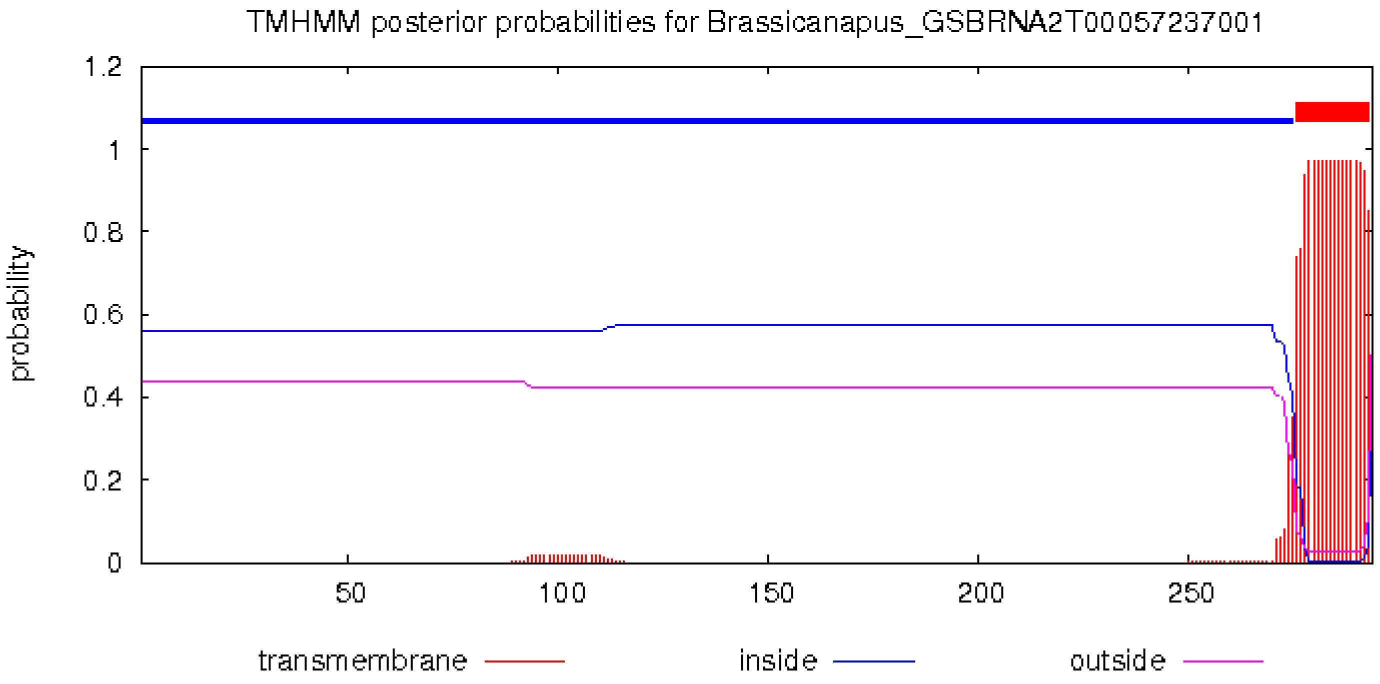

plot in postscript, script for making the plot in gnuplot, data for plot

---

```
# Brassicanapus_GSBRNA2T00057317001 Length: 317
# Brassicanapus_GSBRNA2T00057317001 Number of predicted TMHs:  0
# Brassicanapus_GSBRNA2T00057317001 Exp number of AAs in TMHs: 0.01031
# Brassicanapus_GSBRNA2T00057317001 Exp number, first 60 AAs:  0
# Brassicanapus_GSBRNA2T00057317001 Total prob of N-in:        0.07370
Brassicanapus_GSBRNA2T00057317001	TMHMM2.0	outside	     1   317
```

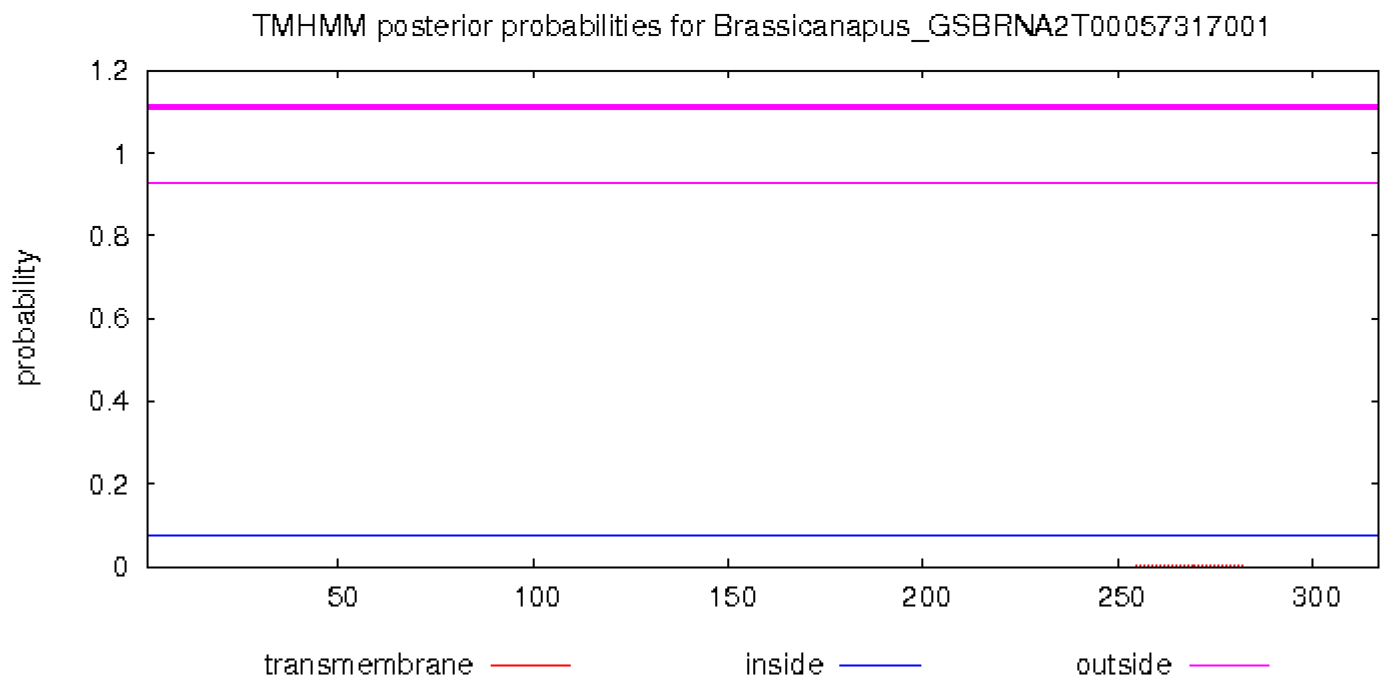

plot in postscript, script for making the plot in gnuplot, data for plot

---

```
# Brassicanapus_GSBRNA2T00057875001 Length: 208
# Brassicanapus_GSBRNA2T00057875001 Number of predicted TMHs:  0
# Brassicanapus_GSBRNA2T00057875001 Exp number of AAs in TMHs: 0
# Brassicanapus_GSBRNA2T00057875001 Exp number, first 60 AAs:  0
# Brassicanapus_GSBRNA2T00057875001 Total prob of N-in:        0.02815
Brassicanapus_GSBRNA2T00057875001	TMHMM2.0	outside	     1   208
```

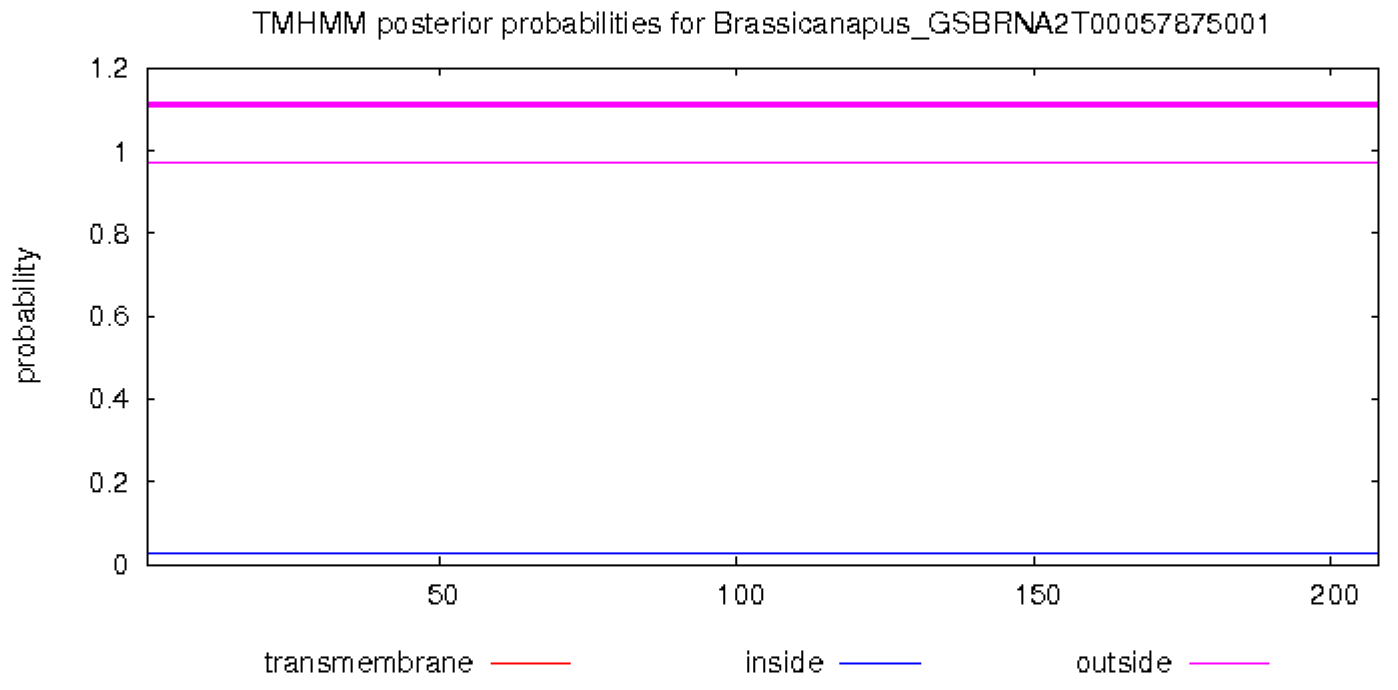

plot in postscript, script for making the plot in gnuplot, data for plot

---

```
# Brassicanapus_GSBRNA2T00058097001 Length: 301
# Brassicanapus_GSBRNA2T00058097001 Number of predicted TMHs:  0
# Brassicanapus_GSBRNA2T00058097001 Exp number of AAs in TMHs: 0.00117
# Brassicanapus_GSBRNA2T00058097001 Exp number, first 60 AAs:  0.00022
# Brassicanapus_GSBRNA2T00058097001 Total prob of N-in:        0.10457
Brassicanapus_GSBRNA2T00058097001	TMHMM2.0	outside	     1   301
```

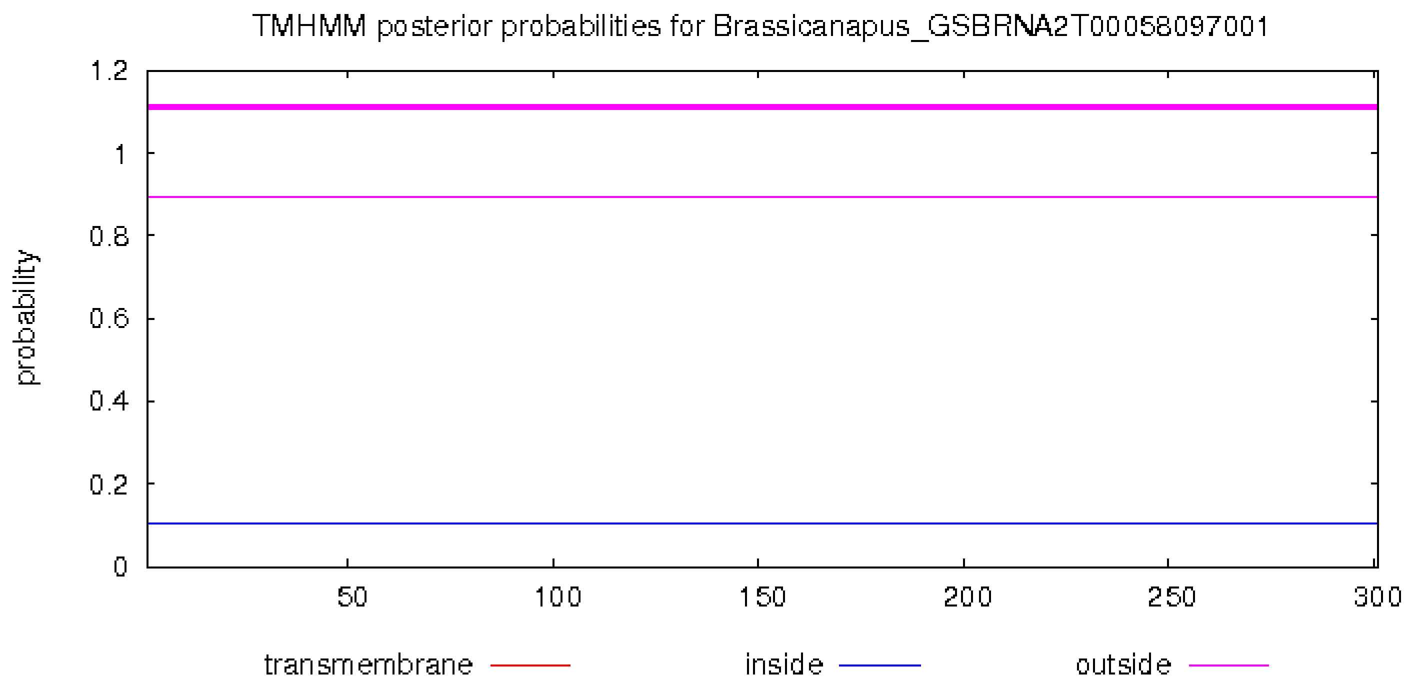

plot in postscript, script for making the plot in gnuplot, data for plot

---

```
# Brassicanapus_GSBRNA2T00058100001 Length: 334
# Brassicanapus_GSBRNA2T00058100001 Number of predicted TMHs:  0
# Brassicanapus_GSBRNA2T00058100001 Exp number of AAs in TMHs: 0
# Brassicanapus_GSBRNA2T00058100001 Exp number, first 60 AAs:  0
# Brassicanapus_GSBRNA2T00058100001 Total prob of N-in:        0.00779
Brassicanapus_GSBRNA2T00058100001	TMHMM2.0	outside	     1   334
```

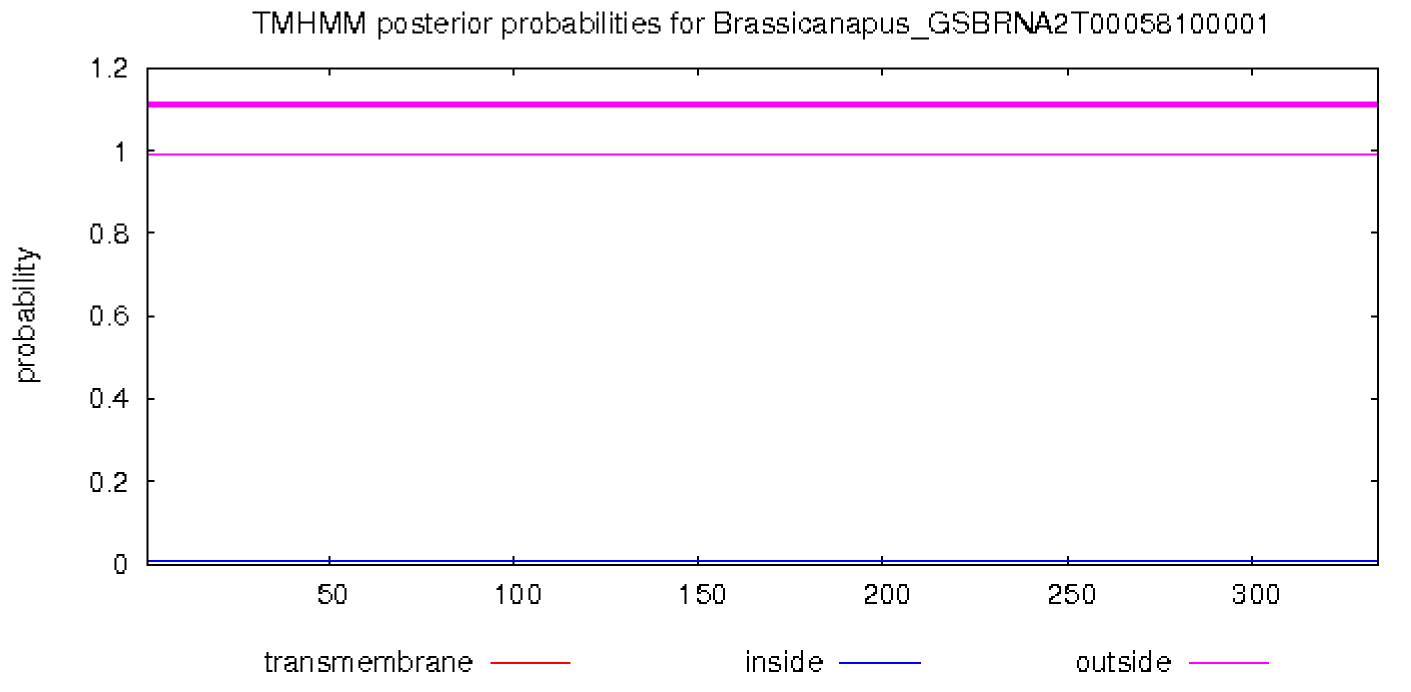

plot in postscript, script for making the plot in gnuplot, data for plot

```
# Brassicanapus_GSBRNA2T00059078001 Length: 343
# Brassicanapus_GSBRNA2T00059078001 Number of predicted TMHs:  0
# Brassicanapus_GSBRNA2T00059078001 Exp number of AAs in TMHs: 12.38583
# Brassicanapus_GSBRNA2T00059078001 Exp number, first 60 AAs:  0.01204
# Brassicanapus_GSBRNA2T00059078001 Total prob of N-in:        0.07612
Brassicanapus_GSBRNA2T00059078001	TMHMM2.0	outside	     1   343
```

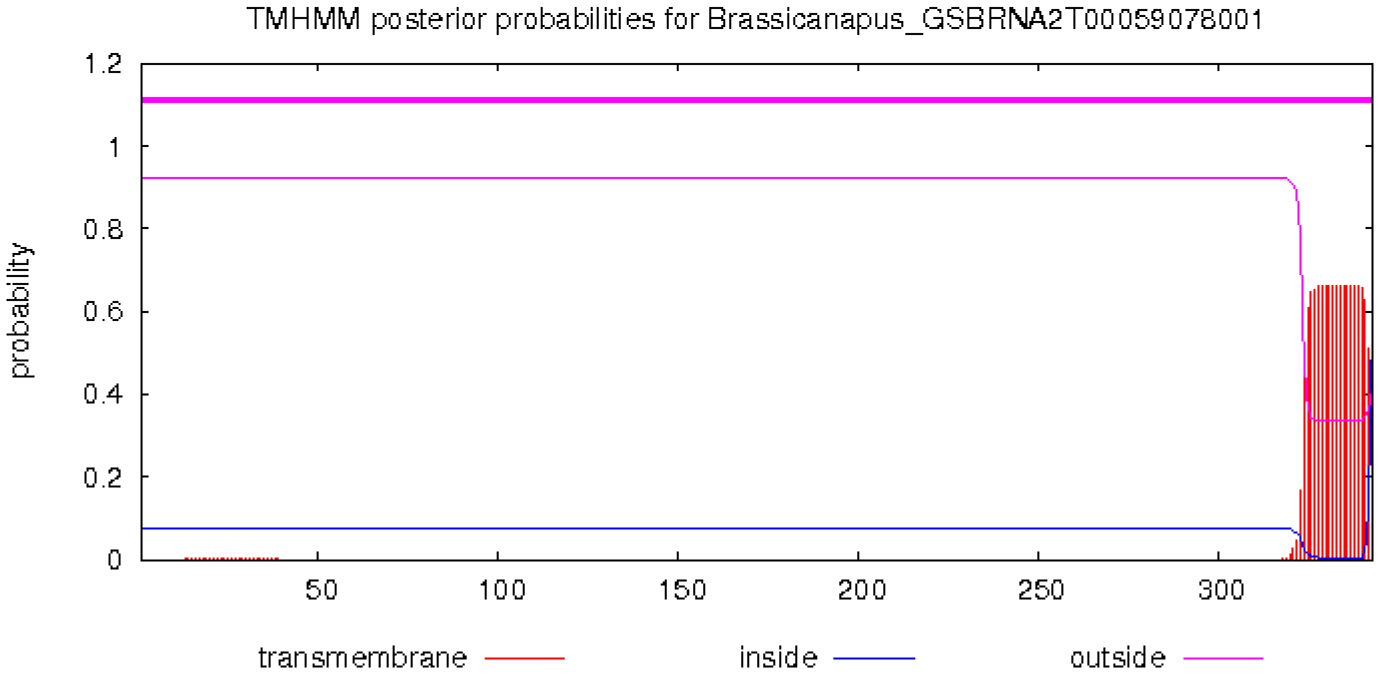

plot in postscript, script for making the plot in gnuplot, data for plot

```
# Brassicanapus_GSBRNA2T00059754001 Length: 286
# Brassicanapus_GSBRNA2T00059754001 Number of predicted TMHs:  0
# Brassicanapus_GSBRNA2T00059754001 Exp number of AAs in TMHs: 0.00029
# Brassicanapus_GSBRNA2T00059754001 Exp number, first 60 AAs:  0
# Brassicanapus_GSBRNA2T00059754001 Total prob of N-in:        0.00682
Brassicanapus_GSBRNA2T00059754001	TMHMM2.0	outside	     1   286
```

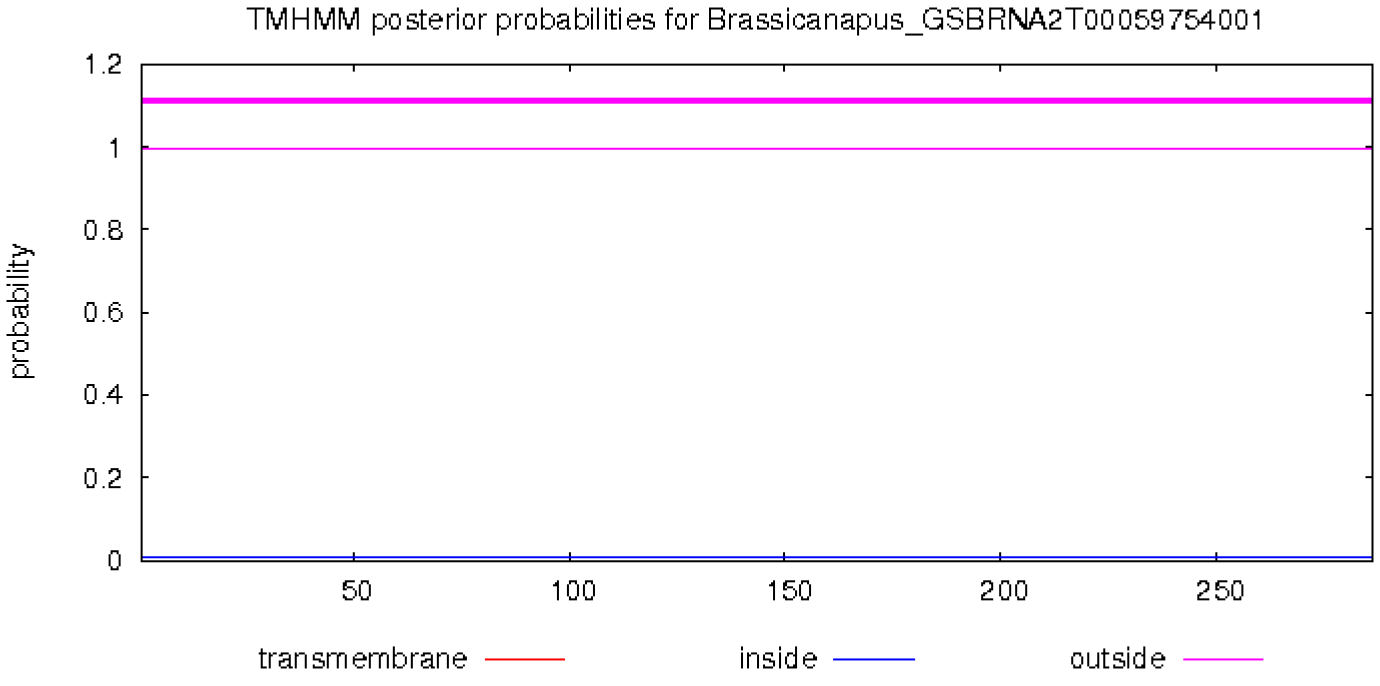

plot in postscript, script for making the plot in gnuplot, data for plot

```
# Brassicanapus_GSBRNA2T00061848001 Length: 545
# Brassicanapus_GSBRNA2T00061848001 Number of predicted TMHs:  1
# Brassicanapus_GSBRNA2T00061848001 Exp number of AAs in TMHs: 21.91236
# Brassicanapus_GSBRNA2T00061848001 Exp number, first 60 AAs:  0.0019
# Brassicanapus_GSBRNA2T00061848001 Total prob of N-in:        0.90687
Brassicanapus_GSBRNA2T00061848001	TMHMM2.0	inside	     1   515
Brassicanapus_GSBRNA2T00061848001	TMHMM2.0	TMhelix	   516   538
Brassicanapus_GSBRNA2T00061848001	TMHMM2.0	outside	   539   545
```

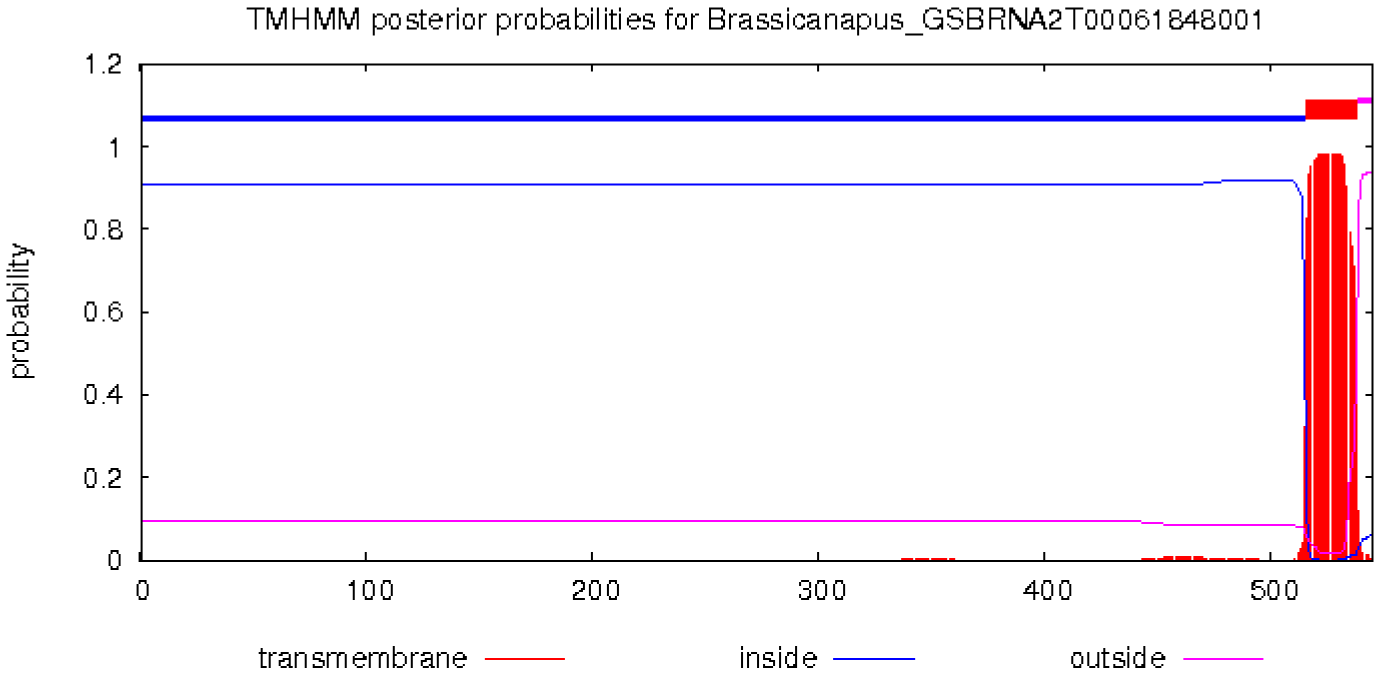

plot in postscript, script for making the plot in gnuplot, data for plot

```
# Brassicanapus_GSBRNA2T00063201001 Length: 288
# Brassicanapus_GSBRNA2T00063201001 Number of predicted TMHs:  0
# Brassicanapus_GSBRNA2T00063201001 Exp number of AAs in TMHs: 0.00026
# Brassicanapus_GSBRNA2T00063201001 Exp number, first 60 AAs:  0.00026
# Brassicanapus_GSBRNA2T00063201001 Total prob of N-in:        0.03061
Brassicanapus_GSBRNA2T00063201001	TMHMM2.0	outside	     1   288
```

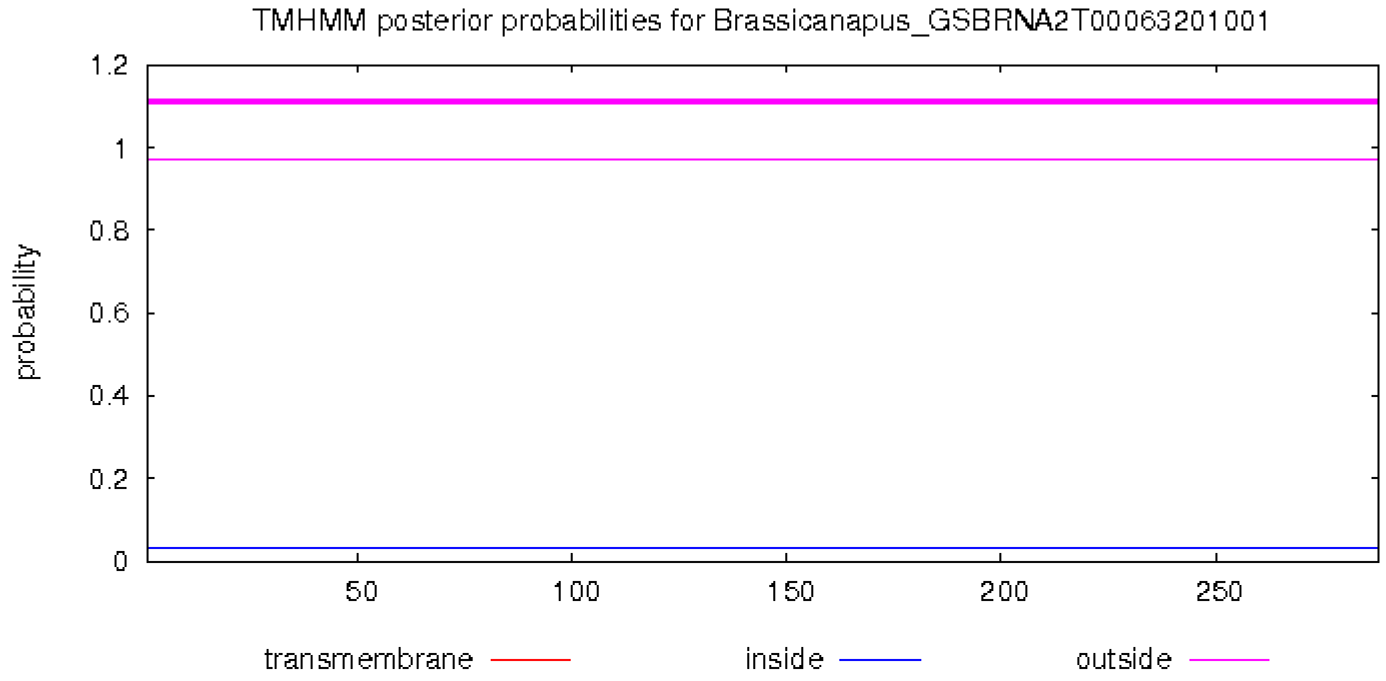

plot in postscript, script for making the plot in gnuplot, data for plot

---

```
# Brassicanapus_GSBRNA2T00064615001 Length: 256
# Brassicanapus_GSBRNA2T00064615001 Number of predicted TMHs:  0
# Brassicanapus_GSBRNA2T00064615001 Exp number of AAs in TMHs: 0.02092
# Brassicanapus_GSBRNA2T00064615001 Exp number, first 60 AAs:  0.0002
# Brassicanapus_GSBRNA2T00064615001 Total prob of N-in:        0.10373
Brassicanapus_GSBRNA2T00064615001	TMHMM2.0	outside	     1   256
```

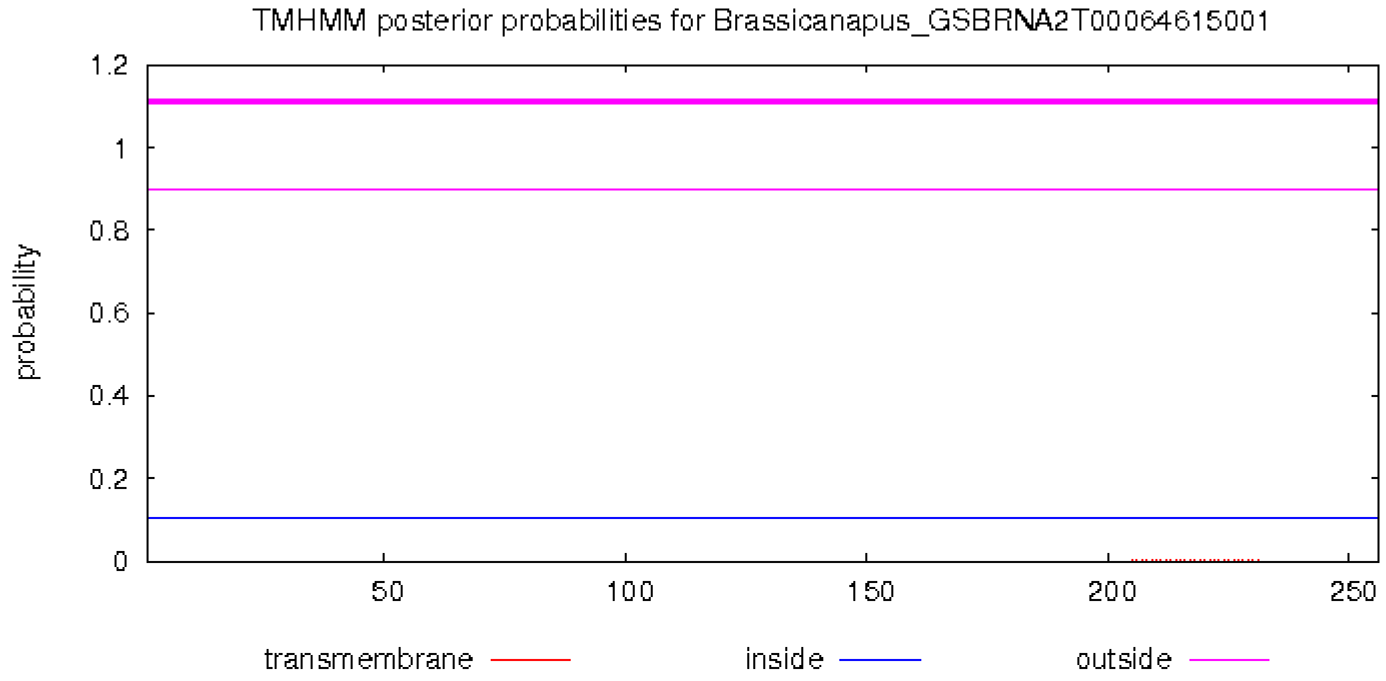

plot in postscript, script for making the plot in gnuplot, data for plot

---

```
# Brassicanapus_GSBRNA2T00065160001 Length: 307
# Brassicanapus_GSBRNA2T00065160001 Number of predicted TMHs:  0
# Brassicanapus_GSBRNA2T00065160001 Exp number of AAs in TMHs: 0.1637
# Brassicanapus_GSBRNA2T00065160001 Exp number, first 60 AAs:  0
# Brassicanapus_GSBRNA2T00065160001 Total prob of N-in:        0.00444
Brassicanapus_GSBRNA2T00065160001	TMHMM2.0	outside	     1   307
```

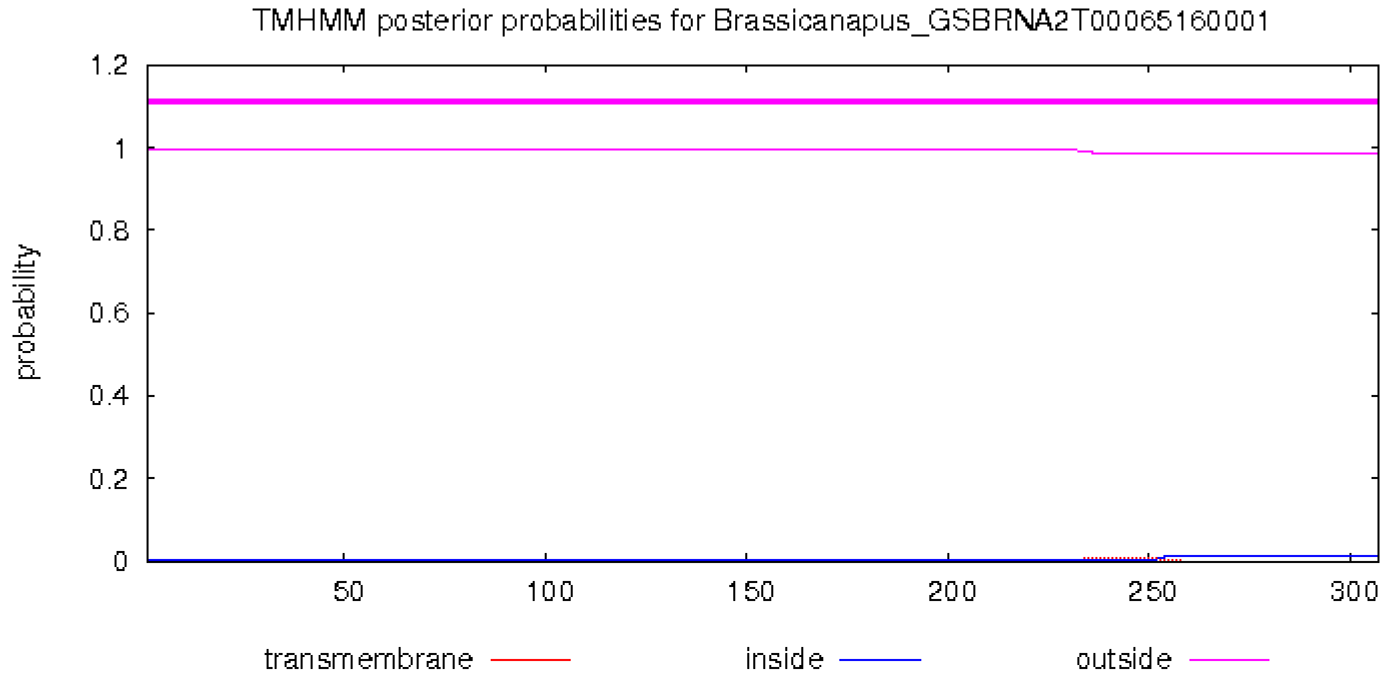

plot in postscript, script for making the plot in gnuplot, data for plot

---

```
# Brassicanapus_GSBRNA2T00065262001 Length: 365
# Brassicanapus_GSBRNA2T00065262001 Number of predicted TMHs:  0
# Brassicanapus_GSBRNA2T00065262001 Exp number of AAs in TMHs: 0.00019
# Brassicanapus_GSBRNA2T00065262001 Exp number, first 60 AAs:  0
# Brassicanapus_GSBRNA2T00065262001 Total prob of N-in:        0.00850
Brassicanapus_GSBRNA2T00065262001       TMHMM2.0        outside      1   365
```

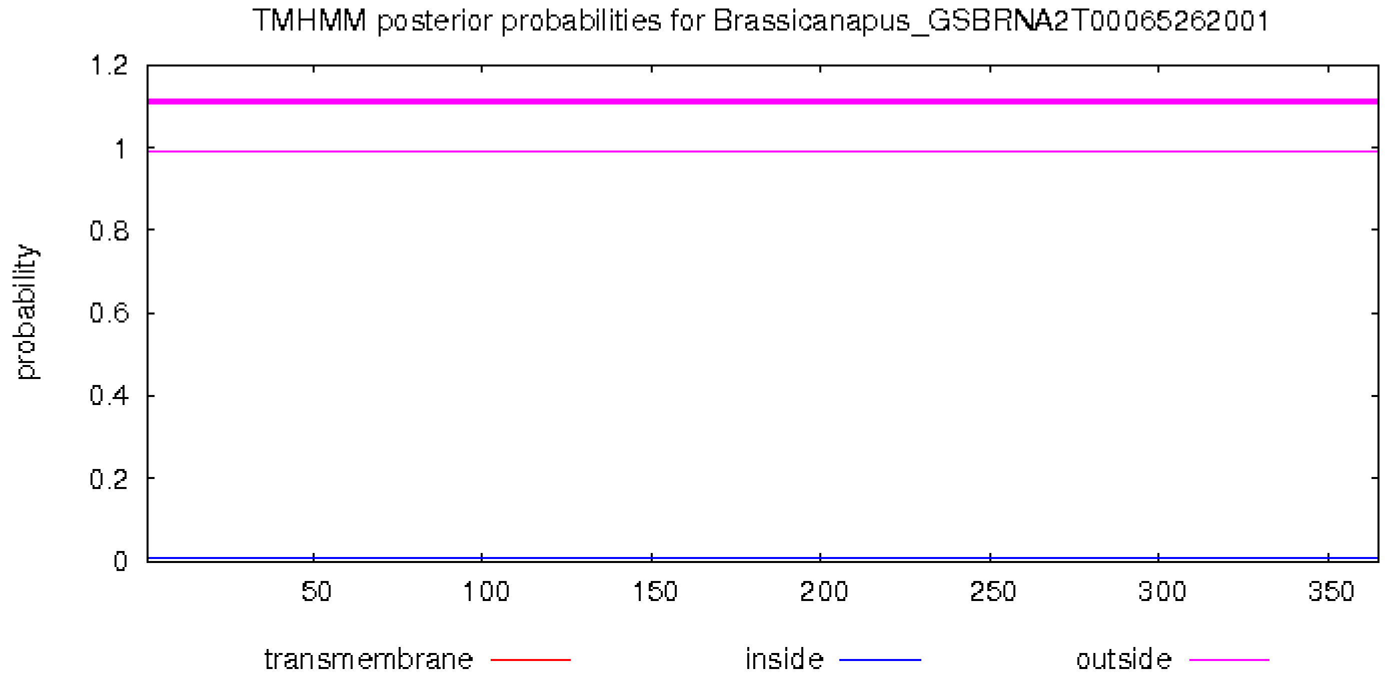

plot in postscript, script for making the plot in gnuplot, data for plot

---

```
# Brassicanapus_GSBRNA2T00065483001 Length: 361
# Brassicanapus_GSBRNA2T00065483001 Number of predicted TMHs:  0
# Brassicanapus_GSBRNA2T00065483001 Exp number of AAs in TMHs: 0
# Brassicanapus_GSBRNA2T00065483001 Exp number, first 60 AAs:  0
# Brassicanapus_GSBRNA2T00065483001 Total prob of N-in:        0.00206
Brassicanapus_GSBRNA2T00065483001       TMHMM2.0        outside      1   361
```

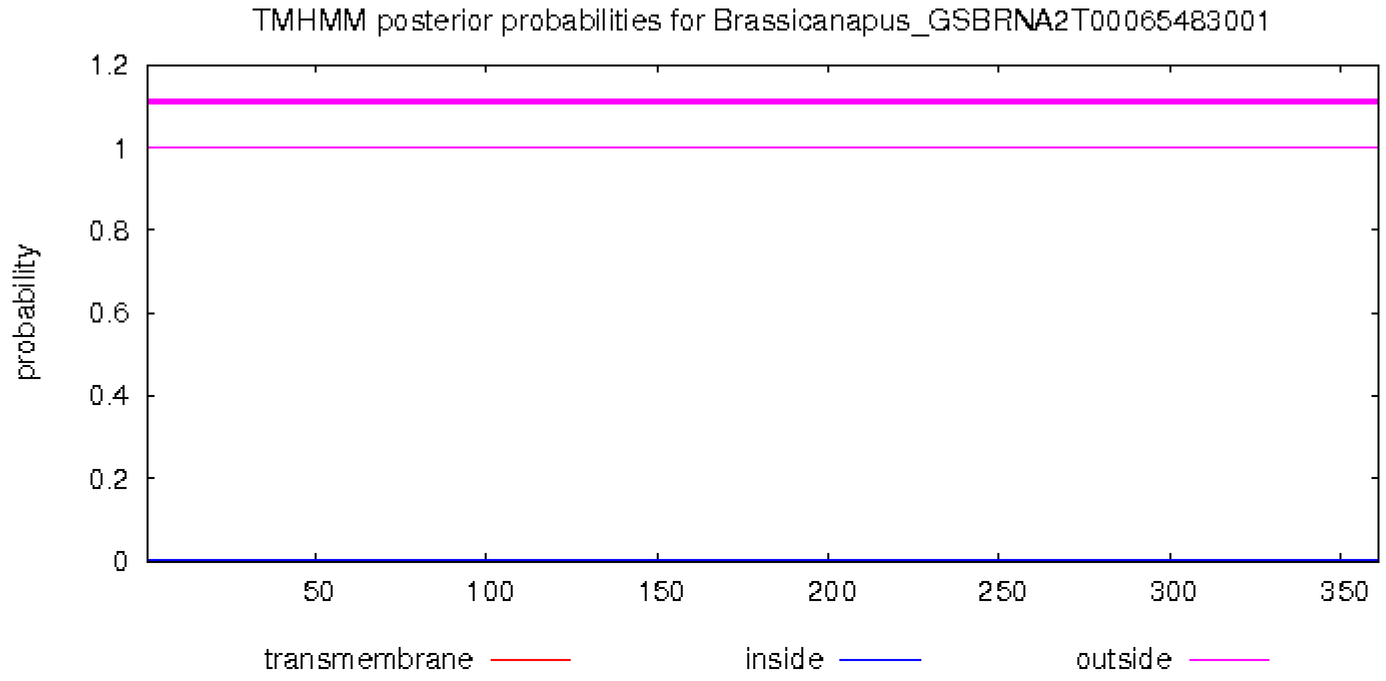

plot in postscript, script for making the plot in gnuplot, data for plot

```
# Brassicanapus_GSBRNA2T00065750001 Length: 260
# Brassicanapus_GSBRNA2T00065750001 Number of predicted TMHs:  0
# Brassicanapus_GSBRNA2T00065750001 Exp number of AAs in TMHs: 0.00061
# Brassicanapus_GSBRNA2T00065750001 Exp number, first 60 AAs:  0.00042
# Brassicanapus_GSBRNA2T00065750001 Total prob of N-in:        0.03308
Brassicanapus_GSBRNA2T00065750001       TMHMM2.0        outside      1   260
```

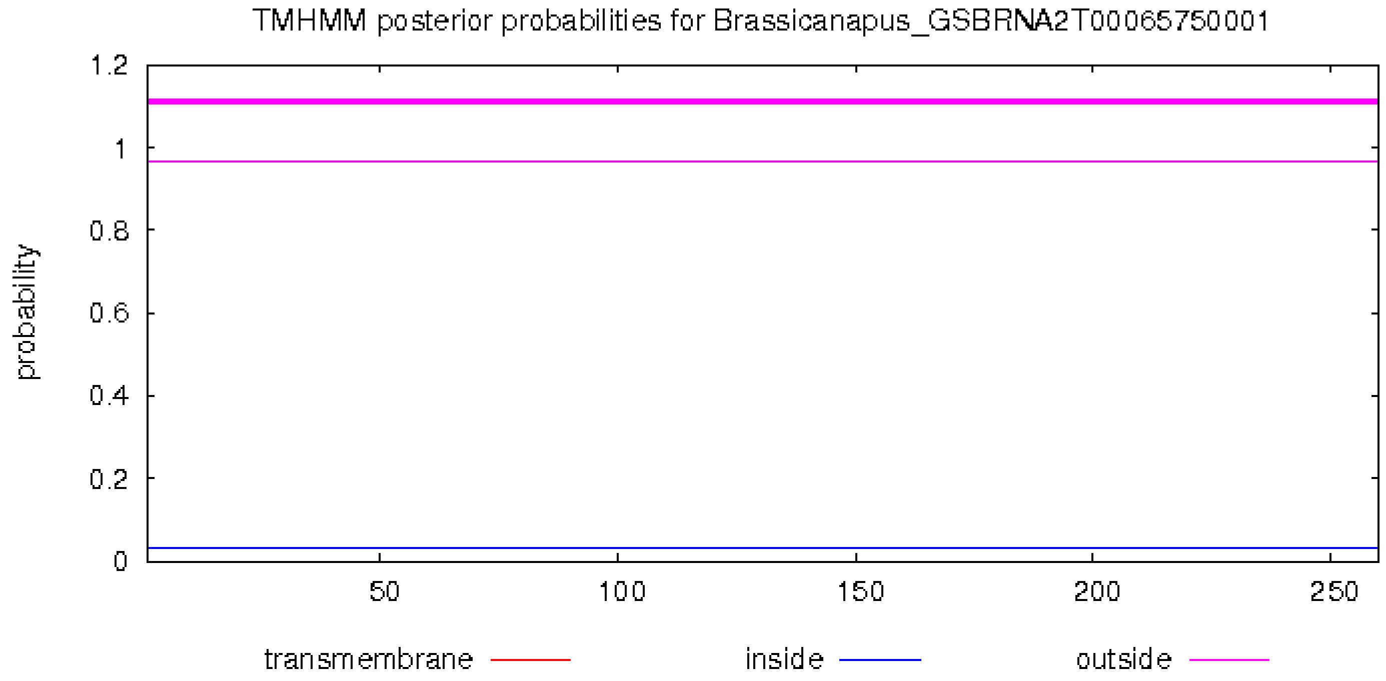

plot in postscript, script for making the plot in gnuplot, data for plot

```
# Brassicanapus_GSBRNA2T00065801001 Length: 344
# Brassicanapus_GSBRNA2T00065801001 Number of predicted TMHs:  0
# Brassicanapus_GSBRNA2T00065801001 Exp number of AAs in TMHs: 0
# Brassicanapus_GSBRNA2T00065801001 Exp number, first 60 AAs:  0
# Brassicanapus_GSBRNA2T00065801001 Total prob of N-in:        0.00689
Brassicanapus_GSBRNA2T00065801001       TMHMM2.0        outside      1   344
```

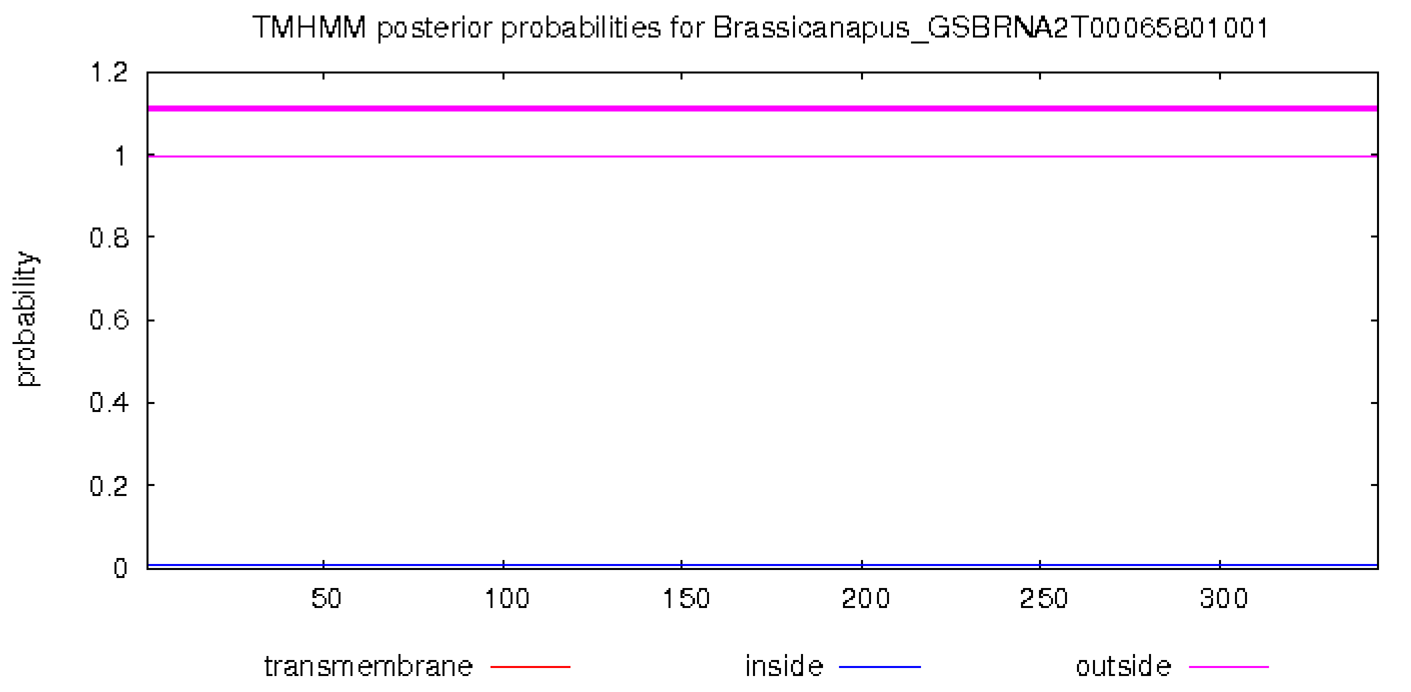

plot in postscript, script for making the plot in gnuplot, data for plot

---

```
# Brassicanapus_GSBRNA2T00065806001 Length: 303
# Brassicanapus_GSBRNA2T00065806001 Number of predicted TMHs:  0
# Brassicanapus_GSBRNA2T00065806001 Exp number of AAs in TMHs: 0.00122
# Brassicanapus_GSBRNA2T00065806001 Exp number, first 60 AAs:  0.0002
# Brassicanapus_GSBRNA2T00065806001 Total prob of N-in:        0.10084
Brassicanapus_GSBRNA2T00065806001       TMHMM2.0        outside      1   303
```

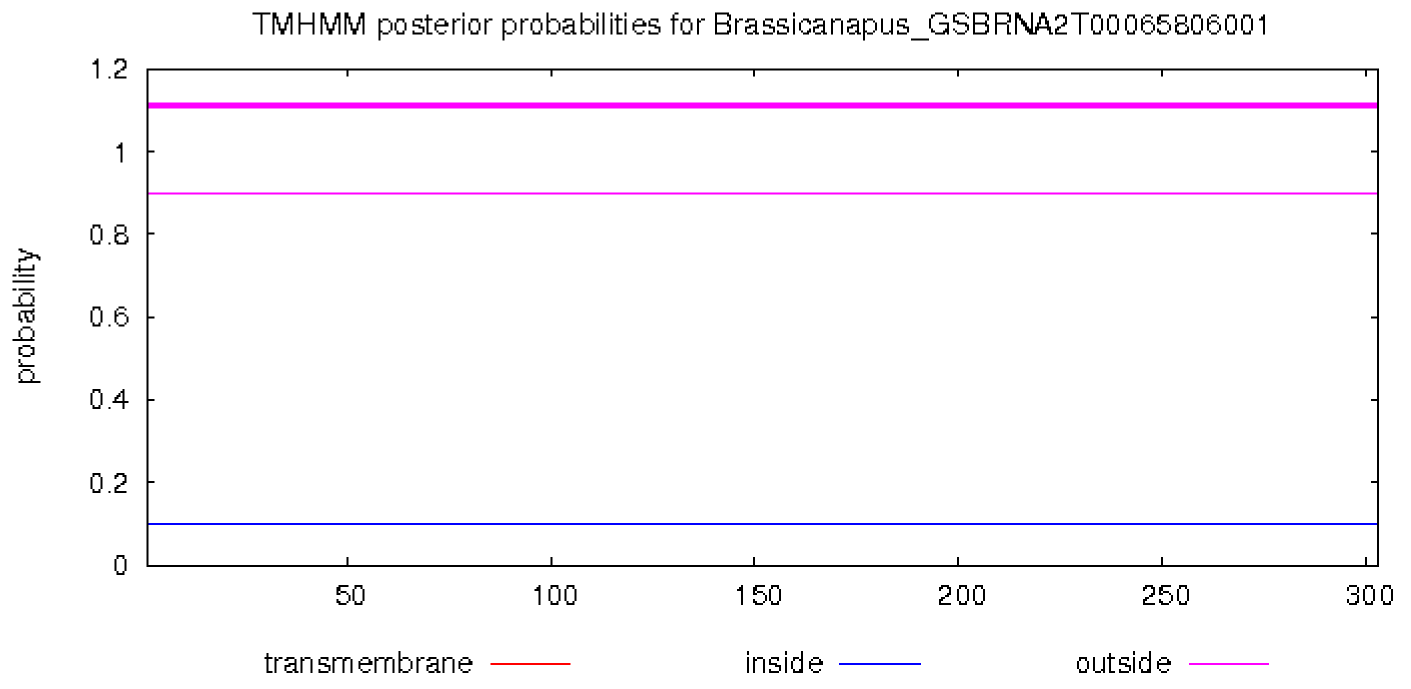

plot in postscript, script for making the plot in gnuplot, data for plot

---

```
# Brassicanapus_GSBRNA2T00067987001 Length: 281
# Brassicanapus_GSBRNA2T00067987001 Number of predicted TMHs:  0
# Brassicanapus_GSBRNA2T00067987001 Exp number of AAs in TMHs: 0.00594
# Brassicanapus_GSBRNA2T00067987001 Exp number, first 60 AAs:  0.00338
# Brassicanapus_GSBRNA2T00067987001 Total prob of N-in:        0.00557
Brassicanapus_GSBRNA2T00067987001       TMHMM2.0        outside      1   281
```

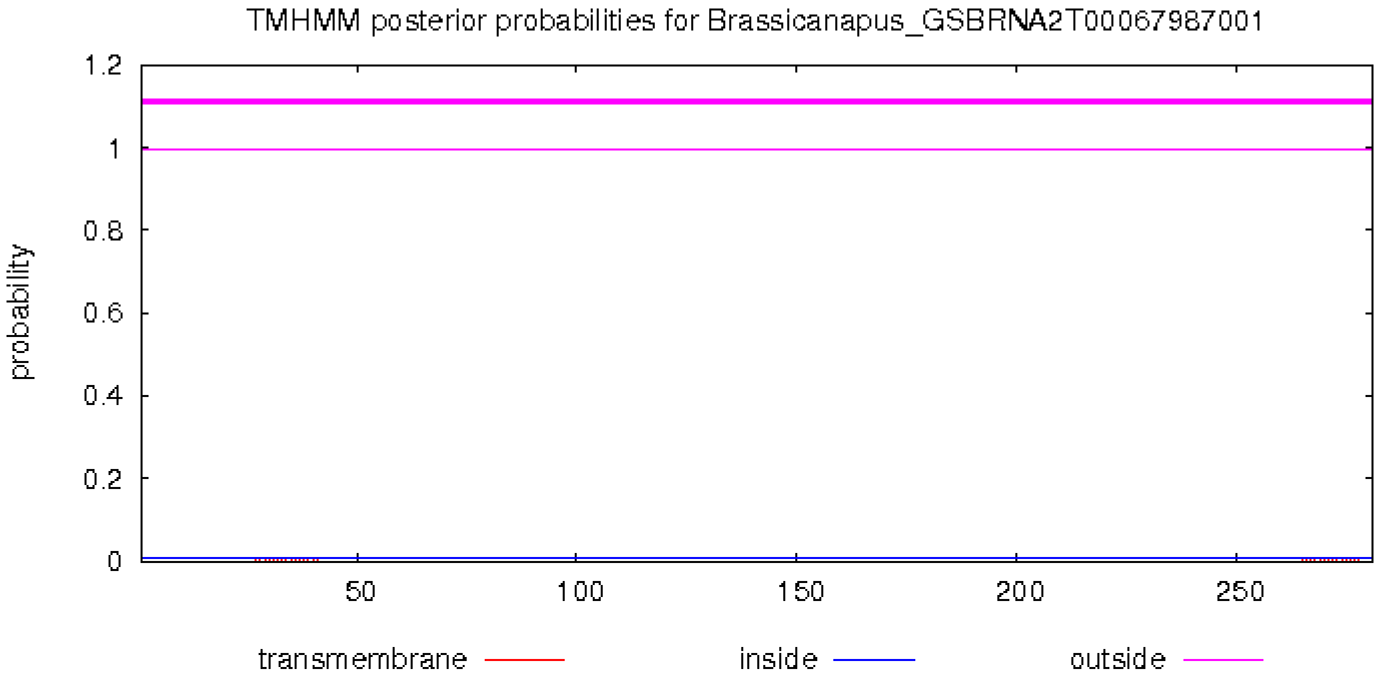

plot in postscript, script for making the plot in gnuplot, data for plot

---

```
# Brassicanapus_GSBRNA2T00068670001 Length: 294
# Brassicanapus_GSBRNA2T00068670001 Number of predicted TMHs:  0
# Brassicanapus_GSBRNA2T00068670001 Exp number of AAs in TMHs: 0.00035
# Brassicanapus_GSBRNA2T00068670001 Exp number, first 60 AAs:  2e-05
# Brassicanapus_GSBRNA2T00068670001 Total prob of N-in:        0.03986
Brassicanapus_GSBRNA2T00068670001       TMHMM2.0        outside      1   294
```

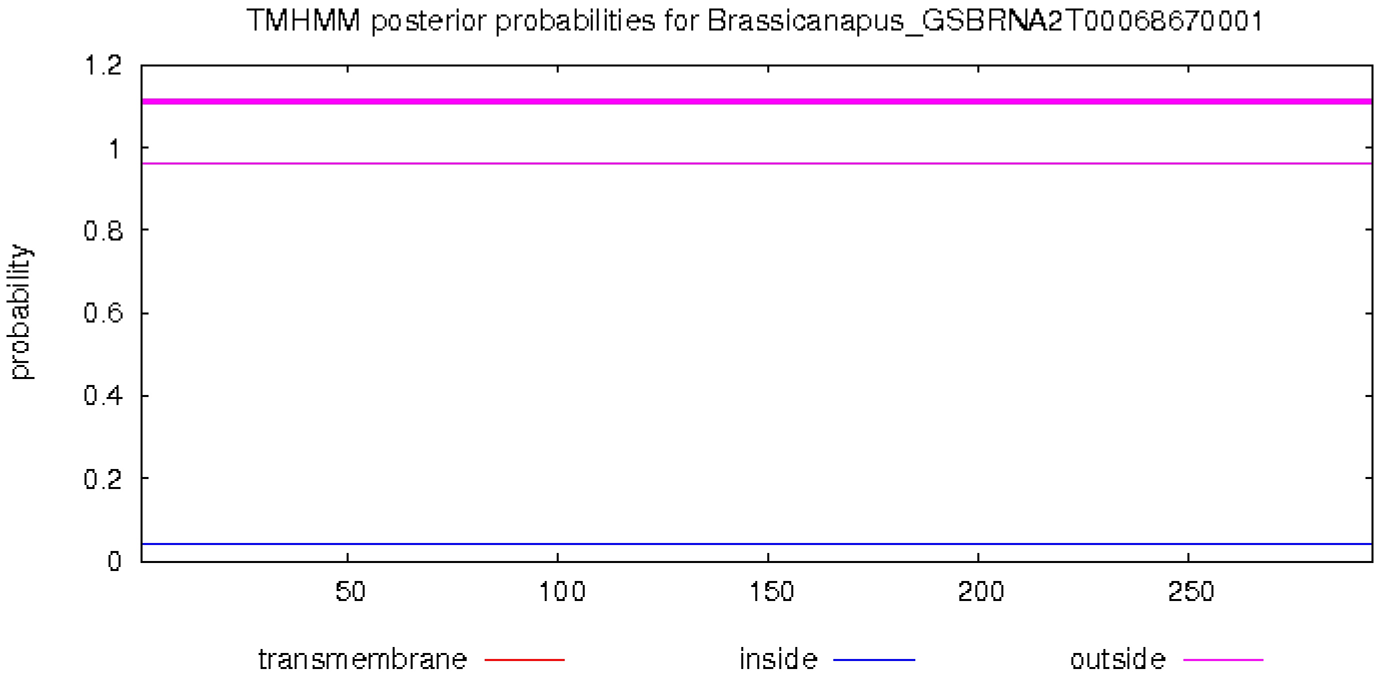

plot in postscript, script for making the plot in gnuplot, data for plot

---

```
# Brassicanapus_GSBRNA2T00070546001 Length: 321
# Brassicanapus_GSBRNA2T00070546001 Number of predicted TMHs:  0
# Brassicanapus_GSBRNA2T00070546001 Exp number of AAs in TMHs: 0.000750000000000000001
# Brassicanapus_GSBRNA2T00070546001 Exp number, first 60 AAs:  0.00054
# Brassicanapus_GSBRNA2T00070546001 Total prob of N-in:        0.01635
Brassicanapus_GSBRNA2T00070546001       TMHMM2.0        outside      1   321
```

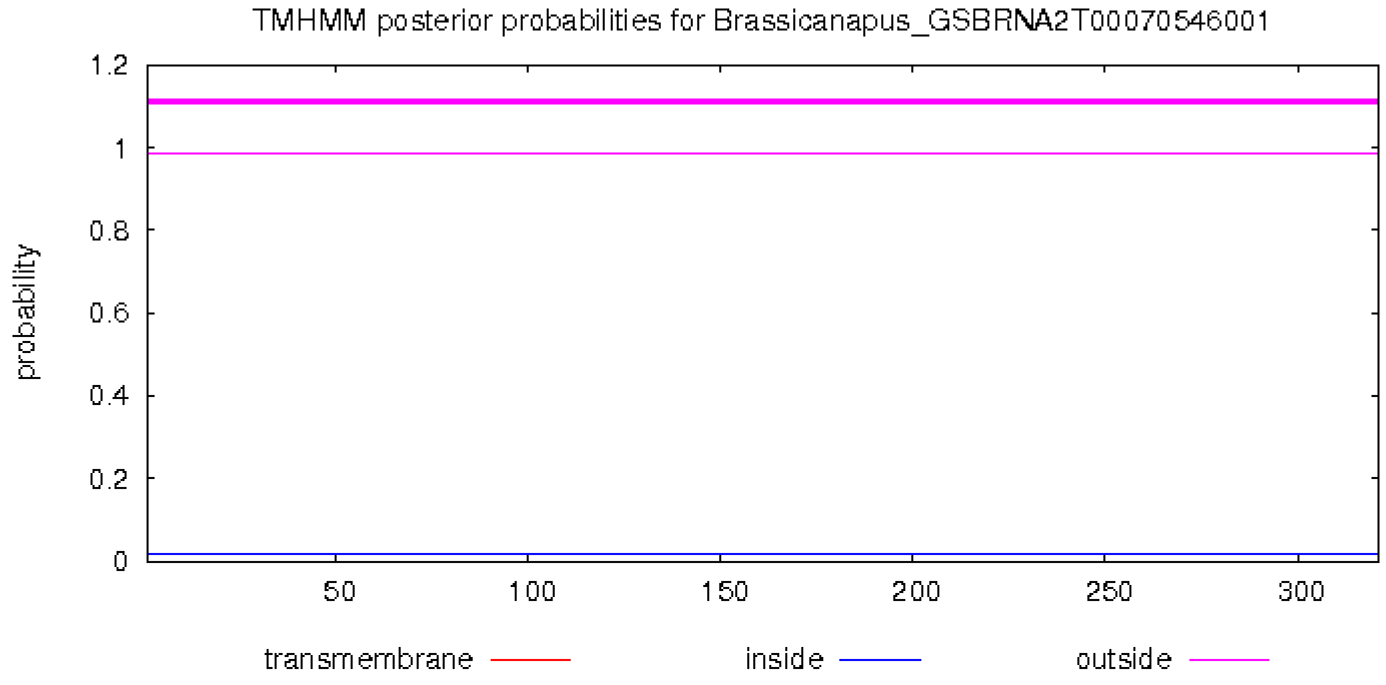

plot in postscript, script for making the plot in gnuplot, data for plot

```
# Brassicanapus_GSBRNA2T00071627001 Length: 258
# Brassicanapus_GSBRNA2T00071627001 Number of predicted TMHs:  0
# Brassicanapus_GSBRNA2T00071627001 Exp number of AAs in TMHs: 0.00019
# Brassicanapus_GSBRNA2T00071627001 Exp number, first 60 AAs:  0
# Brassicanapus_GSBRNA2T00071627001 Total prob of N-in:        0.01136
Brassicanapus_GSBRNA2T00071627001       TMHMM2.0        outside      1   258
```

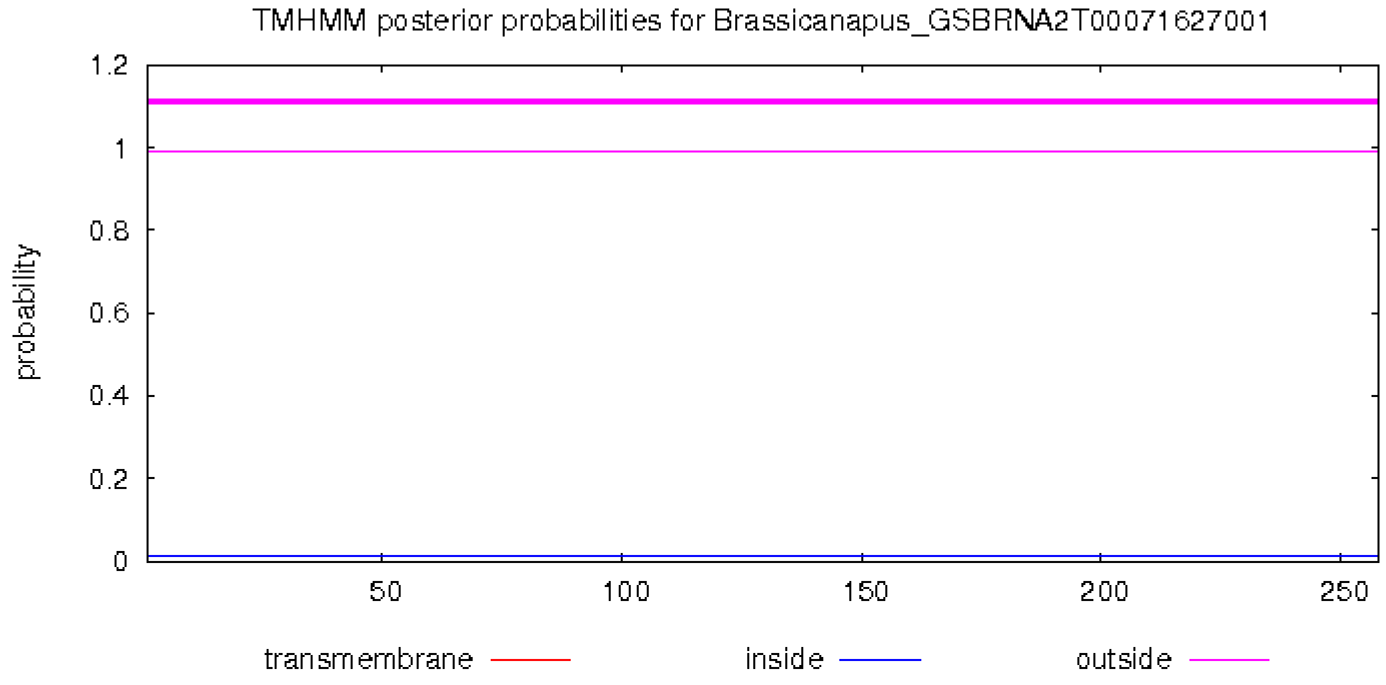

plot in postscript, script for making the plot in gnuplot, data for plot

```
# Brassicanapus_GSBRNA2T00071676001 Length: 324
# Brassicanapus_GSBRNA2T00071676001 Number of predicted TMHs:  0
# Brassicanapus_GSBRNA2T00071676001 Exp number of AAs in TMHs: 0.0002
# Brassicanapus_GSBRNA2T00071676001 Exp number, first 60 AAs:  0
# Brassicanapus_GSBRNA2T00071676001 Total prob of N-in:        0.00521
Brassicanapus_GSBRNA2T00071676001       TMHMM2.0        outside      1   324
```

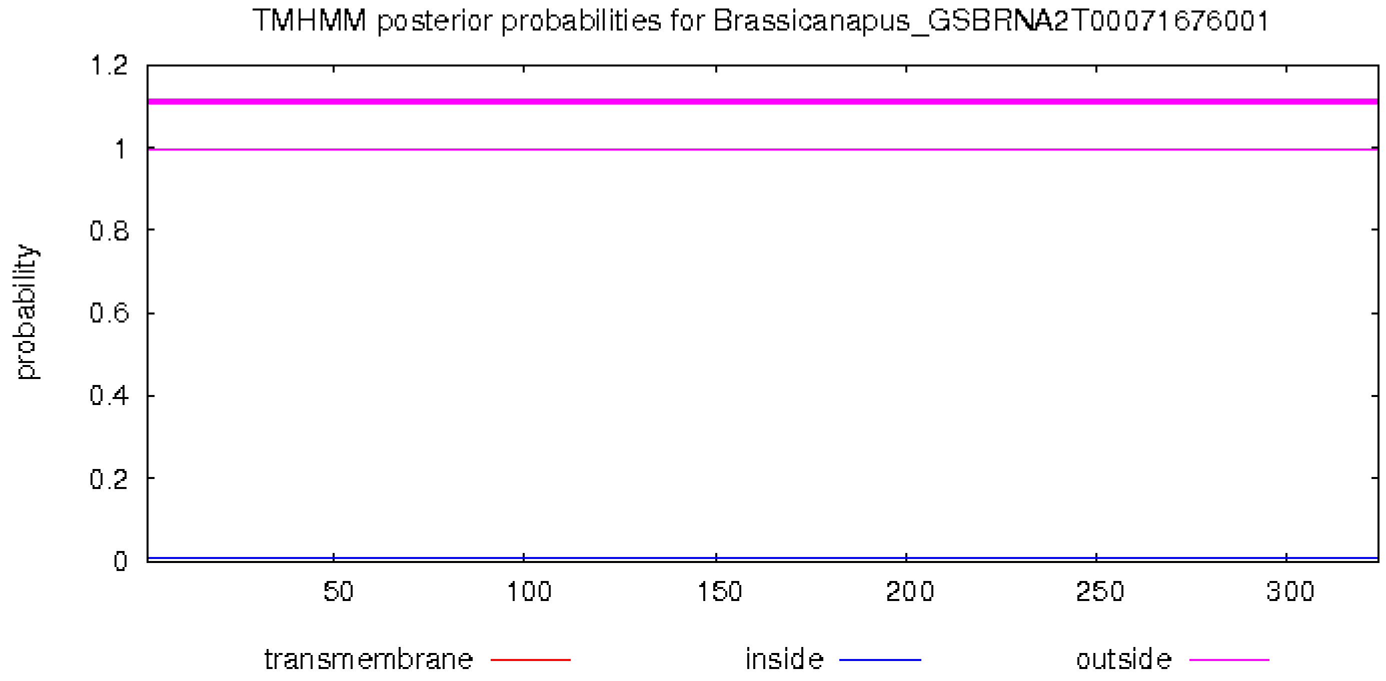

plot in postscript, script for making the plot in gnuplot, data for plot

---

```
# Brassicanapus_GSBRNA2T00071930001 Length: 260
# Brassicanapus_GSBRNA2T00071930001 Number of predicted TMHs:  0
# Brassicanapus_GSBRNA2T00071930001 Exp number of AAs in TMHs: 0.00037
# Brassicanapus_GSBRNA2T00071930001 Exp number, first 60 AAs:  0.00017
# Brassicanapus_GSBRNA2T00071930001 Total prob of N-in:        0.03487
Brassicanapus_GSBRNA2T00071930001       TMHMM2.0        outside      1   260
```

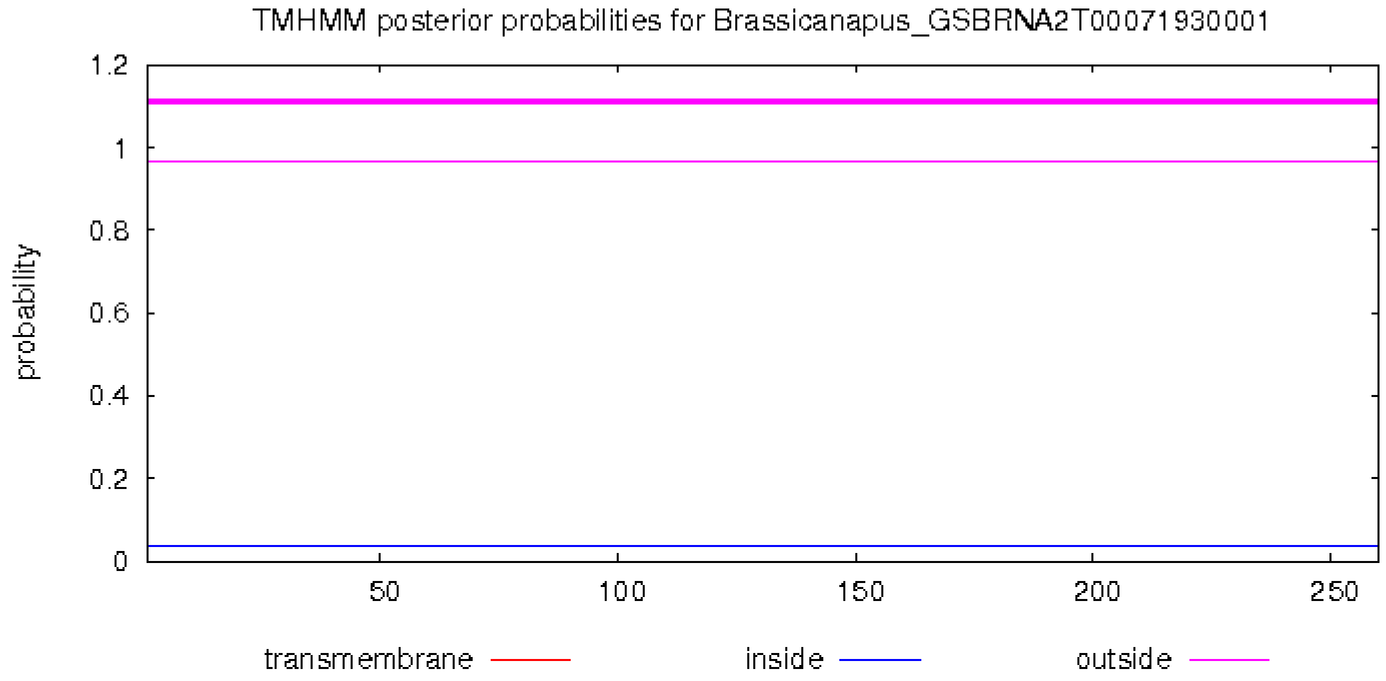

plot in postscript, script for making the plot in gnuplot, data for plot

---

```
# Brassicanapus_GSBRNA2T00071983001 Length: 270
# Brassicanapus_GSBRNA2T00071983001 Number of predicted TMHs:  0
# Brassicanapus_GSBRNA2T00071983001 Exp number of AAs in TMHs: 0.00273
# Brassicanapus_GSBRNA2T00071983001 Exp number, first 60 AAs:  0
# Brassicanapus_GSBRNA2T00071983001 Total prob of N-in:        0.03606
Brassicanapus_GSBRNA2T00071983001       TMHMM2.0        outside      1   270
```

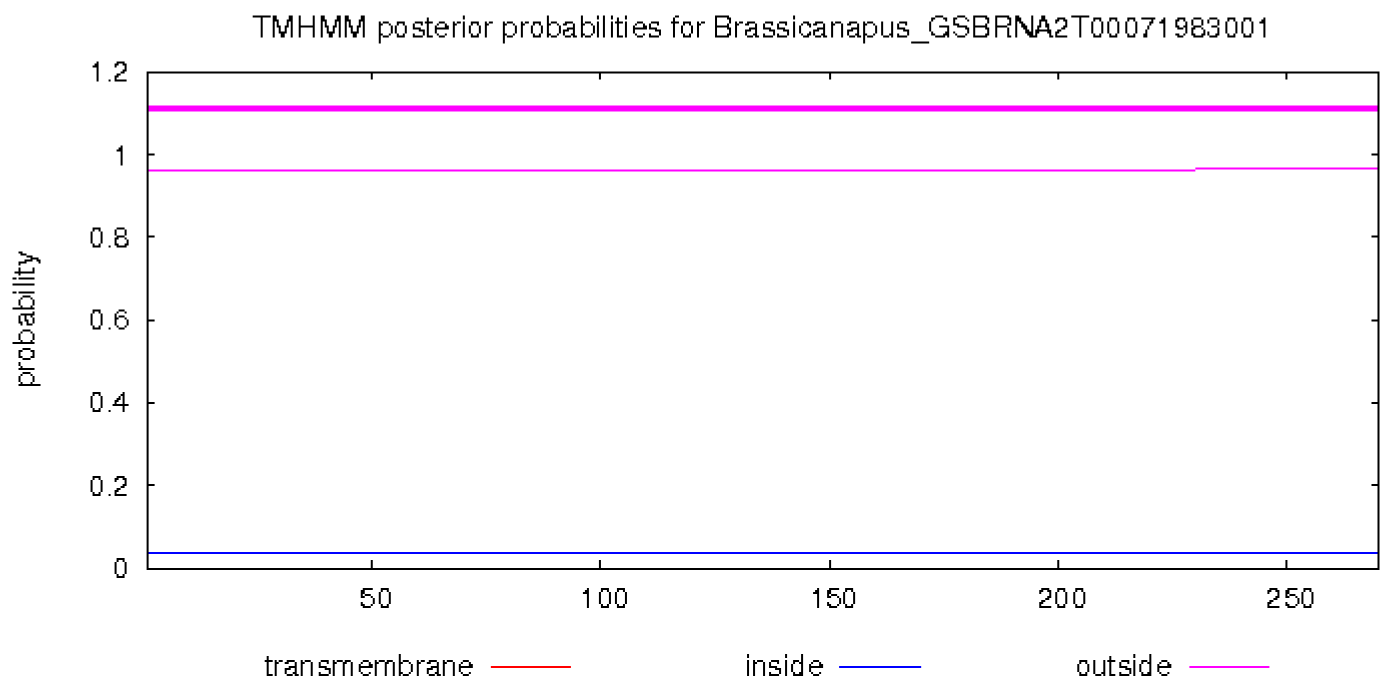

plot in postscript, script for making the plot in gnuplot, data for plot

---

```
# Brassicanapus_GSBRNA2T00072232001 Length: 322
# Brassicanapus_GSBRNA2T00072232001 Number of predicted TMHs:  0
# Brassicanapus_GSBRNA2T00072232001 Exp number of AAs in TMHs: 0.00077000000000000001
# Brassicanapus_GSBRNA2T00072232001 Exp number, first 60 AAs:  0.00054
# Brassicanapus_GSBRNA2T00072232001 Total prob of N-in:        0.01616
Brassicanapus_GSBRNA2T00072232001       TMHMM2.0        outside      1   322
```

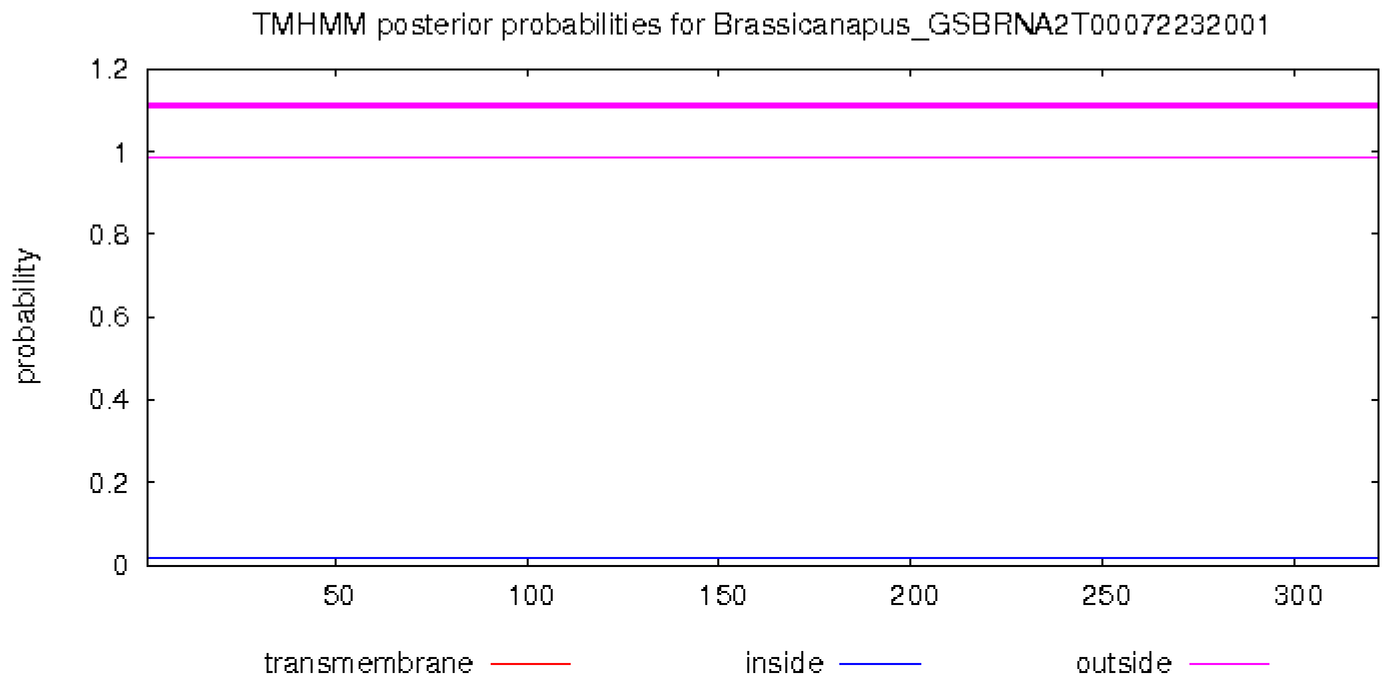

plot in postscript, script for making the plot in gnuplot, data for plot

---

```
# Brassicanapus_GSBRNA2T00072911001 Length: 167
# Brassicanapus_GSBRNA2T00072911001 Number of predicted TMHs:  0
# Brassicanapus_GSBRNA2T00072911001 Exp number of AAs in TMHs: 9.39929
# Brassicanapus_GSBRNA2T00072911001 Exp number, first 60 AAs:  9.39929
# Brassicanapus_GSBRNA2T00072911001 Total prob of N-in:        0.48221
Brassicanapus_GSBRNA2T00072911001       TMHMM2.0        outside      1   167
```

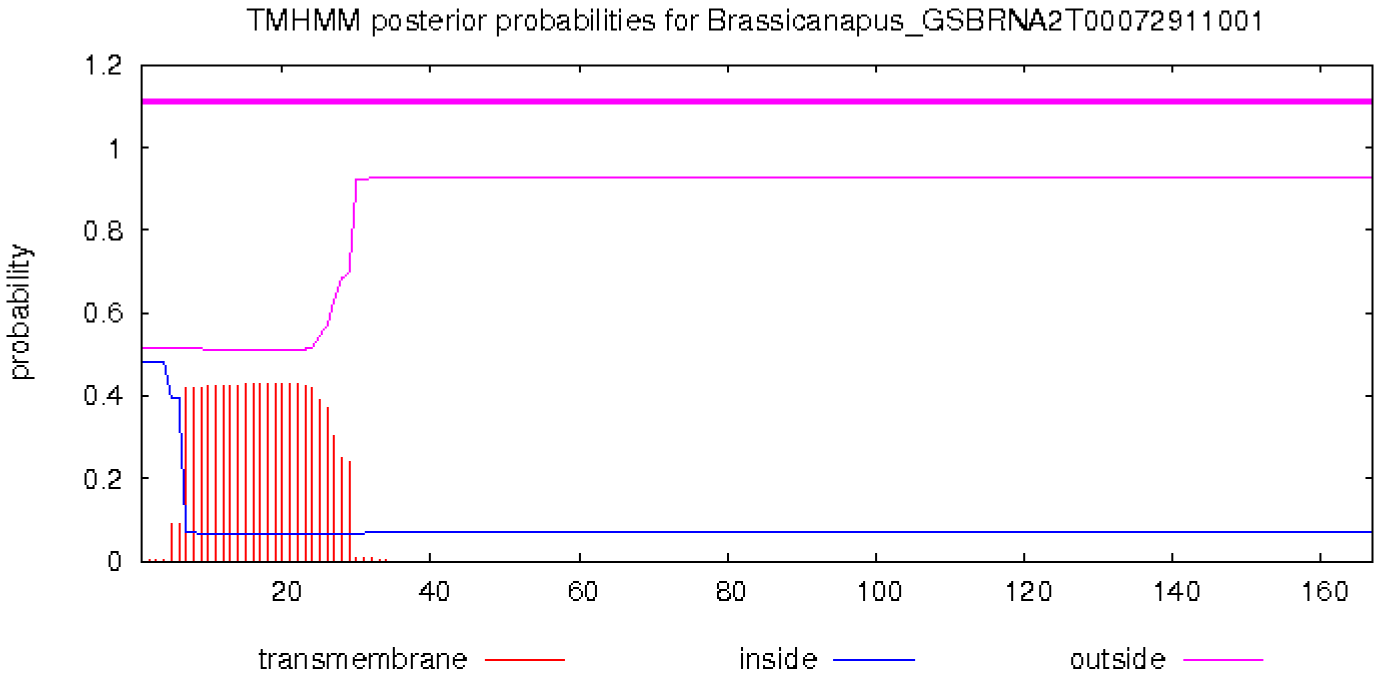

plot in postscript, script for making the plot in gnuplot, data for plot

---

```
# Brassicanapus_GSBRNA2T00072912001 Length: 421
# Brassicanapus_GSBRNA2T00072912001 Number of predicted TMHs:  0
# Brassicanapus_GSBRNA2T00072912001 Exp number of AAs in TMHs: 9.66722999999999999
# Brassicanapus_GSBRNA2T00072912001 Exp number, first 60 AAs:  9.66097
# Brassicanapus_GSBRNA2T00072912001 Total prob of N-in:        0.44253
Brassicanapus_GSBRNA2T00072912001       TMHMM2.0        outside      1   421
```

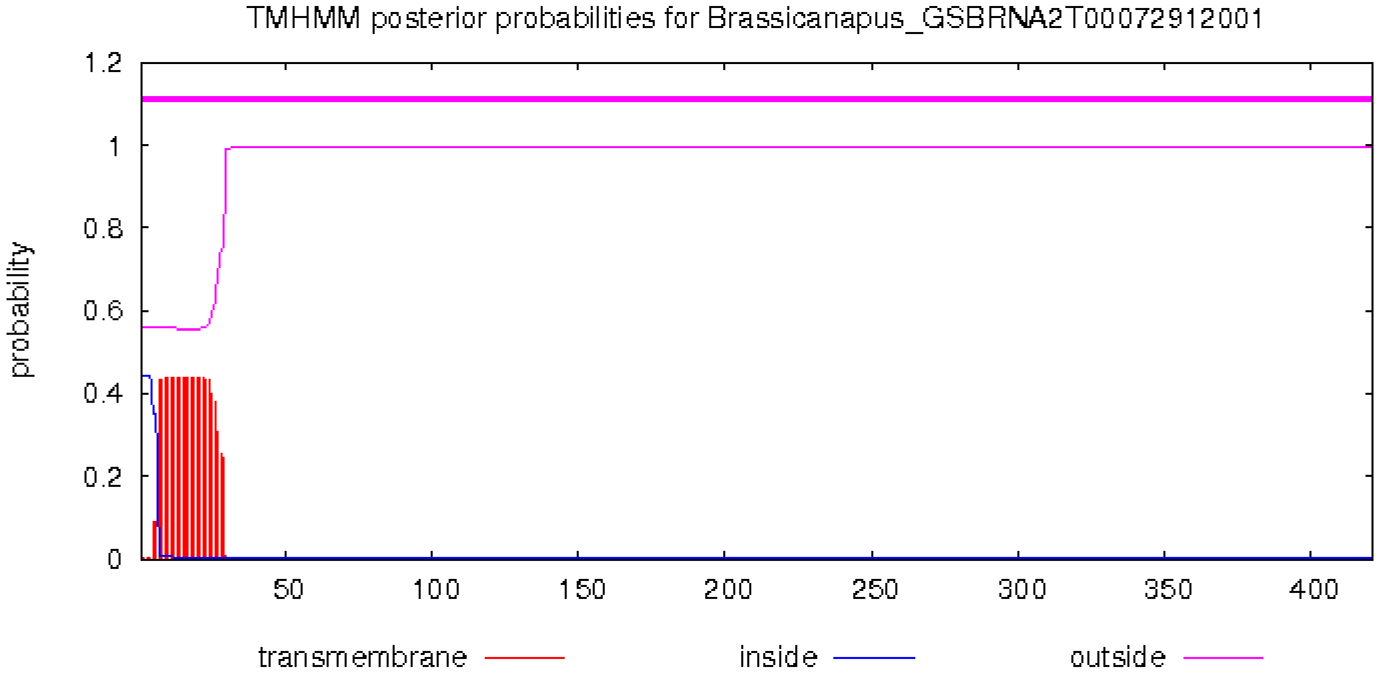

plot in postscript, script for making the plot in gnuplot, data for plot

---

```
# Brassicanapus_GSBRNA2T00072913001 Length: 441
# Brassicanapus_GSBRNA2T00072913001 Number of predicted TMHs:  0
# Brassicanapus_GSBRNA2T00072913001 Exp number of AAs in TMHs: 0.0332
# Brassicanapus_GSBRNA2T00072913001 Exp number, first 60 AAs:  0.02071
# Brassicanapus_GSBRNA2T00072913001 Total prob of N-in:        0.00554
Brassicanapus_GSBRNA2T00072913001       TMHMM2.0        outside      1   441
```

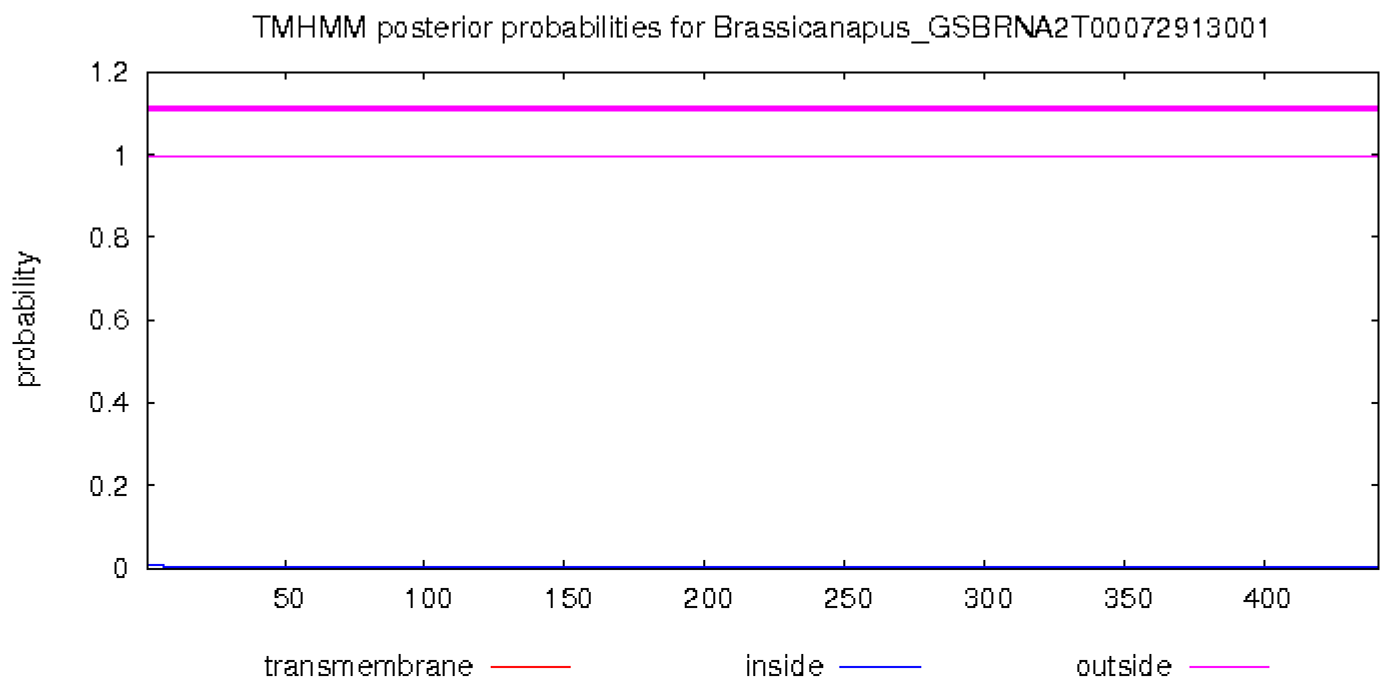

plot in postscript, script for making the plot in gnuplot, data for plot

---

```
# Brassicanapus_GSBRNA2T00072914001 Length: 537
# Brassicanapus_GSBRNA2T00072914001 Number of predicted TMHs:  1
# Brassicanapus_GSBRNA2T00072914001 Exp number of AAs in TMHs: 21.51374
# Brassicanapus_GSBRNA2T00072914001 Exp number, first 60 AAs:  0
# Brassicanapus_GSBRNA2T00072914001 Total prob of N-in:        0.01895
Brassicanapus_GSBRNA2T00072914001	TMHMM2.0	outside	     1   511
Brassicanapus_GSBRNA2T00072914001	TMHMM2.0	TMhelix	   512   534
Brassicanapus_GSBRNA2T00072914001	TMHMM2.0	inside	   535   537
```

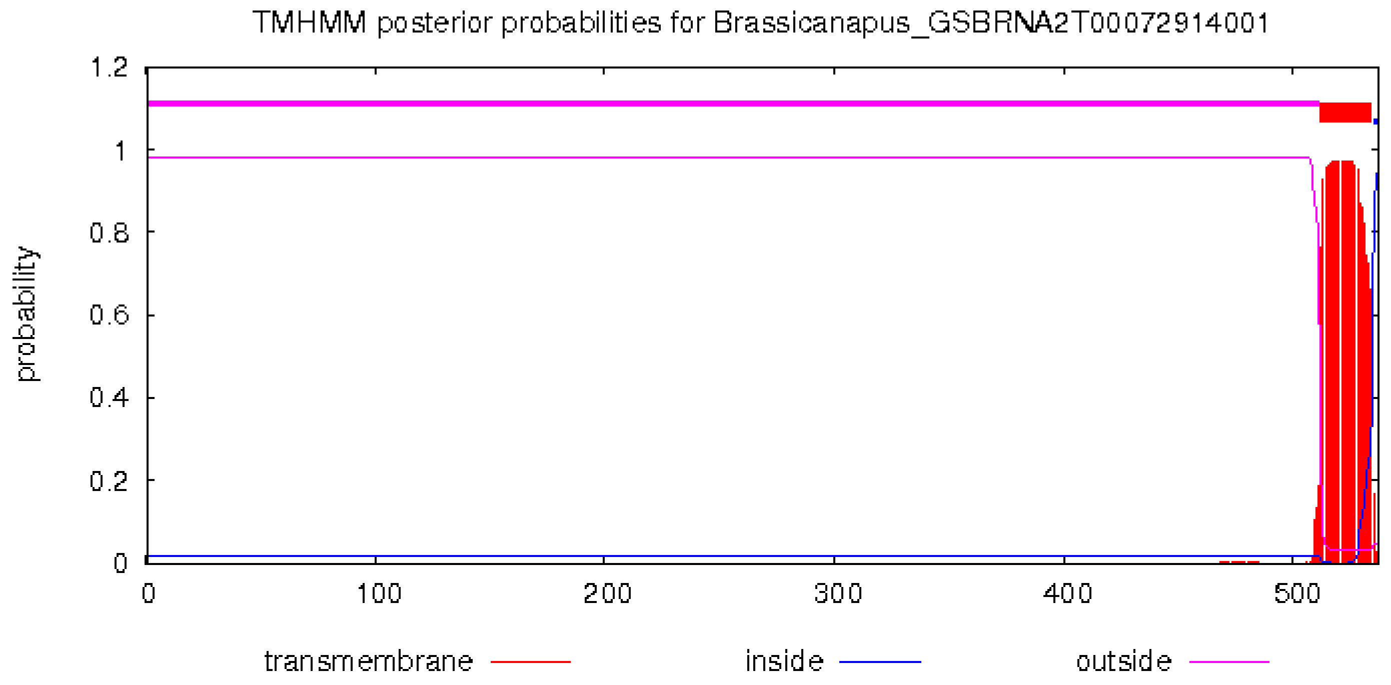

plot in postscript, script for making the plot in gnuplot, data for plot

---

```
# Brassicanapus_GSBRNA2T00073164001 Length: 250
# Brassicanapus_GSBRNA2T00073164001 Number of predicted TMHs:  0
# Brassicanapus_GSBRNA2T00073164001 Exp number of AAs in TMHs: 0.00042
# Brassicanapus_GSBRNA2T00073164001 Exp number, first 60 AAs:  0.00042
```

```
# Brassicanapus_GSBRNA2T00073164001 Total prob of N-in:        0.01339
Brassicanapus_GSBRNA2T00073164001       TMHMM2.0        outside      1   250
```

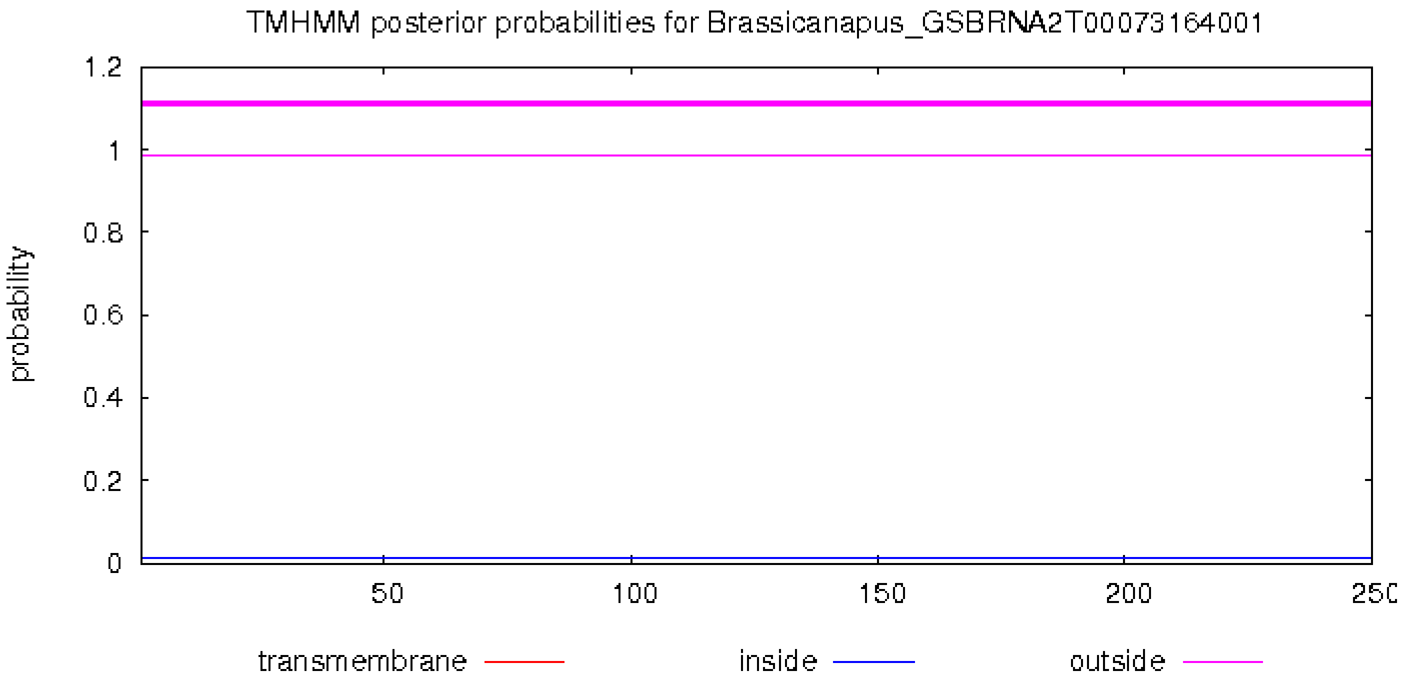

plot in postscript, script for making the plot in gnuplot, data for plot

---

```
# Brassicanapus_GSBRNA2T00073188001 Length: 357
# Brassicanapus_GSBRNA2T00073188001 Number of predicted TMHs:  0
# Brassicanapus_GSBRNA2T00073188001 Exp number of AAs in TMHs: 0.00096
# Brassicanapus_GSBRNA2T00073188001 Exp number, first 60 AAs:  0
# Brassicanapus_GSBRNA2T00073188001 Total prob of N-in:        0.01364
Brassicanapus_GSBRNA2T00073188001       TMHMM2.0        outside      1   357
```

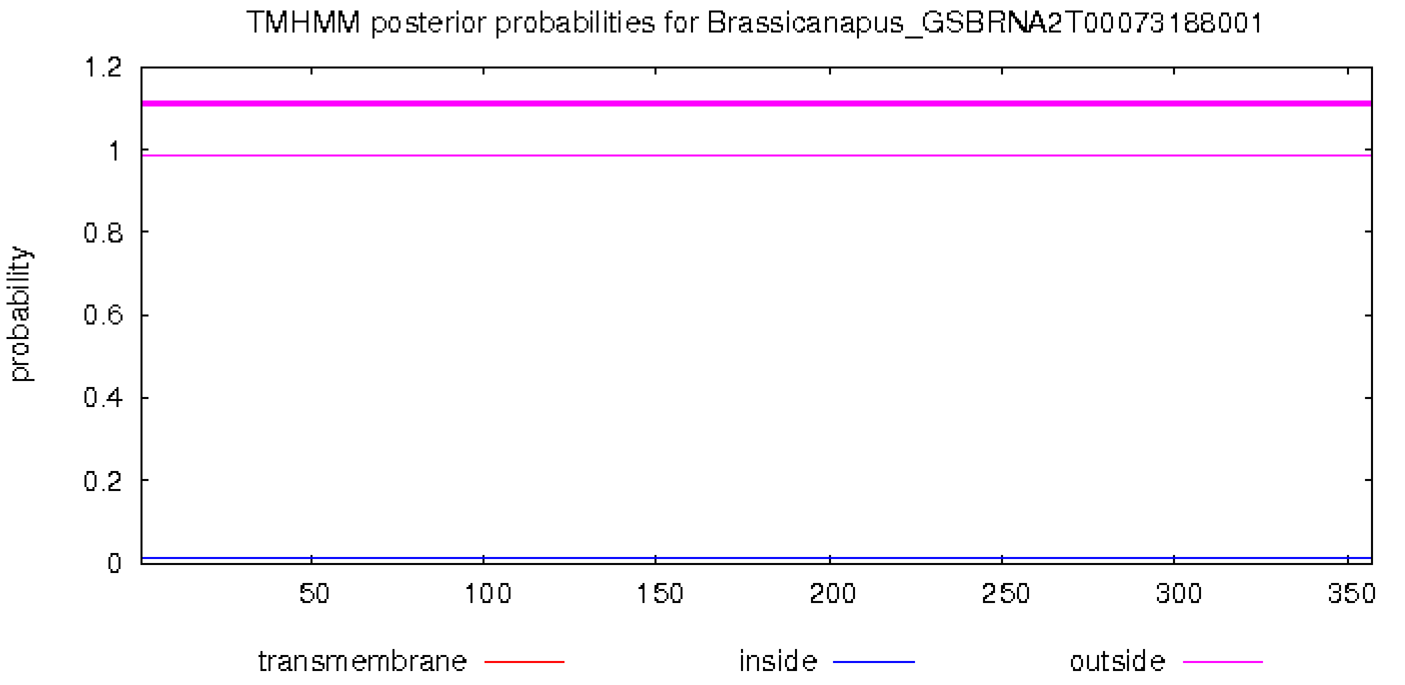

plot in postscript, script for making the plot in gnuplot, data for plot

---

```
# Brassicanapus_GSBRNA2T00074690001 Length: 321
# Brassicanapus_GSBRNA2T00074690001 Number of predicted TMHs:  0
# Brassicanapus_GSBRNA2T00074690001 Exp number of AAs in TMHs: 0.0002
# Brassicanapus_GSBRNA2T00074690001 Exp number, first 60 AAs:  0
```

```
# Brassicanapus_GSBRNA2T00074690001 Total prob of N-in:        0.01548
Brassicanapus_GSBRNA2T00074690001       TMHMM2.0        outside      1   321
```

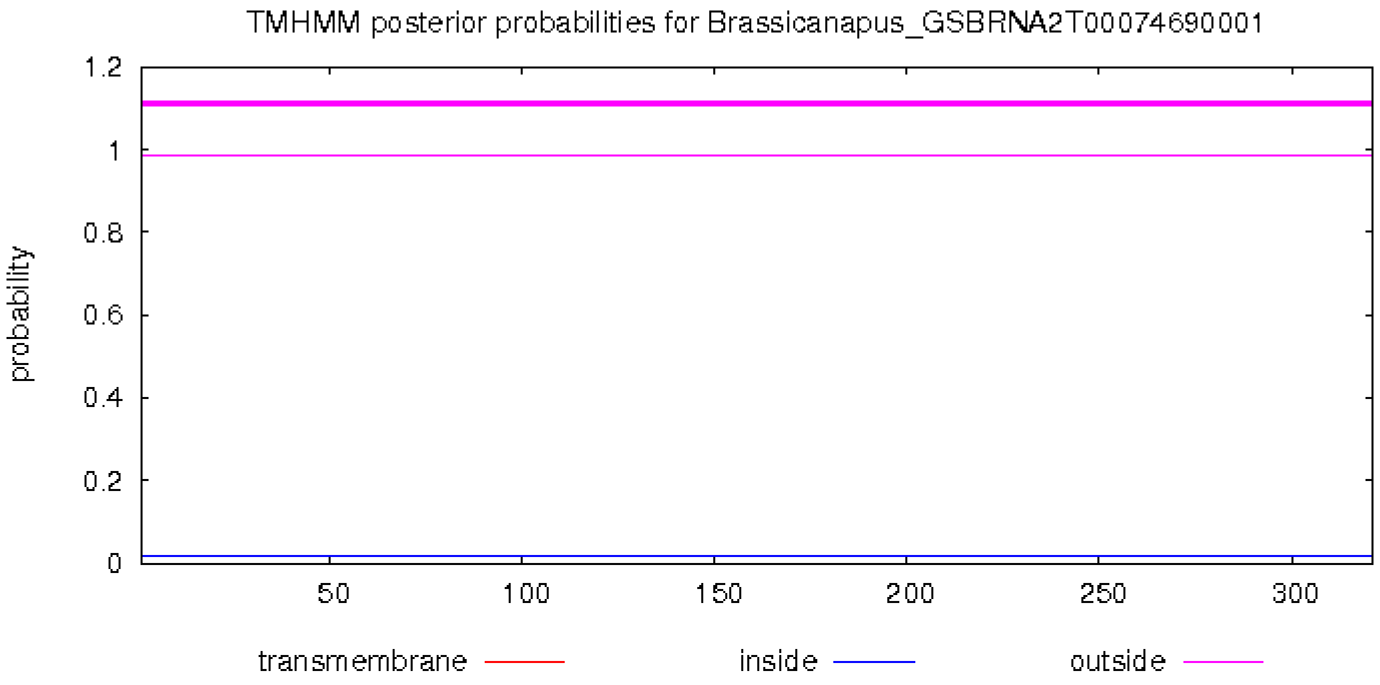

plot in postscript, script for making the plot in gnuplot, data for plot

---

```
# Brassicanapus_GSBRNA2T00074713001 Length: 309
# Brassicanapus_GSBRNA2T00074713001 Number of predicted TMHs:  0
# Brassicanapus_GSBRNA2T00074713001 Exp number of AAs in TMHs: 0
# Brassicanapus_GSBRNA2T00074713001 Exp number, first 60 AAs:  0
# Brassicanapus_GSBRNA2T00074713001 Total prob of N-in:        0.00128
Brassicanapus_GSBRNA2T00074713001       TMHMM2.0        outside      1   309
```

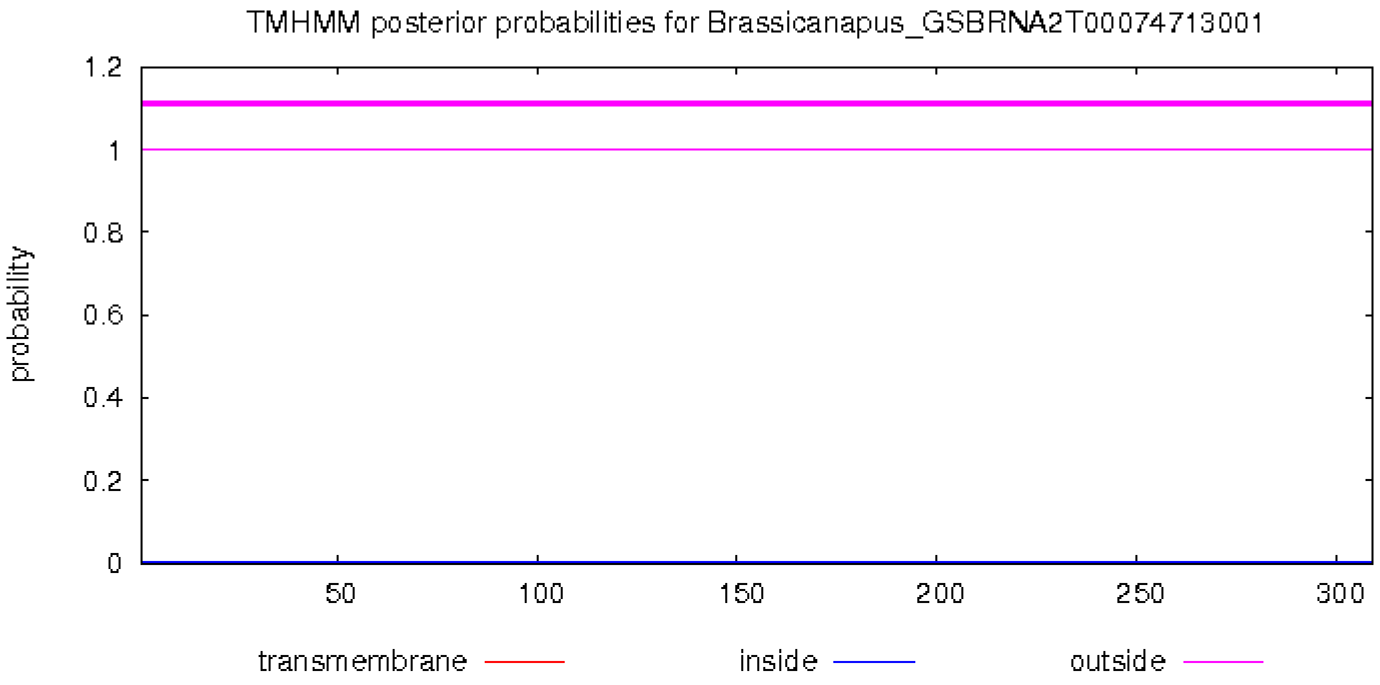

plot in postscript, script for making the plot in gnuplot, data for plot

---

```
# Brassicanapus_GSBRNA2T00075489001 Length: 510
# Brassicanapus_GSBRNA2T00075489001 Number of predicted TMHs:  2
# Brassicanapus_GSBRNA2T00075489001 Exp number of AAs in TMHs: 42.49631
# Brassicanapus_GSBRNA2T00075489001 Exp number, first 60 AAs:  0
```

```
# Brassicanapus_GSBRNA2T00075489001 Total prob of N-in:        0.03368
Brassicanapus_GSBRNA2T00075489001       TMHMM2.0        outside      1   393
Brassicanapus_GSBRNA2T00075489001       TMHMM2.0        TMhelix    394   416
Brassicanapus_GSBRNA2T00075489001       TMHMM2.0        inside     417   422
Brassicanapus_GSBRNA2T00075489001       TMHMM2.0        TMhelix    423   445
Brassicanapus_GSBRNA2T00075489001       TMHMM2.0        outside    446   510
```

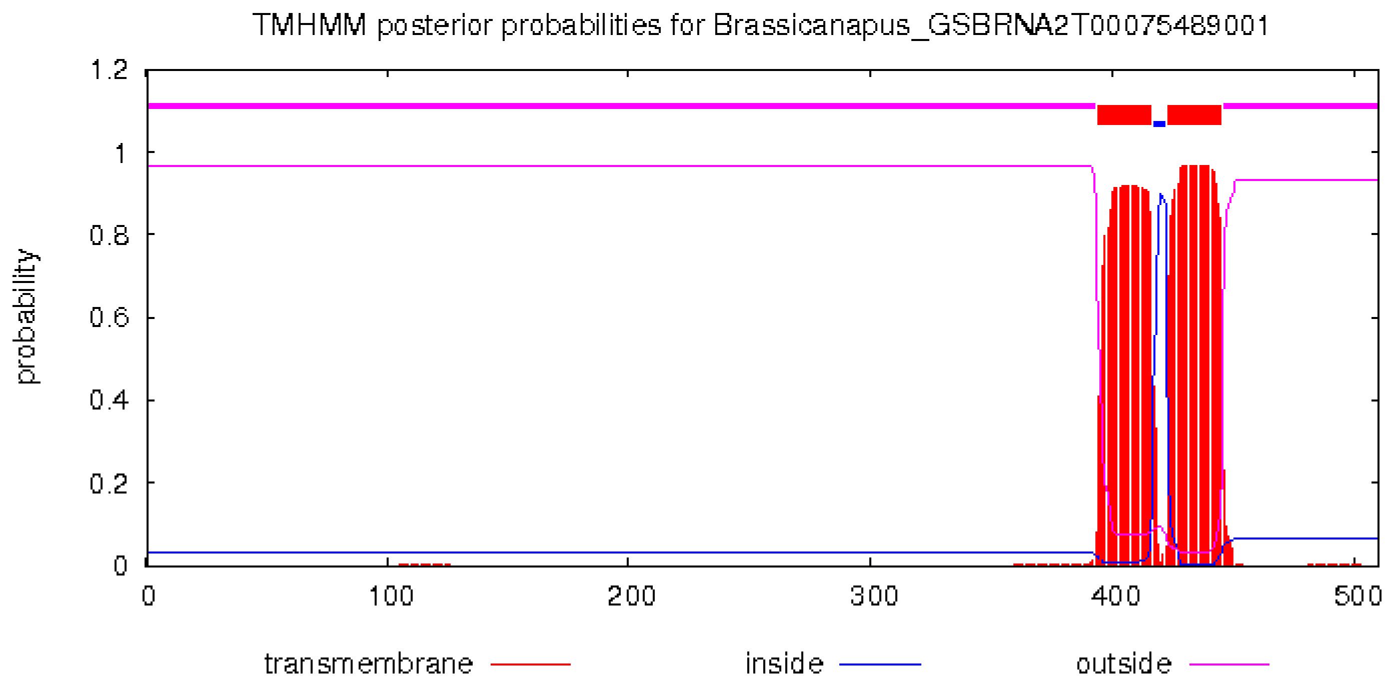

plot in postscript, script for making the plot in gnuplot, data for plot

---

```
# Brassicanapus_GSBRNA2T00075573001 Length: 325
# Brassicanapus_GSBRNA2T00075573001 Number of predicted TMHs:  0
# Brassicanapus_GSBRNA2T00075573001 Exp number of AAs in TMHs: 0.00264
# Brassicanapus_GSBRNA2T00075573001 Exp number, first 60 AAs:  0
# Brassicanapus_GSBRNA2T00075573001 Total prob of N-in:        0.01207
Brassicanapus_GSBRNA2T00075573001       TMHMM2.0        outside      1   325
```

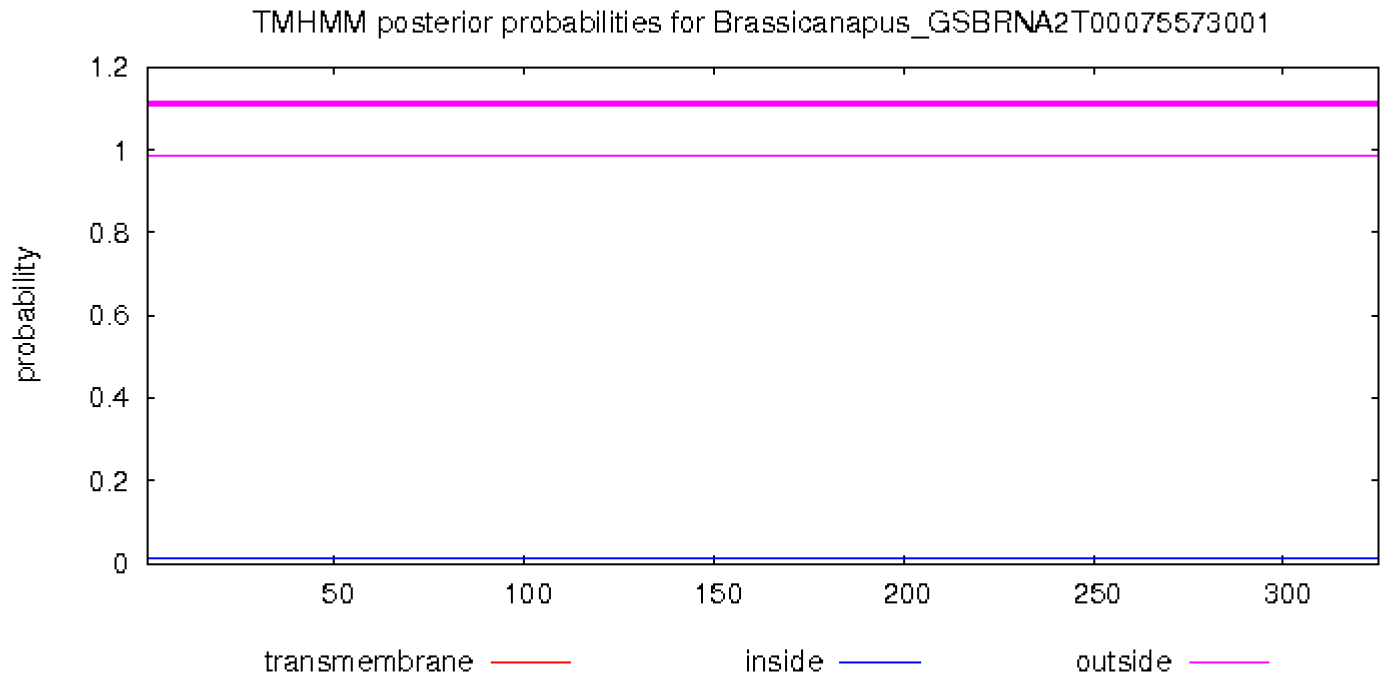

plot in postscript, script for making the plot in gnuplot, data for plot

---

```
# Brassicanapus_GSBRNA2T00075596001 Length: 296
# Brassicanapus_GSBRNA2T00075596001 Number of predicted TMHs:  0
# Brassicanapus_GSBRNA2T00075596001 Exp number of AAs in TMHs: 0.86905
# Brassicanapus_GSBRNA2T00075596001 Exp number, first 60 AAs:  0
# Brassicanapus_GSBRNA2T00075596001 Total prob of N-in:        0.03677
Brassicanapus_GSBRNA2T00075596001	TMHMM2.0	outside	     1   296
```

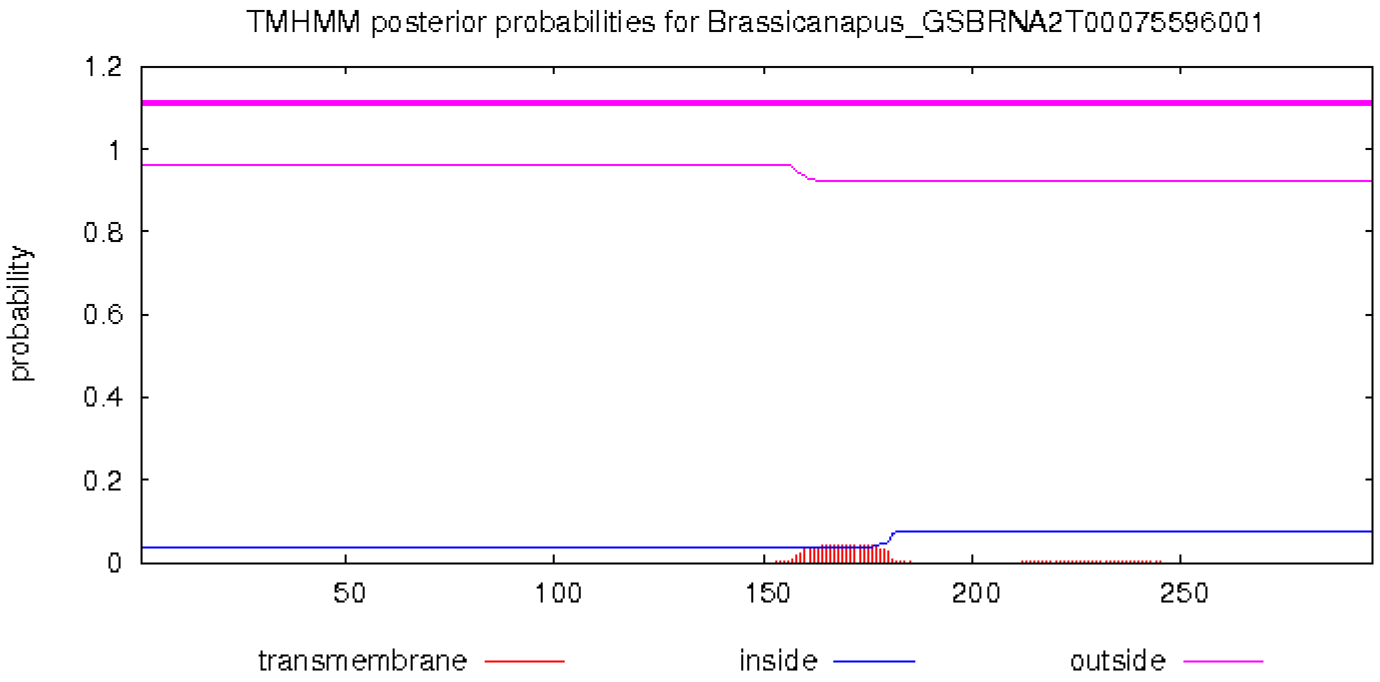

plot in postscript, script for making the plot in gnuplot, data for plot

```
# Brassicanapus_GSBRNA2T00075630001 Length: 344
# Brassicanapus_GSBRNA2T00075630001 Number of predicted TMHs:  0
# Brassicanapus_GSBRNA2T00075630001 Exp number of AAs in TMHs: 0.00097
# Brassicanapus_GSBRNA2T00075630001 Exp number, first 60 AAs:  0
# Brassicanapus_GSBRNA2T00075630001 Total prob of N-in:        0.00699
Brassicanapus_GSBRNA2T00075630001	TMHMM2.0	outside	     1   344
```

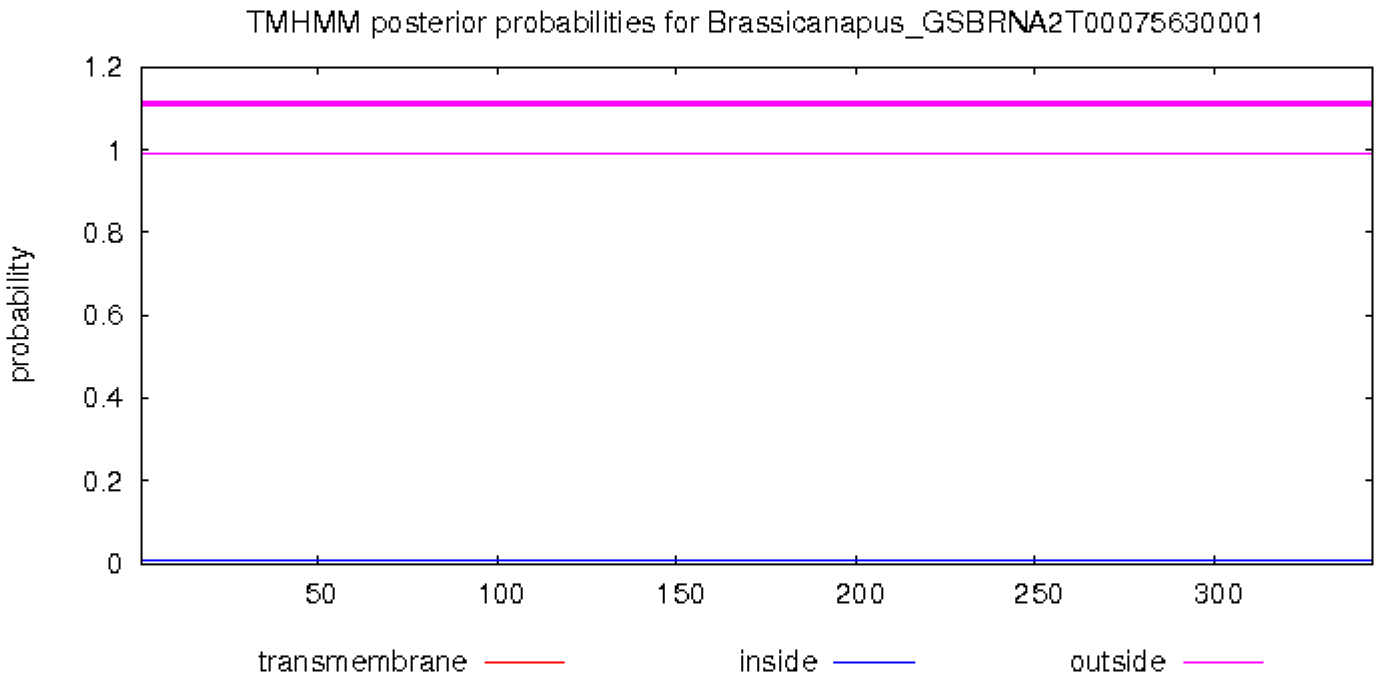

plot in postscript, script for making the plot in gnuplot, data for plot

```
# Brassicanapus_GSBRNA2T00075632001 Length: 280
# Brassicanapus_GSBRNA2T00075632001 Number of predicted TMHs:  0
# Brassicanapus_GSBRNA2T00075632001 Exp number of AAs in TMHs: 0.00058
# Brassicanapus_GSBRNA2T00075632001 Exp number, first 60 AAs:  0.00011
# Brassicanapus_GSBRNA2T00075632001 Total prob of N-in:        0.02514
Brassicanapus_GSBRNA2T00075632001	TMHMM2.0	outside	     1   280
```

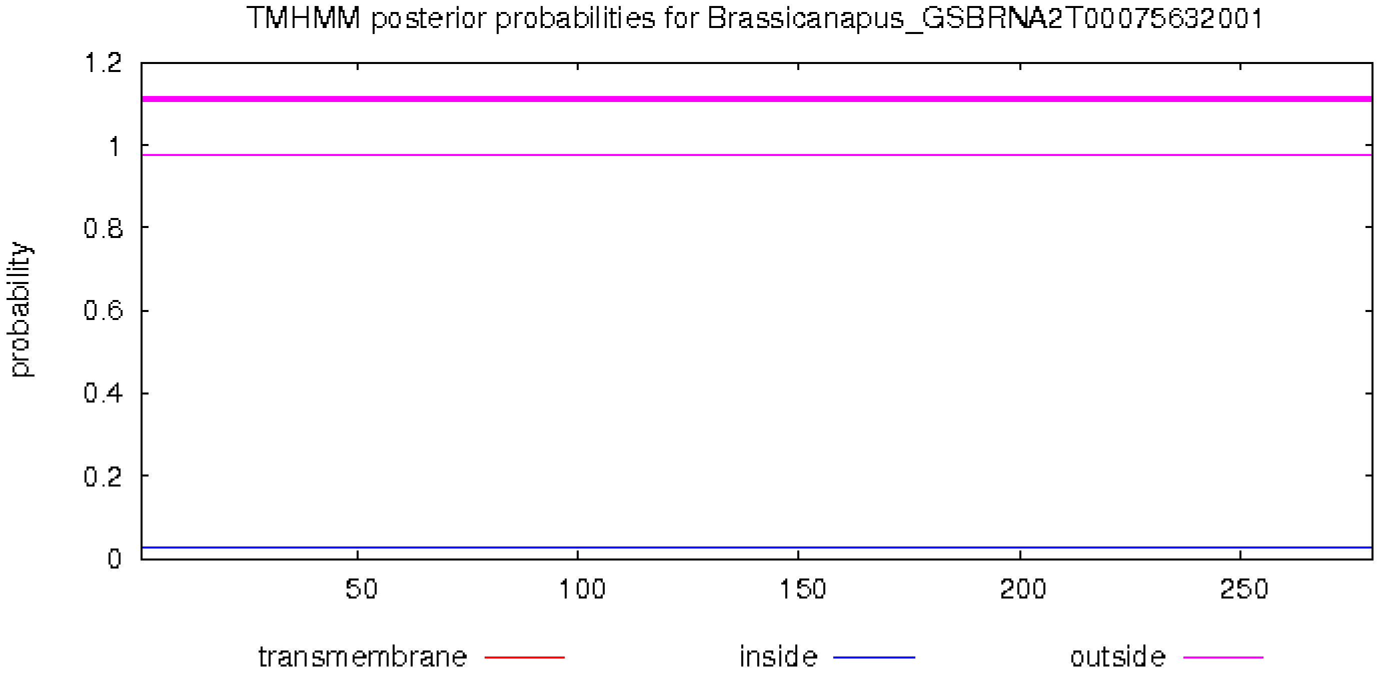

plot in postscript, script for making the plot in gnuplot, data for plot

---

```
# Brassicanapus_GSBRNA2T00076116001 Length: 478
# Brassicanapus_GSBRNA2T00076116001 Number of predicted TMHs:  0
# Brassicanapus_GSBRNA2T00076116001 Exp number of AAs in TMHs: 0.00114
# Brassicanapus_GSBRNA2T00076116001 Exp number, first 60 AAs:  0
# Brassicanapus_GSBRNA2T00076116001 Total prob of N-in:        0.00016
Brassicanapus_GSBRNA2T00076116001	TMHMM2.0	outside	     1   478
```

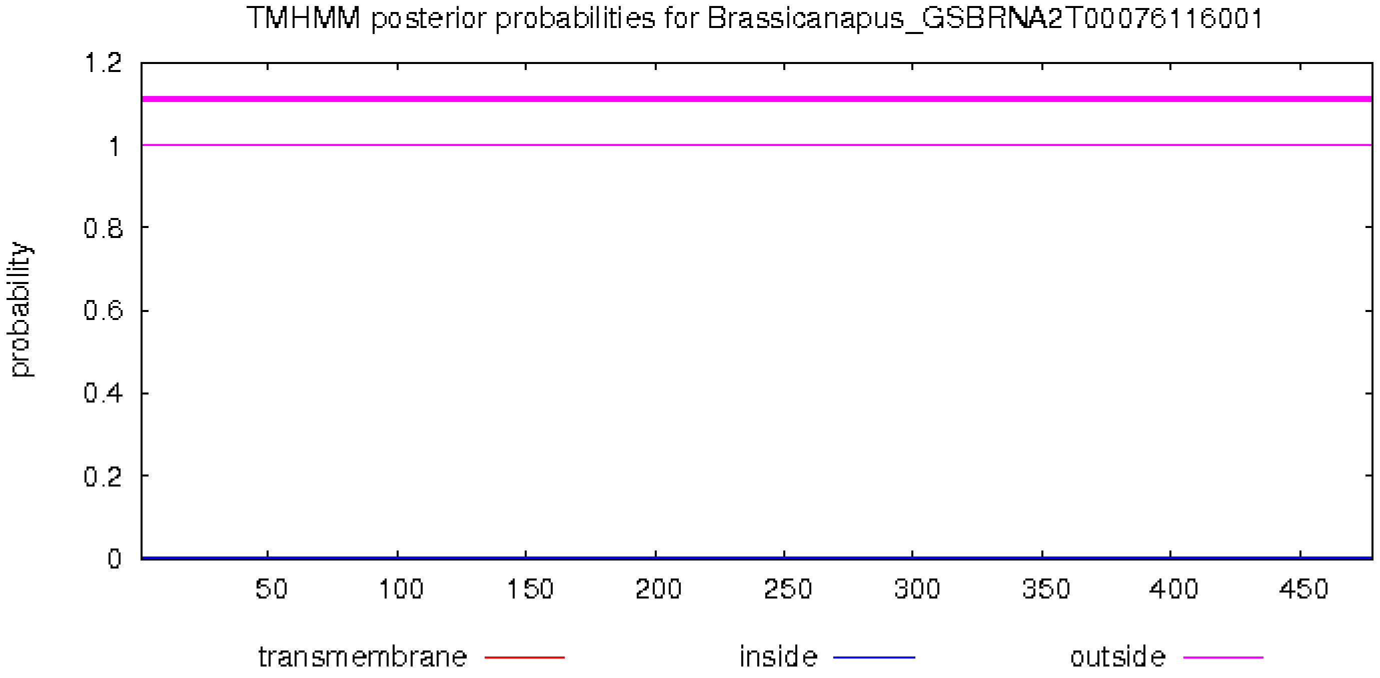

plot in postscript, script for making the plot in gnuplot, data for plot

---

```
# Brassicanapus_GSBRNA2T00076462001 Length: 295
# Brassicanapus_GSBRNA2T00076462001 Number of predicted TMHs:  0
# Brassicanapus_GSBRNA2T00076462001 Exp number of AAs in TMHs: 0.0084
# Brassicanapus_GSBRNA2T00076462001 Exp number, first 60 AAs:  0
# Brassicanapus_GSBRNA2T00076462001 Total prob of N-in:        0.02897
Brassicanapus_GSBRNA2T00076462001       TMHMM2.0        outside      1   295
```

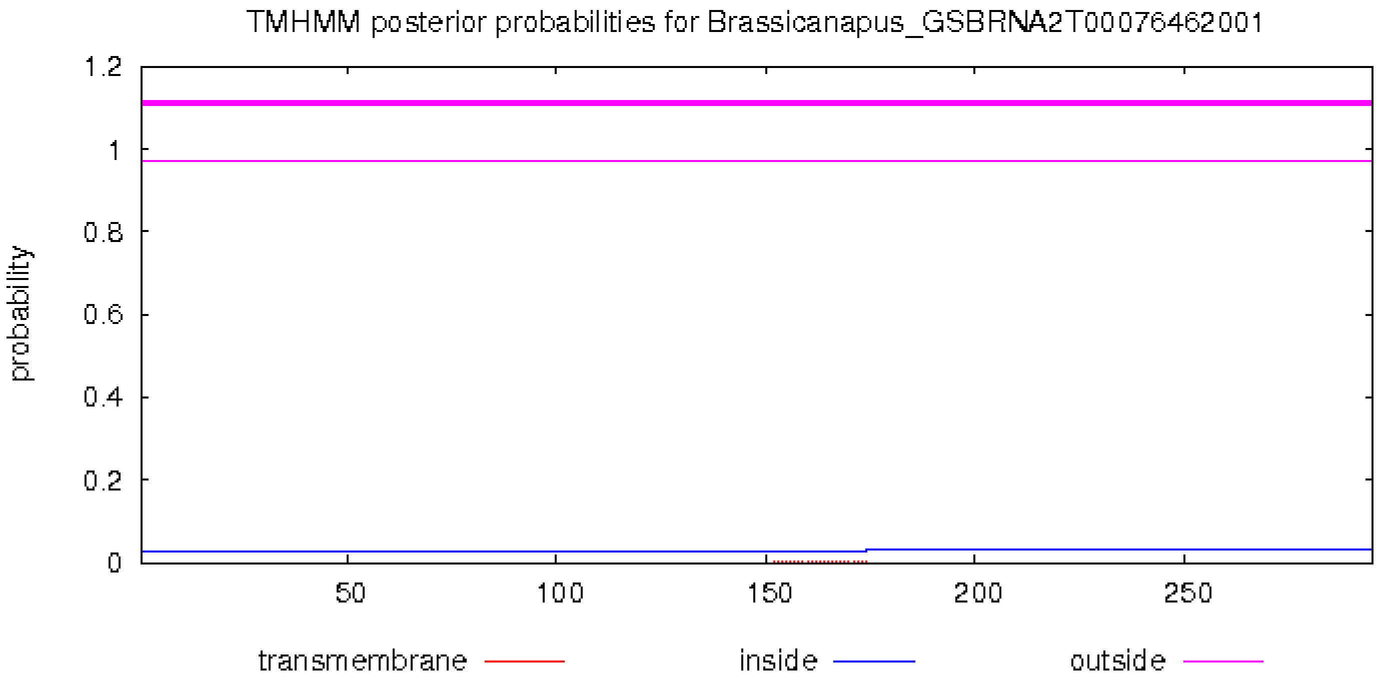

plot in postscript, script for making the plot in gnuplot, data for plot

```
# Brassicanapus_GSBRNA2T00077057001 Length: 210
# Brassicanapus_GSBRNA2T00077057001 Number of predicted TMHs:  0
# Brassicanapus_GSBRNA2T00077057001 Exp number of AAs in TMHs: 0.00053
# Brassicanapus_GSBRNA2T00077057001 Exp number, first 60 AAs:  0
# Brassicanapus_GSBRNA2T00077057001 Total prob of N-in:        0.01922
Brassicanapus_GSBRNA2T00077057001       TMHMM2.0        outside      1   210
```

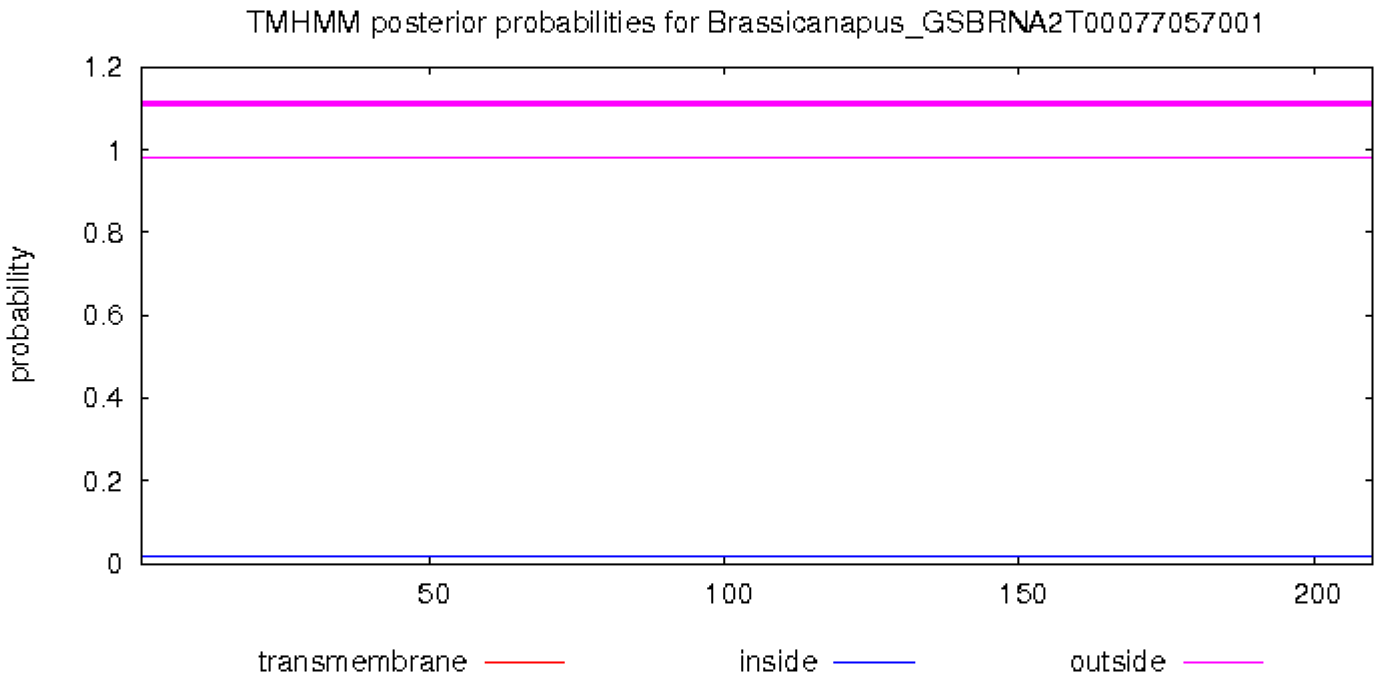

plot in postscript, script for making the plot in gnuplot, data for plot

```
# Brassicanapus_GSBRNA2T00077286001 Length: 344
# Brassicanapus_GSBRNA2T00077286001 Number of predicted TMHs:  0
# Brassicanapus_GSBRNA2T00077286001 Exp number of AAs in TMHs: 0.00018
# Brassicanapus_GSBRNA2T00077286001 Exp number, first 60 AAs:  0.00018
# Brassicanapus_GSBRNA2T00077286001 Total prob of N-in:        0.00343
Brassicanapus_GSBRNA2T00077286001       TMHMM2.0        outside      1   344
```

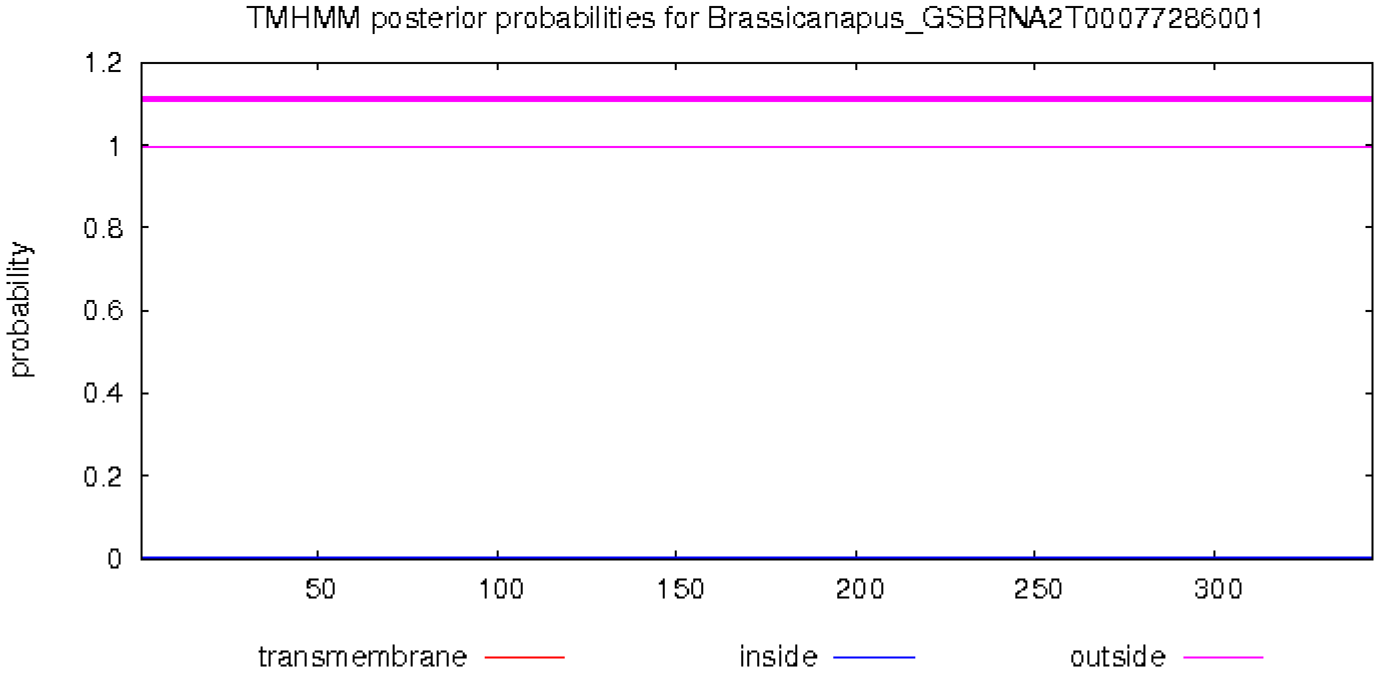

plot in postscript, script for making the plot in gnuplot, data for plot

---

```
# Brassicanapus_GSBRNA2T00078555001 Length: 315
# Brassicanapus_GSBRNA2T00078555001 Number of predicted TMHs:  0
# Brassicanapus_GSBRNA2T00078555001 Exp number of AAs in TMHs: 0.00041
# Brassicanapus_GSBRNA2T00078555001 Exp number, first 60 AAs:  0
# Brassicanapus_GSBRNA2T00078555001 Total prob of N-in:        0.00743
Brassicanapus_GSBRNA2T00078555001       TMHMM2.0        outside      1   315
```

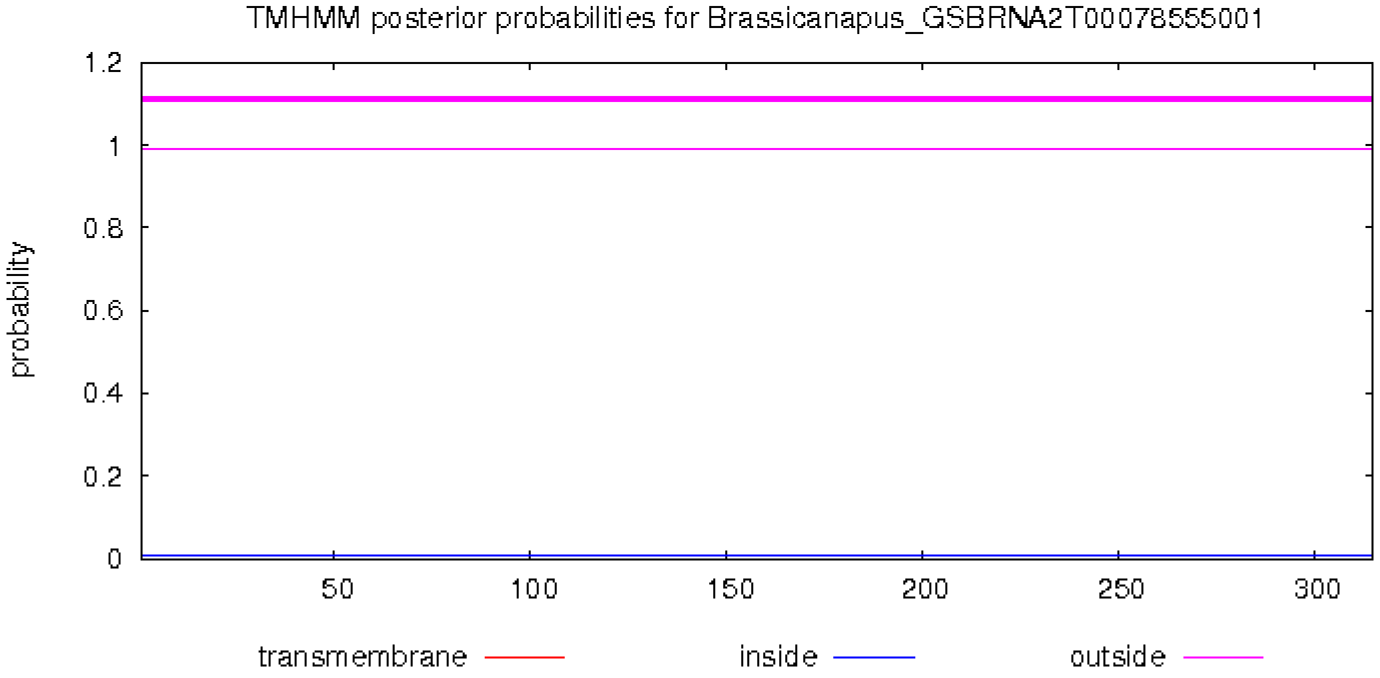

plot in postscript, script for making the plot in gnuplot, data for plot

---

```
# Brassicanapus_GSBRNA2T00078706001 Length: 516
# Brassicanapus_GSBRNA2T00078706001 Number of predicted TMHs:  1
# Brassicanapus_GSBRNA2T00078706001 Exp number of AAs in TMHs: 22.21119
# Brassicanapus_GSBRNA2T00078706001 Exp number, first 60 AAs:  0
# Brassicanapus_GSBRNA2T00078706001 Total prob of N-in:        0.02047
Brassicanapus_GSBRNA2T00078706001	TMHMM2.0	outside	     1   490
Brassicanapus_GSBRNA2T00078706001	TMHMM2.0	TMhelix	   491   513
Brassicanapus_GSBRNA2T00078706001	TMHMM2.0	inside	   514   516
```

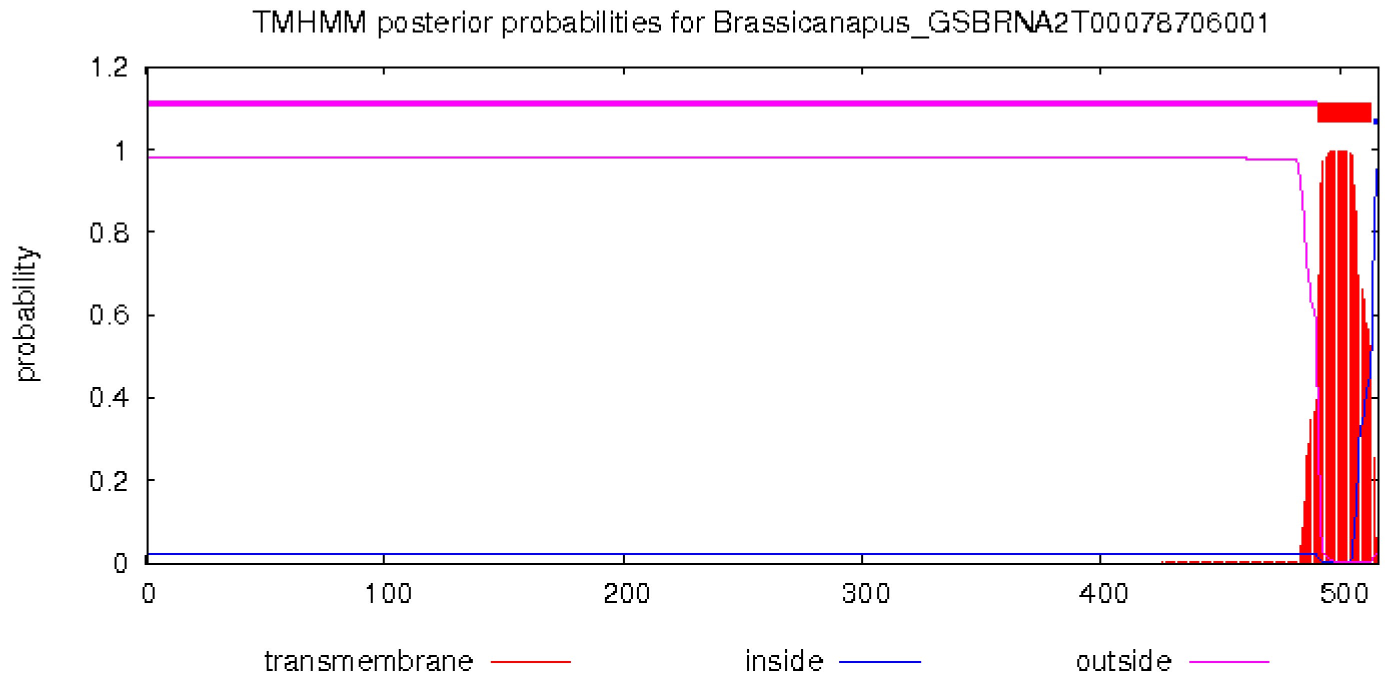

plot in postscript, script for making the plot in gnuplot, data for plot

---

```
# Brassicanapus_GSBRNA2T00078811001 Length: 336
# Brassicanapus_GSBRNA2T00078811001 Number of predicted TMHs:  0
# Brassicanapus_GSBRNA2T00078811001 Exp number of AAs in TMHs: 1e-05
# Brassicanapus_GSBRNA2T00078811001 Exp number, first 60 AAs:  0
# Brassicanapus_GSBRNA2T00078811001 Total prob of N-in:        0.00578
Brassicanapus_GSBRNA2T00078811001	TMHMM2.0	outside	     1   336
```

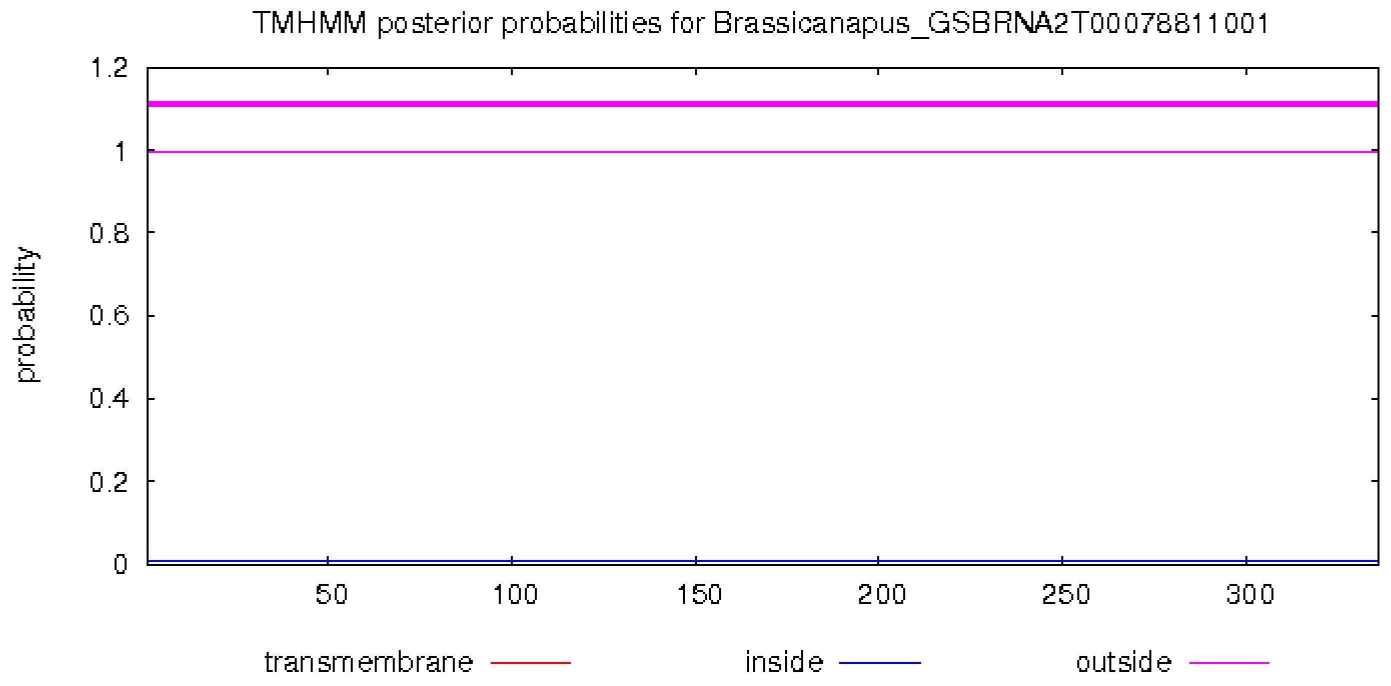

plot in postscript, script for making the plot in gnuplot, data for plot

```
# Brassicanapus_GSBRNA2T00080256001 Length: 292
# Brassicanapus_GSBRNA2T00080256001 Number of predicted TMHs:  0
# Brassicanapus_GSBRNA2T00080256001 Exp number of AAs in TMHs: 0
# Brassicanapus_GSBRNA2T00080256001 Exp number, first 60 AAs:  0
# Brassicanapus_GSBRNA2T00080256001 Total prob of N-in:        0.00489
Brassicanapus_GSBRNA2T00080256001       TMHMM2.0        outside      1   292
```

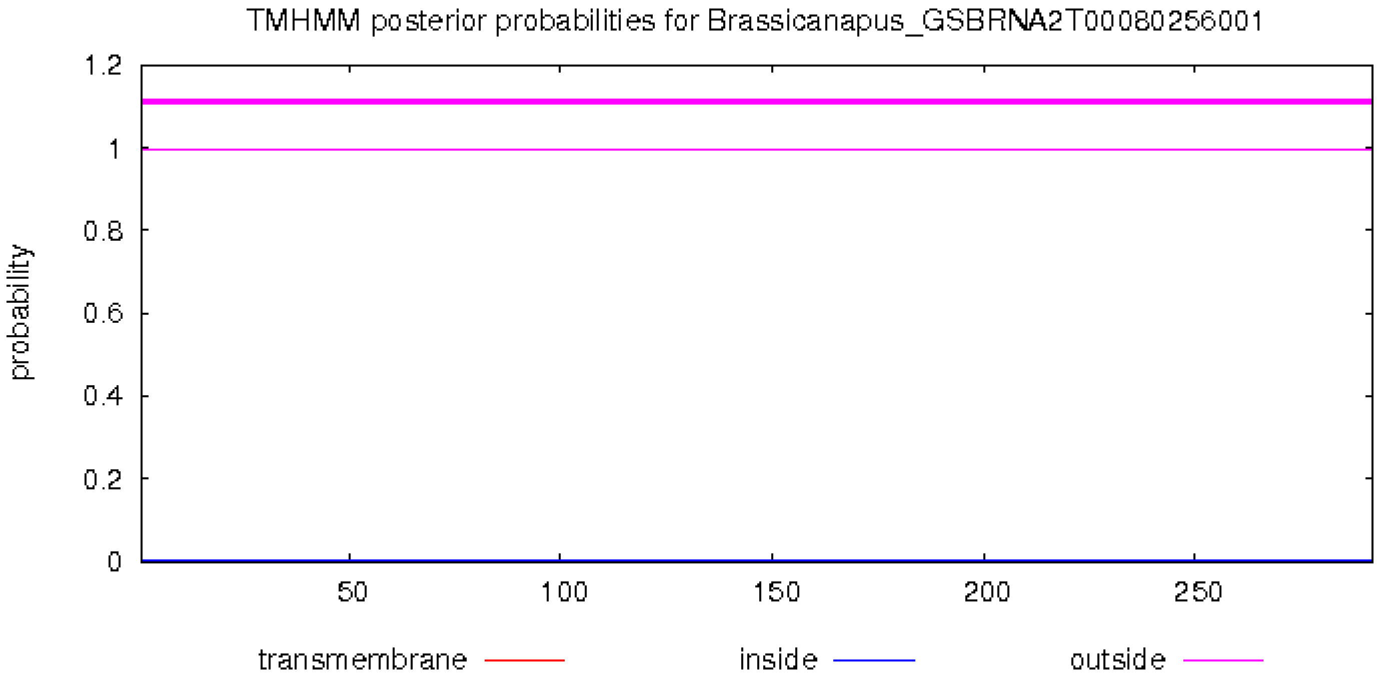

plot in postscript, script for making the plot in gnuplot, data for plot

```
# Brassicanapus_GSBRNA2T00080395001 Length: 235
# Brassicanapus_GSBRNA2T00080395001 Number of predicted TMHs:  0
# Brassicanapus_GSBRNA2T00080395001 Exp number of AAs in TMHs: 0.01048
# Brassicanapus_GSBRNA2T00080395001 Exp number, first 60 AAs:  0
# Brassicanapus_GSBRNA2T00080395001 Total prob of N-in:        0.03757
Brassicanapus_GSBRNA2T00080395001       TMHMM2.0        outside      1   235
```

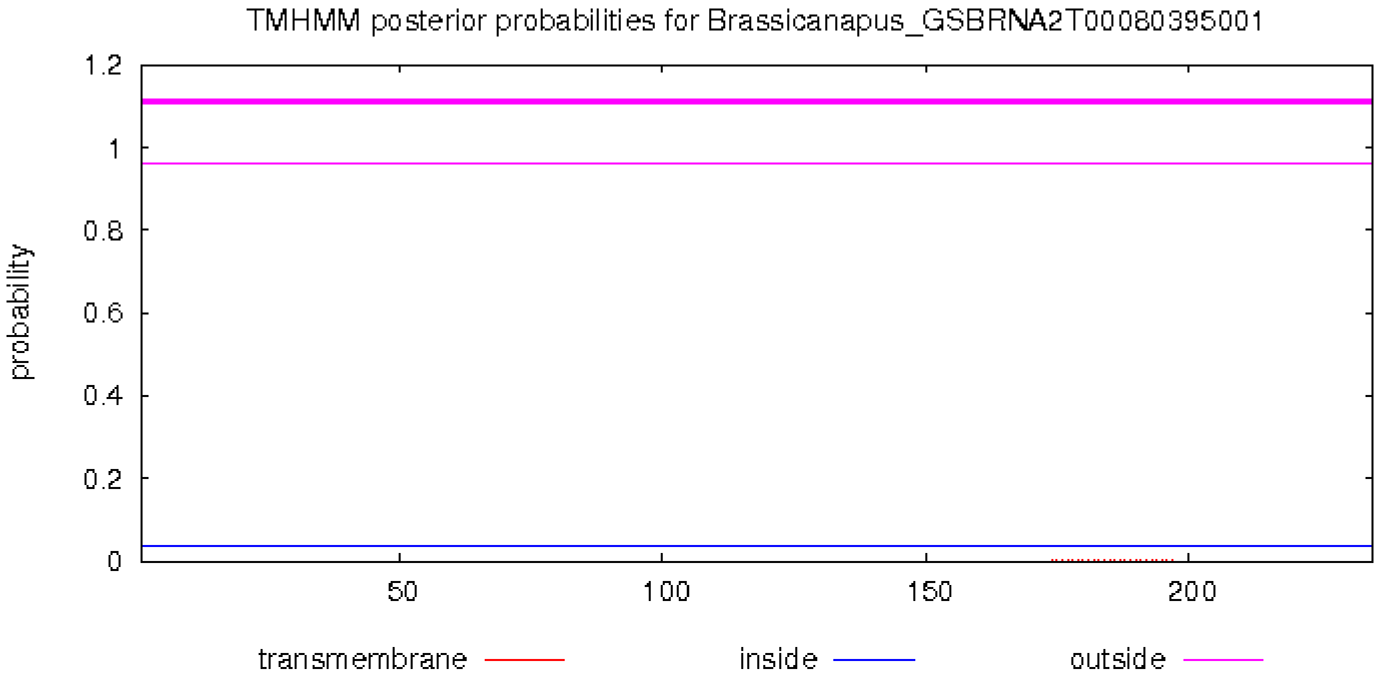

plot in postscript, script for making the plot in gnuplot, data for plot

```
# Brassicanapus_GSBRNA2T00080792001 Length: 262
# Brassicanapus_GSBRNA2T00080792001 Number of predicted TMHs:  0
# Brassicanapus_GSBRNA2T00080792001 Exp number of AAs in TMHs: 0.00019
# Brassicanapus_GSBRNA2T00080792001 Exp number, first 60 AAs:  0
# Brassicanapus_GSBRNA2T00080792001 Total prob of N-in:        0.01082
Brassicanapus_GSBRNA2T00080792001	TMHMM2.0	outside	     1   262
```

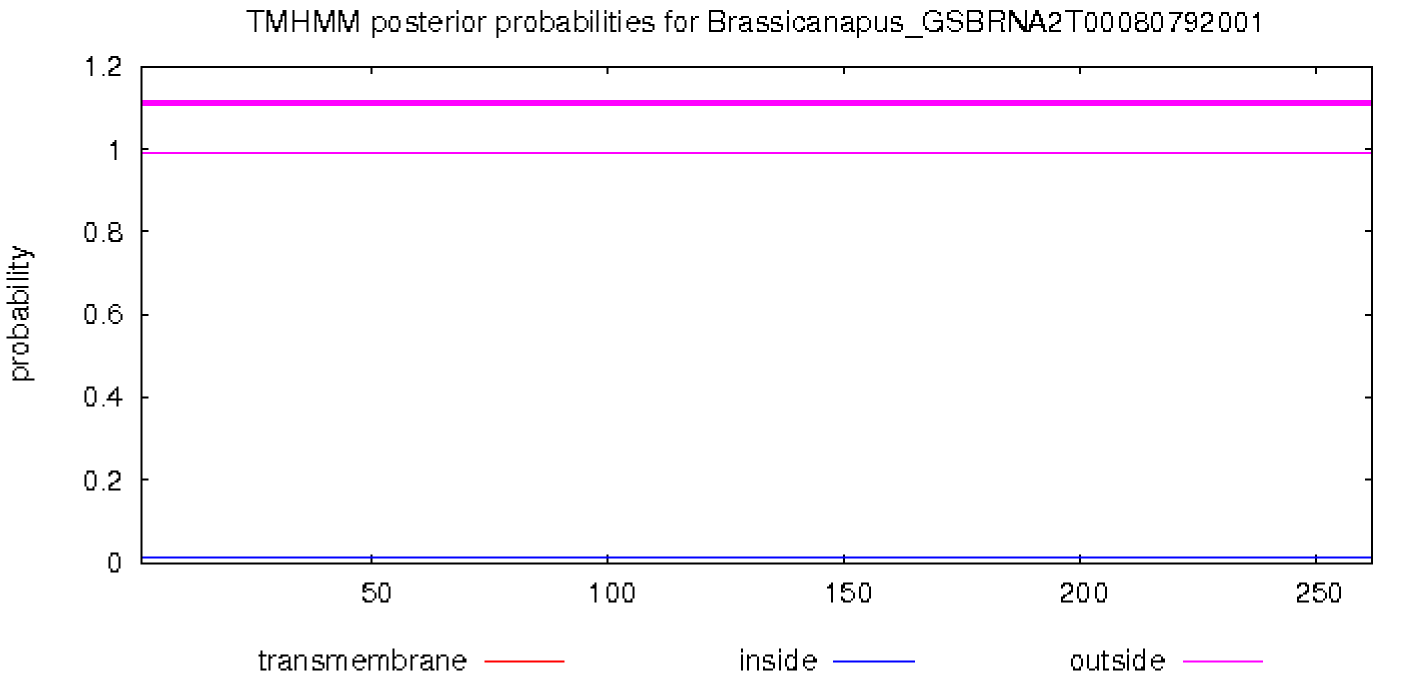

plot in postscript, script for making the plot in gnuplot, data for plot

---

```
# Brassicanapus_GSBRNA2T00080799001 Length: 350
# Brassicanapus_GSBRNA2T00080799001 Number of predicted TMHs:  0
# Brassicanapus_GSBRNA2T00080799001 Exp number of AAs in TMHs: 0
# Brassicanapus_GSBRNA2T00080799001 Exp number, first 60 AAs:  0
# Brassicanapus_GSBRNA2T00080799001 Total prob of N-in:        0.00397
Brassicanapus_GSBRNA2T00080799001	TMHMM2.0	outside	     1   350
```

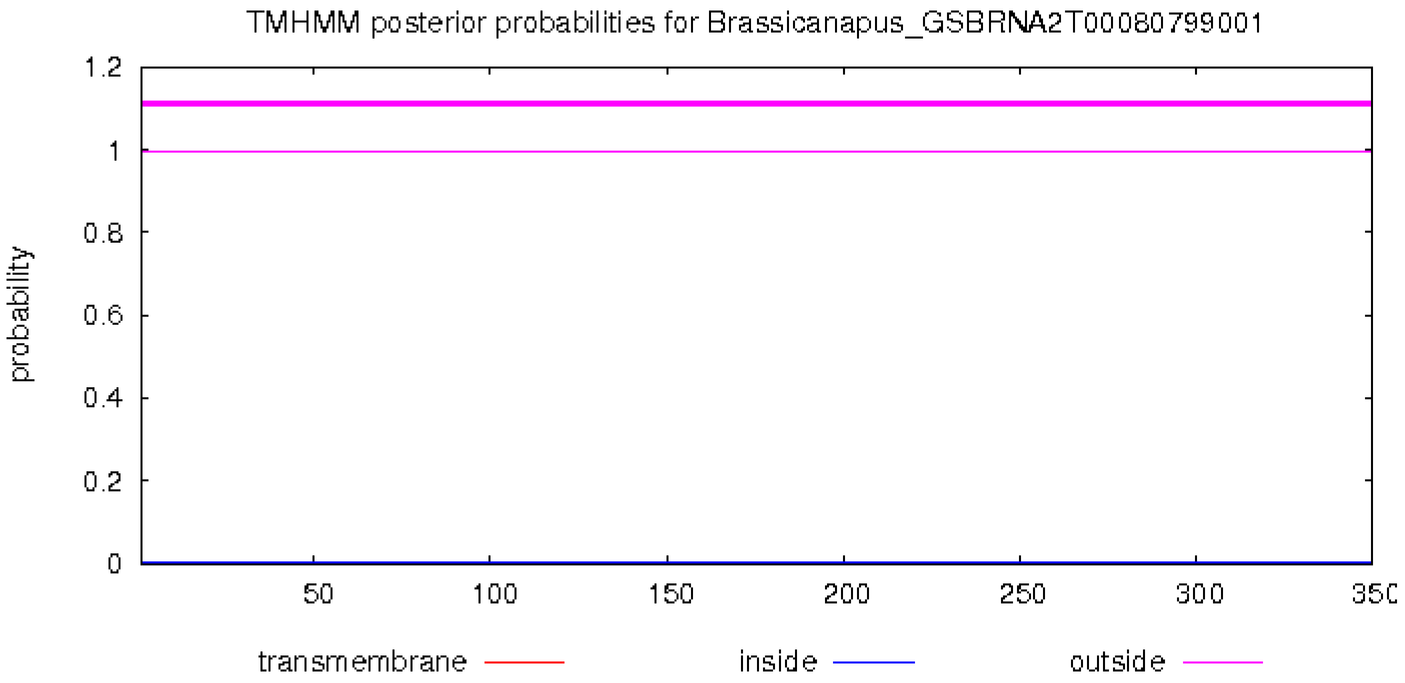

plot in postscript, script for making the plot in gnuplot, data for plot

---

```
# Brassicanapus_GSBRNA2T00080904001 Length: 560
# Brassicanapus_GSBRNA2T00080904001 Number of predicted TMHs:  1
# Brassicanapus_GSBRNA2T00080904001 Exp number of AAs in TMHs: 21.66948
# Brassicanapus_GSBRNA2T00080904001 Exp number, first 60 AAs:  0
# Brassicanapus_GSBRNA2T00080904001 Total prob of N-in:        0.04569
Brassicanapus_GSBRNA2T00080904001	TMHMM2.0	outside	     1   534
Brassicanapus_GSBRNA2T00080904001	TMHMM2.0	TMhelix	   535   557
Brassicanapus_GSBRNA2T00080904001	TMHMM2.0	inside	   558   560
```

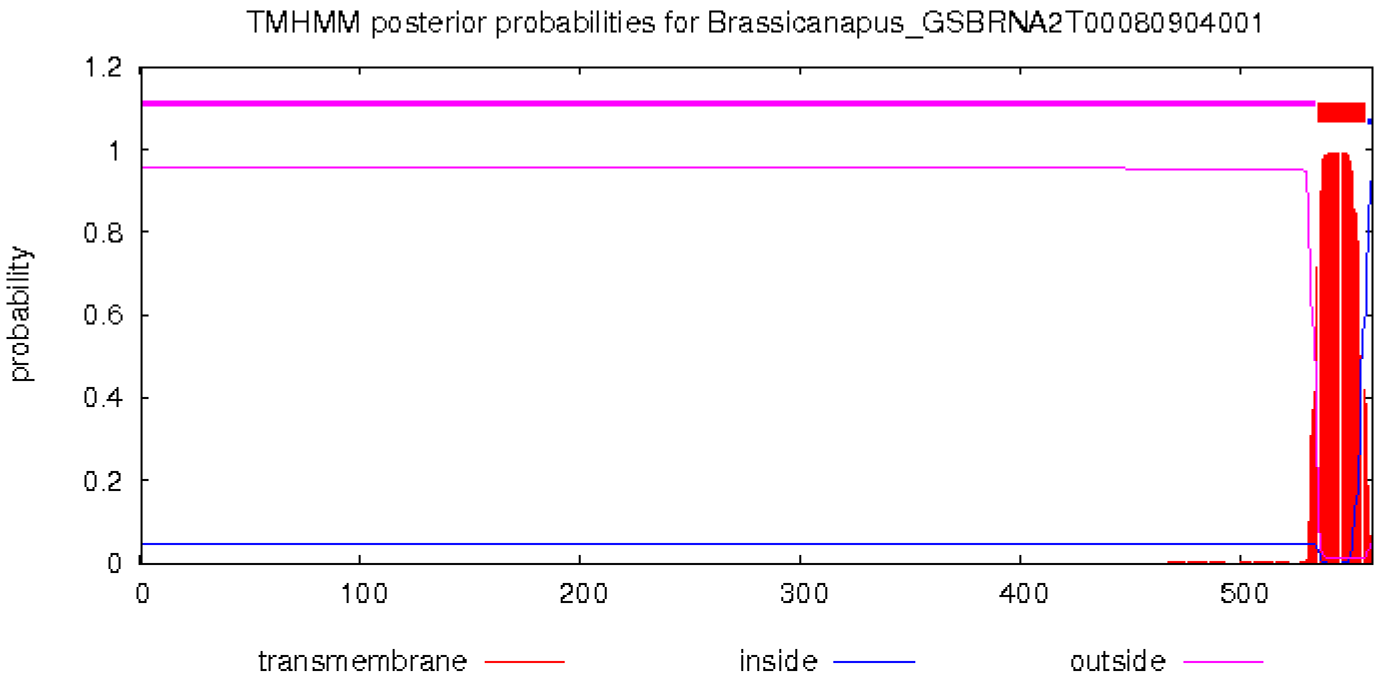

plot in postscript, script for making the plot in gnuplot, data for plot

---

```
# Brassicanapus_GSBRNA2T00081655001 Length: 294
# Brassicanapus_GSBRNA2T00081655001 Number of predicted TMHs:  0
# Brassicanapus_GSBRNA2T00081655001 Exp number of AAs in TMHs: 0.00018
# Brassicanapus_GSBRNA2T00081655001 Exp number, first 60 AAs:  0
# Brassicanapus_GSBRNA2T00081655001 Total prob of N-in:        0.01212
Brassicanapus_GSBRNA2T00081655001	TMHMM2.0	outside	     1   294
```

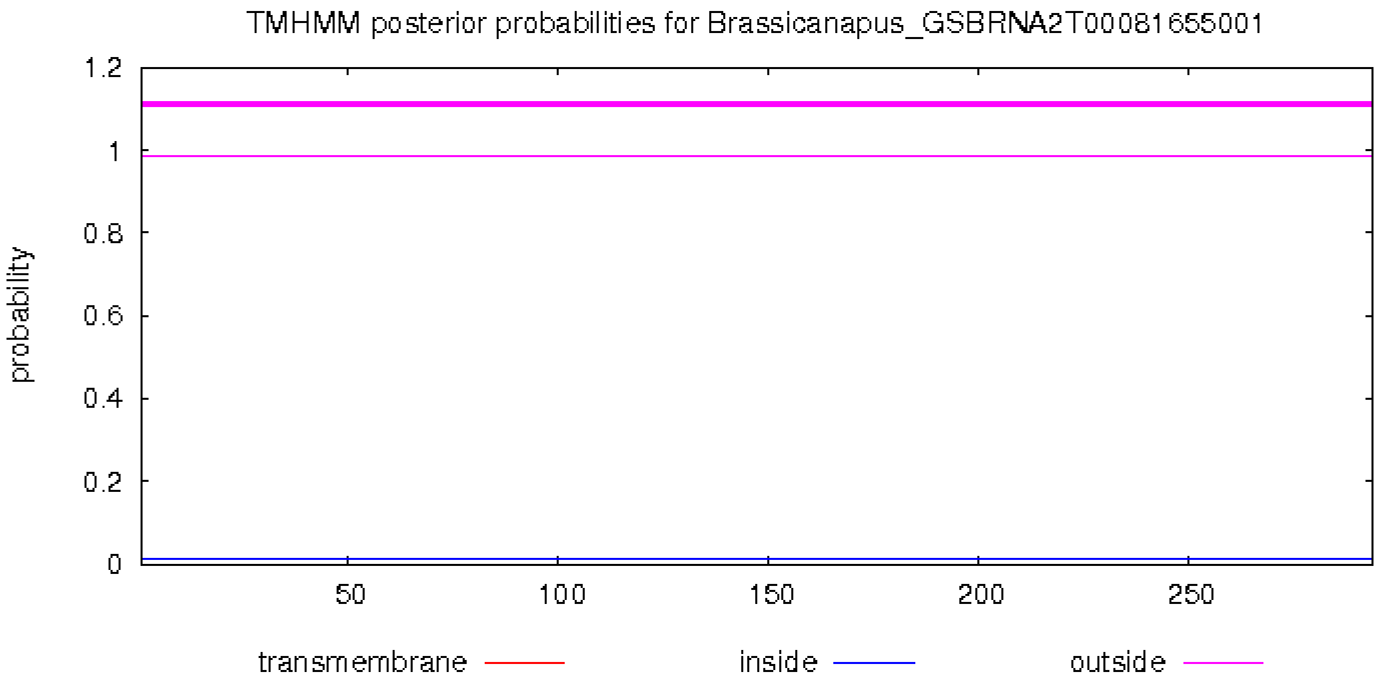

plot in postscript, script for making the plot in gnuplot, data for plot

```
# Brassicanapus_GSBRNA2T00083095001 Length: 320
# Brassicanapus_GSBRNA2T00083095001 Number of predicted TMHs:  0
# Brassicanapus_GSBRNA2T00083095001 Exp number of AAs in TMHs: 0.01391
# Brassicanapus_GSBRNA2T00083095001 Exp number, first 60 AAs:  0.00115
# Brassicanapus_GSBRNA2T00083095001 Total prob of N-in:        0.00441
Brassicanapus_GSBRNA2T00083095001	TMHMM2.0	outside	     1   320
```

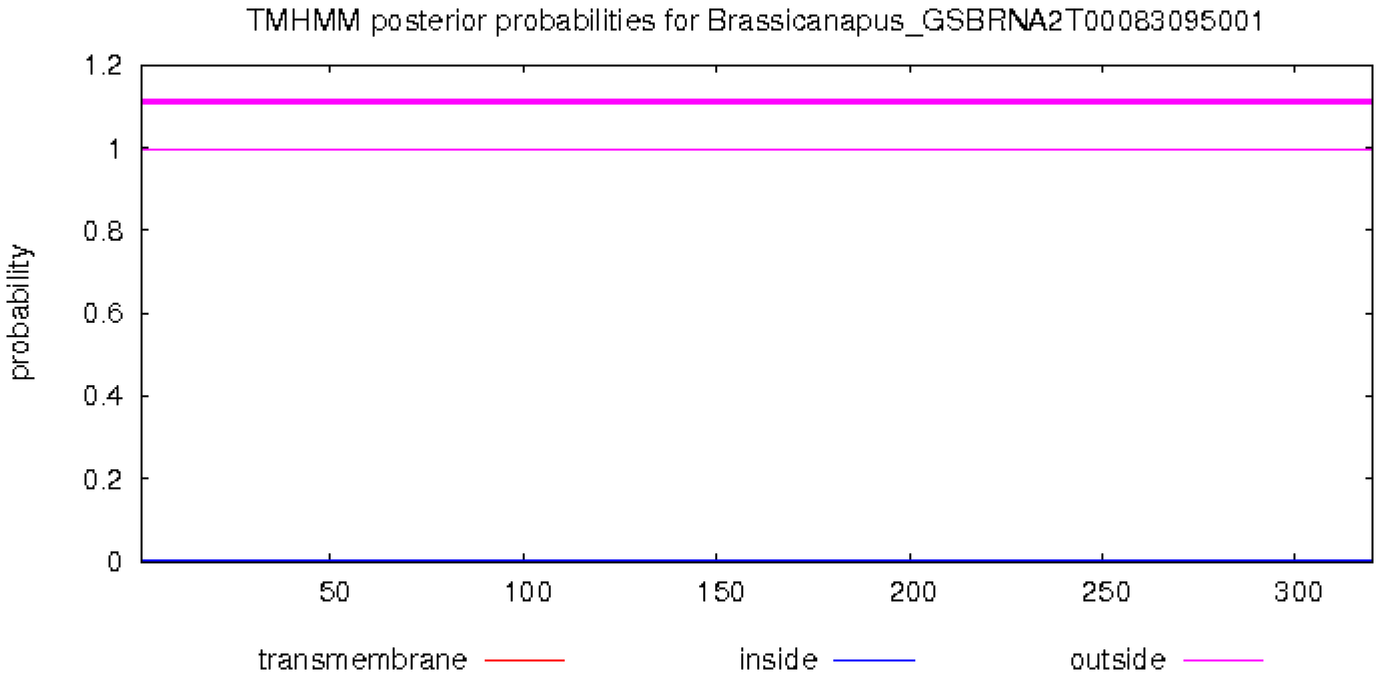

plot in postscript, script for making the plot in gnuplot, data for plot

```
# Brassicanapus_GSBRNA2T00083327001 Length: 251
# Brassicanapus_GSBRNA2T00083327001 Number of predicted TMHs:  0
# Brassicanapus_GSBRNA2T00083327001 Exp number of AAs in TMHs: 0.0019
# Brassicanapus_GSBRNA2T00083327001 Exp number, first 60 AAs:  0.0002
# Brassicanapus_GSBRNA2T00083327001 Total prob of N-in:        0.01559
Brassicanapus_GSBRNA2T00083327001	TMHMM2.0	outside	     1   251
```

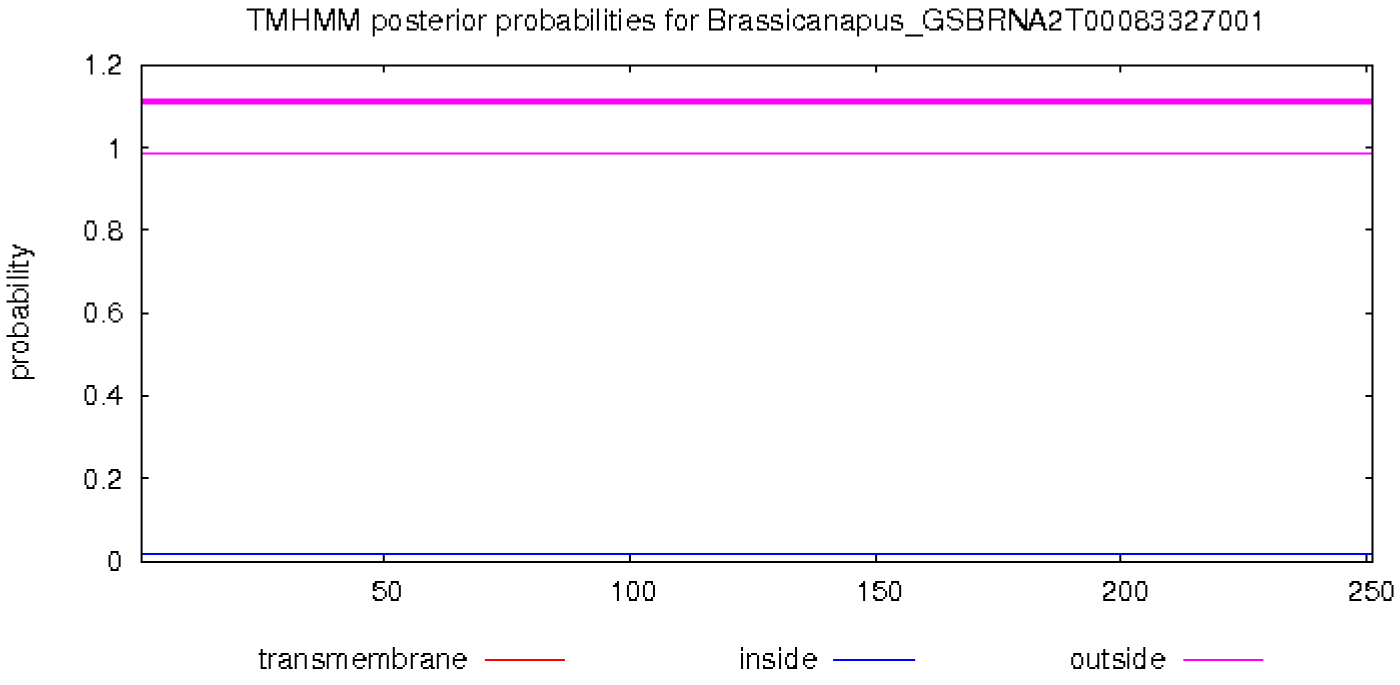

plot in postscript, script for making the plot in gnuplot, data for plot

```
# Brassicanapus_GSBRNA2T00083814001 Length: 220
# Brassicanapus_GSBRNA2T00083814001 Number of predicted TMHs:  0
# Brassicanapus_GSBRNA2T00083814001 Exp number of AAs in TMHs: 0.00021
# Brassicanapus_GSBRNA2T00083814001 Exp number, first 60 AAs:  0
# Brassicanapus_GSBRNA2T00083814001 Total prob of N-in:        0.07264
Brassicanapus_GSBRNA2T00083814001	TMHMM2.0	outside	     1   220
```

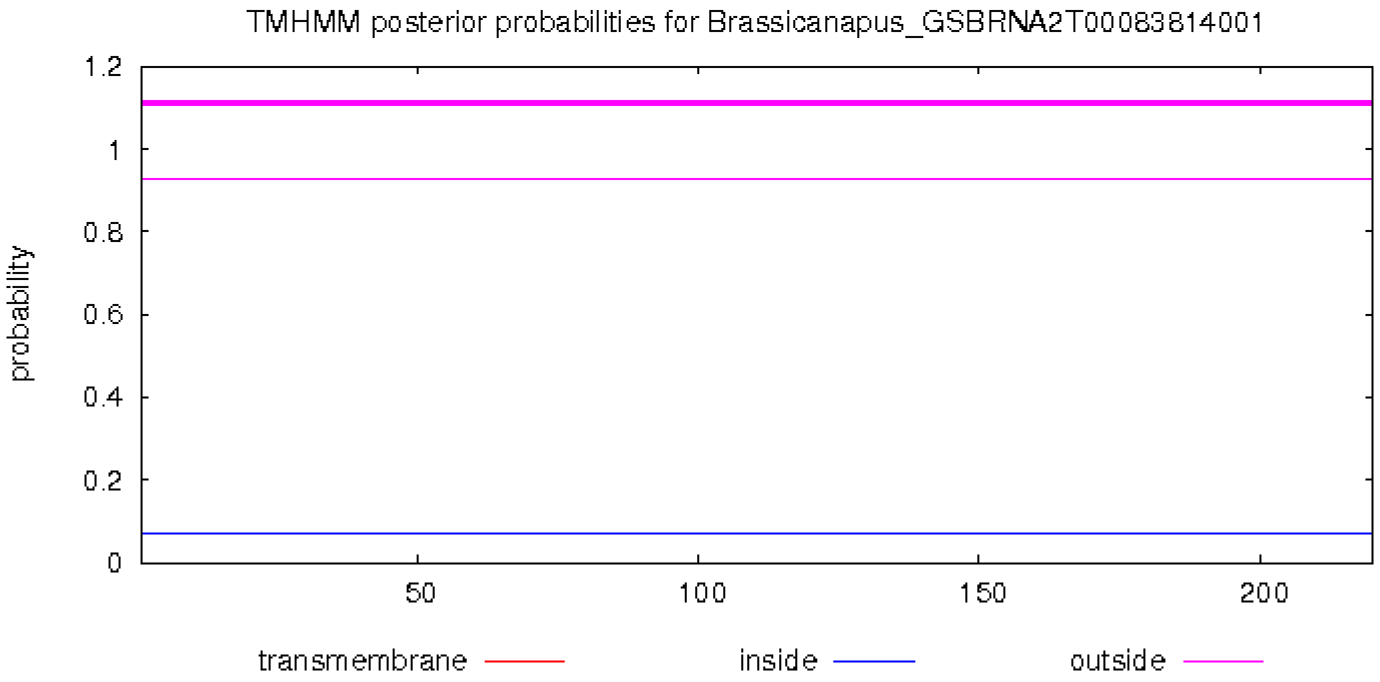

plot in postscript, script for making the plot in gnuplot, data for plot

---

```
# Brassicanapus_GSBRNA2T00083815001 Length: 124
# Brassicanapus_GSBRNA2T00083815001 Number of predicted TMHs:  0
# Brassicanapus_GSBRNA2T00083815001 Exp number of AAs in TMHs: 0.0004
# Brassicanapus_GSBRNA2T00083815001 Exp number, first 60 AAs:  0.00023
# Brassicanapus_GSBRNA2T00083815001 Total prob of N-in:        0.14658
Brassicanapus_GSBRNA2T00083815001	TMHMM2.0	outside	     1   124
```

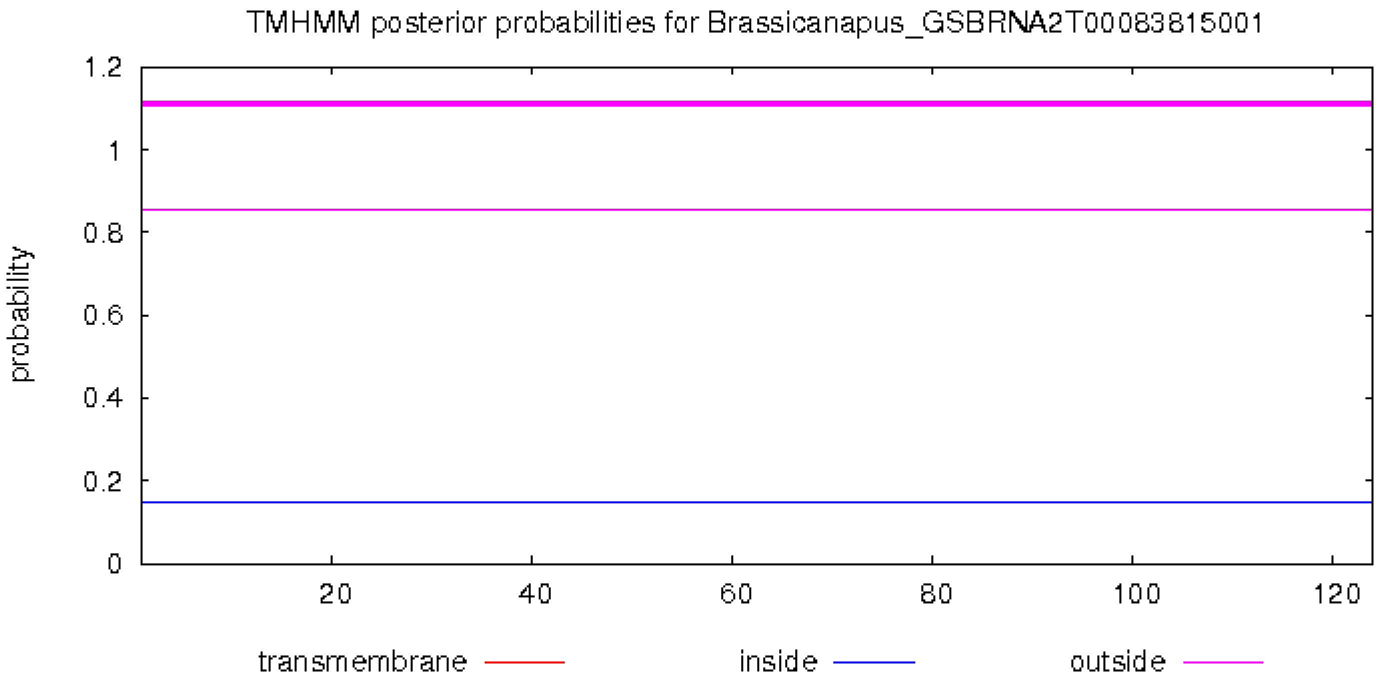

plot in postscript, script for making the plot in gnuplot, data for plot

---

```
# Brassicanapus_GSBRNA2T00083944001 Length: 201
# Brassicanapus_GSBRNA2T00083944001 Number of predicted TMHs:  0
# Brassicanapus_GSBRNA2T00083944001 Exp number of AAs in TMHs: 0.00018
# Brassicanapus_GSBRNA2T00083944001 Exp number, first 60 AAs:  0
# Brassicanapus_GSBRNA2T00083944001 Total prob of N-in:        0.01748
Brassicanapus_GSBRNA2T00083944001       TMHMM2.0        outside      1   201
```

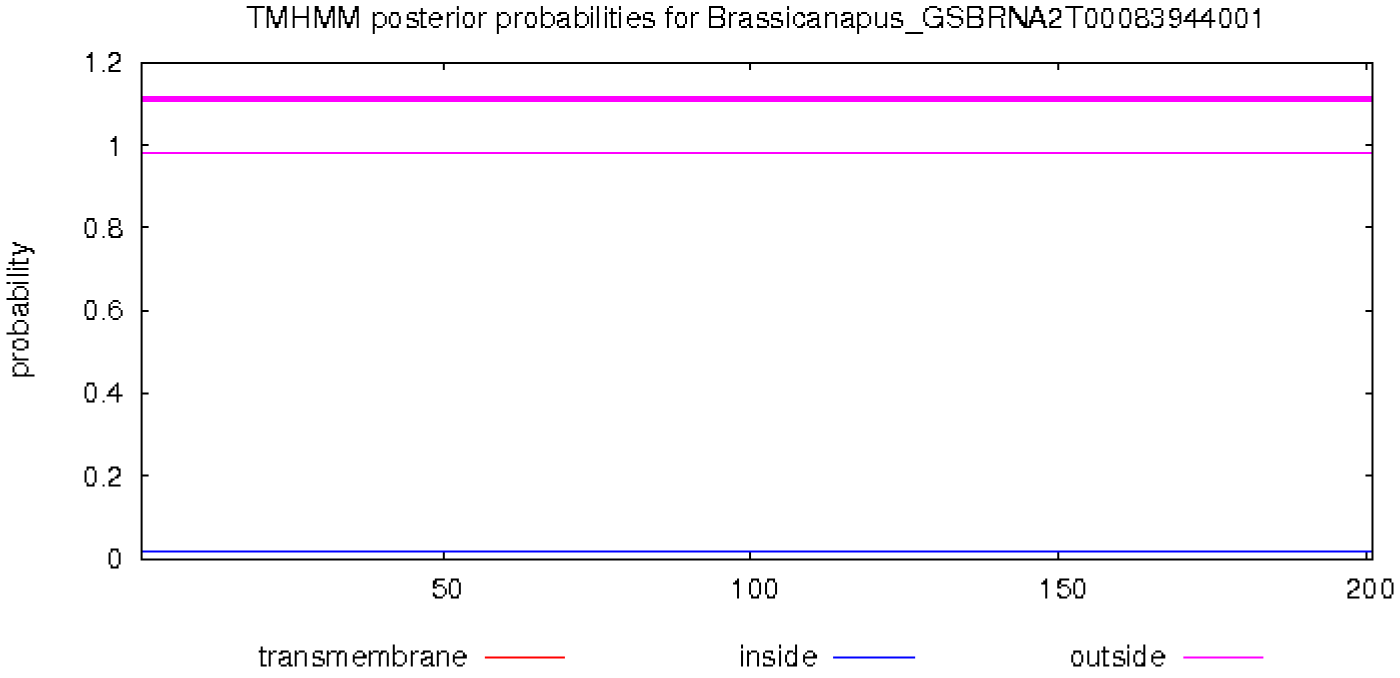

plot in postscript, script for making the plot in gnuplot, data for plot

```
# Brassicanapus_GSBRNA2T00083984001 Length: 412
# Brassicanapus_GSBRNA2T00083984001 Number of predicted TMHs:  0
# Brassicanapus_GSBRNA2T00083984001 Exp number of AAs in TMHs: 0.00019
# Brassicanapus_GSBRNA2T00083984001 Exp number, first 60 AAs:  0
# Brassicanapus_GSBRNA2T00083984001 Total prob of N-in:        0.01564
Brassicanapus_GSBRNA2T00083984001       TMHMM2.0        outside      1   412
```

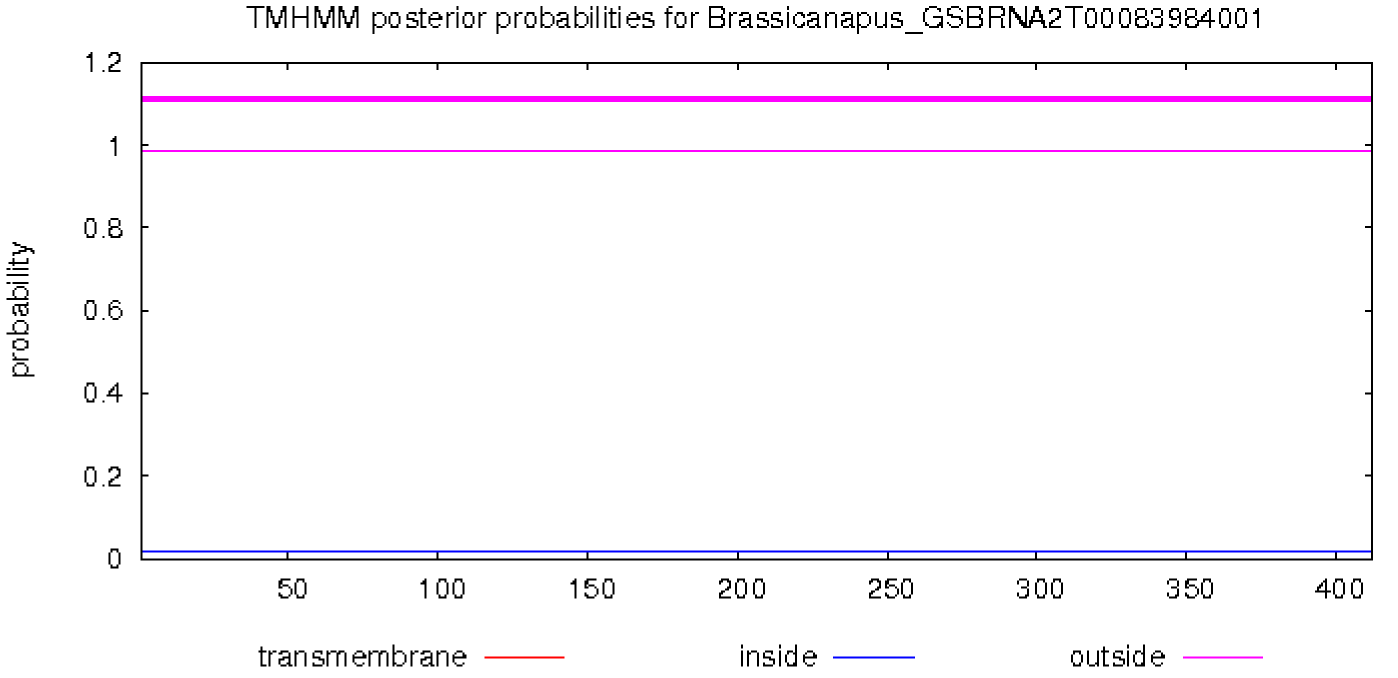

plot in postscript, script for making the plot in gnuplot, data for plot

```
# Brassicanapus_GSBRNA2T00084044001 Length: 304
# Brassicanapus_GSBRNA2T00084044001 Number of predicted TMHs:  0
# Brassicanapus_GSBRNA2T00084044001 Exp number of AAs in TMHs: 0.0003
# Brassicanapus_GSBRNA2T00084044001 Exp number, first 60 AAs:  0.0003
# Brassicanapus_GSBRNA2T00084044001 Total prob of N-in:        0.03675
Brassicanapus_GSBRNA2T00084044001	TMHMM2.0	outside	     1   304
```

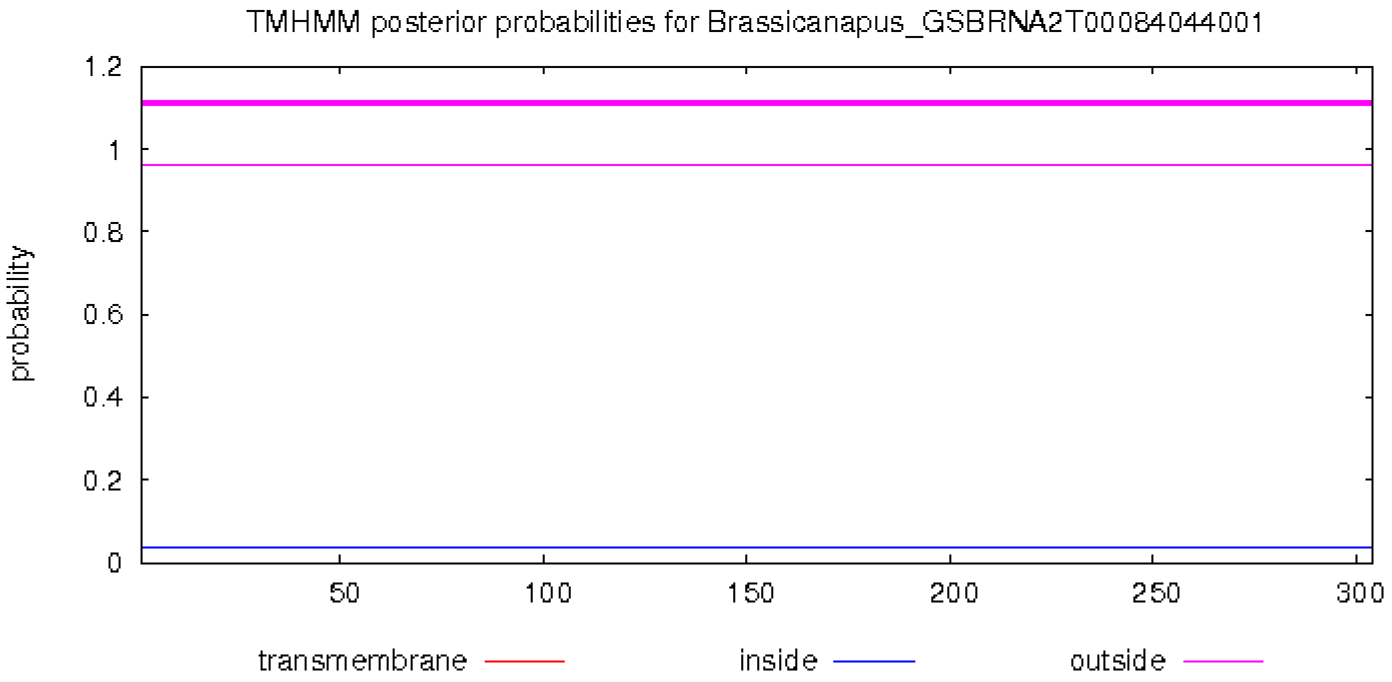

plot in postscript, script for making the plot in gnuplot, data for plot

```
# Brassicanapus_GSBRNA2T00084755001 Length: 277
# Brassicanapus_GSBRNA2T00084755001 Number of predicted TMHs:  0
# Brassicanapus_GSBRNA2T00084755001 Exp number of AAs in TMHs: 0.0008
# Brassicanapus_GSBRNA2T00084755001 Exp number, first 60 AAs:  0
# Brassicanapus_GSBRNA2T00084755001 Total prob of N-in:        0.02399
Brassicanapus_GSBRNA2T00084755001	TMHMM2.0	outside	     1   277
```

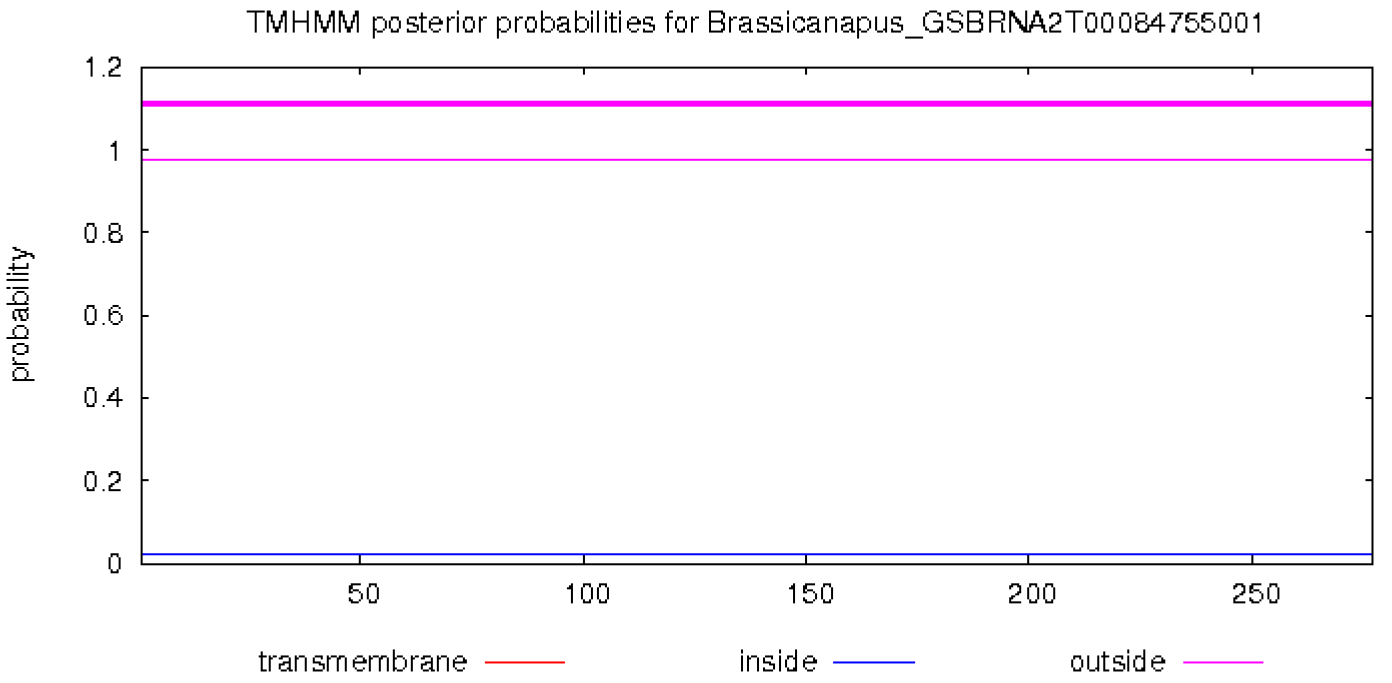

plot in postscript, script for making the plot in gnuplot, data for plot

```
# Brassicanapus_GSBRNA2T00085330001 Length: 286
# Brassicanapus_GSBRNA2T00085330001 Number of predicted TMHs:  0
# Brassicanapus_GSBRNA2T00085330001 Exp number of AAs in TMHs: 0.02442
# Brassicanapus_GSBRNA2T00085330001 Exp number, first 60 AAs:  0.0002
# Brassicanapus_GSBRNA2T00085330001 Total prob of N-in:        0.00269
Brassicanapus_GSBRNA2T00085330001	TMHMM2.0	outside	     1   286
```

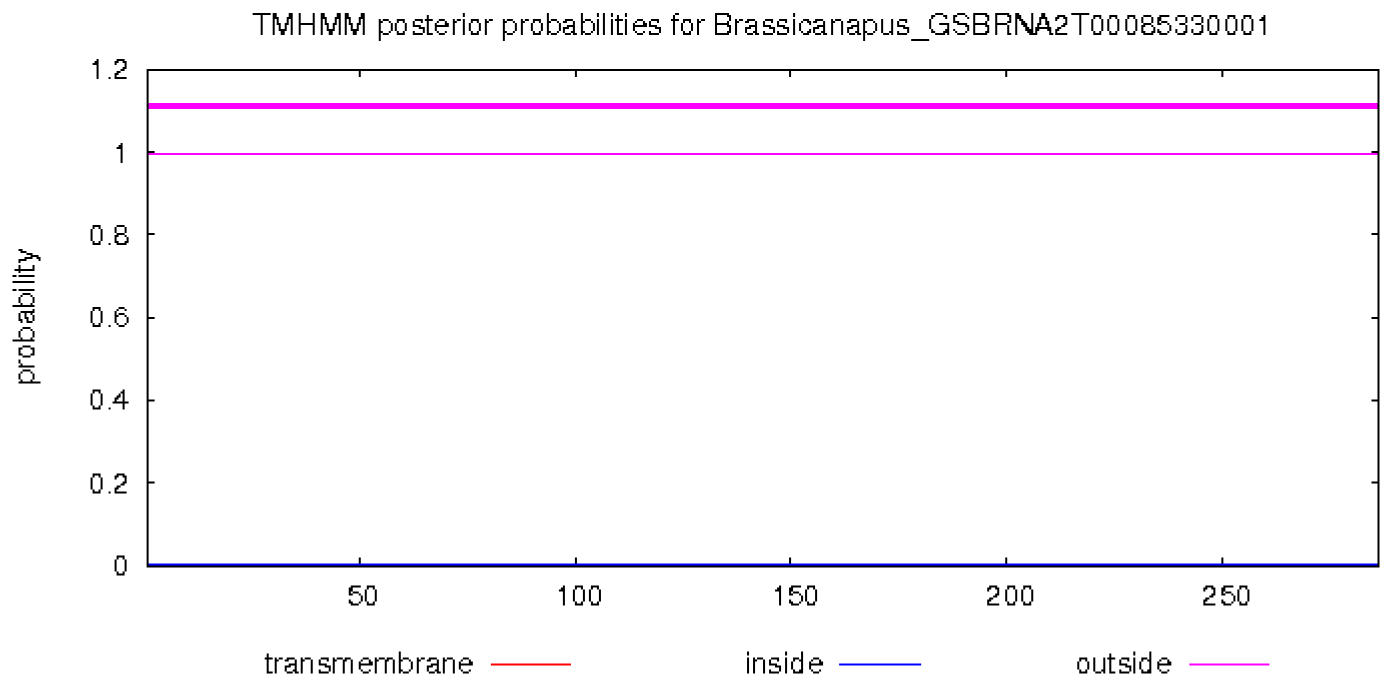

plot in postscript, script for making the plot in gnuplot, data for plot

---

```
# Brassicanapus_GSBRNA2T00085398001 Length: 259
# Brassicanapus_GSBRNA2T00085398001 Number of predicted TMHs:  0
# Brassicanapus_GSBRNA2T00085398001 Exp number of AAs in TMHs: 0.00094
# Brassicanapus_GSBRNA2T00085398001 Exp number, first 60 AAs:  0.00014
# Brassicanapus_GSBRNA2T00085398001 Total prob of N-in:        0.01217
Brassicanapus_GSBRNA2T00085398001	TMHMM2.0	outside	     1   259
```

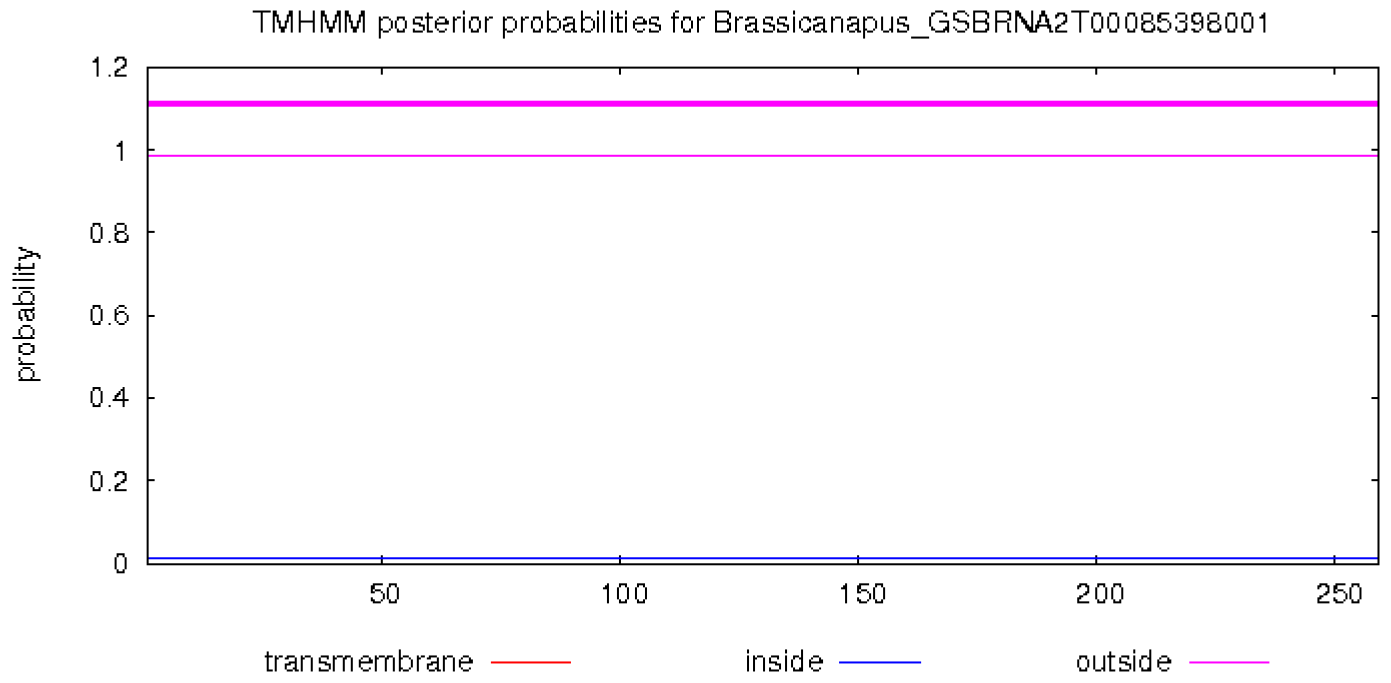

plot in postscript, script for making the plot in gnuplot, data for plot

---

```
# Brassicanapus_GSBRNA2T00085404001 Length: 425
# Brassicanapus_GSBRNA2T00085404001 Number of predicted TMHs:  1
# Brassicanapus_GSBRNA2T00085404001 Exp number of AAs in TMHs: 21.31926
# Brassicanapus_GSBRNA2T00085404001 Exp number, first 60 AAs:  0.00149
# Brassicanapus_GSBRNA2T00085404001 Total prob of N-in:        0.15823
Brassicanapus_GSBRNA2T00085404001	TMHMM2.0	outside	     1   400
Brassicanapus_GSBRNA2T00085404001	TMHMM2.0	TMhelix	   401   423
Brassicanapus_GSBRNA2T00085404001	TMHMM2.0	inside	   424   425
```

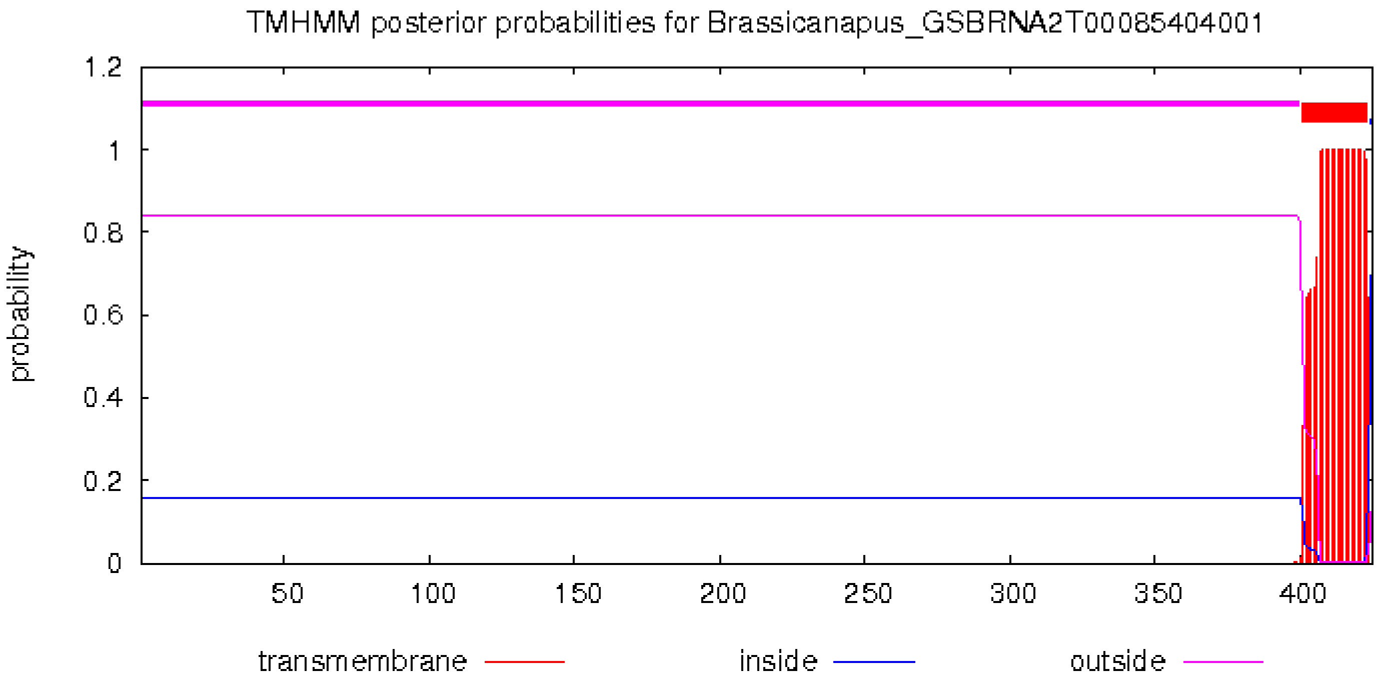

plot in postscript, script for making the plot in gnuplot, data for plot

---

```
# Brassicanapus_GSBRNA2T00085406001 Length: 296
# Brassicanapus_GSBRNA2T00085406001 Number of predicted TMHs:  0
# Brassicanapus_GSBRNA2T00085406001 Exp number of AAs in TMHs: 0
# Brassicanapus_GSBRNA2T00085406001 Exp number, first 60 AAs:  0
# Brassicanapus_GSBRNA2T00085406001 Total prob of N-in:        0.00815
Brassicanapus_GSBRNA2T00085406001	TMHMM2.0	outside	     1   296
```

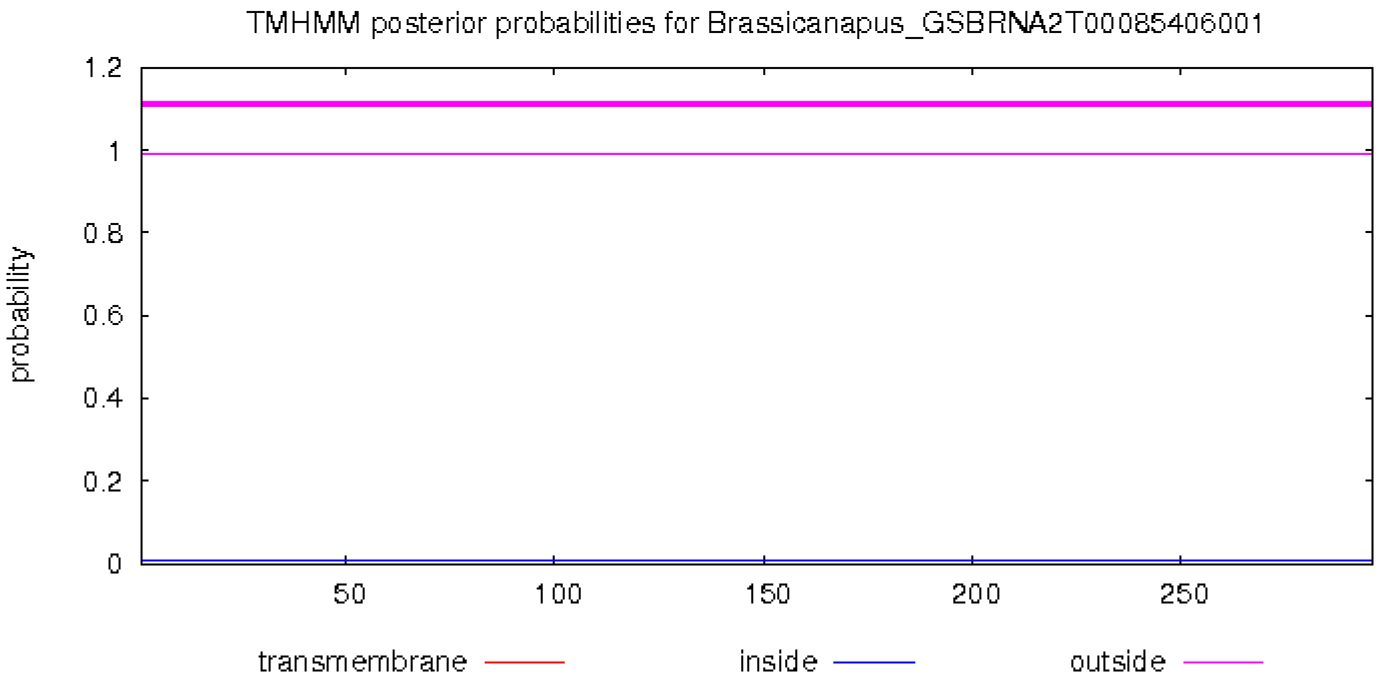

plot in postscript, script for making the plot in gnuplot, data for plot

```
# Brassicanapus_GSBRNA2T00085407001 Length: 425
# Brassicanapus_GSBRNA2T00085407001 Number of predicted TMHs:  1
# Brassicanapus_GSBRNA2T00085407001 Exp number of AAs in TMHs: 22.46599
# Brassicanapus_GSBRNA2T00085407001 Exp number, first 60 AAs:  0.00013
# Brassicanapus_GSBRNA2T00085407001 Total prob of N-in:        0.22629
Brassicanapus_GSBRNA2T00085407001	TMHMM2.0	outside	     1   401
Brassicanapus_GSBRNA2T00085407001	TMHMM2.0	TMhelix	   402   424
Brassicanapus_GSBRNA2T00085407001	TMHMM2.0	inside	   425   425
```

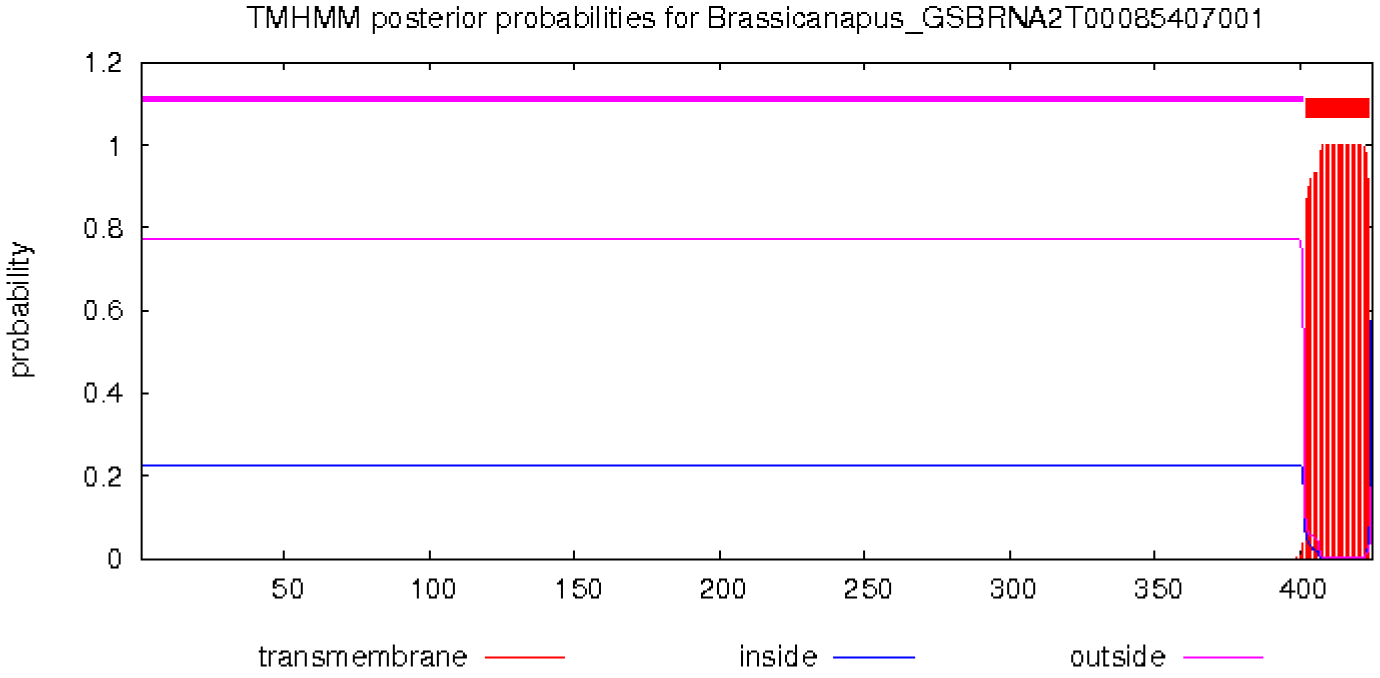

plot in postscript, script for making the plot in gnuplot, data for plot

```
# Brassicanapus_GSBRNA2T00085409001 Length: 349
# Brassicanapus_GSBRNA2T00085409001 Number of predicted TMHs:  0
# Brassicanapus_GSBRNA2T00085409001 Exp number of AAs in TMHs: 0.00154
# Brassicanapus_GSBRNA2T00085409001 Exp number, first 60 AAs:  7e-05
# Brassicanapus_GSBRNA2T00085409001 Total prob of N-in:        0.01404
Brassicanapus_GSBRNA2T00085409001	TMHMM2.0	outside	     1   349
```

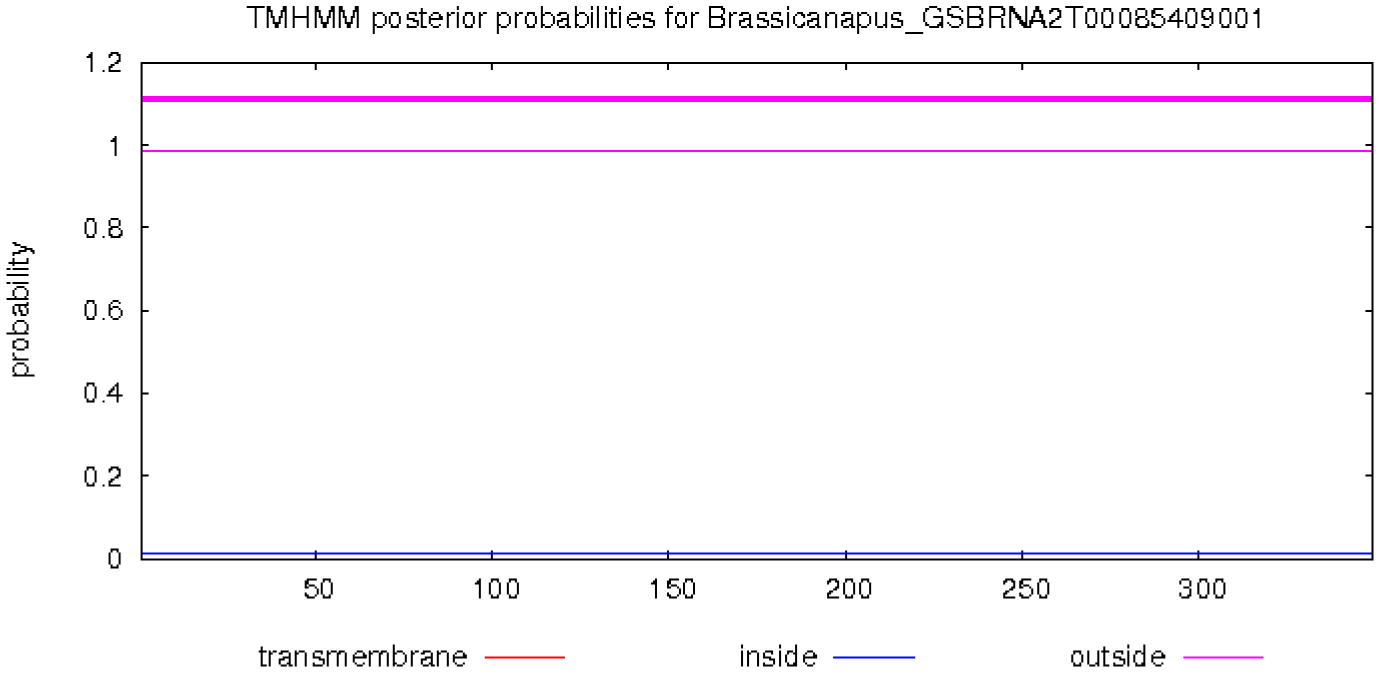

plot in postscript, script for making the plot in gnuplot, data for plot

---

```
# Brassicanapus_GSBRNA2T00085411001 Length: 365
# Brassicanapus_GSBRNA2T00085411001 Number of predicted TMHs:  0
# Brassicanapus_GSBRNA2T00085411001 Exp number of AAs in TMHs: 0.02394
# Brassicanapus_GSBRNA2T00085411001 Exp number, first 60 AAs:  0
# Brassicanapus_GSBRNA2T00085411001 Total prob of N-in:        0.01493
Brassicanapus_GSBRNA2T00085411001	TMHMM2.0	outside	     1   365
```

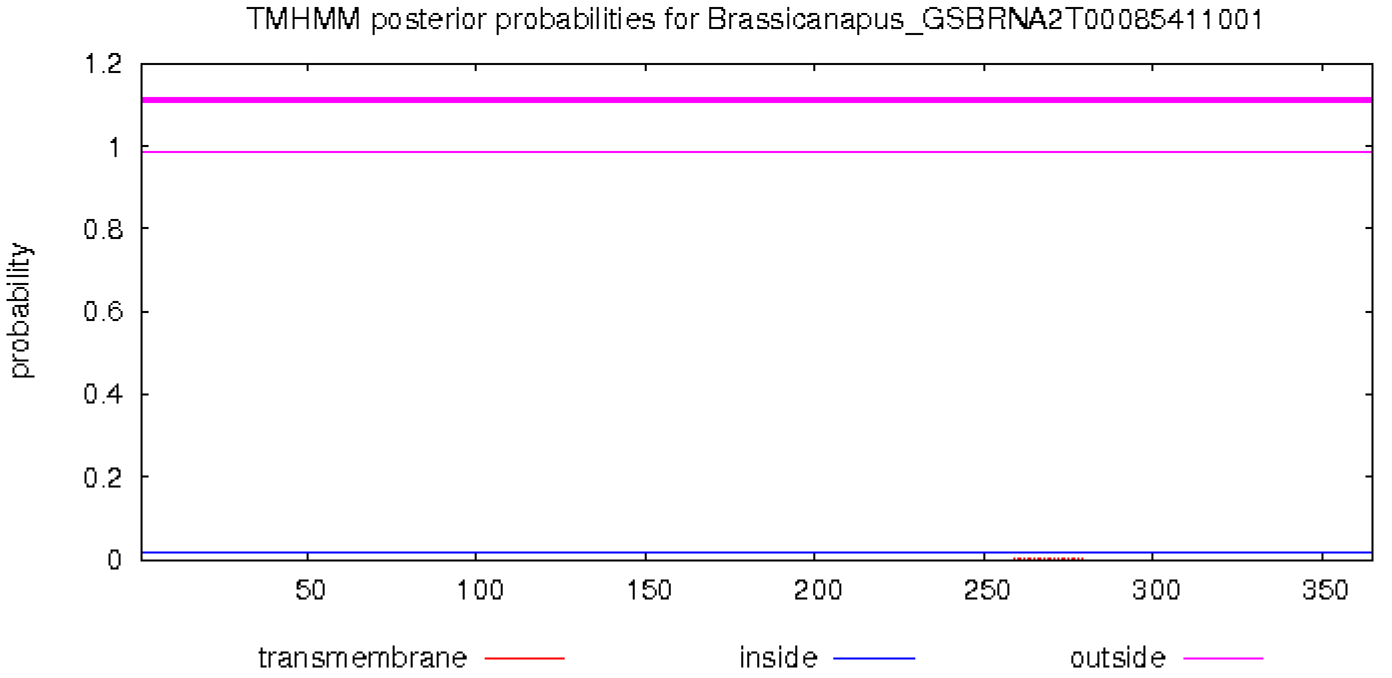

plot in postscript, script for making the plot in gnuplot, data for plot

---

```
# Brassicanapus_GSBRNA2T00085413001 Length: 202
# Brassicanapus_GSBRNA2T00085413001 Number of predicted TMHs:  0
# Brassicanapus_GSBRNA2T00085413001 Exp number of AAs in TMHs: 0
# Brassicanapus_GSBRNA2T00085413001 Exp number, first 60 AAs:  0
# Brassicanapus_GSBRNA2T00085413001 Total prob of N-in:        0.01608
Brassicanapus_GSBRNA2T00085413001	TMHMM2.0	outside	     1   202
```

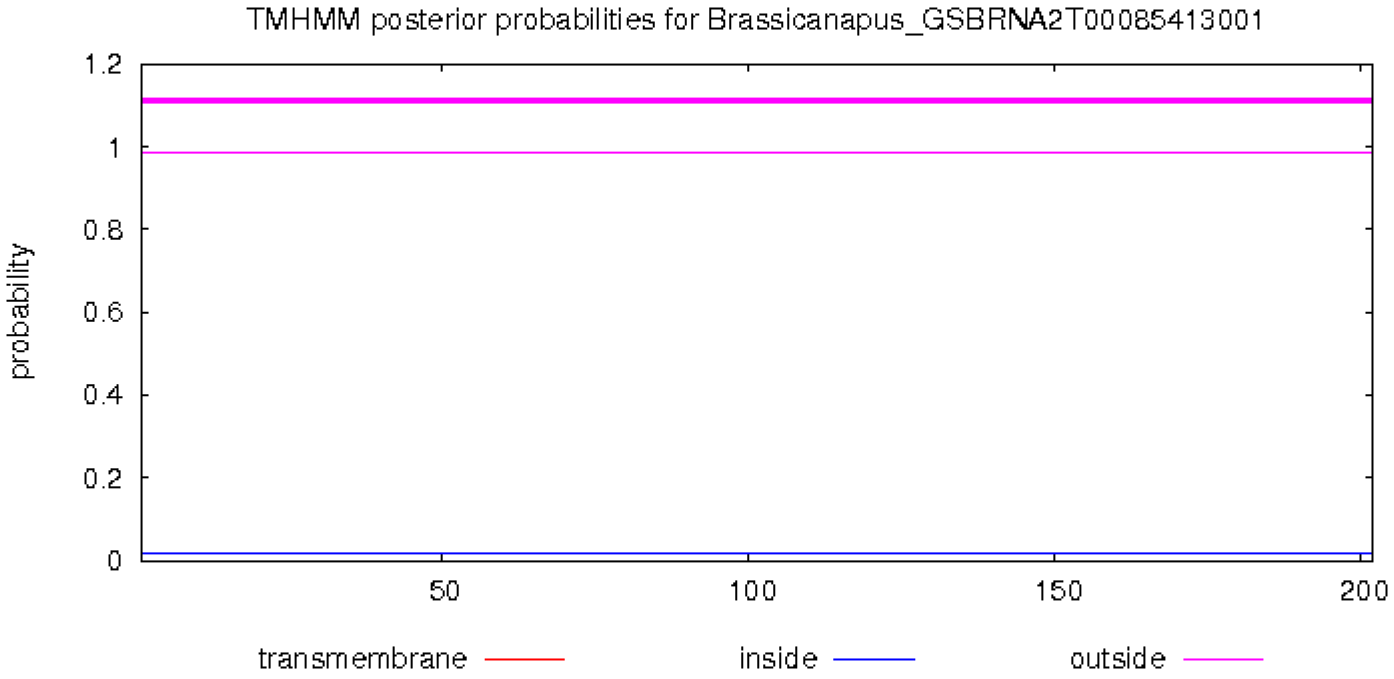

plot in postscript, script for making the plot in gnuplot, data for plot

```
# Brassicanapus_GSBRNA2T00085414001 Length: 425
# Brassicanapus_GSBRNA2T00085414001 Number of predicted TMHs:  0
# Brassicanapus_GSBRNA2T00085414001 Exp number of AAs in TMHs: 0.00106
# Brassicanapus_GSBRNA2T00085414001 Exp number, first 60 AAs:  0
# Brassicanapus_GSBRNA2T00085414001 Total prob of N-in:        0.00594
Brassicanapus_GSBRNA2T00085414001	TMHMM2.0	outside	     1   425
```

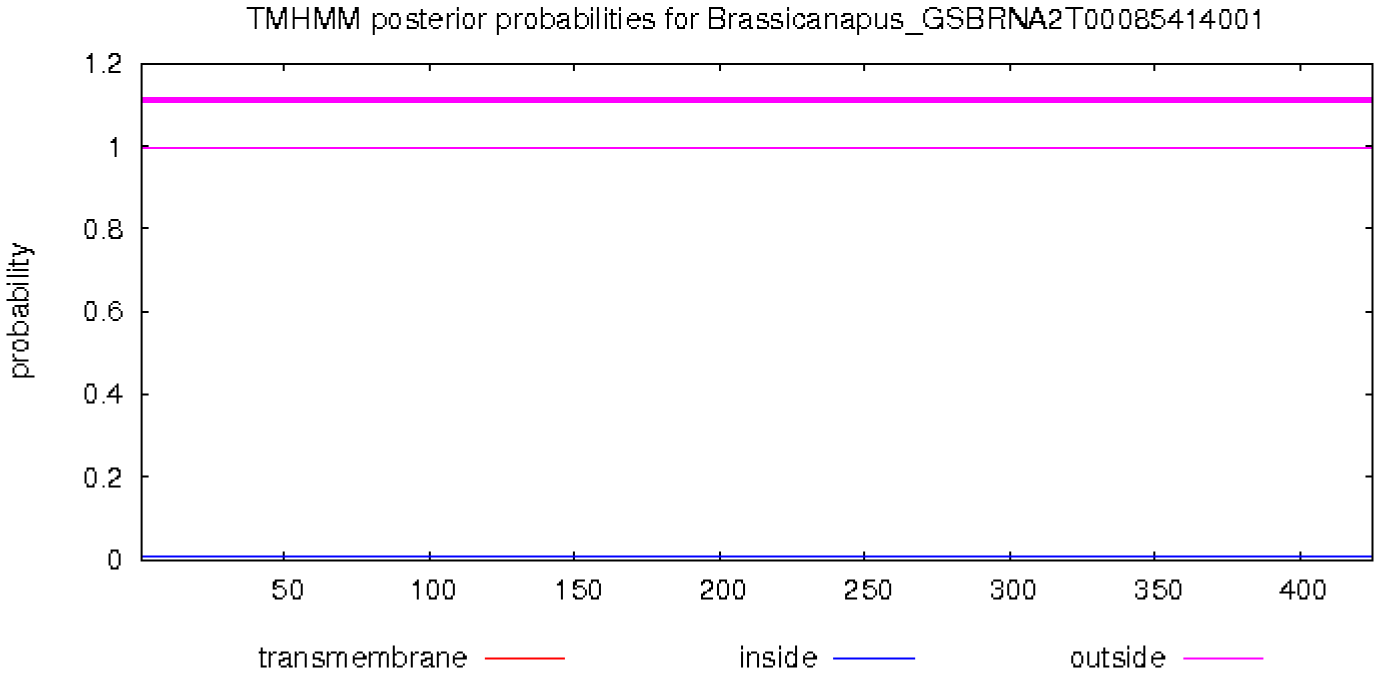

plot in postscript, script for making the plot in gnuplot, data for plot

```
# Brassicanapus_GSBRNA2T00085416001 Length: 212
# Brassicanapus_GSBRNA2T00085416001 Number of predicted TMHs:  0
# Brassicanapus_GSBRNA2T00085416001 Exp number of AAs in TMHs: 0.00082
# Brassicanapus_GSBRNA2T00085416001 Exp number, first 60 AAs:  0
# Brassicanapus_GSBRNA2T00085416001 Total prob of N-in:        0.06505
Brassicanapus_GSBRNA2T00085416001	TMHMM2.0	outside	     1   212
```

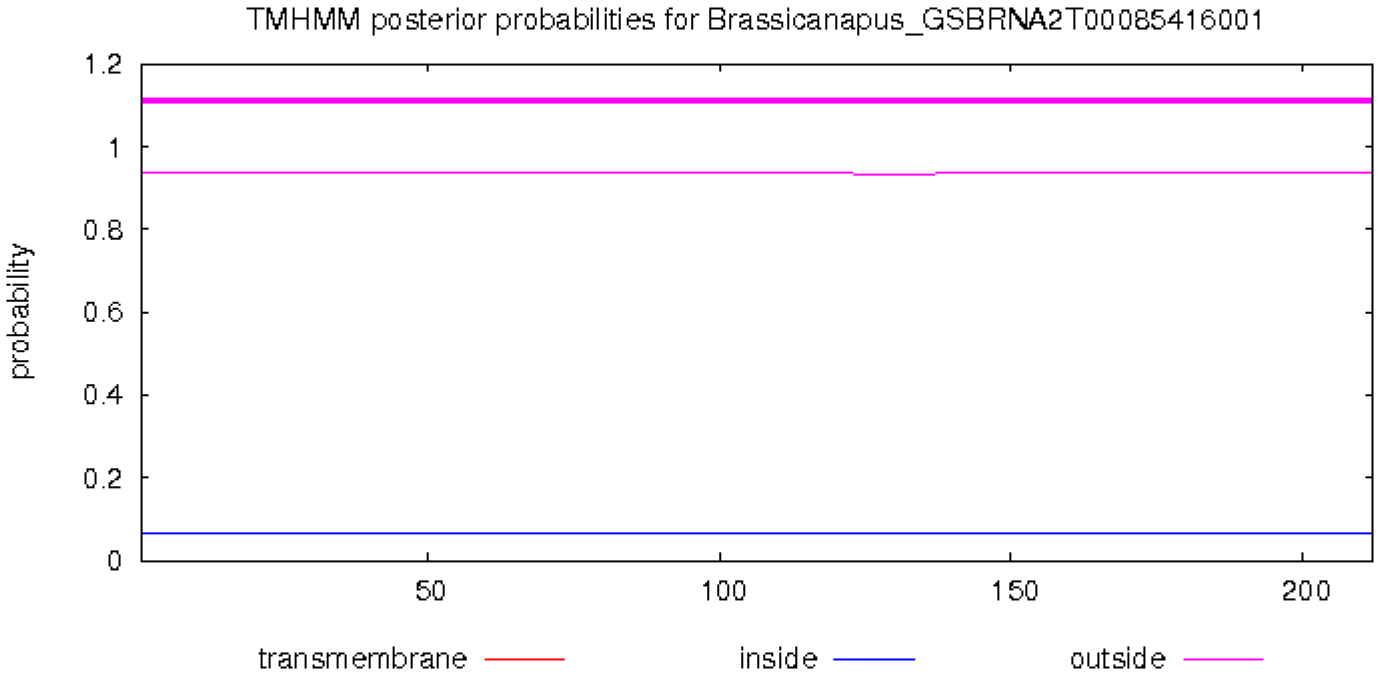

plot in postscript, script for making the plot in gnuplot, data for plot

```
# Brassicanapus_GSBRNA2T00085419001 Length: 374
# Brassicanapus_GSBRNA2T00085419001 Number of predicted TMHs:  0
# Brassicanapus_GSBRNA2T00085419001 Exp number of AAs in TMHs: 0.00635
# Brassicanapus_GSBRNA2T00085419001 Exp number, first 60 AAs:  0
# Brassicanapus_GSBRNA2T00085419001 Total prob of N-in:        0.00359
Brassicanapus_GSBRNA2T00085419001	TMHMM2.0	outside	     1   374
```

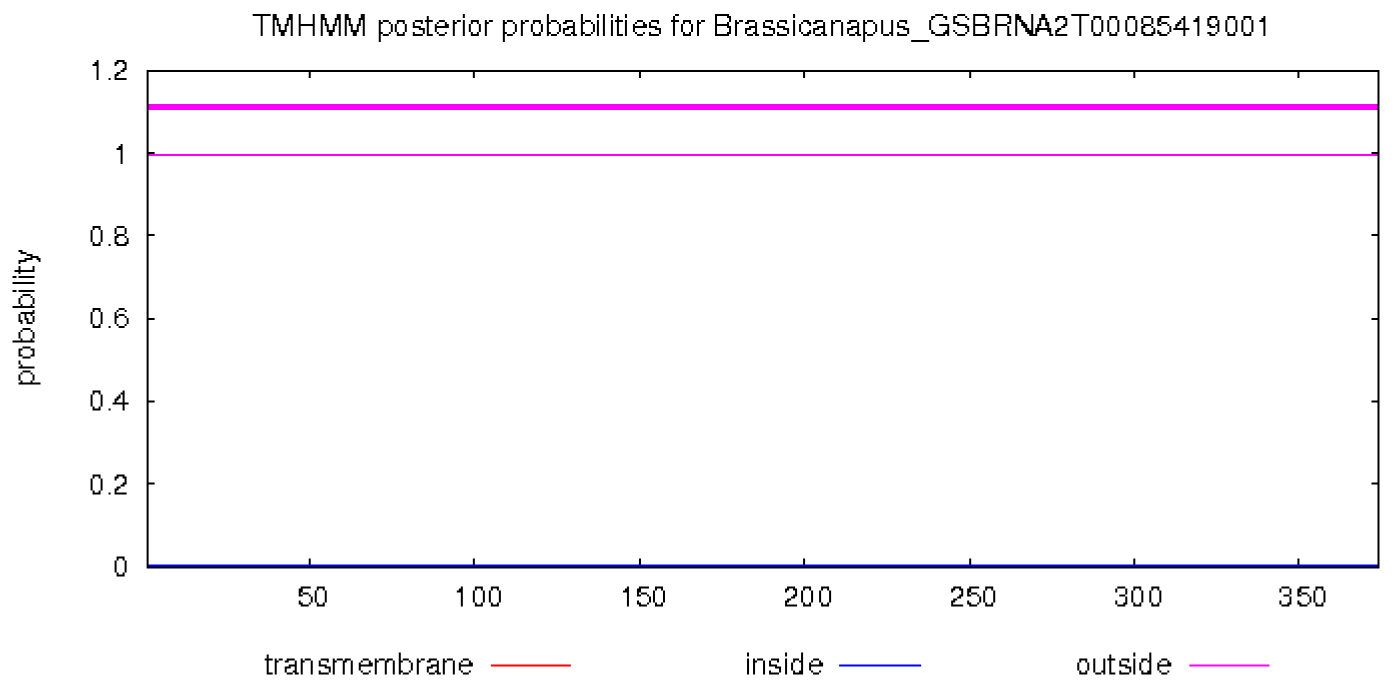

plot in postscript, script for making the plot in gnuplot, data for plot

```
# Brassicanapus_GSBRNA2T00085420001 Length: 399
# Brassicanapus_GSBRNA2T00085420001 Number of predicted TMHs:  0
# Brassicanapus_GSBRNA2T00085420001 Exp number of AAs in TMHs: 0.04238
# Brassicanapus_GSBRNA2T00085420001 Exp number, first 60 AAs:  0.04238
# Brassicanapus_GSBRNA2T00085420001 Total prob of N-in:        0.00511
Brassicanapus_GSBRNA2T00085420001	TMHMM2.0	outside	     1   399
```

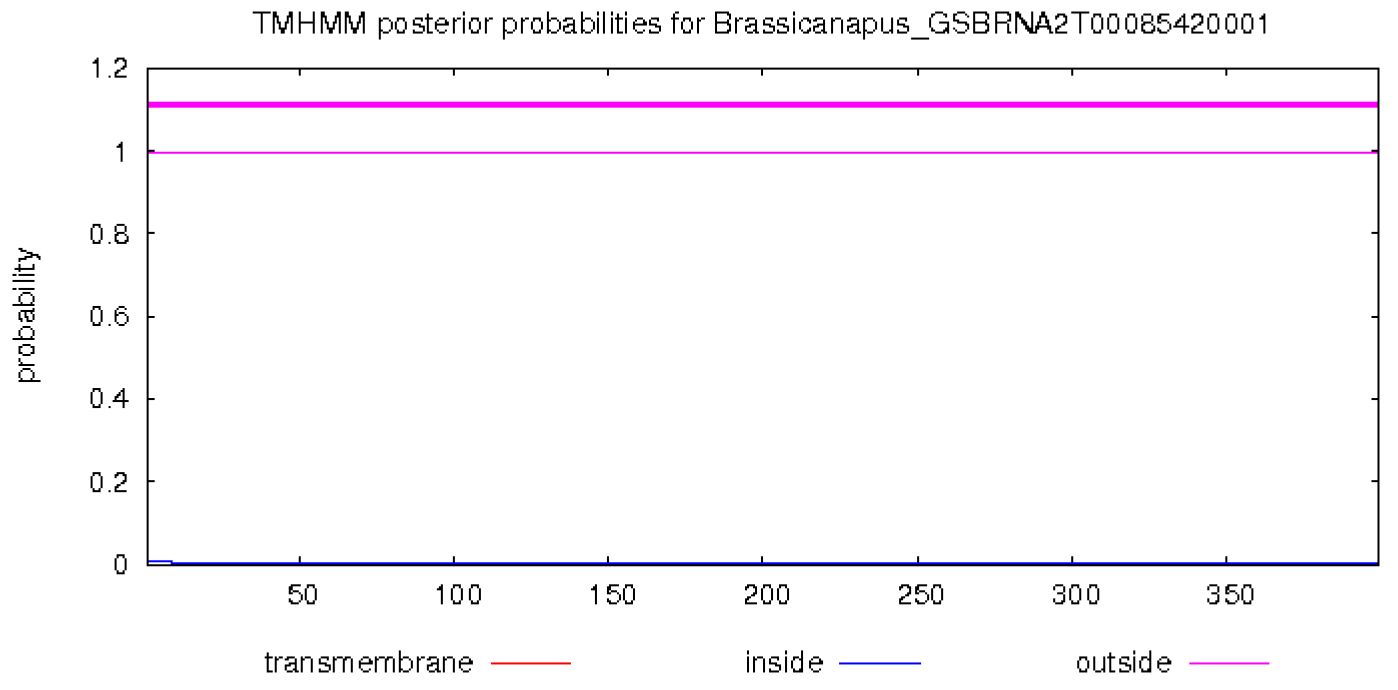

plot in postscript, script for making the plot in gnuplot, data for plot

```
# Brassicanapus_GSBRNA2T00086242001 Length: 678
# Brassicanapus_GSBRNA2T00086242001 Number of predicted TMHs:  2
# Brassicanapus_GSBRNA2T00086242001 Exp number of AAs in TMHs: 62.96296
# Brassicanapus_GSBRNA2T00086242001 Exp number, first 60 AAs:  0
# Brassicanapus_GSBRNA2T00086242001 Total prob of N-in:        0.39592
Brassicanapus_GSBRNA2T00086242001       TMHMM2.0        outside      1   216
Brassicanapus_GSBRNA2T00086242001       TMHMM2.0        TMhelix    217   239
Brassicanapus_GSBRNA2T00086242001       TMHMM2.0        inside     240   492
Brassicanapus_GSBRNA2T00086242001       TMHMM2.0        TMhelix    493   515
Brassicanapus_GSBRNA2T00086242001       TMHMM2.0        outside    516   678
```

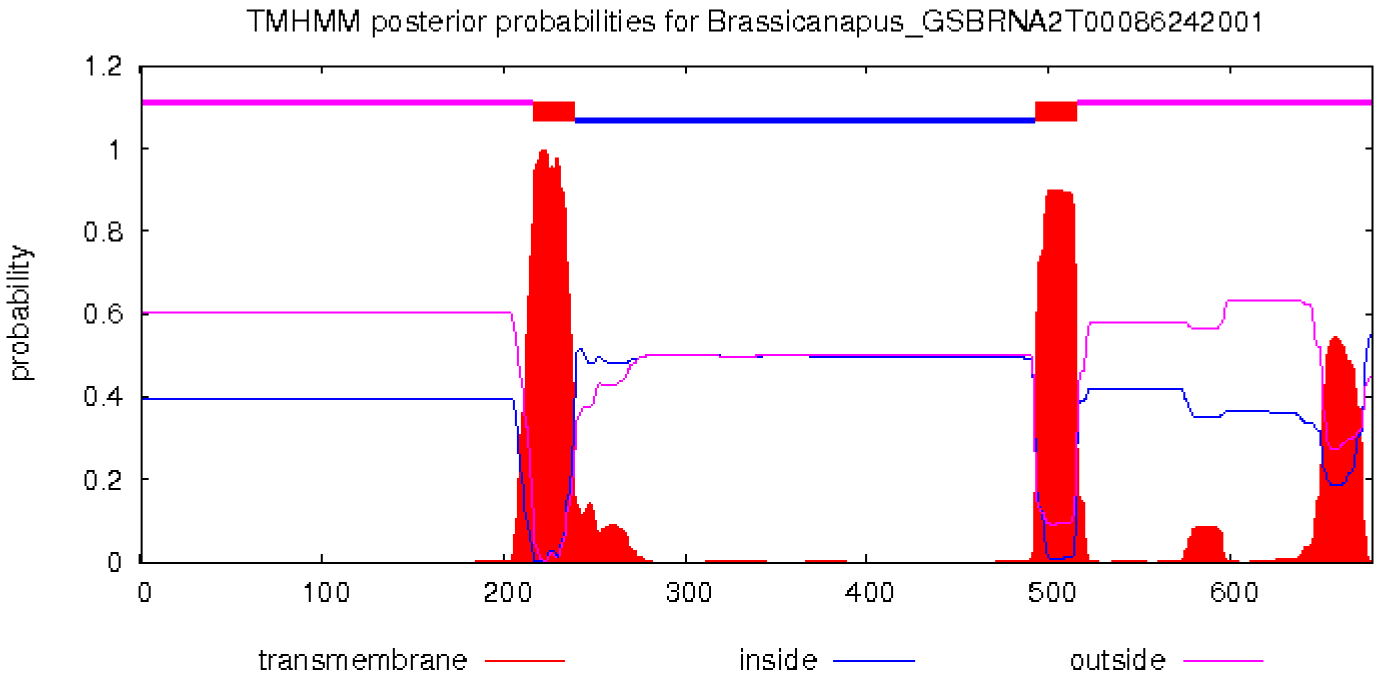

plot in postscript, script for making the plot in gnuplot, data for plot

```
# Brassicanapus_GSBRNA2T00086386001 Length: 361
# Brassicanapus_GSBRNA2T00086386001 Number of predicted TMHs:  0
# Brassicanapus_GSBRNA2T00086386001 Exp number of AAs in TMHs: 0.00018
# Brassicanapus_GSBRNA2T00086386001 Exp number, first 60 AAs:  0
# Brassicanapus_GSBRNA2T00086386001 Total prob of N-in:        0.00255
Brassicanapus_GSBRNA2T00086386001       TMHMM2.0        outside      1   361
```

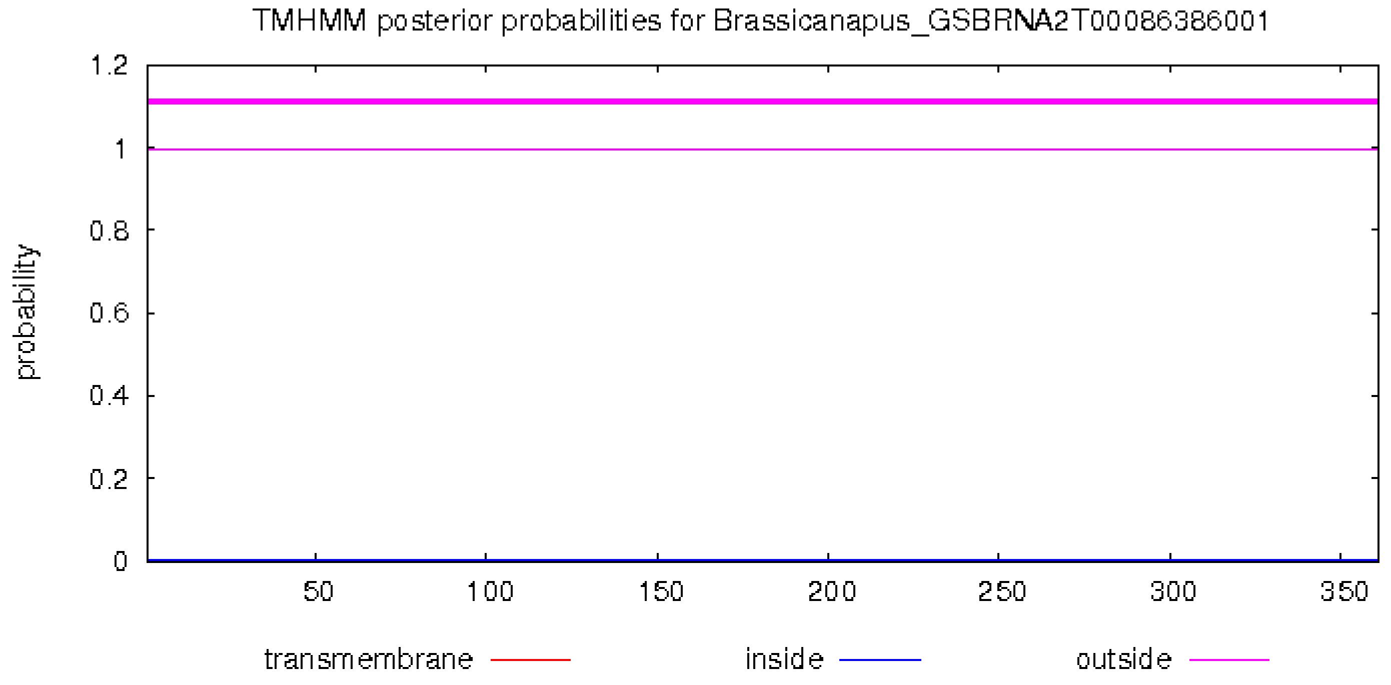

plot in postscript, script for making the plot in gnuplot, data for plot

---

```
# Brassicanapus_GSBRNA2T00086664001 Length: 327
# Brassicanapus_GSBRNA2T00086664001 Number of predicted TMHs:  0
# Brassicanapus_GSBRNA2T00086664001 Exp number of AAs in TMHs: 0
# Brassicanapus_GSBRNA2T00086664001 Exp number, first 60 AAs:  0
# Brassicanapus_GSBRNA2T00086664001 Total prob of N-in:        0.02220
Brassicanapus_GSBRNA2T00086664001       TMHMM2.0        outside      1   327
```

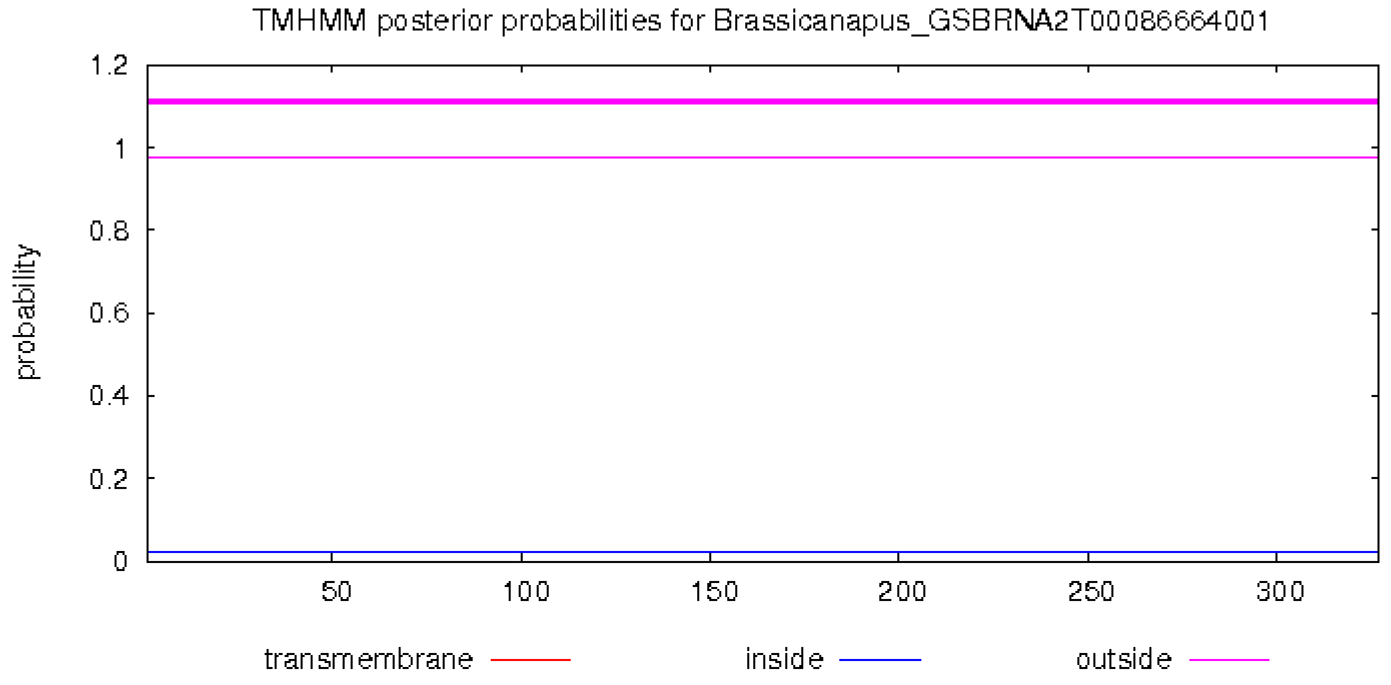

plot in postscript, script for making the plot in gnuplot, data for plot

---

```
# Brassicanapus_GSBRNA2T00087046001 Length: 304
# Brassicanapus_GSBRNA2T00087046001 Number of predicted TMHs:  0
# Brassicanapus_GSBRNA2T00087046001 Exp number of AAs in TMHs: 1.18217
# Brassicanapus_GSBRNA2T00087046001 Exp number, first 60 AAs:  0
# Brassicanapus_GSBRNA2T00087046001 Total prob of N-in:        0.00610
Brassicanapus_GSBRNA2T00087046001       TMHMM2.0        outside      1   304
```

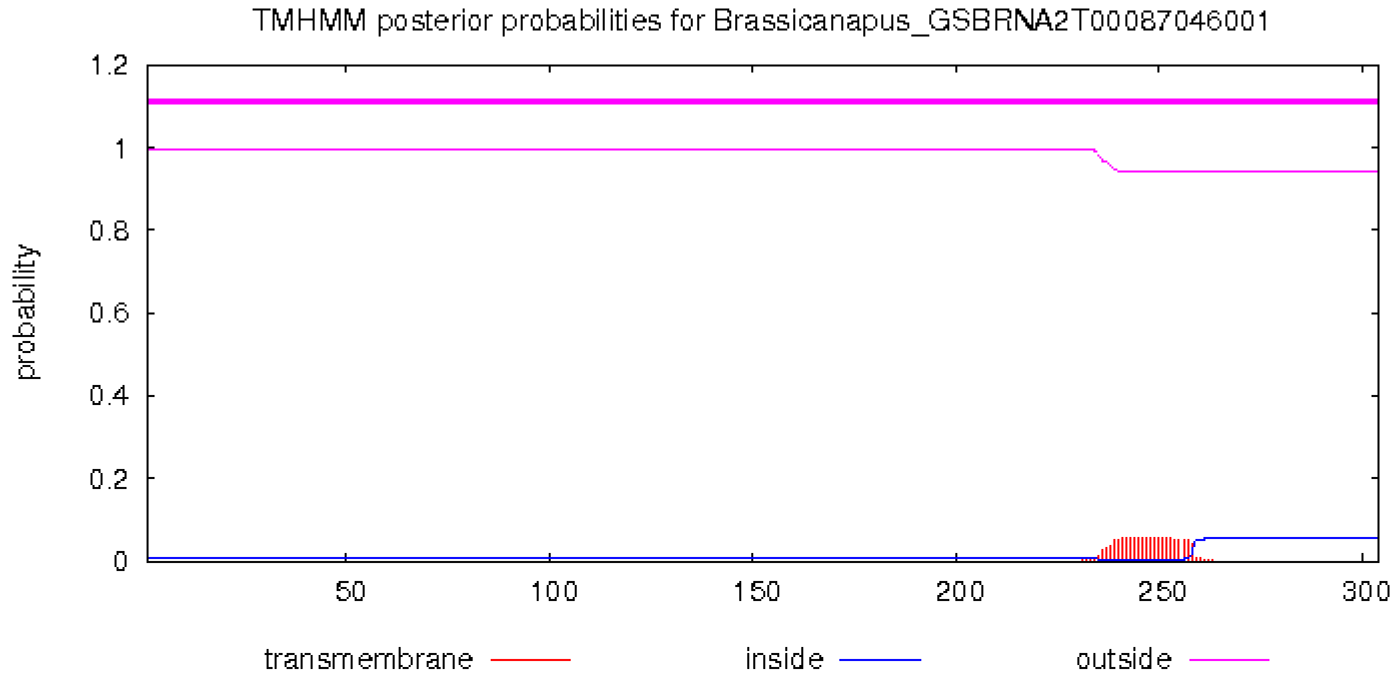

plot in postscript, script for making the plot in gnuplot, data for plot

---

```
# Brassicanapus_GSBRNA2T00087136001 Length: 245
# Brassicanapus_GSBRNA2T00087136001 Number of predicted TMHs:  0
# Brassicanapus_GSBRNA2T00087136001 Exp number of AAs in TMHs: 0.01599
# Brassicanapus_GSBRNA2T00087136001 Exp number, first 60 AAs:  0.01563
# Brassicanapus_GSBRNA2T00087136001 Total prob of N-in:        0.01827
Brassicanapus_GSBRNA2T00087136001	TMHMM2.0	outside	     1   245
```

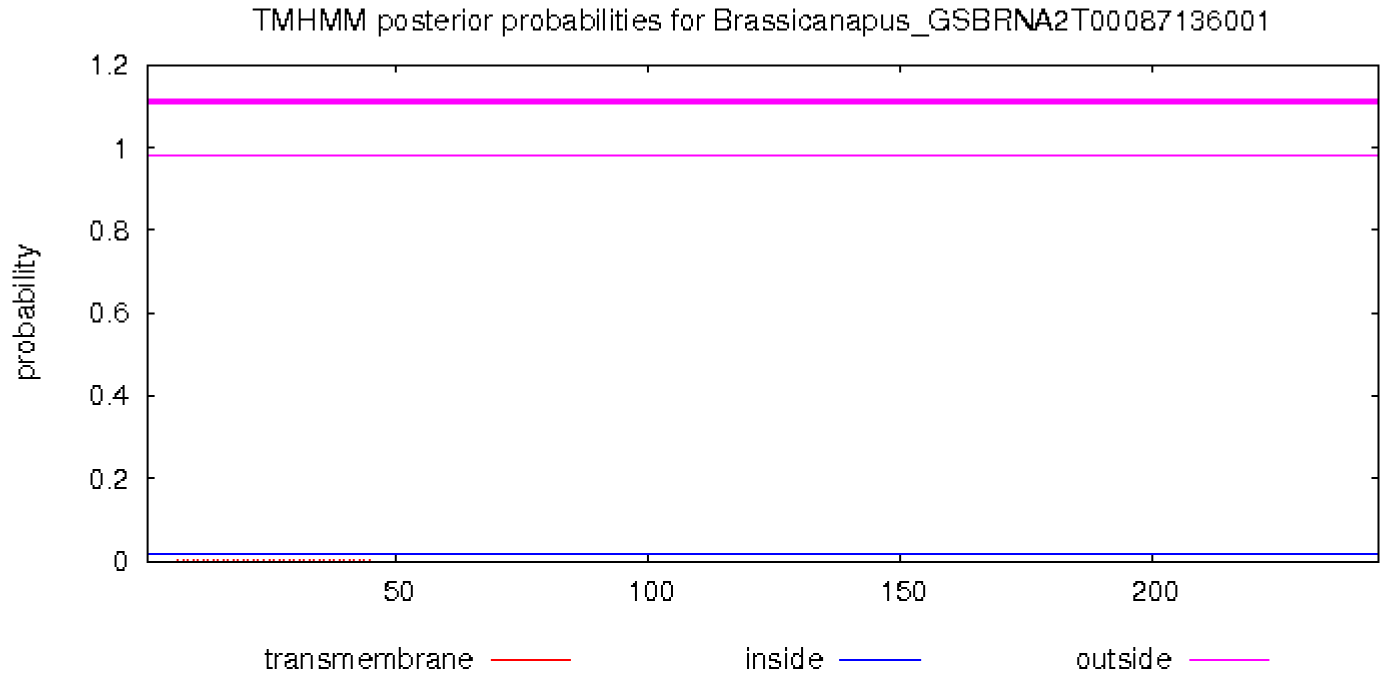

plot in postscript, script for making the plot in gnuplot, data for plot

---

```
# Brassicanapus_GSBRNA2T00087538001 Length: 279
# Brassicanapus_GSBRNA2T00087538001 Number of predicted TMHs:  0
# Brassicanapus_GSBRNA2T00087538001 Exp number of AAs in TMHs: 0.00206
# Brassicanapus_GSBRNA2T00087538001 Exp number, first 60 AAs:  0.00013
# Brassicanapus_GSBRNA2T00087538001 Total prob of N-in:        0.00600
Brassicanapus_GSBRNA2T00087538001	TMHMM2.0	outside	     1   279
```

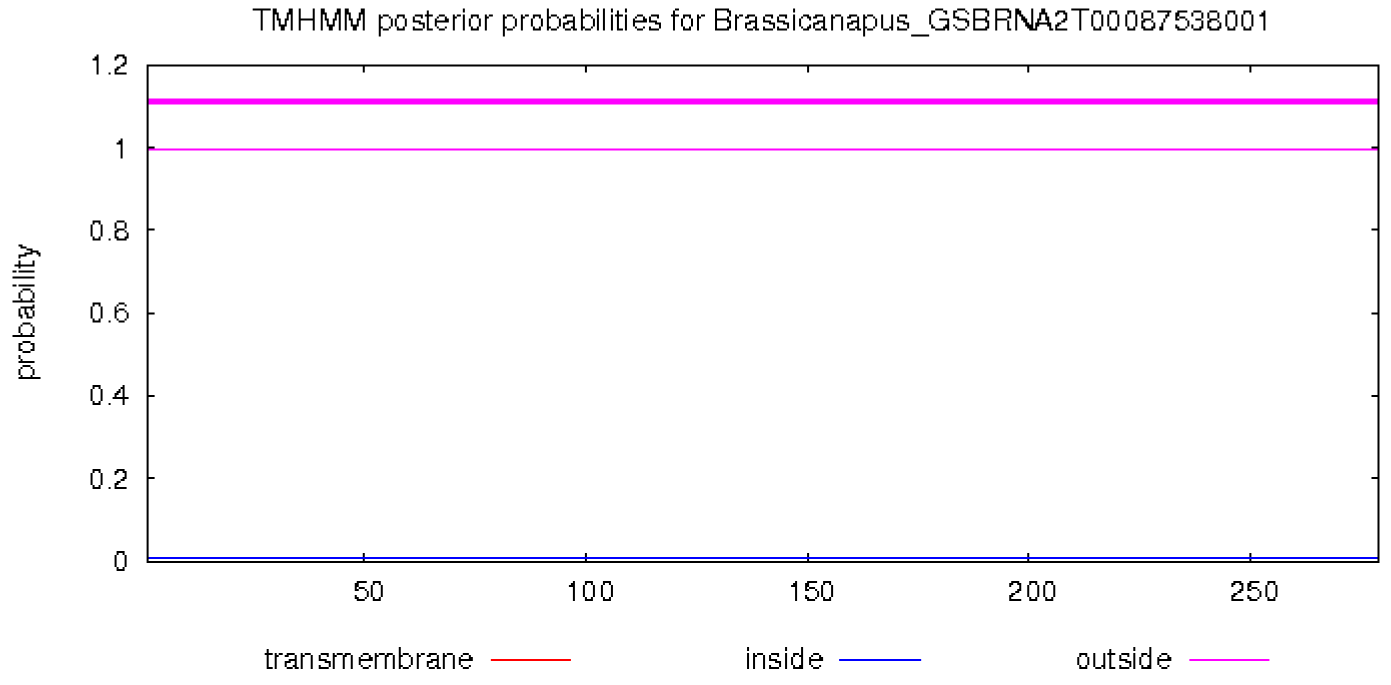

plot in postscript, script for making the plot in gnuplot, data for plot

```
# Brassicanapus_GSBRNA2T00087539001 Length: 279
# Brassicanapus_GSBRNA2T00087539001 Number of predicted TMHs:  0
# Brassicanapus_GSBRNA2T00087539001 Exp number of AAs in TMHs: 0.00206
# Brassicanapus_GSBRNA2T00087539001 Exp number, first 60 AAs:  0.00013
# Brassicanapus_GSBRNA2T00087539001 Total prob of N-in:        0.00600
Brassicanapus_GSBRNA2T00087539001       TMHMM2.0        outside      1   279
```

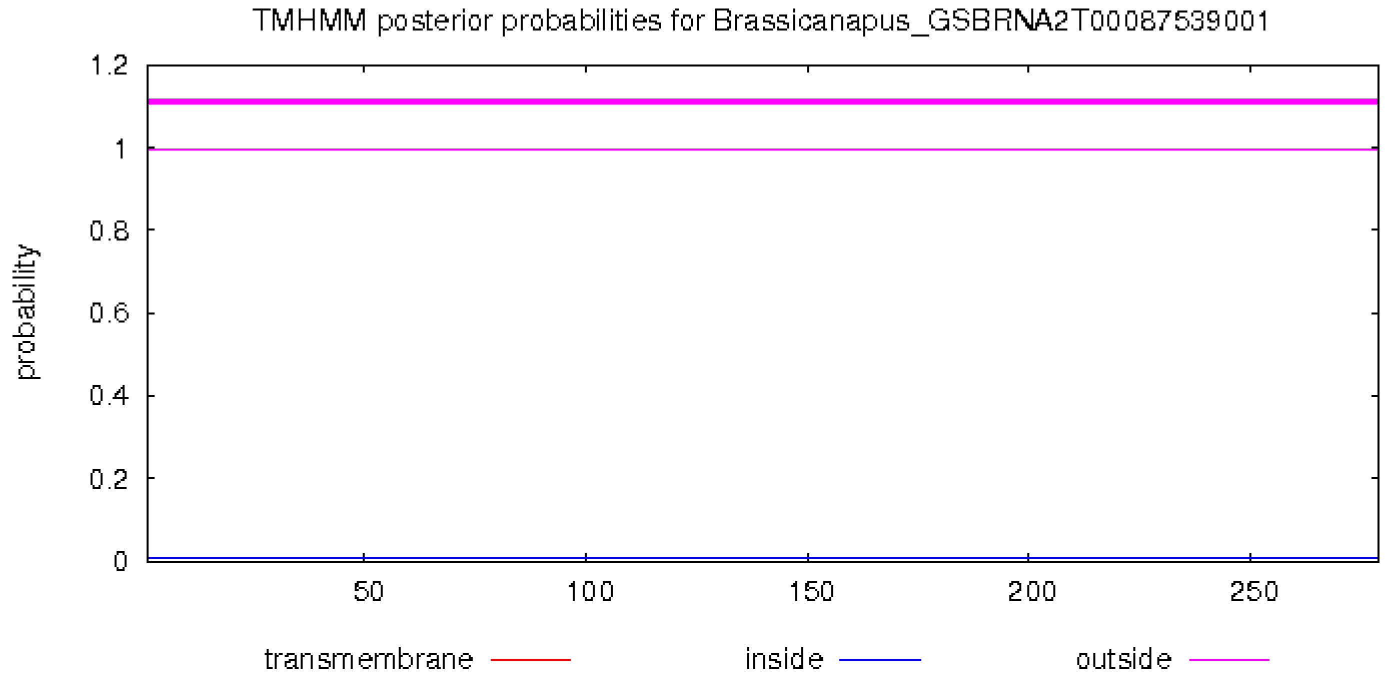

plot in postscript, script for making the plot in gnuplot, data for plot

```
# Brassicanapus_GSBRNA2T00088073001 Length: 454
# Brassicanapus_GSBRNA2T00088073001 Number of predicted TMHs:  0
# Brassicanapus_GSBRNA2T00088073001 Exp number of AAs in TMHs: 0.02887
# Brassicanapus_GSBRNA2T00088073001 Exp number, first 60 AAs:  0
# Brassicanapus_GSBRNA2T00088073001 Total prob of N-in:        0.00666
Brassicanapus_GSBRNA2T00088073001       TMHMM2.0        outside      1   454
```

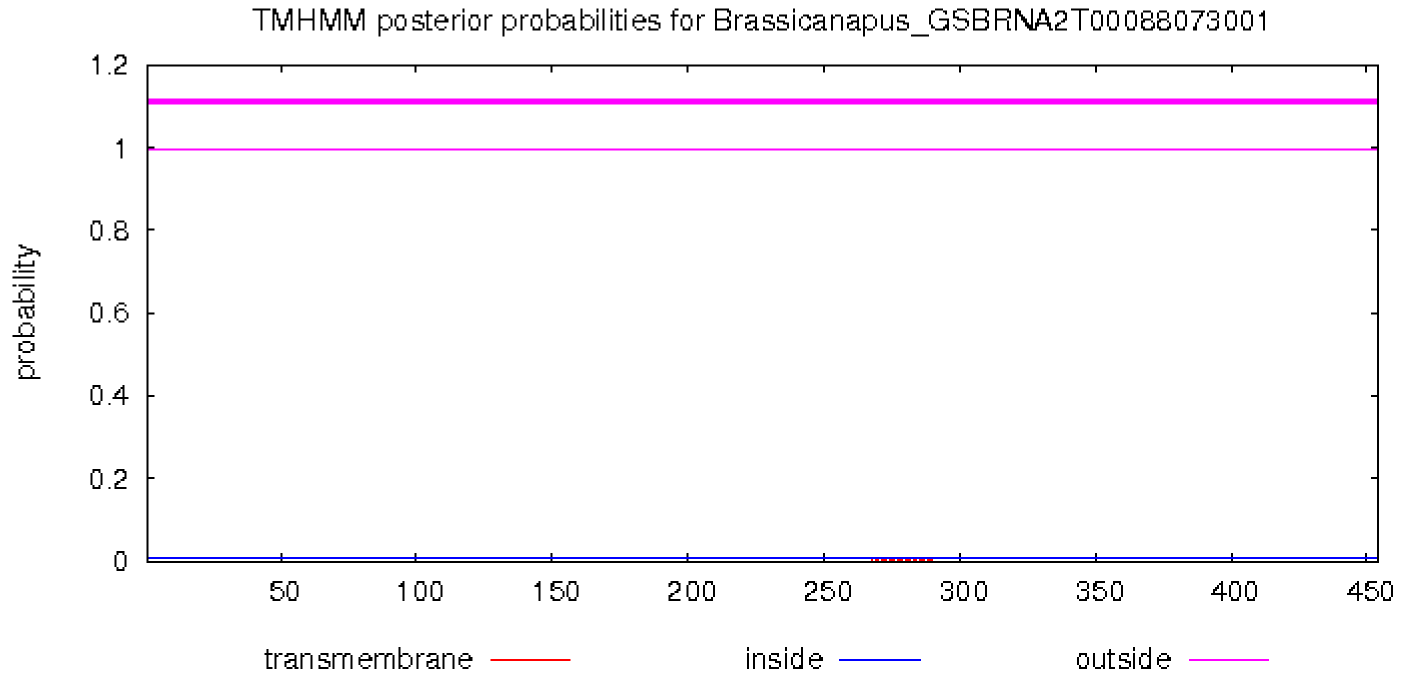

plot in postscript, script for making the plot in gnuplot, data for plot

---

```
# Brassicanapus_GSBRNA2T00088528001 Length: 312
# Brassicanapus_GSBRNA2T00088528001 Number of predicted TMHs:  0
# Brassicanapus_GSBRNA2T00088528001 Exp number of AAs in TMHs: 0
# Brassicanapus_GSBRNA2T00088528001 Exp number, first 60 AAs:  0
# Brassicanapus_GSBRNA2T00088528001 Total prob of N-in:        0.00789
Brassicanapus_GSBRNA2T00088528001       TMHMM2.0        outside      1   312
```

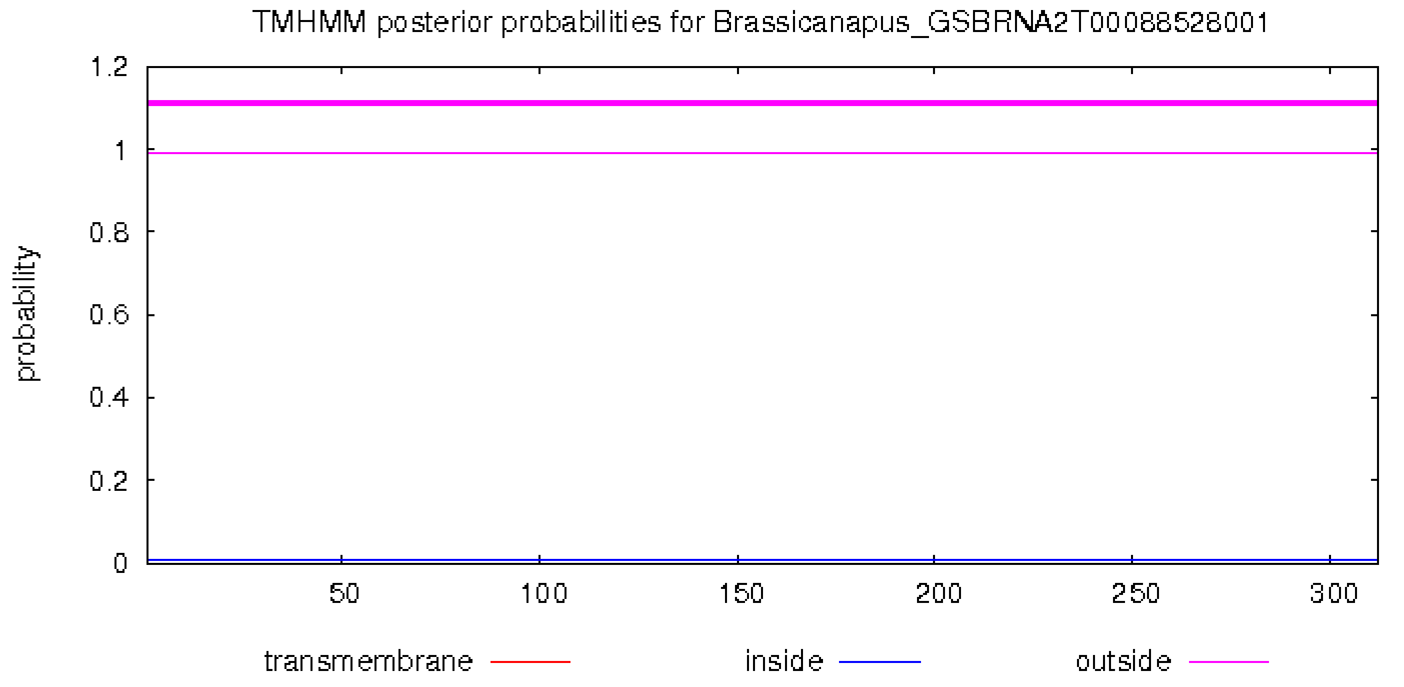

plot in postscript, script for making the plot in gnuplot, data for plot

---

```
# Brassicanapus_GSBRNA2T00089877001 Length: 291
# Brassicanapus_GSBRNA2T00089877001 Number of predicted TMHs:  0
# Brassicanapus_GSBRNA2T00089877001 Exp number of AAs in TMHs: 0.00469
# Brassicanapus_GSBRNA2T00089877001 Exp number, first 60 AAs:  0.00016
# Brassicanapus_GSBRNA2T00089877001 Total prob of N-in:        0.10975
Brassicanapus_GSBRNA2T00089877001       TMHMM2.0        outside      1   291
```

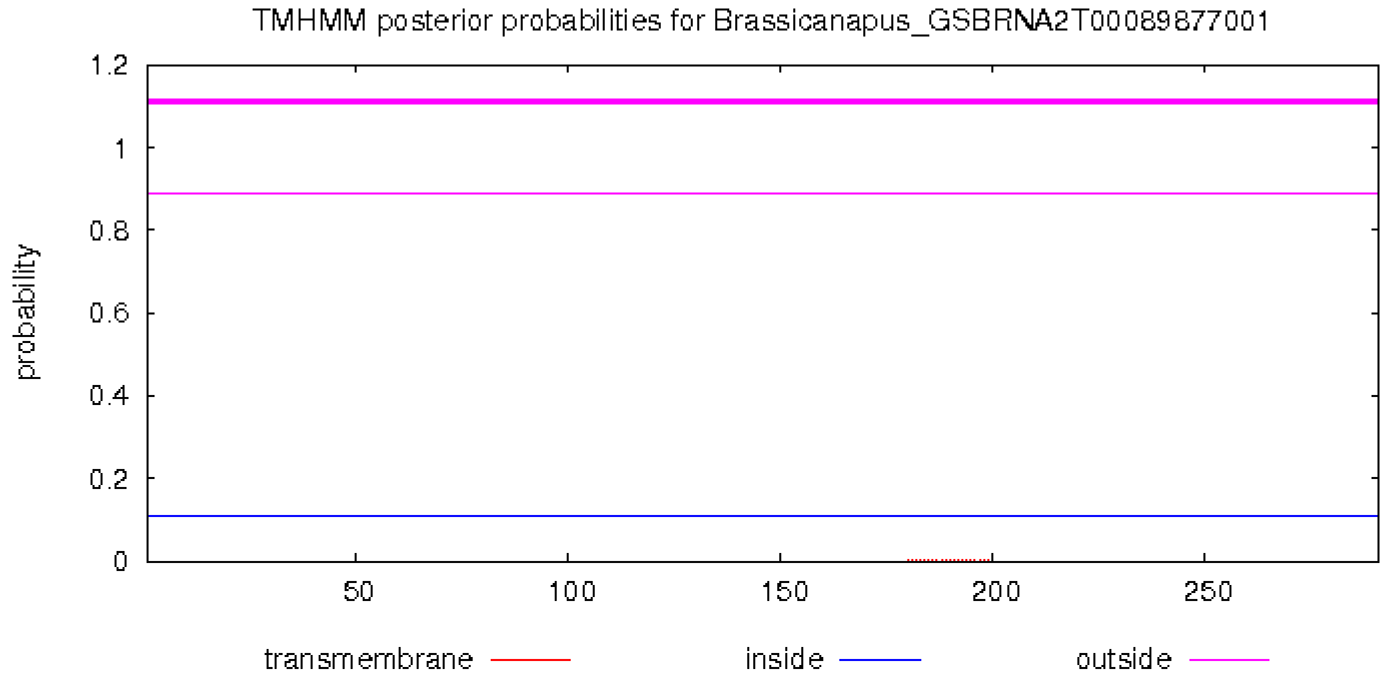

plot in postscript, script for making the plot in gnuplot, data for plot

---

```
# Brassicanapus_GSBRNA2T00090171001 Length: 407
# Brassicanapus_GSBRNA2T00090171001 Number of predicted TMHs:  0
# Brassicanapus_GSBRNA2T00090171001 Exp number of AAs in TMHs: 0.00656
# Brassicanapus_GSBRNA2T00090171001 Exp number, first 60 AAs:  0
# Brassicanapus_GSBRNA2T00090171001 Total prob of N-in:        0.00663
Brassicanapus_GSBRNA2T00090171001       TMHMM2.0        outside      1   407
```

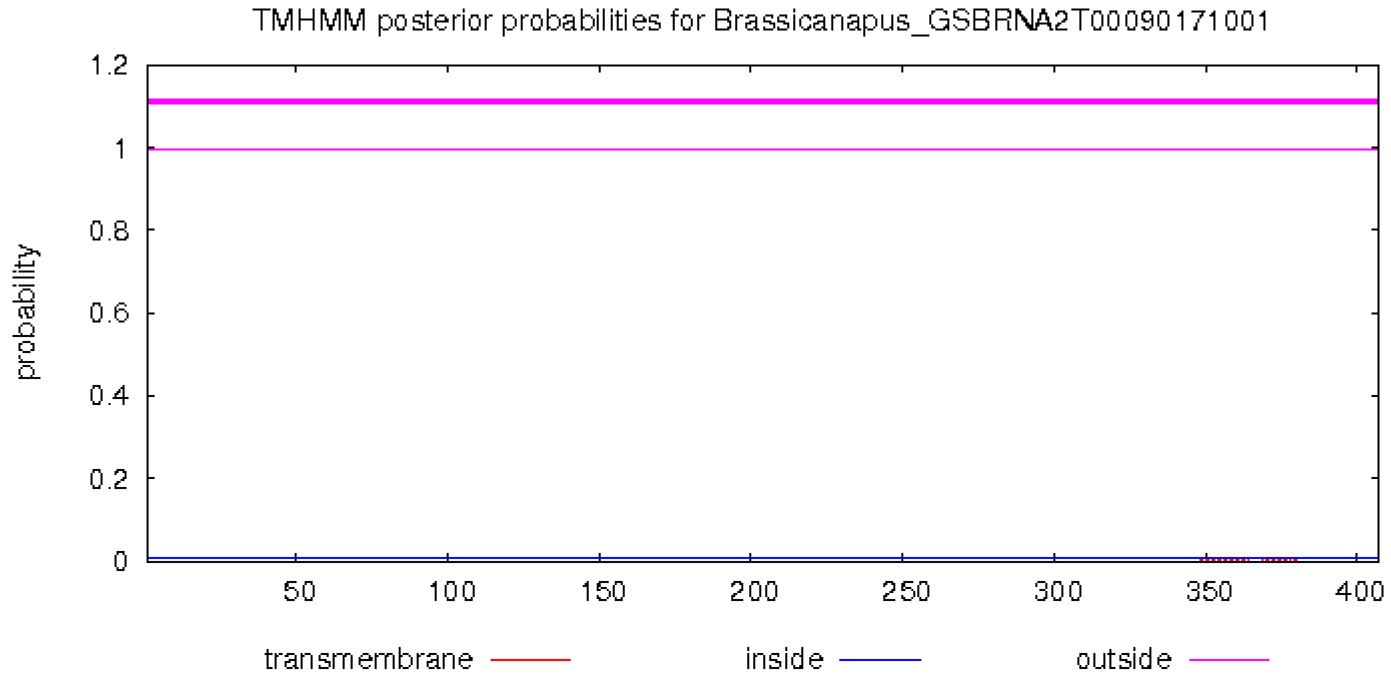

plot in postscript, script for making the plot in gnuplot, data for plot

---

```
# Brassicanapus_GSBRNA2T00090371001 Length: 339
# Brassicanapus_GSBRNA2T00090371001 Number of predicted TMHs:  0
# Brassicanapus_GSBRNA2T00090371001 Exp number of AAs in TMHs: 0.00215
# Brassicanapus_GSBRNA2T00090371001 Exp number, first 60 AAs:  0
# Brassicanapus_GSBRNA2T00090371001 Total prob of N-in:        0.00342
Brassicanapus_GSBRNA2T00090371001       TMHMM2.0        outside      1   339
```

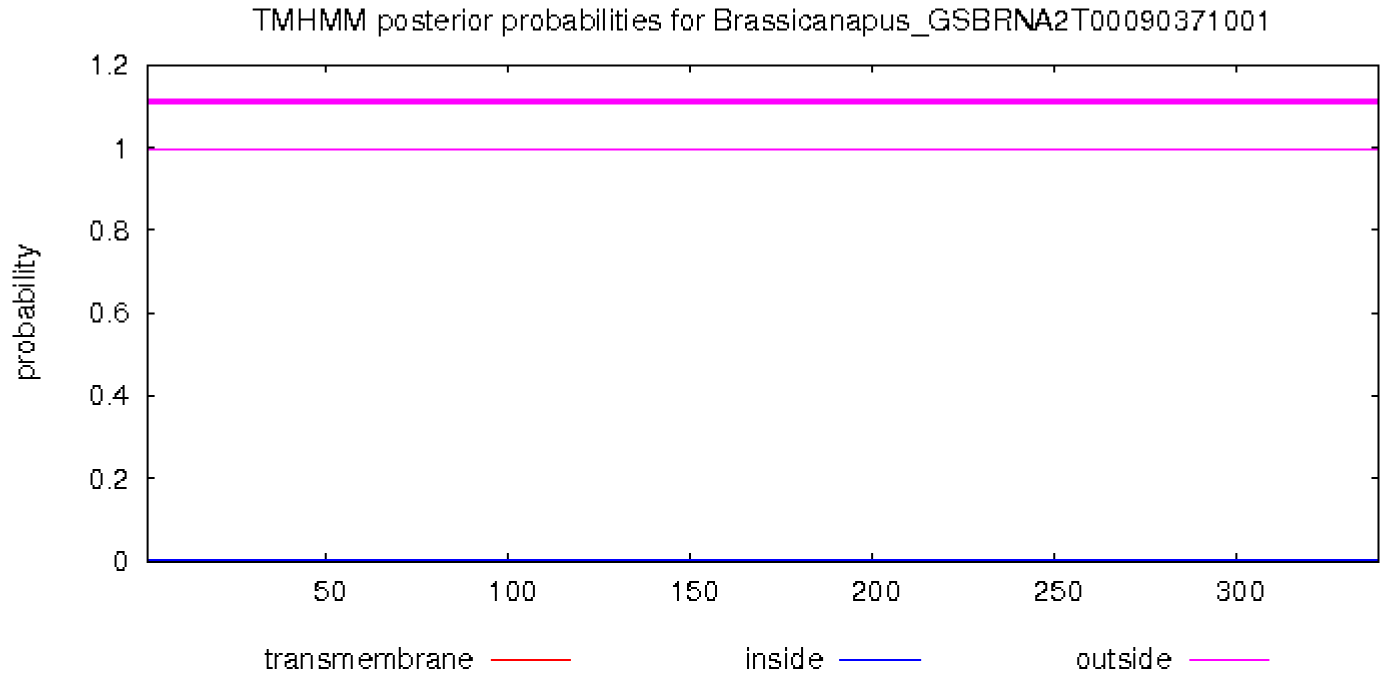

plot in postscript, script for making the plot in gnuplot, data for plot

---

```
# Brassicanapus_GSBRNA2T00090572001 Length: 457
# Brassicanapus_GSBRNA2T00090572001 Number of predicted TMHs:  0
# Brassicanapus_GSBRNA2T00090572001 Exp number of AAs in TMHs: 0.00099
# Brassicanapus_GSBRNA2T00090572001 Exp number, first 60 AAs:  0
# Brassicanapus_GSBRNA2T00090572001 Total prob of N-in:        0.00291
Brassicanapus_GSBRNA2T00090572001       TMHMM2.0        outside      1   457
```

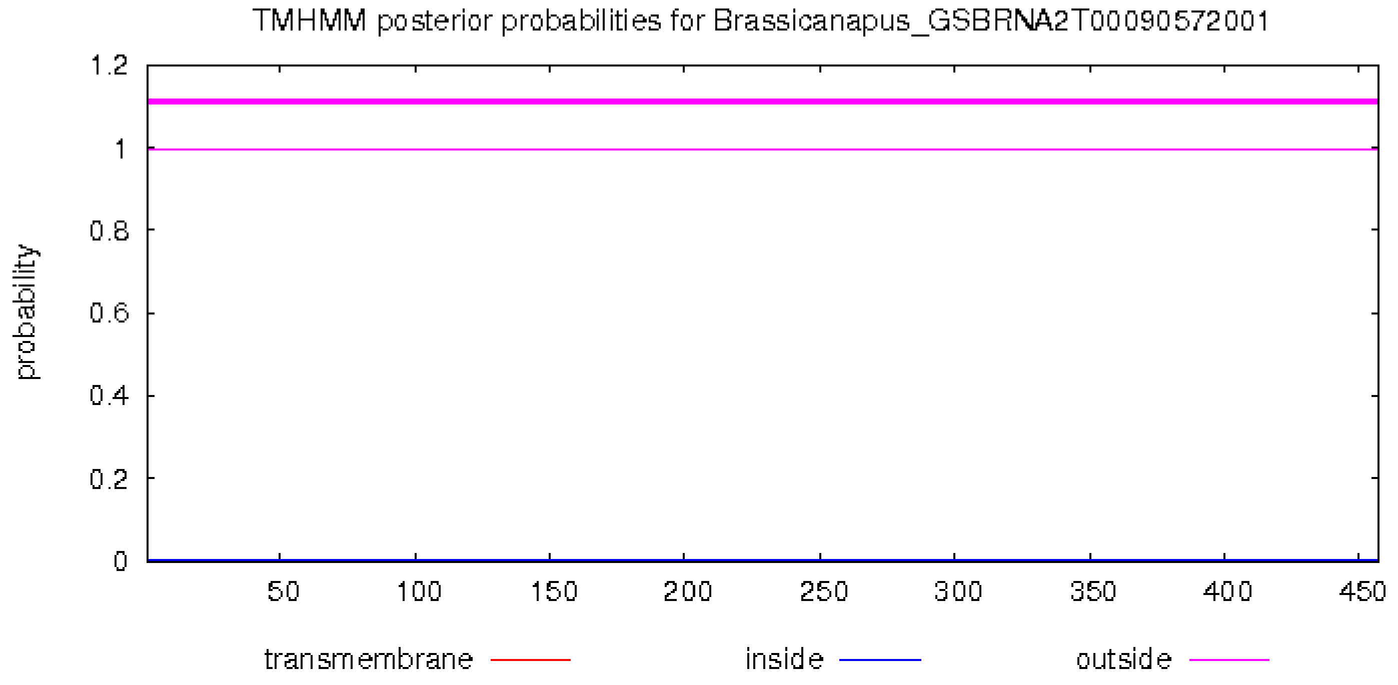

plot in postscript, script for making the plot in gnuplot, data for plot

---

```
# Brassicanapus_GSBRNA2T00090982001 Length: 595
# Brassicanapus_GSBRNA2T00090982001 Number of predicted TMHs:  0
# Brassicanapus_GSBRNA2T00090982001 Exp number of AAs in TMHs: 10.53644
# Brassicanapus_GSBRNA2T00090982001 Exp number, first 60 AAs:  0.00083
# Brassicanapus_GSBRNA2T00090982001 Total prob of N-in:        0.45816
Brassicanapus_GSBRNA2T00090982001       TMHMM2.0        outside      1   595
```

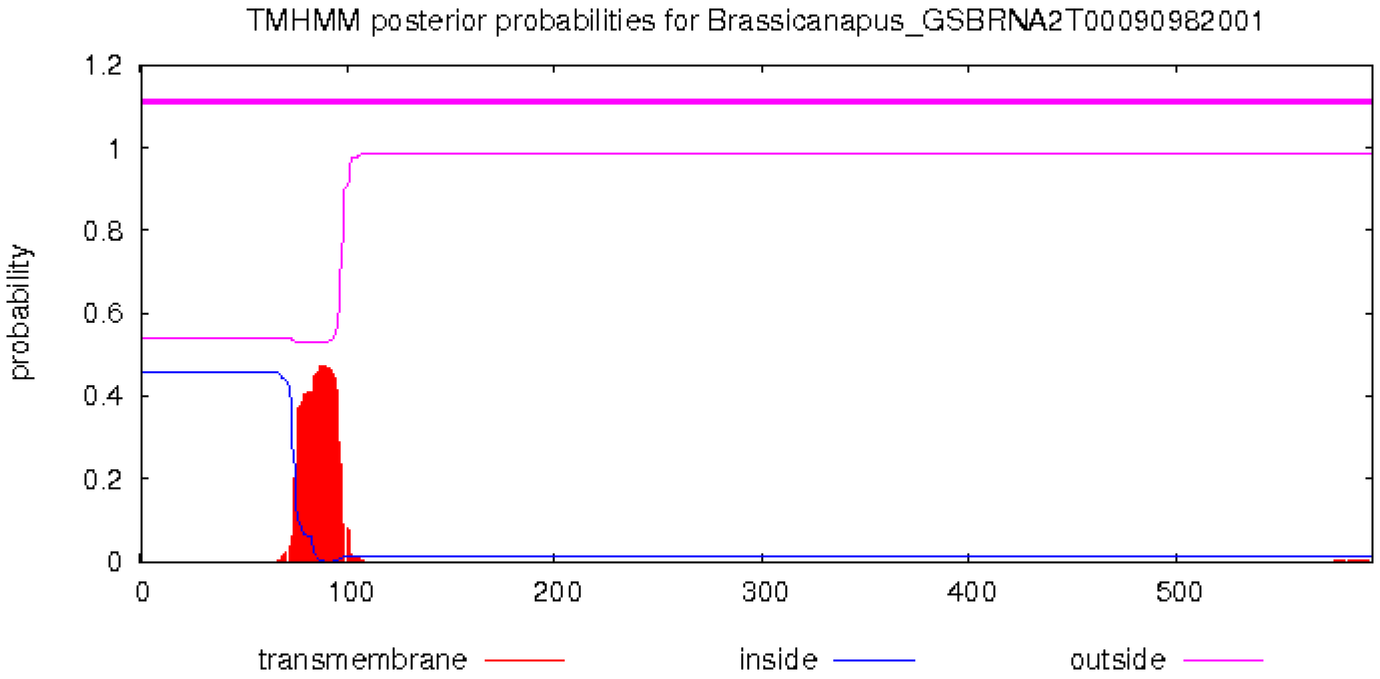

plot in postscript, script for making the plot in gnuplot, data for plot

---

```
# Brassicanapus_GSBRNA2T00091365001 Length: 474
# Brassicanapus_GSBRNA2T00091365001 Number of predicted TMHs:  0
# Brassicanapus_GSBRNA2T00091365001 Exp number of AAs in TMHs: 7.53947
# Brassicanapus_GSBRNA2T00091365001 Exp number, first 60 AAs:  0
# Brassicanapus_GSBRNA2T00091365001 Total prob of N-in:        0.29562
Brassicanapus_GSBRNA2T00091365001	TMHMM2.0	outside	     1   474
```

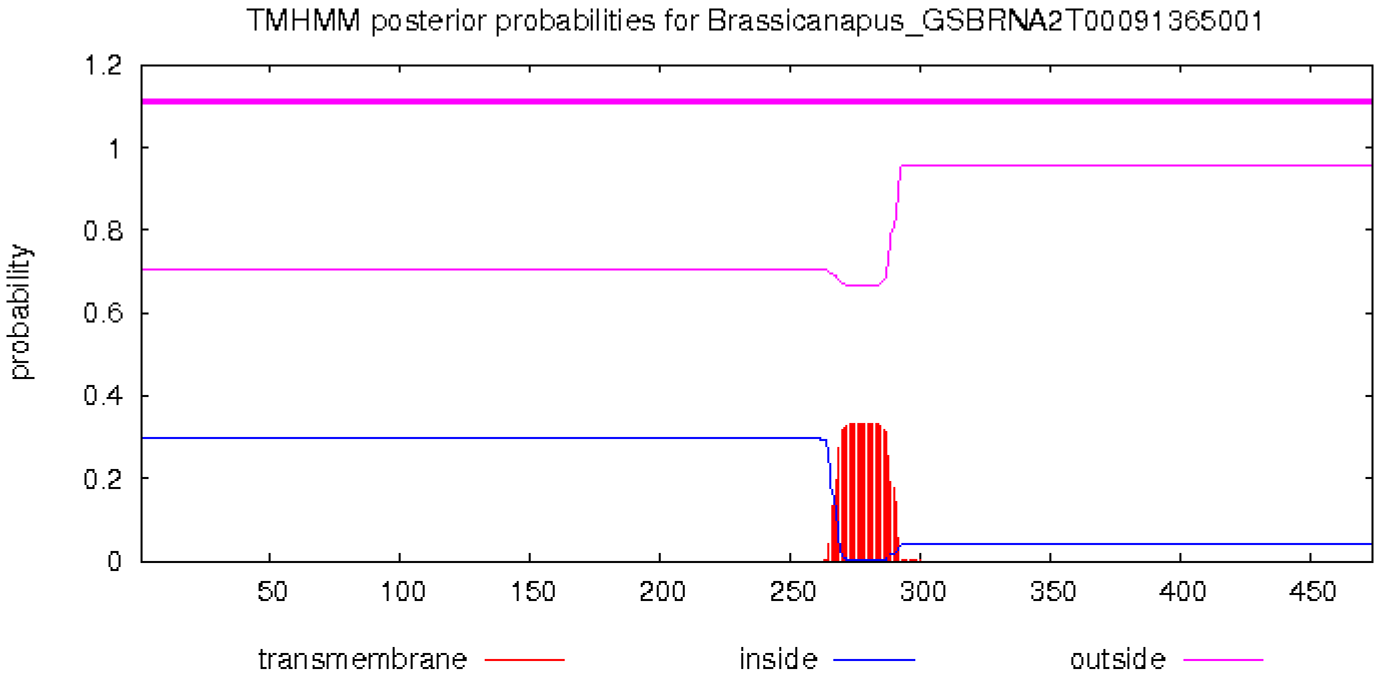

plot in postscript, script for making the plot in gnuplot, data for plot

---

```
# Brassicanapus_GSBRNA2T00091404001 Length: 563
# Brassicanapus_GSBRNA2T00091404001 Number of predicted TMHs:  1
# Brassicanapus_GSBRNA2T00091404001 Exp number of AAs in TMHs: 21.76229
# Brassicanapus_GSBRNA2T00091404001 Exp number, first 60 AAs:  0.00278
# Brassicanapus_GSBRNA2T00091404001 Total prob of N-in:        0.46755
Brassicanapus_GSBRNA2T00091404001	TMHMM2.0	inside	     1   533
```

```
Brassicanapus_GSBRNA2T00091404001       TMHMM2.0        TMhelix     534    556
Brassicanapus_GSBRNA2T00091404001       TMHMM2.0        outside     557    563
```

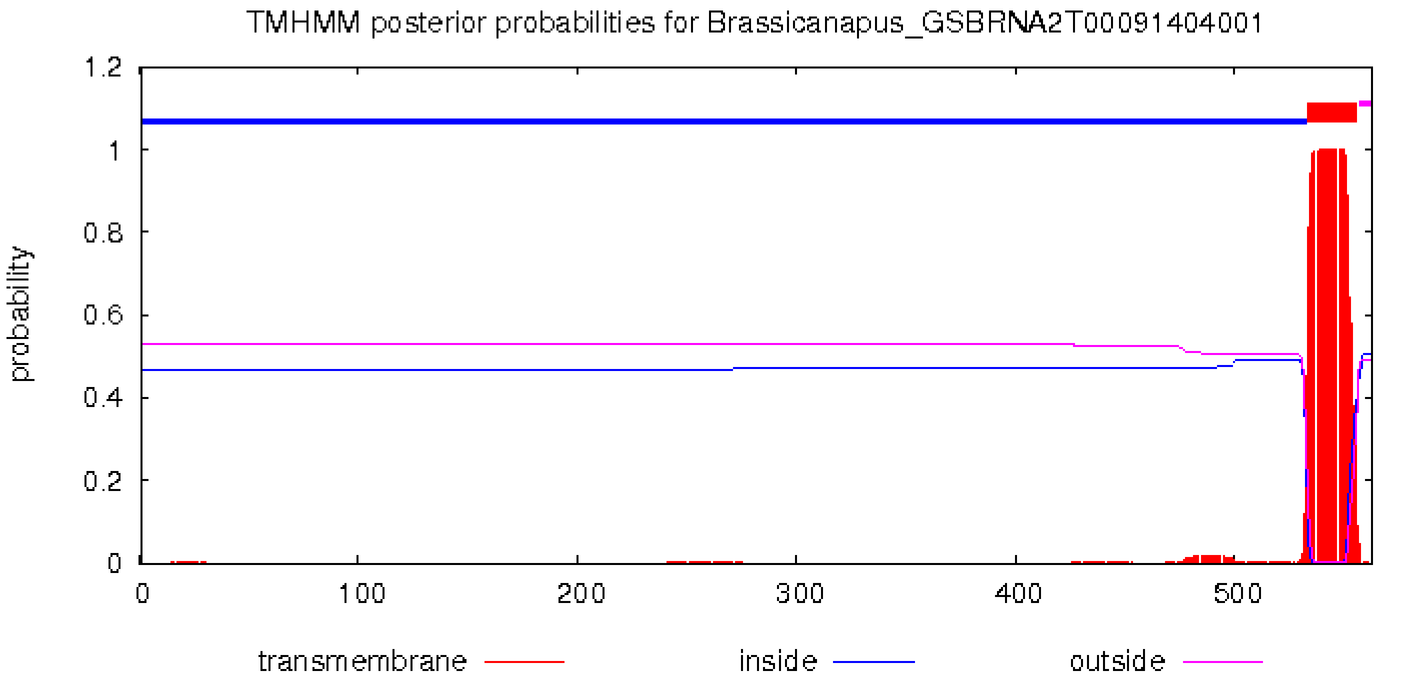

plot in postscript, script for making the plot in gnuplot, data for plot

---

```
# Brassicanapus_GSBRNA2T00092085001 Length: 201
# Brassicanapus_GSBRNA2T00092085001 Number of predicted TMHs:  0
# Brassicanapus_GSBRNA2T00092085001 Exp number of AAs in TMHs: 0.00018
# Brassicanapus_GSBRNA2T00092085001 Exp number, first 60 AAs:  0
# Brassicanapus_GSBRNA2T00092085001 Total prob of N-in:        0.01747
Brassicanapus_GSBRNA2T00092085001       TMHMM2.0        outside      1    201
```

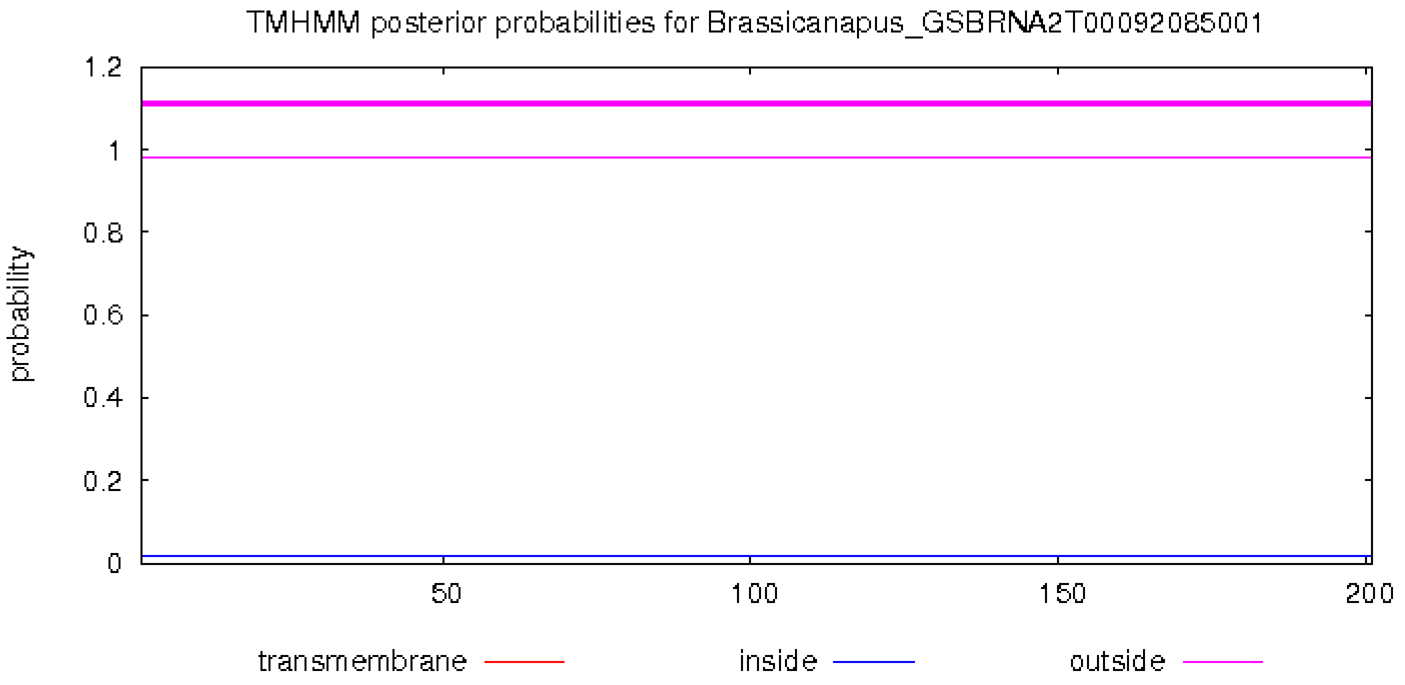

plot in postscript, script for making the plot in gnuplot, data for plot

---

```
# Brassicanapus_GSBRNA2T00093660001 Length: 283
# Brassicanapus_GSBRNA2T00093660001 Number of predicted TMHs:  0
# Brassicanapus_GSBRNA2T00093660001 Exp number of AAs in TMHs: 0.0036
# Brassicanapus_GSBRNA2T00093660001 Exp number, first 60 AAs:  0
```

```
# Brassicanapus_GSBRNA2T00093660001 Total prob of N-in:        0.01750
Brassicanapus_GSBRNA2T00093660001       TMHMM2.0        outside      1   283
```

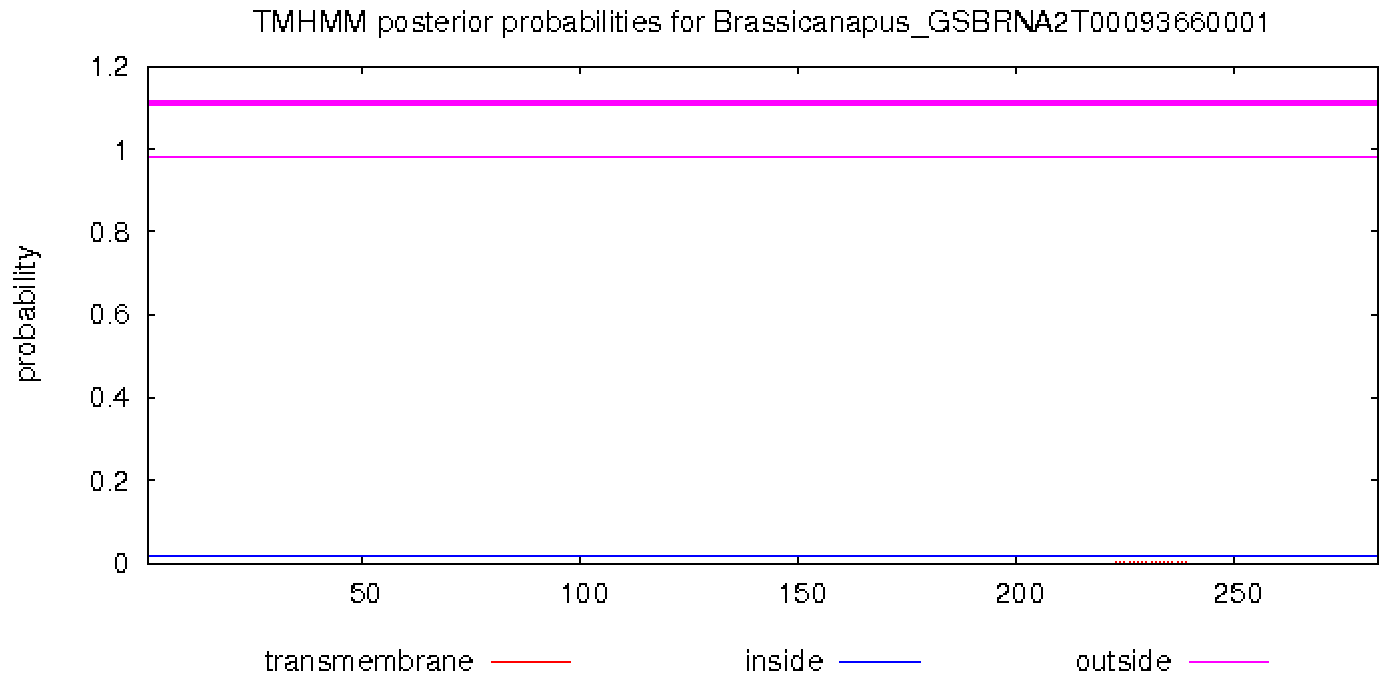

plot in postscript, script for making the plot in gnuplot, data for plot

---

```
# Brassicanapus_GSBRNA2T00094099001 Length: 479
# Brassicanapus_GSBRNA2T00094099001 Number of predicted TMHs:  0
# Brassicanapus_GSBRNA2T00094099001 Exp number of AAs in TMHs: 0.00458
# Brassicanapus_GSBRNA2T00094099001 Exp number, first 60 AAs:  0
# Brassicanapus_GSBRNA2T00094099001 Total prob of N-in:        0.00017
Brassicanapus_GSBRNA2T00094099001       TMHMM2.0        outside      1   479
```

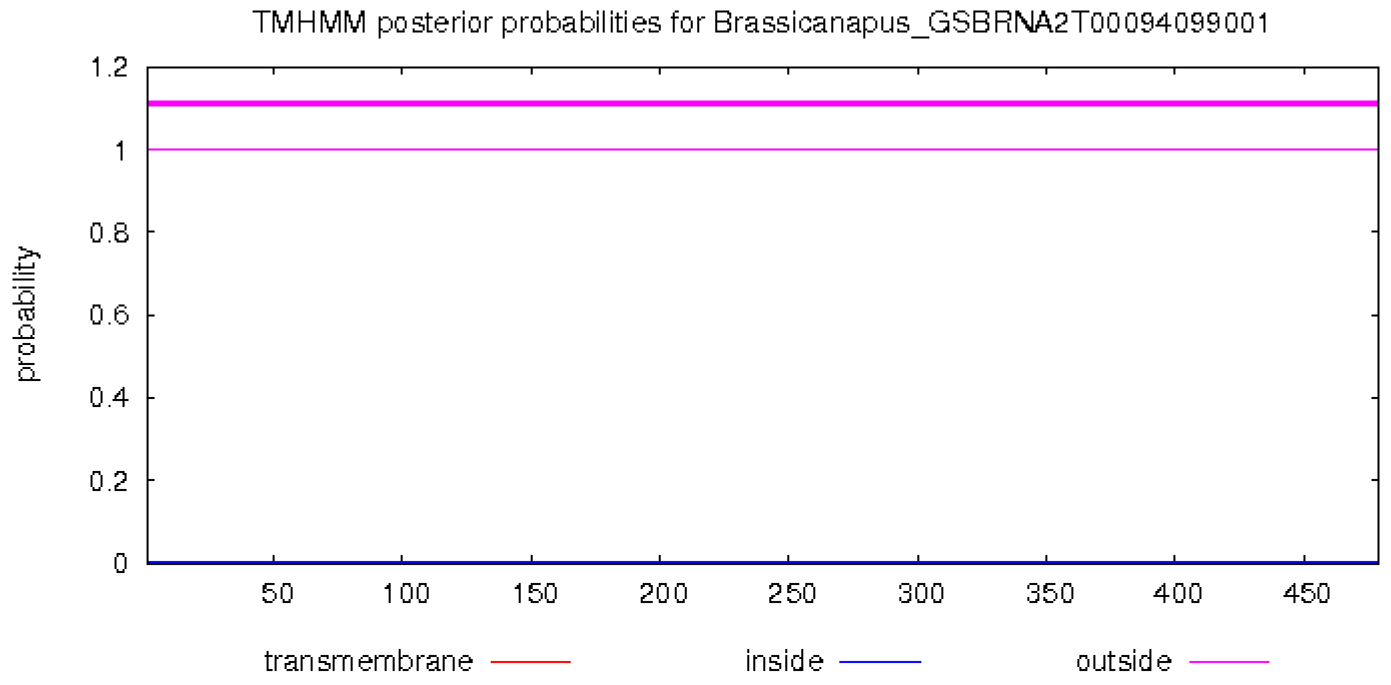

plot in postscript, script for making the plot in gnuplot, data for plot

---

```
# Brassicanapus_GSBRNA2T00094309001 Length: 65
# Brassicanapus_GSBRNA2T00094309001 Number of predicted TMHs:  0
# Brassicanapus_GSBRNA2T00094309001 Exp number of AAs in TMHs: 0.67792
# Brassicanapus_GSBRNA2T00094309001 Exp number, first 60 AAs:  0.67528
```

```
# Brassicanapus_GSBRNA2T00094309001 Total prob of N-in:        0.12563
Brassicanapus_GSBRNA2T00094309001       TMHMM2.0        outside      1    65
```

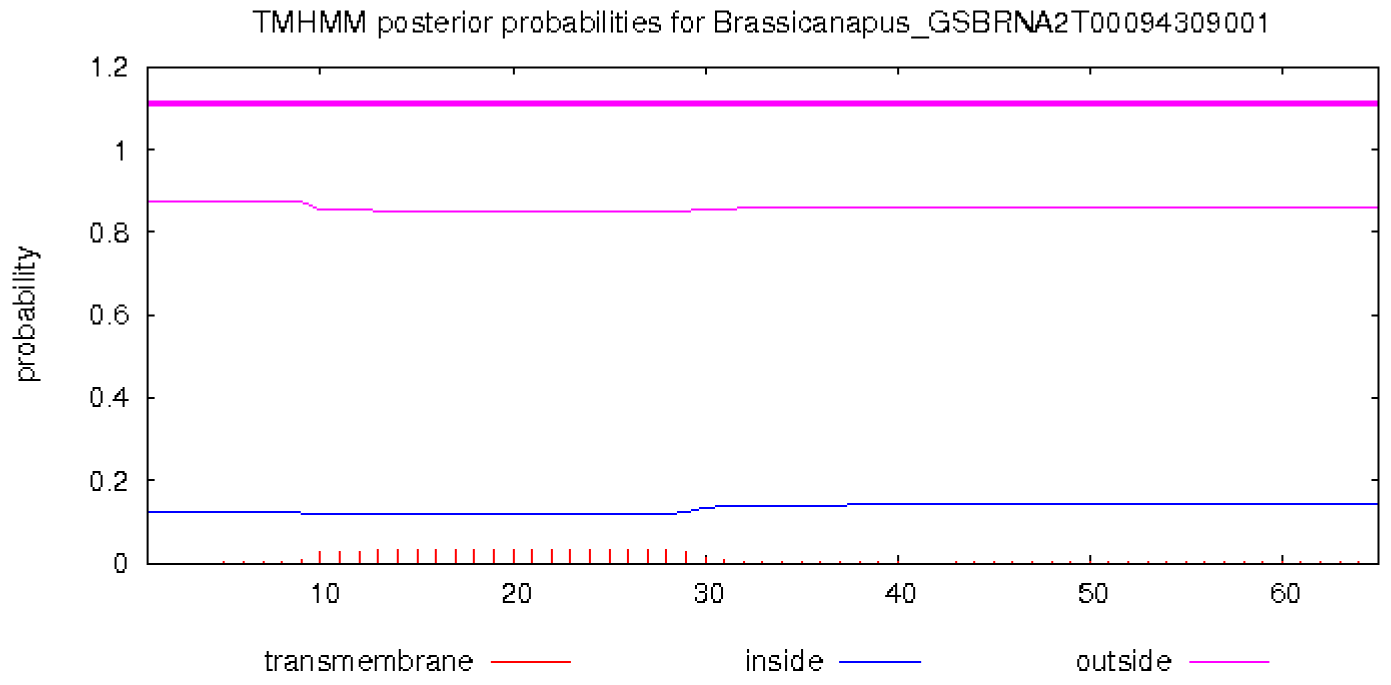

plot in postscript, script for making the plot in gnuplot, data for plot

---

```
# Brassicanapus_GSBRNA2T00094654001 Length: 332
# Brassicanapus_GSBRNA2T00094654001 Number of predicted TMHs:  0
# Brassicanapus_GSBRNA2T00094654001 Exp number of AAs in TMHs: 0
# Brassicanapus_GSBRNA2T00094654001 Exp number, first 60 AAs:  0
# Brassicanapus_GSBRNA2T00094654001 Total prob of N-in:        0.00231
Brassicanapus_GSBRNA2T00094654001       TMHMM2.0        outside      1   332
```

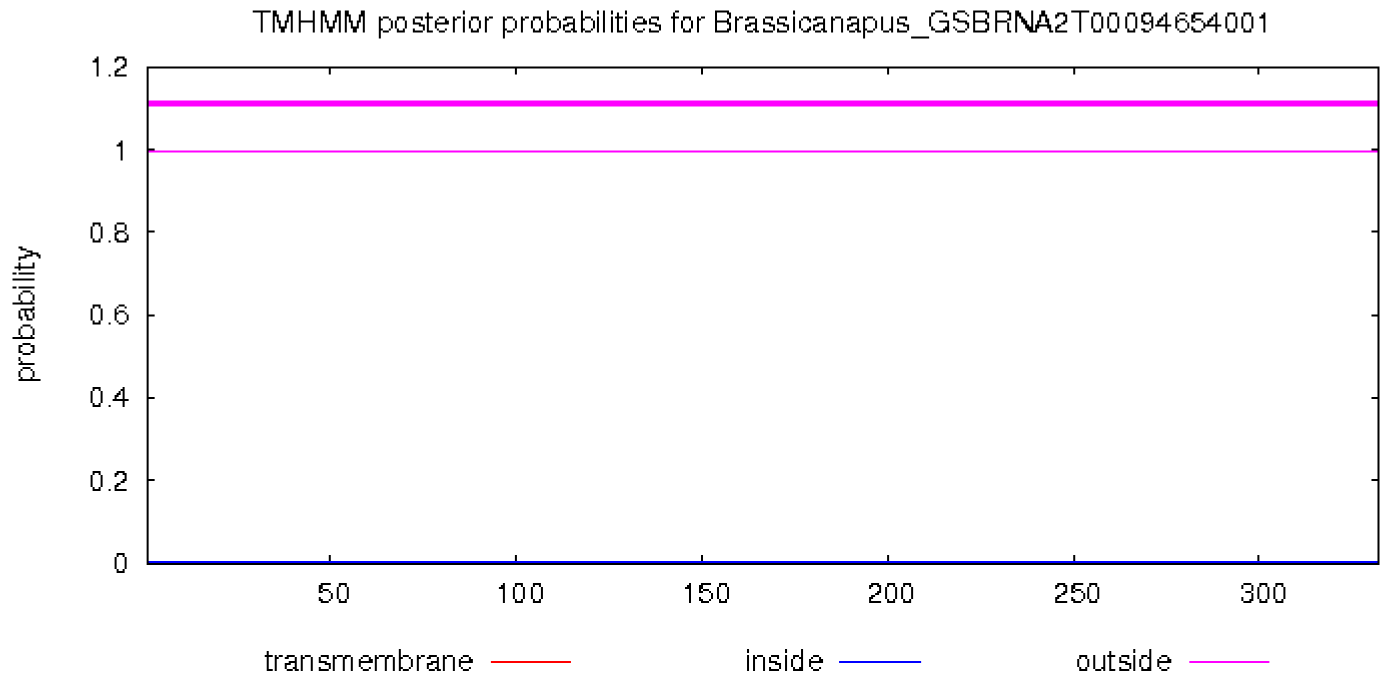

plot in postscript, script for making the plot in gnuplot, data for plot

---

```
# Brassicanapus_GSBRNA2T00094866001 Length: 497
# Brassicanapus_GSBRNA2T00094866001 Number of predicted TMHs:  0
# Brassicanapus_GSBRNA2T00094866001 Exp number of AAs in TMHs: 0.00037
# Brassicanapus_GSBRNA2T00094866001 Exp number, first 60 AAs:  0
```

```
# Brassicanapus_GSBRNA2T00094866001 Total prob of N-in:        0.00013
Brassicanapus_GSBRNA2T00094866001       TMHMM2.0        outside      1   497
```

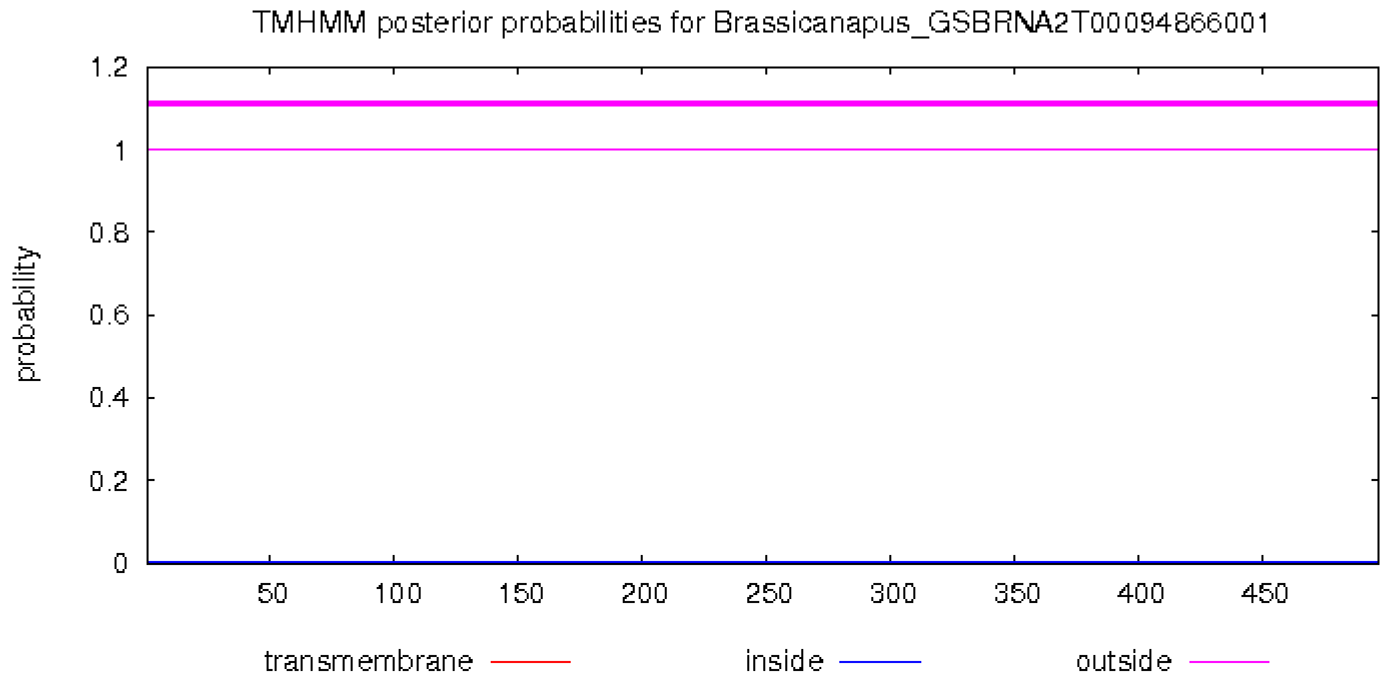

plot in postscript, script for making the plot in gnuplot, data for plot

---

```
# Brassicanapus_GSBRNA2T00095240001 Length: 252
# Brassicanapus_GSBRNA2T00095240001 Number of predicted TMHs:  0
# Brassicanapus_GSBRNA2T00095240001 Exp number of AAs in TMHs: 0.00586
# Brassicanapus_GSBRNA2T00095240001 Exp number, first 60 AAs:  0.00459
# Brassicanapus_GSBRNA2T00095240001 Total prob of N-in:        0.15477
Brassicanapus_GSBRNA2T00095240001       TMHMM2.0        outside      1   252
```

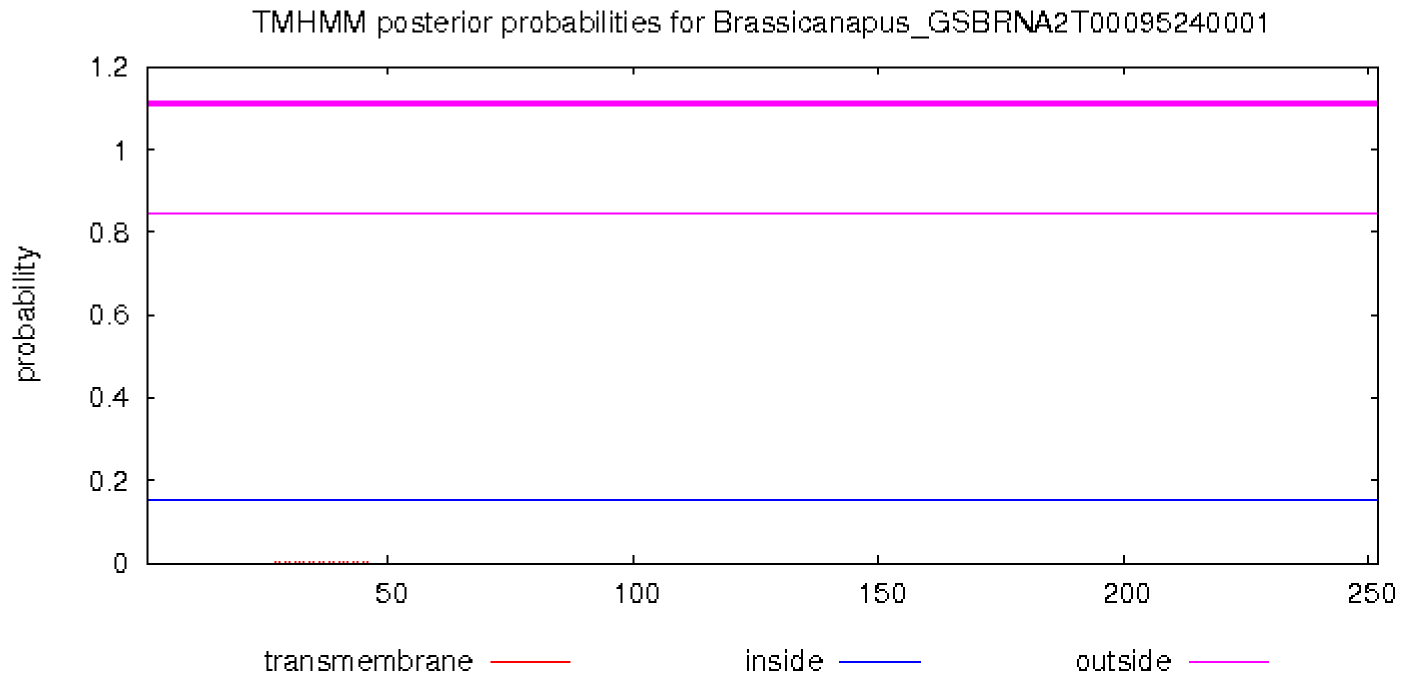

plot in postscript, script for making the plot in gnuplot, data for plot

---

```
# Brassicanapus_GSBRNA2T00095484001 Length: 154
# Brassicanapus_GSBRNA2T00095484001 Number of predicted TMHs:  0
# Brassicanapus_GSBRNA2T00095484001 Exp number of AAs in TMHs: 0
# Brassicanapus_GSBRNA2T00095484001 Exp number, first 60 AAs:  0
```

```
# Brassicanapus_GSBRNA2T00095484001 Total prob of N-in:        0.26085
Brassicanapus_GSBRNA2T00095484001       TMHMM2.0        outside      1   154
```

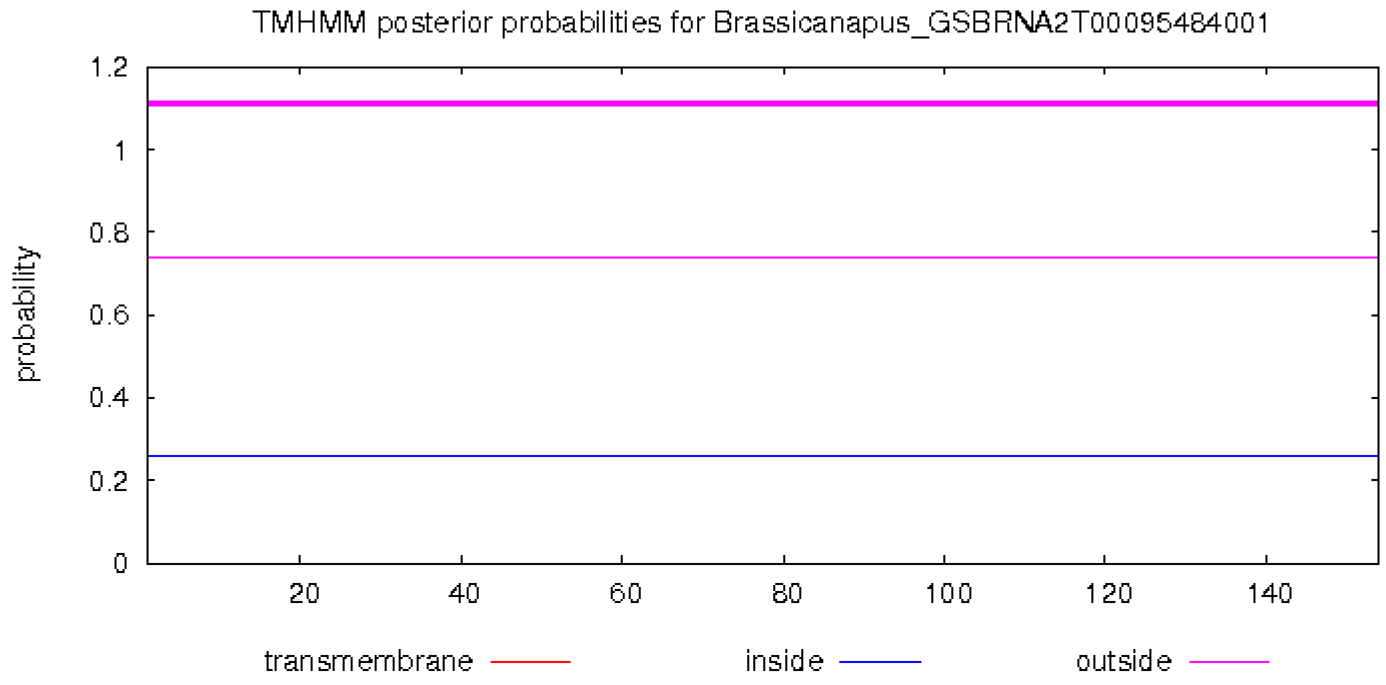

plot in postscript, script for making the plot in gnuplot, data for plot

```
# Brassicanapus_GSBRNA2T00095486001 Length: 324
# Brassicanapus_GSBRNA2T00095486001 Number of predicted TMHs:  0
# Brassicanapus_GSBRNA2T00095486001 Exp number of AAs in TMHs: 0.00186
# Brassicanapus_GSBRNA2T00095486001 Exp number, first 60 AAs:  0.00038
# Brassicanapus_GSBRNA2T00095486001 Total prob of N-in:        0.00888
Brassicanapus_GSBRNA2T00095486001       TMHMM2.0        outside      1   324
```

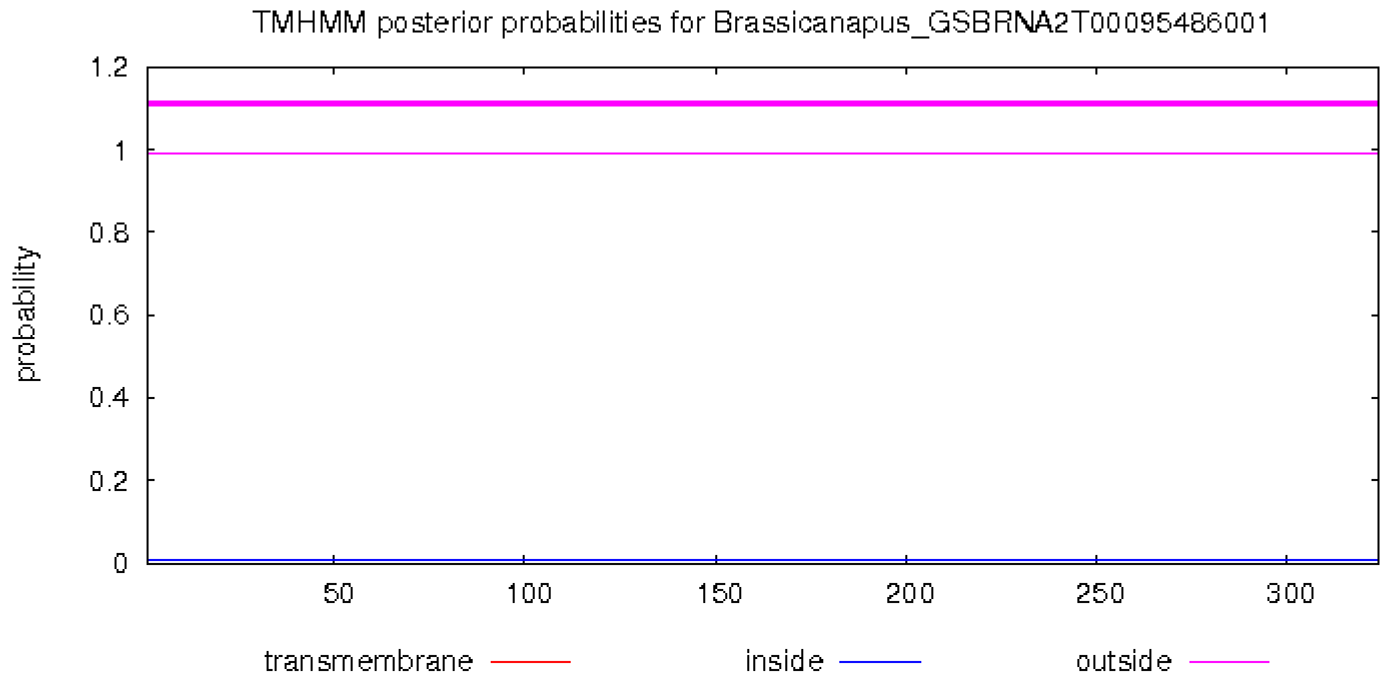

plot in postscript, script for making the plot in gnuplot, data for plot

```
# Brassicanapus_GSBRNA2T00095503001 Length: 323
# Brassicanapus_GSBRNA2T00095503001 Number of predicted TMHs:  0
# Brassicanapus_GSBRNA2T00095503001 Exp number of AAs in TMHs: 0.00475
# Brassicanapus_GSBRNA2T00095503001 Exp number, first 60 AAs:  0
```

```
# Brassicanapus_GSBRNA2T00095503001 Total prob of N-in:        0.03445
Brassicanapus_GSBRNA2T00095503001       TMHMM2.0        outside      1   323
```

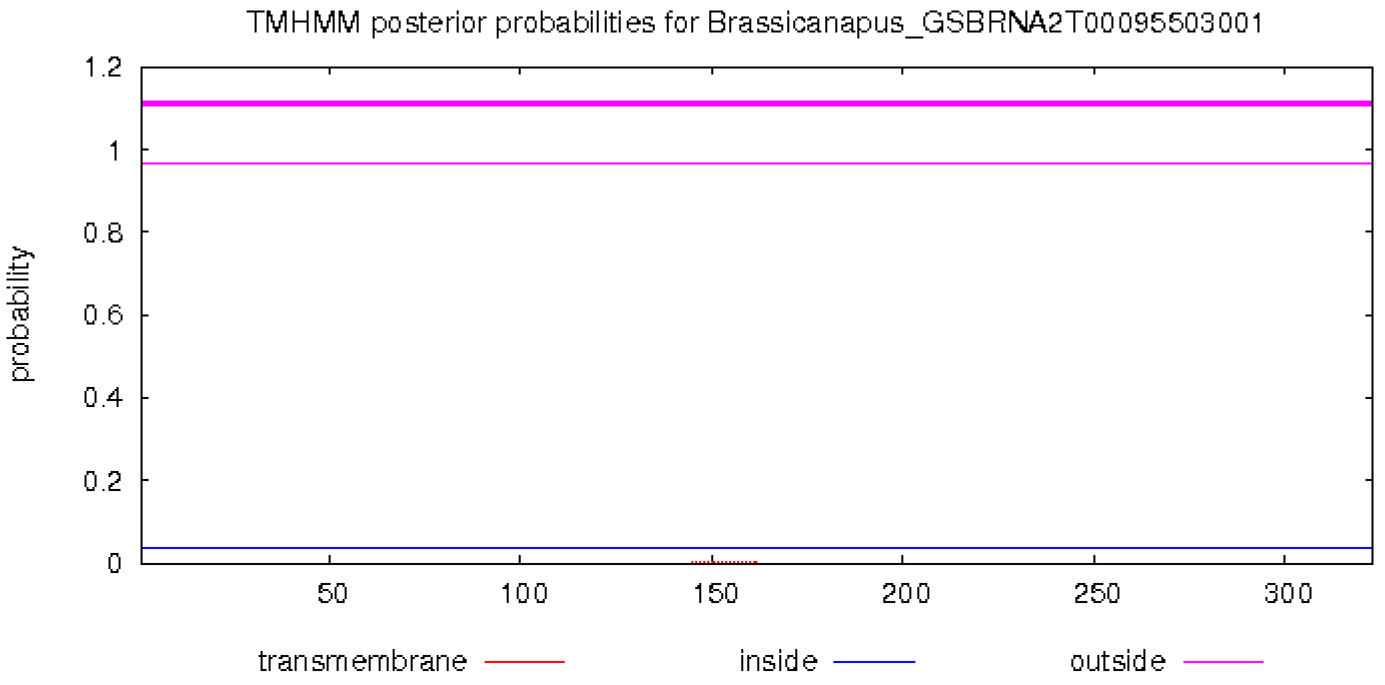

plot in postscript, script for making the plot in gnuplot, data for plot

---

```
# Brassicanapus_GSBRNA2T00095580001 Length: 406
# Brassicanapus_GSBRNA2T00095580001 Number of predicted TMHs:  0
# Brassicanapus_GSBRNA2T00095580001 Exp number of AAs in TMHs: 0.02688
# Brassicanapus_GSBRNA2T00095580001 Exp number, first 60 AAs:  0
# Brassicanapus_GSBRNA2T00095580001 Total prob of N-in:        0.01503
Brassicanapus_GSBRNA2T00095580001       TMHMM2.0        outside      1   406
```

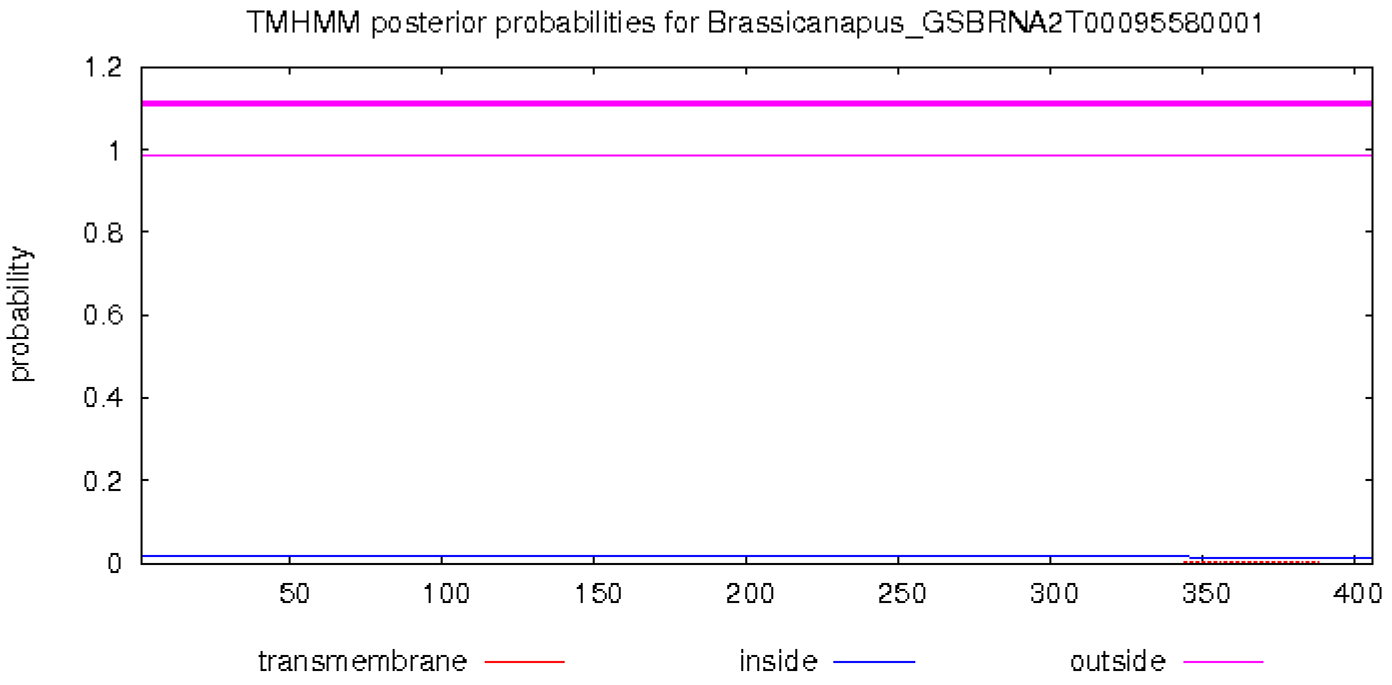

plot in postscript, script for making the plot in gnuplot, data for plot

---

```
# Brassicanapus_GSBRNA2T00095993001 Length: 214
# Brassicanapus_GSBRNA2T00095993001 Number of predicted TMHs:  0
# Brassicanapus_GSBRNA2T00095993001 Exp number of AAs in TMHs: 0.00075
# Brassicanapus_GSBRNA2T00095993001 Exp number, first 60 AAs:  0
```

```
# Brassicanapus_GSBRNA2T00095993001 Total prob of N-in:        0.02457
Brassicanapus_GSBRNA2T00095993001       TMHMM2.0        outside      1   214
```

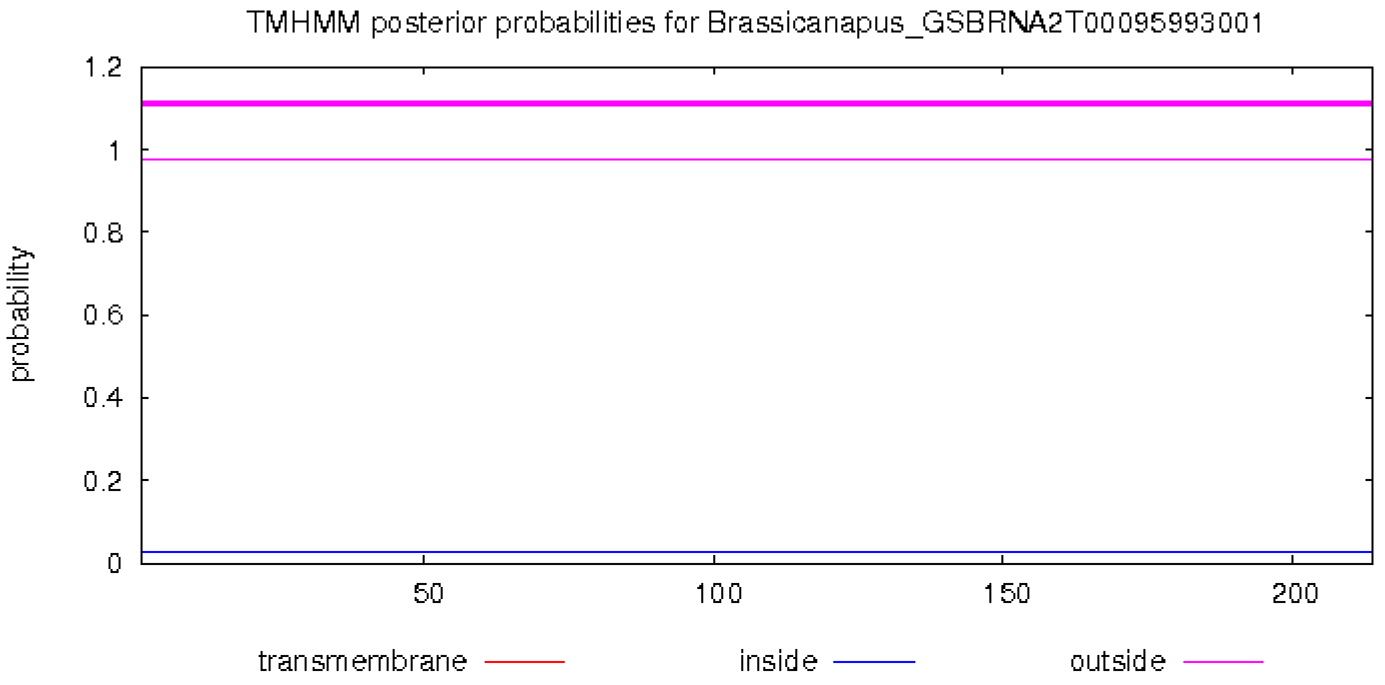

plot in postscript, script for making the plot in gnuplot, data for plot

---

```
# Brassicanapus_GSBRNA2T00096027001 Length: 357
# Brassicanapus_GSBRNA2T00096027001 Number of predicted TMHs:  0
# Brassicanapus_GSBRNA2T00096027001 Exp number of AAs in TMHs: 0.00096
# Brassicanapus_GSBRNA2T00096027001 Exp number, first 60 AAs:  0.00096
# Brassicanapus_GSBRNA2T00096027001 Total prob of N-in:        0.00921
Brassicanapus_GSBRNA2T00096027001       TMHMM2.0        outside      1   357
```

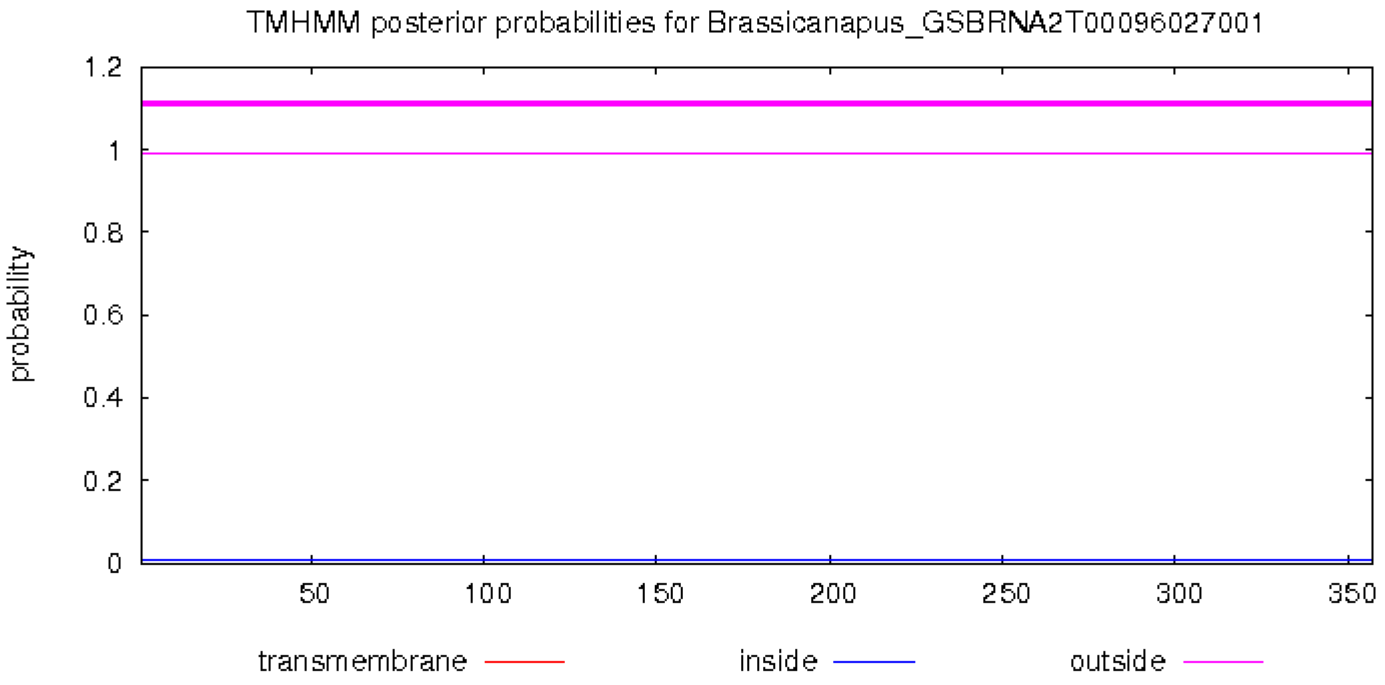

plot in postscript, script for making the plot in gnuplot, data for plot

---

```
# Brassicanapus_GSBRNA2T00097708001 Length: 343
# Brassicanapus_GSBRNA2T00097708001 Number of predicted TMHs:  0
# Brassicanapus_GSBRNA2T00097708001 Exp number of AAs in TMHs: 0.00076
# Brassicanapus_GSBRNA2T00097708001 Exp number, first 60 AAs:  0
```

```
# Brassicanapus_GSBRNA2T00097708001 Total prob of N-in:        0.01168
Brassicanapus_GSBRNA2T00097708001       TMHMM2.0        outside      1   343
```

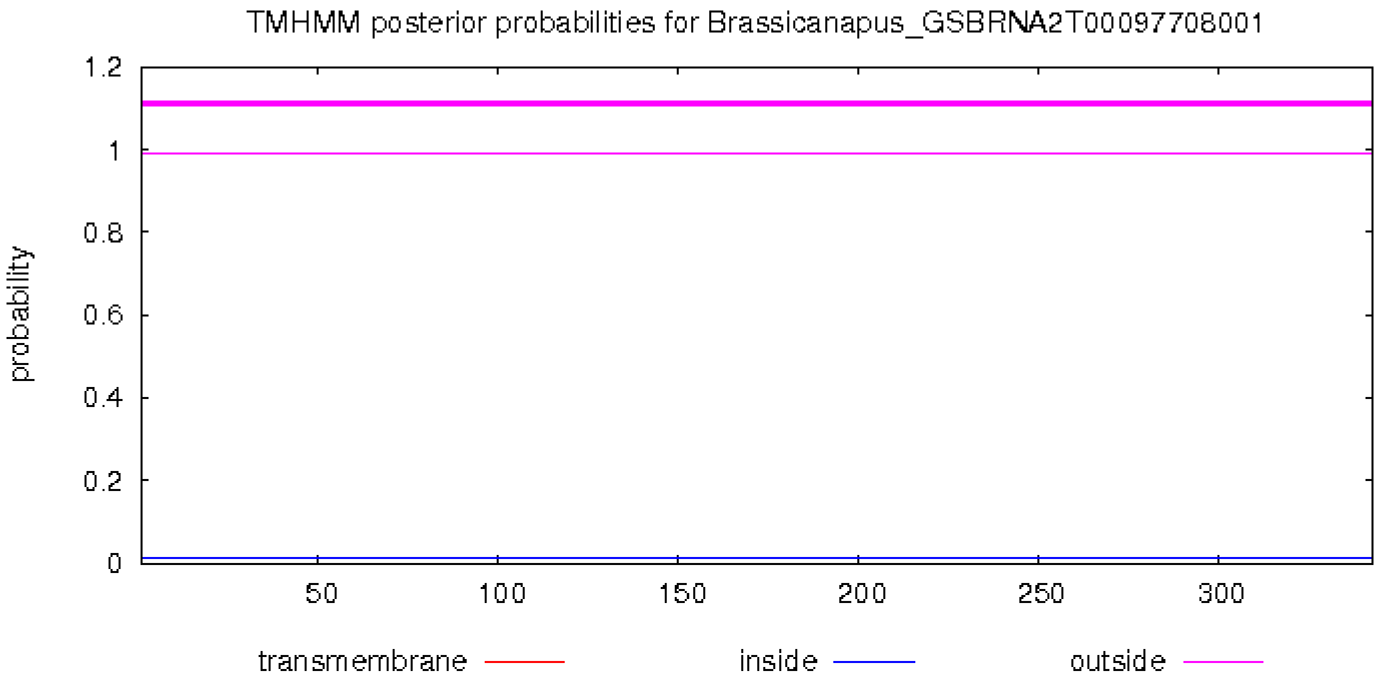

plot in postscript, script for making the plot in gnuplot, data for plot

---

```
# Brassicanapus_GSBRNA2T00097709001 Length: 325
# Brassicanapus_GSBRNA2T00097709001 Number of predicted TMHs:  0
# Brassicanapus_GSBRNA2T00097709001 Exp number of AAs in TMHs: 8e-05
# Brassicanapus_GSBRNA2T00097709001 Exp number, first 60 AAs:  0
# Brassicanapus_GSBRNA2T00097709001 Total prob of N-in:        0.01754
Brassicanapus_GSBRNA2T00097709001       TMHMM2.0        outside      1   325
```

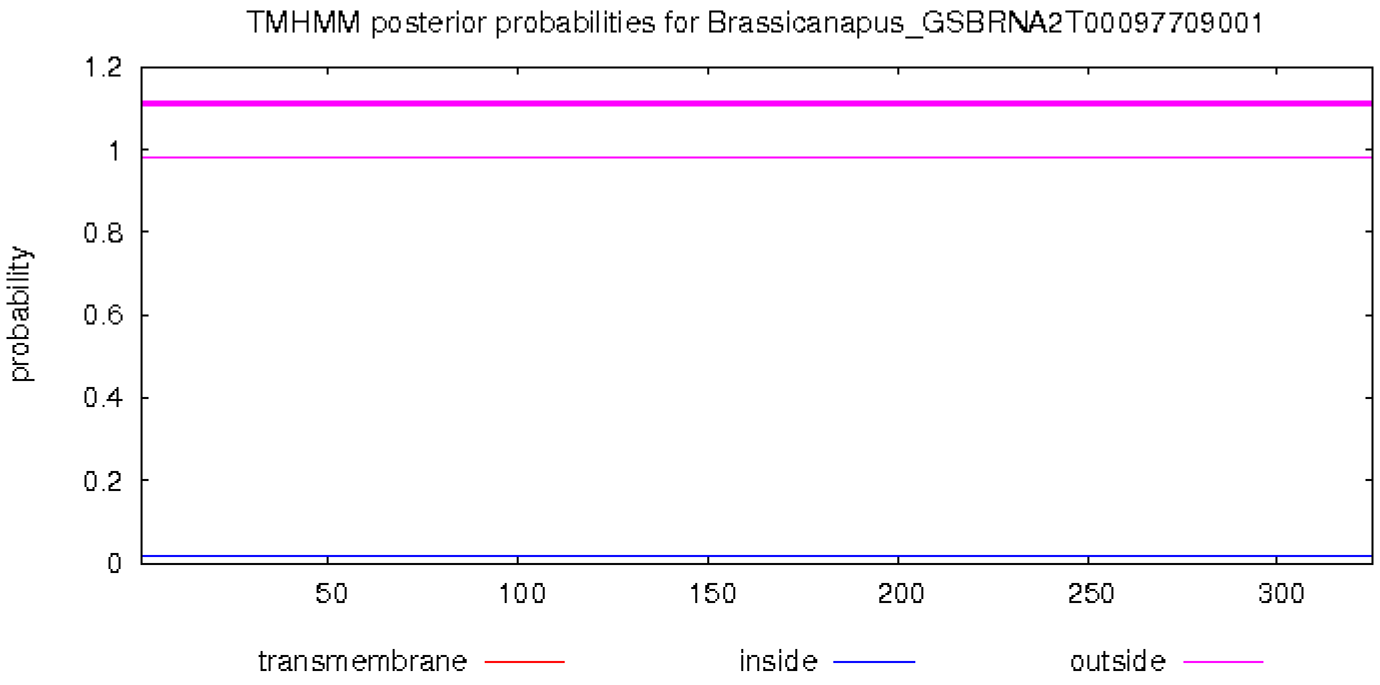

plot in postscript, script for making the plot in gnuplot, data for plot

---

```
# Brassicanapus_GSBRNA2T00098287001 Length: 358
# Brassicanapus_GSBRNA2T00098287001 Number of predicted TMHs:  0
# Brassicanapus_GSBRNA2T00098287001 Exp number of AAs in TMHs: 0.00033
# Brassicanapus_GSBRNA2T00098287001 Exp number, first 60 AAs:  0.00018
```

```
# Brassicanapus_GSBRNA2T00098287001 Total prob of N-in:        0.03319
Brassicanapus_GSBRNA2T00098287001       TMHMM2.0        outside      1   358
```

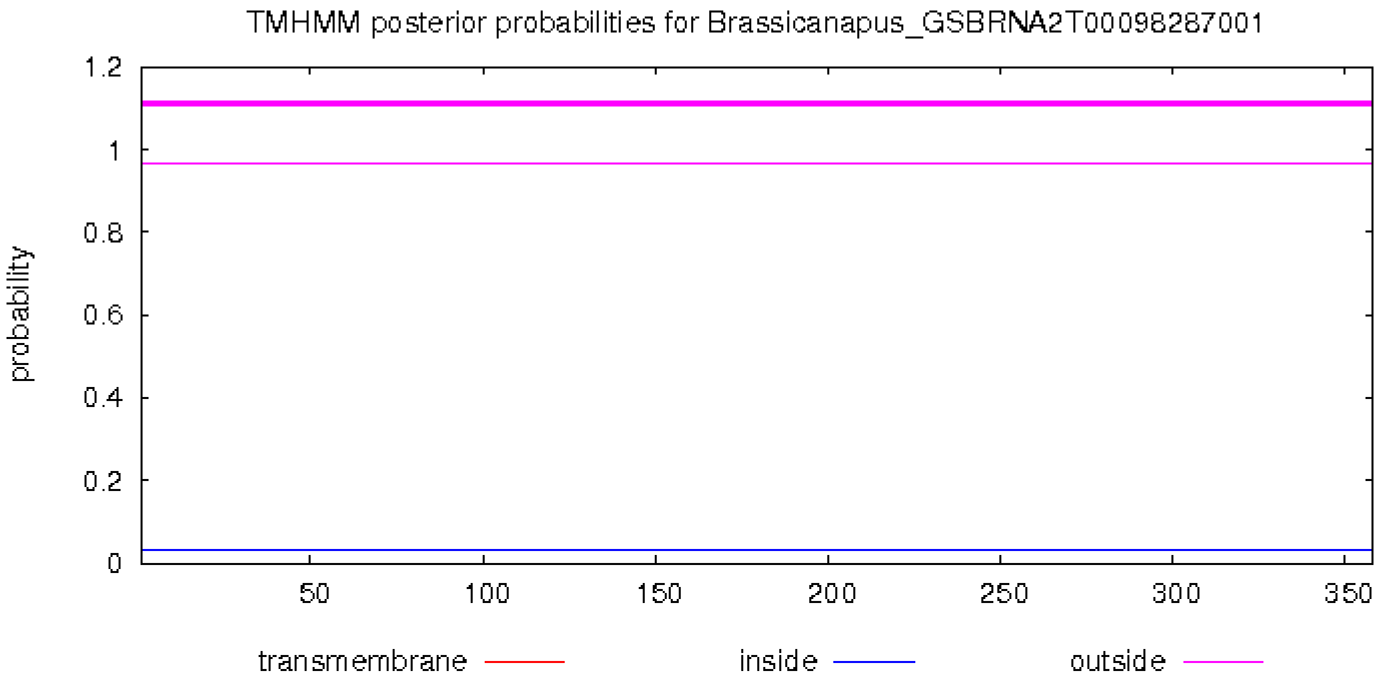

plot in postscript, script for making the plot in gnuplot, data for plot

---

```
# Brassicanapus_GSBRNA2T00098300001 Length: 701
# Brassicanapus_GSBRNA2T00098300001 Number of predicted TMHs:  6
# Brassicanapus_GSBRNA2T00098300001 Exp number of AAs in TMHs: 124.88035
# Brassicanapus_GSBRNA2T00098300001 Exp number, first 60 AAs:  40.27226
# Brassicanapus_GSBRNA2T00098300001 Total prob of N-in:        0.67475
# Brassicanapus_GSBRNA2T00098300001 POSSIBLE N-term signal sequence
Brassicanapus_GSBRNA2T00098300001       TMHMM2.0        inside       1     6
Brassicanapus_GSBRNA2T00098300001       TMHMM2.0        TMhelix      7    28
Brassicanapus_GSBRNA2T00098300001       TMHMM2.0        outside     29    42
Brassicanapus_GSBRNA2T00098300001       TMHMM2.0        TMhelix     43    62
Brassicanapus_GSBRNA2T00098300001       TMHMM2.0        inside      63    74
Brassicanapus_GSBRNA2T00098300001       TMHMM2.0        TMhelix     75    97
Brassicanapus_GSBRNA2T00098300001       TMHMM2.0        outside     98   111
Brassicanapus_GSBRNA2T00098300001       TMHMM2.0        TMhelix    112   134
Brassicanapus_GSBRNA2T00098300001       TMHMM2.0        inside     135   146
Brassicanapus_GSBRNA2T00098300001       TMHMM2.0        TMhelix    147   169
Brassicanapus_GSBRNA2T00098300001       TMHMM2.0        outside    170   188
Brassicanapus_GSBRNA2T00098300001       TMHMM2.0        TMhelix    189   211
Brassicanapus_GSBRNA2T00098300001       TMHMM2.0        inside     212   701
```

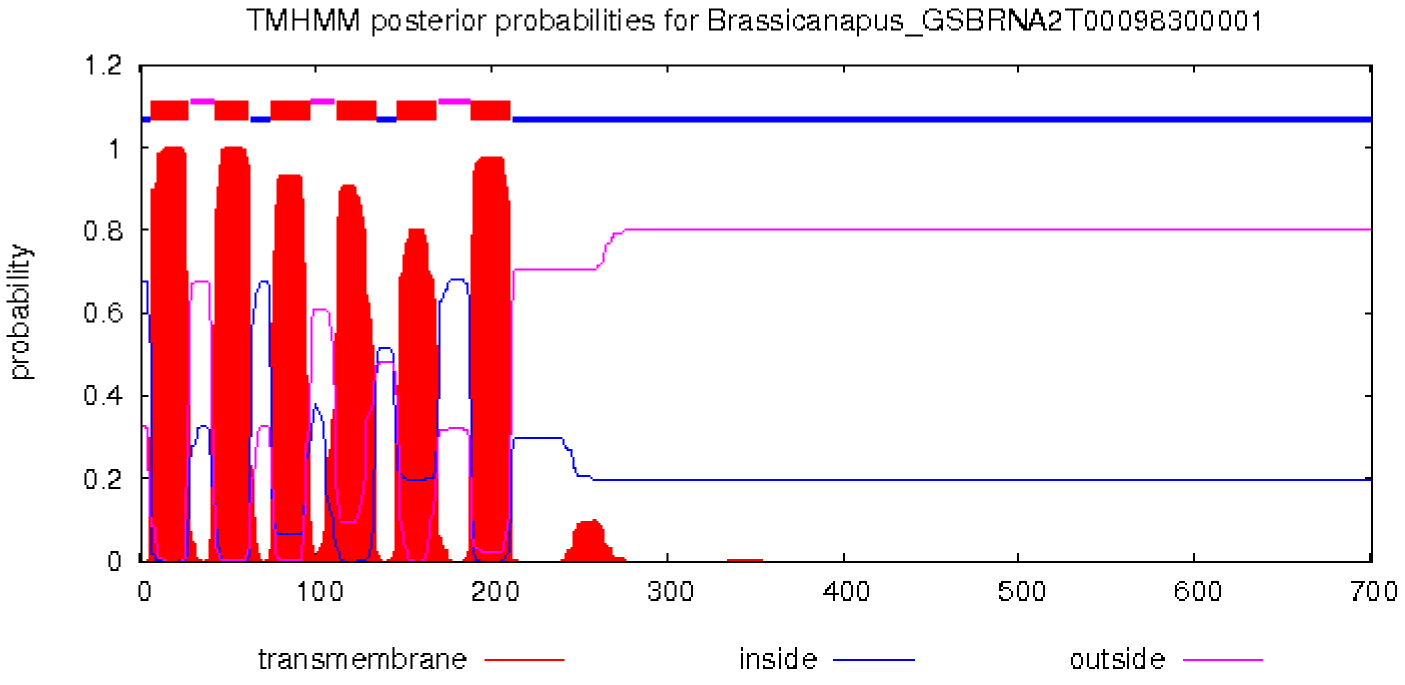

plot in postscript, script for making the plot in gnuplot, data for plot

---

```
# Brassicanapus_GSBRNA2T00098528001 Length: 288
# Brassicanapus_GSBRNA2T00098528001 Number of predicted TMHs:  0
# Brassicanapus_GSBRNA2T00098528001 Exp number of AAs in TMHs: 0.00078
# Brassicanapus_GSBRNA2T00098528001 Exp number, first 60 AAs:  0.00062
# Brassicanapus_GSBRNA2T00098528001 Total prob of N-in:        0.05556
Brassicanapus_GSBRNA2T00098528001       TMHMM2.0        outside      1   288
```

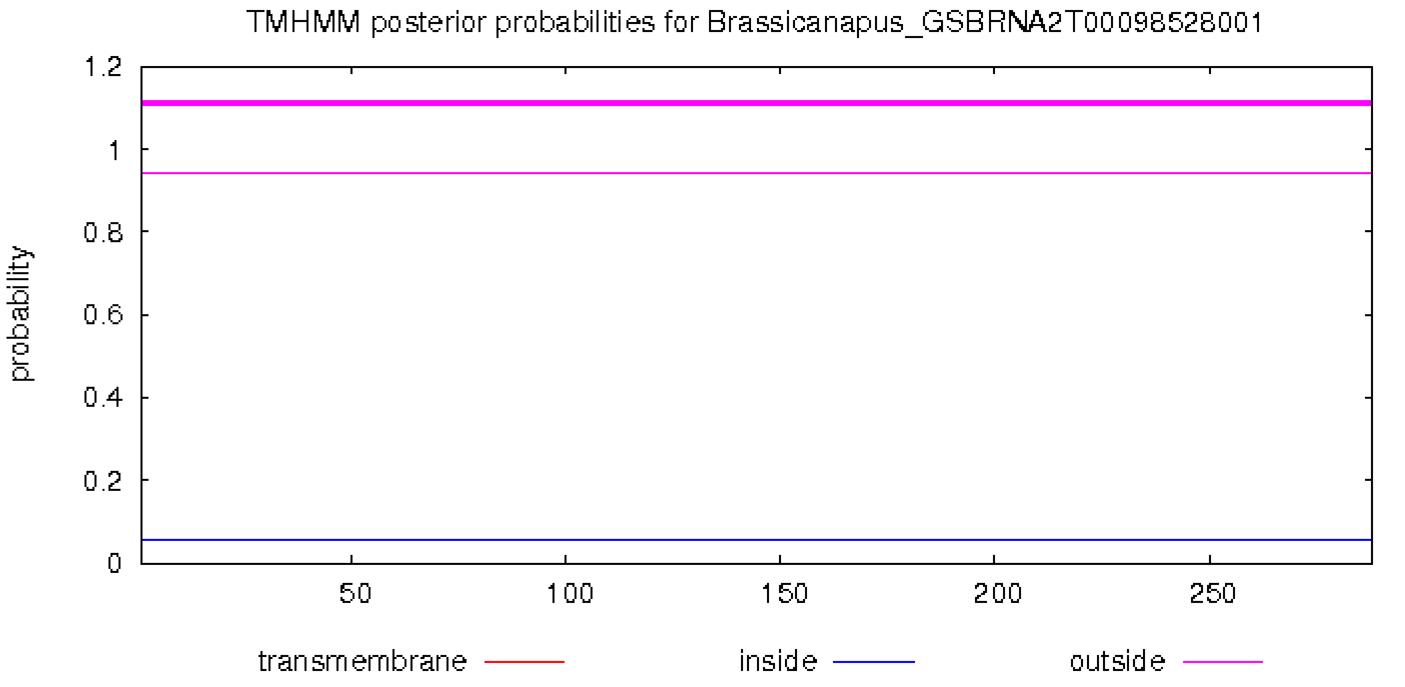

plot in postscript, script for making the plot in gnuplot, data for plot

---

```
# Brassicanapus_GSBRNA2T00098539001 Length: 335
# Brassicanapus_GSBRNA2T00098539001 Number of predicted TMHs:  0
# Brassicanapus_GSBRNA2T00098539001 Exp number of AAs in TMHs: 0.00655
# Brassicanapus_GSBRNA2T00098539001 Exp number, first 60 AAs:  0
# Brassicanapus_GSBRNA2T00098539001 Total prob of N-in:        0.02322
Brassicanapus_GSBRNA2T00098539001       TMHMM2.0        outside      1   335
```

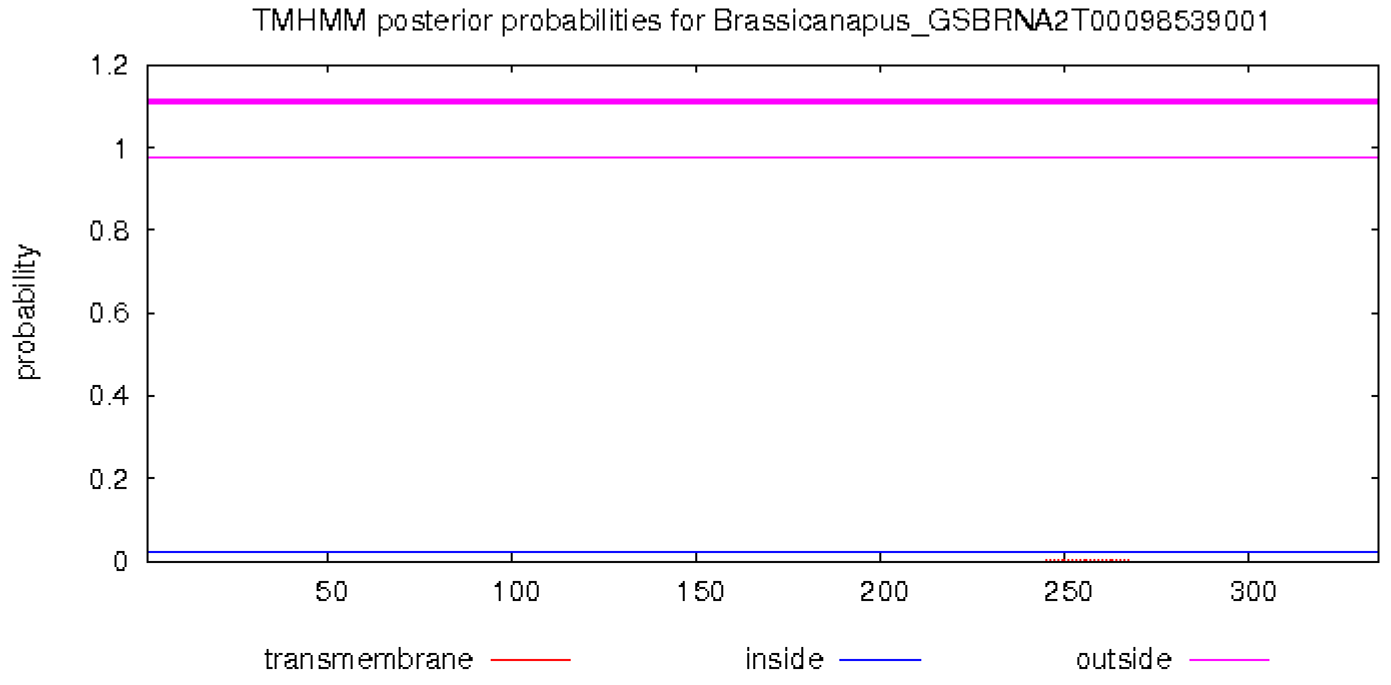

plot in postscript, script for making the plot in gnuplot, data for plot

---

```
# Brassicanapus_GSBRNA2T00098598001 Length: 197
# Brassicanapus_GSBRNA2T00098598001 Number of predicted TMHs:  0
# Brassicanapus_GSBRNA2T00098598001 Exp number of AAs in TMHs: 0.00121
# Brassicanapus_GSBRNA2T00098598001 Exp number, first 60 AAs:  0
# Brassicanapus_GSBRNA2T00098598001 Total prob of N-in:        0.04873
Brassicanapus_GSBRNA2T00098598001       TMHMM2.0        outside      1   197
```

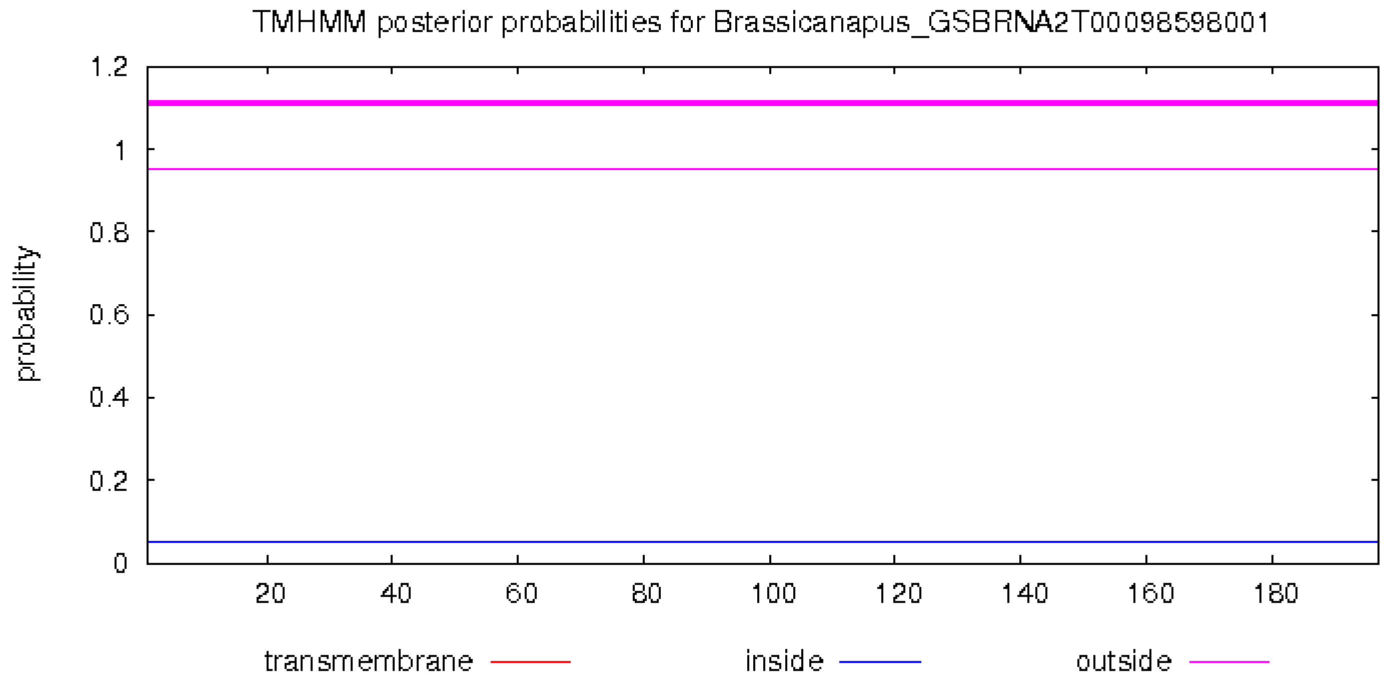

plot in postscript, script for making the plot in gnuplot, data for plot

---

```
# Brassicanapus_GSBRNA2T00098805001 Length: 162
# Brassicanapus_GSBRNA2T00098805001 Number of predicted TMHs:  0
# Brassicanapus_GSBRNA2T00098805001 Exp number of AAs in TMHs: 0.00282
# Brassicanapus_GSBRNA2T00098805001 Exp number, first 60 AAs:  0.00023
# Brassicanapus_GSBRNA2T00098805001 Total prob of N-in:        0.05400
Brassicanapus_GSBRNA2T00098805001       TMHMM2.0        outside      1   162
```

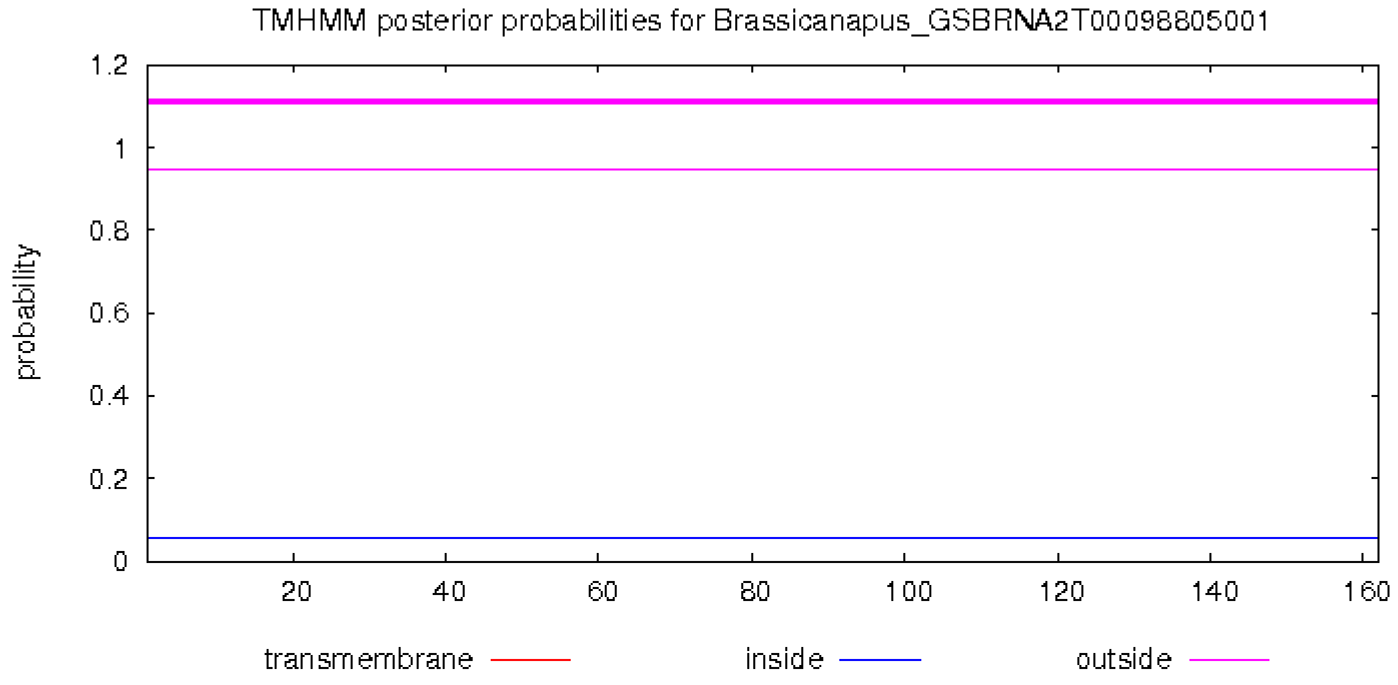

plot in postscript, script for making the plot in gnuplot, data for plot

---

```
# Brassicanapus_GSBRNA2T00098817001 Length: 363
# Brassicanapus_GSBRNA2T00098817001 Number of predicted TMHs:  0
# Brassicanapus_GSBRNA2T00098817001 Exp number of AAs in TMHs: 0.0005
# Brassicanapus_GSBRNA2T00098817001 Exp number, first 60 AAs:  0
# Brassicanapus_GSBRNA2T00098817001 Total prob of N-in:        0.02373
Brassicanapus_GSBRNA2T00098817001       TMHMM2.0        outside      1   363
```

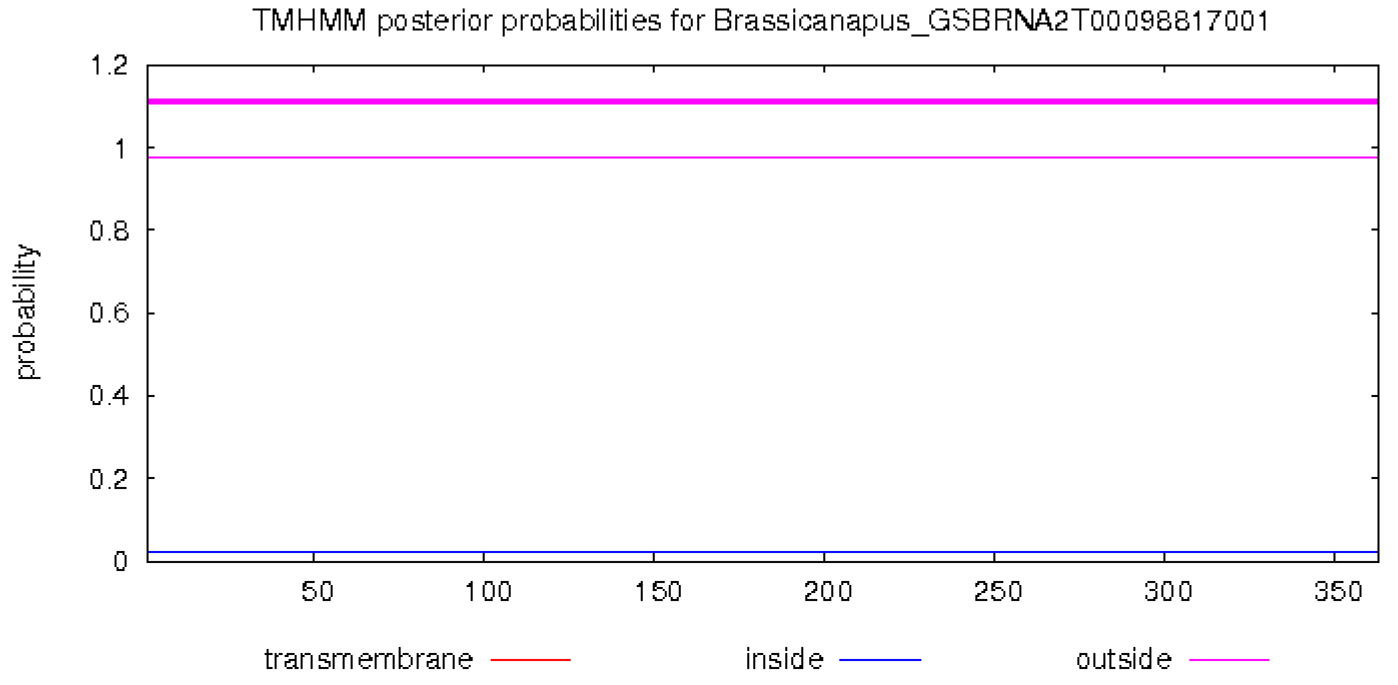

plot in postscript, script for making the plot in gnuplot, data for plot

---

```
# Brassicanapus_GSBRNA2T00098926001 Length: 311
# Brassicanapus_GSBRNA2T00098926001 Number of predicted TMHs:  0
# Brassicanapus_GSBRNA2T00098926001 Exp number of AAs in TMHs: 0
# Brassicanapus_GSBRNA2T00098926001 Exp number, first 60 AAs:  0
# Brassicanapus_GSBRNA2T00098926001 Total prob of N-in:        0.00125
Brassicanapus_GSBRNA2T00098926001       TMHMM2.0        outside      1   311
```

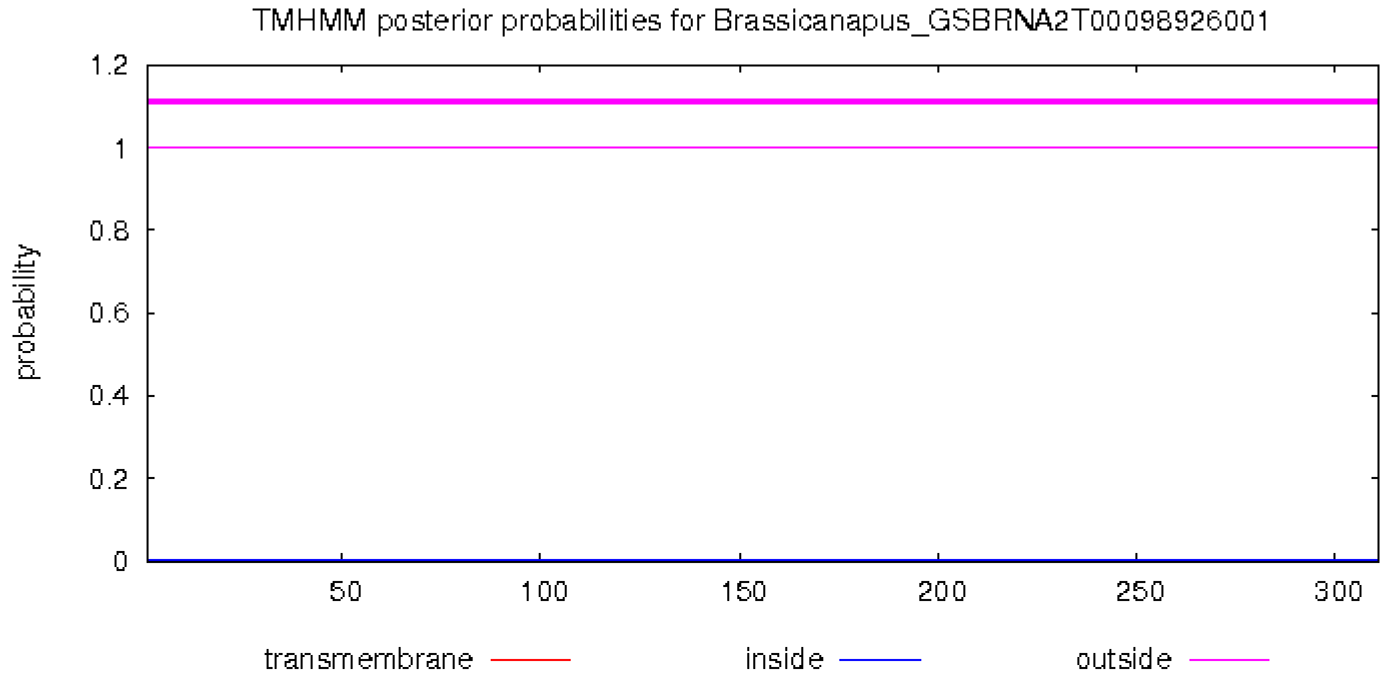

plot in postscript, script for making the plot in gnuplot, data for plot

---

```
# Brassicanapus_GSBRNA2T00099070001 Length: 336
# Brassicanapus_GSBRNA2T00099070001 Number of predicted TMHs:  0
# Brassicanapus_GSBRNA2T00099070001 Exp number of AAs in TMHs: 0.00021
# Brassicanapus_GSBRNA2T00099070001 Exp number, first 60 AAs:  0
# Brassicanapus_GSBRNA2T00099070001 Total prob of N-in:        0.02697
Brassicanapus_GSBRNA2T00099070001       TMHMM2.0        outside      1   336
```

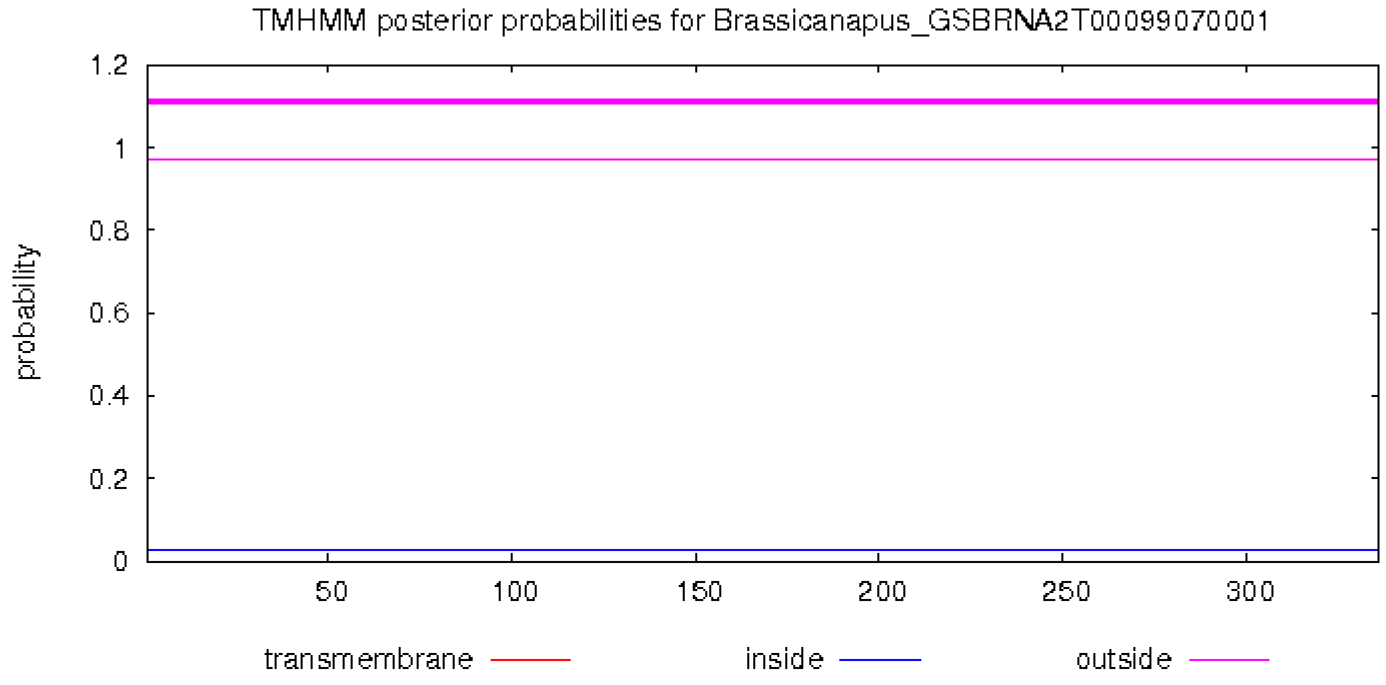

plot in postscript, script for making the plot in gnuplot, data for plot

---

```
# Brassicanapus_GSBRNA2T00100118001 Length: 339
# Brassicanapus_GSBRNA2T00100118001 Number of predicted TMHs:  0
# Brassicanapus_GSBRNA2T00100118001 Exp number of AAs in TMHs: 0
# Brassicanapus_GSBRNA2T00100118001 Exp number, first 60 AAs:  0
# Brassicanapus_GSBRNA2T00100118001 Total prob of N-in:        0.01933
Brassicanapus_GSBRNA2T00100118001       TMHMM2.0        outside      1   339
```

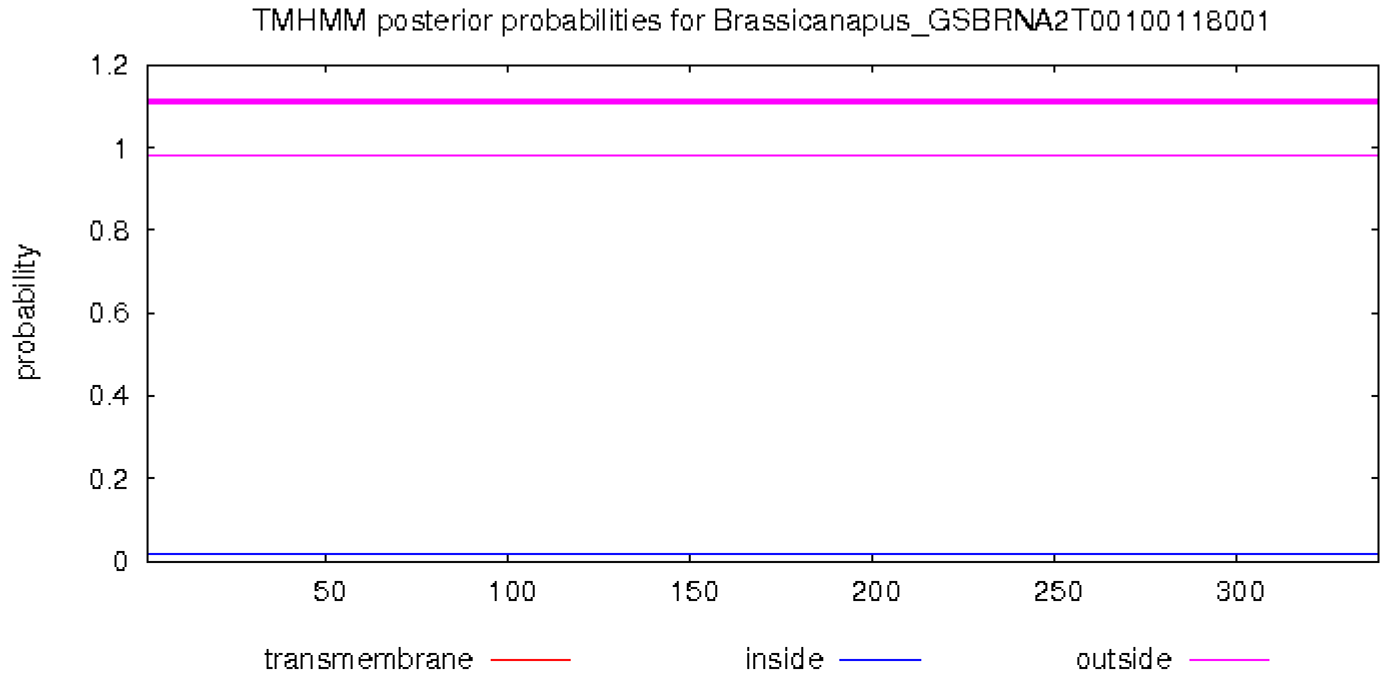

plot in postscript, script for making the plot in gnuplot, data for plot

---

```
# Brassicanapus_GSBRNA2T00100375001 Length: 495
# Brassicanapus_GSBRNA2T00100375001 Number of predicted TMHs:  0
# Brassicanapus_GSBRNA2T00100375001 Exp number of AAs in TMHs: 0.02236
# Brassicanapus_GSBRNA2T00100375001 Exp number, first 60 AAs:  0
# Brassicanapus_GSBRNA2T00100375001 Total prob of N-in:        0.00379
Brassicanapus_GSBRNA2T00100375001	TMHMM2.0	outside	     1   495
```

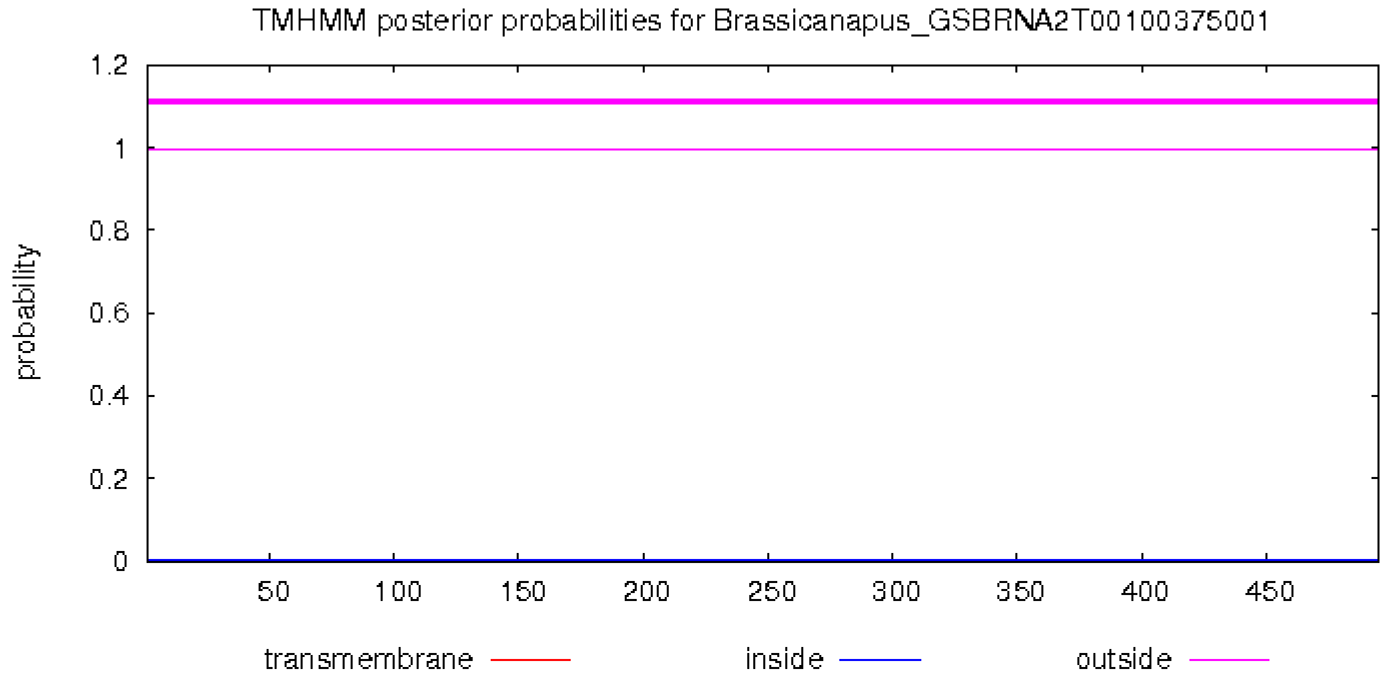

plot in postscript, script for making the plot in gnuplot, data for plot

---

```
# Brassicanapus_GSBRNA2T00101780001 Length: 226
# Brassicanapus_GSBRNA2T00101780001 Number of predicted TMHs:  0
# Brassicanapus_GSBRNA2T00101780001 Exp number of AAs in TMHs: 0
# Brassicanapus_GSBRNA2T00101780001 Exp number, first 60 AAs:  0
# Brassicanapus_GSBRNA2T00101780001 Total prob of N-in:        0.00285
Brassicanapus_GSBRNA2T00101780001	TMHMM2.0	outside	     1   226
```

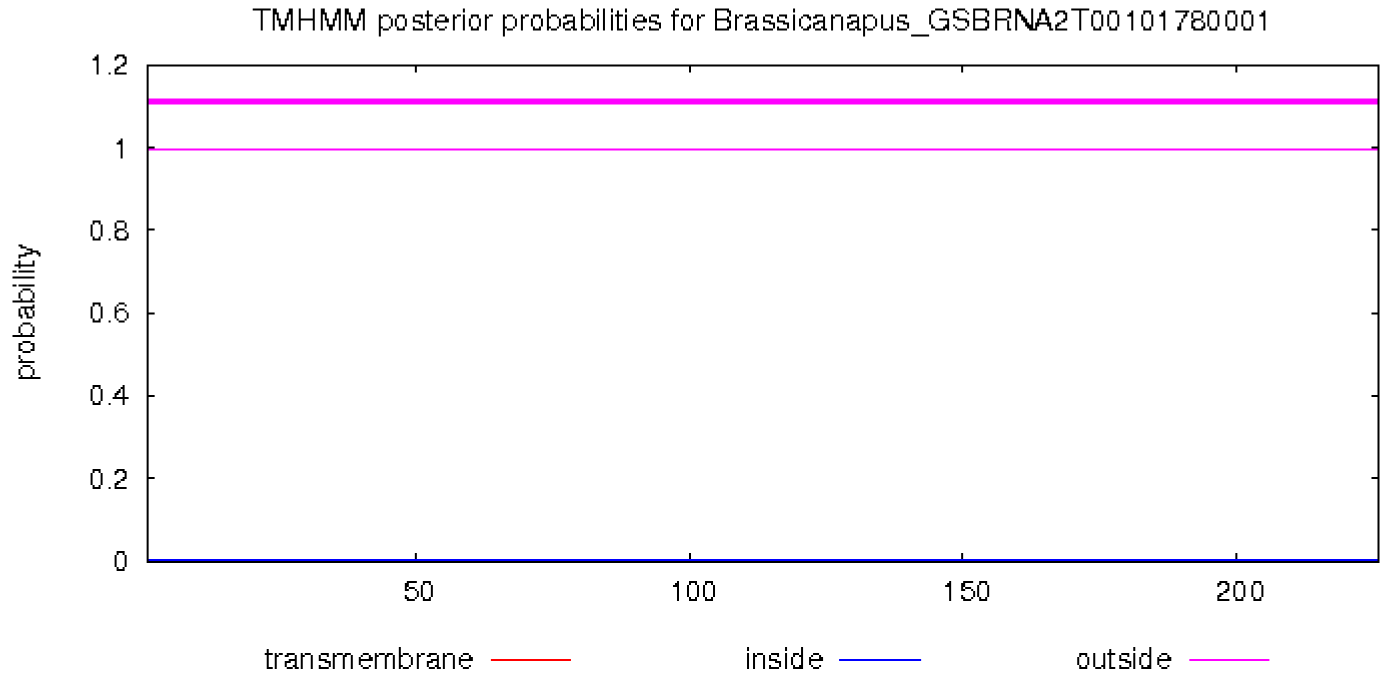

plot in postscript, script for making the plot in gnuplot, data for plot

---

```
# Brassicanapus_GSBRNA2T00101917001 Length: 409
# Brassicanapus_GSBRNA2T00101917001 Number of predicted TMHs:  0
# Brassicanapus_GSBRNA2T00101917001 Exp number of AAs in TMHs: 0.00023
# Brassicanapus_GSBRNA2T00101917001 Exp number, first 60 AAs:  0
# Brassicanapus_GSBRNA2T00101917001 Total prob of N-in:        0.00096
Brassicanapus_GSBRNA2T00101917001       TMHMM2.0        outside      1   409
```

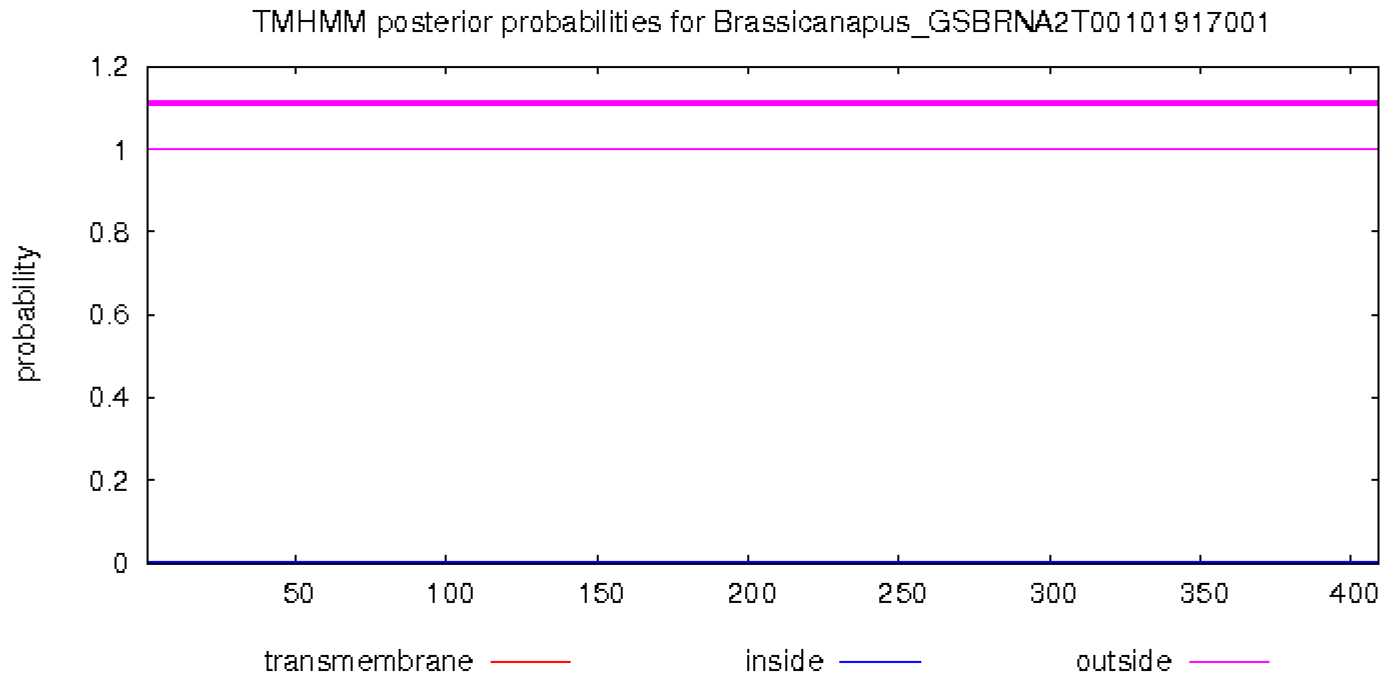

plot in postscript, script for making the plot in gnuplot, data for plot

---

```
# Brassicanapus_GSBRNA2T00101944001 Length: 270
# Brassicanapus_GSBRNA2T00101944001 Number of predicted TMHs:  0
# Brassicanapus_GSBRNA2T00101944001 Exp number of AAs in TMHs: 0.0074
# Brassicanapus_GSBRNA2T00101944001 Exp number, first 60 AAs:  0
# Brassicanapus_GSBRNA2T00101944001 Total prob of N-in:        0.03628
Brassicanapus_GSBRNA2T00101944001       TMHMM2.0        outside      1   270
```

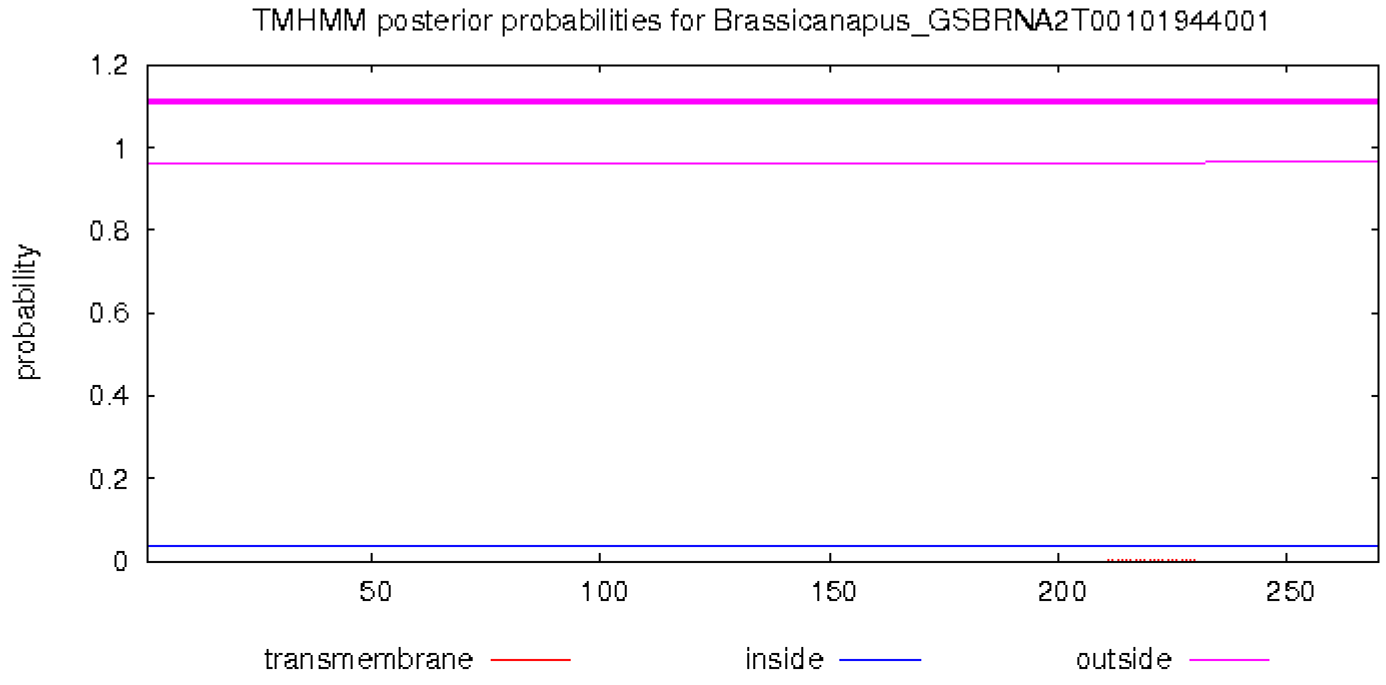

plot in postscript, script for making the plot in gnuplot, data for plot

---

```
# Brassicanapus_GSBRNA2T00102391001 Length: 367
# Brassicanapus_GSBRNA2T00102391001 Number of predicted TMHs:  0
# Brassicanapus_GSBRNA2T00102391001 Exp number of AAs in TMHs: 0.00988999999999999999
# Brassicanapus_GSBRNA2T00102391001 Exp number, first 60 AAs:  0
# Brassicanapus_GSBRNA2T00102391001 Total prob of N-in:        0.01571
Brassicanapus_GSBRNA2T00102391001       TMHMM2.0        outside      1   367
```

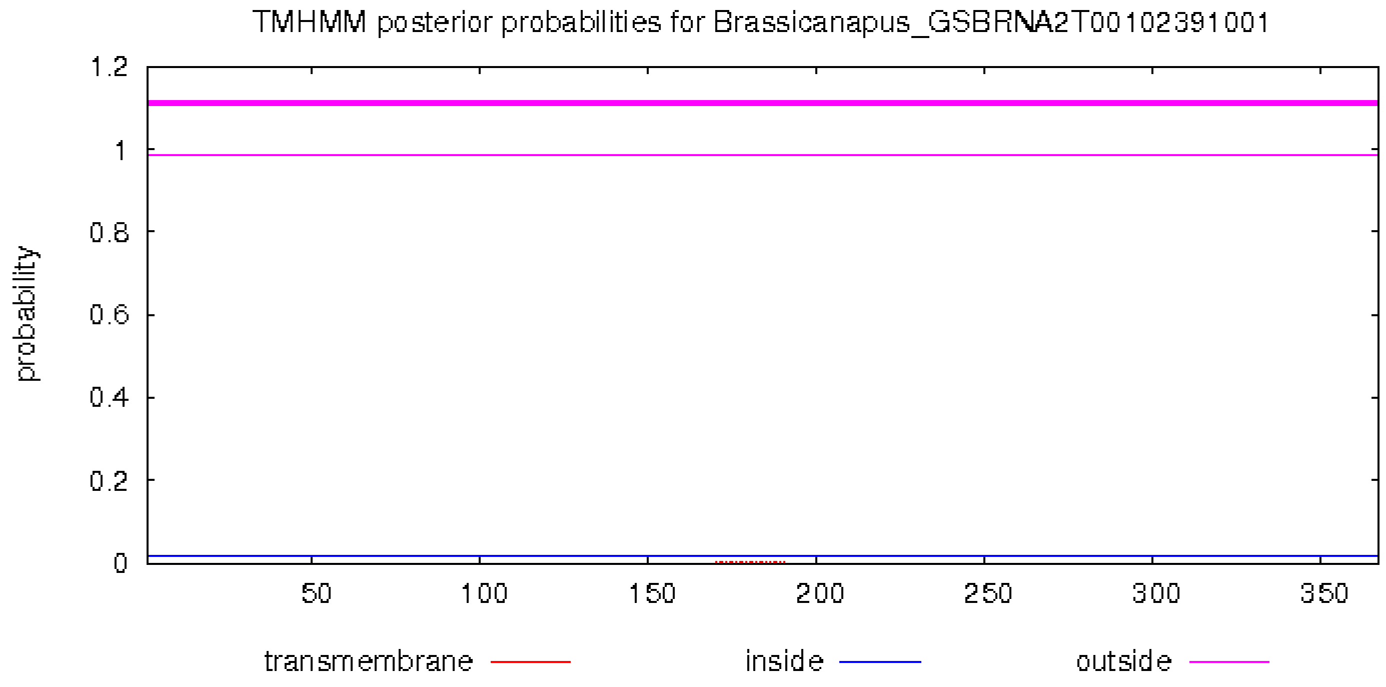

plot in postscript, script for making the plot in gnuplot, data for plot

---

```
# Brassicanapus_GSBRNA2T00102514001 Length: 325
# Brassicanapus_GSBRNA2T00102514001 Number of predicted TMHs:  0
# Brassicanapus_GSBRNA2T00102514001 Exp number of AAs in TMHs: 0
# Brassicanapus_GSBRNA2T00102514001 Exp number, first 60 AAs:  0
# Brassicanapus_GSBRNA2T00102514001 Total prob of N-in:        0.00761
Brassicanapus_GSBRNA2T00102514001       TMHMM2.0        outside      1   325
```

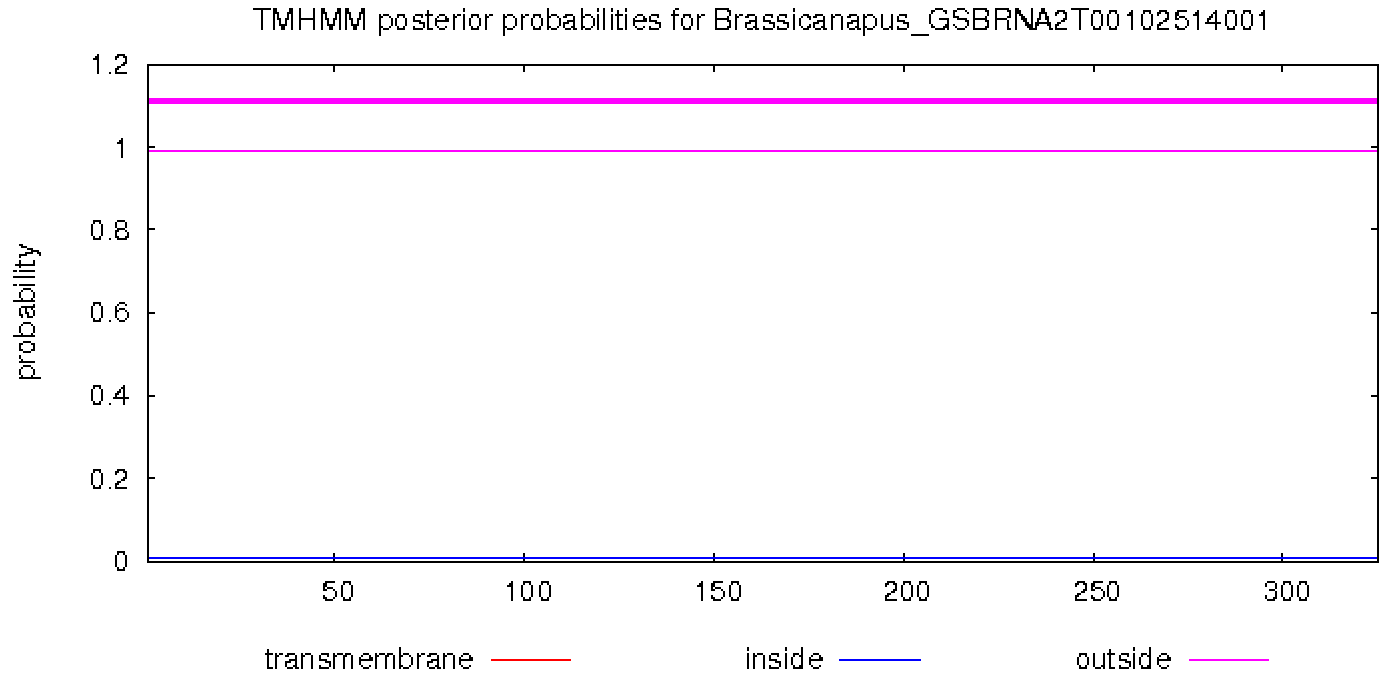

plot in postscript, script for making the plot in gnuplot, data for plot

---

```
# Brassicanapus_GSBRNA2T00103244001 Length: 325
# Brassicanapus_GSBRNA2T00103244001 Number of predicted TMHs:  0
# Brassicanapus_GSBRNA2T00103244001 Exp number of AAs in TMHs: 0
# Brassicanapus_GSBRNA2T00103244001 Exp number, first 60 AAs:  0
# Brassicanapus_GSBRNA2T00103244001 Total prob of N-in:        0.00761
Brassicanapus_GSBRNA2T00103244001       TMHMM2.0        outside      1   325
```

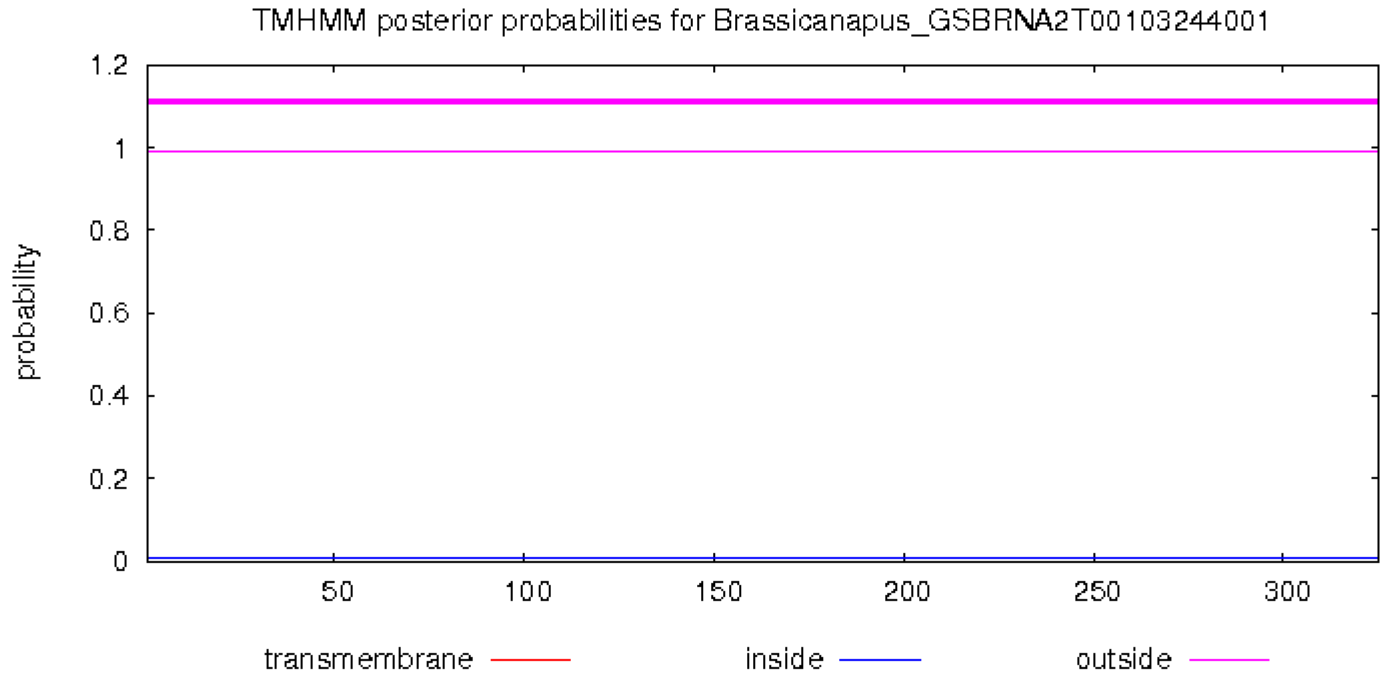

plot in postscript, script for making the plot in gnuplot, data for plot

---

```
# Brassicanapus_GSBRNA2T00104250001 Length: 138
# Brassicanapus_GSBRNA2T00104250001 Number of predicted TMHs:  0
# Brassicanapus_GSBRNA2T00104250001 Exp number of AAs in TMHs: 0.06759
# Brassicanapus_GSBRNA2T00104250001 Exp number, first 60 AAs:  0.0002
# Brassicanapus_GSBRNA2T00104250001 Total prob of N-in:        0.48226
Brassicanapus_GSBRNA2T00104250001       TMHMM2.0        outside      1   138
```

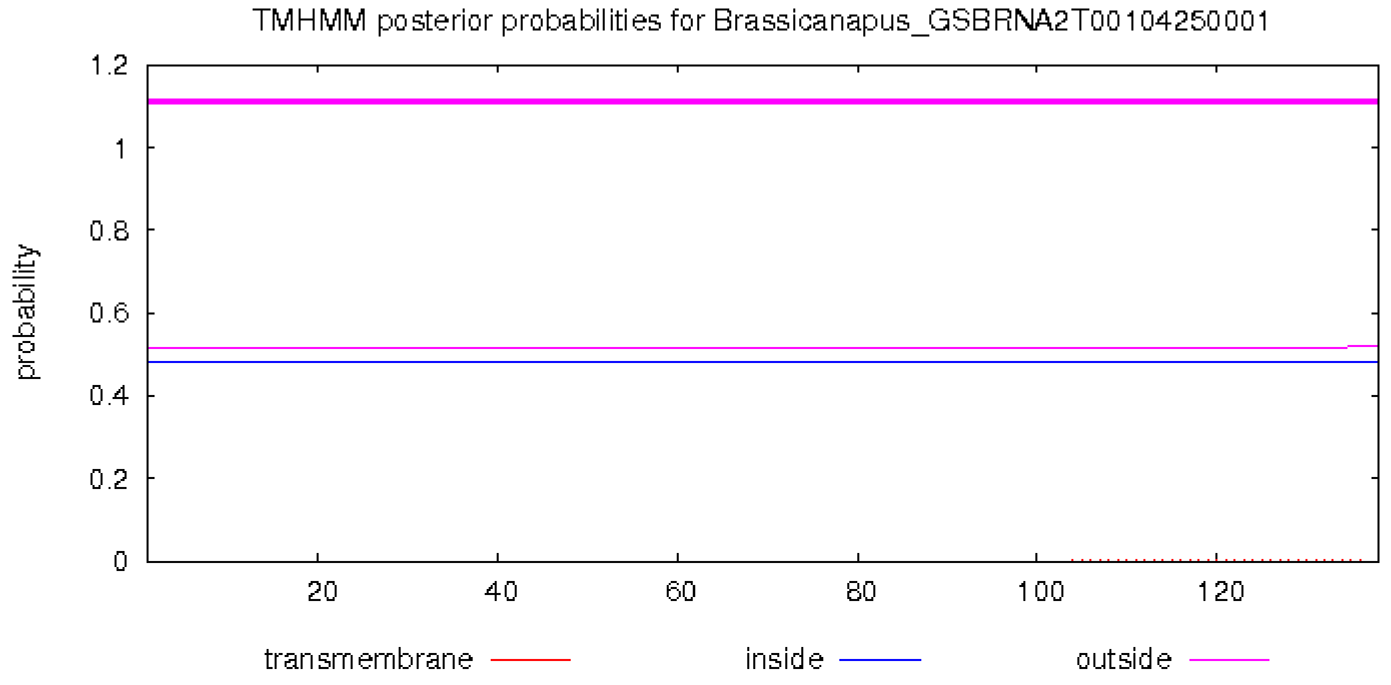

plot in postscript, script for making the plot in gnuplot, data for plot

---

```
# Brassicanapus_GSBRNA2T00104269001 Length: 427
# Brassicanapus_GSBRNA2T00104269001 Number of predicted TMHs:  1
# Brassicanapus_GSBRNA2T00104269001 Exp number of AAs in TMHs: 22.88114
# Brassicanapus_GSBRNA2T00104269001 Exp number, first 60 AAs:  0.00052
# Brassicanapus_GSBRNA2T00104269001 Total prob of N-in:        0.60694
Brassicanapus_GSBRNA2T00104269001       TMHMM2.0        inside       1   392
Brassicanapus_GSBRNA2T00104269001       TMHMM2.0        TMhelix    393   415
Brassicanapus_GSBRNA2T00104269001       TMHMM2.0        outside    416   427
```

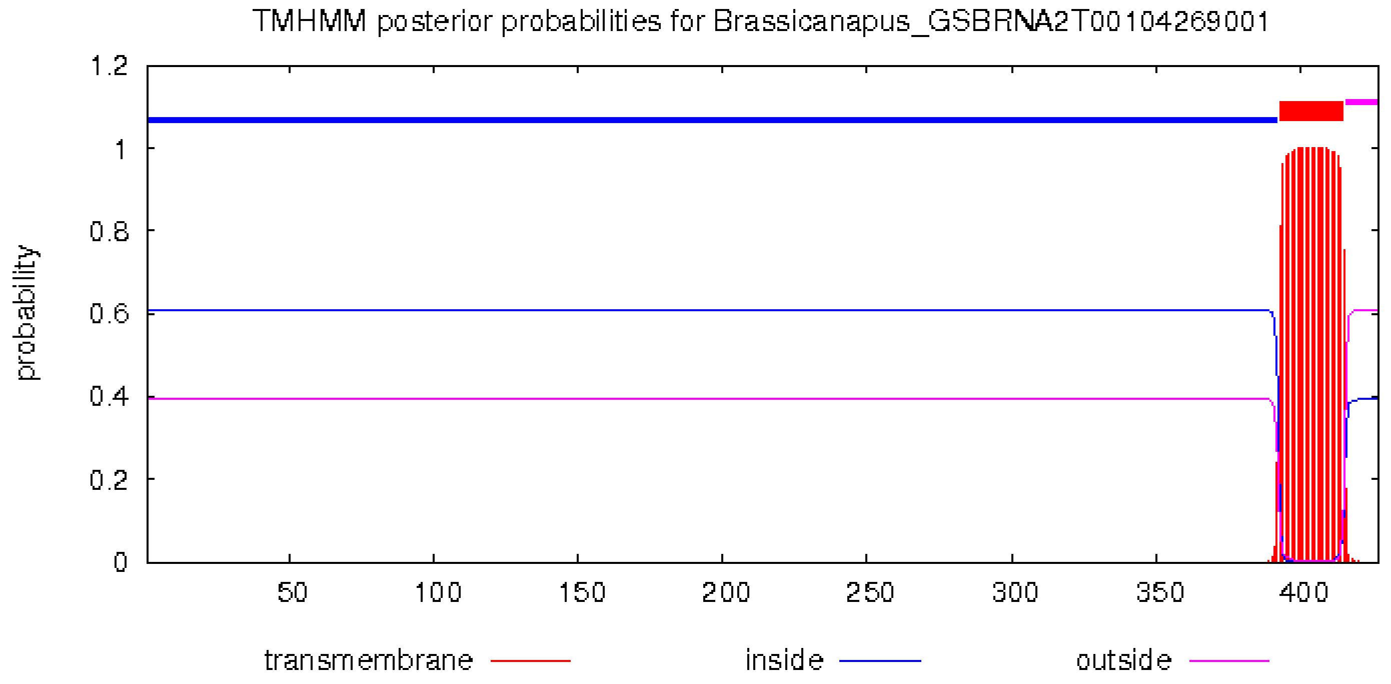

plot in postscript, script for making the plot in gnuplot, data for plot

---

```
# Brassicanapus_GSBRNA2T00104607001 Length: 336
# Brassicanapus_GSBRNA2T00104607001 Number of predicted TMHs:  0
# Brassicanapus_GSBRNA2T00104607001 Exp number of AAs in TMHs: 6e-05
# Brassicanapus_GSBRNA2T00104607001 Exp number, first 60 AAs:  0
```

```
# Brassicanapus_GSBRNA2T00104607001 Total prob of N-in:        0.00578
Brassicanapus_GSBRNA2T00104607001       TMHMM2.0        outside      1   336
```

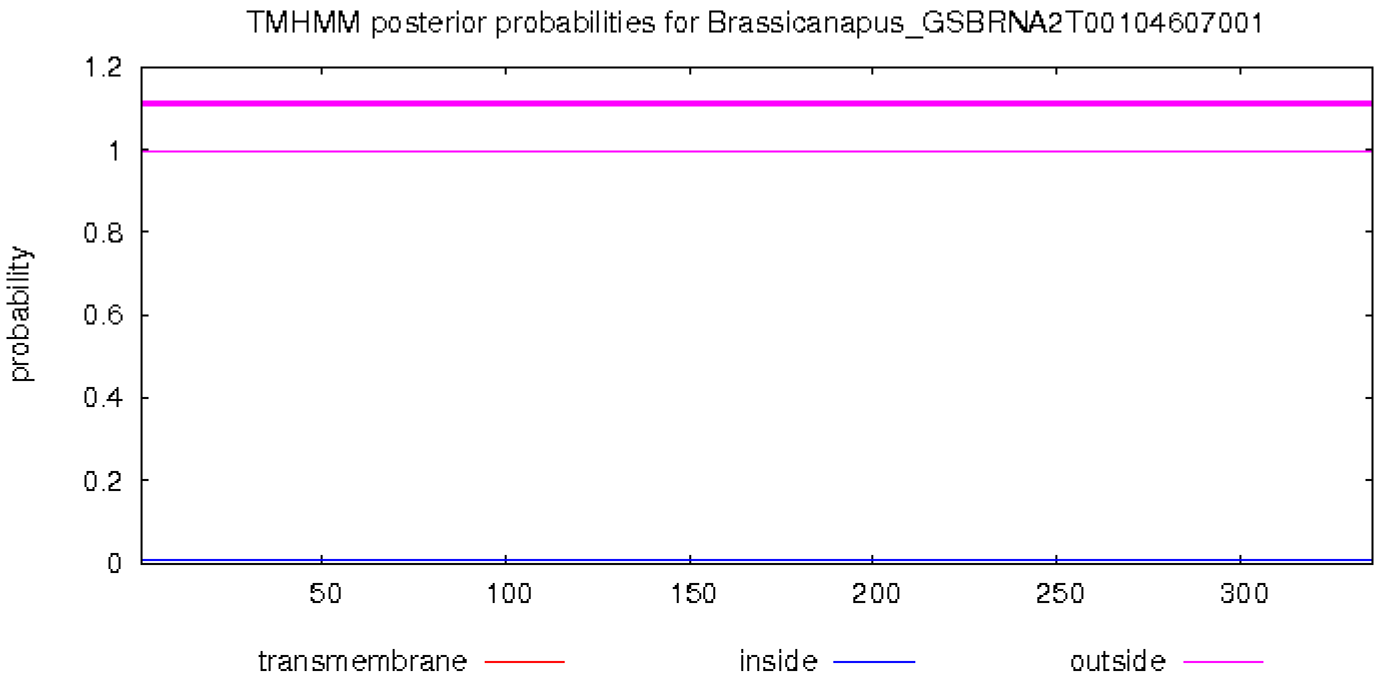

plot in postscript, script for making the plot in gnuplot, data for plot

---

```
# Brassicanapus_GSBRNA2T00104909001 Length: 321
# Brassicanapus_GSBRNA2T00104909001 Number of predicted TMHs:  0
# Brassicanapus_GSBRNA2T00104909001 Exp number of AAs in TMHs: 0
# Brassicanapus_GSBRNA2T00104909001 Exp number, first 60 AAs:  0
# Brassicanapus_GSBRNA2T00104909001 Total prob of N-in:        0.00379
Brassicanapus_GSBRNA2T00104909001       TMHMM2.0        outside      1   321
```

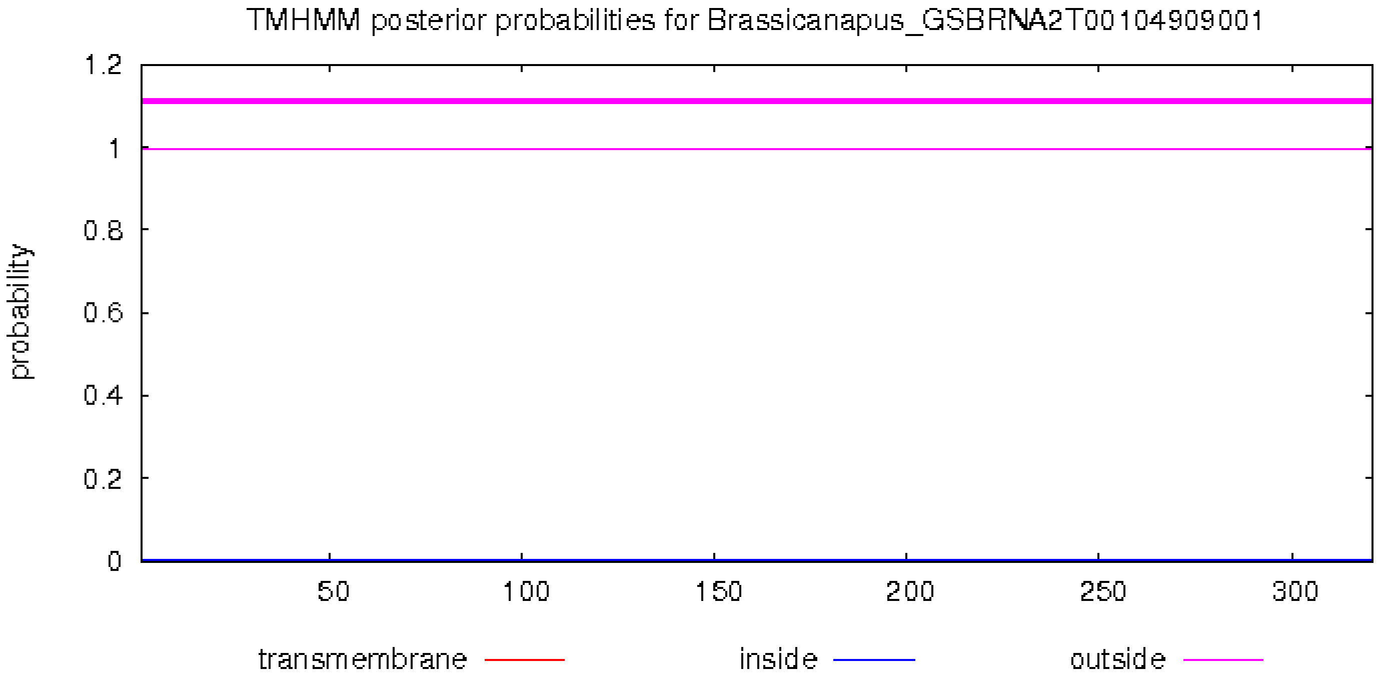

plot in postscript, script for making the plot in gnuplot, data for plot

---

```
# Brassicanapus_GSBRNA2T00104912001 Length: 343
# Brassicanapus_GSBRNA2T00104912001 Number of predicted TMHs:  0
# Brassicanapus_GSBRNA2T00104912001 Exp number of AAs in TMHs: 0.00555
# Brassicanapus_GSBRNA2T00104912001 Exp number, first 60 AAs:  0.00018
```

```
# Brassicanapus_GSBRNA2T00104912001 Total prob of N-in:        0.00541
Brassicanapus_GSBRNA2T00104912001	TMHMM2.0	outside	     1   343
```

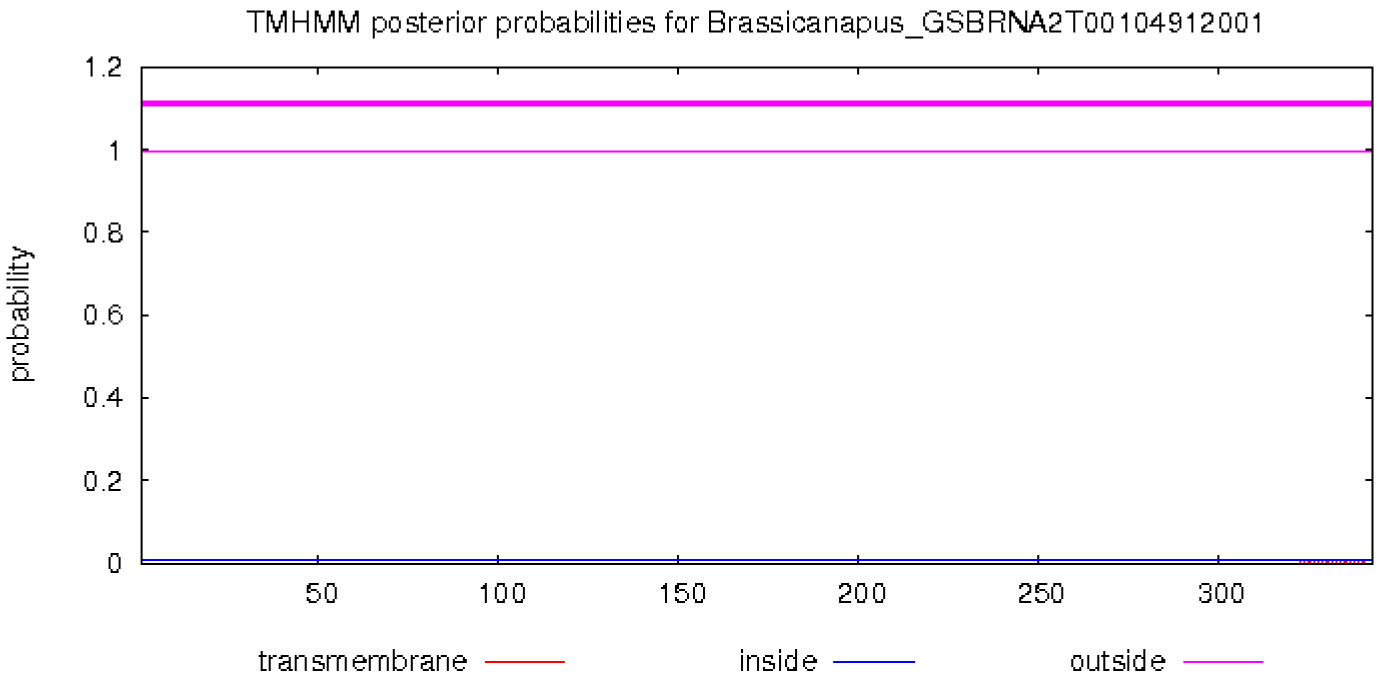

plot in postscript, script for making the plot in gnuplot, data for plot

---

```
# Brassicanapus_GSBRNA2T00105458001 Length: 338
# Brassicanapus_GSBRNA2T00105458001 Number of predicted TMHs:  0
# Brassicanapus_GSBRNA2T00105458001 Exp number of AAs in TMHs: 0.00018
# Brassicanapus_GSBRNA2T00105458001 Exp number, first 60 AAs:  0
# Brassicanapus_GSBRNA2T00105458001 Total prob of N-in:        0.00179
Brassicanapus_GSBRNA2T00105458001	TMHMM2.0	outside	     1   338
```

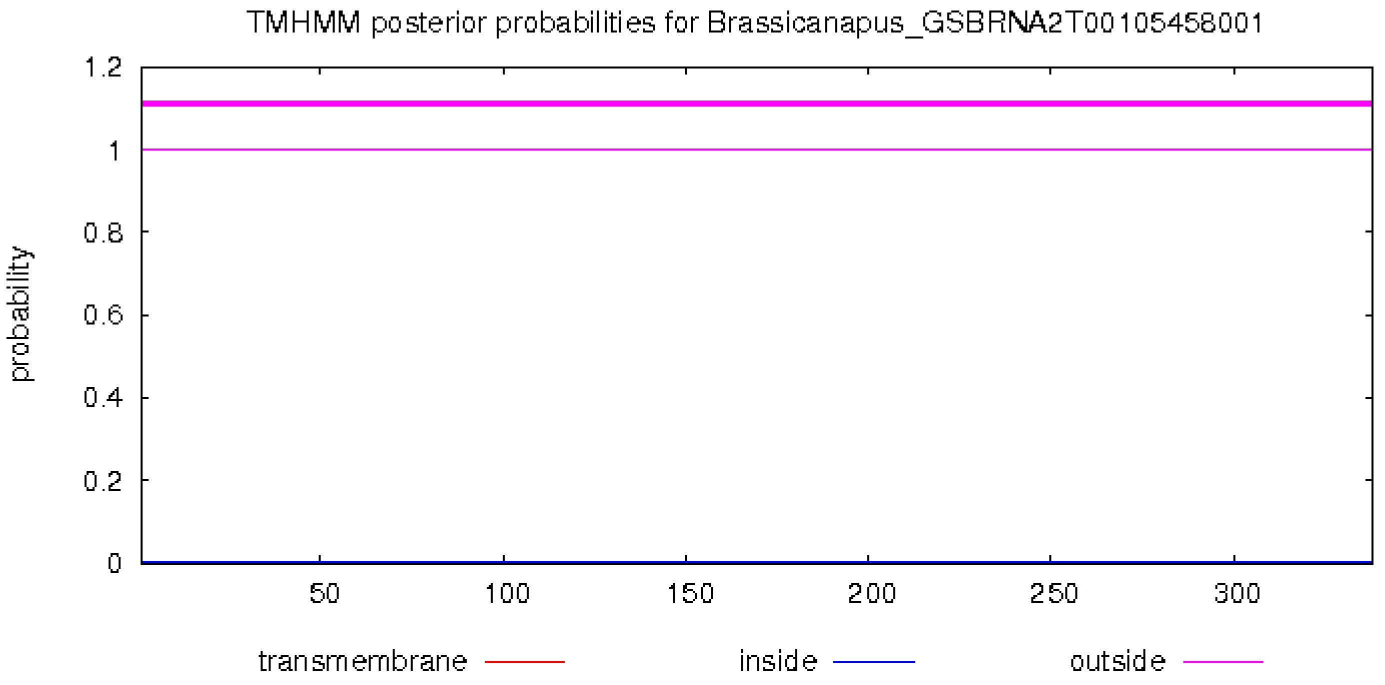

plot in postscript, script for making the plot in gnuplot, data for plot

---

```
# Brassicanapus_GSBRNA2T00106173001 Length: 273
# Brassicanapus_GSBRNA2T00106173001 Number of predicted TMHs:  0
# Brassicanapus_GSBRNA2T00106173001 Exp number of AAs in TMHs: 0.02109
# Brassicanapus_GSBRNA2T00106173001 Exp number, first 60 AAs:  0
```

```
# Brassicanapus_GSBRNA2T00106173001 Total prob of N-in:        0.01805
Brassicanapus_GSBRNA2T00106173001	TMHMM2.0	outside	     1   273
```

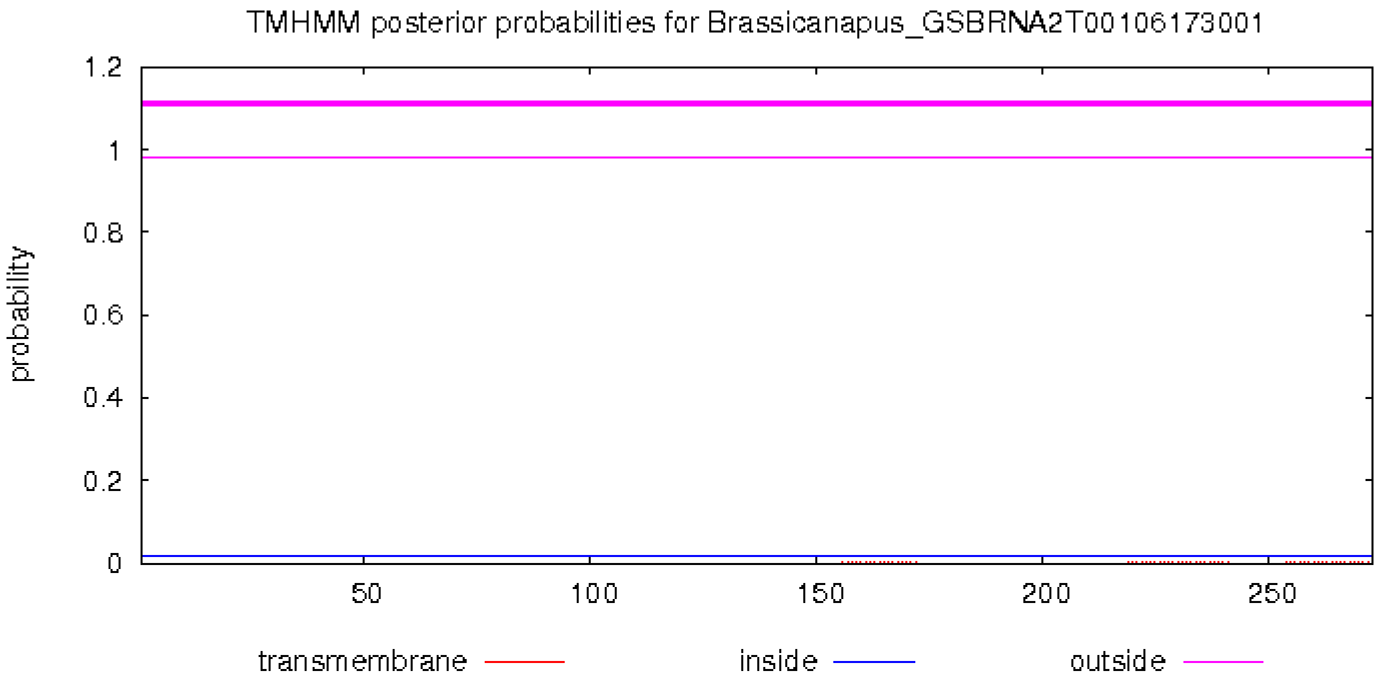

plot in postscript, script for making the plot in gnuplot, data for plot

---

```
# Brassicanapus_GSBRNA2T00106217001 Length: 320
# Brassicanapus_GSBRNA2T00106217001 Number of predicted TMHs:  0
# Brassicanapus_GSBRNA2T00106217001 Exp number of AAs in TMHs: 0
# Brassicanapus_GSBRNA2T00106217001 Exp number, first 60 AAs:  0
# Brassicanapus_GSBRNA2T00106217001 Total prob of N-in:        0.00384
Brassicanapus_GSBRNA2T00106217001	TMHMM2.0	outside	     1   320
```

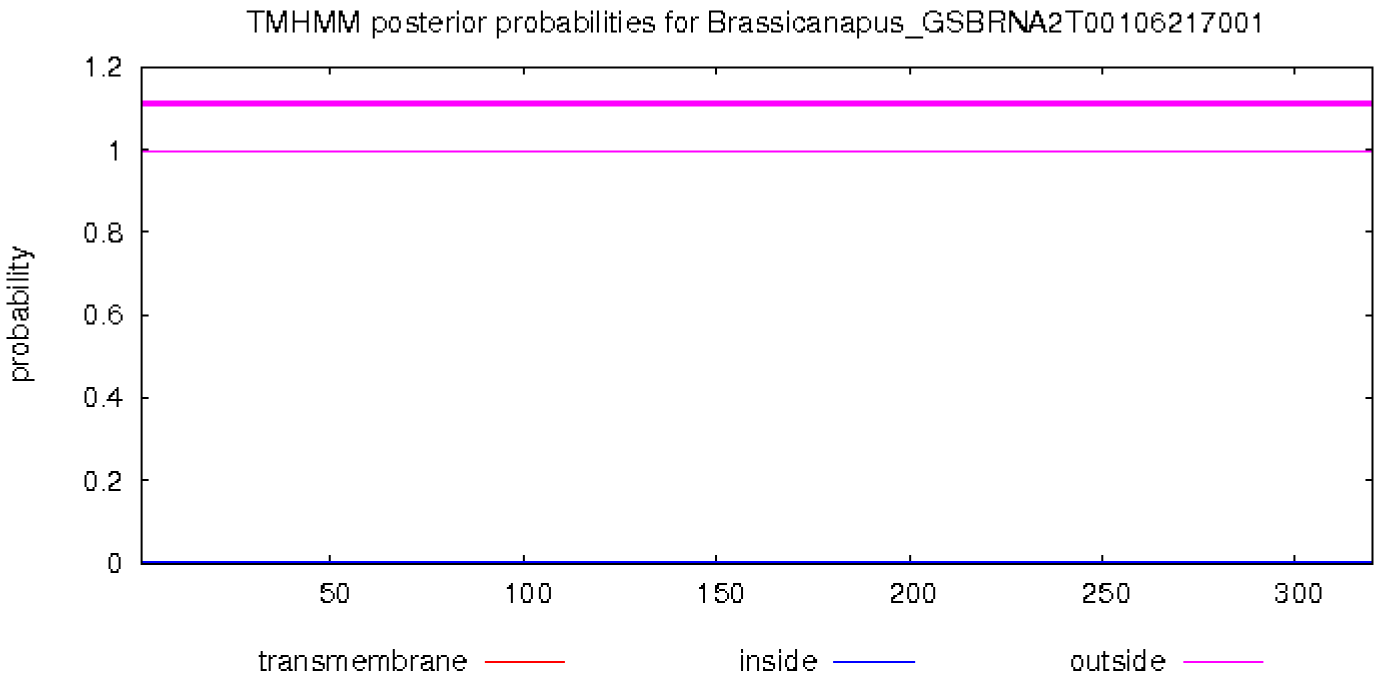

plot in postscript, script for making the plot in gnuplot, data for plot

---

```
# Brassicanapus_GSBRNA2T00106219001 Length: 359
# Brassicanapus_GSBRNA2T00106219001 Number of predicted TMHs:  0
# Brassicanapus_GSBRNA2T00106219001 Exp number of AAs in TMHs: 0.00329
# Brassicanapus_GSBRNA2T00106219001 Exp number, first 60 AAs:  0.00116
```

```
# Brassicanapus_GSBRNA2T00106219001 Total prob of N-in:        0.00300
Brassicanapus_GSBRNA2T00106219001       TMHMM2.0        outside      1   359
```

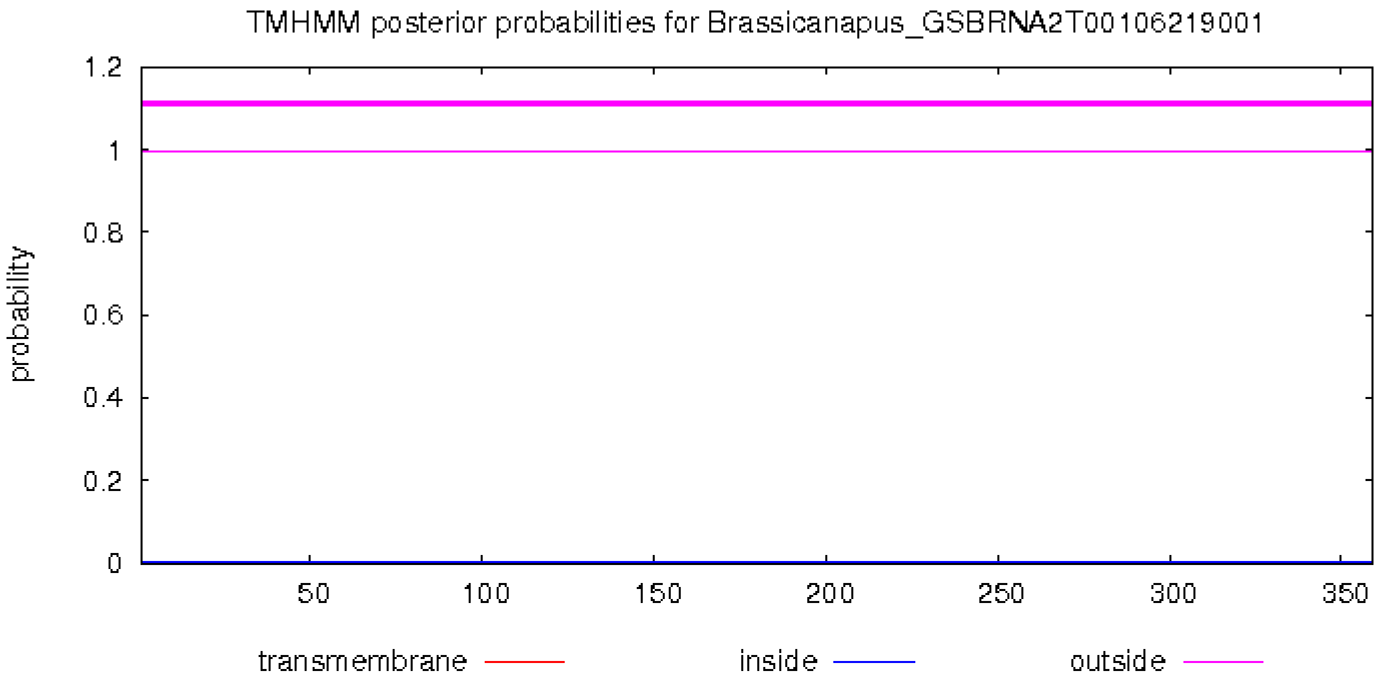

plot in postscript, script for making the plot in gnuplot, data for plot

```
# Brassicanapus_GSBRNA2T00106315001 Length: 325
# Brassicanapus_GSBRNA2T00106315001 Number of predicted TMHs:  0
# Brassicanapus_GSBRNA2T00106315001 Exp number of AAs in TMHs: 0.00553
# Brassicanapus_GSBRNA2T00106315001 Exp number, first 60 AAs:  0
# Brassicanapus_GSBRNA2T00106315001 Total prob of N-in:        0.01967
Brassicanapus_GSBRNA2T00106315001       TMHMM2.0        outside      1   325
```

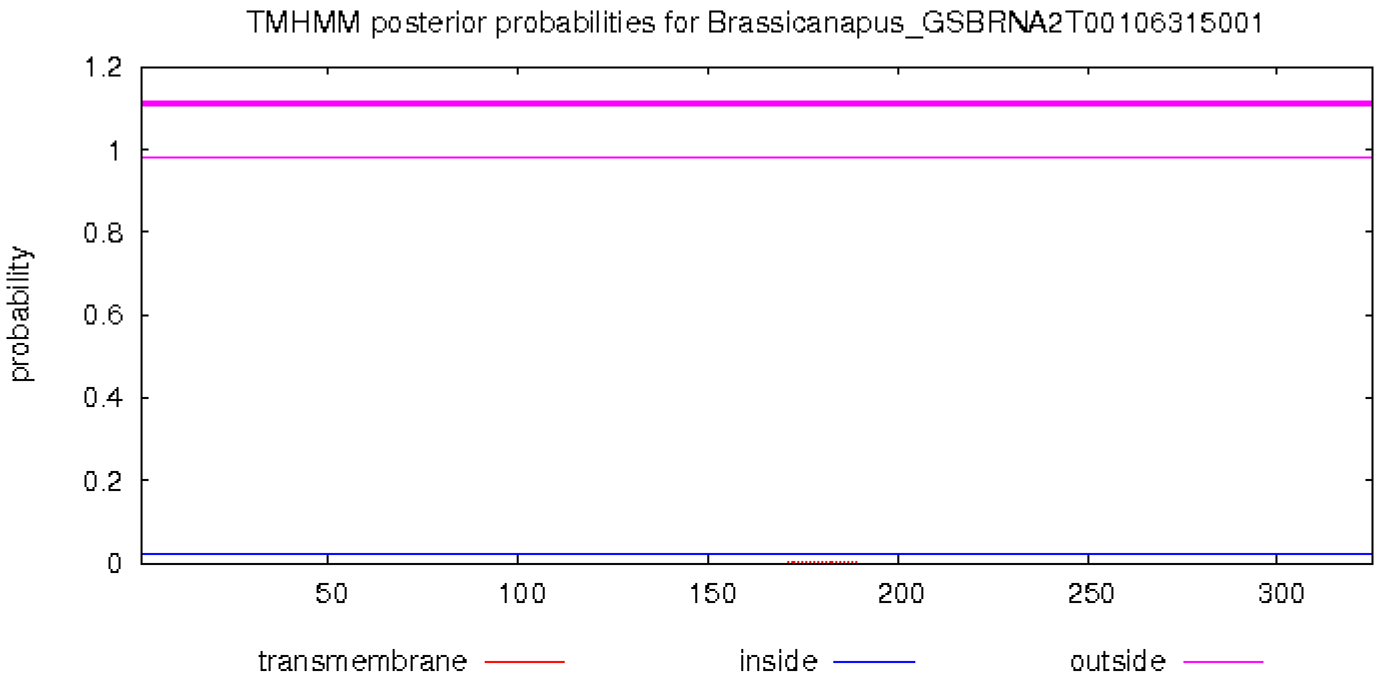

plot in postscript, script for making the plot in gnuplot, data for plot

```
# Brassicanapus_GSBRNA2T00106395001 Length: 583
# Brassicanapus_GSBRNA2T00106395001 Number of predicted TMHs:  1
# Brassicanapus_GSBRNA2T00106395001 Exp number of AAs in TMHs: 22.57124
# Brassicanapus_GSBRNA2T00106395001 Exp number, first 60 AAs:  0.0019
```

```
# Brassicanapus_GSBRNA2T00106395001 Total prob of N-in:        0.11262
Brassicanapus_GSBRNA2T00106395001       TMHMM2.0        outside      1   550
Brassicanapus_GSBRNA2T00106395001       TMHMM2.0        TMhelix    551   573
Brassicanapus_GSBRNA2T00106395001       TMHMM2.0        inside     574   583
```

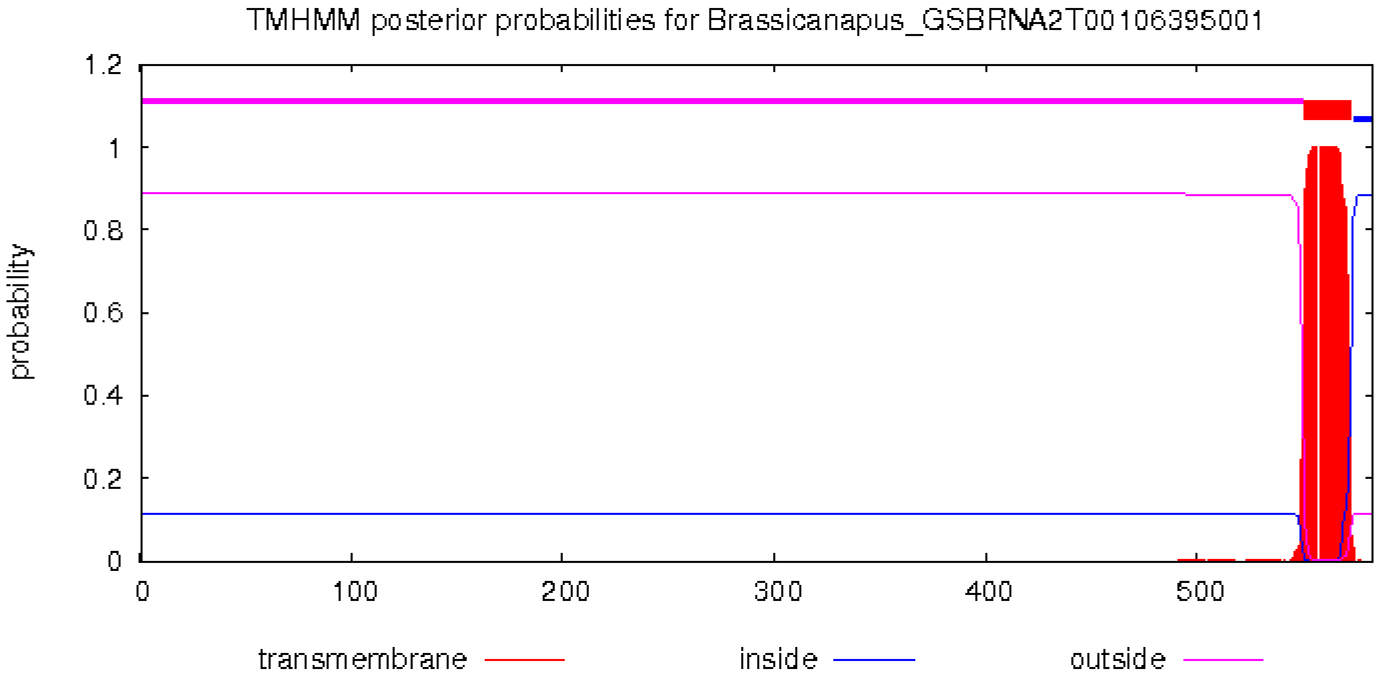

plot in postscript, script for making the plot in gnuplot, data for plot

---

```
# Brassicanapus_GSBRNA2T00106396001 Length: 521
# Brassicanapus_GSBRNA2T00106396001 Number of predicted TMHs:  1
# Brassicanapus_GSBRNA2T00106396001 Exp number of AAs in TMHs: 21.41043
# Brassicanapus_GSBRNA2T00106396001 Exp number, first 60 AAs:  0.03156
# Brassicanapus_GSBRNA2T00106396001 Total prob of N-in:        0.45081
Brassicanapus_GSBRNA2T00106396001       TMHMM2.0        inside       1   491
Brassicanapus_GSBRNA2T00106396001       TMHMM2.0        TMhelix    492   514
Brassicanapus_GSBRNA2T00106396001       TMHMM2.0        outside    515   521
```

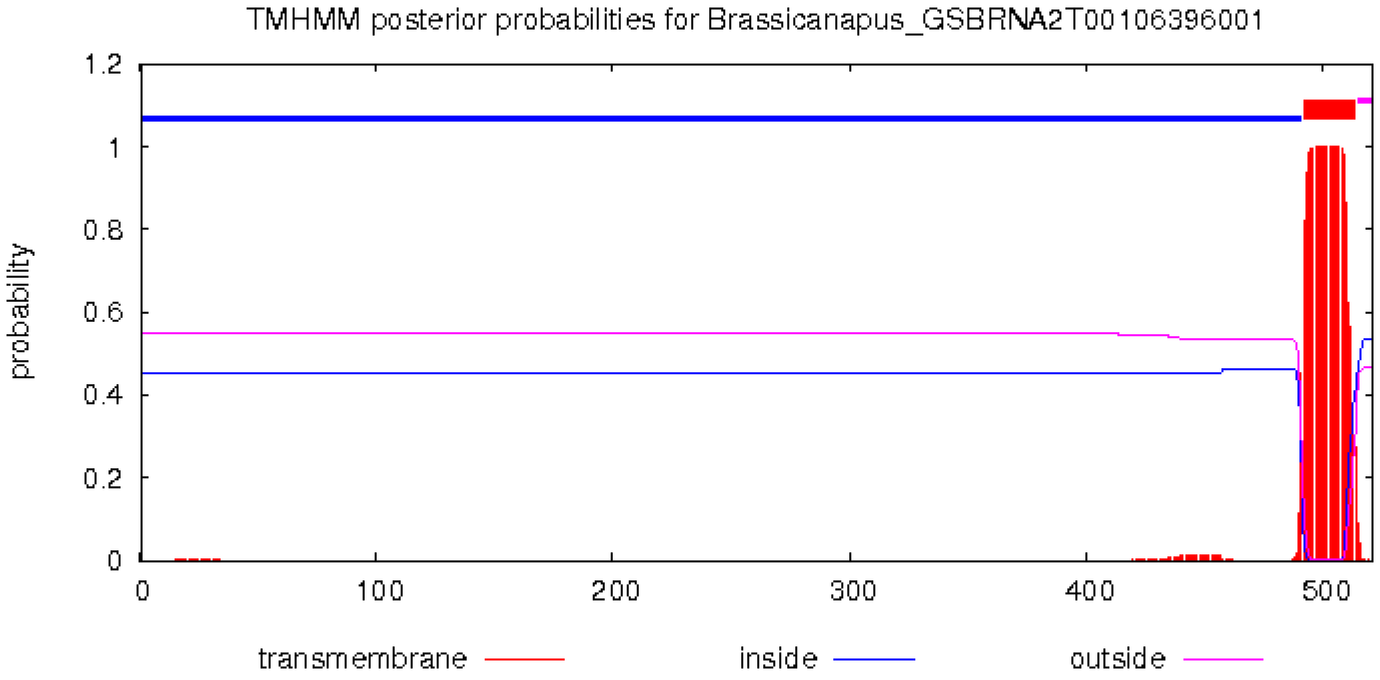

plot in postscript, script for making the plot in gnuplot, data for plot

---

```
# Brassicanapus_GSBRNA2T00108261001 Length: 273
# Brassicanapus_GSBRNA2T00108261001 Number of predicted TMHs:  0
# Brassicanapus_GSBRNA2T00108261001 Exp number of AAs in TMHs: 0.02109
# Brassicanapus_GSBRNA2T00108261001 Exp number, first 60 AAs:  0
# Brassicanapus_GSBRNA2T00108261001 Total prob of N-in:        0.01805
Brassicanapus_GSBRNA2T00108261001       TMHMM2.0        outside      1   273
```

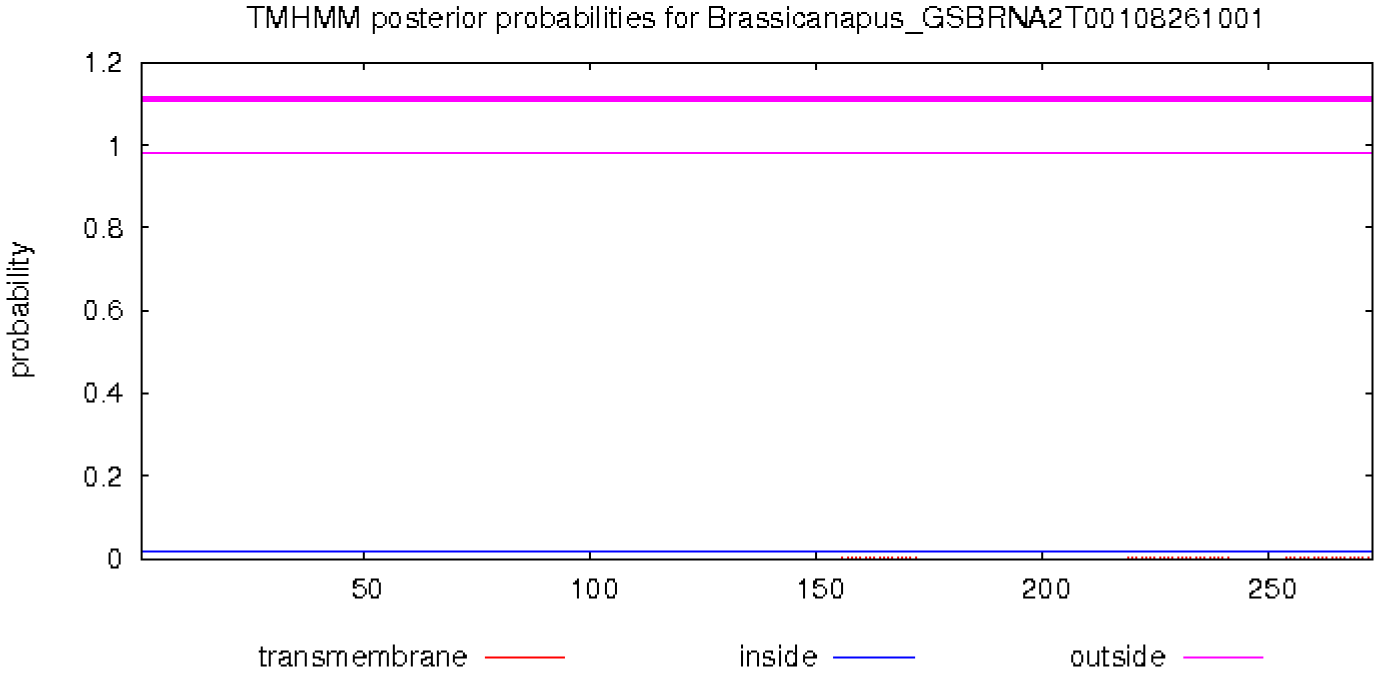

plot in postscript, script for making the plot in gnuplot, data for plot

---

```
# Brassicanapus_GSBRNA2T00109745001 Length: 432
# Brassicanapus_GSBRNA2T00109745001 Number of predicted TMHs:  1
# Brassicanapus_GSBRNA2T00109745001 Exp number of AAs in TMHs: 20.58103
# Brassicanapus_GSBRNA2T00109745001 Exp number, first 60 AAs:  0
# Brassicanapus_GSBRNA2T00109745001 Total prob of N-in:        0.13184
Brassicanapus_GSBRNA2T00109745001       TMHMM2.0        outside      1   399
Brassicanapus_GSBRNA2T00109745001       TMHMM2.0        TMhelix    400   422
Brassicanapus_GSBRNA2T00109745001       TMHMM2.0        inside     423   432
```

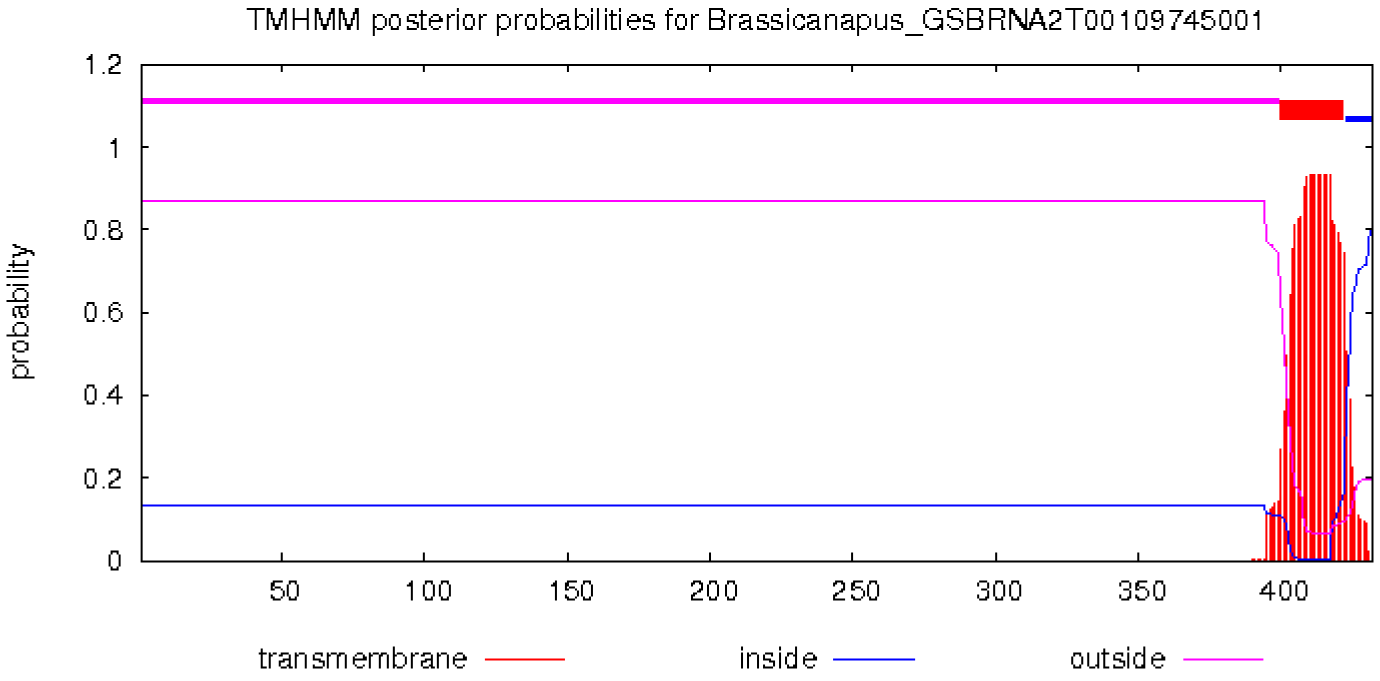

plot in postscript, script for making the plot in gnuplot, data for plot

```
# Brassicanapus_GSBRNA2T00109854001 Length: 260
# Brassicanapus_GSBRNA2T00109854001 Number of predicted TMHs:  0
# Brassicanapus_GSBRNA2T00109854001 Exp number of AAs in TMHs: 0
# Brassicanapus_GSBRNA2T00109854001 Exp number, first 60 AAs:  0
# Brassicanapus_GSBRNA2T00109854001 Total prob of N-in:        0.00337
Brassicanapus_GSBRNA2T00109854001	TMHMM2.0	outside	     1   260
```

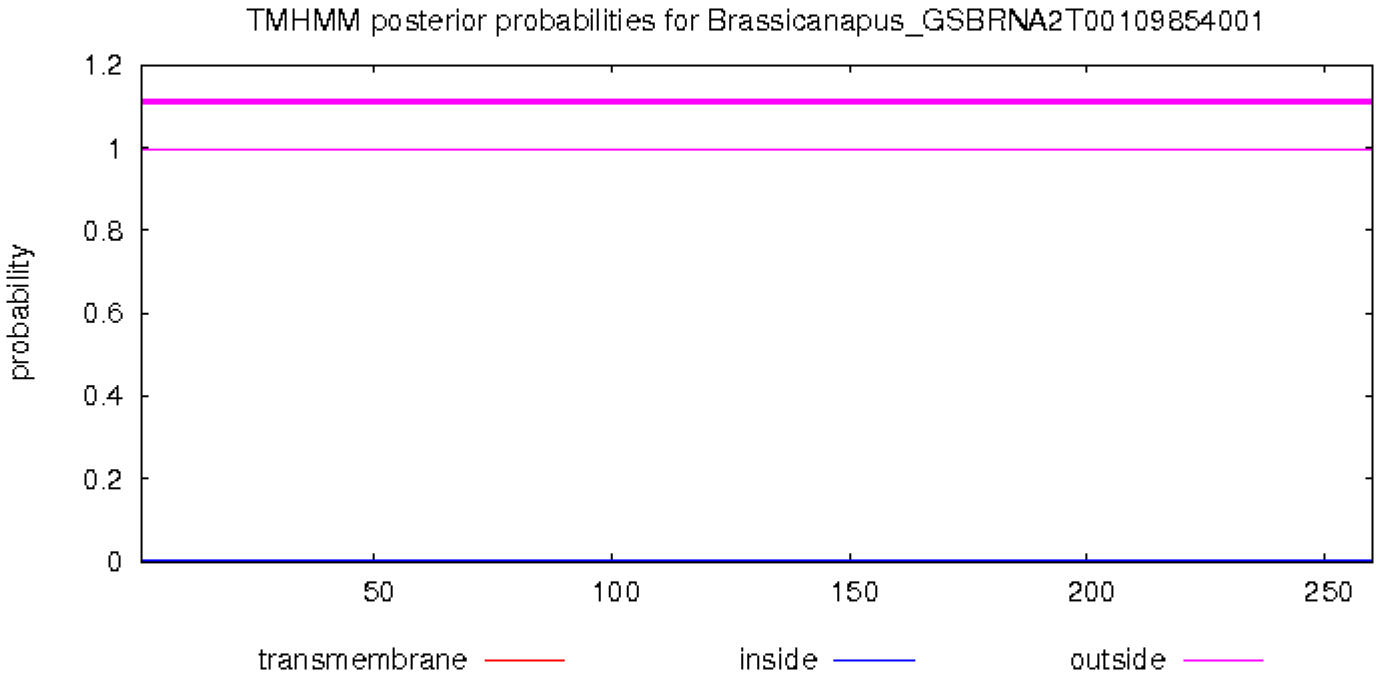

plot in postscript, script for making the plot in gnuplot, data for plot

```
# Brassicanapus_GSBRNA2T00110506001 Length: 428
# Brassicanapus_GSBRNA2T00110506001 Number of predicted TMHs:  0
# Brassicanapus_GSBRNA2T00110506001 Exp number of AAs in TMHs: 0.04037
# Brassicanapus_GSBRNA2T00110506001 Exp number, first 60 AAs:  0.00772
# Brassicanapus_GSBRNA2T00110506001 Total prob of N-in:        0.00568
Brassicanapus_GSBRNA2T00110506001	TMHMM2.0	outside	     1   428
```

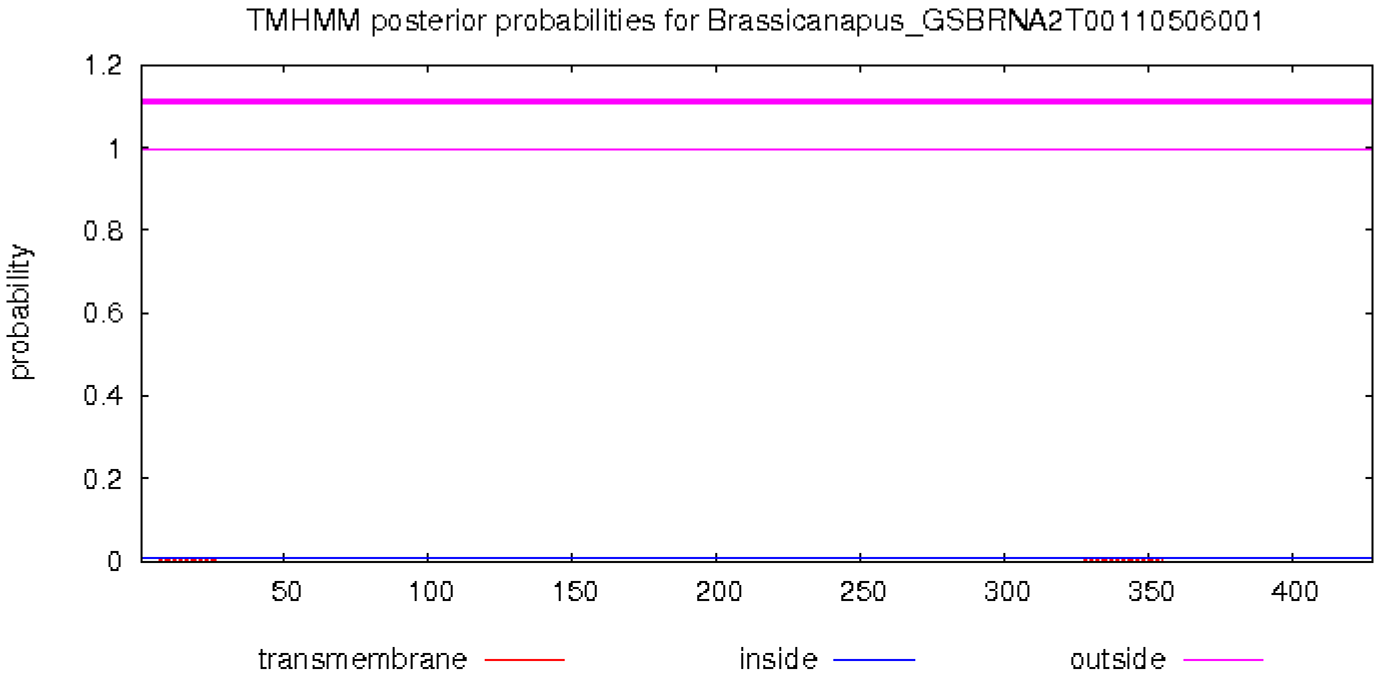

plot in postscript, script for making the plot in gnuplot, data for plot

```
# Brassicanapus_GSBRNA2T00110507001 Length: 445
# Brassicanapus_GSBRNA2T00110507001 Number of predicted TMHs:  0
# Brassicanapus_GSBRNA2T00110507001 Exp number of AAs in TMHs: 0.1371
# Brassicanapus_GSBRNA2T00110507001 Exp number, first 60 AAs:  0.05749
# Brassicanapus_GSBRNA2T00110507001 Total prob of N-in:        0.00848
Brassicanapus_GSBRNA2T00110507001	TMHMM2.0	outside	     1   445
```

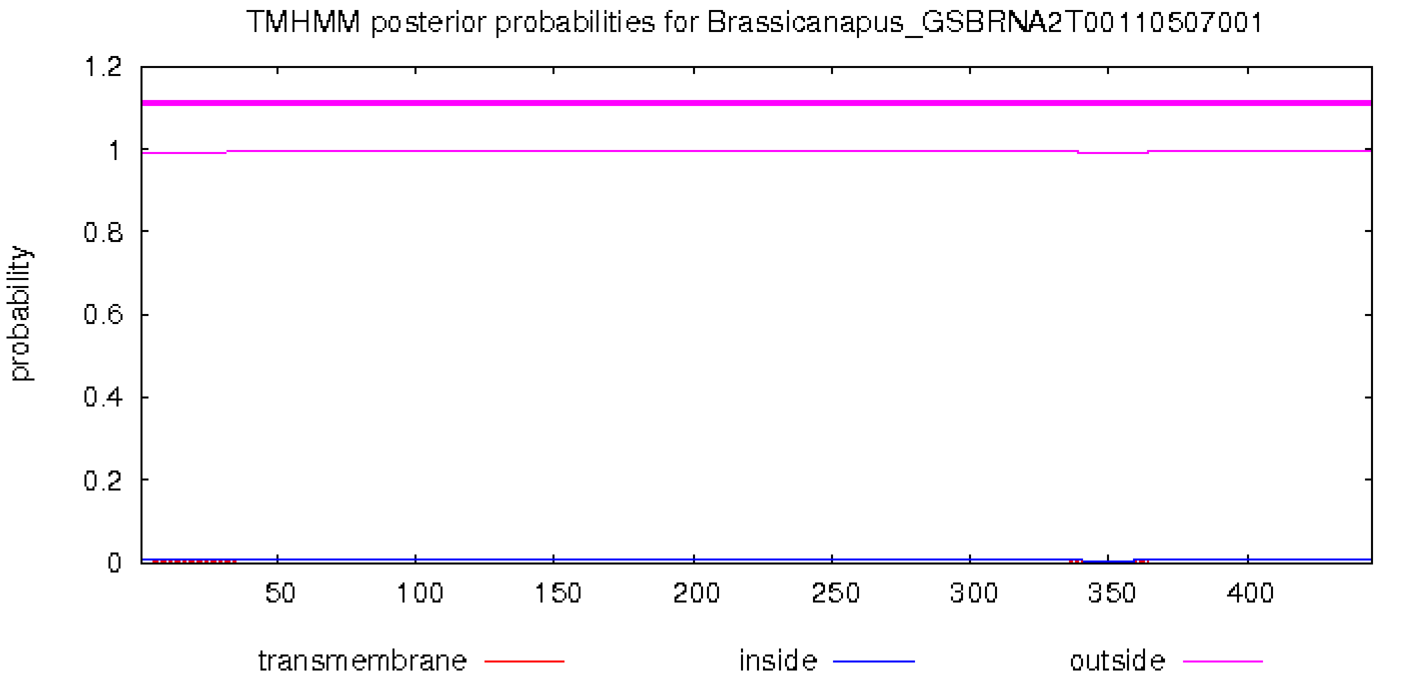

plot in postscript, script for making the plot in gnuplot, data for plot

```
# Brassicanapus_GSBRNA2T00110954001 Length: 451
# Brassicanapus_GSBRNA2T00110954001 Number of predicted TMHs:  0
# Brassicanapus_GSBRNA2T00110954001 Exp number of AAs in TMHs: 0.10329
# Brassicanapus_GSBRNA2T00110954001 Exp number, first 60 AAs:  0.05374
# Brassicanapus_GSBRNA2T00110954001 Total prob of N-in:        0.00644
Brassicanapus_GSBRNA2T00110954001	TMHMM2.0	outside	     1   451
```

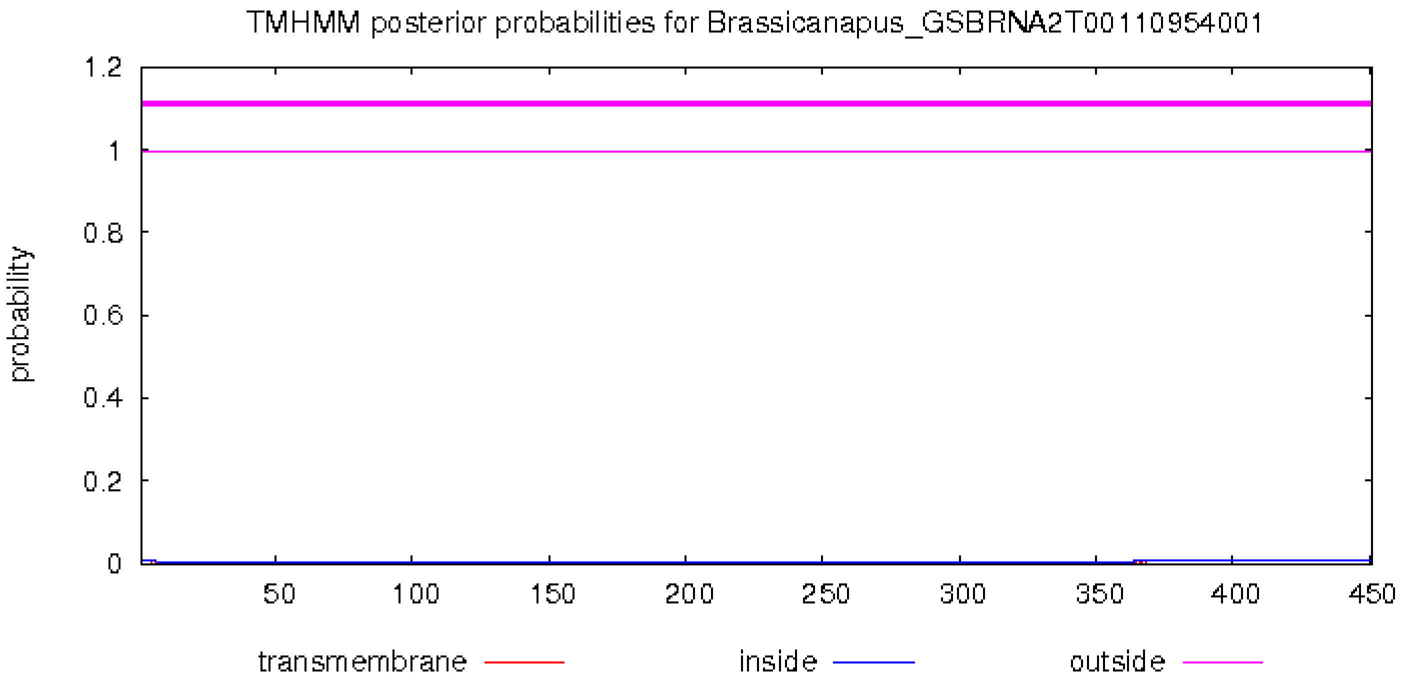

plot in postscript, script for making the plot in gnuplot, data for plot

```
# Brassicanapus_GSBRNA2T00111390001 Length: 371
# Brassicanapus_GSBRNA2T00111390001 Number of predicted TMHs:  0
# Brassicanapus_GSBRNA2T00111390001 Exp number of AAs in TMHs: 0
# Brassicanapus_GSBRNA2T00111390001 Exp number, first 60 AAs:  0
# Brassicanapus_GSBRNA2T00111390001 Total prob of N-in:        0.00257
Brassicanapus_GSBRNA2T00111390001       TMHMM2.0        outside      1   371
```

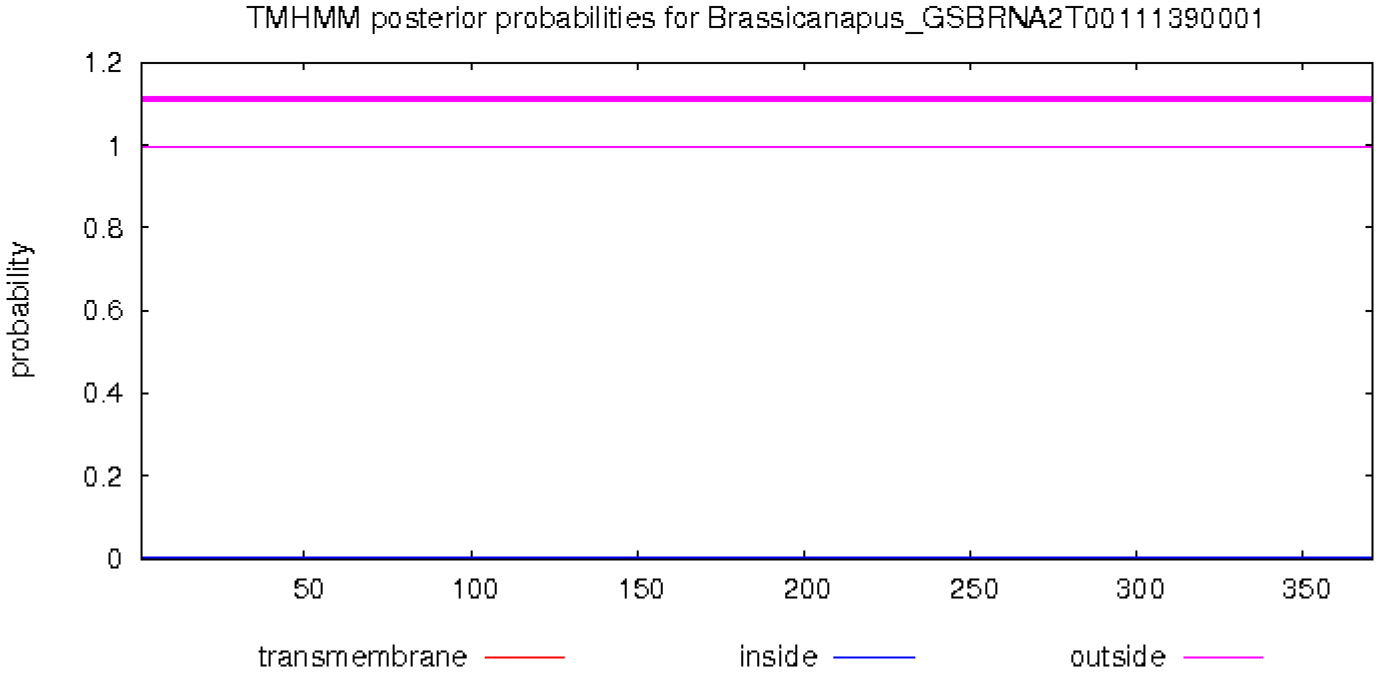

plot in postscript, script for making the plot in gnuplot, data for plot

---

```
# Brassicanapus_GSBRNA2T00111648001 Length: 447
# Brassicanapus_GSBRNA2T00111648001 Number of predicted TMHs:  0
# Brassicanapus_GSBRNA2T00111648001 Exp number of AAs in TMHs: 0.23206
# Brassicanapus_GSBRNA2T00111648001 Exp number, first 60 AAs:  0.15978
# Brassicanapus_GSBRNA2T00111648001 Total prob of N-in:        0.01243
Brassicanapus_GSBRNA2T00111648001       TMHMM2.0        outside      1   447
```

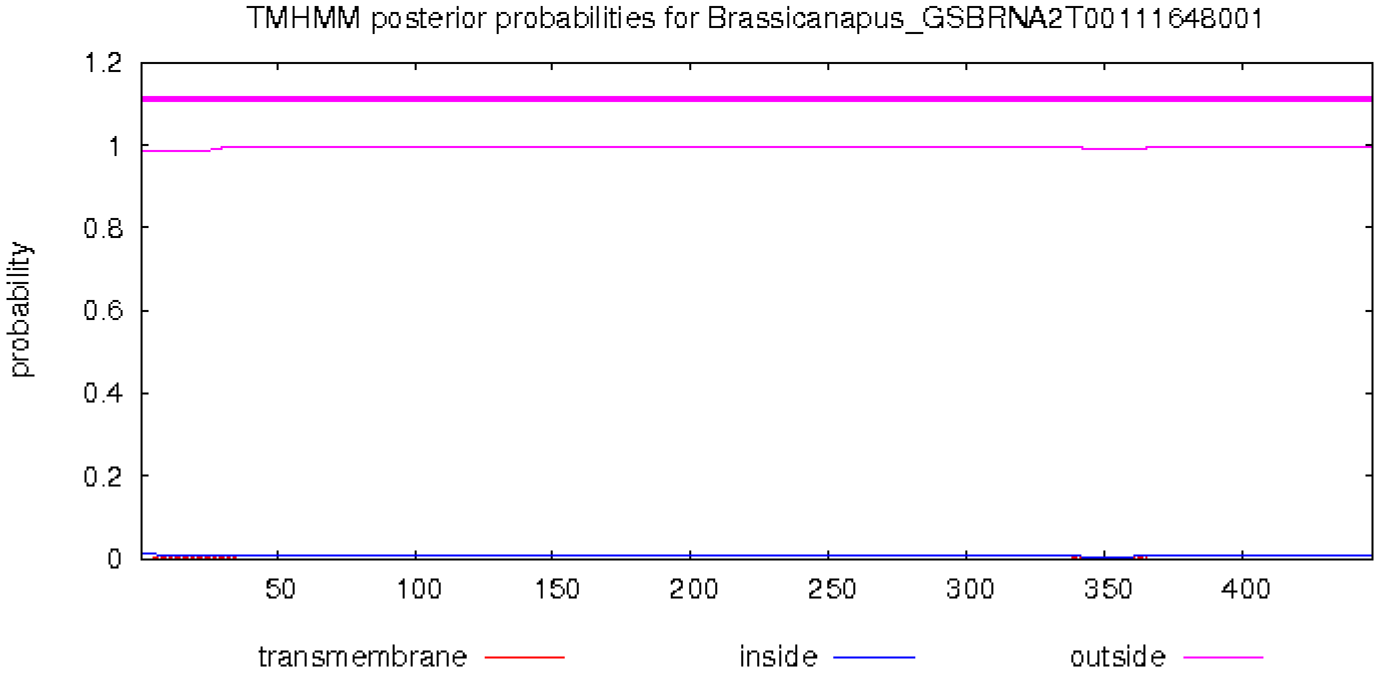

plot in postscript, script for making the plot in gnuplot, data for plot

---

```
# Brassicanapus_GSBRNA2T00111649001 Length: 456
# Brassicanapus_GSBRNA2T00111649001 Number of predicted TMHs:  0
# Brassicanapus_GSBRNA2T00111649001 Exp number of AAs in TMHs: 9.92253999999999999
# Brassicanapus_GSBRNA2T00111649001 Exp number, first 60 AAs:  0.68616
# Brassicanapus_GSBRNA2T00111649001 Total prob of N-in:        0.37287
Brassicanapus_GSBRNA2T00111649001       TMHMM2.0        outside      1   456
```

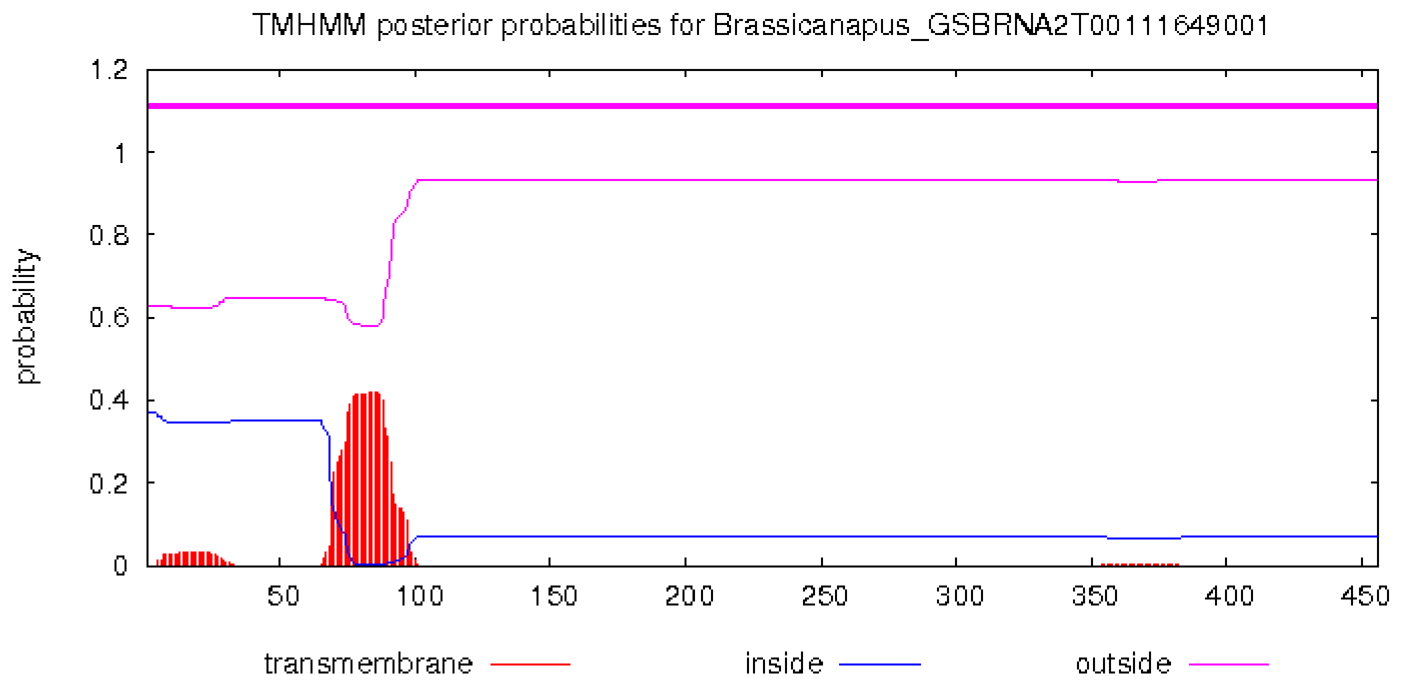

plot in postscript, script for making the plot in gnuplot, data for plot

---

```
# Brassicanapus_GSBRNA2T00112100001 Length: 702
# Brassicanapus_GSBRNA2T00112100001 Number of predicted TMHs:  0
# Brassicanapus_GSBRNA2T00112100001 Exp number of AAs in TMHs: 0.00366
# Brassicanapus_GSBRNA2T00112100001 Exp number, first 60 AAs:  0.00131
# Brassicanapus_GSBRNA2T00112100001 Total prob of N-in:        0.00022
Brassicanapus_GSBRNA2T00112100001       TMHMM2.0        outside      1   702
```

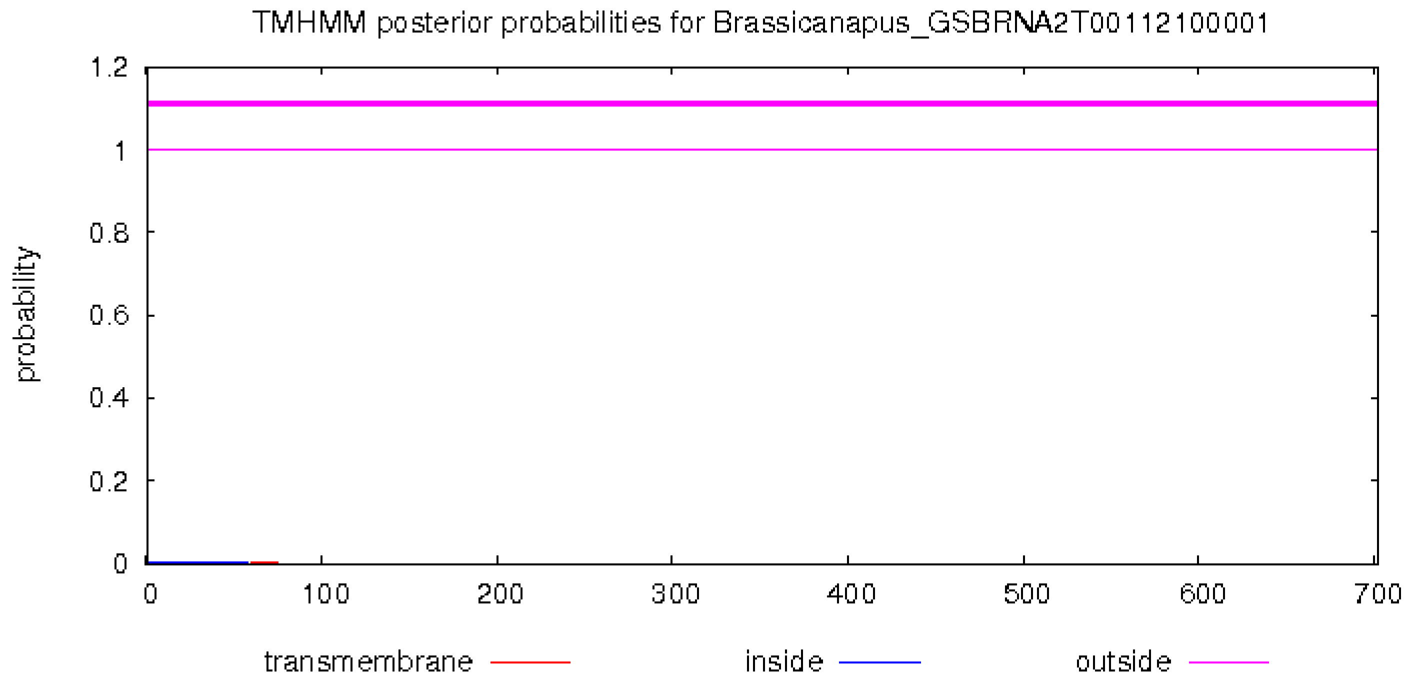

plot in postscript, script for making the plot in gnuplot, data for plot

---

```
# Brassicanapus_GSBRNA2T00112525001 Length: 286
# Brassicanapus_GSBRNA2T00112525001 Number of predicted TMHs:  0
# Brassicanapus_GSBRNA2T00112525001 Exp number of AAs in TMHs: 0
# Brassicanapus_GSBRNA2T00112525001 Exp number, first 60 AAs:  0
# Brassicanapus_GSBRNA2T00112525001 Total prob of N-in:        0.00737
Brassicanapus_GSBRNA2T00112525001	TMHMM2.0	outside	     1   286
```

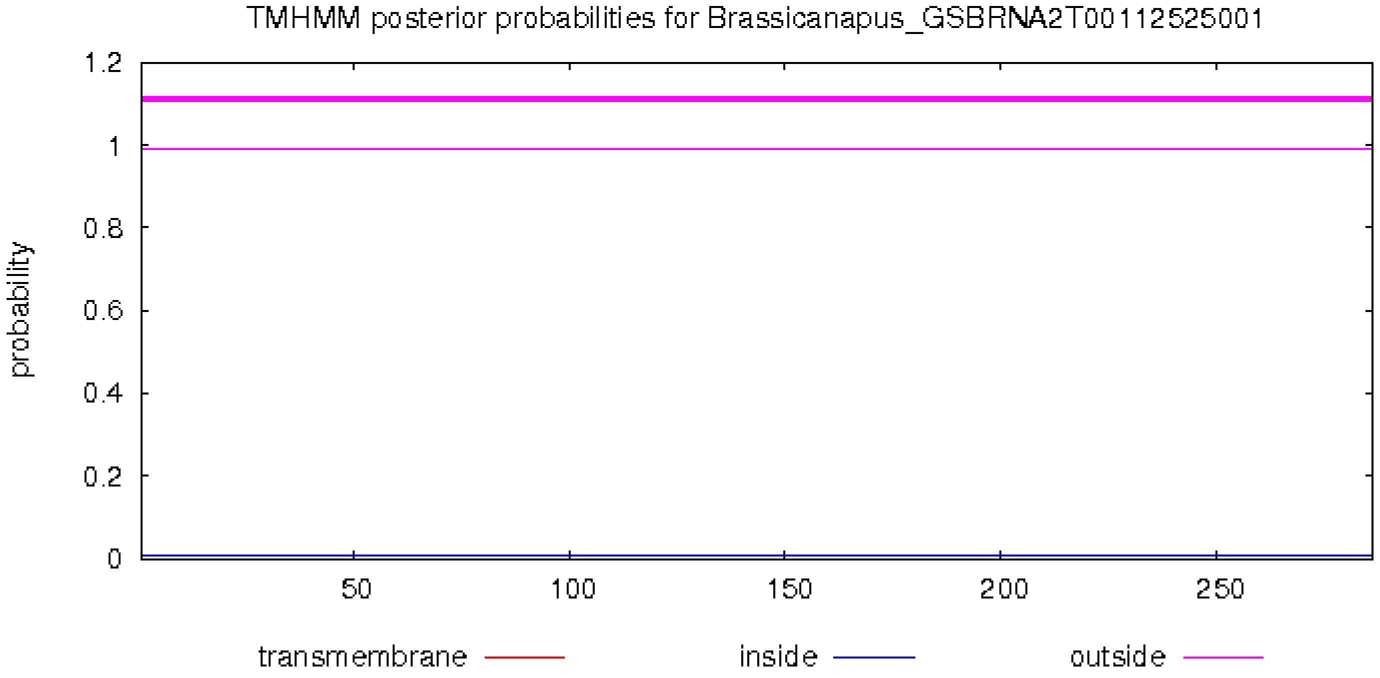

plot in postscript, script for making the plot in gnuplot, data for plot

---

```
# Brassicanapus_GSBRNA2T00112879001 Length: 397
# Brassicanapus_GSBRNA2T00112879001 Number of predicted TMHs:  0
# Brassicanapus_GSBRNA2T00112879001 Exp number of AAs in TMHs: 0.00023
# Brassicanapus_GSBRNA2T00112879001 Exp number, first 60 AAs:  0
# Brassicanapus_GSBRNA2T00112879001 Total prob of N-in:        0.00111
Brassicanapus_GSBRNA2T00112879001	TMHMM2.0	outside	     1   397
```

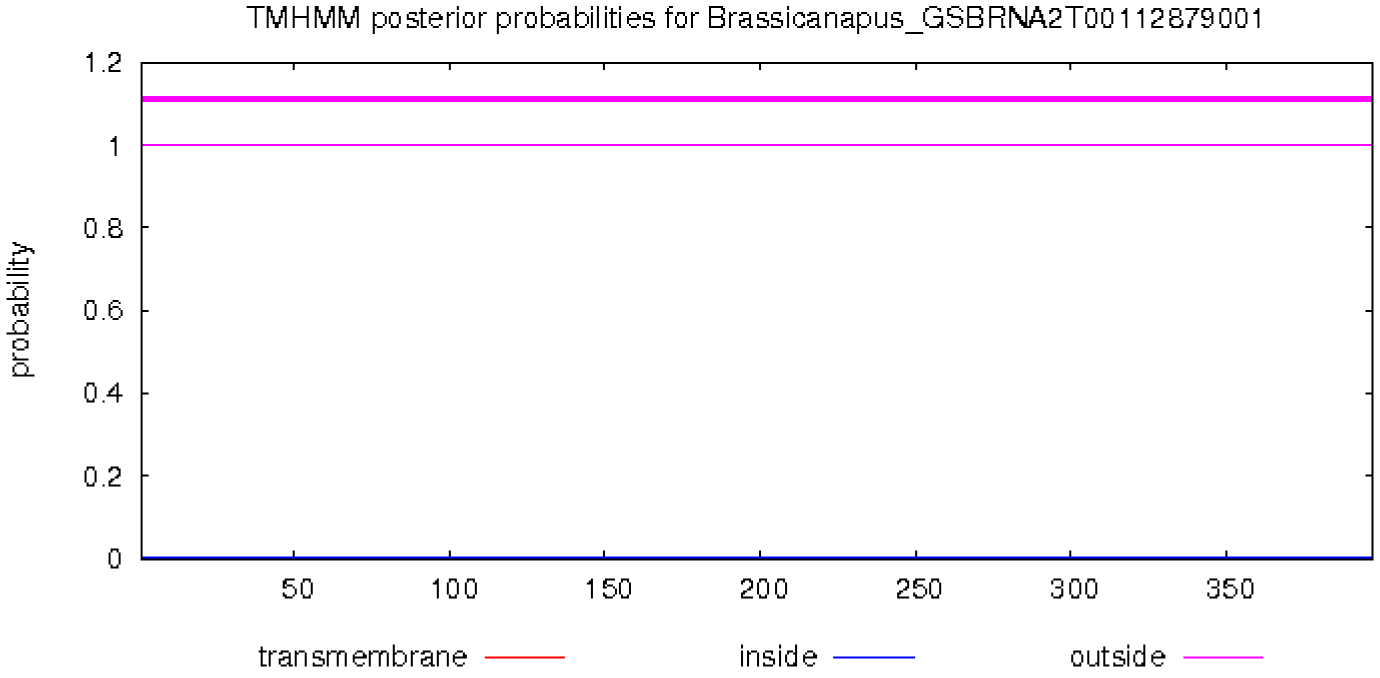

plot in postscript, script for making the plot in gnuplot, data for plot

---

```
# Brassicanapus_GSBRNA2T00113154001 Length: 486
# Brassicanapus_GSBRNA2T00113154001 Number of predicted TMHs:  1
# Brassicanapus_GSBRNA2T00113154001 Exp number of AAs in TMHs: 22.85895
# Brassicanapus_GSBRNA2T00113154001 Exp number, first 60 AAs:  0.00048
# Brassicanapus_GSBRNA2T00113154001 Total prob of N-in:        0.18853
Brassicanapus_GSBRNA2T00113154001       TMHMM2.0        outside      1   457
Brassicanapus_GSBRNA2T00113154001       TMHMM2.0        TMhelix    458   480
Brassicanapus_GSBRNA2T00113154001       TMHMM2.0        inside     481   486
```

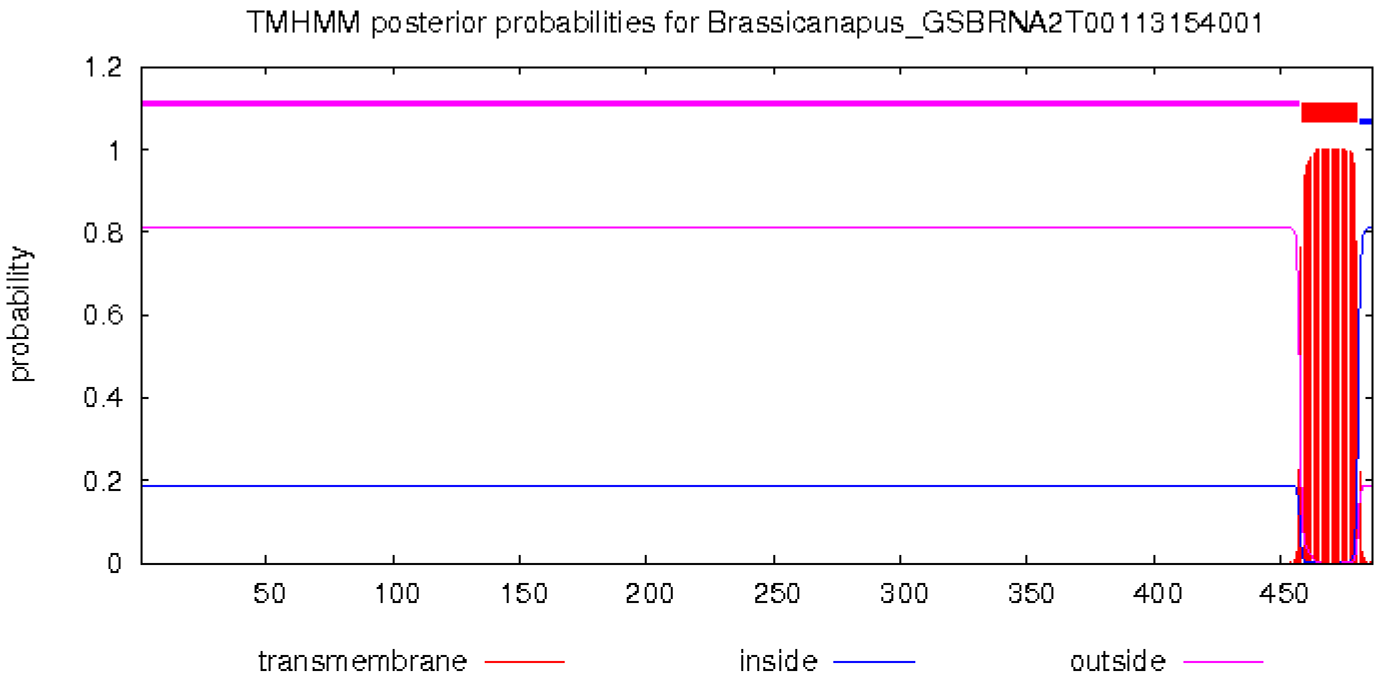

plot in postscript, script for making the plot in gnuplot, data for plot

---

```
# Brassicanapus_GSBRNA2T00113639001 Length: 281
# Brassicanapus_GSBRNA2T00113639001 Number of predicted TMHs:  0
# Brassicanapus_GSBRNA2T00113639001 Exp number of AAs in TMHs: 0.00594
# Brassicanapus_GSBRNA2T00113639001 Exp number, first 60 AAs:  0.00338
# Brassicanapus_GSBRNA2T00113639001 Total prob of N-in:        0.00557
Brassicanapus_GSBRNA2T00113639001       TMHMM2.0        outside      1   281
```

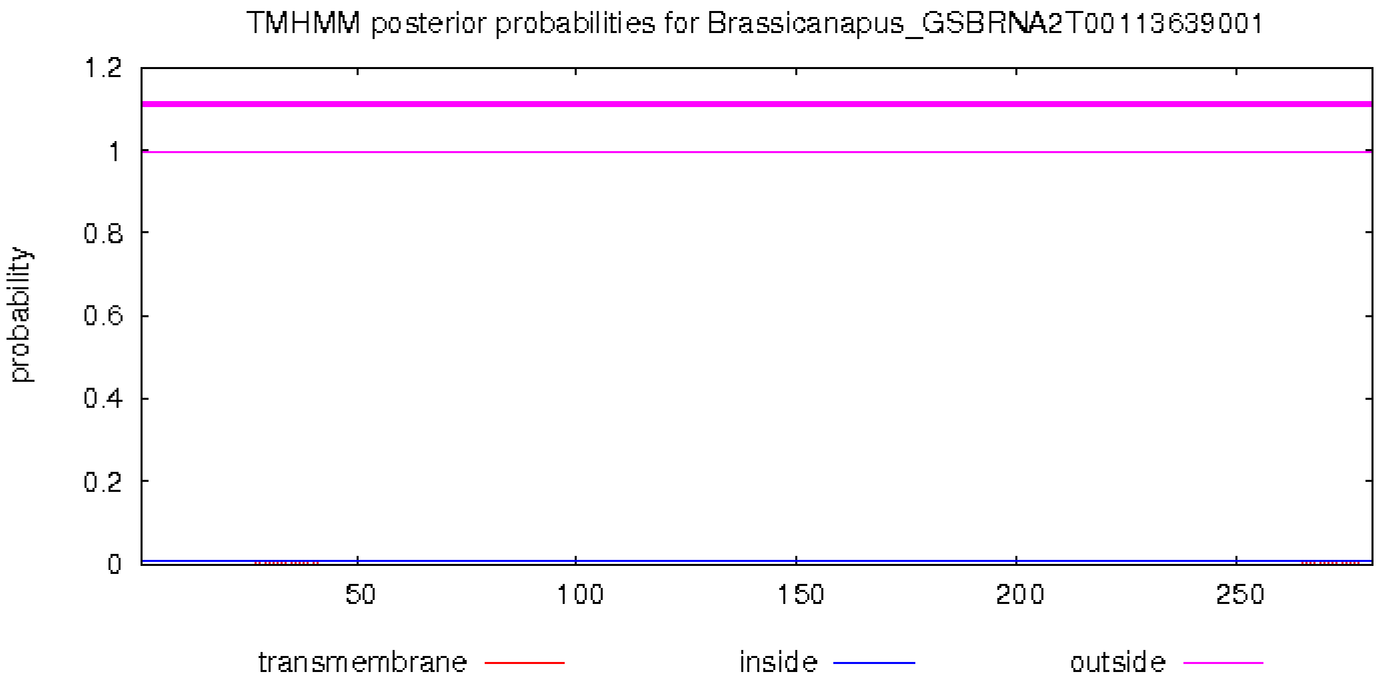

plot in postscript, script for making the plot in gnuplot, data for plot

```
# Brassicanapus_GSBRNA2T00114310001 Length: 289
# Brassicanapus_GSBRNA2T00114310001 Number of predicted TMHs:  0
# Brassicanapus_GSBRNA2T00114310001 Exp number of AAs in TMHs: 0.0008000000000000001
# Brassicanapus_GSBRNA2T00114310001 Exp number, first 60 AAs:  0.00057
# Brassicanapus_GSBRNA2T00114310001 Total prob of N-in:        0.04392
Brassicanapus_GSBRNA2T00114310001	TMHMM2.0	outside	     1   289
```

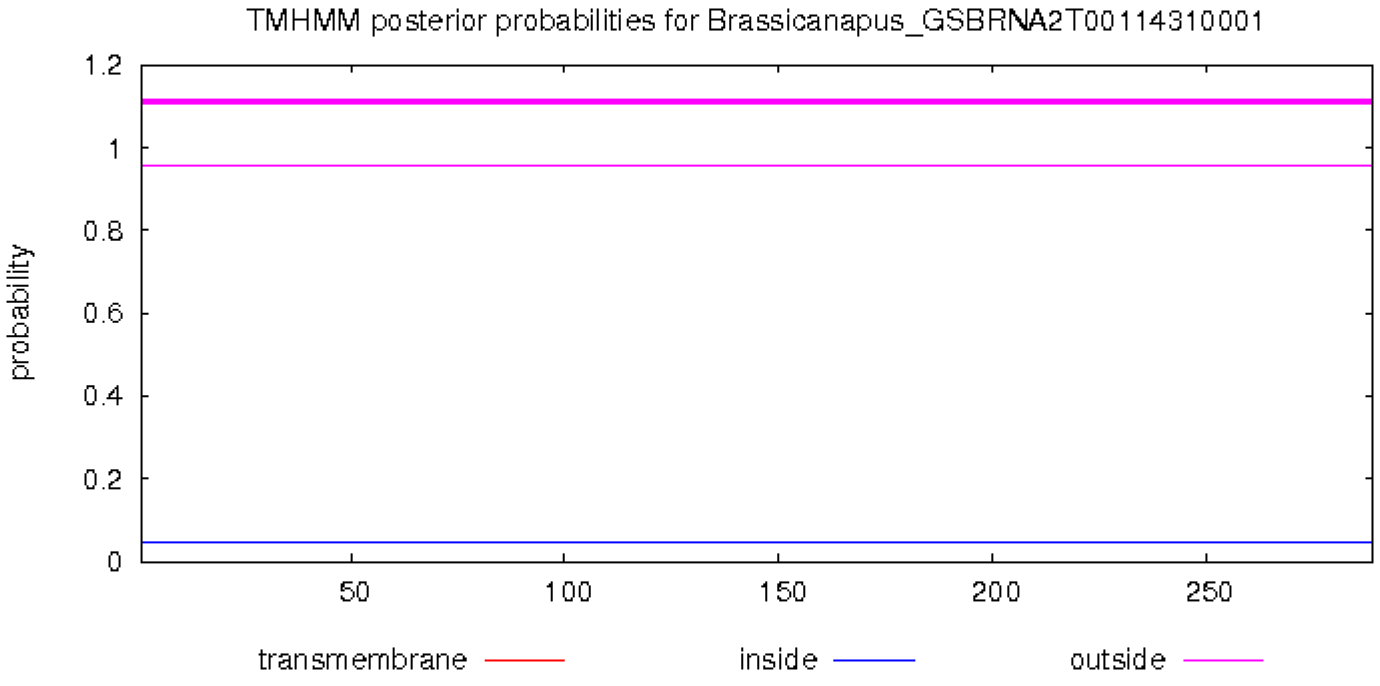

plot in postscript, script for making the plot in gnuplot, data for plot

```
# Brassicanapus_GSBRNA2T00114768001 Length: 337
# Brassicanapus_GSBRNA2T00114768001 Number of predicted TMHs:  0
# Brassicanapus_GSBRNA2T00114768001 Exp number of AAs in TMHs: 0.00017
# Brassicanapus_GSBRNA2T00114768001 Exp number, first 60 AAs:  0
# Brassicanapus_GSBRNA2T00114768001 Total prob of N-in:        0.00864
Brassicanapus_GSBRNA2T00114768001	TMHMM2.0	outside	     1   337
```

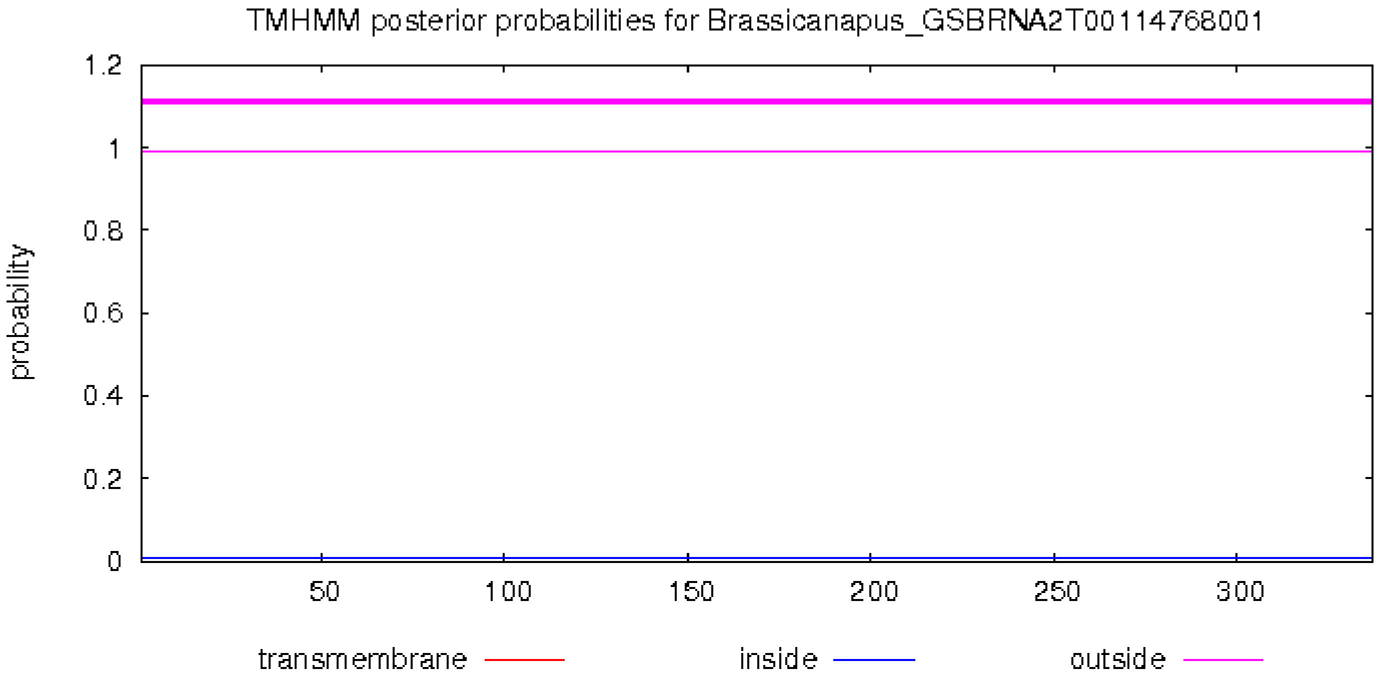

plot in postscript, script for making the plot in gnuplot, data for plot

```
# Brassicanapus_GSBRNA2T00115065001 Length: 292
# Brassicanapus_GSBRNA2T00115065001 Number of predicted TMHs:  0
# Brassicanapus_GSBRNA2T00115065001 Exp number of AAs in TMHs: 0
# Brassicanapus_GSBRNA2T00115065001 Exp number, first 60 AAs:  0
# Brassicanapus_GSBRNA2T00115065001 Total prob of N-in:        0.02308
Brassicanapus_GSBRNA2T00115065001	TMHMM2.0	outside	     1   292
```

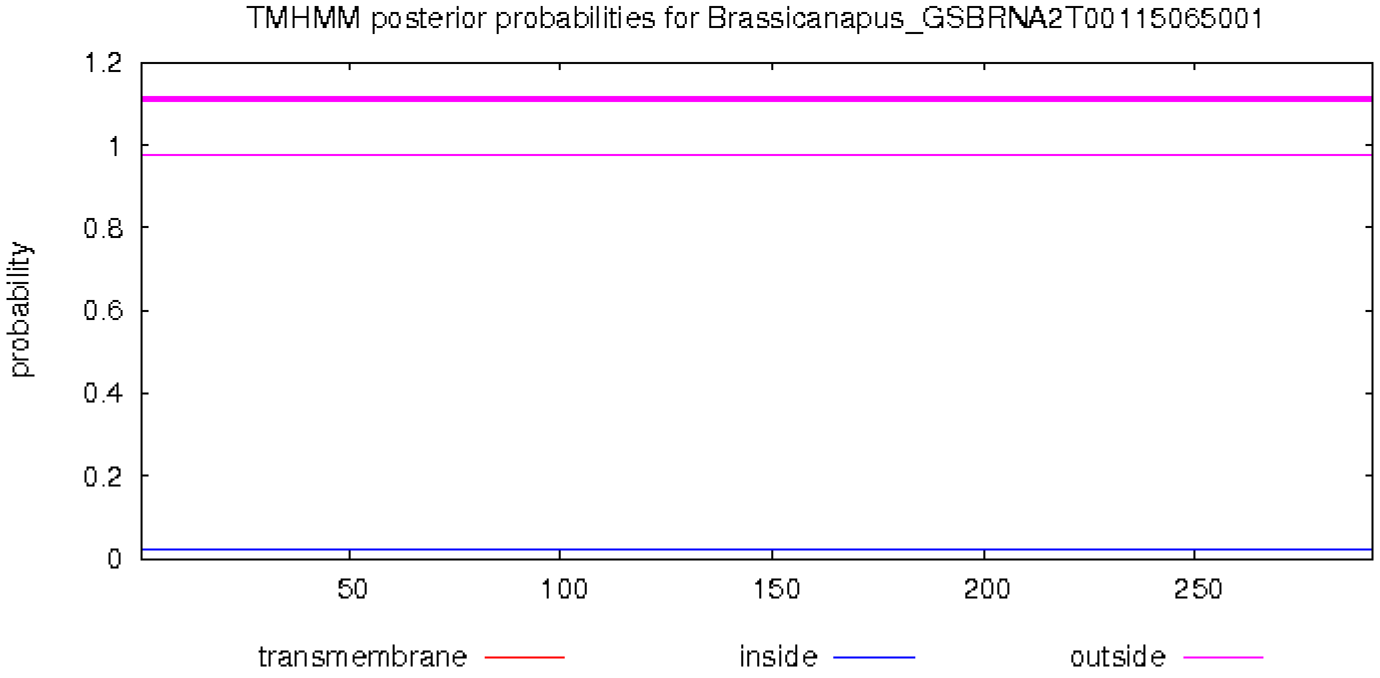

plot in postscript, script for making the plot in gnuplot, data for plot

---

```
# Brassicanapus_GSBRNA2T00115069001 Length: 244
# Brassicanapus_GSBRNA2T00115069001 Number of predicted TMHs:  0
# Brassicanapus_GSBRNA2T00115069001 Exp number of AAs in TMHs: 0.00391
# Brassicanapus_GSBRNA2T00115069001 Exp number, first 60 AAs:  0.00088
# Brassicanapus_GSBRNA2T00115069001 Total prob of N-in:        0.15484
Brassicanapus_GSBRNA2T00115069001	TMHMM2.0	outside	     1   244
```

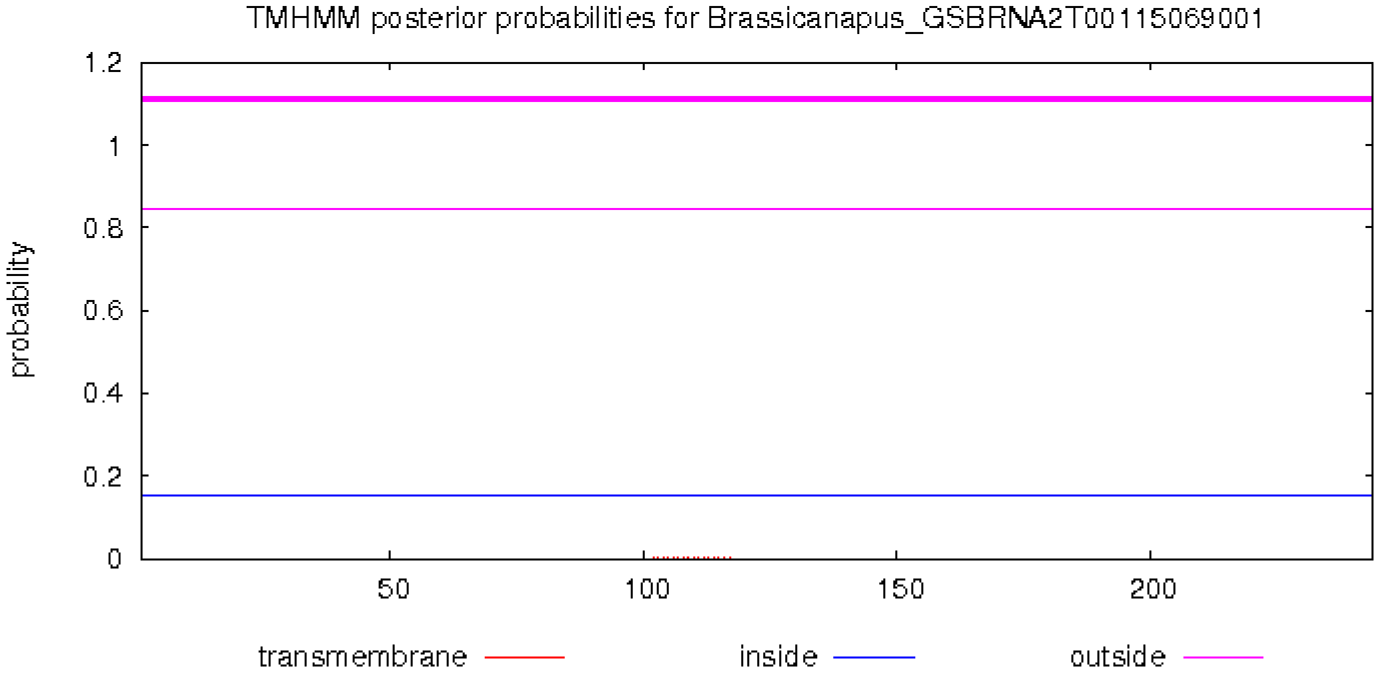

plot in postscript, script for making the plot in gnuplot, data for plot

---

```
# Brassicanapus_GSBRNA2T00115095001 Length: 231
# Brassicanapus_GSBRNA2T00115095001 Number of predicted TMHs:  0
# Brassicanapus_GSBRNA2T00115095001 Exp number of AAs in TMHs: 0.0011
# Brassicanapus_GSBRNA2T00115095001 Exp number, first 60 AAs:  0.00054
# Brassicanapus_GSBRNA2T00115095001 Total prob of N-in:        0.14746
Brassicanapus_GSBRNA2T00115095001	TMHMM2.0	outside	     1   231
```

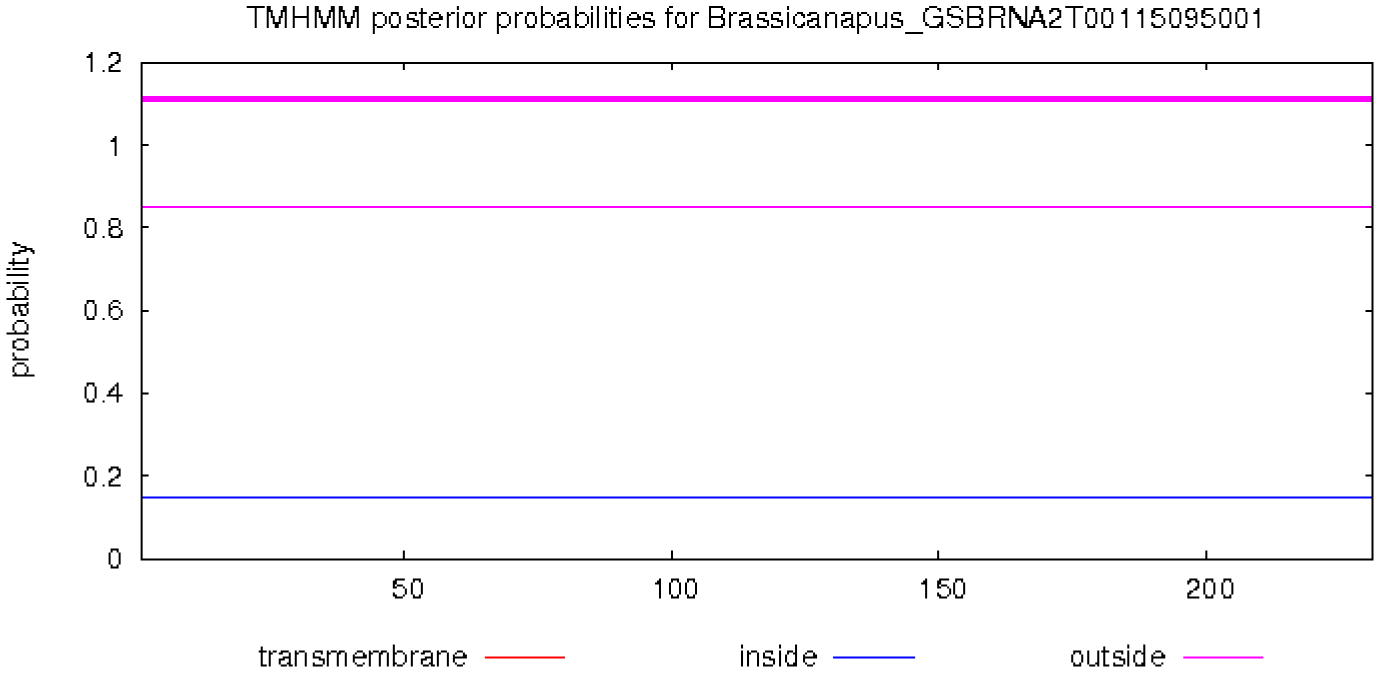

plot in postscript, script for making the plot in gnuplot, data for plot

---

```
# Brassicanapus_GSBRNA2T00115382001 Length: 414
# Brassicanapus_GSBRNA2T00115382001 Number of predicted TMHs:  0
# Brassicanapus_GSBRNA2T00115382001 Exp number of AAs in TMHs: 0.02472
# Brassicanapus_GSBRNA2T00115382001 Exp number, first 60 AAs:  0
# Brassicanapus_GSBRNA2T00115382001 Total prob of N-in:        0.00885
Brassicanapus_GSBRNA2T00115382001	TMHMM2.0	outside	     1   414
```

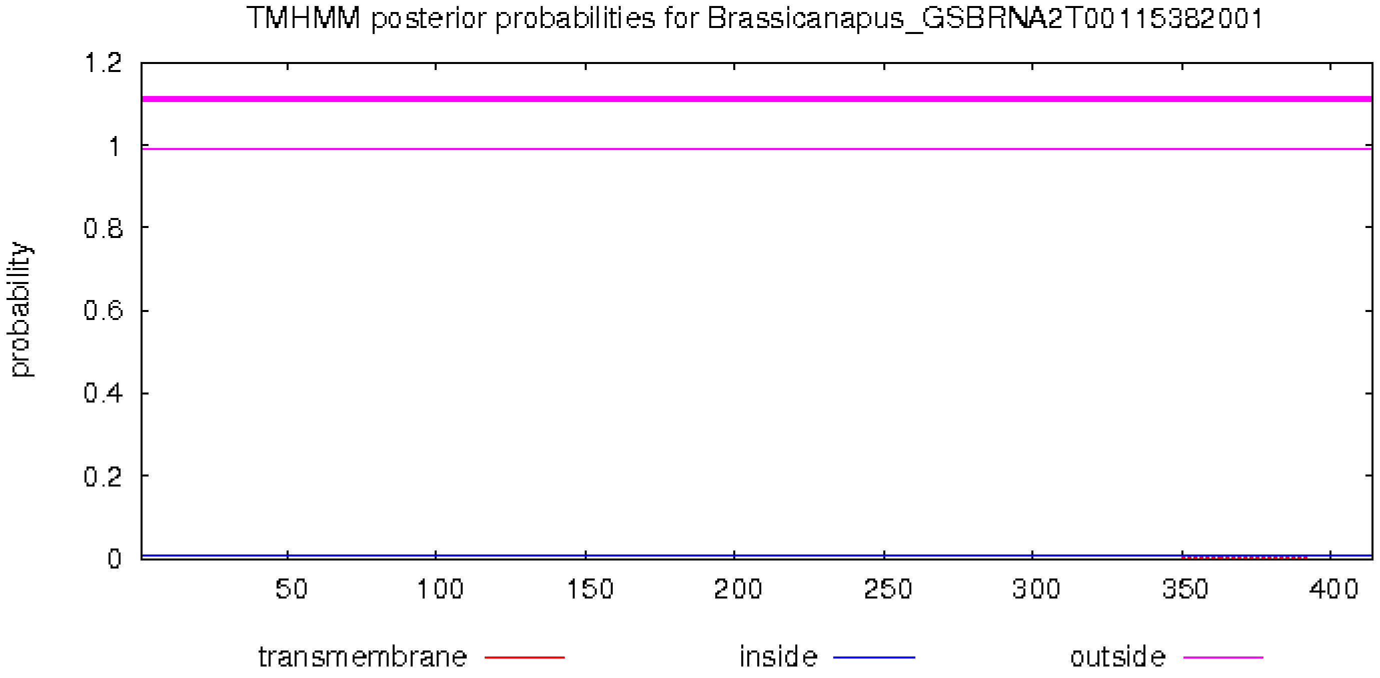

plot in postscript, script for making the plot in gnuplot, data for plot

---

```
# Brassicanapus_GSBRNA2T00116457001 Length: 327
# Brassicanapus_GSBRNA2T00116457001 Number of predicted TMHs:  0
# Brassicanapus_GSBRNA2T00116457001 Exp number of AAs in TMHs: 0
# Brassicanapus_GSBRNA2T00116457001 Exp number, first 60 AAs:  0
# Brassicanapus_GSBRNA2T00116457001 Total prob of N-in:        0.02336
Brassicanapus_GSBRNA2T00116457001       TMHMM2.0        outside      1   327
```

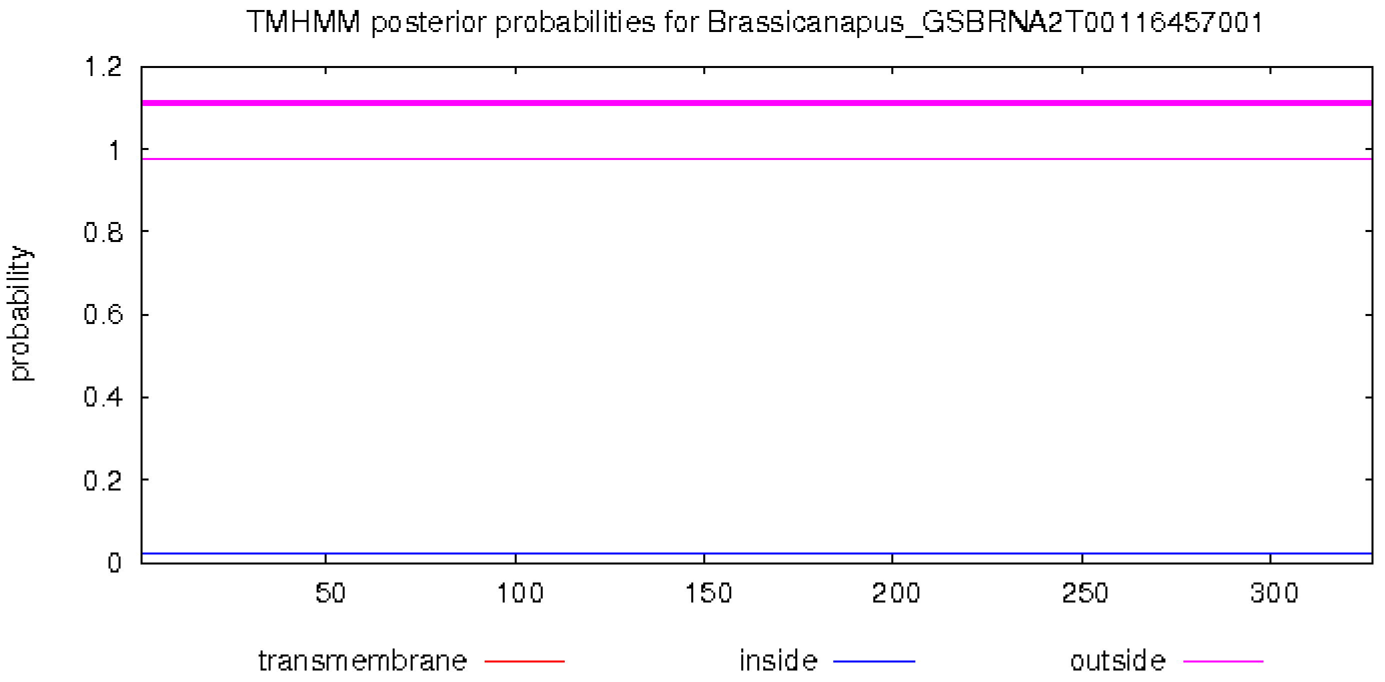

plot in postscript, script for making the plot in gnuplot, data for plot

---

```
# Brassicanapus_GSBRNA2T00117792001 Length: 408
# Brassicanapus_GSBRNA2T00117792001 Number of predicted TMHs:  0
# Brassicanapus_GSBRNA2T00117792001 Exp number of AAs in TMHs: 0.05151
# Brassicanapus_GSBRNA2T00117792001 Exp number, first 60 AAs:  0
# Brassicanapus_GSBRNA2T00117792001 Total prob of N-in:        0.01450
Brassicanapus_GSBRNA2T00117792001       TMHMM2.0        outside      1   408
```

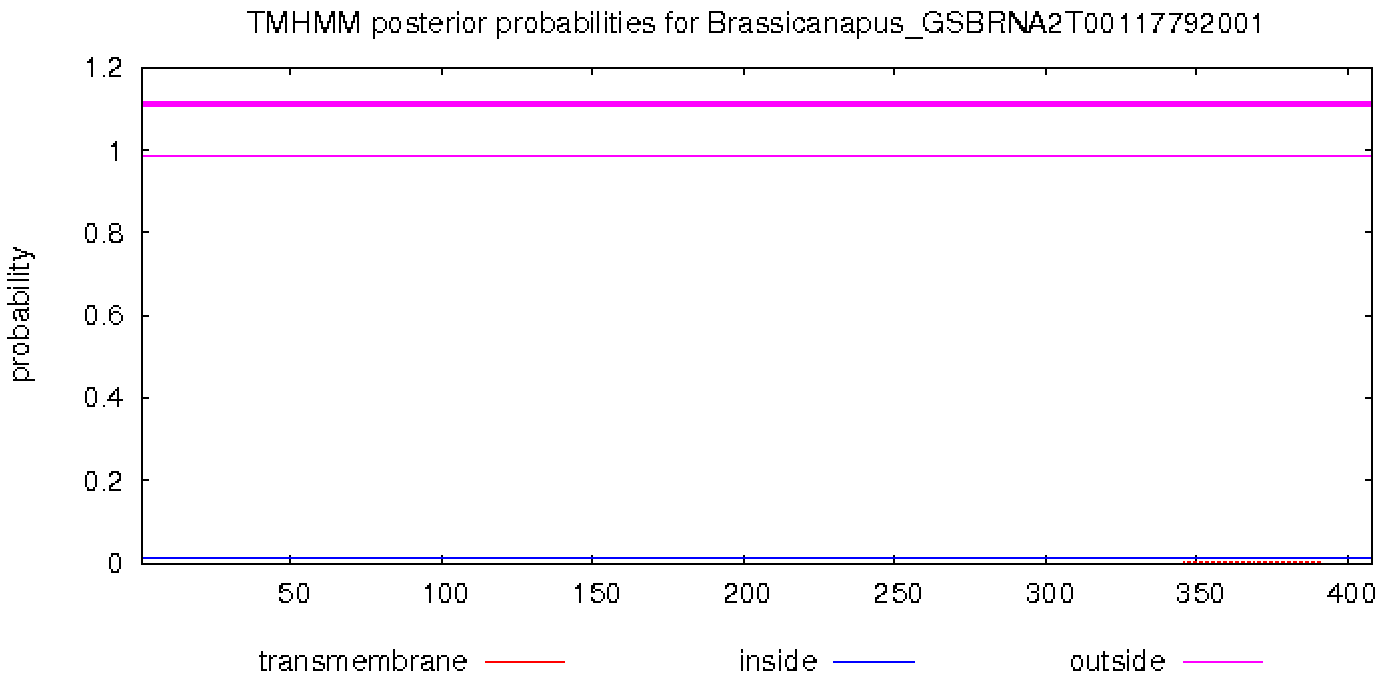

plot in postscript, script for making the plot in gnuplot, data for plot

---

```
# Brassicanapus_GSBRNA2T00118651001 Length: 345
# Brassicanapus_GSBRNA2T00118651001 Number of predicted TMHs:  1
# Brassicanapus_GSBRNA2T00118651001 Exp number of AAs in TMHs: 20.82426
# Brassicanapus_GSBRNA2T00118651001 Exp number, first 60 AAs:  0.10265
# Brassicanapus_GSBRNA2T00118651001 Total prob of N-in:        0.69296
Brassicanapus_GSBRNA2T00118651001       TMHMM2.0        inside       1   319
Brassicanapus_GSBRNA2T00118651001       TMHMM2.0        TMhelix    320   342
Brassicanapus_GSBRNA2T00118651001       TMHMM2.0        outside    343   345
```

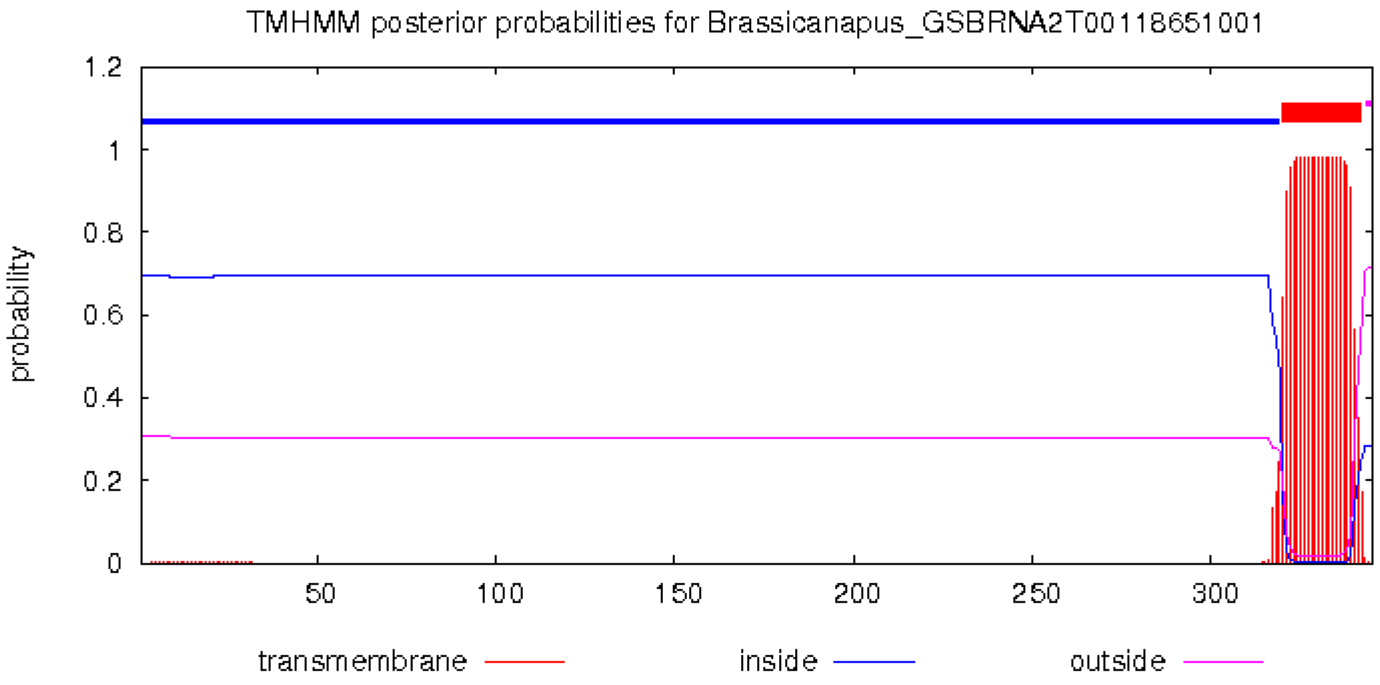

plot in postscript, script for making the plot in gnuplot, data for plot

---

```
# Brassicanapus_GSBRNA2T00119863001 Length: 333
# Brassicanapus_GSBRNA2T00119863001 Number of predicted TMHs:  0
# Brassicanapus_GSBRNA2T00119863001 Exp number of AAs in TMHs: 0
# Brassicanapus_GSBRNA2T00119863001 Exp number, first 60 AAs:  0
# Brassicanapus_GSBRNA2T00119863001 Total prob of N-in:        0.00482
Brassicanapus_GSBRNA2T00119863001       TMHMM2.0        outside      1   333
```

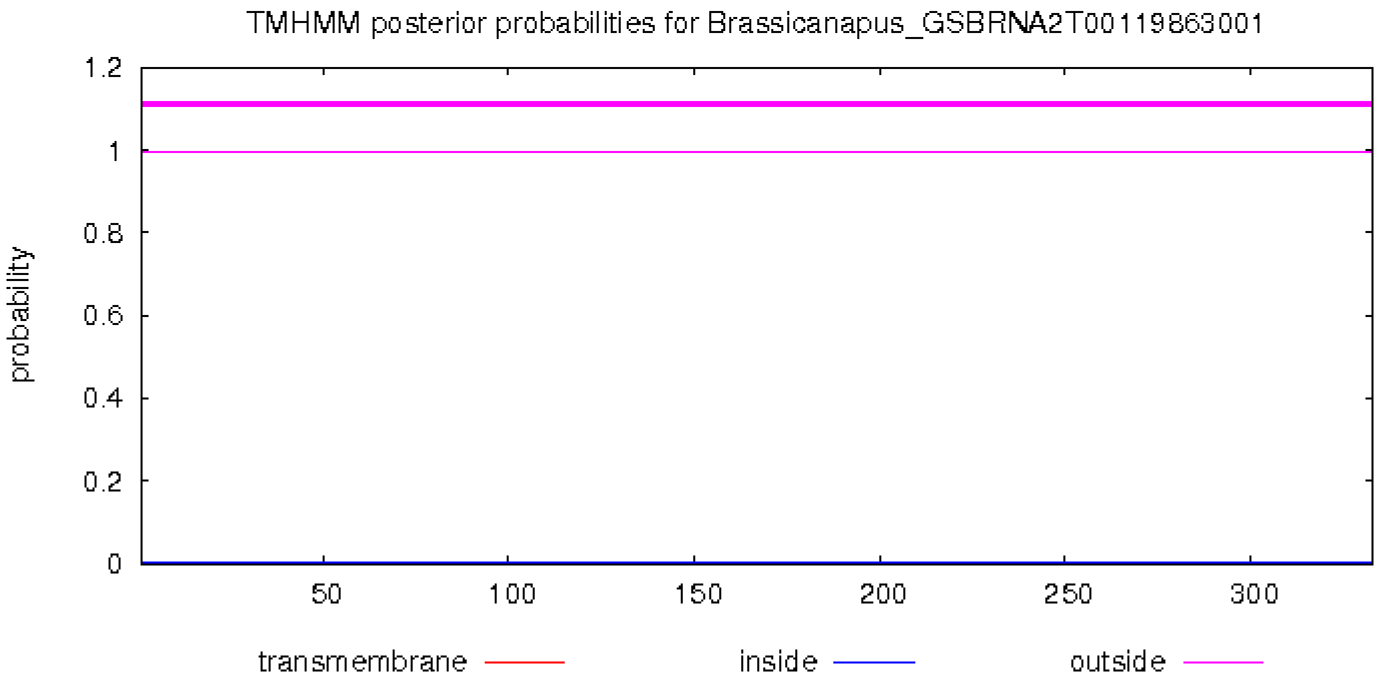

plot in postscript, script for making the plot in gnuplot, data for plot

```
# Brassicanapus_GSBRNA2T00120947001 Length: 193
# Brassicanapus_GSBRNA2T00120947001 Number of predicted TMHs:  0
# Brassicanapus_GSBRNA2T00120947001 Exp number of AAs in TMHs: 0
# Brassicanapus_GSBRNA2T00120947001 Exp number, first 60 AAs:  0
# Brassicanapus_GSBRNA2T00120947001 Total prob of N-in:        0.15545
Brassicanapus_GSBRNA2T00120947001       TMHMM2.0        outside      1   193
```

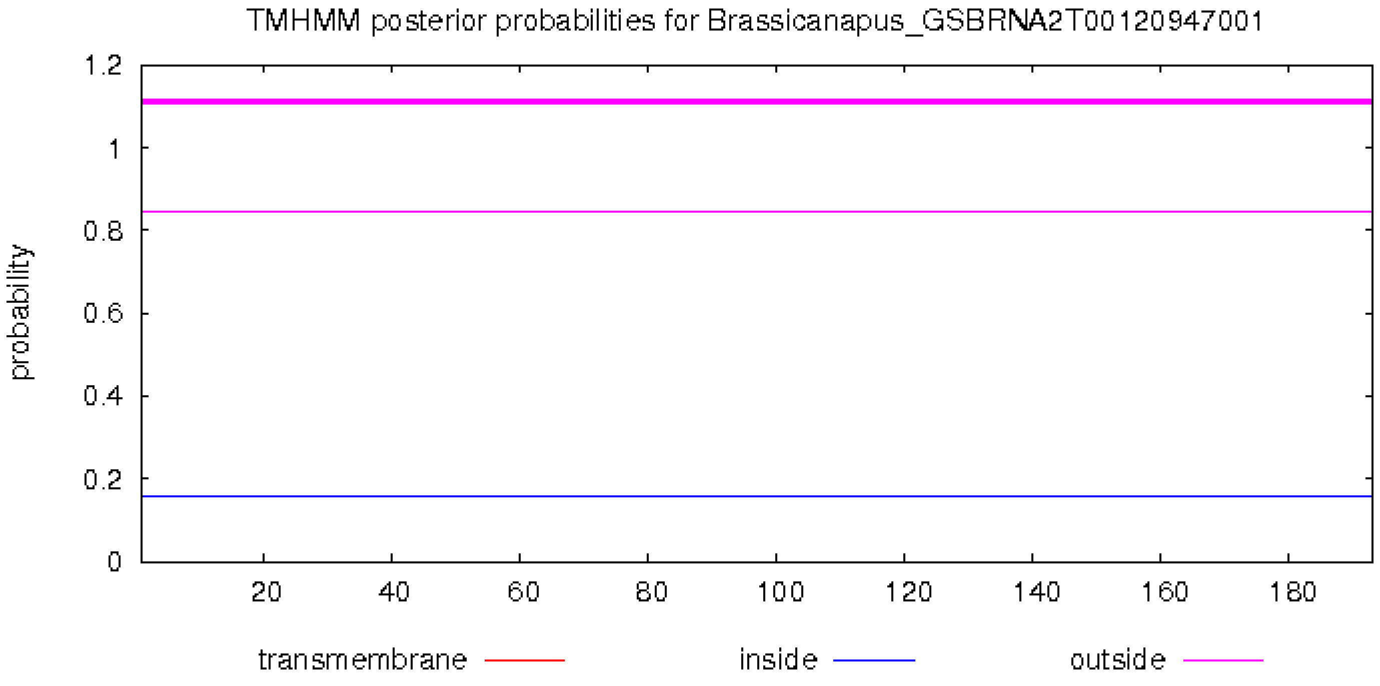

plot in postscript, script for making the plot in gnuplot, data for plot

```
# Brassicanapus_GSBRNA2T00121461001 Length: 343
# Brassicanapus_GSBRNA2T00121461001 Number of predicted TMHs:  0
# Brassicanapus_GSBRNA2T00121461001 Exp number of AAs in TMHs: 0
# Brassicanapus_GSBRNA2T00121461001 Exp number, first 60 AAs:  0
# Brassicanapus_GSBRNA2T00121461001 Total prob of N-in:        0.01882
Brassicanapus_GSBRNA2T00121461001       TMHMM2.0        outside      1   343
```

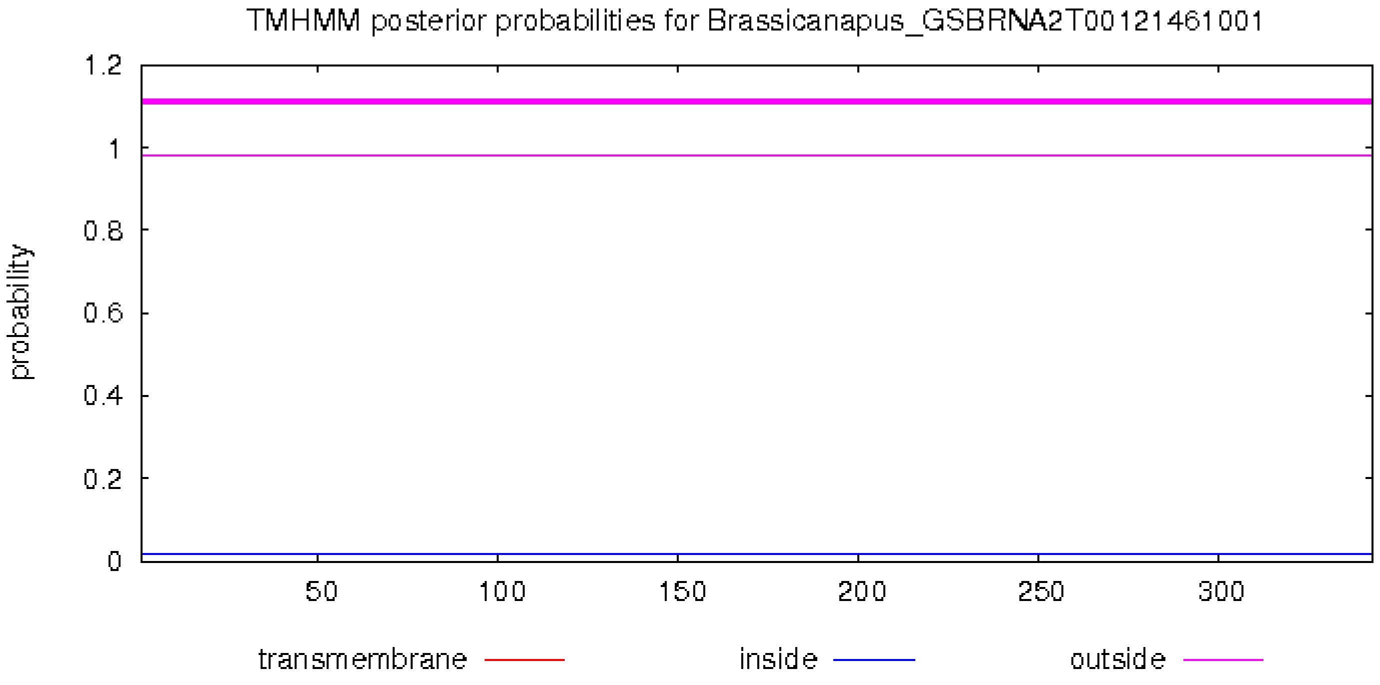

plot in postscript, script for making the plot in gnuplot, data for plot

```
# Brassicanapus_GSBRNA2T00121760001 Length: 463
# Brassicanapus_GSBRNA2T00121760001 Number of predicted TMHs:  0
# Brassicanapus_GSBRNA2T00121760001 Exp number of AAs in TMHs: 17.40184
# Brassicanapus_GSBRNA2T00121760001 Exp number, first 60 AAs:  0.00035
# Brassicanapus_GSBRNA2T00121760001 Total prob of N-in:        0.13160
Brassicanapus_GSBRNA2T00121760001       TMHMM2.0        outside      1   463
```

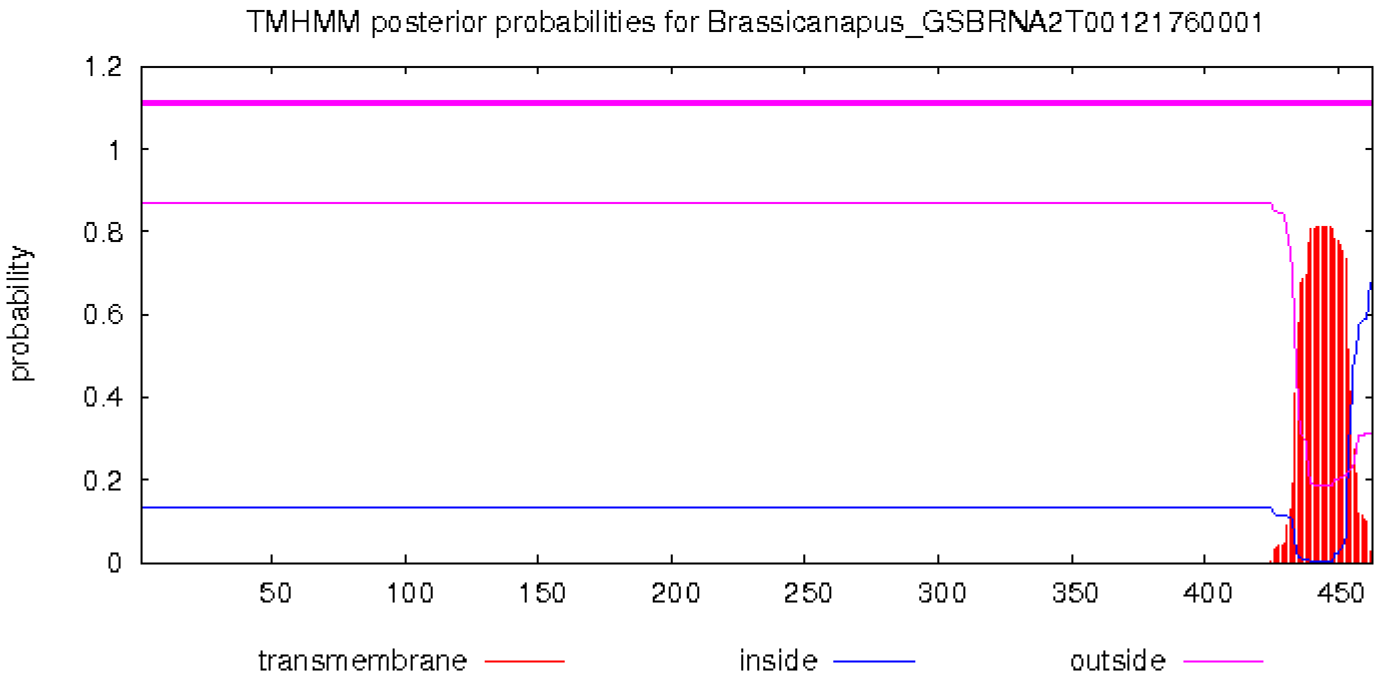

plot in postscript, script for making the plot in gnuplot, data for plot

---

```
# Brassicanapus_GSBRNA2T00123077001 Length: 471
# Brassicanapus_GSBRNA2T00123077001 Number of predicted TMHs:  0
# Brassicanapus_GSBRNA2T00123077001 Exp number of AAs in TMHs: 8.2141
# Brassicanapus_GSBRNA2T00123077001 Exp number, first 60 AAs:  0
# Brassicanapus_GSBRNA2T00123077001 Total prob of N-in:        0.32577
Brassicanapus_GSBRNA2T00123077001       TMHMM2.0        outside      1   471
```

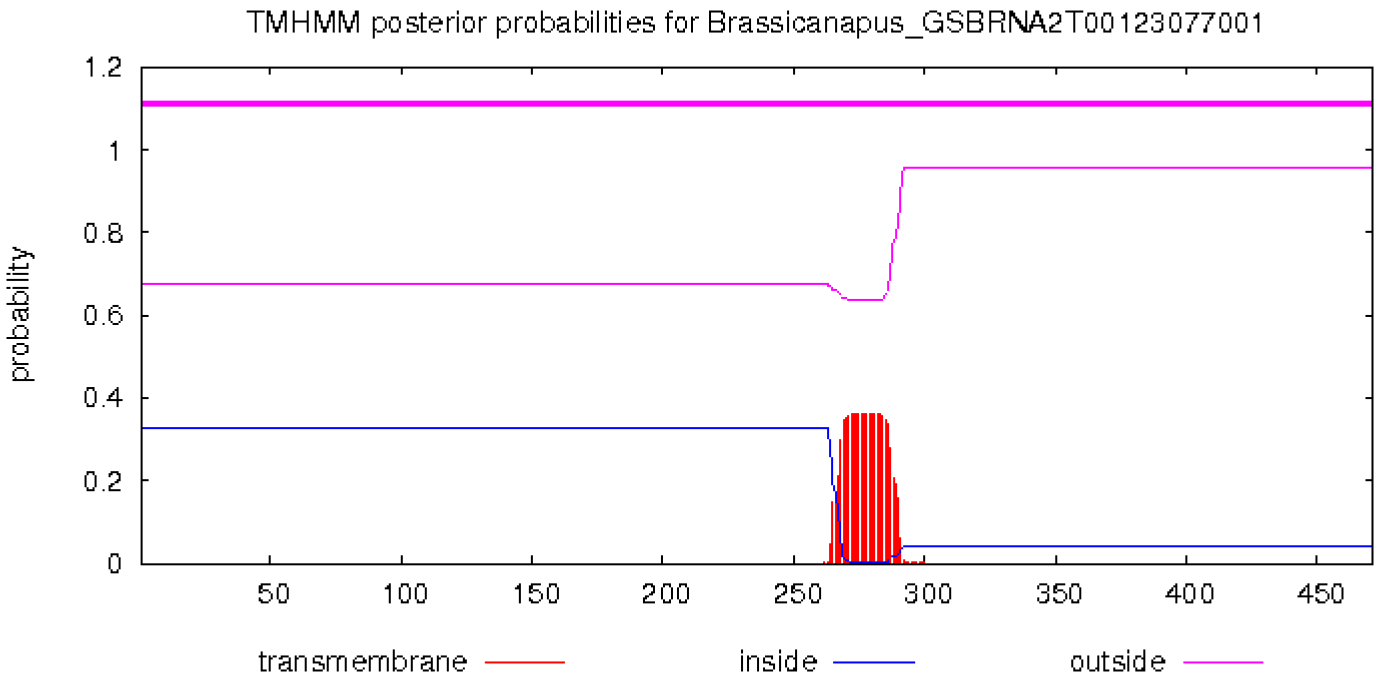

plot in postscript, script for making the plot in gnuplot, data for plot

---

```
# Brassicanapus_GSBRNA2T00123451001 Length: 556
# Brassicanapus_GSBRNA2T00123451001 Number of predicted TMHs:  0
# Brassicanapus_GSBRNA2T00123451001 Exp number of AAs in TMHs: 0.00197
# Brassicanapus_GSBRNA2T00123451001 Exp number, first 60 AAs:  0.00183
# Brassicanapus_GSBRNA2T00123451001 Total prob of N-in:        0.00243
Brassicanapus_GSBRNA2T00123451001       TMHMM2.0        outside      1   556
```

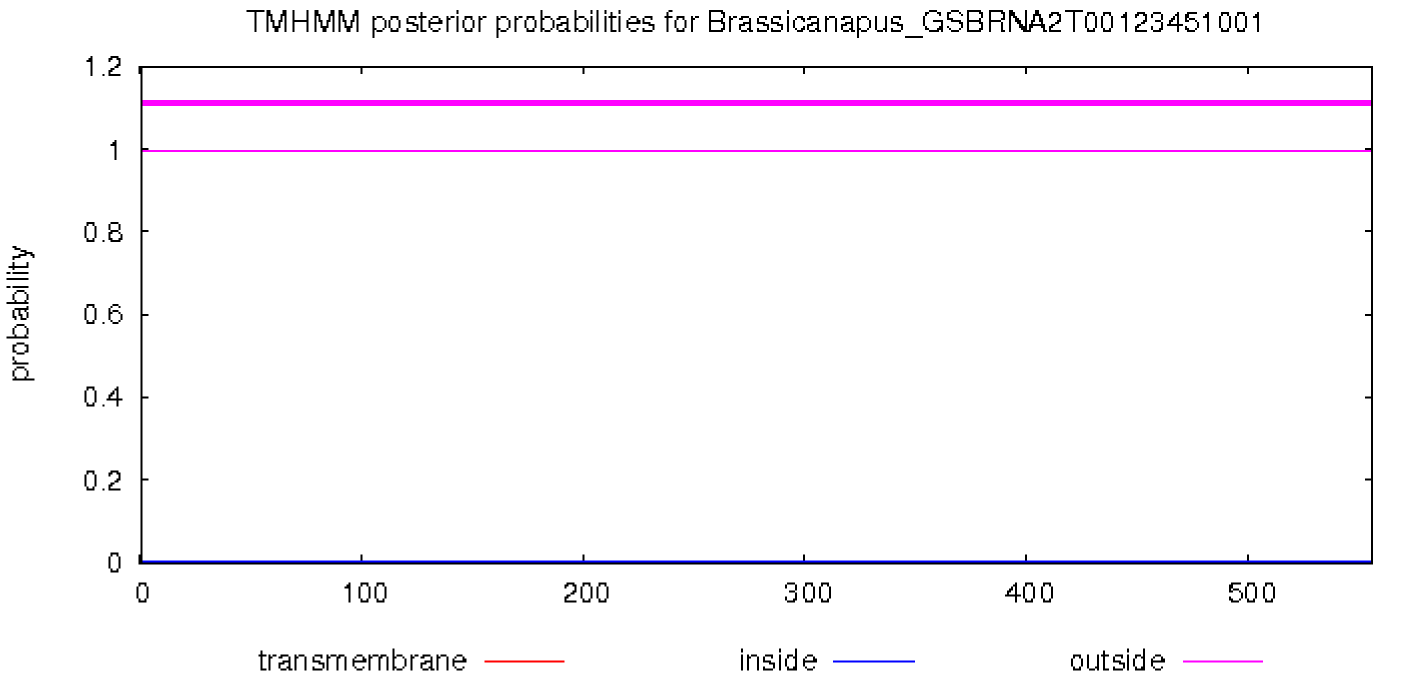

plot in postscript, script for making the plot in gnuplot, data for plot

---

```
# Brassicanapus_GSBRNA2T00123464001 Length: 603
# Brassicanapus_GSBRNA2T00123464001 Number of predicted TMHs:  0
# Brassicanapus_GSBRNA2T00123464001 Exp number of AAs in TMHs: 0
# Brassicanapus_GSBRNA2T00123464001 Exp number, first 60 AAs:  0
# Brassicanapus_GSBRNA2T00123464001 Total prob of N-in:        0.00065
Brassicanapus_GSBRNA2T00123464001       TMHMM2.0        outside      1   603
```

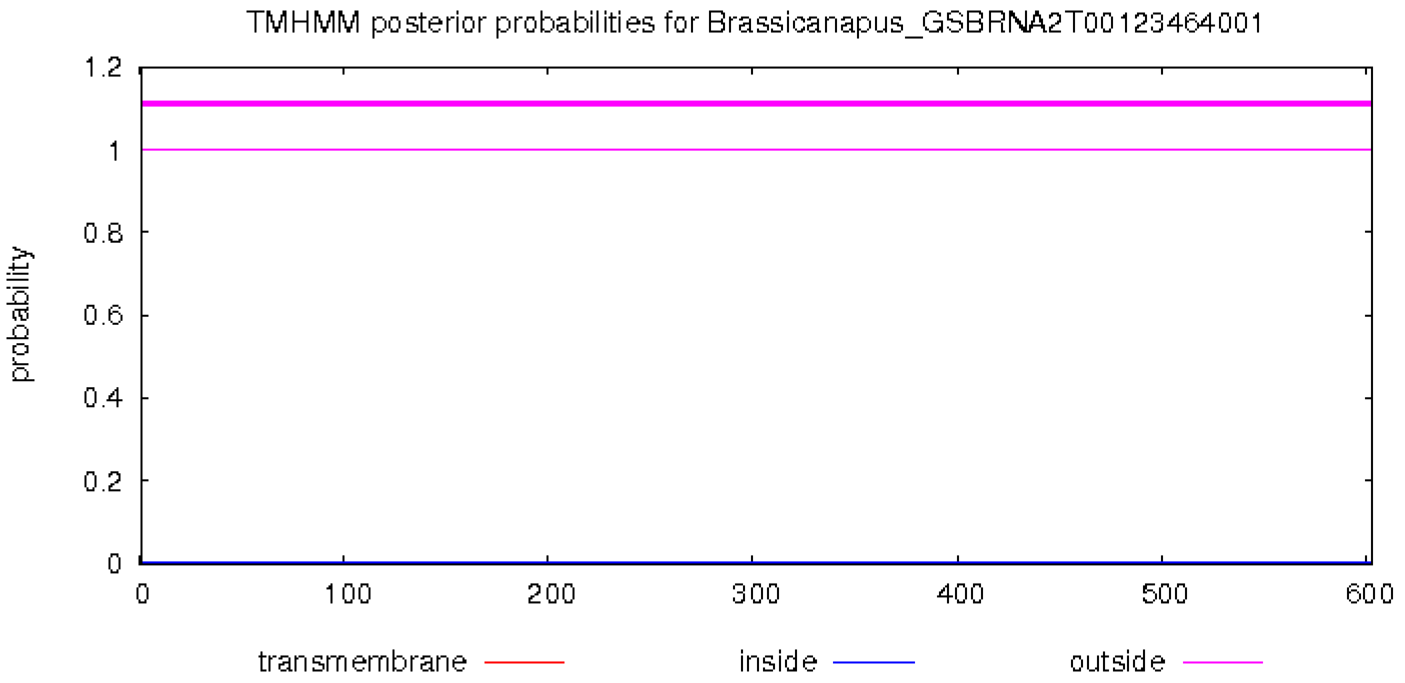

plot in postscript, script for making the plot in gnuplot, data for plot

---

```
# Brassicanapus_GSBRNA2T00123484001 Length: 371
# Brassicanapus_GSBRNA2T00123484001 Number of predicted TMHs:  0
# Brassicanapus_GSBRNA2T00123484001 Exp number of AAs in TMHs: 4.62788
# Brassicanapus_GSBRNA2T00123484001 Exp number, first 60 AAs:  0
# Brassicanapus_GSBRNA2T00123484001 Total prob of N-in:        0.02443
Brassicanapus_GSBRNA2T00123484001       TMHMM2.0        outside      1   371
```

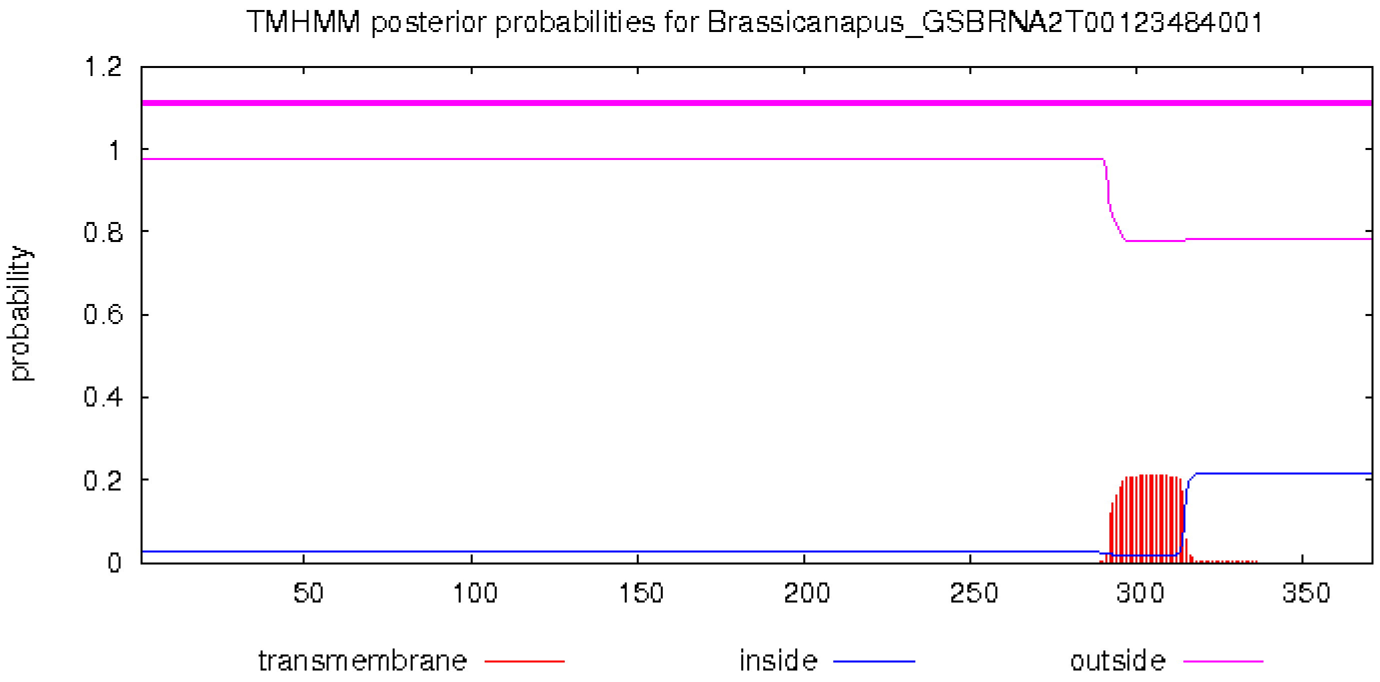

plot in postscript, script for making the plot in gnuplot, data for plot

```
# Brassicanapus_GSBRNA2T00123485001 Length: 426
# Brassicanapus_GSBRNA2T00123485001 Number of predicted TMHs:  1
# Brassicanapus_GSBRNA2T00123485001 Exp number of AAs in TMHs: 21.98136
# Brassicanapus_GSBRNA2T00123485001 Exp number, first 60 AAs:  0.00054
# Brassicanapus_GSBRNA2T00123485001 Total prob of N-in:        0.45378
Brassicanapus_GSBRNA2T00123485001       TMHMM2.0        outside      1   401
Brassicanapus_GSBRNA2T00123485001       TMHMM2.0        TMhelix    402   424
Brassicanapus_GSBRNA2T00123485001       TMHMM2.0        inside     425   426
```

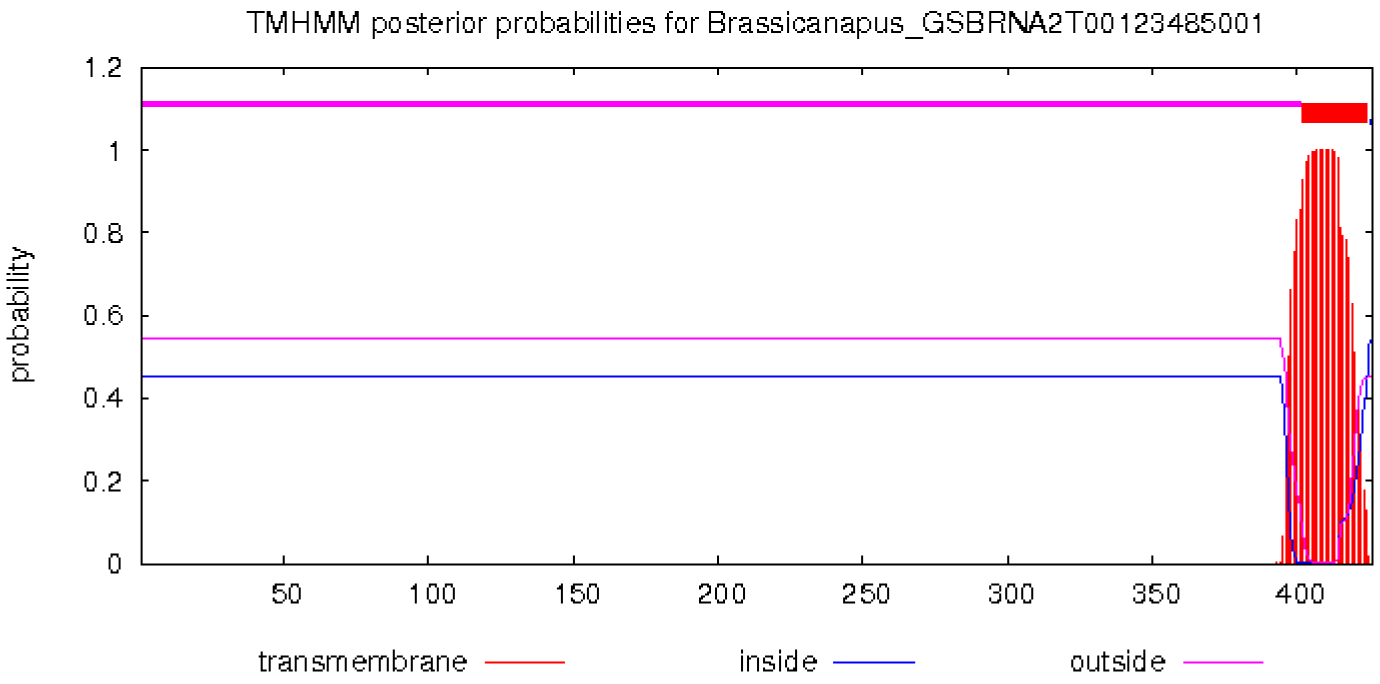

plot in postscript, script for making the plot in gnuplot, data for plot

```
# Brassicanapus_GSBRNA2T00123489001 Length: 288
# Brassicanapus_GSBRNA2T00123489001 Number of predicted TMHs:  0
# Brassicanapus_GSBRNA2T00123489001 Exp number of AAs in TMHs: 0.00018
# Brassicanapus_GSBRNA2T00123489001 Exp number, first 60 AAs:  0
# Brassicanapus_GSBRNA2T00123489001 Total prob of N-in:        0.01880
Brassicanapus_GSBRNA2T00123489001       TMHMM2.0        outside      1   288
```

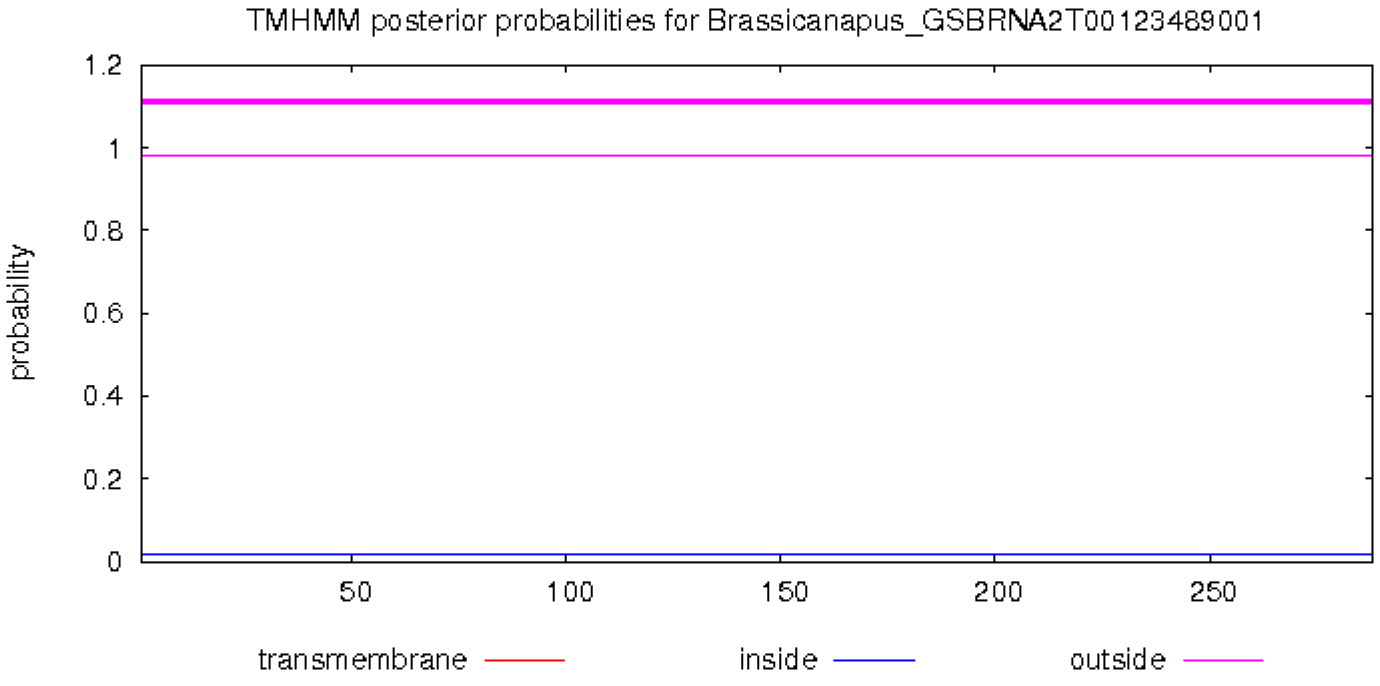

plot in postscript, script for making the plot in gnuplot, data for plot

```
# Brassicanapus_GSBRNA2T00123768001 Length: 308
# Brassicanapus_GSBRNA2T00123768001 Number of predicted TMHs:  0
# Brassicanapus_GSBRNA2T00123768001 Exp number of AAs in TMHs: 0.02152
# Brassicanapus_GSBRNA2T00123768001 Exp number, first 60 AAs:  0.00023
# Brassicanapus_GSBRNA2T00123768001 Total prob of N-in:        0.01439
Brassicanapus_GSBRNA2T00123768001       TMHMM2.0        outside      1   308
```

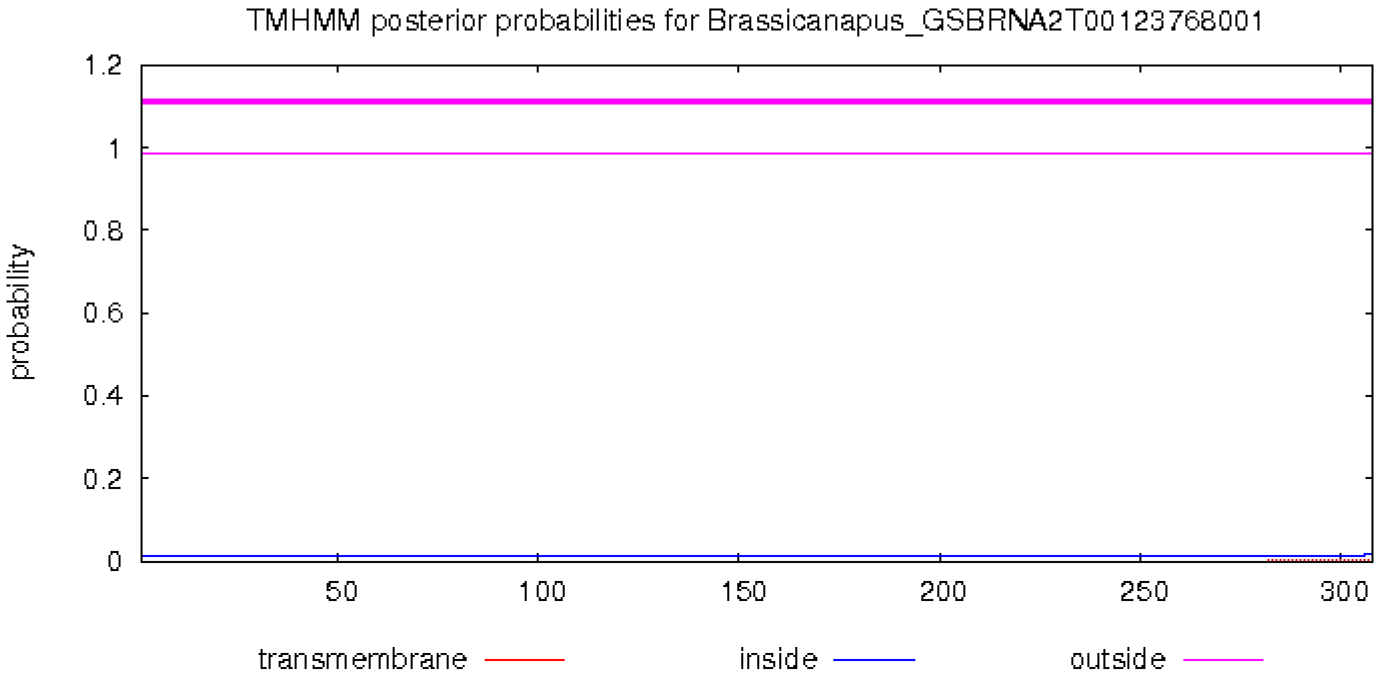

plot in postscript, script for making the plot in gnuplot, data for plot

```
# Brassicanapus_GSBRNA2T00123793001 Length: 297
# Brassicanapus_GSBRNA2T00123793001 Number of predicted TMHs:  0
# Brassicanapus_GSBRNA2T00123793001 Exp number of AAs in TMHs: 0.00039
# Brassicanapus_GSBRNA2T00123793001 Exp number, first 60 AAs:  0.0002
# Brassicanapus_GSBRNA2T00123793001 Total prob of N-in:        0.02152
Brassicanapus_GSBRNA2T00123793001	TMHMM2.0	outside	     1   297
```

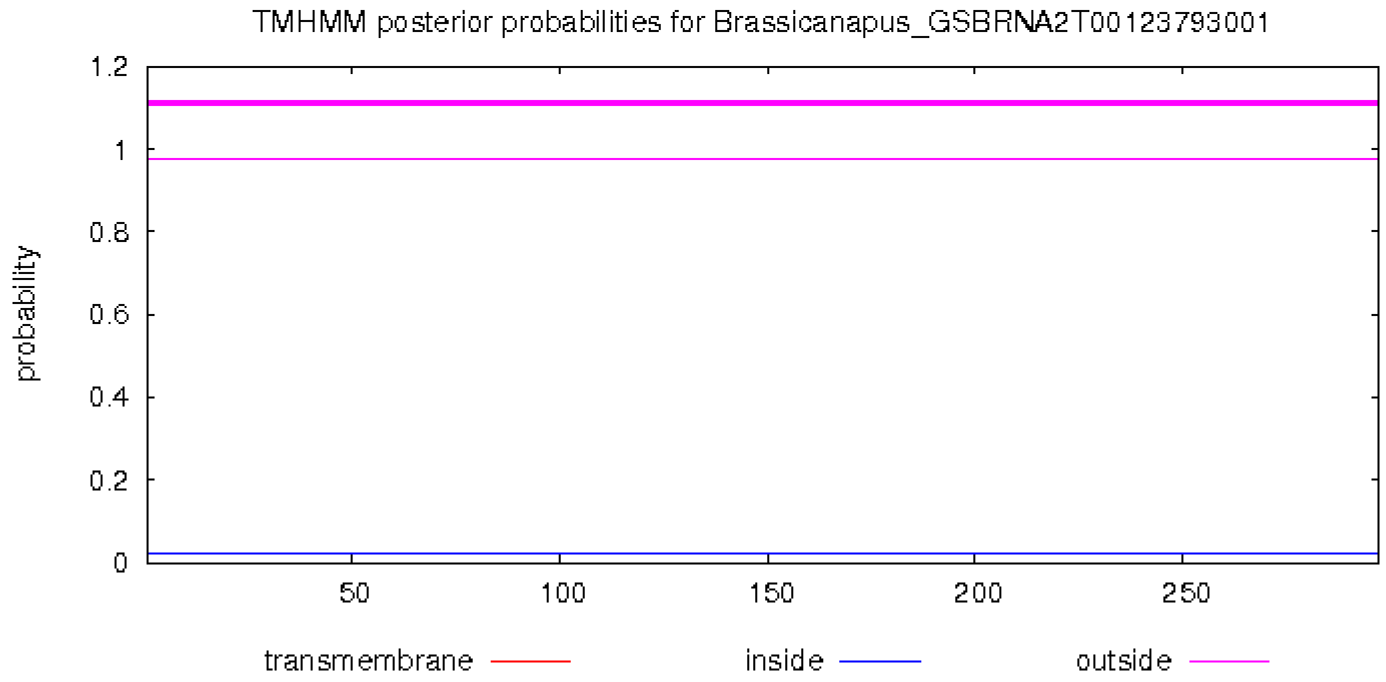

plot in postscript, script for making the plot in gnuplot, data for plot

```
# Brassicanapus_GSBRNA2T00123958001 Length: 67
# Brassicanapus_GSBRNA2T00123958001 Number of predicted TMHs:  0
# Brassicanapus_GSBRNA2T00123958001 Exp number of AAs in TMHs: 0.00018
# Brassicanapus_GSBRNA2T00123958001 Exp number, first 60 AAs:  0.00018
# Brassicanapus_GSBRNA2T00123958001 Total prob of N-in:        0.27975
Brassicanapus_GSBRNA2T00123958001	TMHMM2.0	outside	     1    67
```

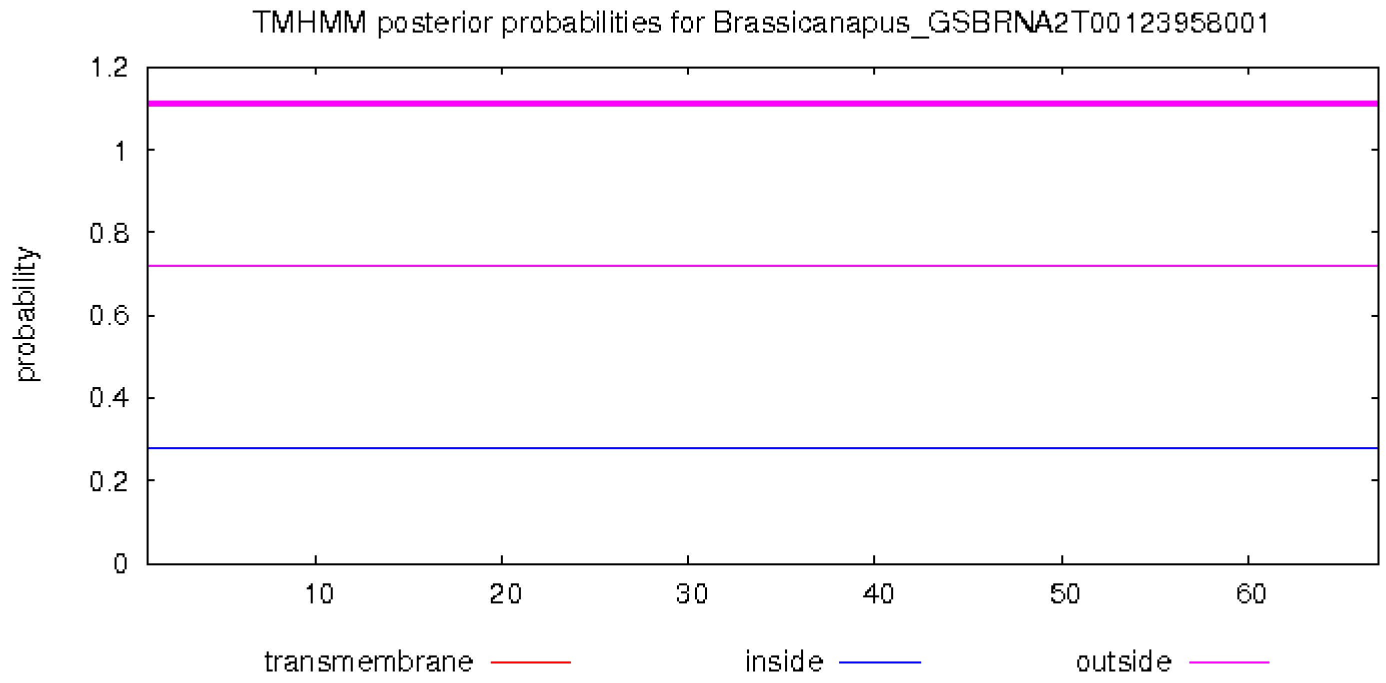

plot in postscript, script for making the plot in gnuplot, data for plot

```
# Brassicanapus_GSBRNA2T00123999001 Length: 281
# Brassicanapus_GSBRNA2T00123999001 Number of predicted TMHs:  0
# Brassicanapus_GSBRNA2T00123999001 Exp number of AAs in TMHs: 0.01805
# Brassicanapus_GSBRNA2T00123999001 Exp number, first 60 AAs:  0
# Brassicanapus_GSBRNA2T00123999001 Total prob of N-in:        0.00679
Brassicanapus_GSBRNA2T00123999001	TMHMM2.0	outside	     1   281
```

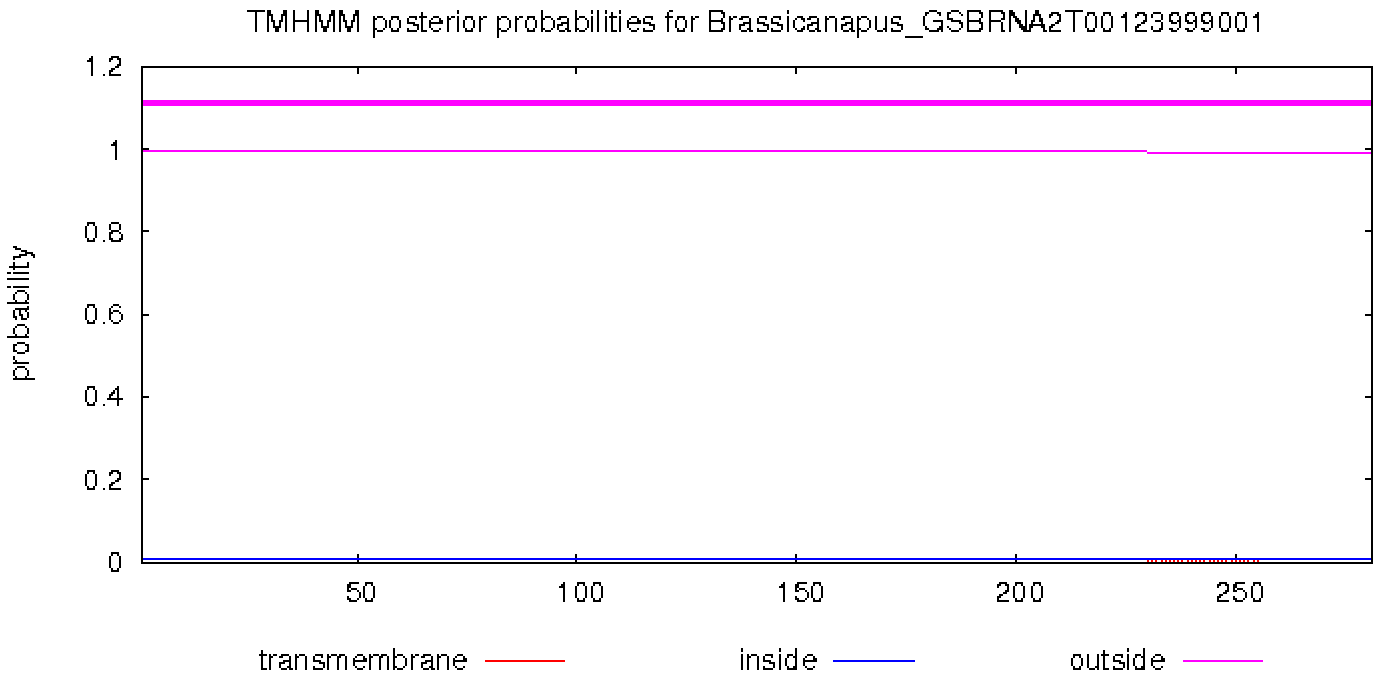

plot in postscript, script for making the plot in gnuplot, data for plot

---

```
# Brassicanapus_GSBRNA2T00124306001 Length: 271
# Brassicanapus_GSBRNA2T00124306001 Number of predicted TMHs:  0
# Brassicanapus_GSBRNA2T00124306001 Exp number of AAs in TMHs: 0.01141
# Brassicanapus_GSBRNA2T00124306001 Exp number, first 60 AAs:  0
# Brassicanapus_GSBRNA2T00124306001 Total prob of N-in:        0.07969
Brassicanapus_GSBRNA2T00124306001	TMHMM2.0	outside	     1   271
```

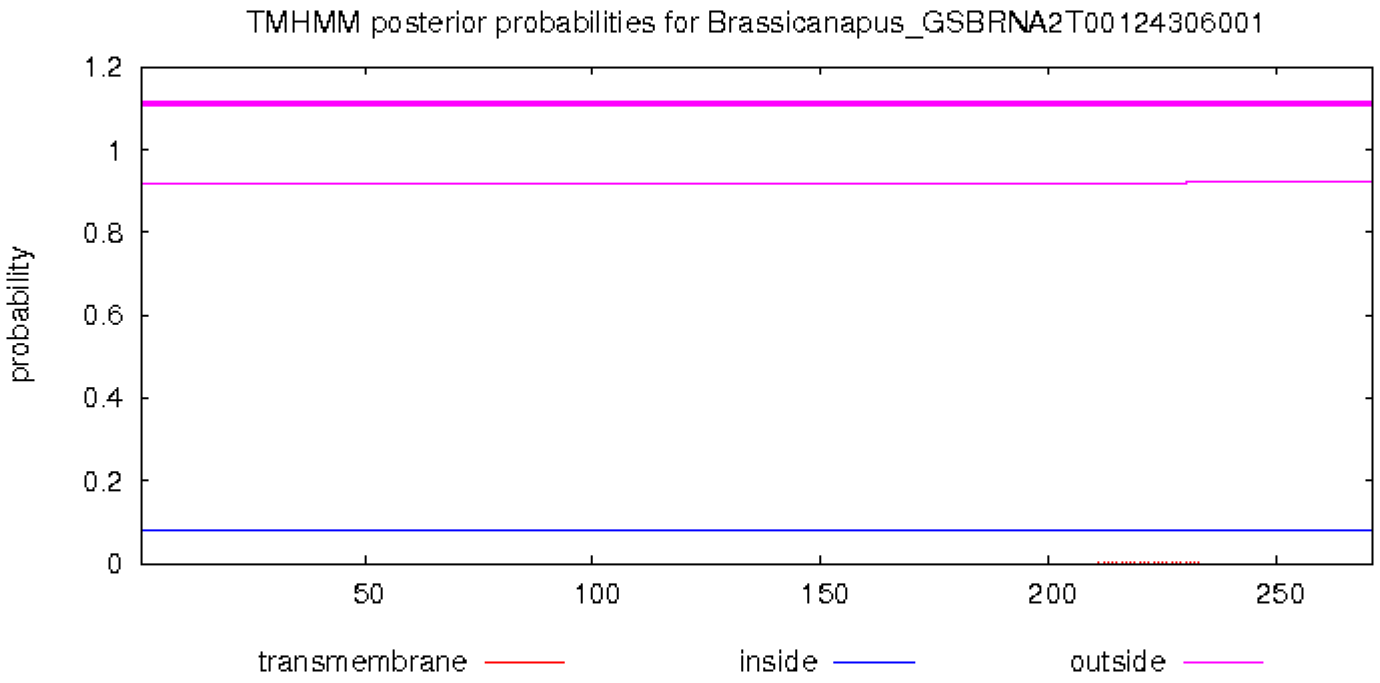

plot in postscript, script for making the plot in gnuplot, data for plot

---

```
# Brassicanapus_GSBRNA2T00124696001 Length: 260
# Brassicanapus_GSBRNA2T00124696001 Number of predicted TMHs:  0
# Brassicanapus_GSBRNA2T00124696001 Exp number of AAs in TMHs: 2.46268
# Brassicanapus_GSBRNA2T00124696001 Exp number, first 60 AAs:  2.45868
# Brassicanapus_GSBRNA2T00124696001 Total prob of N-in:        0.17935
Brassicanapus_GSBRNA2T00124696001       TMHMM2.0        outside      1   260
```

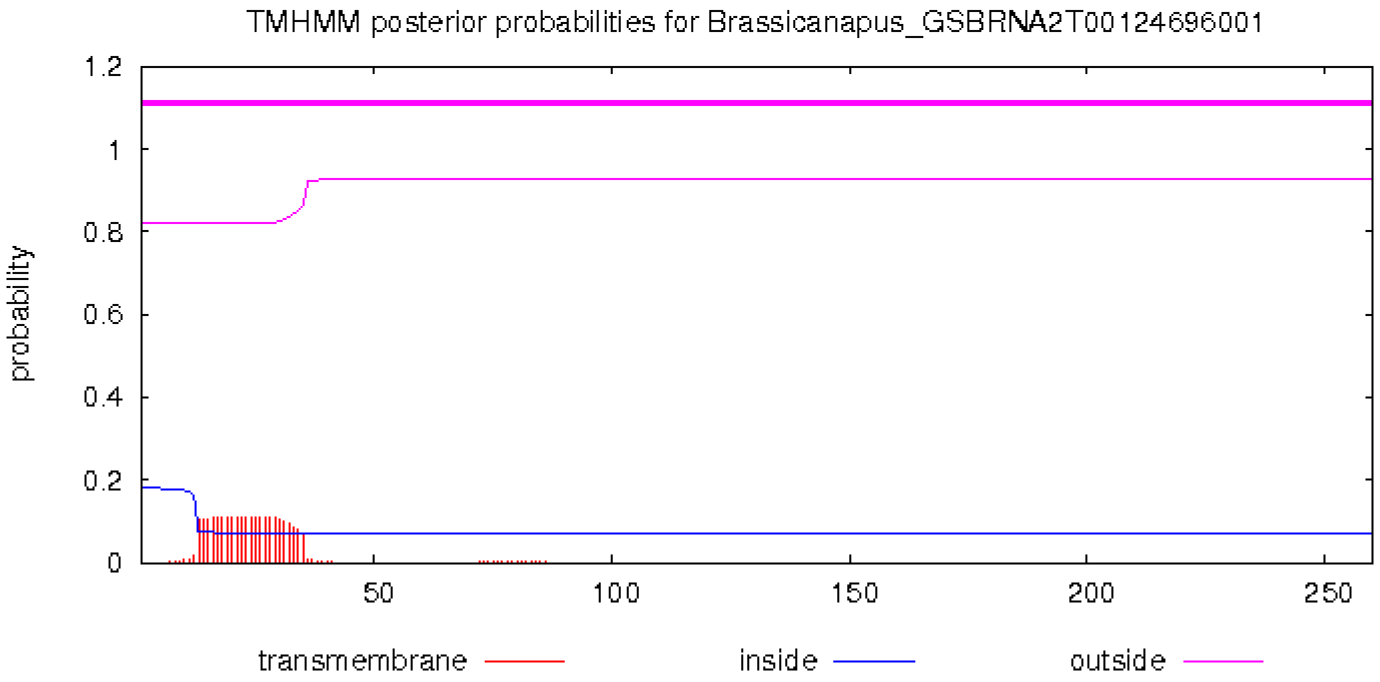

plot in postscript, script for making the plot in gnuplot, data for plot

---

```
# Brassicanapus_GSBRNA2T00125430001 Length: 464
# Brassicanapus_GSBRNA2T00125430001 Number of predicted TMHs:  0
# Brassicanapus_GSBRNA2T00125430001 Exp number of AAs in TMHs: 18.60941
# Brassicanapus_GSBRNA2T00125430001 Exp number, first 60 AAs:  0.00055
# Brassicanapus_GSBRNA2T00125430001 Total prob of N-in:        0.22658
Brassicanapus_GSBRNA2T00125430001       TMHMM2.0        outside      1   464
```

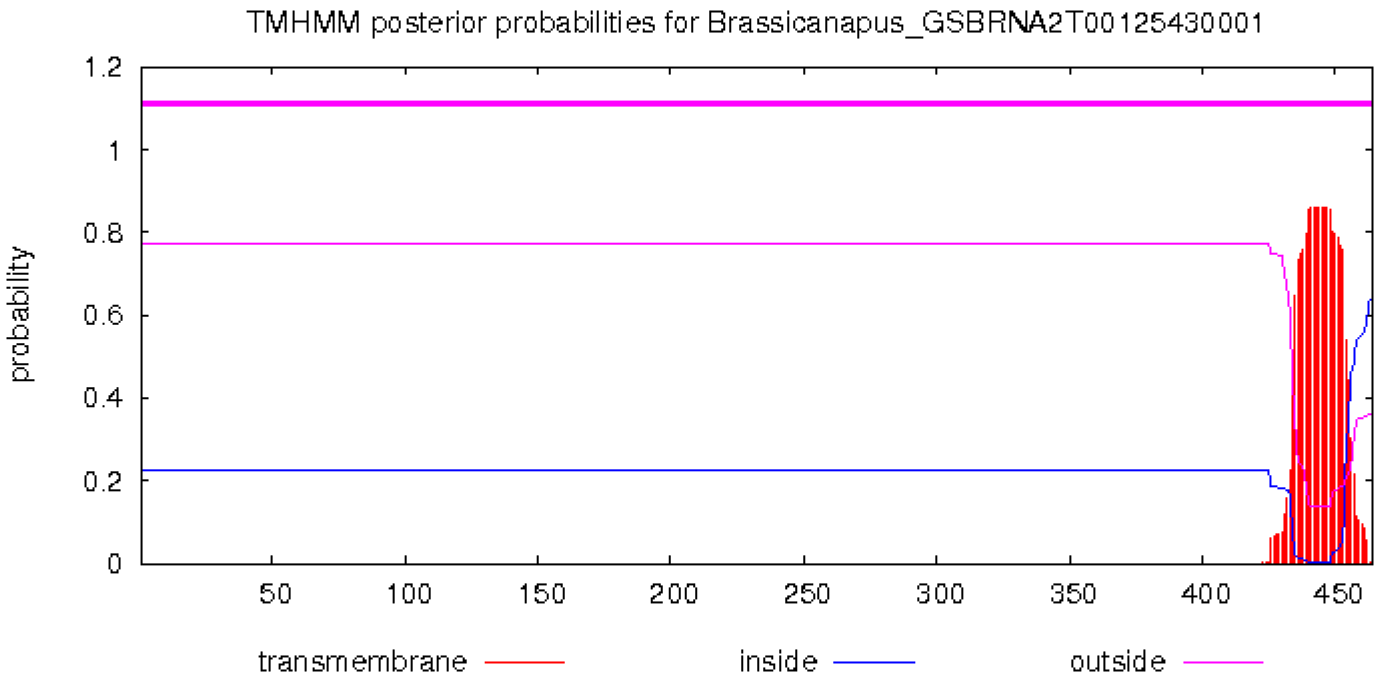

plot in postscript, script for making the plot in gnuplot, data for plot

---

```
# Brassicanapus_GSBRNA2T00125625001 Length: 365
# Brassicanapus_GSBRNA2T00125625001 Number of predicted TMHs:  0
# Brassicanapus_GSBRNA2T00125625001 Exp number of AAs in TMHs: 0.00019
# Brassicanapus_GSBRNA2T00125625001 Exp number, first 60 AAs:  0
# Brassicanapus_GSBRNA2T00125625001 Total prob of N-in:        0.00850
Brassicanapus_GSBRNA2T00125625001	TMHMM2.0	outside	     1   365
```

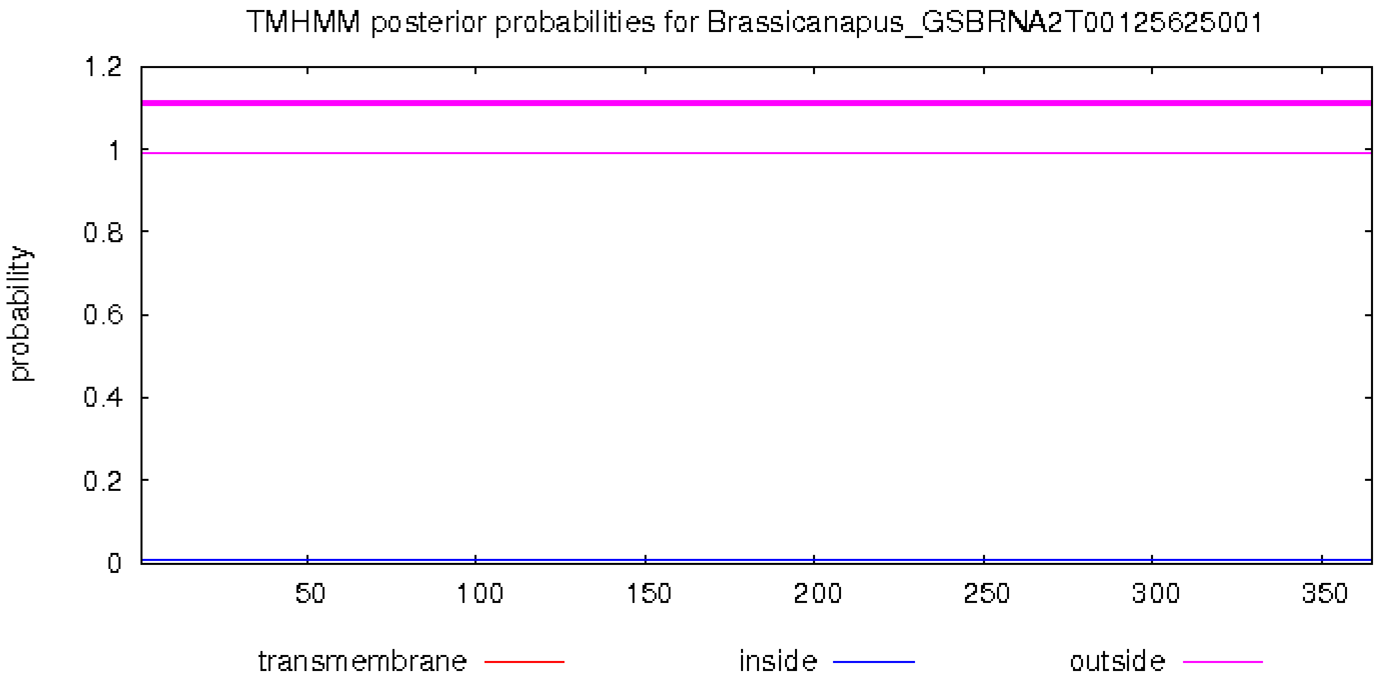

plot in postscript, script for making the plot in gnuplot, data for plot

```
# Brassicanapus_GSBRNA2T00125630001 Length: 361
# Brassicanapus_GSBRNA2T00125630001 Number of predicted TMHs:  0
# Brassicanapus_GSBRNA2T00125630001 Exp number of AAs in TMHs: 0
# Brassicanapus_GSBRNA2T00125630001 Exp number, first 60 AAs:  0
# Brassicanapus_GSBRNA2T00125630001 Total prob of N-in:        0.00902
Brassicanapus_GSBRNA2T00125630001	TMHMM2.0	outside	     1   361
```

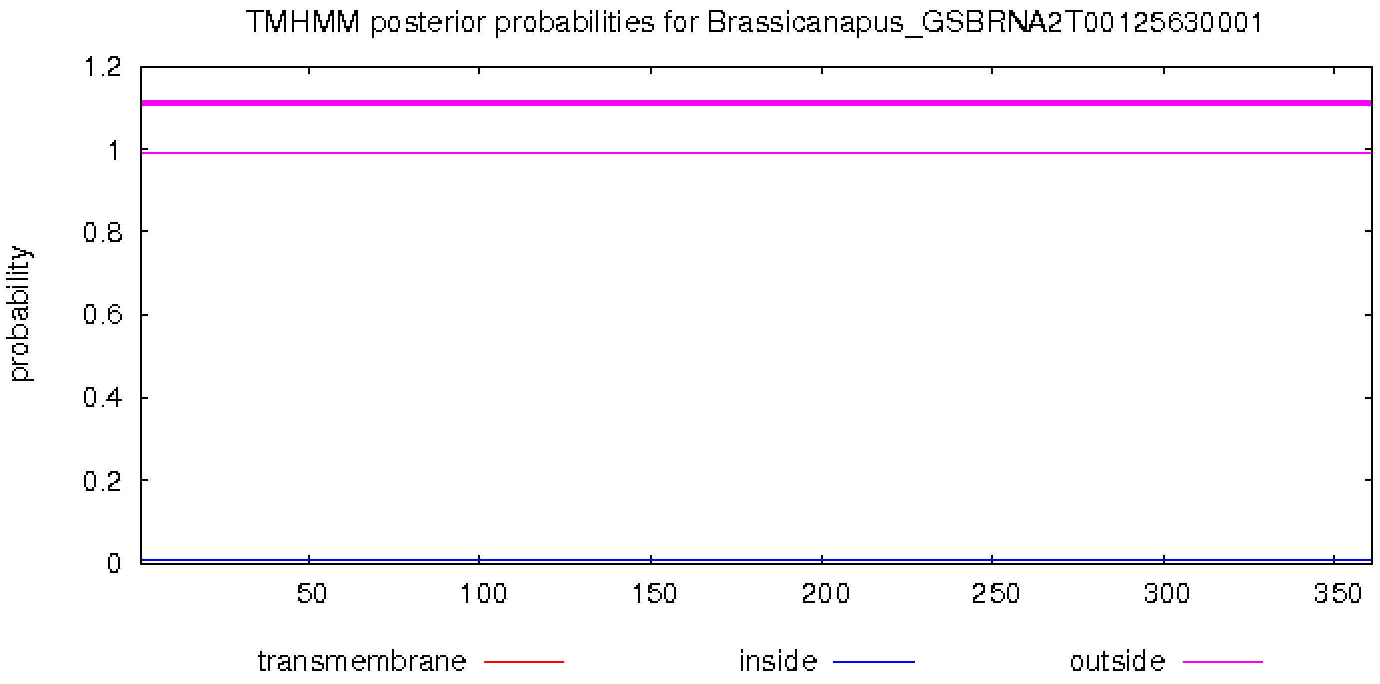

plot in postscript, script for making the plot in gnuplot, data for plot

```
# Brassicanapus_GSBRNA2T00125806001 Length: 235
# Brassicanapus_GSBRNA2T00125806001 Number of predicted TMHs:  0
# Brassicanapus_GSBRNA2T00125806001 Exp number of AAs in TMHs: 0.0014
# Brassicanapus_GSBRNA2T00125806001 Exp number, first 60 AAs:  0
# Brassicanapus_GSBRNA2T00125806001 Total prob of N-in:        0.10267
Brassicanapus_GSBRNA2T00125806001       TMHMM2.0        outside      1   235
```

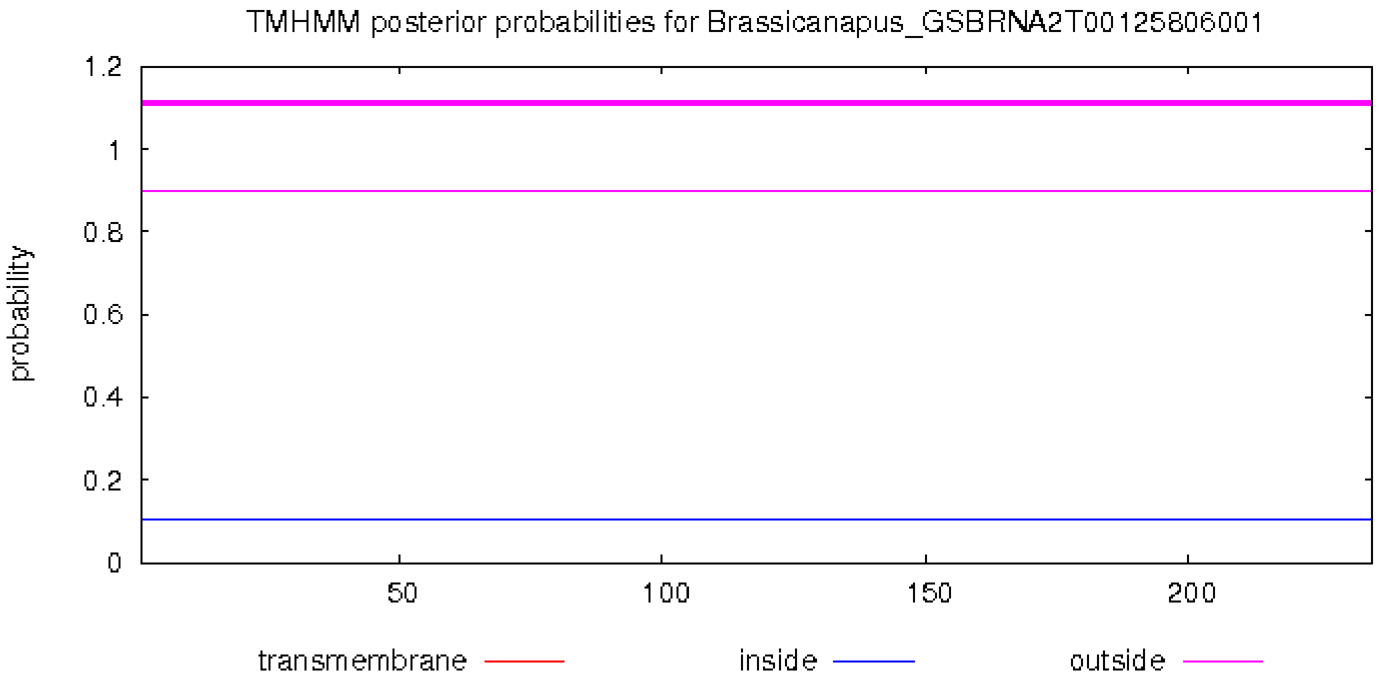

plot in postscript, script for making the plot in gnuplot, data for plot

---

```
# Brassicanapus_GSBRNA2T00126210001 Length: 237
# Brassicanapus_GSBRNA2T00126210001 Number of predicted TMHs:  0
# Brassicanapus_GSBRNA2T00126210001 Exp number of AAs in TMHs: 0.00243
# Brassicanapus_GSBRNA2T00126210001 Exp number, first 60 AAs:  0.00089
# Brassicanapus_GSBRNA2T00126210001 Total prob of N-in:        0.19181
Brassicanapus_GSBRNA2T00126210001       TMHMM2.0        outside      1   237
```

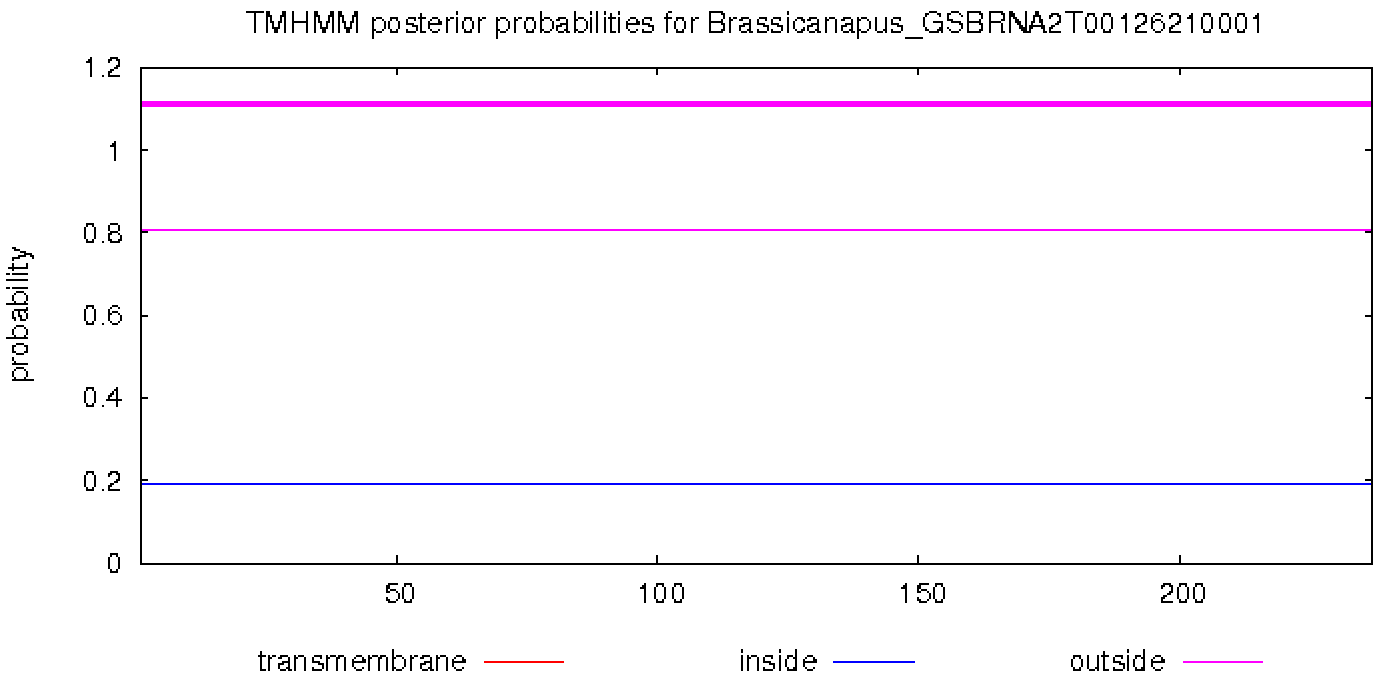

plot in postscript, script for making the plot in gnuplot, data for plot

---

```
# Brassicanapus_GSBRNA2T00127050001 Length: 332
# Brassicanapus_GSBRNA2T00127050001 Number of predicted TMHs:  0
# Brassicanapus_GSBRNA2T00127050001 Exp number of AAs in TMHs: 0
# Brassicanapus_GSBRNA2T00127050001 Exp number, first 60 AAs:  0
# Brassicanapus_GSBRNA2T00127050001 Total prob of N-in:        0.03048
Brassicanapus_GSBRNA2T00127050001	TMHMM2.0	outside	     1   332
```

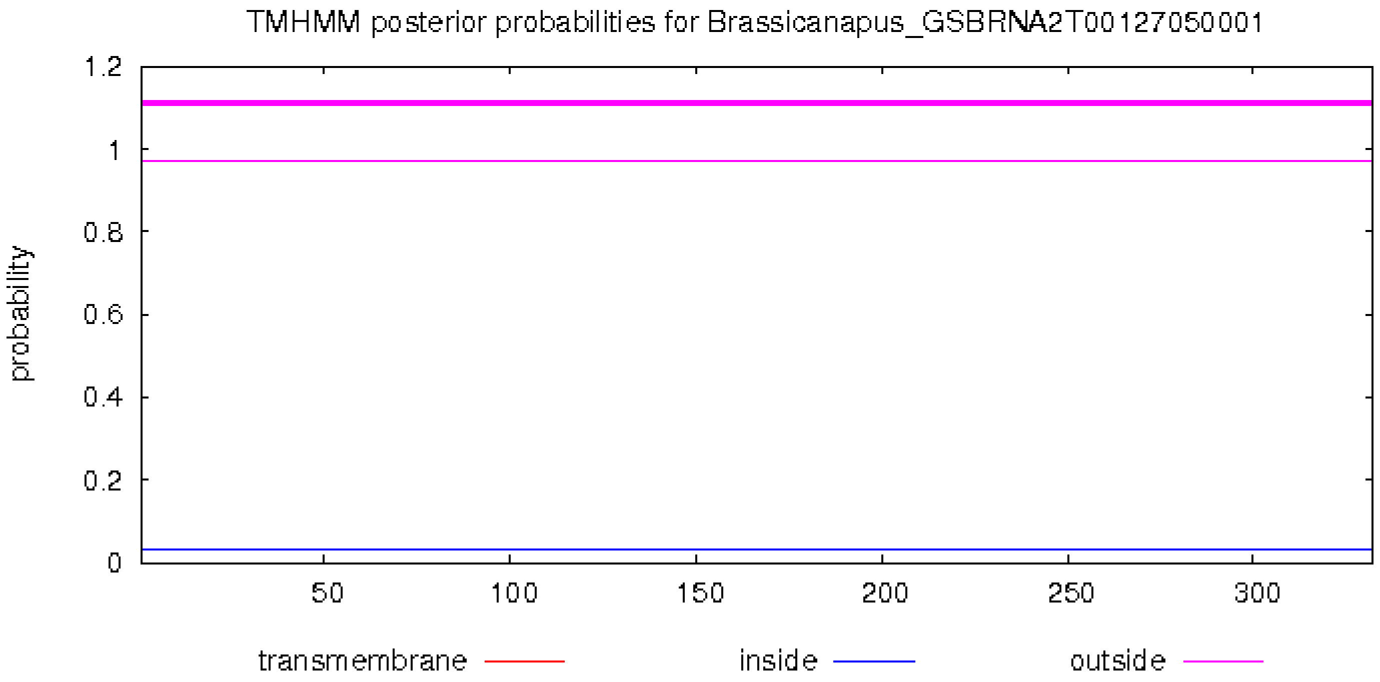

plot in postscript, script for making the plot in gnuplot, data for plot

---

```
# Brassicanapus_GSBRNA2T00127318001 Length: 317
# Brassicanapus_GSBRNA2T00127318001 Number of predicted TMHs:  0
# Brassicanapus_GSBRNA2T00127318001 Exp number of AAs in TMHs: 0.00638
# Brassicanapus_GSBRNA2T00127318001 Exp number, first 60 AAs:  0
# Brassicanapus_GSBRNA2T00127318001 Total prob of N-in:        0.07271
Brassicanapus_GSBRNA2T00127318001	TMHMM2.0	outside	     1   317
```

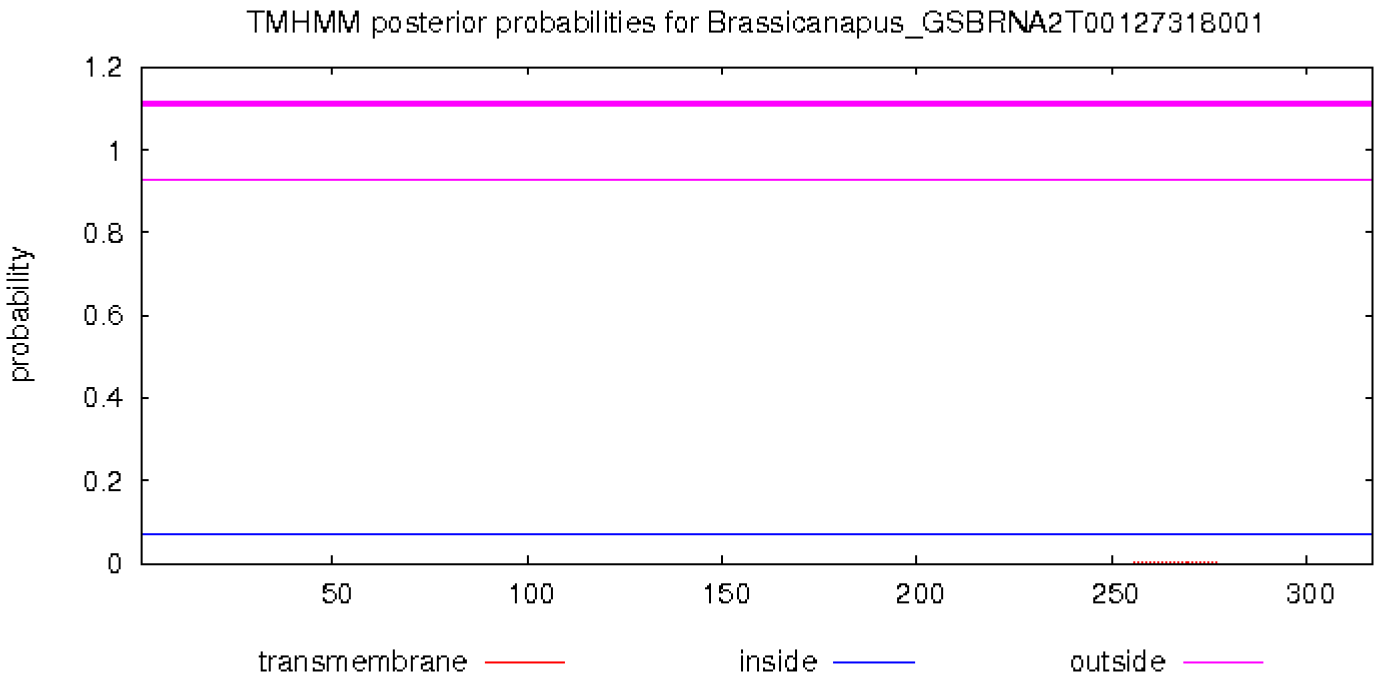

plot in postscript, script for making the plot in gnuplot, data for plot

---

```
# Brassicanapus_GSBRNA2T00128635001 Length: 284
# Brassicanapus_GSBRNA2T00128635001 Number of predicted TMHs:  0
# Brassicanapus_GSBRNA2T00128635001 Exp number of AAs in TMHs: 0
# Brassicanapus_GSBRNA2T00128635001 Exp number, first 60 AAs:  0
# Brassicanapus_GSBRNA2T00128635001 Total prob of N-in:        0.00172
Brassicanapus_GSBRNA2T00128635001	TMHMM2.0	outside	     1   284
```

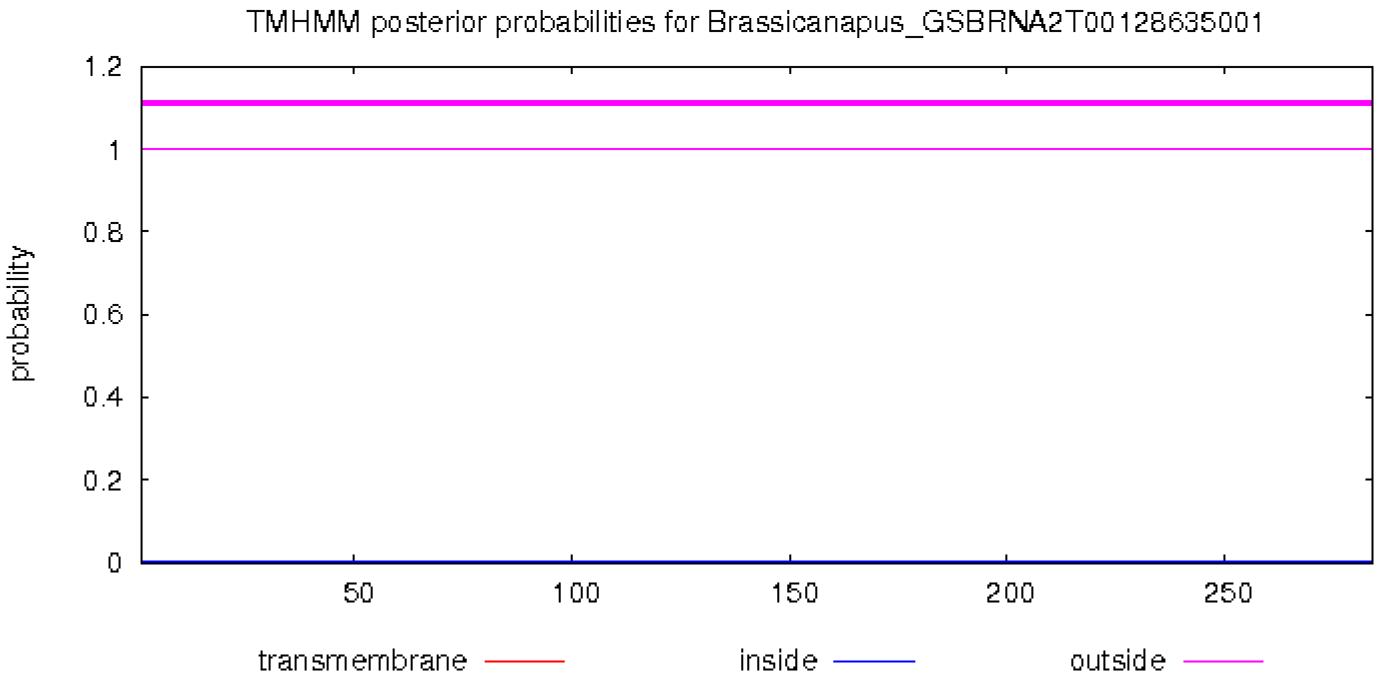

plot in postscript, script for making the plot in gnuplot, data for plot

---

```
# Brassicanapus_GSBRNA2T00129716001 Length: 262
# Brassicanapus_GSBRNA2T00129716001 Number of predicted TMHs:  0
# Brassicanapus_GSBRNA2T00129716001 Exp number of AAs in TMHs: 0.00038
# Brassicanapus_GSBRNA2T00129716001 Exp number, first 60 AAs:  0.00019
# Brassicanapus_GSBRNA2T00129716001 Total prob of N-in:        0.01555
Brassicanapus_GSBRNA2T00129716001	TMHMM2.0	outside	     1   262
```

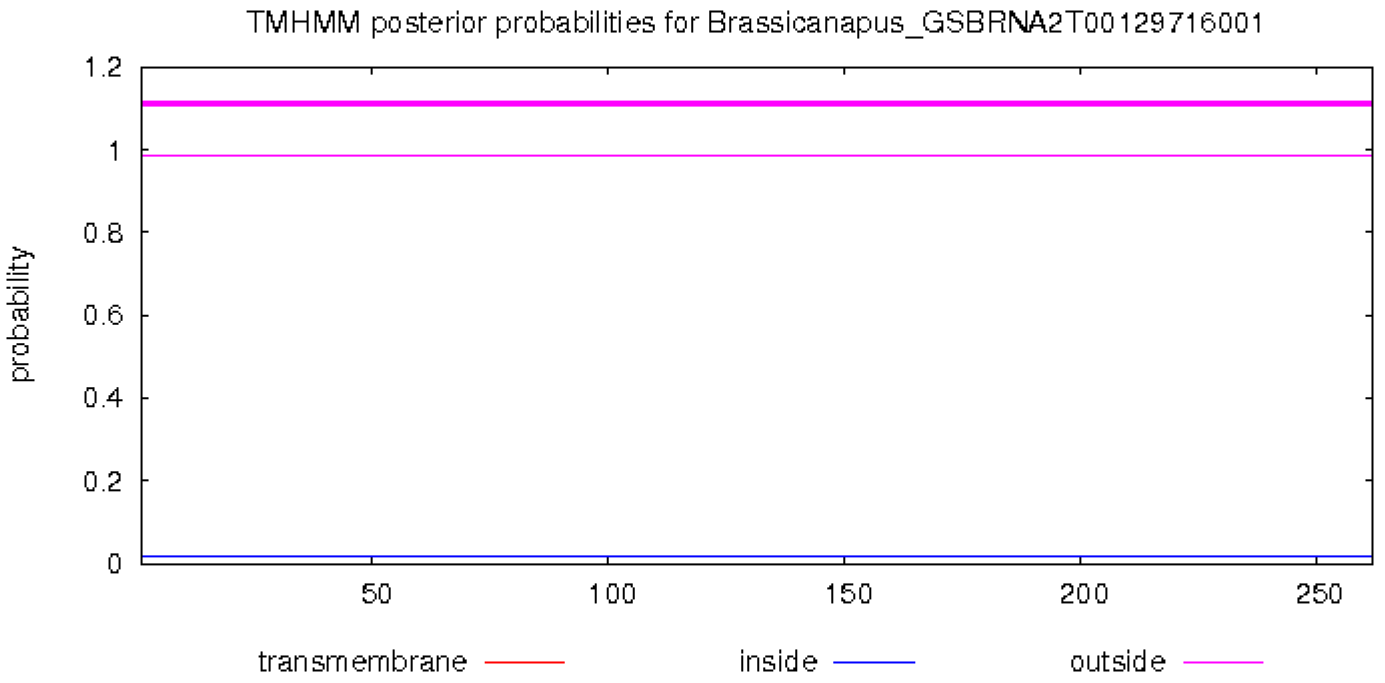

plot in postscript, script for making the plot in gnuplot, data for plot

---

```
# Brassicanapus_GSBRNA2T00129721001 Length: 363
# Brassicanapus_GSBRNA2T00129721001 Number of predicted TMHs:  0
# Brassicanapus_GSBRNA2T00129721001 Exp number of AAs in TMHs: 0.00018
# Brassicanapus_GSBRNA2T00129721001 Exp number, first 60 AAs:  0.00018
# Brassicanapus_GSBRNA2T00129721001 Total prob of N-in:        0.00418
Brassicanapus_GSBRNA2T00129721001	TMHMM2.0	outside	     1   363
```

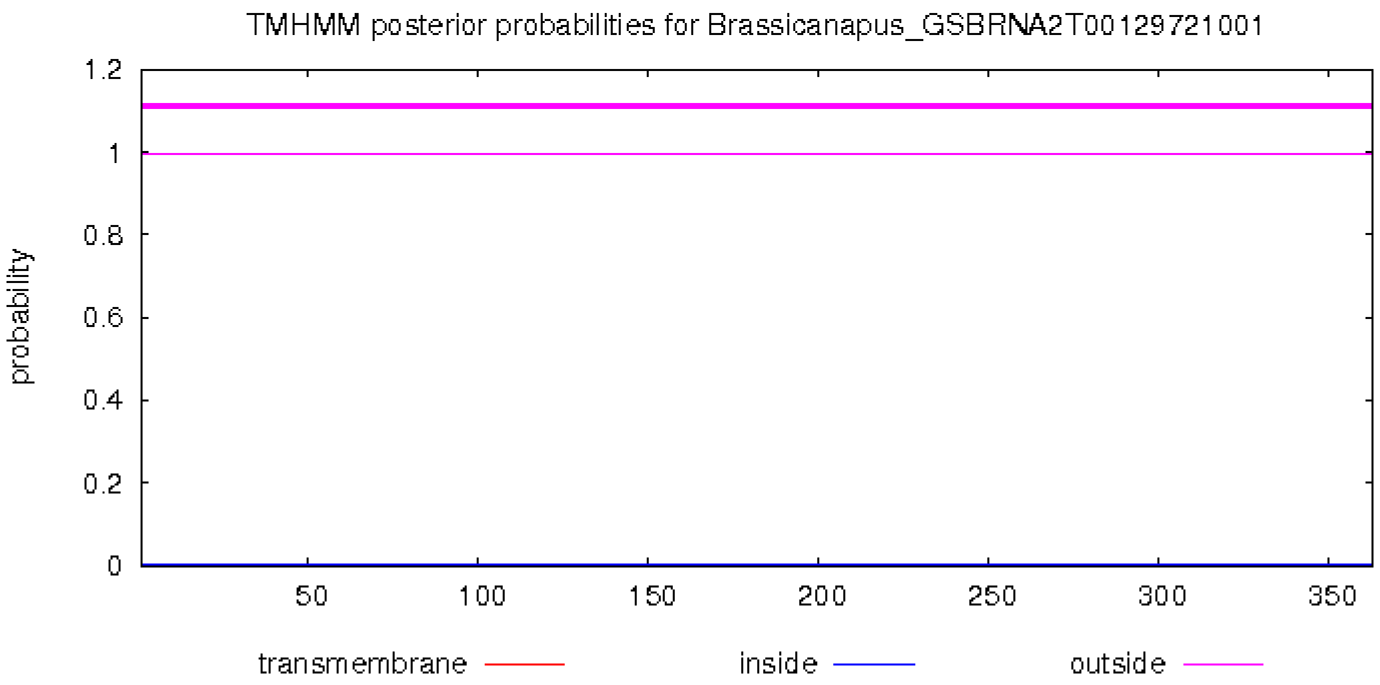

plot in postscript, script for making the plot in gnuplot, data for plot

---

```
# Brassicanapus_GSBRNA2T00129913001 Length: 248
# Brassicanapus_GSBRNA2T00129913001 Number of predicted TMHs:  0
# Brassicanapus_GSBRNA2T00129913001 Exp number of AAs in TMHs: 0.00056
# Brassicanapus_GSBRNA2T00129913001 Exp number, first 60 AAs:  0
# Brassicanapus_GSBRNA2T00129913001 Total prob of N-in:        0.16189
Brassicanapus_GSBRNA2T00129913001	TMHMM2.0	outside	     1   248
```

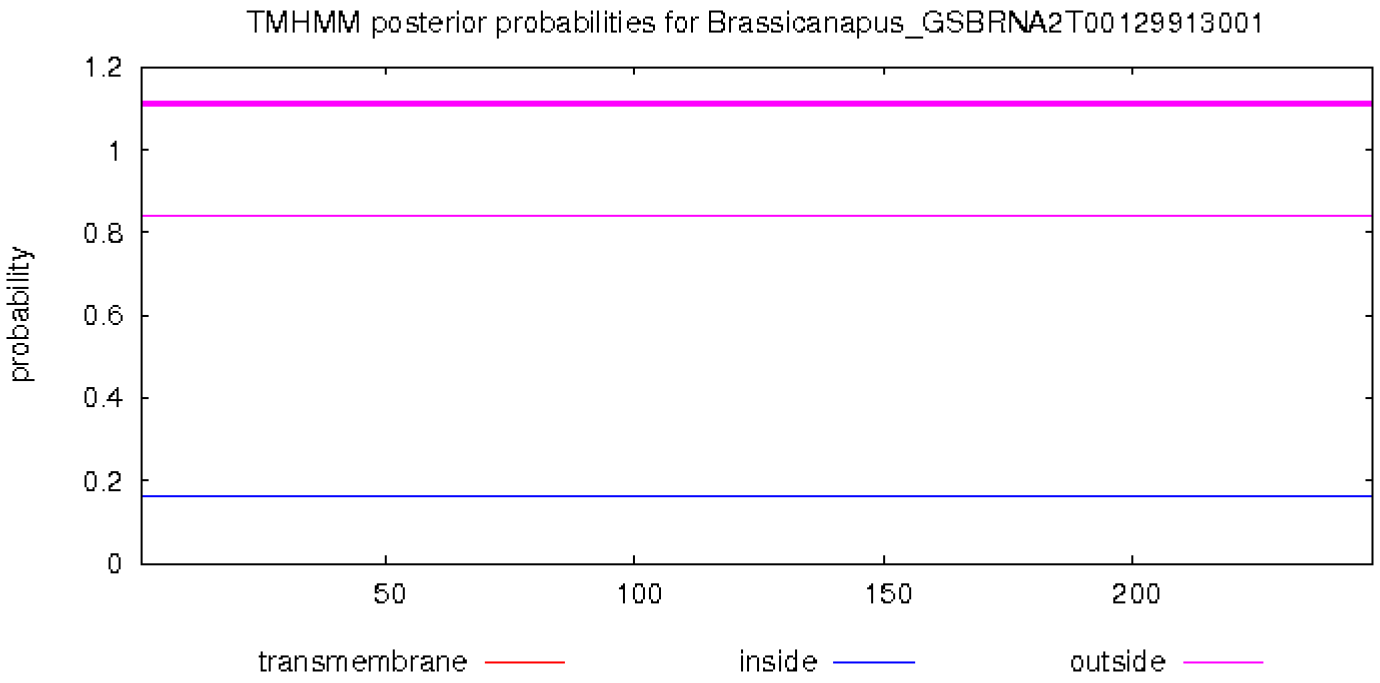

plot in postscript, script for making the plot in gnuplot, data for plot

---

```
# Brassicanapus_GSBRNA2T00129957001 Length: 208
# Brassicanapus_GSBRNA2T00129957001 Number of predicted TMHs:  0
# Brassicanapus_GSBRNA2T00129957001 Exp number of AAs in TMHs: 0.54982
# Brassicanapus_GSBRNA2T00129957001 Exp number, first 60 AAs:  0.51659
# Brassicanapus_GSBRNA2T00129957001 Total prob of N-in:        0.13834
Brassicanapus_GSBRNA2T00129957001	TMHMM2.0	outside	     1   208
```

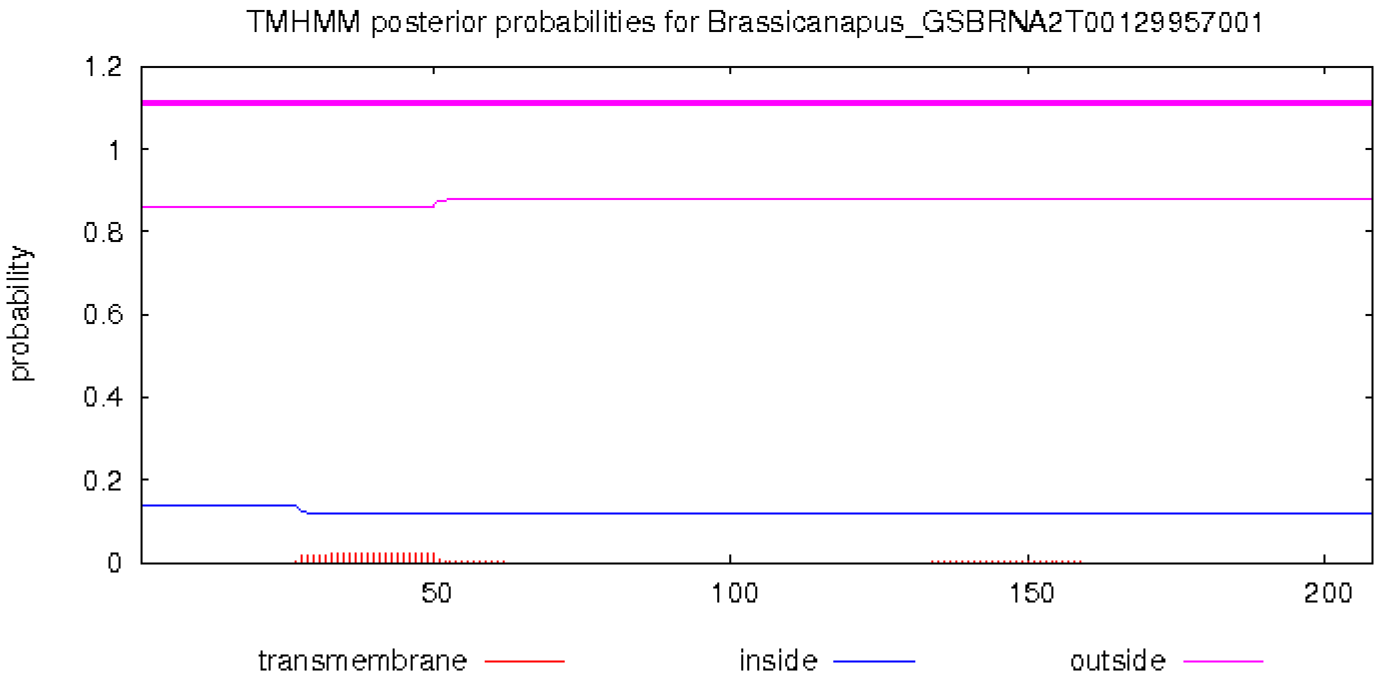

plot in postscript, script for making the plot in gnuplot, data for plot

---

```
# Brassicanapus_GSBRNA2T00129990001 Length: 297
# Brassicanapus_GSBRNA2T00129990001 Number of predicted TMHs:  0
# Brassicanapus_GSBRNA2T00129990001 Exp number of AAs in TMHs: 0.00023
# Brassicanapus_GSBRNA2T00129990001 Exp number, first 60 AAs:  0
# Brassicanapus_GSBRNA2T00129990001 Total prob of N-in:        0.05235
Brassicanapus_GSBRNA2T00129990001	TMHMM2.0	outside	     1   297
```

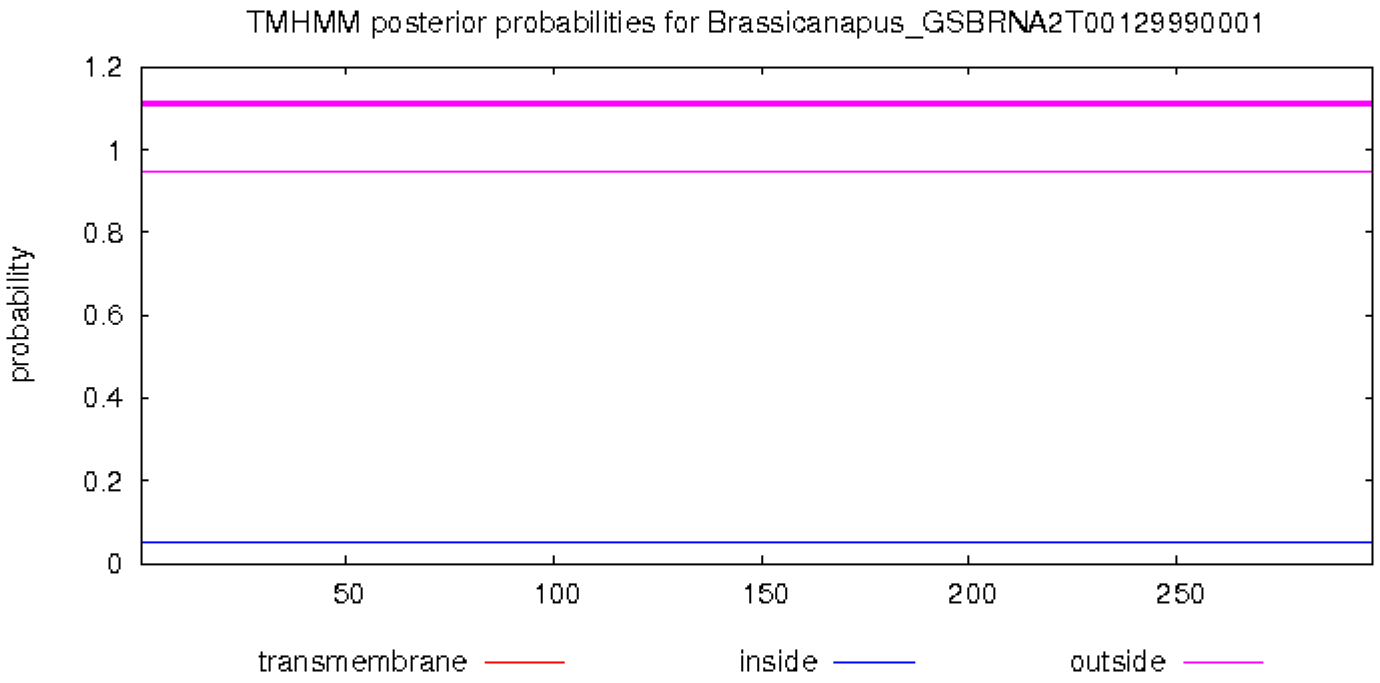

plot in postscript, script for making the plot in gnuplot, data for plot

---

```
# Brassicanapus_GSBRNA2T00130163001 Length: 490
# Brassicanapus_GSBRNA2T00130163001 Number of predicted TMHs:  0
# Brassicanapus_GSBRNA2T00130163001 Exp number of AAs in TMHs: 0
# Brassicanapus_GSBRNA2T00130163001 Exp number, first 60 AAs:  0
# Brassicanapus_GSBRNA2T00130163001 Total prob of N-in:        0.00017
Brassicanapus_GSBRNA2T00130163001	TMHMM2.0	outside	     1   490
```

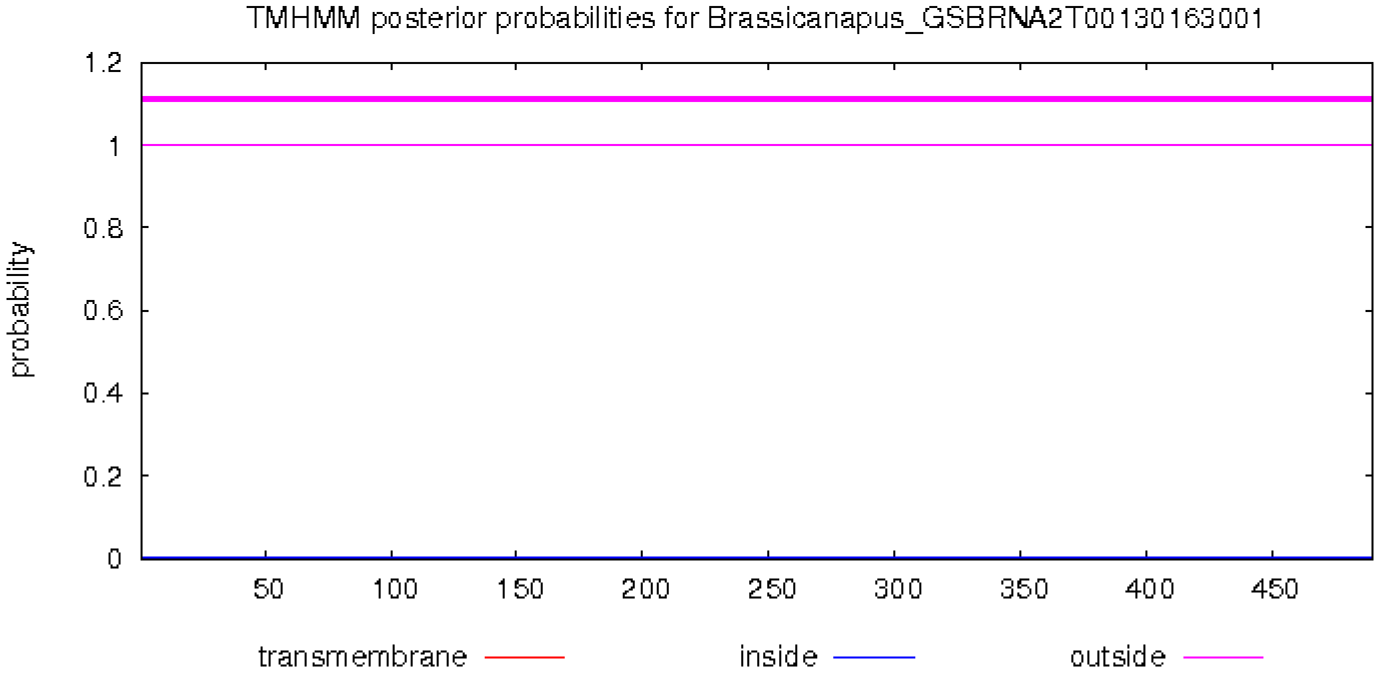

plot in postscript, script for making the plot in gnuplot, data for plot

---

```
# Brassicanapus_GSBRNA2T00131011001 Length: 343
# Brassicanapus_GSBRNA2T00131011001 Number of predicted TMHs:  0
# Brassicanapus_GSBRNA2T00131011001 Exp number of AAs in TMHs: 0
# Brassicanapus_GSBRNA2T00131011001 Exp number, first 60 AAs:  0
# Brassicanapus_GSBRNA2T00131011001 Total prob of N-in:        0.01881
Brassicanapus_GSBRNA2T00131011001	TMHMM2.0	outside	     1   343
```

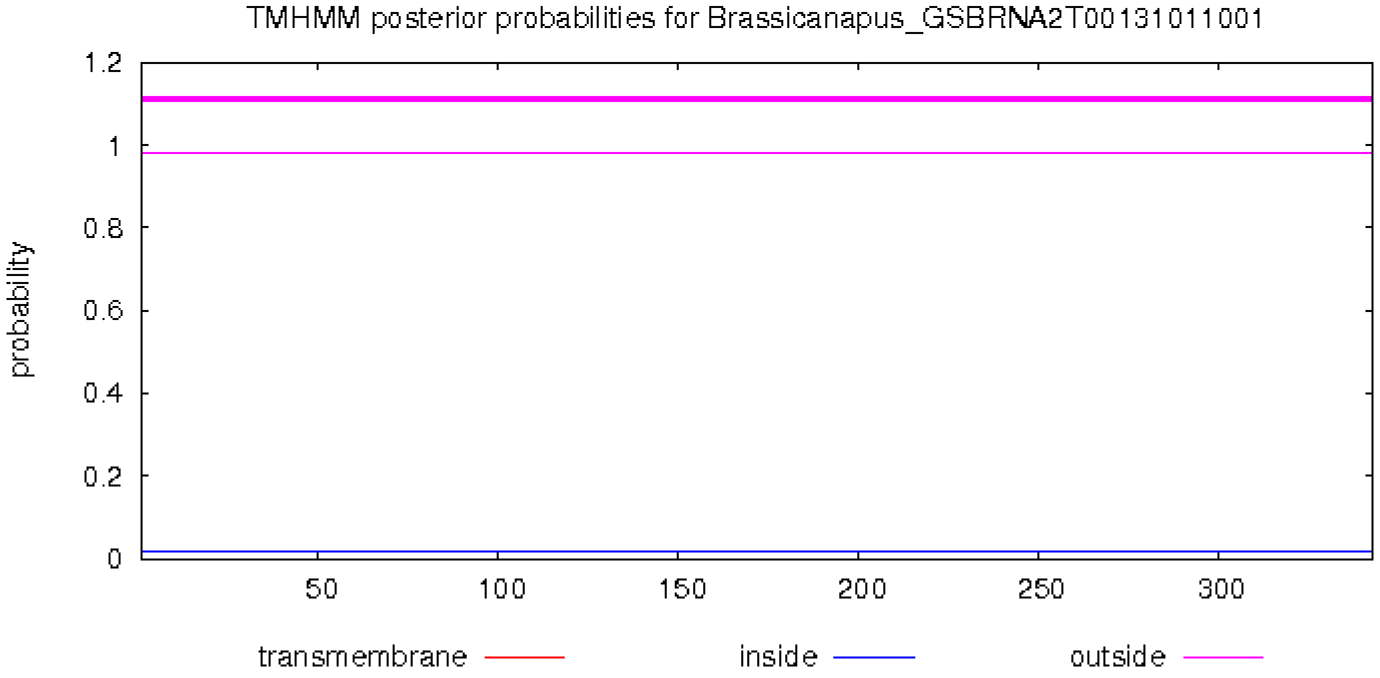

plot in postscript, script for making the plot in gnuplot, data for plot

---

```
# Brassicanapus_GSBRNA2T00131012001 Length: 159
# Brassicanapus_GSBRNA2T00131012001 Number of predicted TMHs:  0
# Brassicanapus_GSBRNA2T00131012001 Exp number of AAs in TMHs: 7e-05
# Brassicanapus_GSBRNA2T00131012001 Exp number, first 60 AAs:  0
# Brassicanapus_GSBRNA2T00131012001 Total prob of N-in:        0.15154
Brassicanapus_GSBRNA2T00131012001	TMHMM2.0	outside	     1   159
```

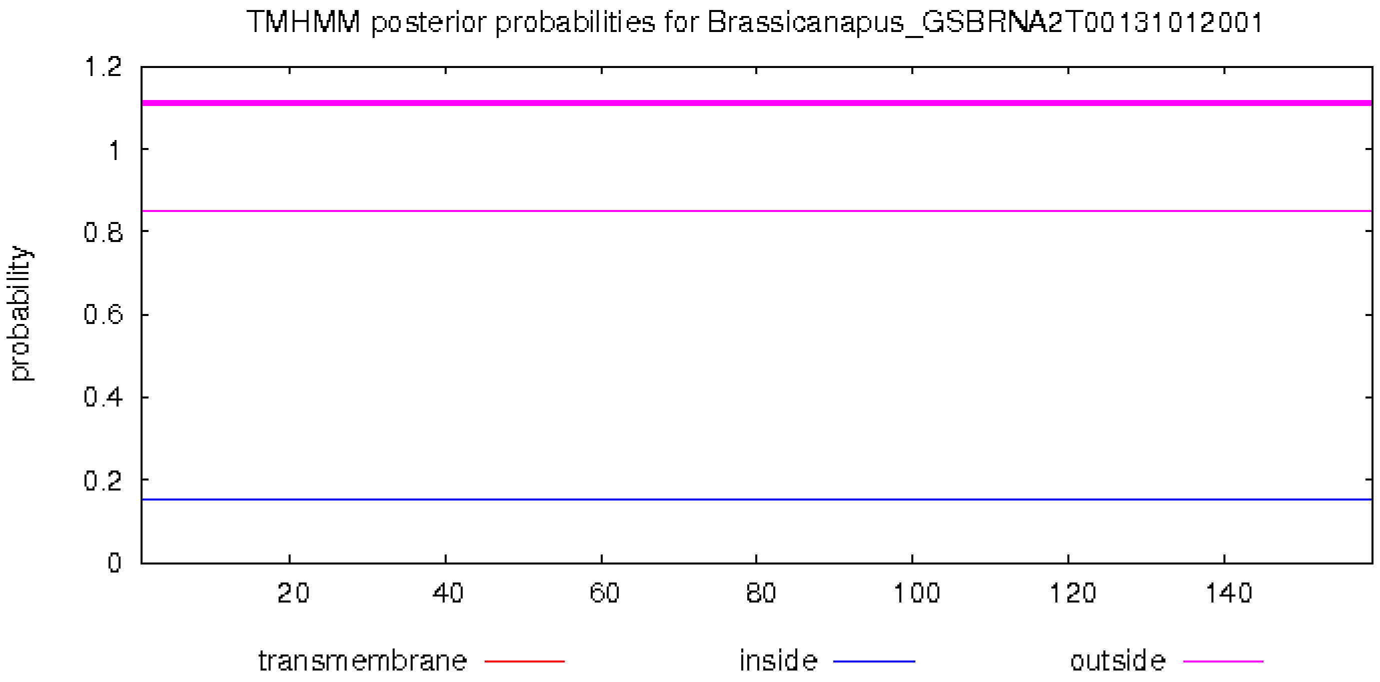

plot in postscript, script for making the plot in gnuplot, data for plot

```
# Brassicanapus_GSBRNA2T00131286001 Length: 292
# Brassicanapus_GSBRNA2T00131286001 Number of predicted TMHs:  0
# Brassicanapus_GSBRNA2T00131286001 Exp number of AAs in TMHs: 0
# Brassicanapus_GSBRNA2T00131286001 Exp number, first 60 AAs:  0
# Brassicanapus_GSBRNA2T00131286001 Total prob of N-in:        0.00489
Brassicanapus_GSBRNA2T00131286001	TMHMM2.0	outside	     1   292
```

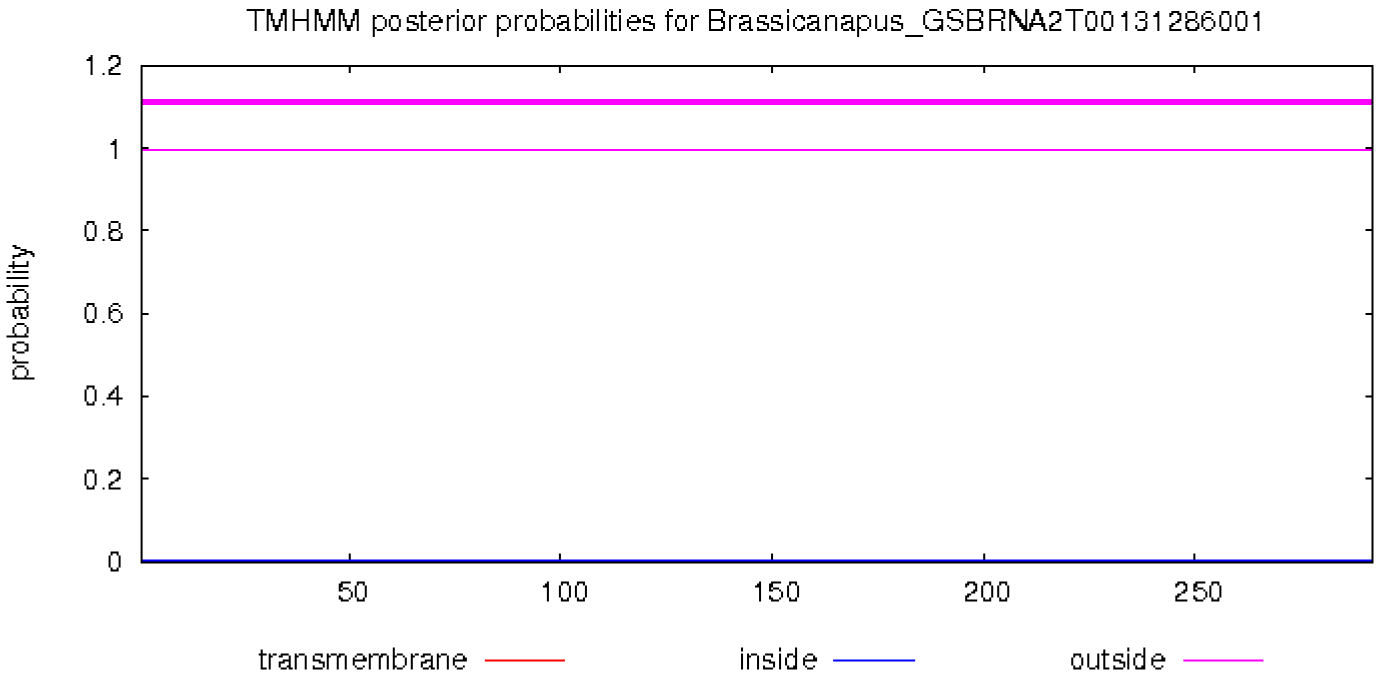

plot in postscript, script for making the plot in gnuplot, data for plot

```
# Brassicanapus_GSBRNA2T00132845001 Length: 455
# Brassicanapus_GSBRNA2T00132845001 Number of predicted TMHs:  0
# Brassicanapus_GSBRNA2T00132845001 Exp number of AAs in TMHs: 0.00017
# Brassicanapus_GSBRNA2T00132845001 Exp number, first 60 AAs:  0
# Brassicanapus_GSBRNA2T00132845001 Total prob of N-in:        0.01915
Brassicanapus_GSBRNA2T00132845001       TMHMM2.0        outside      1   455
```

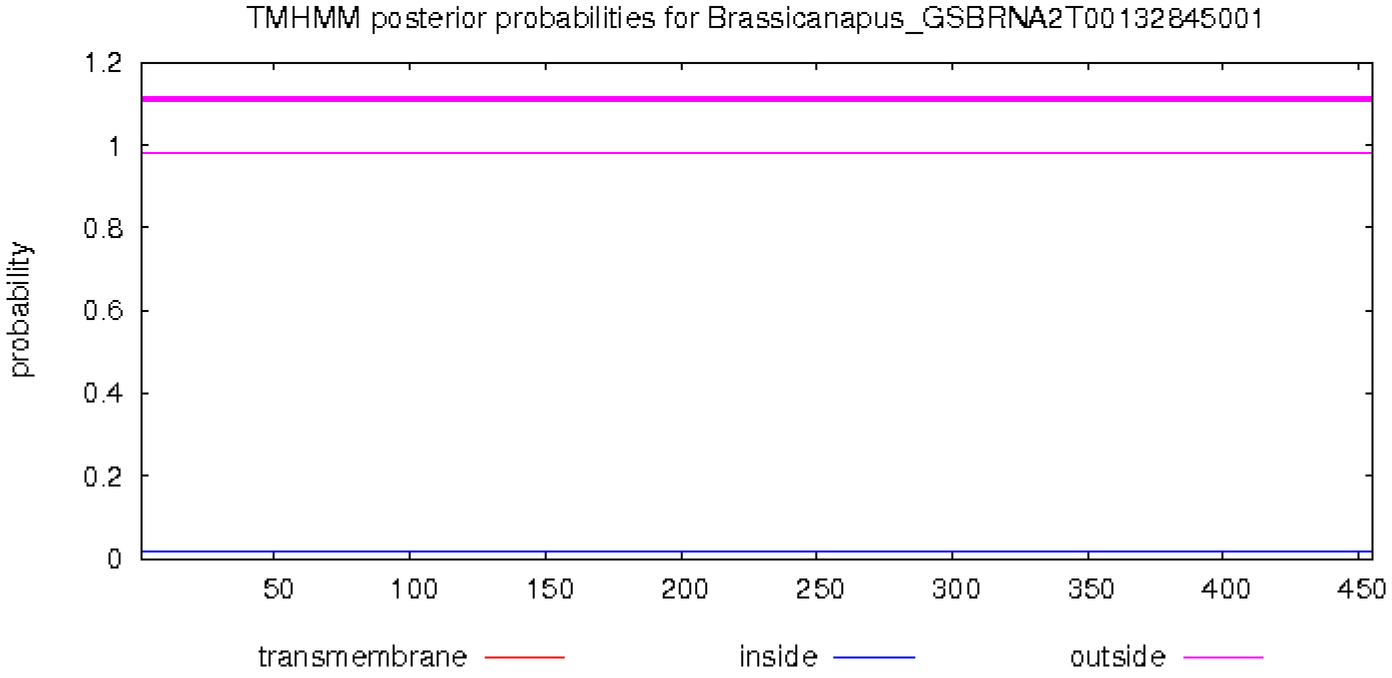

plot in postscript, script for making the plot in gnuplot, data for plot

---

```
# Brassicanapus_GSBRNA2T00132849001 Length: 313
# Brassicanapus_GSBRNA2T00132849001 Number of predicted TMHs:  0
# Brassicanapus_GSBRNA2T00132849001 Exp number of AAs in TMHs: 0.00018
# Brassicanapus_GSBRNA2T00132849001 Exp number, first 60 AAs:  0
# Brassicanapus_GSBRNA2T00132849001 Total prob of N-in:        0.06188
Brassicanapus_GSBRNA2T00132849001       TMHMM2.0        outside      1   313
```

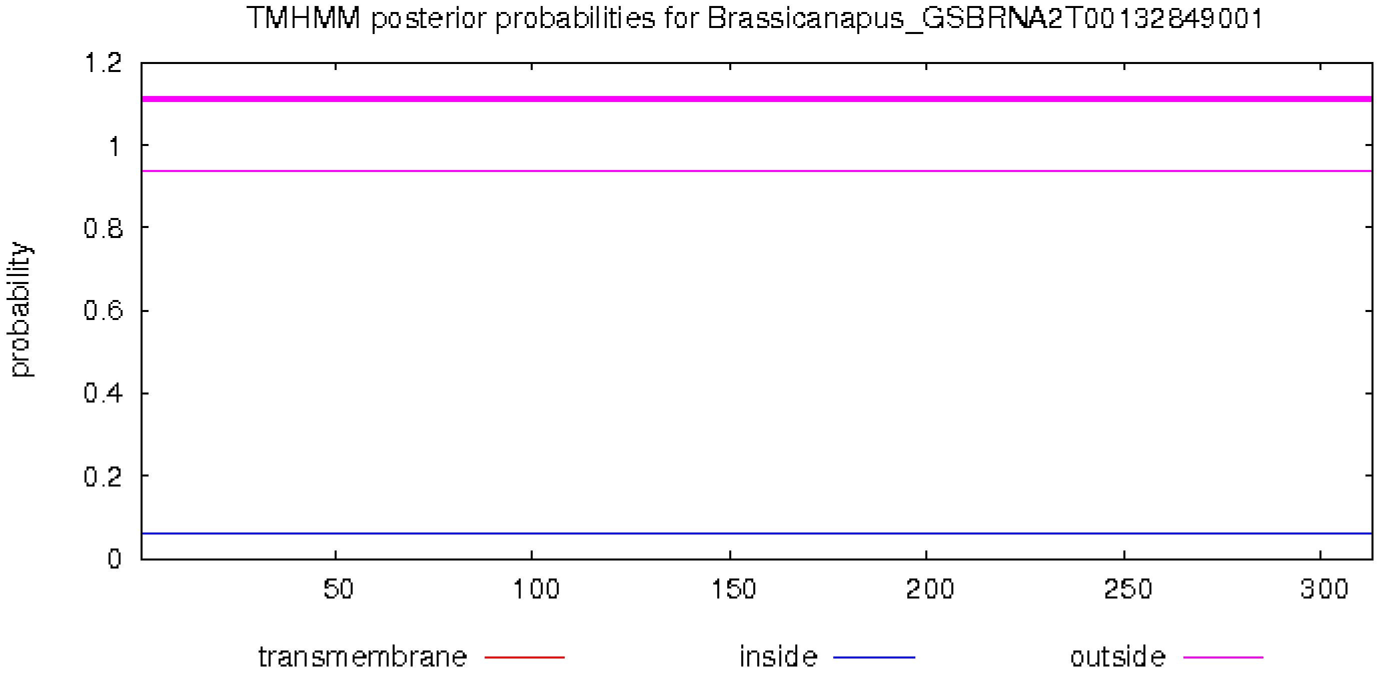

plot in postscript, script for making the plot in gnuplot, data for plot

---

```
# Brassicanapus_GSBRNA2T00132929001 Length: 359
# Brassicanapus_GSBRNA2T00132929001 Number of predicted TMHs:  0
# Brassicanapus_GSBRNA2T00132929001 Exp number of AAs in TMHs: 0.0006
# Brassicanapus_GSBRNA2T00132929001 Exp number, first 60 AAs:  0
# Brassicanapus_GSBRNA2T00132929001 Total prob of N-in:        0.00833
Brassicanapus_GSBRNA2T00132929001	TMHMM2.0	outside	     1   359
```

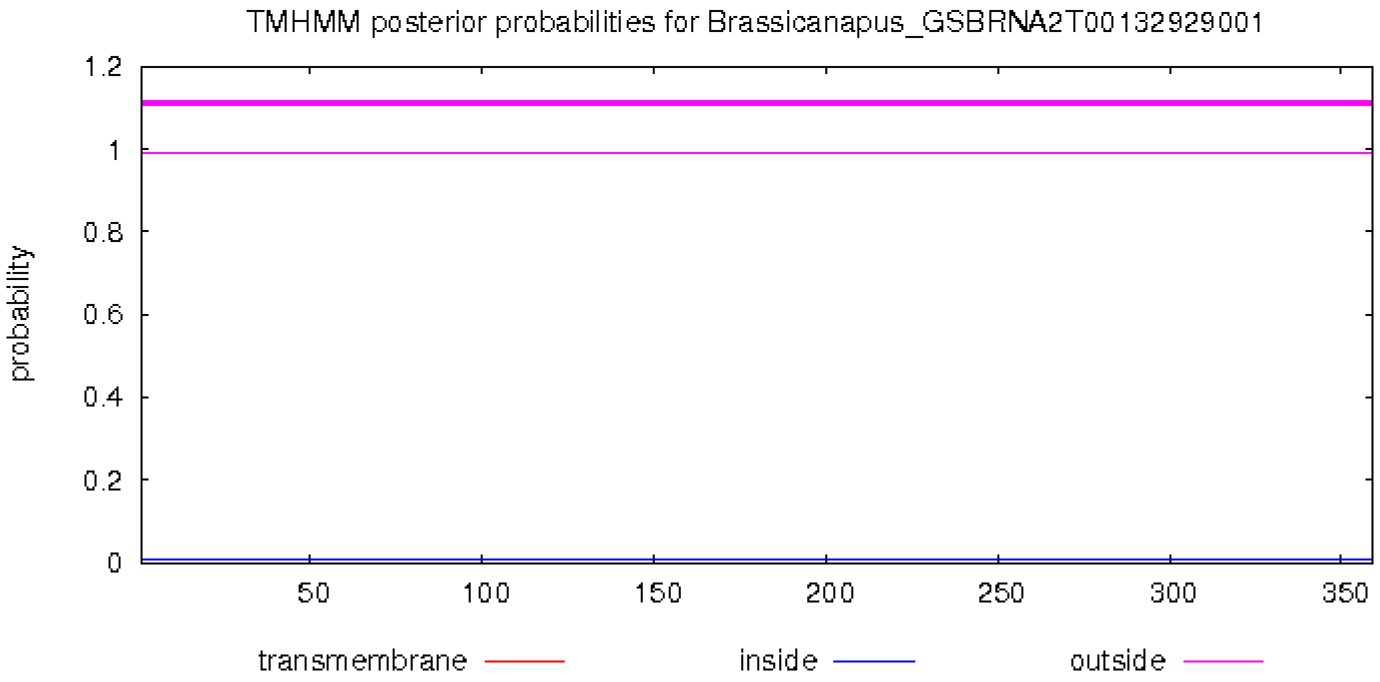

plot in postscript, script for making the plot in gnuplot, data for plot

---

```
# Brassicanapus_GSBRNA2T00133340001 Length: 395
# Brassicanapus_GSBRNA2T00133340001 Number of predicted TMHs:  0
# Brassicanapus_GSBRNA2T00133340001 Exp number of AAs in TMHs: 0.0002
# Brassicanapus_GSBRNA2T00133340001 Exp number, first 60 AAs:  0.00013
# Brassicanapus_GSBRNA2T00133340001 Total prob of N-in:        0.00904
Brassicanapus_GSBRNA2T00133340001	TMHMM2.0	outside	     1   395
```

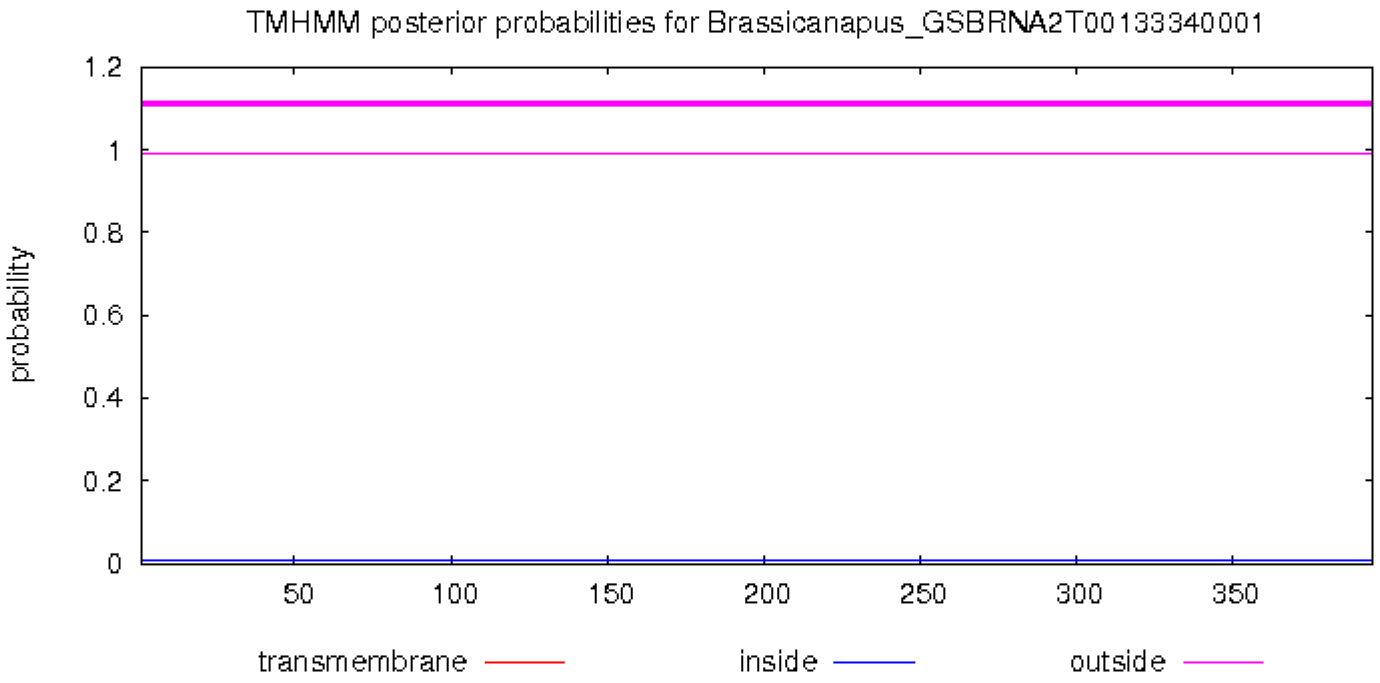

plot in postscript, script for making the plot in gnuplot, data for plot

---

```
# Brassicanapus_GSBRNA2T00133341001 Length: 419
# Brassicanapus_GSBRNA2T00133341001 Number of predicted TMHs:  0
# Brassicanapus_GSBRNA2T00133341001 Exp number of AAs in TMHs: 0
# Brassicanapus_GSBRNA2T00133341001 Exp number, first 60 AAs:  0
# Brassicanapus_GSBRNA2T00133341001 Total prob of N-in:        0.00638
Brassicanapus_GSBRNA2T00133341001	TMHMM2.0	outside	     1   419
```

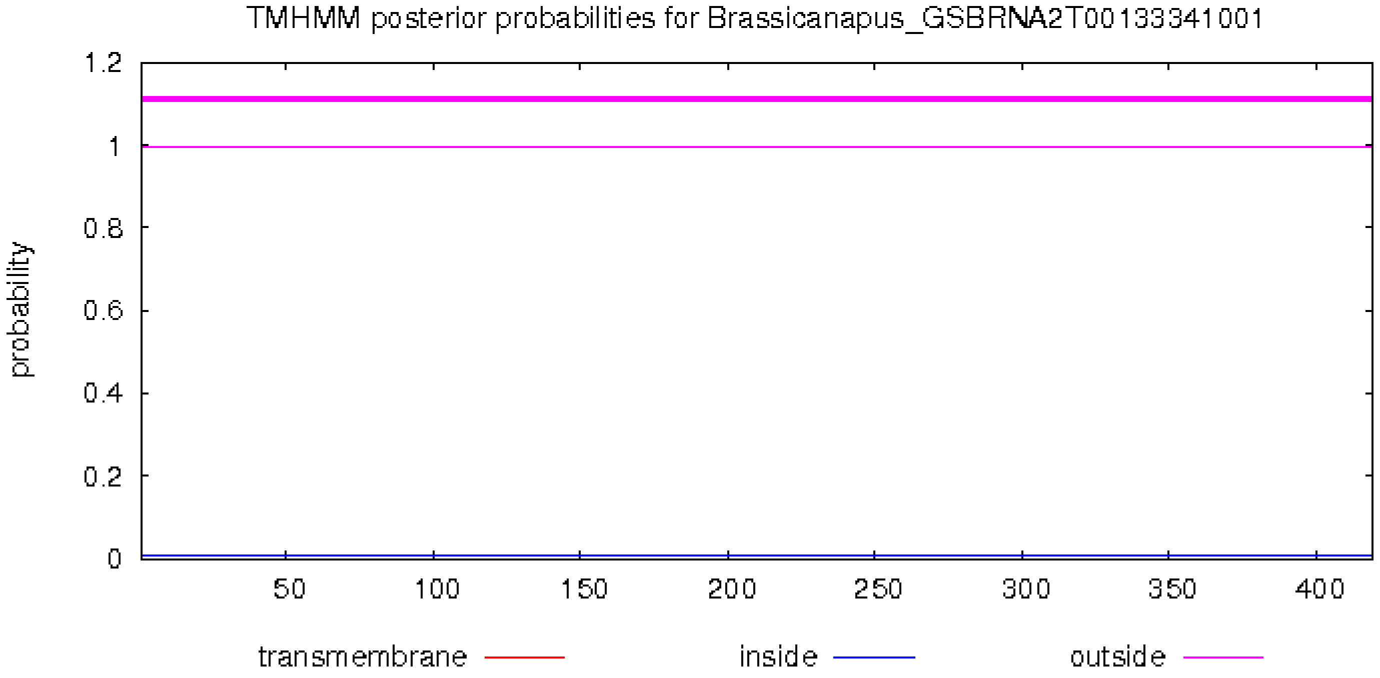

plot in postscript, script for making the plot in gnuplot, data for plot

---

```
# Brassicanapus_GSBRNA2T00133539001 Length: 456
# Brassicanapus_GSBRNA2T00133539001 Number of predicted TMHs:  0
# Brassicanapus_GSBRNA2T00133539001 Exp number of AAs in TMHs: 13.00673
# Brassicanapus_GSBRNA2T00133539001 Exp number, first 60 AAs:  0
# Brassicanapus_GSBRNA2T00133539001 Total prob of N-in:        0.00368
Brassicanapus_GSBRNA2T00133539001	TMHMM2.0	outside	     1   456
```

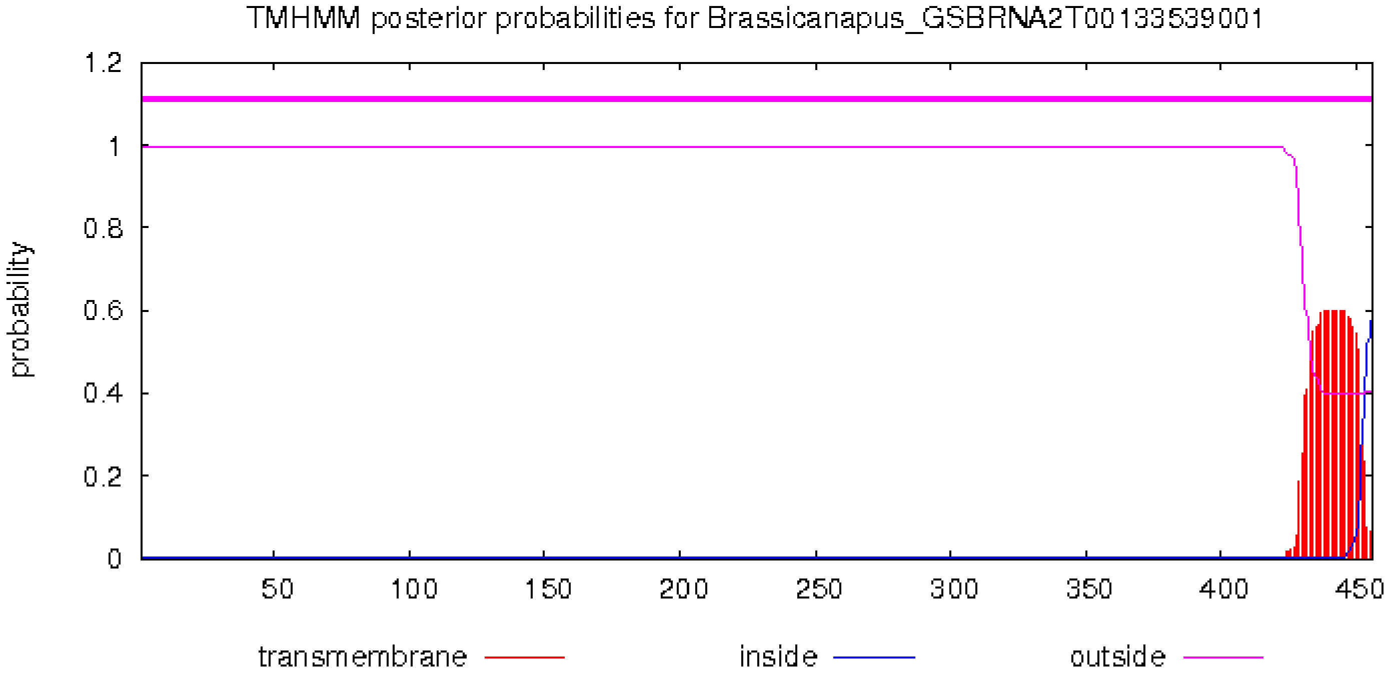

plot in postscript, script for making the plot in gnuplot, data for plot

---

```
# Brassicanapus_GSBRNA2T00134569001 Length: 241
# Brassicanapus_GSBRNA2T00134569001 Number of predicted TMHs:  0
# Brassicanapus_GSBRNA2T00134569001 Exp number of AAs in TMHs: 0.00089
# Brassicanapus_GSBRNA2T00134569001 Exp number, first 60 AAs:  0
# Brassicanapus_GSBRNA2T00134569001 Total prob of N-in:        0.11920
Brassicanapus_GSBRNA2T00134569001       TMHMM2.0        outside      1   241
```

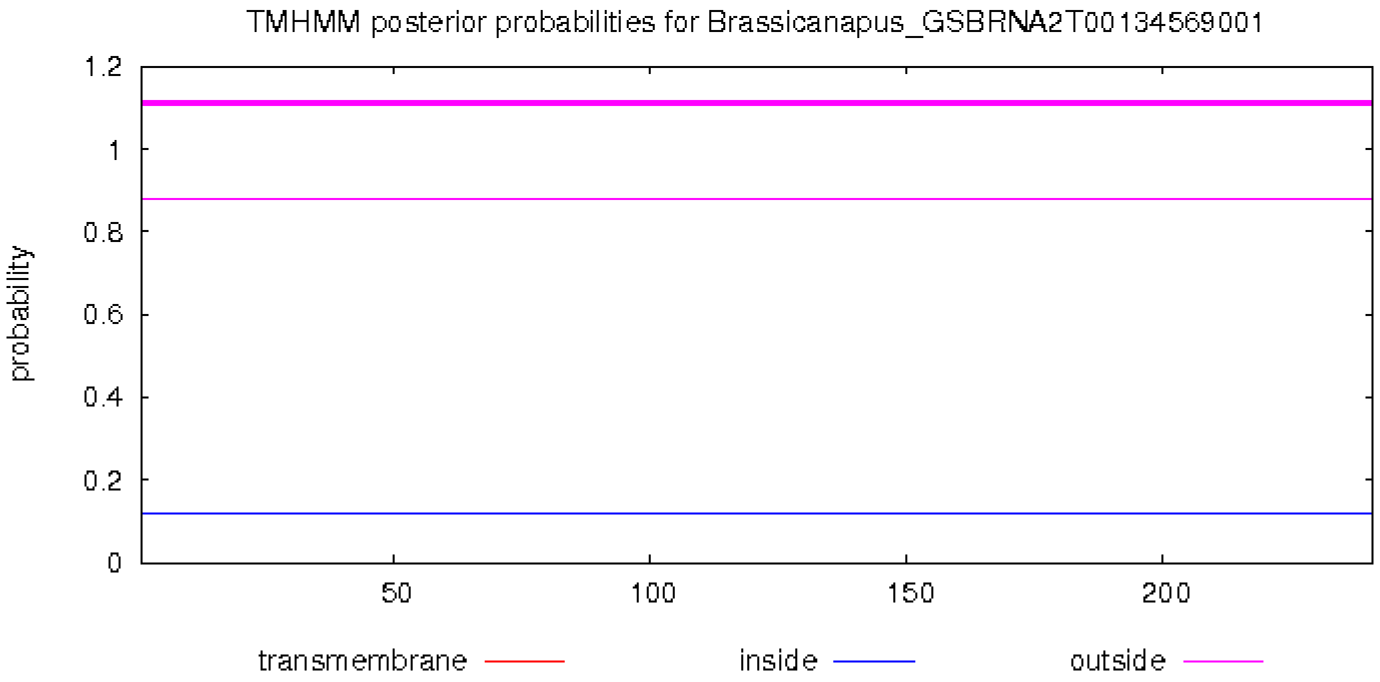

plot in postscript, script for making the plot in gnuplot, data for plot

---

```
# Brassicanapus_GSBRNA2T00134573001 Length: 342
# Brassicanapus_GSBRNA2T00134573001 Number of predicted TMHs:  0
# Brassicanapus_GSBRNA2T00134573001 Exp number of AAs in TMHs: 0.00018
# Brassicanapus_GSBRNA2T00134573001 Exp number, first 60 AAs:  0.00018
# Brassicanapus_GSBRNA2T00134573001 Total prob of N-in:        0.00543
Brassicanapus_GSBRNA2T00134573001       TMHMM2.0        outside      1   342
```

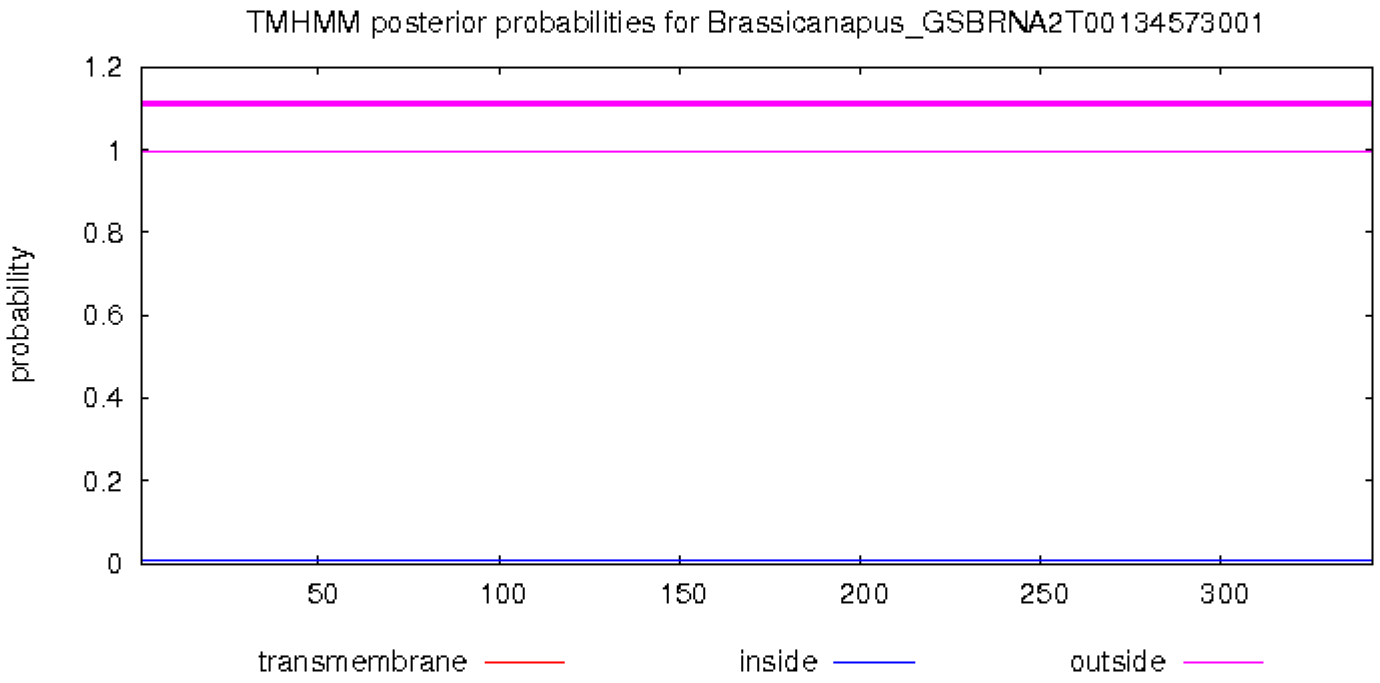

plot in postscript, script for making the plot in gnuplot, data for plot

---

```
# Brassicanapus_GSBRNA2T00134697001 Length: 188
# Brassicanapus_GSBRNA2T00134697001 Number of predicted TMHs:  0
# Brassicanapus_GSBRNA2T00134697001 Exp number of AAs in TMHs: 0
# Brassicanapus_GSBRNA2T00134697001 Exp number, first 60 AAs:  0
# Brassicanapus_GSBRNA2T00134697001 Total prob of N-in:        0.00724
Brassicanapus_GSBRNA2T00134697001       TMHMM2.0        outside      1   188
```

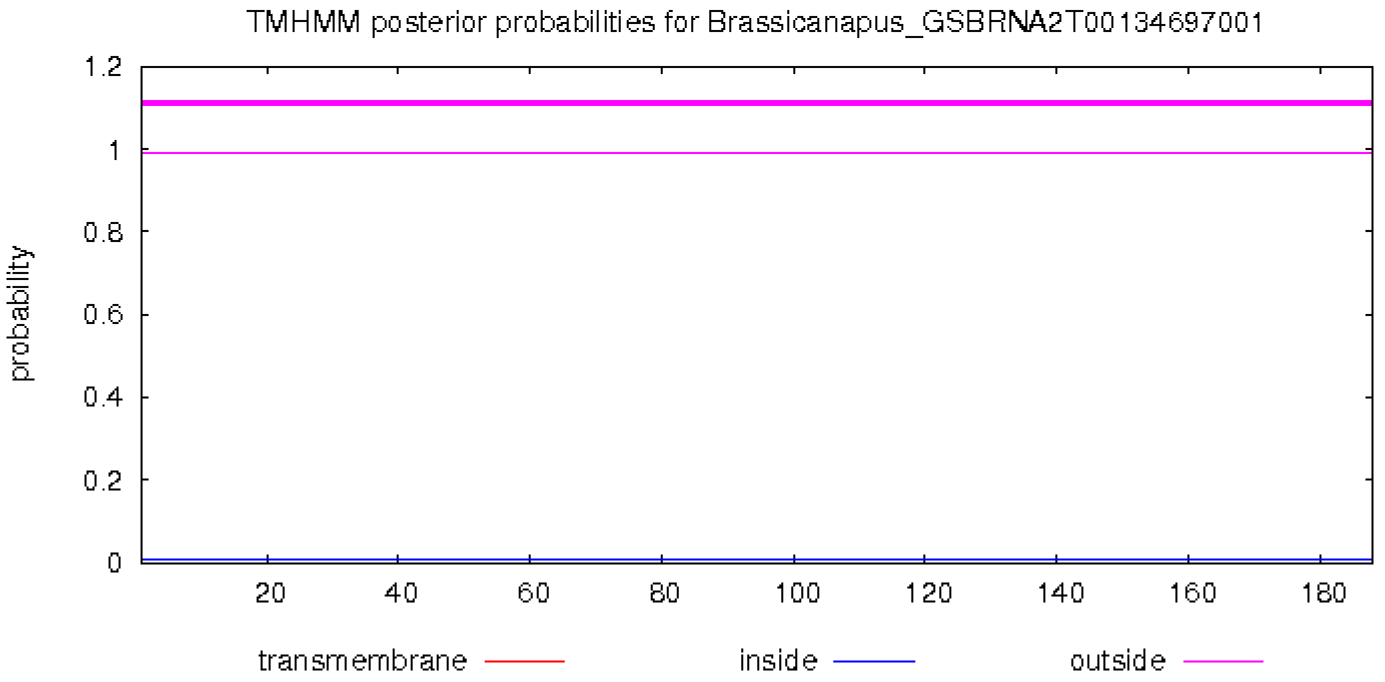

plot in postscript, script for making the plot in gnuplot, data for plot

---

```
# Brassicanapus_GSBRNA2T00135630001 Length: 202
# Brassicanapus_GSBRNA2T00135630001 Number of predicted TMHs:  0
# Brassicanapus_GSBRNA2T00135630001 Exp number of AAs in TMHs: 0.75828
# Brassicanapus_GSBRNA2T00135630001 Exp number, first 60 AAs:  0.73218
# Brassicanapus_GSBRNA2T00135630001 Total prob of N-in:        0.05763
Brassicanapus_GSBRNA2T00135630001       TMHMM2.0        outside      1   202
```

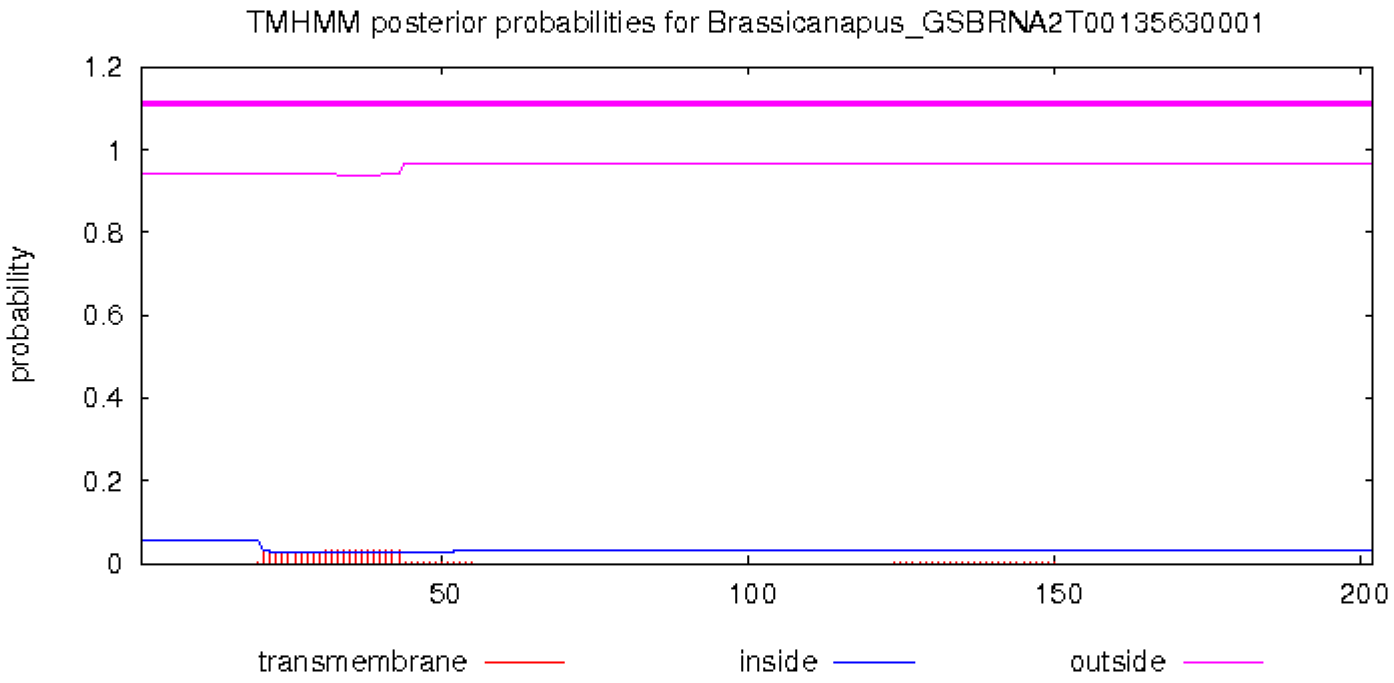

plot in postscript, script for making the plot in gnuplot, data for plot

---

```
# Brassicanapus_GSBRNA2T00135689001 Length: 249
# Brassicanapus_GSBRNA2T00135689001 Number of predicted TMHs:  0
# Brassicanapus_GSBRNA2T00135689001 Exp number of AAs in TMHs: 0.00013
# Brassicanapus_GSBRNA2T00135689001 Exp number, first 60 AAs:  0
# Brassicanapus_GSBRNA2T00135689001 Total prob of N-in:        0.13510
Brassicanapus_GSBRNA2T00135689001	TMHMM2.0	outside	     1   249
```

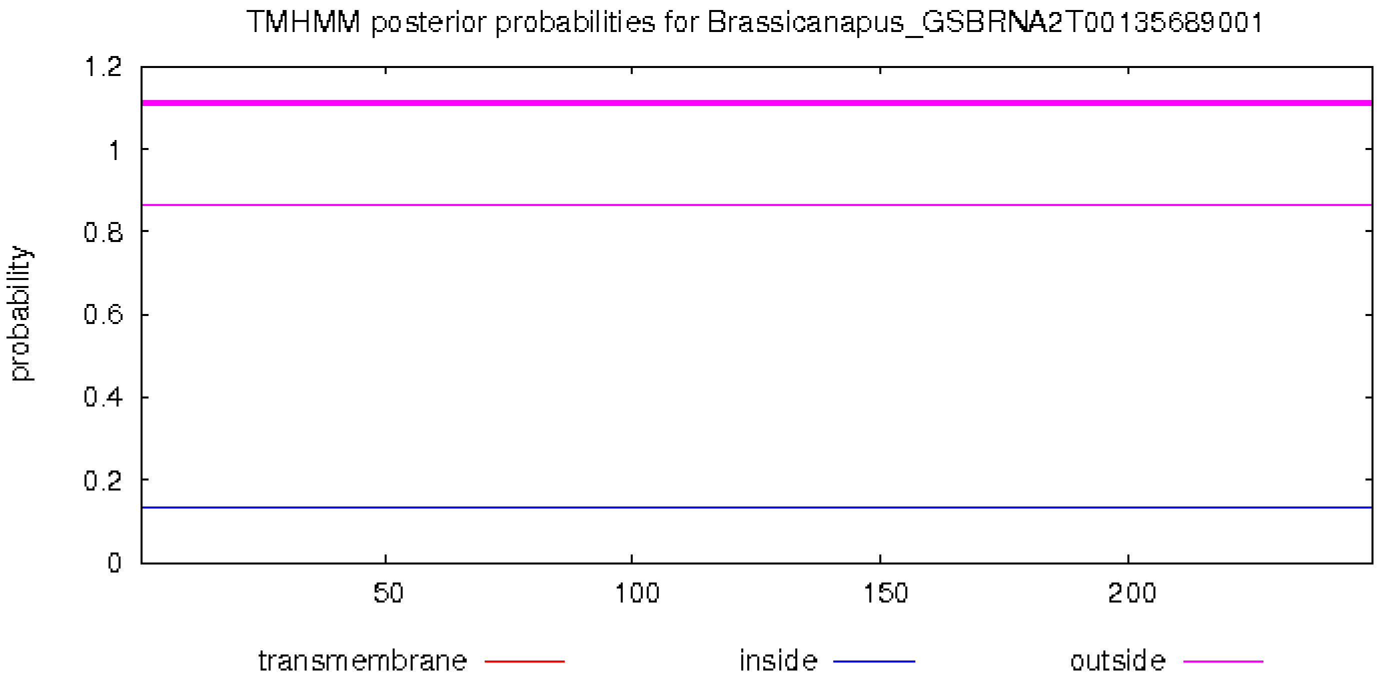

plot in postscript, script for making the plot in gnuplot, data for plot

---

```
# Brassicanapus_GSBRNA2T00135820001 Length: 329
# Brassicanapus_GSBRNA2T00135820001 Number of predicted TMHs:  0
# Brassicanapus_GSBRNA2T00135820001 Exp number of AAs in TMHs: 0.00032
# Brassicanapus_GSBRNA2T00135820001 Exp number, first 60 AAs:  0.0002
# Brassicanapus_GSBRNA2T00135820001 Total prob of N-in:        0.00399
Brassicanapus_GSBRNA2T00135820001	TMHMM2.0	outside	     1   329
```

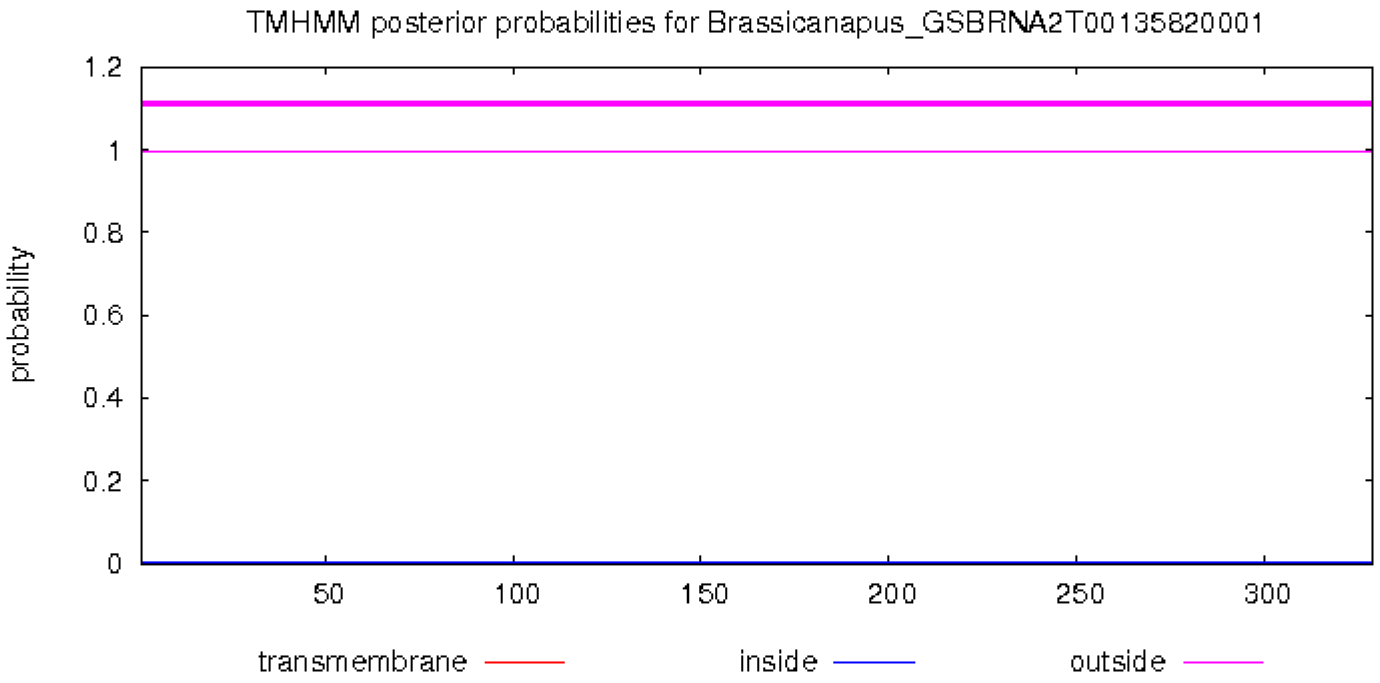

plot in postscript, script for making the plot in gnuplot, data for plot

---

```
# Brassicanapus_GSBRNA2T00135984001 Length: 438
# Brassicanapus_GSBRNA2T00135984001 Number of predicted TMHs:  0
# Brassicanapus_GSBRNA2T00135984001 Exp number of AAs in TMHs: 0
# Brassicanapus_GSBRNA2T00135984001 Exp number, first 60 AAs:  0
# Brassicanapus_GSBRNA2T00135984001 Total prob of N-in:        0.00128
Brassicanapus_GSBRNA2T00135984001	TMHMM2.0	outside	     1   438
```

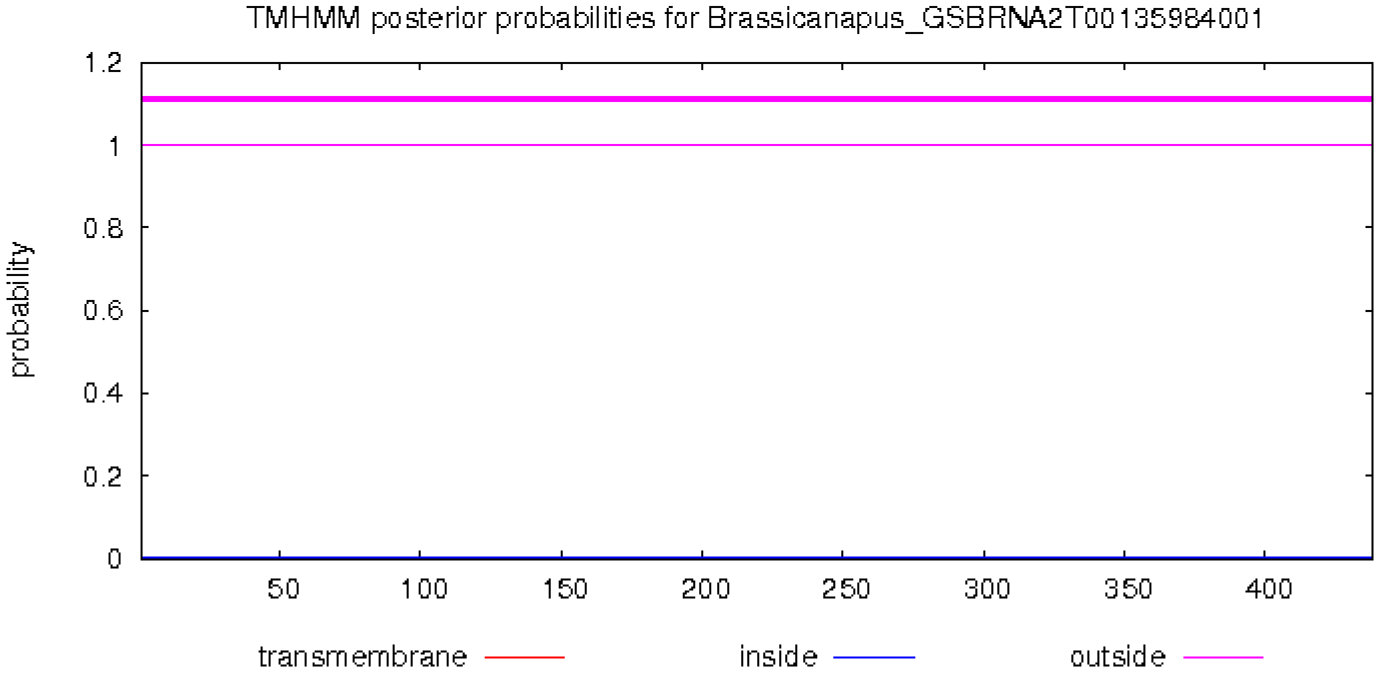

plot in postscript, script for making the plot in gnuplot, data for plot

---

```
# Brassicanapus_GSBRNA2T00135993001 Length: 278
# Brassicanapus_GSBRNA2T00135993001 Number of predicted TMHs:  0
# Brassicanapus_GSBRNA2T00135993001 Exp number of AAs in TMHs: 0.00019
# Brassicanapus_GSBRNA2T00135993001 Exp number, first 60 AAs:  0
# Brassicanapus_GSBRNA2T00135993001 Total prob of N-in:        0.01089
Brassicanapus_GSBRNA2T00135993001	TMHMM2.0	outside	     1   278
```

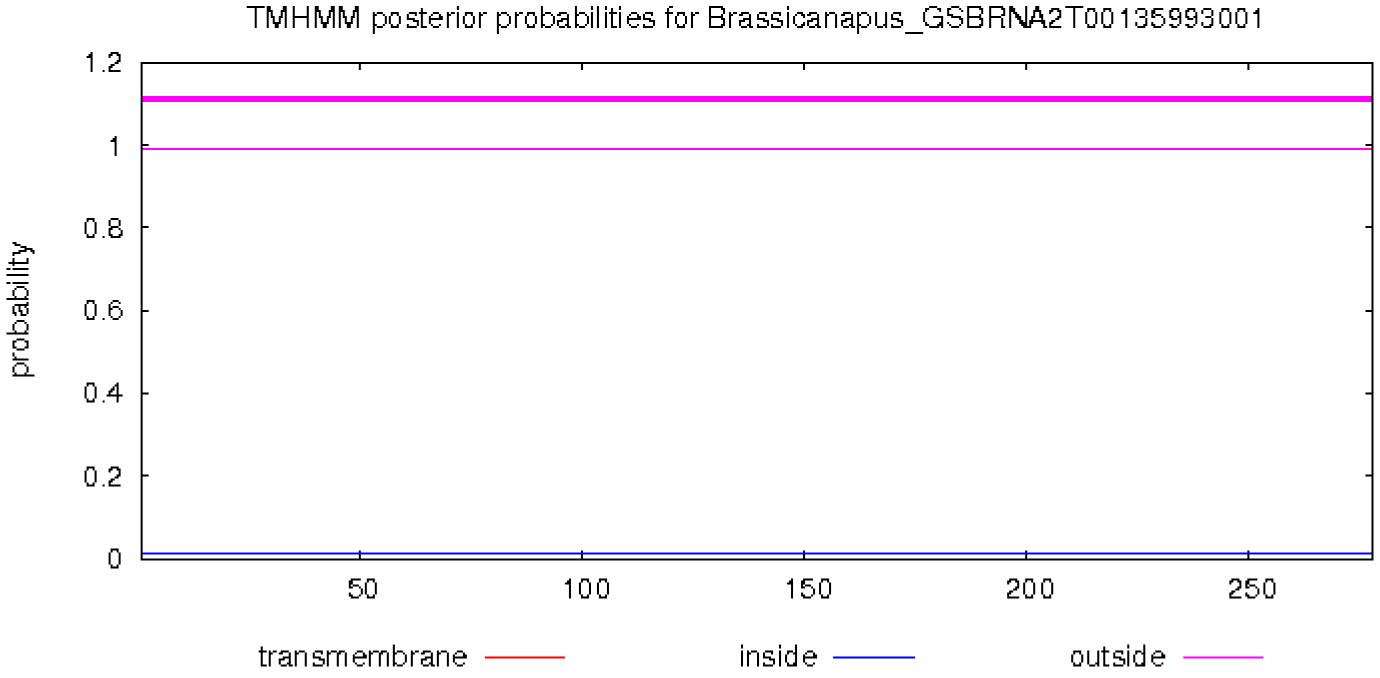

plot in postscript, script for making the plot in gnuplot, data for plot

---

```
# Brassicanapus_GSBRNA2T00136356001 Length: 554
# Brassicanapus_GSBRNA2T00136356001 Number of predicted TMHs:  1
# Brassicanapus_GSBRNA2T00136356001 Exp number of AAs in TMHs: 21.74155
# Brassicanapus_GSBRNA2T00136356001 Exp number, first 60 AAs:  0
# Brassicanapus_GSBRNA2T00136356001 Total prob of N-in:        0.00787
Brassicanapus_GSBRNA2T00136356001	TMHMM2.0	outside	     1   525
Brassicanapus_GSBRNA2T00136356001	TMHMM2.0	TMhelix	   526   548
Brassicanapus_GSBRNA2T00136356001	TMHMM2.0	inside	   549   554
```

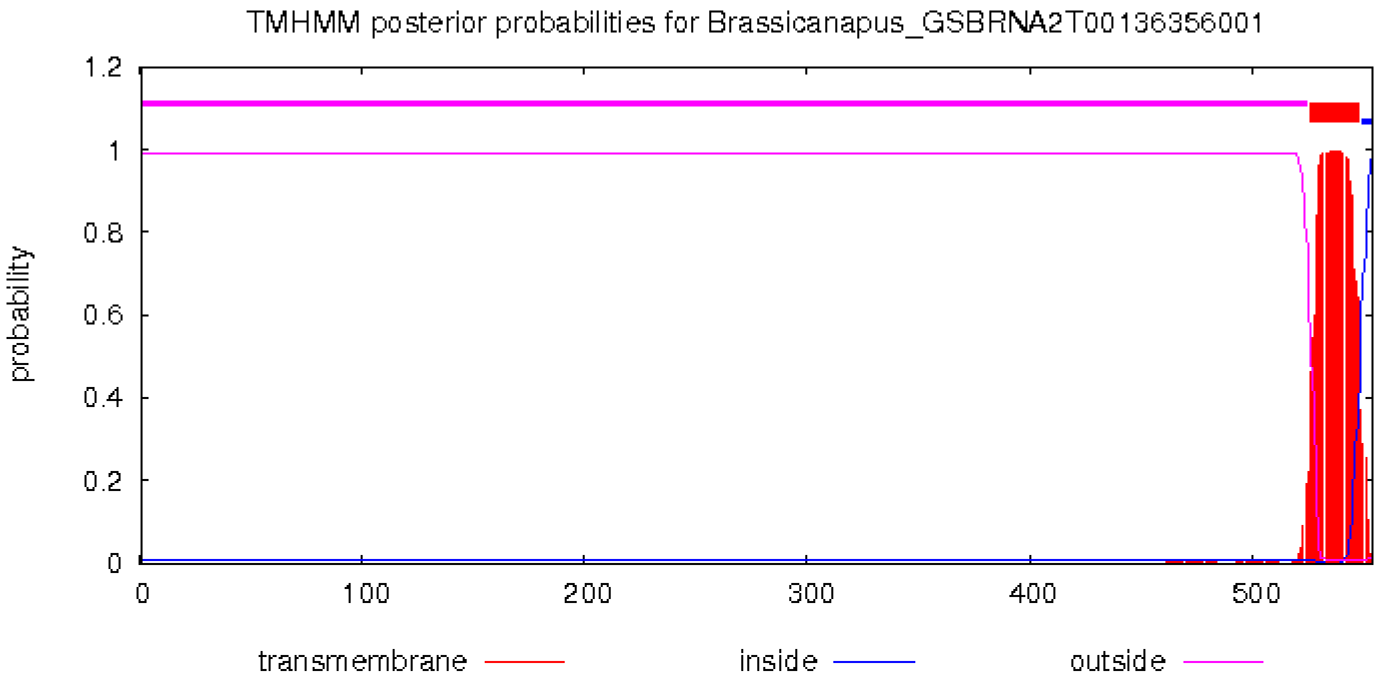

plot in postscript, script for making the plot in gnuplot, data for plot

---

```
# Brassicanapus_GSBRNA2T00137289001 Length: 327
# Brassicanapus_GSBRNA2T00137289001 Number of predicted TMHs:  0
# Brassicanapus_GSBRNA2T00137289001 Exp number of AAs in TMHs: 0.00016
# Brassicanapus_GSBRNA2T00137289001 Exp number, first 60 AAs:  0.00016
# Brassicanapus_GSBRNA2T00137289001 Total prob of N-in:        0.03494
Brassicanapus_GSBRNA2T00137289001	TMHMM2.0	outside	     1   327
```

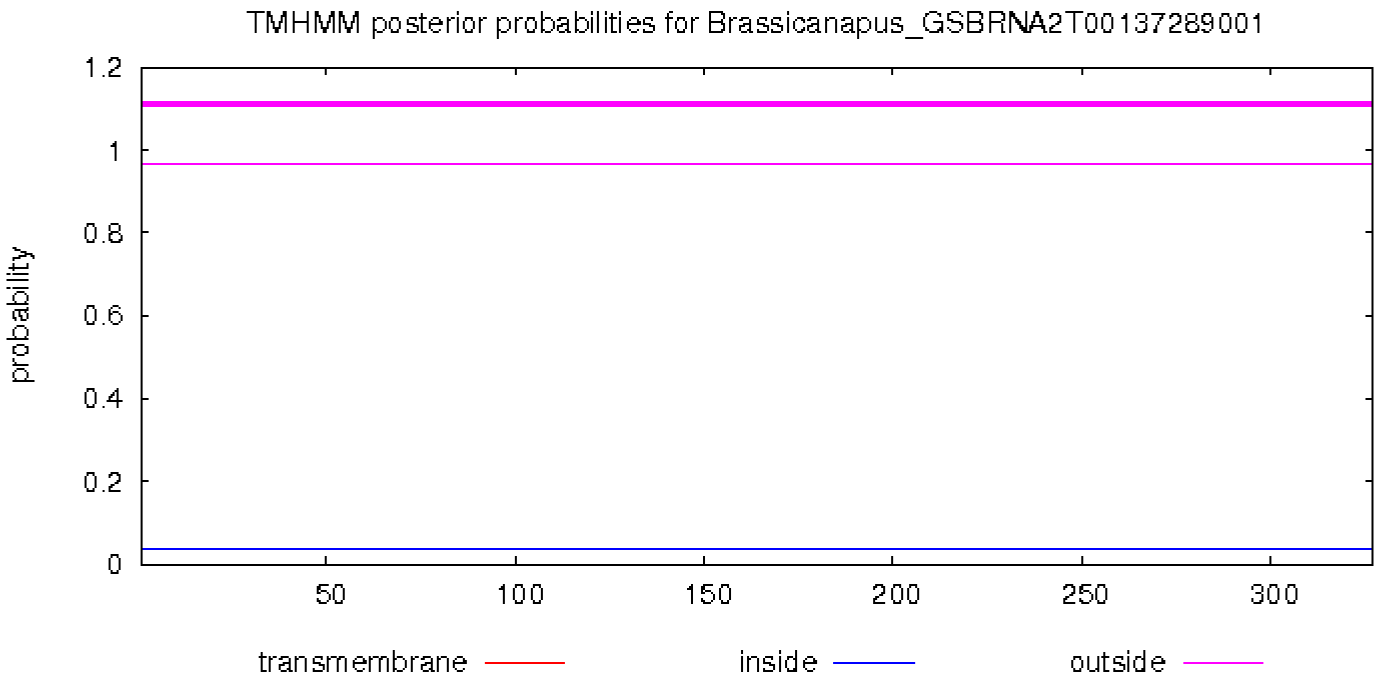

plot in postscript, script for making the plot in gnuplot, data for plot

```
# Brassicanapus_GSBRNA2T00137562001 Length: 199
# Brassicanapus_GSBRNA2T00137562001 Number of predicted TMHs:  0
# Brassicanapus_GSBRNA2T00137562001 Exp number of AAs in TMHs: 0.00074
# Brassicanapus_GSBRNA2T00137562001 Exp number, first 60 AAs:  0.00074
# Brassicanapus_GSBRNA2T00137562001 Total prob of N-in:        0.05687
Brassicanapus_GSBRNA2T00137562001	TMHMM2.0	outside	     1   199
```

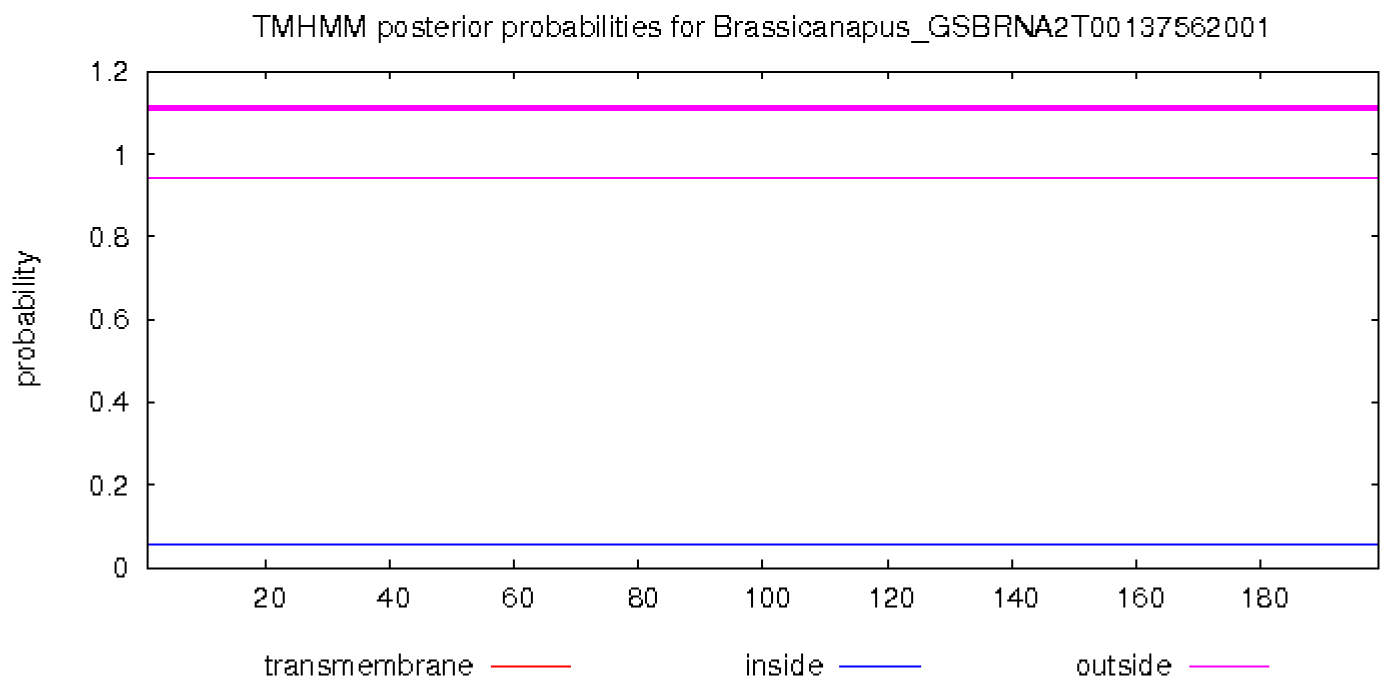

plot in postscript, script for making the plot in gnuplot, data for plot

```
# Brassicanapus_GSBRNA2T00137565001 Length: 247
# Brassicanapus_GSBRNA2T00137565001 Number of predicted TMHs:  0
# Brassicanapus_GSBRNA2T00137565001 Exp number of AAs in TMHs: 0.00022
# Brassicanapus_GSBRNA2T00137565001 Exp number, first 60 AAs:  0.00019
# Brassicanapus_GSBRNA2T00137565001 Total prob of N-in:        0.01209
Brassicanapus_GSBRNA2T00137565001	TMHMM2.0	outside	     1   247
```

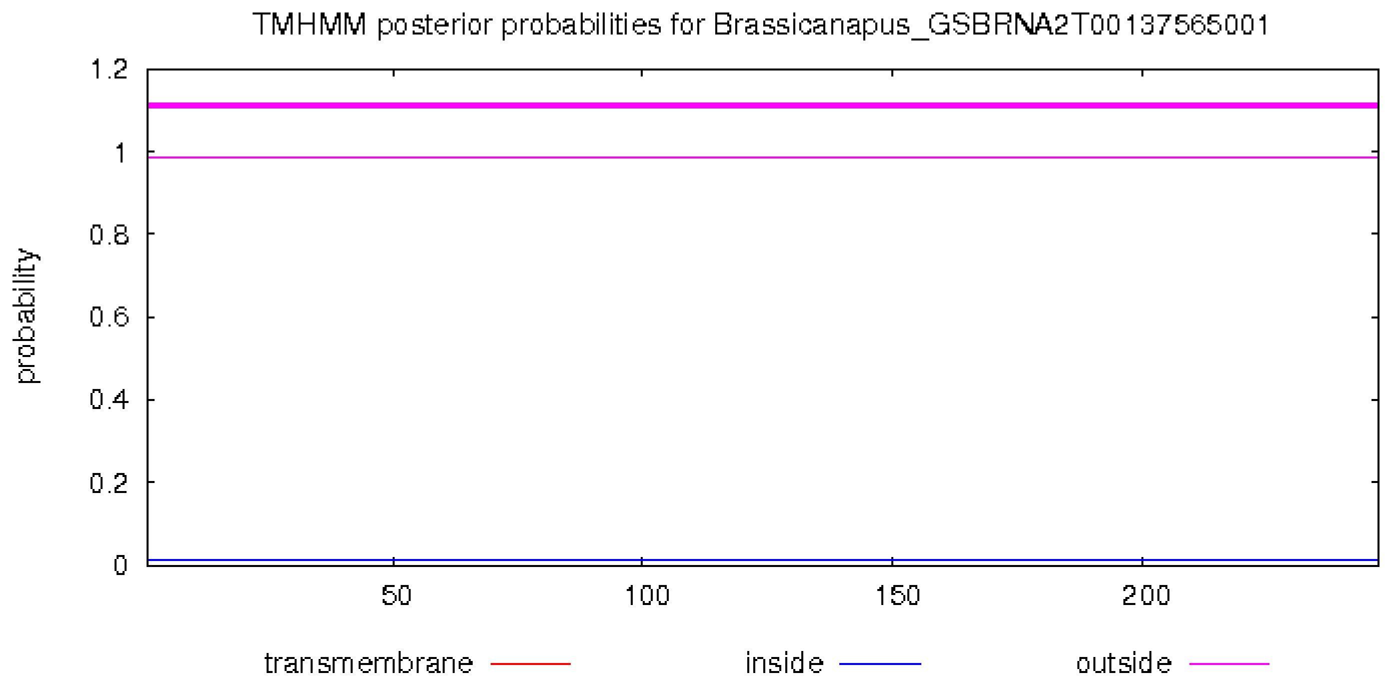

plot in postscript, script for making the plot in gnuplot, data for plot

```
# Brassicanapus_GSBRNA2T00137582001 Length: 309
# Brassicanapus_GSBRNA2T00137582001 Number of predicted TMHs:  0
# Brassicanapus_GSBRNA2T00137582001 Exp number of AAs in TMHs: 0.01231
# Brassicanapus_GSBRNA2T00137582001 Exp number, first 60 AAs:  0.00023
# Brassicanapus_GSBRNA2T00137582001 Total prob of N-in:        0.01419
Brassicanapus_GSBRNA2T00137582001       TMHMM2.0        outside      1   309
```

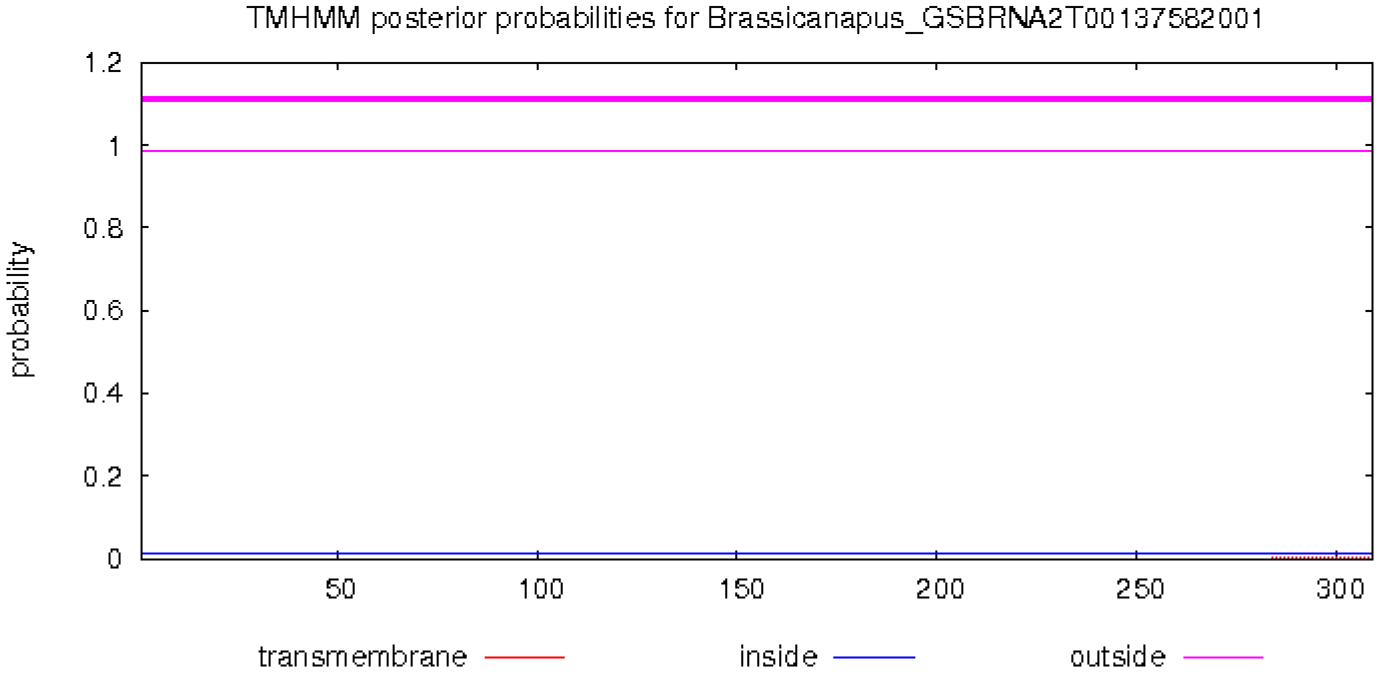

plot in postscript, script for making the plot in gnuplot, data for plot

```
# Brassicanapus_GSBRNA2T00137735001 Length: 277
# Brassicanapus_GSBRNA2T00137735001 Number of predicted TMHs:  0
# Brassicanapus_GSBRNA2T00137735001 Exp number of AAs in TMHs: 0.00018
# Brassicanapus_GSBRNA2T00137735001 Exp number, first 60 AAs:  0
# Brassicanapus_GSBRNA2T00137735001 Total prob of N-in:        0.07714
Brassicanapus_GSBRNA2T00137735001       TMHMM2.0        outside      1   277
```

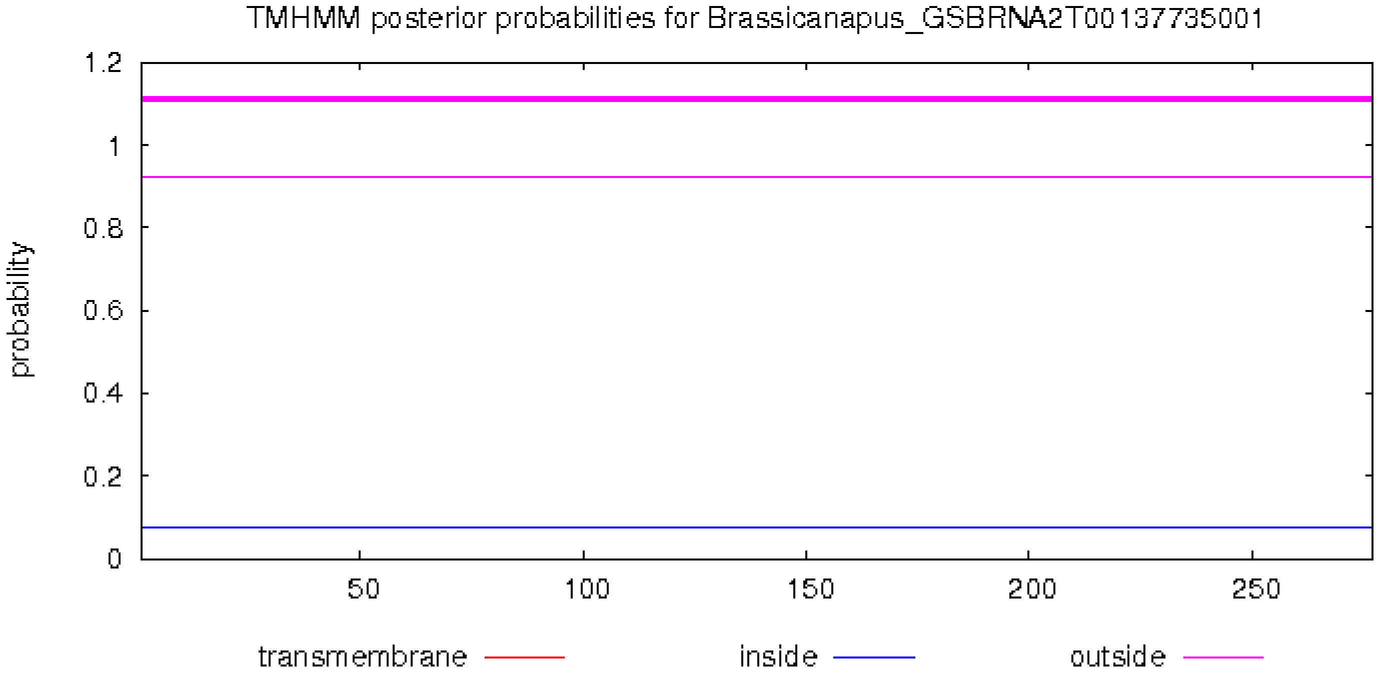

plot in postscript, script for making the plot in gnuplot, data for plot

```
# Brassicanapus_GSBRNA2T00137740001 Length: 302
# Brassicanapus_GSBRNA2T00137740001 Number of predicted TMHs:  0
# Brassicanapus_GSBRNA2T00137740001 Exp number of AAs in TMHs: 0.00095
# Brassicanapus_GSBRNA2T00137740001 Exp number, first 60 AAs:  0
# Brassicanapus_GSBRNA2T00137740001 Total prob of N-in:        0.07866
Brassicanapus_GSBRNA2T00137740001       TMHMM2.0        outside      1   302
```

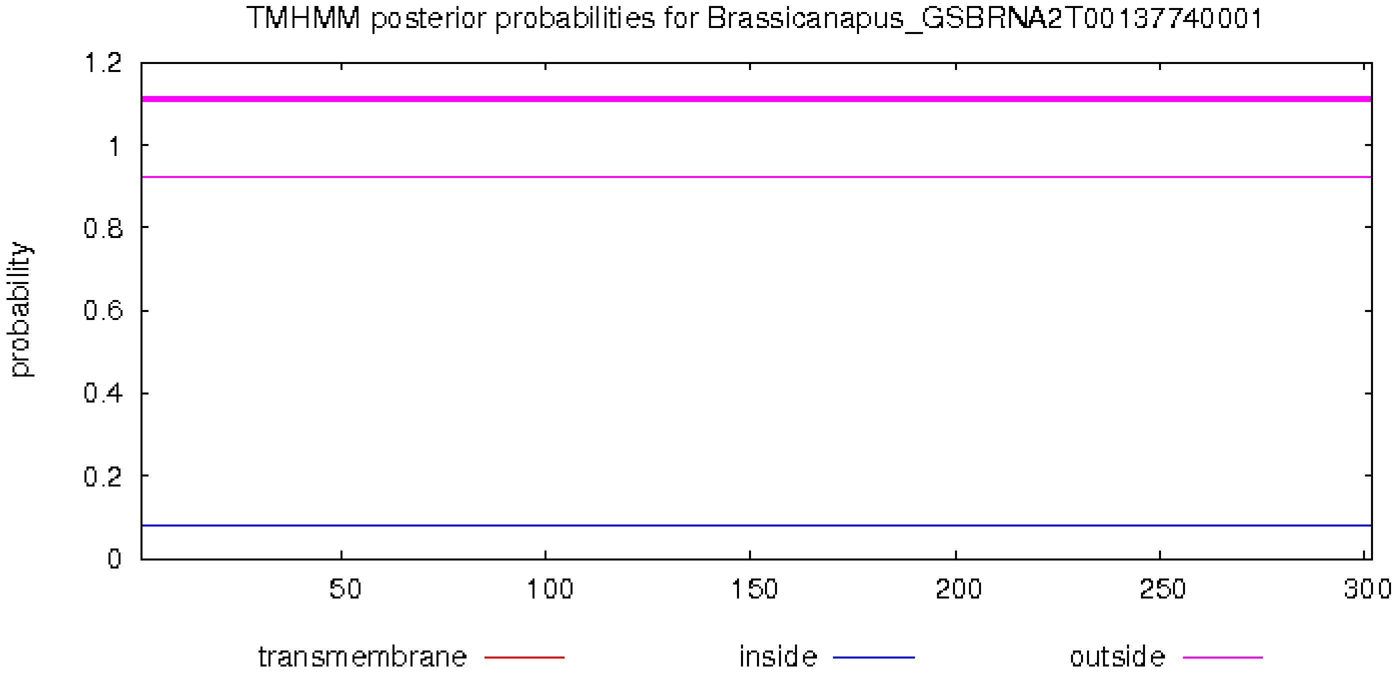

plot in postscript, script for making the plot in gnuplot, data for plot

---

```
# Brassicanapus_GSBRNA2T00139105001 Length: 510
# Brassicanapus_GSBRNA2T00139105001 Number of predicted TMHs:  2
# Brassicanapus_GSBRNA2T00139105001 Exp number of AAs in TMHs: 43.58507
# Brassicanapus_GSBRNA2T00139105001 Exp number, first 60 AAs:  0
# Brassicanapus_GSBRNA2T00139105001 Total prob of N-in:        0.02821
Brassicanapus_GSBRNA2T00139105001       TMHMM2.0        outside      1   393
Brassicanapus_GSBRNA2T00139105001       TMHMM2.0        TMhelix    394   416
Brassicanapus_GSBRNA2T00139105001       TMHMM2.0        inside     417   422
Brassicanapus_GSBRNA2T00139105001       TMHMM2.0        TMhelix    423   445
Brassicanapus_GSBRNA2T00139105001       TMHMM2.0        outside    446   510
```

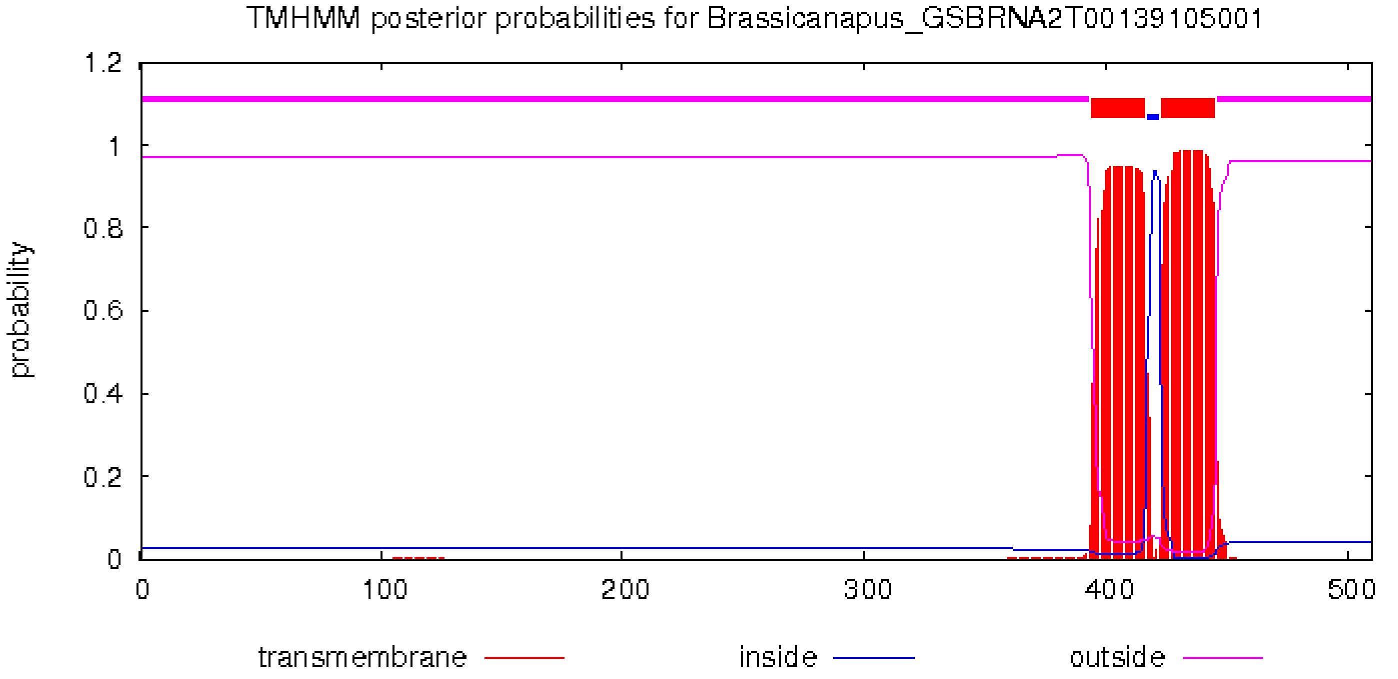

plot in postscript, script for making the plot in gnuplot, data for plot

```
# Brassicanapus_GSBRNA2T00139538001 Length: 145
# Brassicanapus_GSBRNA2T00139538001 Number of predicted TMHs:  0
# Brassicanapus_GSBRNA2T00139538001 Exp number of AAs in TMHs: 0
# Brassicanapus_GSBRNA2T00139538001 Exp number, first 60 AAs:  0
# Brassicanapus_GSBRNA2T00139538001 Total prob of N-in:        0.05412
Brassicanapus_GSBRNA2T00139538001       TMHMM2.0        outside      1   145
```

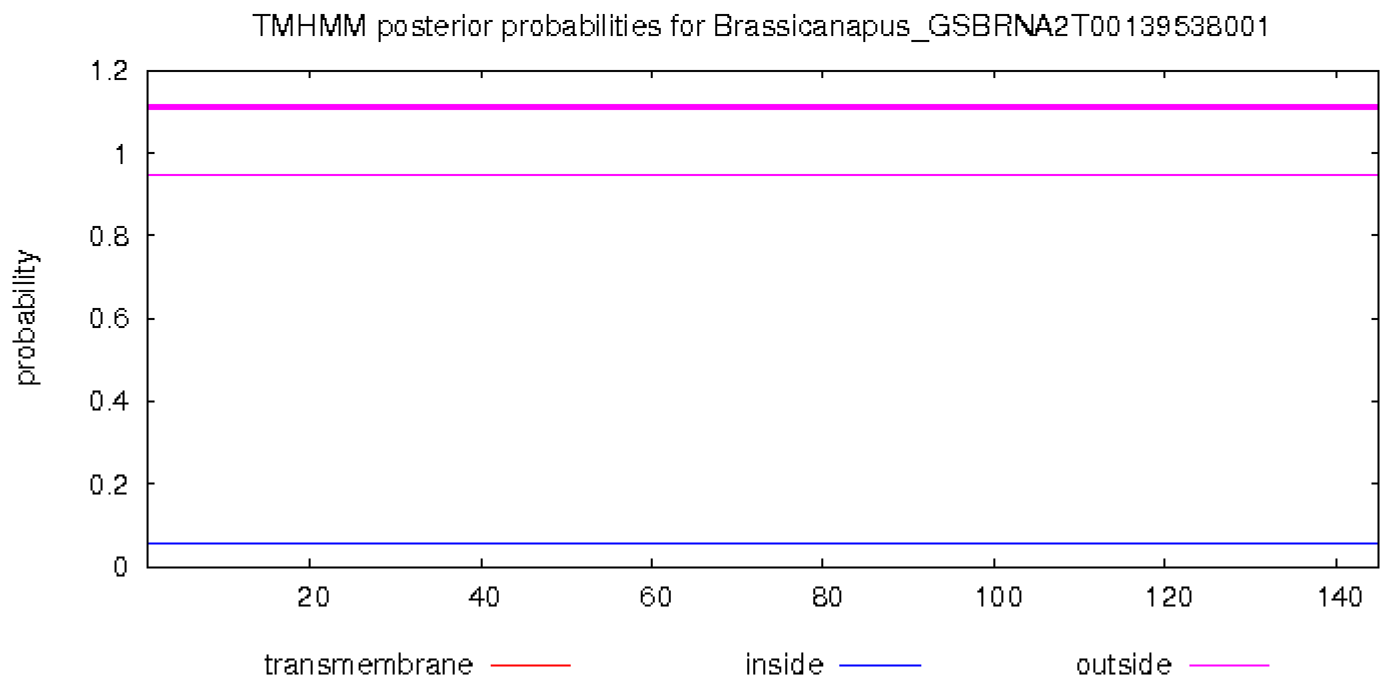

plot in postscript, script for making the plot in gnuplot, data for plot

```
# Brassicanapus_GSBRNA2T00139904001 Length: 193
# Brassicanapus_GSBRNA2T00139904001 Number of predicted TMHs:  0
# Brassicanapus_GSBRNA2T00139904001 Exp number of AAs in TMHs: 0.00053
# Brassicanapus_GSBRNA2T00139904001 Exp number, first 60 AAs:  0
# Brassicanapus_GSBRNA2T00139904001 Total prob of N-in:        0.16083
Brassicanapus_GSBRNA2T00139904001       TMHMM2.0        outside      1   193
```

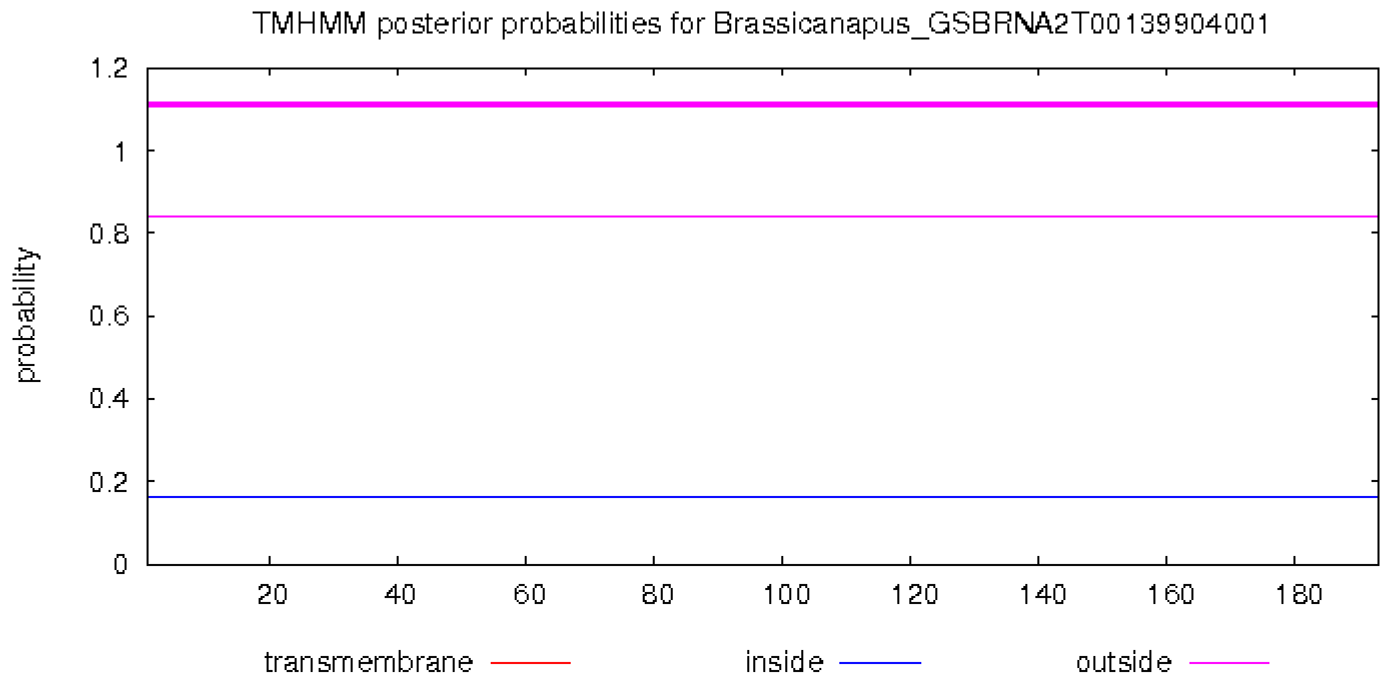

plot in postscript, script for making the plot in gnuplot, data for plot

```
# Brassicanapus_GSBRNA2T00139965001 Length: 283
# Brassicanapus_GSBRNA2T00139965001 Number of predicted TMHs:  0
# Brassicanapus_GSBRNA2T00139965001 Exp number of AAs in TMHs: 0.00025
# Brassicanapus_GSBRNA2T00139965001 Exp number, first 60 AAs:  0
# Brassicanapus_GSBRNA2T00139965001 Total prob of N-in:        0.00643
Brassicanapus_GSBRNA2T00139965001	TMHMM2.0	outside	     1   283
```

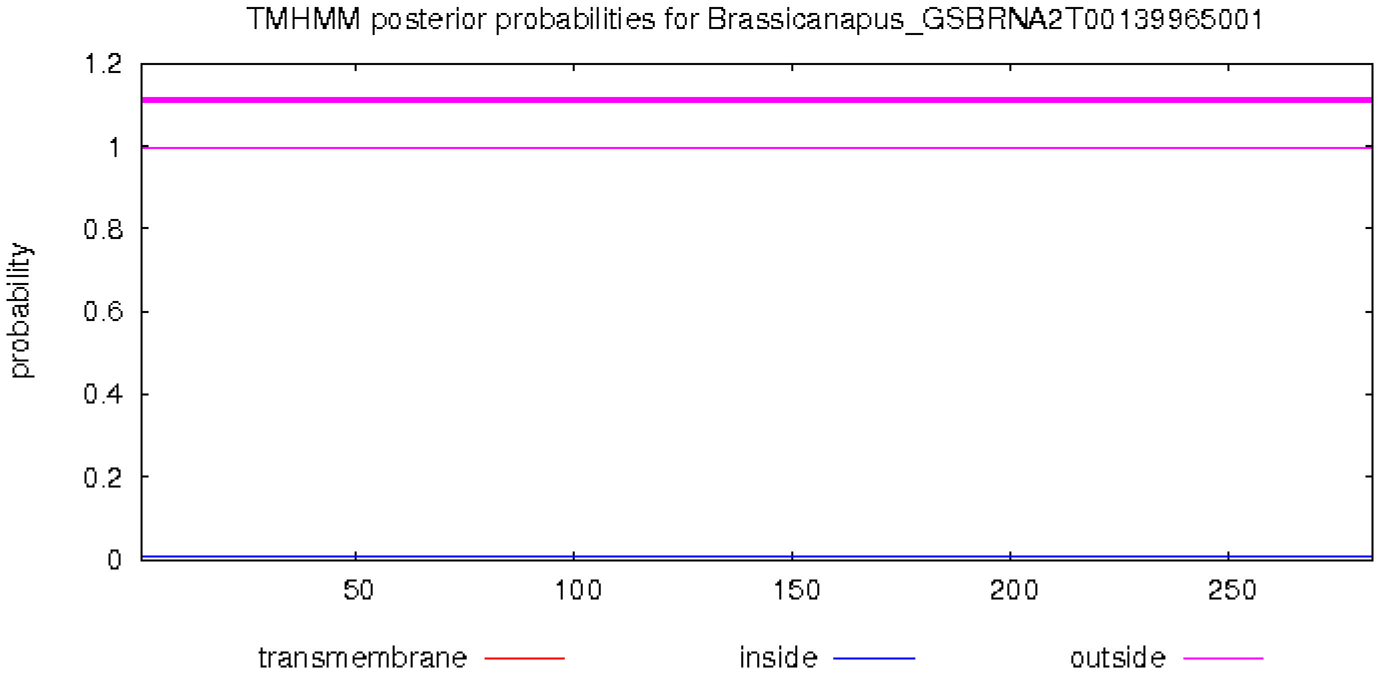

plot in postscript, script for making the plot in gnuplot, data for plot

```
# Brassicanapus_GSBRNA2T00140364001 Length: 208
# Brassicanapus_GSBRNA2T00140364001 Number of predicted TMHs:  0
# Brassicanapus_GSBRNA2T00140364001 Exp number of AAs in TMHs: 0.30125
# Brassicanapus_GSBRNA2T00140364001 Exp number, first 60 AAs:  0.24873
# Brassicanapus_GSBRNA2T00140364001 Total prob of N-in:        0.18529
Brassicanapus_GSBRNA2T00140364001	TMHMM2.0	outside	     1   208
```

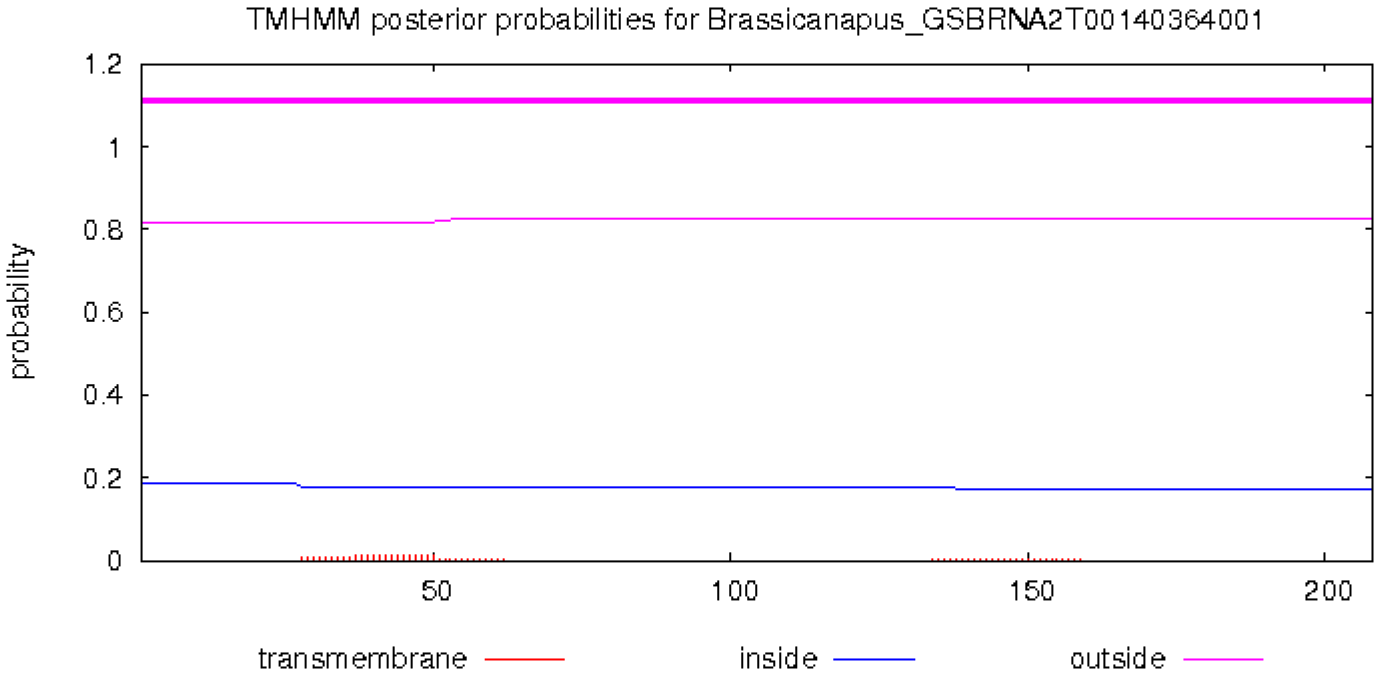

plot in postscript, script for making the plot in gnuplot, data for plot

---

```
# Brassicanapus_GSBRNA2T00140409001 Length: 121
# Brassicanapus_GSBRNA2T00140409001 Number of predicted TMHs:  0
# Brassicanapus_GSBRNA2T00140409001 Exp number of AAs in TMHs: 0
# Brassicanapus_GSBRNA2T00140409001 Exp number, first 60 AAs:  0
# Brassicanapus_GSBRNA2T00140409001 Total prob of N-in:        0.29437
Brassicanapus_GSBRNA2T00140409001       TMHMM2.0        outside      1   121
```

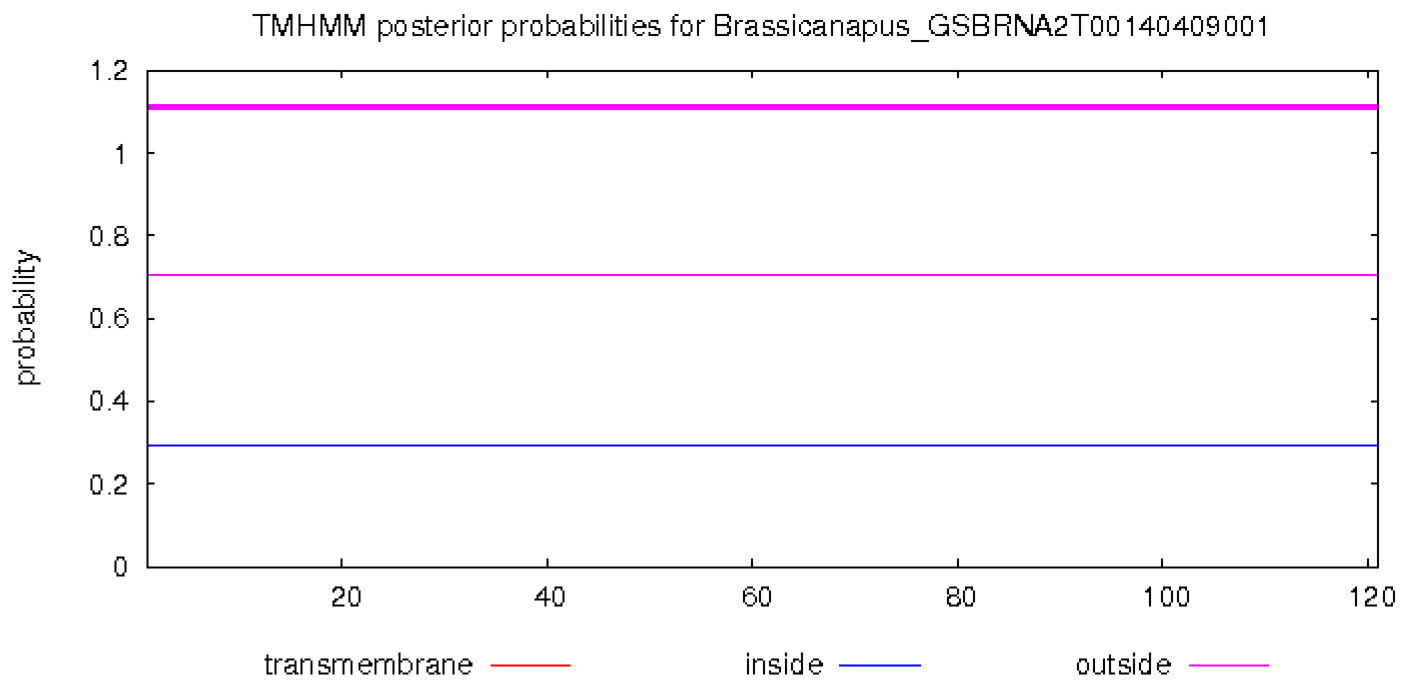

plot in postscript, script for making the plot in gnuplot, data for plot

---

```
# Brassicanapus_GSBRNA2T00140620001 Length: 494
# Brassicanapus_GSBRNA2T00140620001 Number of predicted TMHs:  0
# Brassicanapus_GSBRNA2T00140620001 Exp number of AAs in TMHs: 0.00019
# Brassicanapus_GSBRNA2T00140620001 Exp number, first 60 AAs:  0
# Brassicanapus_GSBRNA2T00140620001 Total prob of N-in:        0.00013
Brassicanapus_GSBRNA2T00140620001       TMHMM2.0        outside      1   494
```

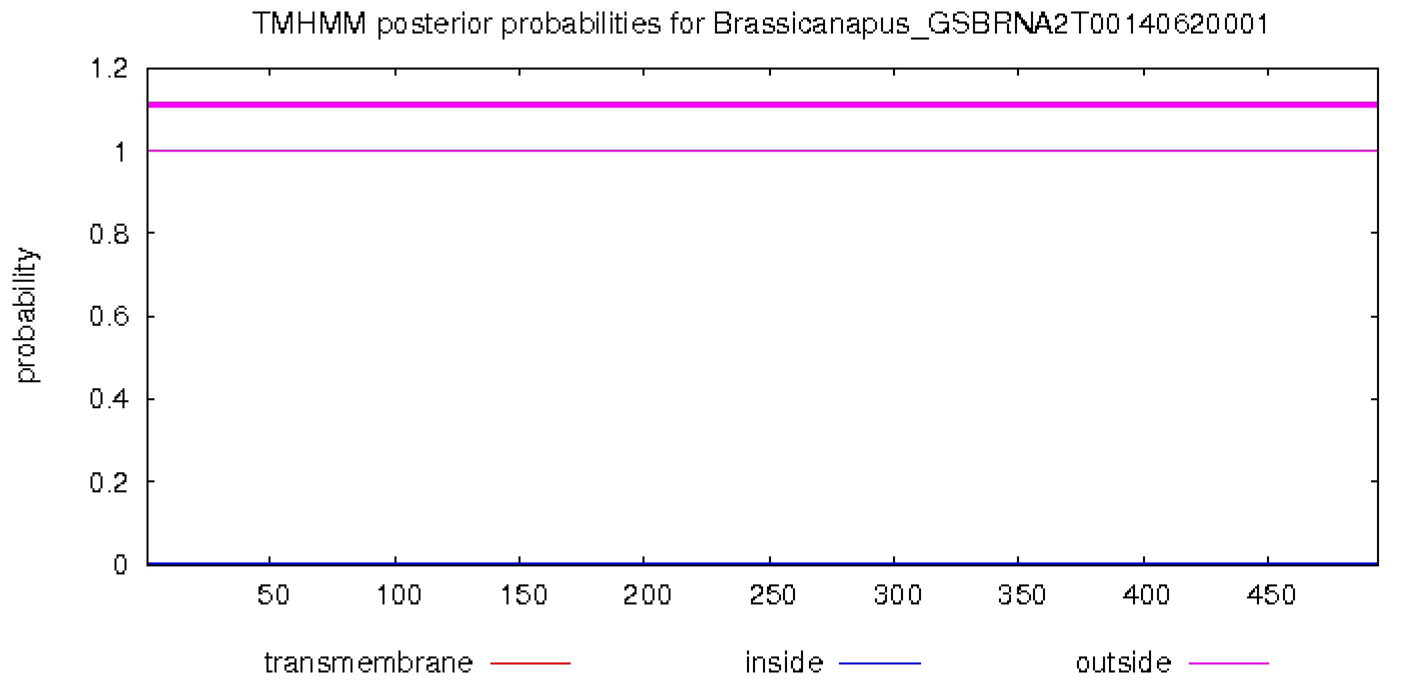

plot in postscript, script for making the plot in gnuplot, data for plot

```
# Brassicanapus_GSBRNA2T00140686001 Length: 337
# Brassicanapus_GSBRNA2T00140686001 Number of predicted TMHs:  0
# Brassicanapus_GSBRNA2T00140686001 Exp number of AAs in TMHs: 0.00037
# Brassicanapus_GSBRNA2T00140686001 Exp number, first 60 AAs:  0
# Brassicanapus_GSBRNA2T00140686001 Total prob of N-in:        0.00891
Brassicanapus_GSBRNA2T00140686001       TMHMM2.0        outside      1   337
```

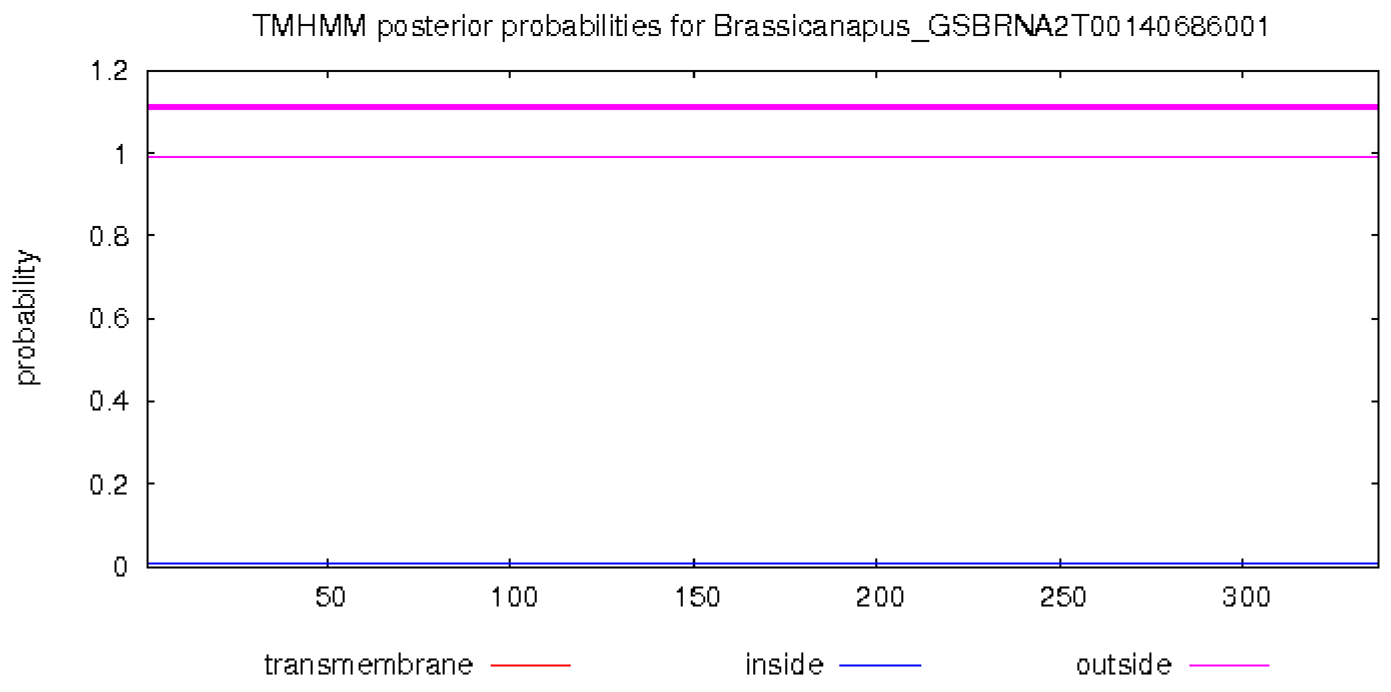

plot in postscript, script for making the plot in gnuplot, data for plot

```
# Brassicanapus_GSBRNA2T00140926001 Length: 334
# Brassicanapus_GSBRNA2T00140926001 Number of predicted TMHs:  0
# Brassicanapus_GSBRNA2T00140926001 Exp number of AAs in TMHs: 10.53305
# Brassicanapus_GSBRNA2T00140926001 Exp number, first 60 AAs:  0.18672
# Brassicanapus_GSBRNA2T00140926001 Total prob of N-in:        0.08751
Brassicanapus_GSBRNA2T00140926001       TMHMM2.0        outside      1   334
```

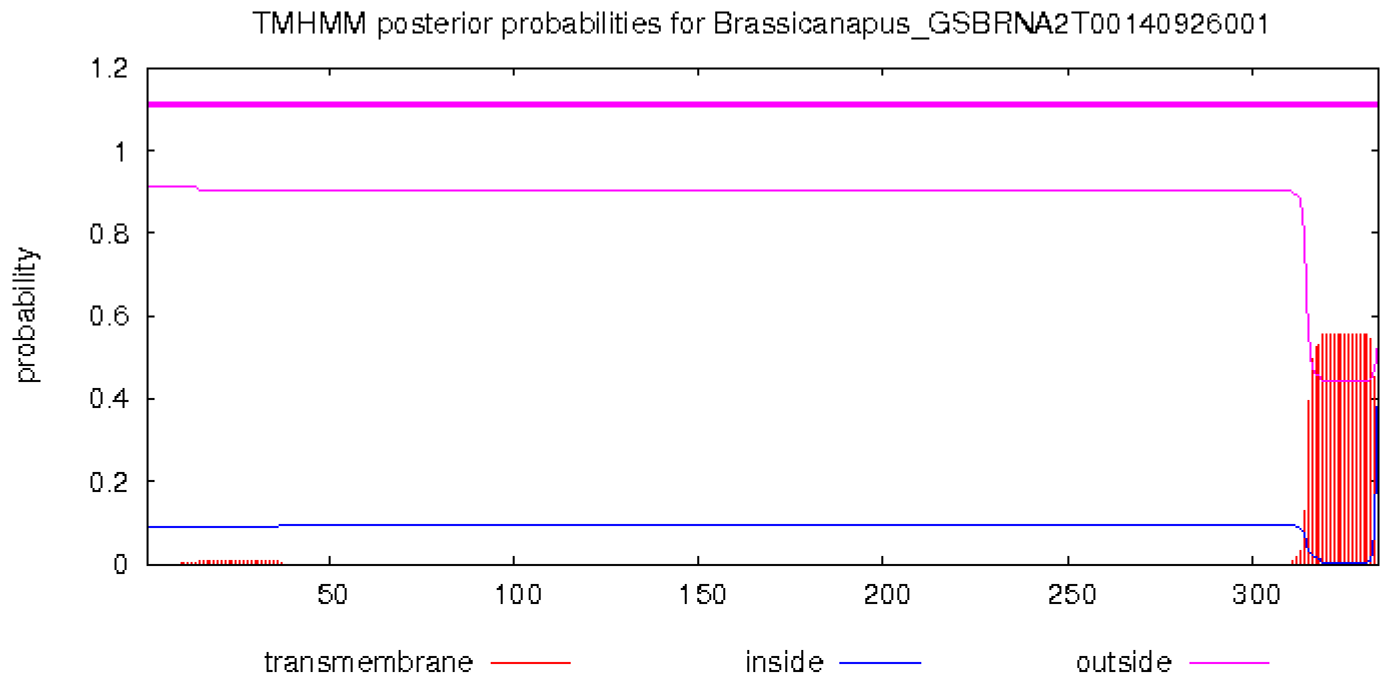

plot in postscript, script for making the plot in gnuplot, data for plot

---

```
# Brassicanapus_GSBRNA2T00141573001 Length: 152
# Brassicanapus_GSBRNA2T00141573001 Number of predicted TMHs:  0
# Brassicanapus_GSBRNA2T00141573001 Exp number of AAs in TMHs: 0
# Brassicanapus_GSBRNA2T00141573001 Exp number, first 60 AAs:  0
# Brassicanapus_GSBRNA2T00141573001 Total prob of N-in:        0.14435
Brassicanapus_GSBRNA2T00141573001       TMHMM2.0        outside      1   152
```

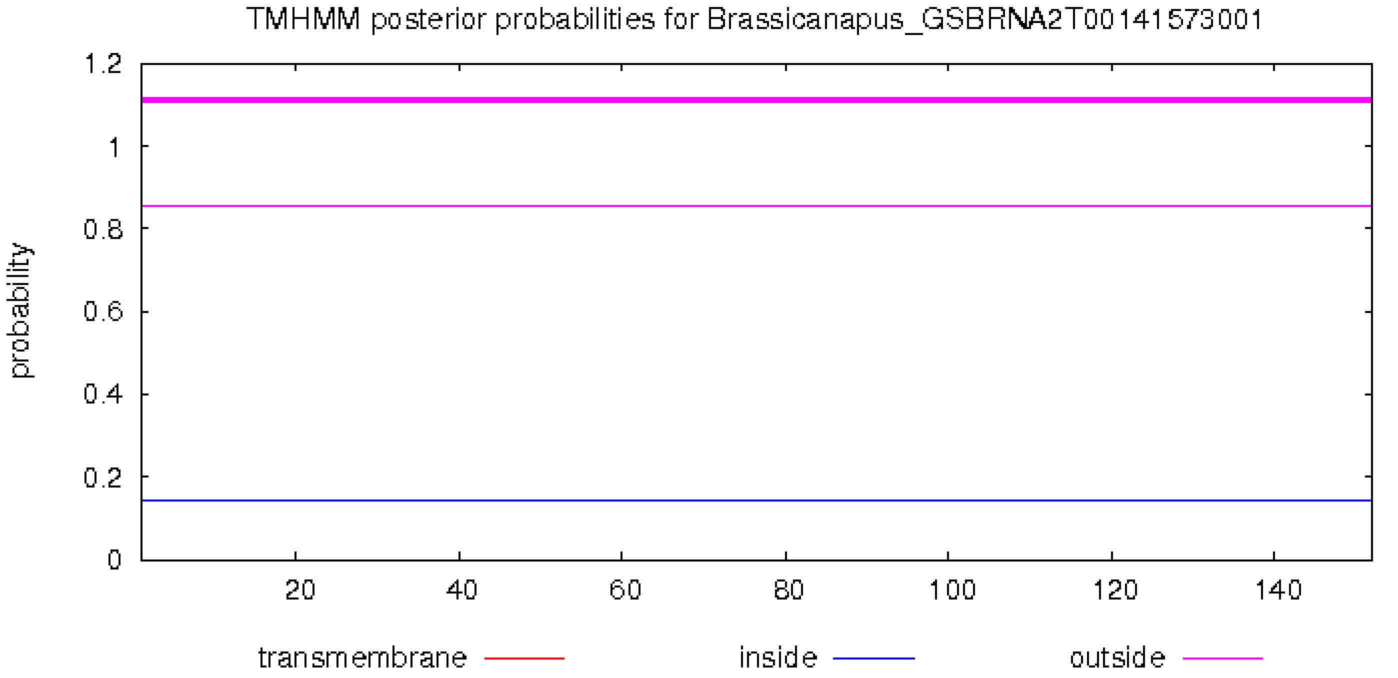

plot in postscript, script for making the plot in gnuplot, data for plot

---

```
# Brassicanapus_GSBRNA2T00141649001 Length: 252
# Brassicanapus_GSBRNA2T00141649001 Number of predicted TMHs:  0
# Brassicanapus_GSBRNA2T00141649001 Exp number of AAs in TMHs: 0
# Brassicanapus_GSBRNA2T00141649001 Exp number, first 60 AAs:  0
# Brassicanapus_GSBRNA2T00141649001 Total prob of N-in:        0.02031
Brassicanapus_GSBRNA2T00141649001       TMHMM2.0        outside      1   252
```

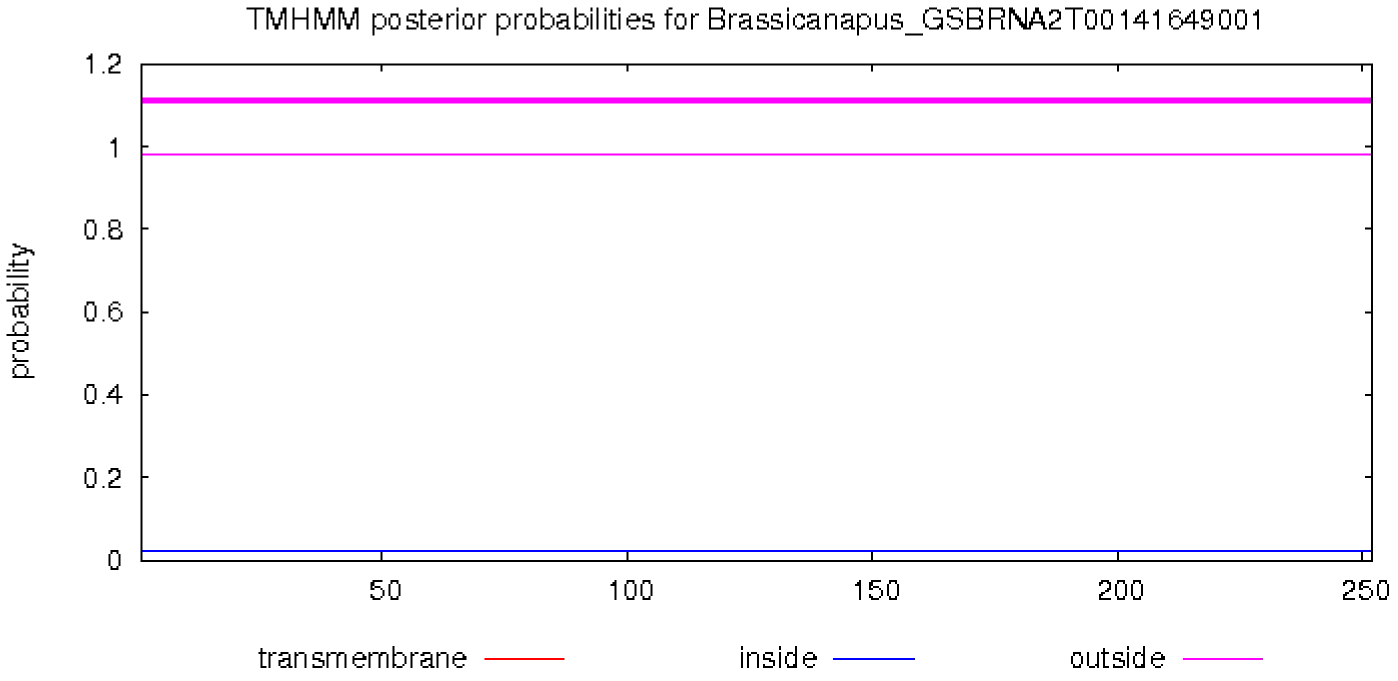

plot in postscript, script for making the plot in gnuplot, data for plot

```
# Brassicanapus_GSBRNA2T00141706001 Length: 346
# Brassicanapus_GSBRNA2T00141706001 Number of predicted TMHs:  0
# Brassicanapus_GSBRNA2T00141706001 Exp number of AAs in TMHs: 0.00018
# Brassicanapus_GSBRNA2T00141706001 Exp number, first 60 AAs:  0.00018
# Brassicanapus_GSBRNA2T00141706001 Total prob of N-in:        0.00335
Brassicanapus_GSBRNA2T00141706001	TMHMM2.0	outside	     1   346
```

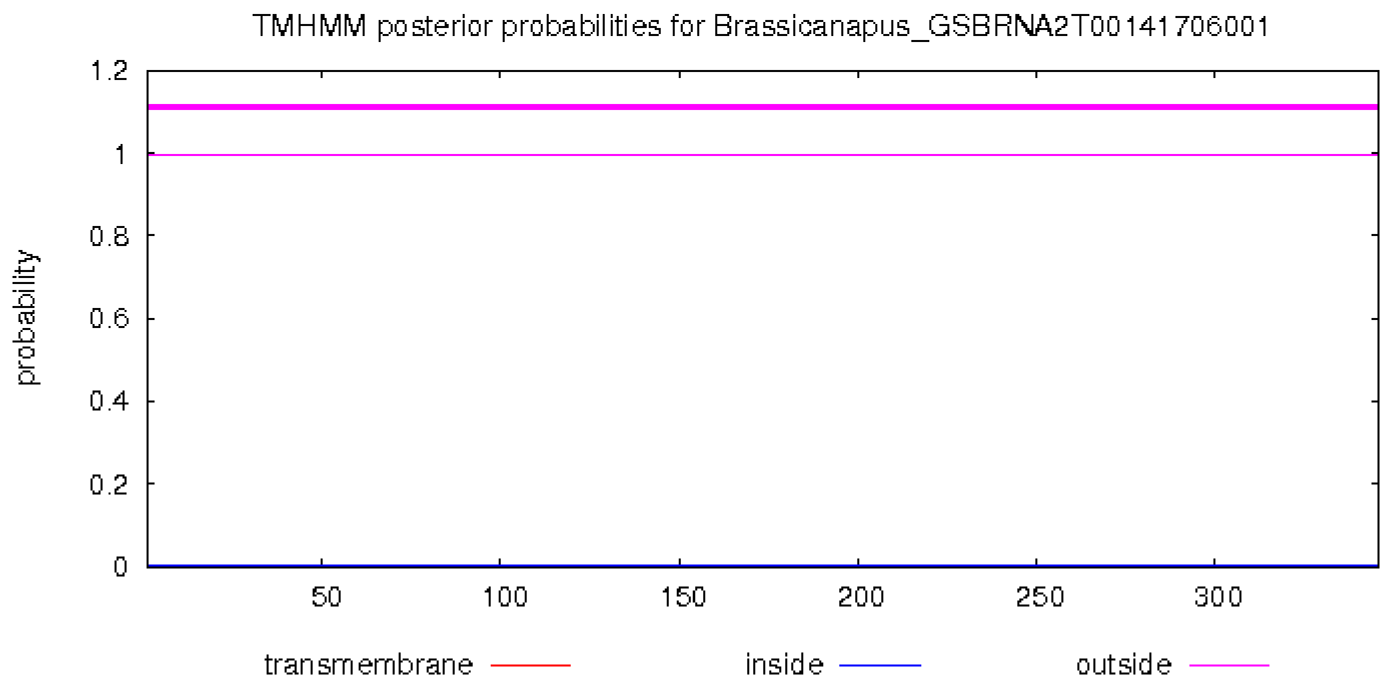

plot in postscript, script for making the plot in gnuplot, data for plot

```
# Brassicanapus_GSBRNA2T00142424001 Length: 448
# Brassicanapus_GSBRNA2T00142424001 Number of predicted TMHs:  0
# Brassicanapus_GSBRNA2T00142424001 Exp number of AAs in TMHs: 0.00158
# Brassicanapus_GSBRNA2T00142424001 Exp number, first 60 AAs:  0
# Brassicanapus_GSBRNA2T00142424001 Total prob of N-in:        0.00328
Brassicanapus_GSBRNA2T00142424001	TMHMM2.0	outside	     1   448
```

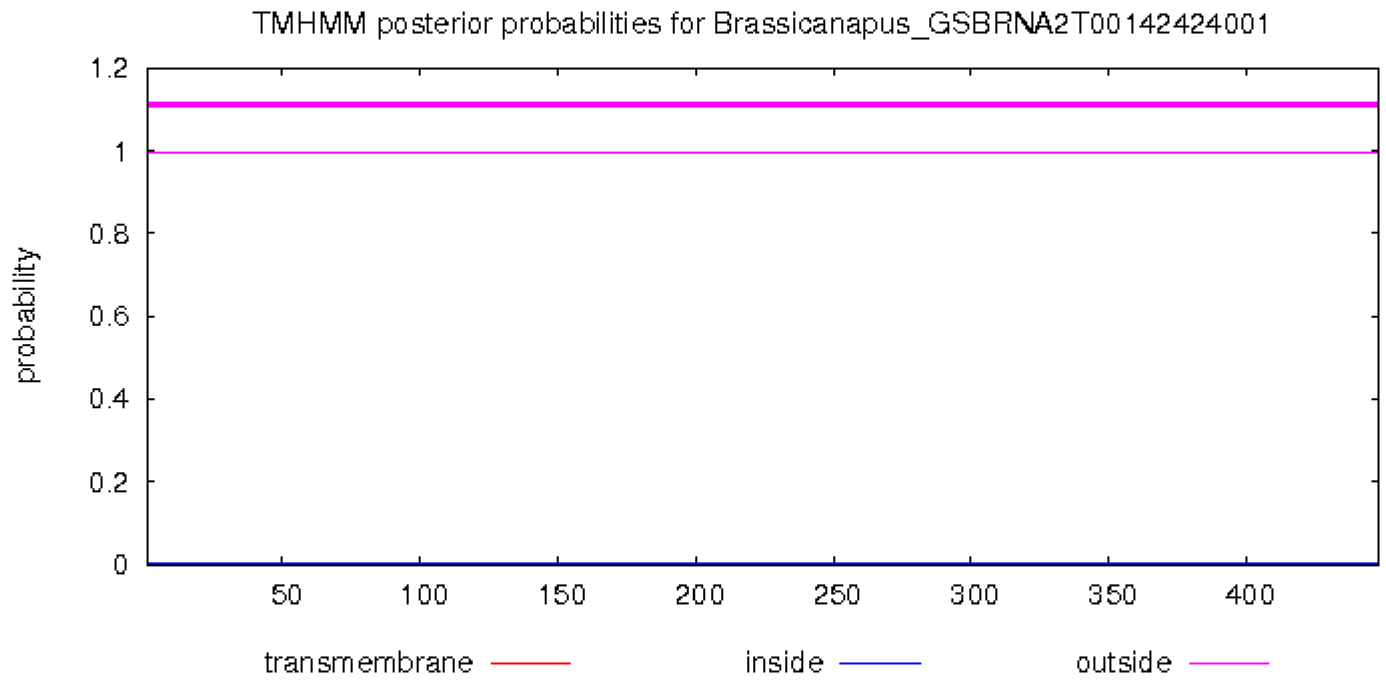

plot in postscript, script for making the plot in gnuplot, data for plot

```
# Brassicanapus_GSBRNA2T00142881001 Length: 321
# Brassicanapus_GSBRNA2T00142881001 Number of predicted TMHs:  0
# Brassicanapus_GSBRNA2T00142881001 Exp number of AAs in TMHs: 0.00915999999999999
# Brassicanapus_GSBRNA2T00142881001 Exp number, first 60 AAs:  0.00036
# Brassicanapus_GSBRNA2T00142881001 Total prob of N-in:        0.03451
Brassicanapus_GSBRNA2T00142881001	TMHMM2.0	outside	     1   321
```

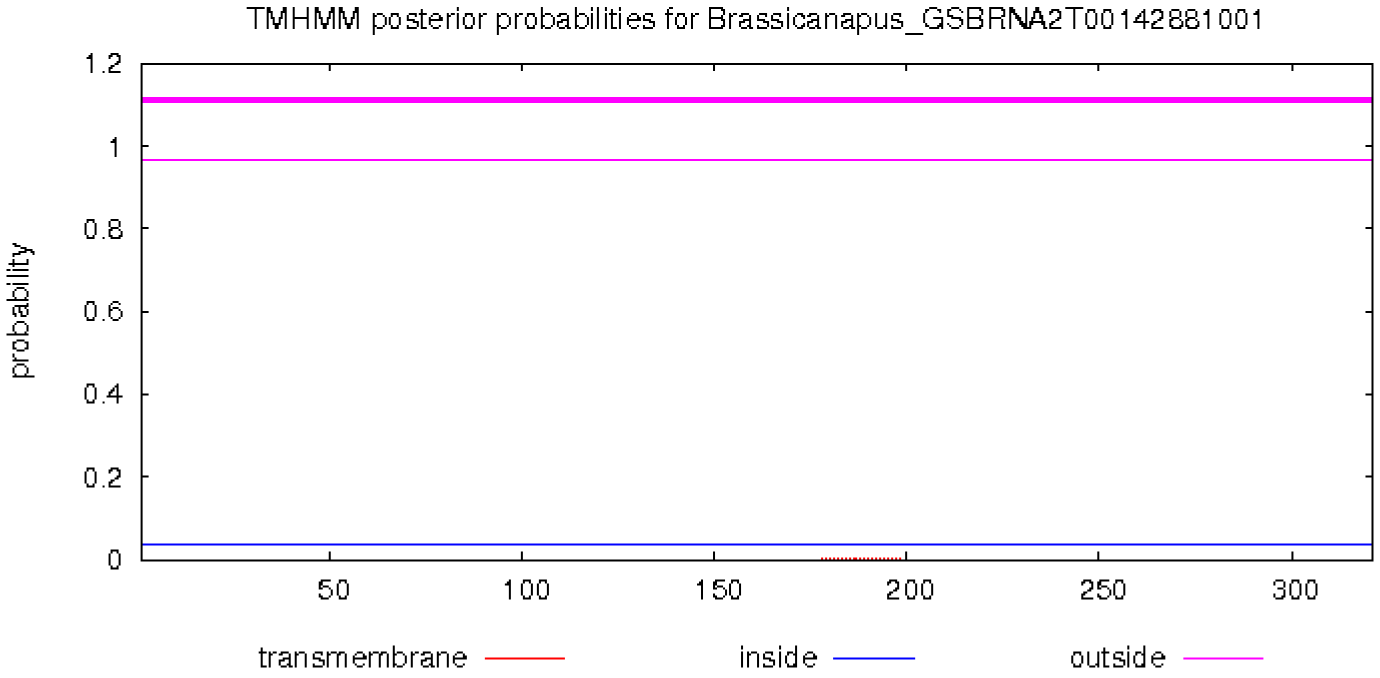

plot in postscript, script for making the plot in gnuplot, data for plot

```
# Brassicanapus_GSBRNA2T00143403001 Length: 247
# Brassicanapus_GSBRNA2T00143403001 Number of predicted TMHs:  0
# Brassicanapus_GSBRNA2T00143403001 Exp number of AAs in TMHs: 0
# Brassicanapus_GSBRNA2T00143403001 Exp number, first 60 AAs:  0
# Brassicanapus_GSBRNA2T00143403001 Total prob of N-in:        0.11741
Brassicanapus_GSBRNA2T00143403001	TMHMM2.0	outside	     1   247
```

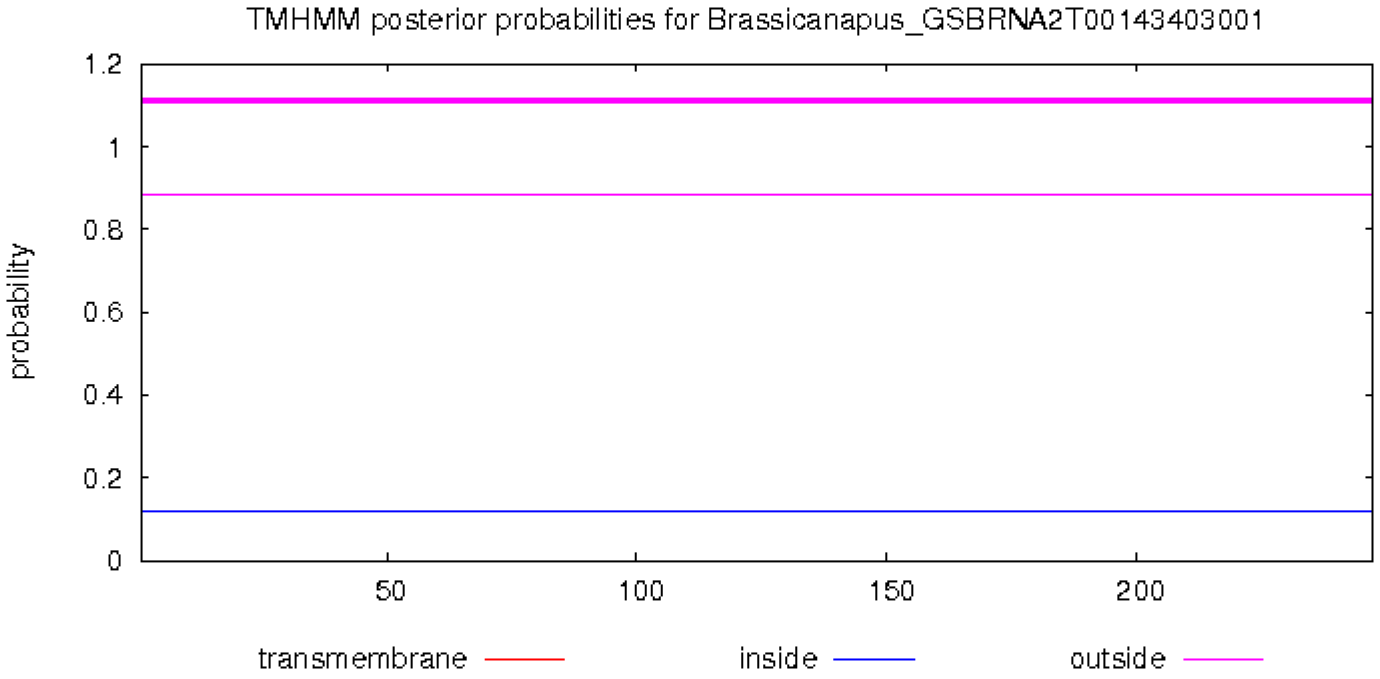

plot in postscript, script for making the plot in gnuplot, data for plot

---

```
# Brassicanapus_GSBRNA2T00143470001 Length: 361
# Brassicanapus_GSBRNA2T00143470001 Number of predicted TMHs:  0
# Brassicanapus_GSBRNA2T00143470001 Exp number of AAs in TMHs: 0.00078
# Brassicanapus_GSBRNA2T00143470001 Exp number, first 60 AAs:  0.0006
# Brassicanapus_GSBRNA2T00143470001 Total prob of N-in:        0.01668
Brassicanapus_GSBRNA2T00143470001	TMHMM2.0	outside	     1   361
```

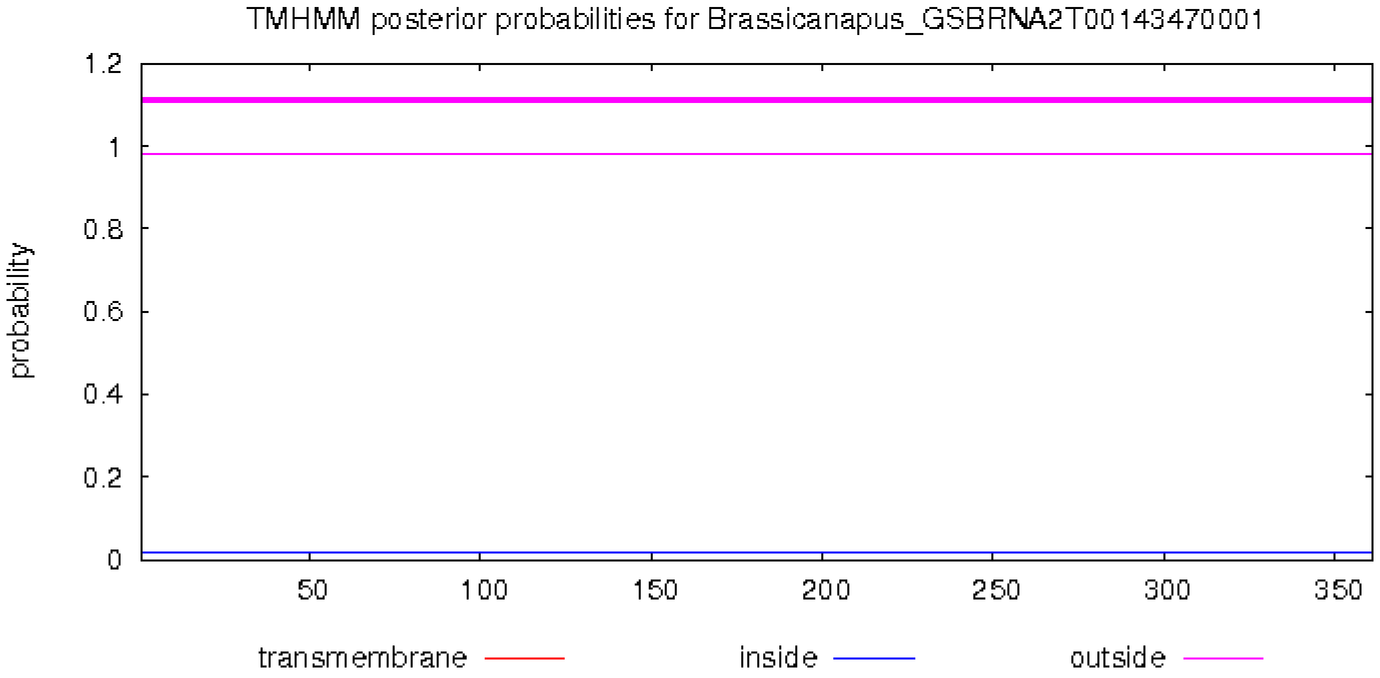

plot in postscript, script for making the plot in gnuplot, data for plot

---

```
# Brassicanapus_GSBRNA2T00144521001 Length: 309
# Brassicanapus_GSBRNA2T00144521001 Number of predicted TMHs:  0
# Brassicanapus_GSBRNA2T00144521001 Exp number of AAs in TMHs: 0.00447
# Brassicanapus_GSBRNA2T00144521001 Exp number, first 60 AAs:  0.00058
# Brassicanapus_GSBRNA2T00144521001 Total prob of N-in:        0.03958
Brassicanapus_GSBRNA2T00144521001	TMHMM2.0	outside	     1   309
```

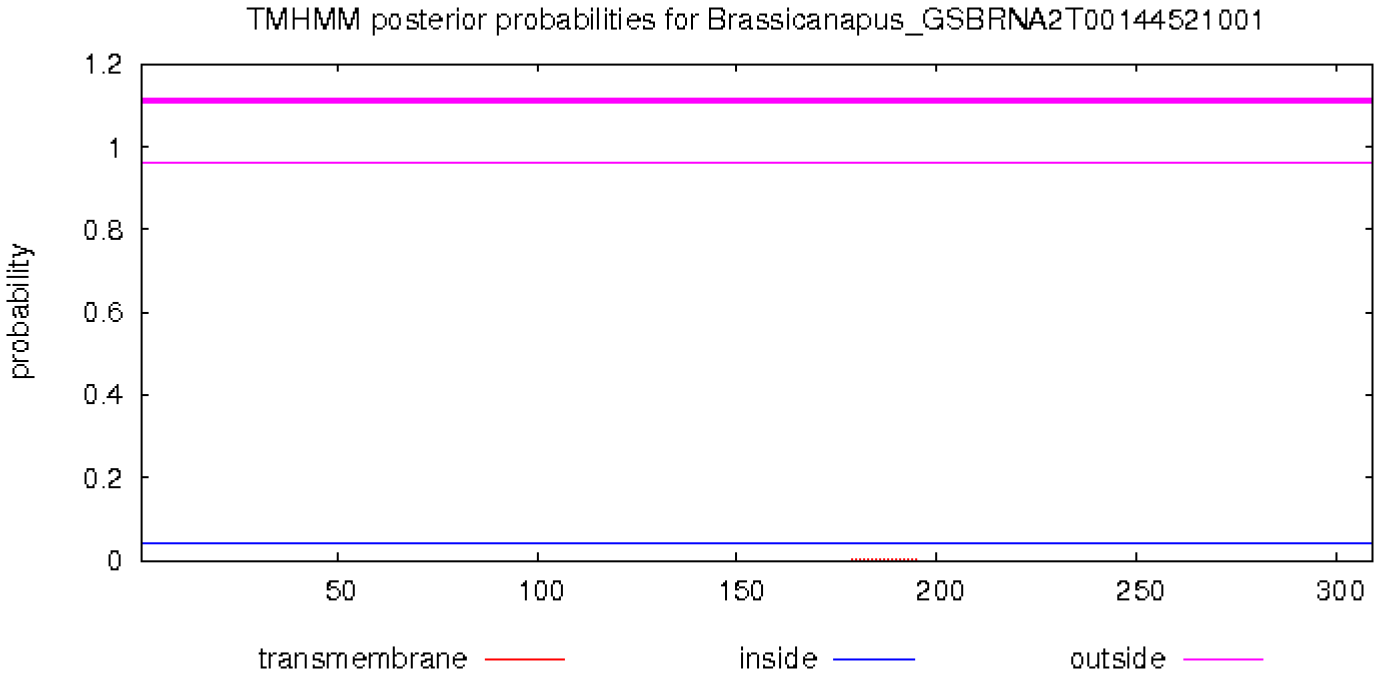

plot in postscript, script for making the plot in gnuplot, data for plot

```
# Brassicanapus_GSBRNA2T00145006001 Length: 307
# Brassicanapus_GSBRNA2T00145006001 Number of predicted TMHs:  0
# Brassicanapus_GSBRNA2T00145006001 Exp number of AAs in TMHs: 0.0522
# Brassicanapus_GSBRNA2T00145006001 Exp number, first 60 AAs:  0.03986
# Brassicanapus_GSBRNA2T00145006001 Total prob of N-in:        0.00479
Brassicanapus_GSBRNA2T00145006001	TMHMM2.0	outside	     1   307
```

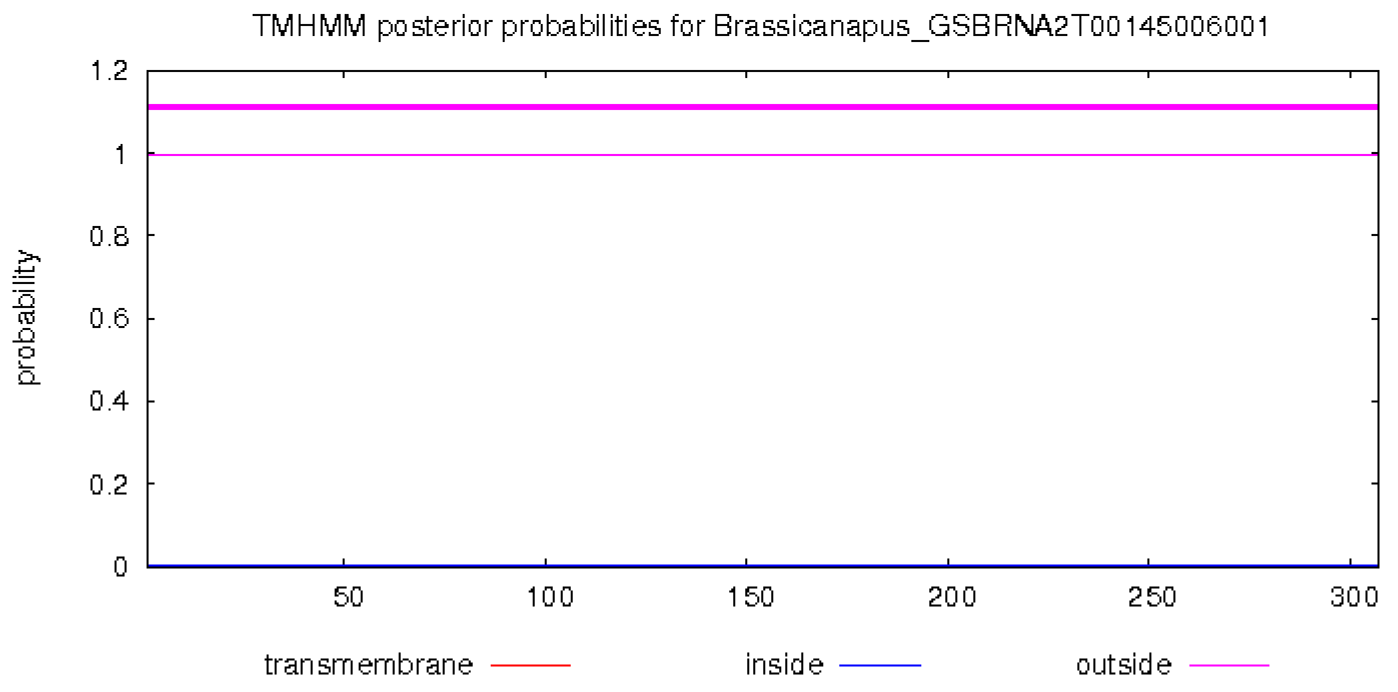

plot in postscript, script for making the plot in gnuplot, data for plot

```
# Brassicanapus_GSBRNA2T00145796001 Length: 316
# Brassicanapus_GSBRNA2T00145796001 Number of predicted TMHs:  0
# Brassicanapus_GSBRNA2T00145796001 Exp number of AAs in TMHs: 0
# Brassicanapus_GSBRNA2T00145796001 Exp number, first 60 AAs:  0
# Brassicanapus_GSBRNA2T00145796001 Total prob of N-in:        0.02152
Brassicanapus_GSBRNA2T00145796001	TMHMM2.0	outside	     1   316
```

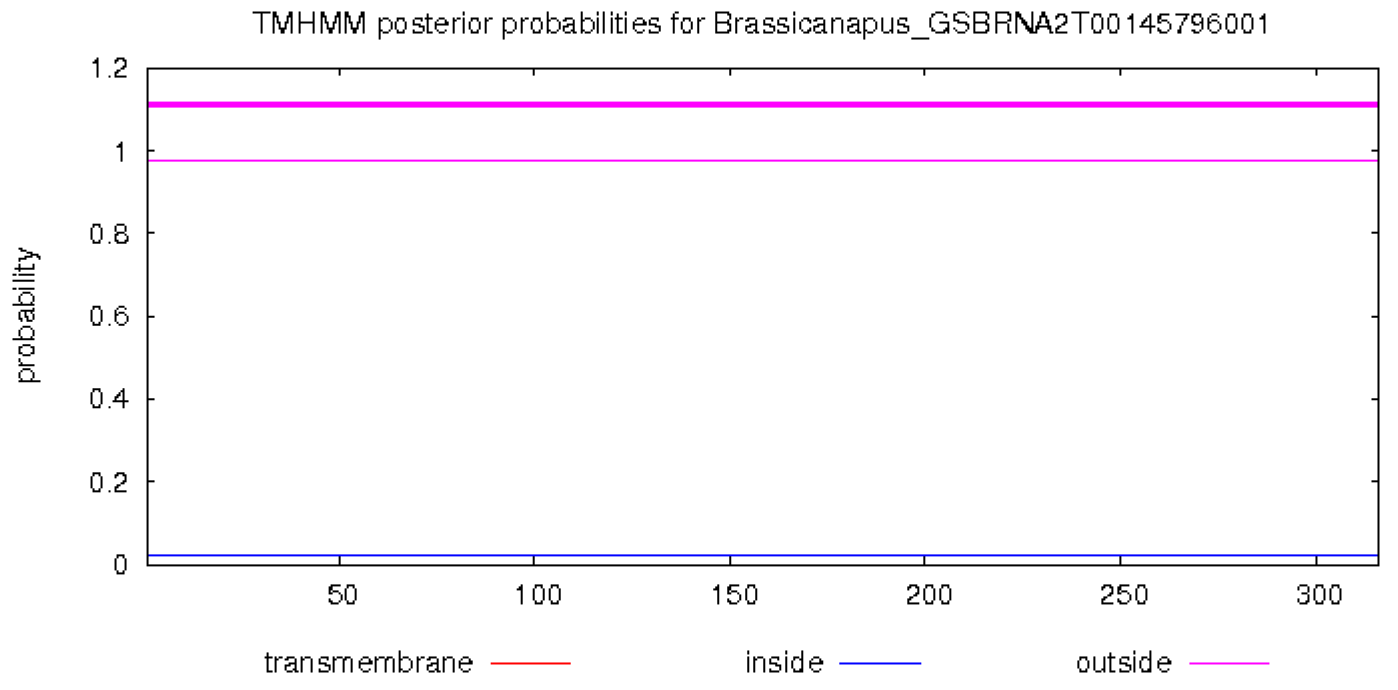

plot in postscript, script for making the plot in gnuplot, data for plot

```
# Brassicanapus_GSBRNA2T00146022001 Length: 832
# Brassicanapus_GSBRNA2T00146022001 Number of predicted TMHs:  0
# Brassicanapus_GSBRNA2T00146022001 Exp number of AAs in TMHs: 9.10537
# Brassicanapus_GSBRNA2T00146022001 Exp number, first 60 AAs:  0
# Brassicanapus_GSBRNA2T00146022001 Total prob of N-in:        0.00111
Brassicanapus_GSBRNA2T00146022001       TMHMM2.0        outside      1   832
```

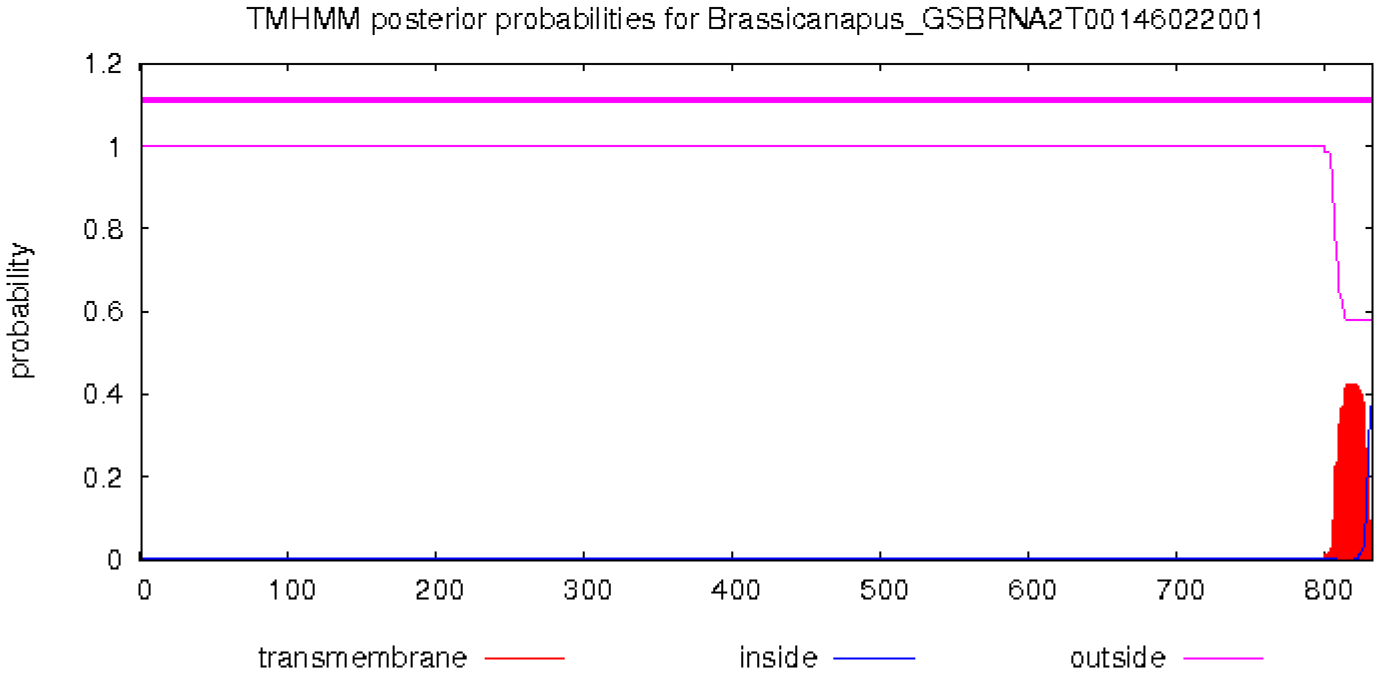

plot in postscript, script for making the plot in gnuplot, data for plot

```
# Brassicanapus_GSBRNA2T00146186001 Length: 336
# Brassicanapus_GSBRNA2T00146186001 Number of predicted TMHs:  0
# Brassicanapus_GSBRNA2T00146186001 Exp number of AAs in TMHs: 0
# Brassicanapus_GSBRNA2T00146186001 Exp number, first 60 AAs:  0
# Brassicanapus_GSBRNA2T00146186001 Total prob of N-in:        0.00220
Brassicanapus_GSBRNA2T00146186001       TMHMM2.0        outside      1   336
```

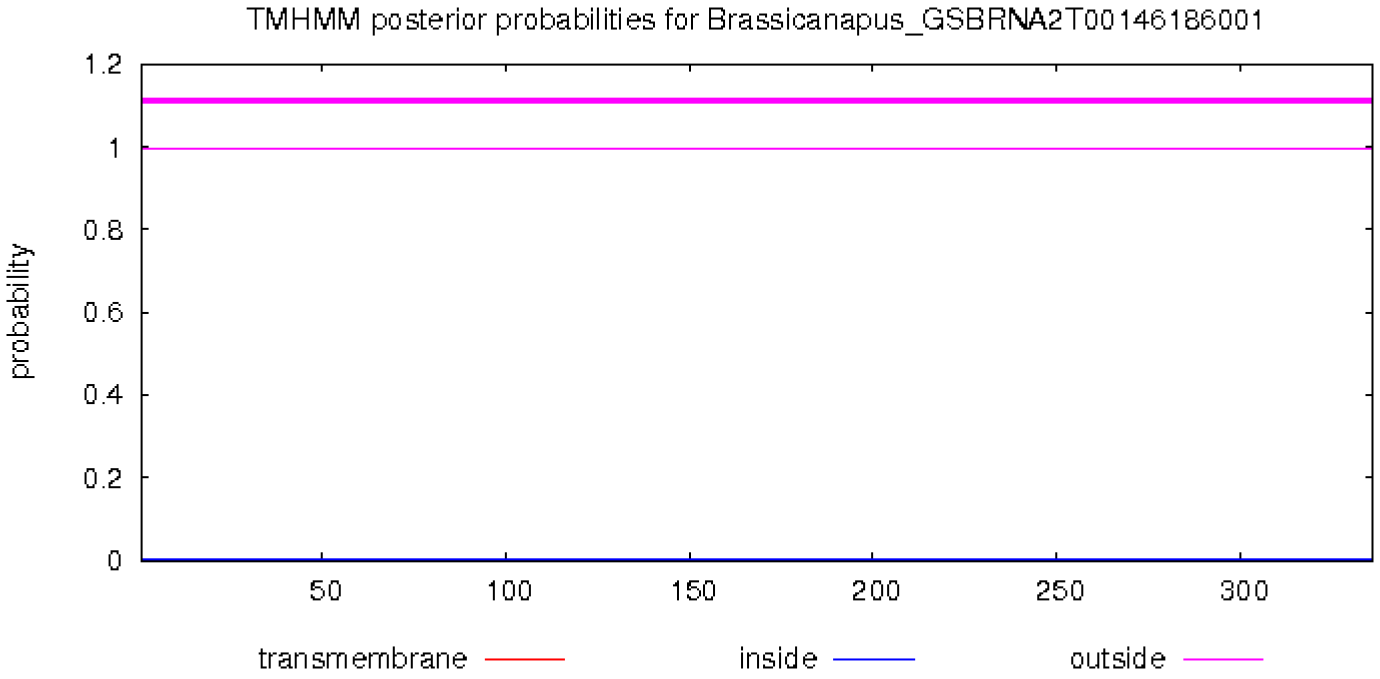

plot in postscript, script for making the plot in gnuplot, data for plot

---

```
# Brassicanapus_GSBRNA2T00146341001 Length: 360
# Brassicanapus_GSBRNA2T00146341001 Number of predicted TMHs:  0
# Brassicanapus_GSBRNA2T00146341001 Exp number of AAs in TMHs: 0.00866
# Brassicanapus_GSBRNA2T00146341001 Exp number, first 60 AAs:  0.00096
# Brassicanapus_GSBRNA2T00146341001 Total prob of N-in:        0.00878
Brassicanapus_GSBRNA2T00146341001	TMHMM2.0	outside	     1   360
```

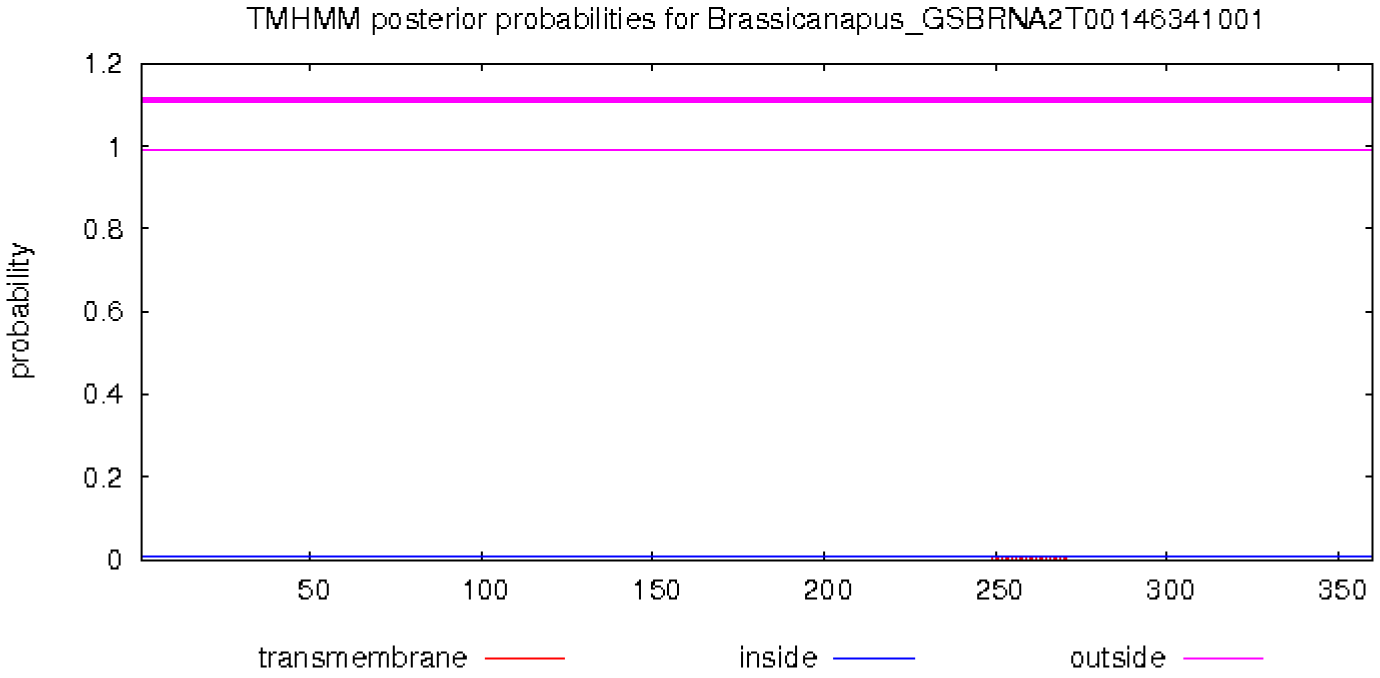

plot in postscript, script for making the plot in gnuplot, data for plot

---

```
# Brassicanapus_GSBRNA2T00146607001 Length: 335
# Brassicanapus_GSBRNA2T00146607001 Number of predicted TMHs:  0
# Brassicanapus_GSBRNA2T00146607001 Exp number of AAs in TMHs: 0.09061
# Brassicanapus_GSBRNA2T00146607001 Exp number, first 60 AAs:  0.02771
# Brassicanapus_GSBRNA2T00146607001 Total prob of N-in:        0.00669
Brassicanapus_GSBRNA2T00146607001	TMHMM2.0	outside	     1   335
```

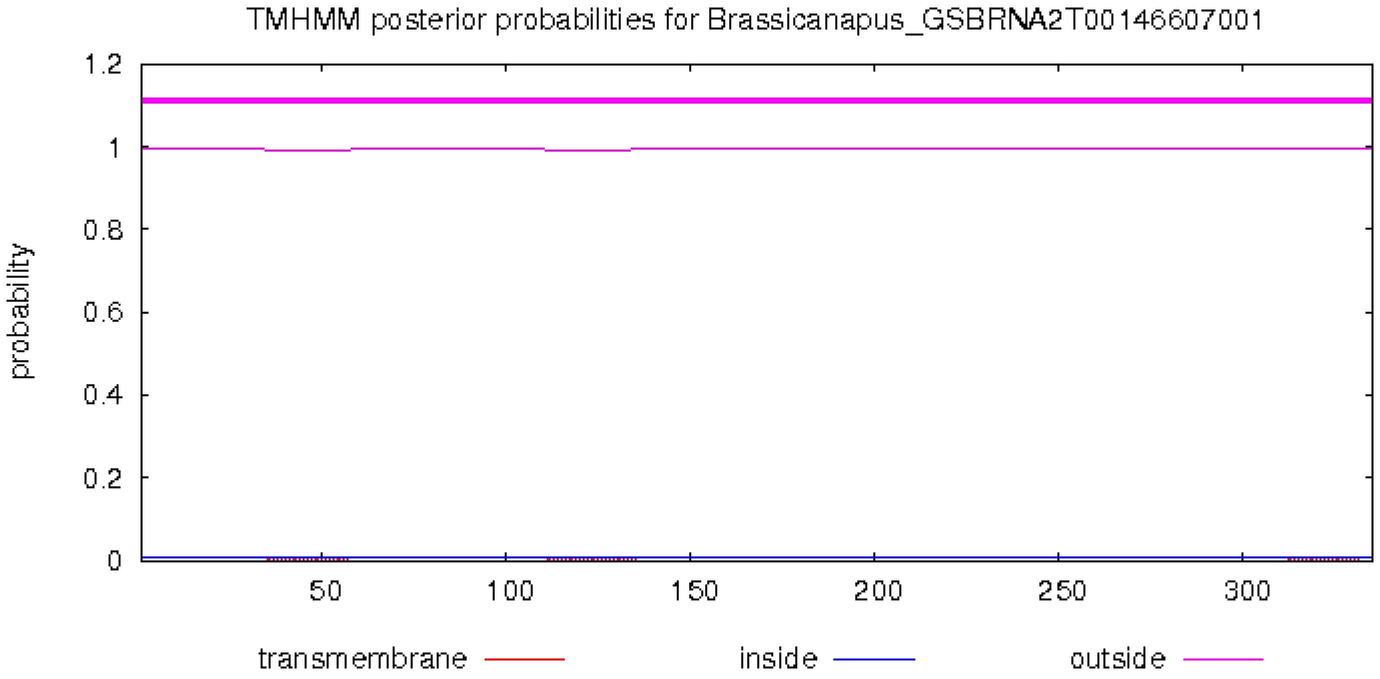

plot in postscript, script for making the plot in gnuplot, data for plot

```
# Brassicanapus_GSBRNA2T00146726001 Length: 294
# Brassicanapus_GSBRNA2T00146726001 Number of predicted TMHs:  0
# Brassicanapus_GSBRNA2T00146726001 Exp number of AAs in TMHs: 0.00055
# Brassicanapus_GSBRNA2T00146726001 Exp number, first 60 AAs:  0
# Brassicanapus_GSBRNA2T00146726001 Total prob of N-in:        0.00206
Brassicanapus_GSBRNA2T00146726001	TMHMM2.0	outside	     1   294
```

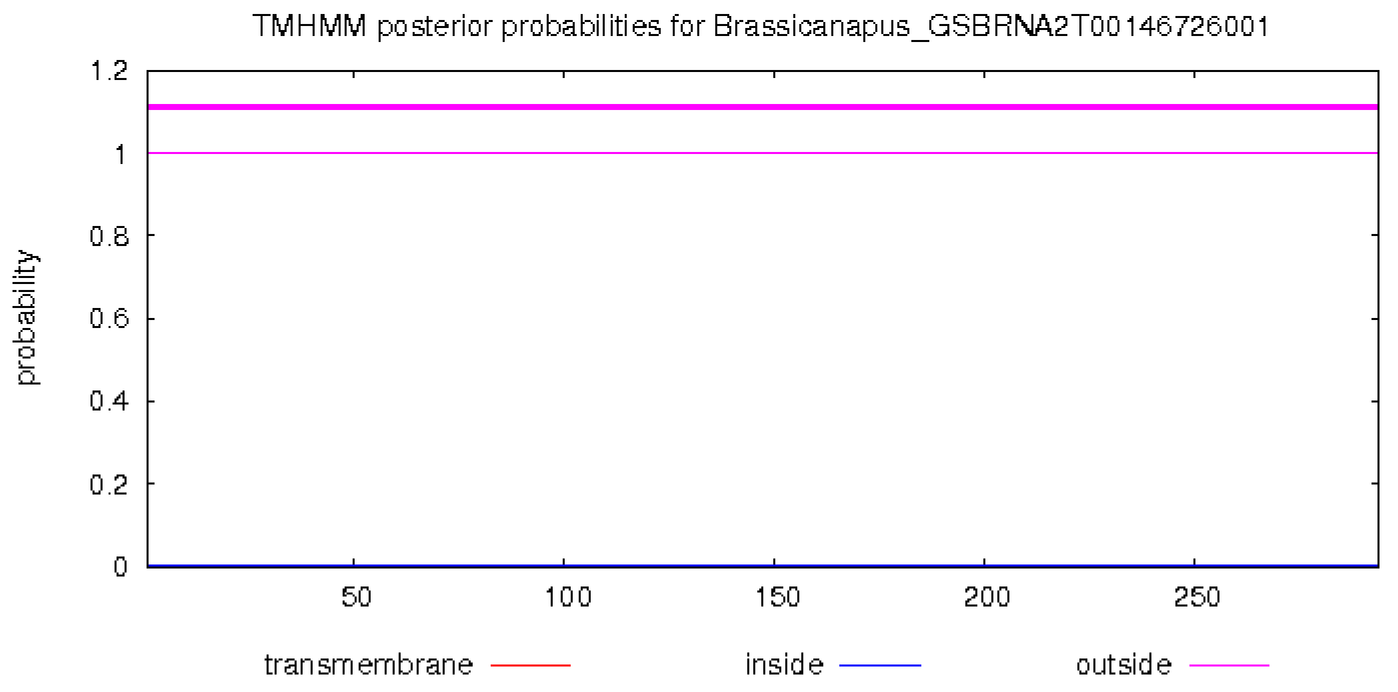

plot in postscript, script for making the plot in gnuplot, data for plot

```
# Brassicanapus_GSBRNA2T00147549001 Length: 443
# Brassicanapus_GSBRNA2T00147549001 Number of predicted TMHs:  0
# Brassicanapus_GSBRNA2T00147549001 Exp number of AAs in TMHs: 11.13711
# Brassicanapus_GSBRNA2T00147549001 Exp number, first 60 AAs:  0
# Brassicanapus_GSBRNA2T00147549001 Total prob of N-in:        0.00365
Brassicanapus_GSBRNA2T00147549001	TMHMM2.0	outside	     1   443
```

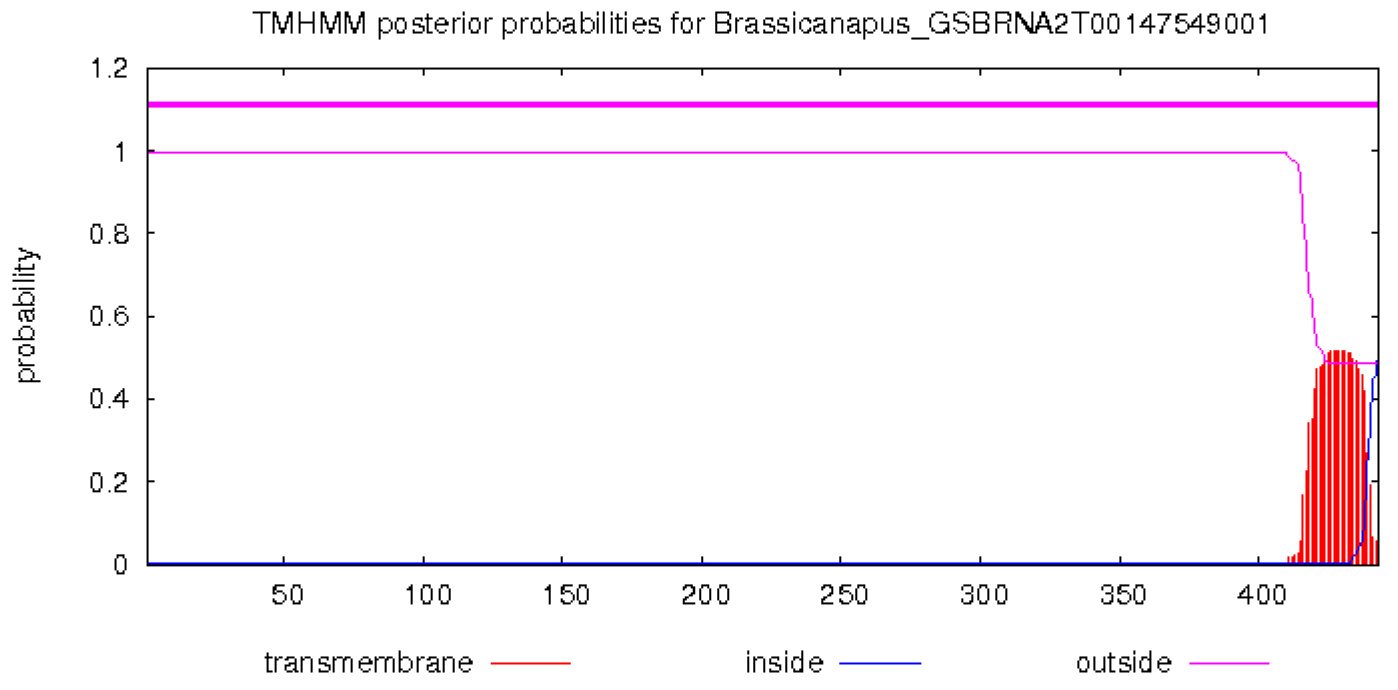

plot in postscript, script for making the plot in gnuplot, data for plot

```
# Brassicanapus_GSBRNA2T00147768001 Length: 337
# Brassicanapus_GSBRNA2T00147768001 Number of predicted TMHs:  0
# Brassicanapus_GSBRNA2T00147768001 Exp number of AAs in TMHs: 0.05491
# Brassicanapus_GSBRNA2T00147768001 Exp number, first 60 AAs:  0.02524
# Brassicanapus_GSBRNA2T00147768001 Total prob of N-in:        0.00589
Brassicanapus_GSBRNA2T00147768001	TMHMM2.0	outside	     1   337
```

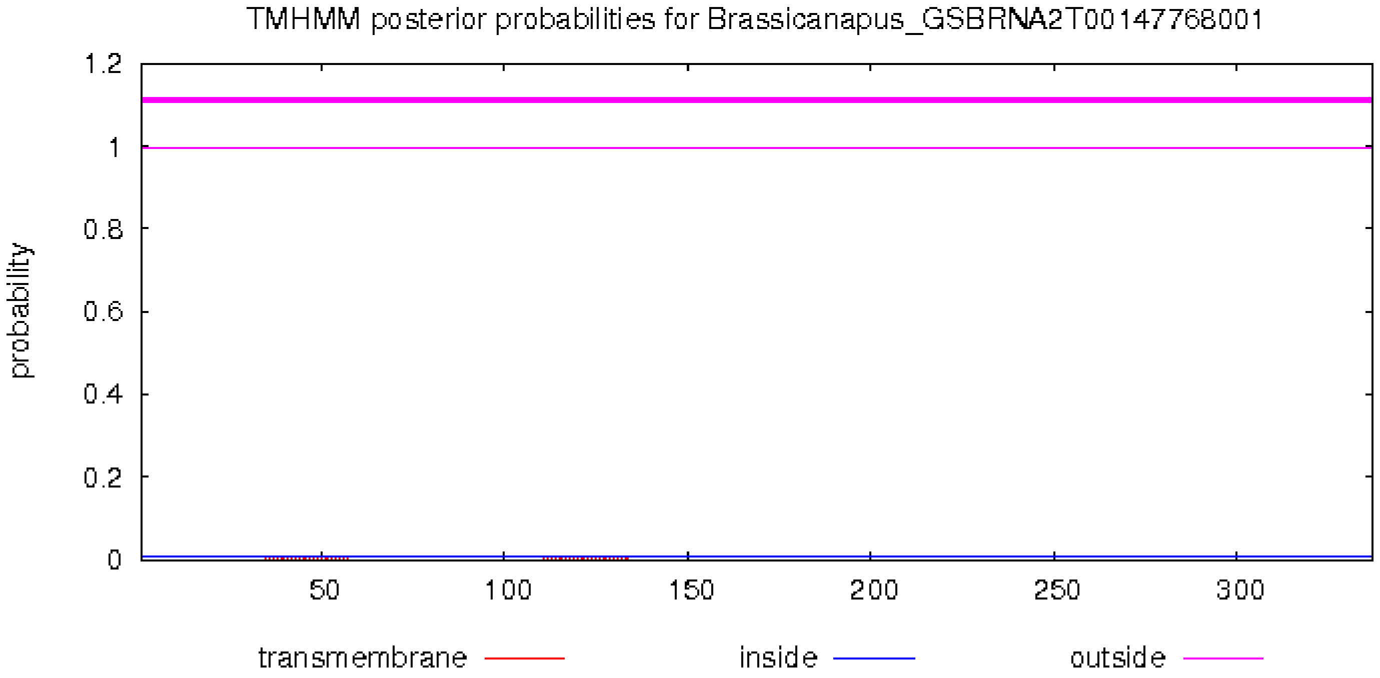

plot in postscript, script for making the plot in gnuplot, data for plot

```
# Brassicanapus_GSBRNA2T00148111001 Length: 363
# Brassicanapus_GSBRNA2T00148111001 Number of predicted TMHs:  0
# Brassicanapus_GSBRNA2T00148111001 Exp number of AAs in TMHs: 0.00834
# Brassicanapus_GSBRNA2T00148111001 Exp number, first 60 AAs:  0.00096
# Brassicanapus_GSBRNA2T00148111001 Total prob of N-in:        0.00846
Brassicanapus_GSBRNA2T00148111001	TMHMM2.0	outside	     1   363
```

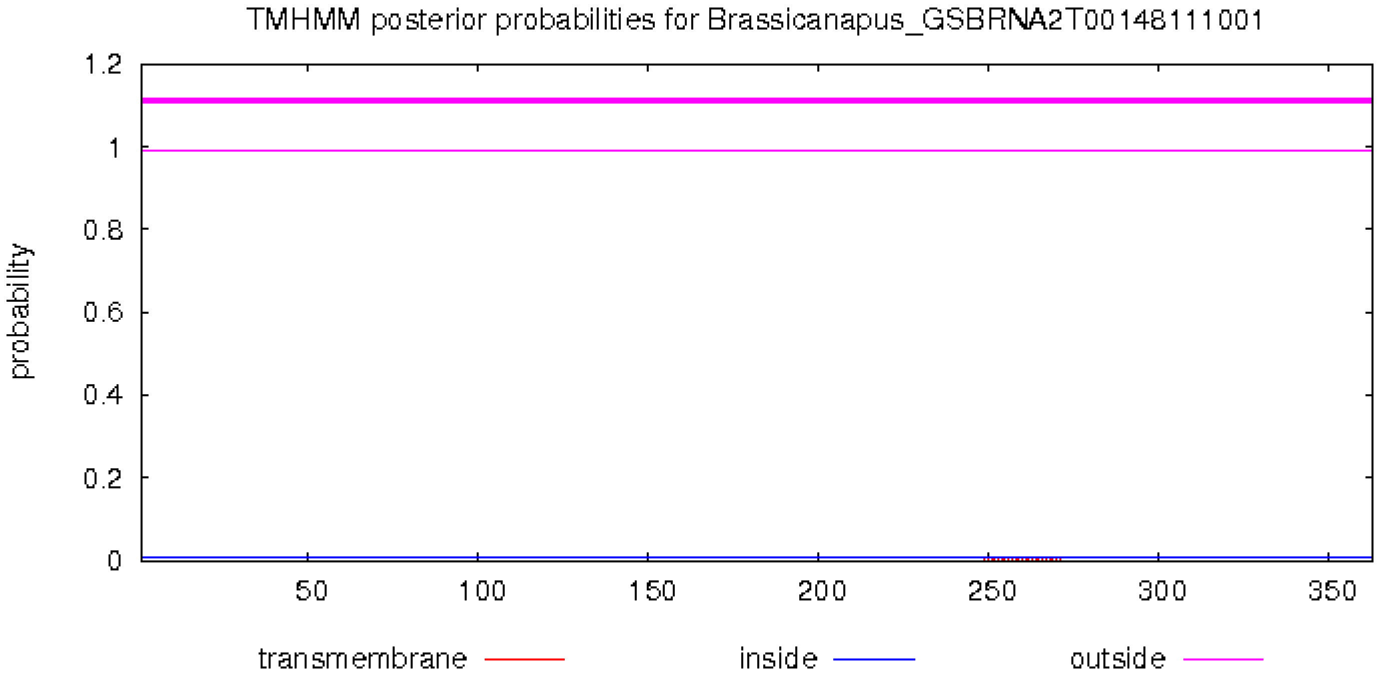

plot in postscript, script for making the plot in gnuplot, data for plot

```
# Brassicanapus_GSBRNA2T00148321001 Length: 345
# Brassicanapus_GSBRNA2T00148321001 Number of predicted TMHs:  1
# Brassicanapus_GSBRNA2T00148321001 Exp number of AAs in TMHs: 20.78902
# Brassicanapus_GSBRNA2T00148321001 Exp number, first 60 AAs:  0.08799
# Brassicanapus_GSBRNA2T00148321001 Total prob of N-in:        0.57540
Brassicanapus_GSBRNA2T00148321001	TMHMM2.0	inside	     1   319
Brassicanapus_GSBRNA2T00148321001	TMHMM2.0	TMhelix	   320   342
Brassicanapus_GSBRNA2T00148321001	TMHMM2.0	outside	   343   345
```

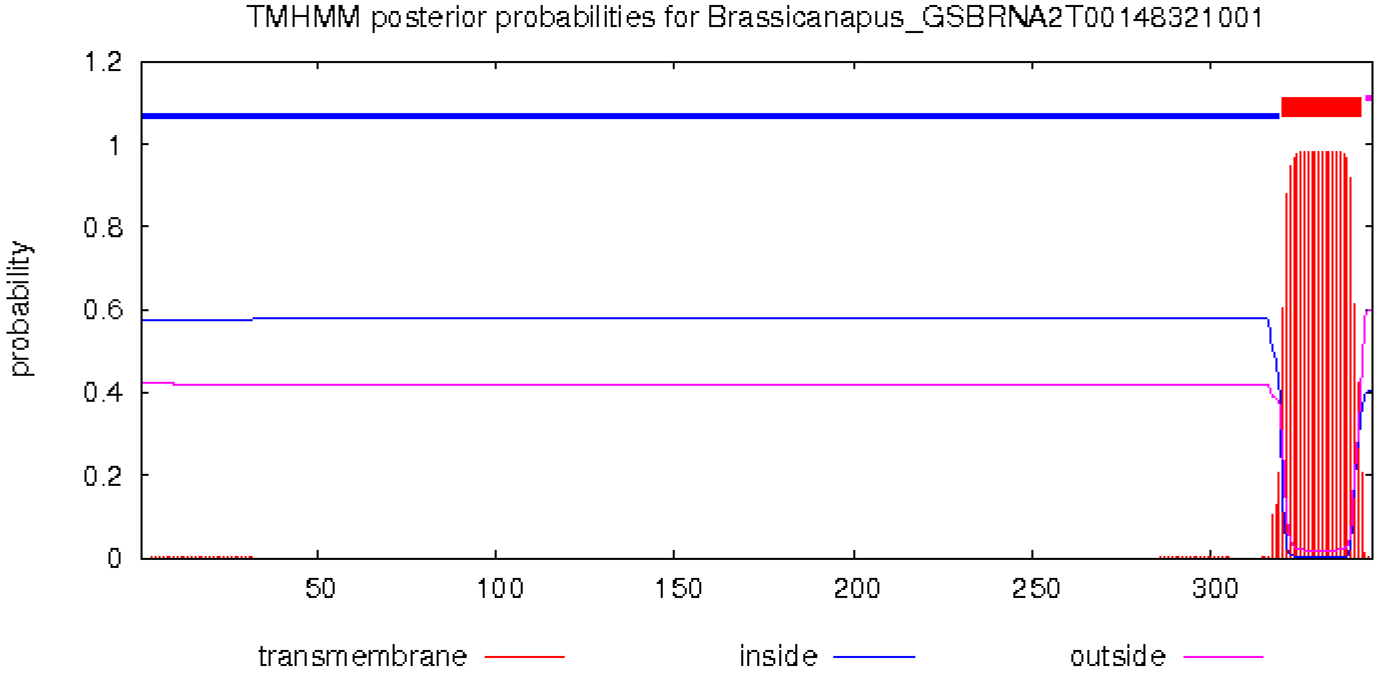

plot in postscript, script for making the plot in gnuplot, data for plot

```
# Brassicanapus_GSBRNA2T00148862001 Length: 235
# Brassicanapus_GSBRNA2T00148862001 Number of predicted TMHs:  0
# Brassicanapus_GSBRNA2T00148862001 Exp number of AAs in TMHs: 0.00026
# Brassicanapus_GSBRNA2T00148862001 Exp number, first 60 AAs:  0
# Brassicanapus_GSBRNA2T00148862001 Total prob of N-in:        0.03726
Brassicanapus_GSBRNA2T00148862001	TMHMM2.0	outside	     1   235
```

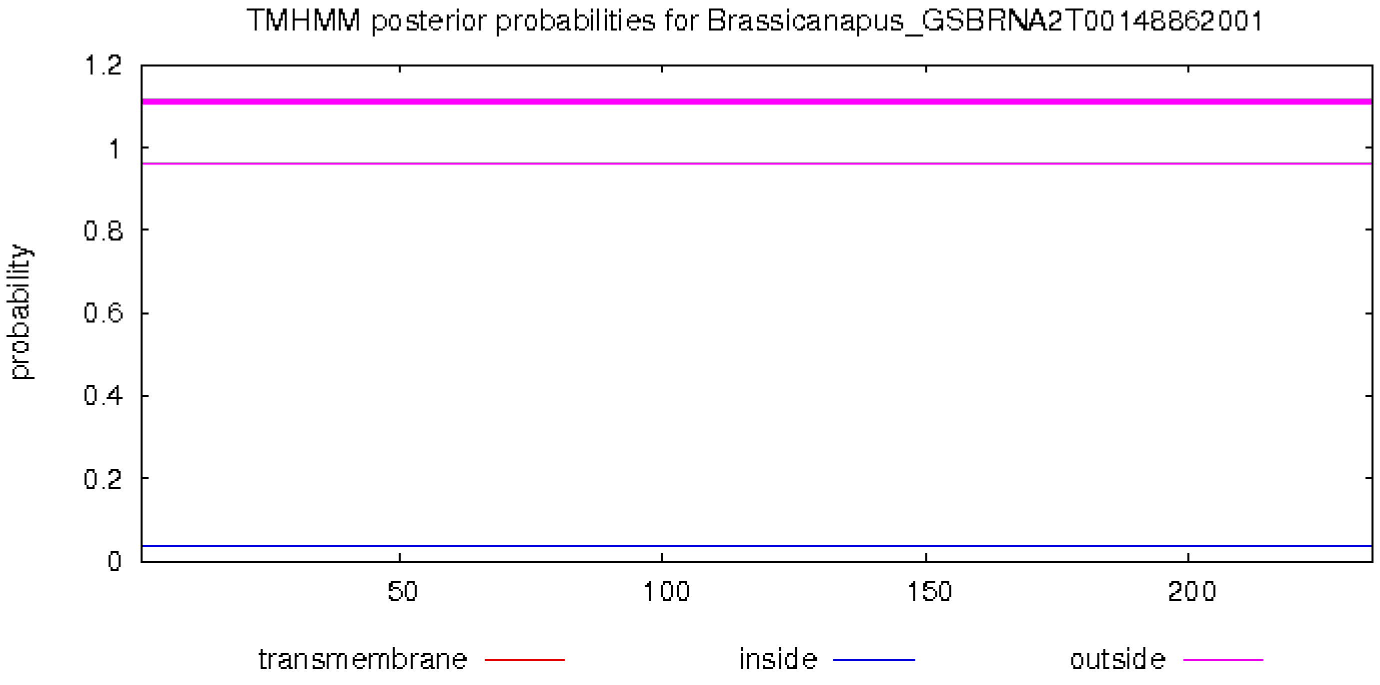

plot in postscript, script for making the plot in gnuplot, data for plot

---

```
# Brassicanapus_GSBRNA2T00148867001 Length: 337
# Brassicanapus_GSBRNA2T00148867001 Number of predicted TMHs:  0
# Brassicanapus_GSBRNA2T00148867001 Exp number of AAs in TMHs: 13.3219
# Brassicanapus_GSBRNA2T00148867001 Exp number, first 60 AAs:  0.0666
# Brassicanapus_GSBRNA2T00148867001 Total prob of N-in:        0.09318
Brassicanapus_GSBRNA2T00148867001       TMHMM2.0        outside      1   337
```

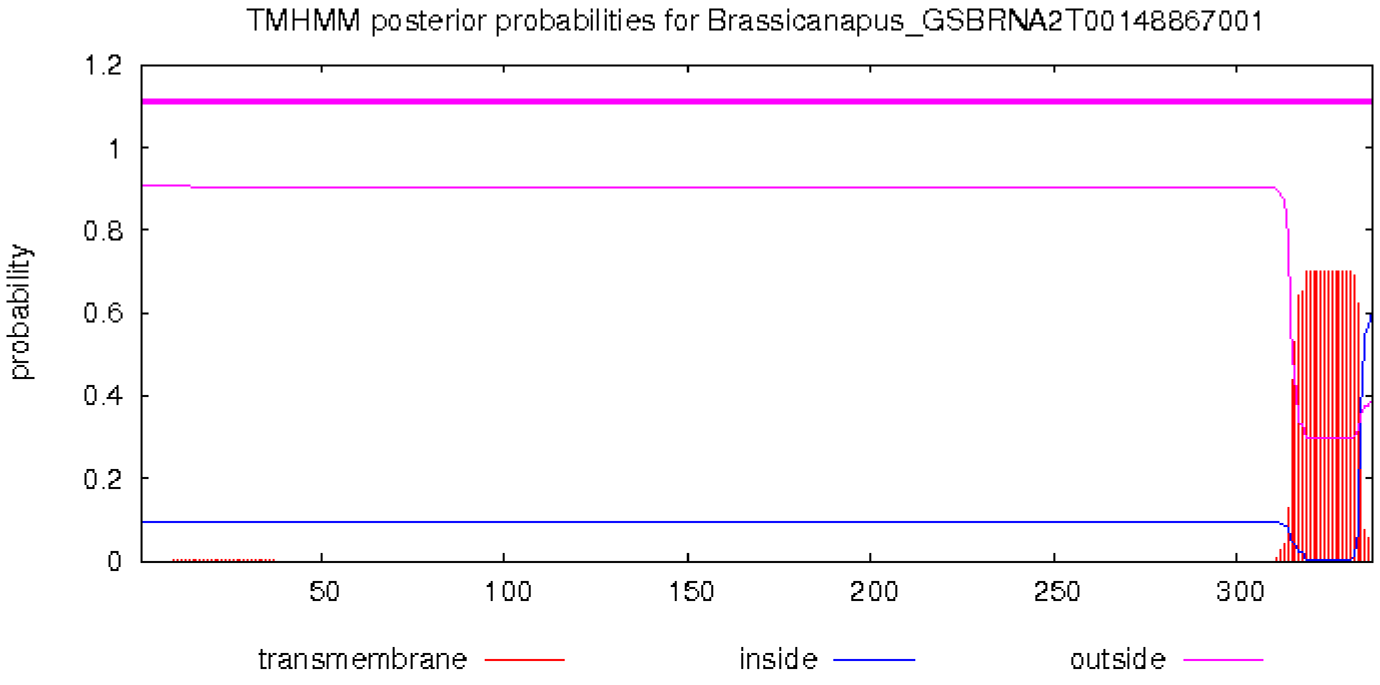

plot in postscript, script for making the plot in gnuplot, data for plot

---

```
# Brassicanapus_GSBRNA2T00149455001 Length: 300
# Brassicanapus_GSBRNA2T00149455001 Number of predicted TMHs:  0
# Brassicanapus_GSBRNA2T00149455001 Exp number of AAs in TMHs: 0.0054
# Brassicanapus_GSBRNA2T00149455001 Exp number, first 60 AAs:  0.00062
# Brassicanapus_GSBRNA2T00149455001 Total prob of N-in:        0.02411
Brassicanapus_GSBRNA2T00149455001       TMHMM2.0        outside      1   300
```

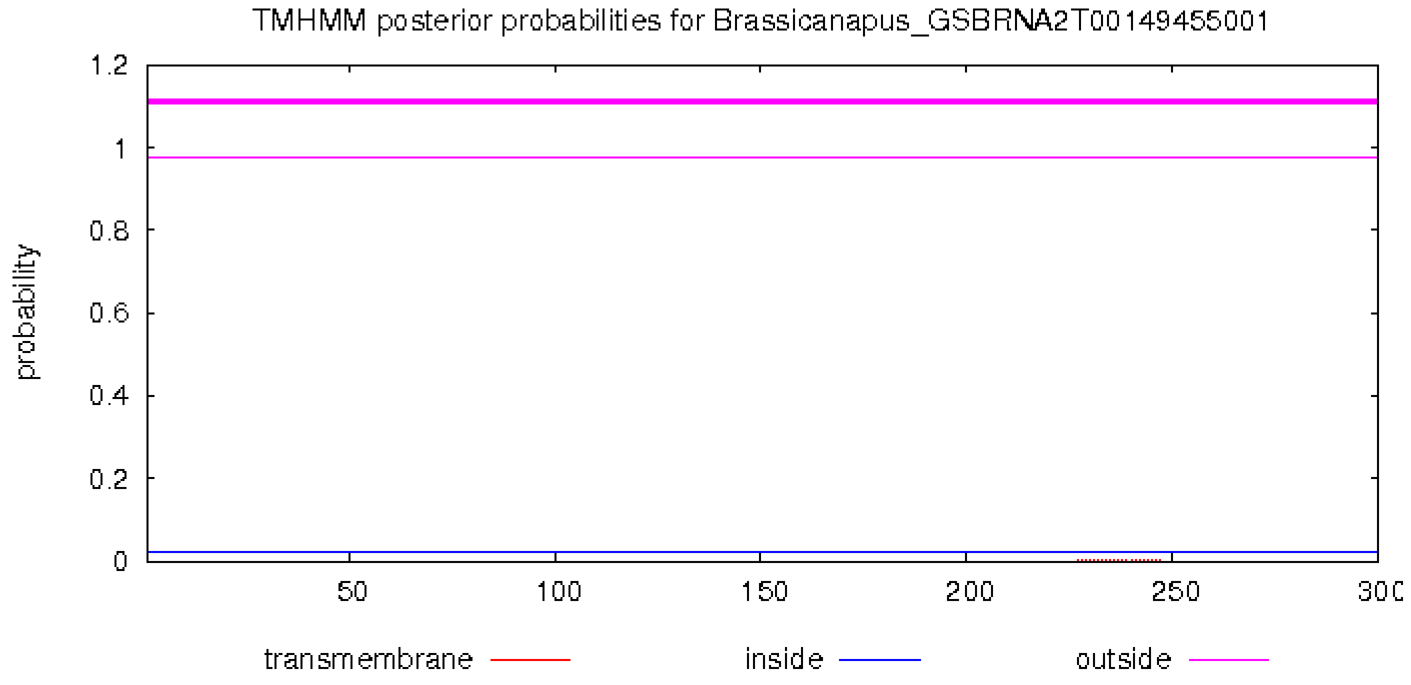

plot in postscript, script for making the plot in gnuplot, data for plot

```
# Brassicanapus_GSBRNA2T00150485001 Length: 396
# Brassicanapus_GSBRNA2T00150485001 Number of predicted TMHs:  0
# Brassicanapus_GSBRNA2T00150485001 Exp number of AAs in TMHs: 0
# Brassicanapus_GSBRNA2T00150485001 Exp number, first 60 AAs:  0
# Brassicanapus_GSBRNA2T00150485001 Total prob of N-in:        0.00289
Brassicanapus_GSBRNA2T00150485001       TMHMM2.0        outside      1   396
```

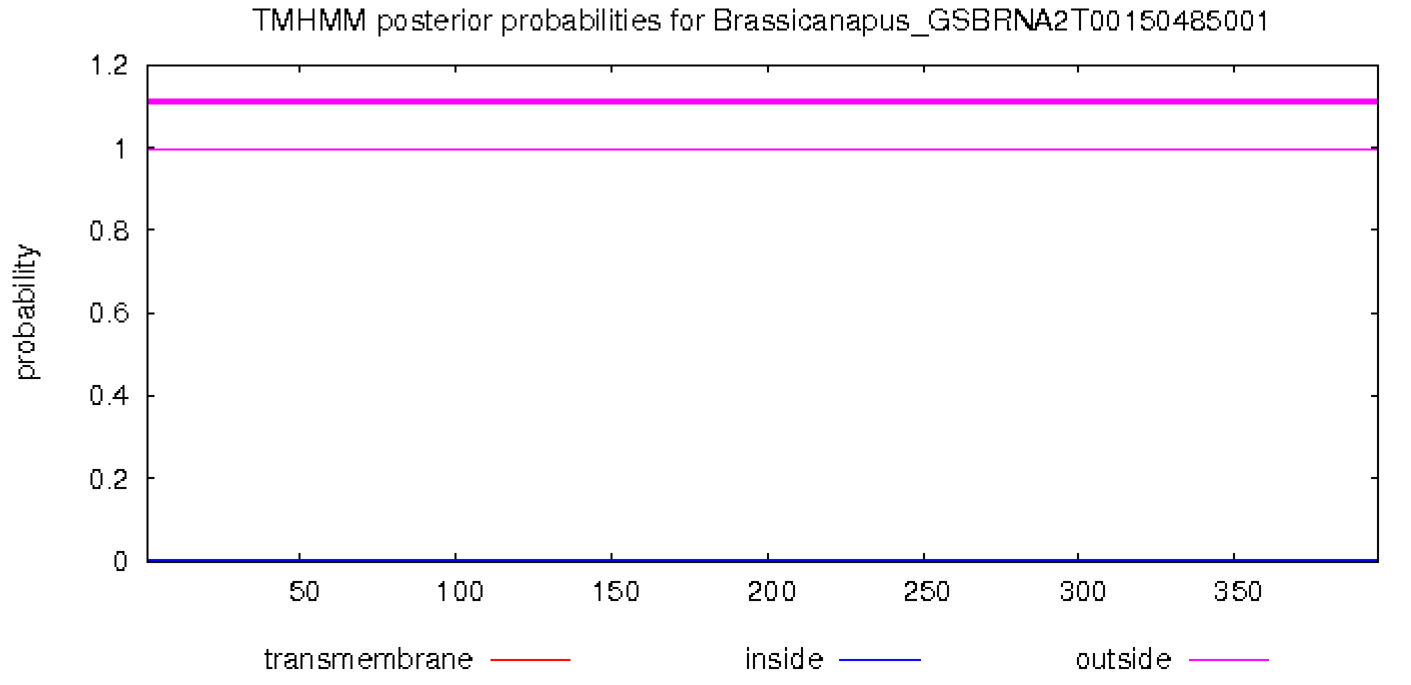

plot in postscript, script for making the plot in gnuplot, data for plot

```
# Brassicanapus_GSBRNA2T00150489001 Length: 269
# Brassicanapus_GSBRNA2T00150489001 Number of predicted TMHs:  0
# Brassicanapus_GSBRNA2T00150489001 Exp number of AAs in TMHs: 0
# Brassicanapus_GSBRNA2T00150489001 Exp number, first 60 AAs:  0
# Brassicanapus_GSBRNA2T00150489001 Total prob of N-in:        0.03035
Brassicanapus_GSBRNA2T00150489001       TMHMM2.0        outside      1   269
```

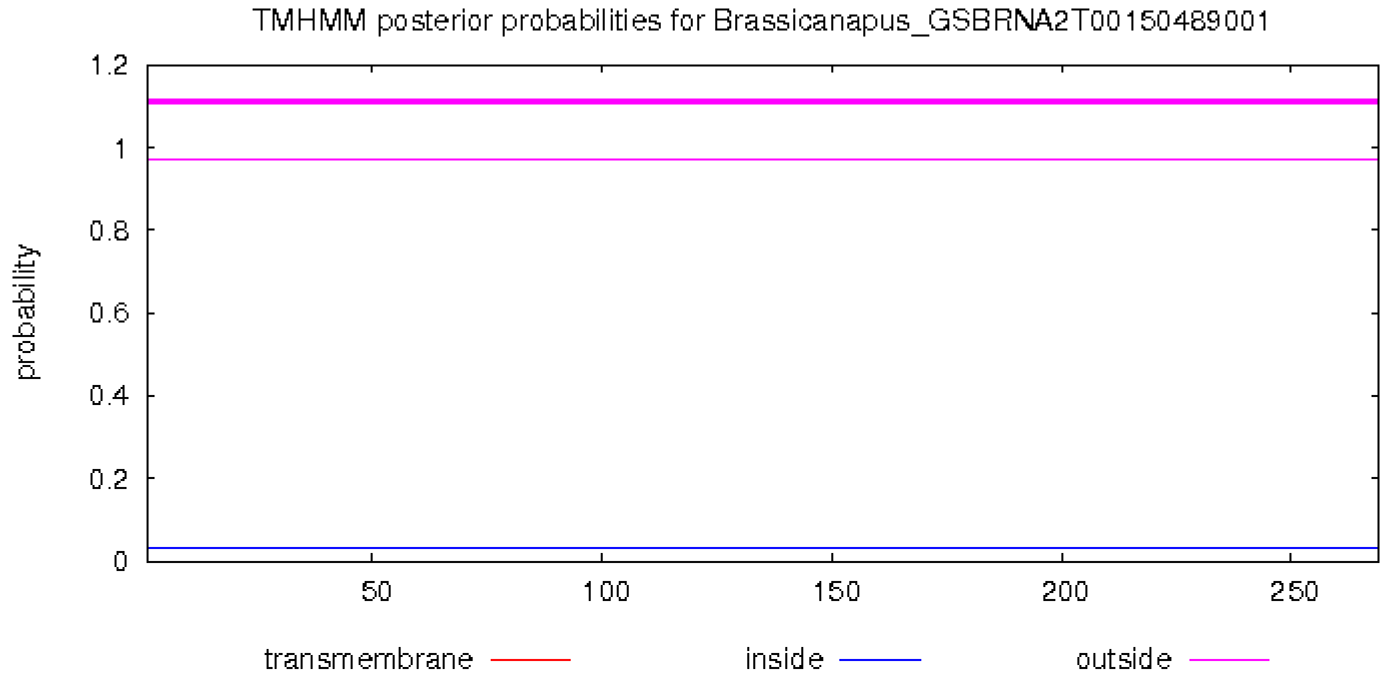

plot in postscript, script for making the plot in gnuplot, data for plot

---

```
# Brassicanapus_GSBRNA2T00150491001 Length: 467
# Brassicanapus_GSBRNA2T00150491001 Number of predicted TMHs:  0
# Brassicanapus_GSBRNA2T00150491001 Exp number of AAs in TMHs: 0.00042
# Brassicanapus_GSBRNA2T00150491001 Exp number, first 60 AAs:  0
# Brassicanapus_GSBRNA2T00150491001 Total prob of N-in:        0.00354
Brassicanapus_GSBRNA2T00150491001       TMHMM2.0        outside      1   467
```

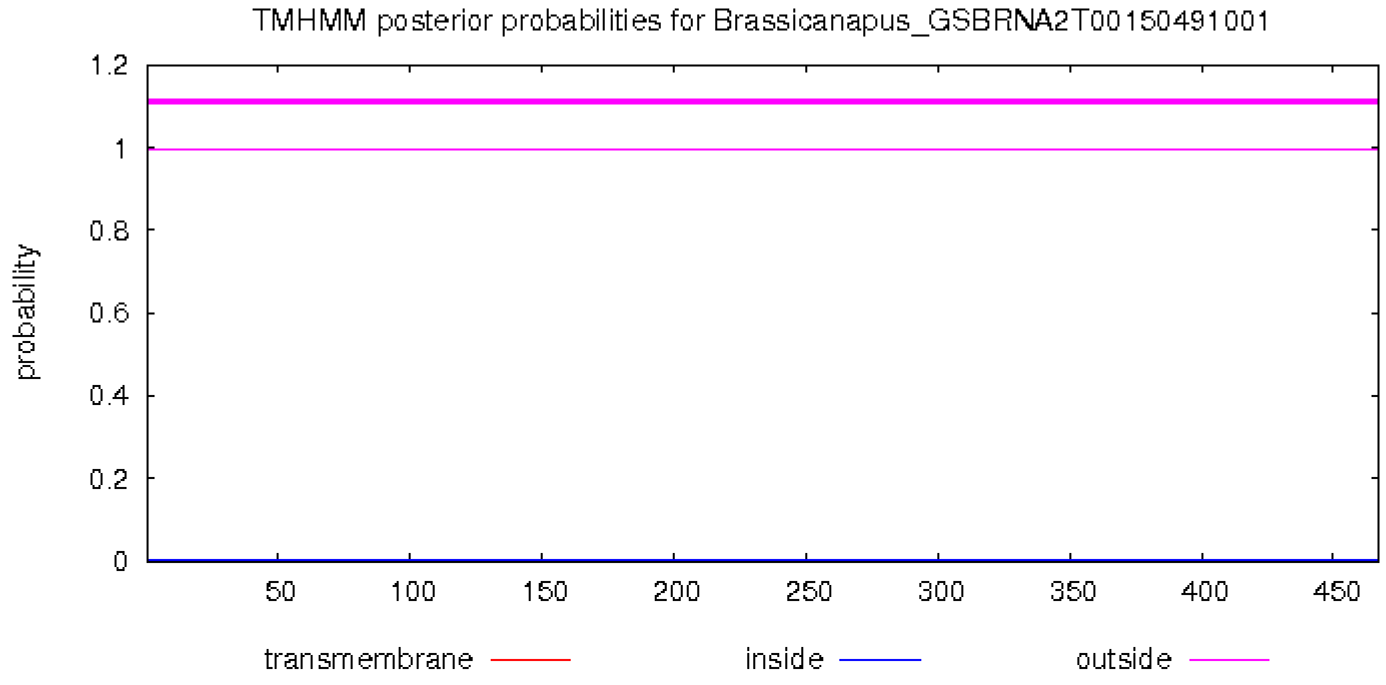

plot in postscript, script for making the plot in gnuplot, data for plot

---

```
# Brassicanapus_GSBRNA2T00150492001 Length: 251
# Brassicanapus_GSBRNA2T00150492001 Number of predicted TMHs:  0
# Brassicanapus_GSBRNA2T00150492001 Exp number of AAs in TMHs: 0
# Brassicanapus_GSBRNA2T00150492001 Exp number, first 60 AAs:  0
# Brassicanapus_GSBRNA2T00150492001 Total prob of N-in:        0.01343
Brassicanapus_GSBRNA2T00150492001       TMHMM2.0        outside      1   251
```

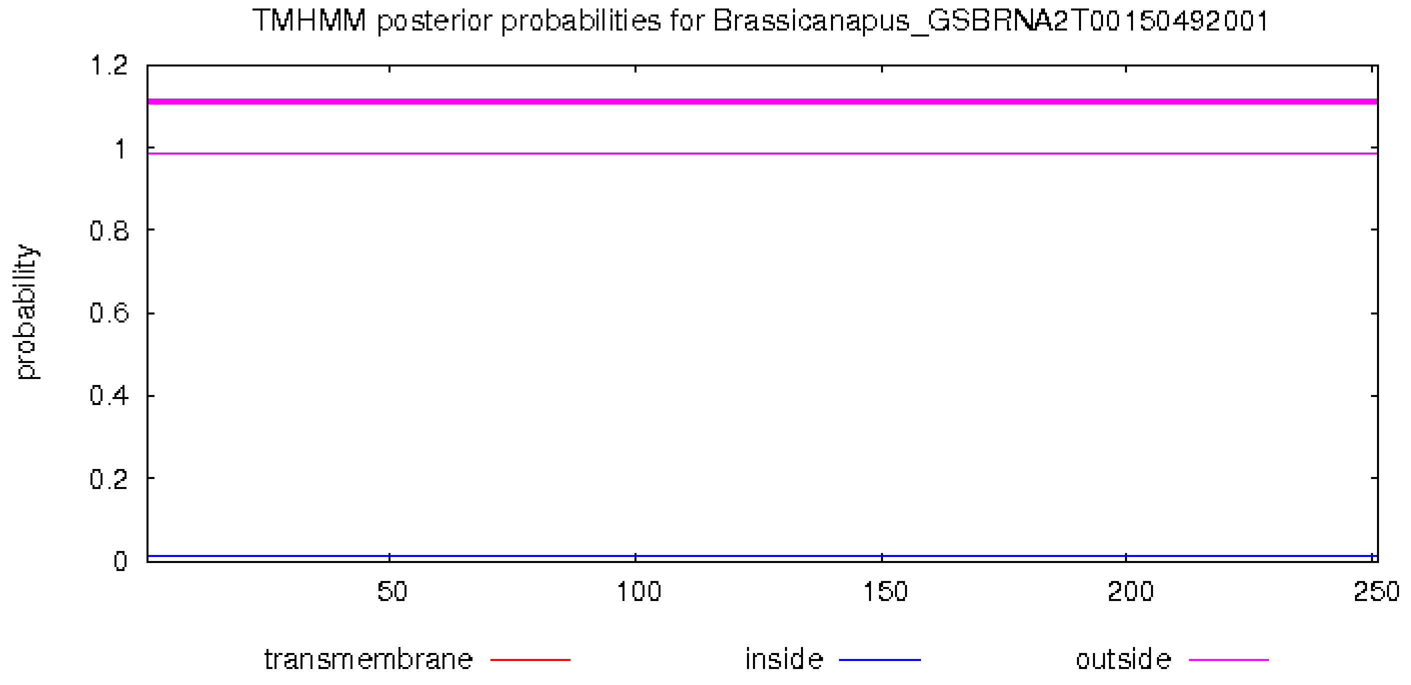

plot in postscript, script for making the plot in gnuplot, data for plot

---

```
# Brassicanapus_GSBRNA2T00150493001 Length: 355
# Brassicanapus_GSBRNA2T00150493001 Number of predicted TMHs:  0
# Brassicanapus_GSBRNA2T00150493001 Exp number of AAs in TMHs: 0.0211
# Brassicanapus_GSBRNA2T00150493001 Exp number, first 60 AAs:  0
# Brassicanapus_GSBRNA2T00150493001 Total prob of N-in:        0.01019
Brassicanapus_GSBRNA2T00150493001       TMHMM2.0        outside      1   355
```

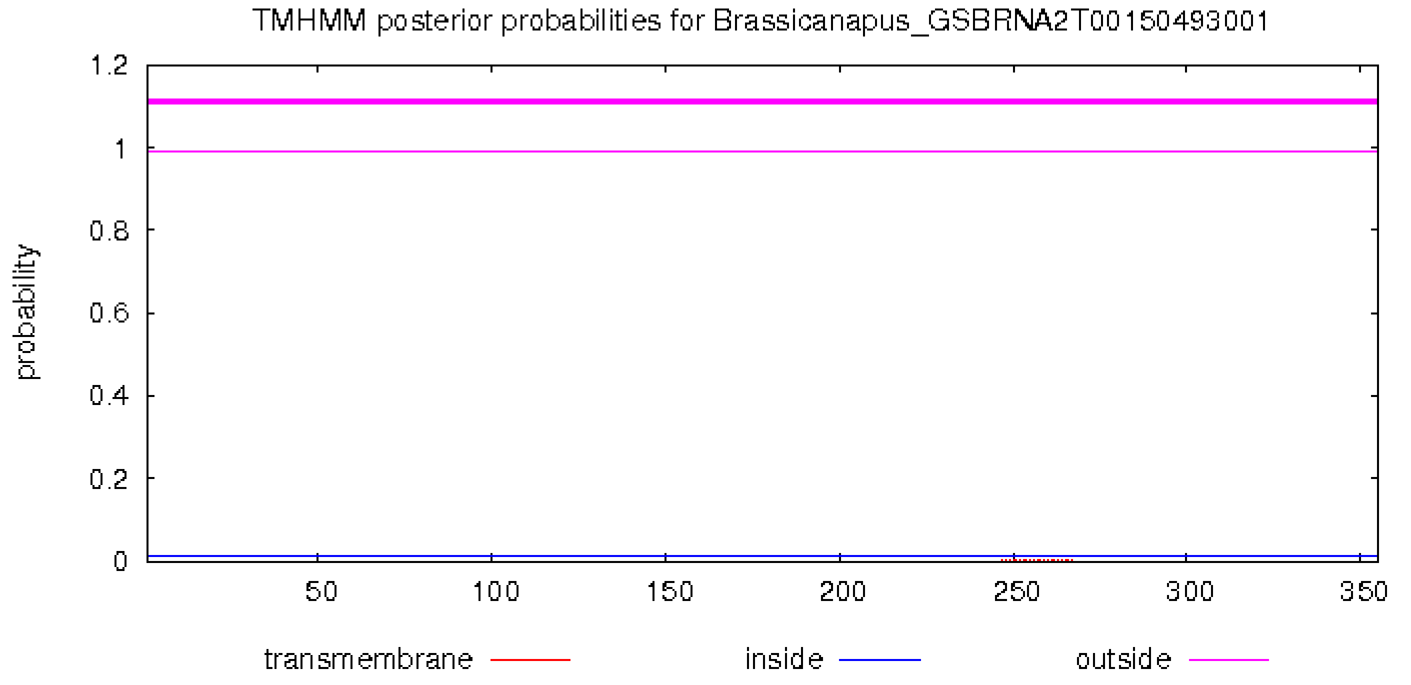

plot in postscript, script for making the plot in gnuplot, data for plot

---

```
# Brassicanapus_GSBRNA2T00150494001 Length: 258
# Brassicanapus_GSBRNA2T00150494001 Number of predicted TMHs:  0
# Brassicanapus_GSBRNA2T00150494001 Exp number of AAs in TMHs: 0.00268
# Brassicanapus_GSBRNA2T00150494001 Exp number, first 60 AAs:  8e-05
# Brassicanapus_GSBRNA2T00150494001 Total prob of N-in:        0.05330
Brassicanapus_GSBRNA2T00150494001       TMHMM2.0        outside      1   258
```

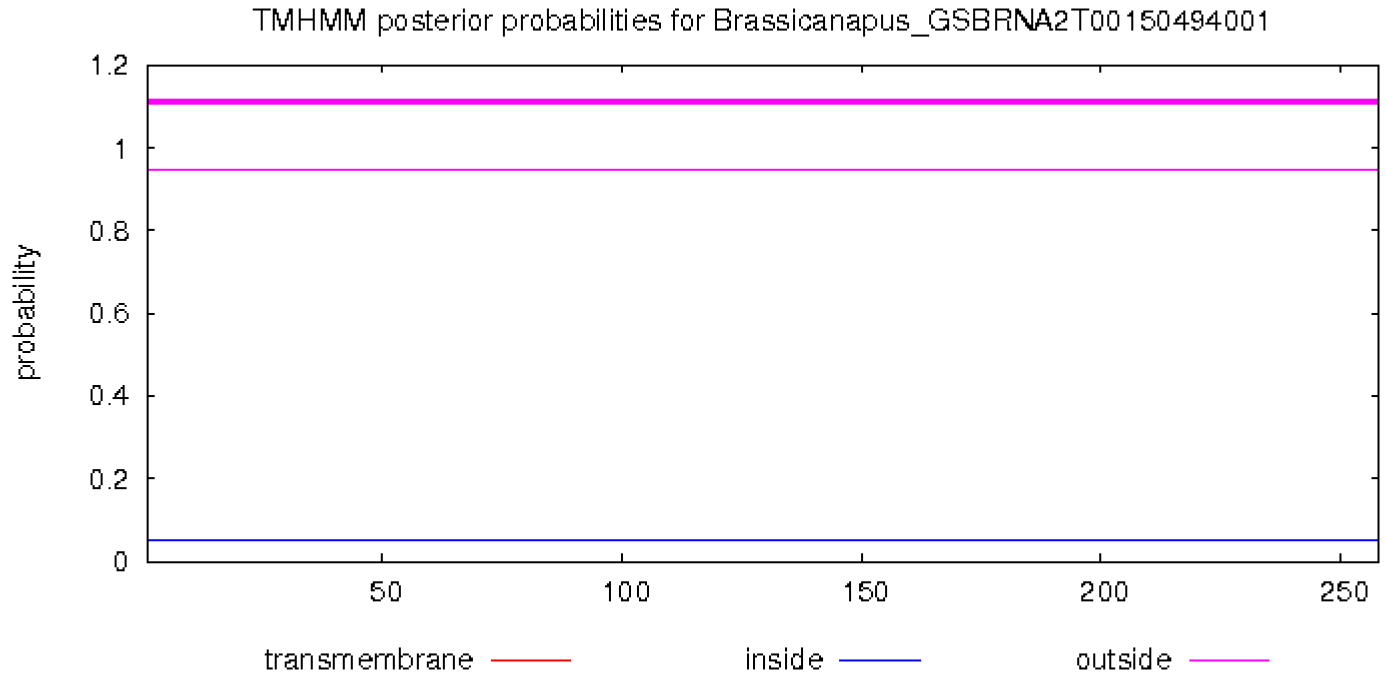

plot in postscript, script for making the plot in gnuplot, data for plot

---

```
# Brassicanapus_GSBRNA2T00150497001 Length: 475
# Brassicanapus_GSBRNA2T00150497001 Number of predicted TMHs:  1
# Brassicanapus_GSBRNA2T00150497001 Exp number of AAs in TMHs: 21.34248
# Brassicanapus_GSBRNA2T00150497001 Exp number, first 60 AAs:  0.00018
# Brassicanapus_GSBRNA2T00150497001 Total prob of N-in:        0.30086
Brassicanapus_GSBRNA2T00150497001	TMHMM2.0	outside	     1   450
Brassicanapus_GSBRNA2T00150497001	TMHMM2.0	TMhelix	   451   473
Brassicanapus_GSBRNA2T00150497001	TMHMM2.0	inside	   474   475
```

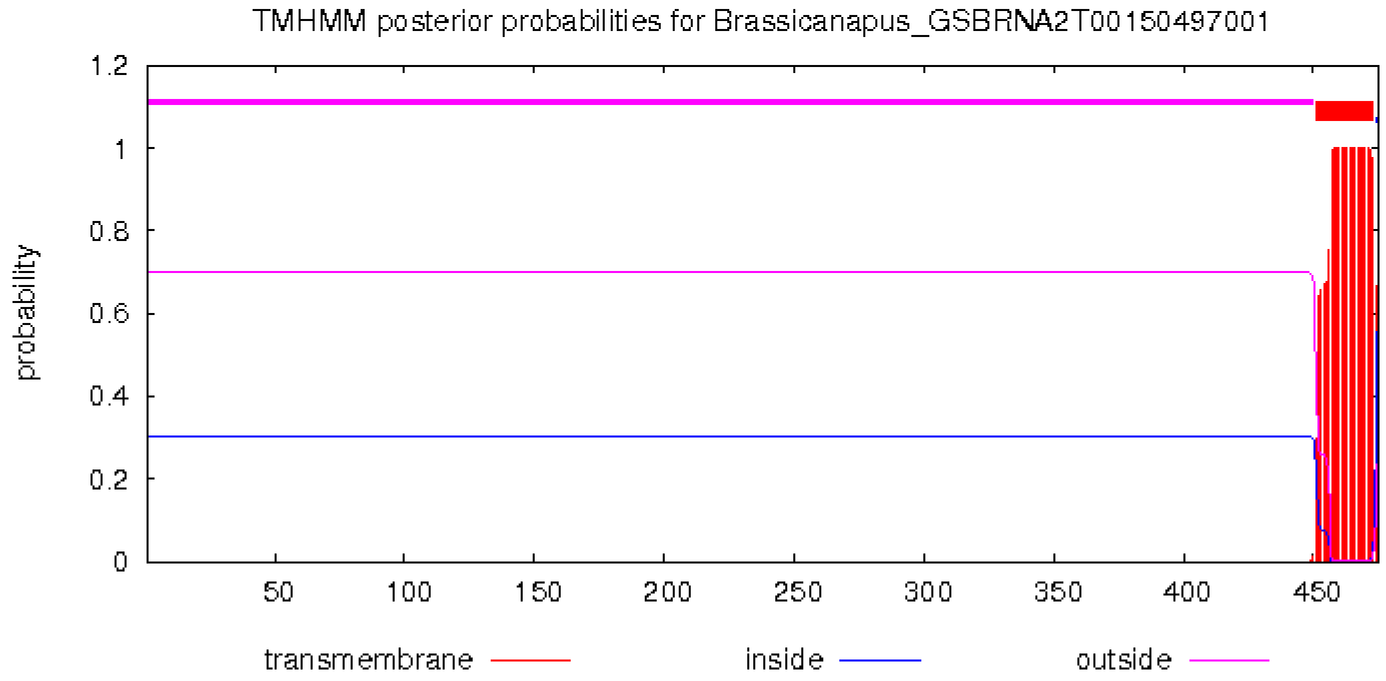

plot in postscript, script for making the plot in gnuplot, data for plot

---

```
# Brassicanapus_GSBRNA2T00150558001 Length: 285
# Brassicanapus_GSBRNA2T00150558001 Number of predicted TMHs:  0
# Brassicanapus_GSBRNA2T00150558001 Exp number of AAs in TMHs: 0.03653
# Brassicanapus_GSBRNA2T00150558001 Exp number, first 60 AAs:  0.00018
```

```
# Brassicanapus_GSBRNA2T00150558001 Total prob of N-in:        0.00266
Brassicanapus_GSBRNA2T00150558001       TMHMM2.0        outside      1   285
```

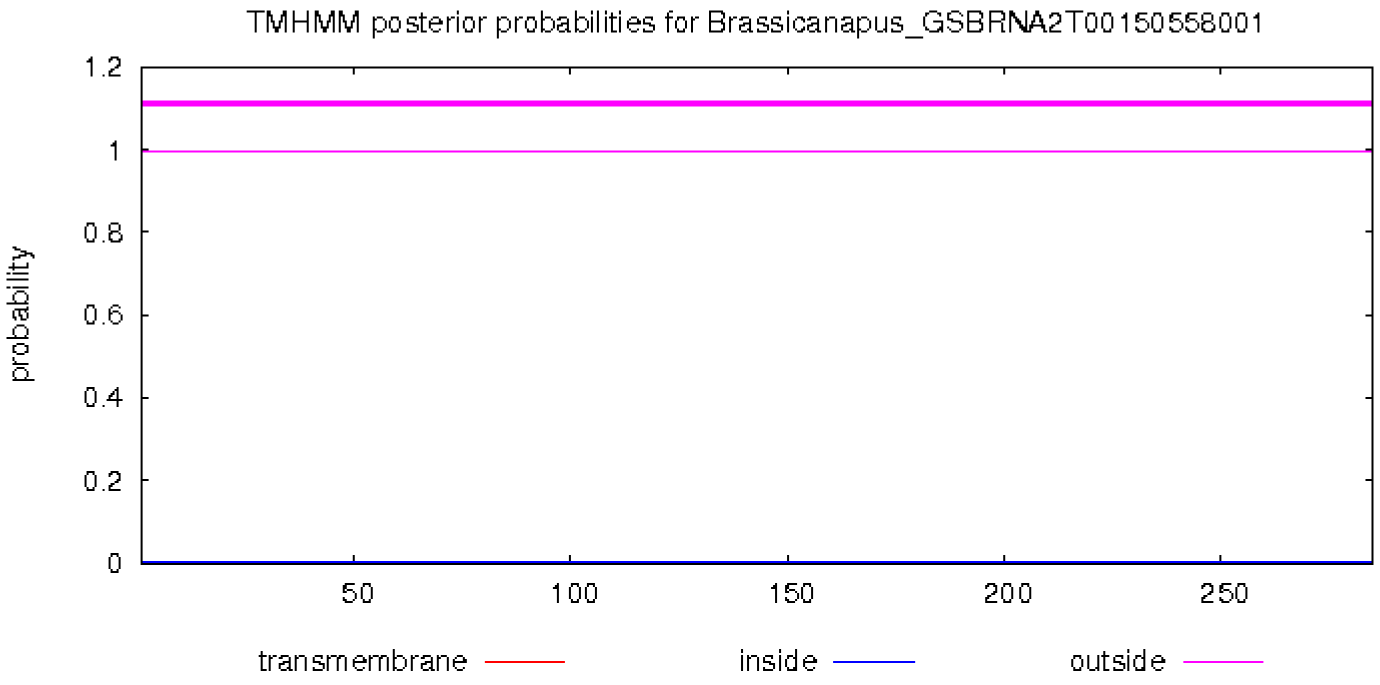

plot in postscript, script for making the plot in gnuplot, data for plot

---

```
# Brassicanapus_GSBRNA2T00150656001 Length: 123
# Brassicanapus_GSBRNA2T00150656001 Number of predicted TMHs:  0
# Brassicanapus_GSBRNA2T00150656001 Exp number of AAs in TMHs: 0.00278
# Brassicanapus_GSBRNA2T00150656001 Exp number, first 60 AAs:  0
# Brassicanapus_GSBRNA2T00150656001 Total prob of N-in:        0.19568
Brassicanapus_GSBRNA2T00150656001       TMHMM2.0        outside      1   123
```

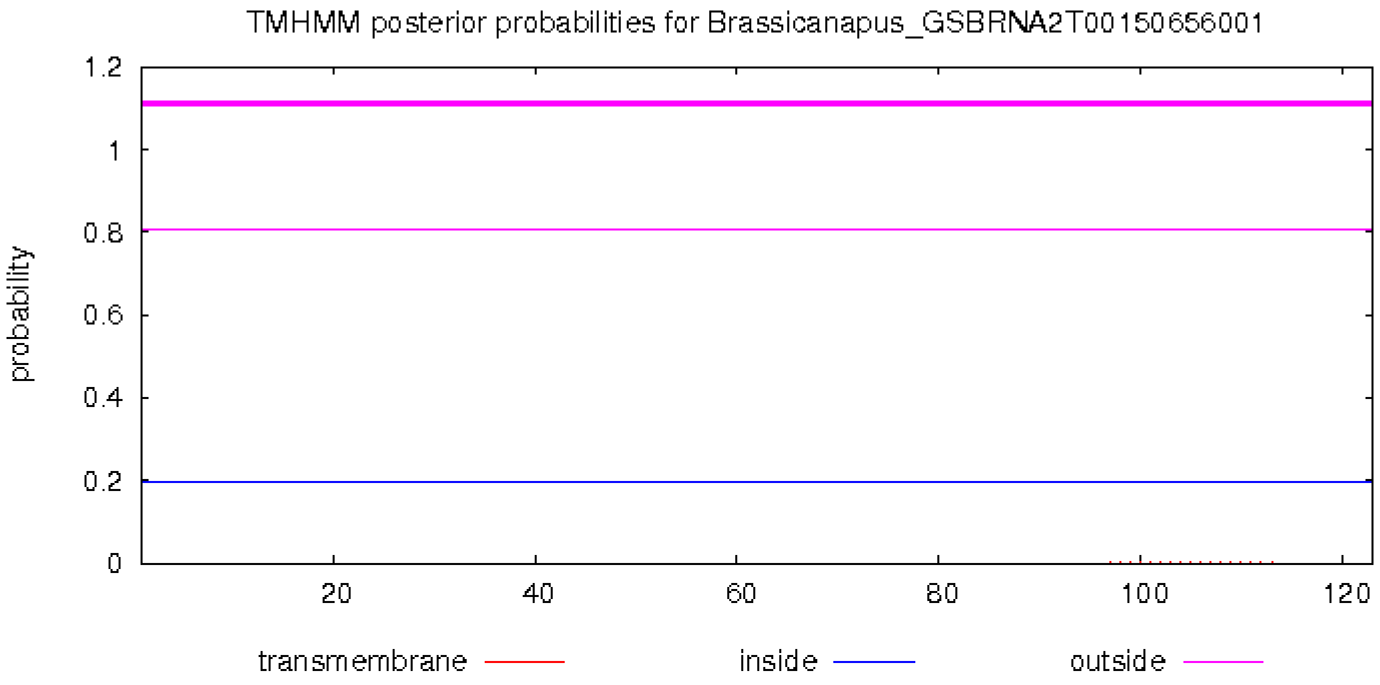

plot in postscript, script for making the plot in gnuplot, data for plot

---

```
# Brassicanapus_GSBRNA2T00151041001 Length: 286
# Brassicanapus_GSBRNA2T00151041001 Number of predicted TMHs:  0
# Brassicanapus_GSBRNA2T00151041001 Exp number of AAs in TMHs: 0.00019
# Brassicanapus_GSBRNA2T00151041001 Exp number, first 60 AAs:  0
```

```
# Brassicanapus_GSBRNA2T00151041001 Total prob of N-in:        0.00738
Brassicanapus_GSBRNA2T00151041001       TMHMM2.0        outside      1   286
```

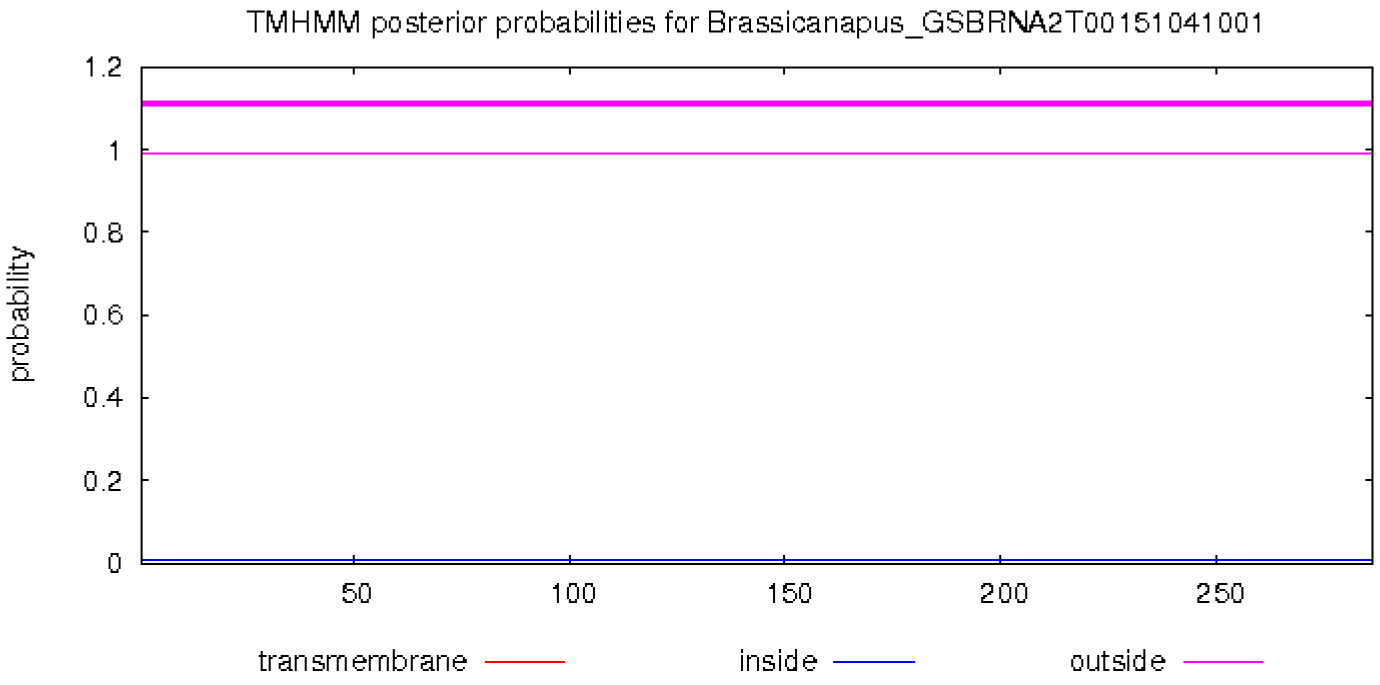

plot in postscript, script for making the plot in gnuplot, data for plot

---

```
# Brassicanapus_GSBRNA2T00151449001 Length: 332
# Brassicanapus_GSBRNA2T00151449001 Number of predicted TMHs:  0
# Brassicanapus_GSBRNA2T00151449001 Exp number of AAs in TMHs: 0
# Brassicanapus_GSBRNA2T00151449001 Exp number, first 60 AAs:  0
# Brassicanapus_GSBRNA2T00151449001 Total prob of N-in:        0.00488
Brassicanapus_GSBRNA2T00151449001       TMHMM2.0        outside      1   332
```

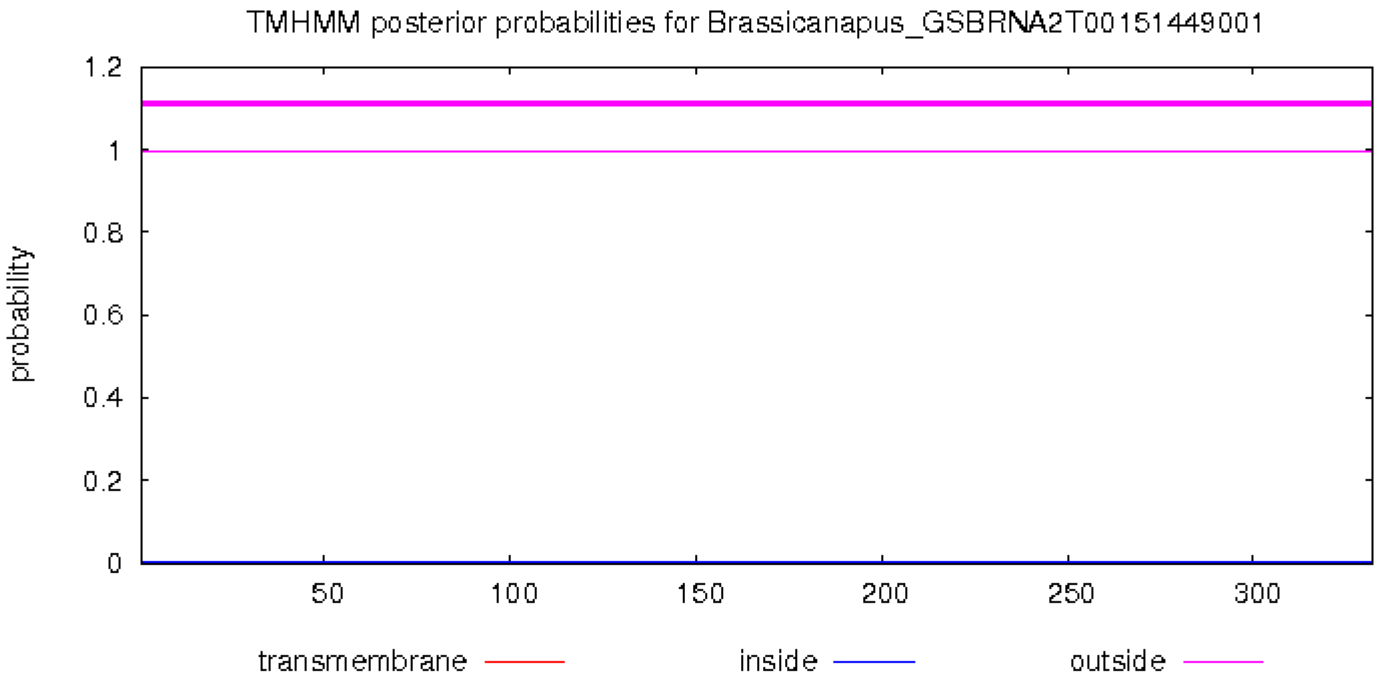

plot in postscript, script for making the plot in gnuplot, data for plot

---

```
# Brassicanapus_GSBRNA2T00151934001 Length: 247
# Brassicanapus_GSBRNA2T00151934001 Number of predicted TMHs:  0
# Brassicanapus_GSBRNA2T00151934001 Exp number of AAs in TMHs: 0
# Brassicanapus_GSBRNA2T00151934001 Exp number, first 60 AAs:  0
```

```
# Brassicanapus_GSBRNA2T00151934001 Total prob of N-in:        0.11741
Brassicanapus_GSBRNA2T00151934001       TMHMM2.0        outside      1   247
```

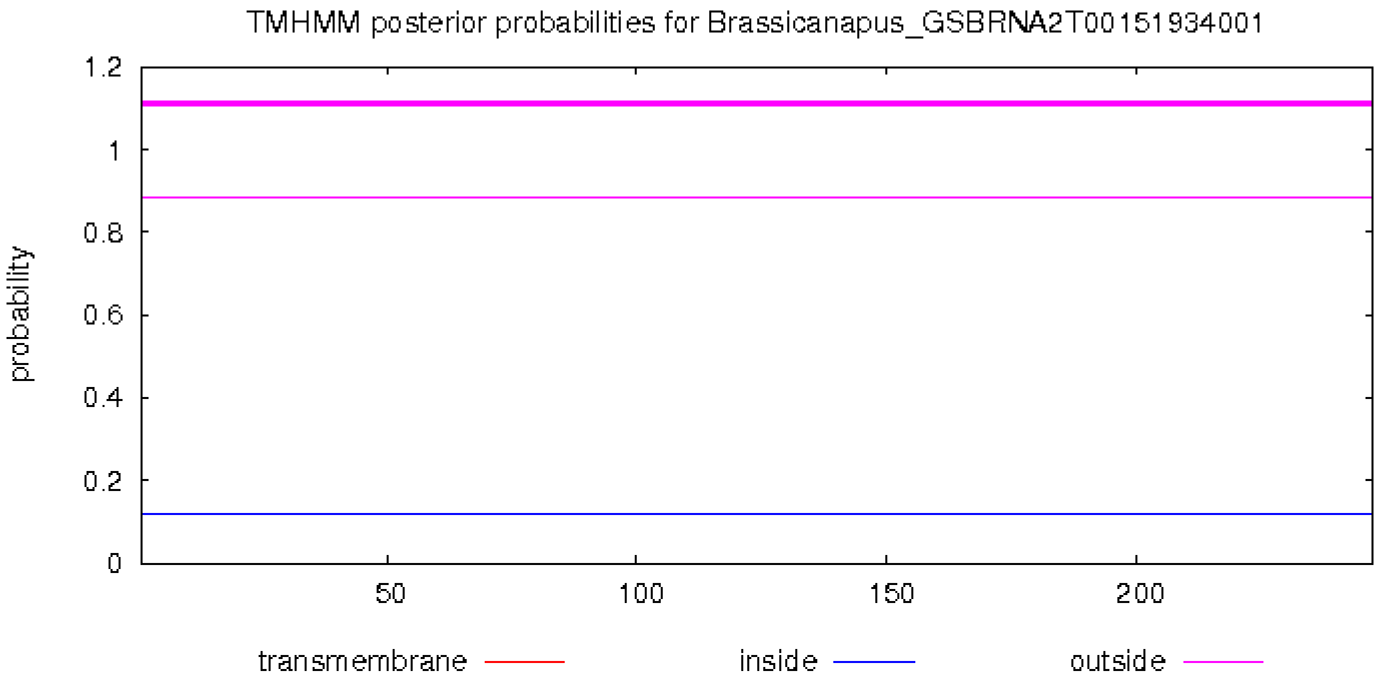

plot in postscript, script for making the plot in gnuplot, data for plot

---

```
# Brassicanapus_GSBRNA2T00152277001 Length: 193
# Brassicanapus_GSBRNA2T00152277001 Number of predicted TMHs:  0
# Brassicanapus_GSBRNA2T00152277001 Exp number of AAs in TMHs: 0.00125
# Brassicanapus_GSBRNA2T00152277001 Exp number, first 60 AAs:  0
# Brassicanapus_GSBRNA2T00152277001 Total prob of N-in:        0.15548
Brassicanapus_GSBRNA2T00152277001       TMHMM2.0        outside      1   193
```

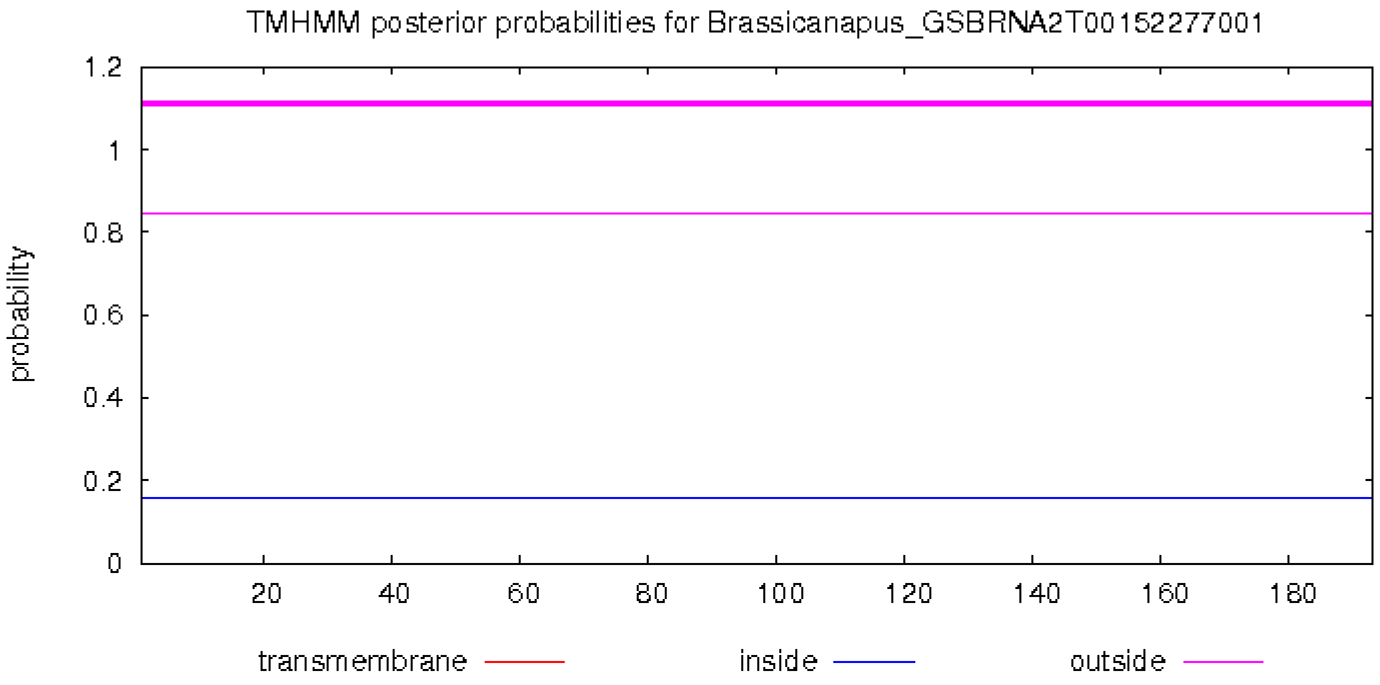

plot in postscript, script for making the plot in gnuplot, data for plot

---

```
# Brassicanapus_GSBRNA2T00152426001 Length: 159
# Brassicanapus_GSBRNA2T00152426001 Number of predicted TMHs:  0
# Brassicanapus_GSBRNA2T00152426001 Exp number of AAs in TMHs: 7.1388
# Brassicanapus_GSBRNA2T00152426001 Exp number, first 60 AAs:  1.08531
```

```
# Brassicanapus_GSBRNA2T00152426001 Total prob of N-in:        0.17034
Brassicanapus_GSBRNA2T00152426001       TMHMM2.0        outside      1   159
```

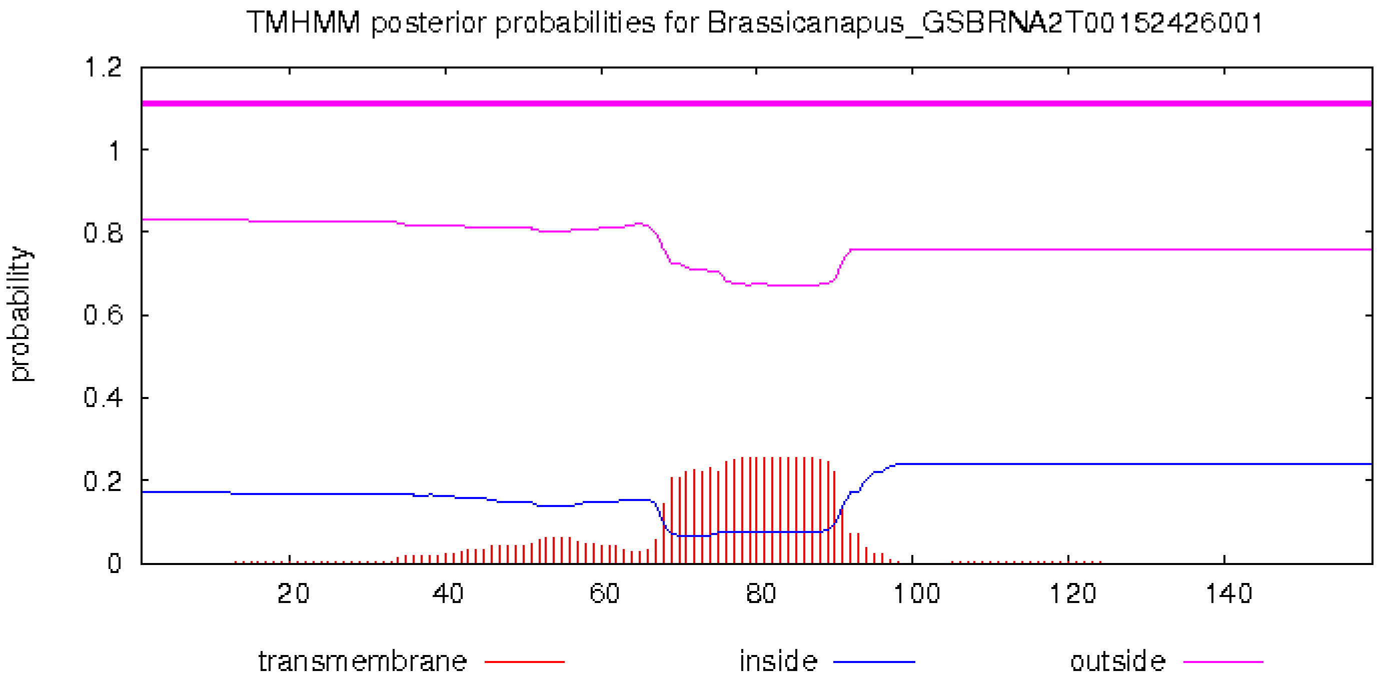

plot in postscript, script for making the plot in gnuplot, data for plot

---

```
# Brassicanapus_GSBRNA2T00153359001 Length: 539
# Brassicanapus_GSBRNA2T00153359001 Number of predicted TMHs:  1
# Brassicanapus_GSBRNA2T00153359001 Exp number of AAs in TMHs: 23.18037
# Brassicanapus_GSBRNA2T00153359001 Exp number, first 60 AAs:  0.00385
# Brassicanapus_GSBRNA2T00153359001 Total prob of N-in:        0.80584
Brassicanapus_GSBRNA2T00153359001       TMHMM2.0        inside       1   508
Brassicanapus_GSBRNA2T00153359001       TMHMM2.0        TMhelix    509   531
Brassicanapus_GSBRNA2T00153359001       TMHMM2.0        outside    532   539
```

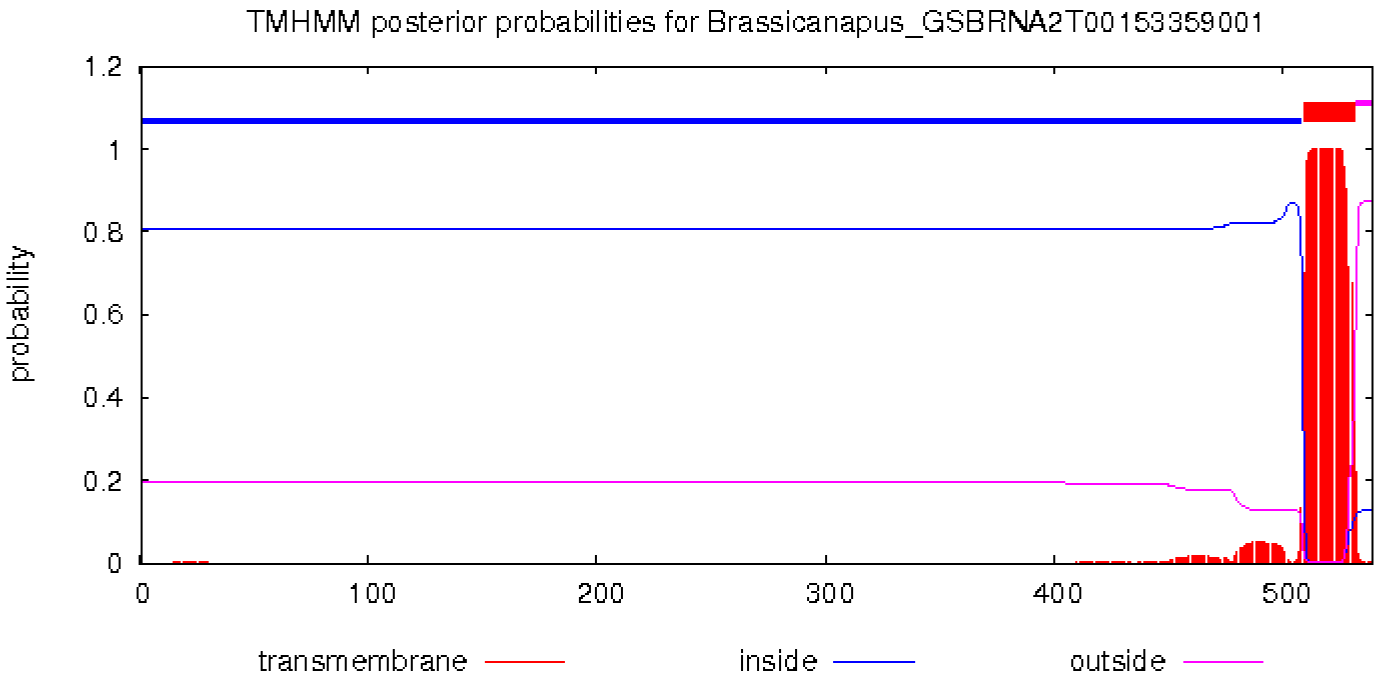

plot in postscript, script for making the plot in gnuplot, data for plot

---

```
# Brassicanapus_GSBRNA2T00153449001 Length: 495
# Brassicanapus_GSBRNA2T00153449001 Number of predicted TMHs:  0
```

```
# Brassicanapus_GSBRNA2T00153449001 Exp number of AAs in TMHs: 0.00074
# Brassicanapus_GSBRNA2T00153449001 Exp number, first 60 AAs:  0.00047
# Brassicanapus_GSBRNA2T00153449001 Total prob of N-in:        0.00085
Brassicanapus_GSBRNA2T00153449001       TMHMM2.0        outside      1   495
```

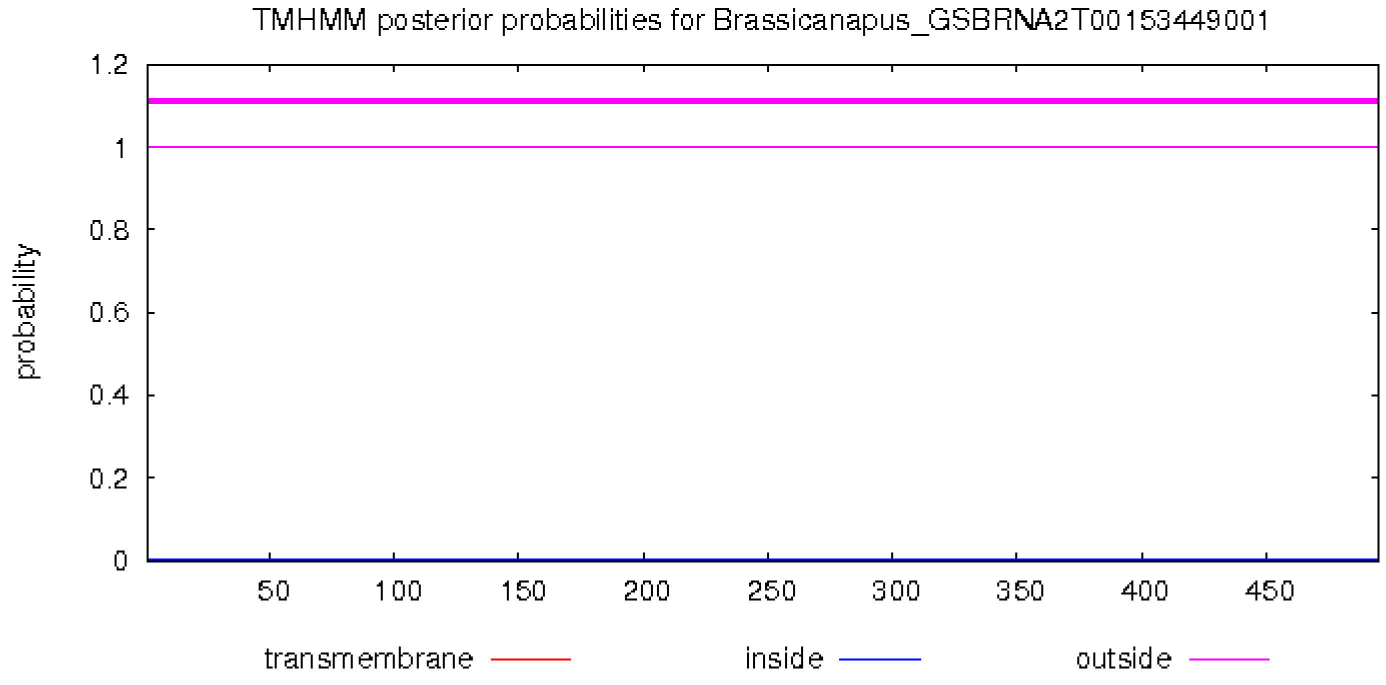

plot in postscript, script for making the plot in gnuplot, data for plot

---

```
# Brassicanapus_GSBRNA2T00153576001 Length: 695
# Brassicanapus_GSBRNA2T00153576001 Number of predicted TMHs:  0
# Brassicanapus_GSBRNA2T00153576001 Exp number of AAs in TMHs: 0.00369
# Brassicanapus_GSBRNA2T00153576001 Exp number, first 60 AAs:  0.00131
# Brassicanapus_GSBRNA2T00153576001 Total prob of N-in:        0.00023
Brassicanapus_GSBRNA2T00153576001       TMHMM2.0        outside      1   695
```

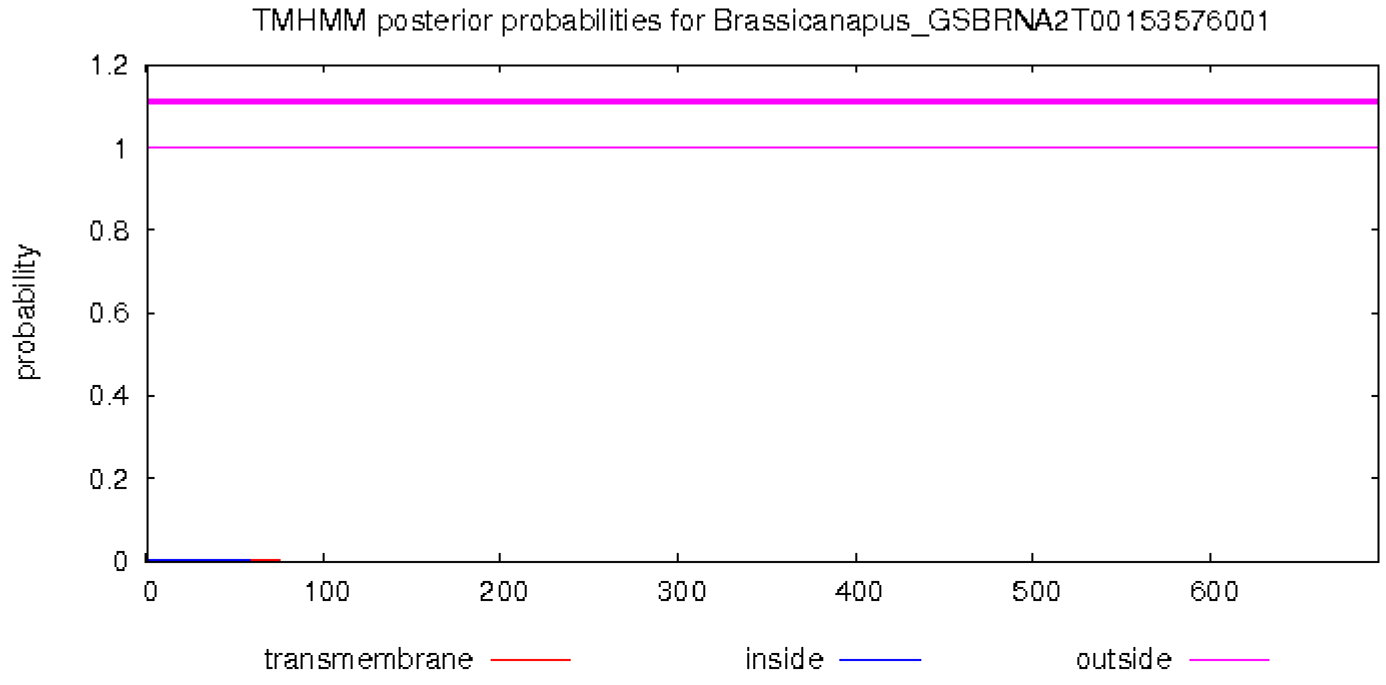

plot in postscript, script for making the plot in gnuplot, data for plot

---

```
# Brassicanapus_GSBRNA2T00153852001 Length: 226
# Brassicanapus_GSBRNA2T00153852001 Number of predicted TMHs:  0
```

```
# Brassicanapus_GSBRNA2T00153852001 Exp number of AAs in TMHs: 0
# Brassicanapus_GSBRNA2T00153852001 Exp number, first 60 AAs:  0
# Brassicanapus_GSBRNA2T00153852001 Total prob of N-in:        0.00285
Brassicanapus_GSBRNA2T00153852001       TMHMM2.0        outside      1   226
```

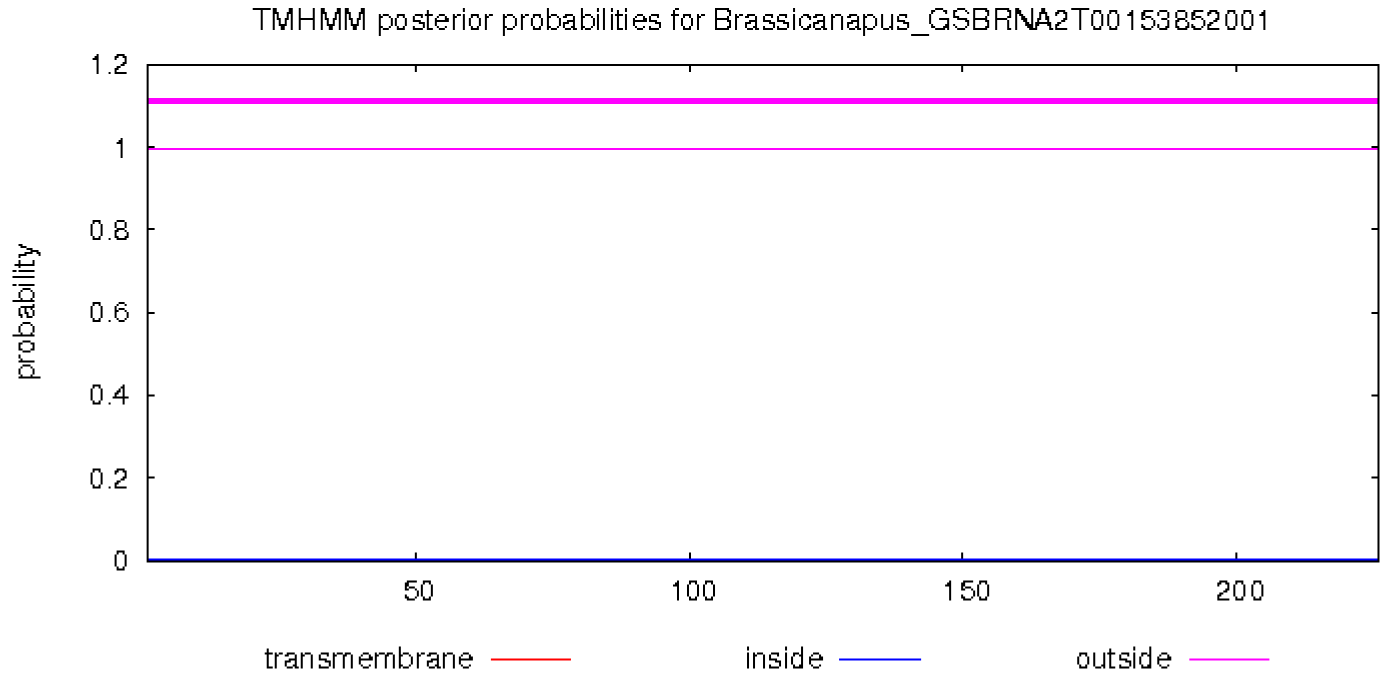

plot in postscript, script for making the plot in gnuplot, data for plot

---

```
# Brassicanapus_GSBRNA2T00154092001 Length: 235
# Brassicanapus_GSBRNA2T00154092001 Number of predicted TMHs:  0
# Brassicanapus_GSBRNA2T00154092001 Exp number of AAs in TMHs: 0.0003
# Brassicanapus_GSBRNA2T00154092001 Exp number, first 60 AAs:  0.00016
# Brassicanapus_GSBRNA2T00154092001 Total prob of N-in:        0.04566
Brassicanapus_GSBRNA2T00154092001       TMHMM2.0        outside      1   235
```

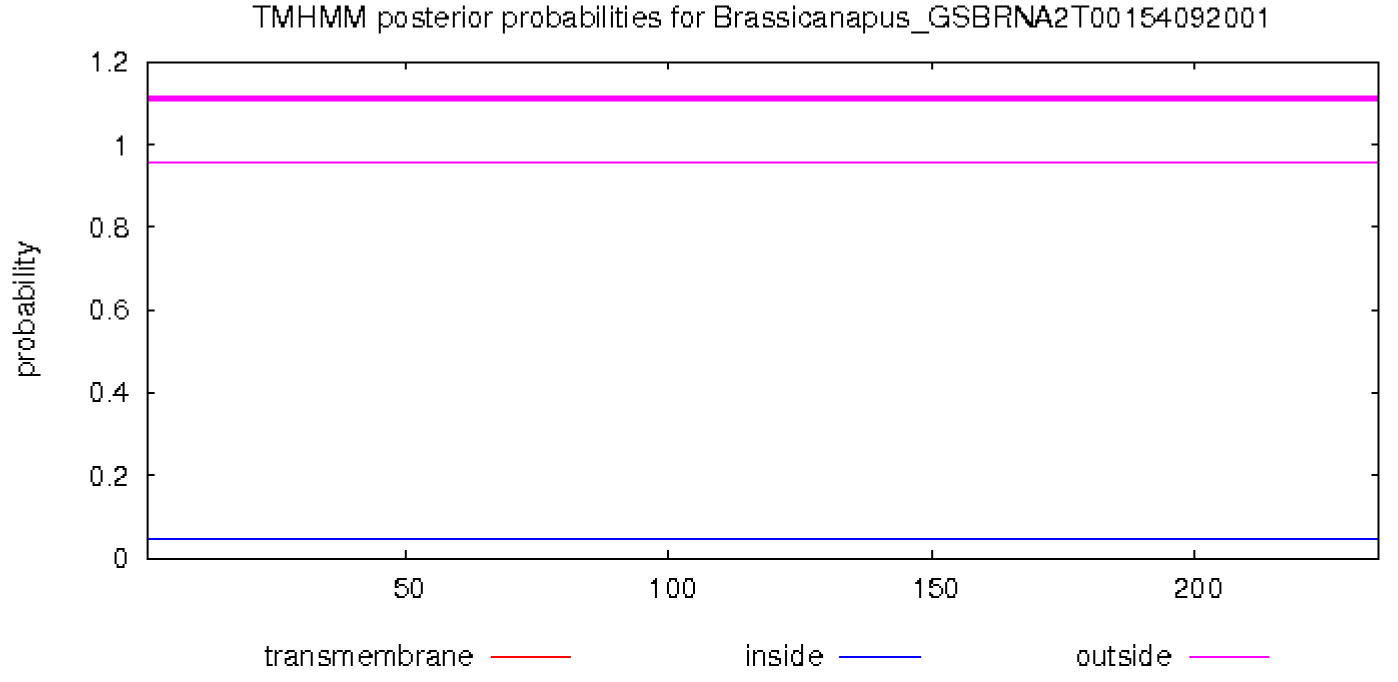

plot in postscript, script for making the plot in gnuplot, data for plot

---

```
# Brassicanapus_GSBRNA2T00154116001 Length: 335
# Brassicanapus_GSBRNA2T00154116001 Number of predicted TMHs:  0
```

```
# Brassicanapus_GSBRNA2T00154116001 Exp number of AAs in TMHs: 13.43245
# Brassicanapus_GSBRNA2T00154116001 Exp number, first 60 AAs:  0.04454
# Brassicanapus_GSBRNA2T00154116001 Total prob of N-in:        0.29742
Brassicanapus_GSBRNA2T00154116001       TMHMM2.0        outside      1   335
```

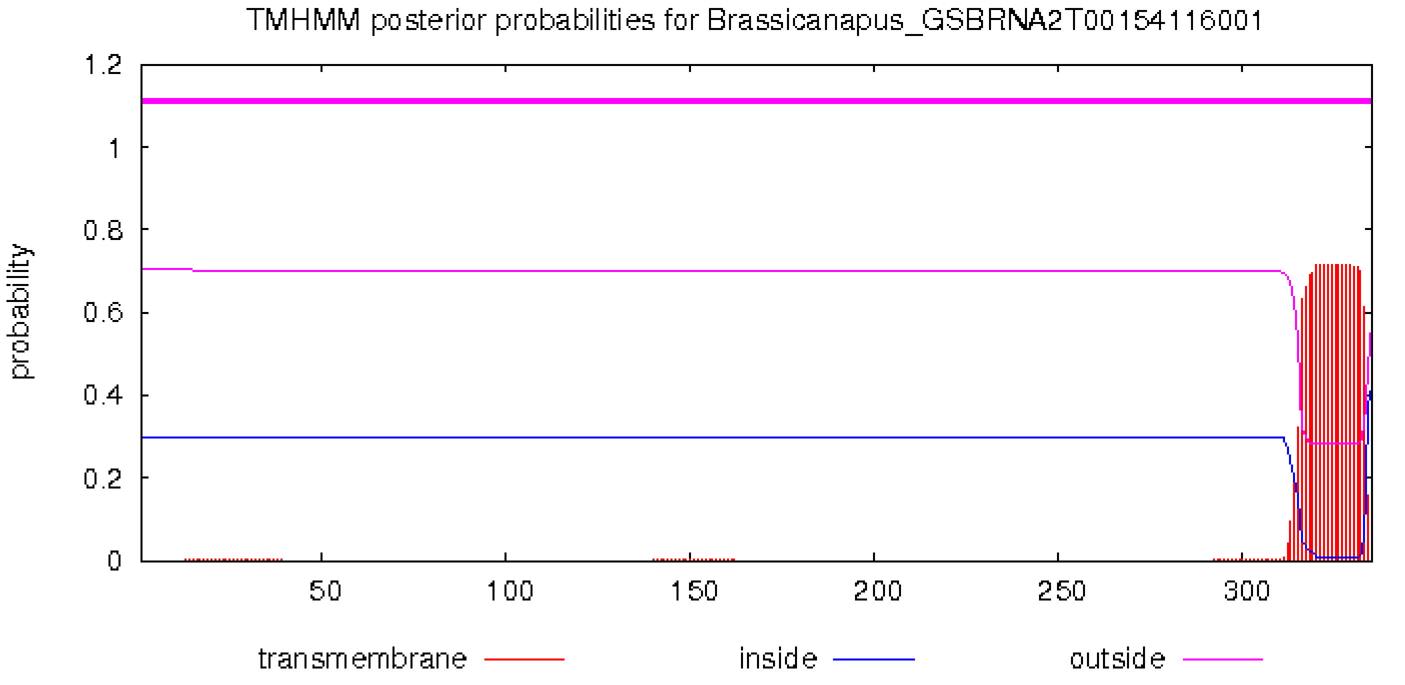

plot in postscript, script for making the plot in gnuplot, data for plot

---

```
# Brassicanapus_GSBRNA2T00154660001 Length: 300
# Brassicanapus_GSBRNA2T00154660001 Number of predicted TMHs:  0
# Brassicanapus_GSBRNA2T00154660001 Exp number of AAs in TMHs: 0.01028
# Brassicanapus_GSBRNA2T00154660001 Exp number, first 60 AAs:  0.00032
# Brassicanapus_GSBRNA2T00154660001 Total prob of N-in:        0.01300
Brassicanapus_GSBRNA2T00154660001       TMHMM2.0        outside      1   300
```

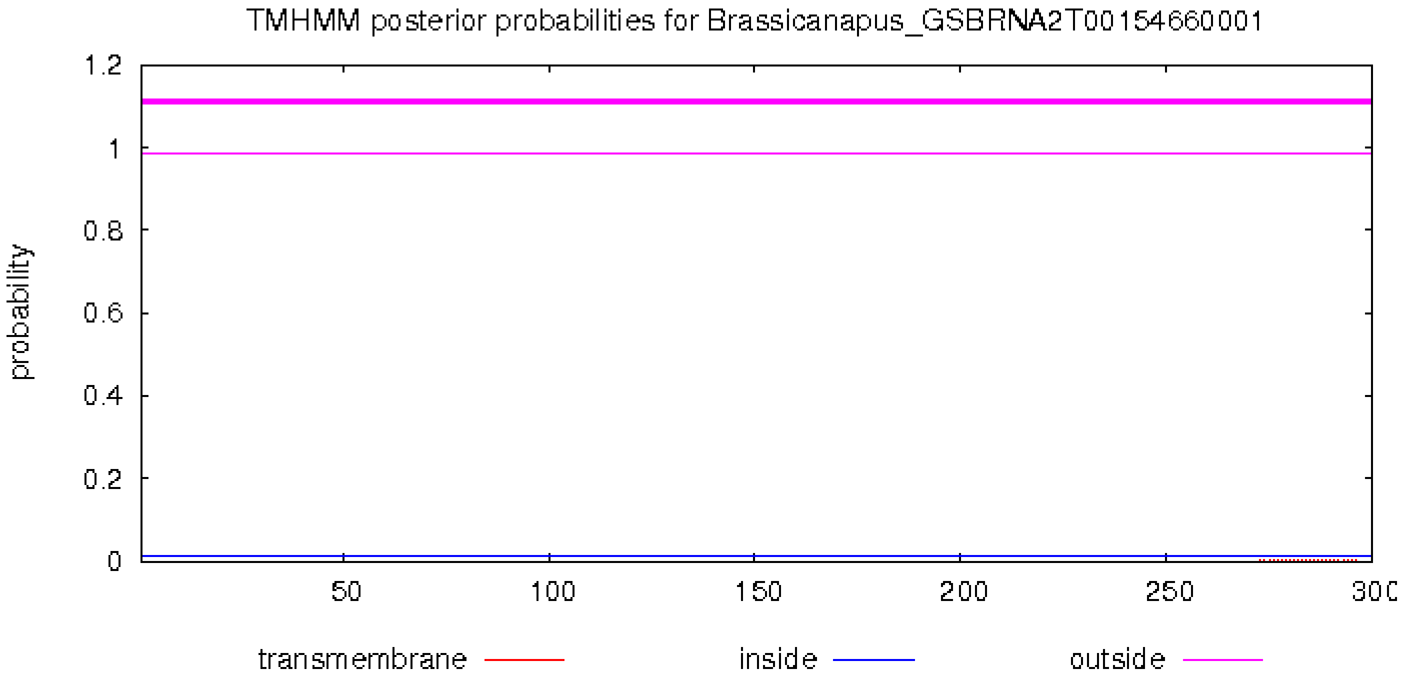

plot in postscript, script for making the plot in gnuplot, data for plot

---

```
# Brassicanapus_GSBRNA2T00154717001 Length: 303
# Brassicanapus_GSBRNA2T00154717001 Number of predicted TMHs:  0
```

```
# Brassicanapus_GSBRNA2T00154717001 Exp number of AAs in TMHs: 0.00022
# Brassicanapus_GSBRNA2T00154717001 Exp number, first 60 AAs:  0
# Brassicanapus_GSBRNA2T00154717001 Total prob of N-in:        0.05667
Brassicanapus_GSBRNA2T00154717001	TMHMM2.0	outside	     1   303
```

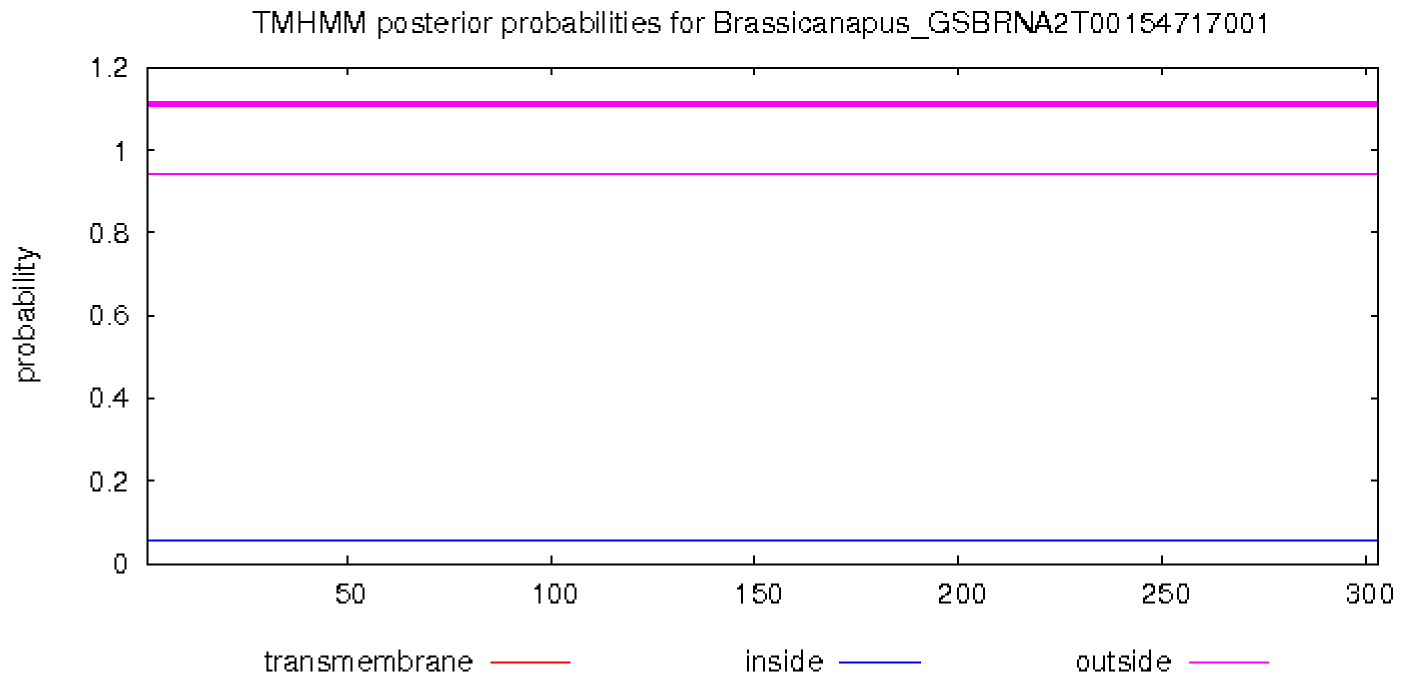

plot in postscript, script for making the plot in gnuplot, data for plot

---

```
# Brassicanapus_GSBRNA2T00154720001 Length: 163
# Brassicanapus_GSBRNA2T00154720001 Number of predicted TMHs:  0
# Brassicanapus_GSBRNA2T00154720001 Exp number of AAs in TMHs: 0.01902
# Brassicanapus_GSBRNA2T00154720001 Exp number, first 60 AAs:  0
# Brassicanapus_GSBRNA2T00154720001 Total prob of N-in:        0.05789
Brassicanapus_GSBRNA2T00154720001	TMHMM2.0	outside	     1   163
```

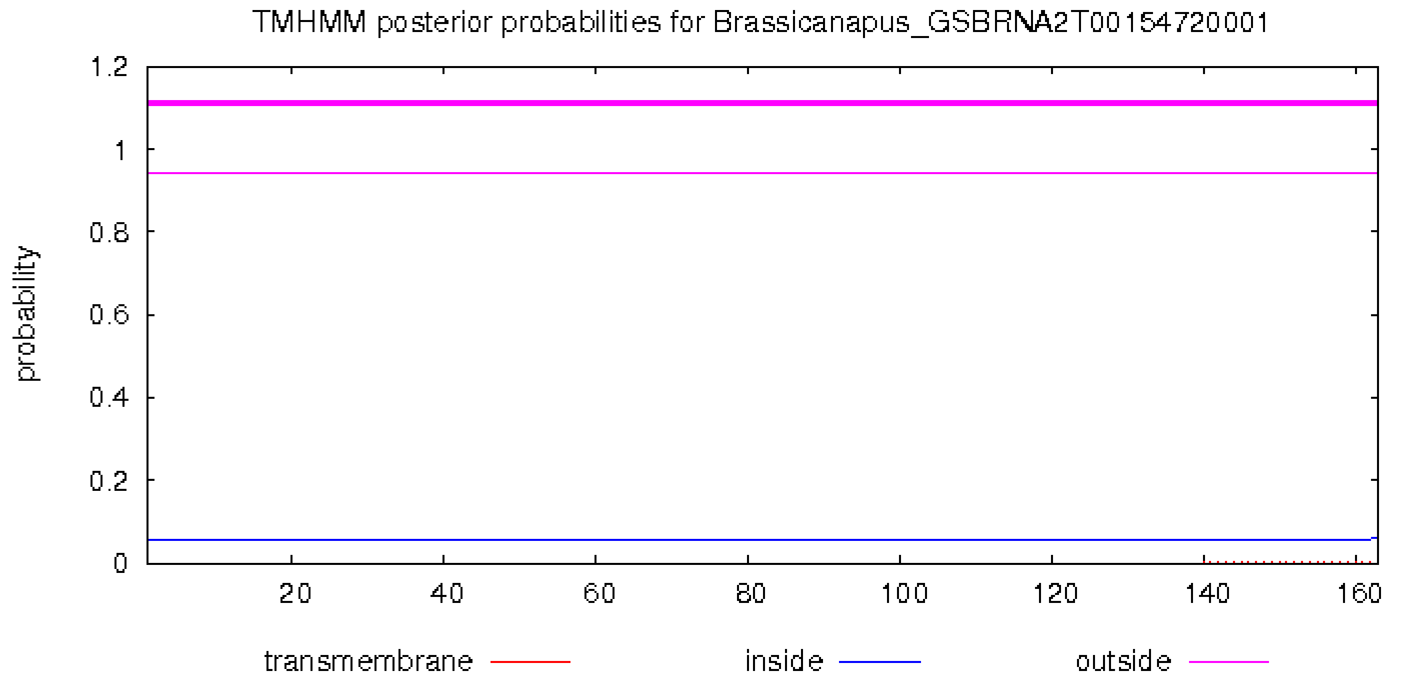

plot in postscript, script for making the plot in gnuplot, data for plot

---

```
# Brassicanapus_GSBRNA2T00155943001 Length: 359
# Brassicanapus_GSBRNA2T00155943001 Number of predicted TMHs:  0
```

```
# Brassicanapus_GSBRNA2T00155943001 Exp number of AAs in TMHs: 0.0004
# Brassicanapus_GSBRNA2T00155943001 Exp number, first 60 AAs:  0
# Brassicanapus_GSBRNA2T00155943001 Total prob of N-in:        0.01670
Brassicanapus_GSBRNA2T00155943001       TMHMM2.0        outside      1   359
```

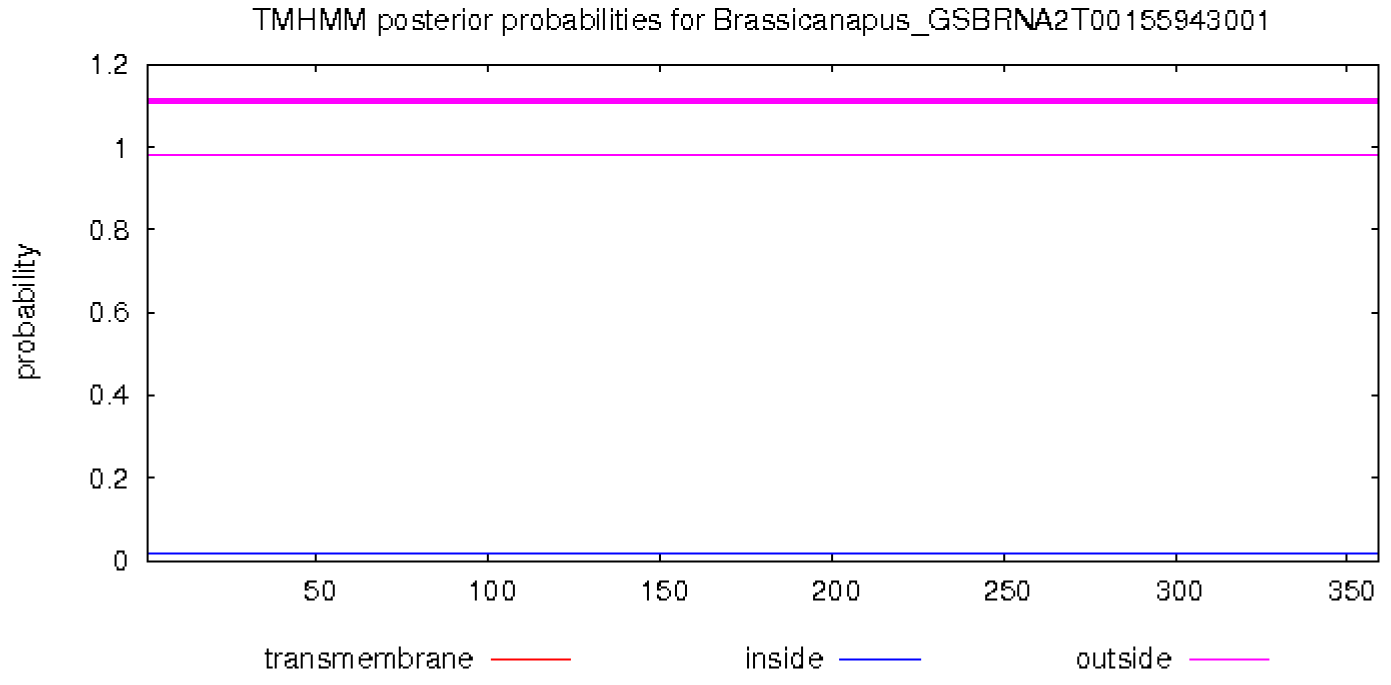

plot in postscript, script for making the plot in gnuplot, data for plot

---

```
# Brassicanapus_GSBRNA2T00156429001 Length: 556
# Brassicanapus_GSBRNA2T00156429001 Number of predicted TMHs:  1
# Brassicanapus_GSBRNA2T00156429001 Exp number of AAs in TMHs: 21.24953
# Brassicanapus_GSBRNA2T00156429001 Exp number, first 60 AAs:  0.00242
# Brassicanapus_GSBRNA2T00156429001 Total prob of N-in:        0.26976
Brassicanapus_GSBRNA2T00156429001       TMHMM2.0        outside      1   525
Brassicanapus_GSBRNA2T00156429001       TMHMM2.0        TMhelix    526   545
Brassicanapus_GSBRNA2T00156429001       TMHMM2.0        inside     546   556
```

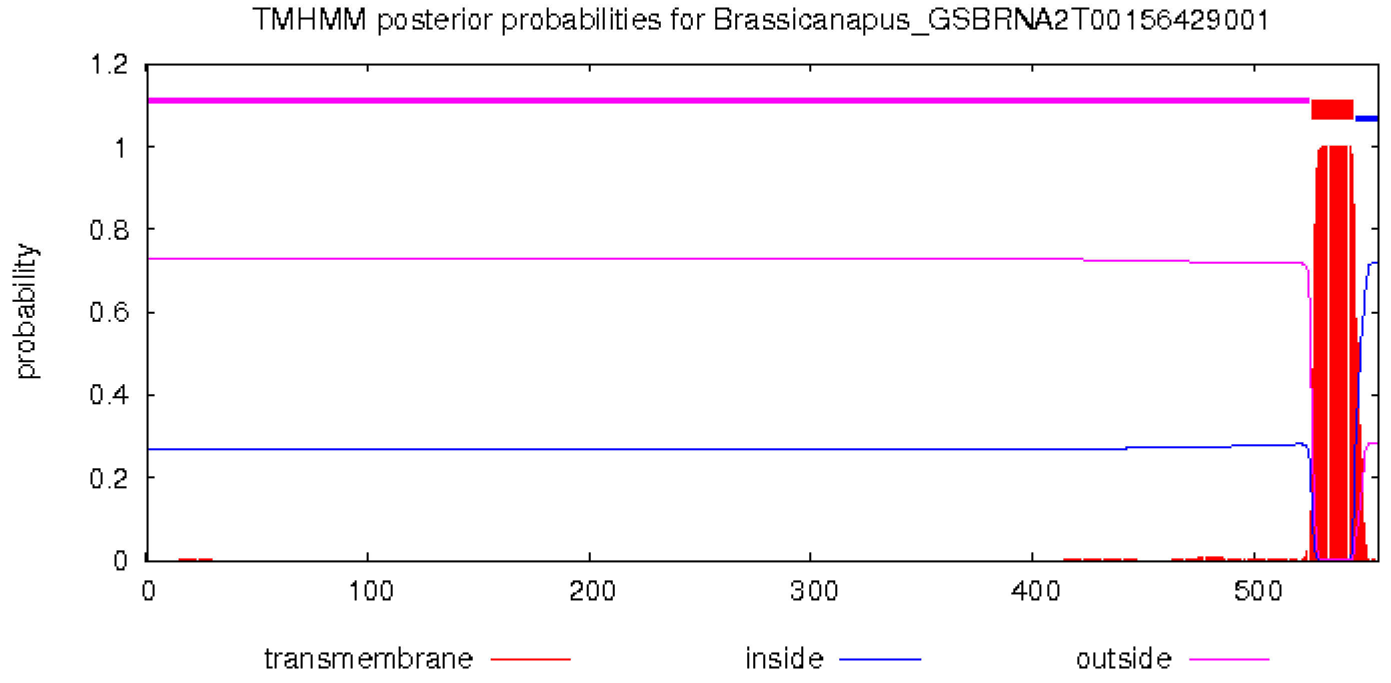

plot in postscript, script for making the plot in gnuplot, data for plot

---

```
# Brassicanapus_GSBRNA2T00156597001 Length: 364
# Brassicanapus_GSBRNA2T00156597001 Number of predicted TMHs:  0
# Brassicanapus_GSBRNA2T00156597001 Exp number of AAs in TMHs: 0
# Brassicanapus_GSBRNA2T00156597001 Exp number, first 60 AAs:  0
# Brassicanapus_GSBRNA2T00156597001 Total prob of N-in:        0.01460
Brassicanapus_GSBRNA2T00156597001       TMHMM2.0        outside      1   364
```

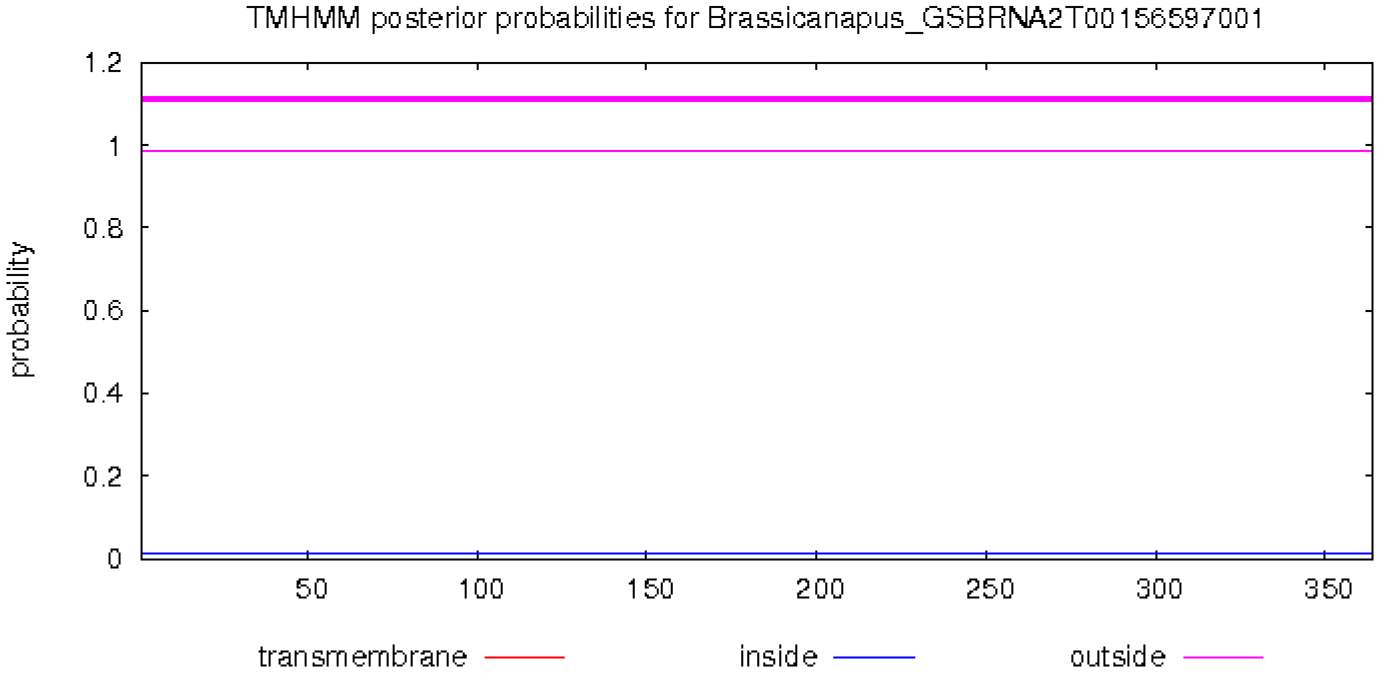

plot in postscript, script for making the plot in gnuplot, data for plot

```
# Brassicanapus_GSBRNA2T00157940001 Length: 309
# Brassicanapus_GSBRNA2T00157940001 Number of predicted TMHs:  0
# Brassicanapus_GSBRNA2T00157940001 Exp number of AAs in TMHs: 0.07236
# Brassicanapus_GSBRNA2T00157940001 Exp number, first 60 AAs:  0.00019
# Brassicanapus_GSBRNA2T00157940001 Total prob of N-in:        0.07883
Brassicanapus_GSBRNA2T00157940001       TMHMM2.0        outside      1   309
```

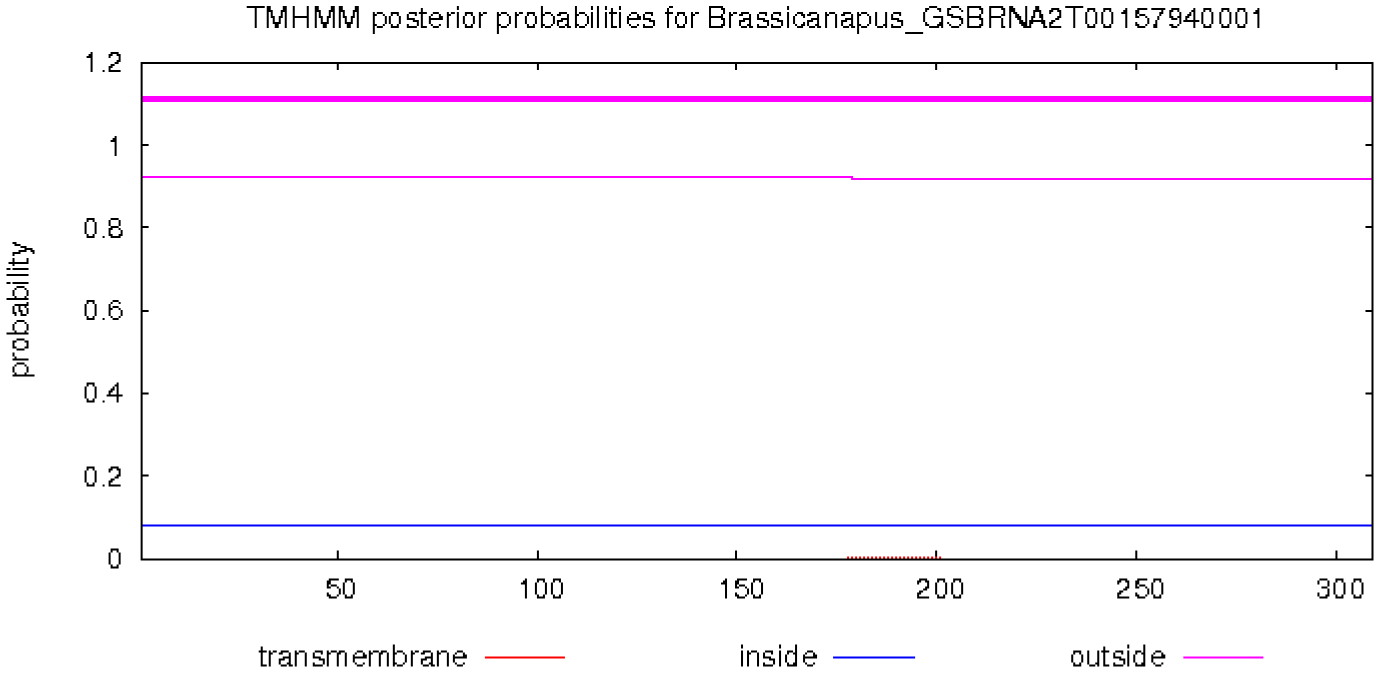

plot in postscript, script for making the plot in gnuplot, data for plot

```
# Brassicaoleracea_XP_013583657.1 Length: 344
# Brassicaoleracea_XP_013583657.1 Number of predicted TMHs:  0
# Brassicaoleracea_XP_013583657.1 Exp number of AAs in TMHs: 0
# Brassicaoleracea_XP_013583657.1 Exp number, first 60 AAs:  0
# Brassicaoleracea_XP_013583657.1 Total prob of N-in:        0.00672
Brassicaoleracea_XP_013583657.1 TMHMM2.0        outside      1   344
```

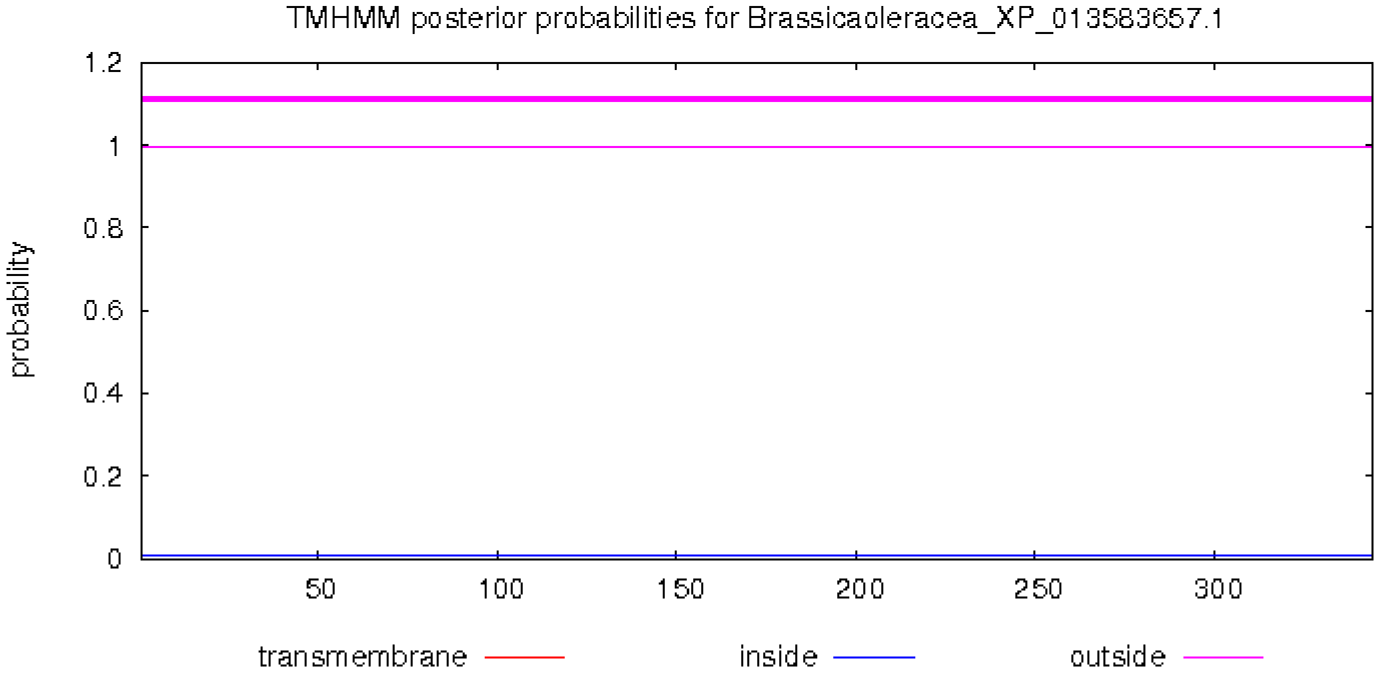

plot in postscript, script for making the plot in gnuplot, data for plot

---

```
# Brassicaoleracea_XP_013583708.1 Length: 359
# Brassicaoleracea_XP_013583708.1 Number of predicted TMHs:  0
# Brassicaoleracea_XP_013583708.1 Exp number of AAs in TMHs: 0.00329
# Brassicaoleracea_XP_013583708.1 Exp number, first 60 AAs:  0.00116
# Brassicaoleracea_XP_013583708.1 Total prob of N-in:        0.00300
Brassicaoleracea_XP_013583708.1 TMHMM2.0        outside      1   359
```

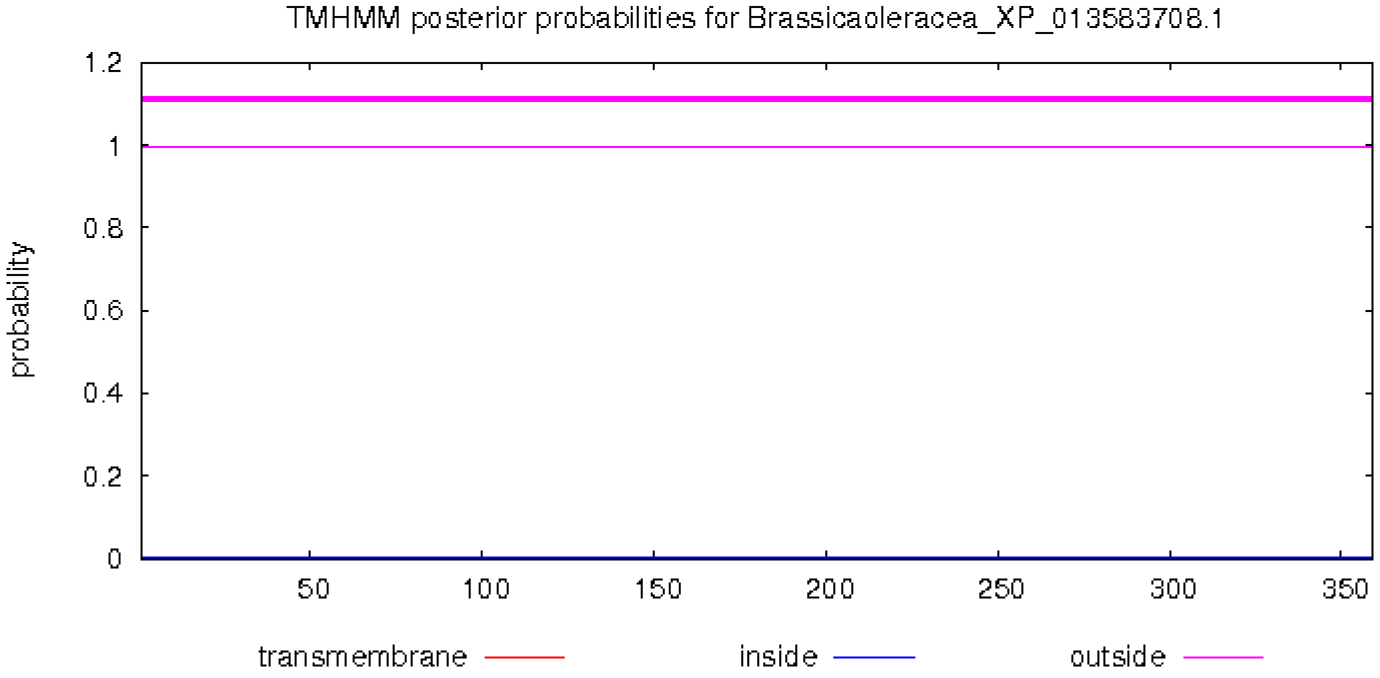

plot in postscript, script for making the plot in gnuplot, data for plot

---

```
# Brassicaoleracea_XP_013584205.1 Length: 317
# Brassicaoleracea_XP_013584205.1 Number of predicted TMHs:  0
# Brassicaoleracea_XP_013584205.1 Exp number of AAs in TMHs: 0
# Brassicaoleracea_XP_013584205.1 Exp number, first 60 AAs:  0
# Brassicaoleracea_XP_013584205.1 Total prob of N-in:        0.00384
Brassicaoleracea_XP_013584205.1 TMHMM2.0        outside      1   317
```

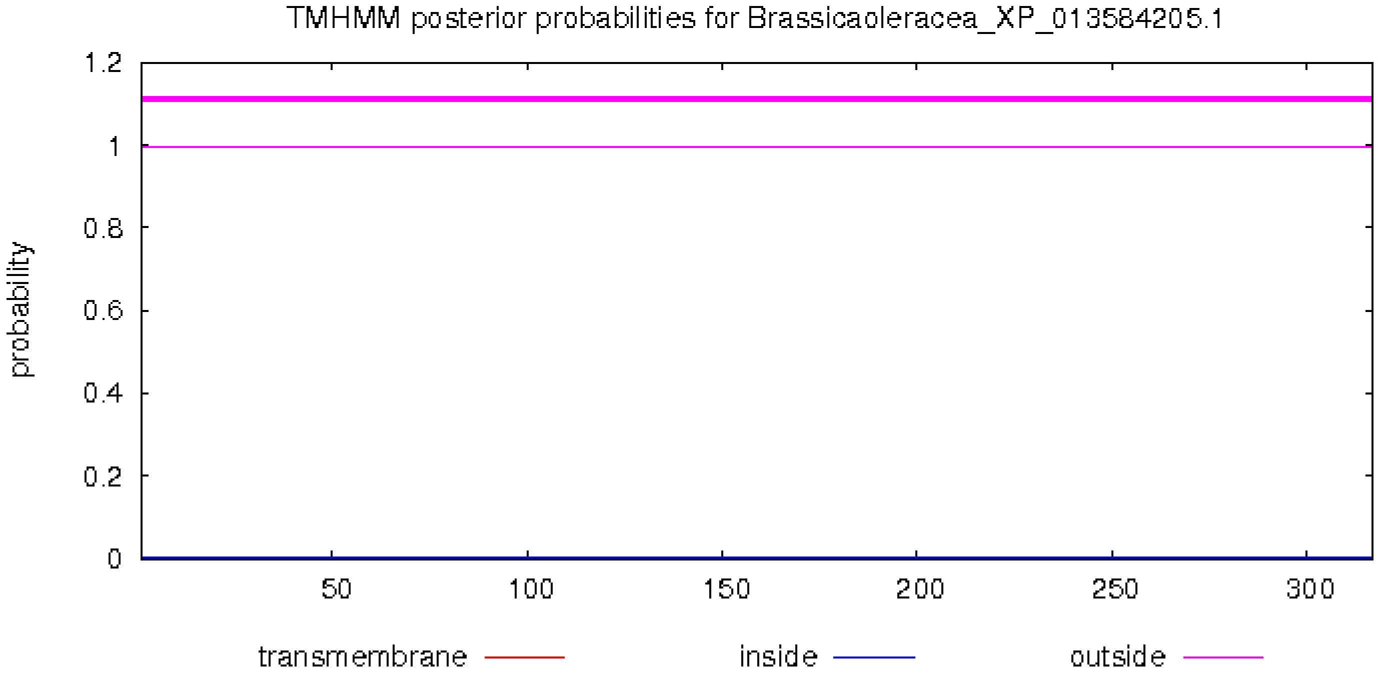

plot in postscript, script for making the plot in gnuplot, data for plot

---

```
# Brassicaoleracea_XP_013584293.1 Length: 394
# Brassicaoleracea_XP_013584293.1 Number of predicted TMHs:  0
# Brassicaoleracea_XP_013584293.1 Exp number of AAs in TMHs: 7e-05
# Brassicaoleracea_XP_013584293.1 Exp number, first 60 AAs:  0
# Brassicaoleracea_XP_013584293.1 Total prob of N-in:        0.00089
Brassicaoleracea_XP_013584293.1 TMHMM2.0        outside      1   394
```

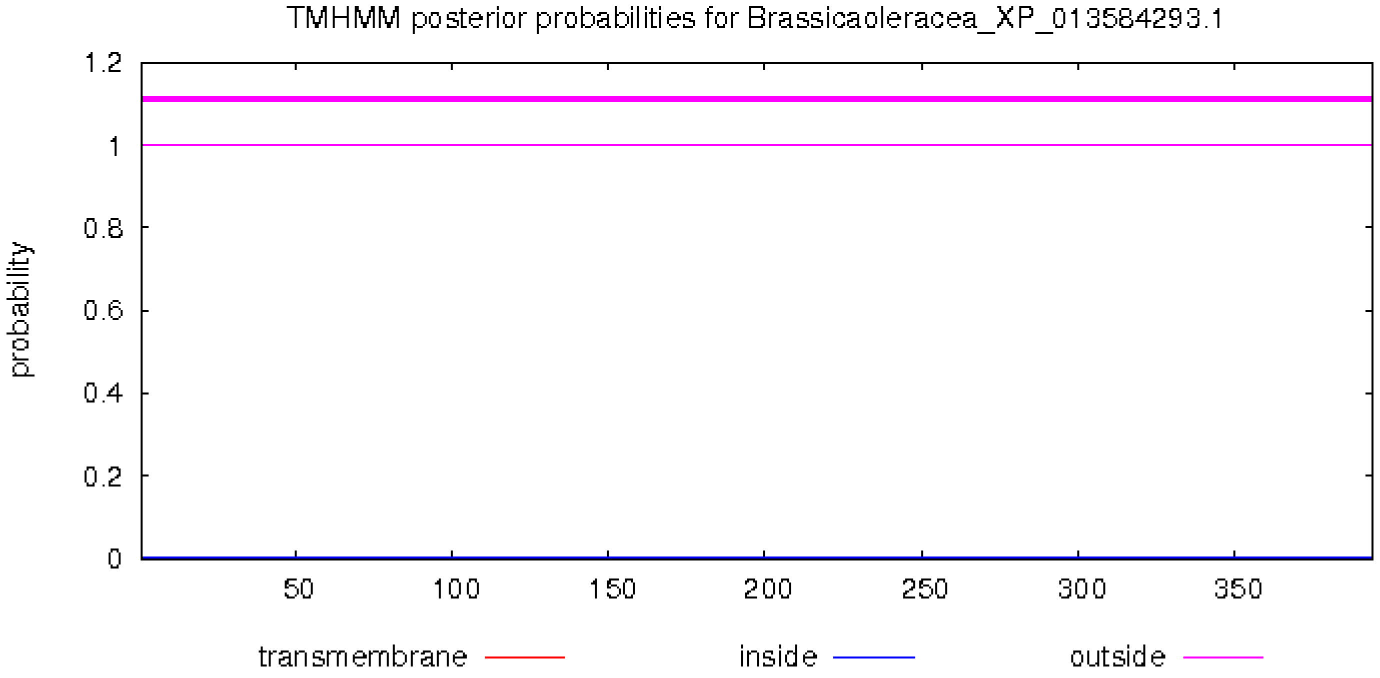

plot in postscript, script for making the plot in gnuplot, data for plot

---

```
# Brassicaoleracea_XP_013584294.1 Length: 342
# Brassicaoleracea_XP_013584294.1 Number of predicted TMHs:  0
# Brassicaoleracea_XP_013584294.1 Exp number of AAs in TMHs: 0.00018
# Brassicaoleracea_XP_013584294.1 Exp number, first 60 AAs:  0
# Brassicaoleracea_XP_013584294.1 Total prob of N-in:        0.00170
Brassicaoleracea_XP_013584294.1 TMHMM2.0        outside      1   342
```

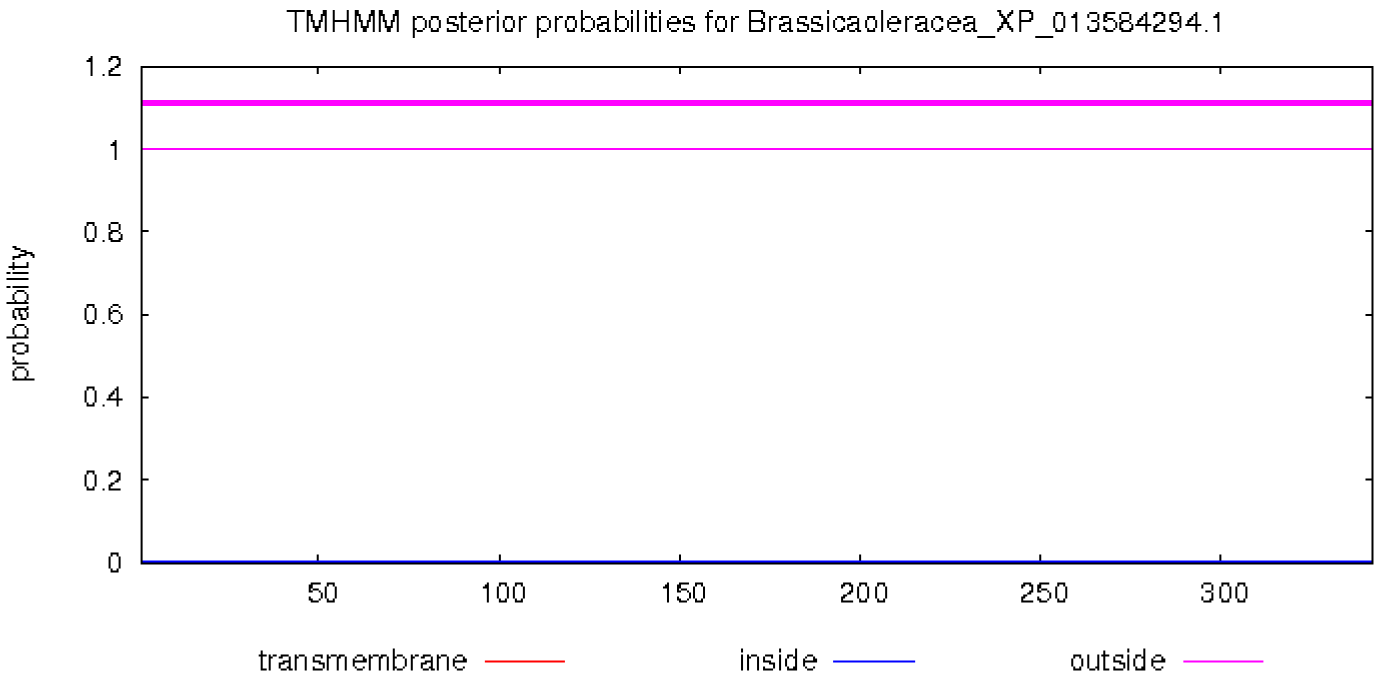

plot in postscript, script for making the plot in gnuplot, data for plot

---

```
# Brassicaoleracea_XP_013584372.1 Length: 274
# Brassicaoleracea_XP_013584372.1 Number of predicted TMHs:  0
# Brassicaoleracea_XP_013584372.1 Exp number of AAs in TMHs: 0.00151
# Brassicaoleracea_XP_013584372.1 Exp number, first 60 AAs:  0
# Brassicaoleracea_XP_013584372.1 Total prob of N-in:        0.04002
Brassicaoleracea_XP_013584372.1 TMHMM2.0        outside      1   274
```

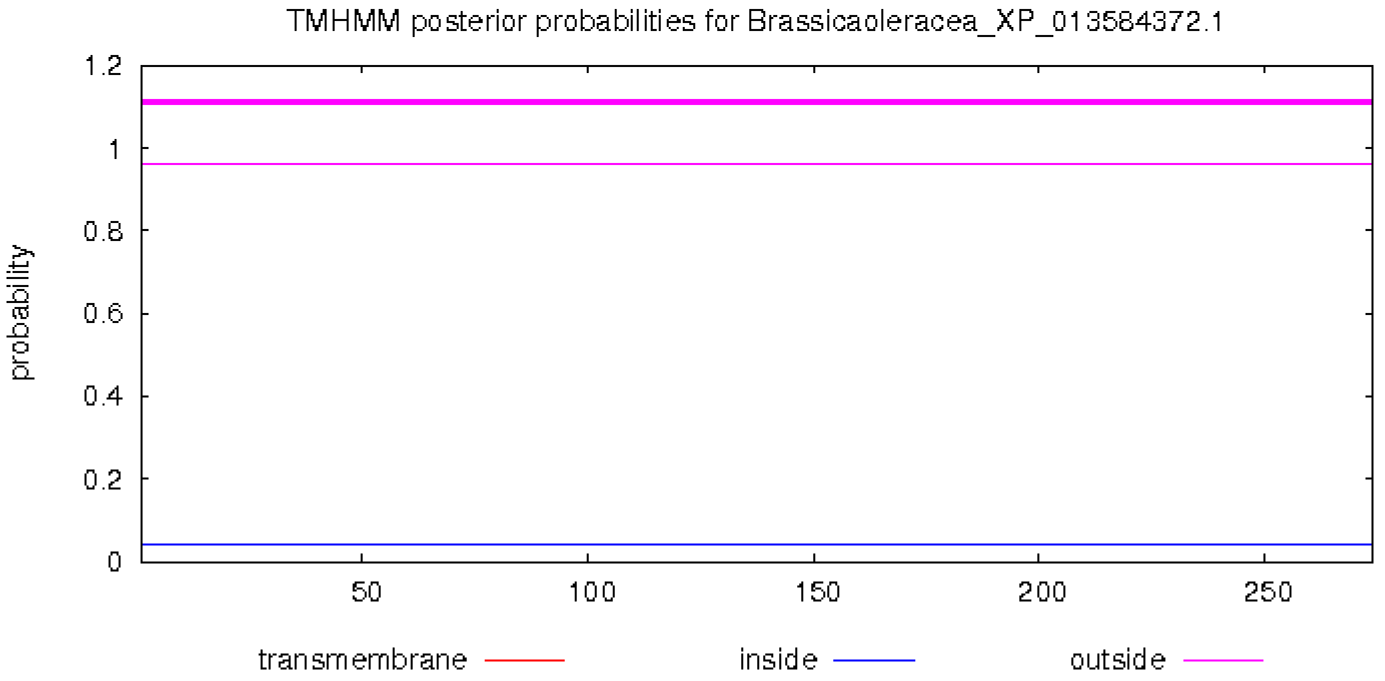

plot in postscript, script for making the plot in gnuplot, data for plot

---

```
# Brassicaoleracea_XP_013584744.1 Length: 371
# Brassicaoleracea_XP_013584744.1 Number of predicted TMHs:  0
# Brassicaoleracea_XP_013584744.1 Exp number of AAs in TMHs: 0
# Brassicaoleracea_XP_013584744.1 Exp number, first 60 AAs:  0
# Brassicaoleracea_XP_013584744.1 Total prob of N-in:        0.00257
Brassicaoleracea_XP_013584744.1 TMHMM2.0        outside      1   371
```

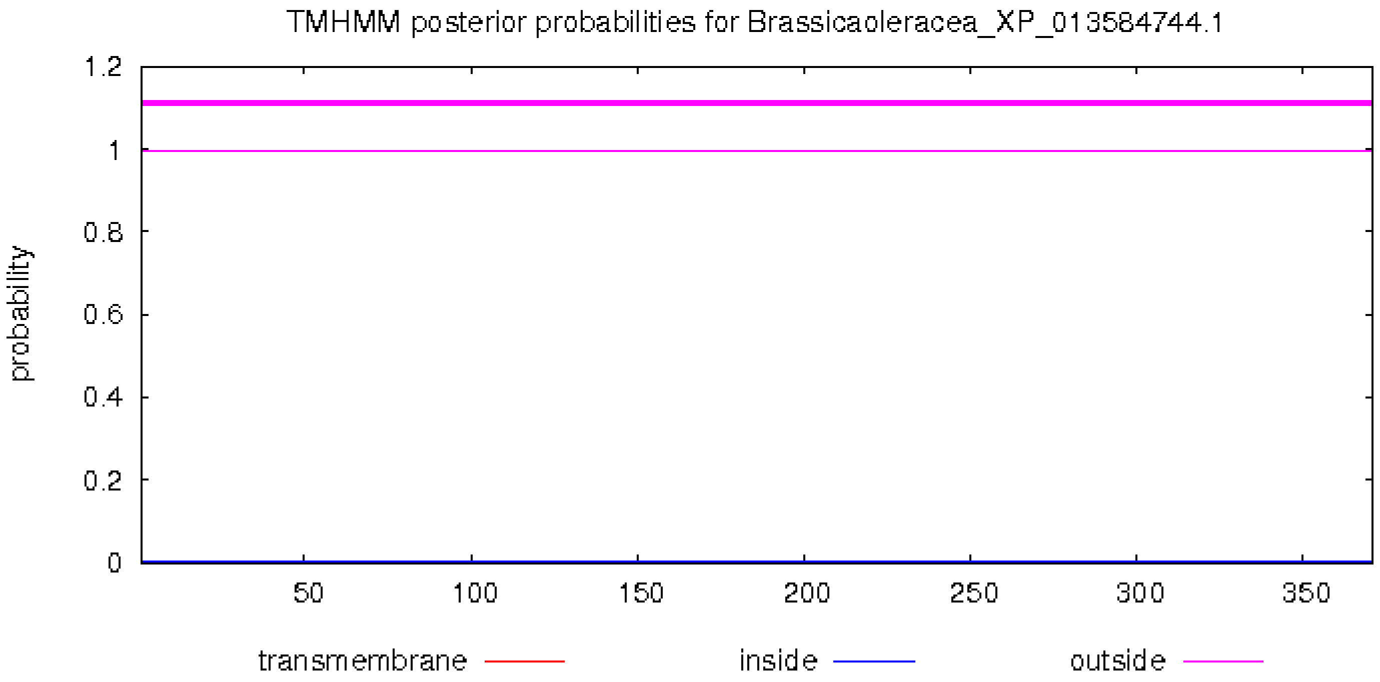

plot in postscript, script for making the plot in gnuplot, data for plot

---

```
# Brassicaoleracea_XP_013584829.1 Length: 478
# Brassicaoleracea_XP_013584829.1 Number of predicted TMHs:  0
# Brassicaoleracea_XP_013584829.1 Exp number of AAs in TMHs: 0.00091
# Brassicaoleracea_XP_013584829.1 Exp number, first 60 AAs:  0
# Brassicaoleracea_XP_013584829.1 Total prob of N-in:        0.00018
Brassicaoleracea_XP_013584829.1 TMHMM2.0        outside      1   478
```

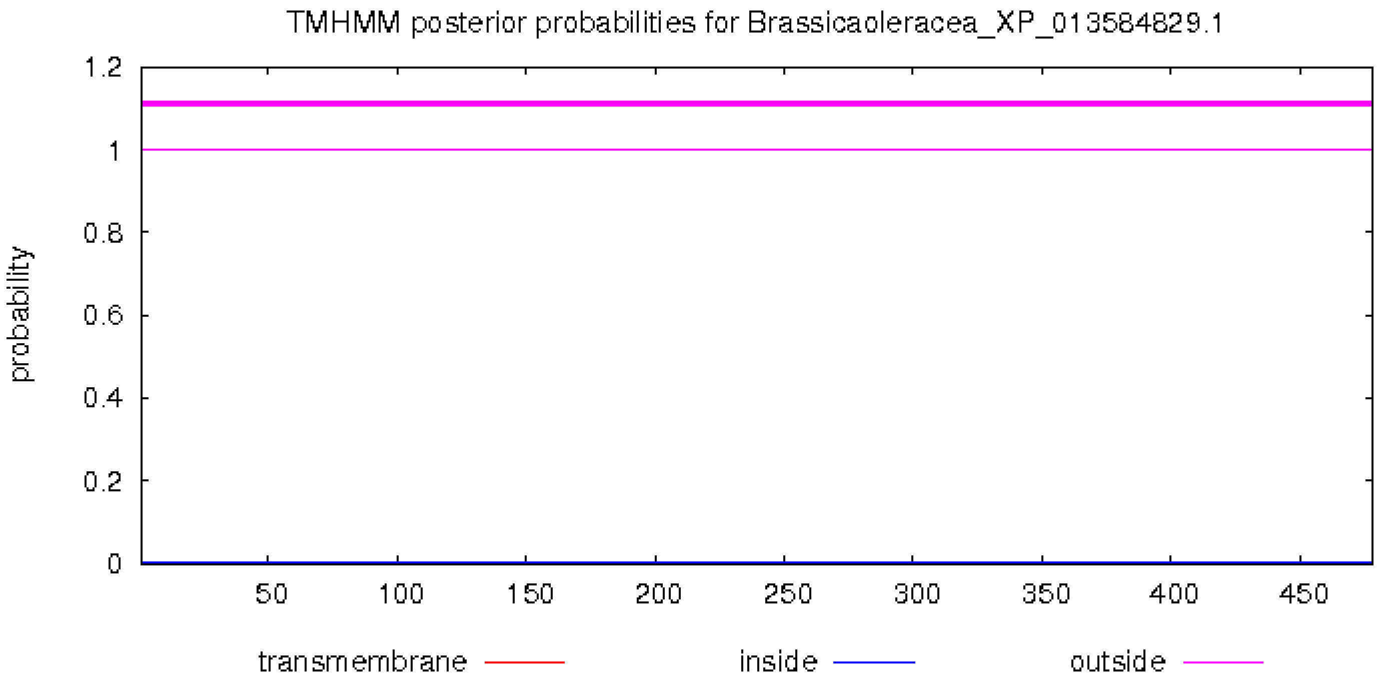

plot in postscript, script for making the plot in gnuplot, data for plot

---

```
# Brassicaoleracea_XP_013585182.1 Length: 306
# Brassicaoleracea_XP_013585182.1 Number of predicted TMHs:  0
# Brassicaoleracea_XP_013585182.1 Exp number of AAs in TMHs: 0.00066
# Brassicaoleracea_XP_013585182.1 Exp number, first 60 AAs:  0
# Brassicaoleracea_XP_013585182.1 Total prob of N-in:        0.02504
Brassicaoleracea_XP_013585182.1 TMHMM2.0        outside      1   306
```

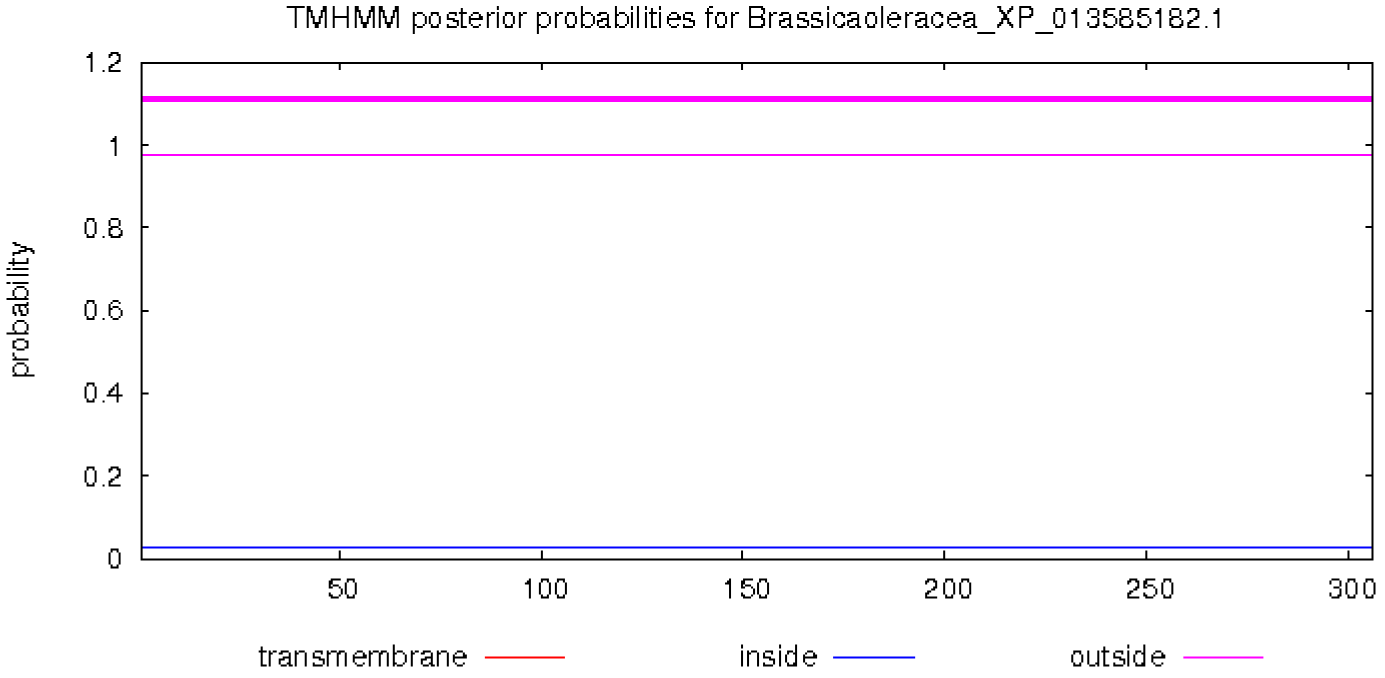

plot in postscript, script for making the plot in gnuplot, data for plot

---

```
# Brassicaoleracea_XP_013585218.1 Length: 424
# Brassicaoleracea_XP_013585218.1 Number of predicted TMHs:  0
# Brassicaoleracea_XP_013585218.1 Exp number of AAs in TMHs: 0
# Brassicaoleracea_XP_013585218.1 Exp number, first 60 AAs:  0
# Brassicaoleracea_XP_013585218.1 Total prob of N-in:        0.00349
Brassicaoleracea_XP_013585218.1 TMHMM2.0        outside      1   424
```

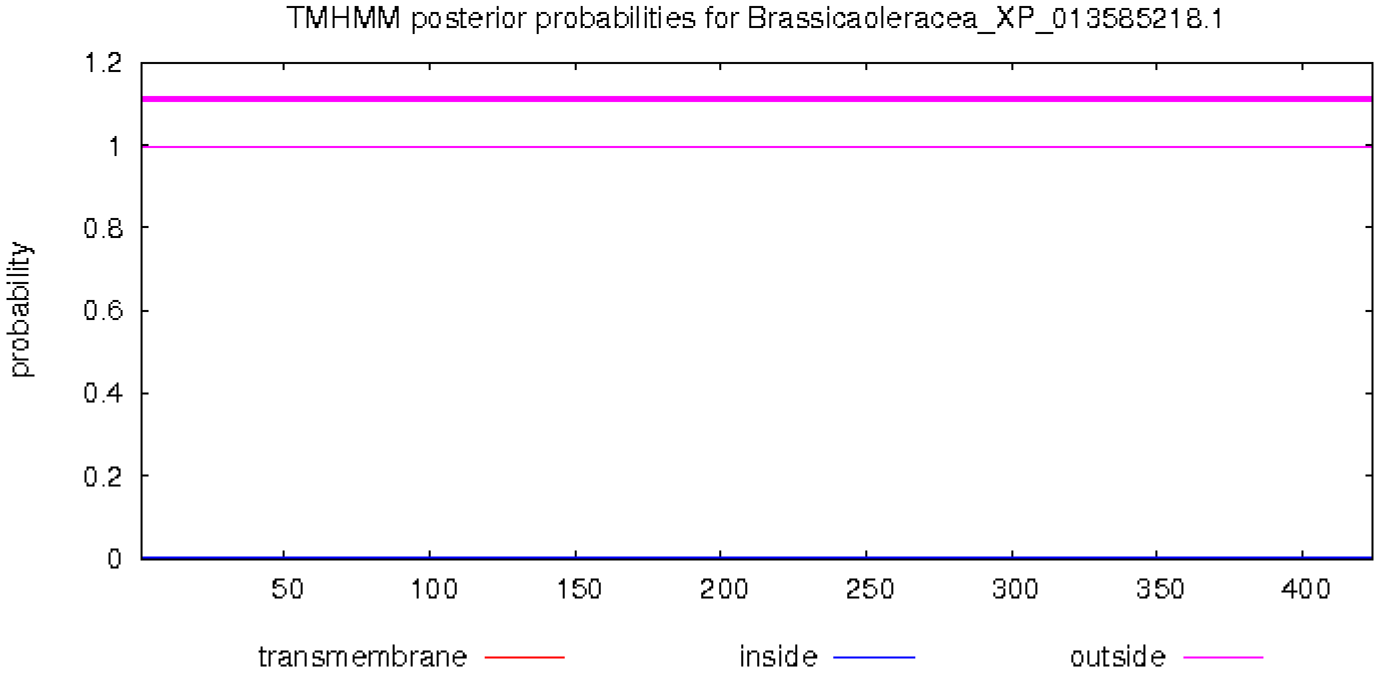

plot in postscript, script for making the plot in gnuplot, data for plot

---

```
# Brassicaoleracea_XP_013585238.1 Length: 301
# Brassicaoleracea_XP_013585238.1 Number of predicted TMHs:  0
# Brassicaoleracea_XP_013585238.1 Exp number of AAs in TMHs: 0.0235
# Brassicaoleracea_XP_013585238.1 Exp number, first 60 AAs:  0.00021
# Brassicaoleracea_XP_013585238.1 Total prob of N-in:        0.02351
Brassicaoleracea_XP_013585238.1 TMHMM2.0        outside      1   301
```

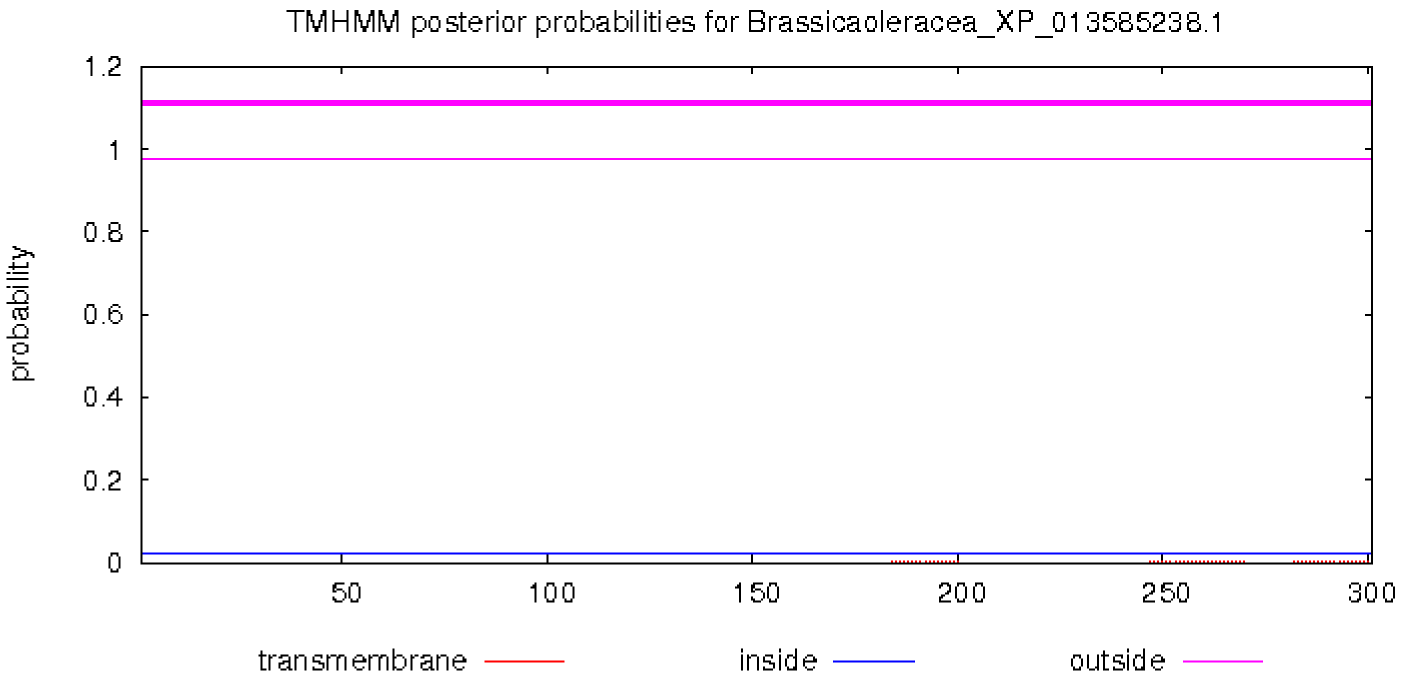

plot in postscript, script for making the plot in gnuplot, data for plot

```
# Brassicaoleracea_XP_013585389.1 Length: 358
# Brassicaoleracea_XP_013585389.1 Number of predicted TMHs:  0
# Brassicaoleracea_XP_013585389.1 Exp number of AAs in TMHs: 0.00031
# Brassicaoleracea_XP_013585389.1 Exp number, first 60 AAs:  0.0001
# Brassicaoleracea_XP_013585389.1 Total prob of N-in:        0.01002
Brassicaoleracea_XP_013585389.1 TMHMM2.0        outside      1   358
```

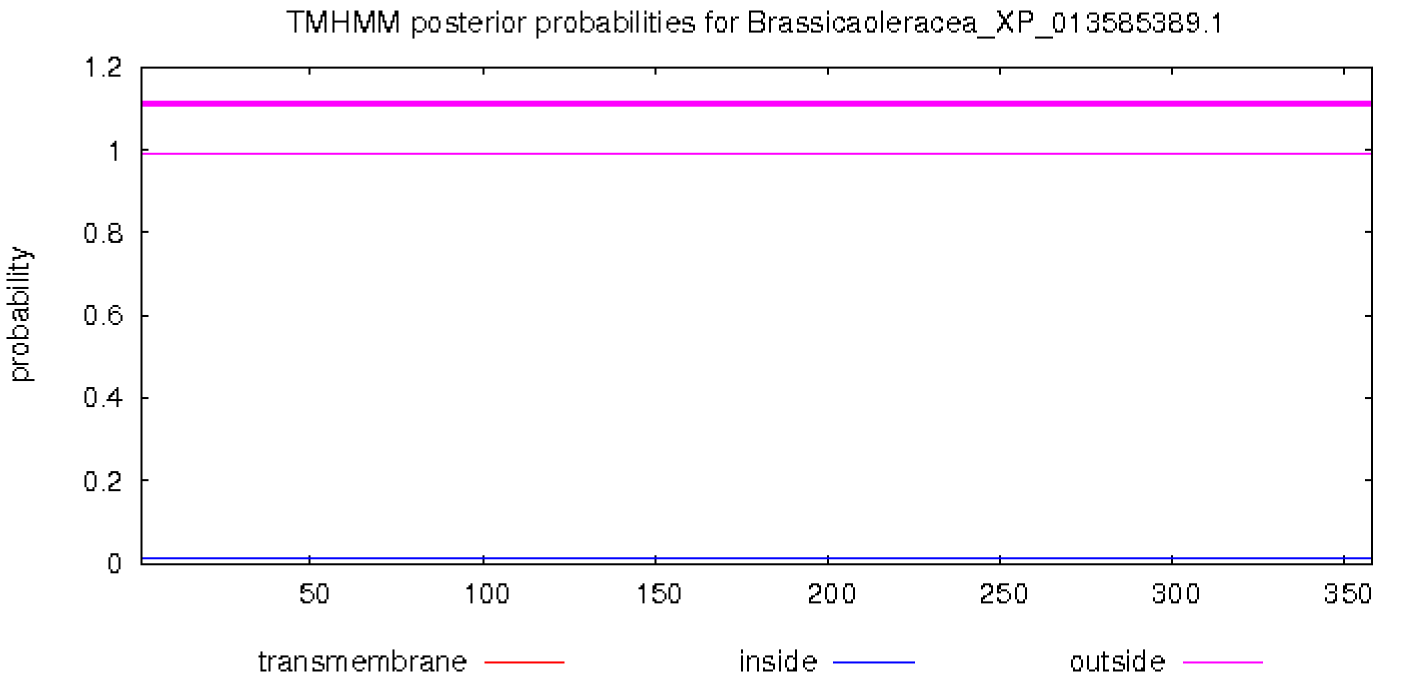

plot in postscript, script for making the plot in gnuplot, data for plot

```
# Brassicaoleracea_XP_013585390.1 Length: 344
# Brassicaoleracea_XP_013585390.1 Number of predicted TMHs:  0
# Brassicaoleracea_XP_013585390.1 Exp number of AAs in TMHs: 0.00097
# Brassicaoleracea_XP_013585390.1 Exp number, first 60 AAs:  0
# Brassicaoleracea_XP_013585390.1 Total prob of N-in:        0.00699
Brassicaoleracea_XP_013585390.1 TMHMM2.0        outside      1   344
```

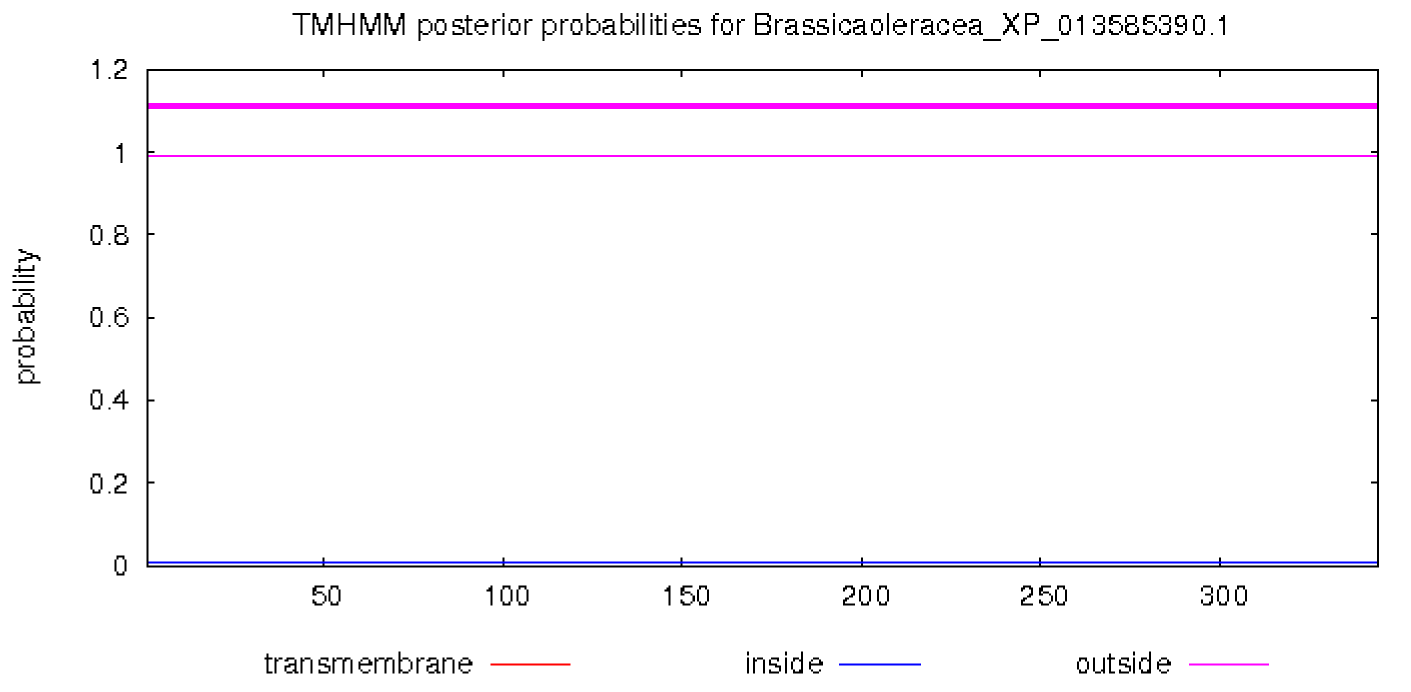

plot in postscript, script for making the plot in gnuplot, data for plot

```
# Brassicaoleracea_XP_013585394.1 Length: 251
# Brassicaoleracea_XP_013585394.1 Number of predicted TMHs:  0
# Brassicaoleracea_XP_013585394.1 Exp number of AAs in TMHs: 0
# Brassicaoleracea_XP_013585394.1 Exp number, first 60 AAs:  0
# Brassicaoleracea_XP_013585394.1 Total prob of N-in:        0.00877
Brassicaoleracea_XP_013585394.1 TMHMM2.0        outside      1   251
```

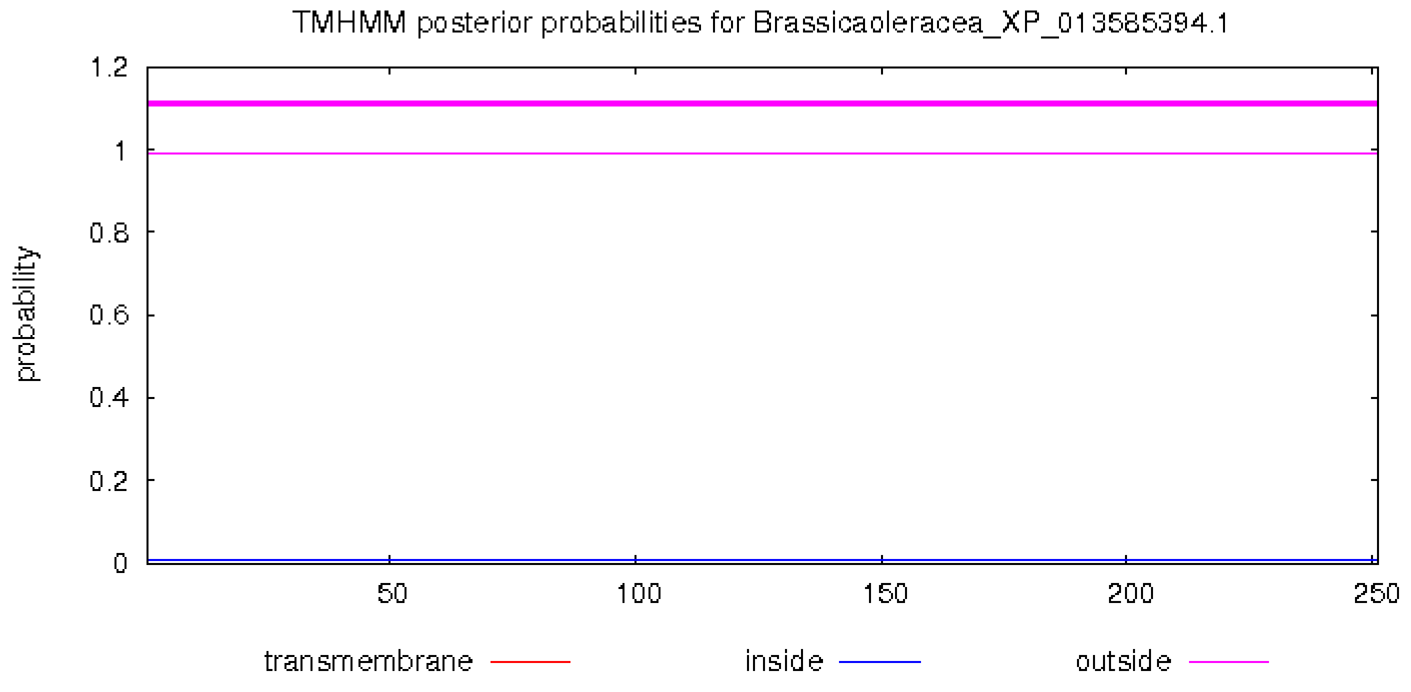

plot in postscript, script for making the plot in gnuplot, data for plot

```
# Brassicaoleracea_XP_013585836.1 Length: 286
# Brassicaoleracea_XP_013585836.1 Number of predicted TMHs:  0
# Brassicaoleracea_XP_013585836.1 Exp number of AAs in TMHs: 0.02442
# Brassicaoleracea_XP_013585836.1 Exp number, first 60 AAs:  0.0002
# Brassicaoleracea_XP_013585836.1 Total prob of N-in:        0.00269
Brassicaoleracea_XP_013585836.1 TMHMM2.0        outside      1   286
```

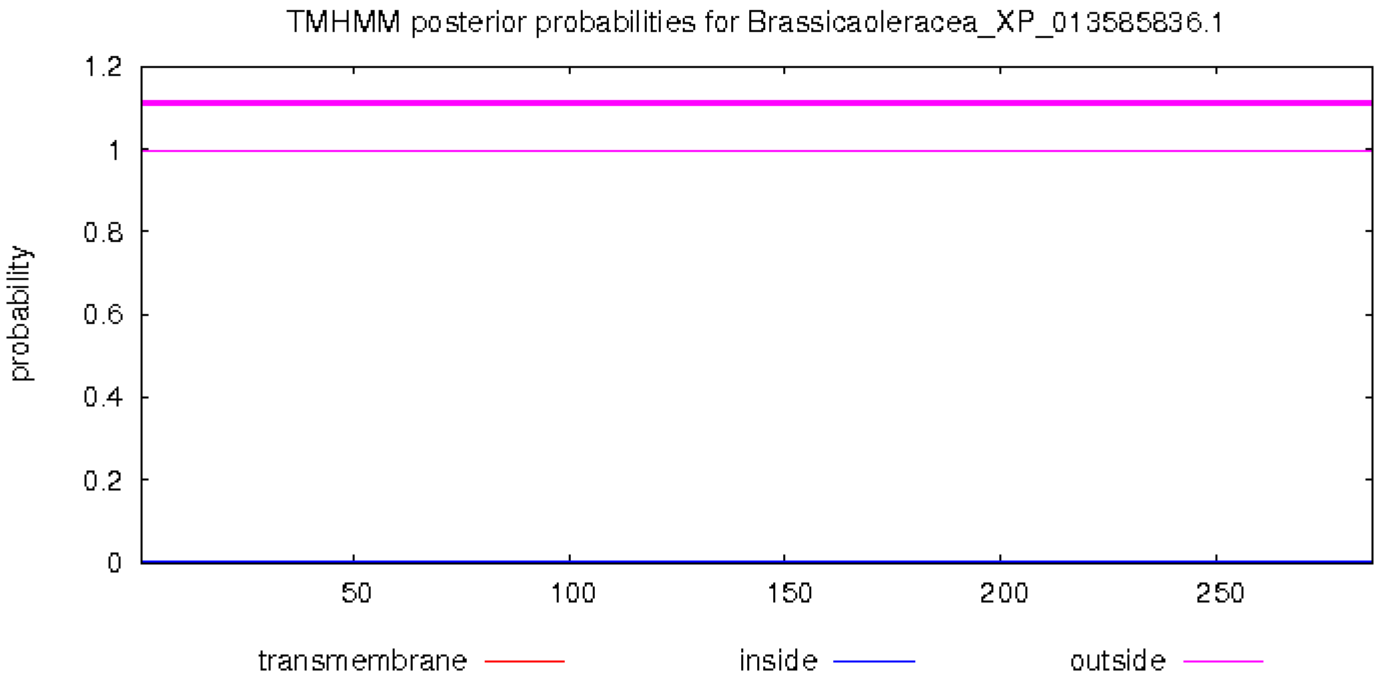

plot in postscript, script for making the plot in gnuplot, data for plot

---

```
# Brassicaoleracea_XP_013586611.1 Length: 425
# Brassicaoleracea_XP_013586611.1 Number of predicted TMHs:  1
# Brassicaoleracea_XP_013586611.1 Exp number of AAs in TMHs: 22.43212
# Brassicaoleracea_XP_013586611.1 Exp number, first 60 AAs:  0.00018
# Brassicaoleracea_XP_013586611.1 Total prob of N-in:        0.19981
Brassicaoleracea_XP_013586611.1 TMHMM2.0        outside      1   401
Brassicaoleracea_XP_013586611.1 TMHMM2.0        TMhelix    402   424
Brassicaoleracea_XP_013586611.1 TMHMM2.0        inside     425   425
```

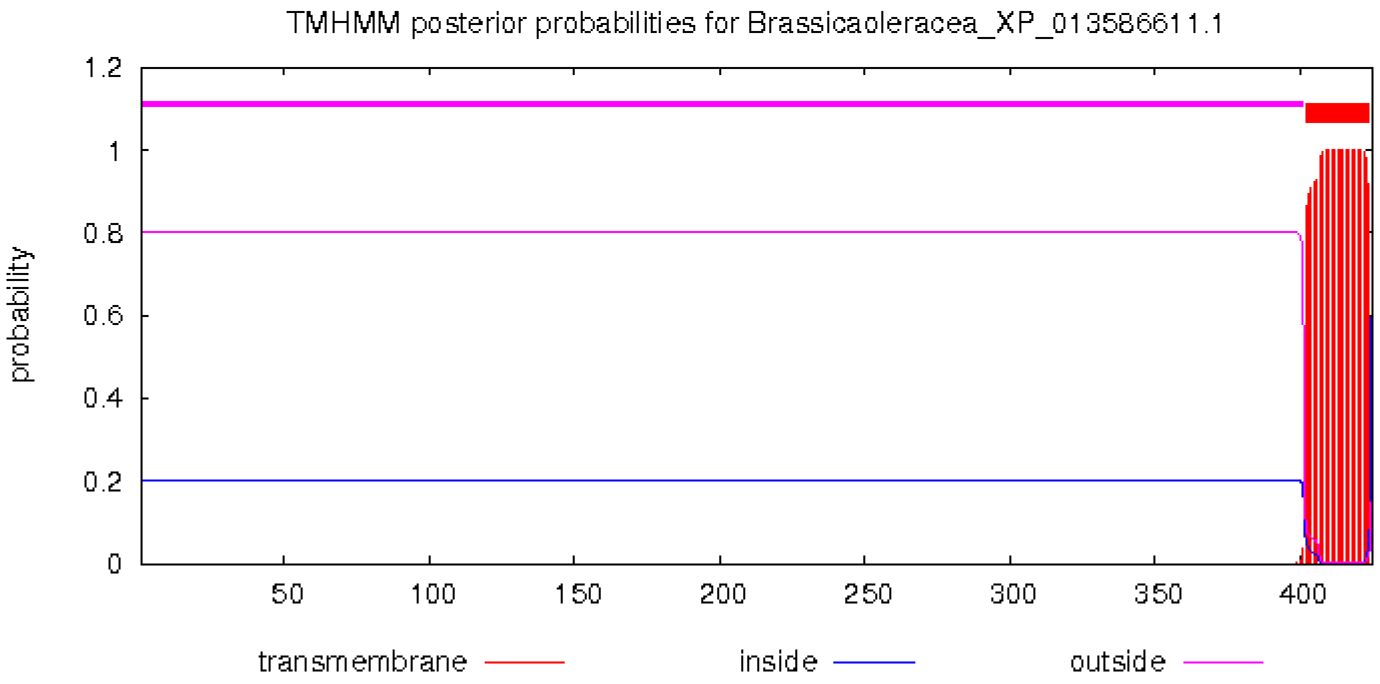

plot in postscript, script for making the plot in gnuplot, data for plot

```
# Brassicaoleracea_XP_013586612.1 Length: 290
# Brassicaoleracea_XP_013586612.1 Number of predicted TMHs:  0
# Brassicaoleracea_XP_013586612.1 Exp number of AAs in TMHs: 0
# Brassicaoleracea_XP_013586612.1 Exp number, first 60 AAs:  0
# Brassicaoleracea_XP_013586612.1 Total prob of N-in:        0.01067
Brassicaoleracea_XP_013586612.1 TMHMM2.0        outside      1   290
```

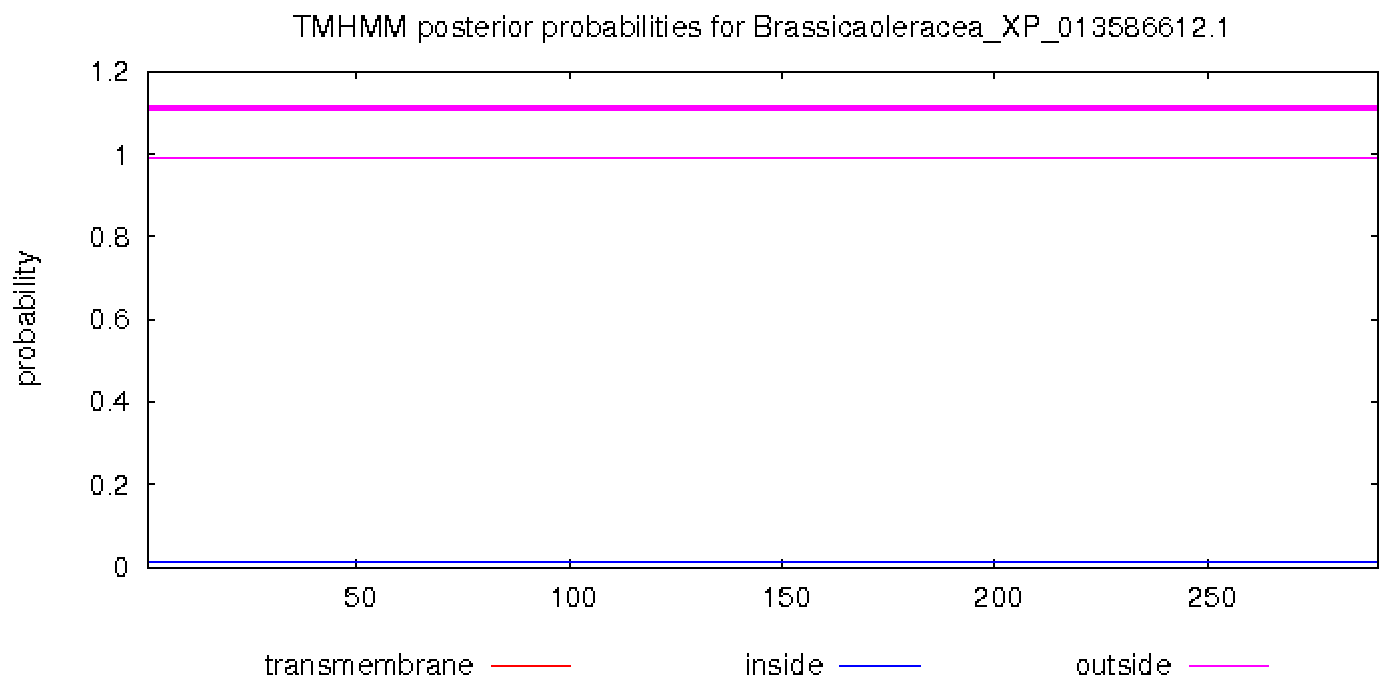

plot in postscript, script for making the plot in gnuplot, data for plot

```
# Brassicaoleracea_XP_013586773.1 Length: 451
# Brassicaoleracea_XP_013586773.1 Number of predicted TMHs:  0
# Brassicaoleracea_XP_013586773.1 Exp number of AAs in TMHs: 0.10329
# Brassicaoleracea_XP_013586773.1 Exp number, first 60 AAs:  0.05374
# Brassicaoleracea_XP_013586773.1 Total prob of N-in:        0.00644
Brassicaoleracea_XP_013586773.1 TMHMM2.0        outside      1   451
```

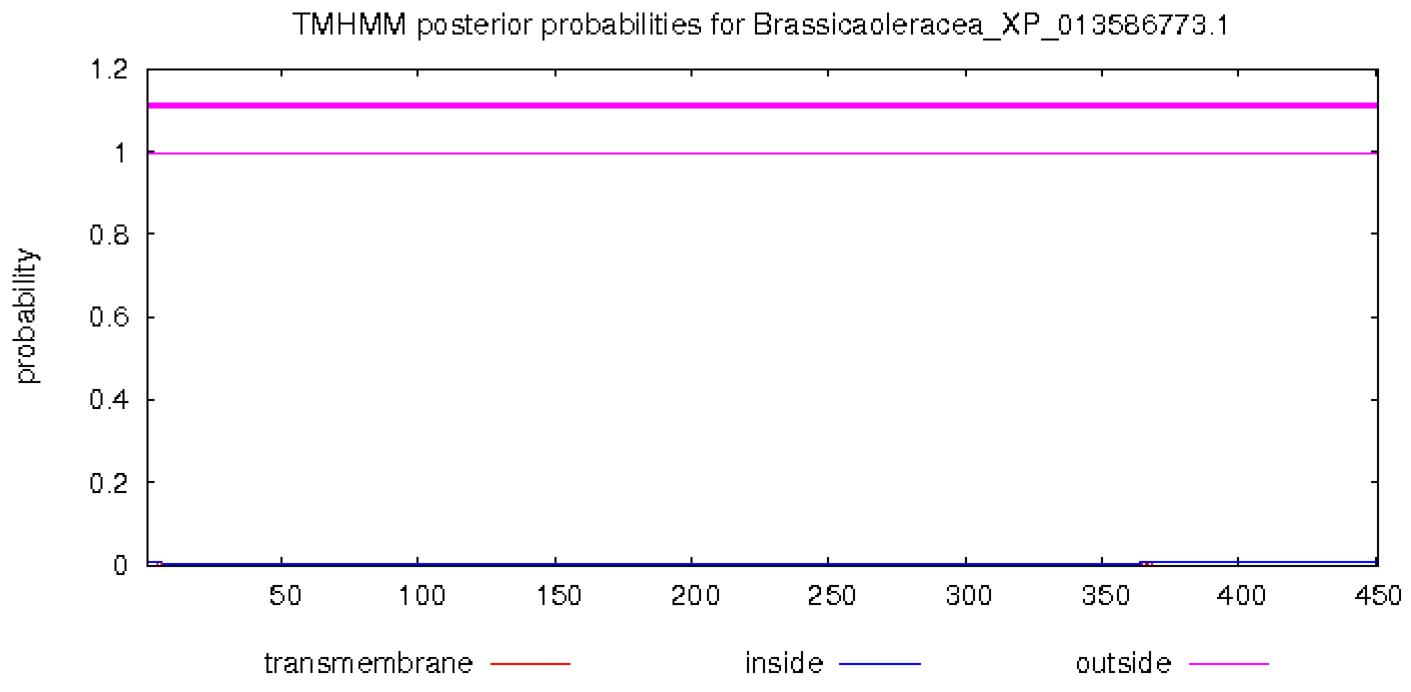

plot in postscript, script for making the plot in gnuplot, data for plot

```
# Brassicaoleracea_XP_013588383.1 Length: 316
# Brassicaoleracea_XP_013588383.1 Number of predicted TMHs:  0
# Brassicaoleracea_XP_013588383.1 Exp number of AAs in TMHs: 0.0072
# Brassicaoleracea_XP_013588383.1 Exp number, first 60 AAs:  0.00021
# Brassicaoleracea_XP_013588383.1 Total prob of N-in:        0.00915
Brassicaoleracea_XP_013588383.1 TMHMM2.0        outside      1   316
```

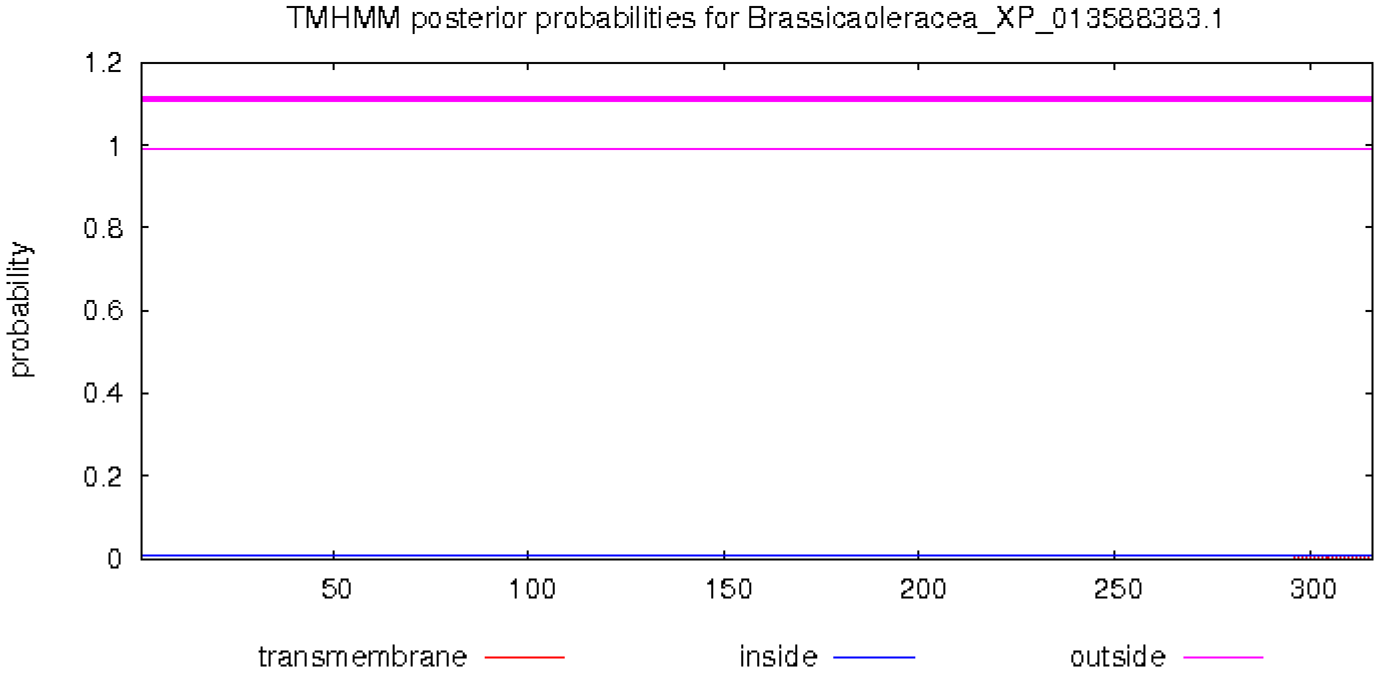

plot in postscript, script for making the plot in gnuplot, data for plot

```
# Brassicaoleracea_XP_013588625.1 Length: 371
# Brassicaoleracea_XP_013588625.1 Number of predicted TMHs:  0
# Brassicaoleracea_XP_013588625.1 Exp number of AAs in TMHs: 0.00278
# Brassicaoleracea_XP_013588625.1 Exp number, first 60 AAs:  0
# Brassicaoleracea_XP_013588625.1 Total prob of N-in:        0.00417
Brassicaoleracea_XP_013588625.1 TMHMM2.0        outside      1   371
```

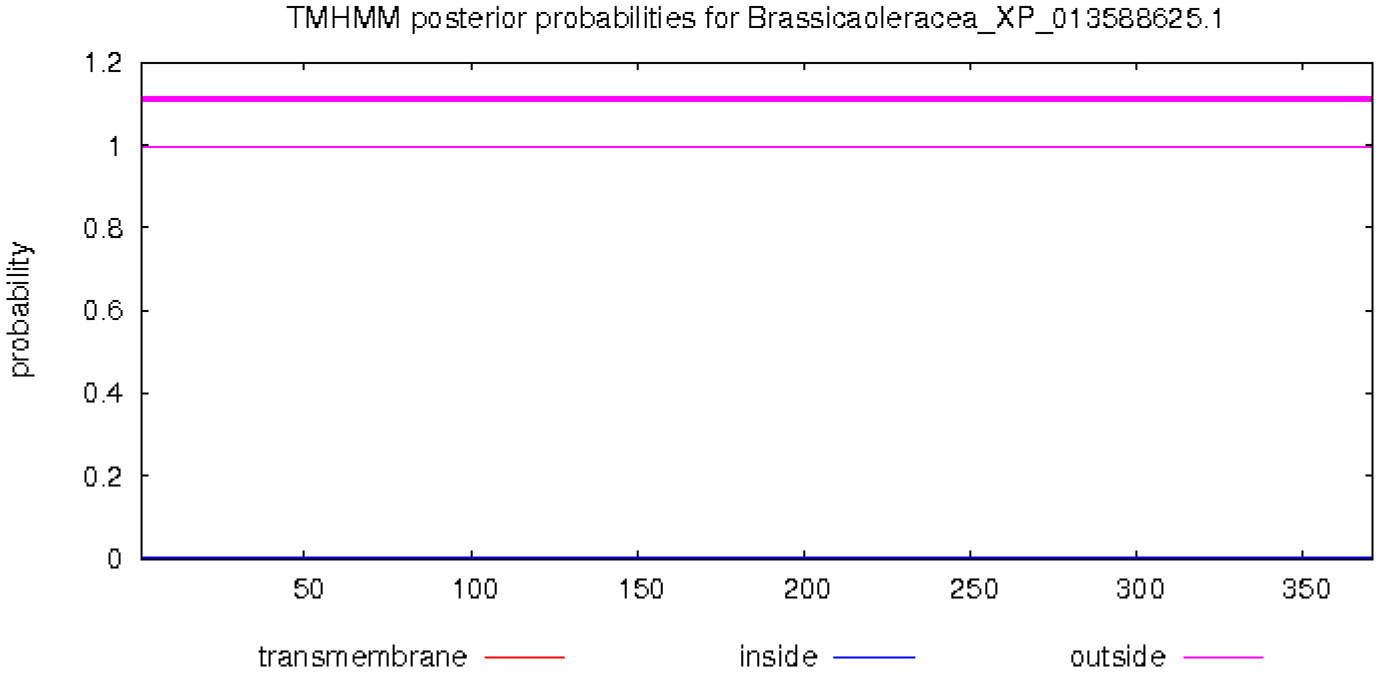

plot in postscript, script for making the plot in gnuplot, data for plot

```
# Brassicaoleracea_XP_013588626.1 Length: 332
# Brassicaoleracea_XP_013588626.1 Number of predicted TMHs:  0
# Brassicaoleracea_XP_013588626.1 Exp number of AAs in TMHs: 0
# Brassicaoleracea_XP_013588626.1 Exp number, first 60 AAs:  0
# Brassicaoleracea_XP_013588626.1 Total prob of N-in:        0.00629
Brassicaoleracea_XP_013588626.1 TMHMM2.0        outside      1   332
```

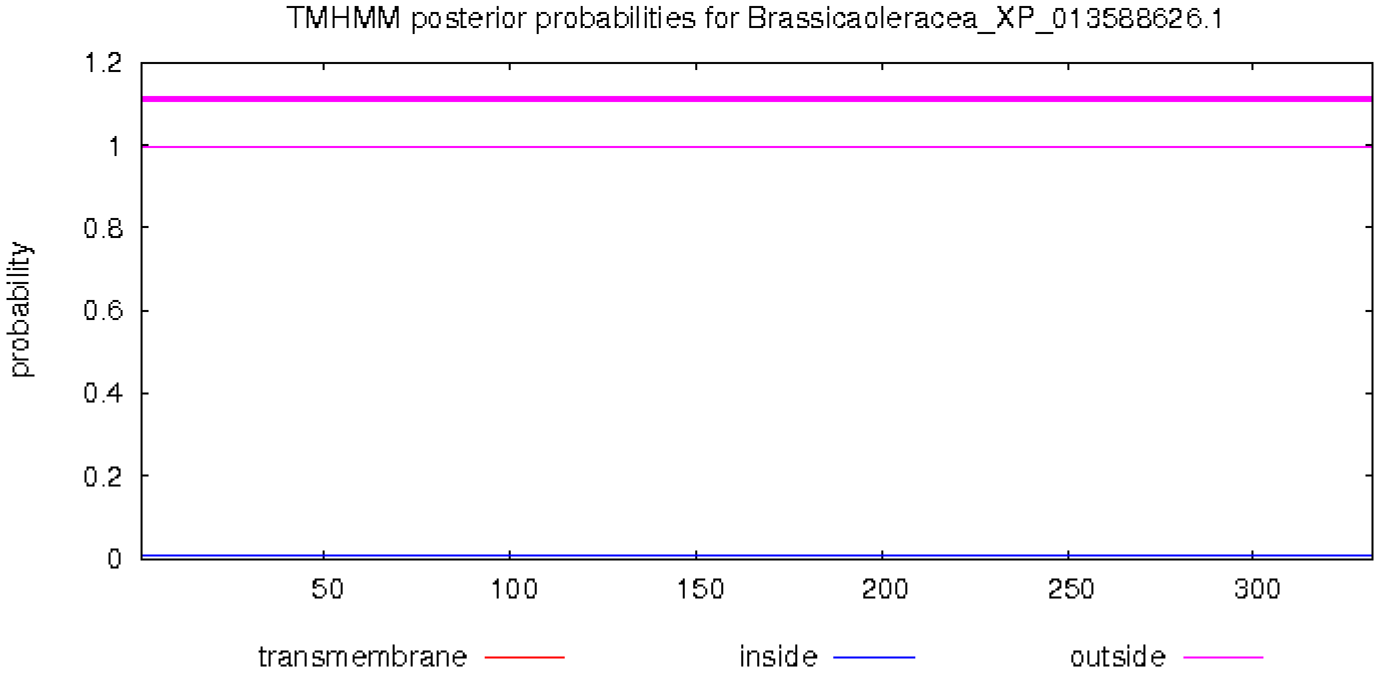

plot in postscript, script for making the plot in gnuplot, data for plot

---

```
# Brassicaoleracea_XP_013588851.1 Length: 337
# Brassicaoleracea_XP_013588851.1 Number of predicted TMHs:  0
# Brassicaoleracea_XP_013588851.1 Exp number of AAs in TMHs: 0.05491
# Brassicaoleracea_XP_013588851.1 Exp number, first 60 AAs:  0.02524
# Brassicaoleracea_XP_013588851.1 Total prob of N-in:        0.00589
Brassicaoleracea_XP_013588851.1 TMHMM2.0        outside      1   337
```

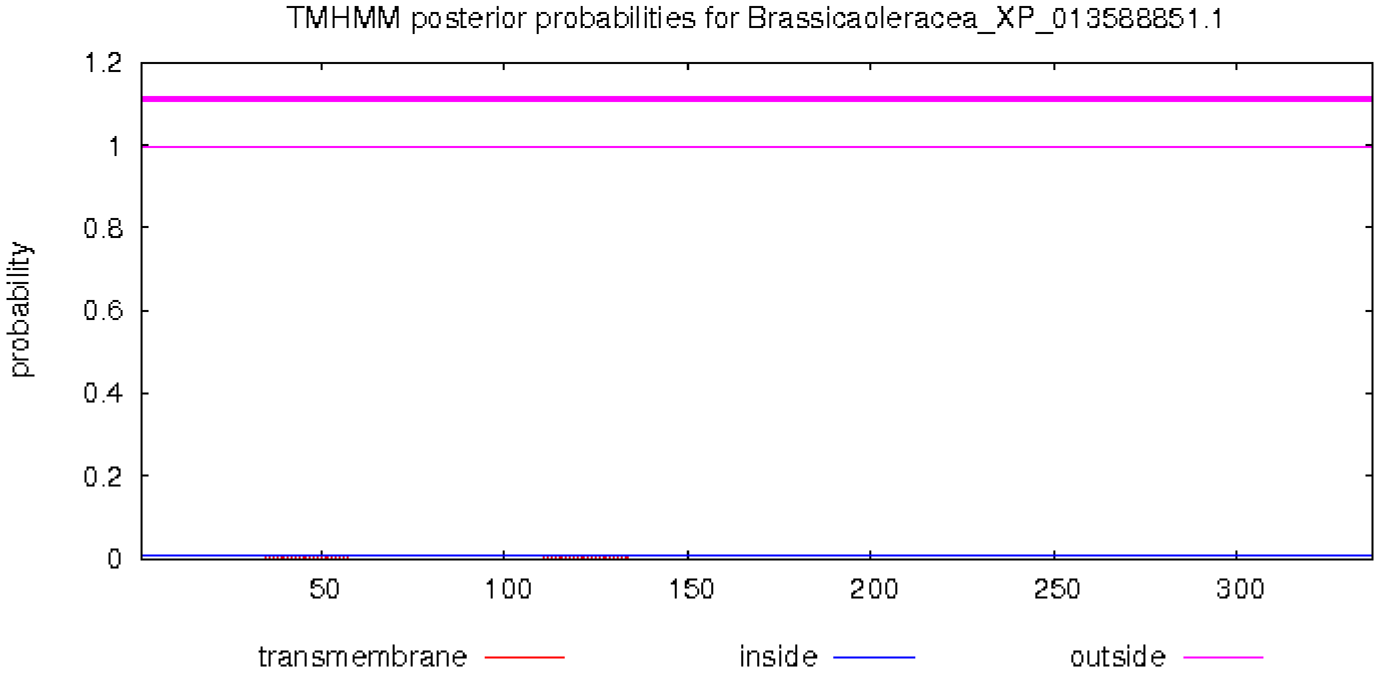

plot in postscript, script for making the plot in gnuplot, data for plot

---

```
# Brassicaoleracea_XP_013589211.1 Length: 304
# Brassicaoleracea_XP_013589211.1 Number of predicted TMHs:  0
# Brassicaoleracea_XP_013589211.1 Exp number of AAs in TMHs: 1.18217
# Brassicaoleracea_XP_013589211.1 Exp number, first 60 AAs:  0
# Brassicaoleracea_XP_013589211.1 Total prob of N-in:        0.00610
Brassicaoleracea_XP_013589211.1 TMHMM2.0        outside      1   304
```

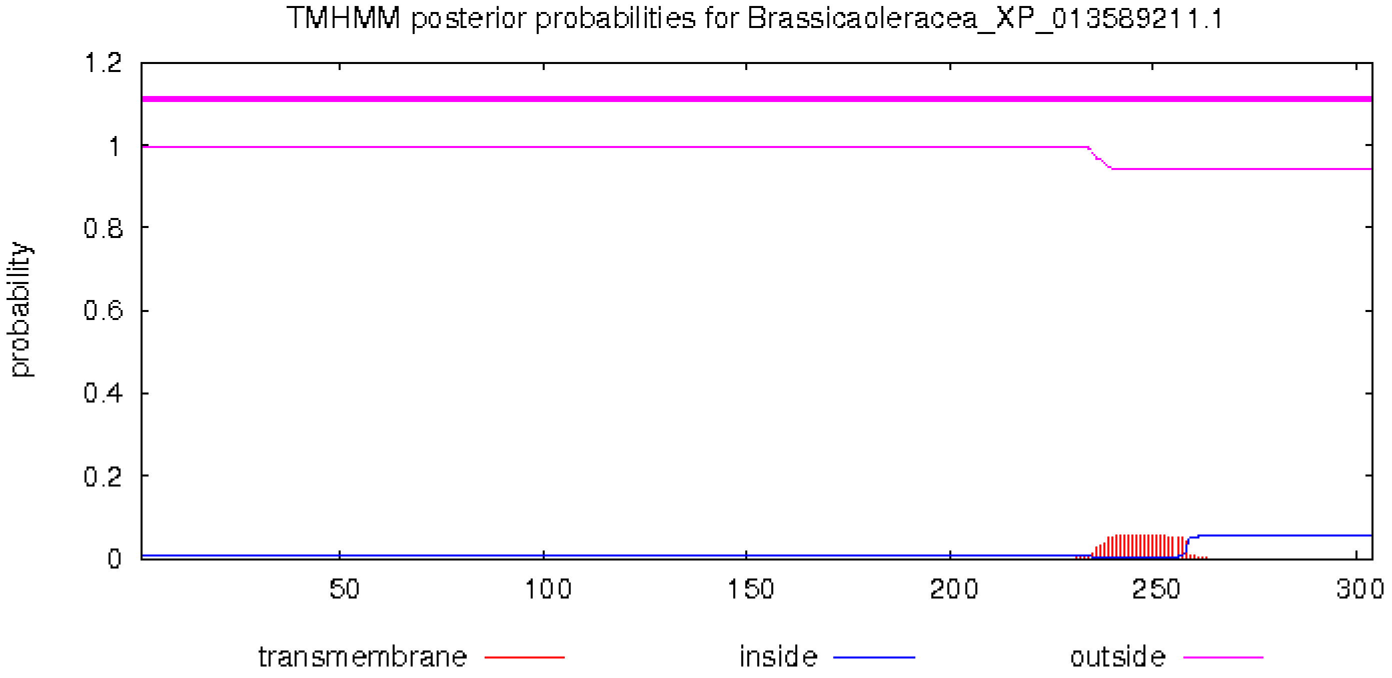

plot in postscript, script for making the plot in gnuplot, data for plot

---

```
# Brassicaoleracea_XP_013589227.1 Length: 365
# Brassicaoleracea_XP_013589227.1 Number of predicted TMHs:  0
# Brassicaoleracea_XP_013589227.1 Exp number of AAs in TMHs: 0.00019
# Brassicaoleracea_XP_013589227.1 Exp number, first 60 AAs:  0
# Brassicaoleracea_XP_013589227.1 Total prob of N-in:        0.00850
Brassicaoleracea_XP_013589227.1 TMHMM2.0        outside      1   365
```

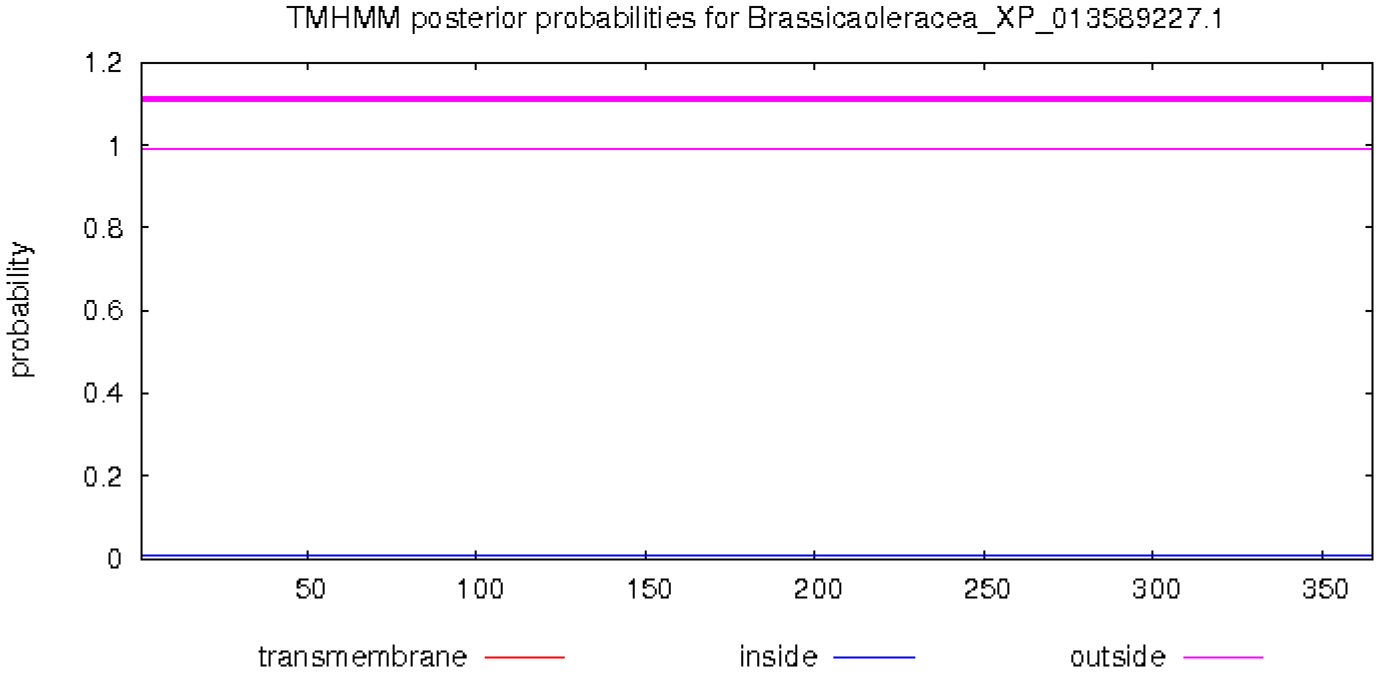

plot in postscript, script for making the plot in gnuplot, data for plot

---

```
# Brassicaoleracea_XP_013589229.1 Length: 316
# Brassicaoleracea_XP_013589229.1 Number of predicted TMHs:  0
# Brassicaoleracea_XP_013589229.1 Exp number of AAs in TMHs: 0
# Brassicaoleracea_XP_013589229.1 Exp number, first 60 AAs:  0
# Brassicaoleracea_XP_013589229.1 Total prob of N-in:        0.01569
Brassicaoleracea_XP_013589229.1 TMHMM2.0        outside      1   316
```

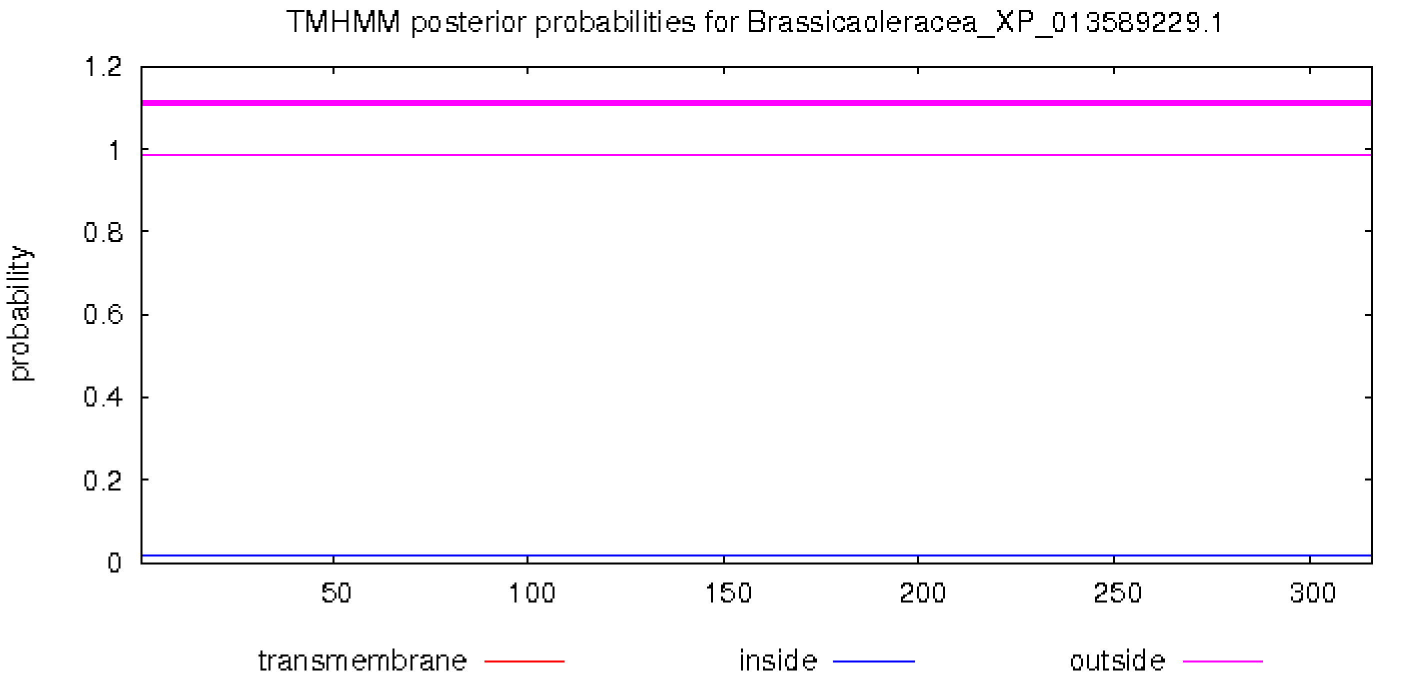

plot in postscript, script for making the plot in gnuplot, data for plot

---

```
# Brassicaoleracea_XP_013589248.1 Length: 300
# Brassicaoleracea_XP_013589248.1 Number of predicted TMHs:  0
# Brassicaoleracea_XP_013589248.1 Exp number of AAs in TMHs: 0.02399
# Brassicaoleracea_XP_013589248.1 Exp number, first 60 AAs:  0.00042
# Brassicaoleracea_XP_013589248.1 Total prob of N-in:        0.01098
Brassicaoleracea_XP_013589248.1 TMHMM2.0        outside      1   300
```

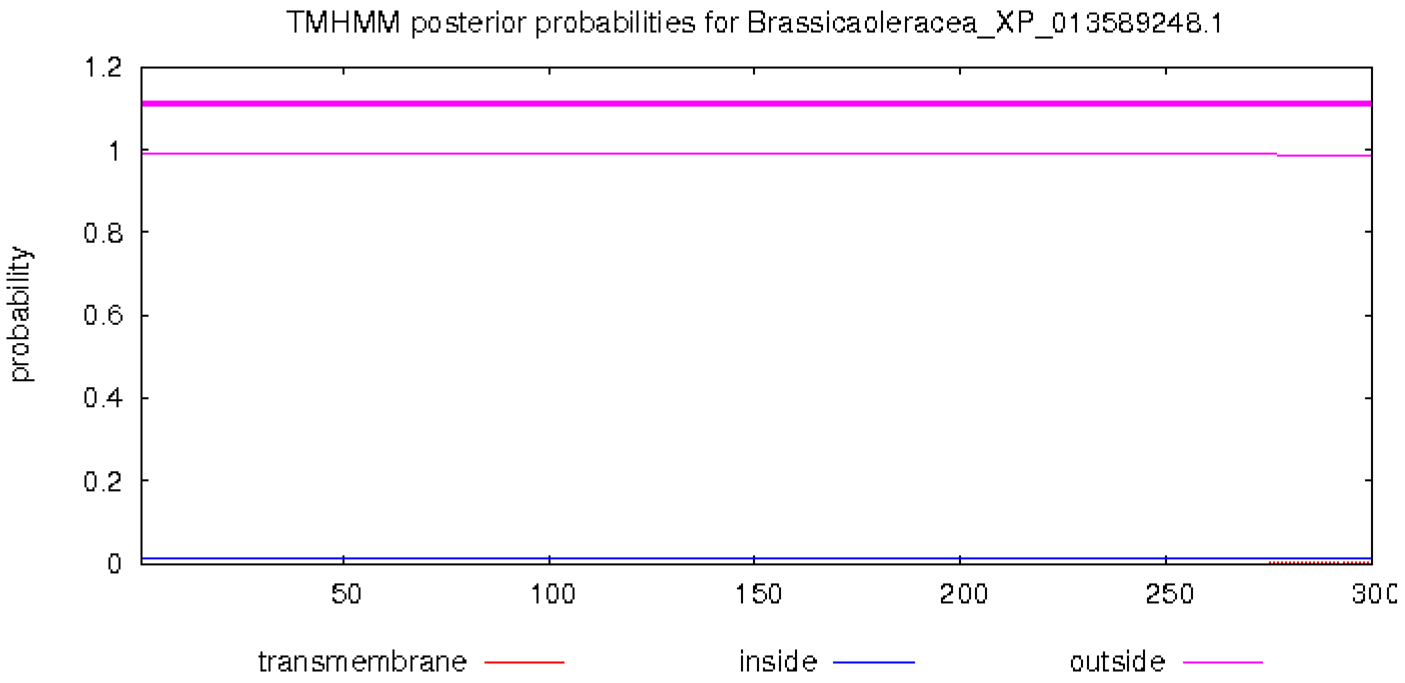

plot in postscript, script for making the plot in gnuplot, data for plot

---

```
# Brassicaoleracea_XP_013589257.1 Length: 335
# Brassicaoleracea_XP_013589257.1 Number of predicted TMHs:  0
# Brassicaoleracea_XP_013589257.1 Exp number of AAs in TMHs: 0.00621
# Brassicaoleracea_XP_013589257.1 Exp number, first 60 AAs:  0
# Brassicaoleracea_XP_013589257.1 Total prob of N-in:        0.02926
Brassicaoleracea_XP_013589257.1 TMHMM2.0        outside      1   335
```

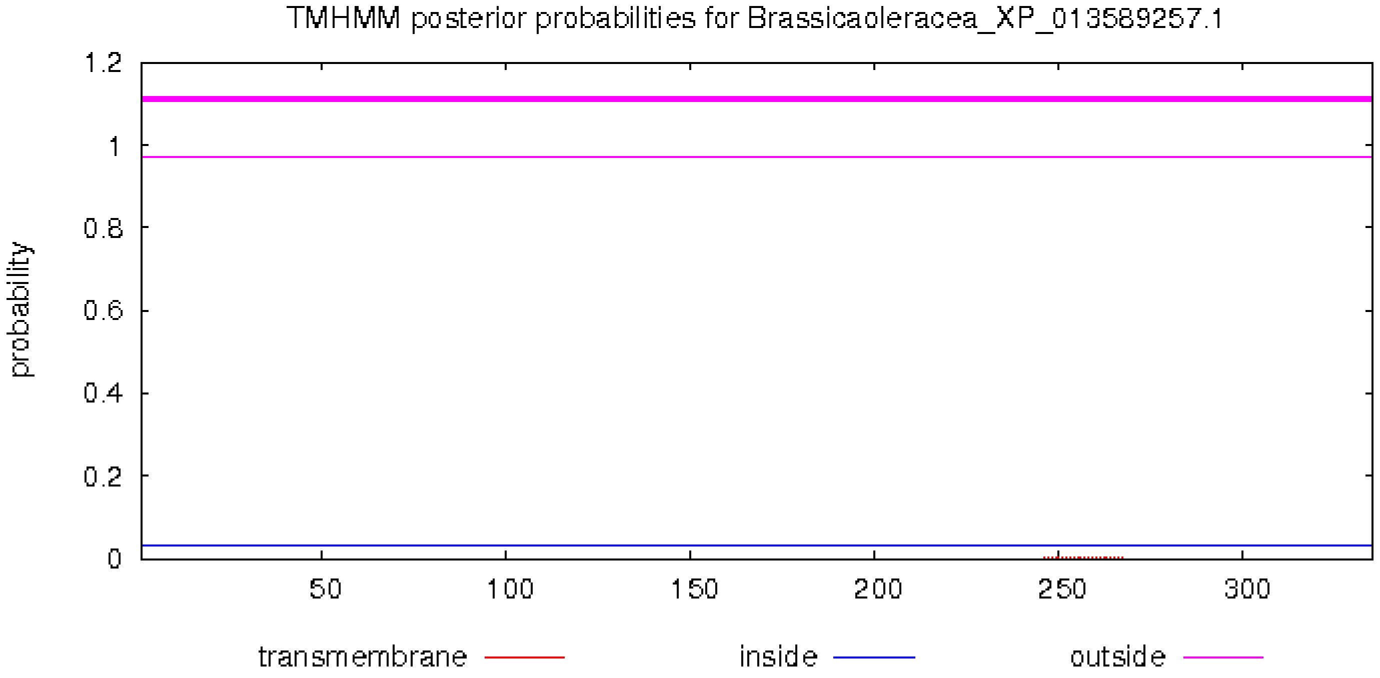

plot in postscript, script for making the plot in gnuplot, data for plot

---

```
# Brassicaoleracea_XP_013589285.1 Length: 278
# Brassicaoleracea_XP_013589285.1 Number of predicted TMHs:  0
# Brassicaoleracea_XP_013589285.1 Exp number of AAs in TMHs: 0.31402
# Brassicaoleracea_XP_013589285.1 Exp number, first 60 AAs:  0
# Brassicaoleracea_XP_013589285.1 Total prob of N-in:        0.04221
Brassicaoleracea_XP_013589285.1 TMHMM2.0        outside      1   278
```

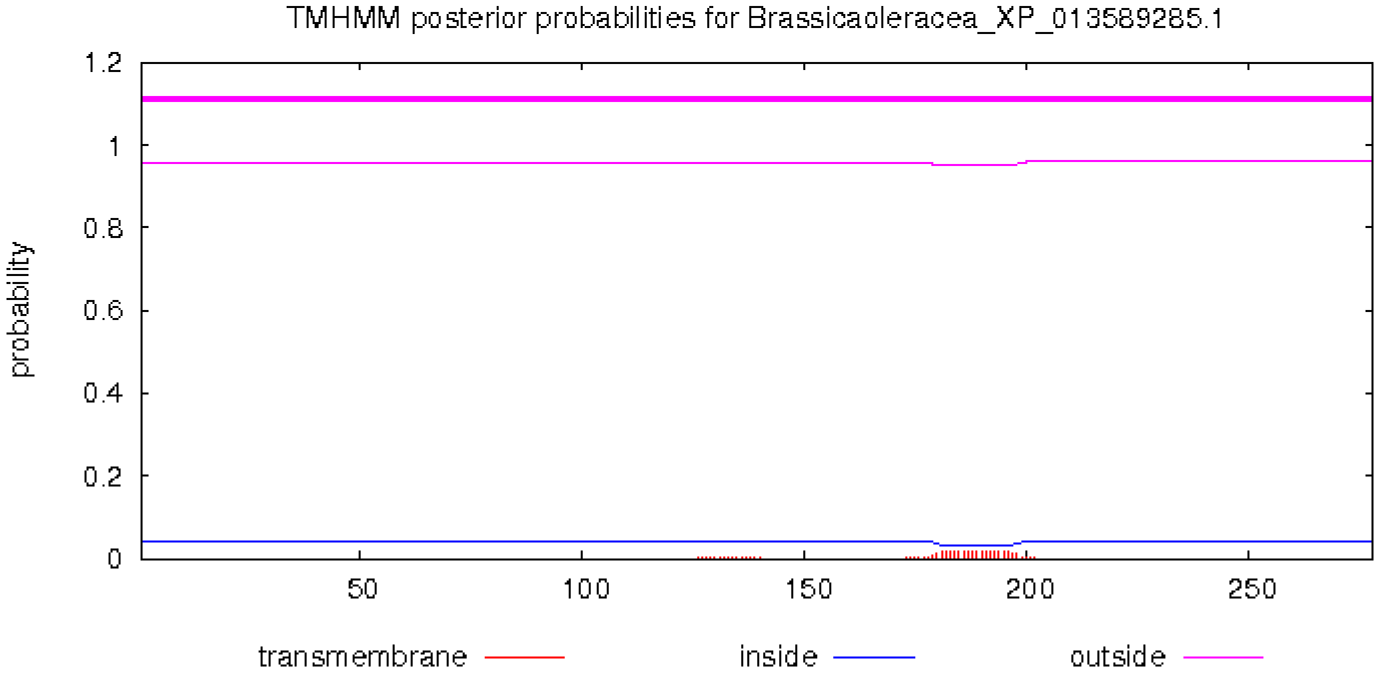

plot in postscript, script for making the plot in gnuplot, data for plot

---

```
# Brassicaoleracea_XP_013589340.1 Length: 375
# Brassicaoleracea_XP_013589340.1 Number of predicted TMHs:  0
# Brassicaoleracea_XP_013589340.1 Exp number of AAs in TMHs: 0
# Brassicaoleracea_XP_013589340.1 Exp number, first 60 AAs:  0
# Brassicaoleracea_XP_013589340.1 Total prob of N-in:        0.02209
Brassicaoleracea_XP_013589340.1 TMHMM2.0        outside      1   375
```

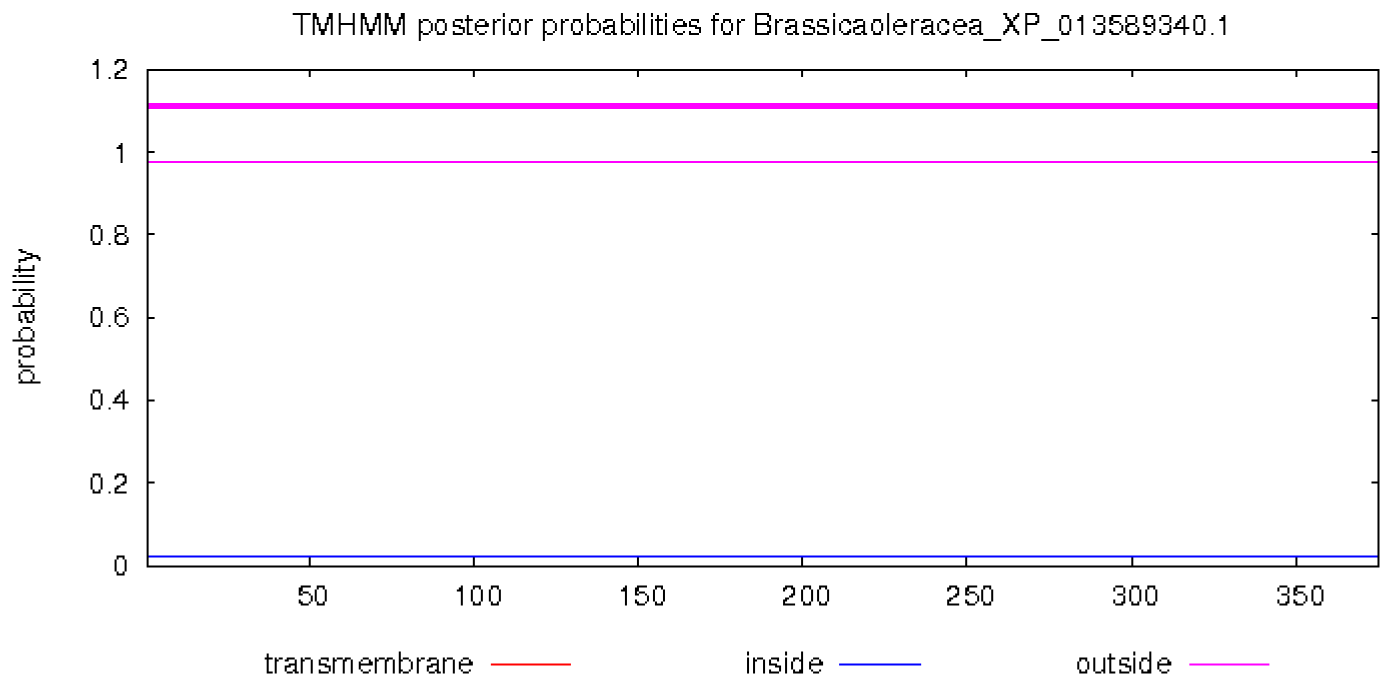

plot in postscript, script for making the plot in gnuplot, data for plot

```
# Brassicaoleracea_XP_013589625.1 Length: 407
# Brassicaoleracea_XP_013589625.1 Number of predicted TMHs:  0
# Brassicaoleracea_XP_013589625.1 Exp number of AAs in TMHs: 0.0006
# Brassicaoleracea_XP_013589625.1 Exp number, first 60 AAs:  0
# Brassicaoleracea_XP_013589625.1 Total prob of N-in:        0.01523
Brassicaoleracea_XP_013589625.1 TMHMM2.0        outside      1   407
```

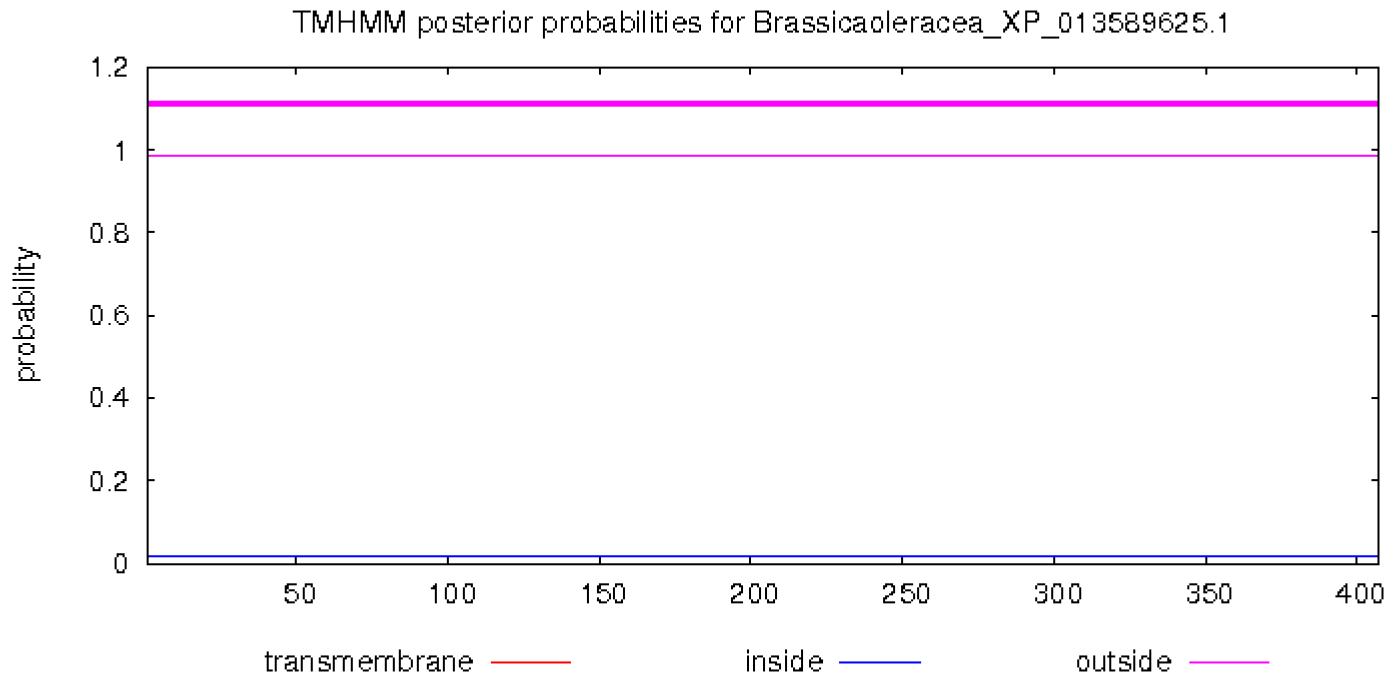

plot in postscript, script for making the plot in gnuplot, data for plot

```
# Brassicaoleracea_XP_013589746.1 Length: 154
# Brassicaoleracea_XP_013589746.1 Number of predicted TMHs:  0
# Brassicaoleracea_XP_013589746.1 Exp number of AAs in TMHs: 0.12181
# Brassicaoleracea_XP_013589746.1 Exp number, first 60 AAs:  0.00054
# Brassicaoleracea_XP_013589746.1 Total prob of N-in:        0.14087
Brassicaoleracea_XP_013589746.1 TMHMM2.0        outside      1   154
```

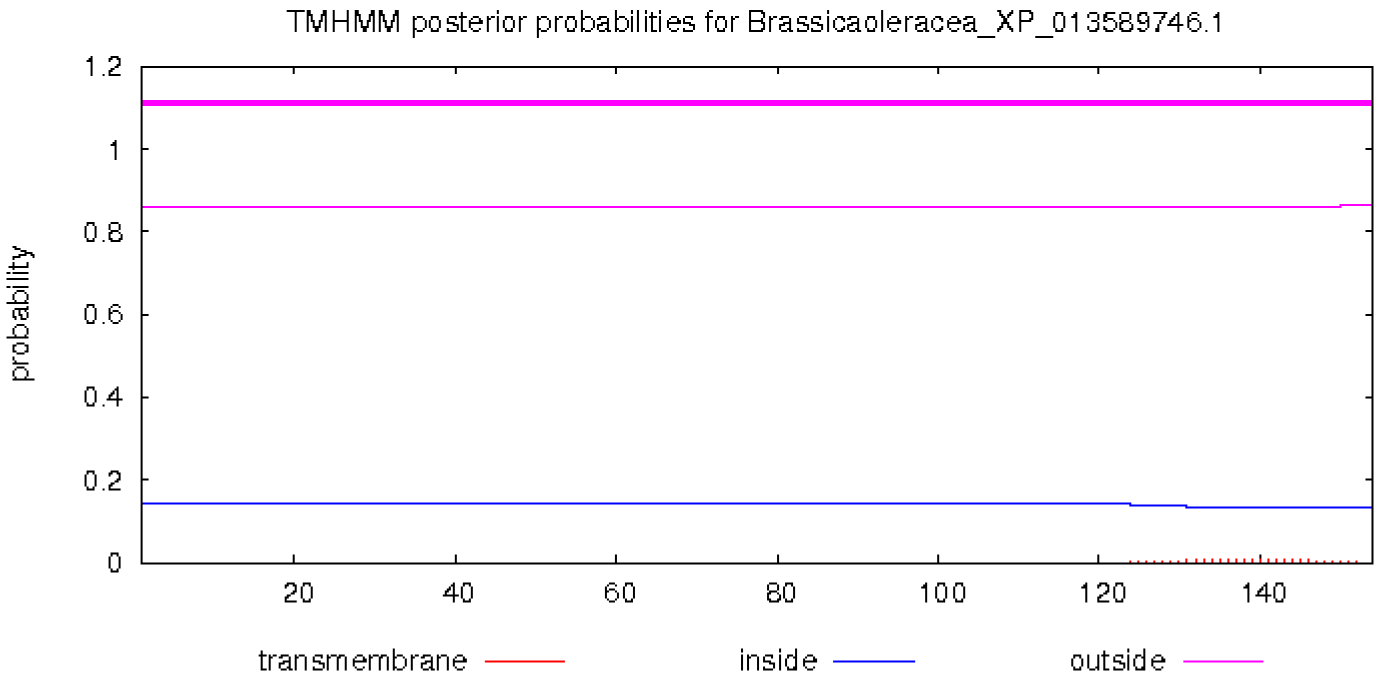

plot in postscript, script for making the plot in gnuplot, data for plot

```
# Brassicaoleracea_XP_013589912.1 Length: 272
# Brassicaoleracea_XP_013589912.1 Number of predicted TMHs:  0
# Brassicaoleracea_XP_013589912.1 Exp number of AAs in TMHs: 0.00802
# Brassicaoleracea_XP_013589912.1 Exp number, first 60 AAs:  0
# Brassicaoleracea_XP_013589912.1 Total prob of N-in:        0.05785
Brassicaoleracea_XP_013589912.1 TMHMM2.0        outside      1   272
```

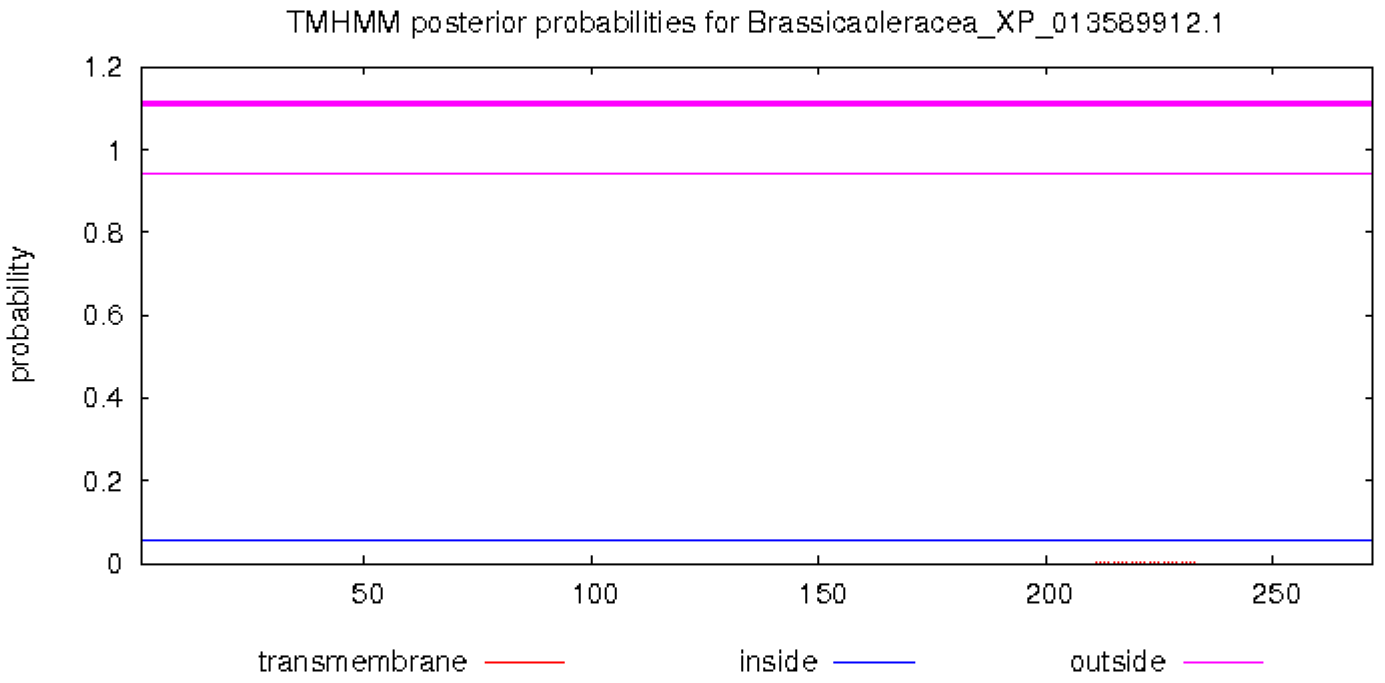

plot in postscript, script for making the plot in gnuplot, data for plot

```
# Brassicaoleracea_XP_013590191.1 Length: 288
# Brassicaoleracea_XP_013590191.1 Number of predicted TMHs:  0
# Brassicaoleracea_XP_013590191.1 Exp number of AAs in TMHs: 0.00026
# Brassicaoleracea_XP_013590191.1 Exp number, first 60 AAs:  0.00026
# Brassicaoleracea_XP_013590191.1 Total prob of N-in:        0.03061
Brassicaoleracea_XP_013590191.1 TMHMM2.0        outside      1   288
```

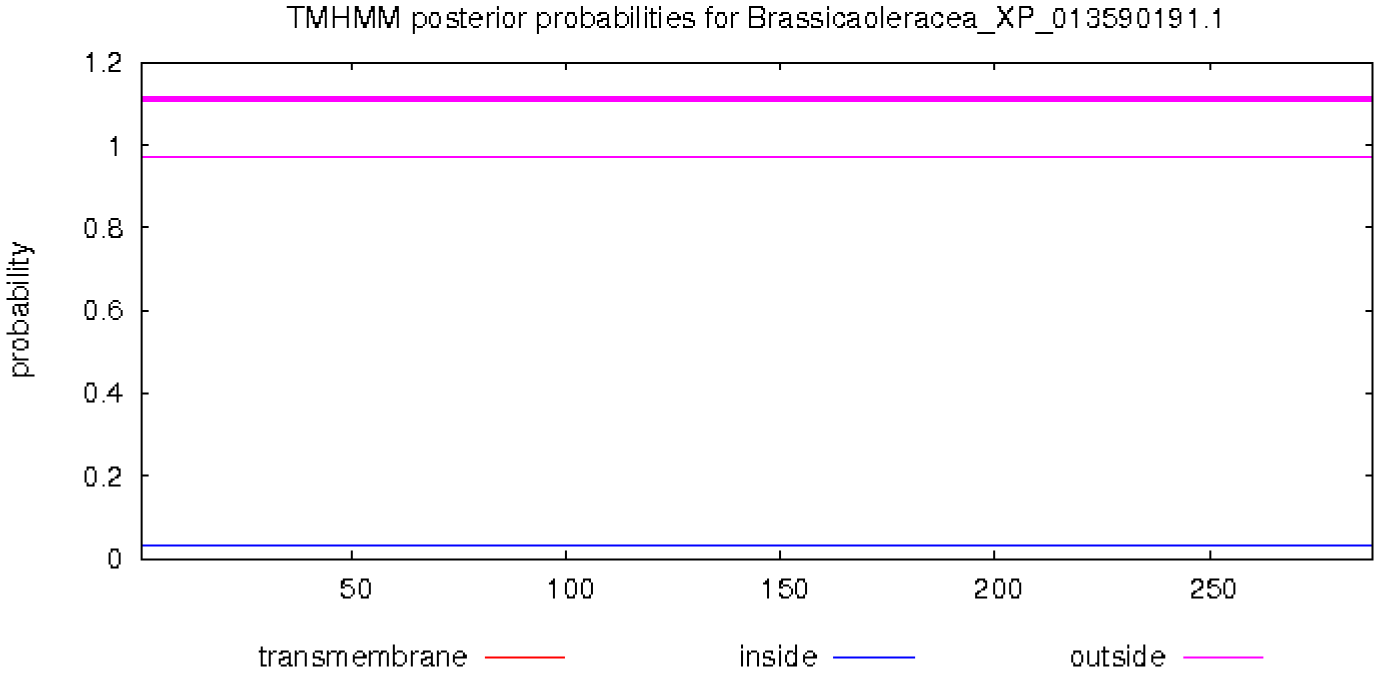

plot in postscript, script for making the plot in gnuplot, data for plot

---

```
# Brassicaoleracea_XP_013590250.1 Length: 311
# Brassicaoleracea_XP_013590250.1 Number of predicted TMHs:  0
# Brassicaoleracea_XP_013590250.1 Exp number of AAs in TMHs: 0
# Brassicaoleracea_XP_013590250.1 Exp number, first 60 AAs:  0
# Brassicaoleracea_XP_013590250.1 Total prob of N-in:        0.00125
Brassicaoleracea_XP_013590250.1 TMHMM2.0        outside      1   311
```

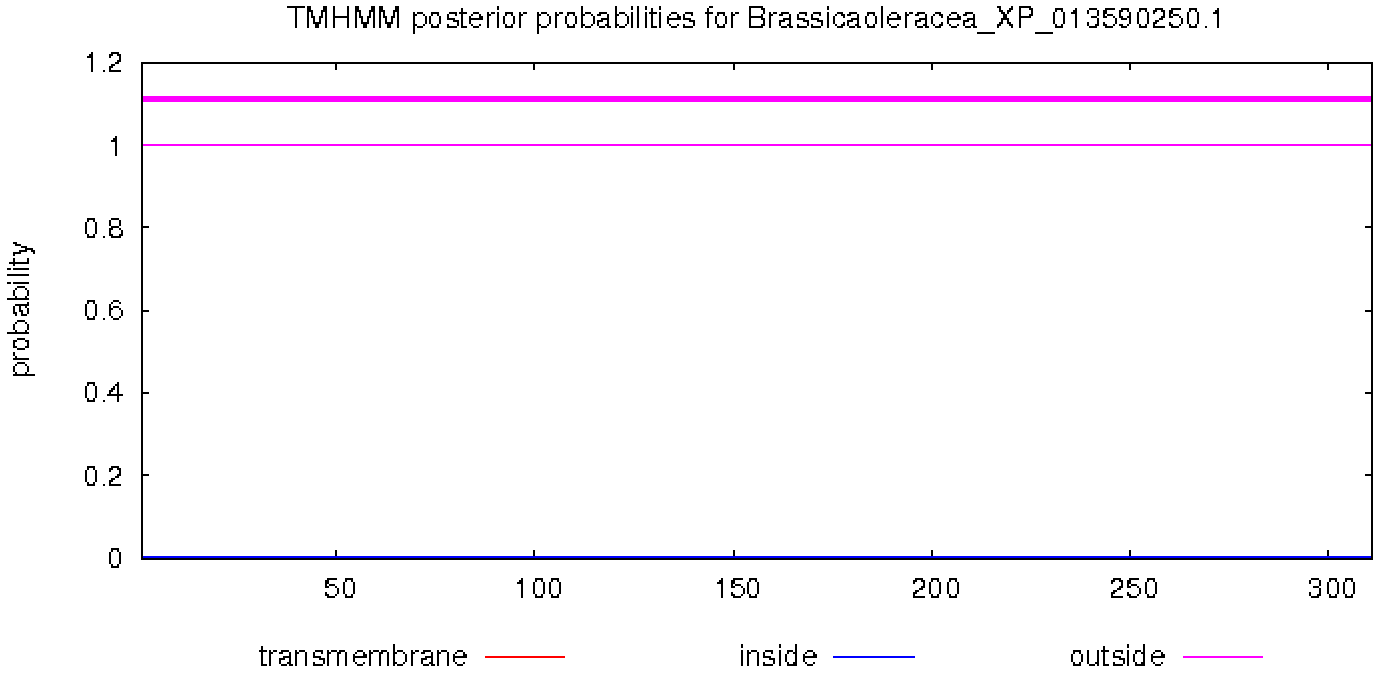

plot in postscript, script for making the plot in gnuplot, data for plot

---

```
# Brassicaoleracea_XP_013590272.1 Length: 270
# Brassicaoleracea_XP_013590272.1 Number of predicted TMHs:  0
# Brassicaoleracea_XP_013590272.1 Exp number of AAs in TMHs: 0.00273
# Brassicaoleracea_XP_013590272.1 Exp number, first 60 AAs:  0
# Brassicaoleracea_XP_013590272.1 Total prob of N-in:        0.03606
Brassicaoleracea_XP_013590272.1 TMHMM2.0        outside      1   270
```

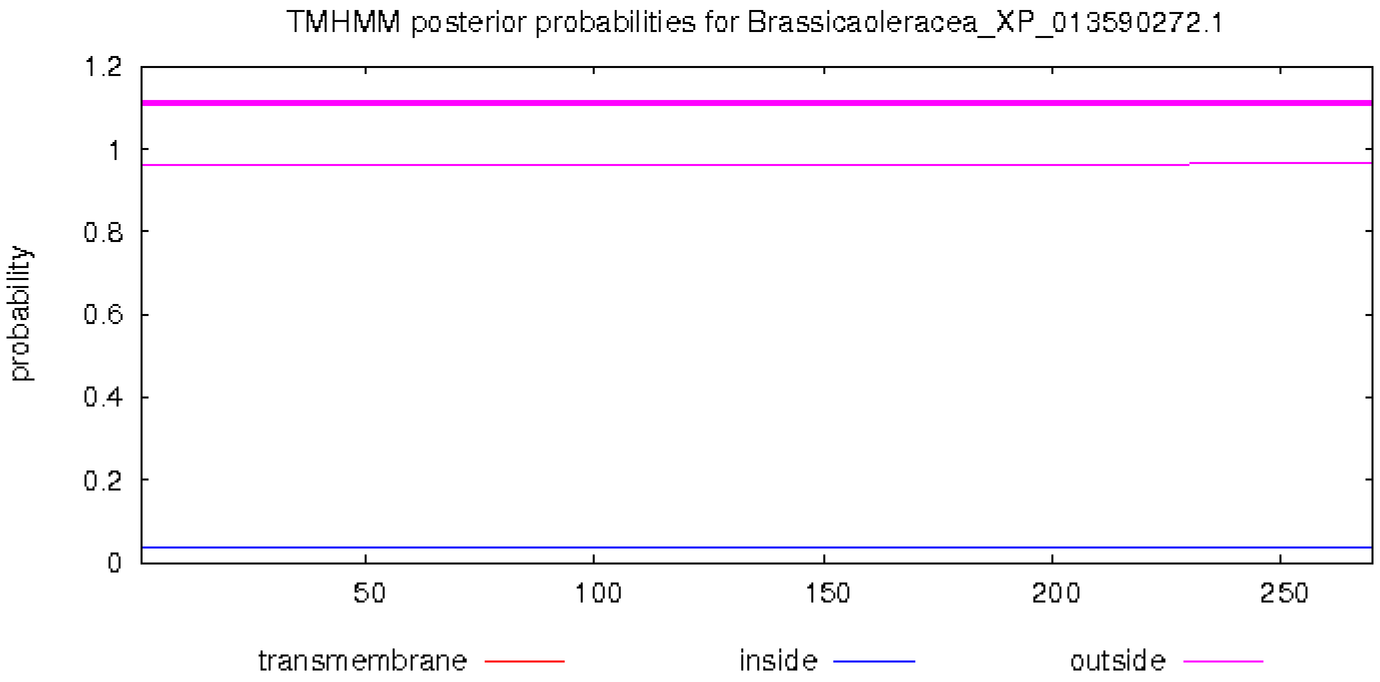

plot in postscript, script for making the plot in gnuplot, data for plot

---

```
# Brassicaoleracea_XP_013590608.1 Length: 325
# Brassicaoleracea_XP_013590608.1 Number of predicted TMHs:  0
# Brassicaoleracea_XP_013590608.1 Exp number of AAs in TMHs: 0
# Brassicaoleracea_XP_013590608.1 Exp number, first 60 AAs:  0
# Brassicaoleracea_XP_013590608.1 Total prob of N-in:        0.00761
Brassicaoleracea_XP_013590608.1 TMHMM2.0        outside      1   325
```

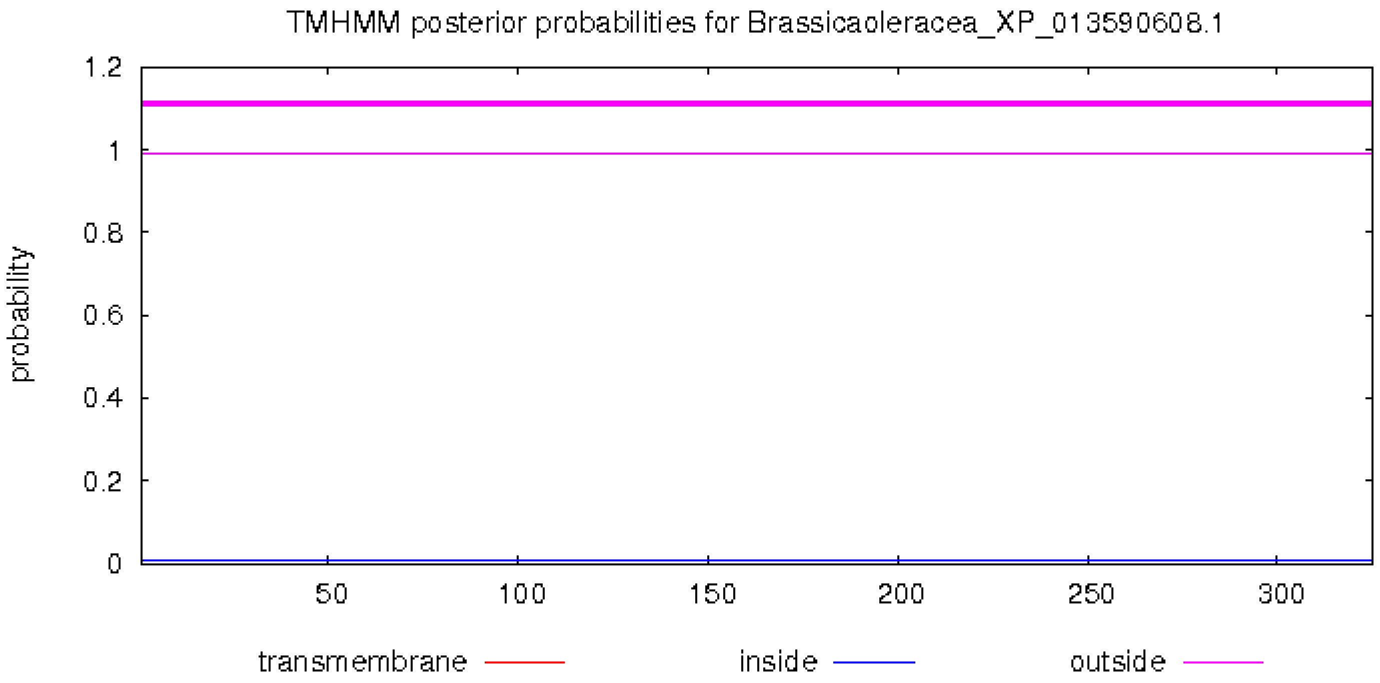

plot in postscript, script for making the plot in gnuplot, data for plot

---

```
# Brassicaoleracea_XP_013590609.1 Length: 325
# Brassicaoleracea_XP_013590609.1 Number of predicted TMHs:  0
# Brassicaoleracea_XP_013590609.1 Exp number of AAs in TMHs: 0
# Brassicaoleracea_XP_013590609.1 Exp number, first 60 AAs:  0
# Brassicaoleracea_XP_013590609.1 Total prob of N-in:        0.00761
Brassicaoleracea_XP_013590609.1 TMHMM2.0        outside      1   325
```

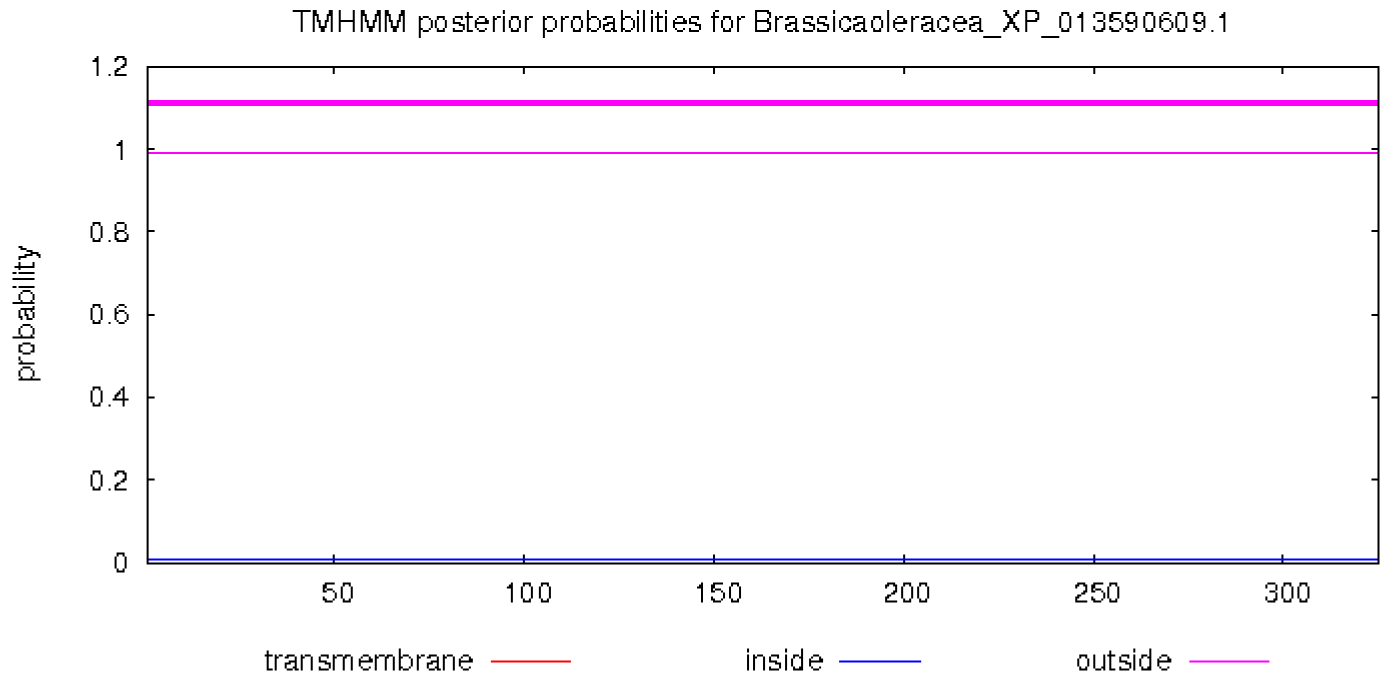

plot in postscript, script for making the plot in gnuplot, data for plot

---

```
# Brassicaoleracea_XP_013590798.1 Length: 432
# Brassicaoleracea_XP_013590798.1 Number of predicted TMHs:  0
# Brassicaoleracea_XP_013590798.1 Exp number of AAs in TMHs: 0.02669
# Brassicaoleracea_XP_013590798.1 Exp number, first 60 AAs:  0.00141
# Brassicaoleracea_XP_013590798.1 Total prob of N-in:        0.00516
Brassicaoleracea_XP_013590798.1 TMHMM2.0        outside      1   432
```

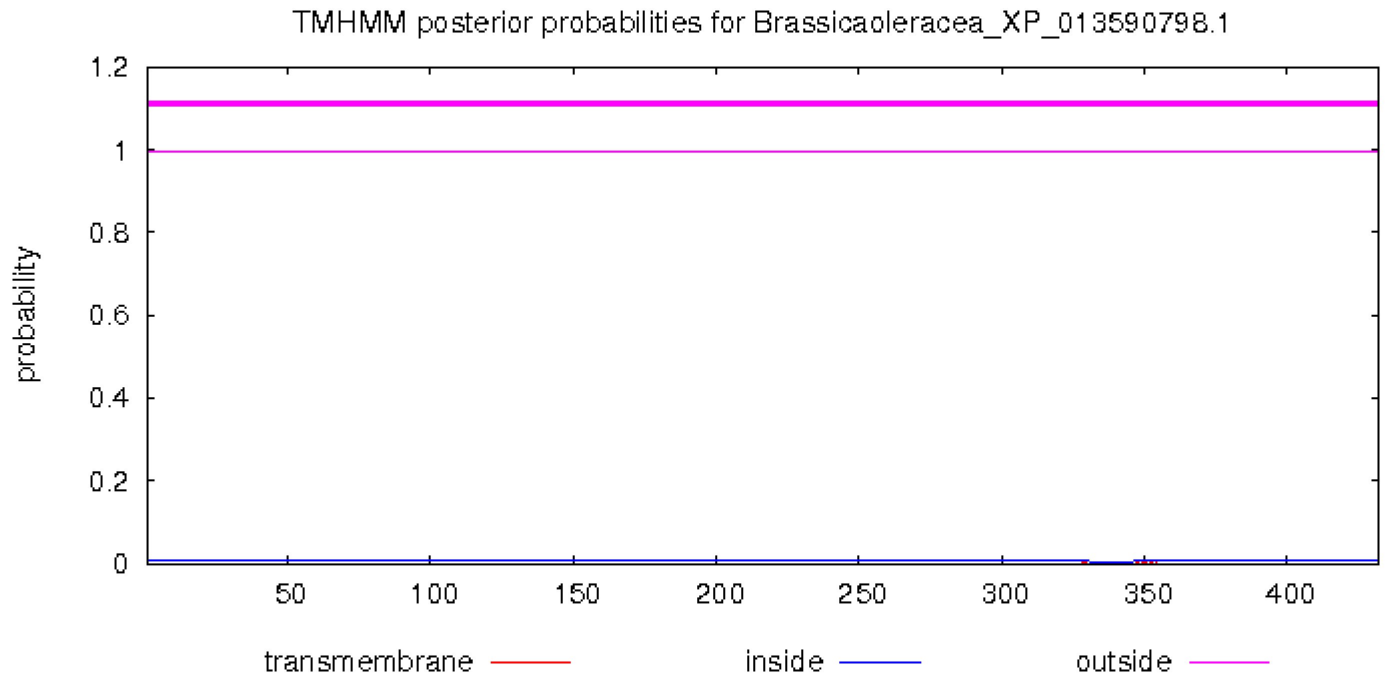

plot in postscript, script for making the plot in gnuplot, data for plot

---

```
# Brassicaoleracea_XP_013590906.1 Length: 326
# Brassicaoleracea_XP_013590906.1 Number of predicted TMHs:  0
# Brassicaoleracea_XP_013590906.1 Exp number of AAs in TMHs: 0
# Brassicaoleracea_XP_013590906.1 Exp number, first 60 AAs:  0
# Brassicaoleracea_XP_013590906.1 Total prob of N-in:        0.03327
Brassicaoleracea_XP_013590906.1 TMHMM2.0        outside      1   326
```

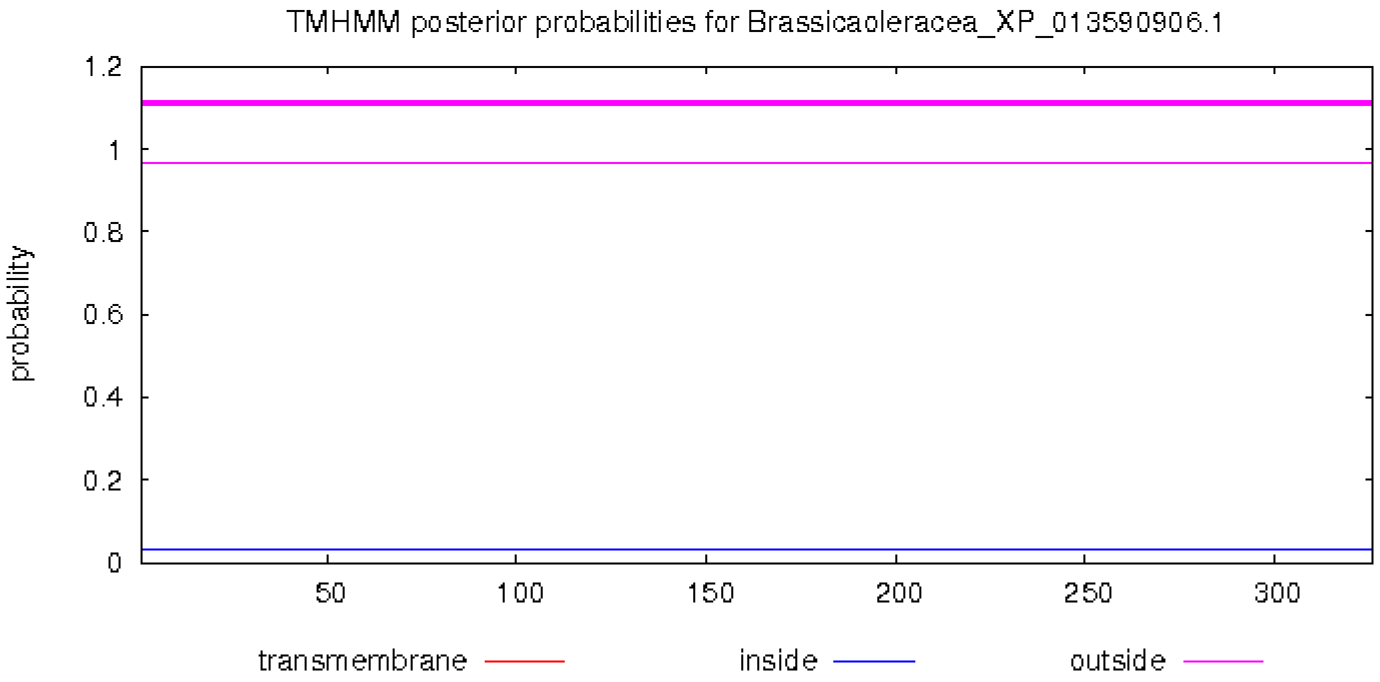

plot in postscript, script for making the plot in gnuplot, data for plot

---

```
# Brassicaoleracea_XP_013590909.1 Length: 198
# Brassicaoleracea_XP_013590909.1 Number of predicted TMHs:  0
# Brassicaoleracea_XP_013590909.1 Exp number of AAs in TMHs: 0
# Brassicaoleracea_XP_013590909.1 Exp number, first 60 AAs:  0
# Brassicaoleracea_XP_013590909.1 Total prob of N-in:        0.04816
Brassicaoleracea_XP_013590909.1 TMHMM2.0        outside      1   198
```

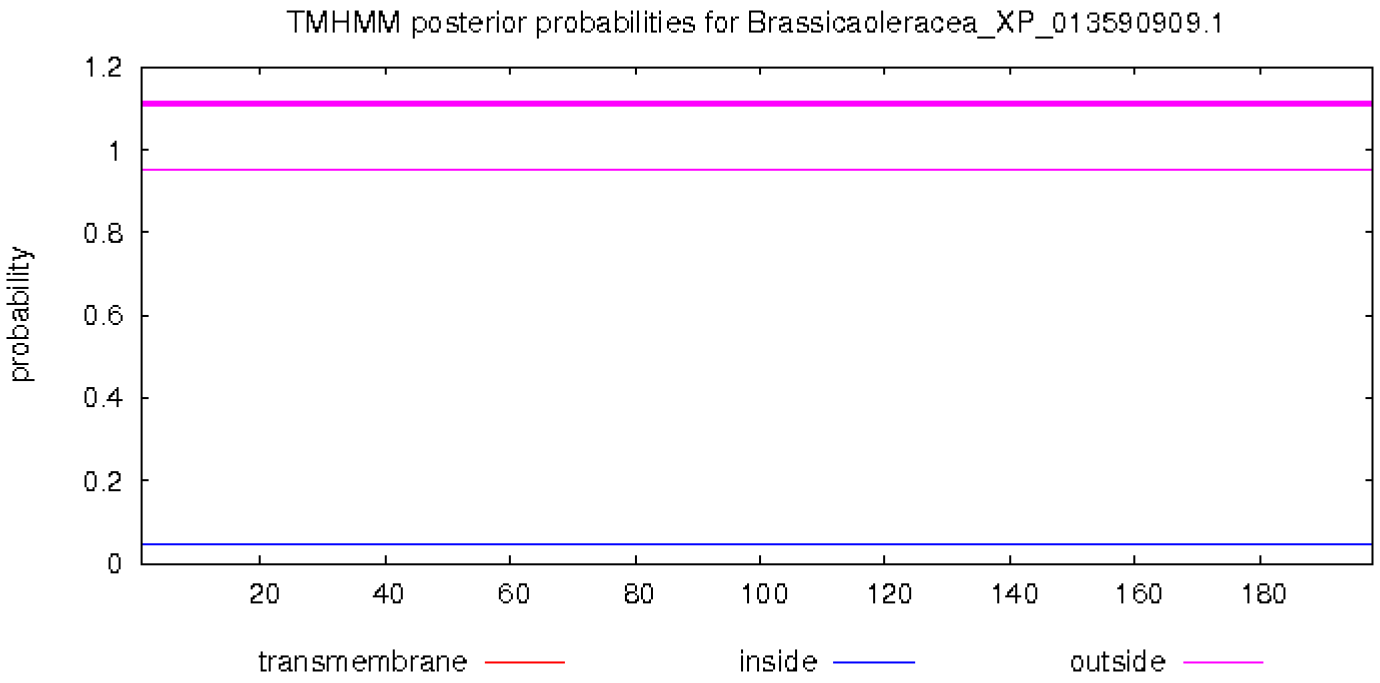

plot in postscript, script for making the plot in gnuplot, data for plot

---

```
# Brassicaoleracea_XP_013590910.1 Length: 359
# Brassicaoleracea_XP_013590910.1 Number of predicted TMHs:  0
# Brassicaoleracea_XP_013590910.1 Exp number of AAs in TMHs: 0.00096
# Brassicaoleracea_XP_013590910.1 Exp number, first 60 AAs:  0.00096
# Brassicaoleracea_XP_013590910.1 Total prob of N-in:        0.00898
Brassicaoleracea_XP_013590910.1 TMHMM2.0        outside      1   359
```

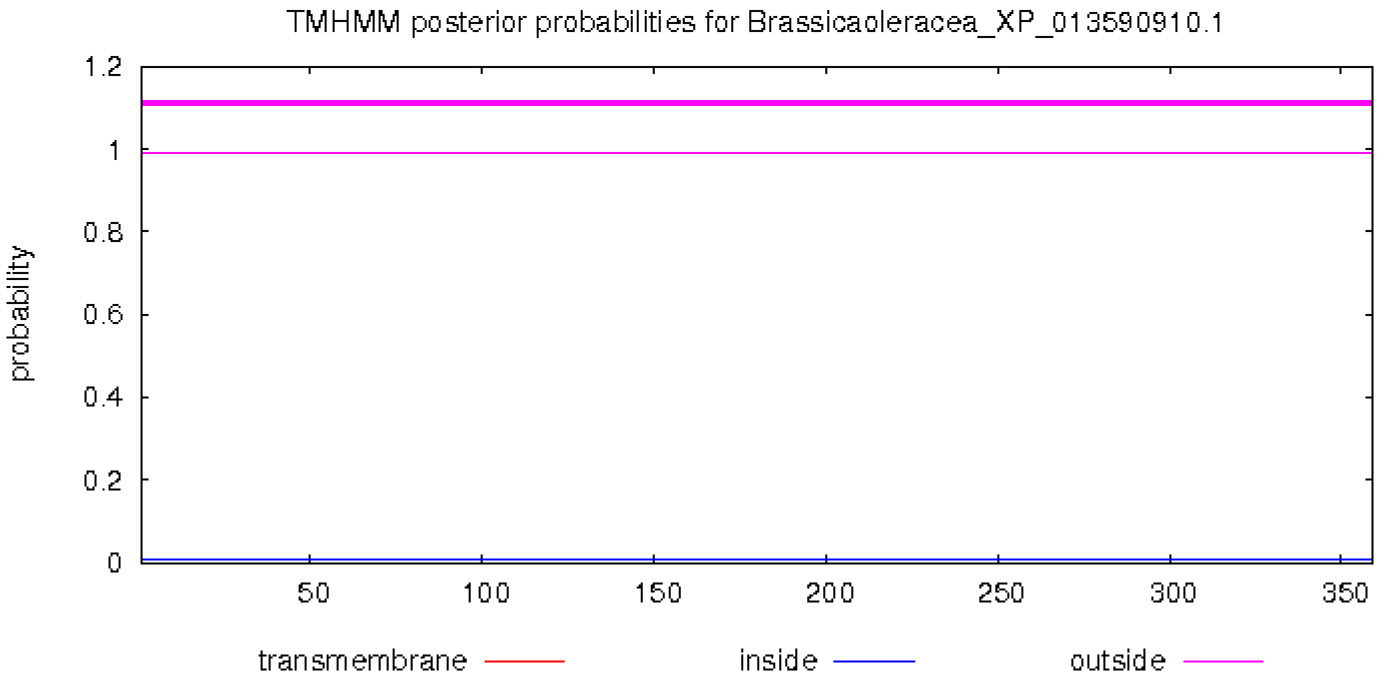

plot in postscript, script for making the plot in gnuplot, data for plot

---

```
# Brassicaoleracea_XP_013591746.1 Length: 418
# Brassicaoleracea_XP_013591746.1 Number of predicted TMHs:  0
# Brassicaoleracea_XP_013591746.1 Exp number of AAs in TMHs: 1.22517
# Brassicaoleracea_XP_013591746.1 Exp number, first 60 AAs:  1.22073
# Brassicaoleracea_XP_013591746.1 Total prob of N-in:        0.06363
Brassicaoleracea_XP_013591746.1 TMHMM2.0        outside      1   418
```

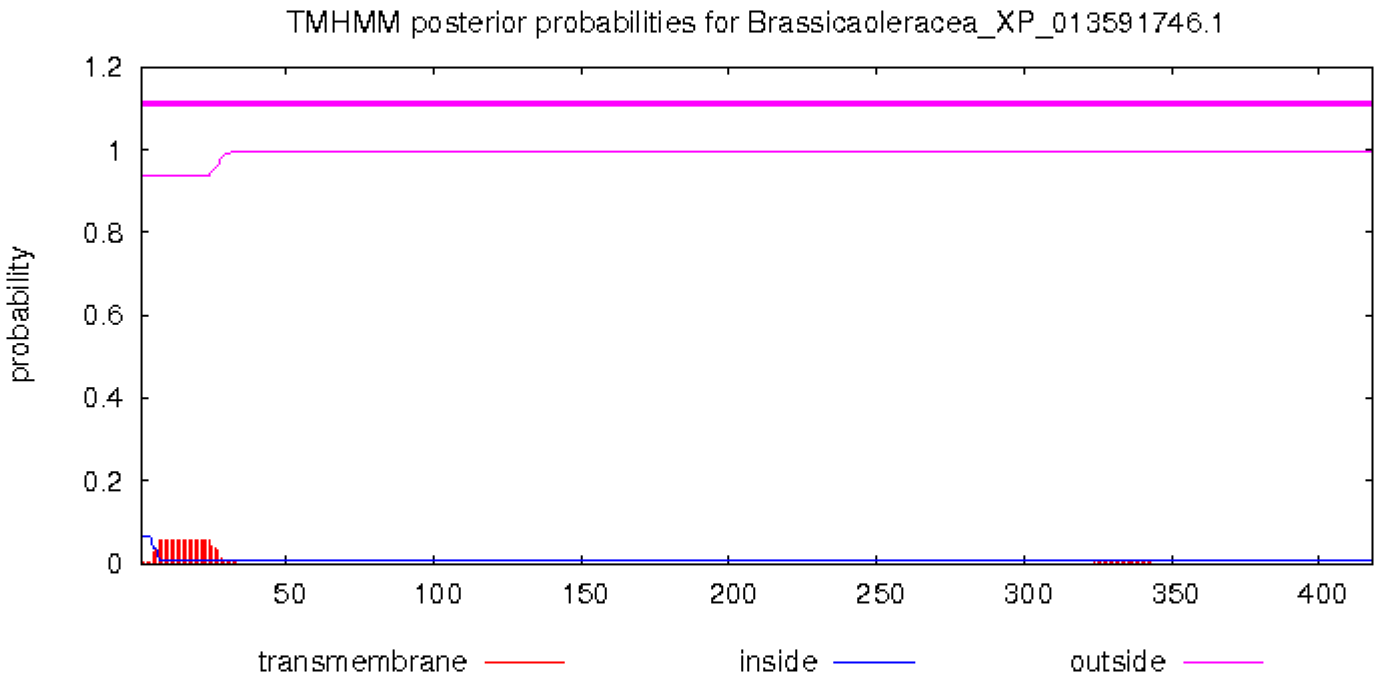

plot in postscript, script for making the plot in gnuplot, data for plot

---

```
# Brassicaoleracea_XP_013592194.1 Length: 560
# Brassicaoleracea_XP_013592194.1 Number of predicted TMHs:  1
# Brassicaoleracea_XP_013592194.1 Exp number of AAs in TMHs: 21.70841
# Brassicaoleracea_XP_013592194.1 Exp number, first 60 AAs:  0.00279
# Brassicaoleracea_XP_013592194.1 Total prob of N-in:        0.47805
Brassicaoleracea_XP_013592194.1 TMHMM2.0        inside       1   530
Brassicaoleracea_XP_013592194.1 TMHMM2.0        TMhelix    531   553
Brassicaoleracea_XP_013592194.1 TMHMM2.0        outside    554   560
```

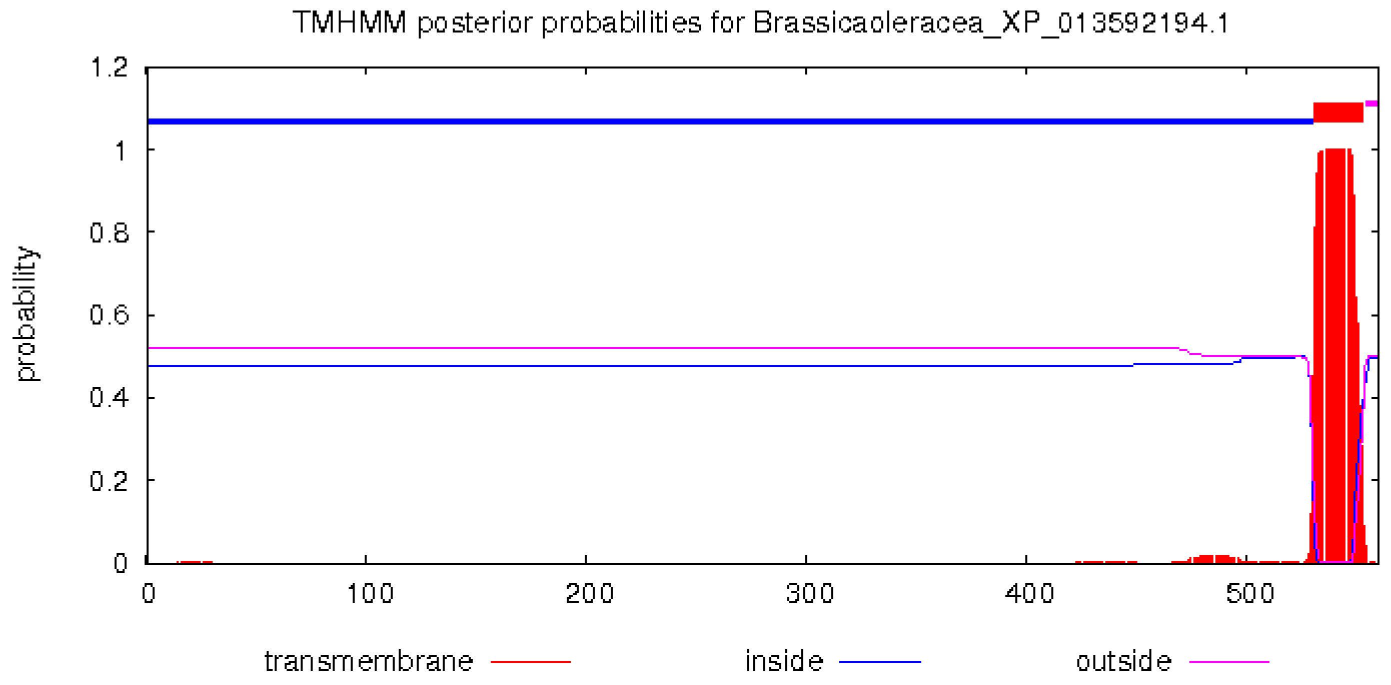

plot in postscript, script for making the plot in gnuplot, data for plot

---

```
# Brassicaoleracea_XP_013592210.1 Length: 362
# Brassicaoleracea_XP_013592210.1 Number of predicted TMHs:  0
# Brassicaoleracea_XP_013592210.1 Exp number of AAs in TMHs: 0.00376
# Brassicaoleracea_XP_013592210.1 Exp number, first 60 AAs:  0.00096
# Brassicaoleracea_XP_013592210.1 Total prob of N-in:        0.00857
Brassicaoleracea_XP_013592210.1 TMHMM2.0        outside      1   362
```

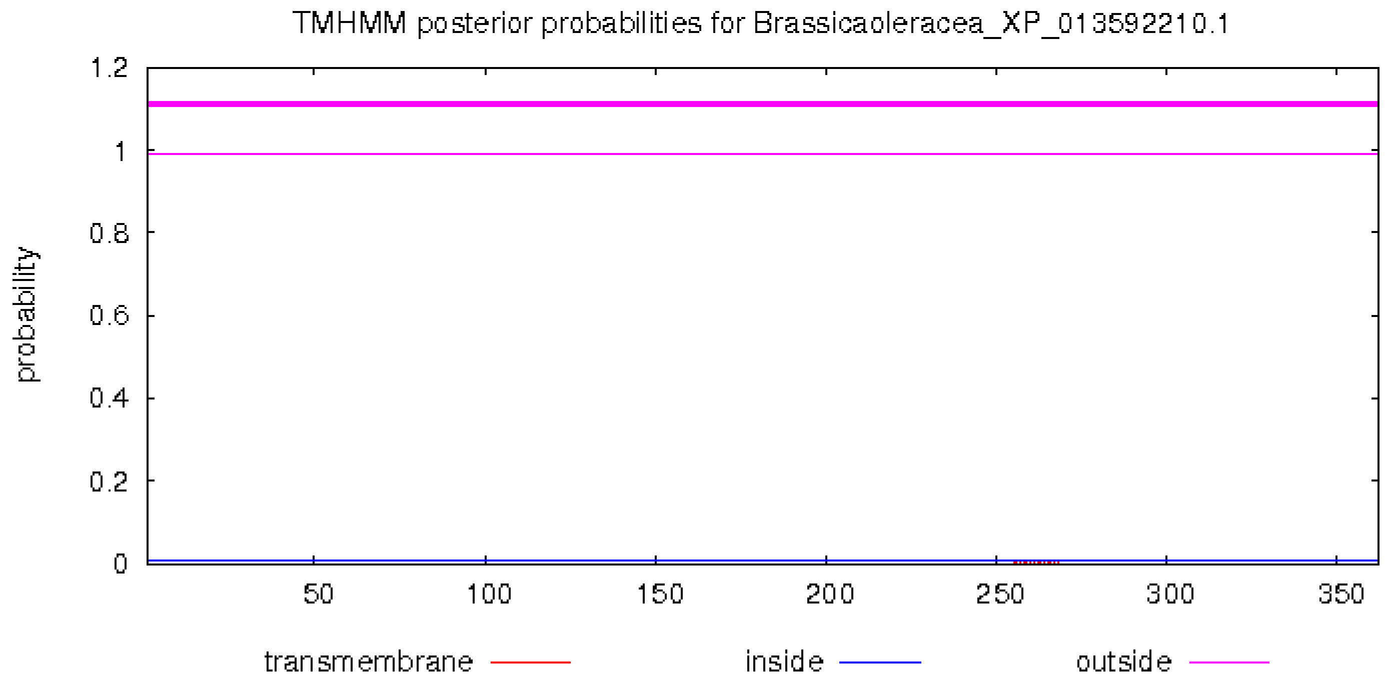

plot in postscript, script for making the plot in gnuplot, data for plot

```
# Brassicaoleracea_XP_013592976.1 Length: 550
# Brassicaoleracea_XP_013592976.1 Number of predicted TMHs:  1
# Brassicaoleracea_XP_013592976.1 Exp number of AAs in TMHs: 21.93773
# Brassicaoleracea_XP_013592976.1 Exp number, first 60 AAs:  0.0019
# Brassicaoleracea_XP_013592976.1 Total prob of N-in:        0.90055
Brassicaoleracea_XP_013592976.1 TMHMM2.0        inside       1   520
Brassicaoleracea_XP_013592976.1 TMHMM2.0        TMhelix    521   543
Brassicaoleracea_XP_013592976.1 TMHMM2.0        outside    544   550
```

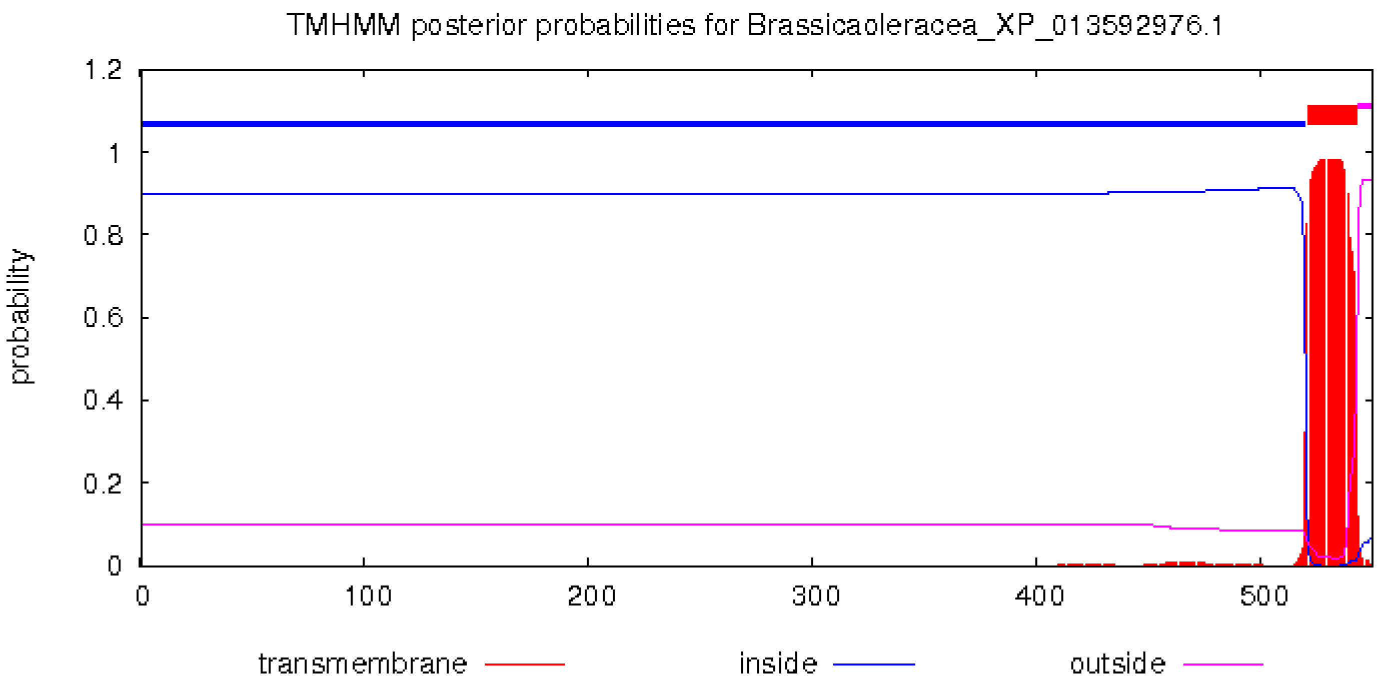

plot in postscript, script for making the plot in gnuplot, data for plot

```
# Brassicaoleracea_XP_013593565.1 Length: 414
# Brassicaoleracea_XP_013593565.1 Number of predicted TMHs:  0
# Brassicaoleracea_XP_013593565.1 Exp number of AAs in TMHs: 0.02472
# Brassicaoleracea_XP_013593565.1 Exp number, first 60 AAs:  0
# Brassicaoleracea_XP_013593565.1 Total prob of N-in:        0.00885
Brassicaoleracea_XP_013593565.1 TMHMM2.0        outside      1   414
```

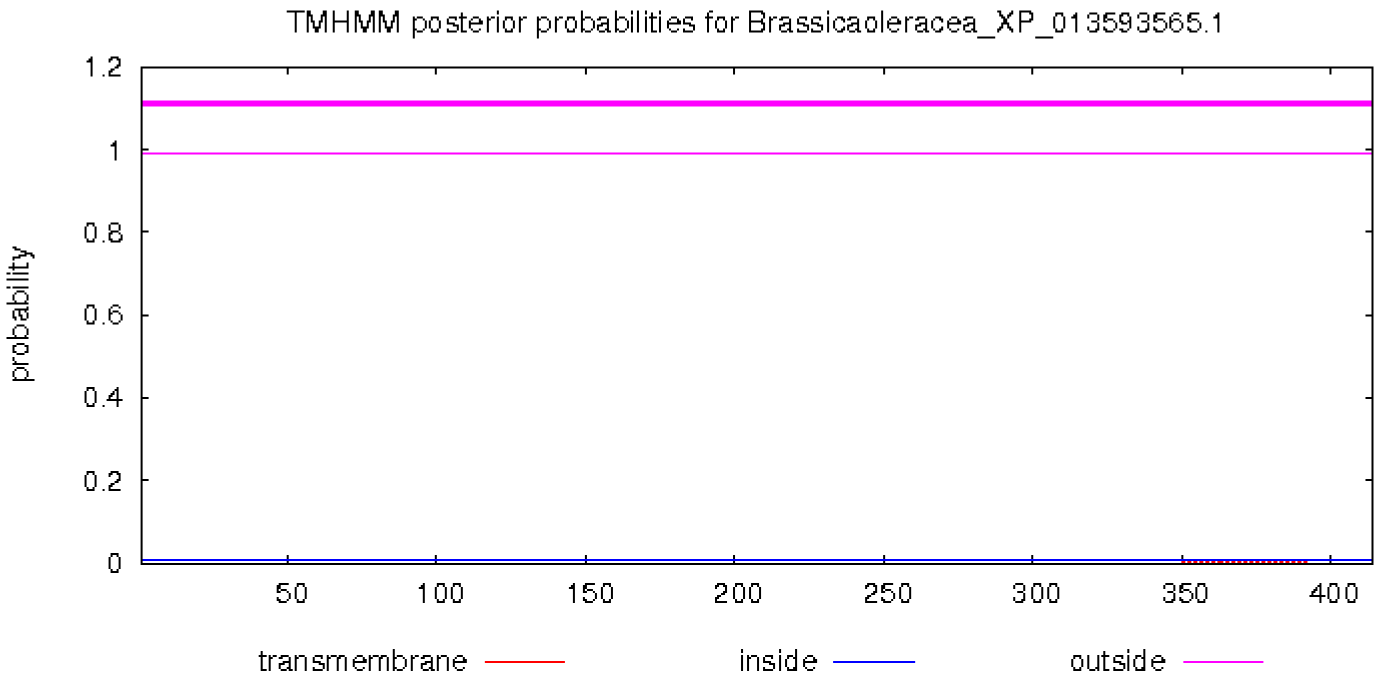

plot in postscript, script for making the plot in gnuplot, data for plot

```
# Brassicaoleracea_XP_013593766.1 Length: 460
# Brassicaoleracea_XP_013593766.1 Number of predicted TMHs:  0
# Brassicaoleracea_XP_013593766.1 Exp number of AAs in TMHs: 0.00115
# Brassicaoleracea_XP_013593766.1 Exp number, first 60 AAs:  0.00018
# Brassicaoleracea_XP_013593766.1 Total prob of N-in:        0.00705
Brassicaoleracea_XP_013593766.1 TMHMM2.0        outside      1   460
```

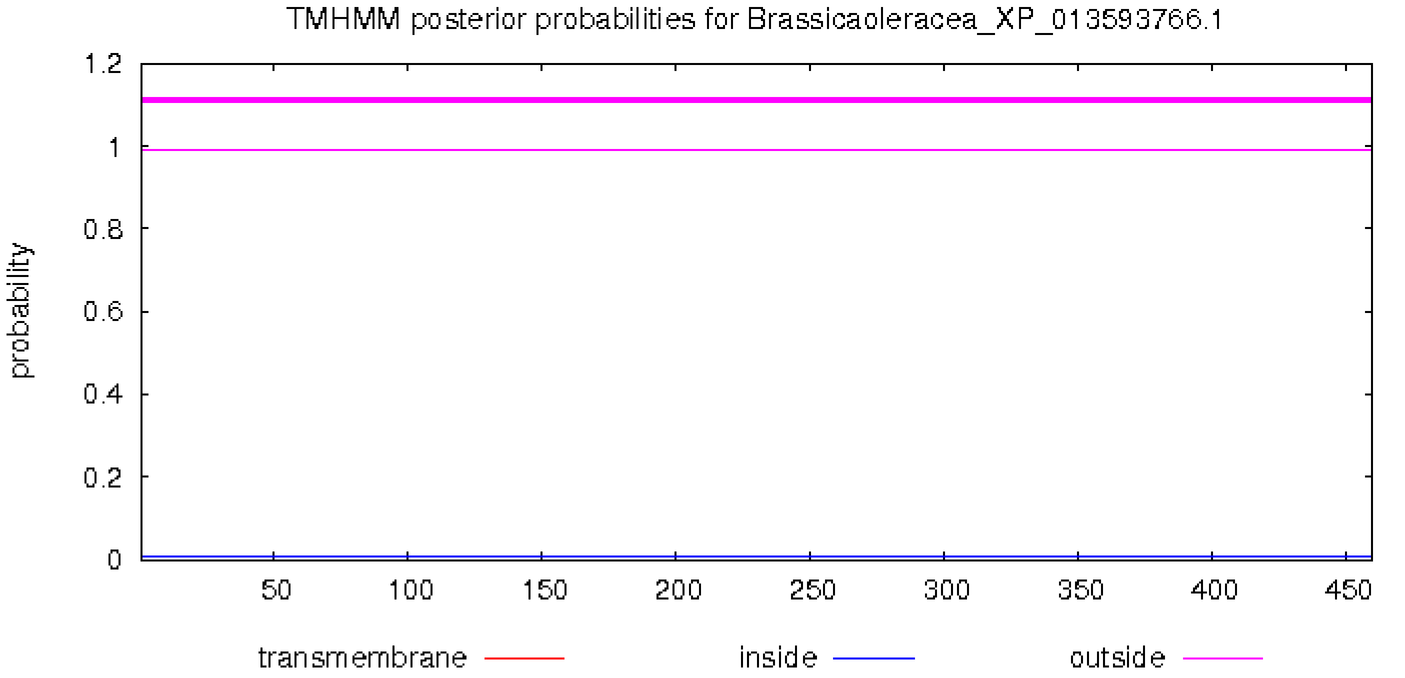

plot in postscript, script for making the plot in gnuplot, data for plot

```
# Brassicaoleracea_XP_013594147.1 Length: 350
# Brassicaoleracea_XP_013594147.1 Number of predicted TMHs:  0
# Brassicaoleracea_XP_013594147.1 Exp number of AAs in TMHs: 0
# Brassicaoleracea_XP_013594147.1 Exp number, first 60 AAs:  0
# Brassicaoleracea_XP_013594147.1 Total prob of N-in:        0.00588
Brassicaoleracea_XP_013594147.1 TMHMM2.0        outside      1   350
```

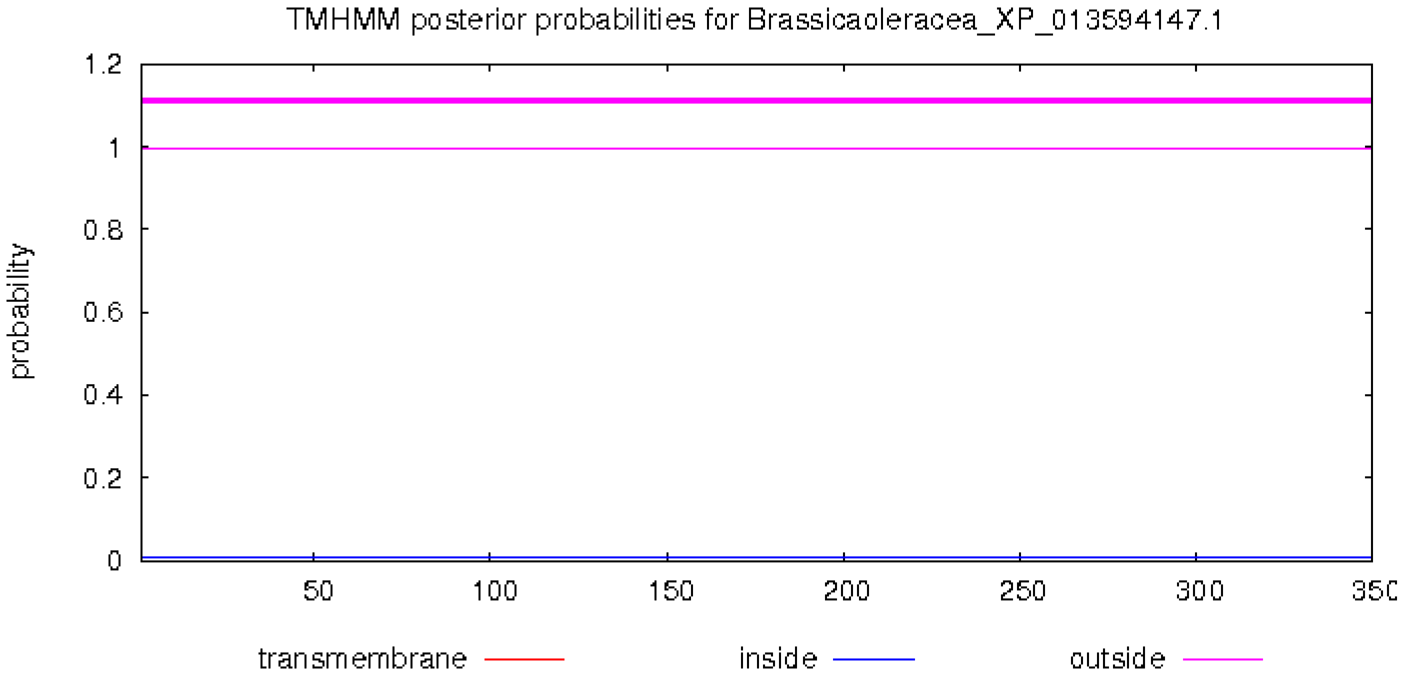

plot in postscript, script for making the plot in gnuplot, data for plot

---

```
# Brassicaoleracea_XP_013594184.1 Length: 496
# Brassicaoleracea_XP_013594184.1 Number of predicted TMHs:  0
# Brassicaoleracea_XP_013594184.1 Exp number of AAs in TMHs: 0.02427
# Brassicaoleracea_XP_013594184.1 Exp number, first 60 AAs:  0
# Brassicaoleracea_XP_013594184.1 Total prob of N-in:        0.00414
Brassicaoleracea_XP_013594184.1 TMHMM2.0        outside      1   496
```

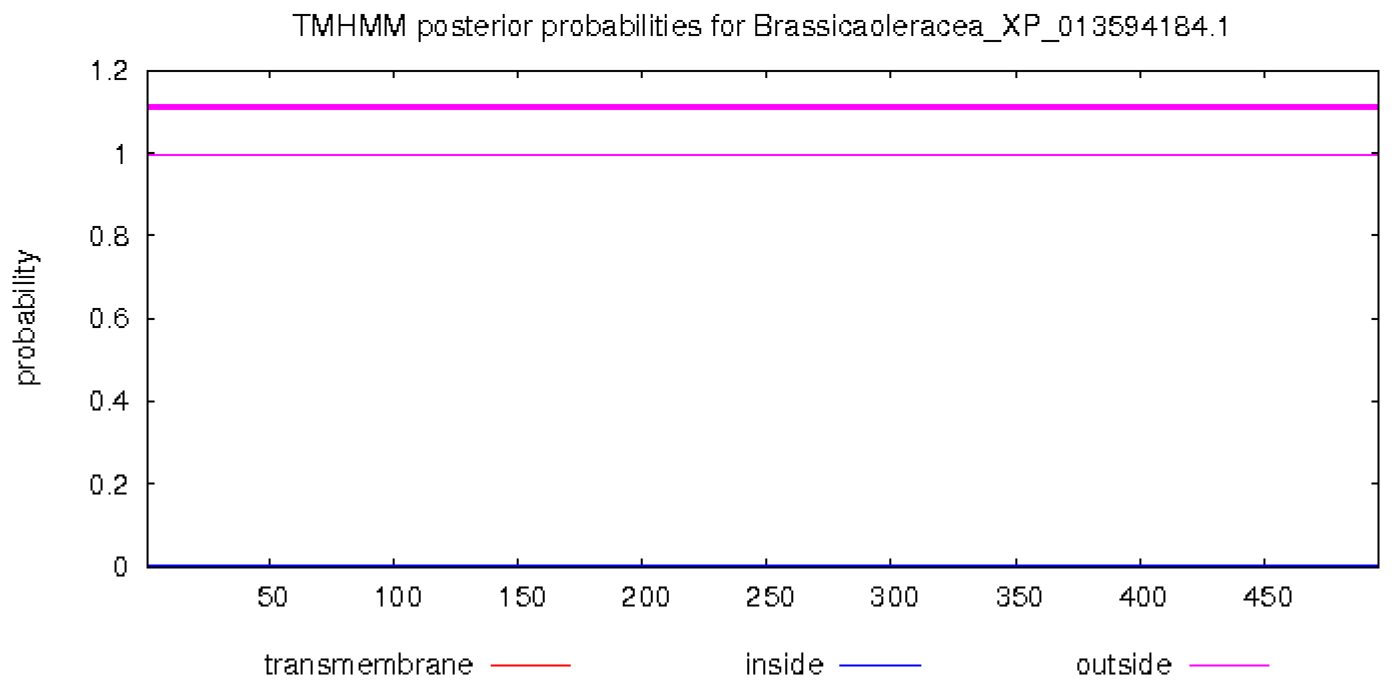

plot in postscript, script for making the plot in gnuplot, data for plot

---

```
# Brassicaoleracea_XP_013594185.1 Length: 495
# Brassicaoleracea_XP_013594185.1 Number of predicted TMHs:  0
# Brassicaoleracea_XP_013594185.1 Exp number of AAs in TMHs: 0.02455
# Brassicaoleracea_XP_013594185.1 Exp number, first 60 AAs:  0
# Brassicaoleracea_XP_013594185.1 Total prob of N-in:        0.00420
Brassicaoleracea_XP_013594185.1 TMHMM2.0        outside      1   495
```

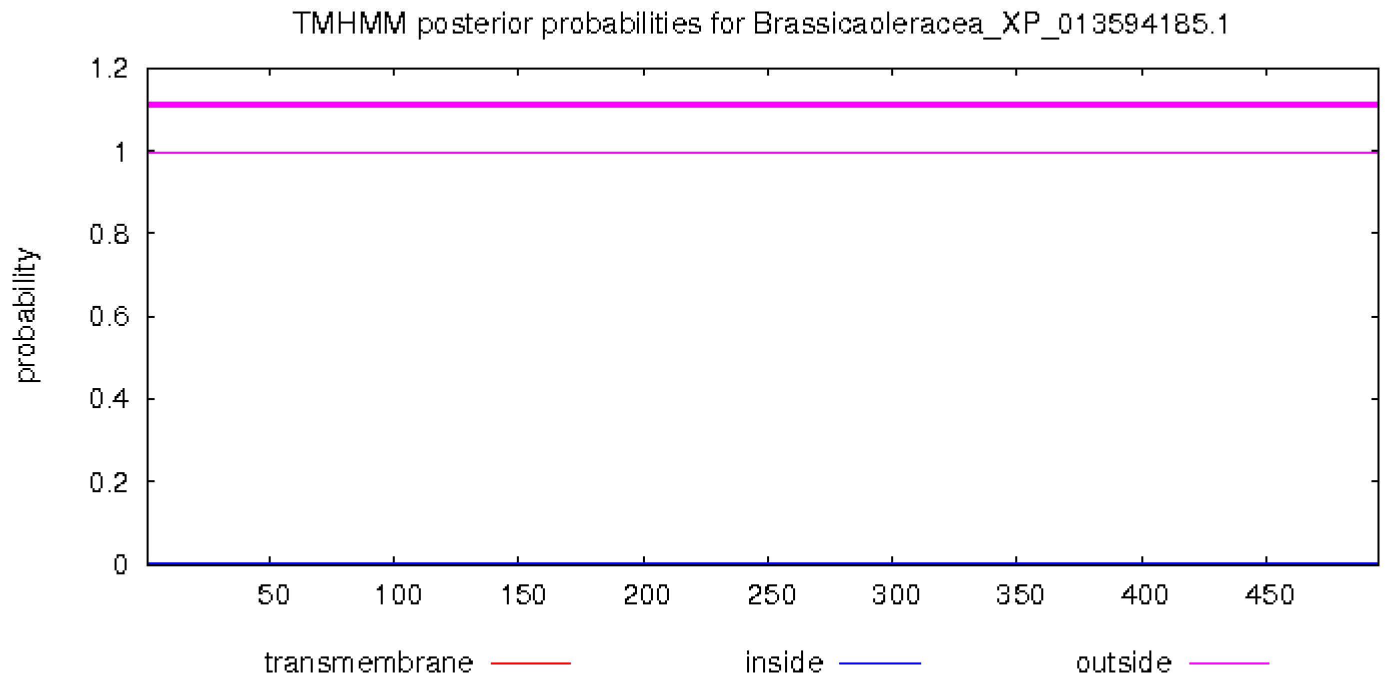

plot in postscript, script for making the plot in gnuplot, data for plot

```
# Brassicaoleracea_XP_013594418.1 Length: 326
# Brassicaoleracea_XP_013594418.1 Number of predicted TMHs:  0
# Brassicaoleracea_XP_013594418.1 Exp number of AAs in TMHs: 0.0004
# Brassicaoleracea_XP_013594418.1 Exp number, first 60 AAs:  0
# Brassicaoleracea_XP_013594418.1 Total prob of N-in:        0.06828
Brassicaoleracea_XP_013594418.1 TMHMM2.0        outside      1   326
```

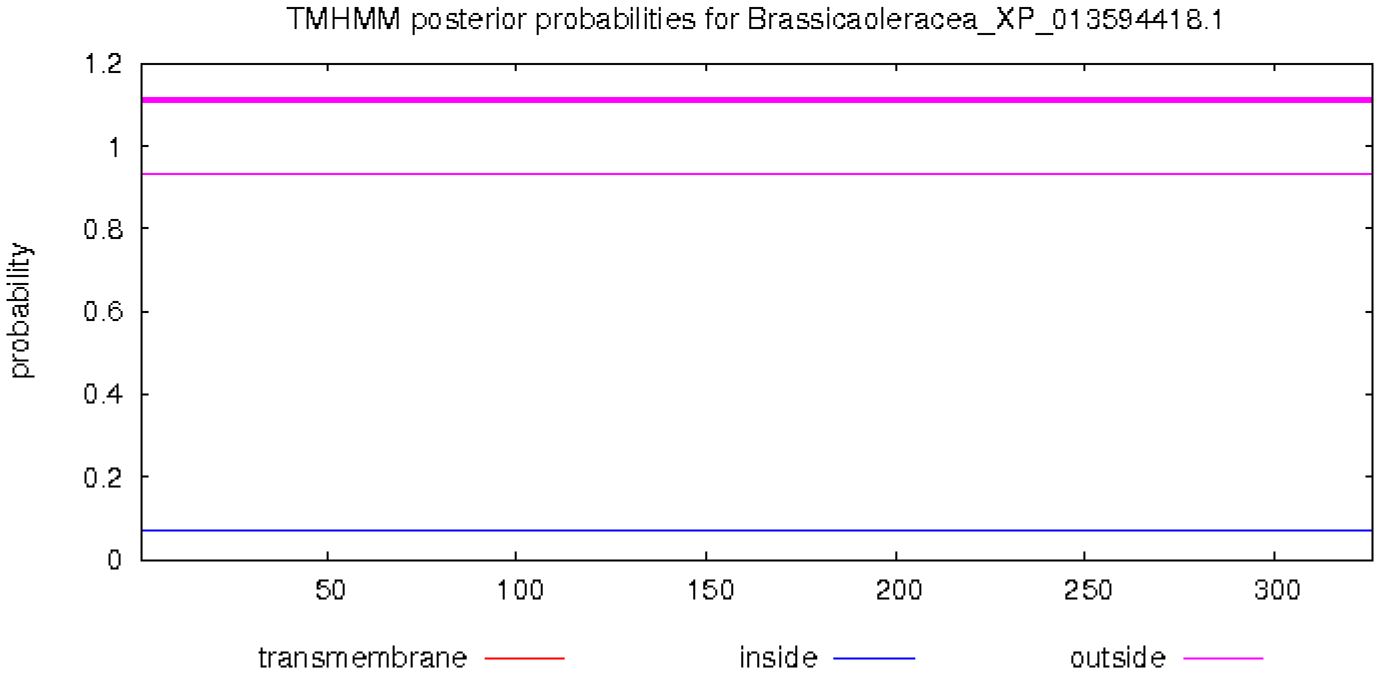

plot in postscript, script for making the plot in gnuplot, data for plot

```
# Brassicaoleracea_XP_013595617.1 Length: 321
# Brassicaoleracea_XP_013595617.1 Number of predicted TMHs:  0
# Brassicaoleracea_XP_013595617.1 Exp number of AAs in TMHs: 0.00785
# Brassicaoleracea_XP_013595617.1 Exp number, first 60 AAs:  0.00036
# Brassicaoleracea_XP_013595617.1 Total prob of N-in:        0.03447
Brassicaoleracea_XP_013595617.1 TMHMM2.0        outside      1   321
```

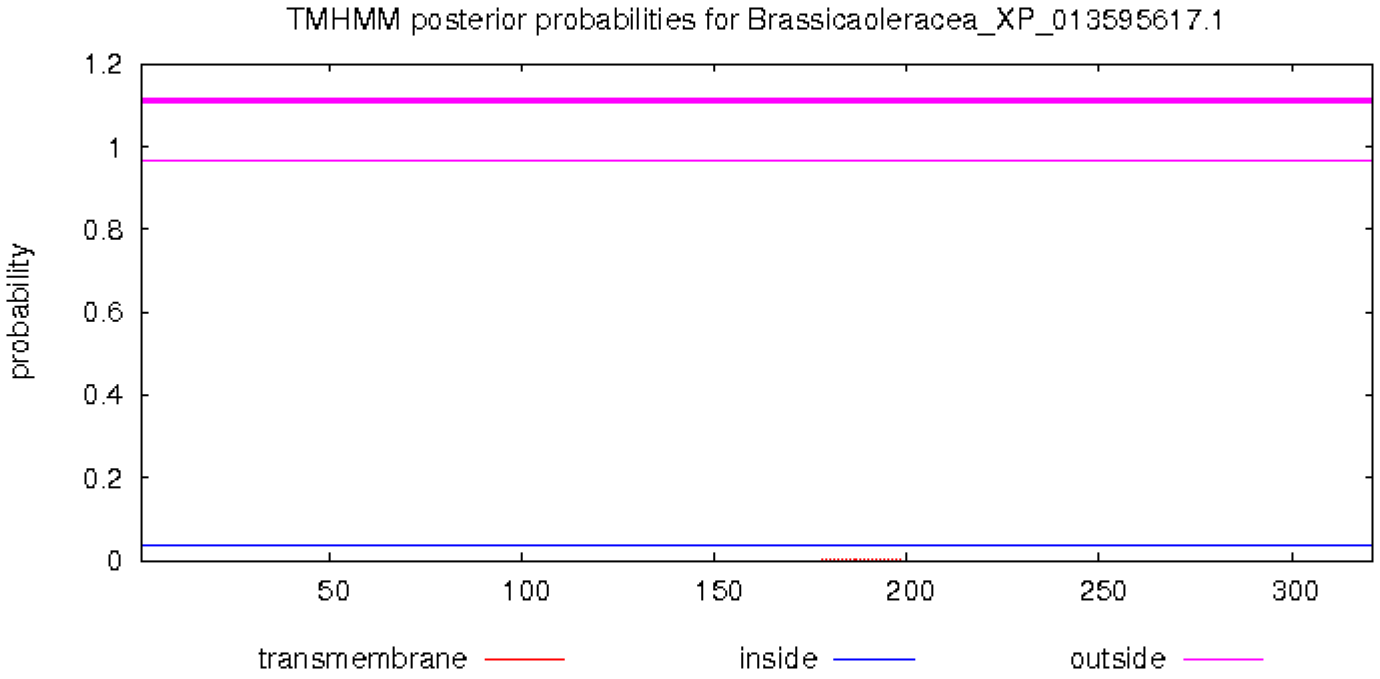

plot in postscript, script for making the plot in gnuplot, data for plot

```
# Brassicaoleracea_XP_013595819.1 Length: 327
# Brassicaoleracea_XP_013595819.1 Number of predicted TMHs:  0
# Brassicaoleracea_XP_013595819.1 Exp number of AAs in TMHs: 0.00023
# Brassicaoleracea_XP_013595819.1 Exp number, first 60 AAs:  0
# Brassicaoleracea_XP_013595819.1 Total prob of N-in:        0.01383
Brassicaoleracea_XP_013595819.1 TMHMM2.0        outside      1   327
```

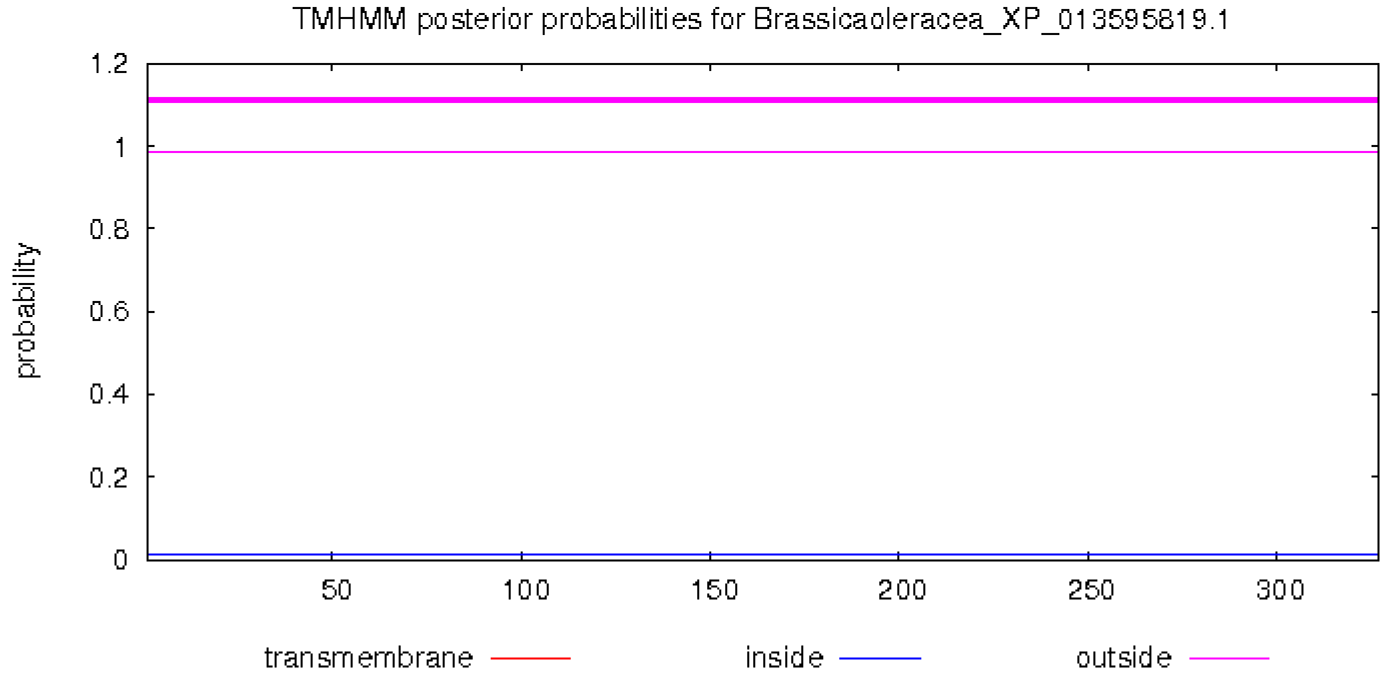

plot in postscript, script for making the plot in gnuplot, data for plot

```
# Brassicaoleracea_XP_013595944.1 Length: 368
# Brassicaoleracea_XP_013595944.1 Number of predicted TMHs:  0
# Brassicaoleracea_XP_013595944.1 Exp number of AAs in TMHs: 0
# Brassicaoleracea_XP_013595944.1 Exp number, first 60 AAs:  0
# Brassicaoleracea_XP_013595944.1 Total prob of N-in:        0.01355
Brassicaoleracea_XP_013595944.1 TMHMM2.0        outside      1   368
```

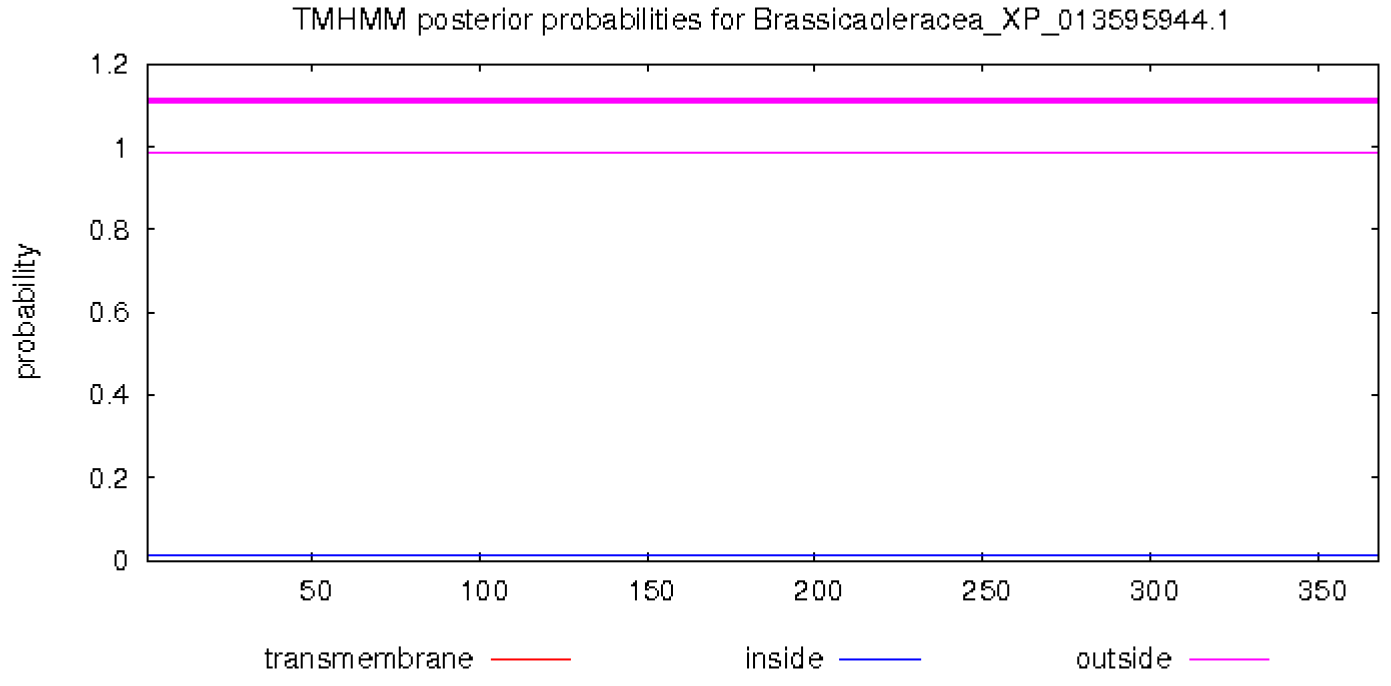

plot in postscript, script for making the plot in gnuplot, data for plot

```
# Brassicaoleracea_XP_013595945.1 Length: 368
# Brassicaoleracea_XP_013595945.1 Number of predicted TMHs:  0
# Brassicaoleracea_XP_013595945.1 Exp number of AAs in TMHs: 0
# Brassicaoleracea_XP_013595945.1 Exp number, first 60 AAs:  0
# Brassicaoleracea_XP_013595945.1 Total prob of N-in:        0.01355
Brassicaoleracea_XP_013595945.1 TMHMM2.0        outside      1   368
```

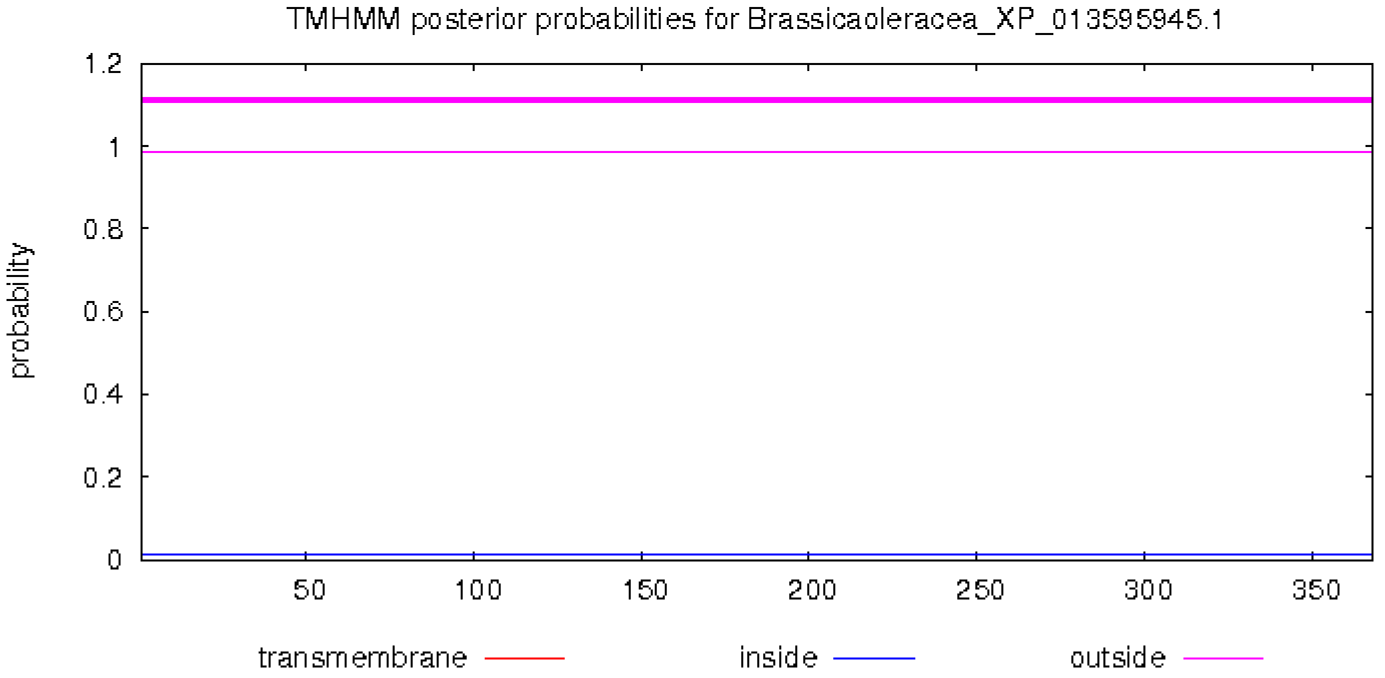

plot in postscript, script for making the plot in gnuplot, data for plot

```
# Brassicaoleracea_XP_013596007.1 Length: 285
# Brassicaoleracea_XP_013596007.1 Number of predicted TMHs:  0
# Brassicaoleracea_XP_013596007.1 Exp number of AAs in TMHs: 0.01237
# Brassicaoleracea_XP_013596007.1 Exp number, first 60 AAs:  0
# Brassicaoleracea_XP_013596007.1 Total prob of N-in:        0.00765
Brassicaoleracea_XP_013596007.1 TMHMM2.0        outside      1   285
```

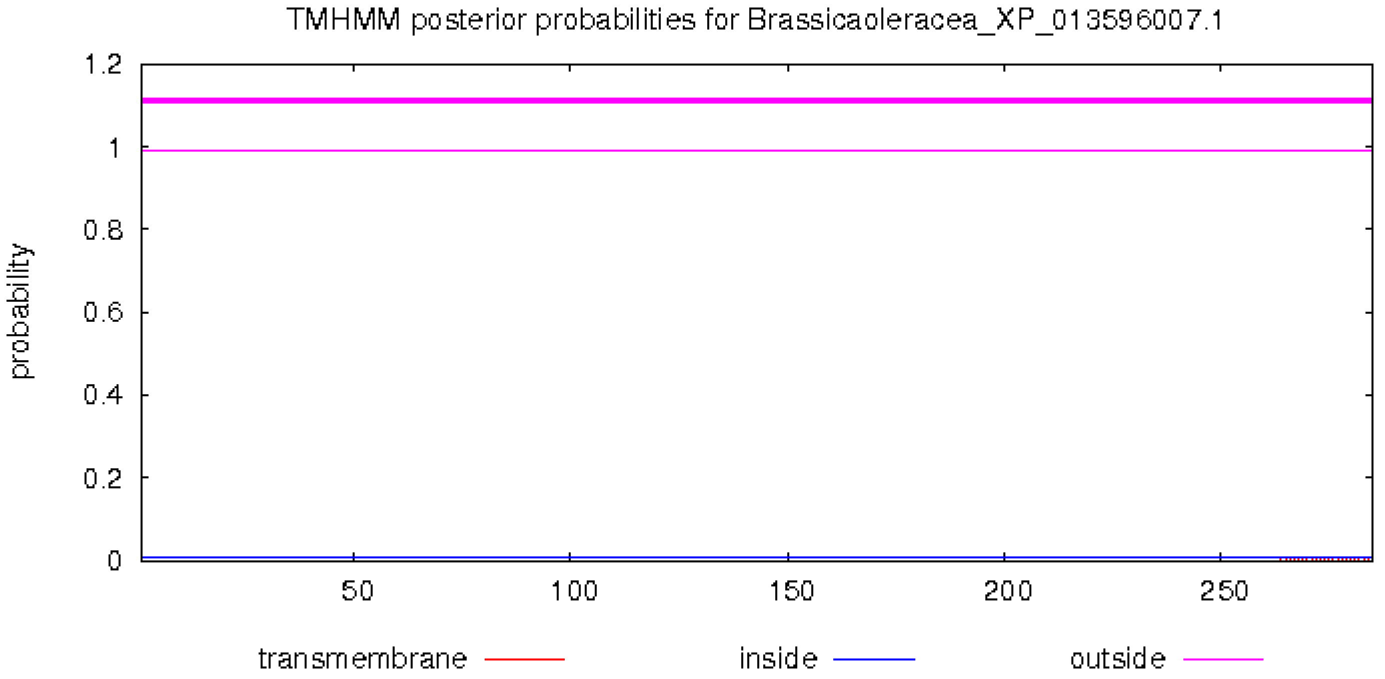

plot in postscript, script for making the plot in gnuplot, data for plot

```
# Brassicaoleracea_XP_013596124.1 Length: 313
# Brassicaoleracea_XP_013596124.1 Number of predicted TMHs:  0
# Brassicaoleracea_XP_013596124.1 Exp number of AAs in TMHs: 0.07
# Brassicaoleracea_XP_013596124.1 Exp number, first 60 AAs:  0.00018
# Brassicaoleracea_XP_013596124.1 Total prob of N-in:        0.07529
Brassicaoleracea_XP_013596124.1 TMHMM2.0        outside      1   313
```

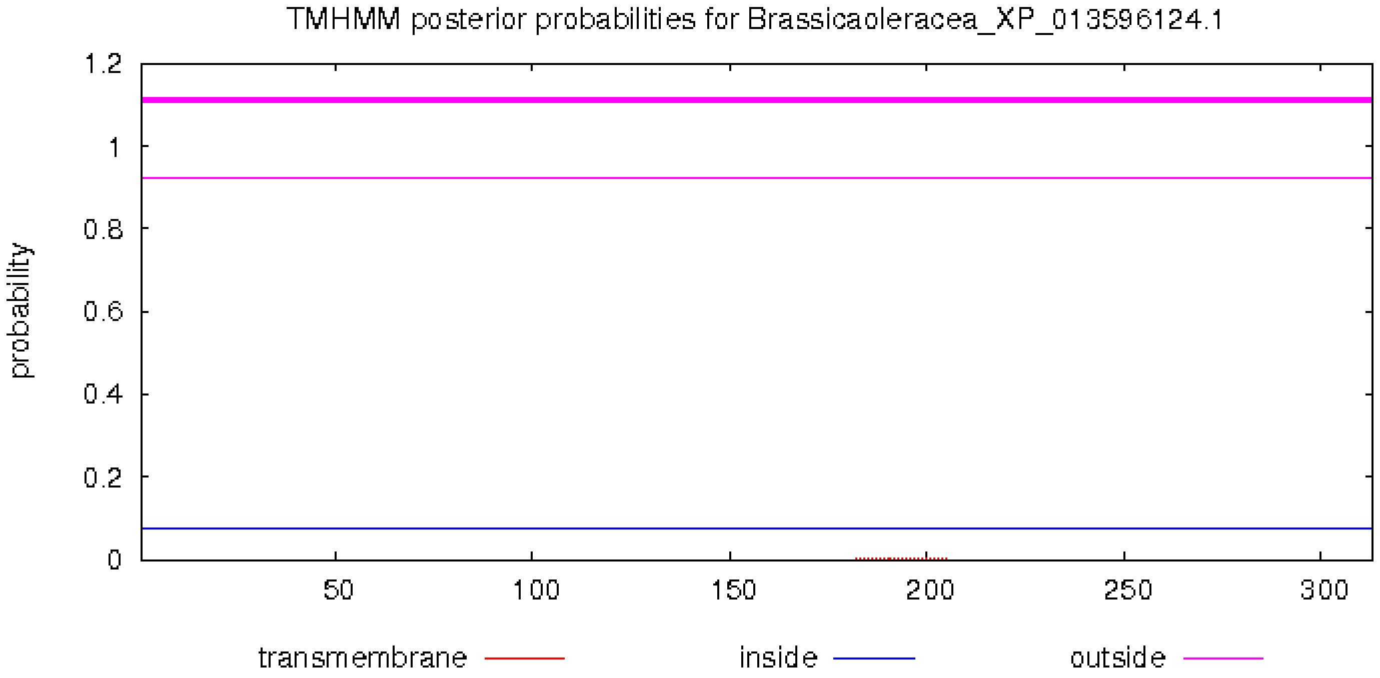

plot in postscript, script for making the plot in gnuplot, data for plot

```
# Brassicaoleracea_XP_013596125.1 Length: 309
# Brassicaoleracea_XP_013596125.1 Number of predicted TMHs:  0
# Brassicaoleracea_XP_013596125.1 Exp number of AAs in TMHs: 0.07236
# Brassicaoleracea_XP_013596125.1 Exp number, first 60 AAs:  0.00019
# Brassicaoleracea_XP_013596125.1 Total prob of N-in:        0.07883
Brassicaoleracea_XP_013596125.1 TMHMM2.0        outside      1   309
```

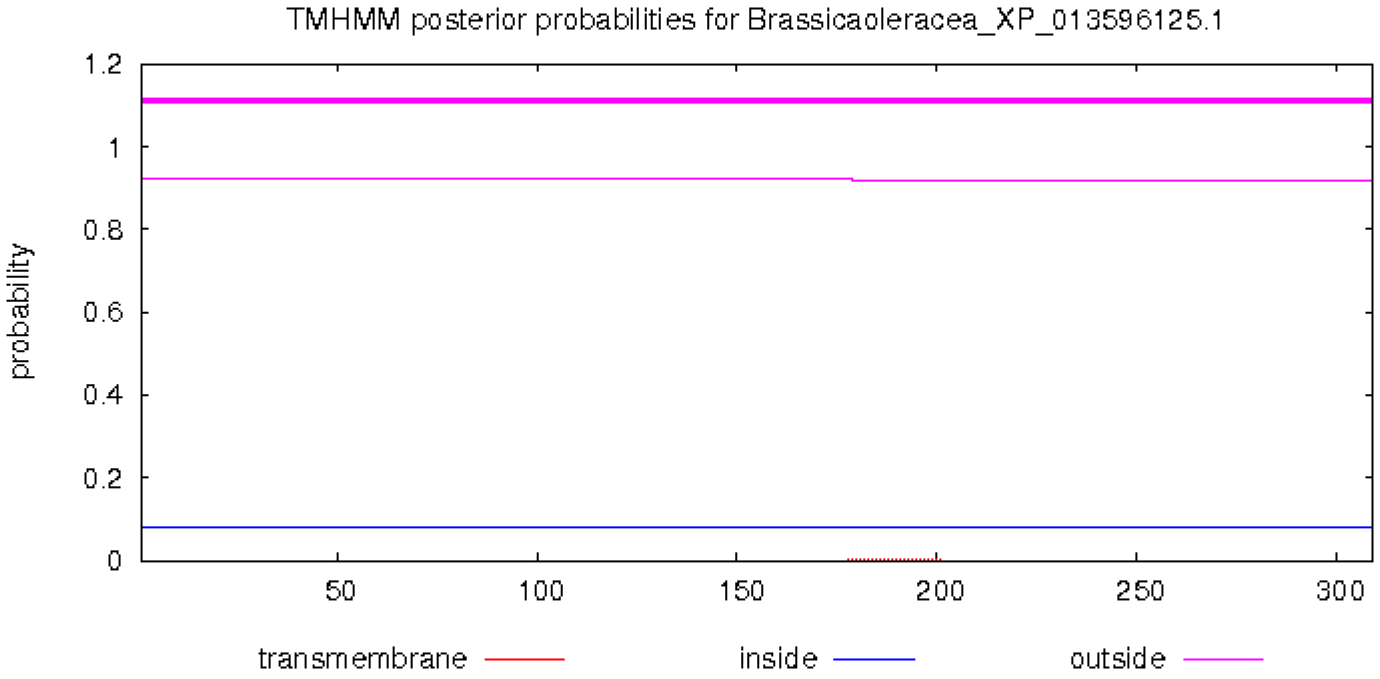

plot in postscript, script for making the plot in gnuplot, data for plot

---

```
# Brassicaoleracea_XP_013596215.1 Length: 302
# Brassicaoleracea_XP_013596215.1 Number of predicted TMHs:  0
# Brassicaoleracea_XP_013596215.1 Exp number of AAs in TMHs: 0.00021
# Brassicaoleracea_XP_013596215.1 Exp number, first 60 AAs:  0
# Brassicaoleracea_XP_013596215.1 Total prob of N-in:        0.04751
Brassicaoleracea_XP_013596215.1 TMHMM2.0        outside      1   302
```

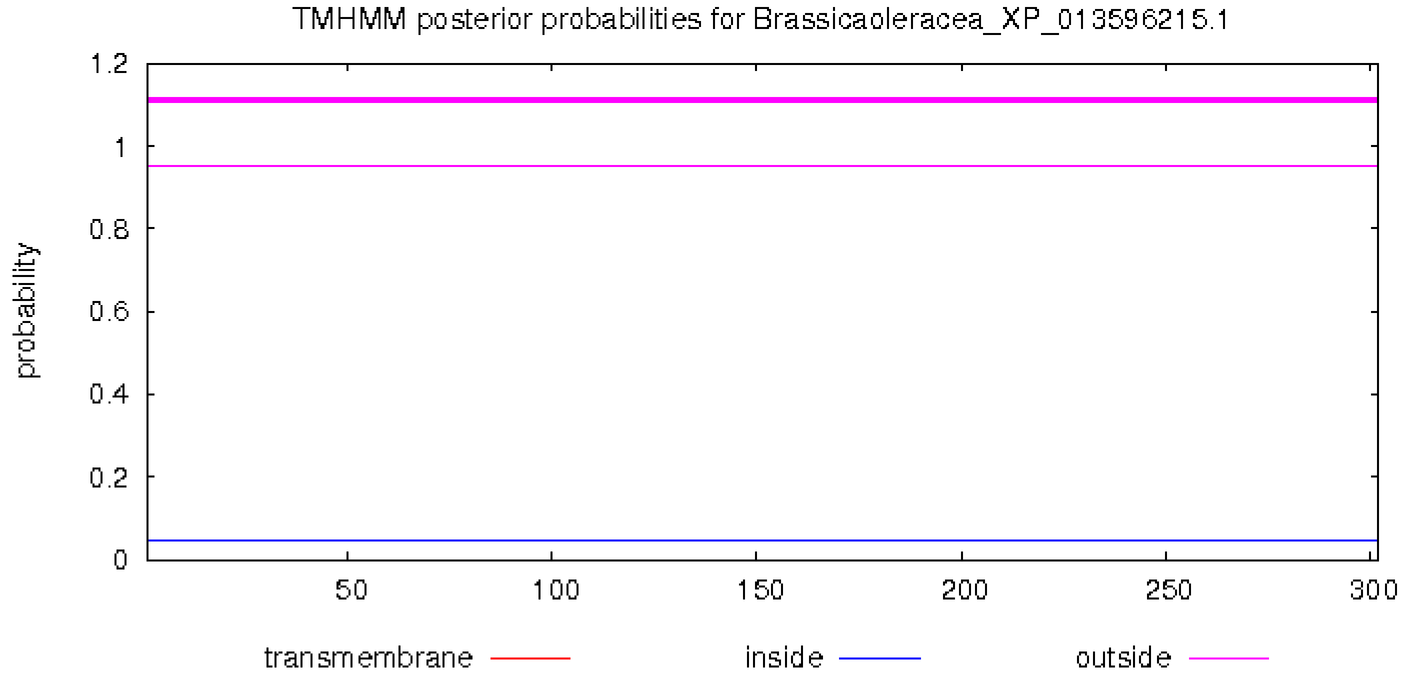

plot in postscript, script for making the plot in gnuplot, data for plot

---

```
# Brassicaoleracea_XP_013596223.1 Length: 295
# Brassicaoleracea_XP_013596223.1 Number of predicted TMHs:  0
# Brassicaoleracea_XP_013596223.1 Exp number of AAs in TMHs: 0.00018
# Brassicaoleracea_XP_013596223.1 Exp number, first 60 AAs:  0
# Brassicaoleracea_XP_013596223.1 Total prob of N-in:        0.01305
Brassicaoleracea_XP_013596223.1 TMHMM2.0        outside      1   295
```

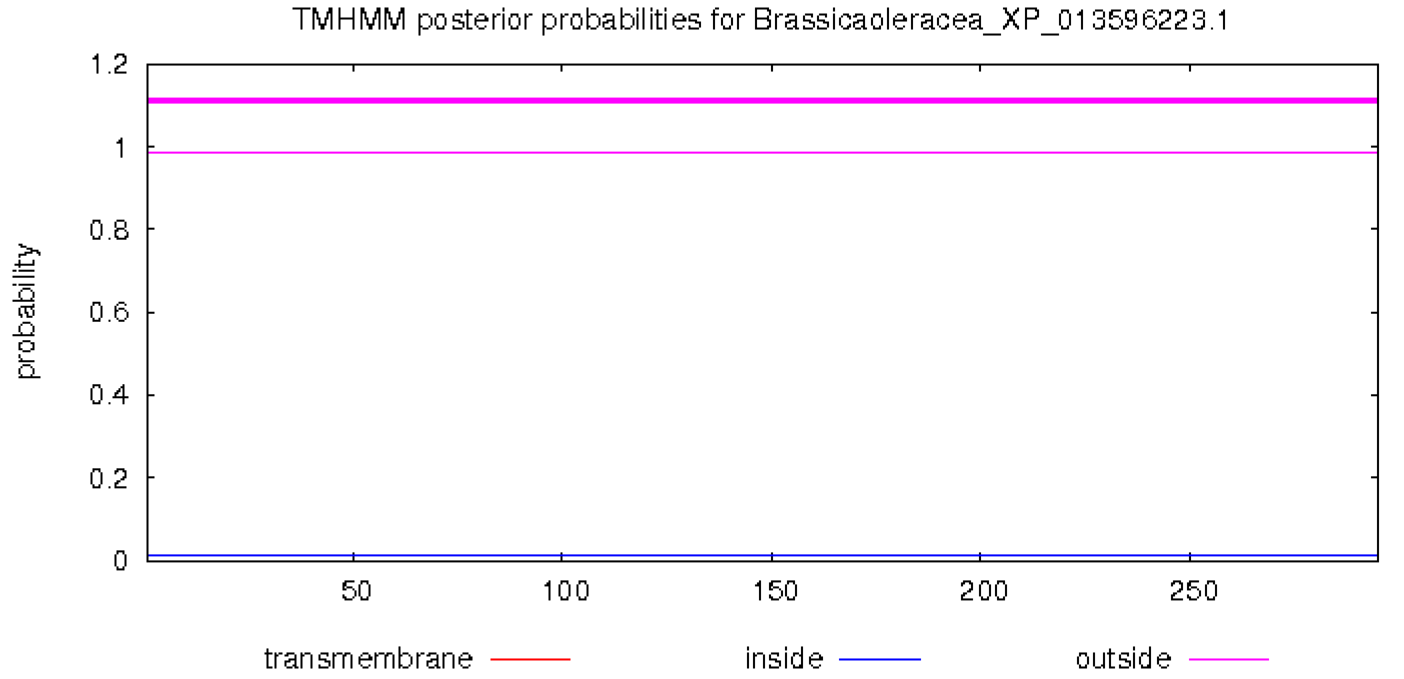

plot in postscript, script for making the plot in gnuplot, data for plot

---

```
# Brassicaoleracea_XP_013596224.1 Length: 294
# Brassicaoleracea_XP_013596224.1 Number of predicted TMHs:  0
# Brassicaoleracea_XP_013596224.1 Exp number of AAs in TMHs: 0.00018
# Brassicaoleracea_XP_013596224.1 Exp number, first 60 AAs:  0
# Brassicaoleracea_XP_013596224.1 Total prob of N-in:        0.01322
Brassicaoleracea_XP_013596224.1 TMHMM2.0        outside      1   294
```

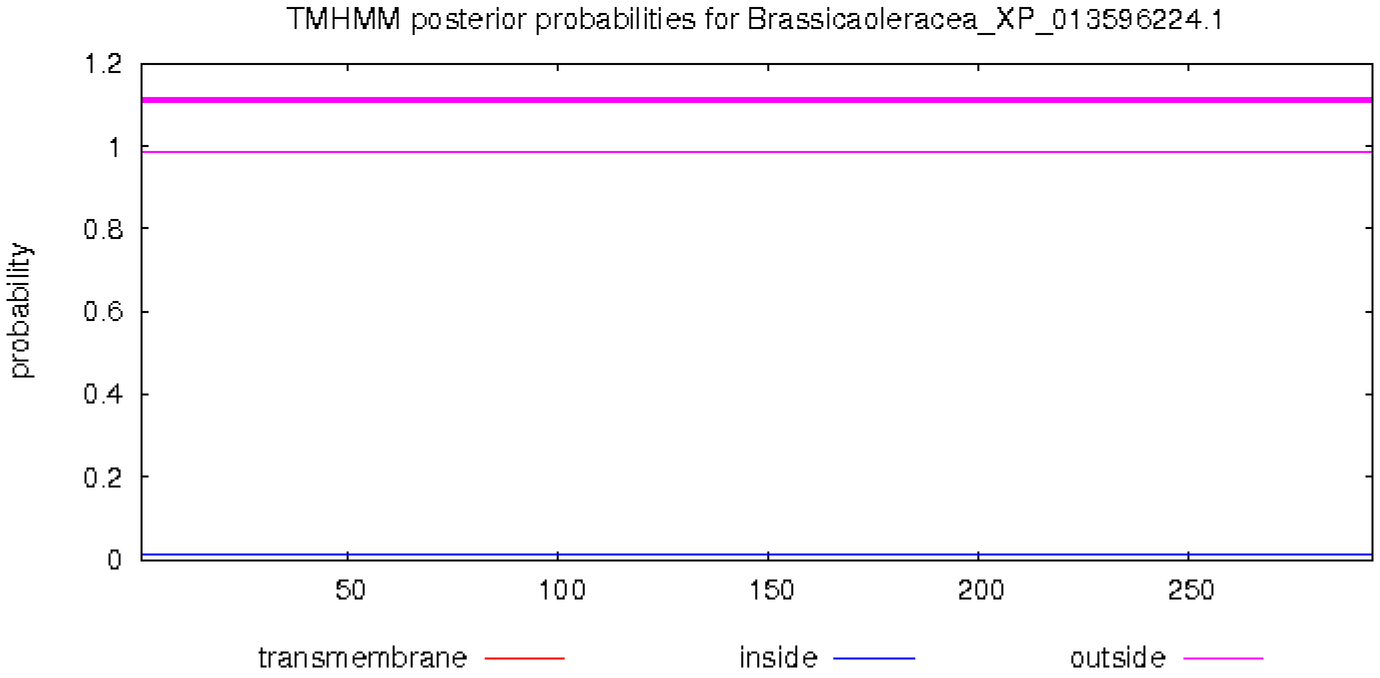

plot in postscript, script for making the plot in gnuplot, data for plot

---

```
# Brassicaoleracea_XP_013596229.1 Length: 361
# Brassicaoleracea_XP_013596229.1 Number of predicted TMHs:  0
# Brassicaoleracea_XP_013596229.1 Exp number of AAs in TMHs: 0
# Brassicaoleracea_XP_013596229.1 Exp number, first 60 AAs:  0
# Brassicaoleracea_XP_013596229.1 Total prob of N-in:        0.00206
Brassicaoleracea_XP_013596229.1 TMHMM2.0        outside      1   361
```

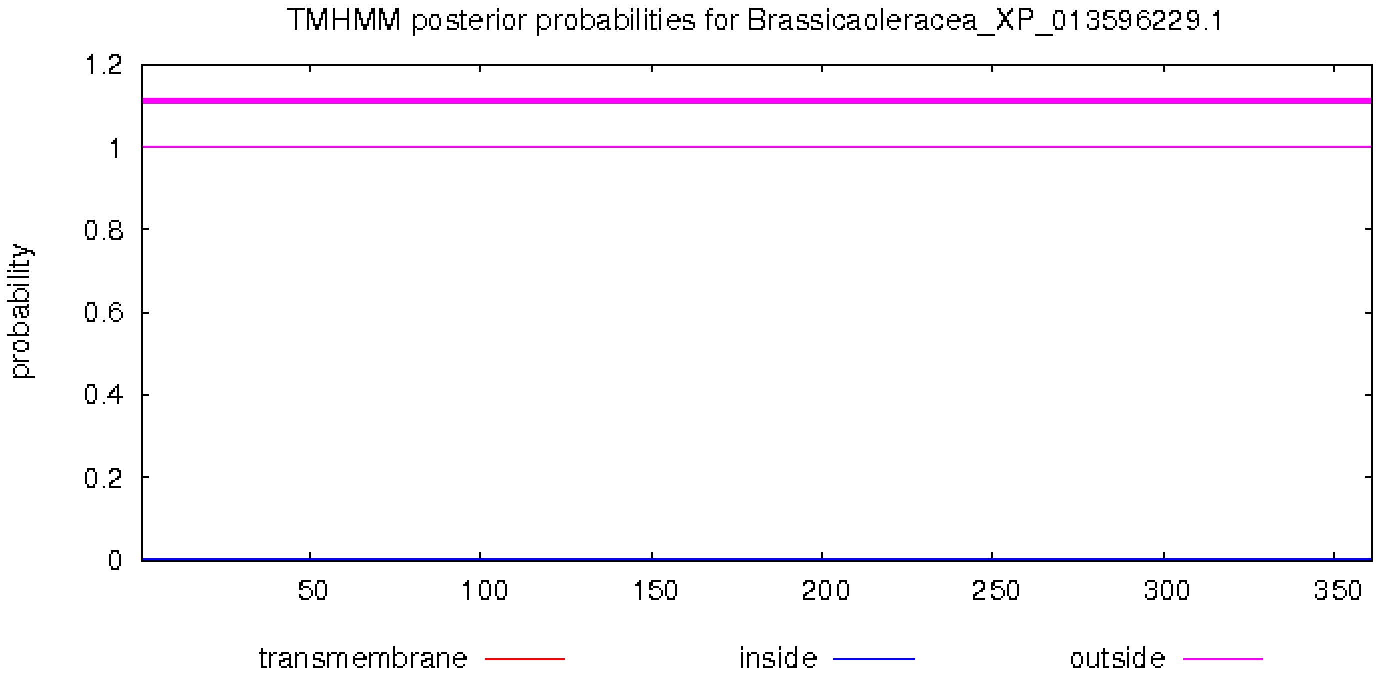

plot in postscript, script for making the plot in gnuplot, data for plot

---

```
# Brassicaoleracea_XP_013596725.1 Length: 286
# Brassicaoleracea_XP_013596725.1 Number of predicted TMHs:  0
# Brassicaoleracea_XP_013596725.1 Exp number of AAs in TMHs: 0
# Brassicaoleracea_XP_013596725.1 Exp number, first 60 AAs:  0
# Brassicaoleracea_XP_013596725.1 Total prob of N-in:        0.00737
Brassicaoleracea_XP_013596725.1 TMHMM2.0        outside      1   286
```

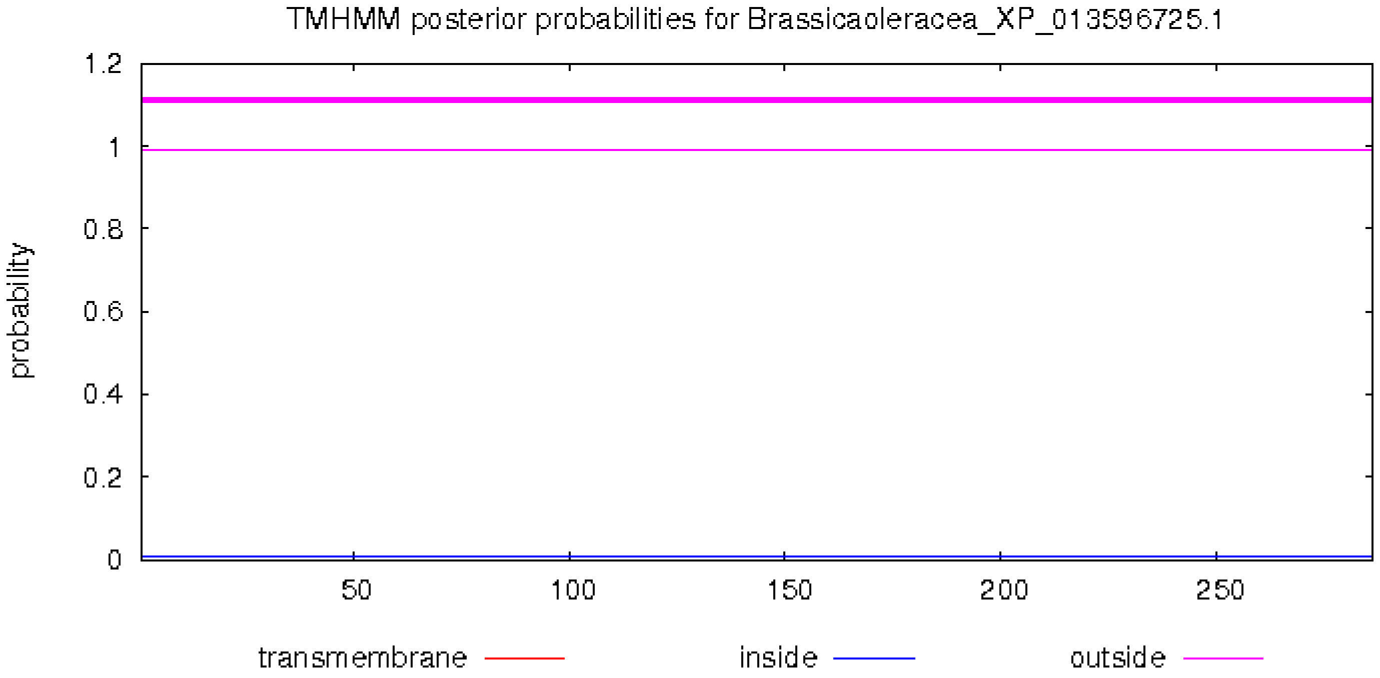

plot in postscript, script for making the plot in gnuplot, data for plot

---

```
# Brassicaoleracea_XP_013596941.1 Length: 284
# Brassicaoleracea_XP_013596941.1 Number of predicted TMHs:  0
# Brassicaoleracea_XP_013596941.1 Exp number of AAs in TMHs: 0
# Brassicaoleracea_XP_013596941.1 Exp number, first 60 AAs:  0
# Brassicaoleracea_XP_013596941.1 Total prob of N-in:        0.00171
Brassicaoleracea_XP_013596941.1 TMHMM2.0        outside      1   284
```

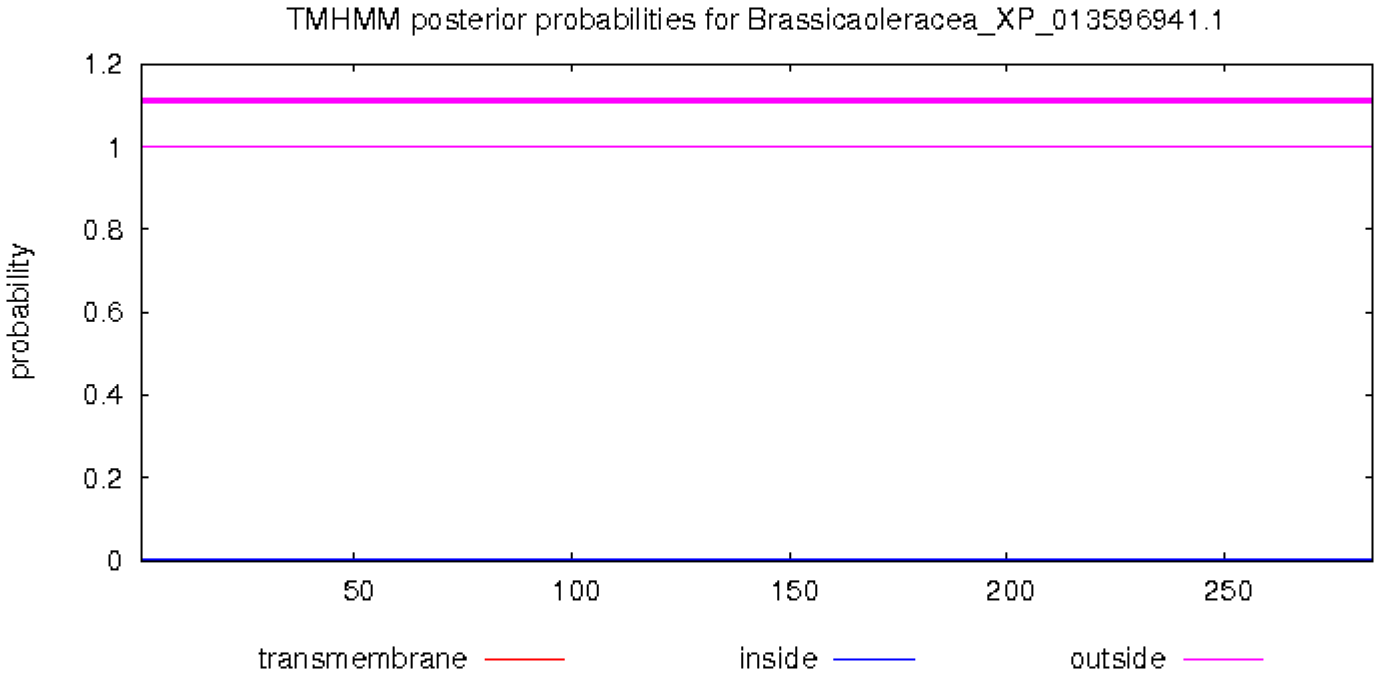

plot in postscript, script for making the plot in gnuplot, data for plot

---

```
# Brassicaoleracea_XP_013597963.1 Length: 365
# Brassicaoleracea_XP_013597963.1 Number of predicted TMHs:  0
# Brassicaoleracea_XP_013597963.1 Exp number of AAs in TMHs: 0.000830000000000000001
# Brassicaoleracea_XP_013597963.1 Exp number, first 60 AAs:  0.00032
# Brassicaoleracea_XP_013597963.1 Total prob of N-in:        0.01018
Brassicaoleracea_XP_013597963.1 TMHMM2.0        outside      1   365
```

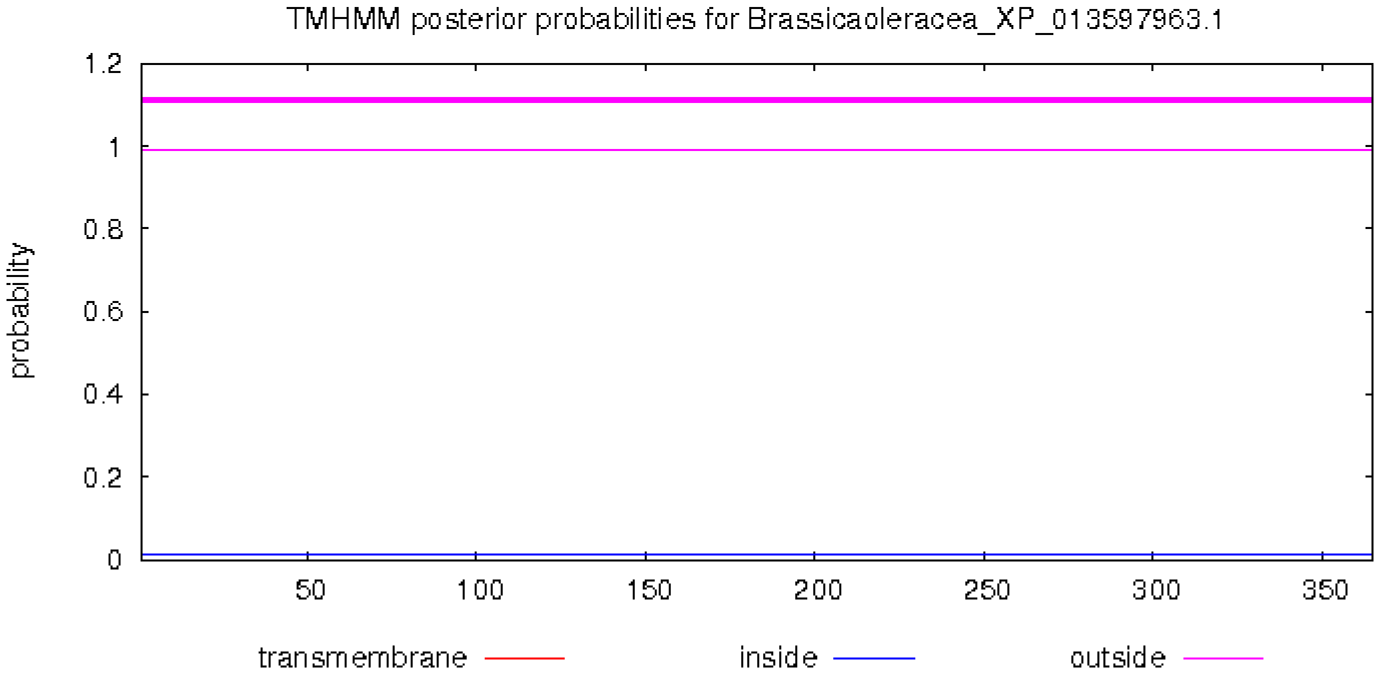

plot in postscript, script for making the plot in gnuplot, data for plot

---

```
# Brassicaoleracea_XP_013597964.1 Length: 365
# Brassicaoleracea_XP_013597964.1 Number of predicted TMHs:  0
# Brassicaoleracea_XP_013597964.1 Exp number of AAs in TMHs: 0.000830000000000000001
# Brassicaoleracea_XP_013597964.1 Exp number, first 60 AAs:  0.00032
# Brassicaoleracea_XP_013597964.1 Total prob of N-in:        0.01018
Brassicaoleracea_XP_013597964.1 TMHMM2.0        outside      1   365
```

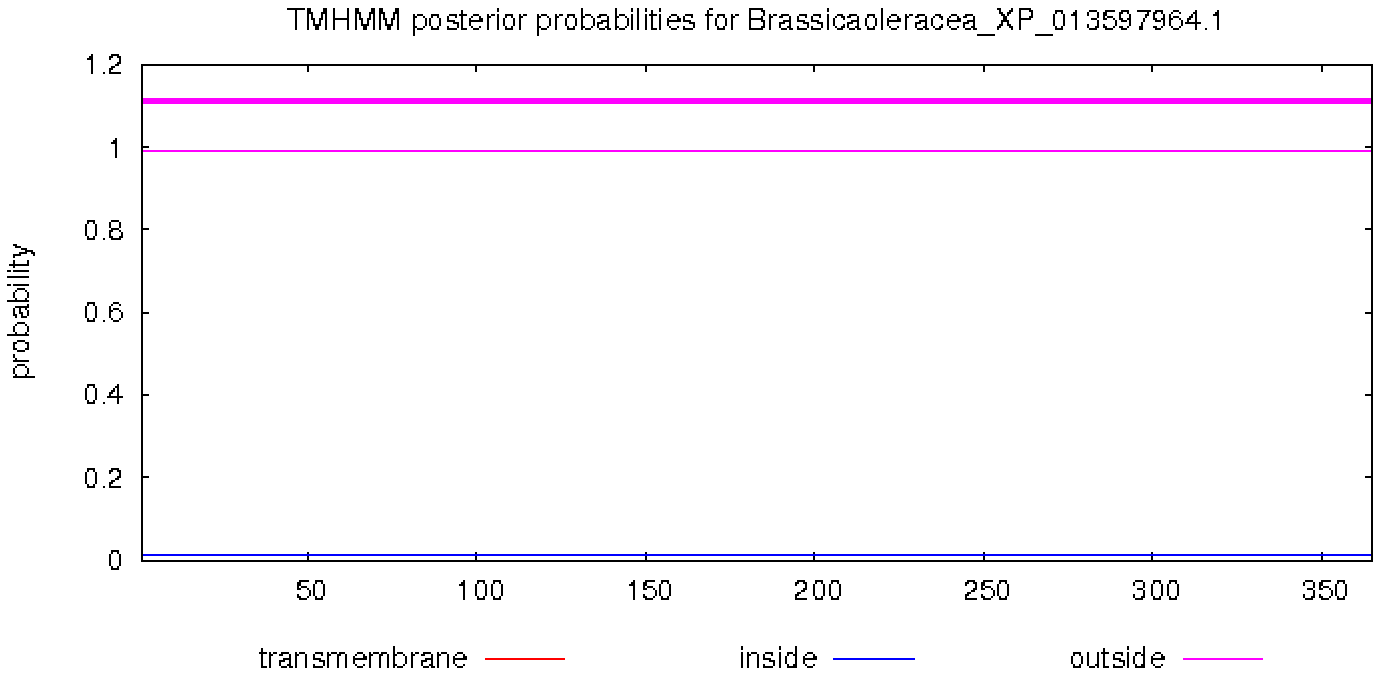

plot in postscript, script for making the plot in gnuplot, data for plot

```
# Brassicaoleracea_XP_013597965.1 Length: 365
# Brassicaoleracea_XP_013597965.1 Number of predicted TMHs:  0
# Brassicaoleracea_XP_013597965.1 Exp number of AAs in TMHs: 0.000830000000000000001
# Brassicaoleracea_XP_013597965.1 Exp number, first 60 AAs:  0.00032
# Brassicaoleracea_XP_013597965.1 Total prob of N-in:        0.01018
Brassicaoleracea_XP_013597965.1 TMHMM2.0        outside      1   365
```

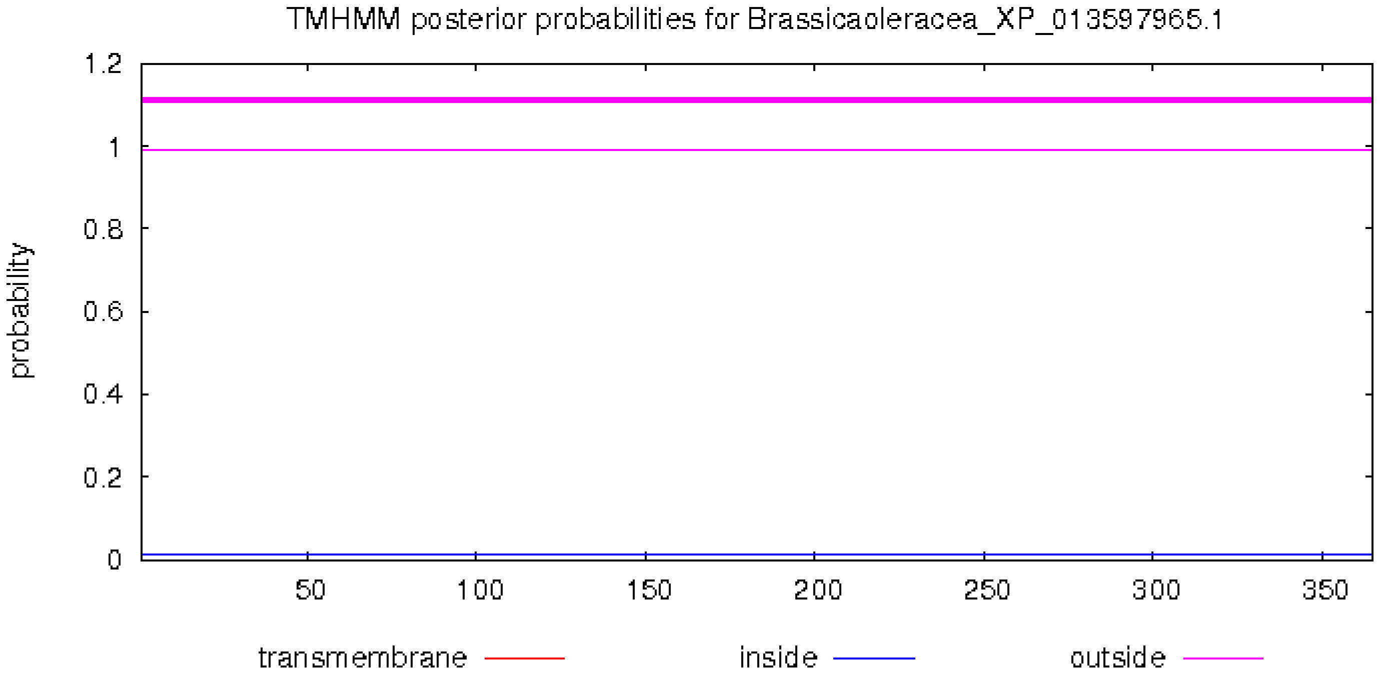

plot in postscript, script for making the plot in gnuplot, data for plot

```
# Brassicaoleracea_XP_013597966.1 Length: 365
# Brassicaoleracea_XP_013597966.1 Number of predicted TMHs:  0
# Brassicaoleracea_XP_013597966.1 Exp number of AAs in TMHs: 0.000830000000000000001
# Brassicaoleracea_XP_013597966.1 Exp number, first 60 AAs:  0.00032
# Brassicaoleracea_XP_013597966.1 Total prob of N-in:        0.01018
Brassicaoleracea_XP_013597966.1 TMHMM2.0        outside      1   365
```

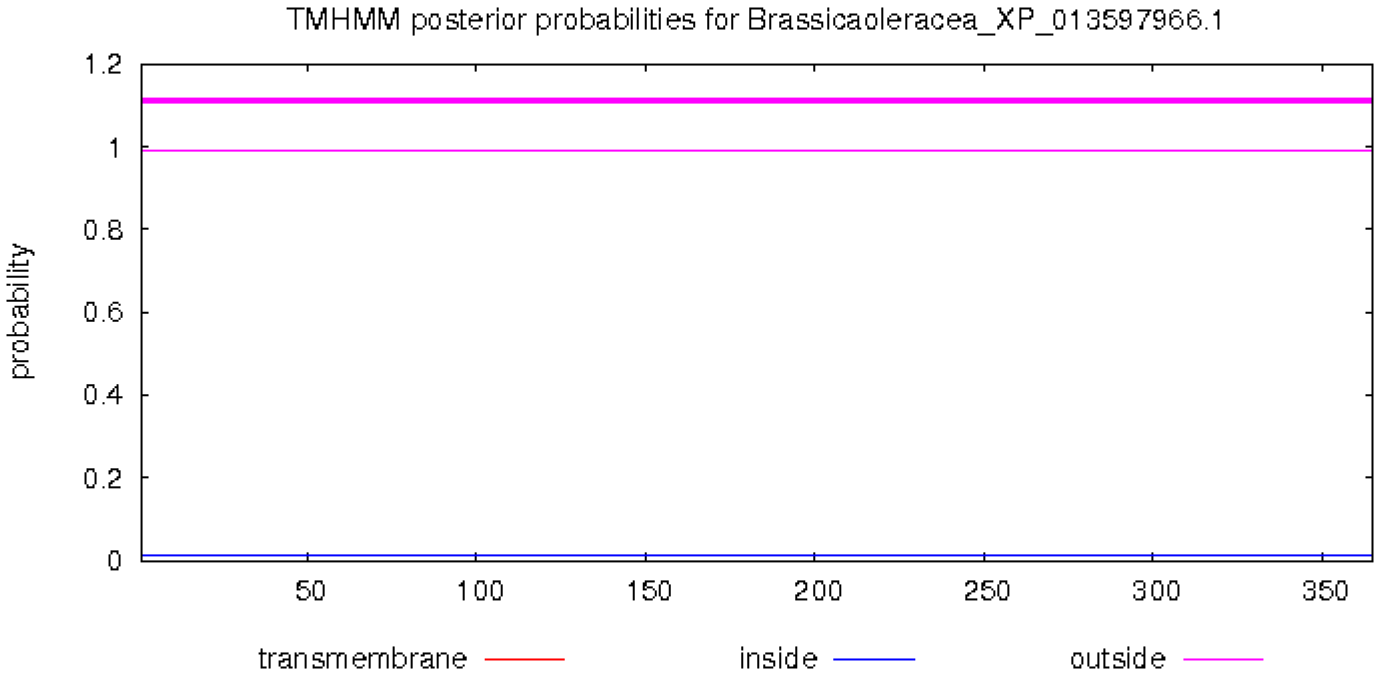

plot in postscript, script for making the plot in gnuplot, data for plot

```
# Brassicaoleracea_XP_013597967.1 Length: 365
# Brassicaoleracea_XP_013597967.1 Number of predicted TMHs:  0
# Brassicaoleracea_XP_013597967.1 Exp number of AAs in TMHs: 0.000830000000000000001
# Brassicaoleracea_XP_013597967.1 Exp number, first 60 AAs:  0.00032
# Brassicaoleracea_XP_013597967.1 Total prob of N-in:        0.01018
Brassicaoleracea_XP_013597967.1 TMHMM2.0        outside      1   365
```

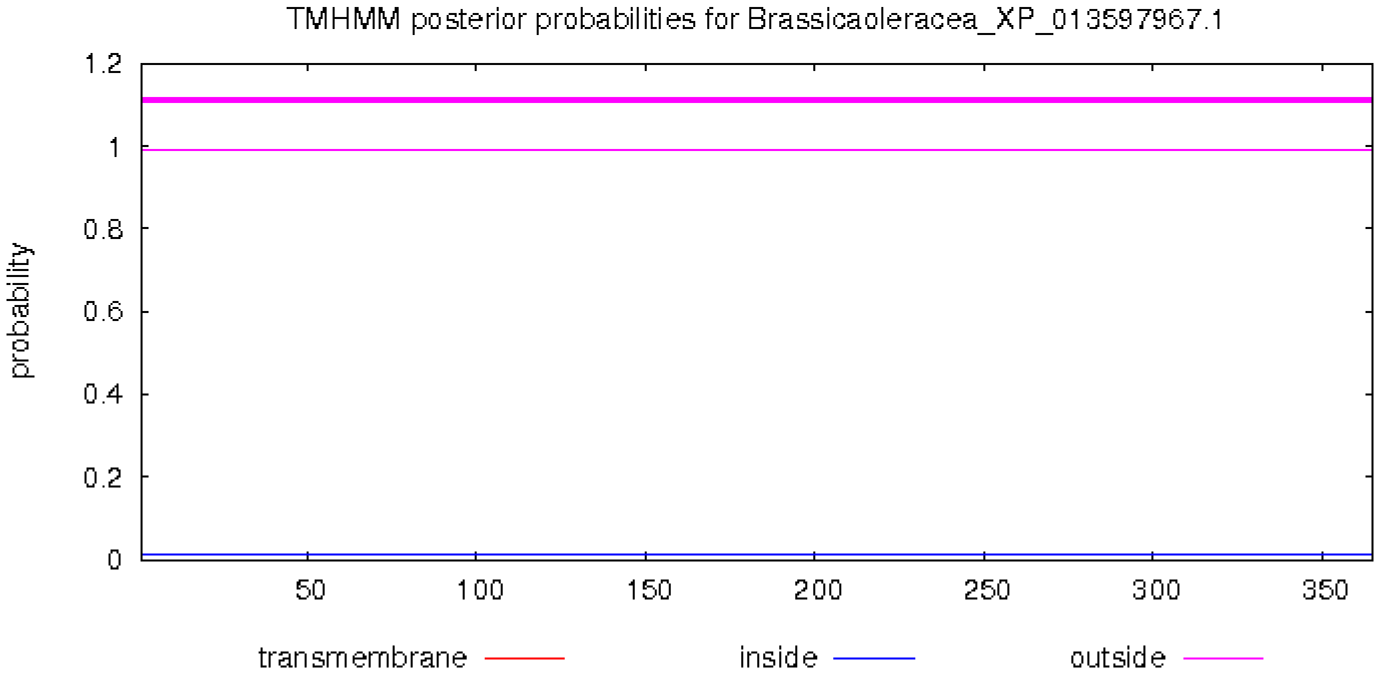

plot in postscript, script for making the plot in gnuplot, data for plot

```
# Brassicaoleracea_XP_013597968.1 Length: 365
# Brassicaoleracea_XP_013597968.1 Number of predicted TMHs:  0
# Brassicaoleracea_XP_013597968.1 Exp number of AAs in TMHs: 0.000830000000000000001
# Brassicaoleracea_XP_013597968.1 Exp number, first 60 AAs:  0.00032
# Brassicaoleracea_XP_013597968.1 Total prob of N-in:        0.01018
Brassicaoleracea_XP_013597968.1 TMHMM2.0        outside      1   365
```

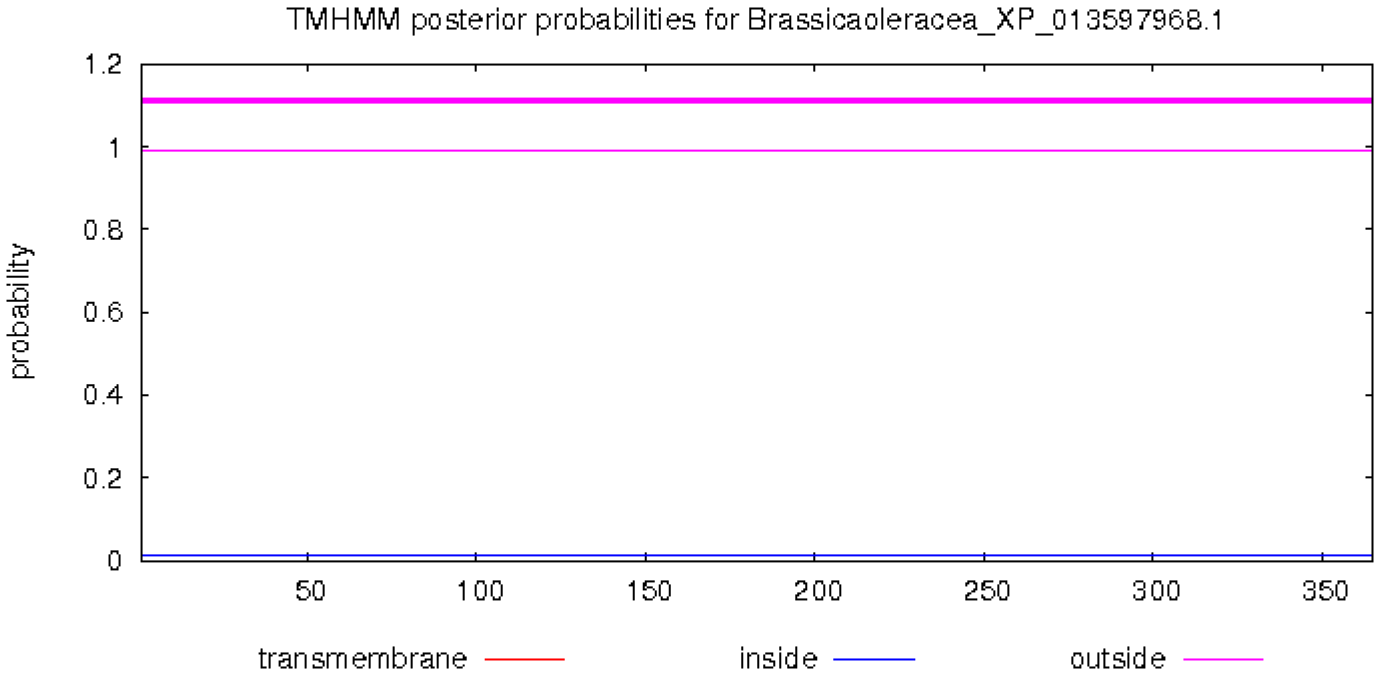

plot in postscript, script for making the plot in gnuplot, data for plot

---

```
# Brassicaoleracea_XP_013597969.1 Length: 365
# Brassicaoleracea_XP_013597969.1 Number of predicted TMHs:  0
# Brassicaoleracea_XP_013597969.1 Exp number of AAs in TMHs: 0.000830000000000000001
# Brassicaoleracea_XP_013597969.1 Exp number, first 60 AAs:  0.00032
# Brassicaoleracea_XP_013597969.1 Total prob of N-in:        0.01018
Brassicaoleracea_XP_013597969.1 TMHMM2.0        outside      1   365
```

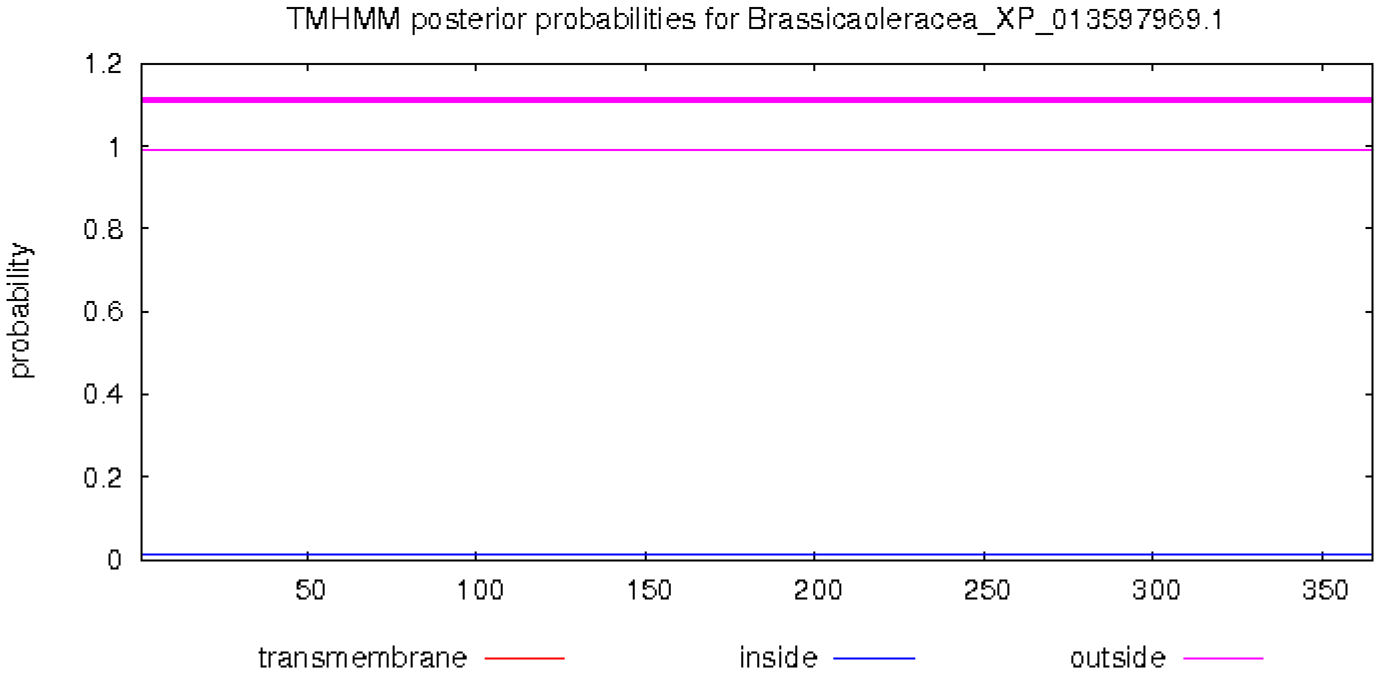

plot in postscript, script for making the plot in gnuplot, data for plot

---

```
# Brassicaoleracea_XP_013597971.1 Length: 365
# Brassicaoleracea_XP_013597971.1 Number of predicted TMHs:  0
# Brassicaoleracea_XP_013597971.1 Exp number of AAs in TMHs: 0.000830000000000000001
# Brassicaoleracea_XP_013597971.1 Exp number, first 60 AAs:  0.00032
# Brassicaoleracea_XP_013597971.1 Total prob of N-in:        0.01018
Brassicaoleracea_XP_013597971.1 TMHMM2.0        outside      1   365
```

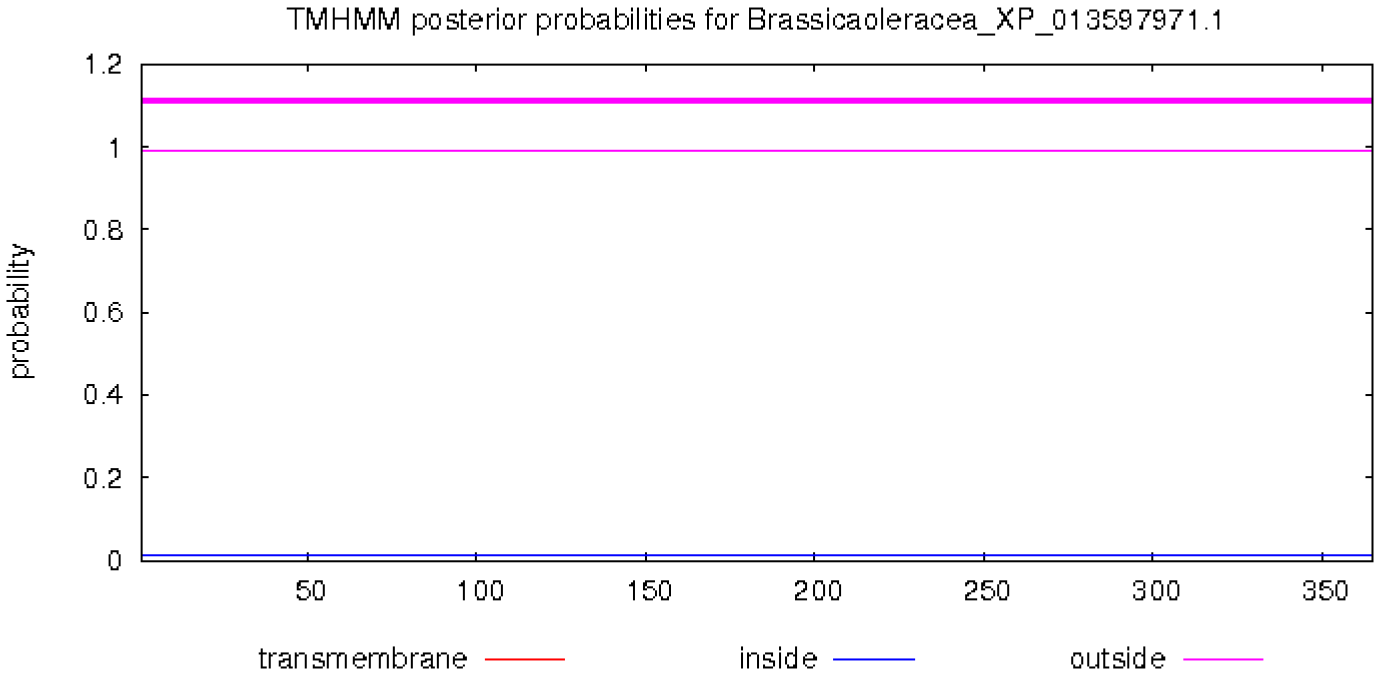

plot in postscript, script for making the plot in gnuplot, data for plot

```
# Brassicaoleracea_XP_013598464.1 Length: 455
# Brassicaoleracea_XP_013598464.1 Number of predicted TMHs:  0
# Brassicaoleracea_XP_013598464.1 Exp number of AAs in TMHs: 13.00706
# Brassicaoleracea_XP_013598464.1 Exp number, first 60 AAs:  0
# Brassicaoleracea_XP_013598464.1 Total prob of N-in:        0.00372
Brassicaoleracea_XP_013598464.1 TMHMM2.0        outside      1   455
```

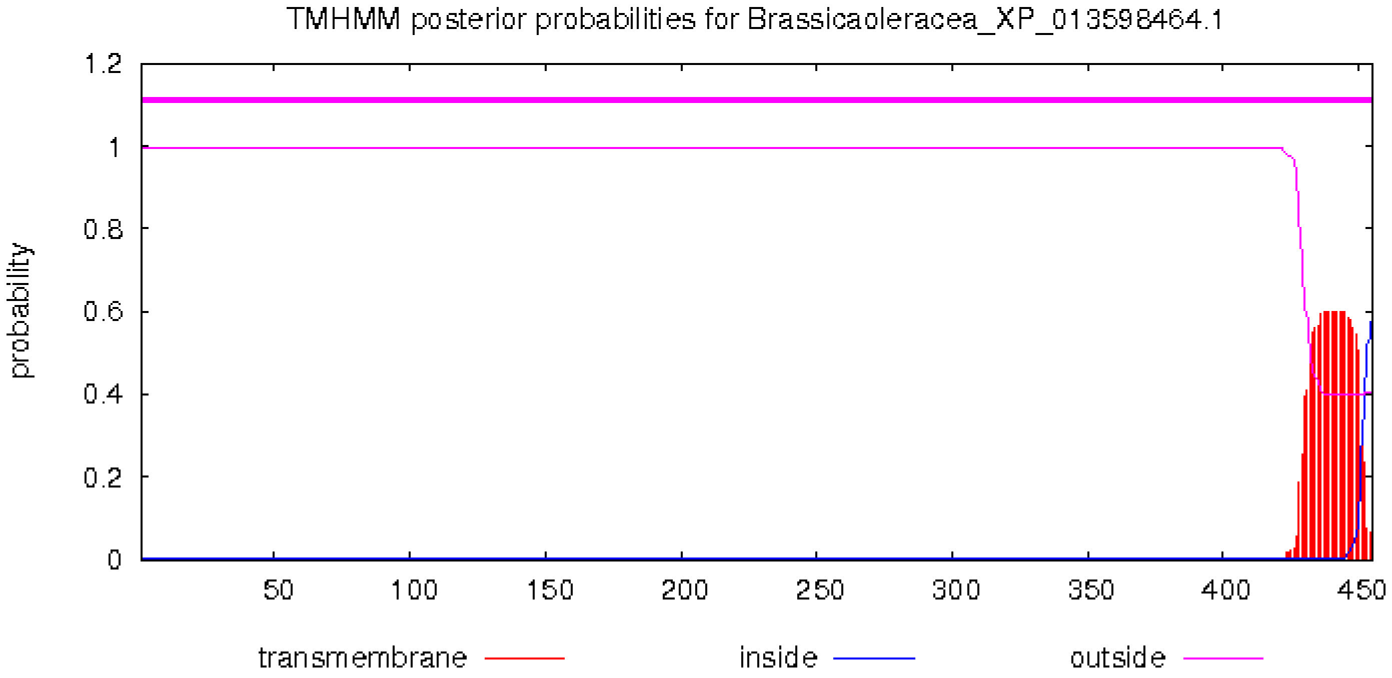

plot in postscript, script for making the plot in gnuplot, data for plot

```
# Brassicaoleracea_XP_013598465.1 Length: 454
# Brassicaoleracea_XP_013598465.1 Number of predicted TMHs:  0
# Brassicaoleracea_XP_013598465.1 Exp number of AAs in TMHs: 13.00743
# Brassicaoleracea_XP_013598465.1 Exp number, first 60 AAs:  0
# Brassicaoleracea_XP_013598465.1 Total prob of N-in:        0.00377
Brassicaoleracea_XP_013598465.1 TMHMM2.0        outside      1   454
```

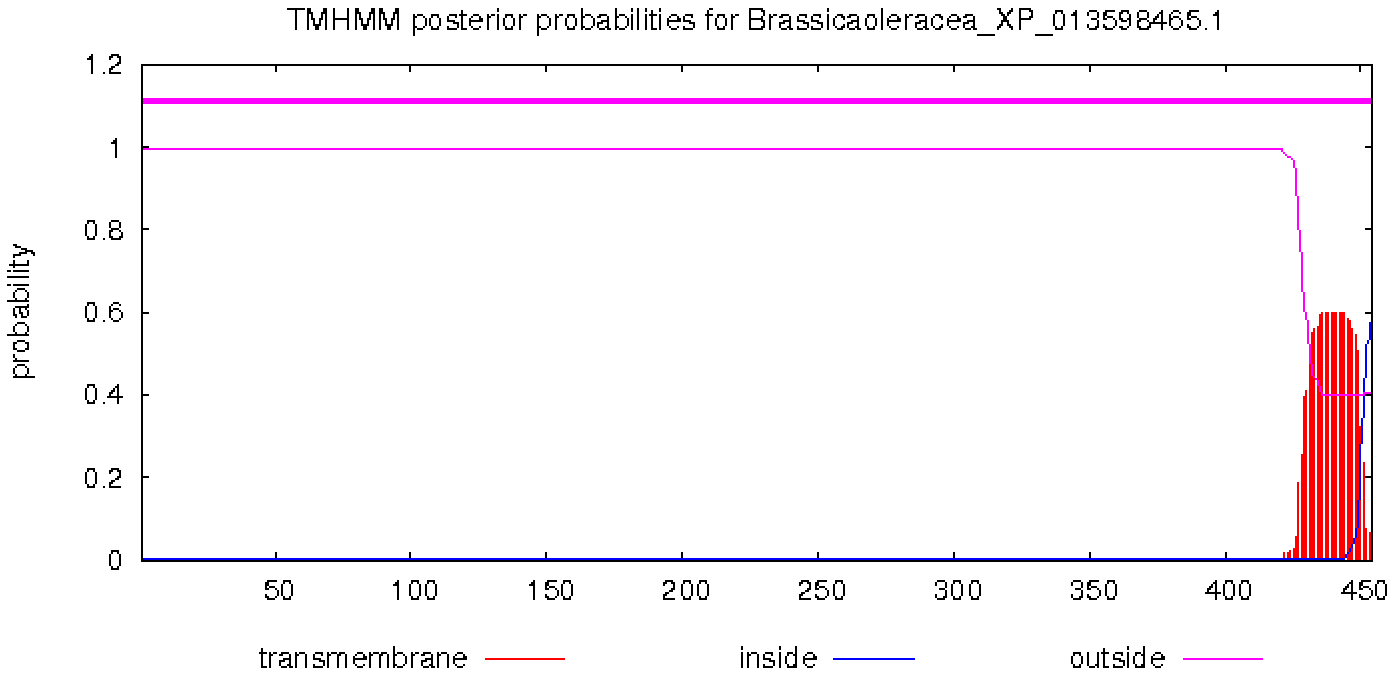

plot in postscript, script for making the plot in gnuplot, data for plot

```
# Brassicaoleracea_XP_013598751.1 Length: 300
# Brassicaoleracea_XP_013598751.1 Number of predicted TMHs:  0
# Brassicaoleracea_XP_013598751.1 Exp number of AAs in TMHs: 0.01028
# Brassicaoleracea_XP_013598751.1 Exp number, first 60 AAs:  0.00032
# Brassicaoleracea_XP_013598751.1 Total prob of N-in:        0.01300
Brassicaoleracea_XP_013598751.1 TMHMM2.0        outside      1   300
```

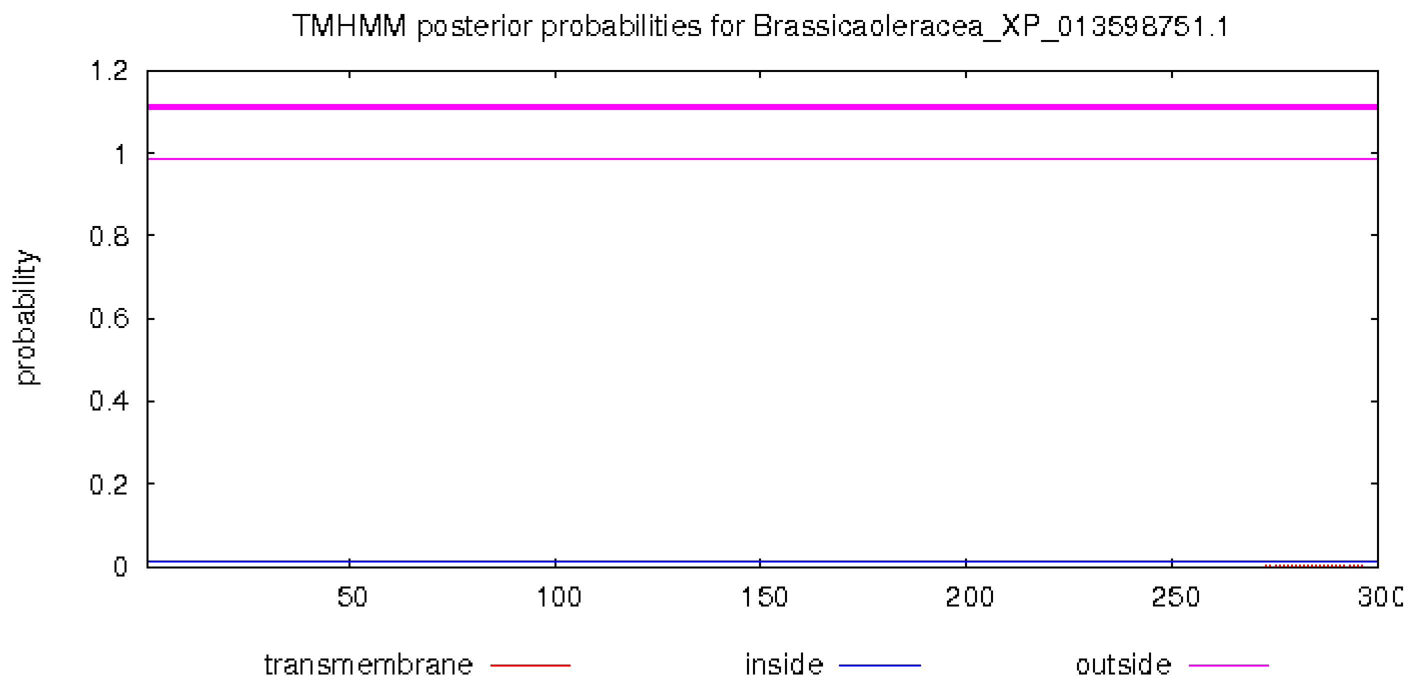

plot in postscript, script for making the plot in gnuplot, data for plot

```
# Brassicaoleracea_XP_013599436.1 Length: 256
# Brassicaoleracea_XP_013599436.1 Number of predicted TMHs:  0
# Brassicaoleracea_XP_013599436.1 Exp number of AAs in TMHs: 0.12716
# Brassicaoleracea_XP_013599436.1 Exp number, first 60 AAs:  0.00038
# Brassicaoleracea_XP_013599436.1 Total prob of N-in:        0.01486
Brassicaoleracea_XP_013599436.1 TMHMM2.0        outside      1   256
```

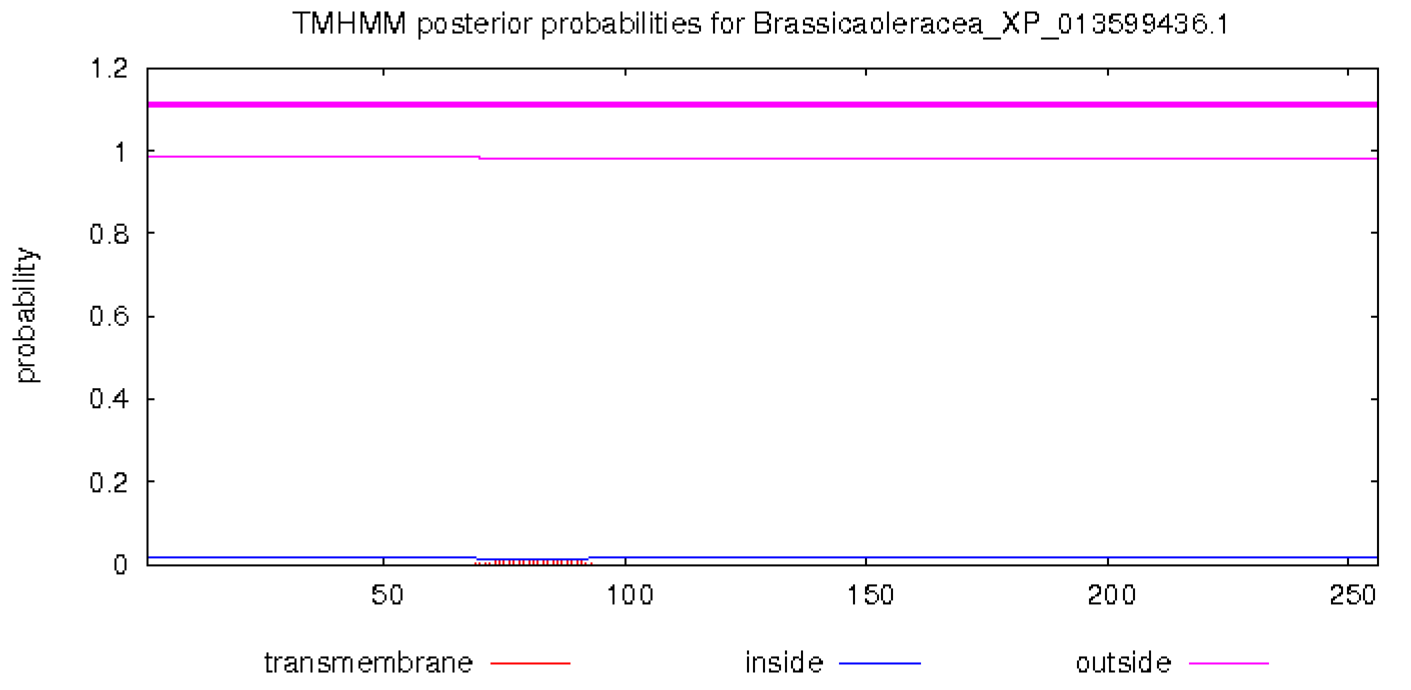

plot in postscript, script for making the plot in gnuplot, data for plot

```
# Brassicaoleracea_XP_013601909.1 Length: 239
# Brassicaoleracea_XP_013601909.1 Number of predicted TMHs:  0
# Brassicaoleracea_XP_013601909.1 Exp number of AAs in TMHs: 0
# Brassicaoleracea_XP_013601909.1 Exp number, first 60 AAs:  0
# Brassicaoleracea_XP_013601909.1 Total prob of N-in:        0.01436
Brassicaoleracea_XP_013601909.1 TMHMM2.0        outside      1   239
```

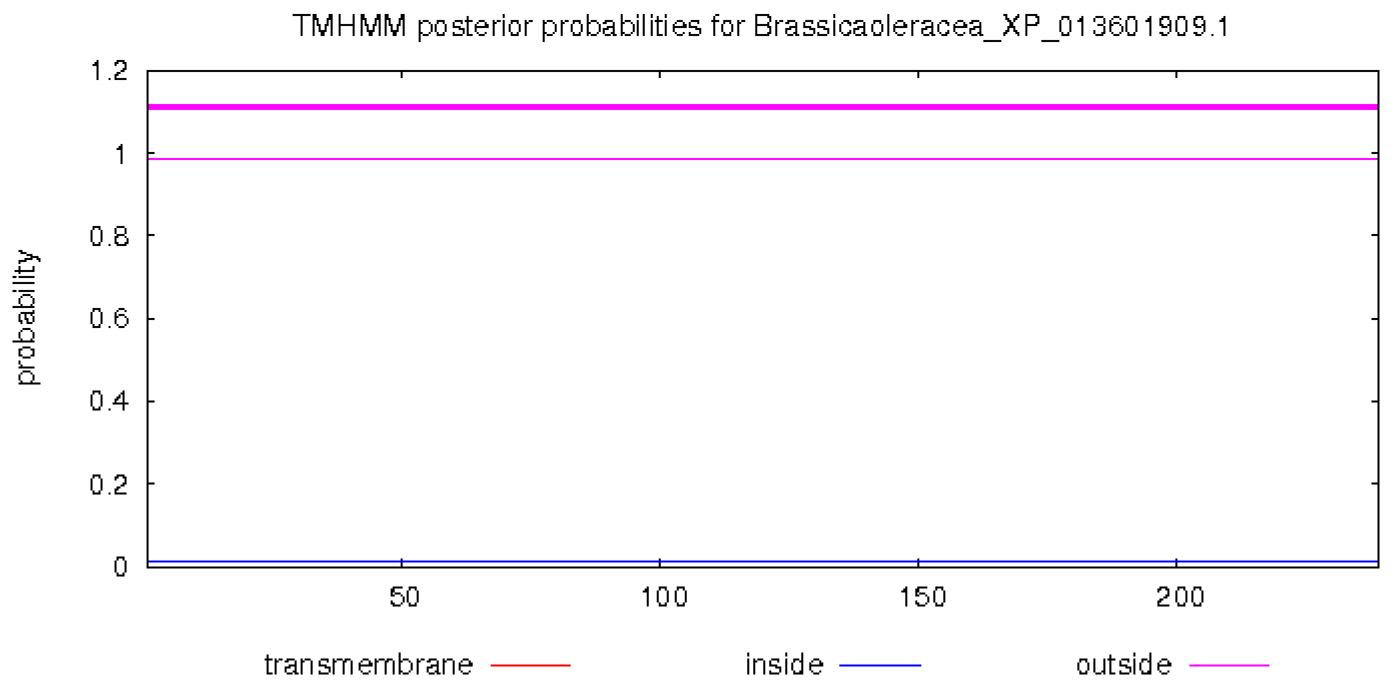

plot in postscript, script for making the plot in gnuplot, data for plot

```
# Brassicaoleracea_XP_013601975.1 Length: 358
# Brassicaoleracea_XP_013601975.1 Number of predicted TMHs:  0
# Brassicaoleracea_XP_013601975.1 Exp number of AAs in TMHs: 0.00152
# Brassicaoleracea_XP_013601975.1 Exp number, first 60 AAs:  0
# Brassicaoleracea_XP_013601975.1 Total prob of N-in:        0.01697
Brassicaoleracea_XP_013601975.1 TMHMM2.0        outside      1   358
```

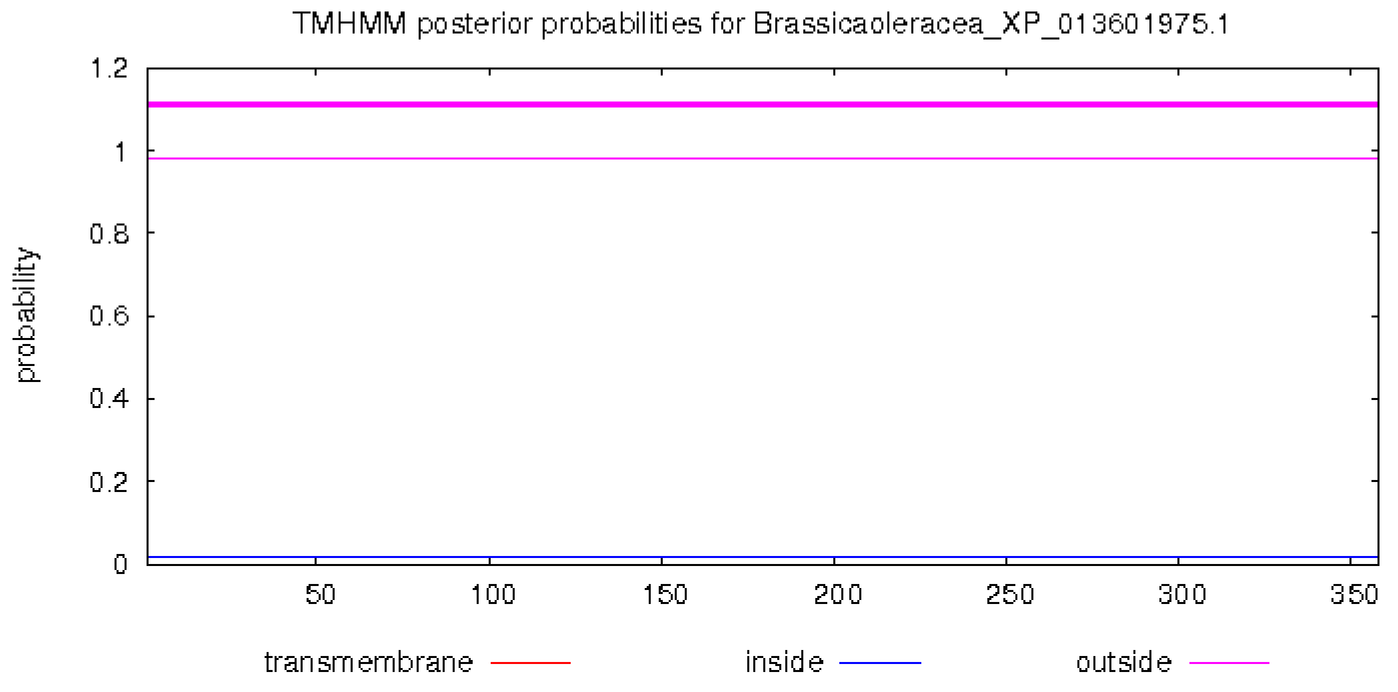

plot in postscript, script for making the plot in gnuplot, data for plot

```
# Brassicaoleracea_XP_013602769.1 Length: 388
# Brassicaoleracea_XP_013602769.1 Number of predicted TMHs:  0
# Brassicaoleracea_XP_013602769.1 Exp number of AAs in TMHs: 0.00018
# Brassicaoleracea_XP_013602769.1 Exp number, first 60 AAs:  0
# Brassicaoleracea_XP_013602769.1 Total prob of N-in:        0.00182
Brassicaoleracea_XP_013602769.1 TMHMM2.0        outside      1   388
```

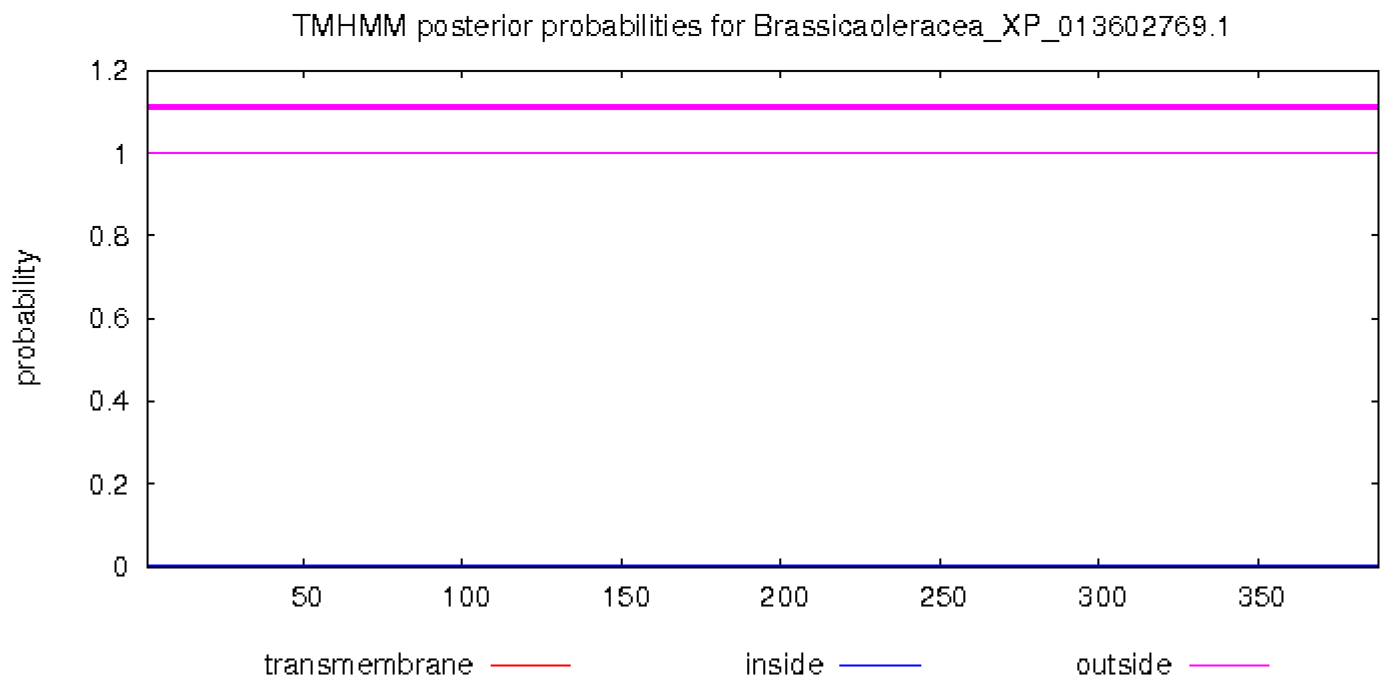

plot in postscript, script for making the plot in gnuplot, data for plot

```
# Brassicaoleracea_XP_013602771.1 Length: 340
# Brassicaoleracea_XP_013602771.1 Number of predicted TMHs:  0
# Brassicaoleracea_XP_013602771.1 Exp number of AAs in TMHs: 0.00018
# Brassicaoleracea_XP_013602771.1 Exp number, first 60 AAs:  0
# Brassicaoleracea_XP_013602771.1 Total prob of N-in:        0.00330
Brassicaoleracea_XP_013602771.1 TMHMM2.0        outside      1   340
```

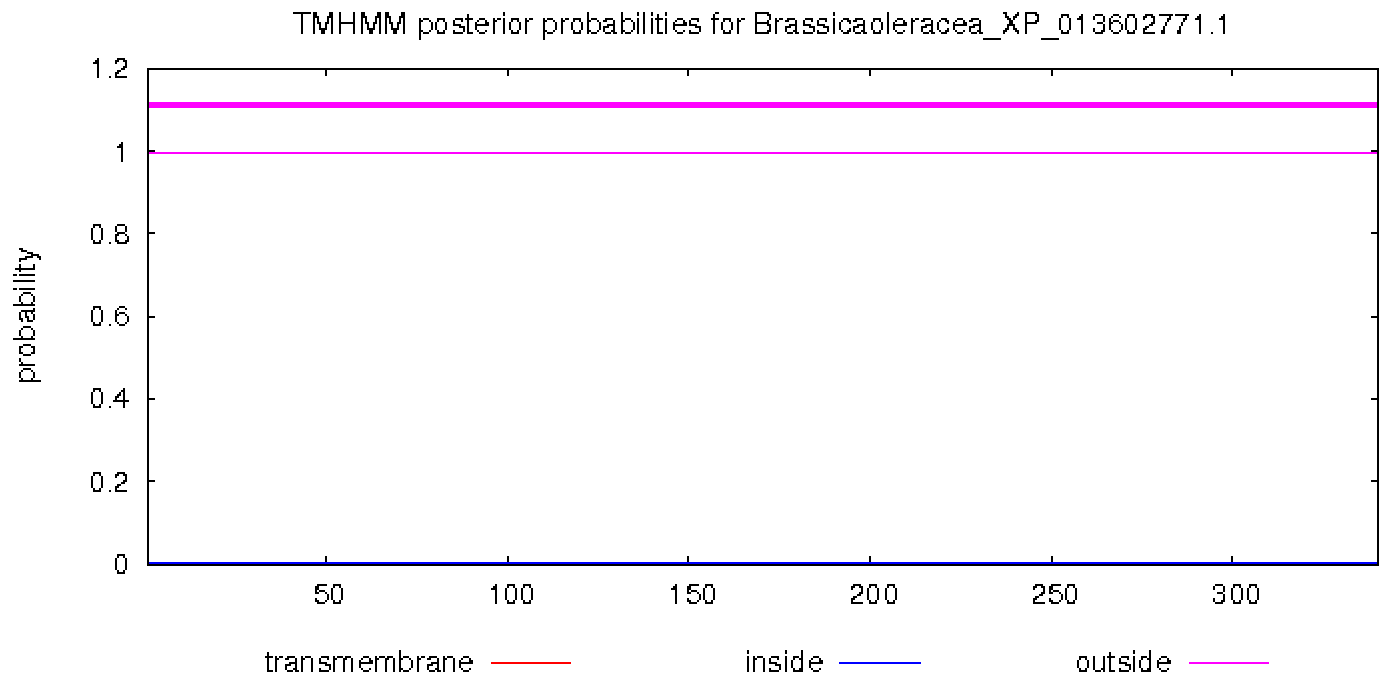

plot in postscript, script for making the plot in gnuplot, data for plot

```
# Brassicaoleracea_XP_013602772.1 Length: 339
# Brassicaoleracea_XP_013602772.1 Number of predicted TMHs:  0
# Brassicaoleracea_XP_013602772.1 Exp number of AAs in TMHs: 0.00018
# Brassicaoleracea_XP_013602772.1 Exp number, first 60 AAs:  0
# Brassicaoleracea_XP_013602772.1 Total prob of N-in:        0.00335
Brassicaoleracea_XP_013602772.1 TMHMM2.0        outside      1   339
```

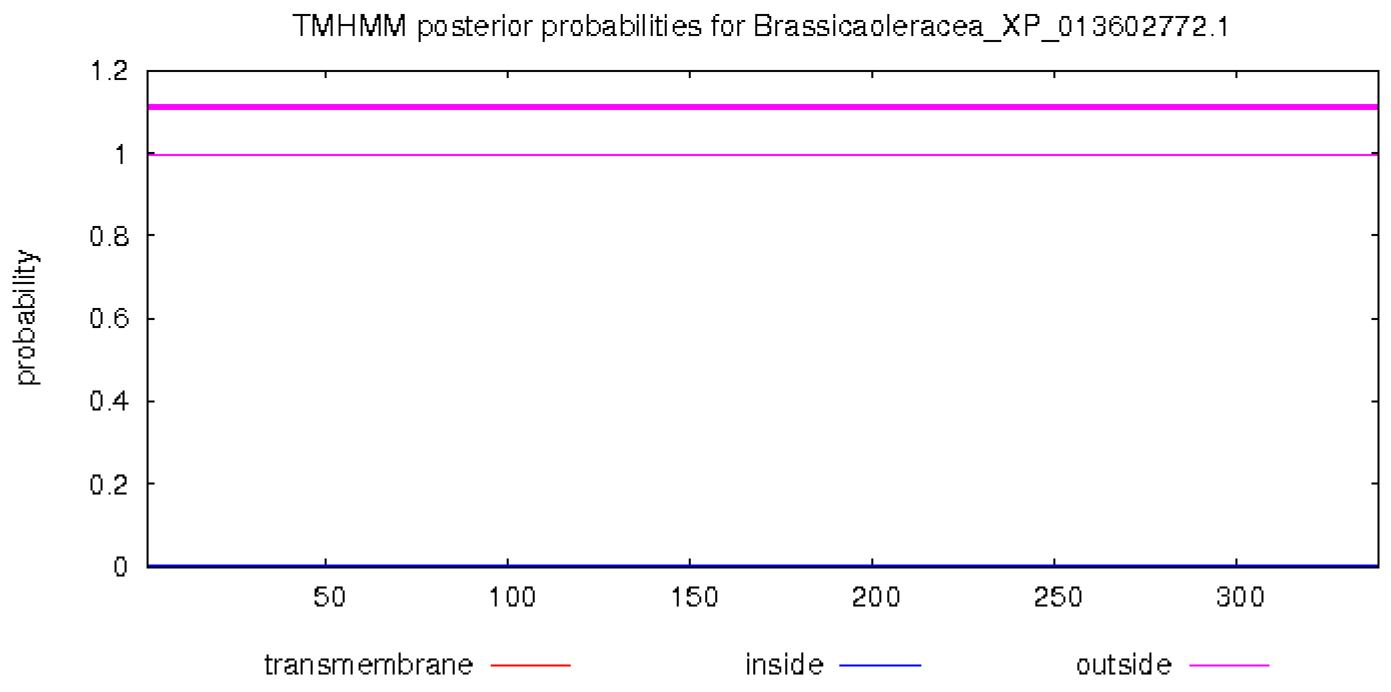

plot in postscript, script for making the plot in gnuplot, data for plot

```
# Brassicaoleracea_XP_013602773.1 Length: 388
# Brassicaoleracea_XP_013602773.1 Number of predicted TMHs:  0
# Brassicaoleracea_XP_013602773.1 Exp number of AAs in TMHs: 0.00018
# Brassicaoleracea_XP_013602773.1 Exp number, first 60 AAs:  0
# Brassicaoleracea_XP_013602773.1 Total prob of N-in:        0.00182
Brassicaoleracea_XP_013602773.1 TMHMM2.0        outside      1   388
```

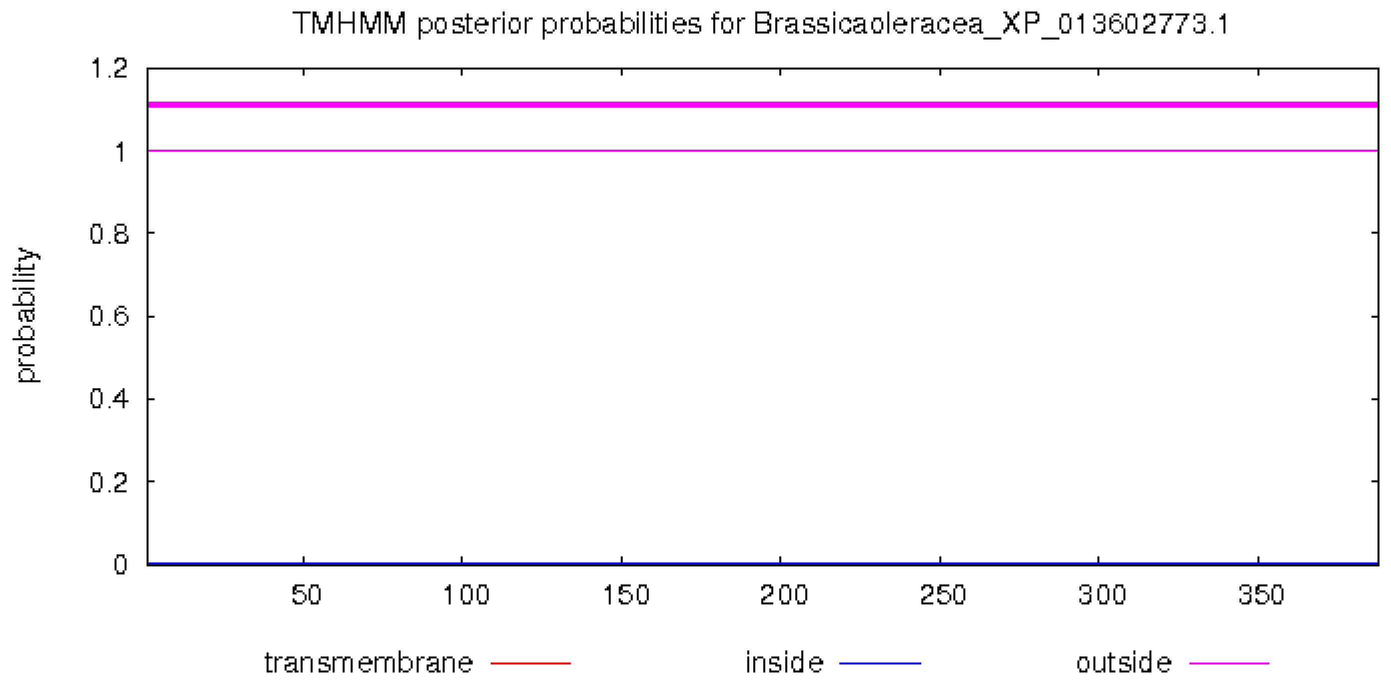

plot in postscript, script for making the plot in gnuplot, data for plot

```
# Brassicaoleracea_XP_013603036.1 Length: 317
# Brassicaoleracea_XP_013603036.1 Number of predicted TMHs:  0
# Brassicaoleracea_XP_013603036.1 Exp number of AAs in TMHs: 0.00638
# Brassicaoleracea_XP_013603036.1 Exp number, first 60 AAs:  0
# Brassicaoleracea_XP_013603036.1 Total prob of N-in:        0.07271
Brassicaoleracea_XP_013603036.1 TMHMM2.0        outside      1   317
```

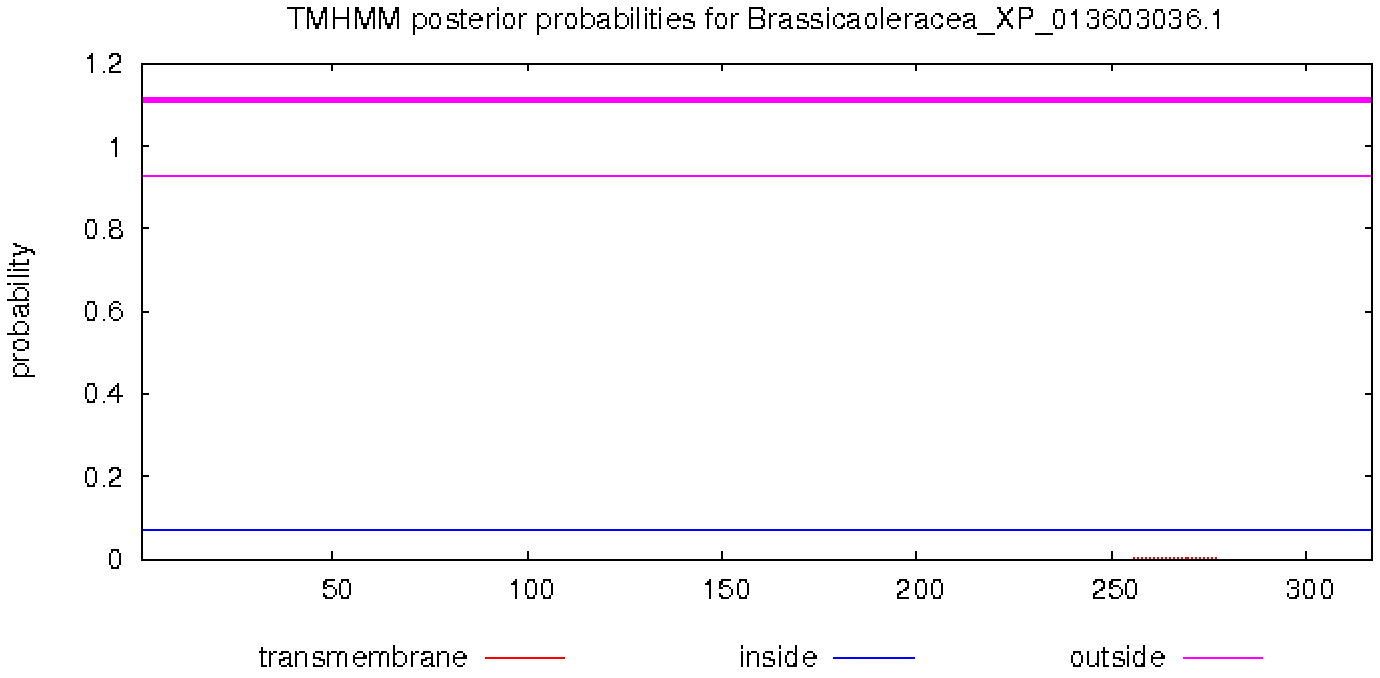

plot in postscript, script for making the plot in gnuplot, data for plot

```
# Brassicaoleracea_XP_013603126.1 Length: 296
# Brassicaoleracea_XP_013603126.1 Number of predicted TMHs:  0
# Brassicaoleracea_XP_013603126.1 Exp number of AAs in TMHs: 0.00025
# Brassicaoleracea_XP_013603126.1 Exp number, first 60 AAs:  0
# Brassicaoleracea_XP_013603126.1 Total prob of N-in:        0.00692
Brassicaoleracea_XP_013603126.1 TMHMM2.0        outside      1   296
```

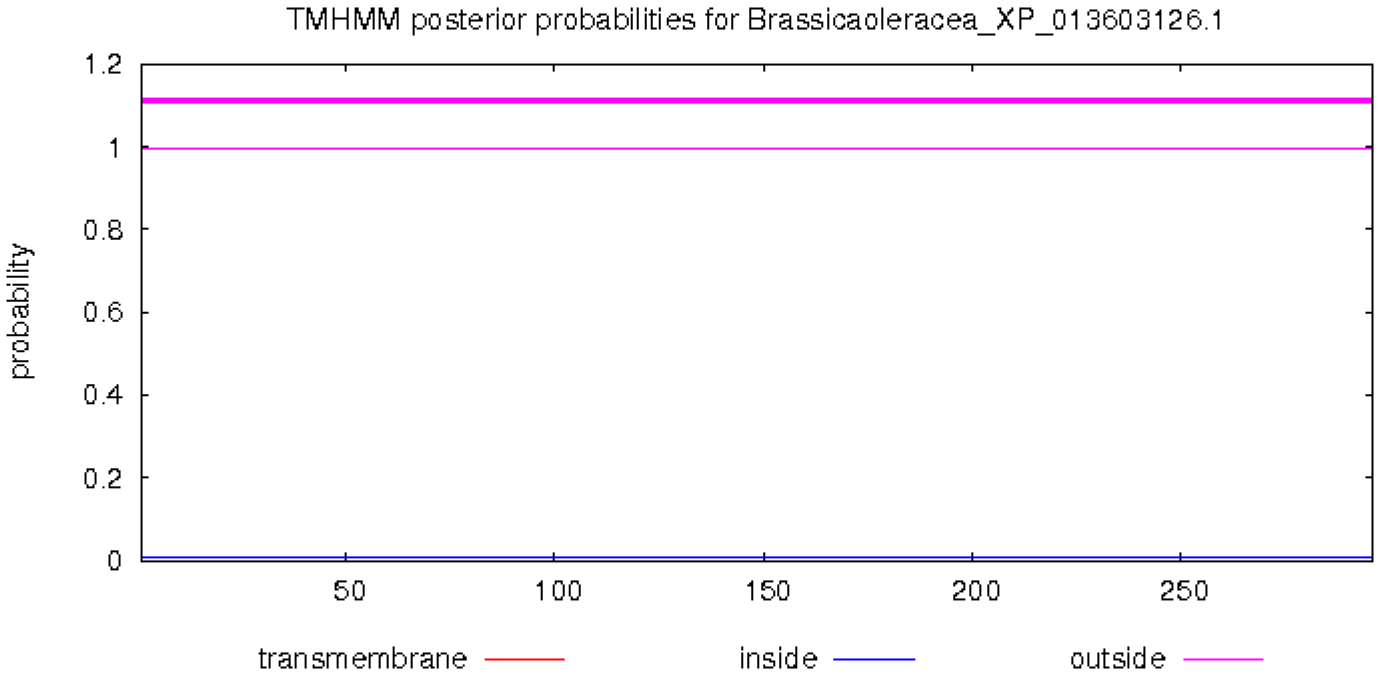

plot in postscript, script for making the plot in gnuplot, data for plot

```
# Brassicaoleracea_XP_013603127.1 Length: 292
# Brassicaoleracea_XP_013603127.1 Number of predicted TMHs:  0
# Brassicaoleracea_XP_013603127.1 Exp number of AAs in TMHs: 0.00025
# Brassicaoleracea_XP_013603127.1 Exp number, first 60 AAs:  0
# Brassicaoleracea_XP_013603127.1 Total prob of N-in:        0.00572
Brassicaoleracea_XP_013603127.1 TMHMM2.0        outside      1   292
```

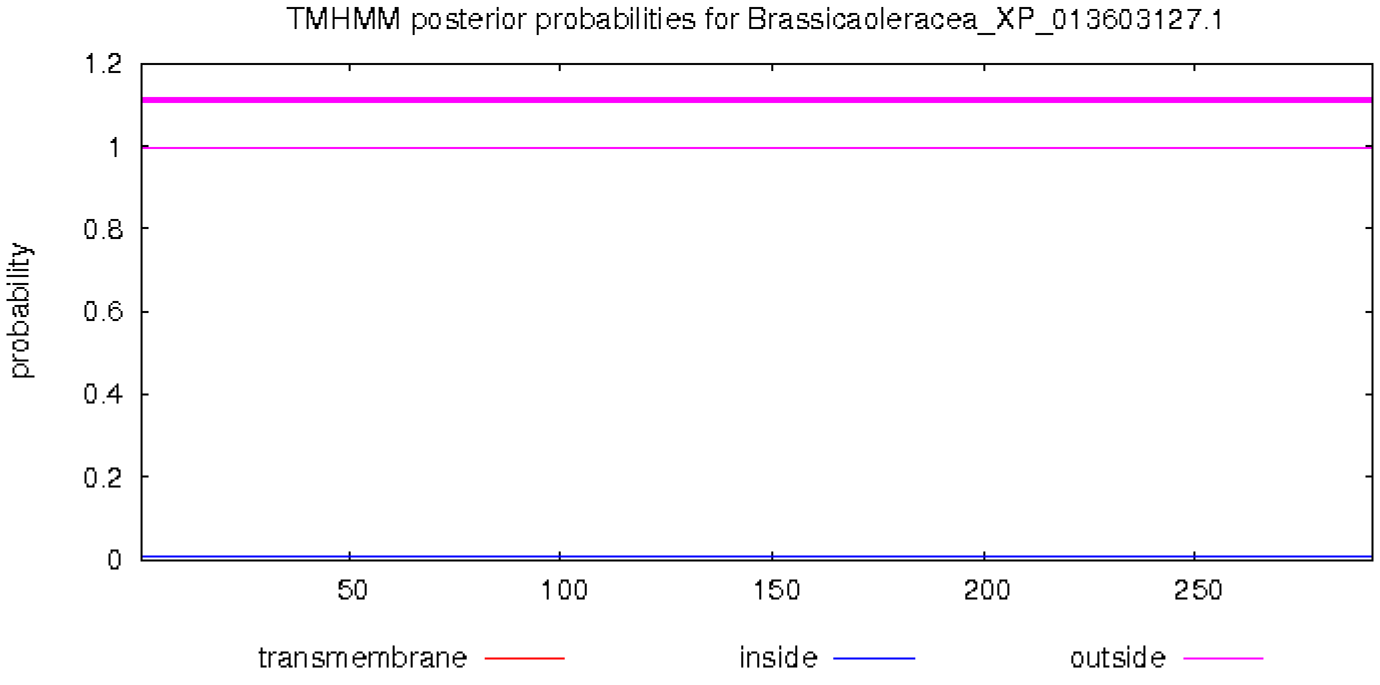

plot in postscript, script for making the plot in gnuplot, data for plot

```
# Brassicaoleracea_XP_013603484.1 Length: 323
# Brassicaoleracea_XP_013603484.1 Number of predicted TMHs:  0
# Brassicaoleracea_XP_013603484.1 Exp number of AAs in TMHs: 0.00036
# Brassicaoleracea_XP_013603484.1 Exp number, first 60 AAs:  0
# Brassicaoleracea_XP_013603484.1 Total prob of N-in:        0.03309
Brassicaoleracea_XP_013603484.1 TMHMM2.0        outside      1   323
```

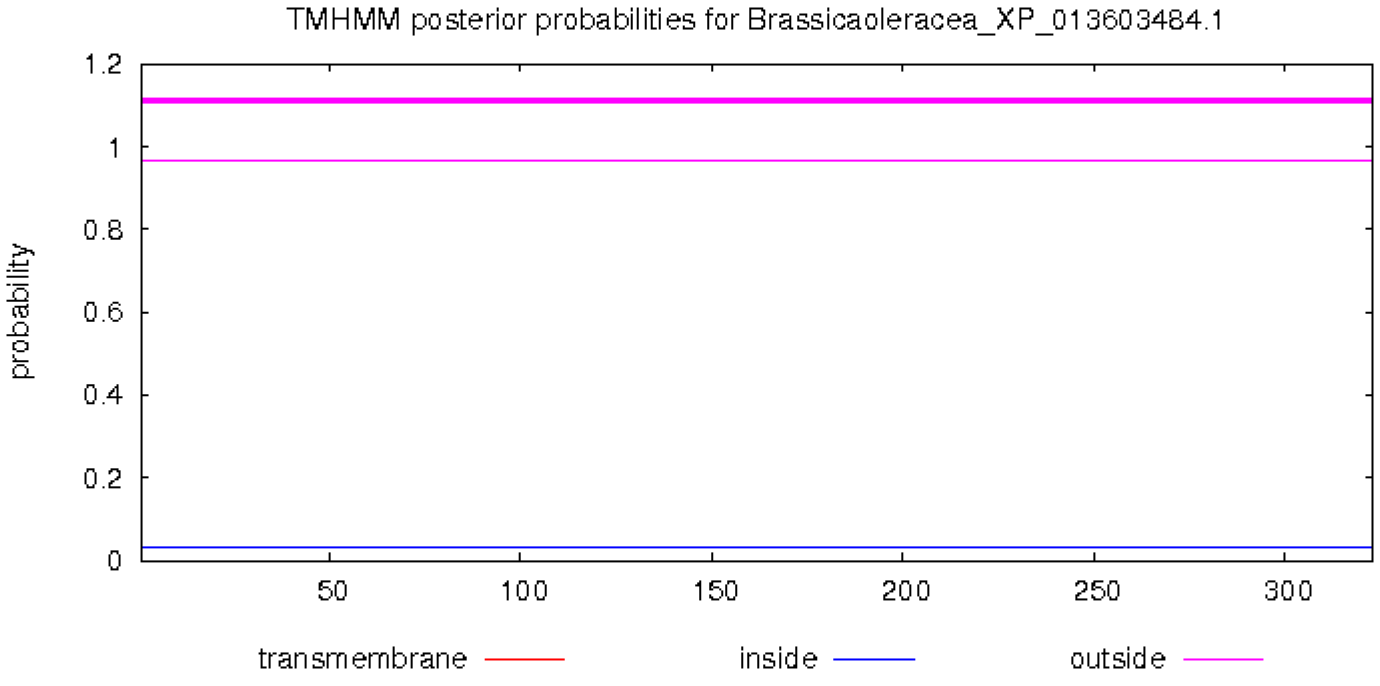

plot in postscript, script for making the plot in gnuplot, data for plot

```
# Brassicaoleracea_XP_013603495.1 Length: 347
# Brassicaoleracea_XP_013603495.1 Number of predicted TMHs:  0
# Brassicaoleracea_XP_013603495.1 Exp number of AAs in TMHs: 0
# Brassicaoleracea_XP_013603495.1 Exp number, first 60 AAs:  0
# Brassicaoleracea_XP_013603495.1 Total prob of N-in:        0.00504
Brassicaoleracea_XP_013603495.1 TMHMM2.0        outside      1   347
```

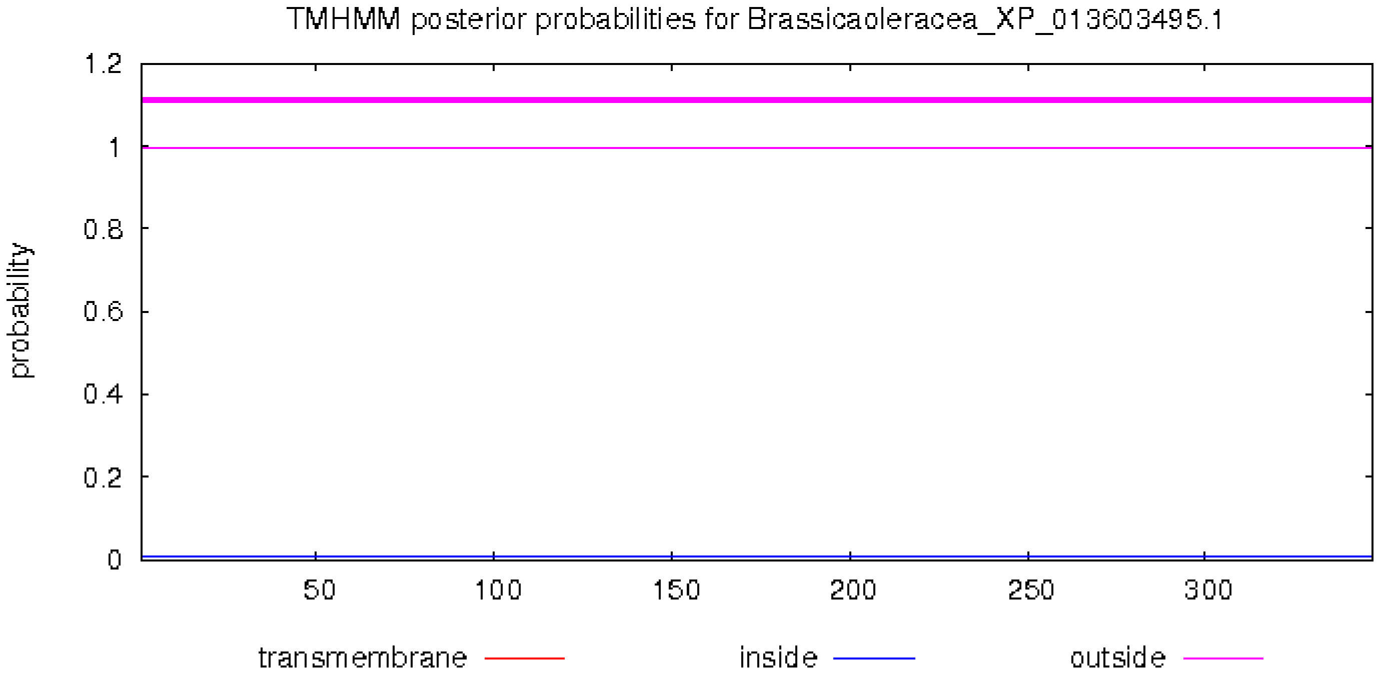

plot in postscript, script for making the plot in gnuplot, data for plot

```
# Brassicaoleracea_XP_013603918.1 Length: 254
# Brassicaoleracea_XP_013603918.1 Number of predicted TMHs:  0
# Brassicaoleracea_XP_013603918.1 Exp number of AAs in TMHs: 0
# Brassicaoleracea_XP_013603918.1 Exp number, first 60 AAs:  0
# Brassicaoleracea_XP_013603918.1 Total prob of N-in:        0.02452
Brassicaoleracea_XP_013603918.1 TMHMM2.0        outside      1   254
```

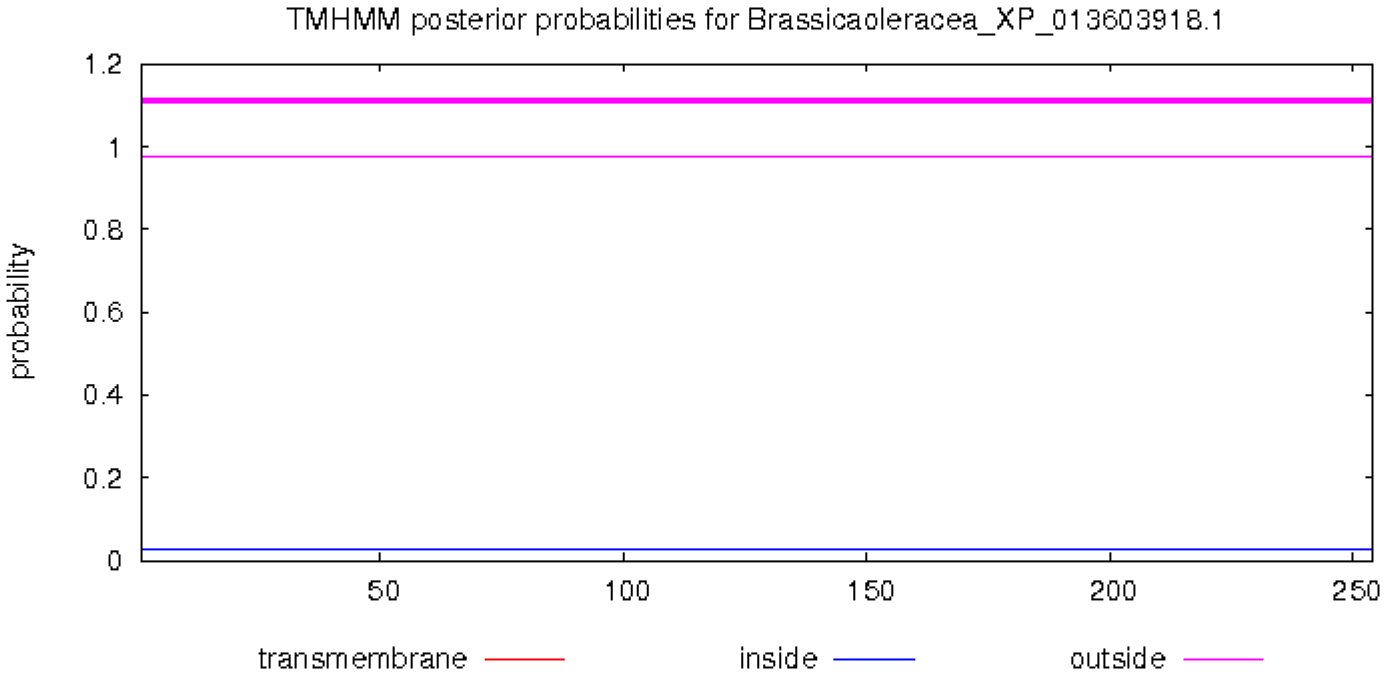

plot in postscript, script for making the plot in gnuplot, data for plot

```
# Brassicaoleracea_XP_013603948.1 Length: 403
# Brassicaoleracea_XP_013603948.1 Number of predicted TMHs:  0
# Brassicaoleracea_XP_013603948.1 Exp number of AAs in TMHs: 0
# Brassicaoleracea_XP_013603948.1 Exp number, first 60 AAs:  0
# Brassicaoleracea_XP_013603948.1 Total prob of N-in:        0.00565
Brassicaoleracea_XP_013603948.1 TMHMM2.0        outside      1   403
```

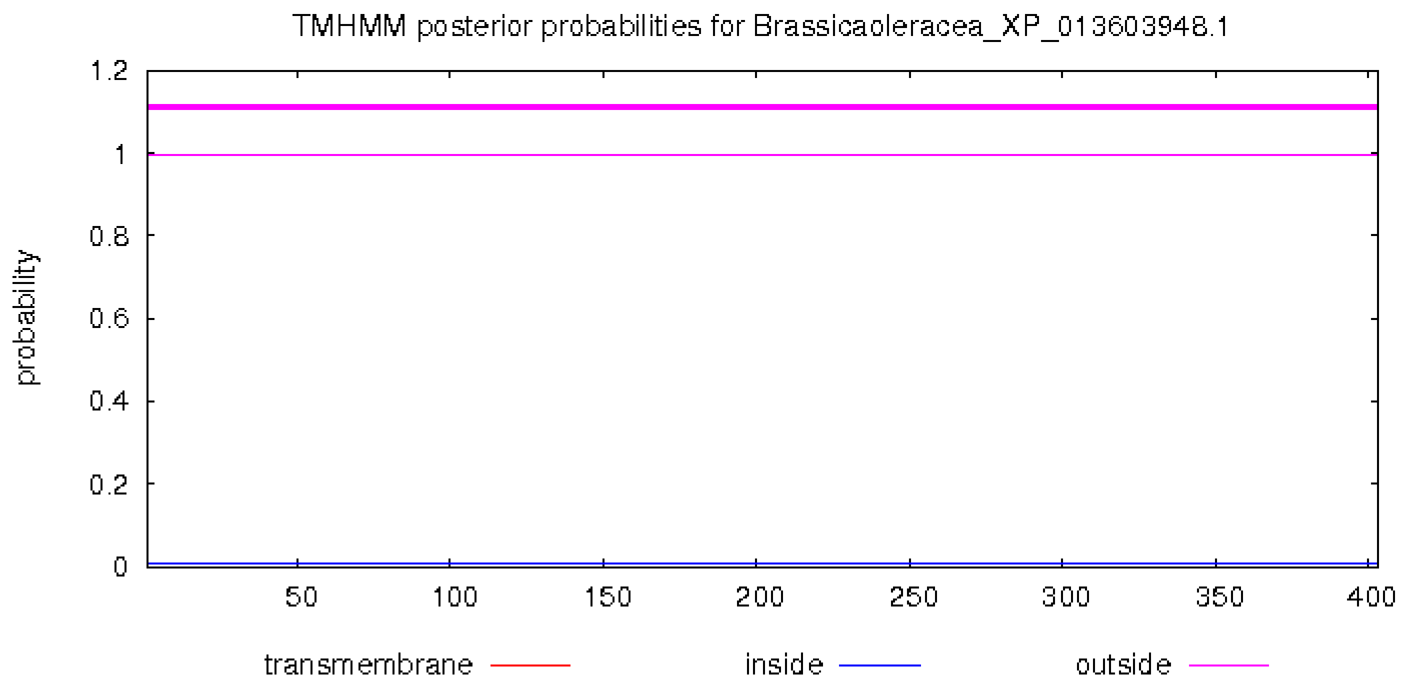

plot in postscript, script for making the plot in gnuplot, data for plot

```
# Brassicaoleracea_XP_013604293.1 Length: 539
# Brassicaoleracea_XP_013604293.1 Number of predicted TMHs:  1
# Brassicaoleracea_XP_013604293.1 Exp number of AAs in TMHs: 23.18037
# Brassicaoleracea_XP_013604293.1 Exp number, first 60 AAs:  0.00385
# Brassicaoleracea_XP_013604293.1 Total prob of N-in:        0.80584
Brassicaoleracea_XP_013604293.1 TMHMM2.0        inside       1   508
Brassicaoleracea_XP_013604293.1 TMHMM2.0        TMhelix    509   531
Brassicaoleracea_XP_013604293.1 TMHMM2.0        outside    532   539
```

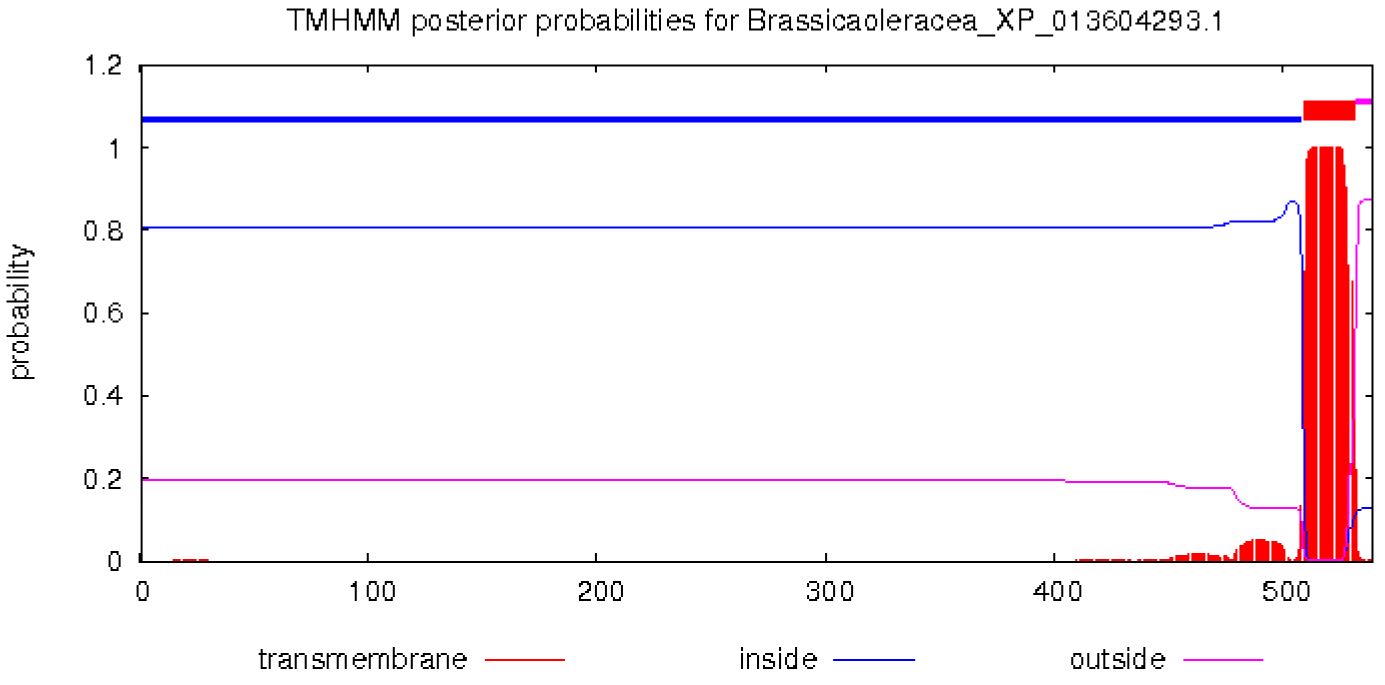

plot in postscript, script for making the plot in gnuplot, data for plot

---

```
# Brassicaoleracea_XP_013605626.1 Length: 463
# Brassicaoleracea_XP_013605626.1 Number of predicted TMHs:  0
# Brassicaoleracea_XP_013605626.1 Exp number of AAs in TMHs: 17.40186
# Brassicaoleracea_XP_013605626.1 Exp number, first 60 AAs:  0.00035
# Brassicaoleracea_XP_013605626.1 Total prob of N-in:        0.13160
Brassicaoleracea_XP_013605626.1 TMHMM2.0        outside      1   463
```

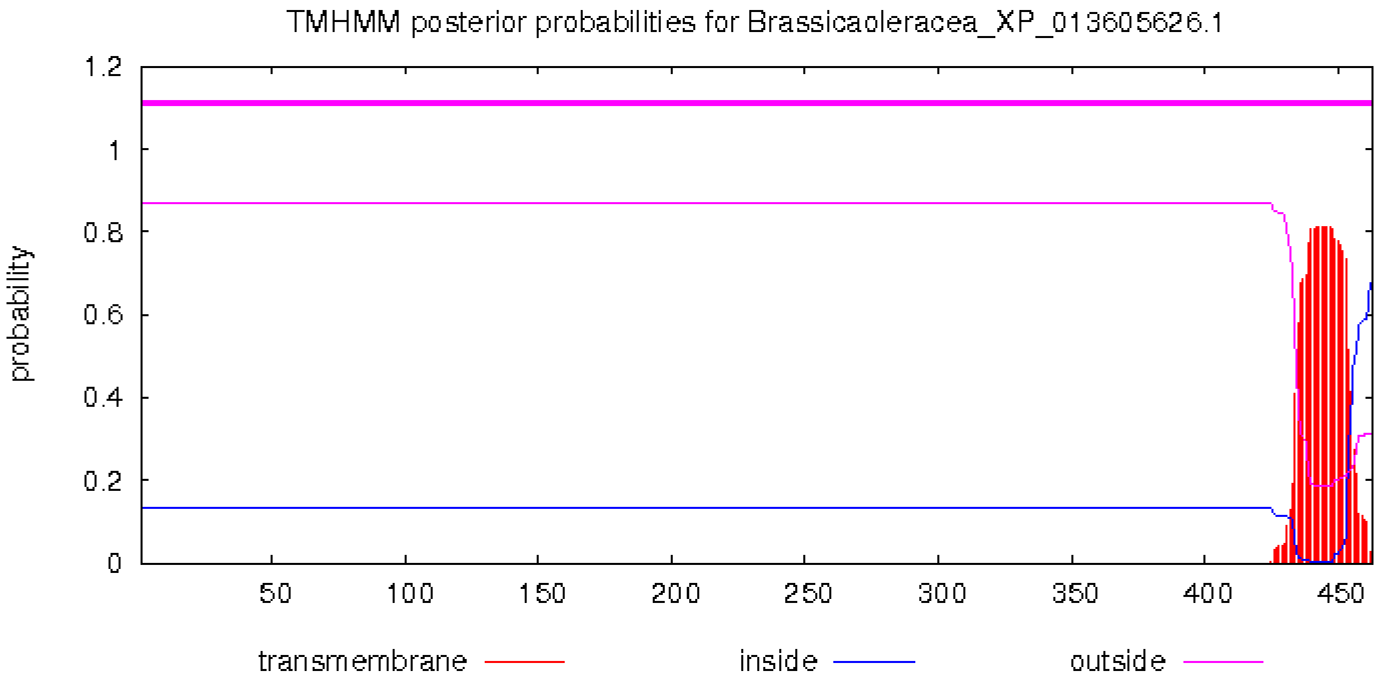

plot in postscript, script for making the plot in gnuplot, data for plot

---

```
# Brassicaoleracea_XP_013605970.1 Length: 426
# Brassicaoleracea_XP_013605970.1 Number of predicted TMHs:  1
# Brassicaoleracea_XP_013605970.1 Exp number of AAs in TMHs: 21.98136
# Brassicaoleracea_XP_013605970.1 Exp number, first 60 AAs:  0.00054
# Brassicaoleracea_XP_013605970.1 Total prob of N-in:        0.45378
Brassicaoleracea_XP_013605970.1 TMHMM2.0        outside      1   401
```

```
Brassicaoleracea_XP_013605970.1 TMHMM2.0        TMhelix    402   424
Brassicaoleracea_XP_013605970.1 TMHMM2.0        inside     425   426
```

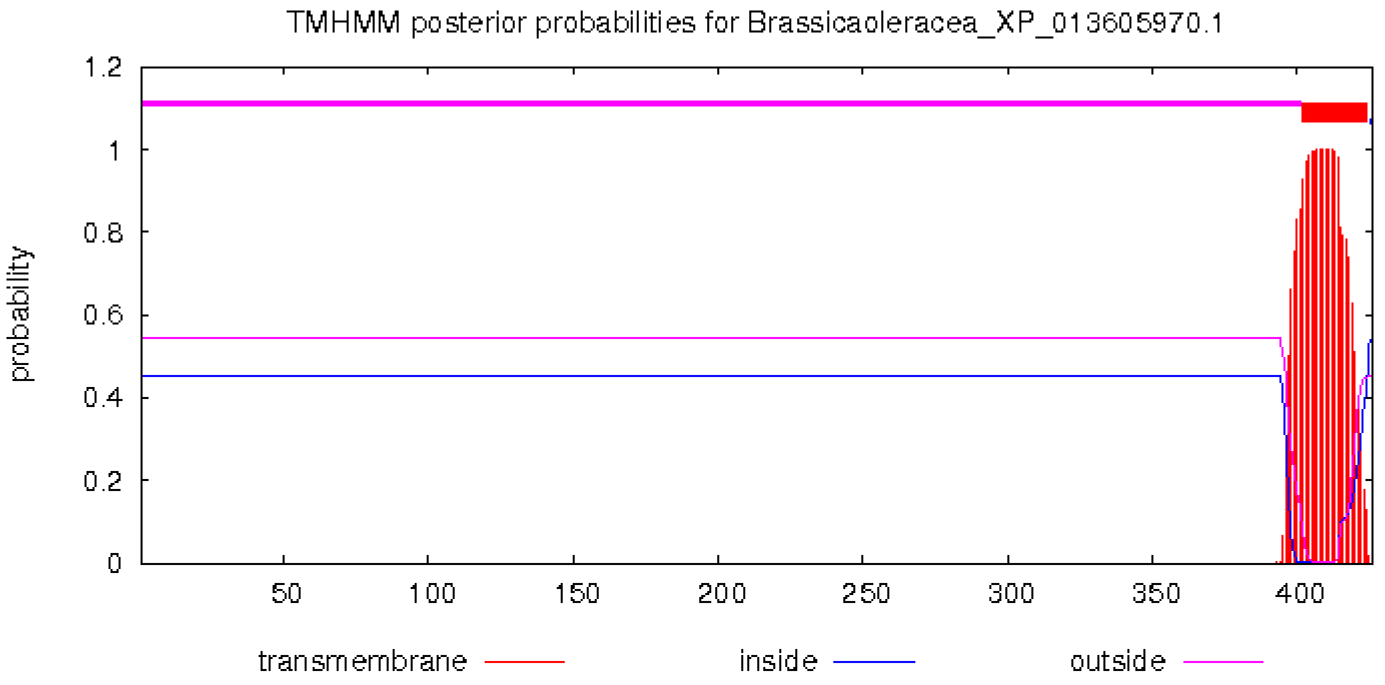

plot in postscript, script for making the plot in gnuplot, data for plot

---

```
# Brassicaoleracea_XP_013605971.1 Length: 420
# Brassicaoleracea_XP_013605971.1 Number of predicted TMHs:  1
# Brassicaoleracea_XP_013605971.1 Exp number of AAs in TMHs: 21.98097
# Brassicaoleracea_XP_013605971.1 Exp number, first 60 AAs:  0.00068
# Brassicaoleracea_XP_013605971.1 Total prob of N-in:        0.47239
Brassicaoleracea_XP_013605971.1 TMHMM2.0        inside       1   391
Brassicaoleracea_XP_013605971.1 TMHMM2.0        TMhelix    392   414
Brassicaoleracea_XP_013605971.1 TMHMM2.0        outside    415   420
```

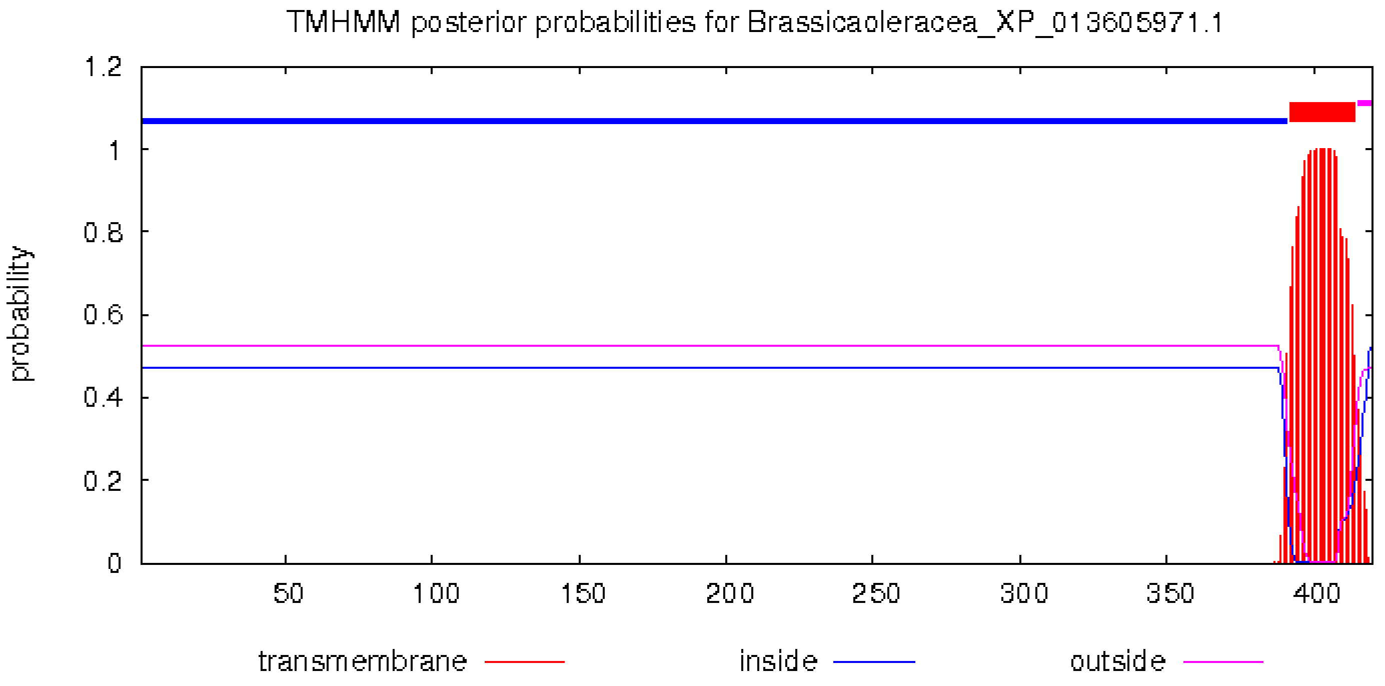

plot in postscript, script for making the plot in gnuplot, data for plot

---

```
# Brassicaoleracea_XP_013606555.1 Length: 435
# Brassicaoleracea_XP_013606555.1 Number of predicted TMHs:  1
```

```
# Brassicaoleracea_XP_013606555.1 Exp number of AAs in TMHs: 22.86722
# Brassicaoleracea_XP_013606555.1 Exp number, first 60 AAs:  0.00036
# Brassicaoleracea_XP_013606555.1 Total prob of N-in:        0.03964
Brassicaoleracea_XP_013606555.1 TMHMM2.0        outside      1   405
Brassicaoleracea_XP_013606555.1 TMHMM2.0        TMhelix    406   428
Brassicaoleracea_XP_013606555.1 TMHMM2.0        inside     429   435
```

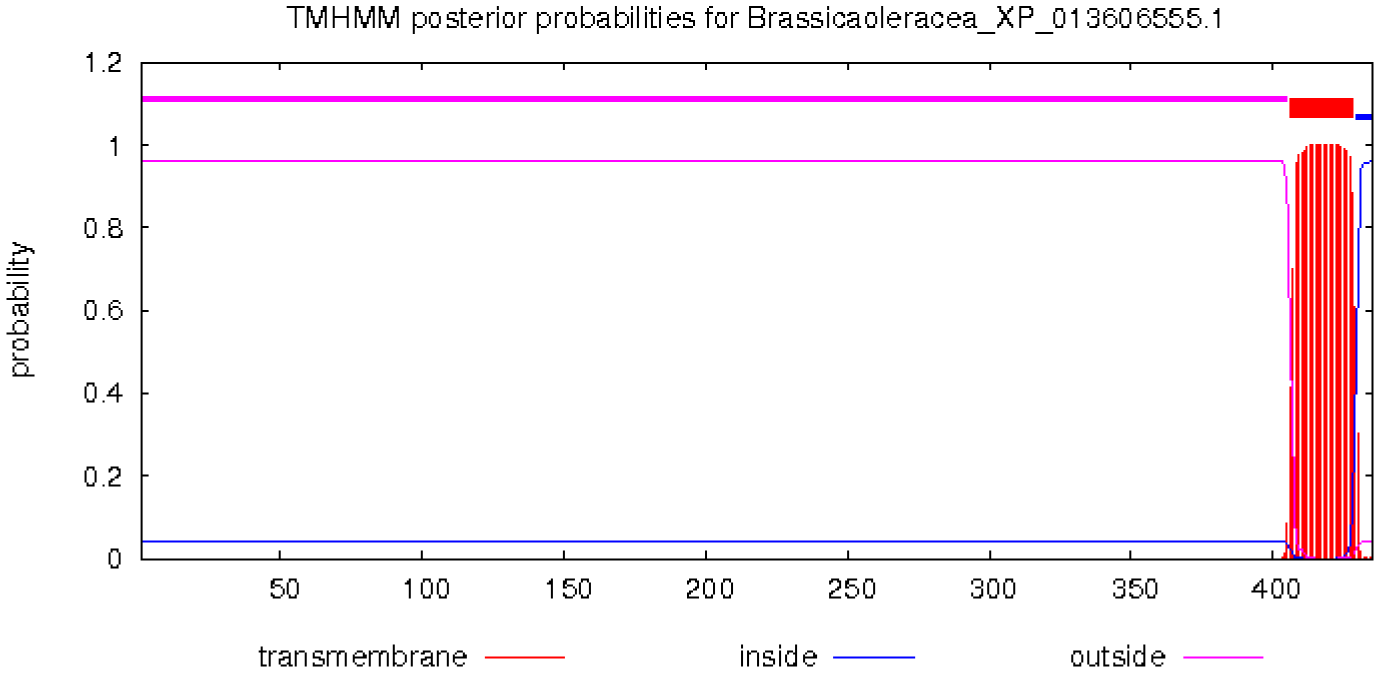

plot in postscript, script for making the plot in gnuplot, data for plot

---

```
# Brassicaoleracea_XP_013606976.1 Length: 367
# Brassicaoleracea_XP_013606976.1 Number of predicted TMHs:  0
# Brassicaoleracea_XP_013606976.1 Exp number of AAs in TMHs: 0.00197
# Brassicaoleracea_XP_013606976.1 Exp number, first 60 AAs:  0
# Brassicaoleracea_XP_013606976.1 Total prob of N-in:        0.00828
Brassicaoleracea_XP_013606976.1 TMHMM2.0        outside      1   367
```

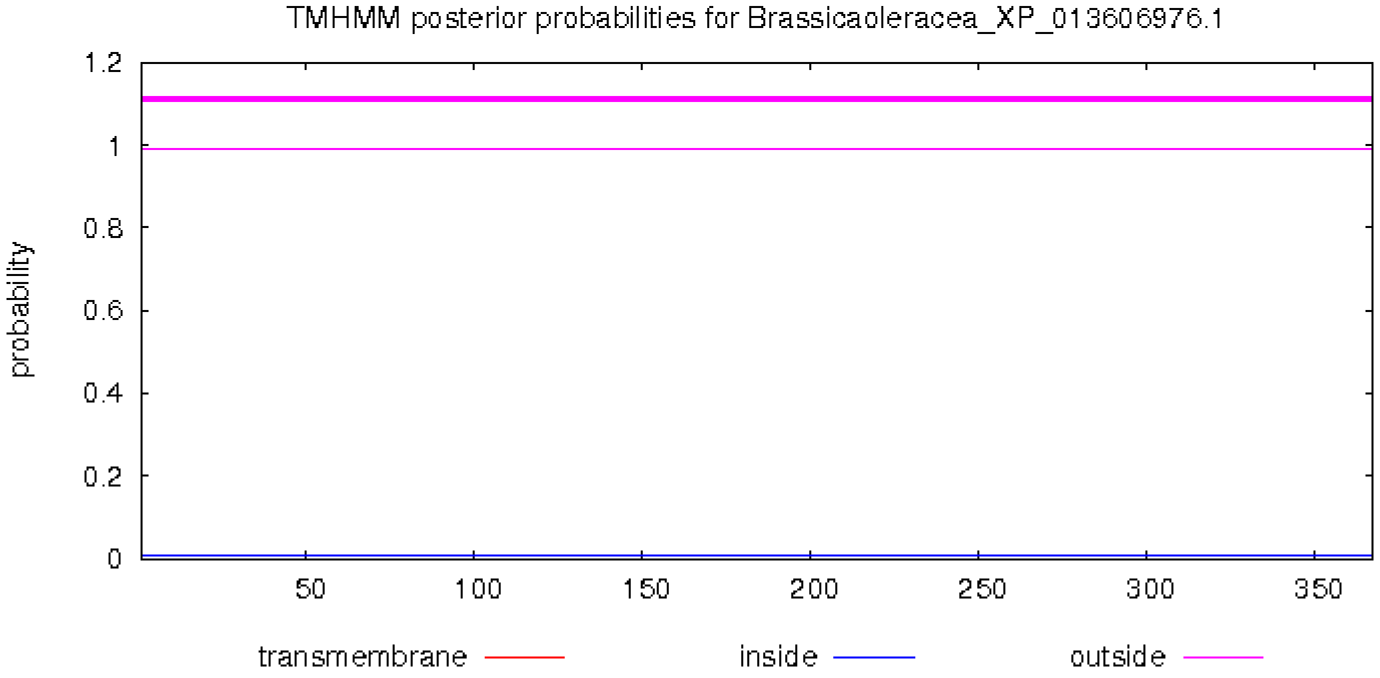

plot in postscript, script for making the plot in gnuplot, data for plot

---

```
# Brassicaoleracea_XP_013607097.1 Length: 406
# Brassicaoleracea_XP_013607097.1 Number of predicted TMHs:  0
# Brassicaoleracea_XP_013607097.1 Exp number of AAs in TMHs: 0.02688
# Brassicaoleracea_XP_013607097.1 Exp number, first 60 AAs:  0
# Brassicaoleracea_XP_013607097.1 Total prob of N-in:        0.01503
Brassicaoleracea_XP_013607097.1 TMHMM2.0        outside      1   406
```

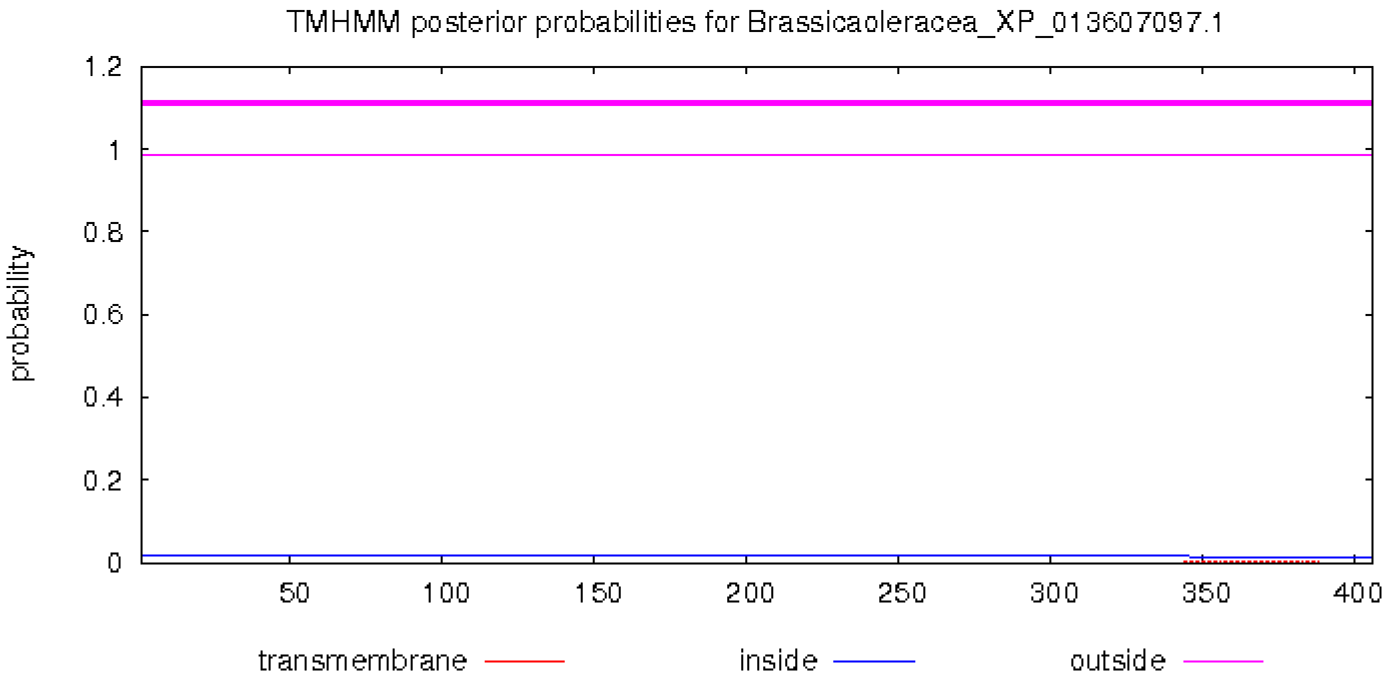

plot in postscript, script for making the plot in gnuplot, data for plot

```
# Brassicaoleracea_XP_013607098.1 Length: 330
# Brassicaoleracea_XP_013607098.1 Number of predicted TMHs:  0
# Brassicaoleracea_XP_013607098.1 Exp number of AAs in TMHs: 0.54698
# Brassicaoleracea_XP_013607098.1 Exp number, first 60 AAs:  0.53147
# Brassicaoleracea_XP_013607098.1 Total prob of N-in:        0.03282
Brassicaoleracea_XP_013607098.1 TMHMM2.0        outside      1   330
```

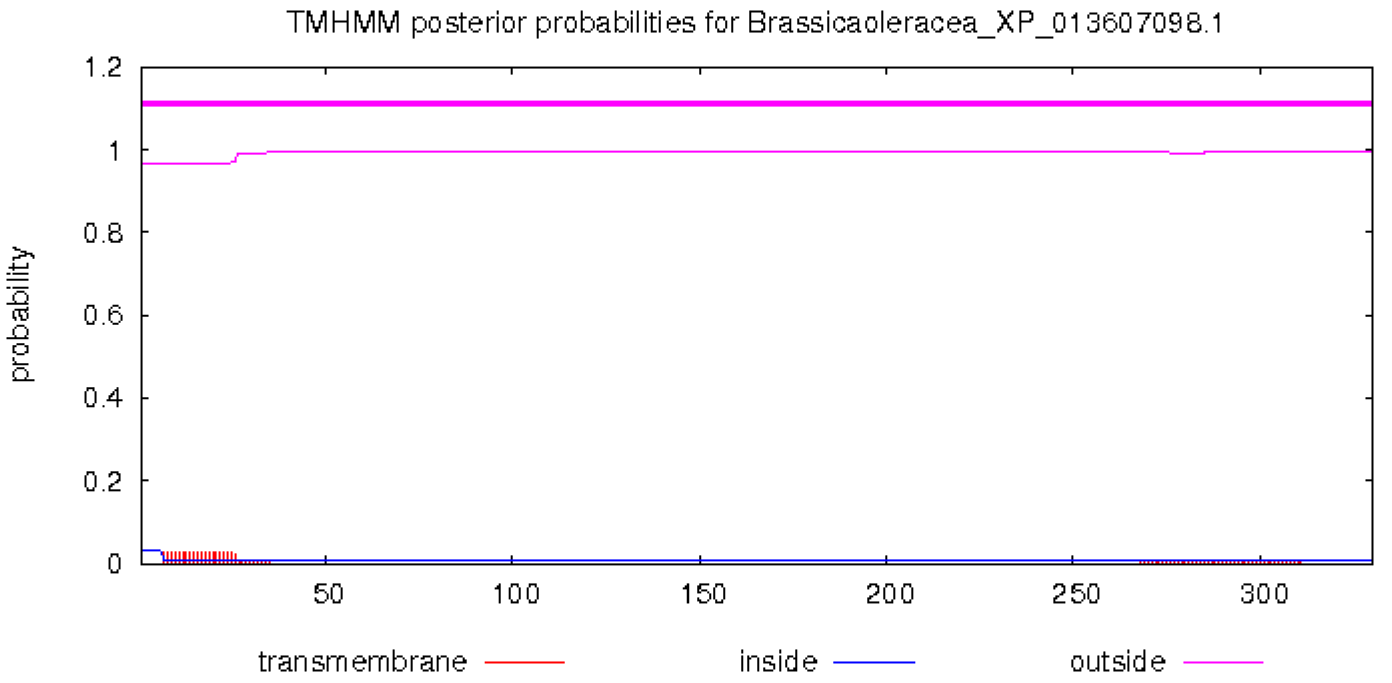

plot in postscript, script for making the plot in gnuplot, data for plot

```
# Brassicaoleracea_XP_013607352.1 Length: 408
# Brassicaoleracea_XP_013607352.1 Number of predicted TMHs:  0
# Brassicaoleracea_XP_013607352.1 Exp number of AAs in TMHs: 0.00095
# Brassicaoleracea_XP_013607352.1 Exp number, first 60 AAs:  0
# Brassicaoleracea_XP_013607352.1 Total prob of N-in:        0.01136
Brassicaoleracea_XP_013607352.1 TMHMM2.0        outside      1   408
```

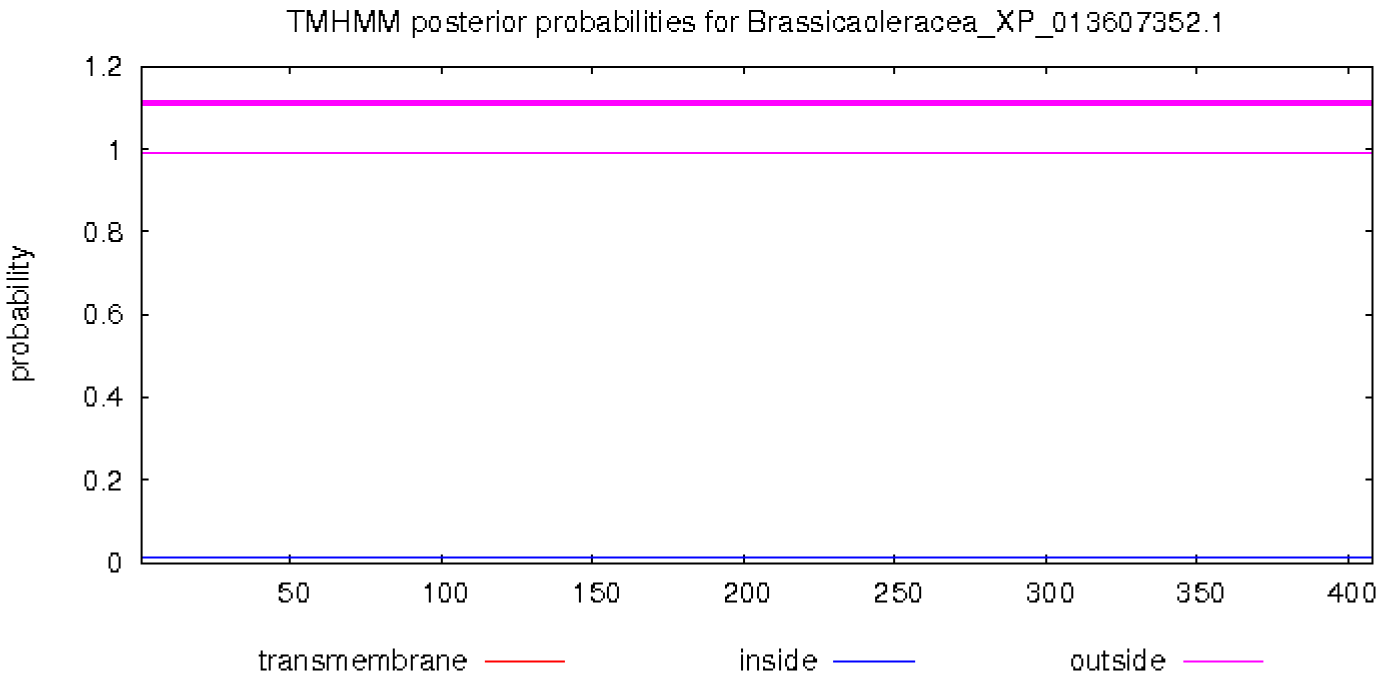

plot in postscript, script for making the plot in gnuplot, data for plot

```
# Brassicaoleracea_XP_013607353.1 Length: 408
# Brassicaoleracea_XP_013607353.1 Number of predicted TMHs:  0
# Brassicaoleracea_XP_013607353.1 Exp number of AAs in TMHs: 0.00095
# Brassicaoleracea_XP_013607353.1 Exp number, first 60 AAs:  0
# Brassicaoleracea_XP_013607353.1 Total prob of N-in:        0.01136
Brassicaoleracea_XP_013607353.1 TMHMM2.0        outside      1   408
```

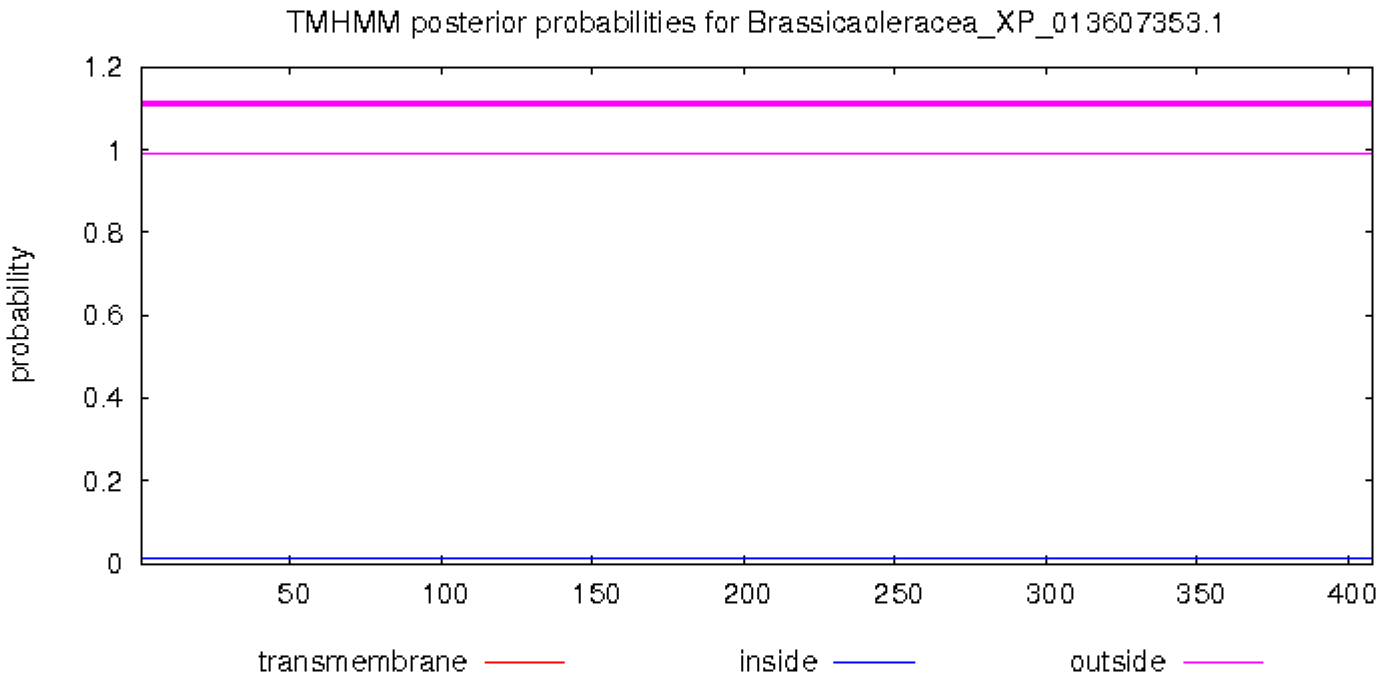

plot in postscript, script for making the plot in gnuplot, data for plot

```
# Brassicaoleracea_XP_013607354.1 Length: 407
# Brassicaoleracea_XP_013607354.1 Number of predicted TMHs:  0
# Brassicaoleracea_XP_013607354.1 Exp number of AAs in TMHs: 0.00097
# Brassicaoleracea_XP_013607354.1 Exp number, first 60 AAs:  0
# Brassicaoleracea_XP_013607354.1 Total prob of N-in:        0.01150
Brassicaoleracea_XP_013607354.1 TMHMM2.0        outside      1   407
```

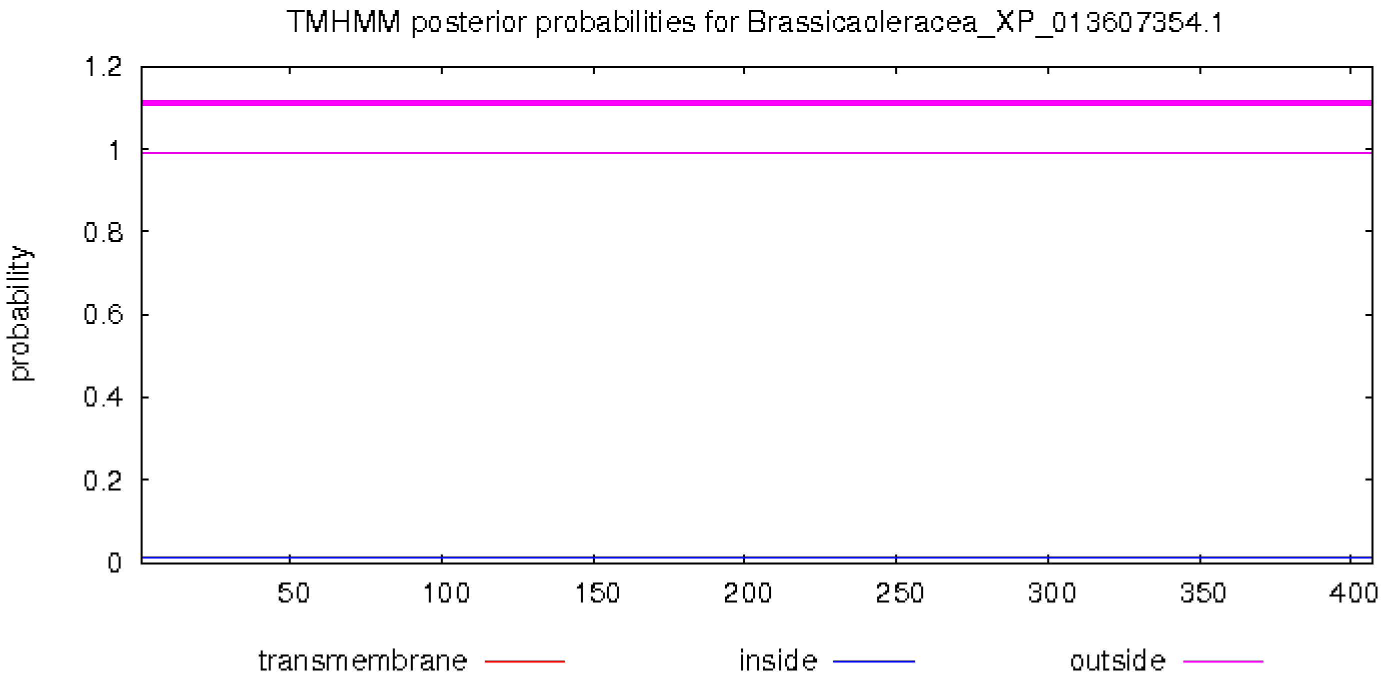

plot in postscript, script for making the plot in gnuplot, data for plot

---

```
# Brassicaoleracea_XP_013607355.1 Length: 427
# Brassicaoleracea_XP_013607355.1 Number of predicted TMHs:  0
# Brassicaoleracea_XP_013607355.1 Exp number of AAs in TMHs: 0.01554
# Brassicaoleracea_XP_013607355.1 Exp number, first 60 AAs:  0
# Brassicaoleracea_XP_013607355.1 Total prob of N-in:        0.00704
Brassicaoleracea_XP_013607355.1 TMHMM2.0        outside      1   427
```

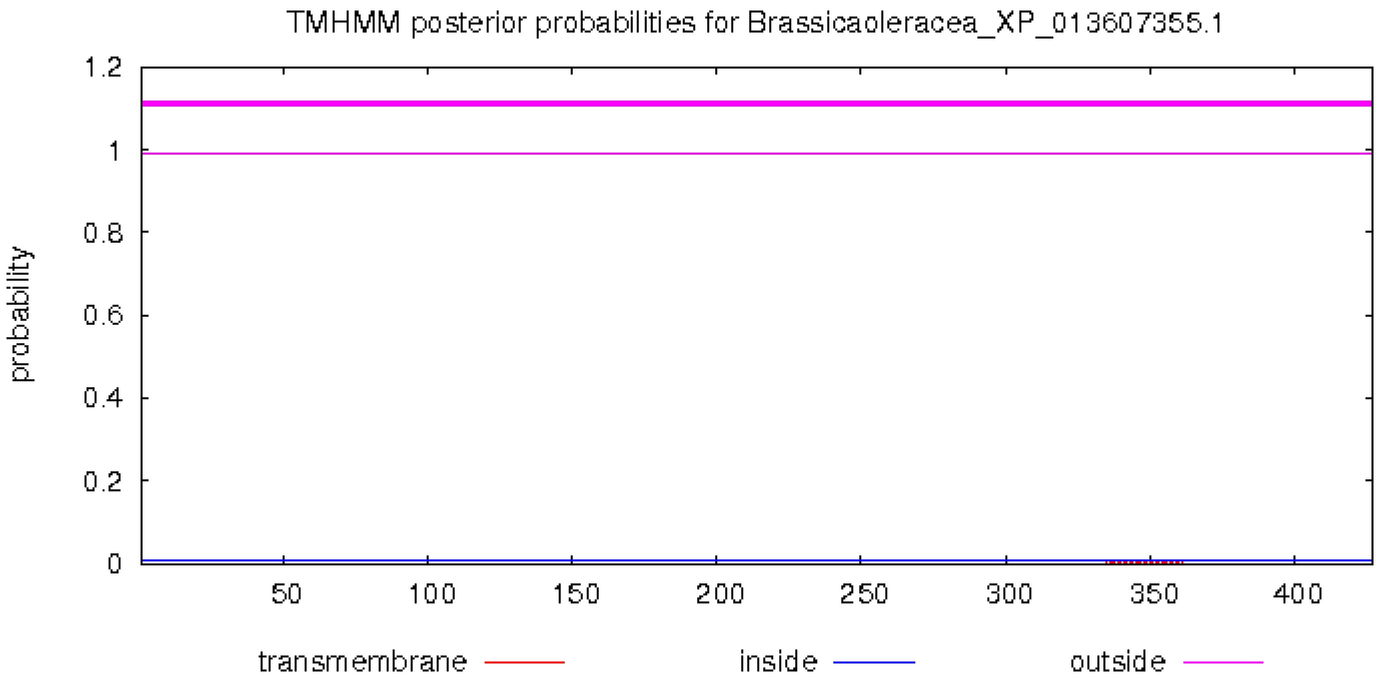

plot in postscript, script for making the plot in gnuplot, data for plot

---

```
# Brassicaoleracea_XP_013607450.1 Length: 171
# Brassicaoleracea_XP_013607450.1 Number of predicted TMHs:  1
# Brassicaoleracea_XP_013607450.1 Exp number of AAs in TMHs: 18.70635
# Brassicaoleracea_XP_013607450.1 Exp number, first 60 AAs:  0.00082
# Brassicaoleracea_XP_013607450.1 Total prob of N-in:        0.26639
Brassicaoleracea_XP_013607450.1 TMHMM2.0        outside      1   145
Brassicaoleracea_XP_013607450.1 TMHMM2.0        TMhelix    146   168
Brassicaoleracea_XP_013607450.1 TMHMM2.0        inside     169   171
```

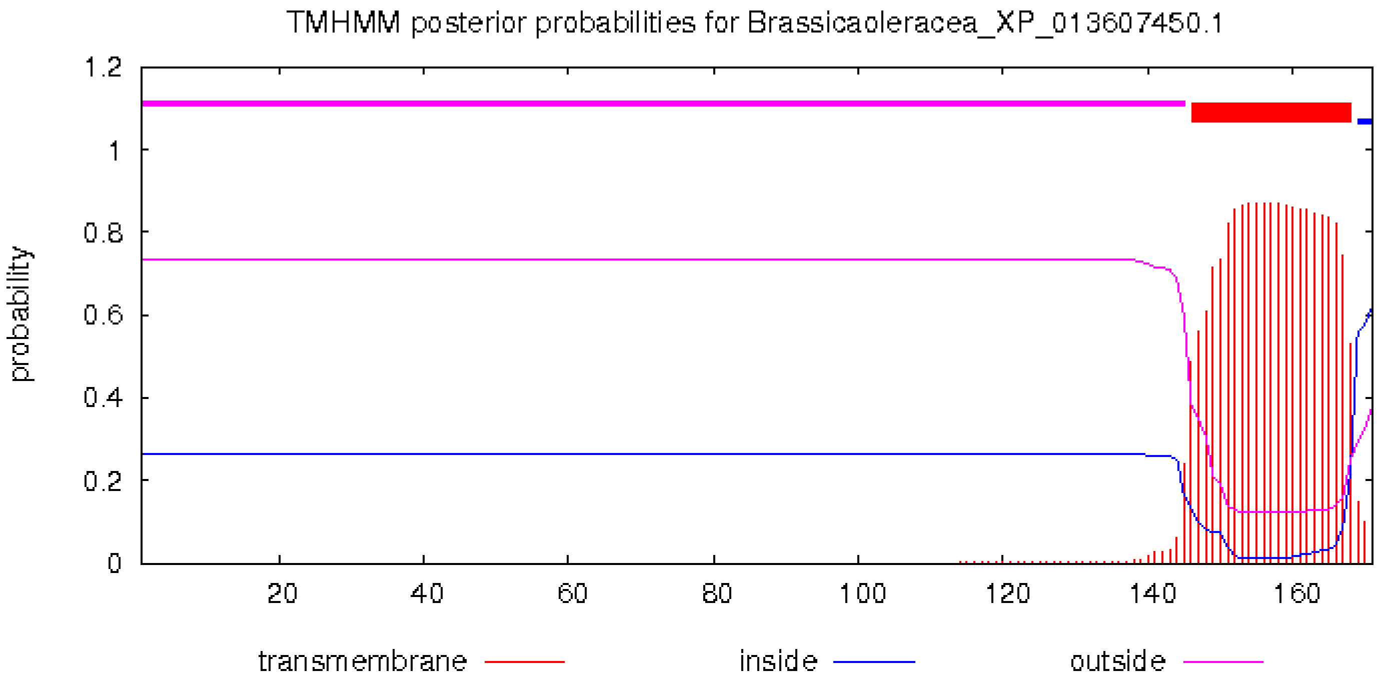

plot in postscript, script for making the plot in gnuplot, data for plot

---

```
# Brassicaoleracea_XP_013607451.1 Length: 373
# Brassicaoleracea_XP_013607451.1 Number of predicted TMHs:  0
# Brassicaoleracea_XP_013607451.1 Exp number of AAs in TMHs: 0.1564
# Brassicaoleracea_XP_013607451.1 Exp number, first 60 AAs:  0
# Brassicaoleracea_XP_013607451.1 Total prob of N-in:        0.01400
Brassicaoleracea_XP_013607451.1 TMHMM2.0        outside      1   373
```

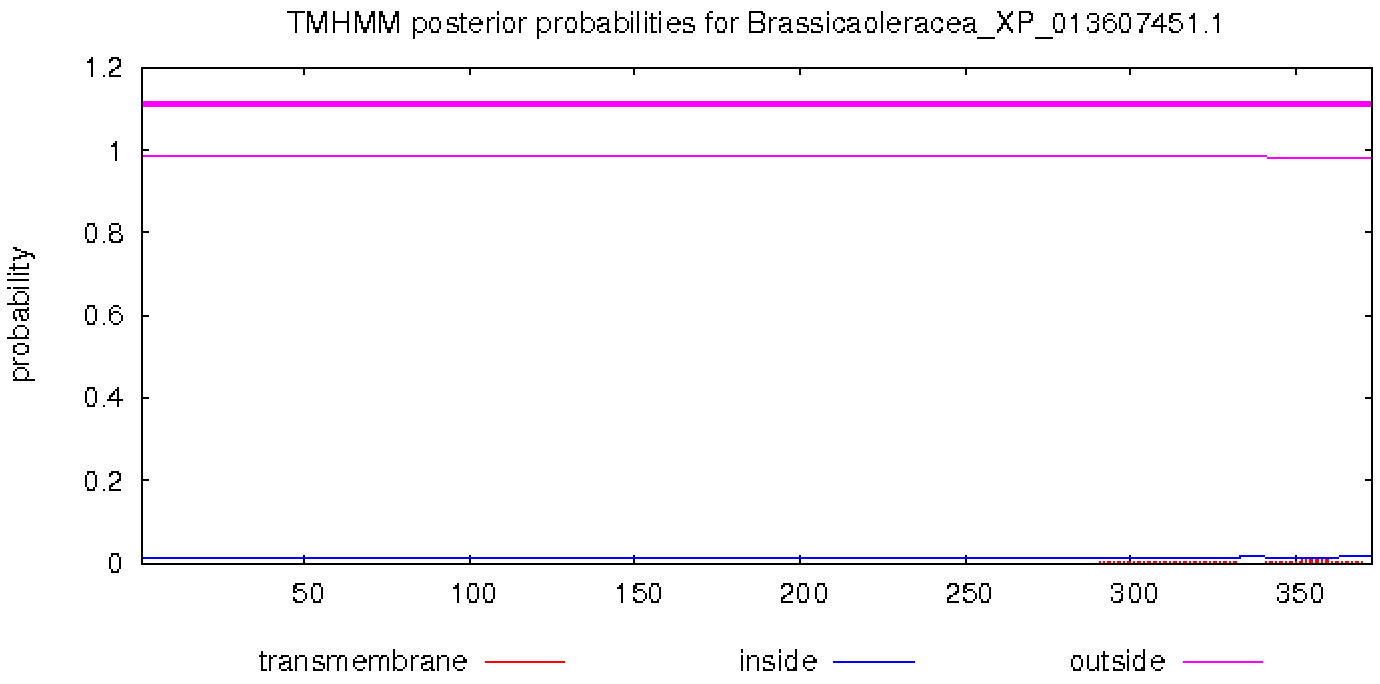

plot in postscript, script for making the plot in gnuplot, data for plot

```
# Brassicaoleracea_XP_013607456.1 Length: 556
# Brassicaoleracea_XP_013607456.1 Number of predicted TMHs:  0
# Brassicaoleracea_XP_013607456.1 Exp number of AAs in TMHs: 0.00197
# Brassicaoleracea_XP_013607456.1 Exp number, first 60 AAs:  0.00183
# Brassicaoleracea_XP_013607456.1 Total prob of N-in:        0.00243
Brassicaoleracea_XP_013607456.1 TMHMM2.0        outside      1   556
```

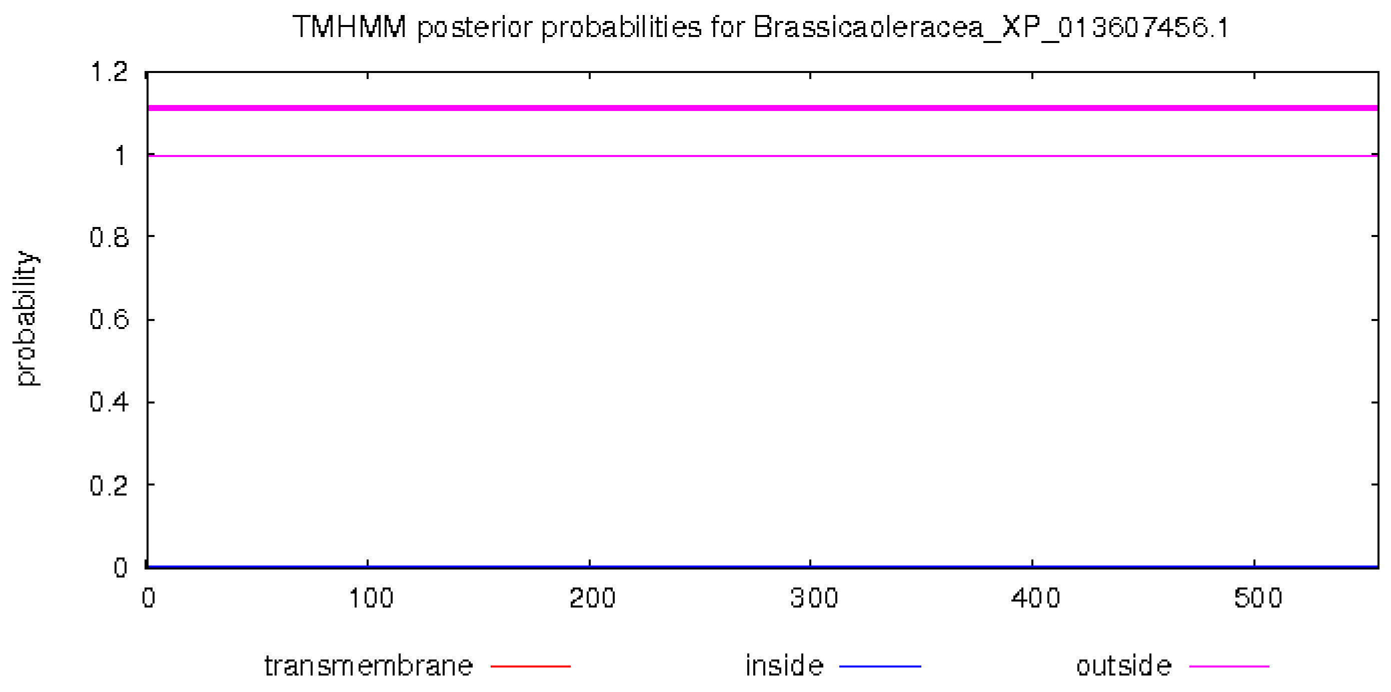

plot in postscript, script for making the plot in gnuplot, data for plot

```
# Brassicaoleracea_XP_013607494.1 Length: 237
# Brassicaoleracea_XP_013607494.1 Number of predicted TMHs:  0
# Brassicaoleracea_XP_013607494.1 Exp number of AAs in TMHs: 0.02749
# Brassicaoleracea_XP_013607494.1 Exp number, first 60 AAs:  0.02745
# Brassicaoleracea_XP_013607494.1 Total prob of N-in:        0.10676
Brassicaoleracea_XP_013607494.1 TMHMM2.0        outside      1   237
```

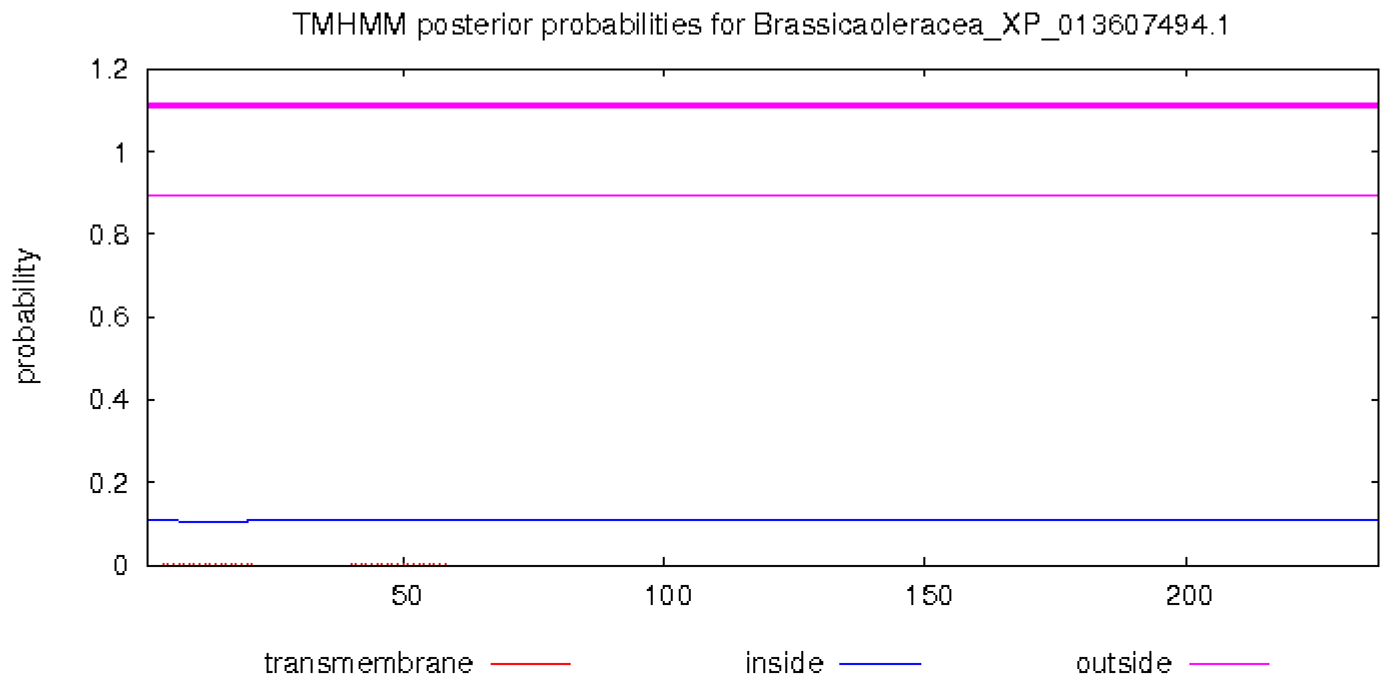

plot in postscript, script for making the plot in gnuplot, data for plot

```
# Brassicaoleracea_XP_013608112.1 Length: 224
# Brassicaoleracea_XP_013608112.1 Number of predicted TMHs:  0
# Brassicaoleracea_XP_013608112.1 Exp number of AAs in TMHs: 8.64613
# Brassicaoleracea_XP_013608112.1 Exp number, first 60 AAs:  0.00464
# Brassicaoleracea_XP_013608112.1 Total prob of N-in:        0.26263
Brassicaoleracea_XP_013608112.1 TMHMM2.0        outside      1   224
```

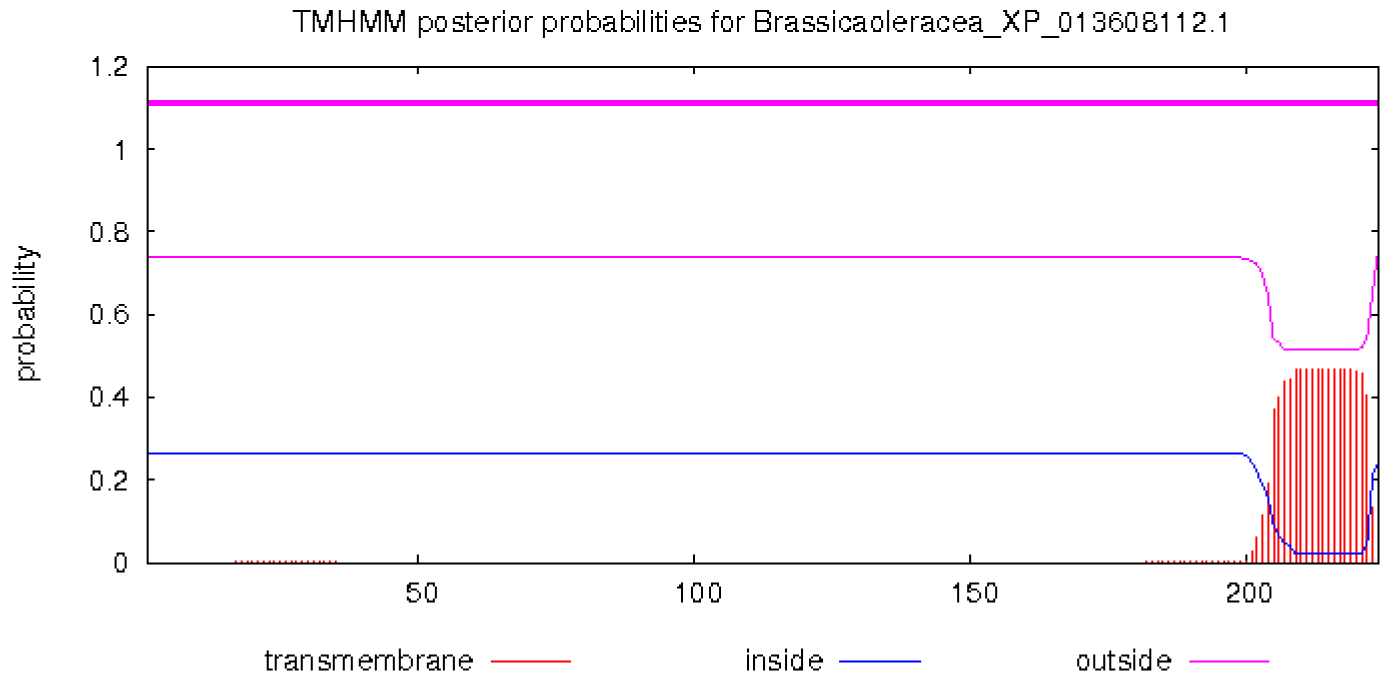

plot in postscript, script for making the plot in gnuplot, data for plot

```
# Brassicaoleracea_XP_013608221.1 Length: 325
# Brassicaoleracea_XP_013608221.1 Number of predicted TMHs:  0
# Brassicaoleracea_XP_013608221.1 Exp number of AAs in TMHs: 0.00273
# Brassicaoleracea_XP_013608221.1 Exp number, first 60 AAs:  0.00039
# Brassicaoleracea_XP_013608221.1 Total prob of N-in:        0.01332
Brassicaoleracea_XP_013608221.1 TMHMM2.0        outside      1   325
```

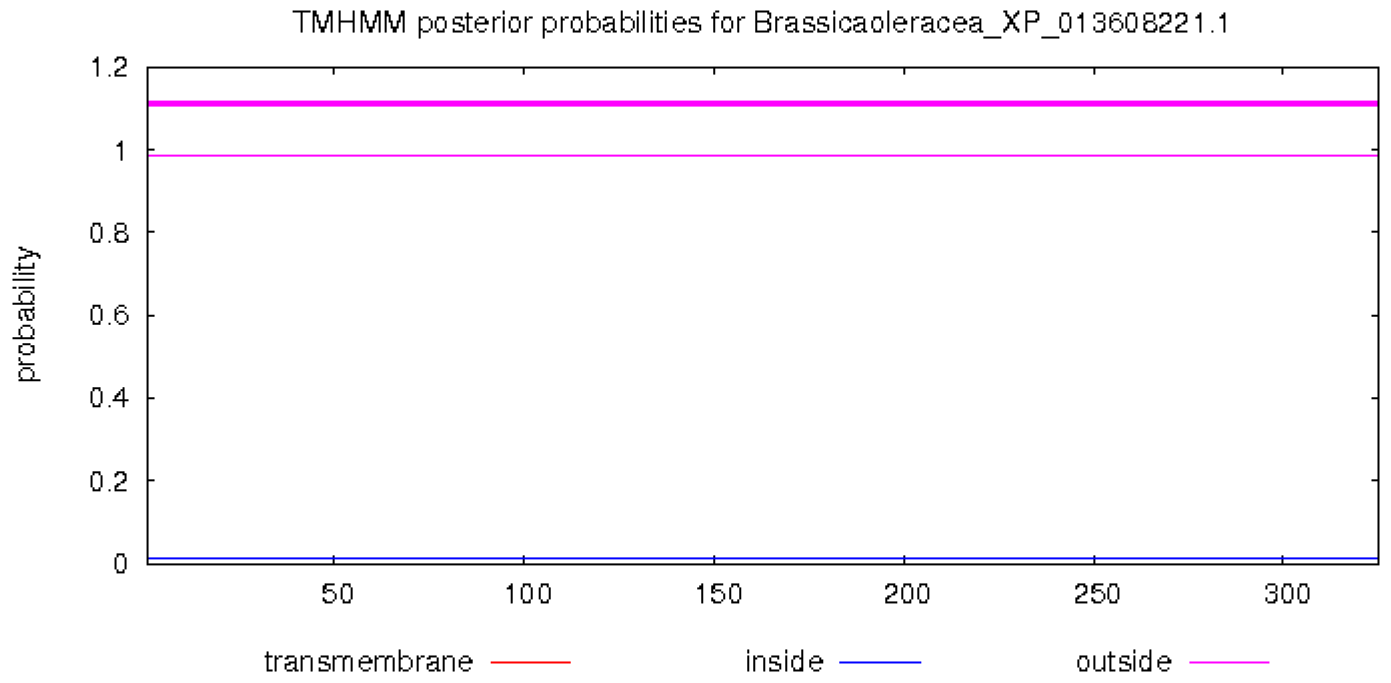

plot in postscript, script for making the plot in gnuplot, data for plot

```
# Brassicaoleracea_XP_013608281.1 Length: 281
# Brassicaoleracea_XP_013608281.1 Number of predicted TMHs:  0
# Brassicaoleracea_XP_013608281.1 Exp number of AAs in TMHs: 1.2745
# Brassicaoleracea_XP_013608281.1 Exp number, first 60 AAs:  0.00051
# Brassicaoleracea_XP_013608281.1 Total prob of N-in:        0.07676
Brassicaoleracea_XP_013608281.1 TMHMM2.0        outside      1   281
```

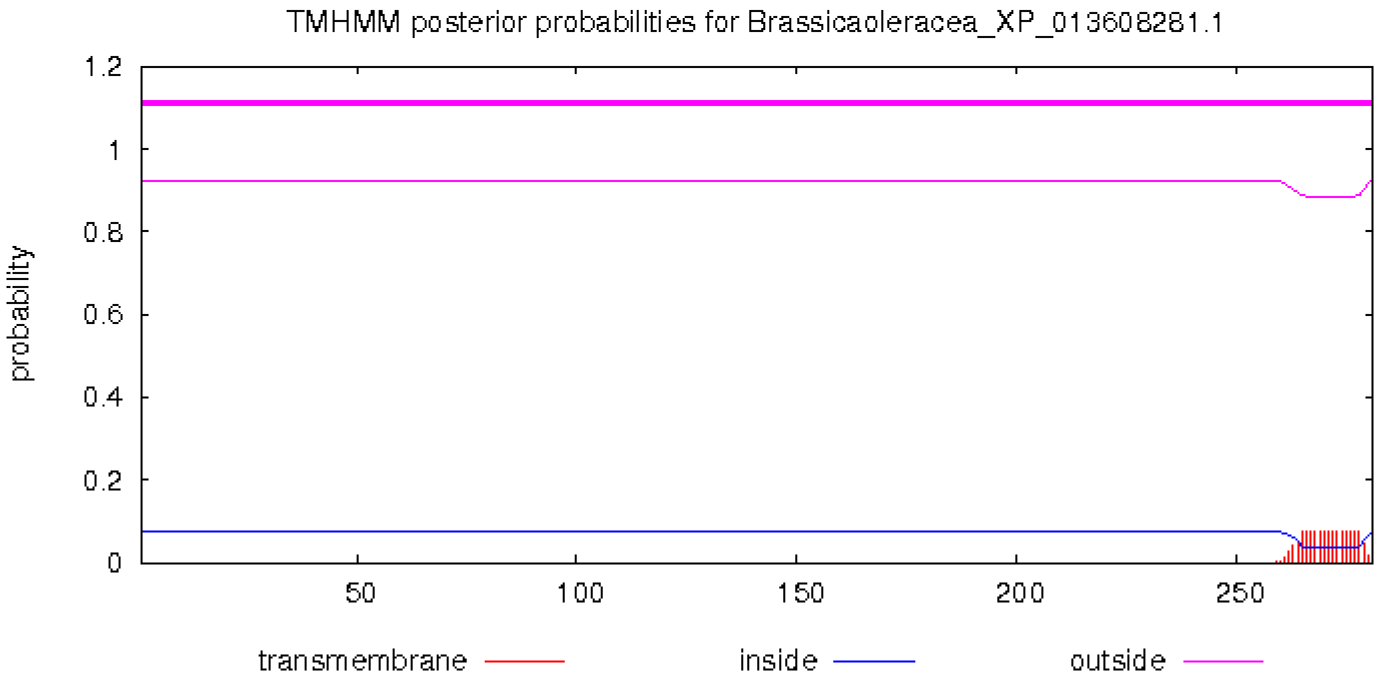

plot in postscript, script for making the plot in gnuplot, data for plot

```
# Brassicaoleracea_XP_013608283.1 Length: 227
# Brassicaoleracea_XP_013608283.1 Number of predicted TMHs:  0
# Brassicaoleracea_XP_013608283.1 Exp number of AAs in TMHs: 0.00094
# Brassicaoleracea_XP_013608283.1 Exp number, first 60 AAs:  0.0005
# Brassicaoleracea_XP_013608283.1 Total prob of N-in:        0.06900
Brassicaoleracea_XP_013608283.1 TMHMM2.0        outside      1   227
```

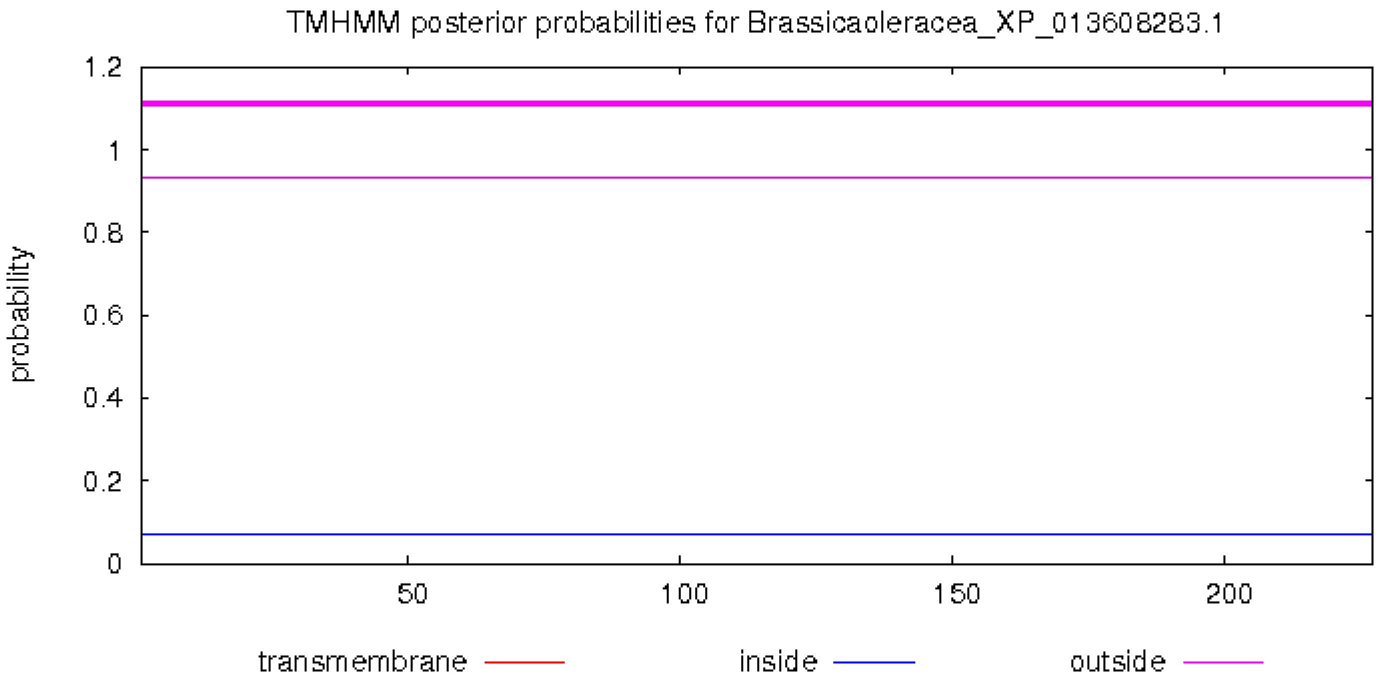

plot in postscript, script for making the plot in gnuplot, data for plot

```
# Brassicaoleracea_XP_013608285.1 Length: 410
# Brassicaoleracea_XP_013608285.1 Number of predicted TMHs:  0
# Brassicaoleracea_XP_013608285.1 Exp number of AAs in TMHs: 0.00058
# Brassicaoleracea_XP_013608285.1 Exp number, first 60 AAs:  0.00013
# Brassicaoleracea_XP_013608285.1 Total prob of N-in:        0.00751
Brassicaoleracea_XP_013608285.1 TMHMM2.0        outside      1   410
```

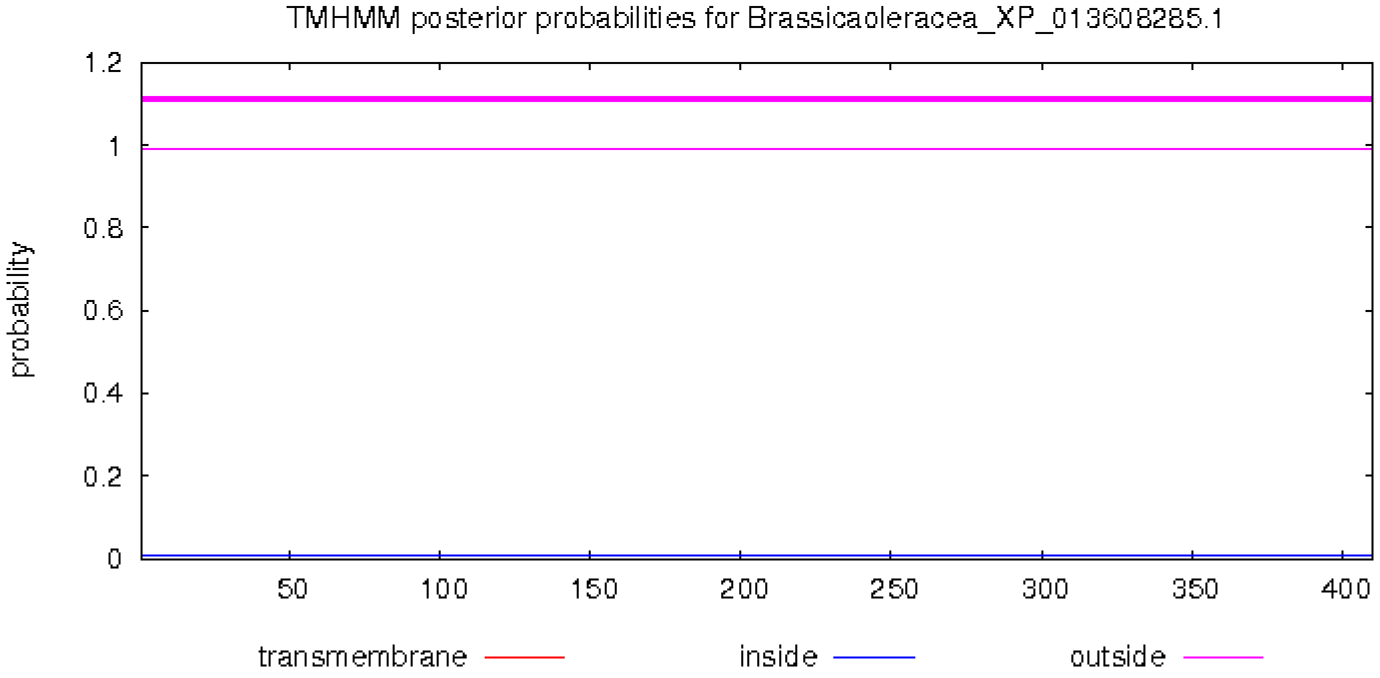

plot in postscript, script for making the plot in gnuplot, data for plot

```
# Brassicaoleracea_XP_013608476.1 Length: 199
# Brassicaoleracea_XP_013608476.1 Number of predicted TMHs:  0
# Brassicaoleracea_XP_013608476.1 Exp number of AAs in TMHs: 0.00147
# Brassicaoleracea_XP_013608476.1 Exp number, first 60 AAs:  0.00147
# Brassicaoleracea_XP_013608476.1 Total prob of N-in:        0.16214
Brassicaoleracea_XP_013608476.1 TMHMM2.0        outside      1   199
```

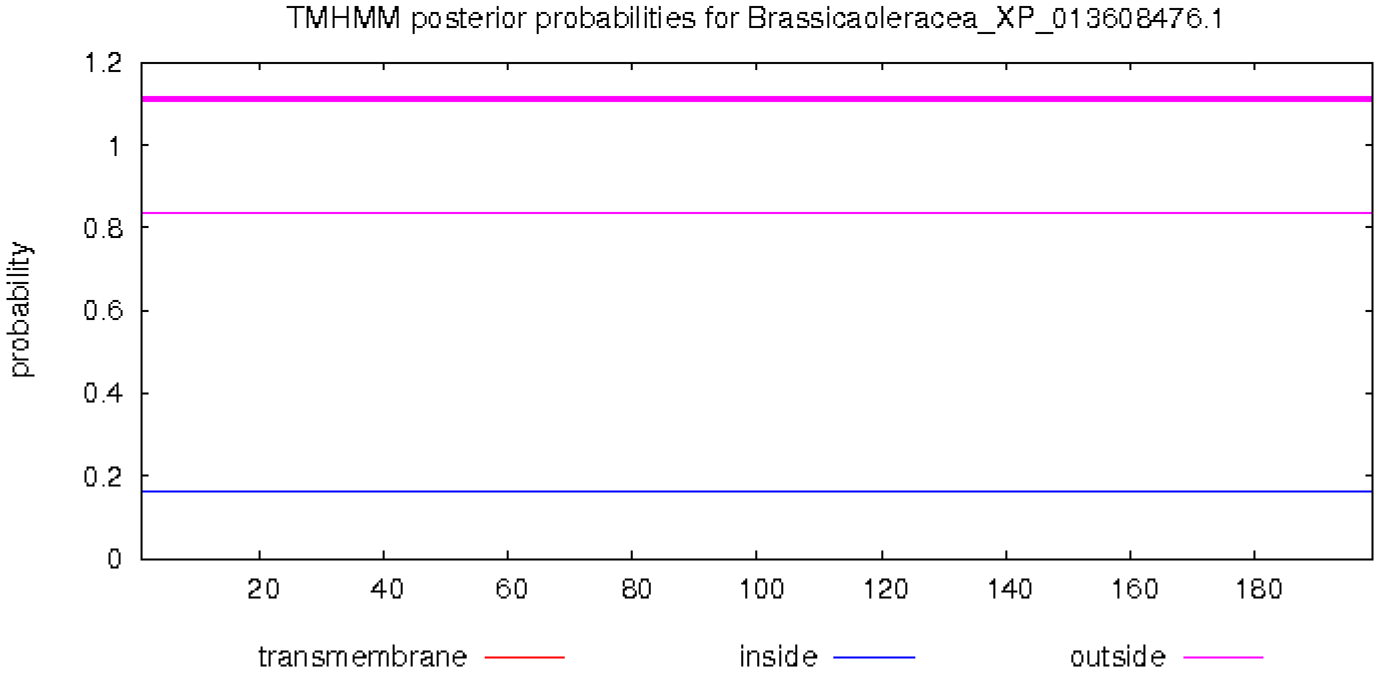

plot in postscript, script for making the plot in gnuplot, data for plot

```
# Brassicaoleracea_XP_013608832.1 Length: 276
# Brassicaoleracea_XP_013608832.1 Number of predicted TMHs:  0
# Brassicaoleracea_XP_013608832.1 Exp number of AAs in TMHs: 0.06065
# Brassicaoleracea_XP_013608832.1 Exp number, first 60 AAs:  0.05986
# Brassicaoleracea_XP_013608832.1 Total prob of N-in:        0.03530
Brassicaoleracea_XP_013608832.1 TMHMM2.0        outside      1   276
```

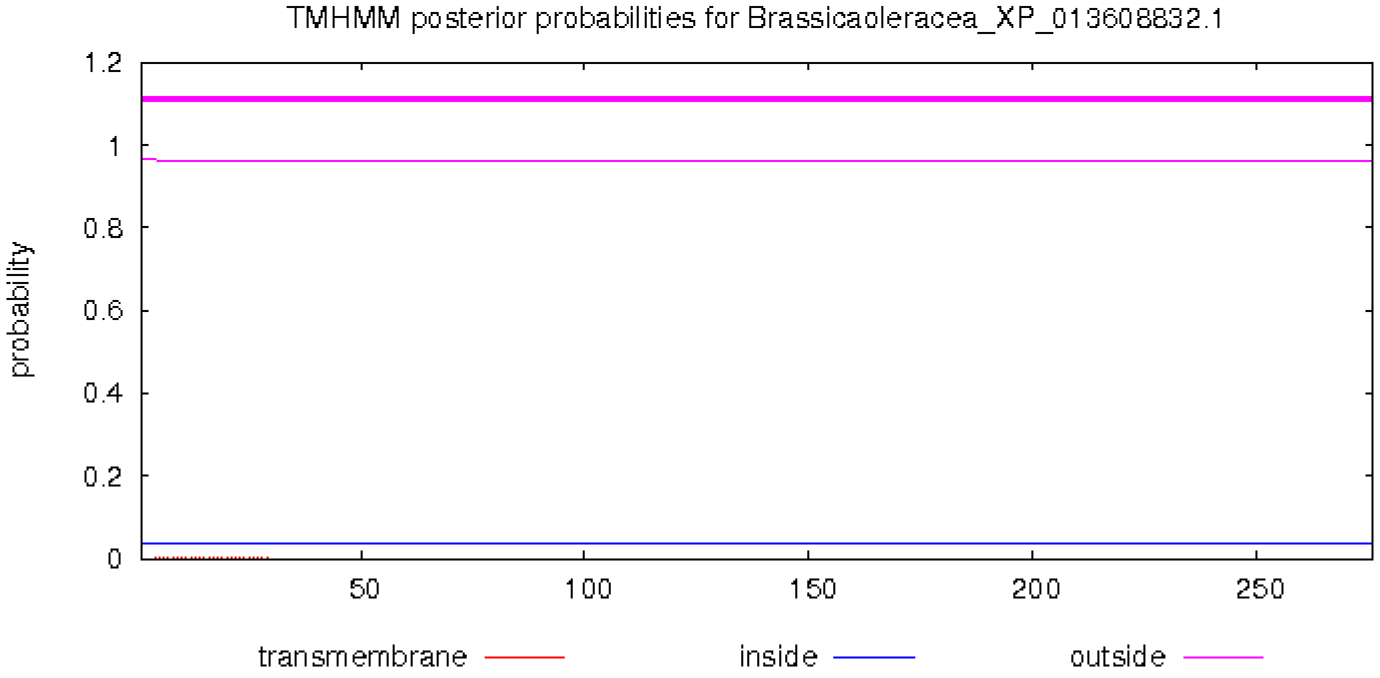

plot in postscript, script for making the plot in gnuplot, data for plot

```
# Brassicaoleracea_XP_013609014.1 Length: 264
# Brassicaoleracea_XP_013609014.1 Number of predicted TMHs:  0
# Brassicaoleracea_XP_013609014.1 Exp number of AAs in TMHs: 0.00094
# Brassicaoleracea_XP_013609014.1 Exp number, first 60 AAs:  0
# Brassicaoleracea_XP_013609014.1 Total prob of N-in:        0.04068
Brassicaoleracea_XP_013609014.1 TMHMM2.0        outside      1   264
```

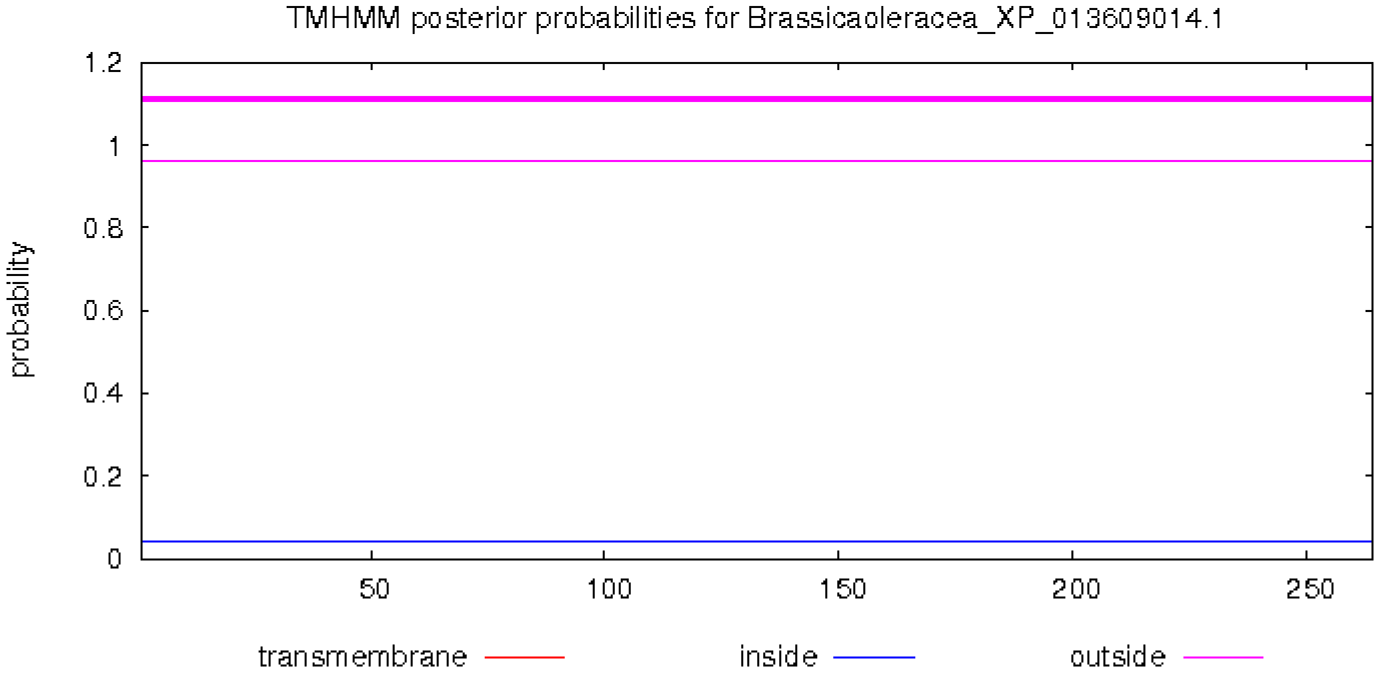

plot in postscript, script for making the plot in gnuplot, data for plot

```
# Brassicaoleracea_XP_013609062.1 Length: 336
# Brassicaoleracea_XP_013609062.1 Number of predicted TMHs:  0
# Brassicaoleracea_XP_013609062.1 Exp number of AAs in TMHs: 0.0002
# Brassicaoleracea_XP_013609062.1 Exp number, first 60 AAs:  0
# Brassicaoleracea_XP_013609062.1 Total prob of N-in:        0.00827
Brassicaoleracea_XP_013609062.1 TMHMM2.0        outside      1   336
```

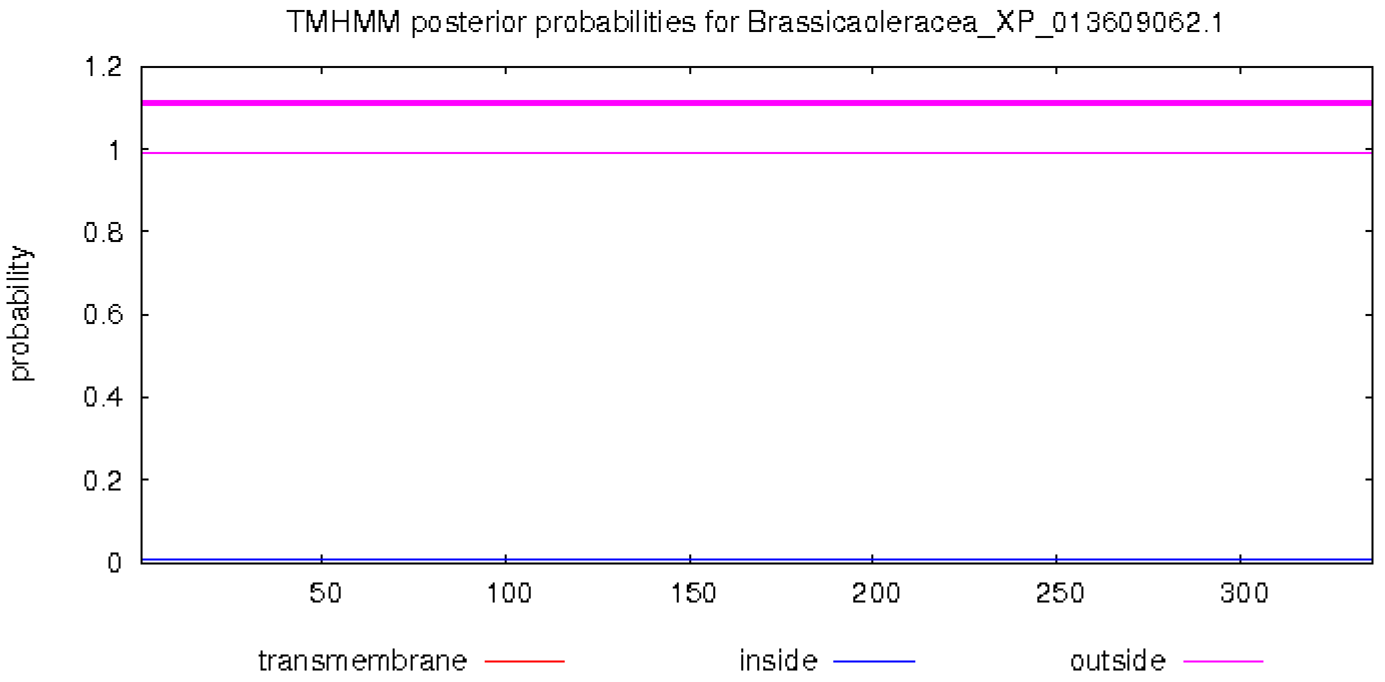

plot in postscript, script for making the plot in gnuplot, data for plot

---

```
# Brassicaoleracea_XP_013609230.1 Length: 279
# Brassicaoleracea_XP_013609230.1 Number of predicted TMHs:  0
# Brassicaoleracea_XP_013609230.1 Exp number of AAs in TMHs: 0.00228
# Brassicaoleracea_XP_013609230.1 Exp number, first 60 AAs:  0.00036
# Brassicaoleracea_XP_013609230.1 Total prob of N-in:        0.00561
Brassicaoleracea_XP_013609230.1 TMHMM2.0        outside      1   279
```

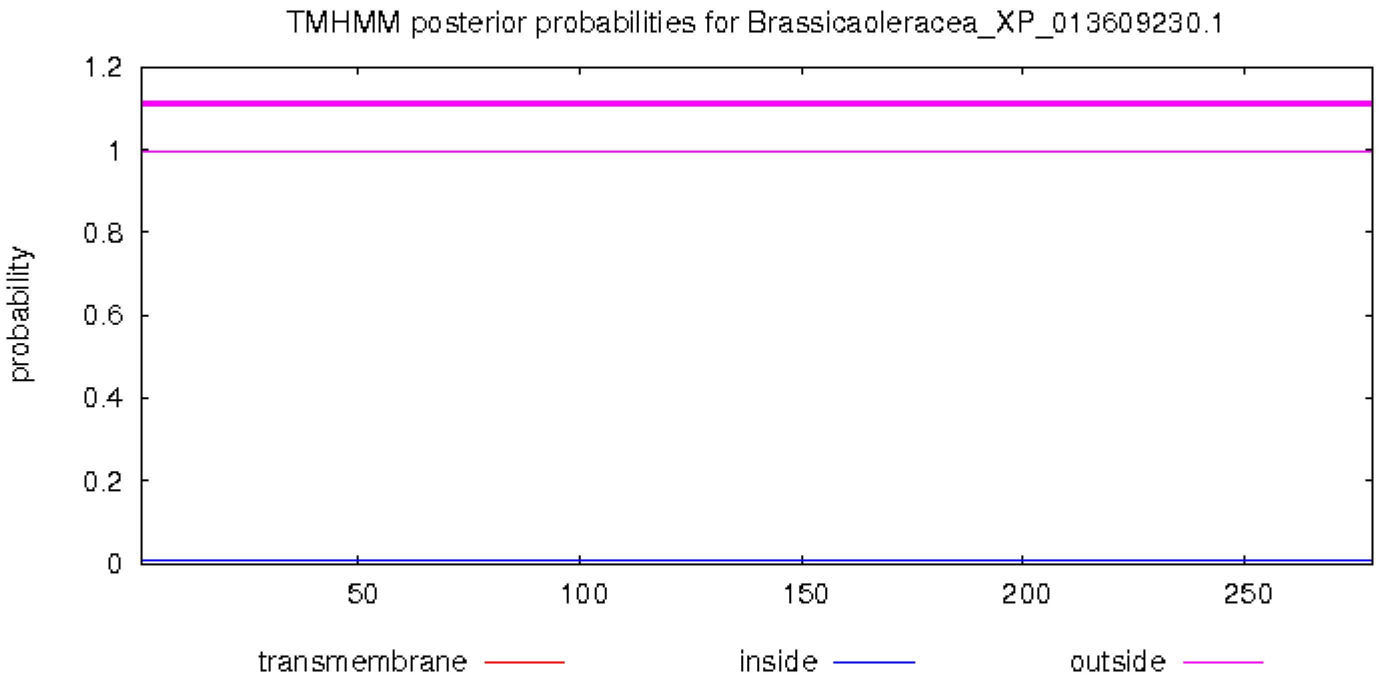

plot in postscript, script for making the plot in gnuplot, data for plot

---

```
# Brassicaoleracea_XP_013609231.1 Length: 328
# Brassicaoleracea_XP_013609231.1 Number of predicted TMHs:  0
# Brassicaoleracea_XP_013609231.1 Exp number of AAs in TMHs: 0.00915
# Brassicaoleracea_XP_013609231.1 Exp number, first 60 AAs:  0.004
# Brassicaoleracea_XP_013609231.1 Total prob of N-in:        0.02381
Brassicaoleracea_XP_013609231.1 TMHMM2.0        outside      1   328
```

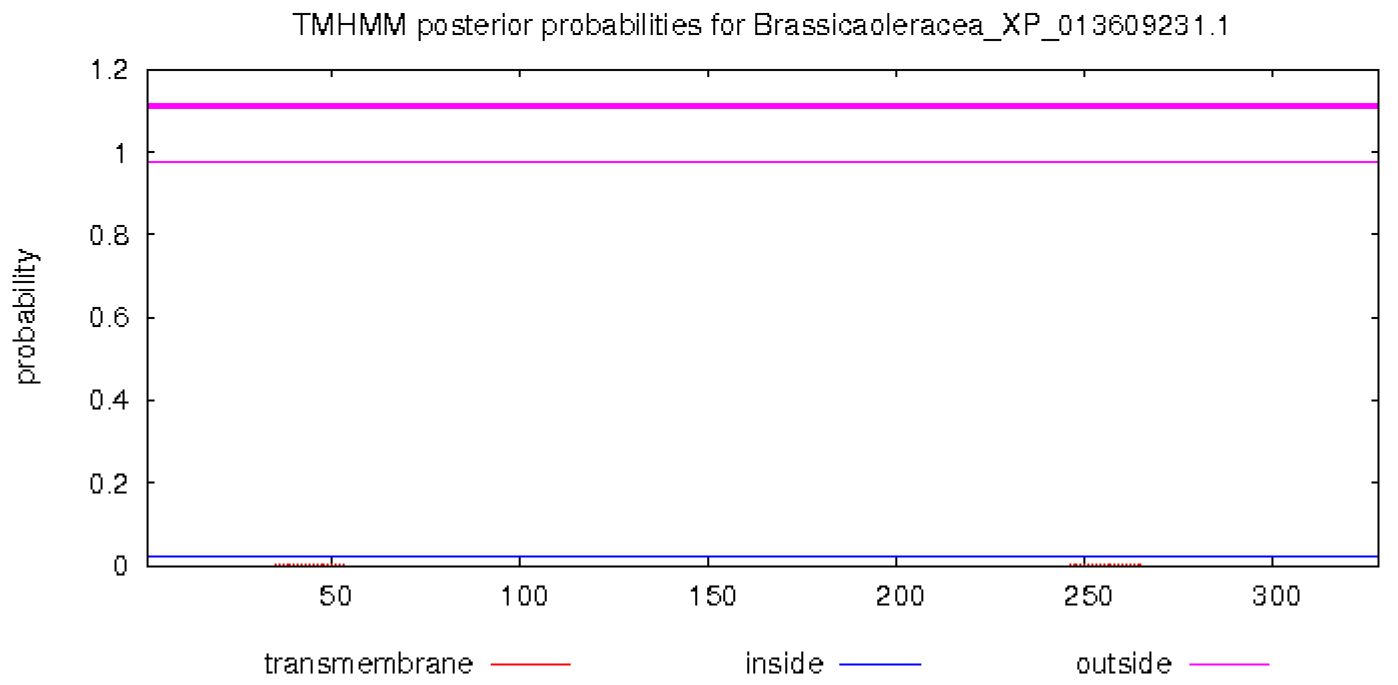

plot in postscript, script for making the plot in gnuplot, data for plot

```
# Brassicaoleracea_XP_013609232.1 Length: 283
# Brassicaoleracea_XP_013609232.1 Number of predicted TMHs:  0
# Brassicaoleracea_XP_013609232.1 Exp number of AAs in TMHs: 0.00529
# Brassicaoleracea_XP_013609232.1 Exp number, first 60 AAs:  0.00432
# Brassicaoleracea_XP_013609232.1 Total prob of N-in:        0.03861
Brassicaoleracea_XP_013609232.1 TMHMM2.0        outside      1   283
```

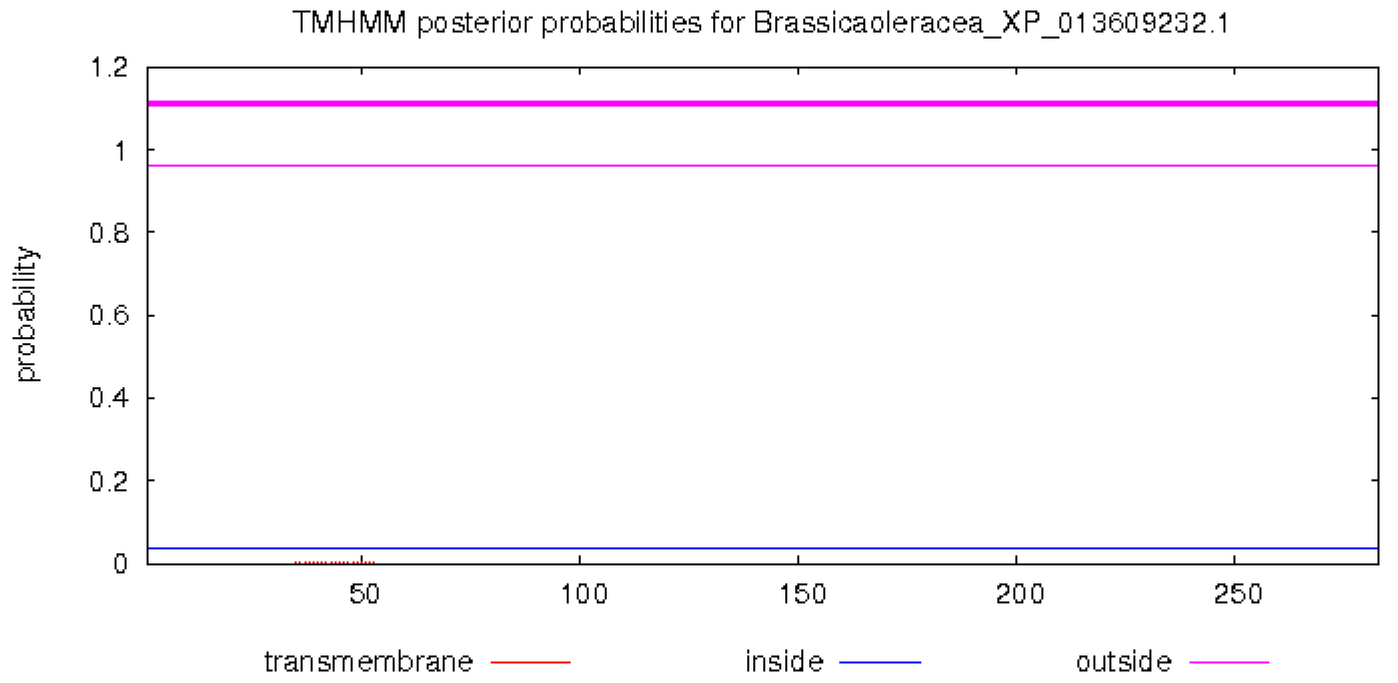

plot in postscript, script for making the plot in gnuplot, data for plot

```
# Brassicaoleracea_XP_013609384.1 Length: 188
# Brassicaoleracea_XP_013609384.1 Number of predicted TMHs:  0
# Brassicaoleracea_XP_013609384.1 Exp number of AAs in TMHs: 0
# Brassicaoleracea_XP_013609384.1 Exp number, first 60 AAs:  0
# Brassicaoleracea_XP_013609384.1 Total prob of N-in:        0.00724
Brassicaoleracea_XP_013609384.1 TMHMM2.0        outside      1   188
```

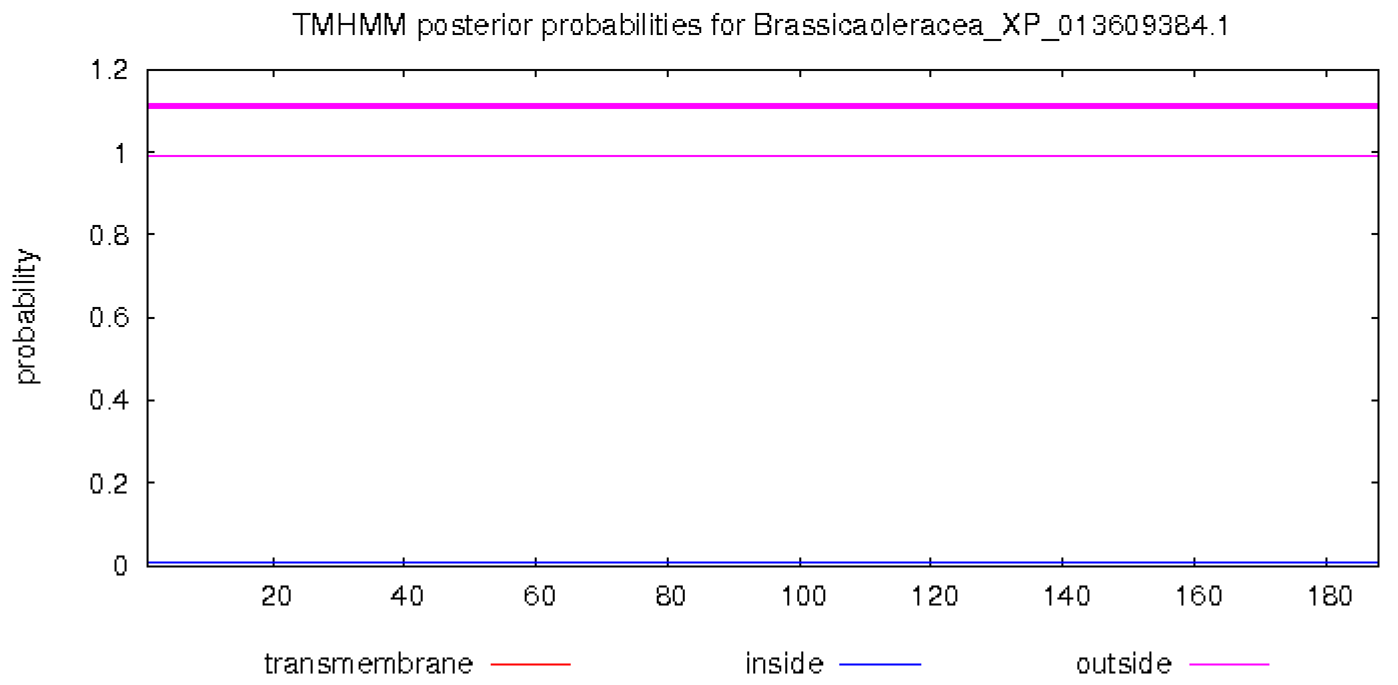

plot in postscript, script for making the plot in gnuplot, data for plot

```
# Brassicaoleracea_XP_013609604.1 Length: 253
# Brassicaoleracea_XP_013609604.1 Number of predicted TMHs:  0
# Brassicaoleracea_XP_013609604.1 Exp number of AAs in TMHs: 0.12126
# Brassicaoleracea_XP_013609604.1 Exp number, first 60 AAs:  0.00055
# Brassicaoleracea_XP_013609604.1 Total prob of N-in:        0.02097
Brassicaoleracea_XP_013609604.1 TMHMM2.0        outside      1   253
```

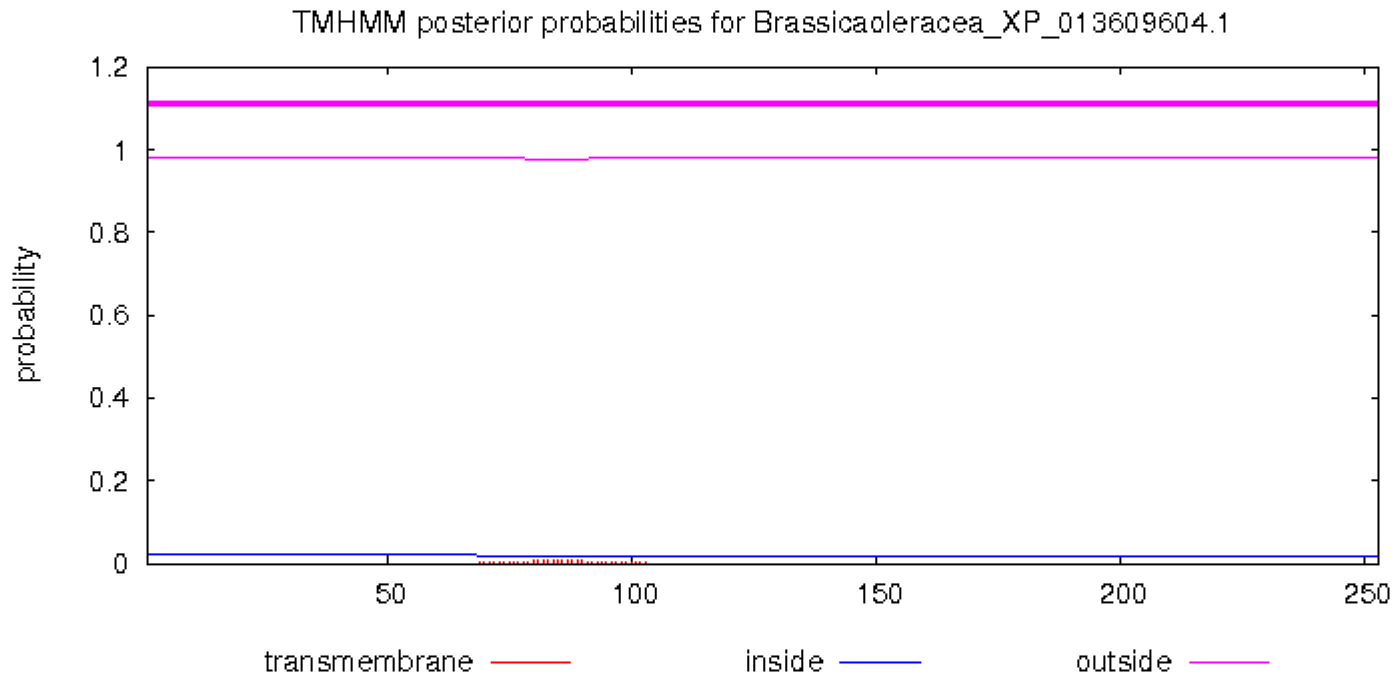

plot in postscript, script for making the plot in gnuplot, data for plot

```
# Brassicaoleracea_XP_013609605.1 Length: 252
# Brassicaoleracea_XP_013609605.1 Number of predicted TMHs:  0
# Brassicaoleracea_XP_013609605.1 Exp number of AAs in TMHs: 0.12865
# Brassicaoleracea_XP_013609605.1 Exp number, first 60 AAs:  0.00055
# Brassicaoleracea_XP_013609605.1 Total prob of N-in:        0.02127
Brassicaoleracea_XP_013609605.1 TMHMM2.0        outside      1   252
```

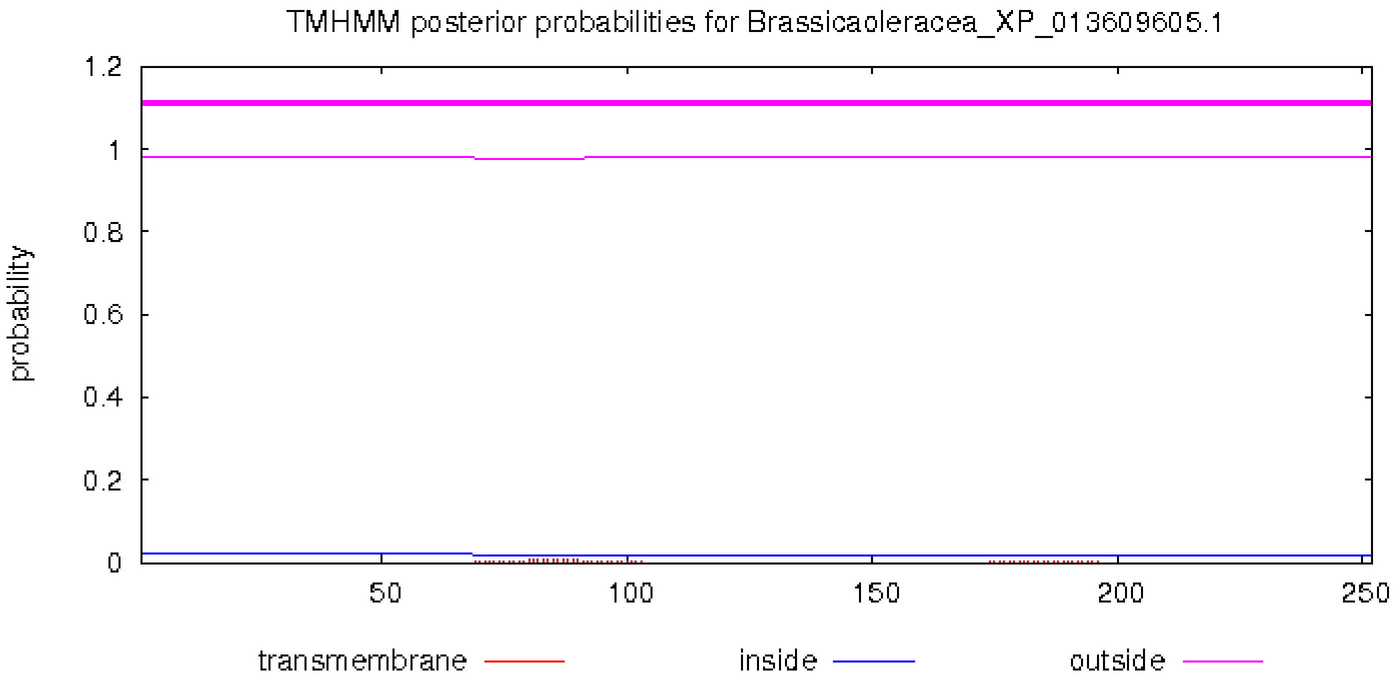

plot in postscript, script for making the plot in gnuplot, data for plot

---

```
# Brassicaoleracea_XP_013609760.1 Length: 501
# Brassicaoleracea_XP_013609760.1 Number of predicted TMHs:  0
# Brassicaoleracea_XP_013609760.1 Exp number of AAs in TMHs: 7.20466
# Brassicaoleracea_XP_013609760.1 Exp number, first 60 AAs:  0
# Brassicaoleracea_XP_013609760.1 Total prob of N-in:        0.28448
Brassicaoleracea_XP_013609760.1 TMHMM2.0        outside      1   501
```

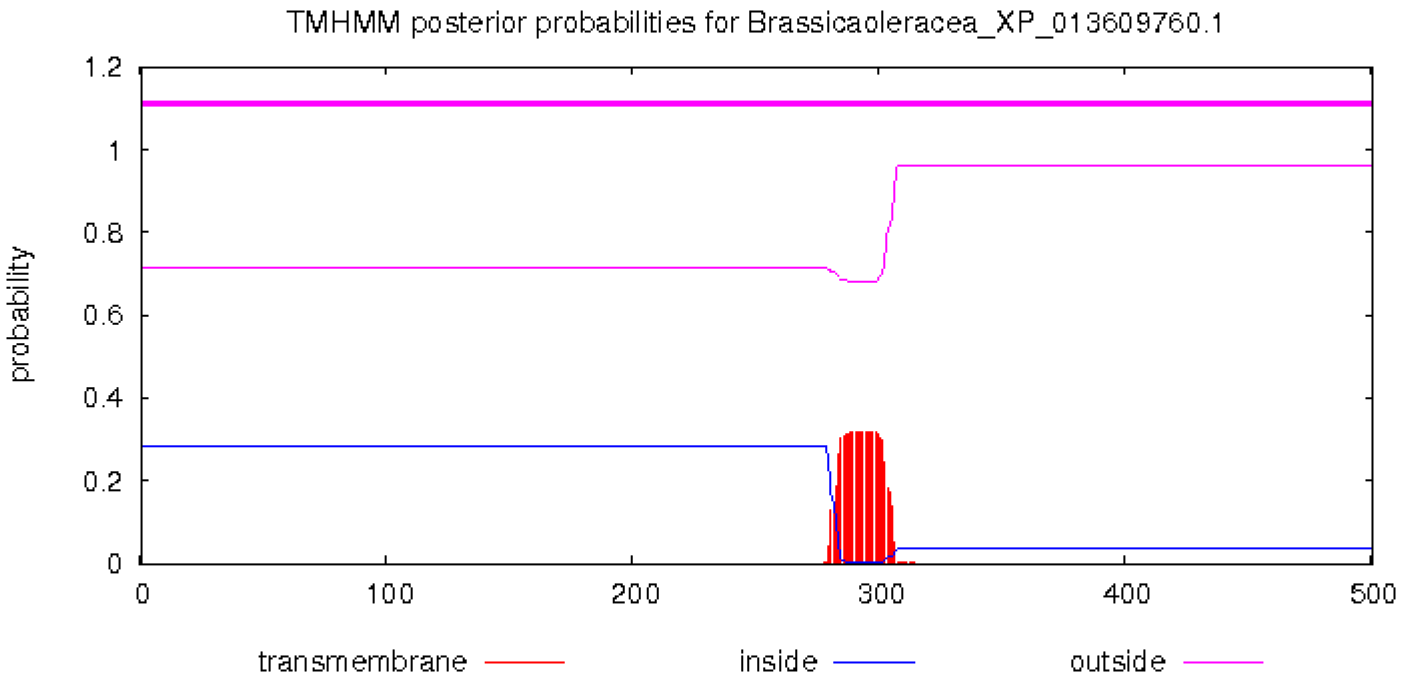

plot in postscript, script for making the plot in gnuplot, data for plot

---

```
# Brassicaoleracea_XP_013609773.1 Length: 501
# Brassicaoleracea_XP_013609773.1 Number of predicted TMHs:  0
# Brassicaoleracea_XP_013609773.1 Exp number of AAs in TMHs: 7.20466
# Brassicaoleracea_XP_013609773.1 Exp number, first 60 AAs:  0
# Brassicaoleracea_XP_013609773.1 Total prob of N-in:        0.28448
Brassicaoleracea_XP_013609773.1 TMHMM2.0        outside      1   501
```

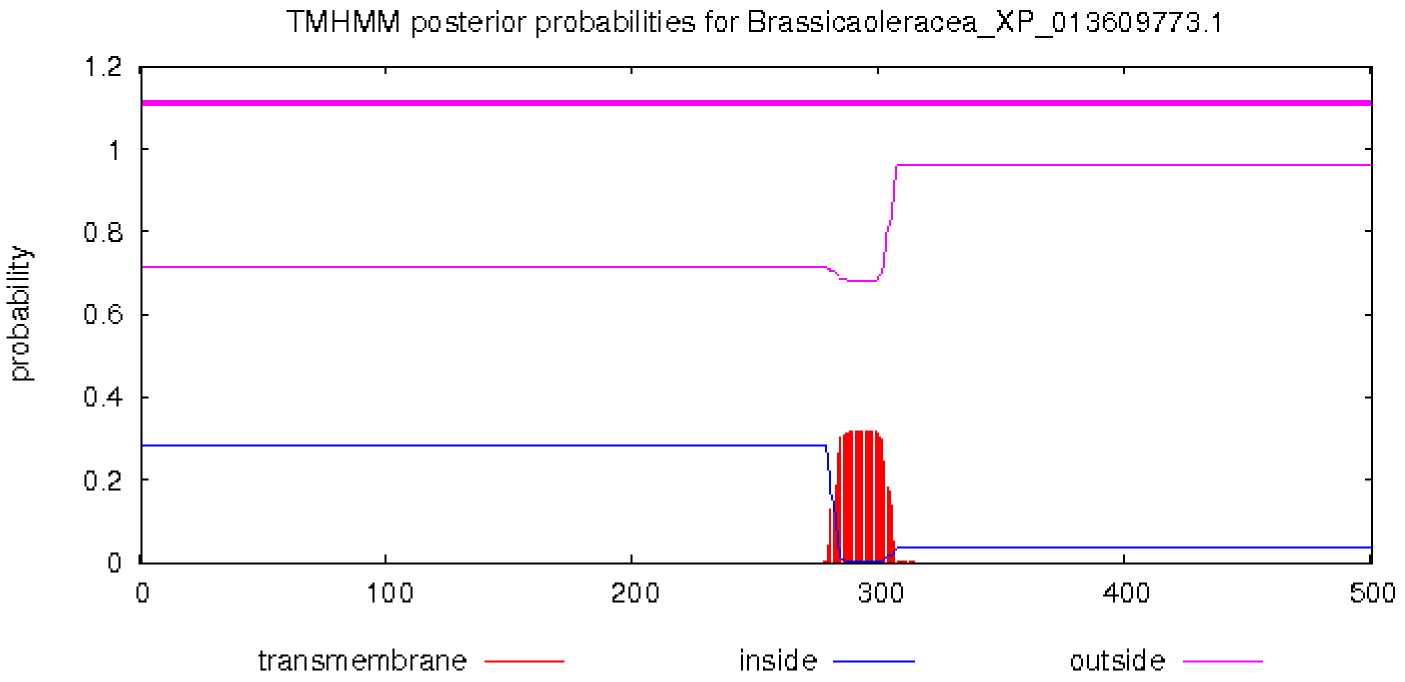

plot in postscript, script for making the plot in gnuplot, data for plot

```
# Brassicaoleracea_XP_013609781.1 Length: 500
# Brassicaoleracea_XP_013609781.1 Number of predicted TMHs:  0
# Brassicaoleracea_XP_013609781.1 Exp number of AAs in TMHs: 7.2594
# Brassicaoleracea_XP_013609781.1 Exp number, first 60 AAs:  0
# Brassicaoleracea_XP_013609781.1 Total prob of N-in:        0.28703
Brassicaoleracea_XP_013609781.1 TMHMM2.0        outside      1   500
```

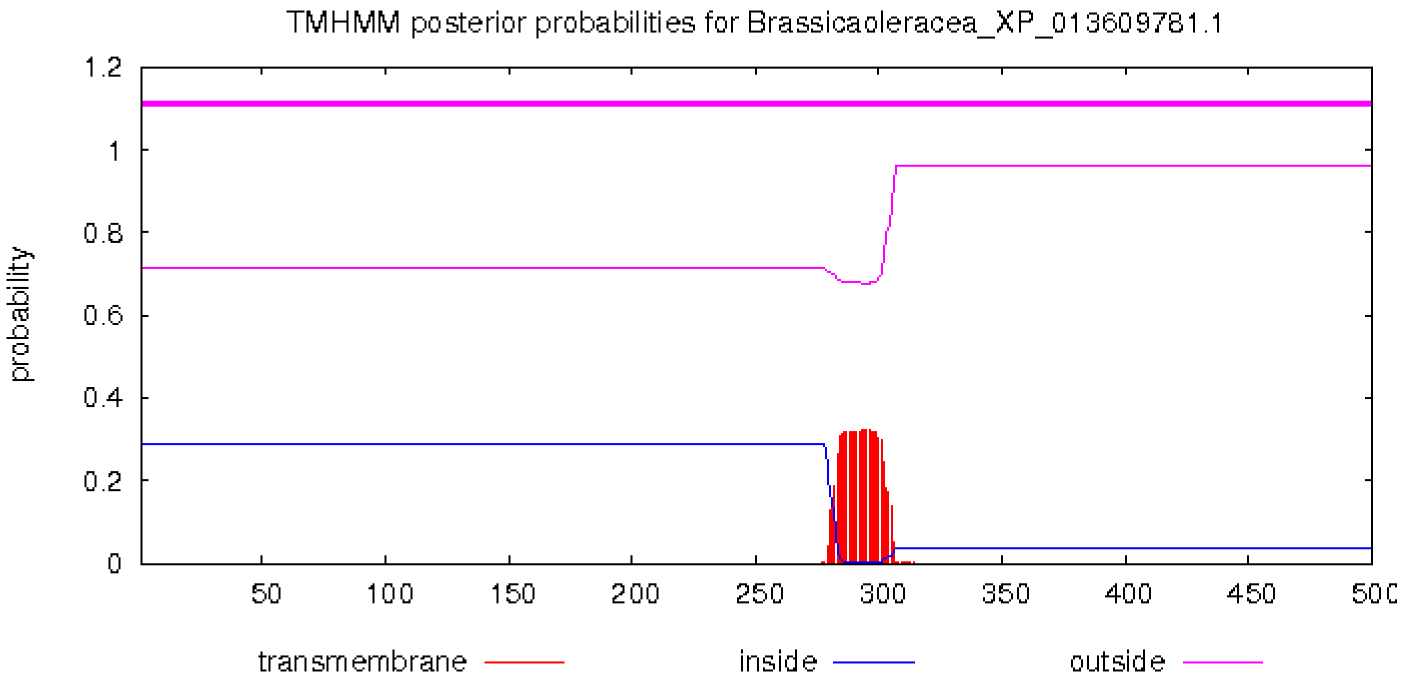

plot in postscript, script for making the plot in gnuplot, data for plot

```
# Brassicaoleracea_XP_013609789.1 Length: 428
# Brassicaoleracea_XP_013609789.1 Number of predicted TMHs:  0
# Brassicaoleracea_XP_013609789.1 Exp number of AAs in TMHs: 4.98503
# Brassicaoleracea_XP_013609789.1 Exp number, first 60 AAs:  0
# Brassicaoleracea_XP_013609789.1 Total prob of N-in:        0.18060
Brassicaoleracea_XP_013609789.1 TMHMM2.0        outside      1   428
```

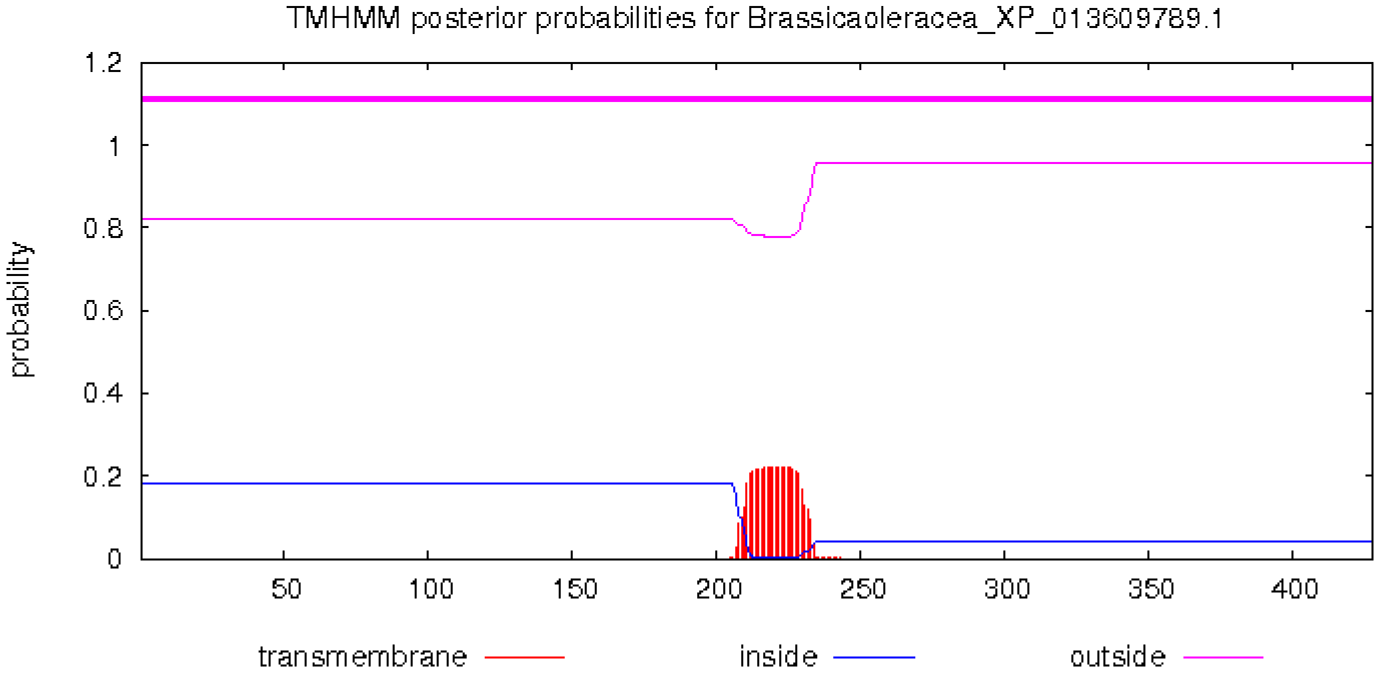

plot in postscript, script for making the plot in gnuplot, data for plot

```
# Brassicaoleracea_XP_013609845.1 Length: 402
# Brassicaoleracea_XP_013609845.1 Number of predicted TMHs:  0
# Brassicaoleracea_XP_013609845.1 Exp number of AAs in TMHs: 0.00017
# Brassicaoleracea_XP_013609845.1 Exp number, first 60 AAs:  0
# Brassicaoleracea_XP_013609845.1 Total prob of N-in:        0.00164
Brassicaoleracea_XP_013609845.1 TMHMM2.0        outside      1   402
```

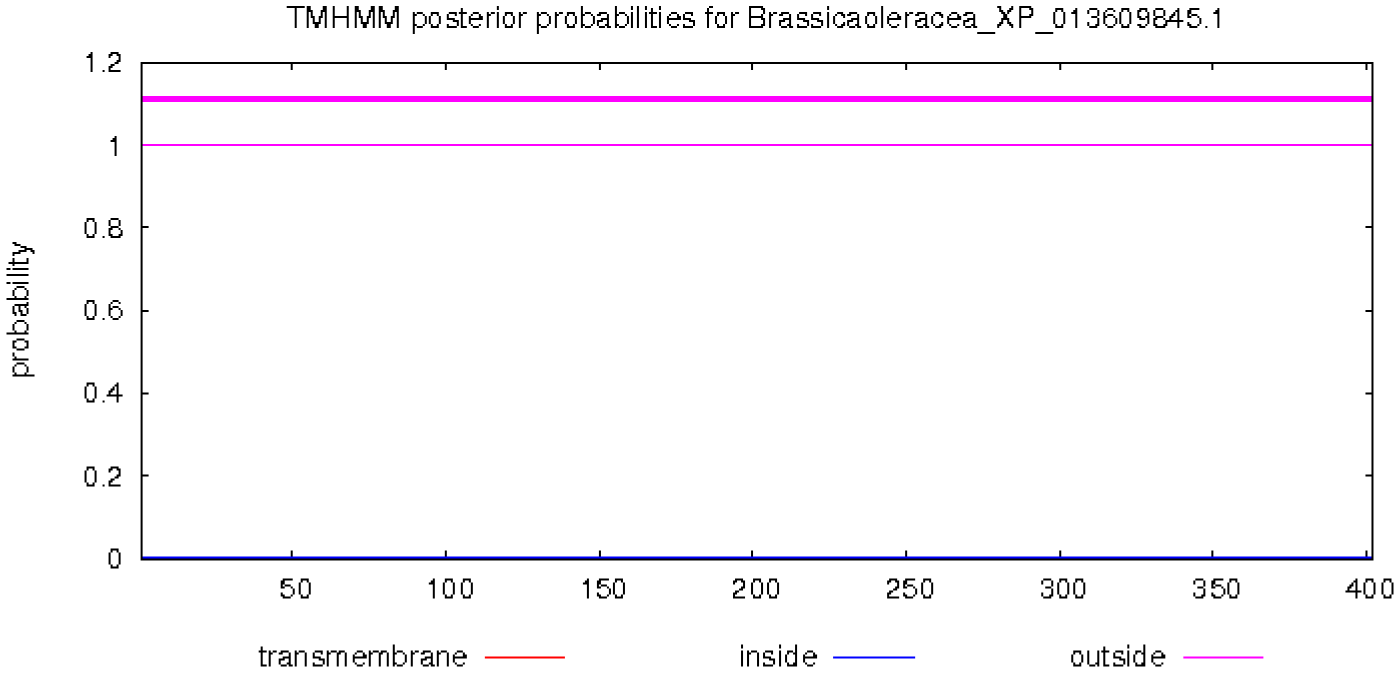

plot in postscript, script for making the plot in gnuplot, data for plot

```
# Brassicaoleracea_XP_013609846.1 Length: 402
# Brassicaoleracea_XP_013609846.1 Number of predicted TMHs:  0
# Brassicaoleracea_XP_013609846.1 Exp number of AAs in TMHs: 0.00017
# Brassicaoleracea_XP_013609846.1 Exp number, first 60 AAs:  0
# Brassicaoleracea_XP_013609846.1 Total prob of N-in:        0.00164
Brassicaoleracea_XP_013609846.1 TMHMM2.0        outside      1   402
```

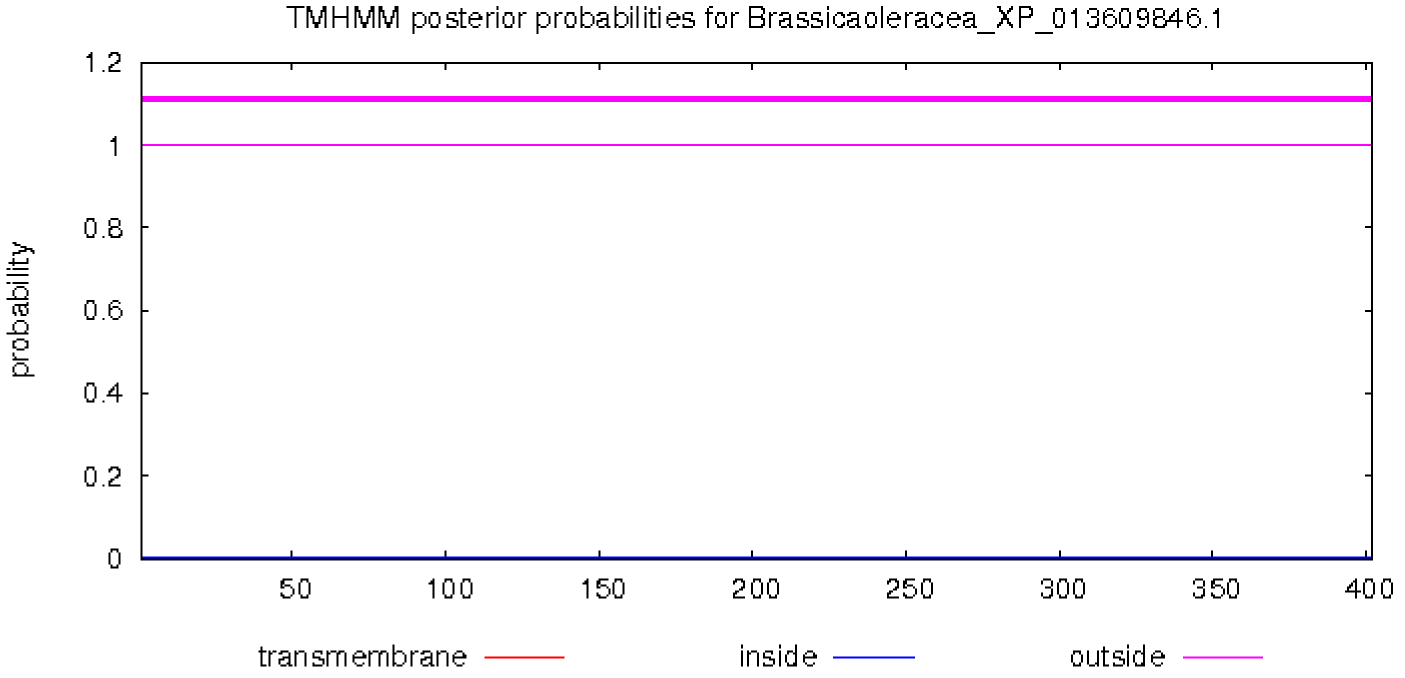

plot in postscript, script for making the plot in gnuplot, data for plot

---

```
# Brassicaoleracea_XP_013609847.1 Length: 402
# Brassicaoleracea_XP_013609847.1 Number of predicted TMHs:  0
# Brassicaoleracea_XP_013609847.1 Exp number of AAs in TMHs: 0.00017
# Brassicaoleracea_XP_013609847.1 Exp number, first 60 AAs:  0
# Brassicaoleracea_XP_013609847.1 Total prob of N-in:        0.00164
Brassicaoleracea_XP_013609847.1 TMHMM2.0        outside      1   402
```

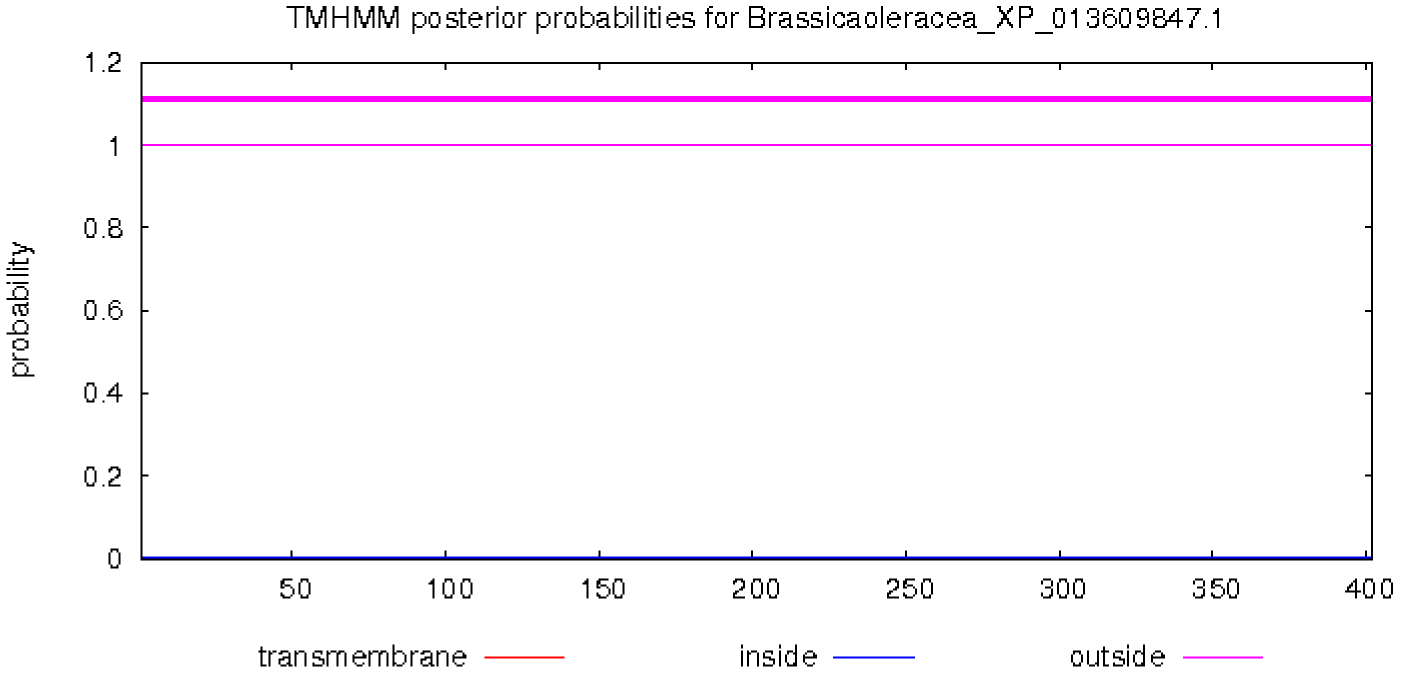

plot in postscript, script for making the plot in gnuplot, data for plot

---

```
# Brassicaoleracea_XP_013609952.1 Length: 303
# Brassicaoleracea_XP_013609952.1 Number of predicted TMHs:  0
# Brassicaoleracea_XP_013609952.1 Exp number of AAs in TMHs: 0.00148
# Brassicaoleracea_XP_013609952.1 Exp number, first 60 AAs:  0.00022
# Brassicaoleracea_XP_013609952.1 Total prob of N-in:        0.10085
Brassicaoleracea_XP_013609952.1 TMHMM2.0        outside      1   303
```

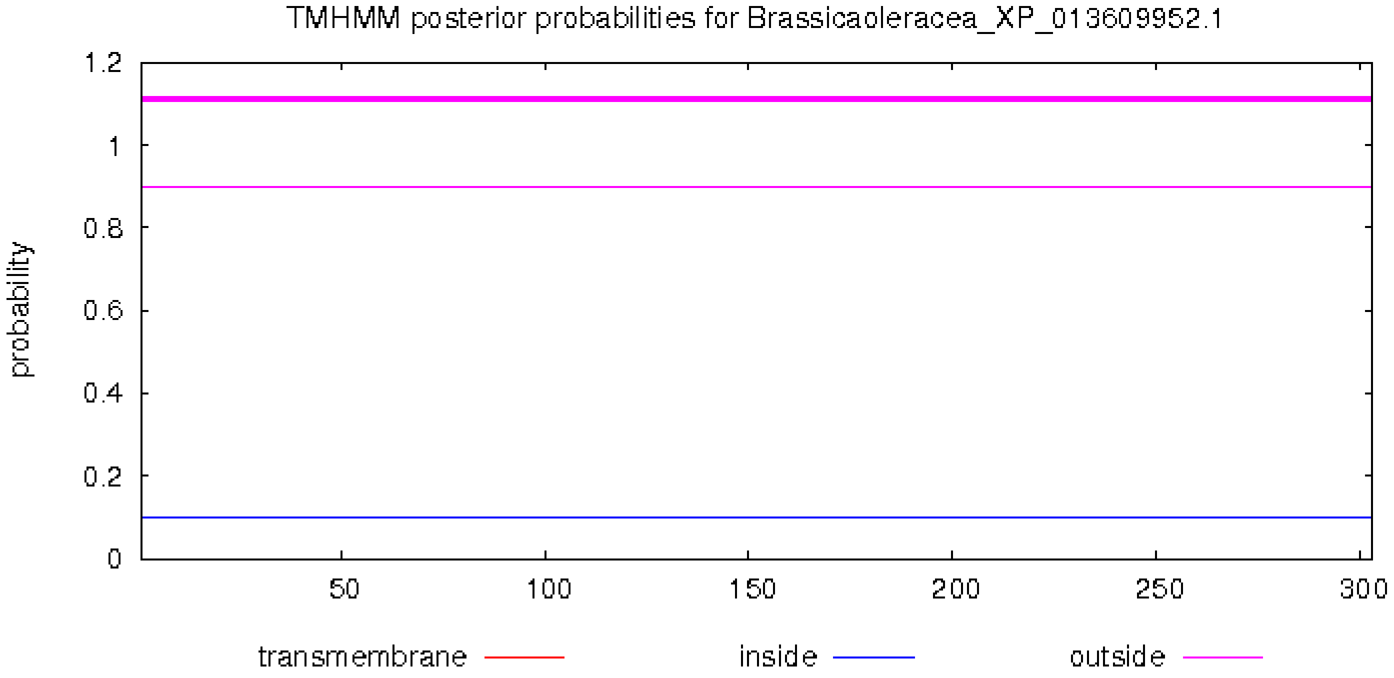

plot in postscript, script for making the plot in gnuplot, data for plot

---

```
# Brassicaoleracea_XP_013610035.1 Length: 283
# Brassicaoleracea_XP_013610035.1 Number of predicted TMHs:  0
# Brassicaoleracea_XP_013610035.1 Exp number of AAs in TMHs: 0.0036
# Brassicaoleracea_XP_013610035.1 Exp number, first 60 AAs:  0
# Brassicaoleracea_XP_013610035.1 Total prob of N-in:        0.01750
Brassicaoleracea_XP_013610035.1 TMHMM2.0        outside      1   283
```

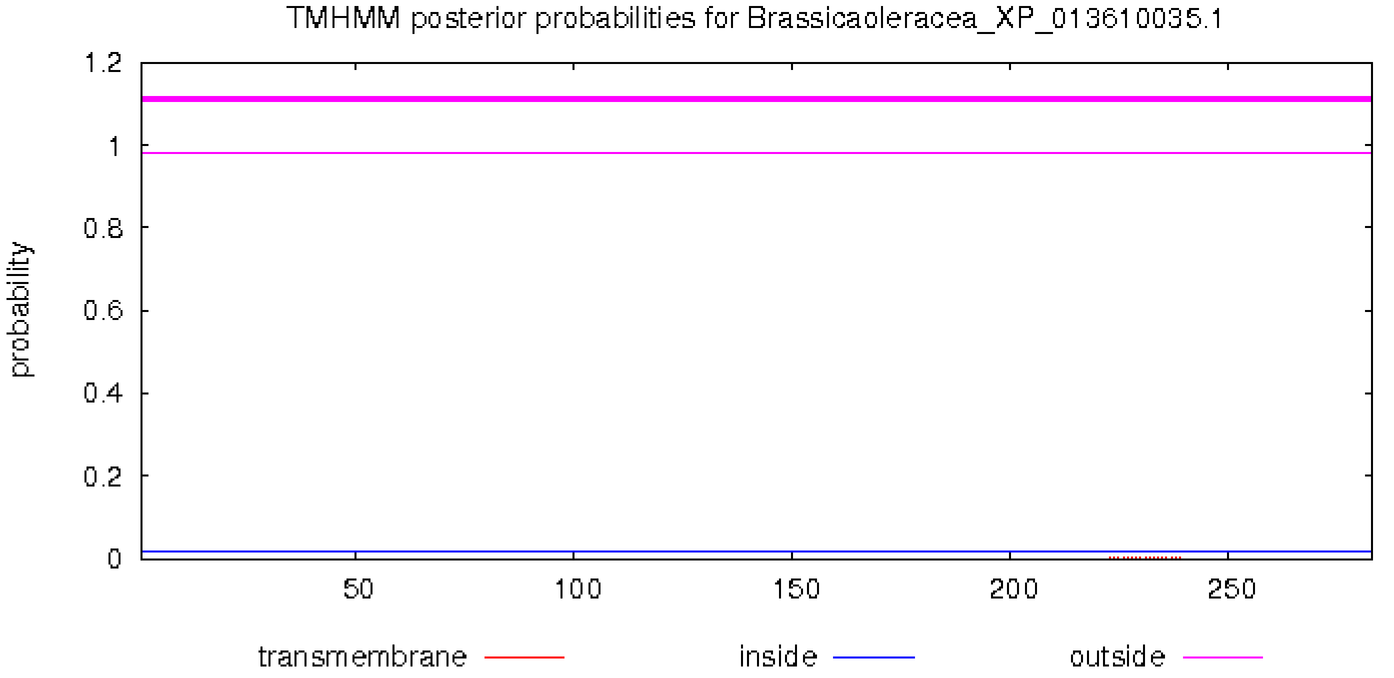

plot in postscript, script for making the plot in gnuplot, data for plot

---

```
# Brassicaoleracea_XP_013610106.1 Length: 496
# Brassicaoleracea_XP_013610106.1 Number of predicted TMHs:  0
# Brassicaoleracea_XP_013610106.1 Exp number of AAs in TMHs: 0.00037
# Brassicaoleracea_XP_013610106.1 Exp number, first 60 AAs:  0
# Brassicaoleracea_XP_013610106.1 Total prob of N-in:        0.00014
Brassicaoleracea_XP_013610106.1 TMHMM2.0        outside      1   496
```

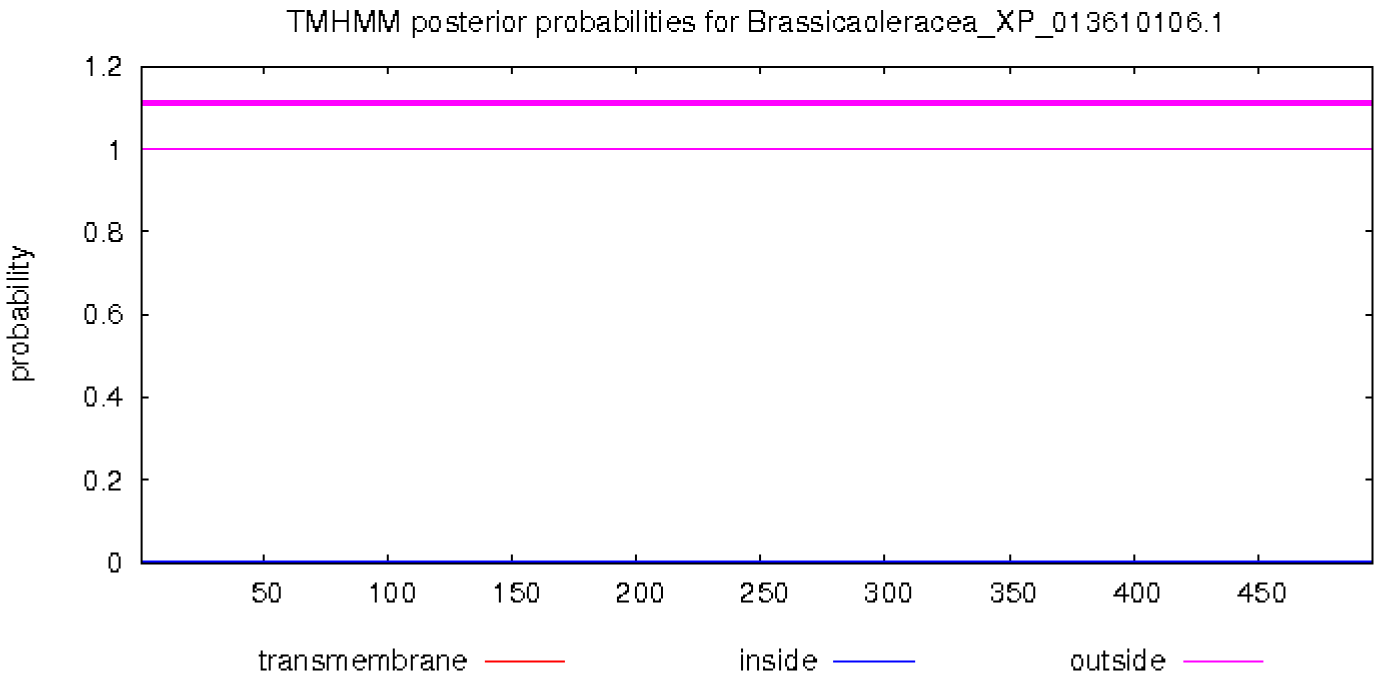

plot in postscript, script for making the plot in gnuplot, data for plot

---

```
# Brassicaoleracea_XP_013610161.1 Length: 193
# Brassicaoleracea_XP_013610161.1 Number of predicted TMHs:  0
# Brassicaoleracea_XP_013610161.1 Exp number of AAs in TMHs: 0.00125
# Brassicaoleracea_XP_013610161.1 Exp number, first 60 AAs:  0
# Brassicaoleracea_XP_013610161.1 Total prob of N-in:        0.15548
Brassicaoleracea_XP_013610161.1 TMHMM2.0        outside      1   193
```

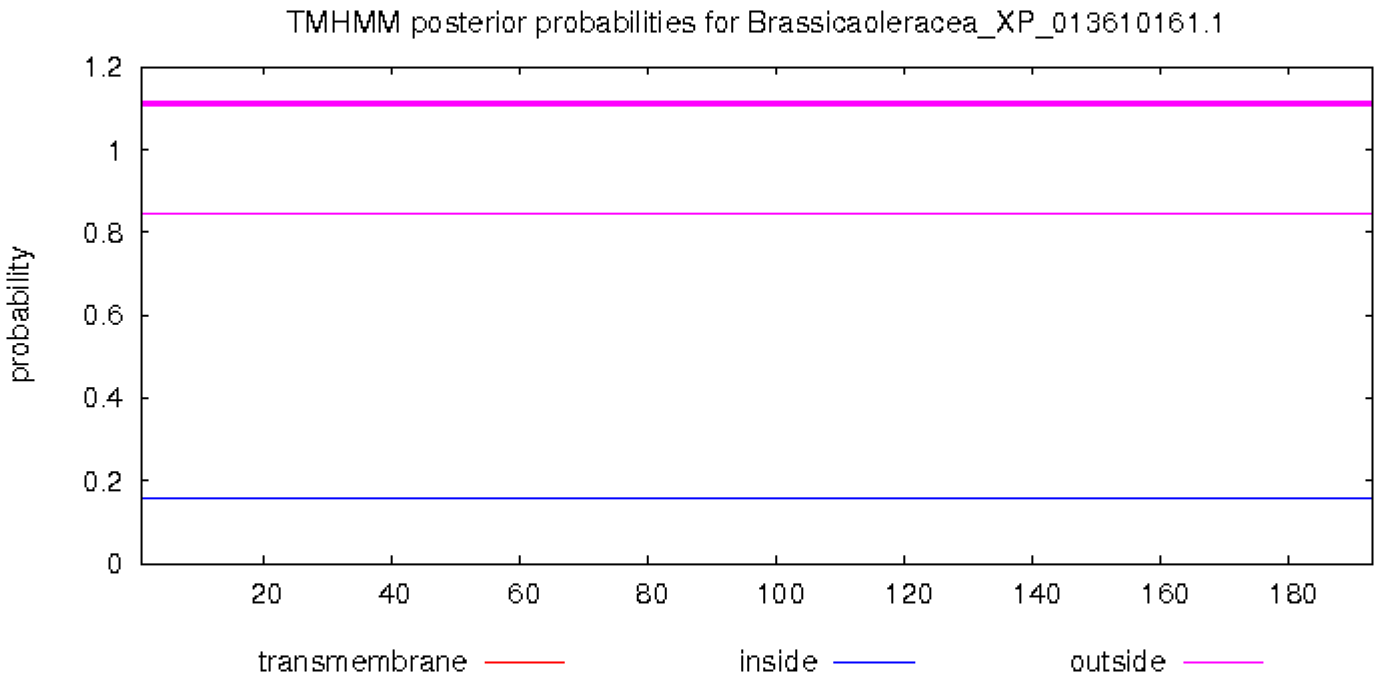

plot in postscript, script for making the plot in gnuplot, data for plot

---

```
# Brassicaoleracea_XP_013610422.1 Length: 338
# Brassicaoleracea_XP_013610422.1 Number of predicted TMHs:  0
# Brassicaoleracea_XP_013610422.1 Exp number of AAs in TMHs: 0
# Brassicaoleracea_XP_013610422.1 Exp number, first 60 AAs:  0
# Brassicaoleracea_XP_013610422.1 Total prob of N-in:        0.00214
Brassicaoleracea_XP_013610422.1 TMHMM2.0        outside      1   338
```

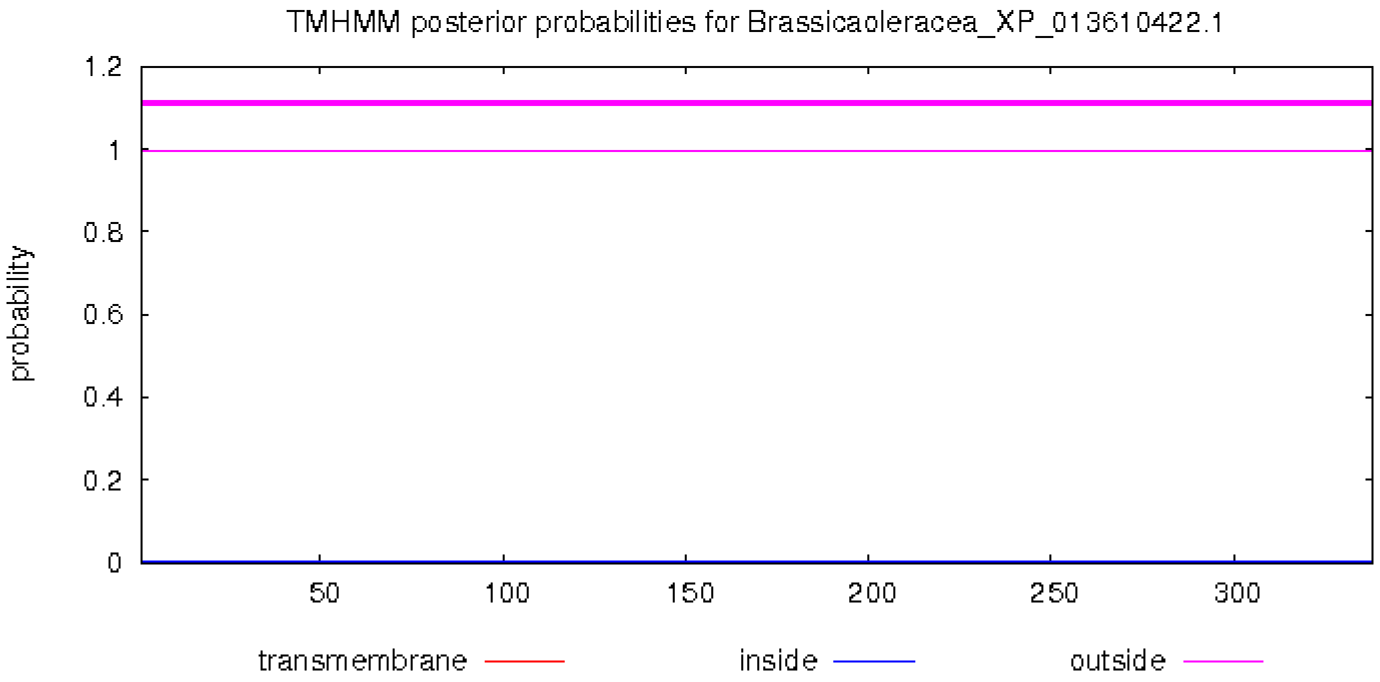

plot in postscript, script for making the plot in gnuplot, data for plot

---

```
# Brassicaoleracea_XP_013610671.1 Length: 325
# Brassicaoleracea_XP_013610671.1 Number of predicted TMHs:  0
# Brassicaoleracea_XP_013610671.1 Exp number of AAs in TMHs: 0.00131
# Brassicaoleracea_XP_013610671.1 Exp number, first 60 AAs:  0
# Brassicaoleracea_XP_013610671.1 Total prob of N-in:        0.02502
Brassicaoleracea_XP_013610671.1 TMHMM2.0        outside      1   325
```

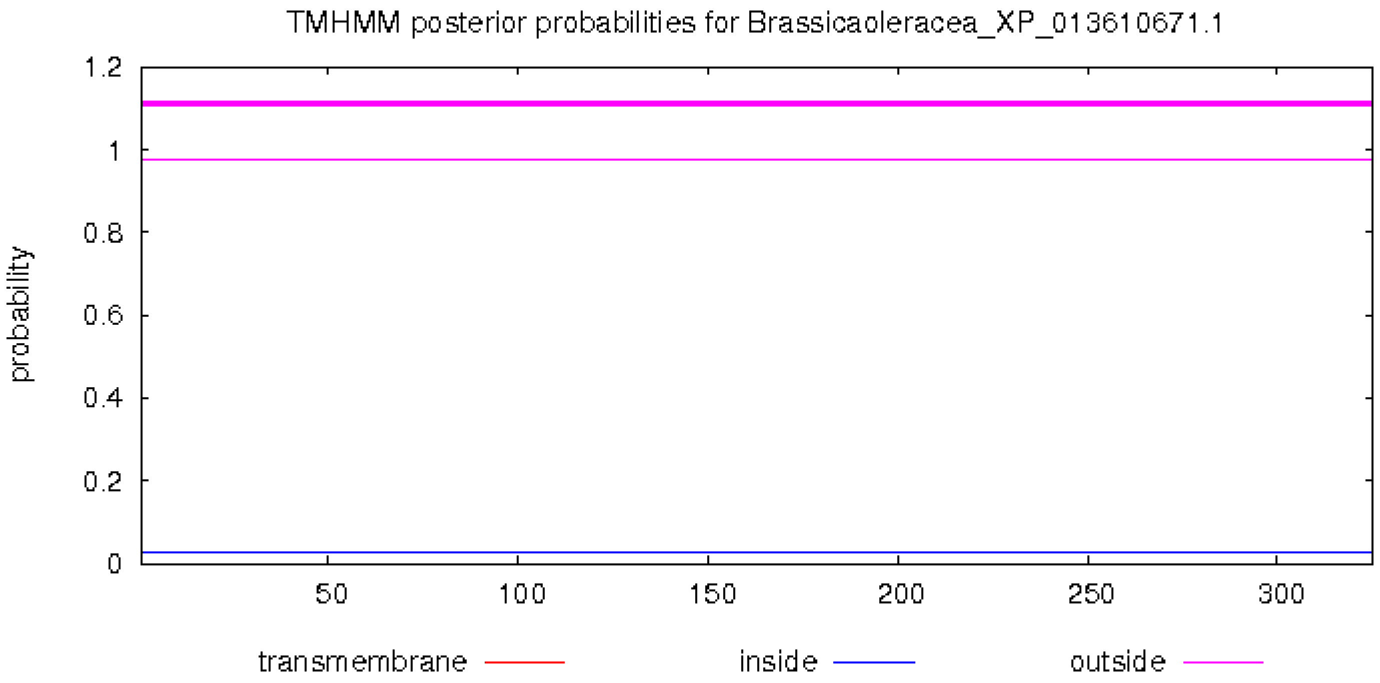

plot in postscript, script for making the plot in gnuplot, data for plot

---

```
# Brassicaoleracea_XP_013610715.1 Length: 234
# Brassicaoleracea_XP_013610715.1 Number of predicted TMHs:  0
# Brassicaoleracea_XP_013610715.1 Exp number of AAs in TMHs: 0.00016
# Brassicaoleracea_XP_013610715.1 Exp number, first 60 AAs:  0.00016
# Brassicaoleracea_XP_013610715.1 Total prob of N-in:        0.04620
Brassicaoleracea_XP_013610715.1 TMHMM2.0        outside      1   234
```

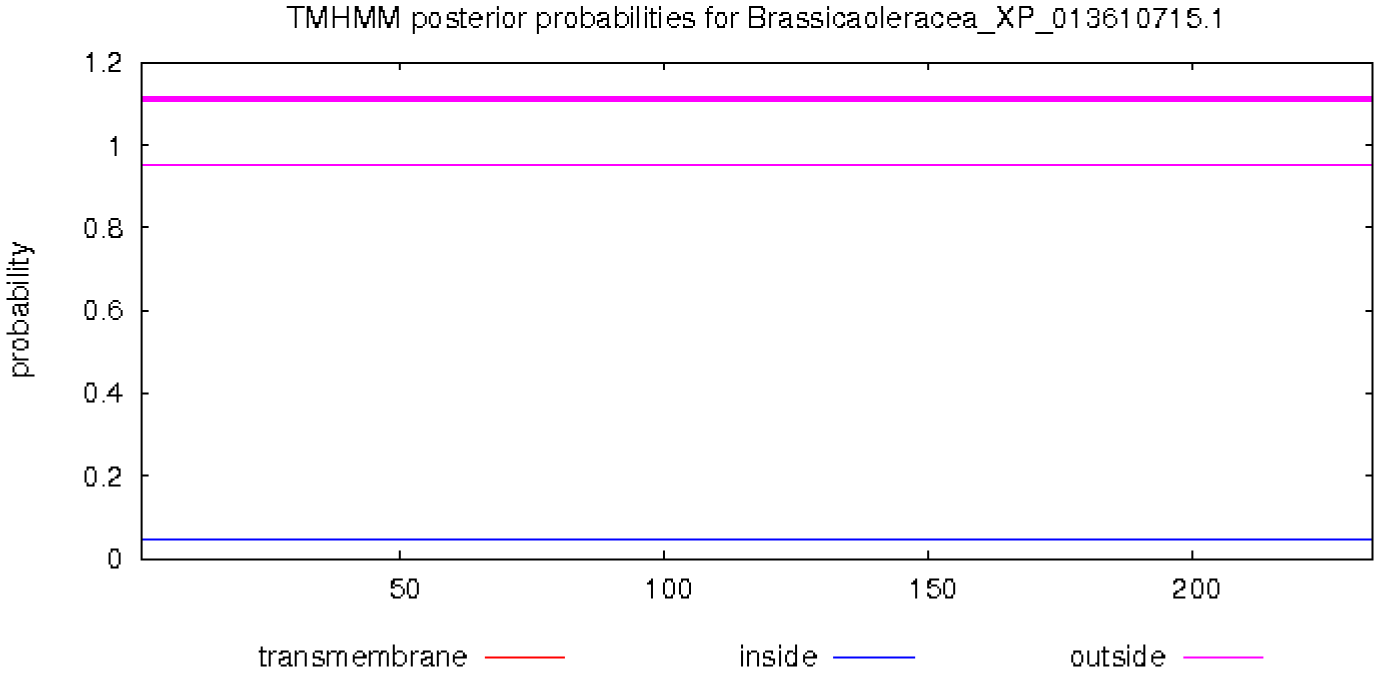

plot in postscript, script for making the plot in gnuplot, data for plot

---

```
# Brassicaoleracea_XP_013611181.1 Length: 307
# Brassicaoleracea_XP_013611181.1 Number of predicted TMHs:  0
# Brassicaoleracea_XP_013611181.1 Exp number of AAs in TMHs: 0.00114
# Brassicaoleracea_XP_013611181.1 Exp number, first 60 AAs:  0
# Brassicaoleracea_XP_013611181.1 Total prob of N-in:        0.02084
Brassicaoleracea_XP_013611181.1 TMHMM2.0        outside      1   307
```

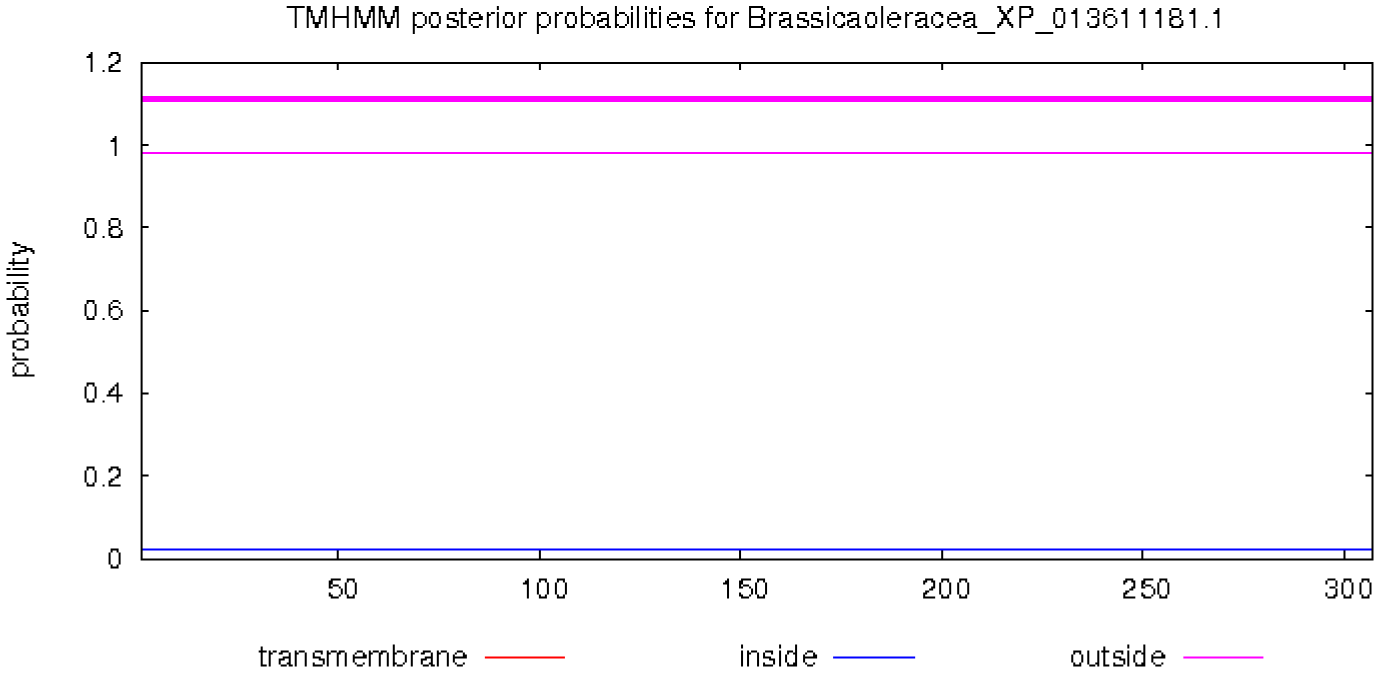

plot in postscript, script for making the plot in gnuplot, data for plot

---

```
# Brassicaoleracea_XP_013611566.1 Length: 434
# Brassicaoleracea_XP_013611566.1 Number of predicted TMHs:  0
# Brassicaoleracea_XP_013611566.1 Exp number of AAs in TMHs: 0
# Brassicaoleracea_XP_013611566.1 Exp number, first 60 AAs:  0
# Brassicaoleracea_XP_013611566.1 Total prob of N-in:        0.00135
Brassicaoleracea_XP_013611566.1 TMHMM2.0        outside      1   434
```

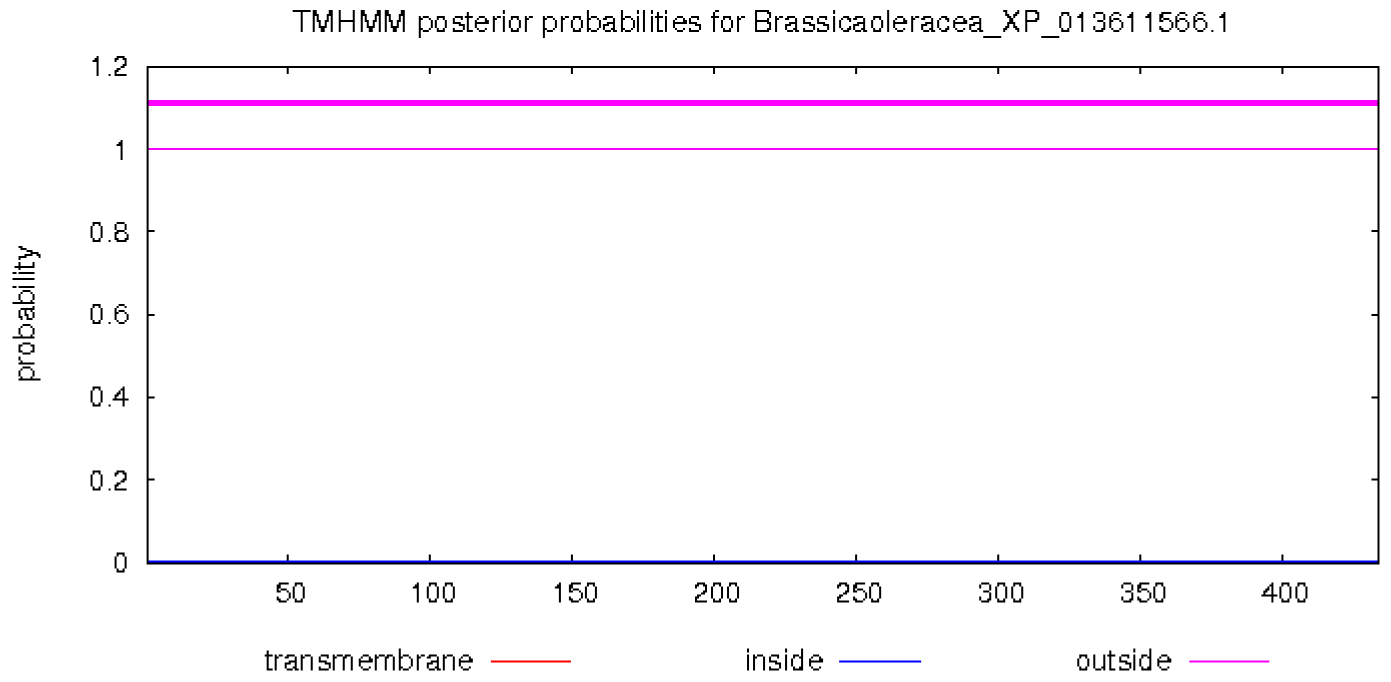

plot in postscript, script for making the plot in gnuplot, data for plot

```
# Brassicaoleracea_XP_013611567.1 Length: 434
# Brassicaoleracea_XP_013611567.1 Number of predicted TMHs:  0
# Brassicaoleracea_XP_013611567.1 Exp number of AAs in TMHs: 0
# Brassicaoleracea_XP_013611567.1 Exp number, first 60 AAs:  0
# Brassicaoleracea_XP_013611567.1 Total prob of N-in:        0.00135
Brassicaoleracea_XP_013611567.1 TMHMM2.0        outside      1   434
```

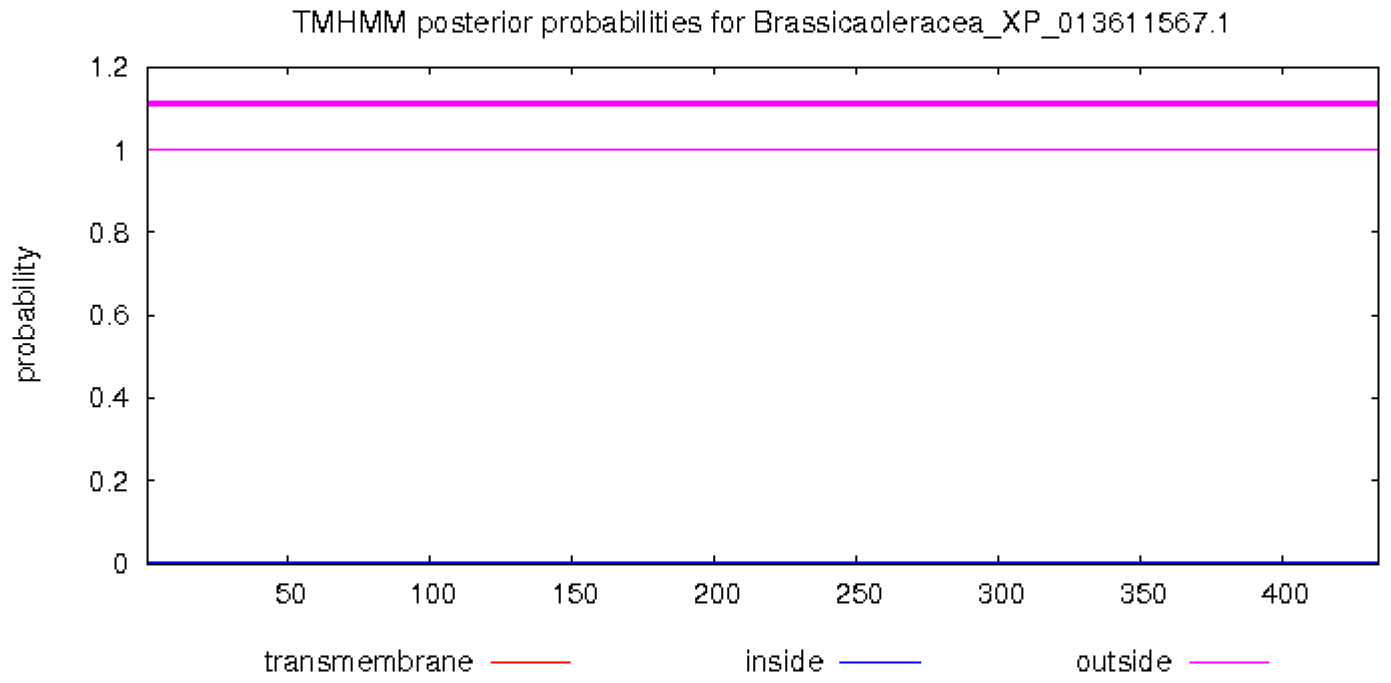

plot in postscript, script for making the plot in gnuplot, data for plot

```
# Brassicaoleracea_XP_013612508.1 Length: 342
# Brassicaoleracea_XP_013612508.1 Number of predicted TMHs:  0
# Brassicaoleracea_XP_013612508.1 Exp number of AAs in TMHs: 0
# Brassicaoleracea_XP_013612508.1 Exp number, first 60 AAs:  0
# Brassicaoleracea_XP_013612508.1 Total prob of N-in:        0.00431
Brassicaoleracea_XP_013612508.1 TMHMM2.0        outside      1   342
```

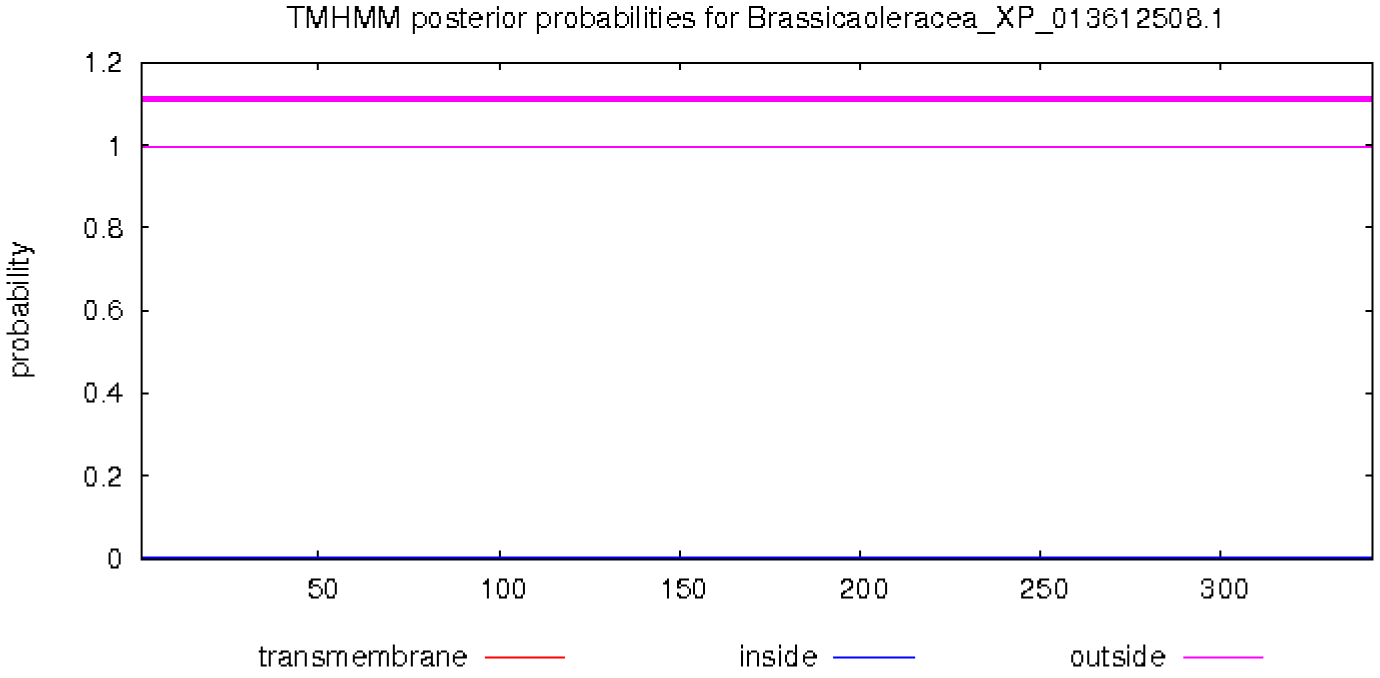

plot in postscript, script for making the plot in gnuplot, data for plot

---

```
# Brassicaoleracea_XP_013612936.1 Length: 456
# Brassicaoleracea_XP_013612936.1 Number of predicted TMHs:  0
# Brassicaoleracea_XP_013612936.1 Exp number of AAs in TMHs: 9.19197
# Brassicaoleracea_XP_013612936.1 Exp number, first 60 AAs:  0
# Brassicaoleracea_XP_013612936.1 Total prob of N-in:        0.00869
Brassicaoleracea_XP_013612936.1 TMHMM2.0        outside      1   456
```

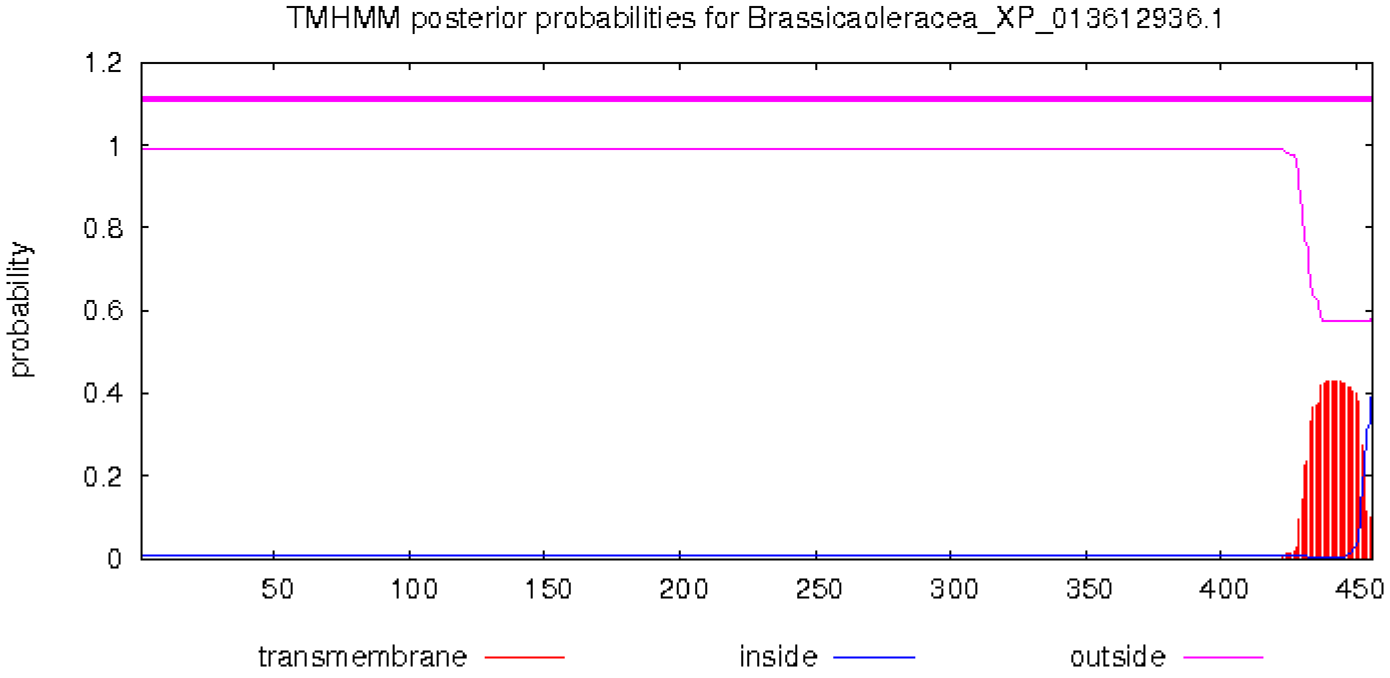

plot in postscript, script for making the plot in gnuplot, data for plot

---

```
# Brassicaoleracea_XP_013612937.1 Length: 455
# Brassicaoleracea_XP_013612937.1 Number of predicted TMHs:  0
# Brassicaoleracea_XP_013612937.1 Exp number of AAs in TMHs: 9.19335
# Brassicaoleracea_XP_013612937.1 Exp number, first 60 AAs:  0
# Brassicaoleracea_XP_013612937.1 Total prob of N-in:        0.00880
Brassicaoleracea_XP_013612937.1 TMHMM2.0        outside      1   455
```

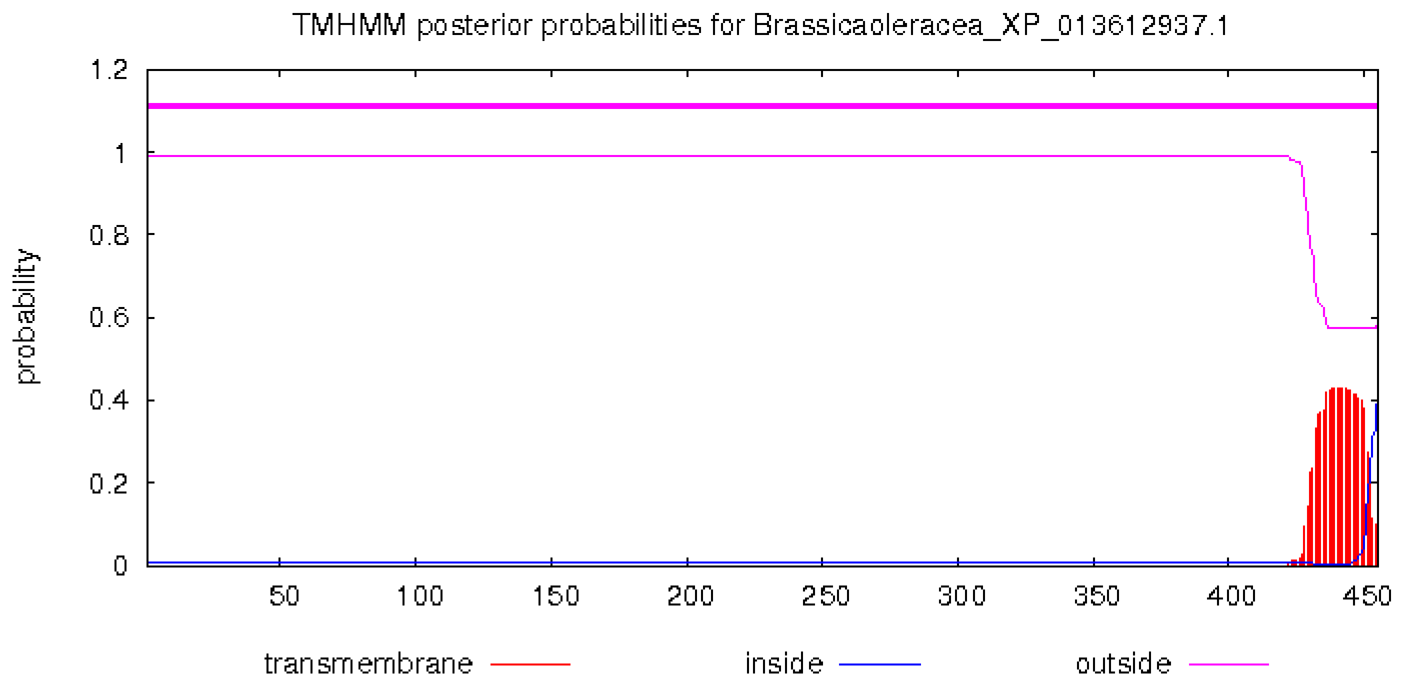

plot in postscript, script for making the plot in gnuplot, data for plot

---

```
# Brassicaoleracea_XP_013613034.1 Length: 278
# Brassicaoleracea_XP_013613034.1 Number of predicted TMHs:  0
# Brassicaoleracea_XP_013613034.1 Exp number of AAs in TMHs: 0.00019
# Brassicaoleracea_XP_013613034.1 Exp number, first 60 AAs:  0
# Brassicaoleracea_XP_013613034.1 Total prob of N-in:        0.01081
Brassicaoleracea_XP_013613034.1 TMHMM2.0        outside      1   278
```

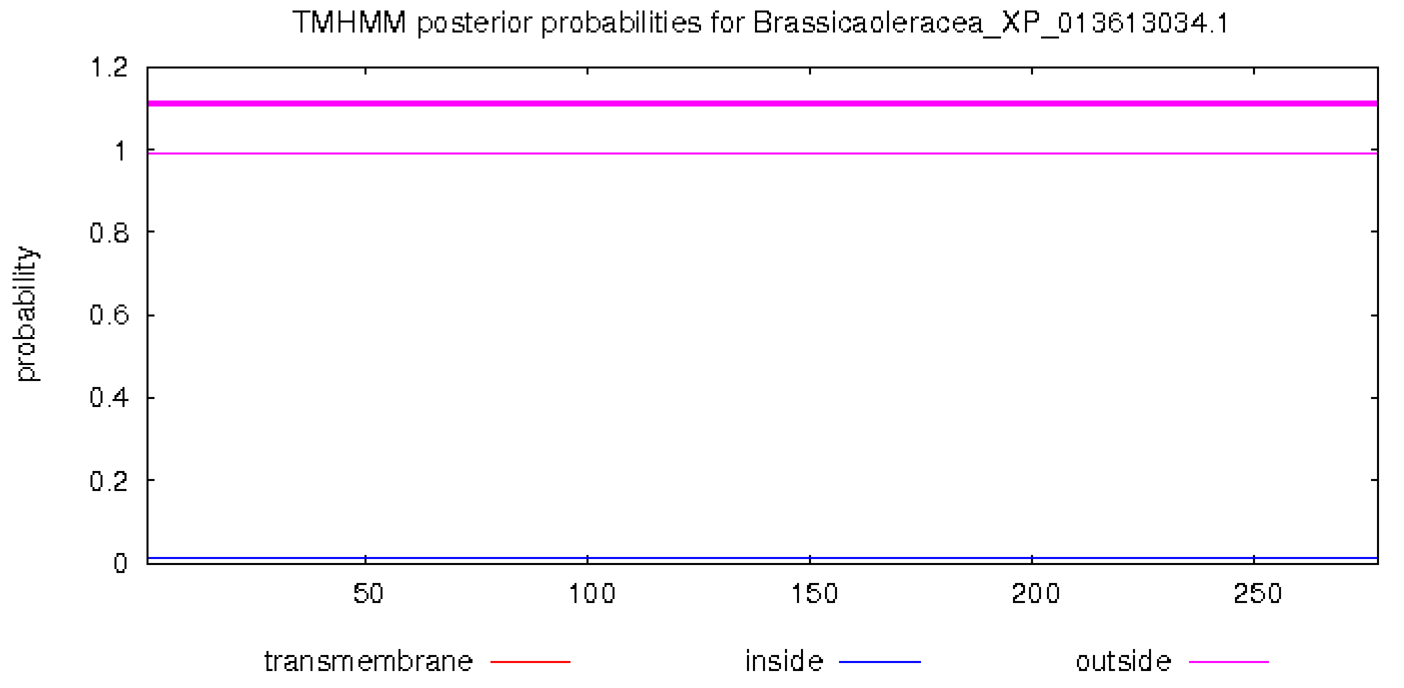

plot in postscript, script for making the plot in gnuplot, data for plot

---

```
# Brassicaoleracea_XP_013613101.1 Length: 561
# Brassicaoleracea_XP_013613101.1 Number of predicted TMHs:  1
# Brassicaoleracea_XP_013613101.1 Exp number of AAs in TMHs: 21.66893
# Brassicaoleracea_XP_013613101.1 Exp number, first 60 AAs:  0
# Brassicaoleracea_XP_013613101.1 Total prob of N-in:        0.04515
Brassicaoleracea_XP_013613101.1 TMHMM2.0        outside      1   535
Brassicaoleracea_XP_013613101.1 TMHMM2.0        TMhelix    536   558
Brassicaoleracea_XP_013613101.1 TMHMM2.0        inside     559   561
```

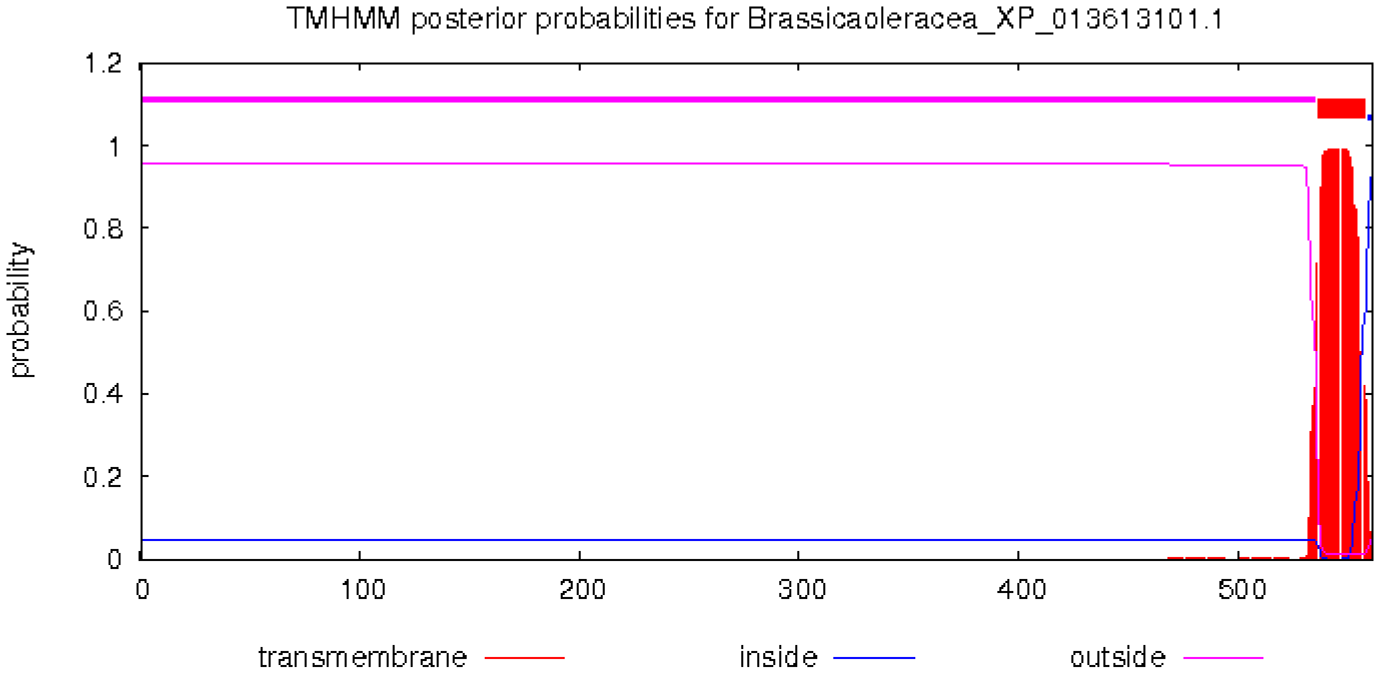

plot in postscript, script for making the plot in gnuplot, data for plot

---

```
# Brassicaoleracea_XP_013613103.1 Length: 560
# Brassicaoleracea_XP_013613103.1 Number of predicted TMHs:  1
# Brassicaoleracea_XP_013613103.1 Exp number of AAs in TMHs: 21.66948
# Brassicaoleracea_XP_013613103.1 Exp number, first 60 AAs:  0
# Brassicaoleracea_XP_013613103.1 Total prob of N-in:        0.04569
Brassicaoleracea_XP_013613103.1 TMHMM2.0        outside      1   534
Brassicaoleracea_XP_013613103.1 TMHMM2.0        TMhelix    535   557
Brassicaoleracea_XP_013613103.1 TMHMM2.0        inside     558   560
```

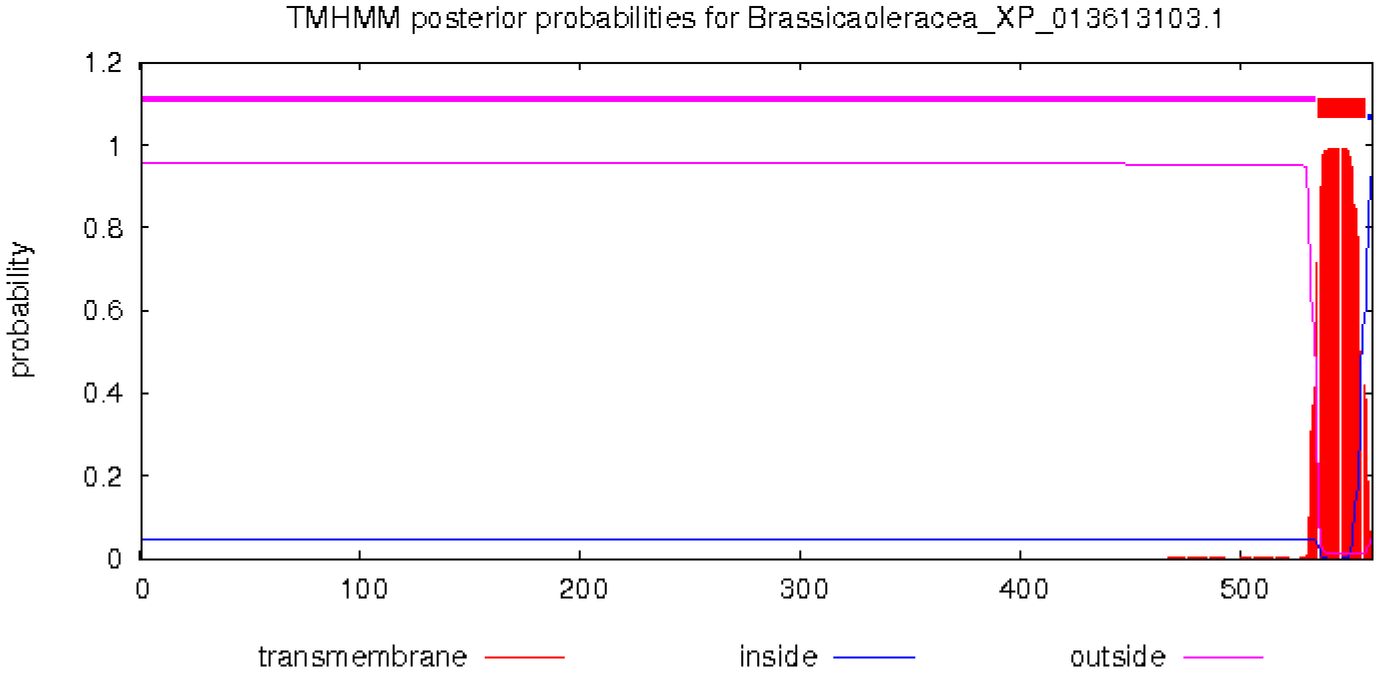

plot in postscript, script for making the plot in gnuplot, data for plot

```
# Brassicaoleracea_XP_013613533.1 Length: 289
# Brassicaoleracea_XP_013613533.1 Number of predicted TMHs:  0
# Brassicaoleracea_XP_013613533.1 Exp number of AAs in TMHs: 0.00057
# Brassicaoleracea_XP_013613533.1 Exp number, first 60 AAs:  0.00057
# Brassicaoleracea_XP_013613533.1 Total prob of N-in:        0.04383
Brassicaoleracea_XP_013613533.1 TMHMM2.0        outside      1   289
```

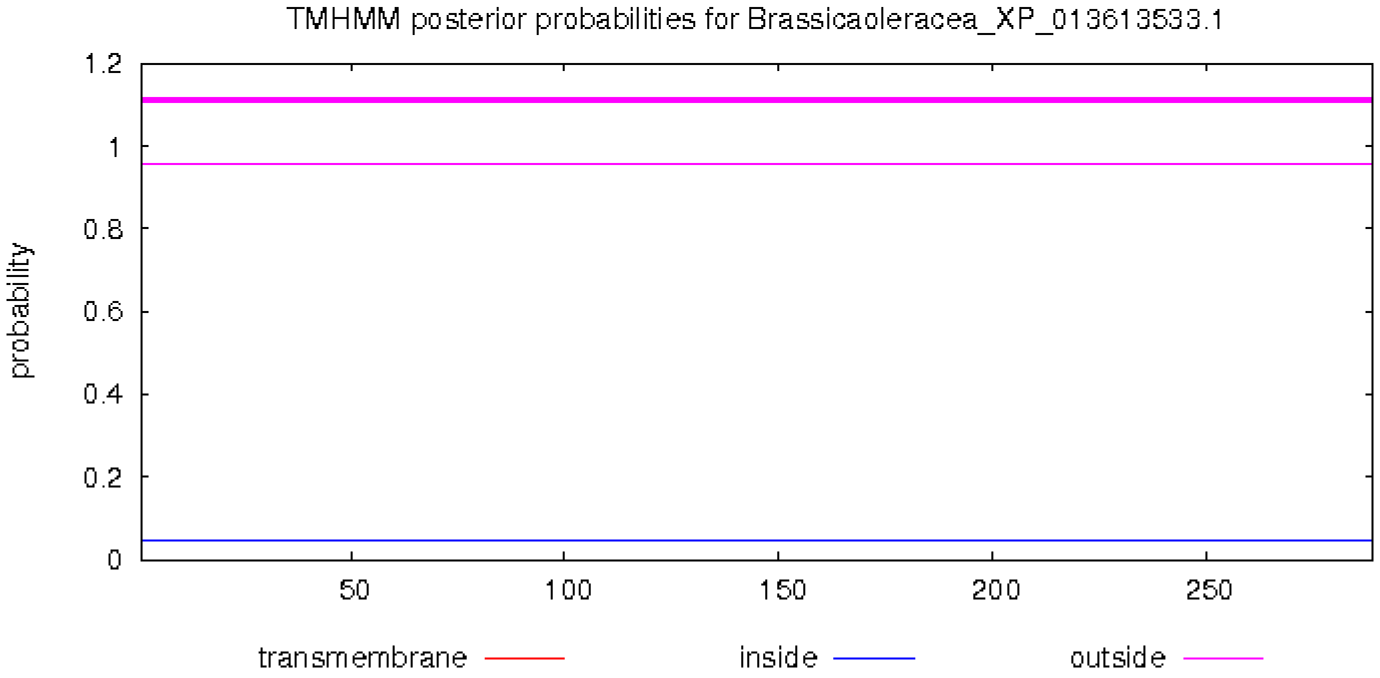

plot in postscript, script for making the plot in gnuplot, data for plot

```
# Brassicaoleracea_XP_013614047.1 Length: 417
# Brassicaoleracea_XP_013614047.1 Number of predicted TMHs:  1
# Brassicaoleracea_XP_013614047.1 Exp number of AAs in TMHs: 20.98948
# Brassicaoleracea_XP_013614047.1 Exp number, first 60 AAs:  0.00141
# Brassicaoleracea_XP_013614047.1 Total prob of N-in:        0.12554
Brassicaoleracea_XP_013614047.1 TMHMM2.0        outside      1   393
Brassicaoleracea_XP_013614047.1 TMHMM2.0        TMhelix    394   416
Brassicaoleracea_XP_013614047.1 TMHMM2.0        inside     417   417
```

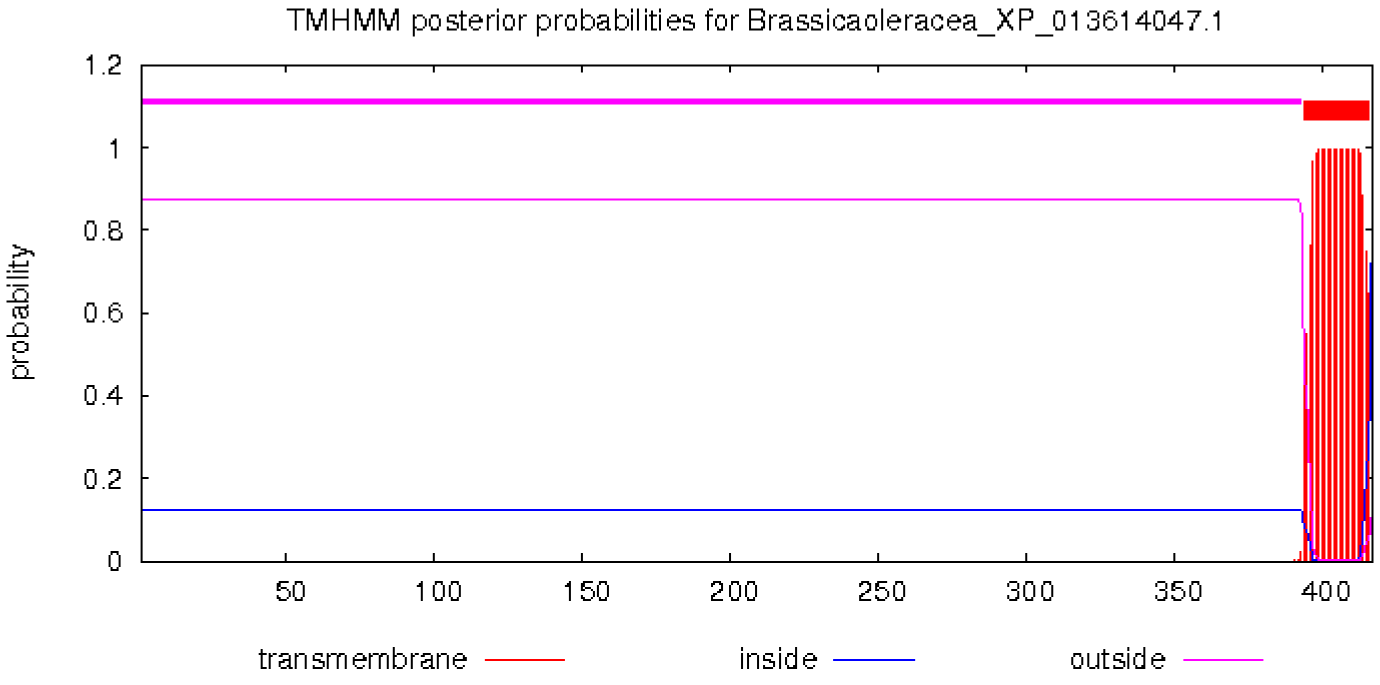

plot in postscript, script for making the plot in gnuplot, data for plot

---

```
# Brassicaoleracea_XP_013614048.1 Length: 415
# Brassicaoleracea_XP_013614048.1 Number of predicted TMHs:  1
# Brassicaoleracea_XP_013614048.1 Exp number of AAs in TMHs: 20.98623
# Brassicaoleracea_XP_013614048.1 Exp number, first 60 AAs:  0.00018
# Brassicaoleracea_XP_013614048.1 Total prob of N-in:        0.12830
Brassicaoleracea_XP_013614048.1 TMHMM2.0        outside      1   391
Brassicaoleracea_XP_013614048.1 TMHMM2.0        TMhelix    392   414
Brassicaoleracea_XP_013614048.1 TMHMM2.0        inside     415   415
```

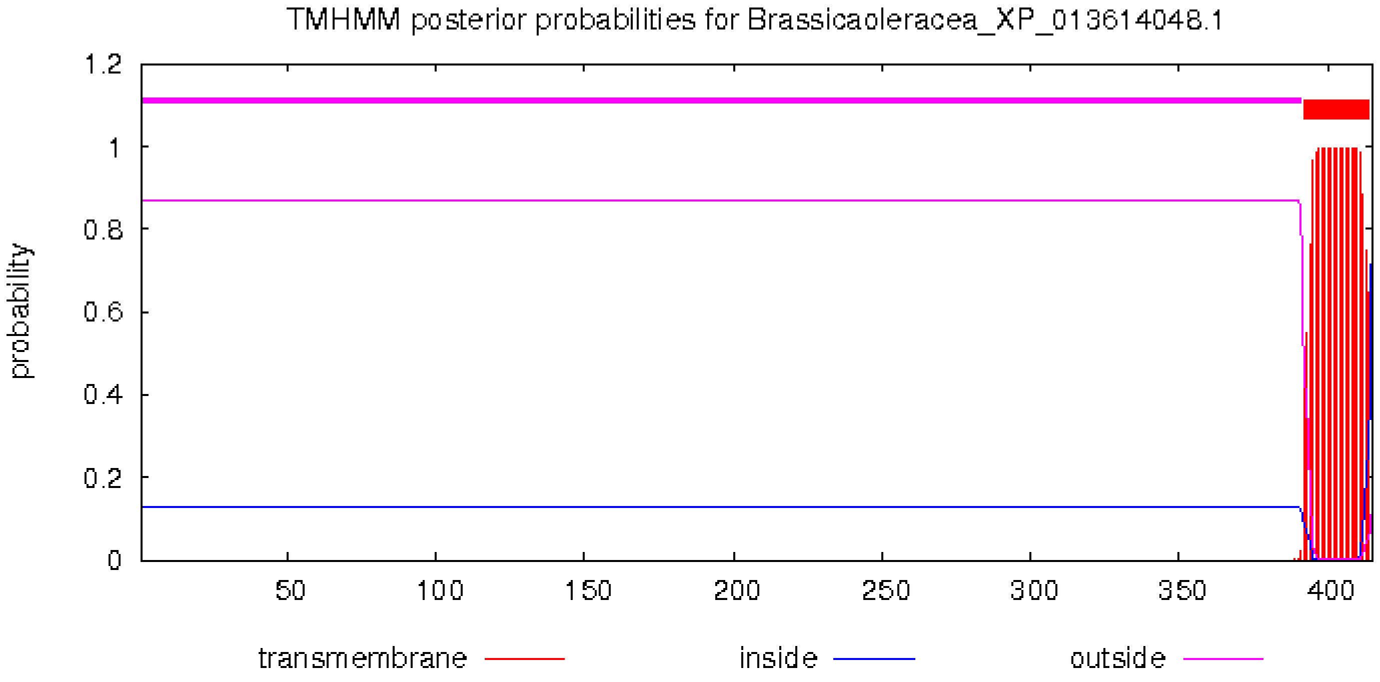

plot in postscript, script for making the plot in gnuplot, data for plot

---

```
# Brassicaoleracea_XP_013614049.1 Length: 391
# Brassicaoleracea_XP_013614049.1 Number of predicted TMHs:  1
# Brassicaoleracea_XP_013614049.1 Exp number of AAs in TMHs: 20.96805
# Brassicaoleracea_XP_013614049.1 Exp number, first 60 AAs:  0.00036
# Brassicaoleracea_XP_013614049.1 Total prob of N-in:        0.16566
```

```
Brassicaoleracea_XP_013614049.1 TMHMM2.0        outside      1   367
Brassicaoleracea_XP_013614049.1 TMHMM2.0        TMhelix    368   390
Brassicaoleracea_XP_013614049.1 TMHMM2.0        inside     391   391
```

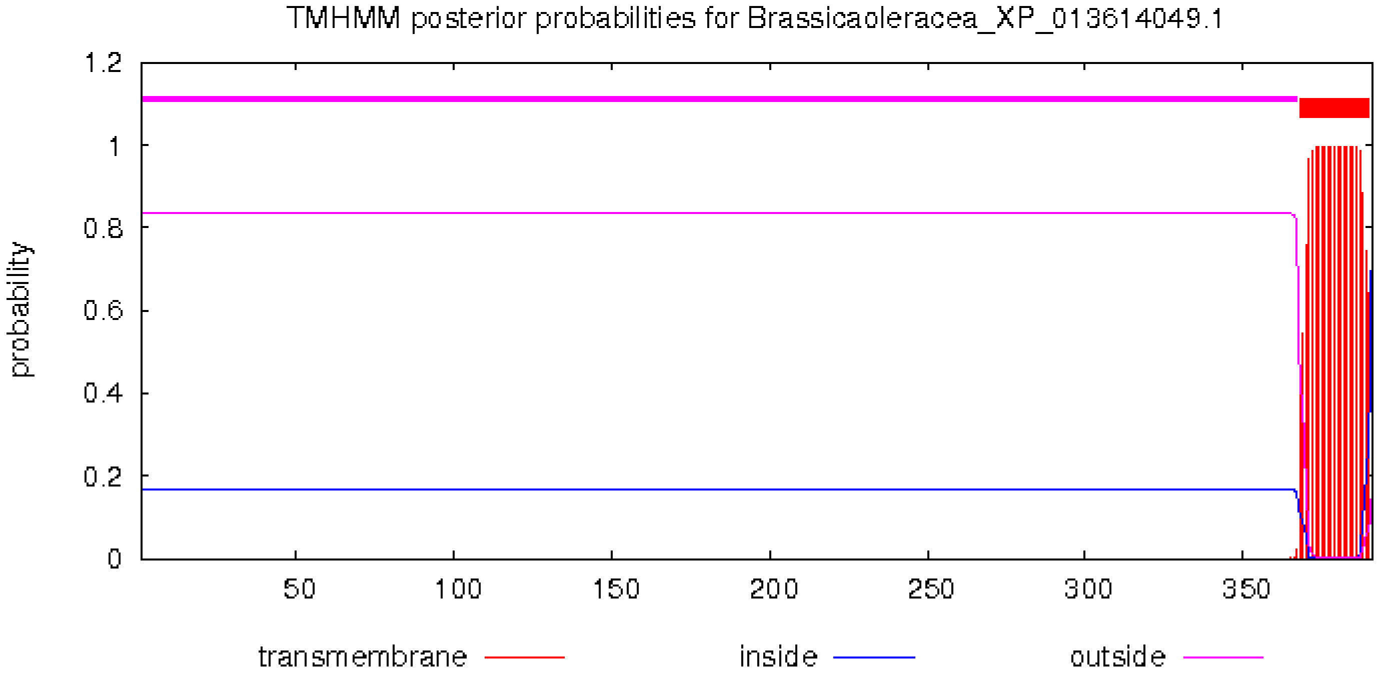

plot in postscript, script for making the plot in gnuplot, data for plot

---

```
# Brassicaoleracea_XP_013614938.1 Length: 322
# Brassicaoleracea_XP_013614938.1 Number of predicted TMHs:  0
# Brassicaoleracea_XP_013614938.1 Exp number of AAs in TMHs: 0.000750000000000000001
# Brassicaoleracea_XP_013614938.1 Exp number, first 60 AAs:  0.00054
# Brassicaoleracea_XP_013614938.1 Total prob of N-in:        0.01616
Brassicaoleracea_XP_013614938.1 TMHMM2.0        outside      1   322
```

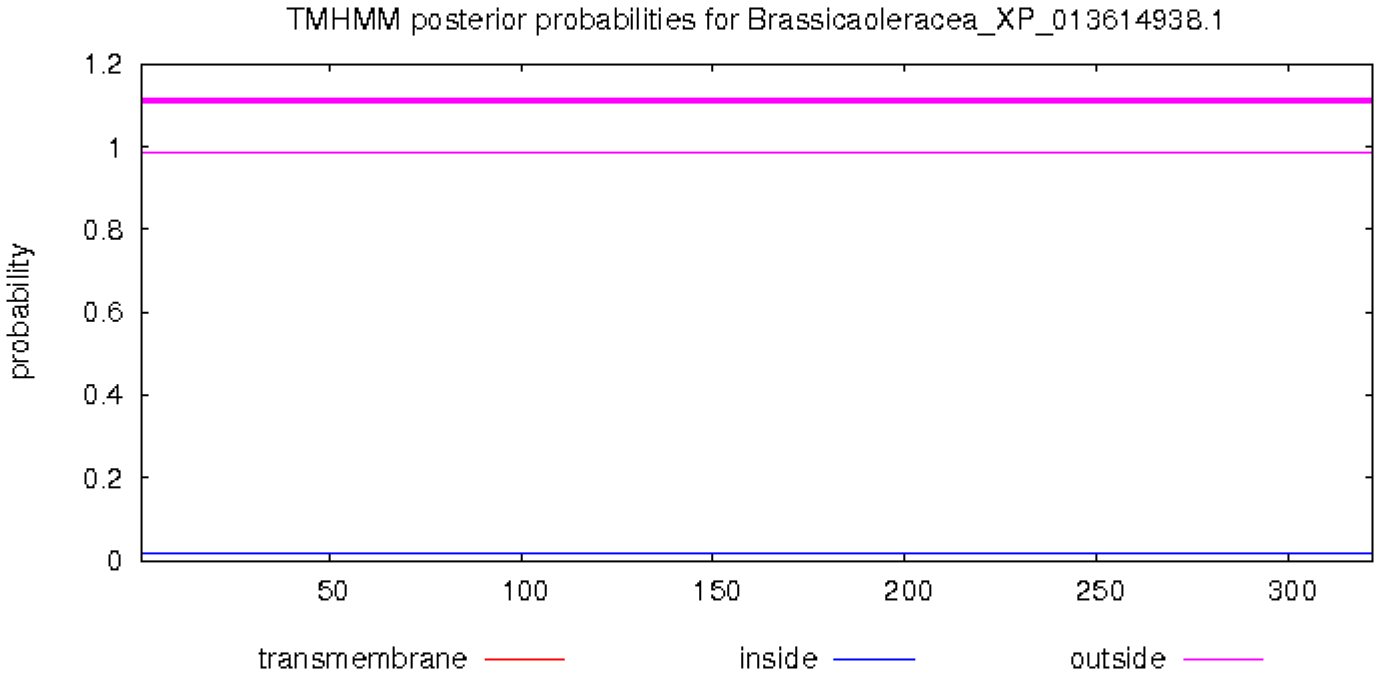

plot in postscript, script for making the plot in gnuplot, data for plot

---

```
# Brassicaoleracea_XP_013614989.1 Length: 311
# Brassicaoleracea_XP_013614989.1 Number of predicted TMHs:  0
# Brassicaoleracea_XP_013614989.1 Exp number of AAs in TMHs: 0.31852
```

```
# Brassicaoleracea_XP_013614989.1 Exp number, first 60 AAs:  0
# Brassicaoleracea_XP_013614989.1 Total prob of N-in:        0.00449
Brassicaoleracea_XP_013614989.1 TMHMM2.0        outside      1   311
```

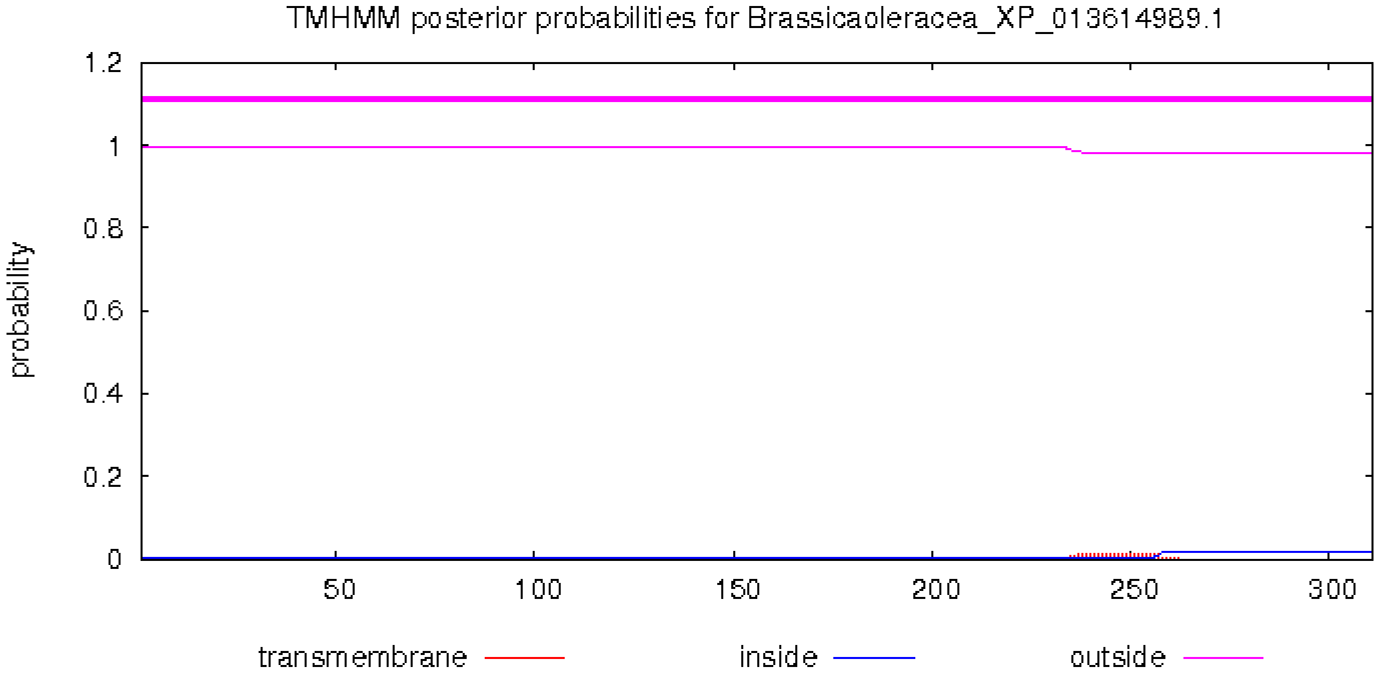

plot in postscript, script for making the plot in gnuplot, data for plot

---

```
# Brassicaoleracea_XP_013615275.1 Length: 166
# Brassicaoleracea_XP_013615275.1 Number of predicted TMHs:  0
# Brassicaoleracea_XP_013615275.1 Exp number of AAs in TMHs: 8.05755
# Brassicaoleracea_XP_013615275.1 Exp number, first 60 AAs:  0.00024
# Brassicaoleracea_XP_013615275.1 Total prob of N-in:        0.29596
Brassicaoleracea_XP_013615275.1 TMHMM2.0        outside      1   166
```

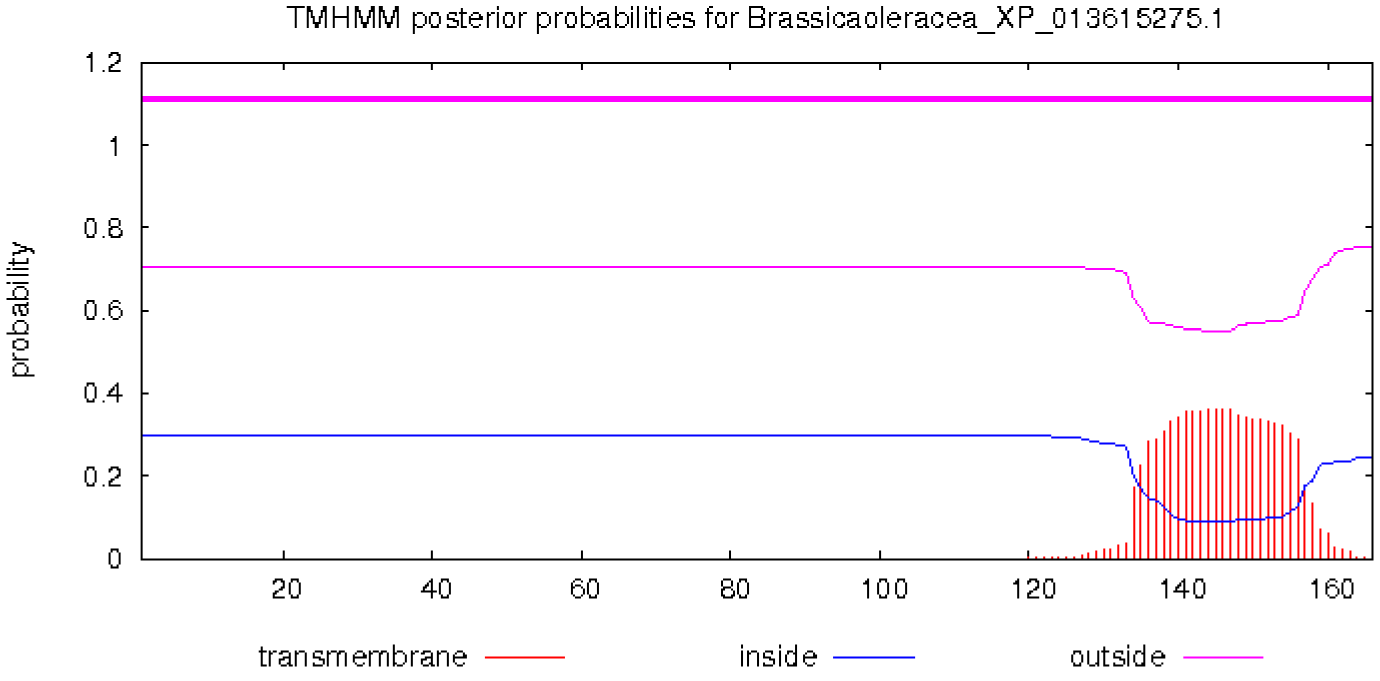

plot in postscript, script for making the plot in gnuplot, data for plot

---

```
# Brassicaoleracea_XP_013615277.1 Length: 129
# Brassicaoleracea_XP_013615277.1 Number of predicted TMHs:  0
# Brassicaoleracea_XP_013615277.1 Exp number of AAs in TMHs: 0.00019
```

```
# Brassicaoleracea_XP_013615277.1 Exp number, first 60 AAs:  0.00019
# Brassicaoleracea_XP_013615277.1 Total prob of N-in:        0.19248
Brassicaoleracea_XP_013615277.1 TMHMM2.0        outside      1   129
```

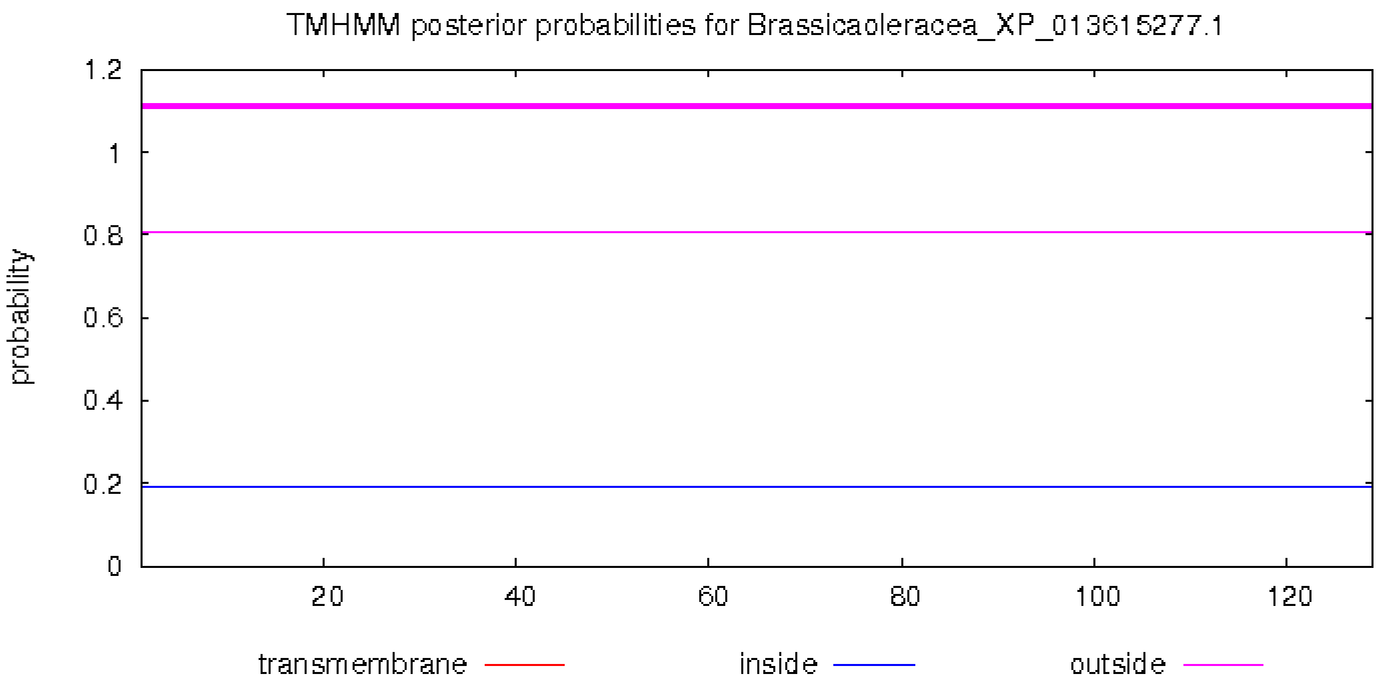

plot in postscript, script for making the plot in gnuplot, data for plot

---

```
# Brassicaoleracea_XP_013615295.1 Length: 277
# Brassicaoleracea_XP_013615295.1 Number of predicted TMHs:  0
# Brassicaoleracea_XP_013615295.1 Exp number of AAs in TMHs: 0.00018
# Brassicaoleracea_XP_013615295.1 Exp number, first 60 AAs:  0
# Brassicaoleracea_XP_013615295.1 Total prob of N-in:        0.00899
Brassicaoleracea_XP_013615295.1 TMHMM2.0        outside      1   277
```

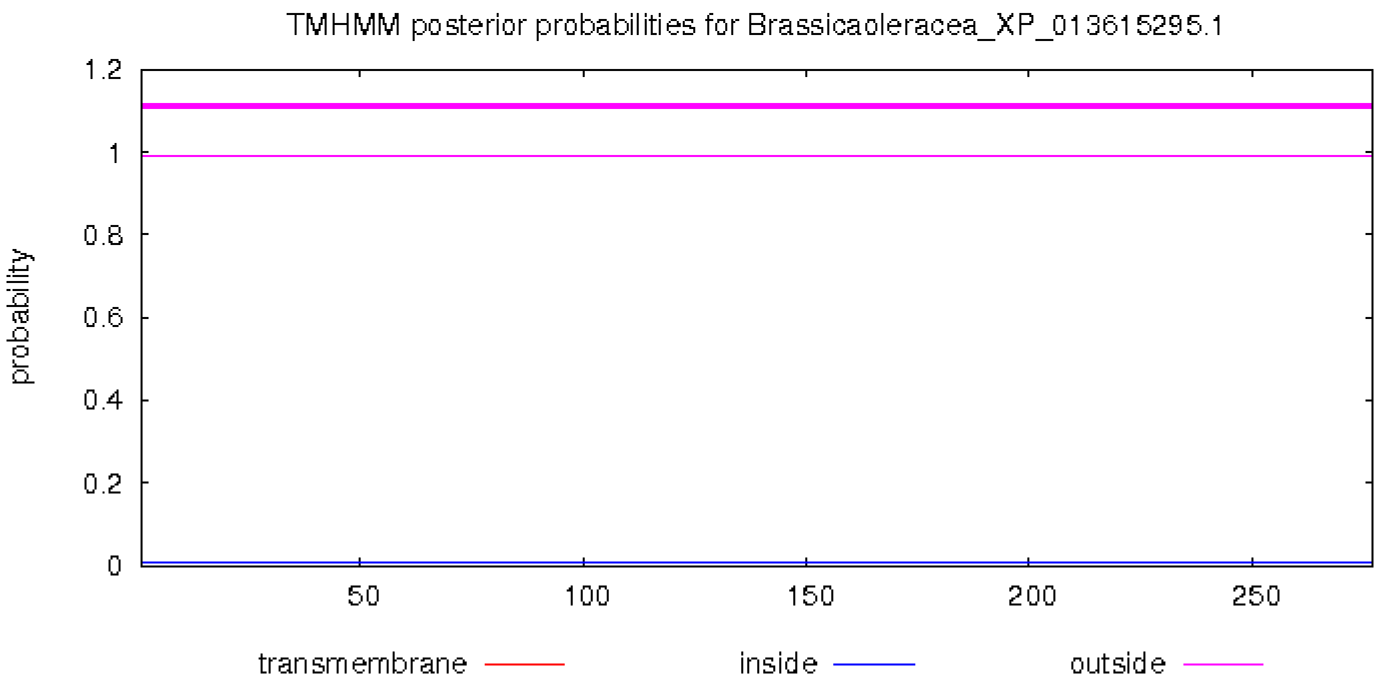

plot in postscript, script for making the plot in gnuplot, data for plot

---

```
# Brassicaoleracea_XP_013615910.1 Length: 350
# Brassicaoleracea_XP_013615910.1 Number of predicted TMHs:  0
# Brassicaoleracea_XP_013615910.1 Exp number of AAs in TMHs: 0
```

```
# Brassicaoleracea_XP_013615910.1 Exp number, first 60 AAs:  0
# Brassicaoleracea_XP_013615910.1 Total prob of N-in:        0.00397
Brassicaoleracea_XP_013615910.1 TMHMM2.0        outside      1   350
```

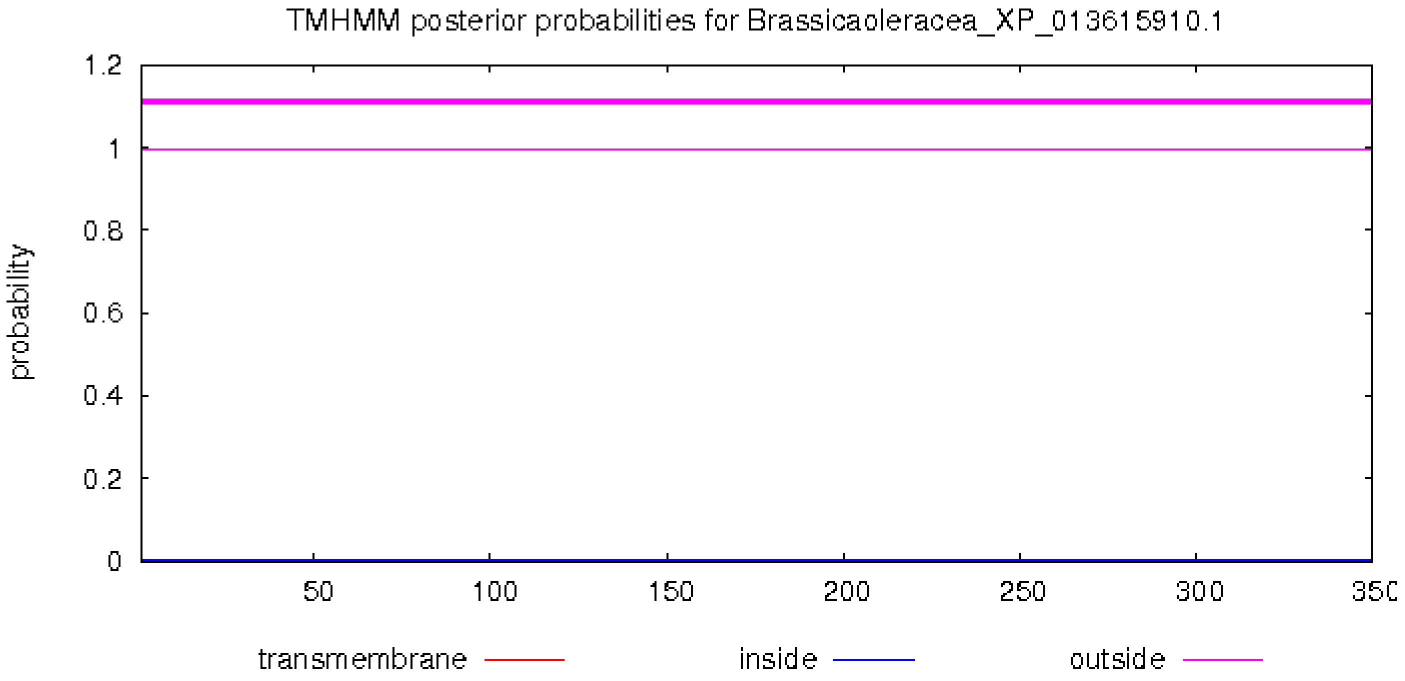

plot in postscript, script for making the plot in gnuplot, data for plot

---

```
# Brassicaoleracea_XP_013615911.1 Length: 350
# Brassicaoleracea_XP_013615911.1 Number of predicted TMHs:  0
# Brassicaoleracea_XP_013615911.1 Exp number of AAs in TMHs: 0
# Brassicaoleracea_XP_013615911.1 Exp number, first 60 AAs:  0
# Brassicaoleracea_XP_013615911.1 Total prob of N-in:        0.00397
Brassicaoleracea_XP_013615911.1 TMHMM2.0        outside      1   350
```

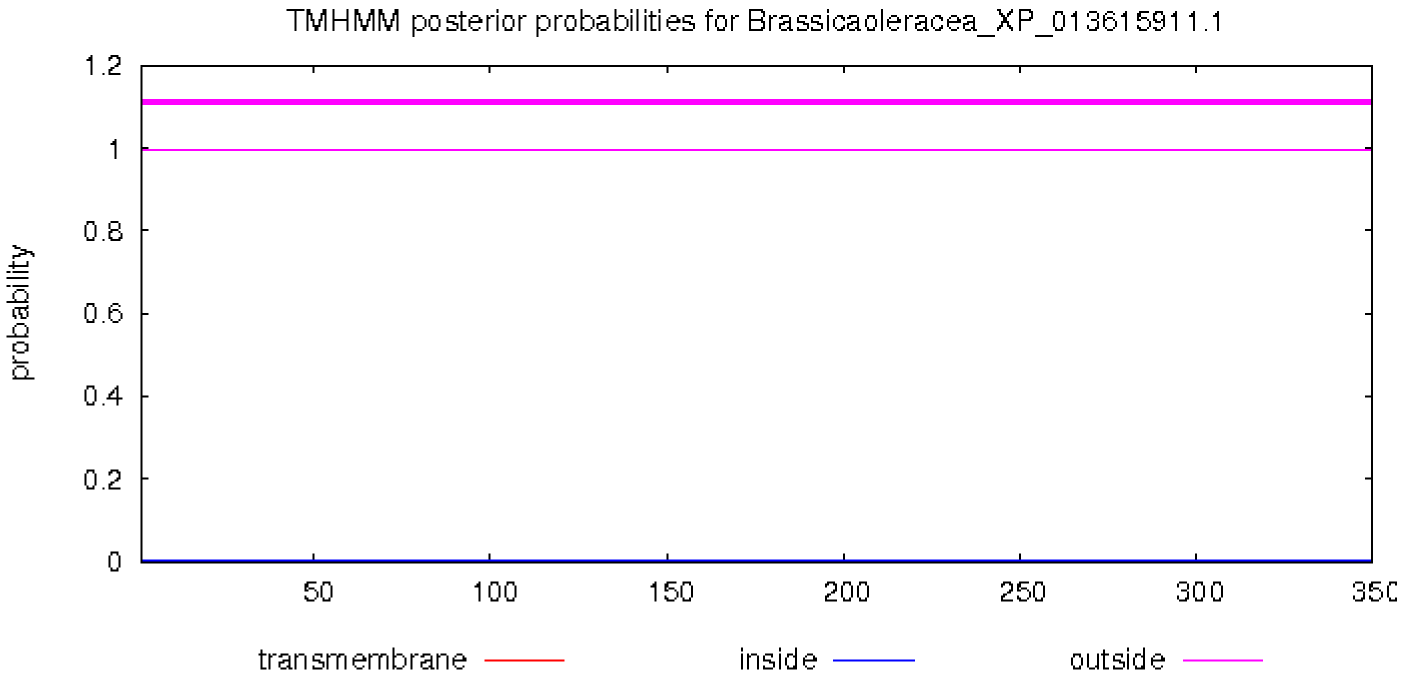

plot in postscript, script for making the plot in gnuplot, data for plot

---

```
# Brassicaoleracea_XP_013616052.1 Length: 277
# Brassicaoleracea_XP_013616052.1 Number of predicted TMHs:  0
# Brassicaoleracea_XP_013616052.1 Exp number of AAs in TMHs: 0.00018
```

```
# Brassicaoleracea_XP_013616052.1 Exp number, first 60 AAs:  0
# Brassicaoleracea_XP_013616052.1 Total prob of N-in:        0.00899
Brassicaoleracea_XP_013616052.1 TMHMM2.0        outside      1   277
```

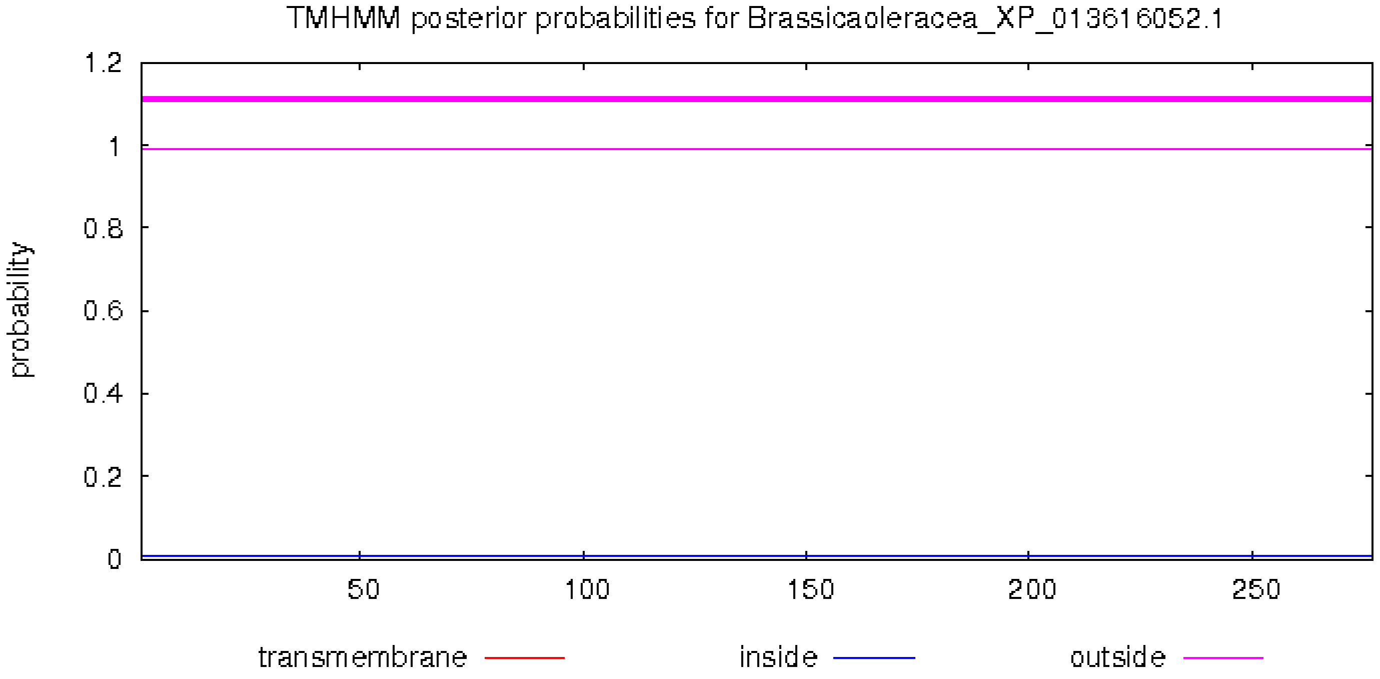

plot in postscript, script for making the plot in gnuplot, data for plot

---

```
# Brassicaoleracea_XP_013616284.1 Length: 247
# Brassicaoleracea_XP_013616284.1 Number of predicted TMHs:  0
# Brassicaoleracea_XP_013616284.1 Exp number of AAs in TMHs: 0
# Brassicaoleracea_XP_013616284.1 Exp number, first 60 AAs:  0
# Brassicaoleracea_XP_013616284.1 Total prob of N-in:        0.11741
Brassicaoleracea_XP_013616284.1 TMHMM2.0        outside      1   247
```

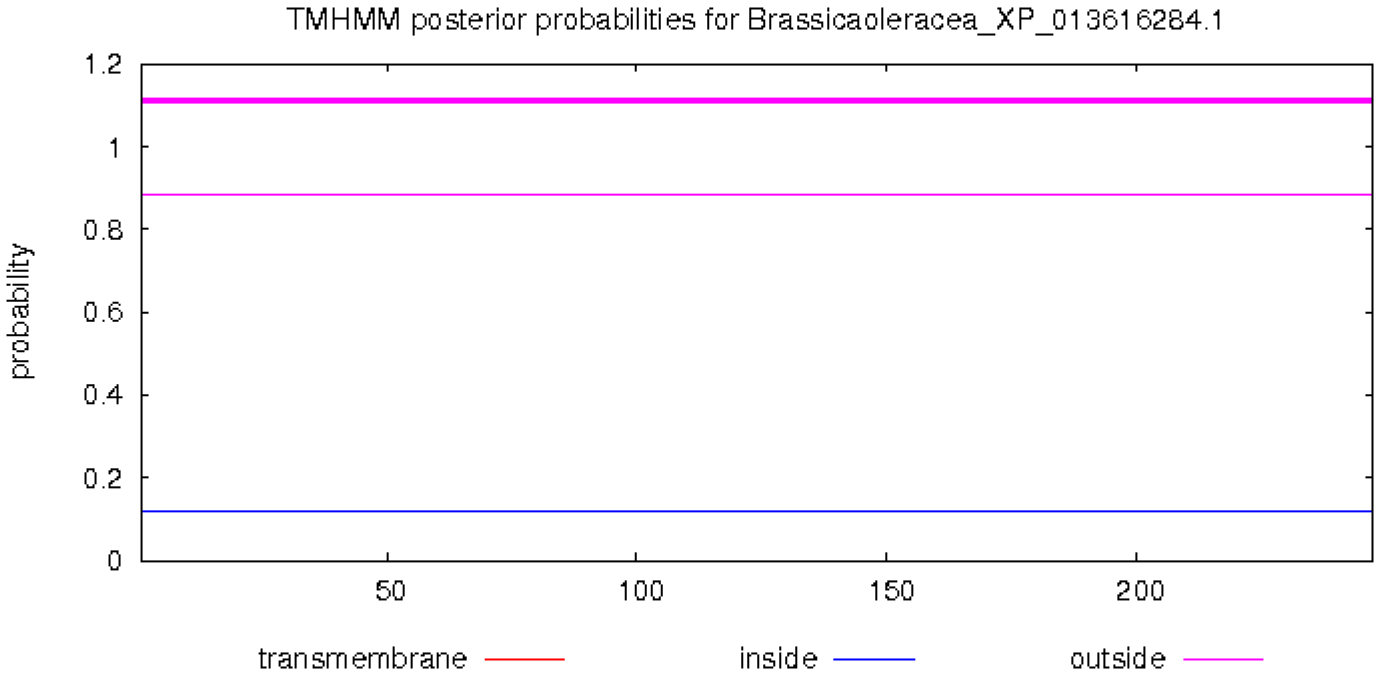

plot in postscript, script for making the plot in gnuplot, data for plot

---

```
# Brassicaoleracea_XP_013616301.1 Length: 407
# Brassicaoleracea_XP_013616301.1 Number of predicted TMHs:  0
# Brassicaoleracea_XP_013616301.1 Exp number of AAs in TMHs: 0.00656
```

```
# Brassicaoleracea_XP_013616301.1 Exp number, first 60 AAs:  0
# Brassicaoleracea_XP_013616301.1 Total prob of N-in:        0.00663
Brassicaoleracea_XP_013616301.1 TMHMM2.0        outside      1   407
```

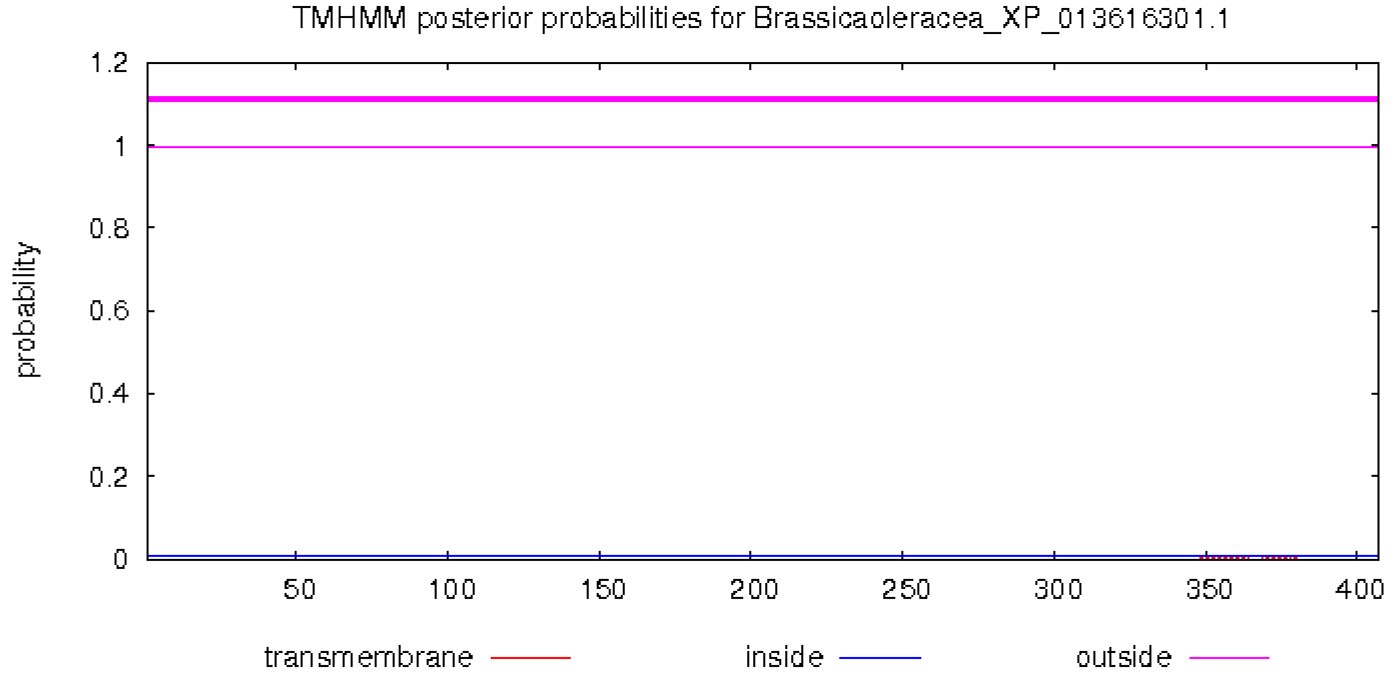

plot in postscript, script for making the plot in gnuplot, data for plot

---

```
# Brassicaoleracea_XP_013616302.1 Length: 377
# Brassicaoleracea_XP_013616302.1 Number of predicted TMHs:  0
# Brassicaoleracea_XP_013616302.1 Exp number of AAs in TMHs: 0.00525
# Brassicaoleracea_XP_013616302.1 Exp number, first 60 AAs:  0
# Brassicaoleracea_XP_013616302.1 Total prob of N-in:        0.00951
Brassicaoleracea_XP_013616302.1 TMHMM2.0        outside      1   377
```

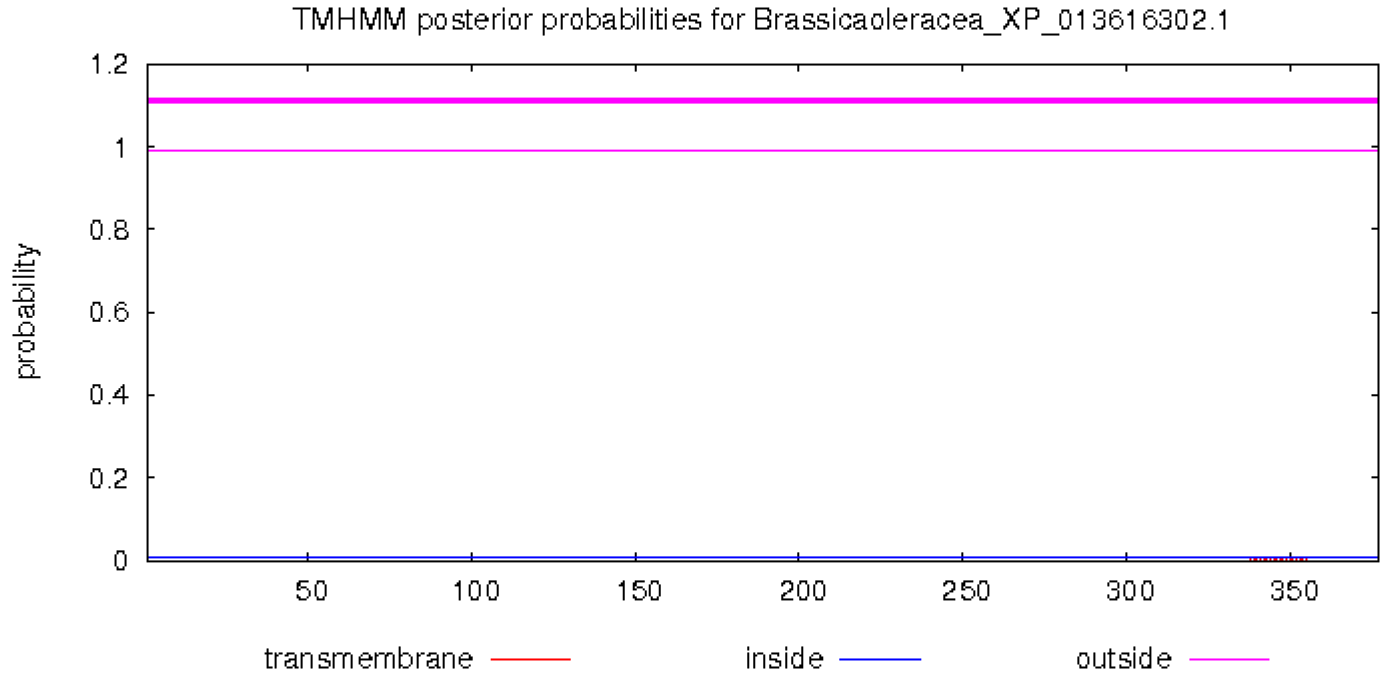

plot in postscript, script for making the plot in gnuplot, data for plot

---

```
# Brassicaoleracea_XP_013616372.1 Length: 357
# Brassicaoleracea_XP_013616372.1 Number of predicted TMHs:  0
# Brassicaoleracea_XP_013616372.1 Exp number of AAs in TMHs: 0.0013
```

```
# Brassicaoleracea_XP_013616372.1 Exp number, first 60 AAs:  0
# Brassicaoleracea_XP_013616372.1 Total prob of N-in:        0.00448
Brassicaoleracea_XP_013616372.1 TMHMM2.0        outside      1   357
```

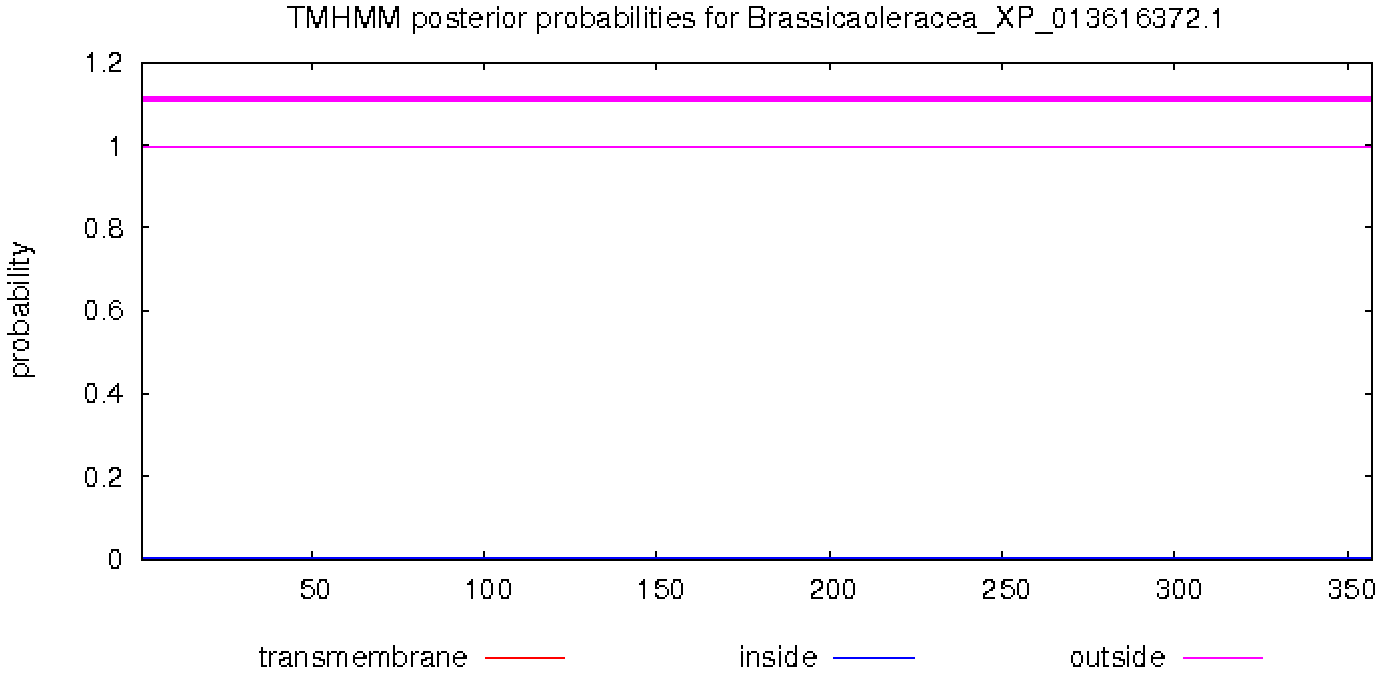

plot in postscript, script for making the plot in gnuplot, data for plot

---

```
# Brassicaoleracea_XP_013616777.1 Length: 362
# Brassicaoleracea_XP_013616777.1 Number of predicted TMHs:  0
# Brassicaoleracea_XP_013616777.1 Exp number of AAs in TMHs: 0.00074
# Brassicaoleracea_XP_013616777.1 Exp number, first 60 AAs:  0.00018
# Brassicaoleracea_XP_013616777.1 Total prob of N-in:        0.03165
Brassicaoleracea_XP_013616777.1 TMHMM2.0        outside      1   362
```

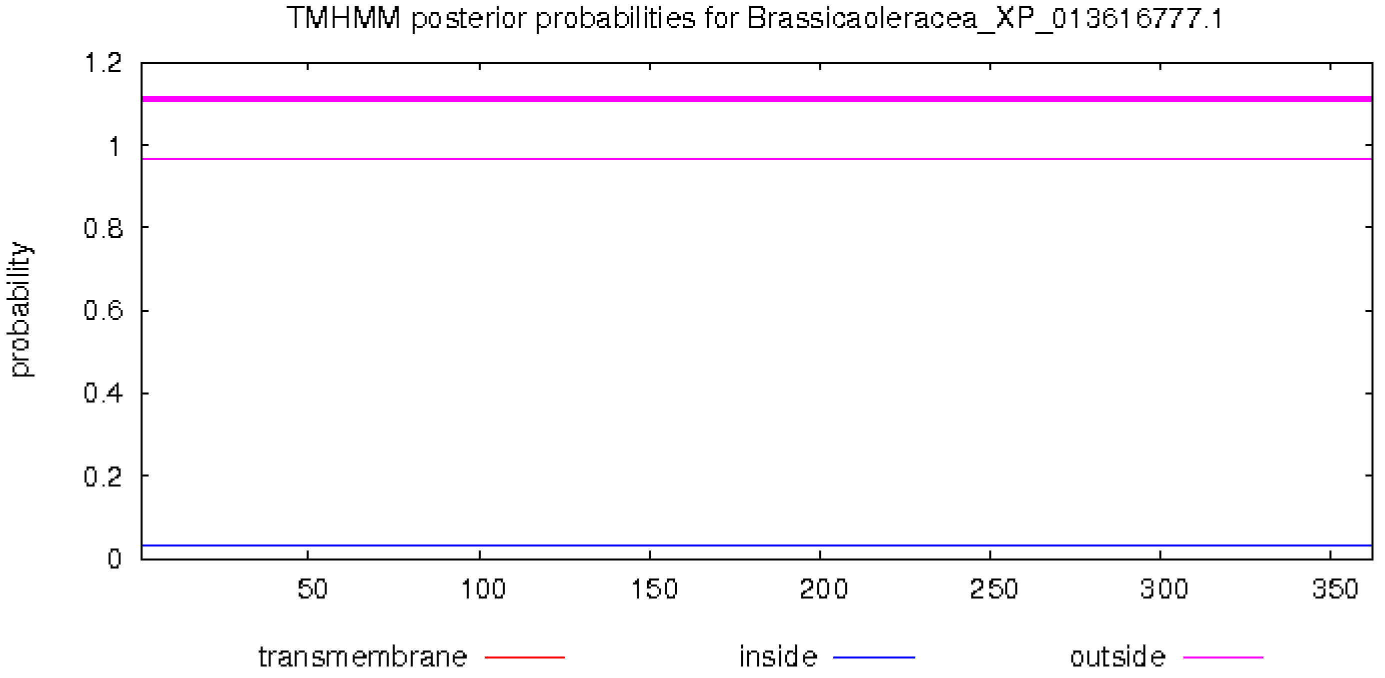

plot in postscript, script for making the plot in gnuplot, data for plot

---

```
# Brassicaoleracea_XP_013616847.1 Length: 337
# Brassicaoleracea_XP_013616847.1 Number of predicted TMHs:  0
# Brassicaoleracea_XP_013616847.1 Exp number of AAs in TMHs: 0.00017
```

```
# Brassicaoleracea_XP_013616847.1 Exp number, first 60 AAs:  0
# Brassicaoleracea_XP_013616847.1 Total prob of N-in:        0.00864
Brassicaoleracea_XP_013616847.1 TMHMM2.0        outside      1   337
```

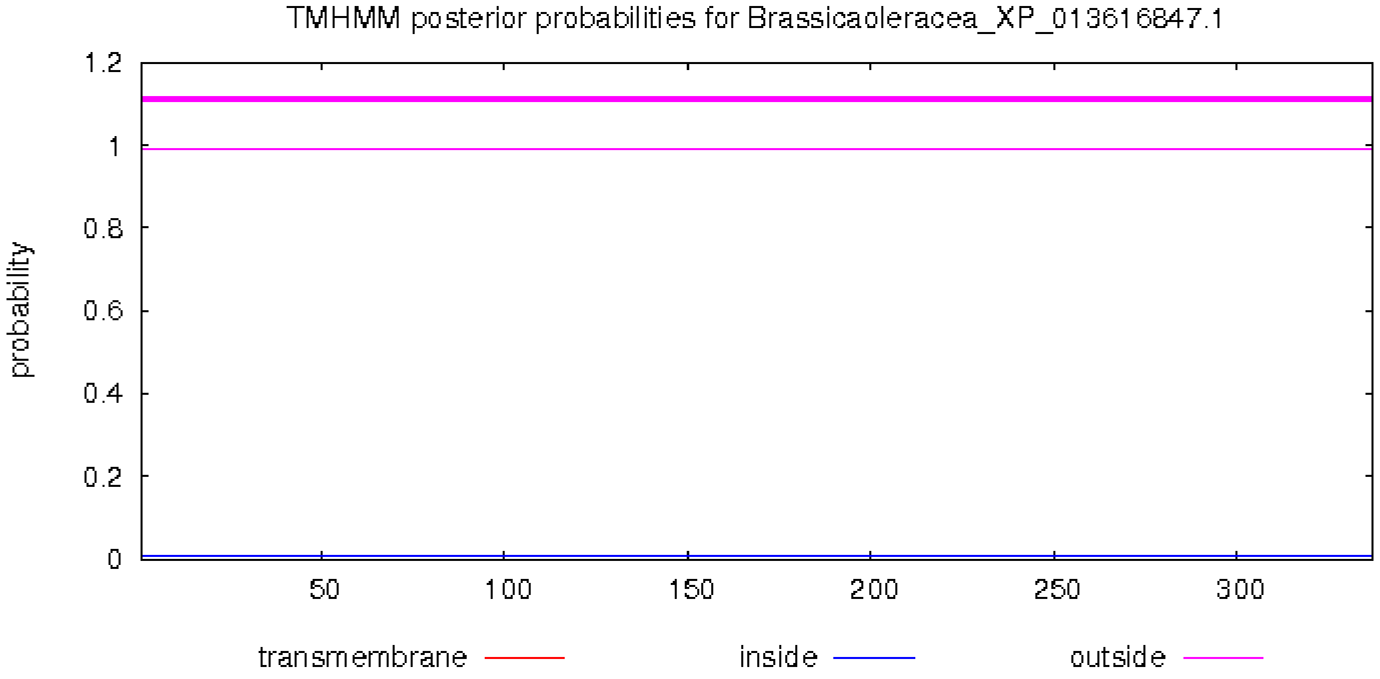

plot in postscript, script for making the plot in gnuplot, data for plot

---

```
# Brassicaoleracea_XP_013616936.1 Length: 294
# Brassicaoleracea_XP_013616936.1 Number of predicted TMHs:  0
# Brassicaoleracea_XP_013616936.1 Exp number of AAs in TMHs: 0.00016
# Brassicaoleracea_XP_013616936.1 Exp number, first 60 AAs:  0
# Brassicaoleracea_XP_013616936.1 Total prob of N-in:        0.02891
Brassicaoleracea_XP_013616936.1 TMHMM2.0        outside      1   294
```

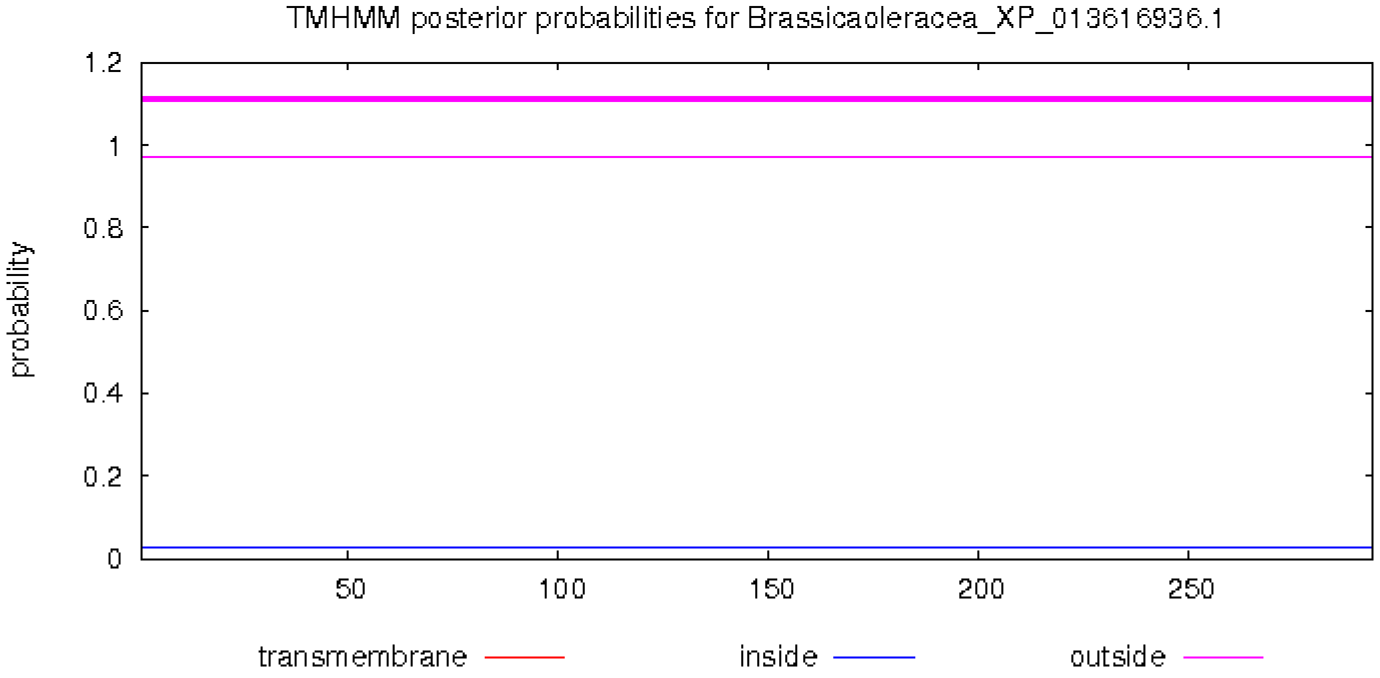

plot in postscript, script for making the plot in gnuplot, data for plot

---

```
# Brassicaoleracea_XP_013616940.1 Length: 133
# Brassicaoleracea_XP_013616940.1 Number of predicted TMHs:  0
# Brassicaoleracea_XP_013616940.1 Exp number of AAs in TMHs: 0.00069
```

```
# Brassicaoleracea_XP_013616940.1 Exp number, first 60 AAs:  0
# Brassicaoleracea_XP_013616940.1 Total prob of N-in:        0.18205
Brassicaoleracea_XP_013616940.1 TMHMM2.0        outside      1   133
```

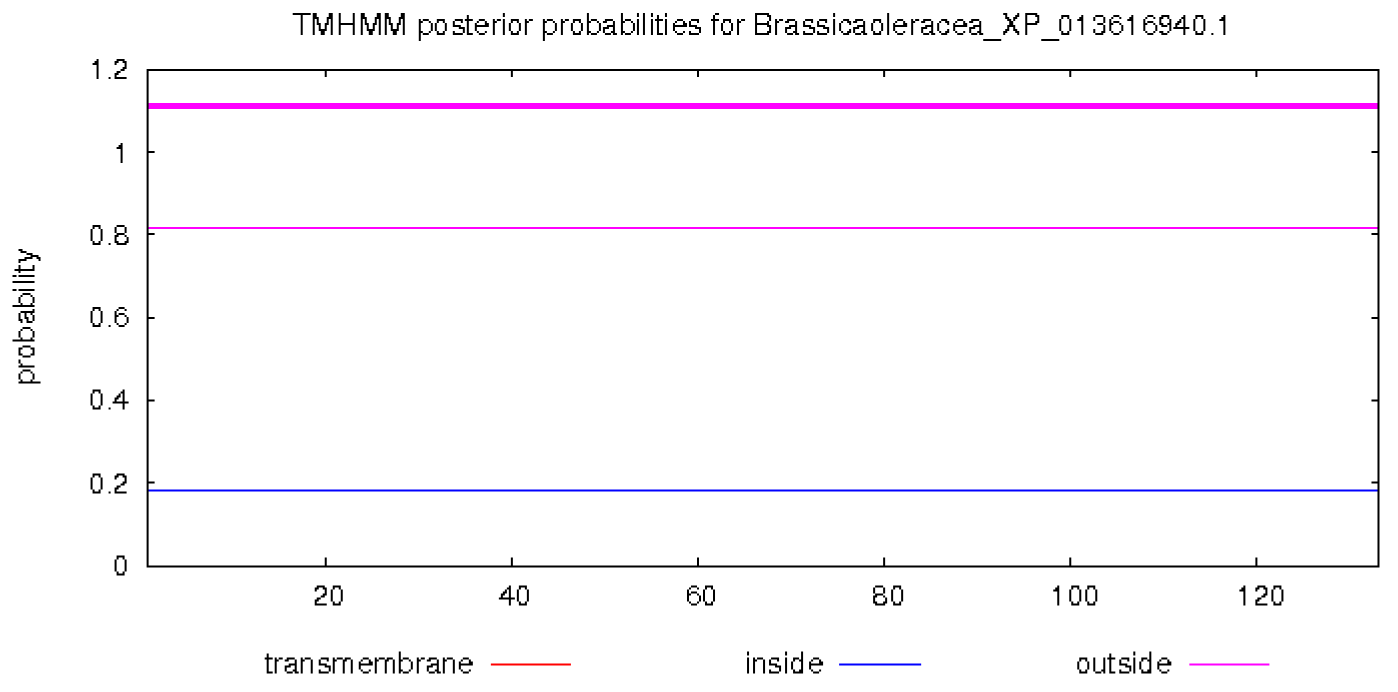

plot in postscript, script for making the plot in gnuplot, data for plot

---

```
# Brassicaoleracea_XP_013616942.1 Length: 204
# Brassicaoleracea_XP_013616942.1 Number of predicted TMHs:  0
# Brassicaoleracea_XP_013616942.1 Exp number of AAs in TMHs: 0.00178
# Brassicaoleracea_XP_013616942.1 Exp number, first 60 AAs:  0.00127
# Brassicaoleracea_XP_013616942.1 Total prob of N-in:        0.19179
Brassicaoleracea_XP_013616942.1 TMHMM2.0        outside      1   204
```

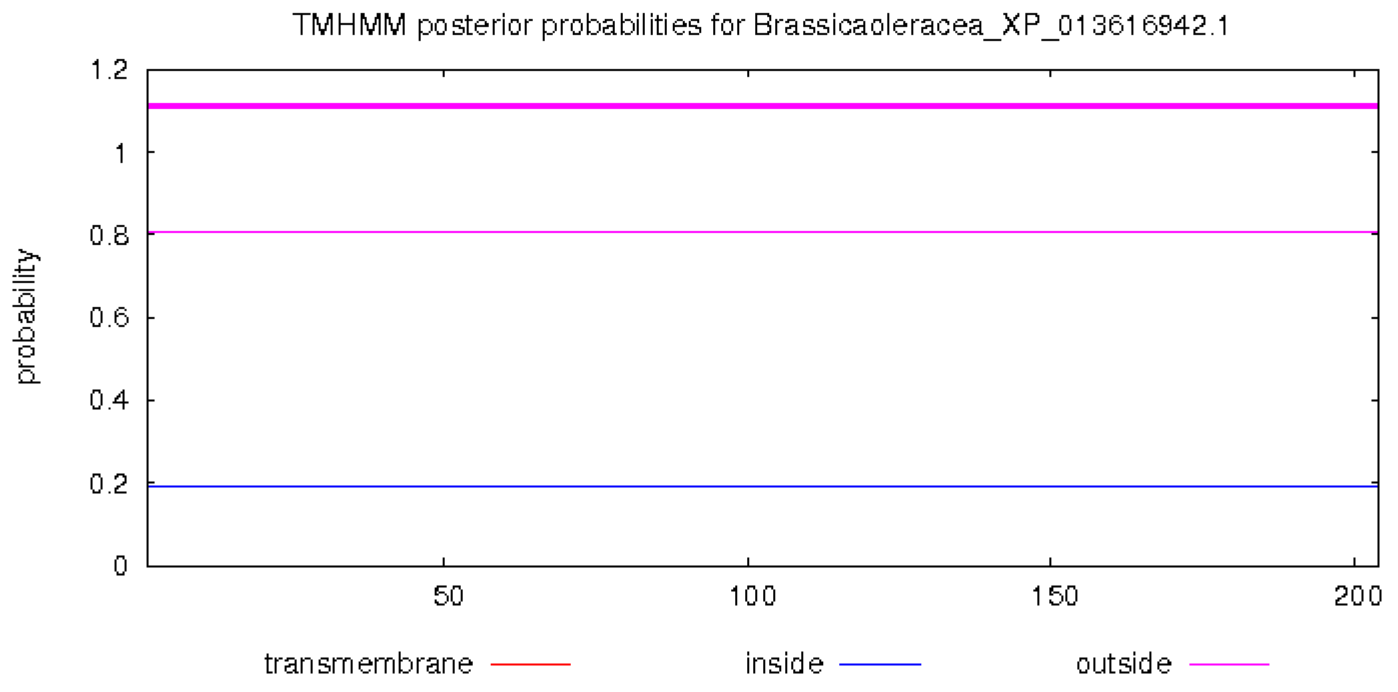

plot in postscript, script for making the plot in gnuplot, data for plot

---

```
# Brassicaoleracea_XP_013616944.1 Length: 202
# Brassicaoleracea_XP_013616944.1 Number of predicted TMHs:  0
# Brassicaoleracea_XP_013616944.1 Exp number of AAs in TMHs: 0.00207
```

```
# Brassicaoleracea_XP_013616944.1 Exp number, first 60 AAs:  0
# Brassicaoleracea_XP_013616944.1 Total prob of N-in:        0.02904
Brassicaoleracea_XP_013616944.1 TMHMM2.0        outside      1   202
```

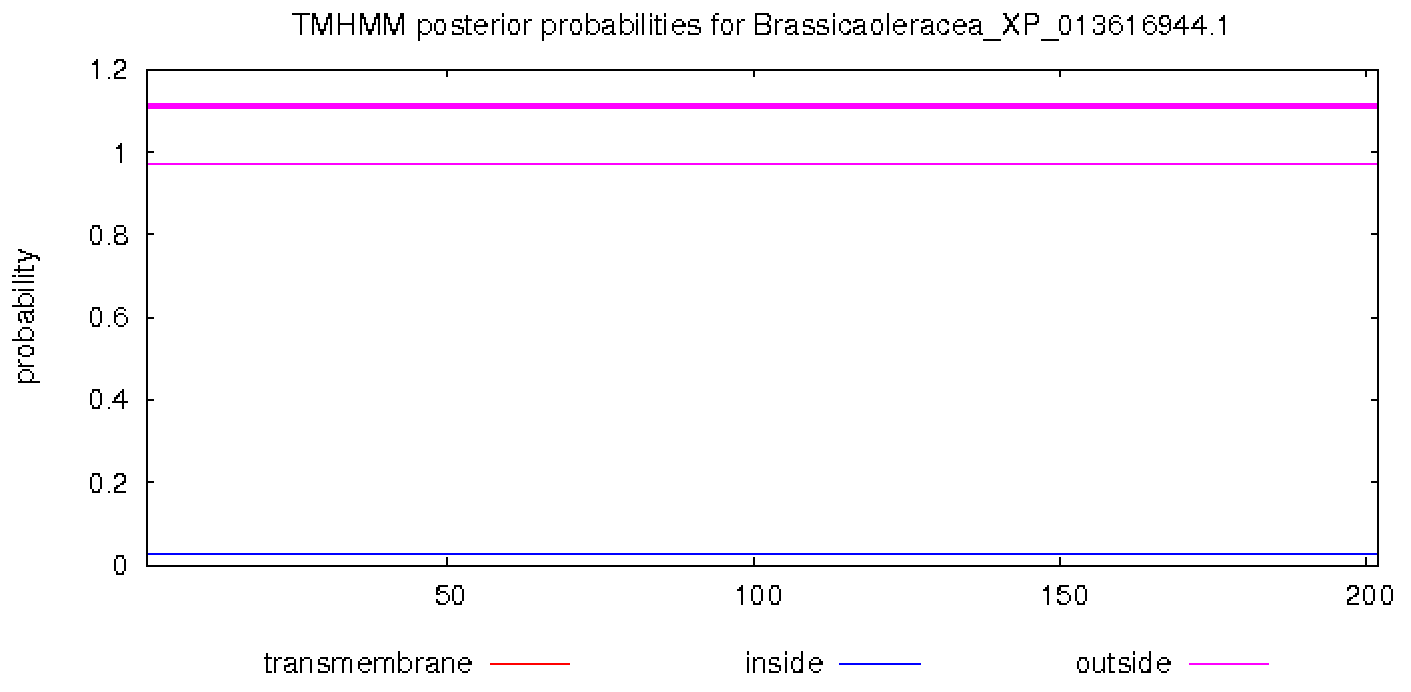

plot in postscript, script for making the plot in gnuplot, data for plot

---

```
# Brassicaoleracea_XP_013617381.1 Length: 333
# Brassicaoleracea_XP_013617381.1 Number of predicted TMHs:  0
# Brassicaoleracea_XP_013617381.1 Exp number of AAs in TMHs: 0.00133
# Brassicaoleracea_XP_013617381.1 Exp number, first 60 AAs:  0.00133
# Brassicaoleracea_XP_013617381.1 Total prob of N-in:        0.00970
Brassicaoleracea_XP_013617381.1 TMHMM2.0        outside      1   333
```

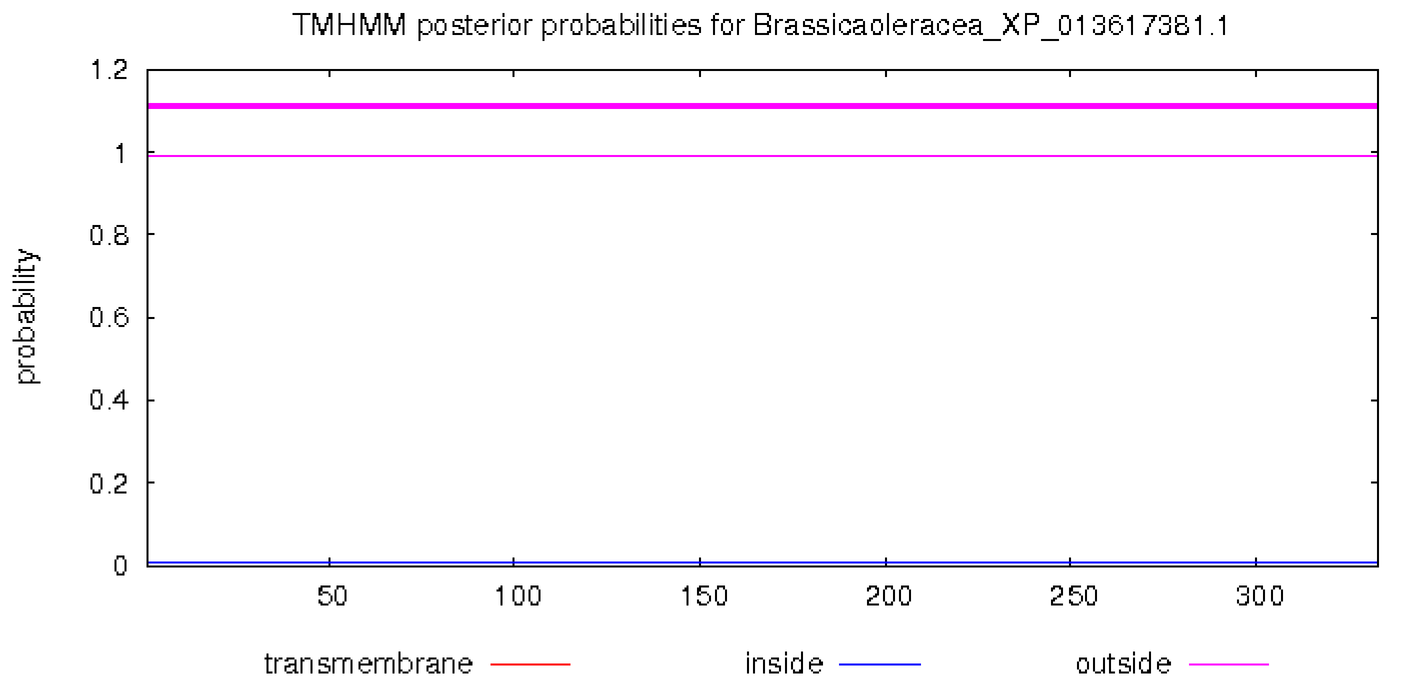

plot in postscript, script for making the plot in gnuplot, data for plot

---

```
# Brassicaoleracea_XP_013617682.1 Length: 300
# Brassicaoleracea_XP_013617682.1 Number of predicted TMHs:  0
# Brassicaoleracea_XP_013617682.1 Exp number of AAs in TMHs: 0.00173
```

```
# Brassicaoleracea_XP_013617682.1 Exp number, first 60 AAs:  0.00038
# Brassicaoleracea_XP_013617682.1 Total prob of N-in:        0.01514
Brassicaoleracea_XP_013617682.1 TMHMM2.0        outside      1   300
```

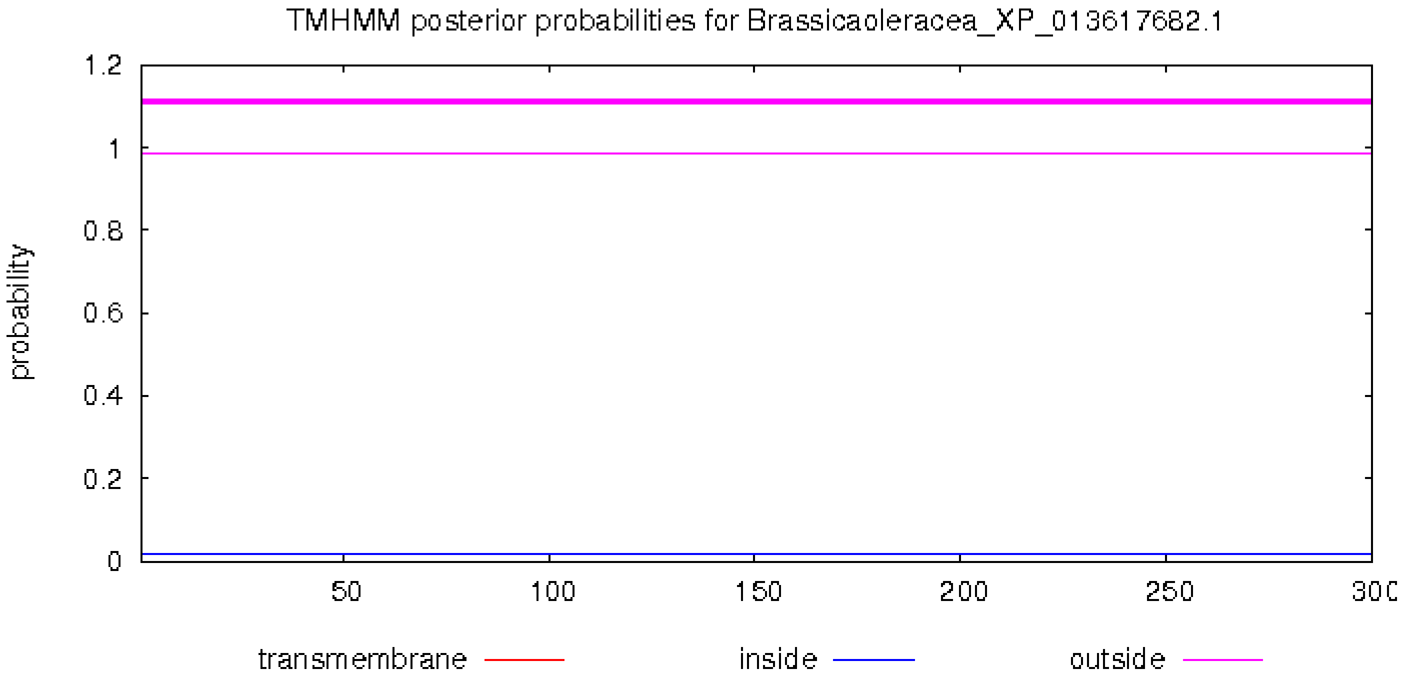

plot in postscript, script for making the plot in gnuplot, data for plot

---

```
# Brassicaoleracea_XP_013617687.1 Length: 293
# Brassicaoleracea_XP_013617687.1 Number of predicted TMHs:  0
# Brassicaoleracea_XP_013617687.1 Exp number of AAs in TMHs: 0.00018
# Brassicaoleracea_XP_013617687.1 Exp number, first 60 AAs:  0
# Brassicaoleracea_XP_013617687.1 Total prob of N-in:        0.00208
Brassicaoleracea_XP_013617687.1 TMHMM2.0        outside      1   293
```

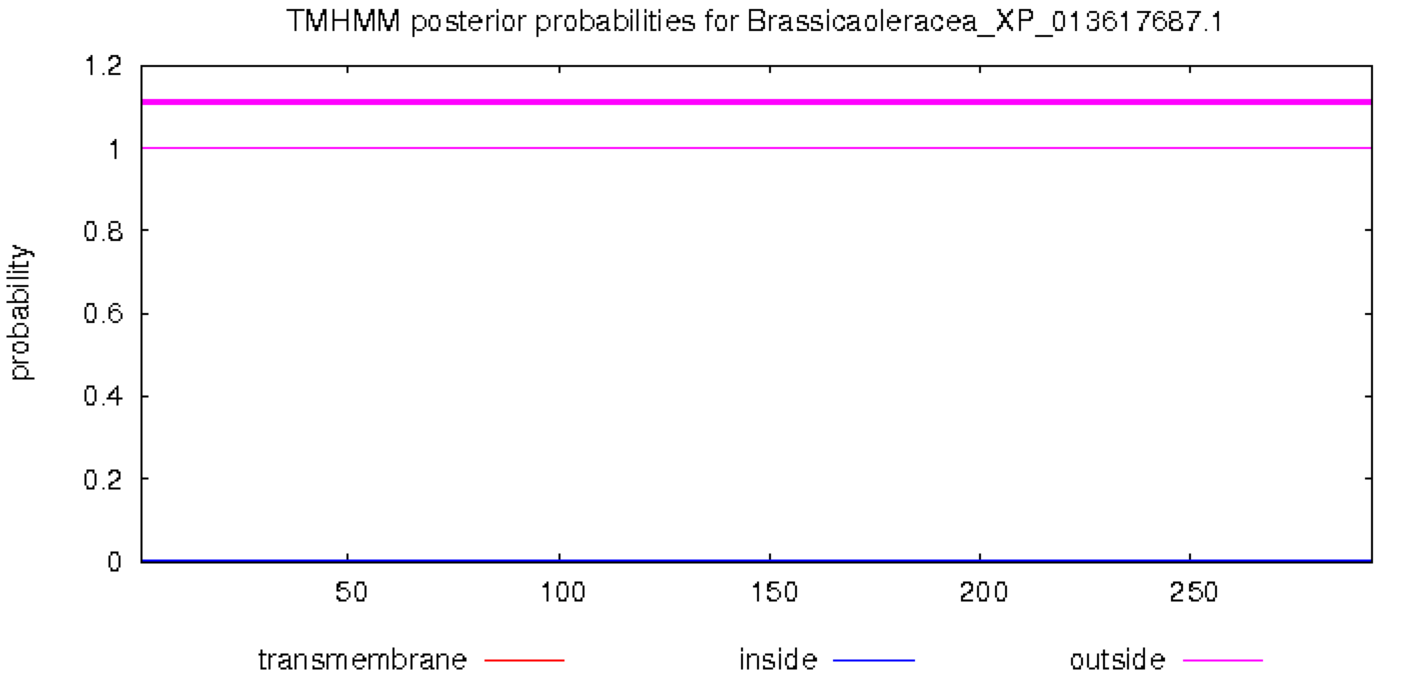

plot in postscript, script for making the plot in gnuplot, data for plot

---

```
# Brassicaoleracea_XP_013618408.1 Length: 259
# Brassicaoleracea_XP_013618408.1 Number of predicted TMHs:  0
# Brassicaoleracea_XP_013618408.1 Exp number of AAs in TMHs: 0.00023
```

```
# Brassicaoleracea_XP_013618408.1 Exp number, first 60 AAs:  0.00023
# Brassicaoleracea_XP_013618408.1 Total prob of N-in:        0.03529
Brassicaoleracea_XP_013618408.1 TMHMM2.0        outside      1   259
```

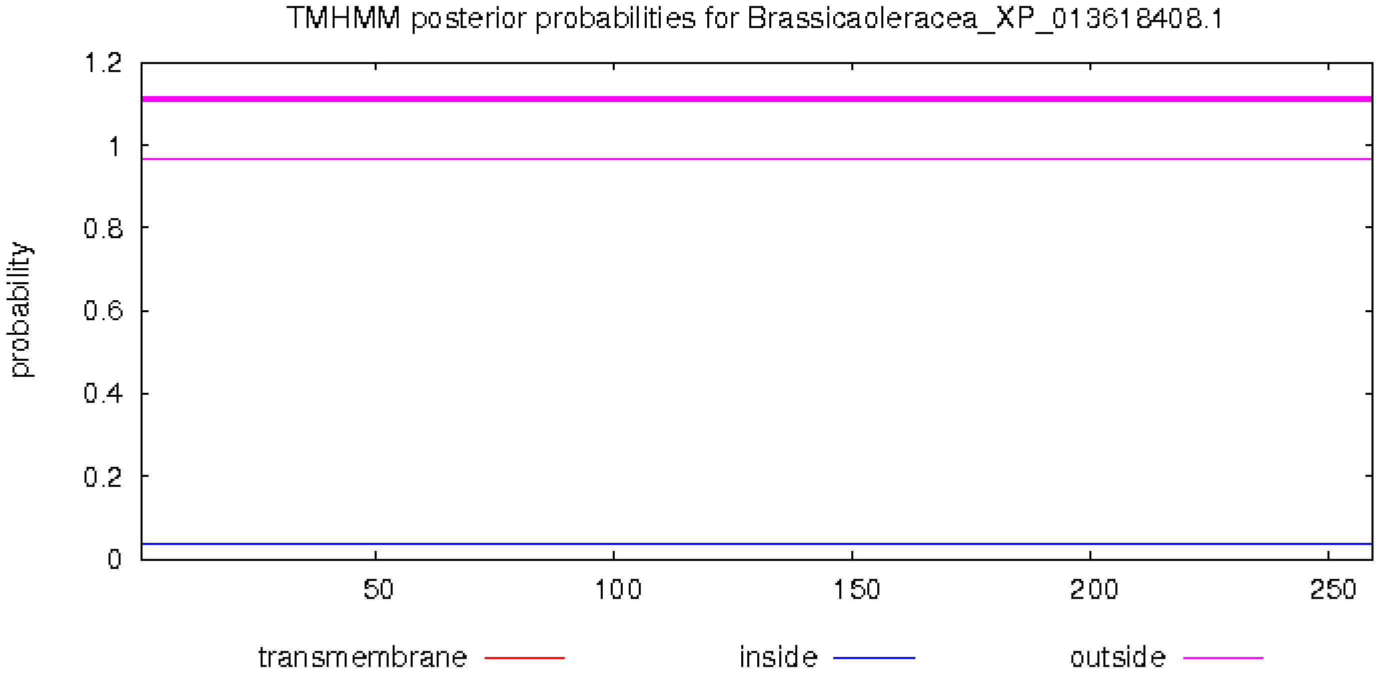

plot in postscript, script for making the plot in gnuplot, data for plot

---

```
# Brassicaoleracea_XP_013618540.1 Length: 325
# Brassicaoleracea_XP_013618540.1 Number of predicted TMHs:  0
# Brassicaoleracea_XP_013618540.1 Exp number of AAs in TMHs: 0.00136
# Brassicaoleracea_XP_013618540.1 Exp number, first 60 AAs:  0
# Brassicaoleracea_XP_013618540.1 Total prob of N-in:        0.01739
Brassicaoleracea_XP_013618540.1 TMHMM2.0        outside      1   325
```

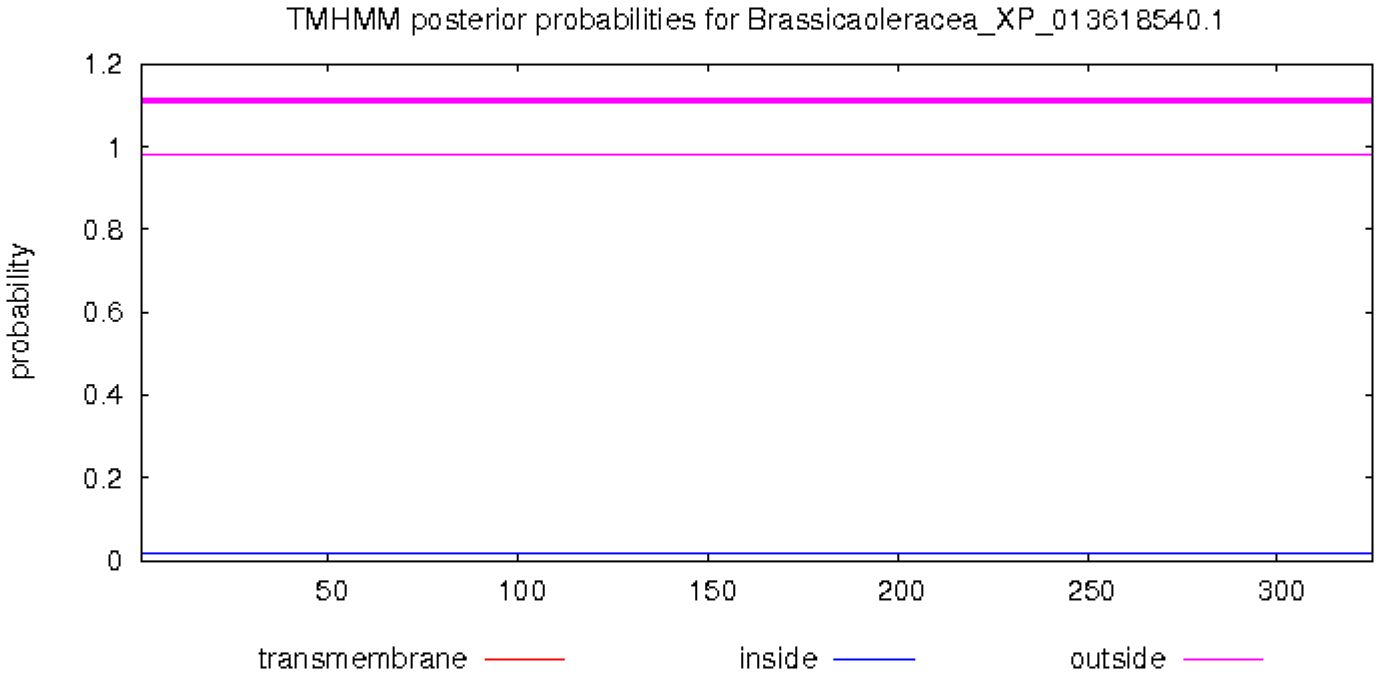

plot in postscript, script for making the plot in gnuplot, data for plot

---

```
# Brassicaoleracea_XP_013618671.1 Length: 315
# Brassicaoleracea_XP_013618671.1 Number of predicted TMHs:  0
# Brassicaoleracea_XP_013618671.1 Exp number of AAs in TMHs: 0.0004
```

```
# Brassicaoleracea_XP_013618671.1 Exp number, first 60 AAs:  0
# Brassicaoleracea_XP_013618671.1 Total prob of N-in:        0.00743
Brassicaoleracea_XP_013618671.1 TMHMM2.0        outside      1   315
```

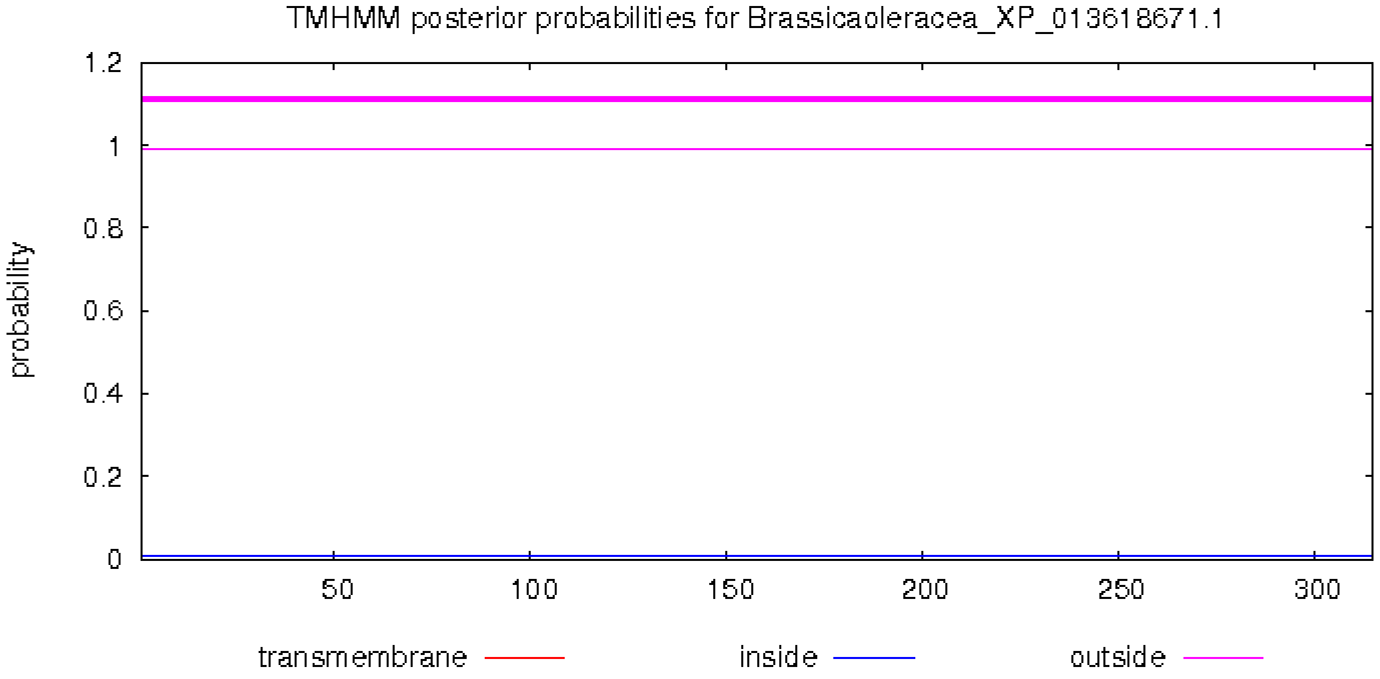

plot in postscript, script for making the plot in gnuplot, data for plot

```
# Brassicaoleracea_XP_013618727.1 Length: 665
# Brassicaoleracea_XP_013618727.1 Number of predicted TMHs:  3
# Brassicaoleracea_XP_013618727.1 Exp number of AAs in TMHs: 50.95343
# Brassicaoleracea_XP_013618727.1 Exp number, first 60 AAs:  0
# Brassicaoleracea_XP_013618727.1 Total prob of N-in:        0.00063
Brassicaoleracea_XP_013618727.1 TMHMM2.0        outside      1   534
Brassicaoleracea_XP_013618727.1 TMHMM2.0        TMhelix    535   552
Brassicaoleracea_XP_013618727.1 TMHMM2.0        inside     553   609
Brassicaoleracea_XP_013618727.1 TMHMM2.0        TMhelix    610   628
Brassicaoleracea_XP_013618727.1 TMHMM2.0        outside    629   642
Brassicaoleracea_XP_013618727.1 TMHMM2.0        TMhelix    643   662
Brassicaoleracea_XP_013618727.1 TMHMM2.0        inside     663   665
```

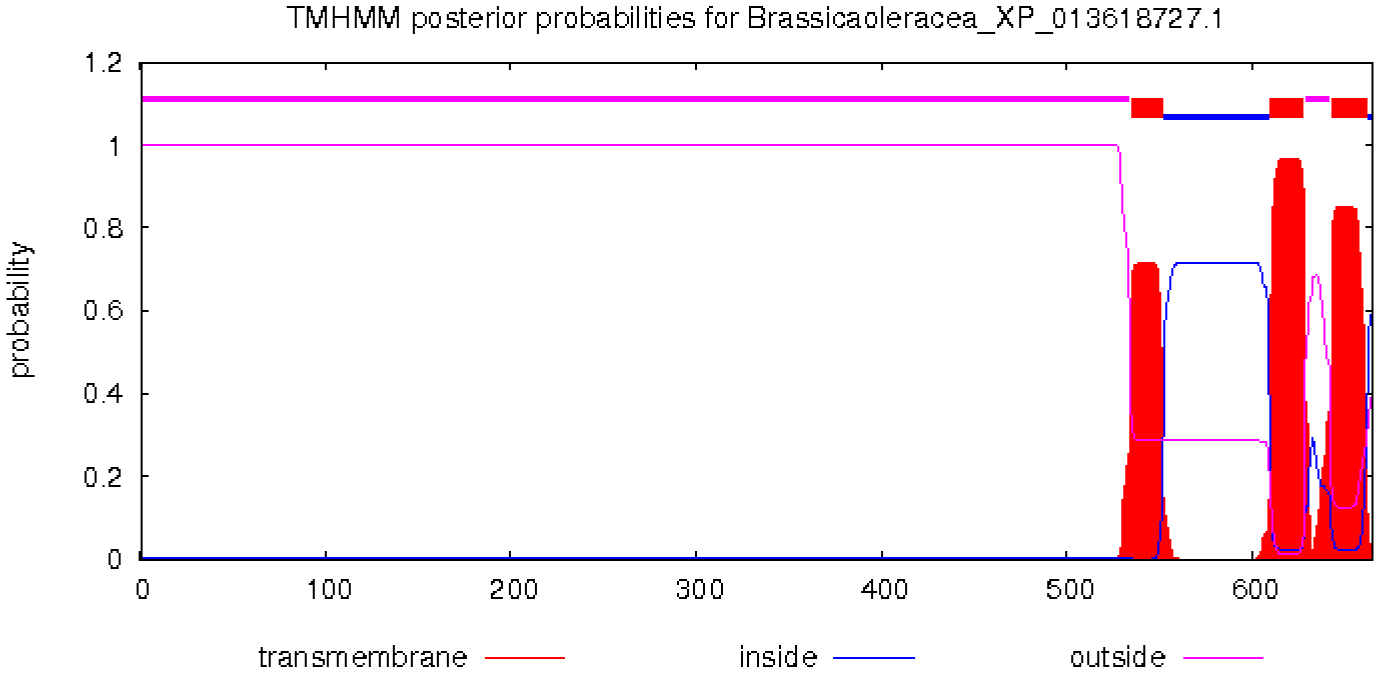

plot in postscript, script for making the plot in gnuplot, data for plot

---

```
# Brassicaoleracea_XP_013618977.1 Length: 367
# Brassicaoleracea_XP_013618977.1 Number of predicted TMHs:  0
# Brassicaoleracea_XP_013618977.1 Exp number of AAs in TMHs: 0.0002
# Brassicaoleracea_XP_013618977.1 Exp number, first 60 AAs:  0
# Brassicaoleracea_XP_013618977.1 Total prob of N-in:        0.00217
Brassicaoleracea_XP_013618977.1 TMHMM2.0        outside      1   367
```

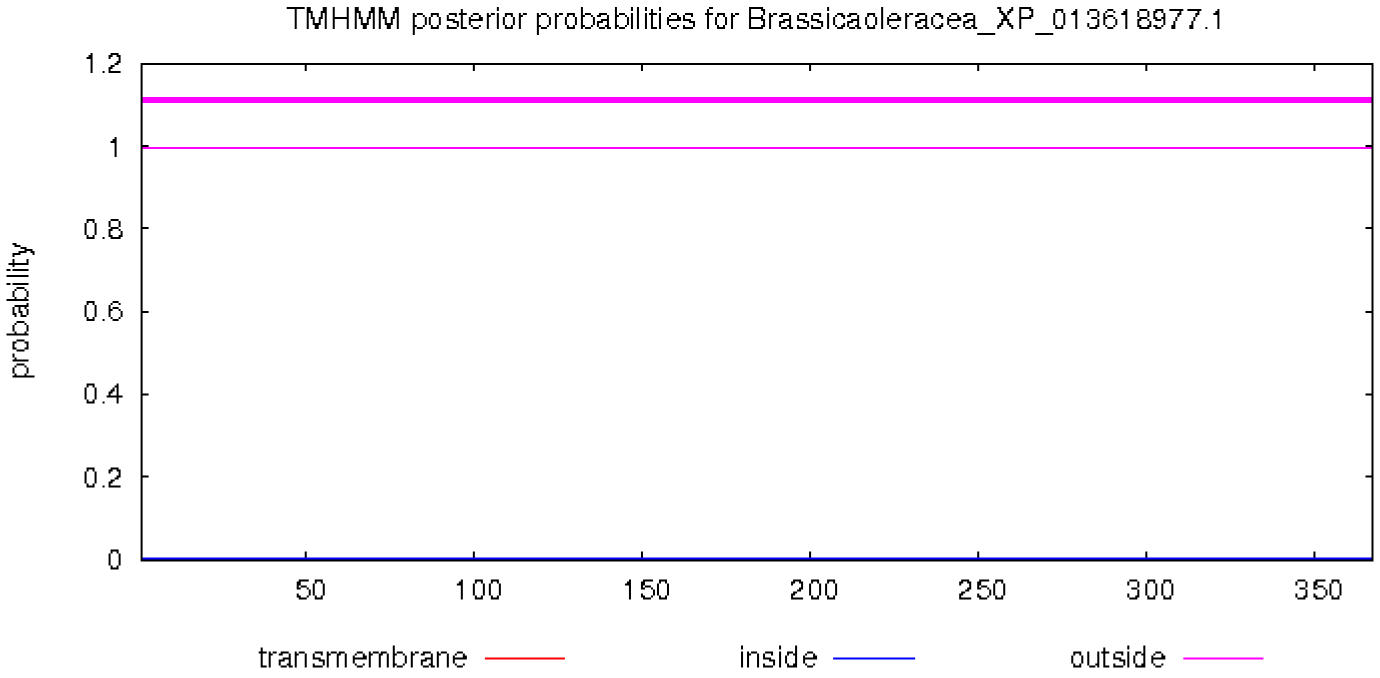

plot in postscript, script for making the plot in gnuplot, data for plot

---

```
# Brassicaoleracea_XP_013618978.1 Length: 367
# Brassicaoleracea_XP_013618978.1 Number of predicted TMHs:  0
# Brassicaoleracea_XP_013618978.1 Exp number of AAs in TMHs: 0.0002
# Brassicaoleracea_XP_013618978.1 Exp number, first 60 AAs:  0
# Brassicaoleracea_XP_013618978.1 Total prob of N-in:        0.00217
Brassicaoleracea_XP_013618978.1 TMHMM2.0        outside      1   367
```

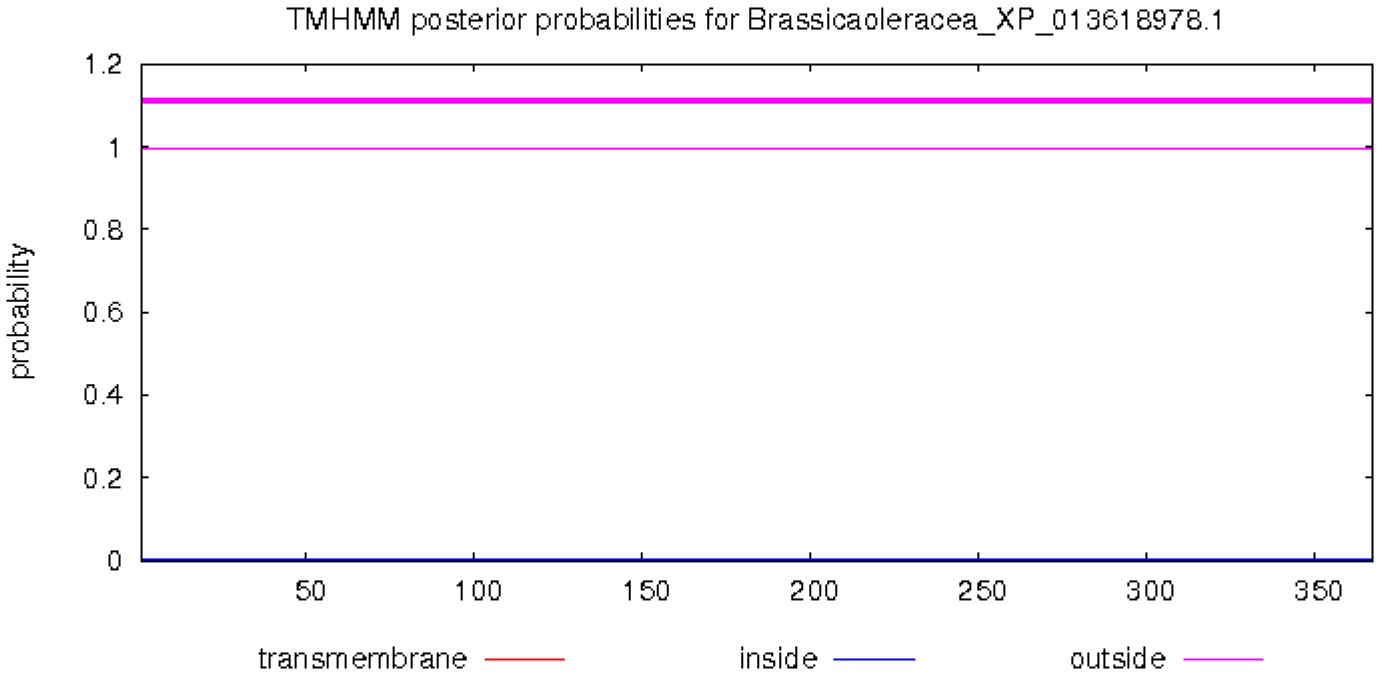

plot in postscript, script for making the plot in gnuplot, data for plot

```
# Brassicaoleracea_XP_013618979.1 Length: 367
# Brassicaoleracea_XP_013618979.1 Number of predicted TMHs:  0
# Brassicaoleracea_XP_013618979.1 Exp number of AAs in TMHs: 0.0002
# Brassicaoleracea_XP_013618979.1 Exp number, first 60 AAs:  0
# Brassicaoleracea_XP_013618979.1 Total prob of N-in:        0.00217
Brassicaoleracea_XP_013618979.1 TMHMM2.0        outside      1   367
```

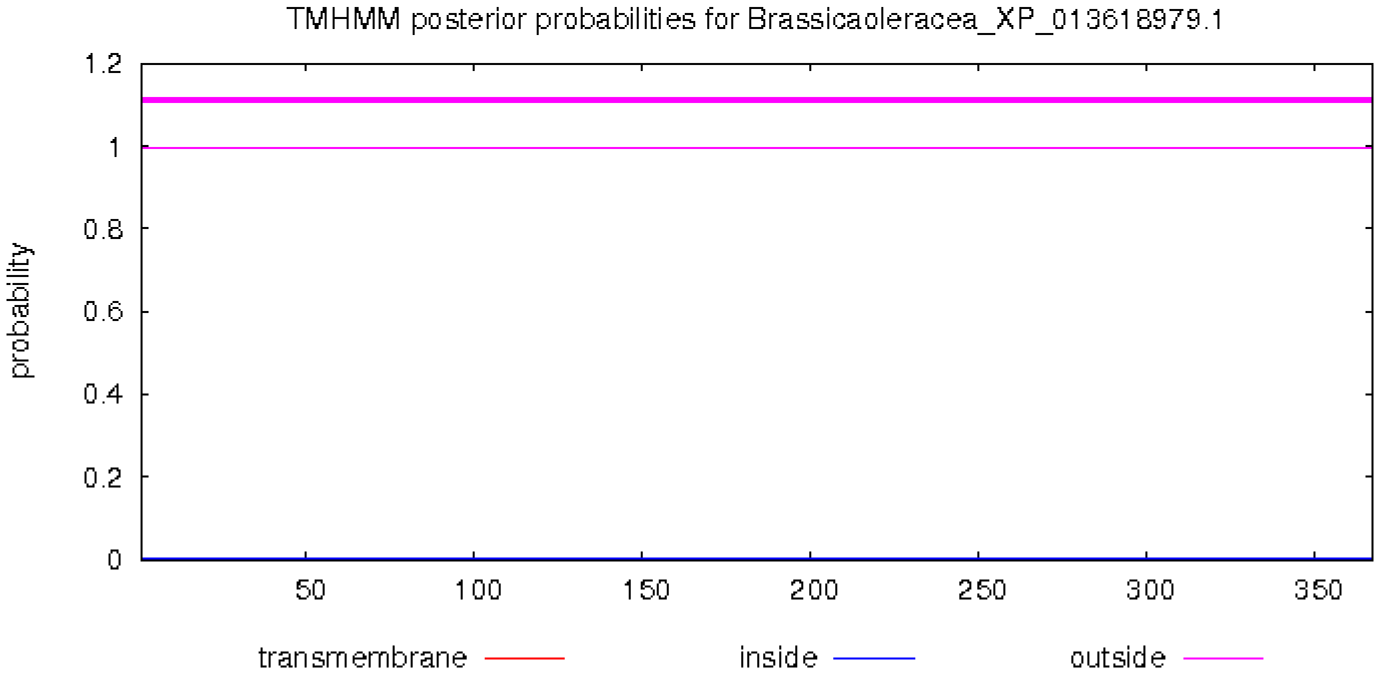

plot in postscript, script for making the plot in gnuplot, data for plot

```
# Brassicaoleracea_XP_013618994.1 Length: 235
# Brassicaoleracea_XP_013618994.1 Number of predicted TMHs:  0
# Brassicaoleracea_XP_013618994.1 Exp number of AAs in TMHs: 0
# Brassicaoleracea_XP_013618994.1 Exp number, first 60 AAs:  0
# Brassicaoleracea_XP_013618994.1 Total prob of N-in:        0.02517
Brassicaoleracea_XP_013618994.1 TMHMM2.0        outside      1   235
```

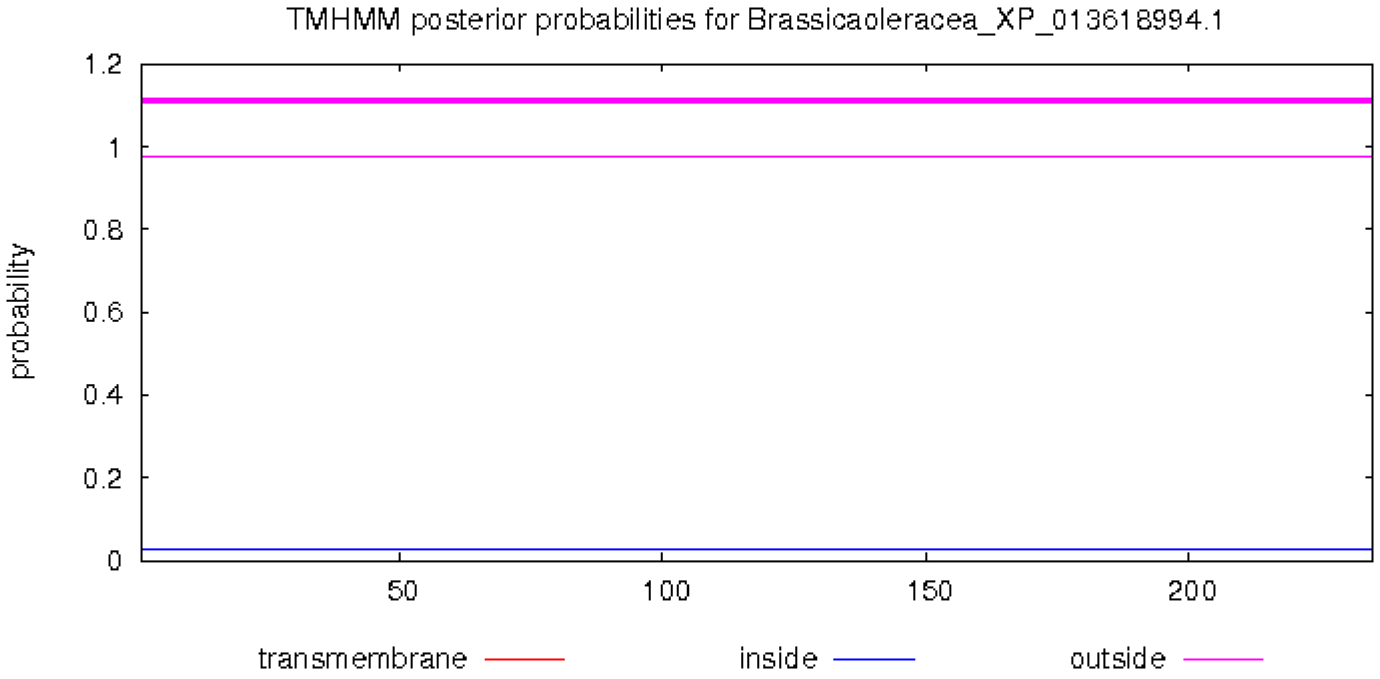

plot in postscript, script for making the plot in gnuplot, data for plot

```
# Brassicaoleracea_XP_013619013.1 Length: 185
# Brassicaoleracea_XP_013619013.1 Number of predicted TMHs:  0
# Brassicaoleracea_XP_013619013.1 Exp number of AAs in TMHs: 0
# Brassicaoleracea_XP_013619013.1 Exp number, first 60 AAs:  0
# Brassicaoleracea_XP_013619013.1 Total prob of N-in:        0.00752
Brassicaoleracea_XP_013619013.1 TMHMM2.0        outside      1   185
```

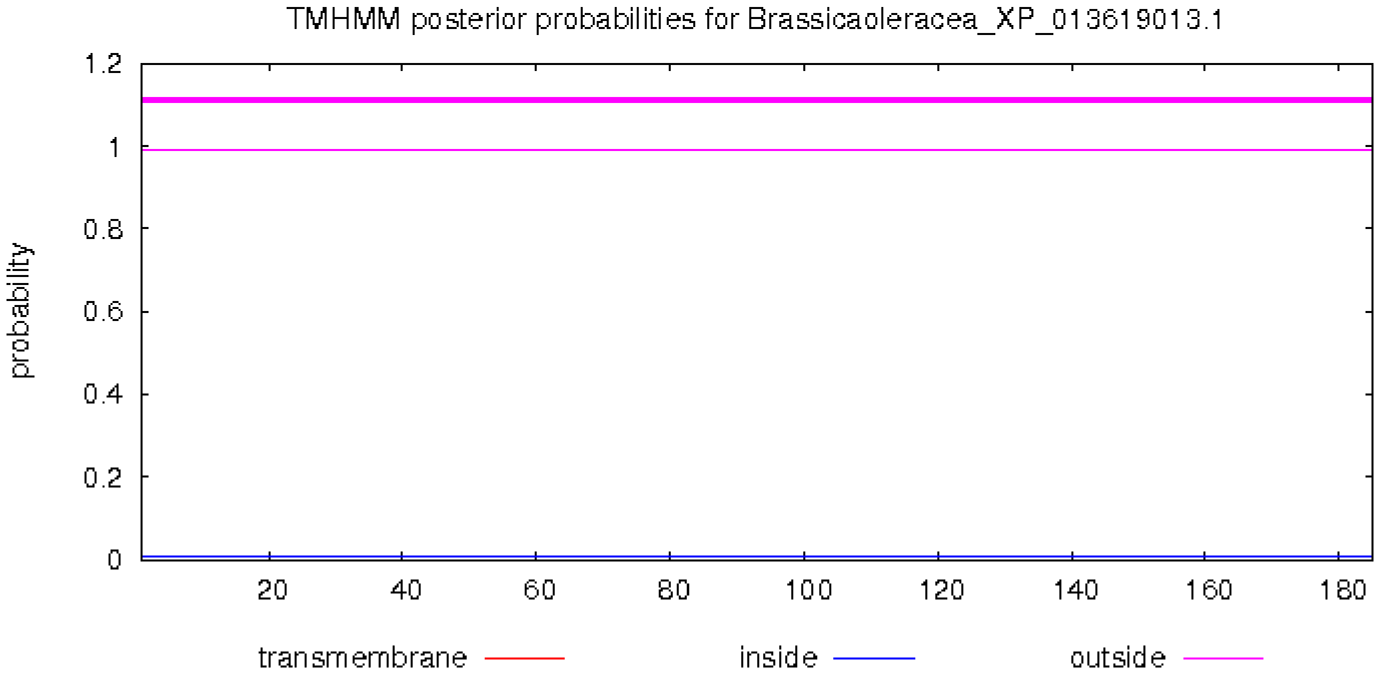

plot in postscript, script for making the plot in gnuplot, data for plot

```
# Brassicaoleracea_XP_013619317.1 Length: 367
# Brassicaoleracea_XP_013619317.1 Number of predicted TMHs:  0
# Brassicaoleracea_XP_013619317.1 Exp number of AAs in TMHs: 0.0141
# Brassicaoleracea_XP_013619317.1 Exp number, first 60 AAs:  0
# Brassicaoleracea_XP_013619317.1 Total prob of N-in:        0.01820
Brassicaoleracea_XP_013619317.1 TMHMM2.0        outside      1   367
```

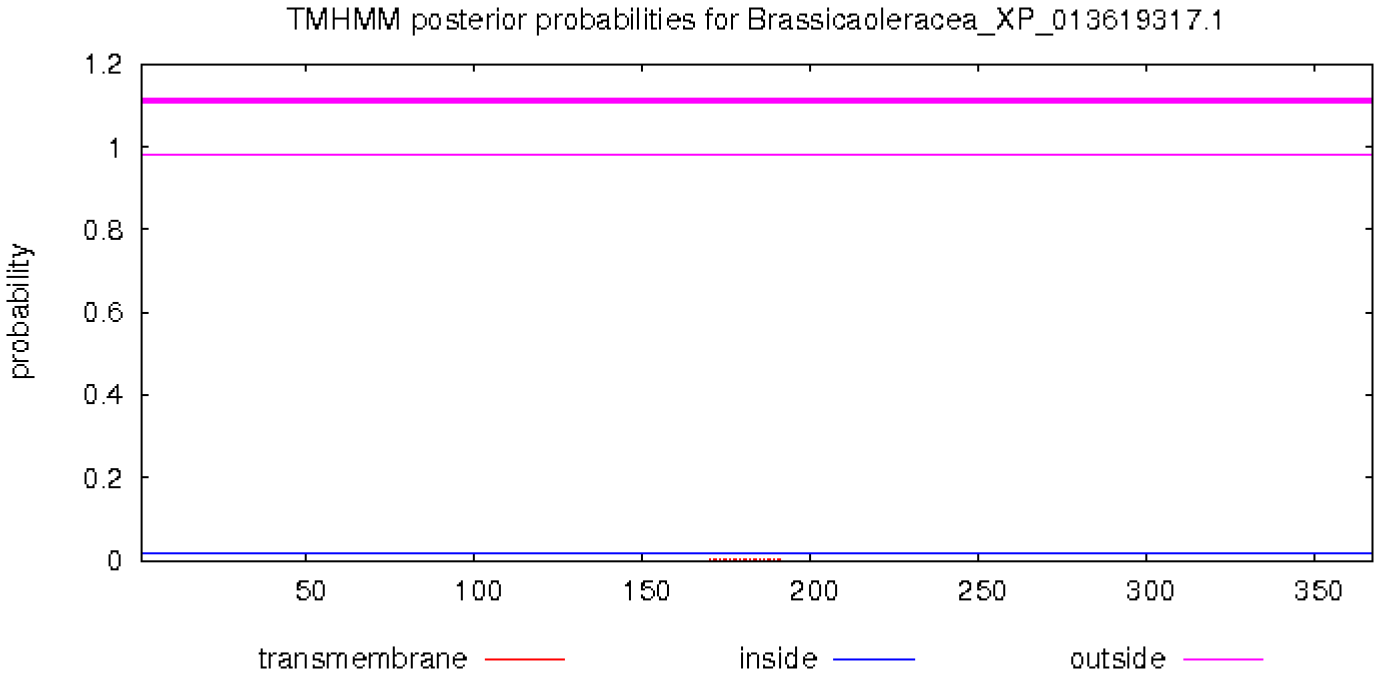

plot in postscript, script for making the plot in gnuplot, data for plot

```
# Brassicaoleracea_XP_013619595.1 Length: 321
# Brassicaoleracea_XP_013619595.1 Number of predicted TMHs:  0
# Brassicaoleracea_XP_013619595.1 Exp number of AAs in TMHs: 0.01338
# Brassicaoleracea_XP_013619595.1 Exp number, first 60 AAs:  0.0006
# Brassicaoleracea_XP_013619595.1 Total prob of N-in:        0.00436
Brassicaoleracea_XP_013619595.1 TMHMM2.0        outside      1   321
```

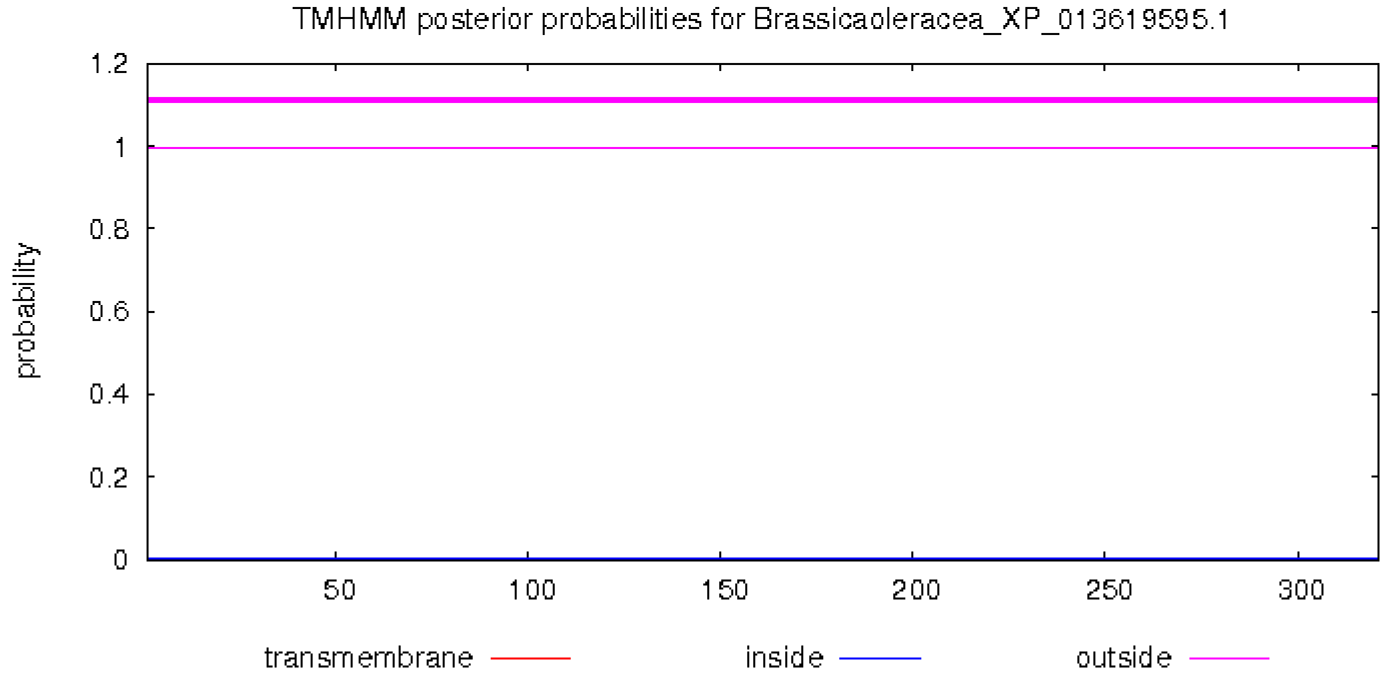

plot in postscript, script for making the plot in gnuplot, data for plot

```
# Brassicaoleracea_XP_013619775.1 Length: 327
# Brassicaoleracea_XP_013619775.1 Number of predicted TMHs:  0
# Brassicaoleracea_XP_013619775.1 Exp number of AAs in TMHs: 0.00016
# Brassicaoleracea_XP_013619775.1 Exp number, first 60 AAs:  0.00016
# Brassicaoleracea_XP_013619775.1 Total prob of N-in:        0.03495
Brassicaoleracea_XP_013619775.1 TMHMM2.0        outside      1   327
```

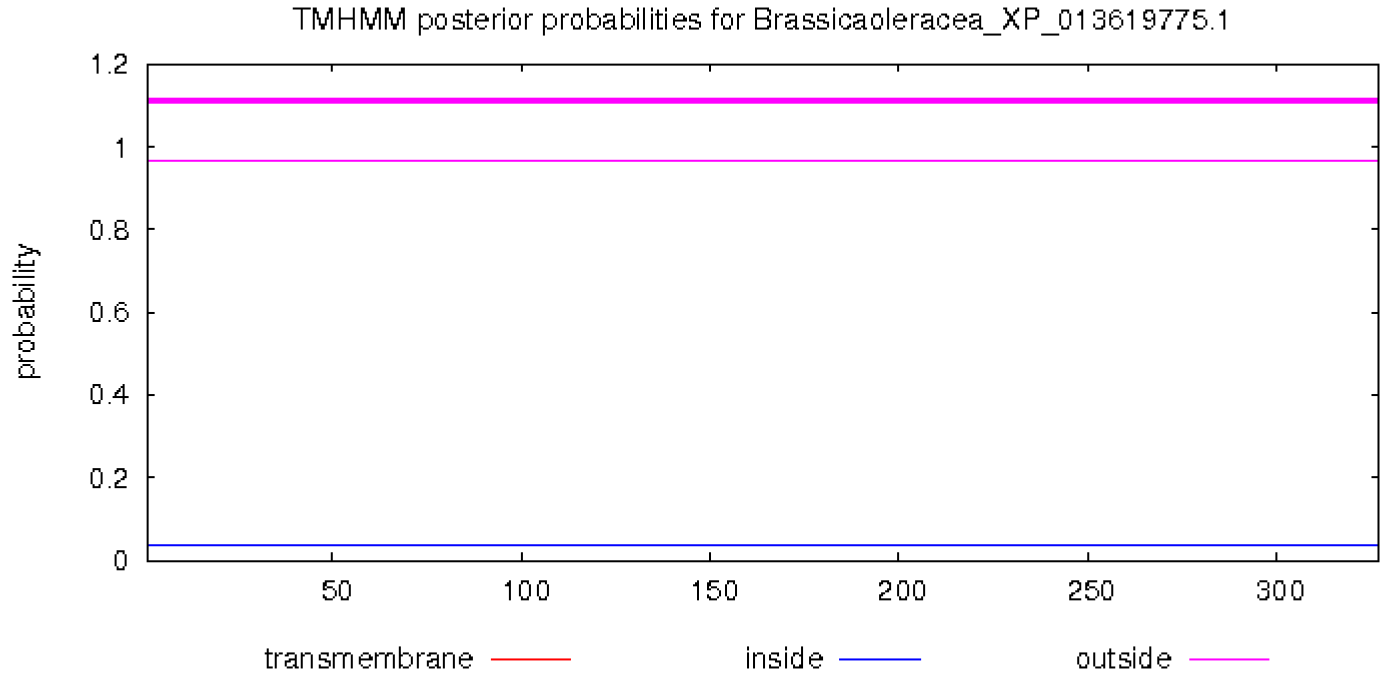

plot in postscript, script for making the plot in gnuplot, data for plot

```
# Brassicaoleracea_XP_013619851.1 Length: 343
# Brassicaoleracea_XP_013619851.1 Number of predicted TMHs:  0
# Brassicaoleracea_XP_013619851.1 Exp number of AAs in TMHs: 12.32386
# Brassicaoleracea_XP_013619851.1 Exp number, first 60 AAs:  0.01178
# Brassicaoleracea_XP_013619851.1 Total prob of N-in:        0.06557
Brassicaoleracea_XP_013619851.1 TMHMM2.0        outside      1   343
```

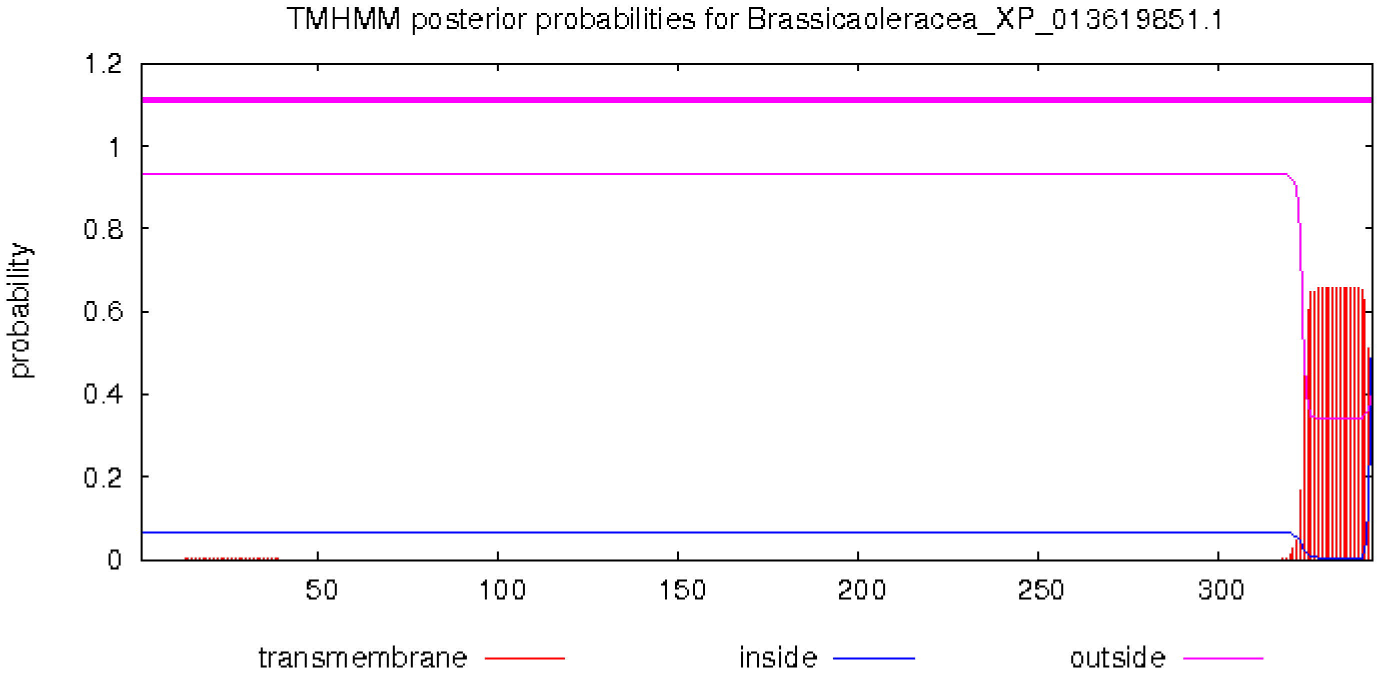

plot in postscript, script for making the plot in gnuplot, data for plot

```
# Brassicaoleracea_XP_013620810.1 Length: 524
# Brassicaoleracea_XP_013620810.1 Number of predicted TMHs:  1
# Brassicaoleracea_XP_013620810.1 Exp number of AAs in TMHs: 21.95928
# Brassicaoleracea_XP_013620810.1 Exp number, first 60 AAs:  0
# Brassicaoleracea_XP_013620810.1 Total prob of N-in:        0.03084
Brassicaoleracea_XP_013620810.1 TMHMM2.0        outside      1   498
Brassicaoleracea_XP_013620810.1 TMHMM2.0        TMhelix    499   521
Brassicaoleracea_XP_013620810.1 TMHMM2.0        inside     522   524
```

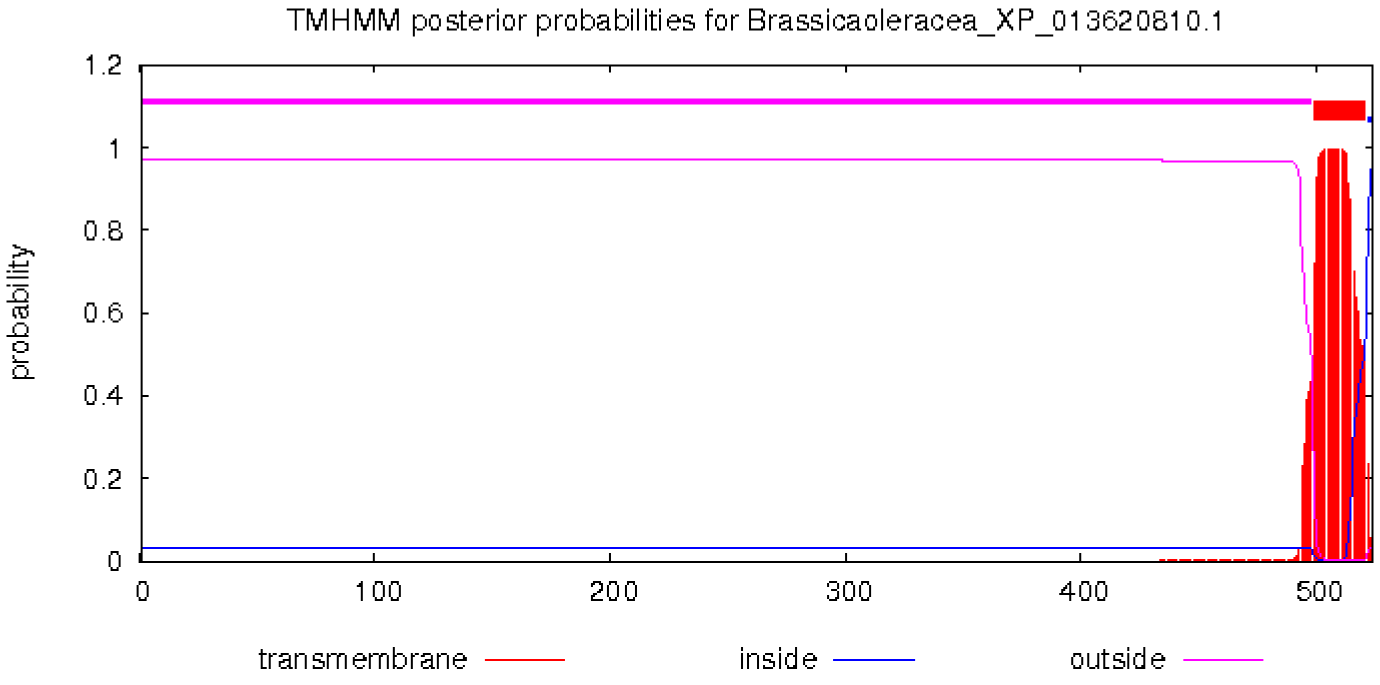

plot in postscript, script for making the plot in gnuplot, data for plot

---

```
# Brassicaoleracea_XP_013620811.1 Length: 523
# Brassicaoleracea_XP_013620811.1 Number of predicted TMHs:  1
# Brassicaoleracea_XP_013620811.1 Exp number of AAs in TMHs: 21.95957
# Brassicaoleracea_XP_013620811.1 Exp number, first 60 AAs:  0
# Brassicaoleracea_XP_013620811.1 Total prob of N-in:        0.03122
Brassicaoleracea_XP_013620811.1 TMHMM2.0        outside      1   497
Brassicaoleracea_XP_013620811.1 TMHMM2.0        TMhelix    498   520
Brassicaoleracea_XP_013620811.1 TMHMM2.0        inside     521   523
```

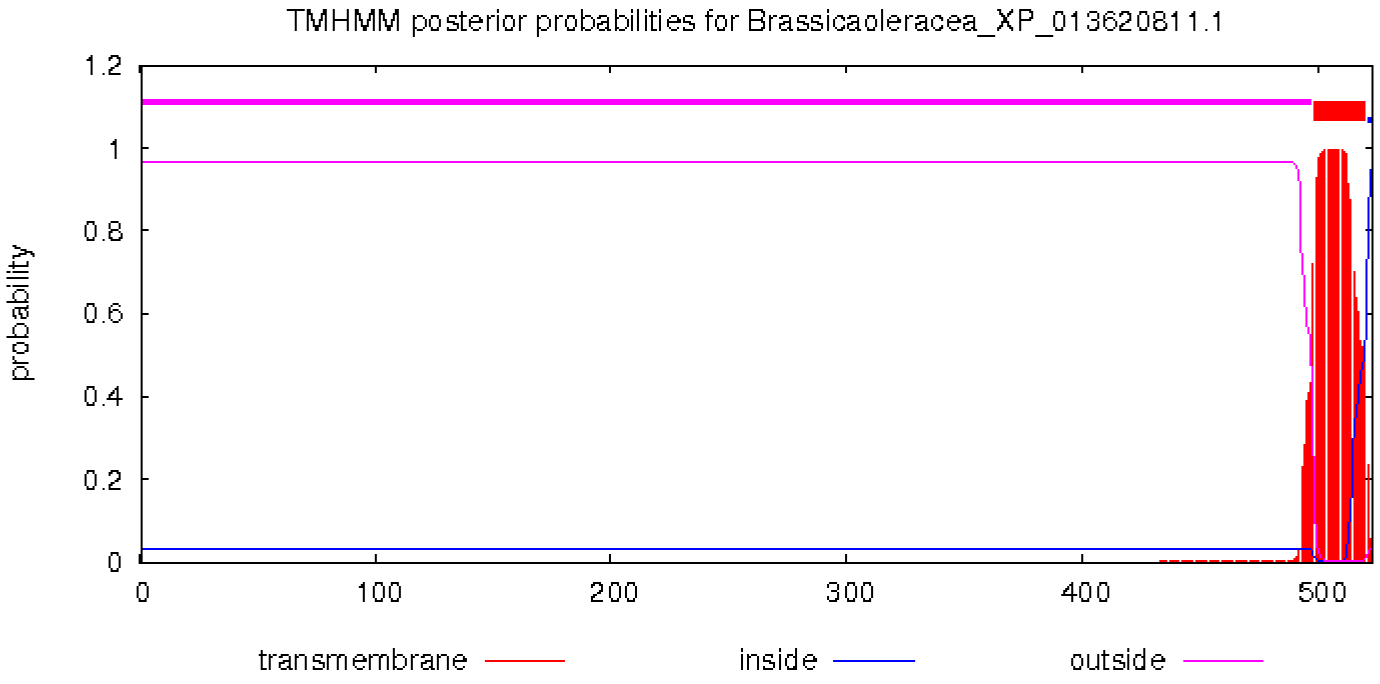

plot in postscript, script for making the plot in gnuplot, data for plot

---

```
# Brassicaoleracea_XP_013620870.1 Length: 264
# Brassicaoleracea_XP_013620870.1 Number of predicted TMHs:  0
# Brassicaoleracea_XP_013620870.1 Exp number of AAs in TMHs: 0
# Brassicaoleracea_XP_013620870.1 Exp number, first 60 AAs:  0
```

```
# Brassicaoleracea_XP_013620870.1 Total prob of N-in:        0.01301
Brassicaoleracea_XP_013620870.1 TMHMM2.0        outside      1   264
```

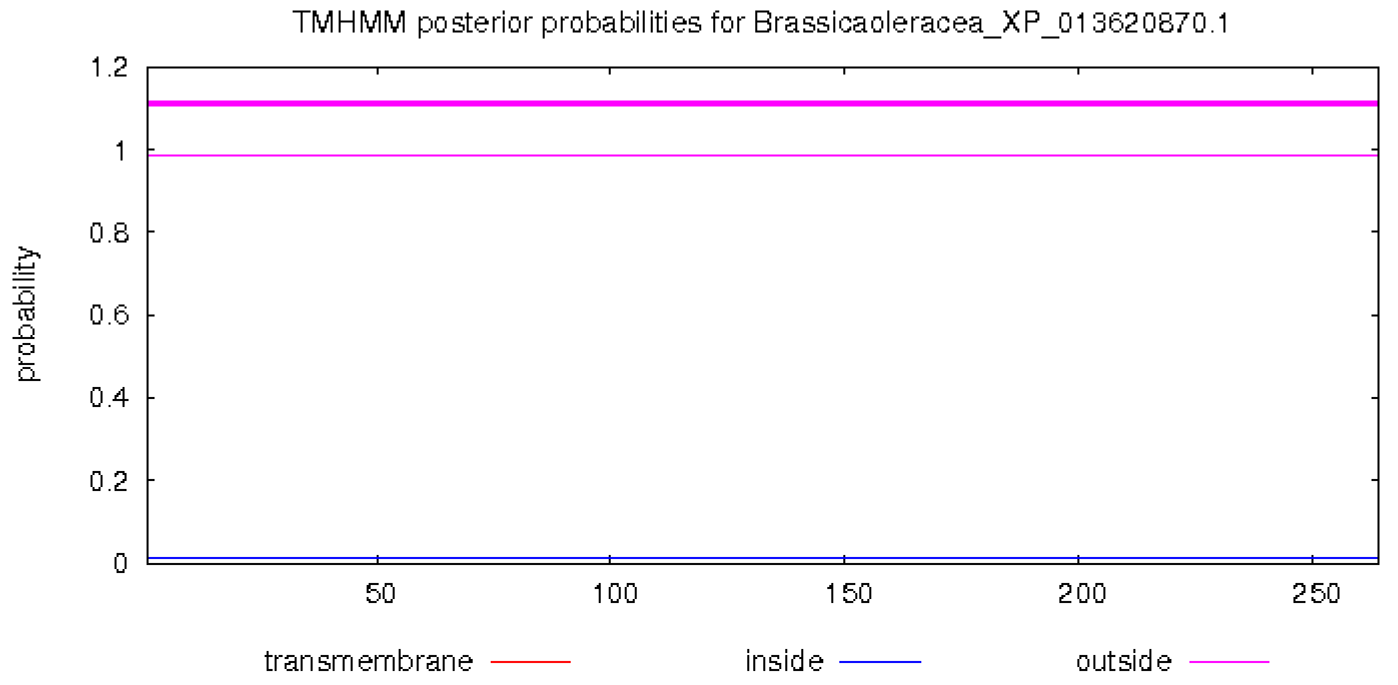

plot in postscript, script for making the plot in gnuplot, data for plot

---

```
# Brassicaoleracea_XP_013621799.1 Length: 479
# Brassicaoleracea_XP_013621799.1 Number of predicted TMHs:  0
# Brassicaoleracea_XP_013621799.1 Exp number of AAs in TMHs: 0.00458
# Brassicaoleracea_XP_013621799.1 Exp number, first 60 AAs:  0
# Brassicaoleracea_XP_013621799.1 Total prob of N-in:        0.00017
Brassicaoleracea_XP_013621799.1 TMHMM2.0        outside      1   479
```

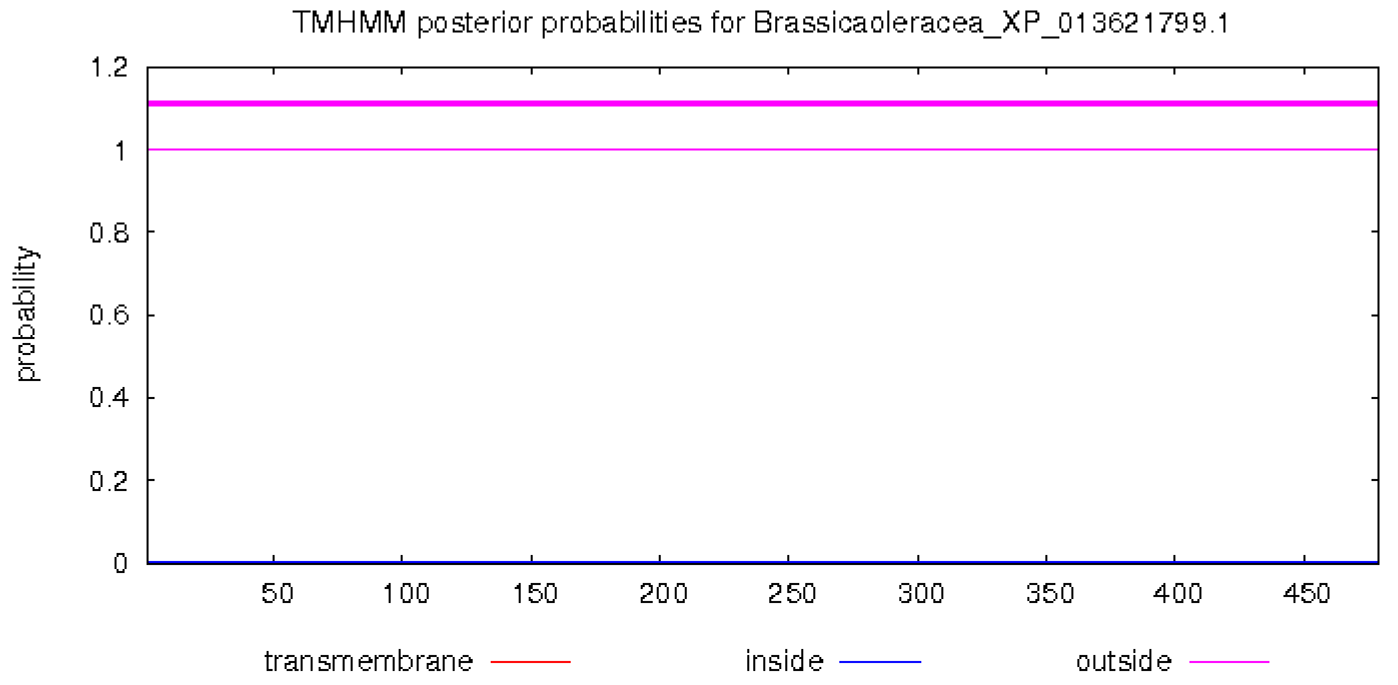

plot in postscript, script for making the plot in gnuplot, data for plot

---

```
# Brassicaoleracea_XP_013621861.1 Length: 384
# Brassicaoleracea_XP_013621861.1 Number of predicted TMHs:  0
# Brassicaoleracea_XP_013621861.1 Exp number of AAs in TMHs: 0.01226
# Brassicaoleracea_XP_013621861.1 Exp number, first 60 AAs:  0
```

```
# Brassicaoleracea_XP_013621861.1 Total prob of N-in:        0.01119
Brassicaoleracea_XP_013621861.1 TMHMM2.0        outside      1   384
```

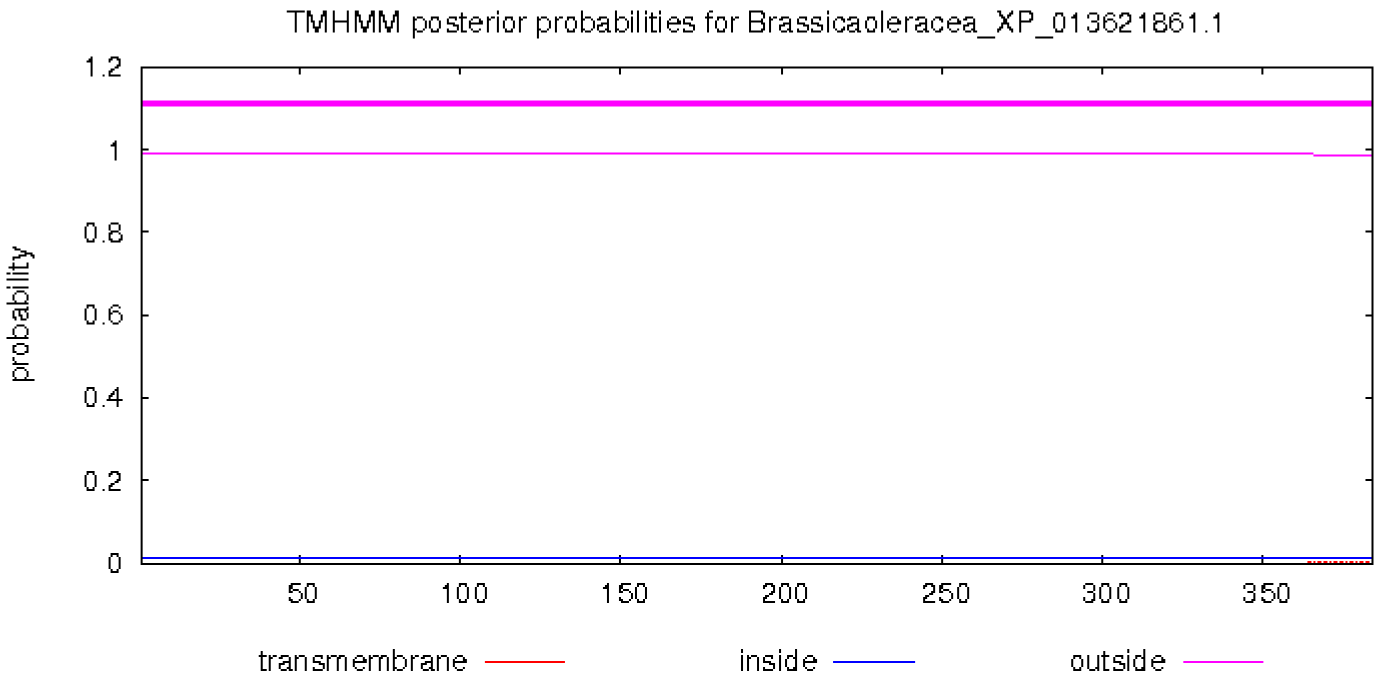

plot in postscript, script for making the plot in gnuplot, data for plot

---

```
# Brassicaoleracea_XP_013622295.1 Length: 337
# Brassicaoleracea_XP_013622295.1 Number of predicted TMHs:  0
# Brassicaoleracea_XP_013622295.1 Exp number of AAs in TMHs: 0.00018
# Brassicaoleracea_XP_013622295.1 Exp number, first 60 AAs:  0.00018
# Brassicaoleracea_XP_013622295.1 Total prob of N-in:        0.00577
Brassicaoleracea_XP_013622295.1 TMHMM2.0        outside      1   337
```

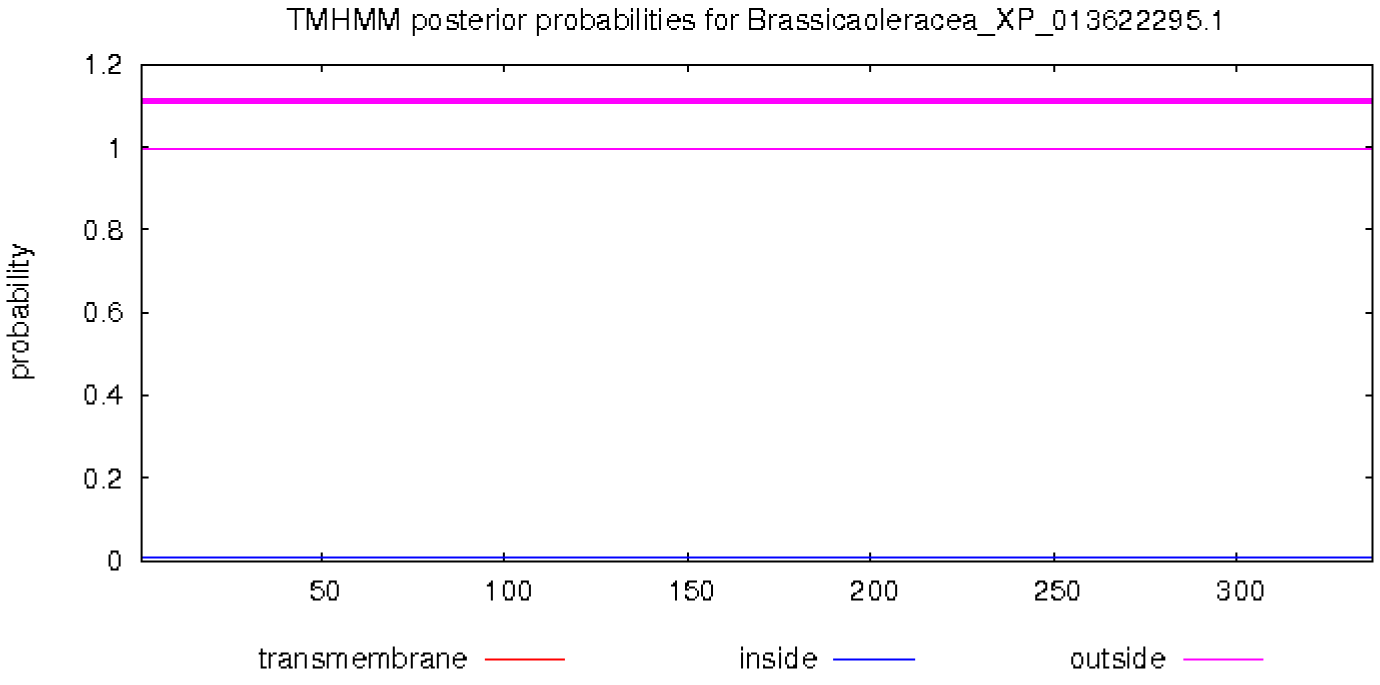

plot in postscript, script for making the plot in gnuplot, data for plot

---

```
# Brassicaoleracea_XP_013622434.1 Length: 436
# Brassicaoleracea_XP_013622434.1 Number of predicted TMHs:  1
# Brassicaoleracea_XP_013622434.1 Exp number of AAs in TMHs: 19.67717
# Brassicaoleracea_XP_013622434.1 Exp number, first 60 AAs:  0
```

```
# Brassicaoleracea_XP_013622434.1 Total prob of N-in:        0.17578
Brassicaoleracea_XP_013622434.1 TMHMM2.0        outside      1   406
Brassicaoleracea_XP_013622434.1 TMHMM2.0        TMhelix    407   426
Brassicaoleracea_XP_013622434.1 TMHMM2.0        inside     427   436
```

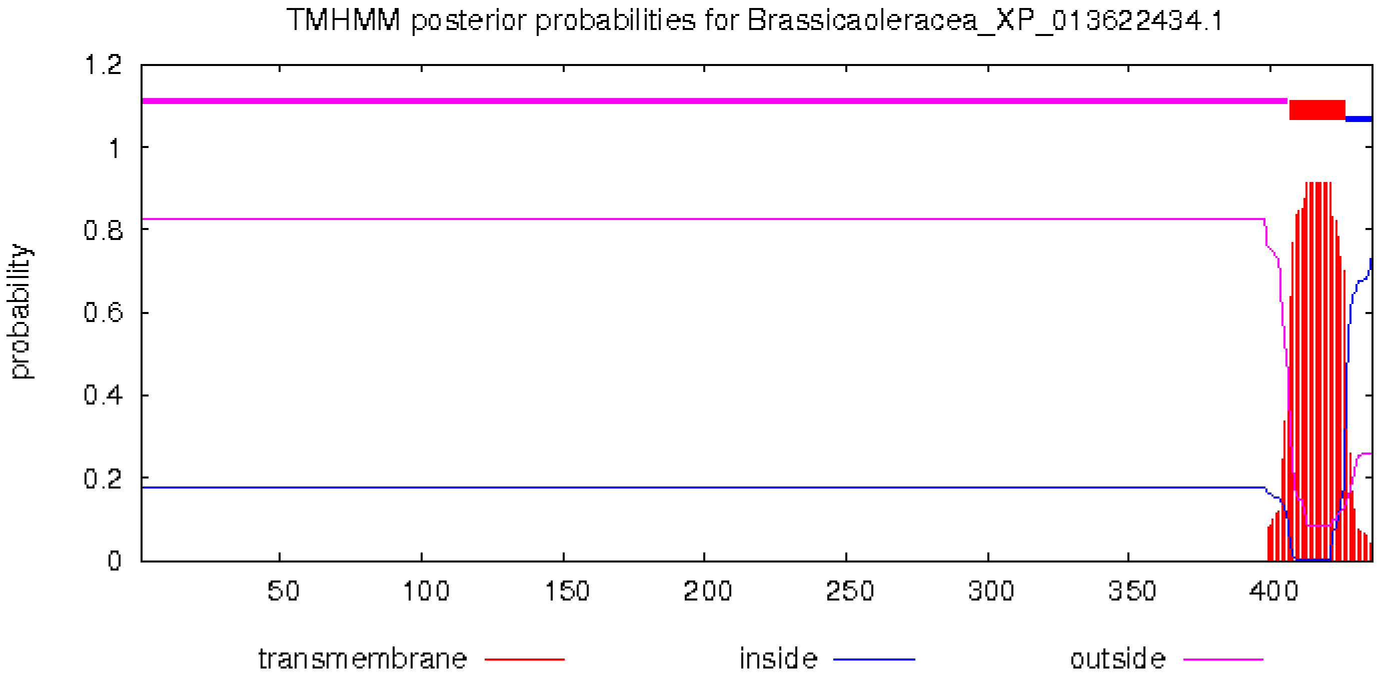

plot in postscript, script for making the plot in gnuplot, data for plot

---

```
# Brassicaoleracea_XP_013622435.1 Length: 436
# Brassicaoleracea_XP_013622435.1 Number of predicted TMHs:  1
# Brassicaoleracea_XP_013622435.1 Exp number of AAs in TMHs: 19.67717
# Brassicaoleracea_XP_013622435.1 Exp number, first 60 AAs:  0
# Brassicaoleracea_XP_013622435.1 Total prob of N-in:        0.17578
Brassicaoleracea_XP_013622435.1 TMHMM2.0        outside      1   406
Brassicaoleracea_XP_013622435.1 TMHMM2.0        TMhelix    407   426
Brassicaoleracea_XP_013622435.1 TMHMM2.0        inside     427   436
```

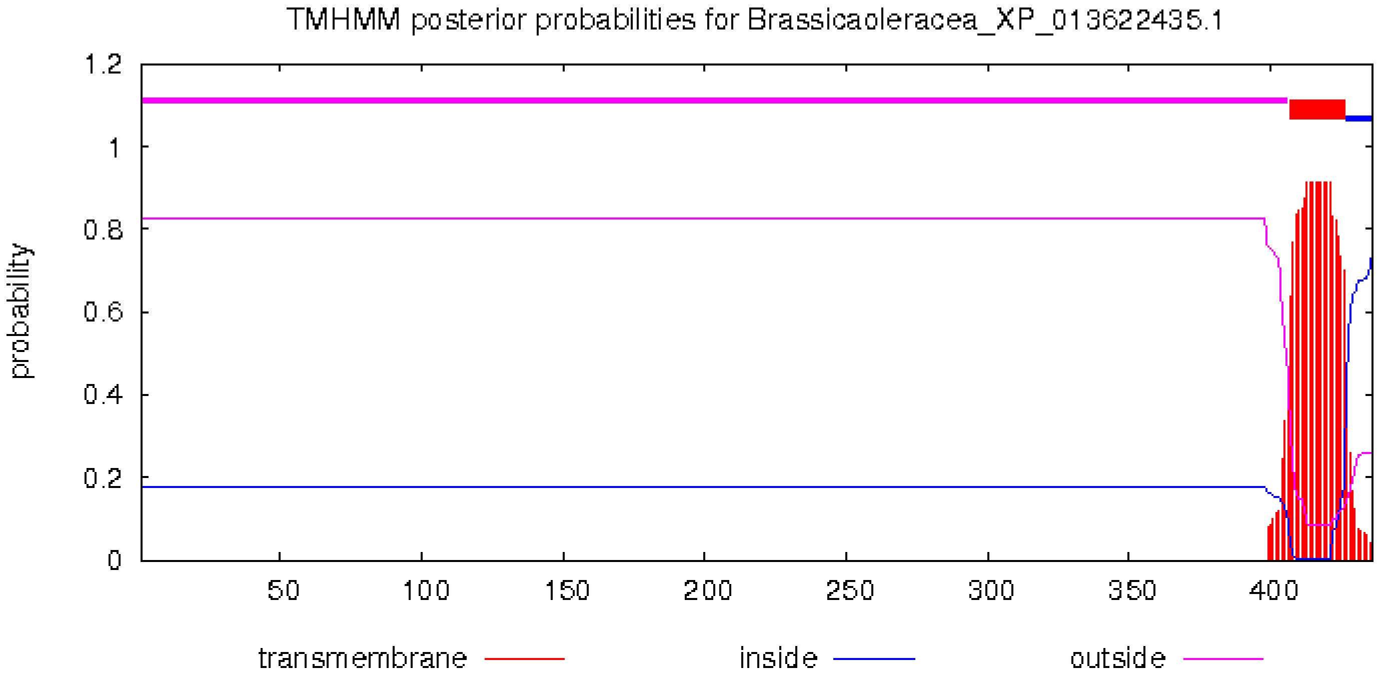

plot in postscript, script for making the plot in gnuplot, data for plot

---

```
# Brassicaoleracea_XP_013622509.1 Length: 177
# Brassicaoleracea_XP_013622509.1 Number of predicted TMHs:  0
# Brassicaoleracea_XP_013622509.1 Exp number of AAs in TMHs: 0.03106
# Brassicaoleracea_XP_013622509.1 Exp number, first 60 AAs:  0
# Brassicaoleracea_XP_013622509.1 Total prob of N-in:        0.10282
Brassicaoleracea_XP_013622509.1 TMHMM2.0        outside      1   177
```

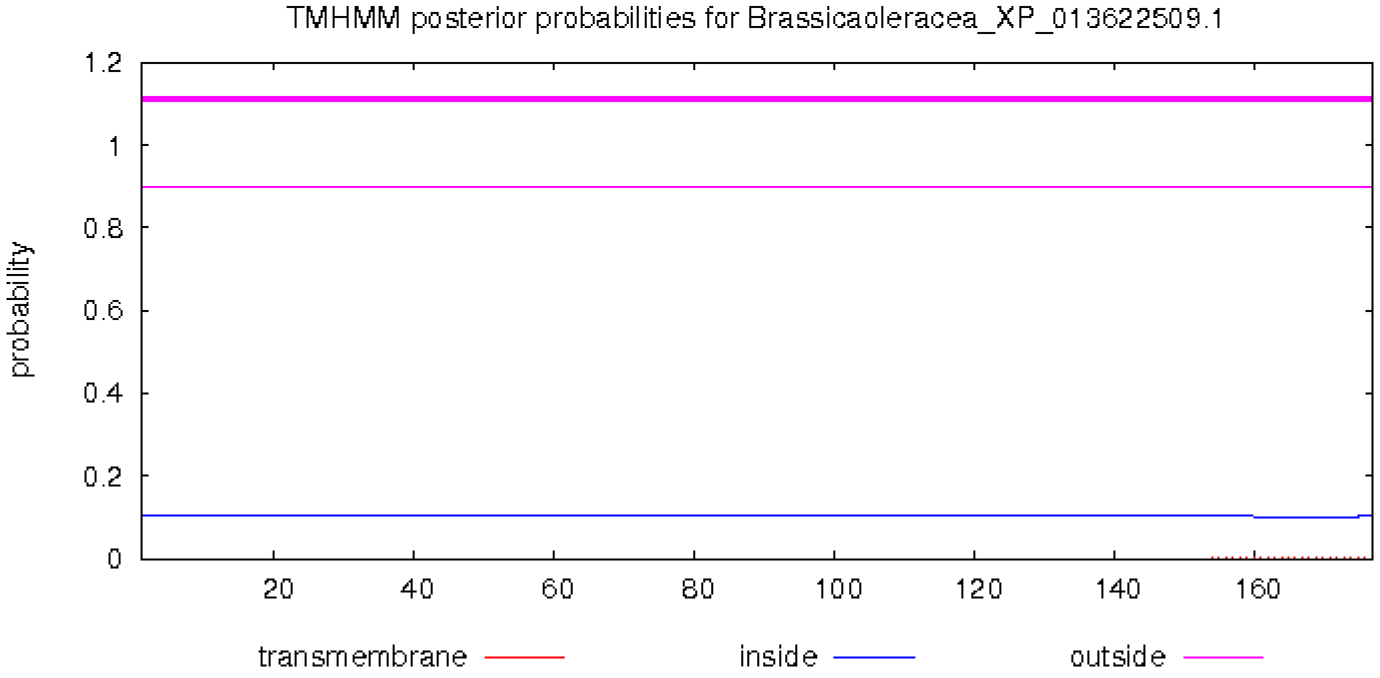

plot in postscript, script for making the plot in gnuplot, data for plot

---

```
# Brassicaoleracea_XP_013622519.1 Length: 421
# Brassicaoleracea_XP_013622519.1 Number of predicted TMHs:  0
# Brassicaoleracea_XP_013622519.1 Exp number of AAs in TMHs: 0.1494
# Brassicaoleracea_XP_013622519.1 Exp number, first 60 AAs:  0.05881
# Brassicaoleracea_XP_013622519.1 Total prob of N-in:        0.01054
Brassicaoleracea_XP_013622519.1 TMHMM2.0        outside      1   421
```

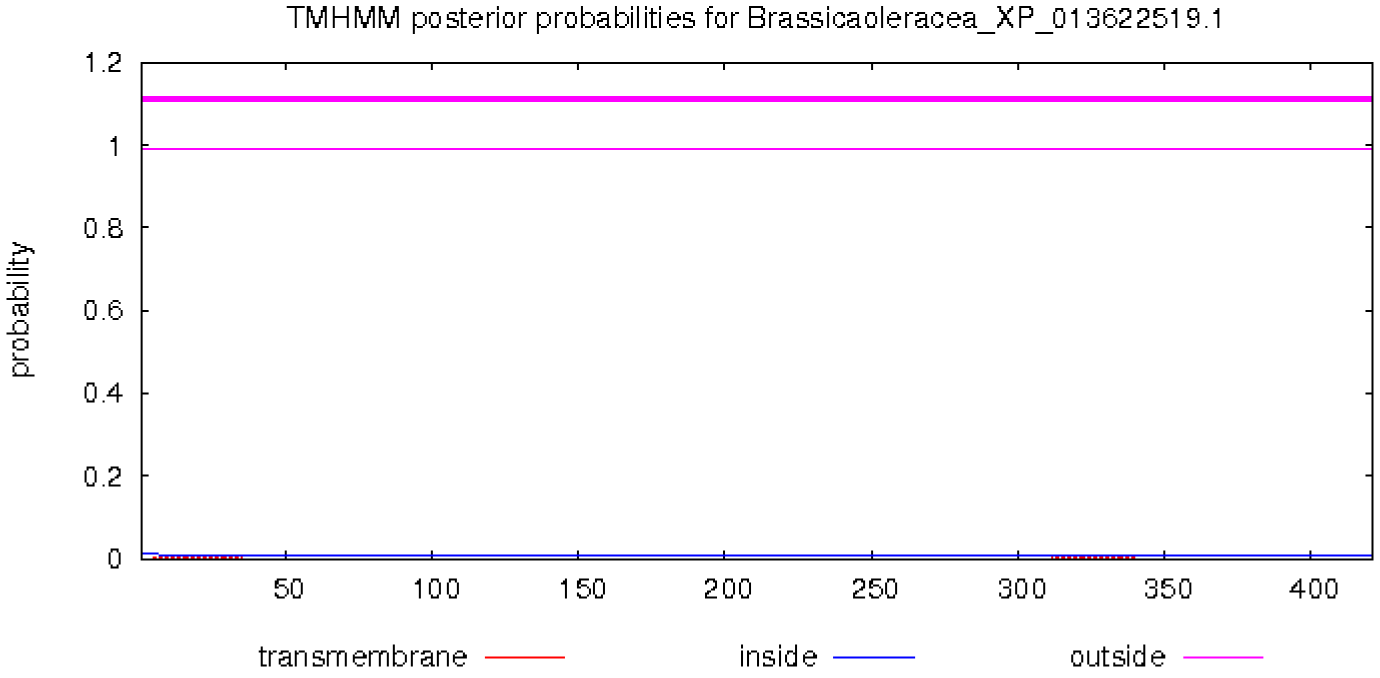

plot in postscript, script for making the plot in gnuplot, data for plot

---

```
# Brassicaoleracea_XP_013622520.1 Length: 352
# Brassicaoleracea_XP_013622520.1 Number of predicted TMHs:  0
# Brassicaoleracea_XP_013622520.1 Exp number of AAs in TMHs: 0.10596
# Brassicaoleracea_XP_013622520.1 Exp number, first 60 AAs:  0
# Brassicaoleracea_XP_013622520.1 Total prob of N-in:        0.01131
Brassicaoleracea_XP_013622520.1 TMHMM2.0        outside      1   352
```

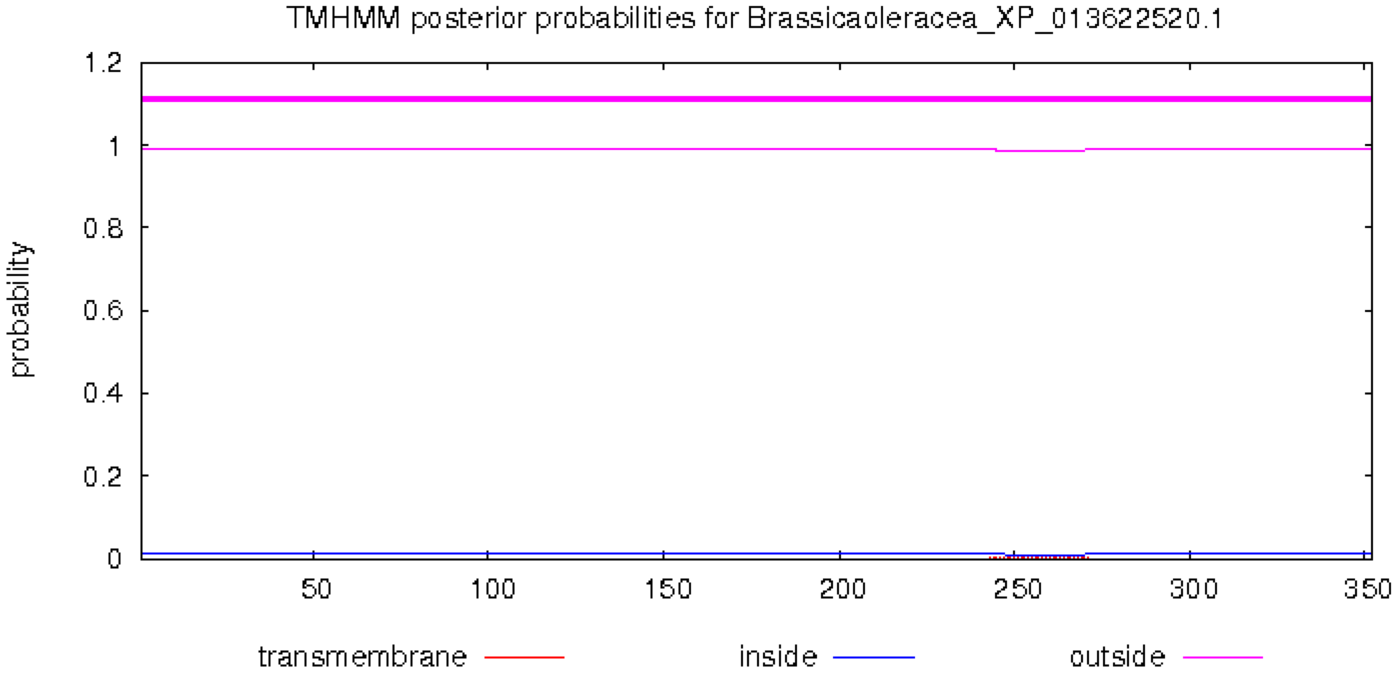

plot in postscript, script for making the plot in gnuplot, data for plot

---

```
# Brassicaoleracea_XP_013623093.1 Length: 428
# Brassicaoleracea_XP_013623093.1 Number of predicted TMHs:  0
# Brassicaoleracea_XP_013623093.1 Exp number of AAs in TMHs: 0.04037
# Brassicaoleracea_XP_013623093.1 Exp number, first 60 AAs:  0.00772
# Brassicaoleracea_XP_013623093.1 Total prob of N-in:        0.00568
Brassicaoleracea_XP_013623093.1 TMHMM2.0        outside      1   428
```

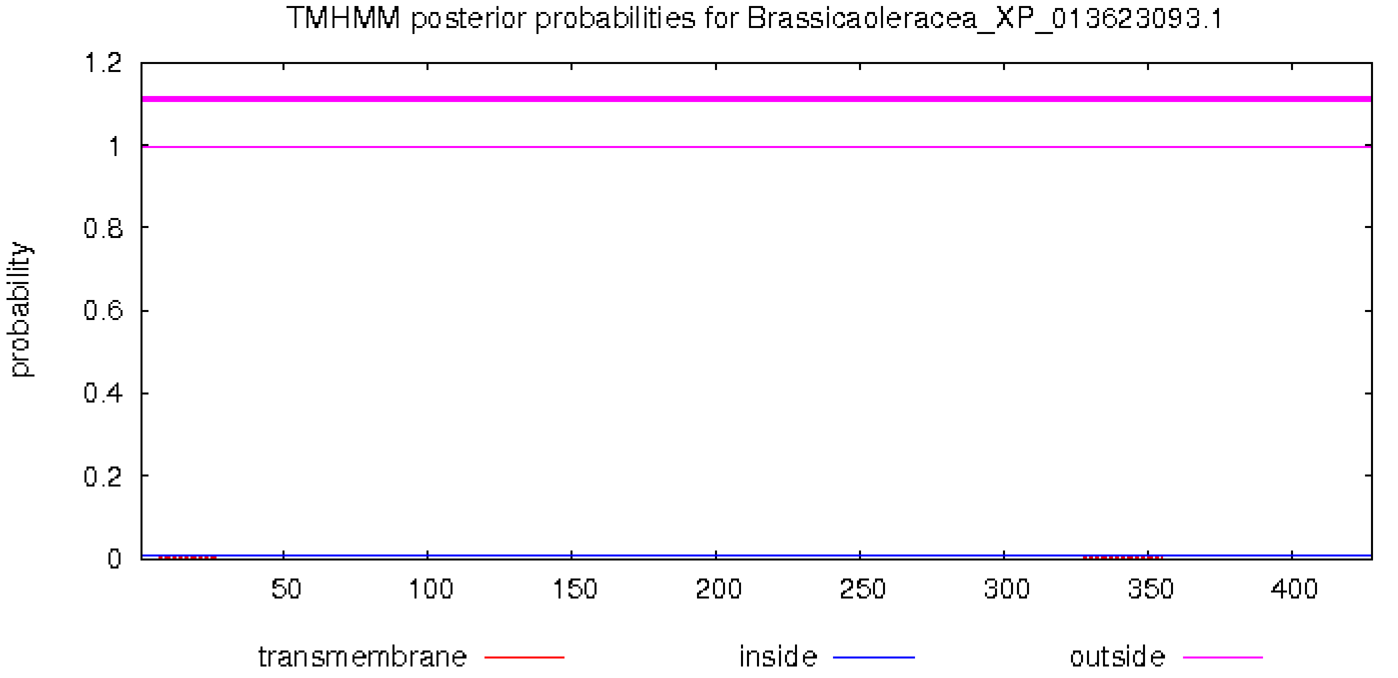

plot in postscript, script for making the plot in gnuplot, data for plot

---

```
# Brassicaoleracea_XP_013624280.1 Length: 175
# Brassicaoleracea_XP_013624280.1 Number of predicted TMHs:  0
# Brassicaoleracea_XP_013624280.1 Exp number of AAs in TMHs: 0.00013
# Brassicaoleracea_XP_013624280.1 Exp number, first 60 AAs:  0
# Brassicaoleracea_XP_013624280.1 Total prob of N-in:        0.06576
Brassicaoleracea_XP_013624280.1 TMHMM2.0        outside      1   175
```

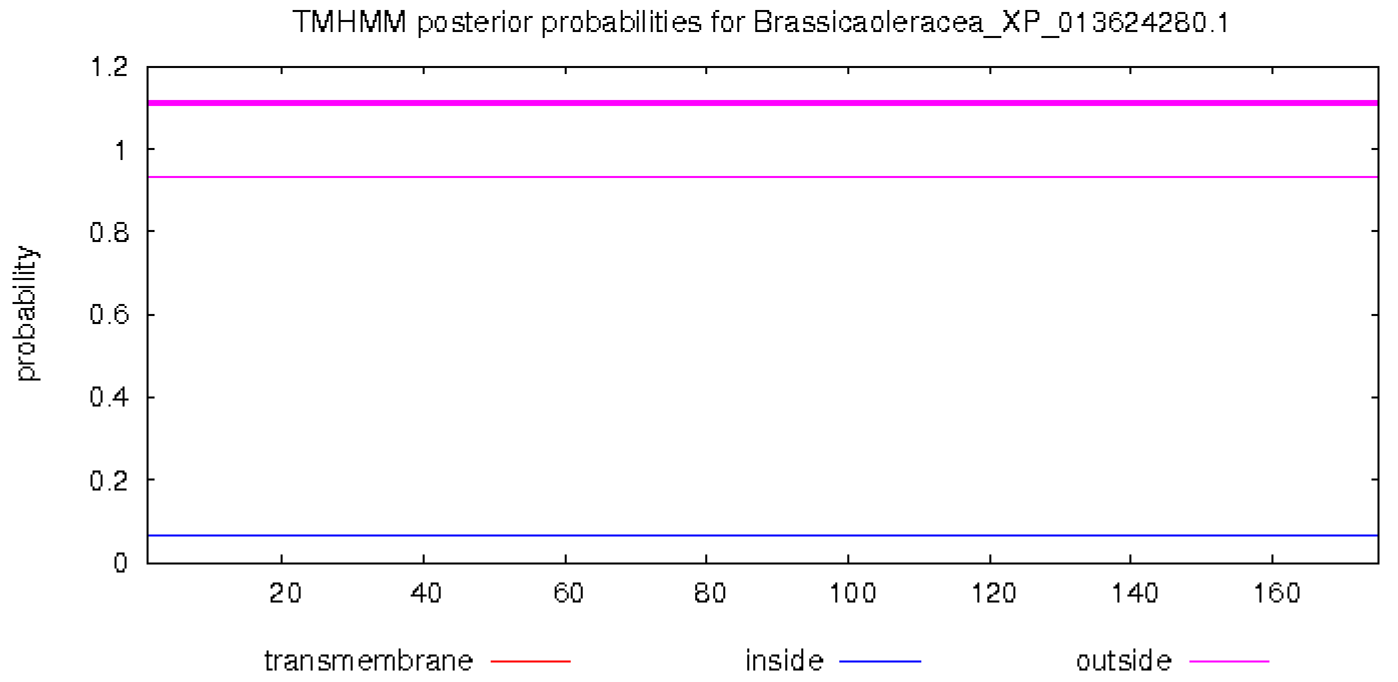

plot in postscript, script for making the plot in gnuplot, data for plot

```
# Brassicaoleracea_XP_013624372.1 Length: 287
# Brassicaoleracea_XP_013624372.1 Number of predicted TMHs:  0
# Brassicaoleracea_XP_013624372.1 Exp number of AAs in TMHs: 0
# Brassicaoleracea_XP_013624372.1 Exp number, first 60 AAs:  0
# Brassicaoleracea_XP_013624372.1 Total prob of N-in:        0.00512
Brassicaoleracea_XP_013624372.1 TMHMM2.0        outside      1   287
```

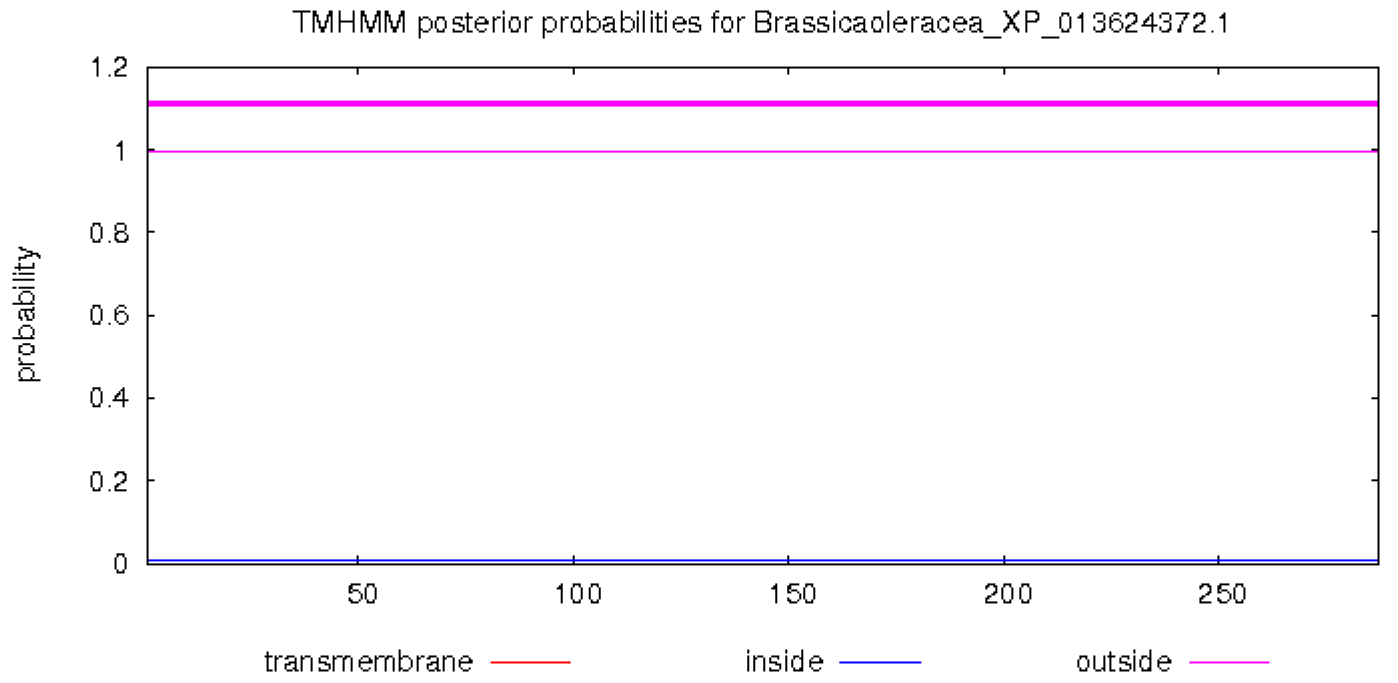

plot in postscript, script for making the plot in gnuplot, data for plot

```
# Brassicaoleracea_XP_013625043.1 Length: 322
# Brassicaoleracea_XP_013625043.1 Number of predicted TMHs:  0
# Brassicaoleracea_XP_013625043.1 Exp number of AAs in TMHs: 0.00041
# Brassicaoleracea_XP_013625043.1 Exp number, first 60 AAs:  0
# Brassicaoleracea_XP_013625043.1 Total prob of N-in:        0.01797
Brassicaoleracea_XP_013625043.1 TMHMM2.0        outside      1   322
```

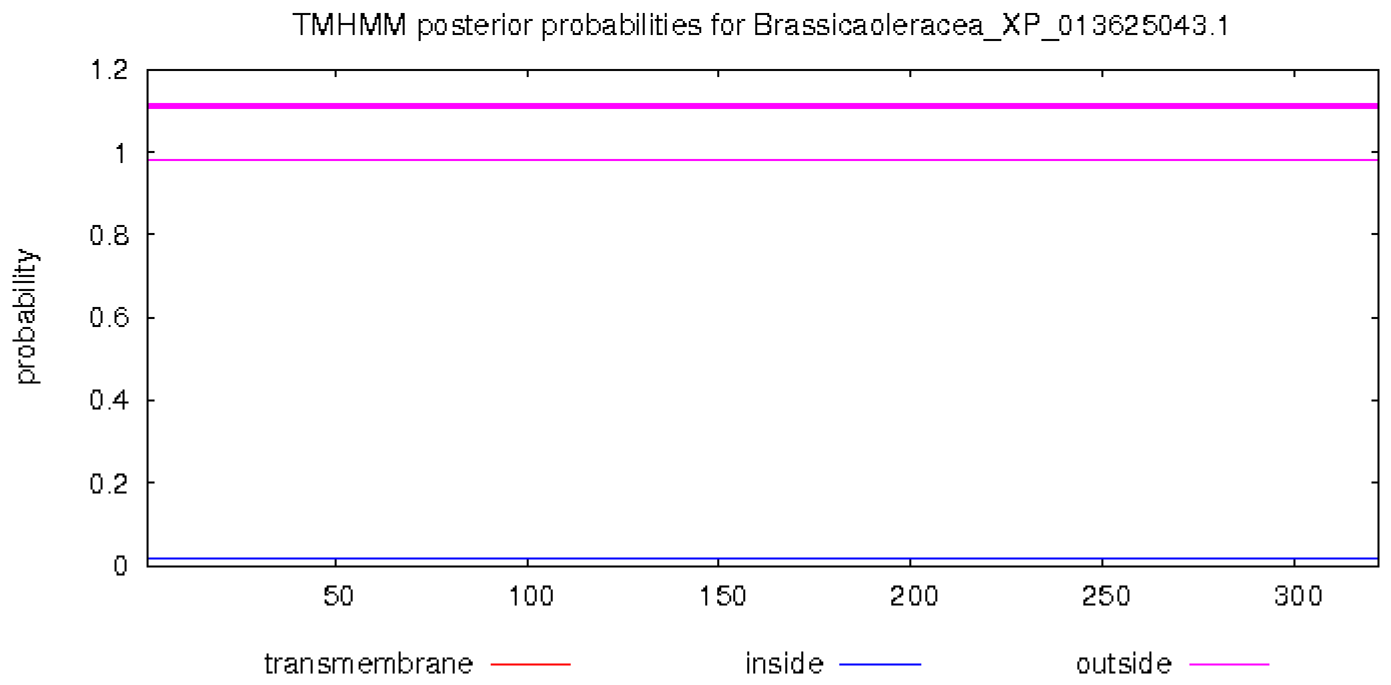

plot in postscript, script for making the plot in gnuplot, data for plot

---

```
# Brassicaoleracea_XP_013625044.1 Length: 307
# Brassicaoleracea_XP_013625044.1 Number of predicted TMHs:  0
# Brassicaoleracea_XP_013625044.1 Exp number of AAs in TMHs: 0
# Brassicaoleracea_XP_013625044.1 Exp number, first 60 AAs:  0
# Brassicaoleracea_XP_013625044.1 Total prob of N-in:        0.00130
Brassicaoleracea_XP_013625044.1 TMHMM2.0        outside      1   307
```

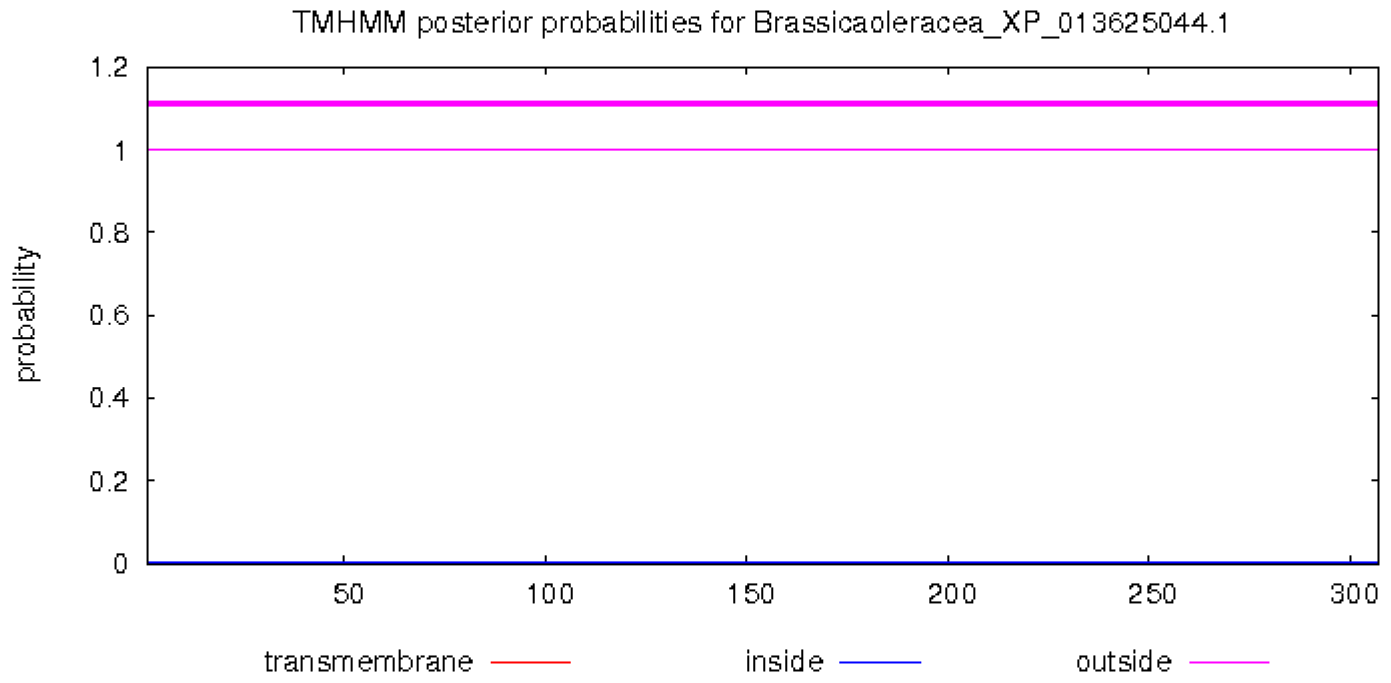

plot in postscript, script for making the plot in gnuplot, data for plot

---

```
# Brassicaoleracea_XP_013625495.1 Length: 346
# Brassicaoleracea_XP_013625495.1 Number of predicted TMHs:  0
# Brassicaoleracea_XP_013625495.1 Exp number of AAs in TMHs: 0.00018
# Brassicaoleracea_XP_013625495.1 Exp number, first 60 AAs:  0.00018
# Brassicaoleracea_XP_013625495.1 Total prob of N-in:        0.00335
Brassicaoleracea_XP_013625495.1 TMHMM2.0        outside      1   346
```

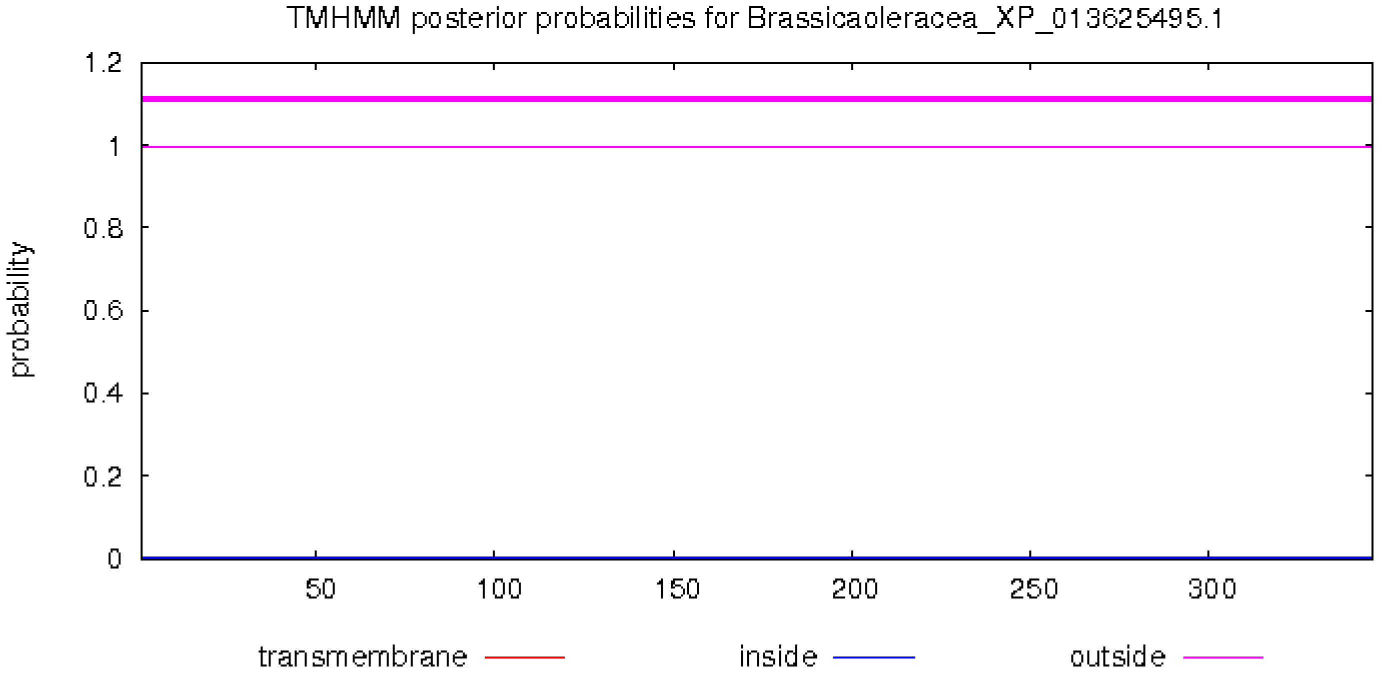

plot in postscript, script for making the plot in gnuplot, data for plot

---

```
# Brassicaoleracea_XP_013625587.1 Length: 248
# Brassicaoleracea_XP_013625587.1 Number of predicted TMHs:  0
# Brassicaoleracea_XP_013625587.1 Exp number of AAs in TMHs: 0.00064
# Brassicaoleracea_XP_013625587.1 Exp number, first 60 AAs:  0
# Brassicaoleracea_XP_013625587.1 Total prob of N-in:        0.16189
Brassicaoleracea_XP_013625587.1 TMHMM2.0        outside      1   248
```

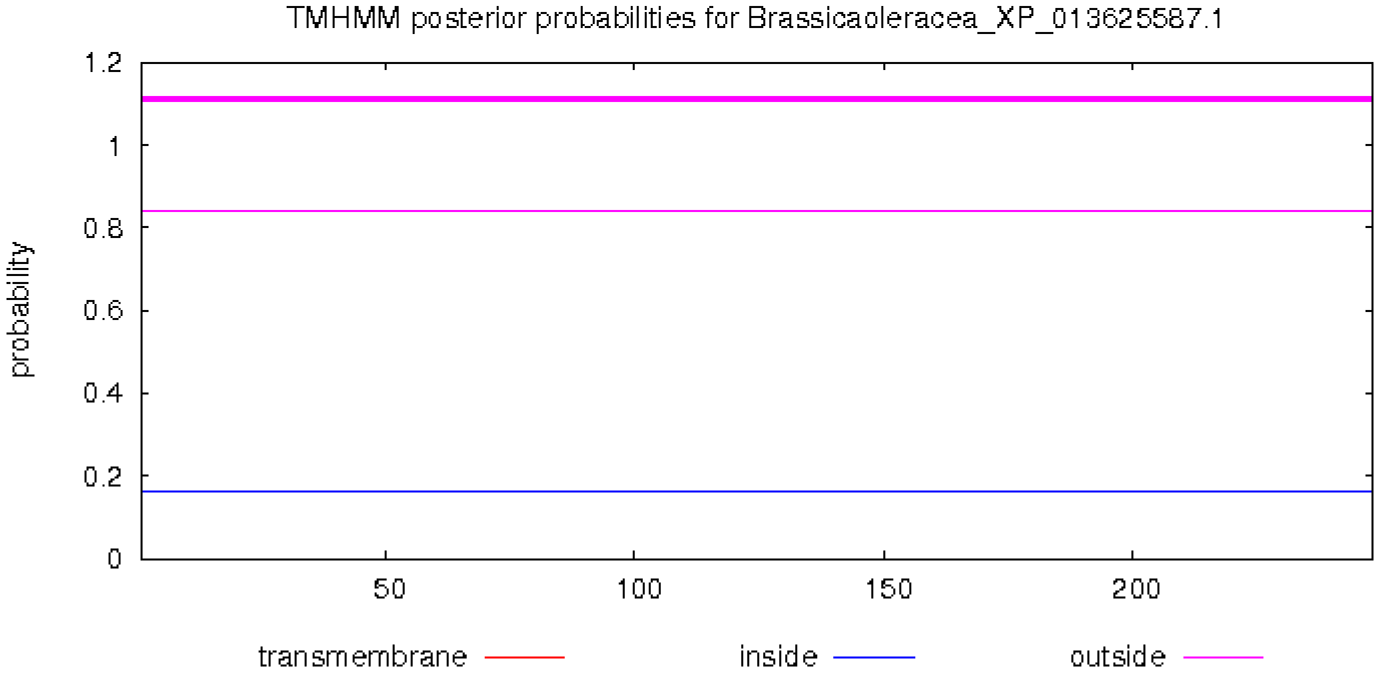

plot in postscript, script for making the plot in gnuplot, data for plot

---

```
# Brassicaoleracea_XP_013625724.1 Length: 281
# Brassicaoleracea_XP_013625724.1 Number of predicted TMHs:  0
# Brassicaoleracea_XP_013625724.1 Exp number of AAs in TMHs: 0.00594
# Brassicaoleracea_XP_013625724.1 Exp number, first 60 AAs:  0.00338
# Brassicaoleracea_XP_013625724.1 Total prob of N-in:        0.00557
Brassicaoleracea_XP_013625724.1 TMHMM2.0        outside      1   281
```

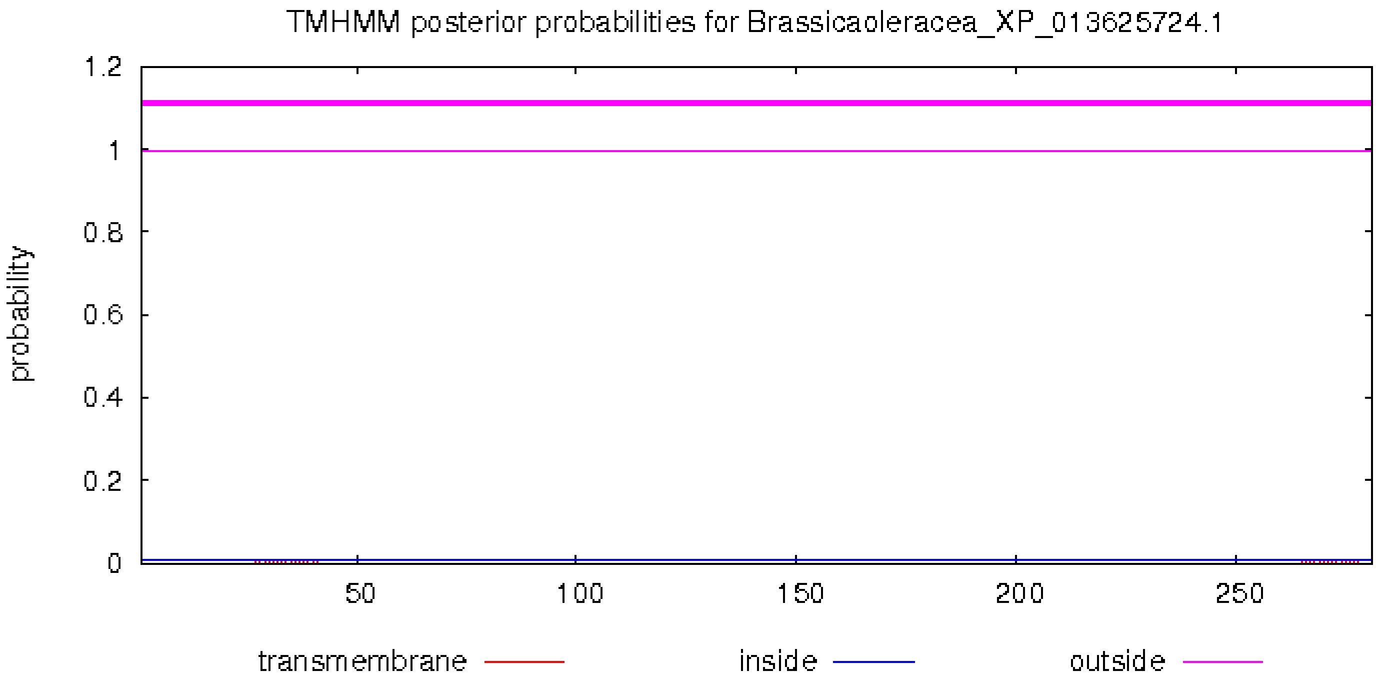

plot in postscript, script for making the plot in gnuplot, data for plot

---

```
# Brassicaoleracea_XP_013625819.1 Length: 336
# Brassicaoleracea_XP_013625819.1 Number of predicted TMHs:  0
# Brassicaoleracea_XP_013625819.1 Exp number of AAs in TMHs: 0.00037
# Brassicaoleracea_XP_013625819.1 Exp number, first 60 AAs:  0
# Brassicaoleracea_XP_013625819.1 Total prob of N-in:        0.00902
Brassicaoleracea_XP_013625819.1 TMHMM2.0        outside      1   336
```

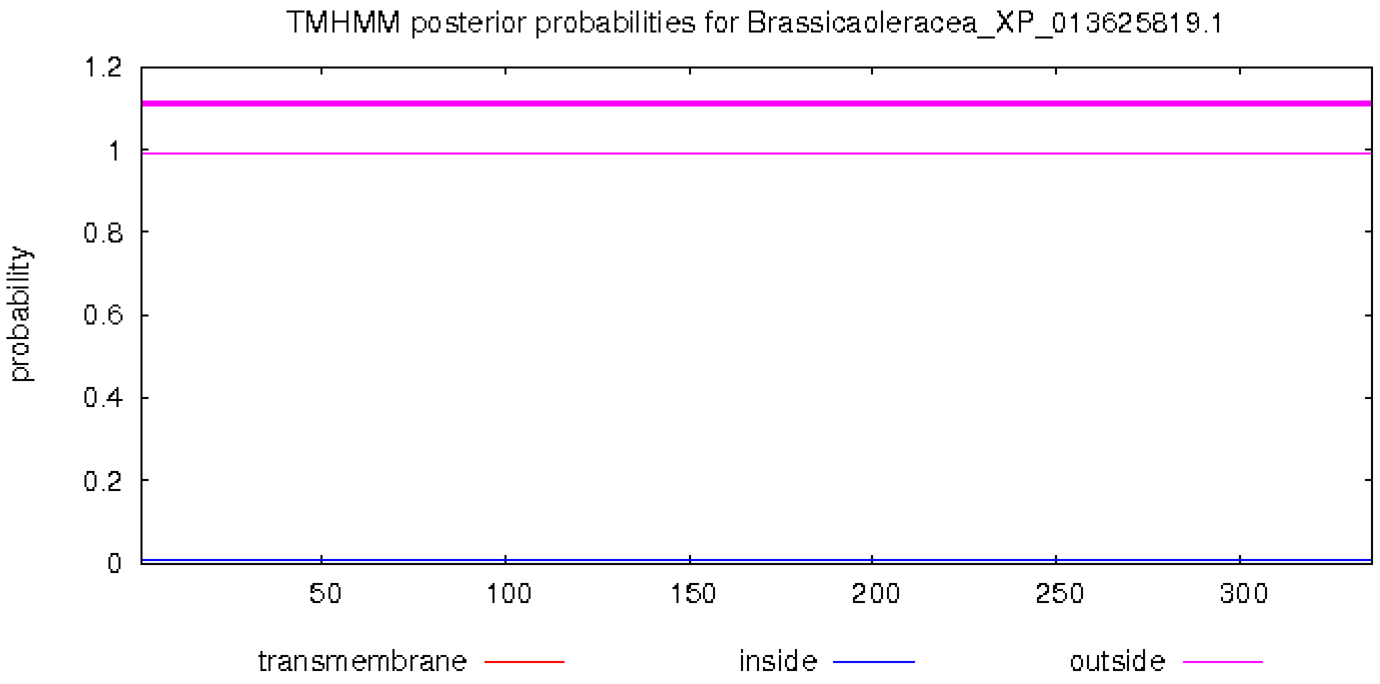

plot in postscript, script for making the plot in gnuplot, data for plot

---

```
# Brassicaoleracea_XP_013626215.1 Length: 361
# Brassicaoleracea_XP_013626215.1 Number of predicted TMHs:  0
# Brassicaoleracea_XP_013626215.1 Exp number of AAs in TMHs: 0
# Brassicaoleracea_XP_013626215.1 Exp number, first 60 AAs:  0
# Brassicaoleracea_XP_013626215.1 Total prob of N-in:        0.00608
Brassicaoleracea_XP_013626215.1 TMHMM2.0        outside      1   361
```

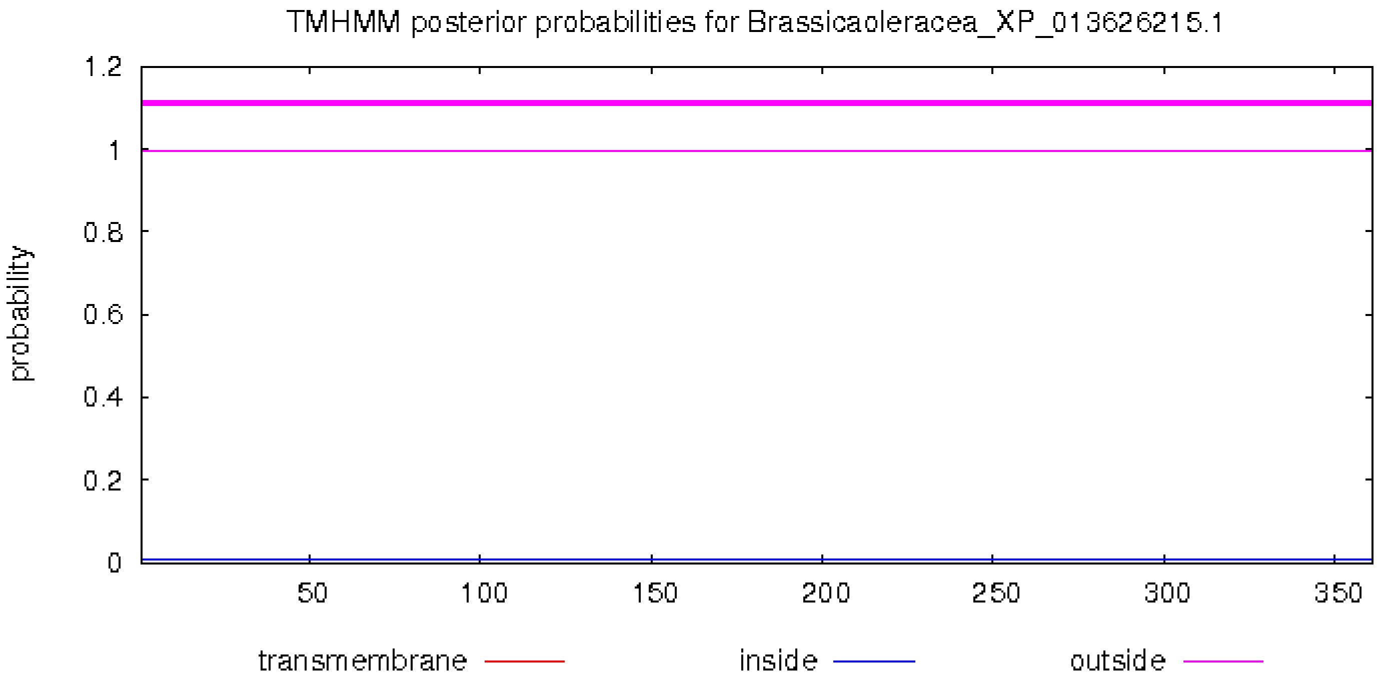

plot in postscript, script for making the plot in gnuplot, data for plot

---

```
# Brassicaoleracea_XP_013626236.1 Length: 274
# Brassicaoleracea_XP_013626236.1 Number of predicted TMHs:  0
# Brassicaoleracea_XP_013626236.1 Exp number of AAs in TMHs: 0.01555
# Brassicaoleracea_XP_013626236.1 Exp number, first 60 AAs:  0.01532
# Brassicaoleracea_XP_013626236.1 Total prob of N-in:        0.01301
Brassicaoleracea_XP_013626236.1 TMHMM2.0        outside      1   274
```

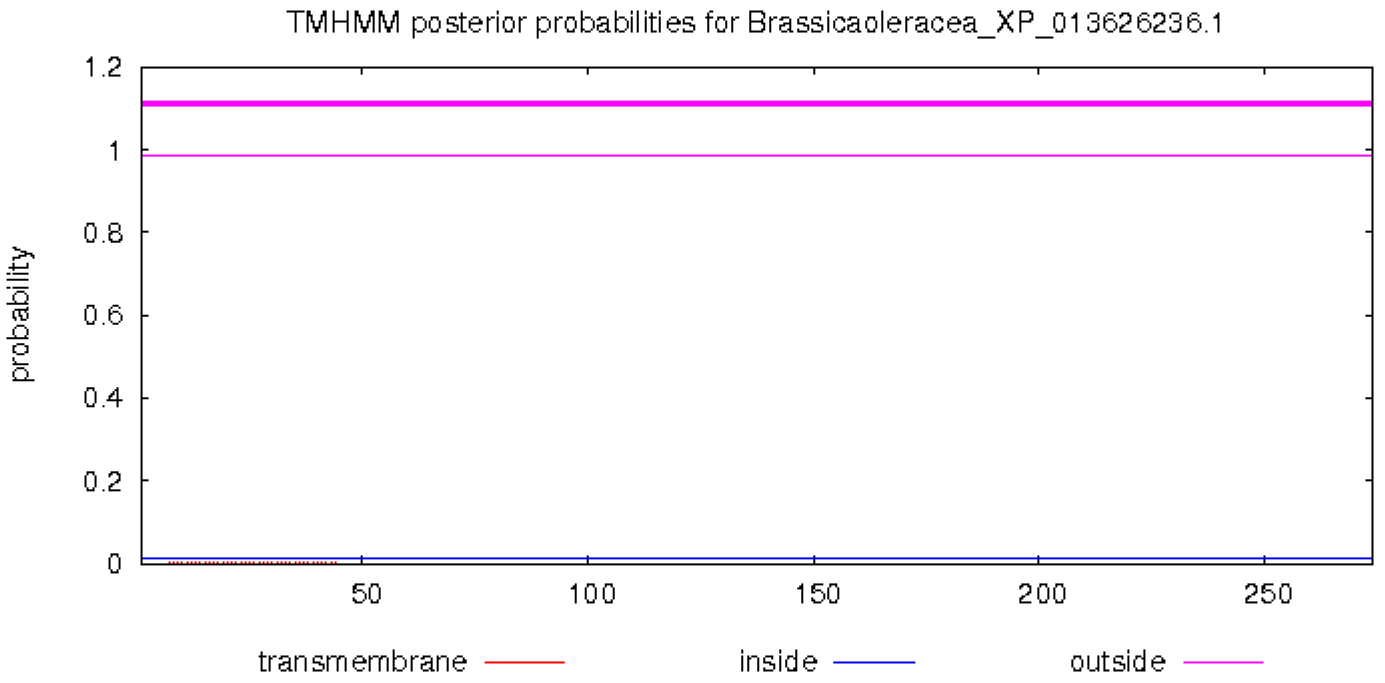

plot in postscript, script for making the plot in gnuplot, data for plot

---

```
# Brassicaoleracea_XP_013626261.1 Length: 188
# Brassicaoleracea_XP_013626261.1 Number of predicted TMHs:  0
# Brassicaoleracea_XP_013626261.1 Exp number of AAs in TMHs: 0
# Brassicaoleracea_XP_013626261.1 Exp number, first 60 AAs:  0
# Brassicaoleracea_XP_013626261.1 Total prob of N-in:        0.00724
Brassicaoleracea_XP_013626261.1 TMHMM2.0        outside      1   188
```

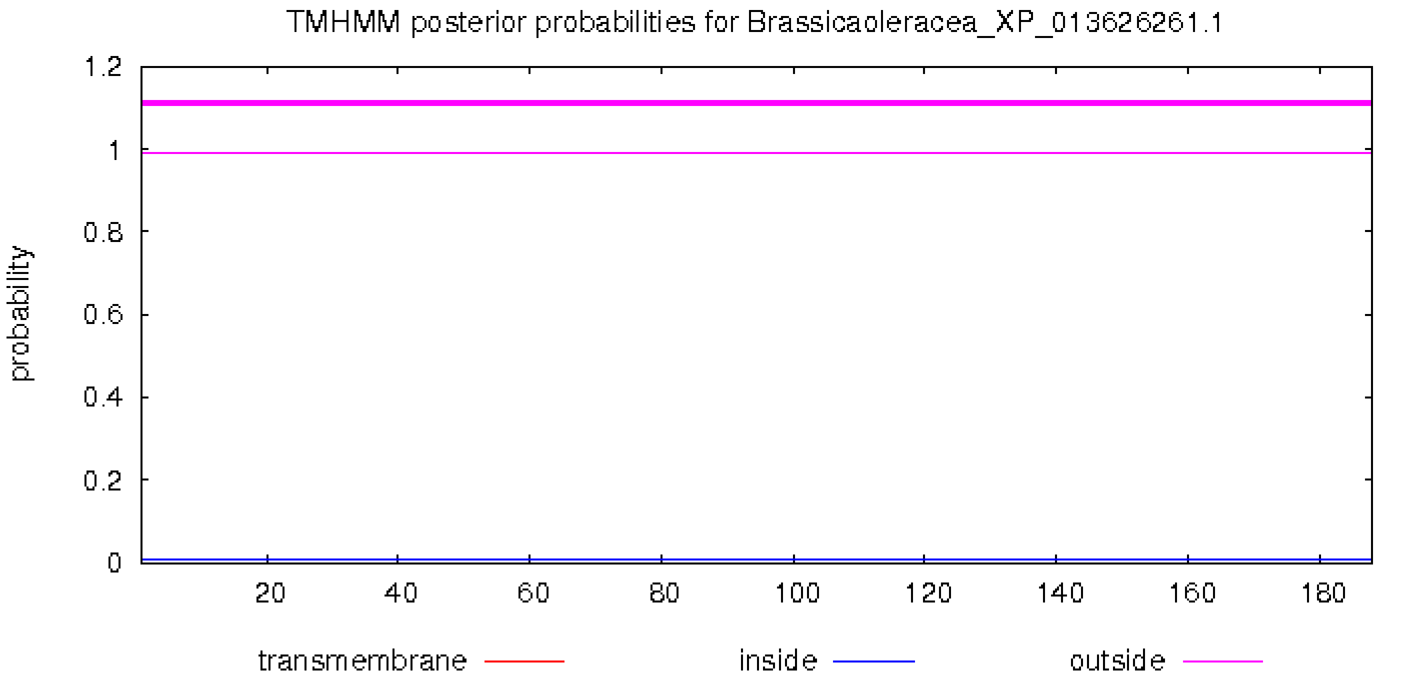

plot in postscript, script for making the plot in gnuplot, data for plot

---

```
# Brassicaoleracea_XP_013626443.1 Length: 350
# Brassicaoleracea_XP_013626443.1 Number of predicted TMHs:  0
# Brassicaoleracea_XP_013626443.1 Exp number of AAs in TMHs: 0.00095
# Brassicaoleracea_XP_013626443.1 Exp number, first 60 AAs:  0.00095
# Brassicaoleracea_XP_013626443.1 Total prob of N-in:        0.00478
Brassicaoleracea_XP_013626443.1 TMHMM2.0        outside      1   350
```

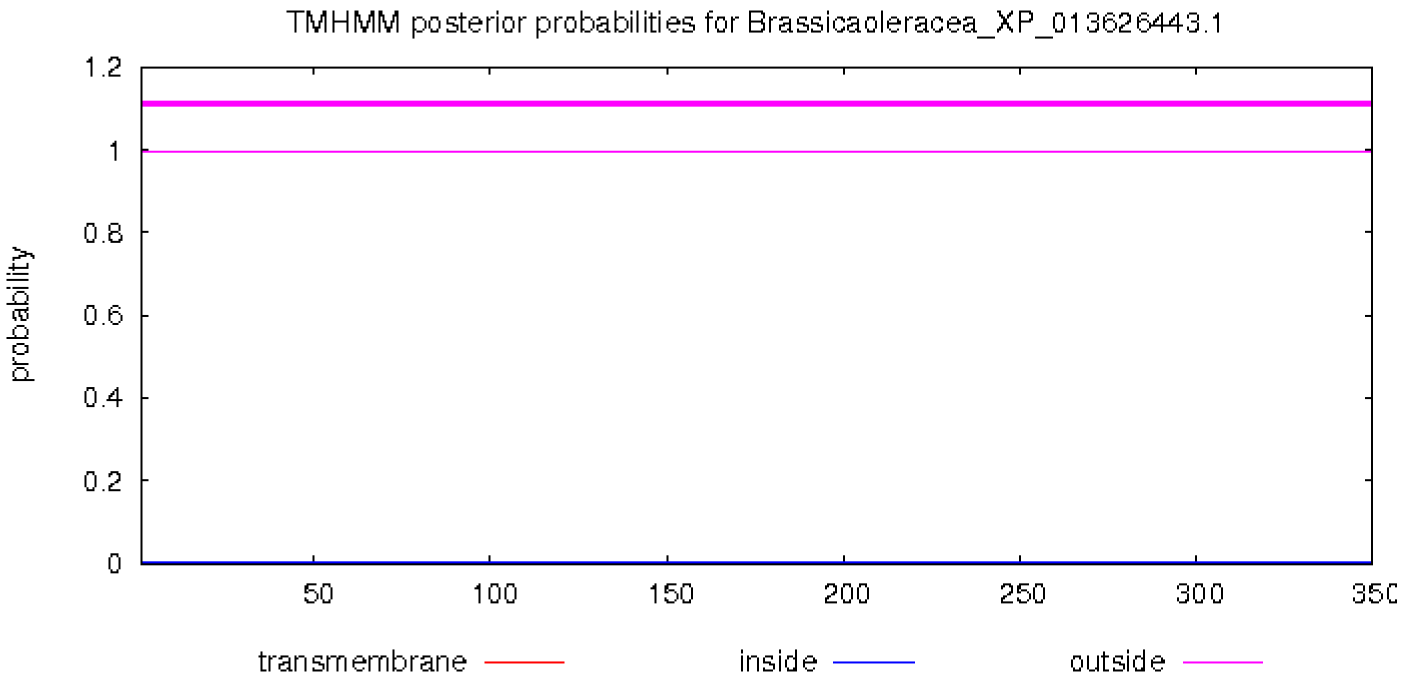

plot in postscript, script for making the plot in gnuplot, data for plot

---

```
# Brassicaoleracea_XP_013626498.1 Length: 250
# Brassicaoleracea_XP_013626498.1 Number of predicted TMHs:  0
# Brassicaoleracea_XP_013626498.1 Exp number of AAs in TMHs: 0.00017
# Brassicaoleracea_XP_013626498.1 Exp number, first 60 AAs:  0
# Brassicaoleracea_XP_013626498.1 Total prob of N-in:        0.08527
Brassicaoleracea_XP_013626498.1 TMHMM2.0        outside      1   250
```

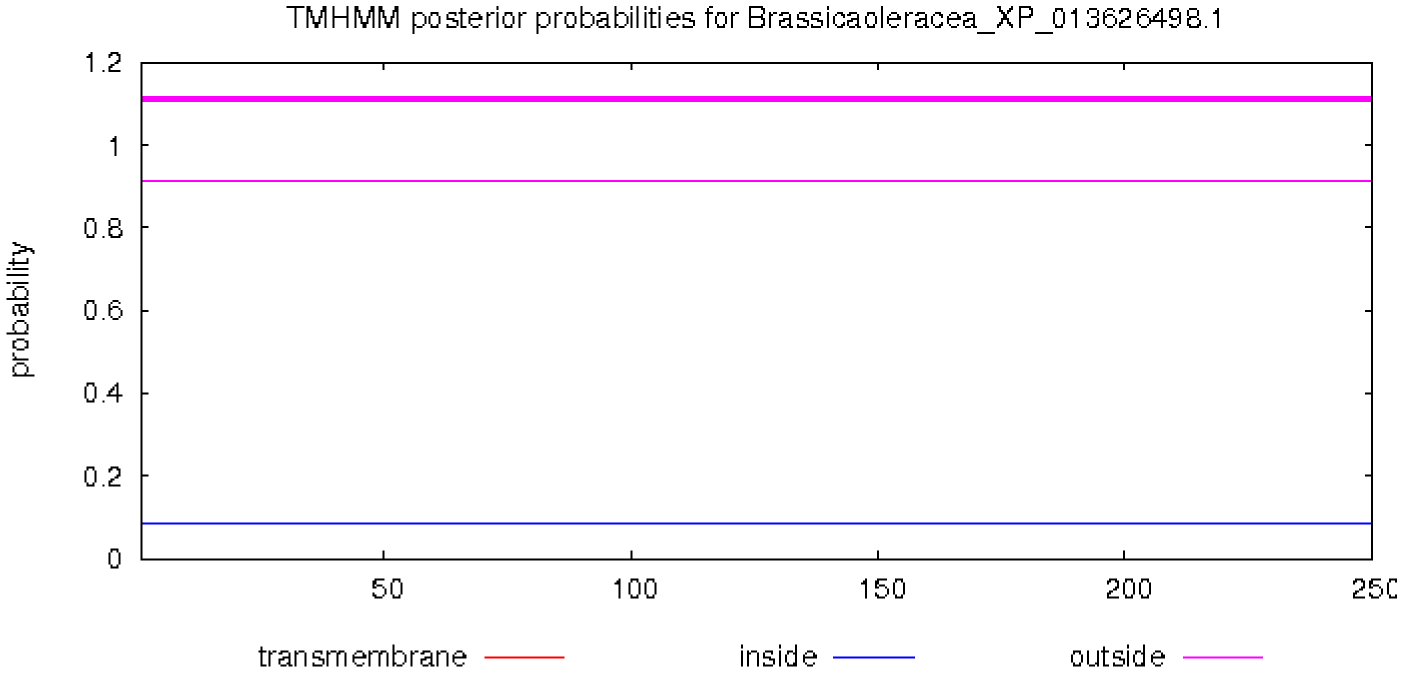

plot in postscript, script for making the plot in gnuplot, data for plot

---

```
# Brassicaoleracea_XP_013626600.1 Length: 298
# Brassicaoleracea_XP_013626600.1 Number of predicted TMHs:  0
# Brassicaoleracea_XP_013626600.1 Exp number of AAs in TMHs: 0.00039
# Brassicaoleracea_XP_013626600.1 Exp number, first 60 AAs:  0.0002
# Brassicaoleracea_XP_013626600.1 Total prob of N-in:        0.02126
Brassicaoleracea_XP_013626600.1 TMHMM2.0        outside      1   298
```

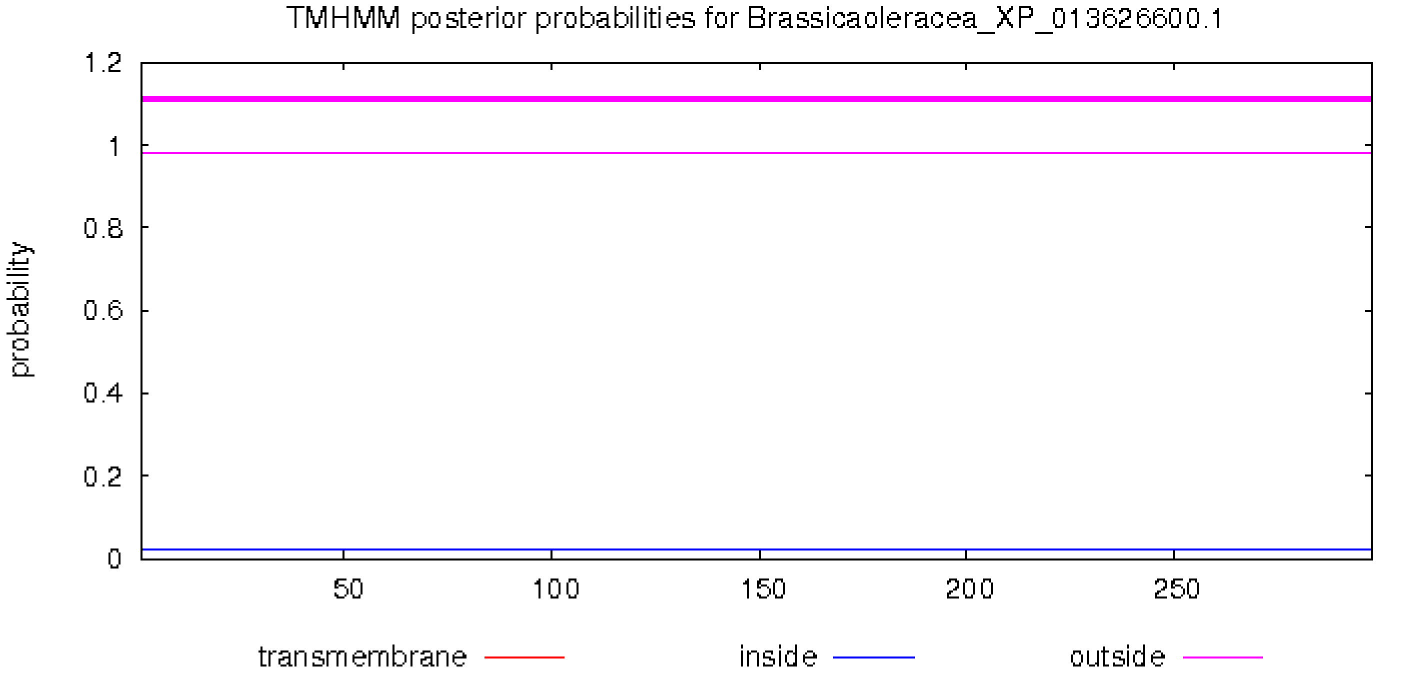

plot in postscript, script for making the plot in gnuplot, data for plot

---

```
# Brassicaoleracea_XP_013626711.1 Length: 308
# Brassicaoleracea_XP_013626711.1 Number of predicted TMHs:  0
# Brassicaoleracea_XP_013626711.1 Exp number of AAs in TMHs: 0.01209
# Brassicaoleracea_XP_013626711.1 Exp number, first 60 AAs:  0.00023
# Brassicaoleracea_XP_013626711.1 Total prob of N-in:        0.01437
Brassicaoleracea_XP_013626711.1 TMHMM2.0        outside      1   308
```

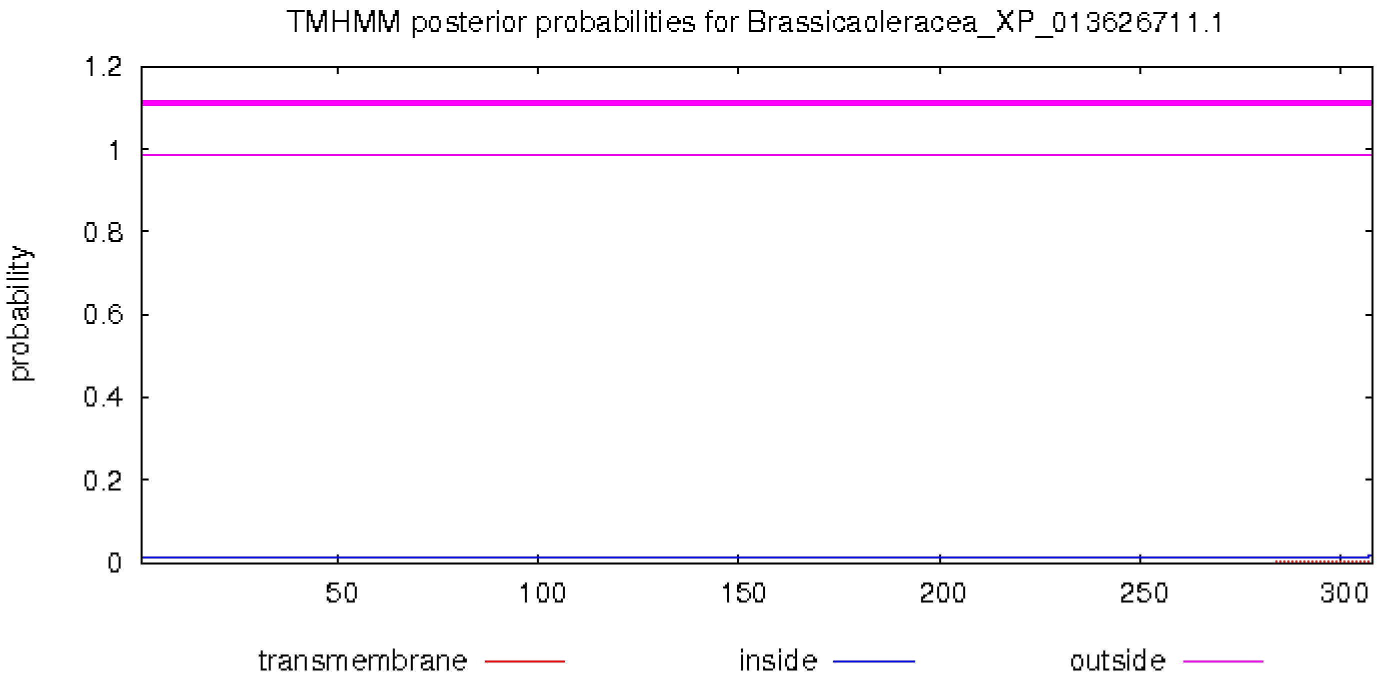

plot in postscript, script for making the plot in gnuplot, data for plot

---

```
# Brassicaoleracea_XP_013626851.1 Length: 272
# Brassicaoleracea_XP_013626851.1 Number of predicted TMHs:  0
# Brassicaoleracea_XP_013626851.1 Exp number of AAs in TMHs: 0.0073
# Brassicaoleracea_XP_013626851.1 Exp number, first 60 AAs:  0
# Brassicaoleracea_XP_013626851.1 Total prob of N-in:        0.04775
Brassicaoleracea_XP_013626851.1 TMHMM2.0        outside      1   272
```

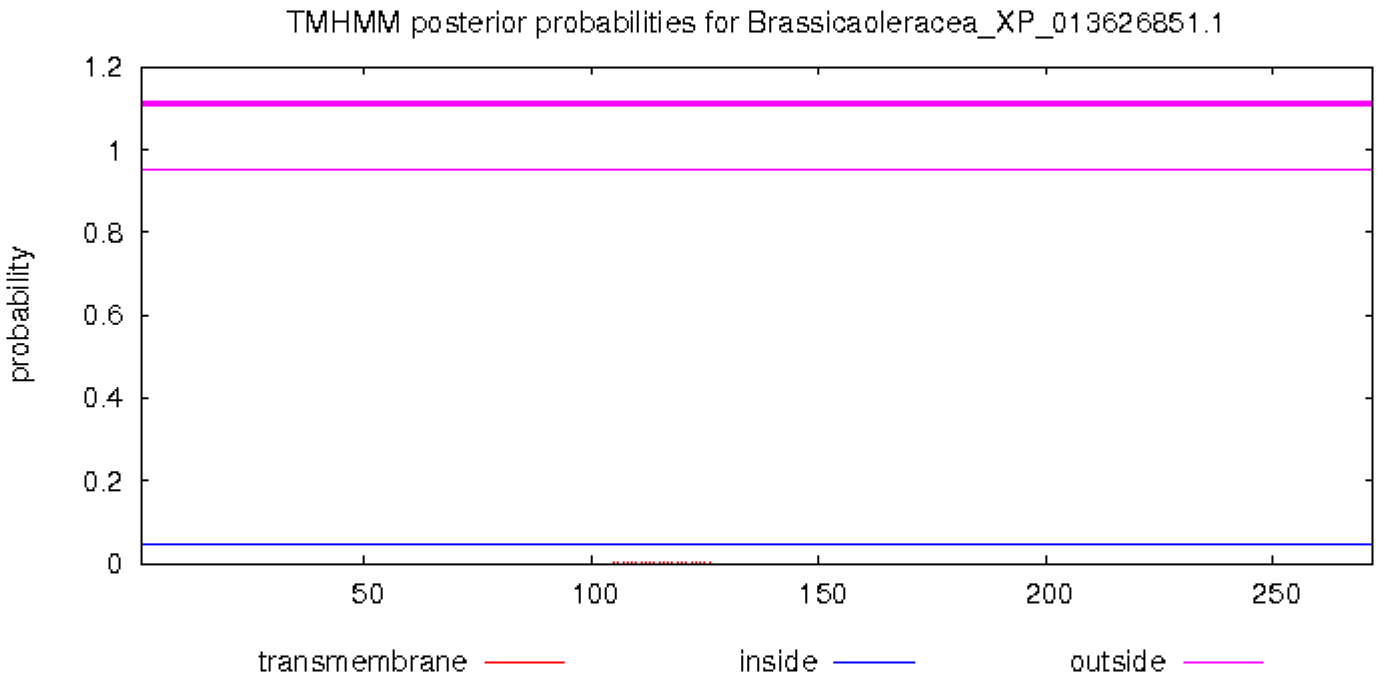

plot in postscript, script for making the plot in gnuplot, data for plot

---

```
# Brassicaoleracea_XP_013626921.1 Length: 334
# Brassicaoleracea_XP_013626921.1 Number of predicted TMHs:  0
# Brassicaoleracea_XP_013626921.1 Exp number of AAs in TMHs: 10.53305
# Brassicaoleracea_XP_013626921.1 Exp number, first 60 AAs:  0.18672
# Brassicaoleracea_XP_013626921.1 Total prob of N-in:        0.08751
Brassicaoleracea_XP_013626921.1 TMHMM2.0        outside      1   334
```

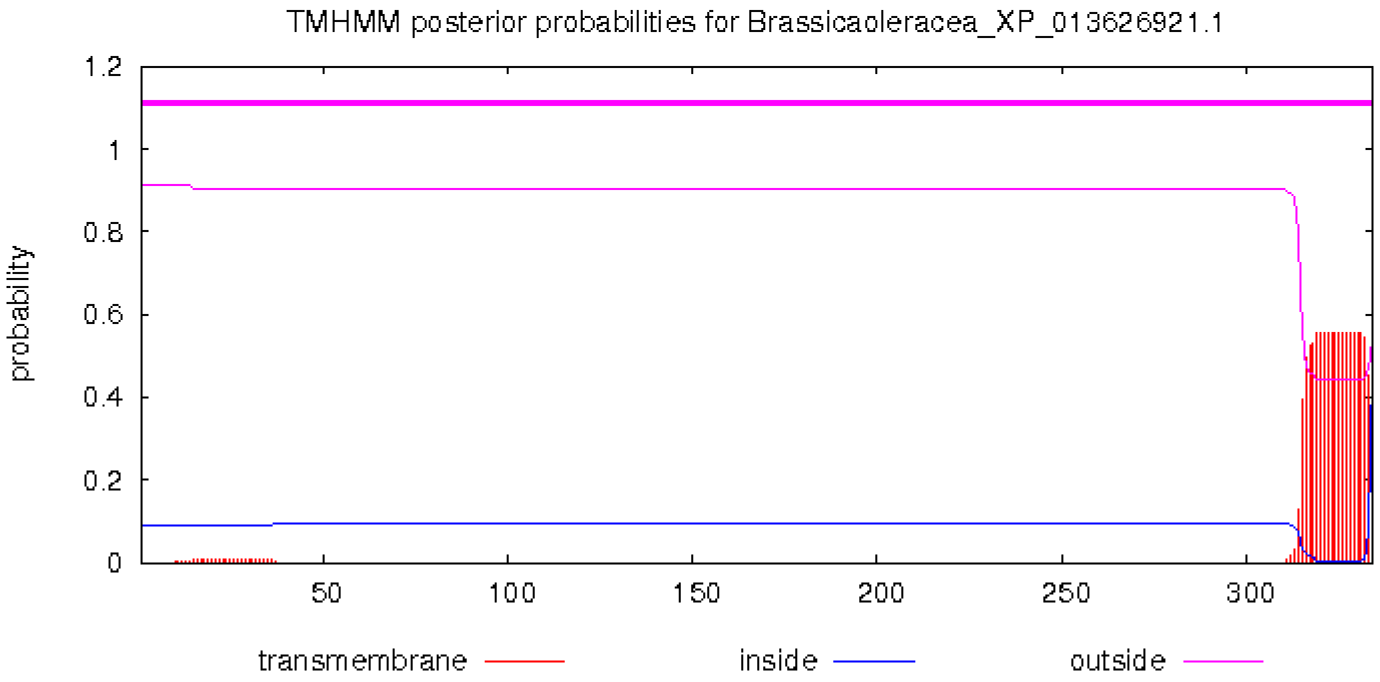

plot in postscript, script for making the plot in gnuplot, data for plot

---

```
# Brassicaoleracea_XP_013626924.1 Length: 348
# Brassicaoleracea_XP_013626924.1 Number of predicted TMHs:  0
# Brassicaoleracea_XP_013626924.1 Exp number of AAs in TMHs: 0.00019
# Brassicaoleracea_XP_013626924.1 Exp number, first 60 AAs:  0
# Brassicaoleracea_XP_013626924.1 Total prob of N-in:        0.00100
Brassicaoleracea_XP_013626924.1 TMHMM2.0        outside      1   348
```

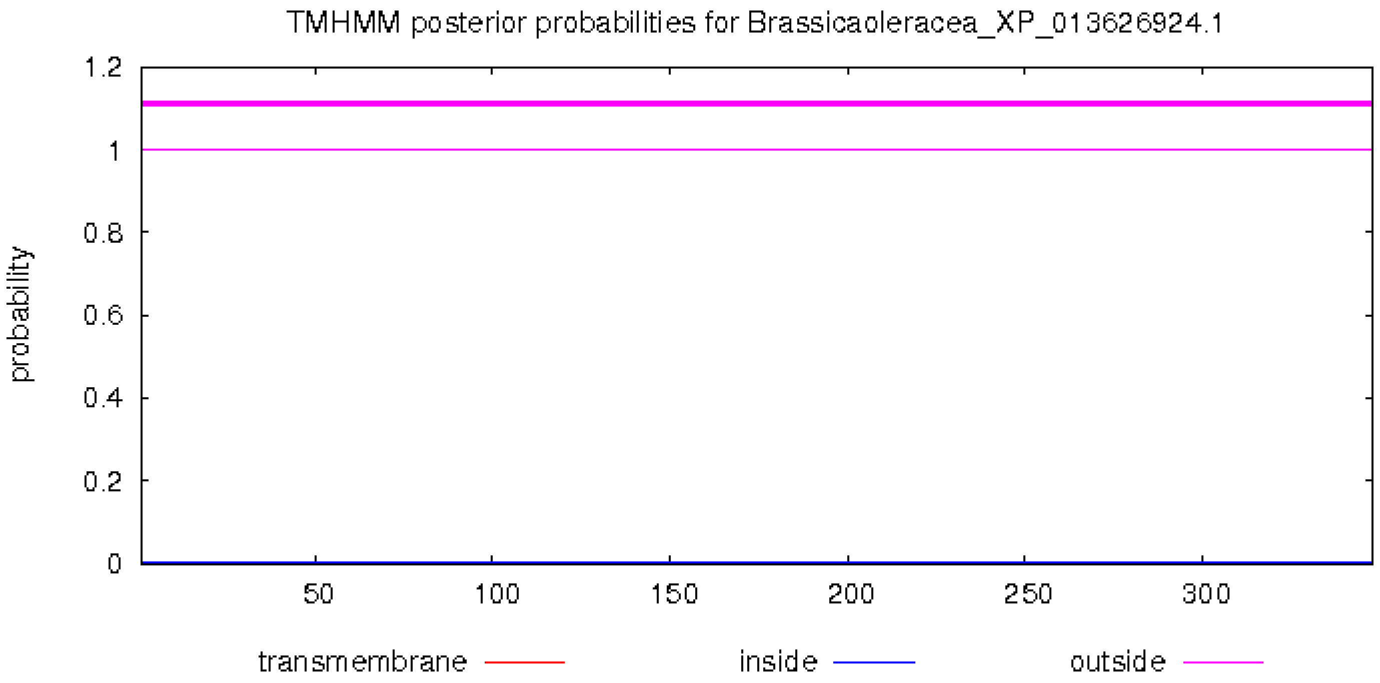

plot in postscript, script for making the plot in gnuplot, data for plot

---

```
# Brassicaoleracea_XP_013626927.1 Length: 494
# Brassicaoleracea_XP_013626927.1 Number of predicted TMHs:  0
# Brassicaoleracea_XP_013626927.1 Exp number of AAs in TMHs: 0.00019
# Brassicaoleracea_XP_013626927.1 Exp number, first 60 AAs:  0
# Brassicaoleracea_XP_013626927.1 Total prob of N-in:        0.00013
Brassicaoleracea_XP_013626927.1 TMHMM2.0        outside      1   494
```

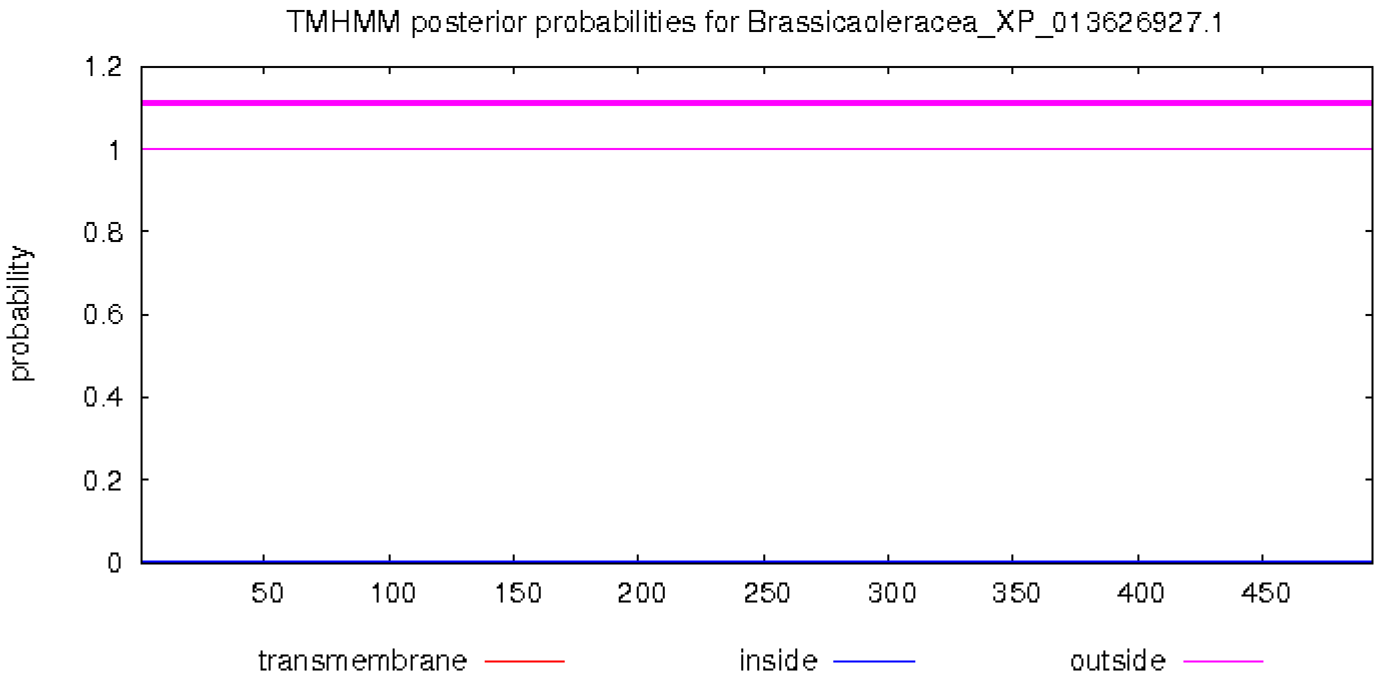

plot in postscript, script for making the plot in gnuplot, data for plot

---

```
# Brassicaoleracea_XP_013626928.1 Length: 493
# Brassicaoleracea_XP_013626928.1 Number of predicted TMHs:  0
# Brassicaoleracea_XP_013626928.1 Exp number of AAs in TMHs: 0.00019
# Brassicaoleracea_XP_013626928.1 Exp number, first 60 AAs:  0
# Brassicaoleracea_XP_013626928.1 Total prob of N-in:        0.00014
Brassicaoleracea_XP_013626928.1 TMHMM2.0        outside      1   493
```

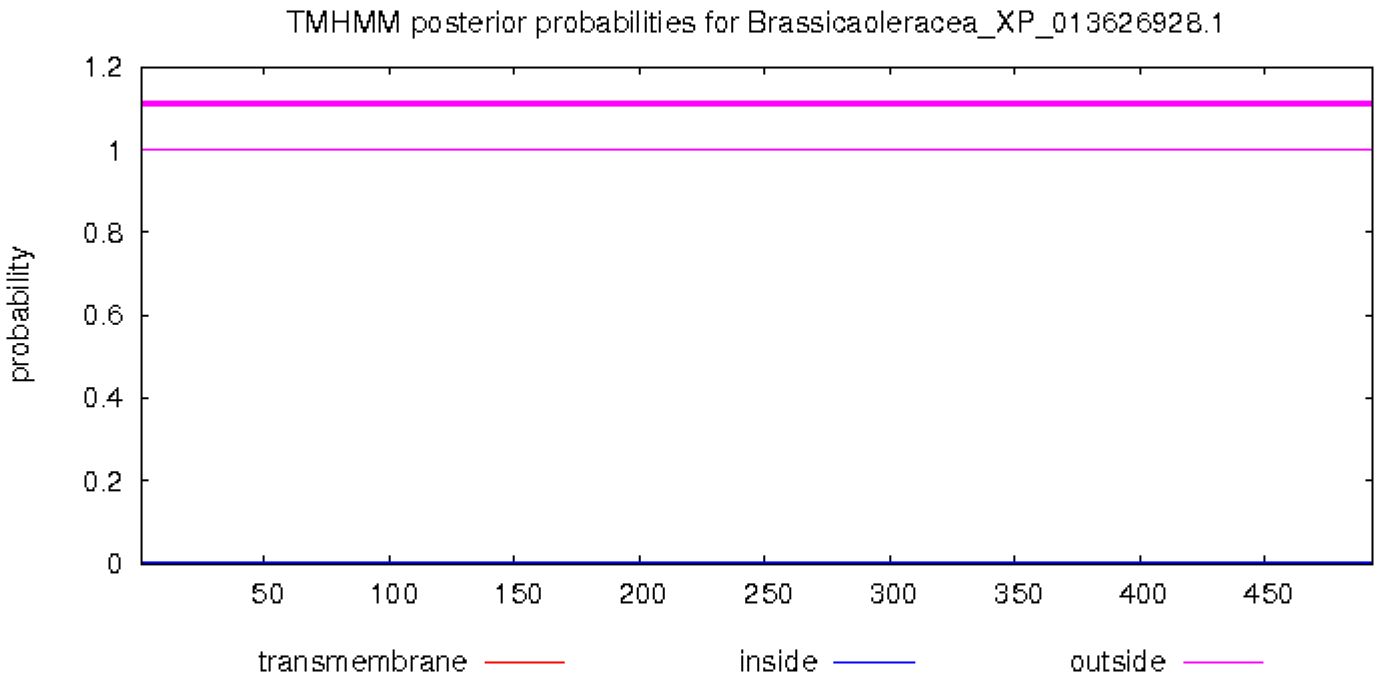

plot in postscript, script for making the plot in gnuplot, data for plot

---

```
# Brassicaoleracea_XP_013627314.1 Length: 235
# Brassicaoleracea_XP_013627314.1 Number of predicted TMHs:  0
# Brassicaoleracea_XP_013627314.1 Exp number of AAs in TMHs: 0.01048
# Brassicaoleracea_XP_013627314.1 Exp number, first 60 AAs:  0
# Brassicaoleracea_XP_013627314.1 Total prob of N-in:        0.03757
Brassicaoleracea_XP_013627314.1 TMHMM2.0        outside      1   235
```

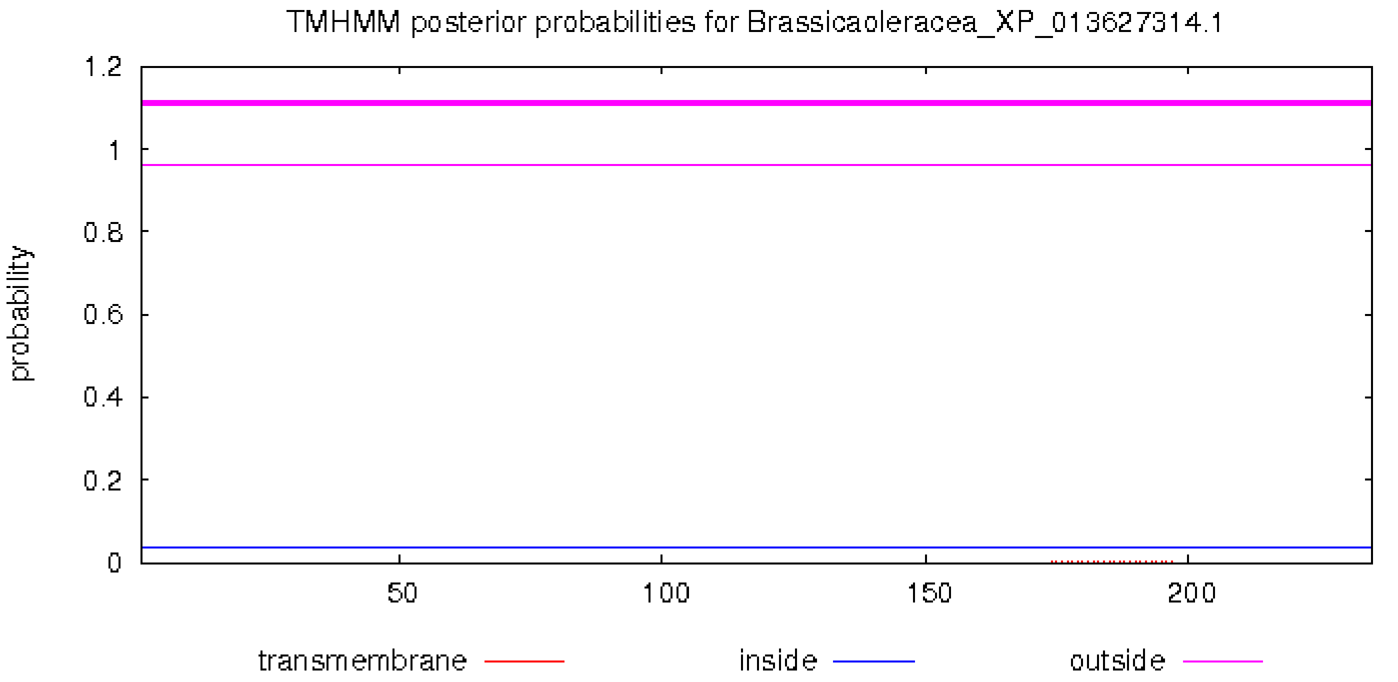

plot in postscript, script for making the plot in gnuplot, data for plot

---

```
# Brassicaoleracea_XP_013627414.1 Length: 303
# Brassicaoleracea_XP_013627414.1 Number of predicted TMHs:  0
# Brassicaoleracea_XP_013627414.1 Exp number of AAs in TMHs: 0.0013
# Brassicaoleracea_XP_013627414.1 Exp number, first 60 AAs:  0
# Brassicaoleracea_XP_013627414.1 Total prob of N-in:        0.07777
Brassicaoleracea_XP_013627414.1 TMHMM2.0        outside      1   303
```

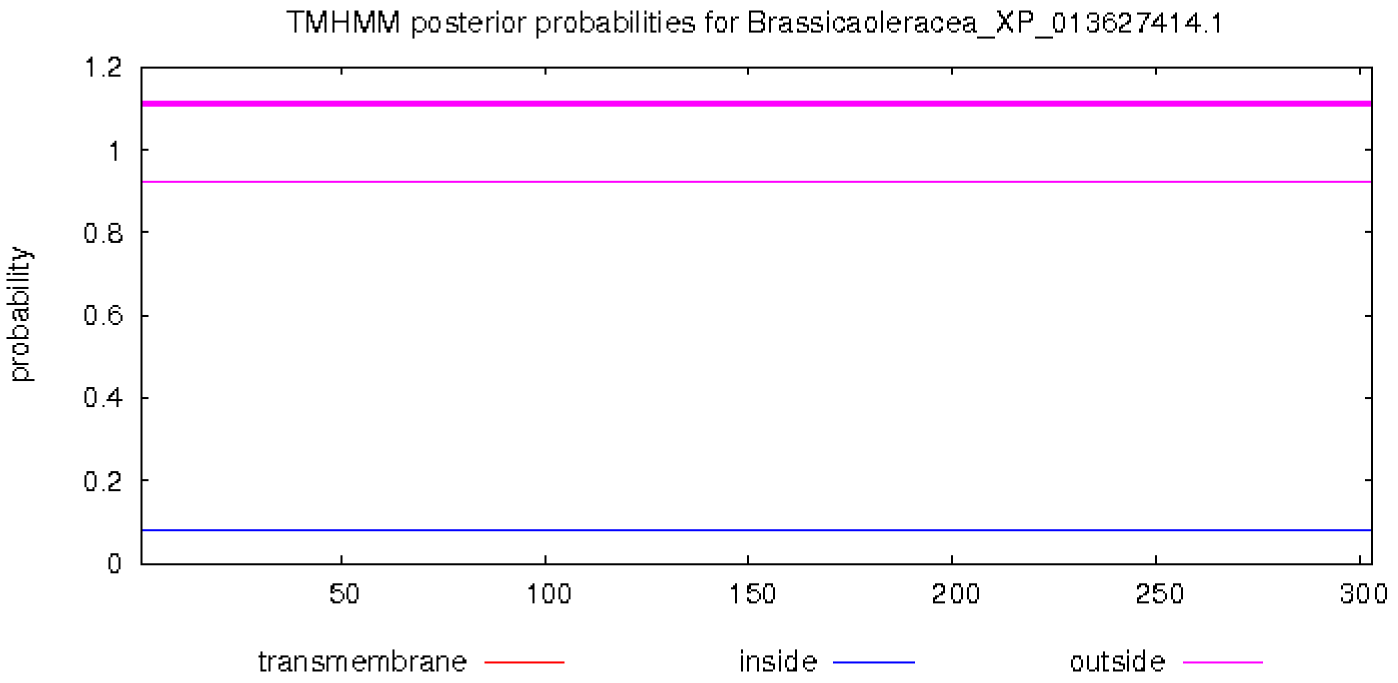

plot in postscript, script for making the plot in gnuplot, data for plot

---

```
# Brassicaoleracea_XP_013627811.1 Length: 341
# Brassicaoleracea_XP_013627811.1 Number of predicted TMHs:  0
# Brassicaoleracea_XP_013627811.1 Exp number of AAs in TMHs: 0
# Brassicaoleracea_XP_013627811.1 Exp number, first 60 AAs:  0
# Brassicaoleracea_XP_013627811.1 Total prob of N-in:        0.00436
Brassicaoleracea_XP_013627811.1 TMHMM2.0        outside      1   341
```

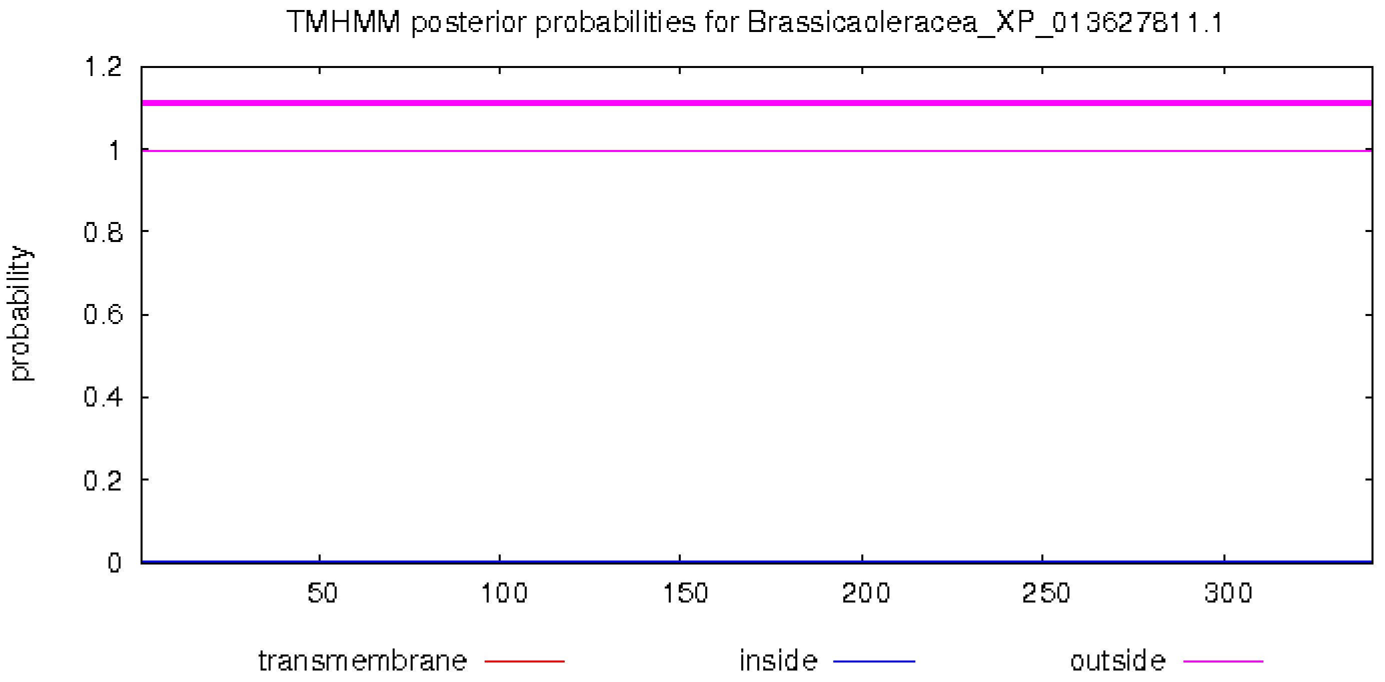

plot in postscript, script for making the plot in gnuplot, data for plot

---

```
# Brassicaoleracea_XP_013628148.1 Length: 233
# Brassicaoleracea_XP_013628148.1 Number of predicted TMHs:  0
# Brassicaoleracea_XP_013628148.1 Exp number of AAs in TMHs: 0.00018
# Brassicaoleracea_XP_013628148.1 Exp number, first 60 AAs:  0.00018
# Brassicaoleracea_XP_013628148.1 Total prob of N-in:        0.02724
Brassicaoleracea_XP_013628148.1 TMHMM2.0        outside      1   233
```

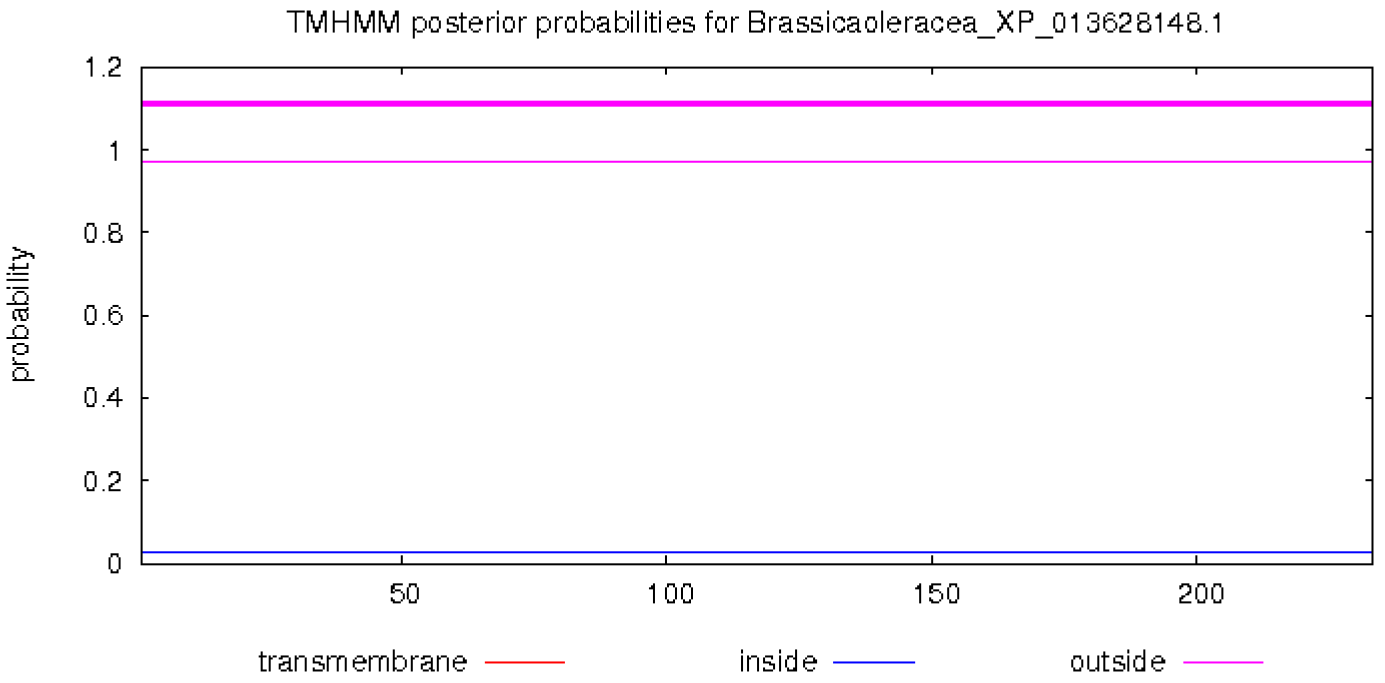

plot in postscript, script for making the plot in gnuplot, data for plot

---

```
# Brassicaoleracea_XP_013628150.1 Length: 233
# Brassicaoleracea_XP_013628150.1 Number of predicted TMHs:  0
# Brassicaoleracea_XP_013628150.1 Exp number of AAs in TMHs: 0.00018
# Brassicaoleracea_XP_013628150.1 Exp number, first 60 AAs:  0.00018
# Brassicaoleracea_XP_013628150.1 Total prob of N-in:        0.02724
Brassicaoleracea_XP_013628150.1 TMHMM2.0        outside      1   233
```

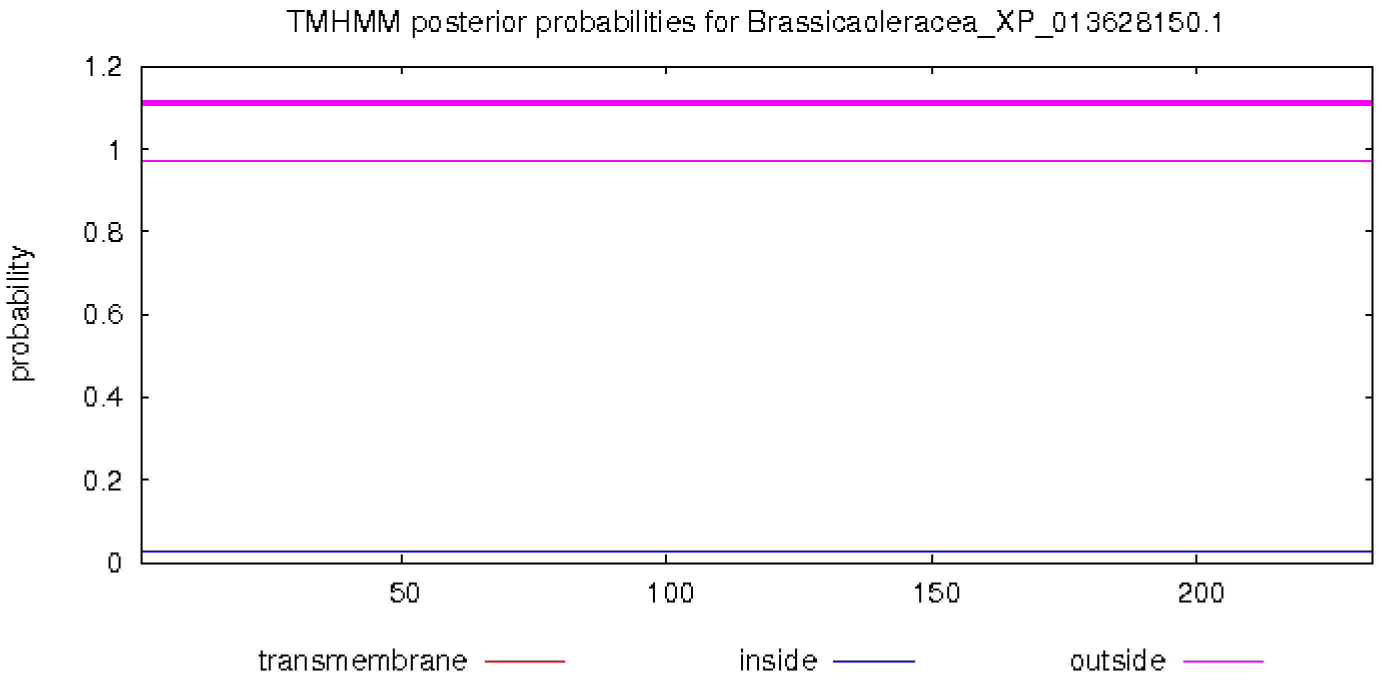

plot in postscript, script for making the plot in gnuplot, data for plot

---

```
# Brassicaoleracea_XP_013628425.1 Length: 208
# Brassicaoleracea_XP_013628425.1 Number of predicted TMHs:  0
# Brassicaoleracea_XP_013628425.1 Exp number of AAs in TMHs: 0.84991
# Brassicaoleracea_XP_013628425.1 Exp number, first 60 AAs:  0.28115
# Brassicaoleracea_XP_013628425.1 Total prob of N-in:        0.19736
Brassicaoleracea_XP_013628425.1 TMHMM2.0        outside      1   208
```

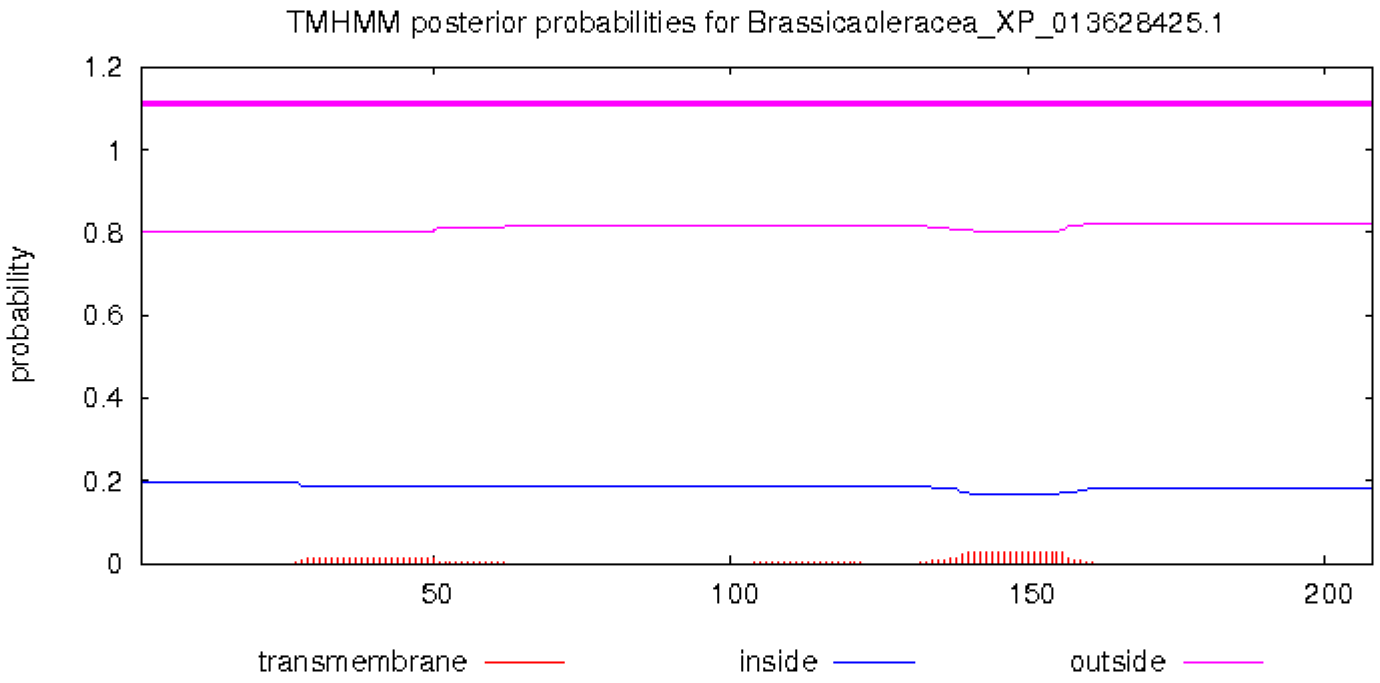

plot in postscript, script for making the plot in gnuplot, data for plot

---

```
# Brassicaoleracea_XP_013628782.1 Length: 298
# Brassicaoleracea_XP_013628782.1 Number of predicted TMHs:  0
# Brassicaoleracea_XP_013628782.1 Exp number of AAs in TMHs: 0.00951
# Brassicaoleracea_XP_013628782.1 Exp number, first 60 AAs:  0
# Brassicaoleracea_XP_013628782.1 Total prob of N-in:        0.00491
Brassicaoleracea_XP_013628782.1 TMHMM2.0        outside      1   298
```

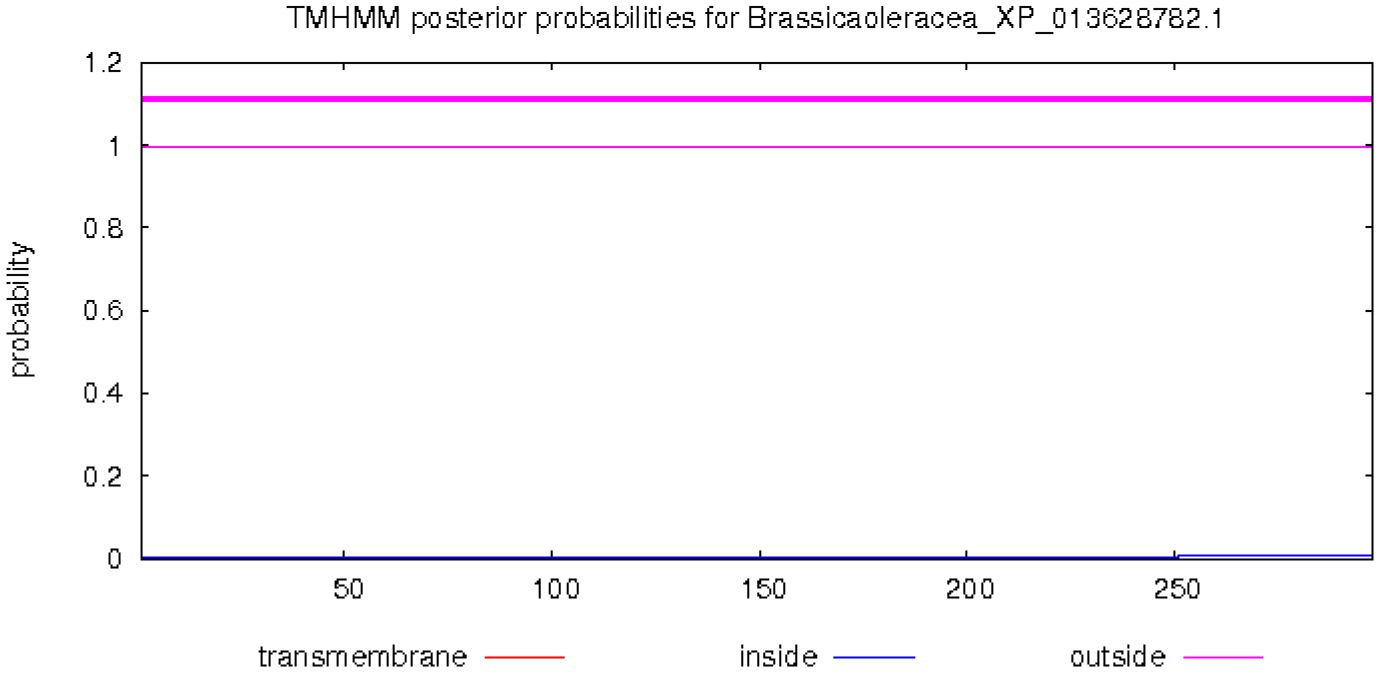

plot in postscript, script for making the plot in gnuplot, data for plot

---

```
# Brassicaoleracea_XP_013628917.1 Length: 557
# Brassicaoleracea_XP_013628917.1 Number of predicted TMHs:  1
# Brassicaoleracea_XP_013628917.1 Exp number of AAs in TMHs: 20.80694
# Brassicaoleracea_XP_013628917.1 Exp number, first 60 AAs:  0
# Brassicaoleracea_XP_013628917.1 Total prob of N-in:        0.01576
Brassicaoleracea_XP_013628917.1 TMHMM2.0        outside      1   531
Brassicaoleracea_XP_013628917.1 TMHMM2.0        TMhelix    532   554
Brassicaoleracea_XP_013628917.1 TMHMM2.0        inside     555   557
```

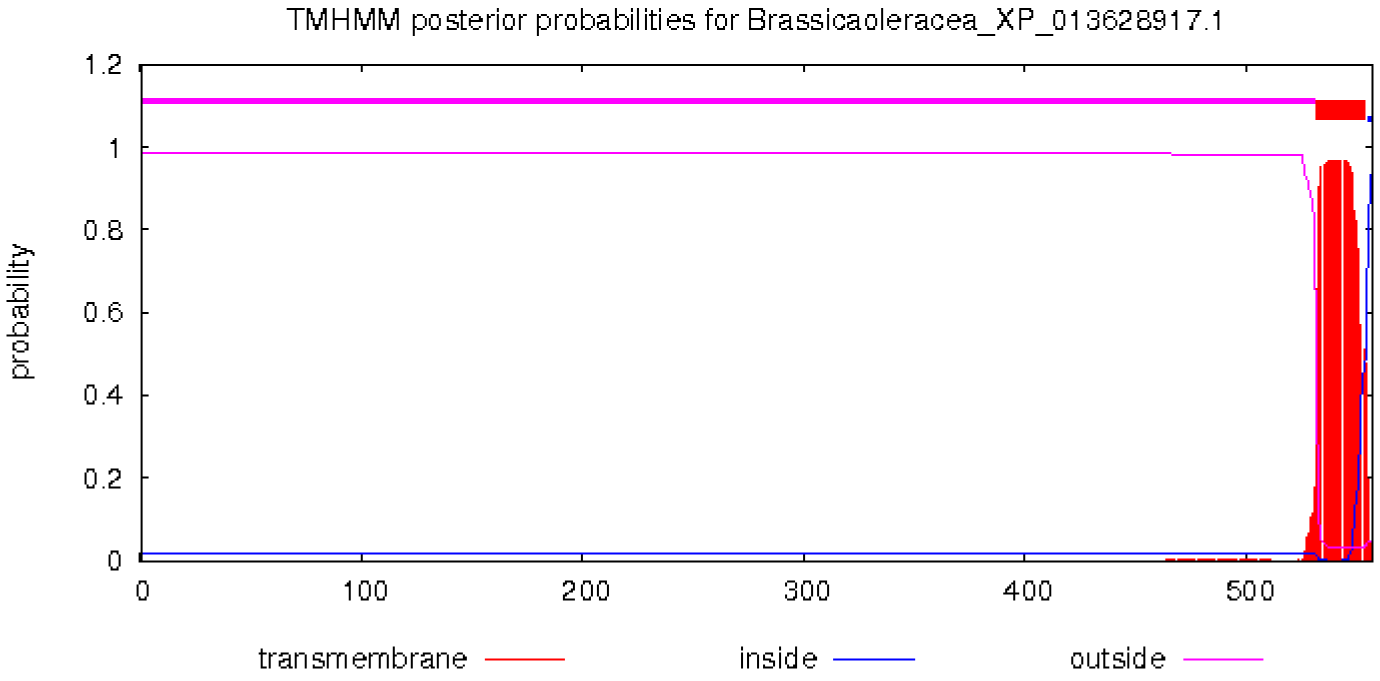

plot in postscript, script for making the plot in gnuplot, data for plot

```
# Brassicaoleracea_XP_013628918.1 Length: 556
# Brassicaoleracea_XP_013628918.1 Number of predicted TMHs:  1
# Brassicaoleracea_XP_013628918.1 Exp number of AAs in TMHs: 20.80731
# Brassicaoleracea_XP_013628918.1 Exp number, first 60 AAs:  0
# Brassicaoleracea_XP_013628918.1 Total prob of N-in:        0.01596
Brassicaoleracea_XP_013628918.1 TMHMM2.0        outside      1   530
Brassicaoleracea_XP_013628918.1 TMHMM2.0        TMhelix    531   553
Brassicaoleracea_XP_013628918.1 TMHMM2.0        inside     554   556
```

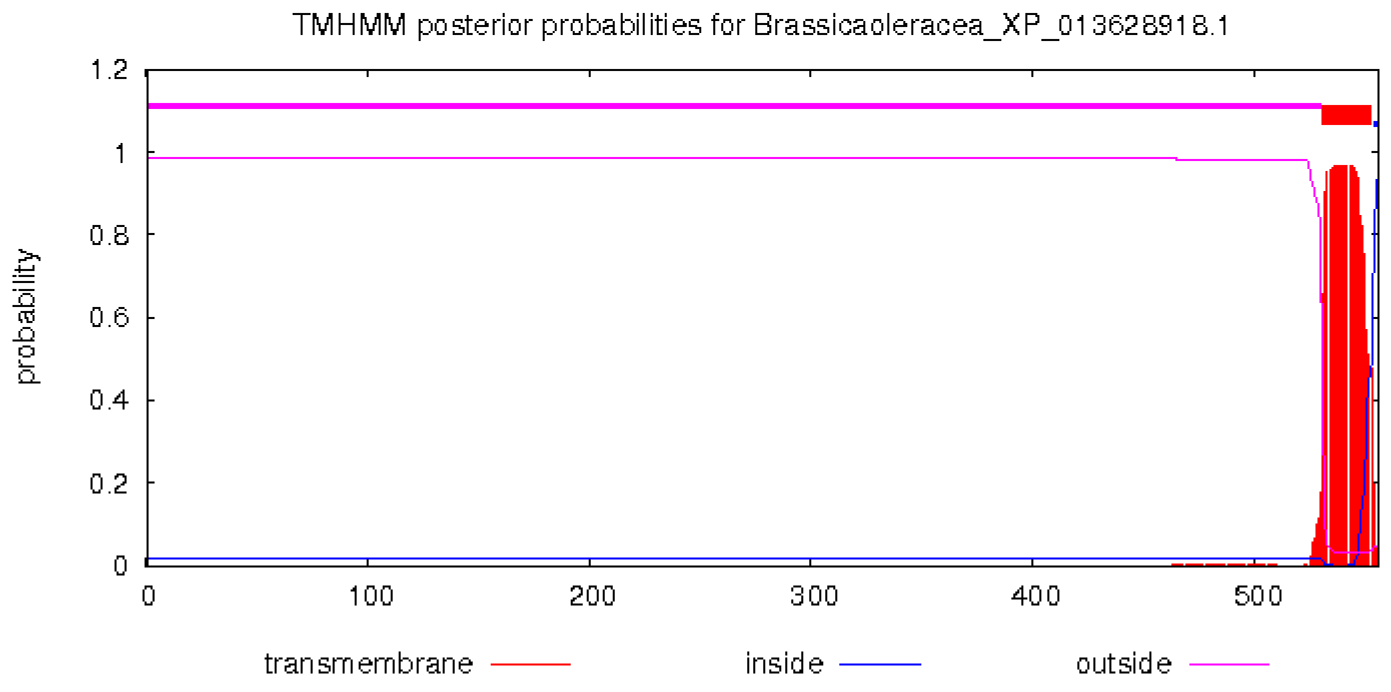

plot in postscript, script for making the plot in gnuplot, data for plot

```
# Brassicaoleracea_XP_013629000.1 Length: 281
# Brassicaoleracea_XP_013629000.1 Number of predicted TMHs:  0
# Brassicaoleracea_XP_013629000.1 Exp number of AAs in TMHs: 0.00036
# Brassicaoleracea_XP_013629000.1 Exp number, first 60 AAs:  0.00017
# Brassicaoleracea_XP_013629000.1 Total prob of N-in:        0.01050
Brassicaoleracea_XP_013629000.1 TMHMM2.0        outside      1   281
```

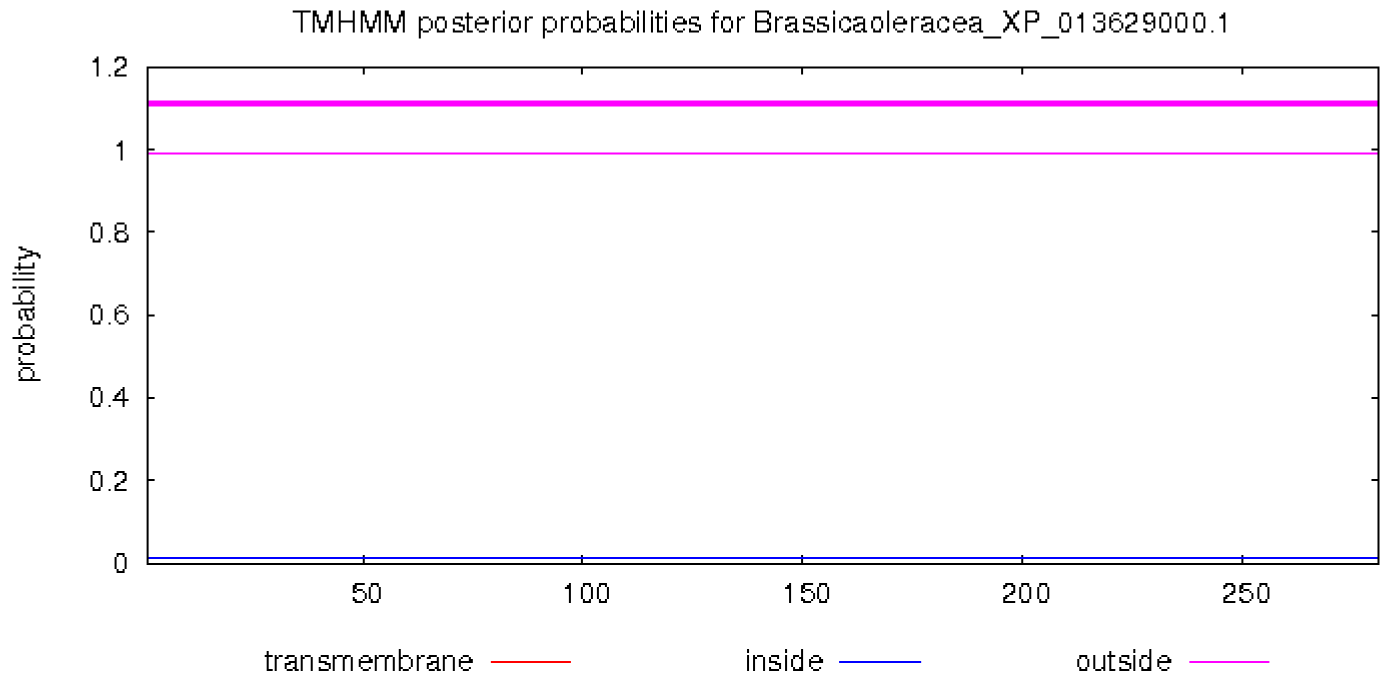

plot in postscript, script for making the plot in gnuplot, data for plot

```
# Brassicaoleracea_XP_013630002.1 Length: 252
# Brassicaoleracea_XP_013630002.1 Number of predicted TMHs:  0
# Brassicaoleracea_XP_013630002.1 Exp number of AAs in TMHs: 0
# Brassicaoleracea_XP_013630002.1 Exp number, first 60 AAs:  0
# Brassicaoleracea_XP_013630002.1 Total prob of N-in:        0.02031
Brassicaoleracea_XP_013630002.1 TMHMM2.0        outside      1   252
```

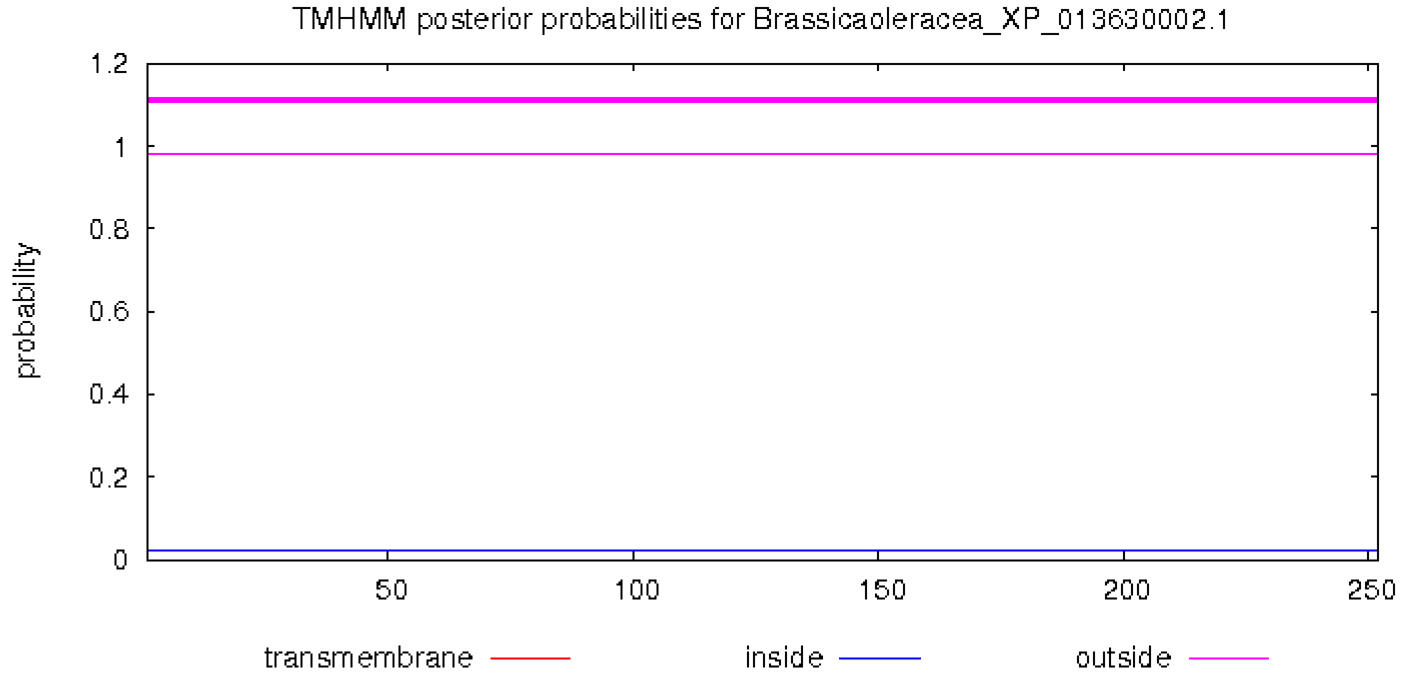

plot in postscript, script for making the plot in gnuplot, data for plot

```
# Brassicaoleracea_XP_013630765.1 Length: 175
# Brassicaoleracea_XP_013630765.1 Number of predicted TMHs:  0
# Brassicaoleracea_XP_013630765.1 Exp number of AAs in TMHs: 0.00013
# Brassicaoleracea_XP_013630765.1 Exp number, first 60 AAs:  0
# Brassicaoleracea_XP_013630765.1 Total prob of N-in:        0.06576
Brassicaoleracea_XP_013630765.1 TMHMM2.0        outside      1   175
```

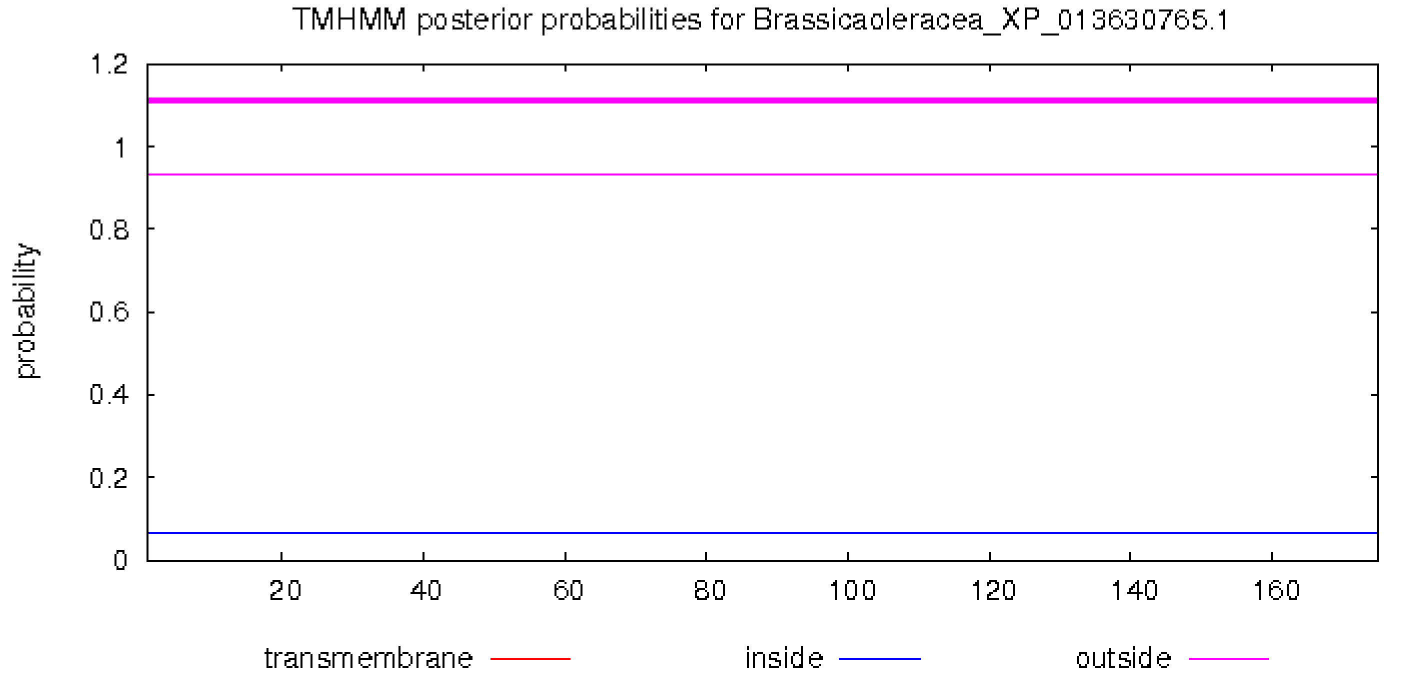

plot in postscript, script for making the plot in gnuplot, data for plot

```
# Brassicaoleracea_XP_013630845.1 Length: 537
# Brassicaoleracea_XP_013630845.1 Number of predicted TMHs:  1
# Brassicaoleracea_XP_013630845.1 Exp number of AAs in TMHs: 21.64331
# Brassicaoleracea_XP_013630845.1 Exp number, first 60 AAs:  0
# Brassicaoleracea_XP_013630845.1 Total prob of N-in:        0.00793
Brassicaoleracea_XP_013630845.1 TMHMM2.0        outside      1   511
Brassicaoleracea_XP_013630845.1 TMHMM2.0        TMhelix    512   534
Brassicaoleracea_XP_013630845.1 TMHMM2.0        inside     535   537
```

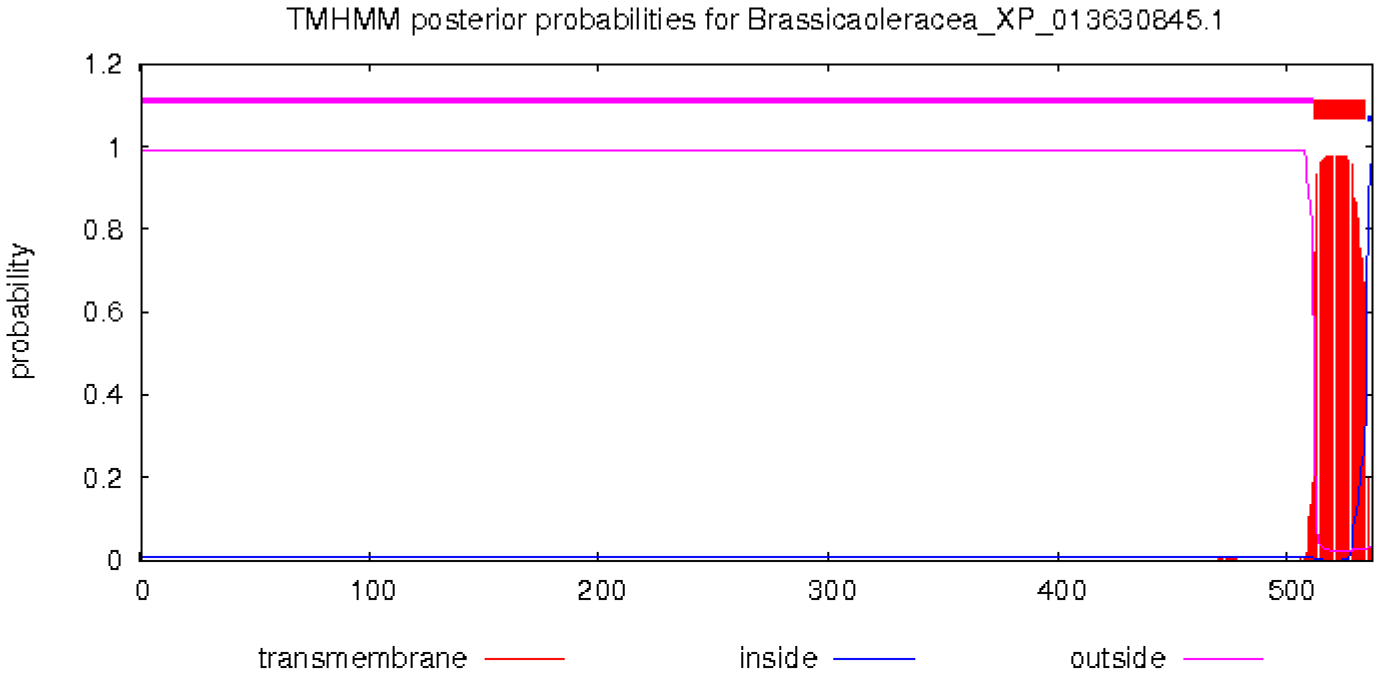

plot in postscript, script for making the plot in gnuplot, data for plot

```
# Brassicaoleracea_XP_013632799.1 Length: 575
# Brassicaoleracea_XP_013632799.1 Number of predicted TMHs:  0
# Brassicaoleracea_XP_013632799.1 Exp number of AAs in TMHs: 0.11966
# Brassicaoleracea_XP_013632799.1 Exp number, first 60 AAs:  0
# Brassicaoleracea_XP_013632799.1 Total prob of N-in:        0.00758
Brassicaoleracea_XP_013632799.1 TMHMM2.0        outside      1   575
```

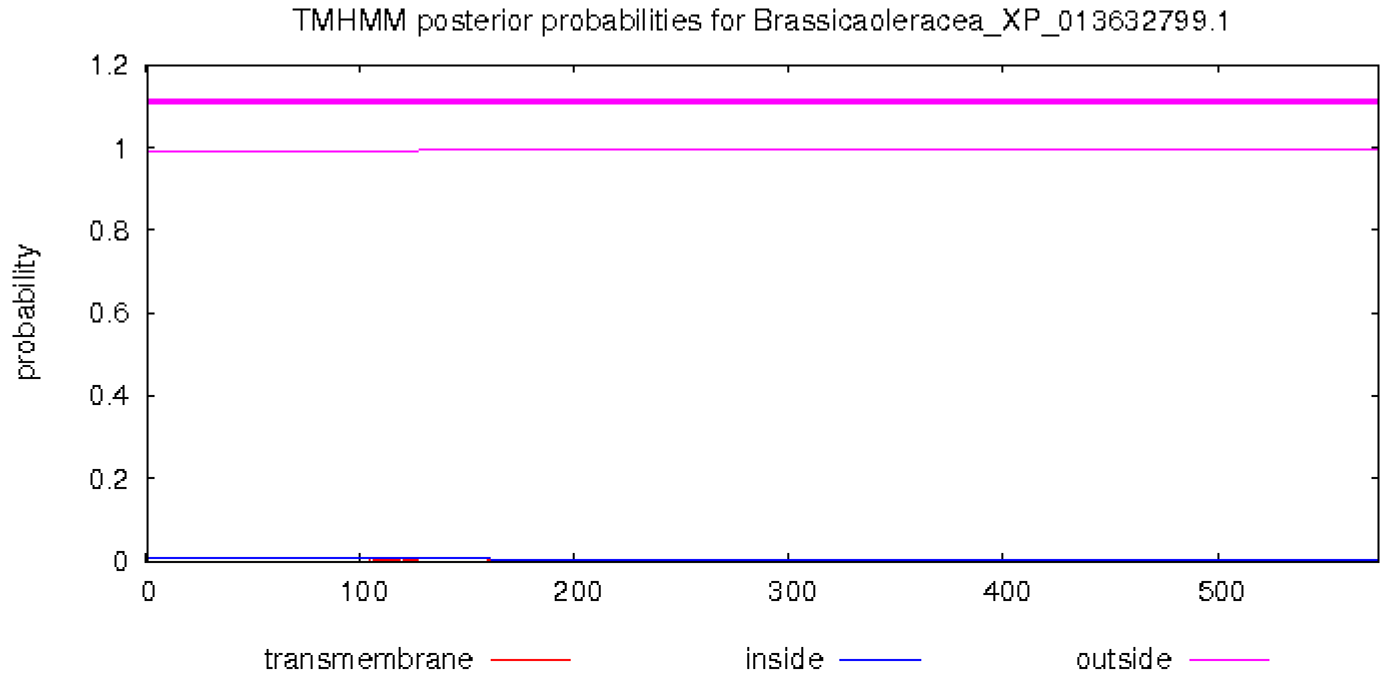

plot in postscript, script for making the plot in gnuplot, data for plot

```
# Brassicaoleracea_XP_013634266.1 Length: 255
# Brassicaoleracea_XP_013634266.1 Number of predicted TMHs:  0
# Brassicaoleracea_XP_013634266.1 Exp number of AAs in TMHs: 0.02487
# Brassicaoleracea_XP_013634266.1 Exp number, first 60 AAs:  0.0002
# Brassicaoleracea_XP_013634266.1 Total prob of N-in:        0.12587
Brassicaoleracea_XP_013634266.1 TMHMM2.0        outside      1   255
```

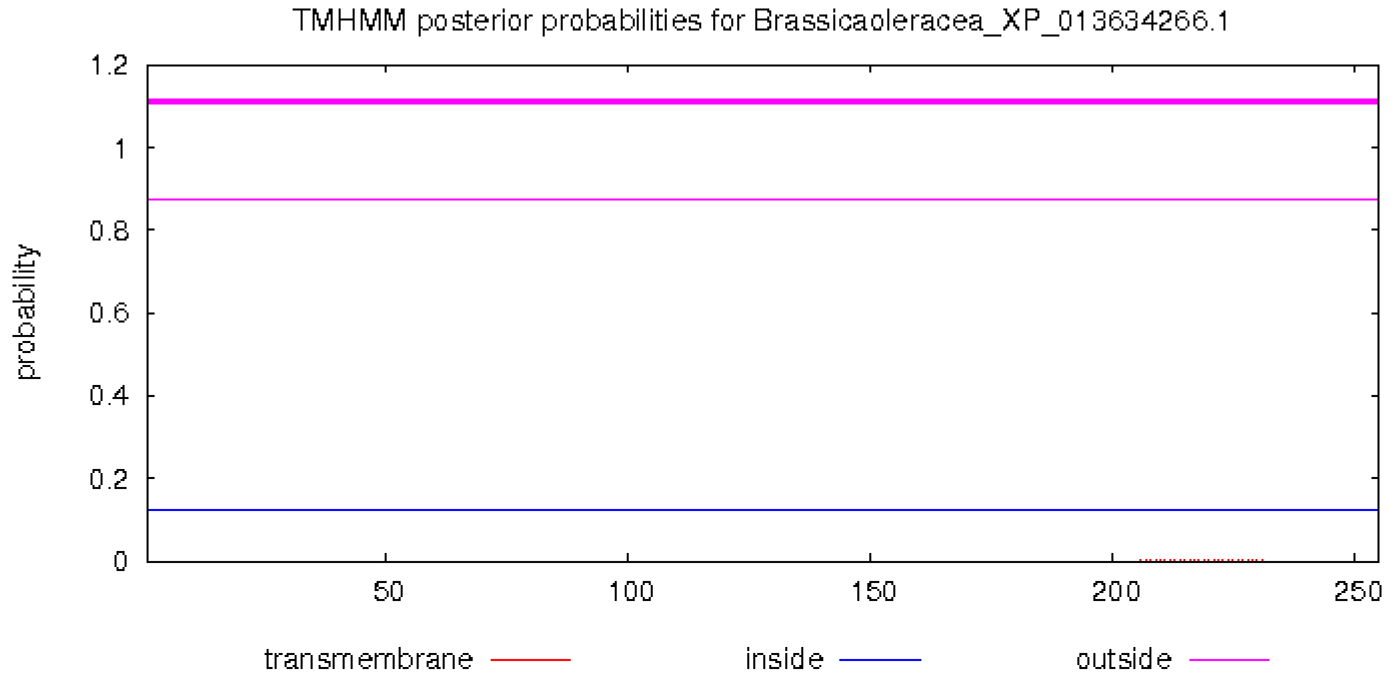

plot in postscript, script for making the plot in gnuplot, data for plot

```
# Brassicaoleracea_XP_013634548.1 Length: 345
# Brassicaoleracea_XP_013634548.1 Number of predicted TMHs:  1
# Brassicaoleracea_XP_013634548.1 Exp number of AAs in TMHs: 20.78927
# Brassicaoleracea_XP_013634548.1 Exp number, first 60 AAs:  0.08799
# Brassicaoleracea_XP_013634548.1 Total prob of N-in:        0.57540
Brassicaoleracea_XP_013634548.1 TMHMM2.0        inside       1   319
```

```
Brassicaoleracea_XP_013634548.1 TMHMM2.0        TMhelix    320   342
Brassicaoleracea_XP_013634548.1 TMHMM2.0        outside    343   345
```

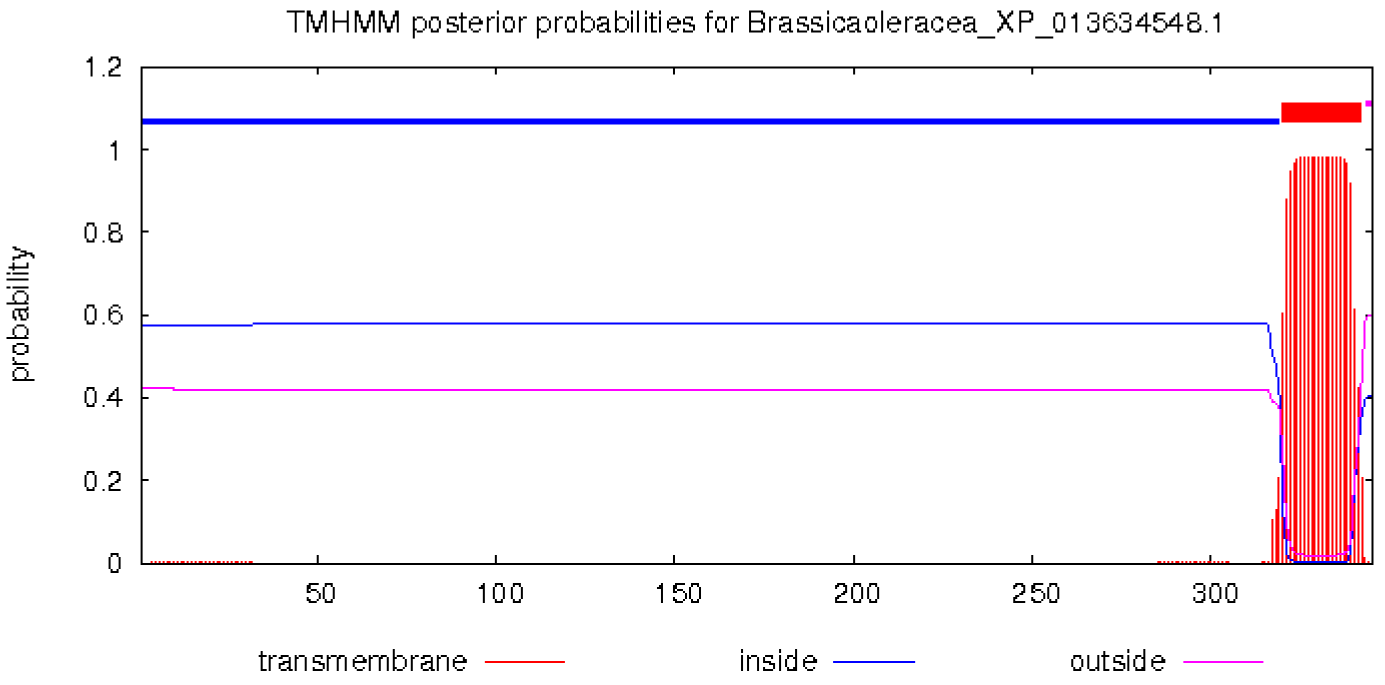

plot in postscript, script for making the plot in gnuplot, data for plot

---

```
# Brassicaoleracea_XP_013634549.1 Length: 343
# Brassicaoleracea_XP_013634549.1 Number of predicted TMHs:  1
# Brassicaoleracea_XP_013634549.1 Exp number of AAs in TMHs: 20.80429
# Brassicaoleracea_XP_013634549.1 Exp number, first 60 AAs:  0.08875
# Brassicaoleracea_XP_013634549.1 Total prob of N-in:        0.58142
Brassicaoleracea_XP_013634549.1 TMHMM2.0        inside       1   317
Brassicaoleracea_XP_013634549.1 TMHMM2.0        TMhelix    318   340
Brassicaoleracea_XP_013634549.1 TMHMM2.0        outside    341   343
```

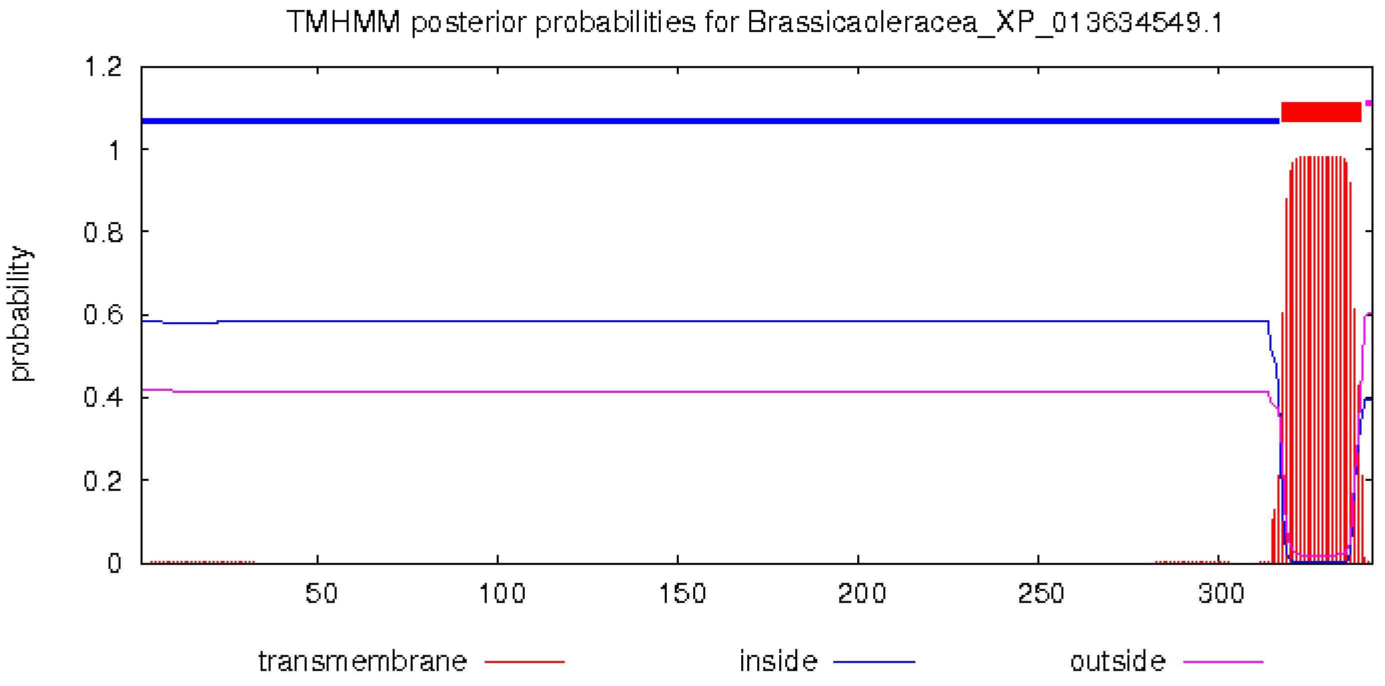

plot in postscript, script for making the plot in gnuplot, data for plot

---

```
# Brassicaoleracea_XP_013634581.1 Length: 252
# Brassicaoleracea_XP_013634581.1 Number of predicted TMHs:  0
```

```
# Brassicaoleracea_XP_013634581.1 Exp number of AAs in TMHs: 0.00541
# Brassicaoleracea_XP_013634581.1 Exp number, first 60 AAs:  0.00459
# Brassicaoleracea_XP_013634581.1 Total prob of N-in:        0.15517
Brassicaoleracea_XP_013634581.1 TMHMM2.0        outside      1   252
```

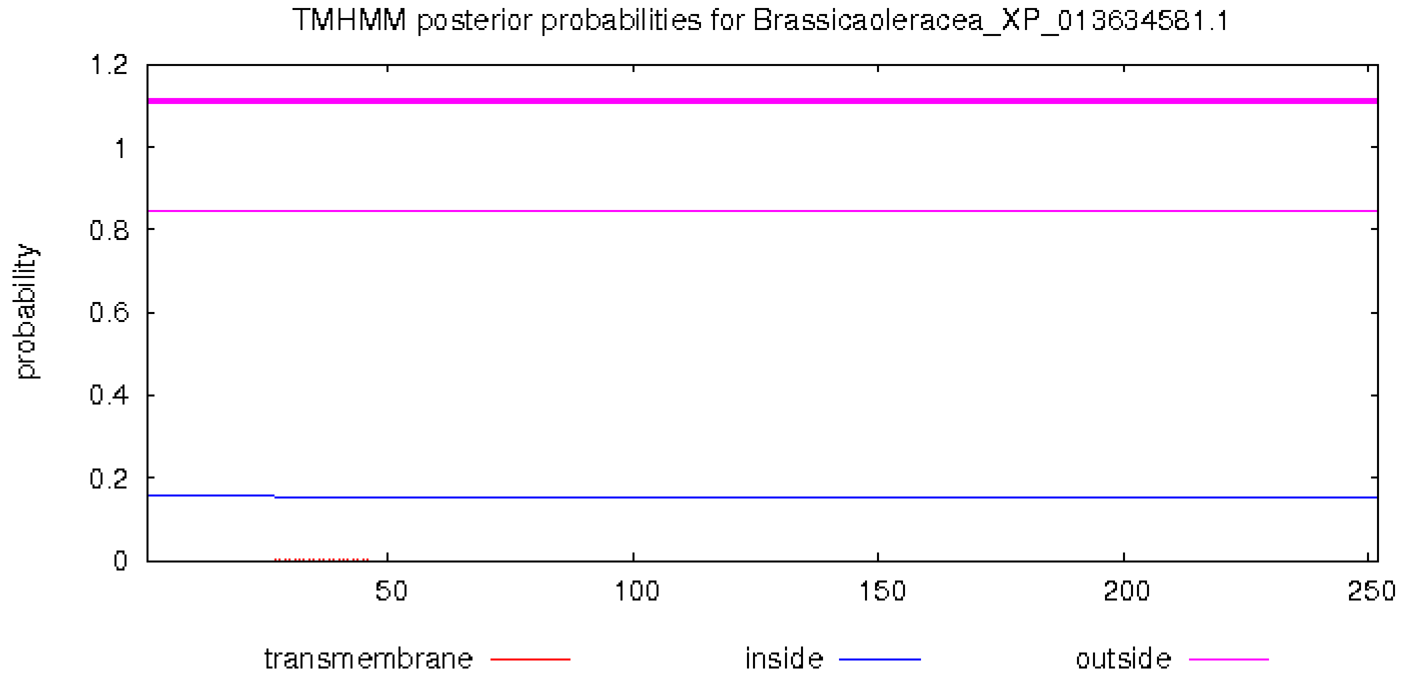

plot in postscript, script for making the plot in gnuplot, data for plot

---

```
# Brassicaoleracea_XP_013634618.1 Length: 277
# Brassicaoleracea_XP_013634618.1 Number of predicted TMHs:  0
# Brassicaoleracea_XP_013634618.1 Exp number of AAs in TMHs: 0.0008
# Brassicaoleracea_XP_013634618.1 Exp number, first 60 AAs:  0
# Brassicaoleracea_XP_013634618.1 Total prob of N-in:        0.02399
Brassicaoleracea_XP_013634618.1 TMHMM2.0        outside      1   277
```

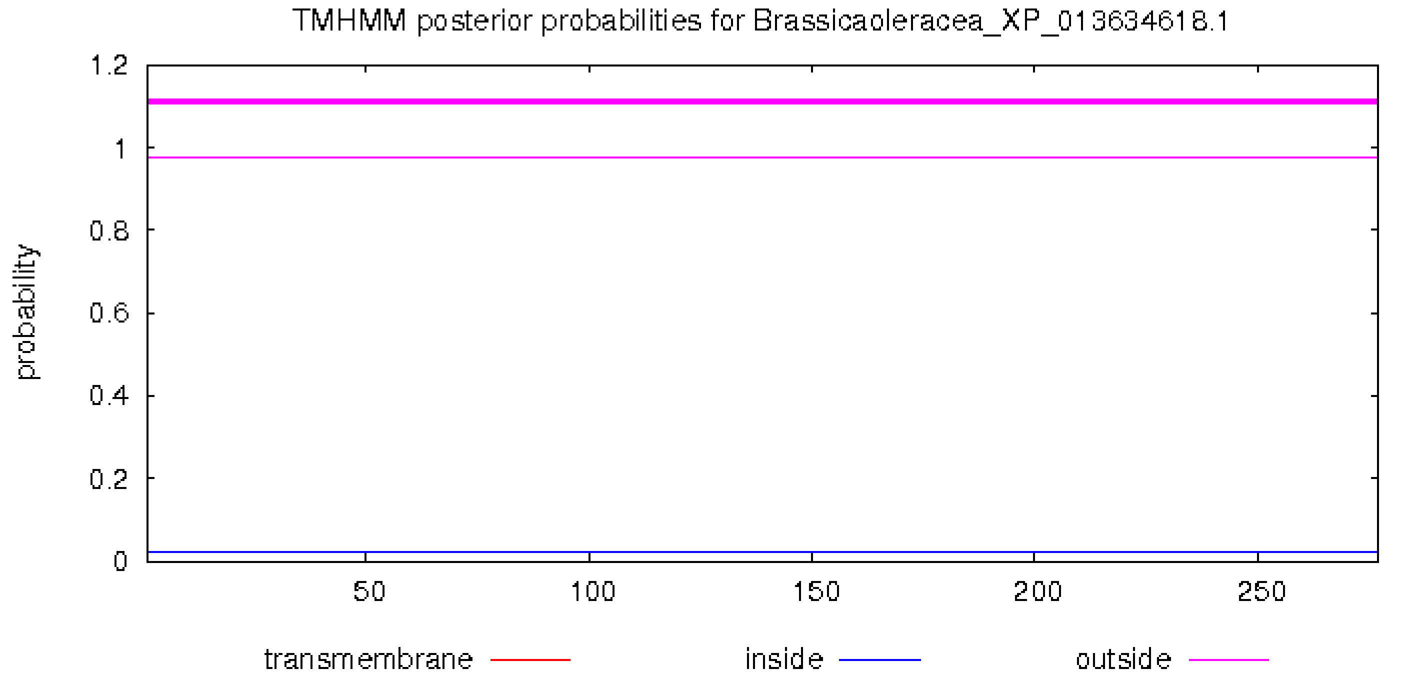

plot in postscript, script for making the plot in gnuplot, data for plot

---

```
# Brassicaoleracea_XP_013634619.1 Length: 277
# Brassicaoleracea_XP_013634619.1 Number of predicted TMHs:  0
```

```
# Brassicaoleracea_XP_013634619.1 Exp number of AAs in TMHs: 0.0008
# Brassicaoleracea_XP_013634619.1 Exp number, first 60 AAs:  0
# Brassicaoleracea_XP_013634619.1 Total prob of N-in:        0.02399
Brassicaoleracea_XP_013634619.1 TMHMM2.0        outside      1   277
```

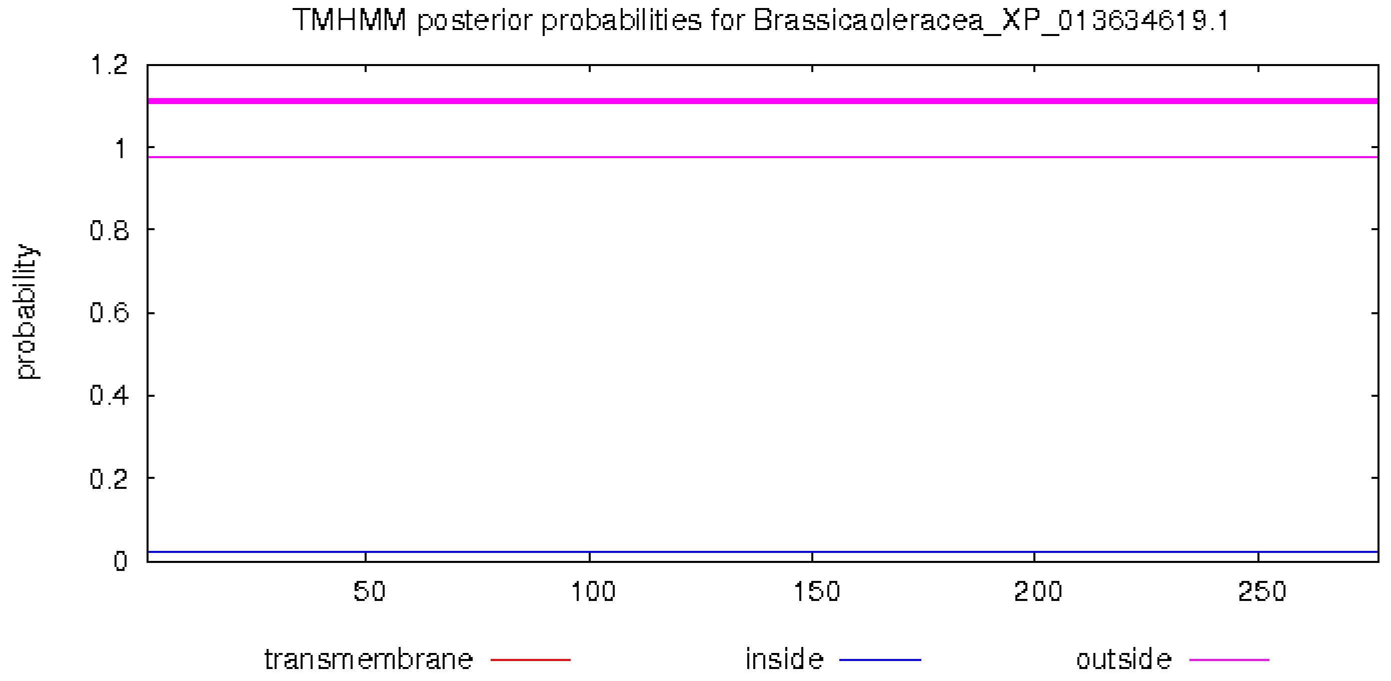

plot in postscript, script for making the plot in gnuplot, data for plot

---

```
# Brassicaoleracea_XP_013634620.1 Length: 277
# Brassicaoleracea_XP_013634620.1 Number of predicted TMHs:  0
# Brassicaoleracea_XP_013634620.1 Exp number of AAs in TMHs: 0.0008
# Brassicaoleracea_XP_013634620.1 Exp number, first 60 AAs:  0
# Brassicaoleracea_XP_013634620.1 Total prob of N-in:        0.02399
Brassicaoleracea_XP_013634620.1 TMHMM2.0        outside      1   277
```

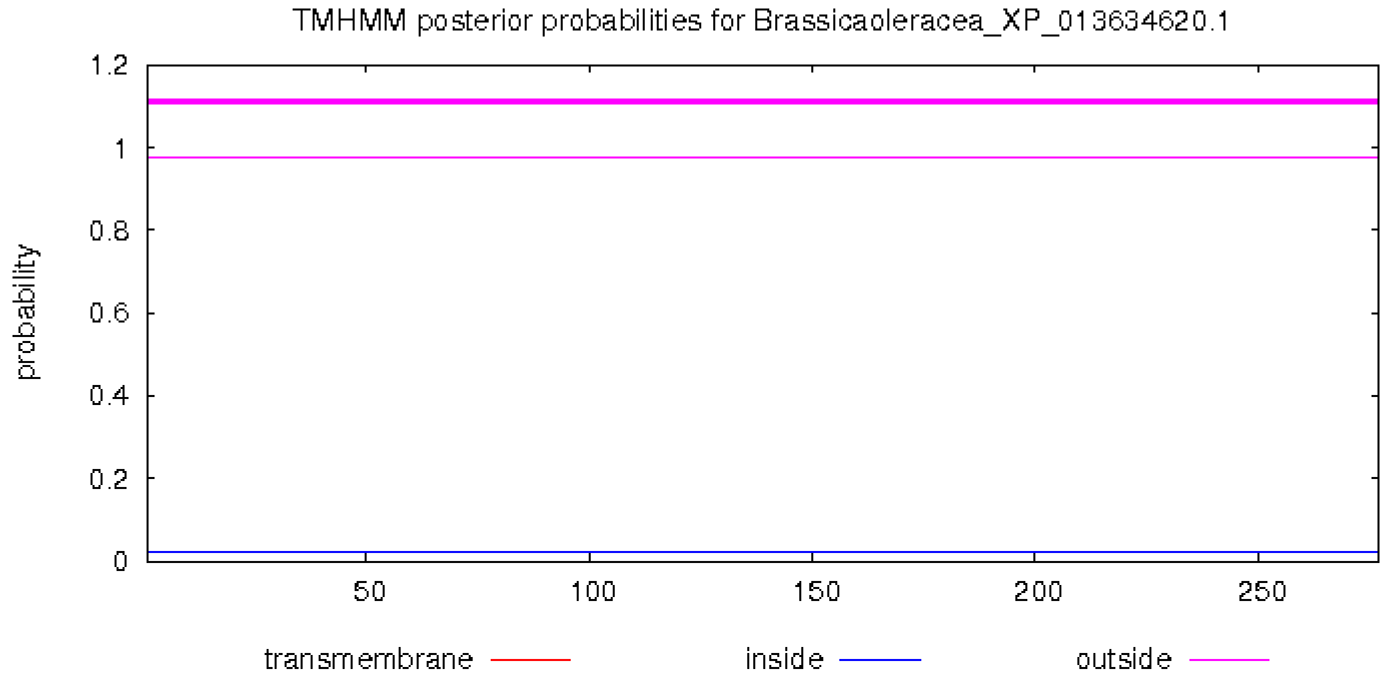

plot in postscript, script for making the plot in gnuplot, data for plot

---

```
# Brassicaoleracea_XP_013634628.1 Length: 364
# Brassicaoleracea_XP_013634628.1 Number of predicted TMHs:  0
```

```
# Brassicaoleracea_XP_013634628.1 Exp number of AAs in TMHs: 0.00039
# Brassicaoleracea_XP_013634628.1 Exp number, first 60 AAs:  0
# Brassicaoleracea_XP_013634628.1 Total prob of N-in:        0.00680
Brassicaoleracea_XP_013634628.1 TMHMM2.0        outside      1   364
```

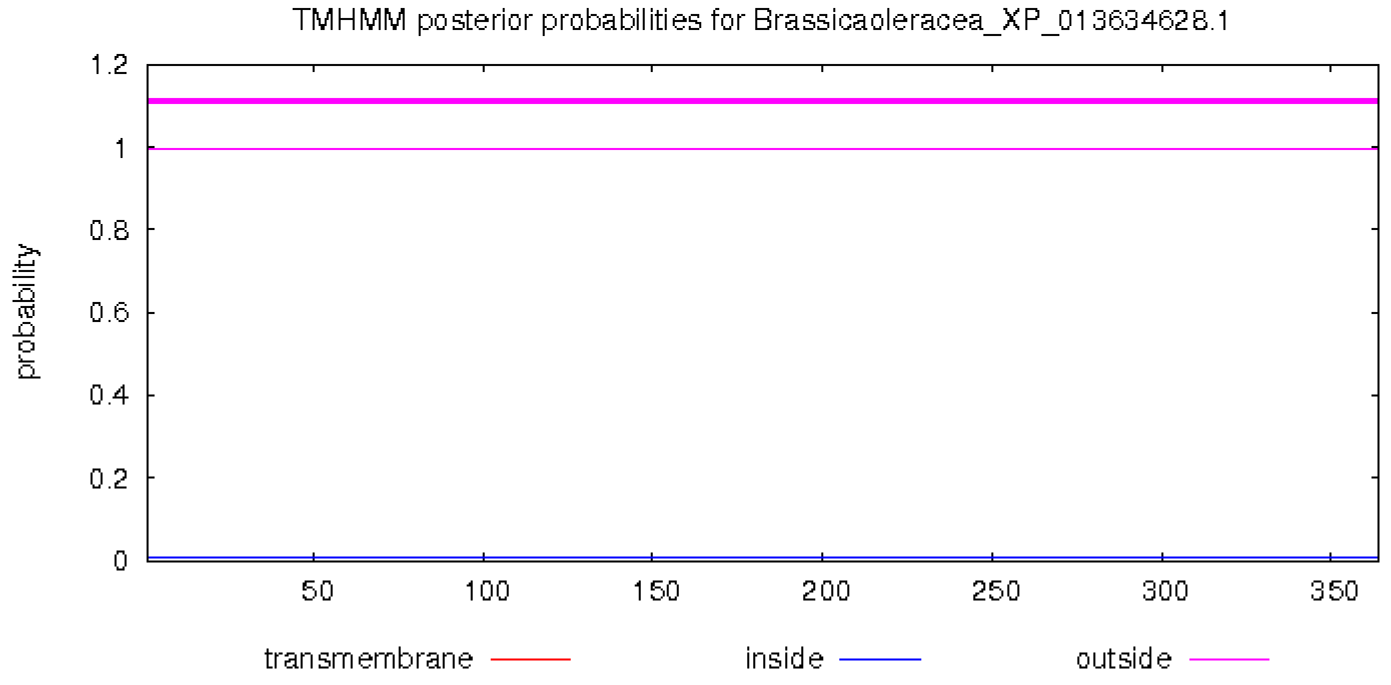

plot in postscript, script for making the plot in gnuplot, data for plot

---

```
# Brassicaoleracea_XP_013634645.1 Length: 317
# Brassicaoleracea_XP_013634645.1 Number of predicted TMHs:  0
# Brassicaoleracea_XP_013634645.1 Exp number of AAs in TMHs: 0.00716
# Brassicaoleracea_XP_013634645.1 Exp number, first 60 AAs:  0
# Brassicaoleracea_XP_013634645.1 Total prob of N-in:        0.07276
Brassicaoleracea_XP_013634645.1 TMHMM2.0        outside      1   317
```

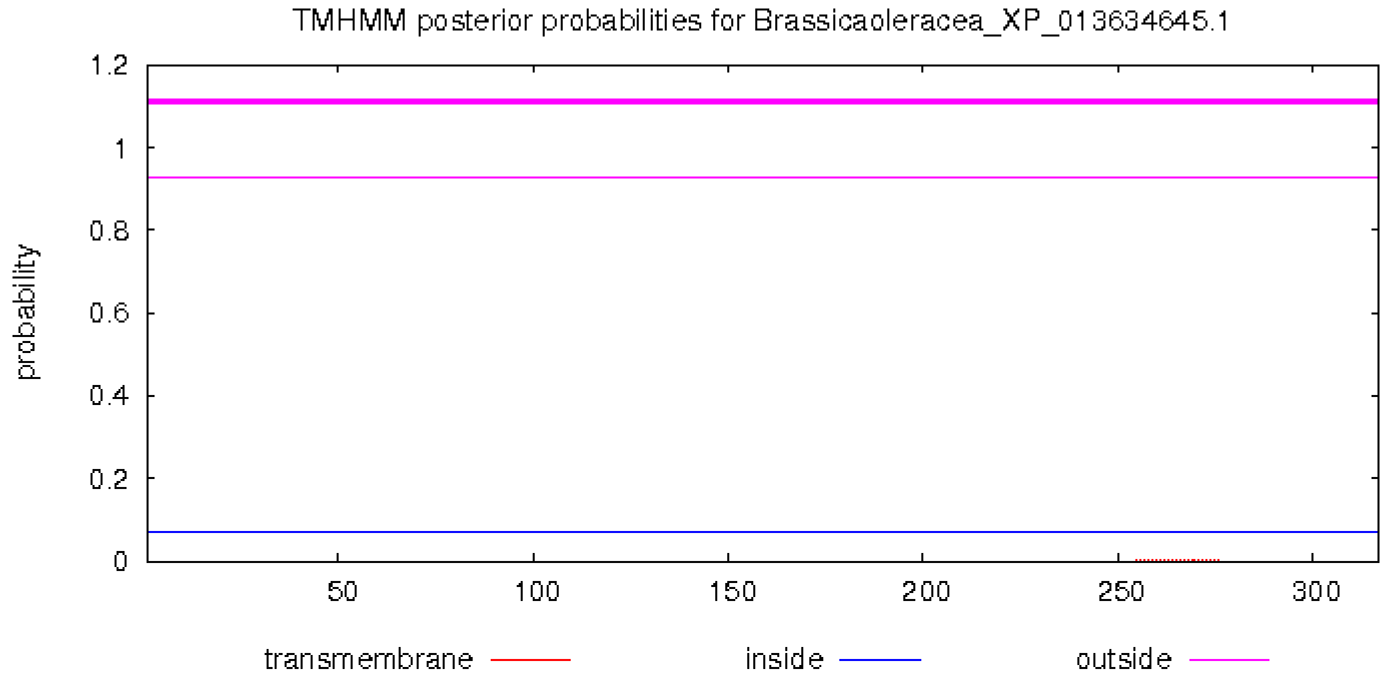

plot in postscript, script for making the plot in gnuplot, data for plot

---

```
# Brassicaoleracea_XP_013634646.1 Length: 317
# Brassicaoleracea_XP_013634646.1 Number of predicted TMHs:  0
```

```
# Brassicaoleracea_XP_013634646.1 Exp number of AAs in TMHs: 0.00716
# Brassicaoleracea_XP_013634646.1 Exp number, first 60 AAs:  0
# Brassicaoleracea_XP_013634646.1 Total prob of N-in:        0.07276
Brassicaoleracea_XP_013634646.1 TMHMM2.0        outside      1   317
```

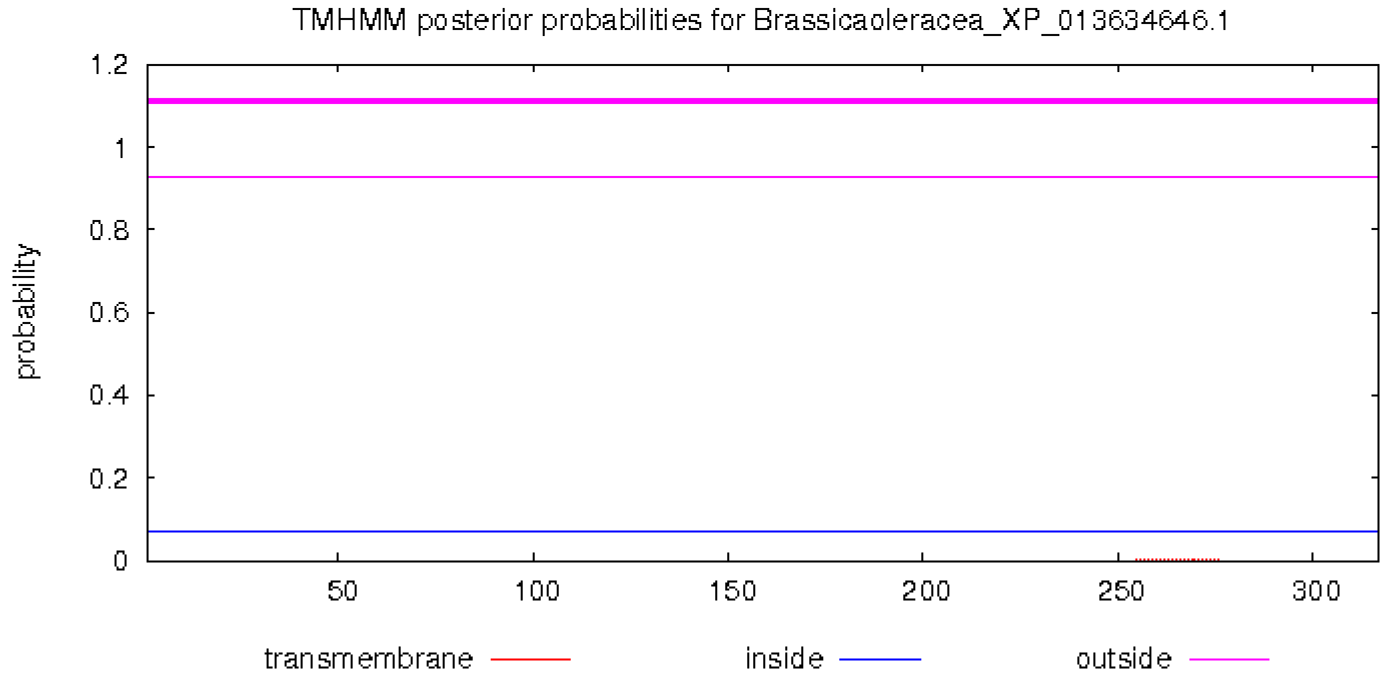

plot in postscript, script for making the plot in gnuplot, data for plot

---

```
# Brassicaoleracea_XP_013634739.1 Length: 357
# Brassicaoleracea_XP_013634739.1 Number of predicted TMHs:  0
# Brassicaoleracea_XP_013634739.1 Exp number of AAs in TMHs: 0.00096
# Brassicaoleracea_XP_013634739.1 Exp number, first 60 AAs:  0
# Brassicaoleracea_XP_013634739.1 Total prob of N-in:        0.01364
Brassicaoleracea_XP_013634739.1 TMHMM2.0        outside      1   357
```

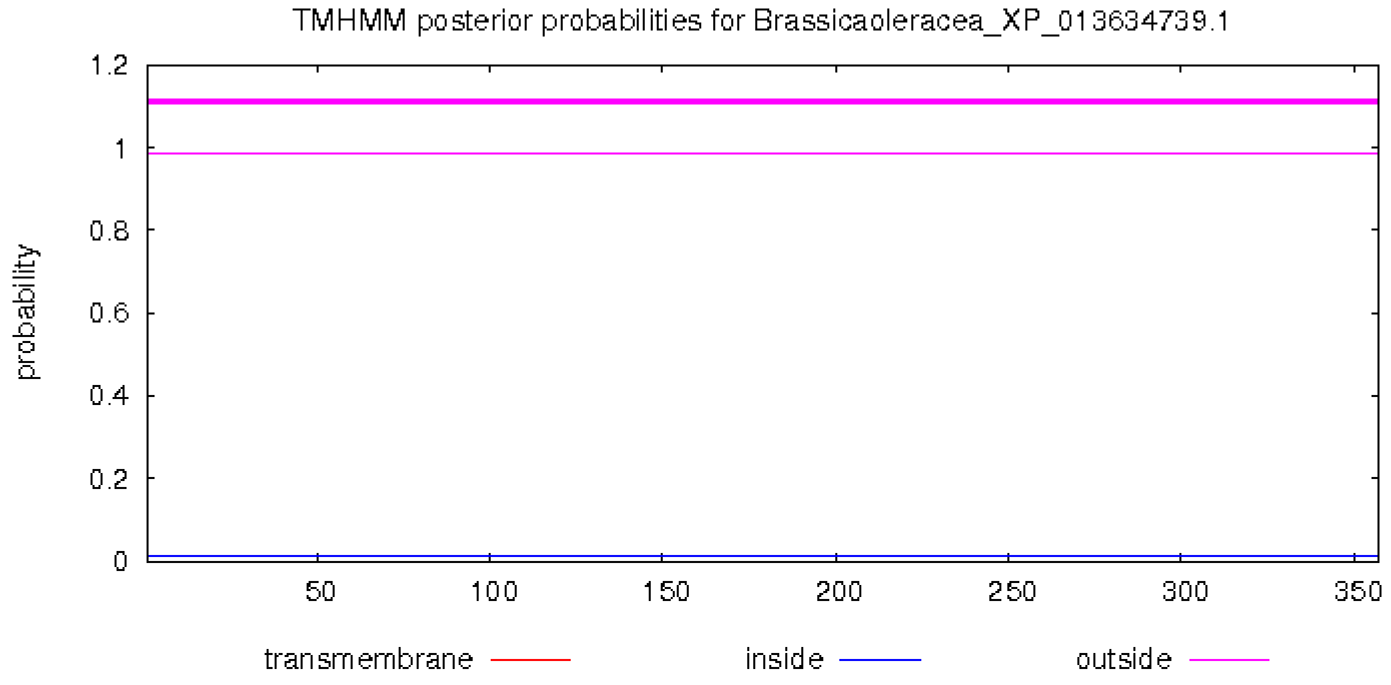

plot in postscript, script for making the plot in gnuplot, data for plot

---

```
# Brassicaoleracea_XP_013634805.1 Length: 279
# Brassicaoleracea_XP_013634805.1 Number of predicted TMHs:  0
```

```
# Brassicaoleracea_XP_013634805.1 Exp number of AAs in TMHs: 0.00119
# Brassicaoleracea_XP_013634805.1 Exp number, first 60 AAs:  0
# Brassicaoleracea_XP_013634805.1 Total prob of N-in:        0.05422
Brassicaoleracea_XP_013634805.1 TMHMM2.0        outside      1   279
```

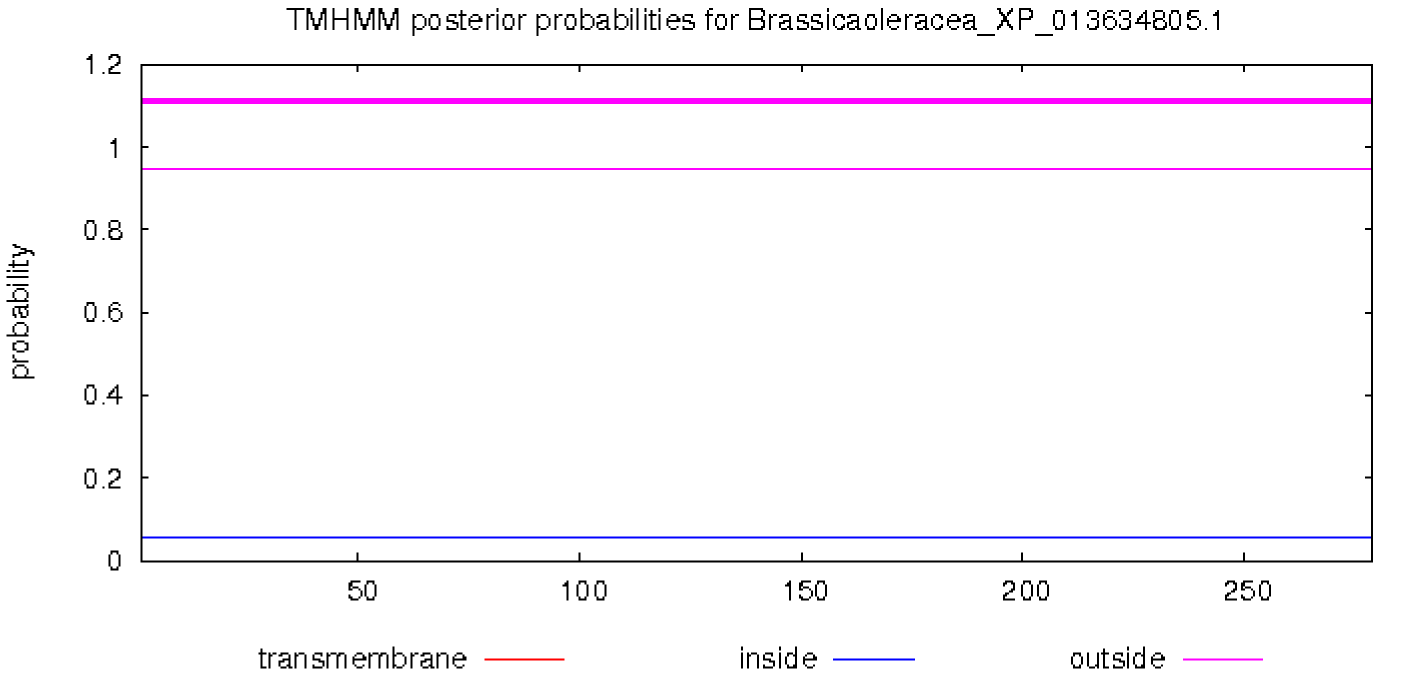

plot in postscript, script for making the plot in gnuplot, data for plot

```
# Brassicaoleracea_XP_013635505.1 Length: 201
# Brassicaoleracea_XP_013635505.1 Number of predicted TMHs:  0
# Brassicaoleracea_XP_013635505.1 Exp number of AAs in TMHs: 0
# Brassicaoleracea_XP_013635505.1 Exp number, first 60 AAs:  0
# Brassicaoleracea_XP_013635505.1 Total prob of N-in:        0.01747
Brassicaoleracea_XP_013635505.1 TMHMM2.0        outside      1   201
```

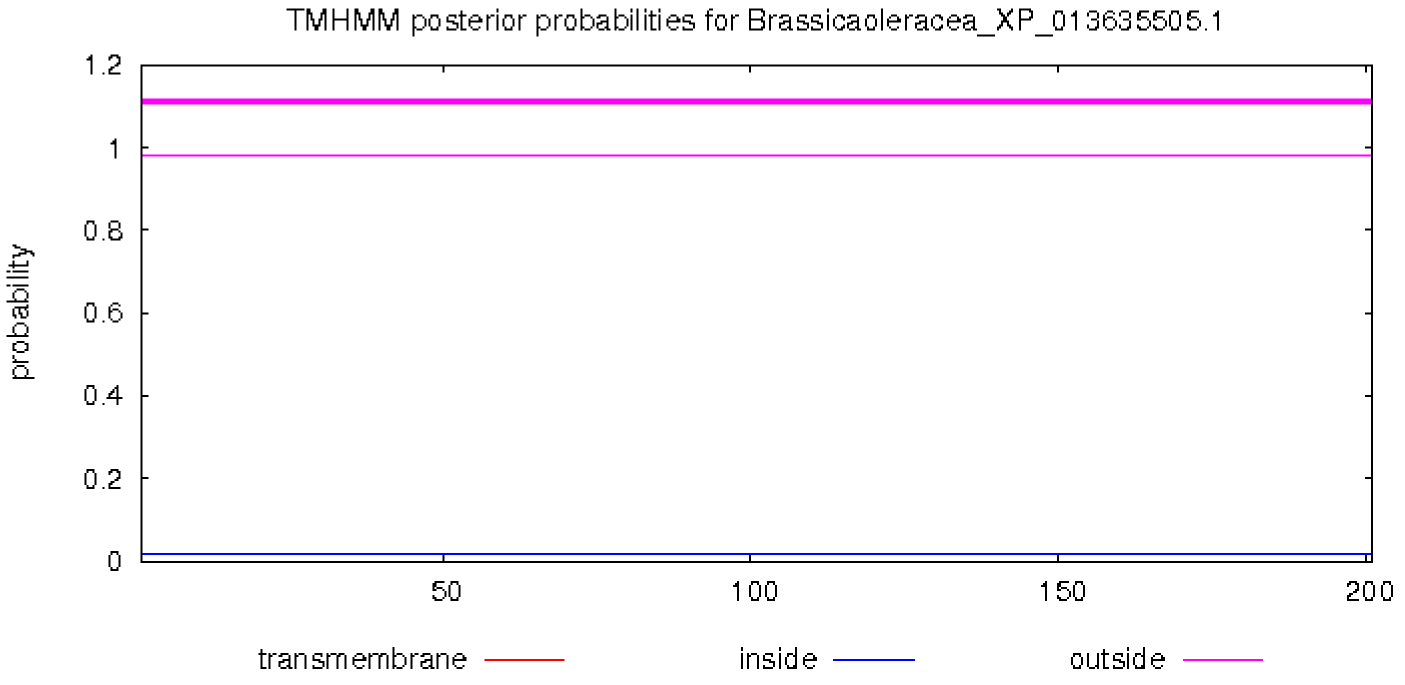

plot in postscript, script for making the plot in gnuplot, data for plot

```
# Brassicaoleracea_XP_013636556.1 Length: 292
# Brassicaoleracea_XP_013636556.1 Number of predicted TMHs:  0
```

```
# Brassicaoleracea_XP_013636556.1 Exp number of AAs in TMHs: 0
# Brassicaoleracea_XP_013636556.1 Exp number, first 60 AAs:  0
# Brassicaoleracea_XP_013636556.1 Total prob of N-in:        0.00489
Brassicaoleracea_XP_013636556.1 TMHMM2.0        outside      1   292
```

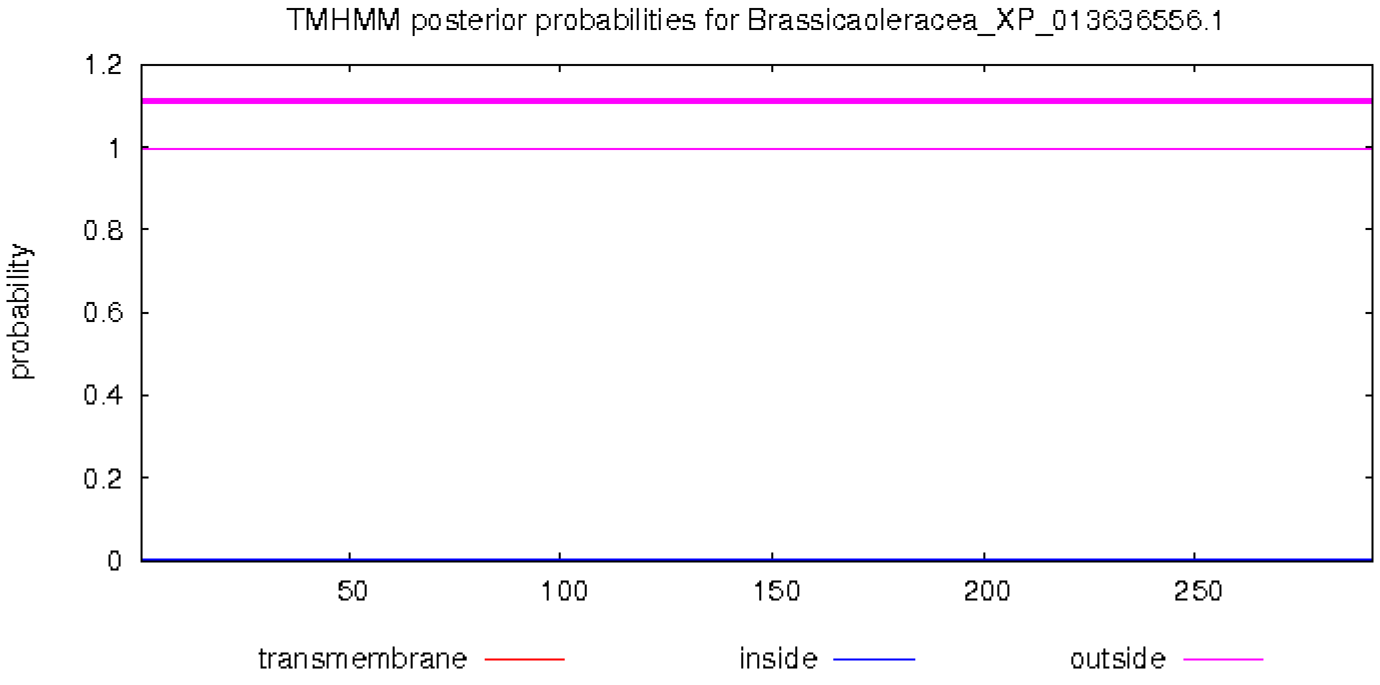

plot in postscript, script for making the plot in gnuplot, data for plot

---

```
# Brassicaoleracea_XP_013637675.1 Length: 653
# Brassicaoleracea_XP_013637675.1 Number of predicted TMHs:  1
# Brassicaoleracea_XP_013637675.1 Exp number of AAs in TMHs: 17.01456
# Brassicaoleracea_XP_013637675.1 Exp number, first 60 AAs:  0
# Brassicaoleracea_XP_013637675.1 Total prob of N-in:        0.07375
Brassicaoleracea_XP_013637675.1 TMHMM2.0        outside      1   594
Brassicaoleracea_XP_013637675.1 TMHMM2.0        TMhelix    595   612
Brassicaoleracea_XP_013637675.1 TMHMM2.0        inside     613   653
```

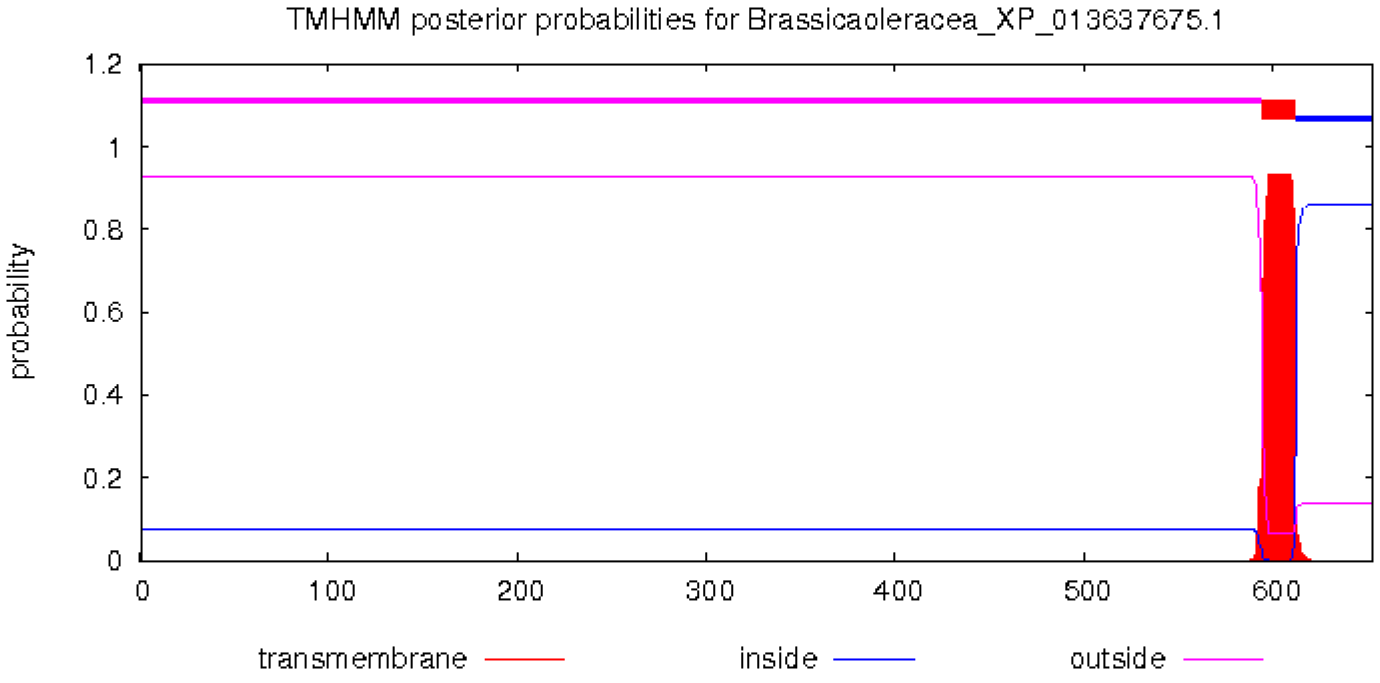

plot in postscript, script for making the plot in gnuplot, data for plot

---

```
# Brassicaoleracea_XP_013637676.1 Length: 635
# Brassicaoleracea_XP_013637676.1 Number of predicted TMHs:  1
# Brassicaoleracea_XP_013637676.1 Exp number of AAs in TMHs: 17.50902
# Brassicaoleracea_XP_013637676.1 Exp number, first 60 AAs:  0
# Brassicaoleracea_XP_013637676.1 Total prob of N-in:        0.10749
Brassicaoleracea_XP_013637676.1 TMHMM2.0        outside      1   594
Brassicaoleracea_XP_013637676.1 TMHMM2.0        TMhelix    595   612
Brassicaoleracea_XP_013637676.1 TMHMM2.0        inside     613   635
```

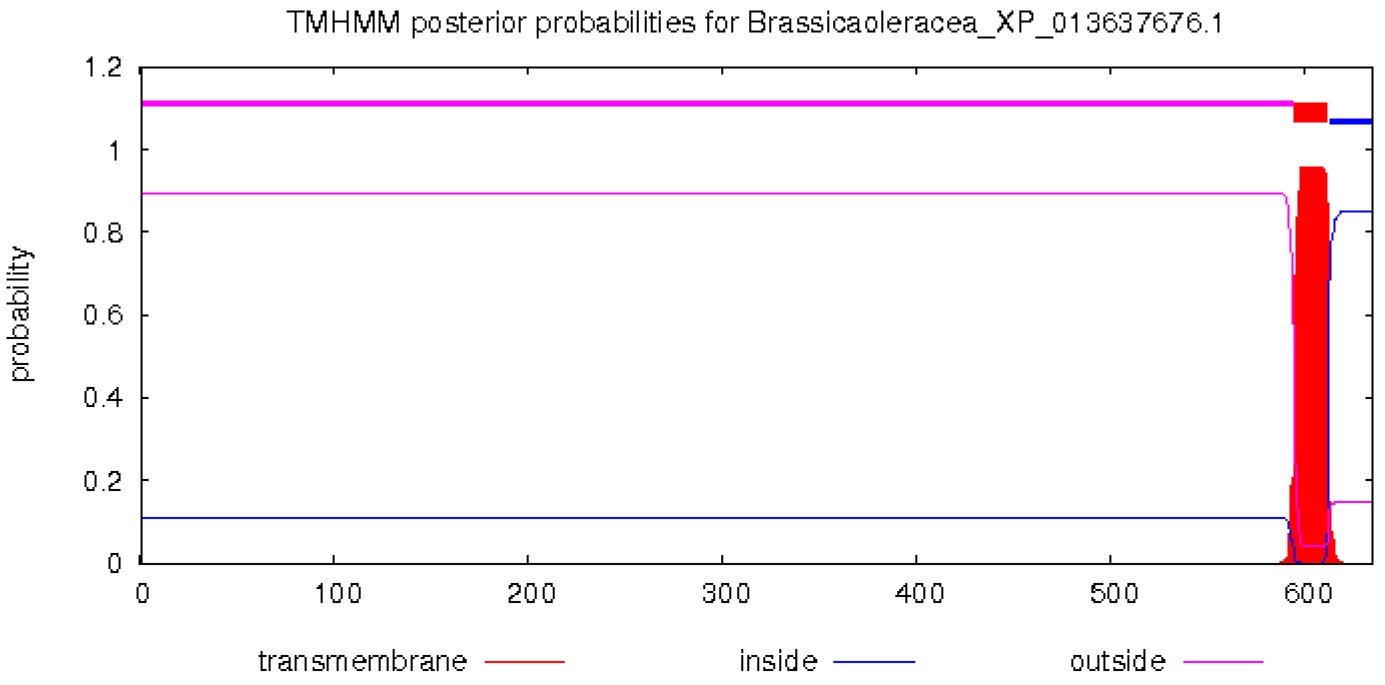

plot in postscript, script for making the plot in gnuplot, data for plot

---

```
# Brassicaoleracea_XP_013638093.1 Length: 457
# Brassicaoleracea_XP_013638093.1 Number of predicted TMHs:  0
# Brassicaoleracea_XP_013638093.1 Exp number of AAs in TMHs: 0.00158
# Brassicaoleracea_XP_013638093.1 Exp number, first 60 AAs:  0
# Brassicaoleracea_XP_013638093.1 Total prob of N-in:        0.00294
Brassicaoleracea_XP_013638093.1 TMHMM2.0        outside      1   457
```

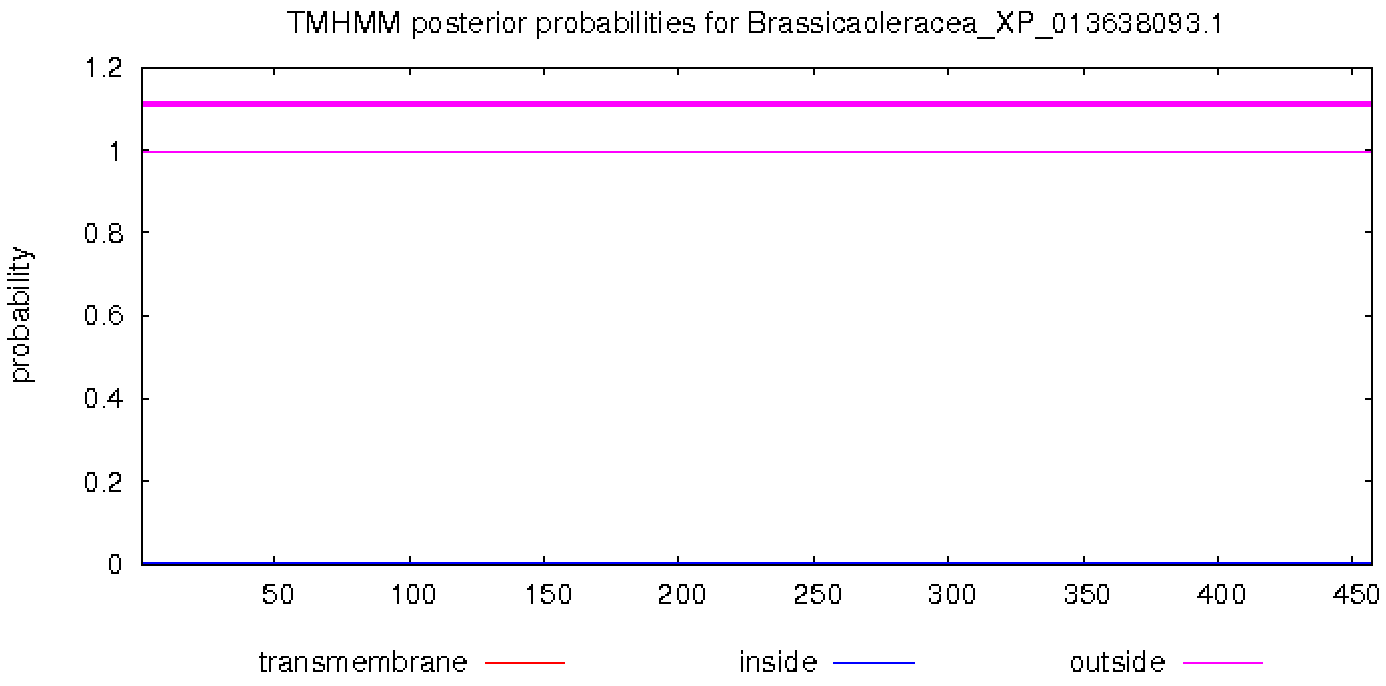

plot in postscript, script for making the plot in gnuplot, data for plot

```
# Brassicaoleracea_XP_013638215.1 Length: 581
# Brassicaoleracea_XP_013638215.1 Number of predicted TMHs:  1
# Brassicaoleracea_XP_013638215.1 Exp number of AAs in TMHs: 22.57199
# Brassicaoleracea_XP_013638215.1 Exp number, first 60 AAs:  0.0019
# Brassicaoleracea_XP_013638215.1 Total prob of N-in:        0.11514
Brassicaoleracea_XP_013638215.1 TMHMM2.0        outside      1   548
Brassicaoleracea_XP_013638215.1 TMHMM2.0        TMhelix    549   571
Brassicaoleracea_XP_013638215.1 TMHMM2.0        inside     572   581
```

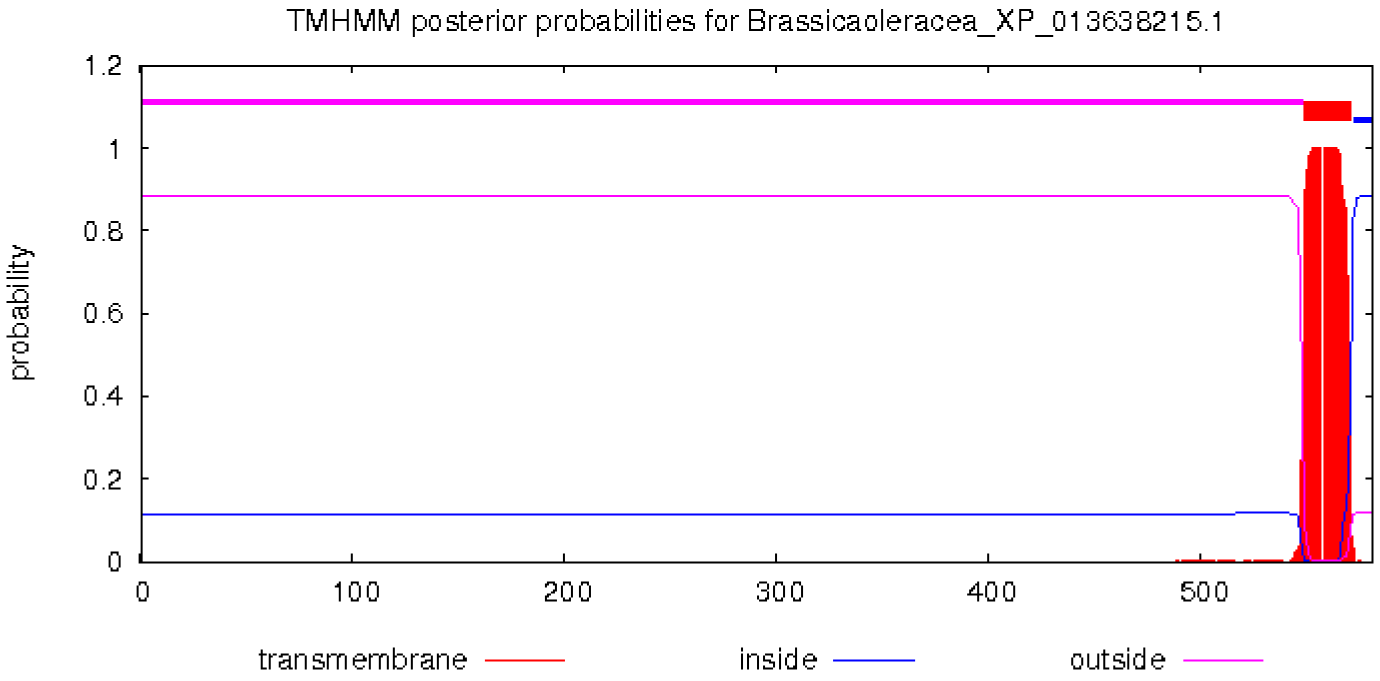

plot in postscript, script for making the plot in gnuplot, data for plot

```
# Brassicaoleracea_XP_013638342.1 Length: 245
# Brassicaoleracea_XP_013638342.1 Number of predicted TMHs:  0
# Brassicaoleracea_XP_013638342.1 Exp number of AAs in TMHs: 0
# Brassicaoleracea_XP_013638342.1 Exp number, first 60 AAs:  0
# Brassicaoleracea_XP_013638342.1 Total prob of N-in:        0.00225
Brassicaoleracea_XP_013638342.1 TMHMM2.0        outside      1   245
```

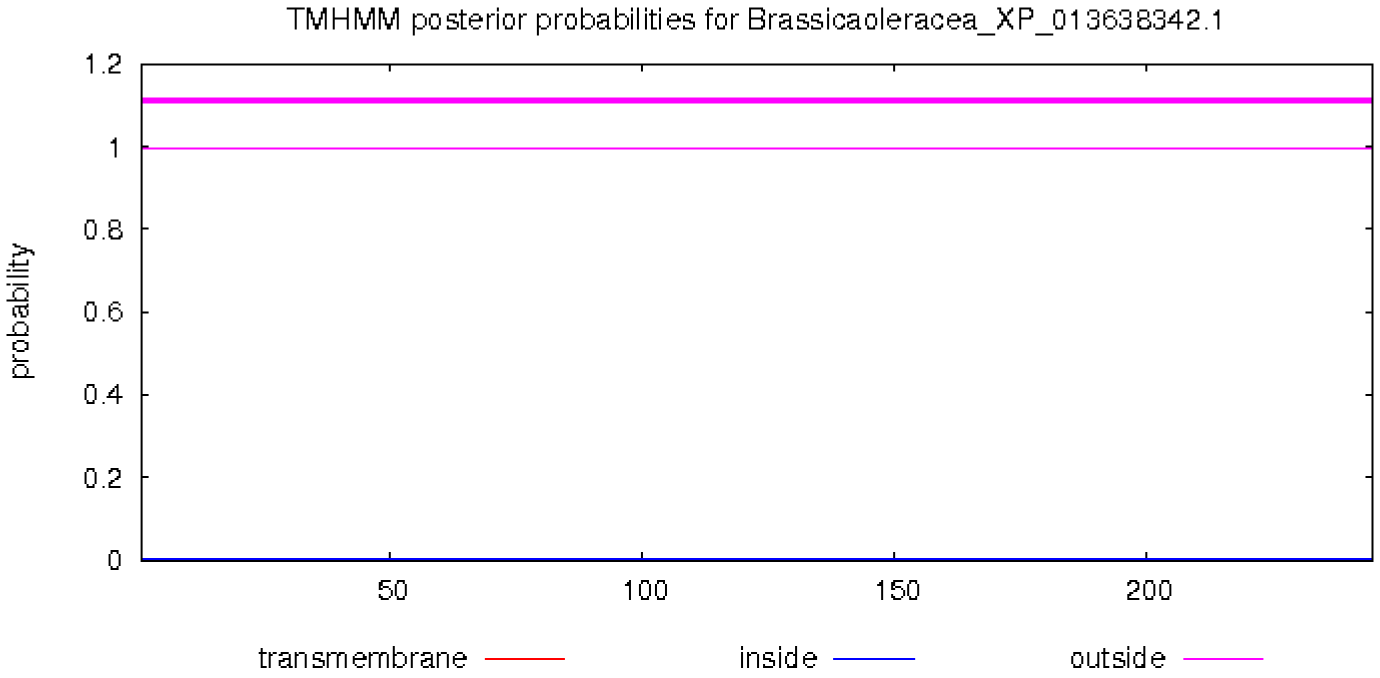

plot in postscript, script for making the plot in gnuplot, data for plot

```
# Brassicaoleracea_XP_013638572.1 Length: 457
# Brassicaoleracea_XP_013638572.1 Number of predicted TMHs:  0
# Brassicaoleracea_XP_013638572.1 Exp number of AAs in TMHs: 0
# Brassicaoleracea_XP_013638572.1 Exp number, first 60 AAs:  0
# Brassicaoleracea_XP_013638572.1 Total prob of N-in:        0.00399
Brassicaoleracea_XP_013638572.1 TMHMM2.0        outside      1   457
```

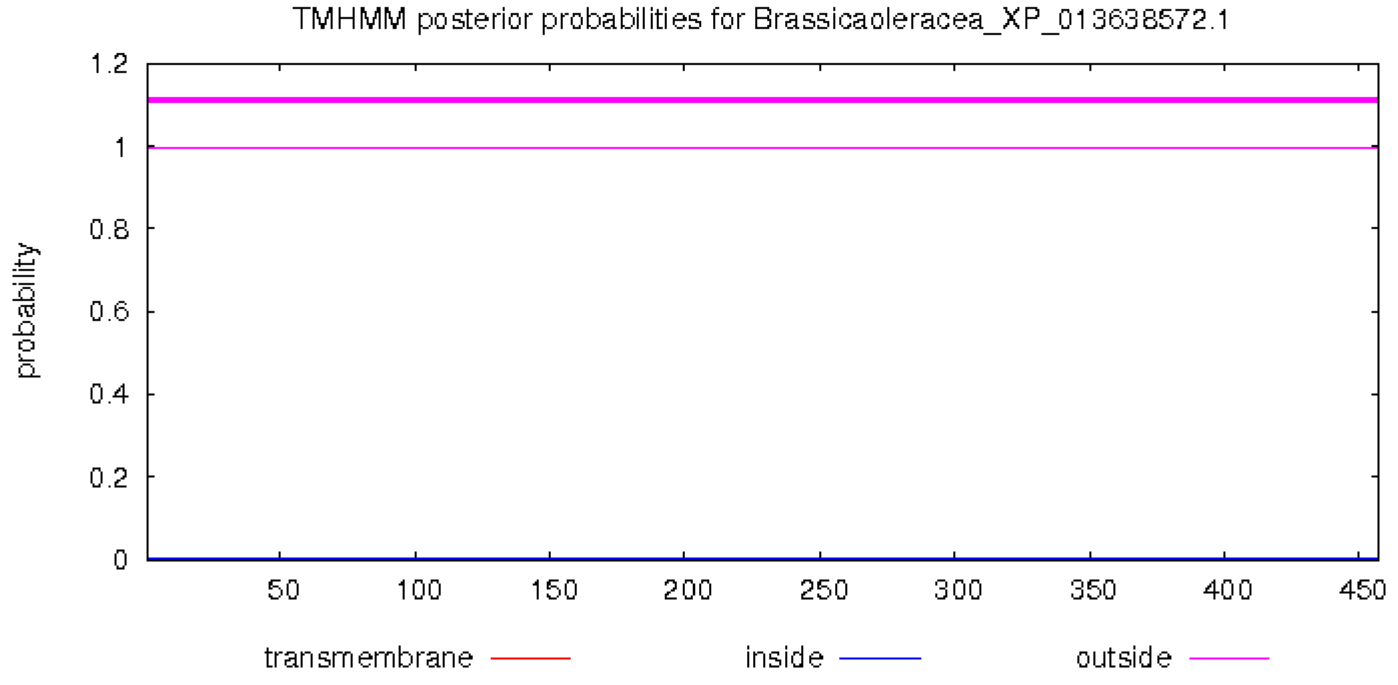

plot in postscript, script for making the plot in gnuplot, data for plot

```
# Brassicaoleracea_XP_013638573.1 Length: 457
# Brassicaoleracea_XP_013638573.1 Number of predicted TMHs:  0
# Brassicaoleracea_XP_013638573.1 Exp number of AAs in TMHs: 0
# Brassicaoleracea_XP_013638573.1 Exp number, first 60 AAs:  0
# Brassicaoleracea_XP_013638573.1 Total prob of N-in:        0.00399
Brassicaoleracea_XP_013638573.1 TMHMM2.0        outside      1   457
```

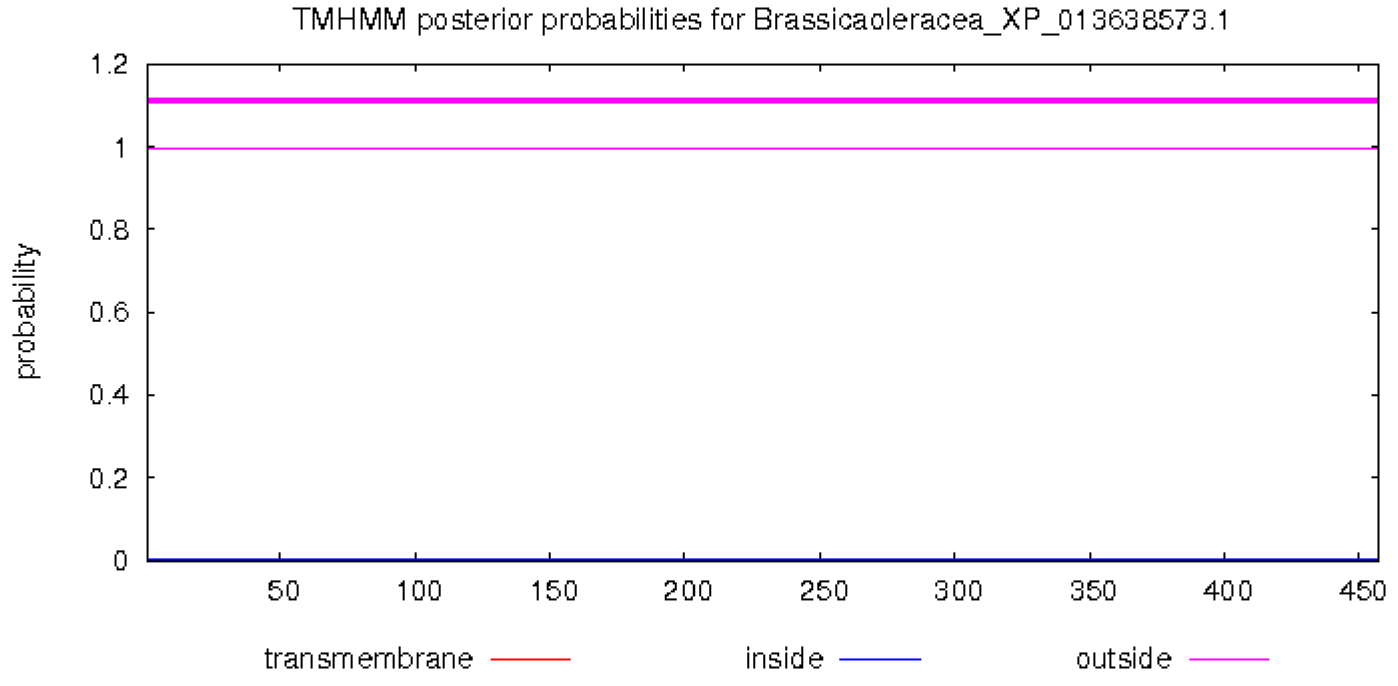

plot in postscript, script for making the plot in gnuplot, data for plot

---

```
# Brassicaoleracea_XP_013638575.1 Length: 456
# Brassicaoleracea_XP_013638575.1 Number of predicted TMHs:  0
# Brassicaoleracea_XP_013638575.1 Exp number of AAs in TMHs: 0
# Brassicaoleracea_XP_013638575.1 Exp number, first 60 AAs:  0
# Brassicaoleracea_XP_013638575.1 Total prob of N-in:        0.00404
Brassicaoleracea_XP_013638575.1 TMHMM2.0        outside      1   456
```

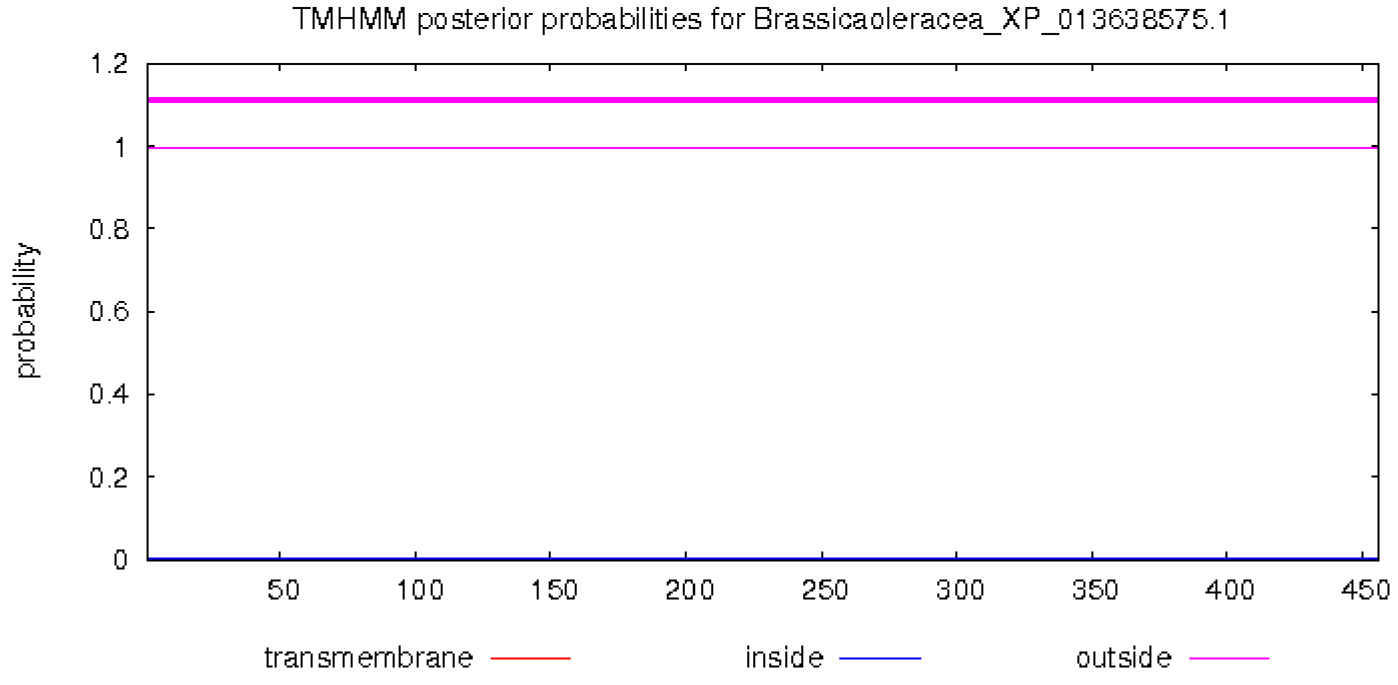

plot in postscript, script for making the plot in gnuplot, data for plot

---

```
# Brassicaoleracea_XP_013638576.1 Length: 456
# Brassicaoleracea_XP_013638576.1 Number of predicted TMHs:  0
# Brassicaoleracea_XP_013638576.1 Exp number of AAs in TMHs: 0
# Brassicaoleracea_XP_013638576.1 Exp number, first 60 AAs:  0
# Brassicaoleracea_XP_013638576.1 Total prob of N-in:        0.00404
Brassicaoleracea_XP_013638576.1 TMHMM2.0        outside      1   456
```

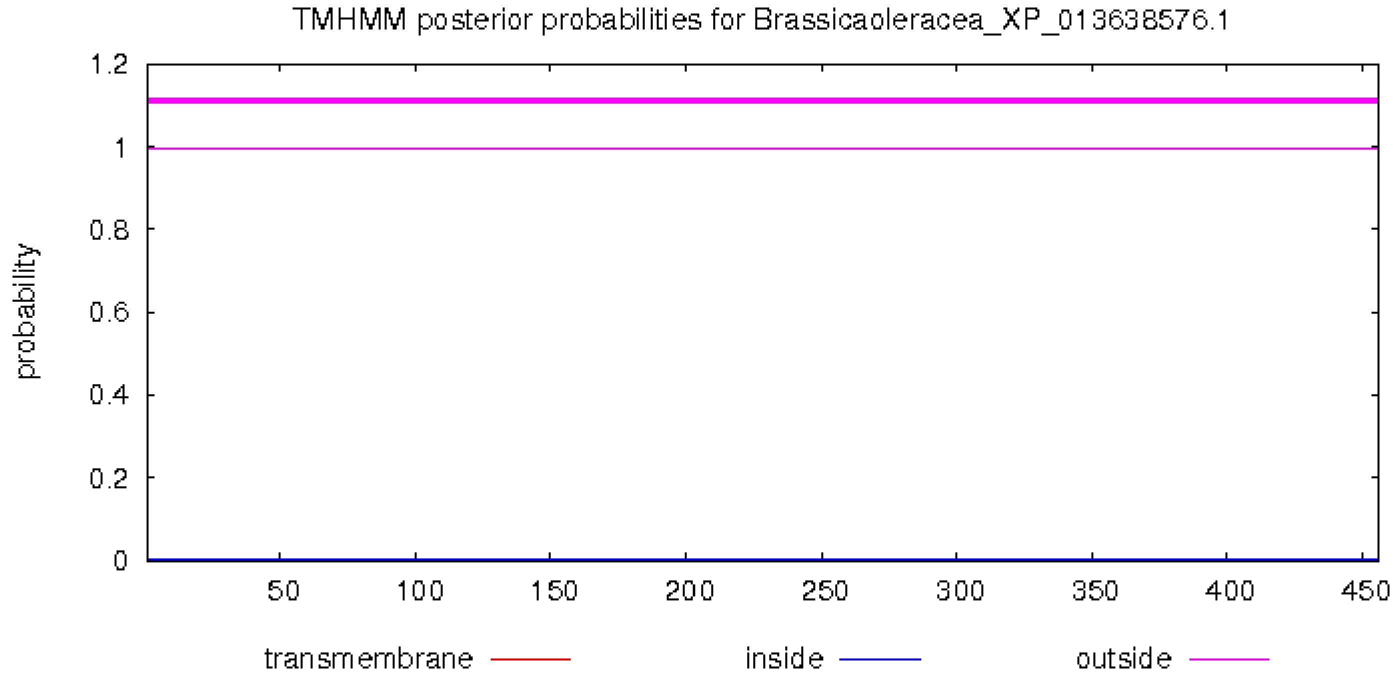

plot in postscript, script for making the plot in gnuplot, data for plot

```
# Brassicaoleracea_XP_013638577.1 Length: 447
# Brassicaoleracea_XP_013638577.1 Number of predicted TMHs:  0
# Brassicaoleracea_XP_013638577.1 Exp number of AAs in TMHs: 0
# Brassicaoleracea_XP_013638577.1 Exp number, first 60 AAs:  0
# Brassicaoleracea_XP_013638577.1 Total prob of N-in:        0.00452
Brassicaoleracea_XP_013638577.1 TMHMM2.0        outside      1   447
```

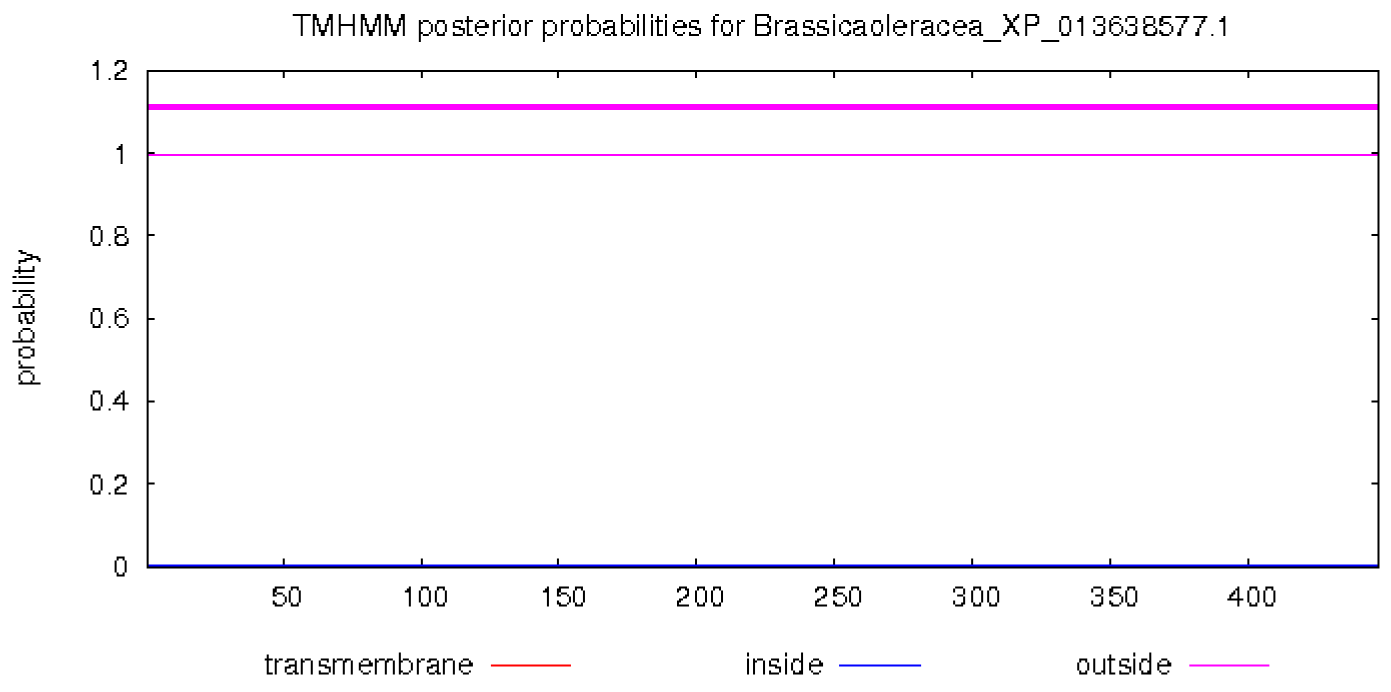

plot in postscript, script for making the plot in gnuplot, data for plot

```
# Brassicaoleracea_XP_013638578.1 Length: 422
# Brassicaoleracea_XP_013638578.1 Number of predicted TMHs:  0
# Brassicaoleracea_XP_013638578.1 Exp number of AAs in TMHs: 0
# Brassicaoleracea_XP_013638578.1 Exp number, first 60 AAs:  0
# Brassicaoleracea_XP_013638578.1 Total prob of N-in:        0.00616
Brassicaoleracea_XP_013638578.1 TMHMM2.0        outside      1   422
```

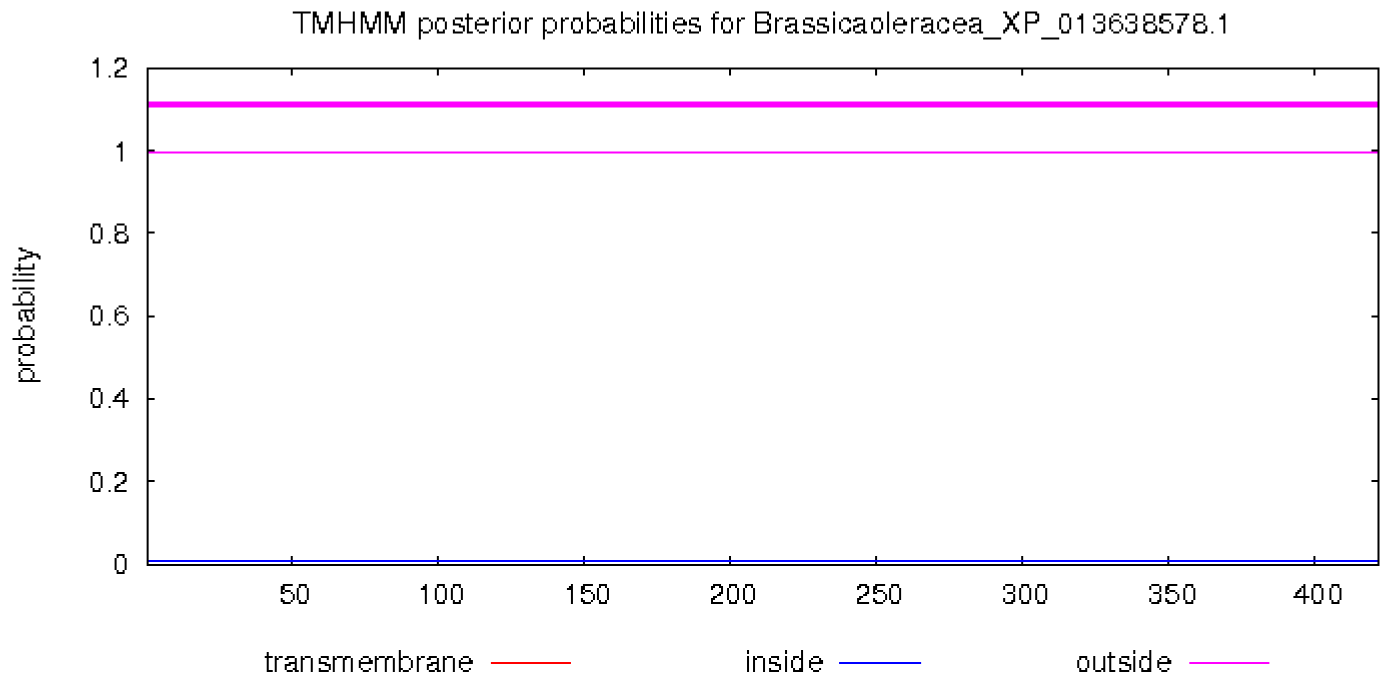

plot in postscript, script for making the plot in gnuplot, data for plot

```
# Brassicaoleracea_XP_013638579.1 Length: 279
# Brassicaoleracea_XP_013638579.1 Number of predicted TMHs:  0
# Brassicaoleracea_XP_013638579.1 Exp number of AAs in TMHs: 0.00134
# Brassicaoleracea_XP_013638579.1 Exp number, first 60 AAs:  0
# Brassicaoleracea_XP_013638579.1 Total prob of N-in:        0.02772
Brassicaoleracea_XP_013638579.1 TMHMM2.0        outside      1   279
```

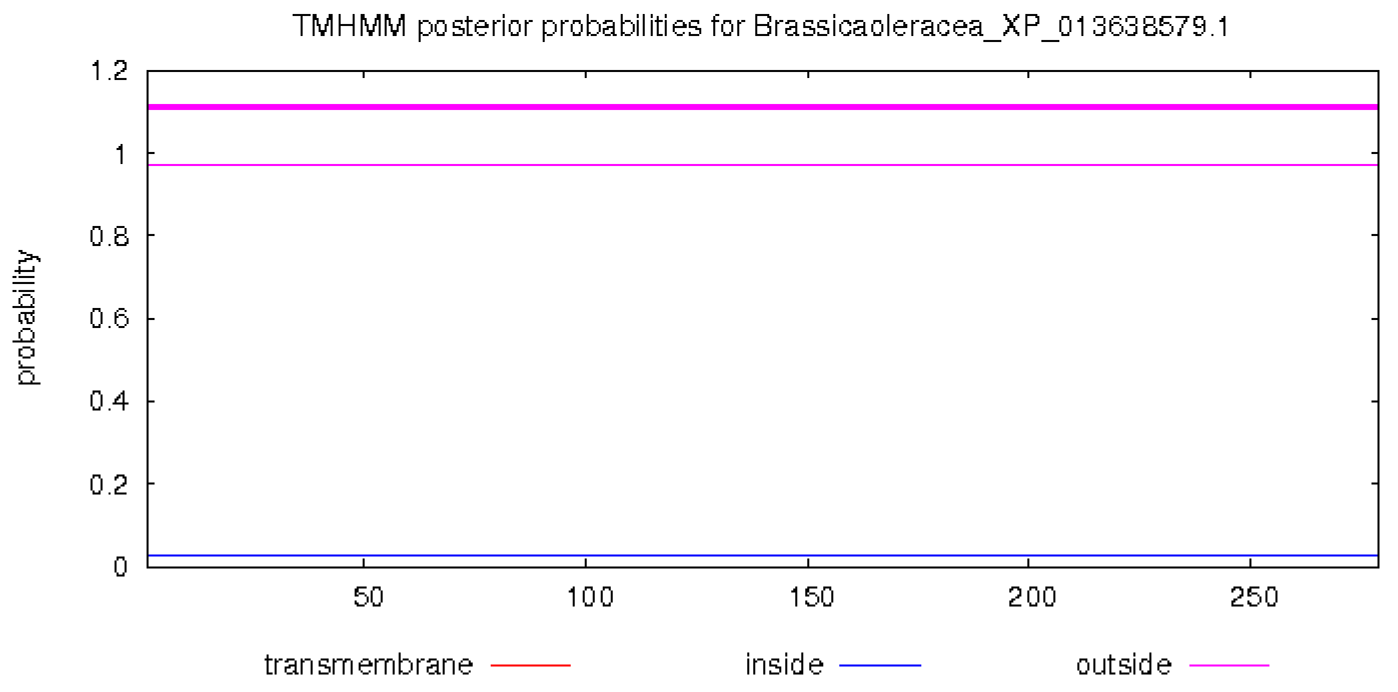

plot in postscript, script for making the plot in gnuplot, data for plot

```
# Brassicaoleracea_XP_013638580.1 Length: 269
# Brassicaoleracea_XP_013638580.1 Number of predicted TMHs:  0
# Brassicaoleracea_XP_013638580.1 Exp number of AAs in TMHs: 0.00138
# Brassicaoleracea_XP_013638580.1 Exp number, first 60 AAs:  0
# Brassicaoleracea_XP_013638580.1 Total prob of N-in:        0.03309
Brassicaoleracea_XP_013638580.1 TMHMM2.0        outside      1   269
```

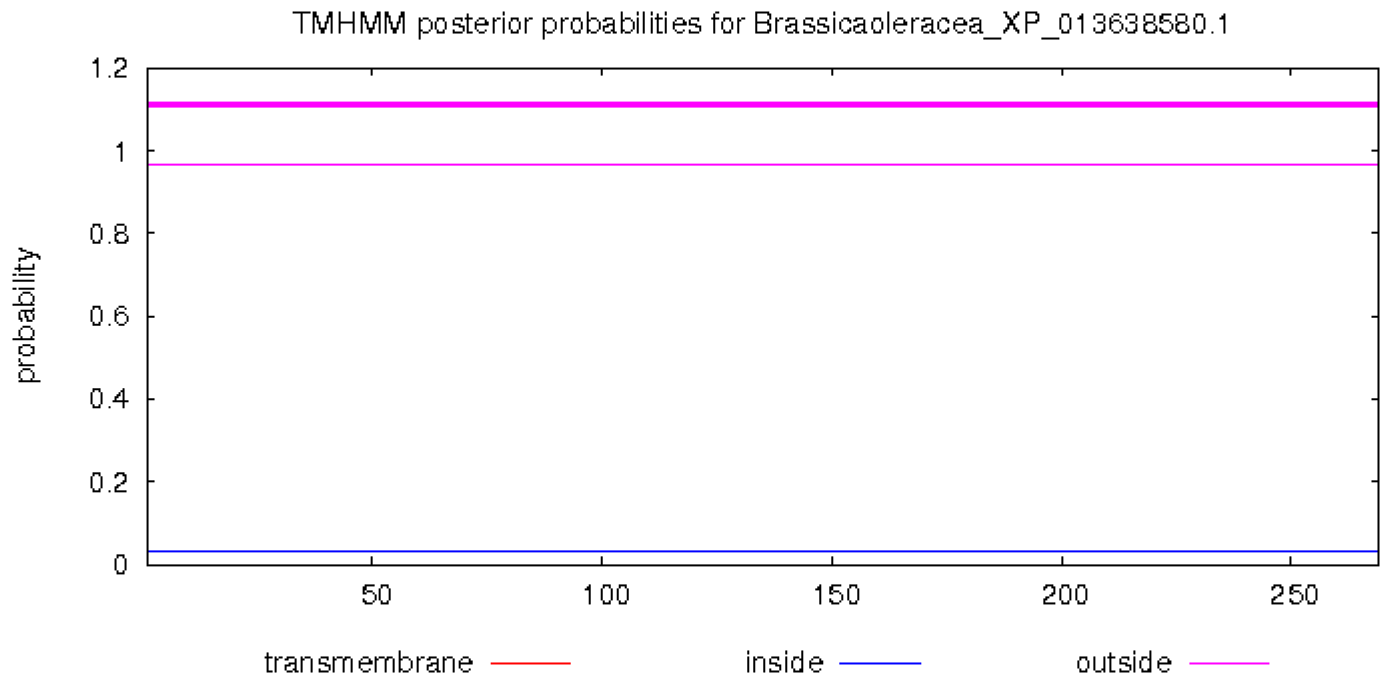

plot in postscript, script for making the plot in gnuplot, data for plot

```
# Brassicaoleracea_XP_013638581.1 Length: 268
# Brassicaoleracea_XP_013638581.1 Number of predicted TMHs:  0
# Brassicaoleracea_XP_013638581.1 Exp number of AAs in TMHs: 0.00151
# Brassicaoleracea_XP_013638581.1 Exp number, first 60 AAs:  0
# Brassicaoleracea_XP_013638581.1 Total prob of N-in:        0.04149
Brassicaoleracea_XP_013638581.1 TMHMM2.0        outside      1   268
```

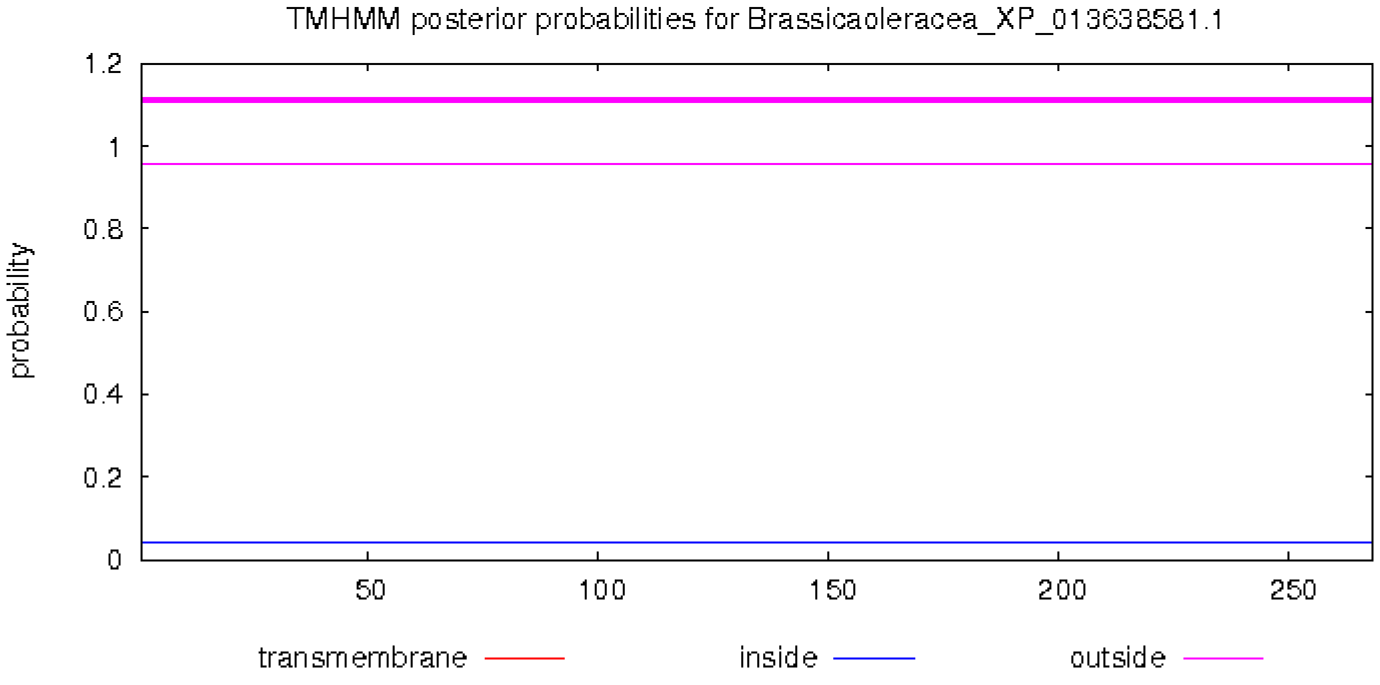

plot in postscript, script for making the plot in gnuplot, data for plot

```
# Brassicaoleracea_XP_013638582.1 Length: 243
# Brassicaoleracea_XP_013638582.1 Number of predicted TMHs:  0
# Brassicaoleracea_XP_013638582.1 Exp number of AAs in TMHs: 0.00161
# Brassicaoleracea_XP_013638582.1 Exp number, first 60 AAs:  0
# Brassicaoleracea_XP_013638582.1 Total prob of N-in:        0.03904
Brassicaoleracea_XP_013638582.1 TMHMM2.0        outside      1   243
```

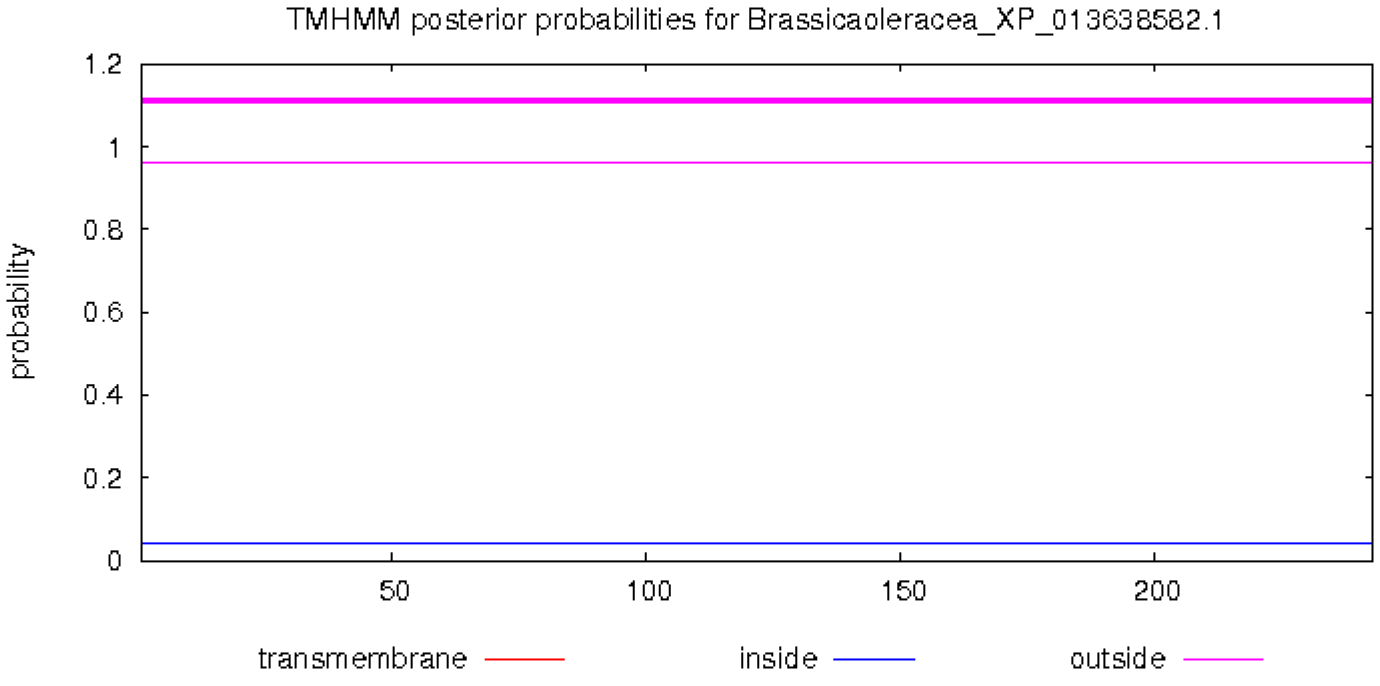

plot in postscript, script for making the plot in gnuplot, data for plot

```
# Brassicaoleracea_XP_013638893.1 Length: 333
# Brassicaoleracea_XP_013638893.1 Number of predicted TMHs:  0
# Brassicaoleracea_XP_013638893.1 Exp number of AAs in TMHs: 0
# Brassicaoleracea_XP_013638893.1 Exp number, first 60 AAs:  0
# Brassicaoleracea_XP_013638893.1 Total prob of N-in:        0.00327
Brassicaoleracea_XP_013638893.1 TMHMM2.0        outside      1   333
```

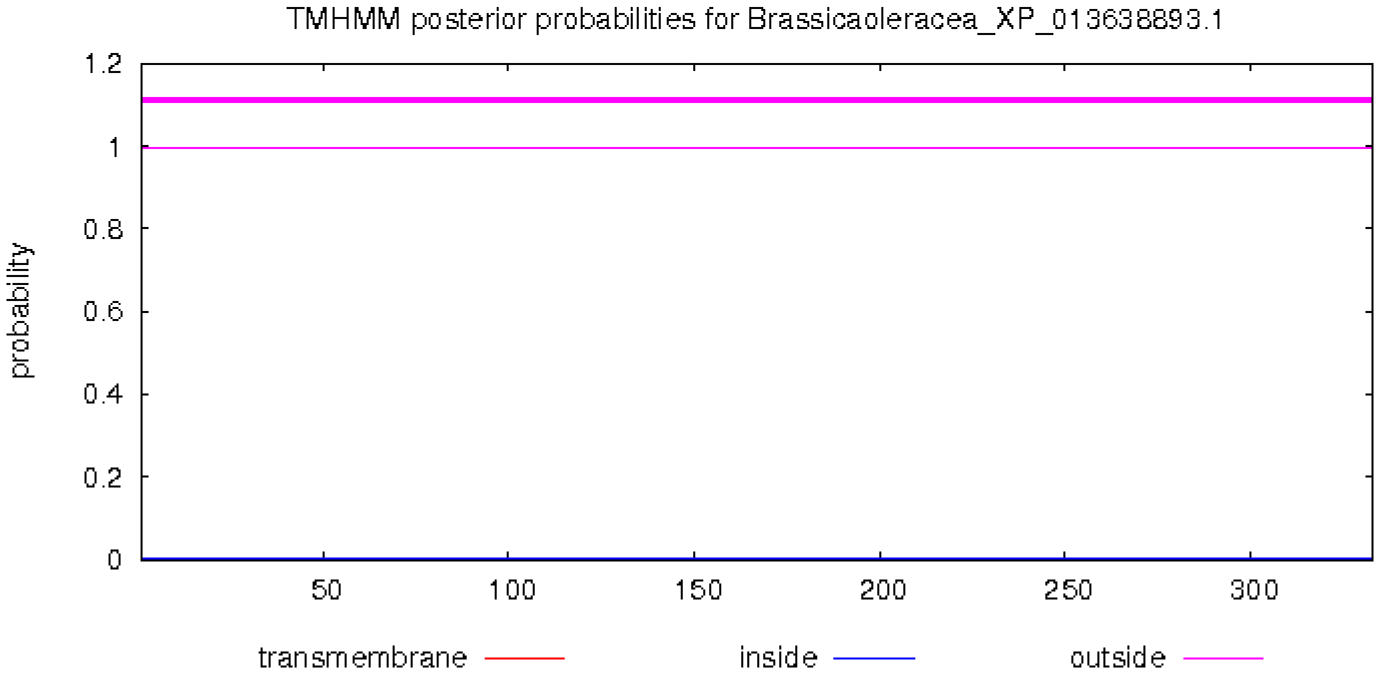

plot in postscript, script for making the plot in gnuplot, data for plot

```
# Brassicaoleracea_XP_013638897.1 Length: 311
# Brassicaoleracea_XP_013638897.1 Number of predicted TMHs:  0
# Brassicaoleracea_XP_013638897.1 Exp number of AAs in TMHs: 0.00021
# Brassicaoleracea_XP_013638897.1 Exp number, first 60 AAs:  0
# Brassicaoleracea_XP_013638897.1 Total prob of N-in:        0.02097
Brassicaoleracea_XP_013638897.1 TMHMM2.0        outside      1   311
```

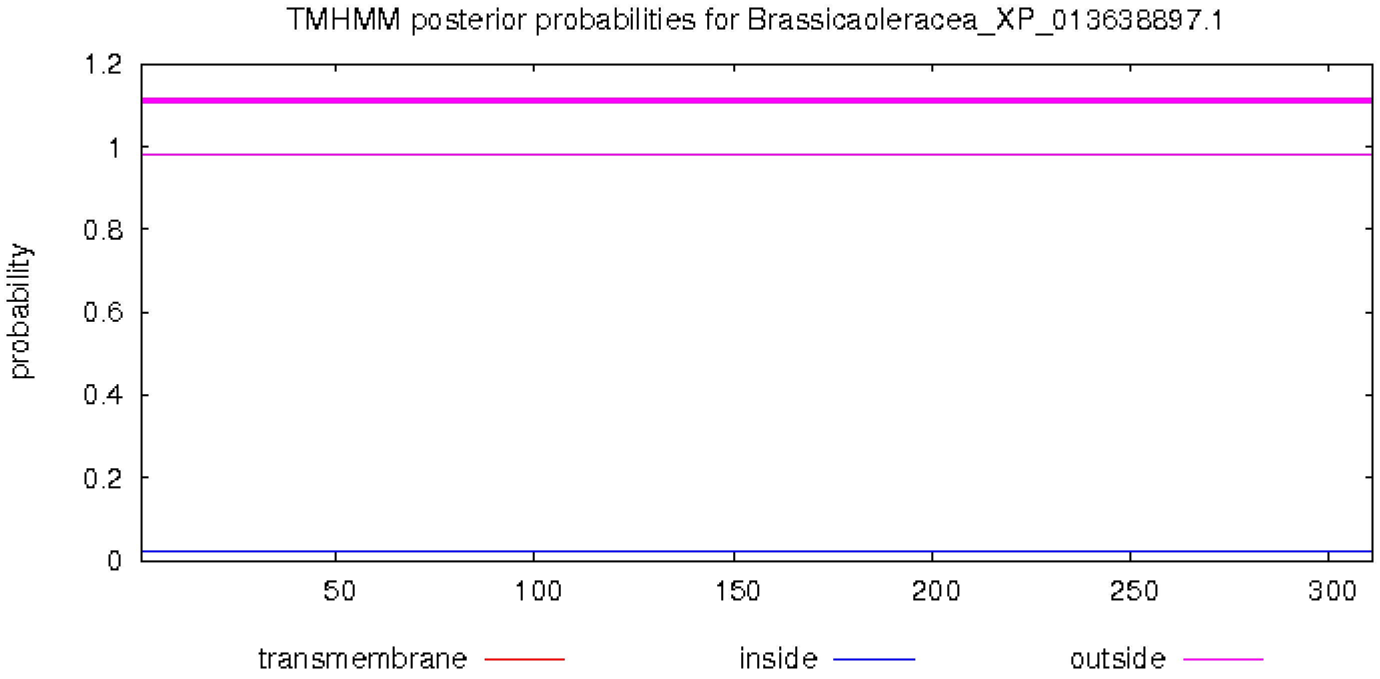

plot in postscript, script for making the plot in gnuplot, data for plot

```
# Brassicaoleracea_XP_013638988.1 Length: 453
# Brassicaoleracea_XP_013638988.1 Number of predicted TMHs:  1
# Brassicaoleracea_XP_013638988.1 Exp number of AAs in TMHs: 21.64588
# Brassicaoleracea_XP_013638988.1 Exp number, first 60 AAs:  0.04558
# Brassicaoleracea_XP_013638988.1 Total prob of N-in:        0.65305
Brassicaoleracea_XP_013638988.1 TMHMM2.0        inside       1   423
Brassicaoleracea_XP_013638988.1 TMHMM2.0        TMhelix    424   446
Brassicaoleracea_XP_013638988.1 TMHMM2.0        outside    447   453
```

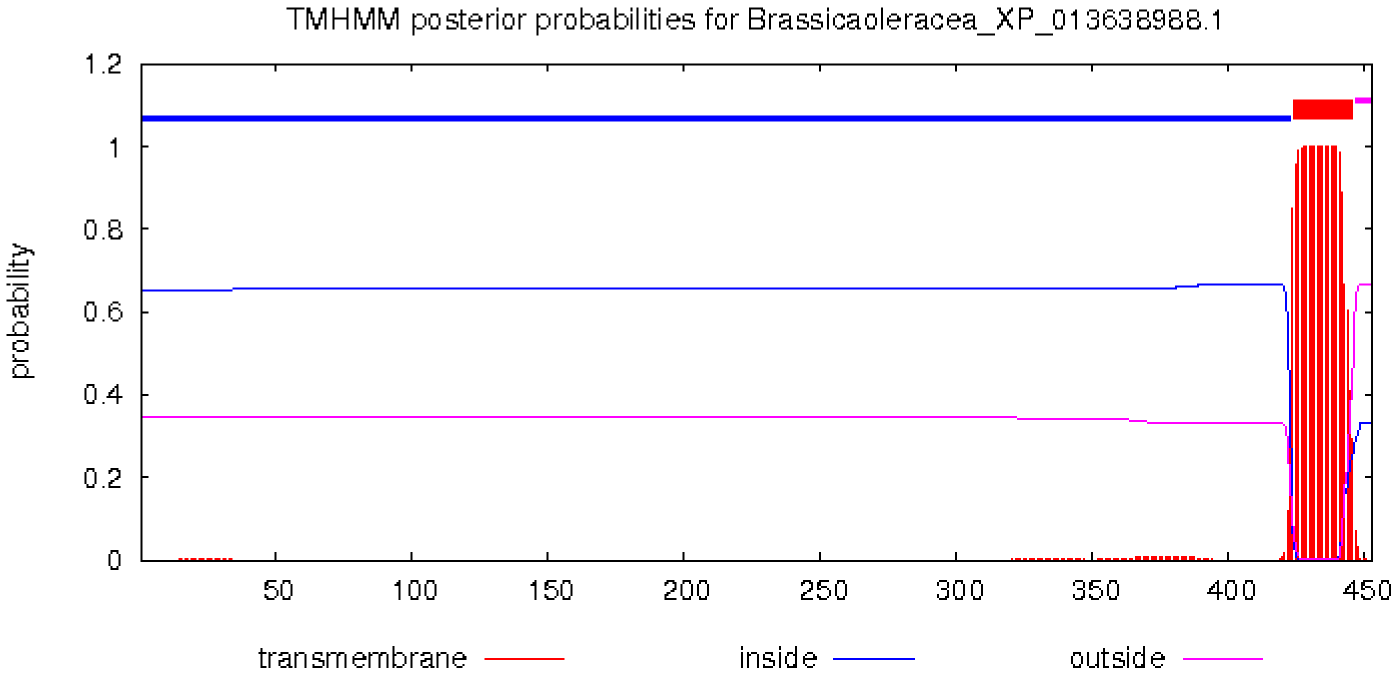

plot in postscript, script for making the plot in gnuplot, data for plot

```
# Brassicaoleracea_XP_013638989.1 Length: 449
# Brassicaoleracea_XP_013638989.1 Number of predicted TMHs:  1
# Brassicaoleracea_XP_013638989.1 Exp number of AAs in TMHs: 21.64904
# Brassicaoleracea_XP_013638989.1 Exp number, first 60 AAs:  0.04633
# Brassicaoleracea_XP_013638989.1 Total prob of N-in:        0.66419
Brassicaoleracea_XP_013638989.1 TMHMM2.0        inside       1   419
Brassicaoleracea_XP_013638989.1 TMHMM2.0        TMhelix    420   442
Brassicaoleracea_XP_013638989.1 TMHMM2.0        outside    443   449
```

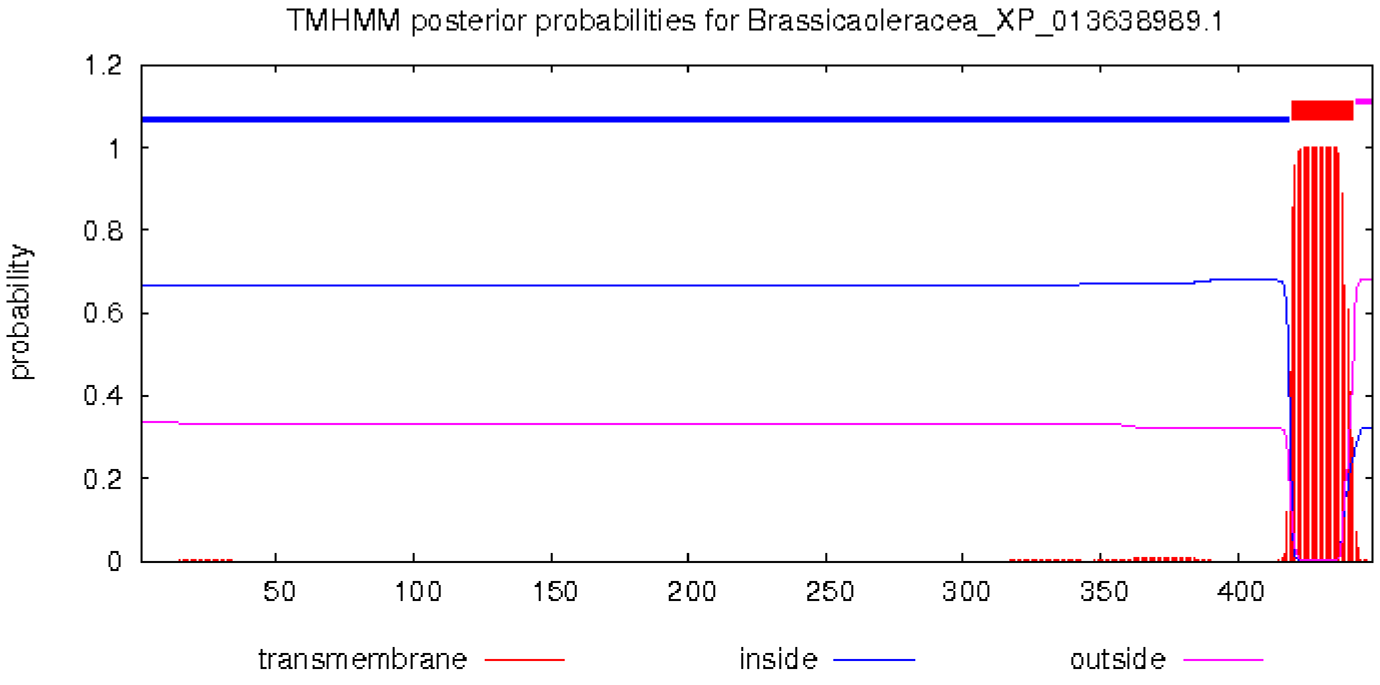

plot in postscript, script for making the plot in gnuplot, data for plot

---

```
# Brassicaoleracea_XP_013639066.1 Length: 259
# Brassicaoleracea_XP_013639066.1 Number of predicted TMHs:  0
# Brassicaoleracea_XP_013639066.1 Exp number of AAs in TMHs: 0.00113
# Brassicaoleracea_XP_013639066.1 Exp number, first 60 AAs:  0.00028
# Brassicaoleracea_XP_013639066.1 Total prob of N-in:        0.01217
Brassicaoleracea_XP_013639066.1 TMHMM2.0        outside      1   259
```

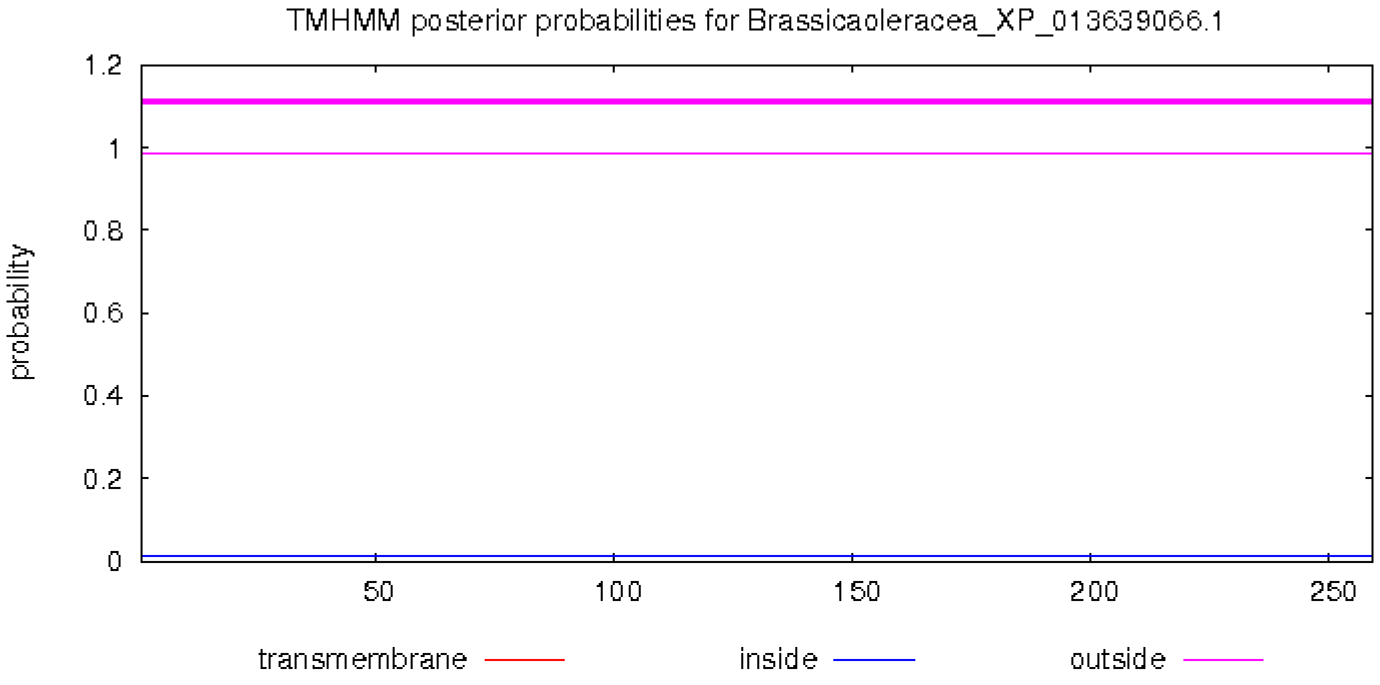

plot in postscript, script for making the plot in gnuplot, data for plot

---

```
# Brassicaoleracea_XP_013639067.1 Length: 365
# Brassicaoleracea_XP_013639067.1 Number of predicted TMHs:  1
# Brassicaoleracea_XP_013639067.1 Exp number of AAs in TMHs: 18.12714
# Brassicaoleracea_XP_013639067.1 Exp number, first 60 AAs:  0
# Brassicaoleracea_XP_013639067.1 Total prob of N-in:        0.55582
Brassicaoleracea_XP_013639067.1 TMHMM2.0        inside       1   343
```

```
Brassicaoleracea_XP_013639067.1 TMHMM2.0        TMhelix    344   363
Brassicaoleracea_XP_013639067.1 TMHMM2.0        outside    364   365
```

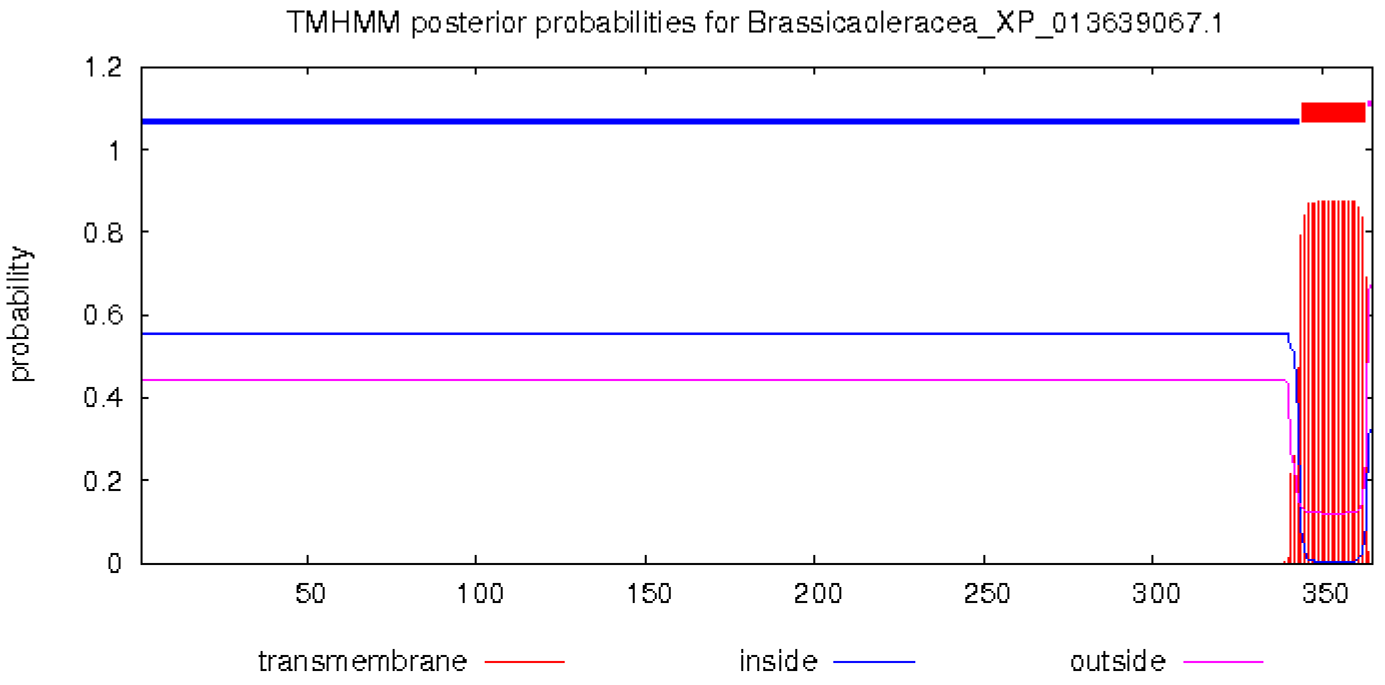

plot in postscript, script for making the plot in gnuplot, data for plot

---

```
# Brassicaoleracea_XP_013639071.1 Length: 233
# Brassicaoleracea_XP_013639071.1 Number of predicted TMHs:  0
# Brassicaoleracea_XP_013639071.1 Exp number of AAs in TMHs: 0.00036
# Brassicaoleracea_XP_013639071.1 Exp number, first 60 AAs:  0.00022
# Brassicaoleracea_XP_013639071.1 Total prob of N-in:        0.05365
Brassicaoleracea_XP_013639071.1 TMHMM2.0        outside      1   233
```

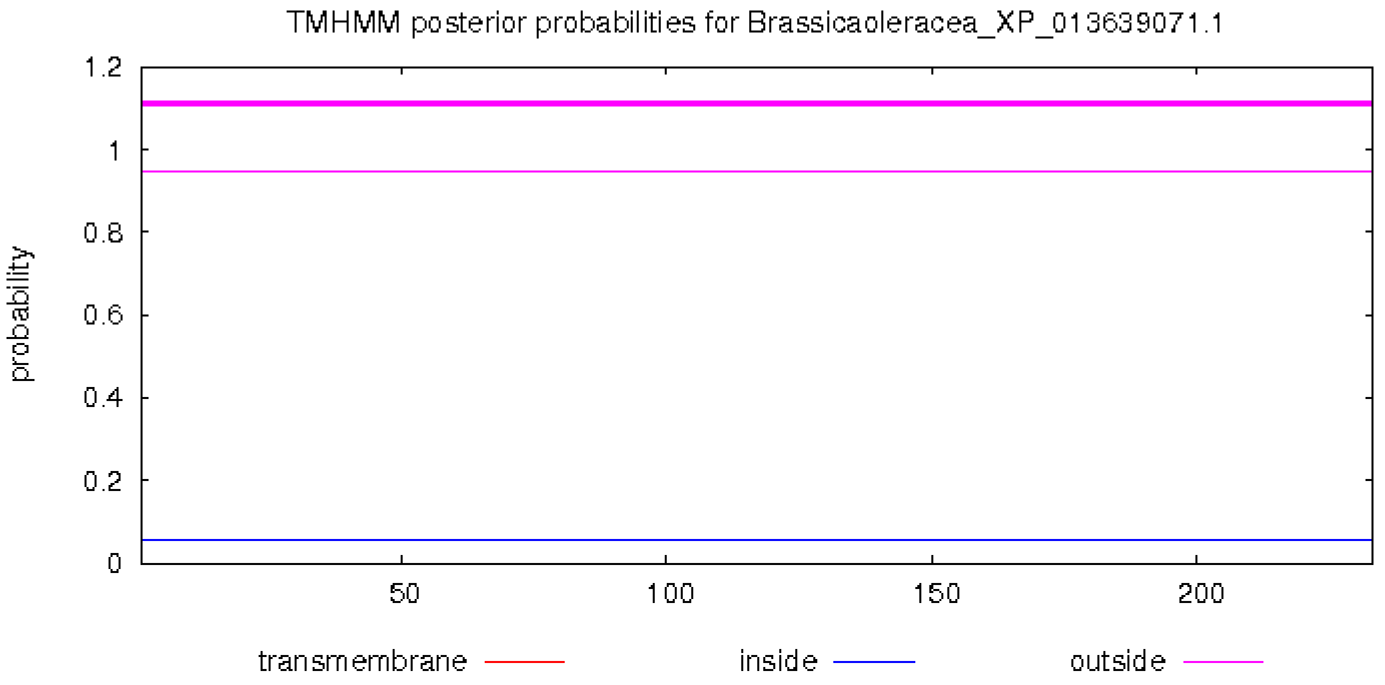

plot in postscript, script for making the plot in gnuplot, data for plot

---

```
# Brassicaoleracea_XP_013639072.1 Length: 378
# Brassicaoleracea_XP_013639072.1 Number of predicted TMHs:  0
# Brassicaoleracea_XP_013639072.1 Exp number of AAs in TMHs: 0.00018
# Brassicaoleracea_XP_013639072.1 Exp number, first 60 AAs:  0
```

```
# Brassicaoleracea_XP_013639072.1 Total prob of N-in:        0.00760
Brassicaoleracea_XP_013639072.1 TMHMM2.0        outside      1   378
```

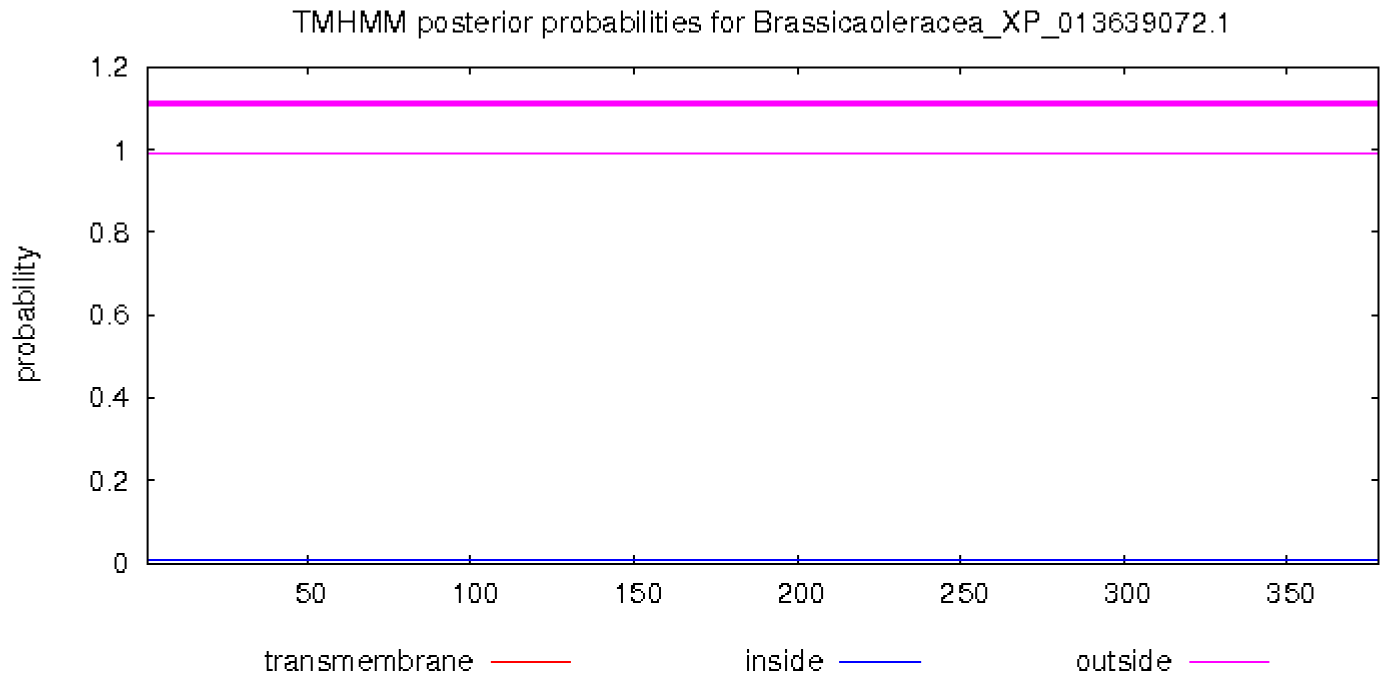

plot in postscript, script for making the plot in gnuplot, data for plot

---

```
# Brassicaoleracea_XP_013639075.1 Length: 155
# Brassicaoleracea_XP_013639075.1 Number of predicted TMHs:  0
# Brassicaoleracea_XP_013639075.1 Exp number of AAs in TMHs: 0.00024
# Brassicaoleracea_XP_013639075.1 Exp number, first 60 AAs:  0
# Brassicaoleracea_XP_013639075.1 Total prob of N-in:        0.05377
Brassicaoleracea_XP_013639075.1 TMHMM2.0        outside      1   155
```

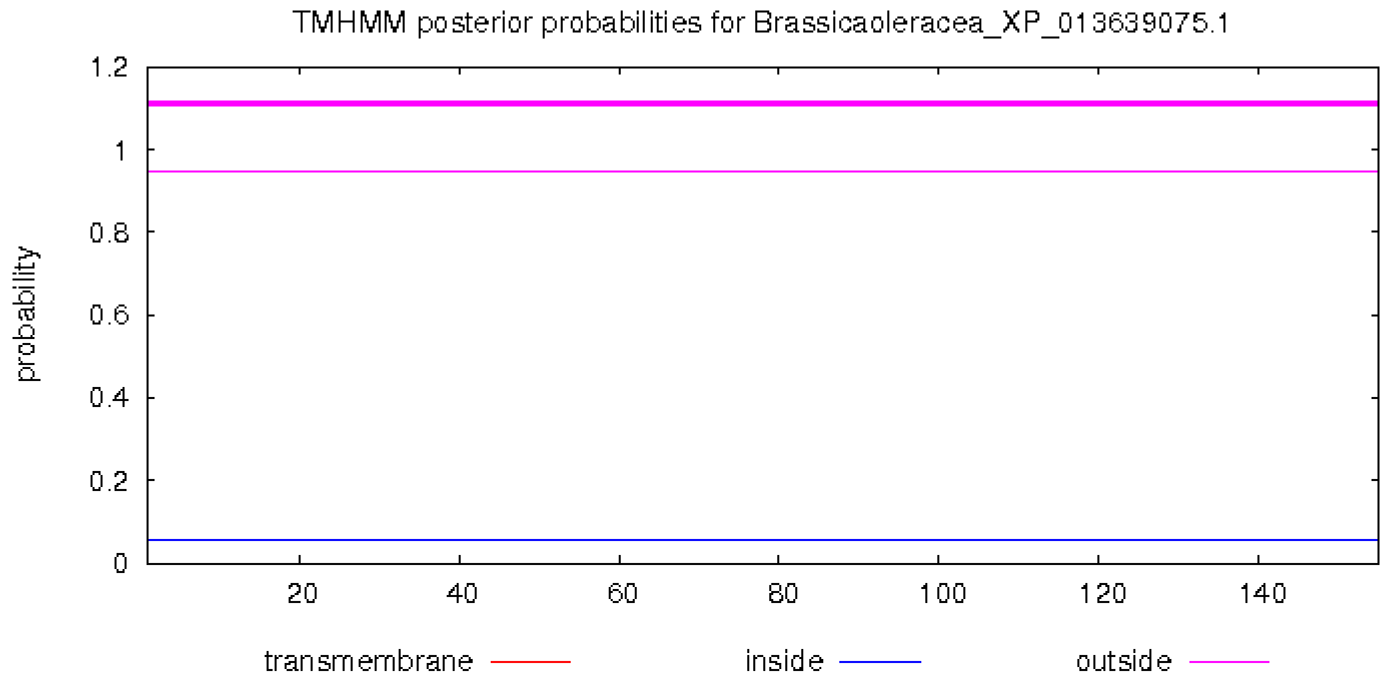

plot in postscript, script for making the plot in gnuplot, data for plot

---

```
# Brassicaoleracea_XP_013639076.1 Length: 394
# Brassicaoleracea_XP_013639076.1 Number of predicted TMHs:  0
# Brassicaoleracea_XP_013639076.1 Exp number of AAs in TMHs: 0.00056
# Brassicaoleracea_XP_013639076.1 Exp number, first 60 AAs:  0.00033
```

```
# Brassicaoleracea_XP_013639076.1 Total prob of N-in:        0.00496
Brassicaoleracea_XP_013639076.1 TMHMM2.0        outside      1   394
```

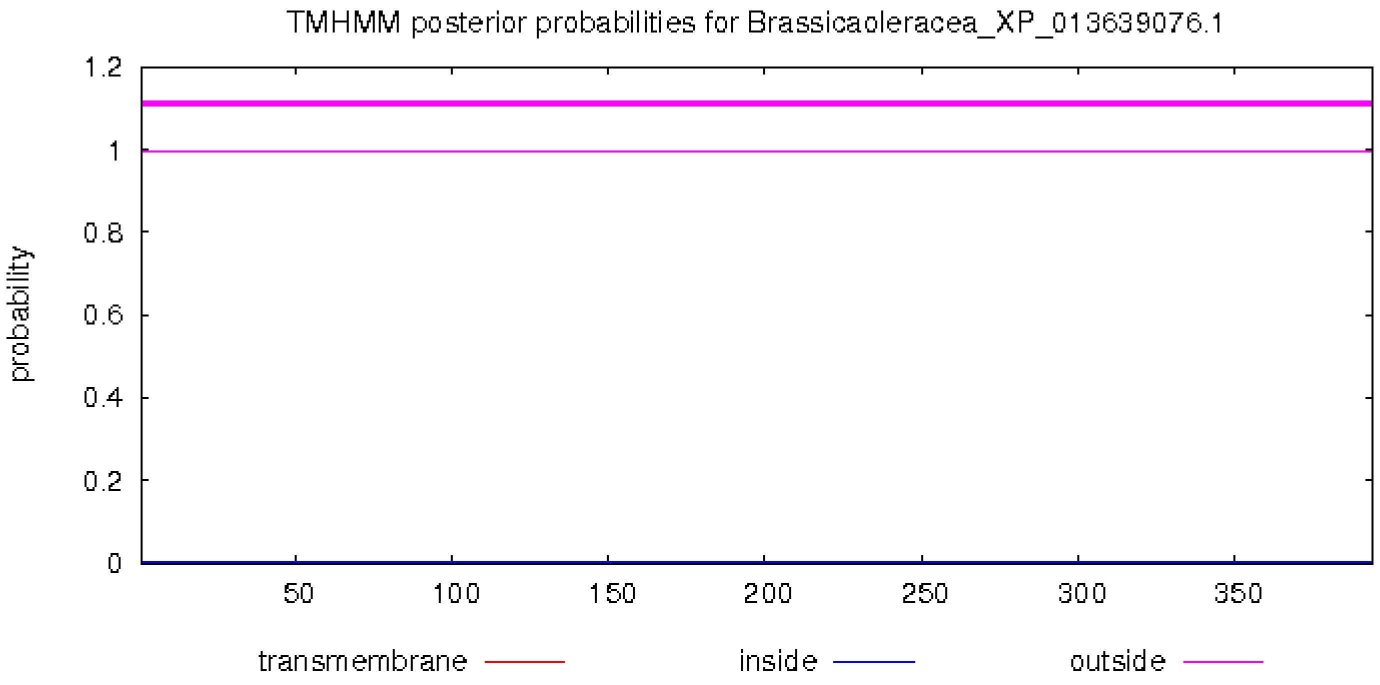

plot in postscript, script for making the plot in gnuplot, data for plot

---

```
# Brassicaoleracea_XP_013639422.1 Length: 339
# Brassicaoleracea_XP_013639422.1 Number of predicted TMHs:  1
# Brassicaoleracea_XP_013639422.1 Exp number of AAs in TMHs: 18.02341
# Brassicaoleracea_XP_013639422.1 Exp number, first 60 AAs:  0.19608
# Brassicaoleracea_XP_013639422.1 Total prob of N-in:        0.30510
Brassicaoleracea_XP_013639422.1 TMHMM2.0        outside      1   320
Brassicaoleracea_XP_013639422.1 TMHMM2.0        TMhelix    321   338
Brassicaoleracea_XP_013639422.1 TMHMM2.0        inside     339   339
```

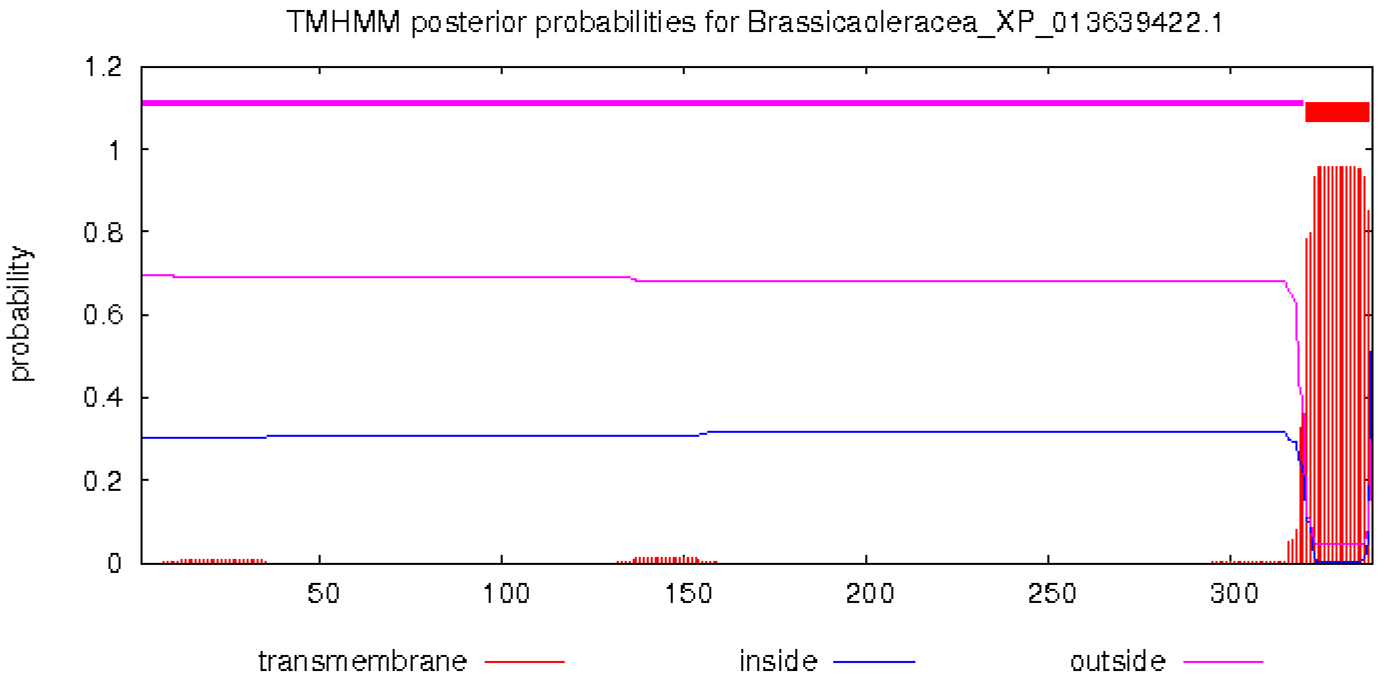

plot in postscript, script for making the plot in gnuplot, data for plot

---

```
# Brassicaoleracea_XP_013639424.1 Length: 336
# Brassicaoleracea_XP_013639424.1 Number of predicted TMHs:  1
```

```
# Brassicaoleracea_XP_013639424.1 Exp number of AAs in TMHs: 17.82496
# Brassicaoleracea_XP_013639424.1 Exp number, first 60 AAs:  0.19742
# Brassicaoleracea_XP_013639424.1 Total prob of N-in:        0.31544
Brassicaoleracea_XP_013639424.1 TMHMM2.0        outside      1   317
Brassicaoleracea_XP_013639424.1 TMHMM2.0        TMhelix    318   335
Brassicaoleracea_XP_013639424.1 TMHMM2.0        inside     336   336
```

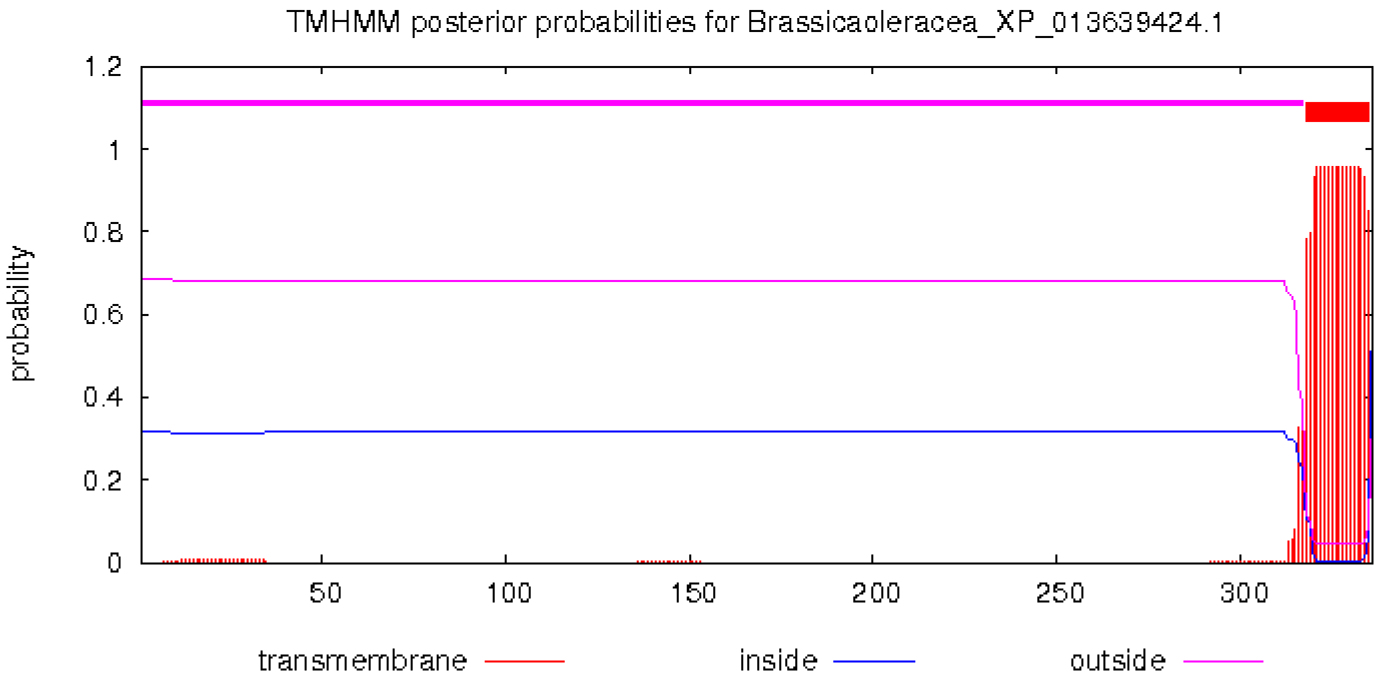

plot in postscript, script for making the plot in gnuplot, data for plot

---

```
# Brassicaoleracea_XP_013639656.1 Length: 538
# Brassicaoleracea_XP_013639656.1 Number of predicted TMHs:  0
# Brassicaoleracea_XP_013639656.1 Exp number of AAs in TMHs: 0.00021
# Brassicaoleracea_XP_013639656.1 Exp number, first 60 AAs:  0
# Brassicaoleracea_XP_013639656.1 Total prob of N-in:        0.00155
Brassicaoleracea_XP_013639656.1 TMHMM2.0        outside      1   538
```

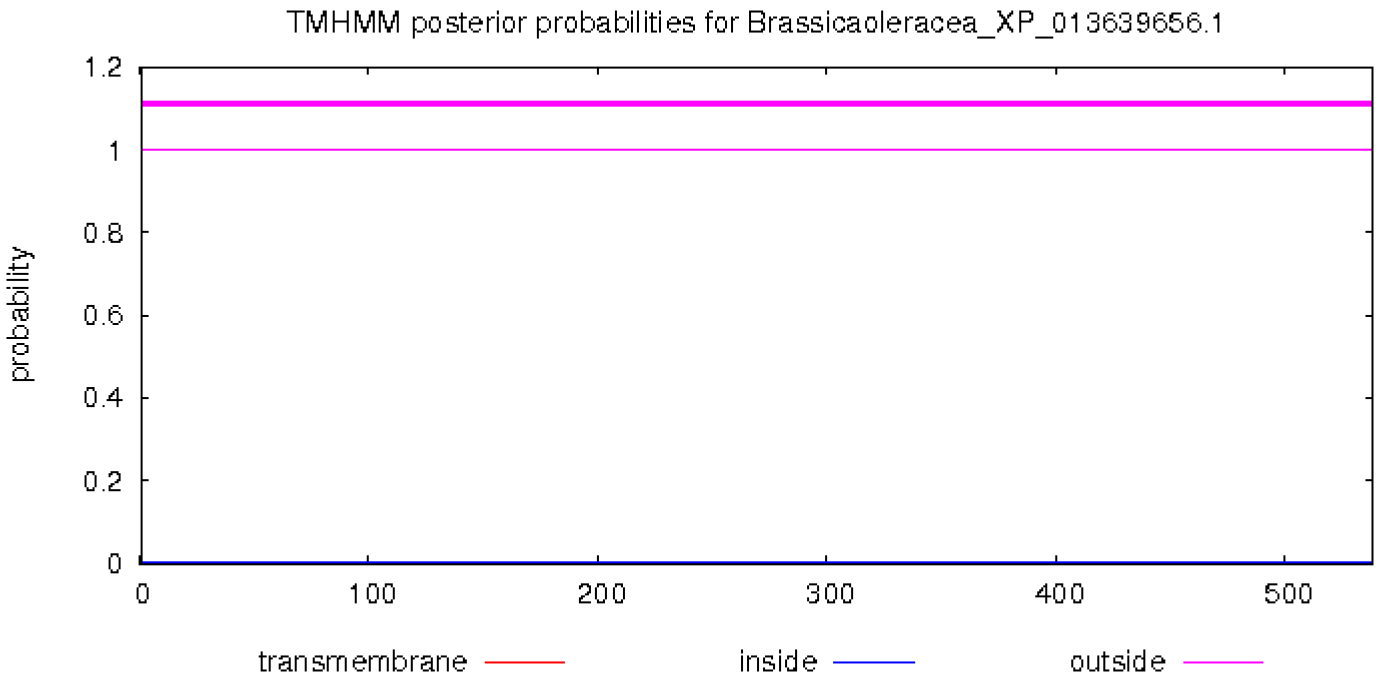

plot in postscript, script for making the plot in gnuplot, data for plot

---

```
# Brassicaoleracea_XP_013639766.1 Length: 355
# Brassicaoleracea_XP_013639766.1 Number of predicted TMHs:  0
# Brassicaoleracea_XP_013639766.1 Exp number of AAs in TMHs: 0.00036
# Brassicaoleracea_XP_013639766.1 Exp number, first 60 AAs:  0.00036
# Brassicaoleracea_XP_013639766.1 Total prob of N-in:        0.01043
Brassicaoleracea_XP_013639766.1 TMHMM2.0        outside      1   355
```

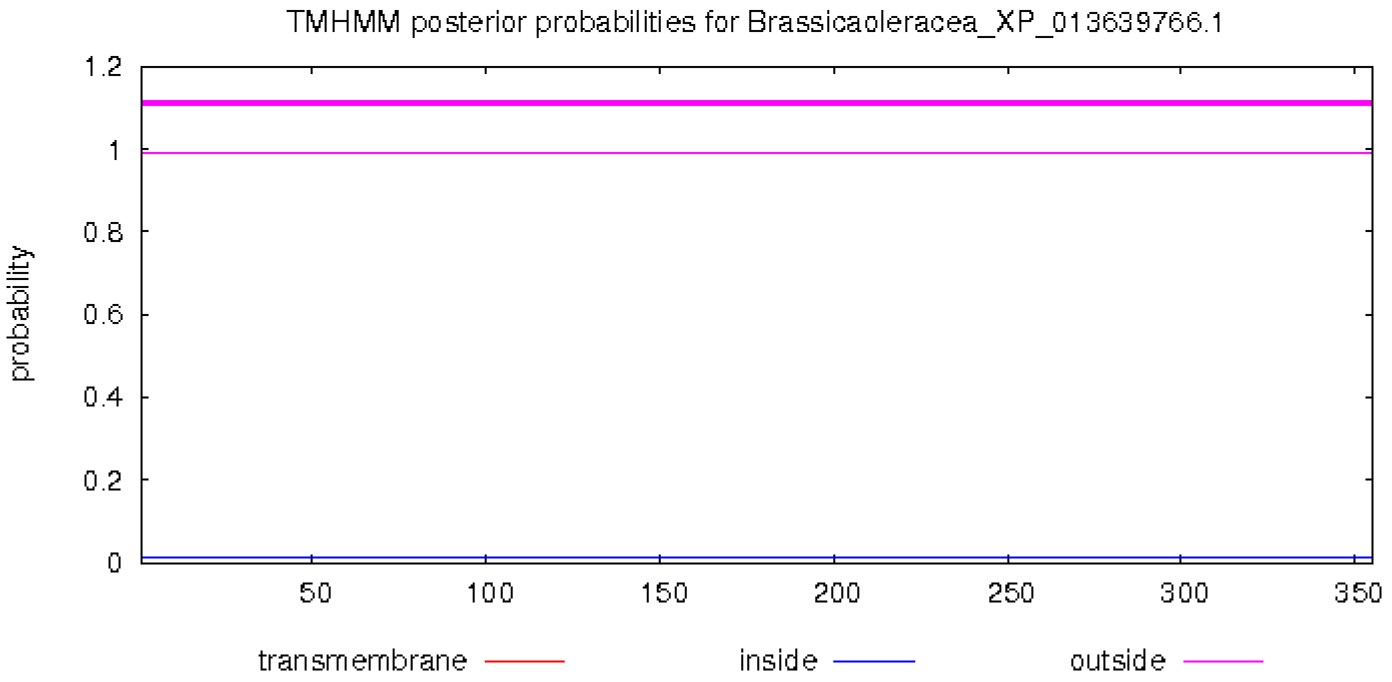

plot in postscript, script for making the plot in gnuplot, data for plot

---

```
# Brassicaoleracea_XP_013639829.1 Length: 344
# Brassicaoleracea_XP_013639829.1 Number of predicted TMHs:  0
# Brassicaoleracea_XP_013639829.1 Exp number of AAs in TMHs: 0.00077
# Brassicaoleracea_XP_013639829.1 Exp number, first 60 AAs:  0
# Brassicaoleracea_XP_013639829.1 Total prob of N-in:        0.00454
Brassicaoleracea_XP_013639829.1 TMHMM2.0        outside      1   344
```

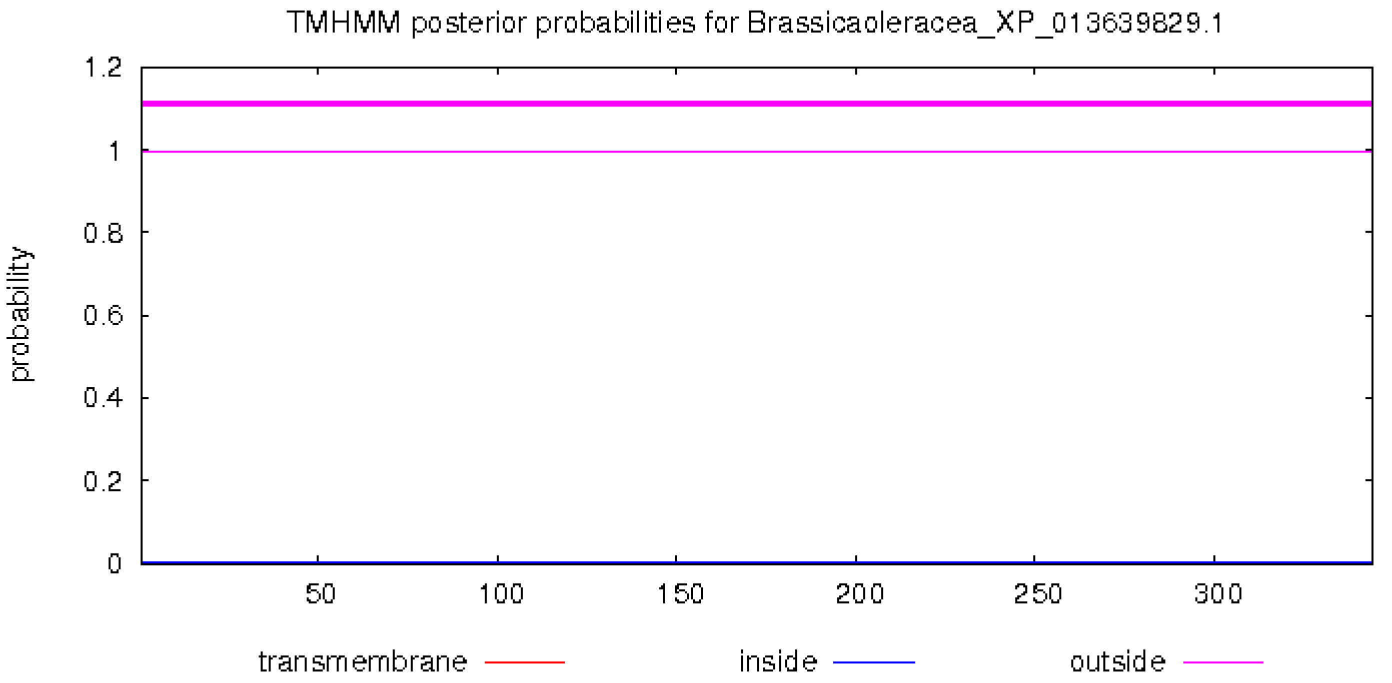

plot in postscript, script for making the plot in gnuplot, data for plot

---

```
# Brassicarapa_XP_009101257.1 Length: 419
# Brassicarapa_XP_009101257.1 Number of predicted TMHs:  0
# Brassicarapa_XP_009101257.1 Exp number of AAs in TMHs: 0.03429
# Brassicarapa_XP_009101257.1 Exp number, first 60 AAs:  0
# Brassicarapa_XP_009101257.1 Total prob of N-in:        0.00857
Brassicarapa_XP_009101257.1     TMHMM2.0        outside      1   419
```

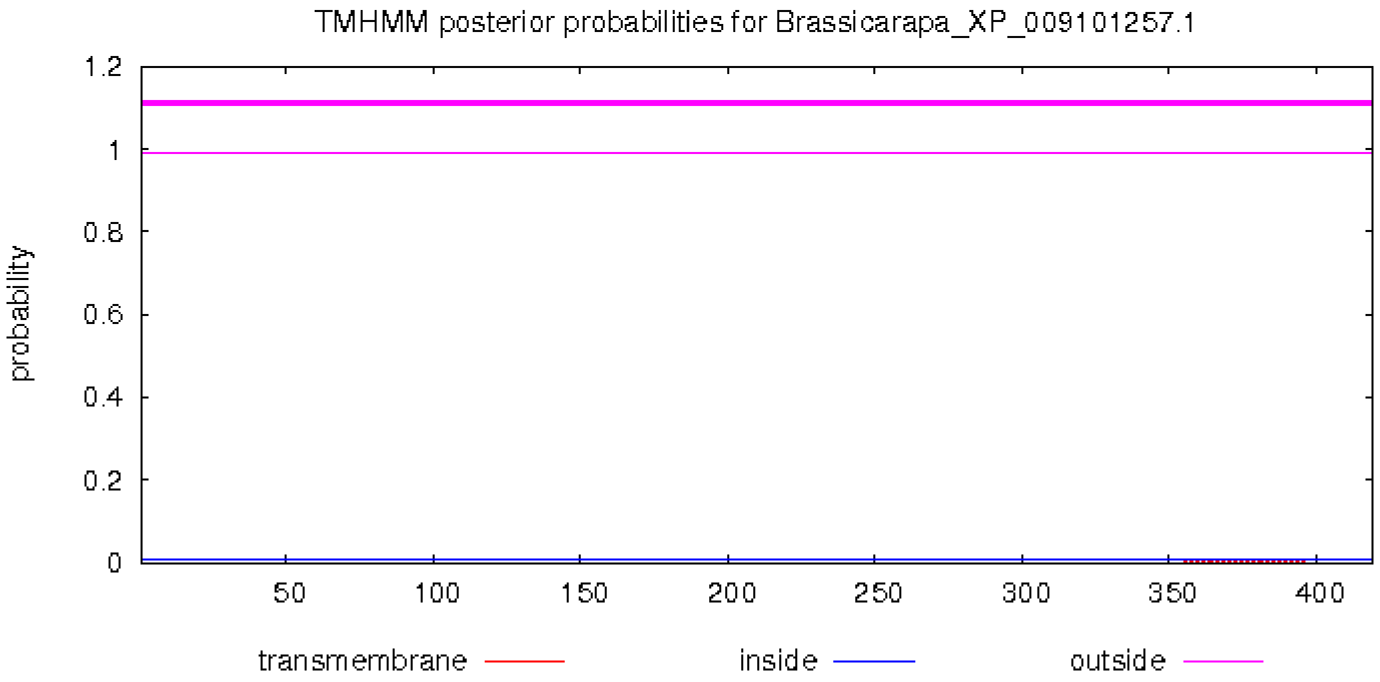

plot in postscript, script for making the plot in gnuplot, data for plot

```
# Brassicarapa_XP_009101506.1 Length: 290
# Brassicarapa_XP_009101506.1 Number of predicted TMHs:  0
# Brassicarapa_XP_009101506.1 Exp number of AAs in TMHs: 0
# Brassicarapa_XP_009101506.1 Exp number, first 60 AAs:  0
# Brassicarapa_XP_009101506.1 Total prob of N-in:        0.00159
Brassicarapa_XP_009101506.1     TMHMM2.0        outside      1   290
```

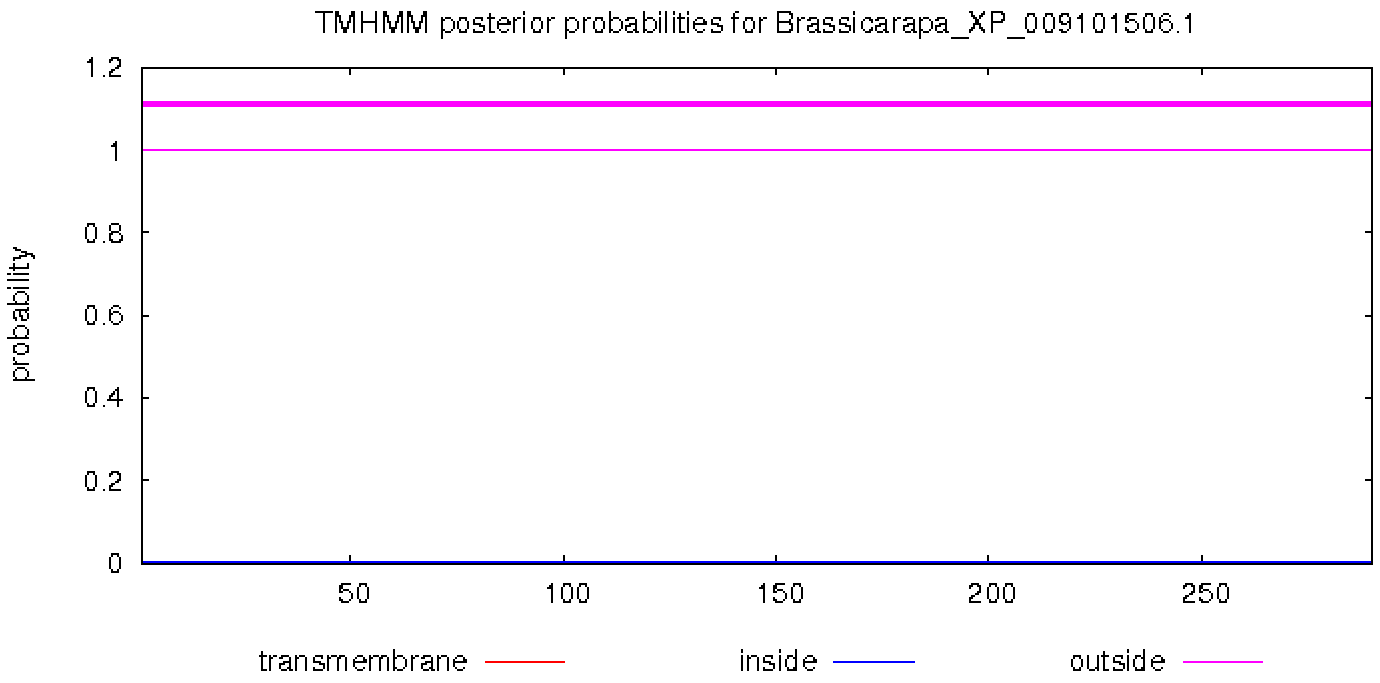

plot in postscript, script for making the plot in gnuplot, data for plot

```
# Brassicarapa_XP_009102282.1 Length: 361
# Brassicarapa_XP_009102282.1 Number of predicted TMHs:  0
# Brassicarapa_XP_009102282.1 Exp number of AAs in TMHs: 0
# Brassicarapa_XP_009102282.1 Exp number, first 60 AAs:  0
# Brassicarapa_XP_009102282.1 Total prob of N-in:        0.00206
Brassicarapa_XP_009102282.1     TMHMM2.0        outside      1   361
```

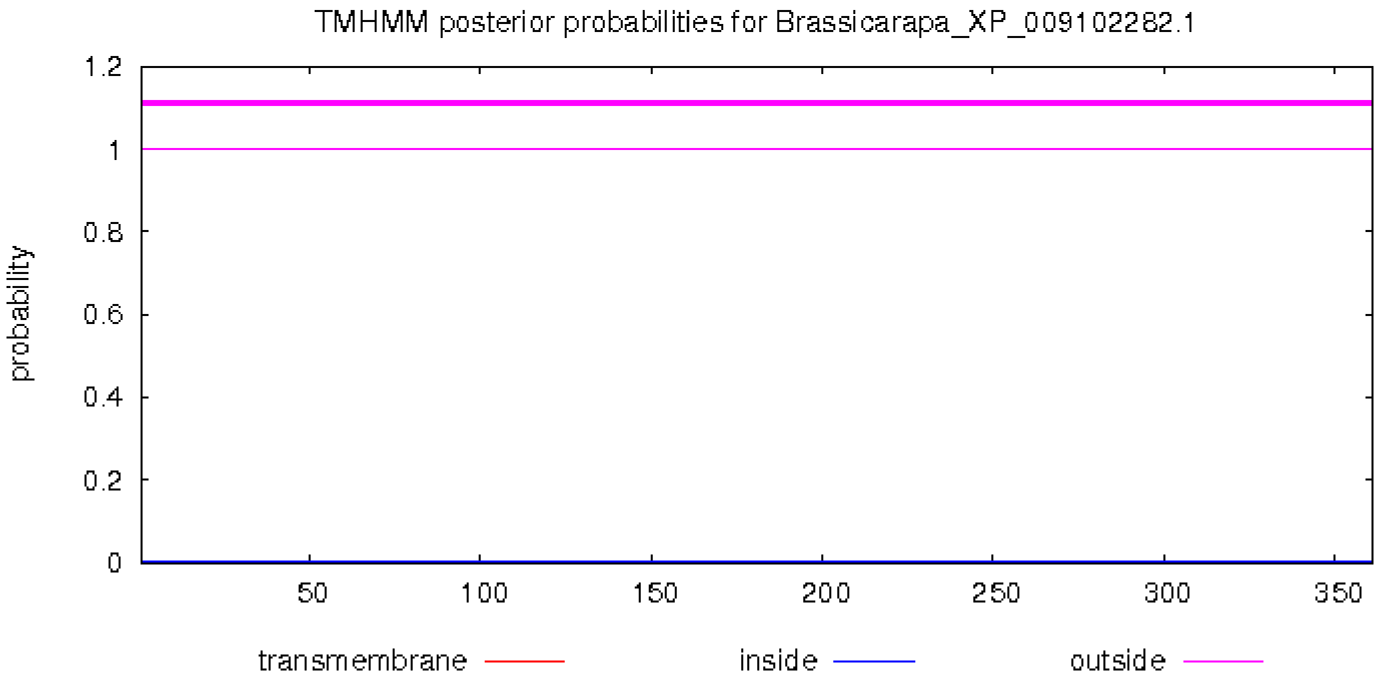

plot in postscript, script for making the plot in gnuplot, data for plot

```
# Brassicarapa_XP_009102422.1 Length: 380
# Brassicarapa_XP_009102422.1 Number of predicted TMHs:  0
# Brassicarapa_XP_009102422.1 Exp number of AAs in TMHs: 0.00048
# Brassicarapa_XP_009102422.1 Exp number, first 60 AAs:  0.00019
# Brassicarapa_XP_009102422.1 Total prob of N-in:        0.00827
Brassicarapa_XP_009102422.1     TMHMM2.0        outside      1   380
```

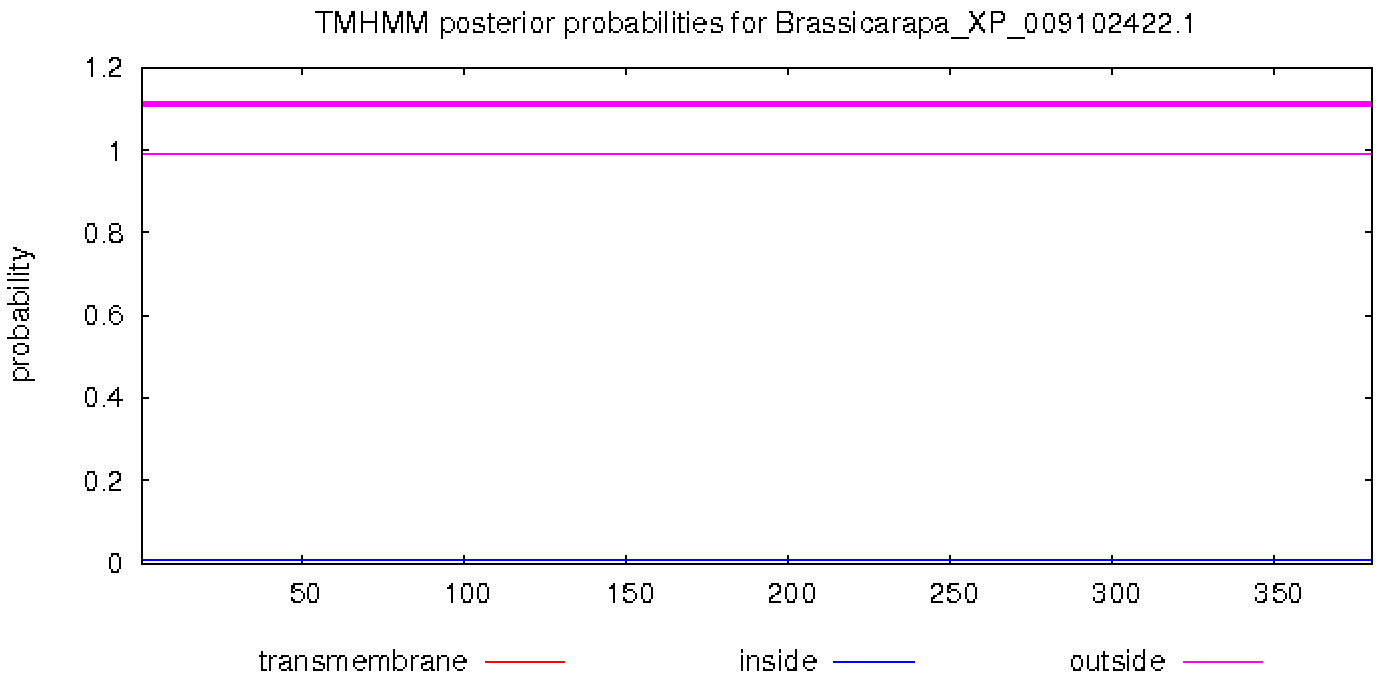

plot in postscript, script for making the plot in gnuplot, data for plot

```
# Brassicarapa_XP_009102423.1 Length: 380
# Brassicarapa_XP_009102423.1 Number of predicted TMHs:  0
# Brassicarapa_XP_009102423.1 Exp number of AAs in TMHs: 0.00048
# Brassicarapa_XP_009102423.1 Exp number, first 60 AAs:  0.00019
# Brassicarapa_XP_009102423.1 Total prob of N-in:        0.00827
Brassicarapa_XP_009102423.1     TMHMM2.0        outside      1   380
```

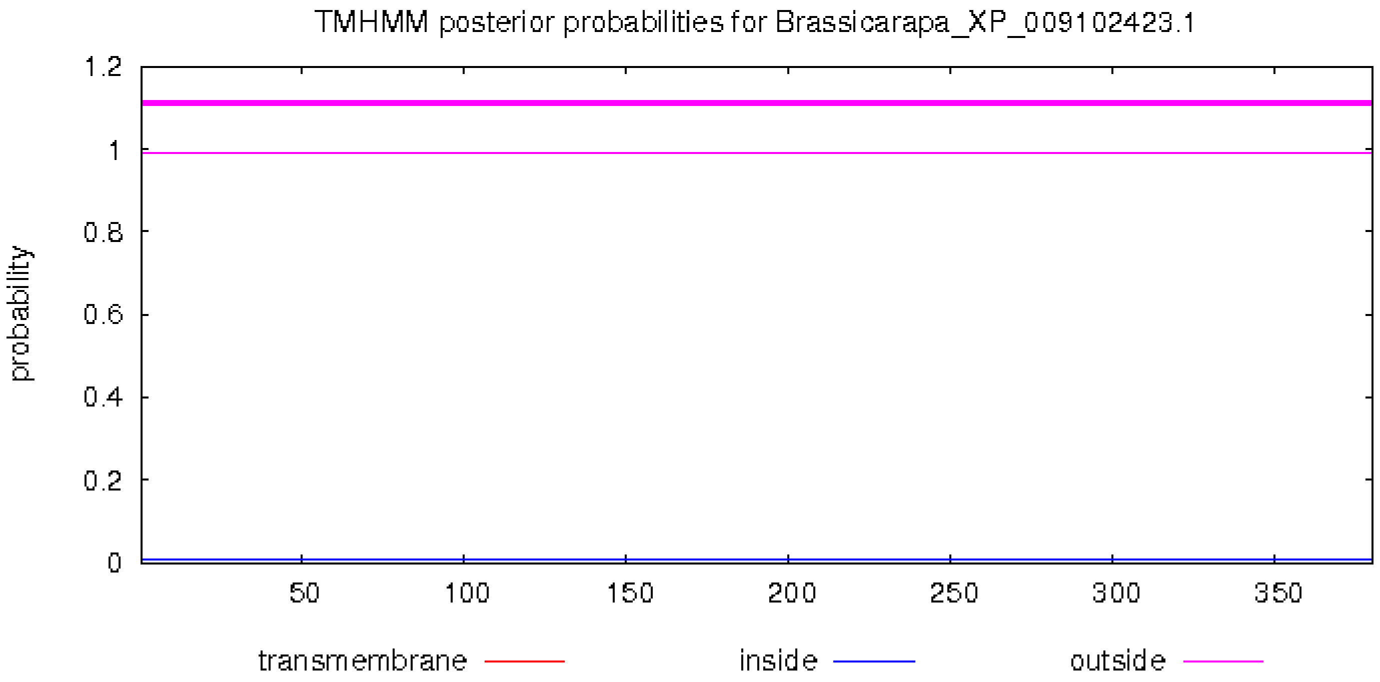

plot in postscript, script for making the plot in gnuplot, data for plot

---

```
# Brassicarapa_XP_009102424.1 Length: 380
# Brassicarapa_XP_009102424.1 Number of predicted TMHs:  0
# Brassicarapa_XP_009102424.1 Exp number of AAs in TMHs: 0.00048
# Brassicarapa_XP_009102424.1 Exp number, first 60 AAs:  0.00019
# Brassicarapa_XP_009102424.1 Total prob of N-in:        0.00827
Brassicarapa_XP_009102424.1     TMHMM2.0        outside      1   380
```

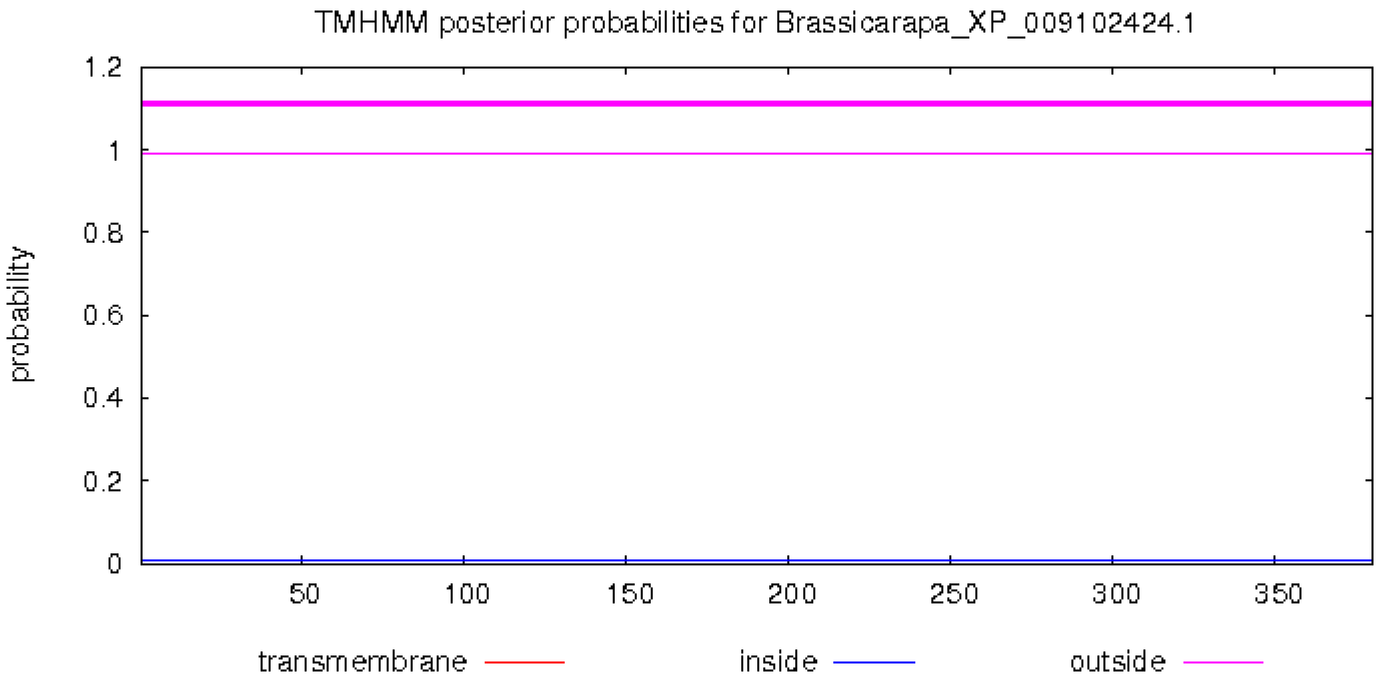

plot in postscript, script for making the plot in gnuplot, data for plot

---

```
# Brassicarapa_XP_009102425.1 Length: 380
# Brassicarapa_XP_009102425.1 Number of predicted TMHs:  0
# Brassicarapa_XP_009102425.1 Exp number of AAs in TMHs: 0.00048
# Brassicarapa_XP_009102425.1 Exp number, first 60 AAs:  0.00019
# Brassicarapa_XP_009102425.1 Total prob of N-in:        0.00827
Brassicarapa_XP_009102425.1     TMHMM2.0        outside      1   380
```

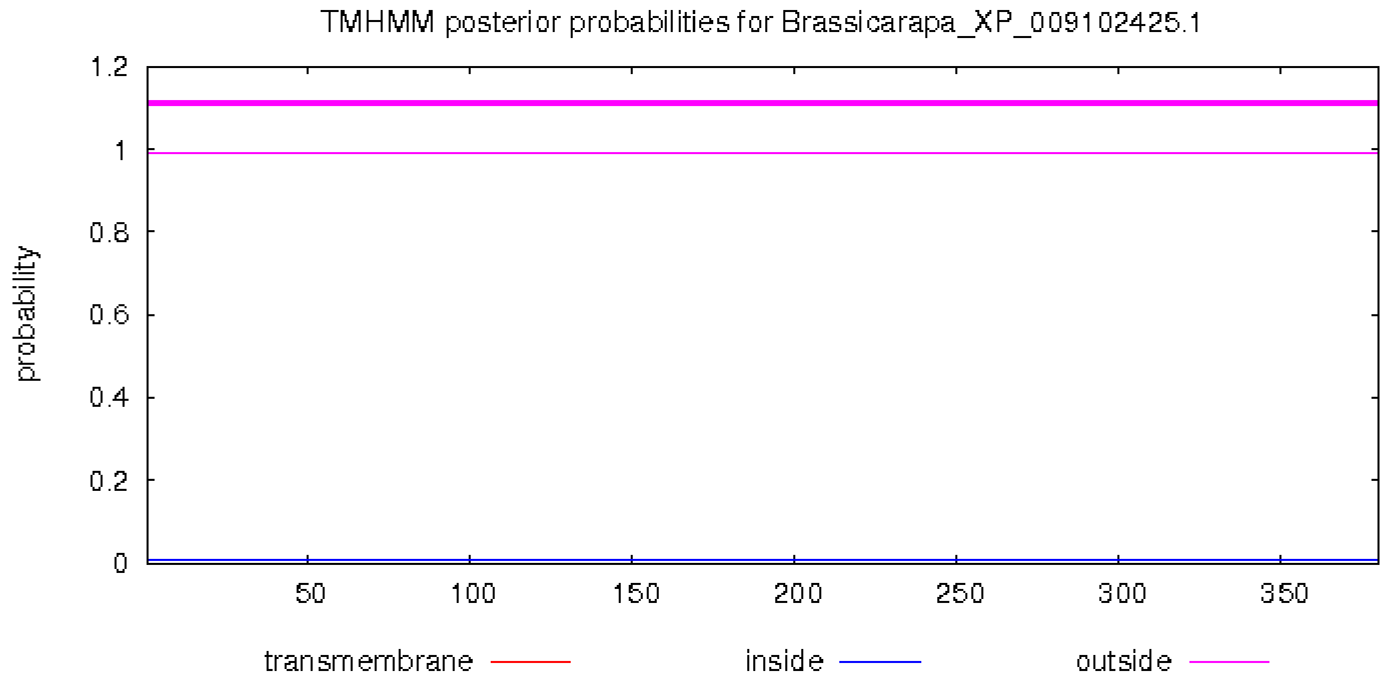

plot in postscript, script for making the plot in gnuplot, data for plot

---

```
# Brassicarapa_XP_009102906.1 Length: 313
# Brassicarapa_XP_009102906.1 Number of predicted TMHs:  0
# Brassicarapa_XP_009102906.1 Exp number of AAs in TMHs: 0.07175
# Brassicarapa_XP_009102906.1 Exp number, first 60 AAs:  0.00015
# Brassicarapa_XP_009102906.1 Total prob of N-in:        0.07531
Brassicarapa_XP_009102906.1     TMHMM2.0        outside      1   313
```

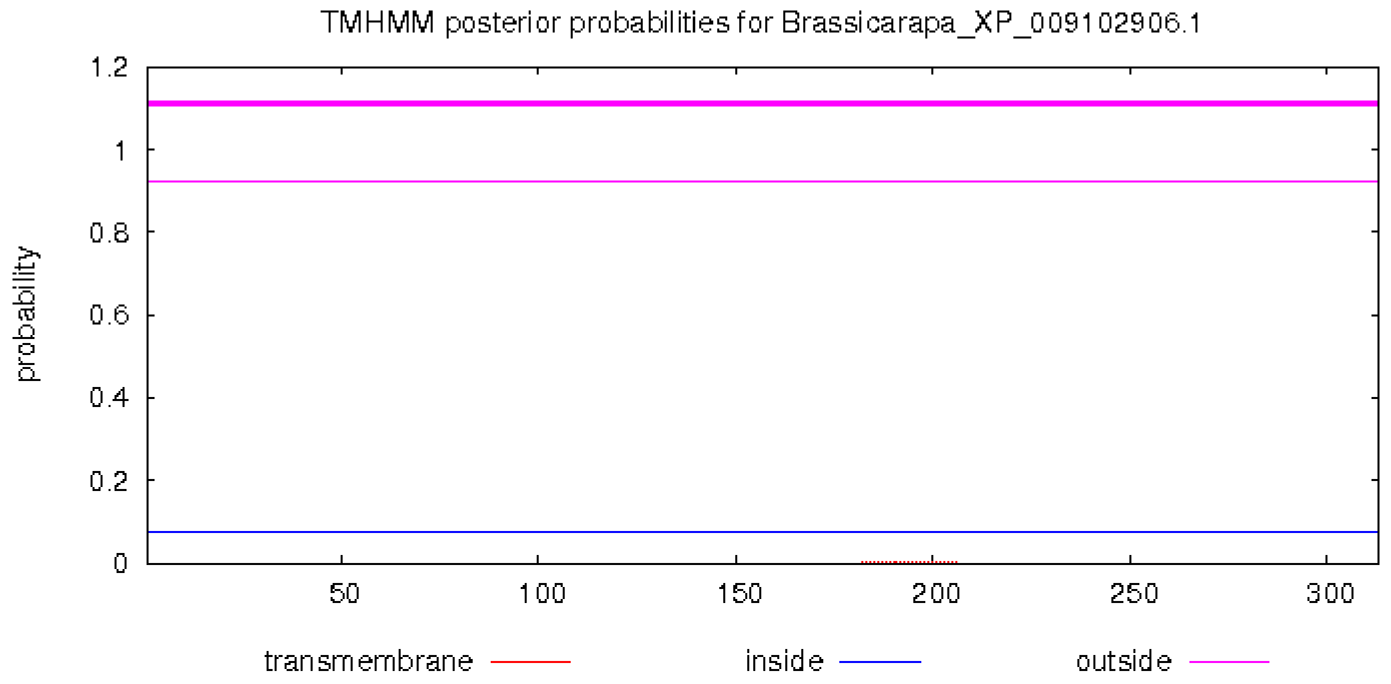

plot in postscript, script for making the plot in gnuplot, data for plot

---

```
# Brassicarapa_XP_009102907.1 Length: 309
# Brassicarapa_XP_009102907.1 Number of predicted TMHs:  0
# Brassicarapa_XP_009102907.1 Exp number of AAs in TMHs: 0.07414
# Brassicarapa_XP_009102907.1 Exp number, first 60 AAs:  0.00015
# Brassicarapa_XP_009102907.1 Total prob of N-in:        0.07885
Brassicarapa_XP_009102907.1	TMHMM2.0	outside	     1   309
```

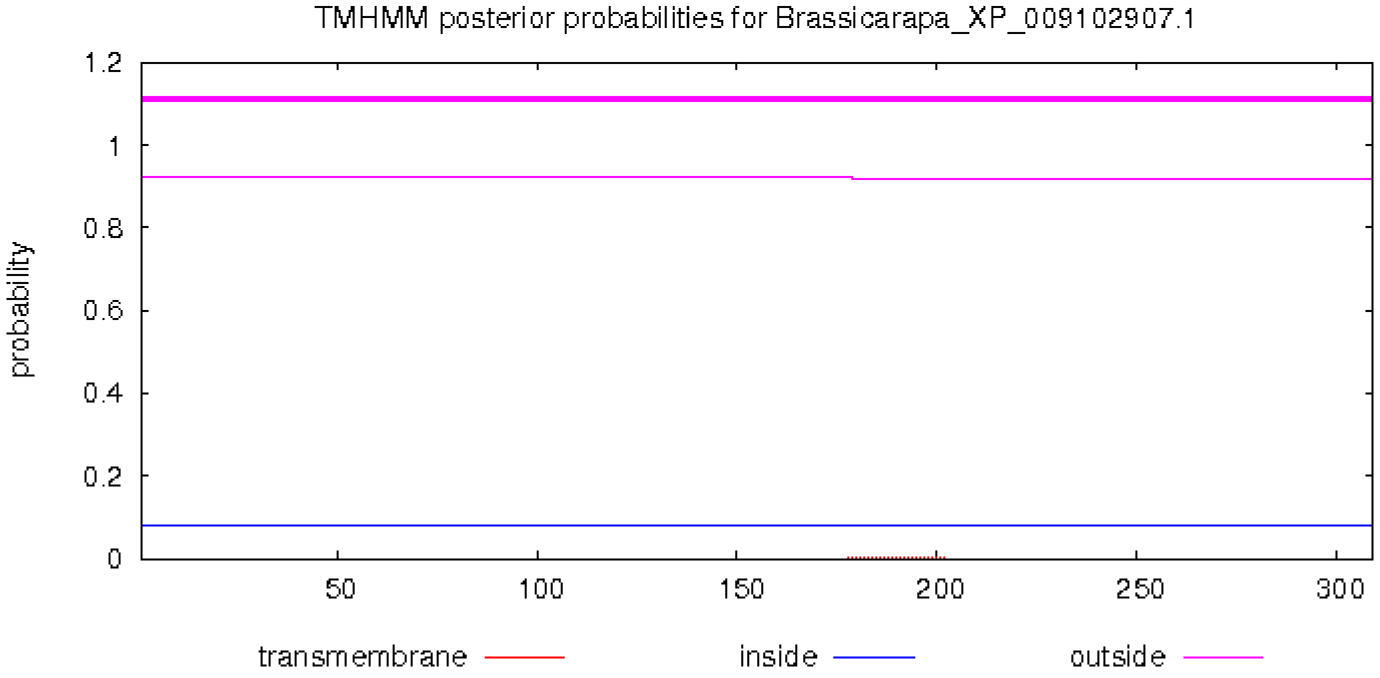

plot in postscript, script for making the plot in gnuplot, data for plot

```
# Brassicarapa_XP_009102908.1 Length: 296
# Brassicarapa_XP_009102908.1 Number of predicted TMHs:  0
# Brassicarapa_XP_009102908.1 Exp number of AAs in TMHs: 0.17069
# Brassicarapa_XP_009102908.1 Exp number, first 60 AAs:  0.00035
# Brassicarapa_XP_009102908.1 Total prob of N-in:        0.20380
Brassicarapa_XP_009102908.1	TMHMM2.0	outside	     1   296
```

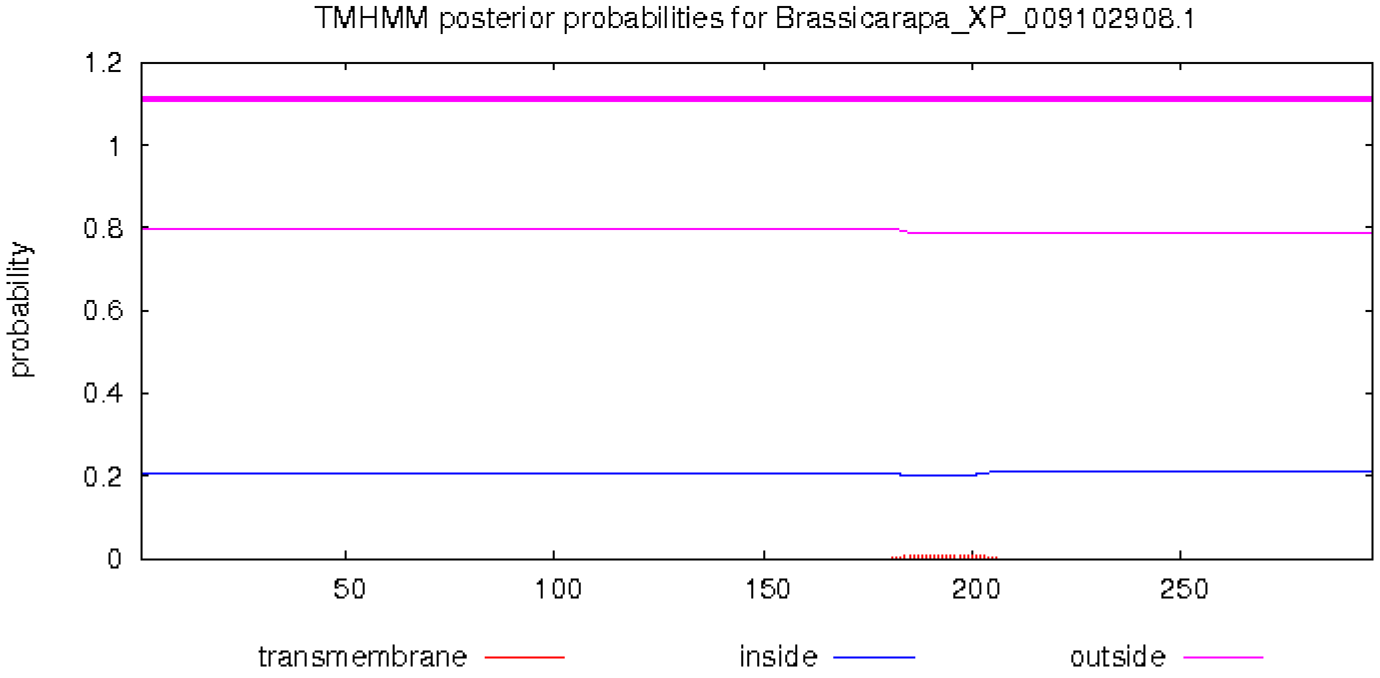

plot in postscript, script for making the plot in gnuplot, data for plot

```
# Brassicarapa_XP_009103075.1 Length: 445
# Brassicarapa_XP_009103075.1 Number of predicted TMHs:  0
# Brassicarapa_XP_009103075.1 Exp number of AAs in TMHs: 0.00078
# Brassicarapa_XP_009103075.1 Exp number, first 60 AAs:  0
# Brassicarapa_XP_009103075.1 Total prob of N-in:        0.00665
Brassicarapa_XP_009103075.1     TMHMM2.0        outside      1   445
```

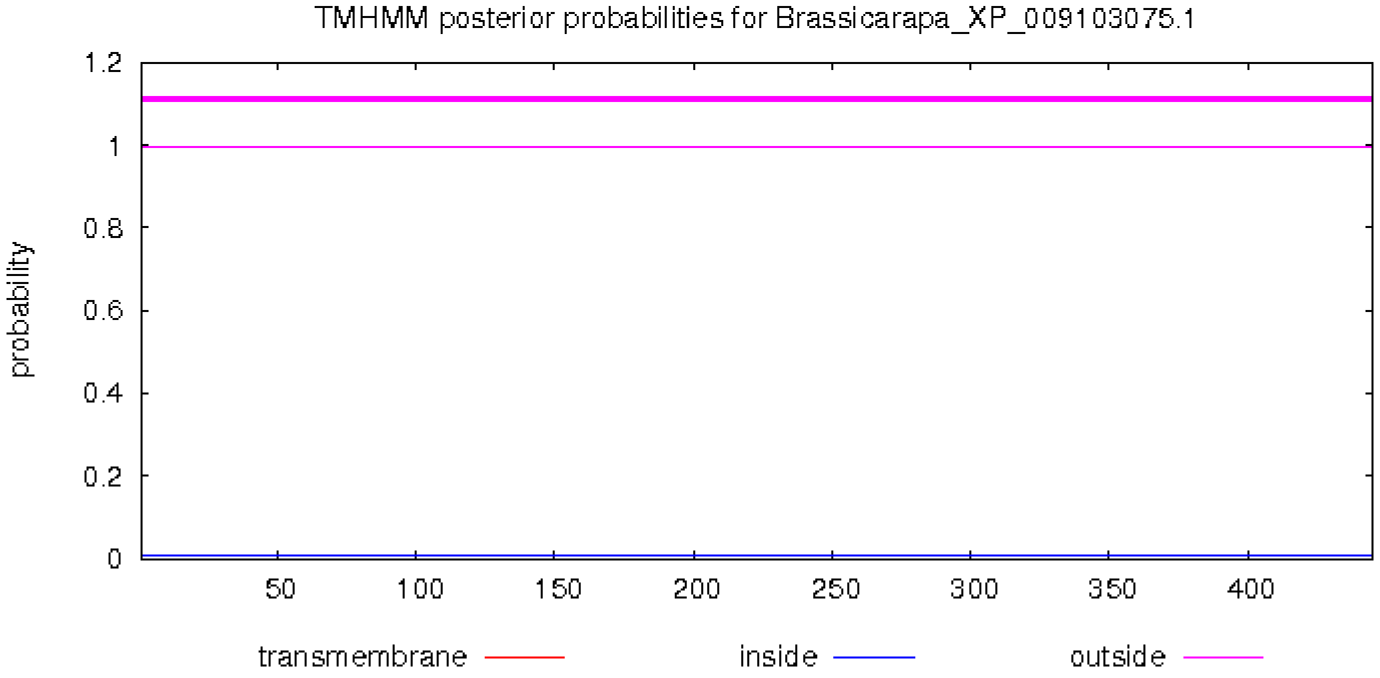

plot in postscript, script for making the plot in gnuplot, data for plot

---

```
# Brassicarapa_XP_009103220.1 Length: 509
# Brassicarapa_XP_009103220.1 Number of predicted TMHs:  1
# Brassicarapa_XP_009103220.1 Exp number of AAs in TMHs: 20.68
# Brassicarapa_XP_009103220.1 Exp number, first 60 AAs:  0.00512
# Brassicarapa_XP_009103220.1 Total prob of N-in:        0.91048
Brassicarapa_XP_009103220.1     TMHMM2.0        inside       1   481
Brassicarapa_XP_009103220.1     TMHMM2.0        TMhelix    482   504
Brassicarapa_XP_009103220.1     TMHMM2.0        outside    505   509
```

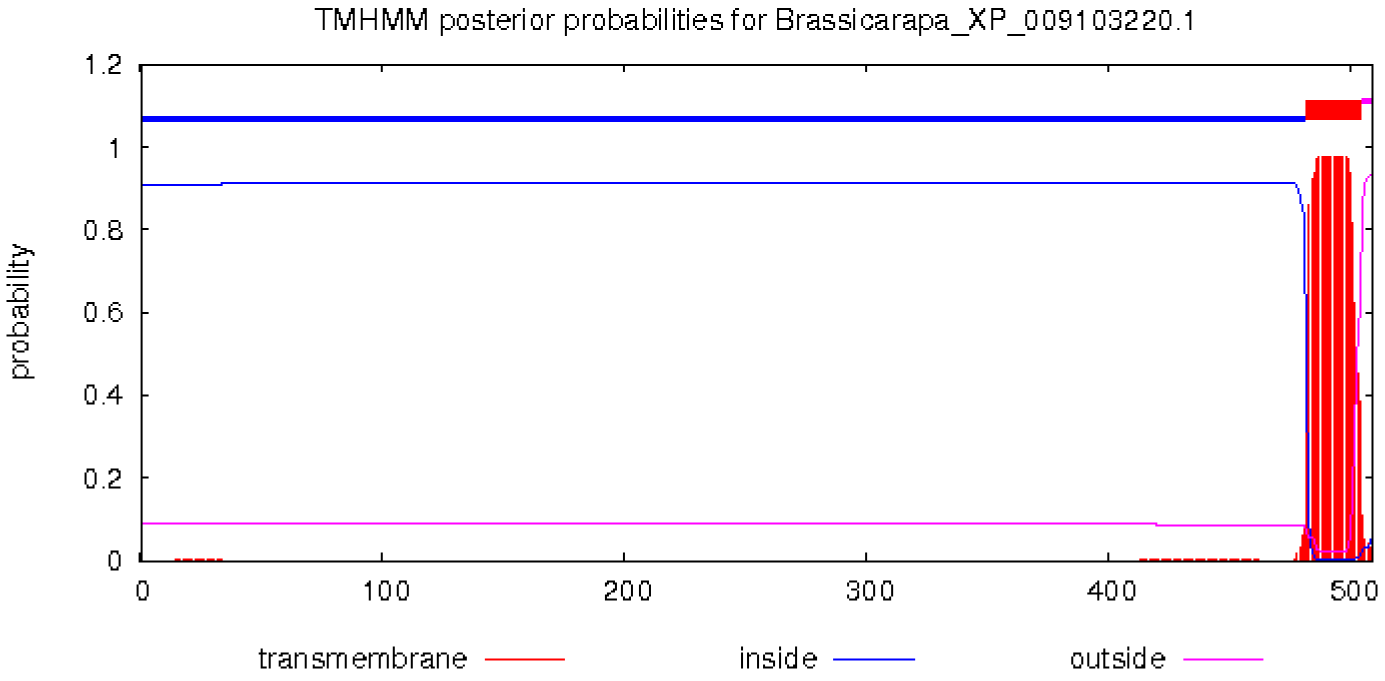

plot in postscript, script for making the plot in gnuplot, data for plot

```
# Brassicarapa_XP_009103221.1 Length: 503
# Brassicarapa_XP_009103221.1 Number of predicted TMHs:  1
# Brassicarapa_XP_009103221.1 Exp number of AAs in TMHs: 20.70055
# Brassicarapa_XP_009103221.1 Exp number, first 60 AAs:  0.00512
# Brassicarapa_XP_009103221.1 Total prob of N-in:        0.91638
Brassicarapa_XP_009103221.1     TMHMM2.0        inside       1   475
Brassicarapa_XP_009103221.1     TMHMM2.0        TMhelix    476   498
Brassicarapa_XP_009103221.1     TMHMM2.0        outside    499   503
```

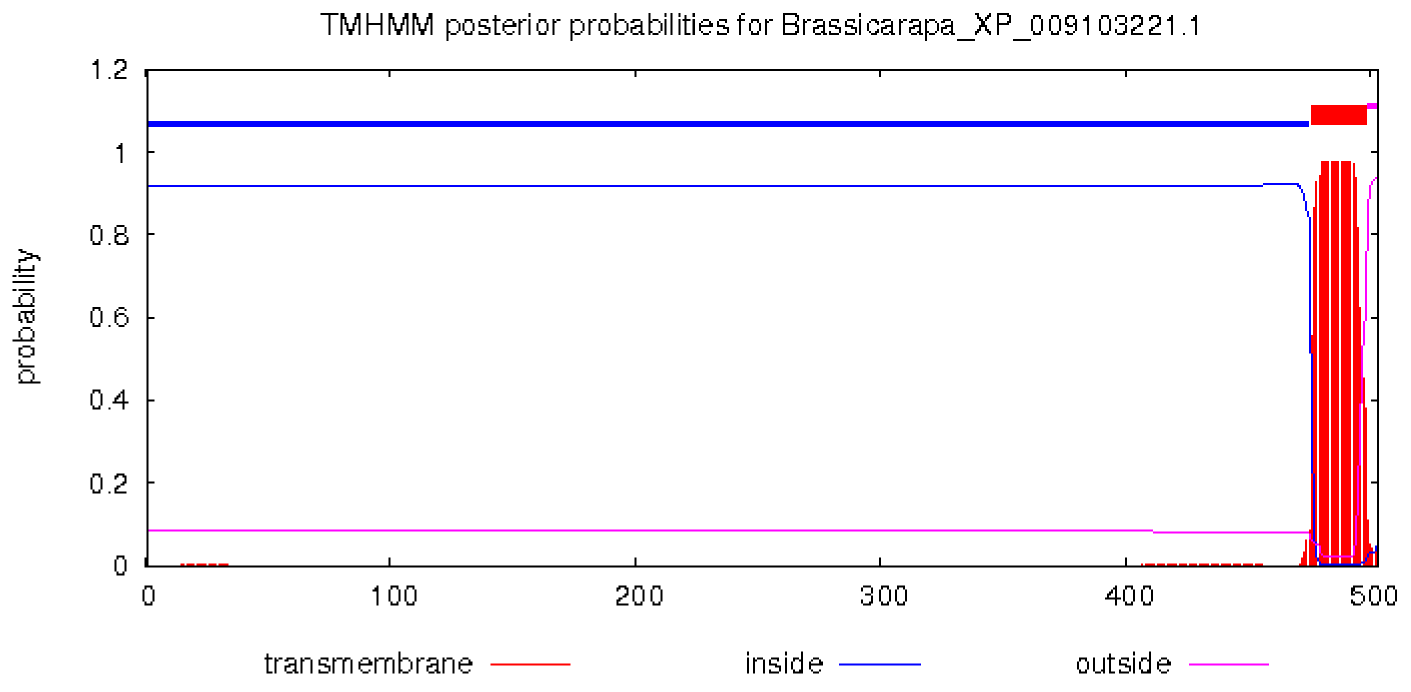

plot in postscript, script for making the plot in gnuplot, data for plot

```
# Brassicarapa_XP_009103300.1 Length: 214
# Brassicarapa_XP_009103300.1 Number of predicted TMHs:  0
# Brassicarapa_XP_009103300.1 Exp number of AAs in TMHs: 0.00036
# Brassicarapa_XP_009103300.1 Exp number, first 60 AAs:  0
# Brassicarapa_XP_009103300.1 Total prob of N-in:        0.33223
Brassicarapa_XP_009103300.1     TMHMM2.0        outside      1   214
```

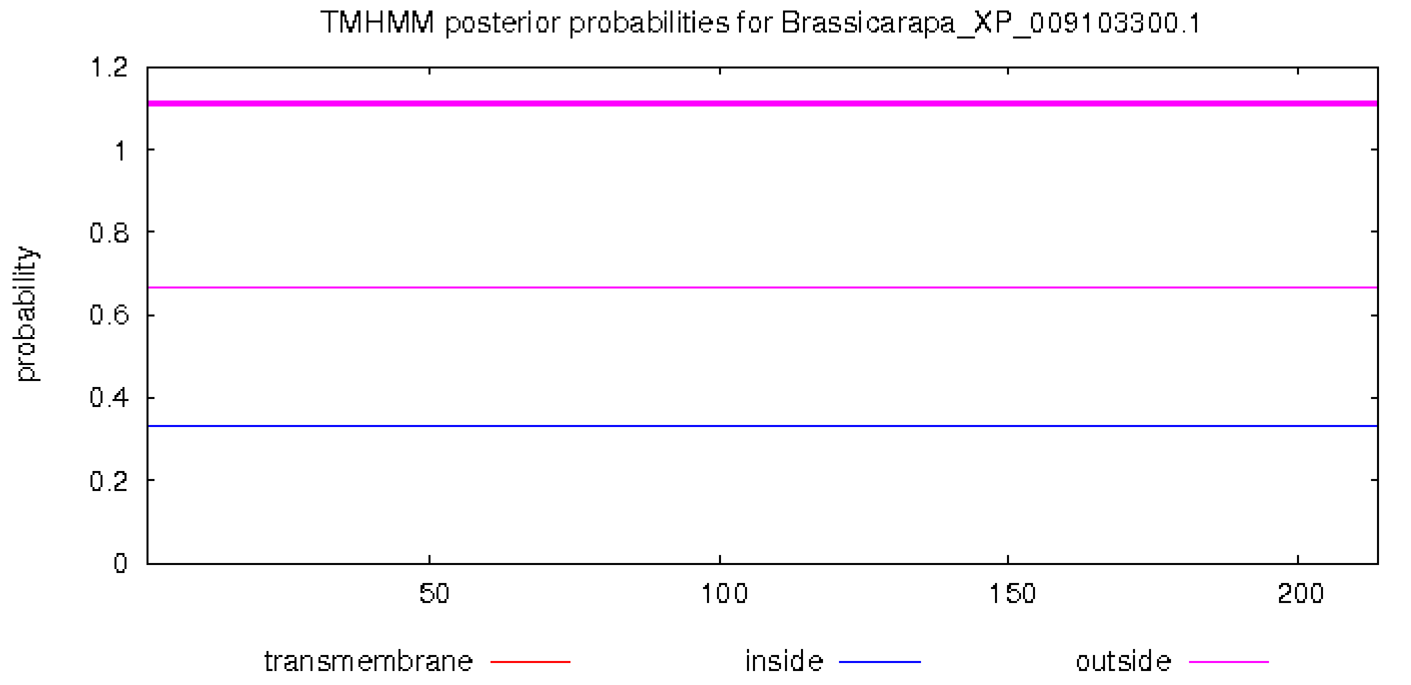

plot in postscript, script for making the plot in gnuplot, data for plot

---

```
# Brassicarapa_XP_009103538.1 Length: 294
# Brassicarapa_XP_009103538.1 Number of predicted TMHs:  0
# Brassicarapa_XP_009103538.1 Exp number of AAs in TMHs: 0.00018
# Brassicarapa_XP_009103538.1 Exp number, first 60 AAs:  0
# Brassicarapa_XP_009103538.1 Total prob of N-in:        0.01212
Brassicarapa_XP_009103538.1     TMHMM2.0        outside      1   294
```

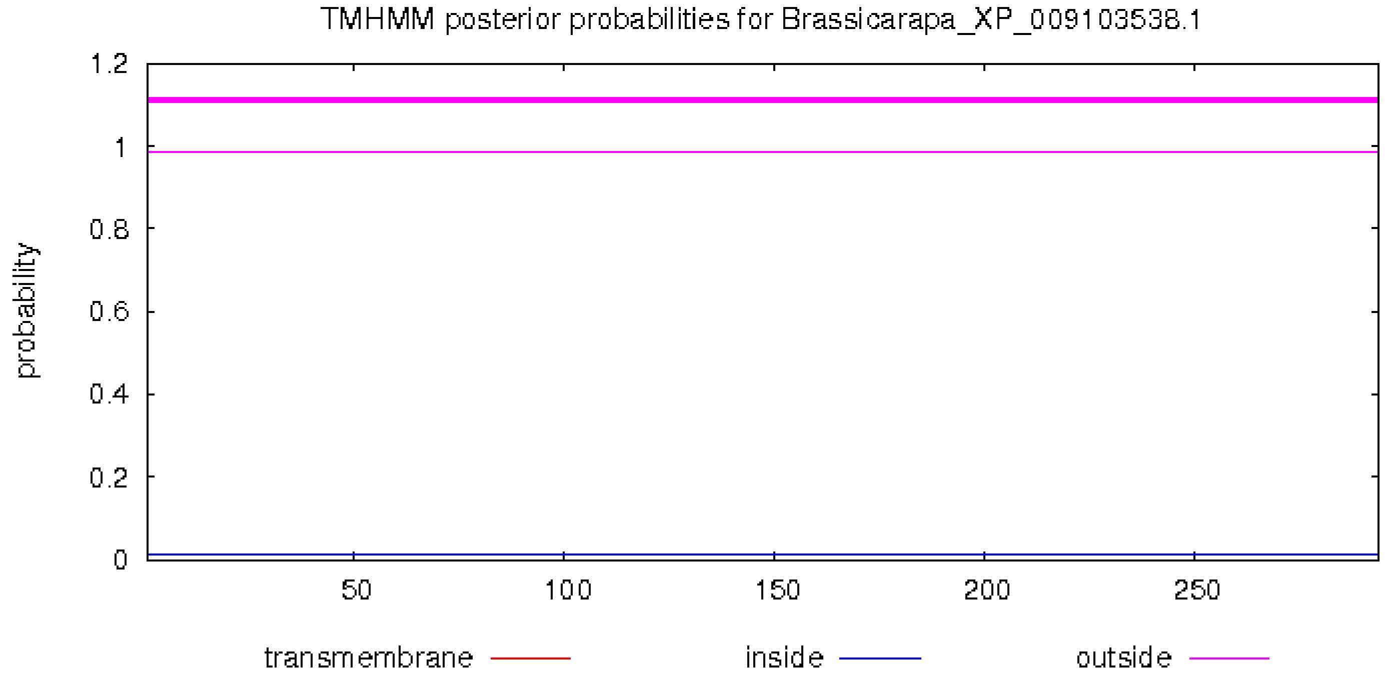

plot in postscript, script for making the plot in gnuplot, data for plot

---

```
# Brassicarapa_XP_009103539.1 Length: 293
# Brassicarapa_XP_009103539.1 Number of predicted TMHs:  0
# Brassicarapa_XP_009103539.1 Exp number of AAs in TMHs: 0.00018
# Brassicarapa_XP_009103539.1 Exp number, first 60 AAs:  0
# Brassicarapa_XP_009103539.1 Total prob of N-in:        0.01227
Brassicarapa_XP_009103539.1     TMHMM2.0        outside      1   293
```

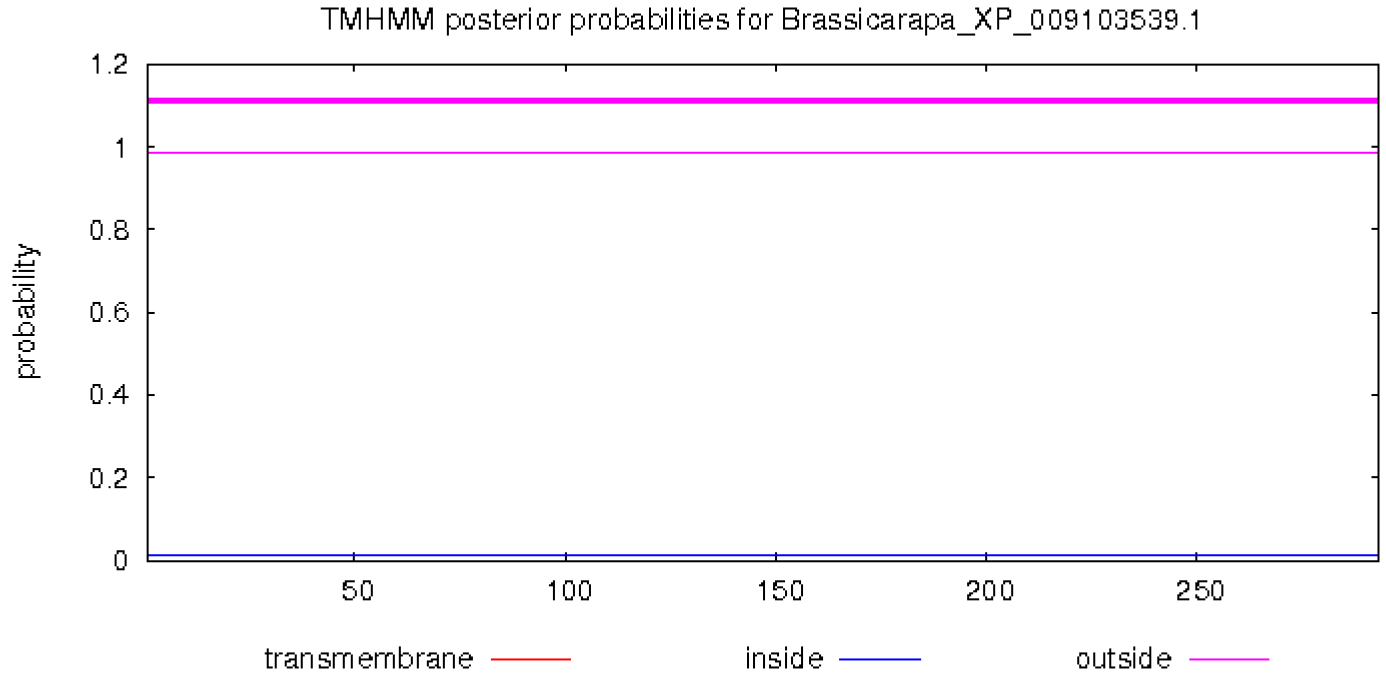

plot in postscript, script for making the plot in gnuplot, data for plot

```
# Brassicarapa_XP_009103591.1 Length: 345
# Brassicarapa_XP_009103591.1 Number of predicted TMHs:  1
# Brassicarapa_XP_009103591.1 Exp number of AAs in TMHs: 20.82426
# Brassicarapa_XP_009103591.1 Exp number, first 60 AAs:  0.10265
# Brassicarapa_XP_009103591.1 Total prob of N-in:        0.69296
Brassicarapa_XP_009103591.1     TMHMM2.0        inside       1   319
Brassicarapa_XP_009103591.1     TMHMM2.0        TMhelix    320   342
Brassicarapa_XP_009103591.1     TMHMM2.0        outside    343   345
```

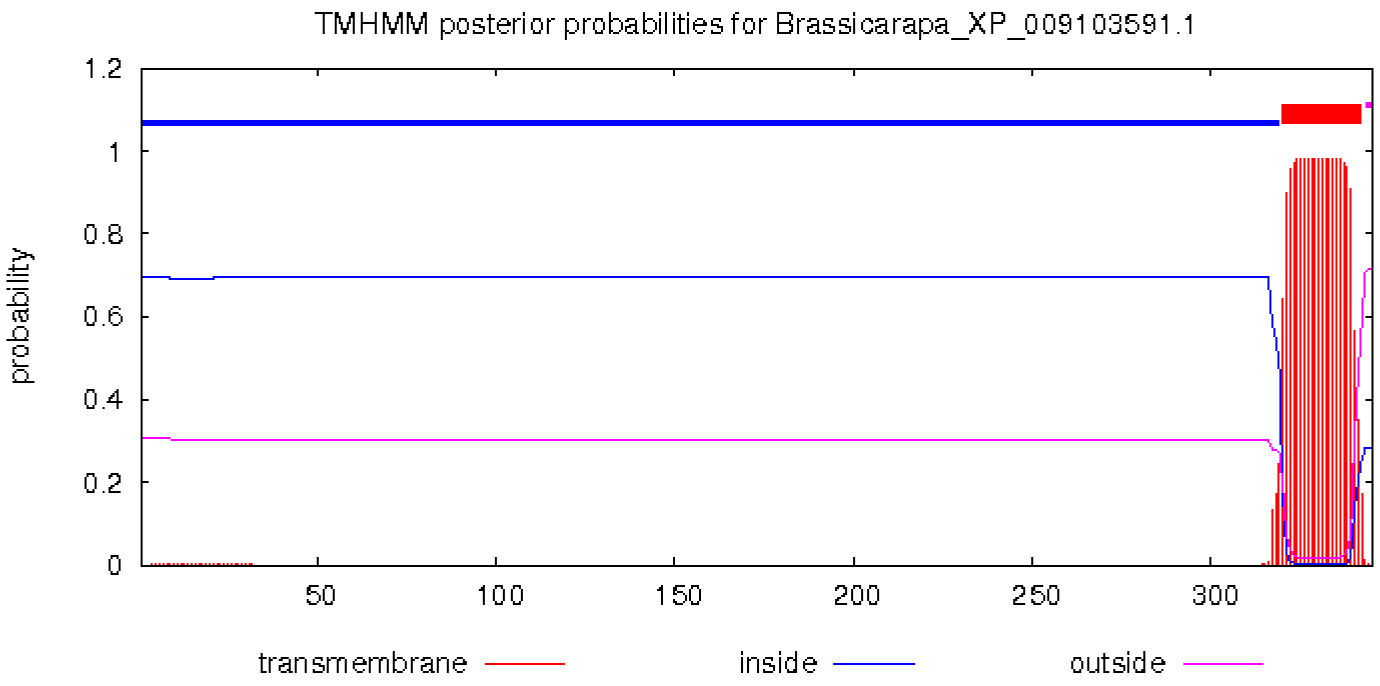

plot in postscript, script for making the plot in gnuplot, data for plot

```
# Brassicarapa_XP_009103592.1 Length: 343
# Brassicarapa_XP_009103592.1 Number of predicted TMHs:  1
# Brassicarapa_XP_009103592.1 Exp number of AAs in TMHs: 20.83764
# Brassicarapa_XP_009103592.1 Exp number, first 60 AAs:  0.10329
# Brassicarapa_XP_009103592.1 Total prob of N-in:        0.69816
Brassicarapa_XP_009103592.1     TMHMM2.0        inside       1   317
Brassicarapa_XP_009103592.1     TMHMM2.0        TMhelix    318   340
Brassicarapa_XP_009103592.1     TMHMM2.0        outside    341   343
```

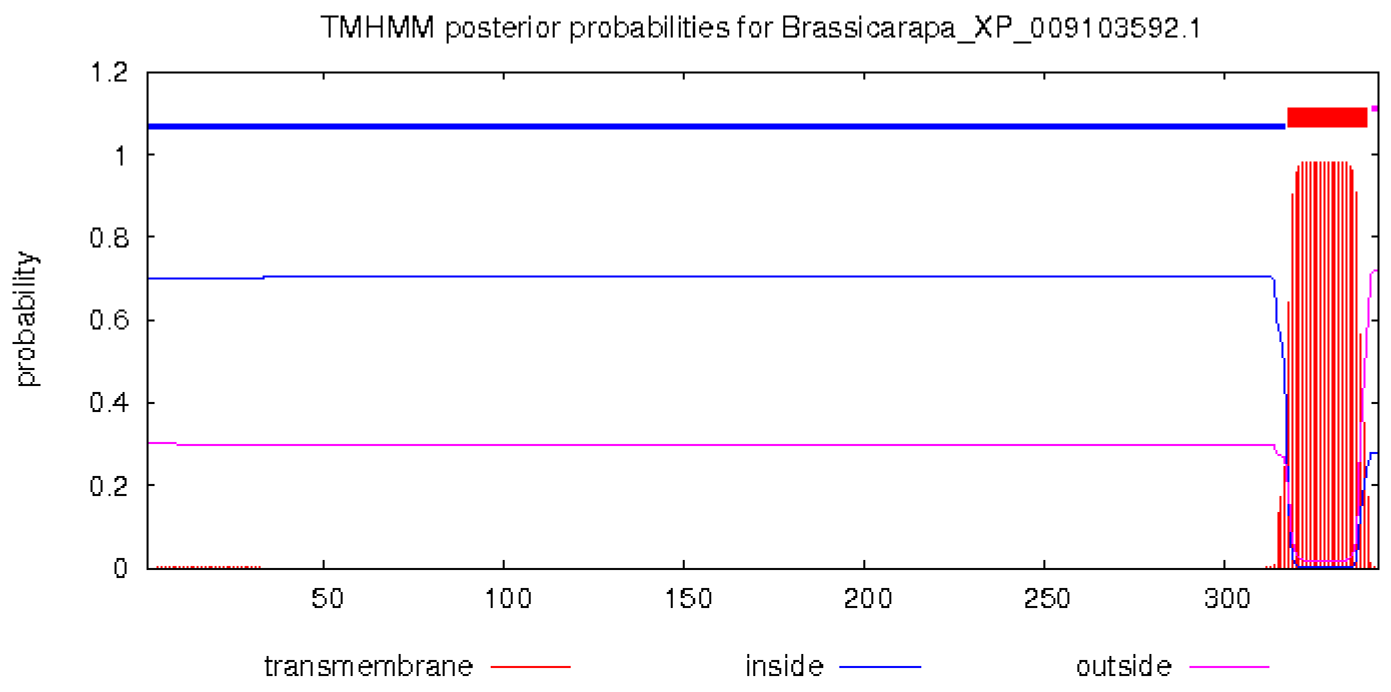

plot in postscript, script for making the plot in gnuplot, data for plot

---

```
# Brassicarapa_XP_009103997.1 Length: 164
# Brassicarapa_XP_009103997.1 Number of predicted TMHs:  0
# Brassicarapa_XP_009103997.1 Exp number of AAs in TMHs: 0.0043
# Brassicarapa_XP_009103997.1 Exp number, first 60 AAs:  0.00393
# Brassicarapa_XP_009103997.1 Total prob of N-in:        0.05282
Brassicarapa_XP_009103997.1     TMHMM2.0        outside      1   164
```

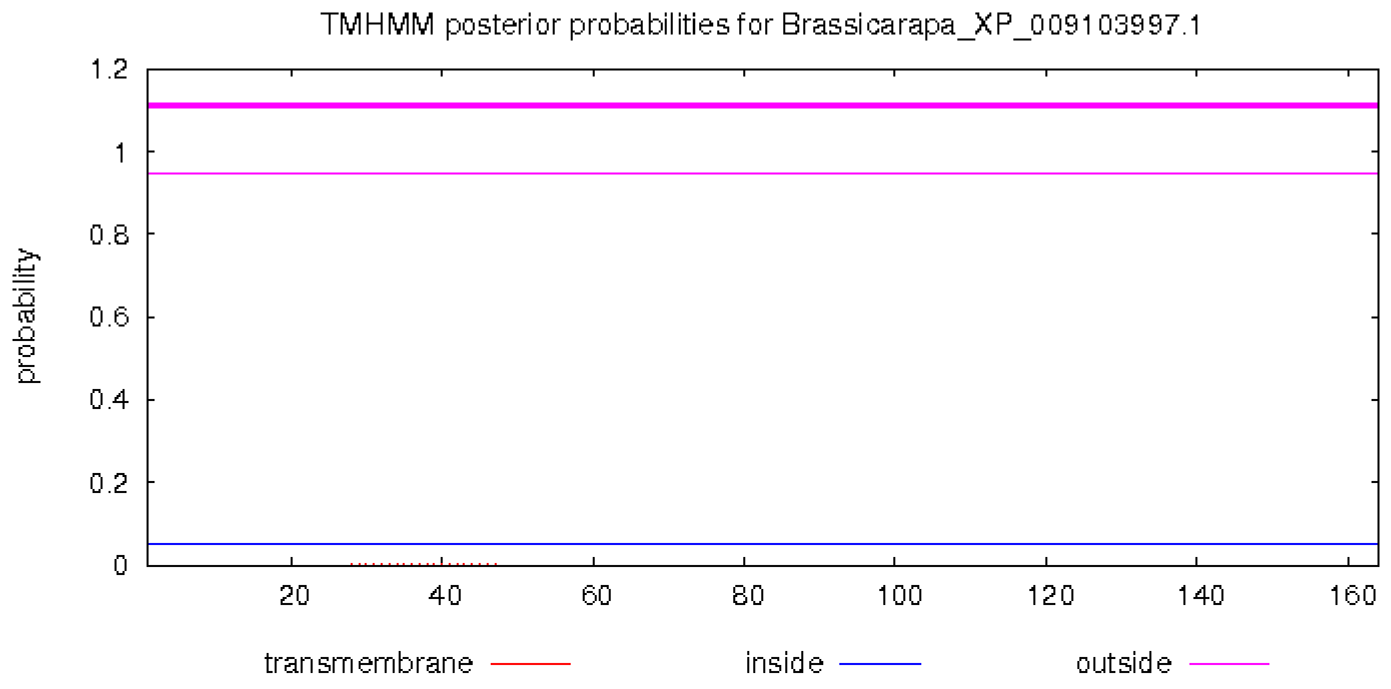

plot in postscript, script for making the plot in gnuplot, data for plot

---

```
# Brassicarapa_XP_009104009.1 Length: 373
# Brassicarapa_XP_009104009.1 Number of predicted TMHs:  0
# Brassicarapa_XP_009104009.1 Exp number of AAs in TMHs: 0.0005
# Brassicarapa_XP_009104009.1 Exp number, first 60 AAs:  0
# Brassicarapa_XP_009104009.1 Total prob of N-in:        0.02100
Brassicarapa_XP_009104009.1     TMHMM2.0        outside      1   373
```

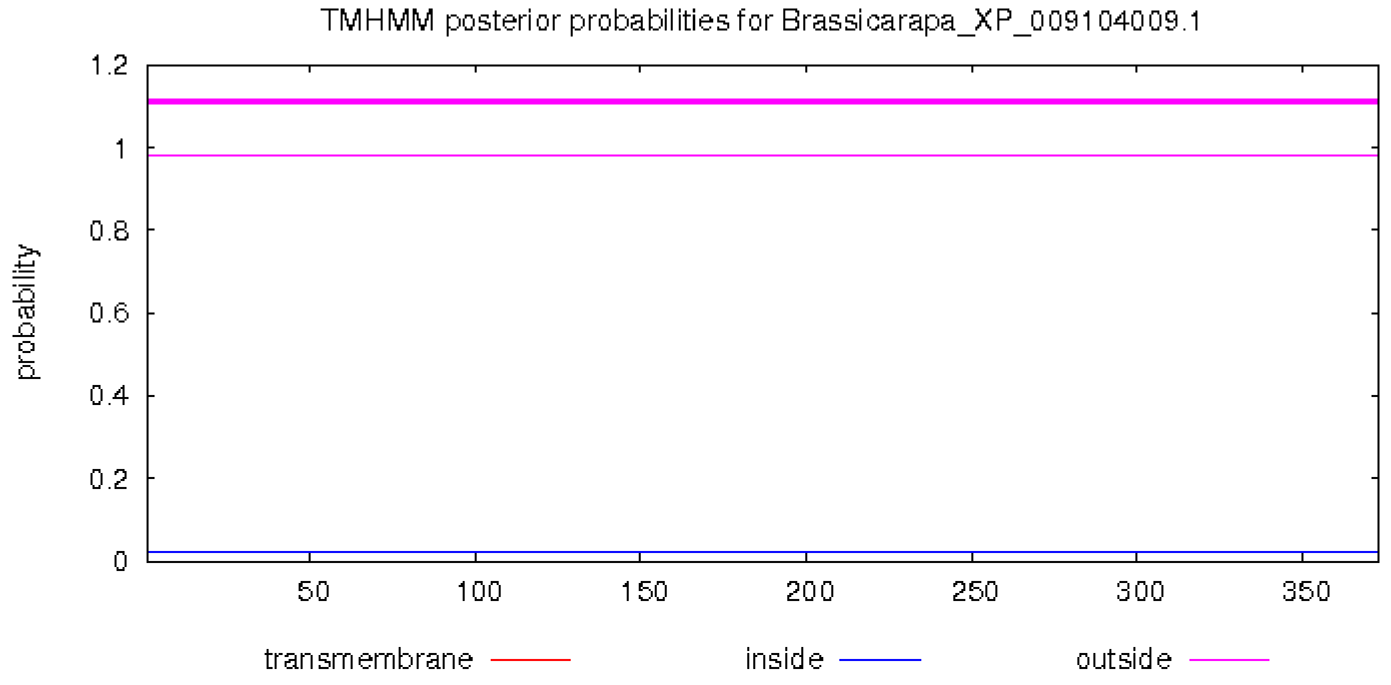

plot in postscript, script for making the plot in gnuplot, data for plot

---

```
# Brassicarapa_XP_009104010.1 Length: 373
# Brassicarapa_XP_009104010.1 Number of predicted TMHs:  0
# Brassicarapa_XP_009104010.1 Exp number of AAs in TMHs: 0.0005
# Brassicarapa_XP_009104010.1 Exp number, first 60 AAs:  0
# Brassicarapa_XP_009104010.1 Total prob of N-in:        0.02100
Brassicarapa_XP_009104010.1     TMHMM2.0        outside      1   373
```

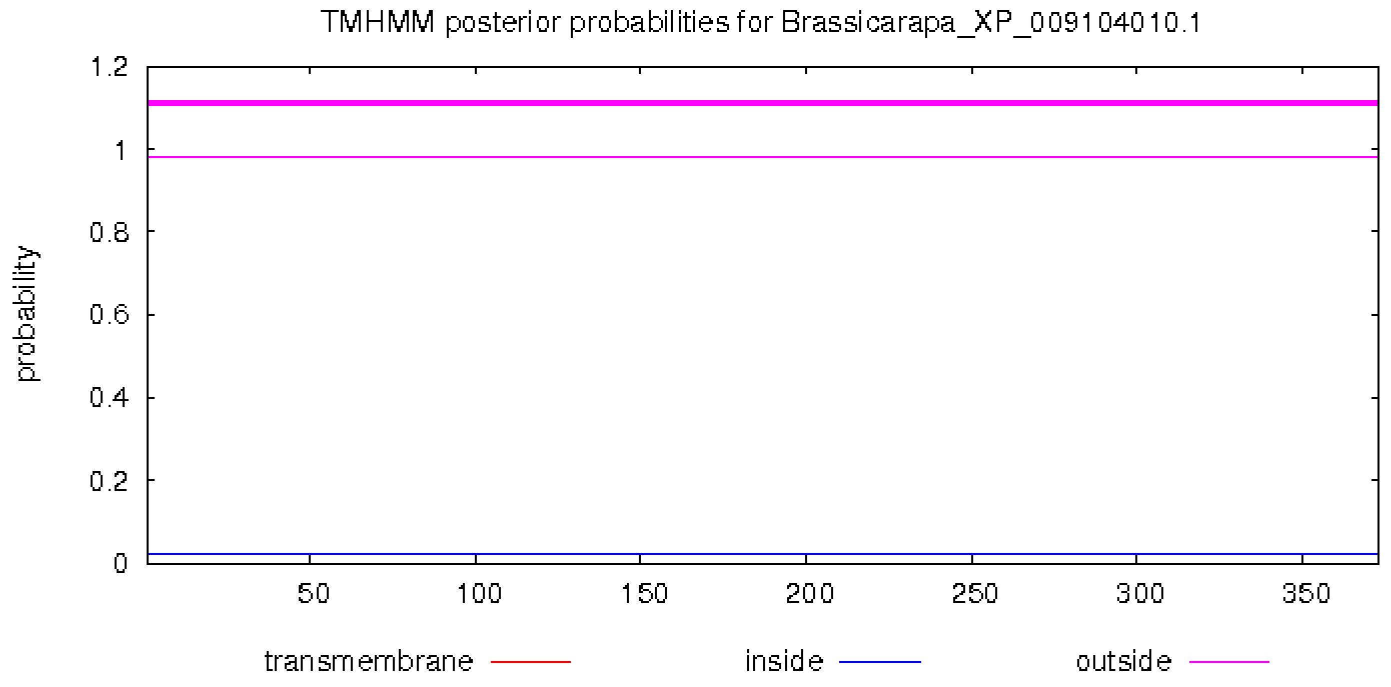

plot in postscript, script for making the plot in gnuplot, data for plot

---

```
# Brassicarapa_XP_009104011.1 Length: 280
# Brassicarapa_XP_009104011.1 Number of predicted TMHs:  0
# Brassicarapa_XP_009104011.1 Exp number of AAs in TMHs: 0.00059
# Brassicarapa_XP_009104011.1 Exp number, first 60 AAs:  0
# Brassicarapa_XP_009104011.1 Total prob of N-in:        0.06573
Brassicarapa_XP_009104011.1     TMHMM2.0        outside      1   280
```

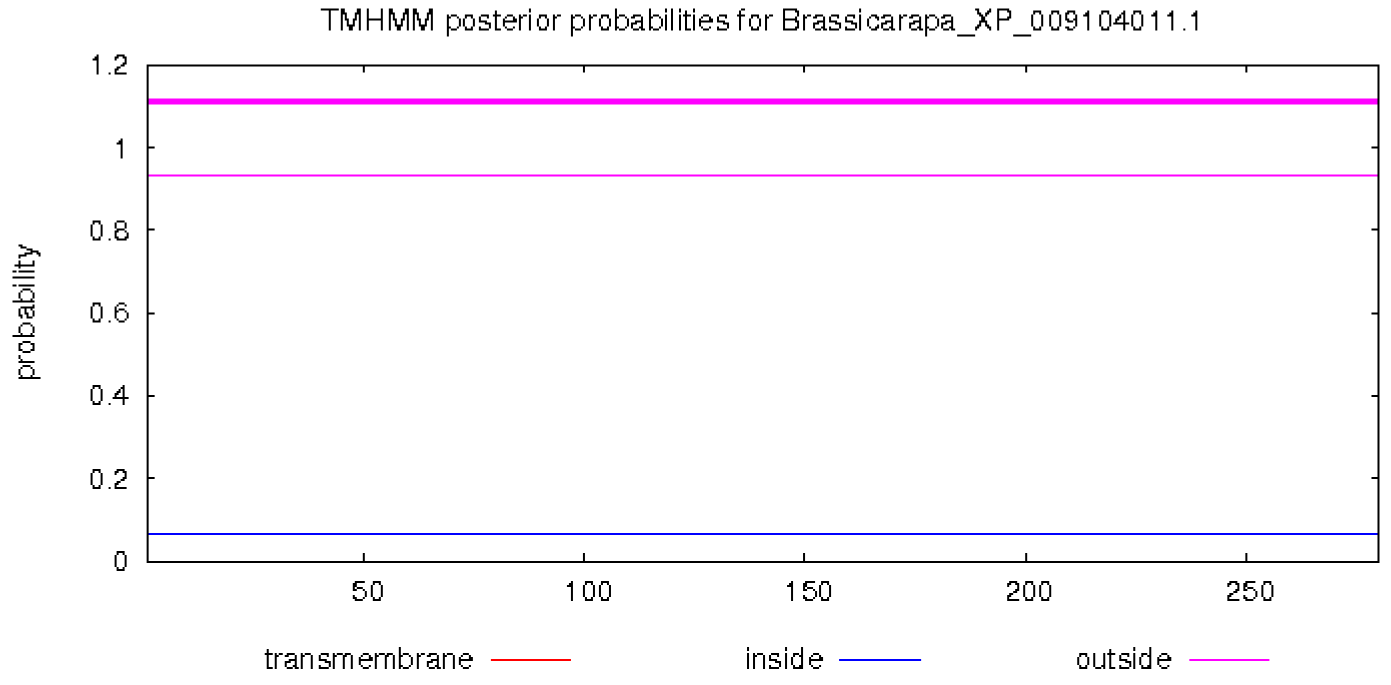

plot in postscript, script for making the plot in gnuplot, data for plot

---

```
# Brassicarapa_XP_009104136.1 Length: 311
# Brassicarapa_XP_009104136.1 Number of predicted TMHs:  0
# Brassicarapa_XP_009104136.1 Exp number of AAs in TMHs: 0
# Brassicarapa_XP_009104136.1 Exp number, first 60 AAs:  0
# Brassicarapa_XP_009104136.1 Total prob of N-in:        0.00125
Brassicarapa_XP_009104136.1     TMHMM2.0        outside      1   311
```

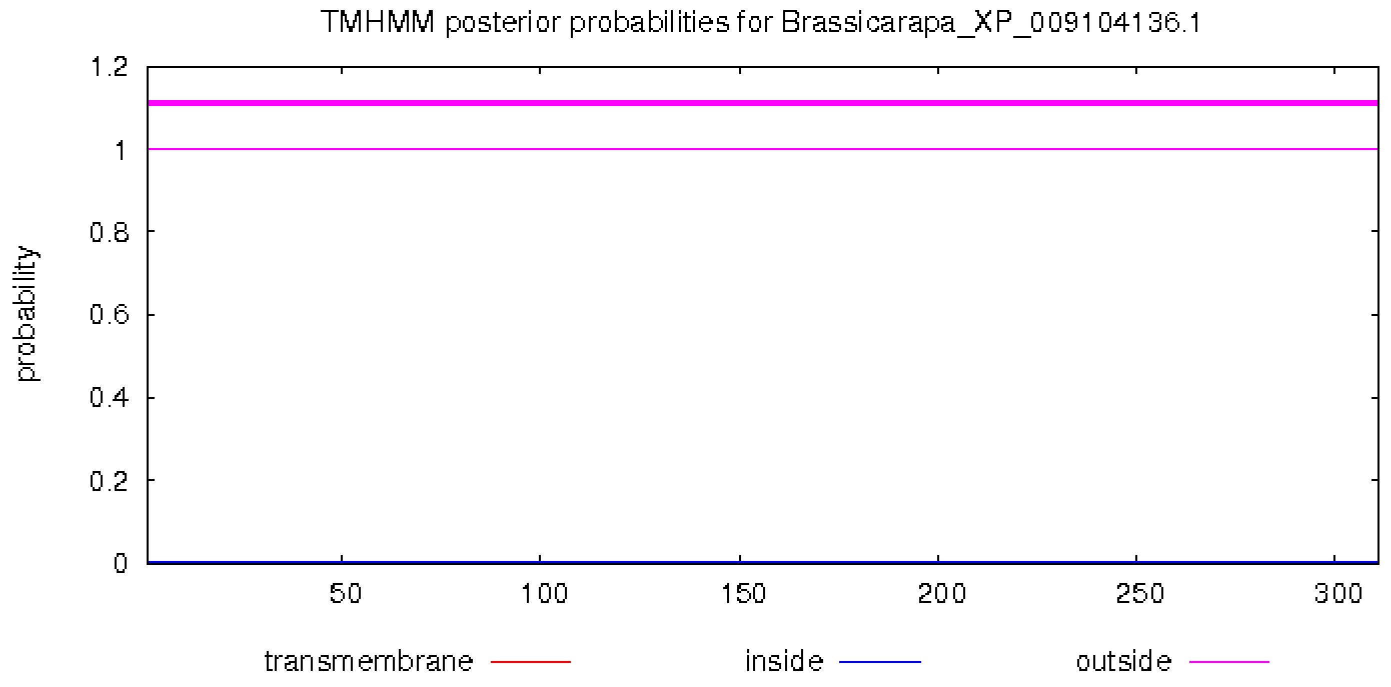

plot in postscript, script for making the plot in gnuplot, data for plot

---

```
# Brassicarapa_XP_009104354.1 Length: 336
# Brassicarapa_XP_009104354.1 Number of predicted TMHs:  0
# Brassicarapa_XP_009104354.1 Exp number of AAs in TMHs: 0.00021
# Brassicarapa_XP_009104354.1 Exp number, first 60 AAs:  0
# Brassicarapa_XP_009104354.1 Total prob of N-in:        0.02697
Brassicarapa_XP_009104354.1     TMHMM2.0        outside      1   336
```

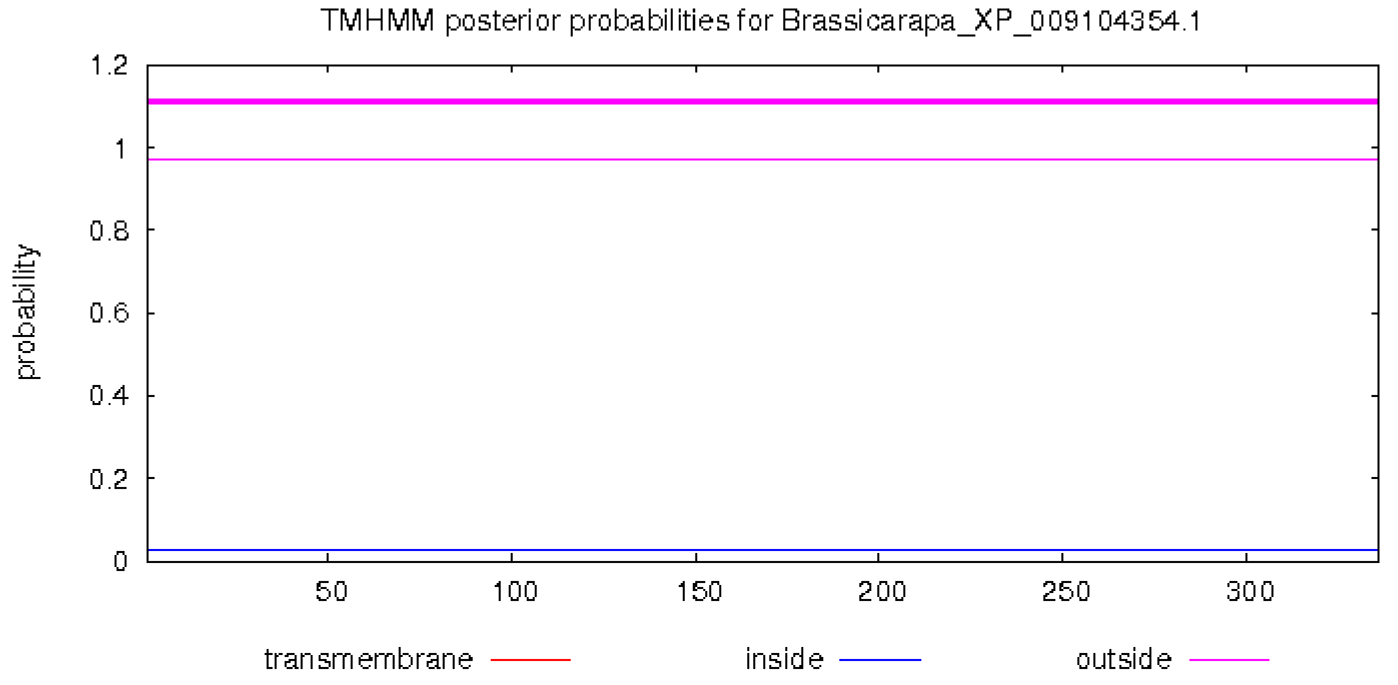

plot in postscript, script for making the plot in gnuplot, data for plot

---

```
# Brassicarapa_XP_009104516.1 Length: 357
# Brassicarapa_XP_009104516.1 Number of predicted TMHs:  0
# Brassicarapa_XP_009104516.1 Exp number of AAs in TMHs: 0.00096
# Brassicarapa_XP_009104516.1 Exp number, first 60 AAs:  0.00096
# Brassicarapa_XP_009104516.1 Total prob of N-in:        0.00921
Brassicarapa_XP_009104516.1     TMHMM2.0        outside      1   357
```

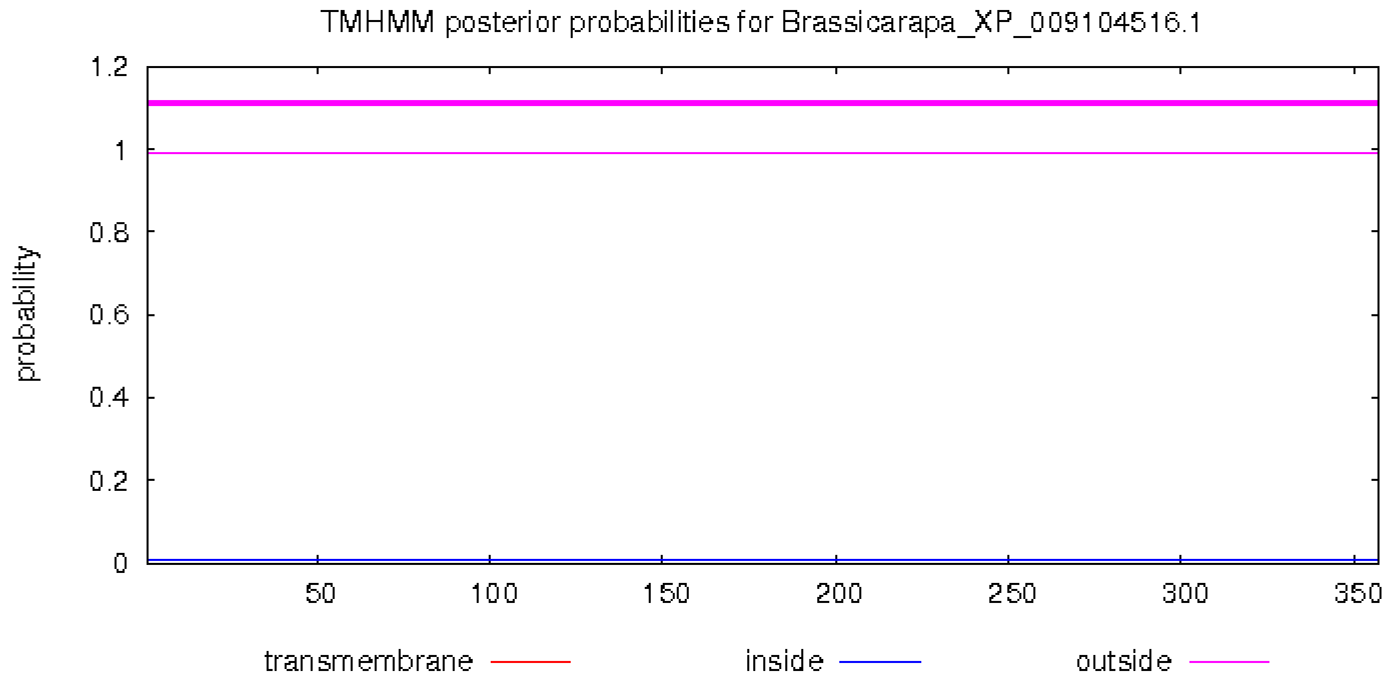

plot in postscript, script for making the plot in gnuplot, data for plot

---

```
# Brassicarapa_XP_009104518.1 Length: 357
# Brassicarapa_XP_009104518.1 Number of predicted TMHs:  0
# Brassicarapa_XP_009104518.1 Exp number of AAs in TMHs: 0.00096
# Brassicarapa_XP_009104518.1 Exp number, first 60 AAs:  0.00096
# Brassicarapa_XP_009104518.1 Total prob of N-in:        0.00921
Brassicarapa_XP_009104518.1     TMHMM2.0        outside      1   357
```

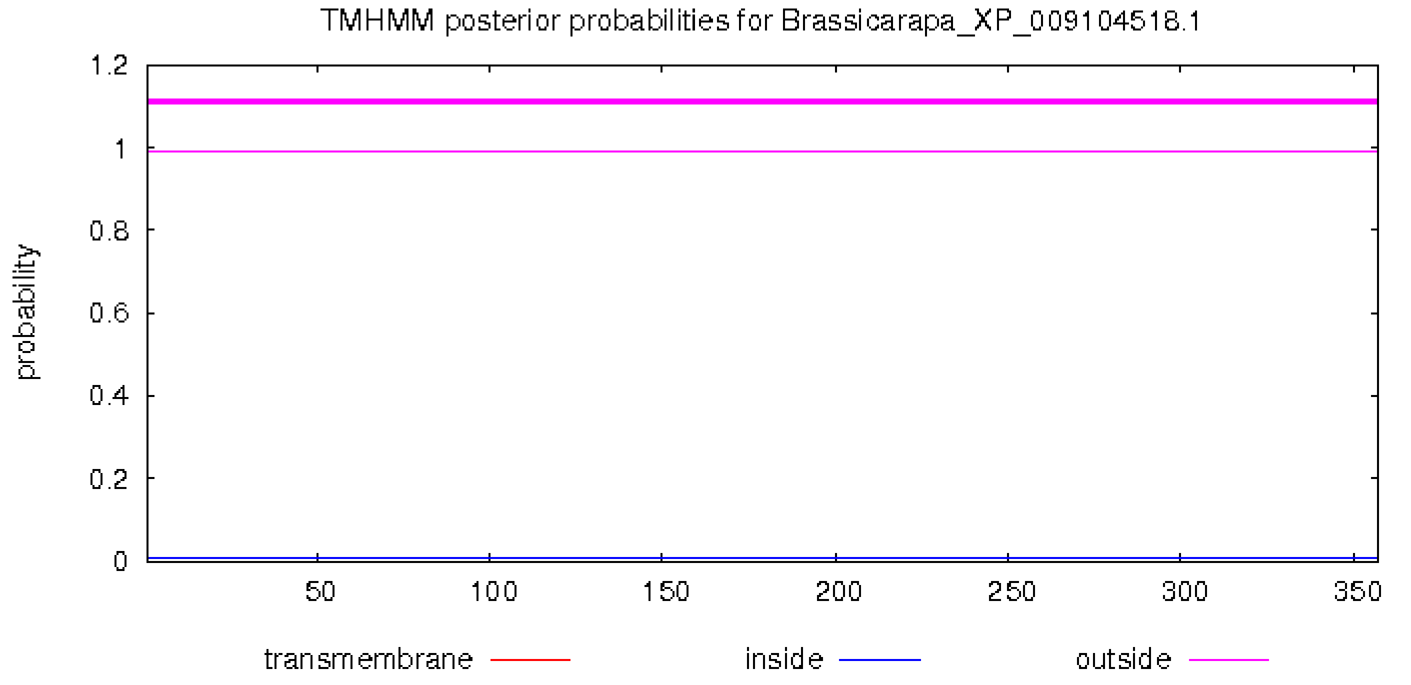

plot in postscript, script for making the plot in gnuplot, data for plot

---

```
# Brassicarapa_XP_009104726.1 Length: 160
# Brassicarapa_XP_009104726.1 Number of predicted TMHs:  0
# Brassicarapa_XP_009104726.1 Exp number of AAs in TMHs: 0.00012
# Brassicarapa_XP_009104726.1 Exp number, first 60 AAs:  0.00012
# Brassicarapa_XP_009104726.1 Total prob of N-in:        0.07722
Brassicarapa_XP_009104726.1     TMHMM2.0        outside      1   160
```

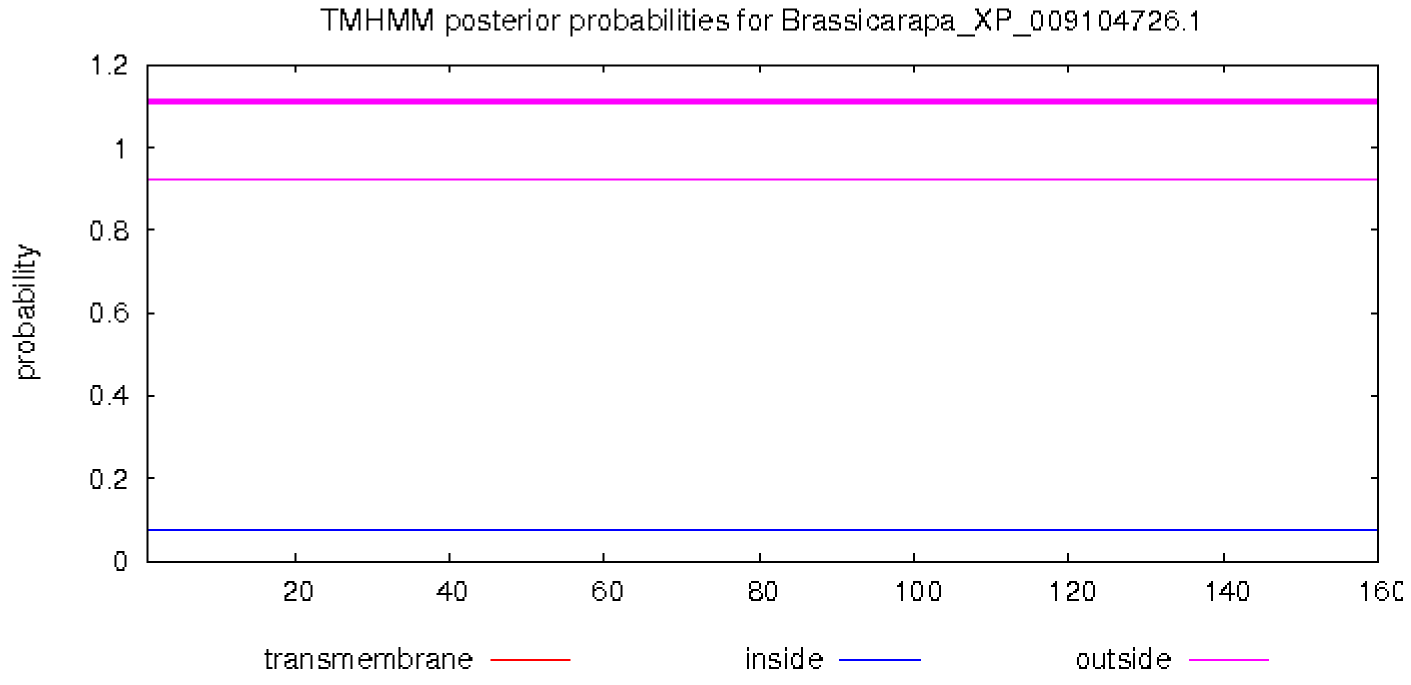

plot in postscript, script for making the plot in gnuplot, data for plot

---

```
# Brassicarapa_XP_009105381.1 Length: 320
# Brassicarapa_XP_009105381.1 Number of predicted TMHs:  0
# Brassicarapa_XP_009105381.1 Exp number of AAs in TMHs: 0.00091
# Brassicarapa_XP_009105381.1 Exp number, first 60 AAs:  0
# Brassicarapa_XP_009105381.1 Total prob of N-in:        0.07307
Brassicarapa_XP_009105381.1     TMHMM2.0        outside      1   320
```

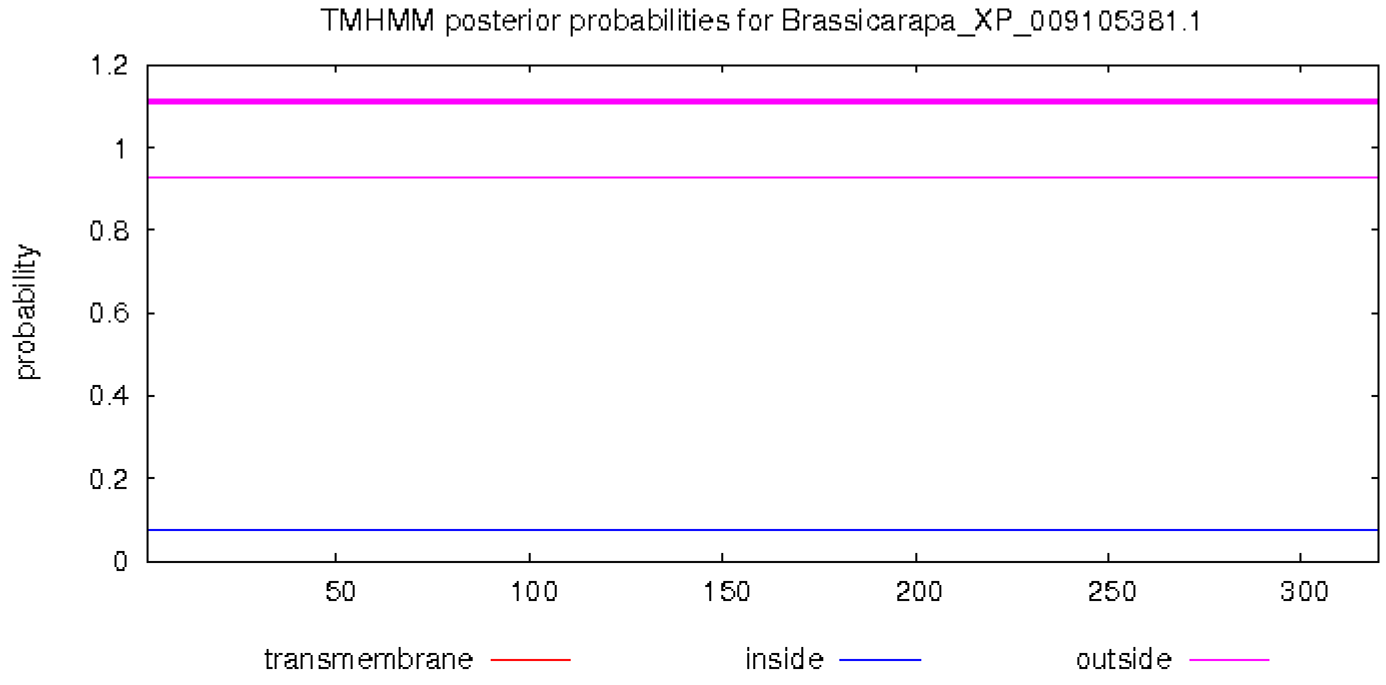

[plot](#) in postscript, [script](#) for making the plot in gnuplot, [data](#) for plot

---

```
# Brassicarapa_XP_009105493.1 Length: 270
# Brassicarapa_XP_009105493.1 Number of predicted TMHs:  0
# Brassicarapa_XP_009105493.1 Exp number of AAs in TMHs: 0.0074
# Brassicarapa_XP_009105493.1 Exp number, first 60 AAs:  0
# Brassicarapa_XP_009105493.1 Total prob of N-in:        0.03628
Brassicarapa_XP_009105493.1     TMHMM2.0        outside      1   270
```

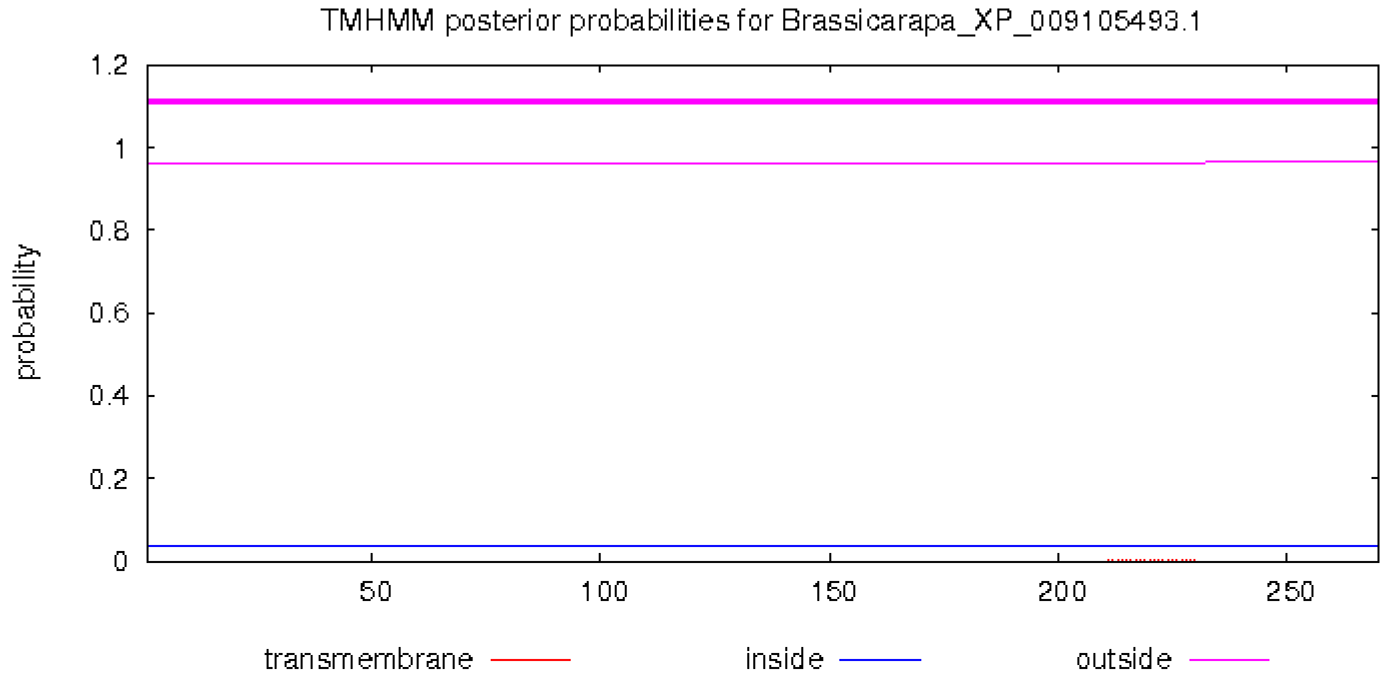

[plot](#) in postscript, [script](#) for making the plot in gnuplot, [data](#) for plot

---

```
# Brassicarapa_XP_009105503.1 Length: 134
# Brassicarapa_XP_009105503.1 Number of predicted TMHs:  0
# Brassicarapa_XP_009105503.1 Exp number of AAs in TMHs: 0
# Brassicarapa_XP_009105503.1 Exp number, first 60 AAs:  0
# Brassicarapa_XP_009105503.1 Total prob of N-in:        0.18103
Brassicarapa_XP_009105503.1     TMHMM2.0        outside      1   134
```

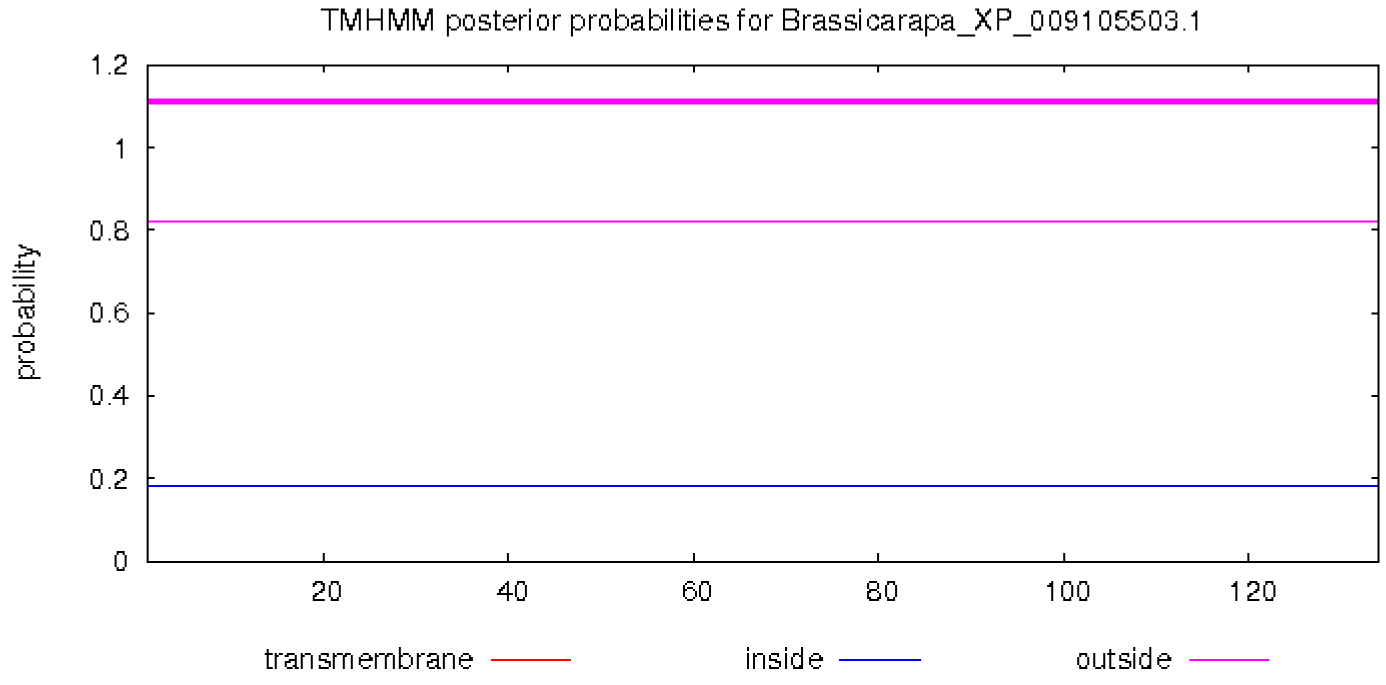

plot in postscript, script for making the plot in gnuplot, data for plot

---

```
# Brassicarapa_XP_009105596.1 Length: 268
# Brassicarapa_XP_009105596.1 Number of predicted TMHs:  0
# Brassicarapa_XP_009105596.1 Exp number of AAs in TMHs: 0.00395
# Brassicarapa_XP_009105596.1 Exp number, first 60 AAs:  0
# Brassicarapa_XP_009105596.1 Total prob of N-in:        0.02132
Brassicarapa_XP_009105596.1     TMHMM2.0        outside      1   268
```

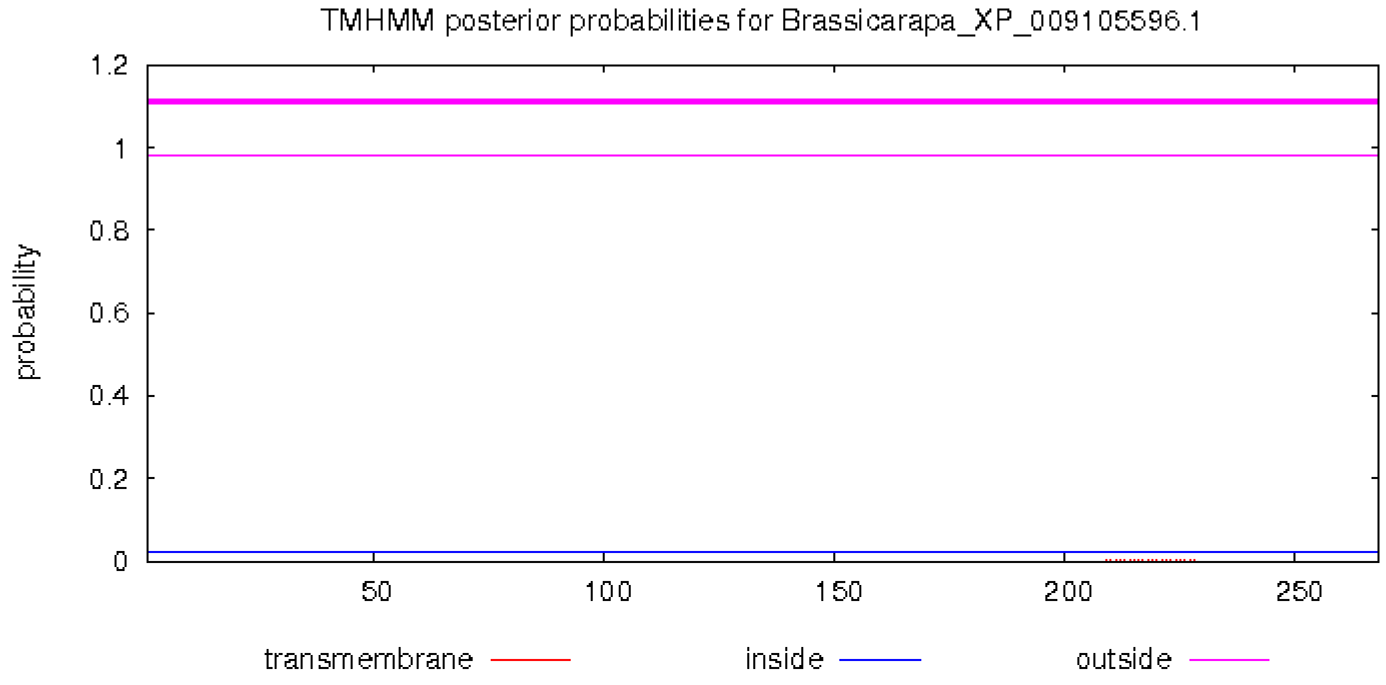

plot in postscript, script for making the plot in gnuplot, data for plot

---

```
# Brassicarapa_XP_009105638.1 Length: 392
# Brassicarapa_XP_009105638.1 Number of predicted TMHs:  0
# Brassicarapa_XP_009105638.1 Exp number of AAs in TMHs: 0.0002
# Brassicarapa_XP_009105638.1 Exp number, first 60 AAs:  0
# Brassicarapa_XP_009105638.1 Total prob of N-in:        0.00118
Brassicarapa_XP_009105638.1     TMHMM2.0        outside      1   392
```

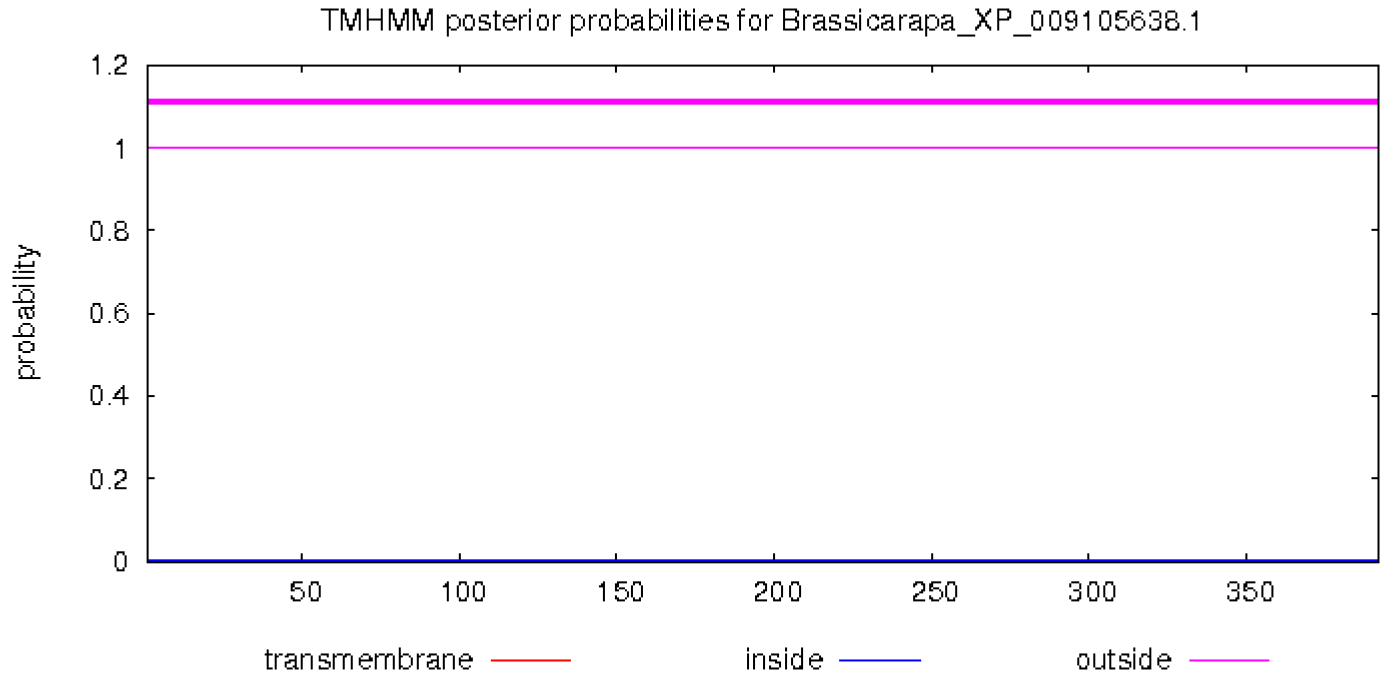

plot in postscript, script for making the plot in gnuplot, data for plot

---

```
# Brassicarapa_XP_009105856.1 Length: 323
# Brassicarapa_XP_009105856.1 Number of predicted TMHs:  0
# Brassicarapa_XP_009105856.1 Exp number of AAs in TMHs: 0
# Brassicarapa_XP_009105856.1 Exp number, first 60 AAs:  0
# Brassicarapa_XP_009105856.1 Total prob of N-in:        0.00781
Brassicarapa_XP_009105856.1     TMHMM2.0        outside      1   323
```

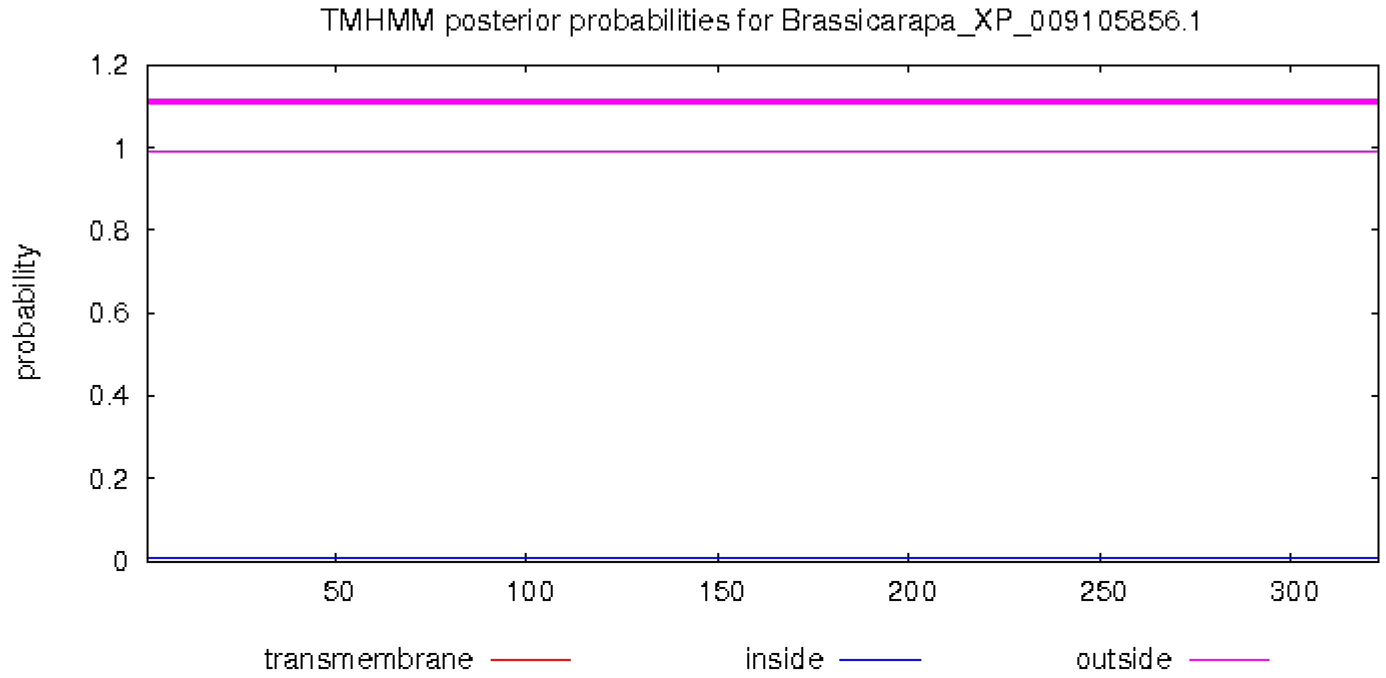

plot in postscript, script for making the plot in gnuplot, data for plot

---

```
# Brassicarapa_XP_009105857.1 Length: 323
# Brassicarapa_XP_009105857.1 Number of predicted TMHs:  0
# Brassicarapa_XP_009105857.1 Exp number of AAs in TMHs: 0
# Brassicarapa_XP_009105857.1 Exp number, first 60 AAs:  0
# Brassicarapa_XP_009105857.1 Total prob of N-in:        0.00781
Brassicarapa_XP_009105857.1     TMHMM2.0        outside      1   323
```

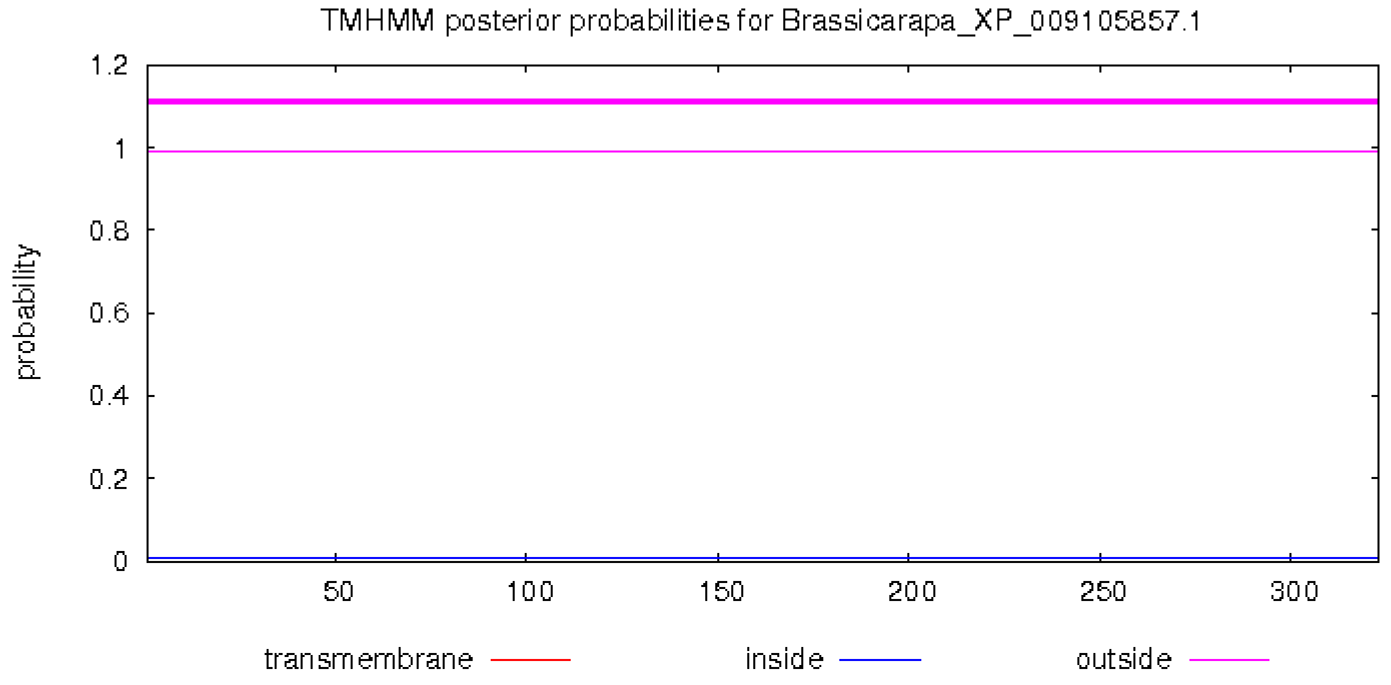

plot in postscript, script for making the plot in gnuplot, data for plot

```
# Brassicarapa_XP_009106303.1 Length: 335
# Brassicarapa_XP_009106303.1 Number of predicted TMHs:  0
# Brassicarapa_XP_009106303.1 Exp number of AAs in TMHs: 0.09061
# Brassicarapa_XP_009106303.1 Exp number, first 60 AAs:  0.02771
# Brassicarapa_XP_009106303.1 Total prob of N-in:        0.00669
Brassicarapa_XP_009106303.1     TMHMM2.0        outside      1   335
```

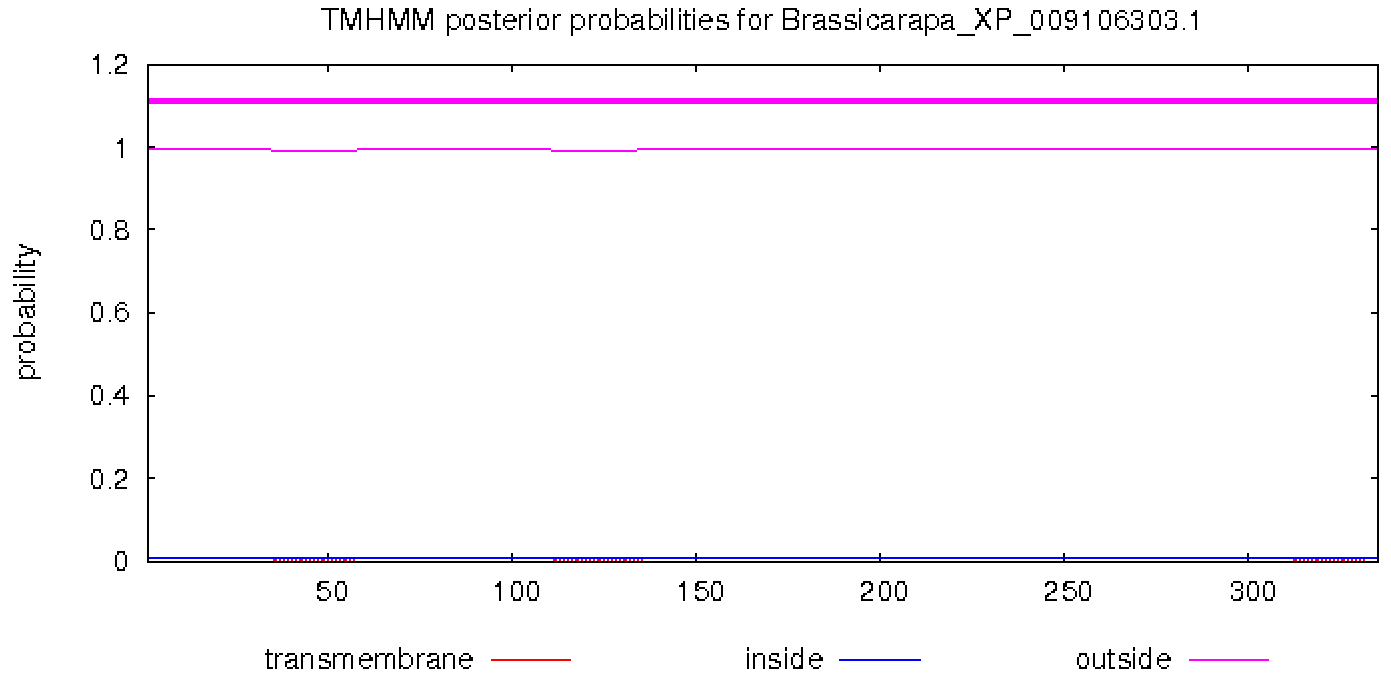

plot in postscript, script for making the plot in gnuplot, data for plot

```
# Brassicarapa_XP_009106681.1 Length: 359
# Brassicarapa_XP_009106681.1 Number of predicted TMHs:  0
# Brassicarapa_XP_009106681.1 Exp number of AAs in TMHs: 0.12979
# Brassicarapa_XP_009106681.1 Exp number, first 60 AAs:  0.00096
# Brassicarapa_XP_009106681.1 Total prob of N-in:        0.00895
Brassicarapa_XP_009106681.1     TMHMM2.0        outside      1   359
```

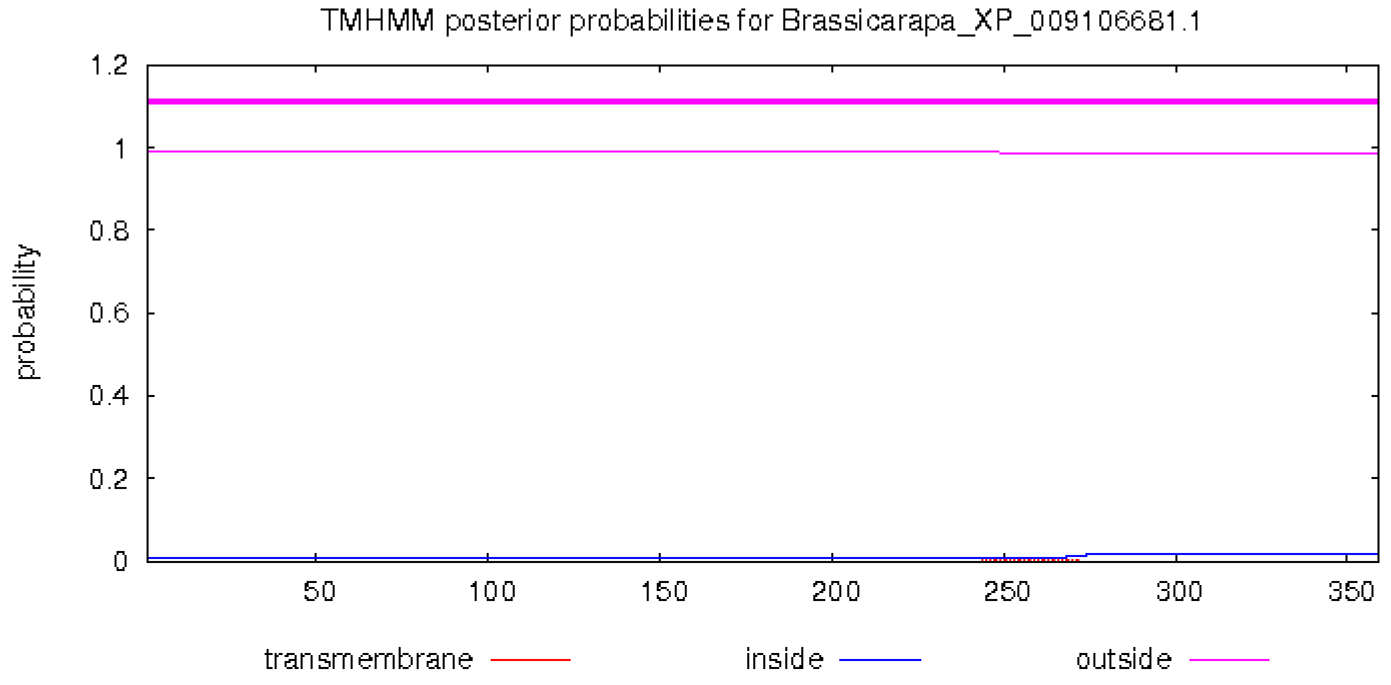

plot in postscript, script for making the plot in gnuplot, data for plot

---

```
# Brassicarapa_XP_009106794.1 Length: 309
# Brassicarapa_XP_009106794.1 Number of predicted TMHs:  0
# Brassicarapa_XP_009106794.1 Exp number of AAs in TMHs: 0
# Brassicarapa_XP_009106794.1 Exp number, first 60 AAs:  0
# Brassicarapa_XP_009106794.1 Total prob of N-in:        0.00128
Brassicarapa_XP_009106794.1     TMHMM2.0        outside      1   309
```

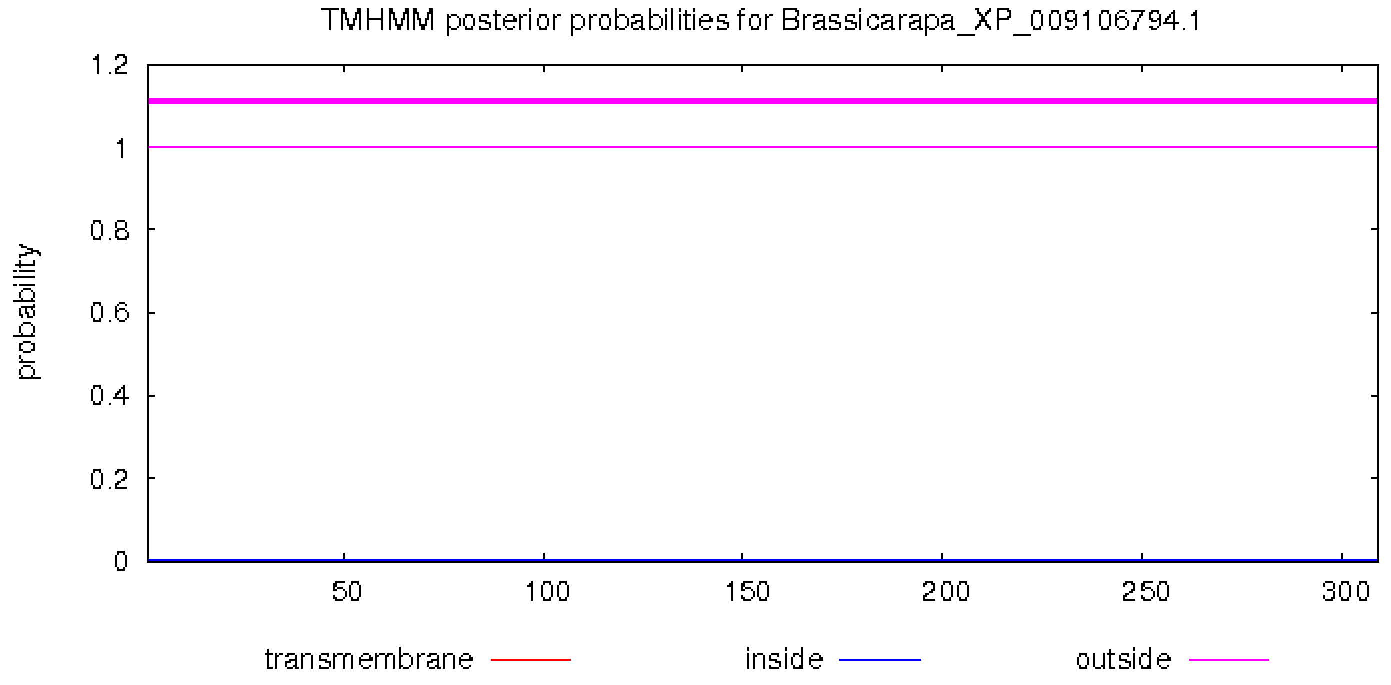

plot in postscript, script for making the plot in gnuplot, data for plot

---

```
# Brassicarapa_XP_009106812.1 Length: 321
# Brassicarapa_XP_009106812.1 Number of predicted TMHs:  0
# Brassicarapa_XP_009106812.1 Exp number of AAs in TMHs: 0.0002
# Brassicarapa_XP_009106812.1 Exp number, first 60 AAs:  0
# Brassicarapa_XP_009106812.1 Total prob of N-in:        0.01548
Brassicarapa_XP_009106812.1     TMHMM2.0        outside      1   321
```

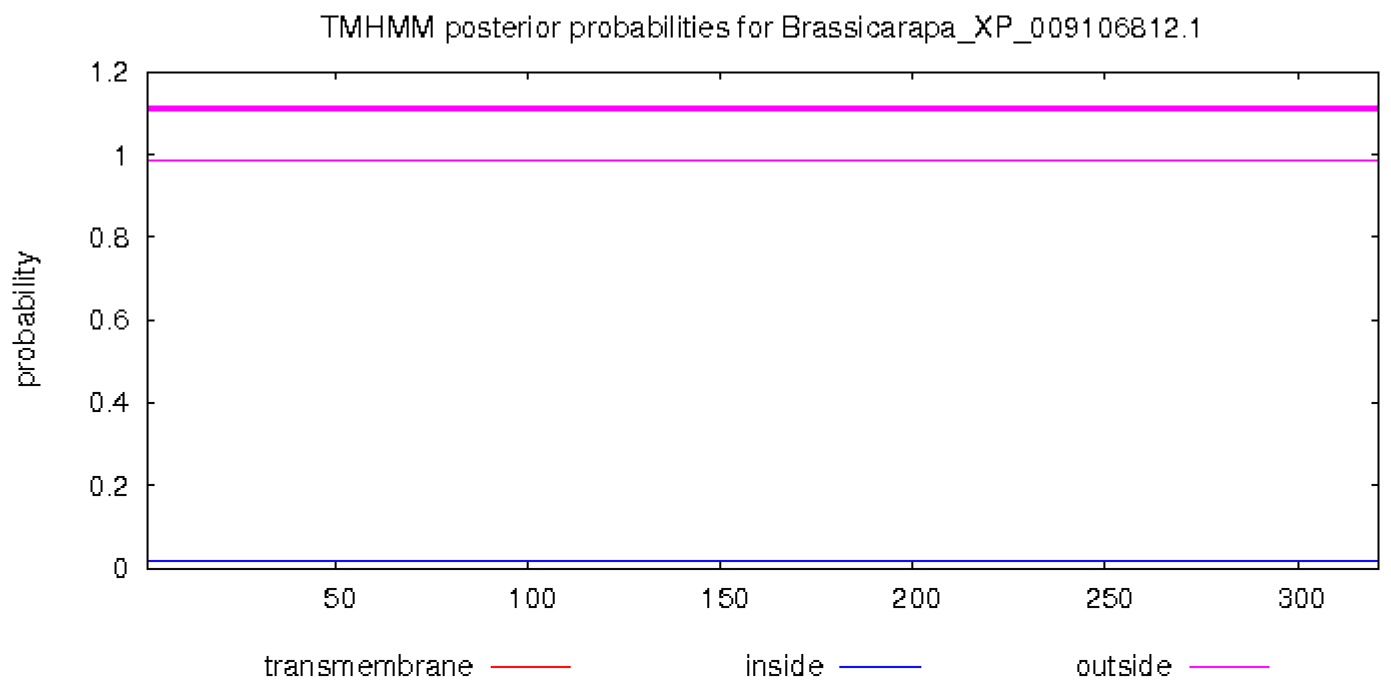

plot in postscript, script for making the plot in gnuplot, data for plot

```
# Brassicarapa_XP_009107934.1 Length: 547
# Brassicarapa_XP_009107934.1 Number of predicted TMHs:  1
# Brassicarapa_XP_009107934.1 Exp number of AAs in TMHs: 23.16592
# Brassicarapa_XP_009107934.1 Exp number, first 60 AAs:  0.00244
# Brassicarapa_XP_009107934.1 Total prob of N-in:        0.77979
Brassicarapa_XP_009107934.1     TMHMM2.0        inside       1   516
Brassicarapa_XP_009107934.1     TMHMM2.0        TMhelix    517   539
Brassicarapa_XP_009107934.1     TMHMM2.0        outside    540   547
```

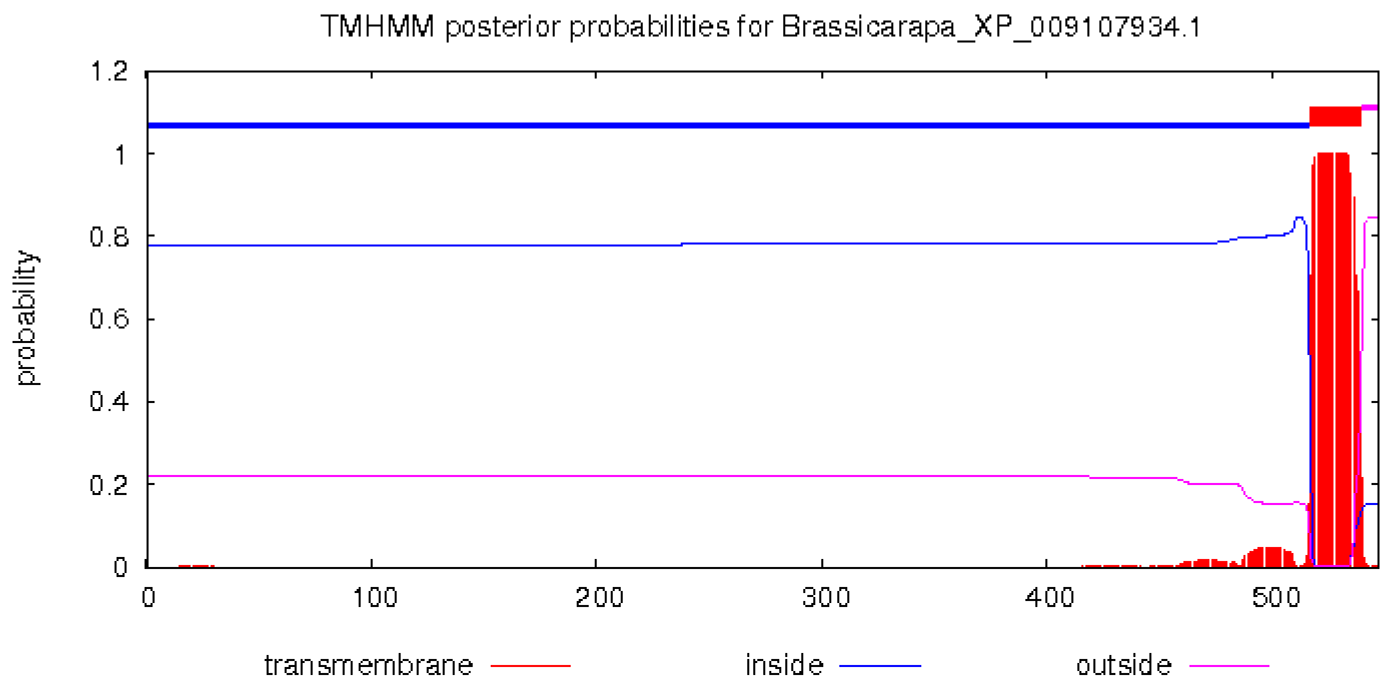

plot in postscript, script for making the plot in gnuplot, data for plot

```
# Brassicarapa_XP_009107969.1 Length: 305
# Brassicarapa_XP_009107969.1 Number of predicted TMHs:  0
# Brassicarapa_XP_009107969.1 Exp number of AAs in TMHs: 0.00126
# Brassicarapa_XP_009107969.1 Exp number, first 60 AAs:  0
```

```
# Brassicarapa_XP_009107969.1 Total prob of N-in:        0.03479
Brassicarapa_XP_009107969.1     TMHMM2.0        outside      1   305
```

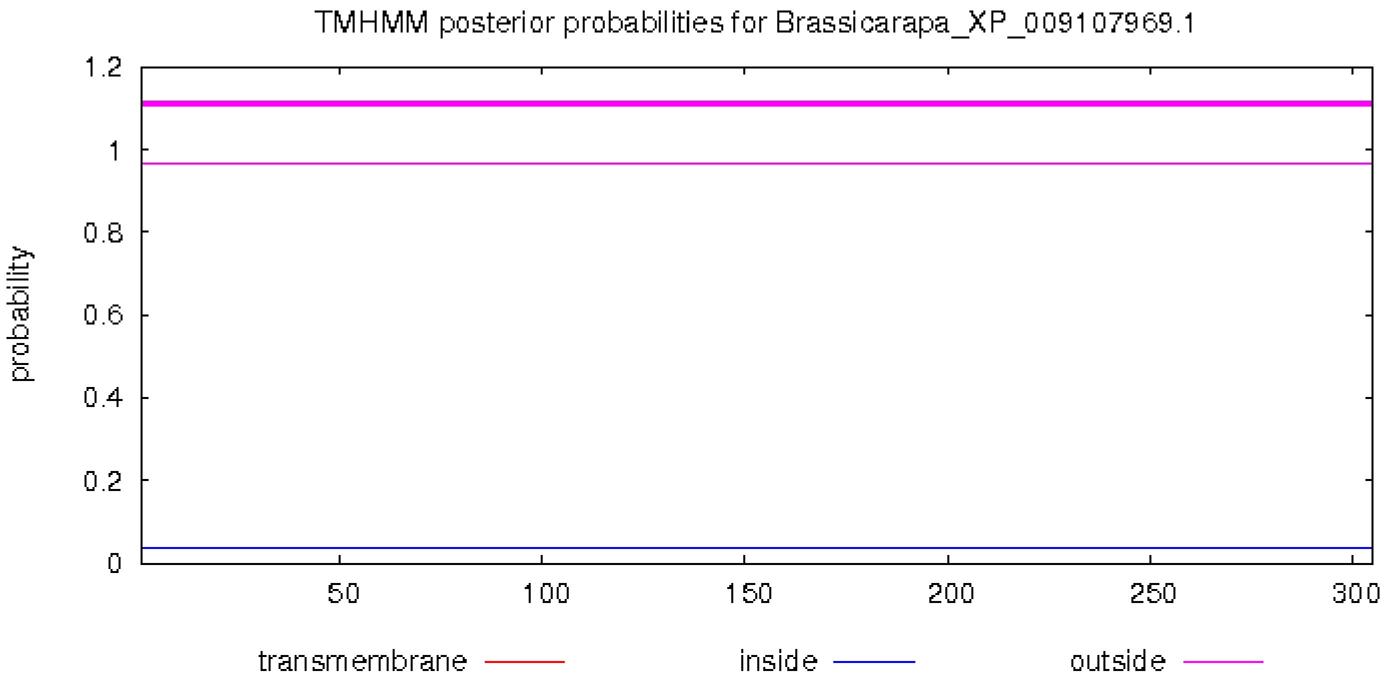

plot in postscript, script for making the plot in gnuplot, data for plot

---

```
# Brassicarapa_XP_009107971.1 Length: 305
# Brassicarapa_XP_009107971.1 Number of predicted TMHs:  0
# Brassicarapa_XP_009107971.1 Exp number of AAs in TMHs: 0.00126
# Brassicarapa_XP_009107971.1 Exp number, first 60 AAs:  0
# Brassicarapa_XP_009107971.1 Total prob of N-in:        0.03479
Brassicarapa_XP_009107971.1     TMHMM2.0        outside      1   305
```

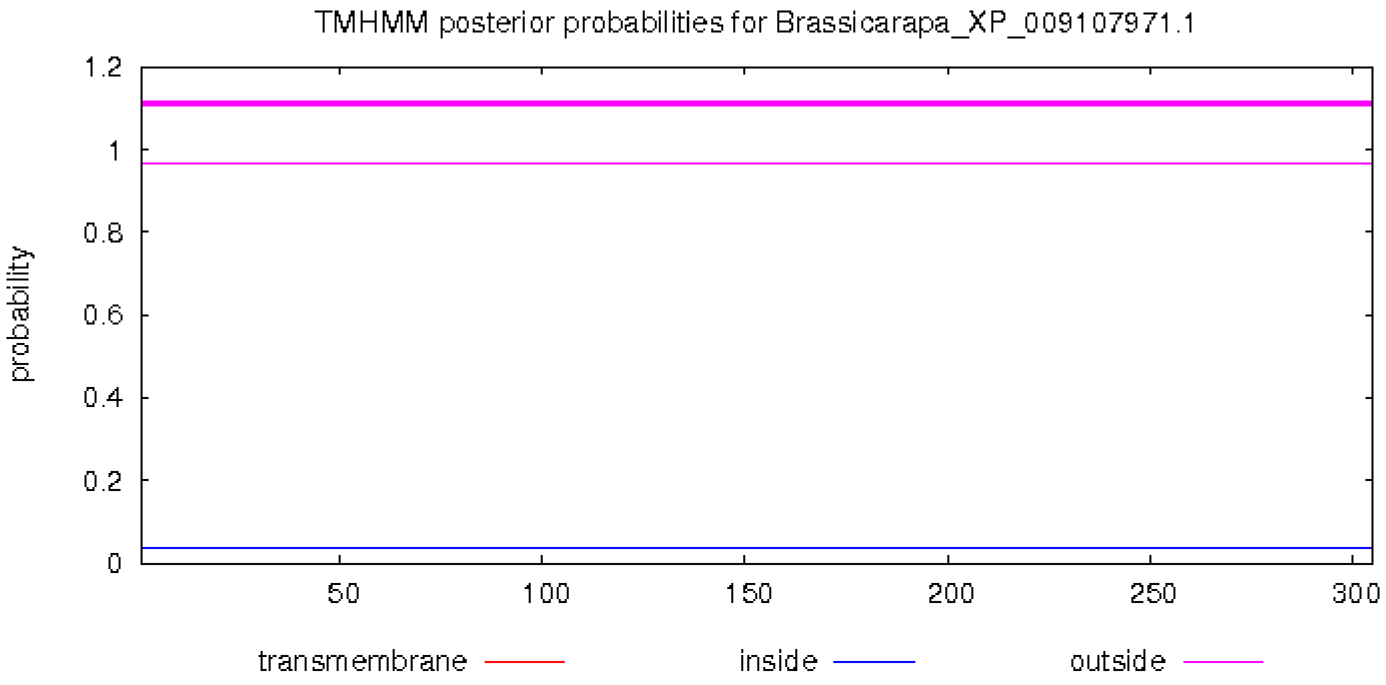

plot in postscript, script for making the plot in gnuplot, data for plot

---

```
# Brassicarapa_XP_009107972.1 Length: 286
# Brassicarapa_XP_009107972.1 Number of predicted TMHs:  0
# Brassicarapa_XP_009107972.1 Exp number of AAs in TMHs: 0.00025
# Brassicarapa_XP_009107972.1 Exp number, first 60 AAs:  0
```

```
# Brassicarapa_XP_009107972.1 Total prob of N-in:        0.00682
Brassicarapa_XP_009107972.1     TMHMM2.0        outside      1   286
```

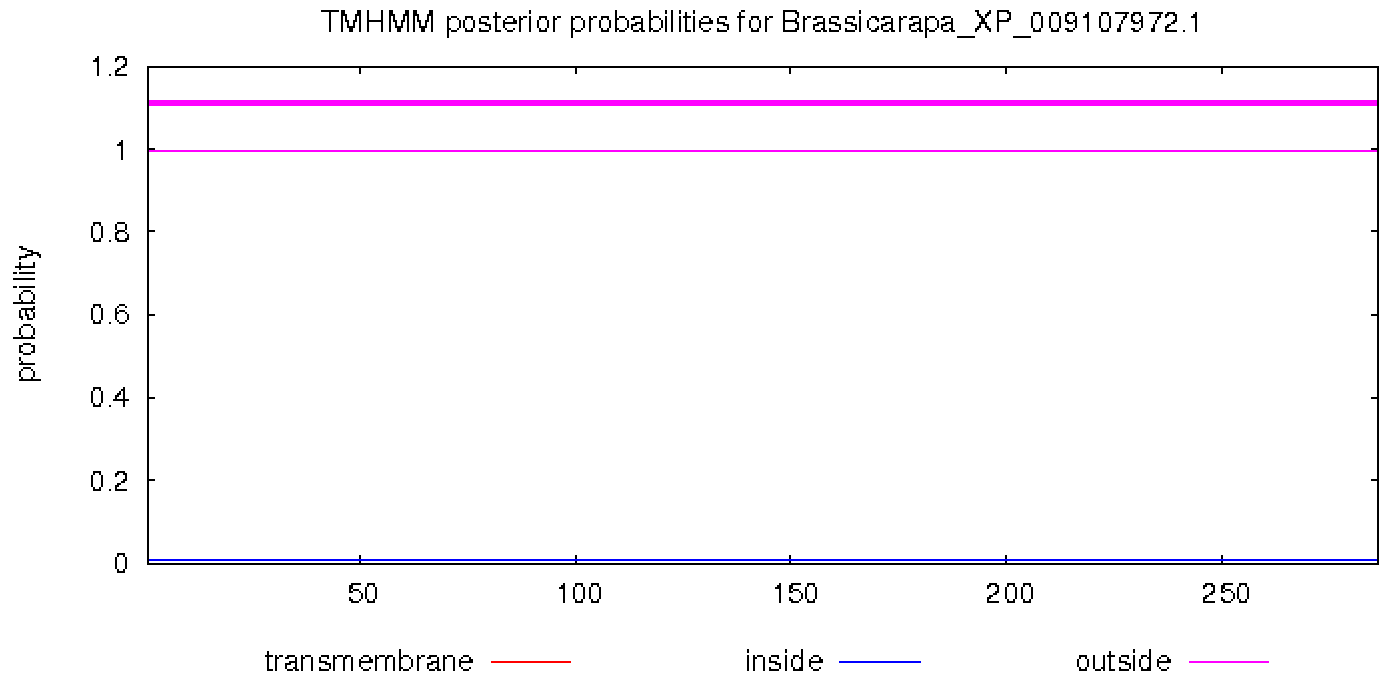

plot in postscript, script for making the plot in gnuplot, data for plot

---

```
# Brassicarapa_XP_009109087.1 Length: 347
# Brassicarapa_XP_009109087.1 Number of predicted TMHs:  0
# Brassicarapa_XP_009109087.1 Exp number of AAs in TMHs: 0
# Brassicarapa_XP_009109087.1 Exp number, first 60 AAs:  0
# Brassicarapa_XP_009109087.1 Total prob of N-in:        0.00510
Brassicarapa_XP_009109087.1     TMHMM2.0        outside      1   347
```

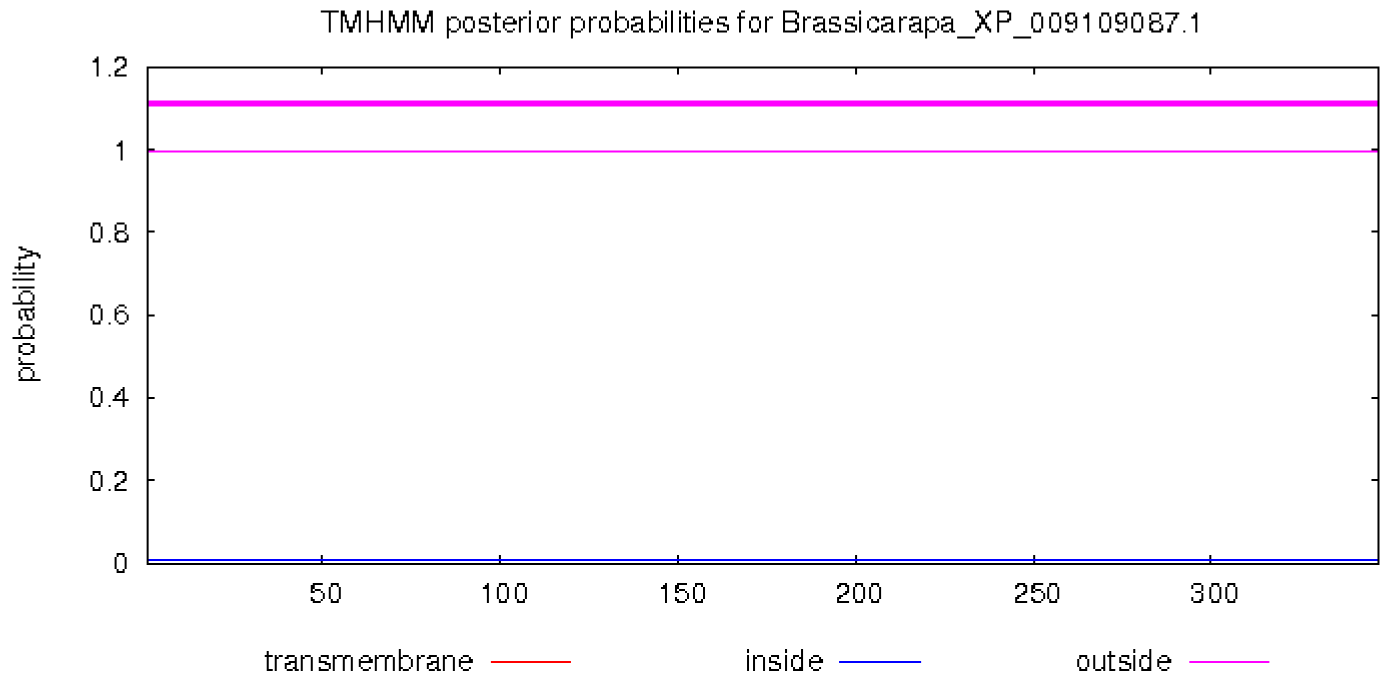

plot in postscript, script for making the plot in gnuplot, data for plot

---

```
# Brassicarapa_XP_009110048.1 Length: 412
# Brassicarapa_XP_009110048.1 Number of predicted TMHs:  0
# Brassicarapa_XP_009110048.1 Exp number of AAs in TMHs: 0.00019
# Brassicarapa_XP_009110048.1 Exp number, first 60 AAs:  0
```

```
# Brassicarapa_XP_009110048.1 Total prob of N-in:        0.01564
Brassicarapa_XP_009110048.1     TMHMM2.0        outside      1   412
```

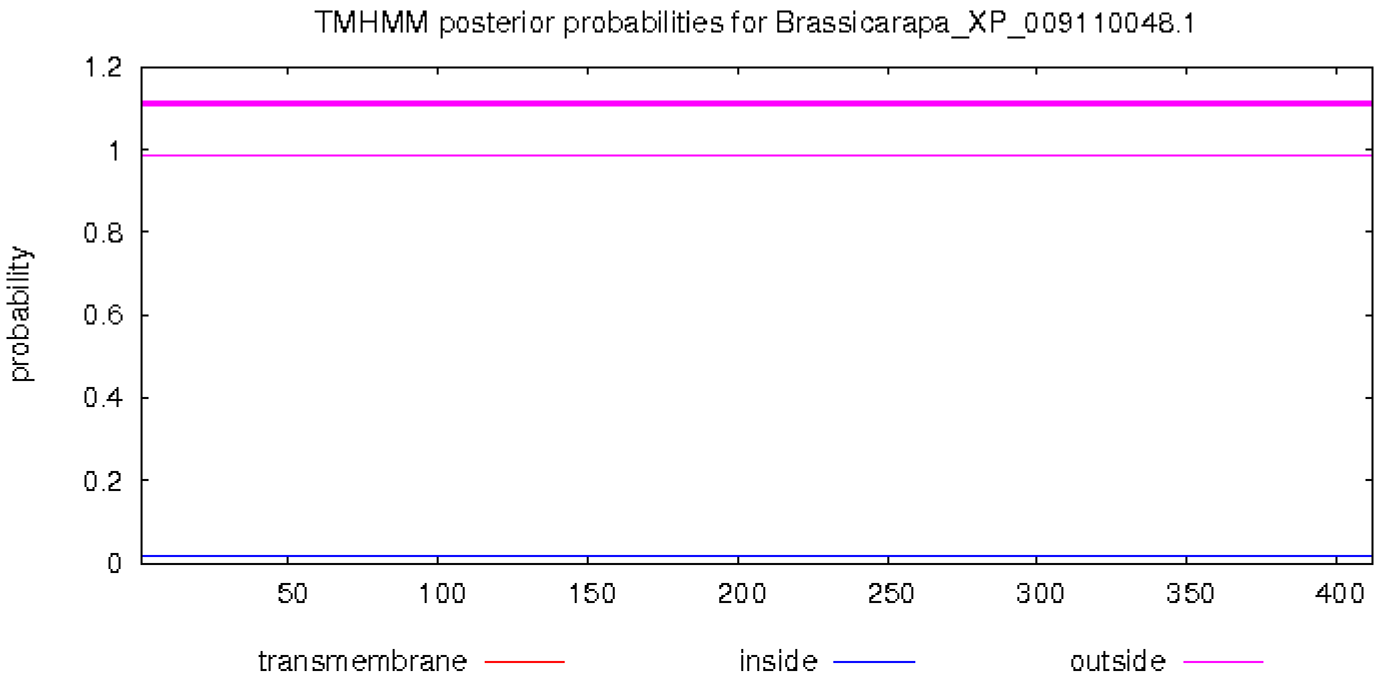

plot in postscript, script for making the plot in gnuplot, data for plot

---

```
# Brassicarapa_XP_009110661.1 Length: 388
# Brassicarapa_XP_009110661.1 Number of predicted TMHs:  0
# Brassicarapa_XP_009110661.1 Exp number of AAs in TMHs: 0.00018
# Brassicarapa_XP_009110661.1 Exp number, first 60 AAs:  0
# Brassicarapa_XP_009110661.1 Total prob of N-in:        0.00182
Brassicarapa_XP_009110661.1     TMHMM2.0        outside      1   388
```

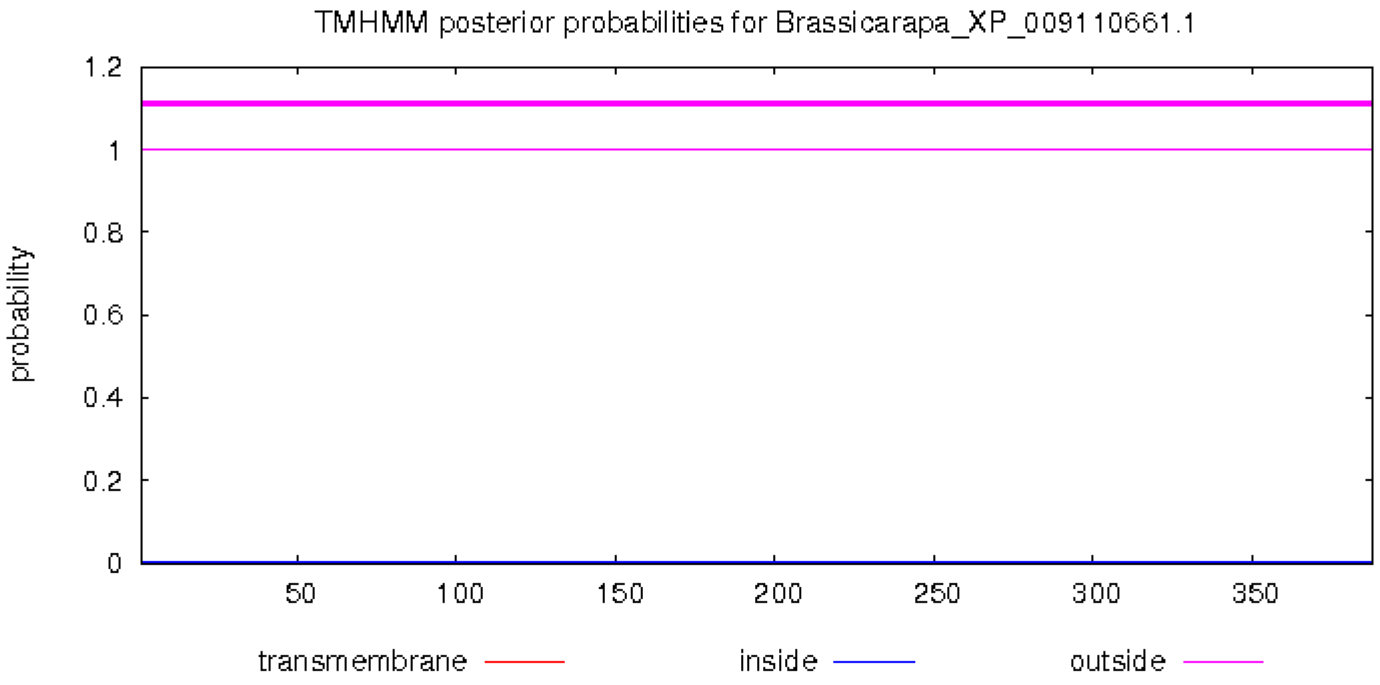

plot in postscript, script for making the plot in gnuplot, data for plot

---

```
# Brassicarapa_XP_009110662.1 Length: 340
# Brassicarapa_XP_009110662.1 Number of predicted TMHs:  0
# Brassicarapa_XP_009110662.1 Exp number of AAs in TMHs: 0.00018
# Brassicarapa_XP_009110662.1 Exp number, first 60 AAs:  0
```

```
# Brassicarapa_XP_009110662.1 Total prob of N-in:        0.00330
Brassicarapa_XP_009110662.1     TMHMM2.0        outside      1   340
```

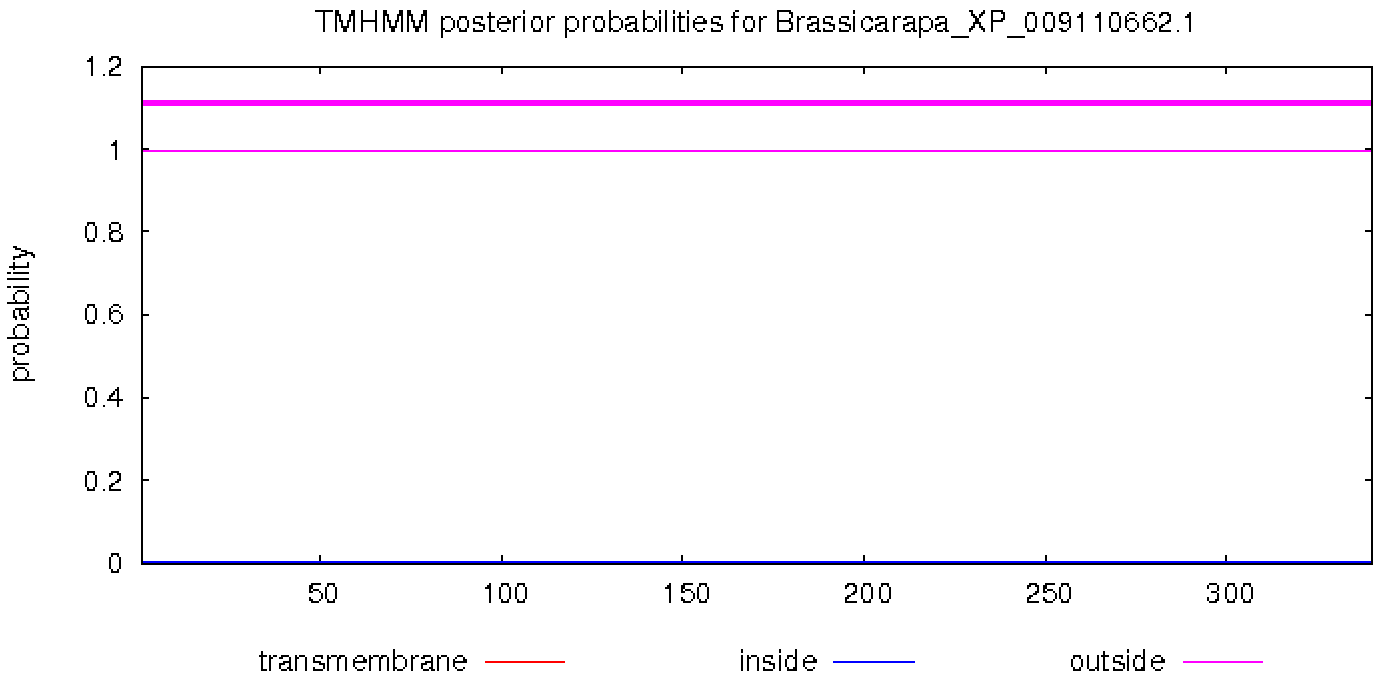

plot in postscript, script for making the plot in gnuplot, data for plot

---

```
# Brassicarapa_XP_009110663.1 Length: 339
# Brassicarapa_XP_009110663.1 Number of predicted TMHs:  0
# Brassicarapa_XP_009110663.1 Exp number of AAs in TMHs: 0.00018
# Brassicarapa_XP_009110663.1 Exp number, first 60 AAs:  0
# Brassicarapa_XP_009110663.1 Total prob of N-in:        0.00335
Brassicarapa_XP_009110663.1     TMHMM2.0        outside      1   339
```

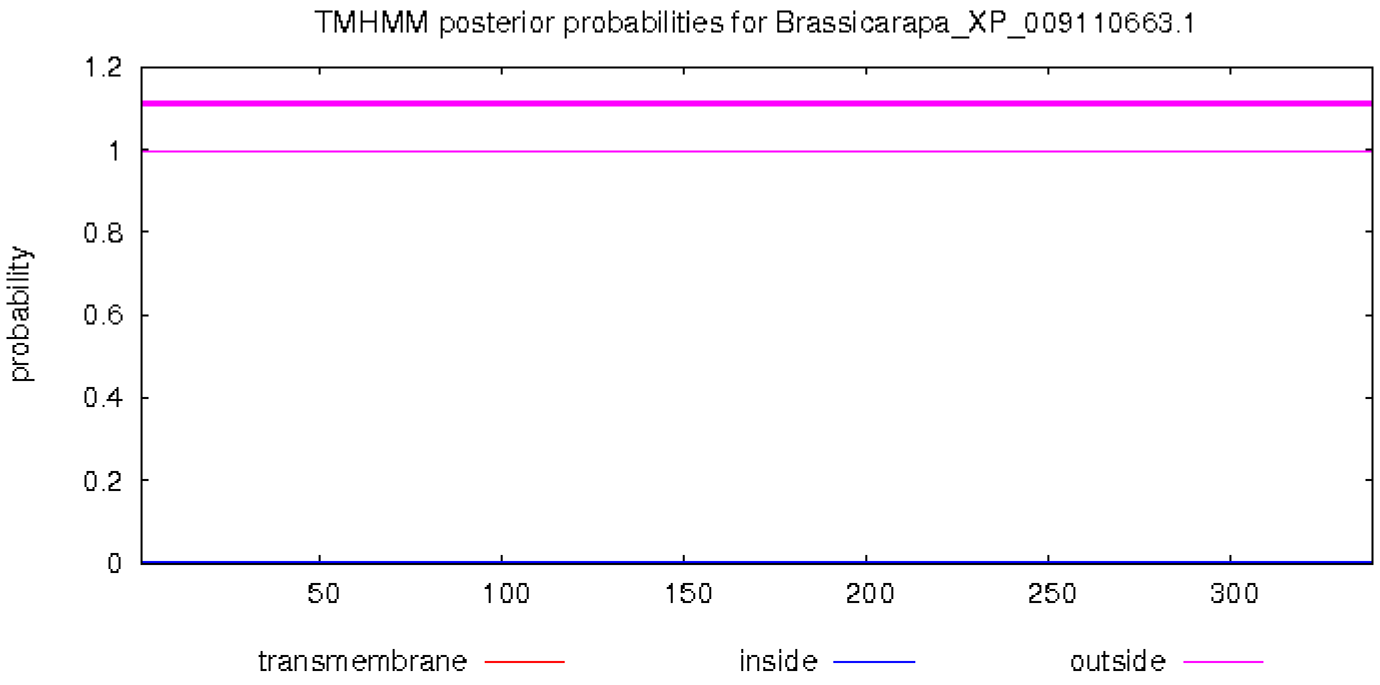

plot in postscript, script for making the plot in gnuplot, data for plot

---

```
# Brassicarapa_XP_009111333.1 Length: 553
# Brassicarapa_XP_009111333.1 Number of predicted TMHs:  0
# Brassicarapa_XP_009111333.1 Exp number of AAs in TMHs: 0.00122
# Brassicarapa_XP_009111333.1 Exp number, first 60 AAs:  0.00088
```

```
# Brassicarapa_XP_009111333.1 Total prob of N-in:        0.00508
Brassicarapa_XP_009111333.1     TMHMM2.0        outside      1   553
```

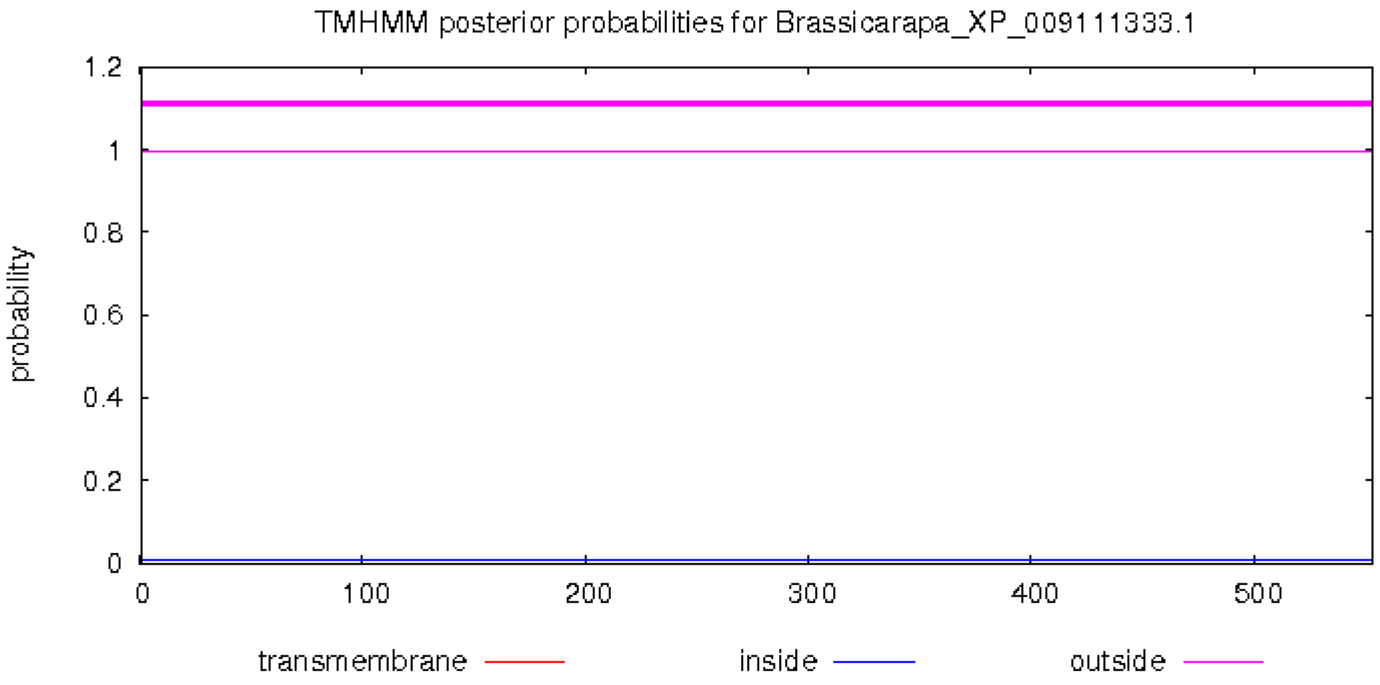

plot in postscript, script for making the plot in gnuplot, data for plot

---

```
# Brassicarapa_XP_009111357.1 Length: 425
# Brassicarapa_XP_009111357.1 Number of predicted TMHs:  1
# Brassicarapa_XP_009111357.1 Exp number of AAs in TMHs: 22.4619
# Brassicarapa_XP_009111357.1 Exp number, first 60 AAs:  0.0009
# Brassicarapa_XP_009111357.1 Total prob of N-in:        0.71591
Brassicarapa_XP_009111357.1     TMHMM2.0        inside       1   396
Brassicarapa_XP_009111357.1     TMHMM2.0        TMhelix    397   419
Brassicarapa_XP_009111357.1     TMHMM2.0        outside    420   425
```

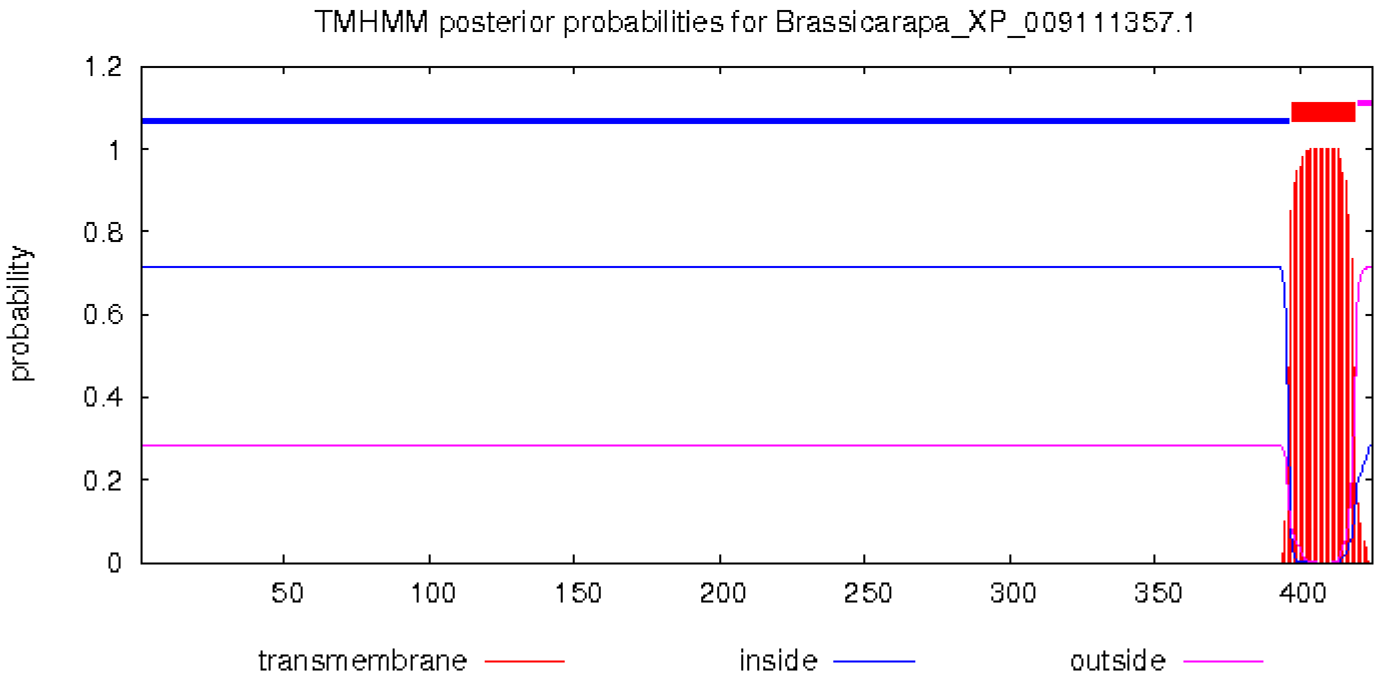

plot in postscript, script for making the plot in gnuplot, data for plot

---

```
# Brassicarapa_XP_009111358.1 Length: 288
# Brassicarapa_XP_009111358.1 Number of predicted TMHs:  0
```

```
# Brassicarapa_XP_009111358.1 Exp number of AAs in TMHs: 0.00055
# Brassicarapa_XP_009111358.1 Exp number, first 60 AAs:  0.00016
# Brassicarapa_XP_009111358.1 Total prob of N-in:        0.01881
Brassicarapa_XP_009111358.1	TMHMM2.0	outside	     1   288
```

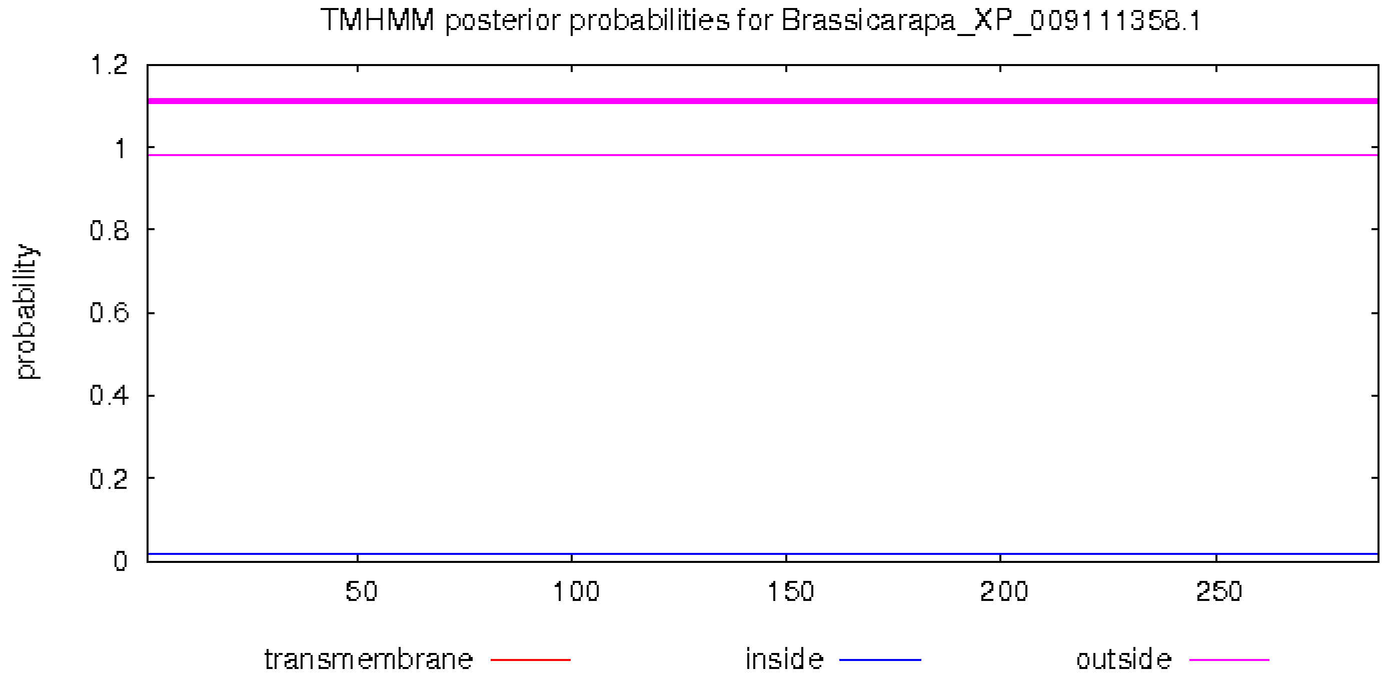

plot in postscript, script for making the plot in gnuplot, data for plot

---

```
# Brassicarapa_XP_009111374.1 Length: 311
# Brassicarapa_XP_009111374.1 Number of predicted TMHs:  0
# Brassicarapa_XP_009111374.1 Exp number of AAs in TMHs: 0.00622
# Brassicarapa_XP_009111374.1 Exp number, first 60 AAs:  0.00088
# Brassicarapa_XP_009111374.1 Total prob of N-in:        0.02457
Brassicarapa_XP_009111374.1	TMHMM2.0	outside	     1   311
```

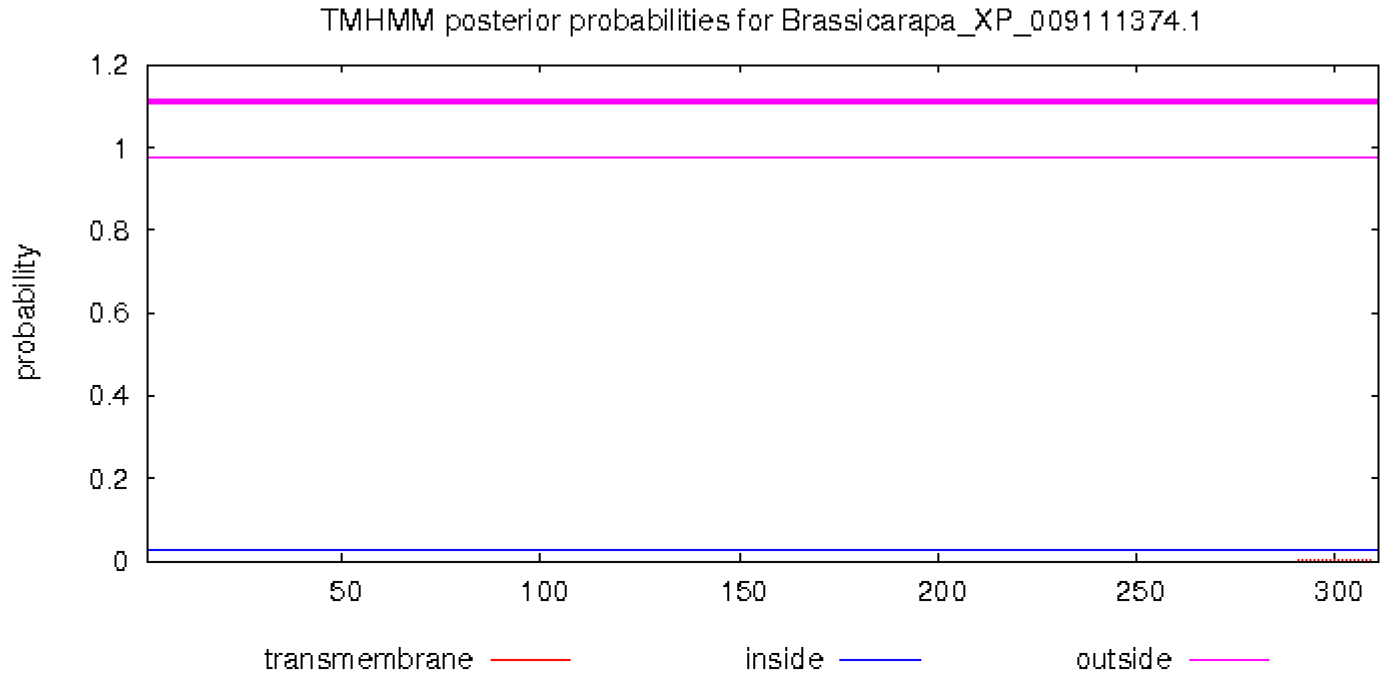

plot in postscript, script for making the plot in gnuplot, data for plot

---

```
# Brassicarapa_XP_009111638.1 Length: 446
# Brassicarapa_XP_009111638.1 Number of predicted TMHs:  0
```

```
# Brassicarapa_XP_009111638.1 Exp number of AAs in TMHs: 10.82317
# Brassicarapa_XP_009111638.1 Exp number, first 60 AAs:  0
# Brassicarapa_XP_009111638.1 Total prob of N-in:        0.00947
Brassicarapa_XP_009111638.1     TMHMM2.0        outside      1   446
```

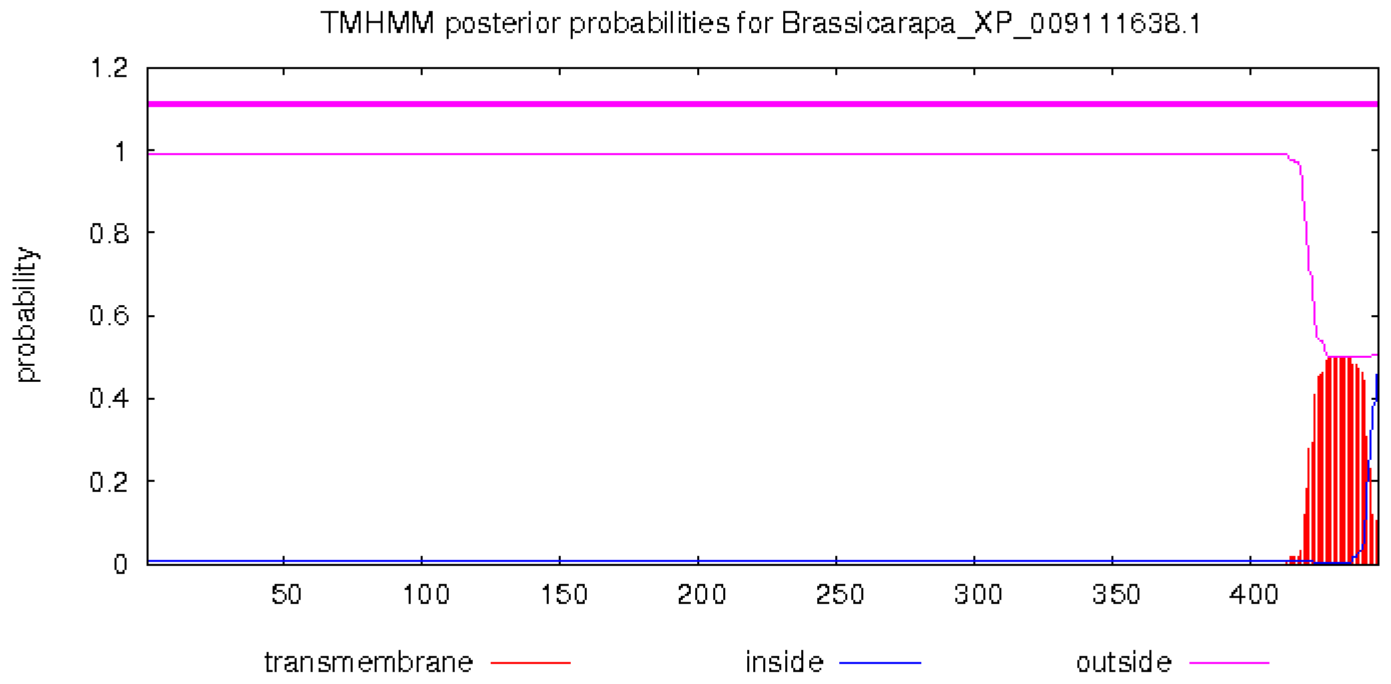

plot in postscript, script for making the plot in gnuplot, data for plot

---

```
# Brassicarapa_XP_009111639.1 Length: 445
# Brassicarapa_XP_009111639.1 Number of predicted TMHs:  0
# Brassicarapa_XP_009111639.1 Exp number of AAs in TMHs: 10.82447
# Brassicarapa_XP_009111639.1 Exp number, first 60 AAs:  0
# Brassicarapa_XP_009111639.1 Total prob of N-in:        0.00959
Brassicarapa_XP_009111639.1     TMHMM2.0        outside      1   445
```

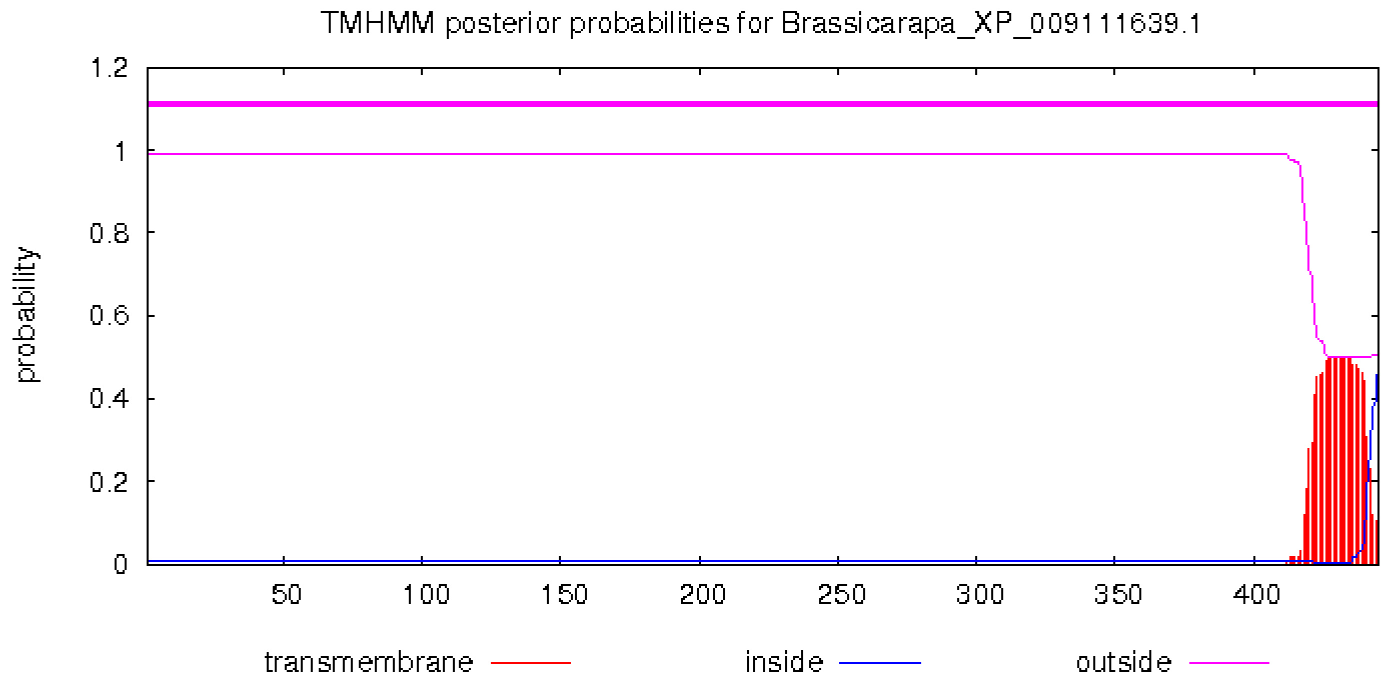

plot in postscript, script for making the plot in gnuplot, data for plot

---

```
# Brassicarapa_XP_009111778.1 Length: 328
# Brassicarapa_XP_009111778.1 Number of predicted TMHs:  0
```

```
# Brassicarapa_XP_009111778.1 Exp number of AAs in TMHs: 0.01078
# Brassicarapa_XP_009111778.1 Exp number, first 60 AAs:  0.00493
# Brassicarapa_XP_009111778.1 Total prob of N-in:        0.02733
Brassicarapa_XP_009111778.1     TMHMM2.0        outside      1   328
```

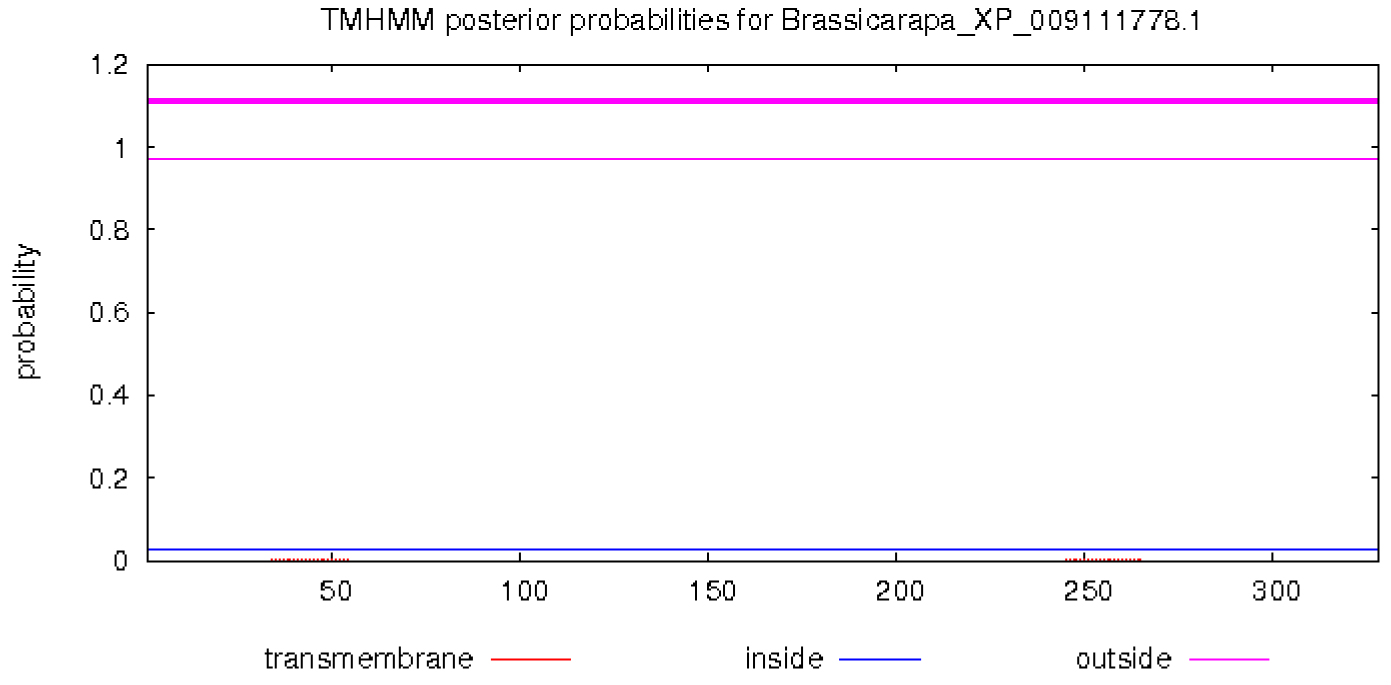

plot in postscript, script for making the plot in gnuplot, data for plot

---

```
# Brassicarapa_XP_009112020.1 Length: 332
# Brassicarapa_XP_009112020.1 Number of predicted TMHs:  0
# Brassicarapa_XP_009112020.1 Exp number of AAs in TMHs: 0
# Brassicarapa_XP_009112020.1 Exp number, first 60 AAs:  0
# Brassicarapa_XP_009112020.1 Total prob of N-in:        0.00231
Brassicarapa_XP_009112020.1     TMHMM2.0        outside      1   332
```

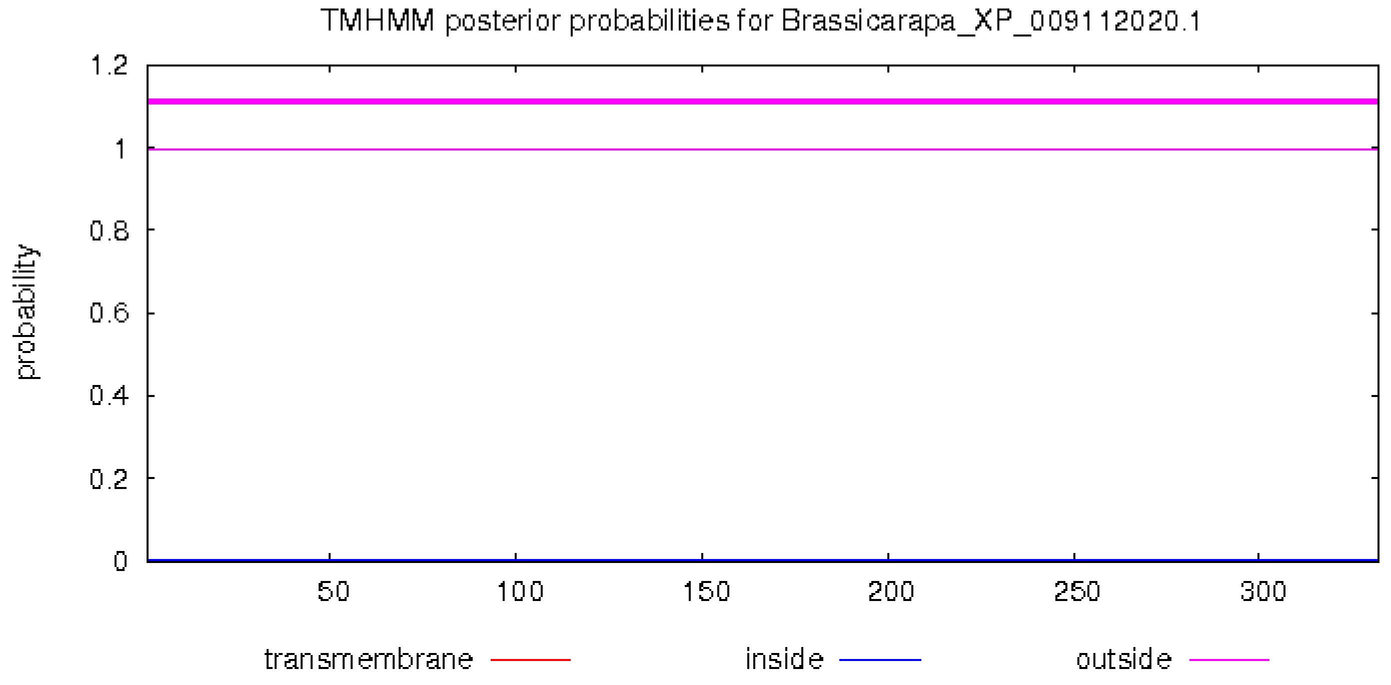

plot in postscript, script for making the plot in gnuplot, data for plot

---

```
# Brassicarapa_XP_009112137.1 Length: 188
# Brassicarapa_XP_009112137.1 Number of predicted TMHs:  0
```

```
# Brassicarapa_XP_009112137.1 Exp number of AAs in TMHs: 0
# Brassicarapa_XP_009112137.1 Exp number, first 60 AAs:  0
# Brassicarapa_XP_009112137.1 Total prob of N-in:        0.00724
Brassicarapa_XP_009112137.1	TMHMM2.0	outside	     1   188
```

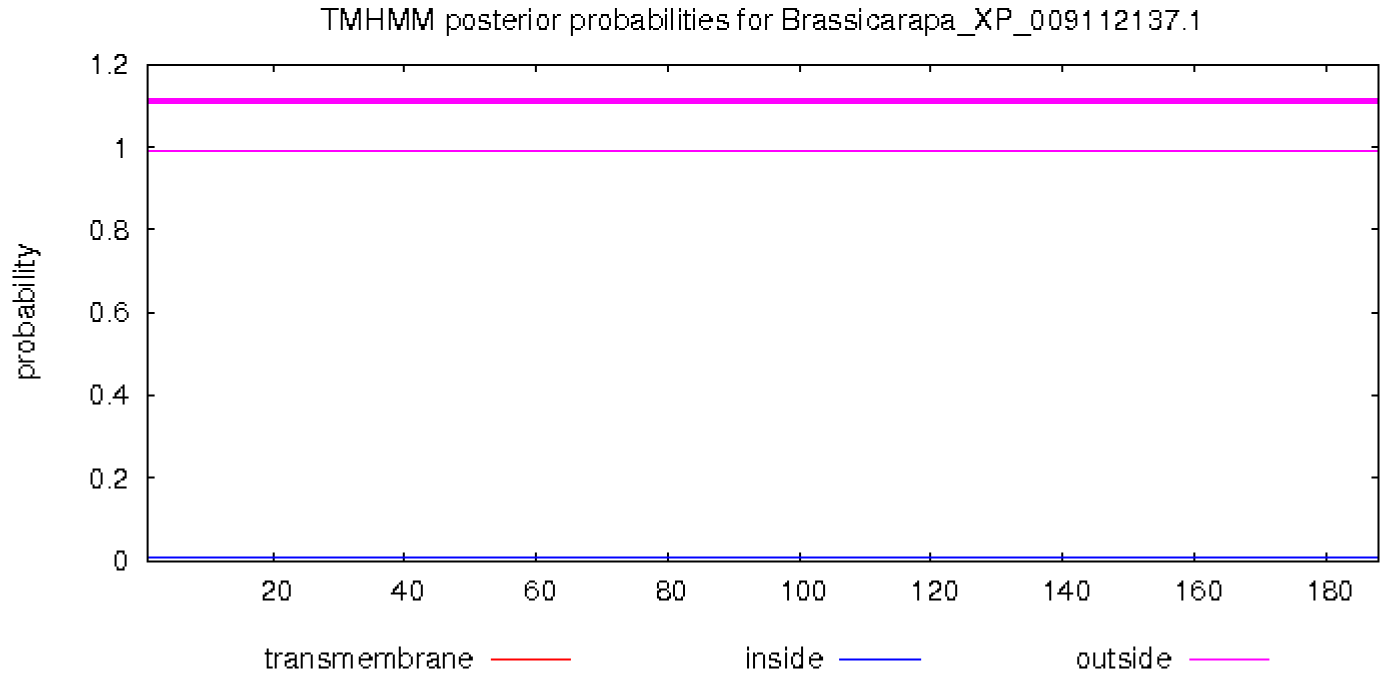

plot in postscript, script for making the plot in gnuplot, data for plot

---

```
# Brassicarapa_XP_009112413.1 Length: 279
# Brassicarapa_XP_009112413.1 Number of predicted TMHs:  0
# Brassicarapa_XP_009112413.1 Exp number of AAs in TMHs: 0.00206
# Brassicarapa_XP_009112413.1 Exp number, first 60 AAs:  0.00013
# Brassicarapa_XP_009112413.1 Total prob of N-in:        0.00600
Brassicarapa_XP_009112413.1	TMHMM2.0	outside	     1   279
```

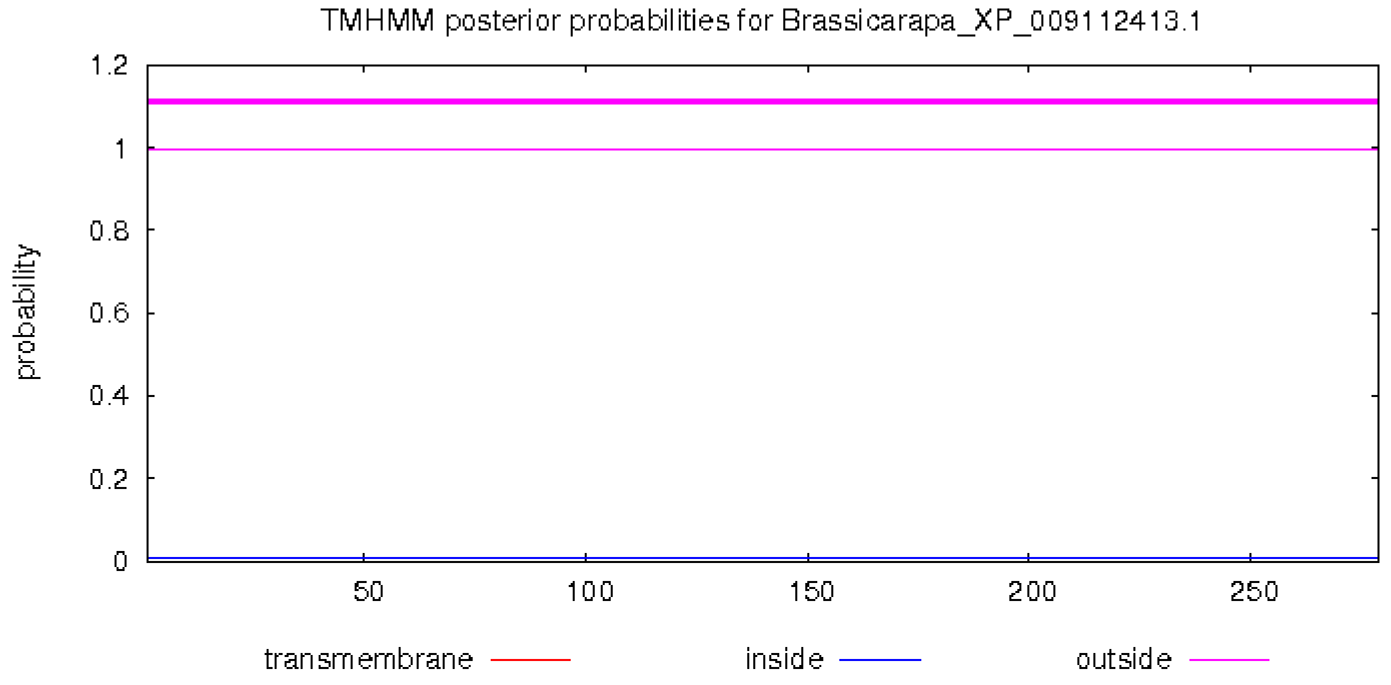

plot in postscript, script for making the plot in gnuplot, data for plot

---

```
# Brassicarapa_XP_009112744.1 Length: 300
# Brassicarapa_XP_009112744.1 Number of predicted TMHs:  0
```

```
# Brassicarapa_XP_009112744.1 Exp number of AAs in TMHs: 0.55261
# Brassicarapa_XP_009112744.1 Exp number, first 60 AAs:  0
# Brassicarapa_XP_009112744.1 Total prob of N-in:        0.00536
Brassicarapa_XP_009112744.1     TMHMM2.0        outside      1   300
```

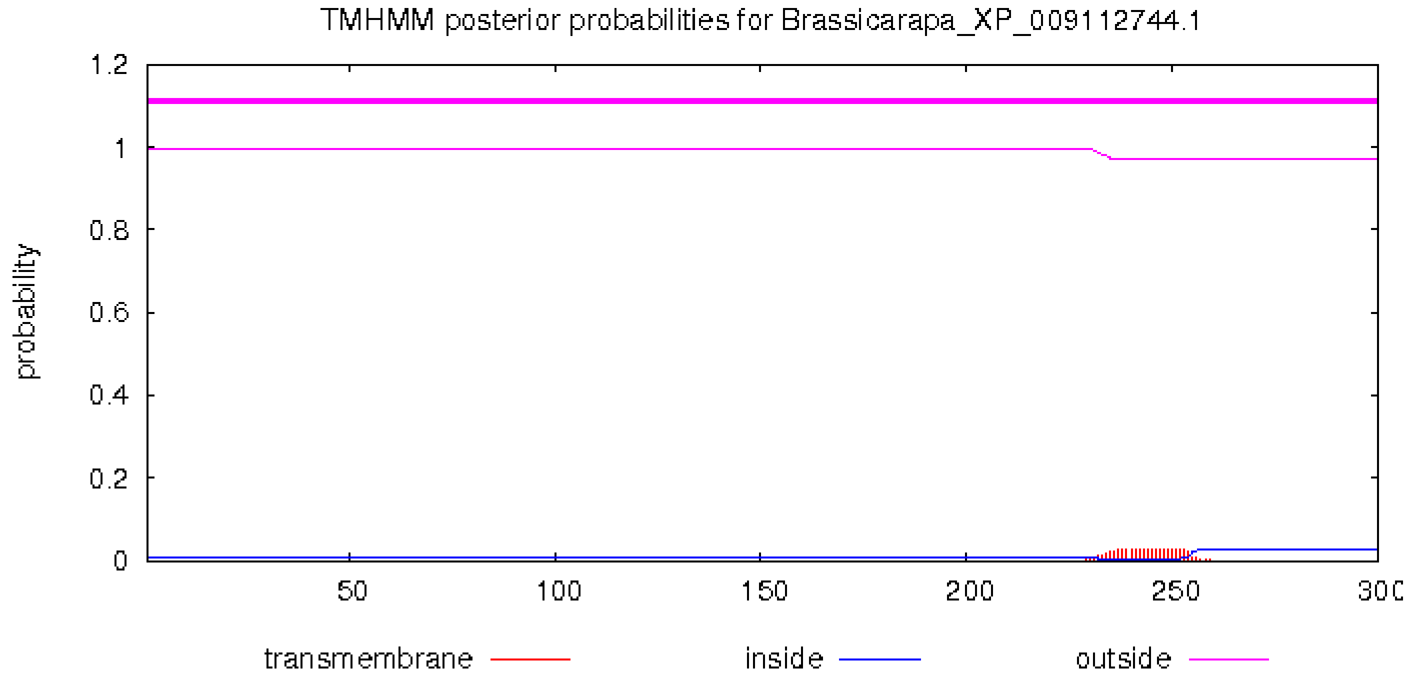

plot in postscript, script for making the plot in gnuplot, data for plot

---

```
# Brassicarapa_XP_009113056.1 Length: 402
# Brassicarapa_XP_009113056.1 Number of predicted TMHs:  0
# Brassicarapa_XP_009113056.1 Exp number of AAs in TMHs: 0.00017
# Brassicarapa_XP_009113056.1 Exp number, first 60 AAs:  0
# Brassicarapa_XP_009113056.1 Total prob of N-in:        0.00153
Brassicarapa_XP_009113056.1     TMHMM2.0        outside      1   402
```

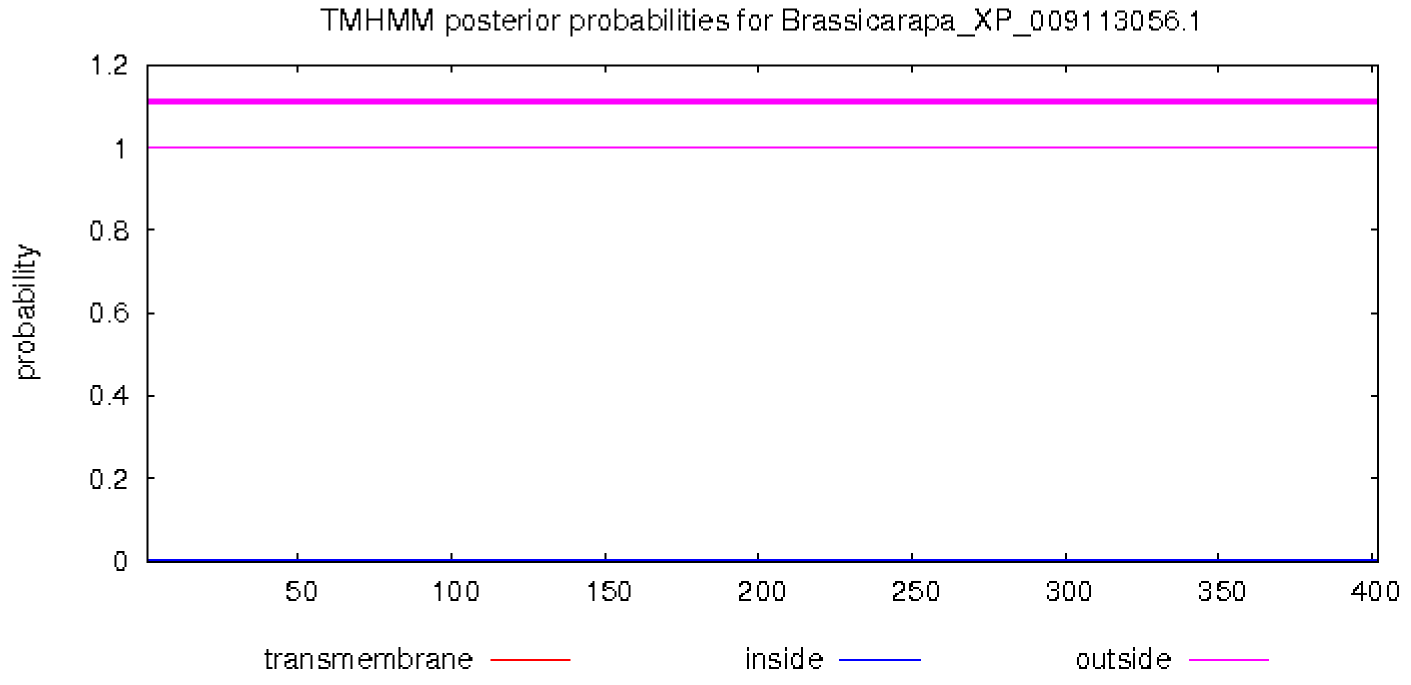

plot in postscript, script for making the plot in gnuplot, data for plot

---

```
# Brassicarapa_XP_009113057.1 Length: 402
# Brassicarapa_XP_009113057.1 Number of predicted TMHs:  0
```

```
# Brassicarapa_XP_009113057.1 Exp number of AAs in TMHs: 0.00017
# Brassicarapa_XP_009113057.1 Exp number, first 60 AAs:  0
# Brassicarapa_XP_009113057.1 Total prob of N-in:        0.00153
Brassicarapa_XP_009113057.1     TMHMM2.0        outside      1   402
```

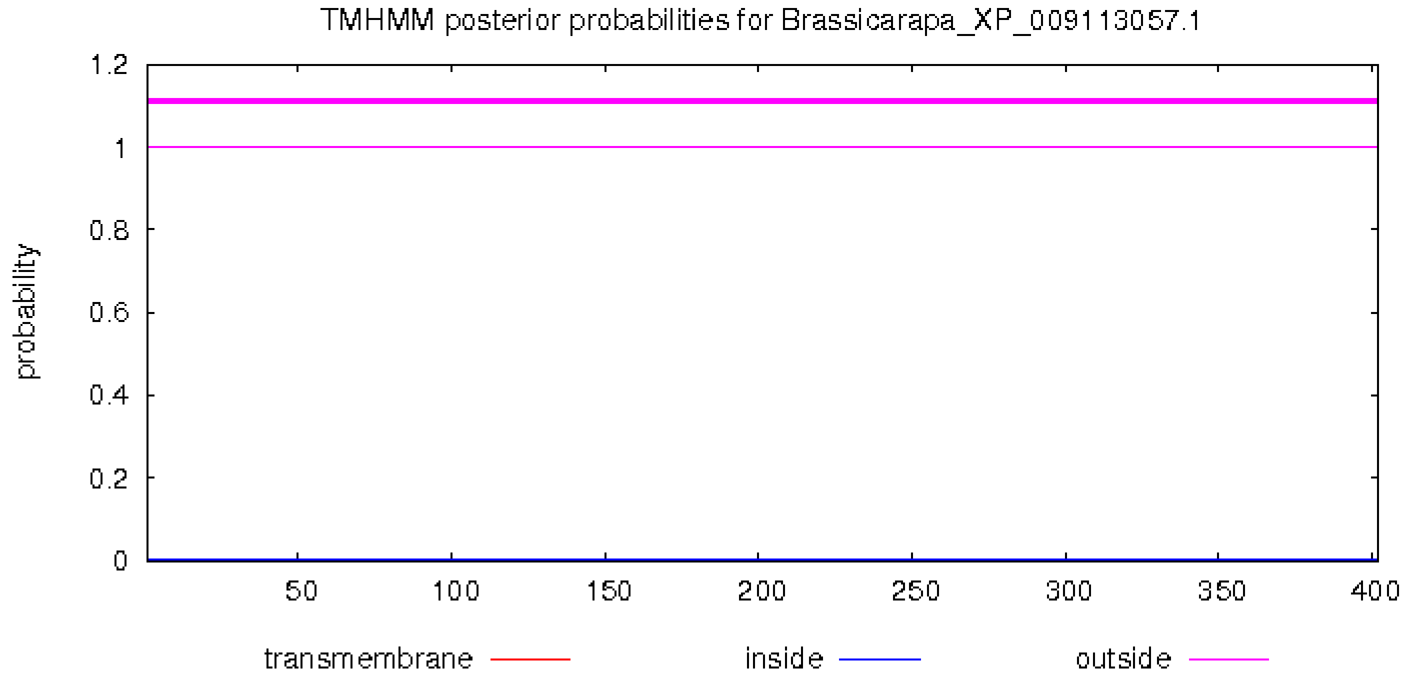

plot in postscript, script for making the plot in gnuplot, data for plot

---

```
# Brassicarapa_XP_009113148.1 Length: 322
# Brassicarapa_XP_009113148.1 Number of predicted TMHs:  0
# Brassicarapa_XP_009113148.1 Exp number of AAs in TMHs: 0.000980000000000000001
# Brassicarapa_XP_009113148.1 Exp number, first 60 AAs:  0.00054
# Brassicarapa_XP_009113148.1 Total prob of N-in:        0.01616
Brassicarapa_XP_009113148.1     TMHMM2.0        outside      1   322
```

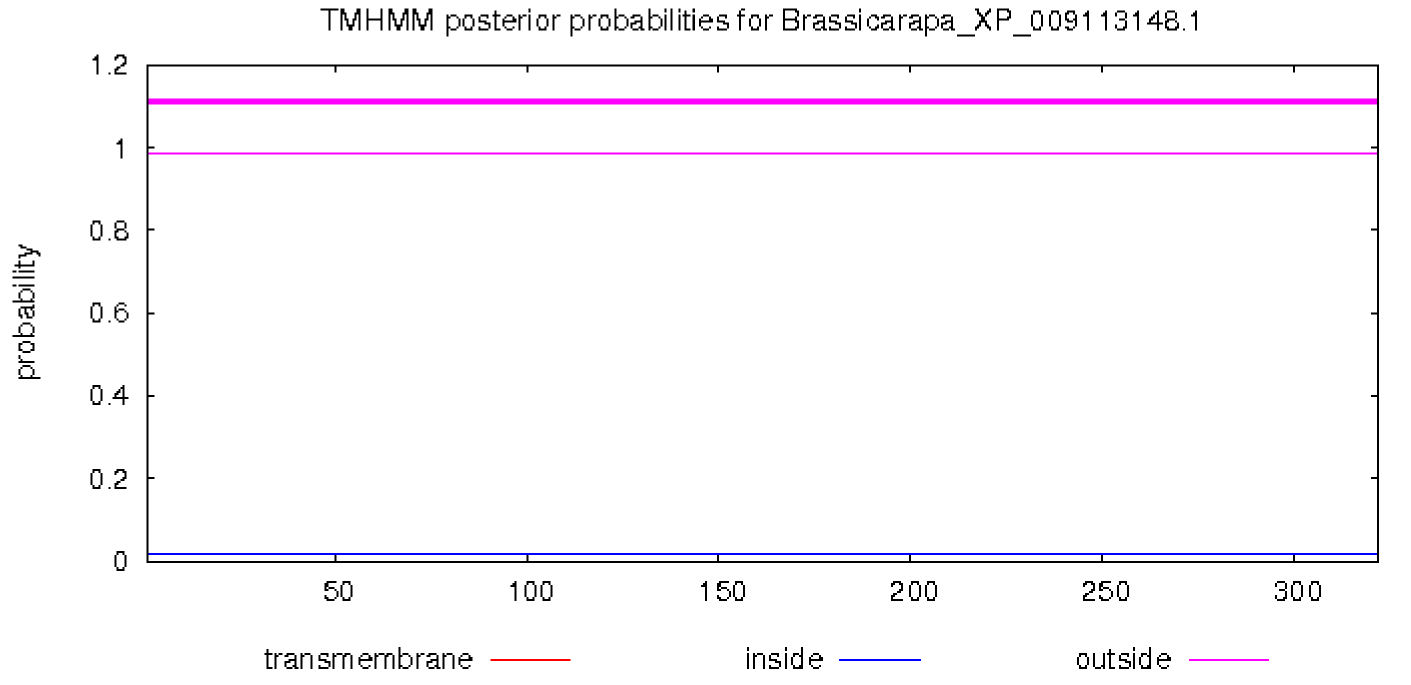

plot in postscript, script for making the plot in gnuplot, data for plot

---

```
# Brassicarapa_XP_009113605.1 Length: 342
# Brassicarapa_XP_009113605.1 Number of predicted TMHs:  0
```

```
# Brassicarapa_XP_009113605.1 Exp number of AAs in TMHs: 0
# Brassicarapa_XP_009113605.1 Exp number, first 60 AAs:  0
# Brassicarapa_XP_009113605.1 Total prob of N-in:        0.00431
Brassicarapa_XP_009113605.1     TMHMM2.0        outside      1   342
```

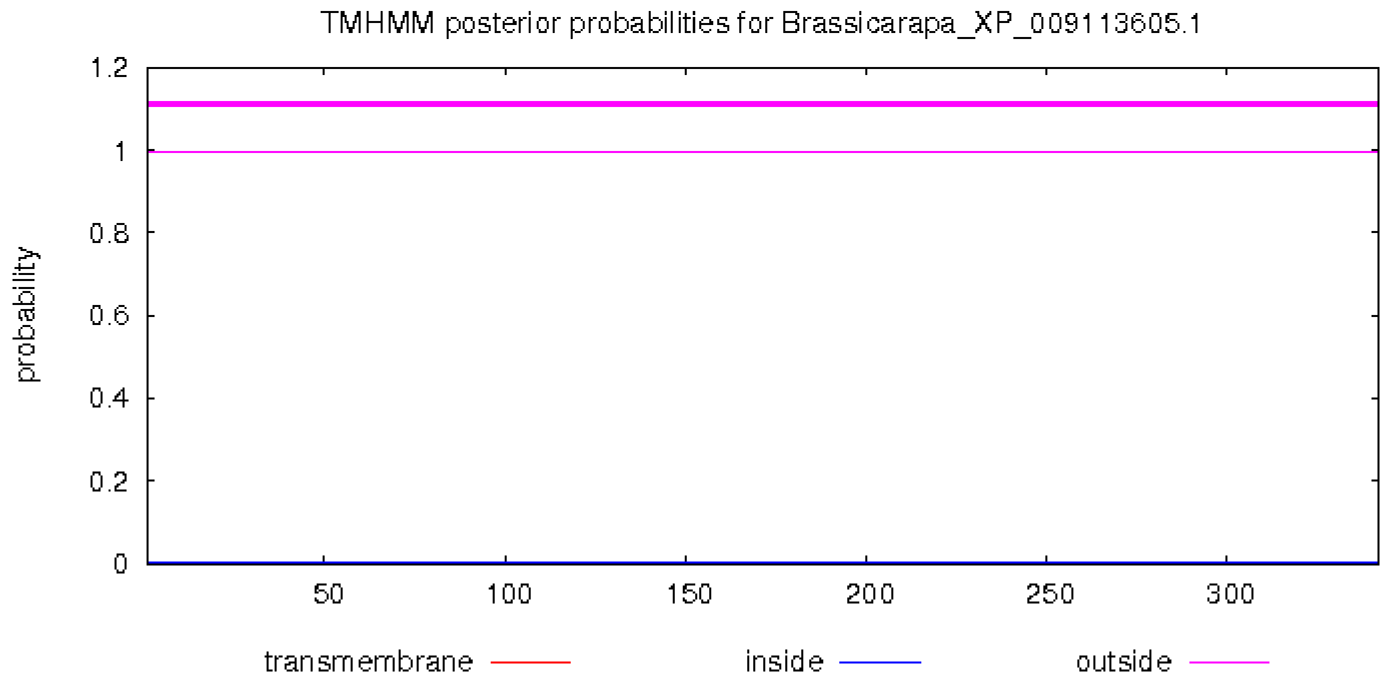

plot in postscript, script for making the plot in gnuplot, data for plot

---

```
# Brassicarapa_XP_009113606.1 Length: 307
# Brassicarapa_XP_009113606.1 Number of predicted TMHs:  0
# Brassicarapa_XP_009113606.1 Exp number of AAs in TMHs: 0
# Brassicarapa_XP_009113606.1 Exp number, first 60 AAs:  0
# Brassicarapa_XP_009113606.1 Total prob of N-in:        0.00665
Brassicarapa_XP_009113606.1     TMHMM2.0        outside      1   307
```

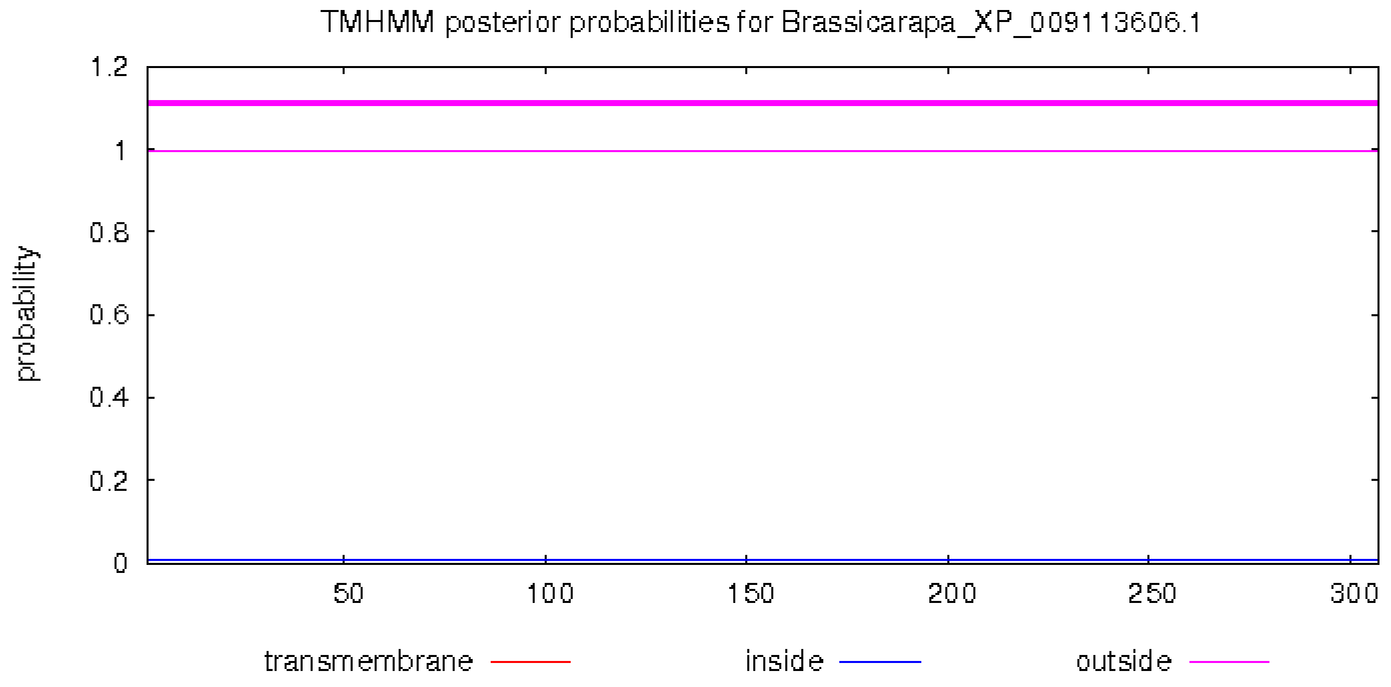

plot in postscript, script for making the plot in gnuplot, data for plot

---

```
# Brassicarapa_XP_009113660.1 Length: 311
# Brassicarapa_XP_009113660.1 Number of predicted TMHs:  0
```

```
# Brassicarapa_XP_009113660.1 Exp number of AAs in TMHs: 0.00021
# Brassicarapa_XP_009113660.1 Exp number, first 60 AAs:  0
# Brassicarapa_XP_009113660.1 Total prob of N-in:        0.02097
Brassicarapa_XP_009113660.1     TMHMM2.0        outside      1   311
```

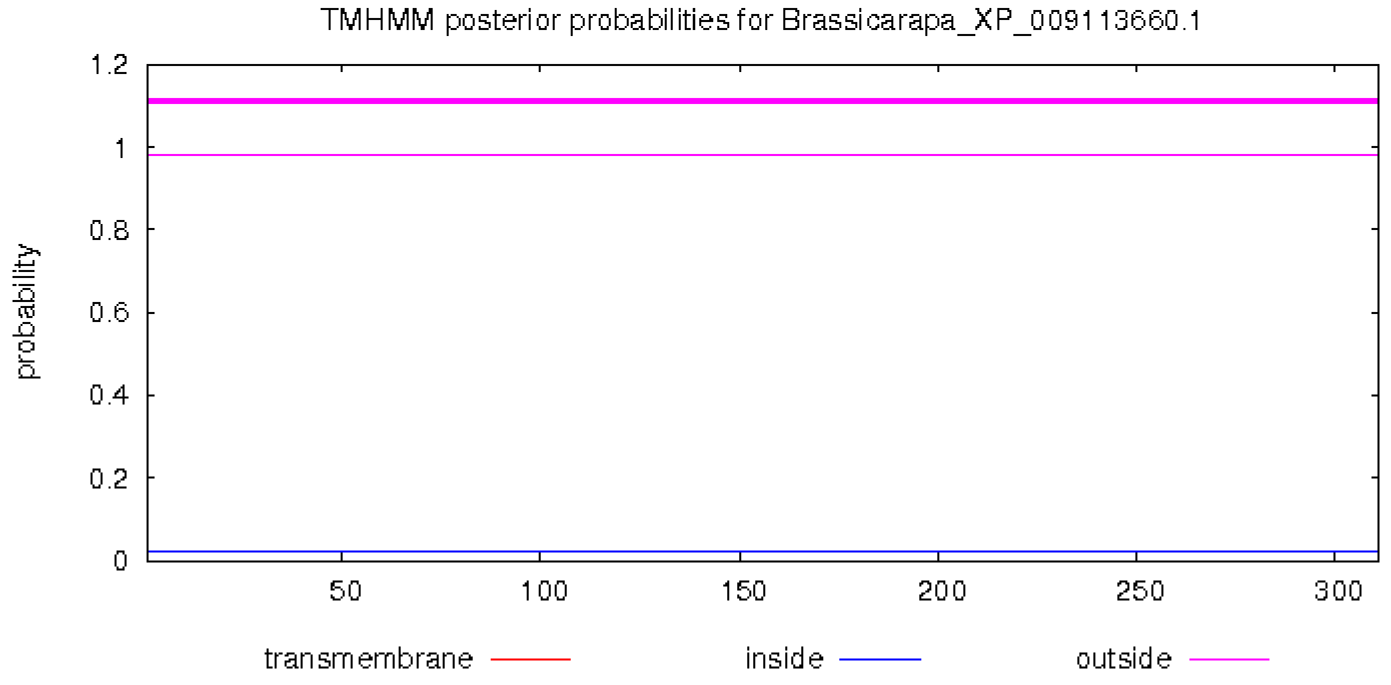

plot in postscript, script for making the plot in gnuplot, data for plot

---

```
# Brassicarapa_XP_009113875.1 Length: 242
# Brassicarapa_XP_009113875.1 Number of predicted TMHs:  0
# Brassicarapa_XP_009113875.1 Exp number of AAs in TMHs: 0.00667
# Brassicarapa_XP_009113875.1 Exp number, first 60 AAs:  0.0056
# Brassicarapa_XP_009113875.1 Total prob of N-in:        0.12068
Brassicarapa_XP_009113875.1     TMHMM2.0        outside      1   242
```

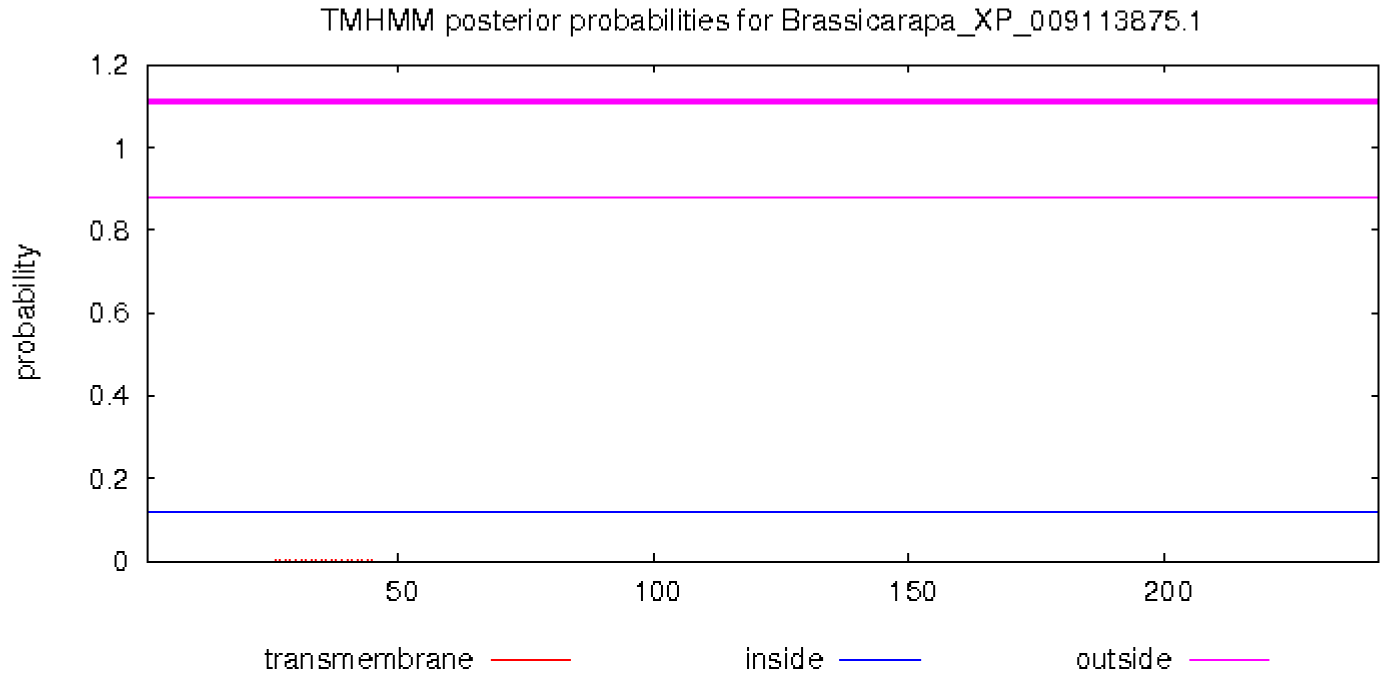

plot in postscript, script for making the plot in gnuplot, data for plot

---

```
# Brassicarapa_XP_009114197.1 Length: 408
# Brassicarapa_XP_009114197.1 Number of predicted TMHs:  0
```

```
# Brassicarapa_XP_009114197.1 Exp number of AAs in TMHs: 0.04585
# Brassicarapa_XP_009114197.1 Exp number, first 60 AAs:  0
# Brassicarapa_XP_009114197.1 Total prob of N-in:        0.01417
Brassicarapa_XP_009114197.1     TMHMM2.0        outside      1   408
```

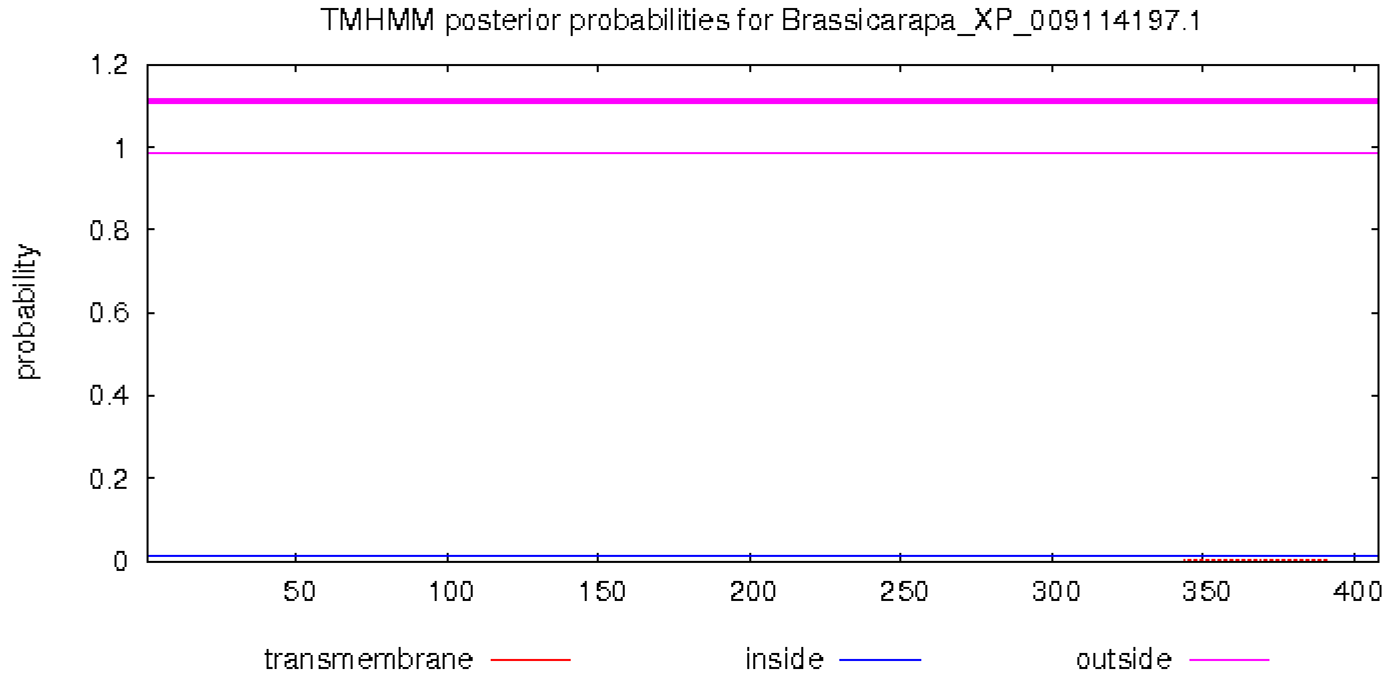

plot in postscript, script for making the plot in gnuplot, data for plot

---

```
# Brassicarapa_XP_009114491.1 Length: 366
# Brassicarapa_XP_009114491.1 Number of predicted TMHs:  0
# Brassicarapa_XP_009114491.1 Exp number of AAs in TMHs: 0.00069
# Brassicarapa_XP_009114491.1 Exp number, first 60 AAs:  0
# Brassicarapa_XP_009114491.1 Total prob of N-in:        0.01360
Brassicarapa_XP_009114491.1     TMHMM2.0        outside      1   366
```

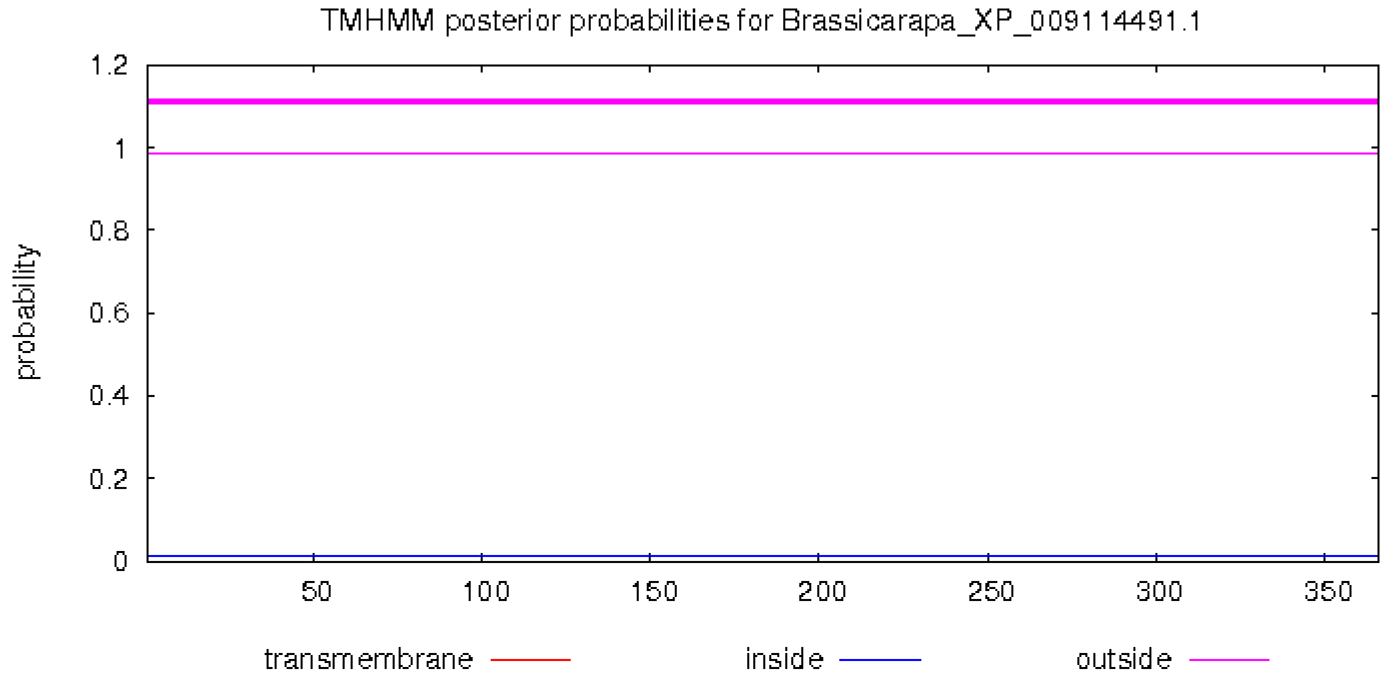

plot in postscript, script for making the plot in gnuplot, data for plot

---

```
# Brassicarapa_XP_009114751.1 Length: 560
# Brassicarapa_XP_009114751.1 Number of predicted TMHs:  1
```

```
# Brassicarapa_XP_009114751.1 Exp number of AAs in TMHs: 21.55516
# Brassicarapa_XP_009114751.1 Exp number, first 60 AAs:  0.00168
# Brassicarapa_XP_009114751.1 Total prob of N-in:        0.36665
Brassicarapa_XP_009114751.1     TMHMM2.0        outside      1   529
Brassicarapa_XP_009114751.1     TMHMM2.0        TMhelix    530   549
Brassicarapa_XP_009114751.1     TMHMM2.0        inside     550   560
```

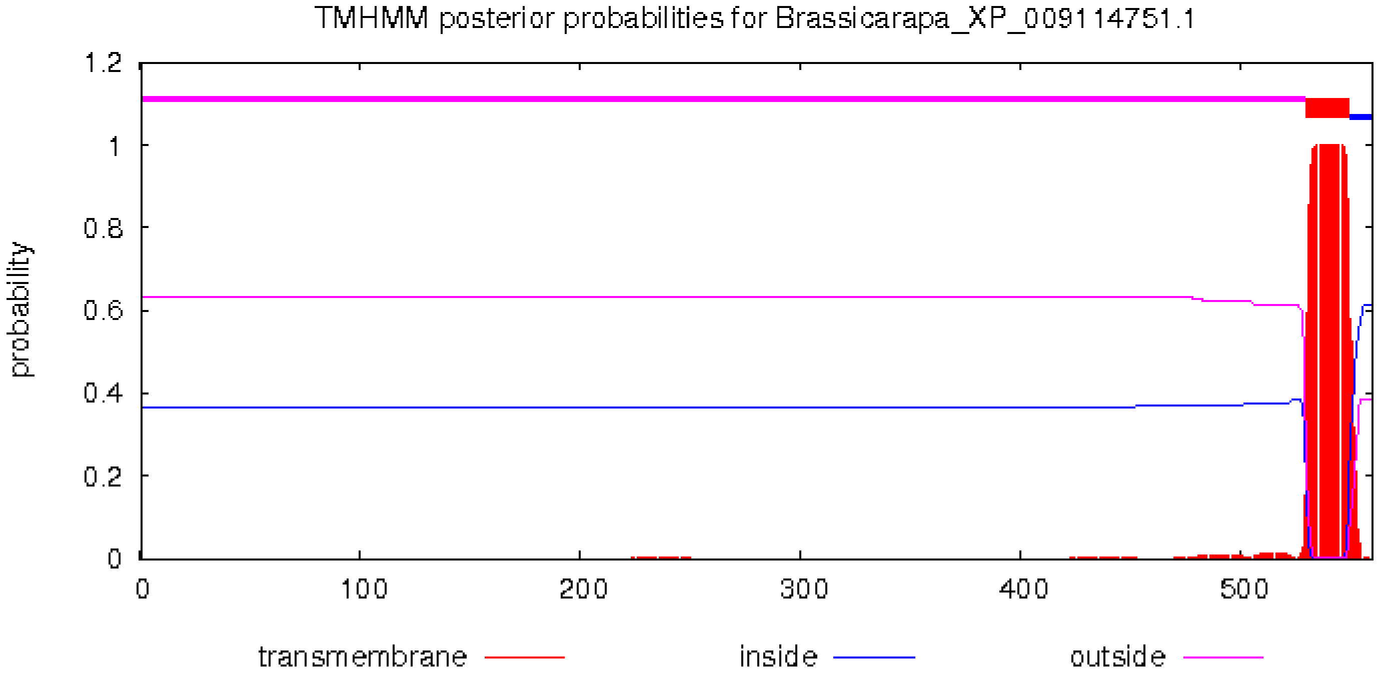

plot in postscript, script for making the plot in gnuplot, data for plot

```
# Brassicarapa_XP_009114752.1 Length: 556
# Brassicarapa_XP_009114752.1 Number of predicted TMHs:  1
# Brassicarapa_XP_009114752.1 Exp number of AAs in TMHs: 21.54928
# Brassicarapa_XP_009114752.1 Exp number, first 60 AAs:  0.00169
# Brassicarapa_XP_009114752.1 Total prob of N-in:        0.37846
Brassicarapa_XP_009114752.1     TMHMM2.0        outside      1   525
Brassicarapa_XP_009114752.1     TMHMM2.0        TMhelix    526   545
Brassicarapa_XP_009114752.1     TMHMM2.0        inside     546   556
```

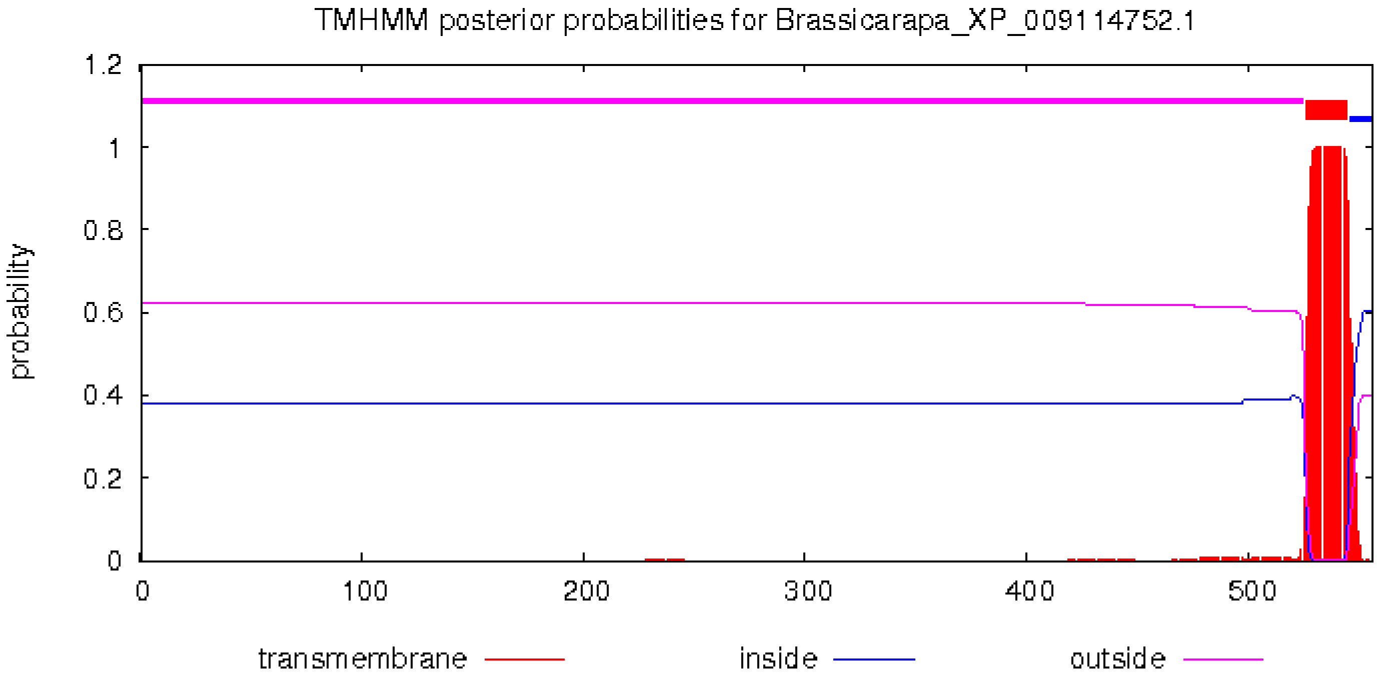

plot in postscript, script for making the plot in gnuplot, data for plot

```
# Brassicarapa_XP_009114859.1 Length: 364
# Brassicarapa_XP_009114859.1 Number of predicted TMHs:  0
# Brassicarapa_XP_009114859.1 Exp number of AAs in TMHs: 0
# Brassicarapa_XP_009114859.1 Exp number, first 60 AAs:  0
# Brassicarapa_XP_009114859.1 Total prob of N-in:        0.01460
Brassicarapa_XP_009114859.1     TMHMM2.0        outside      1   364
```

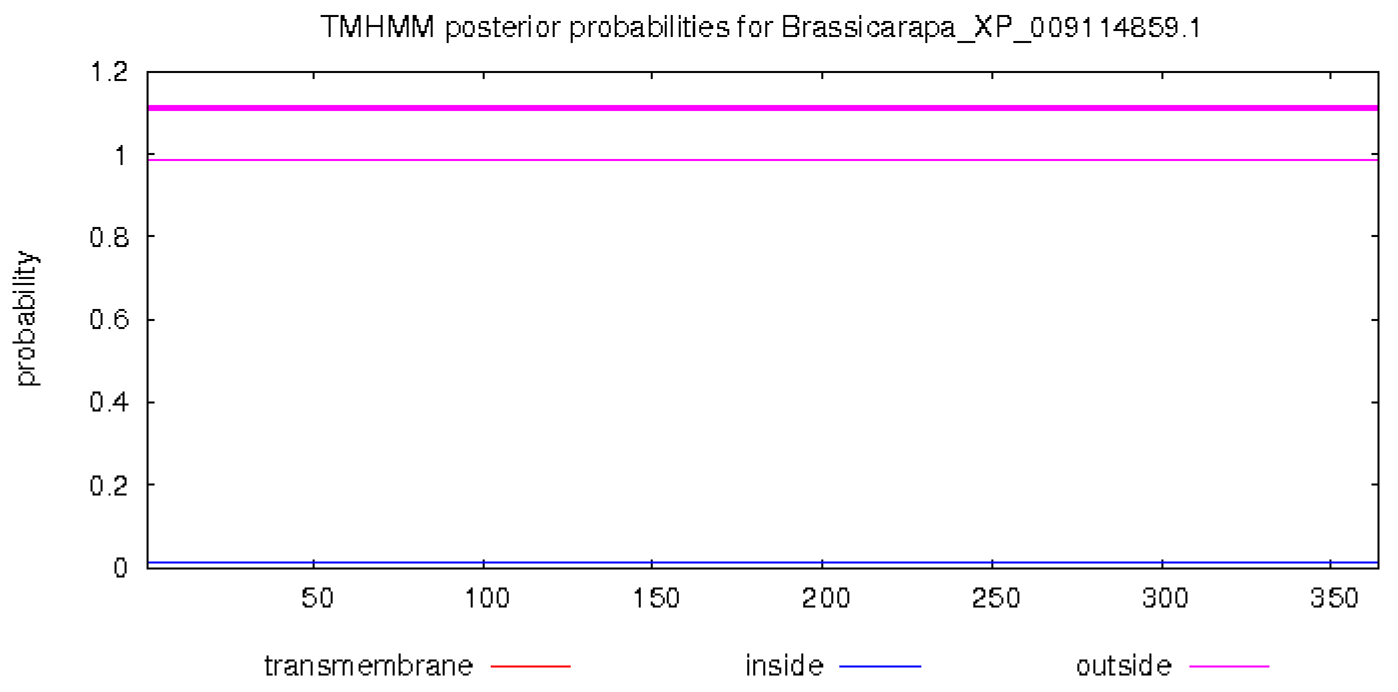

plot in postscript, script for making the plot in gnuplot, data for plot

```
# Brassicarapa_XP_009114860.1 Length: 364
# Brassicarapa_XP_009114860.1 Number of predicted TMHs:  0
# Brassicarapa_XP_009114860.1 Exp number of AAs in TMHs: 0
# Brassicarapa_XP_009114860.1 Exp number, first 60 AAs:  0
# Brassicarapa_XP_009114860.1 Total prob of N-in:        0.01460
Brassicarapa_XP_009114860.1     TMHMM2.0        outside      1   364
```

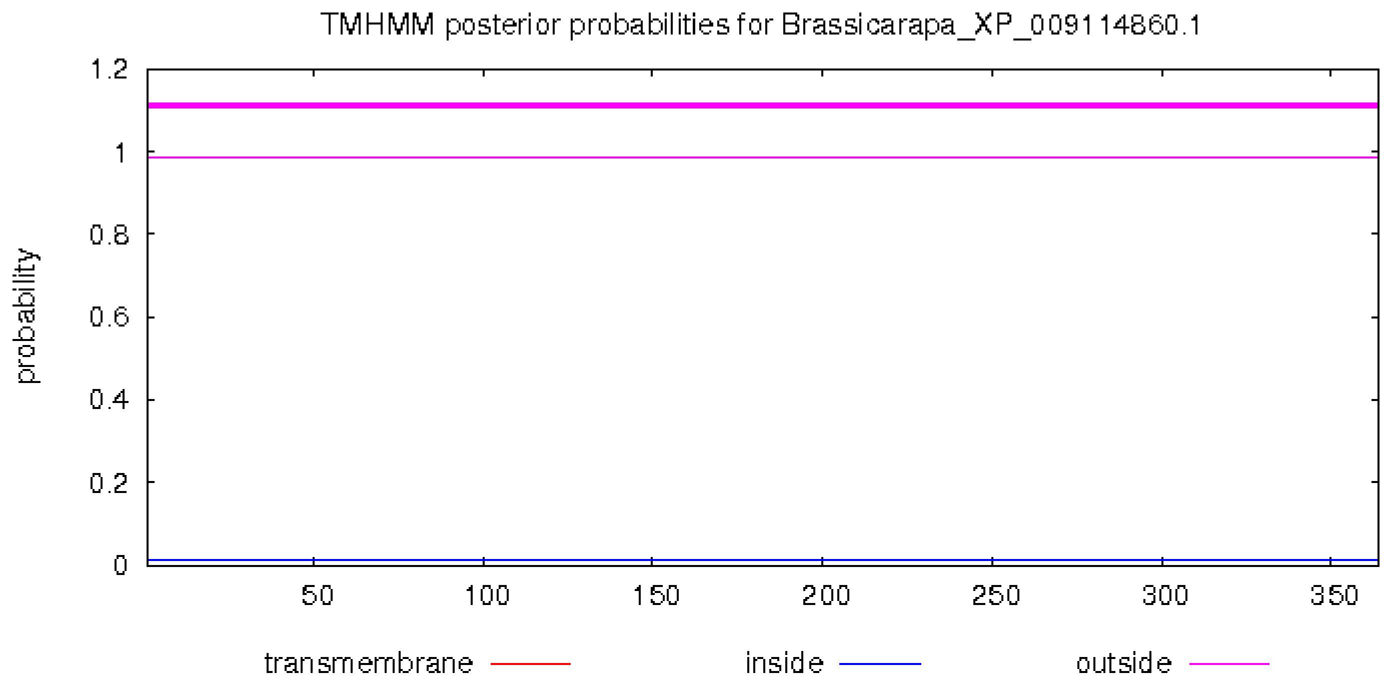

plot in postscript, script for making the plot in gnuplot, data for plot

```
# Brassicarapa_XP_009114861.1 Length: 364
# Brassicarapa_XP_009114861.1 Number of predicted TMHs:  0
# Brassicarapa_XP_009114861.1 Exp number of AAs in TMHs: 0
# Brassicarapa_XP_009114861.1 Exp number, first 60 AAs:  0
# Brassicarapa_XP_009114861.1 Total prob of N-in:        0.01460
Brassicarapa_XP_009114861.1     TMHMM2.0        outside      1   364
```

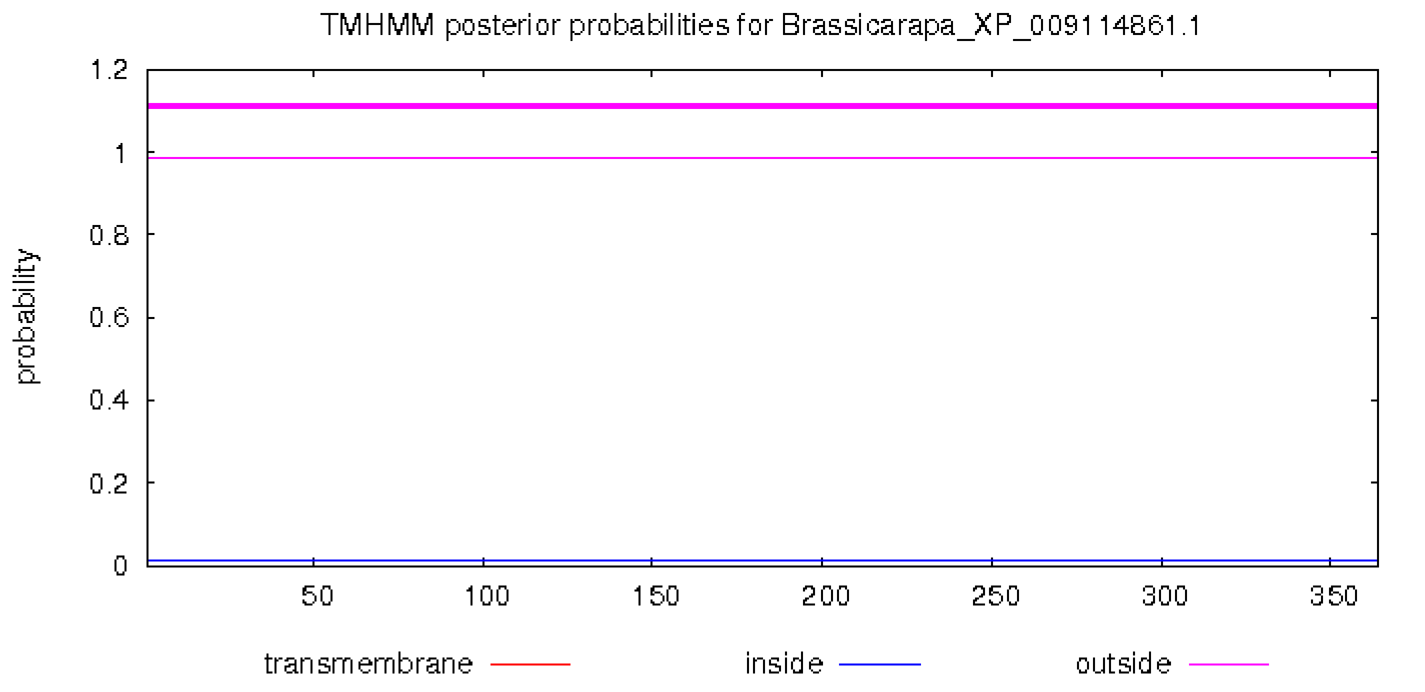

plot in postscript, script for making the plot in gnuplot, data for plot

---

```
# Brassicarapa_XP_009115341.1 Length: 451
# Brassicarapa_XP_009115341.1 Number of predicted TMHs:  0
# Brassicarapa_XP_009115341.1 Exp number of AAs in TMHs: 0.00099
# Brassicarapa_XP_009115341.1 Exp number, first 60 AAs:  0
# Brassicarapa_XP_009115341.1 Total prob of N-in:        0.00313
Brassicarapa_XP_009115341.1     TMHMM2.0        outside      1   451
```

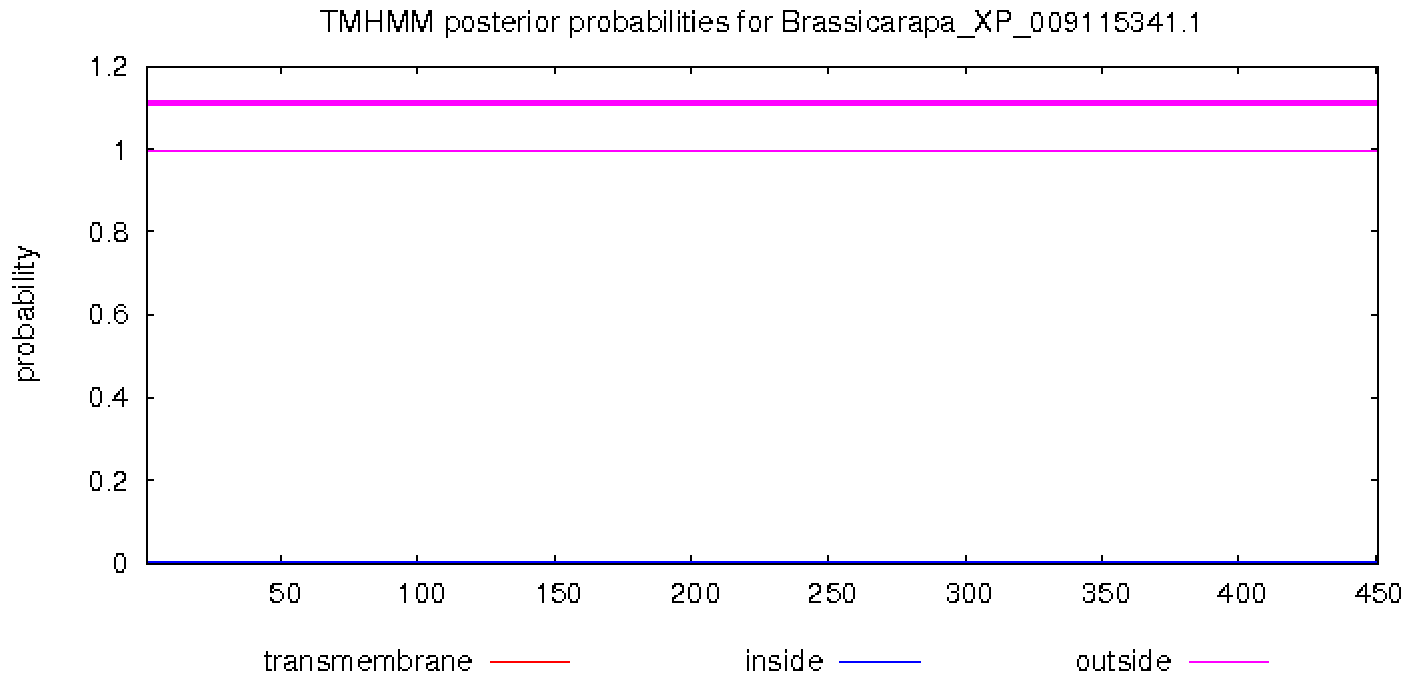

plot in postscript, script for making the plot in gnuplot, data for plot

---

```
# Brassicarapa_XP_009115419.1 Length: 416
# Brassicarapa_XP_009115419.1 Number of predicted TMHs:  0
# Brassicarapa_XP_009115419.1 Exp number of AAs in TMHs: 0.00014
# Brassicarapa_XP_009115419.1 Exp number, first 60 AAs:  0
# Brassicarapa_XP_009115419.1 Total prob of N-in:        0.01044
Brassicarapa_XP_009115419.1	TMHMM2.0	outside	     1   416
```

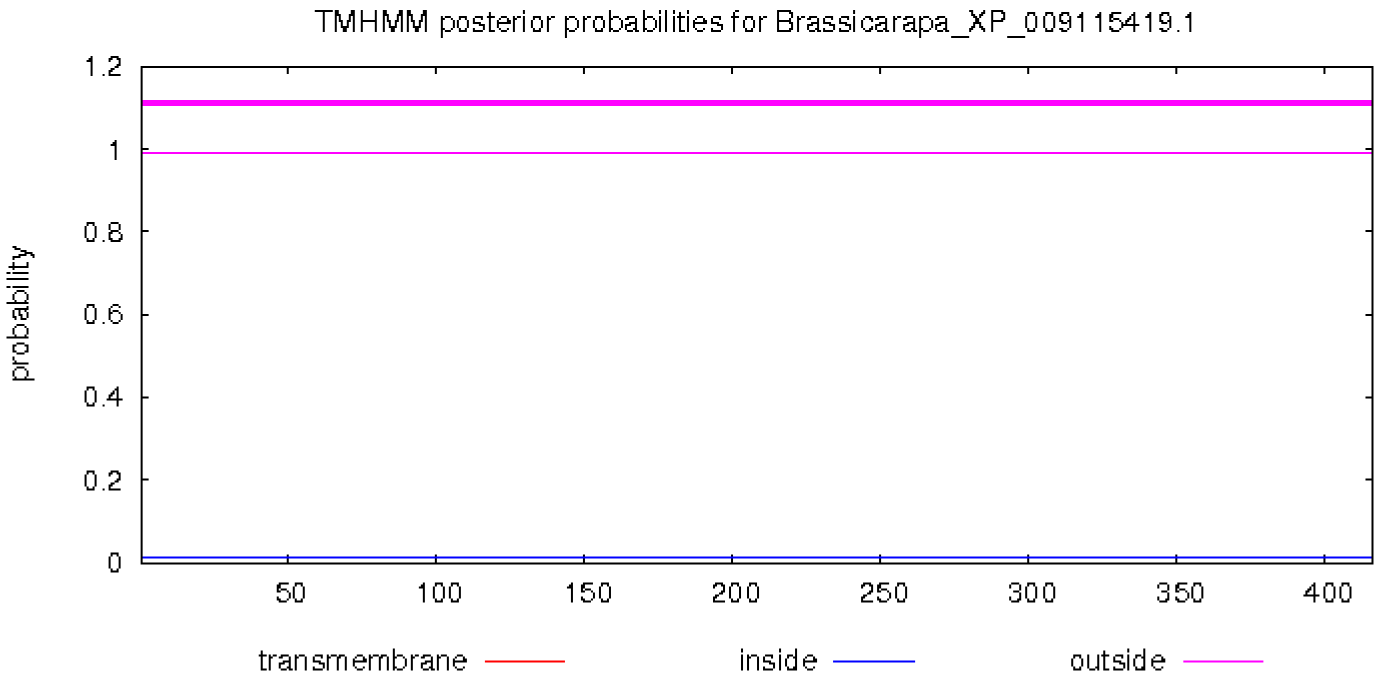

plot in postscript, script for making the plot in gnuplot, data for plot

---

```
# Brassicarapa_XP_009115491.1 Length: 358
# Brassicarapa_XP_009115491.1 Number of predicted TMHs:  0
# Brassicarapa_XP_009115491.1 Exp number of AAs in TMHs: 0.000860000000000000001
# Brassicarapa_XP_009115491.1 Exp number, first 60 AAs:  0
# Brassicarapa_XP_009115491.1 Total prob of N-in:        0.00442
Brassicarapa_XP_009115491.1	TMHMM2.0	outside	     1   358
```

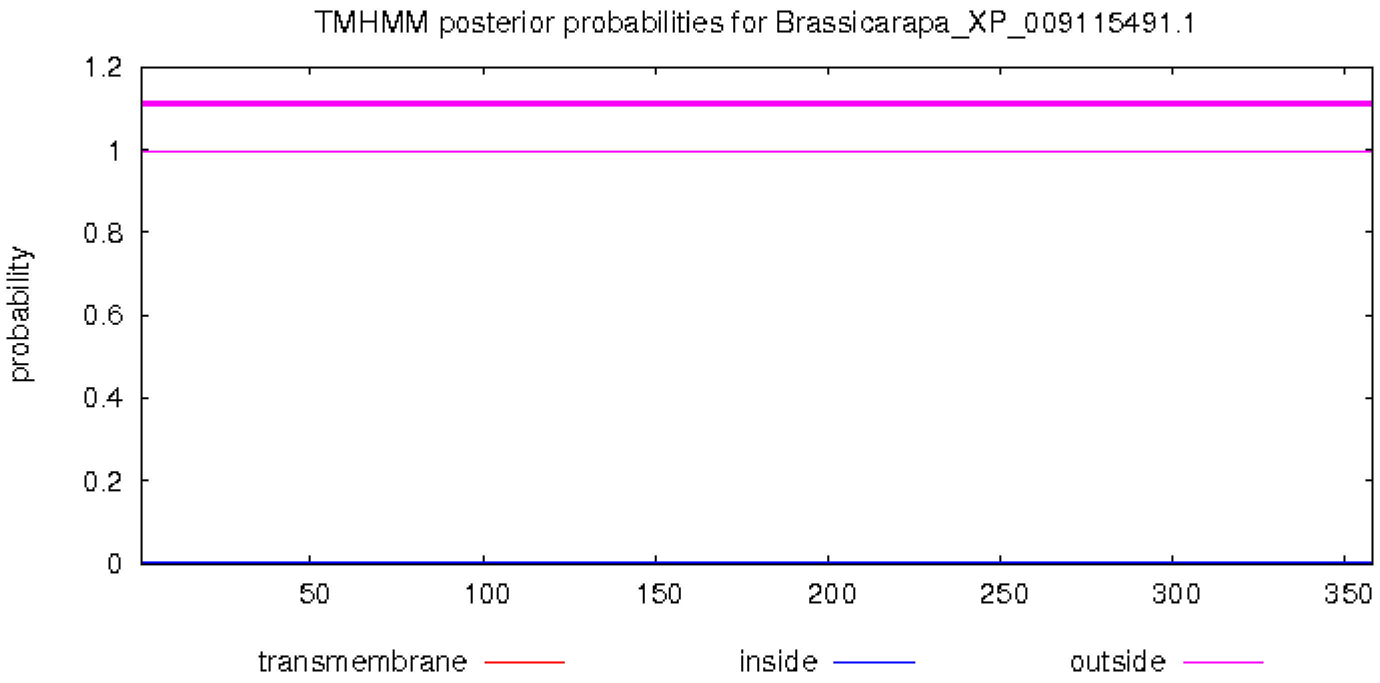

plot in postscript, script for making the plot in gnuplot, data for plot

---

```
# Brassicarapa_XP_009115498.1 Length: 317
# Brassicarapa_XP_009115498.1 Number of predicted TMHs:  0
# Brassicarapa_XP_009115498.1 Exp number of AAs in TMHs: 4e-05
# Brassicarapa_XP_009115498.1 Exp number, first 60 AAs:  0
# Brassicarapa_XP_009115498.1 Total prob of N-in:        0.00384
Brassicarapa_XP_009115498.1     TMHMM2.0        outside      1   317
```

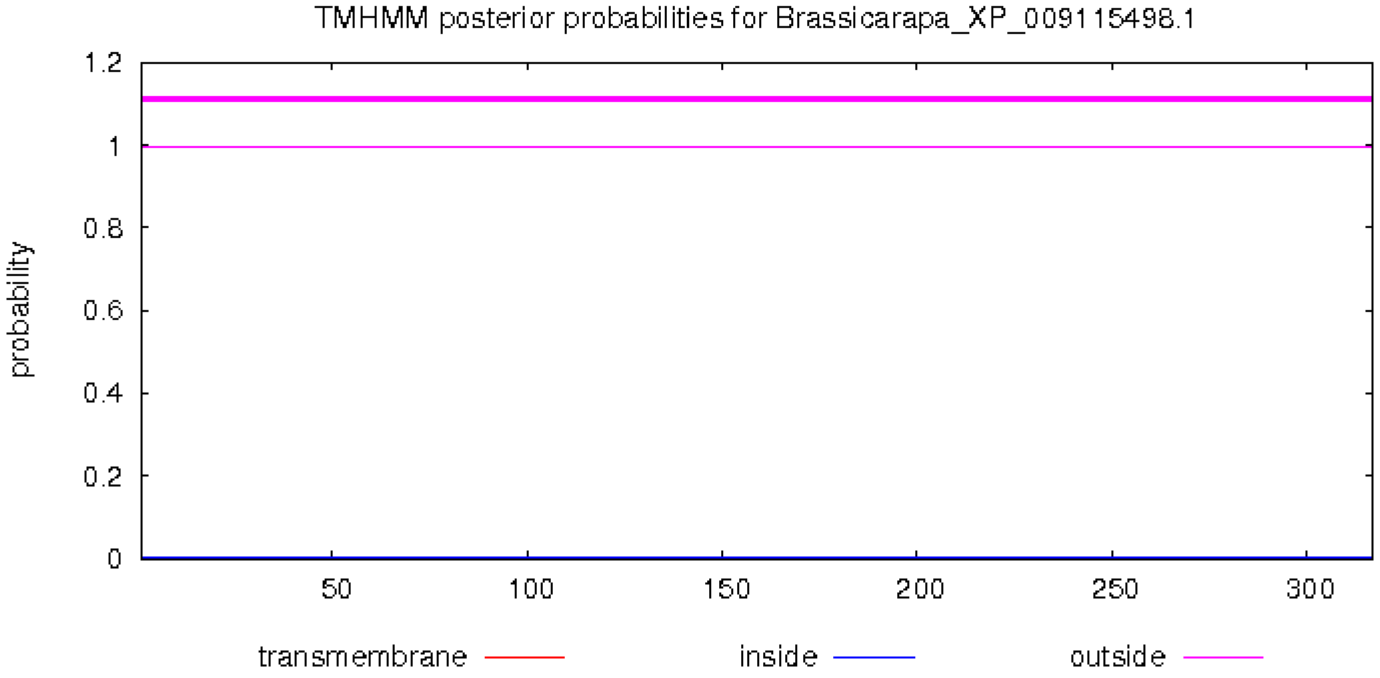

plot in postscript, script for making the plot in gnuplot, data for plot

---

```
# Brassicarapa_XP_009116356.1 Length: 360
# Brassicarapa_XP_009116356.1 Number of predicted TMHs:  0
# Brassicarapa_XP_009116356.1 Exp number of AAs in TMHs: 0.00019
# Brassicarapa_XP_009116356.1 Exp number, first 60 AAs:  0
# Brassicarapa_XP_009116356.1 Total prob of N-in:        0.01980
Brassicarapa_XP_009116356.1     TMHMM2.0        outside      1   360
```

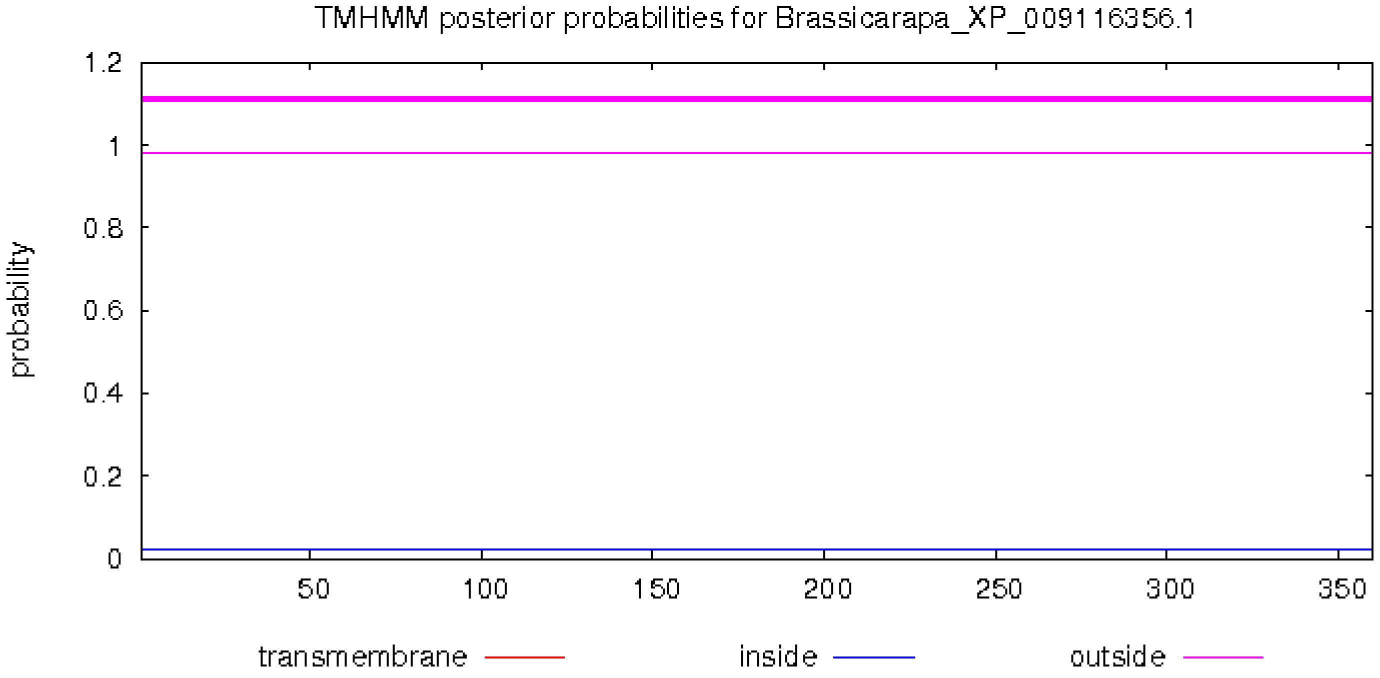

plot in postscript, script for making the plot in gnuplot, data for plot

---

```
# Brassicarapa_XP_009116358.1 Length: 359
# Brassicarapa_XP_009116358.1 Number of predicted TMHs:  0
# Brassicarapa_XP_009116358.1 Exp number of AAs in TMHs: 0.00019
# Brassicarapa_XP_009116358.1 Exp number, first 60 AAs:  0
# Brassicarapa_XP_009116358.1 Total prob of N-in:        0.02004
Brassicarapa_XP_009116358.1     TMHMM2.0        outside      1   359
```

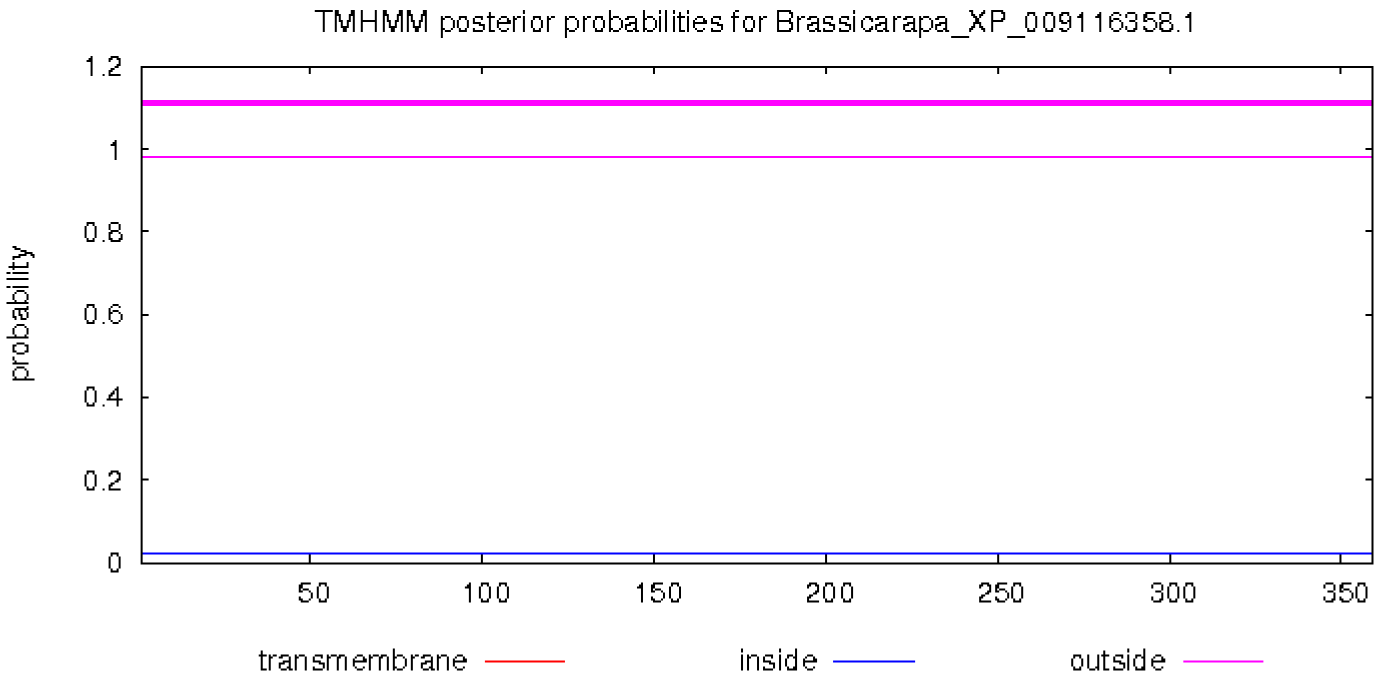

plot in postscript, script for making the plot in gnuplot, data for plot

---

```
# Brassicarapa_XP_009116359.1 Length: 359
# Brassicarapa_XP_009116359.1 Number of predicted TMHs:  0
# Brassicarapa_XP_009116359.1 Exp number of AAs in TMHs: 0.00019
# Brassicarapa_XP_009116359.1 Exp number, first 60 AAs:  0
# Brassicarapa_XP_009116359.1 Total prob of N-in:        0.02005
Brassicarapa_XP_009116359.1     TMHMM2.0        outside      1   359
```

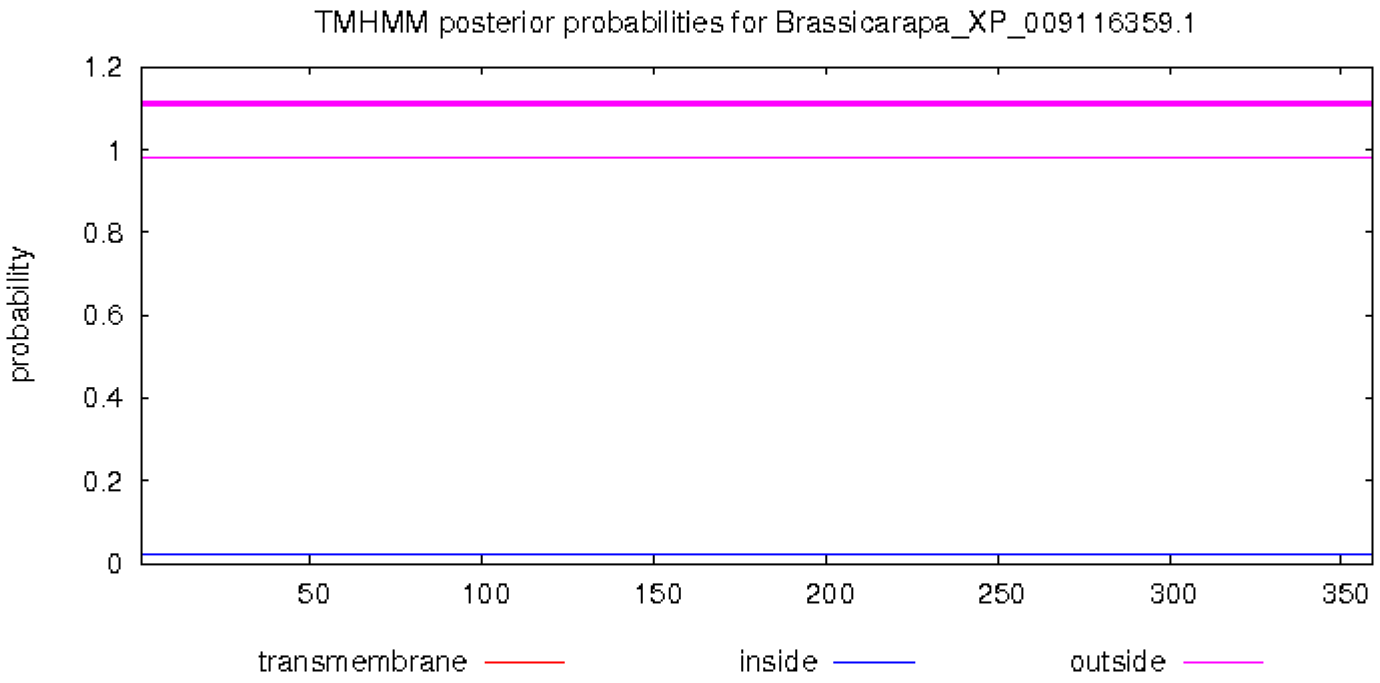

plot in postscript, script for making the plot in gnuplot, data for plot

---

```
# Brassicarapa_XP_009116467.1 Length: 303
# Brassicarapa_XP_009116467.1 Number of predicted TMHs:  0
# Brassicarapa_XP_009116467.1 Exp number of AAs in TMHs: 0.00153
# Brassicarapa_XP_009116467.1 Exp number, first 60 AAs:  0.00042
# Brassicarapa_XP_009116467.1 Total prob of N-in:        0.01052
Brassicarapa_XP_009116467.1     TMHMM2.0        outside      1   303
```

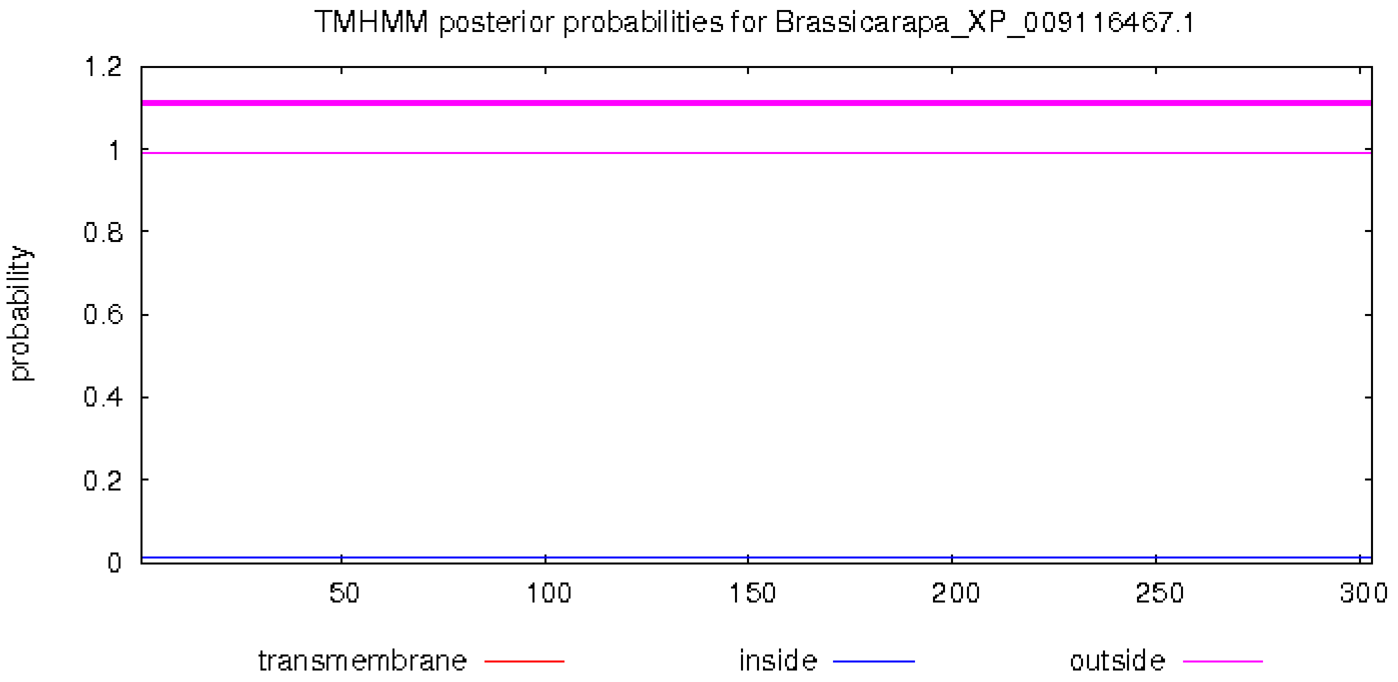

plot in postscript, script for making the plot in gnuplot, data for plot

---

```
# Brassicarapa_XP_009116860.1 Length: 322
# Brassicarapa_XP_009116860.1 Number of predicted TMHs:  0
# Brassicarapa_XP_009116860.1 Exp number of AAs in TMHs: 0
# Brassicarapa_XP_009116860.1 Exp number, first 60 AAs:  0
# Brassicarapa_XP_009116860.1 Total prob of N-in:        0.03347
Brassicarapa_XP_009116860.1     TMHMM2.0        outside      1   322
```

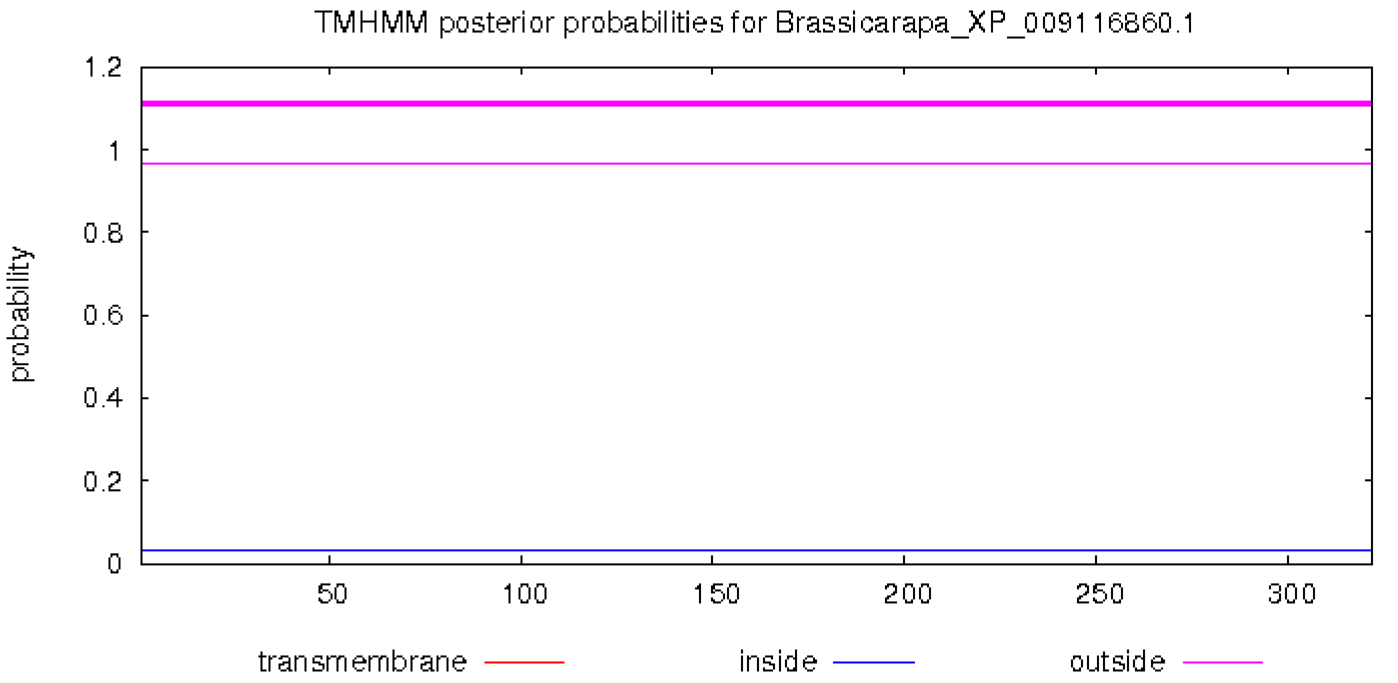

plot in postscript, script for making the plot in gnuplot, data for plot

---

```
# Brassicarapa_XP_009117131.1 Length: 317
# Brassicarapa_XP_009117131.1 Number of predicted TMHs:  0
# Brassicarapa_XP_009117131.1 Exp number of AAs in TMHs: 0.03214
# Brassicarapa_XP_009117131.1 Exp number, first 60 AAs:  0
# Brassicarapa_XP_009117131.1 Total prob of N-in:        0.12493
Brassicarapa_XP_009117131.1     TMHMM2.0        outside      1   317
```

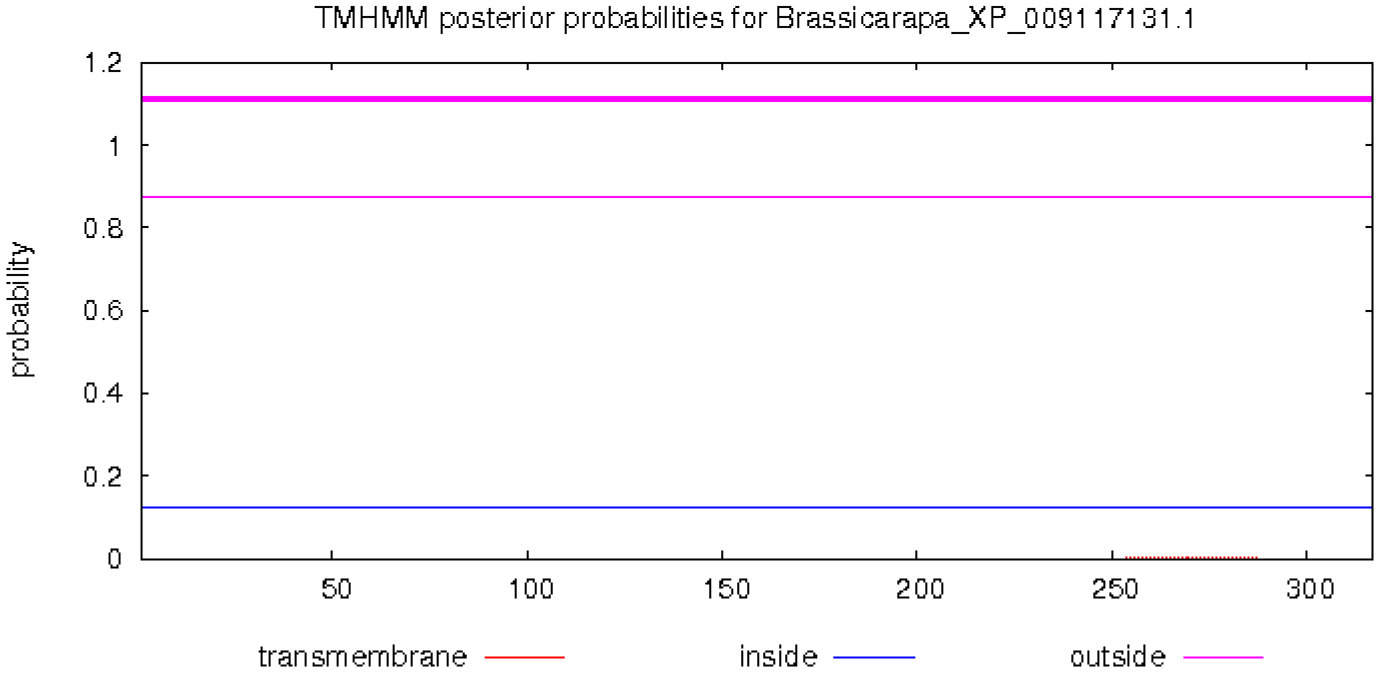

plot in postscript, script for making the plot in gnuplot, data for plot

---

```
# Brassicarapa_XP_009117877.1 Length: 338
# Brassicarapa_XP_009117877.1 Number of predicted TMHs:  1
# Brassicarapa_XP_009117877.1 Exp number of AAs in TMHs: 18.27944
# Brassicarapa_XP_009117877.1 Exp number, first 60 AAs:  0.2641
# Brassicarapa_XP_009117877.1 Total prob of N-in:        0.32583
Brassicarapa_XP_009117877.1     TMHMM2.0        outside      1   319
Brassicarapa_XP_009117877.1     TMHMM2.0        TMhelix    320   337
Brassicarapa_XP_009117877.1     TMHMM2.0        inside     338   338
```

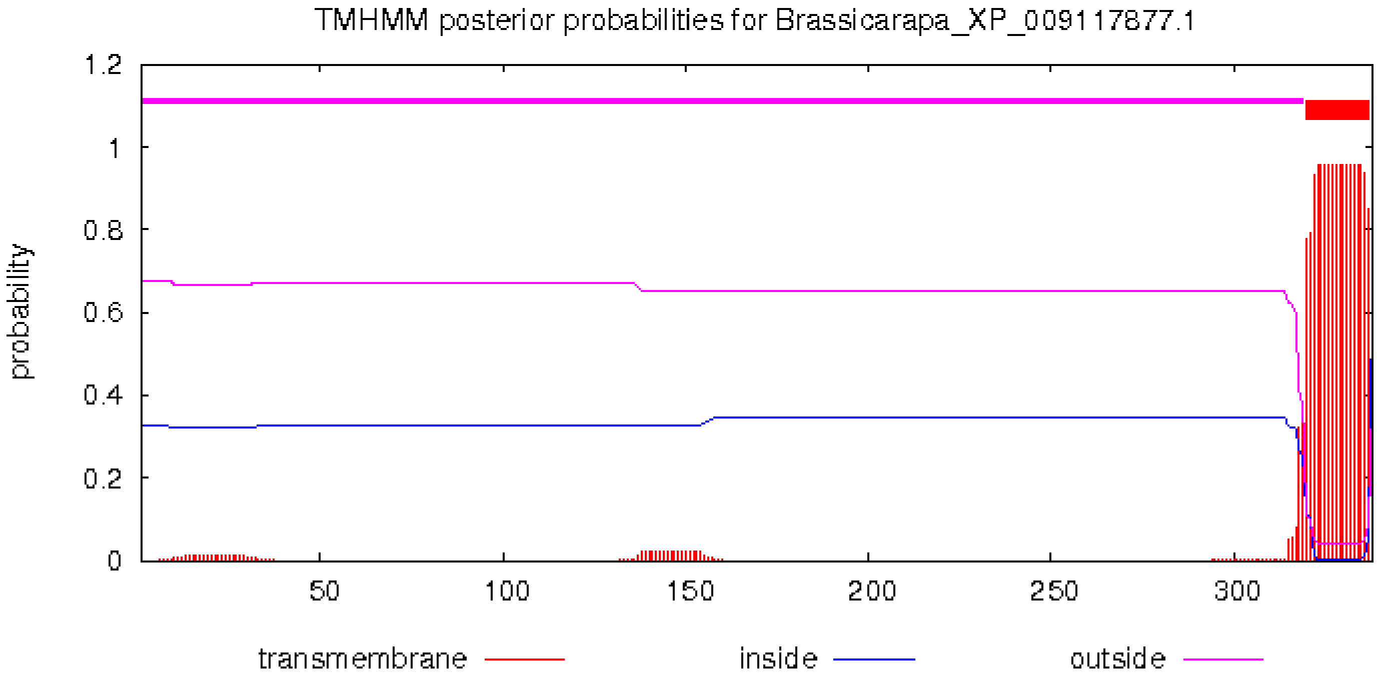

plot in postscript, script for making the plot in gnuplot, data for plot

```
# Brassicarapa_XP_009118784.1 Length: 224
# Brassicarapa_XP_009118784.1 Number of predicted TMHs:  0
# Brassicarapa_XP_009118784.1 Exp number of AAs in TMHs: 0
# Brassicarapa_XP_009118784.1 Exp number, first 60 AAs:  0
# Brassicarapa_XP_009118784.1 Total prob of N-in:        0.03474
Brassicarapa_XP_009118784.1     TMHMM2.0        outside      1   224
```

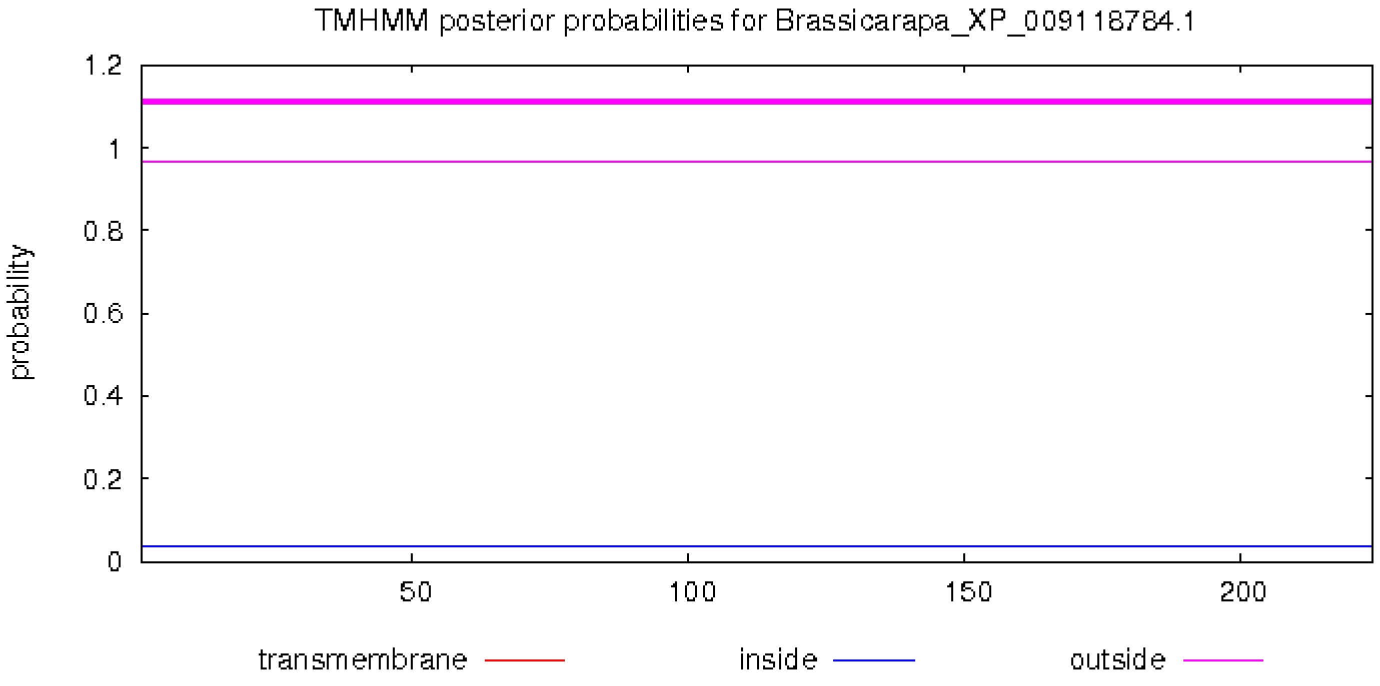

plot in postscript, script for making the plot in gnuplot, data for plot

```
# Brassicarapa_XP_009118785.1 Length: 221
# Brassicarapa_XP_009118785.1 Number of predicted TMHs:  0
# Brassicarapa_XP_009118785.1 Exp number of AAs in TMHs: 0
# Brassicarapa_XP_009118785.1 Exp number, first 60 AAs:  0
# Brassicarapa_XP_009118785.1 Total prob of N-in:        0.03602
Brassicarapa_XP_009118785.1     TMHMM2.0        outside      1   221
```

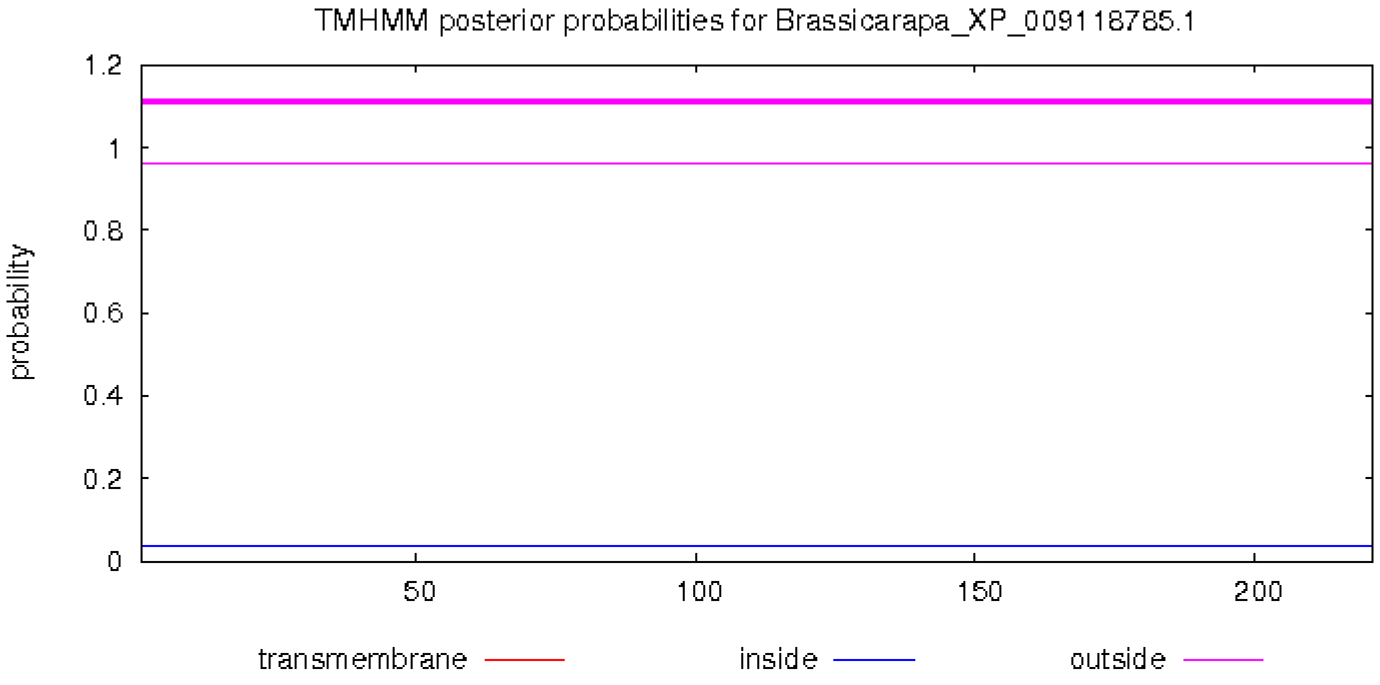

plot in postscript, script for making the plot in gnuplot, data for plot

```
# Brassicarapa_XP_009119137.1 Length: 480
# Brassicarapa_XP_009119137.1 Number of predicted TMHs:  0
# Brassicarapa_XP_009119137.1 Exp number of AAs in TMHs: 0.0043
# Brassicarapa_XP_009119137.1 Exp number, first 60 AAs:  0
# Brassicarapa_XP_009119137.1 Total prob of N-in:        0.00017
Brassicarapa_XP_009119137.1     TMHMM2.0        outside      1   480
```

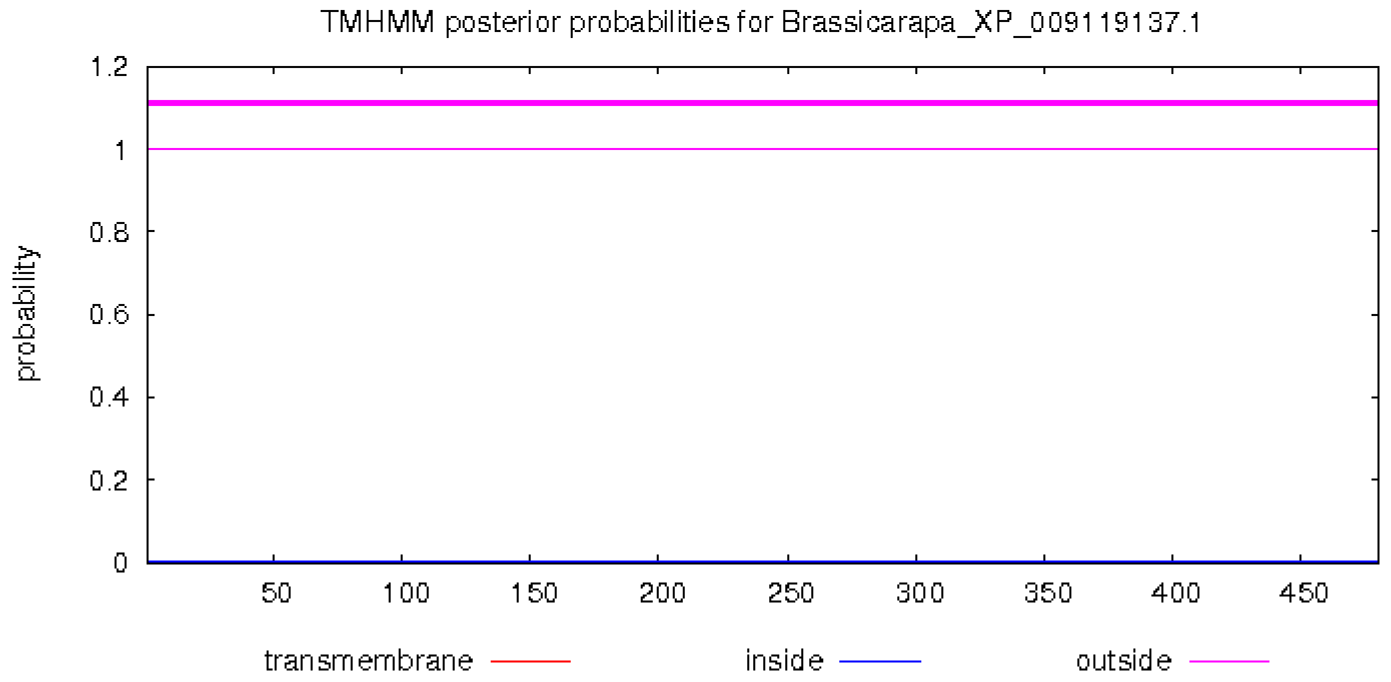

plot in postscript, script for making the plot in gnuplot, data for plot

---

```
# Brassicarapa_XP_009119349.1 Length: 511
# Brassicarapa_XP_009119349.1 Number of predicted TMHs:  0
# Brassicarapa_XP_009119349.1 Exp number of AAs in TMHs: 0.00492
# Brassicarapa_XP_009119349.1 Exp number, first 60 AAs:  0.00492
# Brassicarapa_XP_009119349.1 Total prob of N-in:        0.00466
Brassicarapa_XP_009119349.1     TMHMM2.0        outside      1   511
```

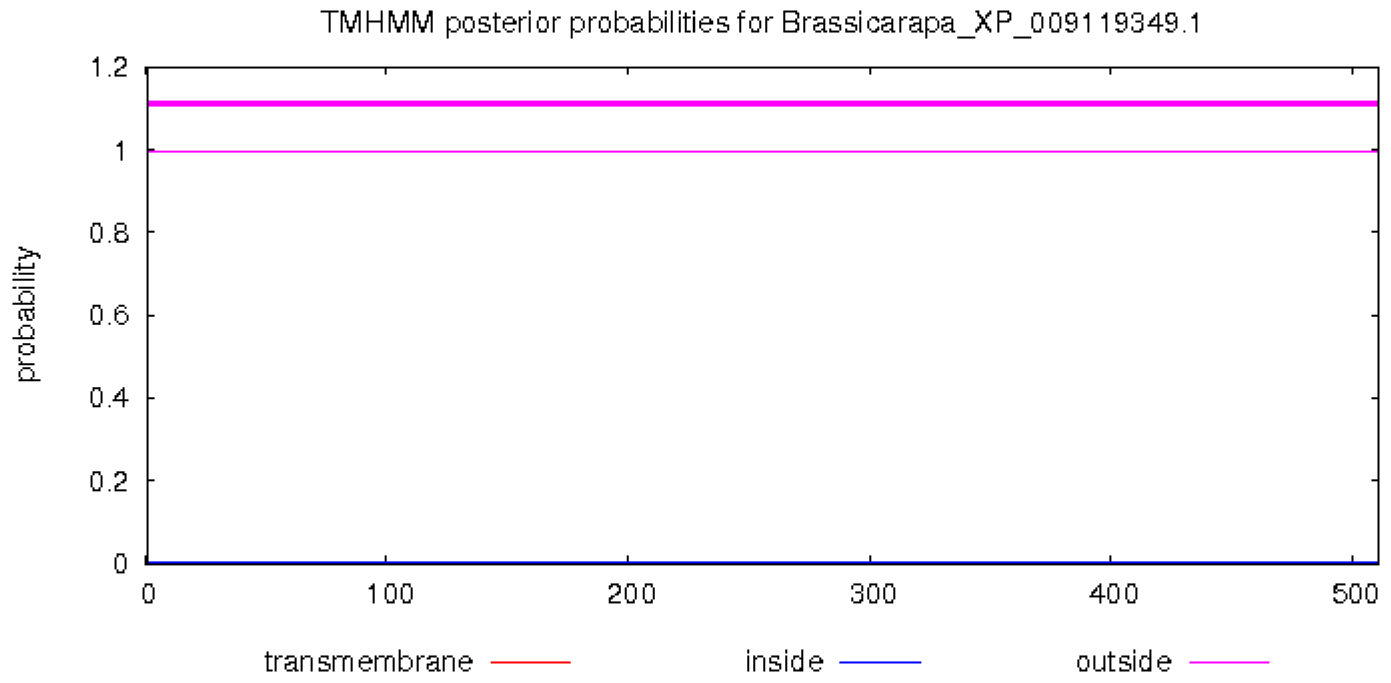

plot in postscript, script for making the plot in gnuplot, data for plot

---

```
# Brassicarapa_XP_009119652.1 Length: 460
# Brassicarapa_XP_009119652.1 Number of predicted TMHs:  0
# Brassicarapa_XP_009119652.1 Exp number of AAs in TMHs: 0.00038
# Brassicarapa_XP_009119652.1 Exp number, first 60 AAs:  0
# Brassicarapa_XP_009119652.1 Total prob of N-in:        0.00304
Brassicarapa_XP_009119652.1     TMHMM2.0        outside      1   460
```

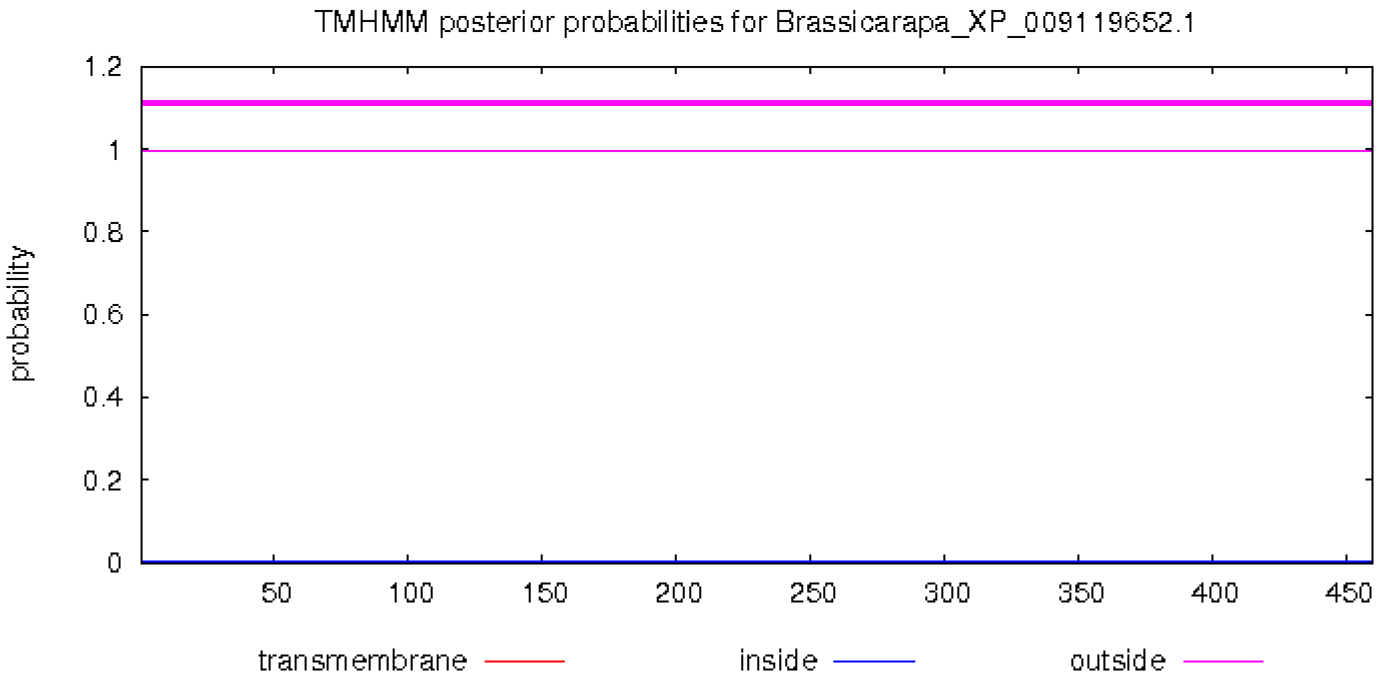

plot in postscript, script for making the plot in gnuplot, data for plot

---

```
# Brassicarapa_XP_009119653.1 Length: 459
# Brassicarapa_XP_009119653.1 Number of predicted TMHs:  0
# Brassicarapa_XP_009119653.1 Exp number of AAs in TMHs: 0.00045
# Brassicarapa_XP_009119653.1 Exp number, first 60 AAs:  0
# Brassicarapa_XP_009119653.1 Total prob of N-in:        0.00392
Brassicarapa_XP_009119653.1     TMHMM2.0        outside      1   459
```

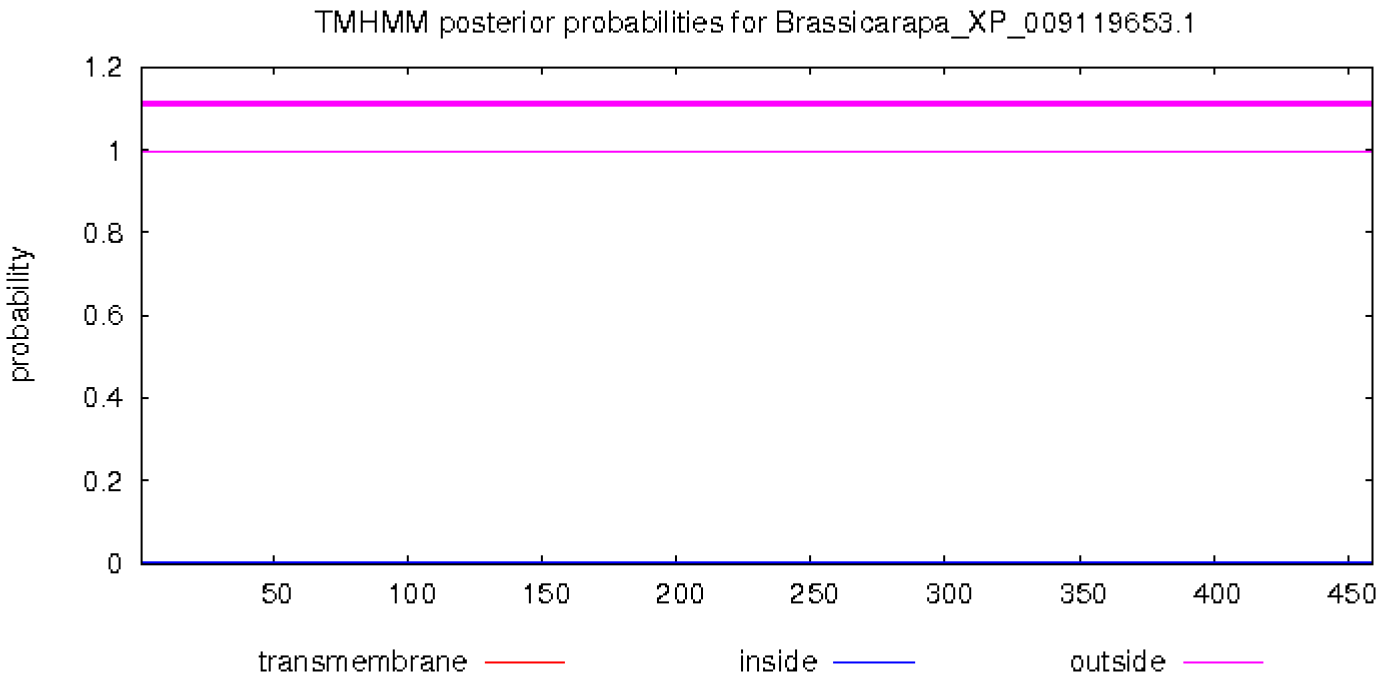

plot in postscript, script for making the plot in gnuplot, data for plot

---

```
# Brassicarapa_XP_009119654.1 Length: 384
# Brassicarapa_XP_009119654.1 Number of predicted TMHs:  0
# Brassicarapa_XP_009119654.1 Exp number of AAs in TMHs: 0.00023
# Brassicarapa_XP_009119654.1 Exp number, first 60 AAs:  0
# Brassicarapa_XP_009119654.1 Total prob of N-in:        0.00484
Brassicarapa_XP_009119654.1     TMHMM2.0        outside      1   384
```

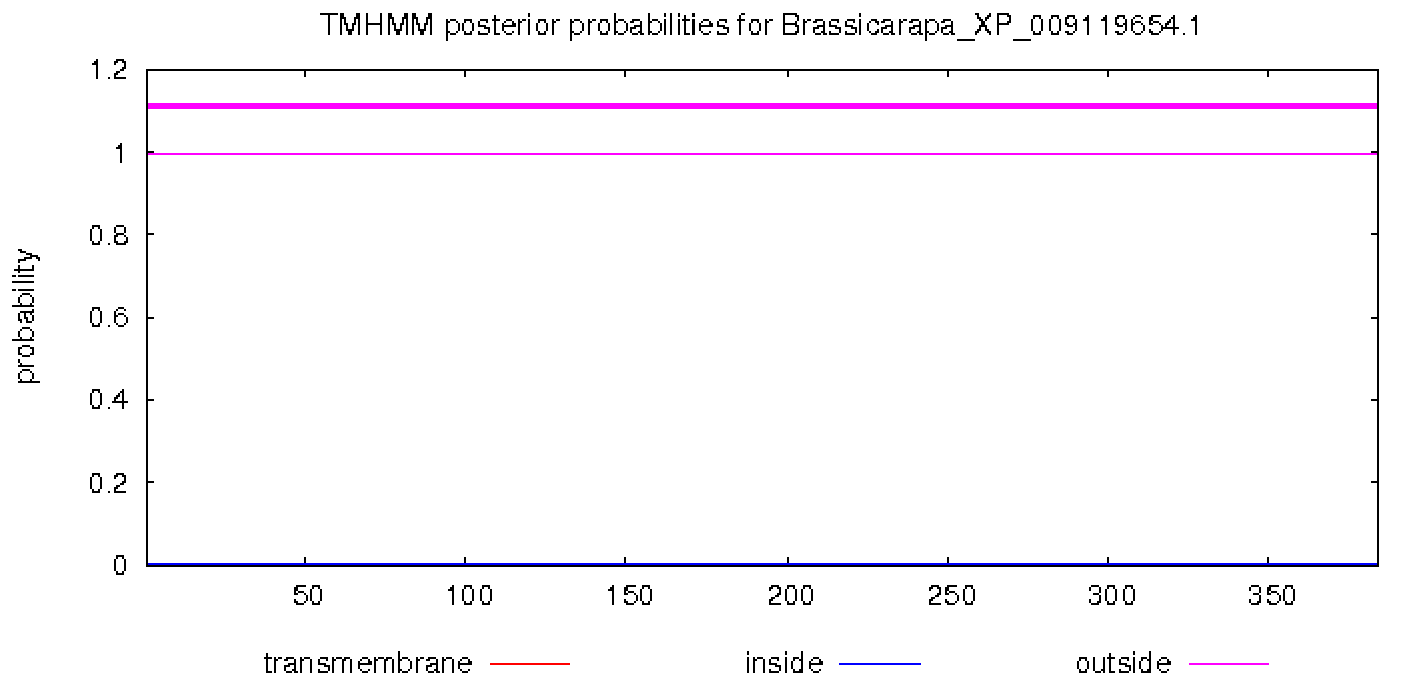

plot in postscript, script for making the plot in gnuplot, data for plot

```
# Brassicarapa_XP_009119656.1 Length: 251
# Brassicarapa_XP_009119656.1 Number of predicted TMHs:  0
# Brassicarapa_XP_009119656.1 Exp number of AAs in TMHs: 0
# Brassicarapa_XP_009119656.1 Exp number, first 60 AAs:  0
# Brassicarapa_XP_009119656.1 Total prob of N-in:        0.01343
Brassicarapa_XP_009119656.1     TMHMM2.0        outside      1   251
```

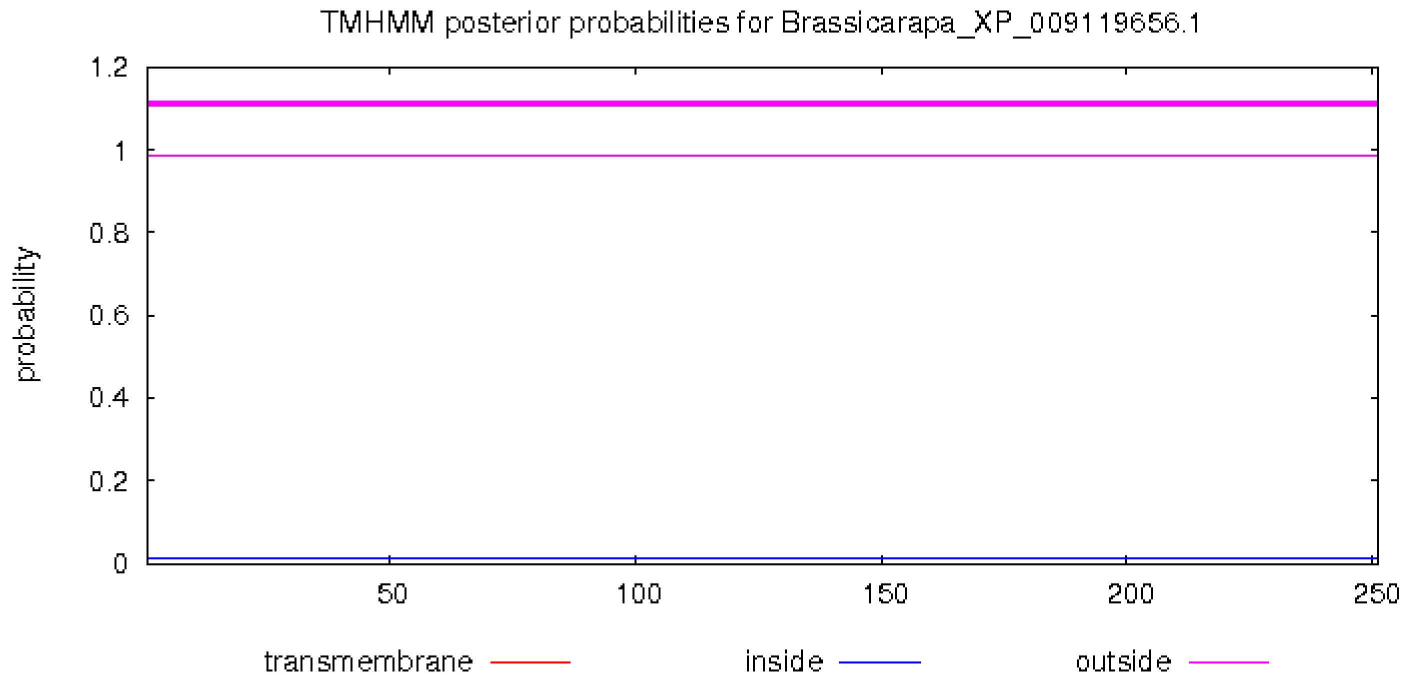

plot in postscript, script for making the plot in gnuplot, data for plot

```
# Brassicarapa_XP_009119657.1 Length: 299
# Brassicarapa_XP_009119657.1 Number of predicted TMHs:  0
# Brassicarapa_XP_009119657.1 Exp number of AAs in TMHs: 0.05502
# Brassicarapa_XP_009119657.1 Exp number, first 60 AAs:  0
# Brassicarapa_XP_009119657.1 Total prob of N-in:        0.02119
Brassicarapa_XP_009119657.1	TMHMM2.0	outside	     1   299
```

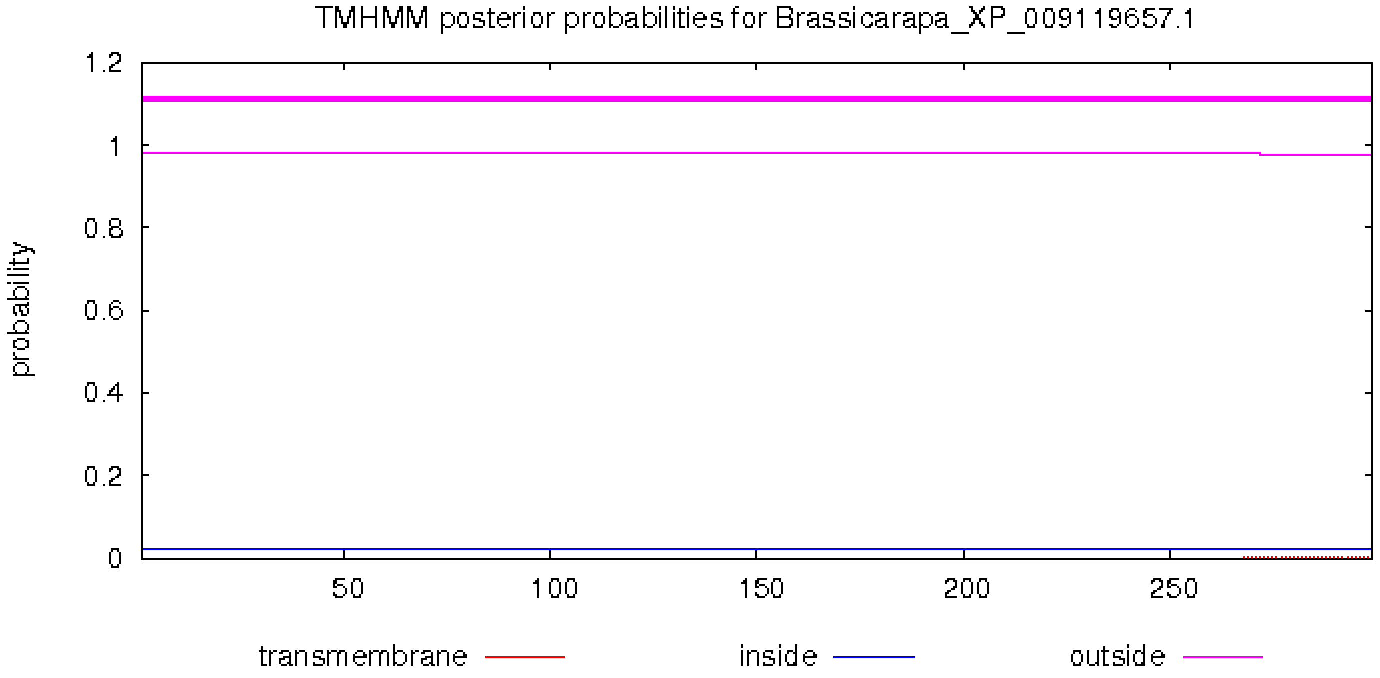

plot in postscript, script for making the plot in gnuplot, data for plot

---

```
# Brassicarapa_XP_009119658.1 Length: 475
# Brassicarapa_XP_009119658.1 Number of predicted TMHs:  1
# Brassicarapa_XP_009119658.1 Exp number of AAs in TMHs: 21.34248
# Brassicarapa_XP_009119658.1 Exp number, first 60 AAs:  0.00018
# Brassicarapa_XP_009119658.1 Total prob of N-in:        0.30086
Brassicarapa_XP_009119658.1	TMHMM2.0	outside	     1   450
Brassicarapa_XP_009119658.1	TMHMM2.0	TMhelix	   451   473
Brassicarapa_XP_009119658.1	TMHMM2.0	inside	   474   475
```

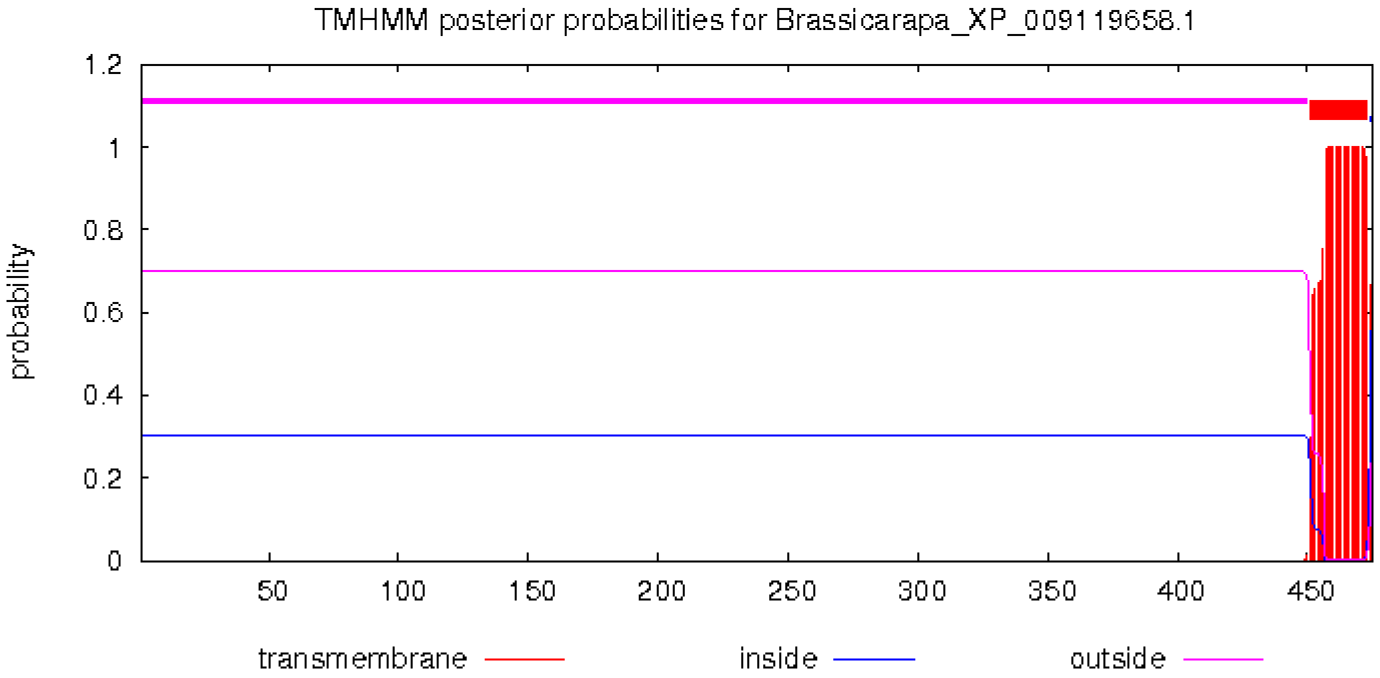

plot in postscript, script for making the plot in gnuplot, data for plot

```
# Brassicarapa_XP_009119659.1 Length: 428
# Brassicarapa_XP_009119659.1 Number of predicted TMHs:  1
# Brassicarapa_XP_009119659.1 Exp number of AAs in TMHs: 21.40913
# Brassicarapa_XP_009119659.1 Exp number, first 60 AAs:  0.00018
# Brassicarapa_XP_009119659.1 Total prob of N-in:        0.43610
Brassicarapa_XP_009119659.1	TMHMM2.0	inside	     1   404
Brassicarapa_XP_009119659.1	TMHMM2.0	TMhelix	   405   427
Brassicarapa_XP_009119659.1	TMHMM2.0	outside	   428   428
```

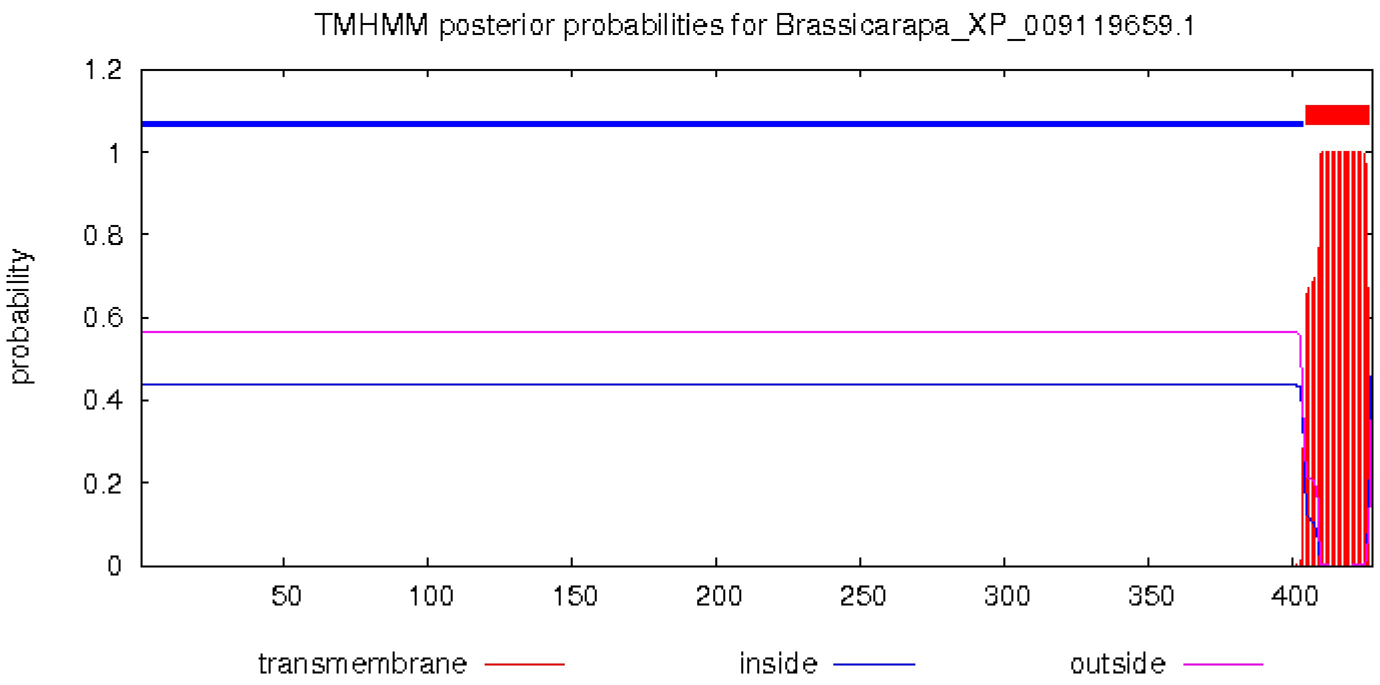

plot in postscript, script for making the plot in gnuplot, data for plot

```
# Brassicarapa_XP_009119728.1 Length: 285
# Brassicarapa_XP_009119728.1 Number of predicted TMHs:  0
# Brassicarapa_XP_009119728.1 Exp number of AAs in TMHs: 0.03653
# Brassicarapa_XP_009119728.1 Exp number, first 60 AAs:  0.00018
# Brassicarapa_XP_009119728.1 Total prob of N-in:        0.00266
Brassicarapa_XP_009119728.1	TMHMM2.0	outside	     1   285
```

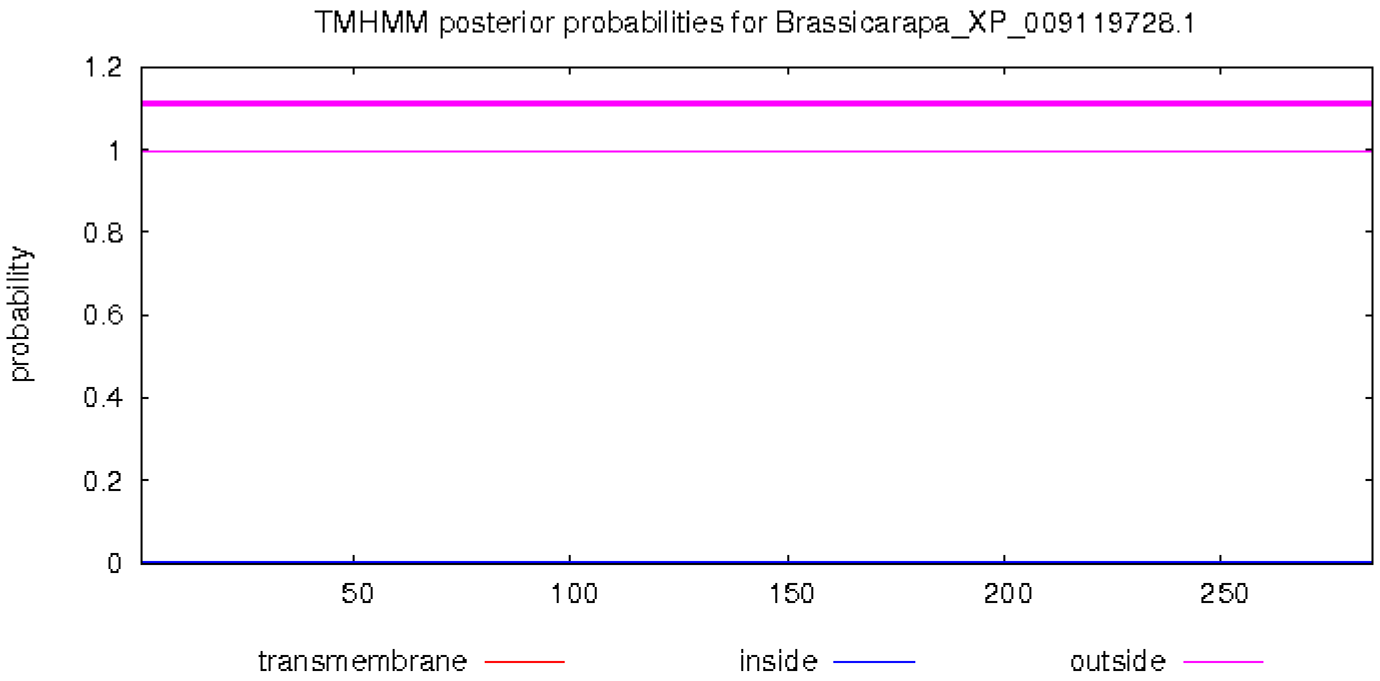

plot in postscript, script for making the plot in gnuplot, data for plot

```
# Brassicarapa_XP_009119767.1 Length: 877
# Brassicarapa_XP_009119767.1 Number of predicted TMHs:  0
# Brassicarapa_XP_009119767.1 Exp number of AAs in TMHs: 0.00492
# Brassicarapa_XP_009119767.1 Exp number, first 60 AAs:  0.00492
# Brassicarapa_XP_009119767.1 Total prob of N-in:        0.00031
Brassicarapa_XP_009119767.1	TMHMM2.0	outside	     1   877
```

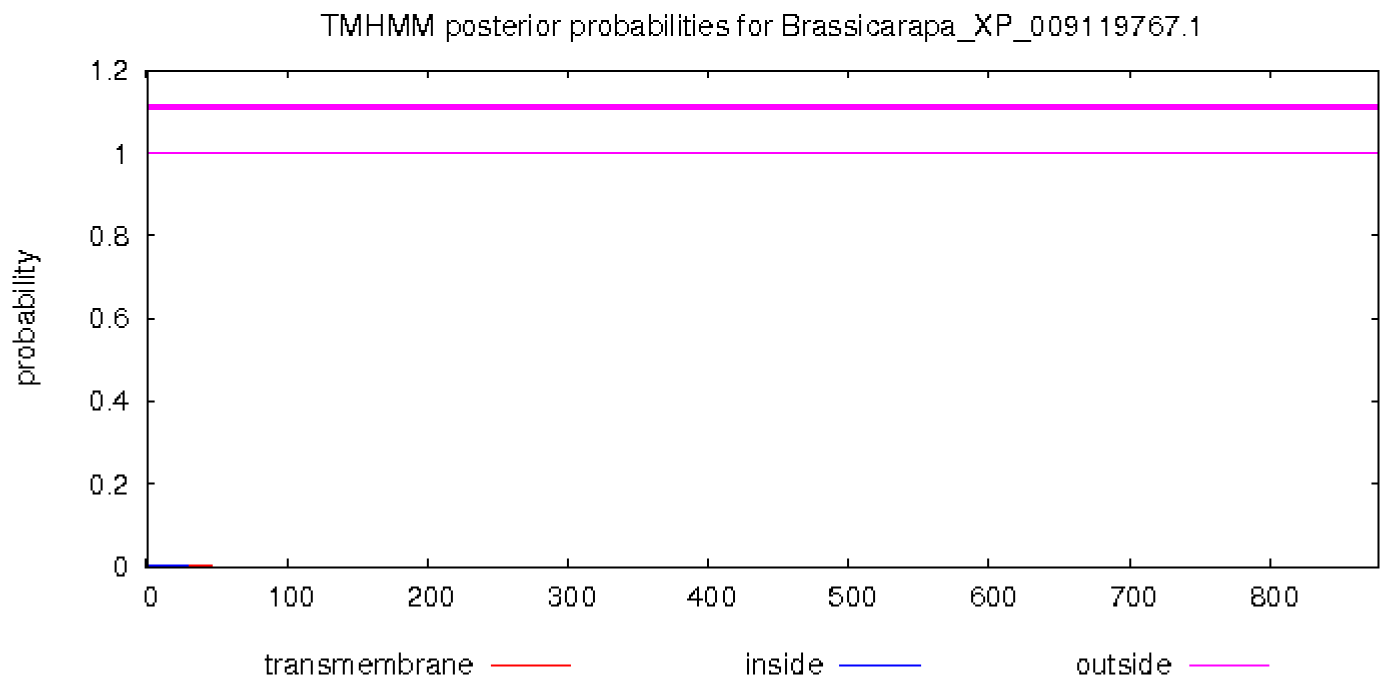

plot in postscript, script for making the plot in gnuplot, data for plot

```
# Brassicarapa_XP_009119784.1 Length: 396
# Brassicarapa_XP_009119784.1 Number of predicted TMHs:  0
# Brassicarapa_XP_009119784.1 Exp number of AAs in TMHs: 0
# Brassicarapa_XP_009119784.1 Exp number, first 60 AAs:  0
# Brassicarapa_XP_009119784.1 Total prob of N-in:        0.00289
Brassicarapa_XP_009119784.1	TMHMM2.0	outside	     1   396
```

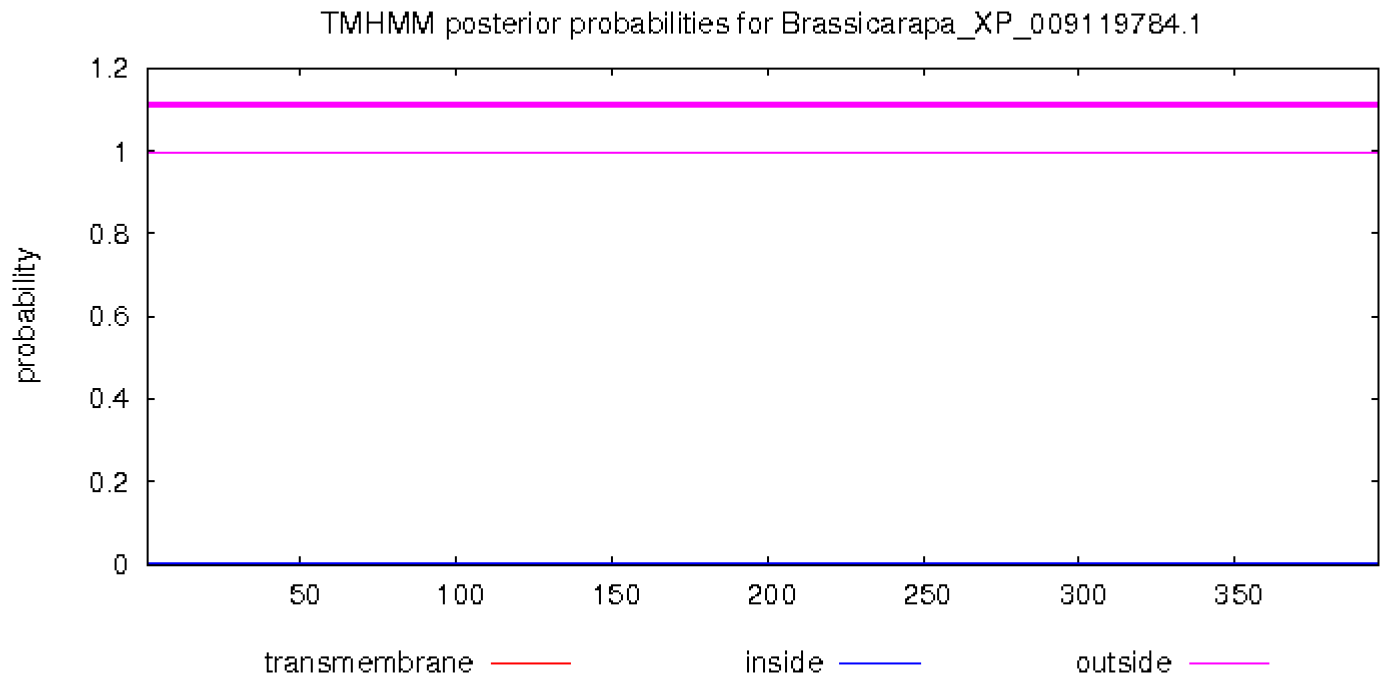

plot in postscript, script for making the plot in gnuplot, data for plot

---

```
# Brassicarapa_XP_009119787.1 Length: 219
# Brassicarapa_XP_009119787.1 Number of predicted TMHs:  0
# Brassicarapa_XP_009119787.1 Exp number of AAs in TMHs: 0.28281
# Brassicarapa_XP_009119787.1 Exp number, first 60 AAs:  0
# Brassicarapa_XP_009119787.1 Total prob of N-in:        0.02309
Brassicarapa_XP_009119787.1     TMHMM2.0        outside      1   219
```

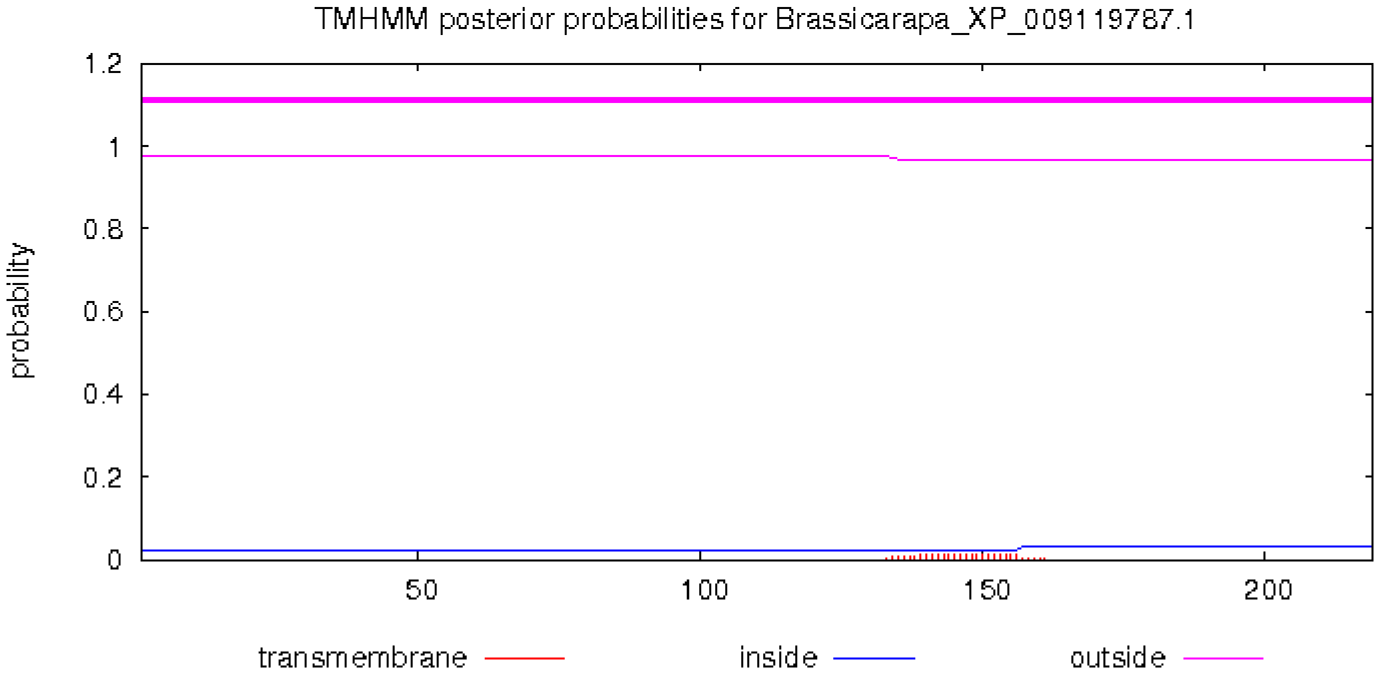

plot in postscript, script for making the plot in gnuplot, data for plot

---

```
# Brassicarapa_XP_009119789.1 Length: 249
# Brassicarapa_XP_009119789.1 Number of predicted TMHs:  1
# Brassicarapa_XP_009119789.1 Exp number of AAs in TMHs: 20.69202
# Brassicarapa_XP_009119789.1 Exp number, first 60 AAs:  20.67986
# Brassicarapa_XP_009119789.1 Total prob of N-in:        0.67771
# Brassicarapa_XP_009119789.1 POSSIBLE N-term signal sequence
Brassicarapa_XP_009119789.1     TMHMM2.0        inside       1    10
Brassicarapa_XP_009119789.1     TMHMM2.0        TMhelix     11    33
Brassicarapa_XP_009119789.1     TMHMM2.0        outside     34   249
```

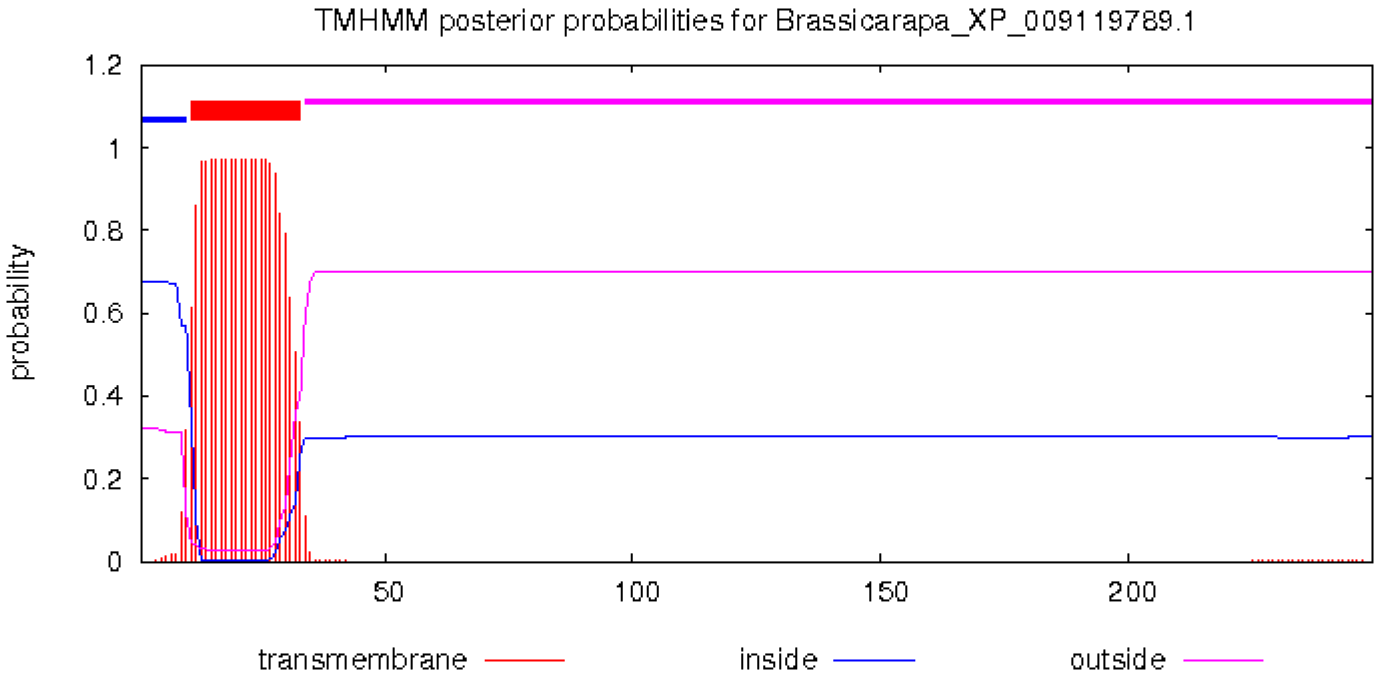

plot in postscript, script for making the plot in gnuplot, data for plot

---

```
# Brassicarapa_XP_009119790.1 Length: 219
# Brassicarapa_XP_009119790.1 Number of predicted TMHs:  0
# Brassicarapa_XP_009119790.1 Exp number of AAs in TMHs: 0.0002
# Brassicarapa_XP_009119790.1 Exp number, first 60 AAs:  0.00011
# Brassicarapa_XP_009119790.1 Total prob of N-in:        0.07269
Brassicarapa_XP_009119790.1     TMHMM2.0        outside      1   219
```

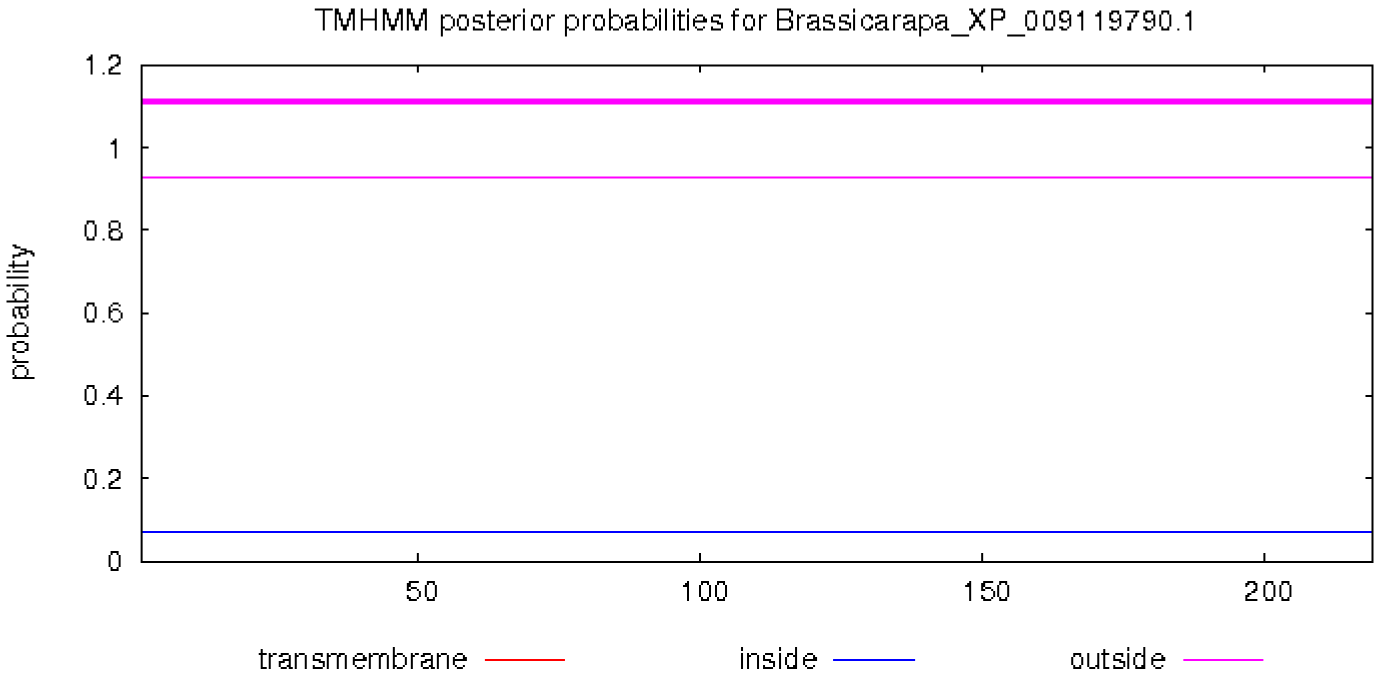

plot in postscript, script for making the plot in gnuplot, data for plot

---

```
# Brassicarapa_XP_009119934.1 Length: 368
# Brassicarapa_XP_009119934.1 Number of predicted TMHs:  0
# Brassicarapa_XP_009119934.1 Exp number of AAs in TMHs: 0.00173
# Brassicarapa_XP_009119934.1 Exp number, first 60 AAs:  0
# Brassicarapa_XP_009119934.1 Total prob of N-in:        0.00817
Brassicarapa_XP_009119934.1     TMHMM2.0        outside      1   368
```

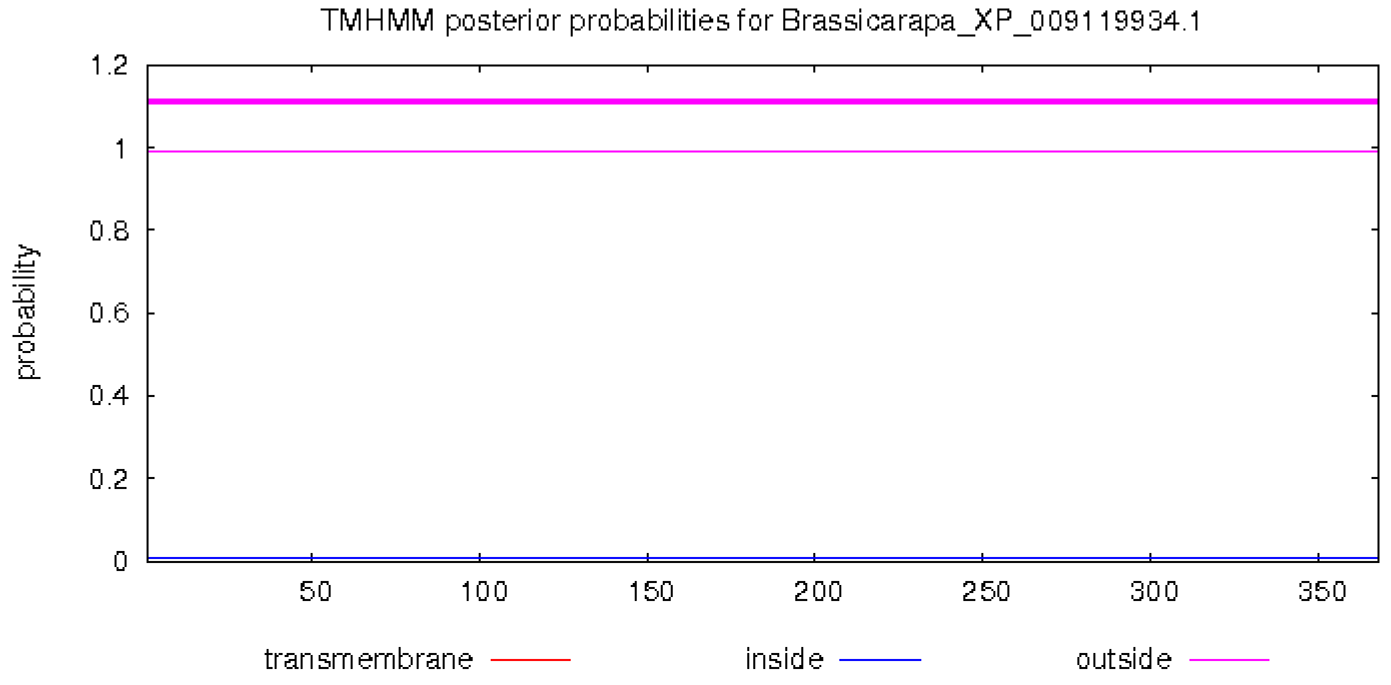

plot in postscript, script for making the plot in gnuplot, data for plot

```
# Brassicarapa_XP_009120111.1 Length: 324
# Brassicarapa_XP_009120111.1 Number of predicted TMHs:  0
# Brassicarapa_XP_009120111.1 Exp number of AAs in TMHs: 0.00191
# Brassicarapa_XP_009120111.1 Exp number, first 60 AAs:  0.00038
# Brassicarapa_XP_009120111.1 Total prob of N-in:        0.00888
Brassicarapa_XP_009120111.1     TMHMM2.0        outside      1   324
```

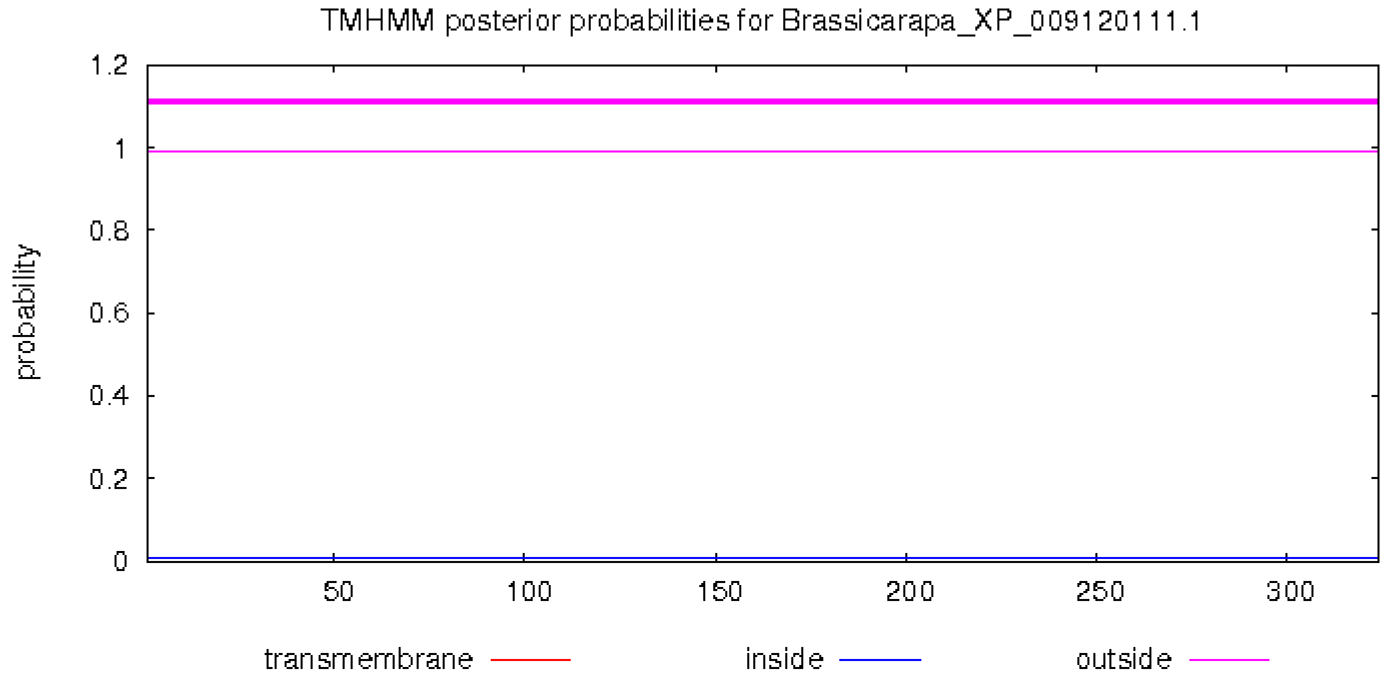

plot in postscript, script for making the plot in gnuplot, data for plot

```
# Brassicarapa_XP_009120122.1 Length: 290
# Brassicarapa_XP_009120122.1 Number of predicted TMHs:  0
# Brassicarapa_XP_009120122.1 Exp number of AAs in TMHs: 0.00566
# Brassicarapa_XP_009120122.1 Exp number, first 60 AAs:  0
# Brassicarapa_XP_009120122.1 Total prob of N-in:        0.05317
Brassicarapa_XP_009120122.1     TMHMM2.0        outside      1   290
```

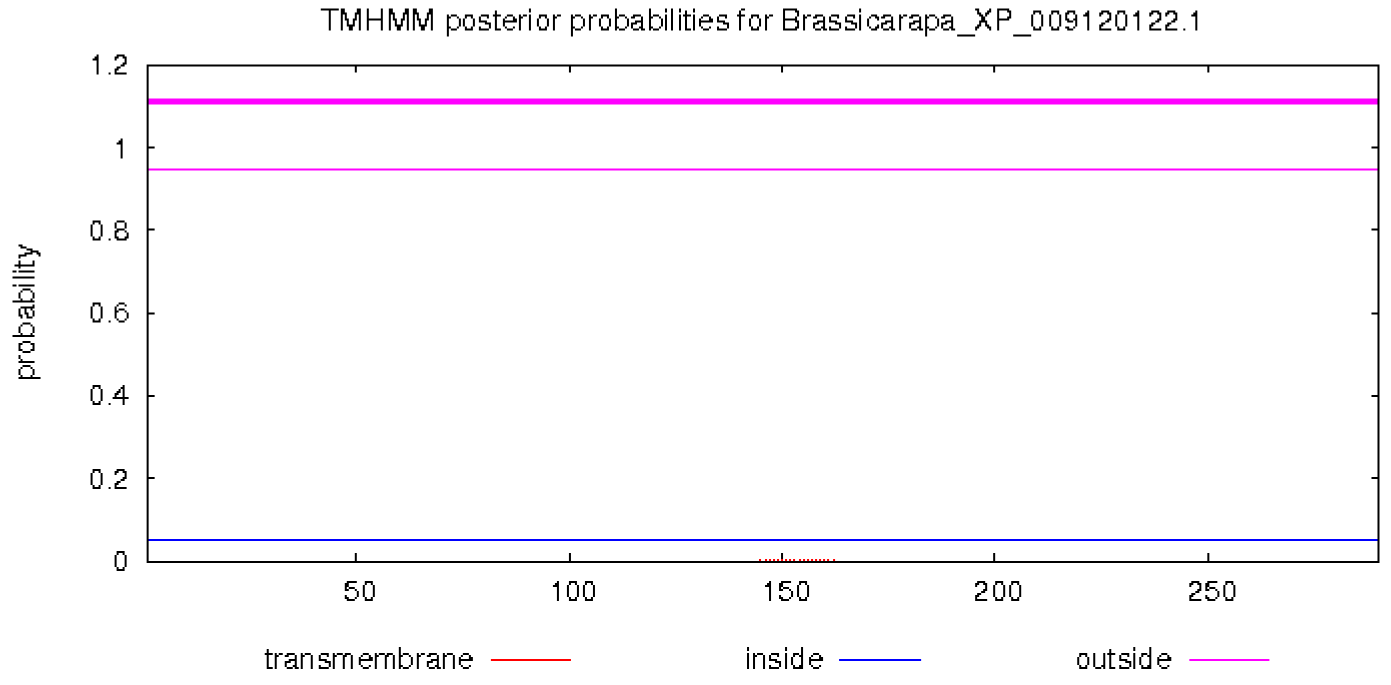

plot in postscript, script for making the plot in gnuplot, data for plot

---

```
# Brassicarapa_XP_009120171.1 Length: 407
# Brassicarapa_XP_009120171.1 Number of predicted TMHs:  0
# Brassicarapa_XP_009120171.1 Exp number of AAs in TMHs: 0.00034
# Brassicarapa_XP_009120171.1 Exp number, first 60 AAs:  0
# Brassicarapa_XP_009120171.1 Total prob of N-in:        0.02610
Brassicarapa_XP_009120171.1     TMHMM2.0        outside      1   407
```

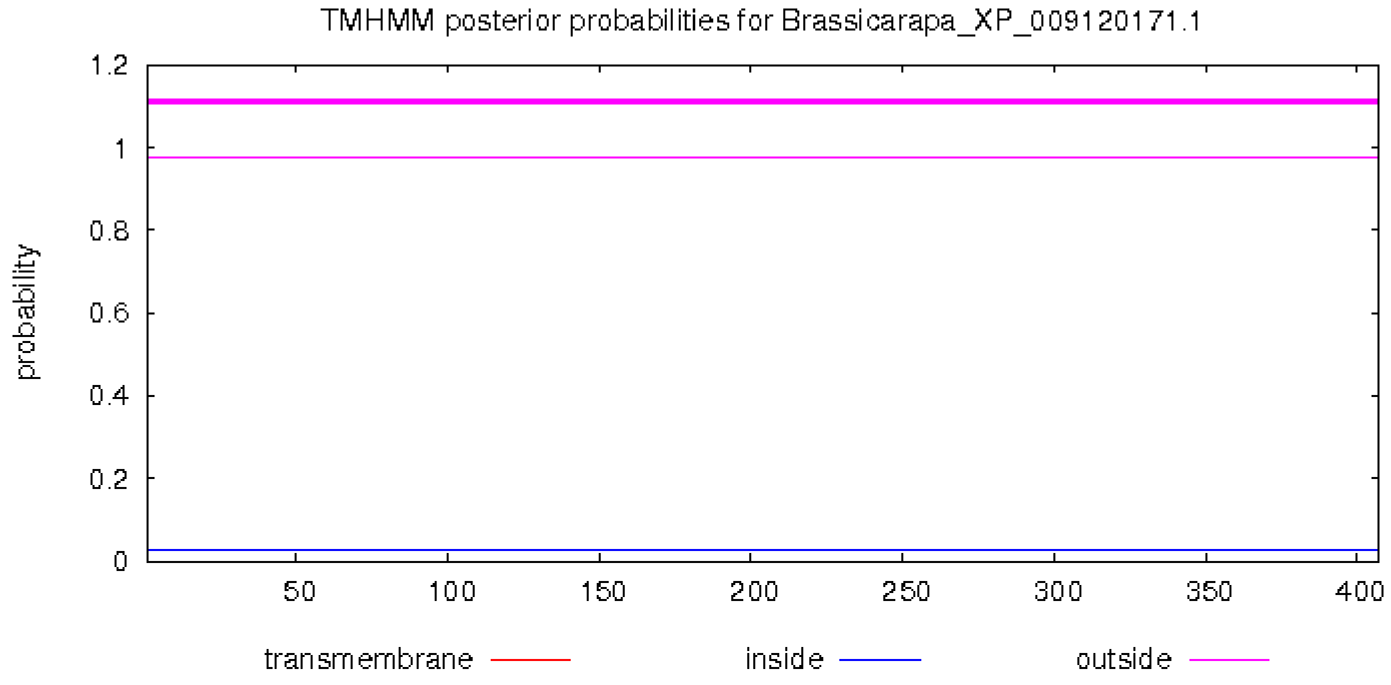

plot in postscript, script for making the plot in gnuplot, data for plot

---

```
# Brassicarapa_XP_009120172.1 Length: 407
# Brassicarapa_XP_009120172.1 Number of predicted TMHs:  0
# Brassicarapa_XP_009120172.1 Exp number of AAs in TMHs: 0.00034
# Brassicarapa_XP_009120172.1 Exp number, first 60 AAs:  0
# Brassicarapa_XP_009120172.1 Total prob of N-in:        0.02610
Brassicarapa_XP_009120172.1     TMHMM2.0        outside      1   407
```

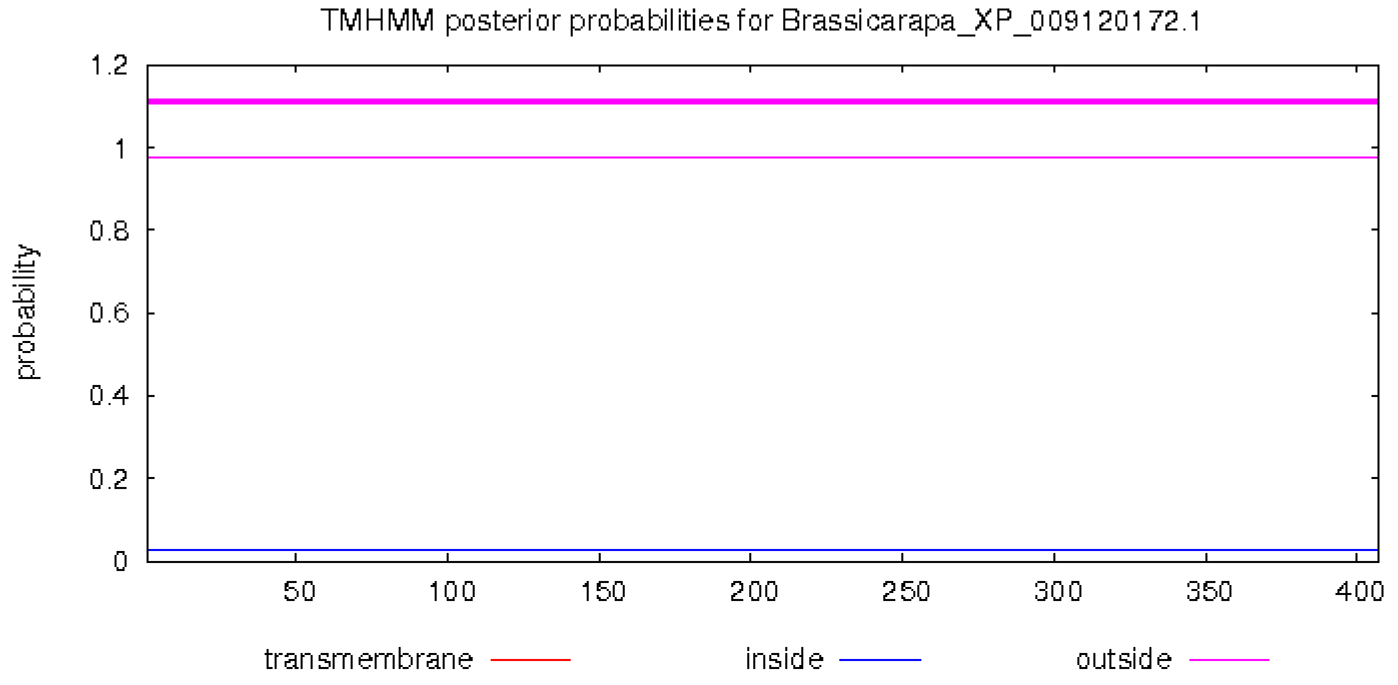

plot in postscript, script for making the plot in gnuplot, data for plot

---

```
# Brassicarapa_XP_009120173.1 Length: 406
# Brassicarapa_XP_009120173.1 Number of predicted TMHs:  0
# Brassicarapa_XP_009120173.1 Exp number of AAs in TMHs: 0.00034
# Brassicarapa_XP_009120173.1 Exp number, first 60 AAs:  0
# Brassicarapa_XP_009120173.1 Total prob of N-in:        0.02641
Brassicarapa_XP_009120173.1     TMHMM2.0        outside      1   406
```

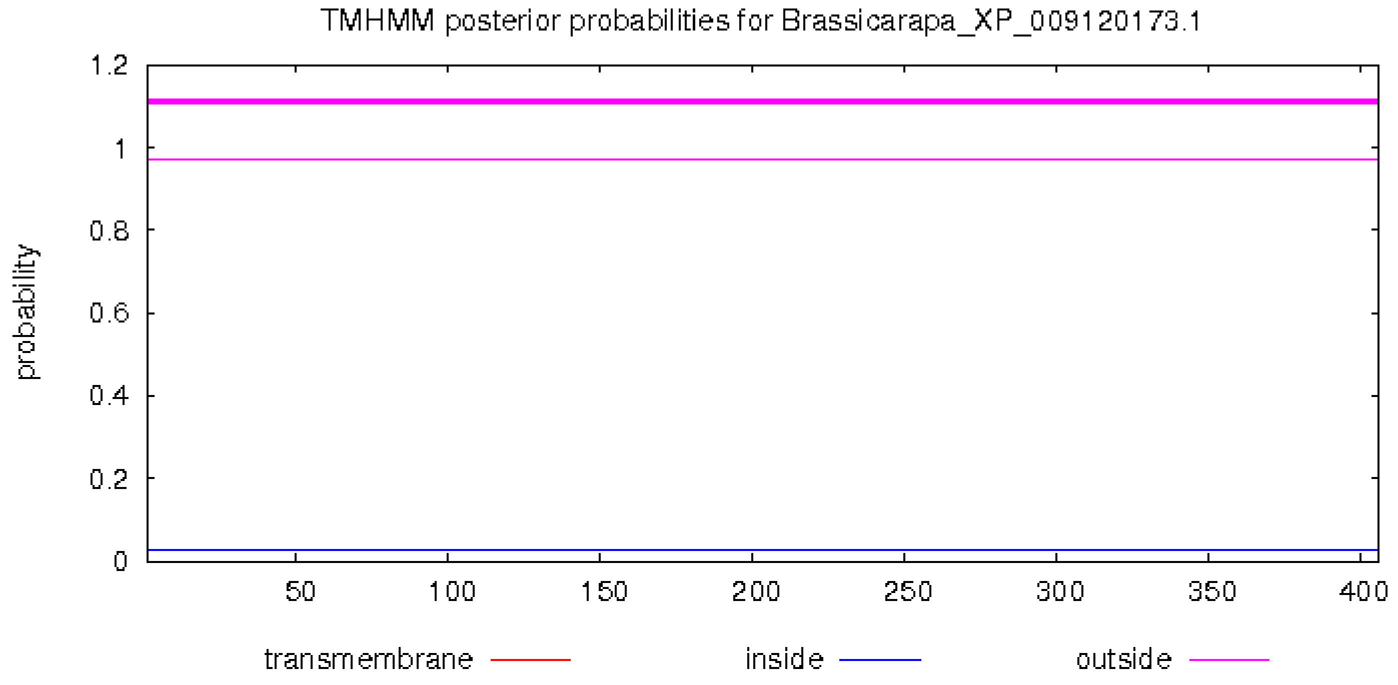

plot in postscript, script for making the plot in gnuplot, data for plot

---

```
# Brassicarapa_XP_009120174.1 Length: 434
# Brassicarapa_XP_009120174.1 Number of predicted TMHs:  0
# Brassicarapa_XP_009120174.1 Exp number of AAs in TMHs: 0.0919
# Brassicarapa_XP_009120174.1 Exp number, first 60 AAs:  0
# Brassicarapa_XP_009120174.1 Total prob of N-in:        0.01391
Brassicarapa_XP_009120174.1     TMHMM2.0        outside      1   434
```

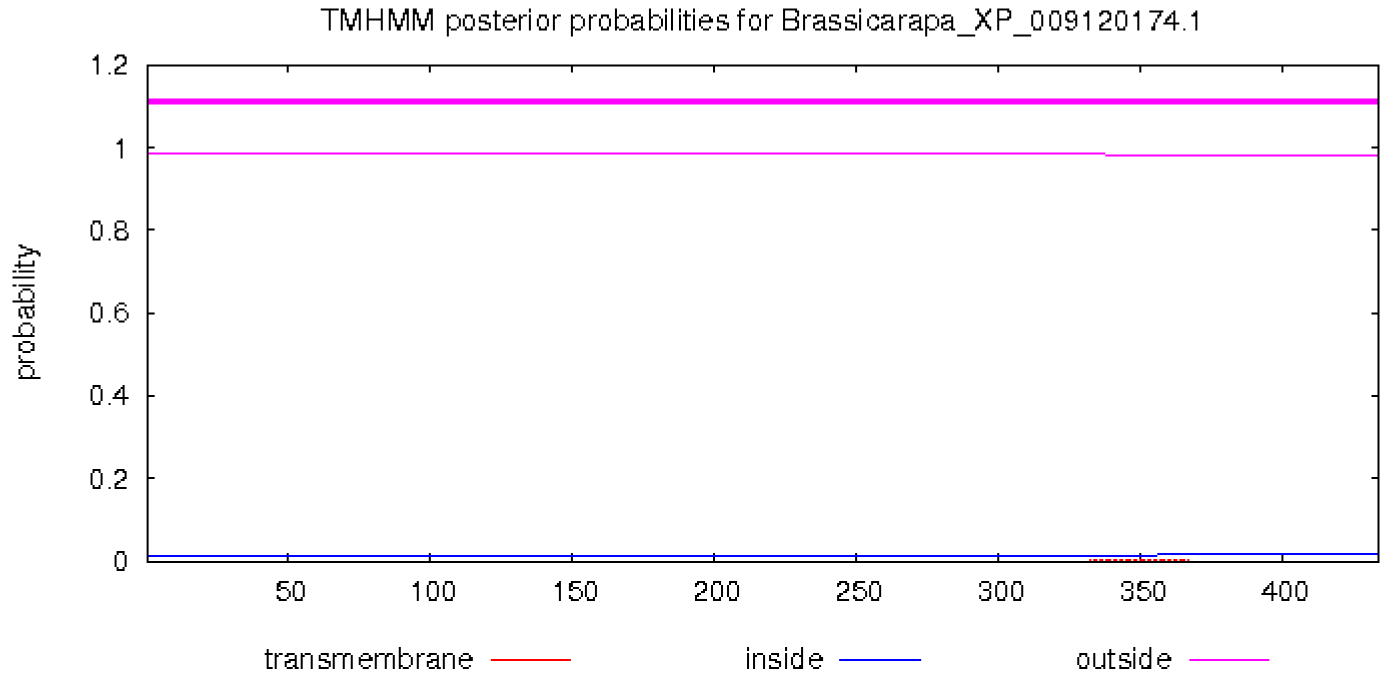

plot in postscript, script for making the plot in gnuplot, data for plot

---

```
# Brassicarapa_XP_009120664.1 Length: 235
# Brassicarapa_XP_009120664.1 Number of predicted TMHs:  0
# Brassicarapa_XP_009120664.1 Exp number of AAs in TMHs: 0.0003
# Brassicarapa_XP_009120664.1 Exp number, first 60 AAs:  0.00016
# Brassicarapa_XP_009120664.1 Total prob of N-in:        0.04565
Brassicarapa_XP_009120664.1	TMHMM2.0	outside	     1   235
```

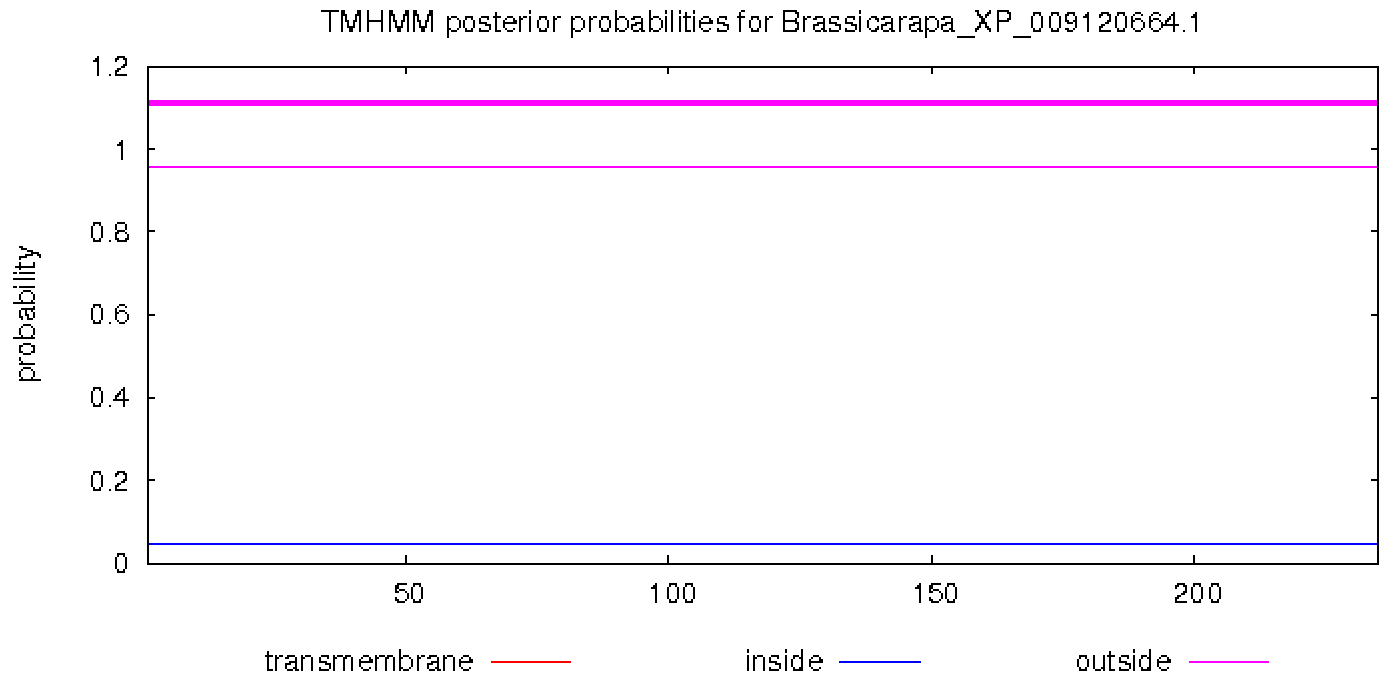

plot in postscript, script for making the plot in gnuplot, data for plot

---

```
# Brassicarapa_XP_009120669.1 Length: 335
# Brassicarapa_XP_009120669.1 Number of predicted TMHs:  0
# Brassicarapa_XP_009120669.1 Exp number of AAs in TMHs: 3.10039
# Brassicarapa_XP_009120669.1 Exp number, first 60 AAs:  0.06967
# Brassicarapa_XP_009120669.1 Total prob of N-in:        0.14606
Brassicarapa_XP_009120669.1	TMHMM2.0	outside	     1   335
```

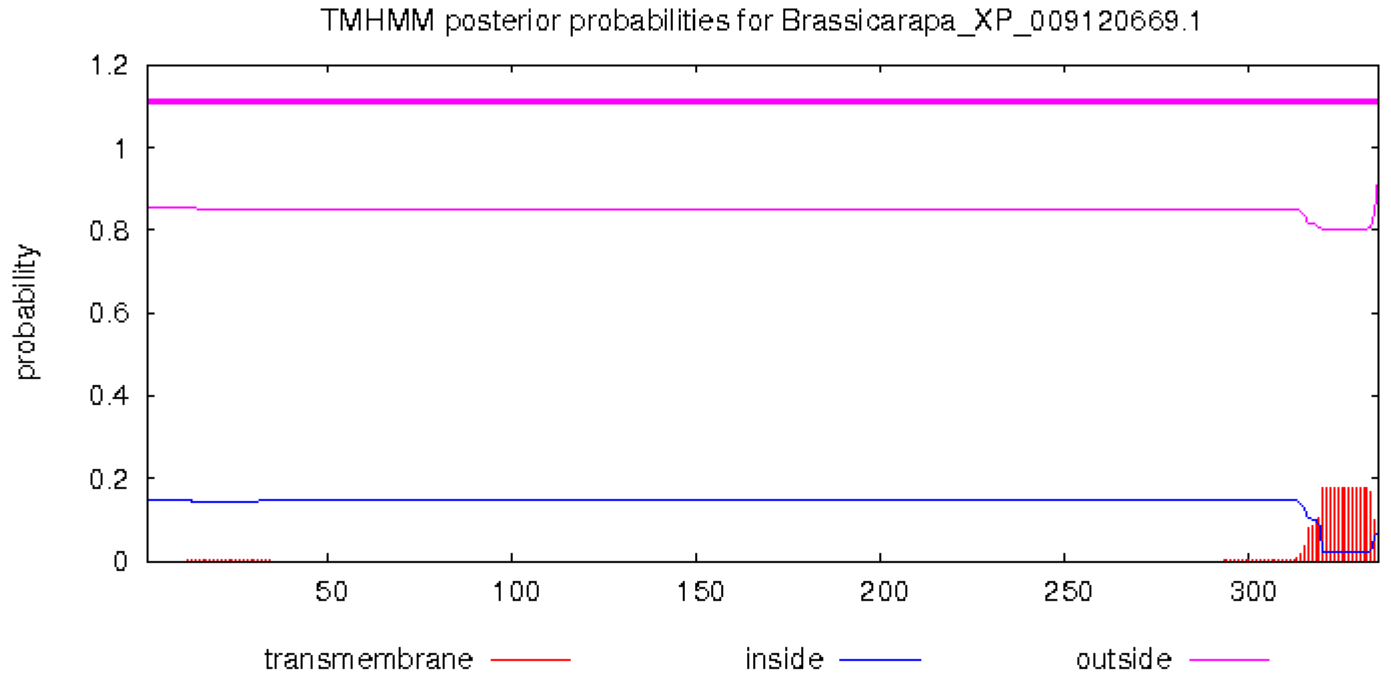

plot in postscript, script for making the plot in gnuplot, data for plot

---

```
# Brassicarapa_XP_009120990.1 Length: 336
# Brassicarapa_XP_009120990.1 Number of predicted TMHs:  0
# Brassicarapa_XP_009120990.1 Exp number of AAs in TMHs: 0.0002
# Brassicarapa_XP_009120990.1 Exp number, first 60 AAs:  0
# Brassicarapa_XP_009120990.1 Total prob of N-in:        0.00873
Brassicarapa_XP_009120990.1     TMHMM2.0        outside      1   336
```

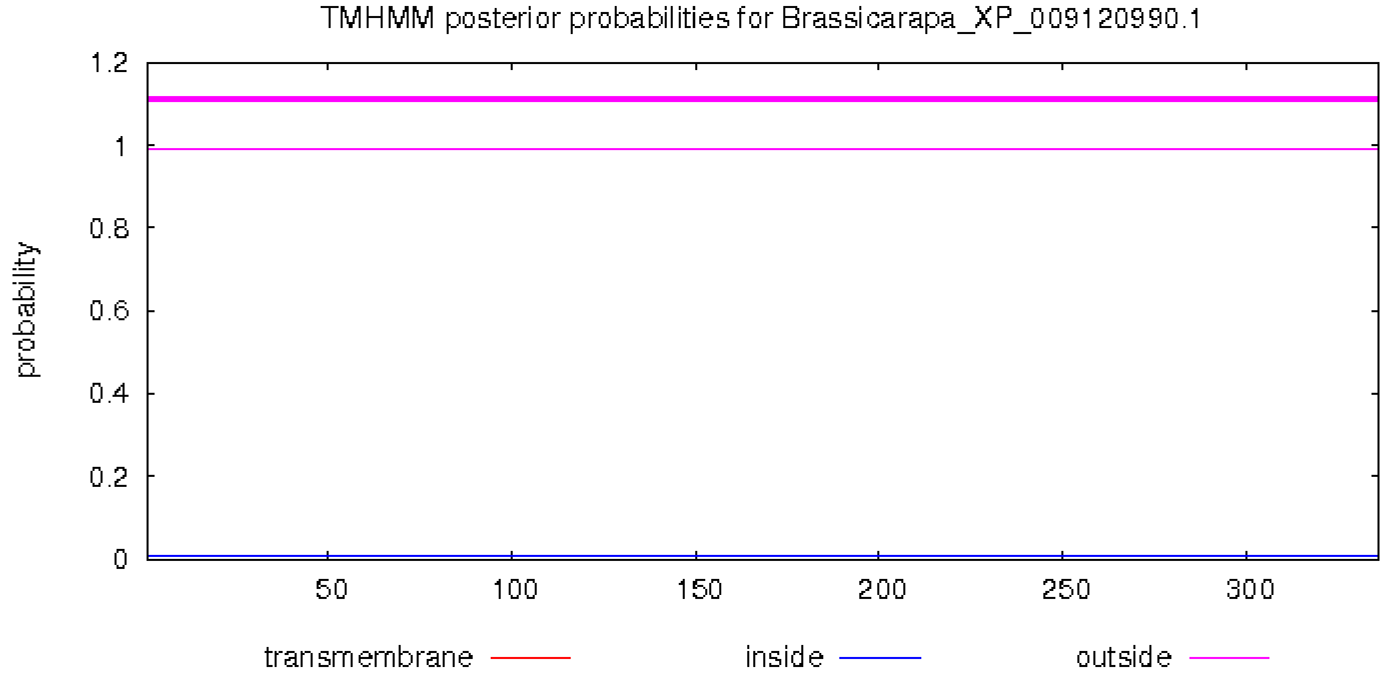

plot in postscript, script for making the plot in gnuplot, data for plot

---

```
# Brassicarapa_XP_009121166.1 Length: 319
# Brassicarapa_XP_009121166.1 Number of predicted TMHs:  0
# Brassicarapa_XP_009121166.1 Exp number of AAs in TMHs: 0.01384
# Brassicarapa_XP_009121166.1 Exp number, first 60 AAs:  0.0015
# Brassicarapa_XP_009121166.1 Total prob of N-in:        0.00256
Brassicarapa_XP_009121166.1     TMHMM2.0        outside      1   319
```

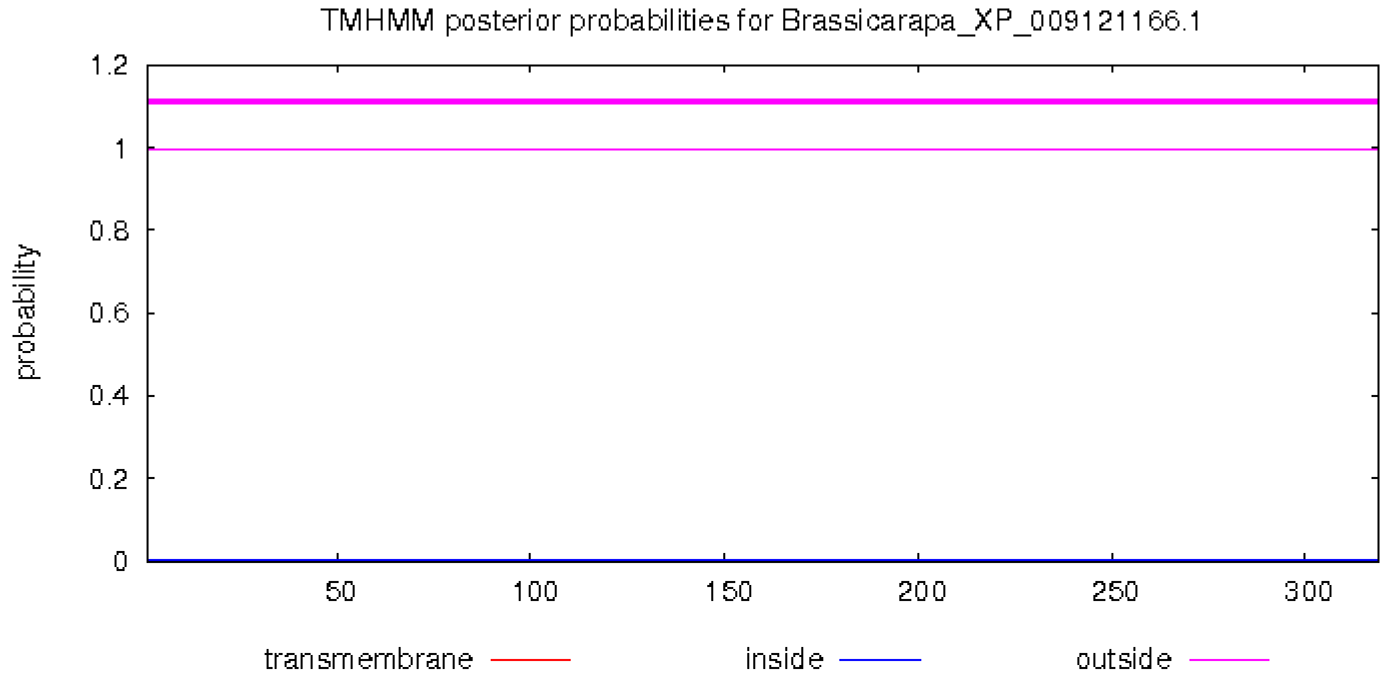

plot in postscript, script for making the plot in gnuplot, data for plot

---

```
# Brassicarapa_XP_009121308.1 Length: 370
# Brassicarapa_XP_009121308.1 Number of predicted TMHs:  0
# Brassicarapa_XP_009121308.1 Exp number of AAs in TMHs: 0.00023
# Brassicarapa_XP_009121308.1 Exp number, first 60 AAs:  0.00013
# Brassicarapa_XP_009121308.1 Total prob of N-in:        0.01176
Brassicarapa_XP_009121308.1	TMHMM2.0	outside	     1   370
```

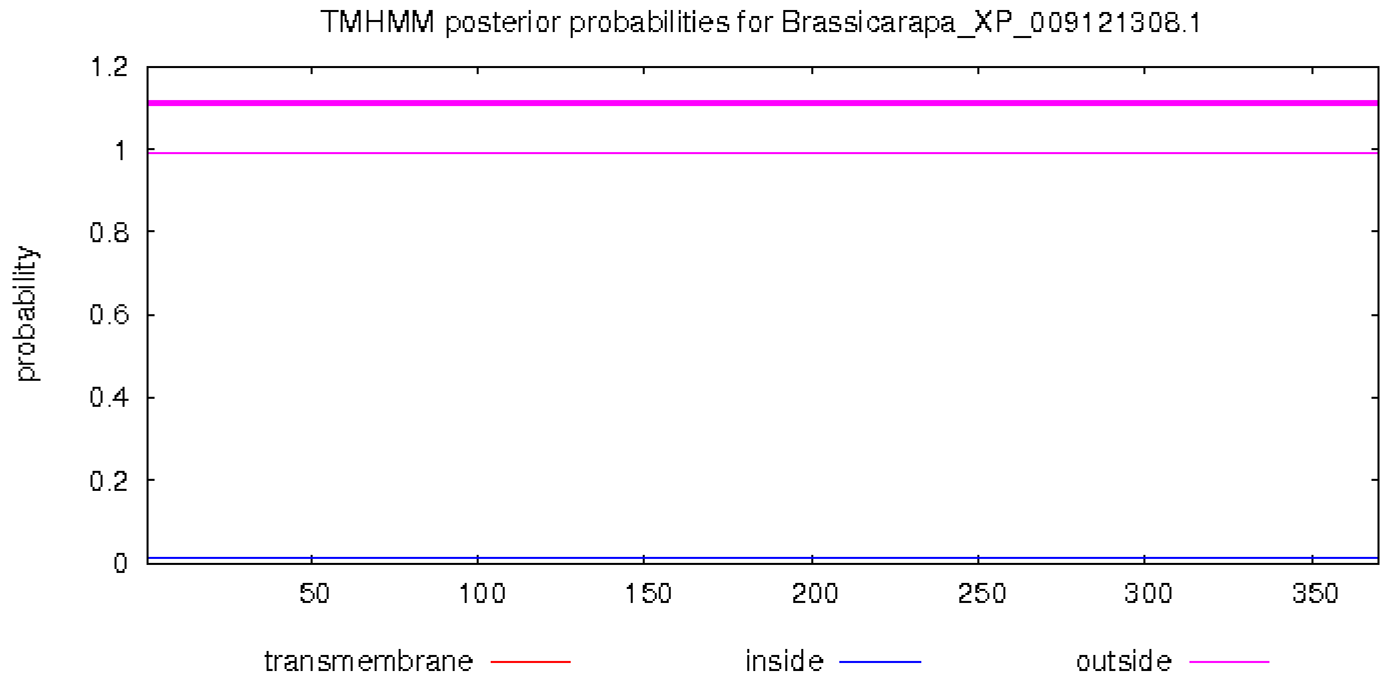

plot in postscript, script for making the plot in gnuplot, data for plot

---

```
# Brassicarapa_XP_009121402.1 Length: 499
# Brassicarapa_XP_009121402.1 Number of predicted TMHs:  0
# Brassicarapa_XP_009121402.1 Exp number of AAs in TMHs: 0.00019
# Brassicarapa_XP_009121402.1 Exp number, first 60 AAs:  0
# Brassicarapa_XP_009121402.1 Total prob of N-in:        0.00012
Brassicarapa_XP_009121402.1	TMHMM2.0	outside	     1   499
```

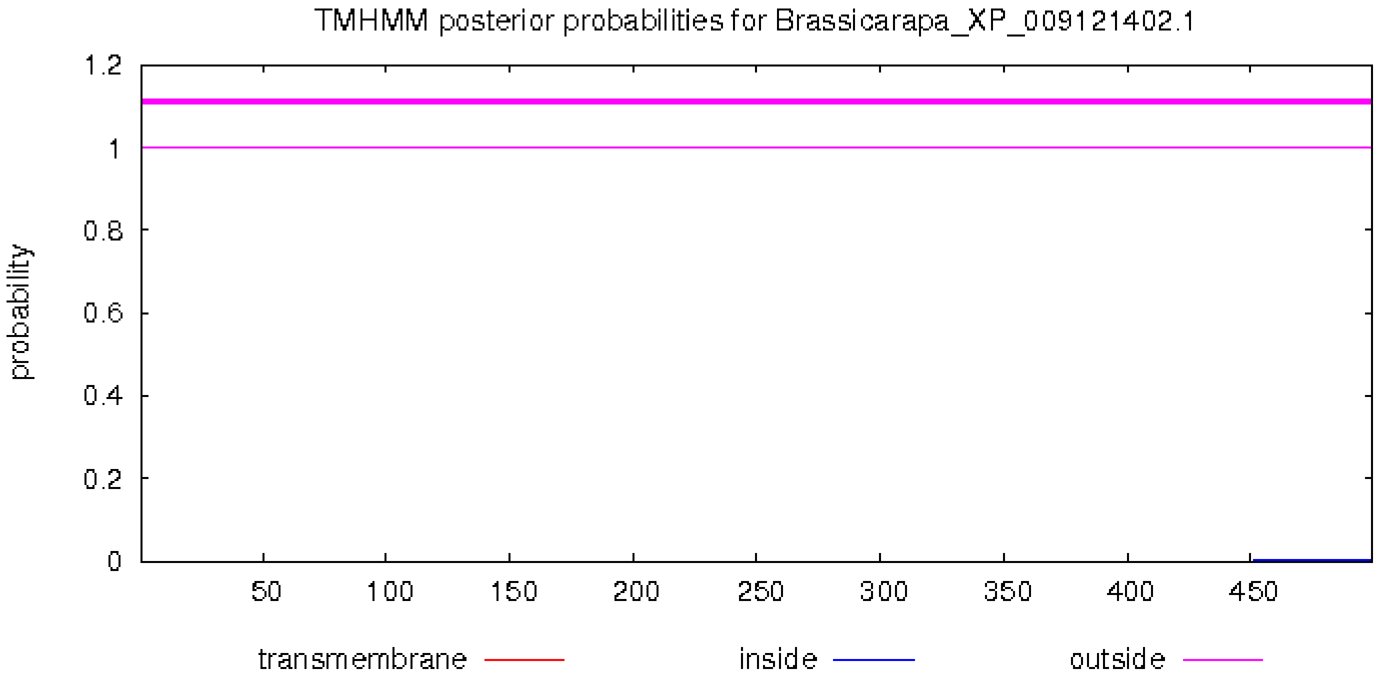

plot in postscript, script for making the plot in gnuplot, data for plot

---

```
# Brassicarapa_XP_009121709.1 Length: 202
# Brassicarapa_XP_009121709.1 Number of predicted TMHs:  0
# Brassicarapa_XP_009121709.1 Exp number of AAs in TMHs: 0.75828
# Brassicarapa_XP_009121709.1 Exp number, first 60 AAs:  0.73218
# Brassicarapa_XP_009121709.1 Total prob of N-in:        0.05763
Brassicarapa_XP_009121709.1     TMHMM2.0        outside      1   202
```

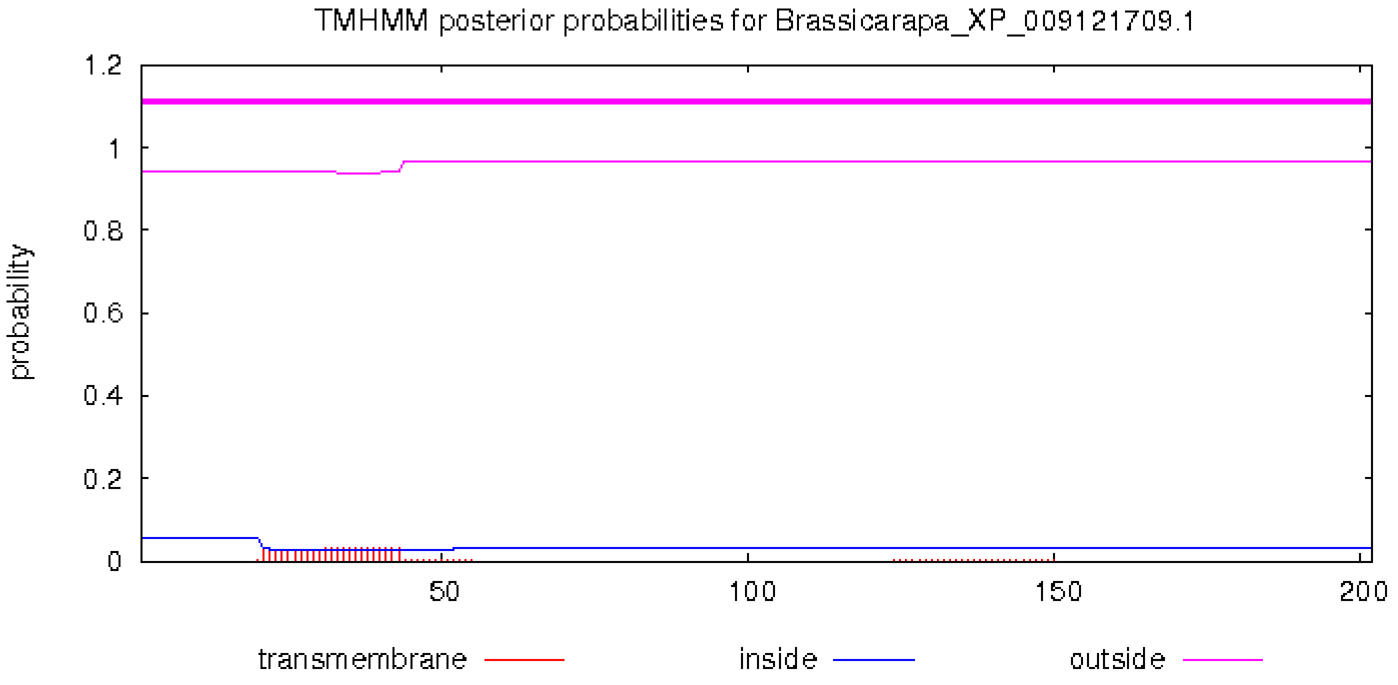

plot in postscript, script for making the plot in gnuplot, data for plot

---

```
# Brassicarapa_XP_009121790.1 Length: 276
# Brassicarapa_XP_009121790.1 Number of predicted TMHs:  0
# Brassicarapa_XP_009121790.1 Exp number of AAs in TMHs: 3.23642
# Brassicarapa_XP_009121790.1 Exp number, first 60 AAs:  3.23629
# Brassicarapa_XP_009121790.1 Total prob of N-in:        0.08297
Brassicarapa_XP_009121790.1     TMHMM2.0        outside      1   276
```

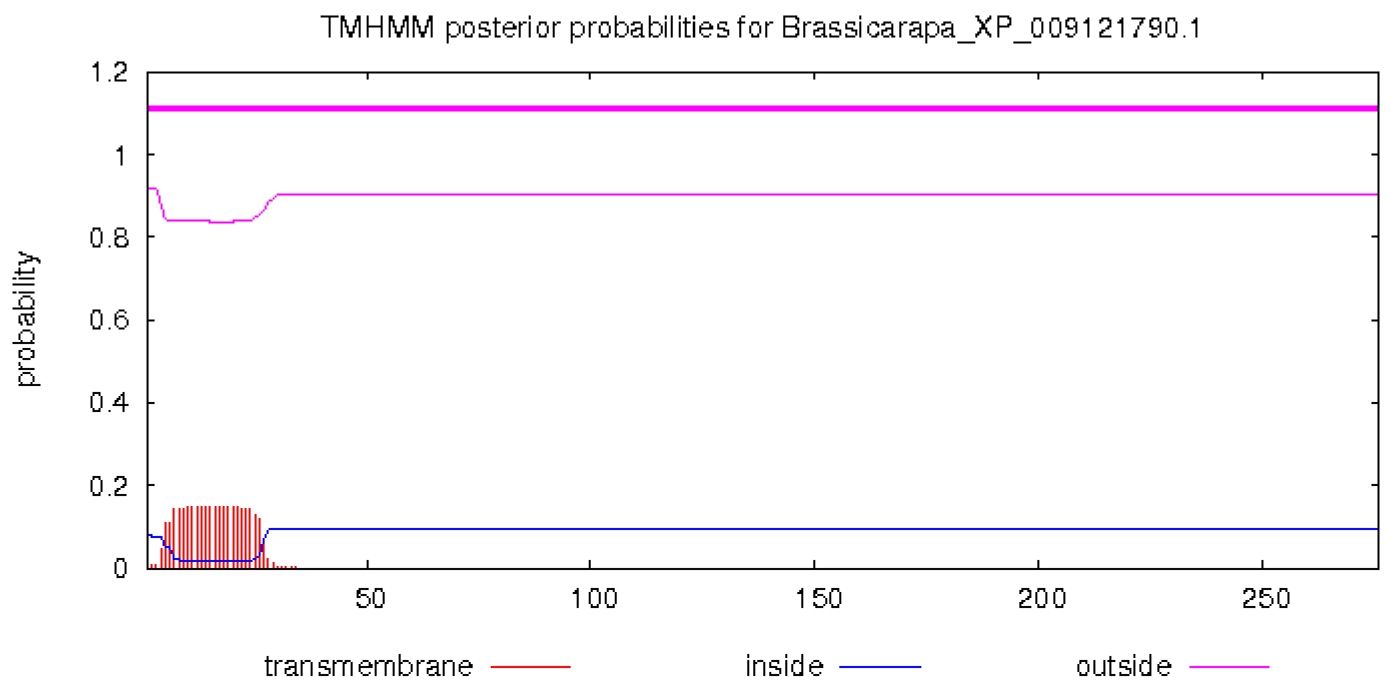

plot in postscript, script for making the plot in gnuplot, data for plot

---

```
# Brassicarapa_XP_009121975.1 Length: 225
# Brassicarapa_XP_009121975.1 Number of predicted TMHs:  0
# Brassicarapa_XP_009121975.1 Exp number of AAs in TMHs: 0.00075
# Brassicarapa_XP_009121975.1 Exp number, first 60 AAs:  0.0004
# Brassicarapa_XP_009121975.1 Total prob of N-in:        0.01249
Brassicarapa_XP_009121975.1     TMHMM2.0        outside      1   225
```

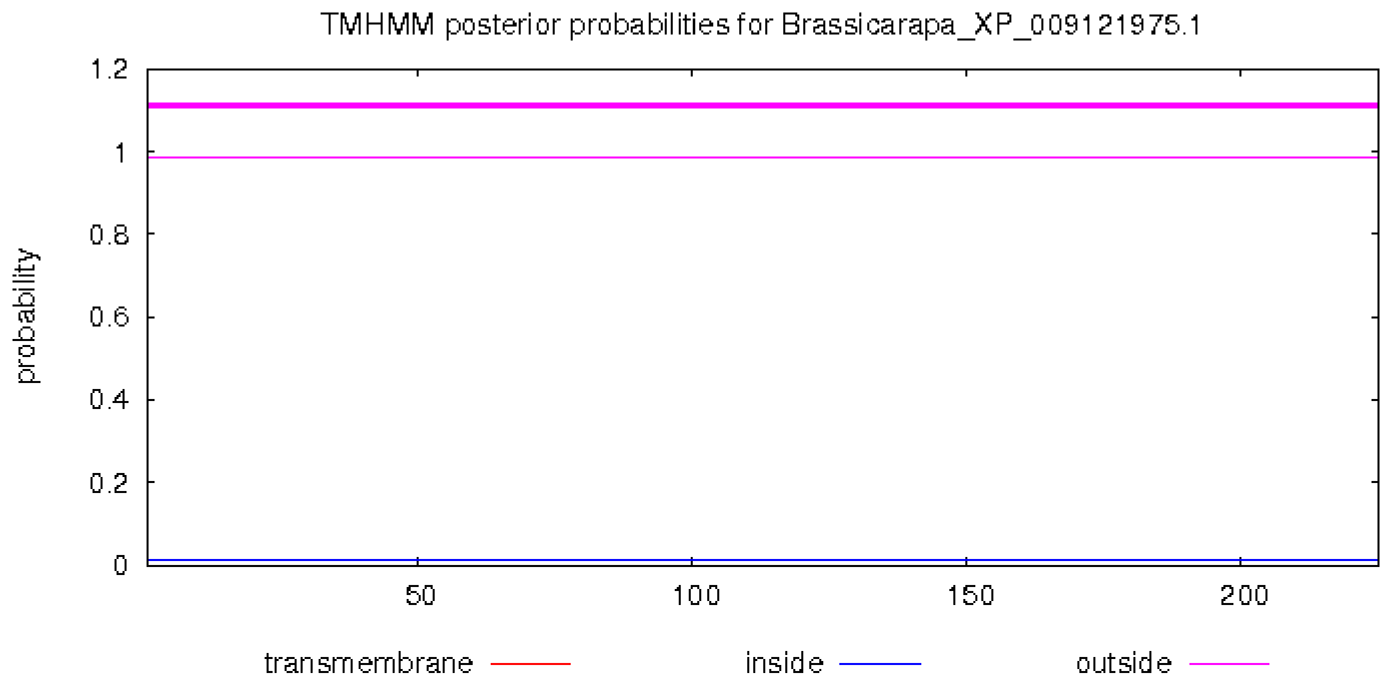

plot in postscript, script for making the plot in gnuplot, data for plot

---

```
# Brassicarapa_XP_009121976.1 Length: 213
# Brassicarapa_XP_009121976.1 Number of predicted TMHs:  0
# Brassicarapa_XP_009121976.1 Exp number of AAs in TMHs: 0.00077
# Brassicarapa_XP_009121976.1 Exp number, first 60 AAs:  0.00041
# Brassicarapa_XP_009121976.1 Total prob of N-in:        0.01448
Brassicarapa_XP_009121976.1     TMHMM2.0        outside      1   213
```

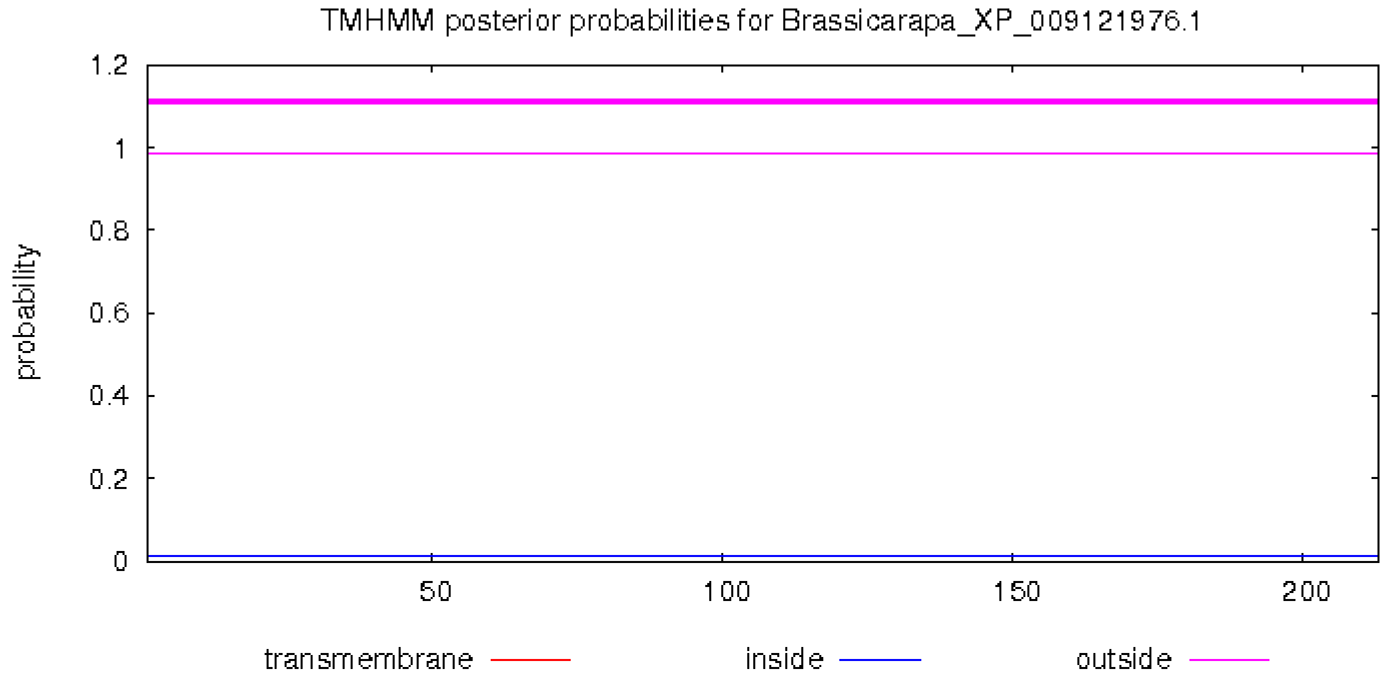

plot in postscript, script for making the plot in gnuplot, data for plot

---

```
# Brassicarapa_XP_009121977.1 Length: 205
# Brassicarapa_XP_009121977.1 Number of predicted TMHs:  0
# Brassicarapa_XP_009121977.1 Exp number of AAs in TMHs: 0.0008
# Brassicarapa_XP_009121977.1 Exp number, first 60 AAs:  0.00043
# Brassicarapa_XP_009121977.1 Total prob of N-in:        0.01597
Brassicarapa_XP_009121977.1     TMHMM2.0        outside      1   205
```

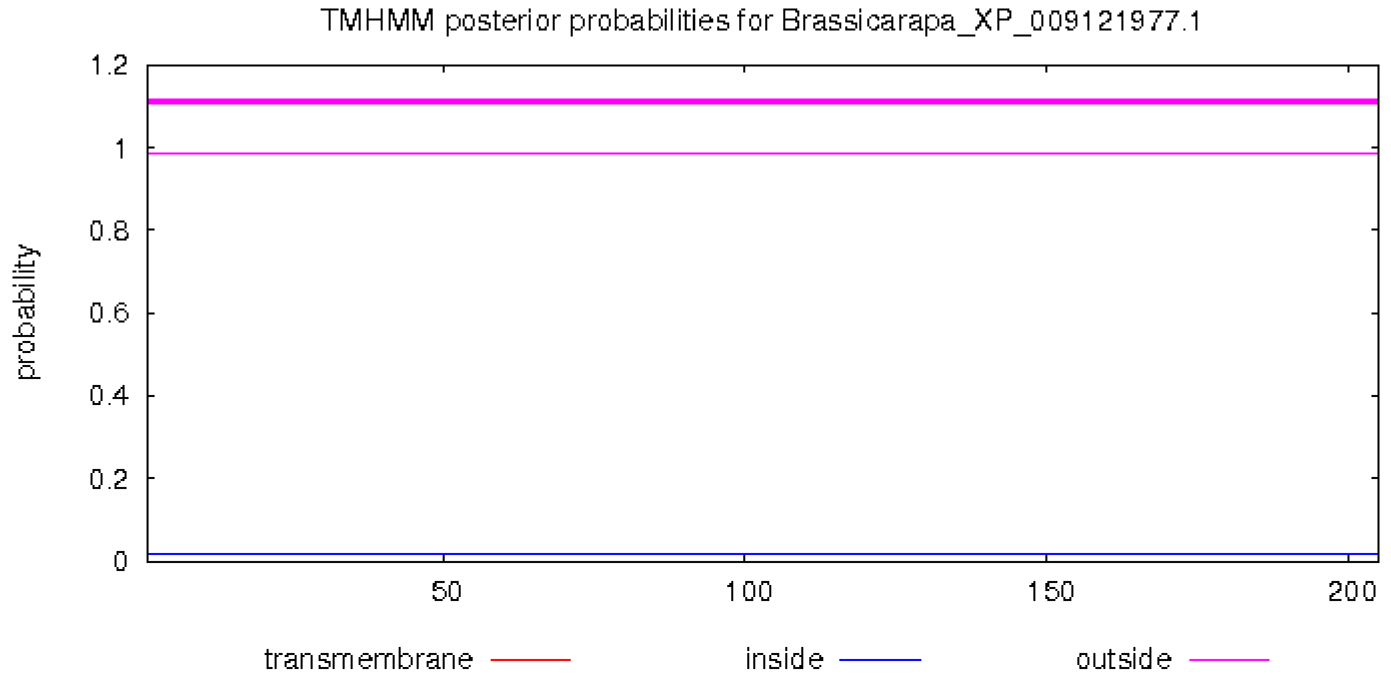

plot in postscript, script for making the plot in gnuplot, data for plot

---

```
# Brassicarapa_XP_009122319.1 Length: 283
# Brassicarapa_XP_009122319.1 Number of predicted TMHs:  0
# Brassicarapa_XP_009122319.1 Exp number of AAs in TMHs: 0.0034
# Brassicarapa_XP_009122319.1 Exp number, first 60 AAs:  0
# Brassicarapa_XP_009122319.1 Total prob of N-in:        0.01731
Brassicarapa_XP_009122319.1     TMHMM2.0        outside      1   283
```

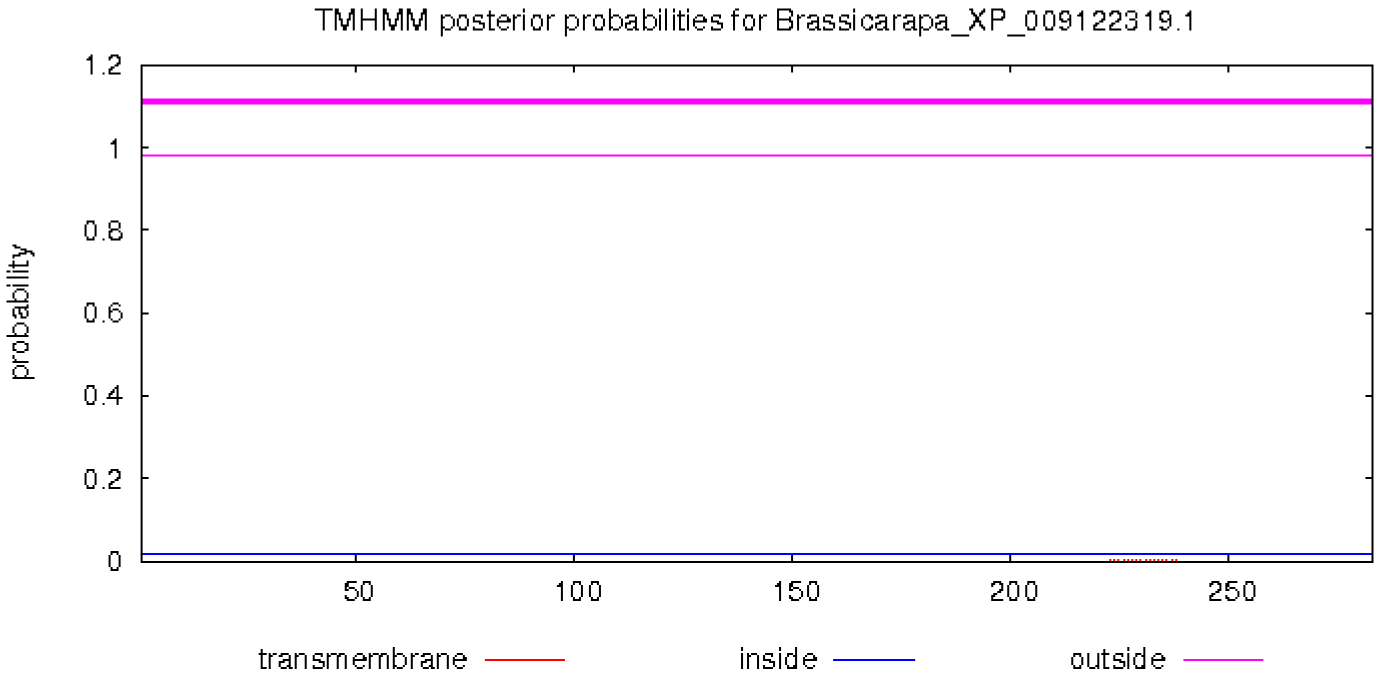

plot in postscript, script for making the plot in gnuplot, data for plot

---

```
# Brassicarapa_XP_009122498.1 Length: 278
# Brassicarapa_XP_009122498.1 Number of predicted TMHs:  0
# Brassicarapa_XP_009122498.1 Exp number of AAs in TMHs: 0.00019
# Brassicarapa_XP_009122498.1 Exp number, first 60 AAs:  0
# Brassicarapa_XP_009122498.1 Total prob of N-in:        0.01089
Brassicarapa_XP_009122498.1     TMHMM2.0        outside      1   278
```

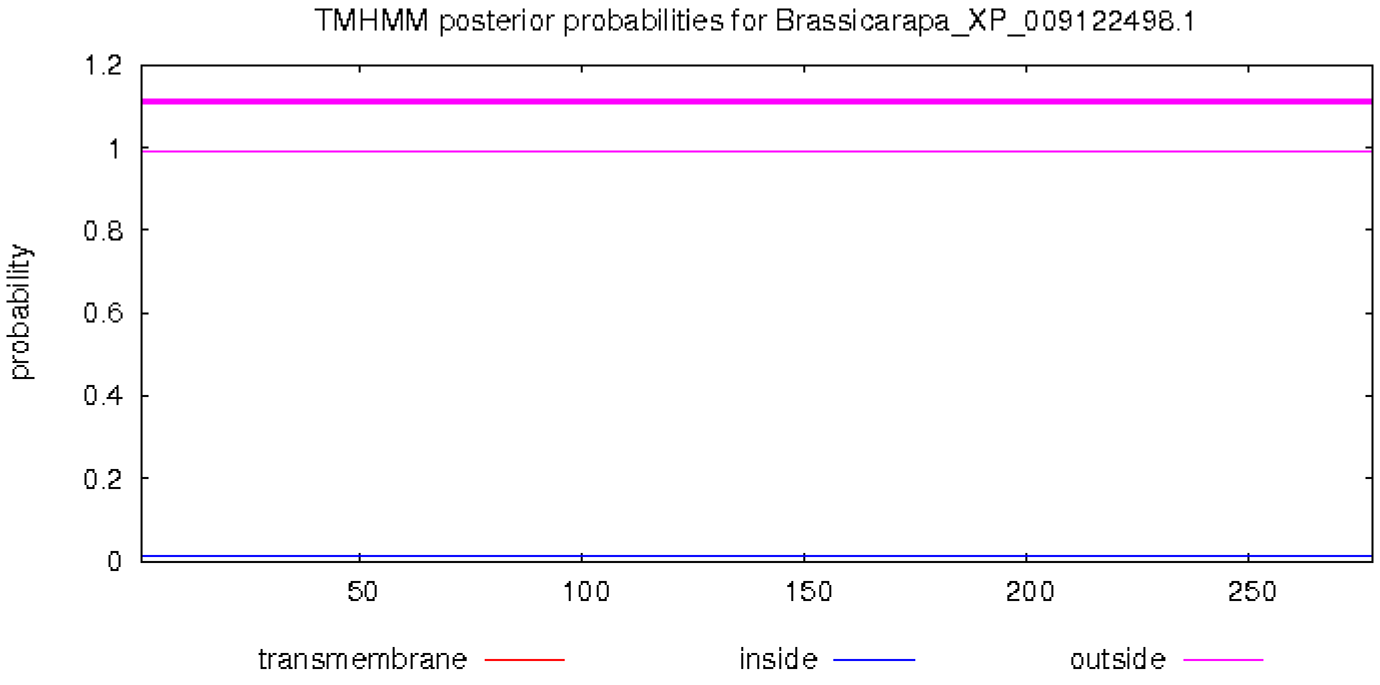

plot in postscript, script for making the plot in gnuplot, data for plot

---

```
# Brassicarapa_XP_009122507.1 Length: 454
# Brassicarapa_XP_009122507.1 Number of predicted TMHs:  0
# Brassicarapa_XP_009122507.1 Exp number of AAs in TMHs: 0
# Brassicarapa_XP_009122507.1 Exp number, first 60 AAs:  0
# Brassicarapa_XP_009122507.1 Total prob of N-in:        0.00105
Brassicarapa_XP_009122507.1     TMHMM2.0        outside      1   454
```

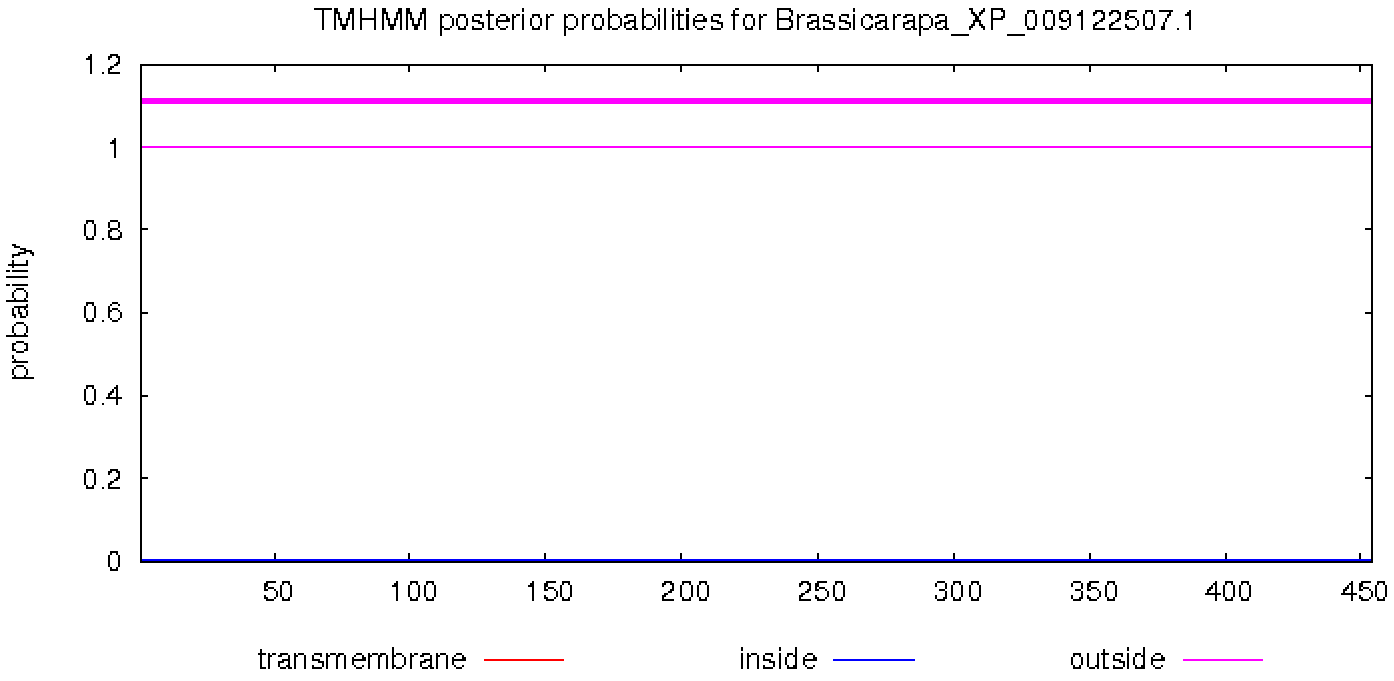

plot in postscript, script for making the plot in gnuplot, data for plot

---

```
# Brassicarapa_XP_009122509.1 Length: 454
# Brassicarapa_XP_009122509.1 Number of predicted TMHs:  0
# Brassicarapa_XP_009122509.1 Exp number of AAs in TMHs: 0
# Brassicarapa_XP_009122509.1 Exp number, first 60 AAs:  0
# Brassicarapa_XP_009122509.1 Total prob of N-in:        0.00105
Brassicarapa_XP_009122509.1     TMHMM2.0        outside      1   454
```

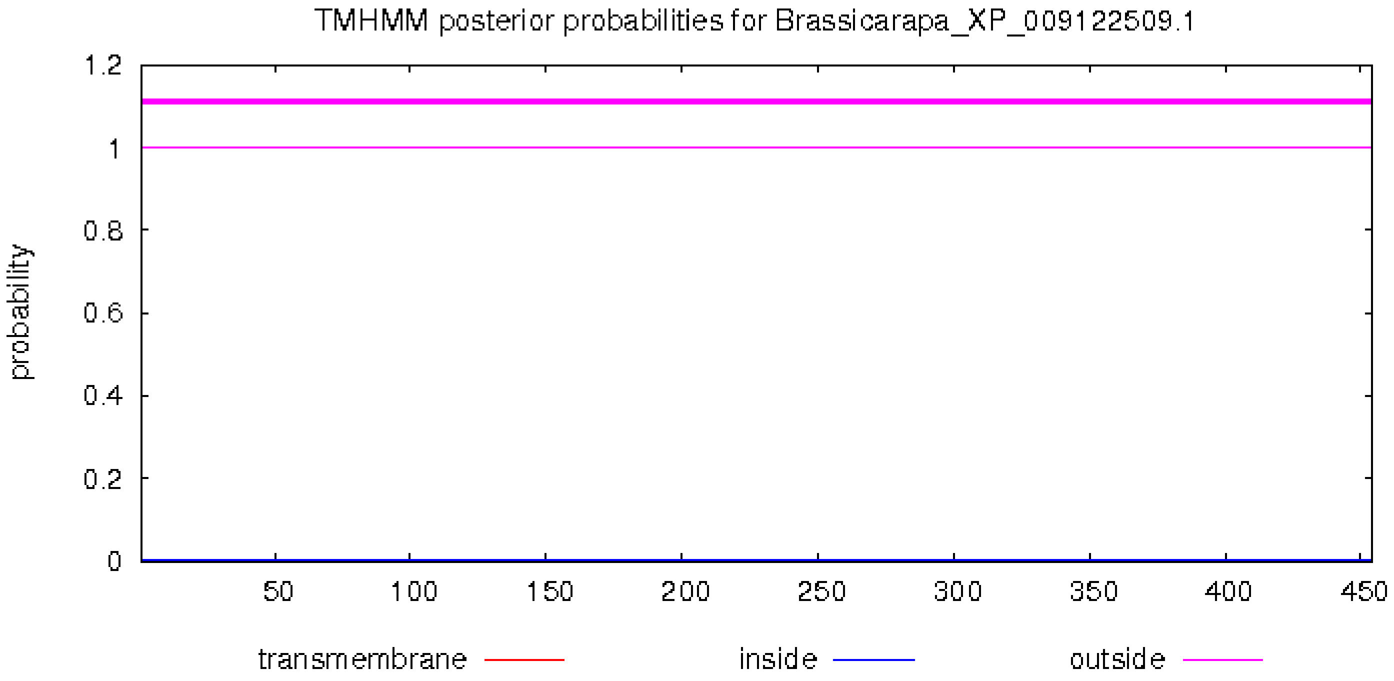

plot in postscript, script for making the plot in gnuplot, data for plot

---

```
# Brassicarapa_XP_009122601.1 Length: 554
# Brassicarapa_XP_009122601.1 Number of predicted TMHs:  1
# Brassicarapa_XP_009122601.1 Exp number of AAs in TMHs: 21.64343
# Brassicarapa_XP_009122601.1 Exp number, first 60 AAs:  0
# Brassicarapa_XP_009122601.1 Total prob of N-in:        0.00405
Brassicarapa_XP_009122601.1     TMHMM2.0        outside      1   525
```

```
Brassicarapa_XP_009122601.1     TMHMM2.0        TMhelix     526   548
Brassicarapa_XP_009122601.1     TMHMM2.0        inside      549   554
```

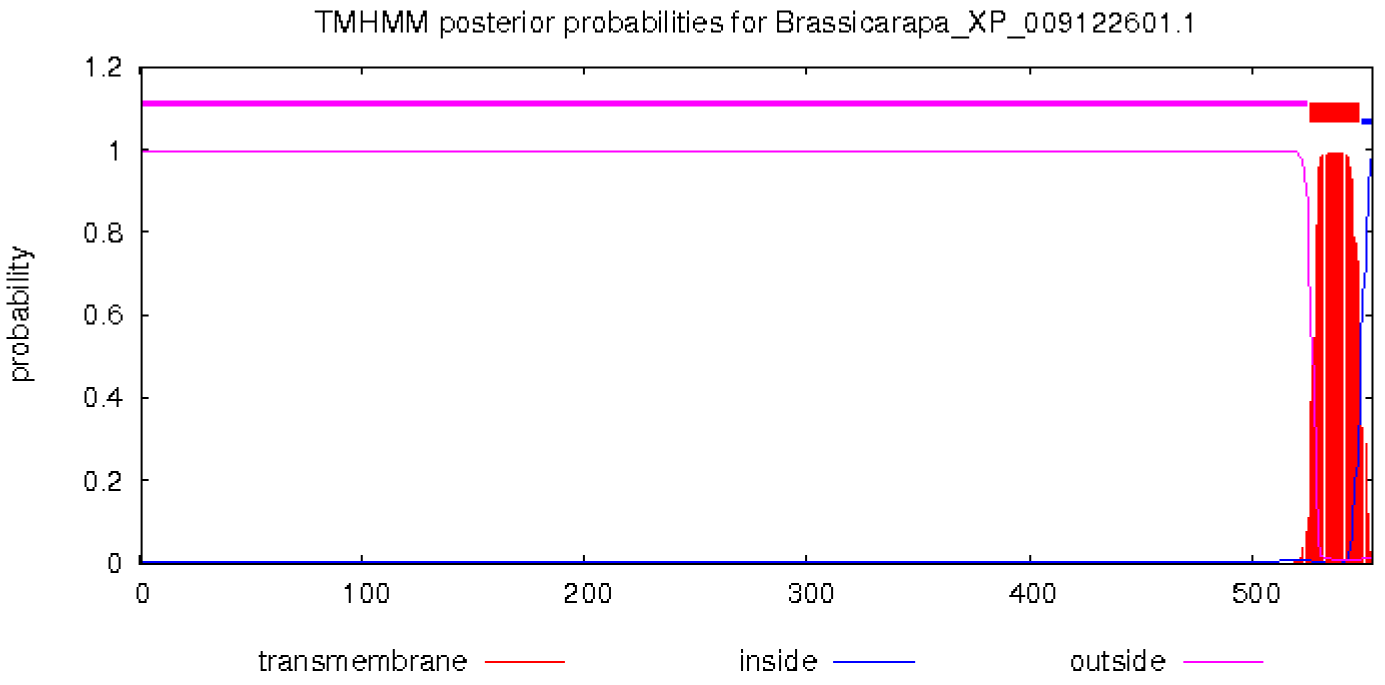

plot in postscript, script for making the plot in gnuplot, data for plot

---

```
# Brassicarapa_XP_009122853.1 Length: 537
# Brassicarapa_XP_009122853.1 Number of predicted TMHs:  1
# Brassicarapa_XP_009122853.1 Exp number of AAs in TMHs: 21.49652
# Brassicarapa_XP_009122853.1 Exp number, first 60 AAs:  0
# Brassicarapa_XP_009122853.1 Total prob of N-in:        0.02530
Brassicarapa_XP_009122853.1     TMHMM2.0        outside       1   511
Brassicarapa_XP_009122853.1     TMHMM2.0        TMhelix     512   534
Brassicarapa_XP_009122853.1     TMHMM2.0        inside      535   537
```

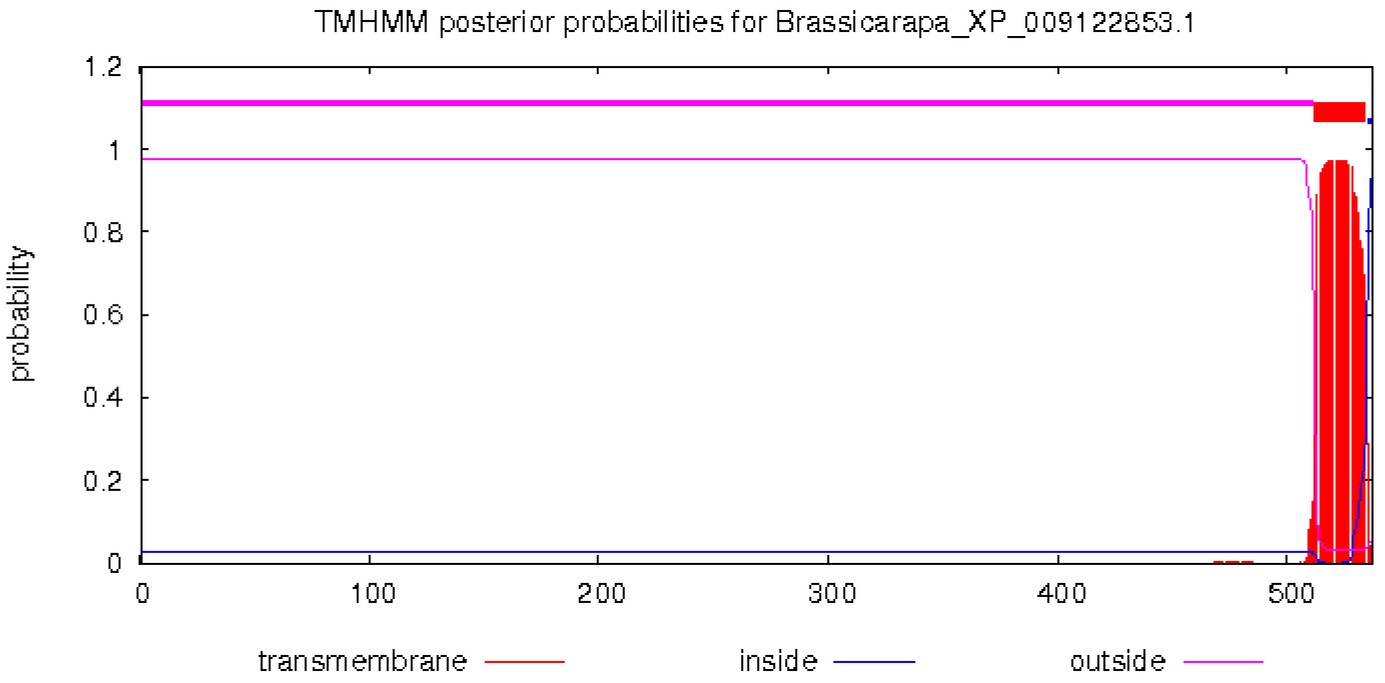

plot in postscript, script for making the plot in gnuplot, data for plot

---

```
# Brassicarapa_XP_009122861.1 Length: 440
# Brassicarapa_XP_009122861.1 Number of predicted TMHs:  0
```

```
# Brassicarapa_XP_009122861.1 Exp number of AAs in TMHs: 0.03647
# Brassicarapa_XP_009122861.1 Exp number, first 60 AAs:  0.02071
# Brassicarapa_XP_009122861.1 Total prob of N-in:        0.00555
Brassicarapa_XP_009122861.1	TMHMM2.0	outside	     1   440
```

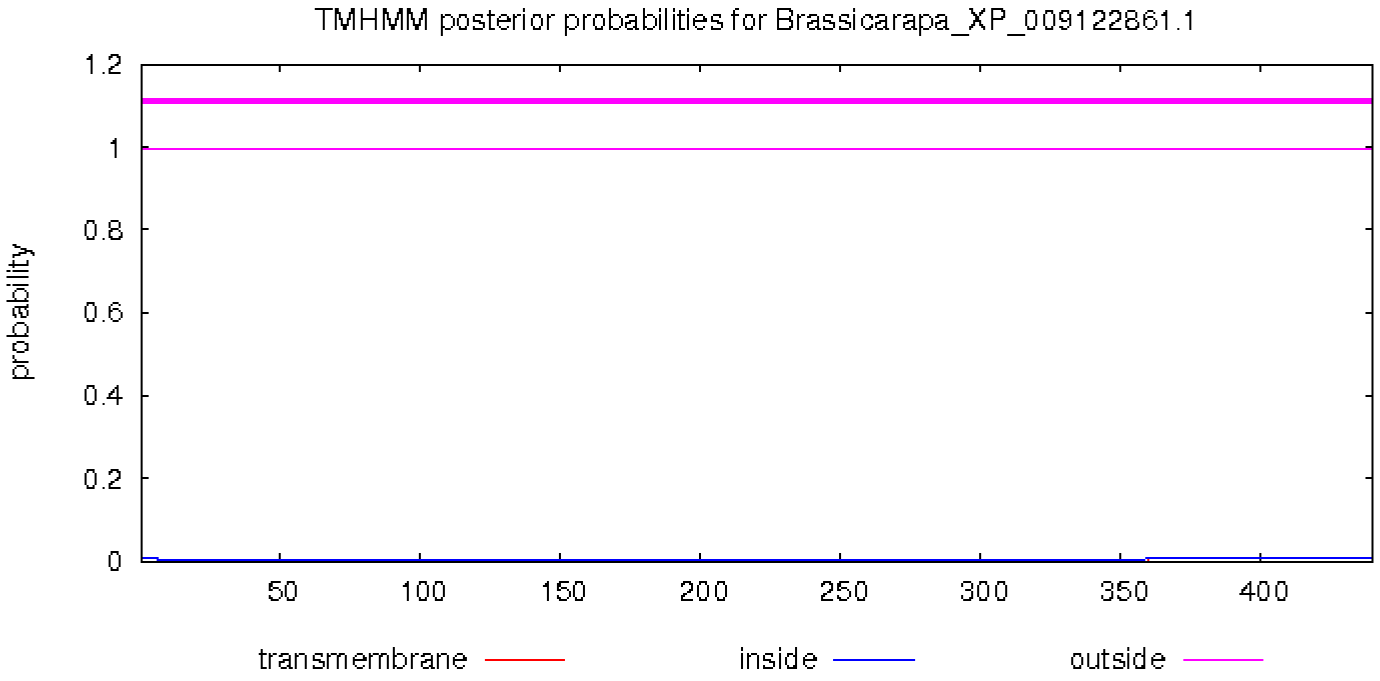

plot in postscript, script for making the plot in gnuplot, data for plot

---

```
# Brassicarapa_XP_009122872.1 Length: 421
# Brassicarapa_XP_009122872.1 Number of predicted TMHs:  0
# Brassicarapa_XP_009122872.1 Exp number of AAs in TMHs: 9.66723999999999999
# Brassicarapa_XP_009122872.1 Exp number, first 60 AAs:  9.66097
# Brassicarapa_XP_009122872.1 Total prob of N-in:        0.44253
Brassicarapa_XP_009122872.1	TMHMM2.0	outside	     1   421
```

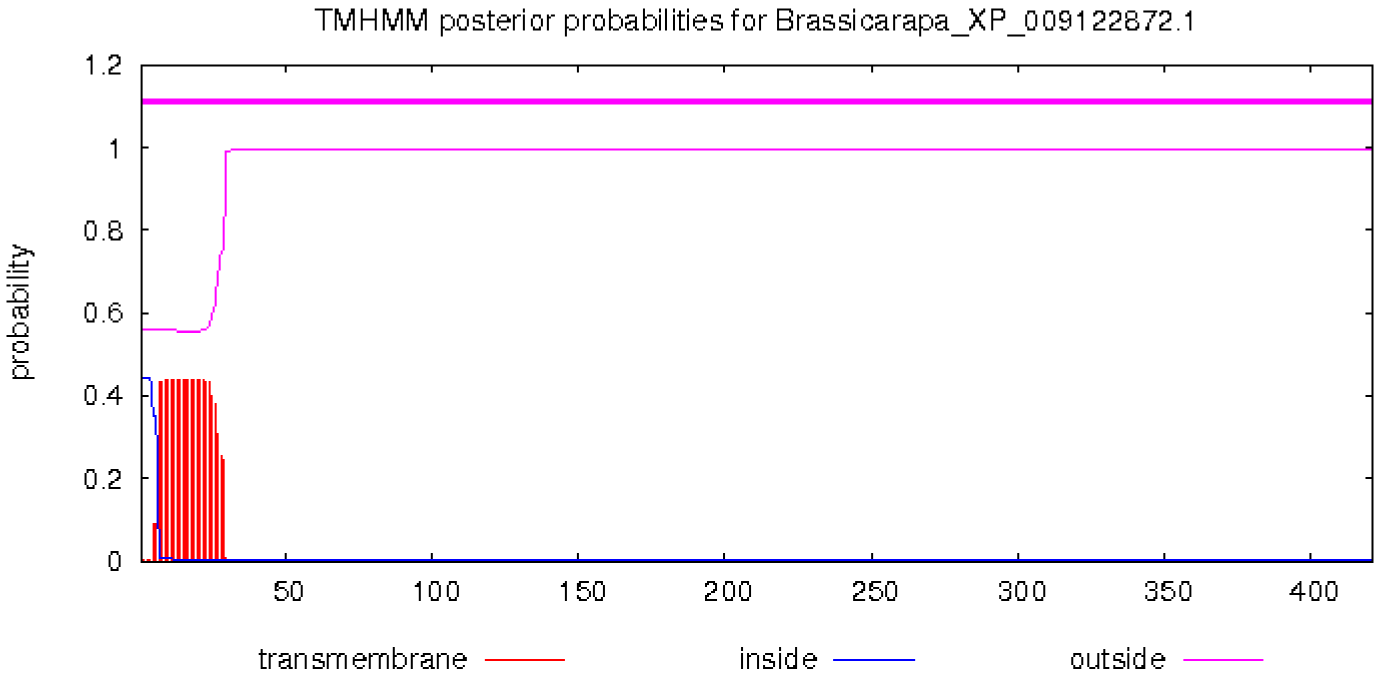

plot in postscript, script for making the plot in gnuplot, data for plot

---

```
# Brassicarapa_XP_009124687.1 Length: 307
# Brassicarapa_XP_009124687.1 Number of predicted TMHs:  0
```

```
# Brassicarapa_XP_009124687.1 Exp number of AAs in TMHs: 0.00144
# Brassicarapa_XP_009124687.1 Exp number, first 60 AAs:  0
# Brassicarapa_XP_009124687.1 Total prob of N-in:        0.02504
Brassicarapa_XP_009124687.1     TMHMM2.0        outside      1   307
```

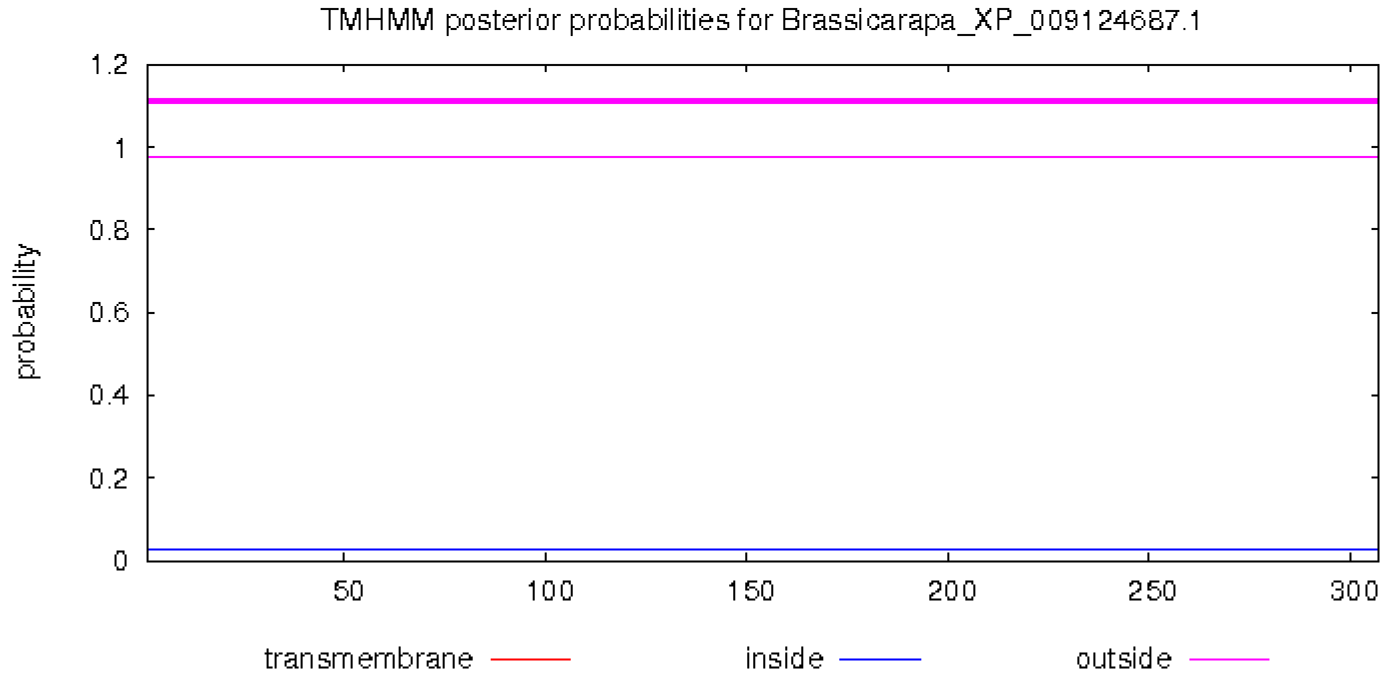

plot in postscript, script for making the plot in gnuplot, data for plot

---

```
# Brassicarapa_XP_009125011.1 Length: 357
# Brassicarapa_XP_009125011.1 Number of predicted TMHs:  0
# Brassicarapa_XP_009125011.1 Exp number of AAs in TMHs: 0.00017
# Brassicarapa_XP_009125011.1 Exp number, first 60 AAs:  0.00017
# Brassicarapa_XP_009125011.1 Total prob of N-in:        0.01055
Brassicarapa_XP_009125011.1     TMHMM2.0        outside      1   357
```

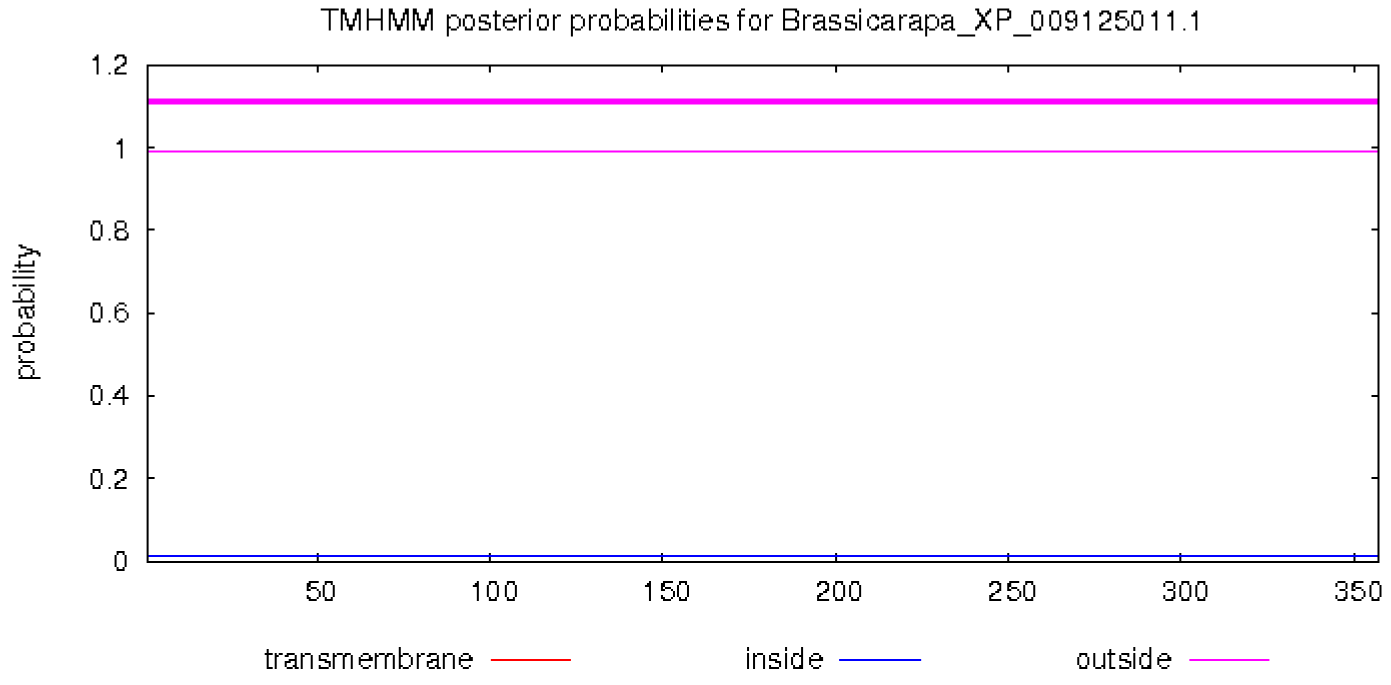

plot in postscript, script for making the plot in gnuplot, data for plot

---

```
# Brassicarapa_XP_009125012.1 Length: 346
# Brassicarapa_XP_009125012.1 Number of predicted TMHs:  0
```

```
# Brassicarapa_XP_009125012.1 Exp number of AAs in TMHs: 0.00078
# Brassicarapa_XP_009125012.1 Exp number, first 60 AAs:  0
# Brassicarapa_XP_009125012.1 Total prob of N-in:        0.00443
Brassicarapa_XP_009125012.1     TMHMM2.0        outside      1   346
```

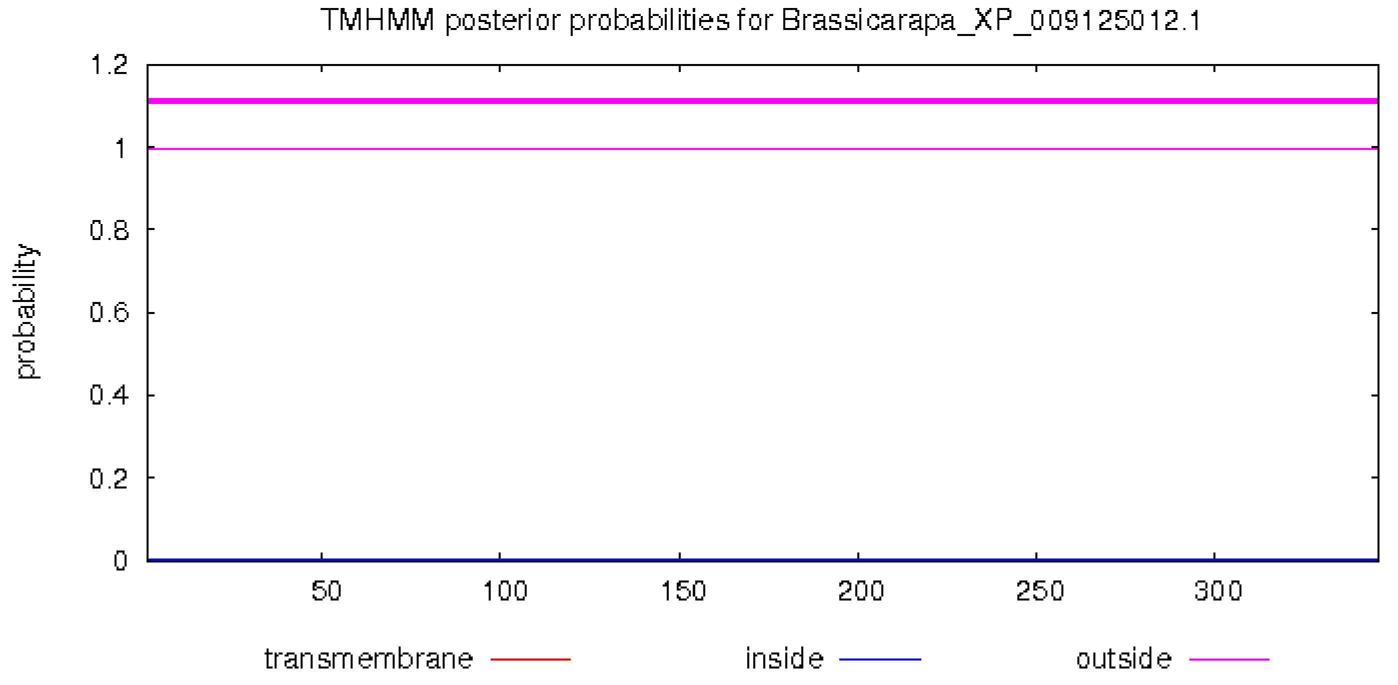

plot in postscript, script for making the plot in gnuplot, data for plot

---

```
# Brassicarapa_XP_009125013.1 Length: 343
# Brassicarapa_XP_009125013.1 Number of predicted TMHs:  0
# Brassicarapa_XP_009125013.1 Exp number of AAs in TMHs: 0.00078
# Brassicarapa_XP_009125013.1 Exp number, first 60 AAs:  0
# Brassicarapa_XP_009125013.1 Total prob of N-in:        0.00460
Brassicarapa_XP_009125013.1     TMHMM2.0        outside      1   343
```

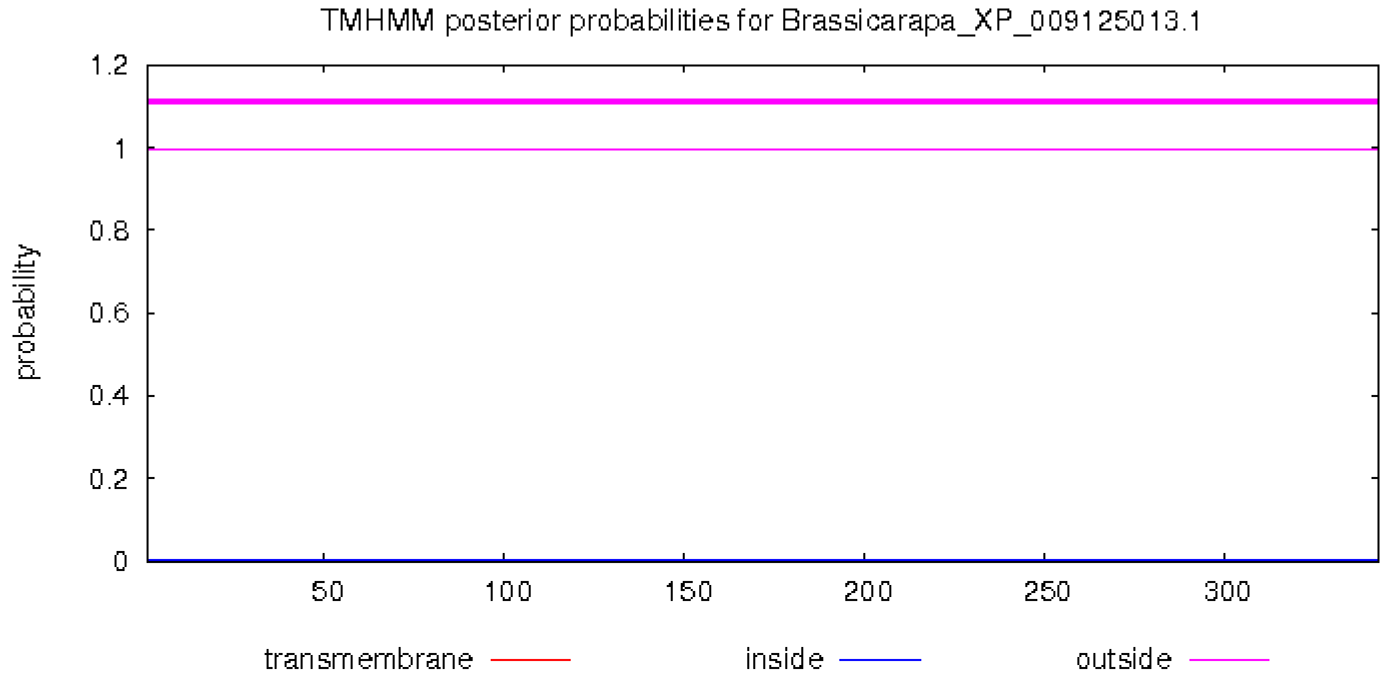

plot in postscript, script for making the plot in gnuplot, data for plot

---

```
# Brassicarapa_XP_009125108.1 Length: 288
# Brassicarapa_XP_009125108.1 Number of predicted TMHs:  0
```

```
# Brassicarapa_XP_009125108.1 Exp number of AAs in TMHs: 0.00078
# Brassicarapa_XP_009125108.1 Exp number, first 60 AAs:  0.00062
# Brassicarapa_XP_009125108.1 Total prob of N-in:        0.05556
Brassicarapa_XP_009125108.1     TMHMM2.0        outside      1   288
```

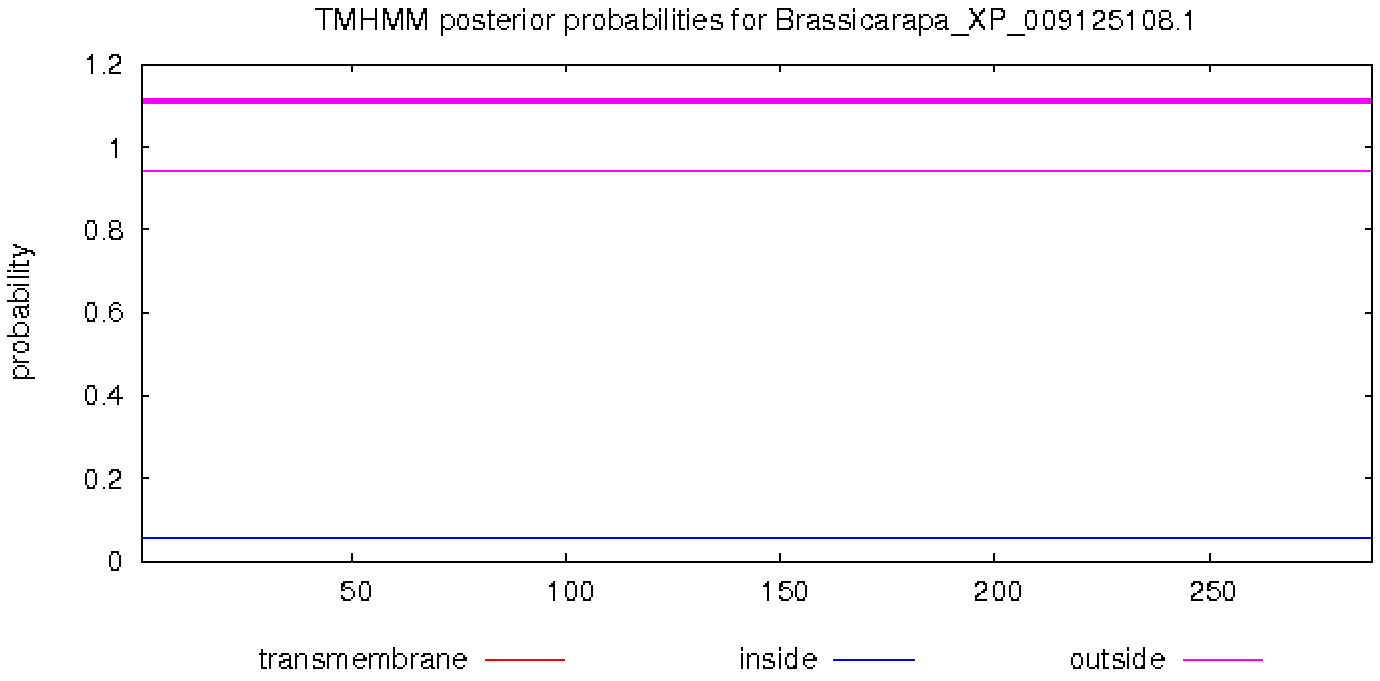

plot in postscript, script for making the plot in gnuplot, data for plot

```
# Brassicarapa_XP_009125115.1 Length: 335
# Brassicarapa_XP_009125115.1 Number of predicted TMHs:  0
# Brassicarapa_XP_009125115.1 Exp number of AAs in TMHs: 0.00572
# Brassicarapa_XP_009125115.1 Exp number, first 60 AAs:  0
# Brassicarapa_XP_009125115.1 Total prob of N-in:        0.02322
Brassicarapa_XP_009125115.1     TMHMM2.0        outside      1   335
```

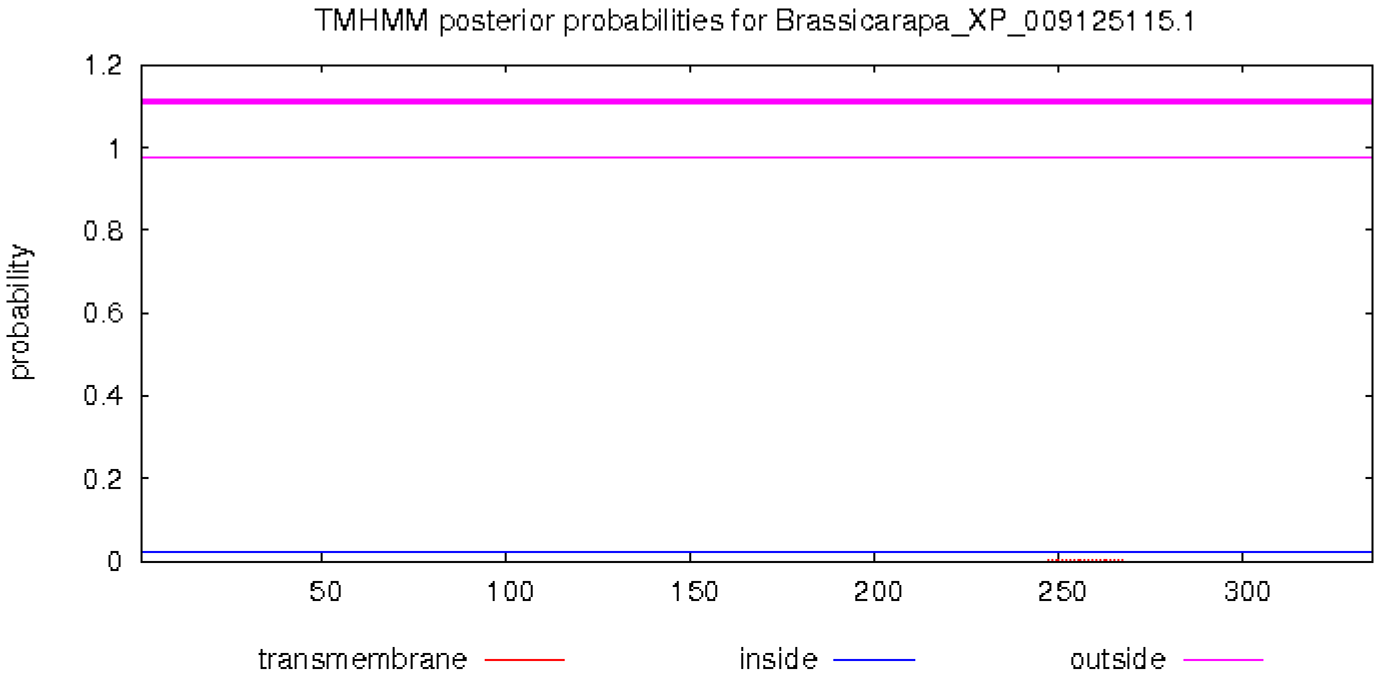

plot in postscript, script for making the plot in gnuplot, data for plot

```
# Brassicarapa_XP_009125174.1 Length: 197
# Brassicarapa_XP_009125174.1 Number of predicted TMHs:  0
```

```
# Brassicarapa_XP_009125174.1 Exp number of AAs in TMHs: 0.00121
# Brassicarapa_XP_009125174.1 Exp number, first 60 AAs:  0
# Brassicarapa_XP_009125174.1 Total prob of N-in:        0.04873
Brassicarapa_XP_009125174.1	TMHMM2.0	outside	     1   197
```

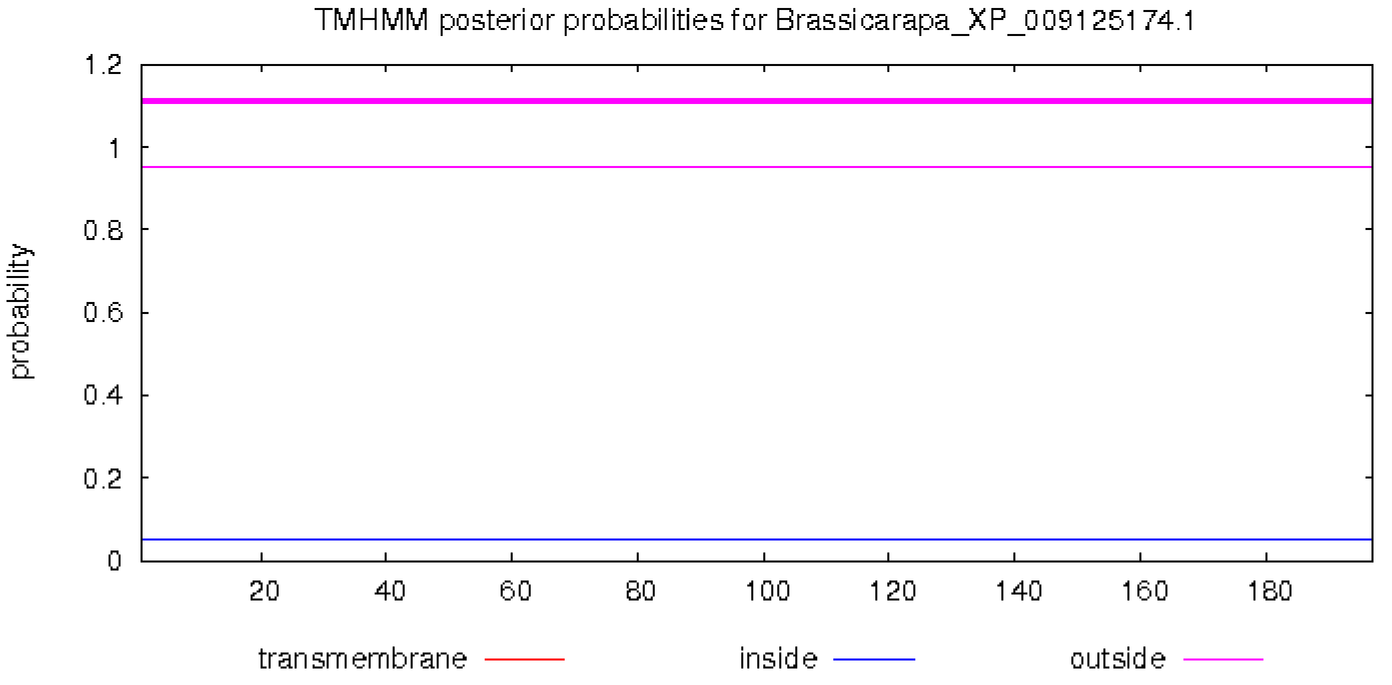

plot in postscript, script for making the plot in gnuplot, data for plot

---

```
# Brassicarapa_XP_009125558.1 Length: 505
# Brassicarapa_XP_009125558.1 Number of predicted TMHs:  1
# Brassicarapa_XP_009125558.1 Exp number of AAs in TMHs: 22.0105
# Brassicarapa_XP_009125558.1 Exp number, first 60 AAs:  0
# Brassicarapa_XP_009125558.1 Total prob of N-in:        0.08895
Brassicarapa_XP_009125558.1	TMHMM2.0	outside	     1   479
Brassicarapa_XP_009125558.1	TMHMM2.0	TMhelix	   480   502
Brassicarapa_XP_009125558.1	TMHMM2.0	inside	   503   505
```

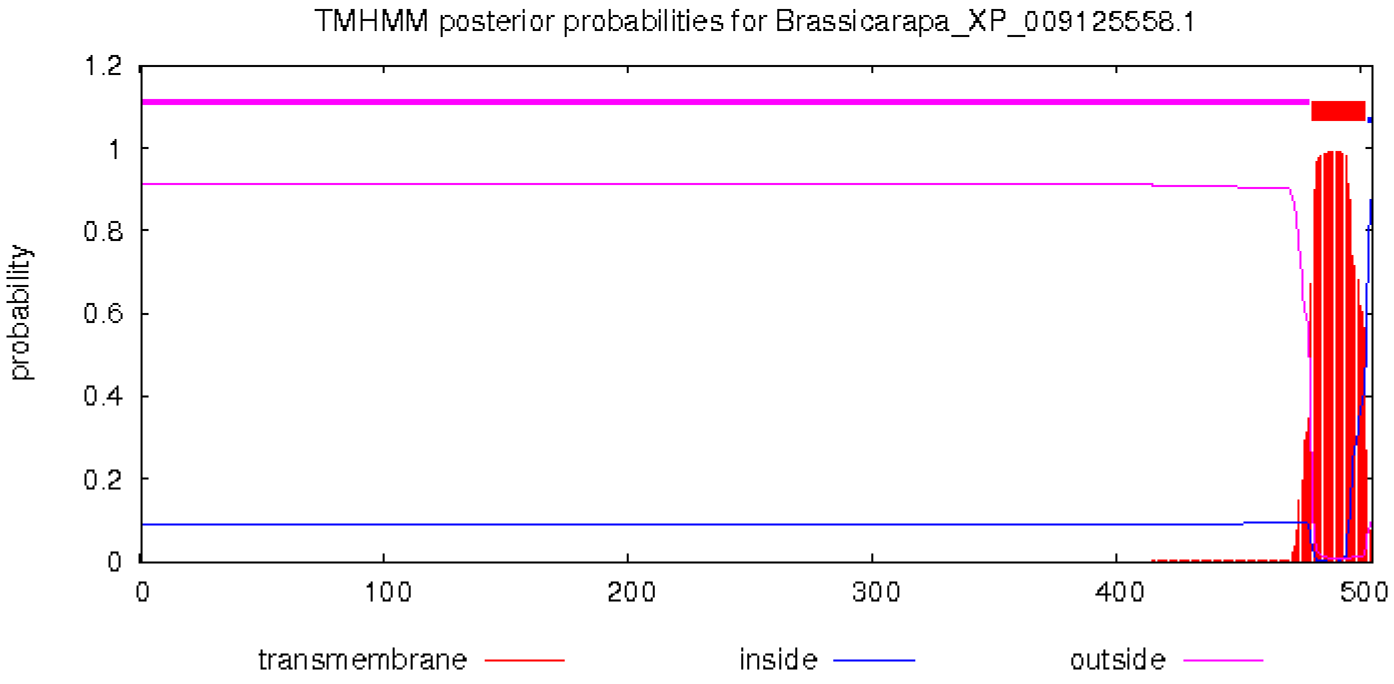

plot in postscript, script for making the plot in gnuplot, data for plot

---

```
# Brassicarapa_XP_009125559.1 Length: 504
# Brassicarapa_XP_009125559.1 Number of predicted TMHs:  1
# Brassicarapa_XP_009125559.1 Exp number of AAs in TMHs: 22.01133
# Brassicarapa_XP_009125559.1 Exp number, first 60 AAs:  0
# Brassicarapa_XP_009125559.1 Total prob of N-in:        0.08996
Brassicarapa_XP_009125559.1     TMHMM2.0        outside      1   478
Brassicarapa_XP_009125559.1     TMHMM2.0        TMhelix    479   501
Brassicarapa_XP_009125559.1     TMHMM2.0        inside     502   504
```

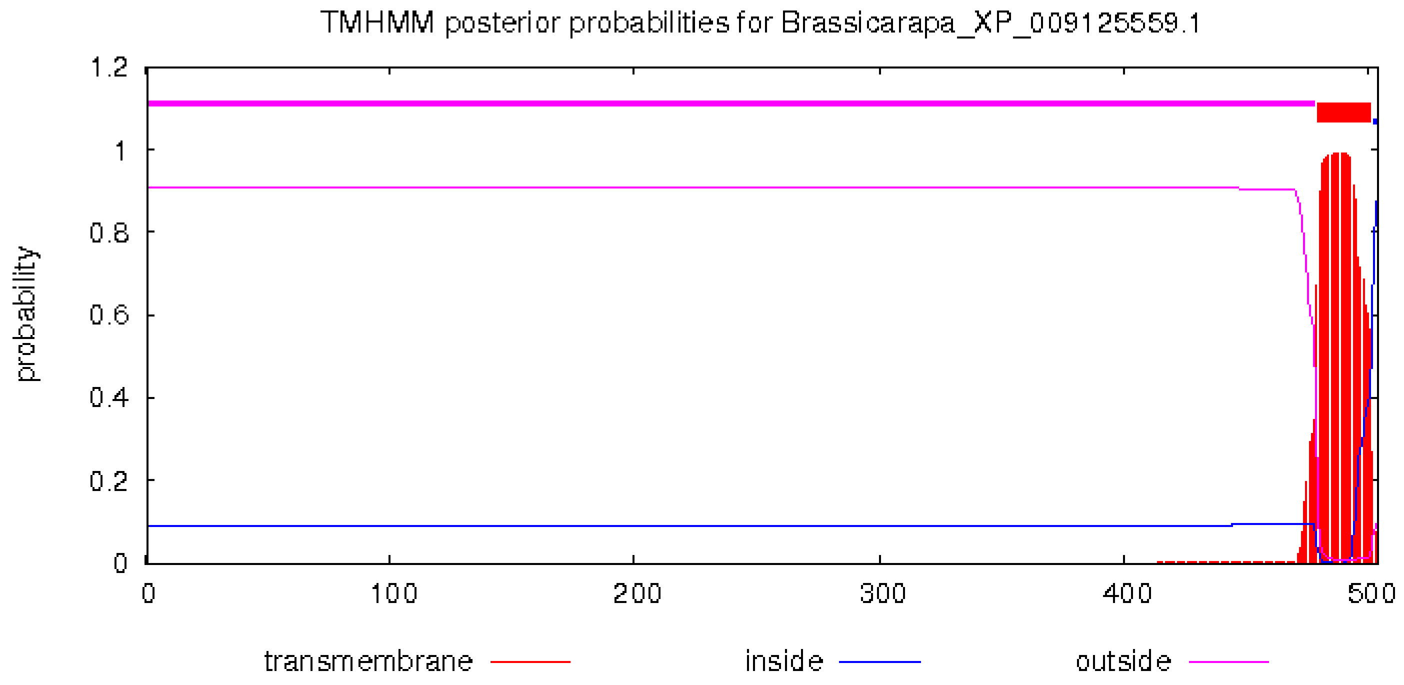

plot in postscript, script for making the plot in gnuplot, data for plot

---

```
# Brassicarapa_XP_009125699.1 Length: 314
# Brassicarapa_XP_009125699.1 Number of predicted TMHs:  0
# Brassicarapa_XP_009125699.1 Exp number of AAs in TMHs: 0.00062
# Brassicarapa_XP_009125699.1 Exp number, first 60 AAs:  0
# Brassicarapa_XP_009125699.1 Total prob of N-in:        0.00591
Brassicarapa_XP_009125699.1     TMHMM2.0        outside      1   314
```

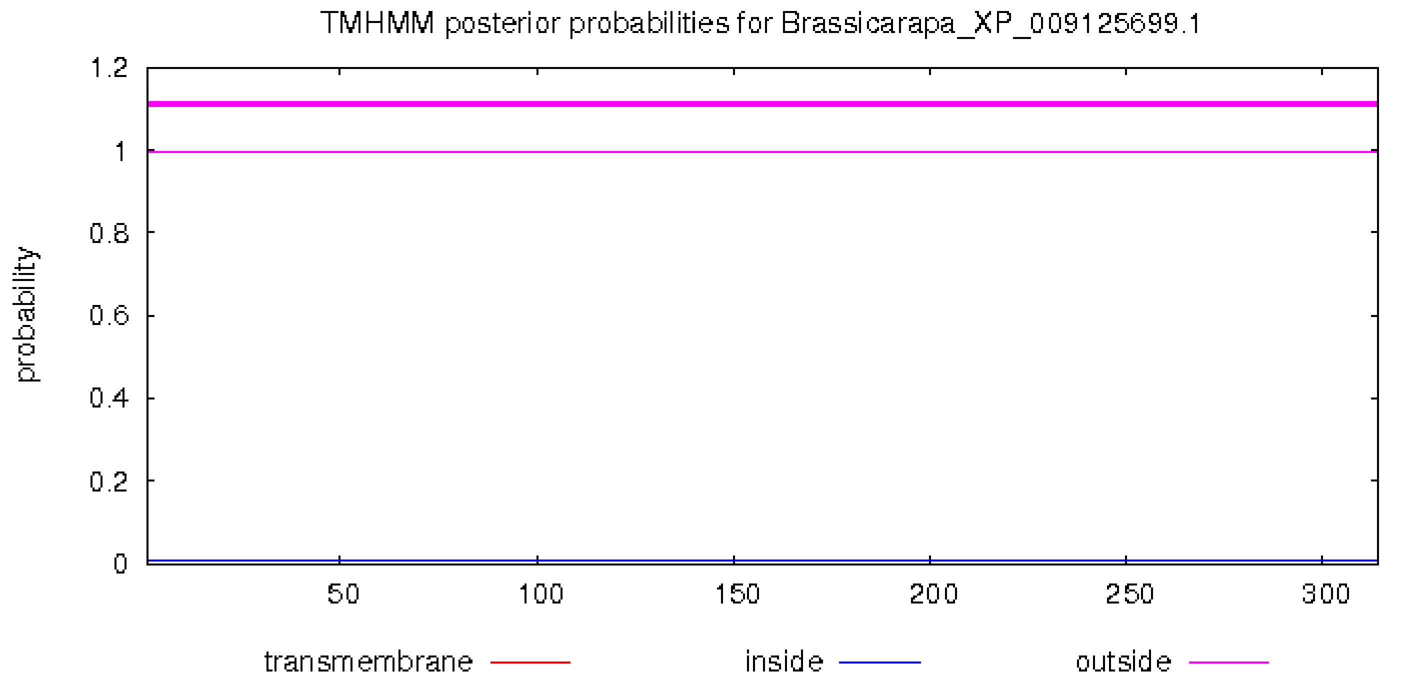

plot in postscript, script for making the plot in gnuplot, data for plot

```
# Brassicarapa_XP_009125745.1 Length: 278
# Brassicarapa_XP_009125745.1 Number of predicted TMHs:  0
# Brassicarapa_XP_009125745.1 Exp number of AAs in TMHs: 0.00018
# Brassicarapa_XP_009125745.1 Exp number, first 60 AAs:  0
# Brassicarapa_XP_009125745.1 Total prob of N-in:        0.00888
Brassicarapa_XP_009125745.1     TMHMM2.0        outside      1   278
```

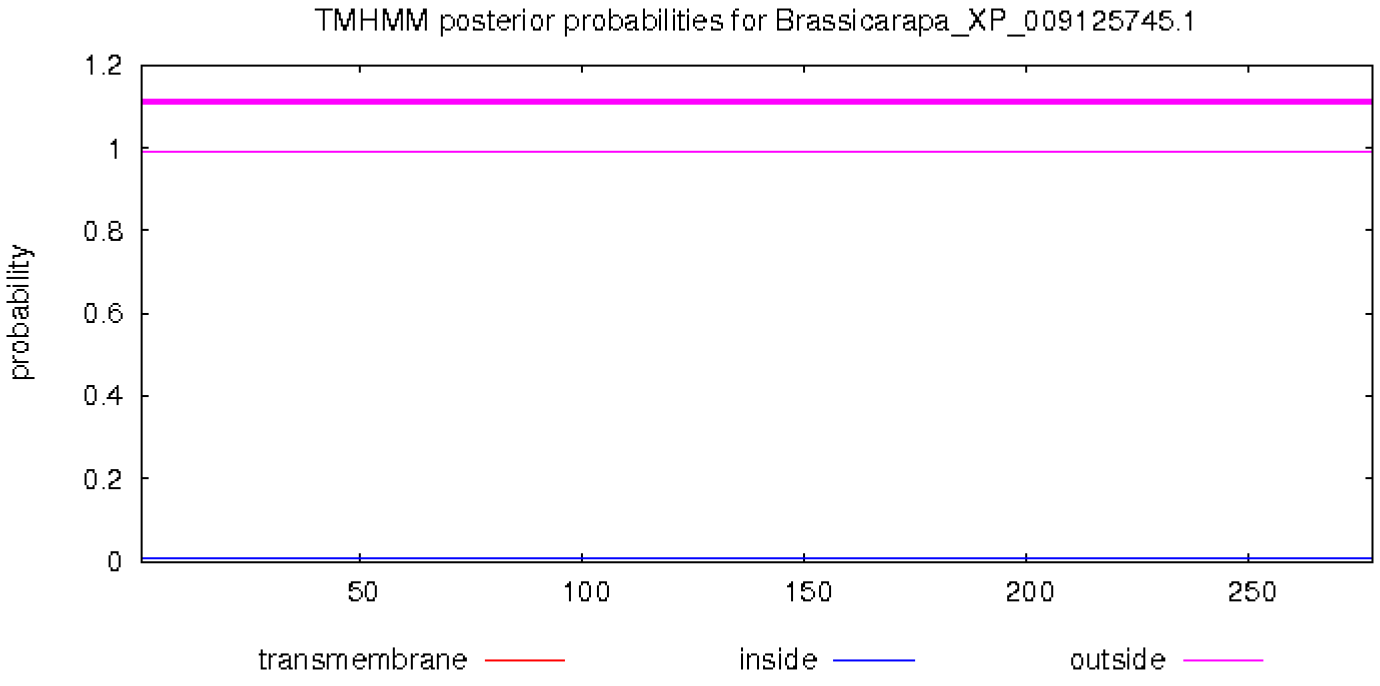

plot in postscript, script for making the plot in gnuplot, data for plot

```
# Brassicarapa_XP_009125756.1 Length: 399
# Brassicarapa_XP_009125756.1 Number of predicted TMHs:  0
# Brassicarapa_XP_009125756.1 Exp number of AAs in TMHs: 0.00018
# Brassicarapa_XP_009125756.1 Exp number, first 60 AAs:  0.00018
# Brassicarapa_XP_009125756.1 Total prob of N-in:        0.00265
Brassicarapa_XP_009125756.1     TMHMM2.0        outside      1   399
```

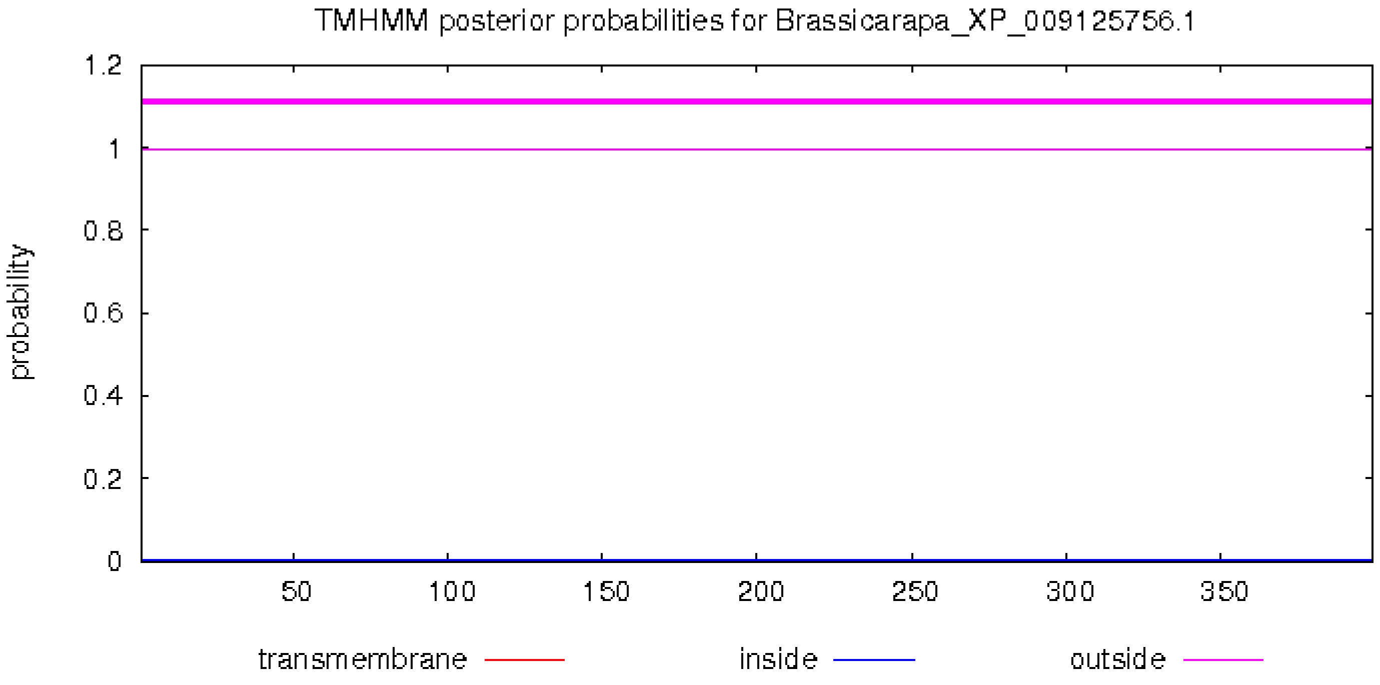

plot in postscript, script for making the plot in gnuplot, data for plot

```
# Brassicarapa_XP_009125795.1 Length: 361
# Brassicarapa_XP_009125795.1 Number of predicted TMHs:  0
# Brassicarapa_XP_009125795.1 Exp number of AAs in TMHs: 0.00255
# Brassicarapa_XP_009125795.1 Exp number, first 60 AAs:  0.00255
# Brassicarapa_XP_009125795.1 Total prob of N-in:        0.00953
Brassicarapa_XP_009125795.1     TMHMM2.0        outside      1   361
```

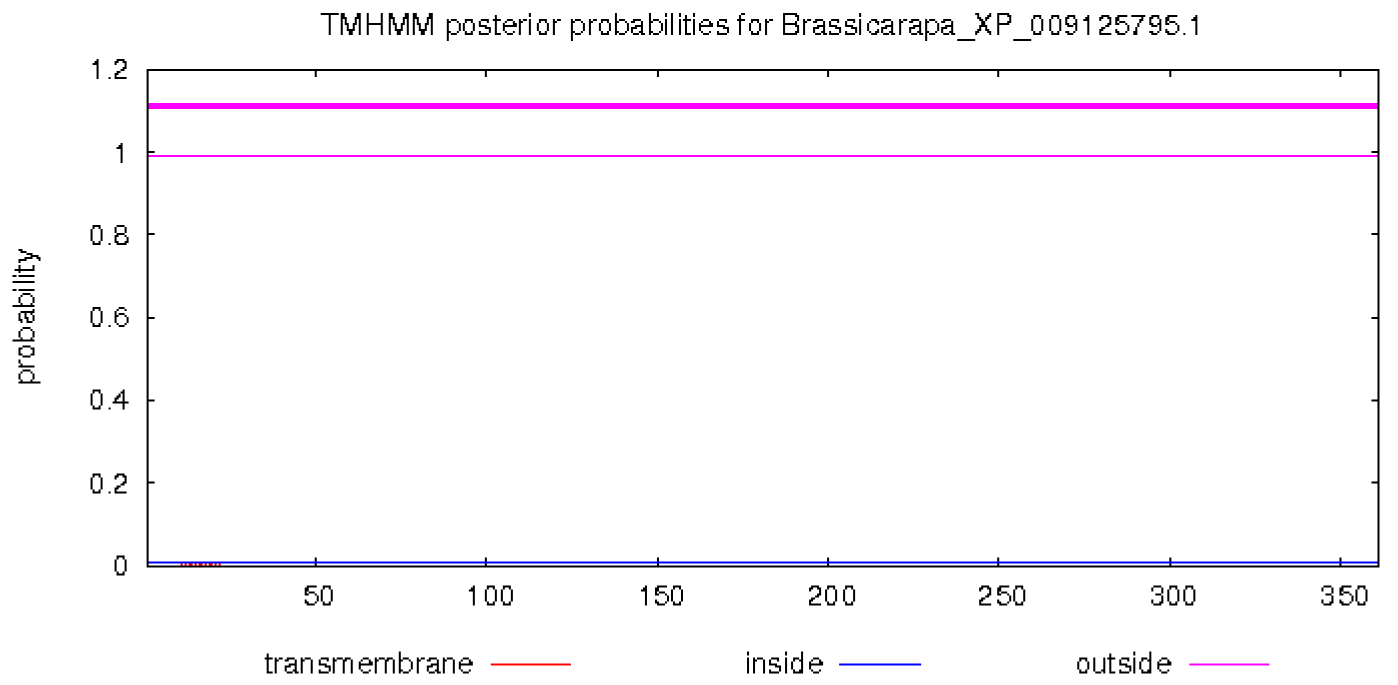

plot in postscript, script for making the plot in gnuplot, data for plot

---

```
# Brassicarapa_XP_009125891.1 Length: 247
# Brassicarapa_XP_009125891.1 Number of predicted TMHs:  0
# Brassicarapa_XP_009125891.1 Exp number of AAs in TMHs: 0
# Brassicarapa_XP_009125891.1 Exp number, first 60 AAs:  0
# Brassicarapa_XP_009125891.1 Total prob of N-in:        0.11741
Brassicarapa_XP_009125891.1     TMHMM2.0        outside      1   247
```

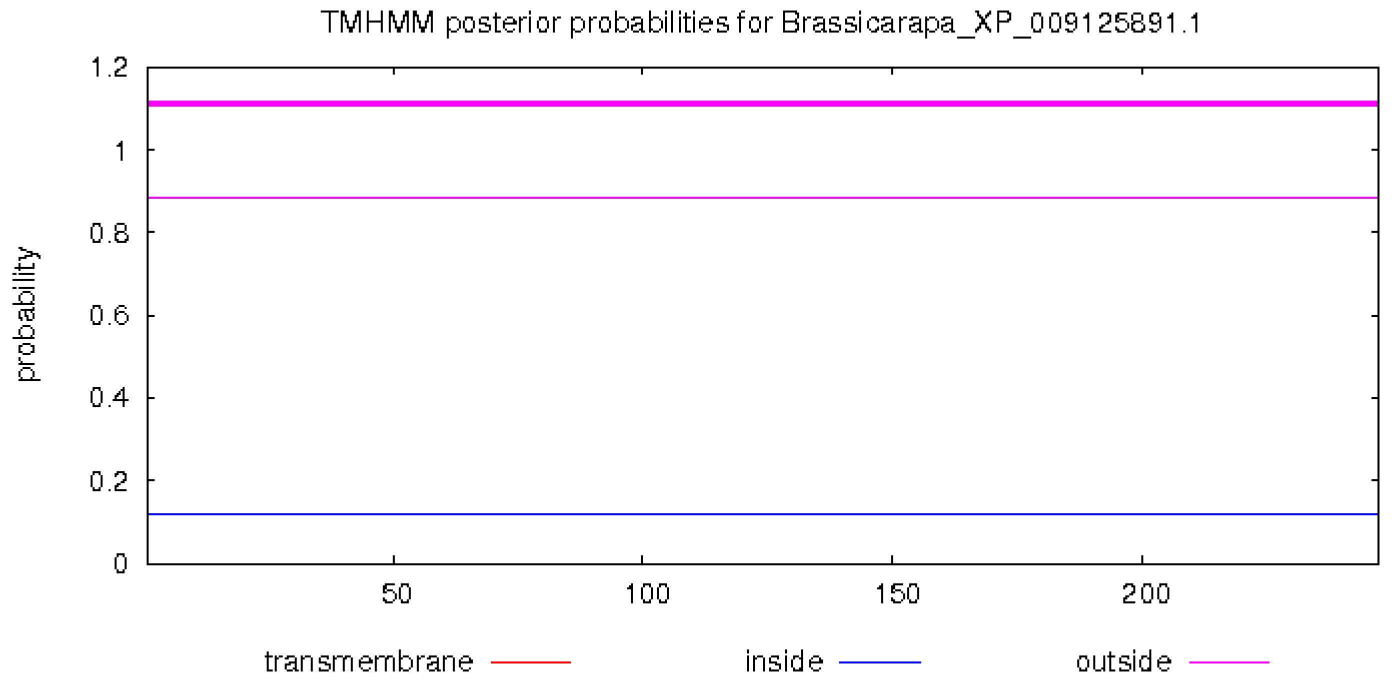

plot in postscript, script for making the plot in gnuplot, data for plot

---

```
# Brassicarapa_XP_009126247.1 Length: 336
# Brassicarapa_XP_009126247.1 Number of predicted TMHs:  0
# Brassicarapa_XP_009126247.1 Exp number of AAs in TMHs: 0.00017
# Brassicarapa_XP_009126247.1 Exp number, first 60 AAs:  0
# Brassicarapa_XP_009126247.1 Total prob of N-in:        0.00866
Brassicarapa_XP_009126247.1     TMHMM2.0        outside      1   336
```

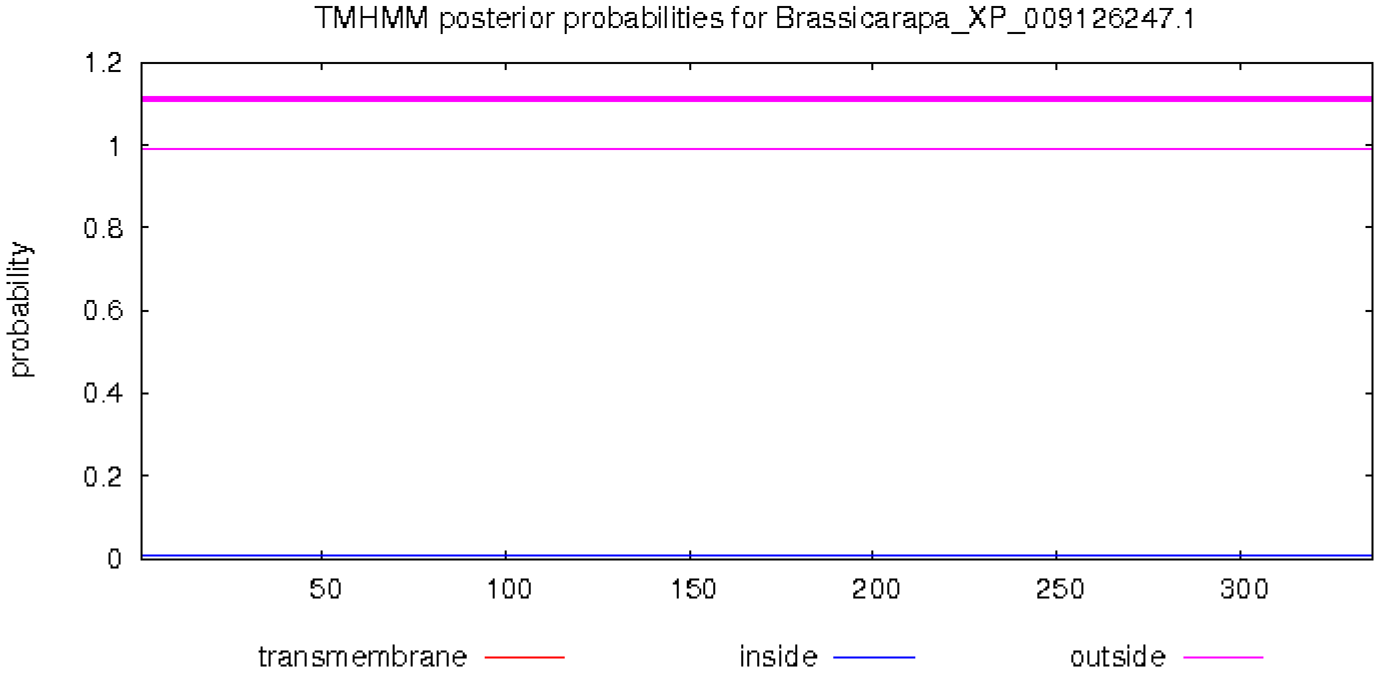

plot in postscript, script for making the plot in gnuplot, data for plot

---

```
# Brassicarapa_XP_009126534.1 Length: 343
# Brassicarapa_XP_009126534.1 Number of predicted TMHs:  0
# Brassicarapa_XP_009126534.1 Exp number of AAs in TMHs: 10.14168
# Brassicarapa_XP_009126534.1 Exp number, first 60 AAs:  0.00982
# Brassicarapa_XP_009126534.1 Total prob of N-in:        0.05612
Brassicarapa_XP_009126534.1     TMHMM2.0        outside      1   343
```

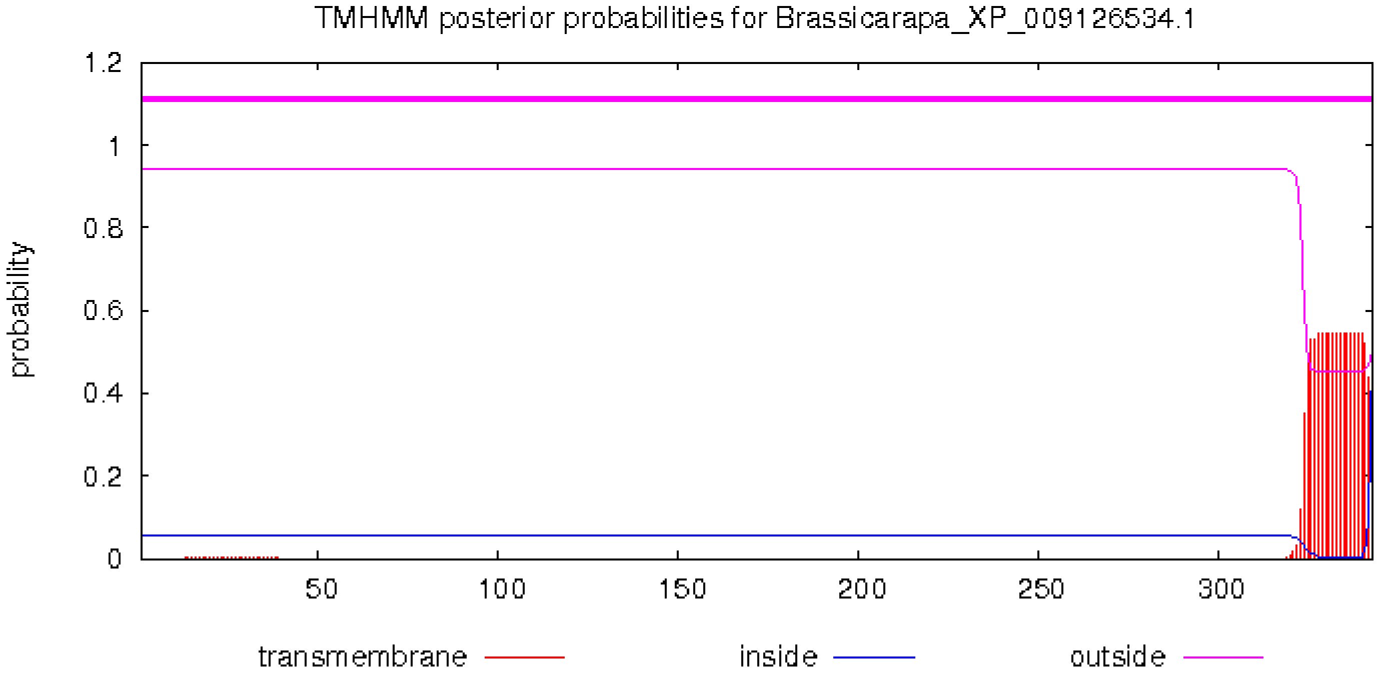

plot in postscript, script for making the plot in gnuplot, data for plot

---

```
# Brassicarapa_XP_009126539.1 Length: 235
# Brassicarapa_XP_009126539.1 Number of predicted TMHs:  0
# Brassicarapa_XP_009126539.1 Exp number of AAs in TMHs: 0
# Brassicarapa_XP_009126539.1 Exp number, first 60 AAs:  0
# Brassicarapa_XP_009126539.1 Total prob of N-in:        0.02517
Brassicarapa_XP_009126539.1     TMHMM2.0        outside      1   235
```

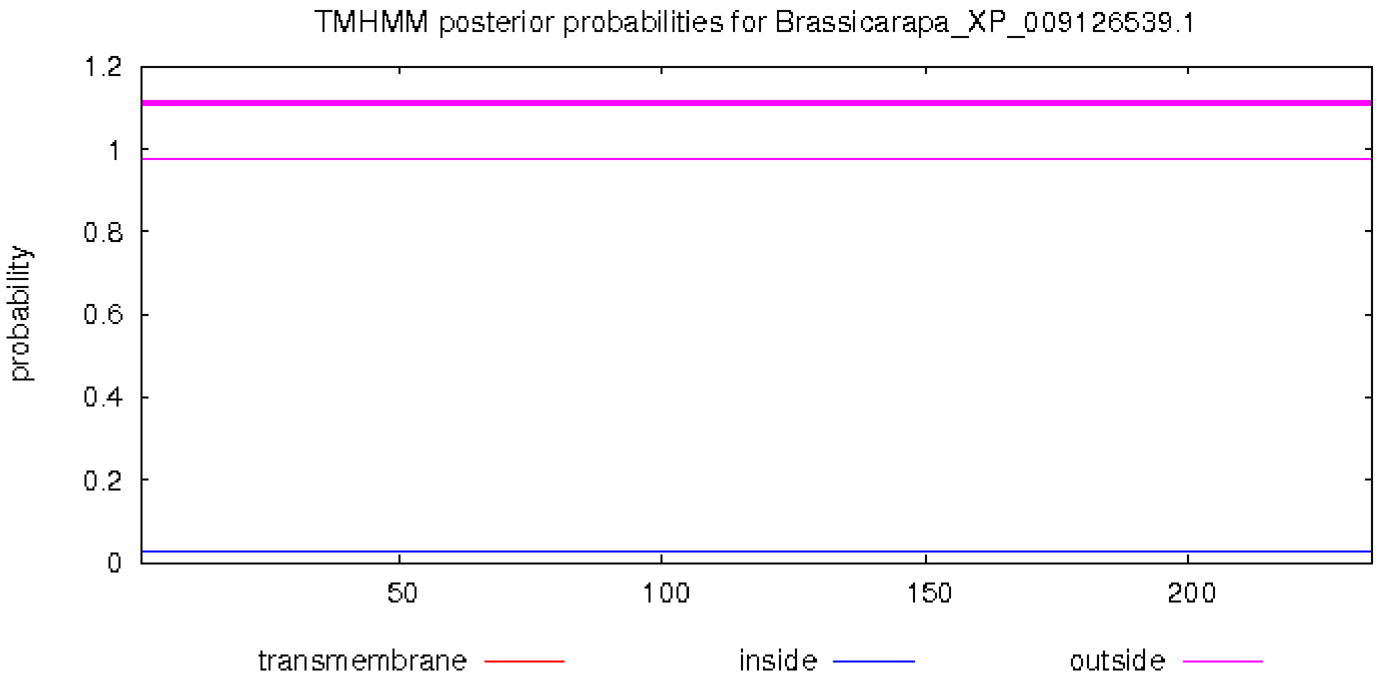

plot in postscript, script for making the plot in gnuplot, data for plot

---

```
# Brassicarapa_XP_009126706.1 Length: 306
# Brassicarapa_XP_009126706.1 Number of predicted TMHs:  0
# Brassicarapa_XP_009126706.1 Exp number of AAs in TMHs: 0.00092
# Brassicarapa_XP_009126706.1 Exp number, first 60 AAs:  0
# Brassicarapa_XP_009126706.1 Total prob of N-in:        0.03917
Brassicarapa_XP_009126706.1     TMHMM2.0        outside      1   306
```

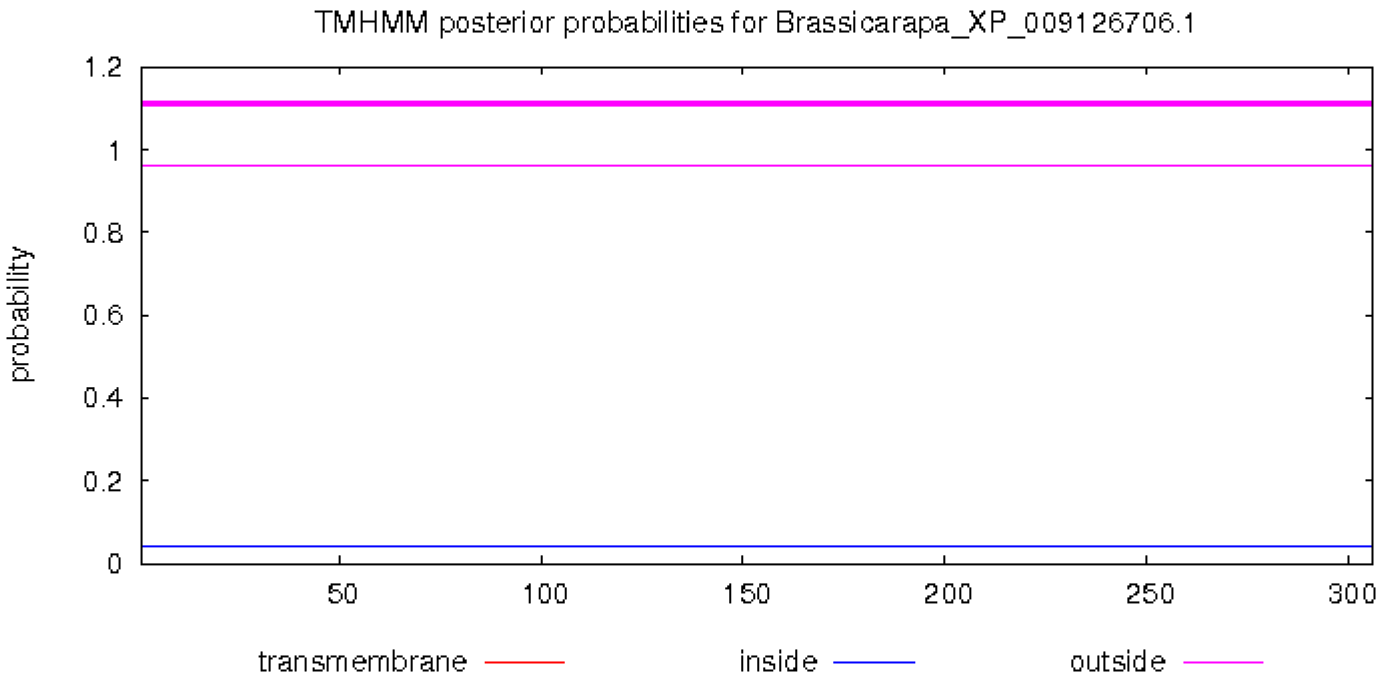

plot in postscript, script for making the plot in gnuplot, data for plot

---

```
# Brassicarapa_XP_009126707.1 Length: 306
# Brassicarapa_XP_009126707.1 Number of predicted TMHs:  0
# Brassicarapa_XP_009126707.1 Exp number of AAs in TMHs: 0.00092
# Brassicarapa_XP_009126707.1 Exp number, first 60 AAs:  0
# Brassicarapa_XP_009126707.1 Total prob of N-in:        0.03917
Brassicarapa_XP_009126707.1	TMHMM2.0	outside	     1   306
```

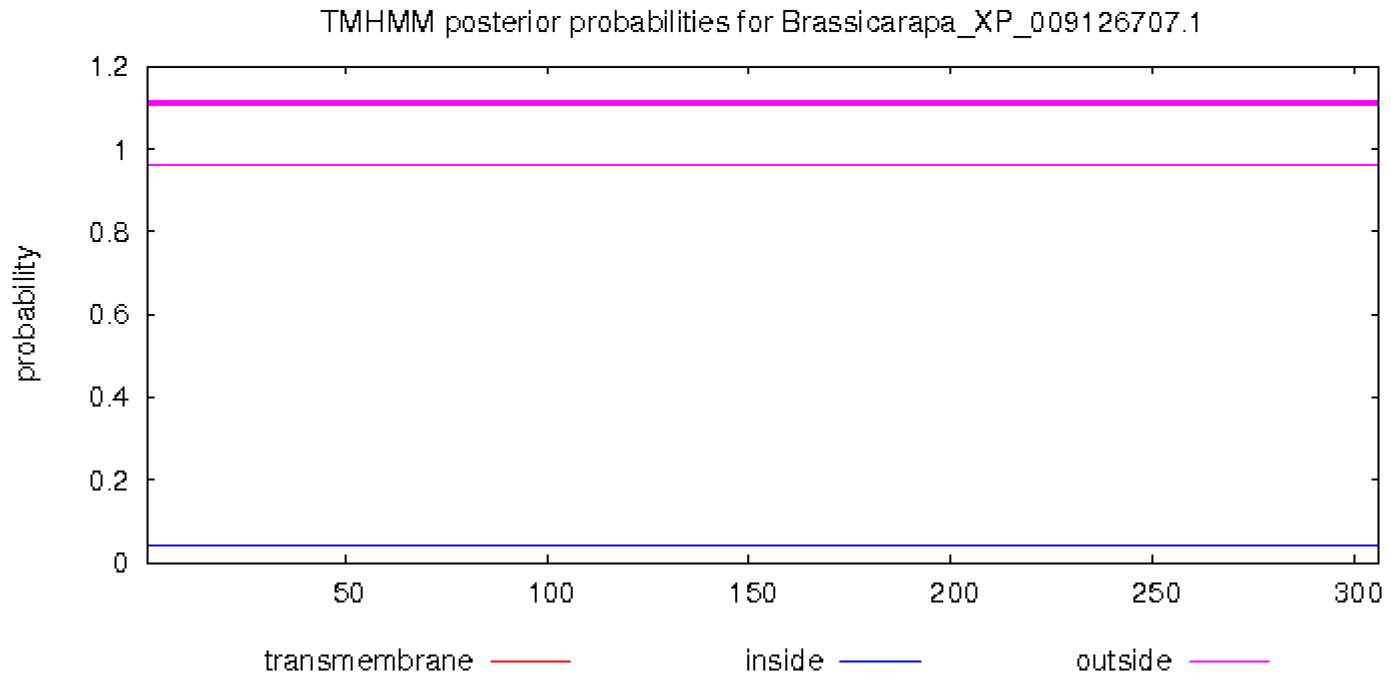

plot in postscript, script for making the plot in gnuplot, data for plot

---

```
# Brassicarapa_XP_009127127.1 Length: 367
# Brassicarapa_XP_009127127.1 Number of predicted TMHs:  0
# Brassicarapa_XP_009127127.1 Exp number of AAs in TMHs: 0.00349
# Brassicarapa_XP_009127127.1 Exp number, first 60 AAs:  0
# Brassicarapa_XP_009127127.1 Total prob of N-in:        0.01798
Brassicarapa_XP_009127127.1	TMHMM2.0	outside	     1   367
```

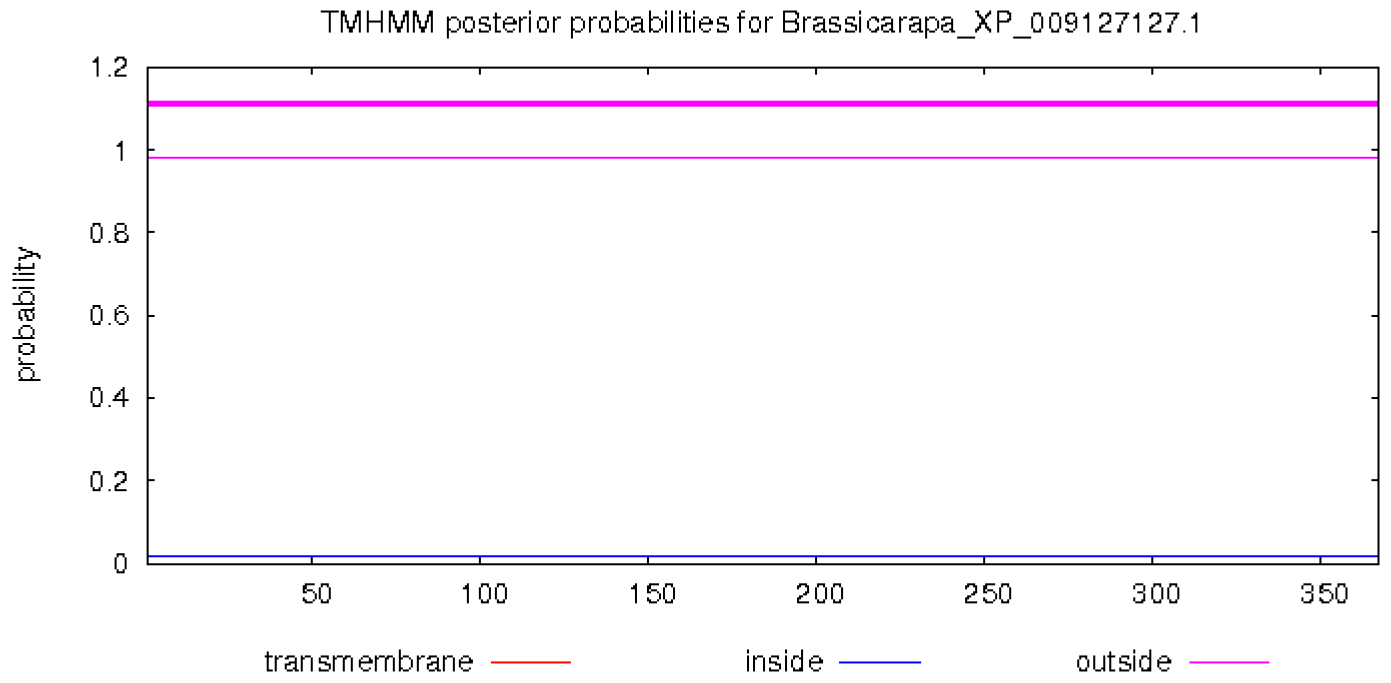

plot in postscript, script for making the plot in gnuplot, data for plot

---

```
# Brassicarapa_XP_009127458.1 Length: 665
# Brassicarapa_XP_009127458.1 Number of predicted TMHs:  3
# Brassicarapa_XP_009127458.1 Exp number of AAs in TMHs: 56.66912
# Brassicarapa_XP_009127458.1 Exp number, first 60 AAs:  0
# Brassicarapa_XP_009127458.1 Total prob of N-in:        0.00033
Brassicarapa_XP_009127458.1     TMHMM2.0        outside      1   534
Brassicarapa_XP_009127458.1     TMHMM2.0        TMhelix    535   552
Brassicarapa_XP_009127458.1     TMHMM2.0        inside     553   609
Brassicarapa_XP_009127458.1     TMHMM2.0        TMhelix    610   629
Brassicarapa_XP_009127458.1     TMHMM2.0        outside    630   643
Brassicarapa_XP_009127458.1     TMHMM2.0        TMhelix    644   663
Brassicarapa_XP_009127458.1     TMHMM2.0        inside     664   665
```

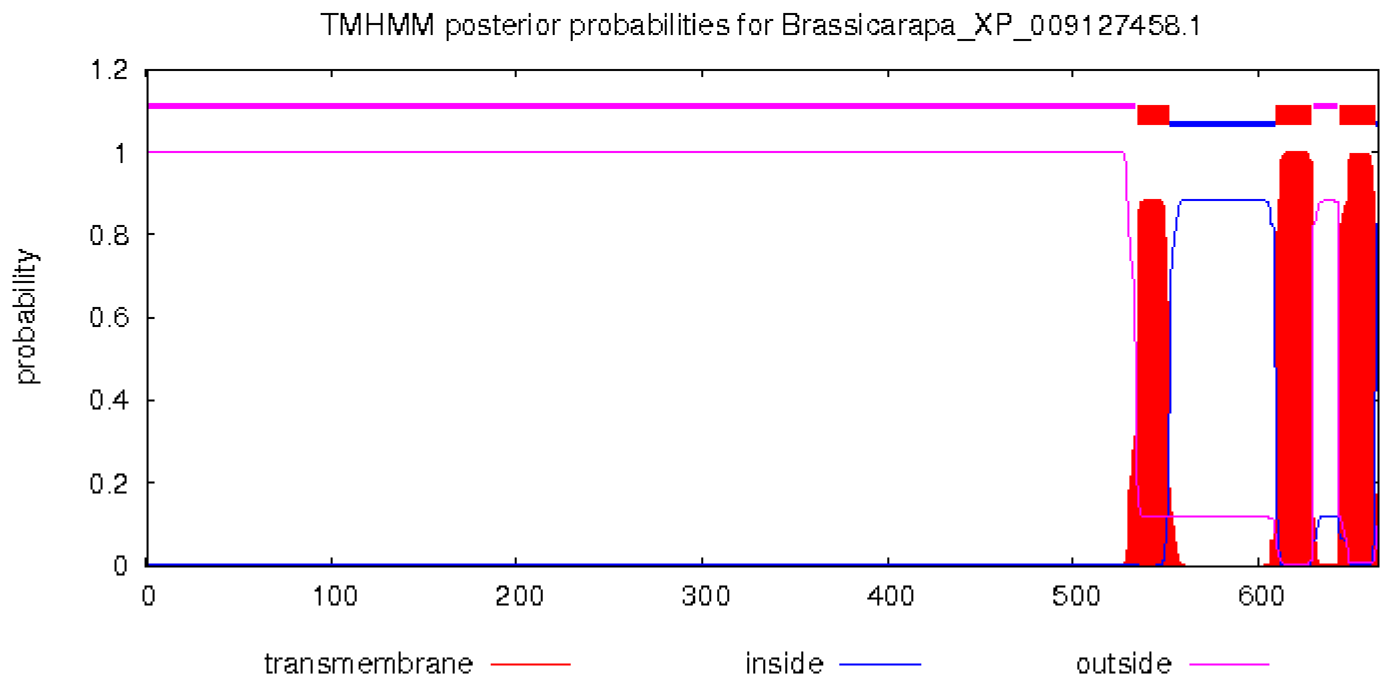

plot in postscript, script for making the plot in gnuplot, data for plot

---

```
# Brassicarapa_XP_009127884.1 Length: 327
# Brassicarapa_XP_009127884.1 Number of predicted TMHs:  0
# Brassicarapa_XP_009127884.1 Exp number of AAs in TMHs: 0
# Brassicarapa_XP_009127884.1 Exp number, first 60 AAs:  0
# Brassicarapa_XP_009127884.1 Total prob of N-in:        0.02336
Brassicarapa_XP_009127884.1     TMHMM2.0        outside      1   327
```

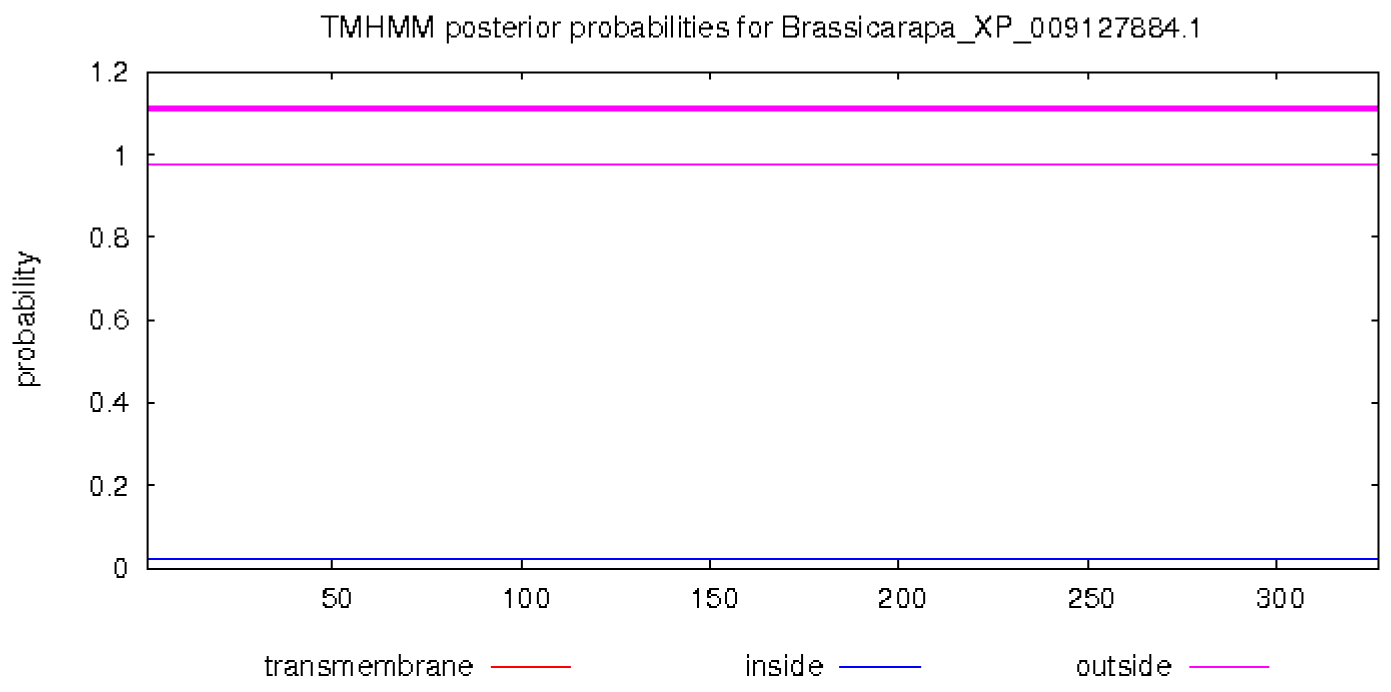

plot in postscript, script for making the plot in gnuplot, data for plot

---

```
# Brassicarapa_XP_009127885.1 Length: 327
# Brassicarapa_XP_009127885.1 Number of predicted TMHs:  0
# Brassicarapa_XP_009127885.1 Exp number of AAs in TMHs: 0
# Brassicarapa_XP_009127885.1 Exp number, first 60 AAs:  0
# Brassicarapa_XP_009127885.1 Total prob of N-in:        0.02336
Brassicarapa_XP_009127885.1     TMHMM2.0        outside      1   327
```

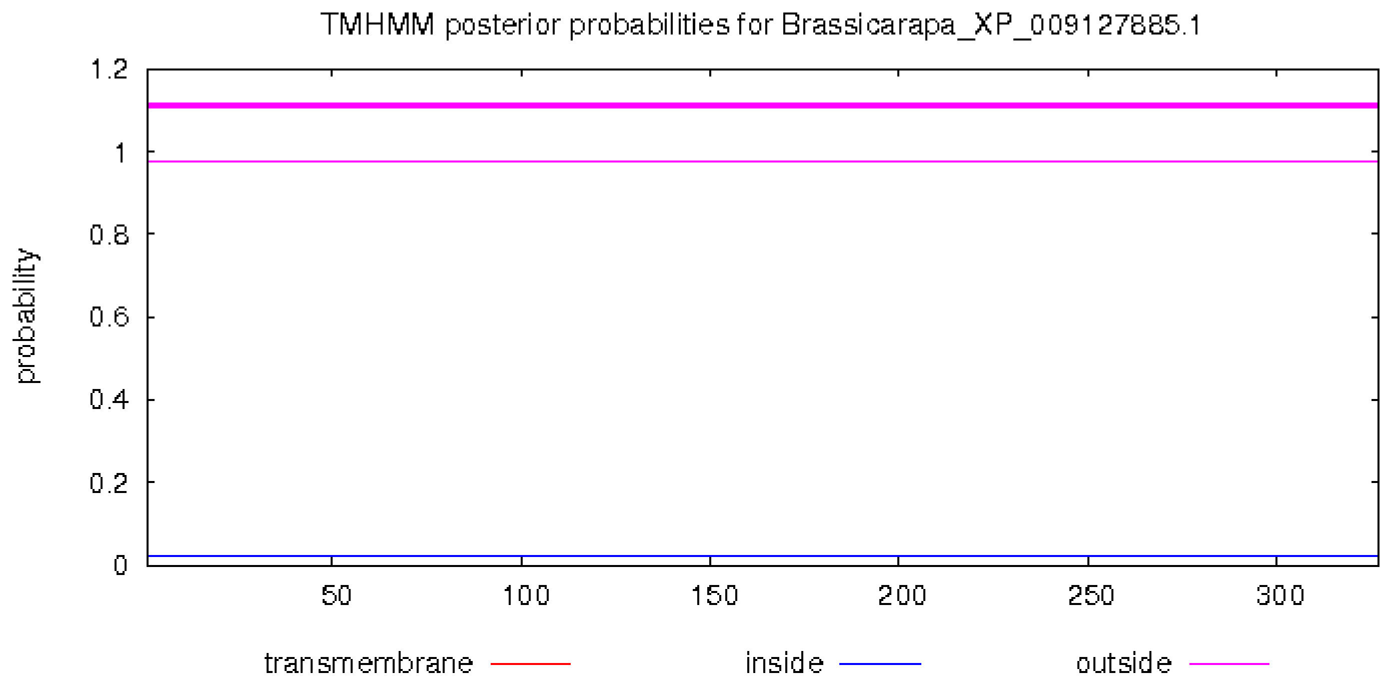

plot in postscript, script for making the plot in gnuplot, data for plot

---

```
# Brassicarapa_XP_009128177.1 Length: 321
# Brassicarapa_XP_009128177.1 Number of predicted TMHs:  0
# Brassicarapa_XP_009128177.1 Exp number of AAs in TMHs: 0.01396
# Brassicarapa_XP_009128177.1 Exp number, first 60 AAs:  0.00115
# Brassicarapa_XP_009128177.1 Total prob of N-in:        0.00436
Brassicarapa_XP_009128177.1     TMHMM2.0        outside      1   321
```

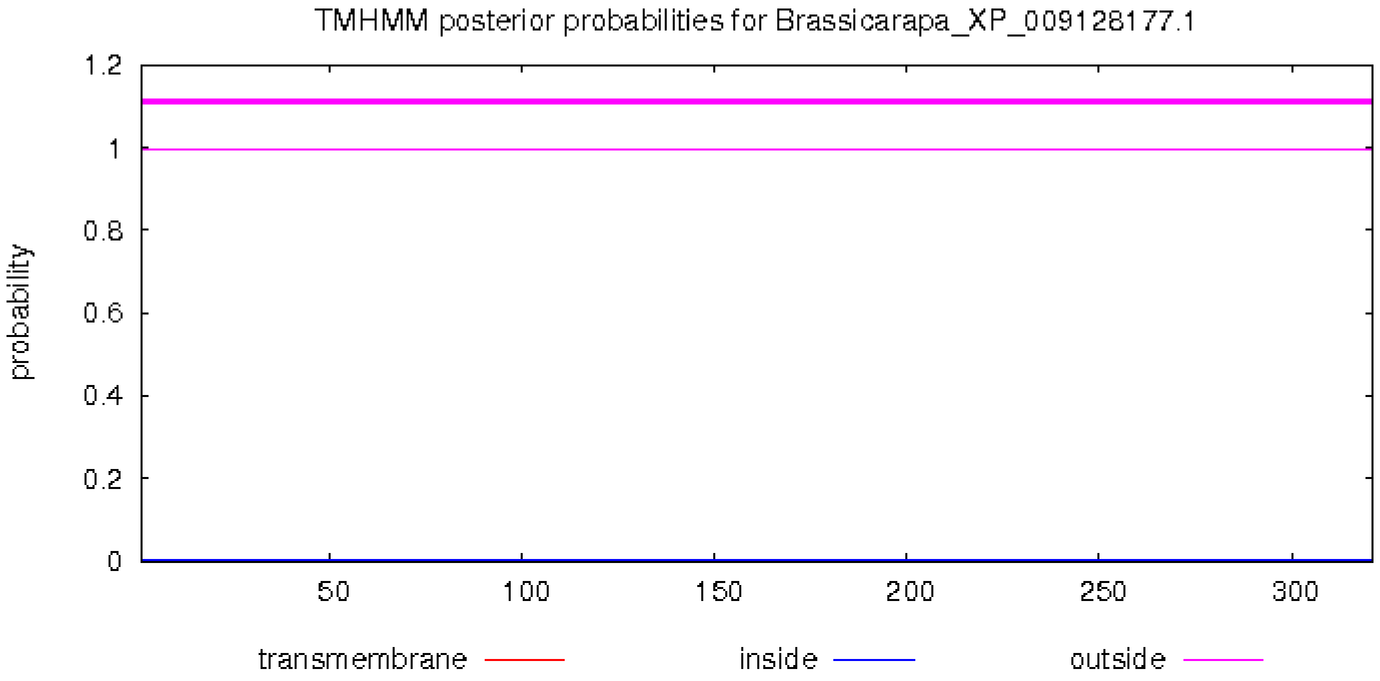

plot in postscript, script for making the plot in gnuplot, data for plot

---

```
# Brassicarapa_XP_009128229.1 Length: 260
# Brassicarapa_XP_009128229.1 Number of predicted TMHs:  0
# Brassicarapa_XP_009128229.1 Exp number of AAs in TMHs: 0.00061
# Brassicarapa_XP_009128229.1 Exp number, first 60 AAs:  0.00042
# Brassicarapa_XP_009128229.1 Total prob of N-in:        0.03308
Brassicarapa_XP_009128229.1     TMHMM2.0        outside      1   260
```

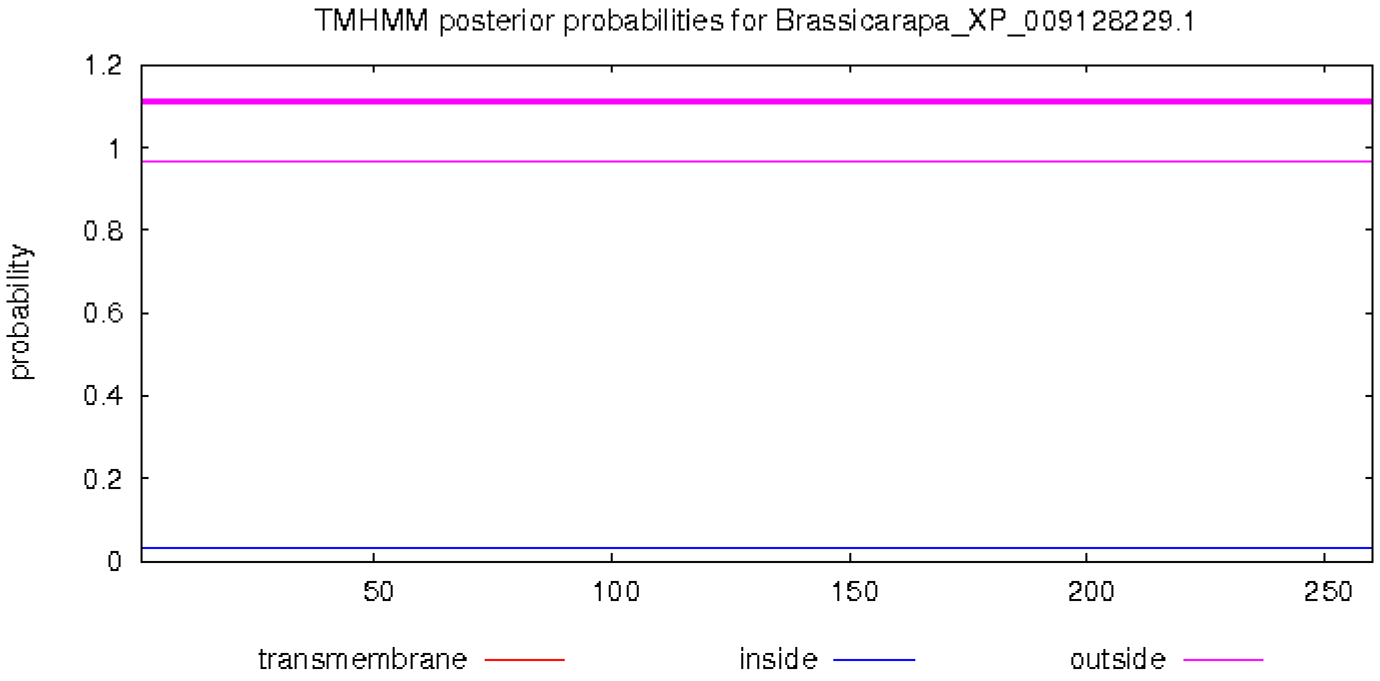

plot in postscript, script for making the plot in gnuplot, data for plot

---

```
# Brassicarapa_XP_009128370.1 Length: 349
# Brassicarapa_XP_009128370.1 Number of predicted TMHs:  0
# Brassicarapa_XP_009128370.1 Exp number of AAs in TMHs: 0.00096
# Brassicarapa_XP_009128370.1 Exp number, first 60 AAs:  0.00096
# Brassicarapa_XP_009128370.1 Total prob of N-in:        0.01005
Brassicarapa_XP_009128370.1     TMHMM2.0        outside      1   349
```

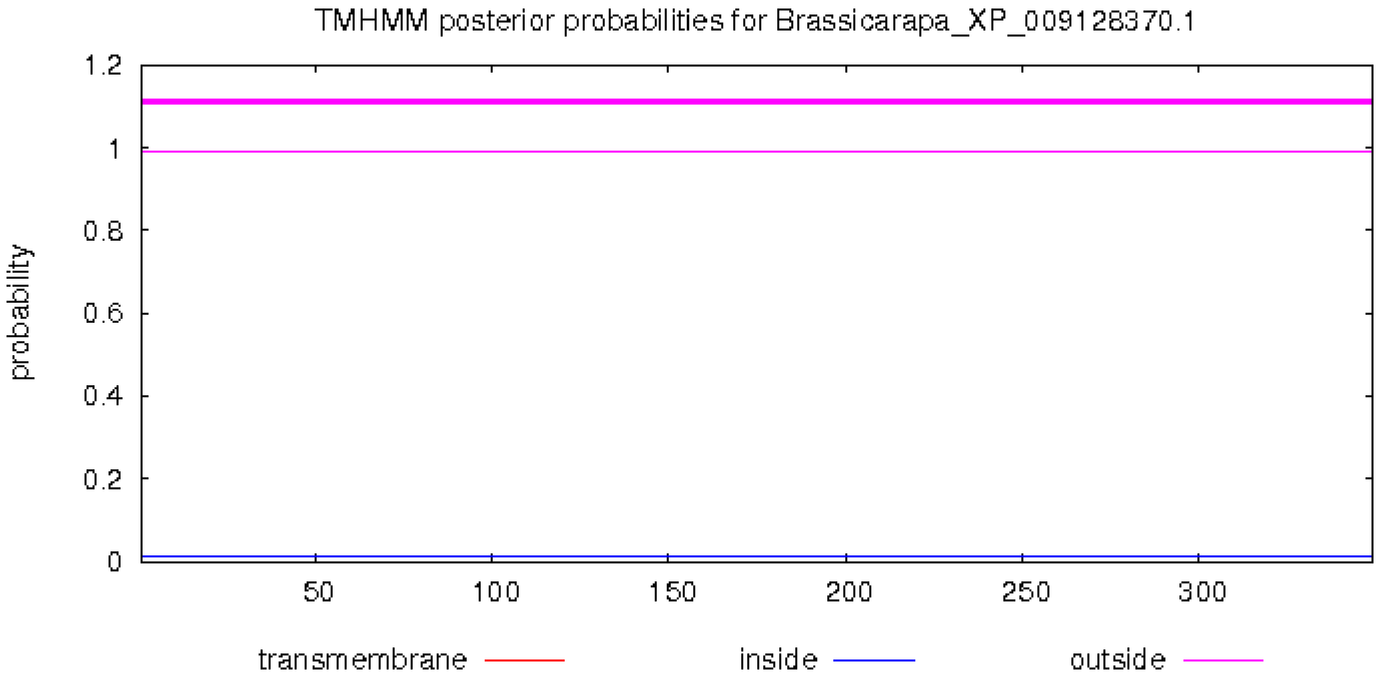

plot in postscript, script for making the plot in gnuplot, data for plot

---

```
# Brassicarapa_XP_009128520.1 Length: 427
# Brassicarapa_XP_009128520.1 Number of predicted TMHs:  1
# Brassicarapa_XP_009128520.1 Exp number of AAs in TMHs: 22.88114
# Brassicarapa_XP_009128520.1 Exp number, first 60 AAs:  0.00052
# Brassicarapa_XP_009128520.1 Total prob of N-in:        0.60694
Brassicarapa_XP_009128520.1     TMHMM2.0        inside       1   392
Brassicarapa_XP_009128520.1     TMHMM2.0        TMhelix    393   415
Brassicarapa_XP_009128520.1     TMHMM2.0        outside    416   427
```

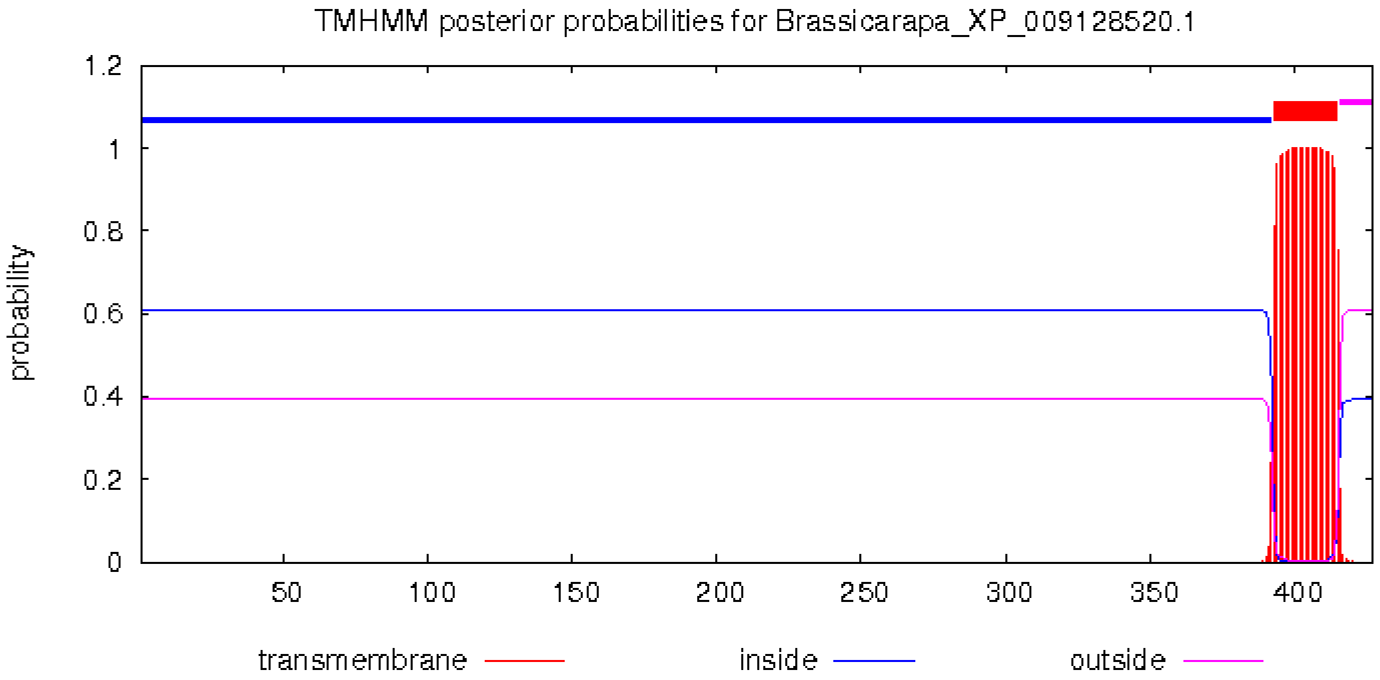

plot in postscript, script for making the plot in gnuplot, data for plot

---

```
# Brassicarapa_XP_009128881.1 Length: 300
# Brassicarapa_XP_009128881.1 Number of predicted TMHs:  0
# Brassicarapa_XP_009128881.1 Exp number of AAs in TMHs: 0
# Brassicarapa_XP_009128881.1 Exp number, first 60 AAs:  0
```

```
# Brassicarapa_XP_009128881.1 Total prob of N-in:        0.00910
Brassicarapa_XP_009128881.1     TMHMM2.0        outside      1   300
```

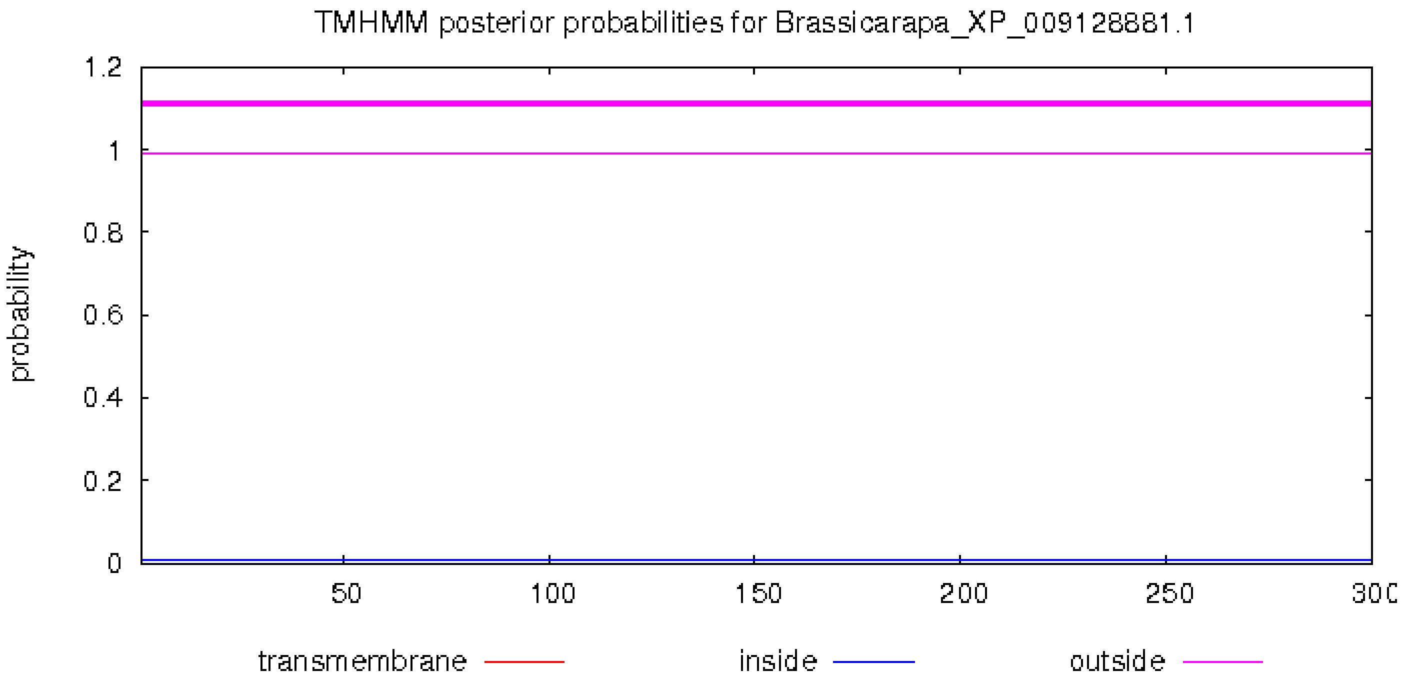

plot in postscript, script for making the plot in gnuplot, data for plot

```
# Brassicarapa_XP_009128919.1 Length: 501
# Brassicarapa_XP_009128919.1 Number of predicted TMHs:  0
# Brassicarapa_XP_009128919.1 Exp number of AAs in TMHs: 8.4653
# Brassicarapa_XP_009128919.1 Exp number, first 60 AAs:  0
# Brassicarapa_XP_009128919.1 Total prob of N-in:        0.34121
Brassicarapa_XP_009128919.1     TMHMM2.0        outside      1   501
```

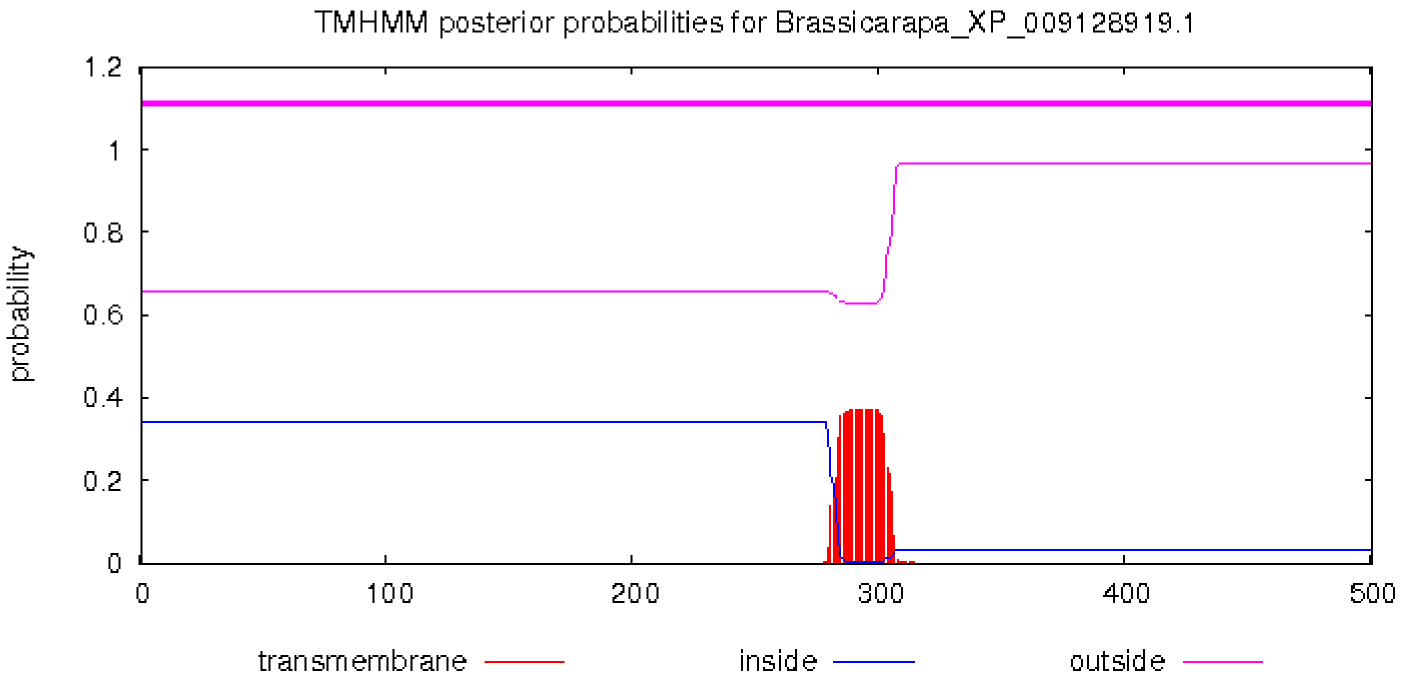

plot in postscript, script for making the plot in gnuplot, data for plot

```
# Brassicarapa_XP_009128925.1 Length: 500
# Brassicarapa_XP_009128925.1 Number of predicted TMHs:  0
# Brassicarapa_XP_009128925.1 Exp number of AAs in TMHs: 8.5262
# Brassicarapa_XP_009128925.1 Exp number, first 60 AAs:  0
```

```
# Brassicarapa_XP_009128925.1 Total prob of N-in:        0.34402
Brassicarapa_XP_009128925.1     TMHMM2.0        outside      1   500
```

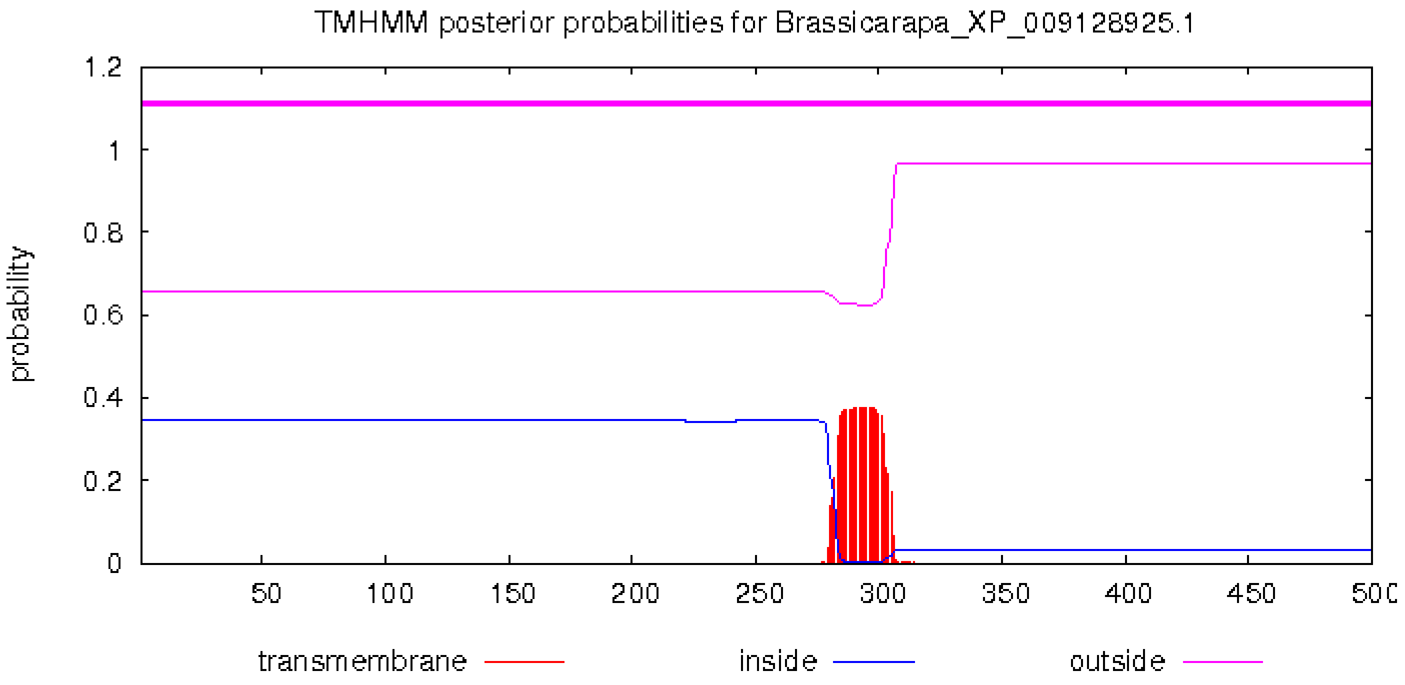

plot in postscript, script for making the plot in gnuplot, data for plot

---

```
# Brassicarapa_XP_009128929.1 Length: 428
# Brassicarapa_XP_009128929.1 Number of predicted TMHs:  0
# Brassicarapa_XP_009128929.1 Exp number of AAs in TMHs: 6.42216
# Brassicarapa_XP_009128929.1 Exp number, first 60 AAs:  0
# Brassicarapa_XP_009128929.1 Total prob of N-in:        0.24635
Brassicarapa_XP_009128929.1     TMHMM2.0        outside      1   428
```

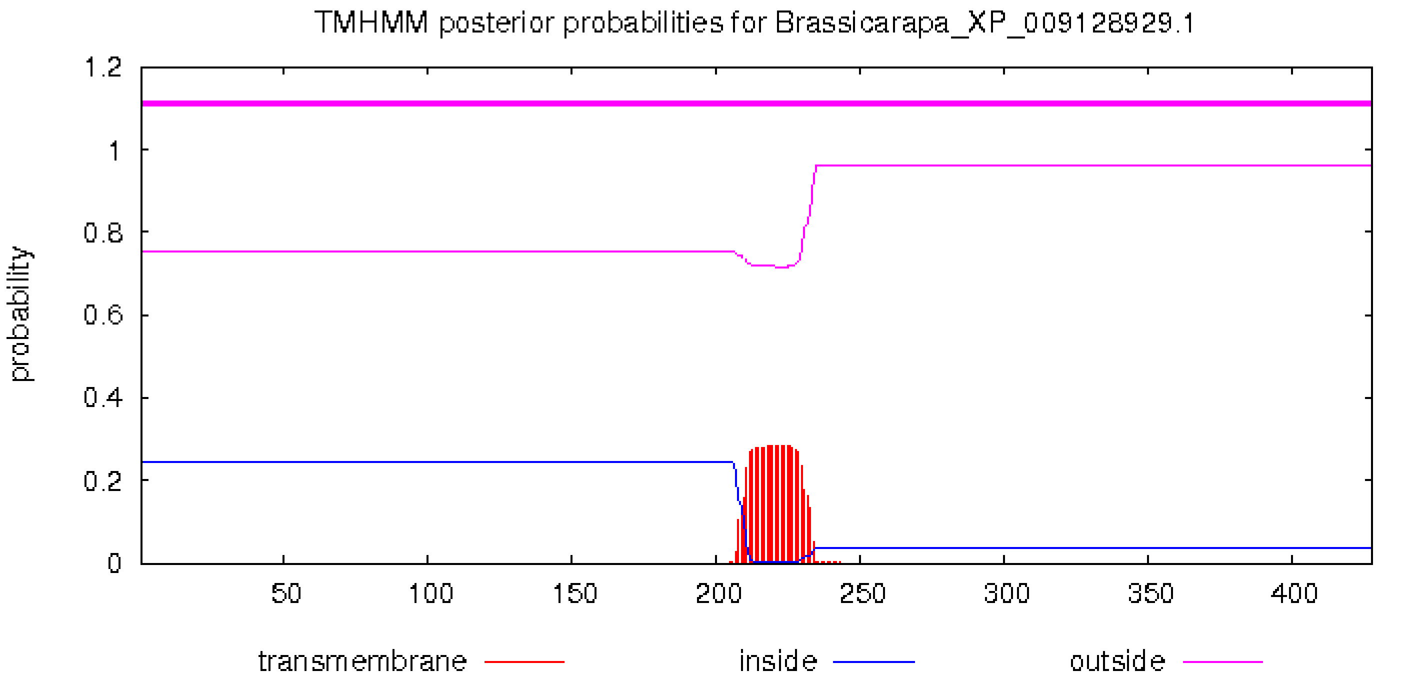

plot in postscript, script for making the plot in gnuplot, data for plot

---

```
# Brassicarapa_XP_009129144.1 Length: 406
# Brassicarapa_XP_009129144.1 Number of predicted TMHs:  0
# Brassicarapa_XP_009129144.1 Exp number of AAs in TMHs: 0.00264
# Brassicarapa_XP_009129144.1 Exp number, first 60 AAs:  0
```

```
# Brassicarapa_XP_009129144.1 Total prob of N-in:        0.00707
Brassicarapa_XP_009129144.1     TMHMM2.0        outside      1   406
```

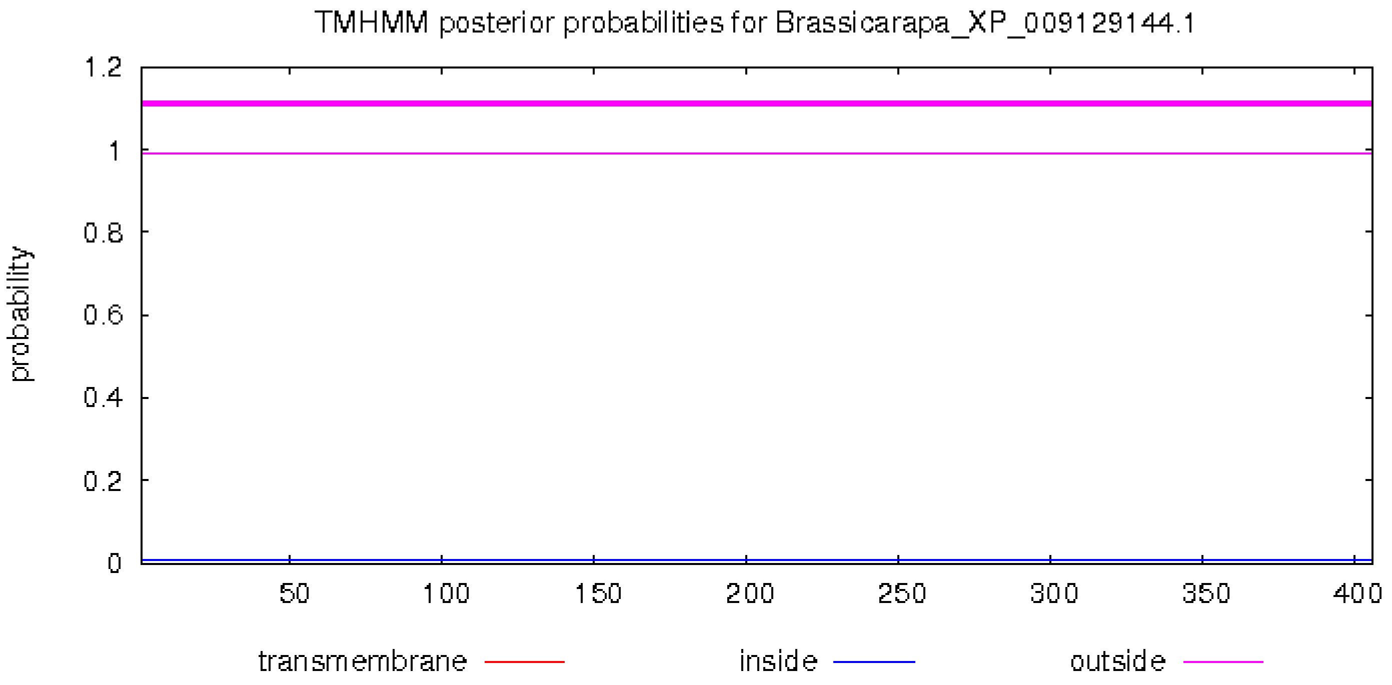

plot in postscript, script for making the plot in gnuplot, data for plot

---

```
# Brassicarapa_XP_009129145.1 Length: 376
# Brassicarapa_XP_009129145.1 Number of predicted TMHs:  0
# Brassicarapa_XP_009129145.1 Exp number of AAs in TMHs: 0.00231
# Brassicarapa_XP_009129145.1 Exp number, first 60 AAs:  0
# Brassicarapa_XP_009129145.1 Total prob of N-in:        0.01022
Brassicarapa_XP_009129145.1     TMHMM2.0        outside      1   376
```

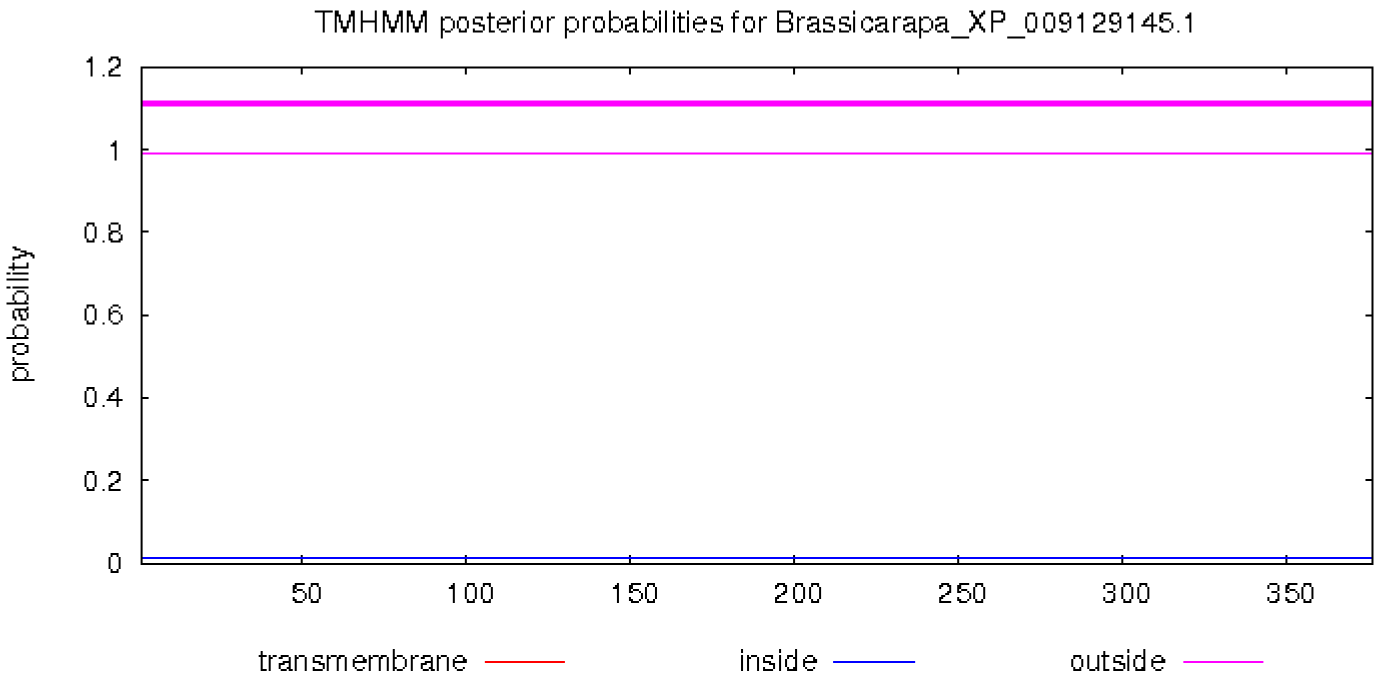

plot in postscript, script for making the plot in gnuplot, data for plot

---

```
# Brassicarapa_XP_009129548.1 Length: 344
# Brassicarapa_XP_009129548.1 Number of predicted TMHs:  0
# Brassicarapa_XP_009129548.1 Exp number of AAs in TMHs: 0
# Brassicarapa_XP_009129548.1 Exp number, first 60 AAs:  0
```

```
# Brassicarapa_XP_009129548.1 Total prob of N-in:        0.00689
Brassicarapa_XP_009129548.1     TMHMM2.0        outside      1   344
```

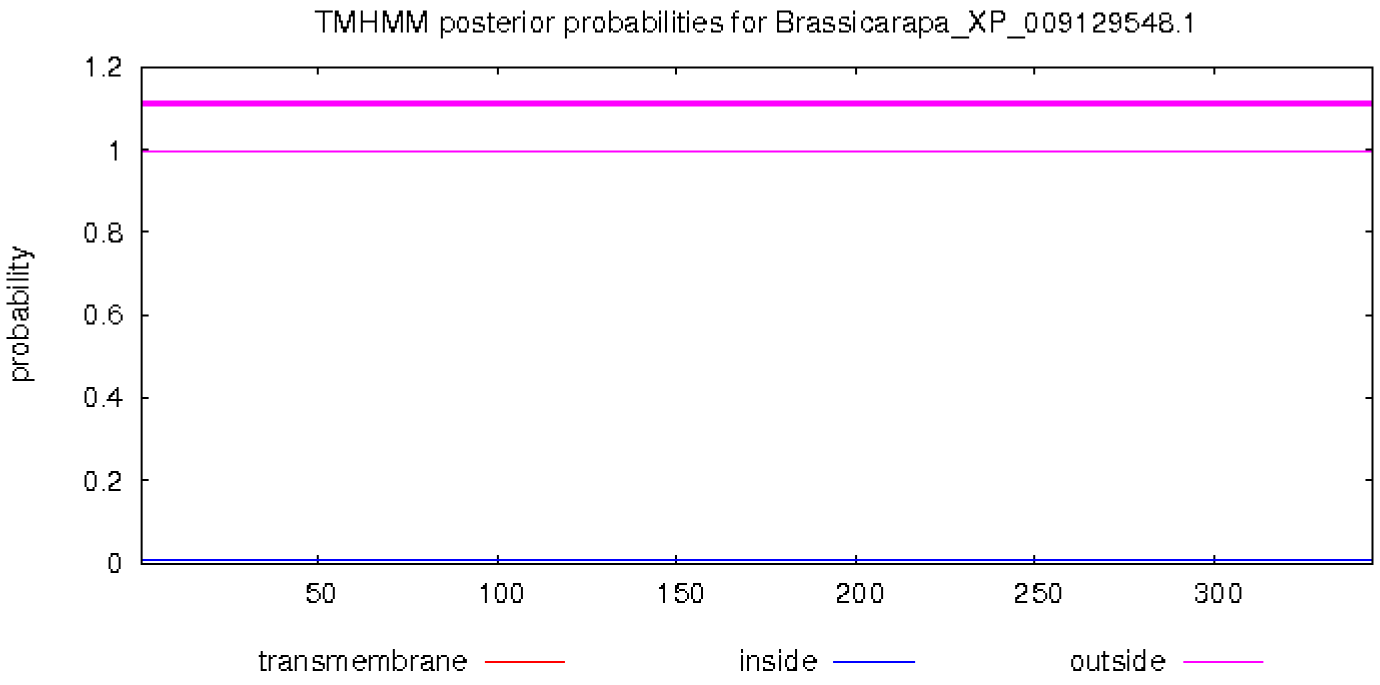

plot in postscript, script for making the plot in gnuplot, data for plot

---

```
# Brassicarapa_XP_009129565.1 Length: 301
# Brassicarapa_XP_009129565.1 Number of predicted TMHs:  0
# Brassicarapa_XP_009129565.1 Exp number of AAs in TMHs: 0.00114
# Brassicarapa_XP_009129565.1 Exp number, first 60 AAs:  0.00019
# Brassicarapa_XP_009129565.1 Total prob of N-in:        0.10457
Brassicarapa_XP_009129565.1     TMHMM2.0        outside      1   301
```

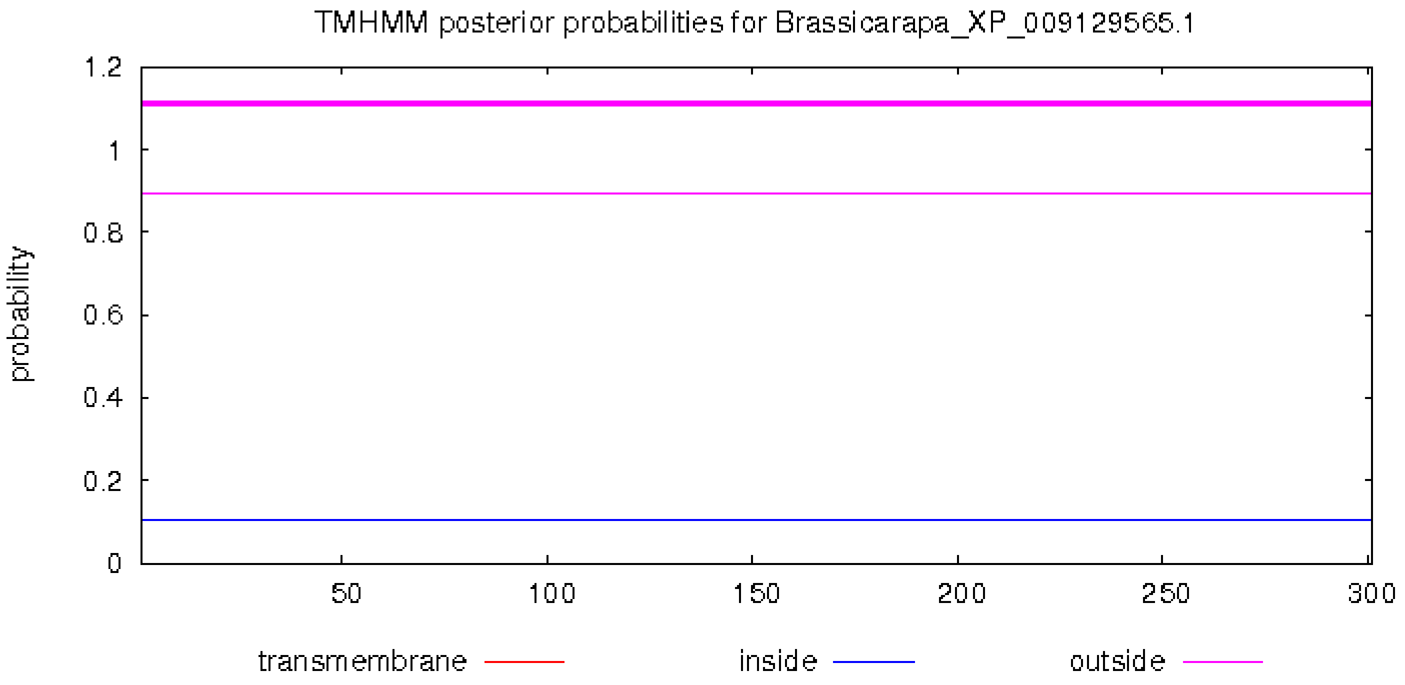

plot in postscript, script for making the plot in gnuplot, data for plot

---

```
# Brassicarapa_XP_009130171.1 Length: 325
# Brassicarapa_XP_009130171.1 Number of predicted TMHs:  0
# Brassicarapa_XP_009130171.1 Exp number of AAs in TMHs: 0.00115
# Brassicarapa_XP_009130171.1 Exp number, first 60 AAs:  0
```

```
# Brassicarapa_XP_009130171.1 Total prob of N-in:        0.01204
Brassicarapa_XP_009130171.1     TMHMM2.0        outside      1   325
```

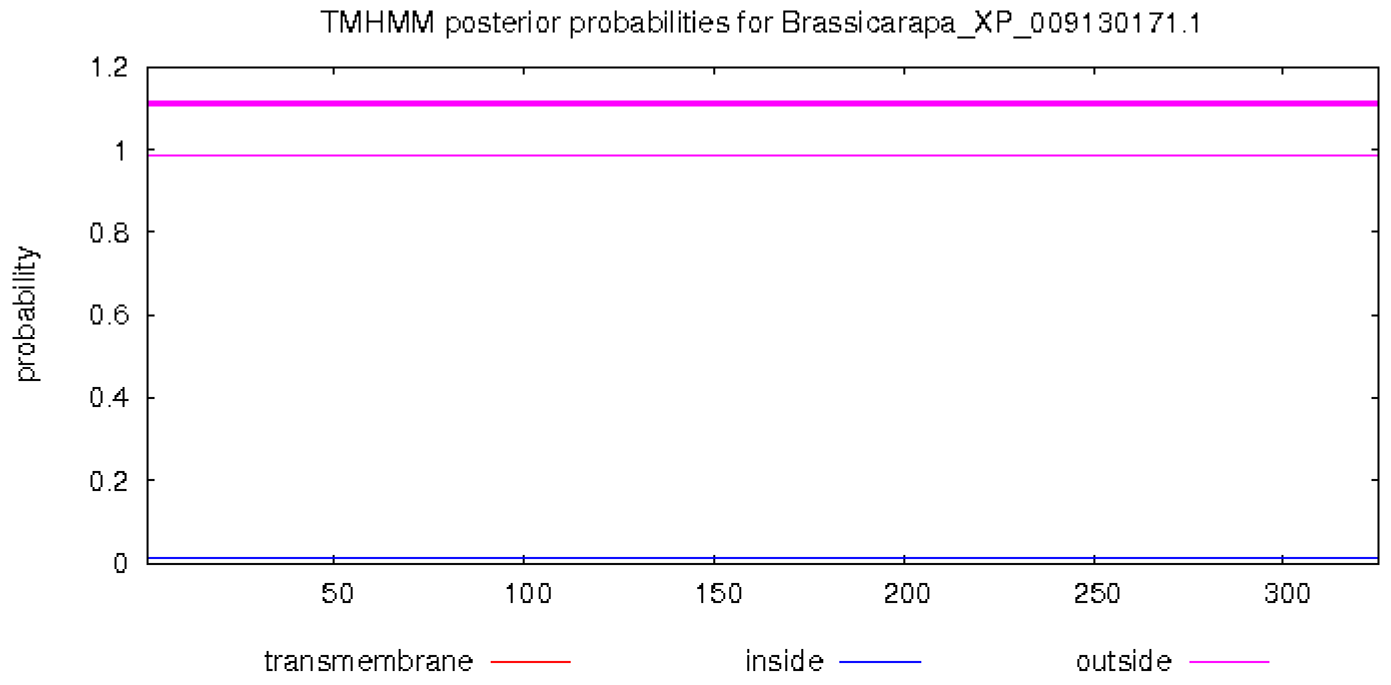

plot in postscript, script for making the plot in gnuplot, data for plot

---

```
# Brassicarapa_XP_009130209.1 Length: 364
# Brassicarapa_XP_009130209.1 Number of predicted TMHs:  0
# Brassicarapa_XP_009130209.1 Exp number of AAs in TMHs: 0.00037
# Brassicarapa_XP_009130209.1 Exp number, first 60 AAs:  0
# Brassicarapa_XP_009130209.1 Total prob of N-in:        0.00226
Brassicarapa_XP_009130209.1     TMHMM2.0        outside      1   364
```

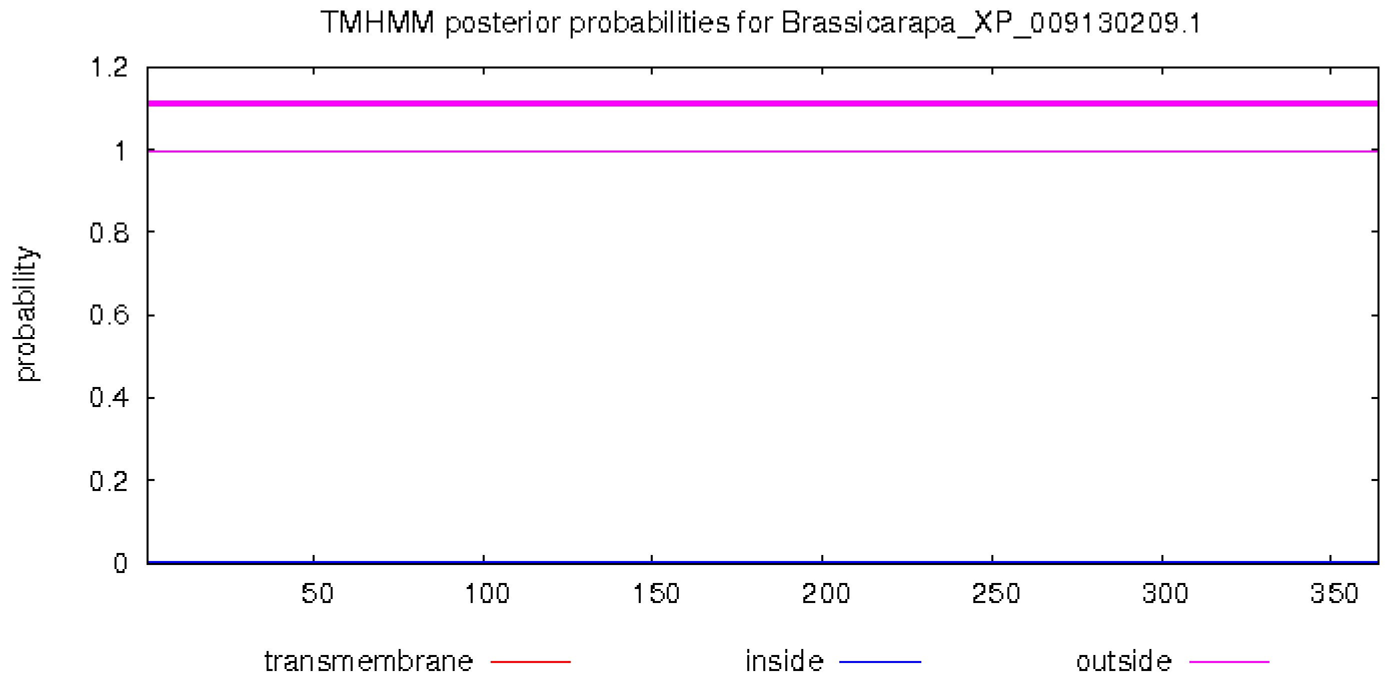

plot in postscript, script for making the plot in gnuplot, data for plot

---

```
# Brassicarapa_XP_009130210.1 Length: 364
# Brassicarapa_XP_009130210.1 Number of predicted TMHs:  0
# Brassicarapa_XP_009130210.1 Exp number of AAs in TMHs: 0.00037
# Brassicarapa_XP_009130210.1 Exp number, first 60 AAs:  0
```

```
# Brassicarapa_XP_009130210.1 Total prob of N-in:        0.00226
Brassicarapa_XP_009130210.1     TMHMM2.0        outside      1   364
```

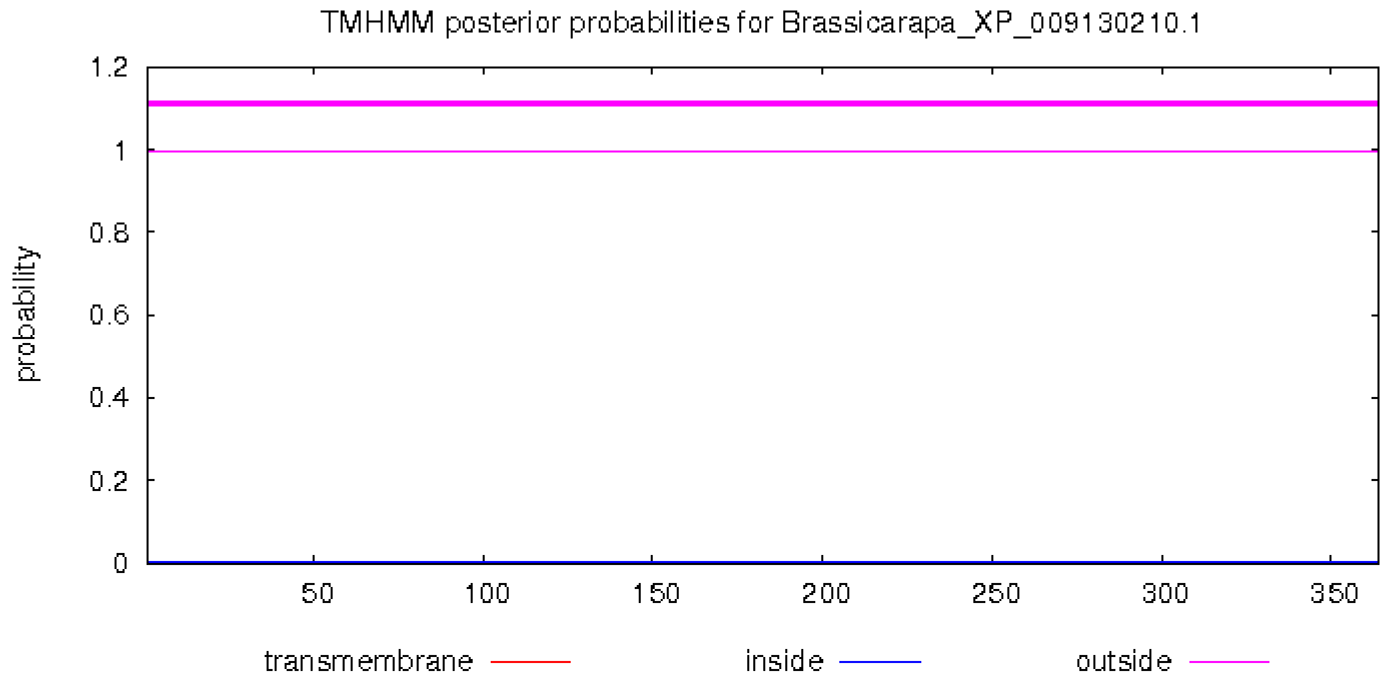

plot in postscript, script for making the plot in gnuplot, data for plot

---

```
# Brassicarapa_XP_009130211.1 Length: 364
# Brassicarapa_XP_009130211.1 Number of predicted TMHs:  0
# Brassicarapa_XP_009130211.1 Exp number of AAs in TMHs: 0.00037
# Brassicarapa_XP_009130211.1 Exp number, first 60 AAs:  0
# Brassicarapa_XP_009130211.1 Total prob of N-in:        0.00226
Brassicarapa_XP_009130211.1     TMHMM2.0        outside      1   364
```

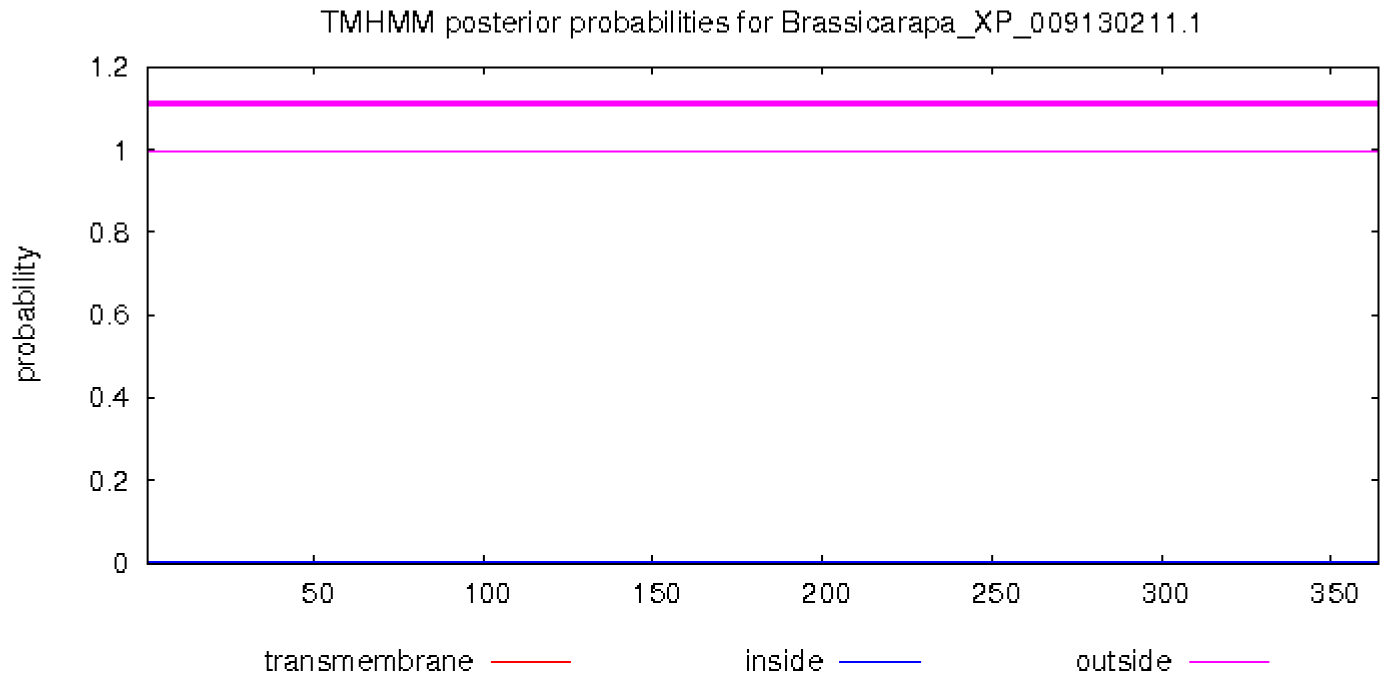

plot in postscript, script for making the plot in gnuplot, data for plot

---

```
# Brassicarapa_XP_009130273.1 Length: 265
# Brassicarapa_XP_009130273.1 Number of predicted TMHs:  0
# Brassicarapa_XP_009130273.1 Exp number of AAs in TMHs: 0
# Brassicarapa_XP_009130273.1 Exp number, first 60 AAs:  0
```

```
# Brassicarapa_XP_009130273.1 Total prob of N-in:        0.01252
Brassicarapa_XP_009130273.1     TMHMM2.0        outside      1   265
```

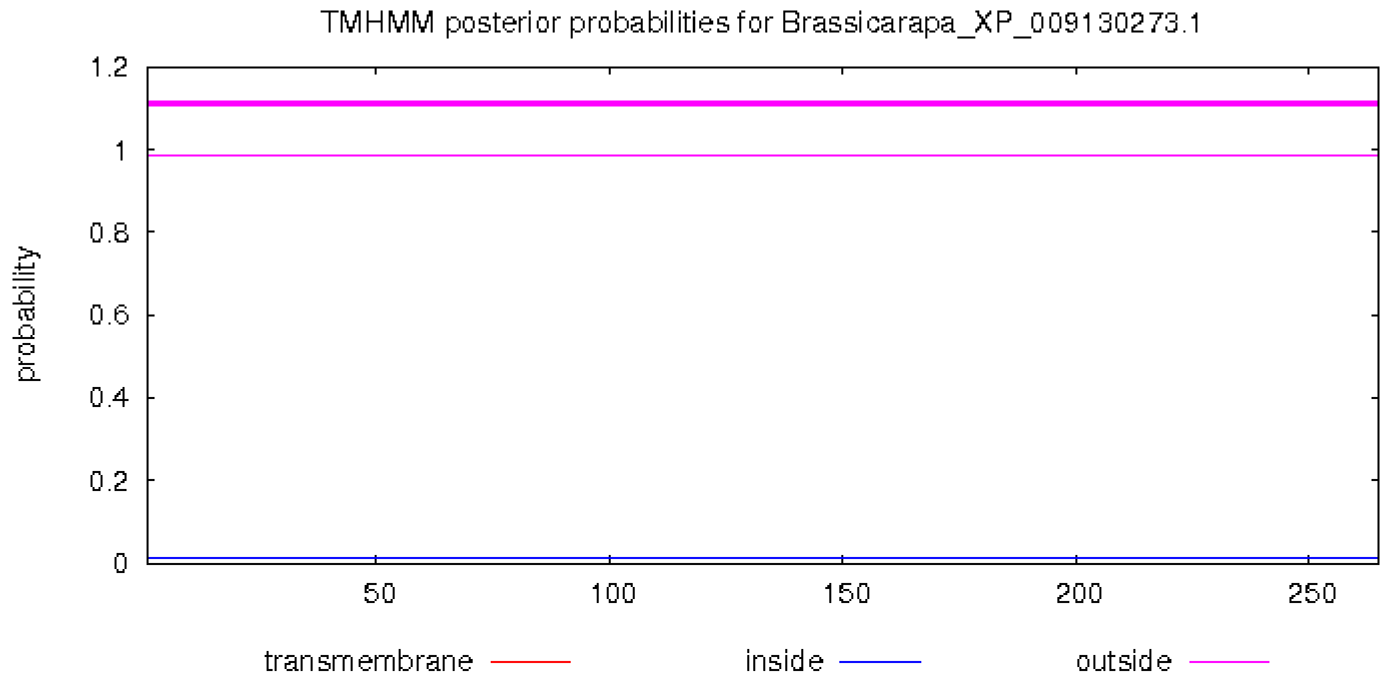

plot in postscript, script for making the plot in gnuplot, data for plot

---

```
# Brassicarapa_XP_009130343.1 Length: 481
# Brassicarapa_XP_009130343.1 Number of predicted TMHs:  0
# Brassicarapa_XP_009130343.1 Exp number of AAs in TMHs: 0.00112
# Brassicarapa_XP_009130343.1 Exp number, first 60 AAs:  0
# Brassicarapa_XP_009130343.1 Total prob of N-in:        0.00016
Brassicarapa_XP_009130343.1     TMHMM2.0        outside      1   481
```

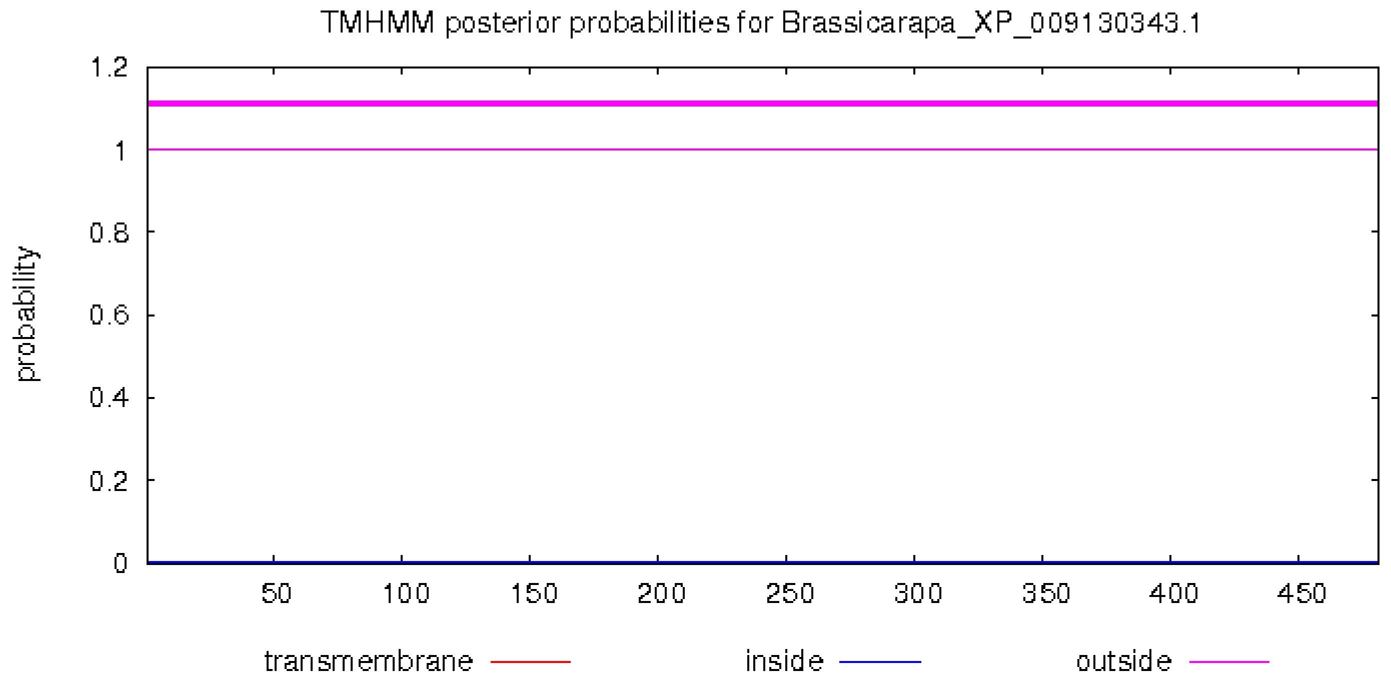

plot in postscript, script for making the plot in gnuplot, data for plot

---

```
# Brassicarapa_XP_009130344.1 Length: 478
# Brassicarapa_XP_009130344.1 Number of predicted TMHs:  0
# Brassicarapa_XP_009130344.1 Exp number of AAs in TMHs: 0.00112
# Brassicarapa_XP_009130344.1 Exp number, first 60 AAs:  0
```

```
# Brassicarapa_XP_009130344.1 Total prob of N-in:        0.00016
Brassicarapa_XP_009130344.1     TMHMM2.0        outside      1   478
```

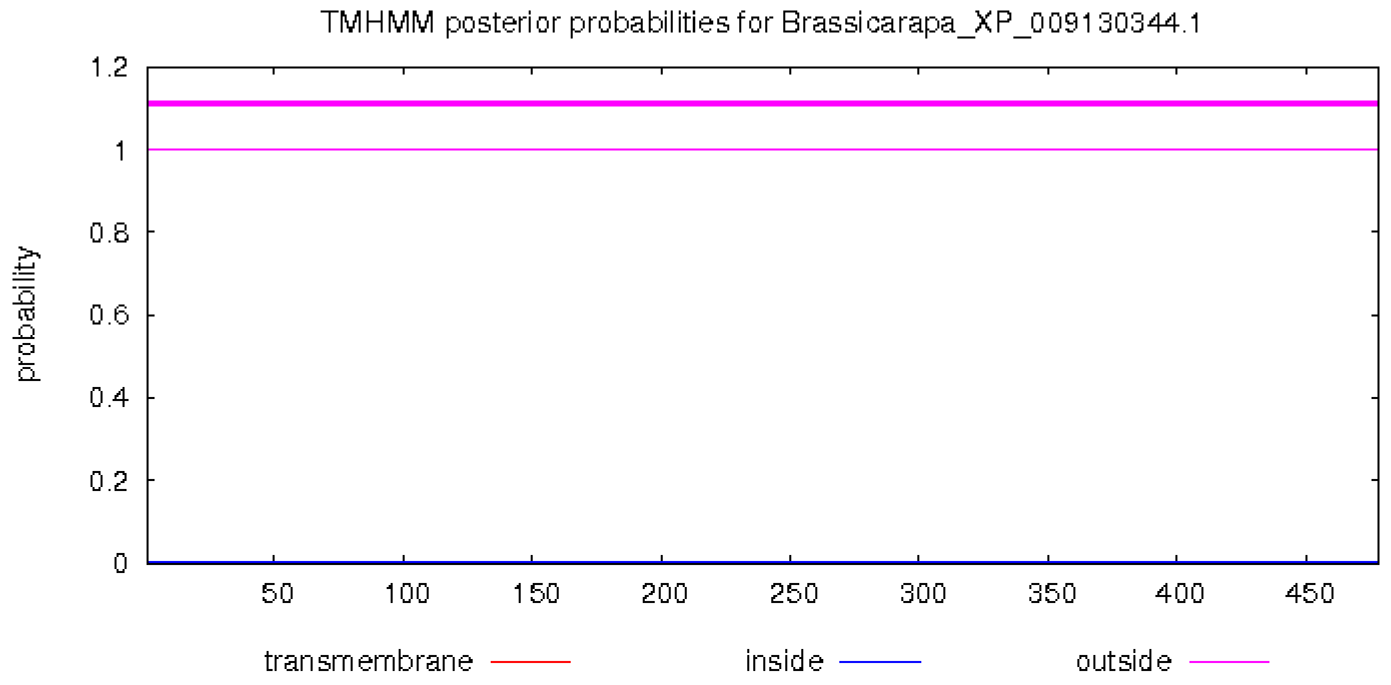

plot in postscript, script for making the plot in gnuplot, data for plot

---

```
# Brassicarapa_XP_009130418.1 Length: 344
# Brassicarapa_XP_009130418.1 Number of predicted TMHs:  0
# Brassicarapa_XP_009130418.1 Exp number of AAs in TMHs: 0.00077
# Brassicarapa_XP_009130418.1 Exp number, first 60 AAs:  0
# Brassicarapa_XP_009130418.1 Total prob of N-in:        0.01154
Brassicarapa_XP_009130418.1     TMHMM2.0        outside      1   344
```

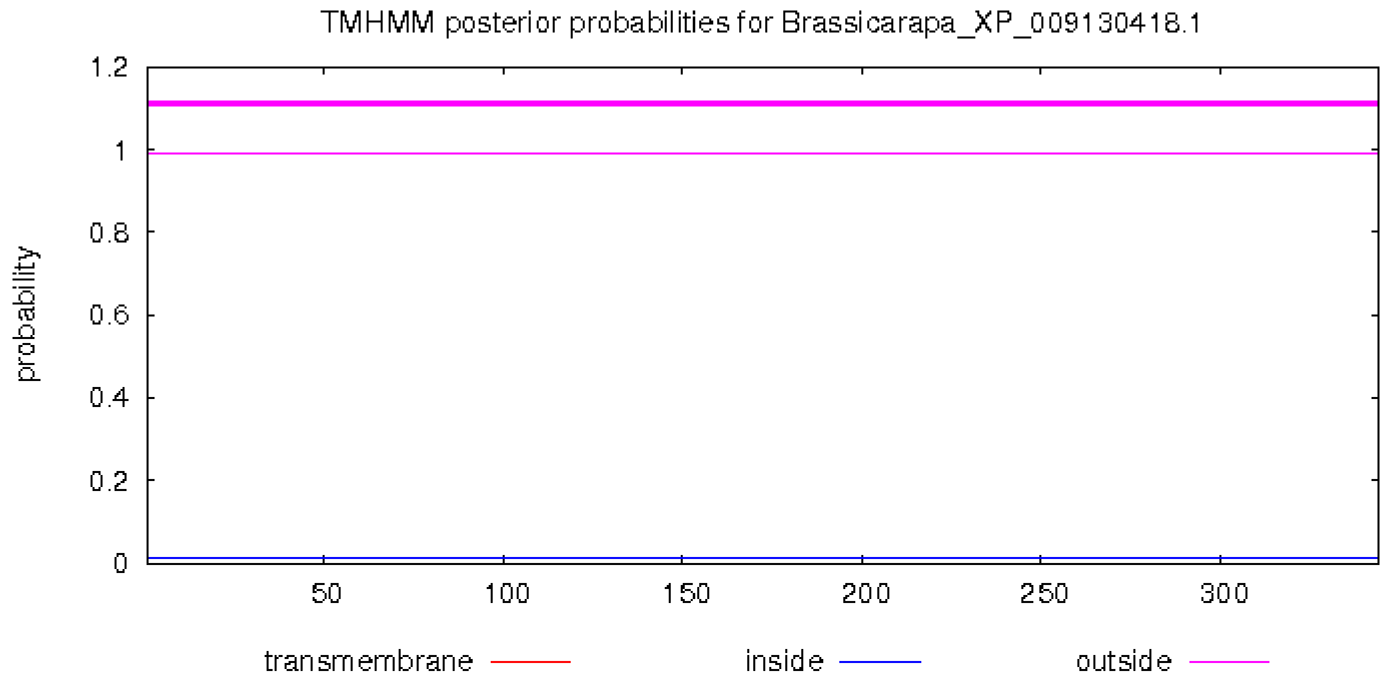

plot in postscript, script for making the plot in gnuplot, data for plot

---

```
# Brassicarapa_XP_009130419.1 Length: 353
# Brassicarapa_XP_009130419.1 Number of predicted TMHs:  0
# Brassicarapa_XP_009130419.1 Exp number of AAs in TMHs: 2e-05
# Brassicarapa_XP_009130419.1 Exp number, first 60 AAs:  0
```

```
# Brassicarapa_XP_009130419.1 Total prob of N-in:        0.01031
Brassicarapa_XP_009130419.1     TMHMM2.0        outside      1   353
```

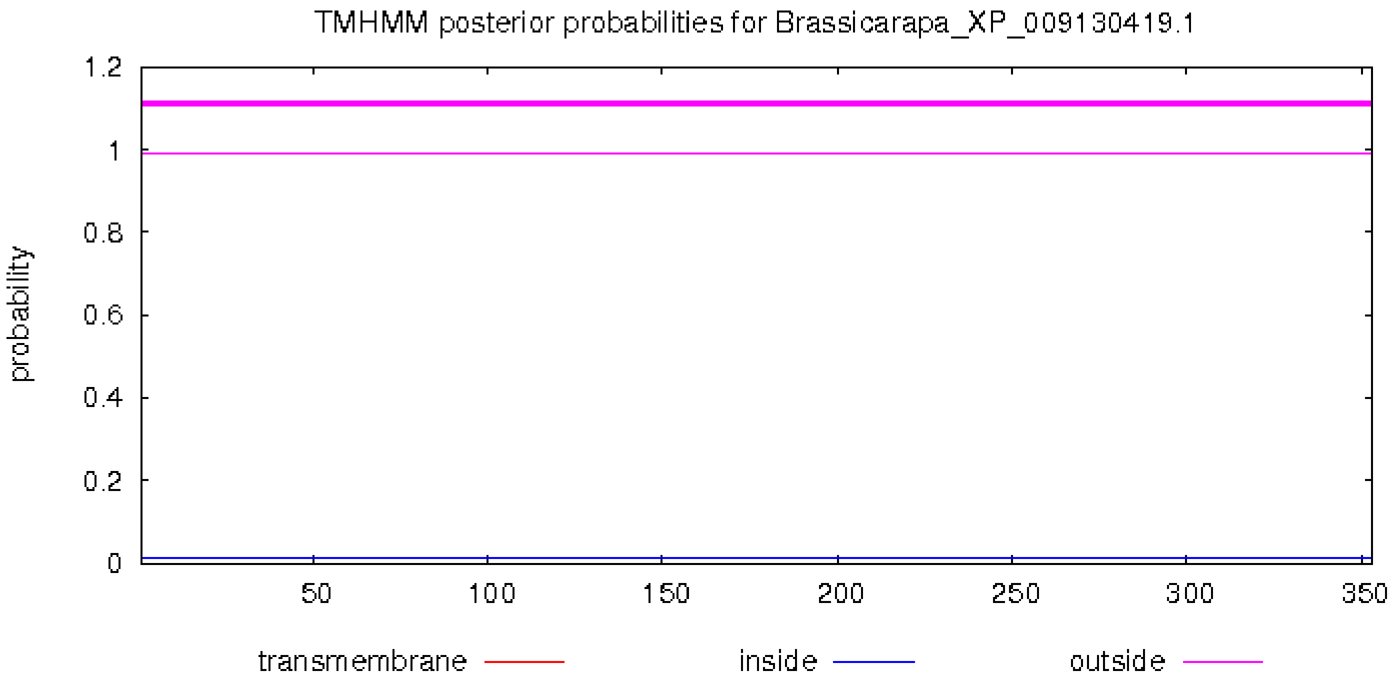

plot in postscript, script for making the plot in gnuplot, data for plot

---

```
# Brassicarapa_XP_009130458.1 Length: 185
# Brassicarapa_XP_009130458.1 Number of predicted TMHs:  0
# Brassicarapa_XP_009130458.1 Exp number of AAs in TMHs: 0.00014
# Brassicarapa_XP_009130458.1 Exp number, first 60 AAs:  0
# Brassicarapa_XP_009130458.1 Total prob of N-in:        0.00752
Brassicarapa_XP_009130458.1     TMHMM2.0        outside      1   185
```

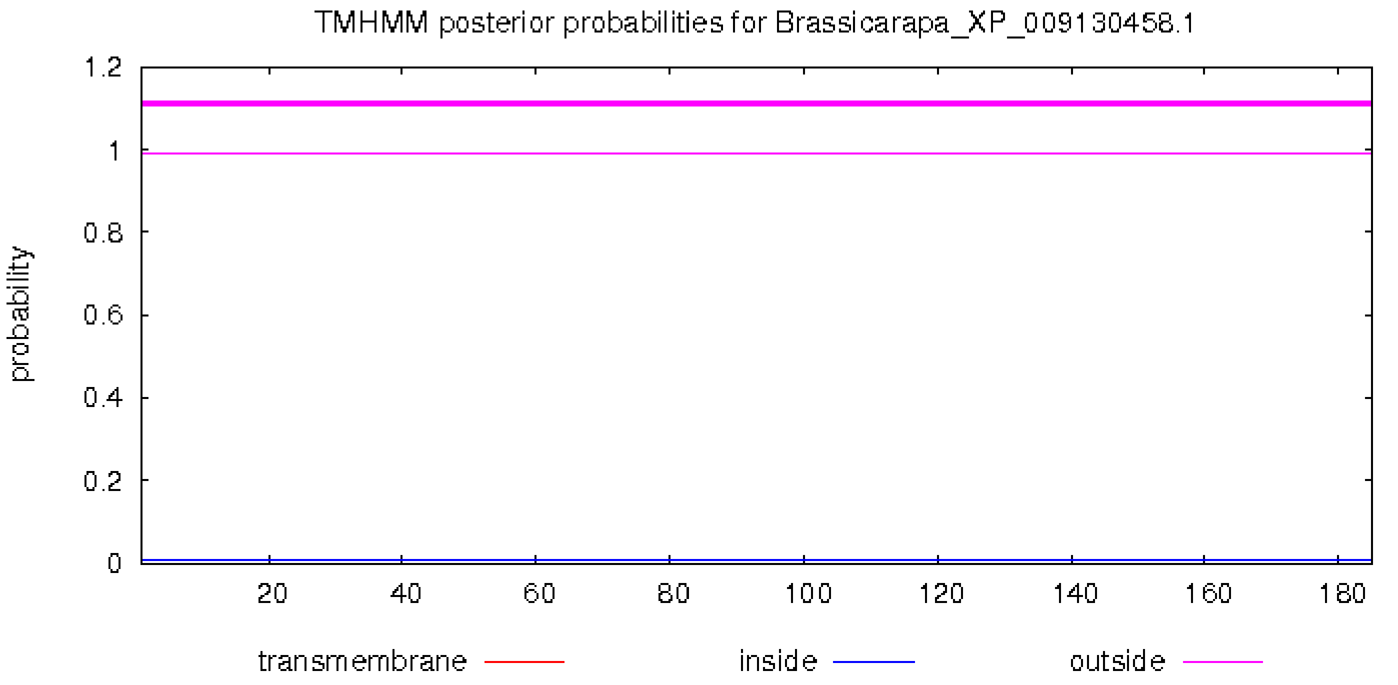

plot in postscript, script for making the plot in gnuplot, data for plot

---

```
# Brassicarapa_XP_009130821.1 Length: 567
# Brassicarapa_XP_009130821.1 Number of predicted TMHs:  1
# Brassicarapa_XP_009130821.1 Exp number of AAs in TMHs: 21.26967
# Brassicarapa_XP_009130821.1 Exp number, first 60 AAs:  0
```

```
# Brassicarapa_XP_009130821.1 Total prob of N-in:        0.00754
Brassicarapa_XP_009130821.1     TMHMM2.0        outside      1   541
Brassicarapa_XP_009130821.1     TMHMM2.0        TMhelix    542   564
Brassicarapa_XP_009130821.1     TMHMM2.0        inside     565   567
```

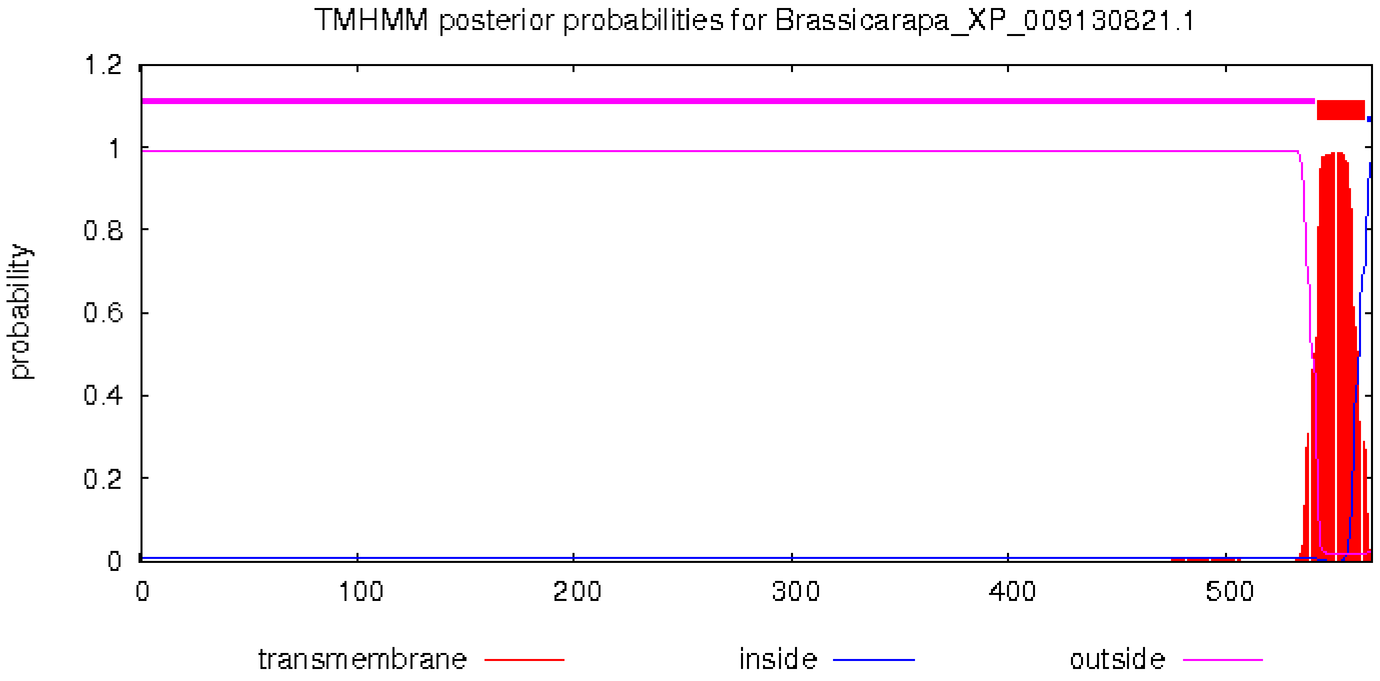

plot in postscript, script for making the plot in gnuplot, data for plot

---

```
# Brassicarapa_XP_009130822.1 Length: 566
# Brassicarapa_XP_009130822.1 Number of predicted TMHs:  1
# Brassicarapa_XP_009130822.1 Exp number of AAs in TMHs: 21.2699
# Brassicarapa_XP_009130822.1 Exp number, first 60 AAs:  0
# Brassicarapa_XP_009130822.1 Total prob of N-in:        0.00763
Brassicarapa_XP_009130822.1     TMHMM2.0        outside      1   540
Brassicarapa_XP_009130822.1     TMHMM2.0        TMhelix    541   563
Brassicarapa_XP_009130822.1     TMHMM2.0        inside     564   566
```

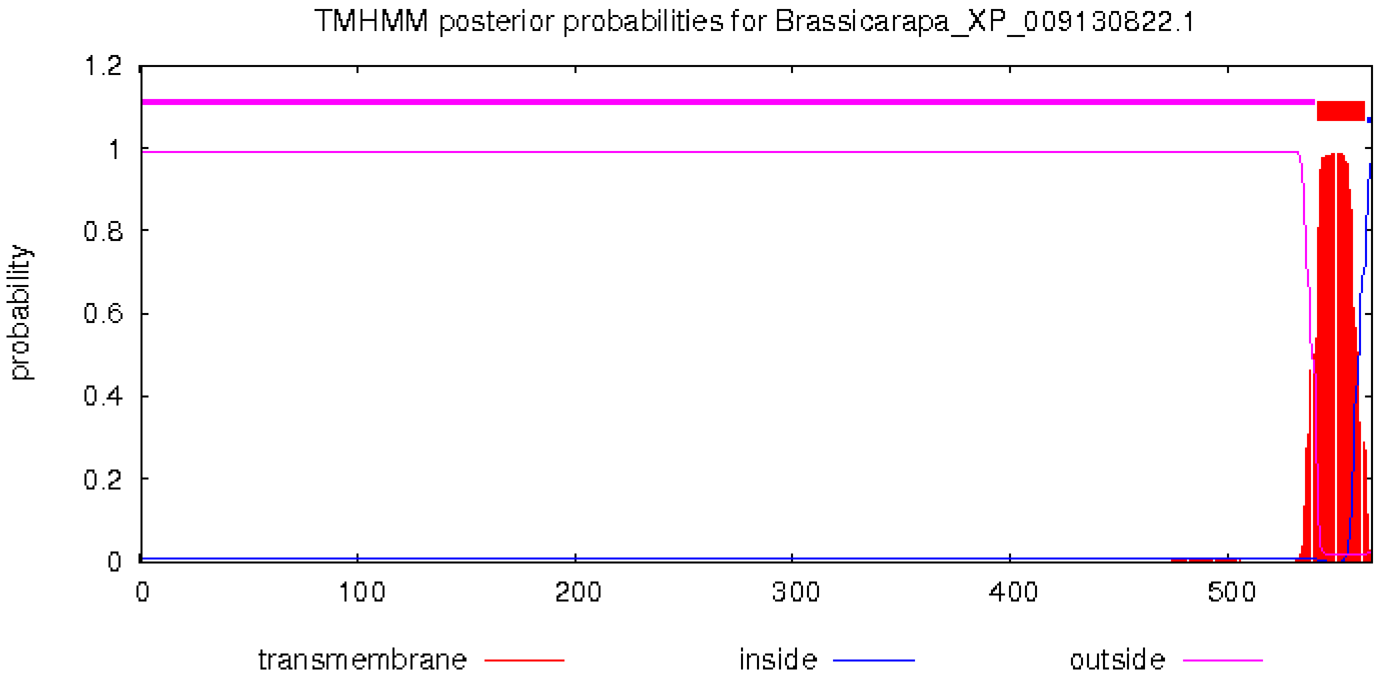

plot in postscript, script for making the plot in gnuplot, data for plot

---

```
# Brassicarapa_XP_009131103.1 Length: 280
# Brassicarapa_XP_009131103.1 Number of predicted TMHs:  0
# Brassicarapa_XP_009131103.1 Exp number of AAs in TMHs: 0.00038
# Brassicarapa_XP_009131103.1 Exp number, first 60 AAs:  0.00019
# Brassicarapa_XP_009131103.1 Total prob of N-in:        0.01246
Brassicarapa_XP_009131103.1	TMHMM2.0	outside	     1   280
```

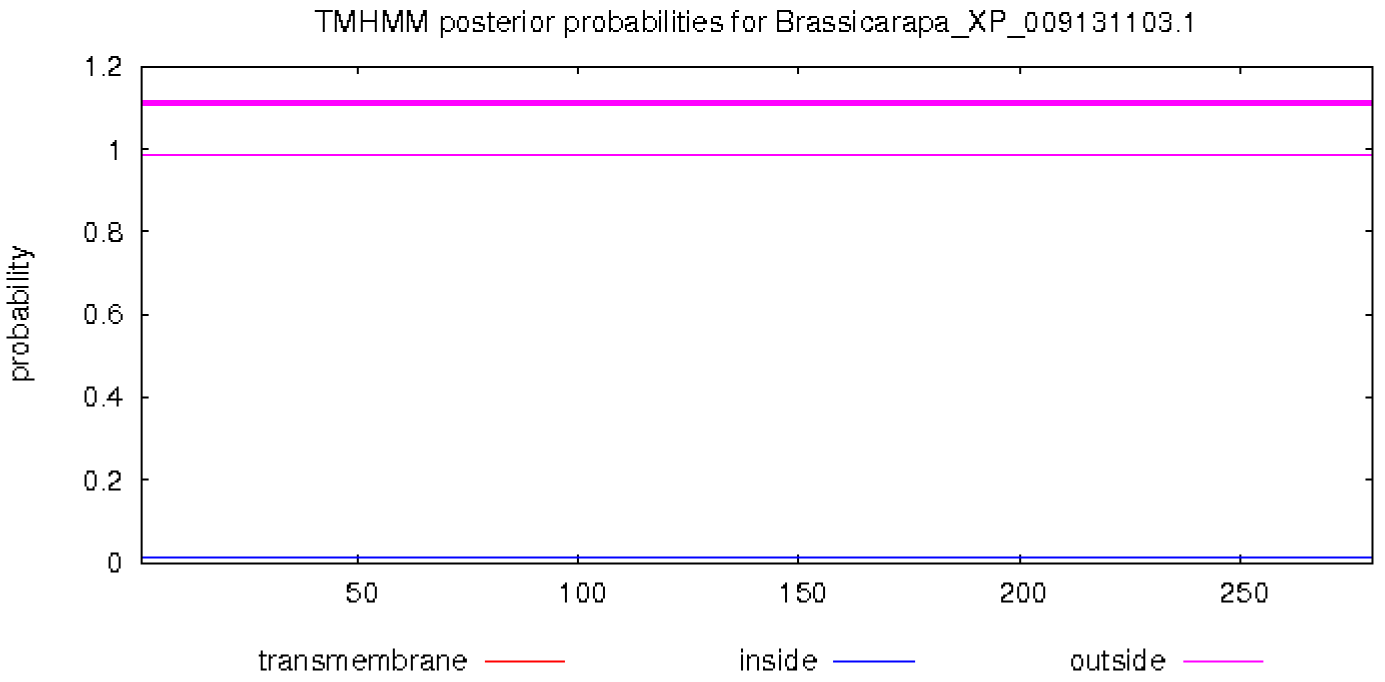

plot in postscript, script for making the plot in gnuplot, data for plot

---

```
# Brassicarapa_XP_009131217.1 Length: 292
# Brassicarapa_XP_009131217.1 Number of predicted TMHs:  0
# Brassicarapa_XP_009131217.1 Exp number of AAs in TMHs: 0
# Brassicarapa_XP_009131217.1 Exp number, first 60 AAs:  0
# Brassicarapa_XP_009131217.1 Total prob of N-in:        0.00489
Brassicarapa_XP_009131217.1	TMHMM2.0	outside	     1   292
```

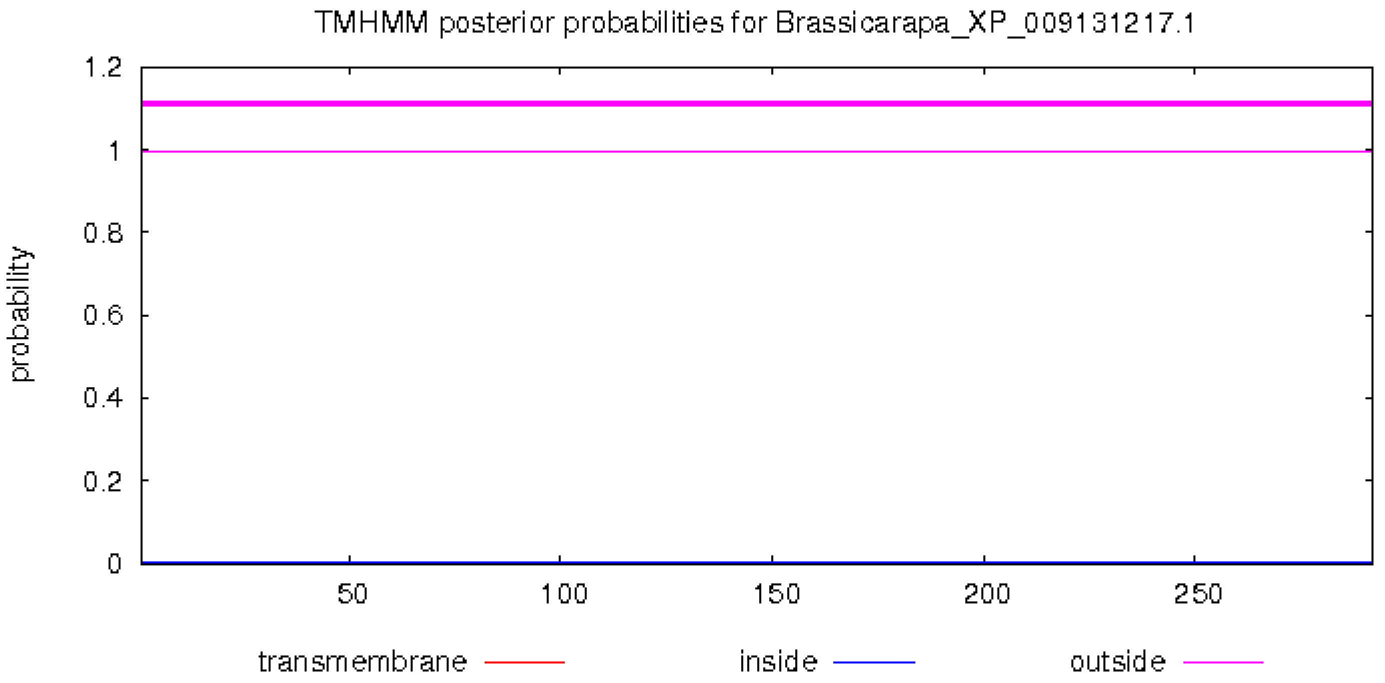

plot in postscript, script for making the plot in gnuplot, data for plot

---

```
# Brassicarapa_XP_009131224.1 Length: 240
# Brassicarapa_XP_009131224.1 Number of predicted TMHs:  0
# Brassicarapa_XP_009131224.1 Exp number of AAs in TMHs: 0
# Brassicarapa_XP_009131224.1 Exp number, first 60 AAs:  0
# Brassicarapa_XP_009131224.1 Total prob of N-in:        0.00948
Brassicarapa_XP_009131224.1     TMHMM2.0        outside      1   240
```

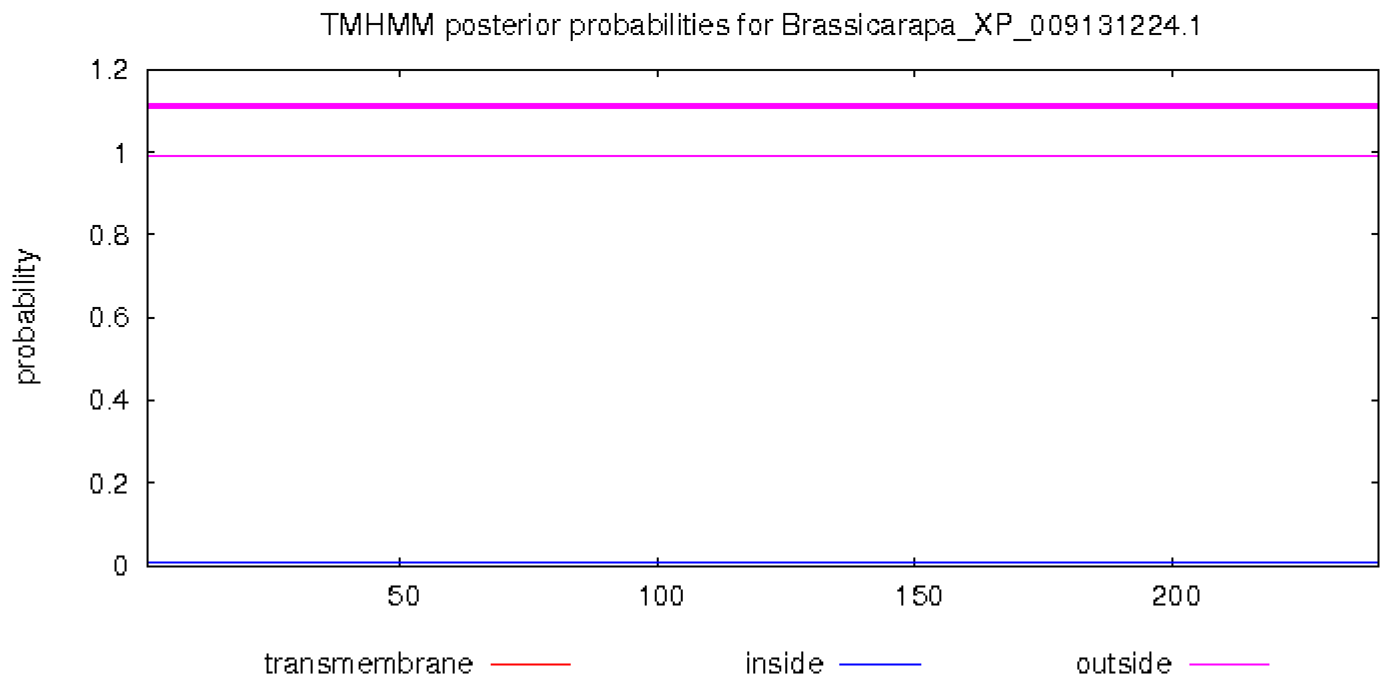

plot in postscript, script for making the plot in gnuplot, data for plot

```
# Brassicarapa_XP_009131323.1 Length: 254
# Brassicarapa_XP_009131323.1 Number of predicted TMHs:  0
# Brassicarapa_XP_009131323.1 Exp number of AAs in TMHs: 0.00369
# Brassicarapa_XP_009131323.1 Exp number, first 60 AAs:  0
# Brassicarapa_XP_009131323.1 Total prob of N-in:        0.15201
Brassicarapa_XP_009131323.1     TMHMM2.0        outside      1   254
```

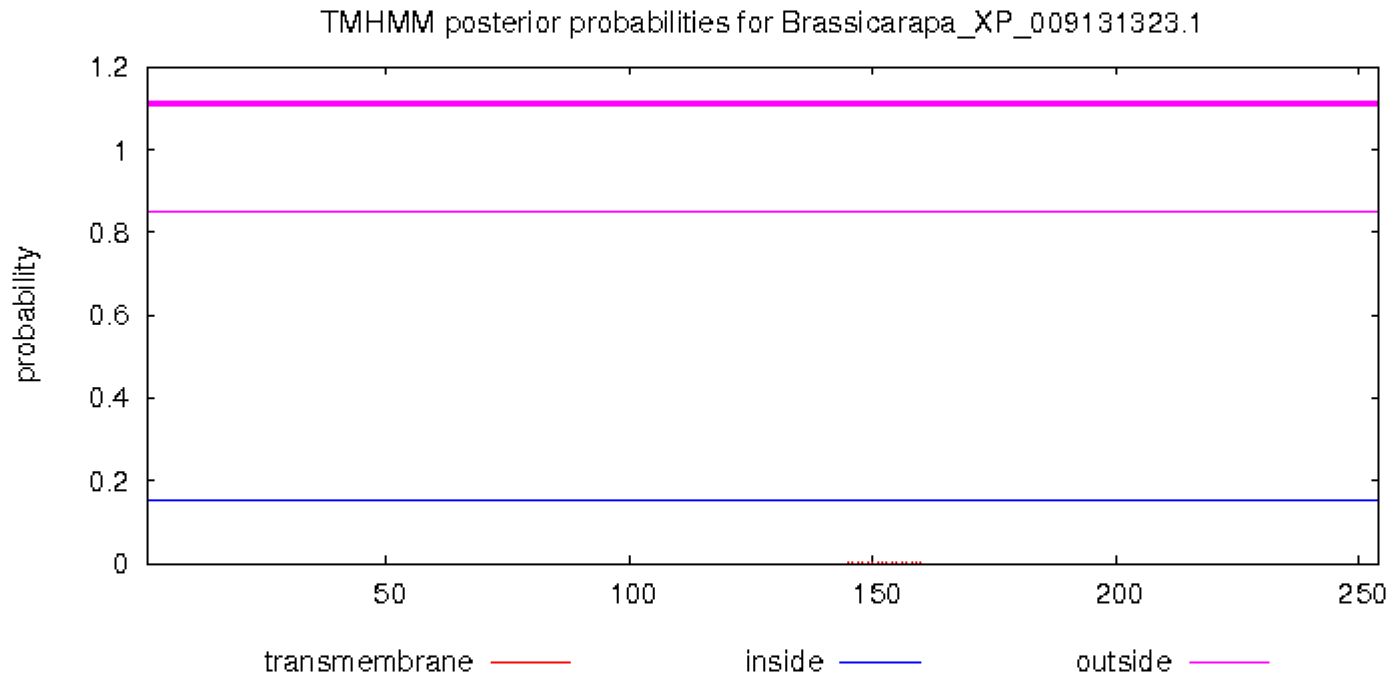

plot in postscript, script for making the plot in gnuplot, data for plot

```
# Brassicarapa_XP_009131324.1 Length: 248
# Brassicarapa_XP_009131324.1 Number of predicted TMHs:  0
# Brassicarapa_XP_009131324.1 Exp number of AAs in TMHs: 0.00056
# Brassicarapa_XP_009131324.1 Exp number, first 60 AAs:  0
# Brassicarapa_XP_009131324.1 Total prob of N-in:        0.16189
Brassicarapa_XP_009131324.1     TMHMM2.0        outside      1   248
```

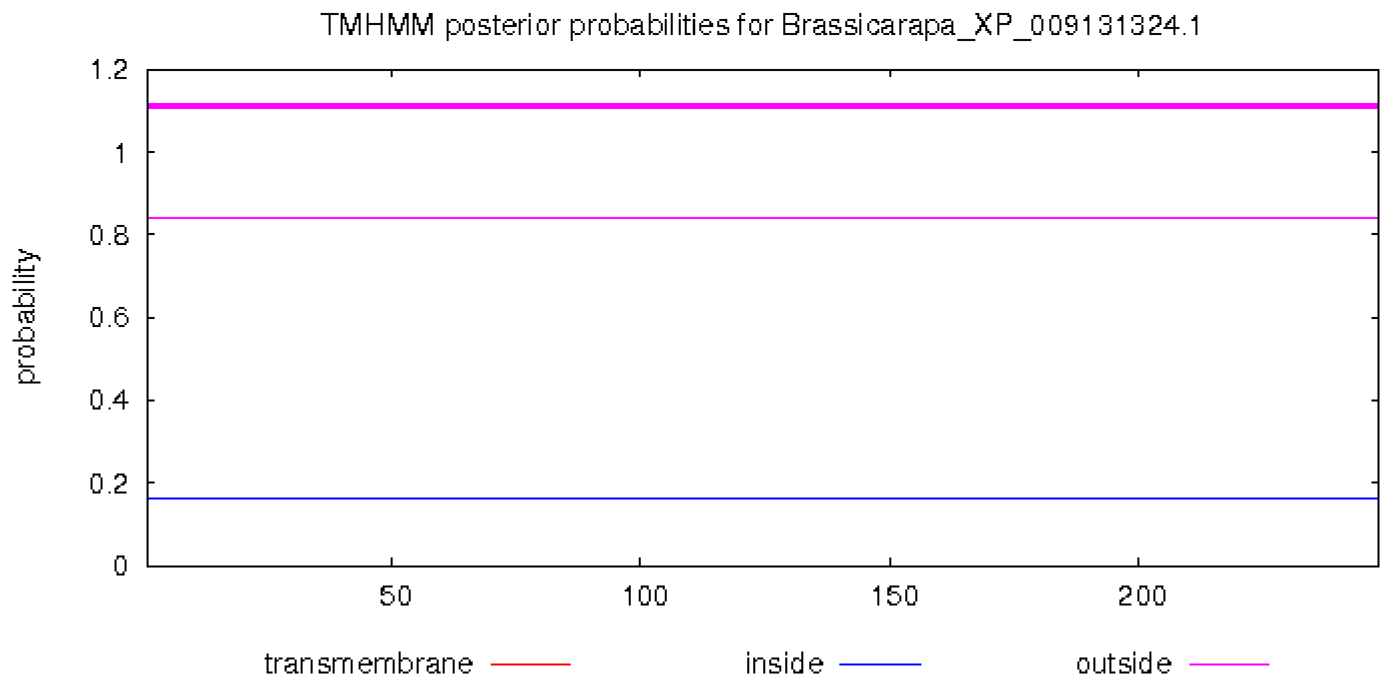

plot in postscript, script for making the plot in gnuplot, data for plot

```
# Brassicarapa_XP_009131374.1 Length: 208
# Brassicarapa_XP_009131374.1 Number of predicted TMHs:  0
# Brassicarapa_XP_009131374.1 Exp number of AAs in TMHs: 0.55888
# Brassicarapa_XP_009131374.1 Exp number, first 60 AAs:  0.521
# Brassicarapa_XP_009131374.1 Total prob of N-in:        0.11786
Brassicarapa_XP_009131374.1     TMHMM2.0        outside      1   208
```

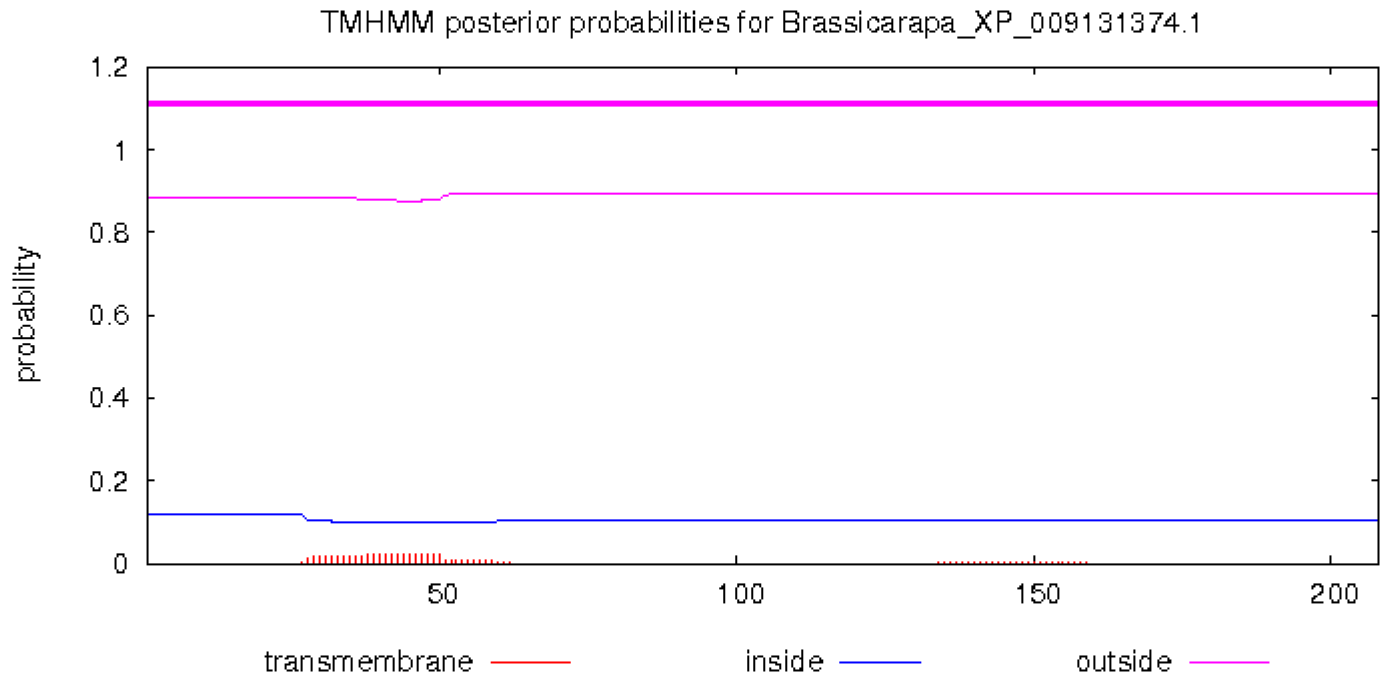

plot in postscript, script for making the plot in gnuplot, data for plot

```
# Brassicarapa_XP_009131585.1 Length: 490
# Brassicarapa_XP_009131585.1 Number of predicted TMHs:  0
# Brassicarapa_XP_009131585.1 Exp number of AAs in TMHs: 0
# Brassicarapa_XP_009131585.1 Exp number, first 60 AAs:  0
# Brassicarapa_XP_009131585.1 Total prob of N-in:        0.00017
Brassicarapa_XP_009131585.1     TMHMM2.0        outside      1   490
```

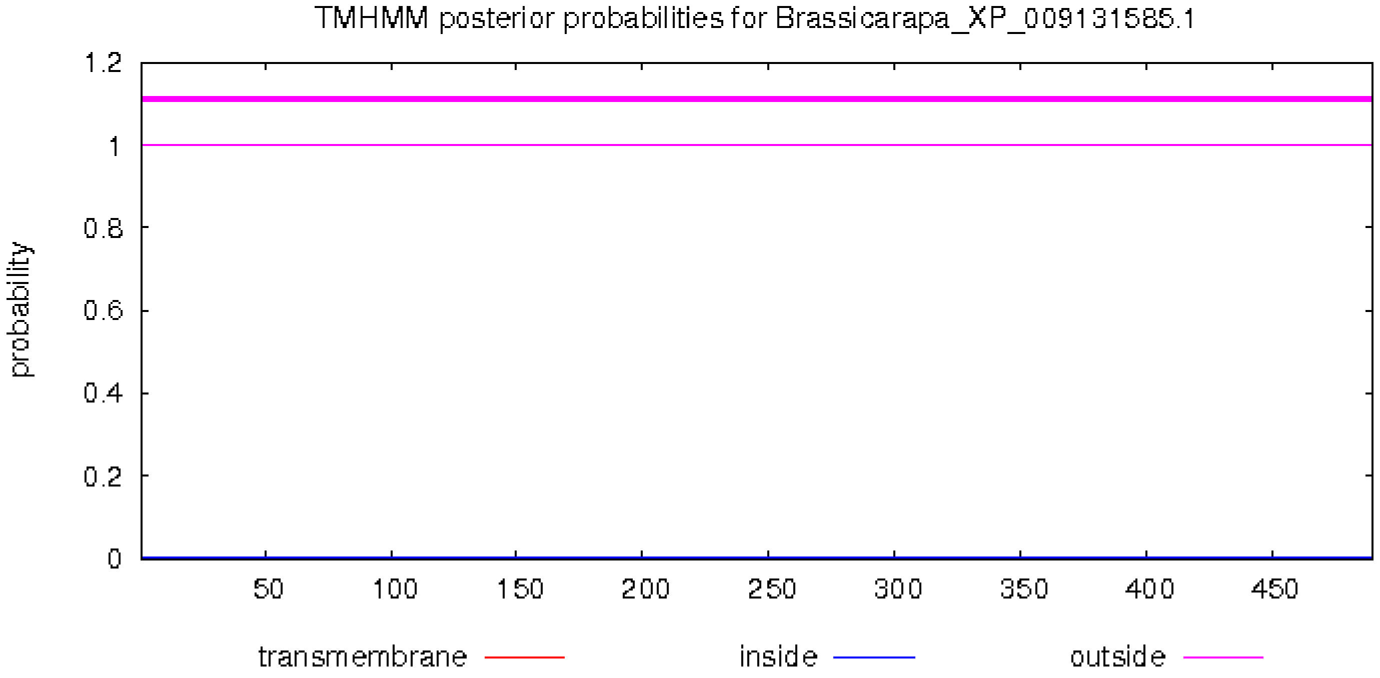

plot in postscript, script for making the plot in gnuplot, data for plot

---

```
# Brassicarapa_XP_009131648.1 Length: 336
# Brassicarapa_XP_009131648.1 Number of predicted TMHs:  0
# Brassicarapa_XP_009131648.1 Exp number of AAs in TMHs: 0.00059
# Brassicarapa_XP_009131648.1 Exp number, first 60 AAs:  0
# Brassicarapa_XP_009131648.1 Total prob of N-in:        0.00902
Brassicarapa_XP_009131648.1     TMHMM2.0        outside      1   336
```

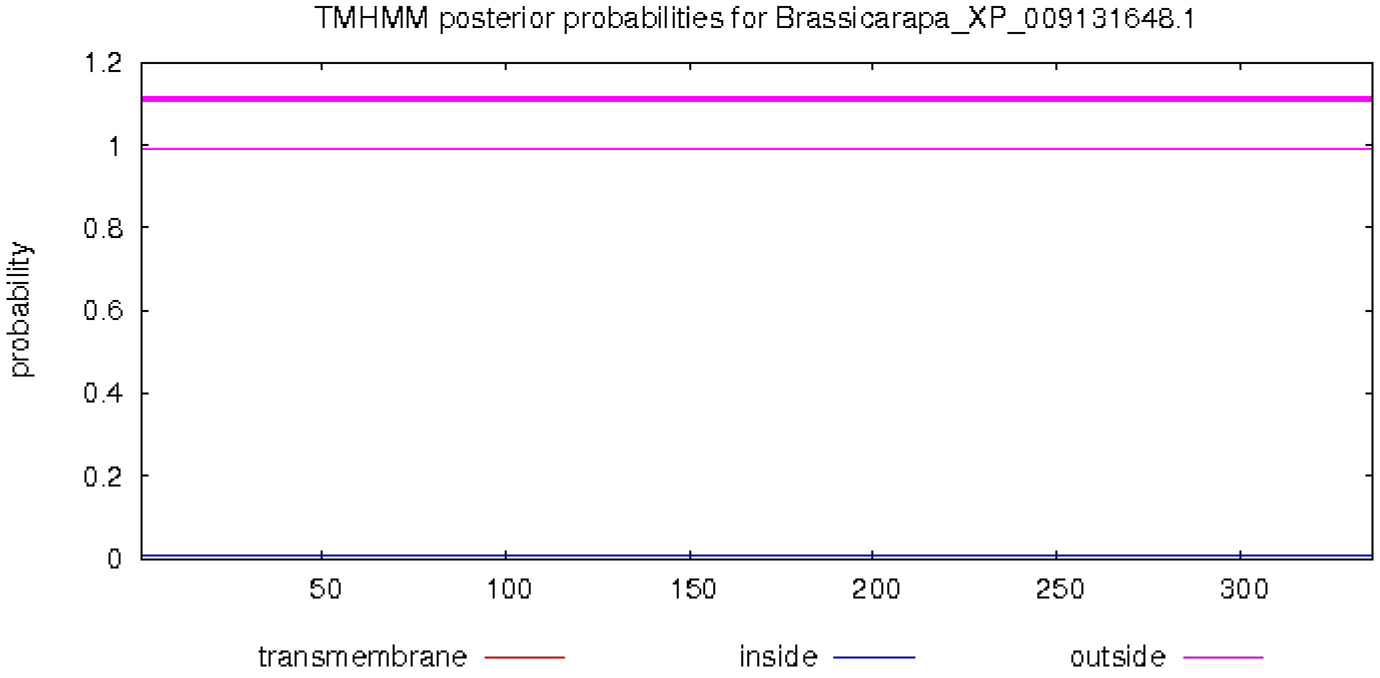

plot in postscript, script for making the plot in gnuplot, data for plot

---

```
# Brassicarapa_XP_009131870.1 Length: 392
# Brassicarapa_XP_009131870.1 Number of predicted TMHs:  0
# Brassicarapa_XP_009131870.1 Exp number of AAs in TMHs: 13.31753
# Brassicarapa_XP_009131870.1 Exp number, first 60 AAs:  0.05523
# Brassicarapa_XP_009131870.1 Total prob of N-in:        0.09415
Brassicarapa_XP_009131870.1     TMHMM2.0        outside      1   392
```

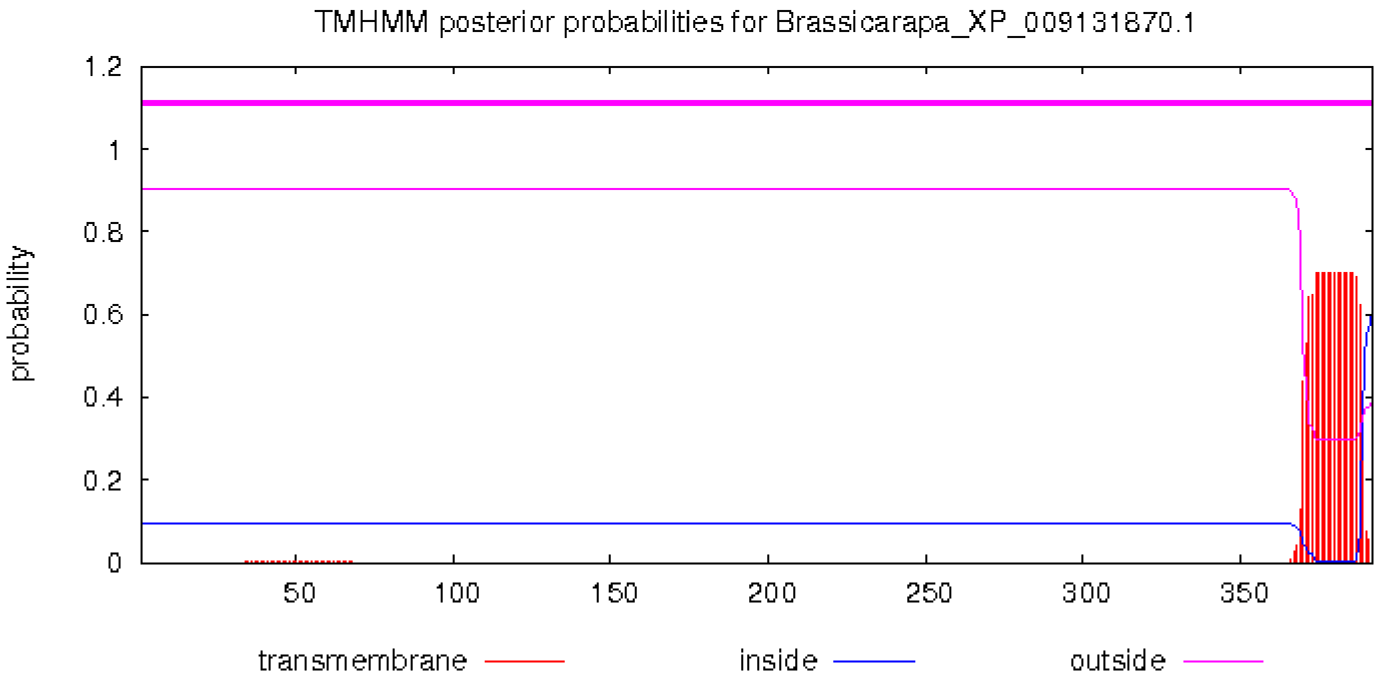

plot in postscript, script for making the plot in gnuplot, data for plot

---

```
# Brassicarapa_XP_009131877.1 Length: 235
# Brassicarapa_XP_009131877.1 Number of predicted TMHs:  0
# Brassicarapa_XP_009131877.1 Exp number of AAs in TMHs: 0.00026
# Brassicarapa_XP_009131877.1 Exp number, first 60 AAs:  0
# Brassicarapa_XP_009131877.1 Total prob of N-in:        0.03726
Brassicarapa_XP_009131877.1     TMHMM2.0        outside      1   235
```

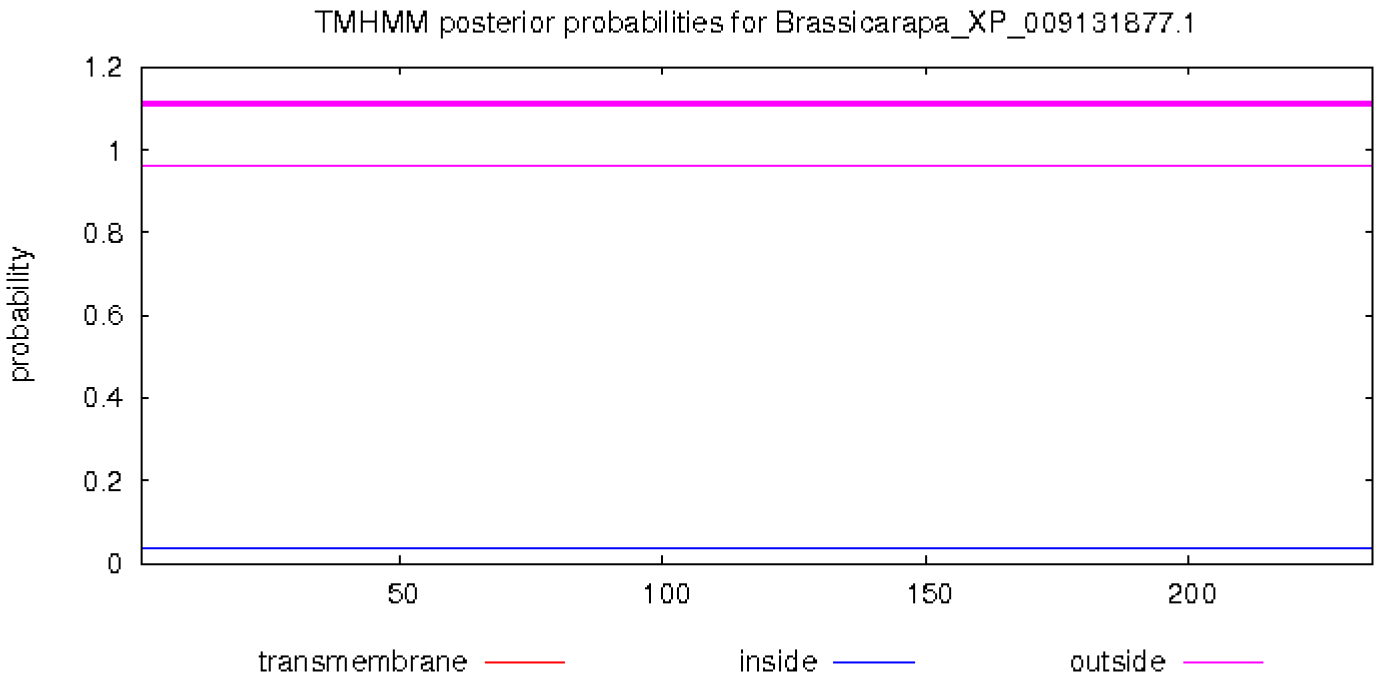

plot in postscript, script for making the plot in gnuplot, data for plot

---

```
# Brassicarapa_XP_009132241.1 Length: 295
# Brassicarapa_XP_009132241.1 Number of predicted TMHs:  0
# Brassicarapa_XP_009132241.1 Exp number of AAs in TMHs: 0.00037
# Brassicarapa_XP_009132241.1 Exp number, first 60 AAs:  0.00037
# Brassicarapa_XP_009132241.1 Total prob of N-in:        0.01098
Brassicarapa_XP_009132241.1	TMHMM2.0	outside	     1   295
```

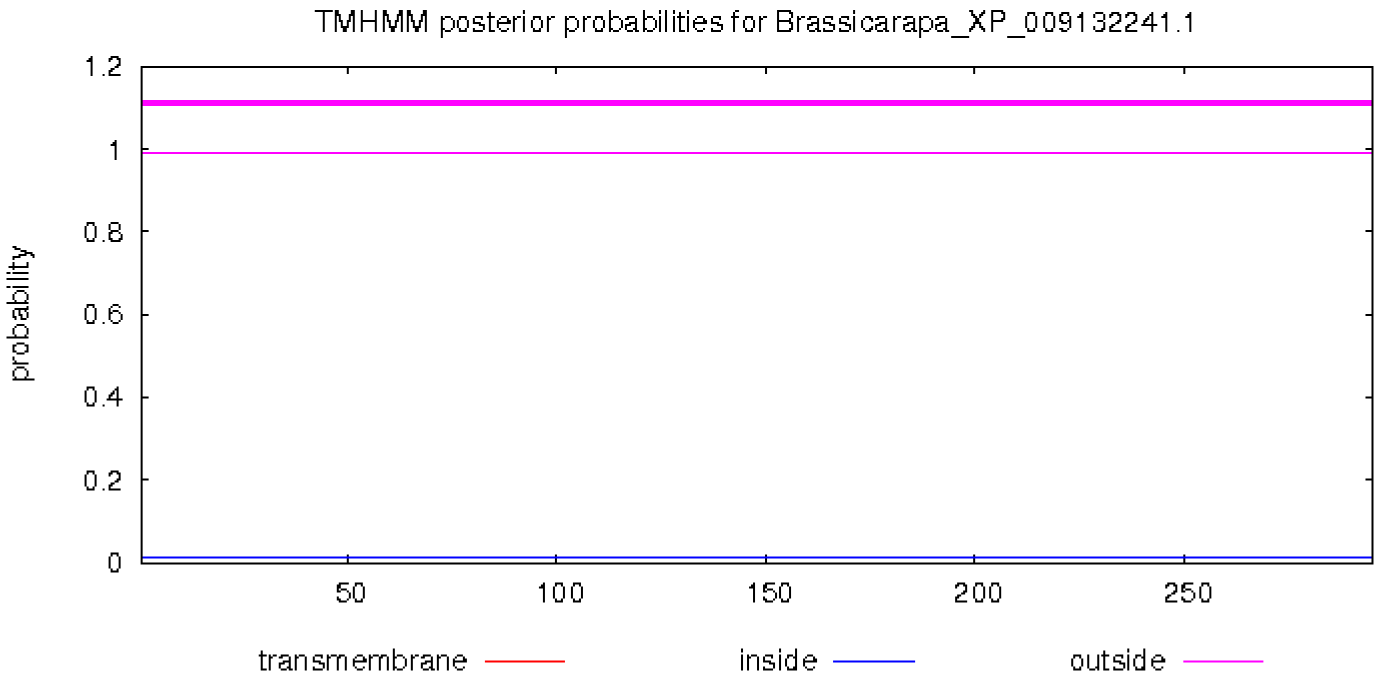

plot in postscript, script for making the plot in gnuplot, data for plot

---

```
# Brassicarapa_XP_009133561.1 Length: 272
# Brassicarapa_XP_009133561.1 Number of predicted TMHs:  0
# Brassicarapa_XP_009133561.1 Exp number of AAs in TMHs: 0.00867
# Brassicarapa_XP_009133561.1 Exp number, first 60 AAs:  0
# Brassicarapa_XP_009133561.1 Total prob of N-in:        0.05785
Brassicarapa_XP_009133561.1	TMHMM2.0	outside	     1   272
```

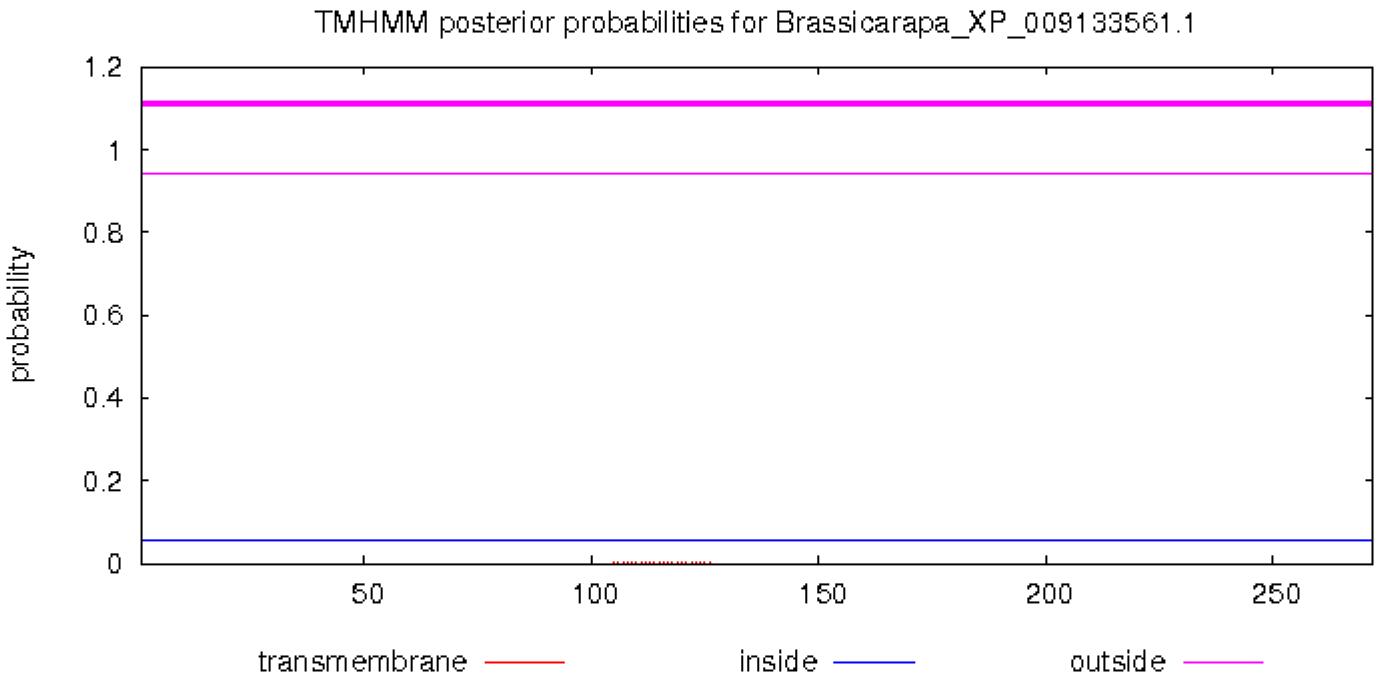

plot in postscript, script for making the plot in gnuplot, data for plot

---

```
# Brassicarapa_XP_009134191.1 Length: 341
# Brassicarapa_XP_009134191.1 Number of predicted TMHs:  0
# Brassicarapa_XP_009134191.1 Exp number of AAs in TMHs: 0
# Brassicarapa_XP_009134191.1 Exp number, first 60 AAs:  0
# Brassicarapa_XP_009134191.1 Total prob of N-in:        0.00436
Brassicarapa_XP_009134191.1     TMHMM2.0        outside      1   341
```

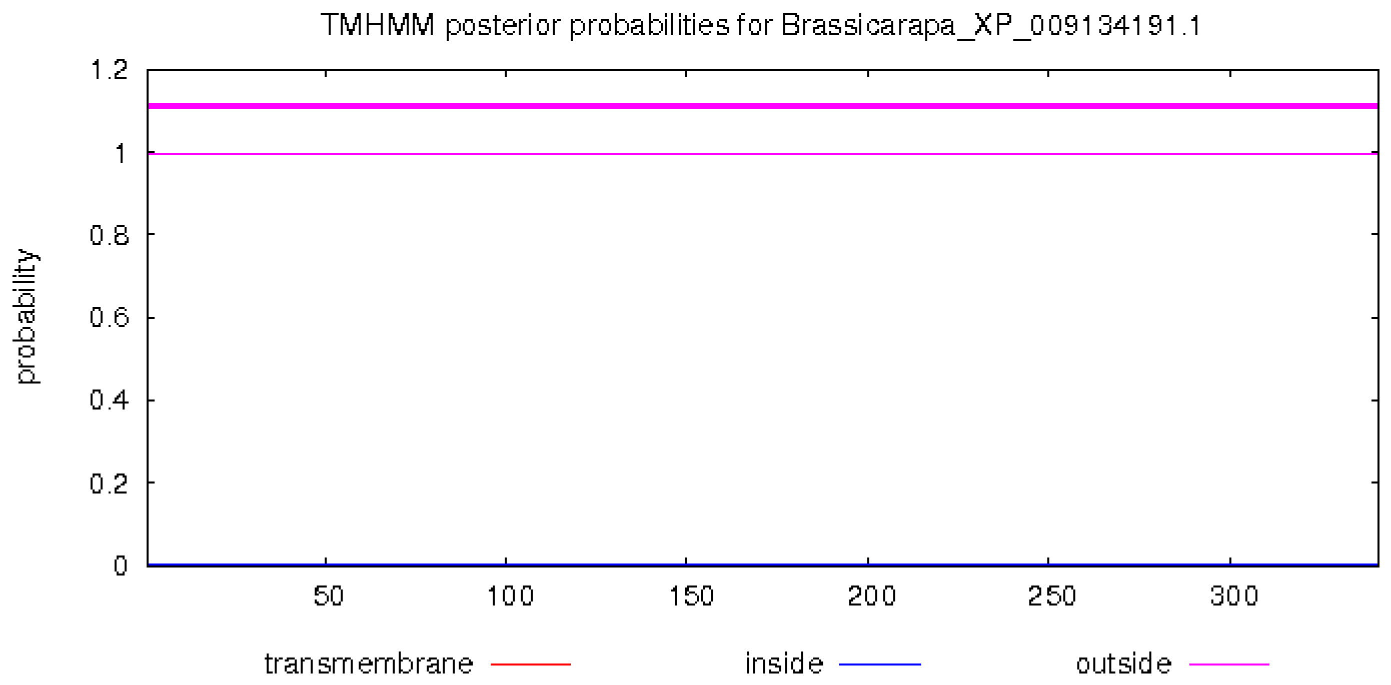

plot in postscript, script for making the plot in gnuplot, data for plot

```
# Brassicarapa_XP_009134601.1 Length: 358
# Brassicarapa_XP_009134601.1 Number of predicted TMHs:  0
# Brassicarapa_XP_009134601.1 Exp number of AAs in TMHs: 0.00015
# Brassicarapa_XP_009134601.1 Exp number, first 60 AAs:  0.00015
# Brassicarapa_XP_009134601.1 Total prob of N-in:        0.01076
Brassicarapa_XP_009134601.1     TMHMM2.0        outside      1   358
```

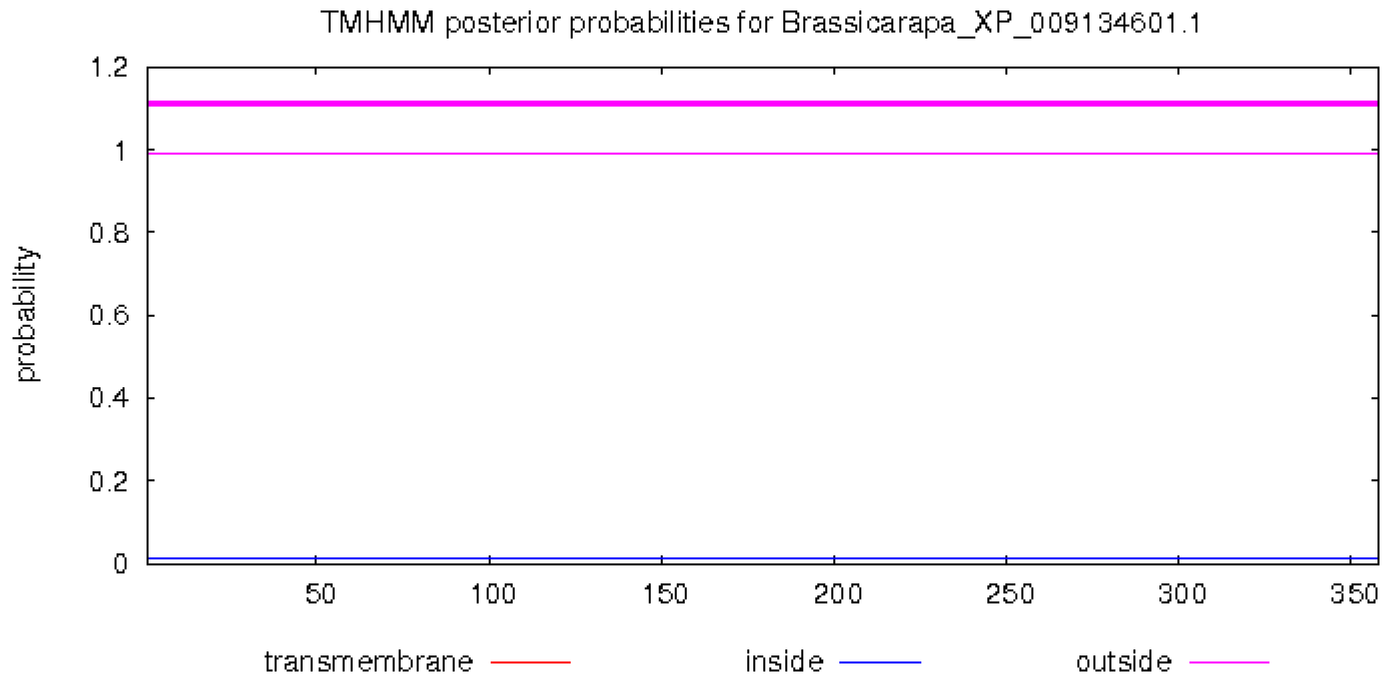

plot in postscript, script for making the plot in gnuplot, data for plot

```
# Brassicarapa_XP_009135111.1 Length: 431
# Brassicarapa_XP_009135111.1 Number of predicted TMHs:  0
# Brassicarapa_XP_009135111.1 Exp number of AAs in TMHs: 0.24788
# Brassicarapa_XP_009135111.1 Exp number, first 60 AAs:  0.22738
# Brassicarapa_XP_009135111.1 Total prob of N-in:        0.01543
Brassicarapa_XP_009135111.1	TMHMM2.0	outside	     1   431
```

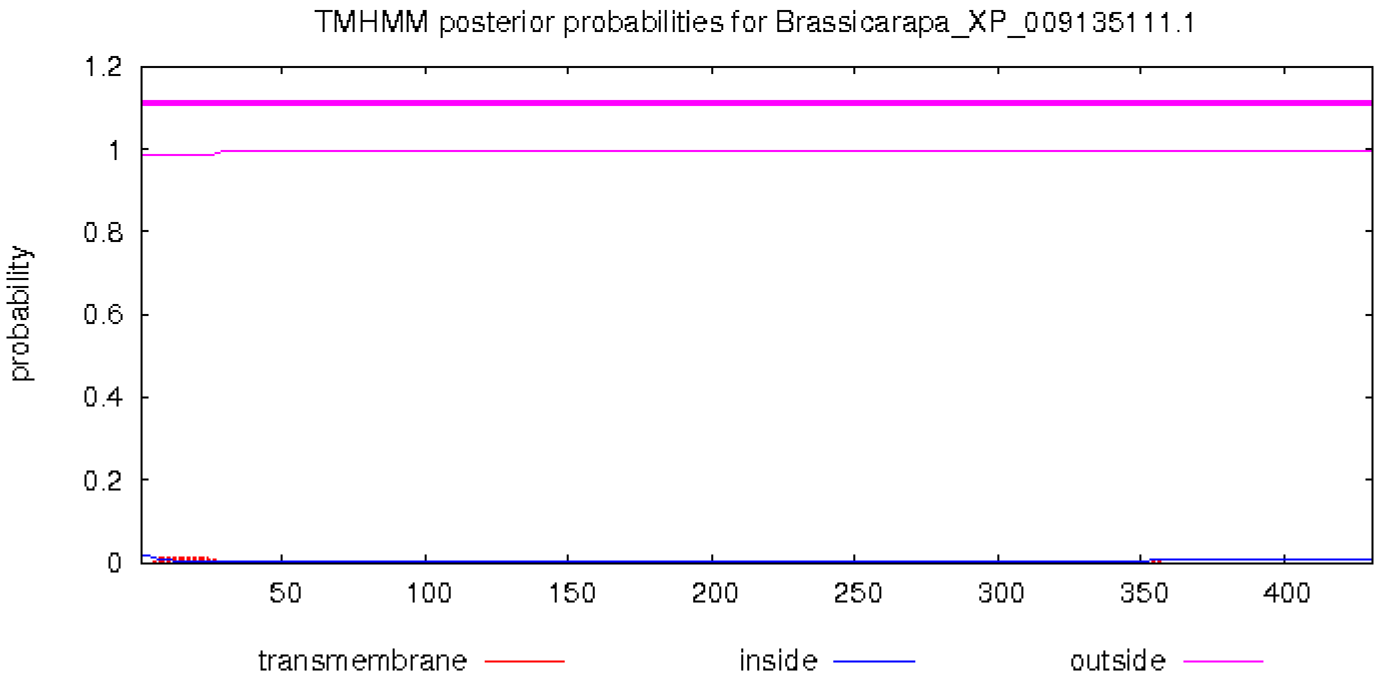

plot in postscript, script for making the plot in gnuplot, data for plot

---

```
# Brassicarapa_XP_009135112.1 Length: 421
# Brassicarapa_XP_009135112.1 Number of predicted TMHs:  0
# Brassicarapa_XP_009135112.1 Exp number of AAs in TMHs: 0.07999
# Brassicarapa_XP_009135112.1 Exp number, first 60 AAs:  0.04885
# Brassicarapa_XP_009135112.1 Total prob of N-in:        0.00929
Brassicarapa_XP_009135112.1	TMHMM2.0	outside	     1   421
```

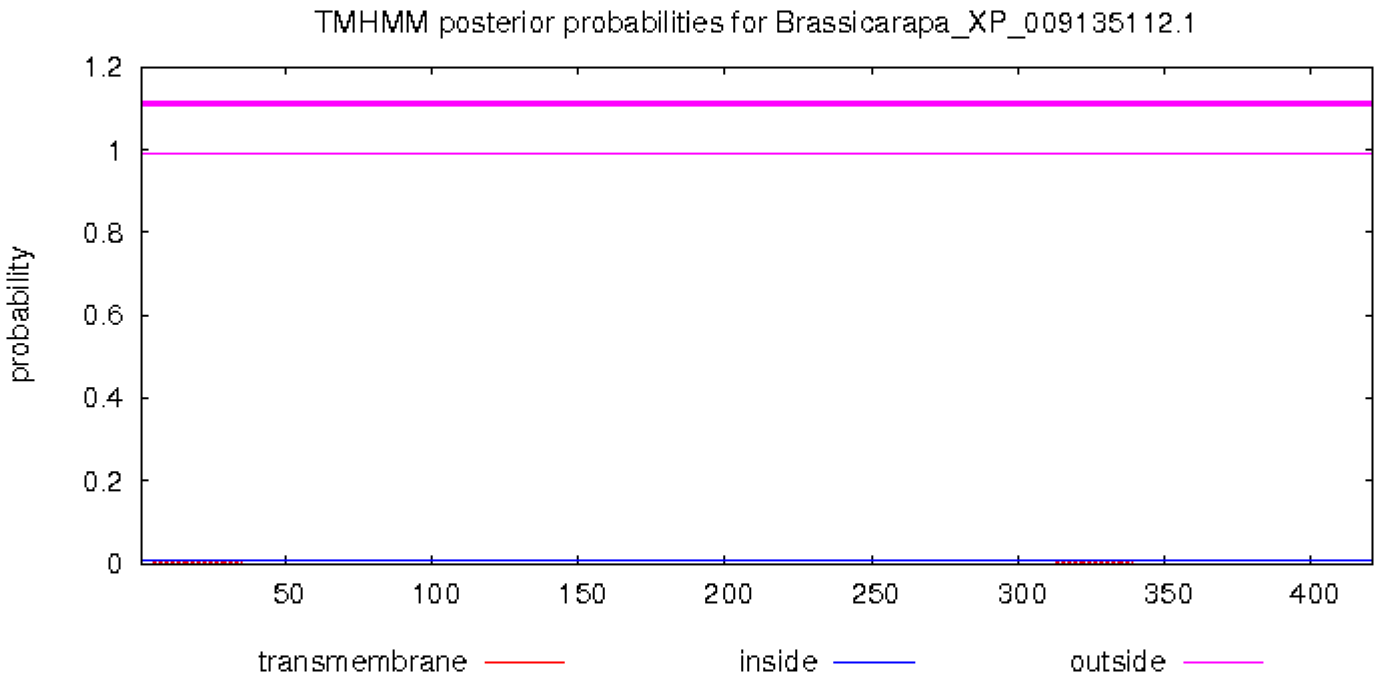

plot in postscript, script for making the plot in gnuplot, data for plot

---

```
# Brassicarapa_XP_009135113.1 Length: 421
# Brassicarapa_XP_009135113.1 Number of predicted TMHs:  0
# Brassicarapa_XP_009135113.1 Exp number of AAs in TMHs: 0.07999
# Brassicarapa_XP_009135113.1 Exp number, first 60 AAs:  0.04885
# Brassicarapa_XP_009135113.1 Total prob of N-in:        0.00929
Brassicarapa_XP_009135113.1     TMHMM2.0        outside      1   421
```

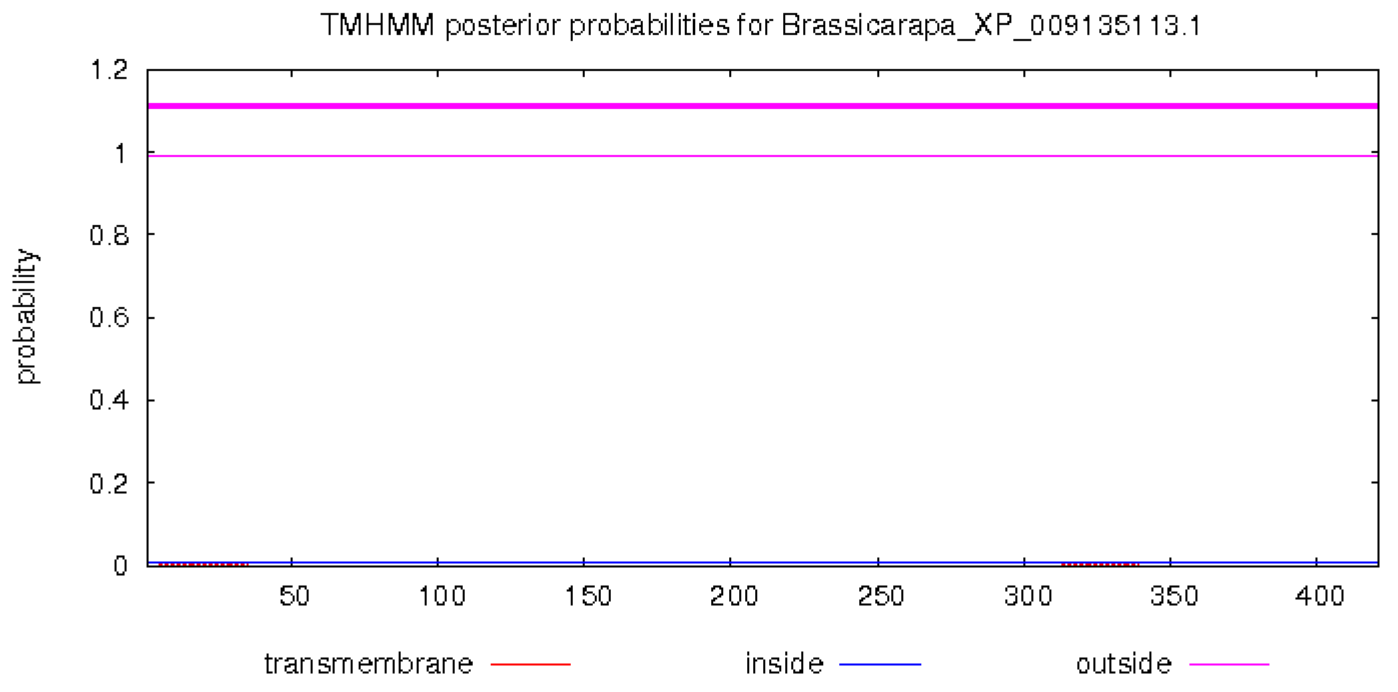

plot in postscript, script for making the plot in gnuplot, data for plot

---

```
# Brassicarapa_XP_009135261.1 Length: 303
# Brassicarapa_XP_009135261.1 Number of predicted TMHs:  0
# Brassicarapa_XP_009135261.1 Exp number of AAs in TMHs: 0.00121
# Brassicarapa_XP_009135261.1 Exp number, first 60 AAs:  0
# Brassicarapa_XP_009135261.1 Total prob of N-in:        0.07777
Brassicarapa_XP_009135261.1     TMHMM2.0        outside      1   303
```

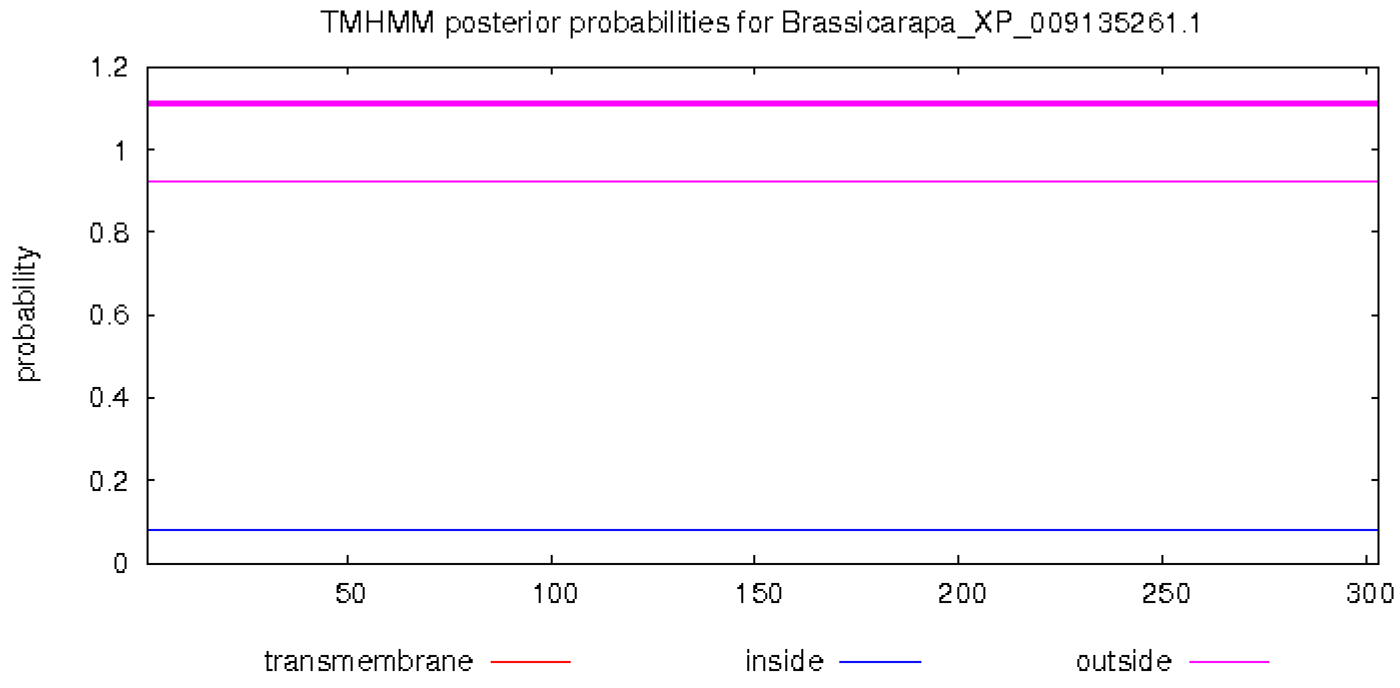

plot in postscript, script for making the plot in gnuplot, data for plot

---

```
# Brassicarapa_XP_009135268.1 Length: 293
# Brassicarapa_XP_009135268.1 Number of predicted TMHs:  0
# Brassicarapa_XP_009135268.1 Exp number of AAs in TMHs: 0.00017
# Brassicarapa_XP_009135268.1 Exp number, first 60 AAs:  0
# Brassicarapa_XP_009135268.1 Total prob of N-in:        0.06800
Brassicarapa_XP_009135268.1     TMHMM2.0        outside      1   293
```

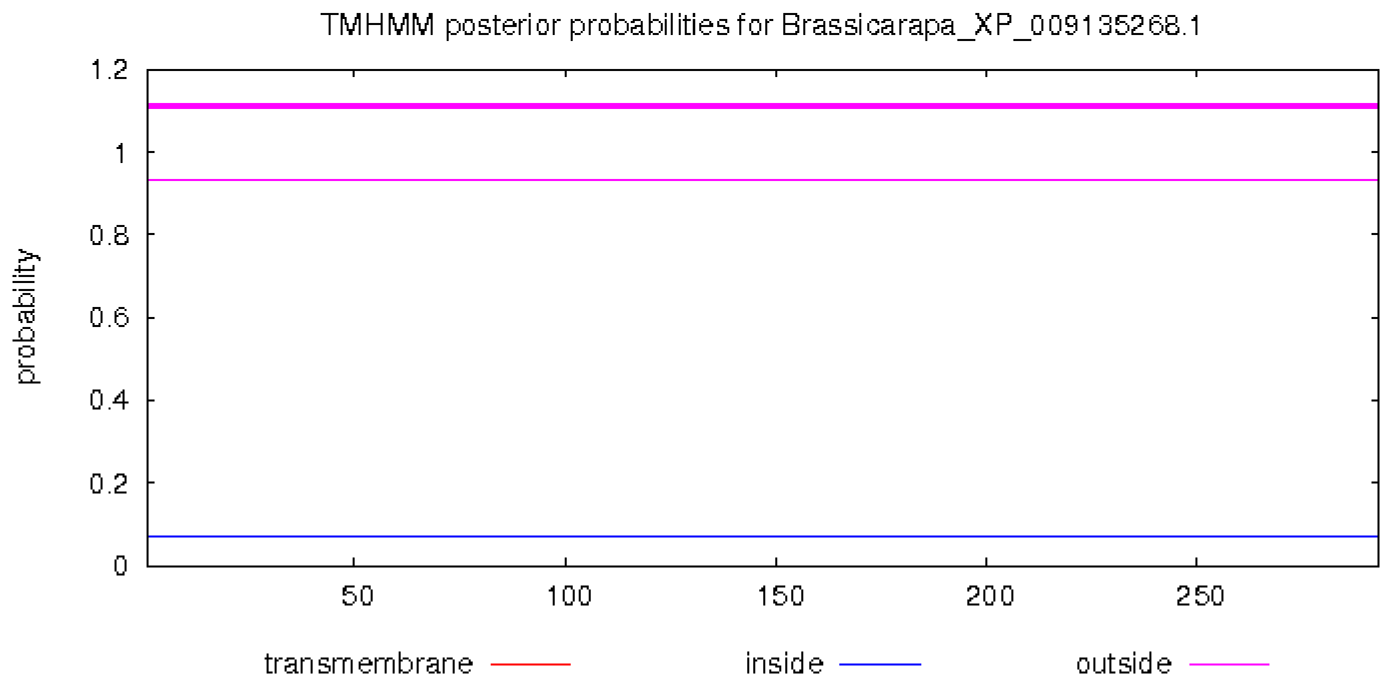

plot in postscript, script for making the plot in gnuplot, data for plot

```
# Brassicarapa_XP_009135429.1 Length: 309
# Brassicarapa_XP_009135429.1 Number of predicted TMHs:  0
# Brassicarapa_XP_009135429.1 Exp number of AAs in TMHs: 0.01293
# Brassicarapa_XP_009135429.1 Exp number, first 60 AAs:  0.00023
# Brassicarapa_XP_009135429.1 Total prob of N-in:        0.01416
Brassicarapa_XP_009135429.1     TMHMM2.0        outside      1   309
```

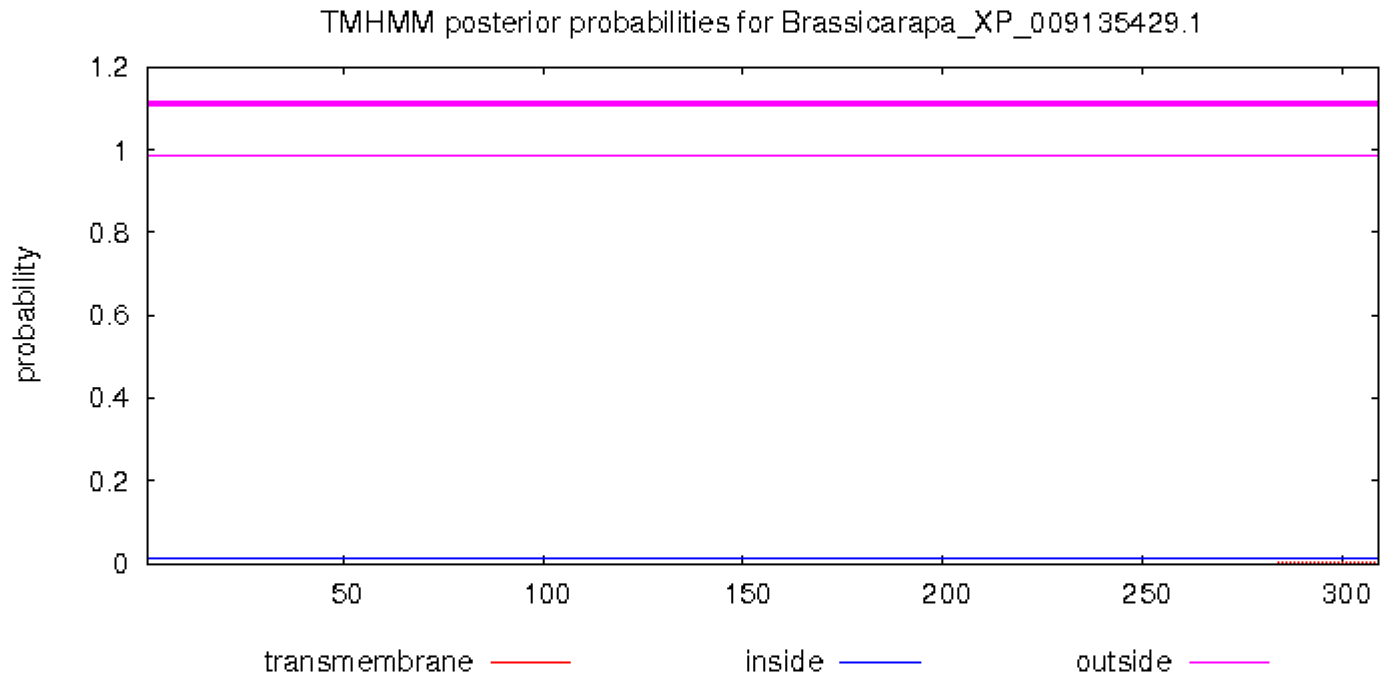

plot in postscript, script for making the plot in gnuplot, data for plot

```
# Brassicarapa_XP_009135445.1 Length: 295
# Brassicarapa_XP_009135445.1 Number of predicted TMHs:  0
# Brassicarapa_XP_009135445.1 Exp number of AAs in TMHs: 0.00017
# Brassicarapa_XP_009135445.1 Exp number, first 60 AAs:  0.00017
# Brassicarapa_XP_009135445.1 Total prob of N-in:        0.00668
Brassicarapa_XP_009135445.1	TMHMM2.0	outside	     1   295
```

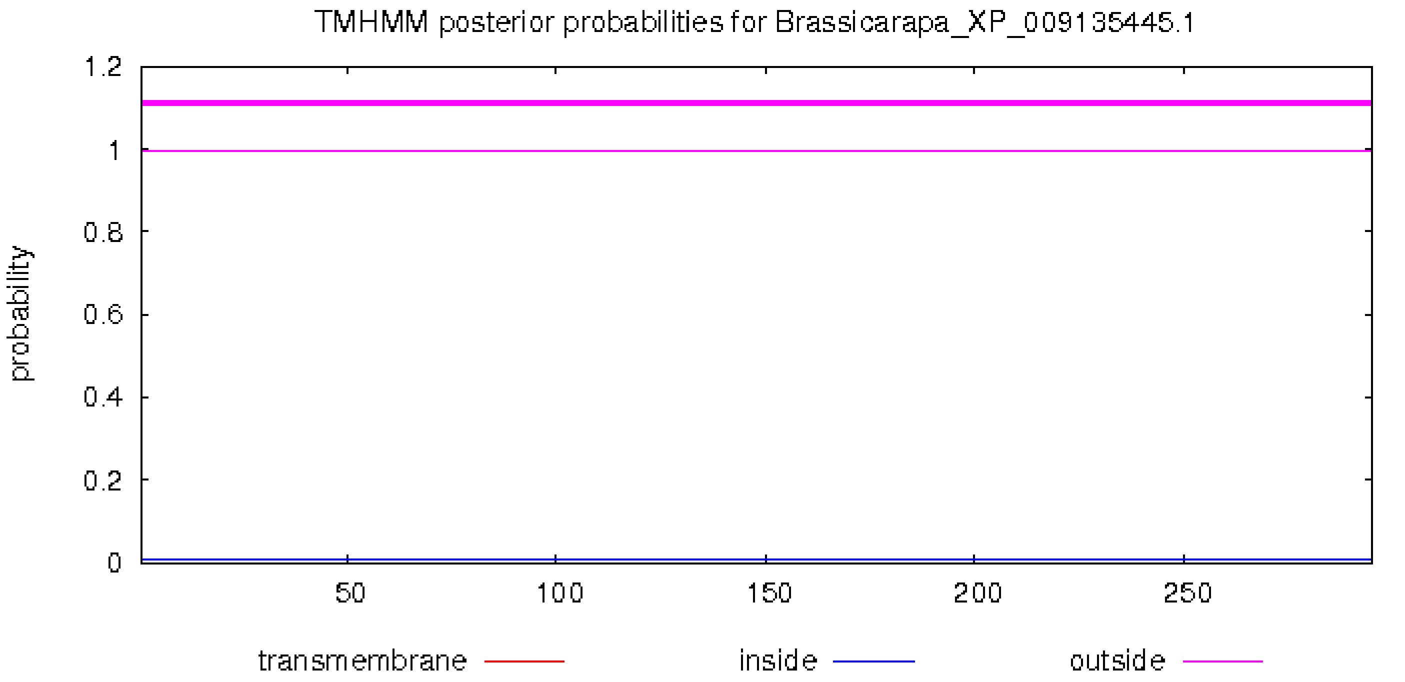

plot in postscript, script for making the plot in gnuplot, data for plot

---

```
# Brassicarapa_XP_009135446.1 Length: 350
# Brassicarapa_XP_009135446.1 Number of predicted TMHs:  0
# Brassicarapa_XP_009135446.1 Exp number of AAs in TMHs: 0.00055
# Brassicarapa_XP_009135446.1 Exp number, first 60 AAs:  0.00027
# Brassicarapa_XP_009135446.1 Total prob of N-in:        0.00479
Brassicarapa_XP_009135446.1	TMHMM2.0	outside	     1   350
```

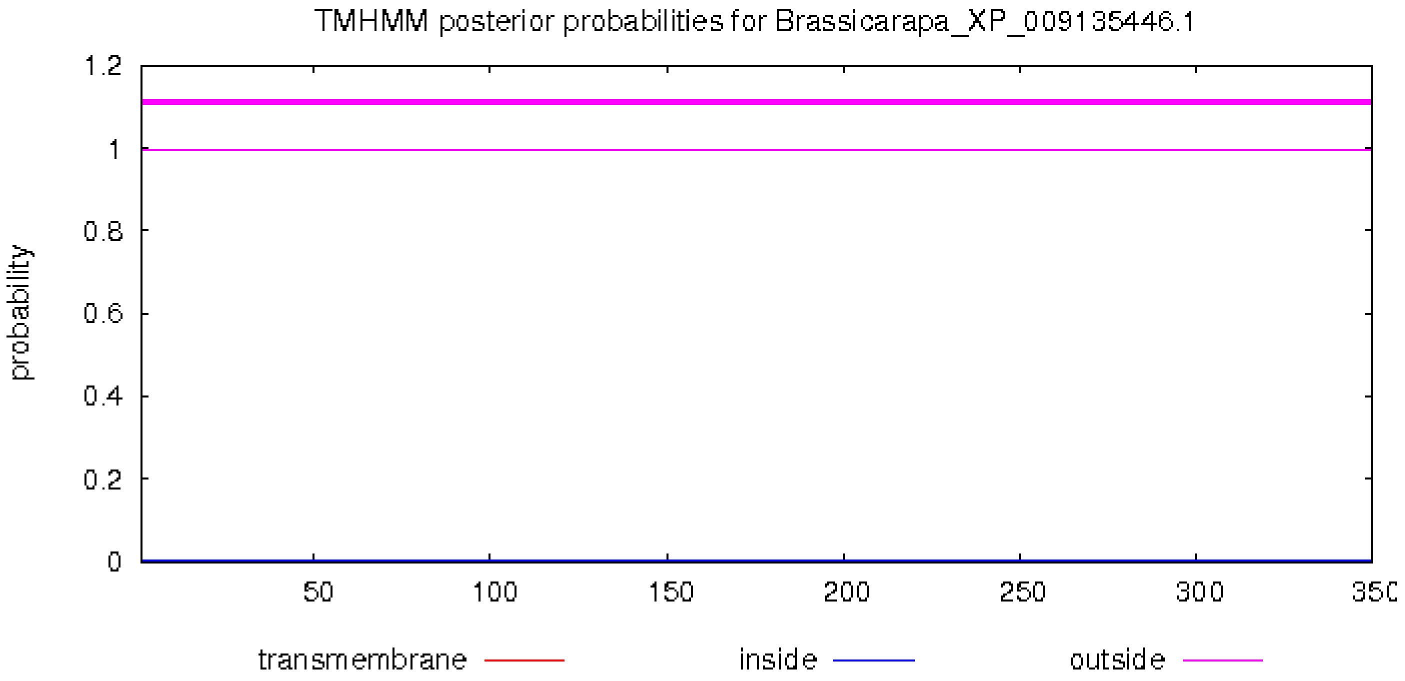

plot in postscript, script for making the plot in gnuplot, data for plot

---

```
# Brassicarapa_XP_009135577.1 Length: 299
# Brassicarapa_XP_009135577.1 Number of predicted TMHs:  0
# Brassicarapa_XP_009135577.1 Exp number of AAs in TMHs: 0.00407
# Brassicarapa_XP_009135577.1 Exp number, first 60 AAs:  0
# Brassicarapa_XP_009135577.1 Total prob of N-in:        0.00484
Brassicarapa_XP_009135577.1     TMHMM2.0        outside      1   299
```

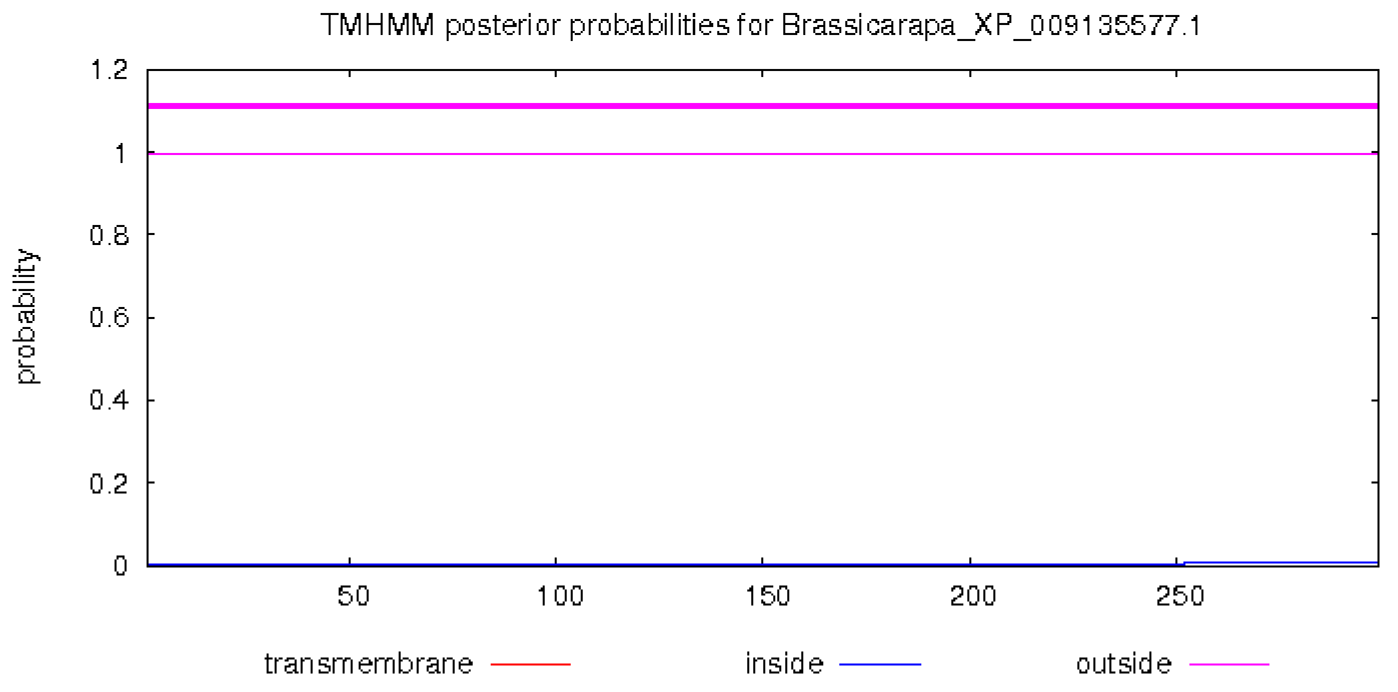

plot in postscript, script for making the plot in gnuplot, data for plot

---

```
# Brassicarapa_XP_009136365.1 Length: 281
# Brassicarapa_XP_009136365.1 Number of predicted TMHs:  0
# Brassicarapa_XP_009136365.1 Exp number of AAs in TMHs: 0.00594
# Brassicarapa_XP_009136365.1 Exp number, first 60 AAs:  0.00338
# Brassicarapa_XP_009136365.1 Total prob of N-in:        0.00557
Brassicarapa_XP_009136365.1     TMHMM2.0        outside      1   281
```

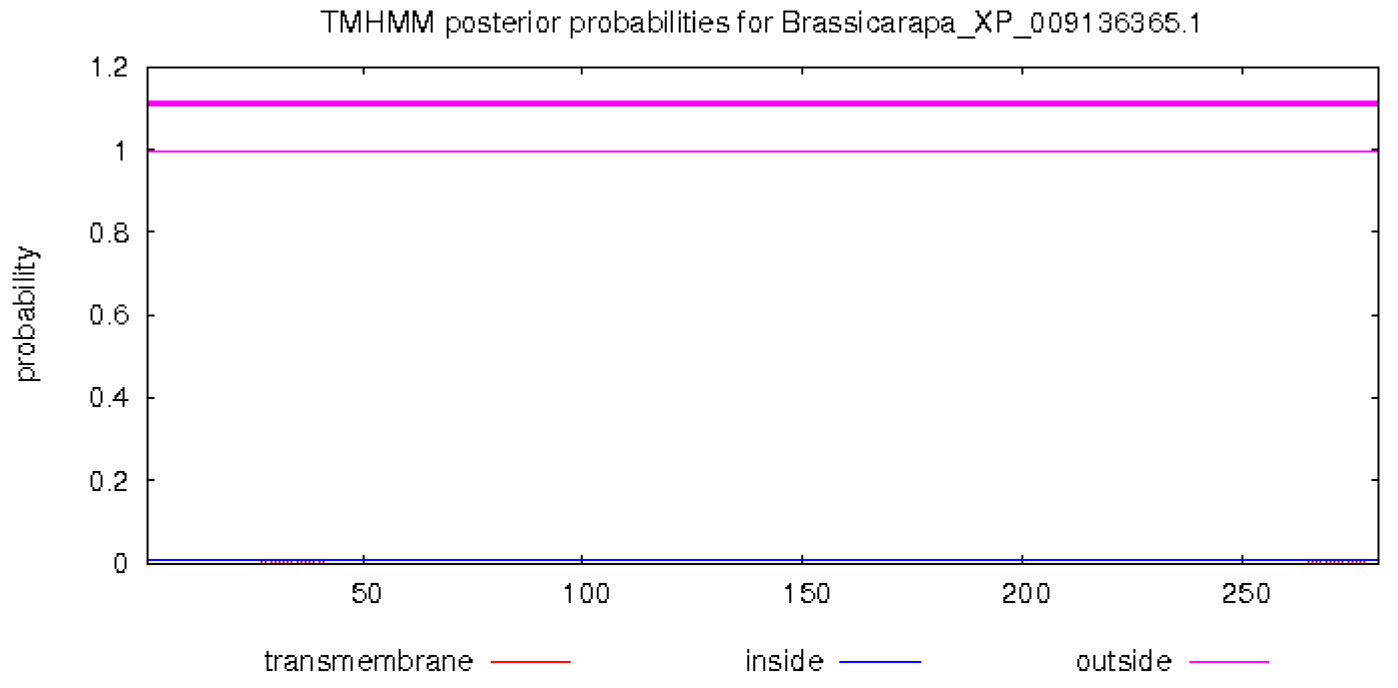

plot in postscript, script for making the plot in gnuplot, data for plot

---

```
# Brassicarapa_XP_009136471.1 Length: 327
# Brassicarapa_XP_009136471.1 Number of predicted TMHs:  0
# Brassicarapa_XP_009136471.1 Exp number of AAs in TMHs: 0.0001
# Brassicarapa_XP_009136471.1 Exp number, first 60 AAs:  0
# Brassicarapa_XP_009136471.1 Total prob of N-in:        0.01382
Brassicarapa_XP_009136471.1     TMHMM2.0        outside      1   327
```

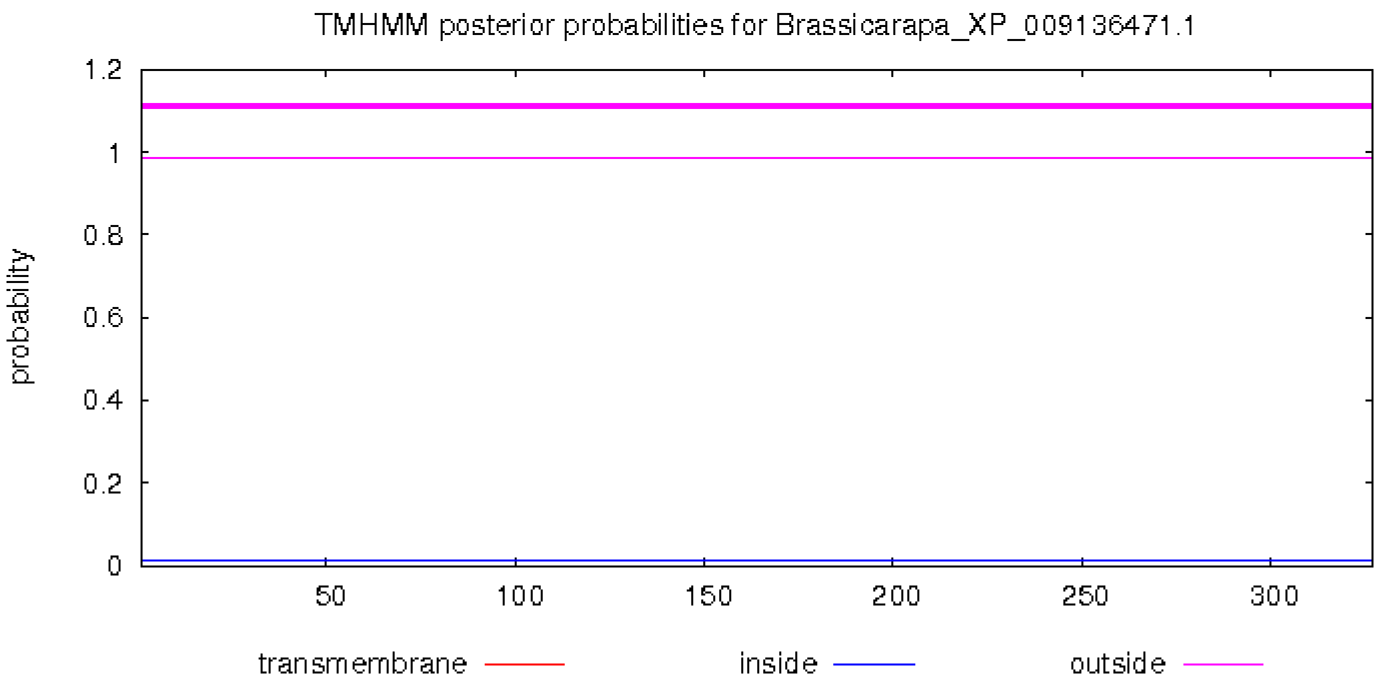

plot in postscript, script for making the plot in gnuplot, data for plot

---

```
# Brassicarapa_XP_009136888.1 Length: 286
# Brassicarapa_XP_009136888.1 Number of predicted TMHs:  0
# Brassicarapa_XP_009136888.1 Exp number of AAs in TMHs: 0.00019
# Brassicarapa_XP_009136888.1 Exp number, first 60 AAs:  0
# Brassicarapa_XP_009136888.1 Total prob of N-in:        0.00738
Brassicarapa_XP_009136888.1     TMHMM2.0        outside      1   286
```

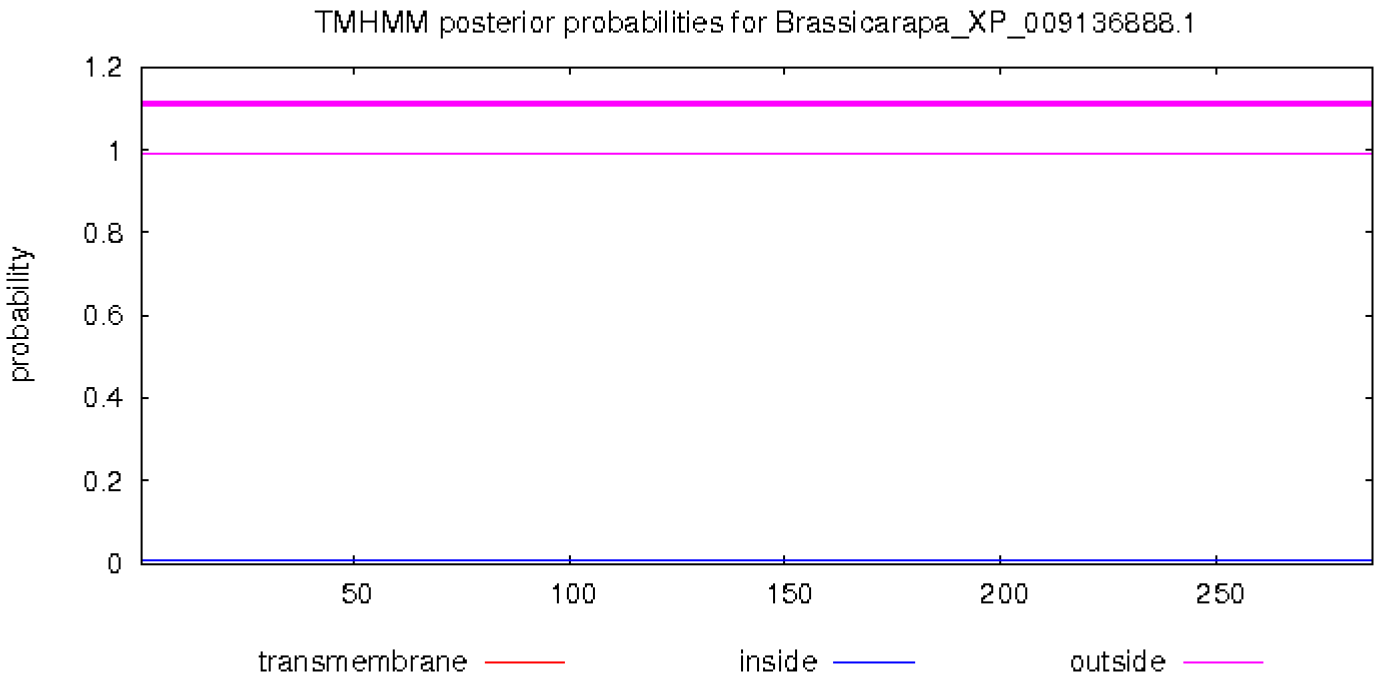

plot in postscript, script for making the plot in gnuplot, data for plot

---

```
# Brassicarapa_XP_009137656.1 Length: 300
# Brassicarapa_XP_009137656.1 Number of predicted TMHs:  0
# Brassicarapa_XP_009137656.1 Exp number of AAs in TMHs: 0.0024
# Brassicarapa_XP_009137656.1 Exp number, first 60 AAs:  0.00032
# Brassicarapa_XP_009137656.1 Total prob of N-in:        0.01333
Brassicarapa_XP_009137656.1     TMHMM2.0        outside      1   300
```

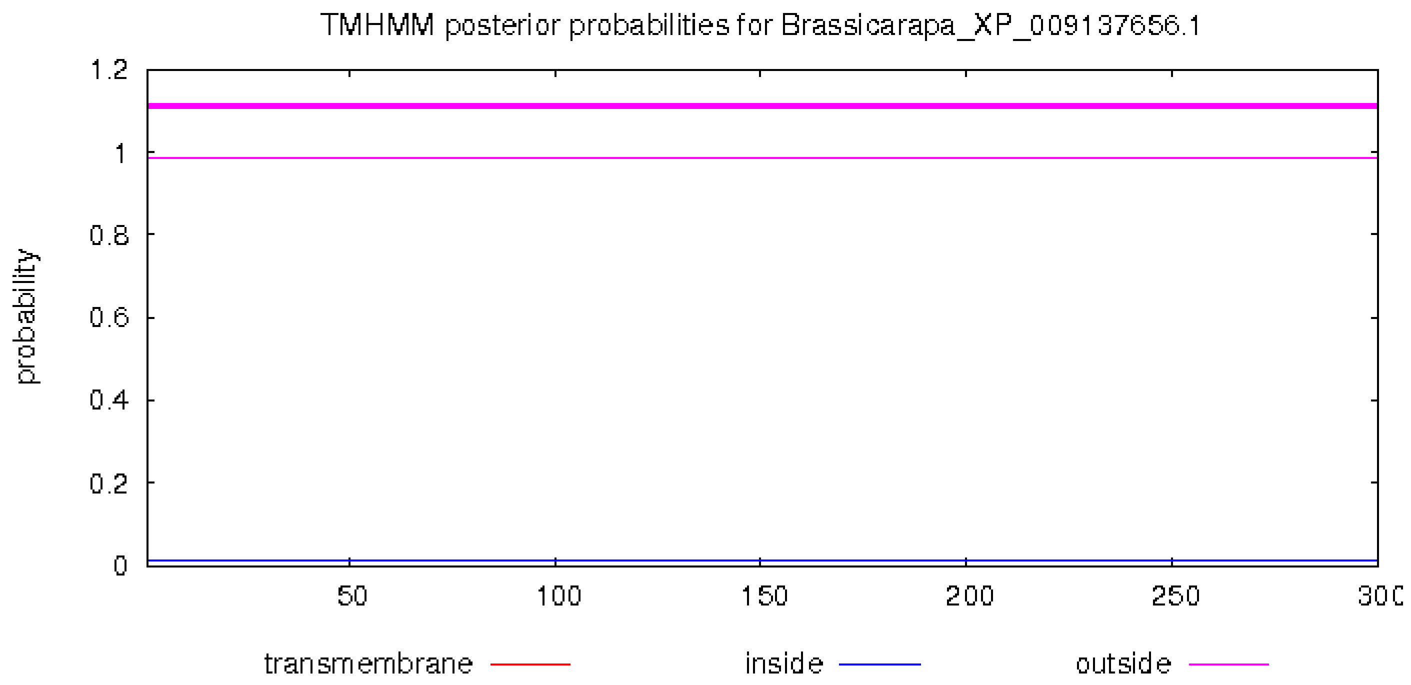

plot in postscript, script for making the plot in gnuplot, data for plot

---

```
# Brassicarapa_XP_009137790.1 Length: 303
# Brassicarapa_XP_009137790.1 Number of predicted TMHs:  0
# Brassicarapa_XP_009137790.1 Exp number of AAs in TMHs: 0.0005
# Brassicarapa_XP_009137790.1 Exp number, first 60 AAs:  0
# Brassicarapa_XP_009137790.1 Total prob of N-in:        0.05667
Brassicarapa_XP_009137790.1     TMHMM2.0        outside      1   303
```

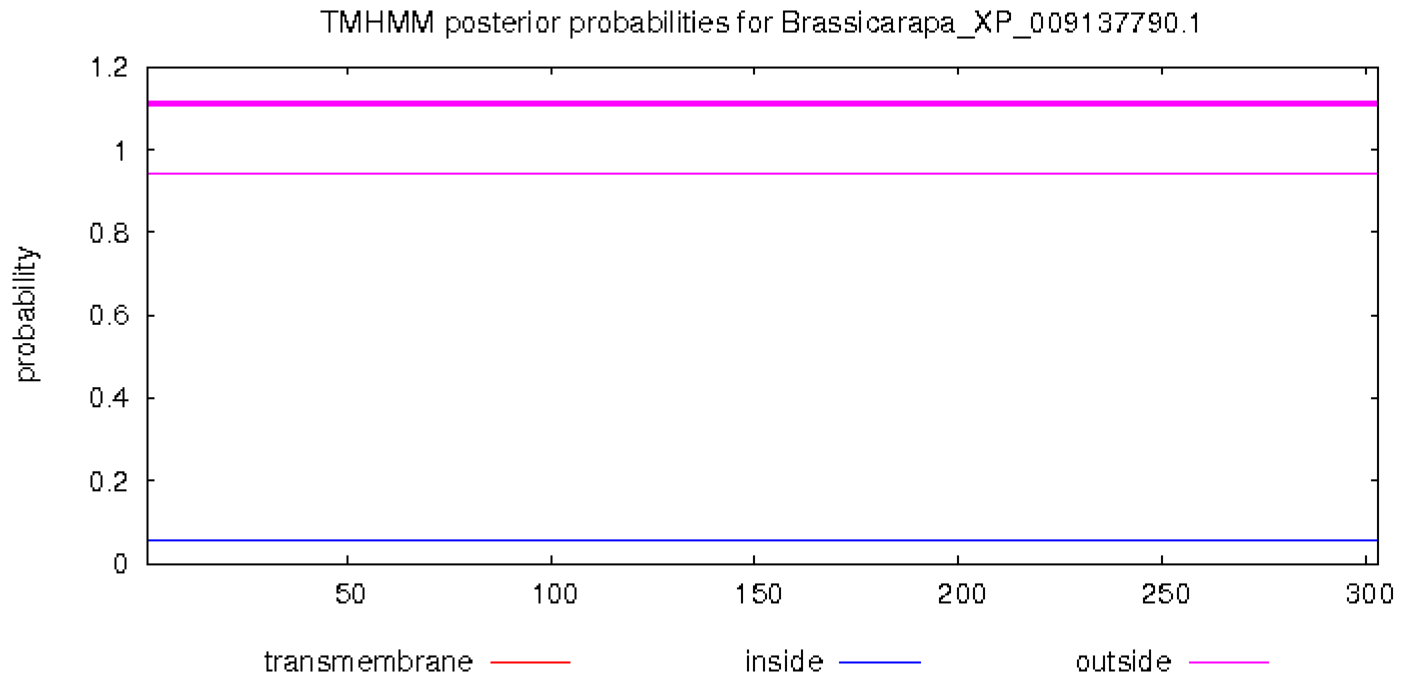

plot in postscript, script for making the plot in gnuplot, data for plot

---

```
# Brassicarapa_XP_009137795.1 Length: 351
# Brassicarapa_XP_009137795.1 Number of predicted TMHs:  0
# Brassicarapa_XP_009137795.1 Exp number of AAs in TMHs: 0
# Brassicarapa_XP_009137795.1 Exp number, first 60 AAs:  0
# Brassicarapa_XP_009137795.1 Total prob of N-in:        0.00581
Brassicarapa_XP_009137795.1     TMHMM2.0        outside      1   351
```

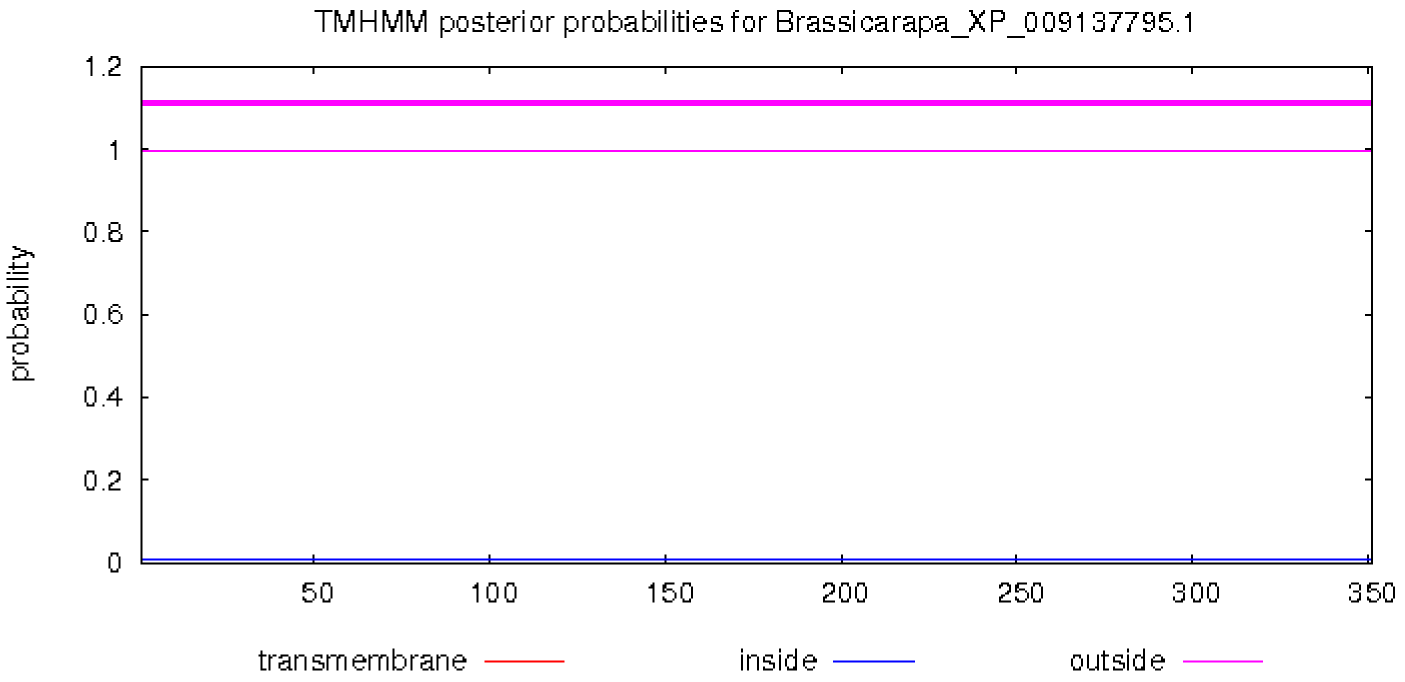

plot in postscript, script for making the plot in gnuplot, data for plot

```
# Brassicarapa_XP_009137840.1 Length: 496
# Brassicarapa_XP_009137840.1 Number of predicted TMHs:  0
# Brassicarapa_XP_009137840.1 Exp number of AAs in TMHs: 0.01433
# Brassicarapa_XP_009137840.1 Exp number, first 60 AAs:  0
# Brassicarapa_XP_009137840.1 Total prob of N-in:        0.00374
Brassicarapa_XP_009137840.1     TMHMM2.0        outside      1   496
```

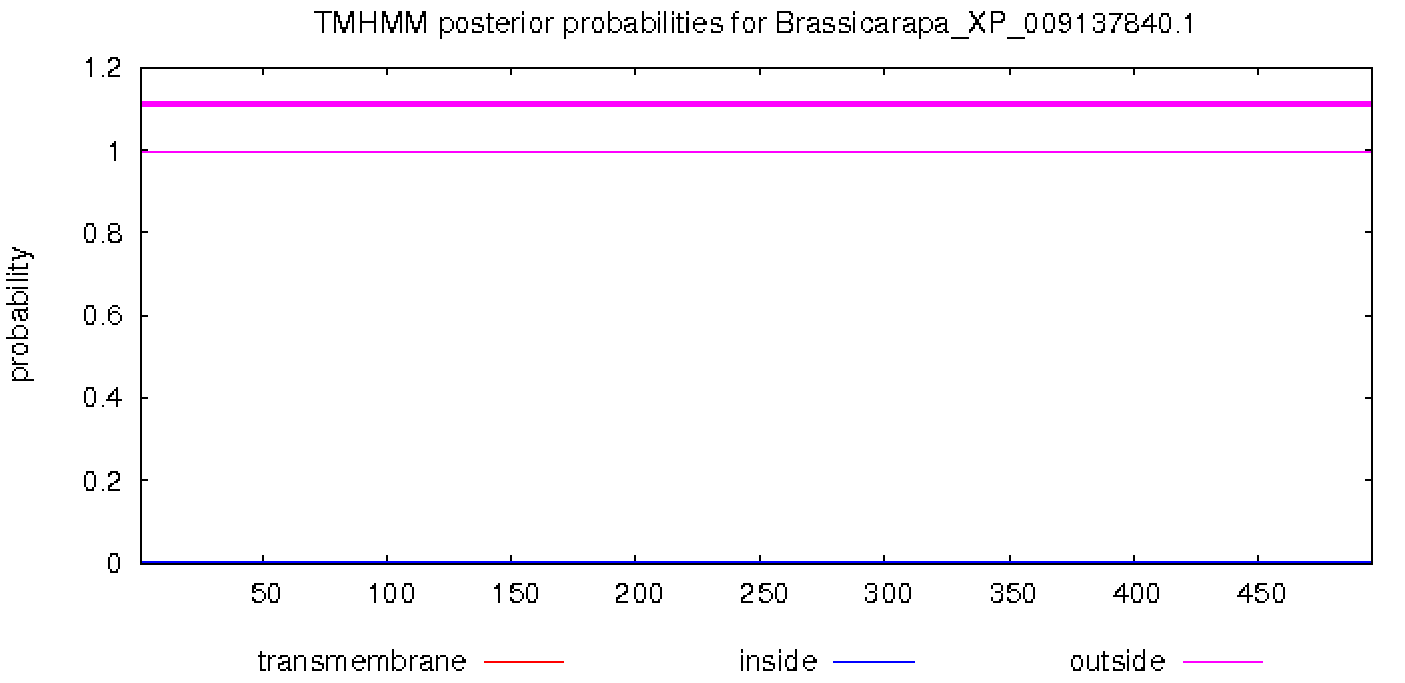

plot in postscript, script for making the plot in gnuplot, data for plot

```
# Brassicarapa_XP_009137841.1 Length: 495
# Brassicarapa_XP_009137841.1 Number of predicted TMHs:  0
# Brassicarapa_XP_009137841.1 Exp number of AAs in TMHs: 0.01451
# Brassicarapa_XP_009137841.1 Exp number, first 60 AAs:  0
# Brassicarapa_XP_009137841.1 Total prob of N-in:        0.00379
Brassicarapa_XP_009137841.1     TMHMM2.0        outside      1   495
```

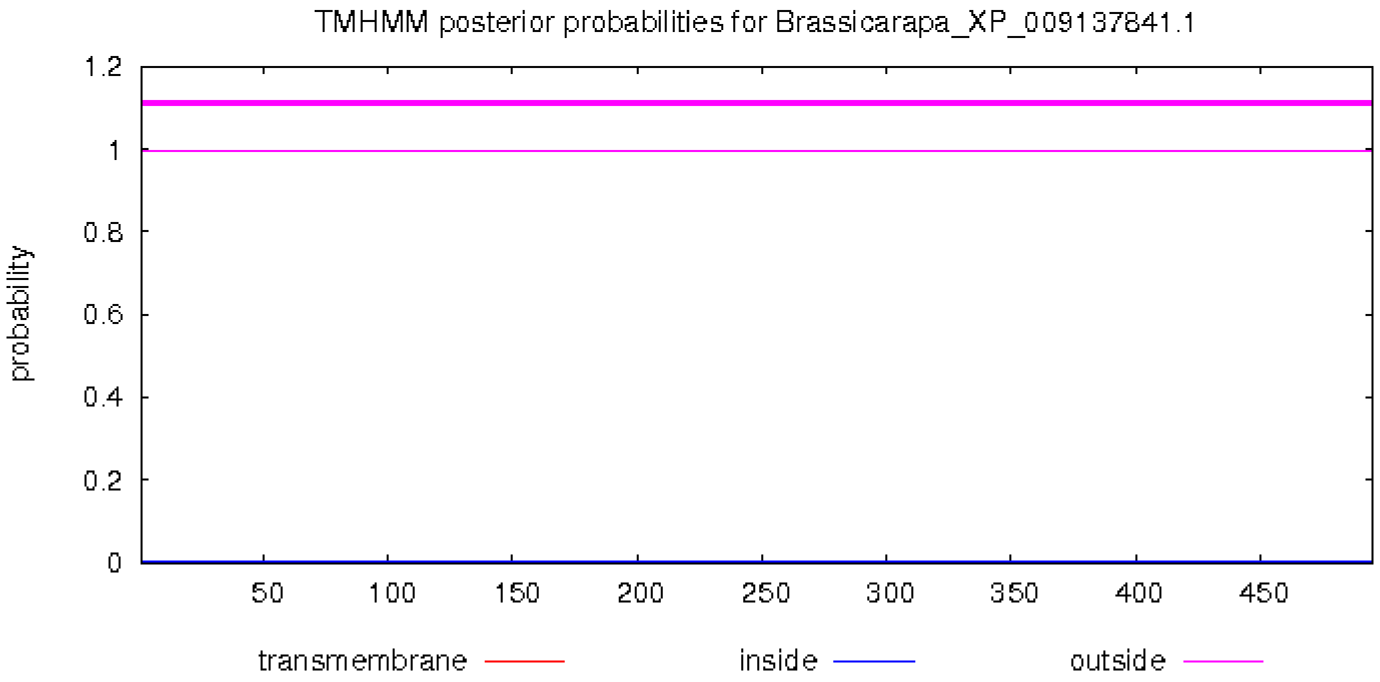

plot in postscript, script for making the plot in gnuplot, data for plot

---

```
# Brassicarapa_XP_009138410.1 Length: 370
# Brassicarapa_XP_009138410.1 Number of predicted TMHs:  0
# Brassicarapa_XP_009138410.1 Exp number of AAs in TMHs: 0
# Brassicarapa_XP_009138410.1 Exp number, first 60 AAs:  0
# Brassicarapa_XP_009138410.1 Total prob of N-in:        0.01311
Brassicarapa_XP_009138410.1     TMHMM2.0        outside      1   370
```

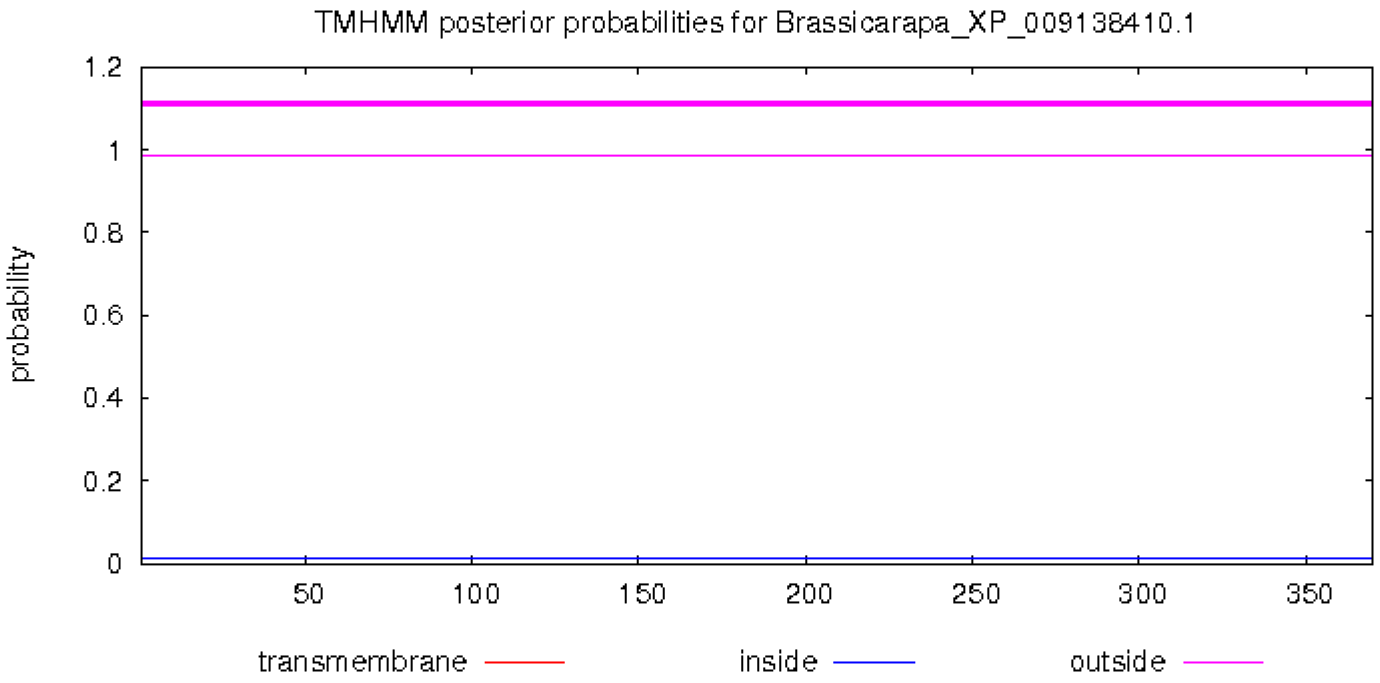

plot in postscript, script for making the plot in gnuplot, data for plot

---

```
# Brassicarapa_XP_009138411.1 Length: 370
# Brassicarapa_XP_009138411.1 Number of predicted TMHs:  0
# Brassicarapa_XP_009138411.1 Exp number of AAs in TMHs: 0
# Brassicarapa_XP_009138411.1 Exp number, first 60 AAs:  0
# Brassicarapa_XP_009138411.1 Total prob of N-in:        0.01311
Brassicarapa_XP_009138411.1     TMHMM2.0        outside      1   370
```

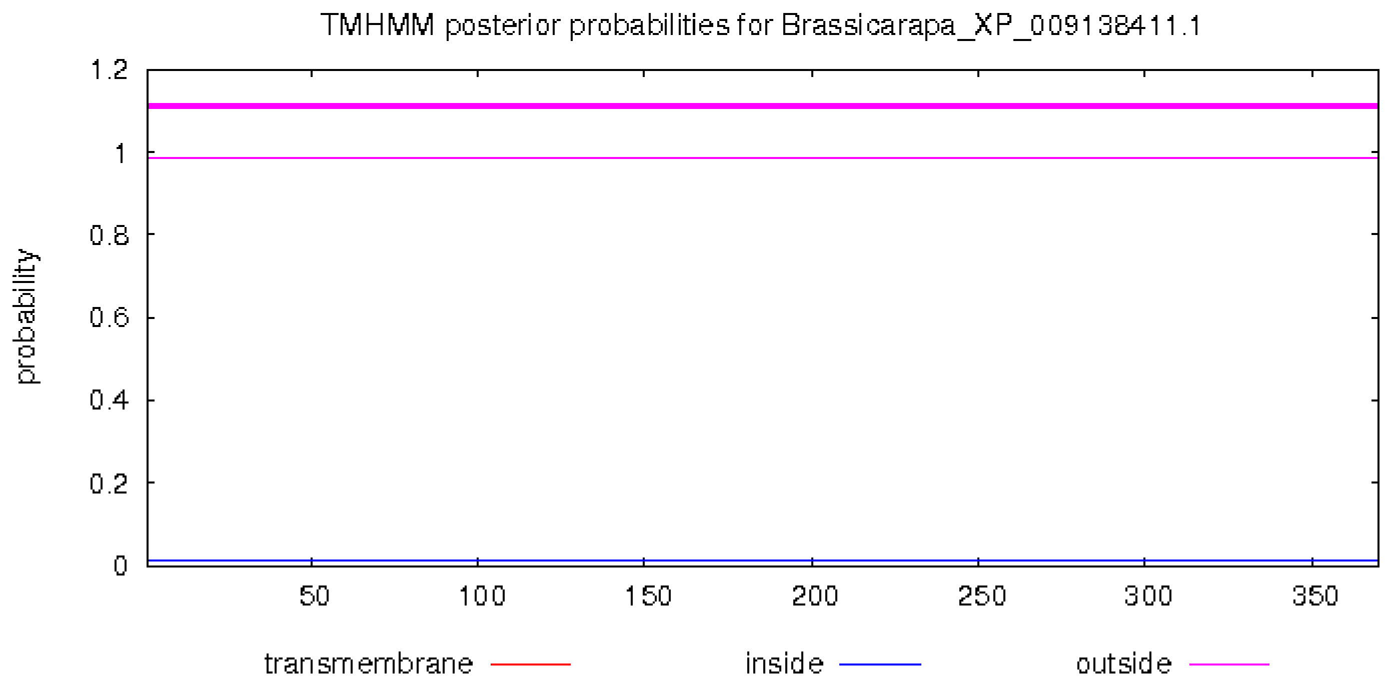

plot in postscript, script for making the plot in gnuplot, data for plot

---

```
# Brassicarapa_XP_009139340.1 Length: 593
# Brassicarapa_XP_009139340.1 Number of predicted TMHs:  0
# Brassicarapa_XP_009139340.1 Exp number of AAs in TMHs: 0.00084
# Brassicarapa_XP_009139340.1 Exp number, first 60 AAs:  0.00069
# Brassicarapa_XP_009139340.1 Total prob of N-in:        0.00285
Brassicarapa_XP_009139340.1     TMHMM2.0        outside      1   593
```

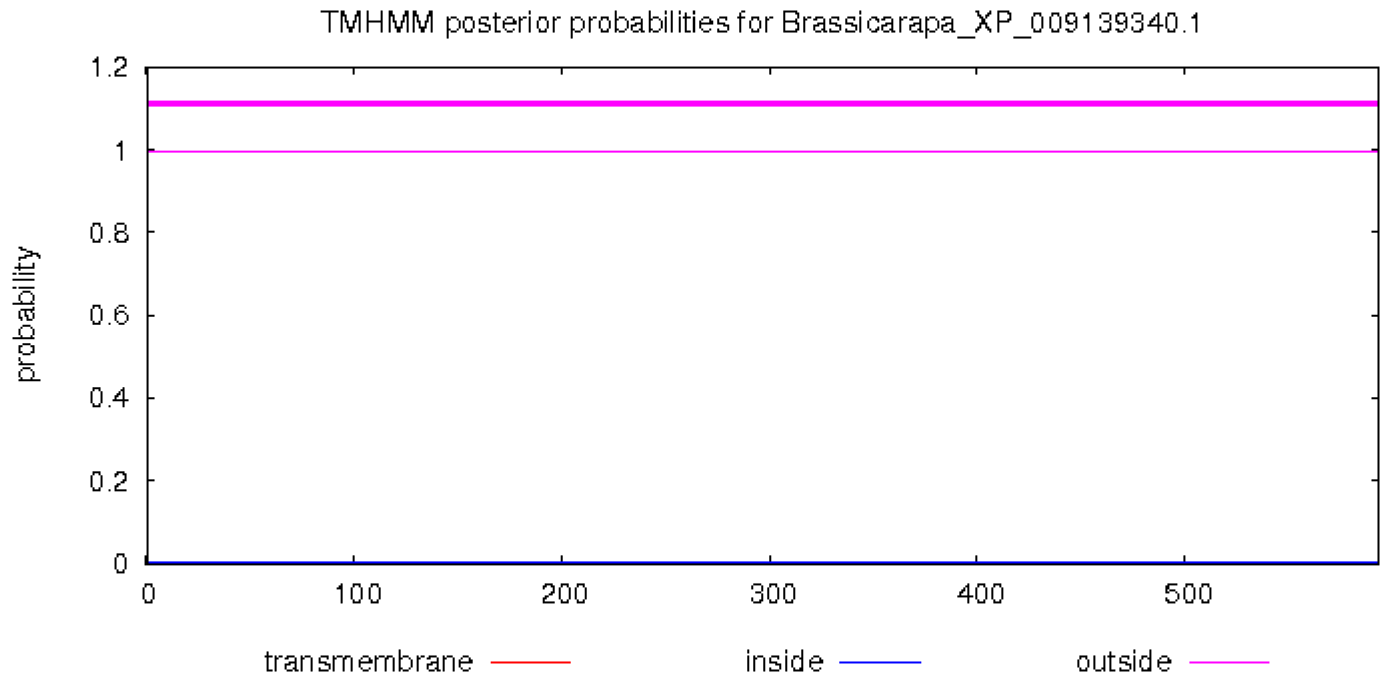

plot in postscript, script for making the plot in gnuplot, data for plot

---

```
# Brassicarapa_XP_009139909.1 Length: 289
# Brassicarapa_XP_009139909.1 Number of predicted TMHs:  0
# Brassicarapa_XP_009139909.1 Exp number of AAs in TMHs: 0.000800000000000000001
# Brassicarapa_XP_009139909.1 Exp number, first 60 AAs:  0.00057
# Brassicarapa_XP_009139909.1 Total prob of N-in:        0.04392
Brassicarapa_XP_009139909.1     TMHMM2.0        outside      1   289
```

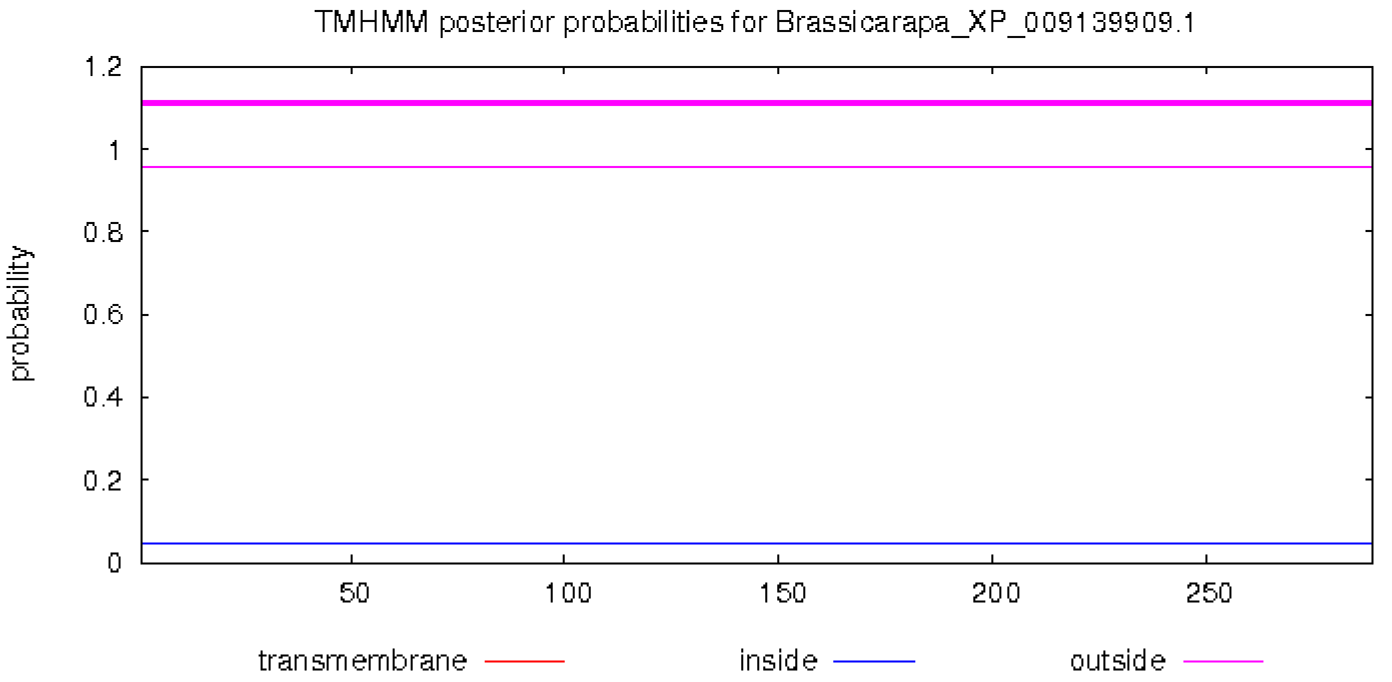

plot in postscript, script for making the plot in gnuplot, data for plot

---

```
# Brassicarapa_XP_009140067.1 Length: 202
# Brassicarapa_XP_009140067.1 Number of predicted TMHs:  0
# Brassicarapa_XP_009140067.1 Exp number of AAs in TMHs: 0
# Brassicarapa_XP_009140067.1 Exp number, first 60 AAs:  0
# Brassicarapa_XP_009140067.1 Total prob of N-in:        0.03275
Brassicarapa_XP_009140067.1     TMHMM2.0        outside      1   202
```

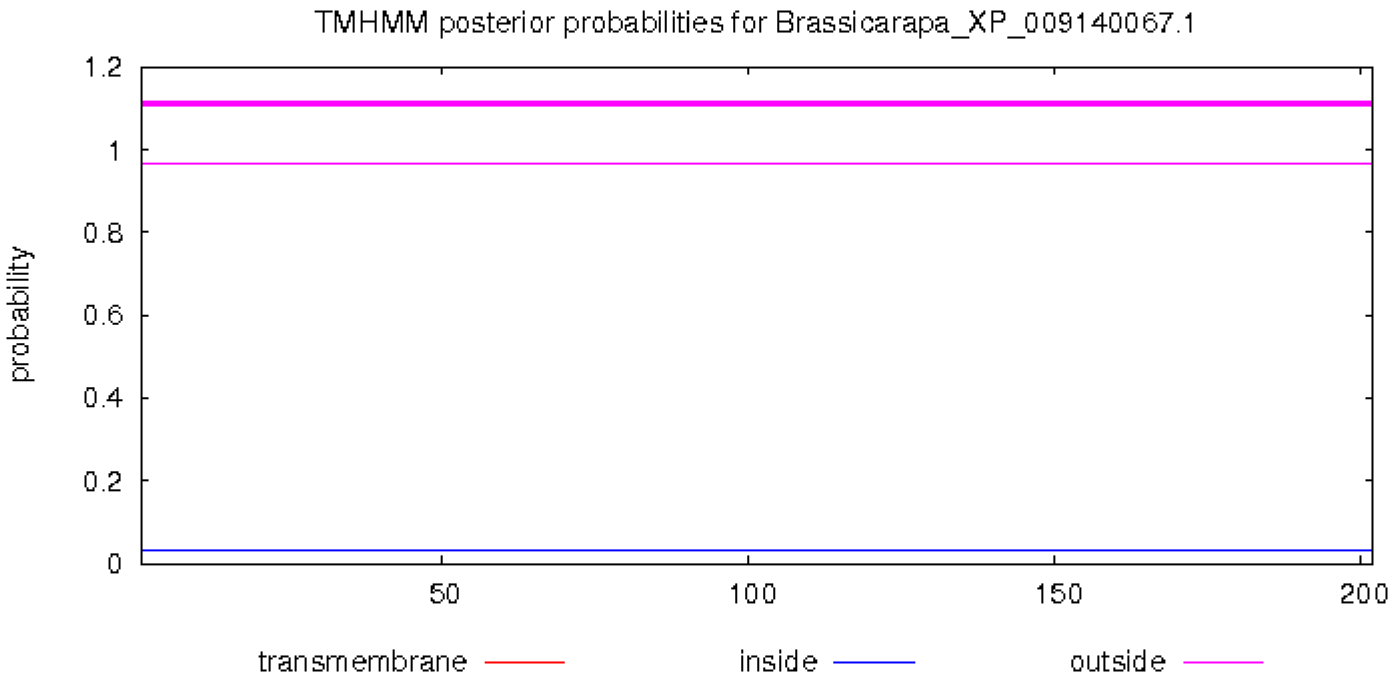

plot in postscript, script for making the plot in gnuplot, data for plot

---

```
# Brassicarapa_XP_009140553.1 Length: 317
# Brassicarapa_XP_009140553.1 Number of predicted TMHs:  0
# Brassicarapa_XP_009140553.1 Exp number of AAs in TMHs: 0.0072
# Brassicarapa_XP_009140553.1 Exp number, first 60 AAs:  0
# Brassicarapa_XP_009140553.1 Total prob of N-in:        0.07276
Brassicarapa_XP_009140553.1	TMHMM2.0	outside	     1   317
```

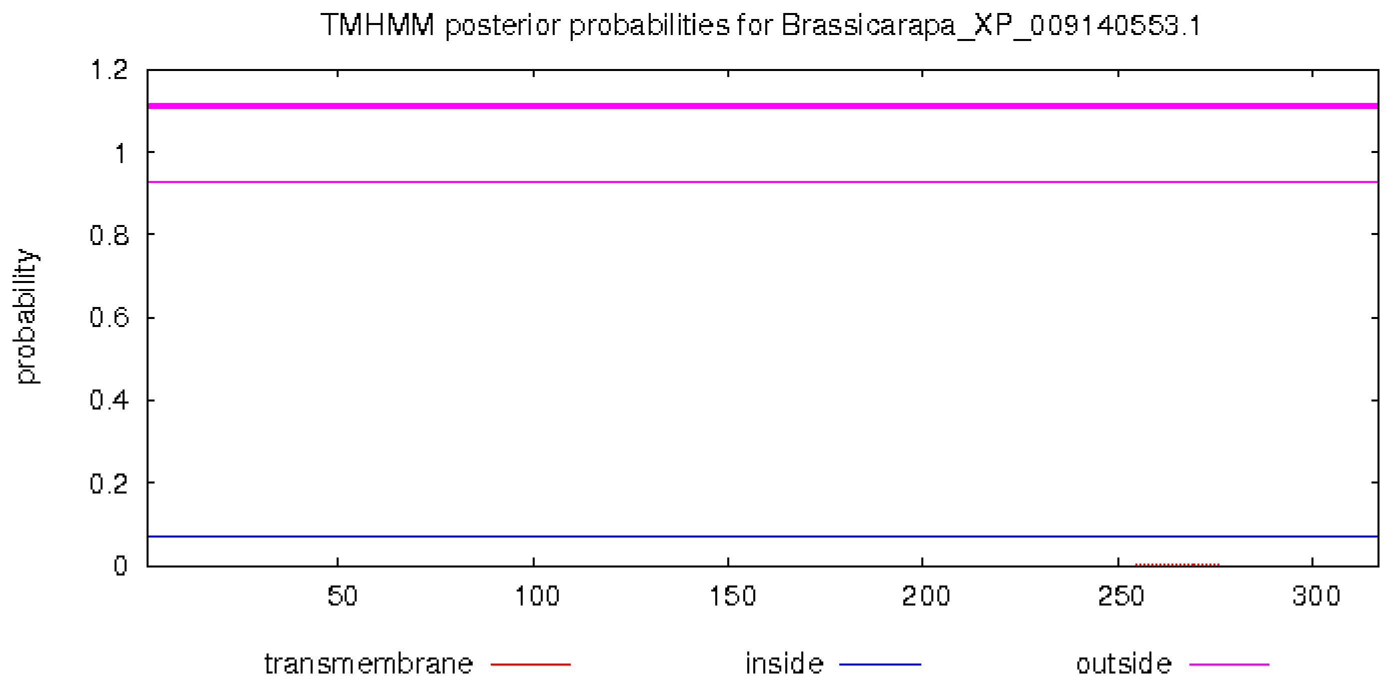

plot in postscript, script for making the plot in gnuplot, data for plot

---

```
# Brassicarapa_XP_009140890.1 Length: 193
# Brassicarapa_XP_009140890.1 Number of predicted TMHs:  0
# Brassicarapa_XP_009140890.1 Exp number of AAs in TMHs: 0.00012
# Brassicarapa_XP_009140890.1 Exp number, first 60 AAs:  0
# Brassicarapa_XP_009140890.1 Total prob of N-in:        0.13918
Brassicarapa_XP_009140890.1	TMHMM2.0	outside	     1   193
```

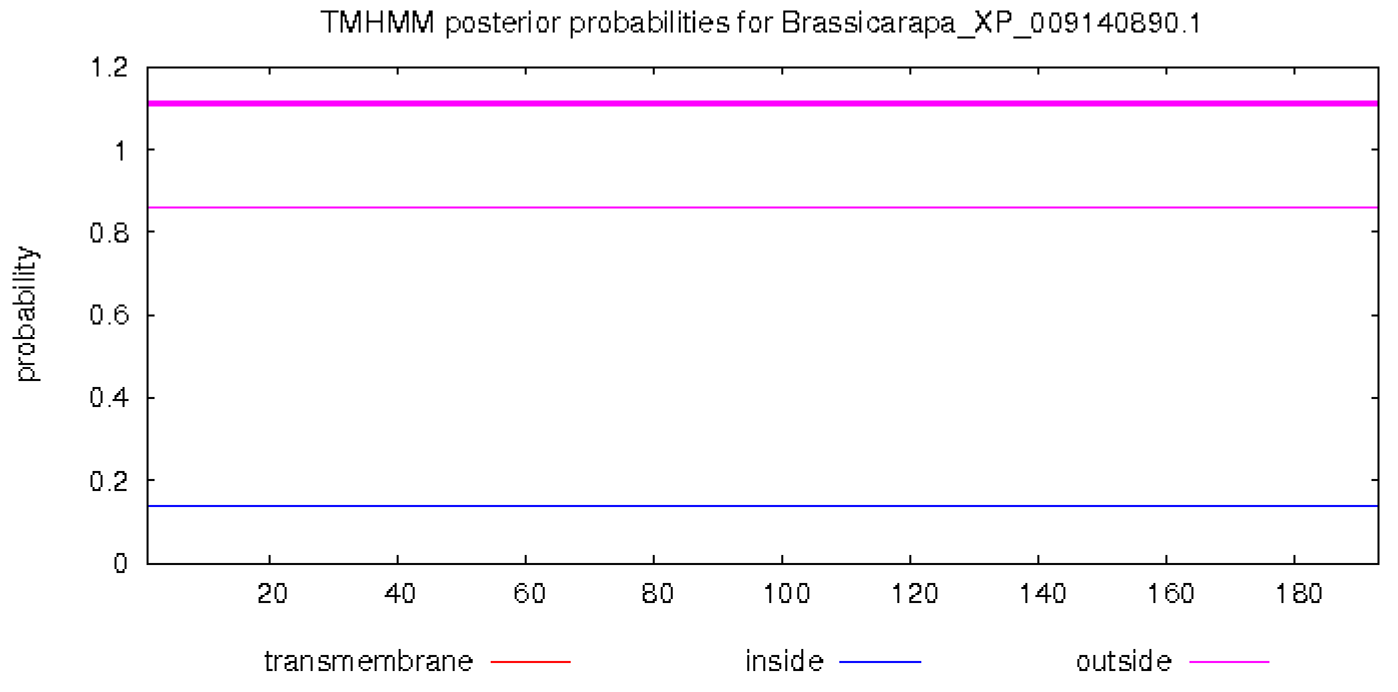

plot in postscript, script for making the plot in gnuplot, data for plot

---

```
# Brassicarapa_XP_009141320.1 Length: 256
# Brassicarapa_XP_009141320.1 Number of predicted TMHs:  0
# Brassicarapa_XP_009141320.1 Exp number of AAs in TMHs: 0.02092
# Brassicarapa_XP_009141320.1 Exp number, first 60 AAs:  0.0002
# Brassicarapa_XP_009141320.1 Total prob of N-in:        0.10373
Brassicarapa_XP_009141320.1     TMHMM2.0        outside      1   256
```

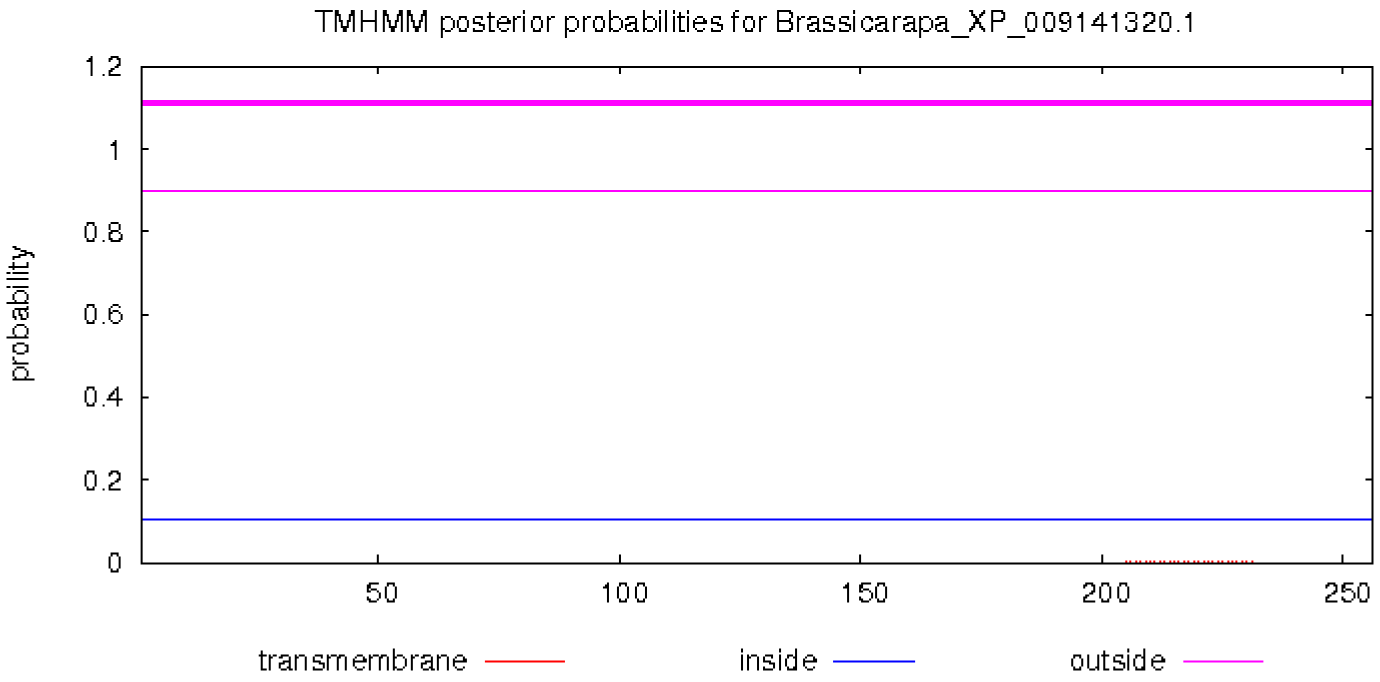

plot in postscript, script for making the plot in gnuplot, data for plot

---

```
# Brassicarapa_XP_009142151.1 Length: 277
# Brassicarapa_XP_009142151.1 Number of predicted TMHs:  0
# Brassicarapa_XP_009142151.1 Exp number of AAs in TMHs: 0.00108
# Brassicarapa_XP_009142151.1 Exp number, first 60 AAs:  0
# Brassicarapa_XP_009142151.1 Total prob of N-in:        0.02400
Brassicarapa_XP_009142151.1     TMHMM2.0        outside      1   277
```

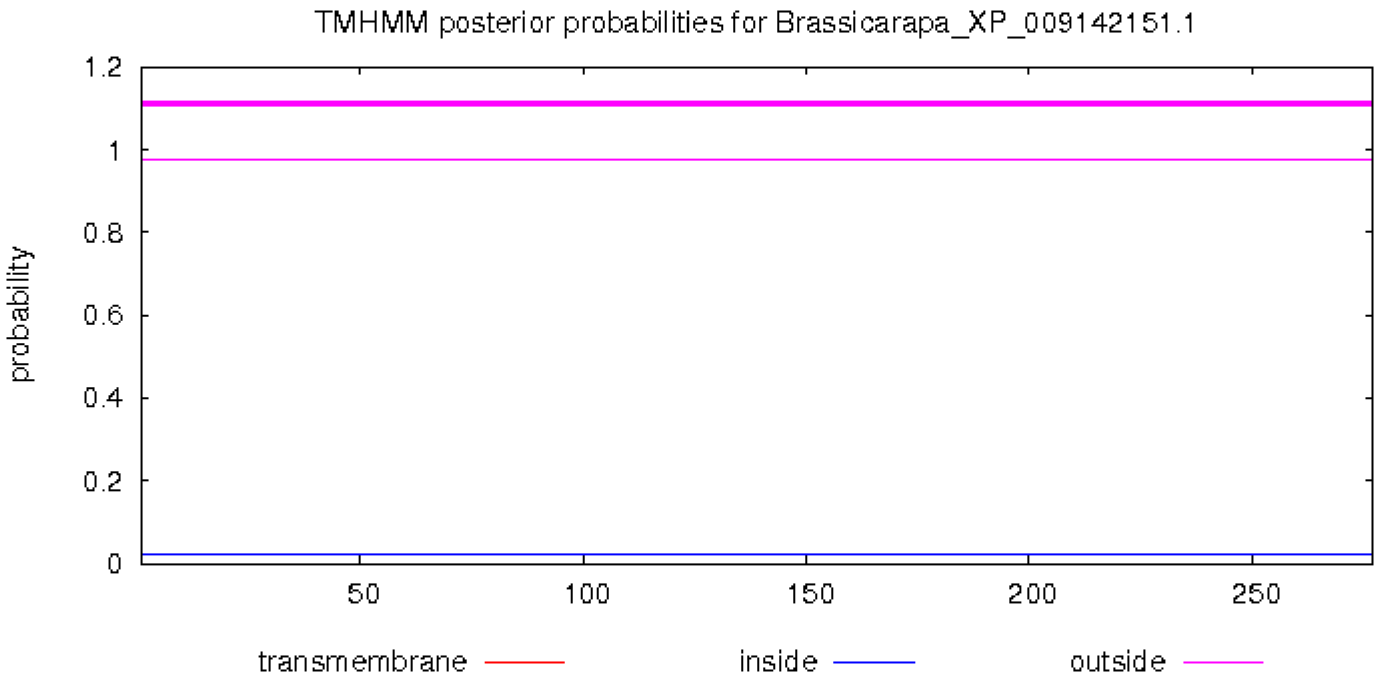

plot in postscript, script for making the plot in gnuplot, data for plot

---

```
# Brassicarapa_XP_009142445.1 Length: 364
# Brassicarapa_XP_009142445.1 Number of predicted TMHs:  0
# Brassicarapa_XP_009142445.1 Exp number of AAs in TMHs: 0
# Brassicarapa_XP_009142445.1 Exp number, first 60 AAs:  0
# Brassicarapa_XP_009142445.1 Total prob of N-in:        0.00679
Brassicarapa_XP_009142445.1     TMHMM2.0        outside      1   364
```

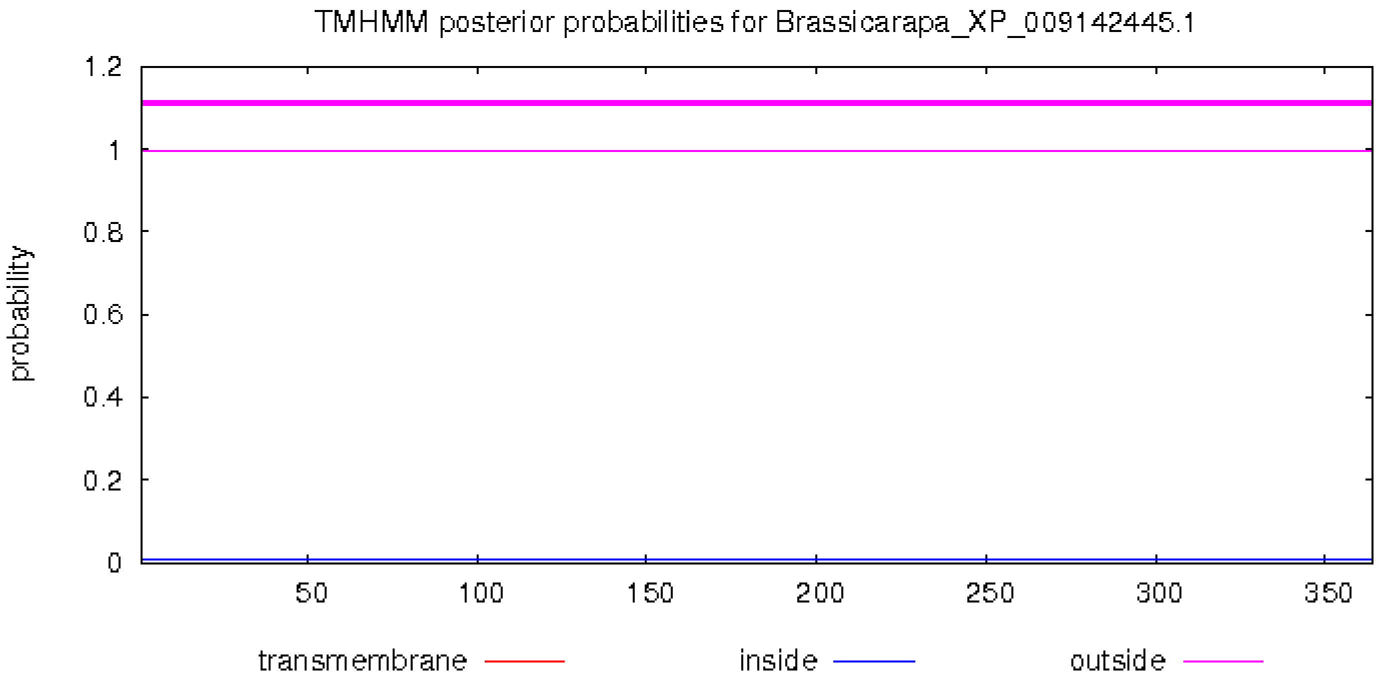

plot in postscript, script for making the plot in gnuplot, data for plot

---

```
# Brassicarapa_XP_009142607.1 Length: 359
# Brassicarapa_XP_009142607.1 Number of predicted TMHs:  0
# Brassicarapa_XP_009142607.1 Exp number of AAs in TMHs: 0.0006
# Brassicarapa_XP_009142607.1 Exp number, first 60 AAs:  0
# Brassicarapa_XP_009142607.1 Total prob of N-in:        0.00833
Brassicarapa_XP_009142607.1     TMHMM2.0        outside      1   359
```

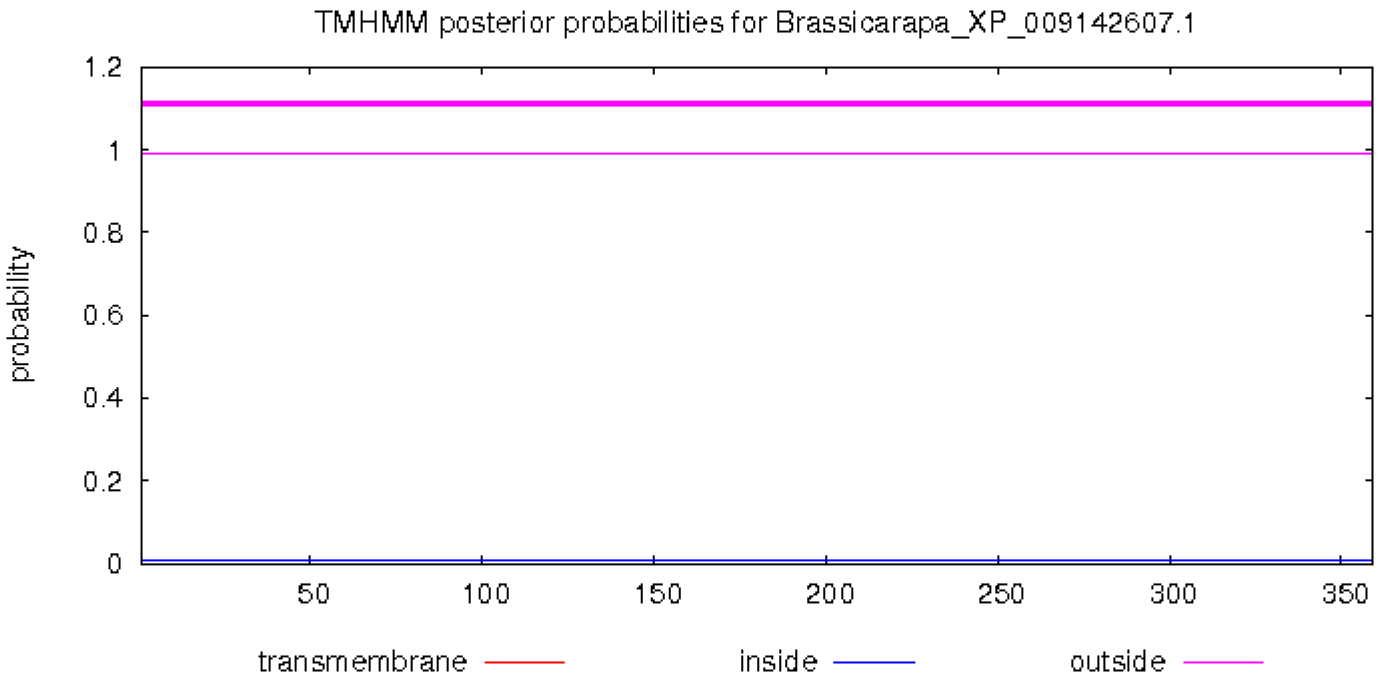

plot in postscript, script for making the plot in gnuplot, data for plot

---

```
# Brassicarapa_XP_009142897.1 Length: 280
# Brassicarapa_XP_009142897.1 Number of predicted TMHs:  0
# Brassicarapa_XP_009142897.1 Exp number of AAs in TMHs: 0.00124
# Brassicarapa_XP_009142897.1 Exp number, first 60 AAs:  0
# Brassicarapa_XP_009142897.1 Total prob of N-in:        0.03749
Brassicarapa_XP_009142897.1     TMHMM2.0        outside      1   280
```

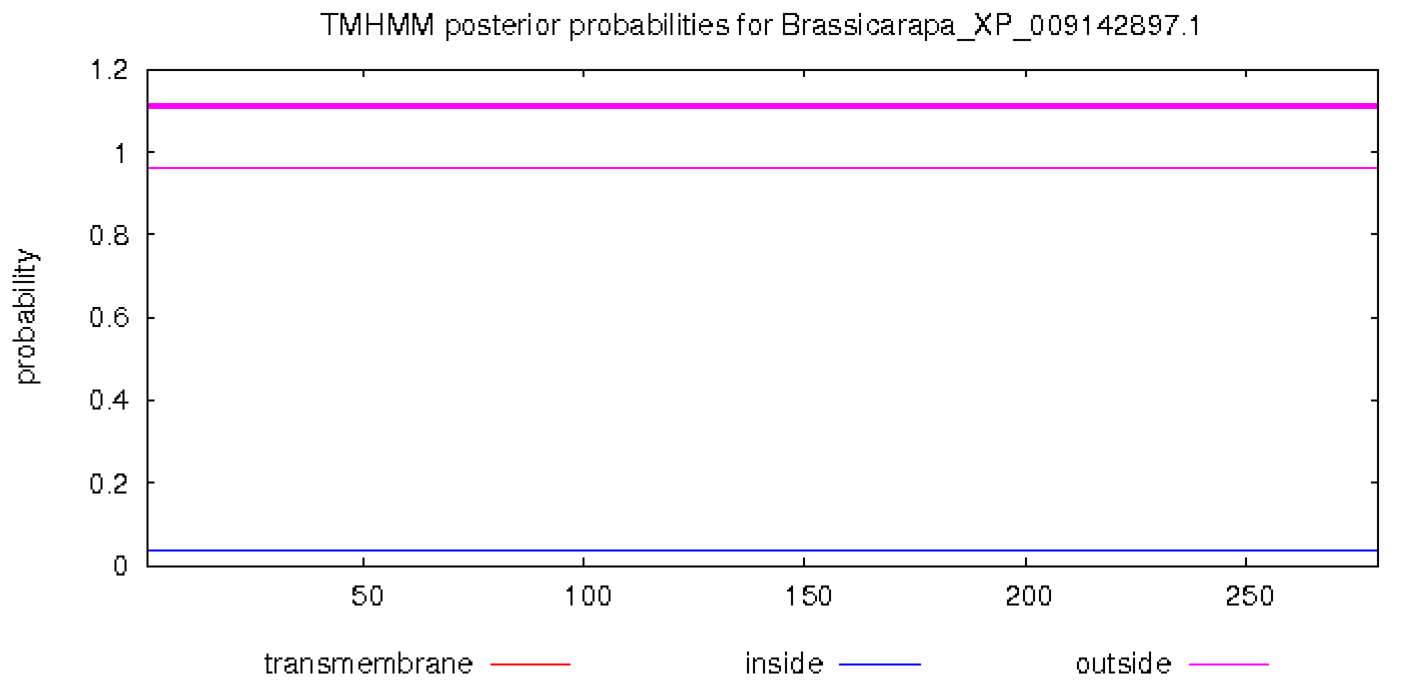

plot in postscript, script for making the plot in gnuplot, data for plot

```
# Brassicarapa_XP_009143398.1 Length: 300
# Brassicarapa_XP_009143398.1 Number of predicted TMHs:  0
# Brassicarapa_XP_009143398.1 Exp number of AAs in TMHs: 0.0054
# Brassicarapa_XP_009143398.1 Exp number, first 60 AAs:  0.00062
# Brassicarapa_XP_009143398.1 Total prob of N-in:        0.02411
Brassicarapa_XP_009143398.1     TMHMM2.0        outside      1   300
```

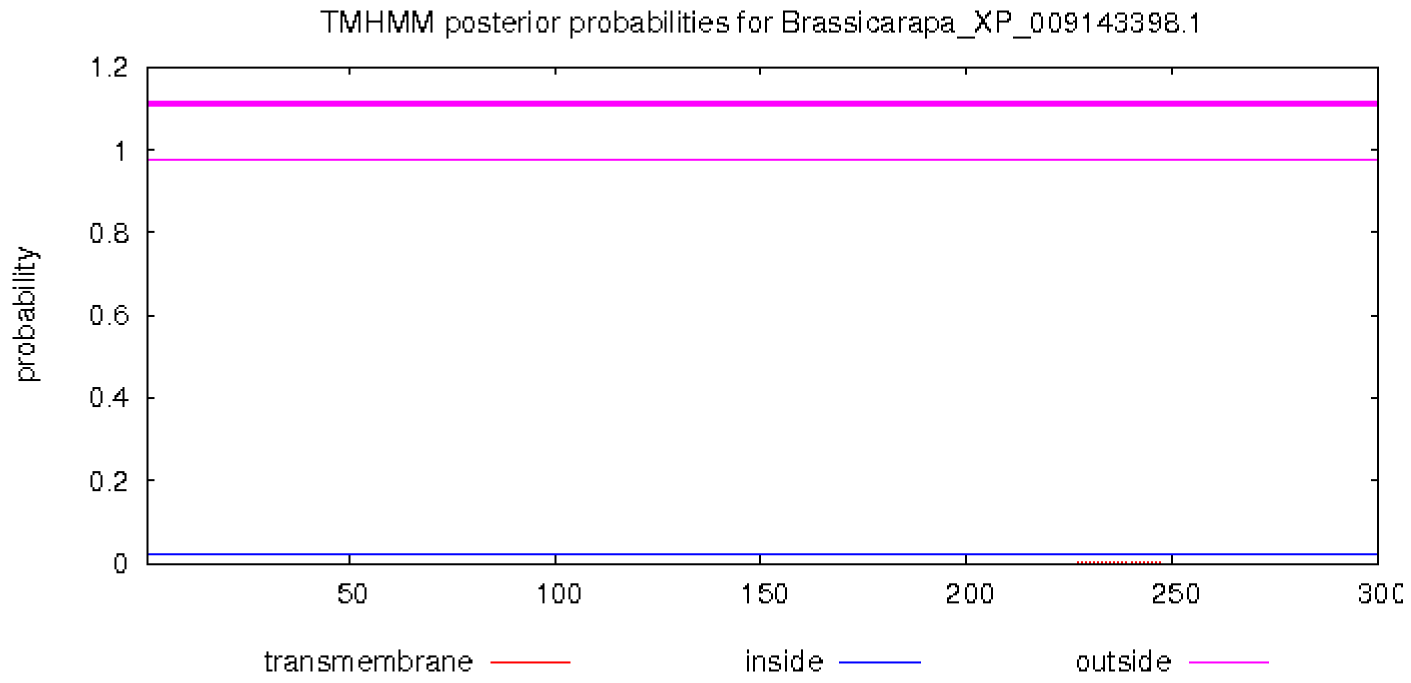

plot in postscript, script for making the plot in gnuplot, data for plot

```
# Brassicarapa_XP_009143857.1 Length: 252
# Brassicarapa_XP_009143857.1 Number of predicted TMHs:  0
# Brassicarapa_XP_009143857.1 Exp number of AAs in TMHs: 0.00586
# Brassicarapa_XP_009143857.1 Exp number, first 60 AAs:  0.00459
# Brassicarapa_XP_009143857.1 Total prob of N-in:        0.15477
Brassicarapa_XP_009143857.1	TMHMM2.0	outside	     1   252
```

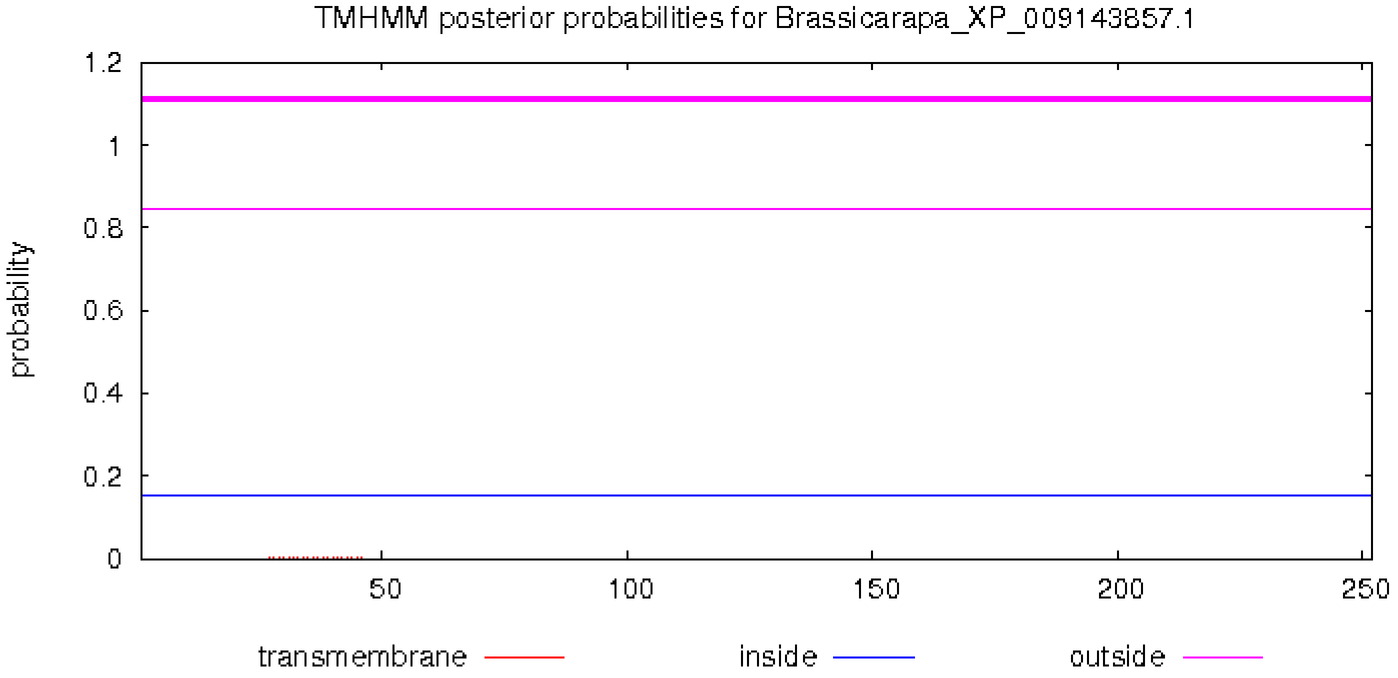

plot in postscript, script for making the plot in gnuplot, data for plot

---

```
# Brassicarapa_XP_009144711.1 Length: 346
# Brassicarapa_XP_009144711.1 Number of predicted TMHs:  0
# Brassicarapa_XP_009144711.1 Exp number of AAs in TMHs: 0
# Brassicarapa_XP_009144711.1 Exp number, first 60 AAs:  0
# Brassicarapa_XP_009144711.1 Total prob of N-in:        0.01085
Brassicarapa_XP_009144711.1	TMHMM2.0	outside	     1   346
```

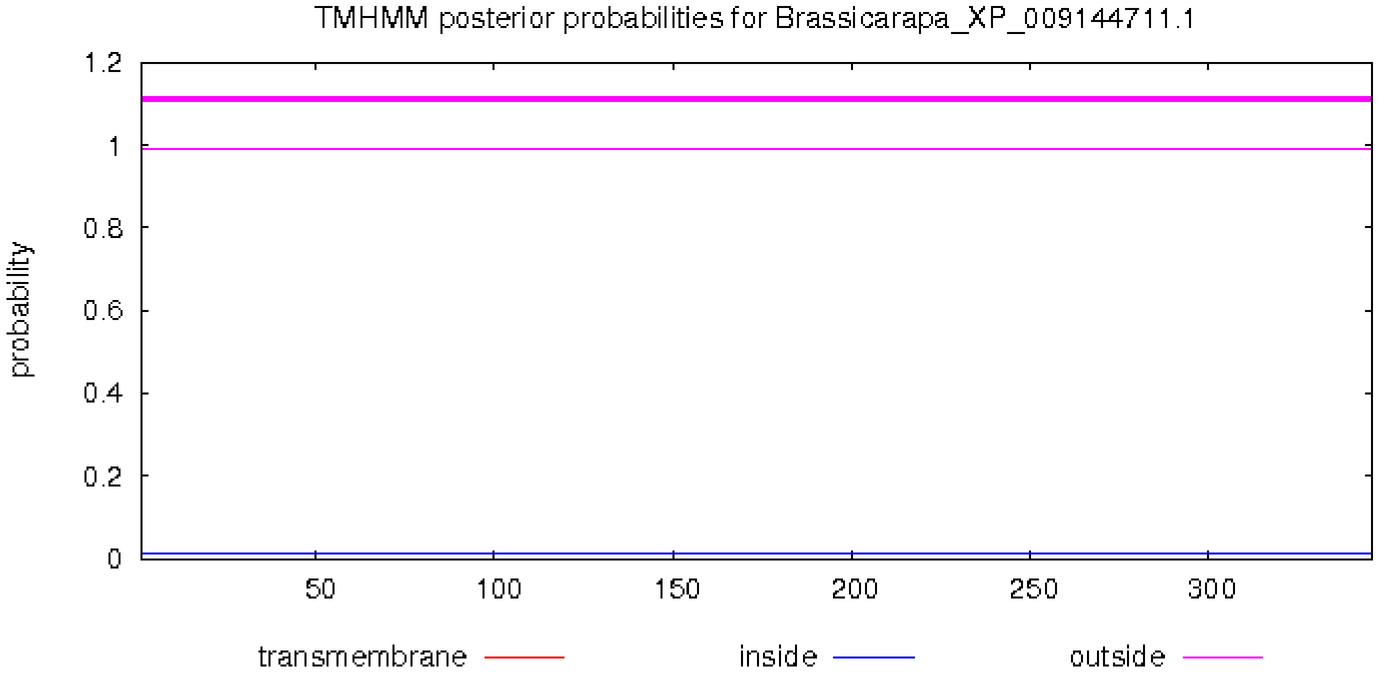

plot in postscript, script for making the plot in gnuplot, data for plot

---

```
# Brassicarapa_XP_009145145.1 Length: 546
# Brassicarapa_XP_009145145.1 Number of predicted TMHs:  1
# Brassicarapa_XP_009145145.1 Exp number of AAs in TMHs: 34.03834
# Brassicarapa_XP_009145145.1 Exp number, first 60 AAs:  0.0186
# Brassicarapa_XP_009145145.1 Total prob of N-in:        0.29331
Brassicarapa_XP_009145145.1     TMHMM2.0        inside       1   516
Brassicarapa_XP_009145145.1     TMHMM2.0        TMhelix    517   539
Brassicarapa_XP_009145145.1     TMHMM2.0        outside    540   546
```

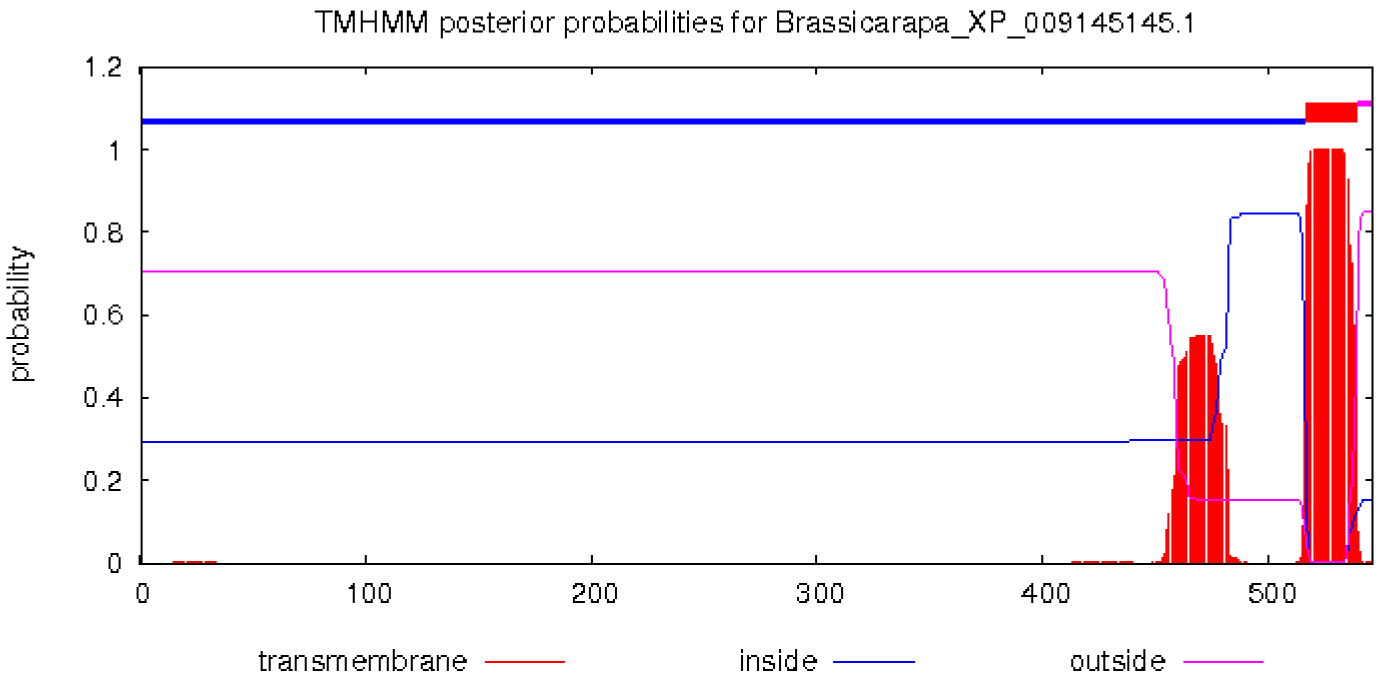

plot in postscript, script for making the plot in gnuplot, data for plot

---

```
# Brassicarapa_XP_009145146.1 Length: 578
# Brassicarapa_XP_009145146.1 Number of predicted TMHs:  1
# Brassicarapa_XP_009145146.1 Exp number of AAs in TMHs: 22.57695
# Brassicarapa_XP_009145146.1 Exp number, first 60 AAs:  0.0022
# Brassicarapa_XP_009145146.1 Total prob of N-in:        0.14245
Brassicarapa_XP_009145146.1     TMHMM2.0        outside      1   545
Brassicarapa_XP_009145146.1     TMHMM2.0        TMhelix    546   568
Brassicarapa_XP_009145146.1     TMHMM2.0        inside     569   578
```

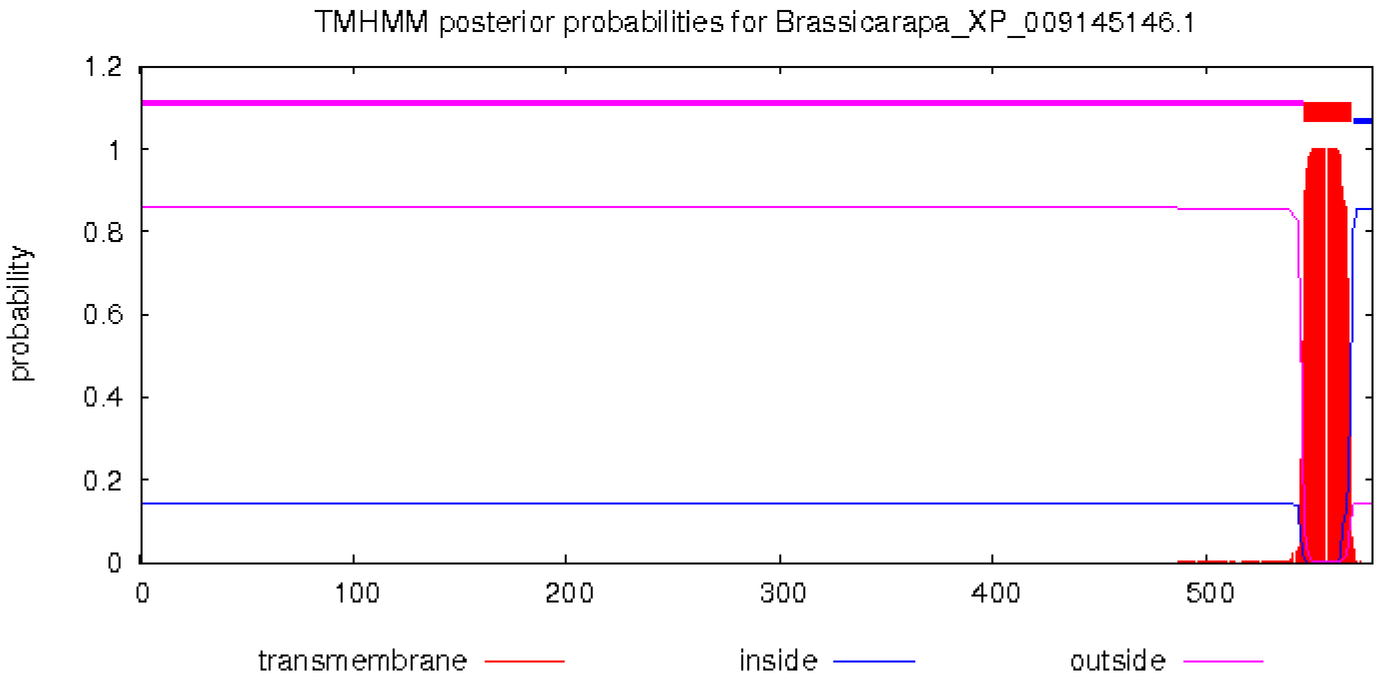

plot in postscript, script for making the plot in gnuplot, data for plot

```
# Brassicarapa_XP_009145148.1 Length: 578
# Brassicarapa_XP_009145148.1 Number of predicted TMHs:  1
# Brassicarapa_XP_009145148.1 Exp number of AAs in TMHs: 22.57695
# Brassicarapa_XP_009145148.1 Exp number, first 60 AAs:  0.0022
# Brassicarapa_XP_009145148.1 Total prob of N-in:        0.14245
Brassicarapa_XP_009145148.1     TMHMM2.0        outside      1   545
Brassicarapa_XP_009145148.1     TMHMM2.0        TMhelix    546   568
Brassicarapa_XP_009145148.1     TMHMM2.0        inside     569   578
```

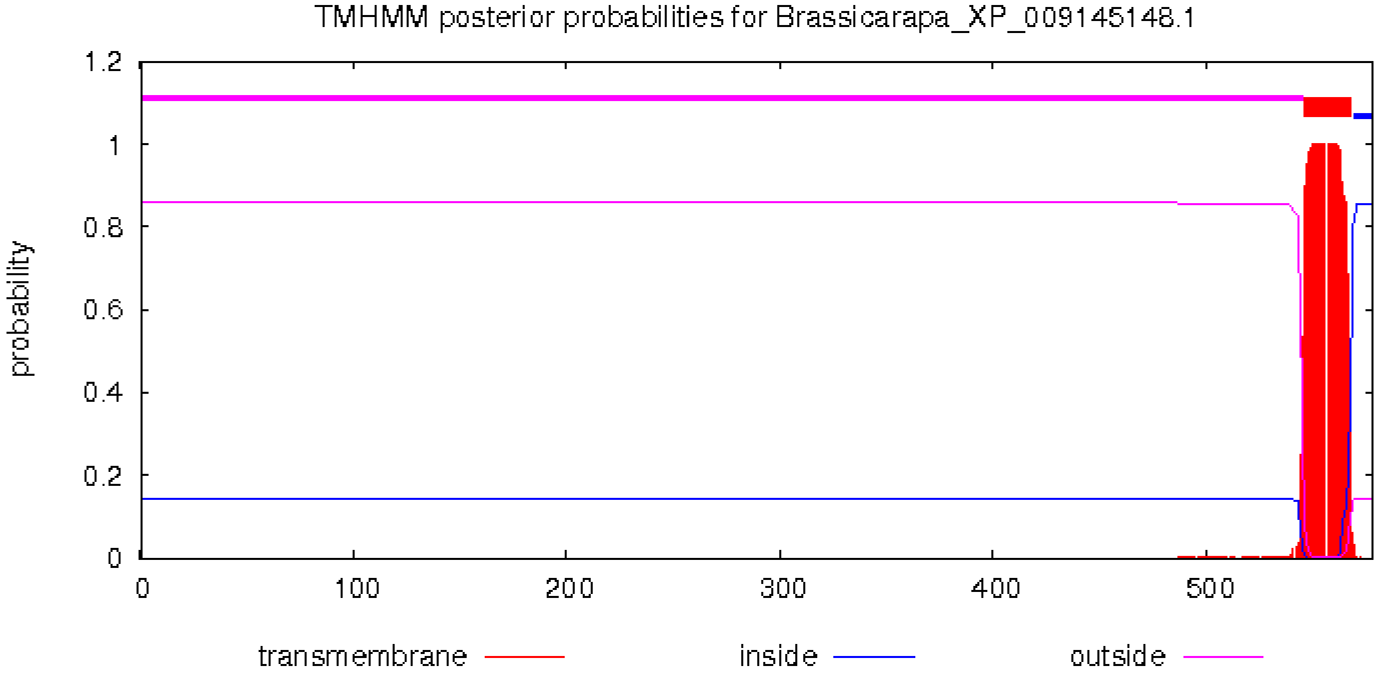

plot in postscript, script for making the plot in gnuplot, data for plot

```
# Brassicarapa_XP_009145149.1 Length: 578
# Brassicarapa_XP_009145149.1 Number of predicted TMHs:  1
# Brassicarapa_XP_009145149.1 Exp number of AAs in TMHs: 22.57695
# Brassicarapa_XP_009145149.1 Exp number, first 60 AAs:  0.0022
# Brassicarapa_XP_009145149.1 Total prob of N-in:        0.14245
Brassicarapa_XP_009145149.1     TMHMM2.0        outside      1   545
Brassicarapa_XP_009145149.1     TMHMM2.0        TMhelix    546   568
Brassicarapa_XP_009145149.1     TMHMM2.0        inside     569   578
```

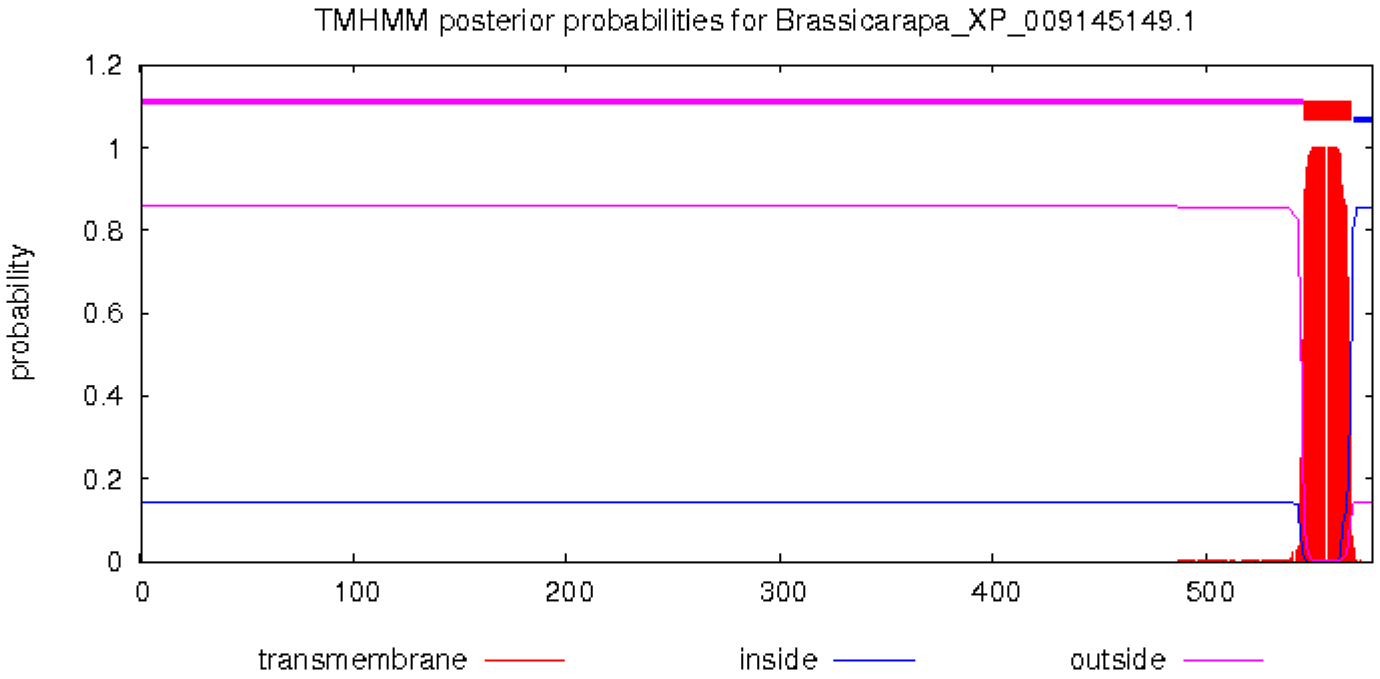

plot in postscript, script for making the plot in gnuplot, data for plot

```
# Brassicarapa_XP_009145150.1 Length: 578
# Brassicarapa_XP_009145150.1 Number of predicted TMHs:  1
# Brassicarapa_XP_009145150.1 Exp number of AAs in TMHs: 22.57695
# Brassicarapa_XP_009145150.1 Exp number, first 60 AAs:  0.0022
# Brassicarapa_XP_009145150.1 Total prob of N-in:        0.14245
Brassicarapa_XP_009145150.1     TMHMM2.0        outside      1   545
Brassicarapa_XP_009145150.1     TMHMM2.0        TMhelix    546   568
Brassicarapa_XP_009145150.1     TMHMM2.0        inside     569   578
```

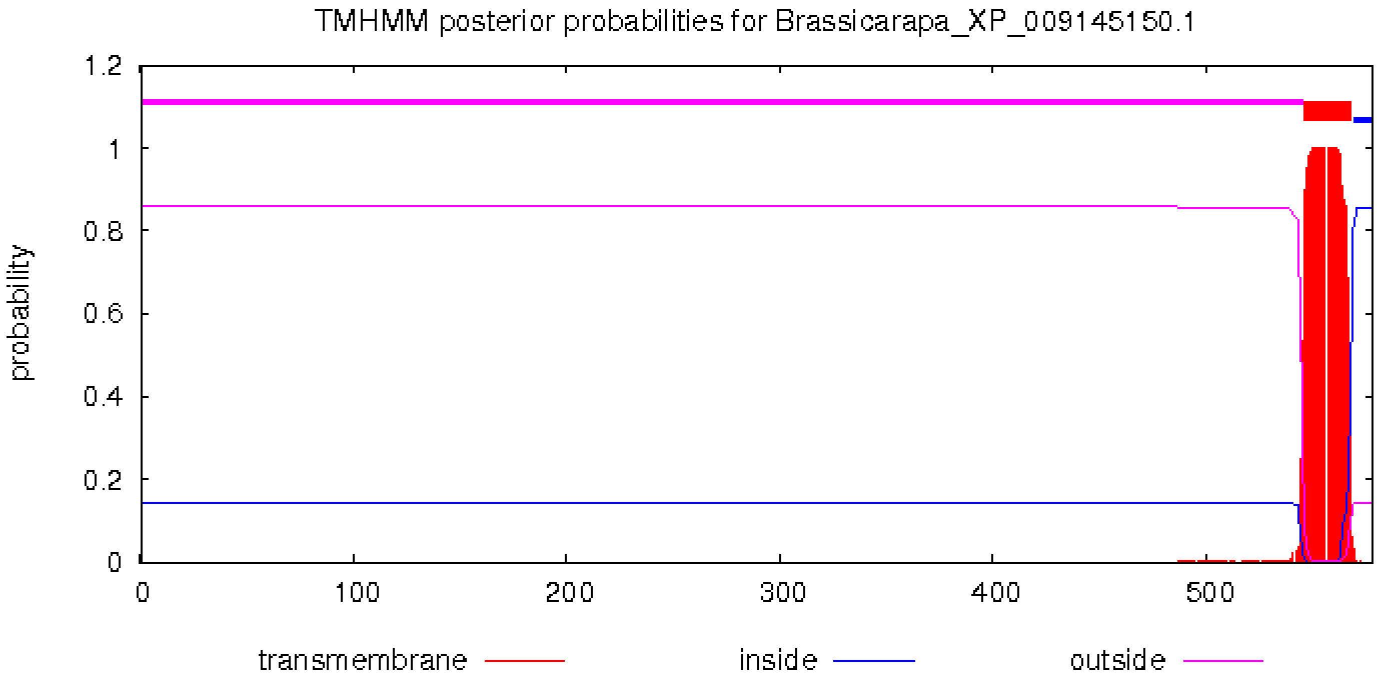

plot in postscript, script for making the plot in gnuplot, data for plot

```
# Brassicarapa_XP_009145151.1 Length: 578
# Brassicarapa_XP_009145151.1 Number of predicted TMHs:  1
# Brassicarapa_XP_009145151.1 Exp number of AAs in TMHs: 22.57695
# Brassicarapa_XP_009145151.1 Exp number, first 60 AAs:  0.0022
# Brassicarapa_XP_009145151.1 Total prob of N-in:        0.14245
```

```
Brassicarapa_XP_009145151.1     TMHMM2.0        outside      1   545
Brassicarapa_XP_009145151.1     TMHMM2.0        TMhelix    546   568
Brassicarapa_XP_009145151.1     TMHMM2.0        inside     569   578
```

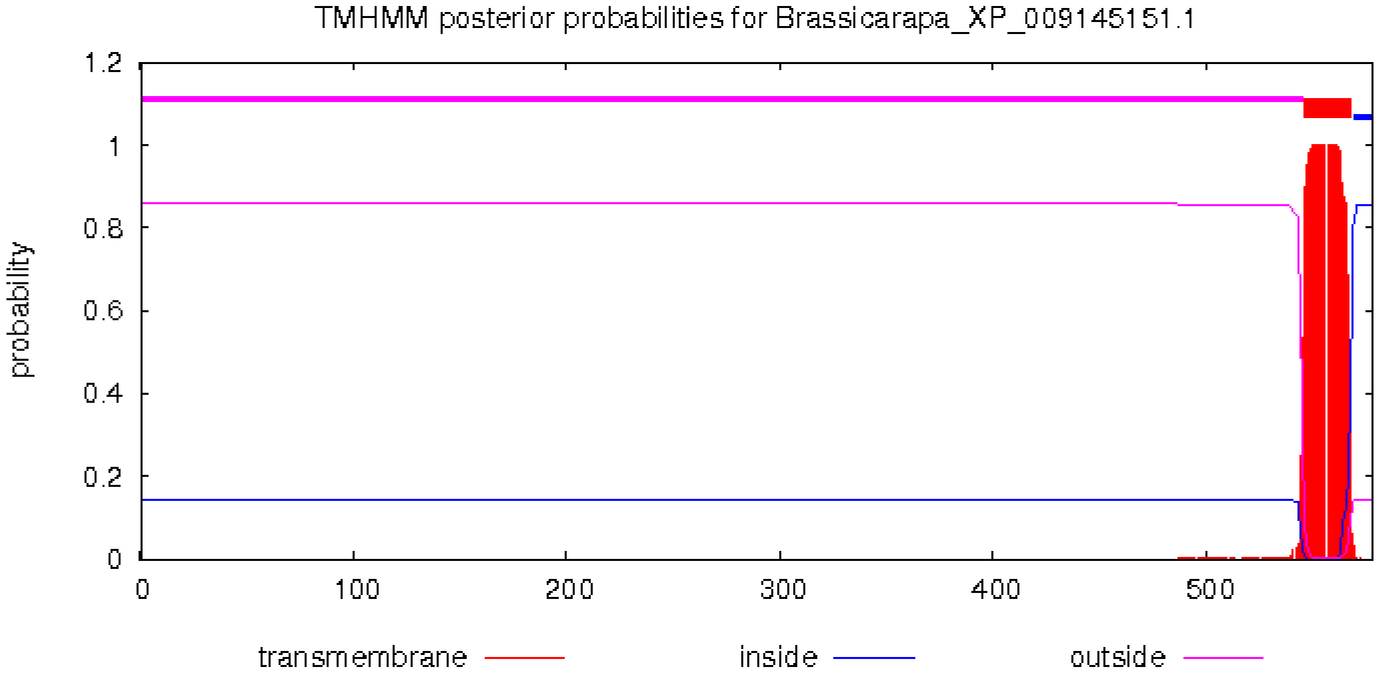

plot in postscript, script for making the plot in gnuplot, data for plot

```
# Brassicarapa_XP_009145896.1 Length: 307
# Brassicarapa_XP_009145896.1 Number of predicted TMHs:  0
# Brassicarapa_XP_009145896.1 Exp number of AAs in TMHs: 0.1637
# Brassicarapa_XP_009145896.1 Exp number, first 60 AAs:  0
# Brassicarapa_XP_009145896.1 Total prob of N-in:        0.00445
Brassicarapa_XP_009145896.1     TMHMM2.0        outside      1   307
```

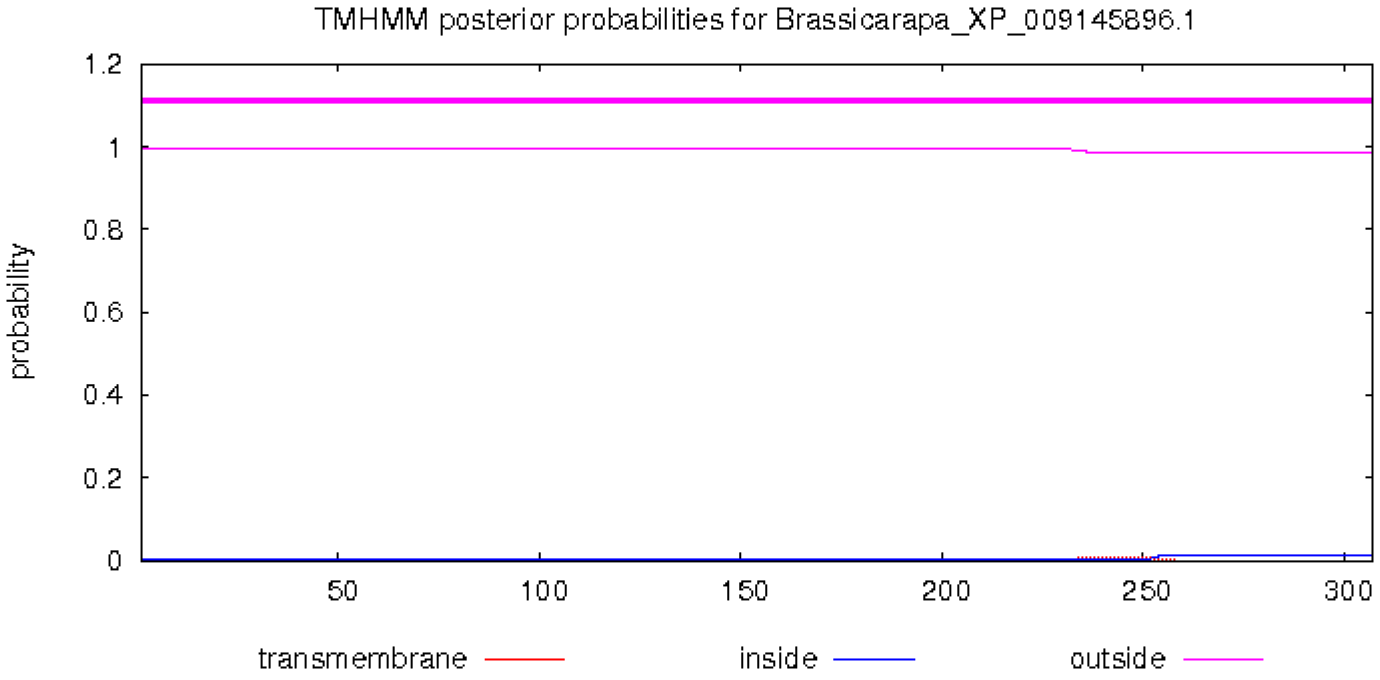

plot in postscript, script for making the plot in gnuplot, data for plot

```
# Brassicarapa_XP_009145897.1 Length: 307
# Brassicarapa_XP_009145897.1 Number of predicted TMHs:  0
# Brassicarapa_XP_009145897.1 Exp number of AAs in TMHs: 0.1637
```

```
# Brassicarapa_XP_009145897.1 Exp number, first 60 AAs:  0
# Brassicarapa_XP_009145897.1 Total prob of N-in:        0.00445
Brassicarapa_XP_009145897.1     TMHMM2.0        outside      1   307
```

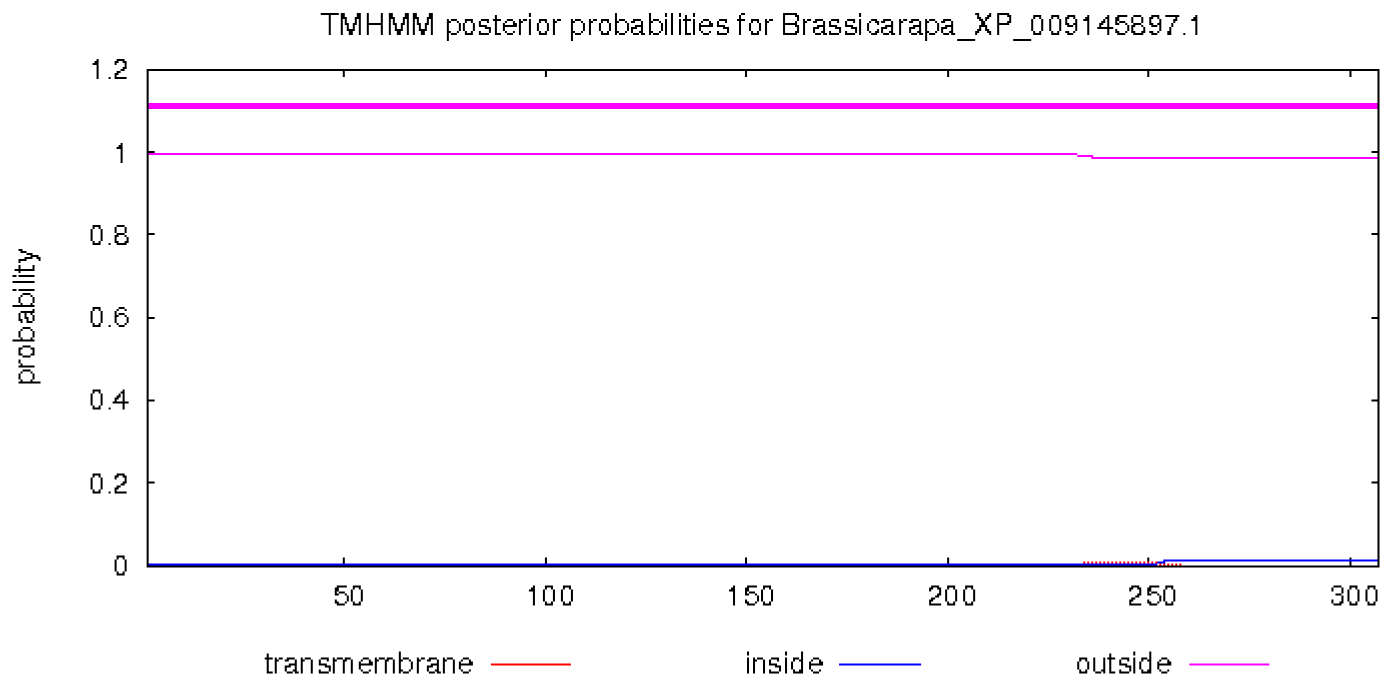

plot in postscript, script for making the plot in gnuplot, data for plot

---

```
# Brassicarapa_XP_009145987.1 Length: 245
# Brassicarapa_XP_009145987.1 Number of predicted TMHs:  0
# Brassicarapa_XP_009145987.1 Exp number of AAs in TMHs: 0
# Brassicarapa_XP_009145987.1 Exp number, first 60 AAs:  0
# Brassicarapa_XP_009145987.1 Total prob of N-in:        0.00225
Brassicarapa_XP_009145987.1     TMHMM2.0        outside      1   245
```

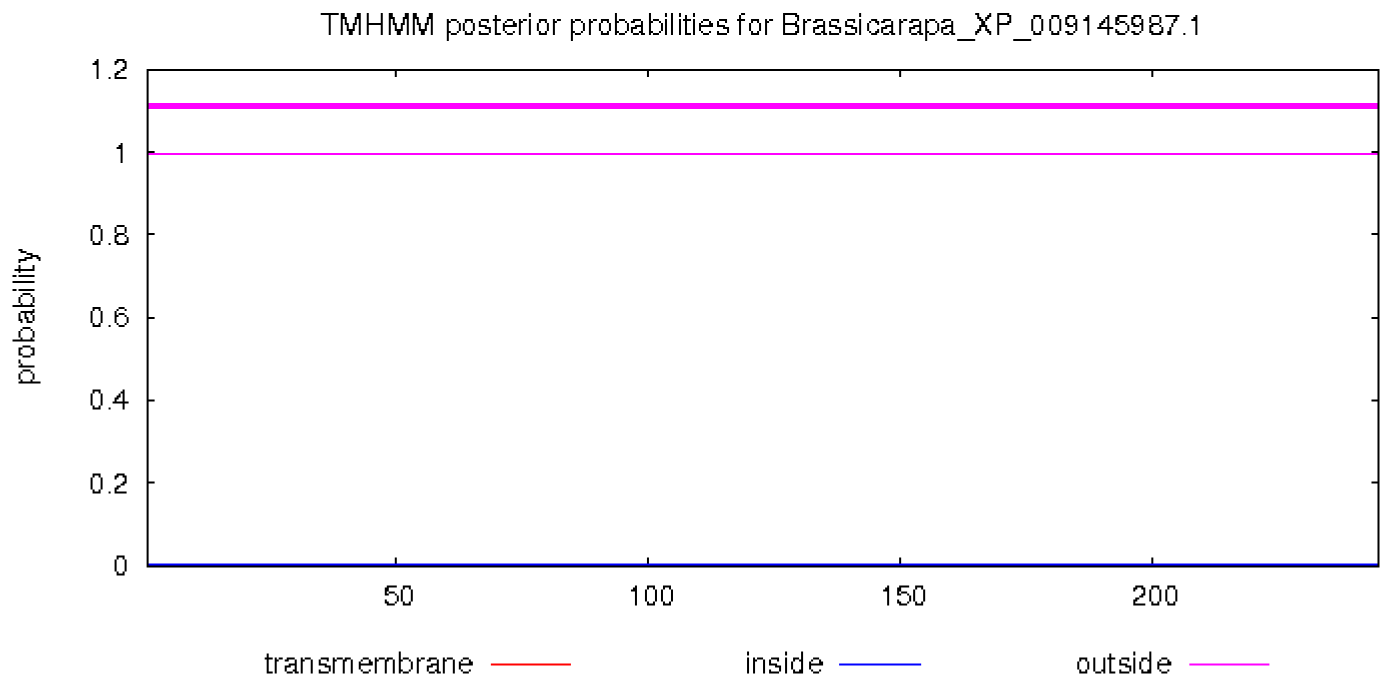

plot in postscript, script for making the plot in gnuplot, data for plot

---

```
# Brassicarapa_XP_009146237.1 Length: 351
# Brassicarapa_XP_009146237.1 Number of predicted TMHs:  0
# Brassicarapa_XP_009146237.1 Exp number of AAs in TMHs: 0.00543
```

```
# Brassicarapa_XP_009146237.1 Exp number, first 60 AAs:  0.00018
# Brassicarapa_XP_009146237.1 Total prob of N-in:        0.00490
Brassicarapa_XP_009146237.1     TMHMM2.0        outside      1   351
```

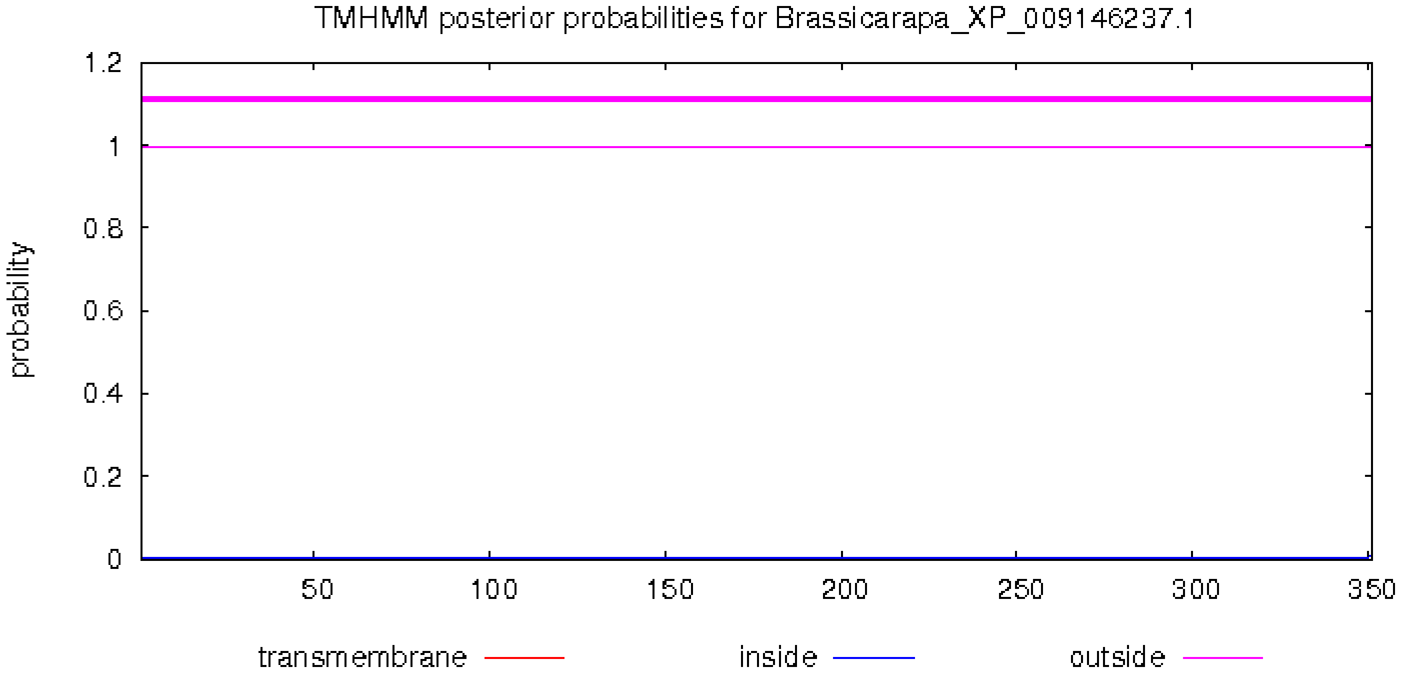

plot in postscript, script for making the plot in gnuplot, data for plot

---

```
# Brassicarapa_XP_009146239.1 Length: 333
# Brassicarapa_XP_009146239.1 Number of predicted TMHs:  0
# Brassicarapa_XP_009146239.1 Exp number of AAs in TMHs: 0
# Brassicarapa_XP_009146239.1 Exp number, first 60 AAs:  0
# Brassicarapa_XP_009146239.1 Total prob of N-in:        0.00327
Brassicarapa_XP_009146239.1     TMHMM2.0        outside      1   333
```

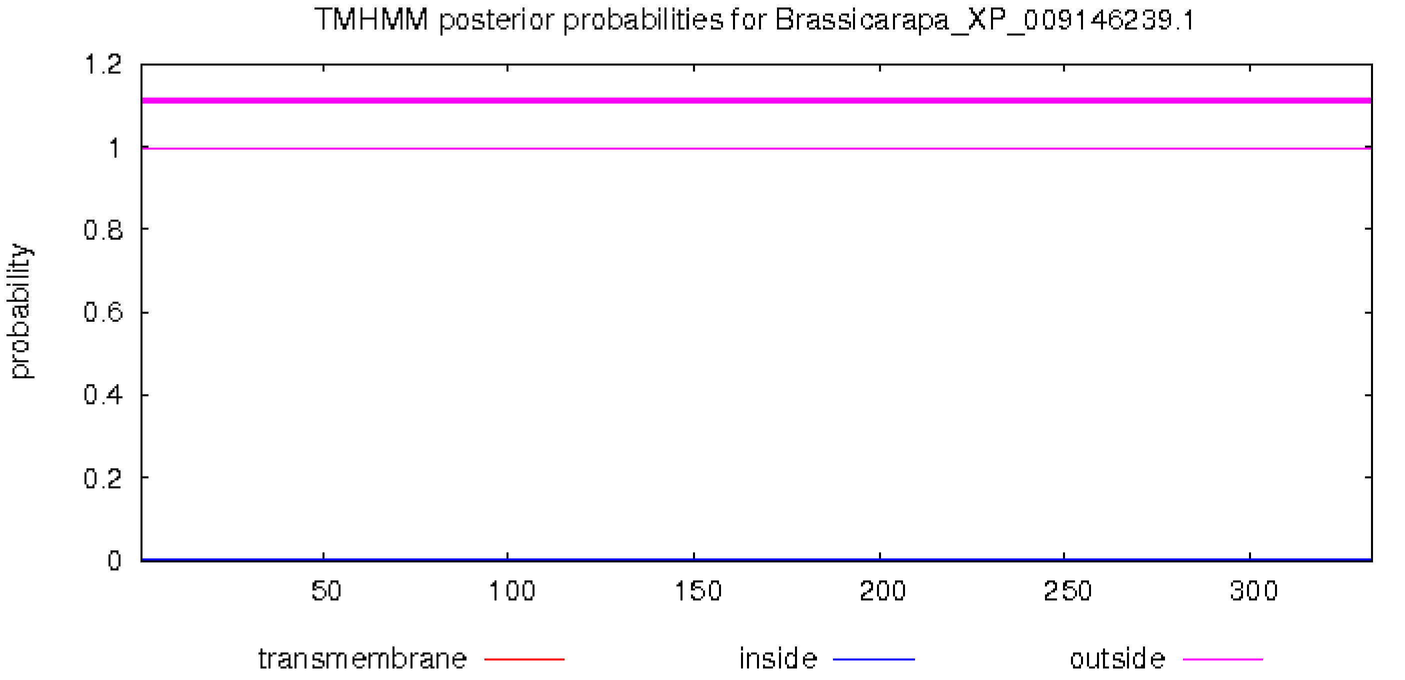

plot in postscript, script for making the plot in gnuplot, data for plot

---

```
# Brassicarapa_XP_009146450.1 Length: 371
# Brassicarapa_XP_009146450.1 Number of predicted TMHs:  0
# Brassicarapa_XP_009146450.1 Exp number of AAs in TMHs: 0
```

```
# Brassicarapa_XP_009146450.1 Exp number, first 60 AAs:  0
# Brassicarapa_XP_009146450.1 Total prob of N-in:        0.01334
Brassicarapa_XP_009146450.1     TMHMM2.0        outside      1   371
```

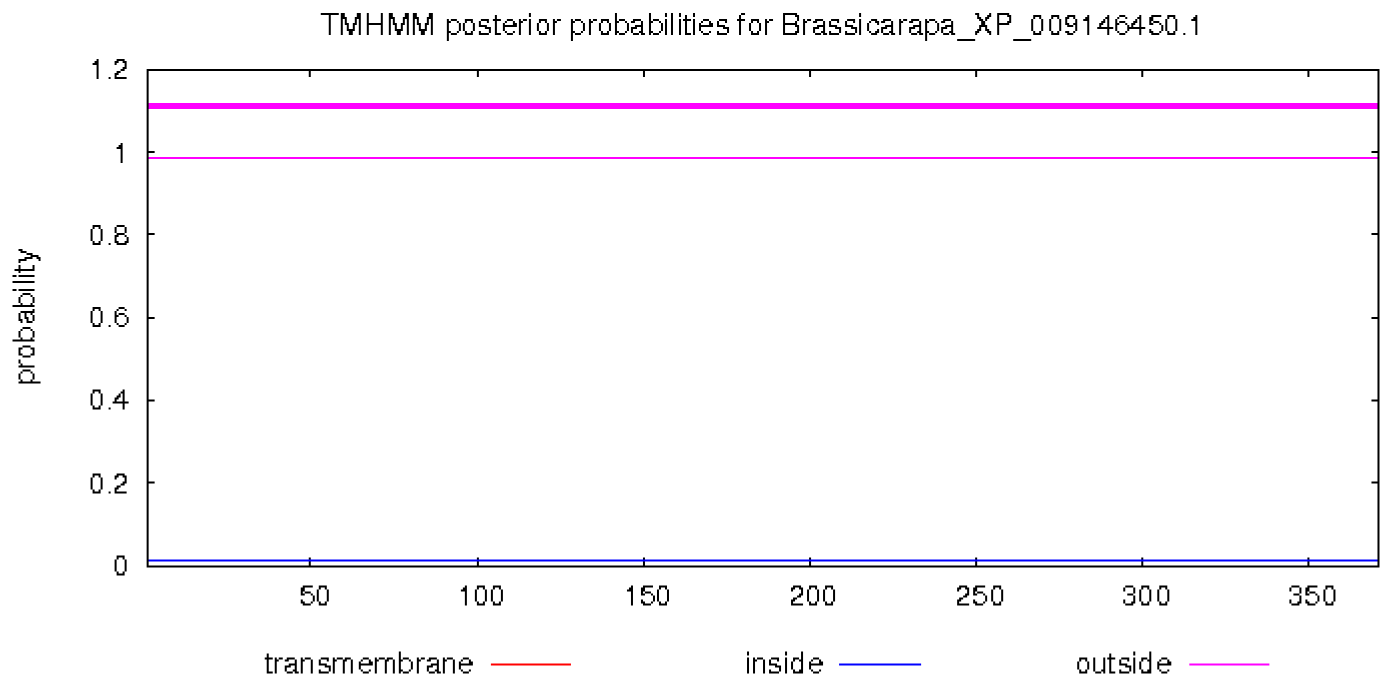

plot in postscript, script for making the plot in gnuplot, data for plot

---

```
# Brassicarapa_XP_009146562.1 Length: 280
# Brassicarapa_XP_009146562.1 Number of predicted TMHs:  0
# Brassicarapa_XP_009146562.1 Exp number of AAs in TMHs: 0.00017
# Brassicarapa_XP_009146562.1 Exp number, first 60 AAs:  0
# Brassicarapa_XP_009146562.1 Total prob of N-in:        0.04037
Brassicarapa_XP_009146562.1     TMHMM2.0        outside      1   280
```

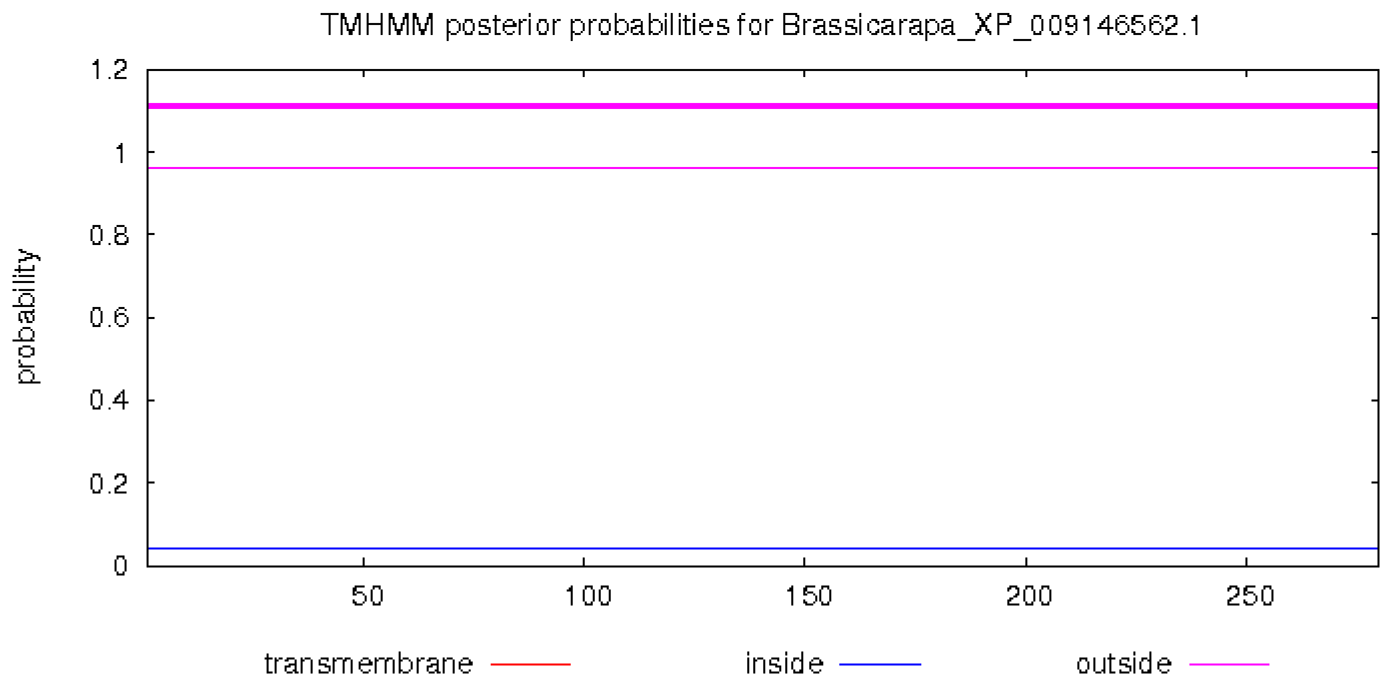

plot in postscript, script for making the plot in gnuplot, data for plot

---

```
# Brassicarapa_XP_009146563.1 Length: 275
# Brassicarapa_XP_009146563.1 Number of predicted TMHs:  0
# Brassicarapa_XP_009146563.1 Exp number of AAs in TMHs: 0.00017
```

```
# Brassicarapa_XP_009146563.1 Exp number, first 60 AAs:  0
# Brassicarapa_XP_009146563.1 Total prob of N-in:        0.04286
Brassicarapa_XP_009146563.1     TMHMM2.0        outside      1   275
```

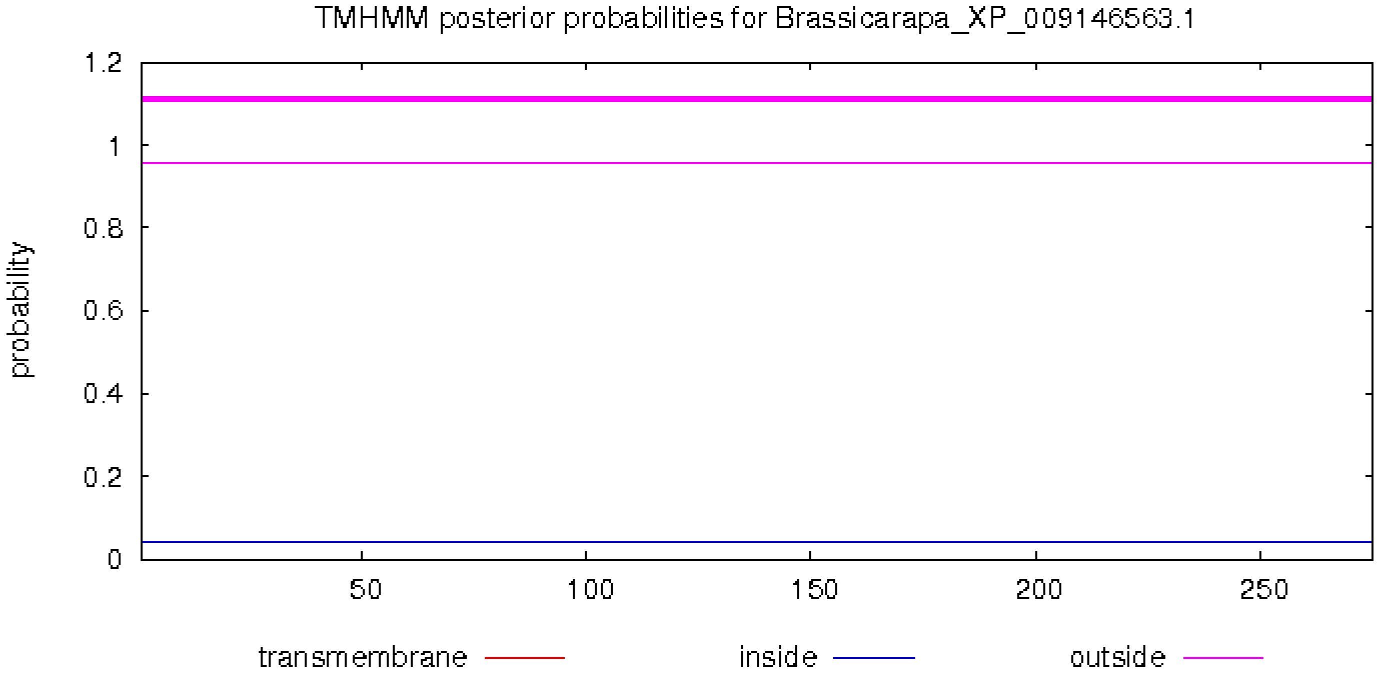

plot in postscript, script for making the plot in gnuplot, data for plot

---

```
# Brassicarapa_XP_009146571.1 Length: 308
# Brassicarapa_XP_009146571.1 Number of predicted TMHs:  0
# Brassicarapa_XP_009146571.1 Exp number of AAs in TMHs: 0.00282
# Brassicarapa_XP_009146571.1 Exp number, first 60 AAs:  0
# Brassicarapa_XP_009146571.1 Total prob of N-in:        0.02450
Brassicarapa_XP_009146571.1     TMHMM2.0        outside      1   308
```

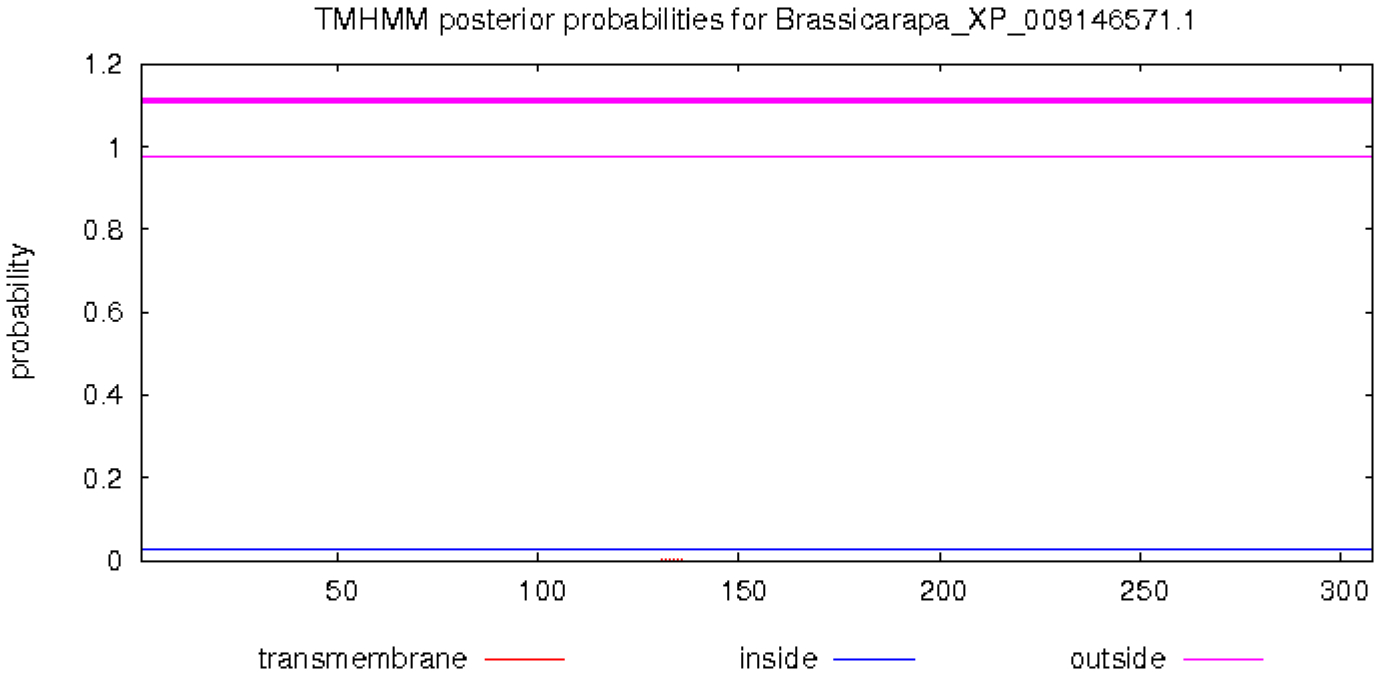

plot in postscript, script for making the plot in gnuplot, data for plot

---

```
# Brassicarapa_XP_009146834.1 Length: 455
# Brassicarapa_XP_009146834.1 Number of predicted TMHs:  0
# Brassicarapa_XP_009146834.1 Exp number of AAs in TMHs: 0.09474
```

```
# Brassicarapa_XP_009146834.1 Exp number, first 60 AAs:  0.06543
# Brassicarapa_XP_009146834.1 Total prob of N-in:        0.00669
Brassicarapa_XP_009146834.1     TMHMM2.0        outside      1   455
```

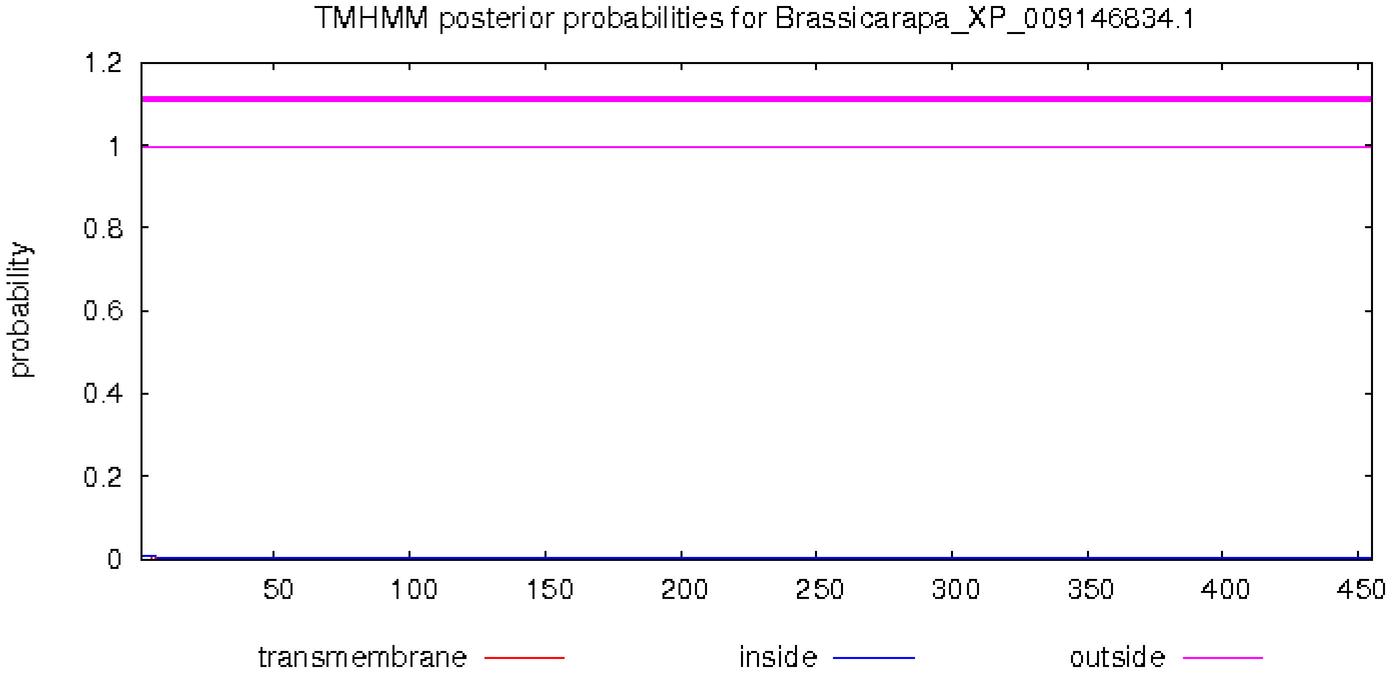

plot in postscript, script for making the plot in gnuplot, data for plot

---

```
# Brassicarapa_XP_009146835.1 Length: 452
# Brassicarapa_XP_009146835.1 Number of predicted TMHs:  0
# Brassicarapa_XP_009146835.1 Exp number of AAs in TMHs: 0.09487
# Brassicarapa_XP_009146835.1 Exp number, first 60 AAs:  0.06544
# Brassicarapa_XP_009146835.1 Total prob of N-in:        0.00683
Brassicarapa_XP_009146835.1     TMHMM2.0        outside      1   452
```

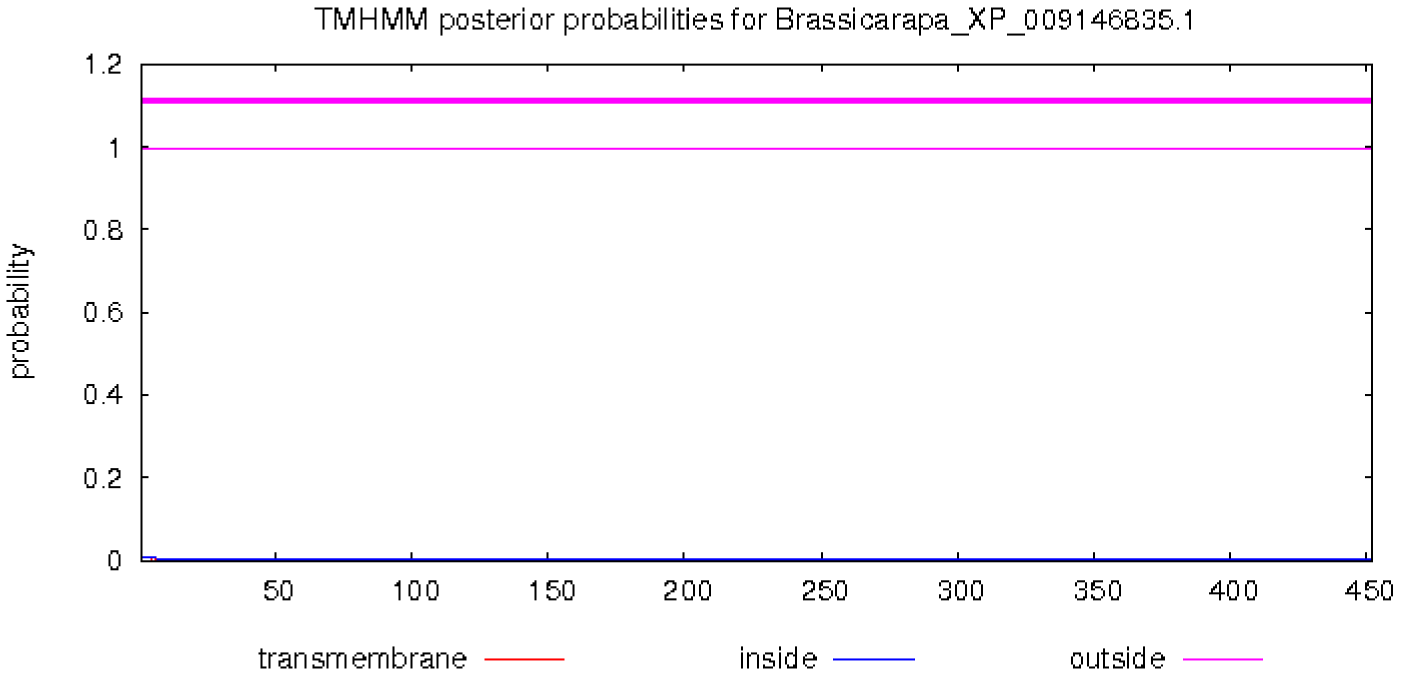

plot in postscript, script for making the plot in gnuplot, data for plot

---

```
# Brassicarapa_XP_009147649.1 Length: 315
# Brassicarapa_XP_009147649.1 Number of predicted TMHs:  0
# Brassicarapa_XP_009147649.1 Exp number of AAs in TMHs: 0.01177
```

```
# Brassicarapa_XP_009147649.1 Exp number, first 60 AAs:  0.00021
# Brassicarapa_XP_009147649.1 Total prob of N-in:        0.00927
Brassicarapa_XP_009147649.1     TMHMM2.0        outside      1   315
```

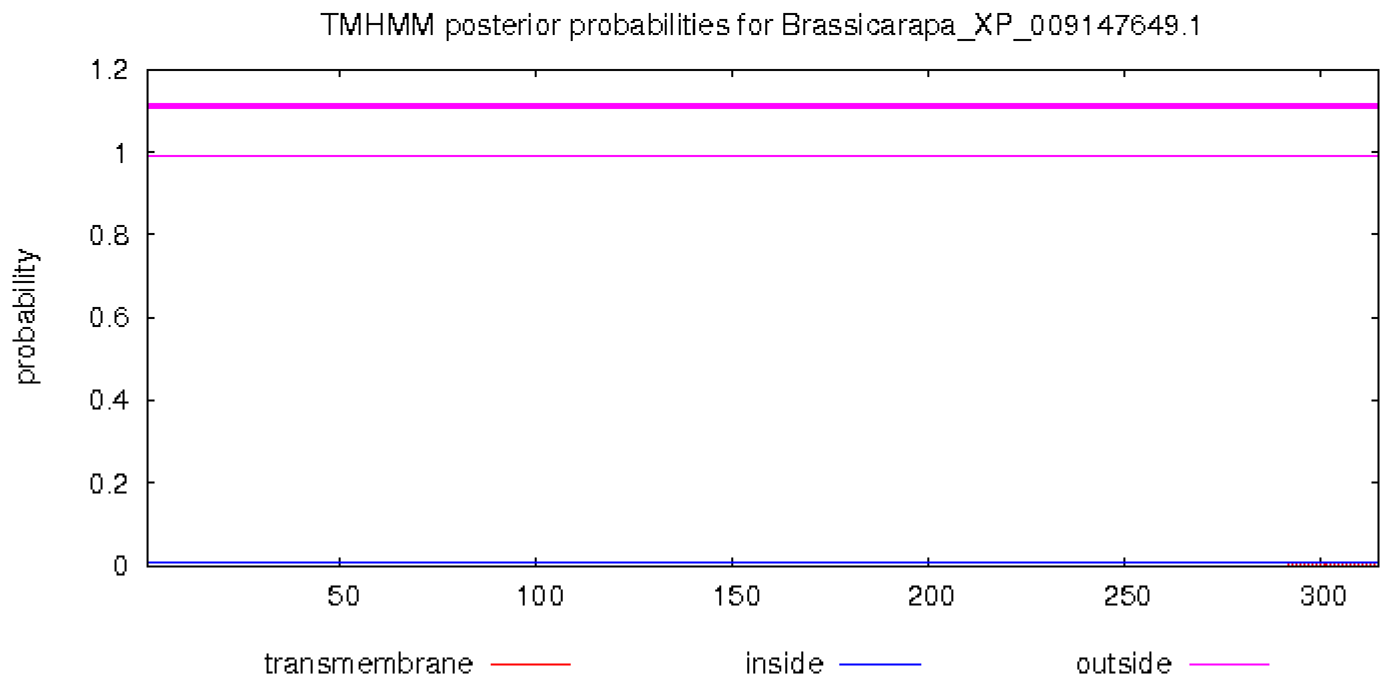

plot in postscript, script for making the plot in gnuplot, data for plot

---

```
# Brassicarapa_XP_009147650.1 Length: 335
# Brassicarapa_XP_009147650.1 Number of predicted TMHs:  0
# Brassicarapa_XP_009147650.1 Exp number of AAs in TMHs: 0.00021
# Brassicarapa_XP_009147650.1 Exp number, first 60 AAs:  0
# Brassicarapa_XP_009147650.1 Total prob of N-in:        0.03210
Brassicarapa_XP_009147650.1     TMHMM2.0        outside      1   335
```

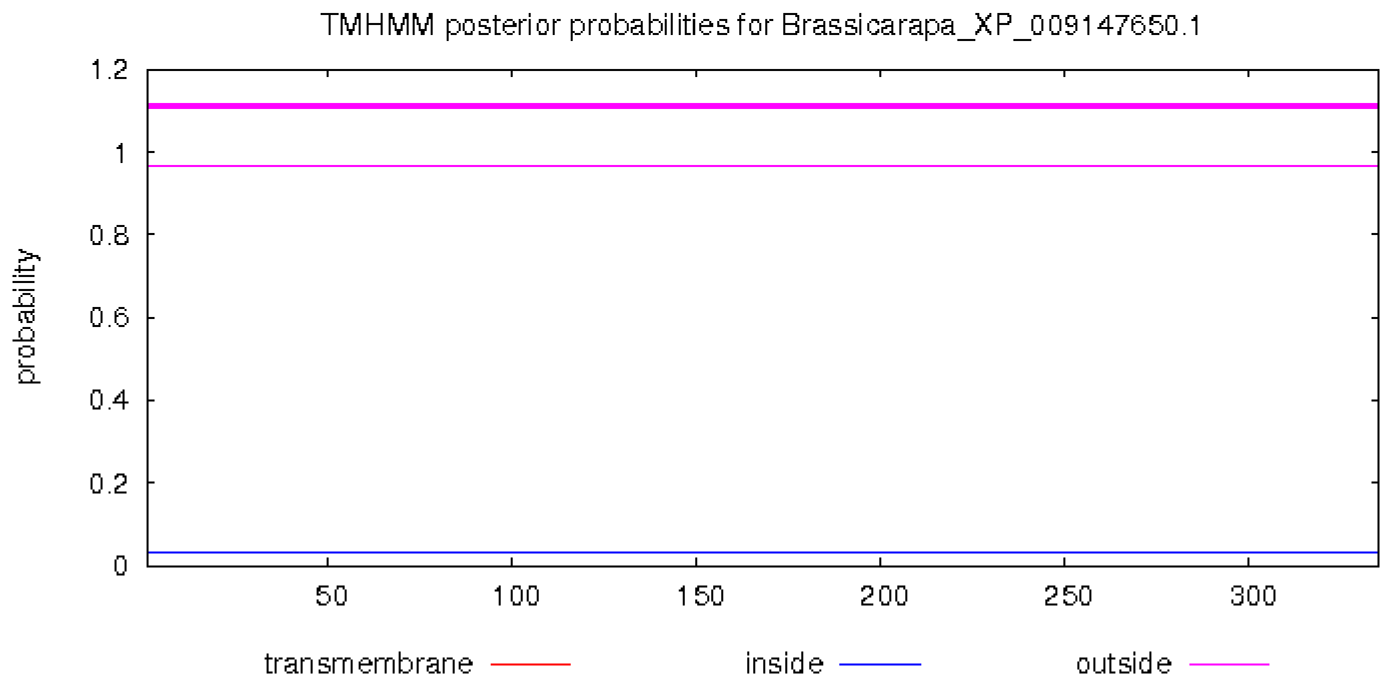

plot in postscript, script for making the plot in gnuplot, data for plot

---

```
# Brassicarapa_XP_009147651.1 Length: 327
# Brassicarapa_XP_009147651.1 Number of predicted TMHs:  0
# Brassicarapa_XP_009147651.1 Exp number of AAs in TMHs: 0.00022
```

```
# Brassicarapa_XP_009147651.1 Exp number, first 60 AAs:  0
# Brassicarapa_XP_009147651.1 Total prob of N-in:        0.03535
Brassicarapa_XP_009147651.1     TMHMM2.0        outside      1   327
```

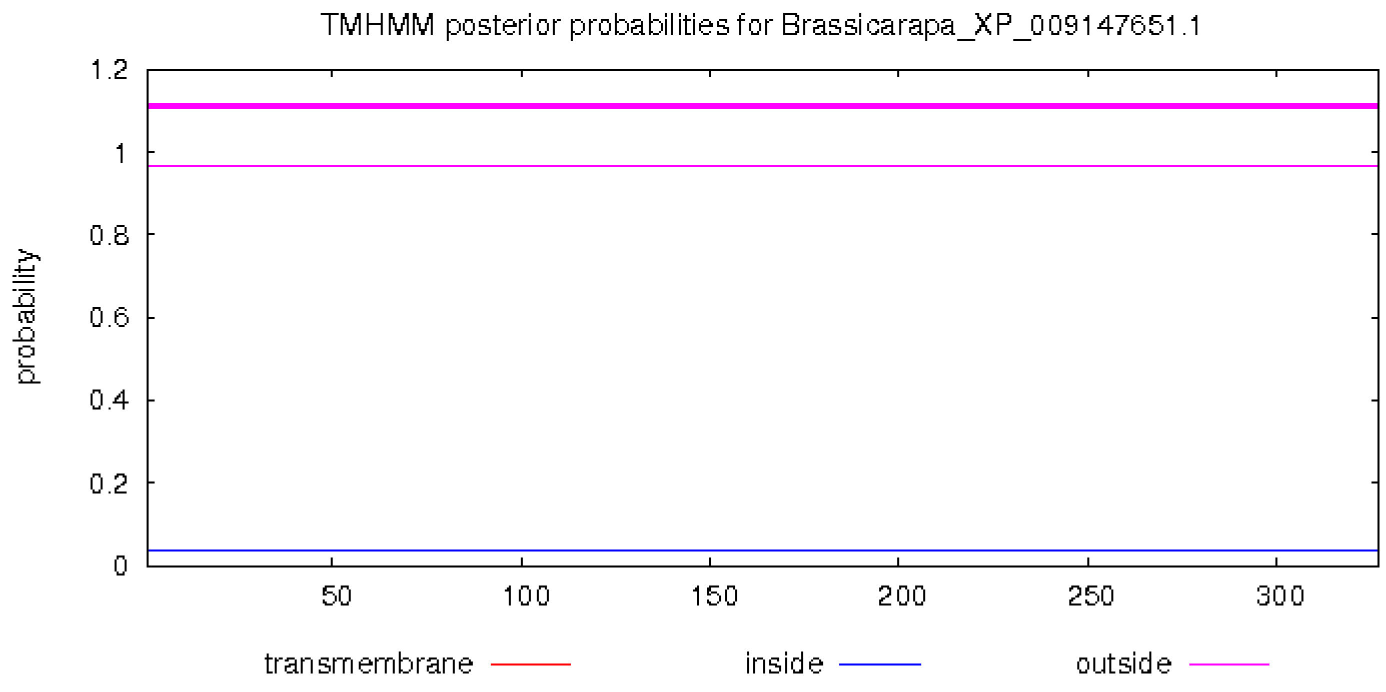

plot in postscript, script for making the plot in gnuplot, data for plot

---

```
# Brassicarapa_XP_009148605.1 Length: 393
# Brassicarapa_XP_009148605.1 Number of predicted TMHs:  0
# Brassicarapa_XP_009148605.1 Exp number of AAs in TMHs: 0
# Brassicarapa_XP_009148605.1 Exp number, first 60 AAs:  0
# Brassicarapa_XP_009148605.1 Total prob of N-in:        0.00090
Brassicarapa_XP_009148605.1     TMHMM2.0        outside      1   393
```

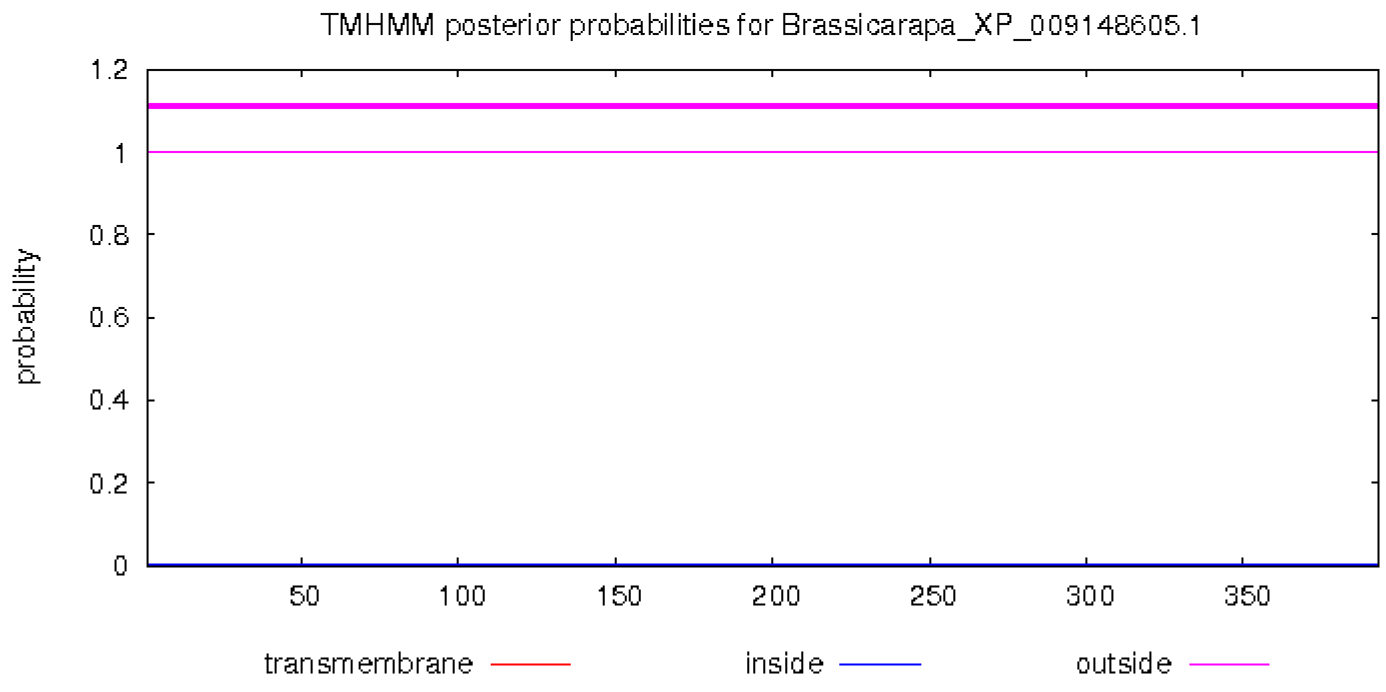

plot in postscript, script for making the plot in gnuplot, data for plot

---

```
# Brassicarapa_XP_009148606.1 Length: 342
# Brassicarapa_XP_009148606.1 Number of predicted TMHs:  0
# Brassicarapa_XP_009148606.1 Exp number of AAs in TMHs: 5e-05
```

```
# Brassicarapa_XP_009148606.1 Exp number, first 60 AAs:  0
# Brassicarapa_XP_009148606.1 Total prob of N-in:        0.00170
Brassicarapa_XP_009148606.1     TMHMM2.0        outside      1   342
```

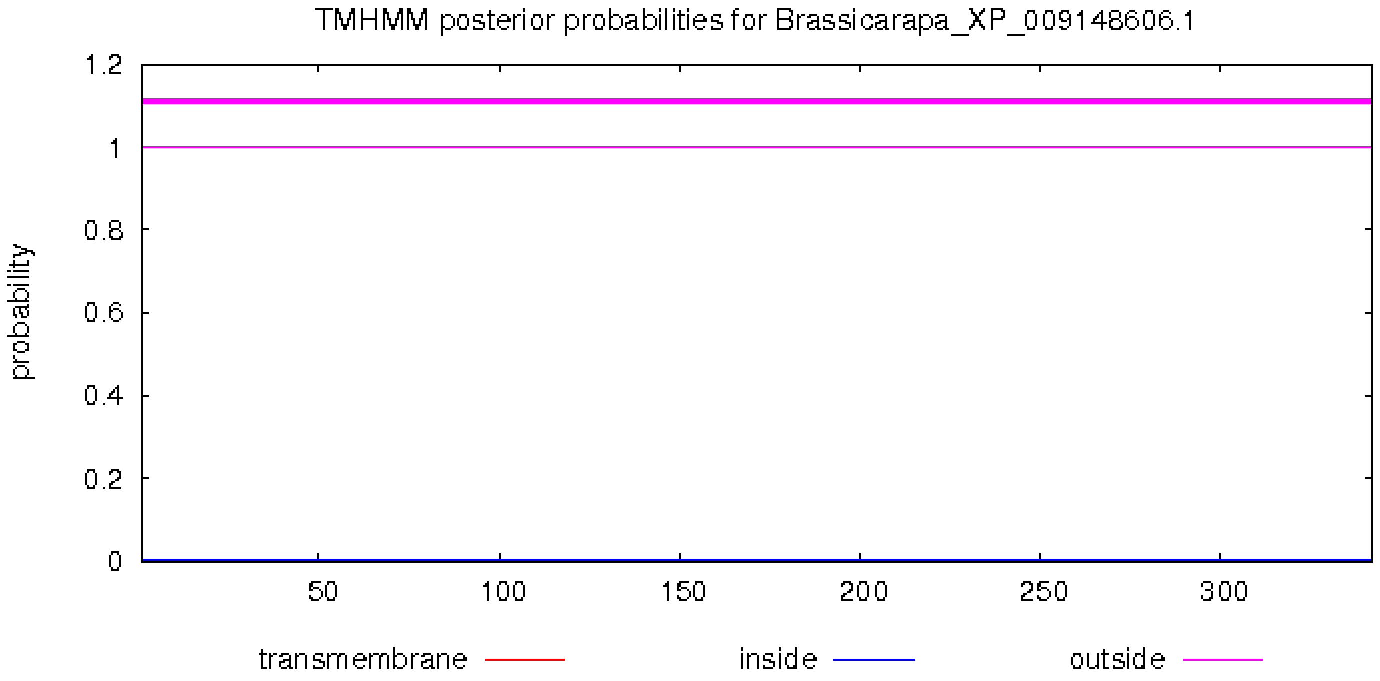

plot in postscript, script for making the plot in gnuplot, data for plot

---

```
# Brassicarapa_XP_009149732.1 Length: 463
# Brassicarapa_XP_009149732.1 Number of predicted TMHs:  0
# Brassicarapa_XP_009149732.1 Exp number of AAs in TMHs: 18.61843
# Brassicarapa_XP_009149732.1 Exp number, first 60 AAs:  0.00055
# Brassicarapa_XP_009149732.1 Total prob of N-in:        0.22874
Brassicarapa_XP_009149732.1     TMHMM2.0        outside      1   463
```

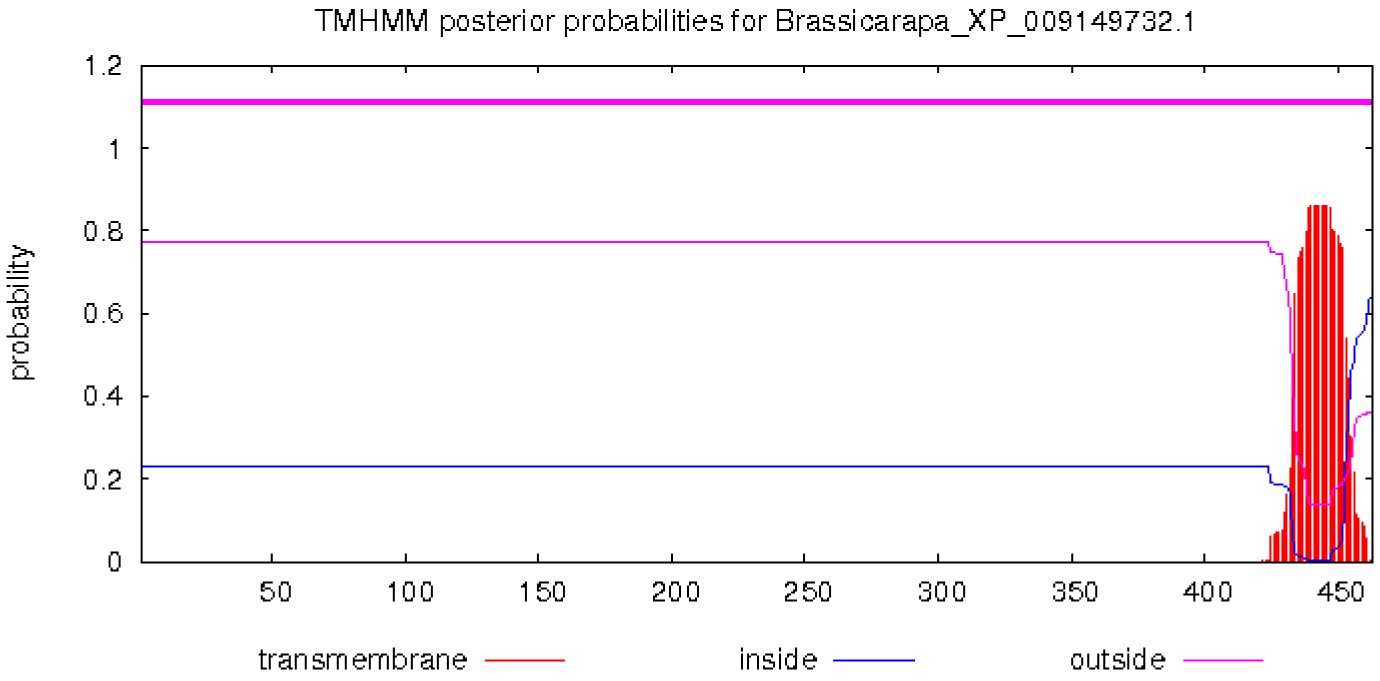

plot in postscript, script for making the plot in gnuplot, data for plot

---

```
# Brassicarapa_XP_009150140.1 Length: 432
# Brassicarapa_XP_009150140.1 Number of predicted TMHs:  1
# Brassicarapa_XP_009150140.1 Exp number of AAs in TMHs: 20.58103
```

```
# Brassicarapa_XP_009150140.1 Exp number, first 60 AAs:  0
# Brassicarapa_XP_009150140.1 Total prob of N-in:        0.13184
Brassicarapa_XP_009150140.1     TMHMM2.0        outside      1   399
Brassicarapa_XP_009150140.1     TMHMM2.0        TMhelix    400   422
Brassicarapa_XP_009150140.1     TMHMM2.0        inside     423   432
```

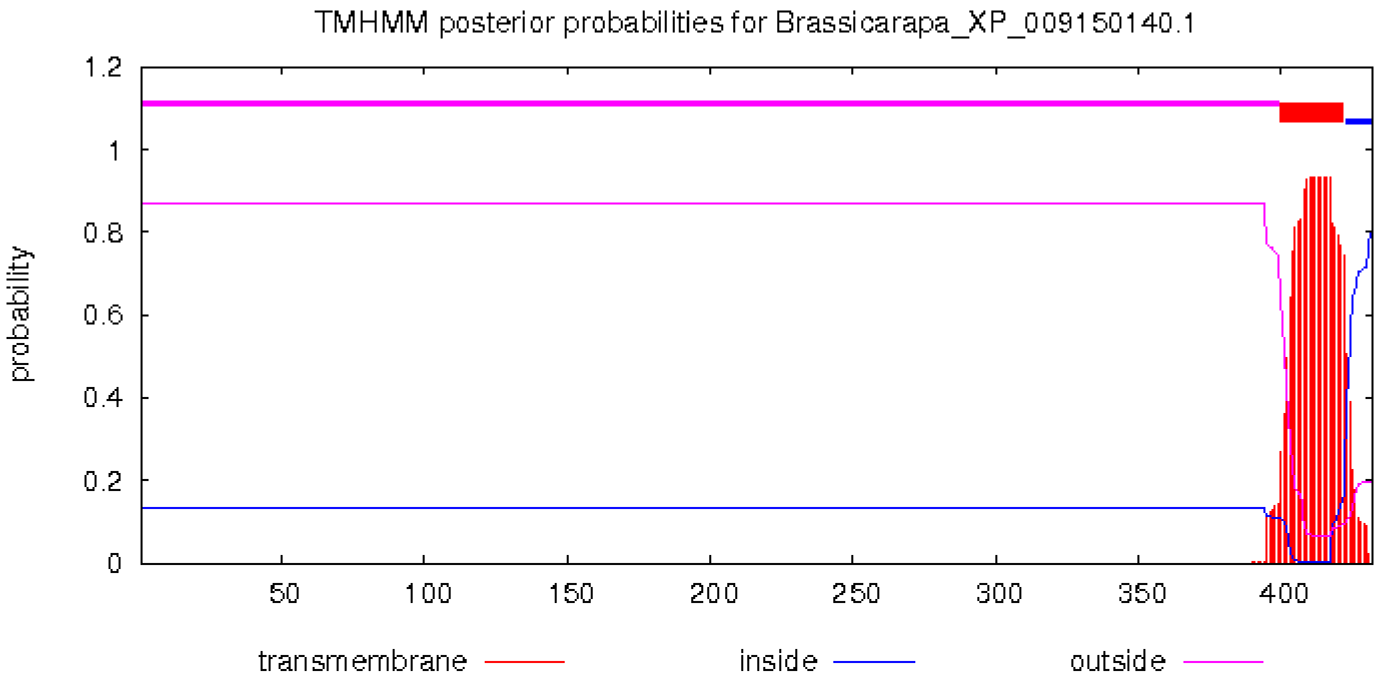

plot in postscript, script for making the plot in gnuplot, data for plot

---

```
# Brassicarapa_XP_009150275.1 Length: 281
# Brassicarapa_XP_009150275.1 Number of predicted TMHs:  0
# Brassicarapa_XP_009150275.1 Exp number of AAs in TMHs: 0.00762
# Brassicarapa_XP_009150275.1 Exp number, first 60 AAs:  0.00762
# Brassicarapa_XP_009150275.1 Total prob of N-in:        0.00249
Brassicarapa_XP_009150275.1     TMHMM2.0        outside      1   281
```

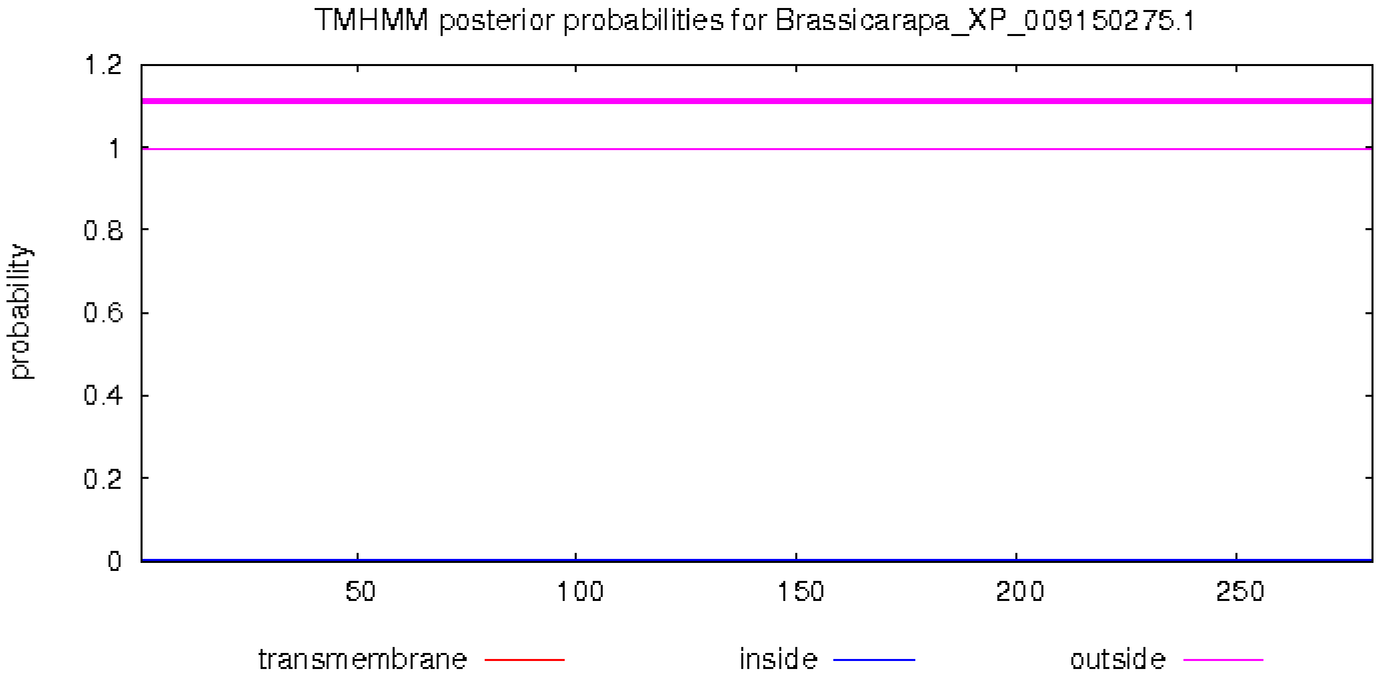

plot in postscript, script for making the plot in gnuplot, data for plot

---

```
# Brassicarapa_XP_009150295.1 Length: 274
# Brassicarapa_XP_009150295.1 Number of predicted TMHs:  0
# Brassicarapa_XP_009150295.1 Exp number of AAs in TMHs: 0.01564
# Brassicarapa_XP_009150295.1 Exp number, first 60 AAs:  0.01532
# Brassicarapa_XP_009150295.1 Total prob of N-in:        0.01301
Brassicarapa_XP_009150295.1     TMHMM2.0        outside      1   274
```

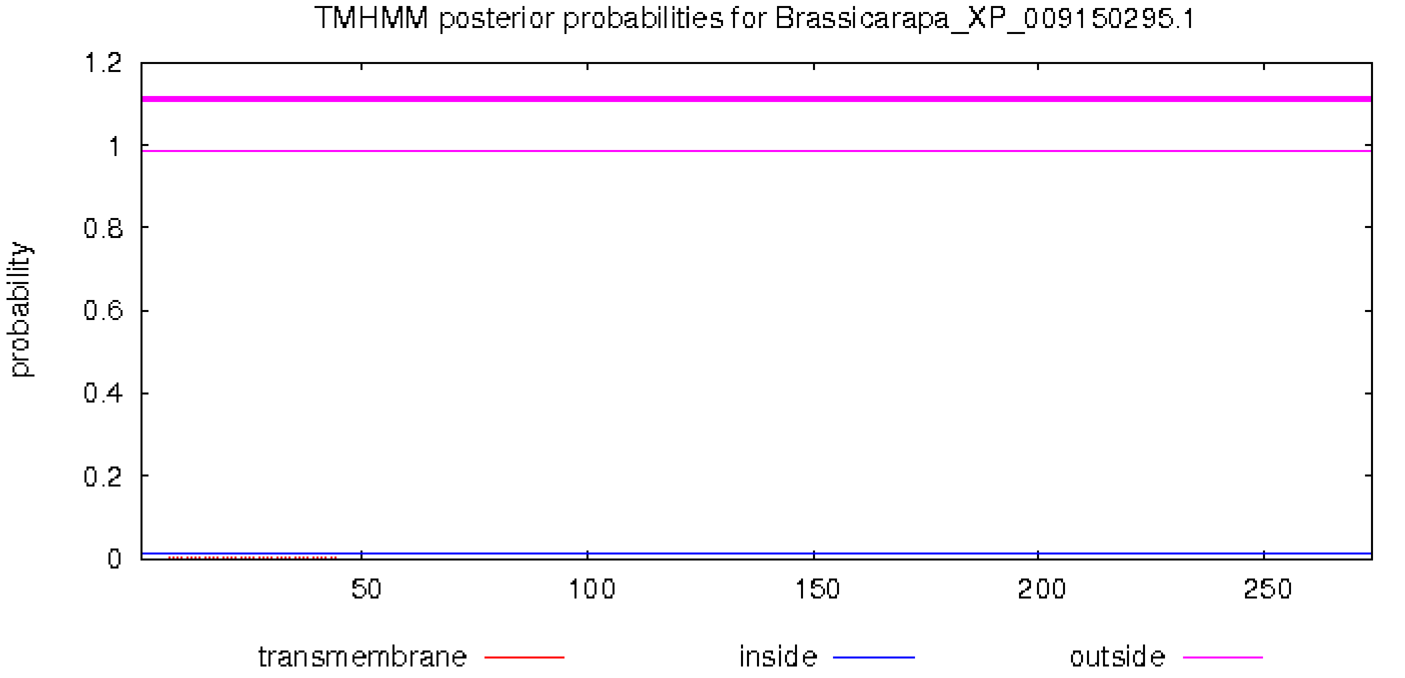

plot in postscript, script for making the plot in gnuplot, data for plot

```
# Brassicarapa_XP_009150429.1 Length: 250
# Brassicarapa_XP_009150429.1 Number of predicted TMHs:  0
# Brassicarapa_XP_009150429.1 Exp number of AAs in TMHs: 0.00042
# Brassicarapa_XP_009150429.1 Exp number, first 60 AAs:  0.00042
# Brassicarapa_XP_009150429.1 Total prob of N-in:        0.01339
Brassicarapa_XP_009150429.1     TMHMM2.0        outside      1   250
```

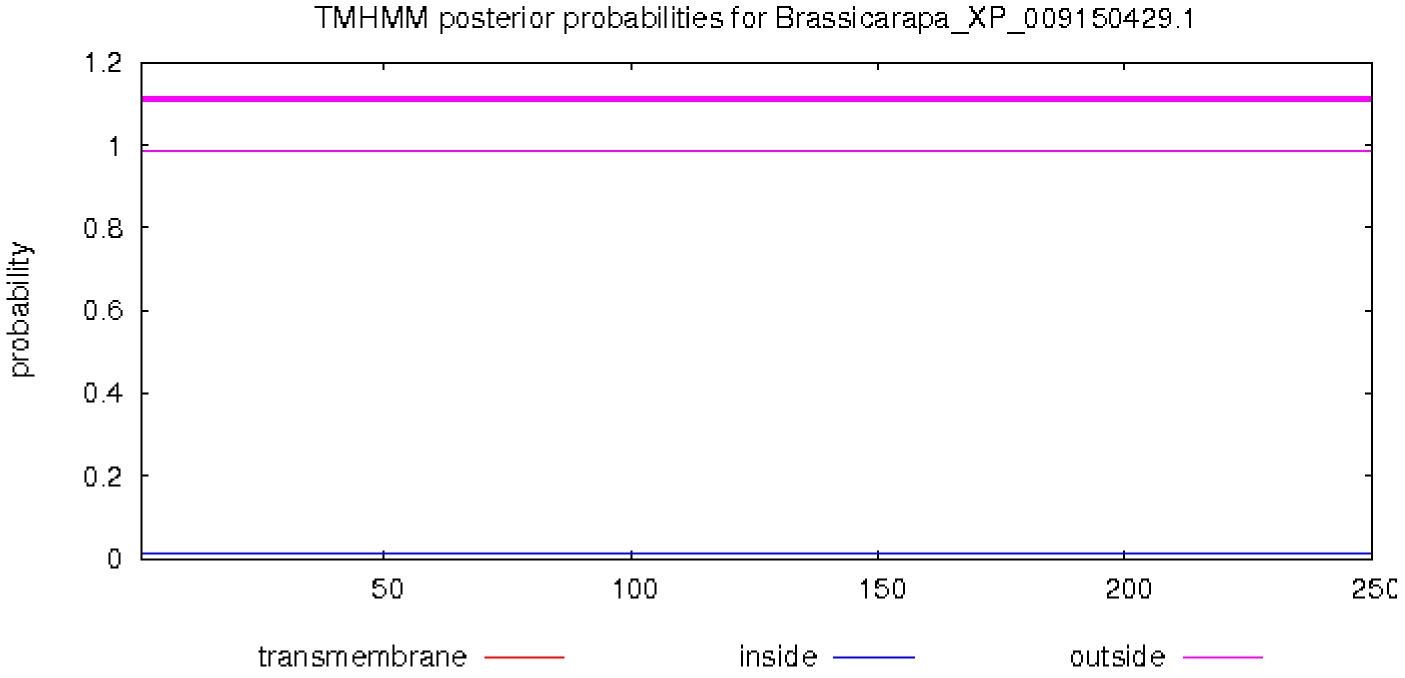

plot in postscript, script for making the plot in gnuplot, data for plot

```
# Brassicarapa_XP_009150476.1 Length: 344
# Brassicarapa_XP_009150476.1 Number of predicted TMHs:  0
# Brassicarapa_XP_009150476.1 Exp number of AAs in TMHs: 0.00018
# Brassicarapa_XP_009150476.1 Exp number, first 60 AAs:  0.00018
# Brassicarapa_XP_009150476.1 Total prob of N-in:        0.00343
Brassicarapa_XP_009150476.1     TMHMM2.0        outside      1   344
```

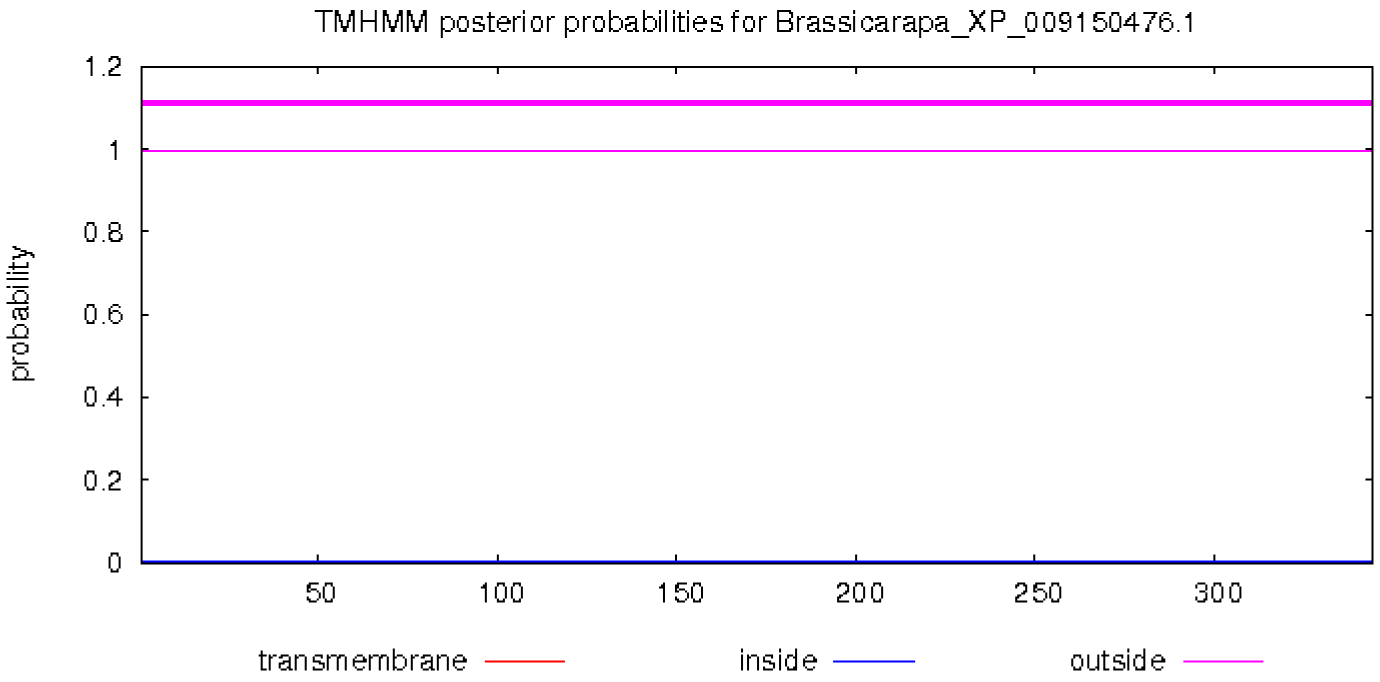

plot in postscript, script for making the plot in gnuplot, data for plot

---

```
# Brassicarapa_XP_009150535.1 Length: 188
# Brassicarapa_XP_009150535.1 Number of predicted TMHs:  0
# Brassicarapa_XP_009150535.1 Exp number of AAs in TMHs: 0
# Brassicarapa_XP_009150535.1 Exp number, first 60 AAs:  0
# Brassicarapa_XP_009150535.1 Total prob of N-in:        0.00724
Brassicarapa_XP_009150535.1     TMHMM2.0        outside      1   188
```

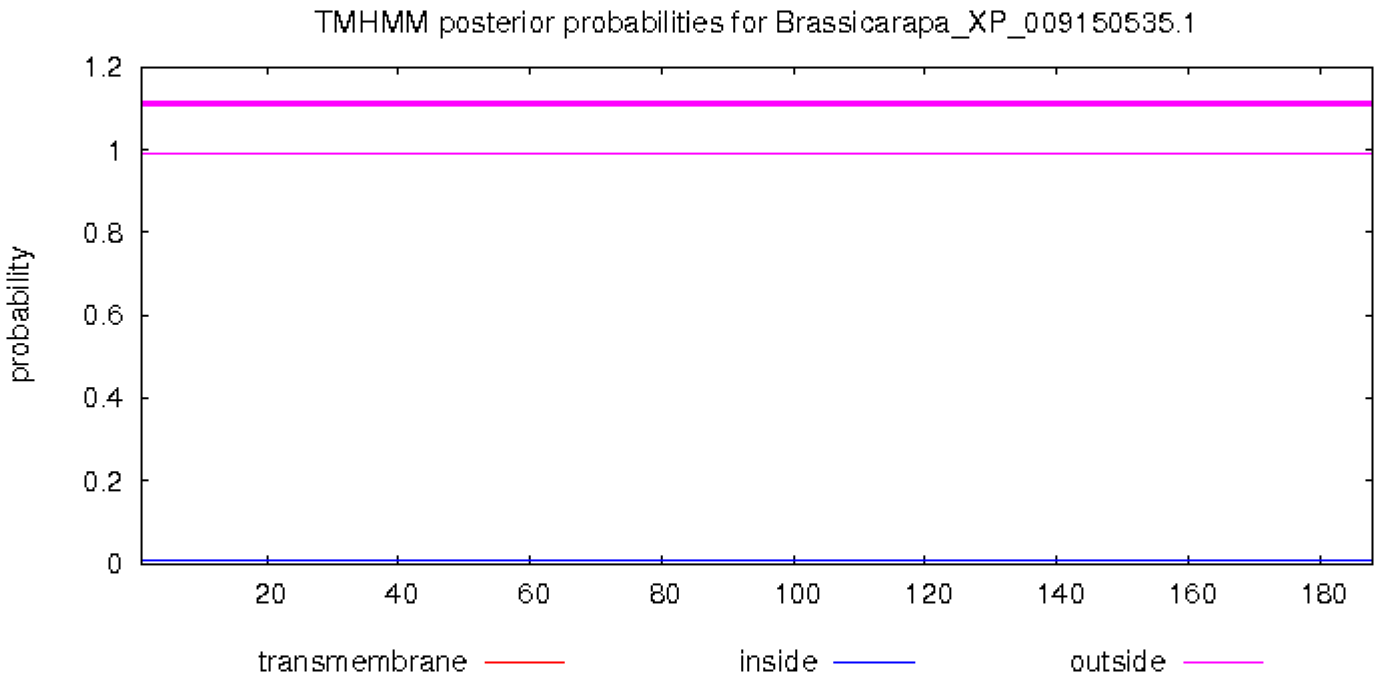

plot in postscript, script for making the plot in gnuplot, data for plot

---

```
# Brassicarapa_XP_009150982.1 Length: 456
# Brassicarapa_XP_009150982.1 Number of predicted TMHs:  0
# Brassicarapa_XP_009150982.1 Exp number of AAs in TMHs: 11.13208
# Brassicarapa_XP_009150982.1 Exp number, first 60 AAs:  0
# Brassicarapa_XP_009150982.1 Total prob of N-in:        0.00310
Brassicarapa_XP_009150982.1	TMHMM2.0	outside	     1   456
```

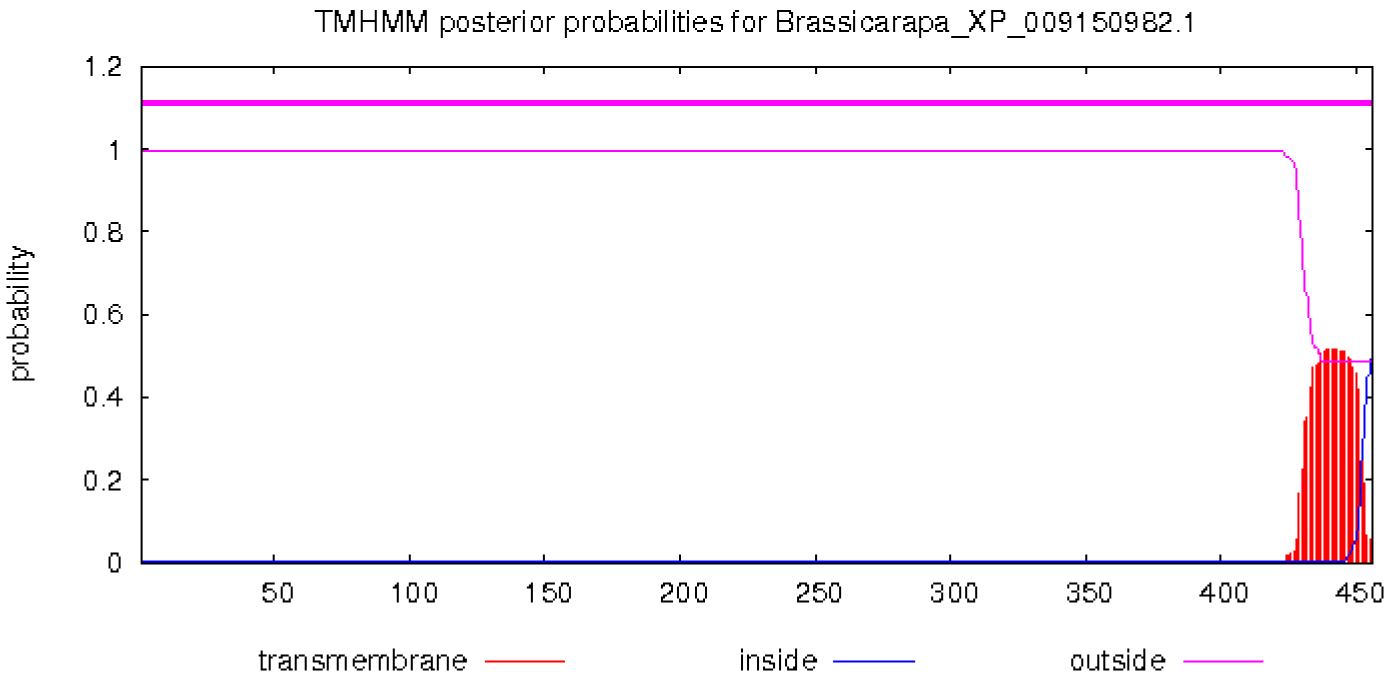

plot in postscript, script for making the plot in gnuplot, data for plot

---

```
# Brassicarapa_XP_009150983.1 Length: 455
# Brassicarapa_XP_009150983.1 Number of predicted TMHs:  0
# Brassicarapa_XP_009150983.1 Exp number of AAs in TMHs: 11.13242
# Brassicarapa_XP_009150983.1 Exp number, first 60 AAs:  0
# Brassicarapa_XP_009150983.1 Total prob of N-in:        0.00314
Brassicarapa_XP_009150983.1	TMHMM2.0	outside	     1   455
```

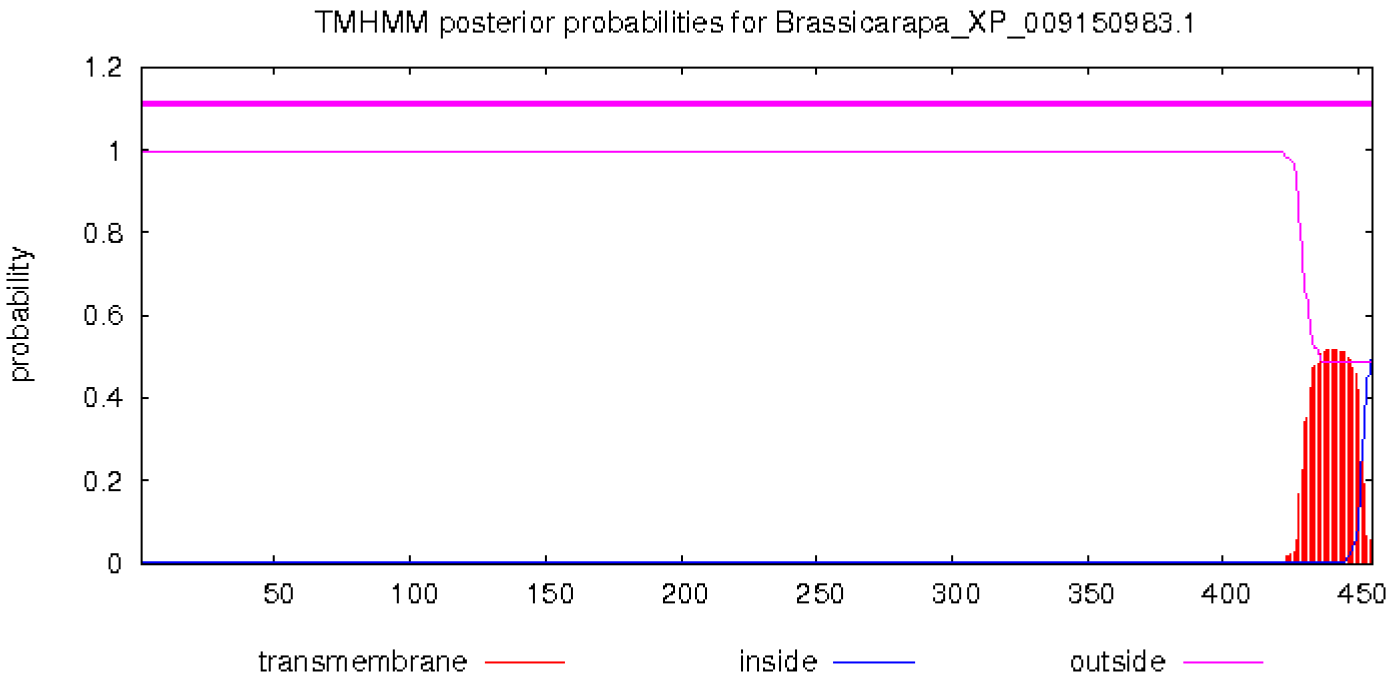

plot in postscript, script for making the plot in gnuplot, data for plot

---

```
# Brassicarapa_XP_009151357.1 Length: 347
# Brassicarapa_XP_009151357.1 Number of predicted TMHs:  0
# Brassicarapa_XP_009151357.1 Exp number of AAs in TMHs: 0.00018
# Brassicarapa_XP_009151357.1 Exp number, first 60 AAs:  0
# Brassicarapa_XP_009151357.1 Total prob of N-in:        0.00074
Brassicarapa_XP_009151357.1     TMHMM2.0        outside      1   347
```

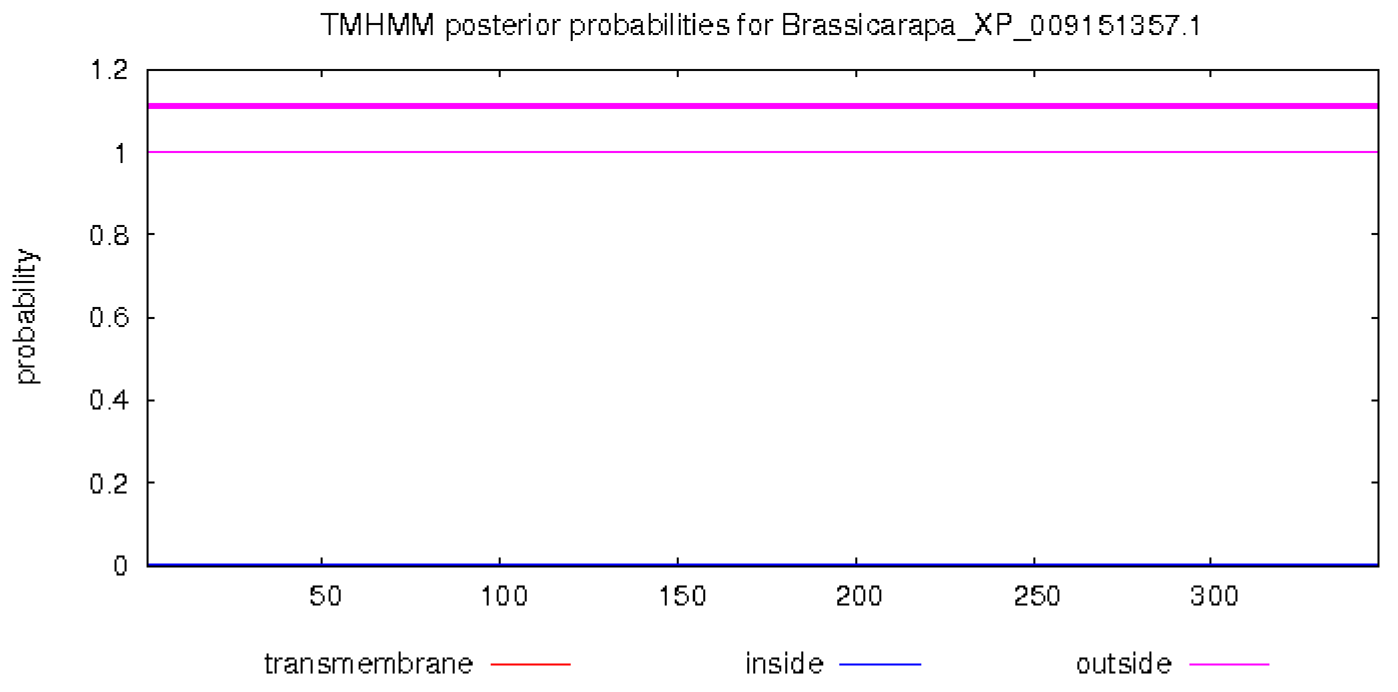

plot in postscript, script for making the plot in gnuplot, data for plot

---

```
# Brassicarapa_XP_009151358.1 Length: 314
# Brassicarapa_XP_009151358.1 Number of predicted TMHs:  0
# Brassicarapa_XP_009151358.1 Exp number of AAs in TMHs: 0.00019
# Brassicarapa_XP_009151358.1 Exp number, first 60 AAs:  0
# Brassicarapa_XP_009151358.1 Total prob of N-in:        0.04567
Brassicarapa_XP_009151358.1     TMHMM2.0        outside      1   314
```

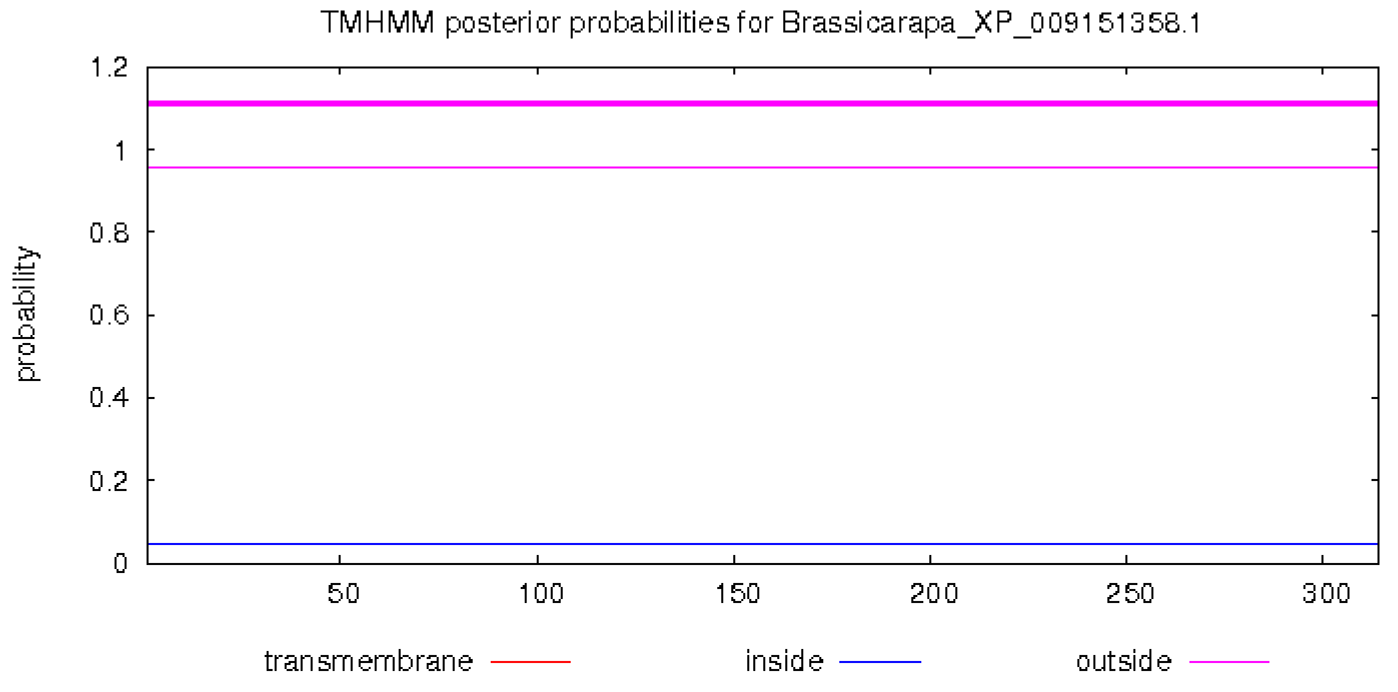

plot in postscript, script for making the plot in gnuplot, data for plot

---

```
# Brassicarapa_XP_009151359.1 Length: 314
# Brassicarapa_XP_009151359.1 Number of predicted TMHs:  0
# Brassicarapa_XP_009151359.1 Exp number of AAs in TMHs: 0.00019
# Brassicarapa_XP_009151359.1 Exp number, first 60 AAs:  0
# Brassicarapa_XP_009151359.1 Total prob of N-in:        0.04567
Brassicarapa_XP_009151359.1     TMHMM2.0        outside      1   314
```

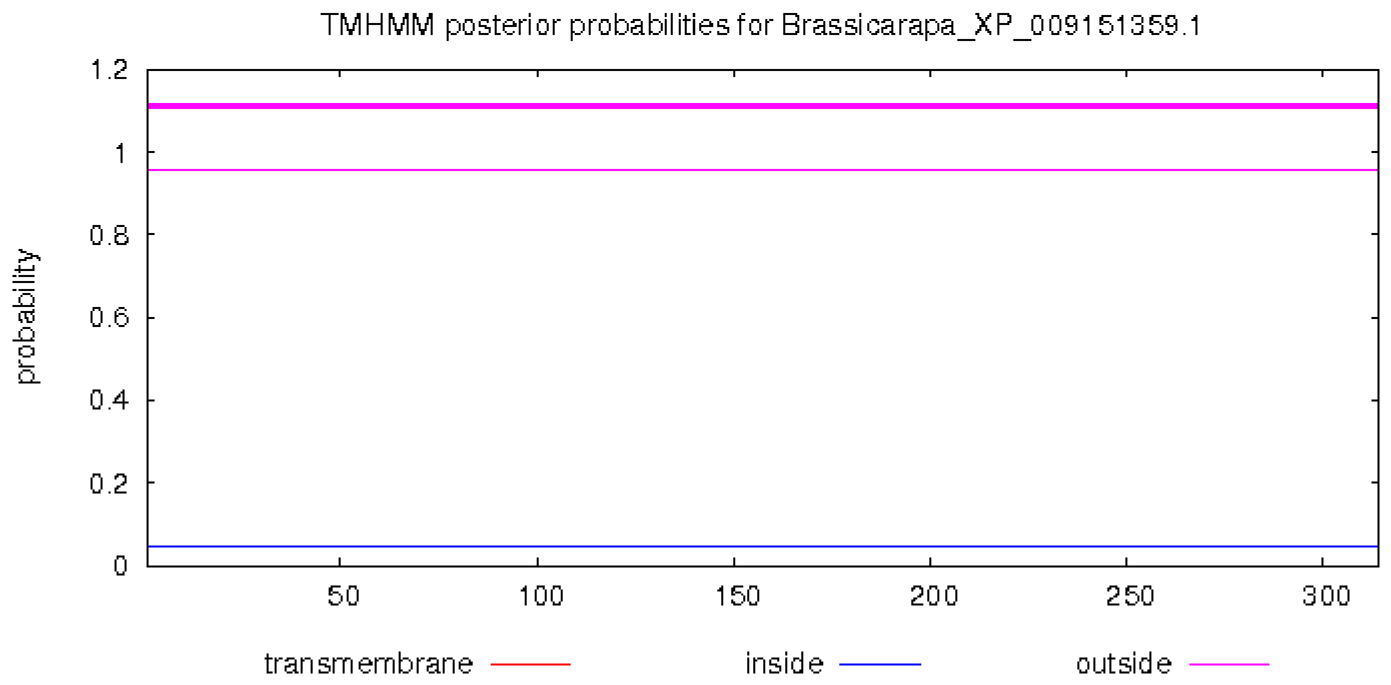

plot in postscript, script for making the plot in gnuplot, data for plot

---

```
# Brassicarapa_XP_009151451.1 Length: 360
# Brassicarapa_XP_009151451.1 Number of predicted TMHs:  0
# Brassicarapa_XP_009151451.1 Exp number of AAs in TMHs: 0
# Brassicarapa_XP_009151451.1 Exp number, first 60 AAs:  0
# Brassicarapa_XP_009151451.1 Total prob of N-in:        0.01534
Brassicarapa_XP_009151451.1     TMHMM2.0        outside      1   360
```

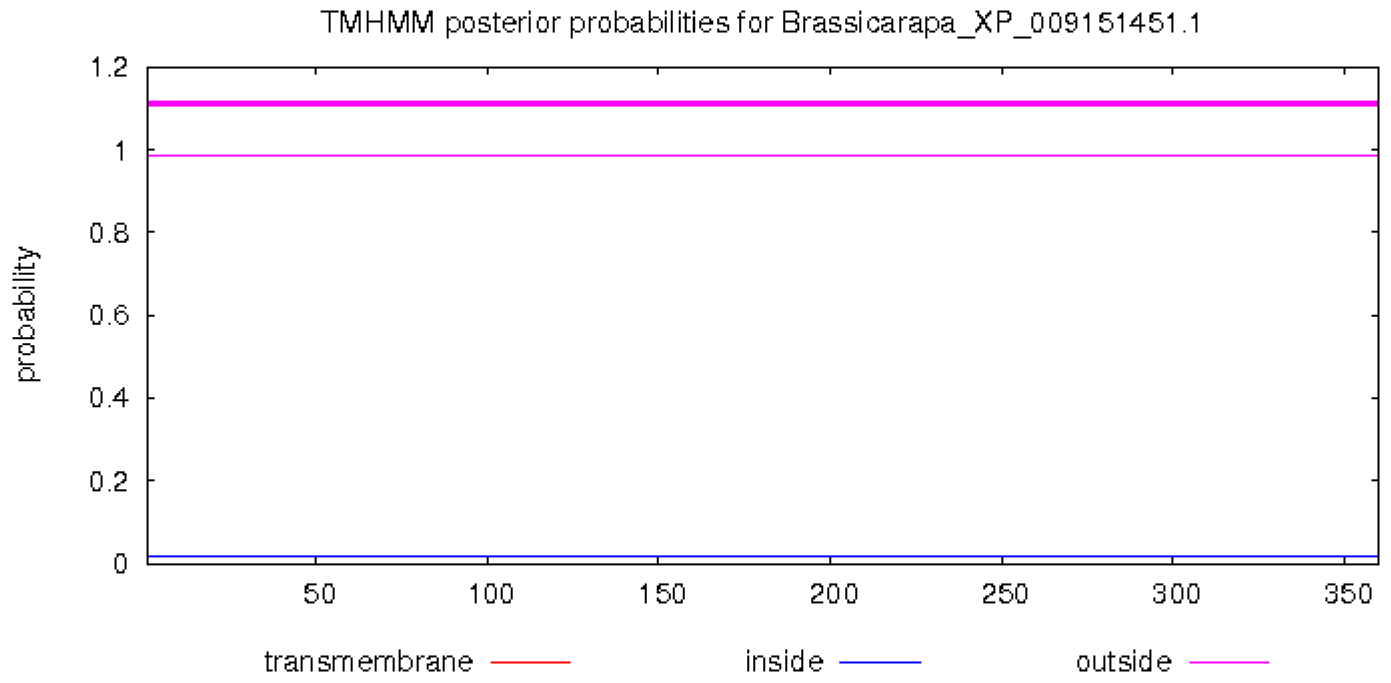

plot in postscript, script for making the plot in gnuplot, data for plot

---

```
# Brassicarapa_XP_009151462.1 Length: 636
# Brassicarapa_XP_009151462.1 Number of predicted TMHs:  1
# Brassicarapa_XP_009151462.1 Exp number of AAs in TMHs: 17.67923
# Brassicarapa_XP_009151462.1 Exp number, first 60 AAs:  0
# Brassicarapa_XP_009151462.1 Total prob of N-in:        0.19341
Brassicarapa_XP_009151462.1     TMHMM2.0        outside      1   595
Brassicarapa_XP_009151462.1     TMHMM2.0        TMhelix    596   613
Brassicarapa_XP_009151462.1     TMHMM2.0        inside     614   636
```

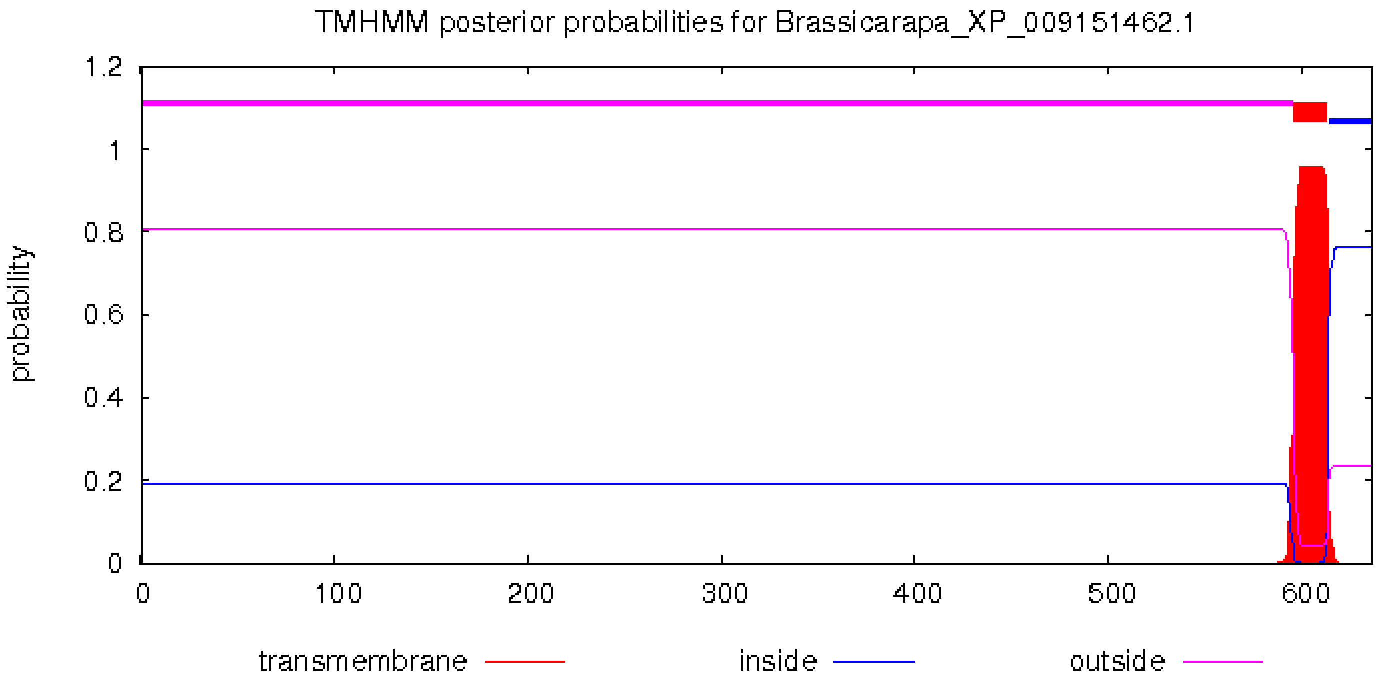

plot in postscript, script for making the plot in gnuplot, data for plot

---

```
# Brassicarapa_XP_009151811.1 Length: 309
# Brassicarapa_XP_009151811.1 Number of predicted TMHs:  0
# Brassicarapa_XP_009151811.1 Exp number of AAs in TMHs: 0.00447
# Brassicarapa_XP_009151811.1 Exp number, first 60 AAs:  0.00058
# Brassicarapa_XP_009151811.1 Total prob of N-in:        0.03958
Brassicarapa_XP_009151811.1     TMHMM2.0        outside      1   309
```

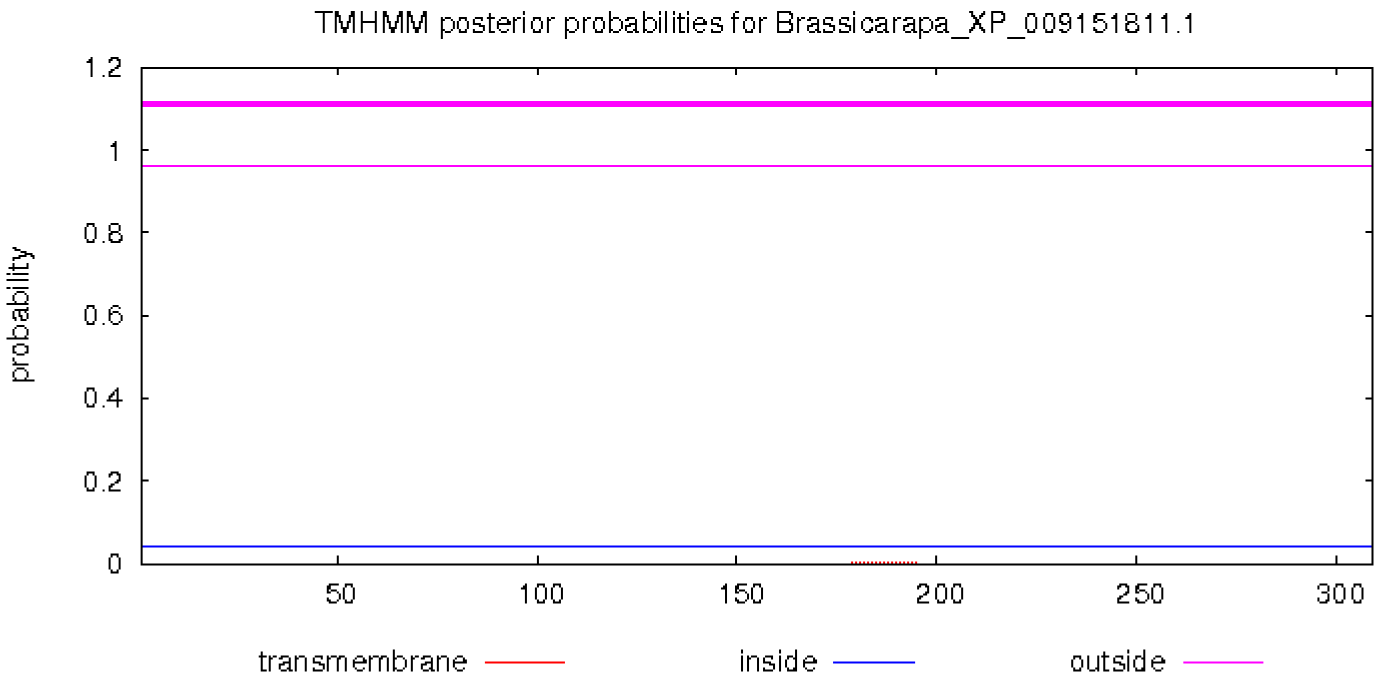

plot in postscript, script for making the plot in gnuplot, data for plot

```
# Cajanuscajan_C.cajan_01304 Length: 271
# Cajanuscajan_C.cajan_01304 Number of predicted TMHs:  0
# Cajanuscajan_C.cajan_01304 Exp number of AAs in TMHs: 0.00018
# Cajanuscajan_C.cajan_01304 Exp number, first 60 AAs:  0
# Cajanuscajan_C.cajan_01304 Total prob of N-in:        0.00429
Cajanuscajan_C.cajan_01304      TMHMM2.0        outside      1   271
```

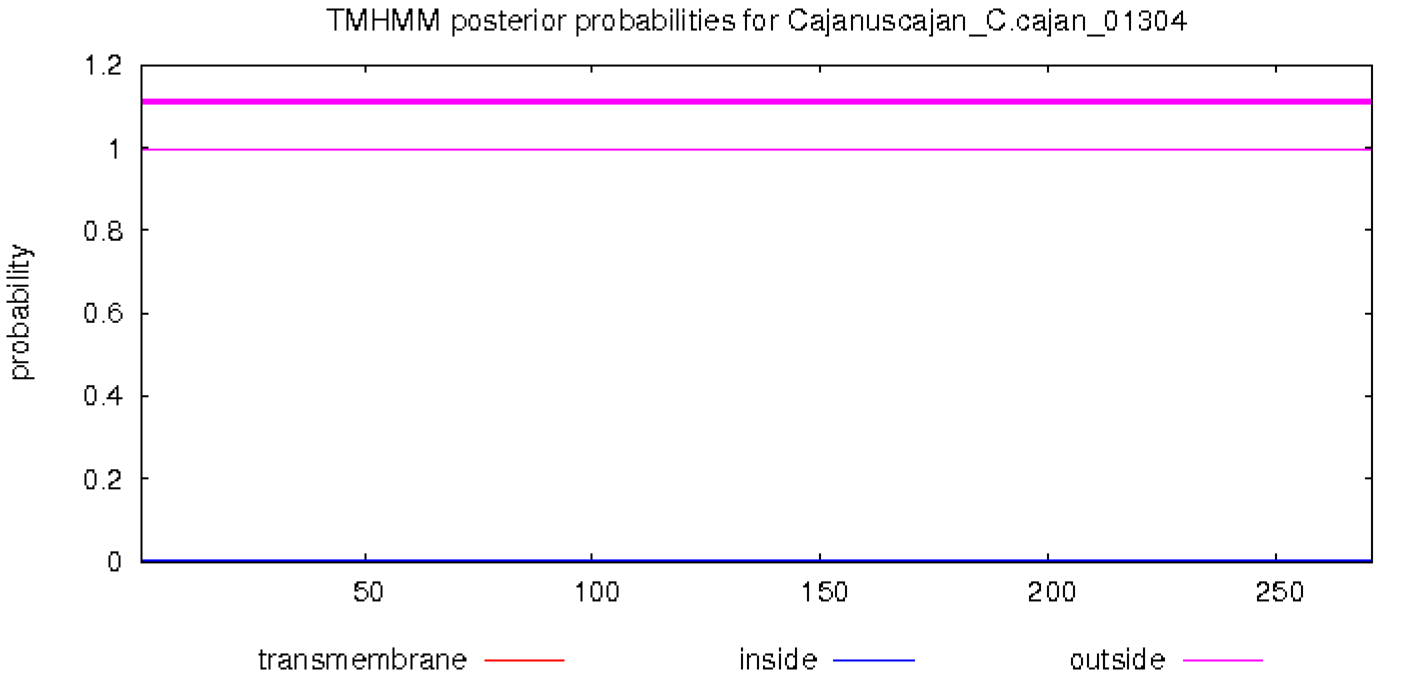

plot in postscript, script for making the plot in gnuplot, data for plot

```
# Cajanuscajan_C.cajan_01440 Length: 350
# Cajanuscajan_C.cajan_01440 Number of predicted TMHs:  0
# Cajanuscajan_C.cajan_01440 Exp number of AAs in TMHs: 0
# Cajanuscajan_C.cajan_01440 Exp number, first 60 AAs:  0
# Cajanuscajan_C.cajan_01440 Total prob of N-in:        0.00066
Cajanuscajan_C.cajan_01440      TMHMM2.0        outside      1   350
```

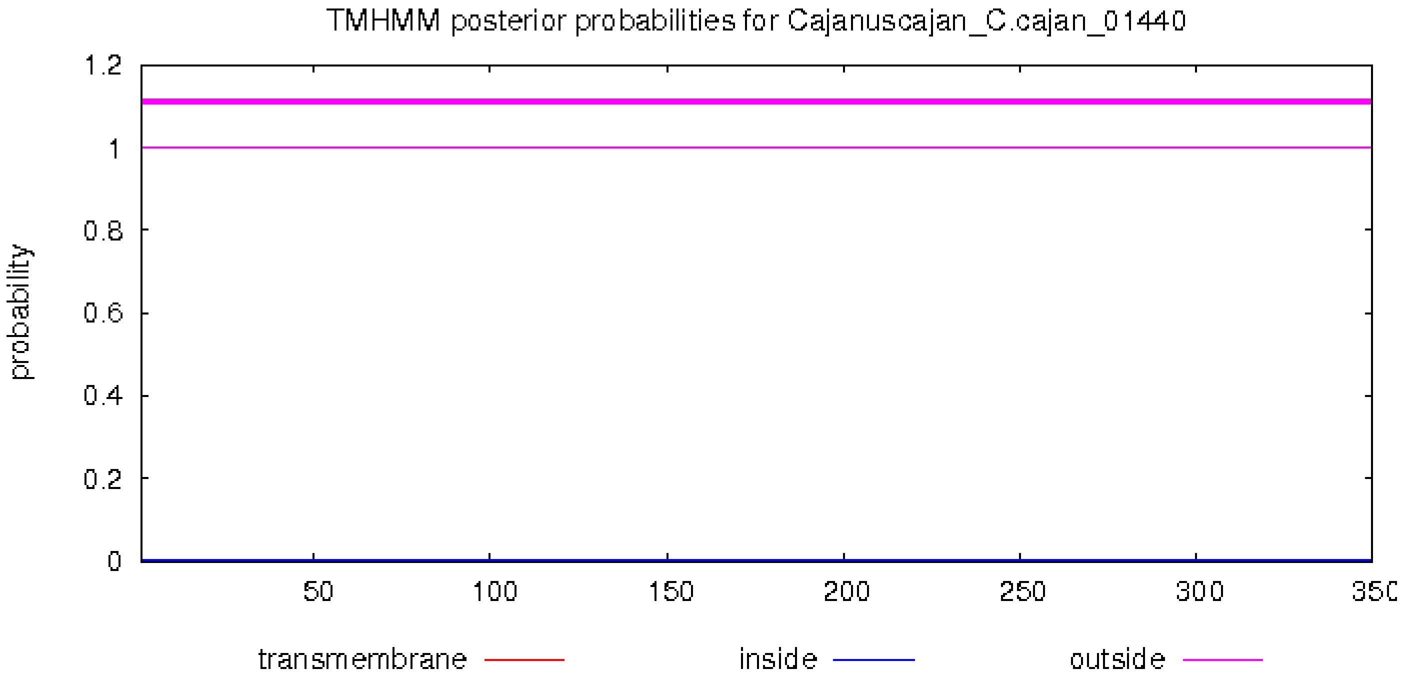

plot in postscript, script for making the plot in gnuplot, data for plot

```
# Cajanuscajan_C.cajan_01567 Length: 360
# Cajanuscajan_C.cajan_01567 Number of predicted TMHs:  0
# Cajanuscajan_C.cajan_01567 Exp number of AAs in TMHs: 0
# Cajanuscajan_C.cajan_01567 Exp number, first 60 AAs:  0
# Cajanuscajan_C.cajan_01567 Total prob of N-in:        0.00375
Cajanuscajan_C.cajan_01567      TMHMM2.0        outside      1   360
```

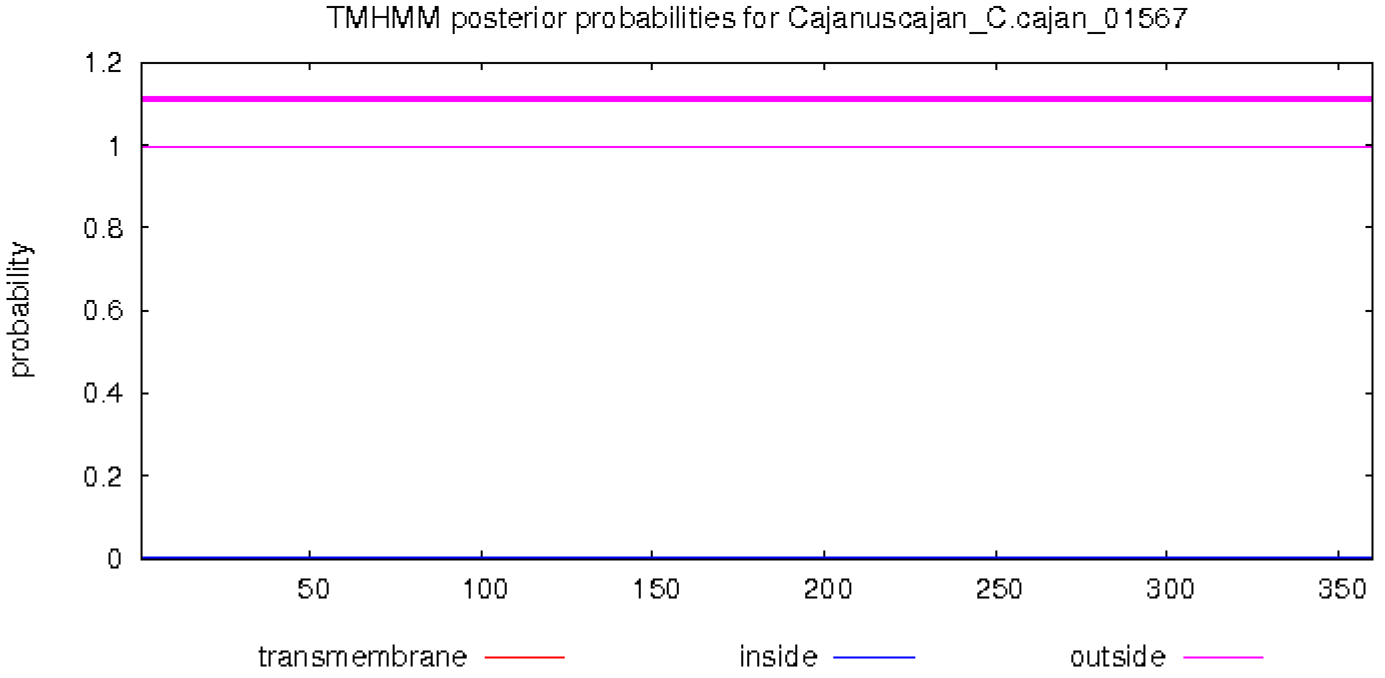

plot in postscript, script for making the plot in gnuplot, data for plot

---

```
# Cajanuscajan_C.cajan_01726 Length: 334
# Cajanuscajan_C.cajan_01726 Number of predicted TMHs:  0
# Cajanuscajan_C.cajan_01726 Exp number of AAs in TMHs: 0
# Cajanuscajan_C.cajan_01726 Exp number, first 60 AAs:  0
# Cajanuscajan_C.cajan_01726 Total prob of N-in:        0.00914
Cajanuscajan_C.cajan_01726      TMHMM2.0        outside      1   334
```

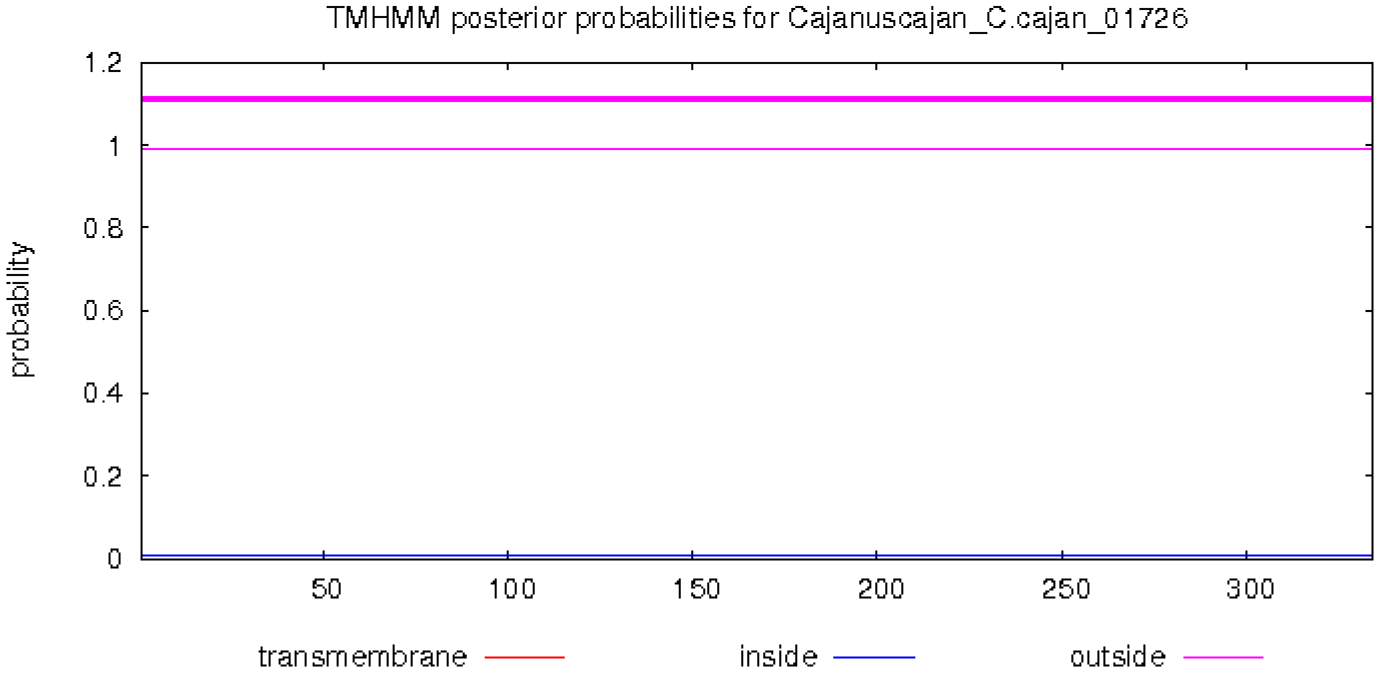

plot in postscript, script for making the plot in gnuplot, data for plot

---

```
# Cajanuscajan_C.cajan_02029 Length: 370
# Cajanuscajan_C.cajan_02029 Number of predicted TMHs:  0
# Cajanuscajan_C.cajan_02029 Exp number of AAs in TMHs: 0.00141
# Cajanuscajan_C.cajan_02029 Exp number, first 60 AAs:  0
# Cajanuscajan_C.cajan_02029 Total prob of N-in:        0.01263
Cajanuscajan_C.cajan_02029      TMHMM2.0        outside      1   370
```

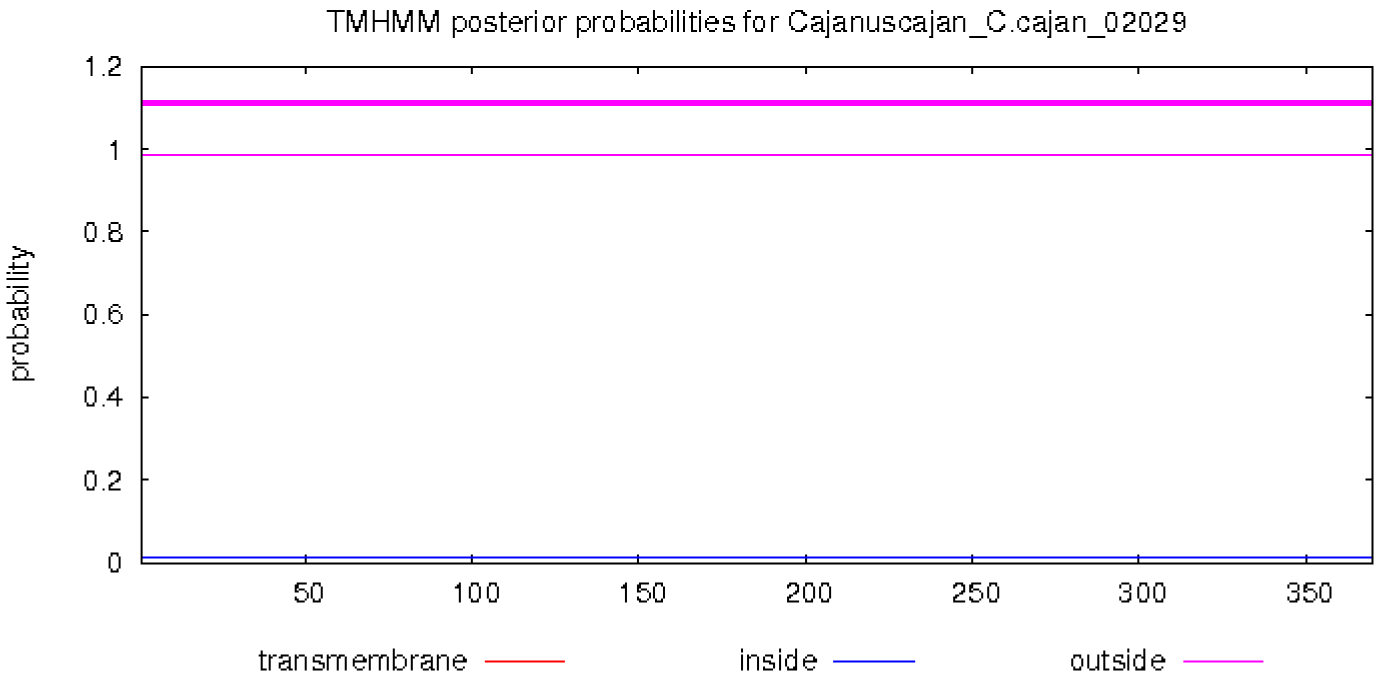

plot in postscript, script for making the plot in gnuplot, data for plot

---

```
# Cajanuscajan_C.cajan_02050 Length: 163
# Cajanuscajan_C.cajan_02050 Number of predicted TMHs:  0
# Cajanuscajan_C.cajan_02050 Exp number of AAs in TMHs: 0.61439
# Cajanuscajan_C.cajan_02050 Exp number, first 60 AAs:  0.00061
# Cajanuscajan_C.cajan_02050 Total prob of N-in:        0.10643
Cajanuscajan_C.cajan_02050      TMHMM2.0        outside      1   163
```

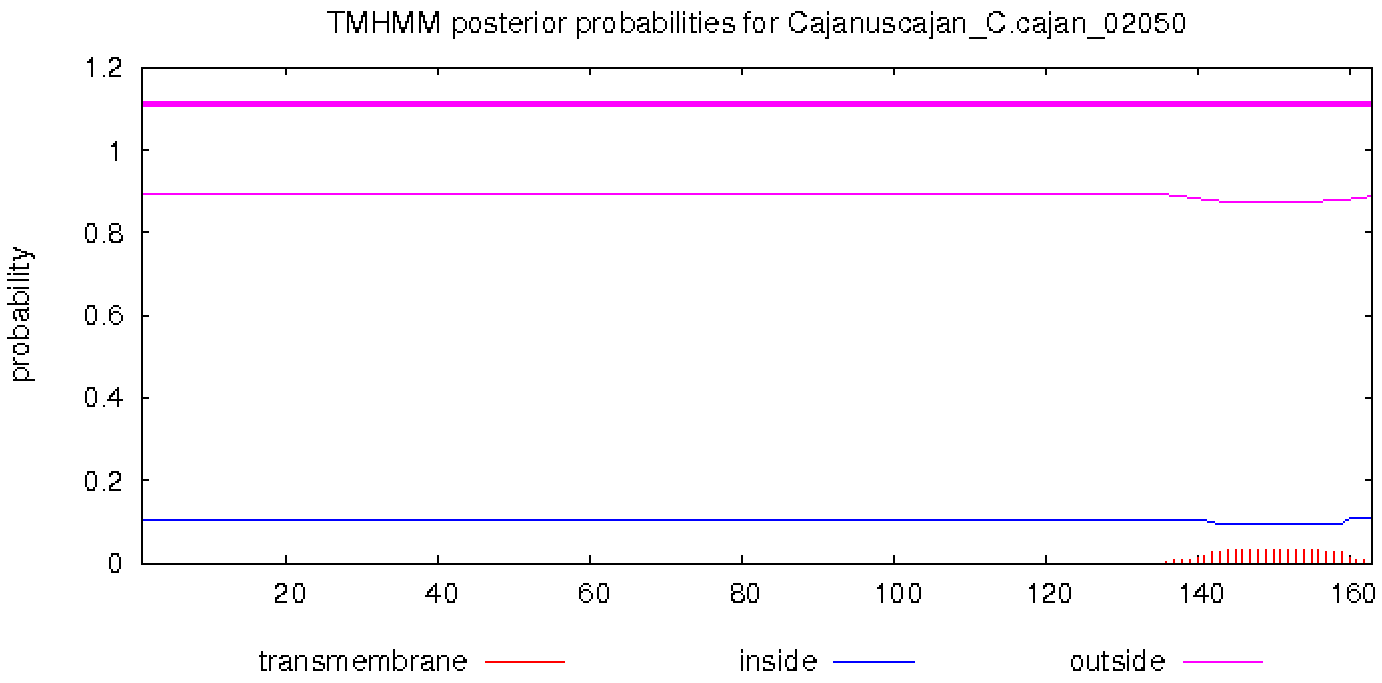

plot in postscript, script for making the plot in gnuplot, data for plot

---

```
# Cajanuscajan_C.cajan_02051 Length: 207
# Cajanuscajan_C.cajan_02051 Number of predicted TMHs:  0
# Cajanuscajan_C.cajan_02051 Exp number of AAs in TMHs: 0.01547
# Cajanuscajan_C.cajan_02051 Exp number, first 60 AAs:  0.00141
# Cajanuscajan_C.cajan_02051 Total prob of N-in:        0.04649
Cajanuscajan_C.cajan_02051      TMHMM2.0        outside      1   207
```

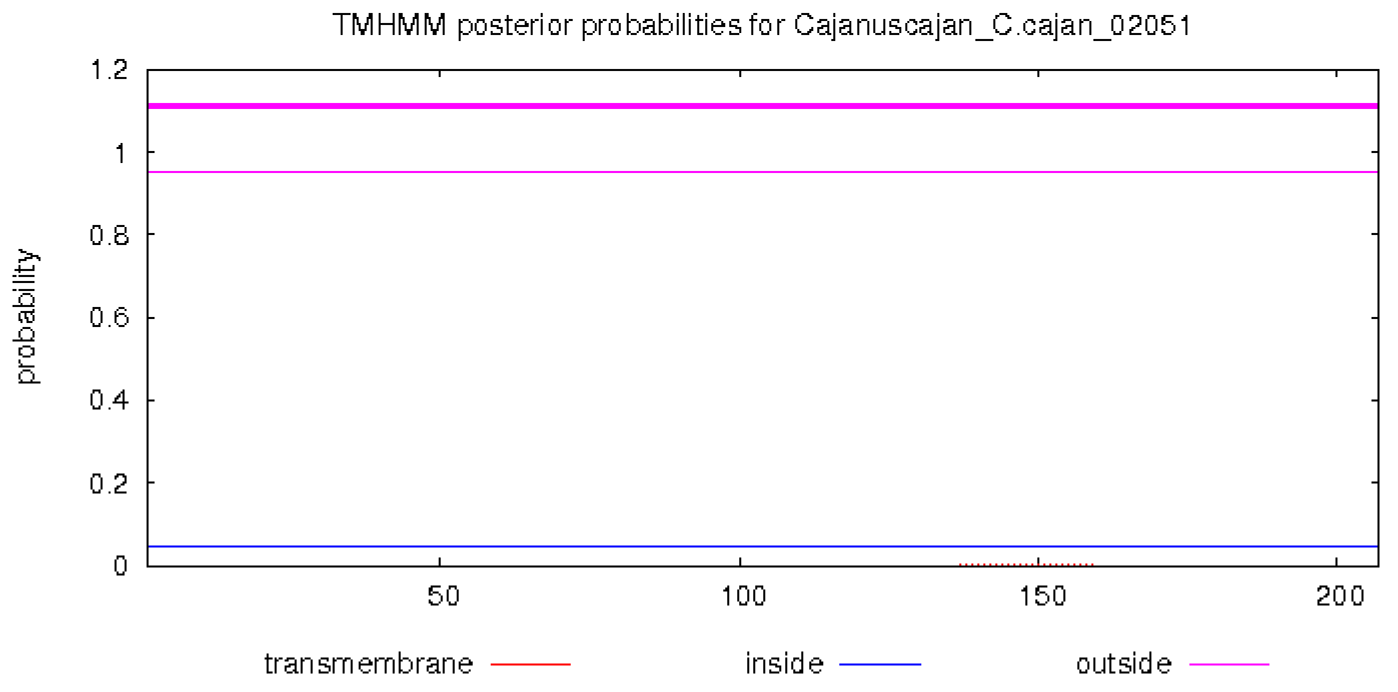

plot in postscript, script for making the plot in gnuplot, data for plot

---

```
# Cajanuscajan_C.cajan_02052 Length: 210
# Cajanuscajan_C.cajan_02052 Number of predicted TMHs:  0
# Cajanuscajan_C.cajan_02052 Exp number of AAs in TMHs: 0.00215
# Cajanuscajan_C.cajan_02052 Exp number, first 60 AAs:  0
# Cajanuscajan_C.cajan_02052 Total prob of N-in:        0.04388
Cajanuscajan_C.cajan_02052      TMHMM2.0        outside      1   210
```

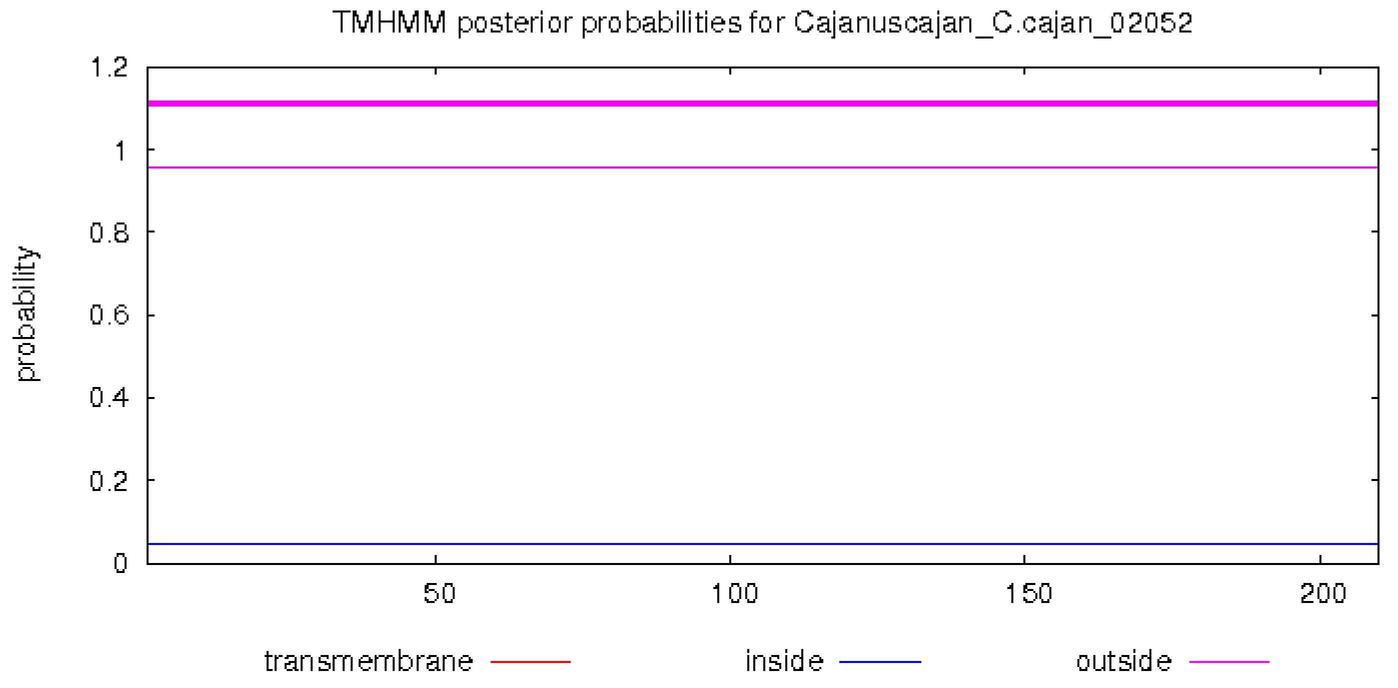

plot in postscript, script for making the plot in gnuplot, data for plot

---

```
# Cajanuscajan_C.cajan_04121 Length: 202
# Cajanuscajan_C.cajan_04121 Number of predicted TMHs:  0
# Cajanuscajan_C.cajan_04121 Exp number of AAs in TMHs: 0.00764
# Cajanuscajan_C.cajan_04121 Exp number, first 60 AAs:  0.00736
# Cajanuscajan_C.cajan_04121 Total prob of N-in:        0.01578
Cajanuscajan_C.cajan_04121      TMHMM2.0        outside      1   202
```

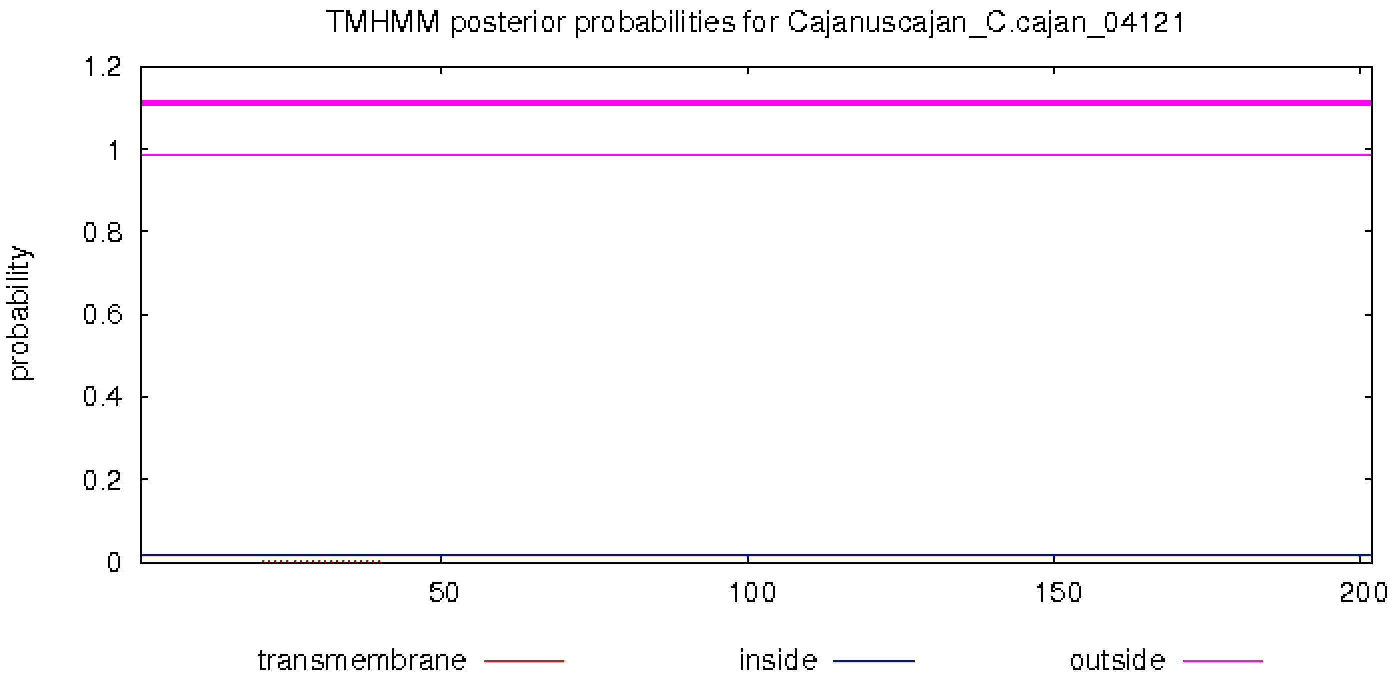

plot in postscript, script for making the plot in gnuplot, data for plot

---

```
# Cajanuscajan_C.cajan_04140 Length: 480
# Cajanuscajan_C.cajan_04140 Number of predicted TMHs:  0
# Cajanuscajan_C.cajan_04140 Exp number of AAs in TMHs: 0.00068
# Cajanuscajan_C.cajan_04140 Exp number, first 60 AAs:  0.00052
# Cajanuscajan_C.cajan_04140 Total prob of N-in:        0.00298
Cajanuscajan_C.cajan_04140      TMHMM2.0        outside      1   480
```

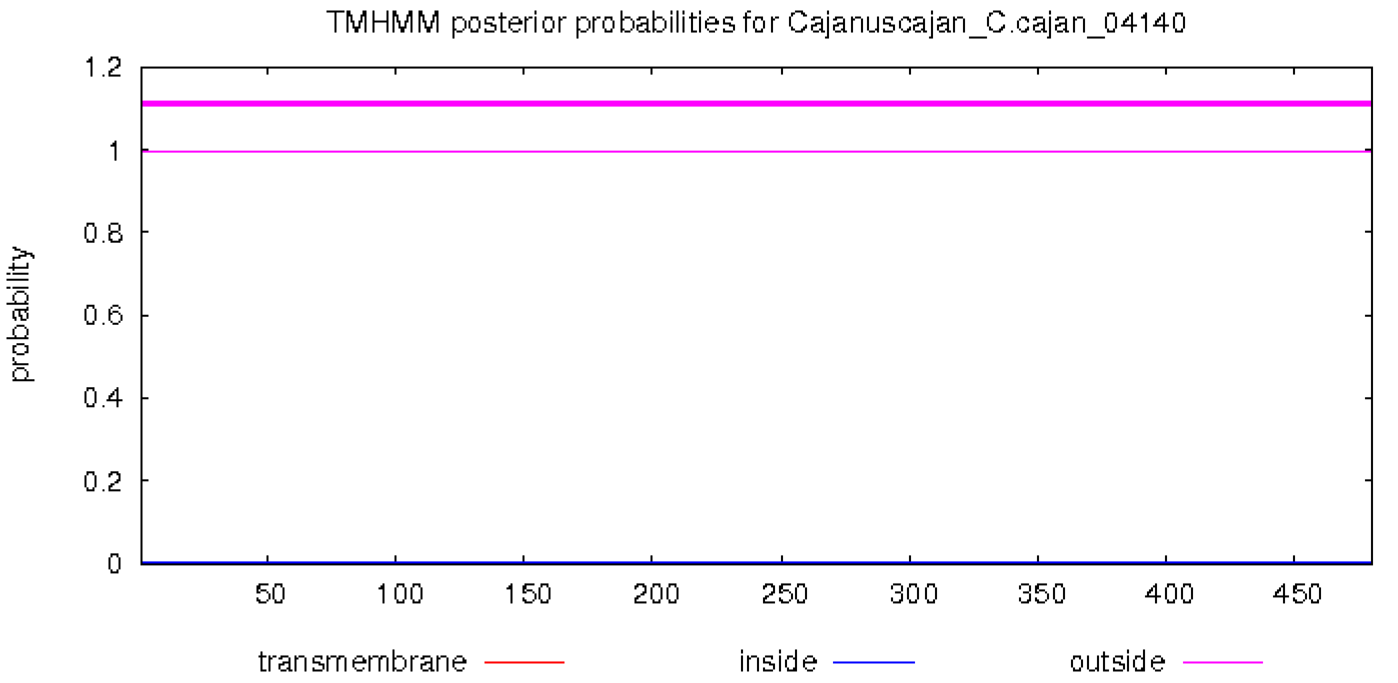

plot in postscript, script for making the plot in gnuplot, data for plot

---

```
# Cajanuscajan_C.cajan_06648 Length: 276
# Cajanuscajan_C.cajan_06648 Number of predicted TMHs:  0
# Cajanuscajan_C.cajan_06648 Exp number of AAs in TMHs: 0
# Cajanuscajan_C.cajan_06648 Exp number, first 60 AAs:  0
# Cajanuscajan_C.cajan_06648 Total prob of N-in:        0.01023
Cajanuscajan_C.cajan_06648      TMHMM2.0        outside      1   276
```

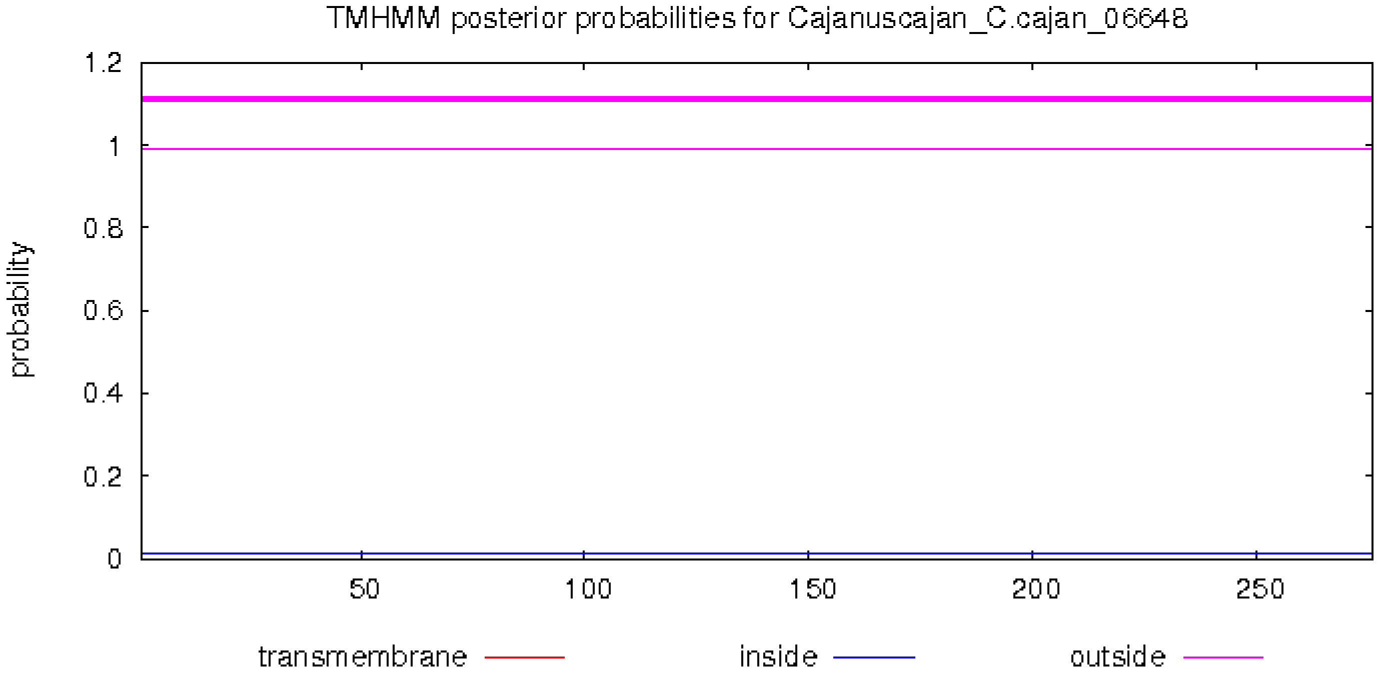

plot in postscript, script for making the plot in gnuplot, data for plot

---

```
# Cajanuscajan_C.cajan_07217 Length: 346
# Cajanuscajan_C.cajan_07217 Number of predicted TMHs:  0
# Cajanuscajan_C.cajan_07217 Exp number of AAs in TMHs: 0
# Cajanuscajan_C.cajan_07217 Exp number, first 60 AAs:  0
# Cajanuscajan_C.cajan_07217 Total prob of N-in:        0.00415
Cajanuscajan_C.cajan_07217      TMHMM2.0        outside      1   346
```

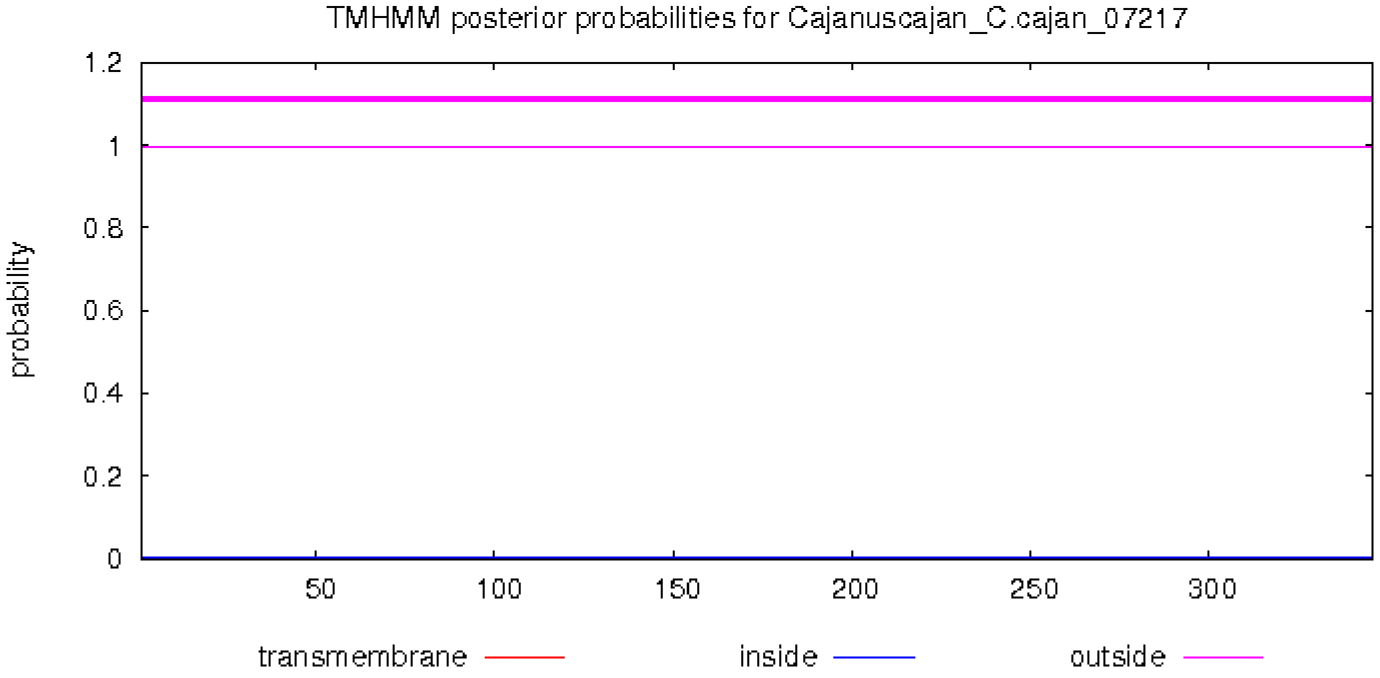

plot in postscript, script for making the plot in gnuplot, data for plot

---

```
# Cajanuscajan_C.cajan_08648 Length: 326
# Cajanuscajan_C.cajan_08648 Number of predicted TMHs:  0
# Cajanuscajan_C.cajan_08648 Exp number of AAs in TMHs: 0.01494
# Cajanuscajan_C.cajan_08648 Exp number, first 60 AAs:  0
# Cajanuscajan_C.cajan_08648 Total prob of N-in:        0.00335
Cajanuscajan_C.cajan_08648      TMHMM2.0        outside      1   326
```

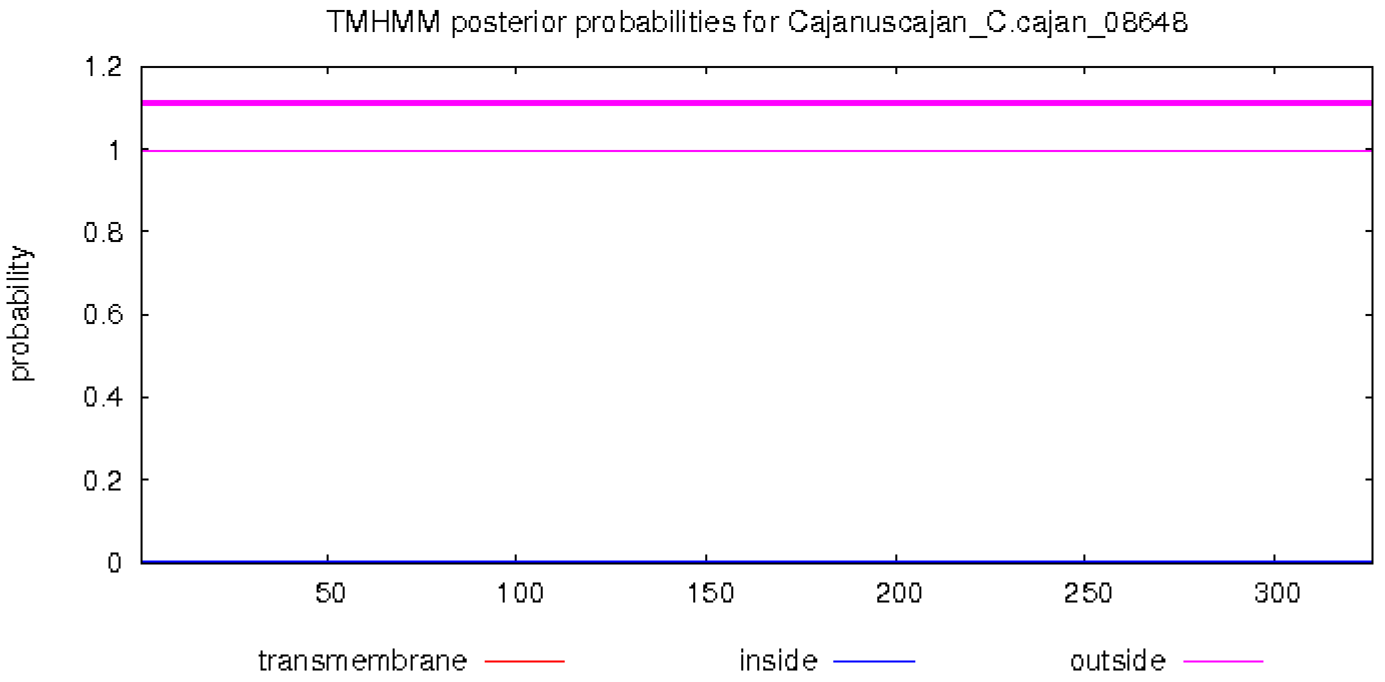

plot in postscript, script for making the plot in gnuplot, data for plot

---

```
# Cajanuscajan_C.cajan_08704 Length: 276
# Cajanuscajan_C.cajan_08704 Number of predicted TMHs:  0
# Cajanuscajan_C.cajan_08704 Exp number of AAs in TMHs: 0.00023
# Cajanuscajan_C.cajan_08704 Exp number, first 60 AAs:  0
# Cajanuscajan_C.cajan_08704 Total prob of N-in:        0.04013
Cajanuscajan_C.cajan_08704      TMHMM2.0        outside      1   276
```

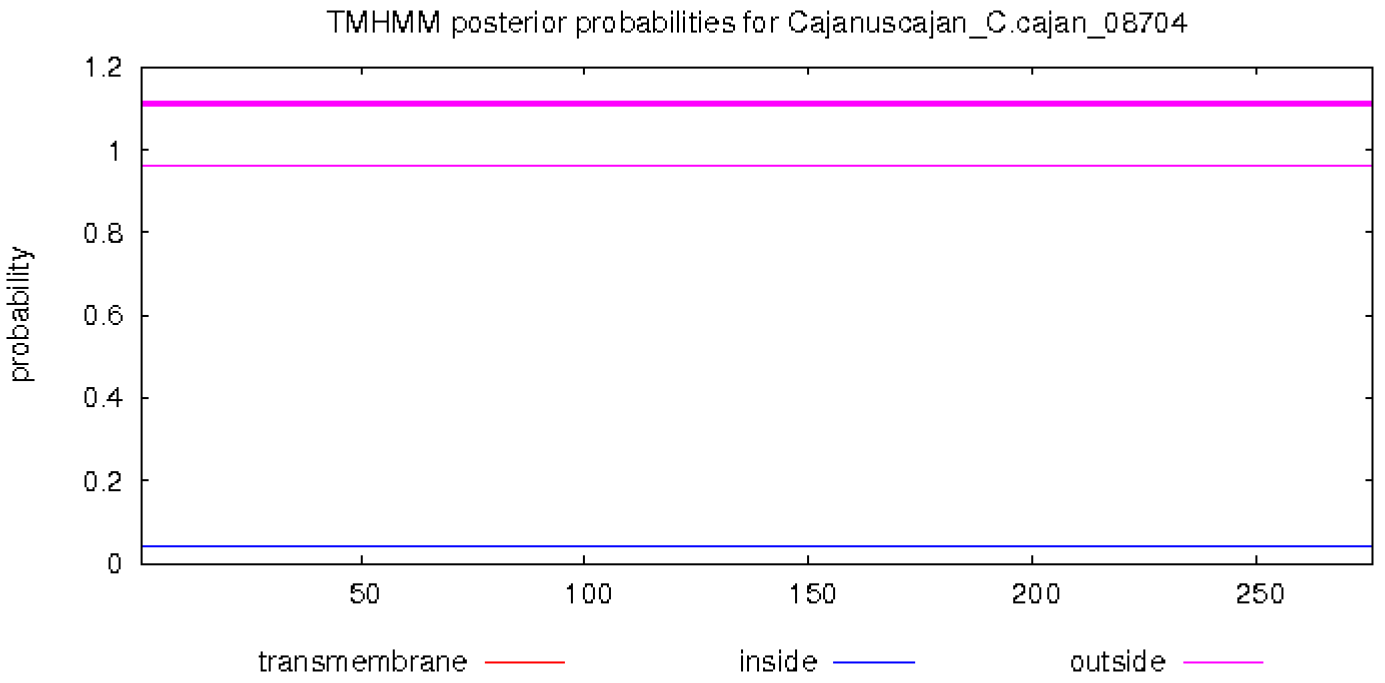

plot in postscript, script for making the plot in gnuplot, data for plot

---

```
# Cajanuscajan_C.cajan_09117 Length: 343
# Cajanuscajan_C.cajan_09117 Number of predicted TMHs:  0
# Cajanuscajan_C.cajan_09117 Exp number of AAs in TMHs: 0.589670000000000001
# Cajanuscajan_C.cajan_09117 Exp number, first 60 AAs:  0
# Cajanuscajan_C.cajan_09117 Total prob of N-in:        0.01537
Cajanuscajan_C.cajan_09117      TMHMM2.0        outside      1   343
```

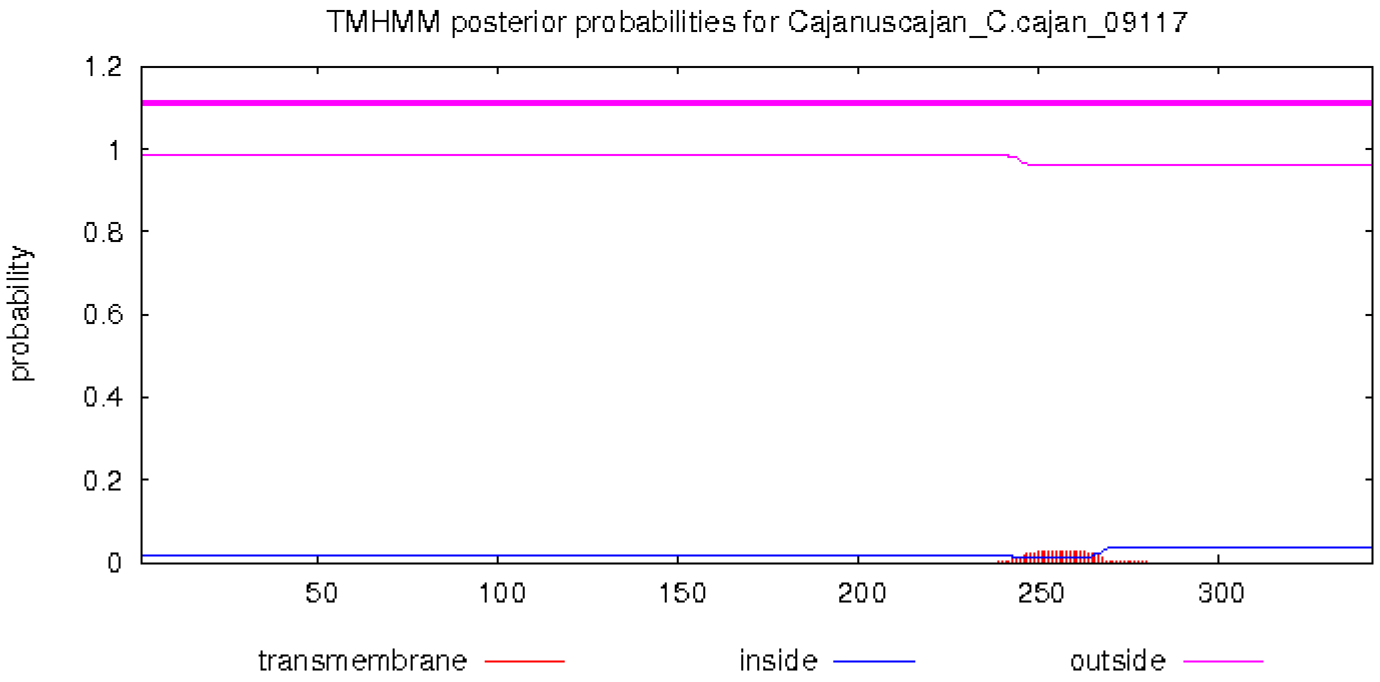

plot in postscript, script for making the plot in gnuplot, data for plot

---

```
# Cajanuscajan_C.cajan_09817 Length: 334
# Cajanuscajan_C.cajan_09817 Number of predicted TMHs:  0
# Cajanuscajan_C.cajan_09817 Exp number of AAs in TMHs: 0.02165
# Cajanuscajan_C.cajan_09817 Exp number, first 60 AAs:  0.00014
# Cajanuscajan_C.cajan_09817 Total prob of N-in:        0.03722
Cajanuscajan_C.cajan_09817      TMHMM2.0        outside      1   334
```

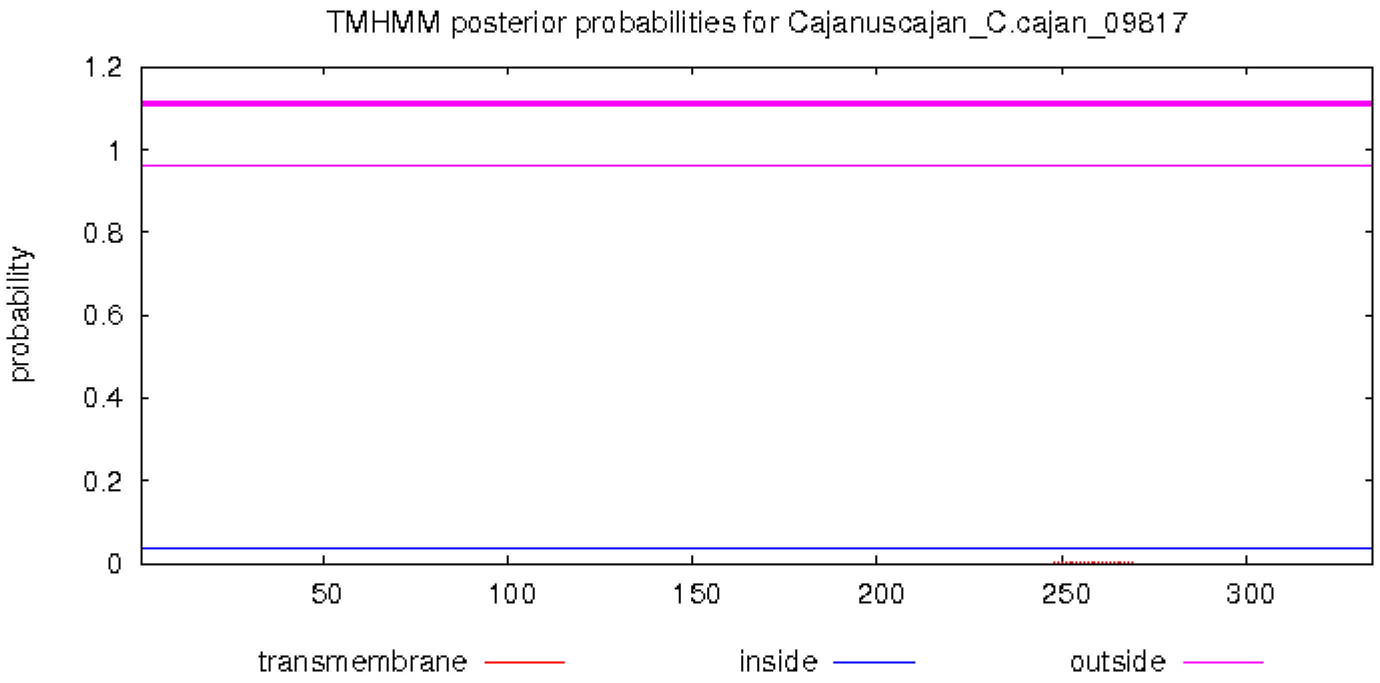

plot in postscript, script for making the plot in gnuplot, data for plot

---

```
# Cajanuscajan_C.cajan_11824 Length: 422
# Cajanuscajan_C.cajan_11824 Number of predicted TMHs:  0
# Cajanuscajan_C.cajan_11824 Exp number of AAs in TMHs: 0
# Cajanuscajan_C.cajan_11824 Exp number, first 60 AAs:  0
# Cajanuscajan_C.cajan_11824 Total prob of N-in:        0.01288
Cajanuscajan_C.cajan_11824      TMHMM2.0        outside      1   422
```

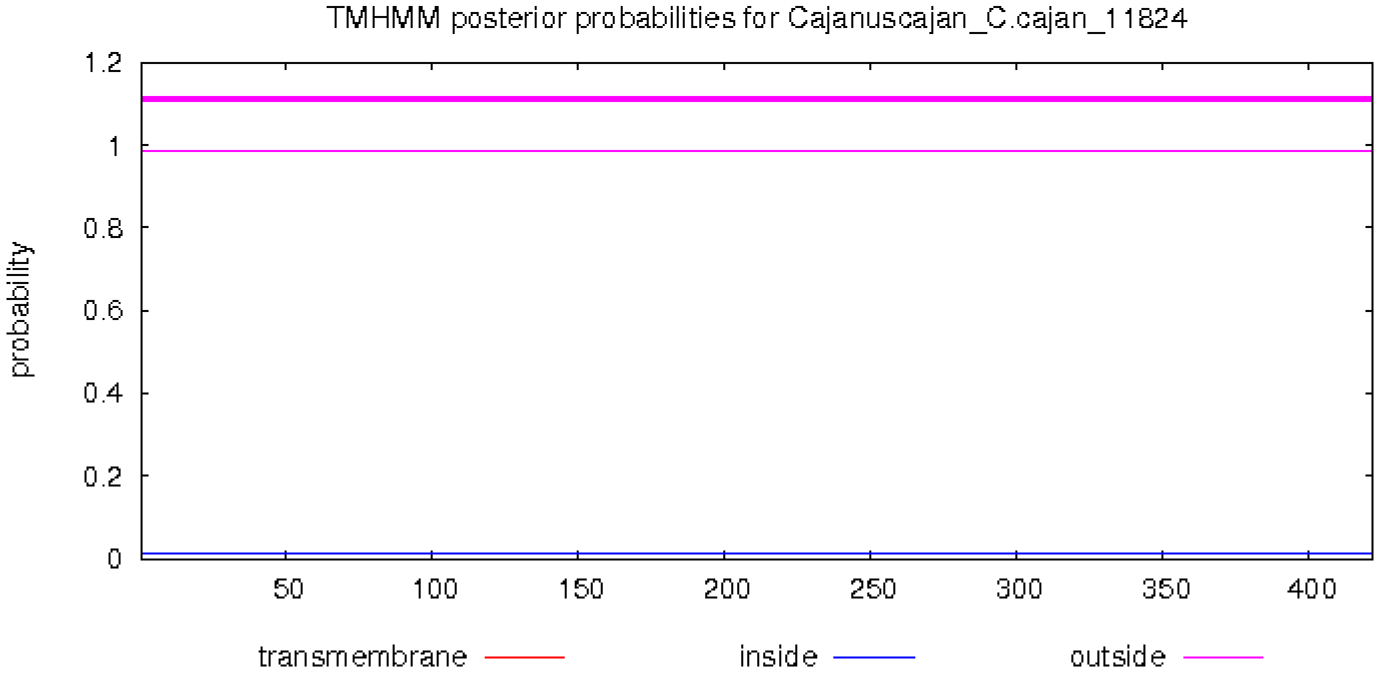

plot in postscript, script for making the plot in gnuplot, data for plot

```
# Cajanuscajan_C.cajan_12803 Length: 263
# Cajanuscajan_C.cajan_12803 Number of predicted TMHs:  0
# Cajanuscajan_C.cajan_12803 Exp number of AAs in TMHs: 0.00159
# Cajanuscajan_C.cajan_12803 Exp number, first 60 AAs:  0.00016
# Cajanuscajan_C.cajan_12803 Total prob of N-in:        0.01459
Cajanuscajan_C.cajan_12803      TMHMM2.0        outside      1   263
```

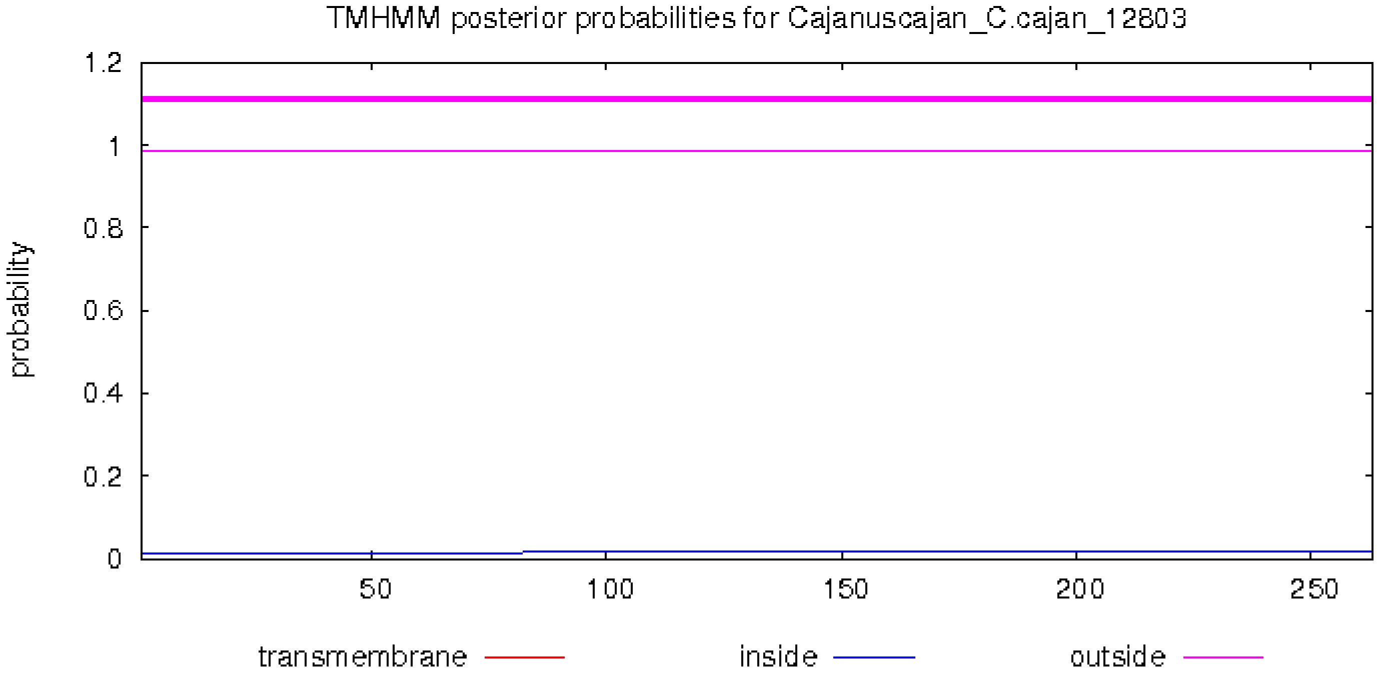

plot in postscript, script for making the plot in gnuplot, data for plot

```
# Cajanuscajan_C.cajan_12845 Length: 263
# Cajanuscajan_C.cajan_12845 Number of predicted TMHs:  0
# Cajanuscajan_C.cajan_12845 Exp number of AAs in TMHs: 0.00159
# Cajanuscajan_C.cajan_12845 Exp number, first 60 AAs:  0.00016
# Cajanuscajan_C.cajan_12845 Total prob of N-in:        0.01459
Cajanuscajan_C.cajan_12845      TMHMM2.0        outside      1   263
```

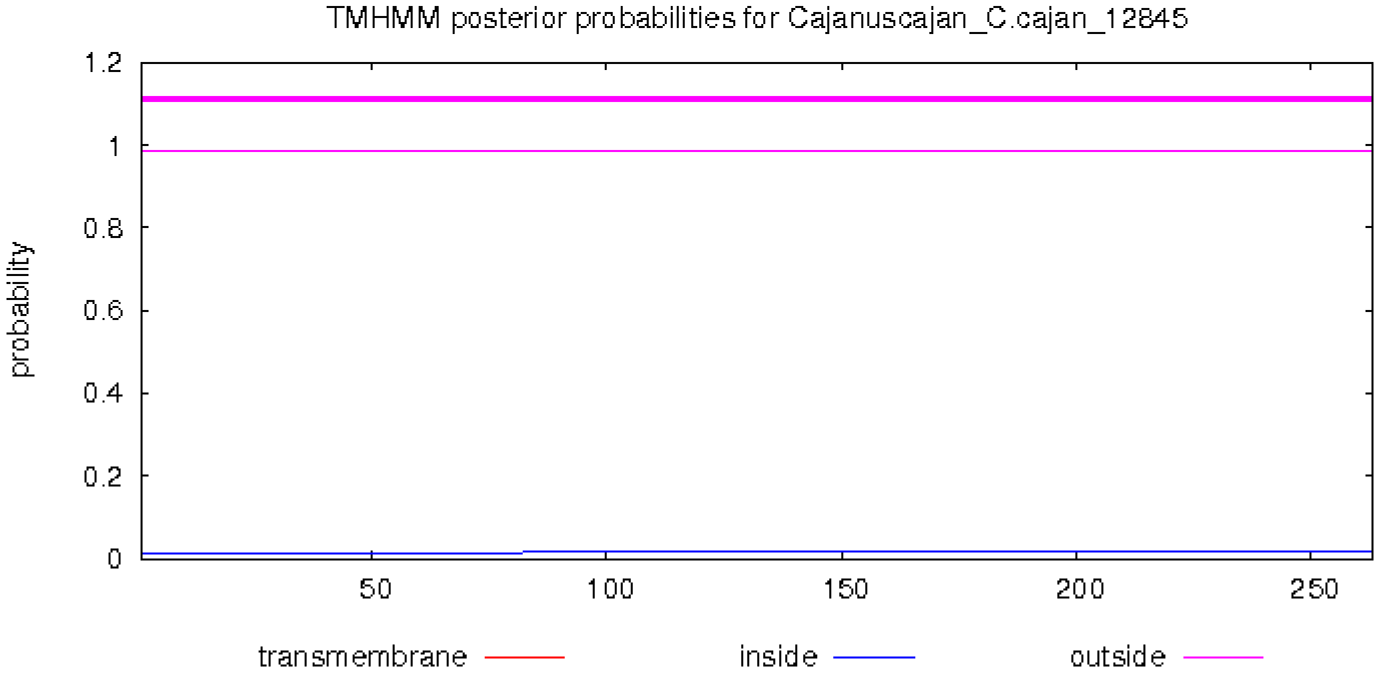

plot in postscript, script for making the plot in gnuplot, data for plot

---

```
# Cajanuscajan_C.cajan_13790 Length: 284
# Cajanuscajan_C.cajan_13790 Number of predicted TMHs:  0
# Cajanuscajan_C.cajan_13790 Exp number of AAs in TMHs: 0
# Cajanuscajan_C.cajan_13790 Exp number, first 60 AAs:  0
# Cajanuscajan_C.cajan_13790 Total prob of N-in:        0.06598
Cajanuscajan_C.cajan_13790      TMHMM2.0        outside      1   284
```

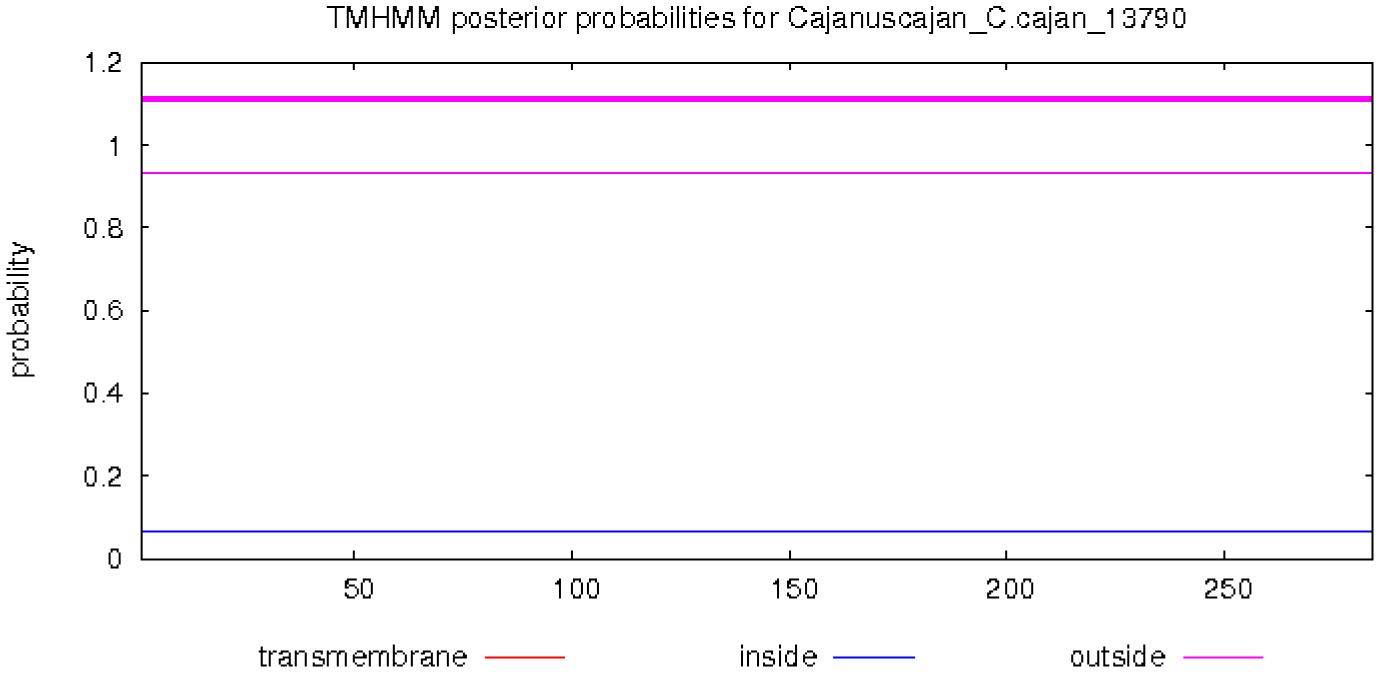

plot in postscript, script for making the plot in gnuplot, data for plot

---

```
# Cajanuscajan_C.cajan_14029 Length: 307
# Cajanuscajan_C.cajan_14029 Number of predicted TMHs:  0
# Cajanuscajan_C.cajan_14029 Exp number of AAs in TMHs: 0.00176
# Cajanuscajan_C.cajan_14029 Exp number, first 60 AAs:  0
# Cajanuscajan_C.cajan_14029 Total prob of N-in:        0.04415
Cajanuscajan_C.cajan_14029      TMHMM2.0        outside      1   307
```

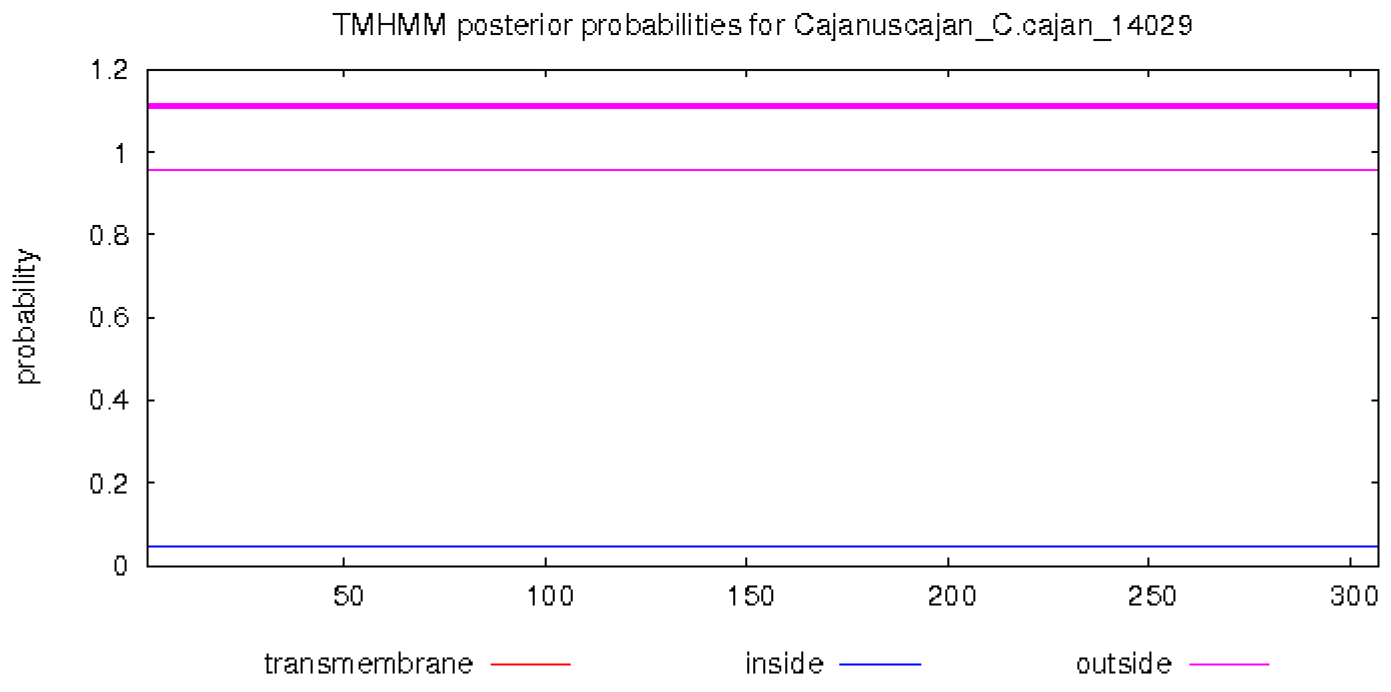

plot in postscript, script for making the plot in gnuplot, data for plot

---

```
# Cajanuscajan_C.cajan_14854 Length: 215
# Cajanuscajan_C.cajan_14854 Number of predicted TMHs:  1
# Cajanuscajan_C.cajan_14854 Exp number of AAs in TMHs: 22.67864
# Cajanuscajan_C.cajan_14854 Exp number, first 60 AAs:  0.00049
# Cajanuscajan_C.cajan_14854 Total prob of N-in:        0.90770
Cajanuscajan_C.cajan_14854      TMHMM2.0        inside       1   150
Cajanuscajan_C.cajan_14854      TMHMM2.0        TMhelix    151   173
Cajanuscajan_C.cajan_14854      TMHMM2.0        outside    174   215
```

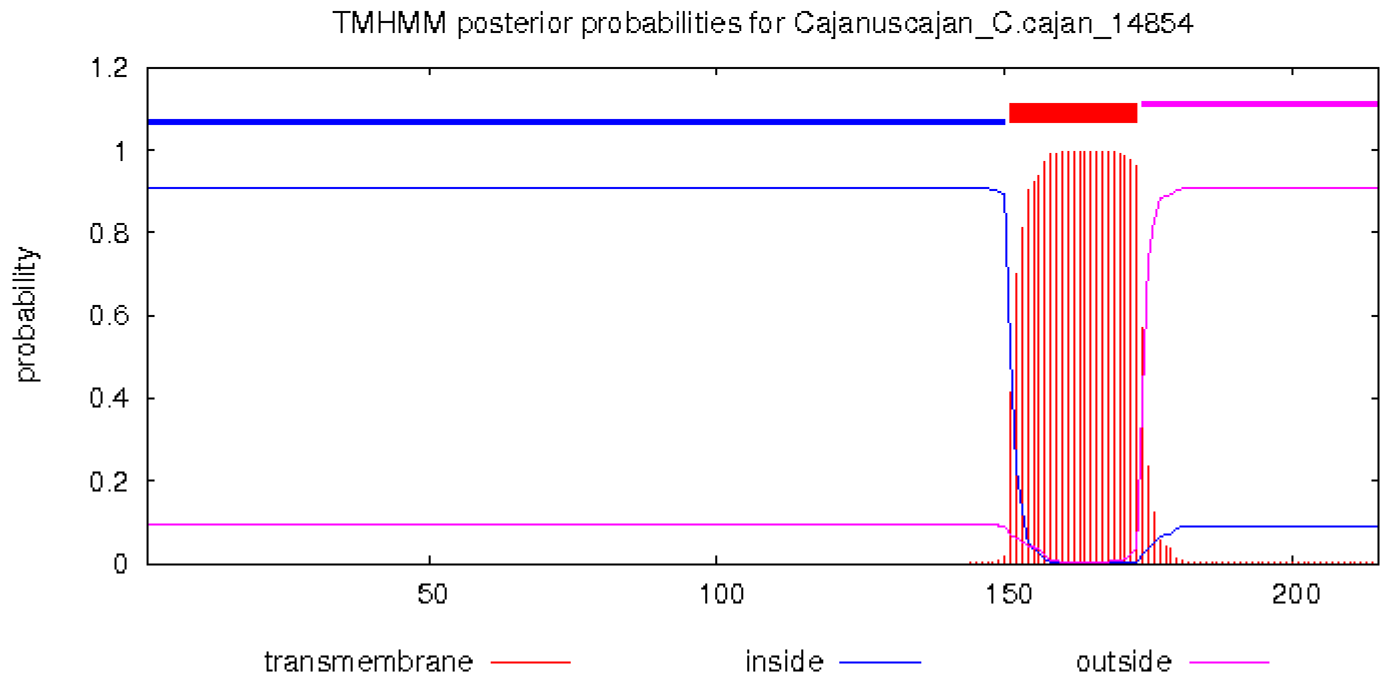

plot in postscript, script for making the plot in gnuplot, data for plot

```
# Cajanuscajan_C.cajan_15439 Length: 452
# Cajanuscajan_C.cajan_15439 Number of predicted TMHs:  0
# Cajanuscajan_C.cajan_15439 Exp number of AAs in TMHs: 0
# Cajanuscajan_C.cajan_15439 Exp number, first 60 AAs:  0
# Cajanuscajan_C.cajan_15439 Total prob of N-in:        0.00346
Cajanuscajan_C.cajan_15439      TMHMM2.0        outside      1   452
```

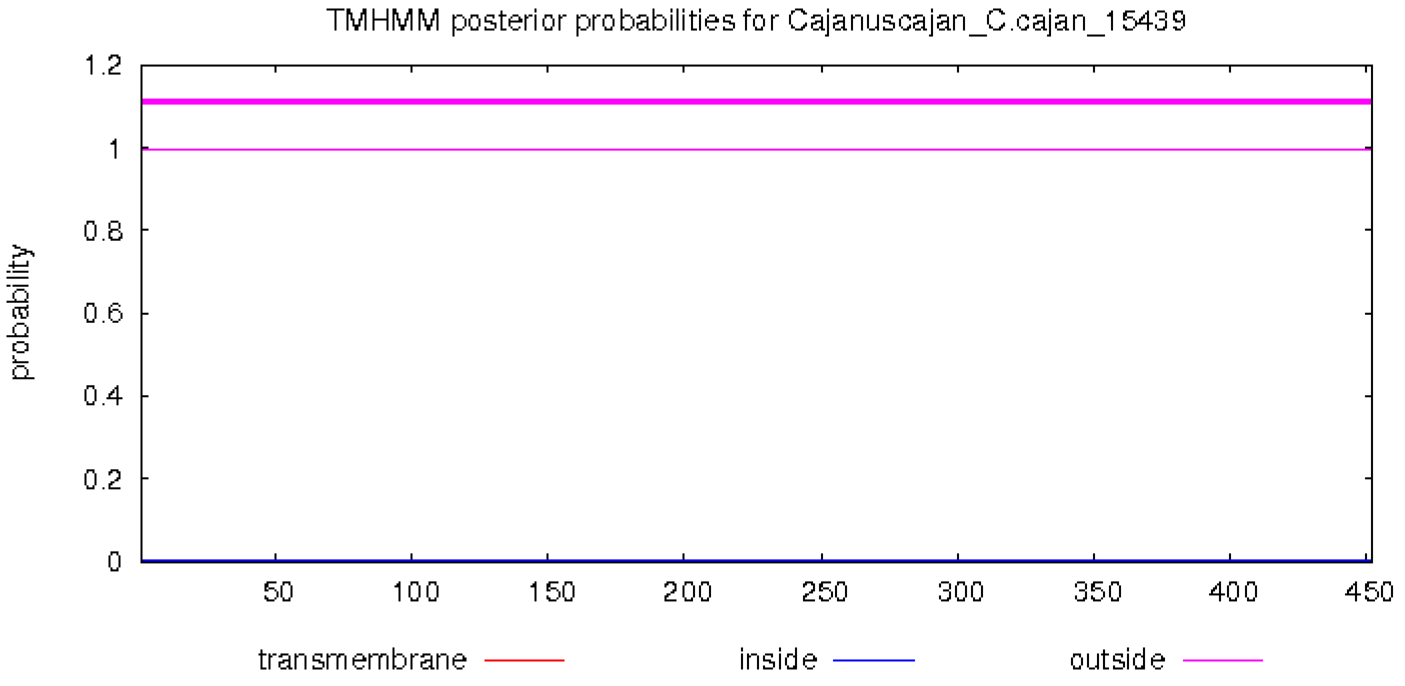

plot in postscript, script for making the plot in gnuplot, data for plot

```
# Cajanuscajan_C.cajan_15622 Length: 299
# Cajanuscajan_C.cajan_15622 Number of predicted TMHs:  0
# Cajanuscajan_C.cajan_15622 Exp number of AAs in TMHs: 0.05806
# Cajanuscajan_C.cajan_15622 Exp number, first 60 AAs:  0
# Cajanuscajan_C.cajan_15622 Total prob of N-in:        0.00777
Cajanuscajan_C.cajan_15622      TMHMM2.0        outside      1   299
```

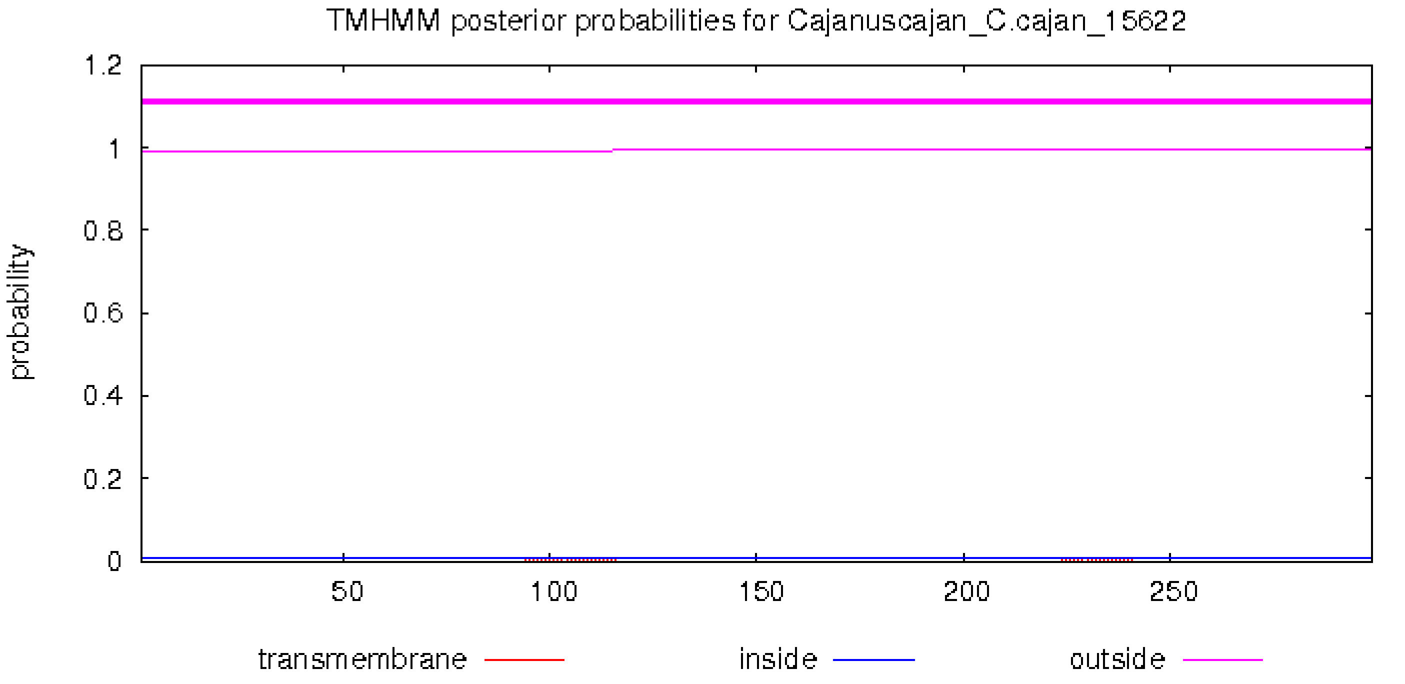

plot in postscript, script for making the plot in gnuplot, data for plot

```
# Cajanuscajan_C.cajan_15737 Length: 238
# Cajanuscajan_C.cajan_15737 Number of predicted TMHs:  0
# Cajanuscajan_C.cajan_15737 Exp number of AAs in TMHs: 0.0002
# Cajanuscajan_C.cajan_15737 Exp number, first 60 AAs:  0
# Cajanuscajan_C.cajan_15737 Total prob of N-in:        0.00221
Cajanuscajan_C.cajan_15737      TMHMM2.0        outside      1   238
```

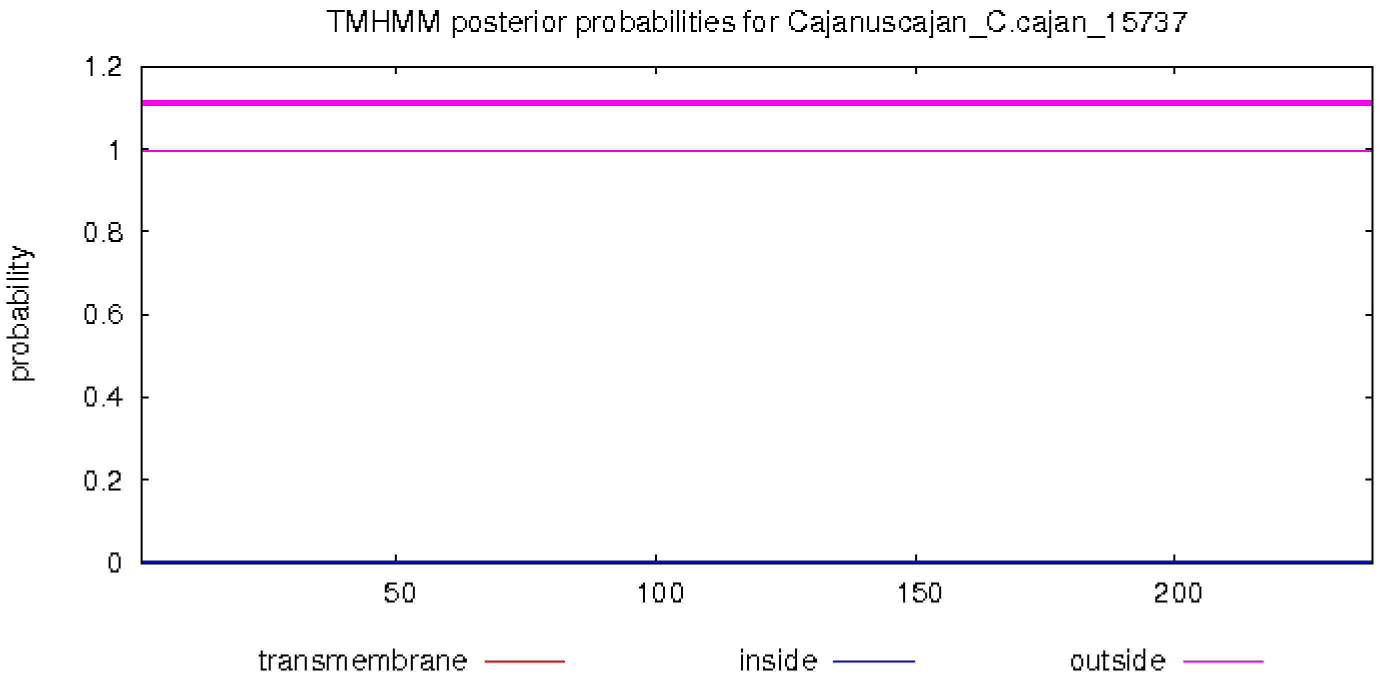

plot in postscript, script for making the plot in gnuplot, data for plot

---

```
# Cajanuscajan_C.cajan_15921 Length: 293
# Cajanuscajan_C.cajan_15921 Number of predicted TMHs:  0
# Cajanuscajan_C.cajan_15921 Exp number of AAs in TMHs: 0
# Cajanuscajan_C.cajan_15921 Exp number, first 60 AAs:  0
# Cajanuscajan_C.cajan_15921 Total prob of N-in:        0.00515
Cajanuscajan_C.cajan_15921      TMHMM2.0        outside      1   293
```

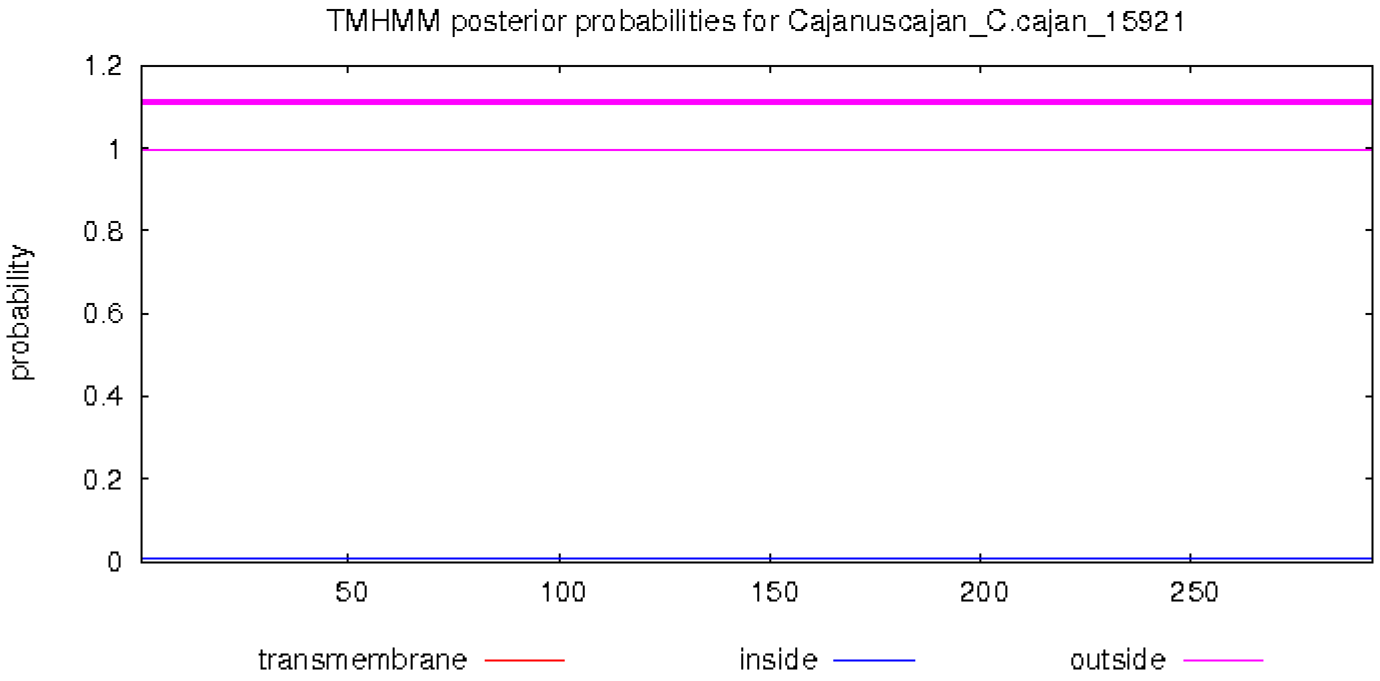

plot in postscript, script for making the plot in gnuplot, data for plot

---

```
# Cajanuscajan_C.cajan_17016 Length: 343
# Cajanuscajan_C.cajan_17016 Number of predicted TMHs:  0
# Cajanuscajan_C.cajan_17016 Exp number of AAs in TMHs: 0.00203
# Cajanuscajan_C.cajan_17016 Exp number, first 60 AAs:  0.00019
# Cajanuscajan_C.cajan_17016 Total prob of N-in:        0.01055
Cajanuscajan_C.cajan_17016      TMHMM2.0        outside      1   343
```

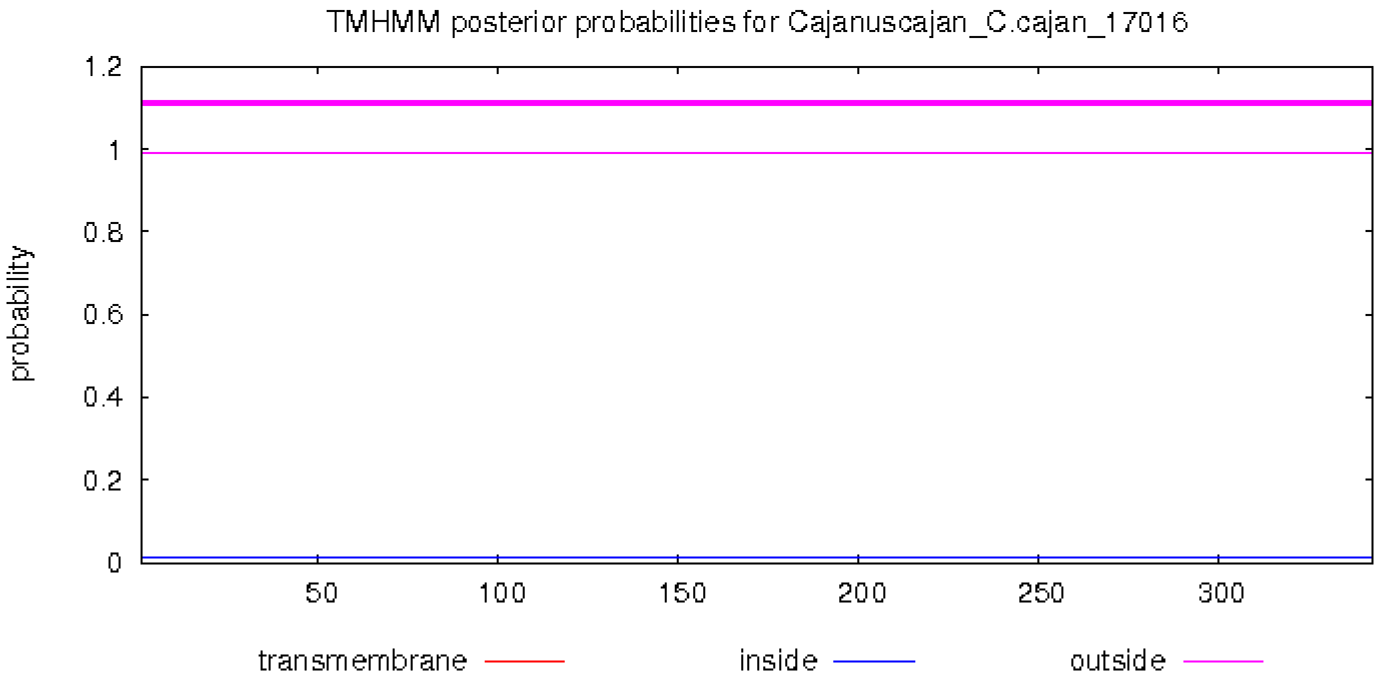

plot in postscript, script for making the plot in gnuplot, data for plot

---

```
# Cajanuscajan_C.cajan_17157 Length: 189
# Cajanuscajan_C.cajan_17157 Number of predicted TMHs:  0
# Cajanuscajan_C.cajan_17157 Exp number of AAs in TMHs: 0.07572
# Cajanuscajan_C.cajan_17157 Exp number, first 60 AAs:  0
# Cajanuscajan_C.cajan_17157 Total prob of N-in:        0.03107
Cajanuscajan_C.cajan_17157      TMHMM2.0        outside      1   189
```

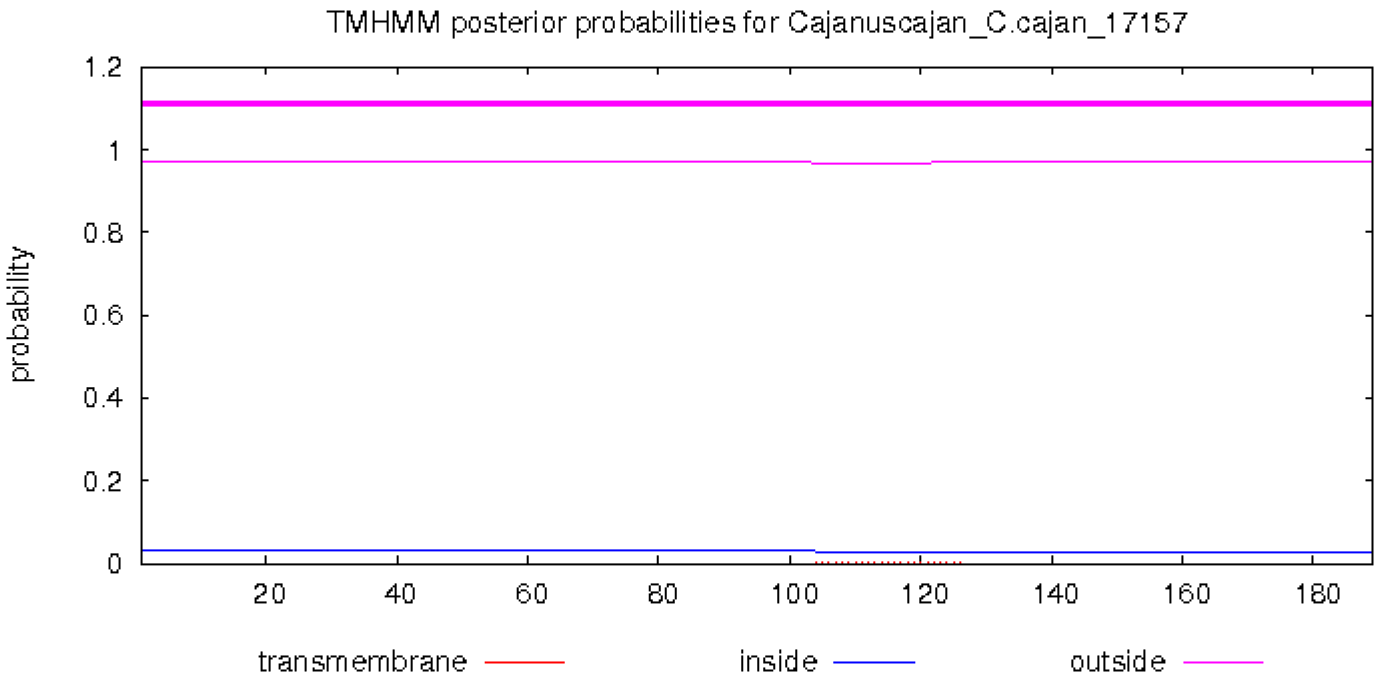

plot in postscript, script for making the plot in gnuplot, data for plot

---

```
# Cajanuscajan_C.cajan_17807 Length: 339
# Cajanuscajan_C.cajan_17807 Number of predicted TMHs:  0
# Cajanuscajan_C.cajan_17807 Exp number of AAs in TMHs: 0.00328
# Cajanuscajan_C.cajan_17807 Exp number, first 60 AAs:  0.00328
# Cajanuscajan_C.cajan_17807 Total prob of N-in:        0.00180
Cajanuscajan_C.cajan_17807      TMHMM2.0        outside      1   339
```

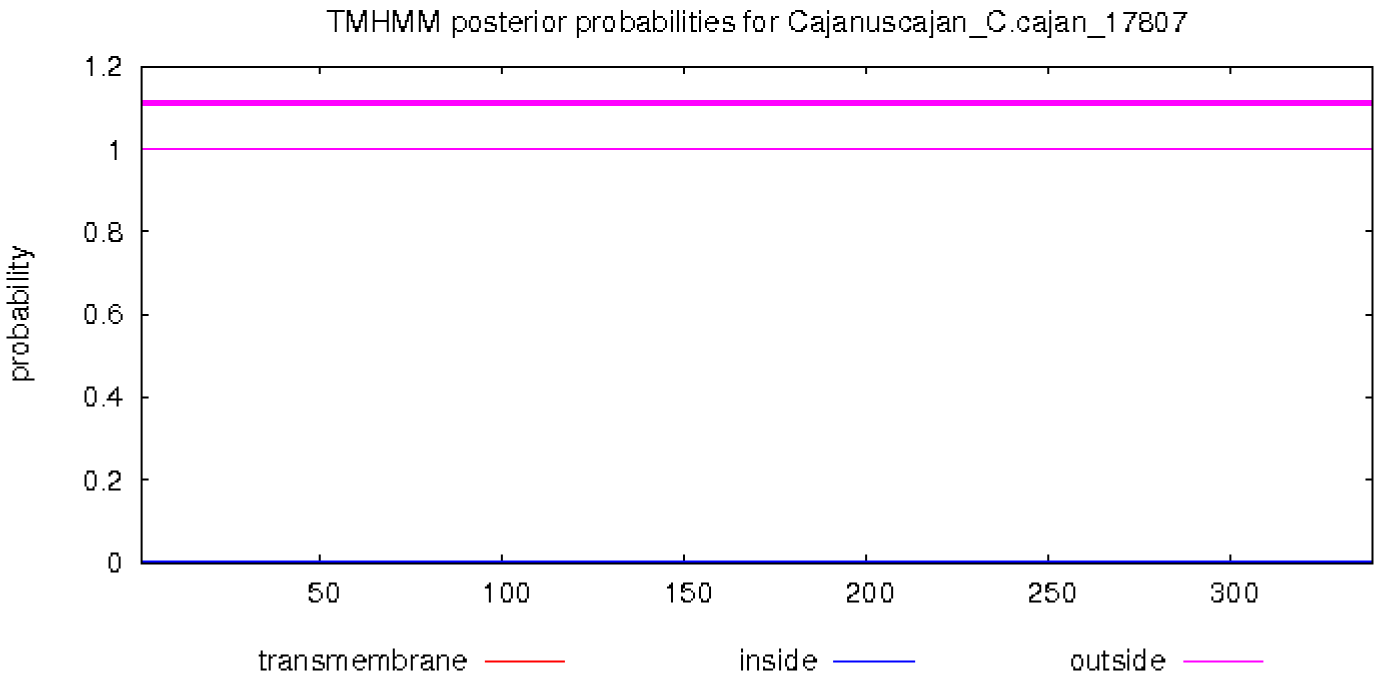

plot in postscript, script for making the plot in gnuplot, data for plot

```
# Cajanuscajan_C.cajan_17814 Length: 410
# Cajanuscajan_C.cajan_17814 Number of predicted TMHs:  0
# Cajanuscajan_C.cajan_17814 Exp number of AAs in TMHs: 0
# Cajanuscajan_C.cajan_17814 Exp number, first 60 AAs:  0
# Cajanuscajan_C.cajan_17814 Total prob of N-in:        0.00266
Cajanuscajan_C.cajan_17814      TMHMM2.0        outside      1   410
```

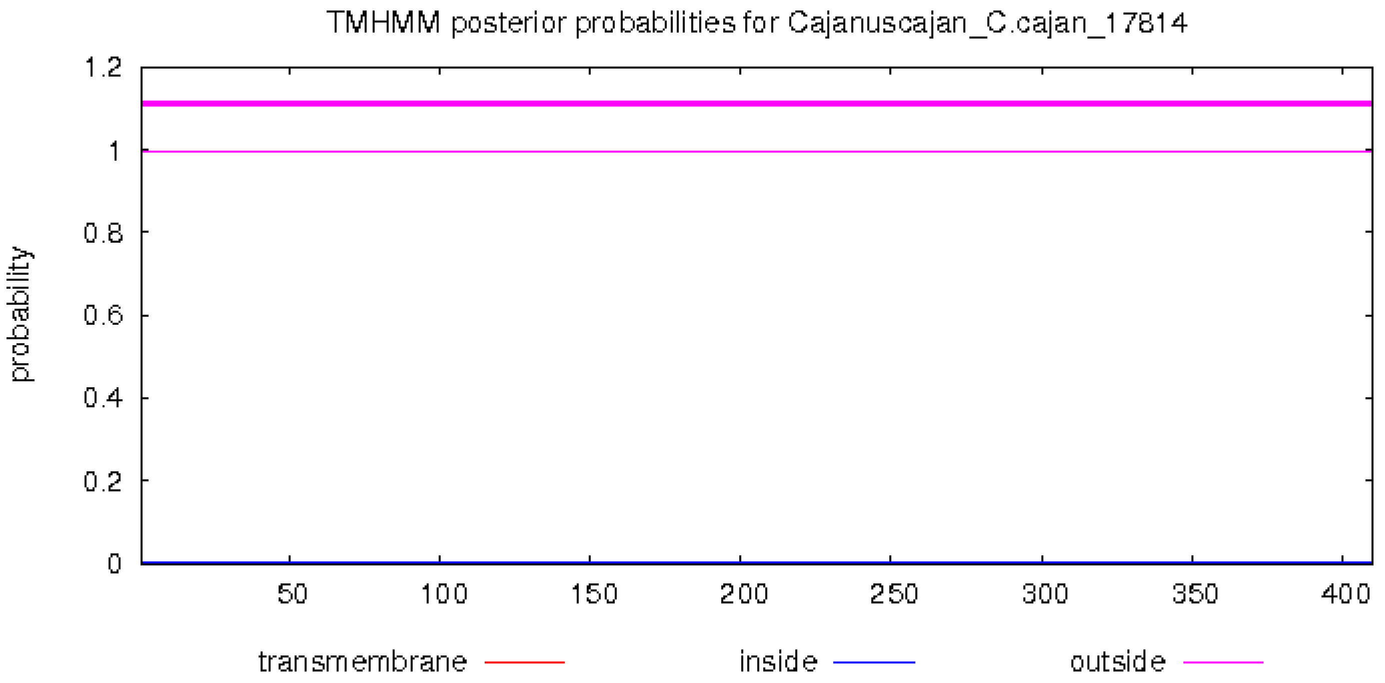

plot in postscript, script for making the plot in gnuplot, data for plot

```
# Cajanuscajan_C.cajan_18402 Length: 322
# Cajanuscajan_C.cajan_18402 Number of predicted TMHs:  0
# Cajanuscajan_C.cajan_18402 Exp number of AAs in TMHs: 0.0004
# Cajanuscajan_C.cajan_18402 Exp number, first 60 AAs:  0
# Cajanuscajan_C.cajan_18402 Total prob of N-in:        0.06076
Cajanuscajan_C.cajan_18402      TMHMM2.0        outside      1   322
```

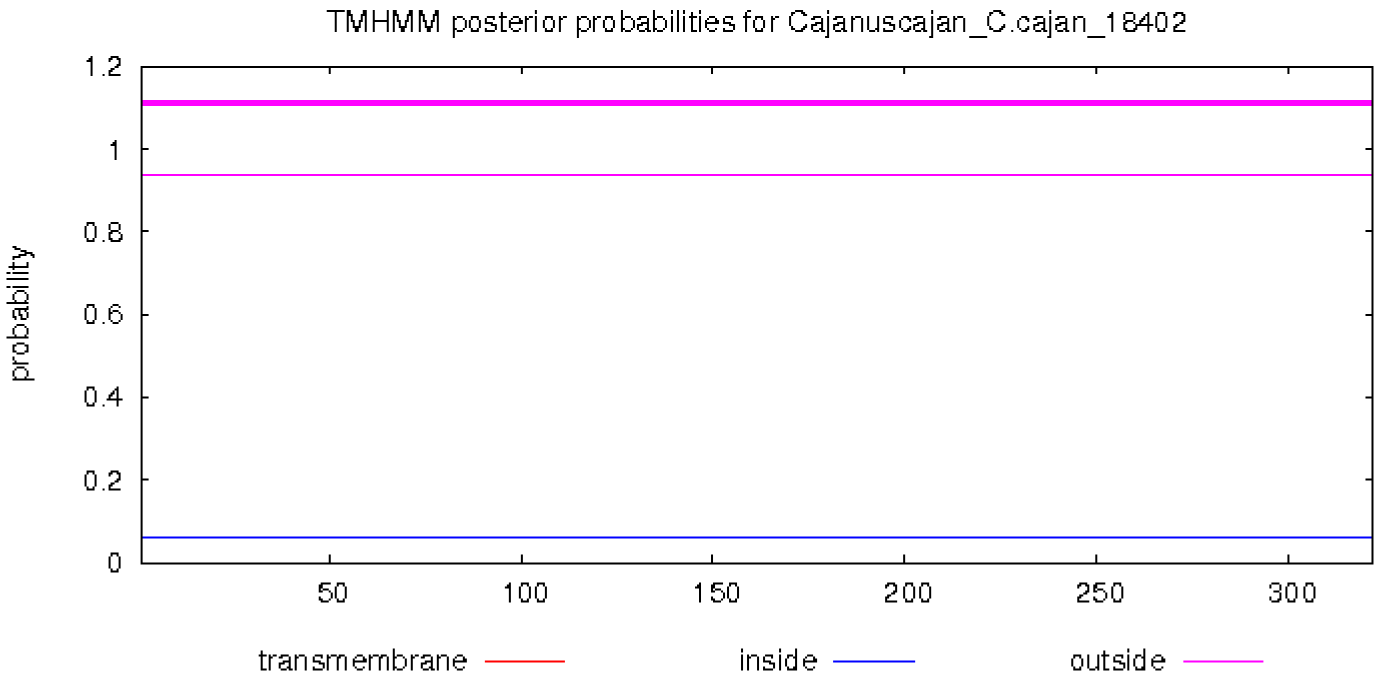

plot in postscript, script for making the plot in gnuplot, data for plot

---

```
# Cajanuscajan_C.cajan_18734 Length: 357
# Cajanuscajan_C.cajan_18734 Number of predicted TMHs:  0
# Cajanuscajan_C.cajan_18734 Exp number of AAs in TMHs: 0.00037
# Cajanuscajan_C.cajan_18734 Exp number, first 60 AAs:  0
# Cajanuscajan_C.cajan_18734 Total prob of N-in:        0.00956
Cajanuscajan_C.cajan_18734      TMHMM2.0        outside      1   357
```

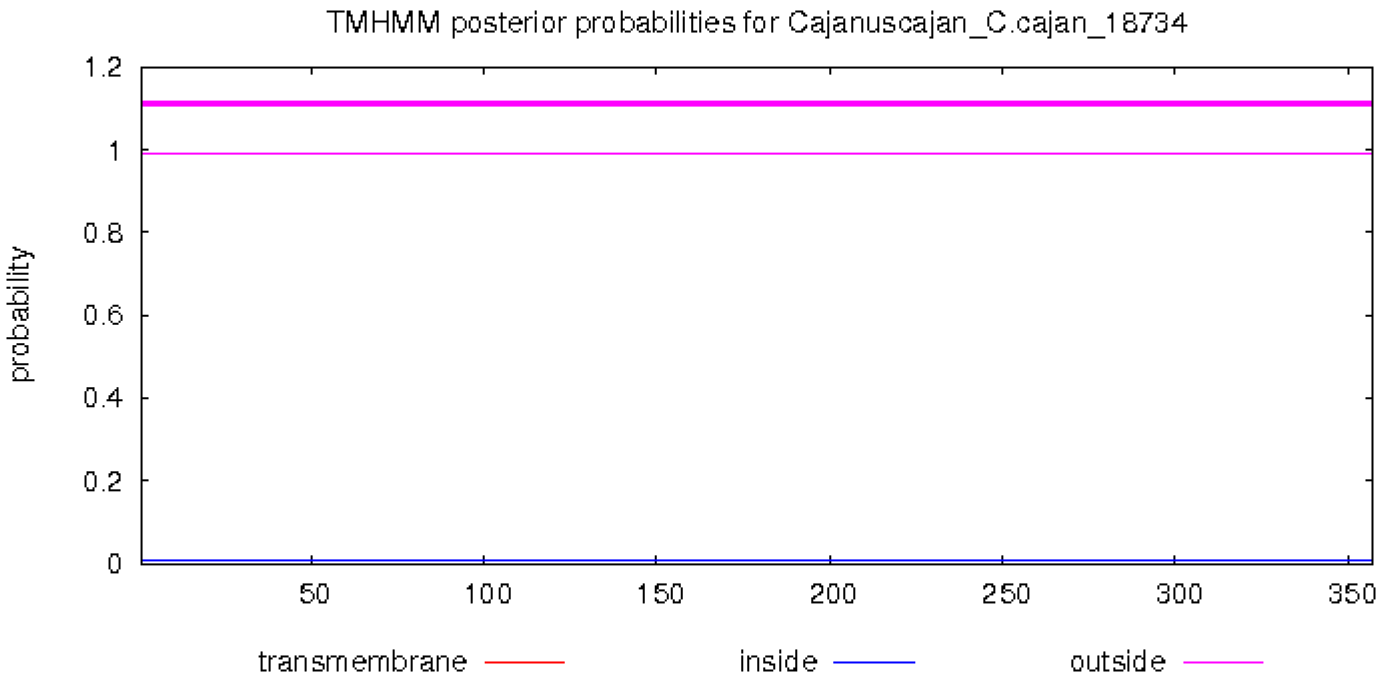

plot in postscript, script for making the plot in gnuplot, data for plot

---

```
# Cajanuscajan_C.cajan_18948 Length: 348
# Cajanuscajan_C.cajan_18948 Number of predicted TMHs:  0
# Cajanuscajan_C.cajan_18948 Exp number of AAs in TMHs: 0.00219
# Cajanuscajan_C.cajan_18948 Exp number, first 60 AAs:  0
# Cajanuscajan_C.cajan_18948 Total prob of N-in:        0.00418
Cajanuscajan_C.cajan_18948      TMHMM2.0        outside      1   348
```

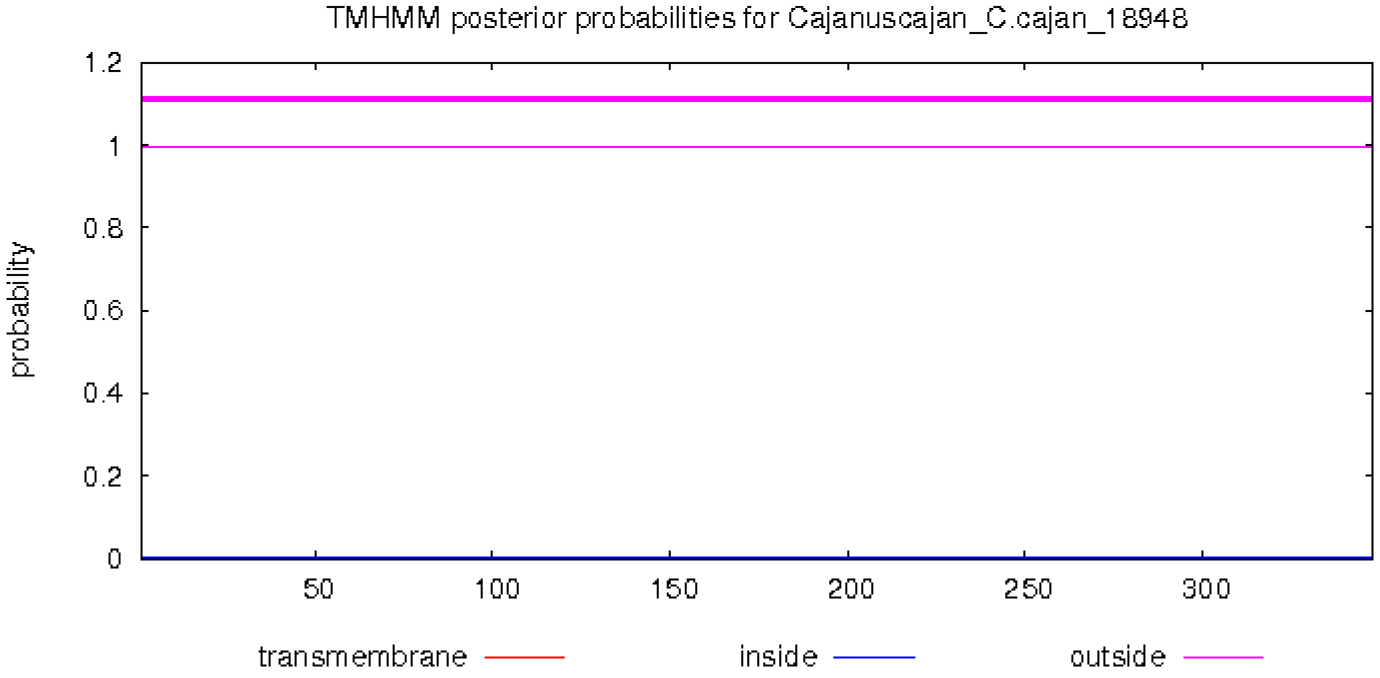

plot in postscript, script for making the plot in gnuplot, data for plot

---

```
# Cajanuscajan_C.cajan_19098 Length: 216
# Cajanuscajan_C.cajan_19098 Number of predicted TMHs:  0
# Cajanuscajan_C.cajan_19098 Exp number of AAs in TMHs: 0
# Cajanuscajan_C.cajan_19098 Exp number, first 60 AAs:  0
# Cajanuscajan_C.cajan_19098 Total prob of N-in:        0.02170
Cajanuscajan_C.cajan_19098      TMHMM2.0        outside      1   216
```

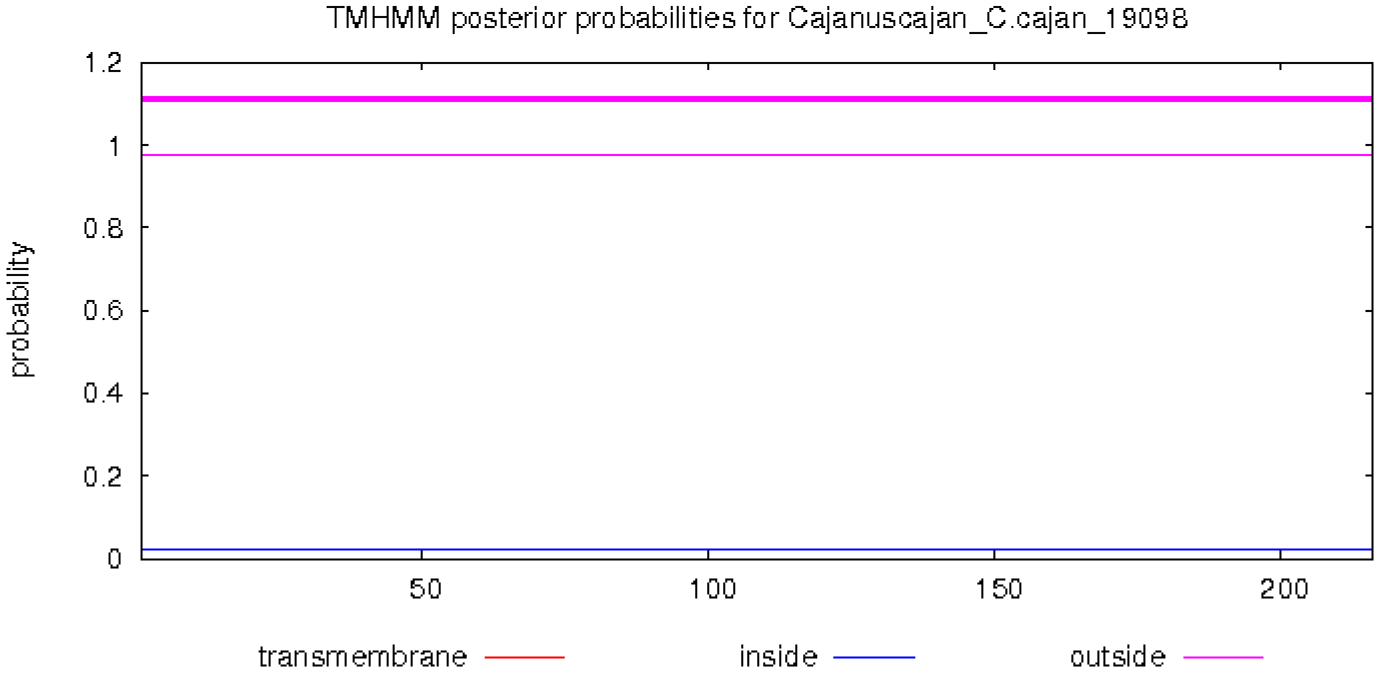

plot in postscript, script for making the plot in gnuplot, data for plot

---

```
# Cajanuscajan_C.cajan_19195 Length: 320
# Cajanuscajan_C.cajan_19195 Number of predicted TMHs:  0
# Cajanuscajan_C.cajan_19195 Exp number of AAs in TMHs: 0
# Cajanuscajan_C.cajan_19195 Exp number, first 60 AAs:  0
# Cajanuscajan_C.cajan_19195 Total prob of N-in:        0.00451
Cajanuscajan_C.cajan_19195      TMHMM2.0        outside      1   320
```

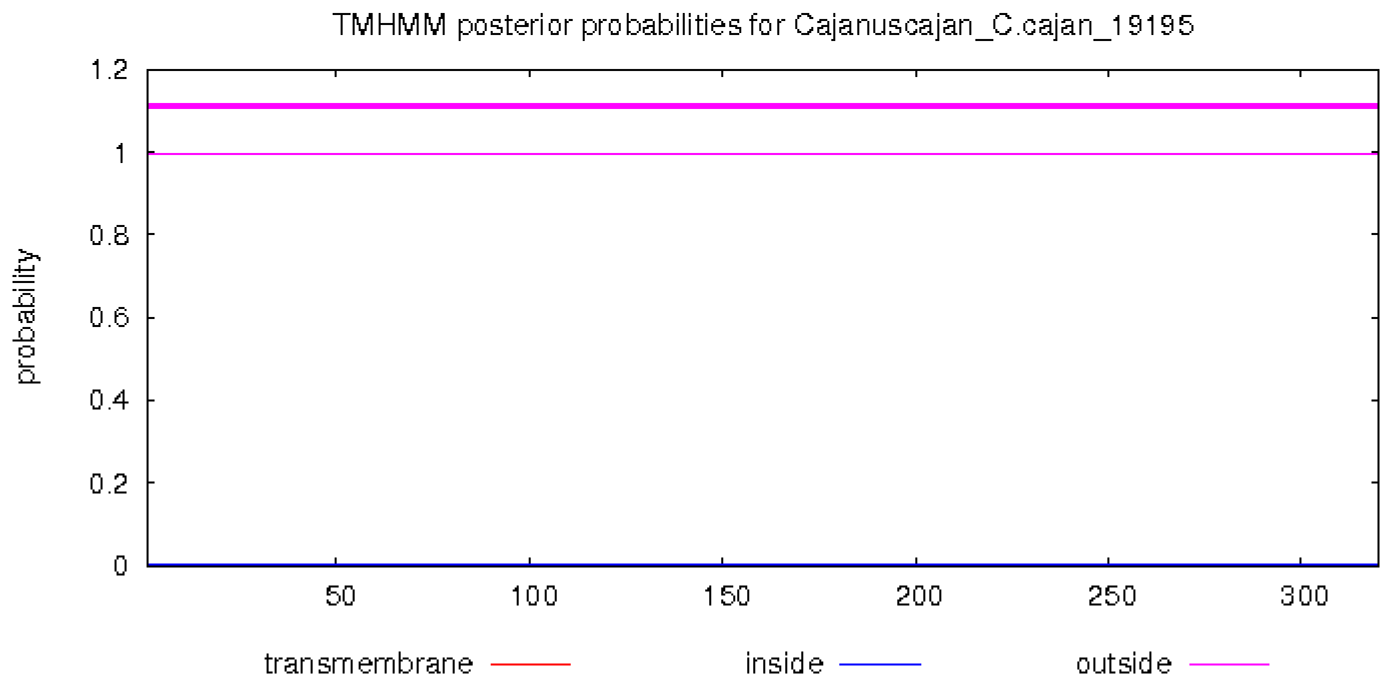

plot in postscript, script for making the plot in gnuplot, data for plot

```
# Cajanuscajan_C.cajan_19966 Length: 323
# Cajanuscajan_C.cajan_19966 Number of predicted TMHs:  0
# Cajanuscajan_C.cajan_19966 Exp number of AAs in TMHs: 0.10212
# Cajanuscajan_C.cajan_19966 Exp number, first 60 AAs:  0.05663
# Cajanuscajan_C.cajan_19966 Total prob of N-in:        0.06560
Cajanuscajan_C.cajan_19966      TMHMM2.0        outside      1   323
```

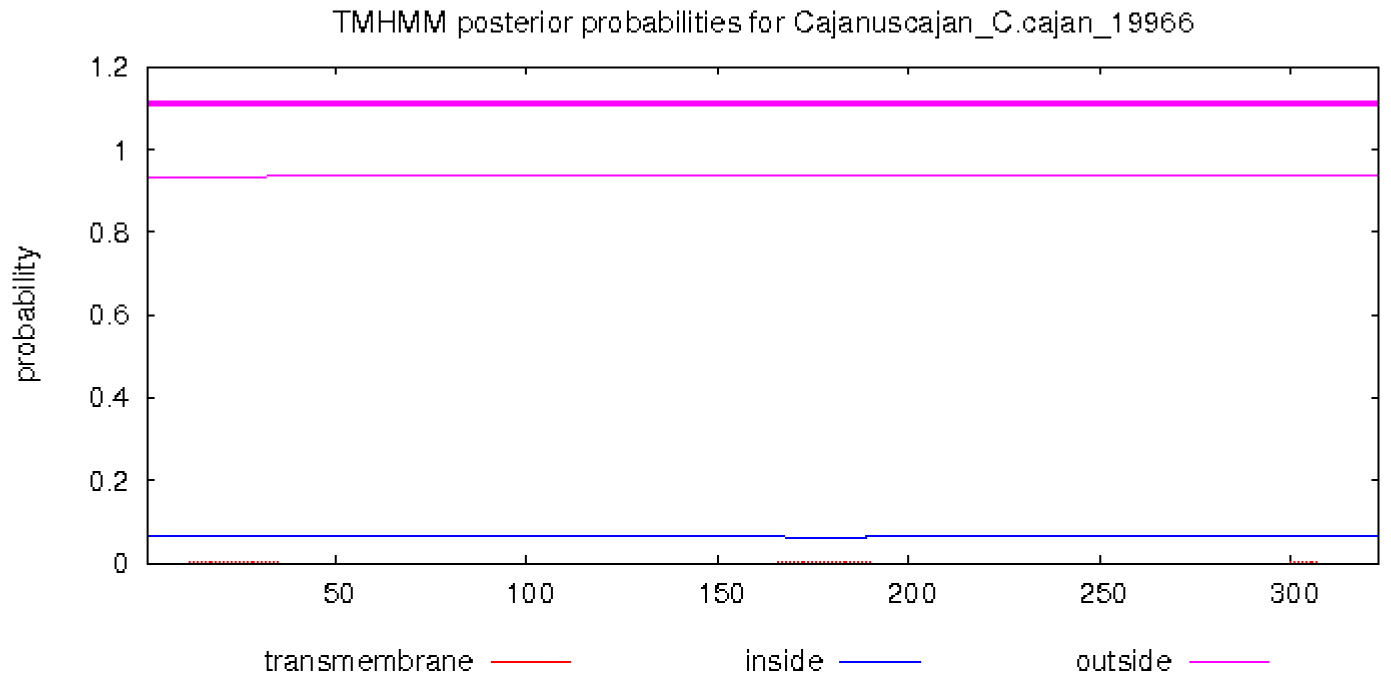

plot in postscript, script for making the plot in gnuplot, data for plot

```
# Cajanuscajan_C.cajan_20225 Length: 278
# Cajanuscajan_C.cajan_20225 Number of predicted TMHs:  0
# Cajanuscajan_C.cajan_20225 Exp number of AAs in TMHs: 0.02658
# Cajanuscajan_C.cajan_20225 Exp number, first 60 AAs:  0.0002
# Cajanuscajan_C.cajan_20225 Total prob of N-in:        0.02119
Cajanuscajan_C.cajan_20225      TMHMM2.0        outside      1   278
```

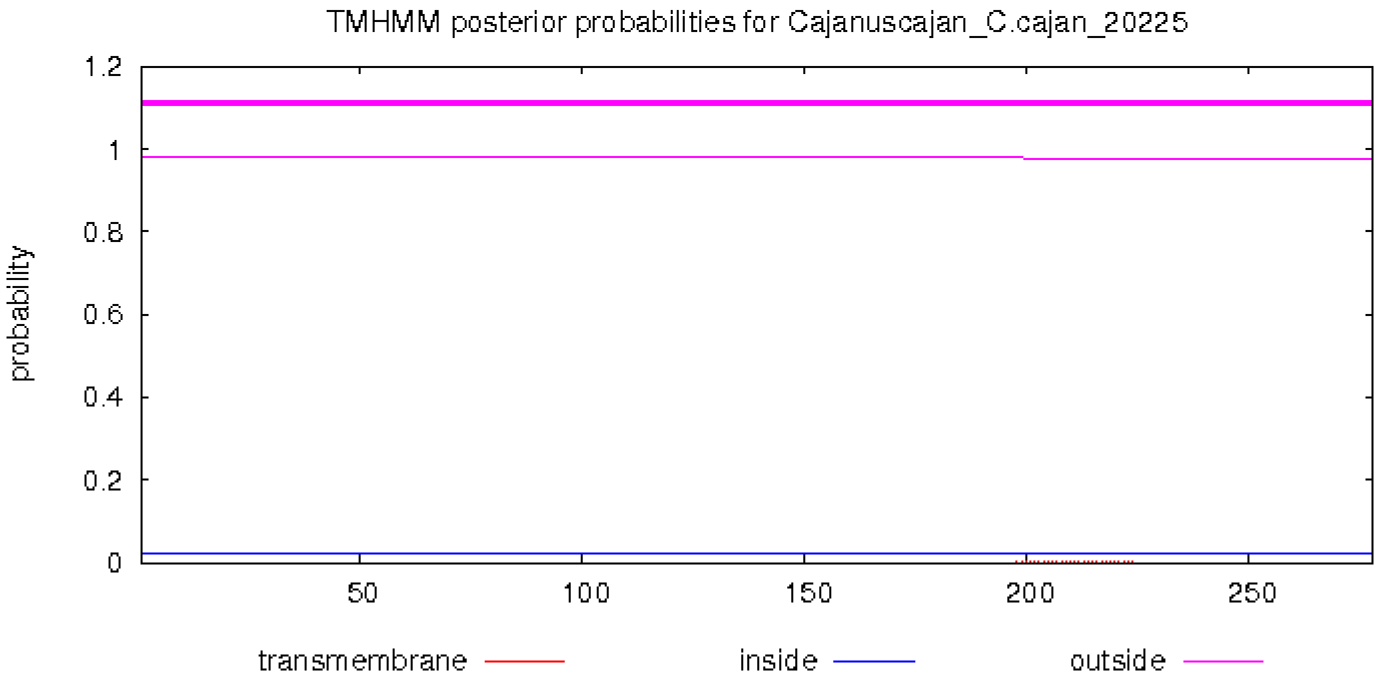

plot in postscript, script for making the plot in gnuplot, data for plot

---

```
# Cajanuscajan_C.cajan_20892 Length: 390
# Cajanuscajan_C.cajan_20892 Number of predicted TMHs:  0
# Cajanuscajan_C.cajan_20892 Exp number of AAs in TMHs: 0.00081
# Cajanuscajan_C.cajan_20892 Exp number, first 60 AAs:  0
# Cajanuscajan_C.cajan_20892 Total prob of N-in:        0.00851
Cajanuscajan_C.cajan_20892      TMHMM2.0        outside      1   390
```

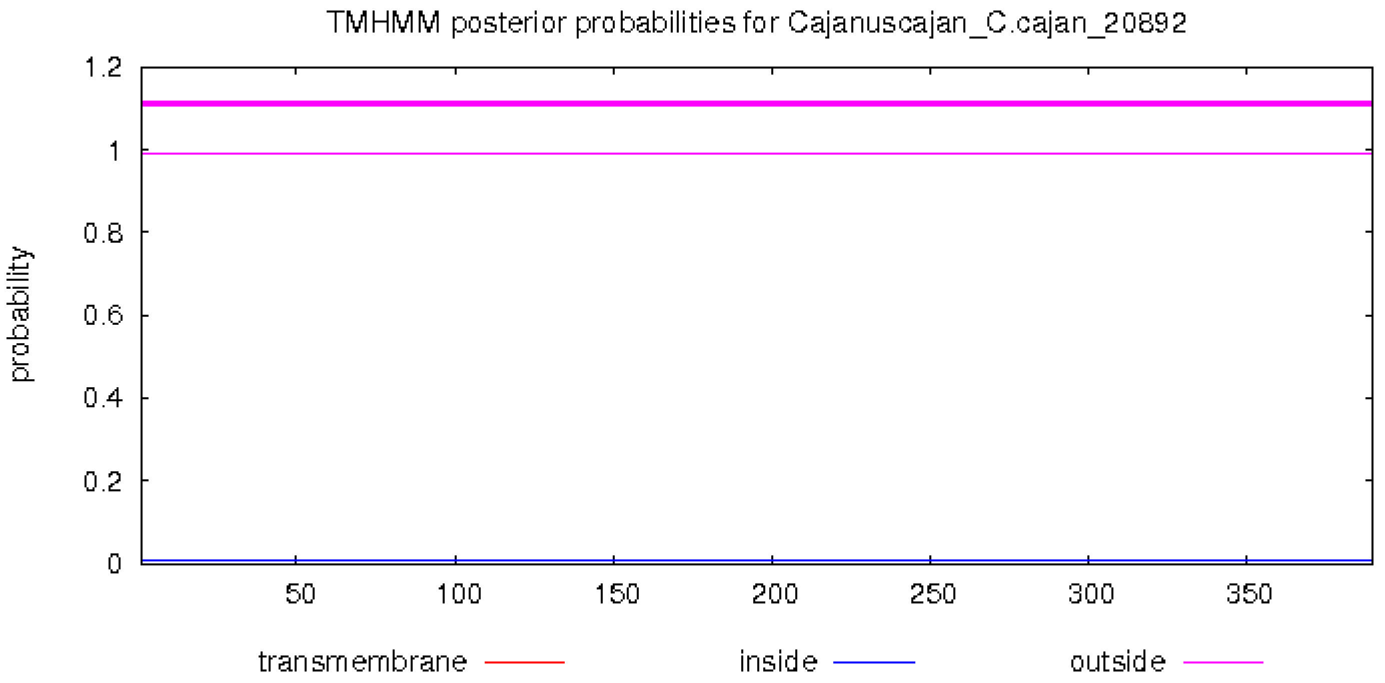

plot in postscript, script for making the plot in gnuplot, data for plot

---

```
# Cajanuscajan_C.cajan_21061 Length: 299
# Cajanuscajan_C.cajan_21061 Number of predicted TMHs:  0
# Cajanuscajan_C.cajan_21061 Exp number of AAs in TMHs: 0.00059
# Cajanuscajan_C.cajan_21061 Exp number, first 60 AAs:  0.00019
# Cajanuscajan_C.cajan_21061 Total prob of N-in:        0.03439
Cajanuscajan_C.cajan_21061      TMHMM2.0        outside      1   299
```

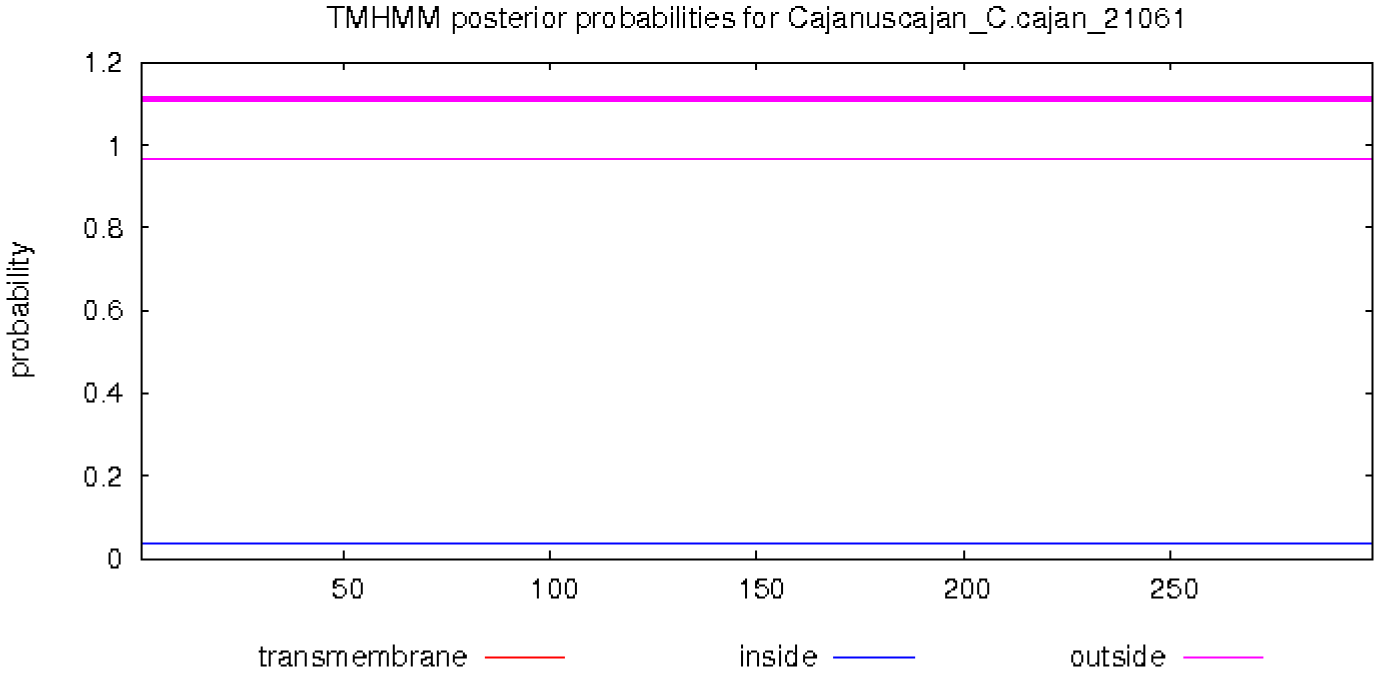

plot in postscript, script for making the plot in gnuplot, data for plot

---

```
# Cajanuscajan_C.cajan_21708 Length: 244
# Cajanuscajan_C.cajan_21708 Number of predicted TMHs:  0
# Cajanuscajan_C.cajan_21708 Exp number of AAs in TMHs: 0.00175
# Cajanuscajan_C.cajan_21708 Exp number, first 60 AAs:  0.00041
# Cajanuscajan_C.cajan_21708 Total prob of N-in:        0.00948
Cajanuscajan_C.cajan_21708      TMHMM2.0        outside      1   244
```

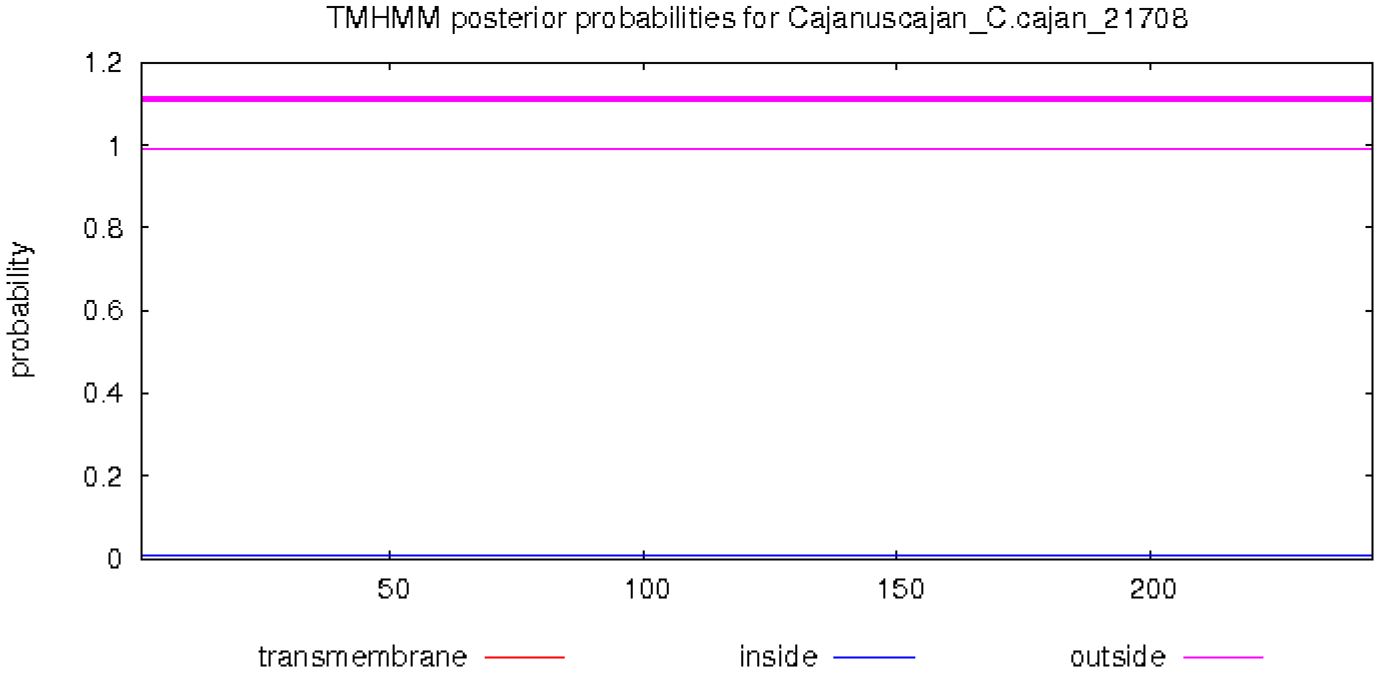

plot in postscript, script for making the plot in gnuplot, data for plot

---

```
# Cajanuscajan_C.cajan_21774 Length: 332
# Cajanuscajan_C.cajan_21774 Number of predicted TMHs:  0
# Cajanuscajan_C.cajan_21774 Exp number of AAs in TMHs: 0
# Cajanuscajan_C.cajan_21774 Exp number, first 60 AAs:  0
# Cajanuscajan_C.cajan_21774 Total prob of N-in:        0.00338
Cajanuscajan_C.cajan_21774      TMHMM2.0        outside      1   332
```

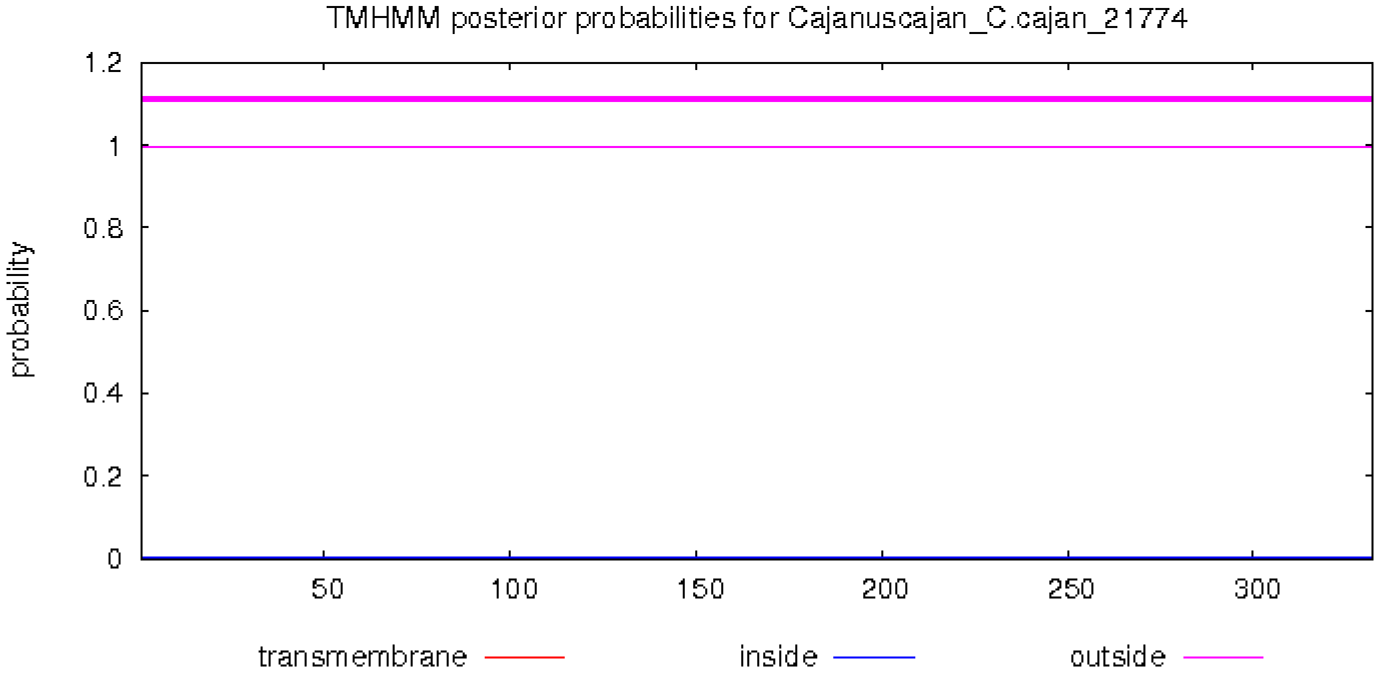

plot in postscript, script for making the plot in gnuplot, data for plot

---

```
# Cajanuscajan_C.cajan_22429 Length: 589
# Cajanuscajan_C.cajan_22429 Number of predicted TMHs:  1
# Cajanuscajan_C.cajan_22429 Exp number of AAs in TMHs: 21.13549
# Cajanuscajan_C.cajan_22429 Exp number, first 60 AAs:  0
# Cajanuscajan_C.cajan_22429 Total prob of N-in:        0.00116
Cajanuscajan_C.cajan_22429      TMHMM2.0        outside      1   563
Cajanuscajan_C.cajan_22429      TMHMM2.0        TMhelix    564   586
Cajanuscajan_C.cajan_22429      TMHMM2.0        inside     587   589
```

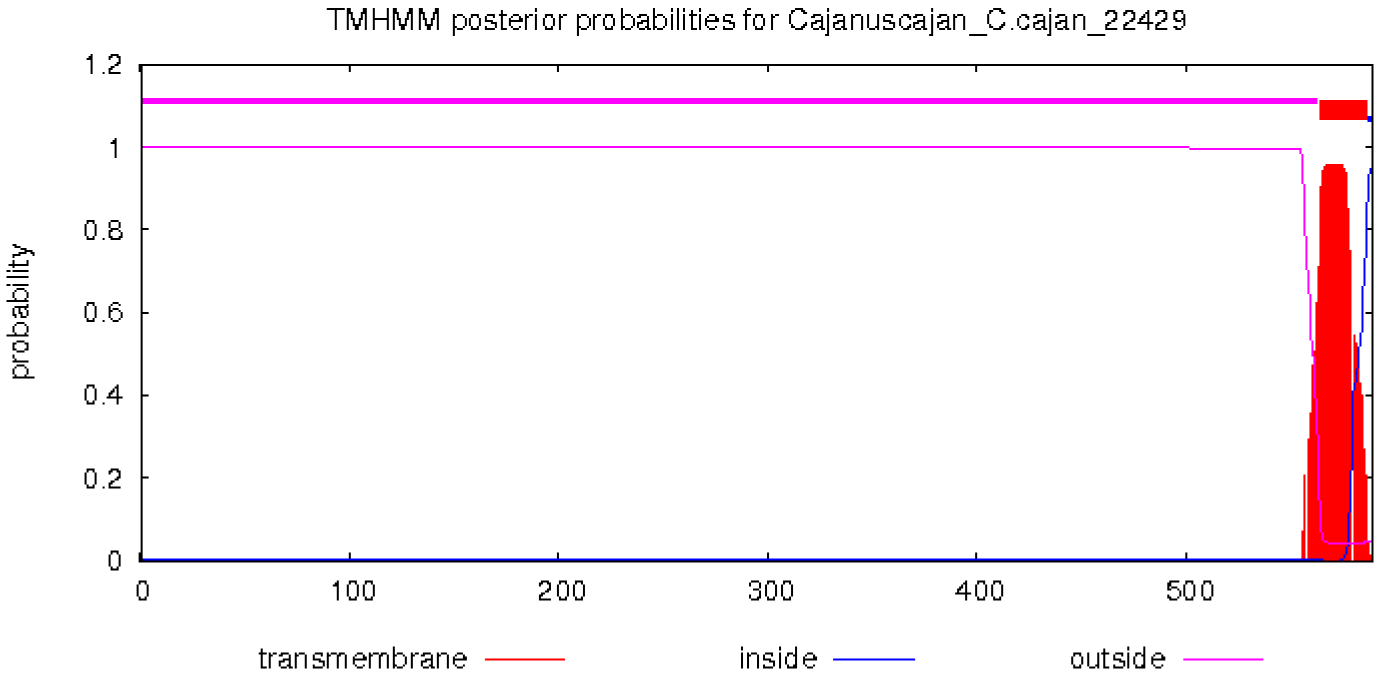

plot in postscript, script for making the plot in gnuplot, data for plot

```
# Cajanuscajan_C.cajan_22430 Length: 500
# Cajanuscajan_C.cajan_22430 Number of predicted TMHs:  0
# Cajanuscajan_C.cajan_22430 Exp number of AAs in TMHs: 0.06035
# Cajanuscajan_C.cajan_22430 Exp number, first 60 AAs:  0.02364
# Cajanuscajan_C.cajan_22430 Total prob of N-in:        0.00223
Cajanuscajan_C.cajan_22430      TMHMM2.0        outside      1   500
```

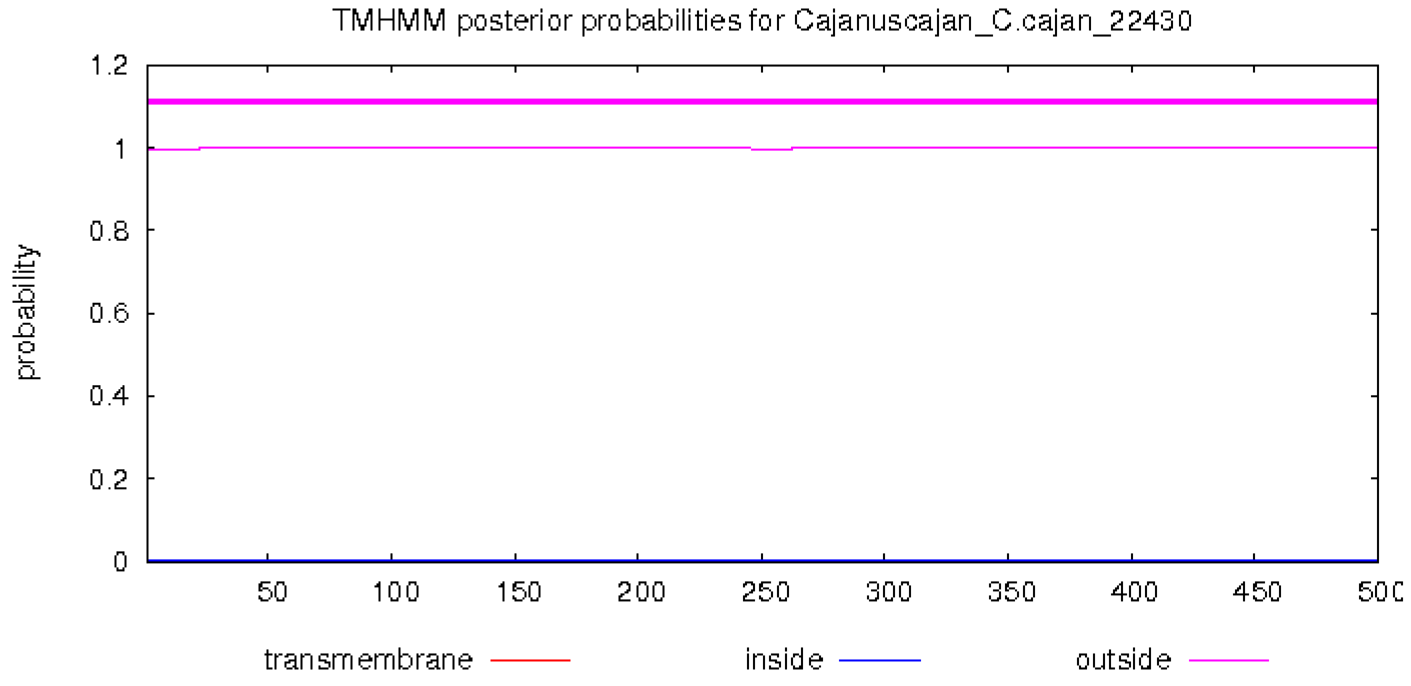

plot in postscript, script for making the plot in gnuplot, data for plot

```
# Cajanuscajan_C.cajan_22489 Length: 172
# Cajanuscajan_C.cajan_22489 Number of predicted TMHs:  0
# Cajanuscajan_C.cajan_22489 Exp number of AAs in TMHs: 0.02479
# Cajanuscajan_C.cajan_22489 Exp number, first 60 AAs:  0.02479
# Cajanuscajan_C.cajan_22489 Total prob of N-in:        0.03905
Cajanuscajan_C.cajan_22489      TMHMM2.0        outside      1   172
```

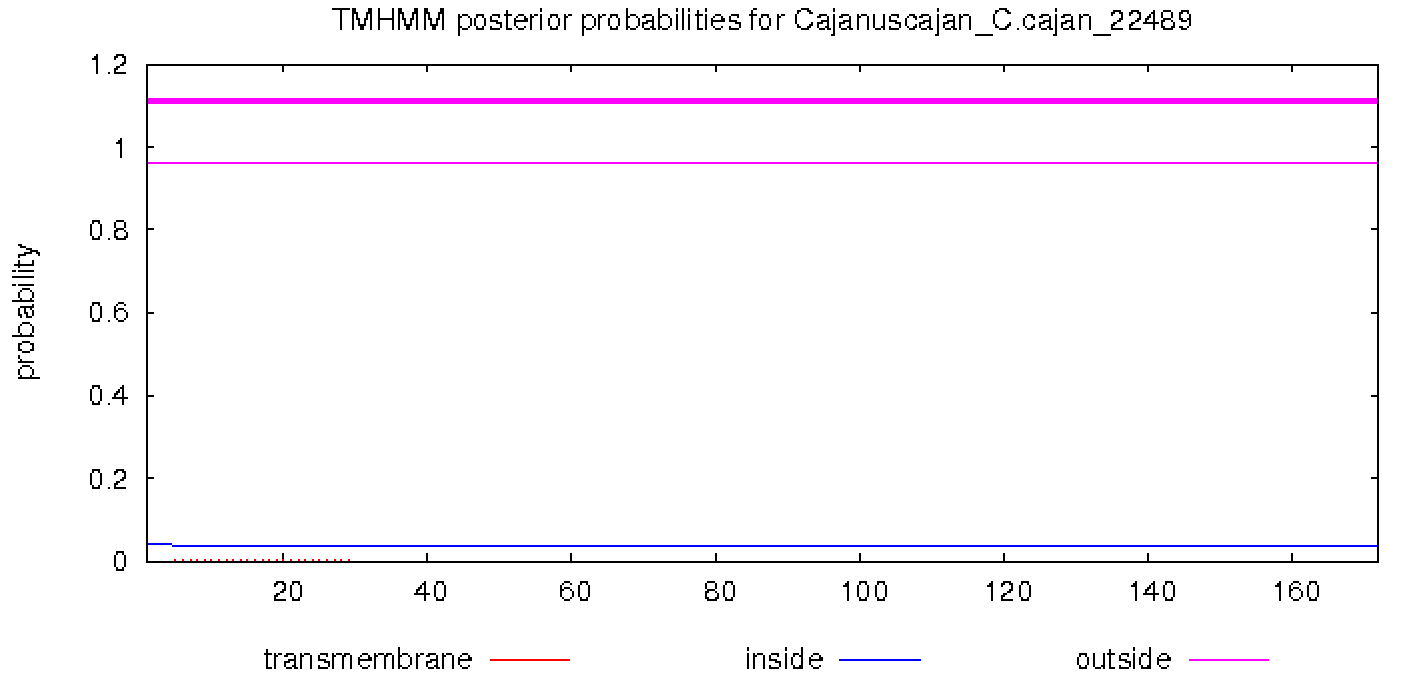

plot in postscript, script for making the plot in gnuplot, data for plot

```
# Cajanuscajan_C.cajan_22852 Length: 349
# Cajanuscajan_C.cajan_22852 Number of predicted TMHs:  0
# Cajanuscajan_C.cajan_22852 Exp number of AAs in TMHs: 0.00017
# Cajanuscajan_C.cajan_22852 Exp number, first 60 AAs:  0.00017
# Cajanuscajan_C.cajan_22852 Total prob of N-in:        0.01249
Cajanuscajan_C.cajan_22852      TMHMM2.0        outside      1   349
```

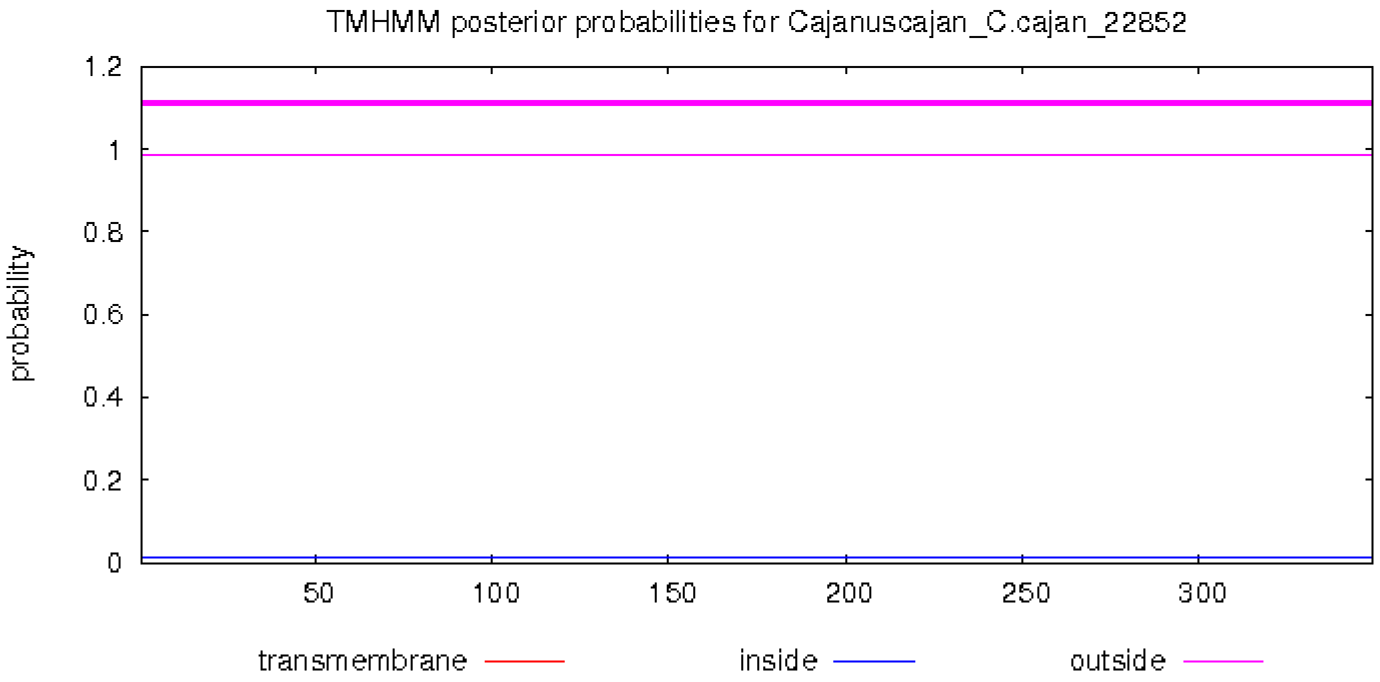

plot in postscript, script for making the plot in gnuplot, data for plot

---

```
# Cajanuscajan_C.cajan_22870 Length: 194
# Cajanuscajan_C.cajan_22870 Number of predicted TMHs:  0
# Cajanuscajan_C.cajan_22870 Exp number of AAs in TMHs: 0.00171
# Cajanuscajan_C.cajan_22870 Exp number, first 60 AAs:  0
# Cajanuscajan_C.cajan_22870 Total prob of N-in:        0.07877
Cajanuscajan_C.cajan_22870      TMHMM2.0        outside      1   194
```

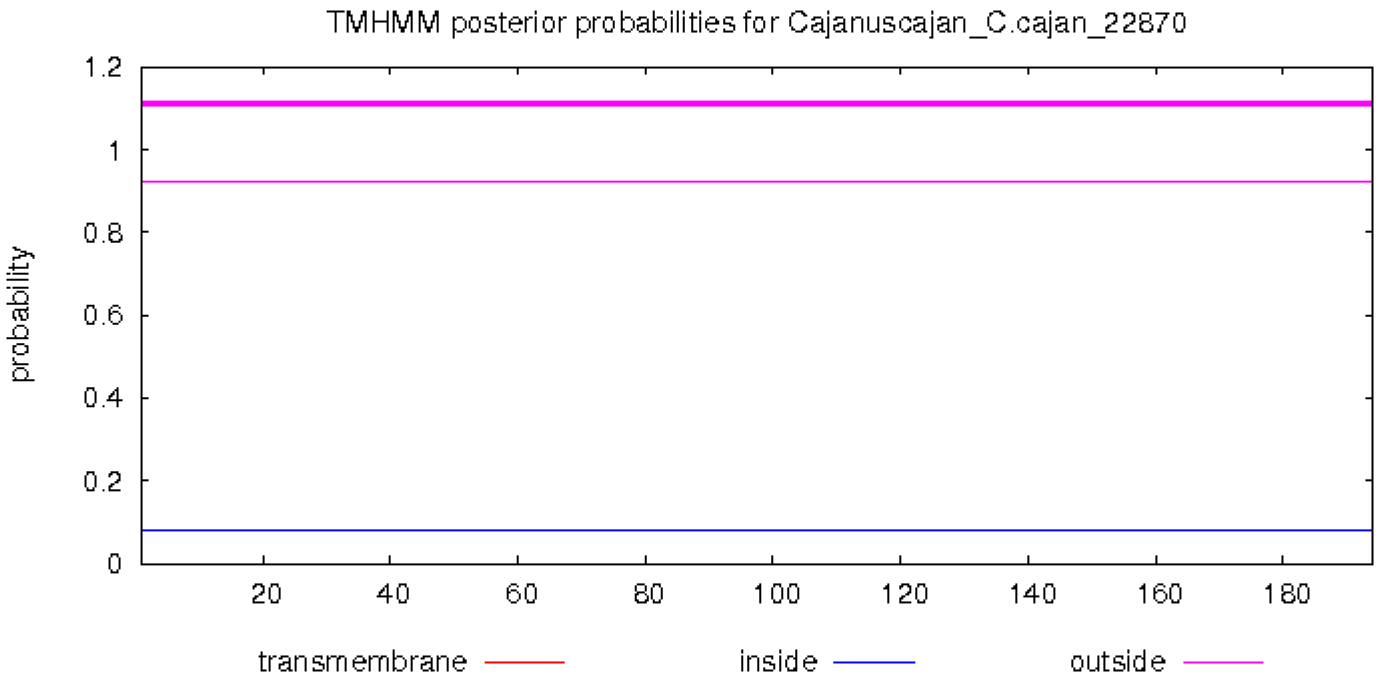

plot in postscript, script for making the plot in gnuplot, data for plot

---

```
# Cajanuscajan_C.cajan_23161 Length: 443
# Cajanuscajan_C.cajan_23161 Number of predicted TMHs:  0
# Cajanuscajan_C.cajan_23161 Exp number of AAs in TMHs: 0.00062
# Cajanuscajan_C.cajan_23161 Exp number, first 60 AAs:  0
# Cajanuscajan_C.cajan_23161 Total prob of N-in:        0.00821
Cajanuscajan_C.cajan_23161      TMHMM2.0        outside      1   443
```

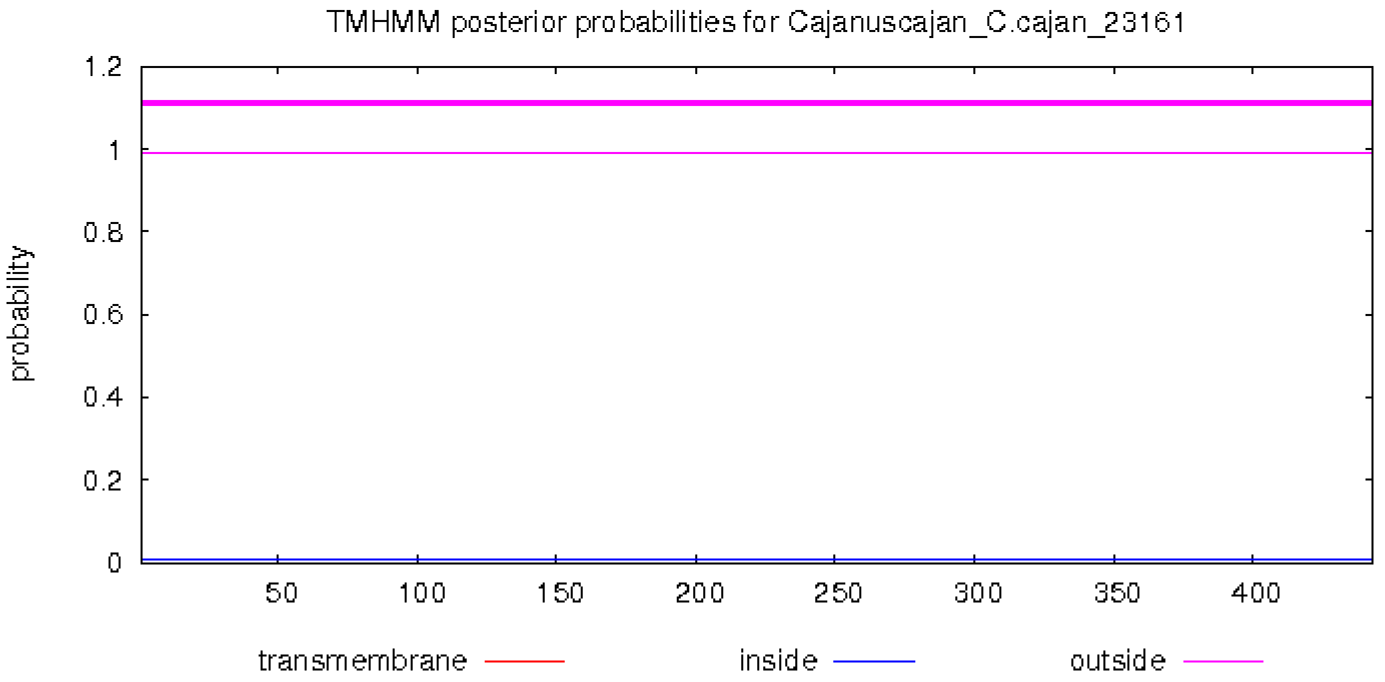

plot in postscript, script for making the plot in gnuplot, data for plot

---

```
# Cajanuscajan_C.cajan_23518 Length: 338
# Cajanuscajan_C.cajan_23518 Number of predicted TMHs:  0
# Cajanuscajan_C.cajan_23518 Exp number of AAs in TMHs: 0.24033
# Cajanuscajan_C.cajan_23518 Exp number, first 60 AAs:  0.2391
# Cajanuscajan_C.cajan_23518 Total prob of N-in:        0.02356
Cajanuscajan_C.cajan_23518      TMHMM2.0        outside      1   338
```

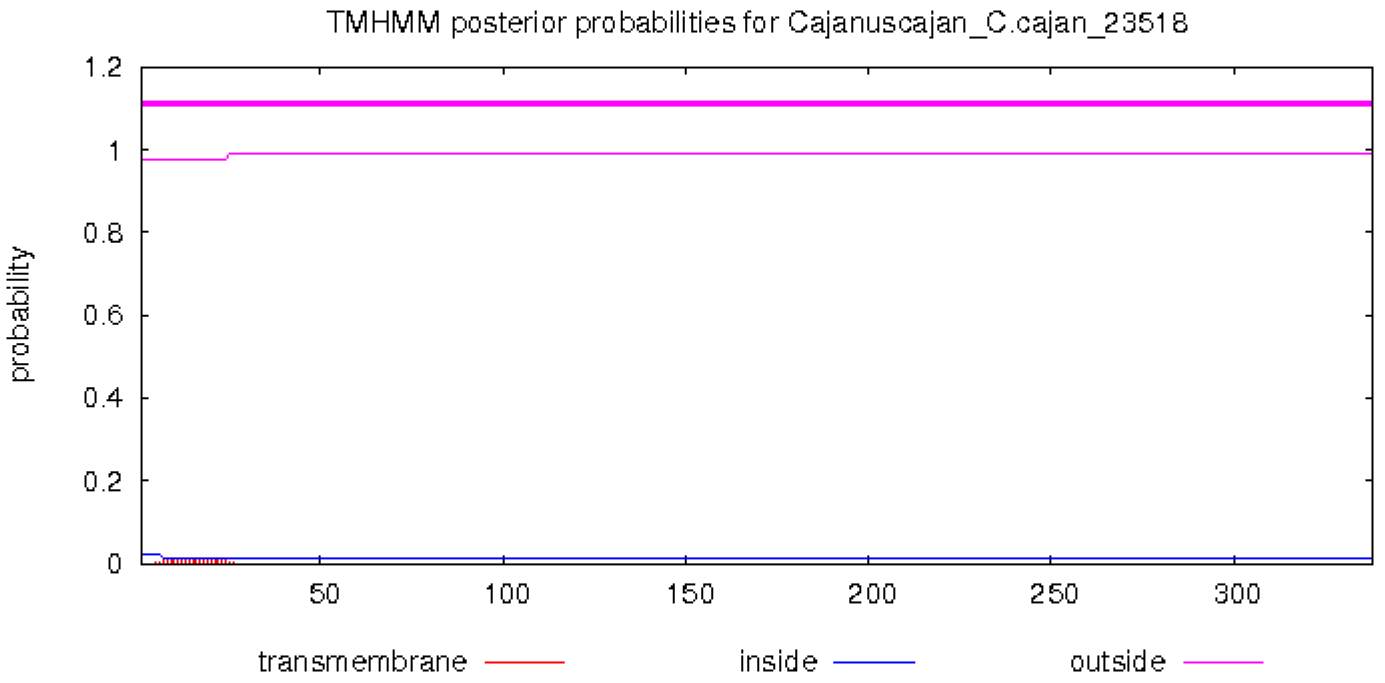

plot in postscript, script for making the plot in gnuplot, data for plot

---

```
# Cajanuscajan_C.cajan_23529 Length: 204
# Cajanuscajan_C.cajan_23529 Number of predicted TMHs:  0
# Cajanuscajan_C.cajan_23529 Exp number of AAs in TMHs: 0.00065
# Cajanuscajan_C.cajan_23529 Exp number, first 60 AAs:  0.00023
# Cajanuscajan_C.cajan_23529 Total prob of N-in:        0.03669
Cajanuscajan_C.cajan_23529      TMHMM2.0        outside      1   204
```

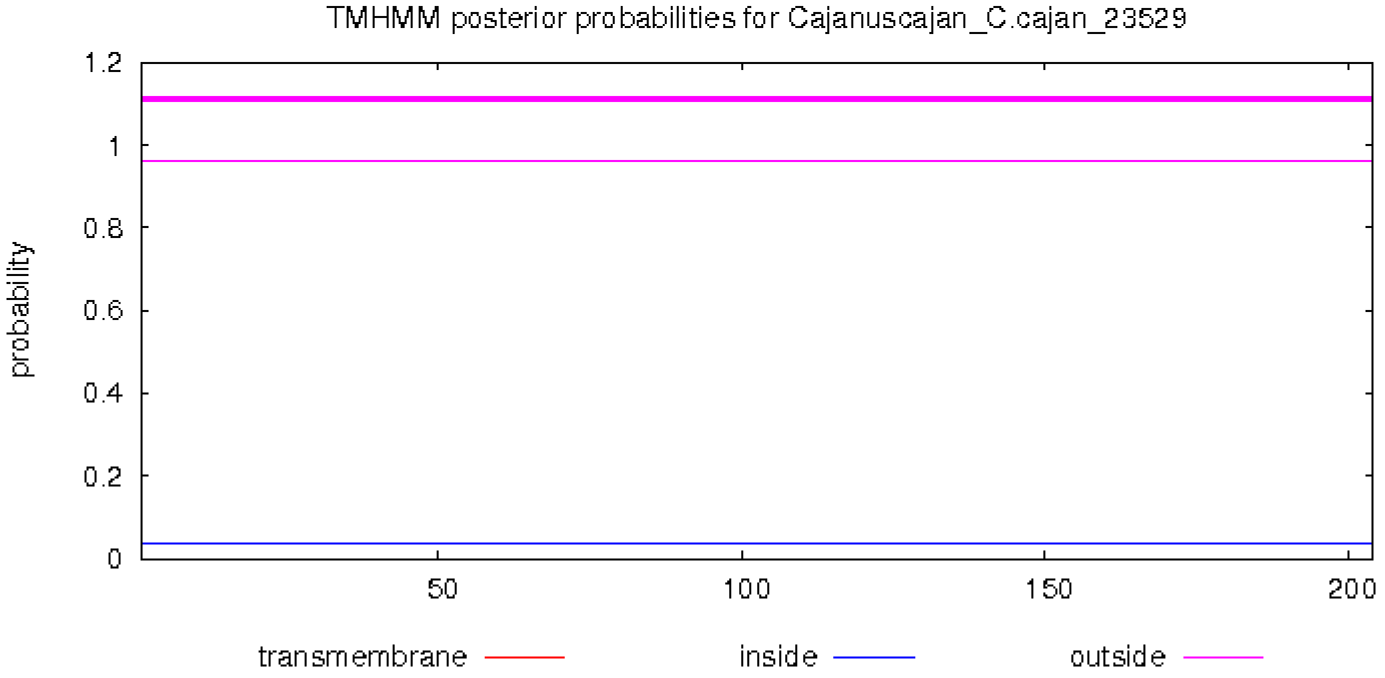

plot in postscript, script for making the plot in gnuplot, data for plot

```
# Cajanuscajan_C.cajan_23928 Length: 279
# Cajanuscajan_C.cajan_23928 Number of predicted TMHs:  0
# Cajanuscajan_C.cajan_23928 Exp number of AAs in TMHs: 0.0008
# Cajanuscajan_C.cajan_23928 Exp number, first 60 AAs:  0
# Cajanuscajan_C.cajan_23928 Total prob of N-in:        0.08228
Cajanuscajan_C.cajan_23928      TMHMM2.0        outside      1   279
```

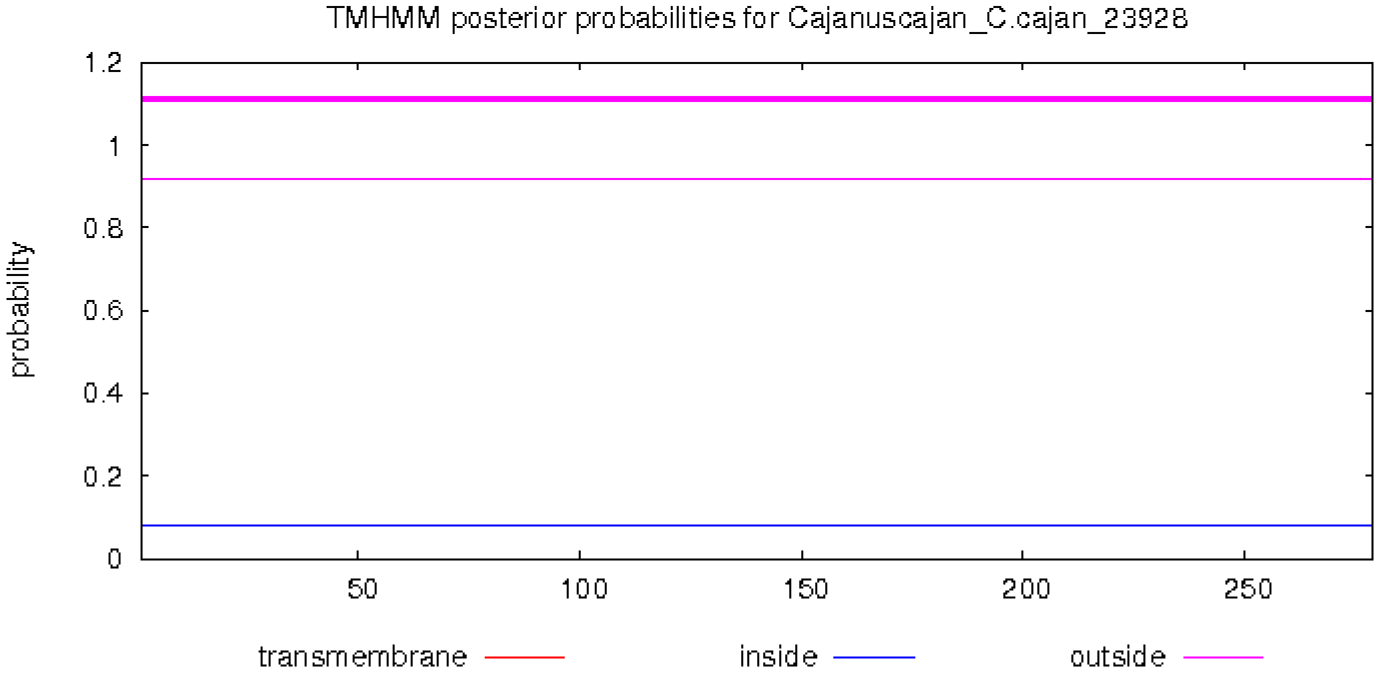

plot in postscript, script for making the plot in gnuplot, data for plot

```
# Cajanuscajan_C.cajan_24591 Length: 403
# Cajanuscajan_C.cajan_24591 Number of predicted TMHs:  0
# Cajanuscajan_C.cajan_24591 Exp number of AAs in TMHs: 0.11515
# Cajanuscajan_C.cajan_24591 Exp number, first 60 AAs:  0
# Cajanuscajan_C.cajan_24591 Total prob of N-in:        0.00977
Cajanuscajan_C.cajan_24591      TMHMM2.0        outside      1   403
```

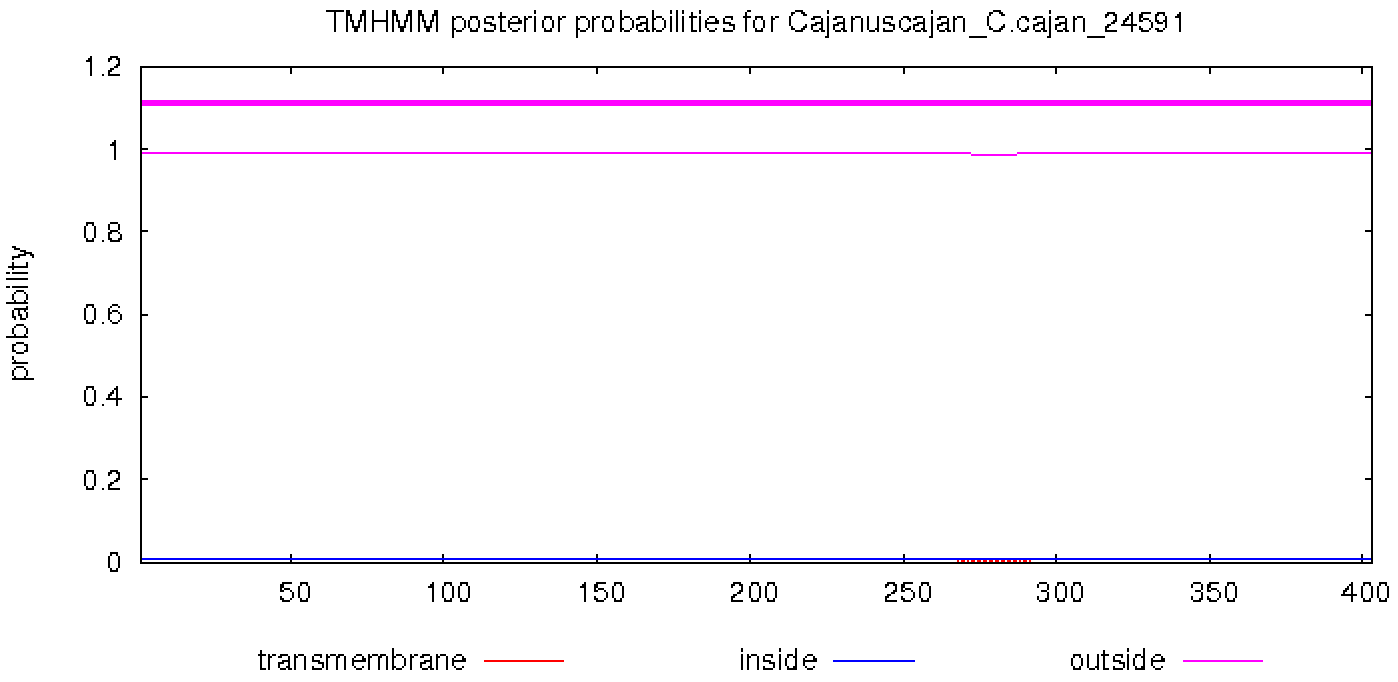

plot in postscript, script for making the plot in gnuplot, data for plot

---

```
# Cajanuscajan_C.cajan_24657 Length: 439
# Cajanuscajan_C.cajan_24657 Number of predicted TMHs:  0
# Cajanuscajan_C.cajan_24657 Exp number of AAs in TMHs: 0.04359
# Cajanuscajan_C.cajan_24657 Exp number, first 60 AAs:  0
# Cajanuscajan_C.cajan_24657 Total prob of N-in:        0.00509
Cajanuscajan_C.cajan_24657      TMHMM2.0        outside      1   439
```

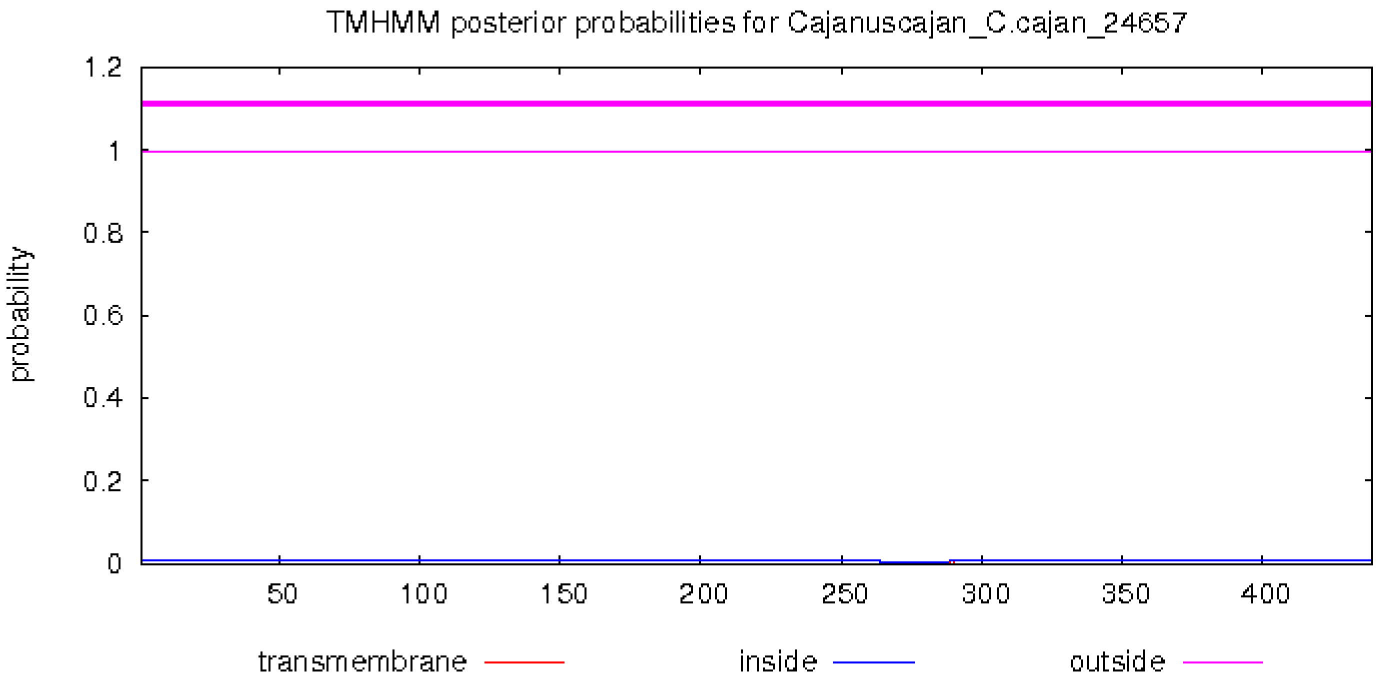

plot in postscript, script for making the plot in gnuplot, data for plot

---

```
# Cajanuscajan_C.cajan_24755 Length: 321
# Cajanuscajan_C.cajan_24755 Number of predicted TMHs:  0
# Cajanuscajan_C.cajan_24755 Exp number of AAs in TMHs: 0.25285
# Cajanuscajan_C.cajan_24755 Exp number, first 60 AAs:  0
# Cajanuscajan_C.cajan_24755 Total prob of N-in:        0.05323
Cajanuscajan_C.cajan_24755      TMHMM2.0        outside      1   321
```

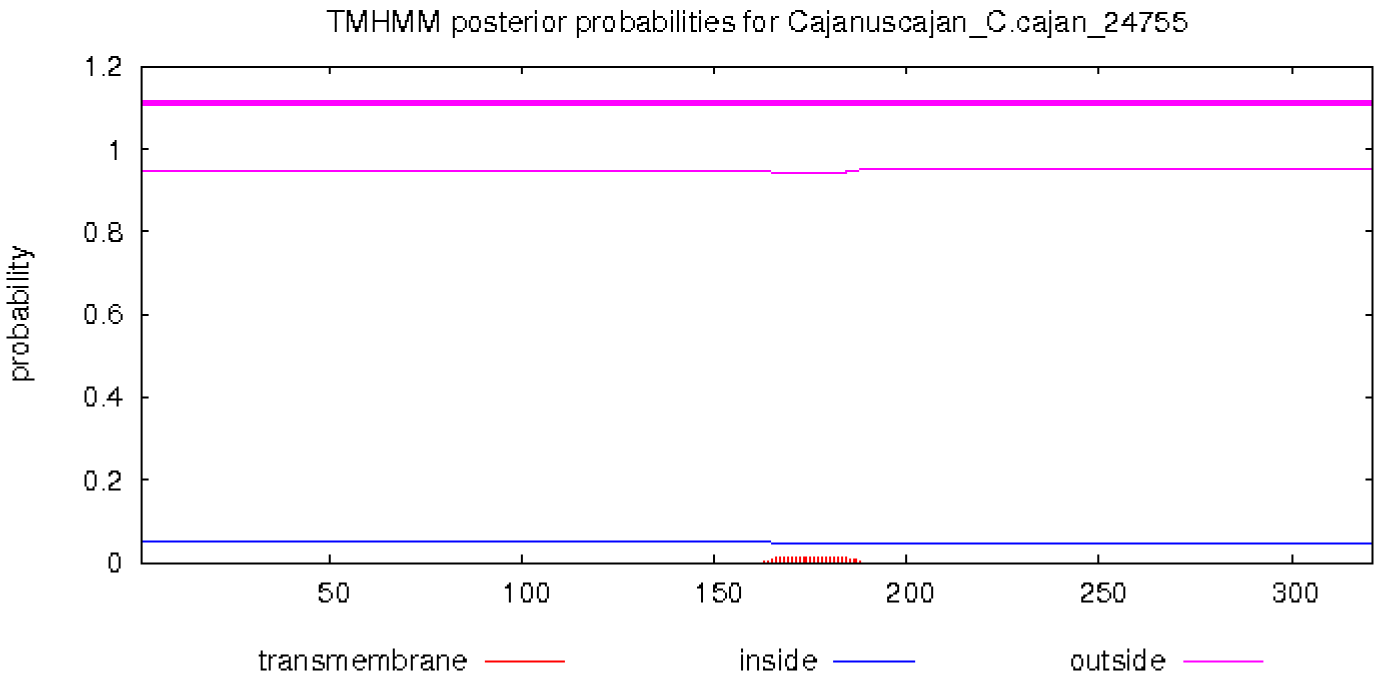

plot in postscript, script for making the plot in gnuplot, data for plot

---

```
# Cajanuscajan_C.cajan_26125 Length: 567
# Cajanuscajan_C.cajan_26125 Number of predicted TMHs:  1
# Cajanuscajan_C.cajan_26125 Exp number of AAs in TMHs: 22.04413
# Cajanuscajan_C.cajan_26125 Exp number, first 60 AAs:  0.00047
# Cajanuscajan_C.cajan_26125 Total prob of N-in:        0.01329
Cajanuscajan_C.cajan_26125      TMHMM2.0        outside      1   537
Cajanuscajan_C.cajan_26125      TMHMM2.0        TMhelix    538   560
Cajanuscajan_C.cajan_26125      TMHMM2.0        inside     561   567
```

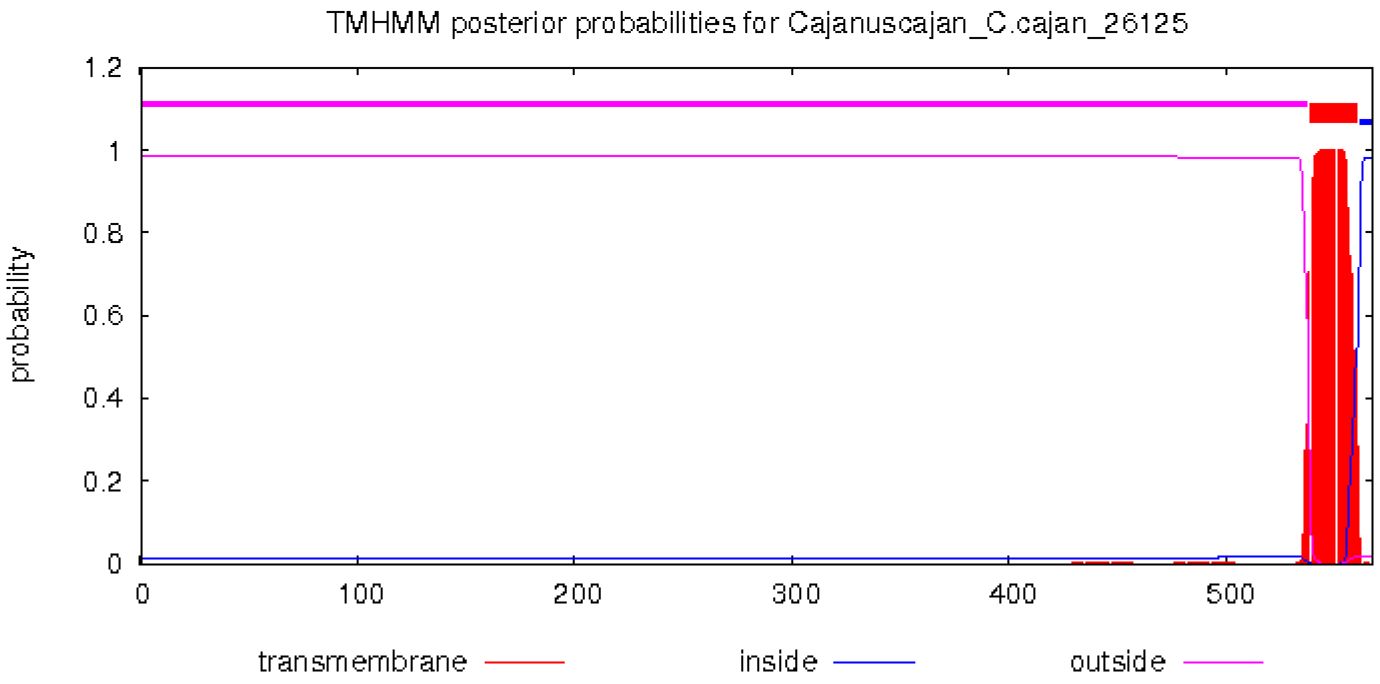

plot in postscript, script for making the plot in gnuplot, data for plot

```
# Cajanuscajan_C.cajan_26764 Length: 358
# Cajanuscajan_C.cajan_26764 Number of predicted TMHs:  0
# Cajanuscajan_C.cajan_26764 Exp number of AAs in TMHs: 0.000960000000000000001
# Cajanuscajan_C.cajan_26764 Exp number, first 60 AAs:  0.00071
# Cajanuscajan_C.cajan_26764 Total prob of N-in:        0.00683
Cajanuscajan_C.cajan_26764      TMHMM2.0        outside      1   358
```

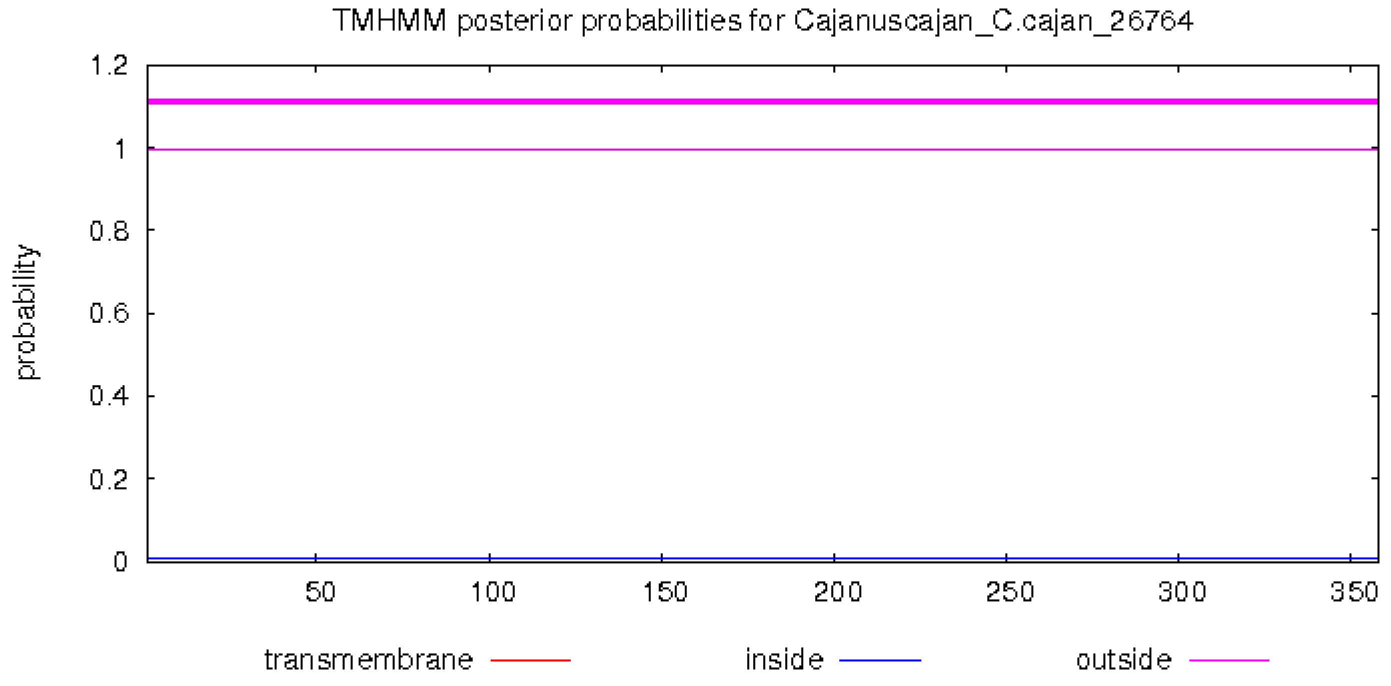

plot in postscript, script for making the plot in gnuplot, data for plot

```
# Cajanuscajan_C.cajan_28603 Length: 244
# Cajanuscajan_C.cajan_28603 Number of predicted TMHs:  0
# Cajanuscajan_C.cajan_28603 Exp number of AAs in TMHs: 0.00023
# Cajanuscajan_C.cajan_28603 Exp number, first 60 AAs:  0.00023
# Cajanuscajan_C.cajan_28603 Total prob of N-in:        0.05100
Cajanuscajan_C.cajan_28603      TMHMM2.0        outside      1   244
```

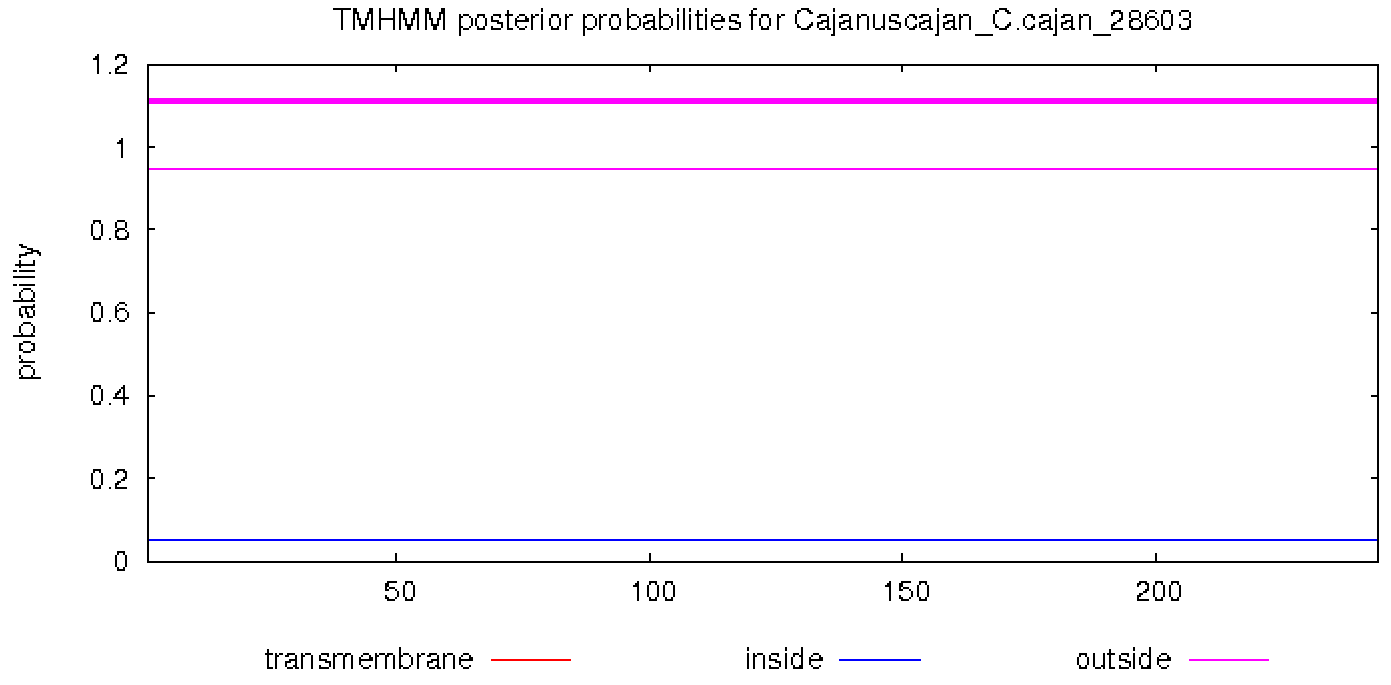

plot in postscript, script for making the plot in gnuplot, data for plot

```
# Cajanuscajan_C.cajan_29425 Length: 625
# Cajanuscajan_C.cajan_29425 Number of predicted TMHs:  1
# Cajanuscajan_C.cajan_29425 Exp number of AAs in TMHs: 35.48482
# Cajanuscajan_C.cajan_29425 Exp number, first 60 AAs:  0
# Cajanuscajan_C.cajan_29425 Total prob of N-in:        0.00042
Cajanuscajan_C.cajan_29425      TMHMM2.0        outside      1   532
Cajanuscajan_C.cajan_29425      TMHMM2.0        TMhelix    533   552
Cajanuscajan_C.cajan_29425      TMHMM2.0        inside     553   625
```

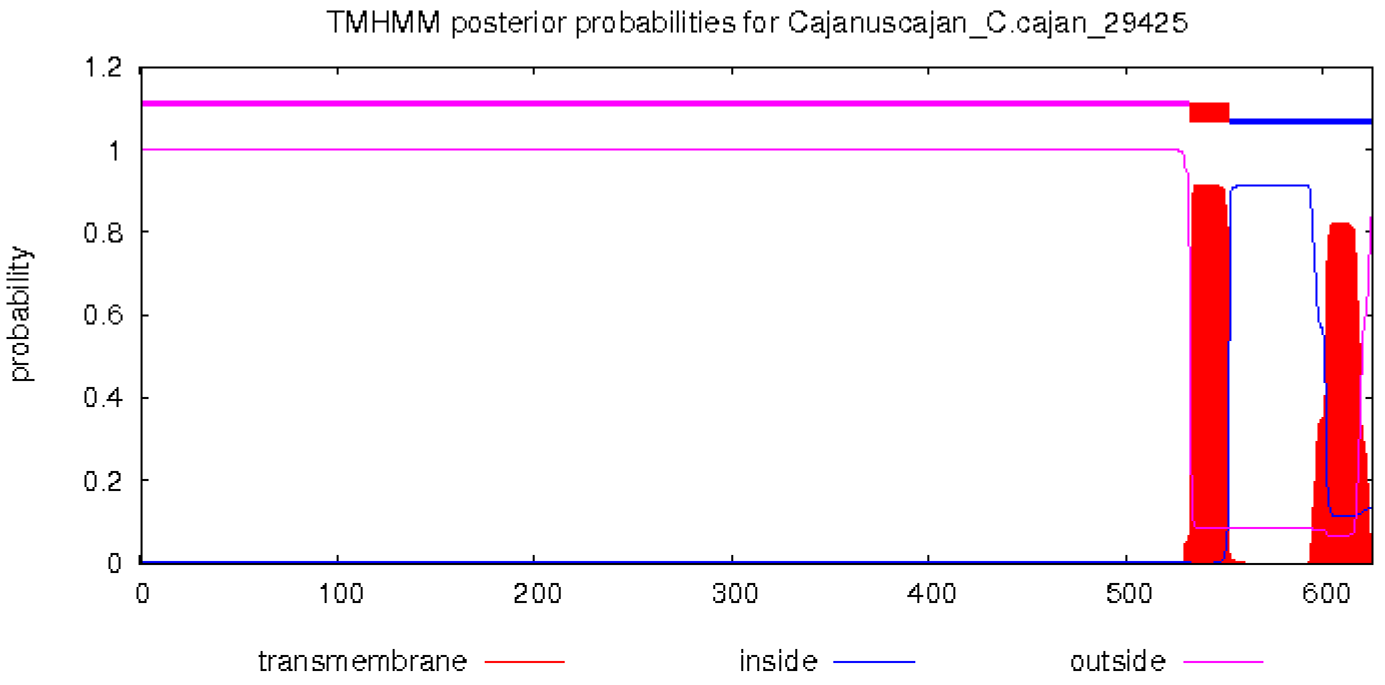

plot in postscript, script for making the plot in gnuplot, data for plot

---

```
# Cajanuscajan_C.cajan_29427 Length: 627
# Cajanuscajan_C.cajan_29427 Number of predicted TMHs:  1
# Cajanuscajan_C.cajan_29427 Exp number of AAs in TMHs: 34.48751
# Cajanuscajan_C.cajan_29427 Exp number, first 60 AAs:  0
# Cajanuscajan_C.cajan_29427 Total prob of N-in:        0.00070
Cajanuscajan_C.cajan_29427      TMHMM2.0        outside      1   534
Cajanuscajan_C.cajan_29427      TMHMM2.0        TMhelix    535   554
Cajanuscajan_C.cajan_29427      TMHMM2.0        inside     555   627
```

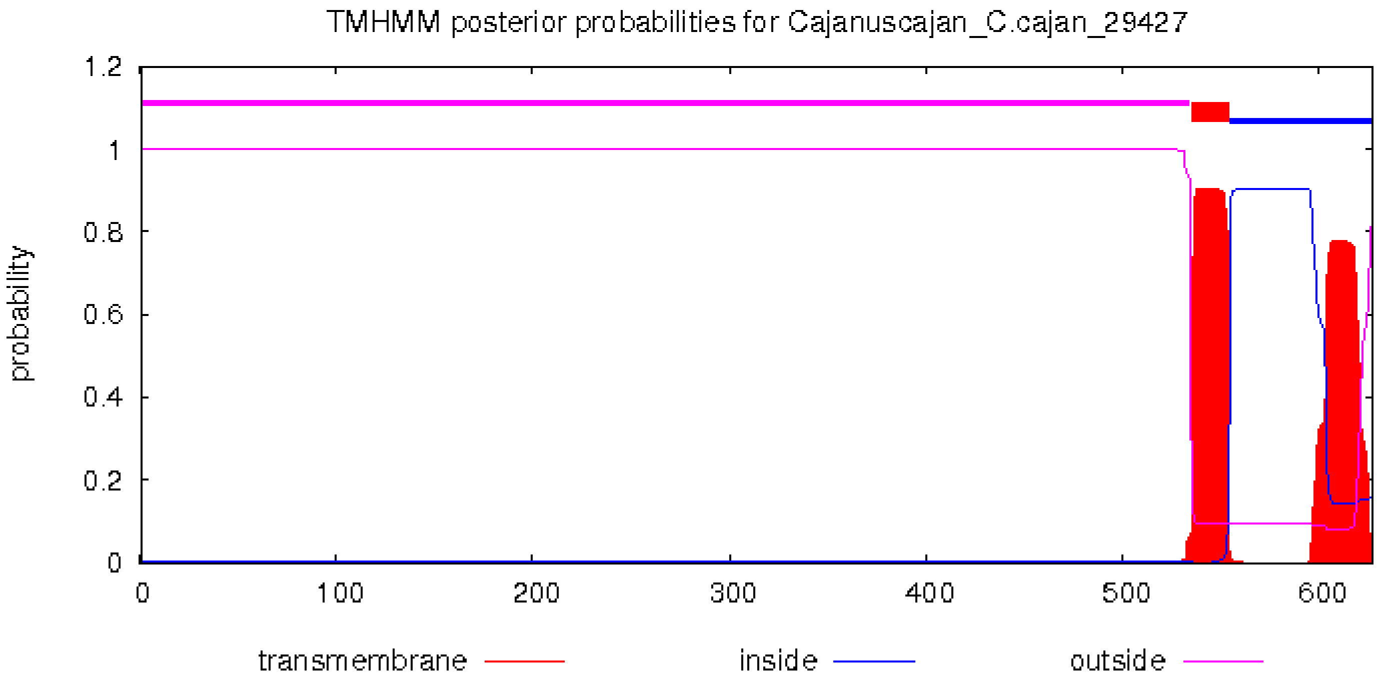

plot in postscript, script for making the plot in gnuplot, data for plot

```
# Cajanuscajan_C.cajan_29871 Length: 271
# Cajanuscajan_C.cajan_29871 Number of predicted TMHs:  0
# Cajanuscajan_C.cajan_29871 Exp number of AAs in TMHs: 0.00066
# Cajanuscajan_C.cajan_29871 Exp number, first 60 AAs:  0.00066
# Cajanuscajan_C.cajan_29871 Total prob of N-in:        0.01503
Cajanuscajan_C.cajan_29871      TMHMM2.0        outside      1   271
```

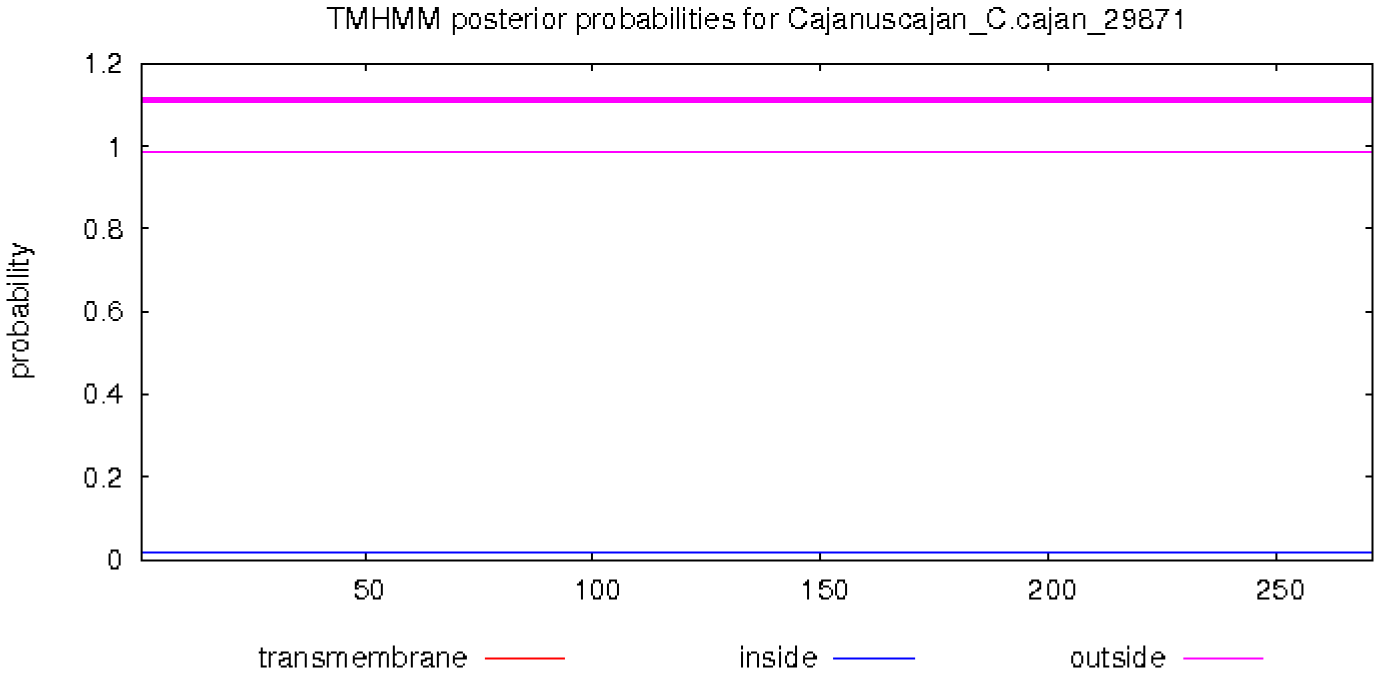

plot in postscript, script for making the plot in gnuplot, data for plot

```
# Cajanuscajan_C.cajan_30472 Length: 285
# Cajanuscajan_C.cajan_30472 Number of predicted TMHs:  0
# Cajanuscajan_C.cajan_30472 Exp number of AAs in TMHs: 0.00057
# Cajanuscajan_C.cajan_30472 Exp number, first 60 AAs:  0.0002
# Cajanuscajan_C.cajan_30472 Total prob of N-in:        0.04877
Cajanuscajan_C.cajan_30472      TMHMM2.0        outside      1   285
```

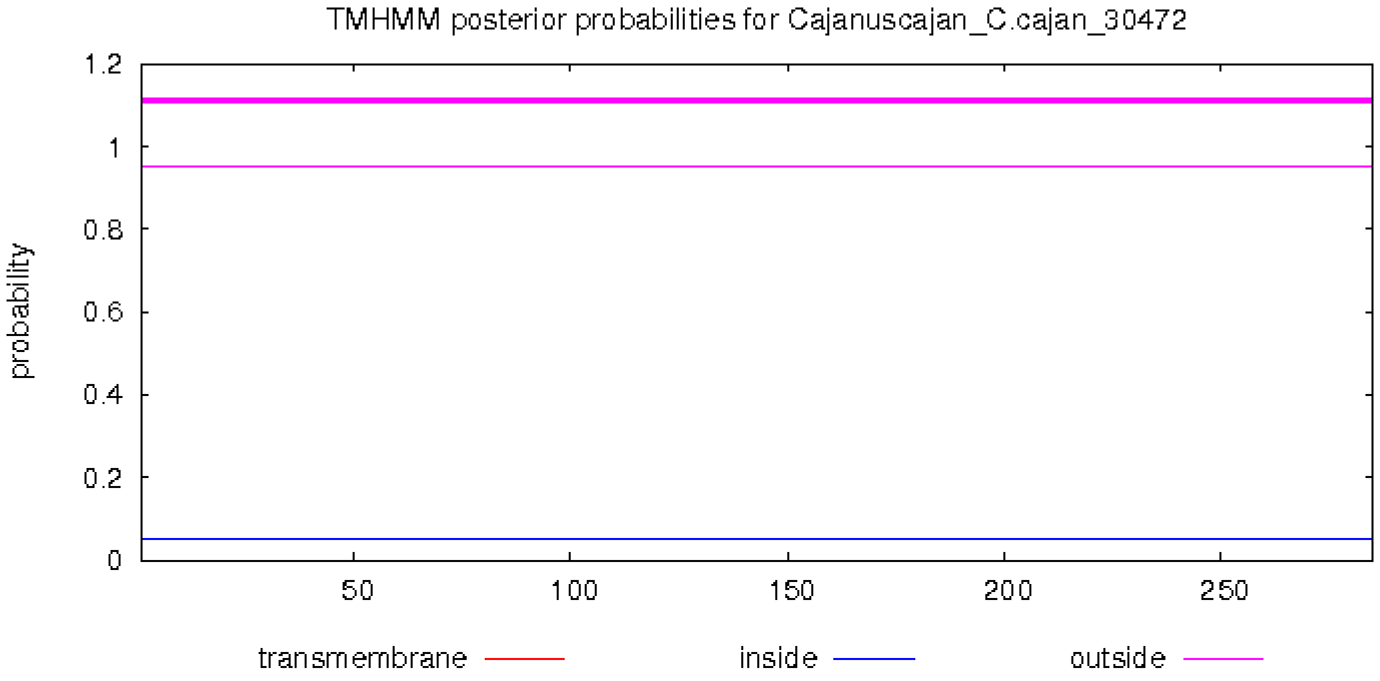

plot in postscript, script for making the plot in gnuplot, data for plot

```
# Cajanuscajan_C.cajan_30485 Length: 225
# Cajanuscajan_C.cajan_30485 Number of predicted TMHs:  0
# Cajanuscajan_C.cajan_30485 Exp number of AAs in TMHs: 0.0004
# Cajanuscajan_C.cajan_30485 Exp number, first 60 AAs:  0.00018
# Cajanuscajan_C.cajan_30485 Total prob of N-in:        0.01403
Cajanuscajan_C.cajan_30485      TMHMM2.0        outside      1   225
```

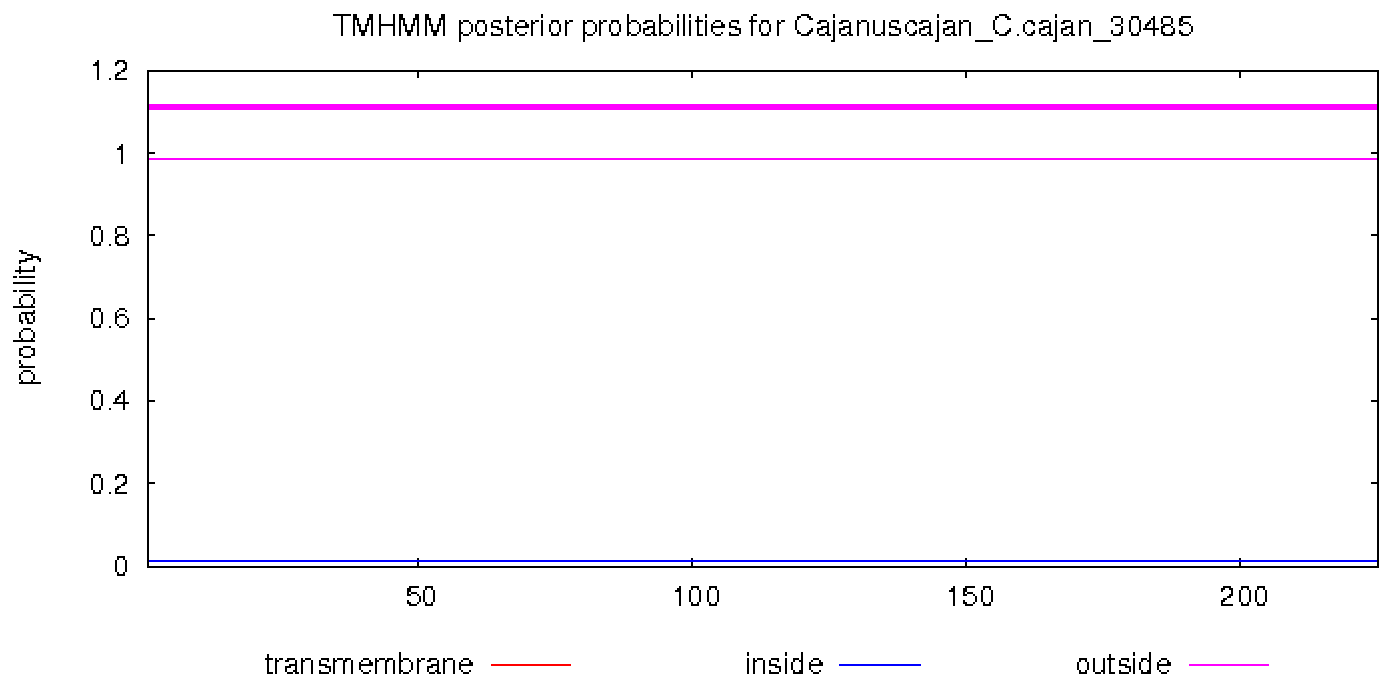

plot in postscript, script for making the plot in gnuplot, data for plot

```
# Cajanuscajan_C.cajan_30687 Length: 242
# Cajanuscajan_C.cajan_30687 Number of predicted TMHs:  0
# Cajanuscajan_C.cajan_30687 Exp number of AAs in TMHs: 0.00117
# Cajanuscajan_C.cajan_30687 Exp number, first 60 AAs:  0.00043
# Cajanuscajan_C.cajan_30687 Total prob of N-in:        0.05495
Cajanuscajan_C.cajan_30687      TMHMM2.0        outside      1   242
```

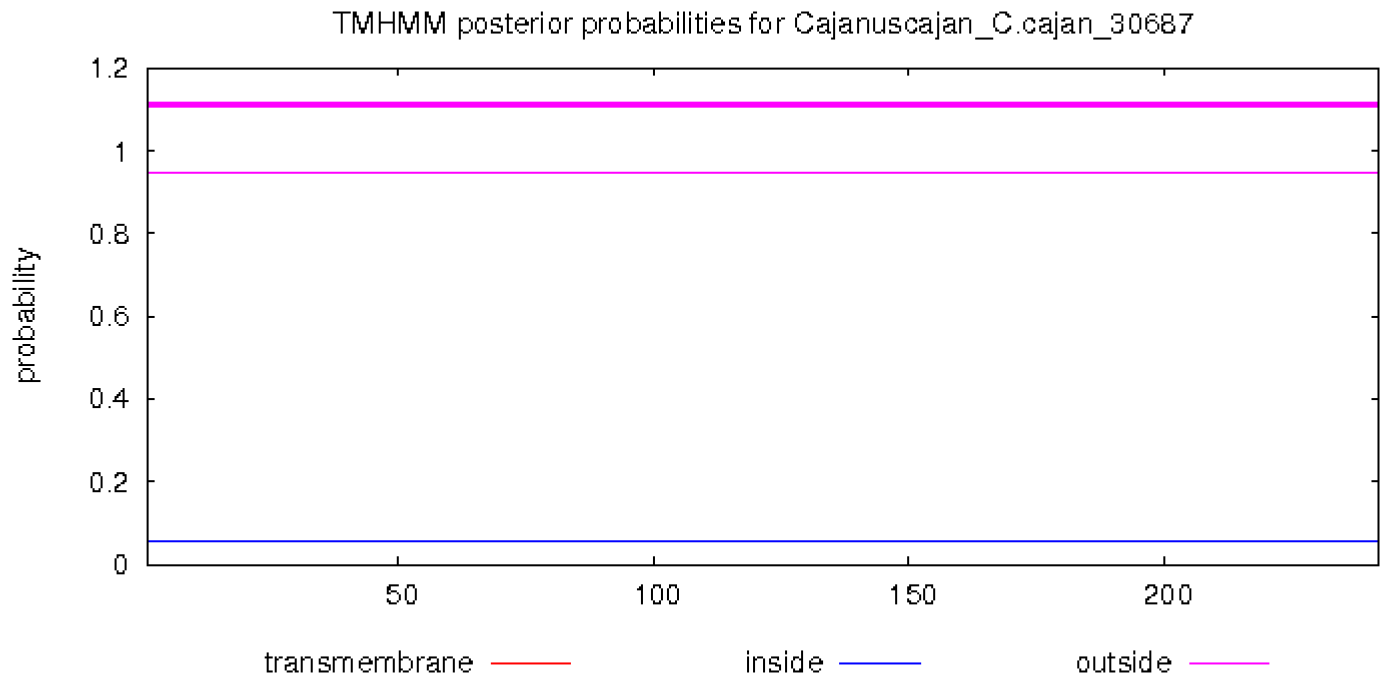

plot in postscript, script for making the plot in gnuplot, data for plot

```
# Cajanuscajan_C.cajan_31437 Length: 412
# Cajanuscajan_C.cajan_31437 Number of predicted TMHs:  0
# Cajanuscajan_C.cajan_31437 Exp number of AAs in TMHs: 11.17642
# Cajanuscajan_C.cajan_31437 Exp number, first 60 AAs:  11.17315
# Cajanuscajan_C.cajan_31437 Total prob of N-in:        0.46290
# Cajanuscajan_C.cajan_31437 POSSIBLE N-term signal sequence
Cajanuscajan_C.cajan_31437      TMHMM2.0        outside      1   412
```

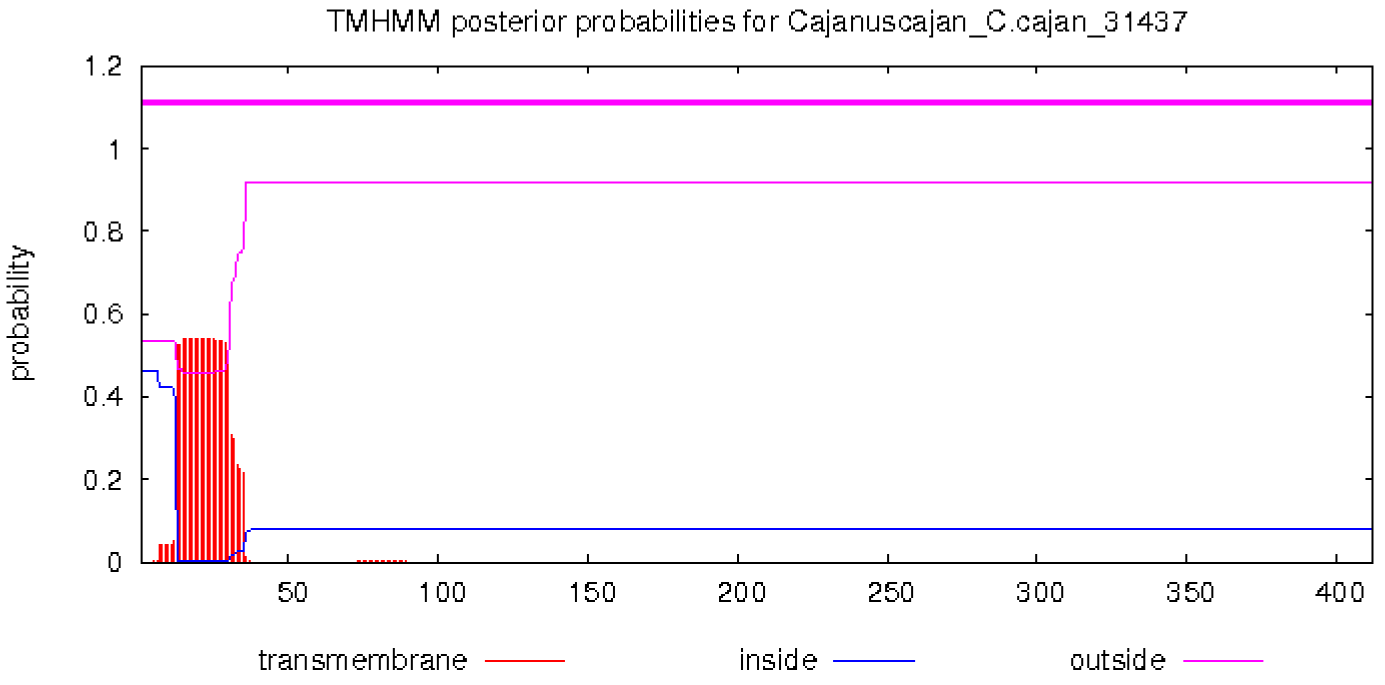

plot in postscript, script for making the plot in gnuplot, data for plot

```
# Cajanuscajan_C.cajan_31741 Length: 254
# Cajanuscajan_C.cajan_31741 Number of predicted TMHs:  0
# Cajanuscajan_C.cajan_31741 Exp number of AAs in TMHs: 0.00058
# Cajanuscajan_C.cajan_31741 Exp number, first 60 AAs:  0.00017
# Cajanuscajan_C.cajan_31741 Total prob of N-in:        0.01315
Cajanuscajan_C.cajan_31741      TMHMM2.0        outside      1   254
```

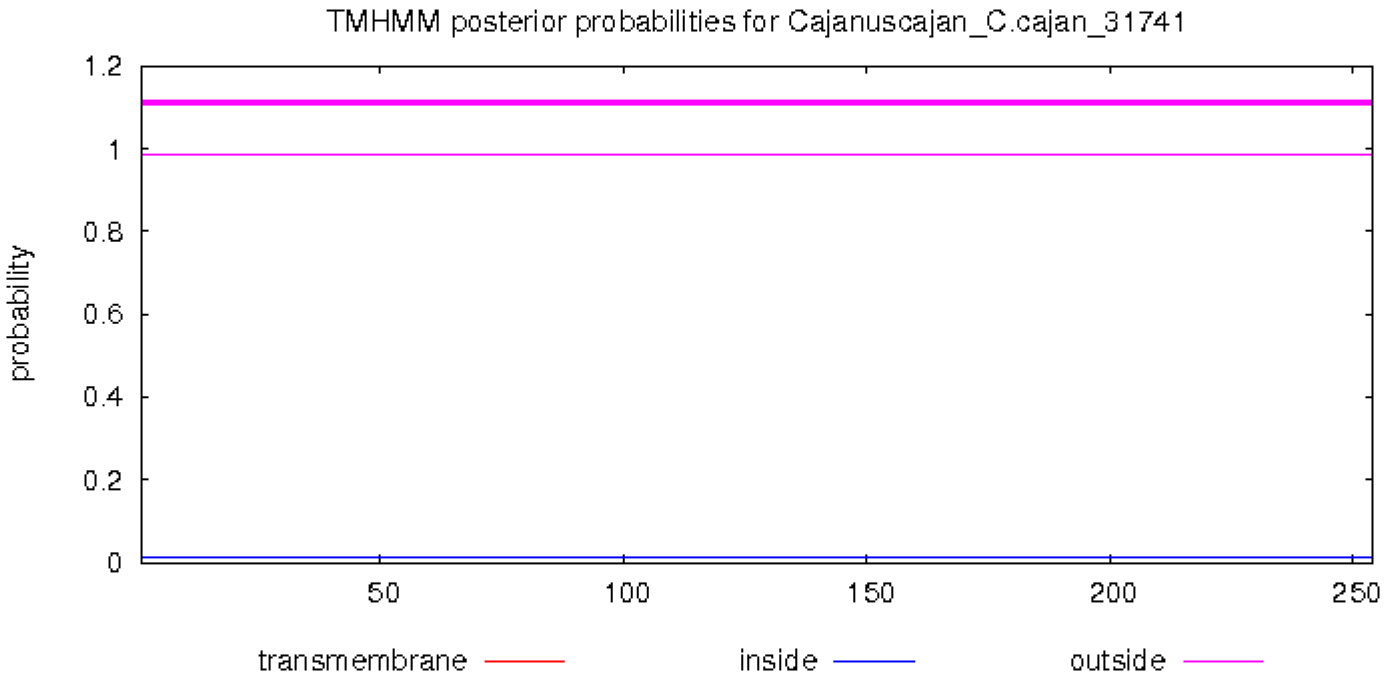

plot in postscript, script for making the plot in gnuplot, data for plot

```
# Cajanuscajan_C.cajan_32331 Length: 404
# Cajanuscajan_C.cajan_32331 Number of predicted TMHs:  0
# Cajanuscajan_C.cajan_32331 Exp number of AAs in TMHs: 0.00055
# Cajanuscajan_C.cajan_32331 Exp number, first 60 AAs:  0
# Cajanuscajan_C.cajan_32331 Total prob of N-in:        0.00872
Cajanuscajan_C.cajan_32331      TMHMM2.0        outside      1   404
```

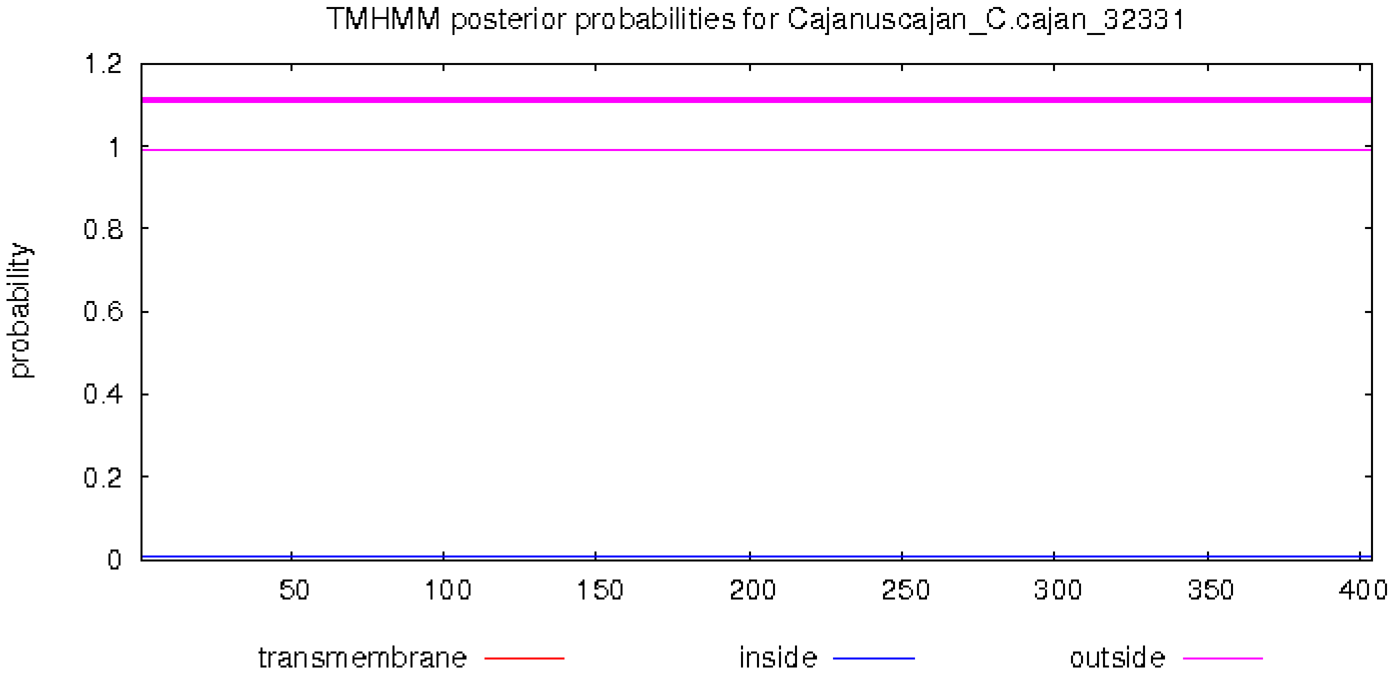

plot in postscript, script for making the plot in gnuplot, data for plot

```
# Cajanuscajan_C.cajan_32993 Length: 350
# Cajanuscajan_C.cajan_32993 Number of predicted TMHs:  0
# Cajanuscajan_C.cajan_32993 Exp number of AAs in TMHs: 0.02333
# Cajanuscajan_C.cajan_32993 Exp number, first 60 AAs:  0.0002
# Cajanuscajan_C.cajan_32993 Total prob of N-in:        0.02072
Cajanuscajan_C.cajan_32993      TMHMM2.0        outside      1   350
```

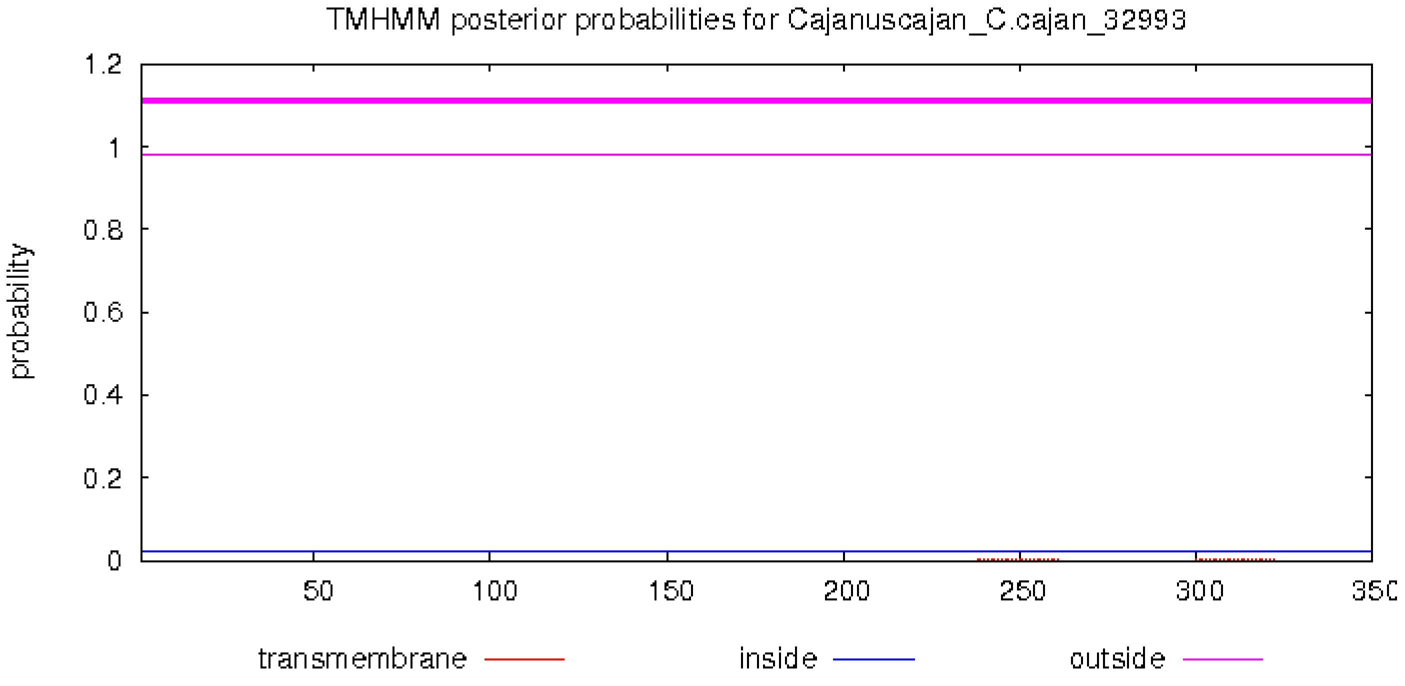

plot in postscript, script for making the plot in gnuplot, data for plot

---

```
# Cajanuscajan_C.cajan_34115 Length: 340
# Cajanuscajan_C.cajan_34115 Number of predicted TMHs:  0
# Cajanuscajan_C.cajan_34115 Exp number of AAs in TMHs: 0.00162
# Cajanuscajan_C.cajan_34115 Exp number, first 60 AAs:  0
# Cajanuscajan_C.cajan_34115 Total prob of N-in:        0.01913
Cajanuscajan_C.cajan_34115      TMHMM2.0        outside      1   340
```

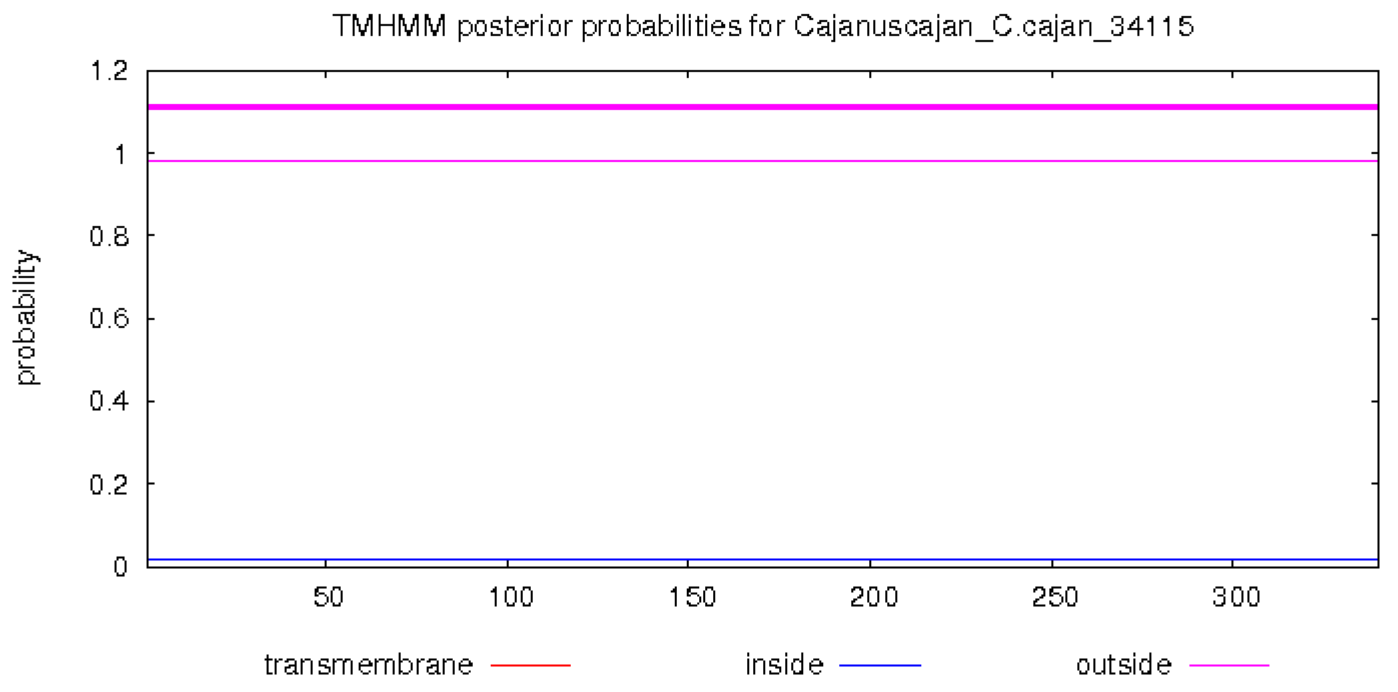

plot in postscript, script for making the plot in gnuplot, data for plot

---

```
# Cajanuscajan_C.cajan_34162 Length: 346
# Cajanuscajan_C.cajan_34162 Number of predicted TMHs:  0
# Cajanuscajan_C.cajan_34162 Exp number of AAs in TMHs: 0
# Cajanuscajan_C.cajan_34162 Exp number, first 60 AAs:  0
# Cajanuscajan_C.cajan_34162 Total prob of N-in:        0.00487
Cajanuscajan_C.cajan_34162      TMHMM2.0        outside      1   346
```

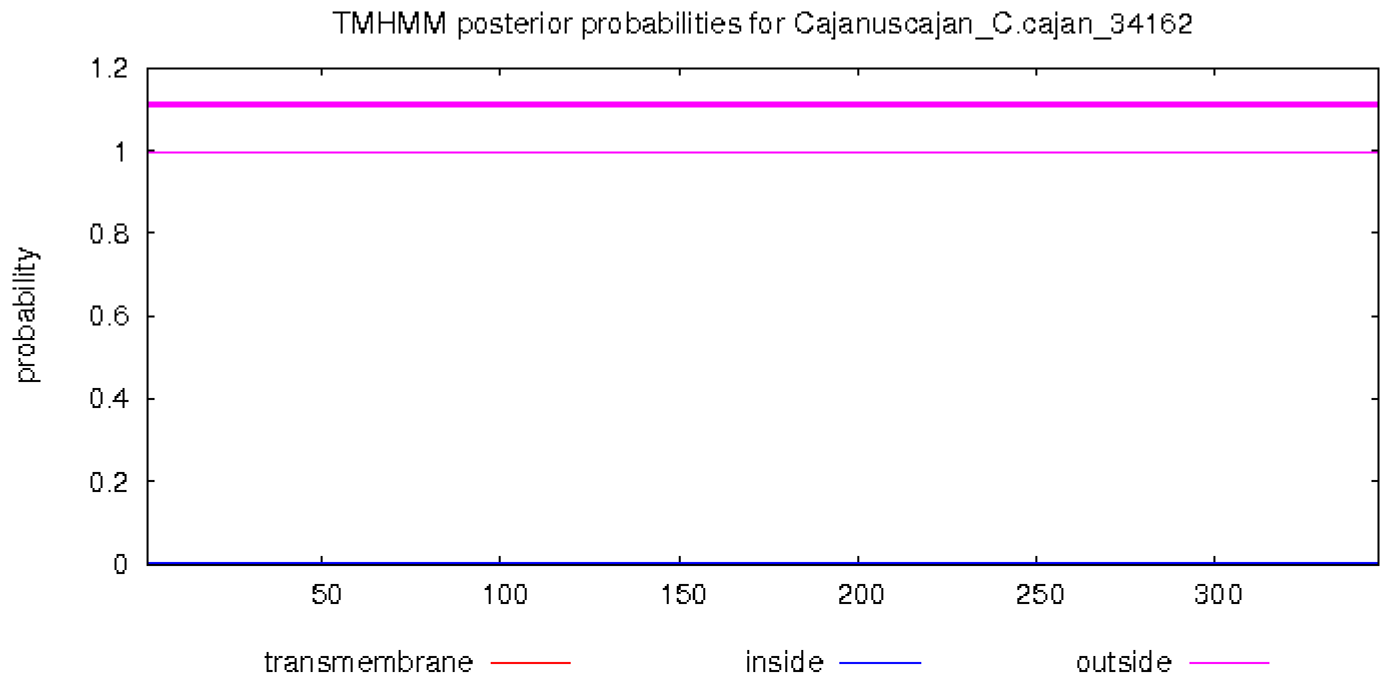

plot in postscript, script for making the plot in gnuplot, data for plot

```
# Cajanuscajan_C.cajan_35648 Length: 398
# Cajanuscajan_C.cajan_35648 Number of predicted TMHs:  0
# Cajanuscajan_C.cajan_35648 Exp number of AAs in TMHs: 0
# Cajanuscajan_C.cajan_35648 Exp number, first 60 AAs:  0
# Cajanuscajan_C.cajan_35648 Total prob of N-in:        0.00472
Cajanuscajan_C.cajan_35648      TMHMM2.0        outside      1   398
```

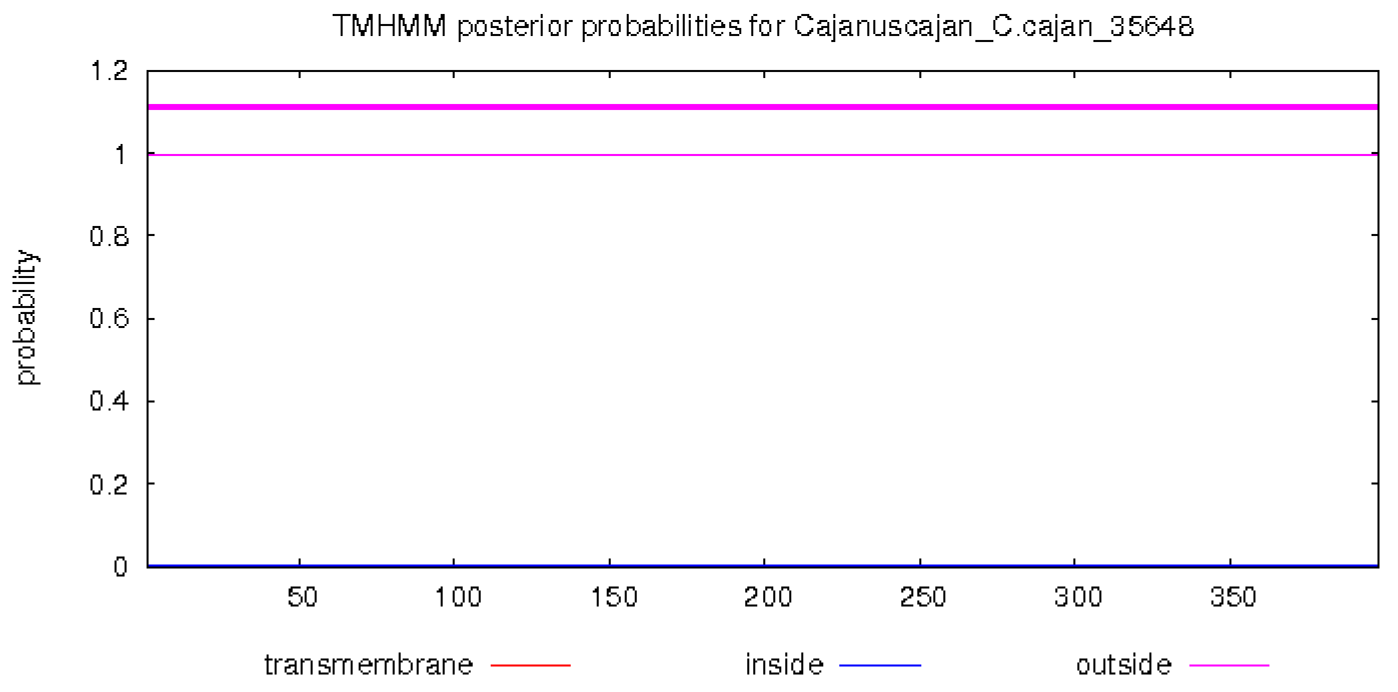

plot in postscript, script for making the plot in gnuplot, data for plot

```
# Cajanuscajan_C.cajan_36091 Length: 368
# Cajanuscajan_C.cajan_36091 Number of predicted TMHs:  0
# Cajanuscajan_C.cajan_36091 Exp number of AAs in TMHs: 0.00147
# Cajanuscajan_C.cajan_36091 Exp number, first 60 AAs:  0
# Cajanuscajan_C.cajan_36091 Total prob of N-in:        0.01300
Cajanuscajan_C.cajan_36091      TMHMM2.0        outside      1   368
```

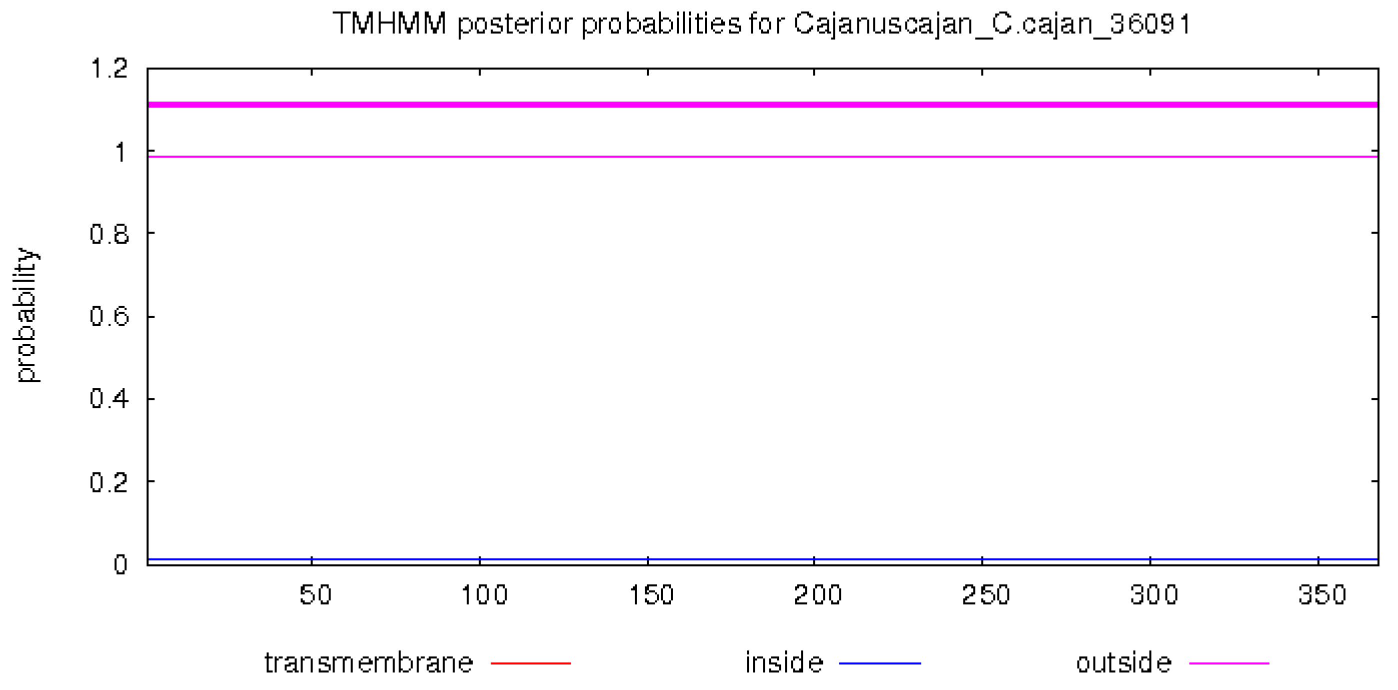

plot in postscript, script for making the plot in gnuplot, data for plot

```
# Cajanuscajan_C.cajan_36992 Length: 373
# Cajanuscajan_C.cajan_36992 Number of predicted TMHs:  0
# Cajanuscajan_C.cajan_36992 Exp number of AAs in TMHs: 0.00021
# Cajanuscajan_C.cajan_36992 Exp number, first 60 AAs:  0
# Cajanuscajan_C.cajan_36992 Total prob of N-in:        0.02942
Cajanuscajan_C.cajan_36992      TMHMM2.0        outside      1   373
```

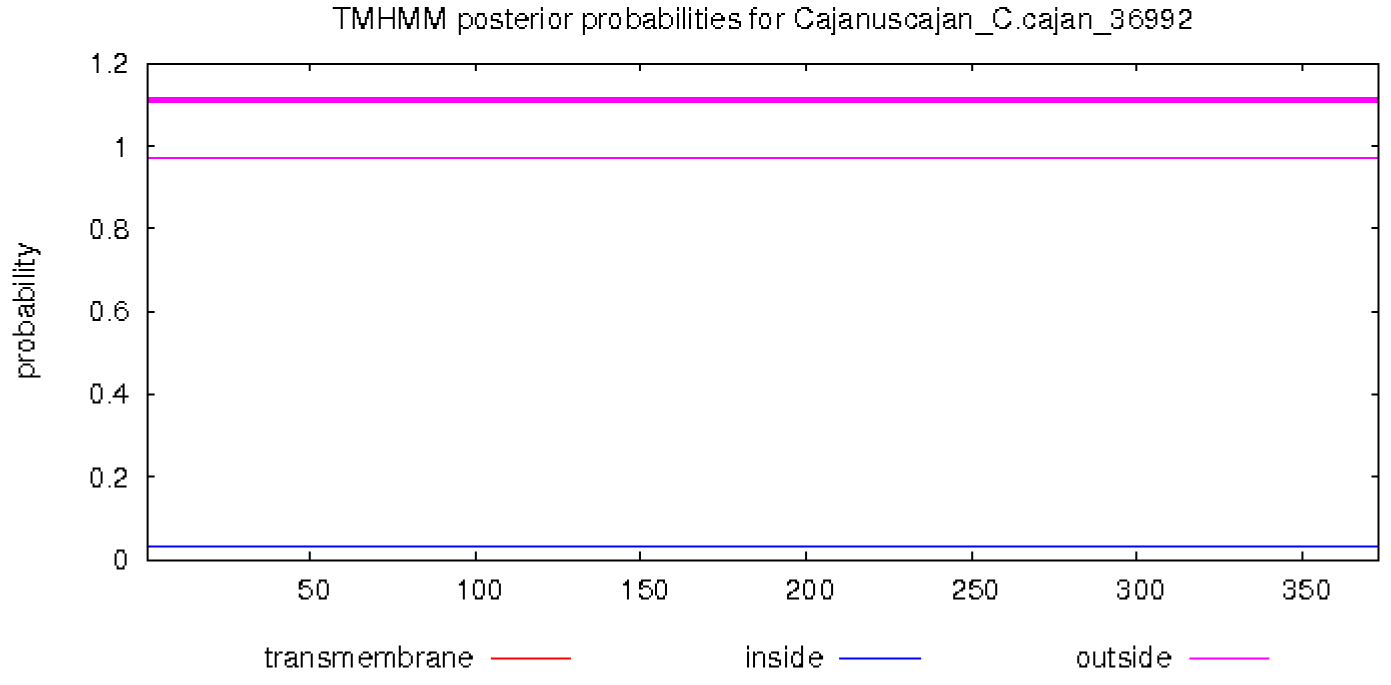

plot in postscript, script for making the plot in gnuplot, data for plot

```
# Cajanuscajan_C.cajan_36997 Length: 78
# Cajanuscajan_C.cajan_36997 Number of predicted TMHs:  0
# Cajanuscajan_C.cajan_36997 Exp number of AAs in TMHs: 9.66002
# Cajanuscajan_C.cajan_36997 Exp number, first 60 AAs:  0.68886
# Cajanuscajan_C.cajan_36997 Total prob of N-in:        0.14562
Cajanuscajan_C.cajan_36997      TMHMM2.0        outside      1    78
```

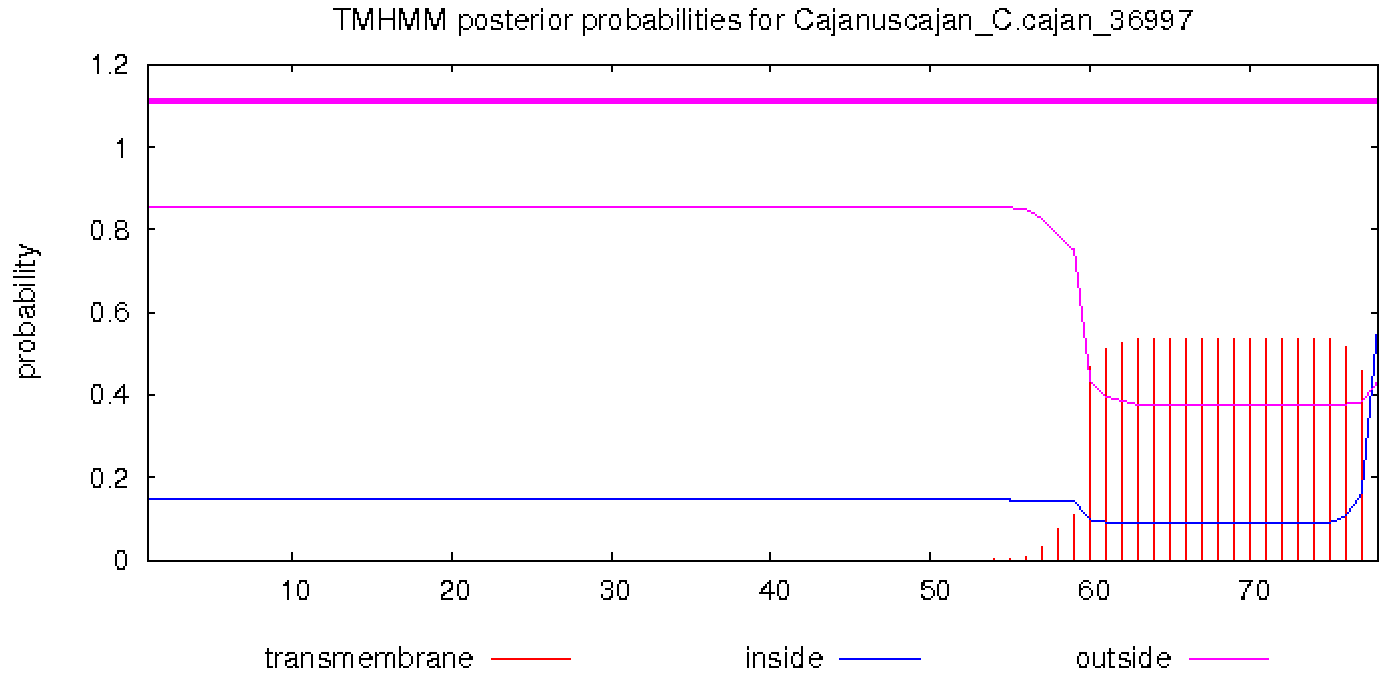

plot in postscript, script for making the plot in gnuplot, data for plot

```
# Cajanuscajan_C.cajan_37341 Length: 405
# Cajanuscajan_C.cajan_37341 Number of predicted TMHs:  0
# Cajanuscajan_C.cajan_37341 Exp number of AAs in TMHs: 0
# Cajanuscajan_C.cajan_37341 Exp number, first 60 AAs:  0
# Cajanuscajan_C.cajan_37341 Total prob of N-in:        0.00590
Cajanuscajan_C.cajan_37341      TMHMM2.0        outside      1   405
```

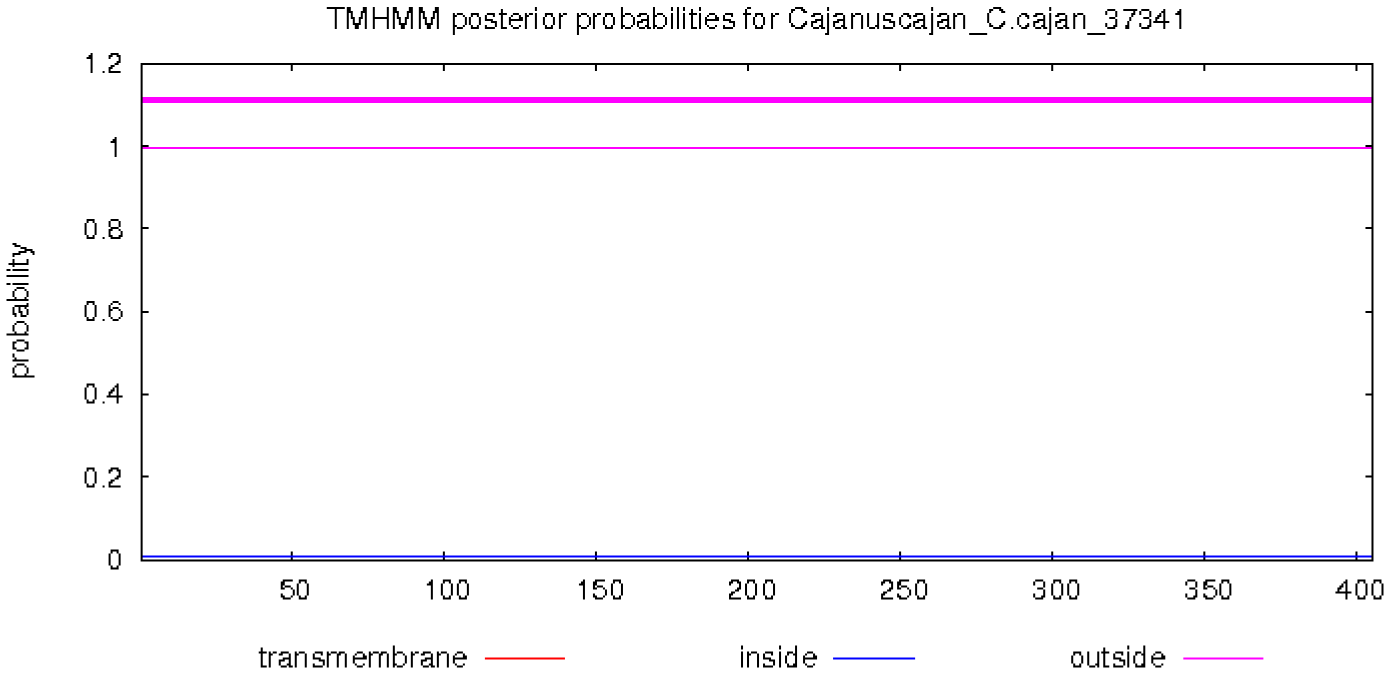

plot in postscript, script for making the plot in gnuplot, data for plot

```
# Cajanuscajan_C.cajan_37342 Length: 471
# Cajanuscajan_C.cajan_37342 Number of predicted TMHs:  0
# Cajanuscajan_C.cajan_37342 Exp number of AAs in TMHs: 0
# Cajanuscajan_C.cajan_37342 Exp number, first 60 AAs:  0
# Cajanuscajan_C.cajan_37342 Total prob of N-in:        0.00165
Cajanuscajan_C.cajan_37342      TMHMM2.0        outside      1   471
```

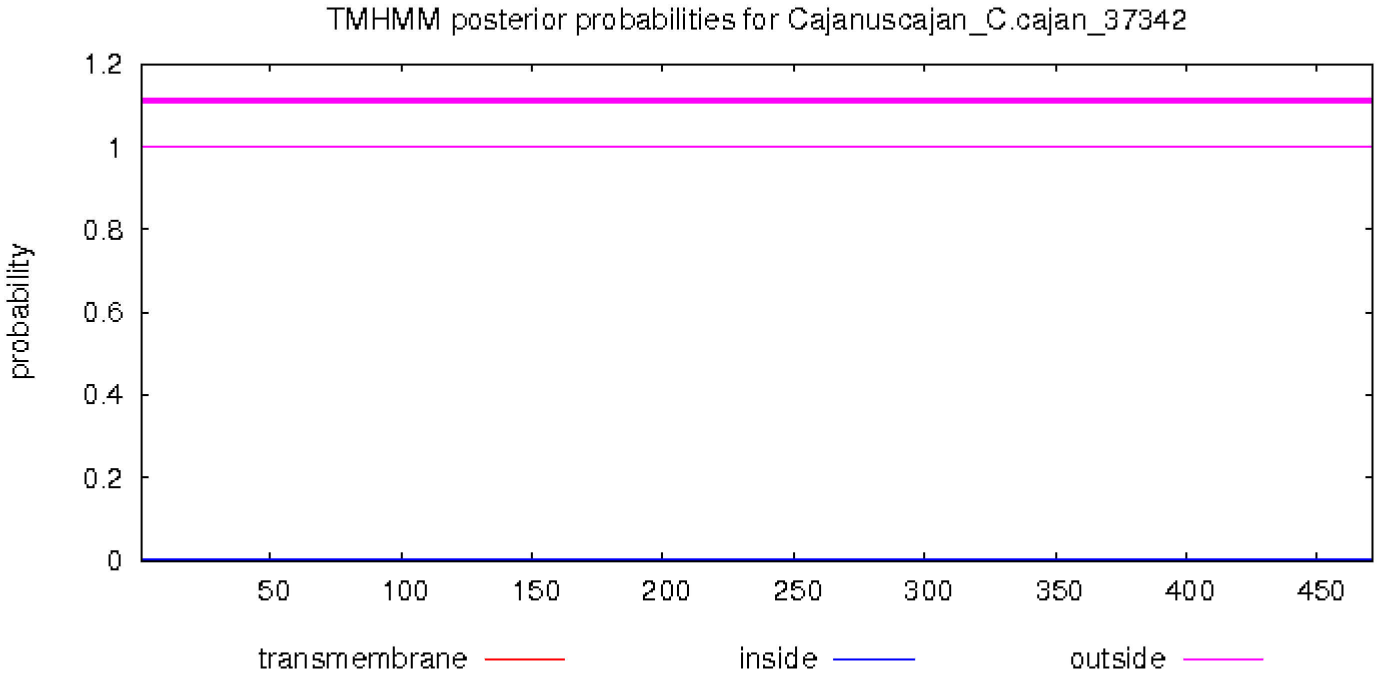

plot in postscript, script for making the plot in gnuplot, data for plot

---

```
# Cajanuscajan_C.cajan_37971 Length: 364
# Cajanuscajan_C.cajan_37971 Number of predicted TMHs:  0
# Cajanuscajan_C.cajan_37971 Exp number of AAs in TMHs: 0.00076
# Cajanuscajan_C.cajan_37971 Exp number, first 60 AAs:  0.00056
# Cajanuscajan_C.cajan_37971 Total prob of N-in:        0.01248
Cajanuscajan_C.cajan_37971      TMHMM2.0        outside      1   364
```

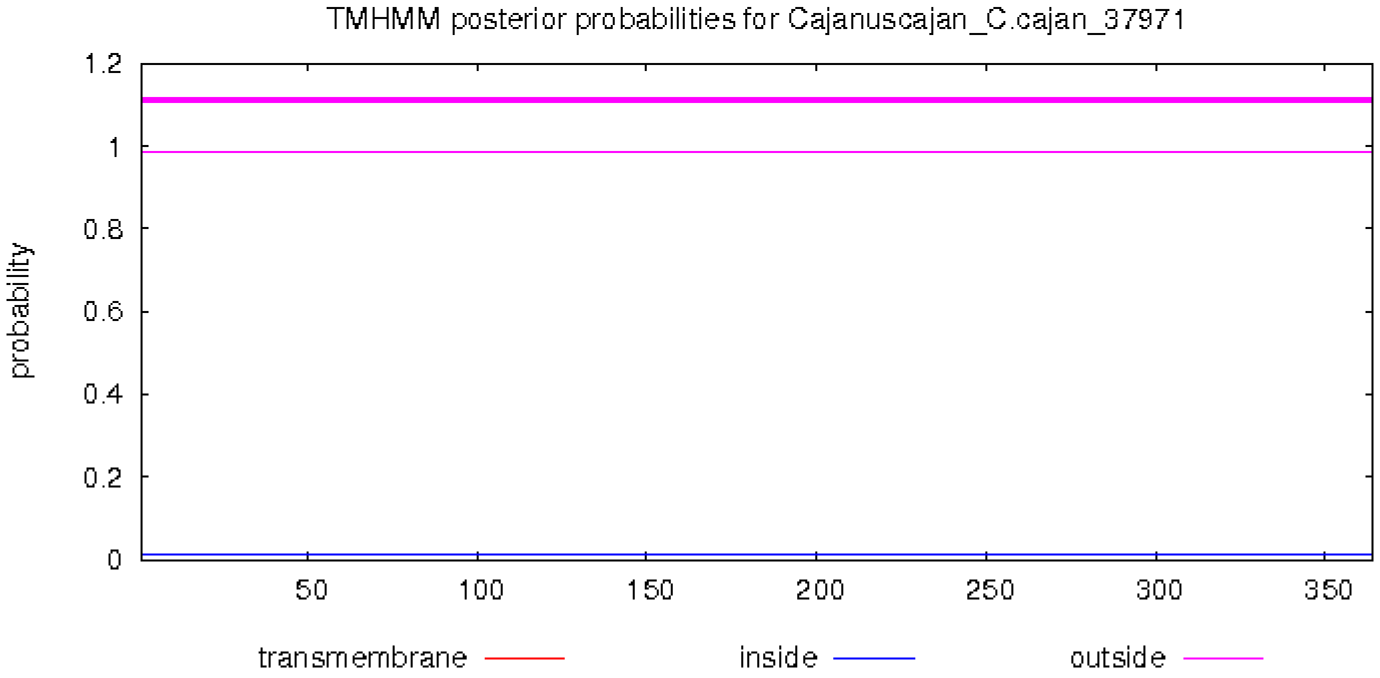

plot in postscript, script for making the plot in gnuplot, data for plot

---

```
# Cajanuscajan_C.cajan_38151 Length: 297
# Cajanuscajan_C.cajan_38151 Number of predicted TMHs:  0
# Cajanuscajan_C.cajan_38151 Exp number of AAs in TMHs: 0.0002
# Cajanuscajan_C.cajan_38151 Exp number, first 60 AAs:  0.0002
# Cajanuscajan_C.cajan_38151 Total prob of N-in:        0.00302
Cajanuscajan_C.cajan_38151      TMHMM2.0        outside      1   297
```

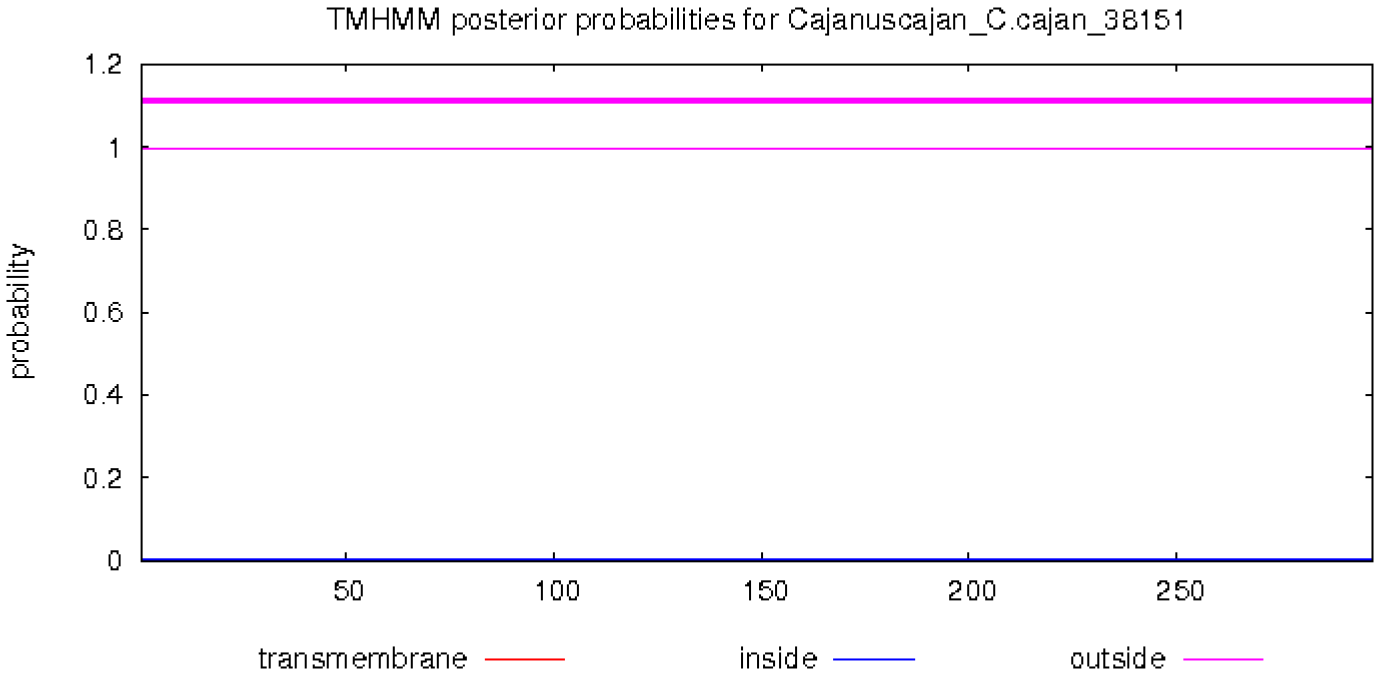

plot in postscript, script for making the plot in gnuplot, data for plot

```
# Cajanuscajan_C.cajan_38763 Length: 271
# Cajanuscajan_C.cajan_38763 Number of predicted TMHs:  0
# Cajanuscajan_C.cajan_38763 Exp number of AAs in TMHs: 0
# Cajanuscajan_C.cajan_38763 Exp number, first 60 AAs:  0
# Cajanuscajan_C.cajan_38763 Total prob of N-in:        0.01181
Cajanuscajan_C.cajan_38763      TMHMM2.0        outside      1   271
```

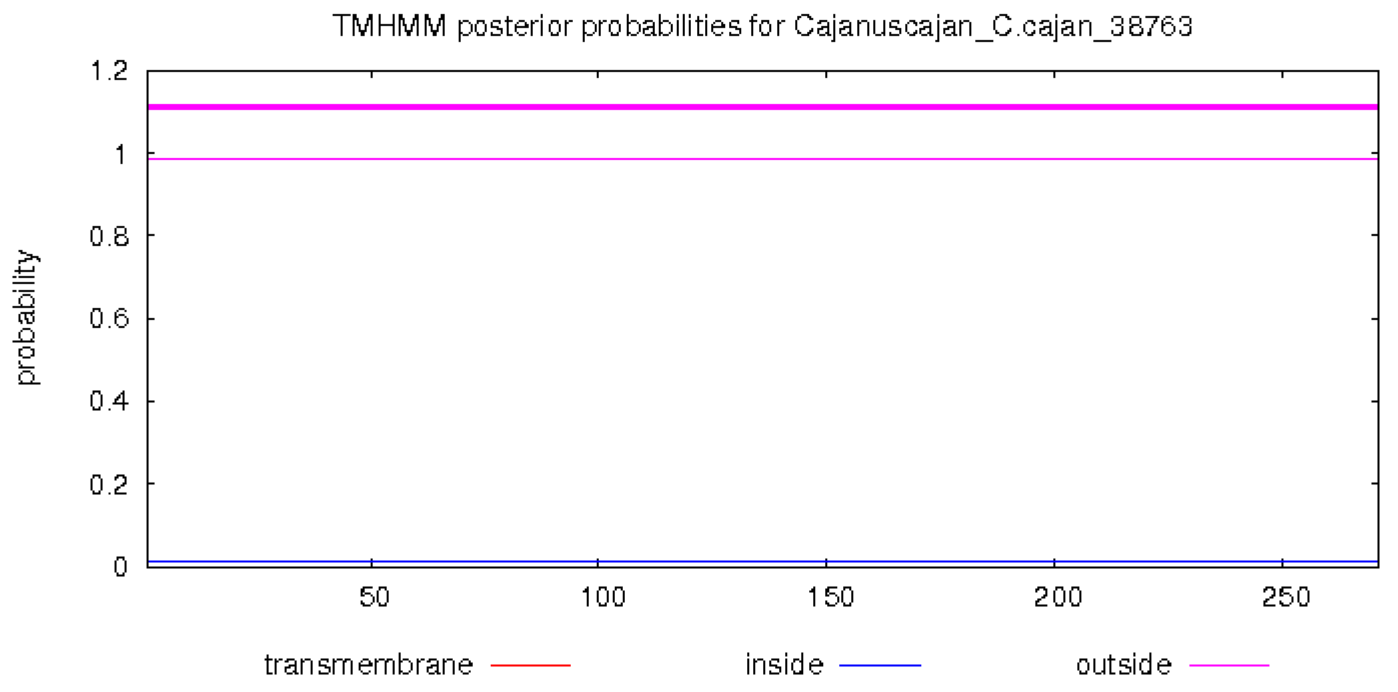

plot in postscript, script for making the plot in gnuplot, data for plot

```
# Cajanuscajan_C.cajan_39141 Length: 346
# Cajanuscajan_C.cajan_39141 Number of predicted TMHs:  0
# Cajanuscajan_C.cajan_39141 Exp number of AAs in TMHs: 0
# Cajanuscajan_C.cajan_39141 Exp number, first 60 AAs:  0
# Cajanuscajan_C.cajan_39141 Total prob of N-in:        0.00081
Cajanuscajan_C.cajan_39141      TMHMM2.0        outside      1   346
```

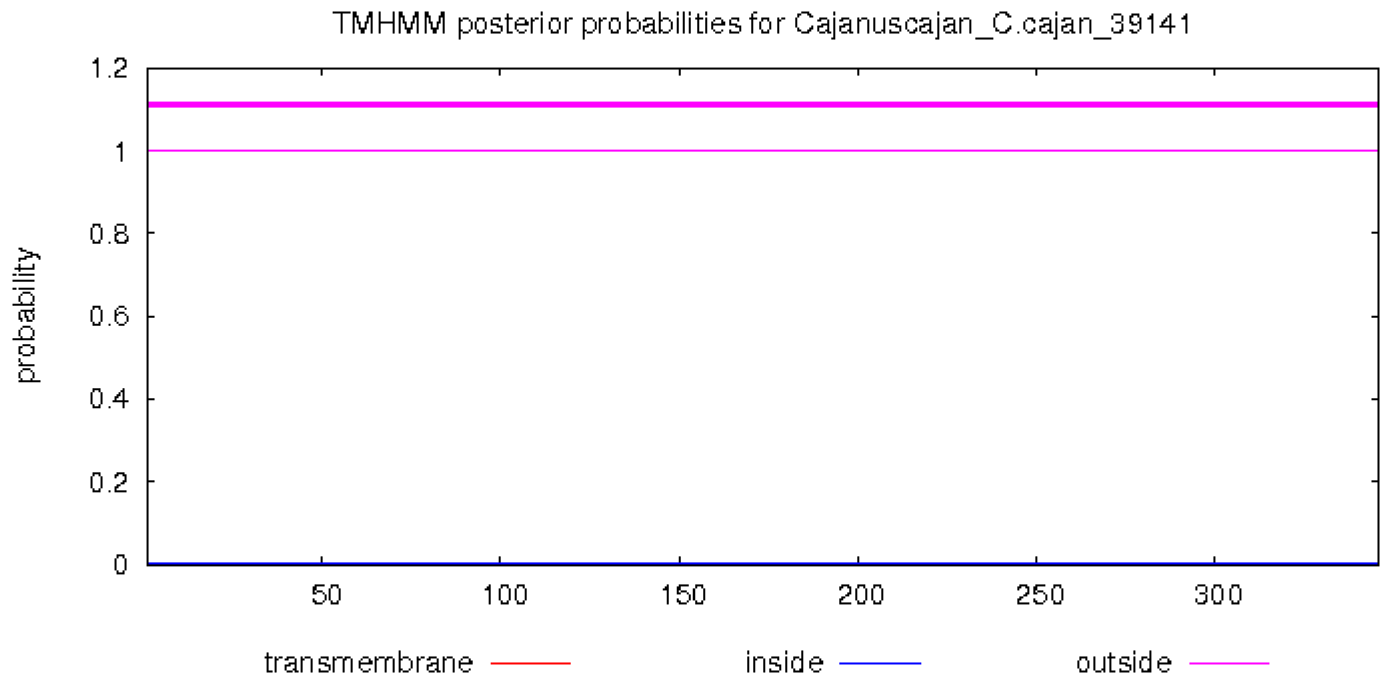

plot in postscript, script for making the plot in gnuplot, data for plot

```
# Cajanuscajan_C.cajan_39689 Length: 248
# Cajanuscajan_C.cajan_39689 Number of predicted TMHs:  0
# Cajanuscajan_C.cajan_39689 Exp number of AAs in TMHs: 0
# Cajanuscajan_C.cajan_39689 Exp number, first 60 AAs:  0
# Cajanuscajan_C.cajan_39689 Total prob of N-in:        0.02021
Cajanuscajan_C.cajan_39689      TMHMM2.0        outside      1   248
```

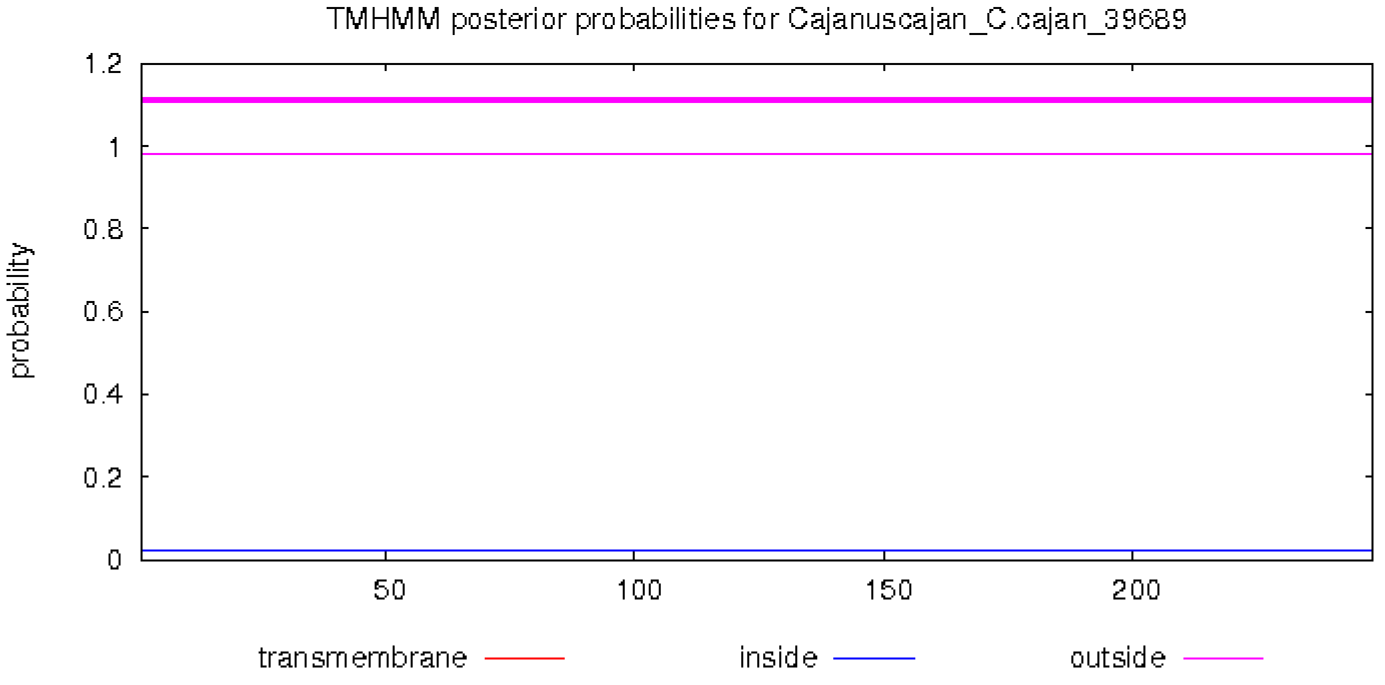

plot in postscript, script for making the plot in gnuplot, data for plot

```
# Cajanuscajan_C.cajan_40054 Length: 308
# Cajanuscajan_C.cajan_40054 Number of predicted TMHs:  0
# Cajanuscajan_C.cajan_40054 Exp number of AAs in TMHs: 0
# Cajanuscajan_C.cajan_40054 Exp number, first 60 AAs:  0
# Cajanuscajan_C.cajan_40054 Total prob of N-in:        0.04335
Cajanuscajan_C.cajan_40054      TMHMM2.0        outside      1   308
```

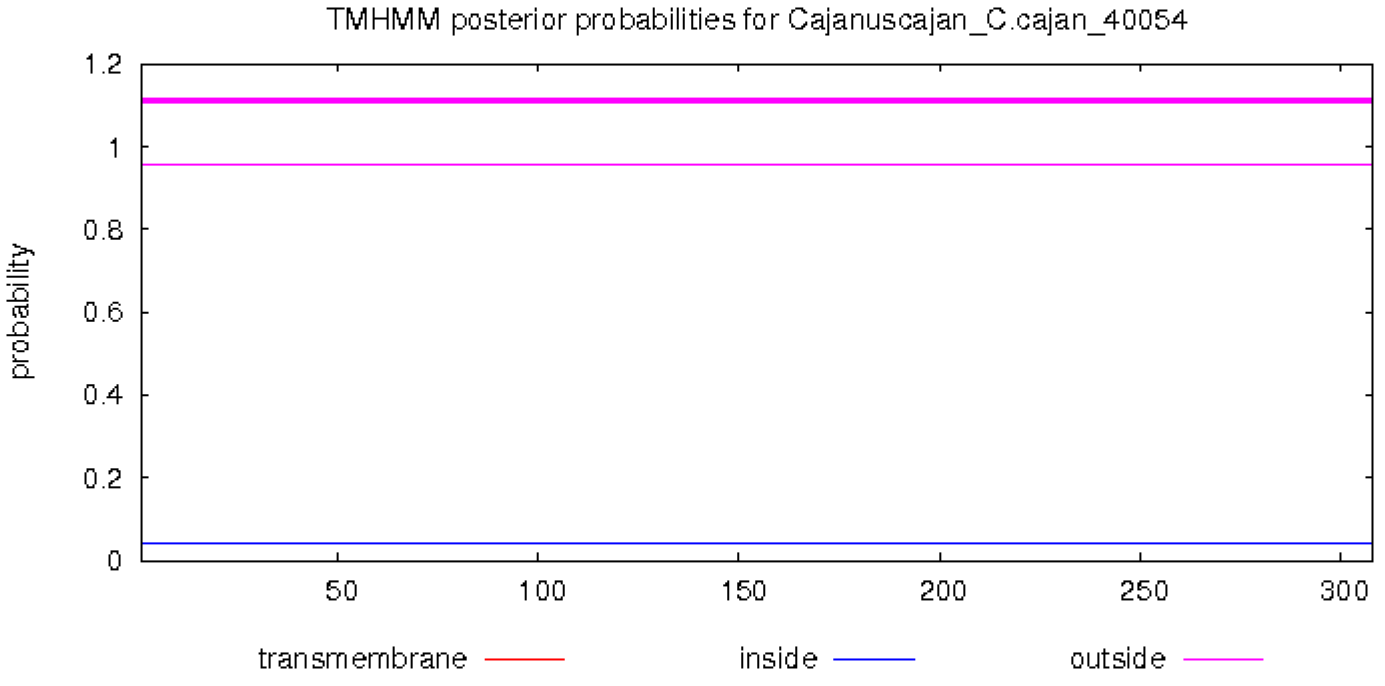

plot in postscript, script for making the plot in gnuplot, data for plot

```
# Cajanuscajan_C.cajan_40311 Length: 572
# Cajanuscajan_C.cajan_40311 Number of predicted TMHs:  1
# Cajanuscajan_C.cajan_40311 Exp number of AAs in TMHs: 22.73951
# Cajanuscajan_C.cajan_40311 Exp number, first 60 AAs:  0.00529
# Cajanuscajan_C.cajan_40311 Total prob of N-in:        0.03352
Cajanuscajan_C.cajan_40311      TMHMM2.0        outside      1   547
Cajanuscajan_C.cajan_40311      TMHMM2.0        TMhelix    548   570
Cajanuscajan_C.cajan_40311      TMHMM2.0        inside     571   572
```

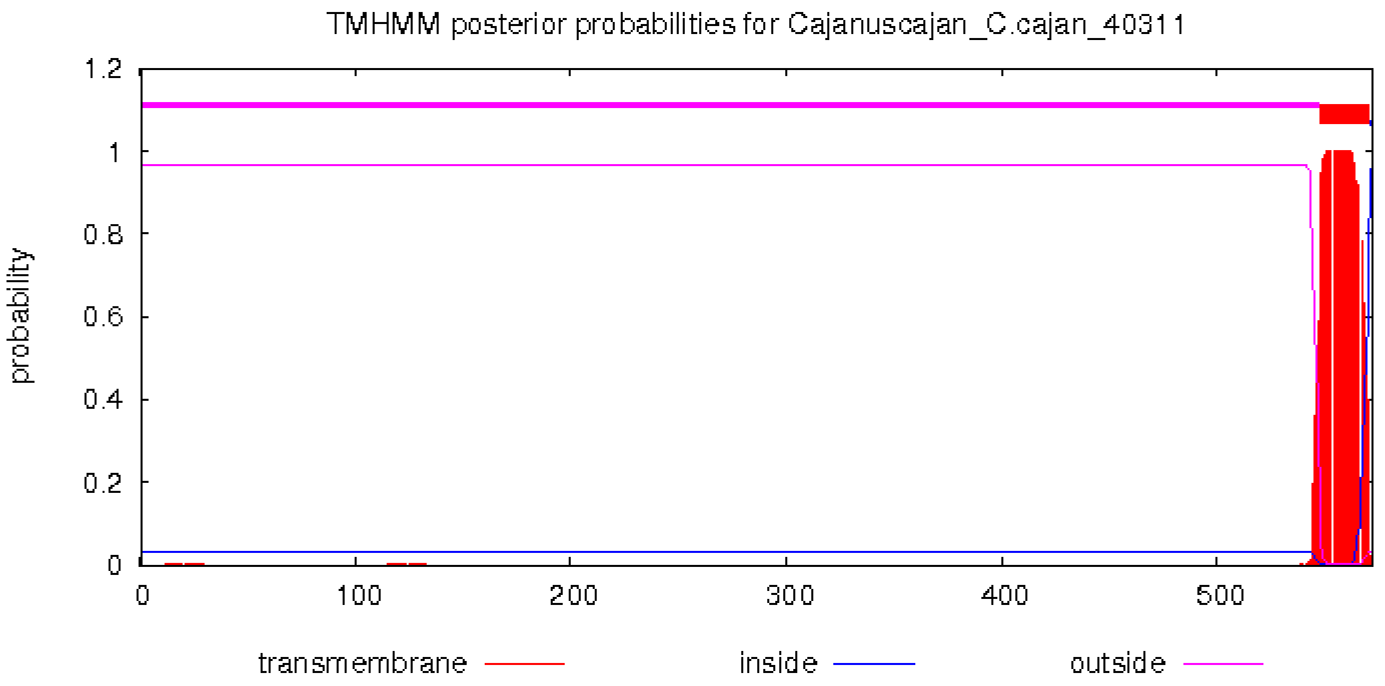

plot in postscript, script for making the plot in gnuplot, data for plot

```
# Cajanuscajan_C.cajan_40755 Length: 296
# Cajanuscajan_C.cajan_40755 Number of predicted TMHs:  0
# Cajanuscajan_C.cajan_40755 Exp number of AAs in TMHs: 0.00036
# Cajanuscajan_C.cajan_40755 Exp number, first 60 AAs:  0.00016
# Cajanuscajan_C.cajan_40755 Total prob of N-in:        0.03503
Cajanuscajan_C.cajan_40755      TMHMM2.0        outside      1   296
```

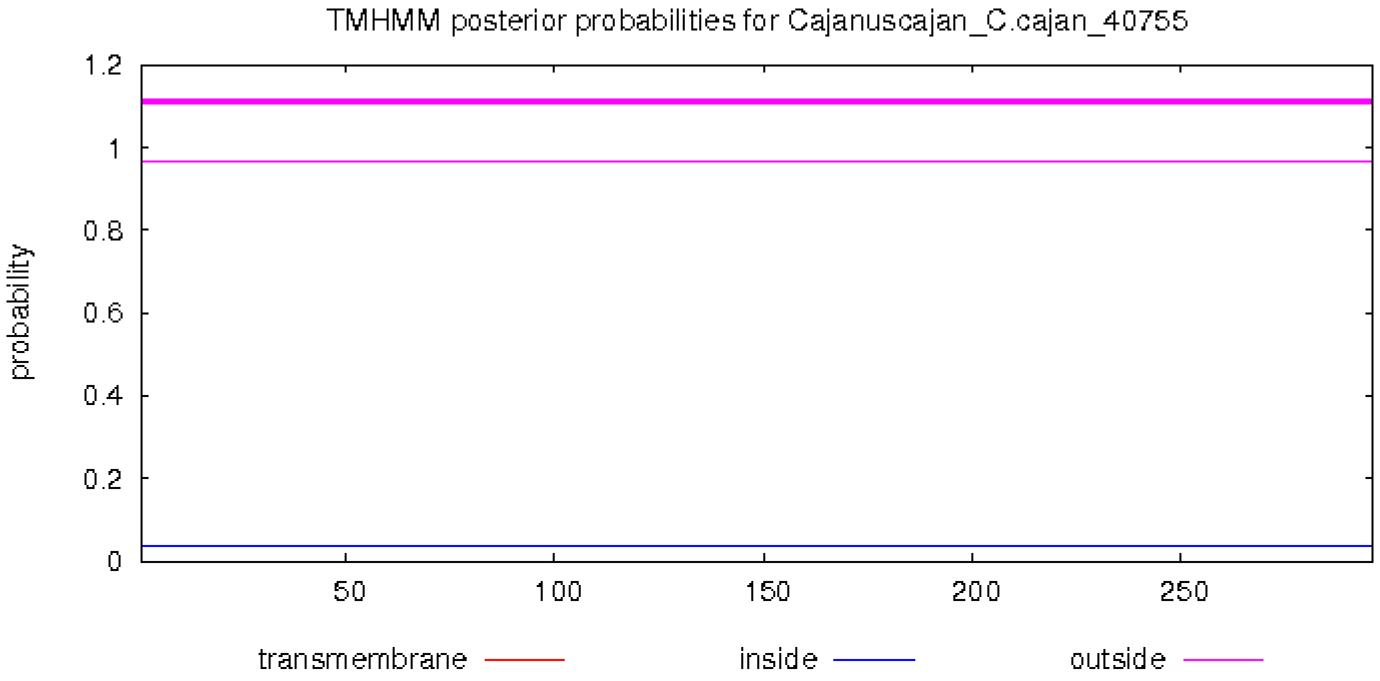

plot in postscript, script for making the plot in gnuplot, data for plot

---

```
# Cajanuscajan_C.cajan_40877 Length: 348
# Cajanuscajan_C.cajan_40877 Number of predicted TMHs:  0
# Cajanuscajan_C.cajan_40877 Exp number of AAs in TMHs: 0.00219
# Cajanuscajan_C.cajan_40877 Exp number, first 60 AAs:  0
# Cajanuscajan_C.cajan_40877 Total prob of N-in:        0.00418
Cajanuscajan_C.cajan_40877      TMHMM2.0        outside      1   348
```

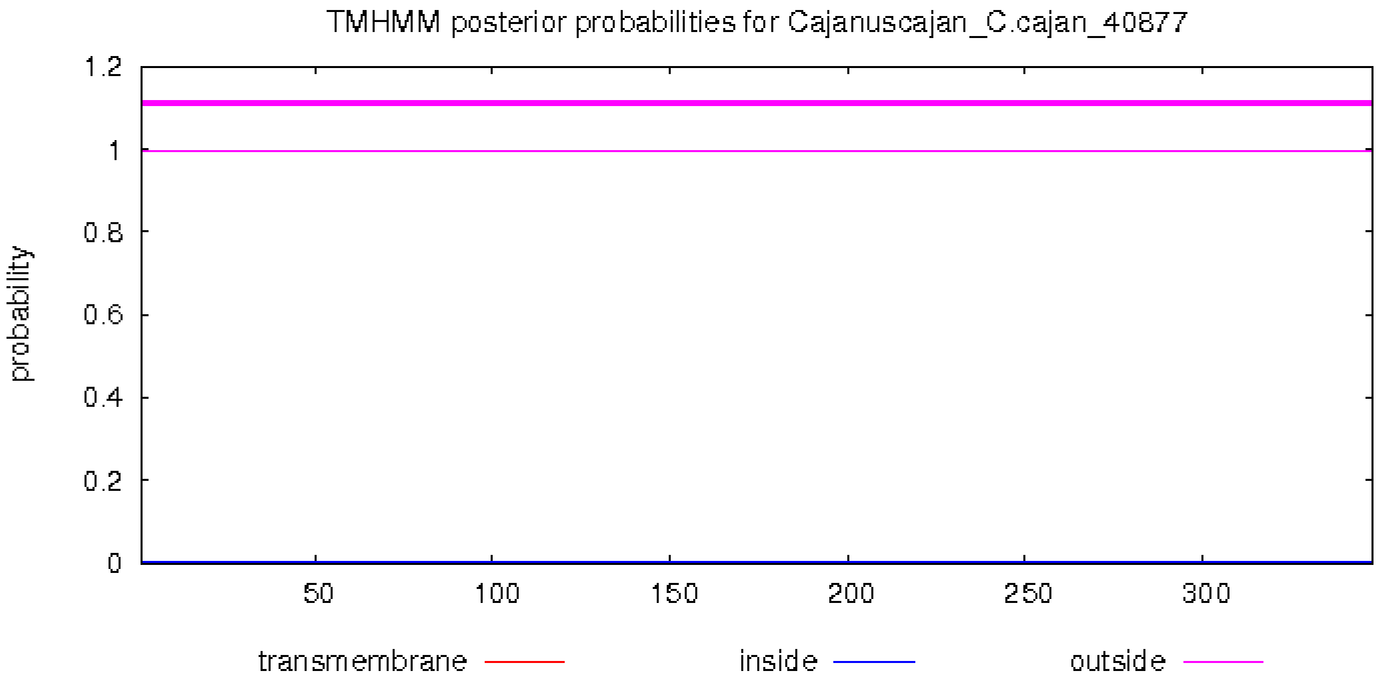

plot in postscript, script for making the plot in gnuplot, data for plot

---

```
# Cajanuscajan_C.cajan_41044 Length: 349
# Cajanuscajan_C.cajan_41044 Number of predicted TMHs:  1
# Cajanuscajan_C.cajan_41044 Exp number of AAs in TMHs: 21.84267
# Cajanuscajan_C.cajan_41044 Exp number, first 60 AAs:  0.0007
# Cajanuscajan_C.cajan_41044 Total prob of N-in:        0.08532
Cajanuscajan_C.cajan_41044      TMHMM2.0        outside      1   325
```

```
Cajanuscajan_C.cajan_41044      TMHMM2.0        TMhelix    326   348
Cajanuscajan_C.cajan_41044      TMHMM2.0        inside     349   349
```

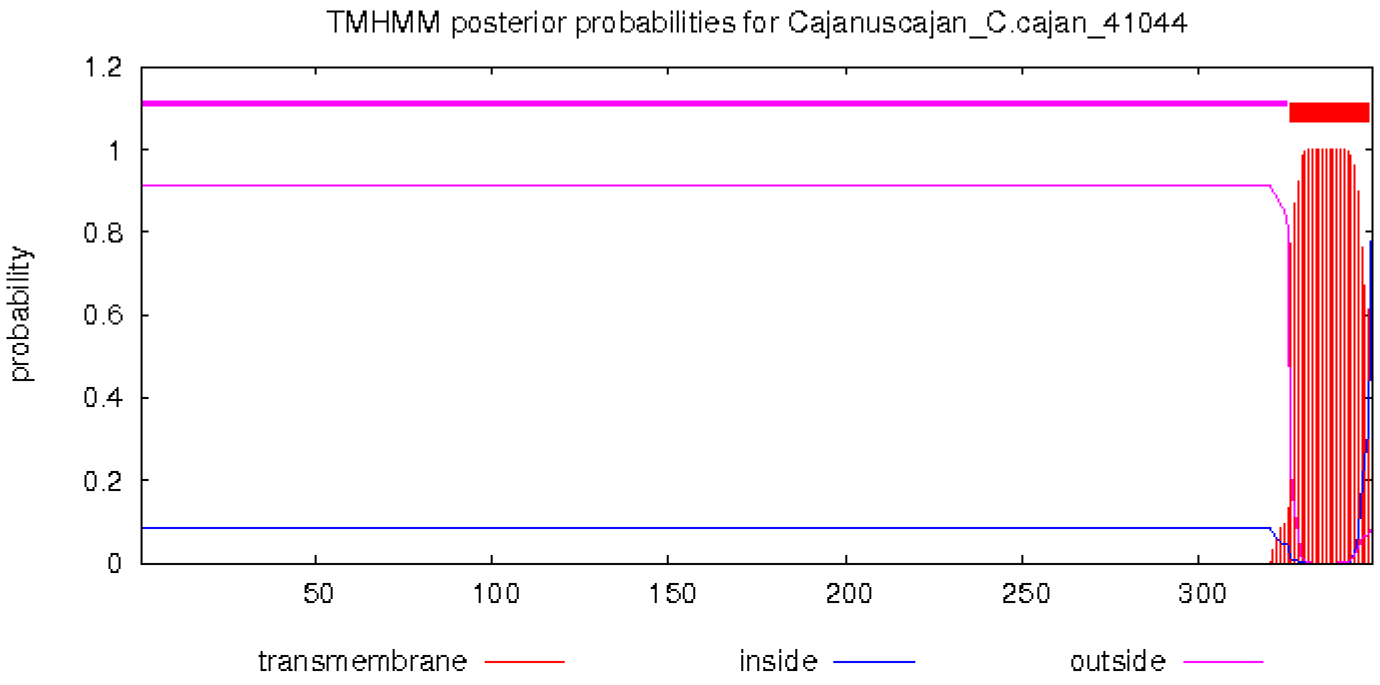

plot in postscript, script for making the plot in gnuplot, data for plot

---

```
# Cajanuscajan_C.cajan_42082 Length: 480
# Cajanuscajan_C.cajan_42082 Number of predicted TMHs:  1
# Cajanuscajan_C.cajan_42082 Exp number of AAs in TMHs: 22.28638
# Cajanuscajan_C.cajan_42082 Exp number, first 60 AAs:  0.00195
# Cajanuscajan_C.cajan_42082 Total prob of N-in:        0.52978
Cajanuscajan_C.cajan_42082      TMHMM2.0        outside      1   455
Cajanuscajan_C.cajan_42082      TMHMM2.0        TMhelix    456   478
Cajanuscajan_C.cajan_42082      TMHMM2.0        inside     479   480
```

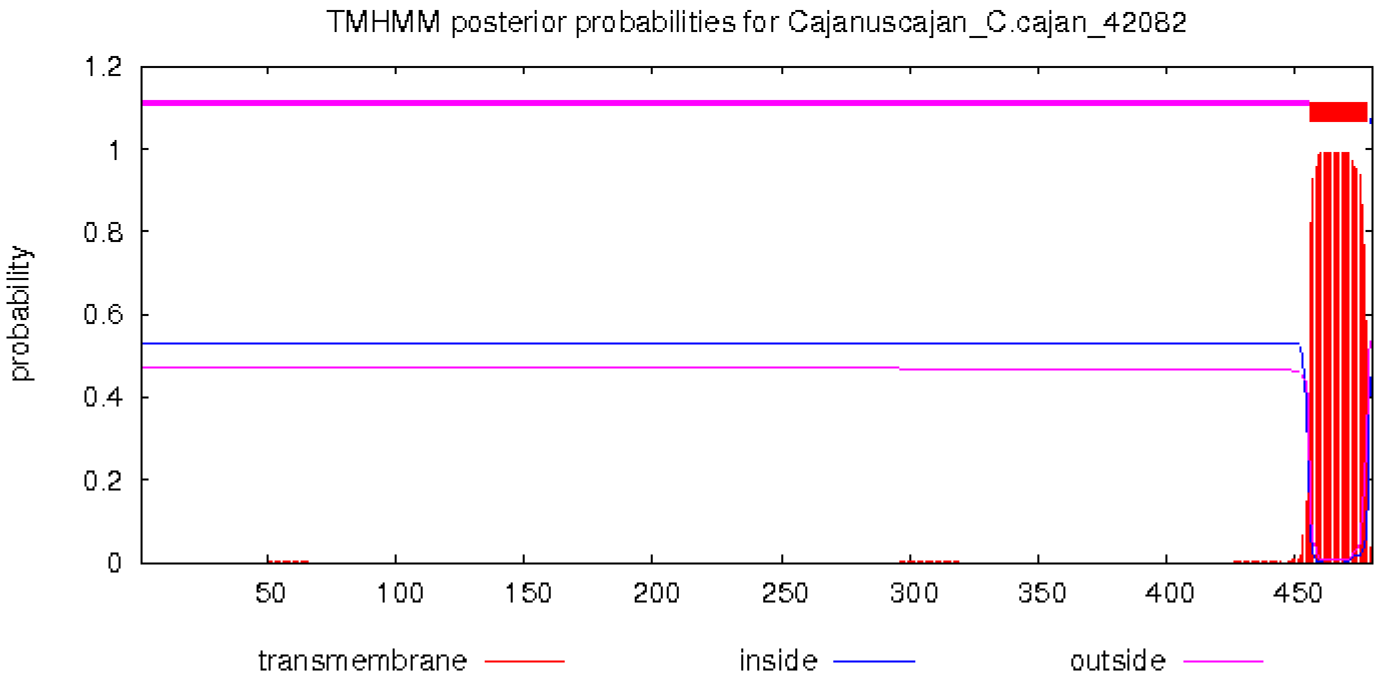

plot in postscript, script for making the plot in gnuplot, data for plot

---

```
# Cajanuscajan_C.cajan_42309 Length: 305
# Cajanuscajan_C.cajan_42309 Number of predicted TMHs:  0
```

```
# Cajanuscajan_C.cajan_42309 Exp number of AAs in TMHs: 0.00041
# Cajanuscajan_C.cajan_42309 Exp number, first 60 AAs:  0.00018
# Cajanuscajan_C.cajan_42309 Total prob of N-in:        0.06888
Cajanuscajan_C.cajan_42309      TMHMM2.0        outside      1   305
```

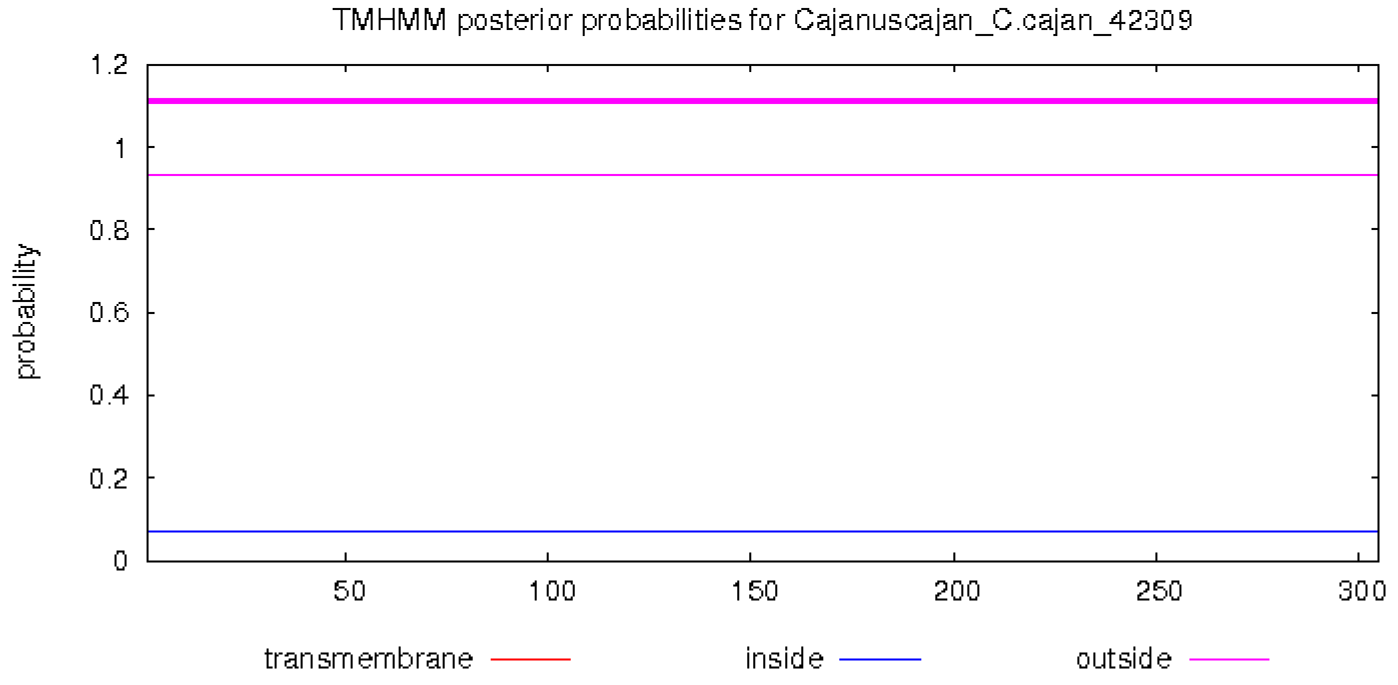

plot in postscript, script for making the plot in gnuplot, data for plot

---

```
# Cajanuscajan_C.cajan_43030 Length: 338
# Cajanuscajan_C.cajan_43030 Number of predicted TMHs:  0
# Cajanuscajan_C.cajan_43030 Exp number of AAs in TMHs: 0.00111
# Cajanuscajan_C.cajan_43030 Exp number, first 60 AAs:  6e-05
# Cajanuscajan_C.cajan_43030 Total prob of N-in:        0.00431
Cajanuscajan_C.cajan_43030      TMHMM2.0        outside      1   338
```

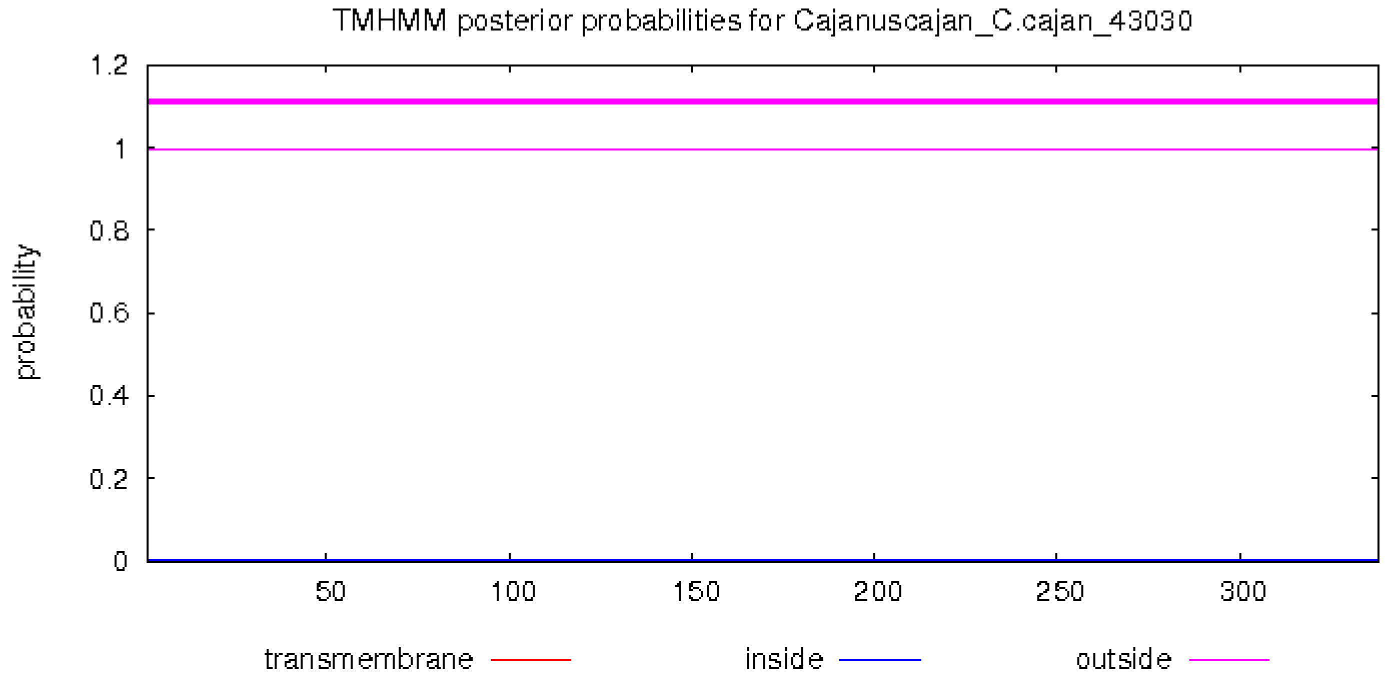

plot in postscript, script for making the plot in gnuplot, data for plot

---

```
# Cajanuscajan_C.cajan_43785 Length: 325
# Cajanuscajan_C.cajan_43785 Number of predicted TMHs:  0
```

```
# Cajanuscajan_C.cajan_43785 Exp number of AAs in TMHs: 0.001
# Cajanuscajan_C.cajan_43785 Exp number, first 60 AAs:  0.00039
# Cajanuscajan_C.cajan_43785 Total prob of N-in:        0.01279
Cajanuscajan_C.cajan_43785      TMHMM2.0        outside      1   325
```

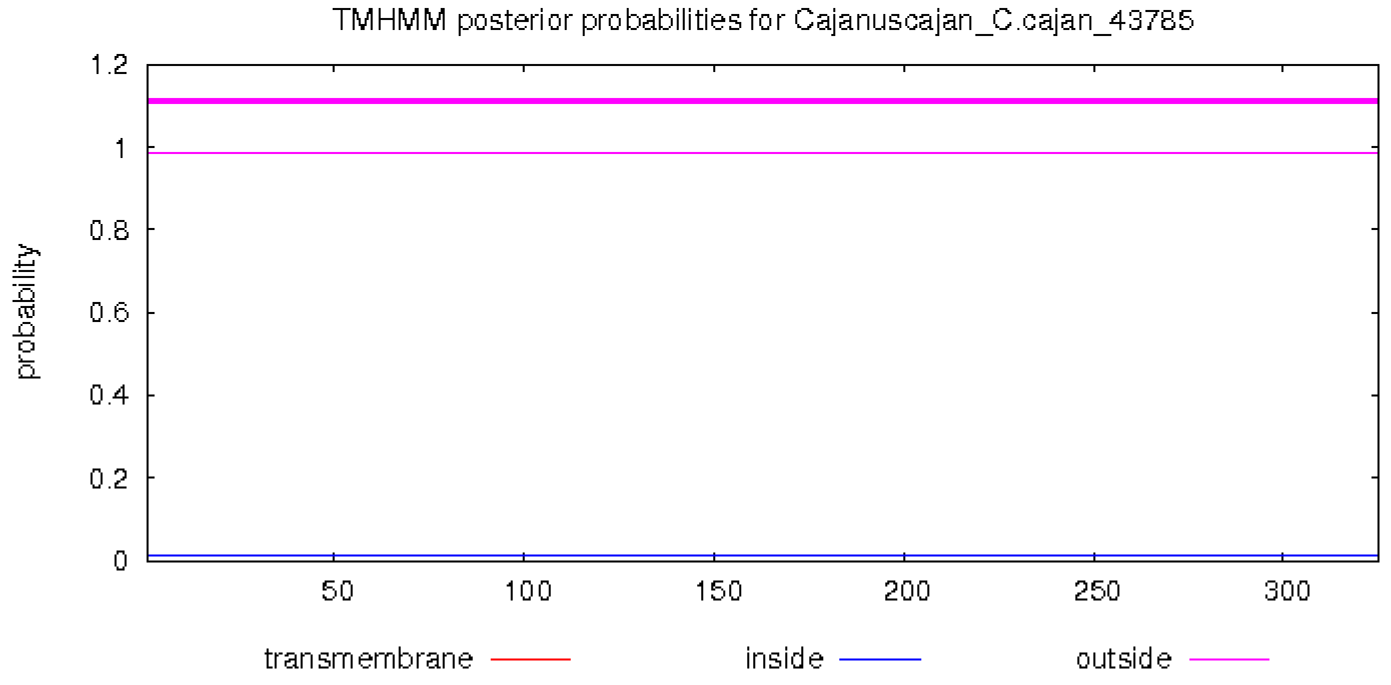

plot in postscript, script for making the plot in gnuplot, data for plot

---

```
# Cajanuscajan_C.cajan_43786 Length: 330
# Cajanuscajan_C.cajan_43786 Number of predicted TMHs:  0
# Cajanuscajan_C.cajan_43786 Exp number of AAs in TMHs: 0.00041
# Cajanuscajan_C.cajan_43786 Exp number, first 60 AAs:  0.00022
# Cajanuscajan_C.cajan_43786 Total prob of N-in:        0.00480
Cajanuscajan_C.cajan_43786      TMHMM2.0        outside      1   330
```

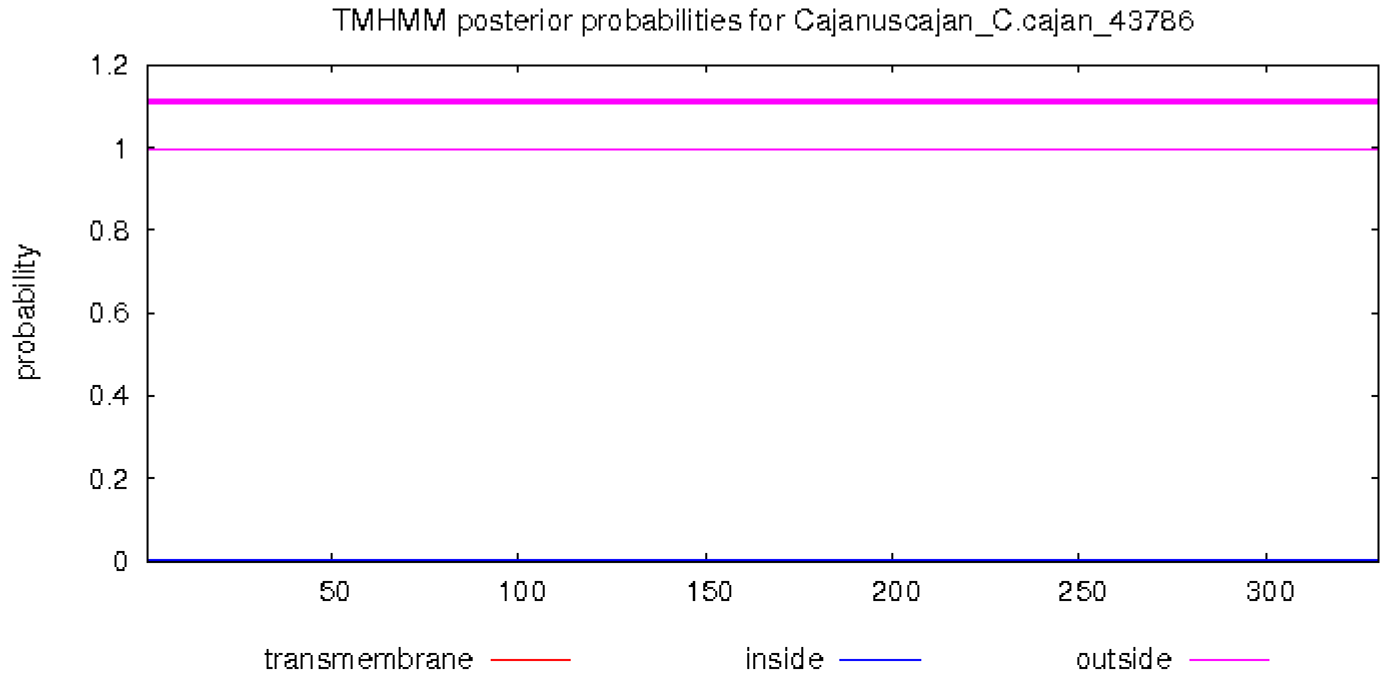

plot in postscript, script for making the plot in gnuplot, data for plot

---

```
# Cajanuscajan_C.cajan_44182 Length: 276
# Cajanuscajan_C.cajan_44182 Number of predicted TMHs:  0
```

```
# Cajanuscajan_C.cajan_44182 Exp number of AAs in TMHs: 0.00086
# Cajanuscajan_C.cajan_44182 Exp number, first 60 AAs:  0
# Cajanuscajan_C.cajan_44182 Total prob of N-in:        0.02167
Cajanuscajan_C.cajan_44182      TMHMM2.0        outside      1   276
```

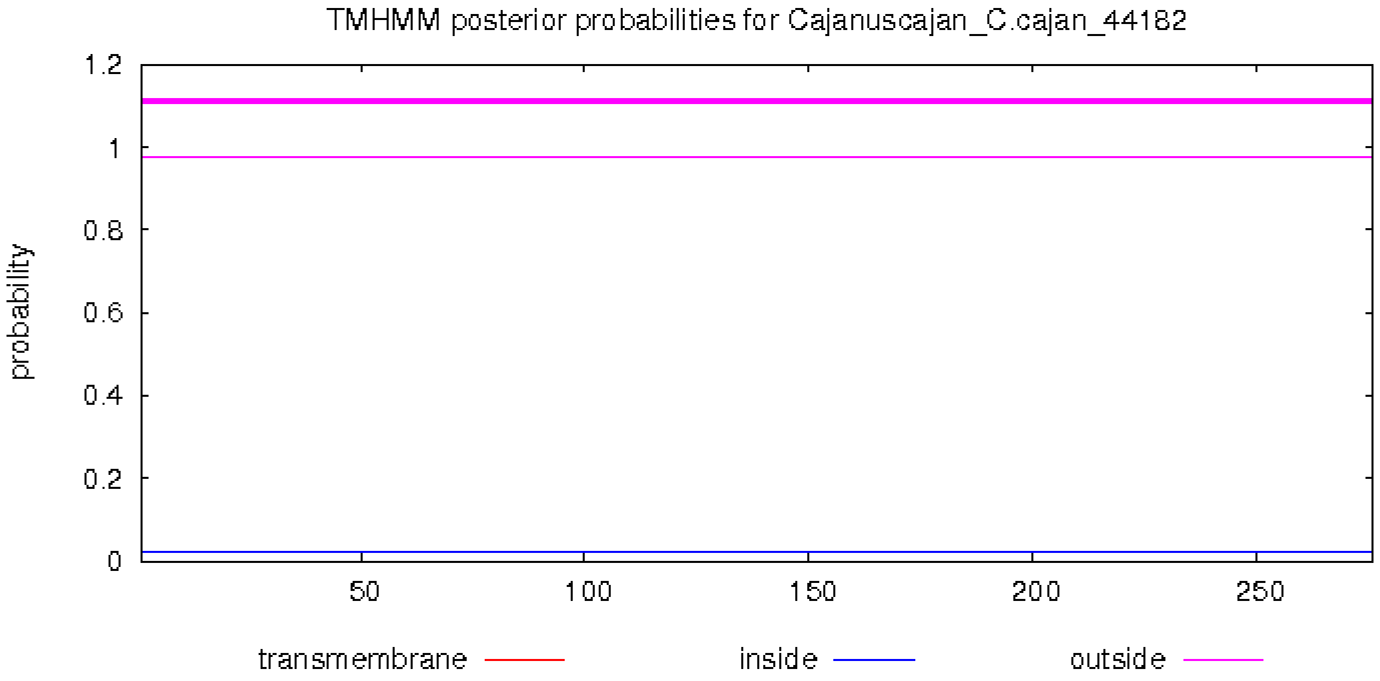

plot in postscript, script for making the plot in gnuplot, data for plot

---

```
# Cajanuscajan_C.cajan_44515 Length: 324
# Cajanuscajan_C.cajan_44515 Number of predicted TMHs:  0
# Cajanuscajan_C.cajan_44515 Exp number of AAs in TMHs: 0.0003
# Cajanuscajan_C.cajan_44515 Exp number, first 60 AAs:  0.00015
# Cajanuscajan_C.cajan_44515 Total prob of N-in:        0.05795
Cajanuscajan_C.cajan_44515      TMHMM2.0        outside      1   324
```

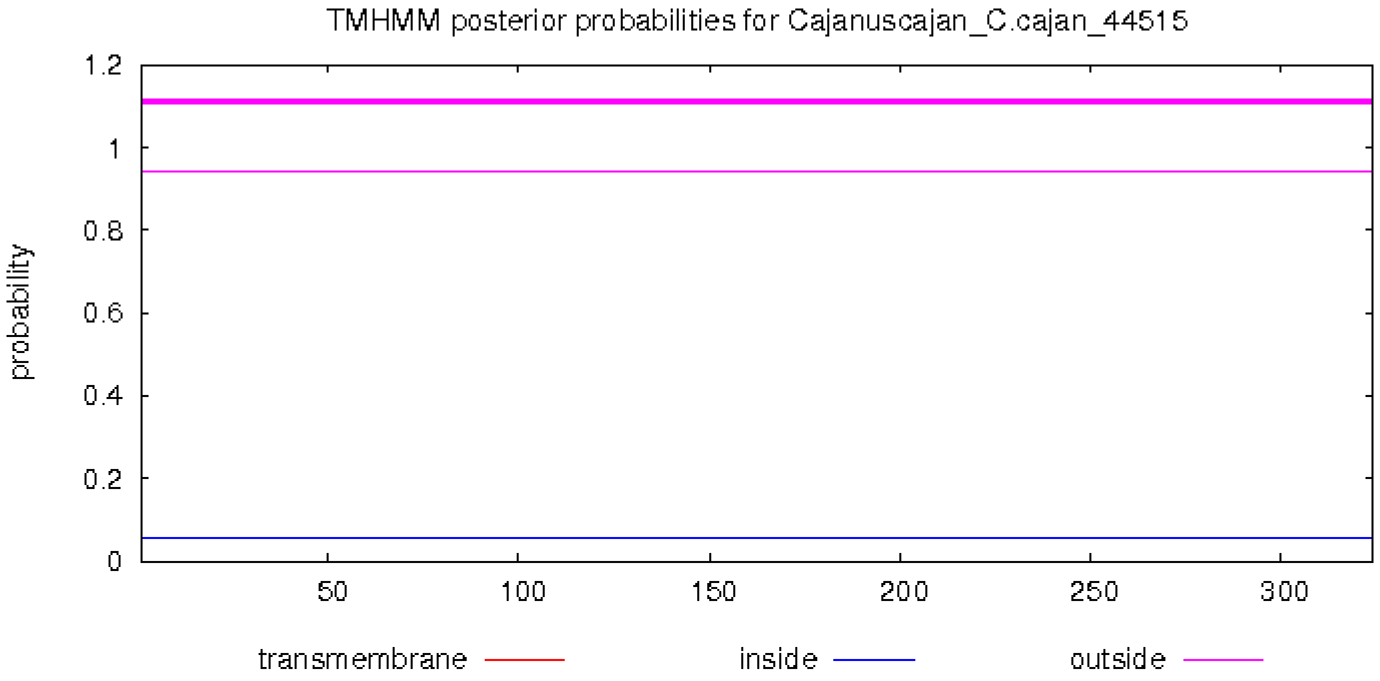

plot in postscript, script for making the plot in gnuplot, data for plot

---

```
# Cajanuscajan_C.cajan_44516 Length: 324
# Cajanuscajan_C.cajan_44516 Number of predicted TMHs:  0
```

```
# Cajanuscajan_C.cajan_44516 Exp number of AAs in TMHs: 0.0003
# Cajanuscajan_C.cajan_44516 Exp number, first 60 AAs:  0.00015
# Cajanuscajan_C.cajan_44516 Total prob of N-in:        0.05795
Cajanuscajan_C.cajan_44516      TMHMM2.0        outside      1   324
```

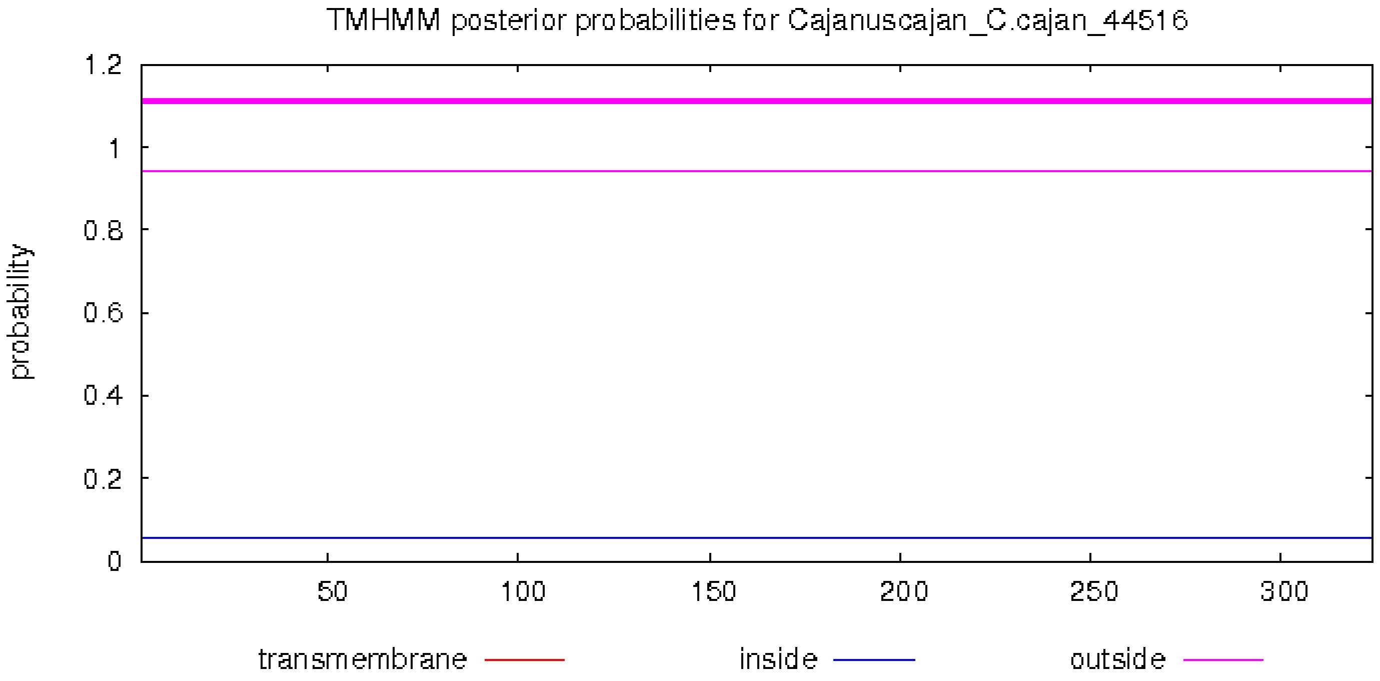

plot in postscript, script for making the plot in gnuplot, data for plot

---

```
# Cajanuscajan_C.cajan_46578 Length: 356
# Cajanuscajan_C.cajan_46578 Number of predicted TMHs:  0
# Cajanuscajan_C.cajan_46578 Exp number of AAs in TMHs: 0.43505
# Cajanuscajan_C.cajan_46578 Exp number, first 60 AAs:  0
# Cajanuscajan_C.cajan_46578 Total prob of N-in:        0.01480
Cajanuscajan_C.cajan_46578      TMHMM2.0        outside      1   356
```

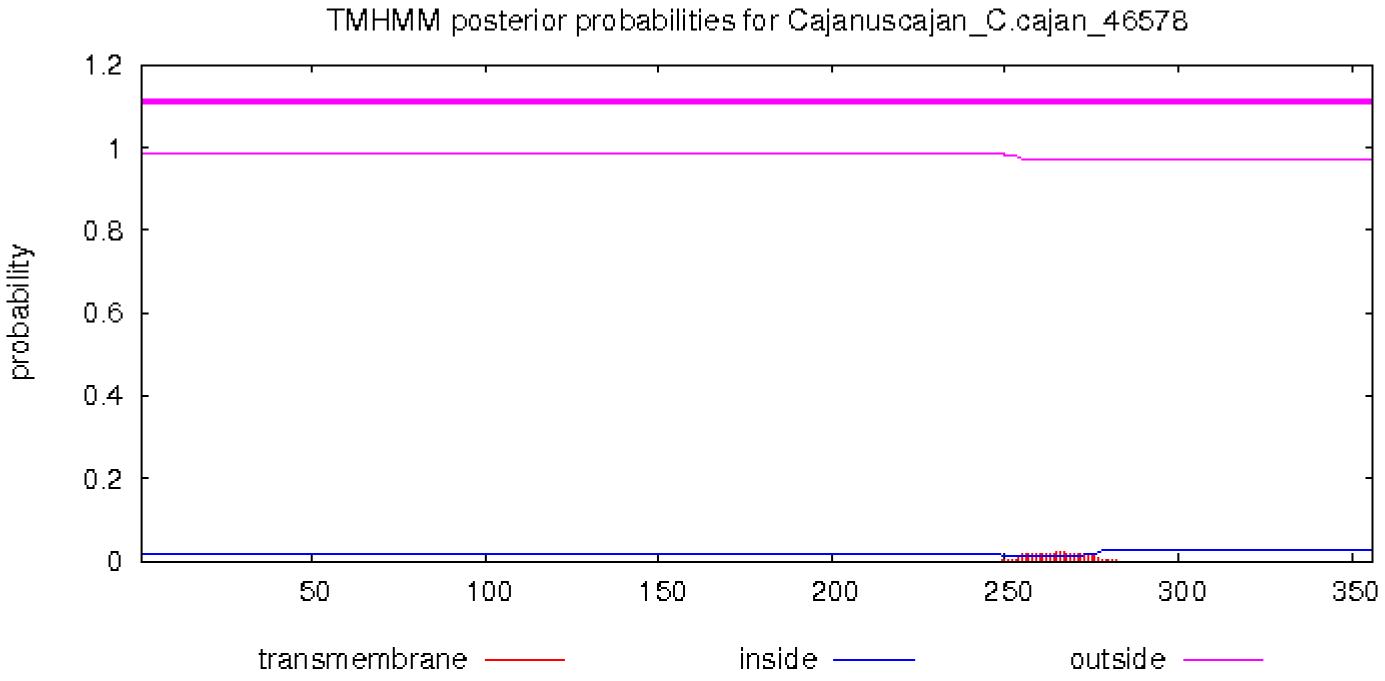

plot in postscript, script for making the plot in gnuplot, data for plot

---

```
# Cajanuscajan_C.cajan_46953 Length: 444
# Cajanuscajan_C.cajan_46953 Number of predicted TMHs:  0
```

```
# Cajanuscajan_C.cajan_46953 Exp number of AAs in TMHs: 0
# Cajanuscajan_C.cajan_46953 Exp number, first 60 AAs:  0
# Cajanuscajan_C.cajan_46953 Total prob of N-in:        0.00396
Cajanuscajan_C.cajan_46953      TMHMM2.0        outside      1   444
```

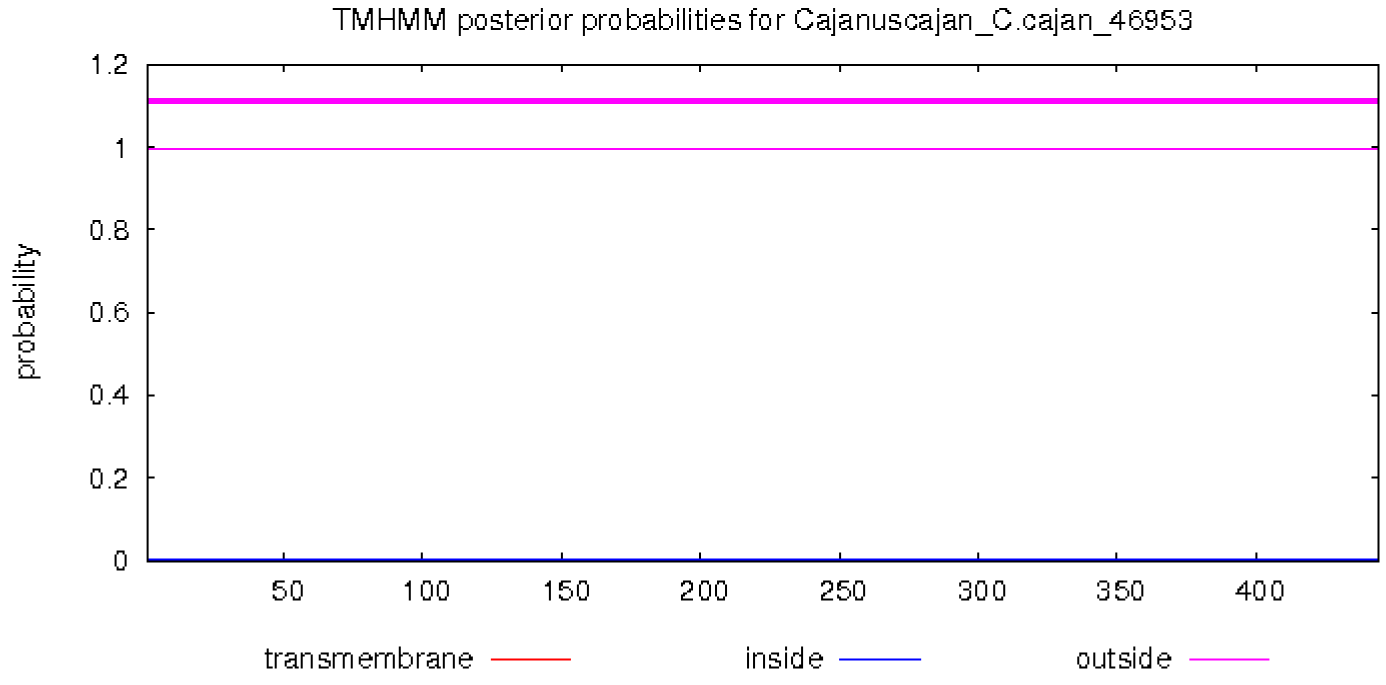

plot in postscript, script for making the plot in gnuplot, data for plot

---

```
# Cajanuscajan_C.cajan_48342 Length: 346
# Cajanuscajan_C.cajan_48342 Number of predicted TMHs:  0
# Cajanuscajan_C.cajan_48342 Exp number of AAs in TMHs: 0
# Cajanuscajan_C.cajan_48342 Exp number, first 60 AAs:  0
# Cajanuscajan_C.cajan_48342 Total prob of N-in:        0.00443
Cajanuscajan_C.cajan_48342      TMHMM2.0        outside      1   346
```

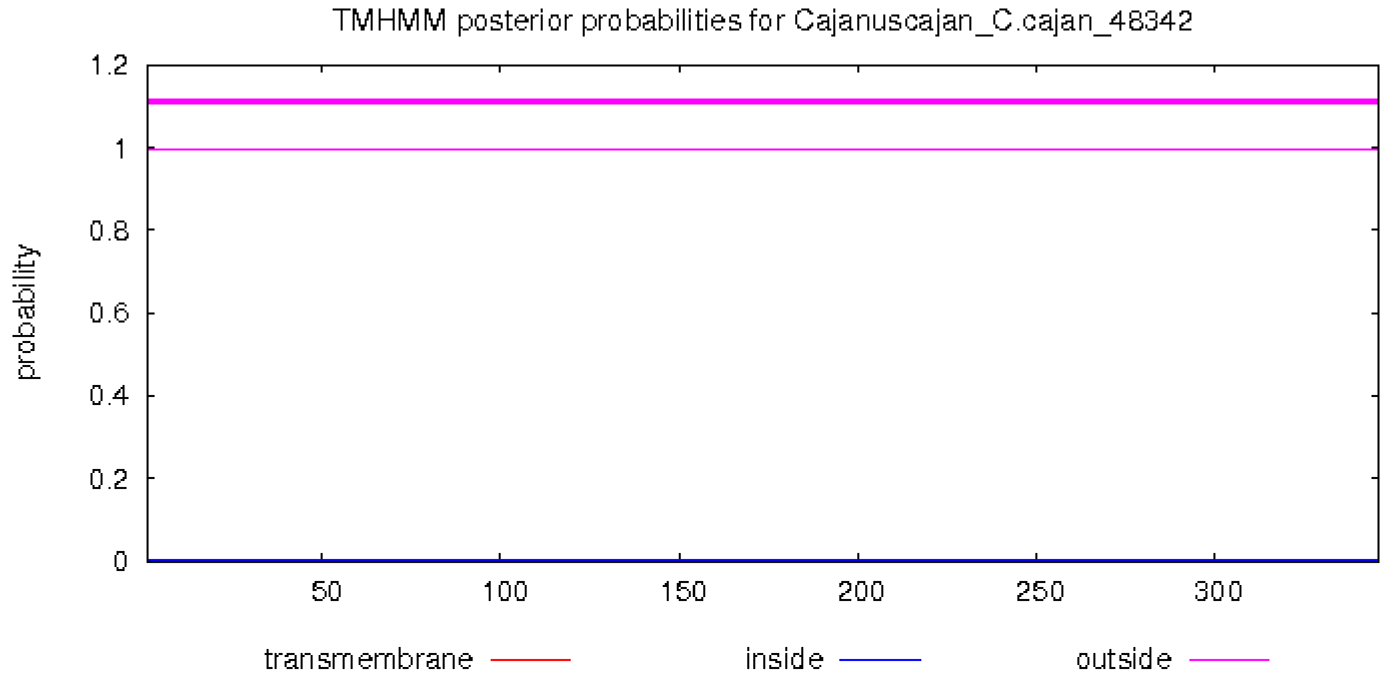

plot in postscript, script for making the plot in gnuplot, data for plot

---

```
# Cajanuscajan_C.cajan_48539 Length: 56
# Cajanuscajan_C.cajan_48539 Number of predicted TMHs:  0
```

```
# Cajanuscajan_C.cajan_48539 Exp number of AAs in TMHs: 0.00588
# Cajanuscajan_C.cajan_48539 Exp number, first 60 AAs:  0.00588
# Cajanuscajan_C.cajan_48539 Total prob of N-in:        0.04511
Cajanuscajan_C.cajan_48539      TMHMM2.0        outside      1    56
```

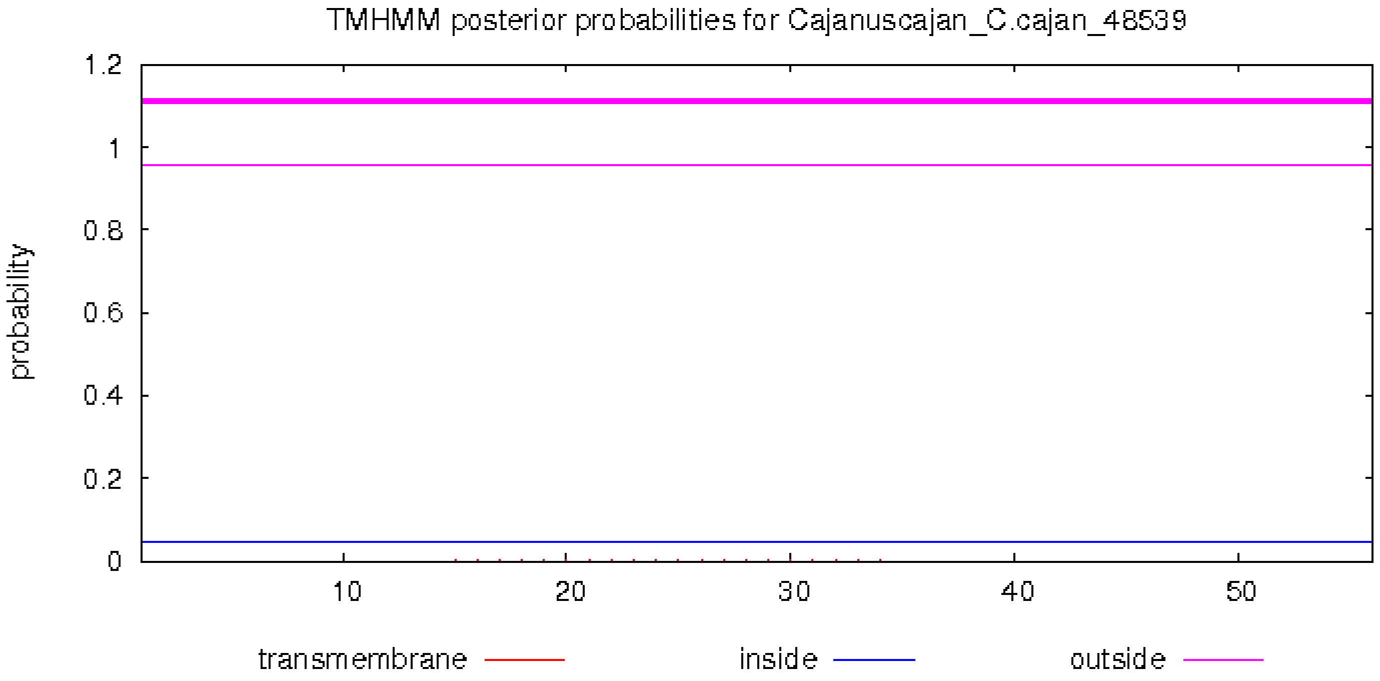

plot in postscript, script for making the plot in gnuplot, data for plot

---

```
# Camelinasativa_Csa00532s170.1 Length: 363
# Camelinasativa_Csa00532s170.1 Number of predicted TMHs:  0
# Camelinasativa_Csa00532s170.1 Exp number of AAs in TMHs: 0.00073
# Camelinasativa_Csa00532s170.1 Exp number, first 60 AAs:  0
# Camelinasativa_Csa00532s170.1 Total prob of N-in:        0.02835
Camelinasativa_Csa00532s170.1   TMHMM2.0        outside      1   363
```

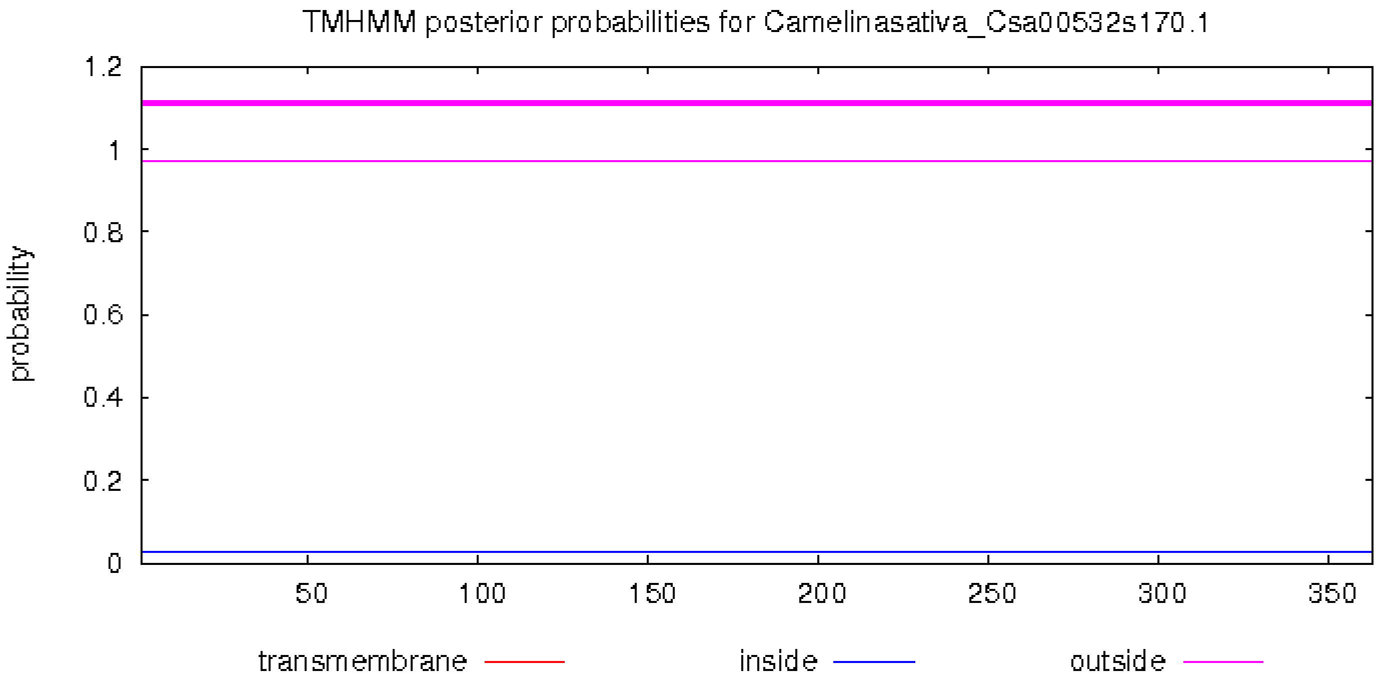

plot in postscript, script for making the plot in gnuplot, data for plot

---

```
# Camelinasativa_Csa01g002880.1 Length: 476
# Camelinasativa_Csa01g002880.1 Number of predicted TMHs:  0
```

```
# Camelinasativa_Csa01g002880.1 Exp number of AAs in TMHs: 0.00056
# Camelinasativa_Csa01g002880.1 Exp number, first 60 AAs:  0
# Camelinasativa_Csa01g002880.1 Total prob of N-in:        0.00017
Camelinasativa_Csa01g002880.1   TMHMM2.0        outside      1   476
```

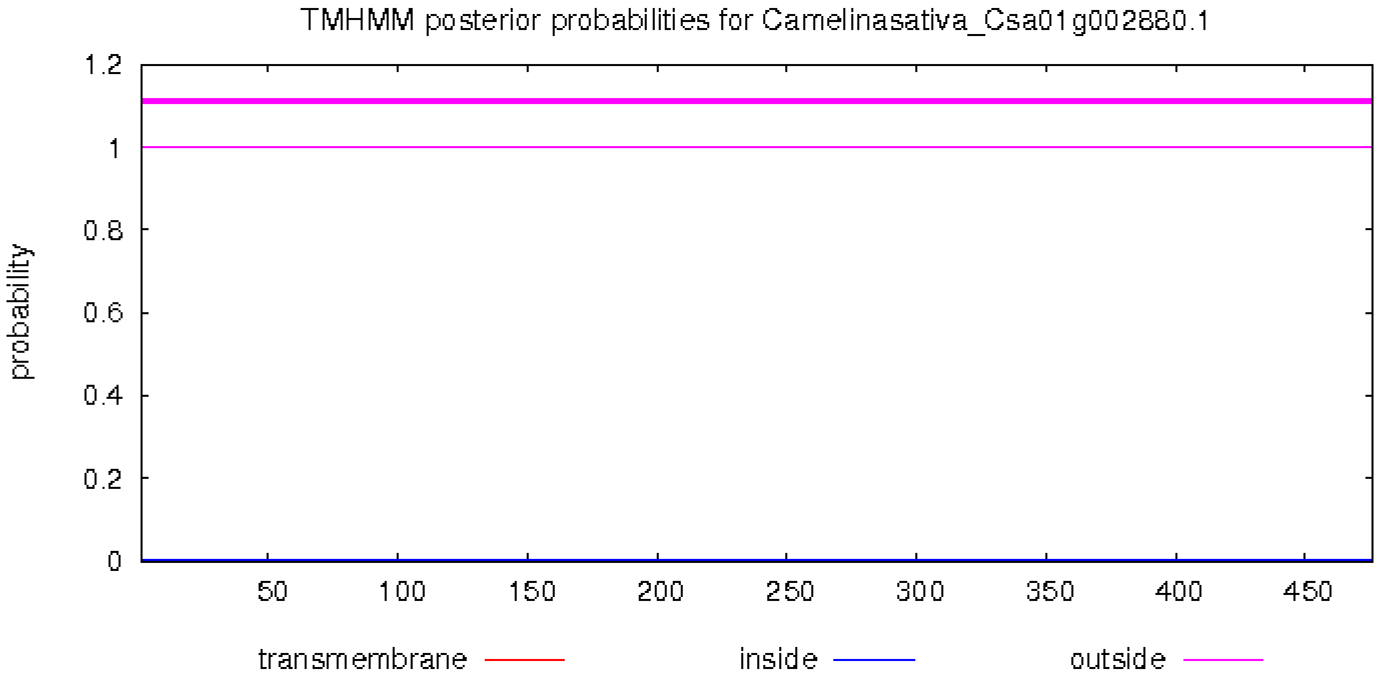

plot in postscript, script for making the plot in gnuplot, data for plot

---

```
# Camelinasativa_Csa01g004850.1 Length: 341
# Camelinasativa_Csa01g004850.1 Number of predicted TMHs:  0
# Camelinasativa_Csa01g004850.1 Exp number of AAs in TMHs: 0.00042
# Camelinasativa_Csa01g004850.1 Exp number, first 60 AAs:  0.00016
# Camelinasativa_Csa01g004850.1 Total prob of N-in:        0.01102
Camelinasativa_Csa01g004850.1   TMHMM2.0        outside      1   341
```

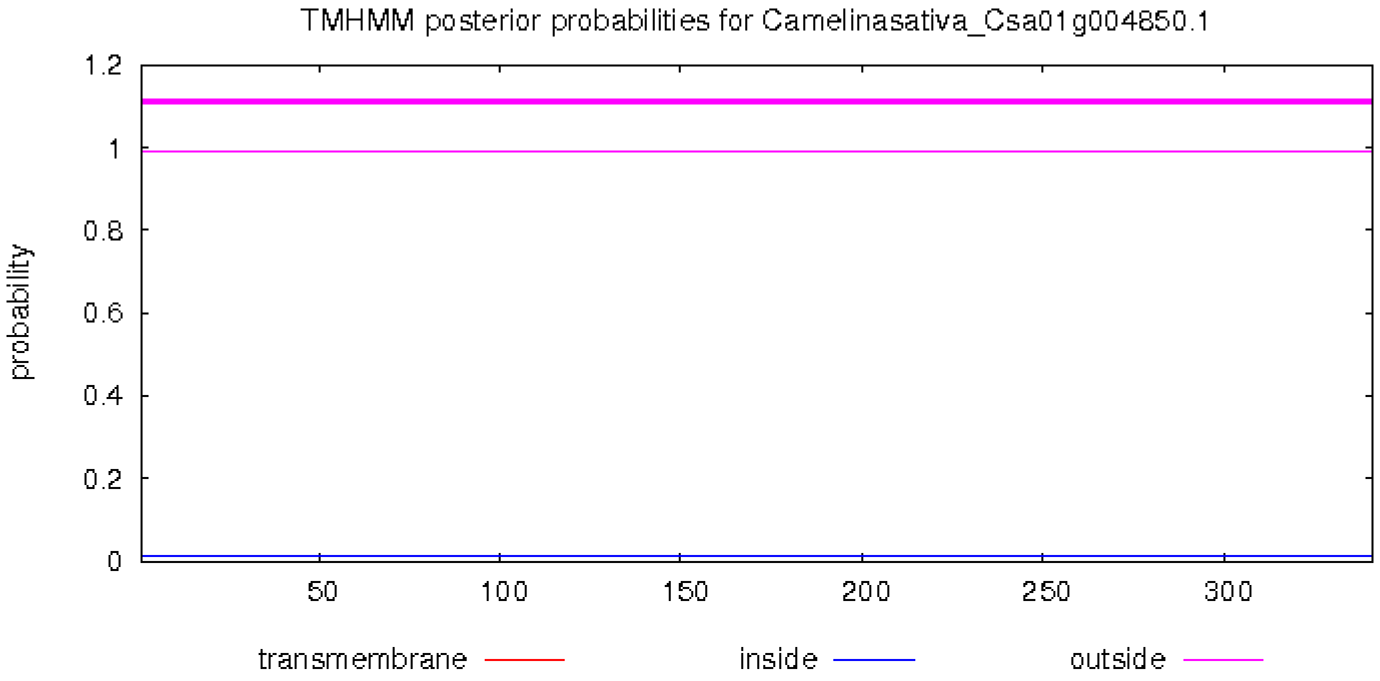

plot in postscript, script for making the plot in gnuplot, data for plot

---

```
# Camelinasativa_Csa01g004860.1 Length: 394
# Camelinasativa_Csa01g004860.1 Number of predicted TMHs:  0
```

```
# Camelinasativa_Csa01g004860.1 Exp number of AAs in TMHs: 1.21407
# Camelinasativa_Csa01g004860.1 Exp number, first 60 AAs:  1.21372
# Camelinasativa_Csa01g004860.1 Total prob of N-in:        0.03626
Camelinasativa_Csa01g004860.1   TMHMM2.0        outside      1   394
```

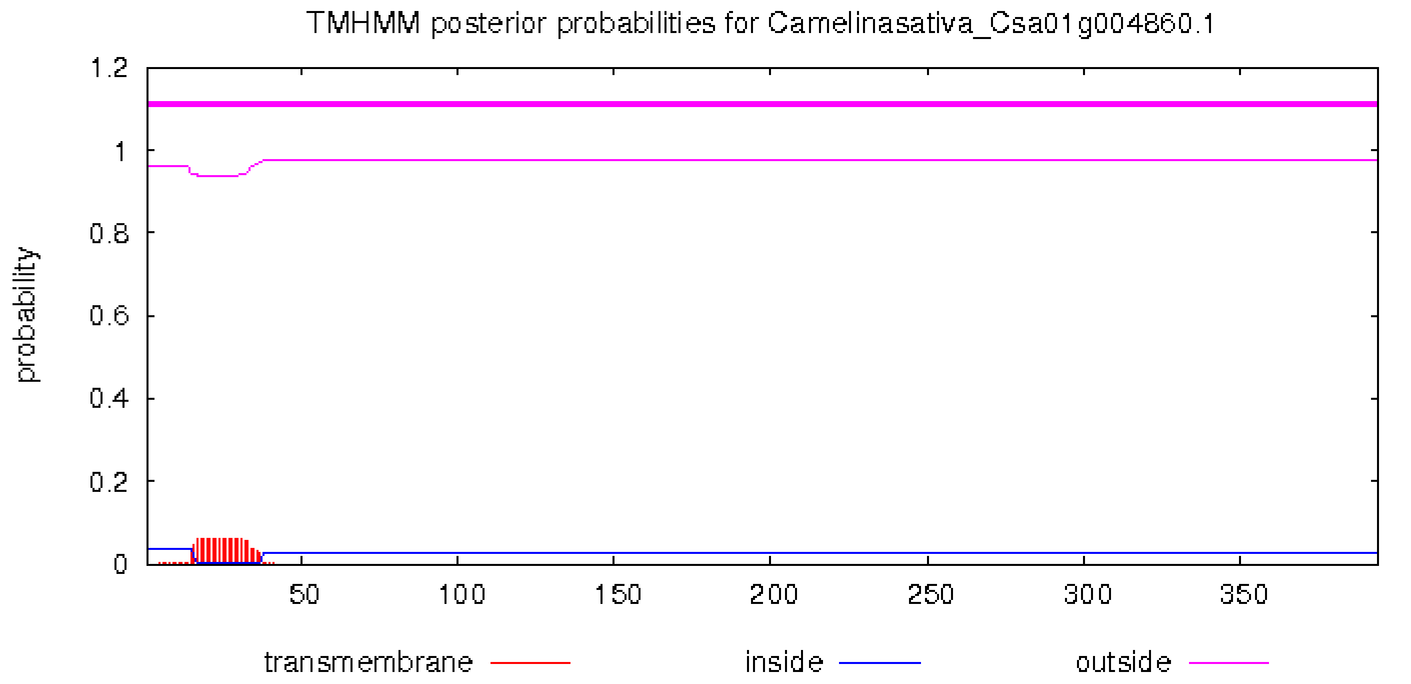

plot in postscript, script for making the plot in gnuplot, data for plot

```
# Camelinasativa_Csa01g011360.1 Length: 450
# Camelinasativa_Csa01g011360.1 Number of predicted TMHs:  0
# Camelinasativa_Csa01g011360.1 Exp number of AAs in TMHs: 0.02894
# Camelinasativa_Csa01g011360.1 Exp number, first 60 AAs:  0.00362
# Camelinasativa_Csa01g011360.1 Total prob of N-in:        0.00168
Camelinasativa_Csa01g011360.1   TMHMM2.0        outside      1   450
```

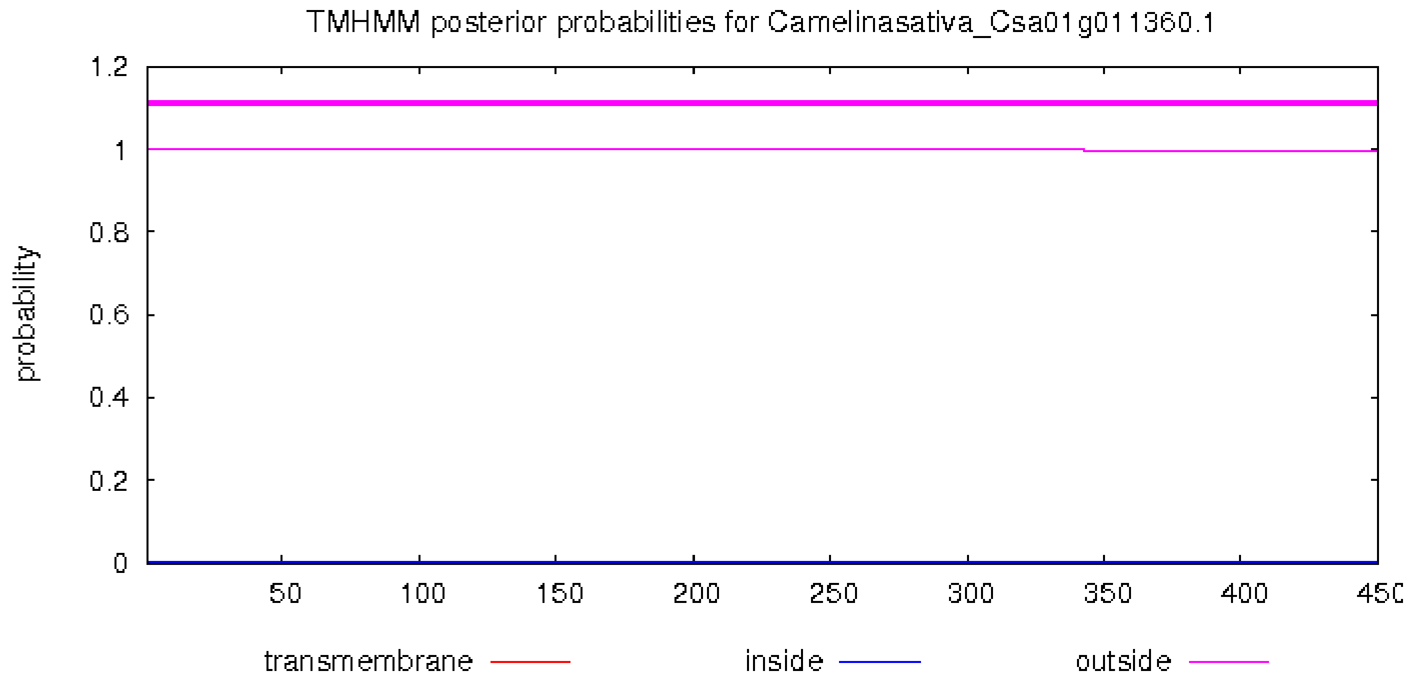

plot in postscript, script for making the plot in gnuplot, data for plot

```
# Camelinasativa_Csa01g011370.1 Length: 462
# Camelinasativa_Csa01g011370.1 Number of predicted TMHs:  0
```

```
# Camelinasativa_Csa01g011370.1 Exp number of AAs in TMHs: 0.02194
# Camelinasativa_Csa01g011370.1 Exp number, first 60 AAs:  0.00136
# Camelinasativa_Csa01g011370.1 Total prob of N-in:        0.00591
Camelinasativa_Csa01g011370.1   TMHMM2.0        outside      1   462
```

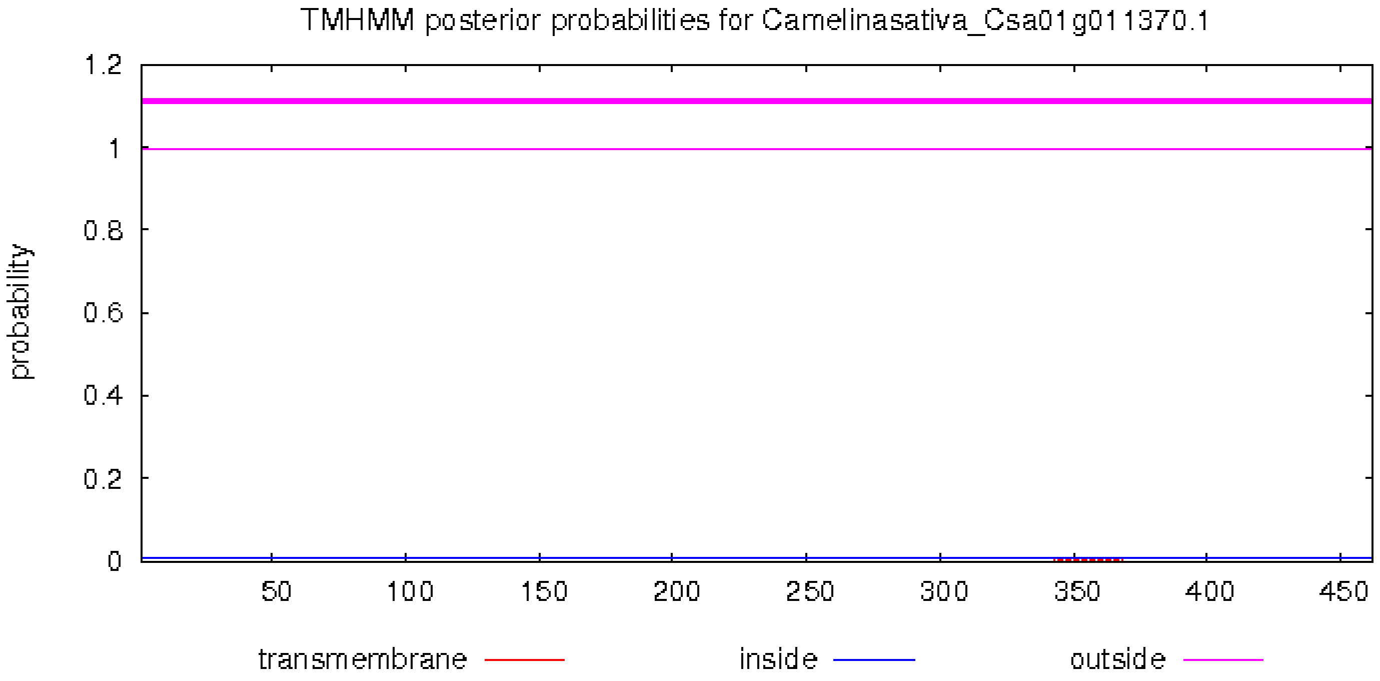

plot in postscript, script for making the plot in gnuplot, data for plot

---

```
# Camelinasativa_Csa01g011380.1 Length: 770
# Camelinasativa_Csa01g011380.1 Number of predicted TMHs:  2
# Camelinasativa_Csa01g011380.1 Exp number of AAs in TMHs: 40.98524
# Camelinasativa_Csa01g011380.1 Exp number, first 60 AAs:  0
# Camelinasativa_Csa01g011380.1 Total prob of N-in:        0.00058
Camelinasativa_Csa01g011380.1   TMHMM2.0        outside      1   604
Camelinasativa_Csa01g011380.1   TMHMM2.0        TMhelix    605   624
Camelinasativa_Csa01g011380.1   TMHMM2.0        inside     625   744
Camelinasativa_Csa01g011380.1   TMHMM2.0        TMhelix    745   767
Camelinasativa_Csa01g011380.1   TMHMM2.0        outside    768   770
```

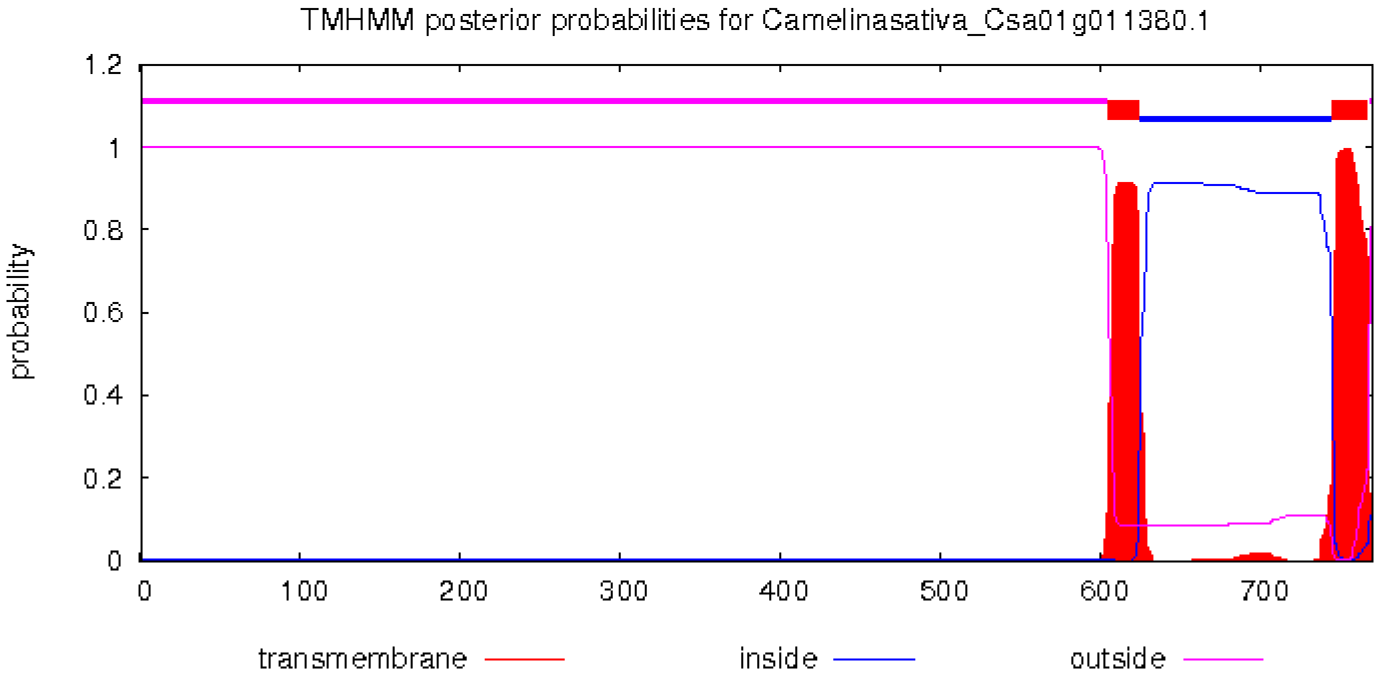

plot in postscript, script for making the plot in gnuplot, data for plot

```
# Camelinasativa_Csa01g014020.1 Length: 302
# Camelinasativa_Csa01g014020.1 Number of predicted TMHs:  0
# Camelinasativa_Csa01g014020.1 Exp number of AAs in TMHs: 0.00072
# Camelinasativa_Csa01g014020.1 Exp number, first 60 AAs:  0
# Camelinasativa_Csa01g014020.1 Total prob of N-in:        0.05216
Camelinasativa_Csa01g014020.1	TMHMM2.0	outside	     1   302
```

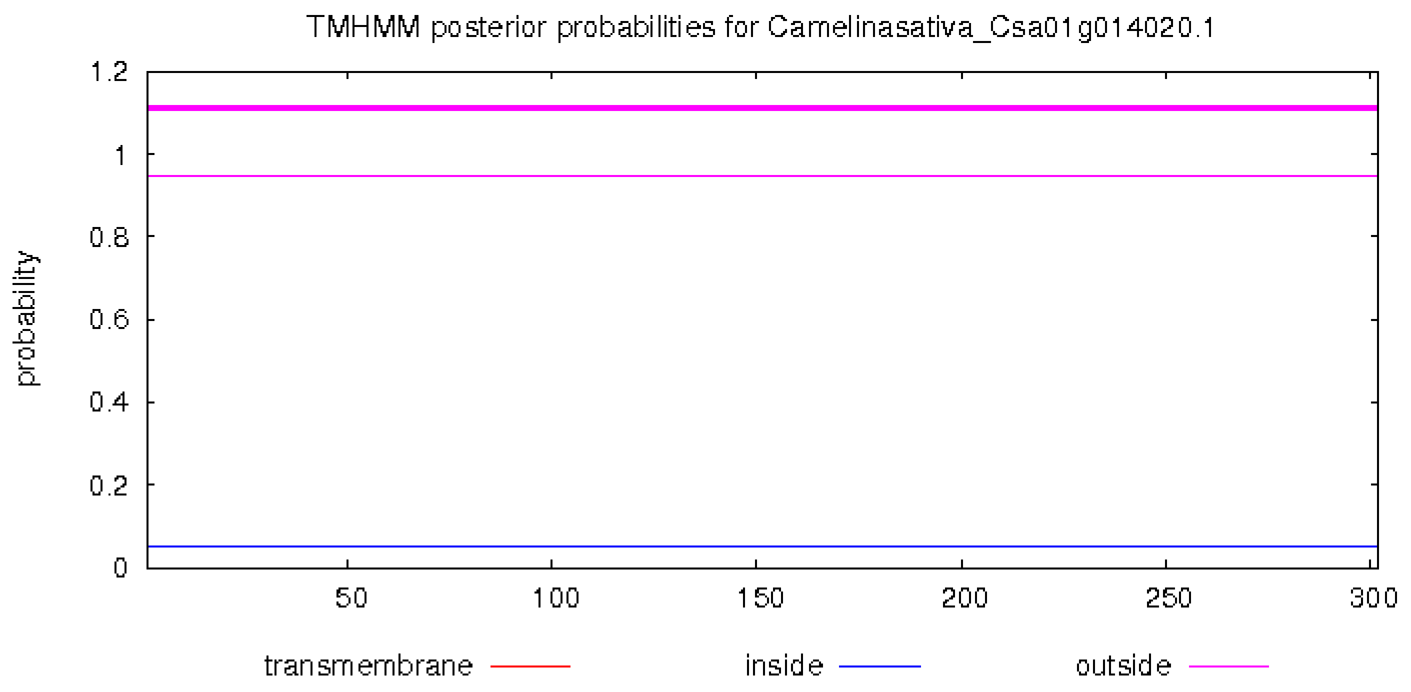

plot in postscript, script for making the plot in gnuplot, data for plot

```
# Camelinasativa_Csa01g014150.1 Length: 264
# Camelinasativa_Csa01g014150.1 Number of predicted TMHs:  0
# Camelinasativa_Csa01g014150.1 Exp number of AAs in TMHs: 0.00097
# Camelinasativa_Csa01g014150.1 Exp number, first 60 AAs:  0
# Camelinasativa_Csa01g014150.1 Total prob of N-in:        0.13312
Camelinasativa_Csa01g014150.1	TMHMM2.0	outside	     1   264
```

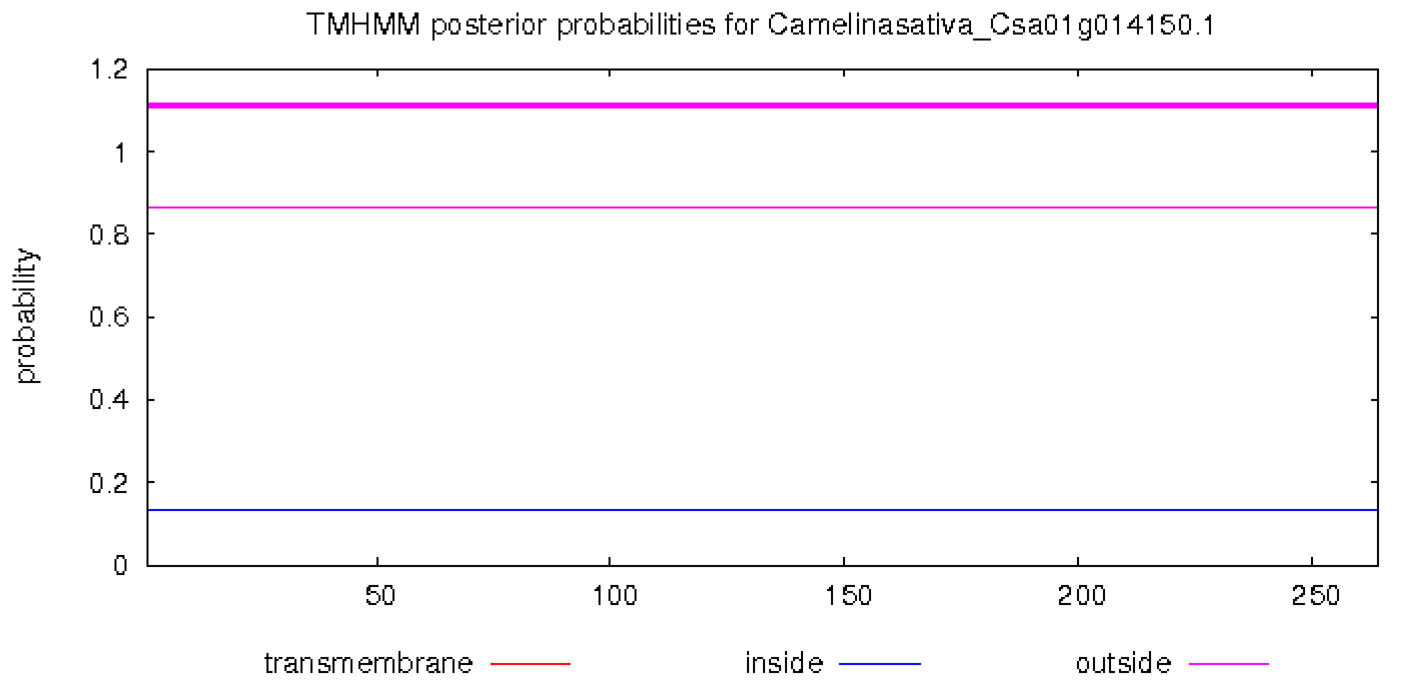

plot in postscript, script for making the plot in gnuplot, data for plot

```
# Camelinasativa_Csa01g017710.1 Length: 307
# Camelinasativa_Csa01g017710.1 Number of predicted TMHs:  0
# Camelinasativa_Csa01g017710.1 Exp number of AAs in TMHs: 0.17877
# Camelinasativa_Csa01g017710.1 Exp number, first 60 AAs:  0.00017
# Camelinasativa_Csa01g017710.1 Total prob of N-in:        0.01307
Camelinasativa_Csa01g017710.1   TMHMM2.0        outside      1   307
```

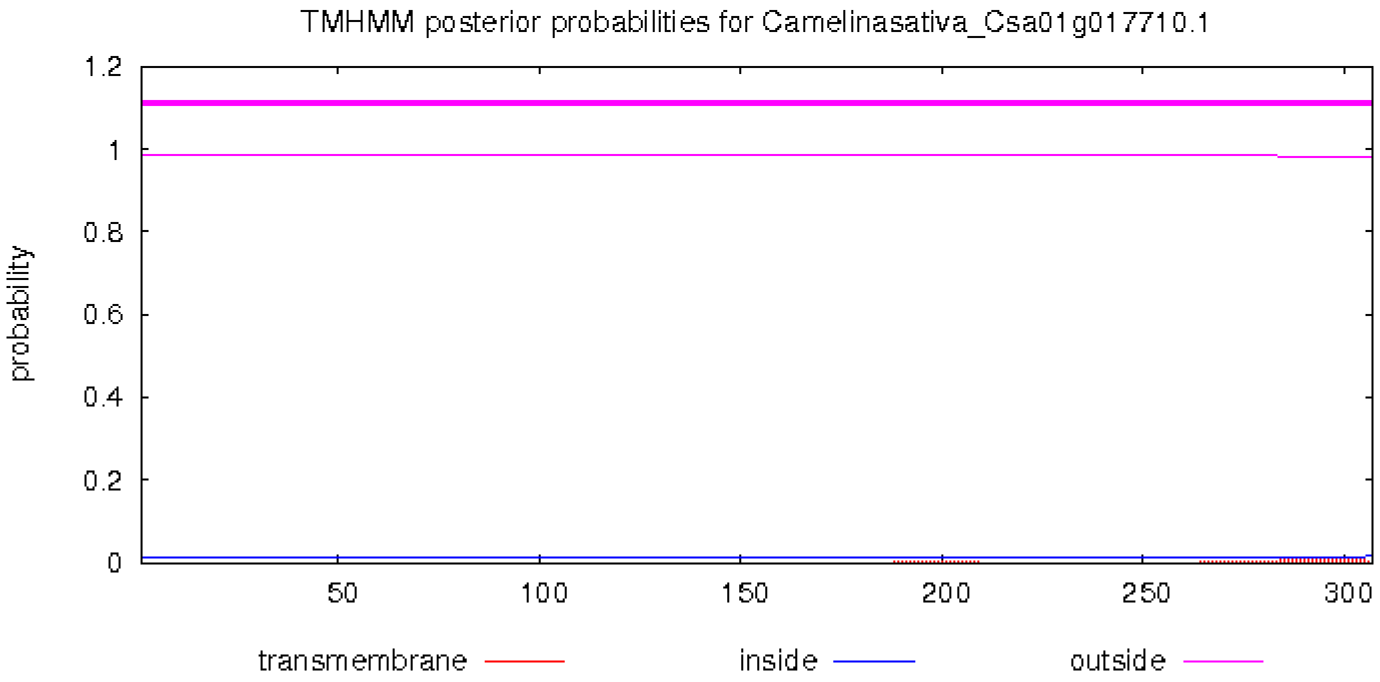

plot in postscript, script for making the plot in gnuplot, data for plot

---

```
# Camelinasativa_Csa01g018090.1 Length: 319
# Camelinasativa_Csa01g018090.1 Number of predicted TMHs:  0
# Camelinasativa_Csa01g018090.1 Exp number of AAs in TMHs: 0
# Camelinasativa_Csa01g018090.1 Exp number, first 60 AAs:  0
# Camelinasativa_Csa01g018090.1 Total prob of N-in:        0.00235
Camelinasativa_Csa01g018090.1   TMHMM2.0        outside      1   319
```

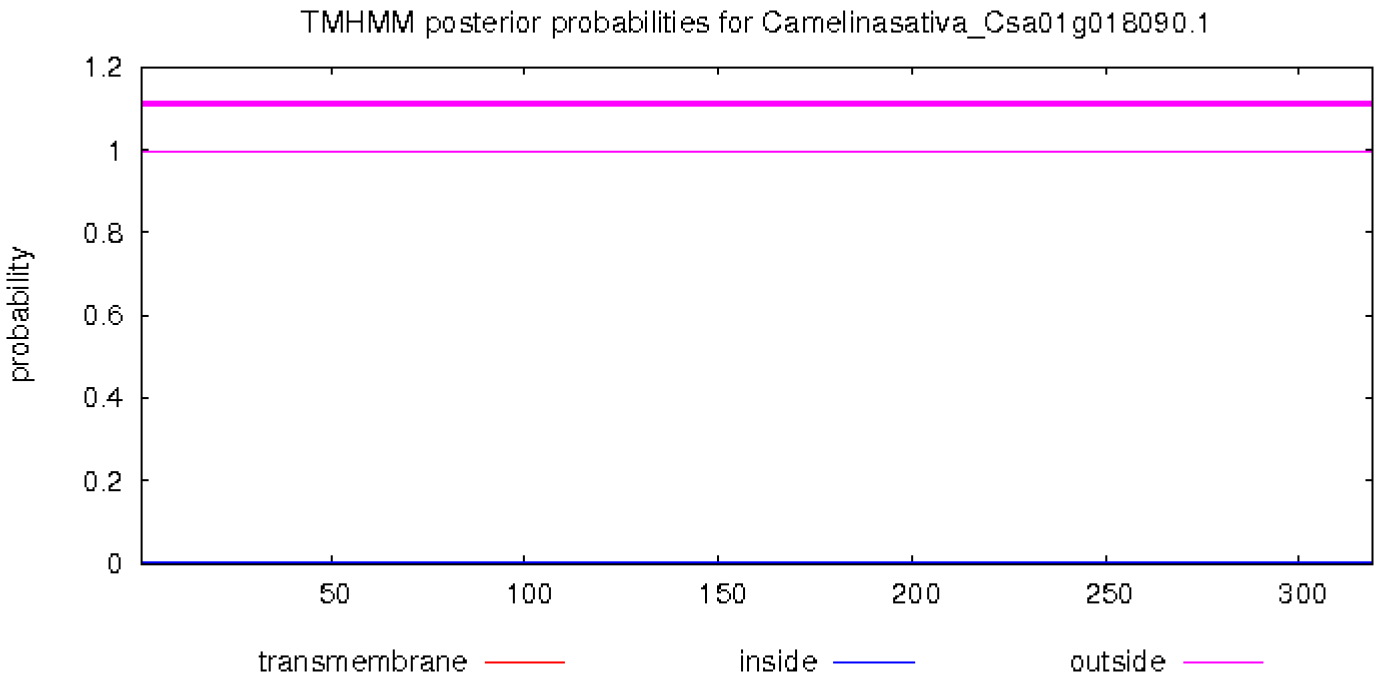

plot in postscript, script for making the plot in gnuplot, data for plot

---

```
# Camelinasativa_Csa01g018100.1 Length: 344
# Camelinasativa_Csa01g018100.1 Number of predicted TMHs:  0
# Camelinasativa_Csa01g018100.1 Exp number of AAs in TMHs: 0.00236
# Camelinasativa_Csa01g018100.1 Exp number, first 60 AAs:  0.00018
# Camelinasativa_Csa01g018100.1 Total prob of N-in:        0.00520
Camelinasativa_Csa01g018100.1   TMHMM2.0        outside      1   344
```

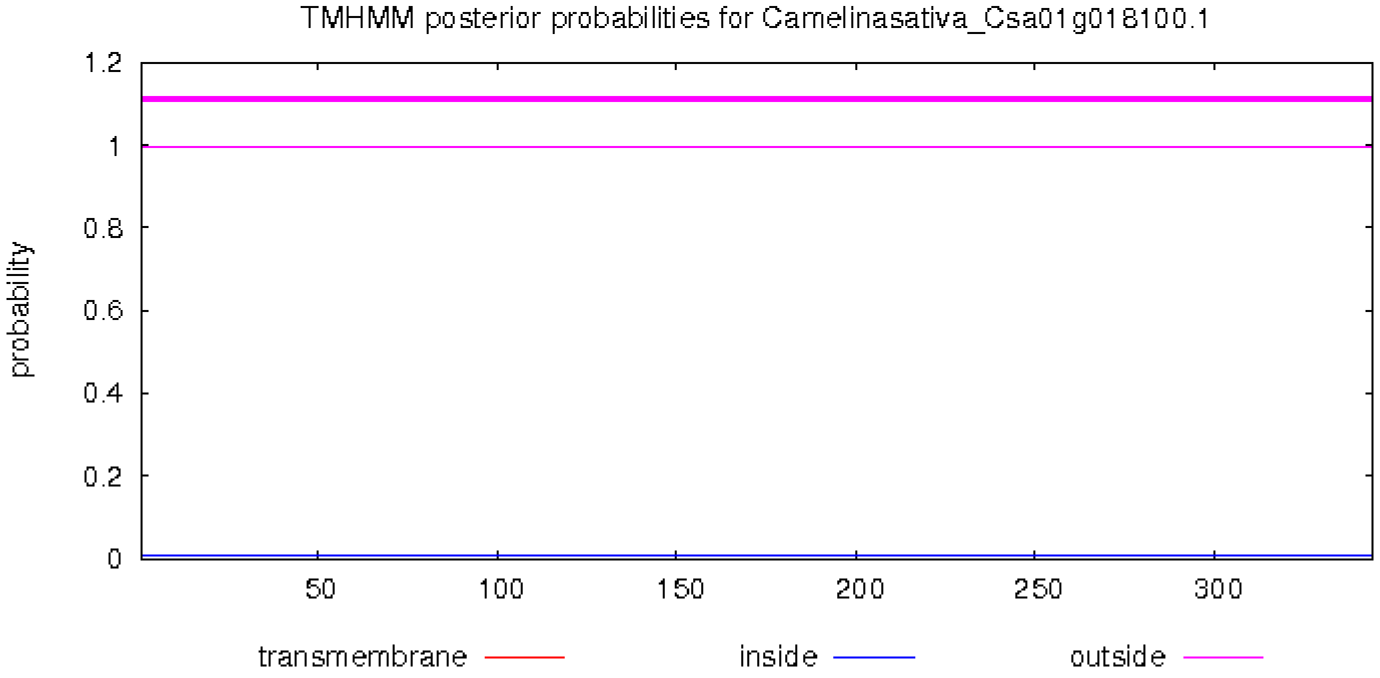

plot in postscript, script for making the plot in gnuplot, data for plot

---

```
# Camelinasativa_Csa01g020430.1 Length: 247
# Camelinasativa_Csa01g020430.1 Number of predicted TMHs:  0
# Camelinasativa_Csa01g020430.1 Exp number of AAs in TMHs: 0
# Camelinasativa_Csa01g020430.1 Exp number, first 60 AAs:  0
# Camelinasativa_Csa01g020430.1 Total prob of N-in:        0.00220
Camelinasativa_Csa01g020430.1   TMHMM2.0        outside      1   247
```

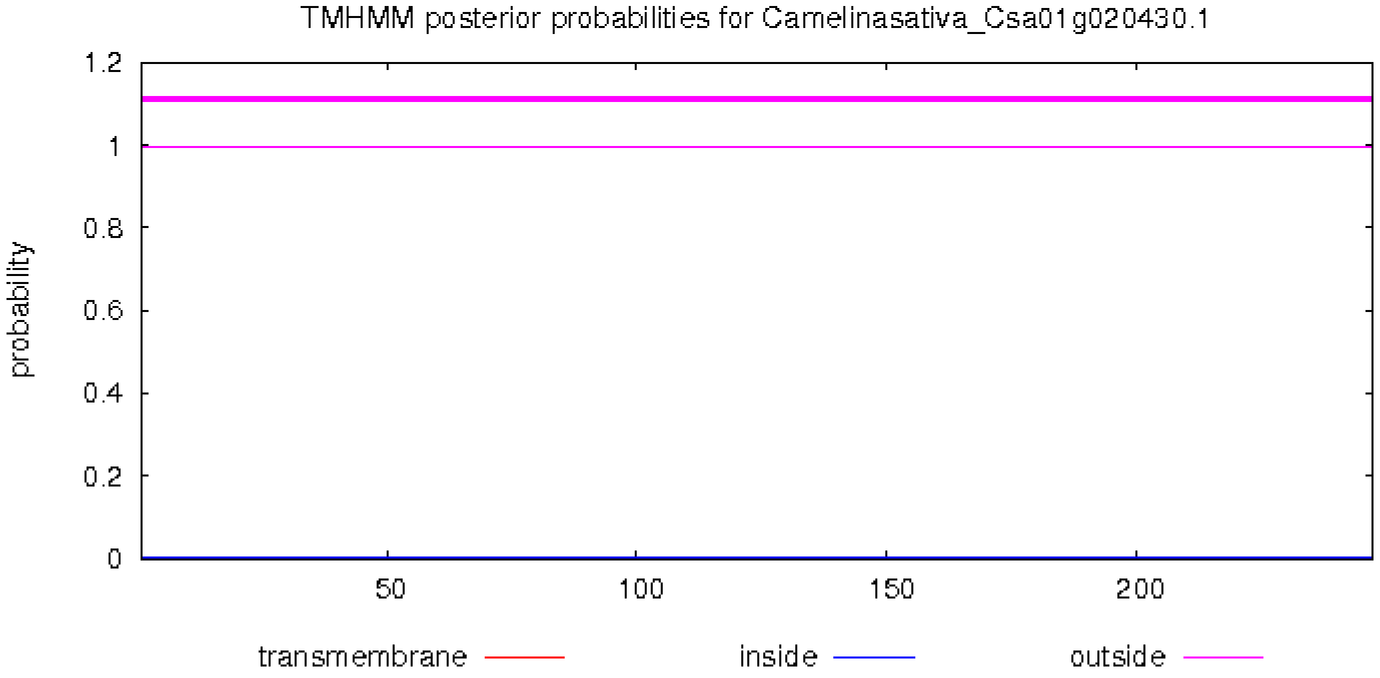

plot in postscript, script for making the plot in gnuplot, data for plot

---

```
# Camelinasativa_Csa01g021240.1 Length: 351
# Camelinasativa_Csa01g021240.1 Number of predicted TMHs:  0
# Camelinasativa_Csa01g021240.1 Exp number of AAs in TMHs: 0.09105
# Camelinasativa_Csa01g021240.1 Exp number, first 60 AAs:  0
# Camelinasativa_Csa01g021240.1 Total prob of N-in:        0.00444
Camelinasativa_Csa01g021240.1	TMHMM2.0	outside	     1   351
```

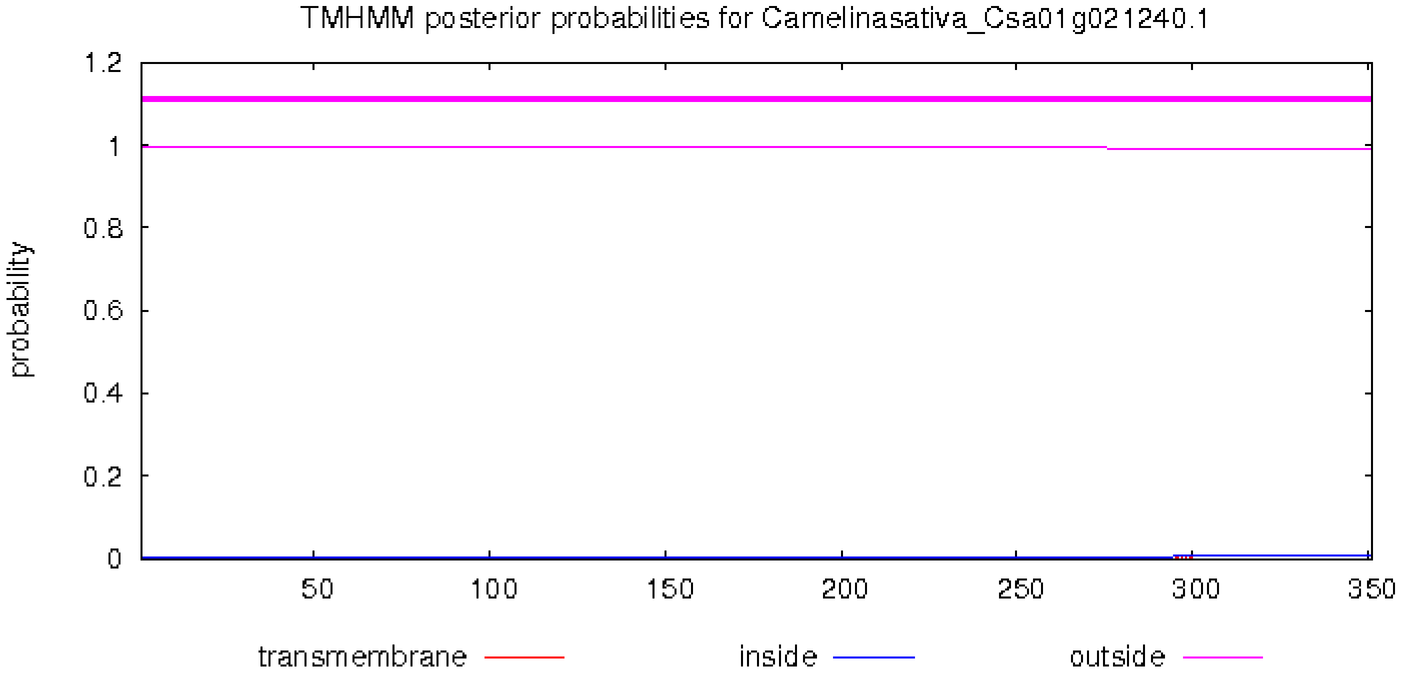

plot in postscript, script for making the plot in gnuplot, data for plot

---

```
# Camelinasativa_Csa01g032870.1 Length: 281
# Camelinasativa_Csa01g032870.1 Number of predicted TMHs:  0
# Camelinasativa_Csa01g032870.1 Exp number of AAs in TMHs: 0.00083
# Camelinasativa_Csa01g032870.1 Exp number, first 60 AAs:  0
# Camelinasativa_Csa01g032870.1 Total prob of N-in:        0.00640
Camelinasativa_Csa01g032870.1	TMHMM2.0	outside	     1   281
```

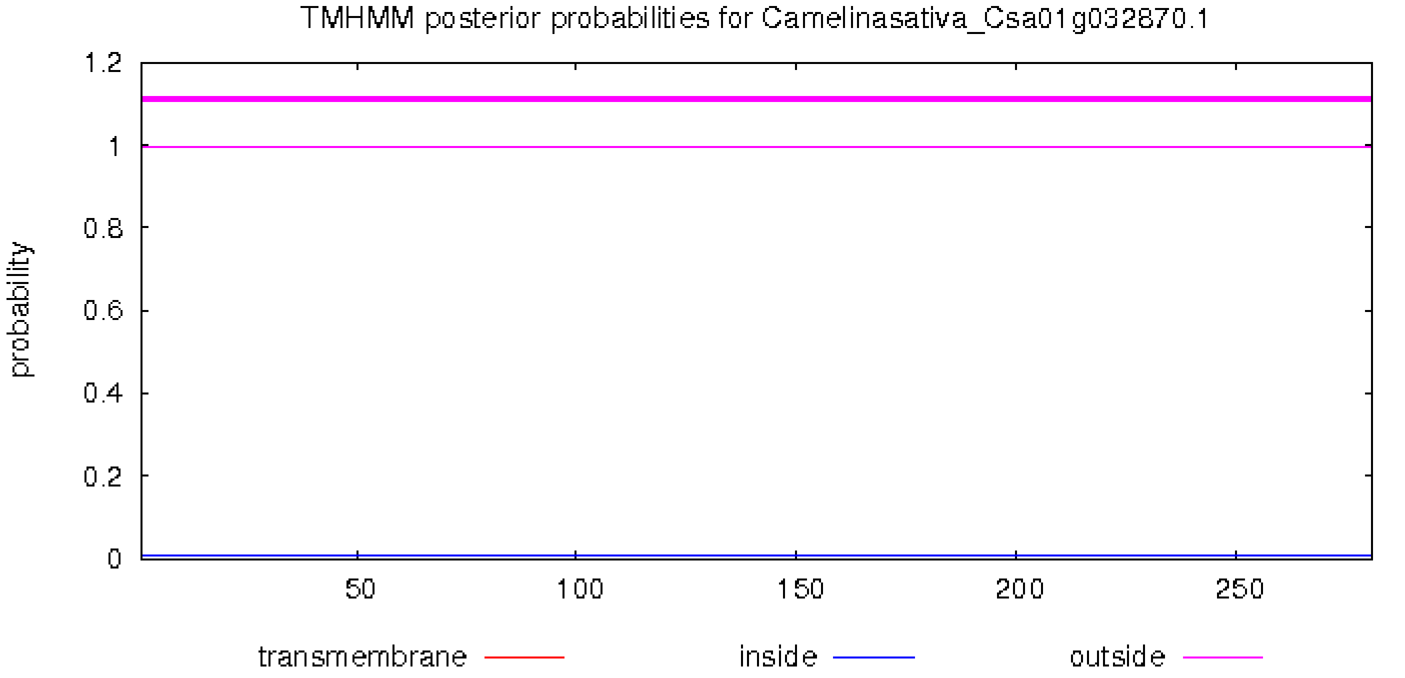

plot in postscript, script for making the plot in gnuplot, data for plot

---

```
# Camelinasativa_Csa01g035930.1 Length: 315
# Camelinasativa_Csa01g035930.1 Number of predicted TMHs:  0
# Camelinasativa_Csa01g035930.1 Exp number of AAs in TMHs: 0.00033
# Camelinasativa_Csa01g035930.1 Exp number, first 60 AAs:  0
# Camelinasativa_Csa01g035930.1 Total prob of N-in:        0.16579
Camelinasativa_Csa01g035930.1   TMHMM2.0        outside      1   315
```

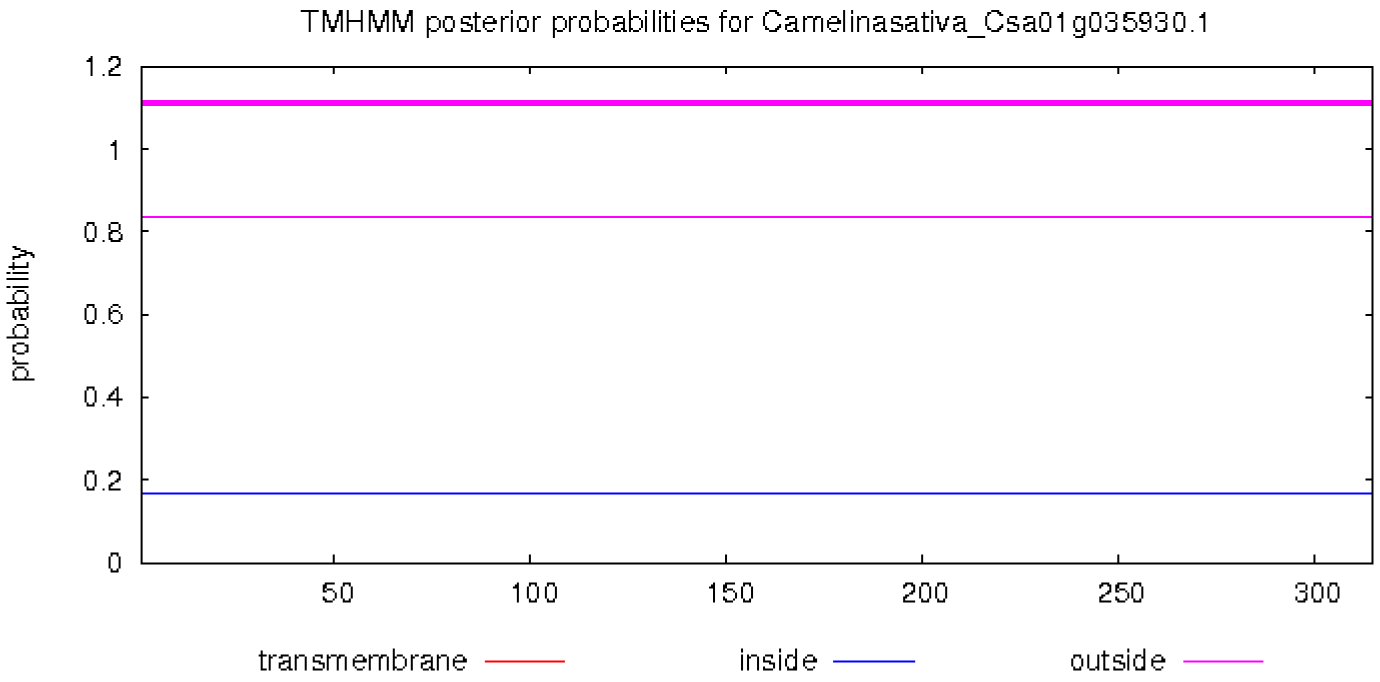

plot in postscript, script for making the plot in gnuplot, data for plot

---

```
# Camelinasativa_Csa01g035940.1 Length: 315
# Camelinasativa_Csa01g035940.1 Number of predicted TMHs:  0
# Camelinasativa_Csa01g035940.1 Exp number of AAs in TMHs: 0.00033
# Camelinasativa_Csa01g035940.1 Exp number, first 60 AAs:  0
# Camelinasativa_Csa01g035940.1 Total prob of N-in:        0.16579
Camelinasativa_Csa01g035940.1   TMHMM2.0        outside      1   315
```

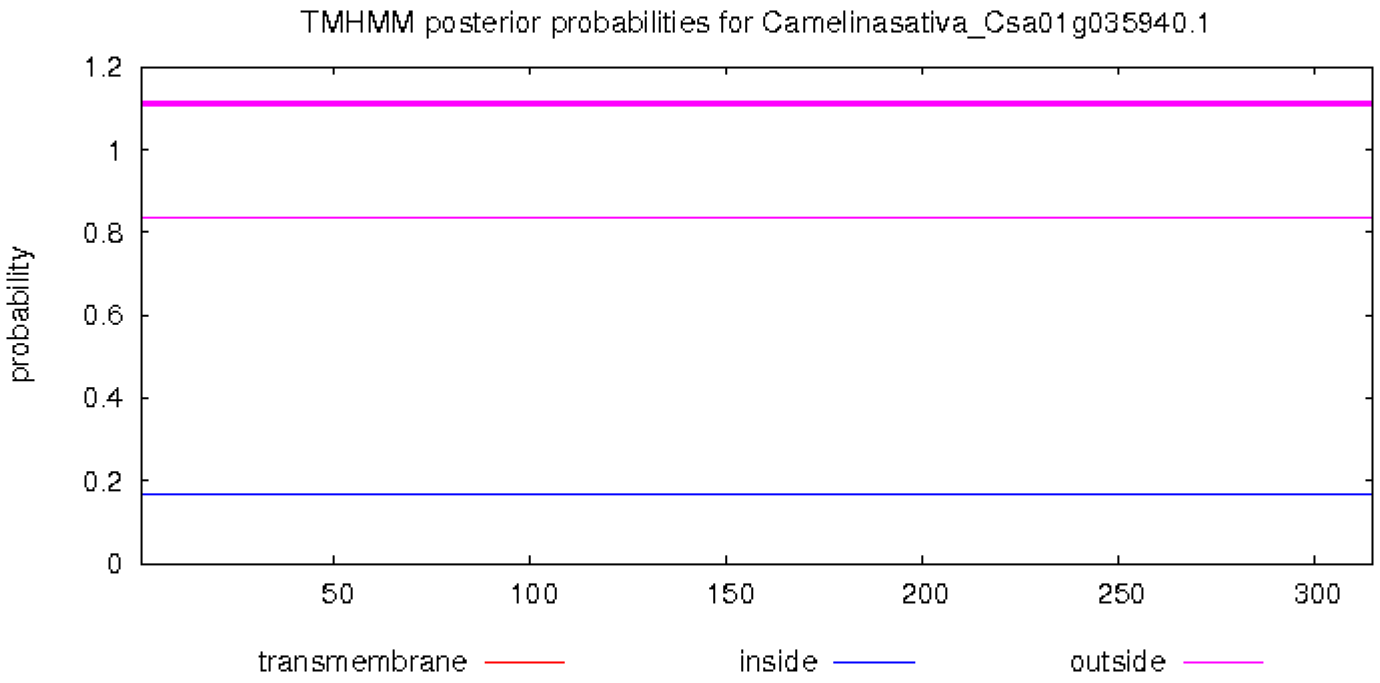

plot in postscript, script for making the plot in gnuplot, data for plot

---

```
# Camelinasativa_Csa02g004650.1 Length: 528
# Camelinasativa_Csa02g004650.1 Number of predicted TMHs:  0
# Camelinasativa_Csa02g004650.1 Exp number of AAs in TMHs: 0.33172
# Camelinasativa_Csa02g004650.1 Exp number, first 60 AAs:  0.00126
# Camelinasativa_Csa02g004650.1 Total prob of N-in:        0.00605
Camelinasativa_Csa02g004650.1   TMHMM2.0        outside      1   528
```

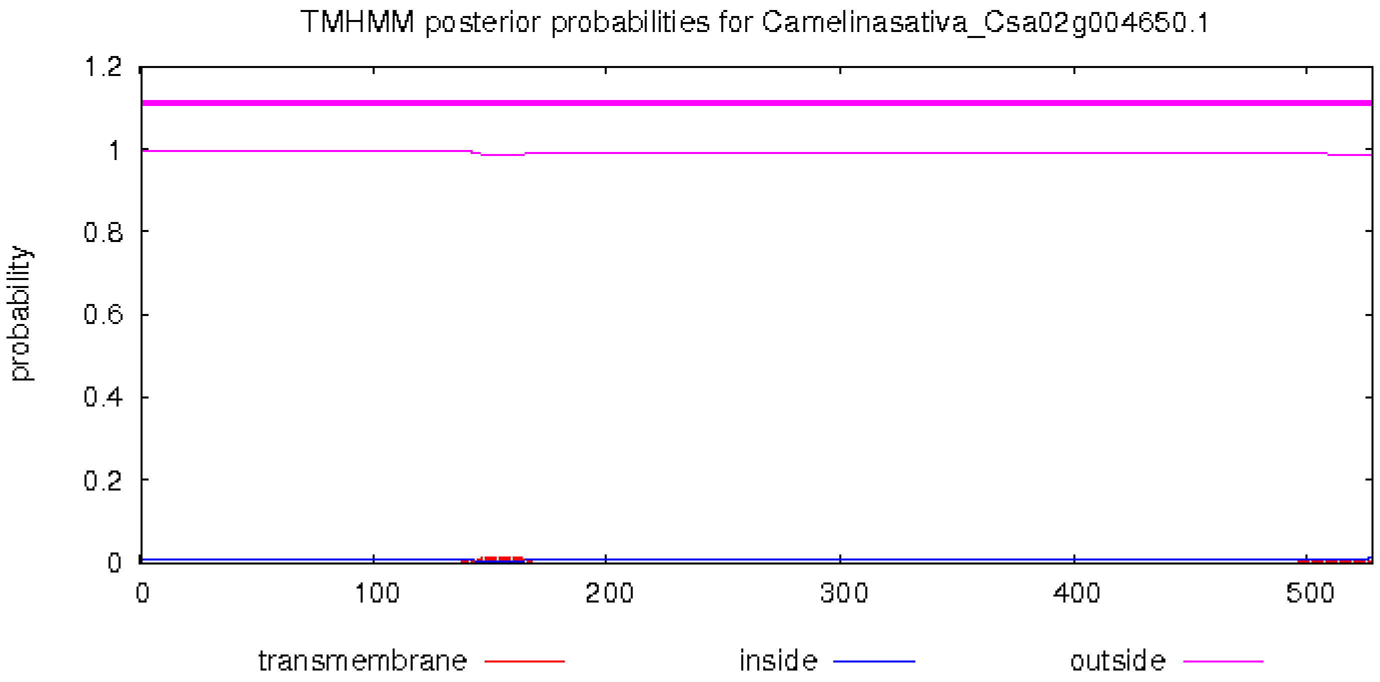

plot in postscript, script for making the plot in gnuplot, data for plot

---

```
# Camelinasativa_Csa02g004670.1 Length: 436
# Camelinasativa_Csa02g004670.1 Number of predicted TMHs:  1
# Camelinasativa_Csa02g004670.1 Exp number of AAs in TMHs: 22.8053
# Camelinasativa_Csa02g004670.1 Exp number, first 60 AAs:  0.00279
# Camelinasativa_Csa02g004670.1 Total prob of N-in:        0.57199
Camelinasativa_Csa02g004670.1   TMHMM2.0        inside       1   407
Camelinasativa_Csa02g004670.1   TMHMM2.0        TMhelix    408   430
Camelinasativa_Csa02g004670.1   TMHMM2.0        outside    431   436
```

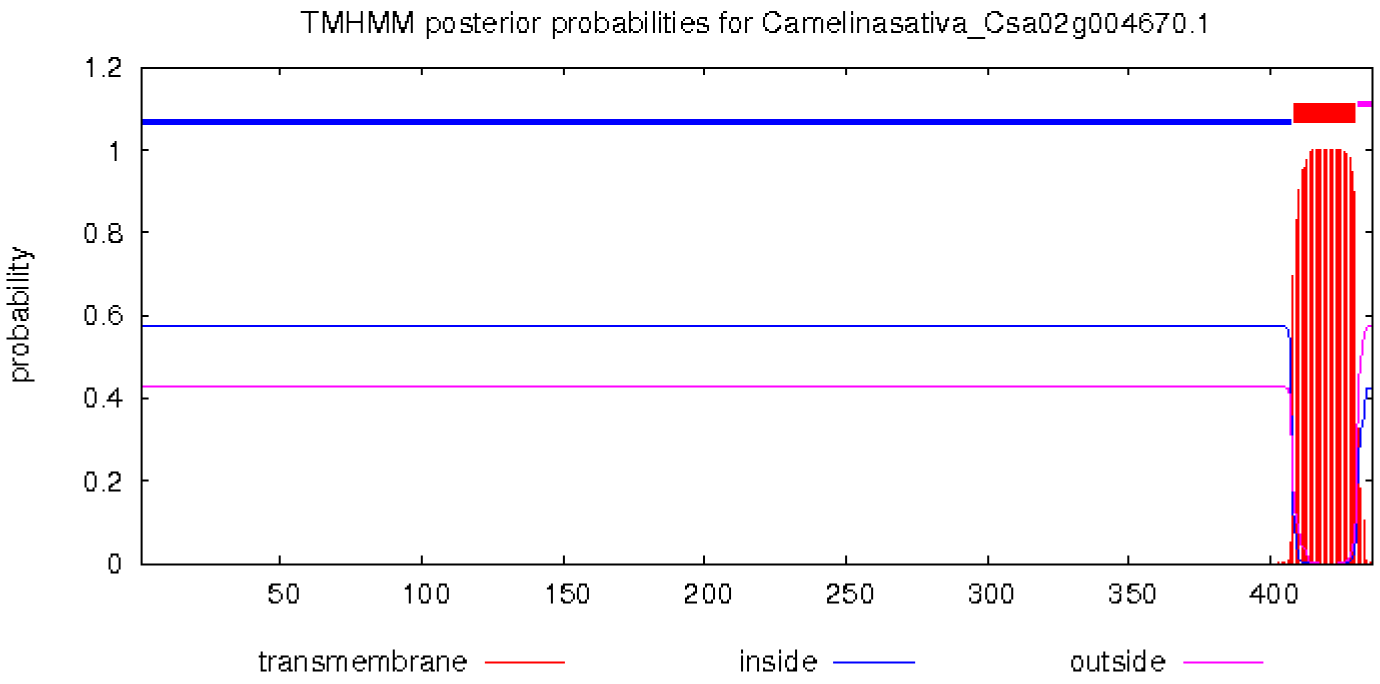

plot in postscript, script for making the plot in gnuplot, data for plot

```
# Camelinasativa_Csa02g004860.1 Length: 525
# Camelinasativa_Csa02g004860.1 Number of predicted TMHs:  0
# Camelinasativa_Csa02g004860.1 Exp number of AAs in TMHs: 0.00093
# Camelinasativa_Csa02g004860.1 Exp number, first 60 AAs:  0.00015
# Camelinasativa_Csa02g004860.1 Total prob of N-in:        0.00364
Camelinasativa_Csa02g004860.1	TMHMM2.0	outside	     1   525
```

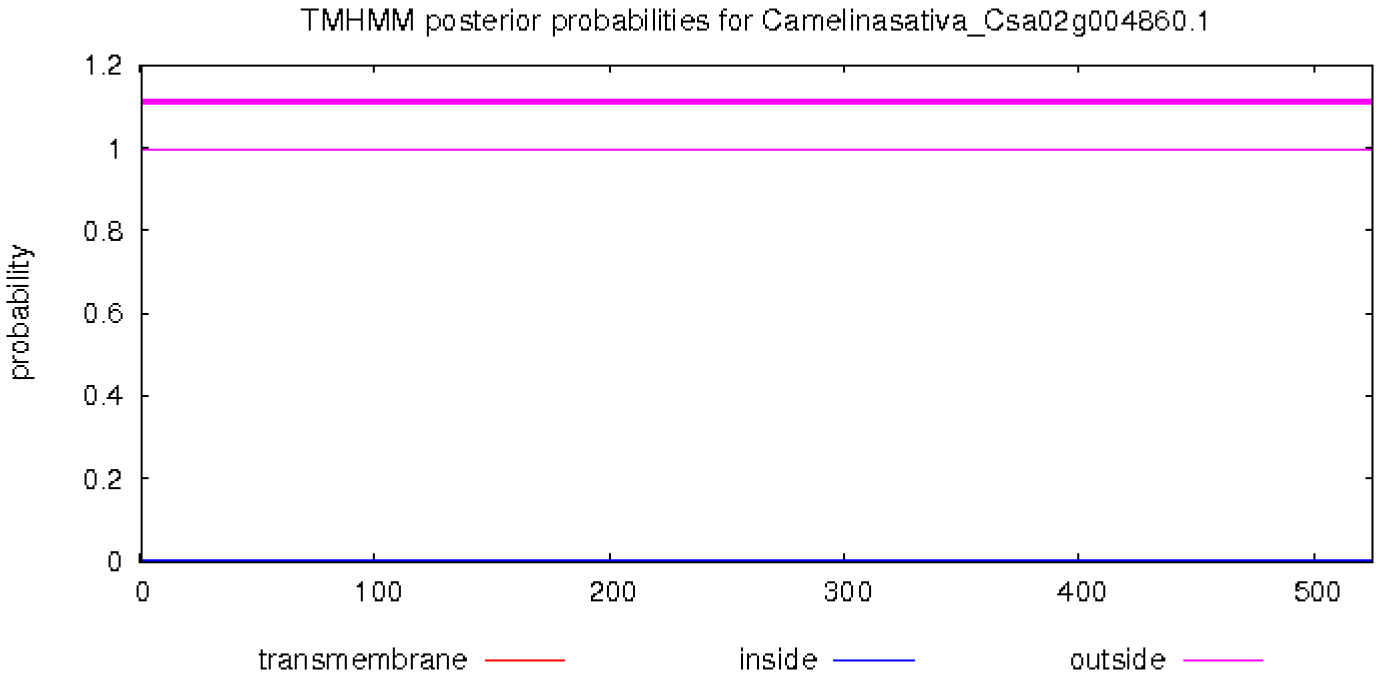

plot in postscript, script for making the plot in gnuplot, data for plot

```
# Camelinasativa_Csa02g011430.1 Length: 454
# Camelinasativa_Csa02g011430.1 Number of predicted TMHs:  0
# Camelinasativa_Csa02g011430.1 Exp number of AAs in TMHs: 0.00307
# Camelinasativa_Csa02g011430.1 Exp number, first 60 AAs:  0
# Camelinasativa_Csa02g011430.1 Total prob of N-in:        0.00197
Camelinasativa_Csa02g011430.1	TMHMM2.0	outside	     1   454
```

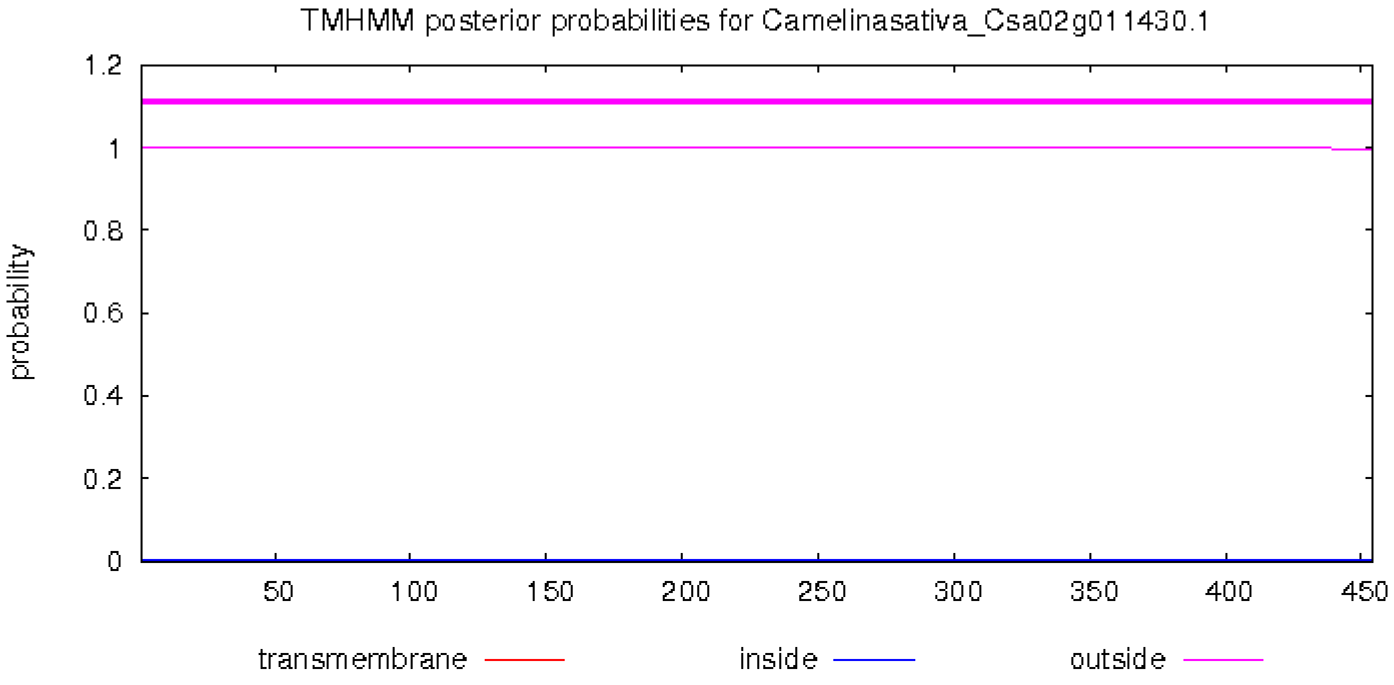

plot in postscript, script for making the plot in gnuplot, data for plot

```
# Camelinasativa_Csa02g026430.1 Length: 340
# Camelinasativa_Csa02g026430.1 Number of predicted TMHs:  0
# Camelinasativa_Csa02g026430.1 Exp number of AAs in TMHs: 0
# Camelinasativa_Csa02g026430.1 Exp number, first 60 AAs:  0
# Camelinasativa_Csa02g026430.1 Total prob of N-in:        0.00442
Camelinasativa_Csa02g026430.1   TMHMM2.0        outside      1   340
```

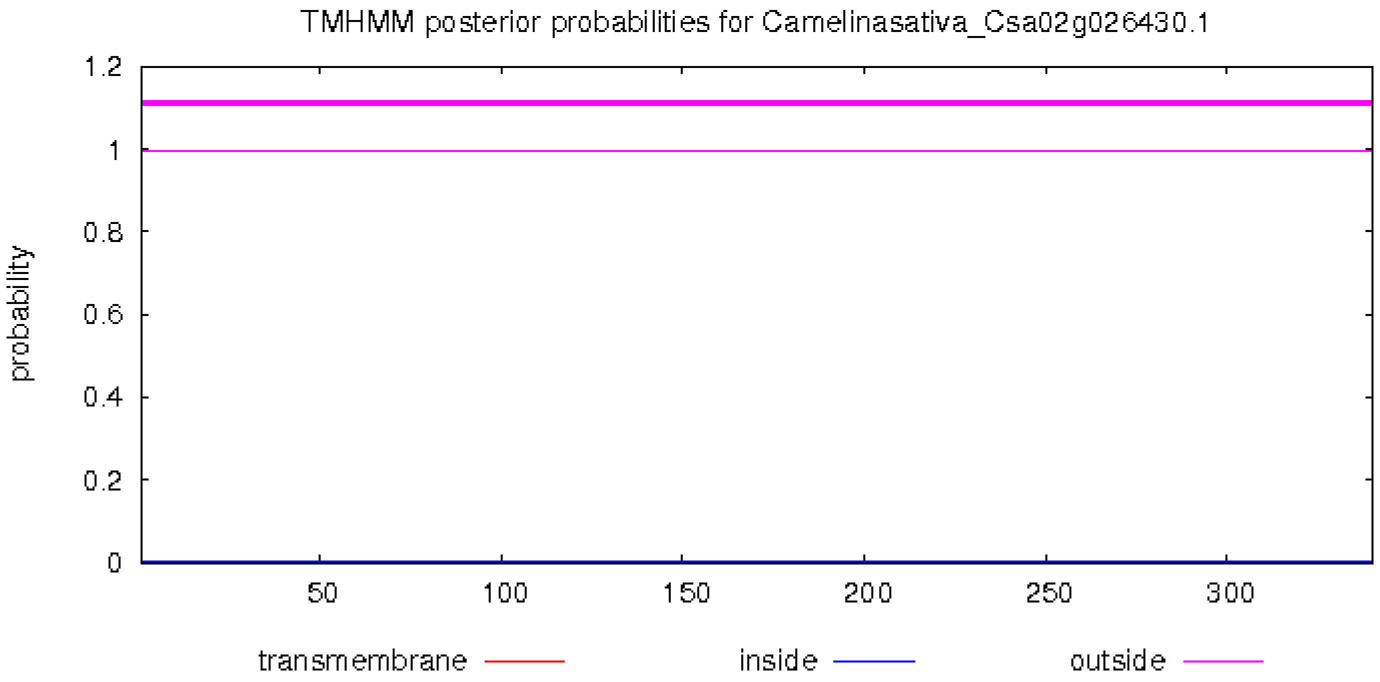

plot in postscript, script for making the plot in gnuplot, data for plot

---

```
# Camelinasativa_Csa02g039410.1 Length: 193
# Camelinasativa_Csa02g039410.1 Number of predicted TMHs:  0
# Camelinasativa_Csa02g039410.1 Exp number of AAs in TMHs: 0.00134
# Camelinasativa_Csa02g039410.1 Exp number, first 60 AAs:  0
# Camelinasativa_Csa02g039410.1 Total prob of N-in:        0.12748
Camelinasativa_Csa02g039410.1   TMHMM2.0        outside      1   193
```

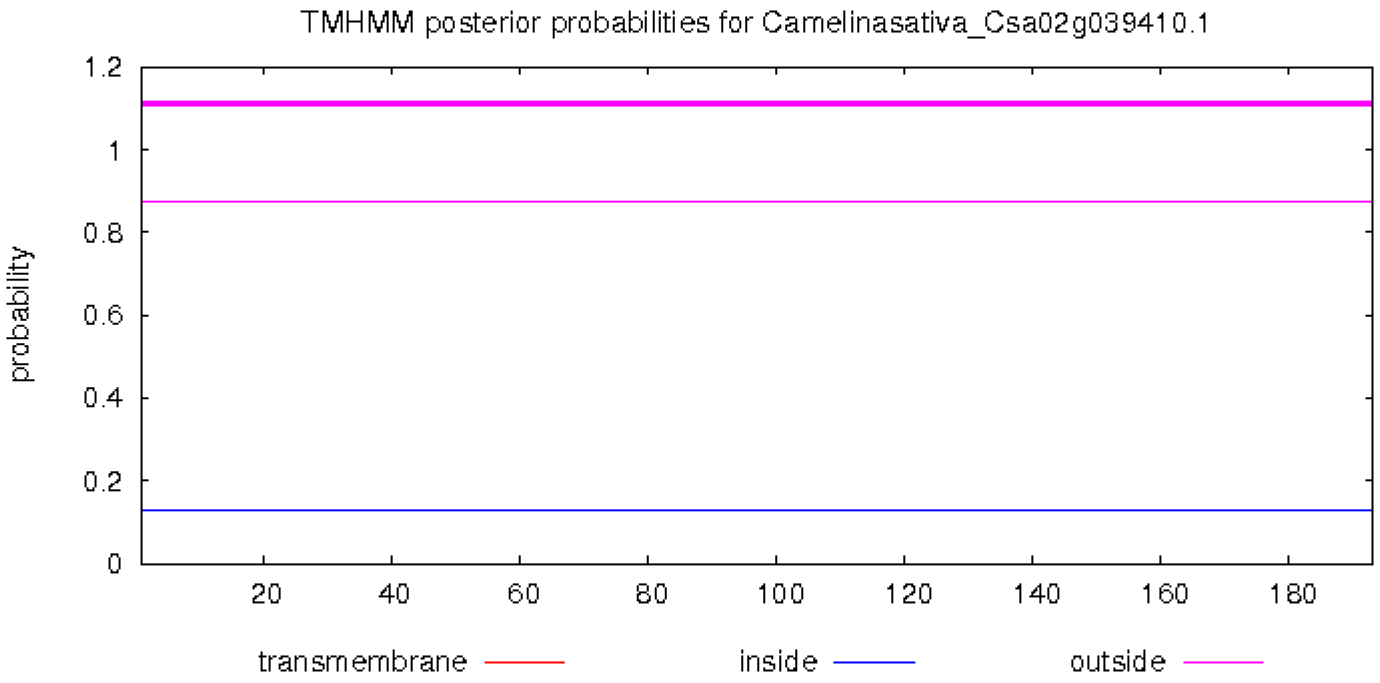

plot in postscript, script for making the plot in gnuplot, data for plot

---

```
# Camelinasativa_Csa02g051570.1 Length: 374
# Camelinasativa_Csa02g051570.1 Number of predicted TMHs:  0
# Camelinasativa_Csa02g051570.1 Exp number of AAs in TMHs: 0.00408
# Camelinasativa_Csa02g051570.1 Exp number, first 60 AAs:  0
# Camelinasativa_Csa02g051570.1 Total prob of N-in:        0.01196
Camelinasativa_Csa02g051570.1   TMHMM2.0        outside      1   374
```

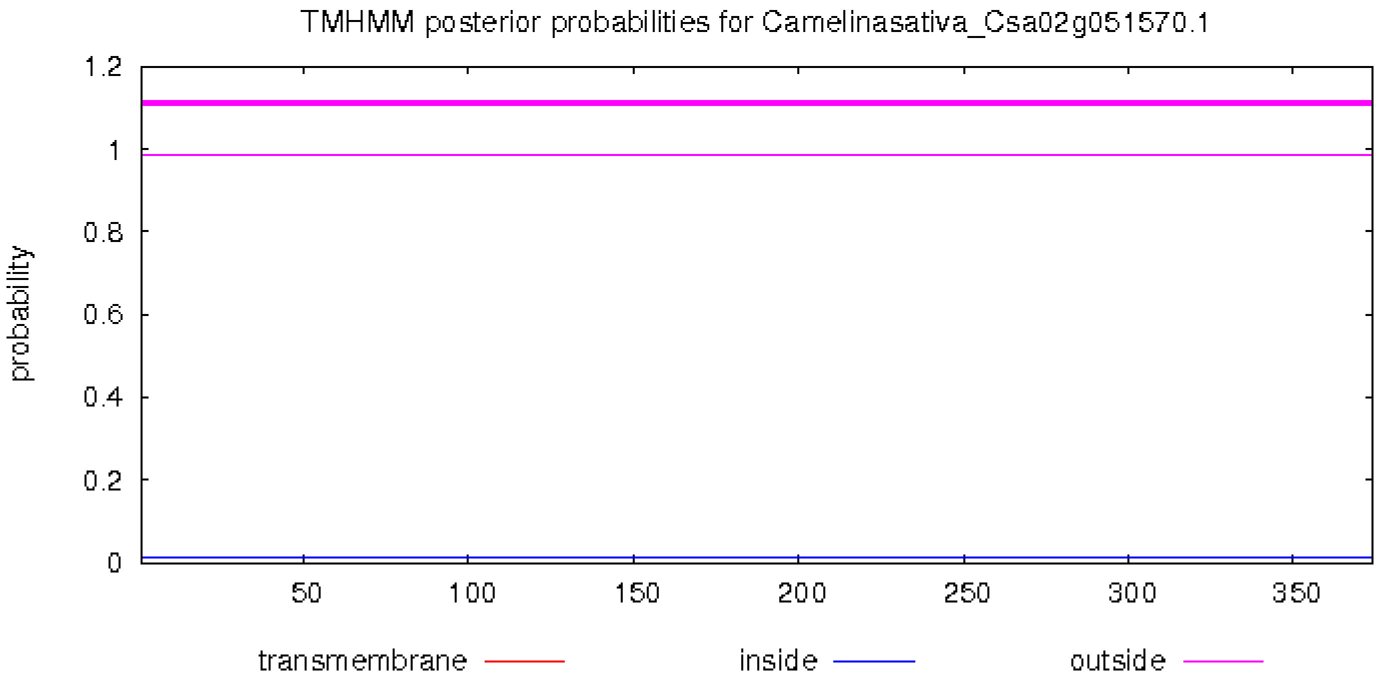

plot in postscript, script for making the plot in gnuplot, data for plot

---

```
# Camelinasativa_Csa02g059890.1 Length: 277
# Camelinasativa_Csa02g059890.1 Number of predicted TMHs:  0
# Camelinasativa_Csa02g059890.1 Exp number of AAs in TMHs: 0.00076
# Camelinasativa_Csa02g059890.1 Exp number, first 60 AAs:  0
# Camelinasativa_Csa02g059890.1 Total prob of N-in:        0.03587
Camelinasativa_Csa02g059890.1   TMHMM2.0        outside      1   277
```

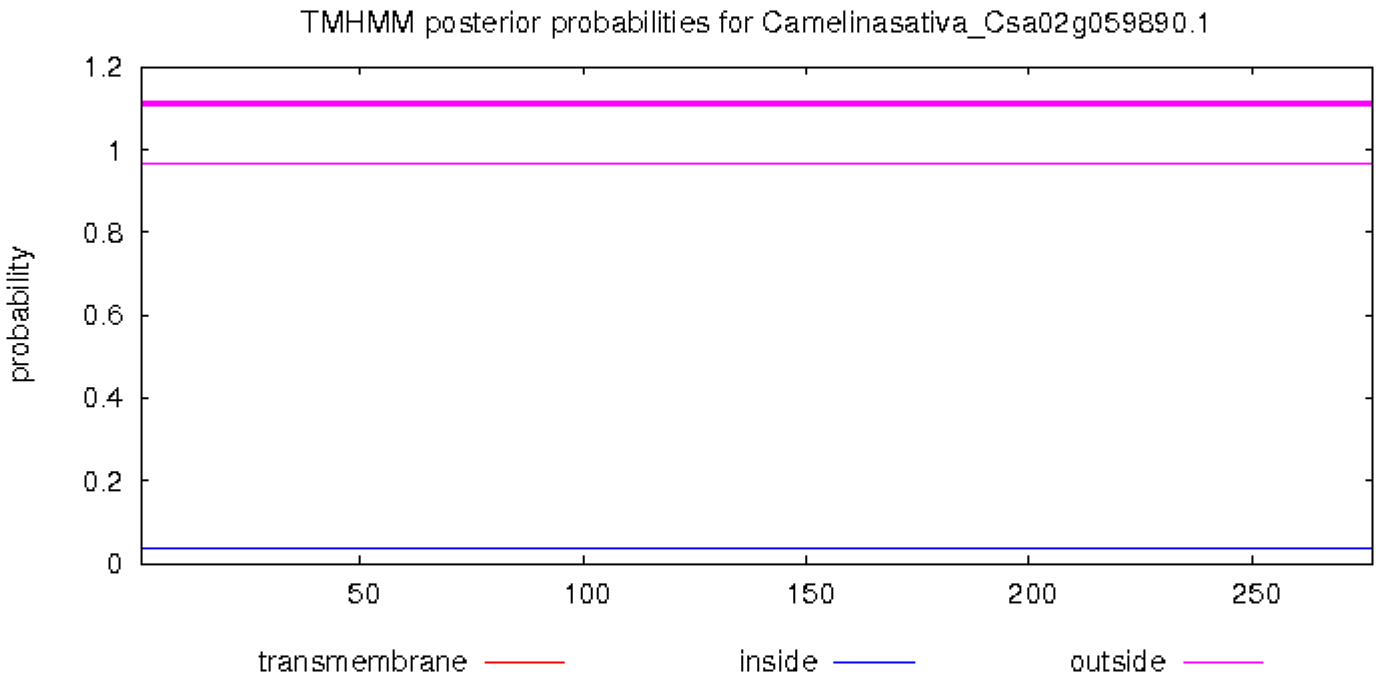

plot in postscript, script for making the plot in gnuplot, data for plot

---

```
# Camelinasativa_Csa02g064730.1 Length: 307
# Camelinasativa_Csa02g064730.1 Number of predicted TMHs:  0
# Camelinasativa_Csa02g064730.1 Exp number of AAs in TMHs: 0
# Camelinasativa_Csa02g064730.1 Exp number, first 60 AAs:  0
# Camelinasativa_Csa02g064730.1 Total prob of N-in:        0.06662
Camelinasativa_Csa02g064730.1   TMHMM2.0        outside      1   307
```

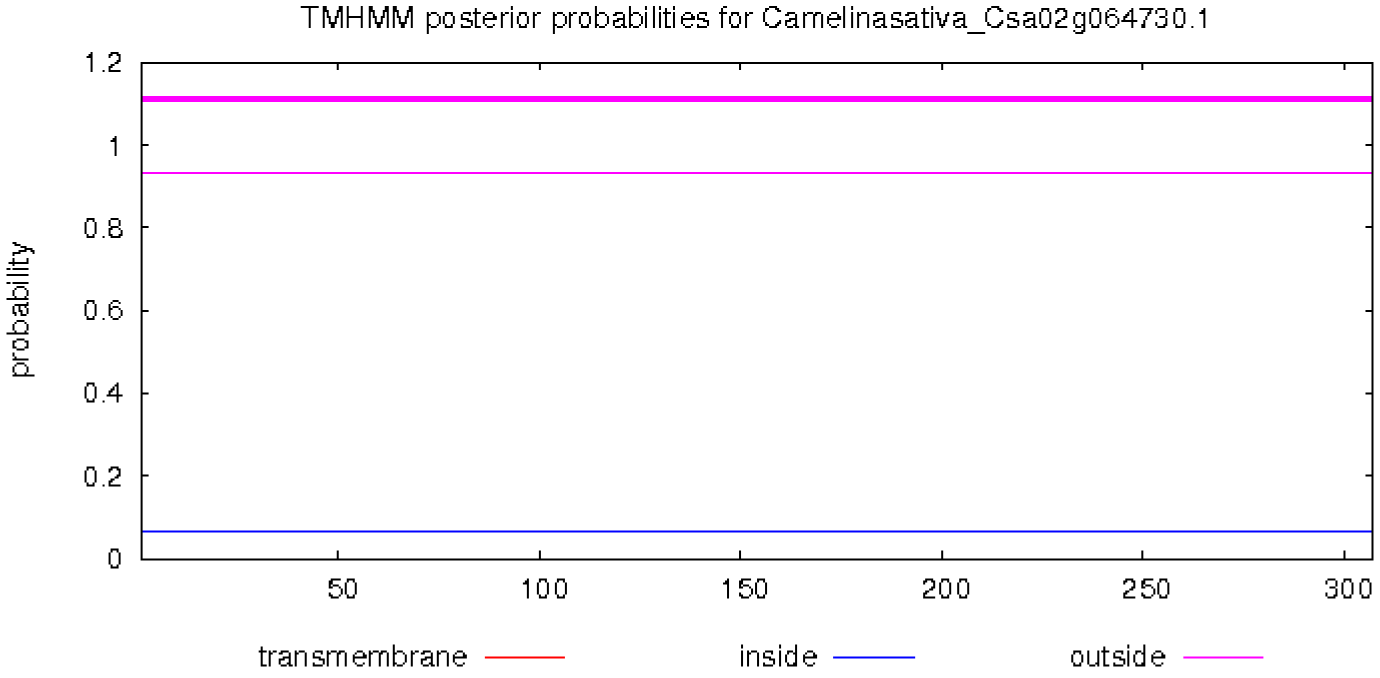

plot in postscript, script for making the plot in gnuplot, data for plot

---

```
# Camelinasativa_Csa02g064950.1 Length: 308
# Camelinasativa_Csa02g064950.1 Number of predicted TMHs:  0
# Camelinasativa_Csa02g064950.1 Exp number of AAs in TMHs: 0
# Camelinasativa_Csa02g064950.1 Exp number, first 60 AAs:  0
# Camelinasativa_Csa02g064950.1 Total prob of N-in:        0.06617
Camelinasativa_Csa02g064950.1   TMHMM2.0        outside      1   308
```

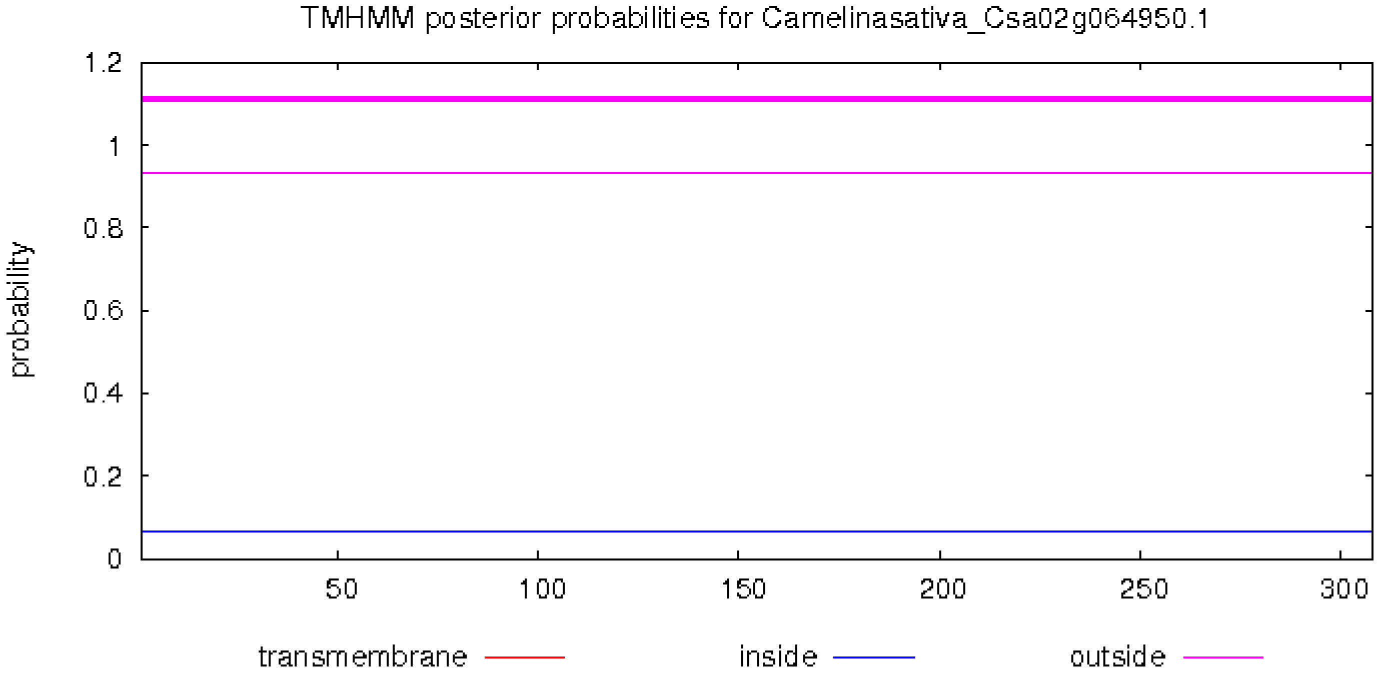

plot in postscript, script for making the plot in gnuplot, data for plot

---

```
# Camelinasativa_Csa02g068390.1 Length: 339
# Camelinasativa_Csa02g068390.1 Number of predicted TMHs:  0
# Camelinasativa_Csa02g068390.1 Exp number of AAs in TMHs: 0.00028
# Camelinasativa_Csa02g068390.1 Exp number, first 60 AAs:  0
# Camelinasativa_Csa02g068390.1 Total prob of N-in:        0.01193
Camelinasativa_Csa02g068390.1   TMHMM2.0        outside      1   339
```

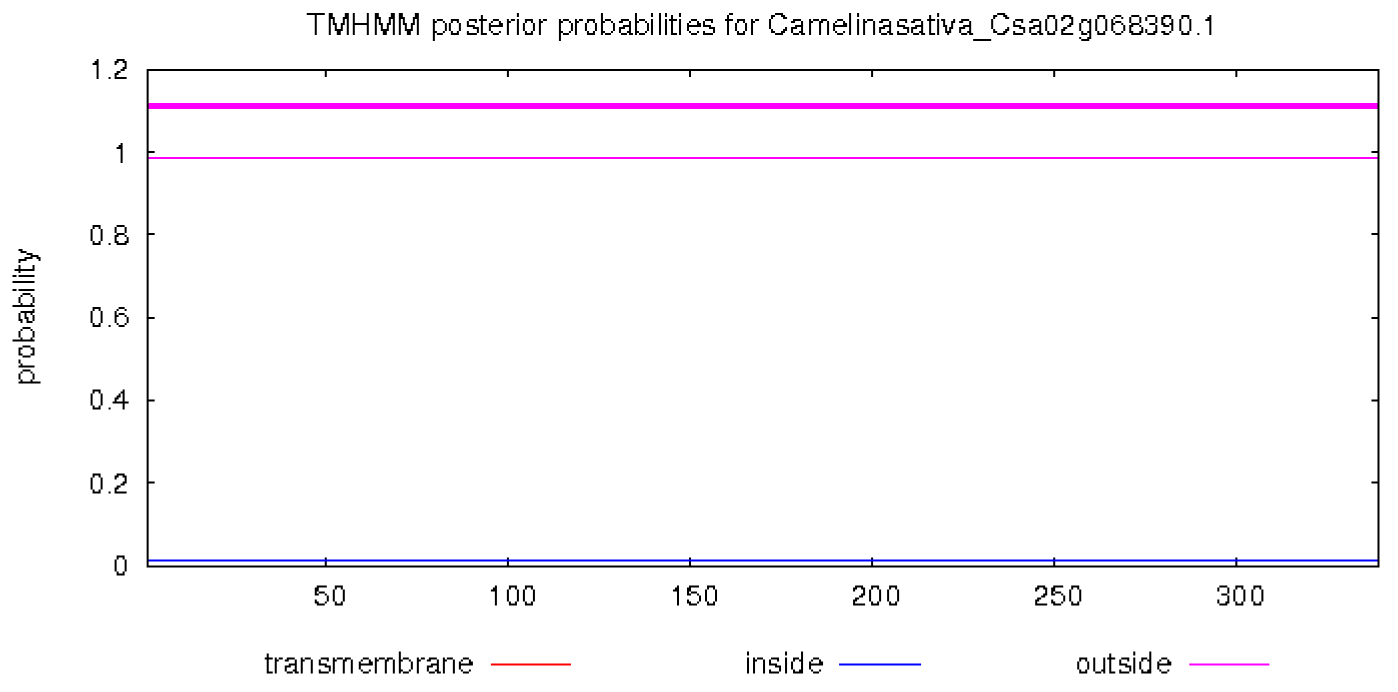

plot in postscript, script for making the plot in gnuplot, data for plot

```
# Camelinasativa_Csa02g070520.1 Length: 344
# Camelinasativa_Csa02g070520.1 Number of predicted TMHs:  0
# Camelinasativa_Csa02g070520.1 Exp number of AAs in TMHs: 0.0008
# Camelinasativa_Csa02g070520.1 Exp number, first 60 AAs:  0
# Camelinasativa_Csa02g070520.1 Total prob of N-in:        0.00190
Camelinasativa_Csa02g070520.1   TMHMM2.0        outside      1   344
```

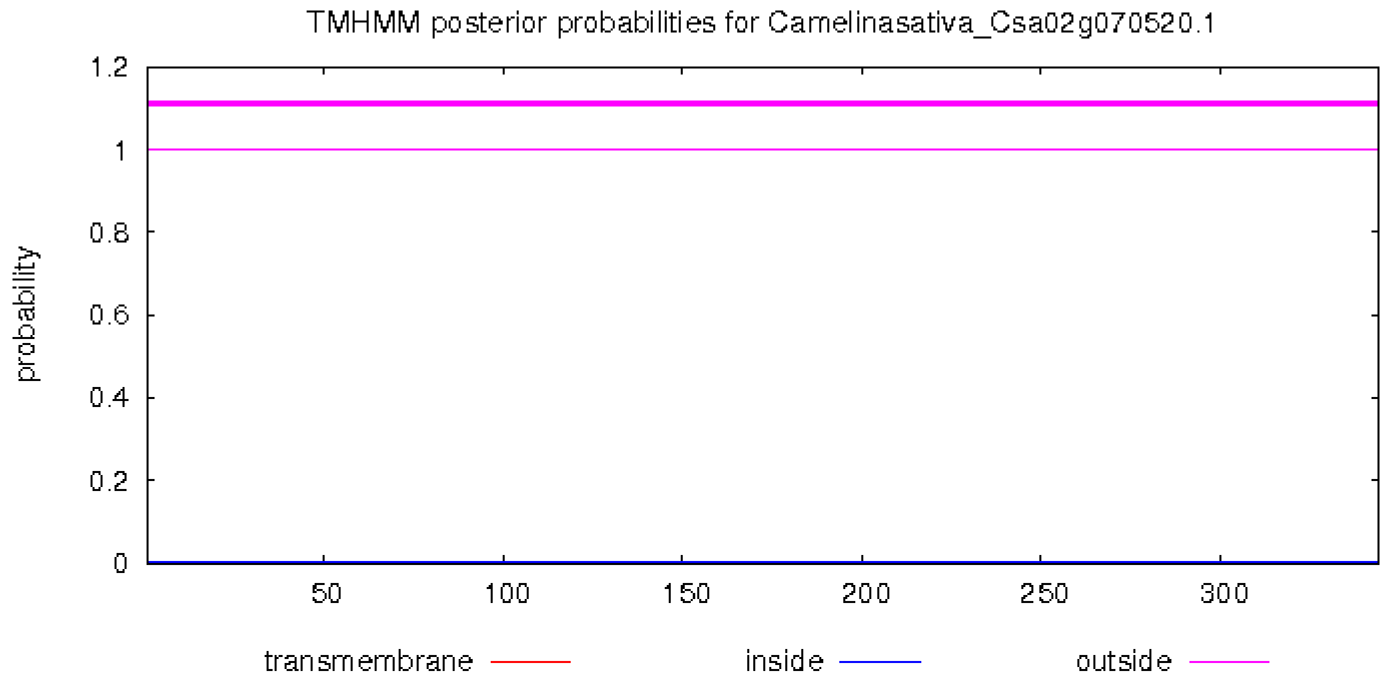

plot in postscript, script for making the plot in gnuplot, data for plot

```
# Camelinasativa_Csa02g072480.1 Length: 283
# Camelinasativa_Csa02g072480.1 Number of predicted TMHs:  0
# Camelinasativa_Csa02g072480.1 Exp number of AAs in TMHs: 0.00031
# Camelinasativa_Csa02g072480.1 Exp number, first 60 AAs:  0
# Camelinasativa_Csa02g072480.1 Total prob of N-in:        0.01021
Camelinasativa_Csa02g072480.1	TMHMM2.0	outside	     1   283
```

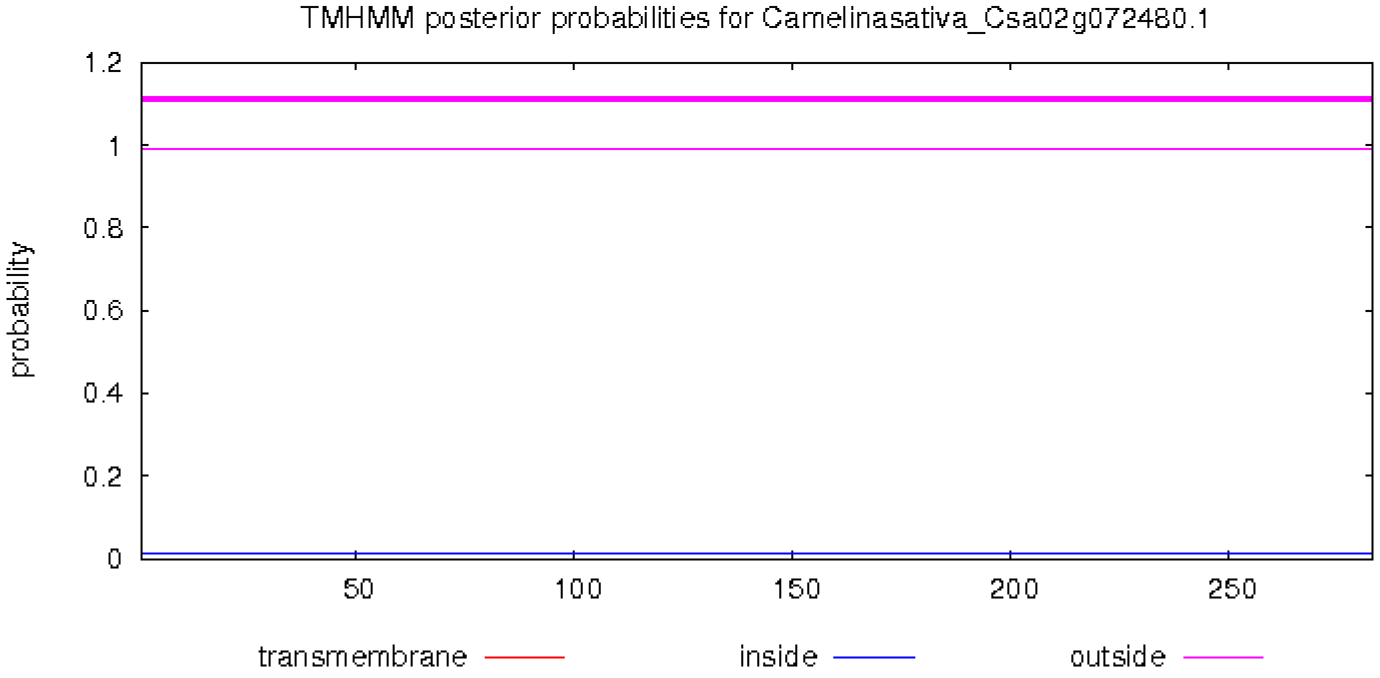

plot in postscript, script for making the plot in gnuplot, data for plot

---

```
# Camelinasativa_Csa02g072750.1 Length: 367
# Camelinasativa_Csa02g072750.1 Number of predicted TMHs:  0
# Camelinasativa_Csa02g072750.1 Exp number of AAs in TMHs: 0.00037
# Camelinasativa_Csa02g072750.1 Exp number, first 60 AAs:  0.00018
# Camelinasativa_Csa02g072750.1 Total prob of N-in:        0.00520
Camelinasativa_Csa02g072750.1	TMHMM2.0	outside	     1   367
```

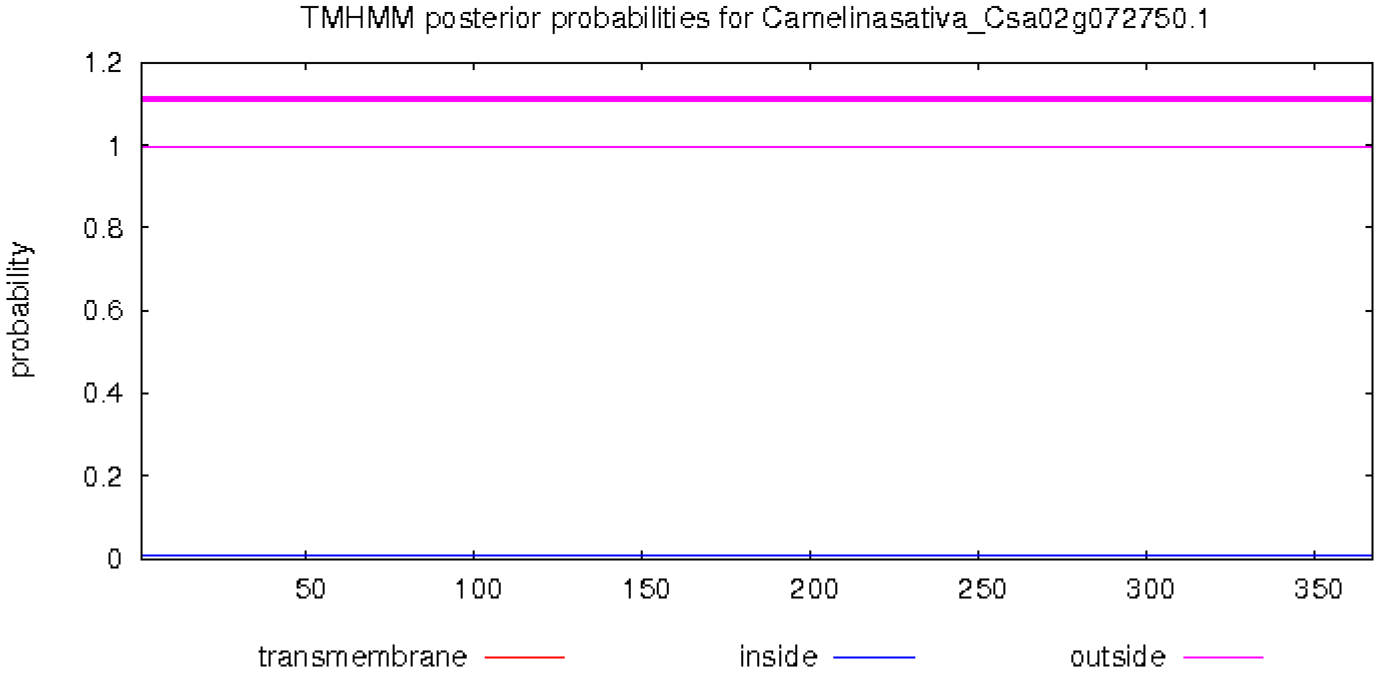

plot in postscript, script for making the plot in gnuplot, data for plot

---

```
# Camelinasativa_Csa02g073300.1 Length: 233
# Camelinasativa_Csa02g073300.1 Number of predicted TMHs:  0
# Camelinasativa_Csa02g073300.1 Exp number of AAs in TMHs: 0.00345
# Camelinasativa_Csa02g073300.1 Exp number, first 60 AAs:  0.00345
# Camelinasativa_Csa02g073300.1 Total prob of N-in:        0.00656
Camelinasativa_Csa02g073300.1	TMHMM2.0	outside	     1   233
```

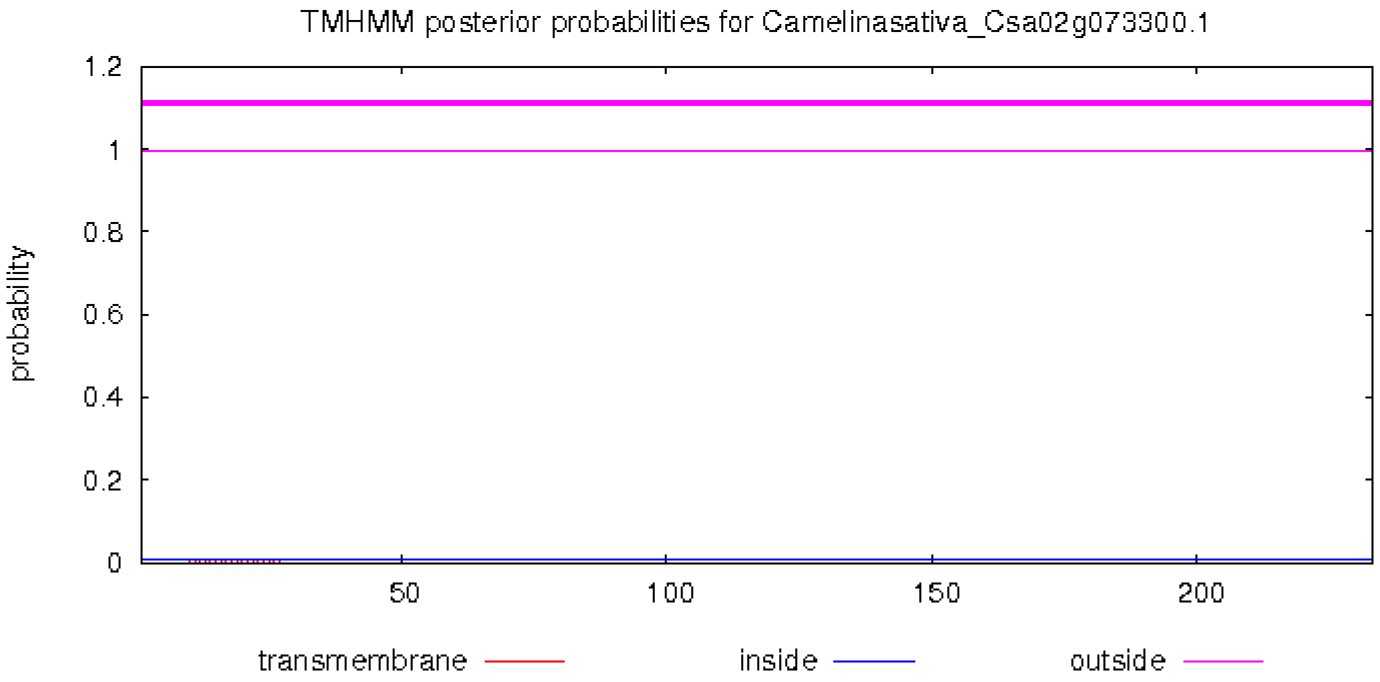

plot in postscript, script for making the plot in gnuplot, data for plot

```
# Camelinasativa_Csa02g076500.1 Length: 296
# Camelinasativa_Csa02g076500.1 Number of predicted TMHs:  0
# Camelinasativa_Csa02g076500.1 Exp number of AAs in TMHs: 0.00085
# Camelinasativa_Csa02g076500.1 Exp number, first 60 AAs:  0
# Camelinasativa_Csa02g076500.1 Total prob of N-in:        0.07311
Camelinasativa_Csa02g076500.1	TMHMM2.0	outside	     1   296
```

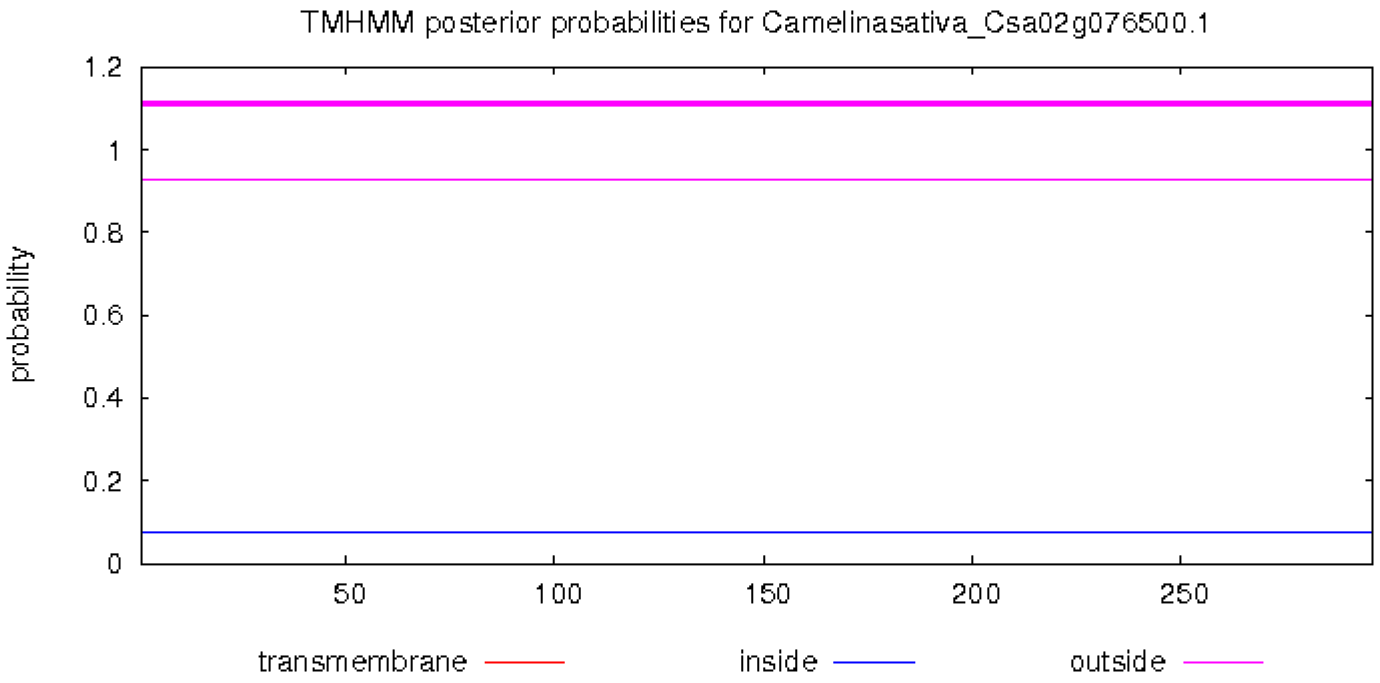

plot in postscript, script for making the plot in gnuplot, data for plot

```
# Camelinasativa_Csa03g002220.1 Length: 954
# Camelinasativa_Csa03g002220.1 Number of predicted TMHs:  1
# Camelinasativa_Csa03g002220.1 Exp number of AAs in TMHs: 30.44178
# Camelinasativa_Csa03g002220.1 Exp number, first 60 AAs:  3e-05
# Camelinasativa_Csa03g002220.1 Total prob of N-in:        0.23858
Camelinasativa_Csa03g002220.1   TMHMM2.0        outside      1   930
Camelinasativa_Csa03g002220.1   TMHMM2.0        TMhelix    931   953
Camelinasativa_Csa03g002220.1   TMHMM2.0        inside     954   954
```

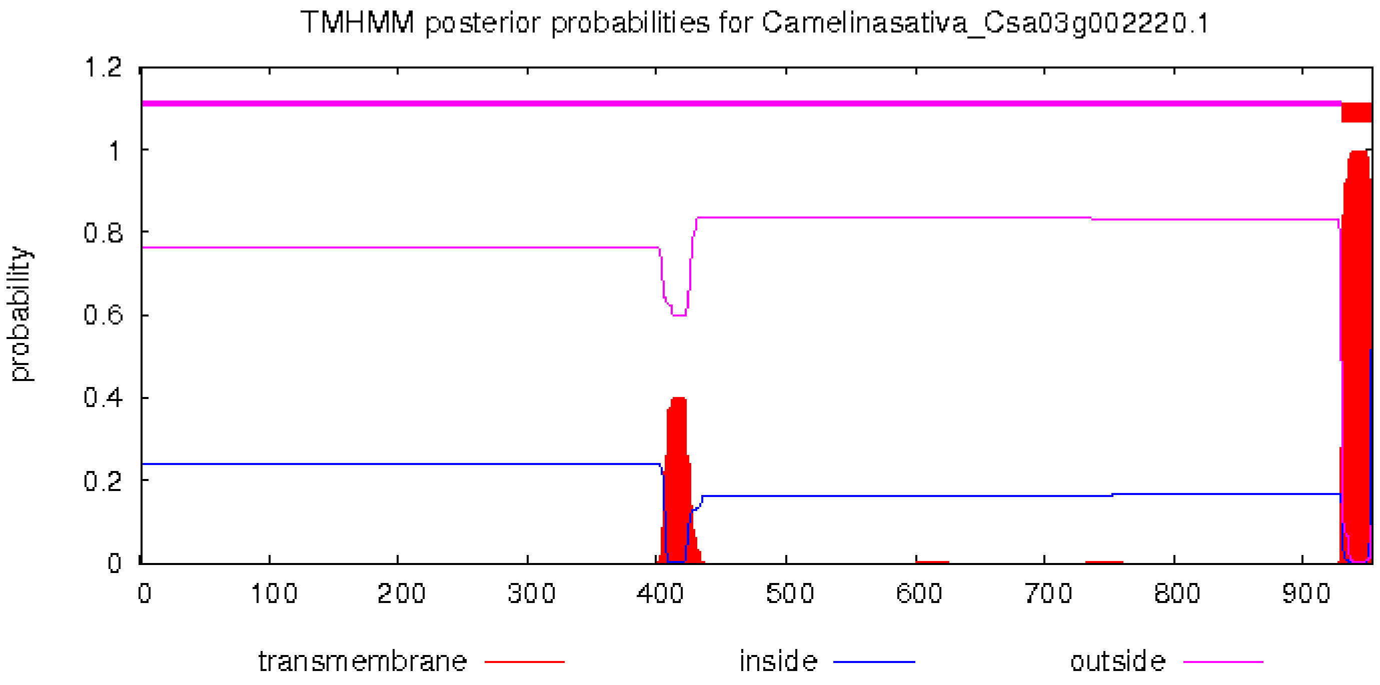

plot in postscript, script for making the plot in gnuplot, data for plot

---

```
# Camelinasativa_Csa03g002230.1 Length: 557
# Camelinasativa_Csa03g002230.1 Number of predicted TMHs:  0
# Camelinasativa_Csa03g002230.1 Exp number of AAs in TMHs: 0.00074
# Camelinasativa_Csa03g002230.1 Exp number, first 60 AAs:  0
# Camelinasativa_Csa03g002230.1 Total prob of N-in:        0.00054
Camelinasativa_Csa03g002230.1   TMHMM2.0        outside      1   557
```

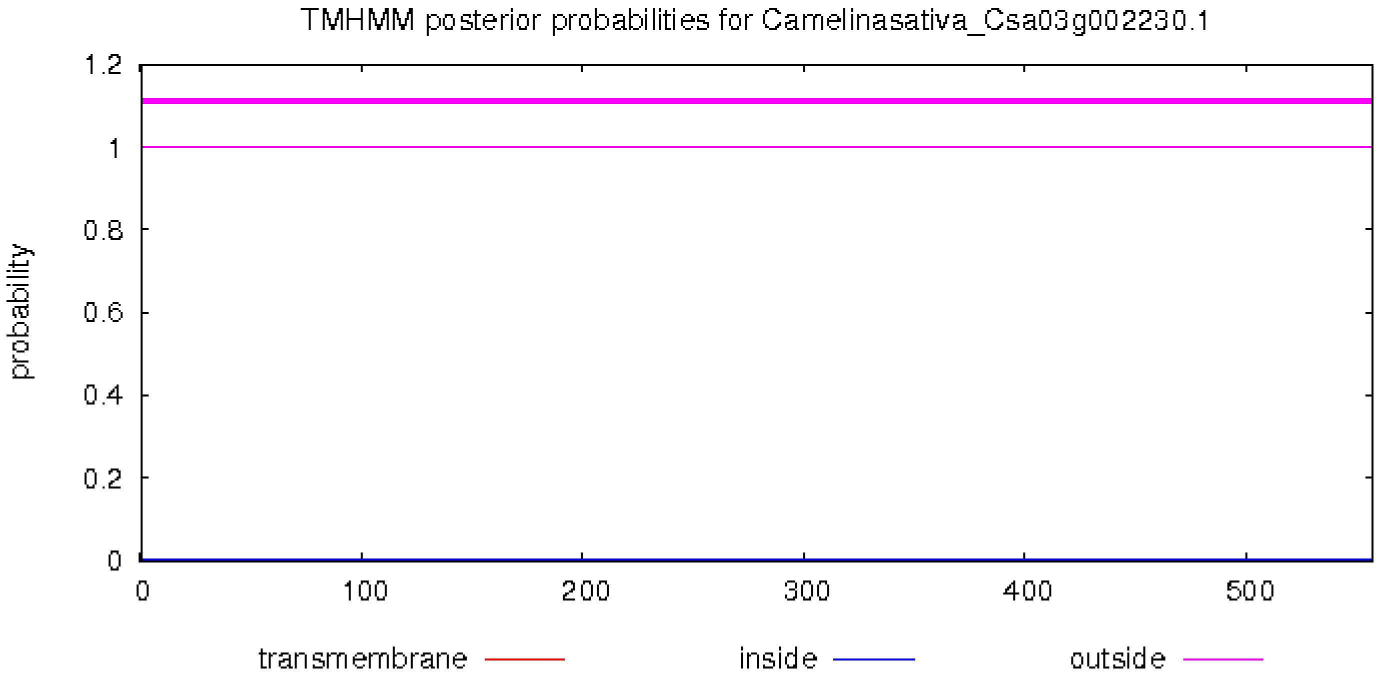

plot in postscript, script for making the plot in gnuplot, data for plot

```
# Camelinasativa_Csa03g002250.1 Length: 694
# Camelinasativa_Csa03g002250.1 Number of predicted TMHs:  0
# Camelinasativa_Csa03g002250.1 Exp number of AAs in TMHs: 0.01034
# Camelinasativa_Csa03g002250.1 Exp number, first 60 AAs:  0.00377
# Camelinasativa_Csa03g002250.1 Total prob of N-in:        0.00049
Camelinasativa_Csa03g002250.1	TMHMM2.0	outside	     1   694
```

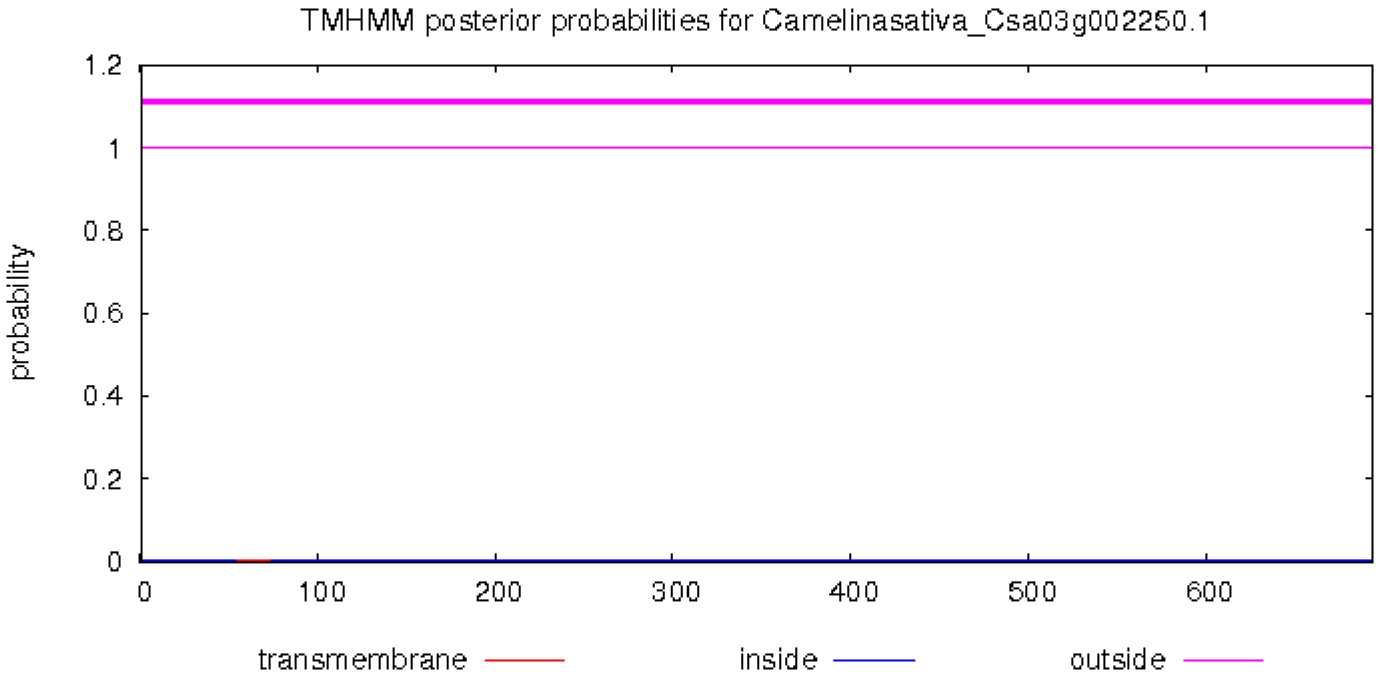

plot in postscript, script for making the plot in gnuplot, data for plot

```
# Camelinasativa_Csa03g002570.1 Length: 729
# Camelinasativa_Csa03g002570.1 Number of predicted TMHs:  0
# Camelinasativa_Csa03g002570.1 Exp number of AAs in TMHs: 0.0037
# Camelinasativa_Csa03g002570.1 Exp number, first 60 AAs:  0
# Camelinasativa_Csa03g002570.1 Total prob of N-in:        0.00031
Camelinasativa_Csa03g002570.1	TMHMM2.0	outside	     1   729
```

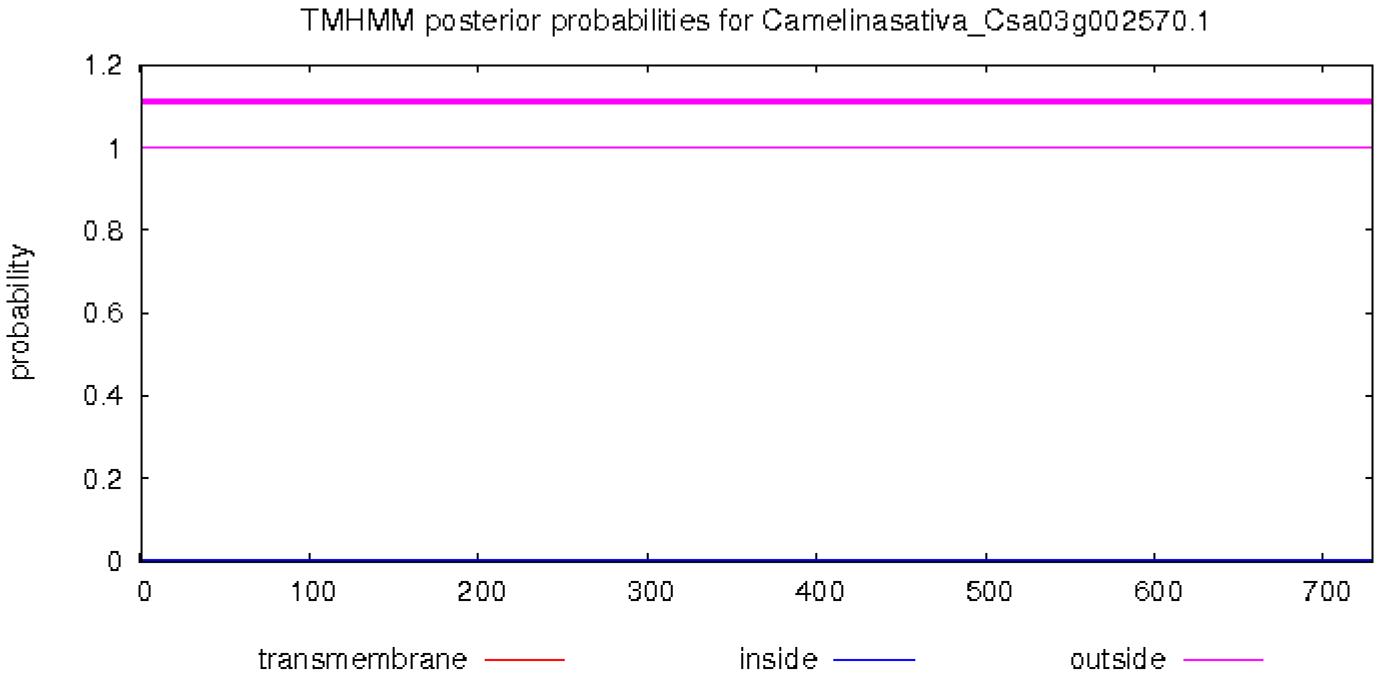

plot in postscript, script for making the plot in gnuplot, data for plot

```
# Camelinasativa_Csa03g004610.1 Length: 301
# Camelinasativa_Csa03g004610.1 Number of predicted TMHs:  0
# Camelinasativa_Csa03g004610.1 Exp number of AAs in TMHs: 0.00235
# Camelinasativa_Csa03g004610.1 Exp number, first 60 AAs:  0.00217
# Camelinasativa_Csa03g004610.1 Total prob of N-in:        0.01126
Camelinasativa_Csa03g004610.1	TMHMM2.0	outside	     1   301
```

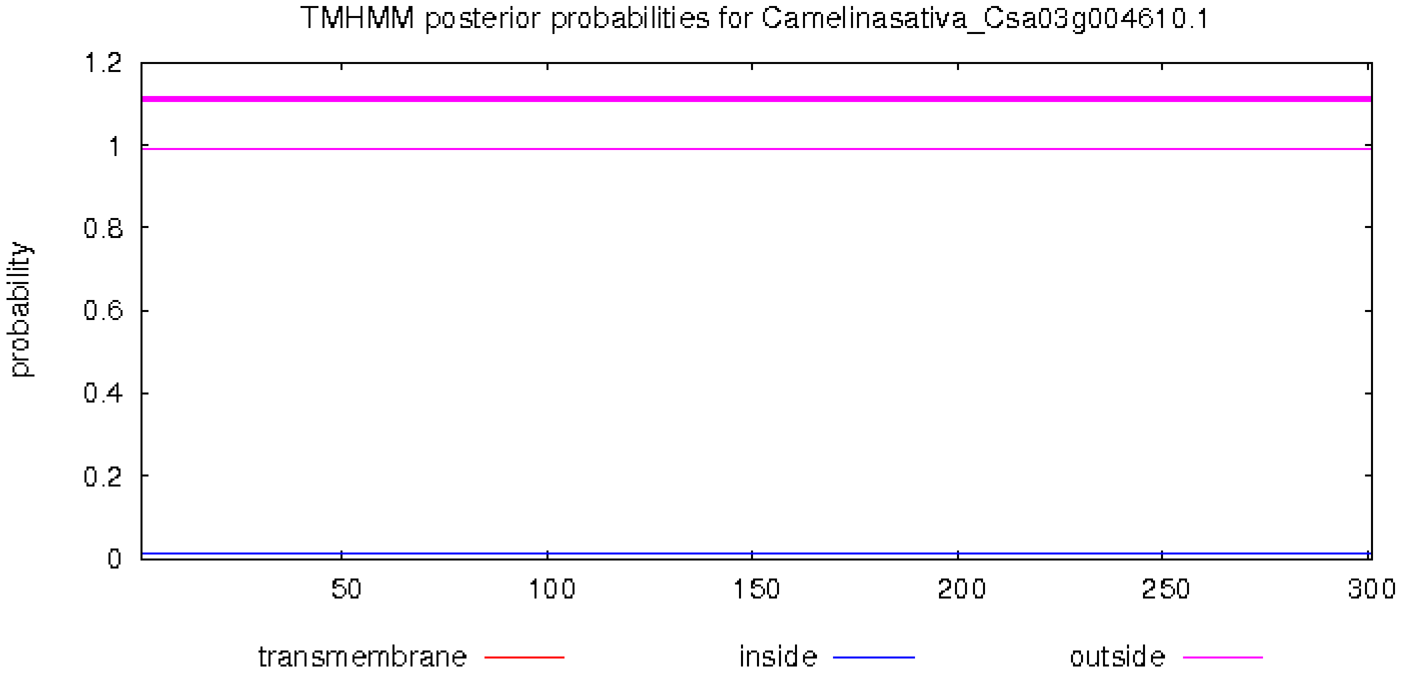

plot in postscript, script for making the plot in gnuplot, data for plot

---

```
# Camelinasativa_Csa03g016020.1 Length: 395
# Camelinasativa_Csa03g016020.1 Number of predicted TMHs:  0
# Camelinasativa_Csa03g016020.1 Exp number of AAs in TMHs: 0.00018
# Camelinasativa_Csa03g016020.1 Exp number, first 60 AAs:  0
# Camelinasativa_Csa03g016020.1 Total prob of N-in:        0.00167
Camelinasativa_Csa03g016020.1	TMHMM2.0	outside	     1   395
```

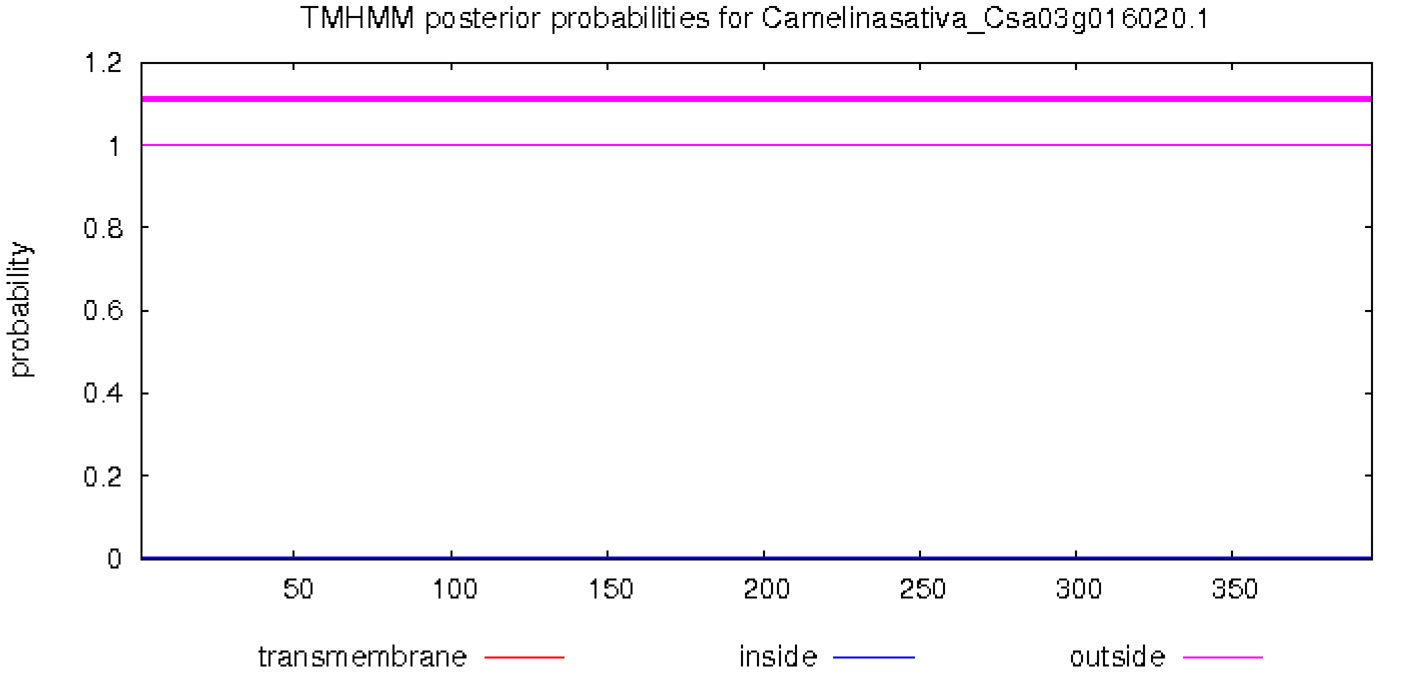

plot in postscript, script for making the plot in gnuplot, data for plot

---

```
# Camelinasativa_Csa03g028620.1 Length: 422
# Camelinasativa_Csa03g028620.1 Number of predicted TMHs:  0
# Camelinasativa_Csa03g028620.1 Exp number of AAs in TMHs: 0.0004
# Camelinasativa_Csa03g028620.1 Exp number, first 60 AAs:  0
# Camelinasativa_Csa03g028620.1 Total prob of N-in:        0.01091
Camelinasativa_Csa03g028620.1   TMHMM2.0        outside      1   422
```

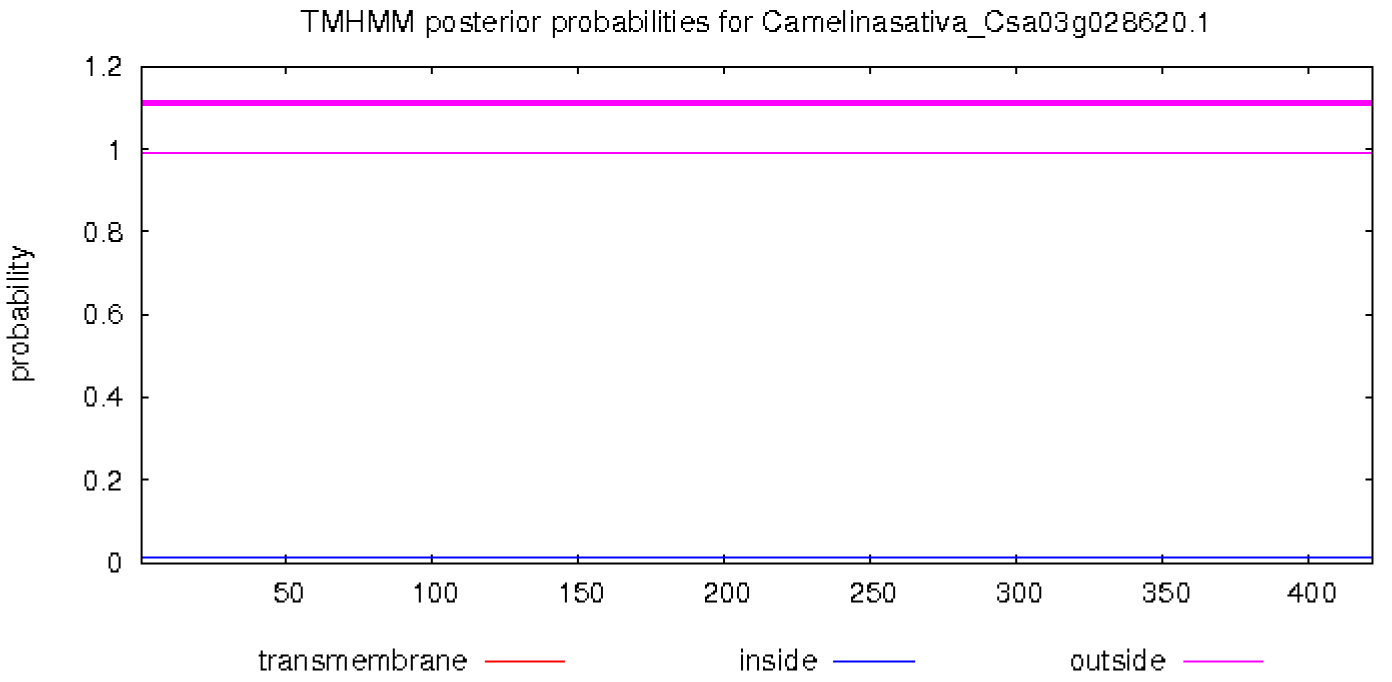

plot in postscript, script for making the plot in gnuplot, data for plot

```
# Camelinasativa_Csa03g029490.1 Length: 457
# Camelinasativa_Csa03g029490.1 Number of predicted TMHs:  0
# Camelinasativa_Csa03g029490.1 Exp number of AAs in TMHs: 0.0002
# Camelinasativa_Csa03g029490.1 Exp number, first 60 AAs:  0
# Camelinasativa_Csa03g029490.1 Total prob of N-in:        0.00272
Camelinasativa_Csa03g029490.1   TMHMM2.0        outside      1   457
```

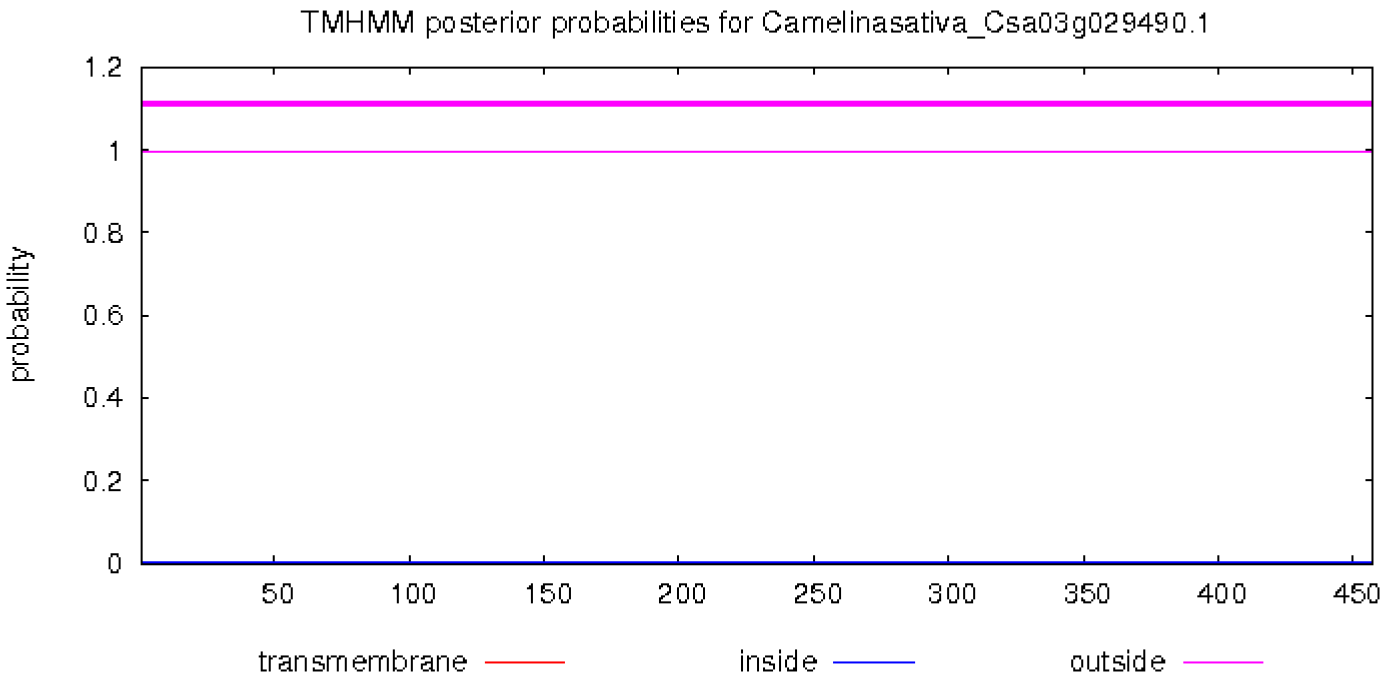

plot in postscript, script for making the plot in gnuplot, data for plot

```
# Camelinasativa_Csa03g031810.1 Length: 266
# Camelinasativa_Csa03g031810.1 Number of predicted TMHs:  0
# Camelinasativa_Csa03g031810.1 Exp number of AAs in TMHs: 0.00056
# Camelinasativa_Csa03g031810.1 Exp number, first 60 AAs:  0
# Camelinasativa_Csa03g031810.1 Total prob of N-in:        0.07984
Camelinasativa_Csa03g031810.1   TMHMM2.0        outside      1   266
```

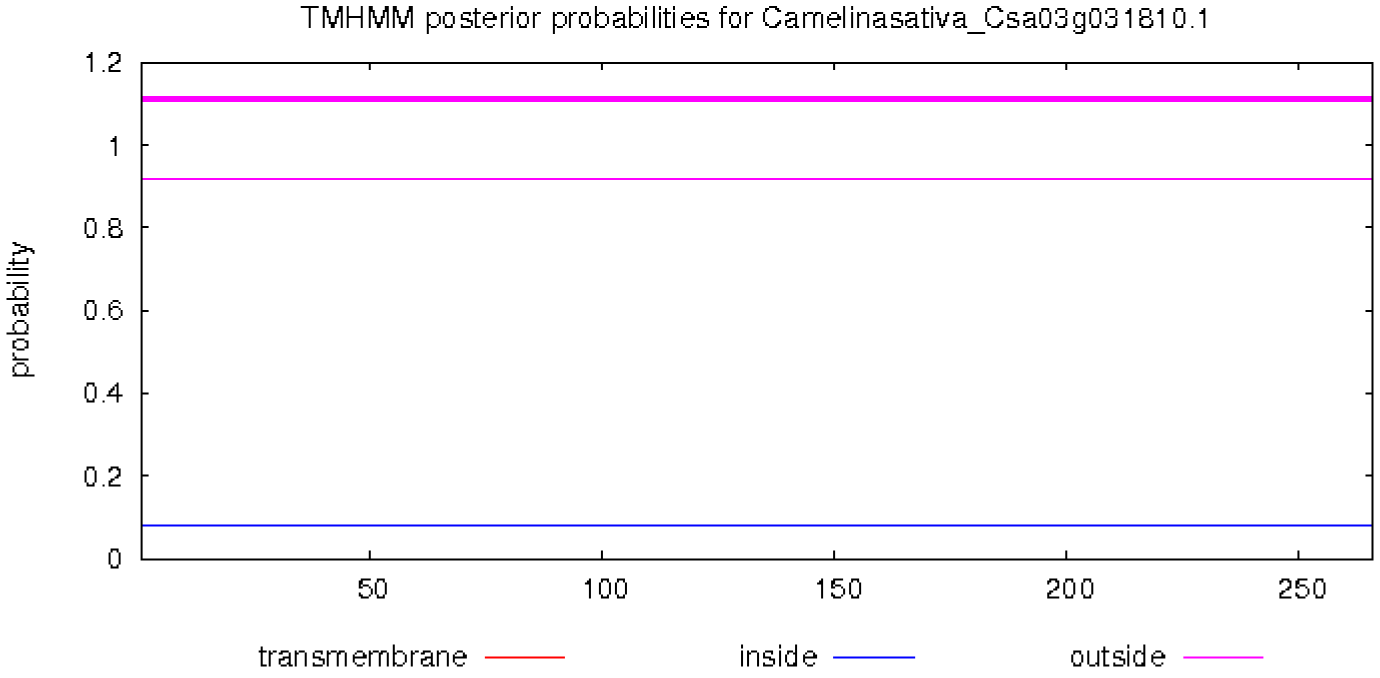

plot in postscript, script for making the plot in gnuplot, data for plot

```
# Camelinasativa_Csa03g037070.1 Length: 668
# Camelinasativa_Csa03g037070.1 Number of predicted TMHs:  0
# Camelinasativa_Csa03g037070.1 Exp number of AAs in TMHs: 0
# Camelinasativa_Csa03g037070.1 Exp number, first 60 AAs:  0
# Camelinasativa_Csa03g037070.1 Total prob of N-in:        0.00041
Camelinasativa_Csa03g037070.1   TMHMM2.0        outside      1   668
```

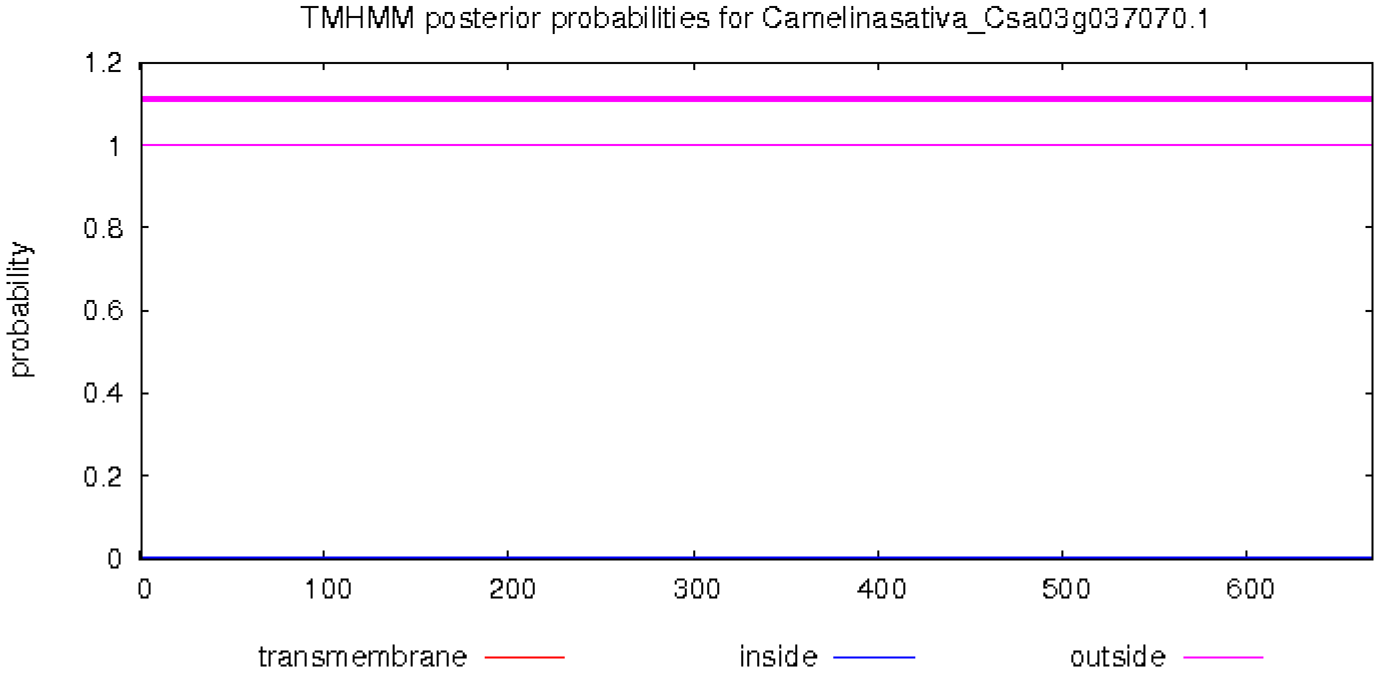

plot in postscript, script for making the plot in gnuplot, data for plot

```
# Camelinasativa_Csa03g037360.1 Length: 698
# Camelinasativa_Csa03g037360.1 Number of predicted TMHs:  1
# Camelinasativa_Csa03g037360.1 Exp number of AAs in TMHs: 21.8679
# Camelinasativa_Csa03g037360.1 Exp number, first 60 AAs:  0
# Camelinasativa_Csa03g037360.1 Total prob of N-in:        0.00568
Camelinasativa_Csa03g037360.1   TMHMM2.0        outside      1   652
Camelinasativa_Csa03g037360.1   TMHMM2.0        TMhelix    653   675
Camelinasativa_Csa03g037360.1   TMHMM2.0        inside     676   698
```

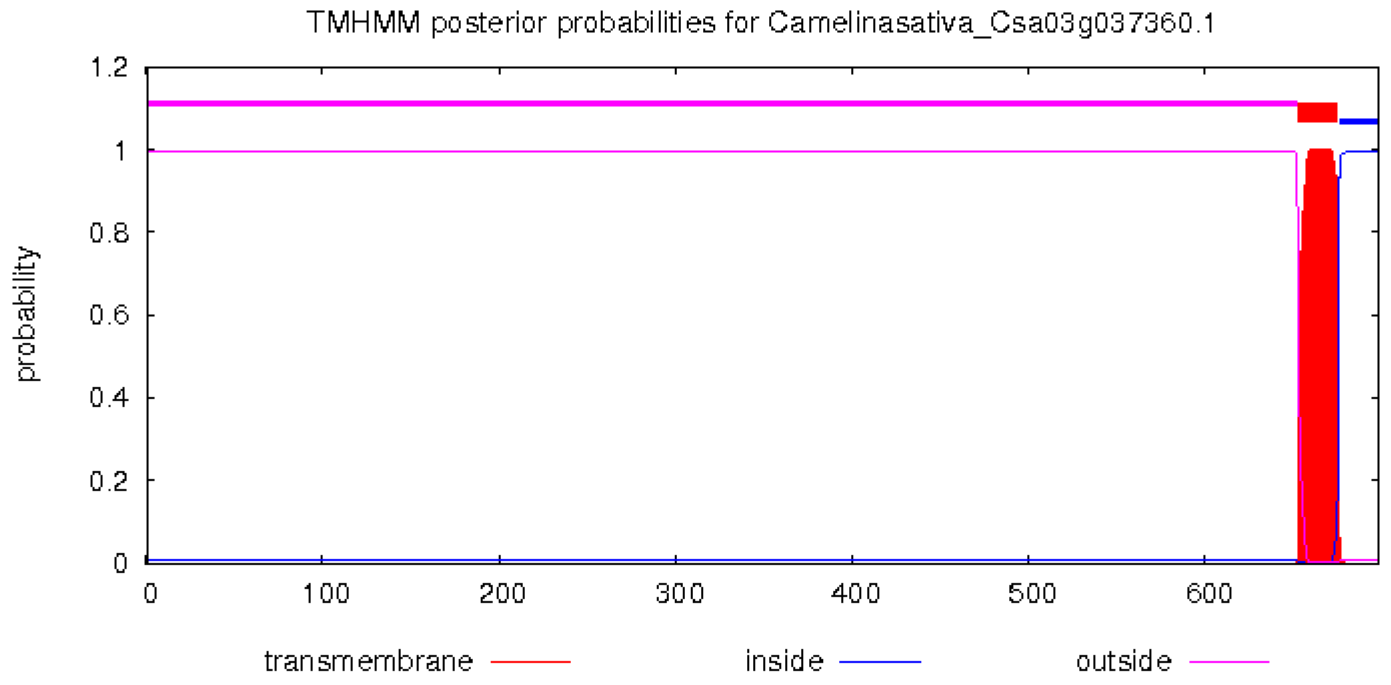

plot in postscript, script for making the plot in gnuplot, data for plot

---

```
# Camelinasativa_Csa03g037530.1 Length: 308
# Camelinasativa_Csa03g037530.1 Number of predicted TMHs:  0
# Camelinasativa_Csa03g037530.1 Exp number of AAs in TMHs: 0
# Camelinasativa_Csa03g037530.1 Exp number, first 60 AAs:  0
# Camelinasativa_Csa03g037530.1 Total prob of N-in:        0.00478
Camelinasativa_Csa03g037530.1   TMHMM2.0        outside      1   308
```

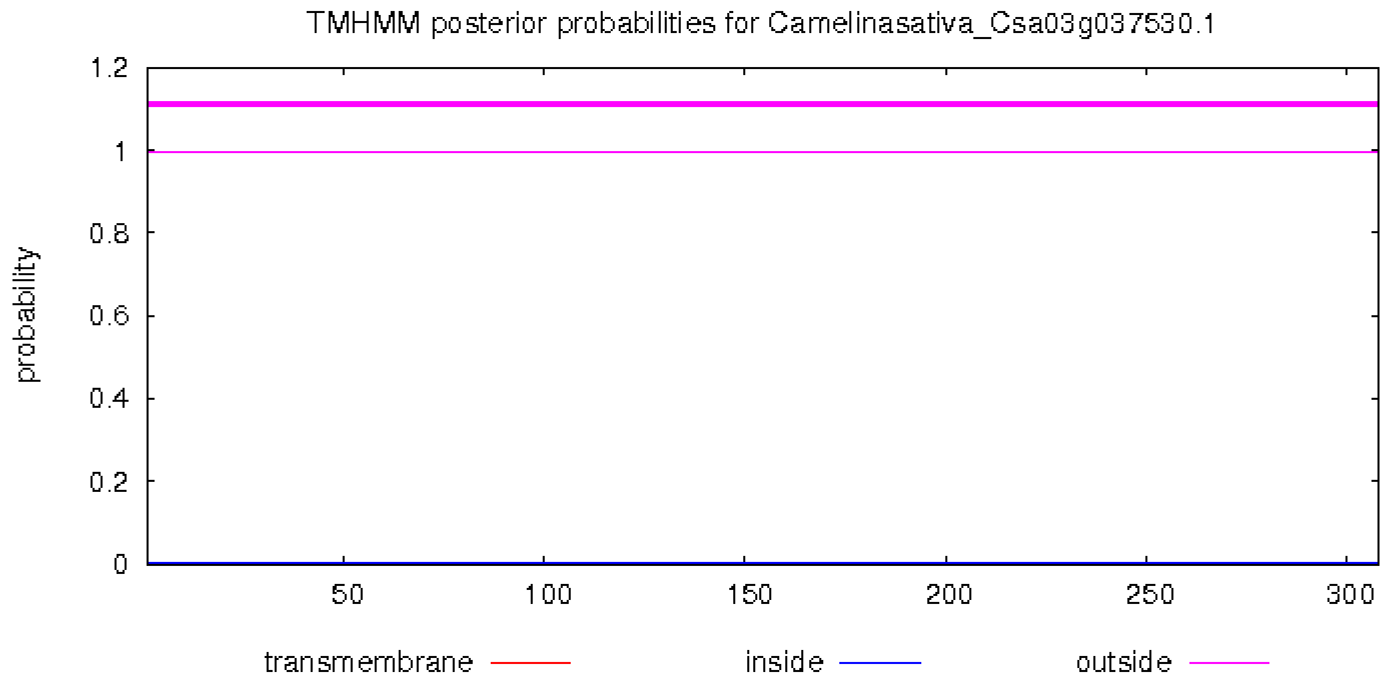

plot in postscript, script for making the plot in gnuplot, data for plot

```
# Camelinasativa_Csa03g038330.1 Length: 628
# Camelinasativa_Csa03g038330.1 Number of predicted TMHs:  1
# Camelinasativa_Csa03g038330.1 Exp number of AAs in TMHs: 22.49719
# Camelinasativa_Csa03g038330.1 Exp number, first 60 AAs:  0.00253
# Camelinasativa_Csa03g038330.1 Total prob of N-in:        0.24343
Camelinasativa_Csa03g038330.1   TMHMM2.0        outside      1   598
Camelinasativa_Csa03g038330.1   TMHMM2.0        TMhelix    599   621
Camelinasativa_Csa03g038330.1   TMHMM2.0        inside     622   628
```

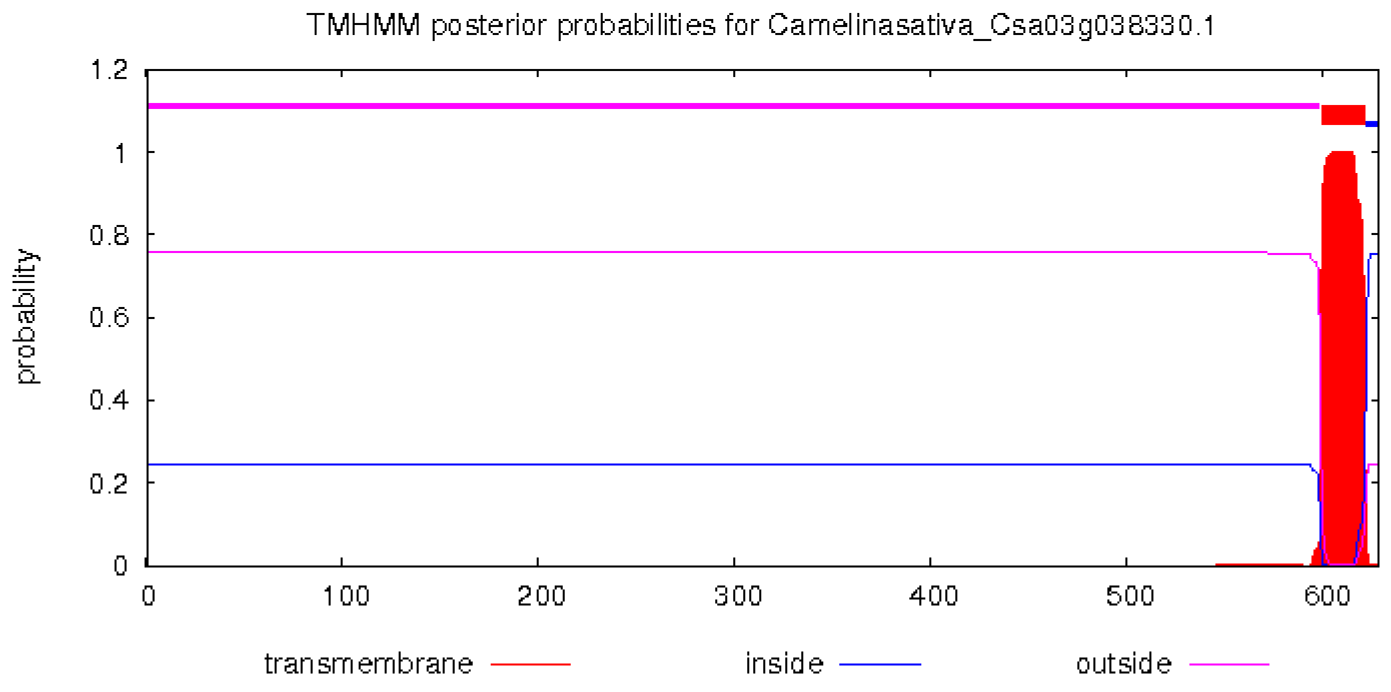

plot in postscript, script for making the plot in gnuplot, data for plot

```
# Camelinasativa_Csa03g038340.1 Length: 545
# Camelinasativa_Csa03g038340.1 Number of predicted TMHs:  1
# Camelinasativa_Csa03g038340.1 Exp number of AAs in TMHs: 21.37659
# Camelinasativa_Csa03g038340.1 Exp number, first 60 AAs:  0.00422
# Camelinasativa_Csa03g038340.1 Total prob of N-in:        0.06721
Camelinasativa_Csa03g038340.1   TMHMM2.0        outside      1   515
Camelinasativa_Csa03g038340.1   TMHMM2.0        TMhelix    516   538
Camelinasativa_Csa03g038340.1   TMHMM2.0        inside     539   545
```

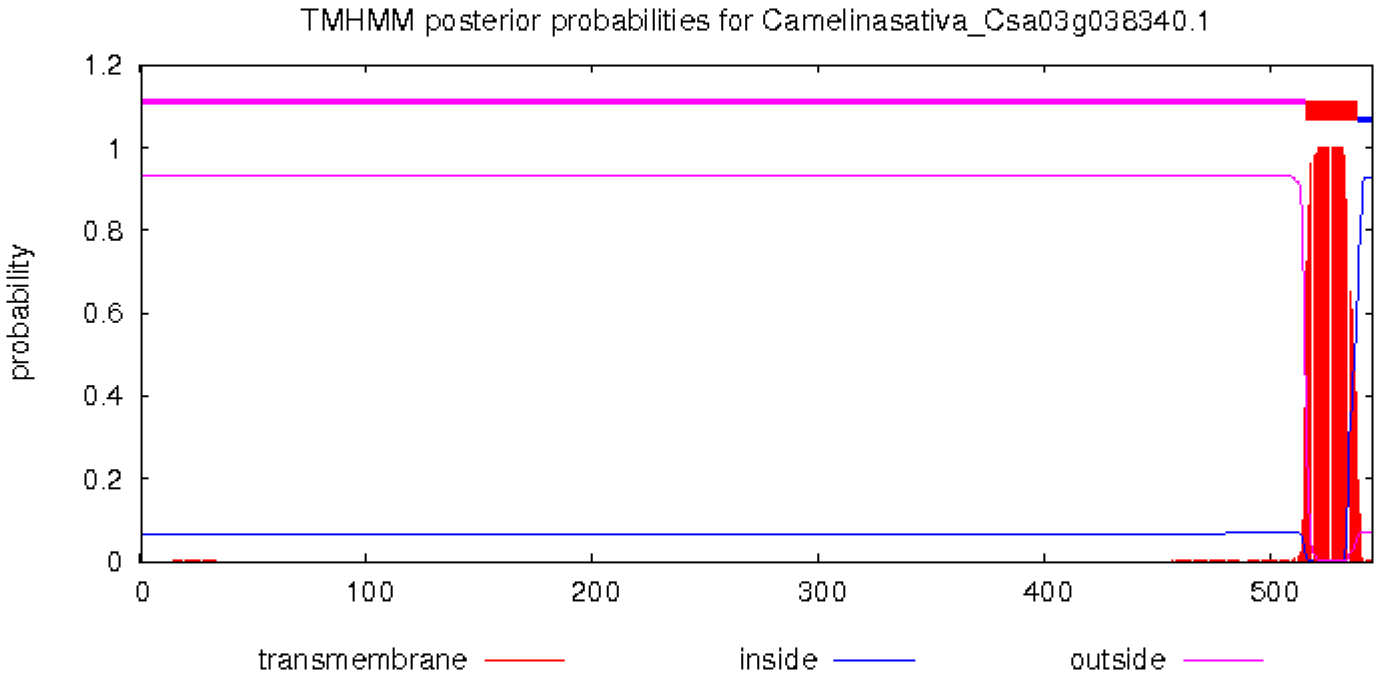

plot in postscript, script for making the plot in gnuplot, data for plot

```
# Camelinasativa_Csa03g059990.1 Length: 322
# Camelinasativa_Csa03g059990.1 Number of predicted TMHs:  0
# Camelinasativa_Csa03g059990.1 Exp number of AAs in TMHs: 0.00111
# Camelinasativa_Csa03g059990.1 Exp number, first 60 AAs:  0
# Camelinasativa_Csa03g059990.1 Total prob of N-in:        0.00698
Camelinasativa_Csa03g059990.1   TMHMM2.0        outside      1   322
```

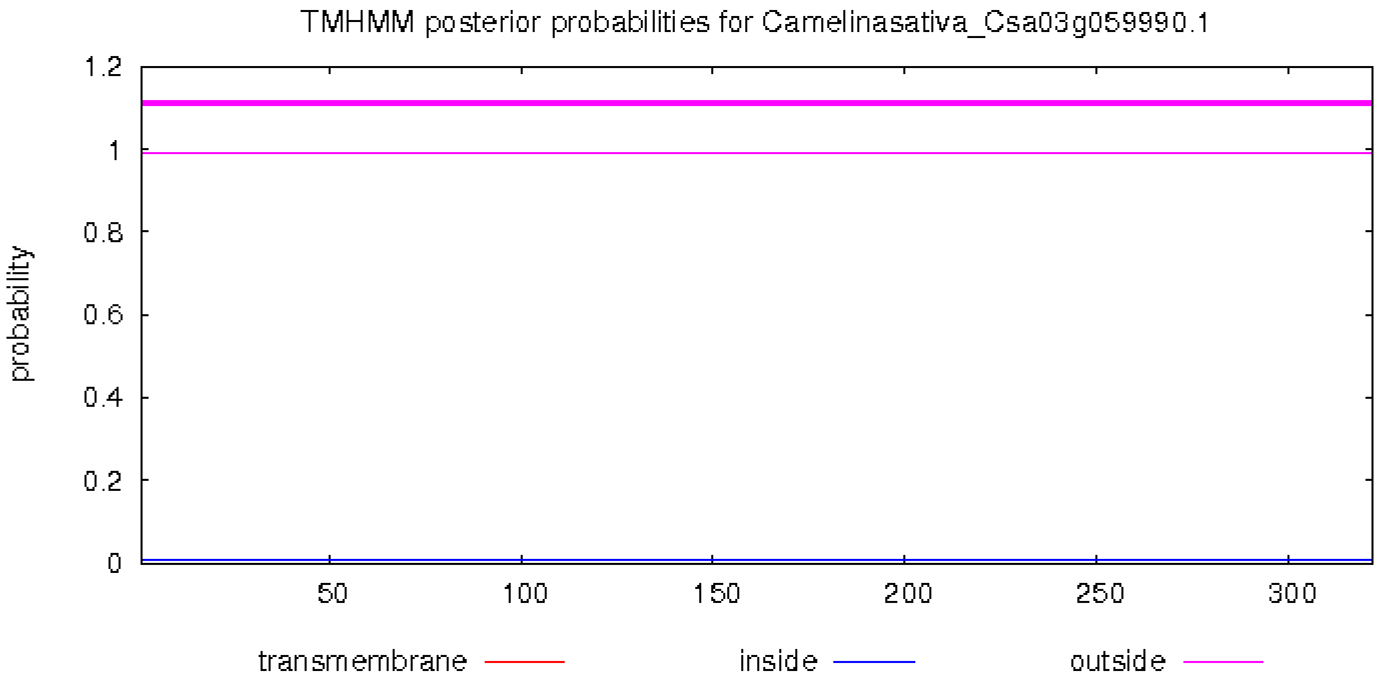

plot in postscript, script for making the plot in gnuplot, data for plot

```
# Camelinasativa_Csa03g060000.1 Length: 306
# Camelinasativa_Csa03g060000.1 Number of predicted TMHs:  0
# Camelinasativa_Csa03g060000.1 Exp number of AAs in TMHs: 0.00093
# Camelinasativa_Csa03g060000.1 Exp number, first 60 AAs:  0.00023
# Camelinasativa_Csa03g060000.1 Total prob of N-in:        0.01519
Camelinasativa_Csa03g060000.1   TMHMM2.0        outside      1   306
```

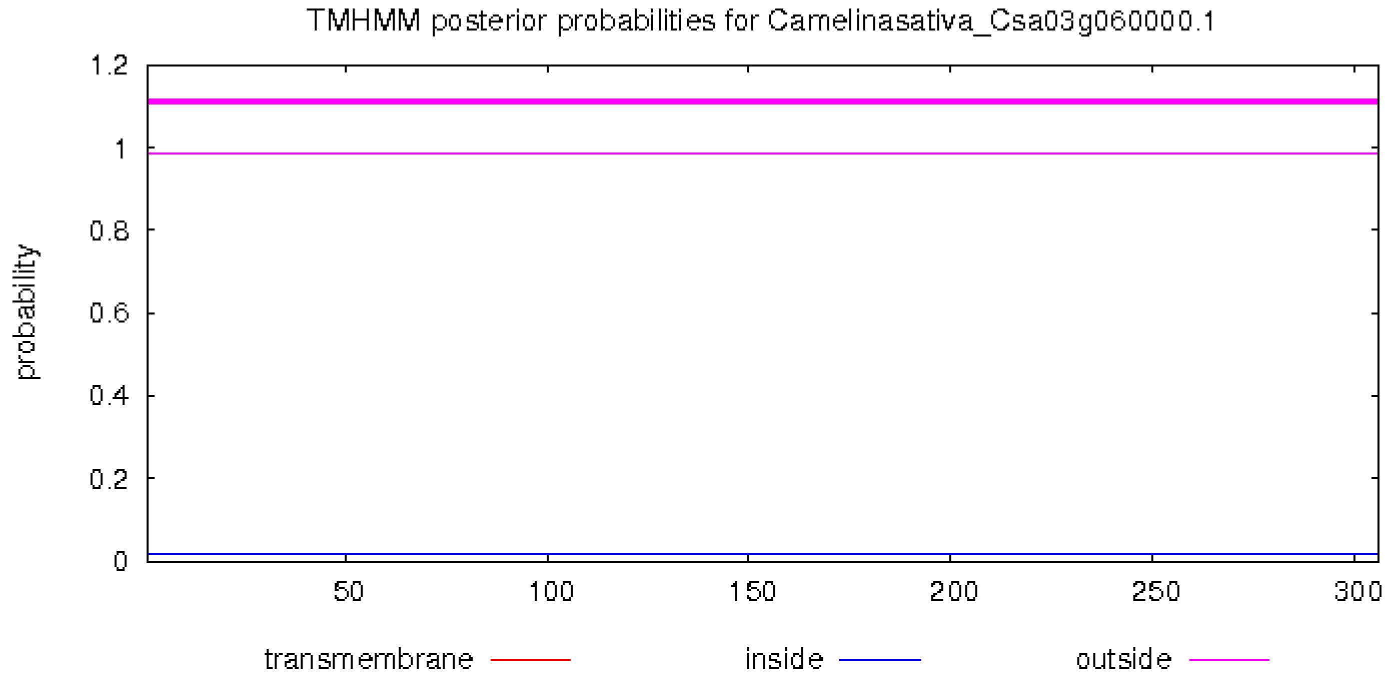

plot in postscript, script for making the plot in gnuplot, data for plot

---

```
# Camelinasativa_Csa03g062660.1 Length: 323
# Camelinasativa_Csa03g062660.1 Number of predicted TMHs:  0
# Camelinasativa_Csa03g062660.1 Exp number of AAs in TMHs: 0.00037
# Camelinasativa_Csa03g062660.1 Exp number, first 60 AAs:  0
# Camelinasativa_Csa03g062660.1 Total prob of N-in:        0.02371
Camelinasativa_Csa03g062660.1   TMHMM2.0        outside      1   323
```

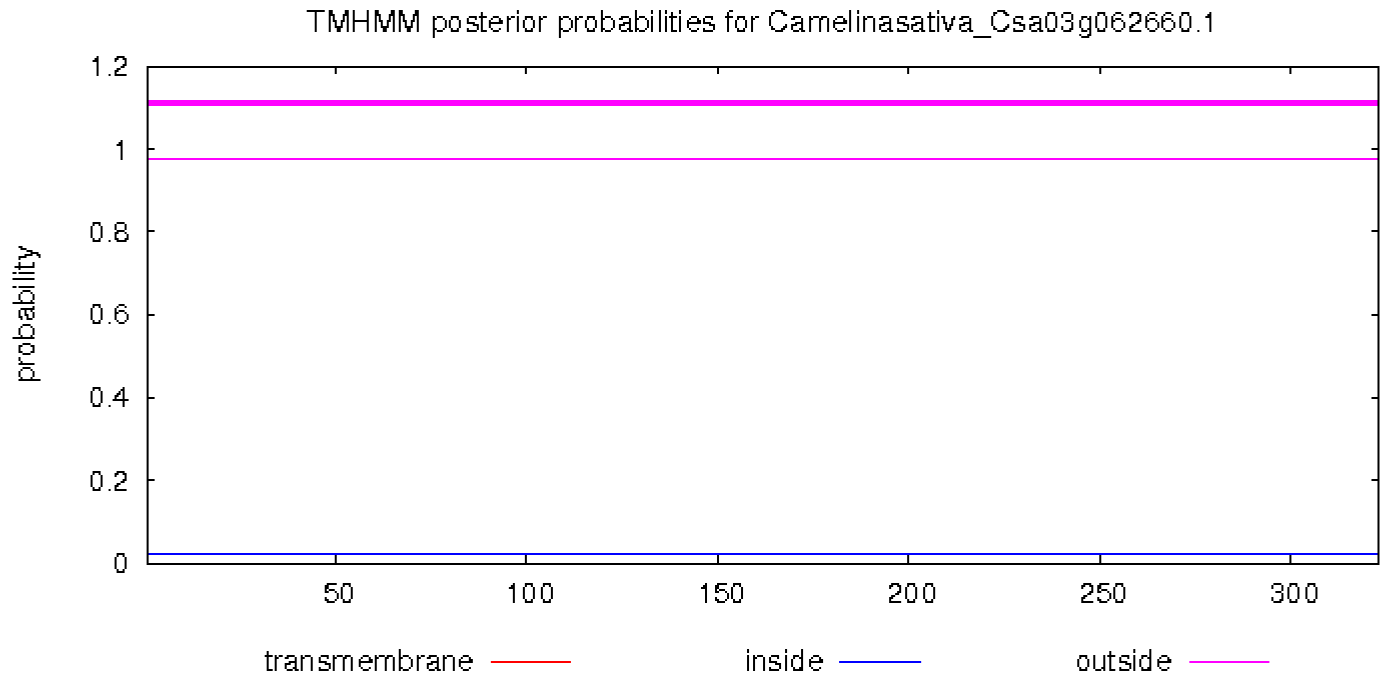

plot in postscript, script for making the plot in gnuplot, data for plot

---

```
# Camelinasativa_Csa03g062870.1 Length: 303
# Camelinasativa_Csa03g062870.1 Number of predicted TMHs:  0
# Camelinasativa_Csa03g062870.1 Exp number of AAs in TMHs: 0
# Camelinasativa_Csa03g062870.1 Exp number, first 60 AAs:  0
# Camelinasativa_Csa03g062870.1 Total prob of N-in:        0.00238
Camelinasativa_Csa03g062870.1   TMHMM2.0        outside      1   303
```

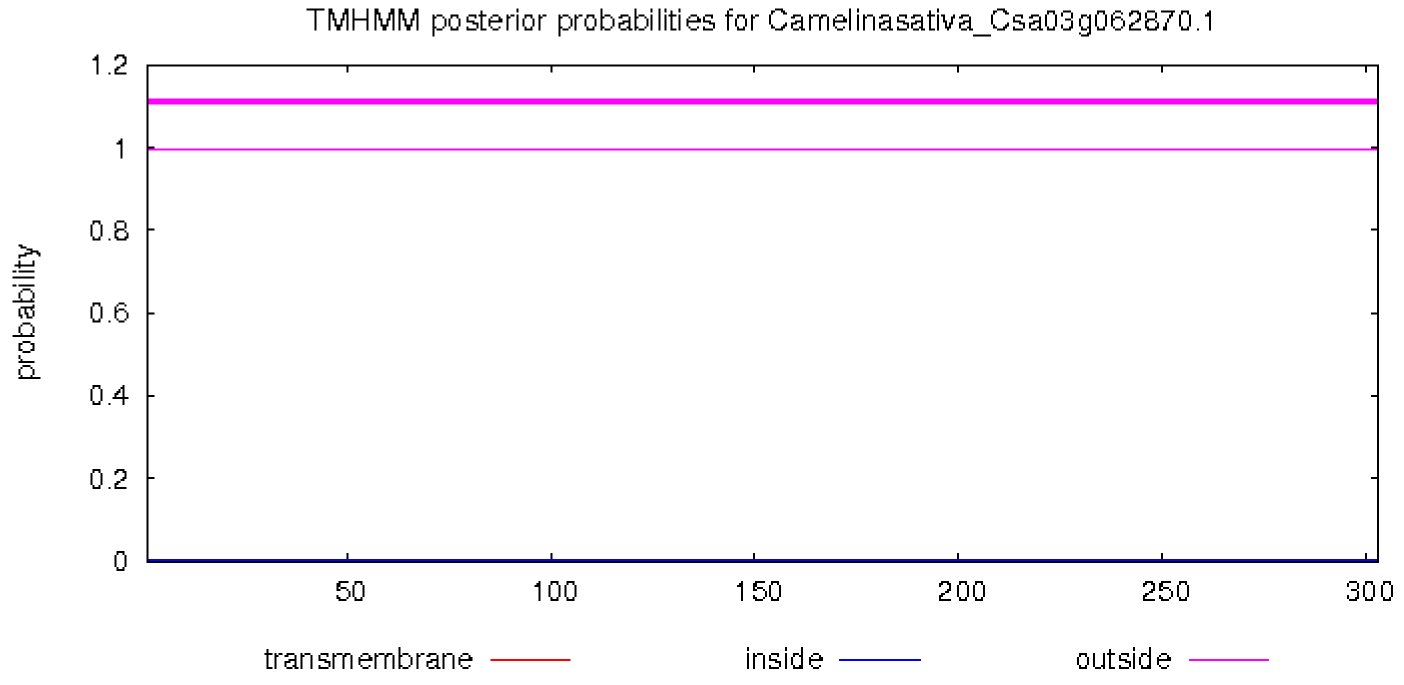

plot in postscript, script for making the plot in gnuplot, data for plot

---

```
# Camelinasativa_Csa04g008000.1 Length: 380
# Camelinasativa_Csa04g008000.1 Number of predicted TMHs:  0
# Camelinasativa_Csa04g008000.1 Exp number of AAs in TMHs: 0.00971
# Camelinasativa_Csa04g008000.1 Exp number, first 60 AAs:  0
# Camelinasativa_Csa04g008000.1 Total prob of N-in:        0.01355
Camelinasativa_Csa04g008000.1	TMHMM2.0	outside	     1   380
```

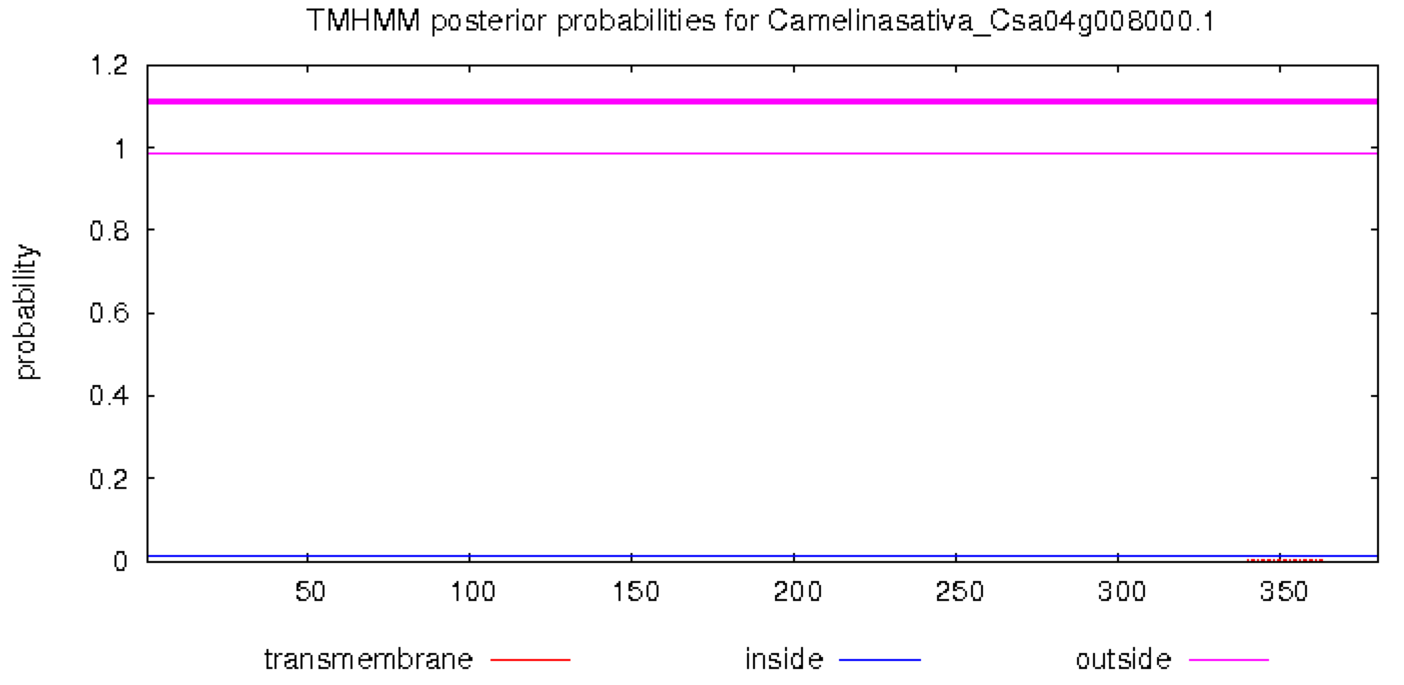

plot in postscript, script for making the plot in gnuplot, data for plot

---

```
# Camelinasativa_Csa04g015120.1 Length: 174
# Camelinasativa_Csa04g015120.1 Number of predicted TMHs:  0
# Camelinasativa_Csa04g015120.1 Exp number of AAs in TMHs: 0.00104
# Camelinasativa_Csa04g015120.1 Exp number, first 60 AAs:  0.00104
# Camelinasativa_Csa04g015120.1 Total prob of N-in:        0.02822
Camelinasativa_Csa04g015120.1	TMHMM2.0	outside	     1   174
```

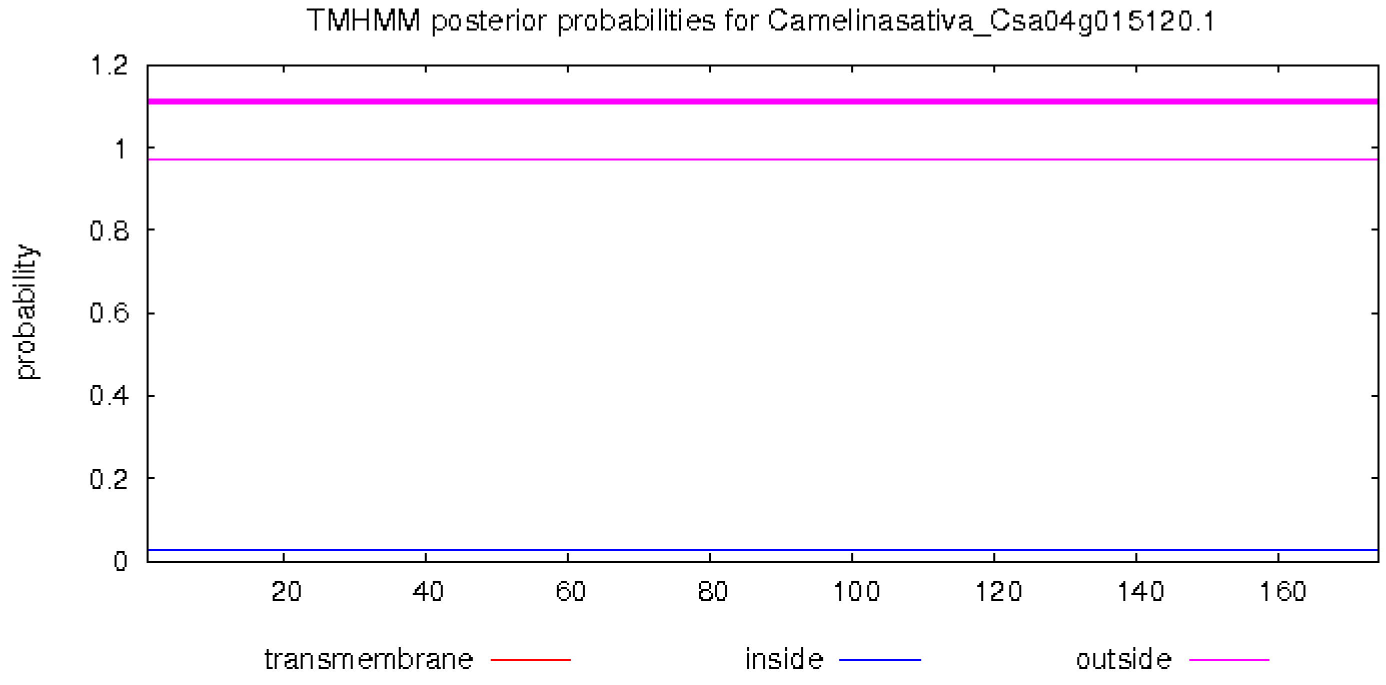

plot in postscript, script for making the plot in gnuplot, data for plot

---

```
# Camelinasativa_Csa04g018350.1 Length: 322
# Camelinasativa_Csa04g018350.1 Number of predicted TMHs:  0
# Camelinasativa_Csa04g018350.1 Exp number of AAs in TMHs: 0.00206
# Camelinasativa_Csa04g018350.1 Exp number, first 60 AAs:  0.0014
# Camelinasativa_Csa04g018350.1 Total prob of N-in:        0.04003
Camelinasativa_Csa04g018350.1	TMHMM2.0	outside	     1   322
```

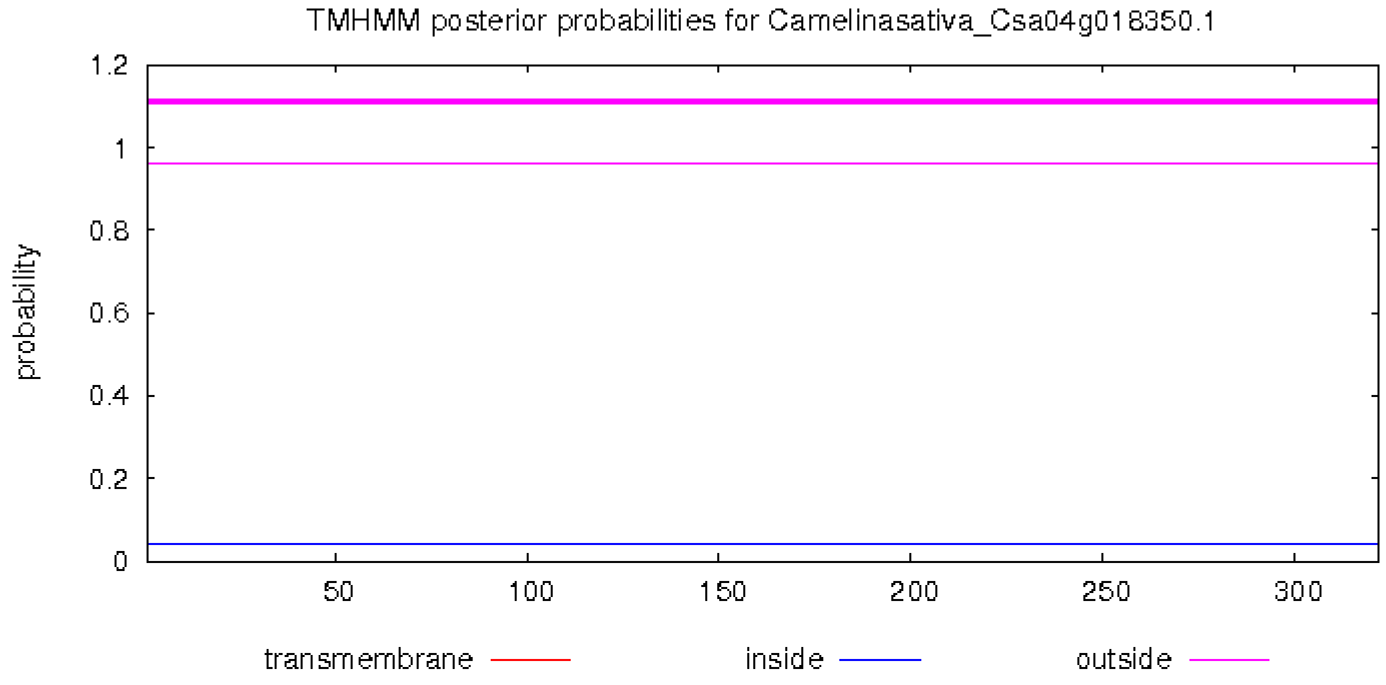

plot in postscript, script for making the plot in gnuplot, data for plot

---

```
# Camelinasativa_Csa04g023980.1 Length: 268
# Camelinasativa_Csa04g023980.1 Number of predicted TMHs:  1
# Camelinasativa_Csa04g023980.1 Exp number of AAs in TMHs: 29.71401
# Camelinasativa_Csa04g023980.1 Exp number, first 60 AAs:  29.31983
# Camelinasativa_Csa04g023980.1 Total prob of N-in:        0.65297
# Camelinasativa_Csa04g023980.1 POSSIBLE N-term signal sequence
Camelinasativa_Csa04g023980.1	TMHMM2.0	inside	     1     1
```

```
Camelinasativa_Csa04g023980.1   TMHMM2.0        TMhelix      2    24
Camelinasativa_Csa04g023980.1   TMHMM2.0        outside     25   268
```

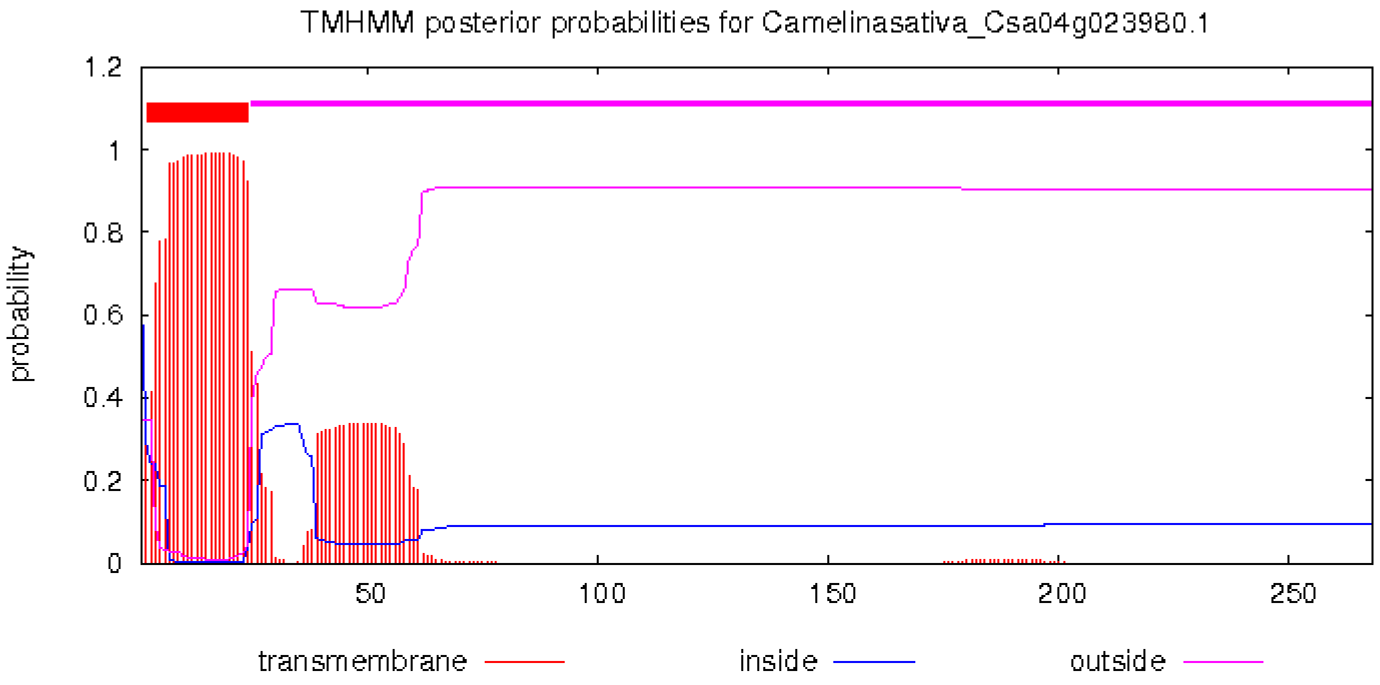

plot in postscript, script for making the plot in gnuplot, data for plot

```
# Camelinasativa_Csa04g024130.1 Length: 241
# Camelinasativa_Csa04g024130.1 Number of predicted TMHs:  0
# Camelinasativa_Csa04g024130.1 Exp number of AAs in TMHs: 0.01856
# Camelinasativa_Csa04g024130.1 Exp number, first 60 AAs:  0.01854
# Camelinasativa_Csa04g024130.1 Total prob of N-in:        0.02742
Camelinasativa_Csa04g024130.1   TMHMM2.0        outside      1   241
```

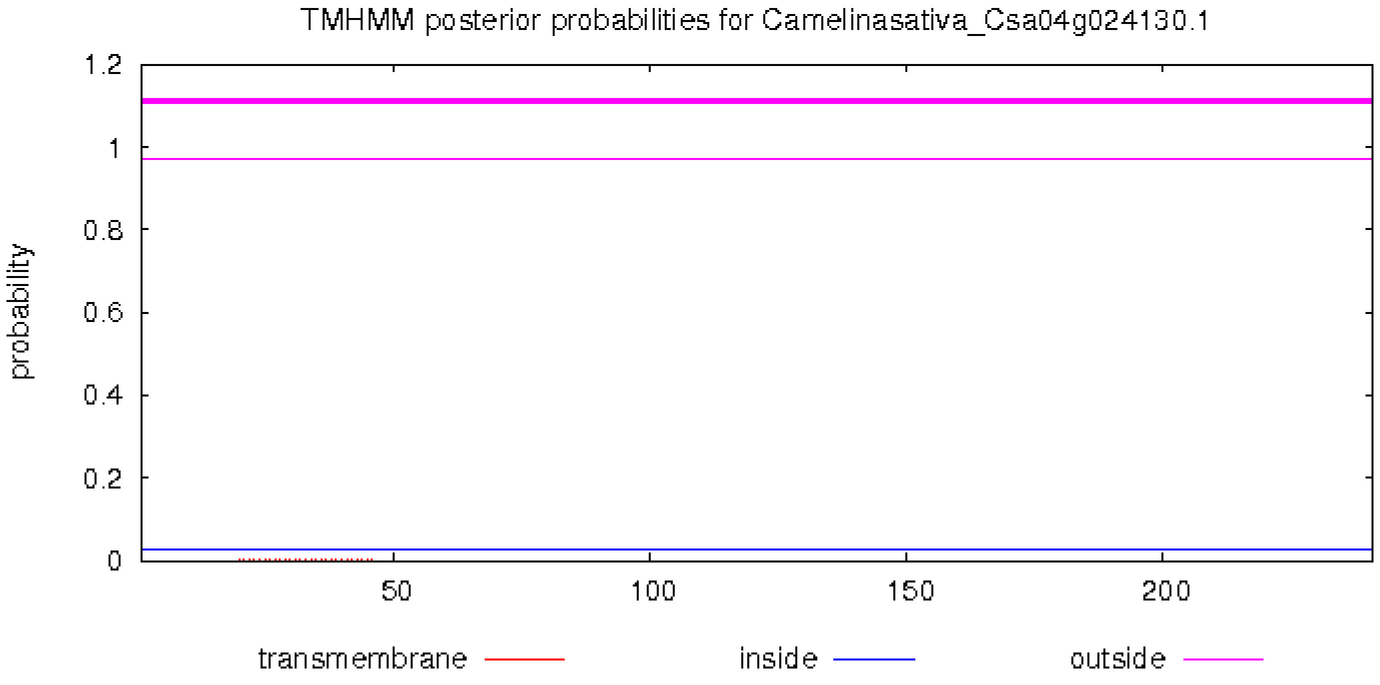

plot in postscript, script for making the plot in gnuplot, data for plot

```
# Camelinasativa_Csa04g035000.1 Length: 455
# Camelinasativa_Csa04g035000.1 Number of predicted TMHs:  1
# Camelinasativa_Csa04g035000.1 Exp number of AAs in TMHs: 20.946
# Camelinasativa_Csa04g035000.1 Exp number, first 60 AAs:  0
```

```
# Camelinasativa_Csa04g035000.1 Total prob of N-in:        0.03704
Camelinasativa_Csa04g035000.1   TMHMM2.0        outside      1   424
Camelinasativa_Csa04g035000.1   TMHMM2.0        TMhelix    425   447
Camelinasativa_Csa04g035000.1   TMHMM2.0        inside     448   455
```

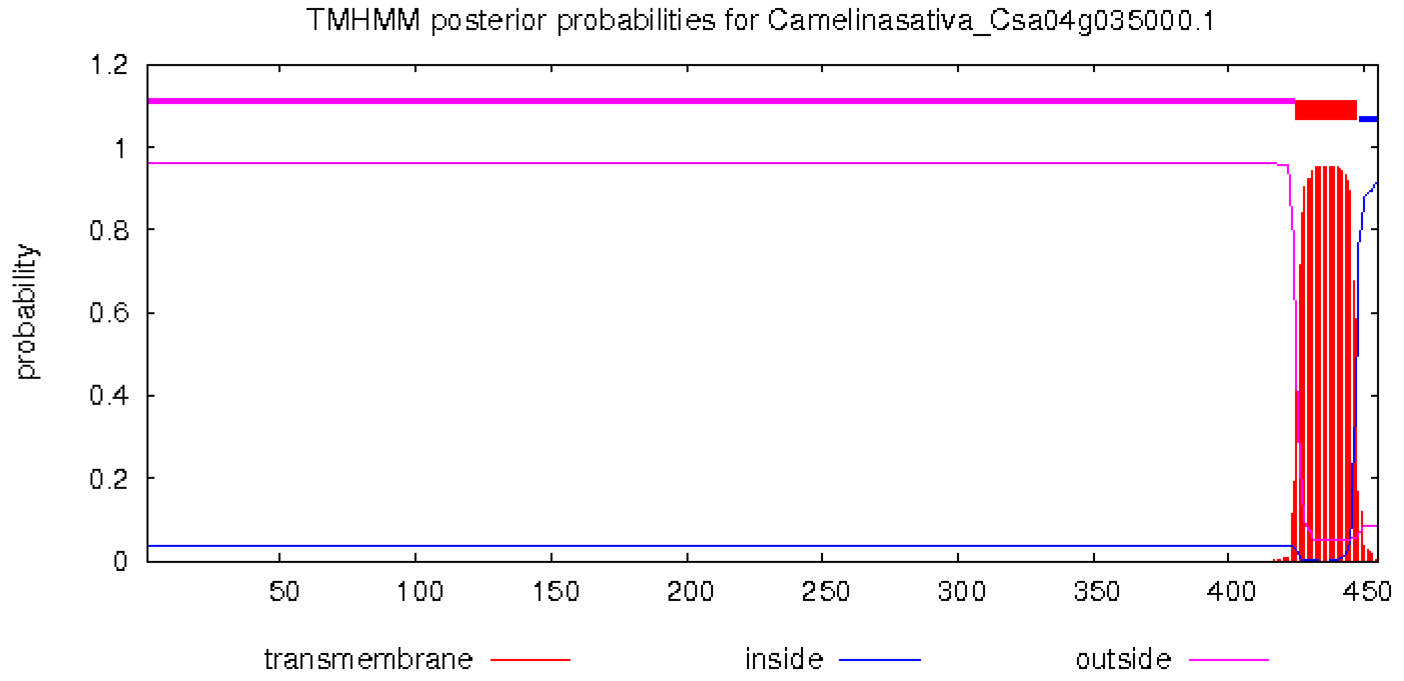

plot in postscript, script for making the plot in gnuplot, data for plot

---

```
# Camelinasativa_Csa04g040470.1 Length: 156
# Camelinasativa_Csa04g040470.1 Number of predicted TMHs:  0
# Camelinasativa_Csa04g040470.1 Exp number of AAs in TMHs: 0.00207
# Camelinasativa_Csa04g040470.1 Exp number, first 60 AAs:  0
# Camelinasativa_Csa04g040470.1 Total prob of N-in:        0.30808
Camelinasativa_Csa04g040470.1   TMHMM2.0        outside      1   156
```

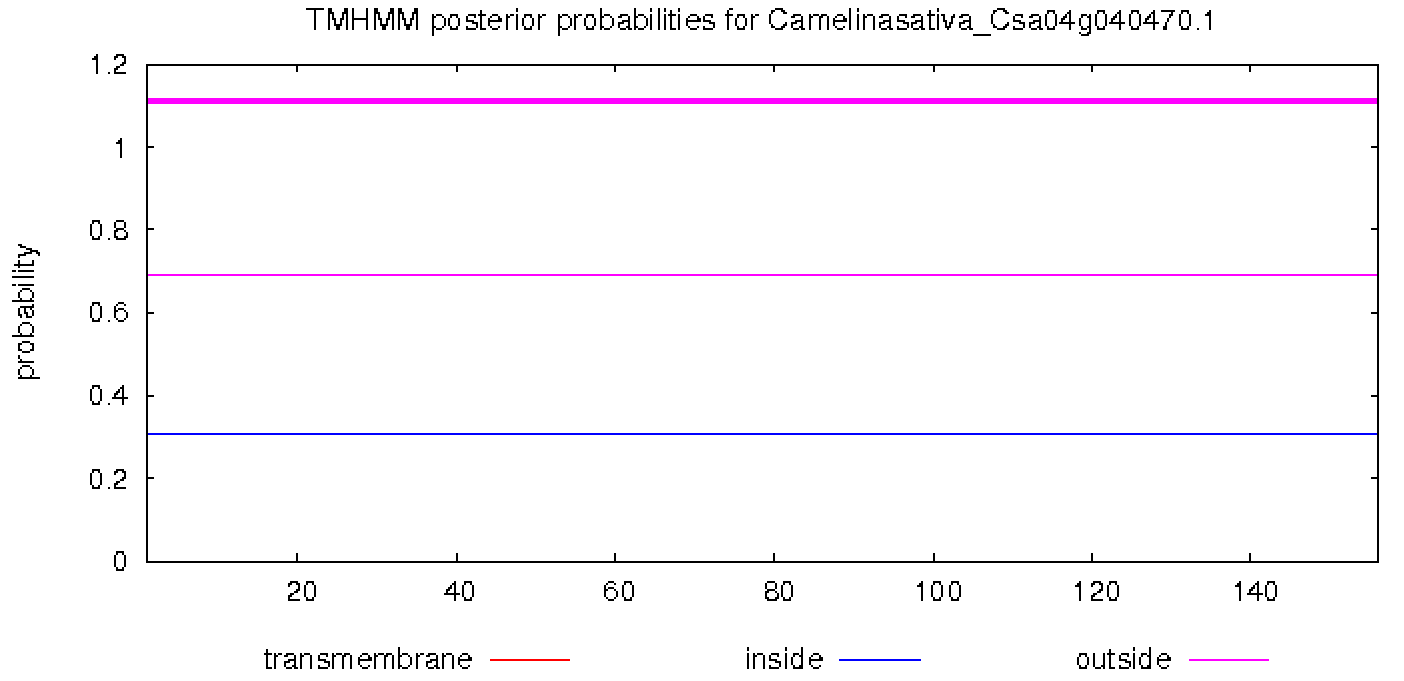

plot in postscript, script for making the plot in gnuplot, data for plot

---

```
# Camelinasativa_Csa04g042950.1 Length: 280
# Camelinasativa_Csa04g042950.1 Number of predicted TMHs:  0
```

```
# Camelinasativa_Csa04g042950.1 Exp number of AAs in TMHs: 0.00016
# Camelinasativa_Csa04g042950.1 Exp number, first 60 AAs:  0
# Camelinasativa_Csa04g042950.1 Total prob of N-in:        0.09452
Camelinasativa_Csa04g042950.1	TMHMM2.0	outside	     1   280
```

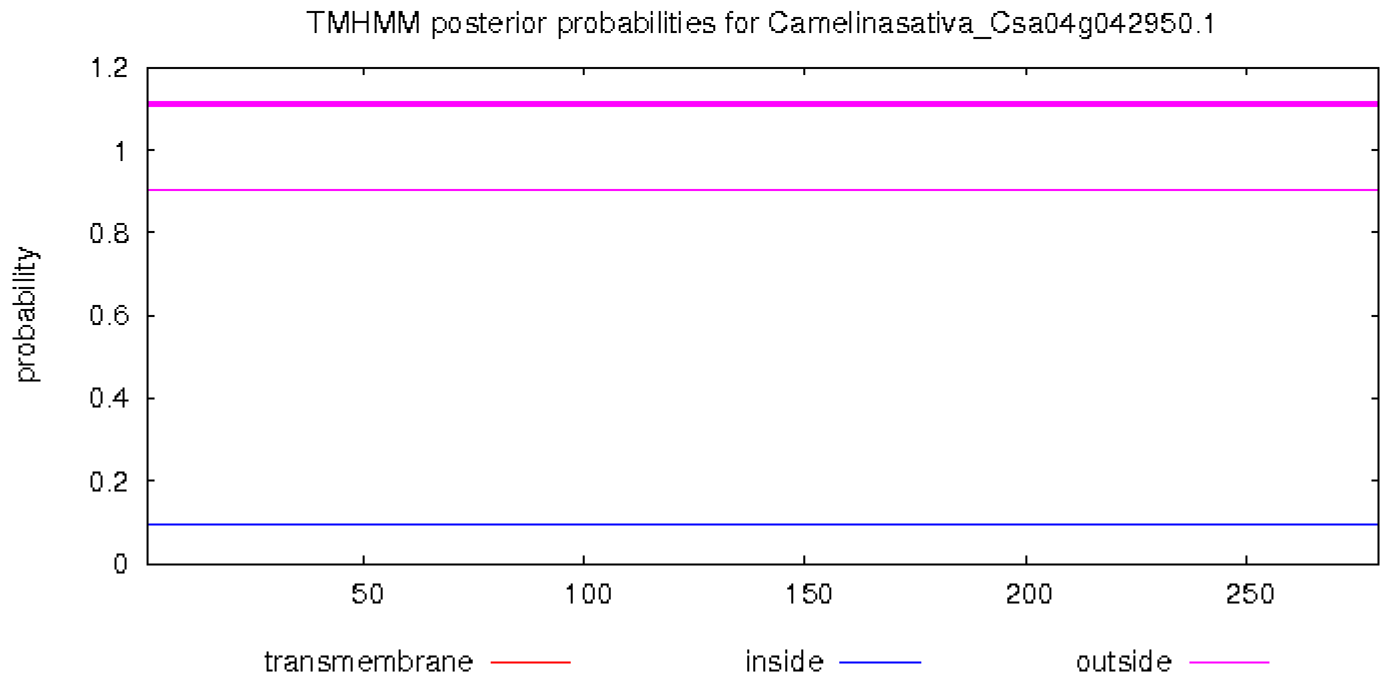

plot in postscript, script for making the plot in gnuplot, data for plot

```
# Camelinasativa_Csa04g042970.1 Length: 427
# Camelinasativa_Csa04g042970.1 Number of predicted TMHs:  0
# Camelinasativa_Csa04g042970.1 Exp number of AAs in TMHs: 0.00106
# Camelinasativa_Csa04g042970.1 Exp number, first 60 AAs:  0
# Camelinasativa_Csa04g042970.1 Total prob of N-in:        0.02429
Camelinasativa_Csa04g042970.1	TMHMM2.0	outside	     1   427
```

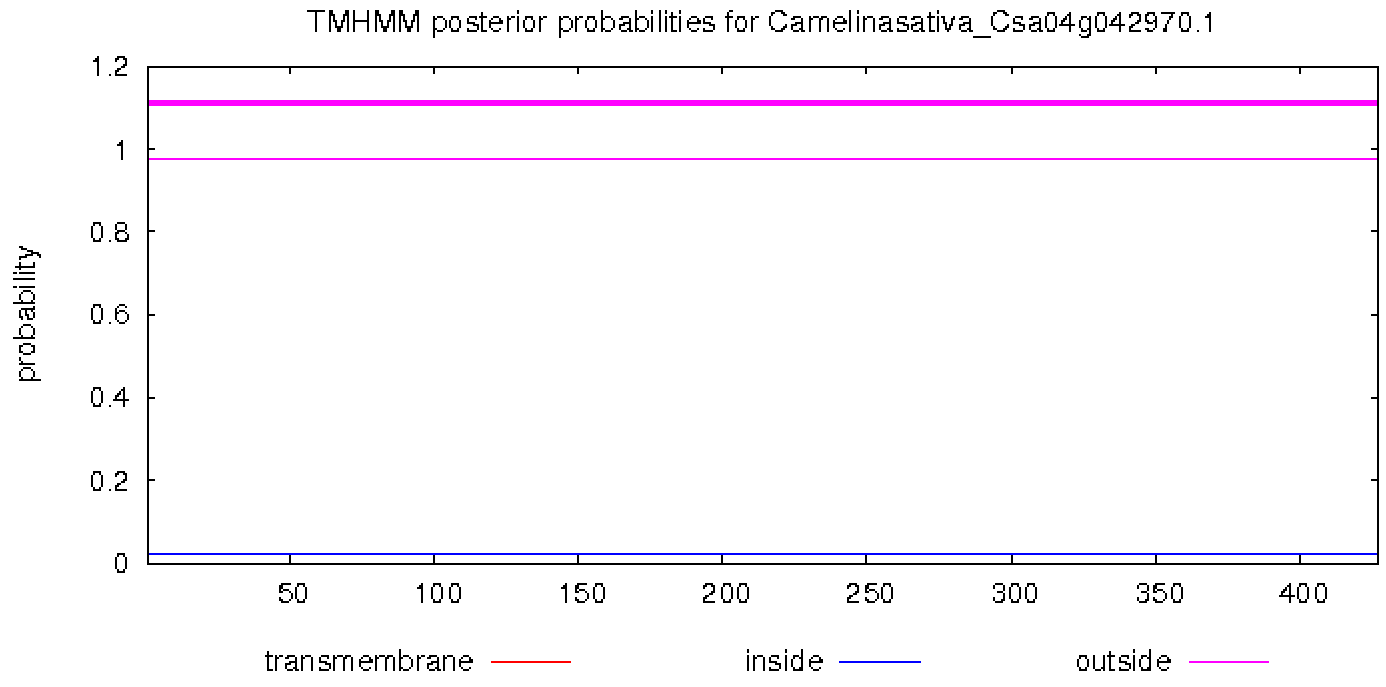

plot in postscript, script for making the plot in gnuplot, data for plot

```
# Camelinasativa_Csa04g049120.1 Length: 267
# Camelinasativa_Csa04g049120.1 Number of predicted TMHs:  0
```

```
# Camelinasativa_Csa04g049120.1 Exp number of AAs in TMHs: 0.00243
# Camelinasativa_Csa04g049120.1 Exp number, first 60 AAs:  0
# Camelinasativa_Csa04g049120.1 Total prob of N-in:        0.24208
Camelinasativa_Csa04g049120.1	TMHMM2.0	outside	     1   267
```

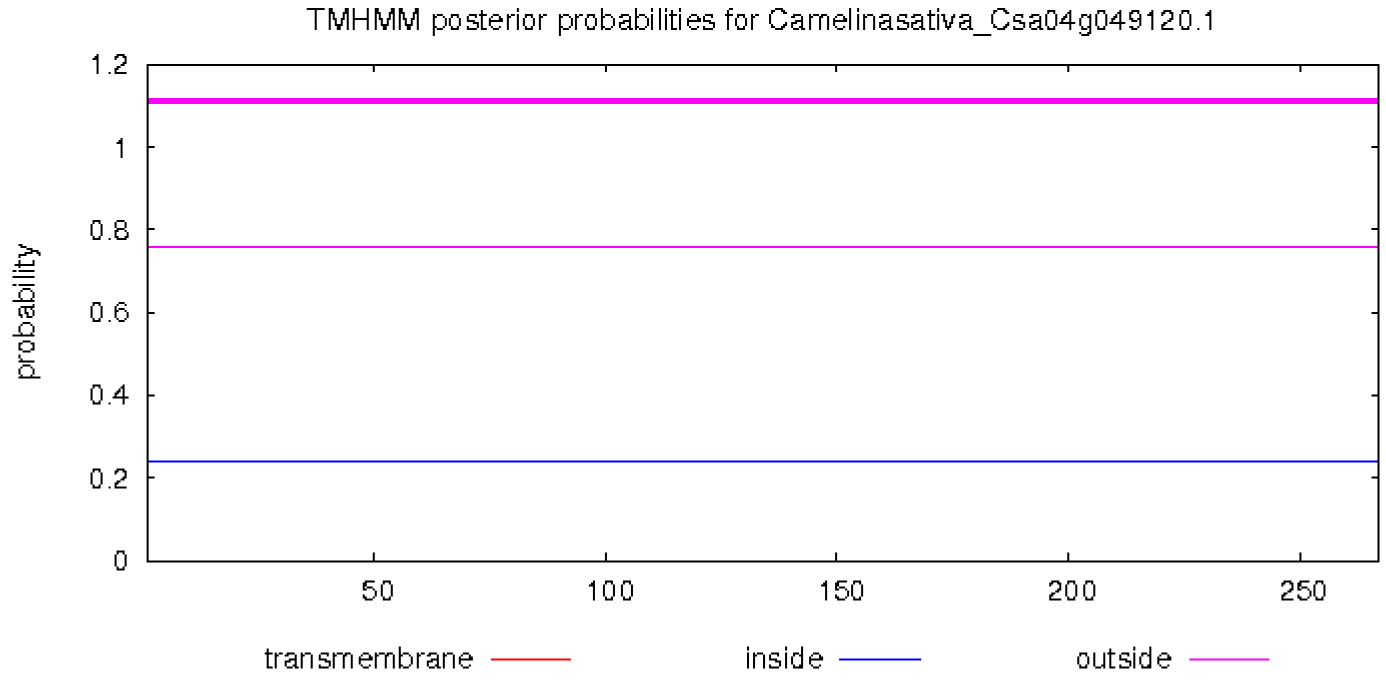

plot in postscript, script for making the plot in gnuplot, data for plot

---

```
# Camelinasativa_Csa04g060600.1 Length: 277
# Camelinasativa_Csa04g060600.1 Number of predicted TMHs:  0
# Camelinasativa_Csa04g060600.1 Exp number of AAs in TMHs: 0.00033
# Camelinasativa_Csa04g060600.1 Exp number, first 60 AAs:  0
# Camelinasativa_Csa04g060600.1 Total prob of N-in:        0.02508
Camelinasativa_Csa04g060600.1	TMHMM2.0	outside	     1   277
```

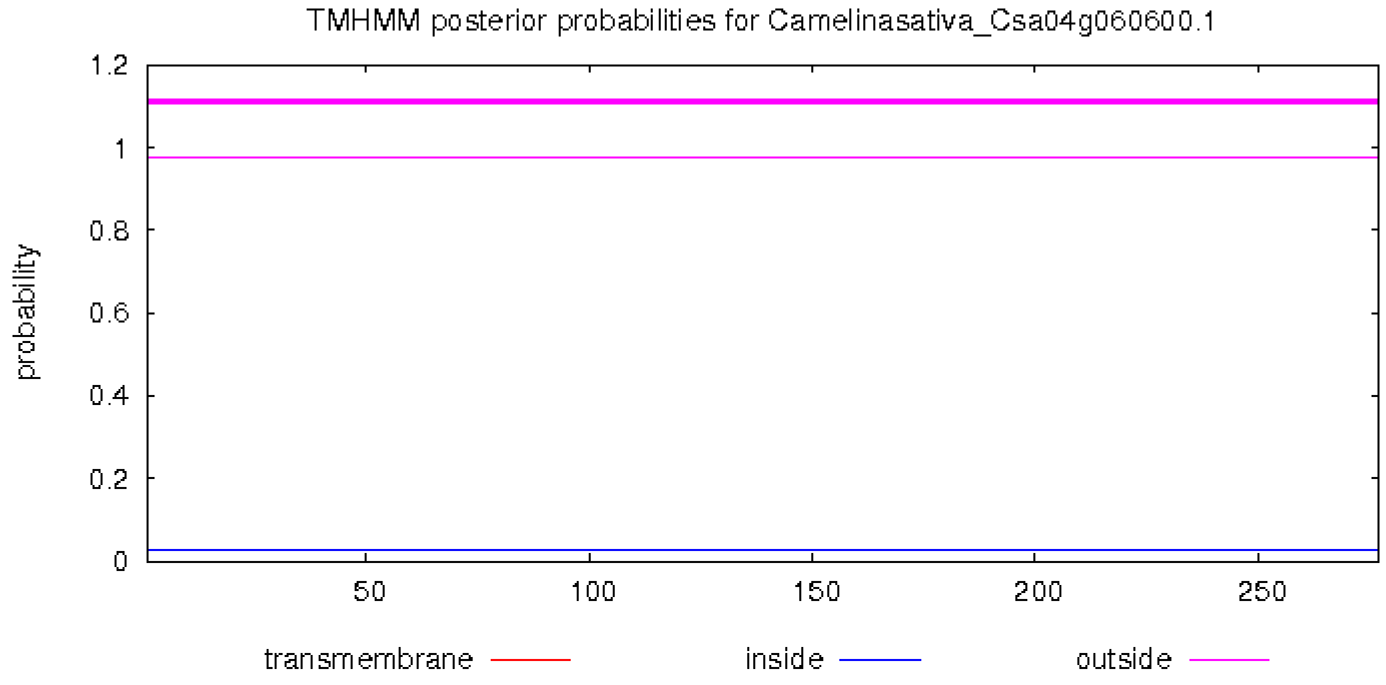

plot in postscript, script for making the plot in gnuplot, data for plot

---

```
# Camelinasativa_Csa04g065900.1 Length: 413
# Camelinasativa_Csa04g065900.1 Number of predicted TMHs:  0
```

```
# Camelinasativa_Csa04g065900.1 Exp number of AAs in TMHs: 0.00018
# Camelinasativa_Csa04g065900.1 Exp number, first 60 AAs:  0
# Camelinasativa_Csa04g065900.1 Total prob of N-in:        0.00666
Camelinasativa_Csa04g065900.1   TMHMM2.0        outside      1   413
```

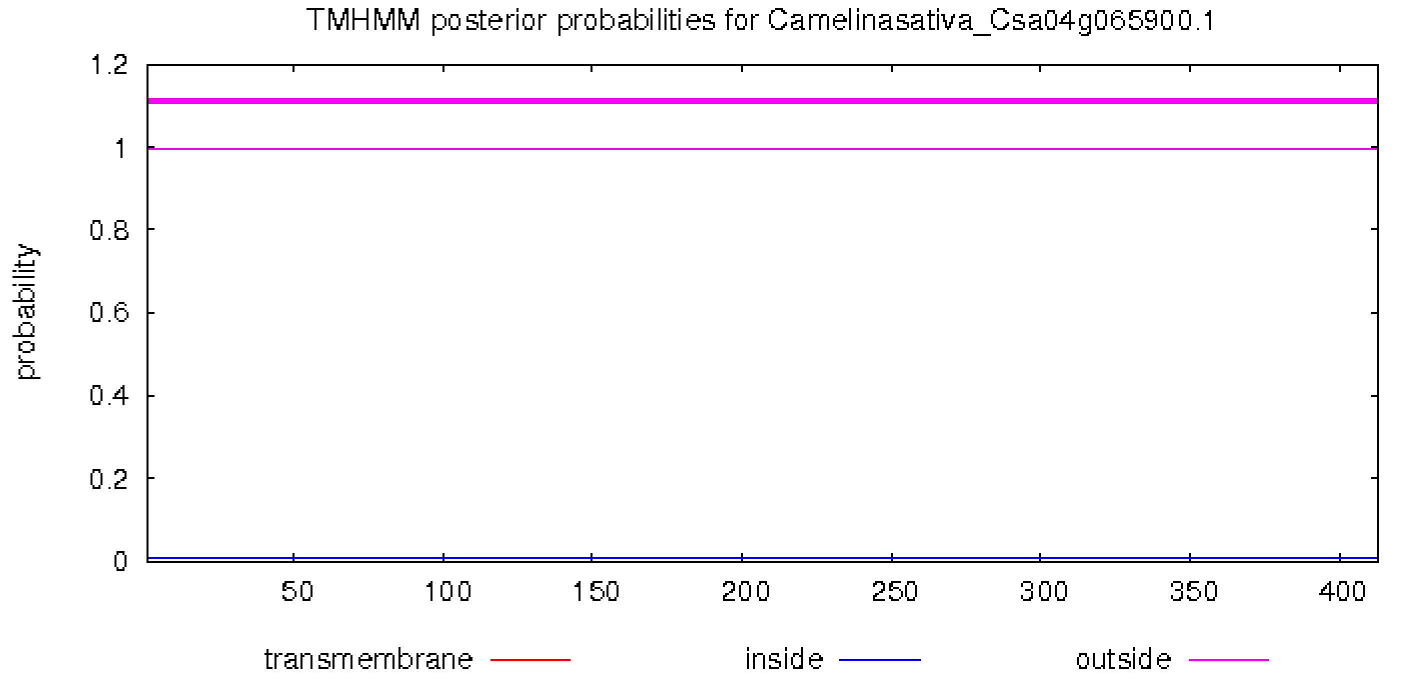

plot in postscript, script for making the plot in gnuplot, data for plot

---

```
# Camelinasativa_Csa05g002390.1 Length: 353
# Camelinasativa_Csa05g002390.1 Number of predicted TMHs:  0
# Camelinasativa_Csa05g002390.1 Exp number of AAs in TMHs: 0.0004
# Camelinasativa_Csa05g002390.1 Exp number, first 60 AAs:  0
# Camelinasativa_Csa05g002390.1 Total prob of N-in:        0.01397
Camelinasativa_Csa05g002390.1   TMHMM2.0        outside      1   353
```

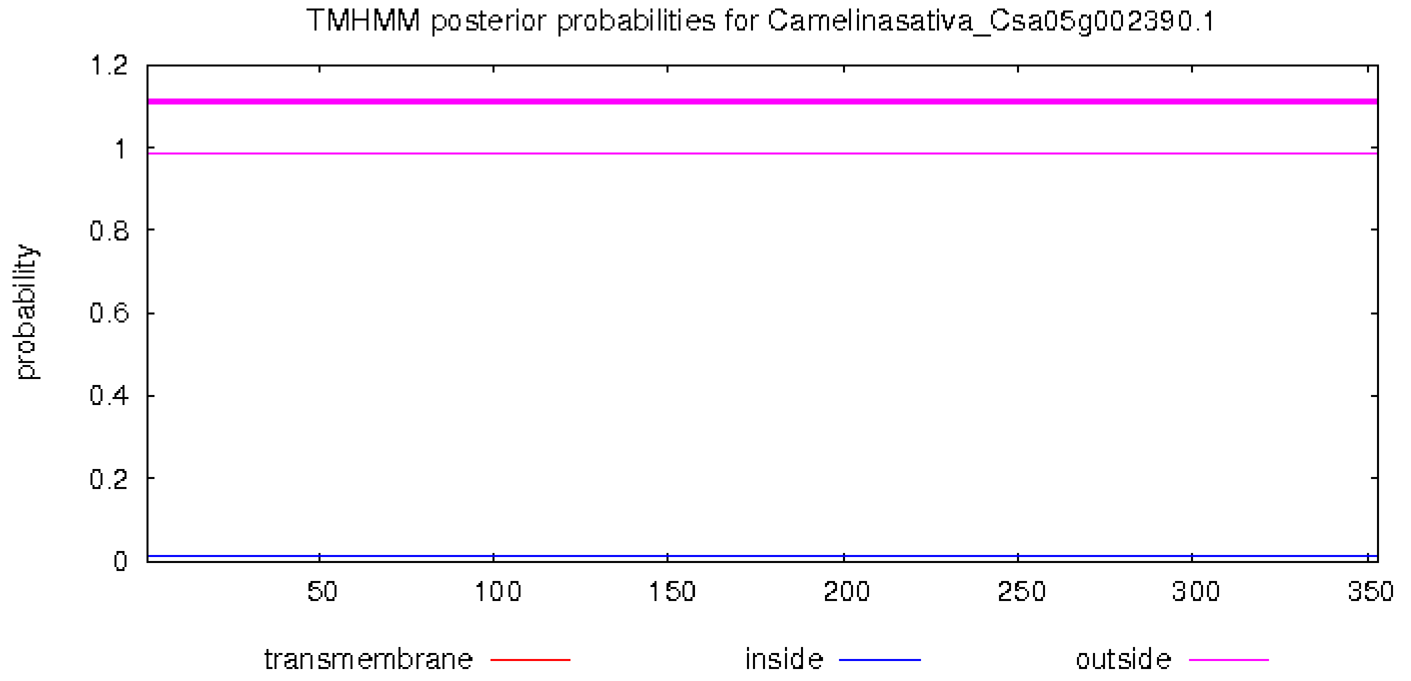

plot in postscript, script for making the plot in gnuplot, data for plot

---

```
# Camelinasativa_Csa05g002930.1 Length: 156
# Camelinasativa_Csa05g002930.1 Number of predicted TMHs:  0
```

```
# Camelinasativa_Csa05g002930.1 Exp number of AAs in TMHs: 0.00054
# Camelinasativa_Csa05g002930.1 Exp number, first 60 AAs:  0
# Camelinasativa_Csa05g002930.1 Total prob of N-in:        0.19192
Camelinasativa_Csa05g002930.1	TMHMM2.0	outside	     1   156
```

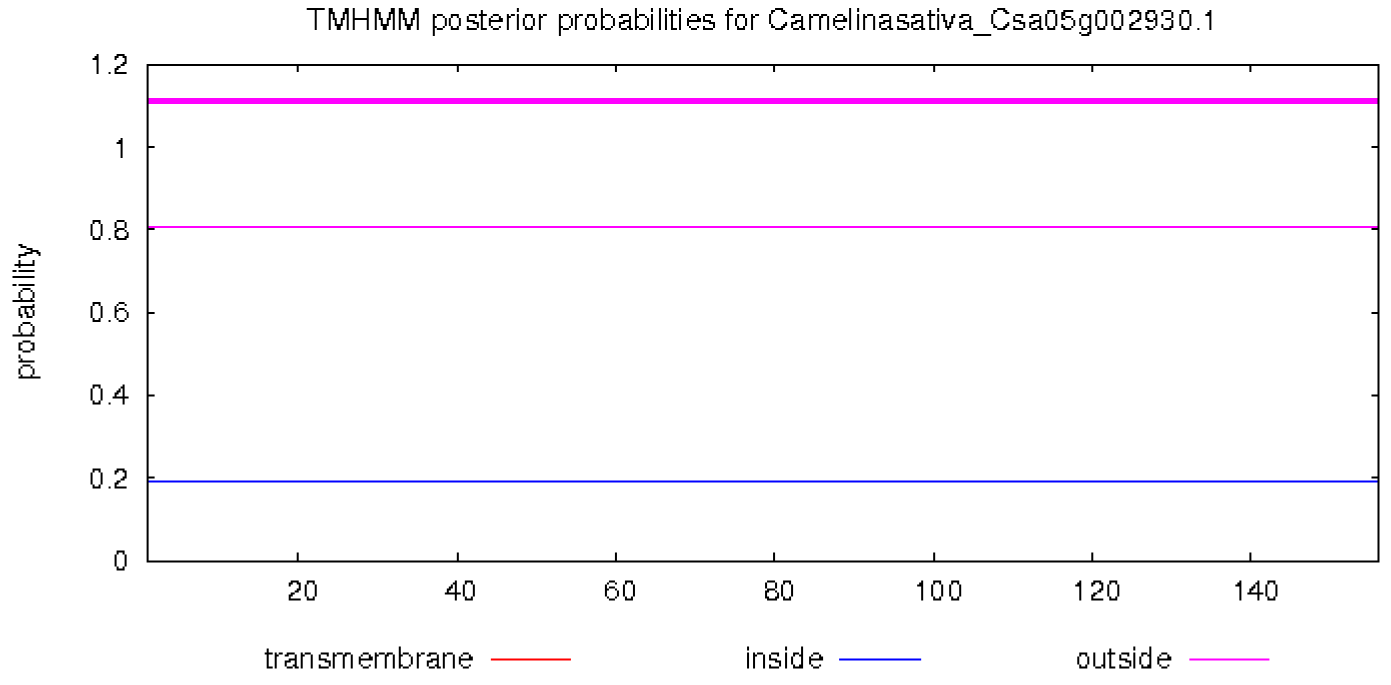

plot in postscript, script for making the plot in gnuplot, data for plot

---

```
# Camelinasativa_Csa05g007460.1 Length: 275
# Camelinasativa_Csa05g007460.1 Number of predicted TMHs:  0
# Camelinasativa_Csa05g007460.1 Exp number of AAs in TMHs: 0.0007
# Camelinasativa_Csa05g007460.1 Exp number, first 60 AAs:  0
# Camelinasativa_Csa05g007460.1 Total prob of N-in:        0.02909
Camelinasativa_Csa05g007460.1	TMHMM2.0	outside	     1   275
```

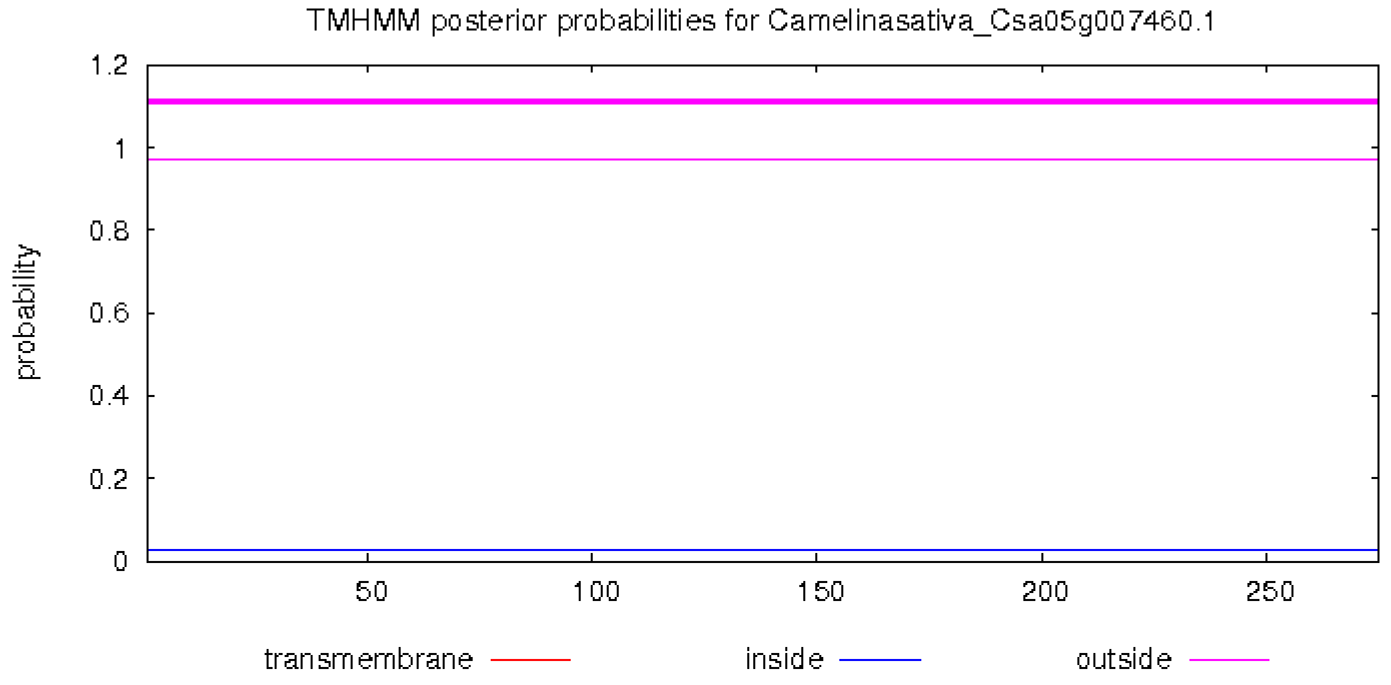

plot in postscript, script for making the plot in gnuplot, data for plot

---

```
# Camelinasativa_Csa05g018490.1 Length: 445
# Camelinasativa_Csa05g018490.1 Number of predicted TMHs:  0
```

```
# Camelinasativa_Csa05g018490.1 Exp number of AAs in TMHs: 0.00208
# Camelinasativa_Csa05g018490.1 Exp number, first 60 AAs:  0
# Camelinasativa_Csa05g018490.1 Total prob of N-in:        0.02146
Camelinasativa_Csa05g018490.1	TMHMM2.0	outside	     1   445
```

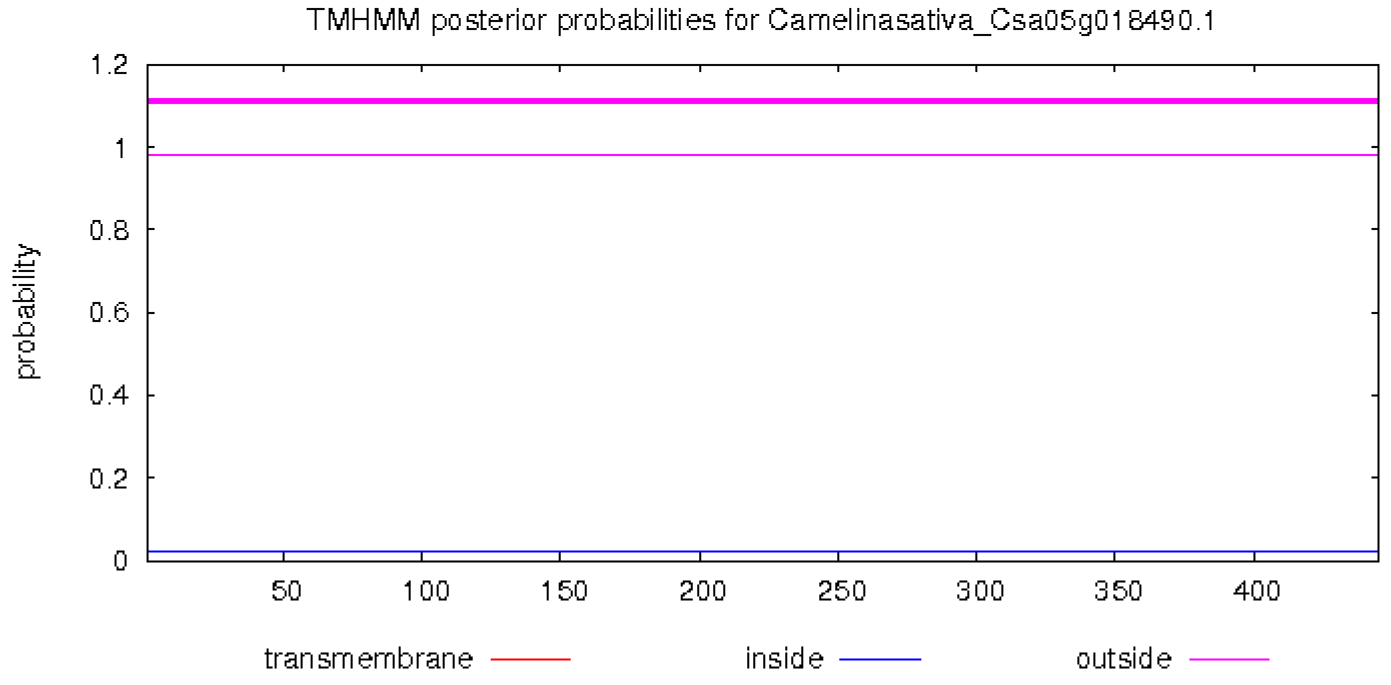

plot in postscript, script for making the plot in gnuplot, data for plot

---

```
# Camelinasativa_Csa05g022930.1 Length: 256
# Camelinasativa_Csa05g022930.1 Number of predicted TMHs:  0
# Camelinasativa_Csa05g022930.1 Exp number of AAs in TMHs: 0.07171
# Camelinasativa_Csa05g022930.1 Exp number, first 60 AAs:  0
# Camelinasativa_Csa05g022930.1 Total prob of N-in:        0.34183
Camelinasativa_Csa05g022930.1	TMHMM2.0	outside	     1   256
```

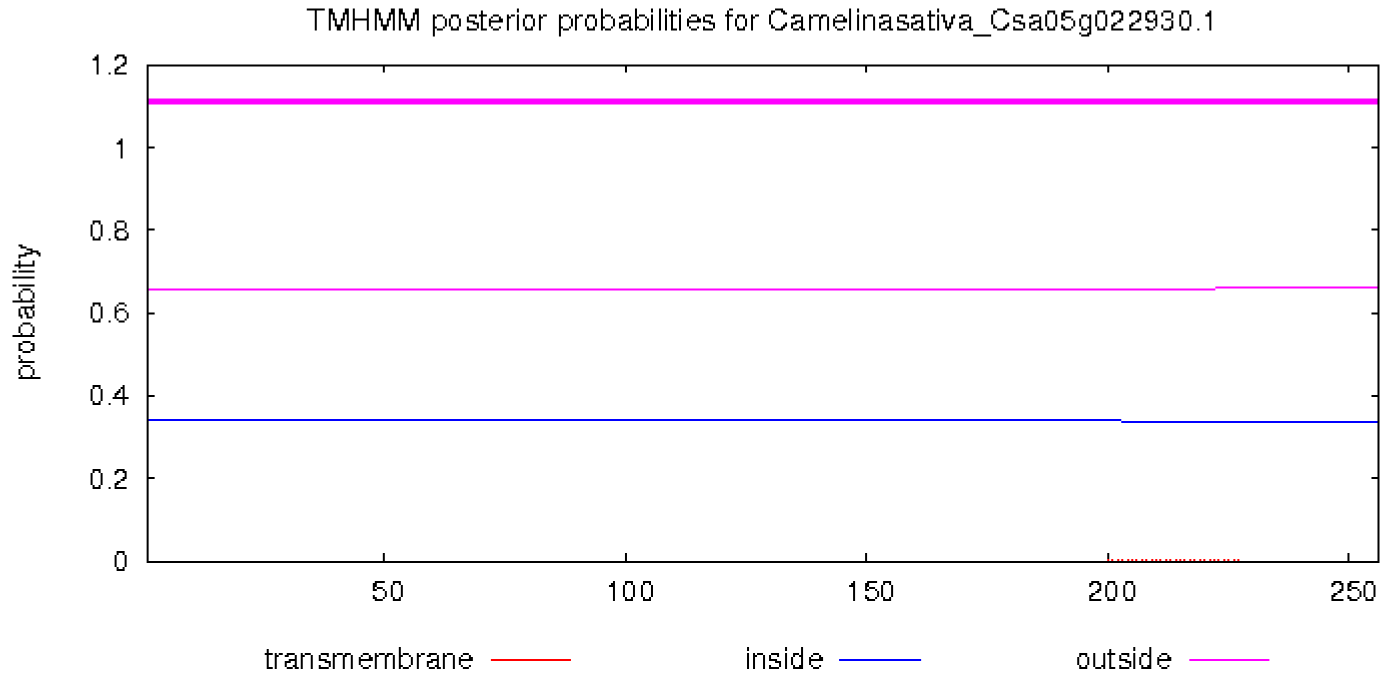

plot in postscript, script for making the plot in gnuplot, data for plot

---

```
# Camelinasativa_Csa05g041670.1 Length: 340
# Camelinasativa_Csa05g041670.1 Number of predicted TMHs:  1
```

```
# Camelinasativa_Csa05g041670.1 Exp number of AAs in TMHs: 21.29635
# Camelinasativa_Csa05g041670.1 Exp number, first 60 AAs:  0.09216
# Camelinasativa_Csa05g041670.1 Total prob of N-in:        0.55513
Camelinasativa_Csa05g041670.1   TMHMM2.0        outside      1   315
Camelinasativa_Csa05g041670.1   TMHMM2.0        TMhelix    316   335
Camelinasativa_Csa05g041670.1   TMHMM2.0        inside     336   340
```

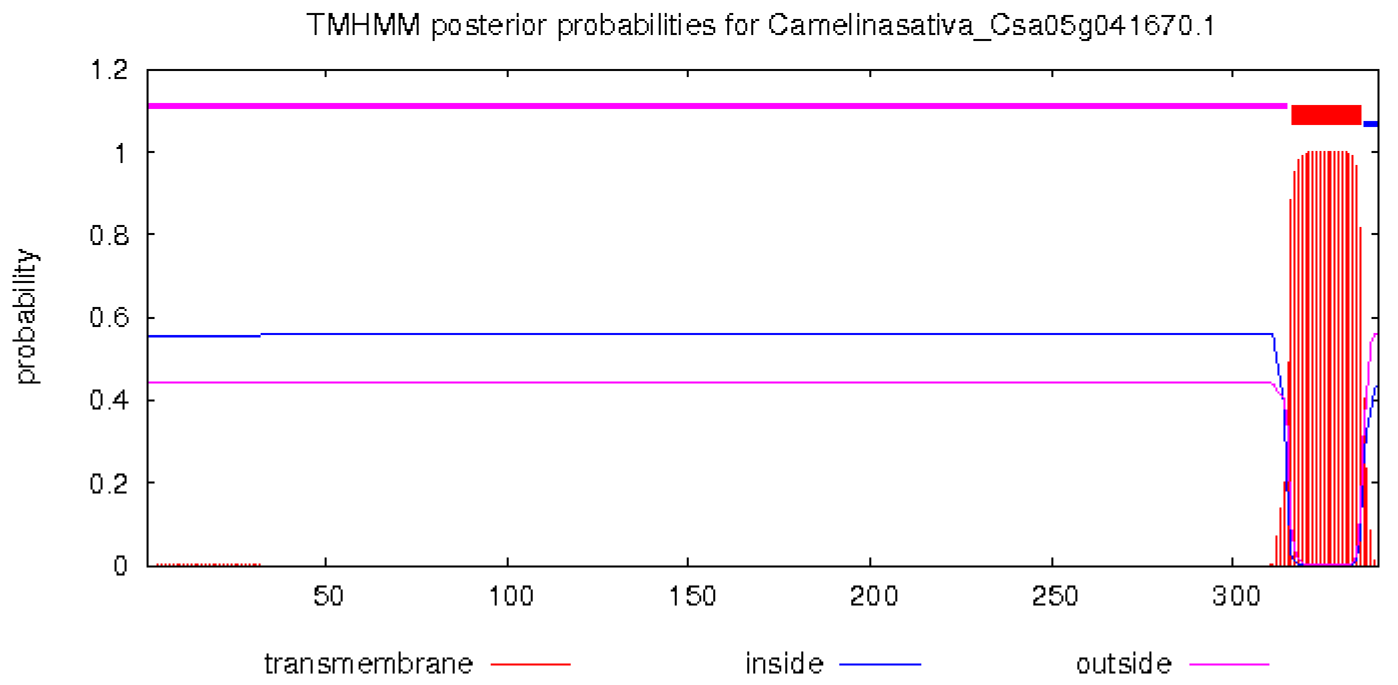

plot in postscript, script for making the plot in gnuplot, data for plot

---

```
# Camelinasativa_Csa05g042090.1 Length: 170
# Camelinasativa_Csa05g042090.1 Number of predicted TMHs:  0
# Camelinasativa_Csa05g042090.1 Exp number of AAs in TMHs: 0.00019
# Camelinasativa_Csa05g042090.1 Exp number, first 60 AAs:  0
# Camelinasativa_Csa05g042090.1 Total prob of N-in:        0.52052
Camelinasativa_Csa05g042090.1   TMHMM2.0        inside       1   170
```

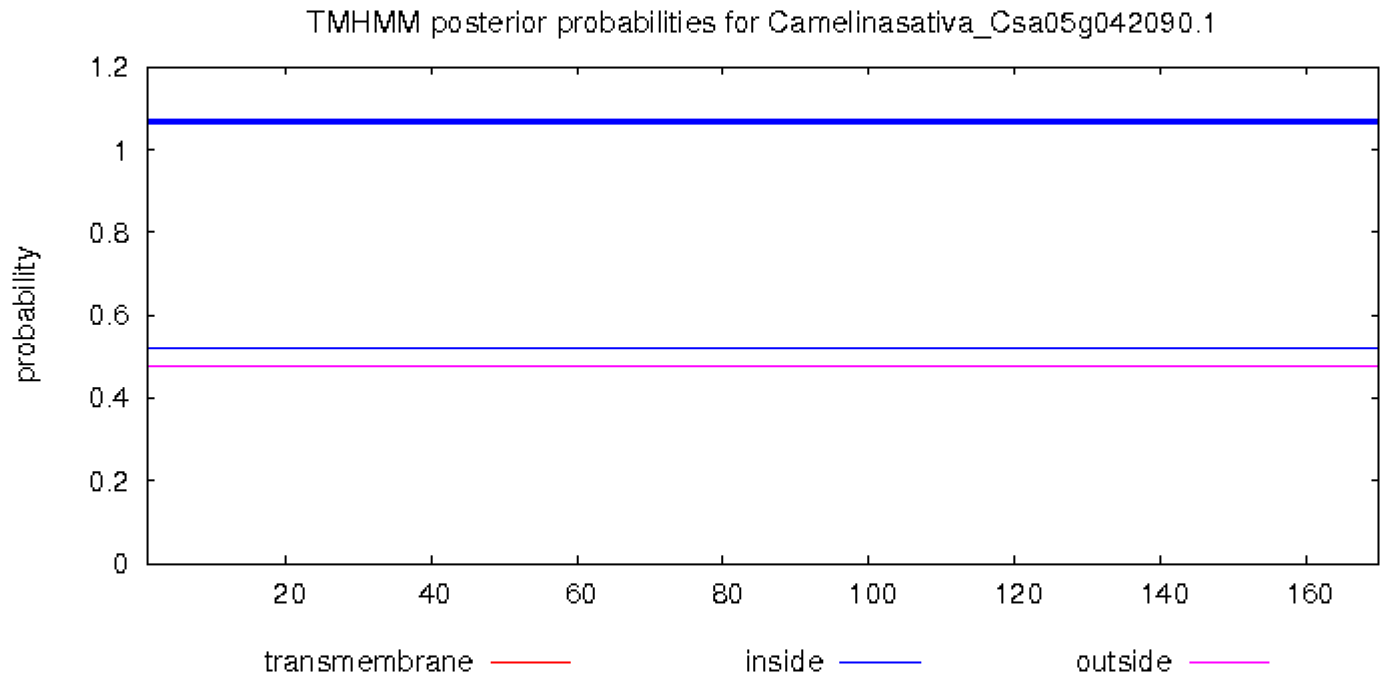

plot in postscript, script for making the plot in gnuplot, data for plot

---

```
# Camelinasativa_Csa05g042120.1 Length: 290
# Camelinasativa_Csa05g042120.1 Number of predicted TMHs:  0
# Camelinasativa_Csa05g042120.1 Exp number of AAs in TMHs: 0
# Camelinasativa_Csa05g042120.1 Exp number, first 60 AAs:  0
# Camelinasativa_Csa05g042120.1 Total prob of N-in:        0.09037
Camelinasativa_Csa05g042120.1   TMHMM2.0        outside      1   290
```

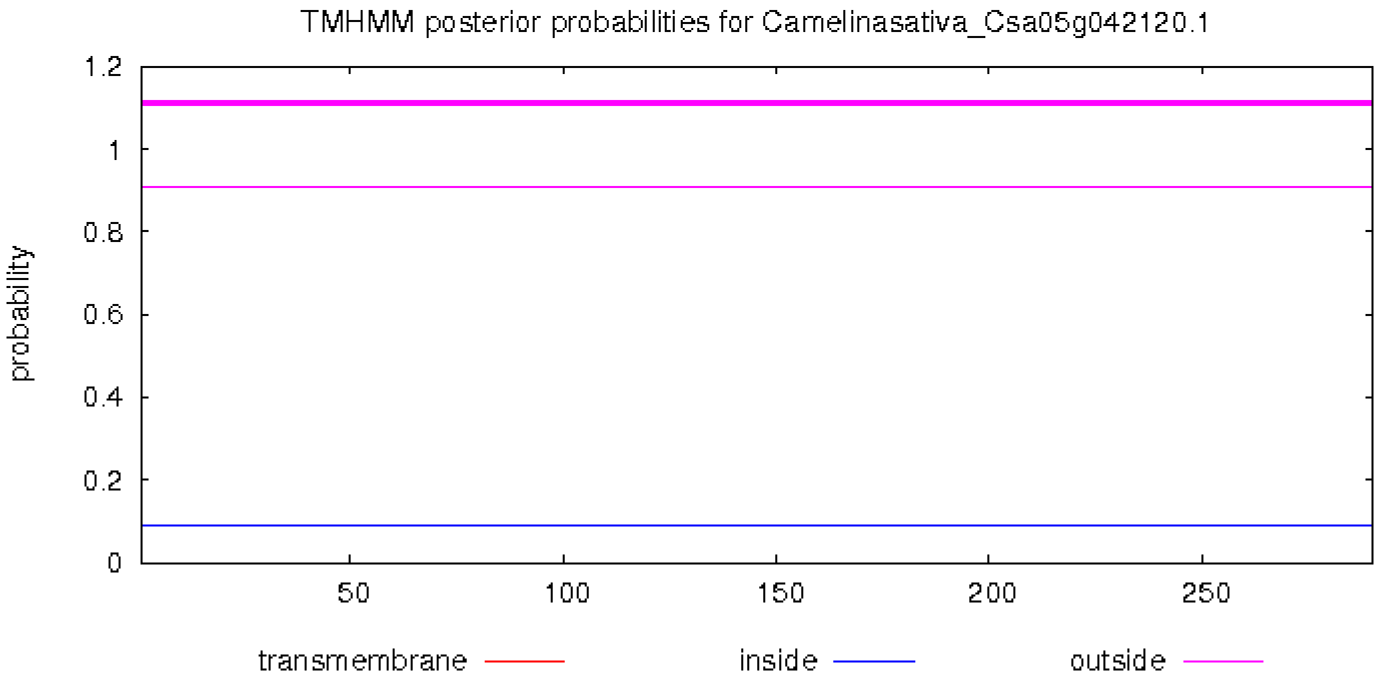

plot in postscript, script for making the plot in gnuplot, data for plot

---

```
# Camelinasativa_Csa05g047220.1 Length: 234
# Camelinasativa_Csa05g047220.1 Number of predicted TMHs:  0
# Camelinasativa_Csa05g047220.1 Exp number of AAs in TMHs: 0.20031
# Camelinasativa_Csa05g047220.1 Exp number, first 60 AAs:  0.0026
# Camelinasativa_Csa05g047220.1 Total prob of N-in:        0.09392
Camelinasativa_Csa05g047220.1   TMHMM2.0        outside      1   234
```

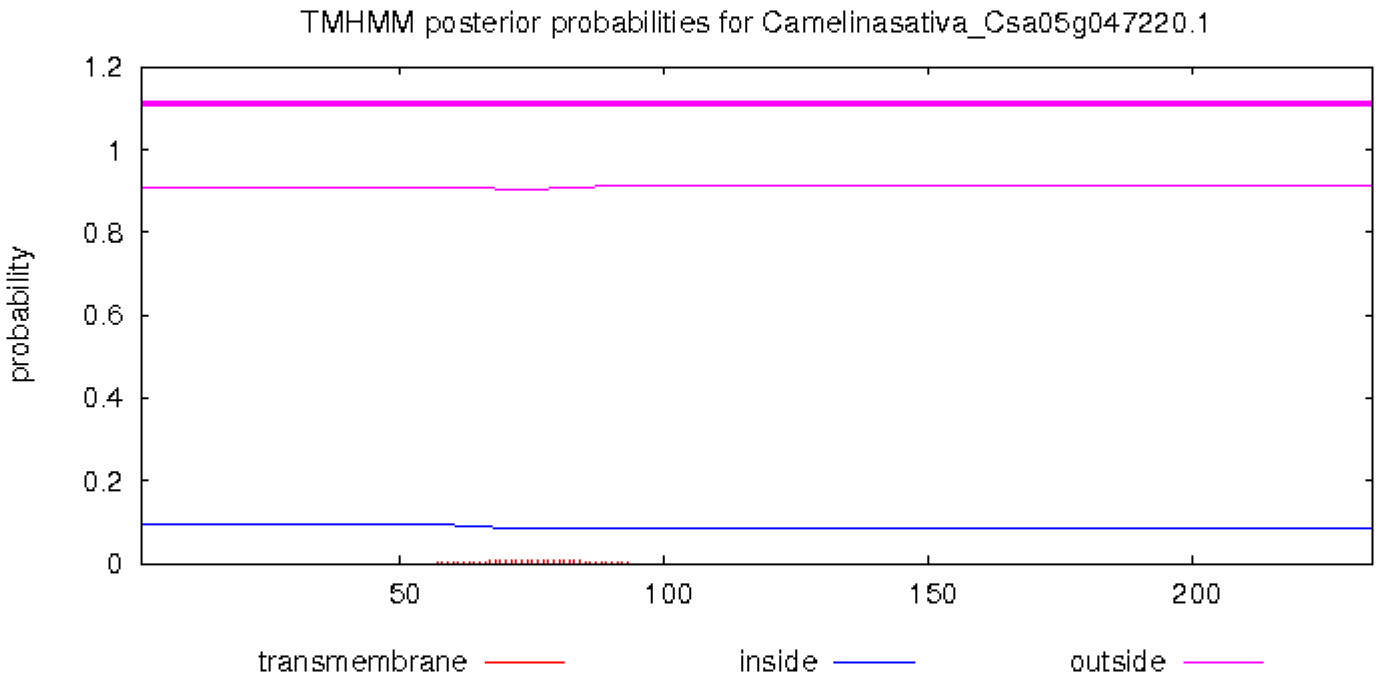

plot in postscript, script for making the plot in gnuplot, data for plot

---

```
# Camelinasativa_Csa05g047230.1 Length: 344
# Camelinasativa_Csa05g047230.1 Number of predicted TMHs:  0
# Camelinasativa_Csa05g047230.1 Exp number of AAs in TMHs: 0.0049
# Camelinasativa_Csa05g047230.1 Exp number, first 60 AAs:  0
# Camelinasativa_Csa05g047230.1 Total prob of N-in:        0.00842
Camelinasativa_Csa05g047230.1   TMHMM2.0        outside      1   344
```

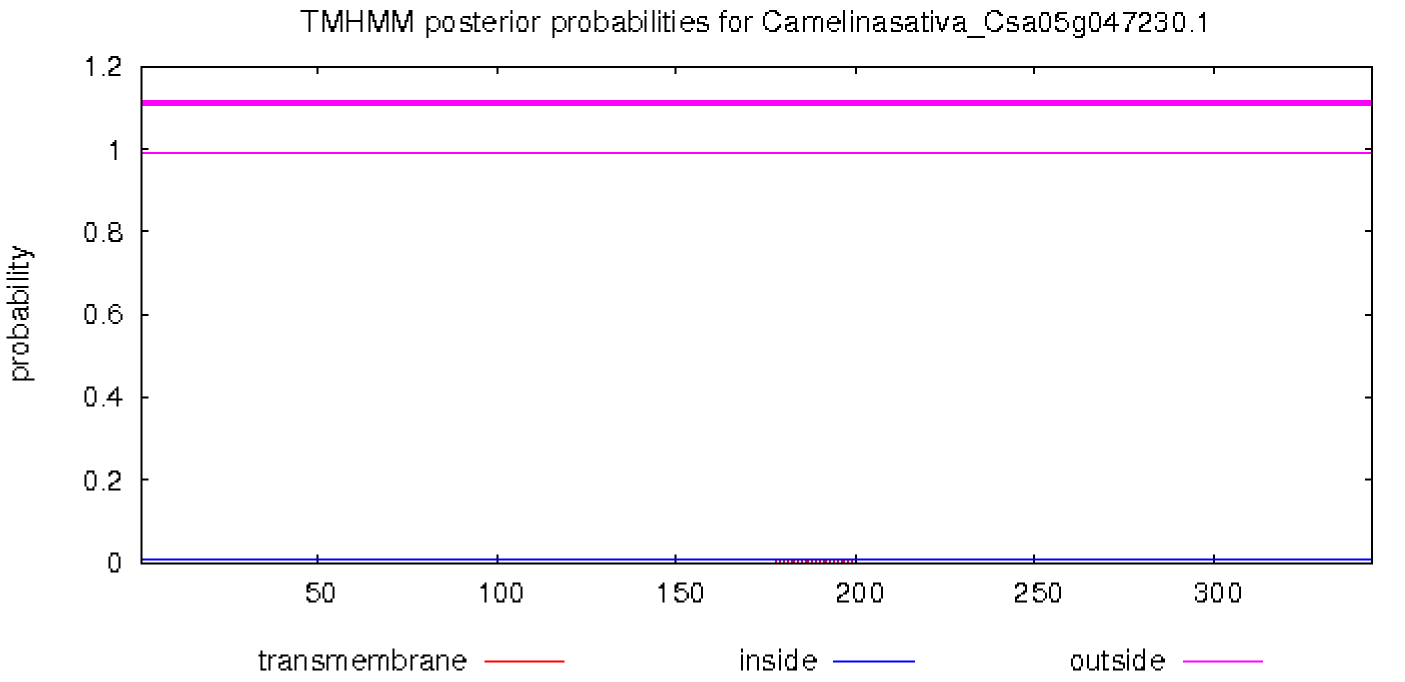

plot in postscript, script for making the plot in gnuplot, data for plot

```
# Camelinasativa_Csa05g047250.1 Length: 161
# Camelinasativa_Csa05g047250.1 Number of predicted TMHs:  0
# Camelinasativa_Csa05g047250.1 Exp number of AAs in TMHs: 0.00108
# Camelinasativa_Csa05g047250.1 Exp number, first 60 AAs:  0.00019
# Camelinasativa_Csa05g047250.1 Total prob of N-in:        0.28536
Camelinasativa_Csa05g047250.1   TMHMM2.0        outside      1   161
```

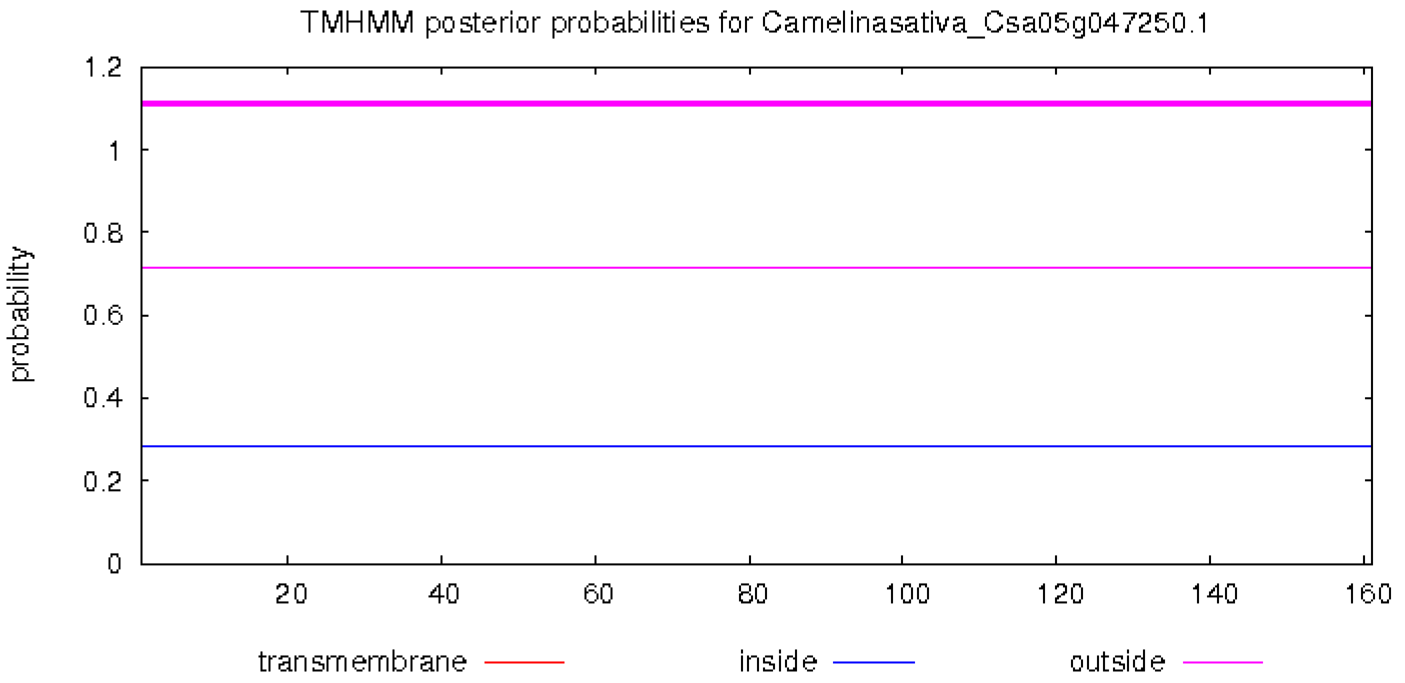

plot in postscript, script for making the plot in gnuplot, data for plot

```
# Camelinasativa_Csa05g070860.1 Length: 178
# Camelinasativa_Csa05g070860.1 Number of predicted TMHs:  0
# Camelinasativa_Csa05g070860.1 Exp number of AAs in TMHs: 0.00039
# Camelinasativa_Csa05g070860.1 Exp number, first 60 AAs:  0.00039
# Camelinasativa_Csa05g070860.1 Total prob of N-in:        0.42738
Camelinasativa_Csa05g070860.1   TMHMM2.0        outside      1   178
```

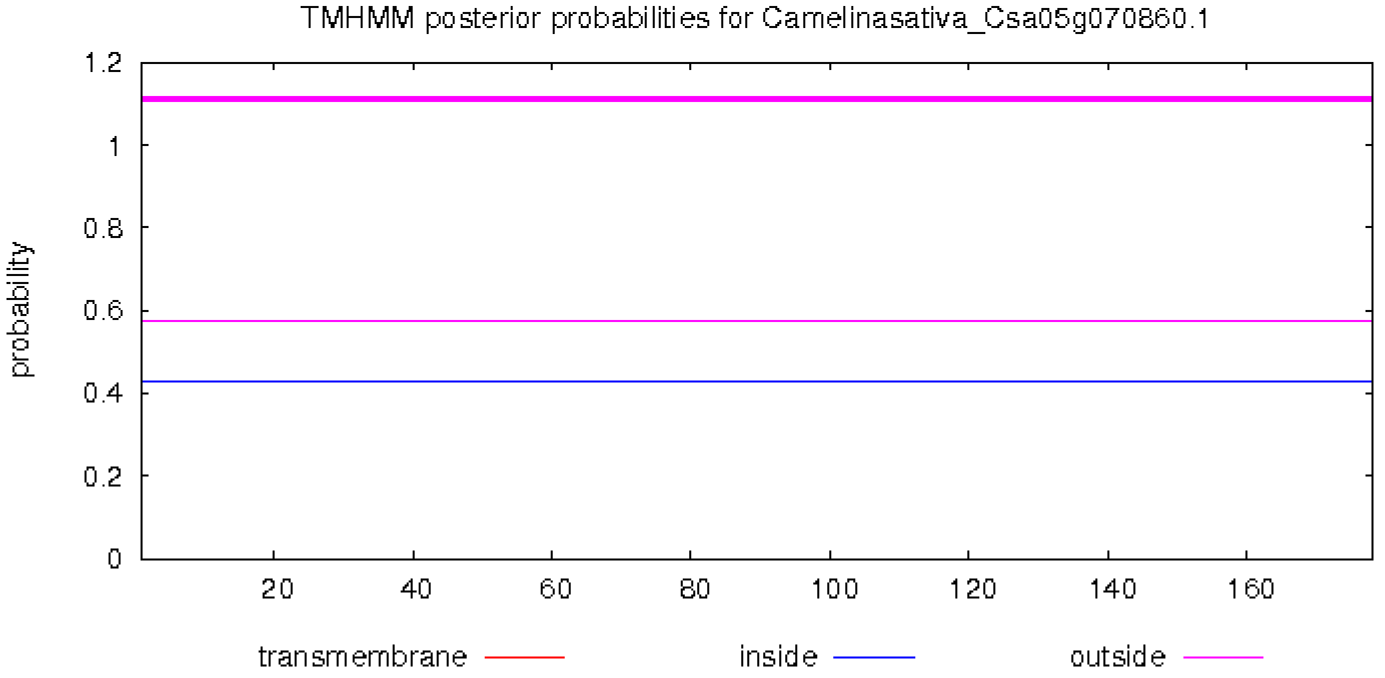

plot in postscript, script for making the plot in gnuplot, data for plot

```
# Camelinasativa_Csa05g073600.1 Length: 628
# Camelinasativa_Csa05g073600.1 Number of predicted TMHs:  0
# Camelinasativa_Csa05g073600.1 Exp number of AAs in TMHs: 7.2114
# Camelinasativa_Csa05g073600.1 Exp number, first 60 AAs:  0
# Camelinasativa_Csa05g073600.1 Total prob of N-in:        0.00008
Camelinasativa_Csa05g073600.1   TMHMM2.0        outside      1   628
```

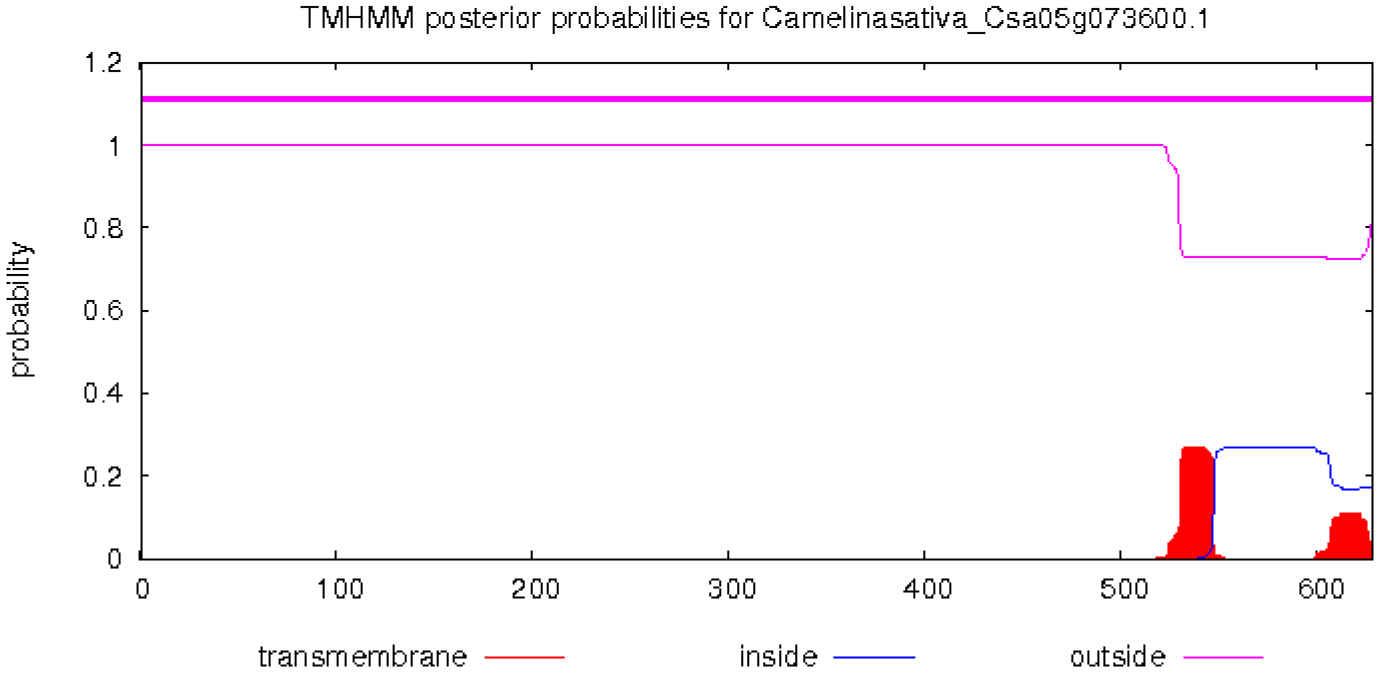

plot in postscript, script for making the plot in gnuplot, data for plot

```
# Camelinasativa_Csa05g086200.1 Length: 273
# Camelinasativa_Csa05g086200.1 Number of predicted TMHs:  0
# Camelinasativa_Csa05g086200.1 Exp number of AAs in TMHs: 0.00046
# Camelinasativa_Csa05g086200.1 Exp number, first 60 AAs:  0
# Camelinasativa_Csa05g086200.1 Total prob of N-in:        0.01800
Camelinasativa_Csa05g086200.1	TMHMM2.0	outside	     1   273
```

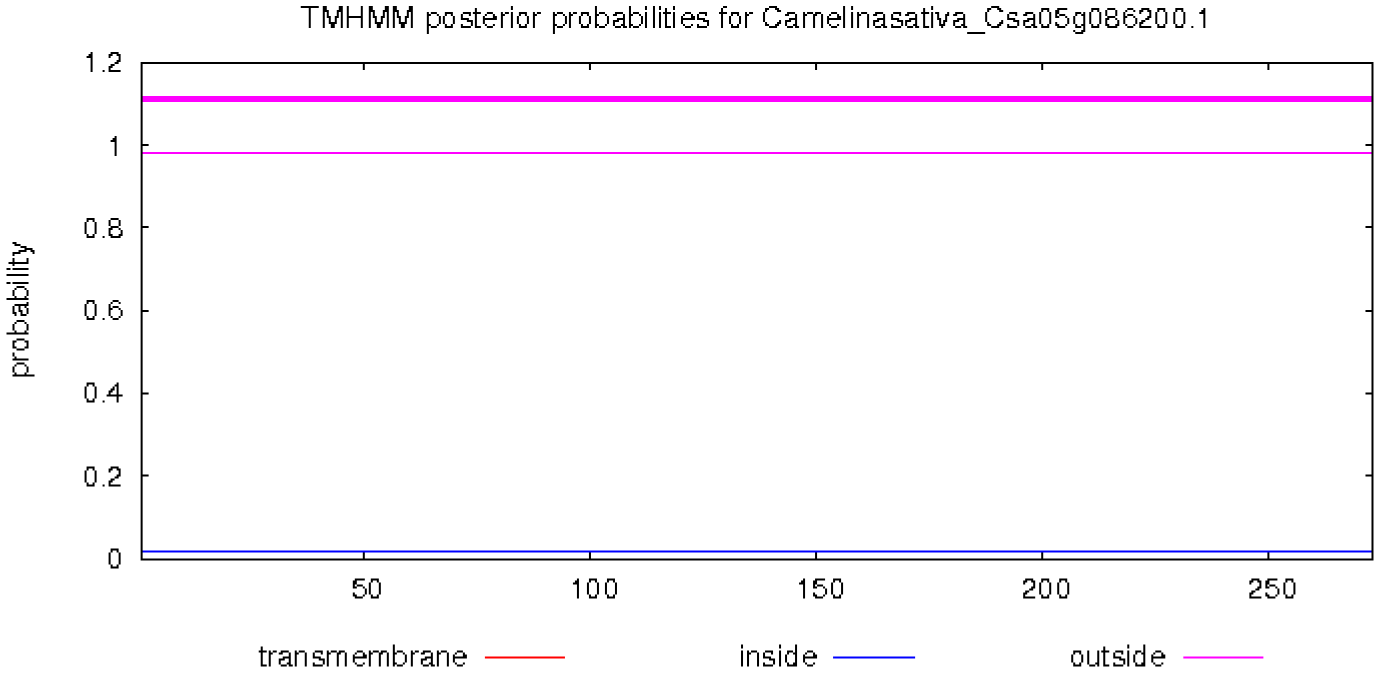

plot in postscript, script for making the plot in gnuplot, data for plot

```
# Camelinasativa_Csa05g086390.1 Length: 353
# Camelinasativa_Csa05g086390.1 Number of predicted TMHs:  0
# Camelinasativa_Csa05g086390.1 Exp number of AAs in TMHs: 0.00022
# Camelinasativa_Csa05g086390.1 Exp number, first 60 AAs:  0
# Camelinasativa_Csa05g086390.1 Total prob of N-in:        0.01092
Camelinasativa_Csa05g086390.1	TMHMM2.0	outside	     1   353
```

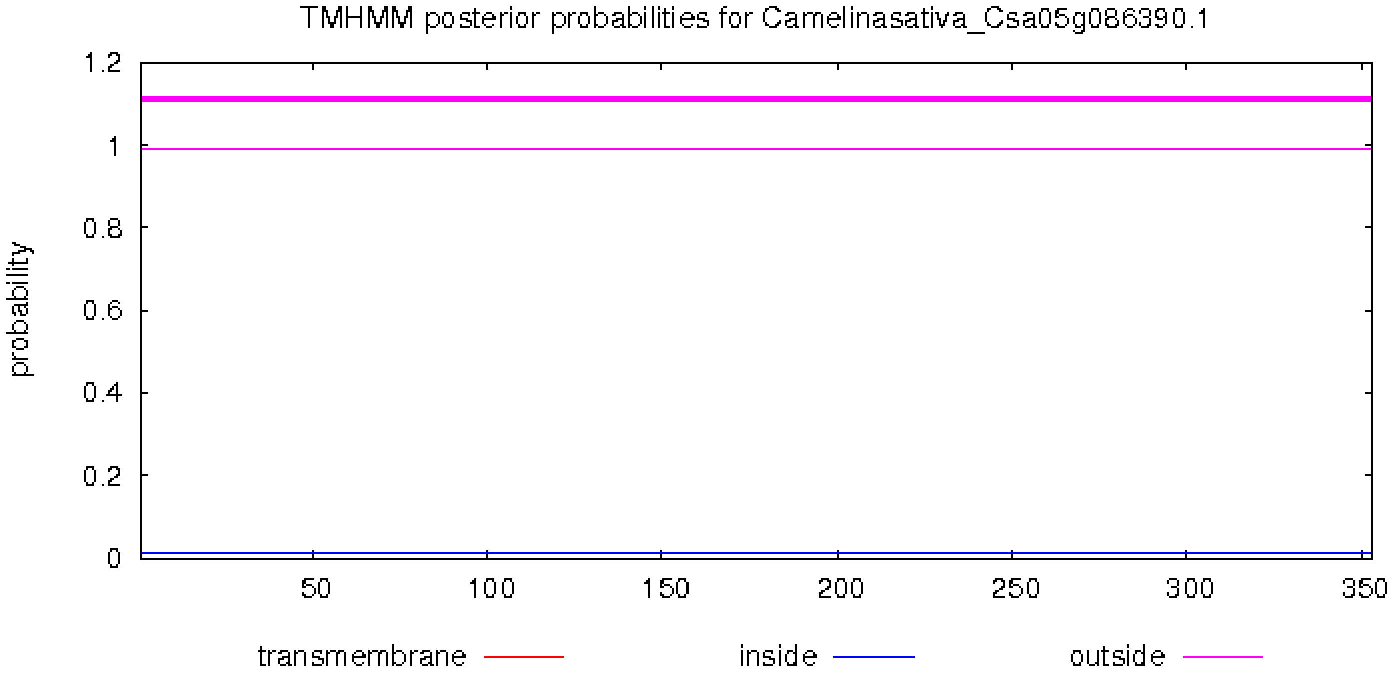

plot in postscript, script for making the plot in gnuplot, data for plot

```
# Camelinasativa_Csa05g094110.1 Length: 336
# Camelinasativa_Csa05g094110.1 Number of predicted TMHs:  0
# Camelinasativa_Csa05g094110.1 Exp number of AAs in TMHs: 0
# Camelinasativa_Csa05g094110.1 Exp number, first 60 AAs:  0
# Camelinasativa_Csa05g094110.1 Total prob of N-in:        0.02904
Camelinasativa_Csa05g094110.1   TMHMM2.0        outside      1   336
```

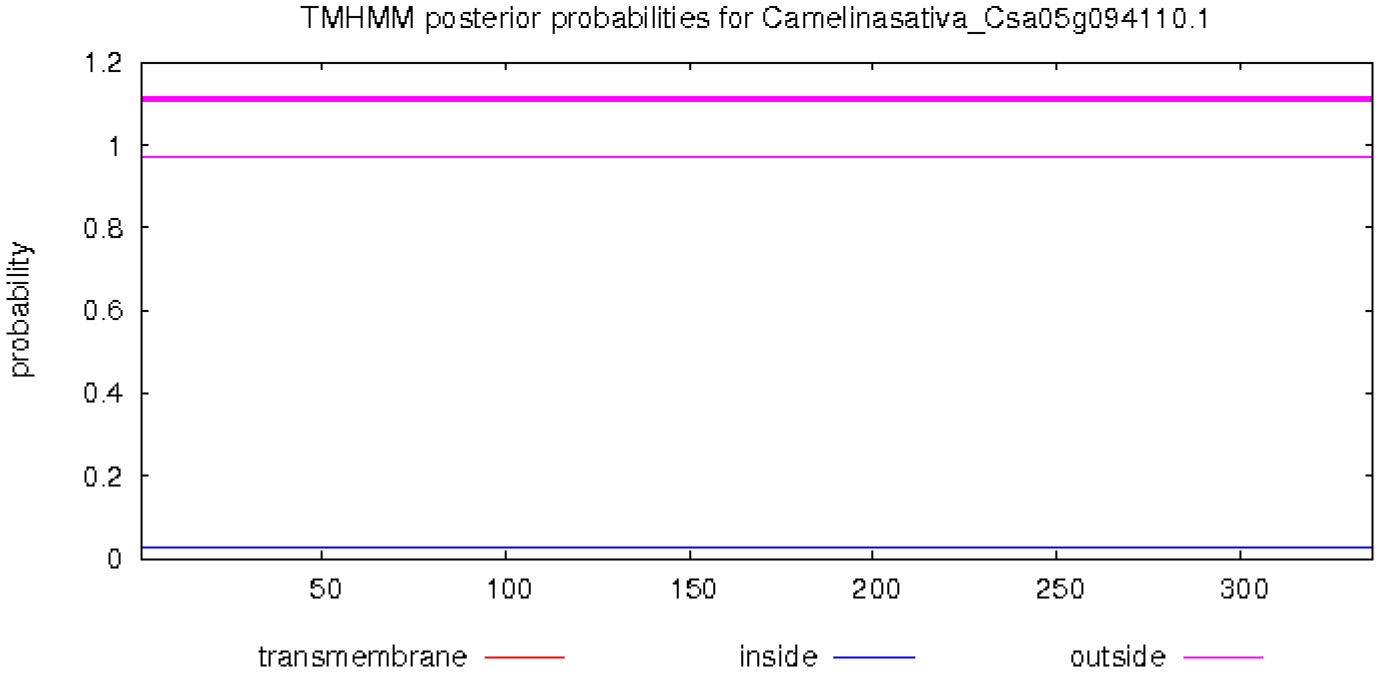

plot in postscript, script for making the plot in gnuplot, data for plot

---

```
# Camelinasativa_Csa06g002590.1 Length: 377
# Camelinasativa_Csa06g002590.1 Number of predicted TMHs:  0
# Camelinasativa_Csa06g002590.1 Exp number of AAs in TMHs: 0.04186
# Camelinasativa_Csa06g002590.1 Exp number, first 60 AAs:  0
# Camelinasativa_Csa06g002590.1 Total prob of N-in:        0.01494
Camelinasativa_Csa06g002590.1   TMHMM2.0        outside      1   377
```

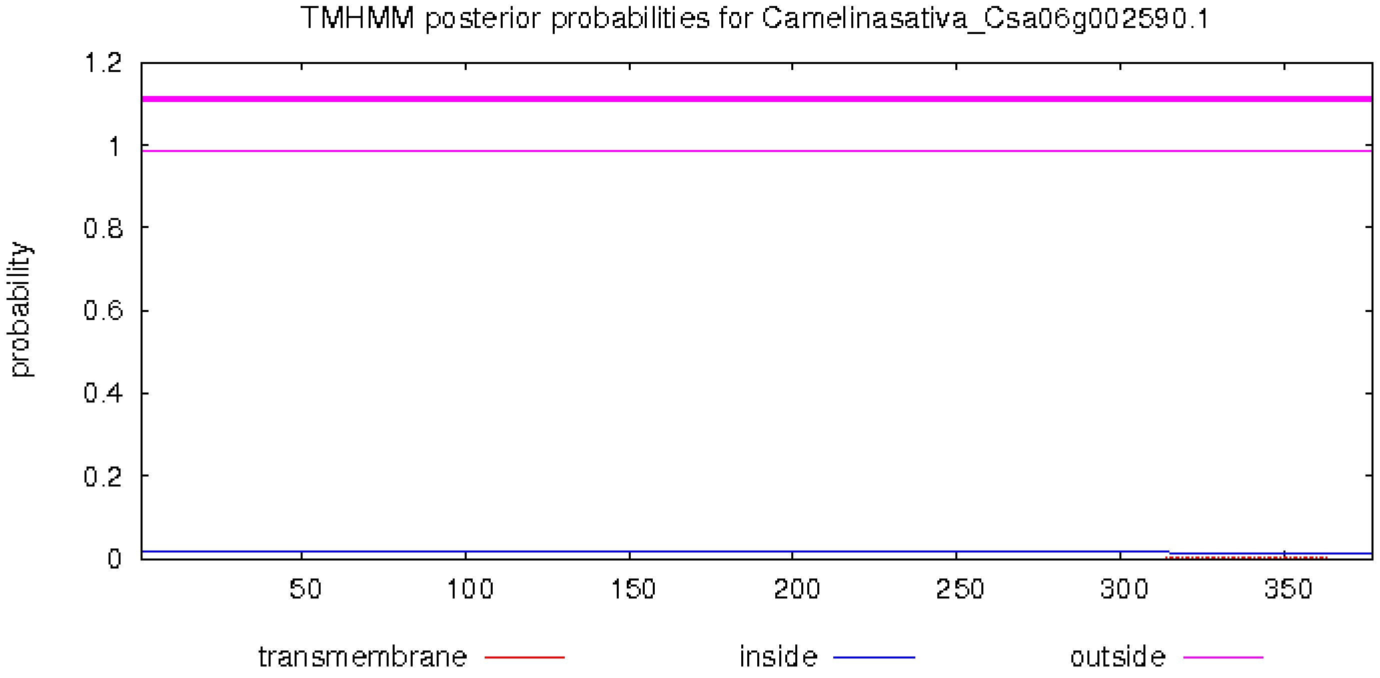

plot in postscript, script for making the plot in gnuplot, data for plot

---

```
# Camelinasativa_Csa06g006850.1 Length: 175
# Camelinasativa_Csa06g006850.1 Number of predicted TMHs:  0
# Camelinasativa_Csa06g006850.1 Exp number of AAs in TMHs: 0.00086
# Camelinasativa_Csa06g006850.1 Exp number, first 60 AAs:  0.00086
# Camelinasativa_Csa06g006850.1 Total prob of N-in:        0.02788
Camelinasativa_Csa06g006850.1   TMHMM2.0        outside      1   175
```

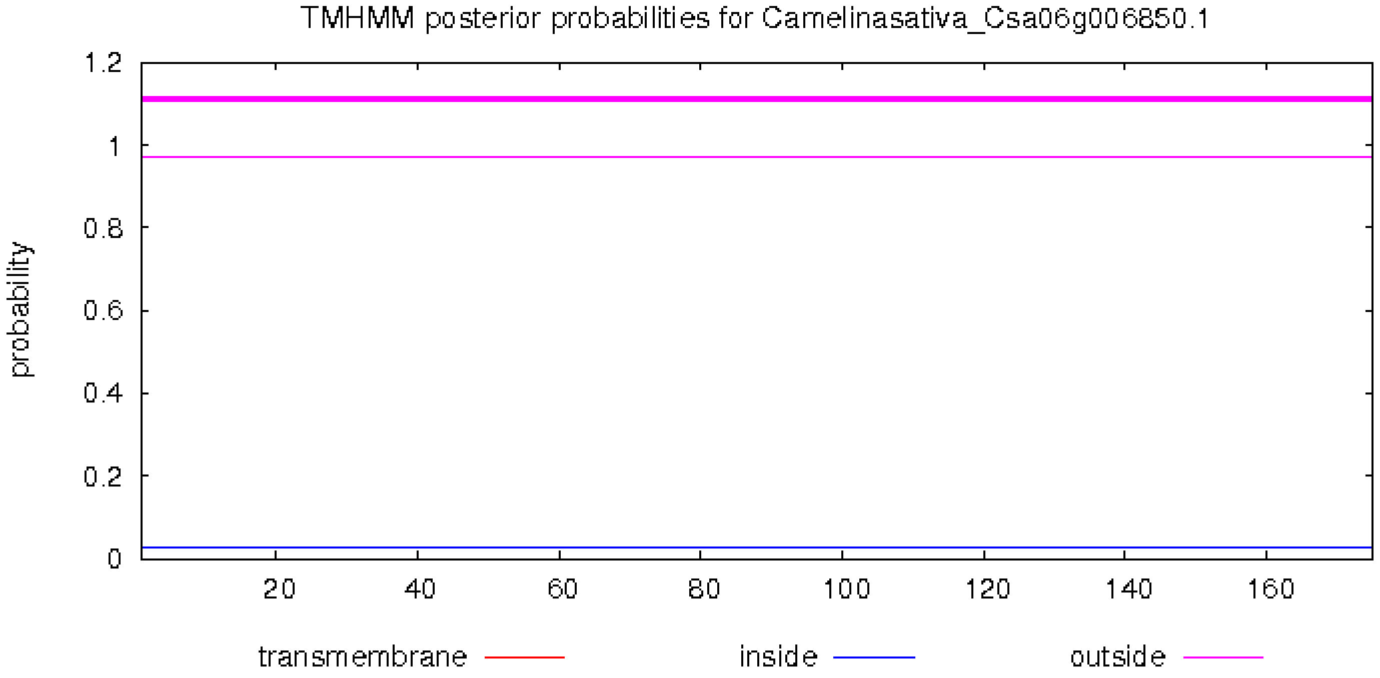

plot in postscript, script for making the plot in gnuplot, data for plot

---

```
# Camelinasativa_Csa06g007190.1 Length: 337
# Camelinasativa_Csa06g007190.1 Number of predicted TMHs:  0
# Camelinasativa_Csa06g007190.1 Exp number of AAs in TMHs: 0.1693
# Camelinasativa_Csa06g007190.1 Exp number, first 60 AAs:  0
# Camelinasativa_Csa06g007190.1 Total prob of N-in:        0.02173
Camelinasativa_Csa06g007190.1   TMHMM2.0        outside      1   337
```

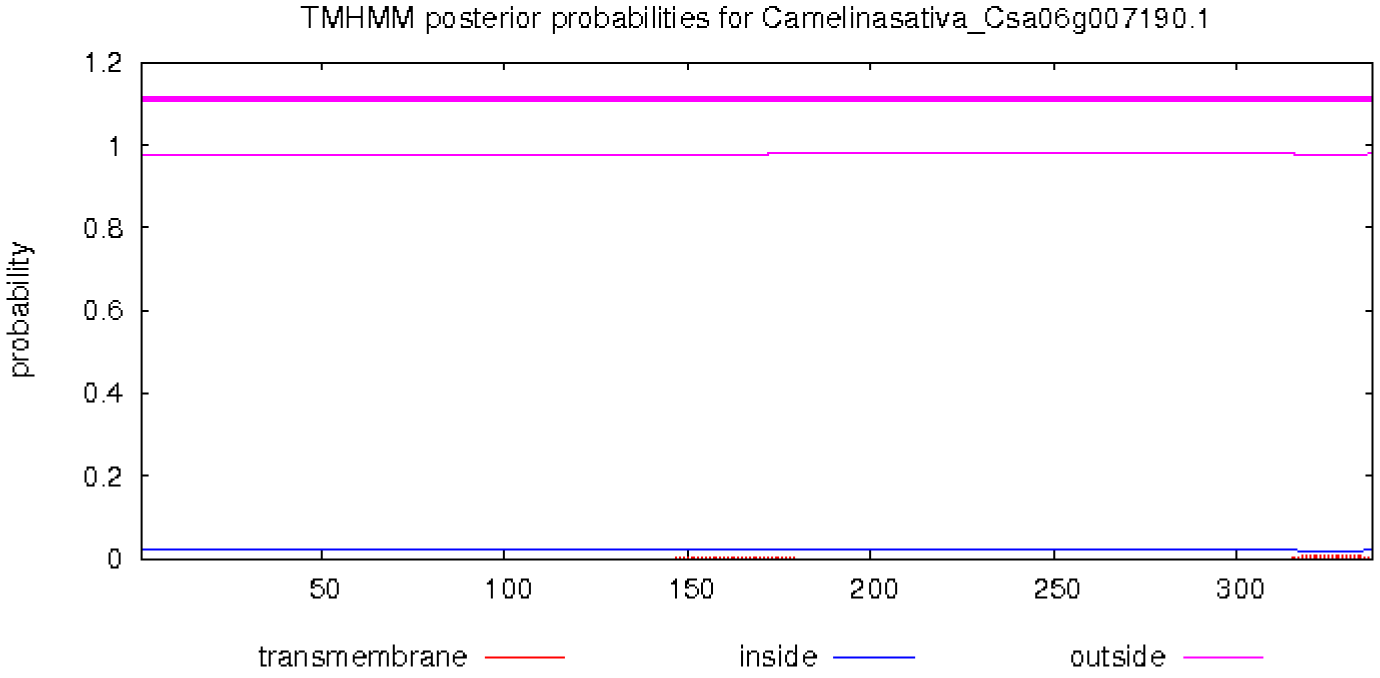

plot in postscript, script for making the plot in gnuplot, data for plot

---

```
# Camelinasativa_Csa06g011390.1 Length: 333
# Camelinasativa_Csa06g011390.1 Number of predicted TMHs:  0
# Camelinasativa_Csa06g011390.1 Exp number of AAs in TMHs: 0.00186
# Camelinasativa_Csa06g011390.1 Exp number, first 60 AAs:  0.0002
# Camelinasativa_Csa06g011390.1 Total prob of N-in:        0.03530
Camelinasativa_Csa06g011390.1   TMHMM2.0        outside      1   333
```

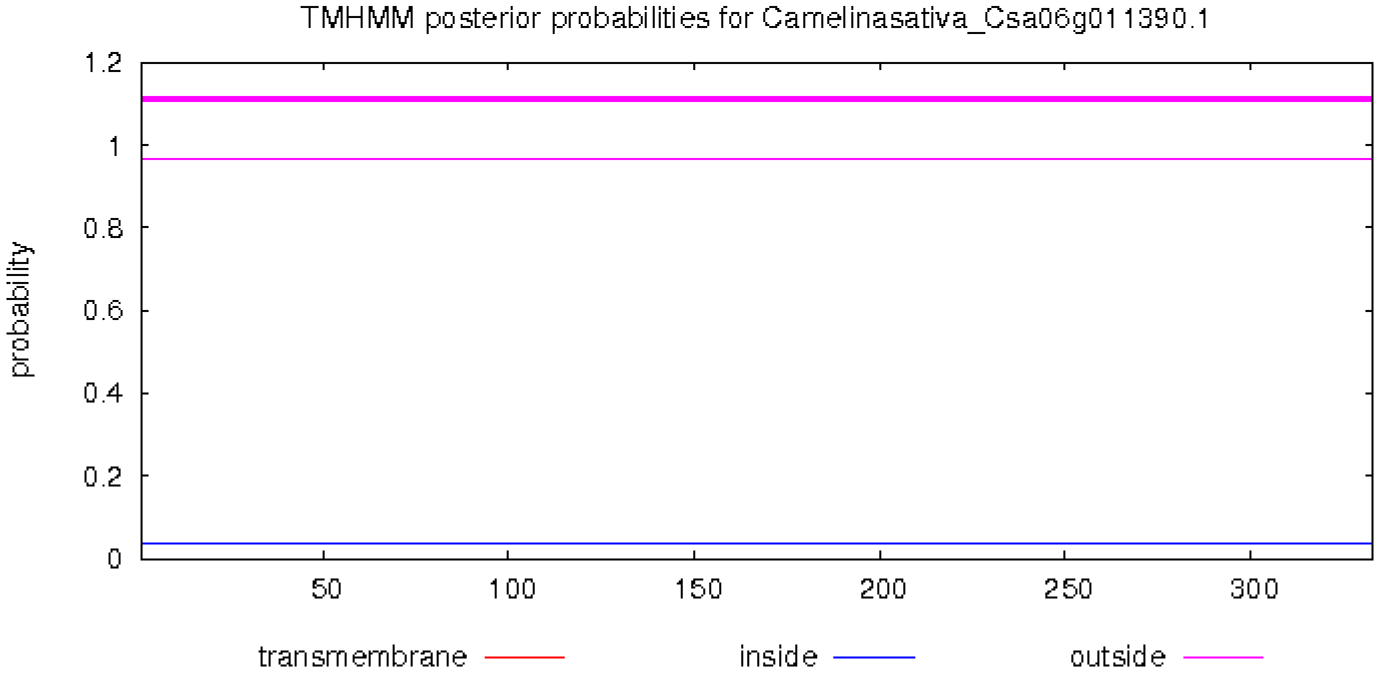

plot in postscript, script for making the plot in gnuplot, data for plot

---

```
# Camelinasativa_Csa06g016930.1 Length: 357
# Camelinasativa_Csa06g016930.1 Number of predicted TMHs:  1
# Camelinasativa_Csa06g016930.1 Exp number of AAs in TMHs: 22.33175
# Camelinasativa_Csa06g016930.1 Exp number, first 60 AAs:  4.33629
# Camelinasativa_Csa06g016930.1 Total prob of N-in:        0.36532
Camelinasativa_Csa06g016930.1   TMHMM2.0        outside      1   338
Camelinasativa_Csa06g016930.1   TMHMM2.0        TMhelix    339   356
Camelinasativa_Csa06g016930.1   TMHMM2.0        inside     357   357
```

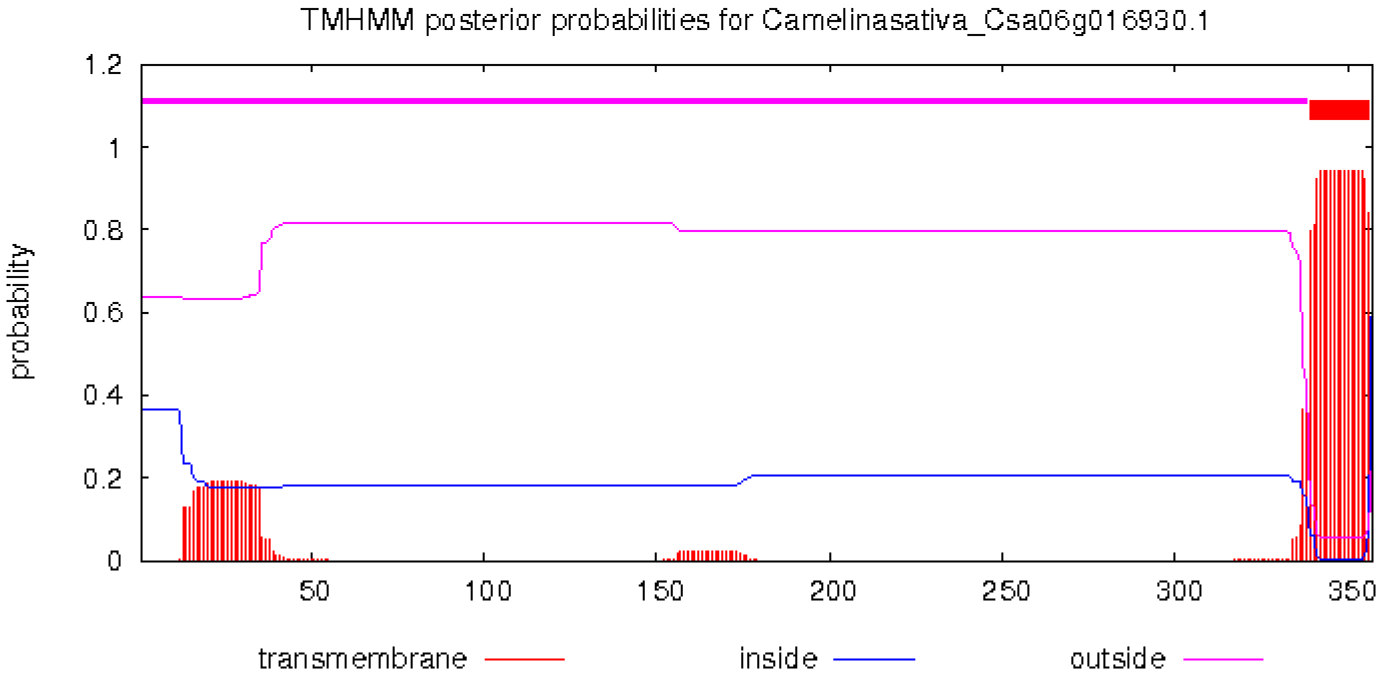

plot in postscript, script for making the plot in gnuplot, data for plot

```
# Camelinasativa_Csa06g017030.1 Length: 242
# Camelinasativa_Csa06g017030.1 Number of predicted TMHs:  0
# Camelinasativa_Csa06g017030.1 Exp number of AAs in TMHs: 0.01873
# Camelinasativa_Csa06g017030.1 Exp number, first 60 AAs:  0.01847
# Camelinasativa_Csa06g017030.1 Total prob of N-in:        0.02469
Camelinasativa_Csa06g017030.1   TMHMM2.0        outside      1   242
```

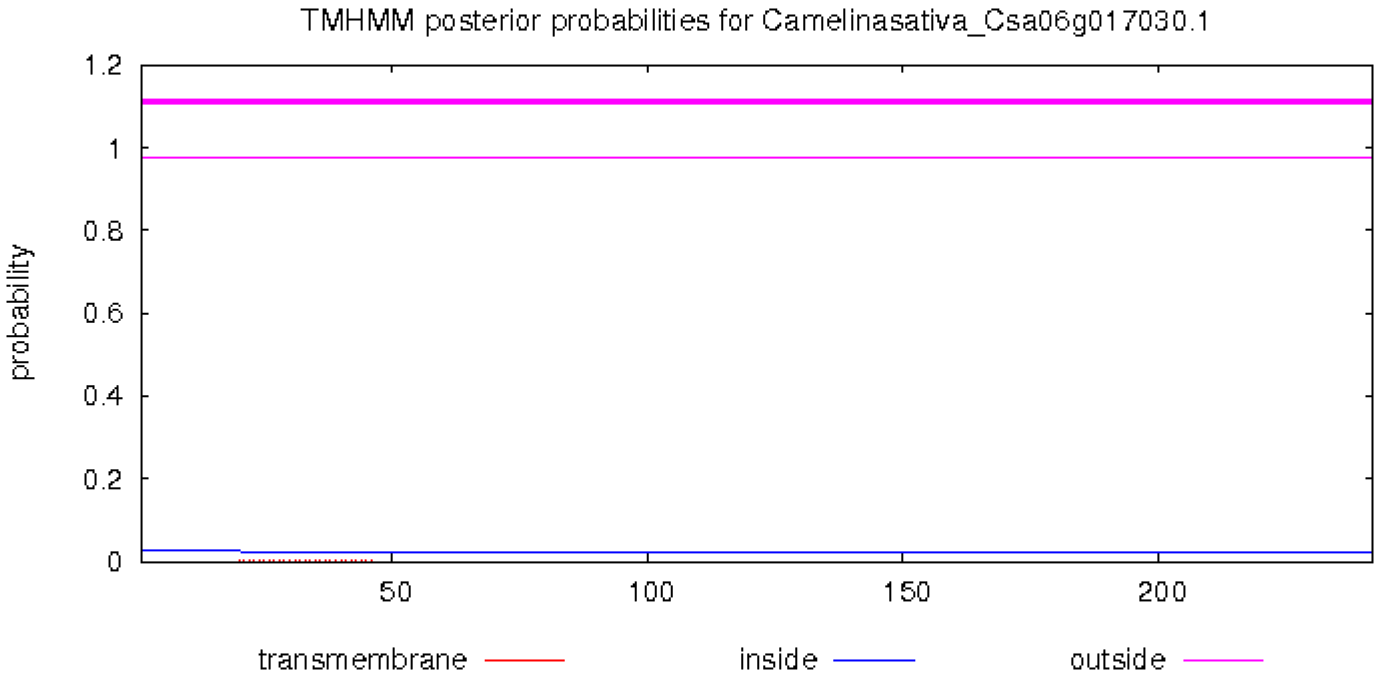

plot in postscript, script for making the plot in gnuplot, data for plot

```
# Camelinasativa_Csa06g023540.1 Length: 453
# Camelinasativa_Csa06g023540.1 Number of predicted TMHs:  1
# Camelinasativa_Csa06g023540.1 Exp number of AAs in TMHs: 20.48777
# Camelinasativa_Csa06g023540.1 Exp number, first 60 AAs:  0
# Camelinasativa_Csa06g023540.1 Total prob of N-in:        0.06011
Camelinasativa_Csa06g023540.1   TMHMM2.0        outside      1   422
Camelinasativa_Csa06g023540.1   TMHMM2.0        TMhelix    423   445
Camelinasativa_Csa06g023540.1   TMHMM2.0        inside     446   453
```

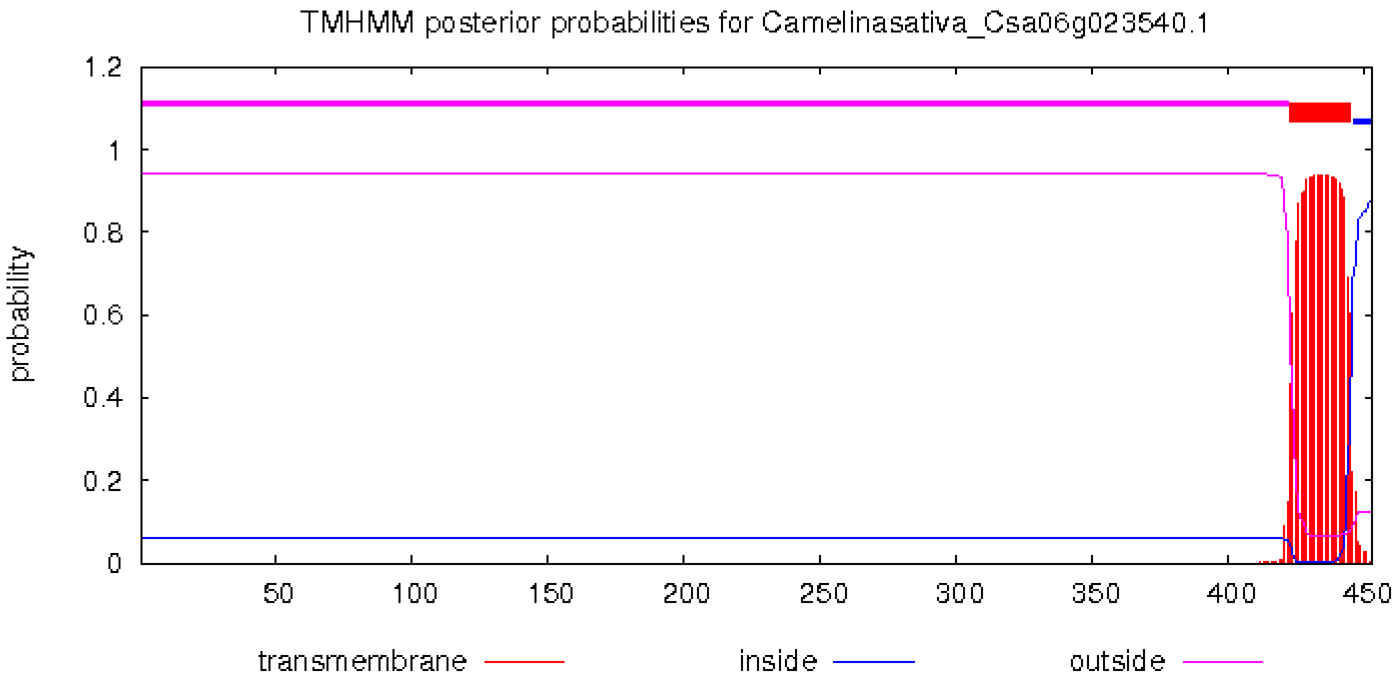

plot in postscript, script for making the plot in gnuplot, data for plot

```
# Camelinasativa_Csa06g031260.1 Length: 580
# Camelinasativa_Csa06g031260.1 Number of predicted TMHs:  0
# Camelinasativa_Csa06g031260.1 Exp number of AAs in TMHs: 0.00781
# Camelinasativa_Csa06g031260.1 Exp number, first 60 AAs:  0
# Camelinasativa_Csa06g031260.1 Total prob of N-in:        0.00443
Camelinasativa_Csa06g031260.1	TMHMM2.0	outside	     1   580
```

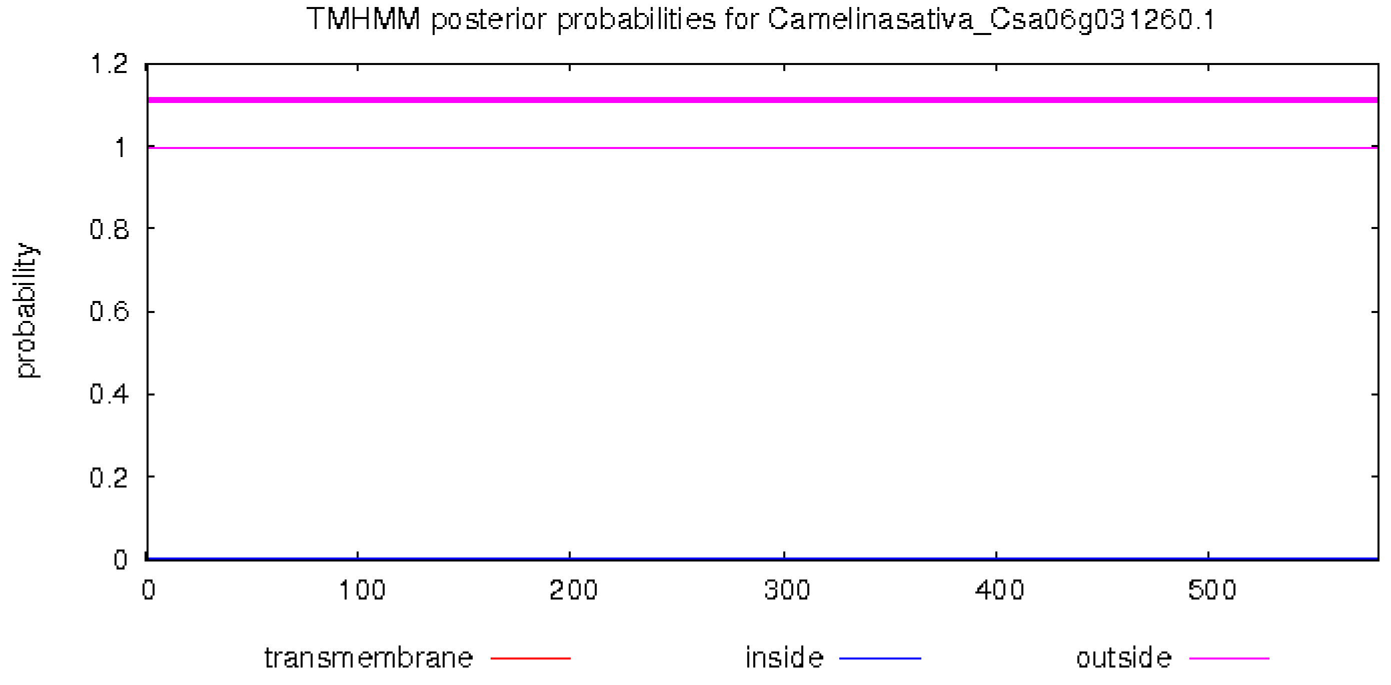

plot in postscript, script for making the plot in gnuplot, data for plot

```
# Camelinasativa_Csa06g031280.1 Length: 585
# Camelinasativa_Csa06g031280.1 Number of predicted TMHs:  0
# Camelinasativa_Csa06g031280.1 Exp number of AAs in TMHs: 0.00019
# Camelinasativa_Csa06g031280.1 Exp number, first 60 AAs:  0
# Camelinasativa_Csa06g031280.1 Total prob of N-in:        0.00219
Camelinasativa_Csa06g031280.1	TMHMM2.0	outside	     1   585
```

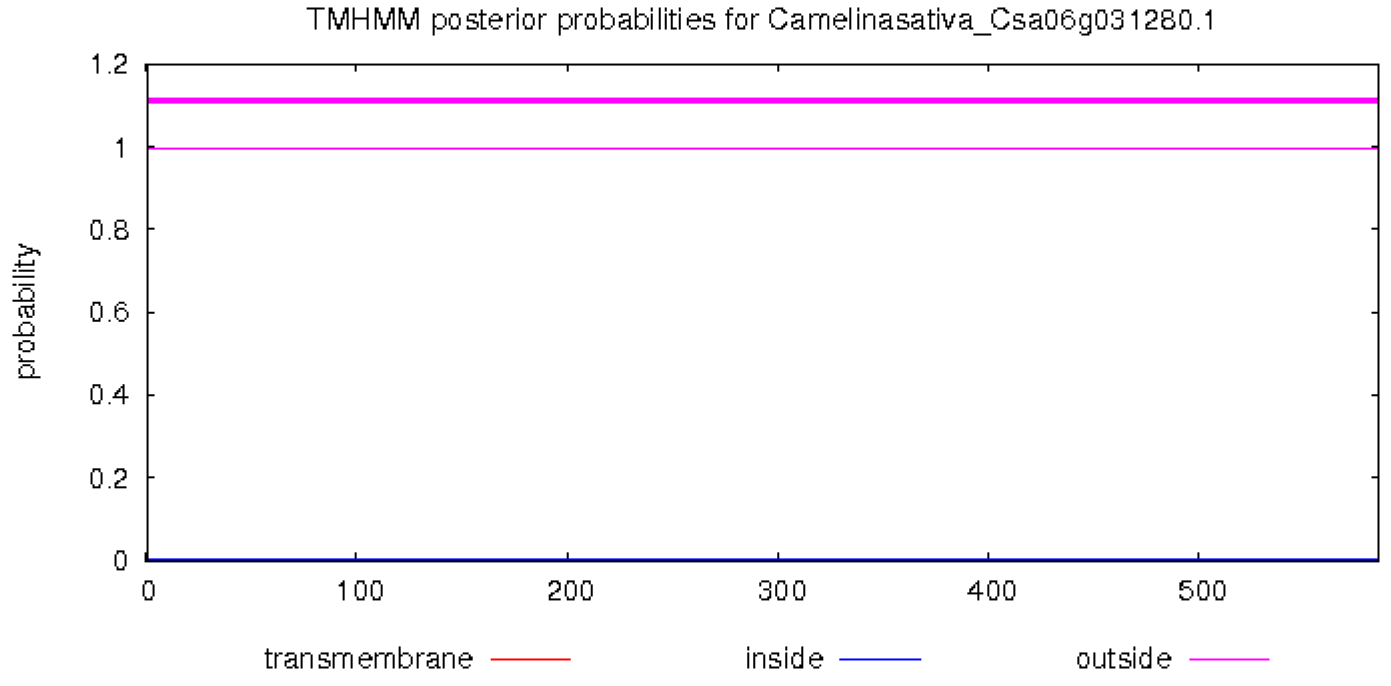

plot in postscript, script for making the plot in gnuplot, data for plot

```
# Camelinasativa_Csa06g038290.1 Length: 398
# Camelinasativa_Csa06g038290.1 Number of predicted TMHs:  0
# Camelinasativa_Csa06g038290.1 Exp number of AAs in TMHs: 0.00334
# Camelinasativa_Csa06g038290.1 Exp number, first 60 AAs:  0
# Camelinasativa_Csa06g038290.1 Total prob of N-in:        0.03671
Camelinasativa_Csa06g038290.1	TMHMM2.0	outside	     1   398
```

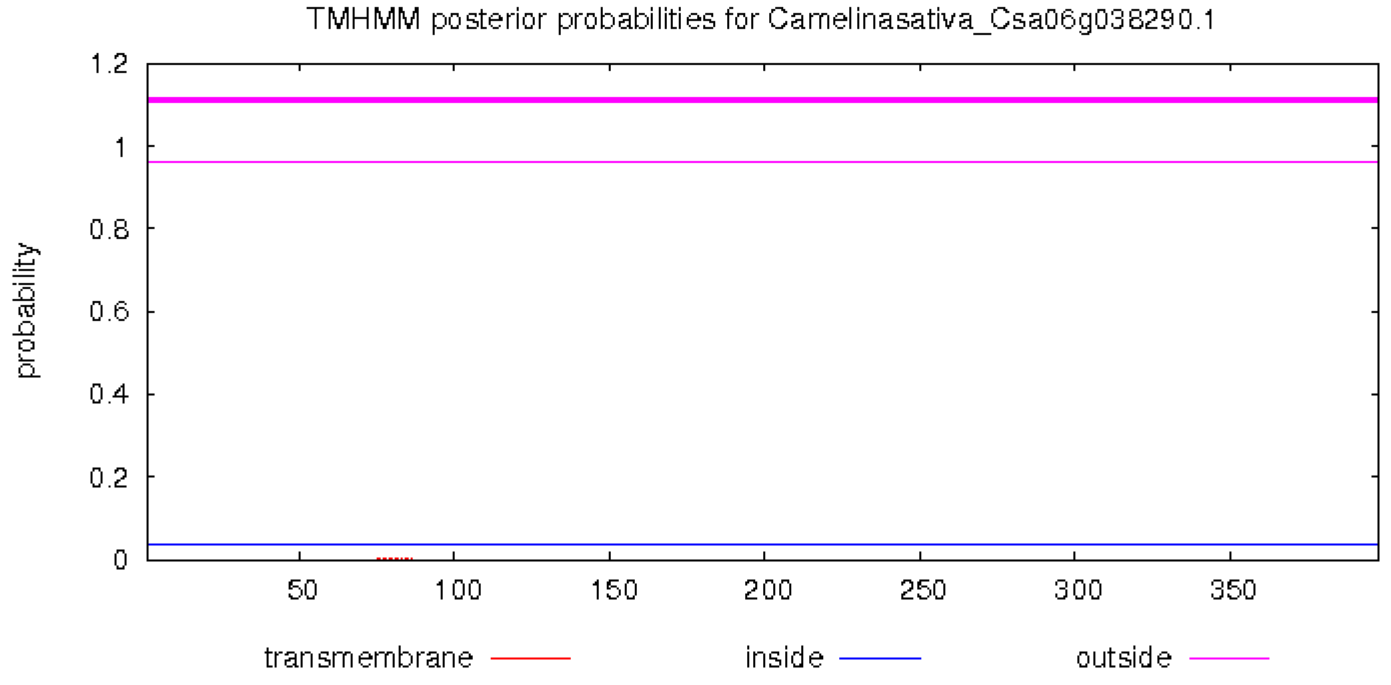

plot in postscript, script for making the plot in gnuplot, data for plot

```
# Camelinasativa_Csa06g049190.1 Length: 276
# Camelinasativa_Csa06g049190.1 Number of predicted TMHs:  0
# Camelinasativa_Csa06g049190.1 Exp number of AAs in TMHs: 0.00062
# Camelinasativa_Csa06g049190.1 Exp number, first 60 AAs:  0
# Camelinasativa_Csa06g049190.1 Total prob of N-in:        0.03073
Camelinasativa_Csa06g049190.1	TMHMM2.0	outside	     1   276
```

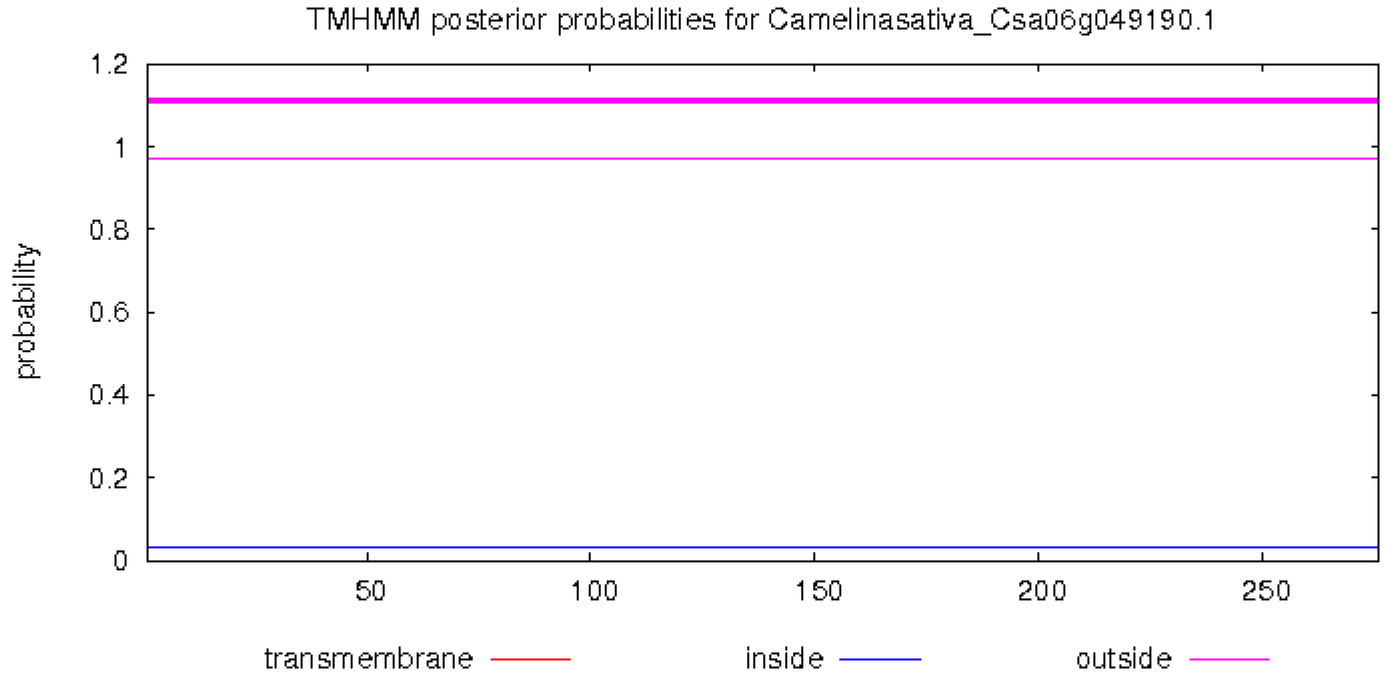

plot in postscript, script for making the plot in gnuplot, data for plot

```
# Camelinasativa_Csa06g053280.1 Length: 353
# Camelinasativa_Csa06g053280.1 Number of predicted TMHs:  0
# Camelinasativa_Csa06g053280.1 Exp number of AAs in TMHs: 0.00039
# Camelinasativa_Csa06g053280.1 Exp number, first 60 AAs:  0
# Camelinasativa_Csa06g053280.1 Total prob of N-in:        0.01397
Camelinasativa_Csa06g053280.1	TMHMM2.0	outside	     1   353
```

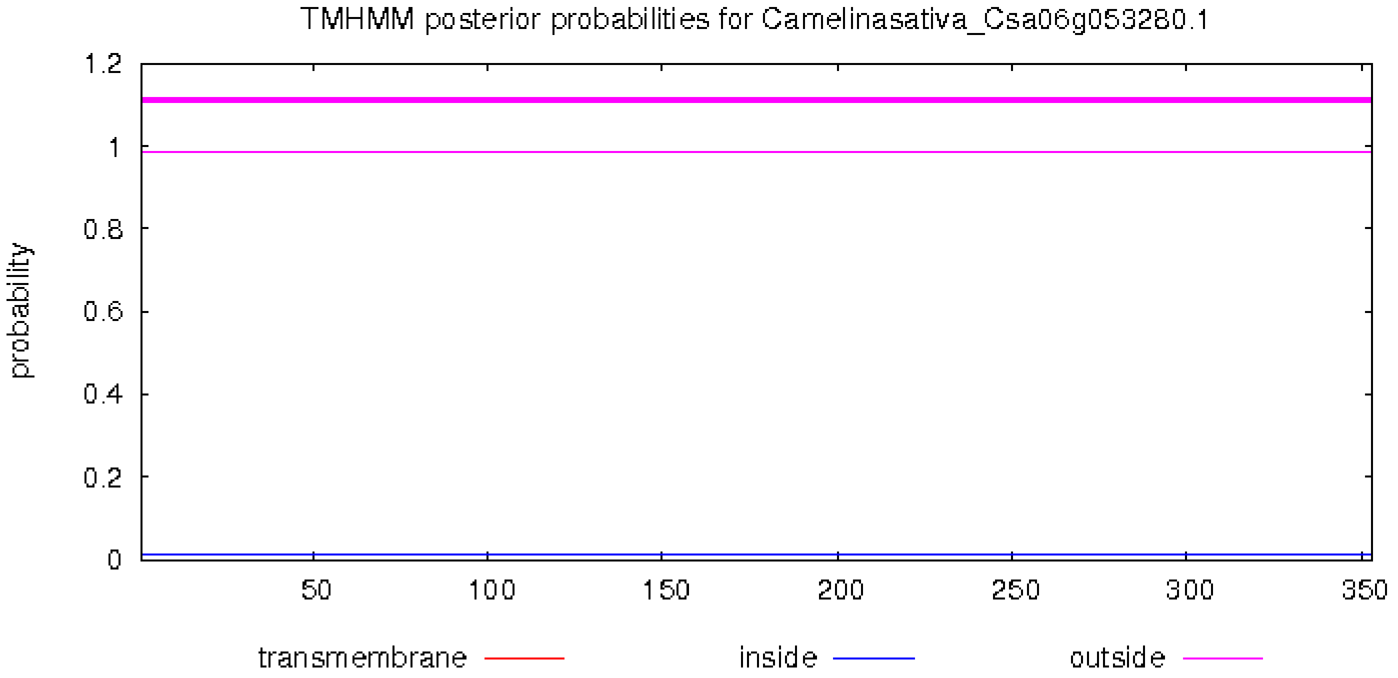

plot in postscript, script for making the plot in gnuplot, data for plot

```
# Camelinasativa_Csa07482s010.1 Length: 82
# Camelinasativa_Csa07482s010.1 Number of predicted TMHs:  0
# Camelinasativa_Csa07482s010.1 Exp number of AAs in TMHs: 0.46019
# Camelinasativa_Csa07482s010.1 Exp number, first 60 AAs:  0.46019
# Camelinasativa_Csa07482s010.1 Total prob of N-in:        0.44063
Camelinasativa_Csa07482s010.1	TMHMM2.0	outside	     1    82
```

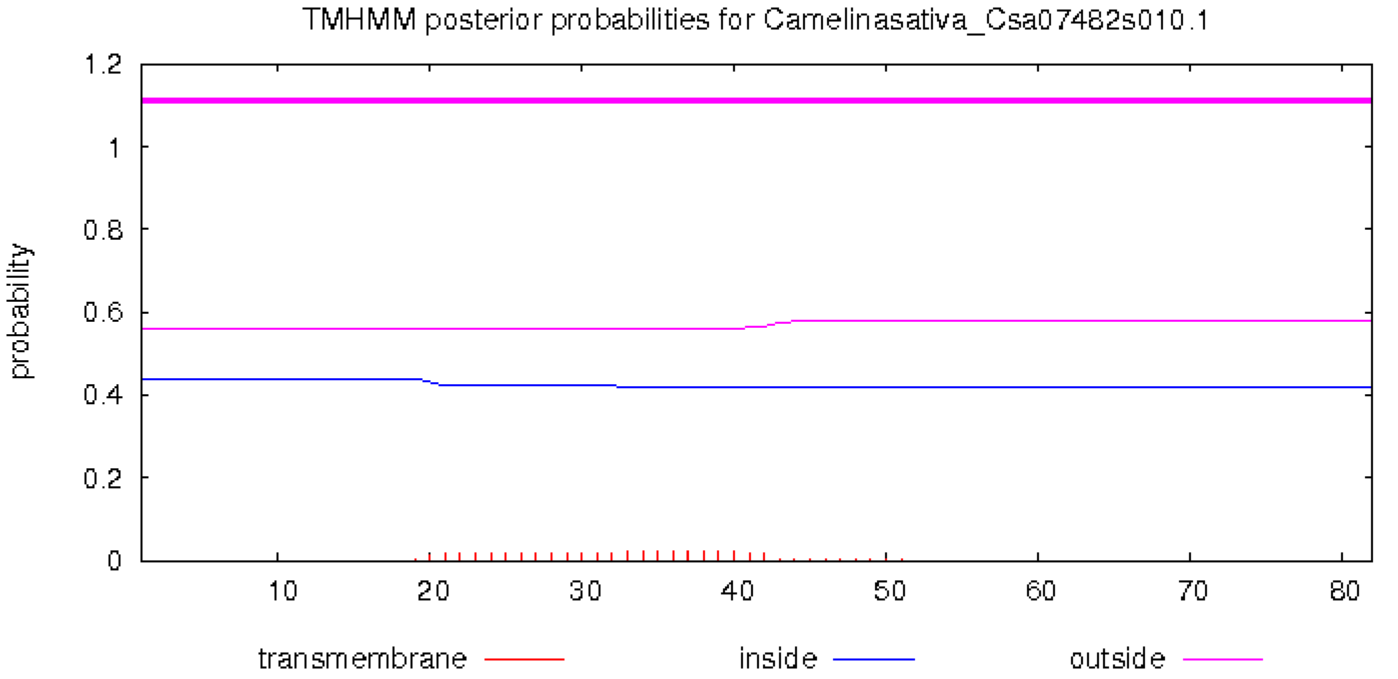

plot in postscript, script for making the plot in gnuplot, data for plot

```
# Camelinasativa_Csa07g002690.1 Length: 335
# Camelinasativa_Csa07g002690.1 Number of predicted TMHs:  0
# Camelinasativa_Csa07g002690.1 Exp number of AAs in TMHs: 0
# Camelinasativa_Csa07g002690.1 Exp number, first 60 AAs:  0
# Camelinasativa_Csa07g002690.1 Total prob of N-in:        0.02940
Camelinasativa_Csa07g002690.1	TMHMM2.0	outside	     1   335
```

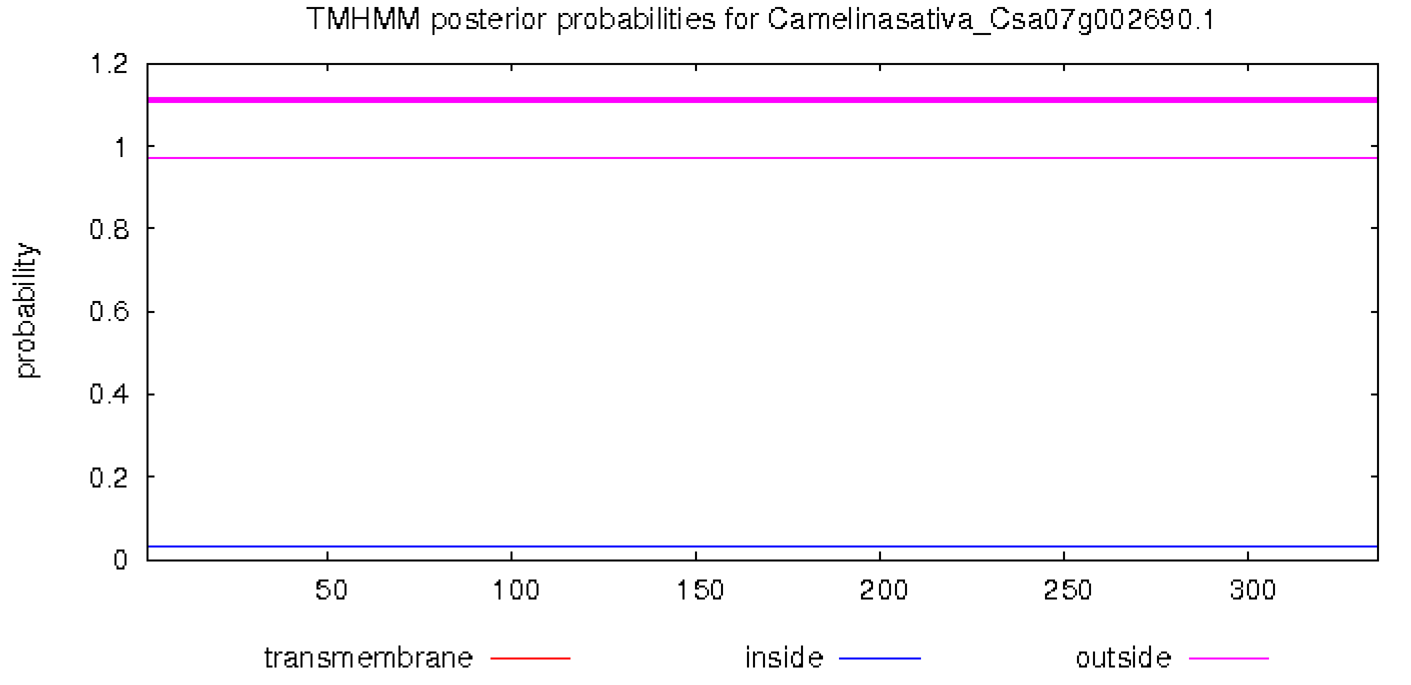

plot in postscript, script for making the plot in gnuplot, data for plot

```
# Camelinasativa_Csa07g007900.1 Length: 160
# Camelinasativa_Csa07g007900.1 Number of predicted TMHs:  0
# Camelinasativa_Csa07g007900.1 Exp number of AAs in TMHs: 0
# Camelinasativa_Csa07g007900.1 Exp number, first 60 AAs:  0
# Camelinasativa_Csa07g007900.1 Total prob of N-in:        0.57564
Camelinasativa_Csa07g007900.1	TMHMM2.0	inside	     1   160
```

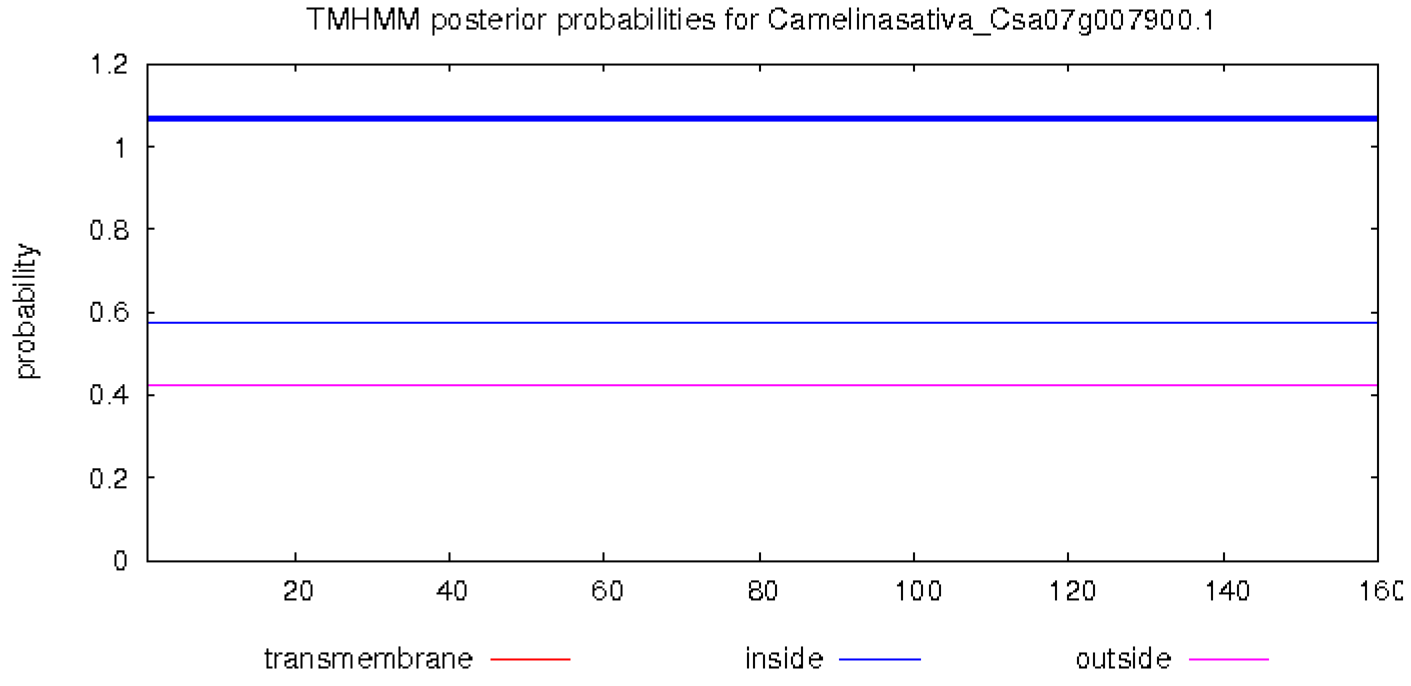

plot in postscript, script for making the plot in gnuplot, data for plot

```
# Camelinasativa_Csa07g036200.1 Length: 272
# Camelinasativa_Csa07g036200.1 Number of predicted TMHs:  0
# Camelinasativa_Csa07g036200.1 Exp number of AAs in TMHs: 0.00049
# Camelinasativa_Csa07g036200.1 Exp number, first 60 AAs:  0
# Camelinasativa_Csa07g036200.1 Total prob of N-in:        0.01821
Camelinasativa_Csa07g036200.1	TMHMM2.0	outside	     1   272
```

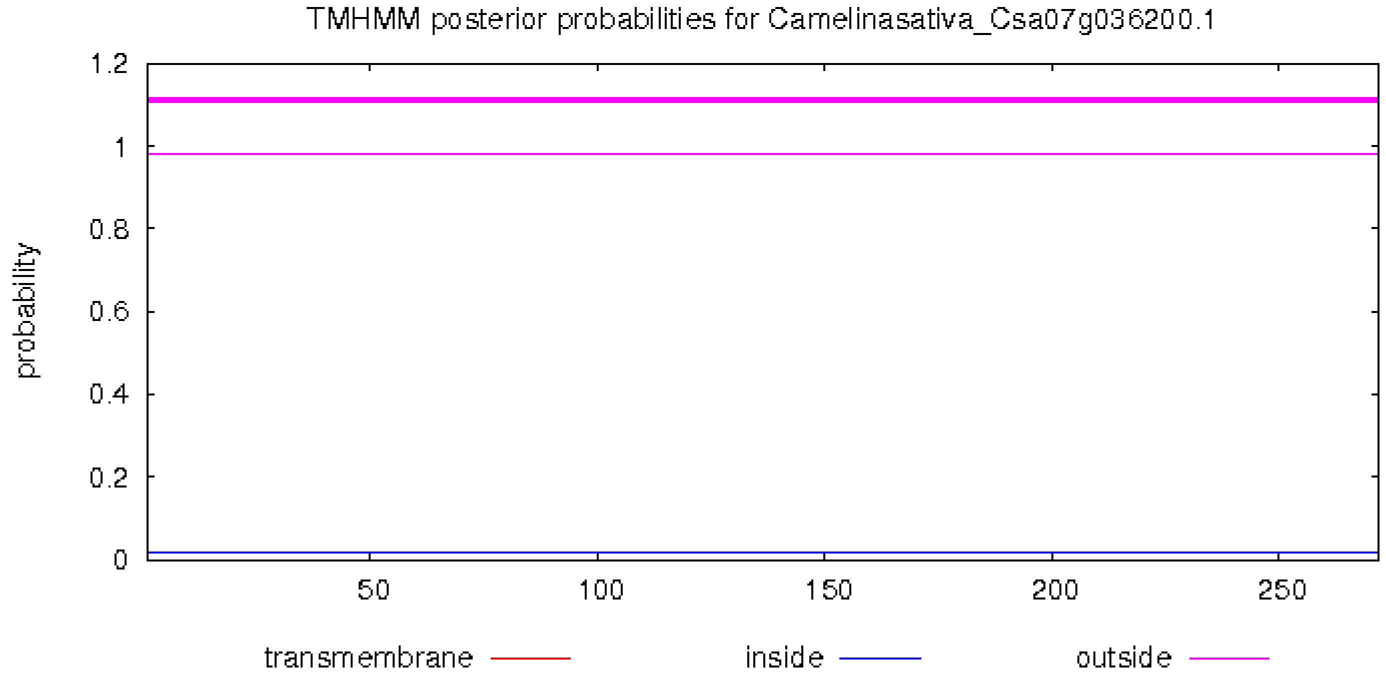

plot in postscript, script for making the plot in gnuplot, data for plot

```
# Camelinasativa_Csa07g036390.1 Length: 323
# Camelinasativa_Csa07g036390.1 Number of predicted TMHs:  0
# Camelinasativa_Csa07g036390.1 Exp number of AAs in TMHs: 0.00066
# Camelinasativa_Csa07g036390.1 Exp number, first 60 AAs:  0.00029
# Camelinasativa_Csa07g036390.1 Total prob of N-in:        0.02459
Camelinasativa_Csa07g036390.1	TMHMM2.0	outside	     1   323
```

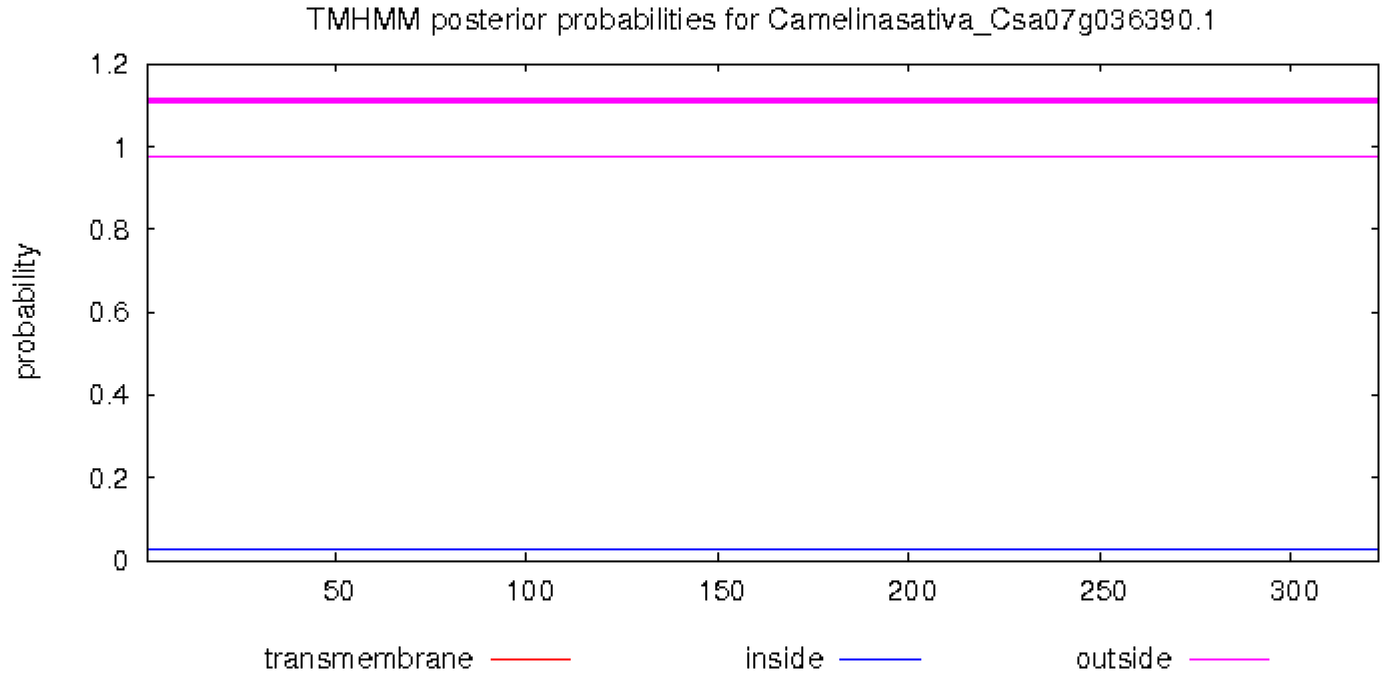

plot in postscript, script for making the plot in gnuplot, data for plot

```
# Camelinasativa_Csa07g038870.1 Length: 326
# Camelinasativa_Csa07g038870.1 Number of predicted TMHs:  0
# Camelinasativa_Csa07g038870.1 Exp number of AAs in TMHs: 0
# Camelinasativa_Csa07g038870.1 Exp number, first 60 AAs:  0
# Camelinasativa_Csa07g038870.1 Total prob of N-in:        0.01705
Camelinasativa_Csa07g038870.1   TMHMM2.0        outside      1   326
```

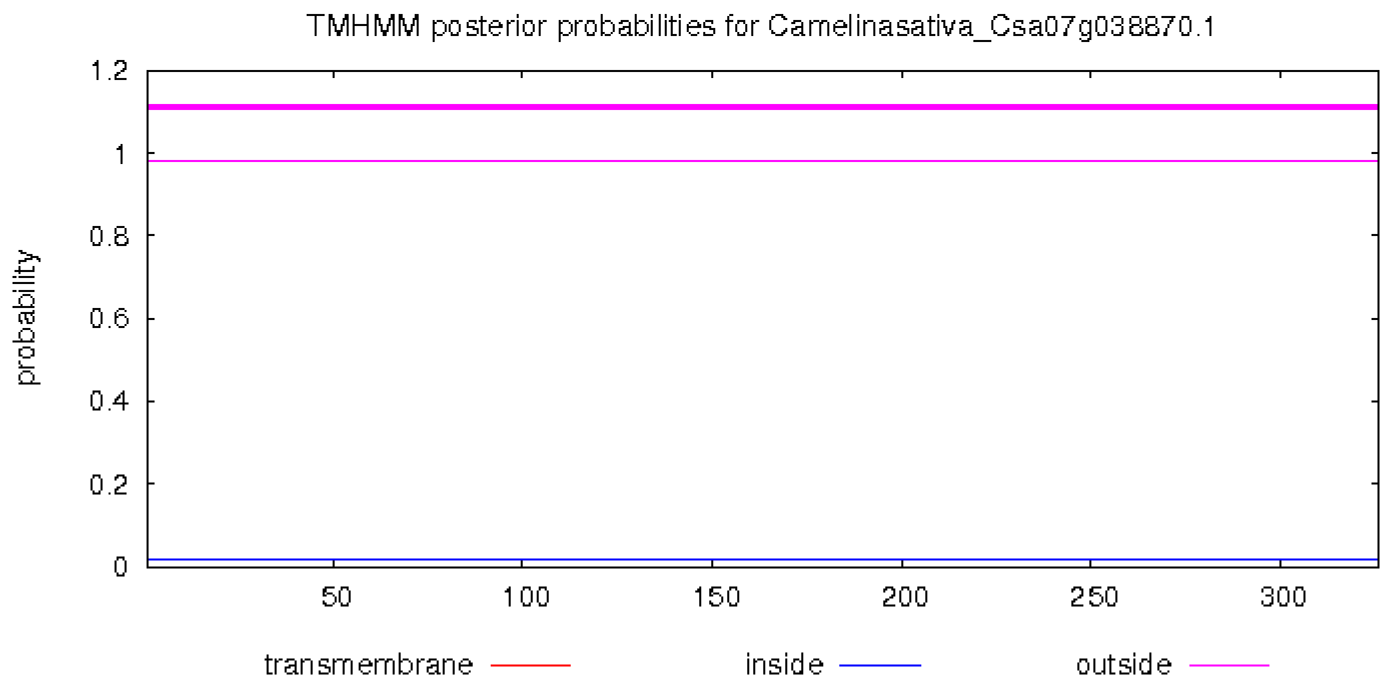

plot in postscript, script for making the plot in gnuplot, data for plot

```
# Camelinasativa_Csa07g047980.1 Length: 337
# Camelinasativa_Csa07g047980.1 Number of predicted TMHs:  0
# Camelinasativa_Csa07g047980.1 Exp number of AAs in TMHs: 0.01378
# Camelinasativa_Csa07g047980.1 Exp number, first 60 AAs:  0.00986
# Camelinasativa_Csa07g047980.1 Total prob of N-in:        0.00498
Camelinasativa_Csa07g047980.1   TMHMM2.0        outside      1   337
```

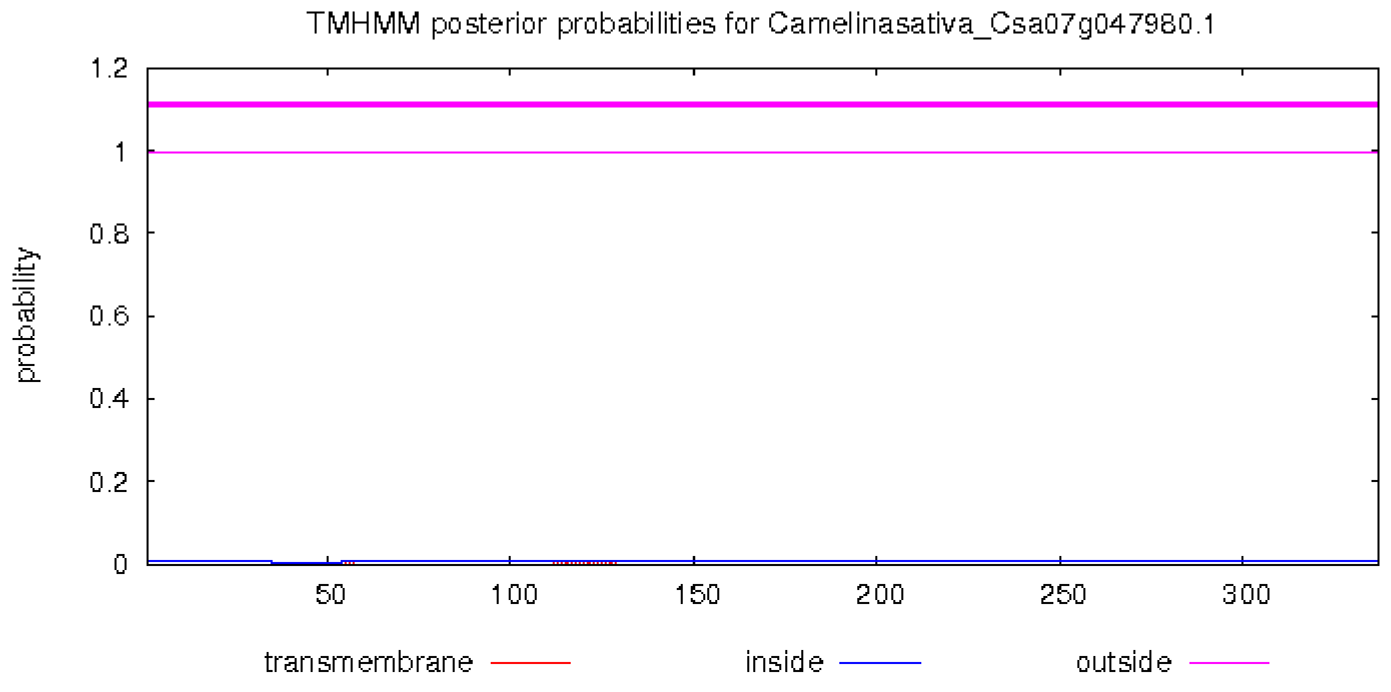

plot in postscript, script for making the plot in gnuplot, data for plot

```
# Camelinasativa_Csa07g050100.1 Length: 252
# Camelinasativa_Csa07g050100.1 Number of predicted TMHs:  0
# Camelinasativa_Csa07g050100.1 Exp number of AAs in TMHs: 0.00021
# Camelinasativa_Csa07g050100.1 Exp number, first 60 AAs:  0
# Camelinasativa_Csa07g050100.1 Total prob of N-in:        0.02032
Camelinasativa_Csa07g050100.1	TMHMM2.0	outside	     1   252
```

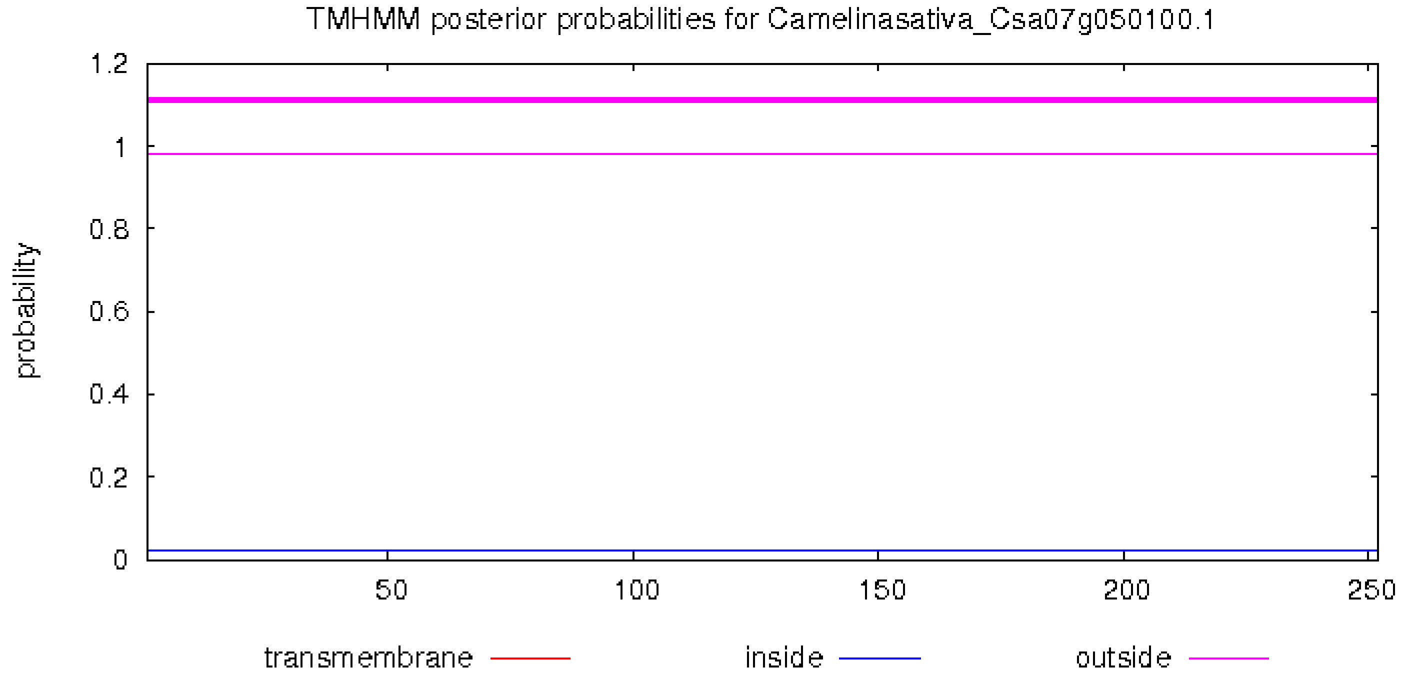

plot in postscript, script for making the plot in gnuplot, data for plot

```
# Camelinasativa_Csa07g054410.1 Length: 317
# Camelinasativa_Csa07g054410.1 Number of predicted TMHs:  0
# Camelinasativa_Csa07g054410.1 Exp number of AAs in TMHs: 0.0287
# Camelinasativa_Csa07g054410.1 Exp number, first 60 AAs:  0
# Camelinasativa_Csa07g054410.1 Total prob of N-in:        0.07369
Camelinasativa_Csa07g054410.1	TMHMM2.0	outside	     1   317
```

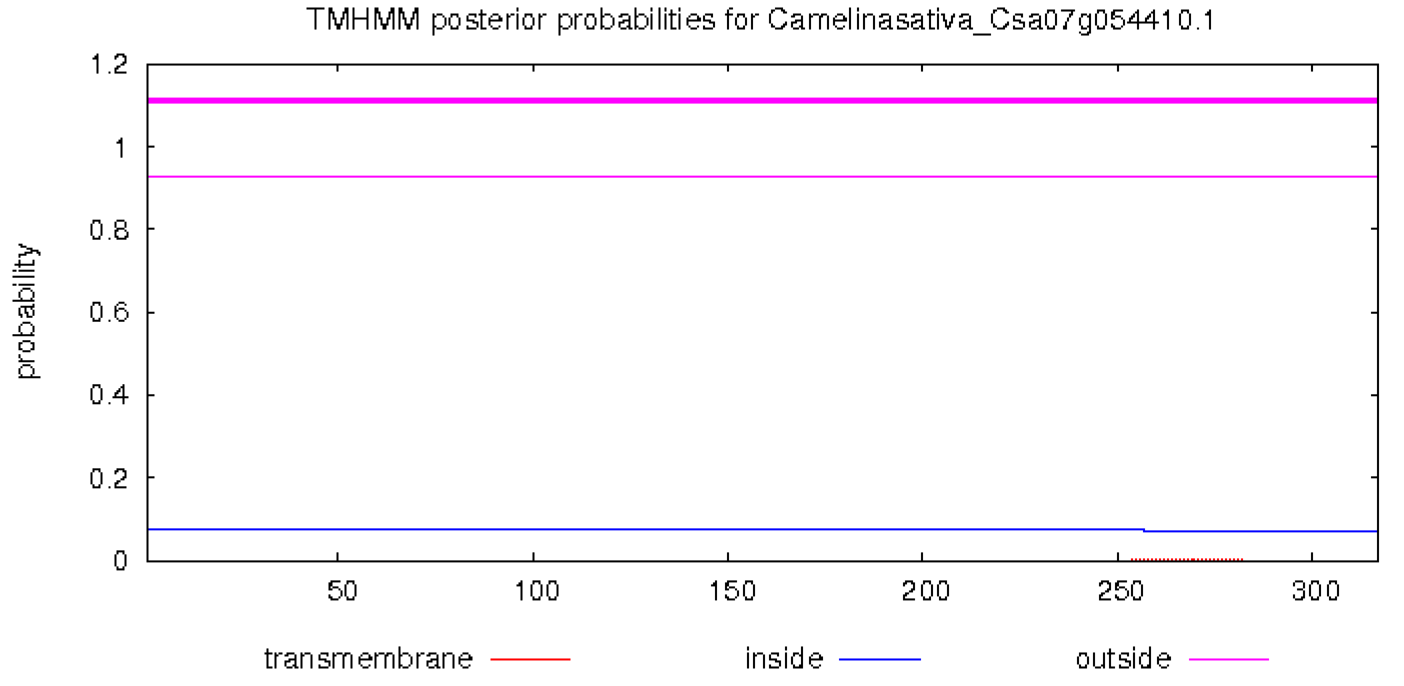

plot in postscript, script for making the plot in gnuplot, data for plot

```
# Camelinasativa_Csa07g058710.1 Length: 372
# Camelinasativa_Csa07g058710.1 Number of predicted TMHs:  0
# Camelinasativa_Csa07g058710.1 Exp number of AAs in TMHs: 0.00497
# Camelinasativa_Csa07g058710.1 Exp number, first 60 AAs:  0.00096
# Camelinasativa_Csa07g058710.1 Total prob of N-in:        0.00758
Camelinasativa_Csa07g058710.1   TMHMM2.0        outside      1   372
```

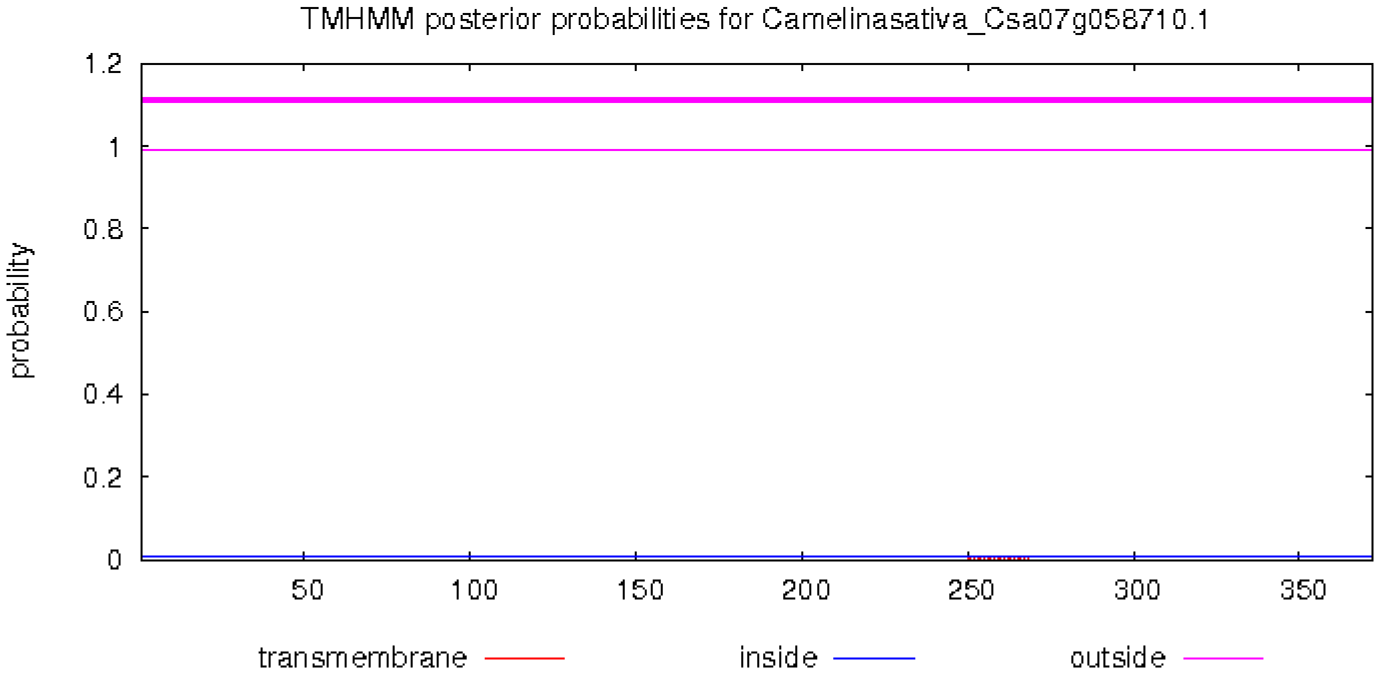

plot in postscript, script for making the plot in gnuplot, data for plot

```
# Camelinasativa_Csa07g060910.1 Length: 340
# Camelinasativa_Csa07g060910.1 Number of predicted TMHs:  1
# Camelinasativa_Csa07g060910.1 Exp number of AAs in TMHs: 21.29562
# Camelinasativa_Csa07g060910.1 Exp number, first 60 AAs:  0.09216
# Camelinasativa_Csa07g060910.1 Total prob of N-in:        0.55515
Camelinasativa_Csa07g060910.1   TMHMM2.0        outside      1   315
Camelinasativa_Csa07g060910.1   TMHMM2.0        TMhelix    316   335
Camelinasativa_Csa07g060910.1   TMHMM2.0        inside     336   340
```

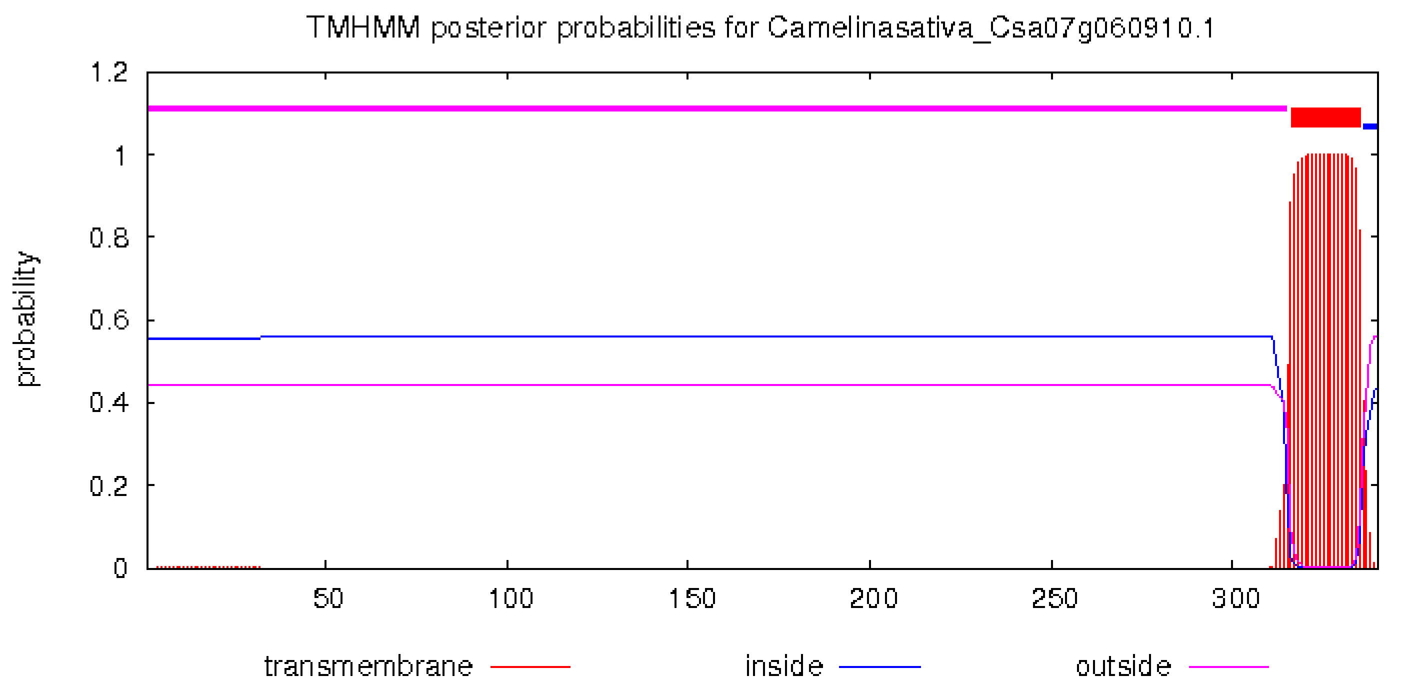

plot in postscript, script for making the plot in gnuplot, data for plot

---

```
# Camelinasativa_Csa07g061560.1 Length: 180
# Camelinasativa_Csa07g061560.1 Number of predicted TMHs:  0
# Camelinasativa_Csa07g061560.1 Exp number of AAs in TMHs: 0.05101
# Camelinasativa_Csa07g061560.1 Exp number, first 60 AAs:  0.03514
# Camelinasativa_Csa07g061560.1 Total prob of N-in:        0.31246
Camelinasativa_Csa07g061560.1   TMHMM2.0        outside      1   180
```

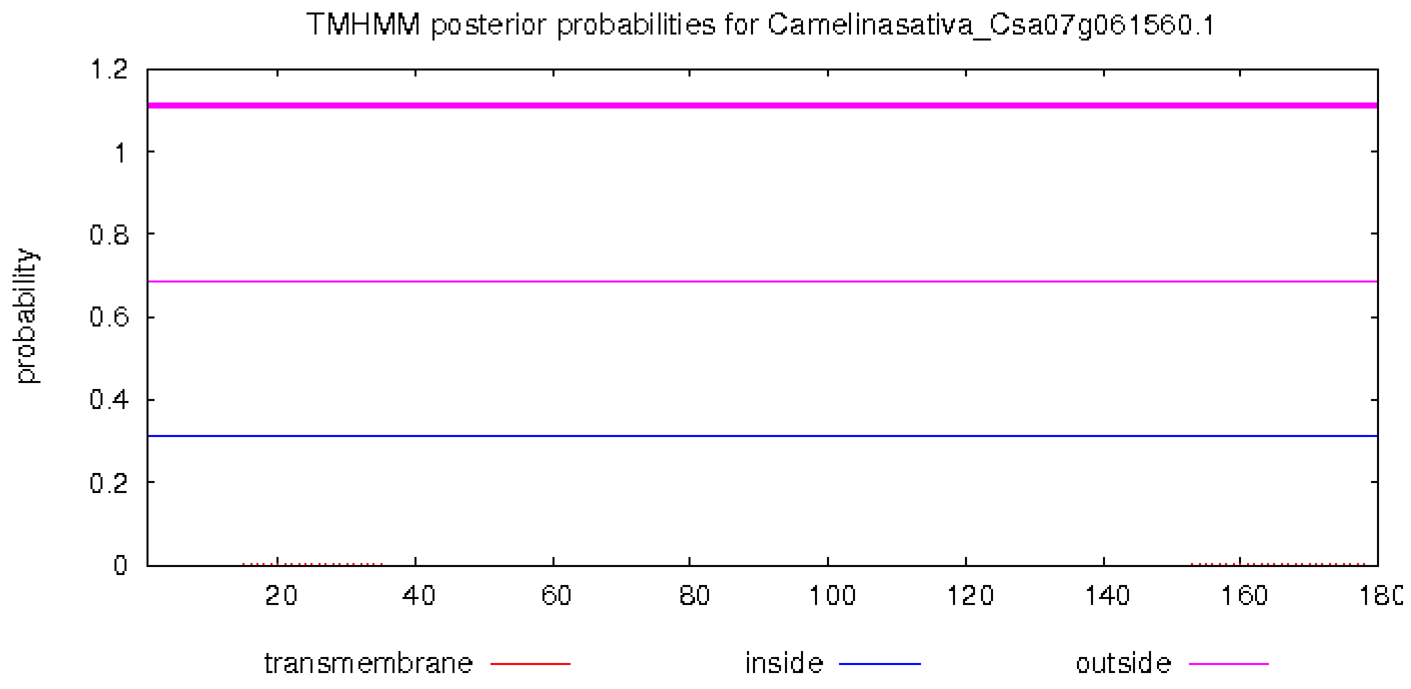

plot in postscript, script for making the plot in gnuplot, data for plot

---

```
# Camelinasativa_Csa07g061590.1 Length: 98
# Camelinasativa_Csa07g061590.1 Number of predicted TMHs:  0
# Camelinasativa_Csa07g061590.1 Exp number of AAs in TMHs: 0.00147
# Camelinasativa_Csa07g061590.1 Exp number, first 60 AAs:  0.00147
# Camelinasativa_Csa07g061590.1 Total prob of N-in:        0.05844
Camelinasativa_Csa07g061590.1   TMHMM2.0        outside      1    98
```

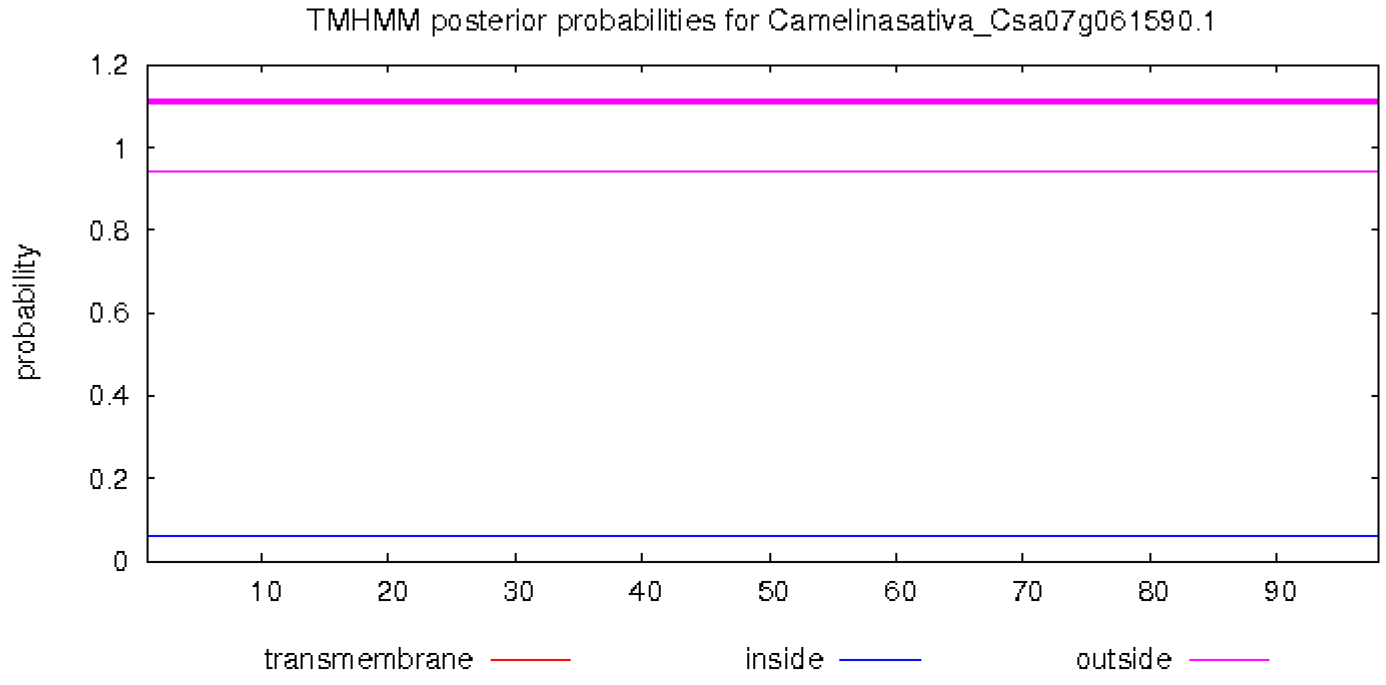

plot in postscript, script for making the plot in gnuplot, data for plot

---

```
# Camelinasativa_Csa07g061600.1 Length: 489
# Camelinasativa_Csa07g061600.1 Number of predicted TMHs:  0
# Camelinasativa_Csa07g061600.1 Exp number of AAs in TMHs: 1.57757
# Camelinasativa_Csa07g061600.1 Exp number, first 60 AAs:  0.00062
# Camelinasativa_Csa07g061600.1 Total prob of N-in:        0.00815
Camelinasativa_Csa07g061600.1   TMHMM2.0        outside      1   489
```

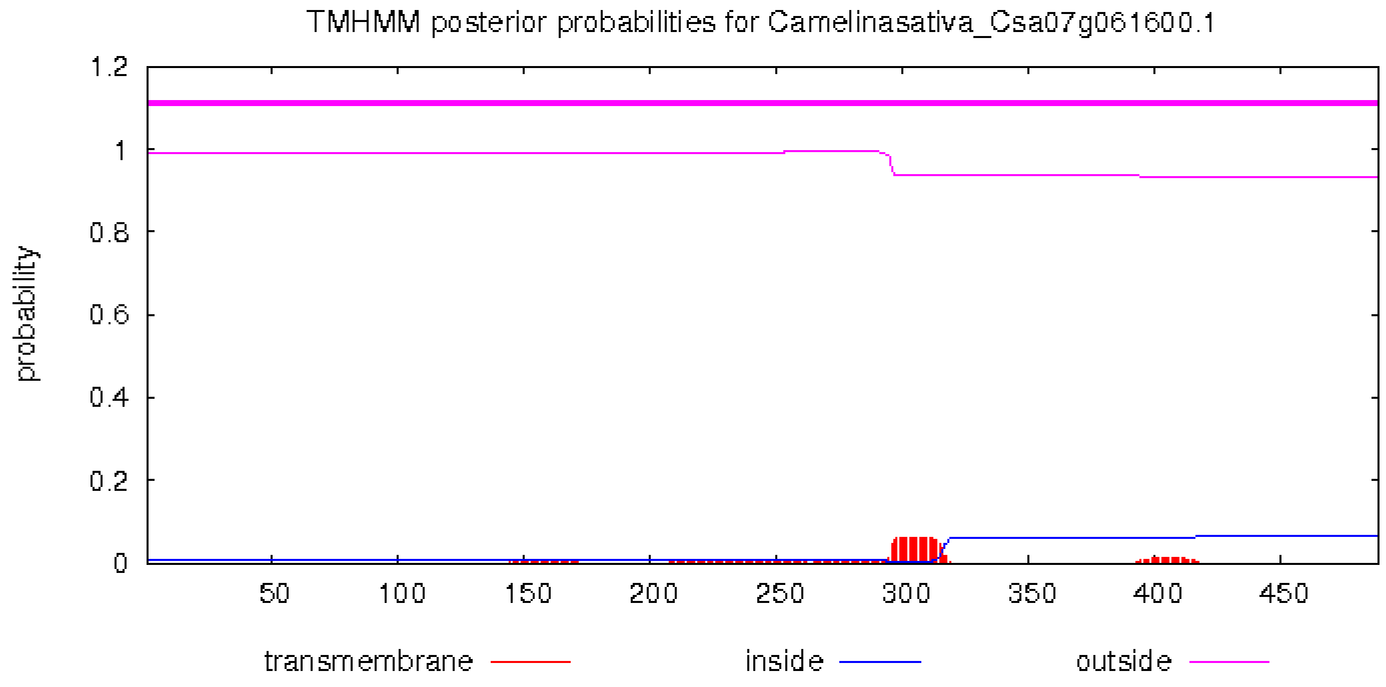

plot in postscript, script for making the plot in gnuplot, data for plot

---

```
# Camelinasativa_Csa07g062260.1 Length: 340
# Camelinasativa_Csa07g062260.1 Number of predicted TMHs:  0
# Camelinasativa_Csa07g062260.1 Exp number of AAs in TMHs: 0.00036
# Camelinasativa_Csa07g062260.1 Exp number, first 60 AAs:  0.00016
# Camelinasativa_Csa07g062260.1 Total prob of N-in:        0.00926
Camelinasativa_Csa07g062260.1   TMHMM2.0        outside      1   340
```

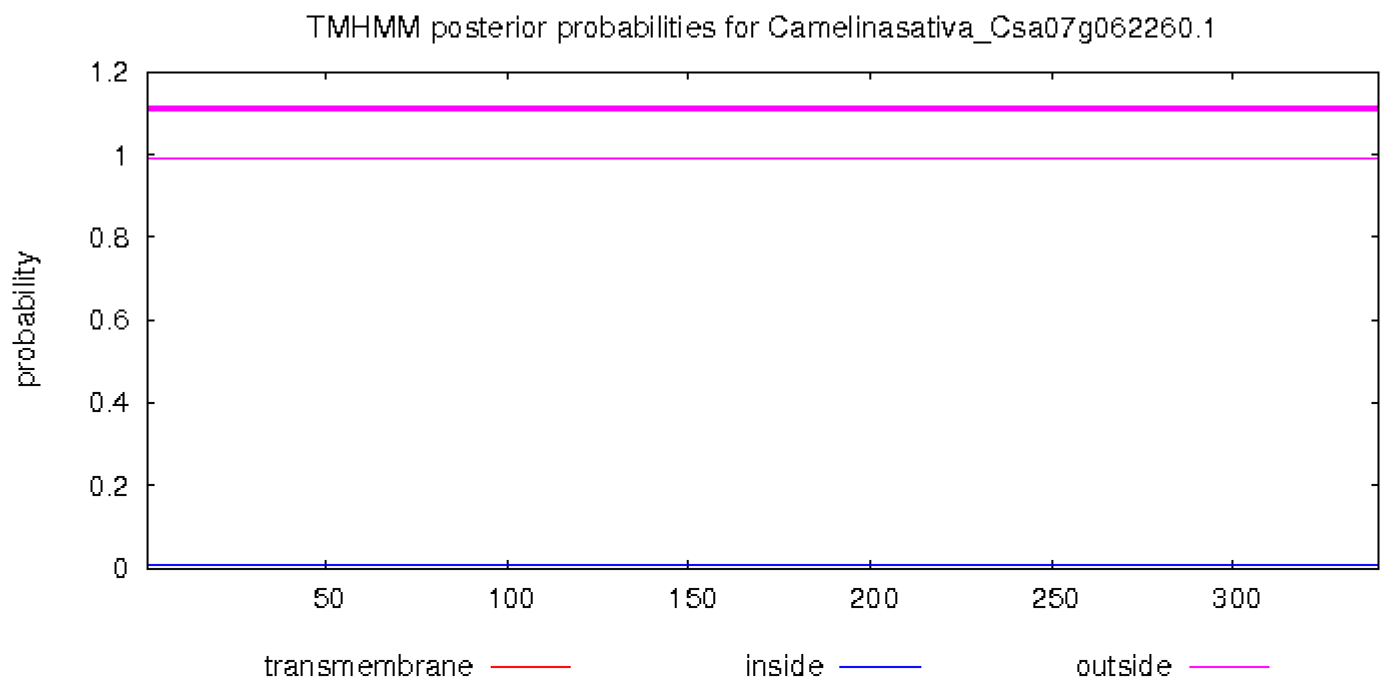

plot in postscript, script for making the plot in gnuplot, data for plot

---

```
# Camelinasativa_Csa07g064020.1 Length: 405
# Camelinasativa_Csa07g064020.1 Number of predicted TMHs:  0
# Camelinasativa_Csa07g064020.1 Exp number of AAs in TMHs: 0.00018
# Camelinasativa_Csa07g064020.1 Exp number, first 60 AAs:  0
# Camelinasativa_Csa07g064020.1 Total prob of N-in:        0.00257
Camelinasativa_Csa07g064020.1   TMHMM2.0        outside      1   405
```

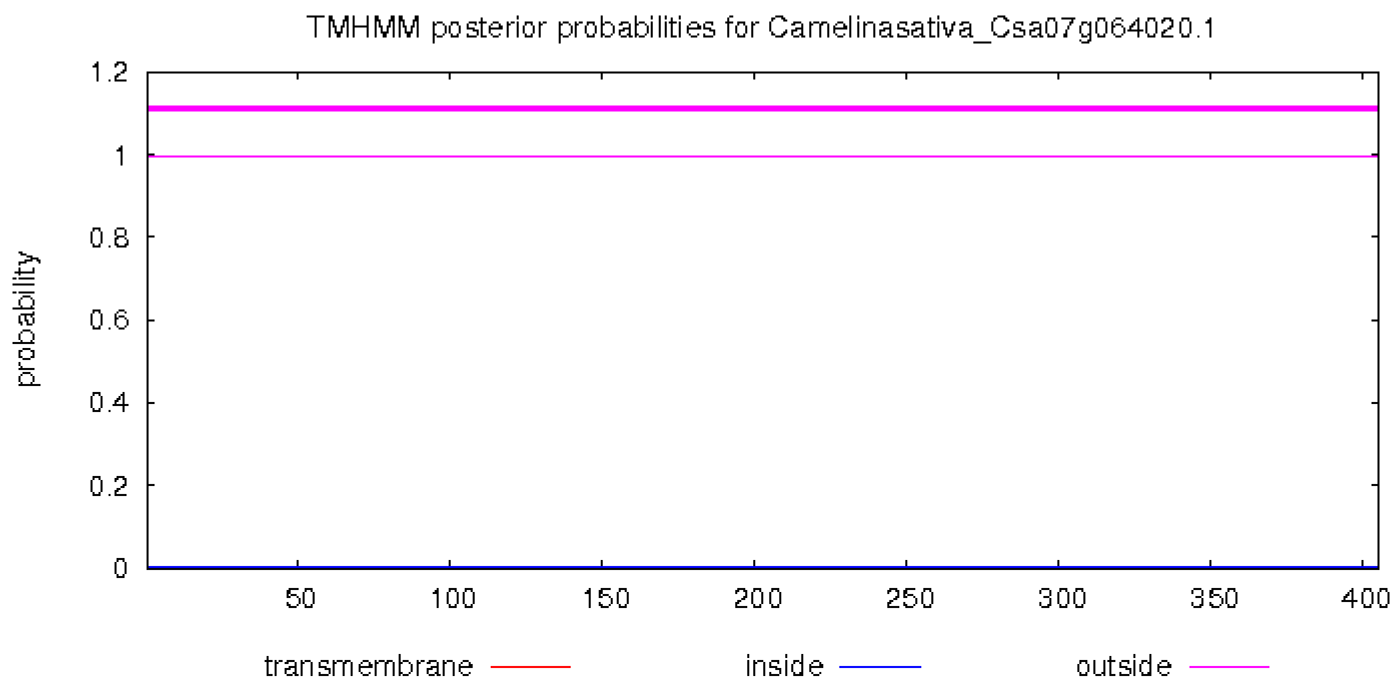

plot in postscript, script for making the plot in gnuplot, data for plot

---

```
# Camelinasativa_Csa08945s010.1 Length: 87
# Camelinasativa_Csa08945s010.1 Number of predicted TMHs:  0
# Camelinasativa_Csa08945s010.1 Exp number of AAs in TMHs: 0.00086
# Camelinasativa_Csa08945s010.1 Exp number, first 60 AAs:  0.00079
# Camelinasativa_Csa08945s010.1 Total prob of N-in:        0.42356
Camelinasativa_Csa08945s010.1   TMHMM2.0        outside      1    87
```

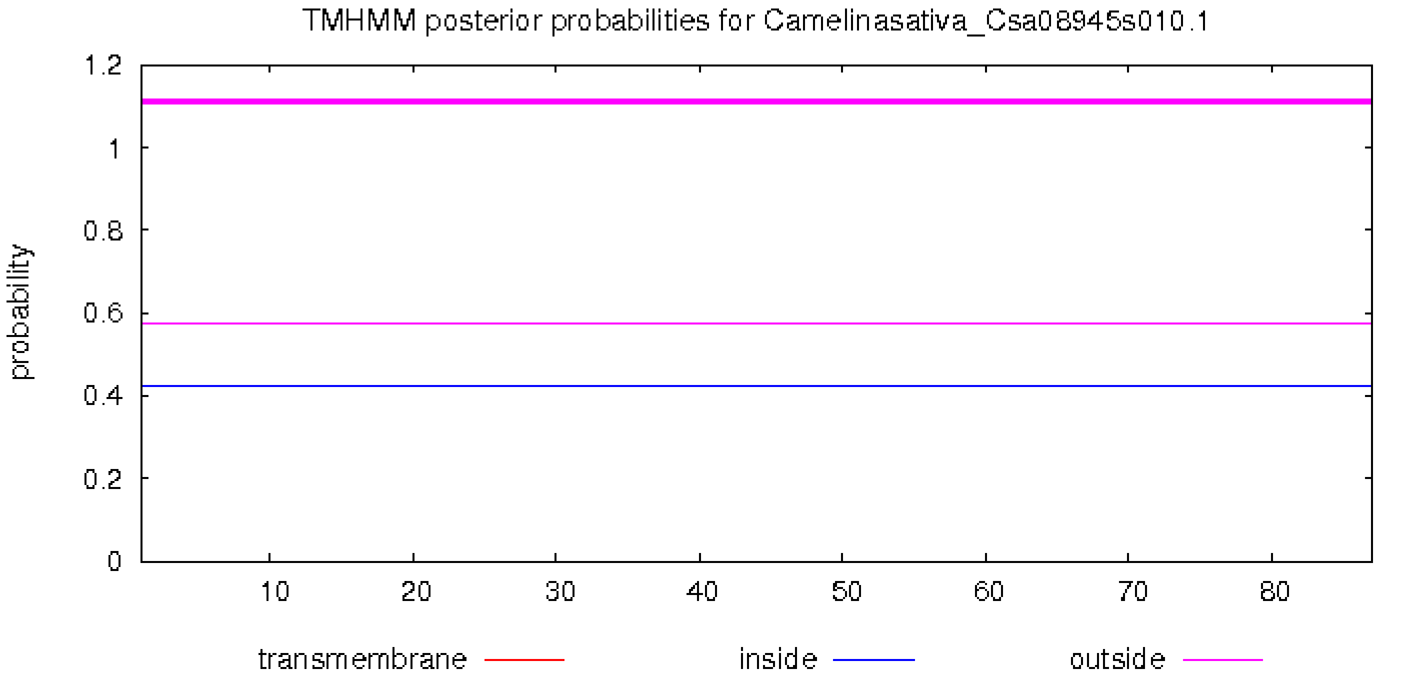

plot in postscript, script for making the plot in gnuplot, data for plot

---

```
# Camelinasativa_Csa08g005060.1 Length: 300
# Camelinasativa_Csa08g005060.1 Number of predicted TMHs:  1
# Camelinasativa_Csa08g005060.1 Exp number of AAs in TMHs: 22.03904
# Camelinasativa_Csa08g005060.1 Exp number, first 60 AAs:  22.03807
# Camelinasativa_Csa08g005060.1 Total prob of N-in:        0.63456
# Camelinasativa_Csa08g005060.1 POSSIBLE N-term signal sequence
Camelinasativa_Csa08g005060.1   TMHMM2.0        inside       1    20
Camelinasativa_Csa08g005060.1   TMHMM2.0        TMhelix     21    43
Camelinasativa_Csa08g005060.1   TMHMM2.0        outside     44   300
```

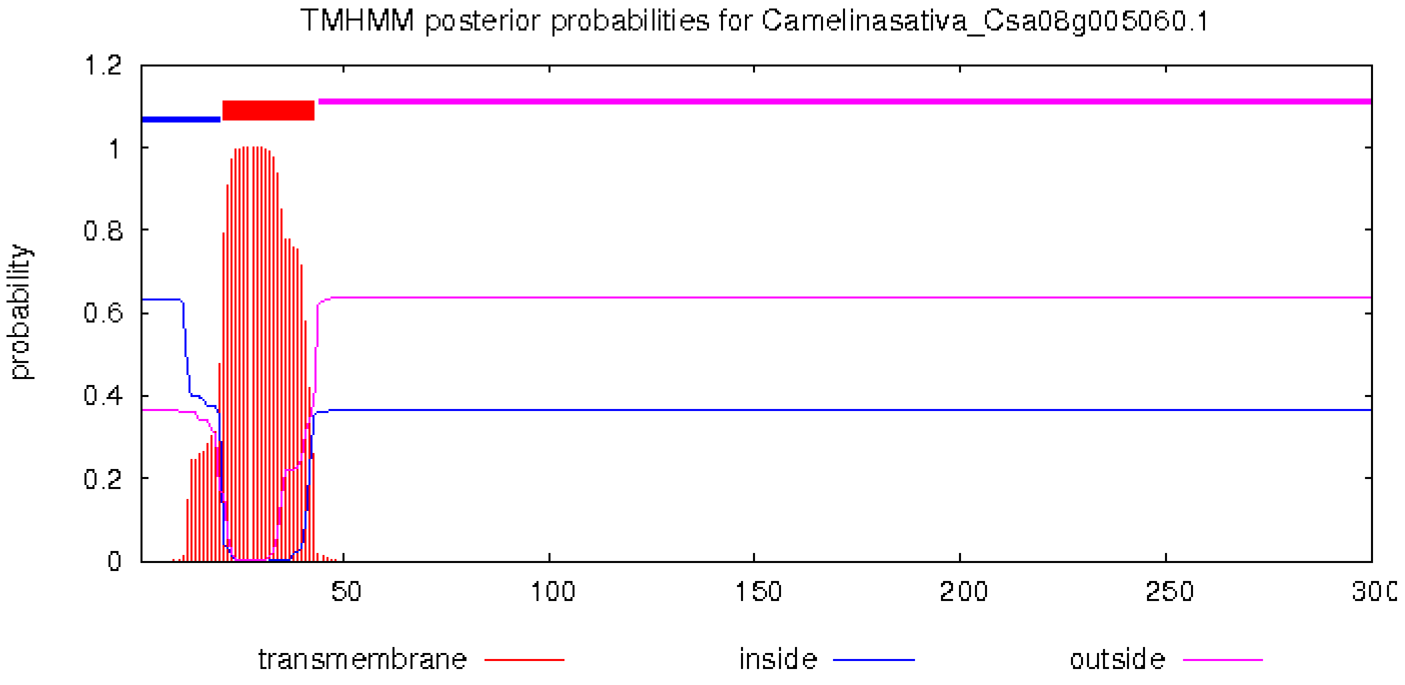

plot in postscript, script for making the plot in gnuplot, data for plot

---

```
# Camelinasativa_Csa08g006010.1 Length: 330
# Camelinasativa_Csa08g006010.1 Number of predicted TMHs:  0
# Camelinasativa_Csa08g006010.1 Exp number of AAs in TMHs: 0.80302
# Camelinasativa_Csa08g006010.1 Exp number, first 60 AAs:  0.0041
```

```
# Camelinasativa_Csa08g006010.1 Total prob of N-in:        0.08022
Camelinasativa_Csa08g006010.1   TMHMM2.0        outside      1   330
```

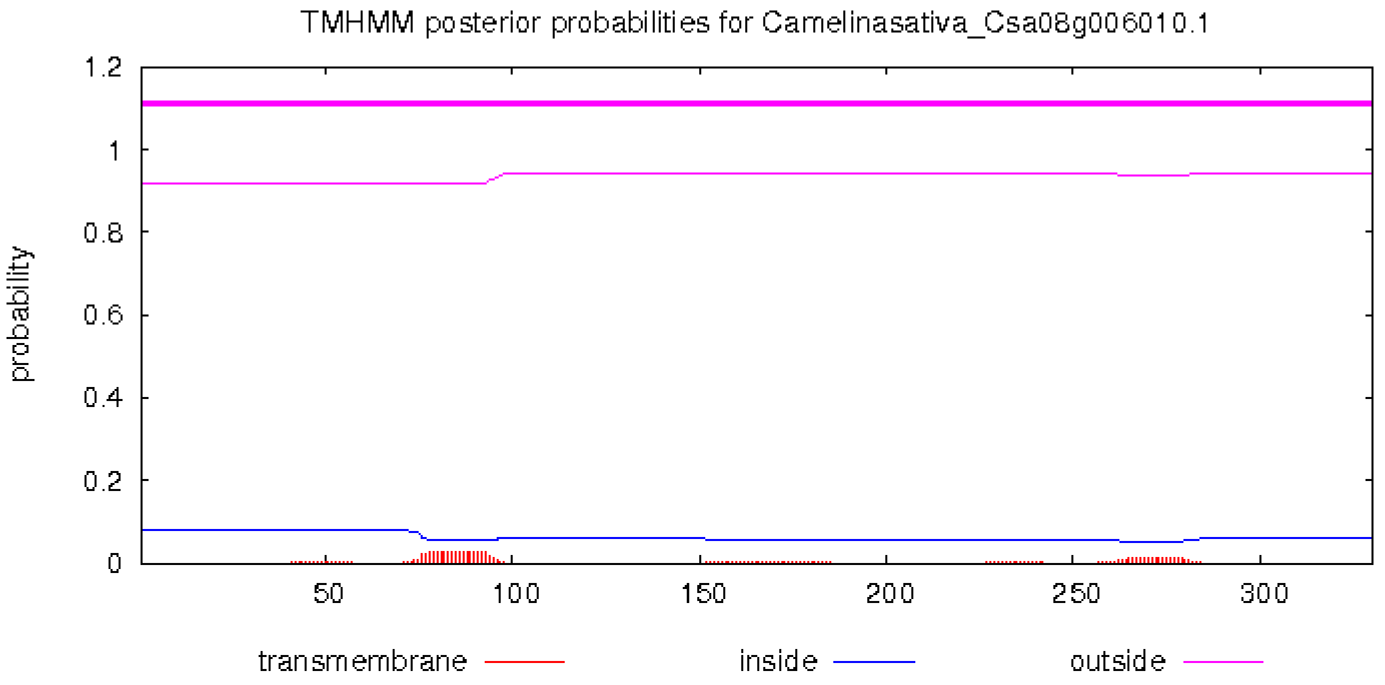

plot in postscript, script for making the plot in gnuplot, data for plot

---

```
# Camelinasativa_Csa08g006520.1 Length: 357
# Camelinasativa_Csa08g006520.1 Number of predicted TMHs:  0
# Camelinasativa_Csa08g006520.1 Exp number of AAs in TMHs: 0
# Camelinasativa_Csa08g006520.1 Exp number, first 60 AAs:  0
# Camelinasativa_Csa08g006520.1 Total prob of N-in:        0.01007
Camelinasativa_Csa08g006520.1   TMHMM2.0        outside      1   357
```

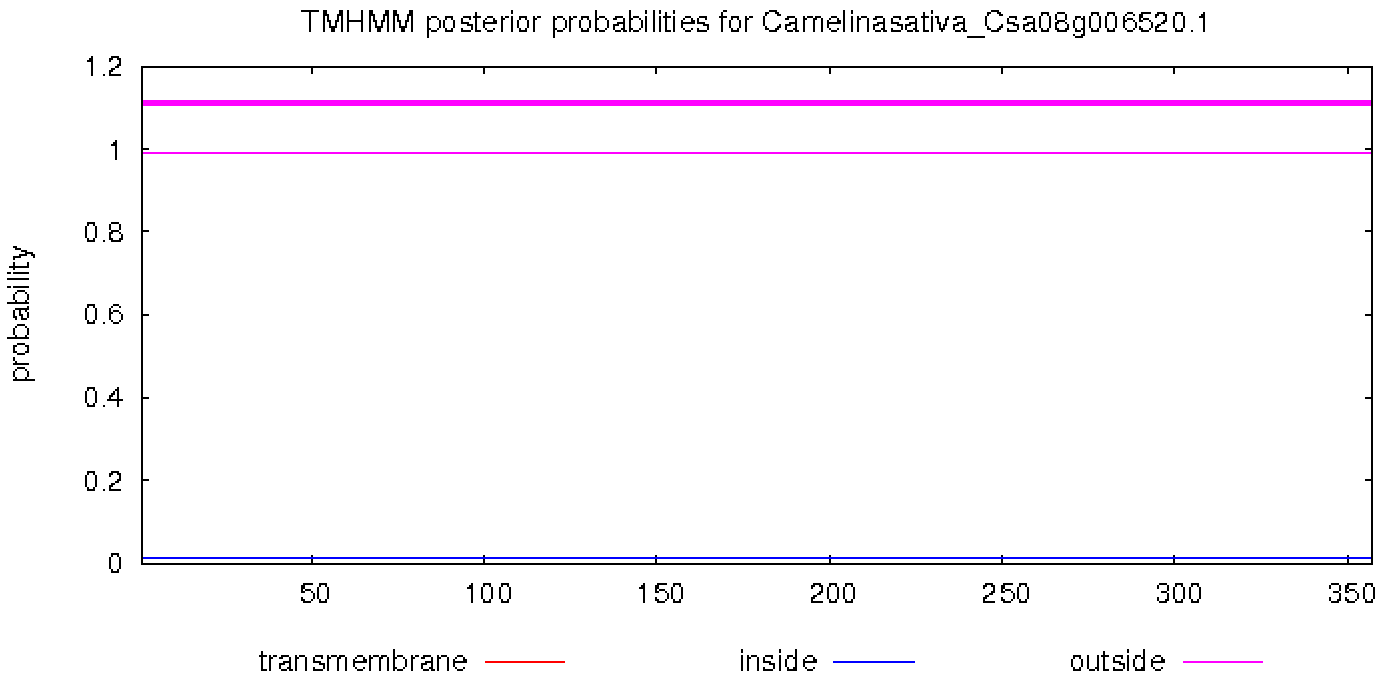

plot in postscript, script for making the plot in gnuplot, data for plot

---

```
# Camelinasativa_Csa08g009420.1 Length: 506
# Camelinasativa_Csa08g009420.1 Number of predicted TMHs:  0
# Camelinasativa_Csa08g009420.1 Exp number of AAs in TMHs: 0.00036
# Camelinasativa_Csa08g009420.1 Exp number, first 60 AAs:  0
```

```
# Camelinasativa_Csa08g009420.1 Total prob of N-in:        0.00012
Camelinasativa_Csa08g009420.1   TMHMM2.0        outside      1   506
```

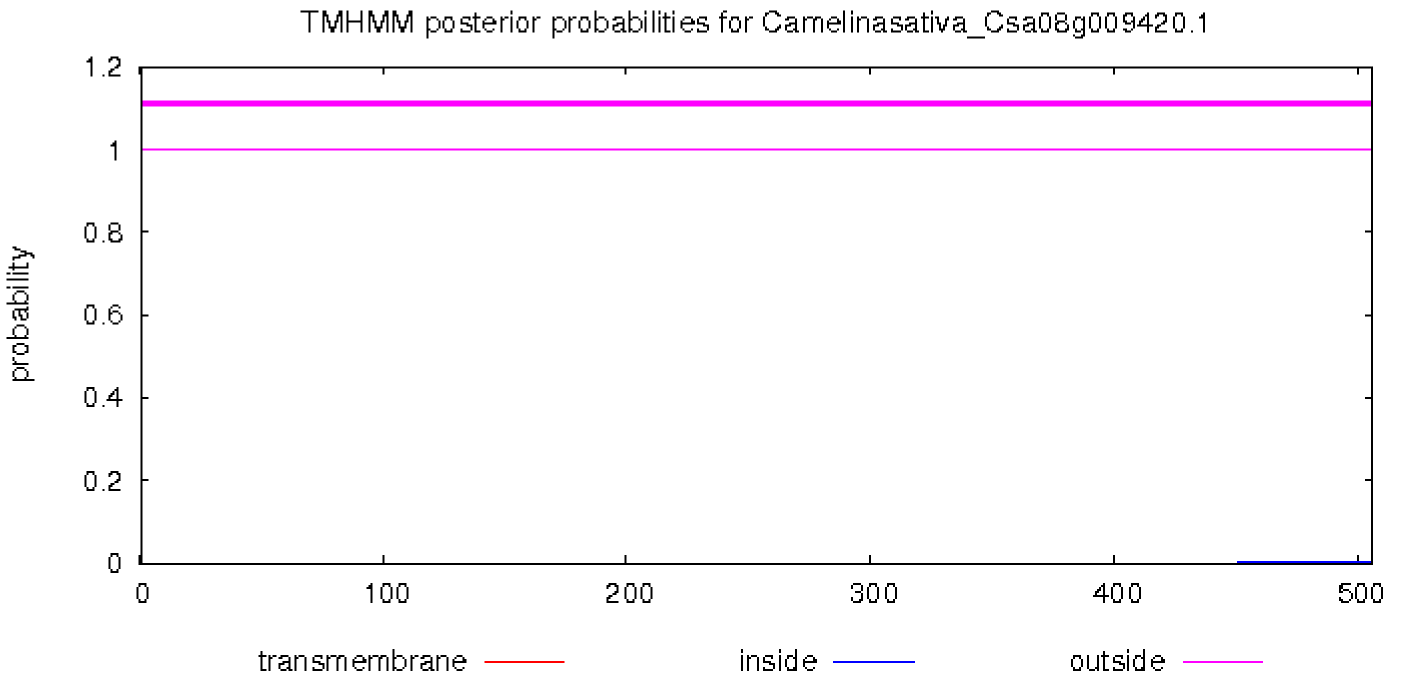

plot in postscript, script for making the plot in gnuplot, data for plot

---

```
# Camelinasativa_Csa08g010450.1 Length: 160
# Camelinasativa_Csa08g010450.1 Number of predicted TMHs:  0
# Camelinasativa_Csa08g010450.1 Exp number of AAs in TMHs: 0.02211
# Camelinasativa_Csa08g010450.1 Exp number, first 60 AAs:  0
# Camelinasativa_Csa08g010450.1 Total prob of N-in:        0.28556
Camelinasativa_Csa08g010450.1   TMHMM2.0        outside      1   160
```

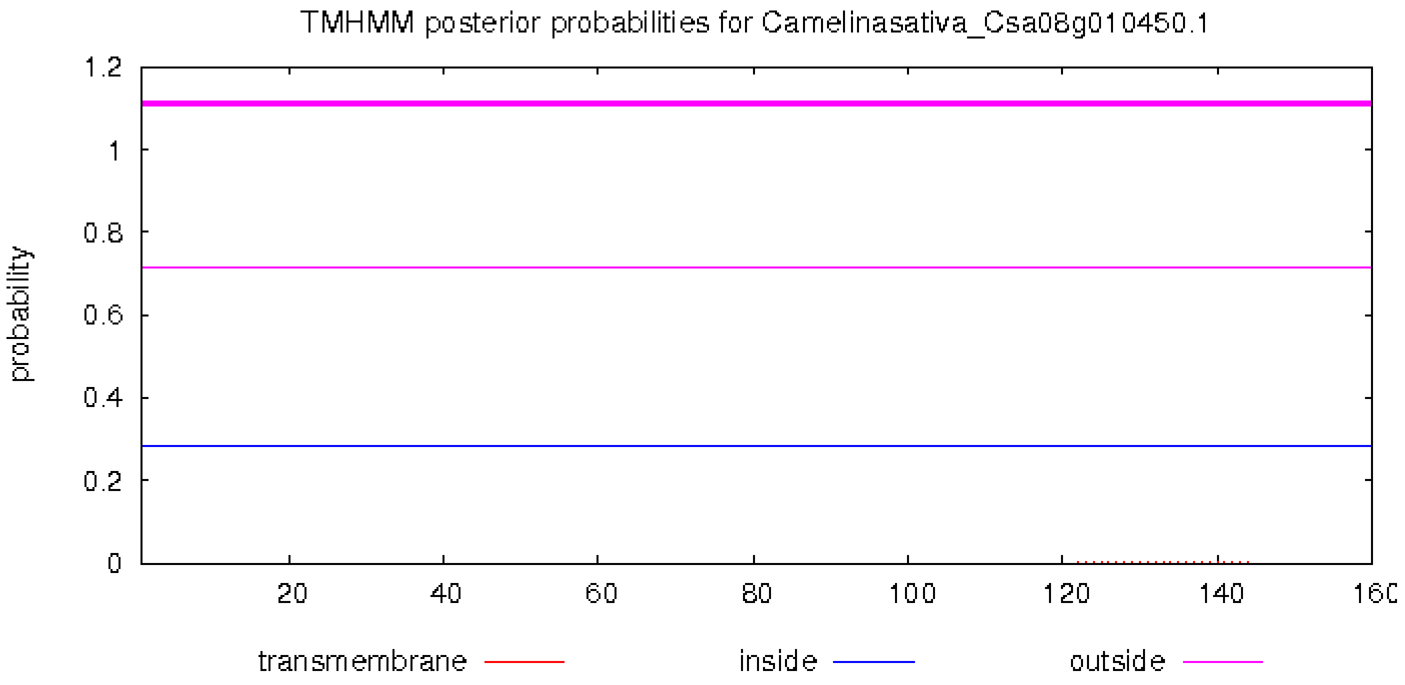

plot in postscript, script for making the plot in gnuplot, data for plot

---

```
# Camelinasativa_Csa08g010510.1 Length: 157
# Camelinasativa_Csa08g010510.1 Number of predicted TMHs:  0
# Camelinasativa_Csa08g010510.1 Exp number of AAs in TMHs: 0.02197
# Camelinasativa_Csa08g010510.1 Exp number, first 60 AAs:  0
```

```
# Camelinasativa_Csa08g010510.1 Total prob of N-in:        0.33404
Camelinasativa_Csa08g010510.1   TMHMM2.0        outside      1   157
```

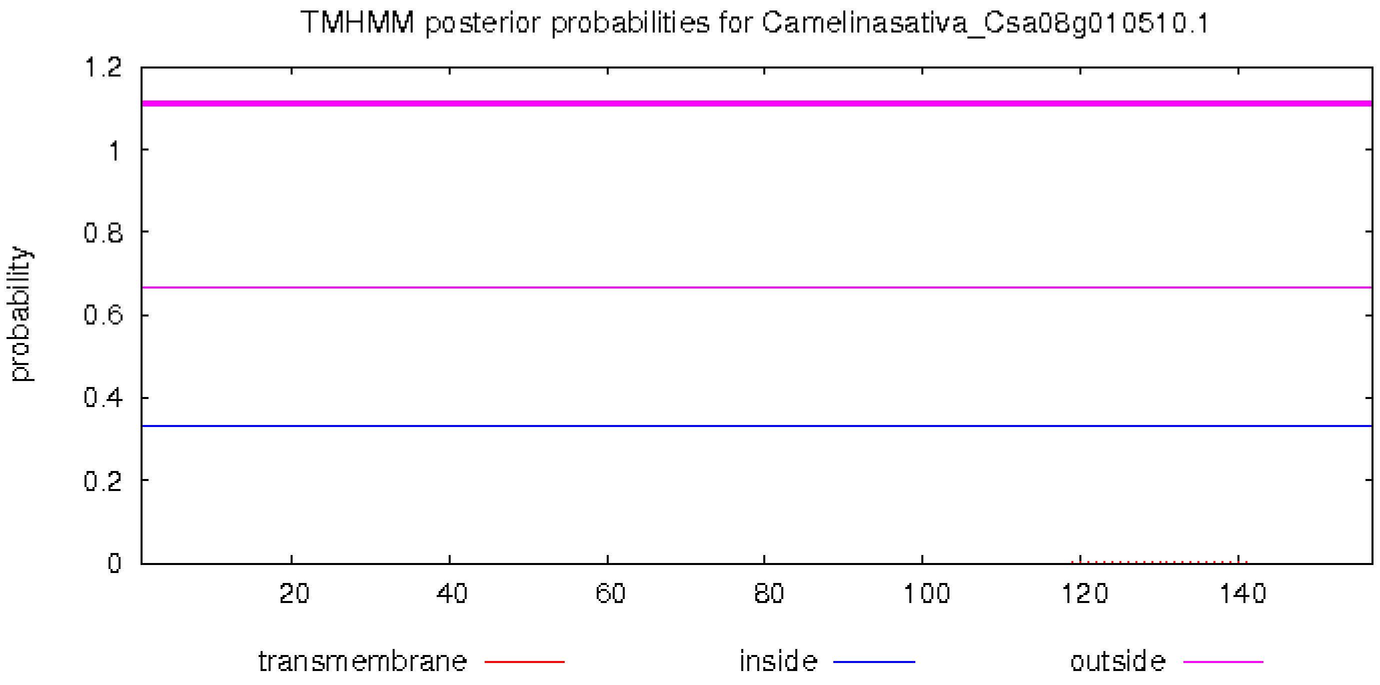

plot in postscript, script for making the plot in gnuplot, data for plot

---

```
# Camelinasativa_Csa08g010750.1 Length: 337
# Camelinasativa_Csa08g010750.1 Number of predicted TMHs:  0
# Camelinasativa_Csa08g010750.1 Exp number of AAs in TMHs: 0.00039
# Camelinasativa_Csa08g010750.1 Exp number, first 60 AAs:  0
# Camelinasativa_Csa08g010750.1 Total prob of N-in:        0.00863
Camelinasativa_Csa08g010750.1   TMHMM2.0        outside      1   337
```

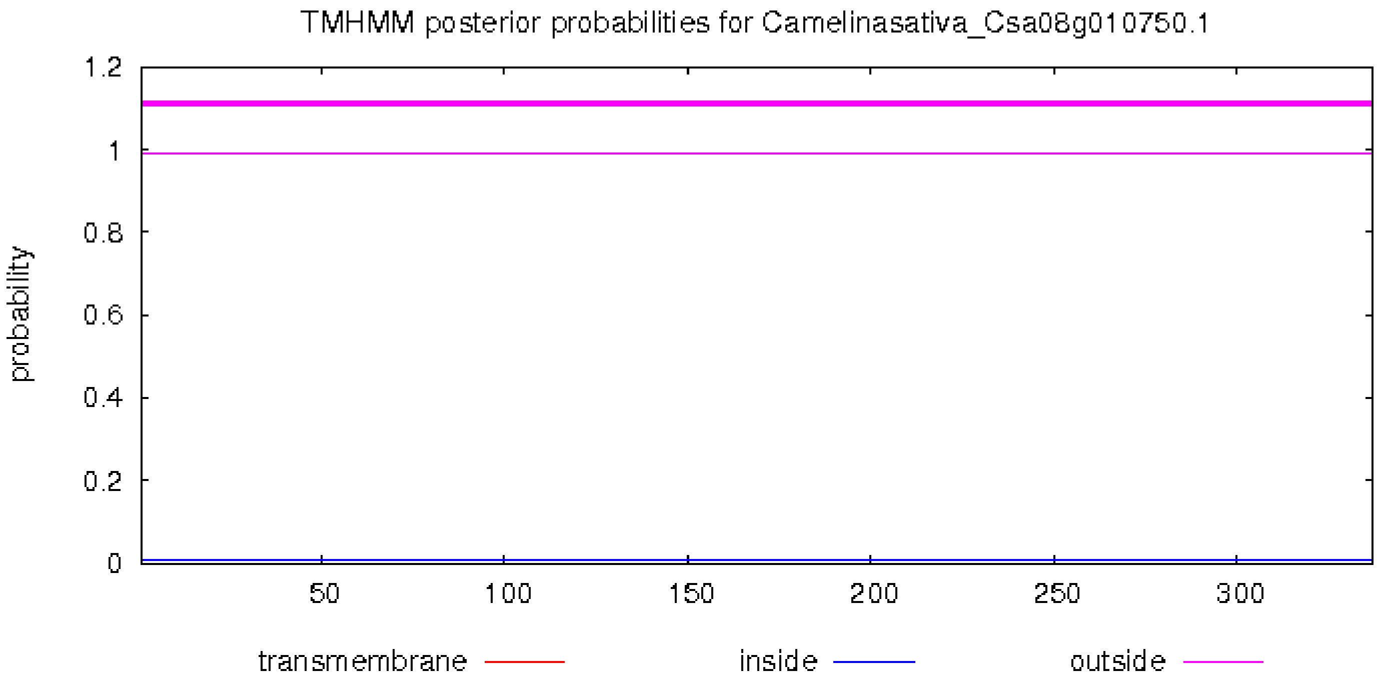

plot in postscript, script for making the plot in gnuplot, data for plot

---

```
# Camelinasativa_Csa08g015450.1 Length: 340
# Camelinasativa_Csa08g015450.1 Number of predicted TMHs:  0
# Camelinasativa_Csa08g015450.1 Exp number of AAs in TMHs: 12.36485
# Camelinasativa_Csa08g015450.1 Exp number, first 60 AAs:  0.0084
```

```
# Camelinasativa_Csa08g015450.1 Total prob of N-in:        0.11814
Camelinasativa_Csa08g015450.1   TMHMM2.0        outside      1   340
```

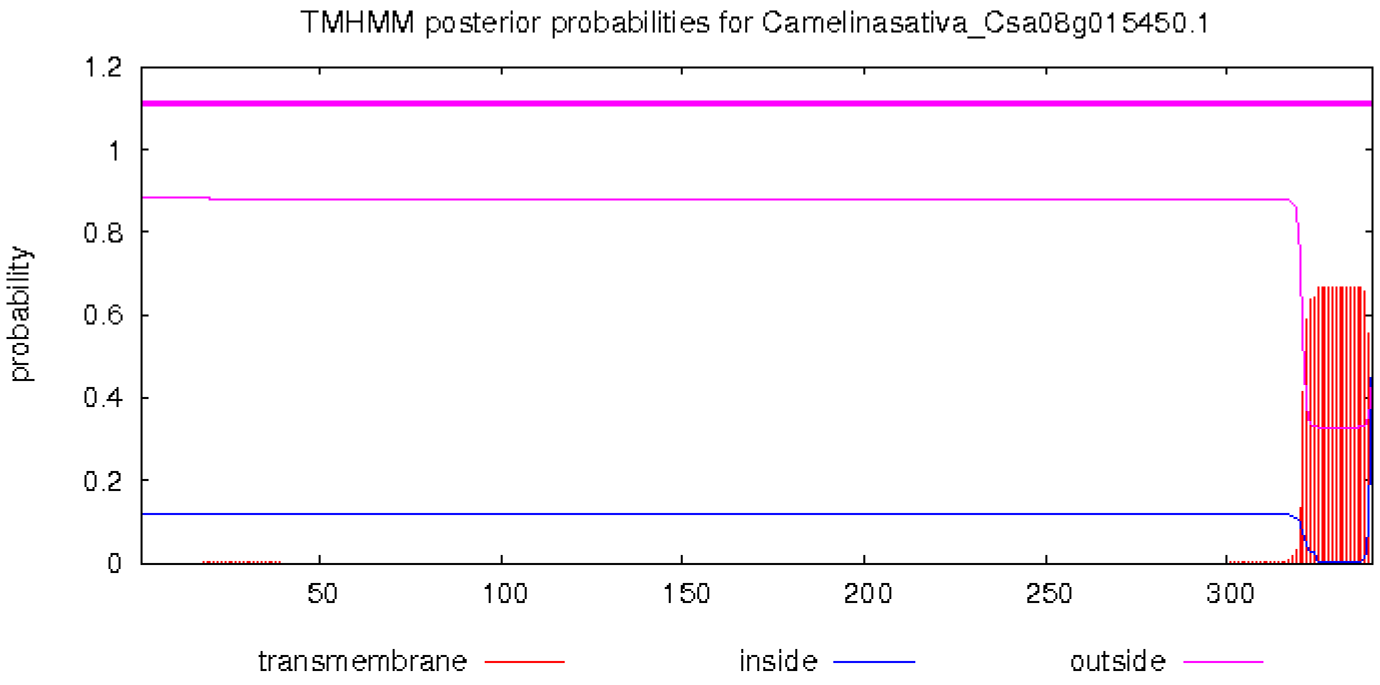

[plot](#) in postscript, [script](#) for making the plot in gnuplot, [data](#) for plot

---

```
# Camelinasativa_Csa08g015570.1 Length: 184
# Camelinasativa_Csa08g015570.1 Number of predicted TMHs:  0
# Camelinasativa_Csa08g015570.1 Exp number of AAs in TMHs: 0.00033
# Camelinasativa_Csa08g015570.1 Exp number, first 60 AAs:  0.00022
# Camelinasativa_Csa08g015570.1 Total prob of N-in:        0.16362
Camelinasativa_Csa08g015570.1   TMHMM2.0        outside      1   184
```

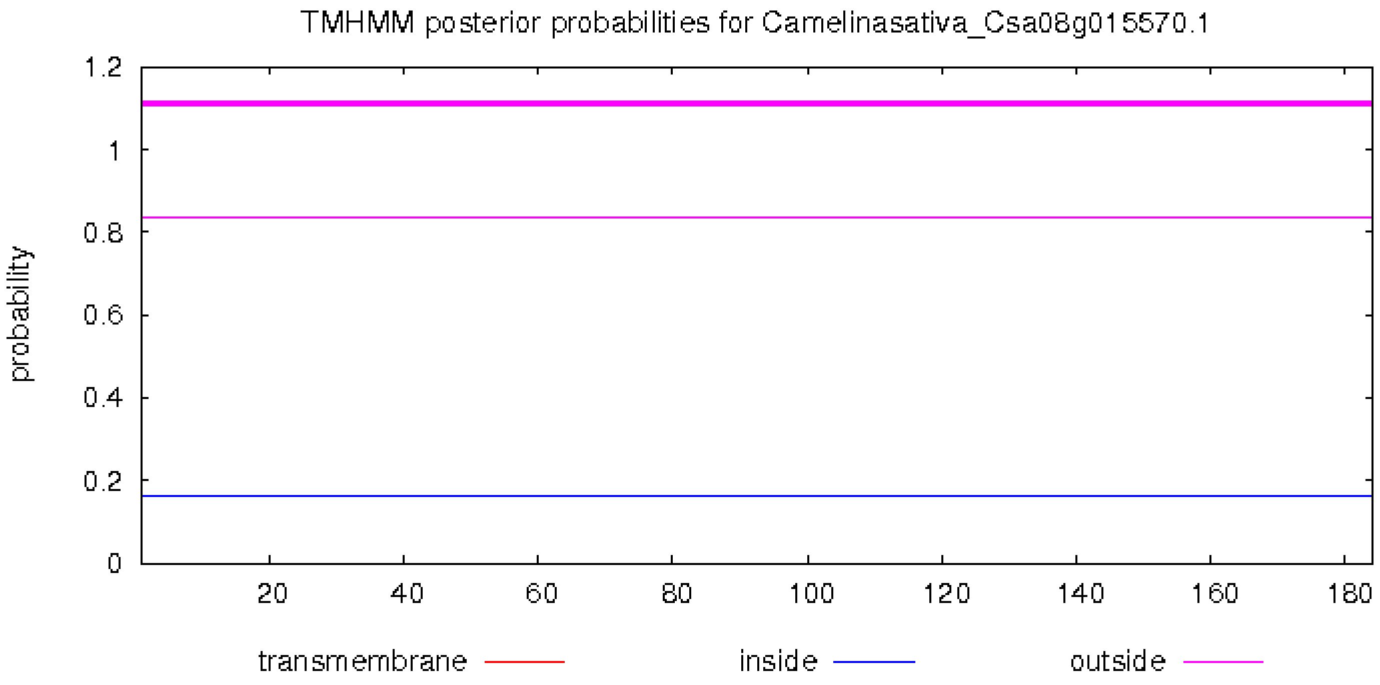

[plot](#) in postscript, [script](#) for making the plot in gnuplot, [data](#) for plot

---

```
# Camelinasativa_Csa08g020160.1 Length: 467
# Camelinasativa_Csa08g020160.1 Number of predicted TMHs:  1
# Camelinasativa_Csa08g020160.1 Exp number of AAs in TMHs: 17.23197
# Camelinasativa_Csa08g020160.1 Exp number, first 60 AAs:  0
```

```
# Camelinasativa_Csa08g020160.1 Total prob of N-in:        0.00730
Camelinasativa_Csa08g020160.1   TMHMM2.0        outside      1   446
Camelinasativa_Csa08g020160.1   TMHMM2.0        TMhelix    447   466
Camelinasativa_Csa08g020160.1   TMHMM2.0        inside     467   467
```

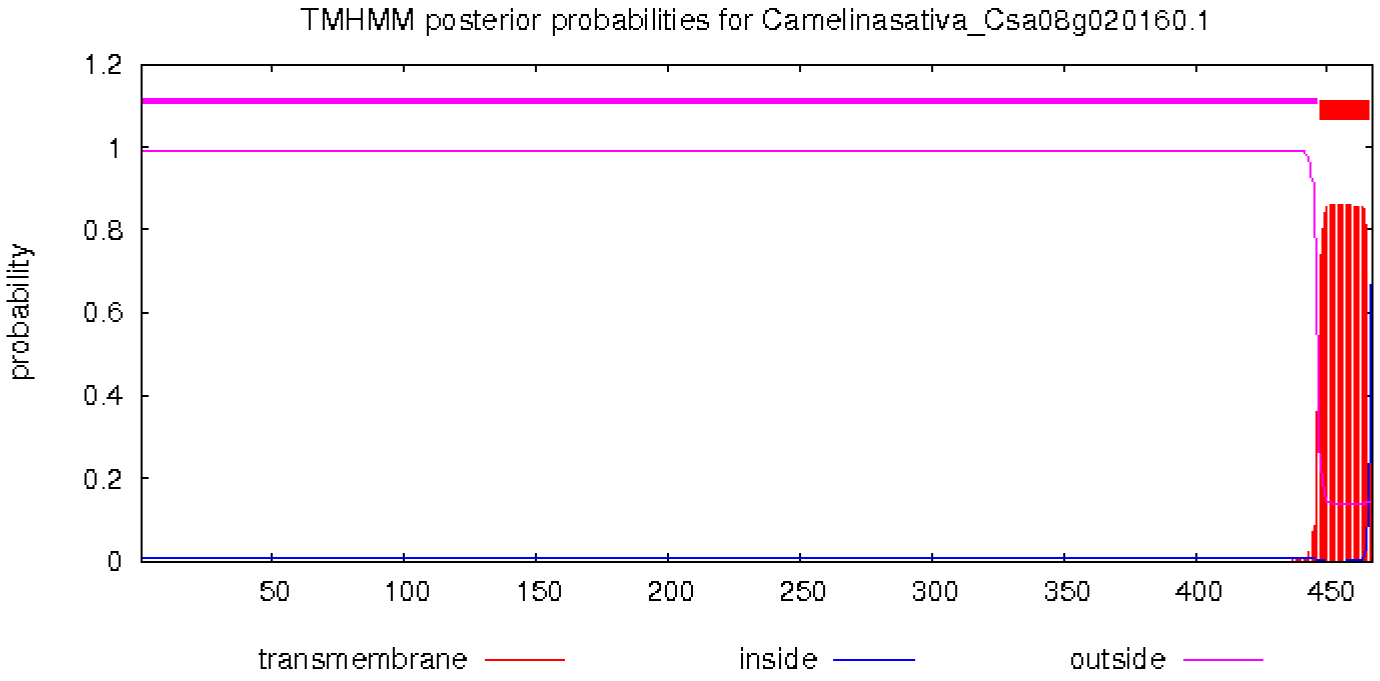

plot in postscript, script for making the plot in gnuplot, data for plot

---

```
# Camelinasativa_Csa08g040330.1 Length: 341
# Camelinasativa_Csa08g040330.1 Number of predicted TMHs:  0
# Camelinasativa_Csa08g040330.1 Exp number of AAs in TMHs: 0
# Camelinasativa_Csa08g040330.1 Exp number, first 60 AAs:  0
# Camelinasativa_Csa08g040330.1 Total prob of N-in:        0.00436
Camelinasativa_Csa08g040330.1   TMHMM2.0        outside      1   341
```

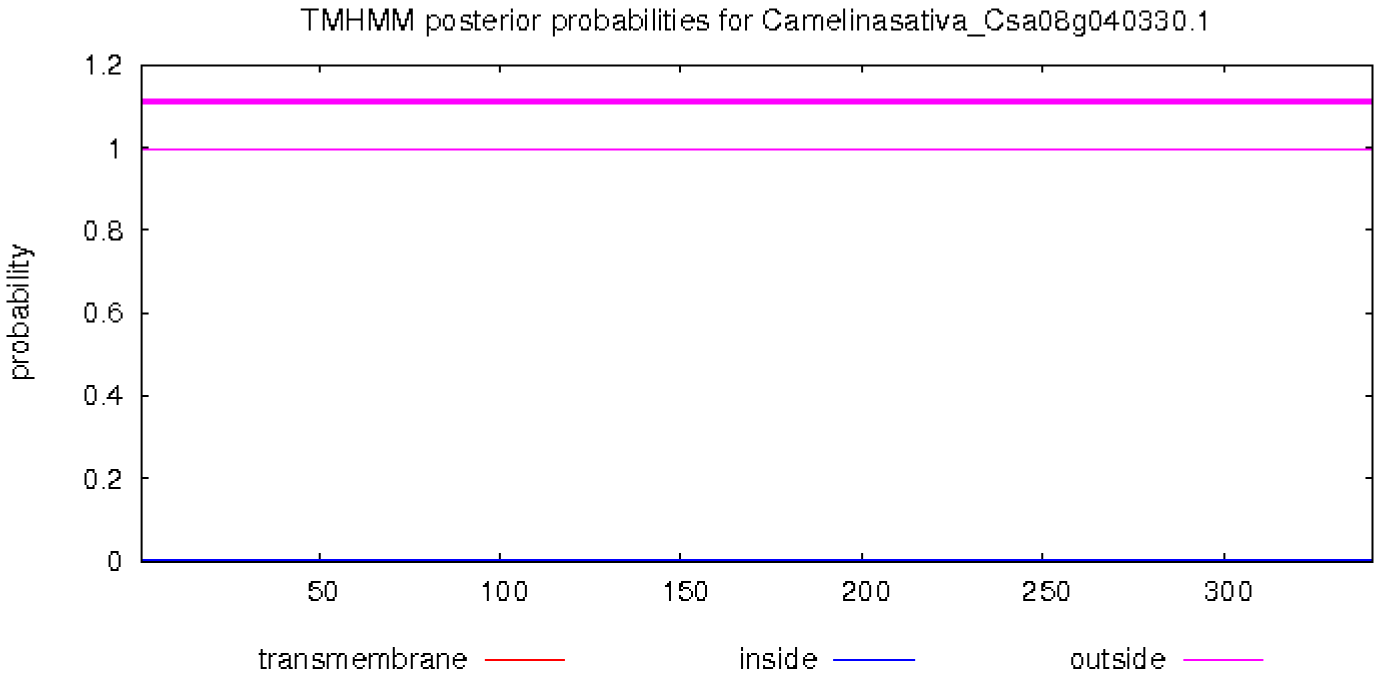

plot in postscript, script for making the plot in gnuplot, data for plot

---

```
# Camelinasativa_Csa08g052780.1 Length: 604
# Camelinasativa_Csa08g052780.1 Number of predicted TMHs:  0
```

```
# Camelinasativa_Csa08g052780.1 Exp number of AAs in TMHs: 0.00095
# Camelinasativa_Csa08g052780.1 Exp number, first 60 AAs:  0.0002
# Camelinasativa_Csa08g052780.1 Total prob of N-in:        0.00455
Camelinasativa_Csa08g052780.1   TMHMM2.0        outside      1   604
```

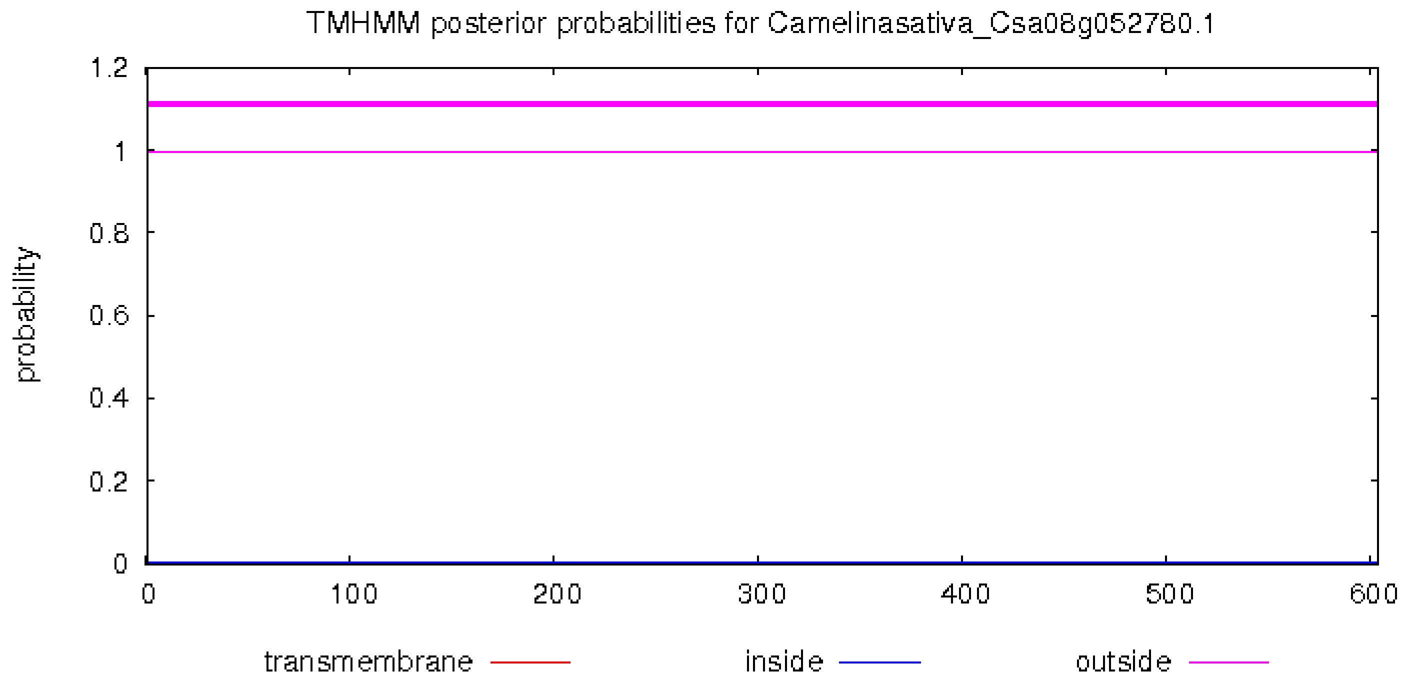

plot in postscript, script for making the plot in gnuplot, data for plot

---

```
# Camelinasativa_Csa08g052950.1 Length: 443
# Camelinasativa_Csa08g052950.1 Number of predicted TMHs:  1
# Camelinasativa_Csa08g052950.1 Exp number of AAs in TMHs: 22.82262
# Camelinasativa_Csa08g052950.1 Exp number, first 60 AAs:  0.01574
# Camelinasativa_Csa08g052950.1 Total prob of N-in:        0.12965
Camelinasativa_Csa08g052950.1   TMHMM2.0        outside      1   408
Camelinasativa_Csa08g052950.1   TMHMM2.0        TMhelix    409   431
Camelinasativa_Csa08g052950.1   TMHMM2.0        inside     432   443
```

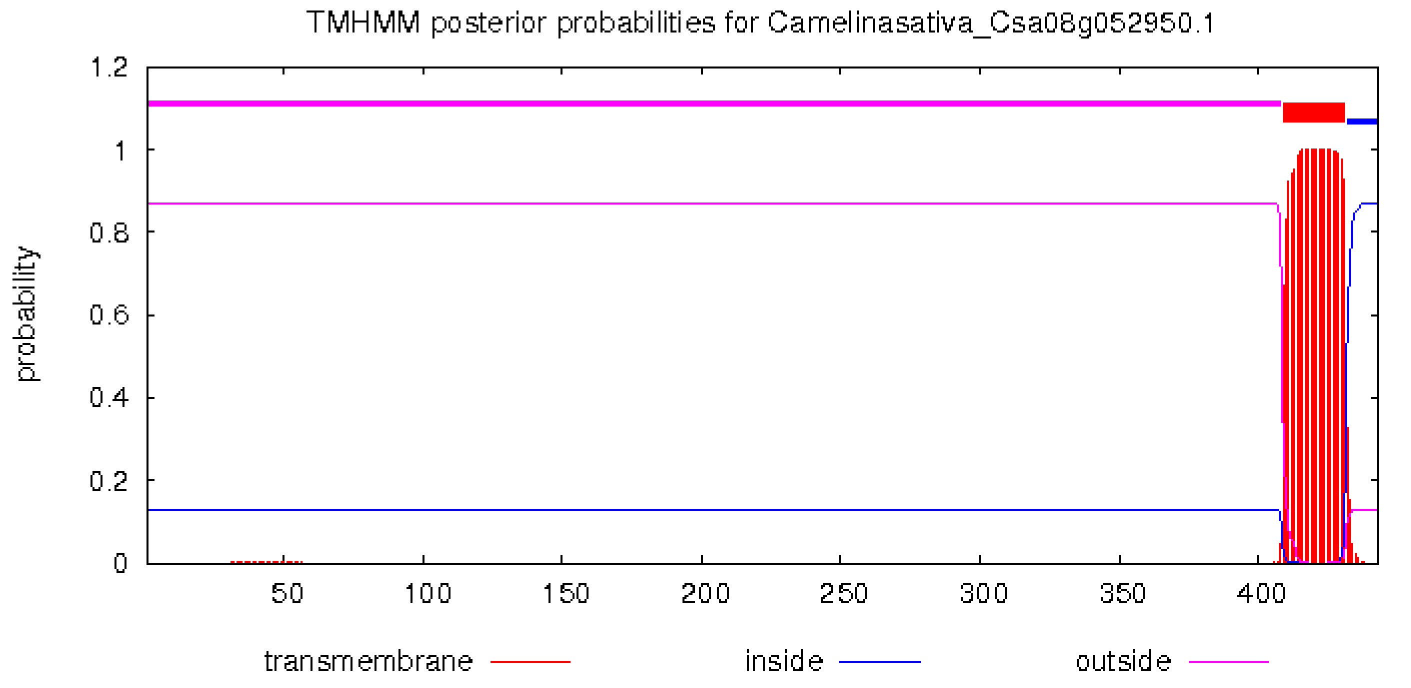

plot in postscript, script for making the plot in gnuplot, data for plot

---

```
# Camelinasativa_Csa08g055310.1 Length: 422
# Camelinasativa_Csa08g055310.1 Number of predicted TMHs:  0
# Camelinasativa_Csa08g055310.1 Exp number of AAs in TMHs: 0.00018
# Camelinasativa_Csa08g055310.1 Exp number, first 60 AAs:  0.00018
# Camelinasativa_Csa08g055310.1 Total prob of N-in:        0.00273
Camelinasativa_Csa08g055310.1	TMHMM2.0	outside	     1   422
```

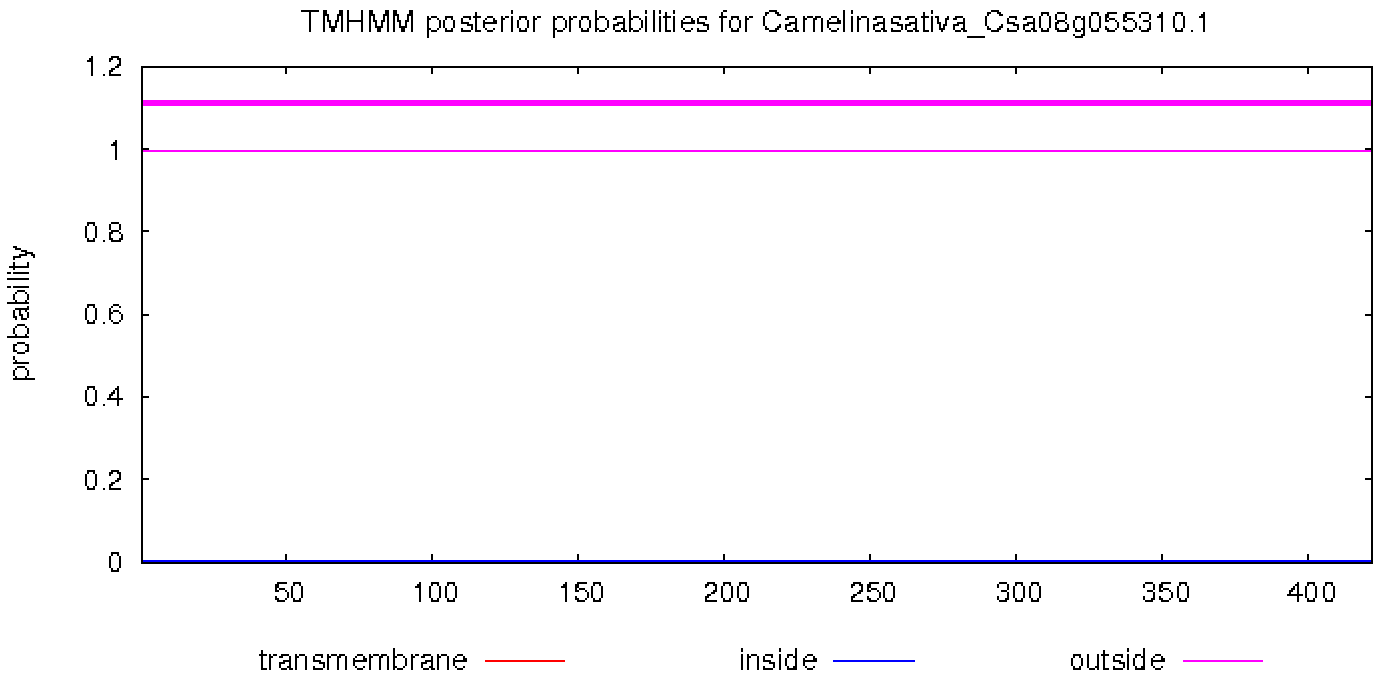

plot in postscript, script for making the plot in gnuplot, data for plot

---

```
# Camelinasativa_Csa08g055440.1 Length: 285
# Camelinasativa_Csa08g055440.1 Number of predicted TMHs:  0
# Camelinasativa_Csa08g055440.1 Exp number of AAs in TMHs: 0.00019
# Camelinasativa_Csa08g055440.1 Exp number, first 60 AAs:  0
# Camelinasativa_Csa08g055440.1 Total prob of N-in:        0.01006
Camelinasativa_Csa08g055440.1	TMHMM2.0	outside	     1   285
```

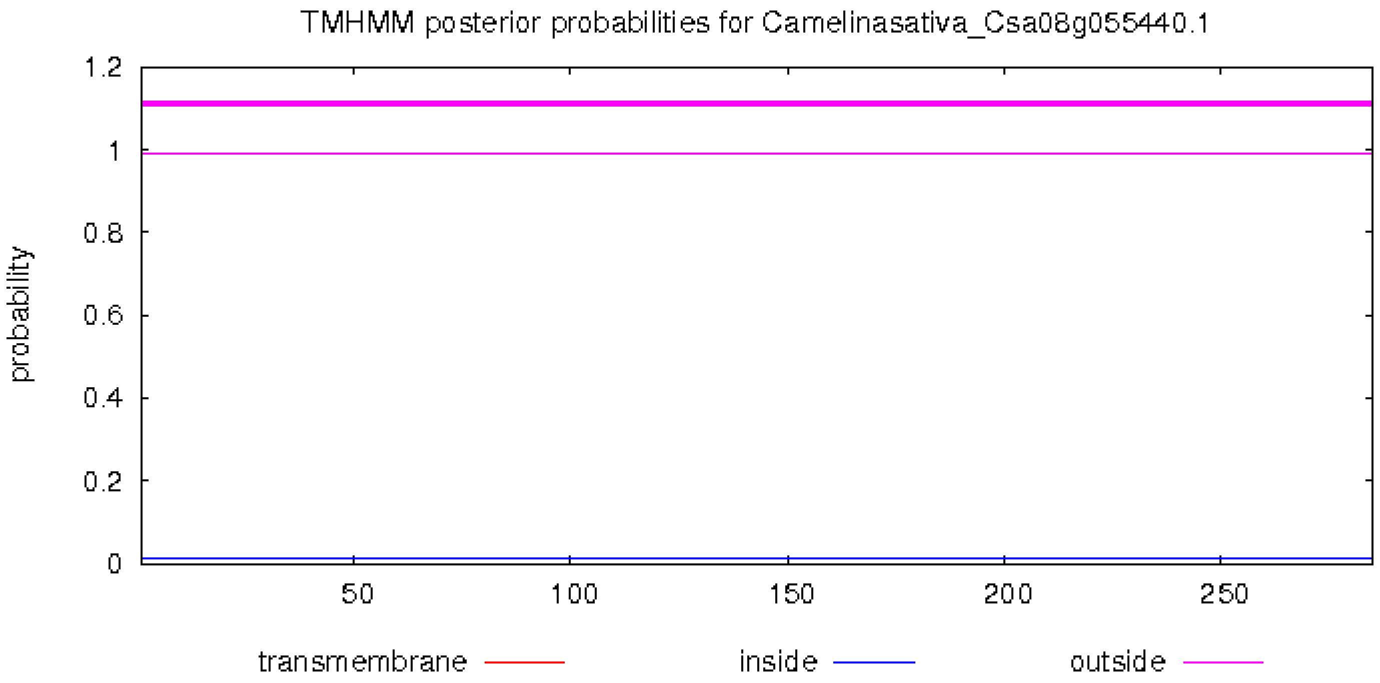

plot in postscript, script for making the plot in gnuplot, data for plot

---

```
# Camelinasativa_Csa08g056700.1 Length: 336
# Camelinasativa_Csa08g056700.1 Number of predicted TMHs:  0
# Camelinasativa_Csa08g056700.1 Exp number of AAs in TMHs: 0.00021
# Camelinasativa_Csa08g056700.1 Exp number, first 60 AAs:  0
# Camelinasativa_Csa08g056700.1 Total prob of N-in:        0.00459
Camelinasativa_Csa08g056700.1   TMHMM2.0        outside      1   336
```

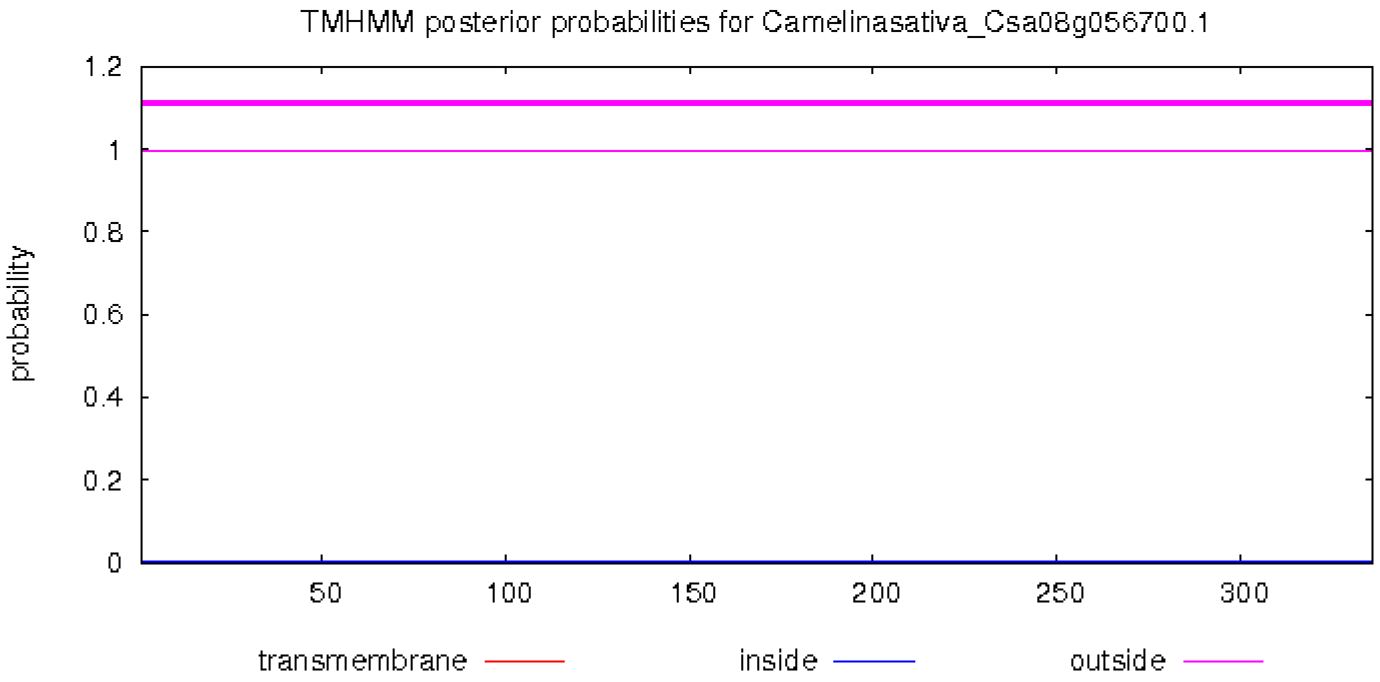

plot in postscript, script for making the plot in gnuplot, data for plot

```
# Camelinasativa_Csa08g056710.1 Length: 147
# Camelinasativa_Csa08g056710.1 Number of predicted TMHs:  0
# Camelinasativa_Csa08g056710.1 Exp number of AAs in TMHs: 0.00021
# Camelinasativa_Csa08g056710.1 Exp number, first 60 AAs:  0
# Camelinasativa_Csa08g056710.1 Total prob of N-in:        0.04993
Camelinasativa_Csa08g056710.1   TMHMM2.0        outside      1   147
```

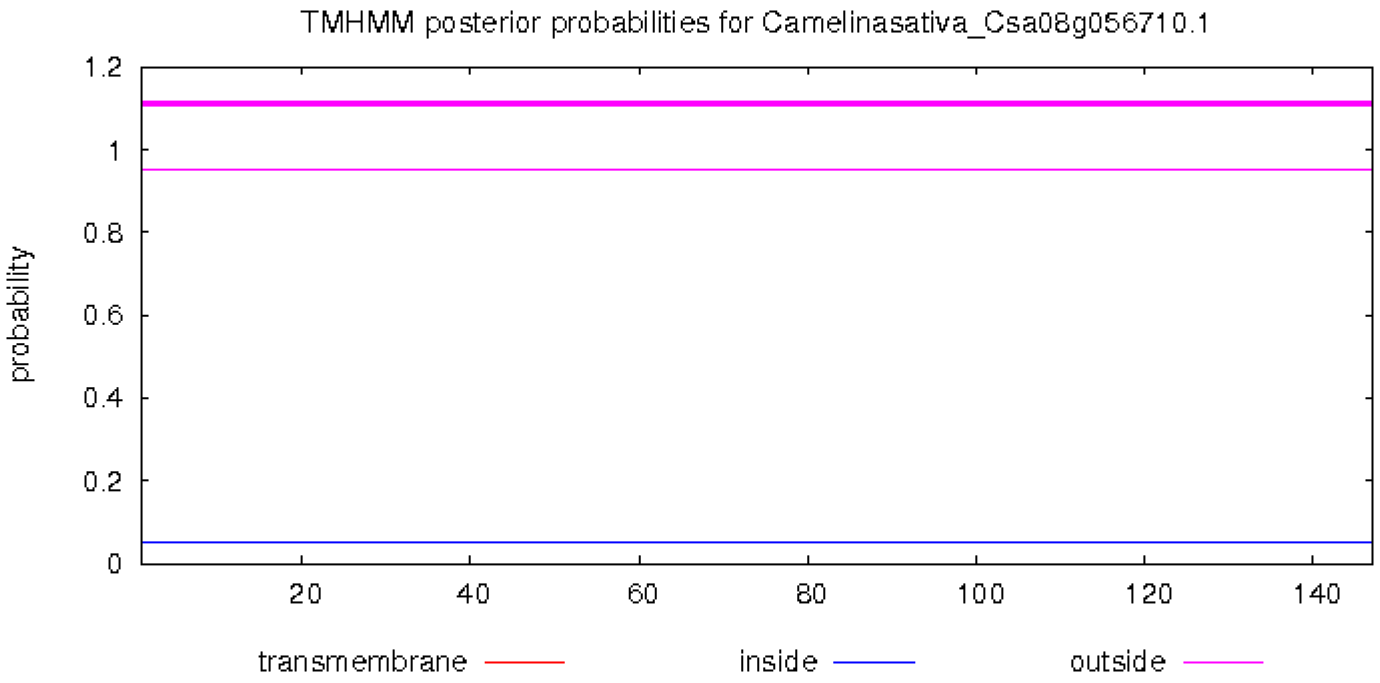

plot in postscript, script for making the plot in gnuplot, data for plot

```
# Camelinasativa_Csa08g060070.1 Length: 651
# Camelinasativa_Csa08g060070.1 Number of predicted TMHs:  1
# Camelinasativa_Csa08g060070.1 Exp number of AAs in TMHs: 21.39433
# Camelinasativa_Csa08g060070.1 Exp number, first 60 AAs:  0
# Camelinasativa_Csa08g060070.1 Total prob of N-in:        0.01090
Camelinasativa_Csa08g060070.1   TMHMM2.0        outside      1   625
Camelinasativa_Csa08g060070.1   TMHMM2.0        TMhelix    626   648
Camelinasativa_Csa08g060070.1   TMHMM2.0        inside     649   651
```

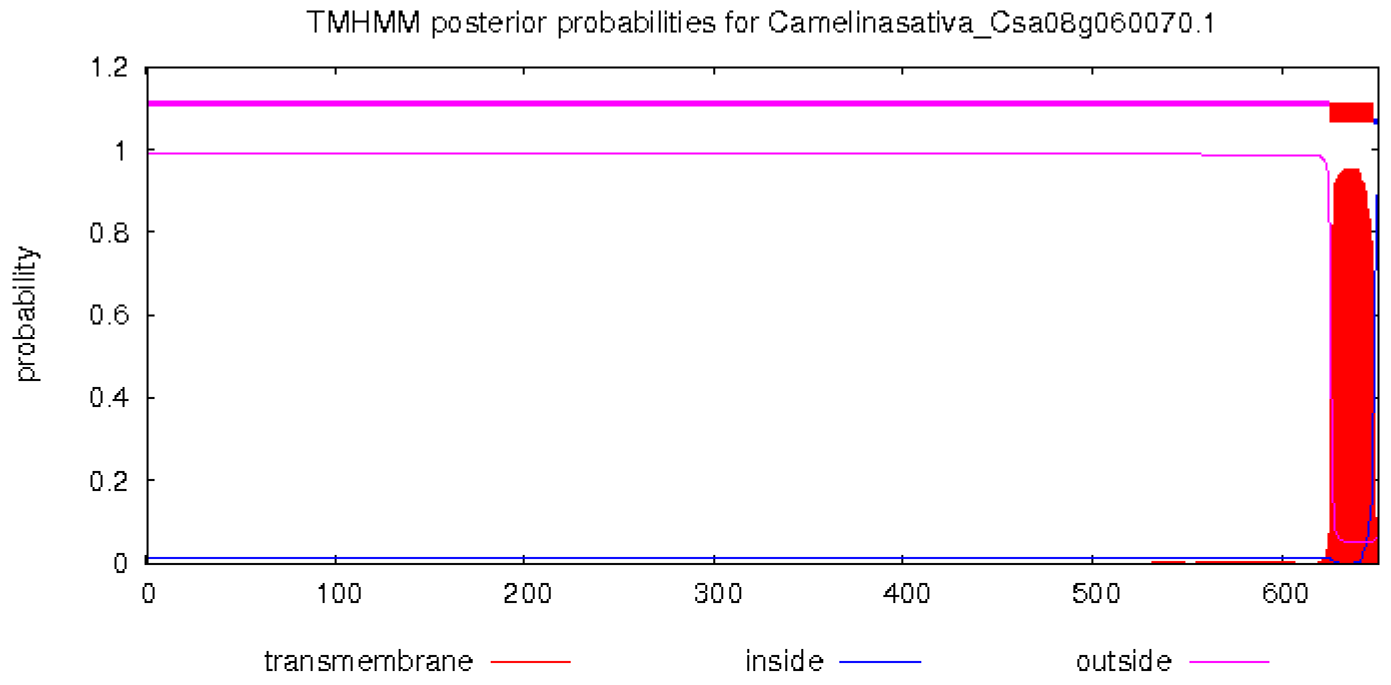

plot in postscript, script for making the plot in gnuplot, data for plot

---

```
# Camelinasativa_Csa08g060080.1 Length: 397
# Camelinasativa_Csa08g060080.1 Number of predicted TMHs:  0
# Camelinasativa_Csa08g060080.1 Exp number of AAs in TMHs: 0.0108
# Camelinasativa_Csa08g060080.1 Exp number, first 60 AAs:  0.00659
# Camelinasativa_Csa08g060080.1 Total prob of N-in:        0.07109
Camelinasativa_Csa08g060080.1   TMHMM2.0        outside      1   397
```

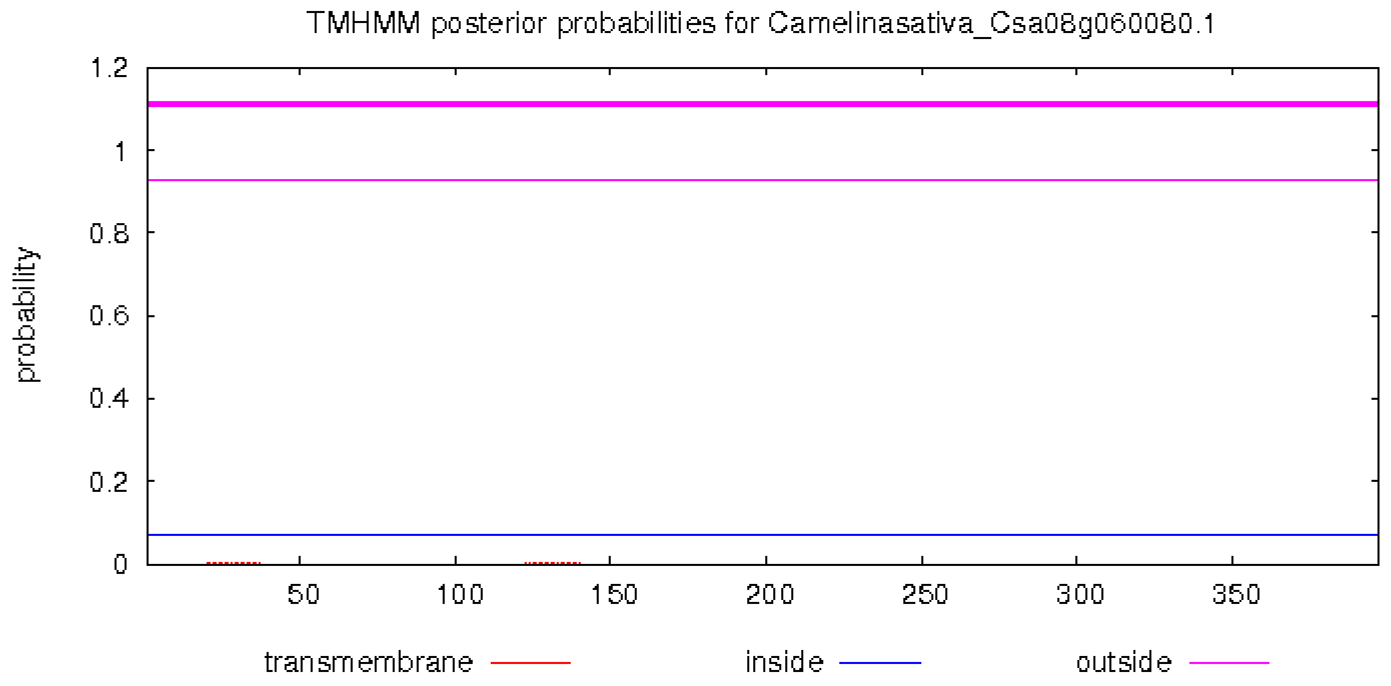

plot in postscript, script for making the plot in gnuplot, data for plot

```
# Camelinasativa_Csa09g004780.1 Length: 380
# Camelinasativa_Csa09g004780.1 Number of predicted TMHs:  0
# Camelinasativa_Csa09g004780.1 Exp number of AAs in TMHs: 0.00913
# Camelinasativa_Csa09g004780.1 Exp number, first 60 AAs:  0
# Camelinasativa_Csa09g004780.1 Total prob of N-in:        0.01302
Camelinasativa_Csa09g004780.1	TMHMM2.0	outside	     1   380
```

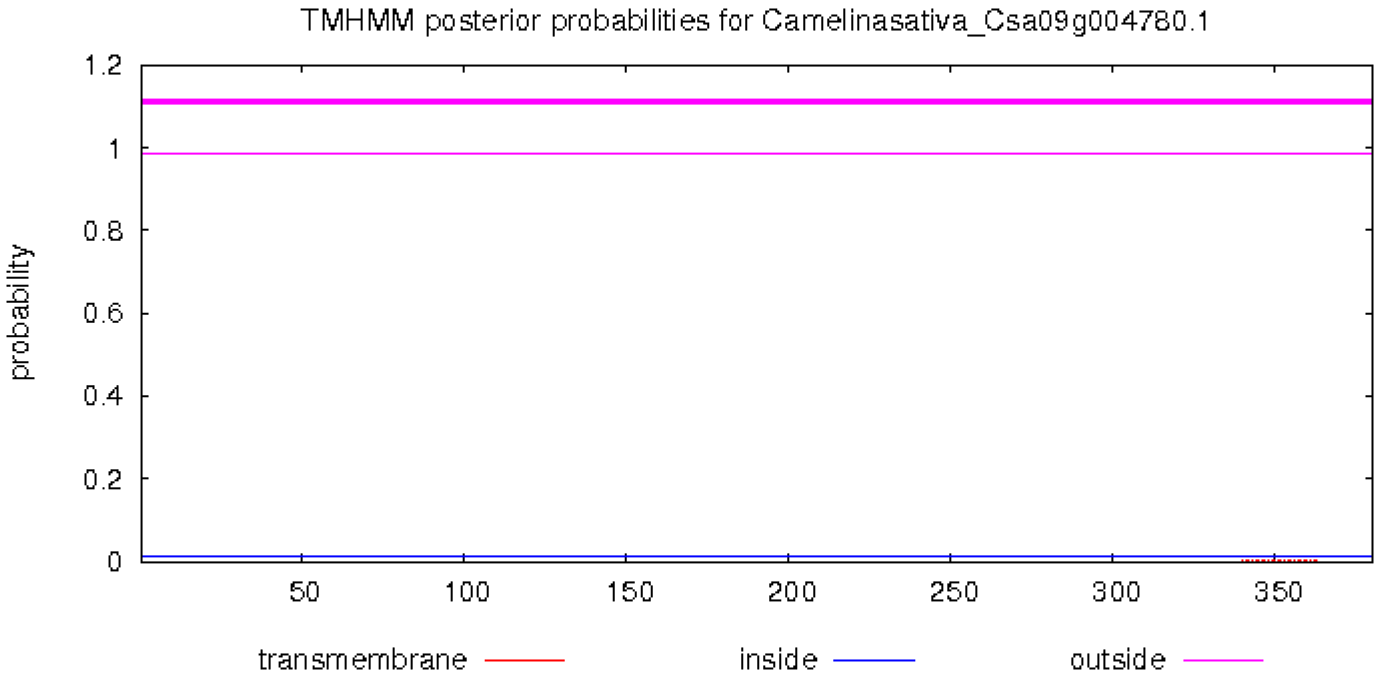

plot in postscript, script for making the plot in gnuplot, data for plot

```
# Camelinasativa_Csa09g013080.1 Length: 336
# Camelinasativa_Csa09g013080.1 Number of predicted TMHs:  0
# Camelinasativa_Csa09g013080.1 Exp number of AAs in TMHs: 0.20071
# Camelinasativa_Csa09g013080.1 Exp number, first 60 AAs:  0
# Camelinasativa_Csa09g013080.1 Total prob of N-in:        0.02990
Camelinasativa_Csa09g013080.1	TMHMM2.0	outside	     1   336
```

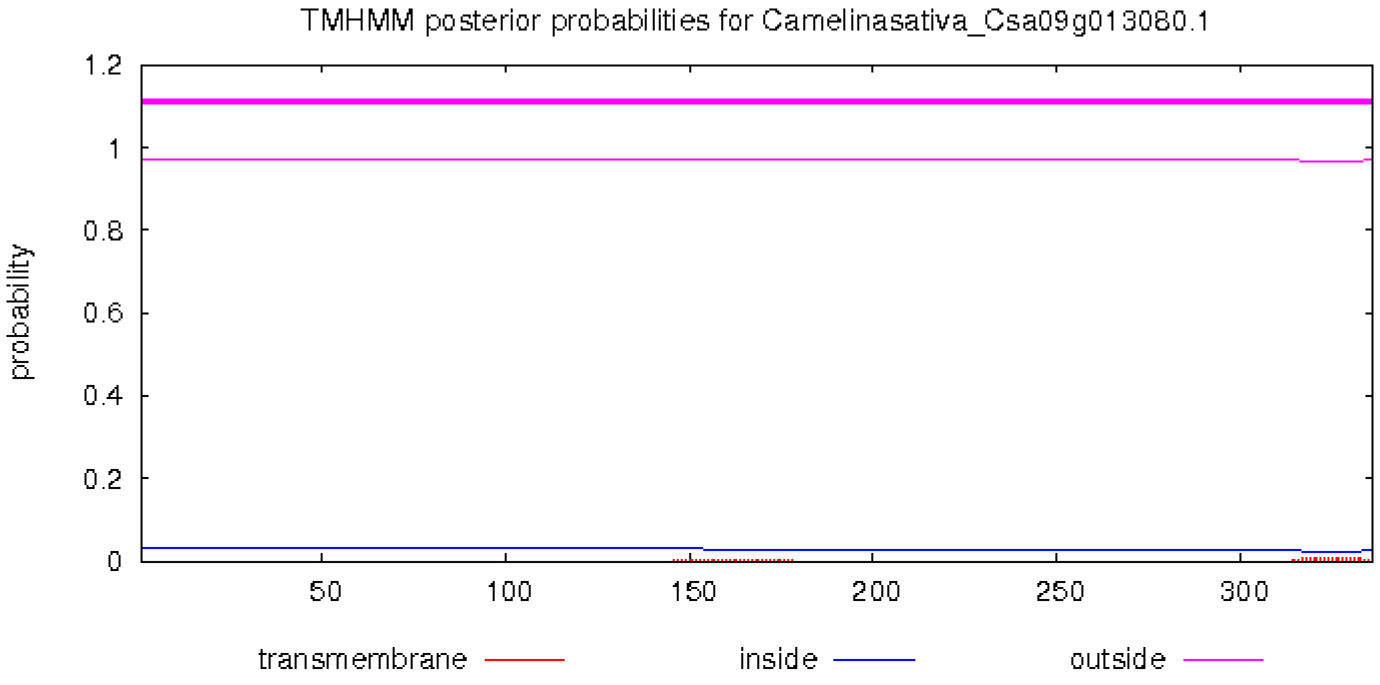

plot in postscript, script for making the plot in gnuplot, data for plot

```
# Camelinasativa_Csa09g022510.1 Length: 337
# Camelinasativa_Csa09g022510.1 Number of predicted TMHs:  0
# Camelinasativa_Csa09g022510.1 Exp number of AAs in TMHs: 0.00357
# Camelinasativa_Csa09g022510.1 Exp number, first 60 AAs:  0.00066
# Camelinasativa_Csa09g022510.1 Total prob of N-in:        0.03346
Camelinasativa_Csa09g022510.1   TMHMM2.0        outside      1   337
```

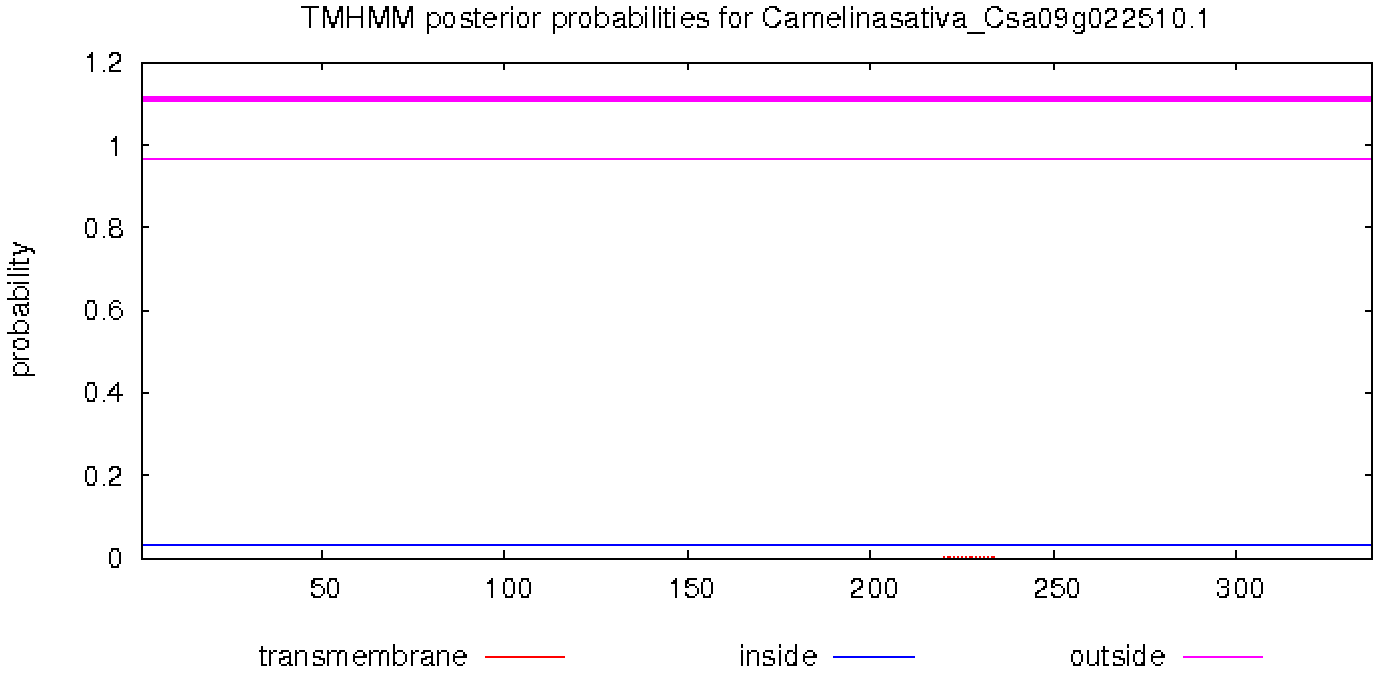

plot in postscript, script for making the plot in gnuplot, data for plot

```
# Camelinasativa_Csa09g034120.1 Length: 209
# Camelinasativa_Csa09g034120.1 Number of predicted TMHs:  0
# Camelinasativa_Csa09g034120.1 Exp number of AAs in TMHs: 30.06517
# Camelinasativa_Csa09g034120.1 Exp number, first 60 AAs:  28.68935
# Camelinasativa_Csa09g034120.1 Total prob of N-in:        0.26773
# Camelinasativa_Csa09g034120.1 POSSIBLE N-term signal sequence
Camelinasativa_Csa09g034120.1   TMHMM2.0        outside      1   209
```

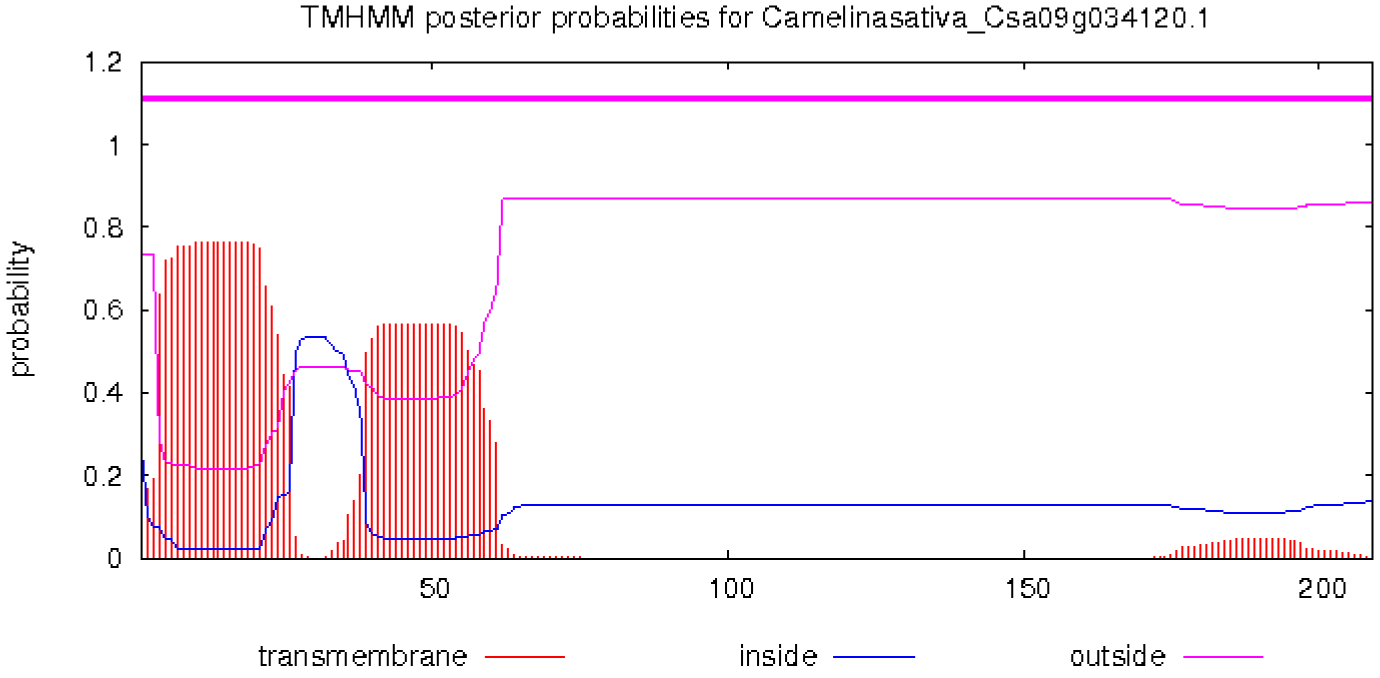

plot in postscript, script for making the plot in gnuplot, data for plot

```
# Camelinasativa_Csa09g034210.1 Length: 243
# Camelinasativa_Csa09g034210.1 Number of predicted TMHs:  0
# Camelinasativa_Csa09g034210.1 Exp number of AAs in TMHs: 0.01844
# Camelinasativa_Csa09g034210.1 Exp number, first 60 AAs:  0.01844
# Camelinasativa_Csa09g034210.1 Total prob of N-in:        0.02369
Camelinasativa_Csa09g034210.1   TMHMM2.0        outside      1   243
```

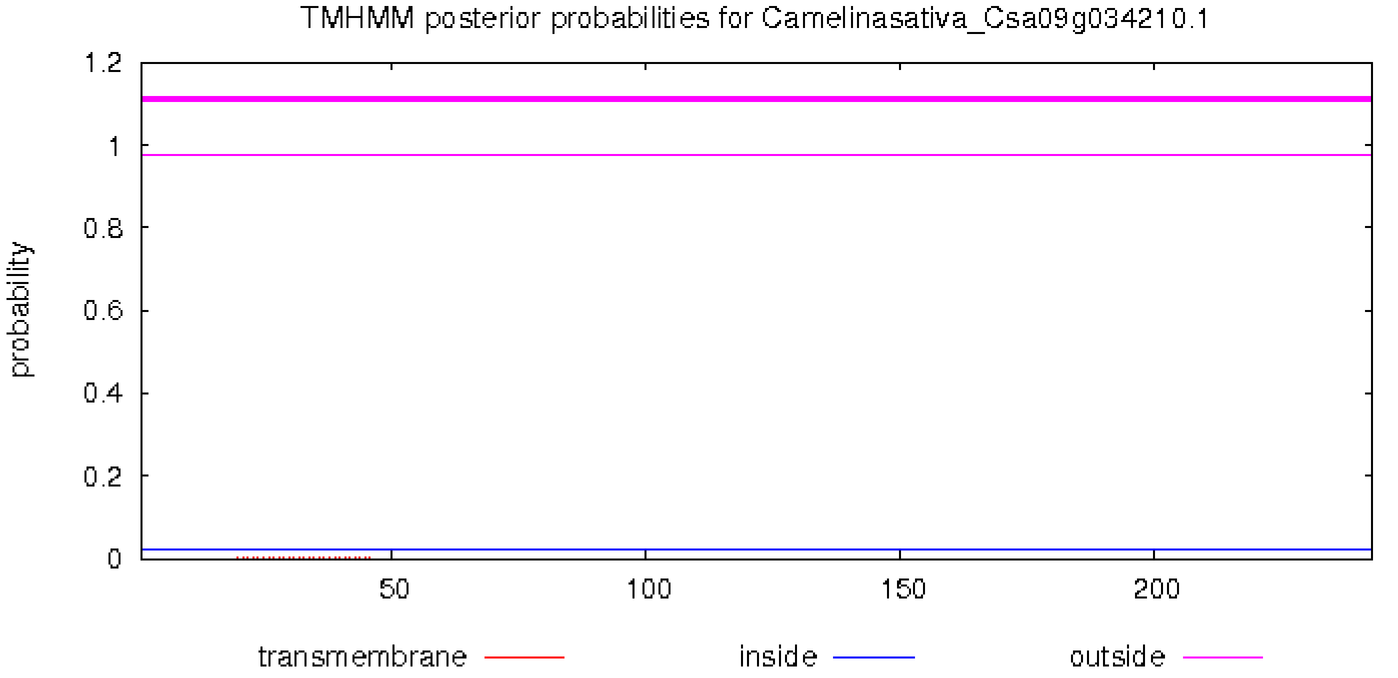

plot in postscript, script for making the plot in gnuplot, data for plot

---

```
# Camelinasativa_Csa09g052140.1 Length: 453
# Camelinasativa_Csa09g052140.1 Number of predicted TMHs:  1
# Camelinasativa_Csa09g052140.1 Exp number of AAs in TMHs: 20.75397
# Camelinasativa_Csa09g052140.1 Exp number, first 60 AAs:  0
# Camelinasativa_Csa09g052140.1 Total prob of N-in:        0.08744
Camelinasativa_Csa09g052140.1   TMHMM2.0        outside      1   422
Camelinasativa_Csa09g052140.1   TMHMM2.0        TMhelix    423   445
Camelinasativa_Csa09g052140.1   TMHMM2.0        inside     446   453
```

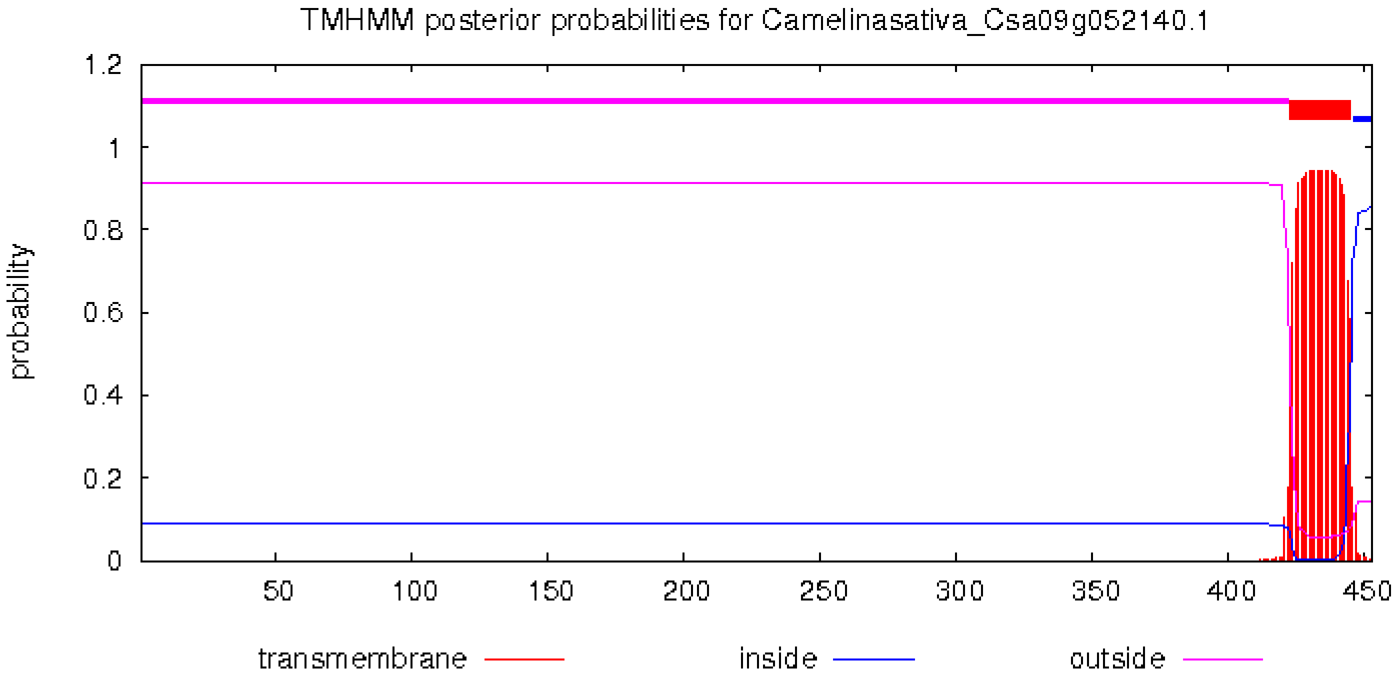

plot in postscript, script for making the plot in gnuplot, data for plot

```
# Camelinasativa_Csa09g067970.1 Length: 354
# Camelinasativa_Csa09g067970.1 Number of predicted TMHs:  0
# Camelinasativa_Csa09g067970.1 Exp number of AAs in TMHs: 0.00467
# Camelinasativa_Csa09g067970.1 Exp number, first 60 AAs:  0
# Camelinasativa_Csa09g067970.1 Total prob of N-in:        0.04936
Camelinasativa_Csa09g067970.1	TMHMM2.0	outside	     1   354
```

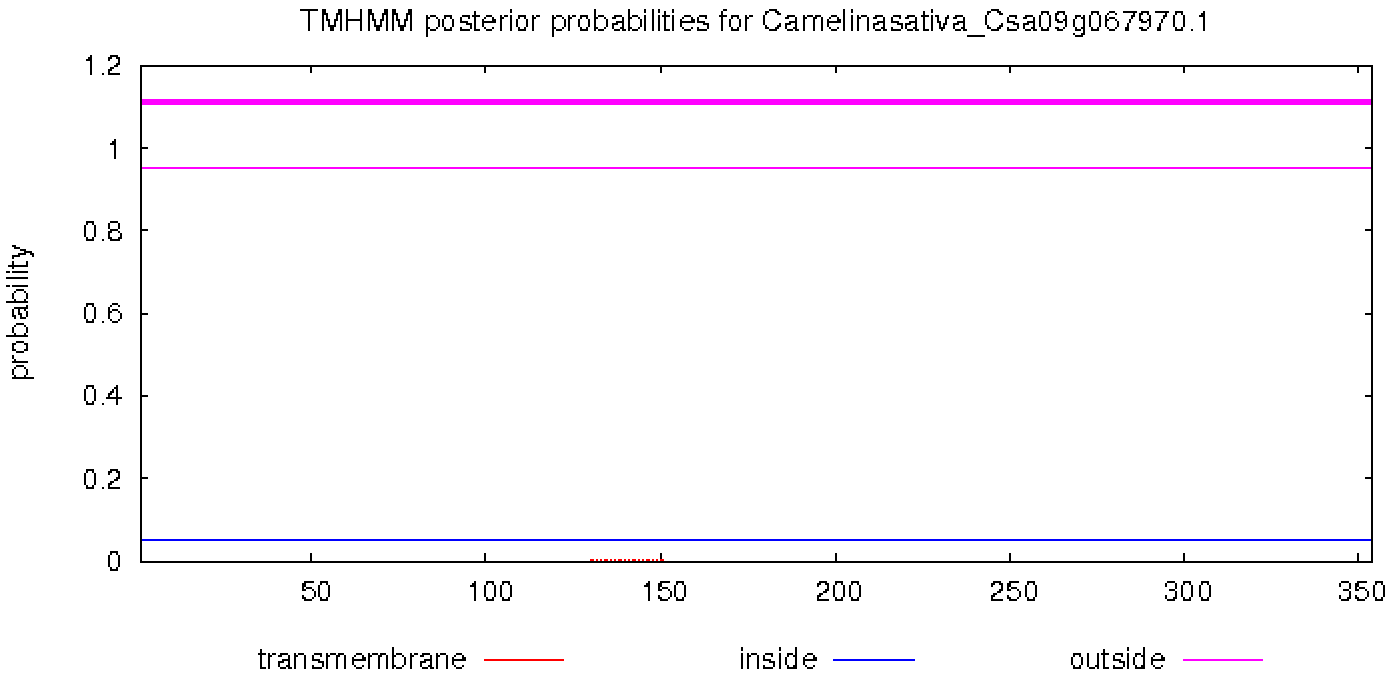

plot in postscript, script for making the plot in gnuplot, data for plot

```
# Camelinasativa_Csa09g068810.1 Length: 590
# Camelinasativa_Csa09g068810.1 Number of predicted TMHs:  0
# Camelinasativa_Csa09g068810.1 Exp number of AAs in TMHs: 0.00394
# Camelinasativa_Csa09g068810.1 Exp number, first 60 AAs:  0
# Camelinasativa_Csa09g068810.1 Total prob of N-in:        0.00403
Camelinasativa_Csa09g068810.1	TMHMM2.0	outside	     1   590
```

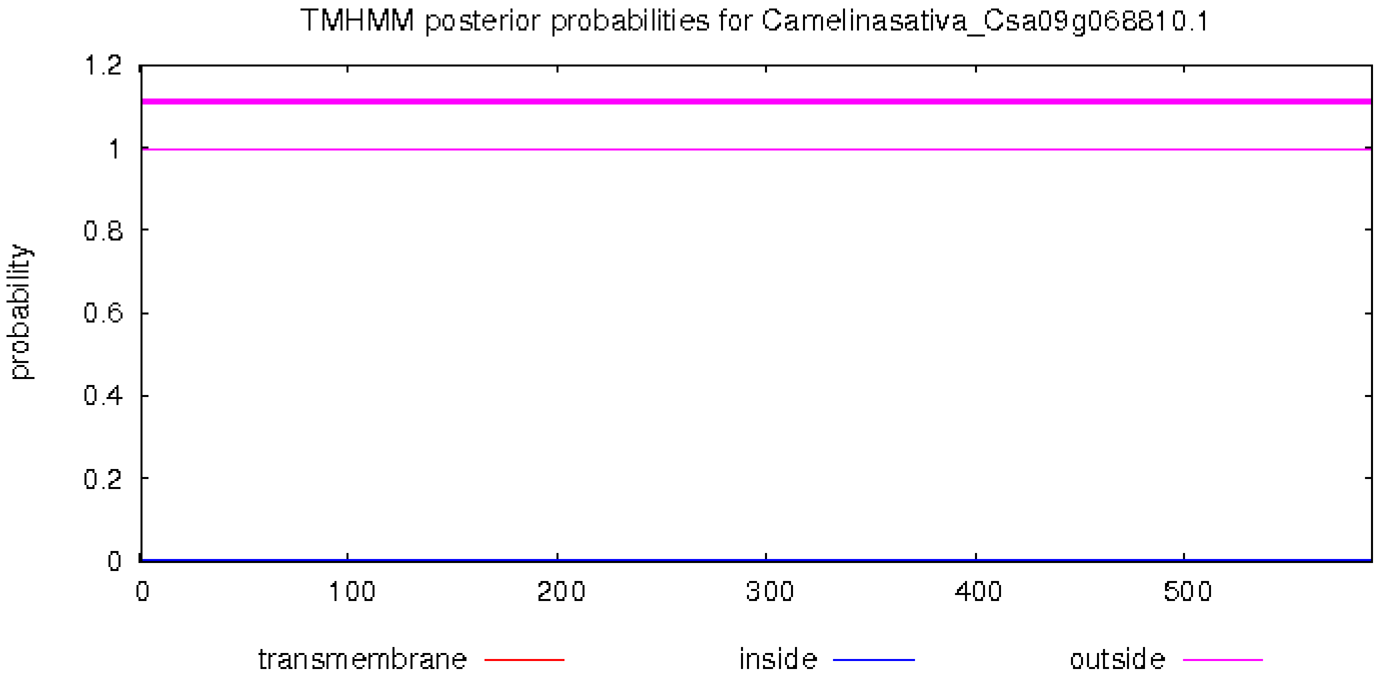

plot in postscript, script for making the plot in gnuplot, data for plot

```
# Camelinasativa_Csa09g068820.1 Length: 188
# Camelinasativa_Csa09g068820.1 Number of predicted TMHs:  0
# Camelinasativa_Csa09g068820.1 Exp number of AAs in TMHs: 0.00212
# Camelinasativa_Csa09g068820.1 Exp number, first 60 AAs:  0.00212
# Camelinasativa_Csa09g068820.1 Total prob of N-in:        0.01525
Camelinasativa_Csa09g068820.1	TMHMM2.0	outside	     1   188
```

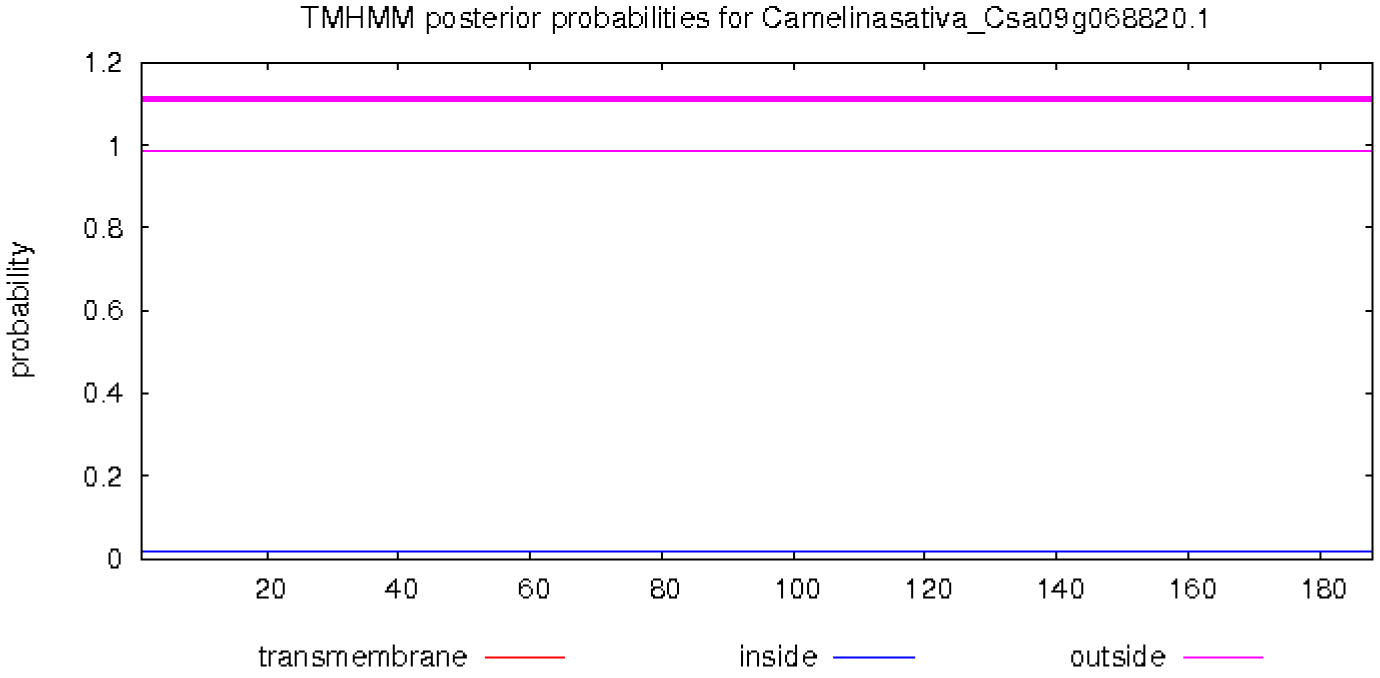

plot in postscript, script for making the plot in gnuplot, data for plot

---

```
# Camelinasativa_Csa09g068830.1 Length: 317
# Camelinasativa_Csa09g068830.1 Number of predicted TMHs:  0
# Camelinasativa_Csa09g068830.1 Exp number of AAs in TMHs: 0.02624
# Camelinasativa_Csa09g068830.1 Exp number, first 60 AAs:  0
# Camelinasativa_Csa09g068830.1 Total prob of N-in:        0.08303
Camelinasativa_Csa09g068830.1	TMHMM2.0	outside	     1   317
```

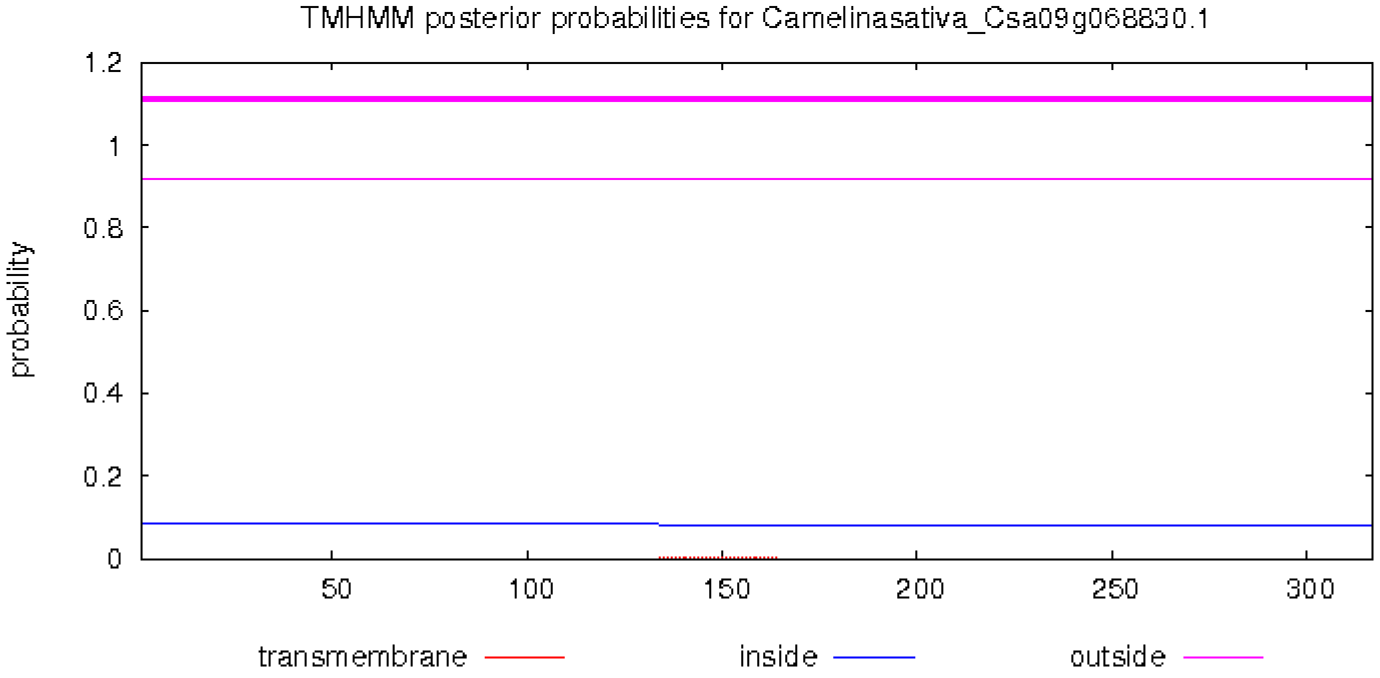

plot in postscript, script for making the plot in gnuplot, data for plot

---

```
# Camelinasativa_Csa09g068840.1 Length: 521
# Camelinasativa_Csa09g068840.1 Number of predicted TMHs:  0
# Camelinasativa_Csa09g068840.1 Exp number of AAs in TMHs: 0.00041
# Camelinasativa_Csa09g068840.1 Exp number, first 60 AAs:  0
# Camelinasativa_Csa09g068840.1 Total prob of N-in:        0.00495
Camelinasativa_Csa09g068840.1	TMHMM2.0	outside	     1   521
```

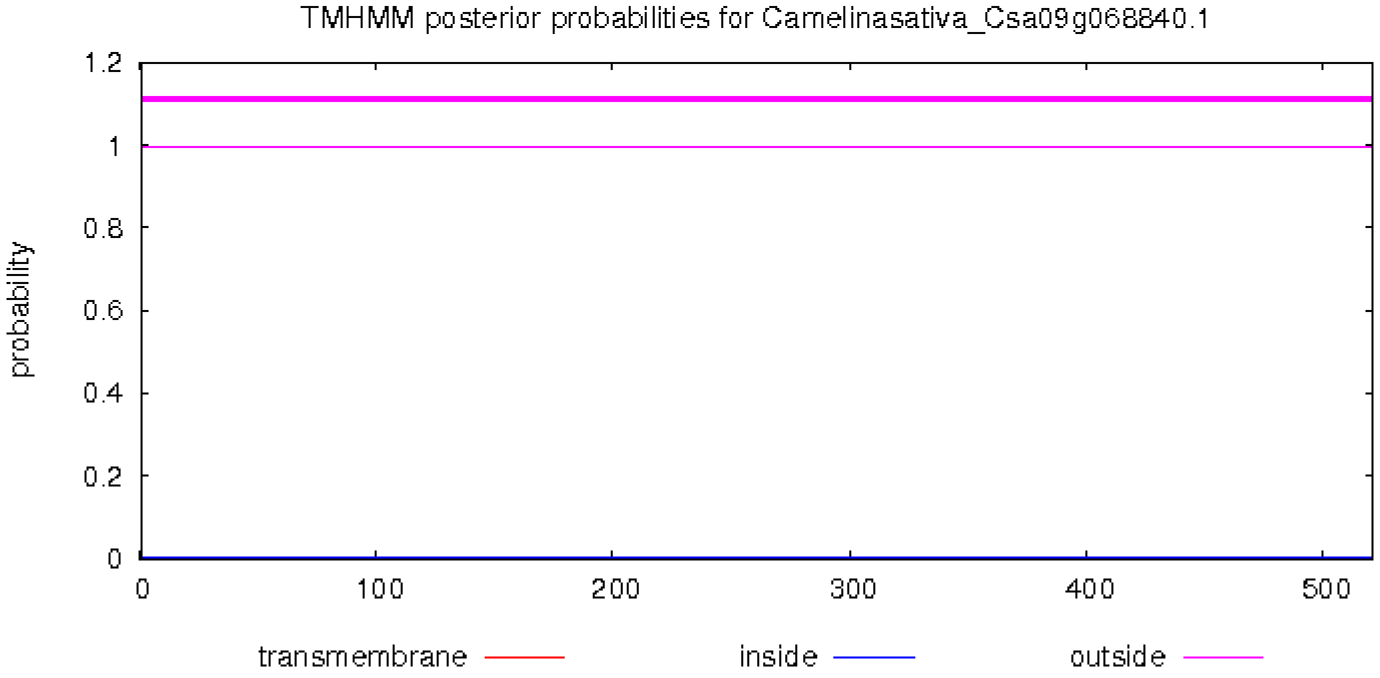

plot in postscript, script for making the plot in gnuplot, data for plot

---

```
# Camelinasativa_Csa09g068850.1 Length: 371
# Camelinasativa_Csa09g068850.1 Number of predicted TMHs:  0
# Camelinasativa_Csa09g068850.1 Exp number of AAs in TMHs: 0.0016
# Camelinasativa_Csa09g068850.1 Exp number, first 60 AAs:  0
# Camelinasativa_Csa09g068850.1 Total prob of N-in:        0.03761
Camelinasativa_Csa09g068850.1	TMHMM2.0	outside	     1   371
```

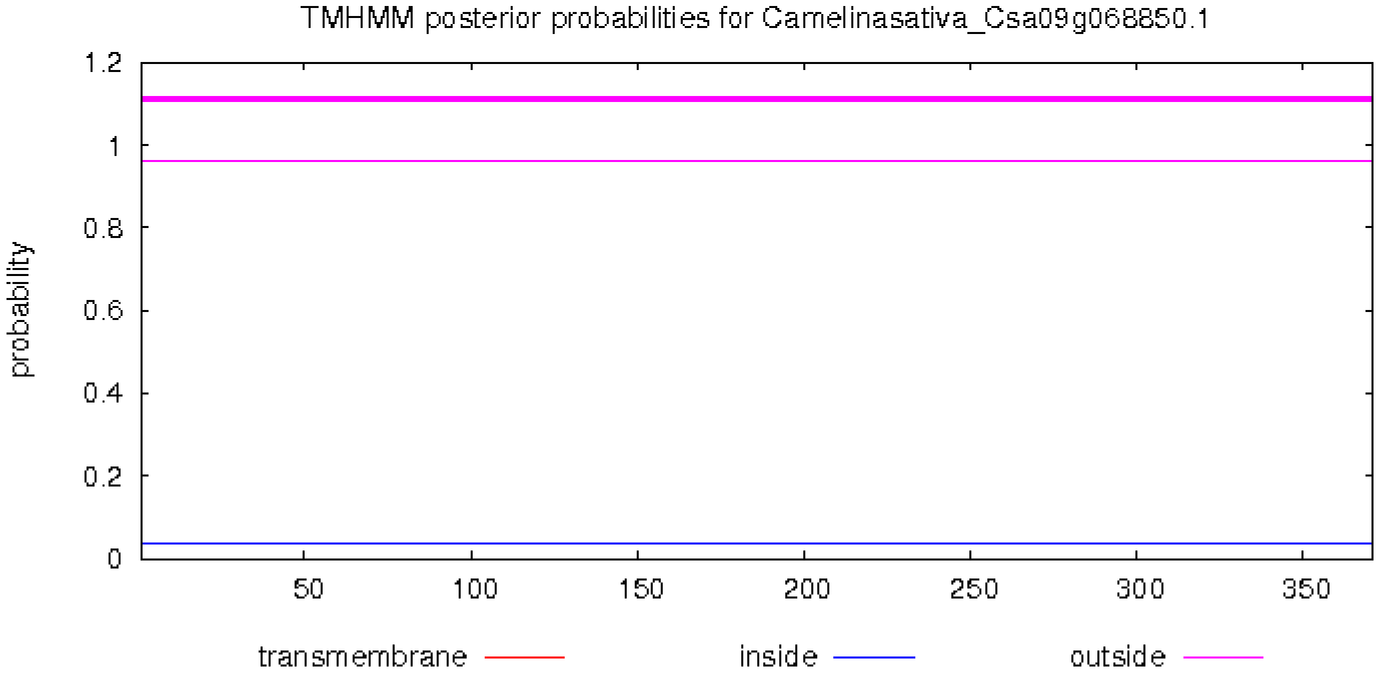

plot in postscript, script for making the plot in gnuplot, data for plot

---

```
# Camelinasativa_Csa09g068880.1 Length: 571
# Camelinasativa_Csa09g068880.1 Number of predicted TMHs:  0
# Camelinasativa_Csa09g068880.1 Exp number of AAs in TMHs: 0.00304
# Camelinasativa_Csa09g068880.1 Exp number, first 60 AAs:  0
# Camelinasativa_Csa09g068880.1 Total prob of N-in:        0.00768
Camelinasativa_Csa09g068880.1	TMHMM2.0	outside	     1   571
```

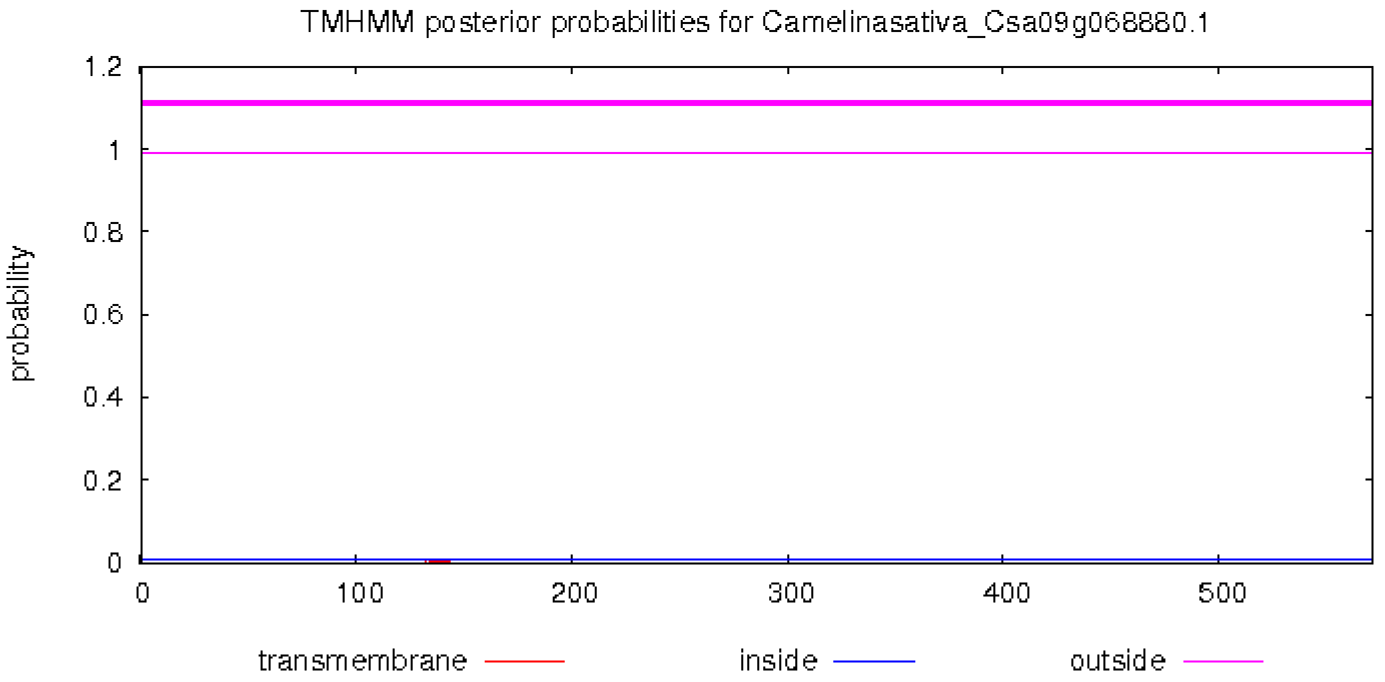

plot in postscript, script for making the plot in gnuplot, data for plot

```
# Camelinasativa_Csa09g068890.1 Length: 394
# Camelinasativa_Csa09g068890.1 Number of predicted TMHs:  0
# Camelinasativa_Csa09g068890.1 Exp number of AAs in TMHs: 0.0237
# Camelinasativa_Csa09g068890.1 Exp number, first 60 AAs:  0
# Camelinasativa_Csa09g068890.1 Total prob of N-in:        0.03312
Camelinasativa_Csa09g068890.1	TMHMM2.0	outside	     1   394
```

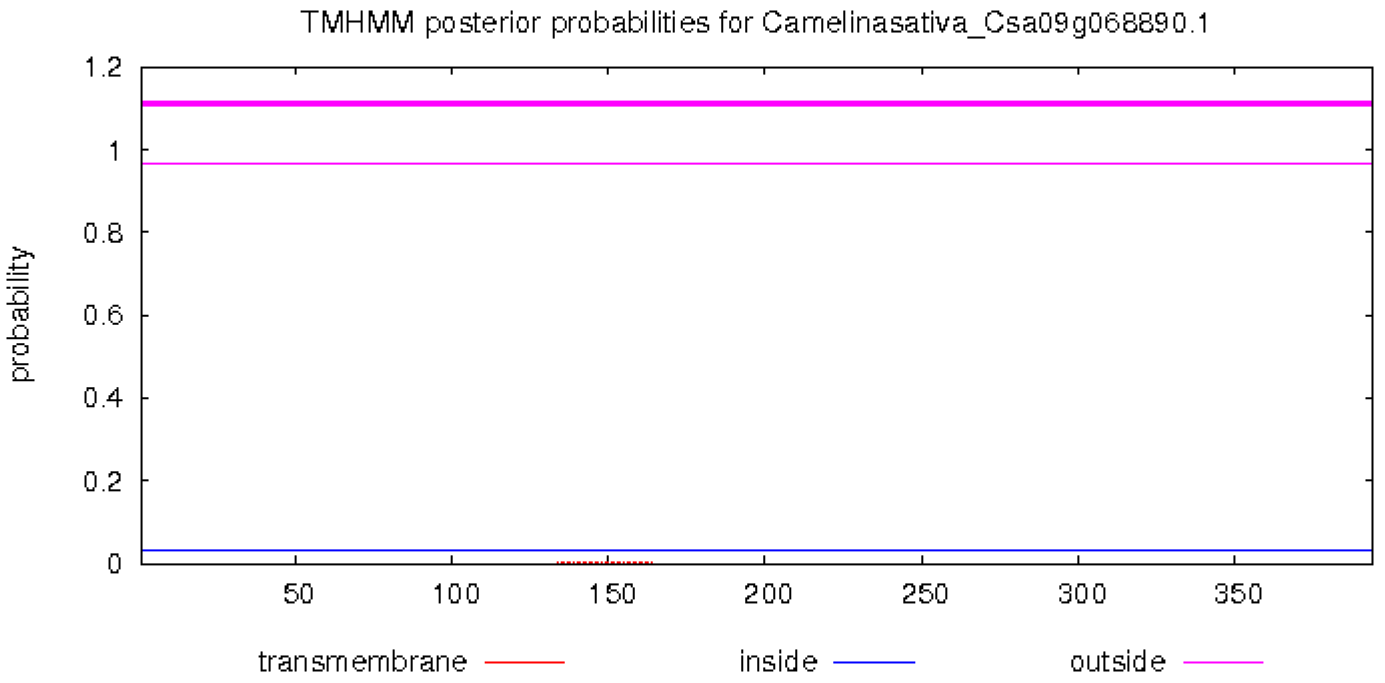

plot in postscript, script for making the plot in gnuplot, data for plot

```
# Camelinasativa_Csa09g068910.1 Length: 397
# Camelinasativa_Csa09g068910.1 Number of predicted TMHs:  1
# Camelinasativa_Csa09g068910.1 Exp number of AAs in TMHs: 19.88141
# Camelinasativa_Csa09g068910.1 Exp number, first 60 AAs:  0
# Camelinasativa_Csa09g068910.1 Total prob of N-in:        0.28613
Camelinasativa_Csa09g068910.1   TMHMM2.0        outside      1   367
Camelinasativa_Csa09g068910.1   TMHMM2.0        TMhelix    368   390
Camelinasativa_Csa09g068910.1   TMHMM2.0        inside     391   397
```

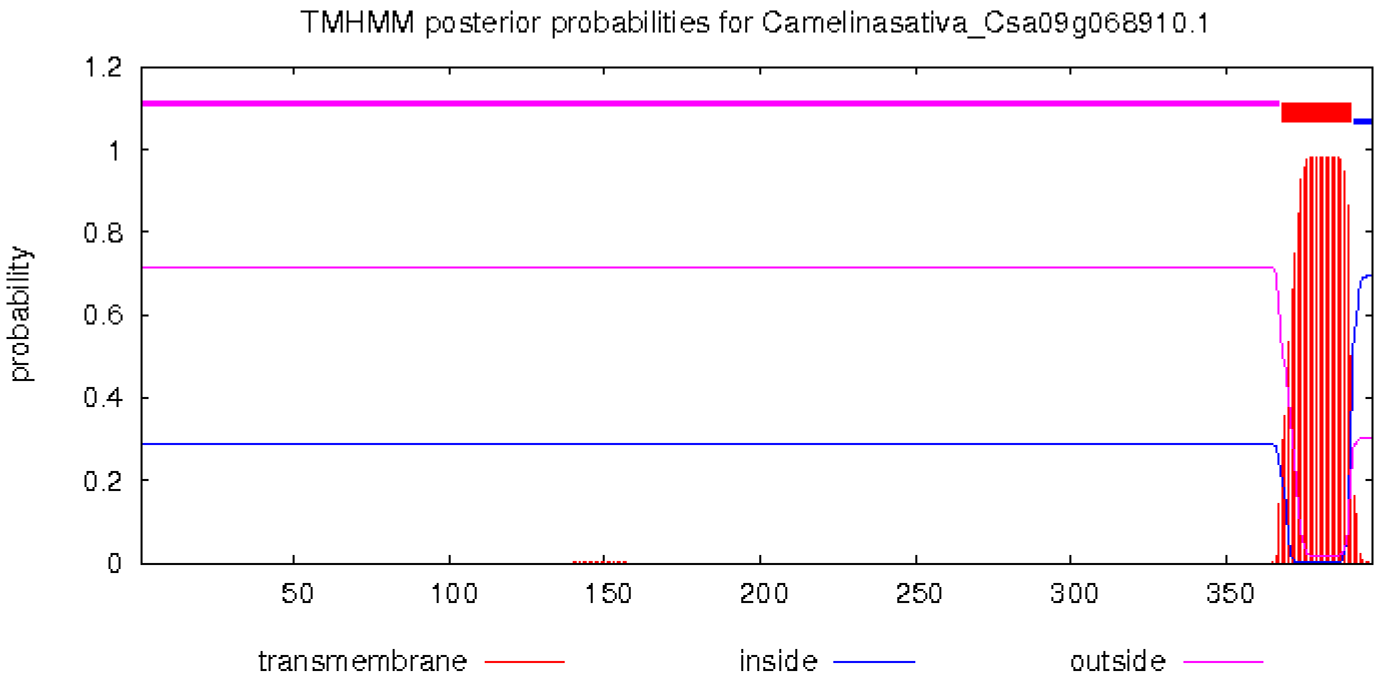

plot in postscript, script for making the plot in gnuplot, data for plot

---

```
# Camelinasativa_Csa09g069310.1 Length: 565
# Camelinasativa_Csa09g069310.1 Number of predicted TMHs:  0
# Camelinasativa_Csa09g069310.1 Exp number of AAs in TMHs: 0.00078
# Camelinasativa_Csa09g069310.1 Exp number, first 60 AAs:  0
# Camelinasativa_Csa09g069310.1 Total prob of N-in:        0.00438
Camelinasativa_Csa09g069310.1   TMHMM2.0        outside      1   565
```

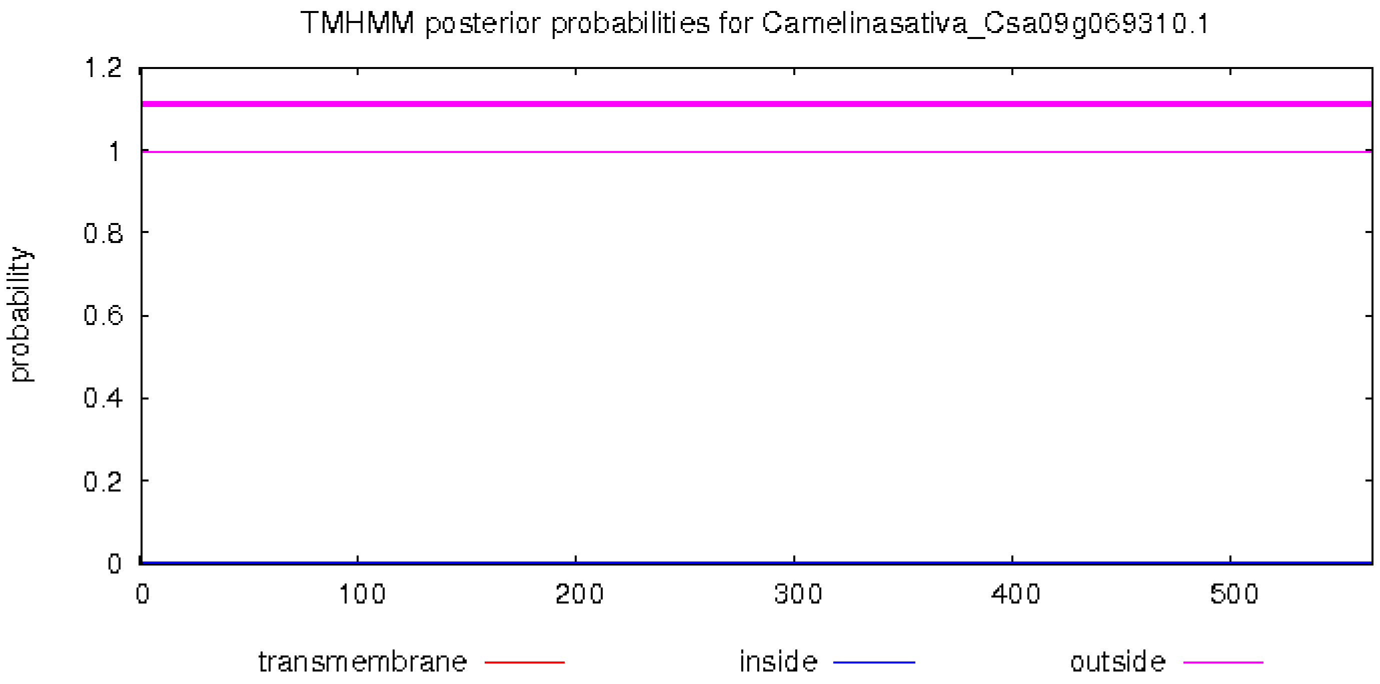

plot in postscript, script for making the plot in gnuplot, data for plot

```
# Camelinasativa_Csa09g070900.1 Length: 184
# Camelinasativa_Csa09g070900.1 Number of predicted TMHs:  0
# Camelinasativa_Csa09g070900.1 Exp number of AAs in TMHs: 0.00227
# Camelinasativa_Csa09g070900.1 Exp number, first 60 AAs:  0.00227
# Camelinasativa_Csa09g070900.1 Total prob of N-in:        0.23744
Camelinasativa_Csa09g070900.1	TMHMM2.0	outside	     1   184
```

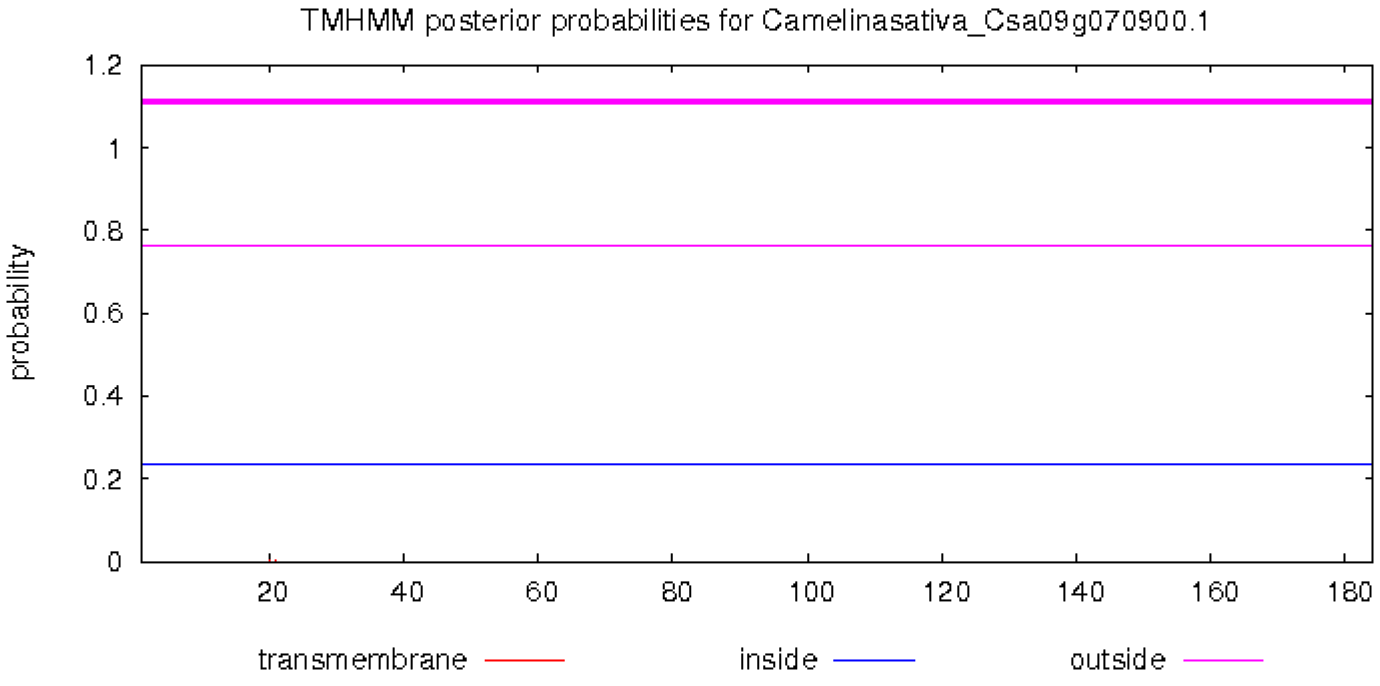

plot in postscript, script for making the plot in gnuplot, data for plot

```
# Camelinasativa_Csa09g075270.1 Length: 359
# Camelinasativa_Csa09g075270.1 Number of predicted TMHs:  0
# Camelinasativa_Csa09g075270.1 Exp number of AAs in TMHs: 0.14737
# Camelinasativa_Csa09g075270.1 Exp number, first 60 AAs:  0.14737
# Camelinasativa_Csa09g075270.1 Total prob of N-in:        0.00828
Camelinasativa_Csa09g075270.1	TMHMM2.0	outside	     1   359
```

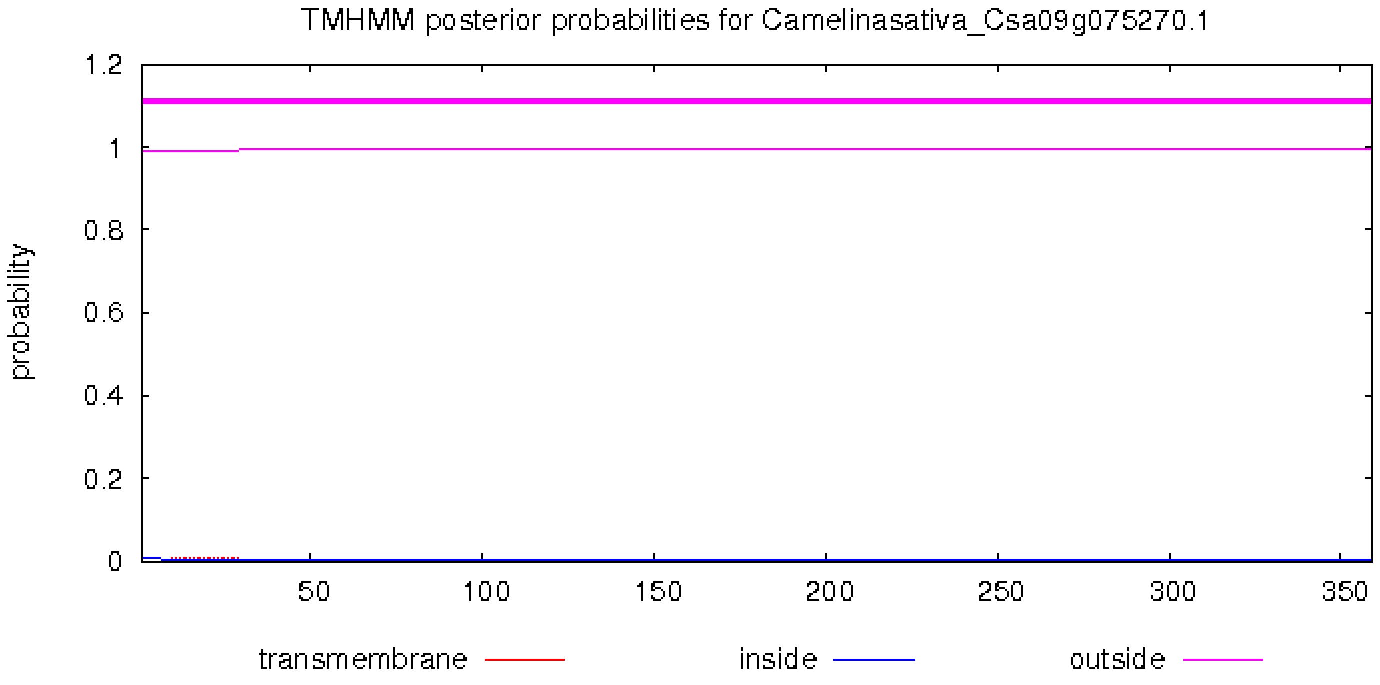

plot in postscript, script for making the plot in gnuplot, data for plot

```
# Camelinasativa_Csa09g081150.1 Length: 270
# Camelinasativa_Csa09g081150.1 Number of predicted TMHs:  0
# Camelinasativa_Csa09g081150.1 Exp number of AAs in TMHs: 0.0178
# Camelinasativa_Csa09g081150.1 Exp number, first 60 AAs:  0.01155
# Camelinasativa_Csa09g081150.1 Total prob of N-in:        0.04350
Camelinasativa_Csa09g081150.1   TMHMM2.0        outside      1   270
```

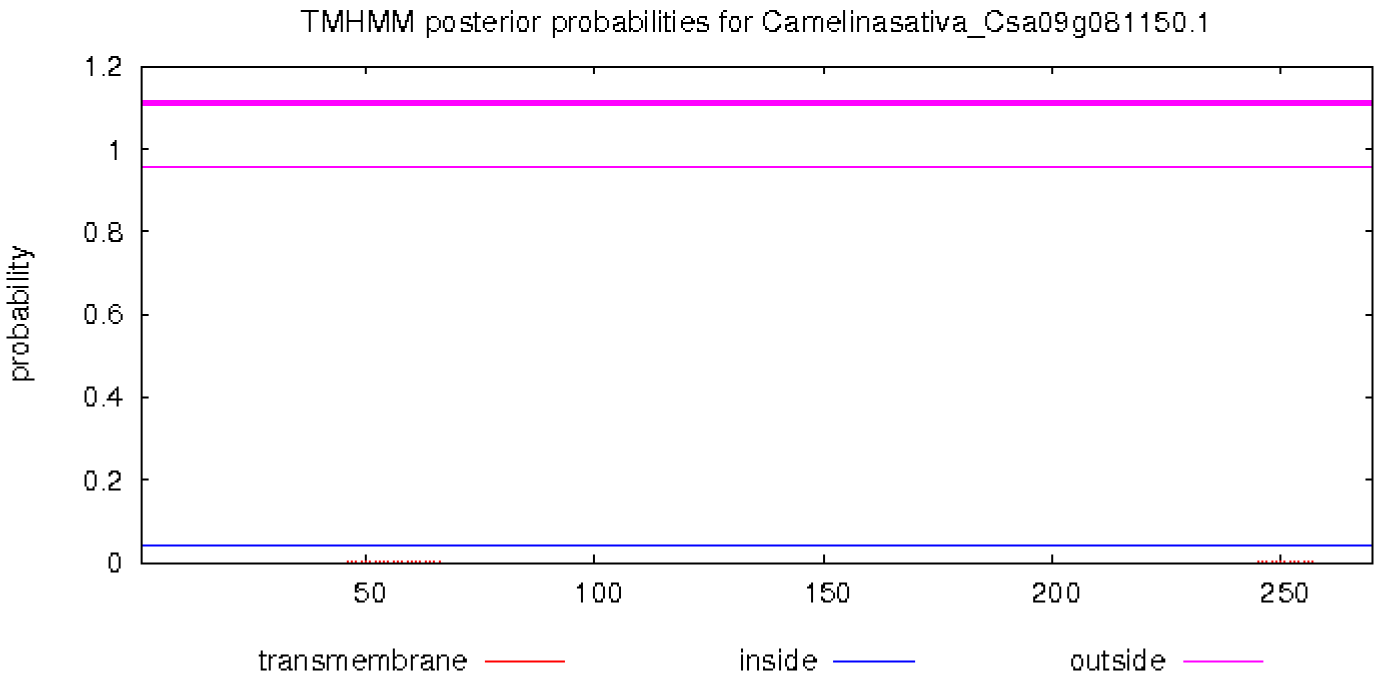

plot in postscript, script for making the plot in gnuplot, data for plot

```
# Camelinasativa_Csa09g081160.1 Length: 394
# Camelinasativa_Csa09g081160.1 Number of predicted TMHs:  0
# Camelinasativa_Csa09g081160.1 Exp number of AAs in TMHs: 10.79328
# Camelinasativa_Csa09g081160.1 Exp number, first 60 AAs:  10.68359
# Camelinasativa_Csa09g081160.1 Total prob of N-in:        0.46790
# Camelinasativa_Csa09g081160.1 POSSIBLE N-term signal sequence
Camelinasativa_Csa09g081160.1   TMHMM2.0        outside      1   394
```

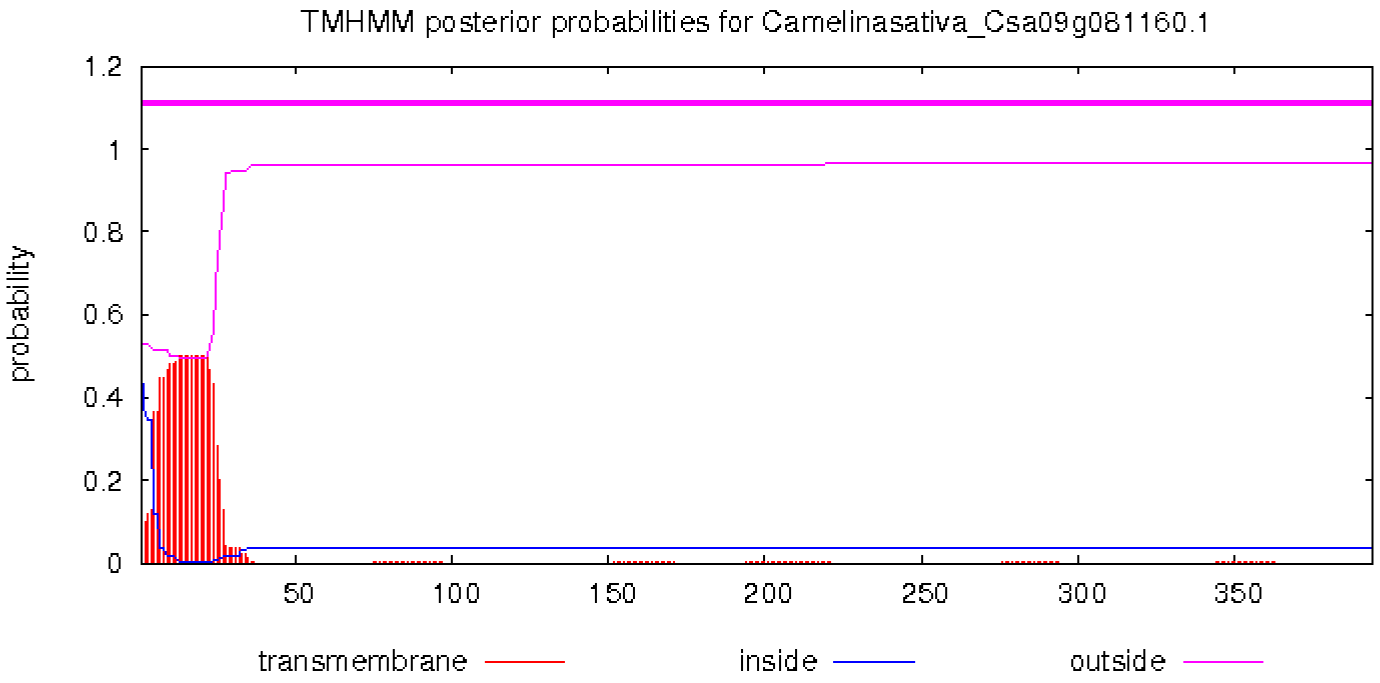

plot in postscript, script for making the plot in gnuplot, data for plot

```
# Camelinasativa_Csa09g084210.1 Length: 250
# Camelinasativa_Csa09g084210.1 Number of predicted TMHs:  0
# Camelinasativa_Csa09g084210.1 Exp number of AAs in TMHs: 0.00021
# Camelinasativa_Csa09g084210.1 Exp number, first 60 AAs:  0
# Camelinasativa_Csa09g084210.1 Total prob of N-in:        0.02270
Camelinasativa_Csa09g084210.1   TMHMM2.0        outside      1   250
```

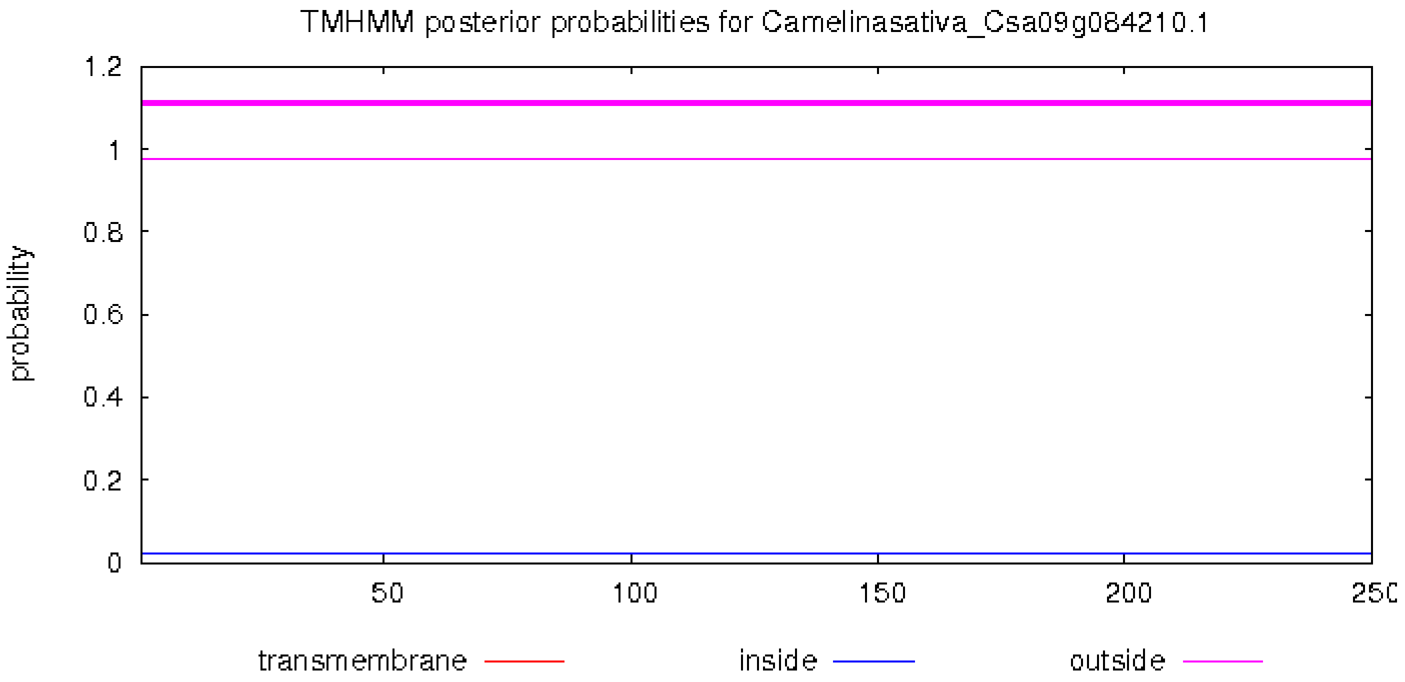

plot in postscript, script for making the plot in gnuplot, data for plot

```
# Camelinasativa_Csa09g088620.1 Length: 317
# Camelinasativa_Csa09g088620.1 Number of predicted TMHs:  0
# Camelinasativa_Csa09g088620.1 Exp number of AAs in TMHs: 0.02703
# Camelinasativa_Csa09g088620.1 Exp number, first 60 AAs:  0
# Camelinasativa_Csa09g088620.1 Total prob of N-in:        0.07579
Camelinasativa_Csa09g088620.1   TMHMM2.0        outside      1   317
```

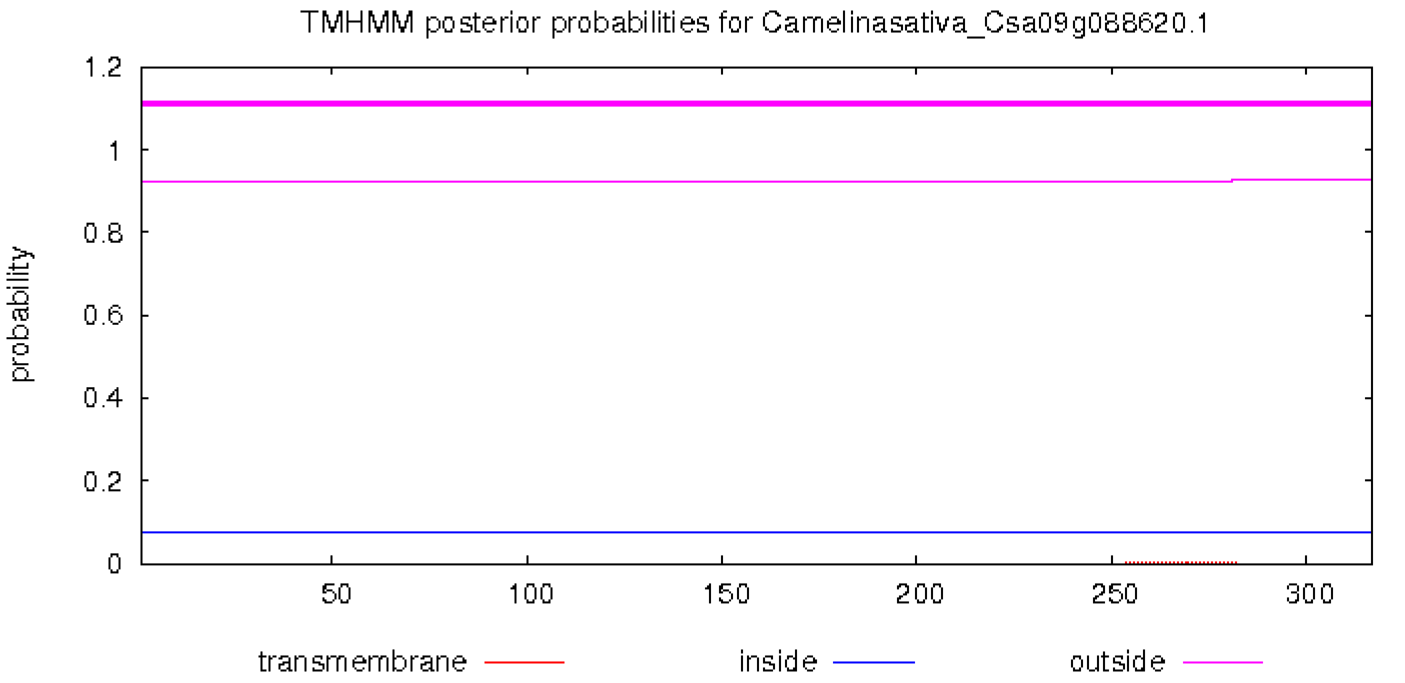

plot in postscript, script for making the plot in gnuplot, data for plot

```
# Camelinasativa_Csa09g093850.1 Length: 376
# Camelinasativa_Csa09g093850.1 Number of predicted TMHs:  0
# Camelinasativa_Csa09g093850.1 Exp number of AAs in TMHs: 0.00495
# Camelinasativa_Csa09g093850.1 Exp number, first 60 AAs:  0.00096
# Camelinasativa_Csa09g093850.1 Total prob of N-in:        0.00721
Camelinasativa_Csa09g093850.1   TMHMM2.0        outside      1   376
```

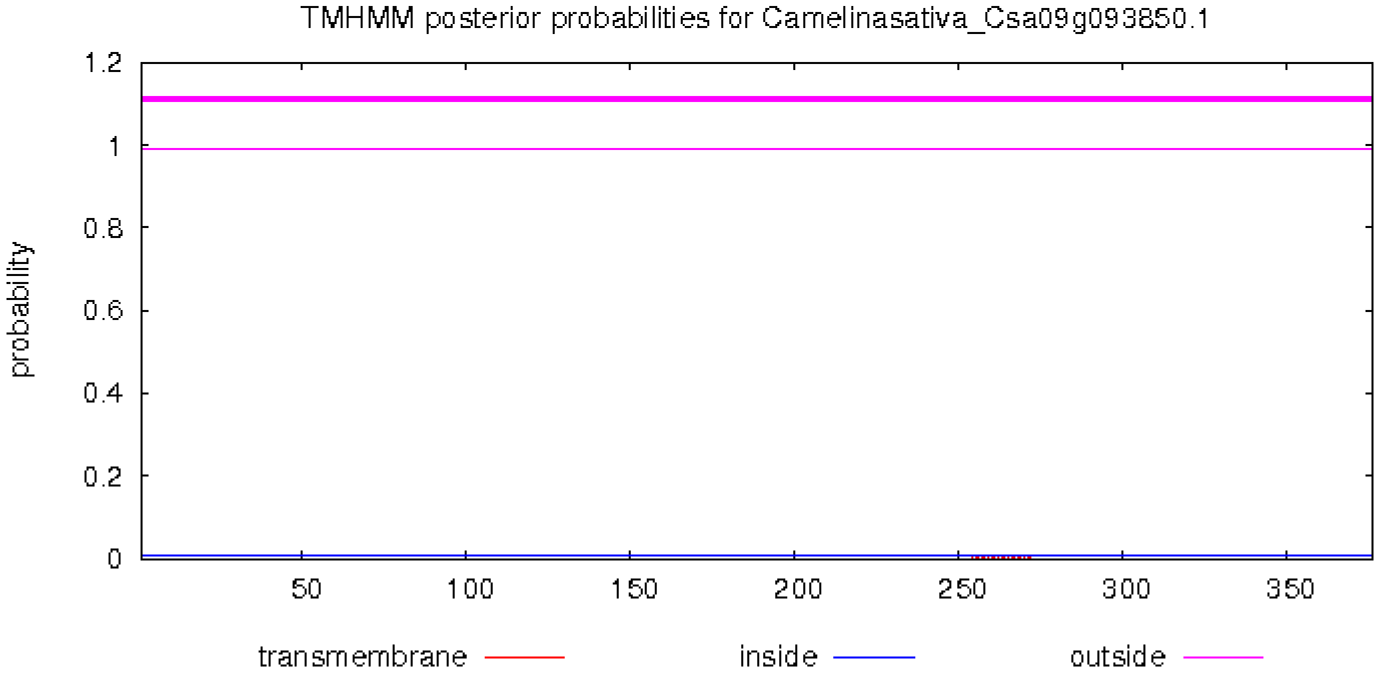

plot in postscript, script for making the plot in gnuplot, data for plot

---

```
# Camelinasativa_Csa09g095630.1 Length: 324
# Camelinasativa_Csa09g095630.1 Number of predicted TMHs:  0
# Camelinasativa_Csa09g095630.1 Exp number of AAs in TMHs: 0.00038
# Camelinasativa_Csa09g095630.1 Exp number, first 60 AAs:  0.00016
# Camelinasativa_Csa09g095630.1 Total prob of N-in:        0.01127
Camelinasativa_Csa09g095630.1   TMHMM2.0        outside      1   324
```

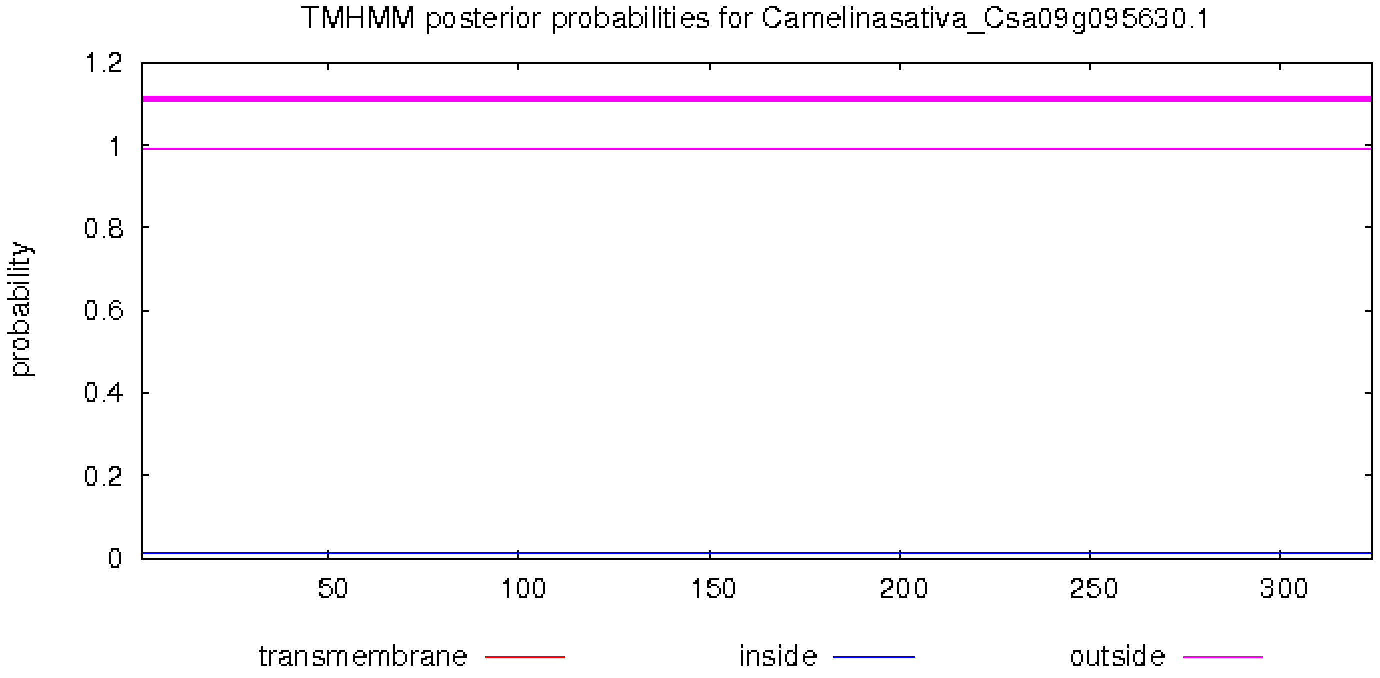

plot in postscript, script for making the plot in gnuplot, data for plot

---

```
# Camelinasativa_Csa09g097350.1 Length: 397
# Camelinasativa_Csa09g097350.1 Number of predicted TMHs:  0
# Camelinasativa_Csa09g097350.1 Exp number of AAs in TMHs: 0
# Camelinasativa_Csa09g097350.1 Exp number, first 60 AAs:  0
# Camelinasativa_Csa09g097350.1 Total prob of N-in:        0.00284
Camelinasativa_Csa09g097350.1   TMHMM2.0        outside      1   397
```

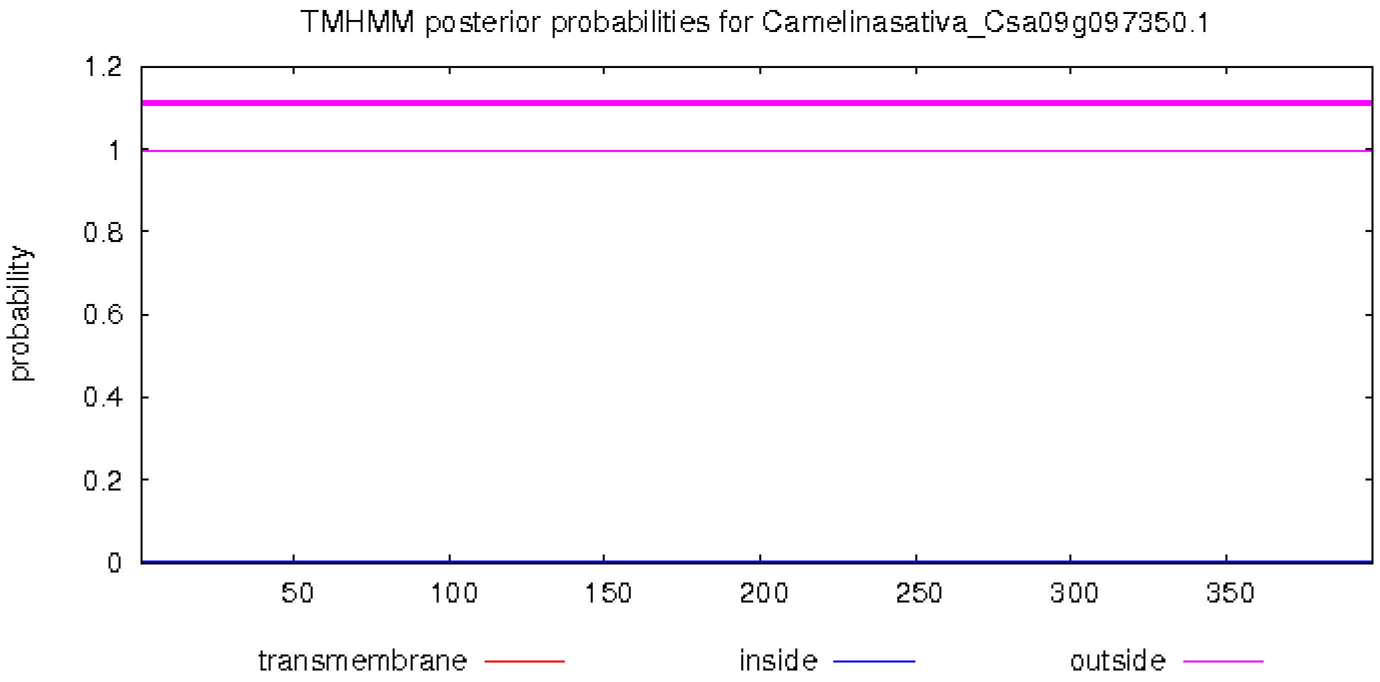

plot in postscript, script for making the plot in gnuplot, data for plot

```
# Camelinasativa_Csa10g004970.1 Length: 376
# Camelinasativa_Csa10g004970.1 Number of predicted TMHs:  0
# Camelinasativa_Csa10g004970.1 Exp number of AAs in TMHs: 0
# Camelinasativa_Csa10g004970.1 Exp number, first 60 AAs:  0
# Camelinasativa_Csa10g004970.1 Total prob of N-in:        0.01338
Camelinasativa_Csa10g004970.1   TMHMM2.0        outside      1   376
```

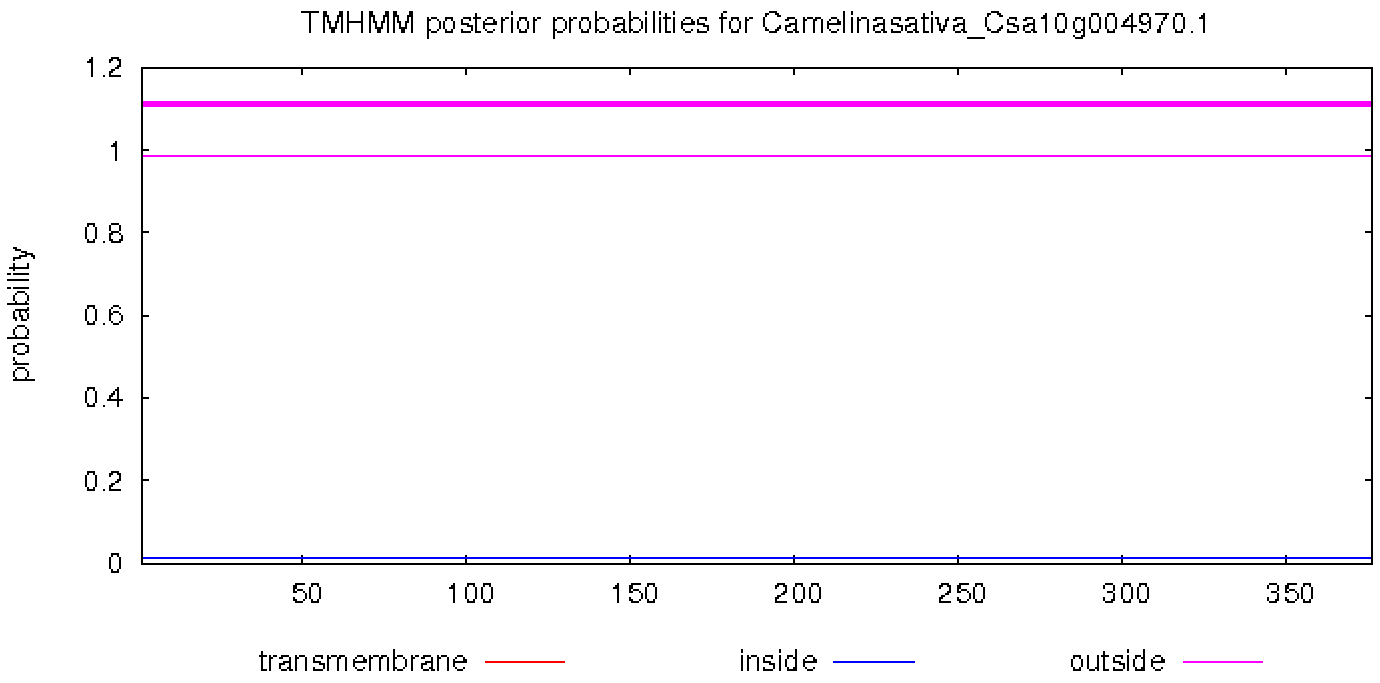

plot in postscript, script for making the plot in gnuplot, data for plot

```
# Camelinasativa_Csa10g006570.1 Length: 522
# Camelinasativa_Csa10g006570.1 Number of predicted TMHs:  1
# Camelinasativa_Csa10g006570.1 Exp number of AAs in TMHs: 21.78612
# Camelinasativa_Csa10g006570.1 Exp number, first 60 AAs:  0
# Camelinasativa_Csa10g006570.1 Total prob of N-in:        0.36192
Camelinasativa_Csa10g006570.1   TMHMM2.0        outside      1   498
Camelinasativa_Csa10g006570.1   TMHMM2.0        TMhelix    499   521
Camelinasativa_Csa10g006570.1   TMHMM2.0        inside     522   522
```

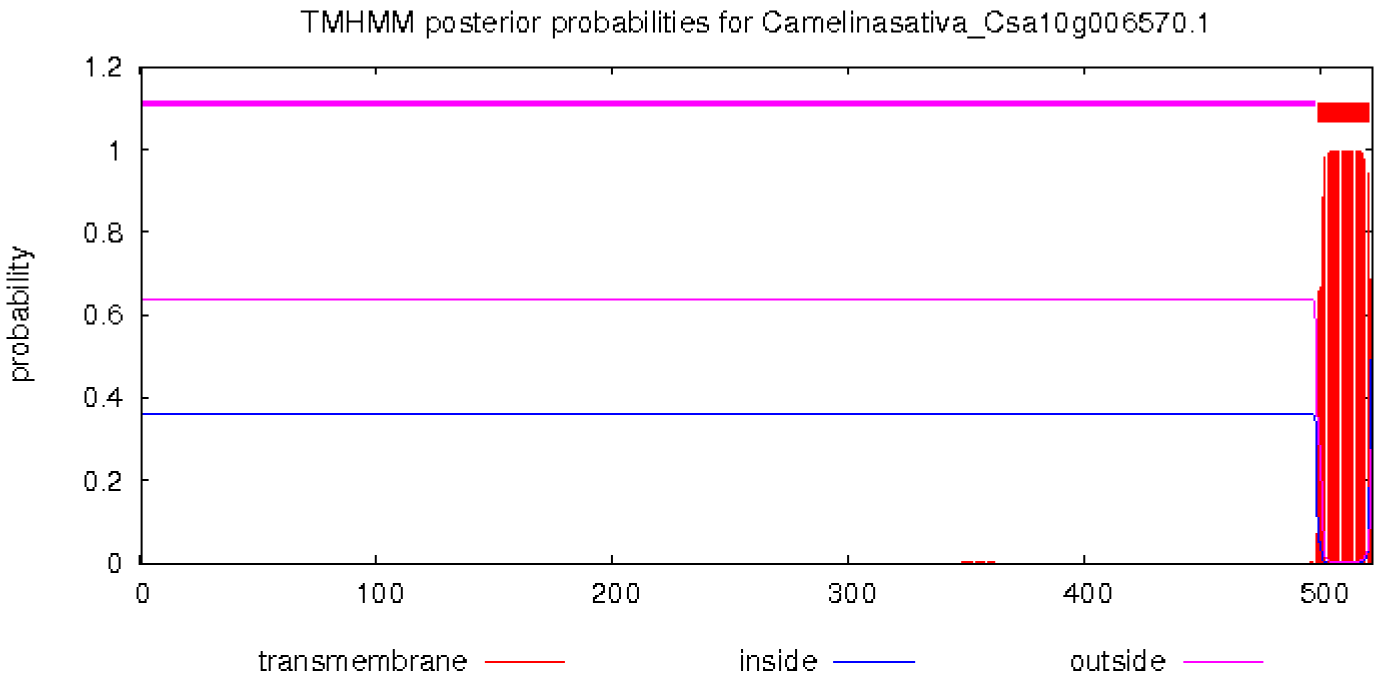

plot in postscript, script for making the plot in gnuplot, data for plot

```
# Camelinasativa_Csa10g013350.1 Length: 495
# Camelinasativa_Csa10g013350.1 Number of predicted TMHs:  0
# Camelinasativa_Csa10g013350.1 Exp number of AAs in TMHs: 5.80045
# Camelinasativa_Csa10g013350.1 Exp number, first 60 AAs:  0
# Camelinasativa_Csa10g013350.1 Total prob of N-in:        0.22595
Camelinasativa_Csa10g013350.1   TMHMM2.0        outside      1   495
```

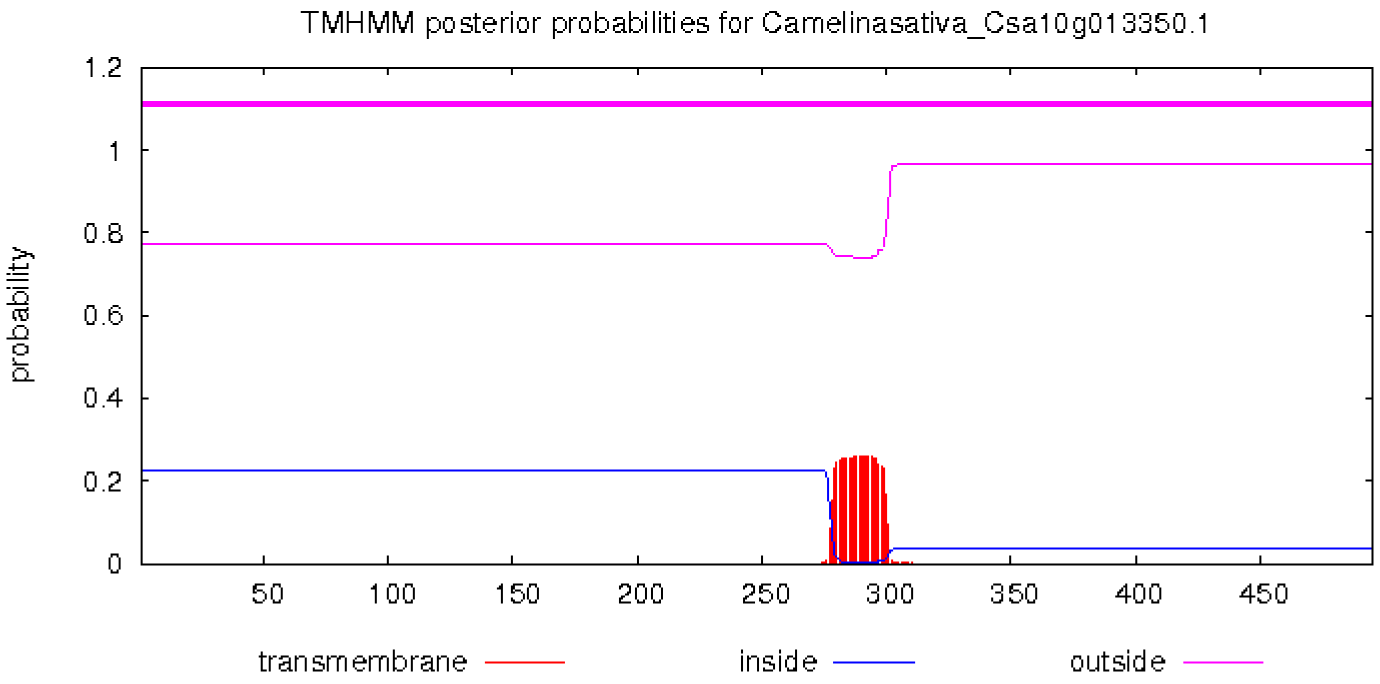

plot in postscript, script for making the plot in gnuplot, data for plot

```
# Camelinasativa_Csa10g014090.1 Length: 352
# Camelinasativa_Csa10g014090.1 Number of predicted TMHs:  0
# Camelinasativa_Csa10g014090.1 Exp number of AAs in TMHs: 0
# Camelinasativa_Csa10g014090.1 Exp number, first 60 AAs:  0
# Camelinasativa_Csa10g014090.1 Total prob of N-in:        0.00564
Camelinasativa_Csa10g014090.1	TMHMM2.0	outside	     1   352
```

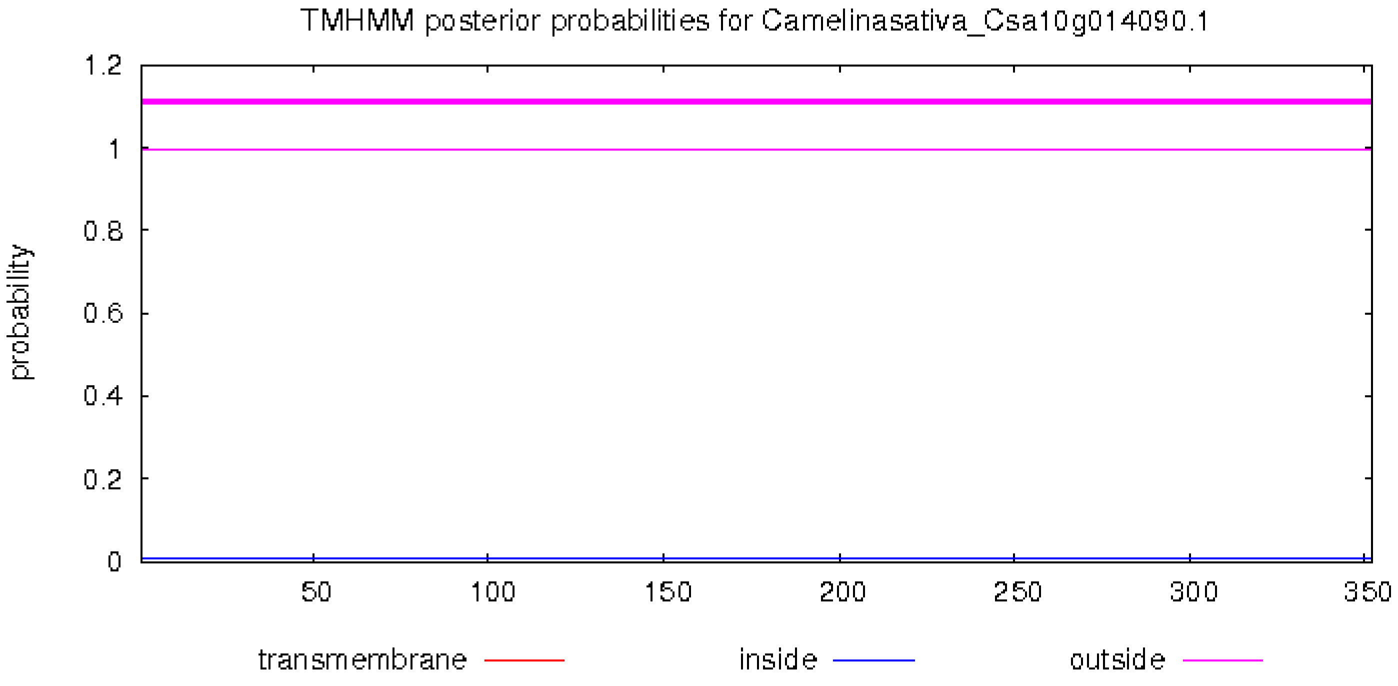

plot in postscript, script for making the plot in gnuplot, data for plot

```
# Camelinasativa_Csa10g014120.1 Length: 306
# Camelinasativa_Csa10g014120.1 Number of predicted TMHs:  0
# Camelinasativa_Csa10g014120.1 Exp number of AAs in TMHs: 0.00148
# Camelinasativa_Csa10g014120.1 Exp number, first 60 AAs:  0
# Camelinasativa_Csa10g014120.1 Total prob of N-in:        0.09757
Camelinasativa_Csa10g014120.1	TMHMM2.0	outside	     1   306
```

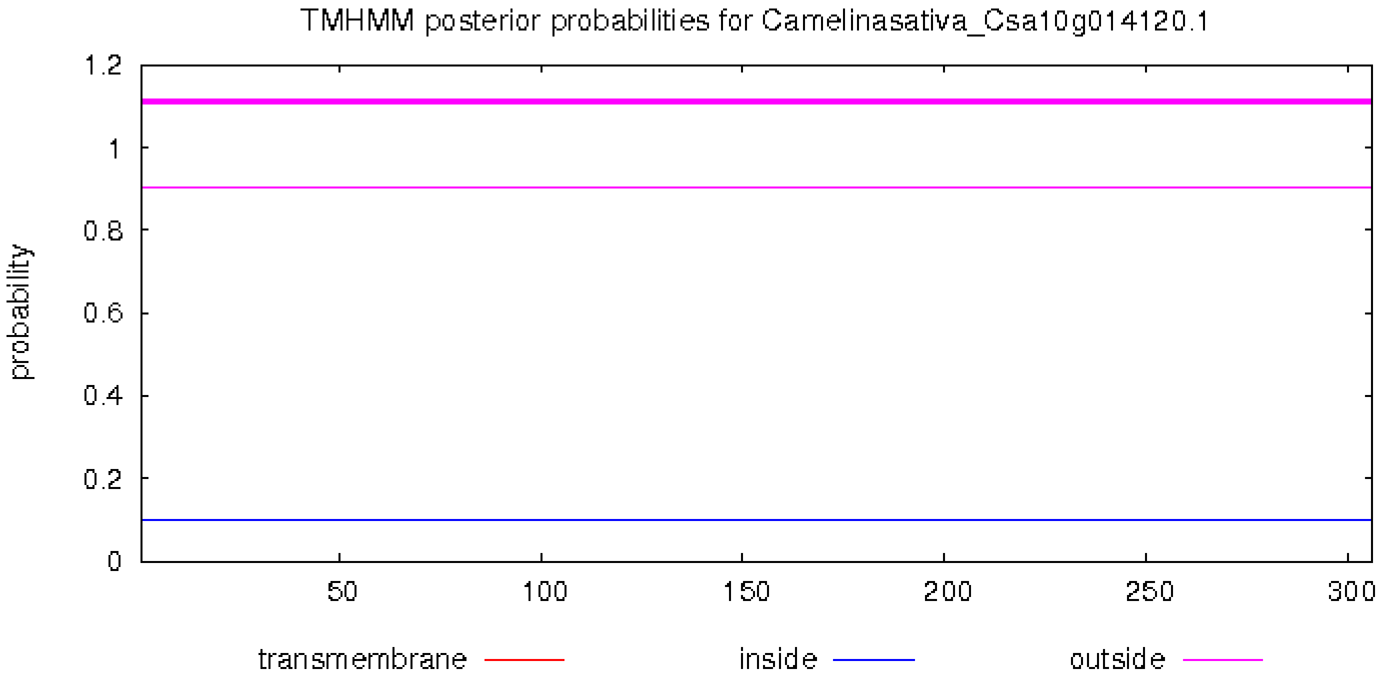

plot in postscript, script for making the plot in gnuplot, data for plot

```
# Camelinasativa_Csa10g015440.1 Length: 300
# Camelinasativa_Csa10g015440.1 Number of predicted TMHs:  0
# Camelinasativa_Csa10g015440.1 Exp number of AAs in TMHs: 0.00261
# Camelinasativa_Csa10g015440.1 Exp number, first 60 AAs:  0.00039
# Camelinasativa_Csa10g015440.1 Total prob of N-in:        0.02120
Camelinasativa_Csa10g015440.1   TMHMM2.0        outside      1   300
```

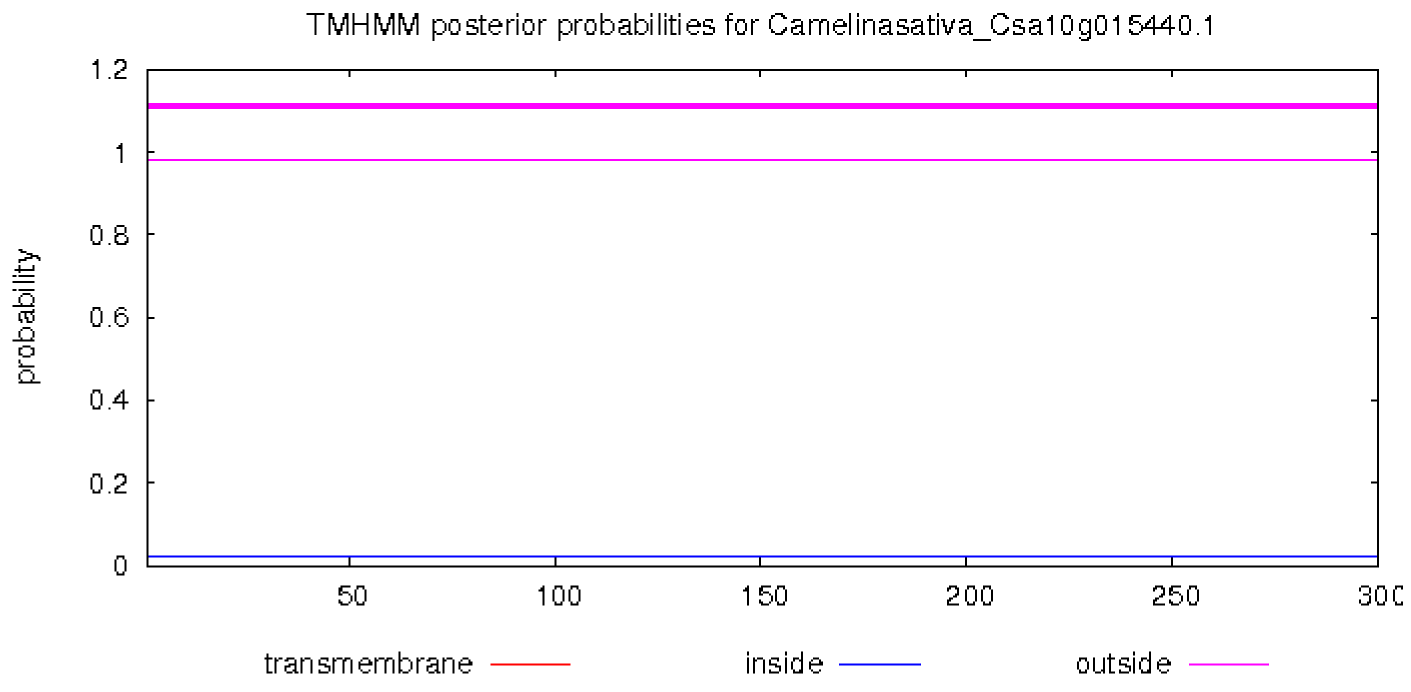

plot in postscript, script for making the plot in gnuplot, data for plot

```
# Camelinasativa_Csa10g027250.1 Length: 287
# Camelinasativa_Csa10g027250.1 Number of predicted TMHs:  0
# Camelinasativa_Csa10g027250.1 Exp number of AAs in TMHs: 0
# Camelinasativa_Csa10g027250.1 Exp number, first 60 AAs:  0
# Camelinasativa_Csa10g027250.1 Total prob of N-in:        0.00172
Camelinasativa_Csa10g027250.1   TMHMM2.0        outside      1   287
```

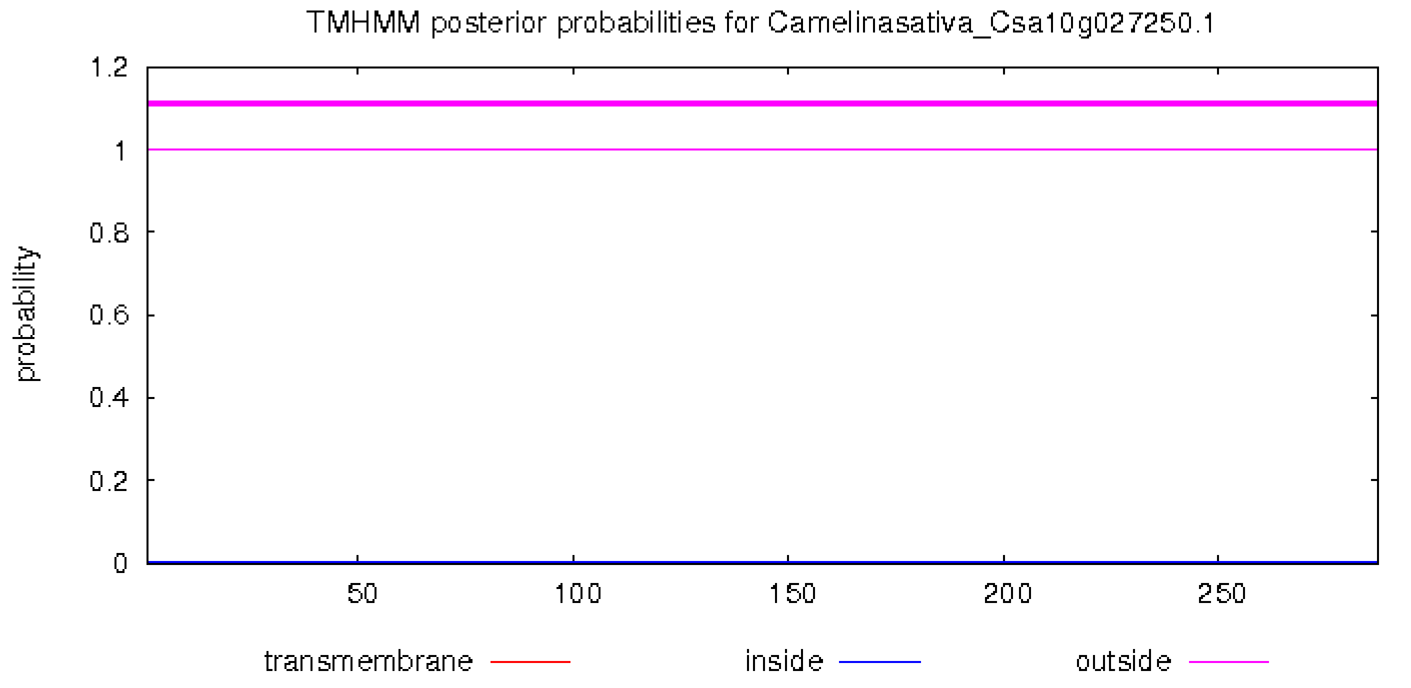

plot in postscript, script for making the plot in gnuplot, data for plot

```
# Camelinasativa_Csa10g042450.1 Length: 363
# Camelinasativa_Csa10g042450.1 Number of predicted TMHs:  0
# Camelinasativa_Csa10g042450.1 Exp number of AAs in TMHs: 0.00018
# Camelinasativa_Csa10g042450.1 Exp number, first 60 AAs:  0
# Camelinasativa_Csa10g042450.1 Total prob of N-in:        0.02832
Camelinasativa_Csa10g042450.1	TMHMM2.0	outside	     1   363
```

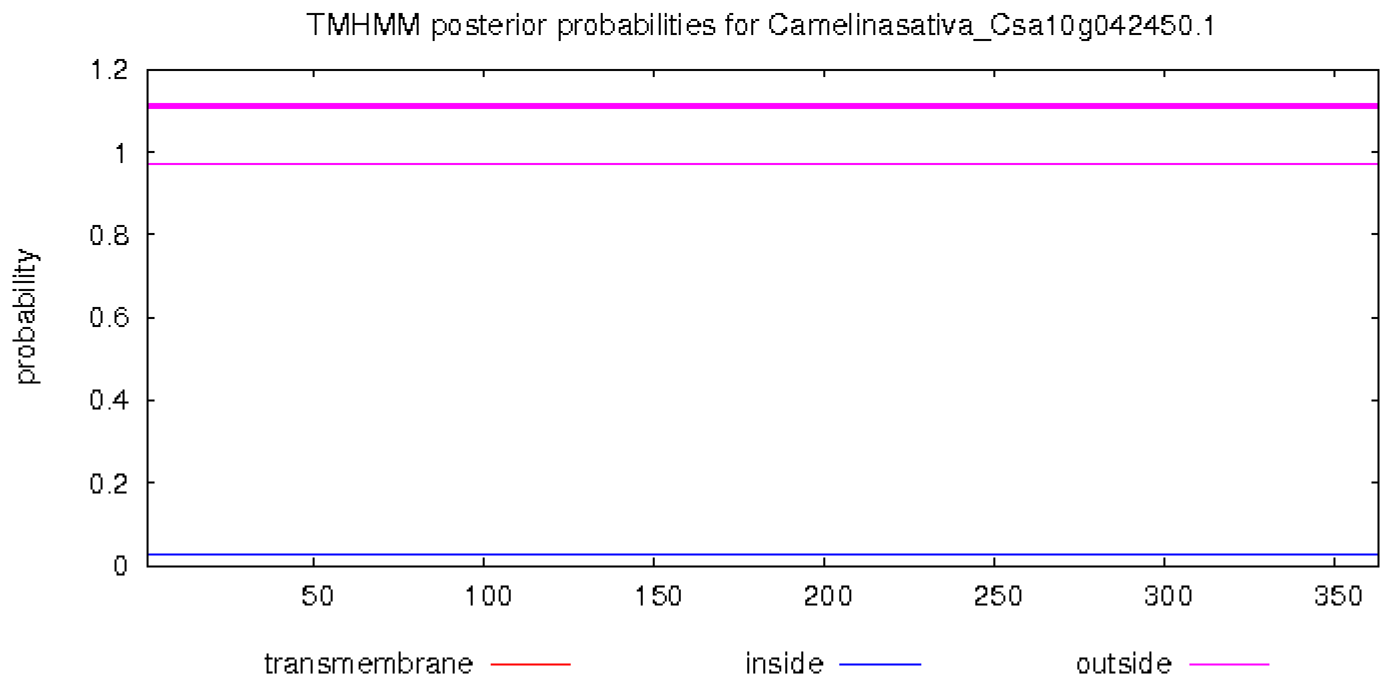

plot in postscript, script for making the plot in gnuplot, data for plot

---

```
# Camelinasativa_Csa10g046660.1 Length: 217
# Camelinasativa_Csa10g046660.1 Number of predicted TMHs:  0
# Camelinasativa_Csa10g046660.1 Exp number of AAs in TMHs: 0.01259
# Camelinasativa_Csa10g046660.1 Exp number, first 60 AAs:  0.0111
# Camelinasativa_Csa10g046660.1 Total prob of N-in:        0.02544
Camelinasativa_Csa10g046660.1	TMHMM2.0	outside	     1   217
```

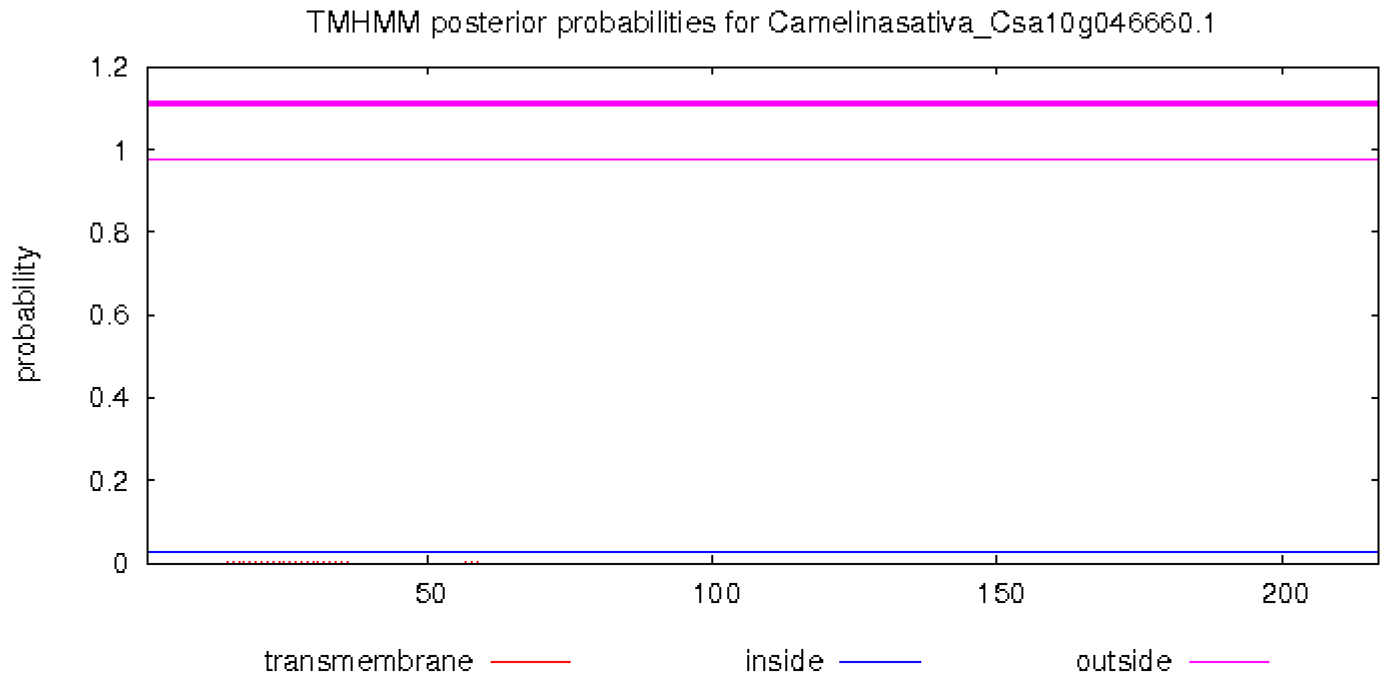

plot in postscript, script for making the plot in gnuplot, data for plot

---

```
# Camelinasativa_Csa10g046670.1 Length: 172
# Camelinasativa_Csa10g046670.1 Number of predicted TMHs:  0
# Camelinasativa_Csa10g046670.1 Exp number of AAs in TMHs: 0.00021
# Camelinasativa_Csa10g046670.1 Exp number, first 60 AAs:  0
# Camelinasativa_Csa10g046670.1 Total prob of N-in:        0.08648
Camelinasativa_Csa10g046670.1	TMHMM2.0	outside	     1   172
```

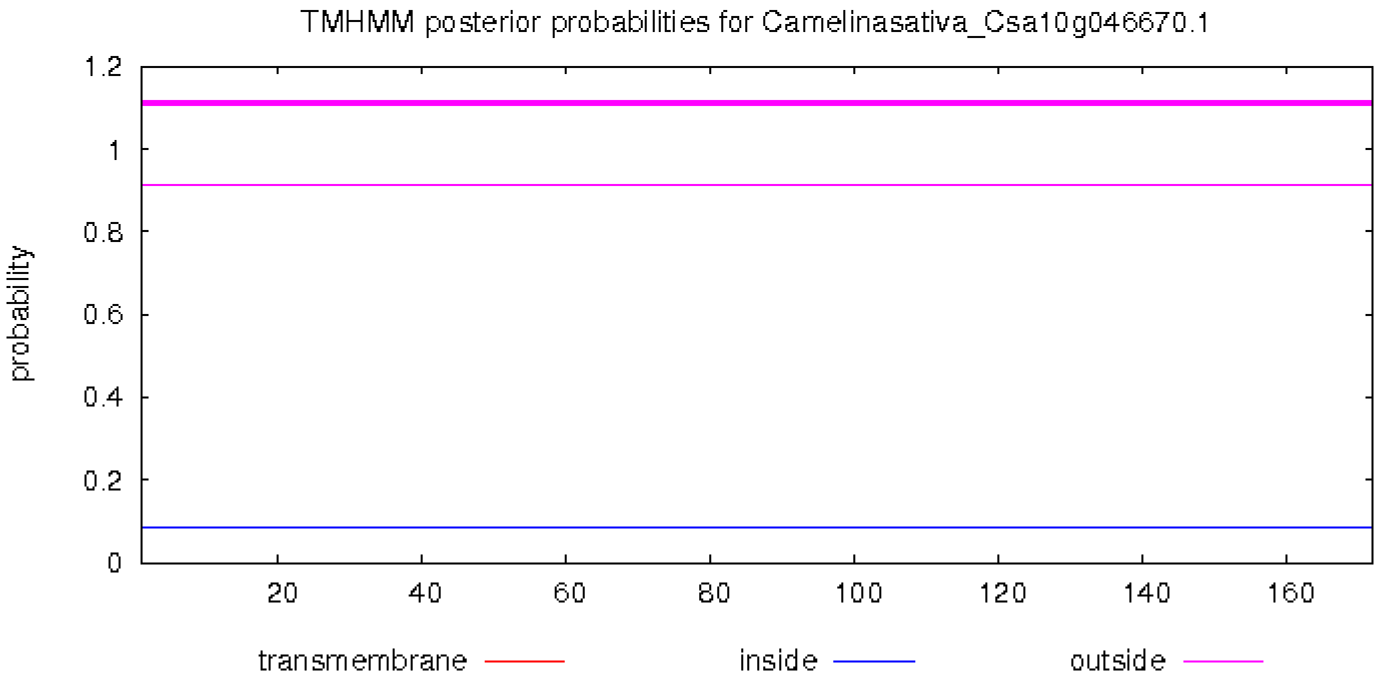

plot in postscript, script for making the plot in gnuplot, data for plot

---

```
# Camelinasativa_Csa10g046680.1 Length: 267
# Camelinasativa_Csa10g046680.1 Number of predicted TMHs:  0
# Camelinasativa_Csa10g046680.1 Exp number of AAs in TMHs: 0.00251
# Camelinasativa_Csa10g046680.1 Exp number, first 60 AAs:  0.0005
# Camelinasativa_Csa10g046680.1 Total prob of N-in:        0.04796
Camelinasativa_Csa10g046680.1	TMHMM2.0	outside	     1   267
```

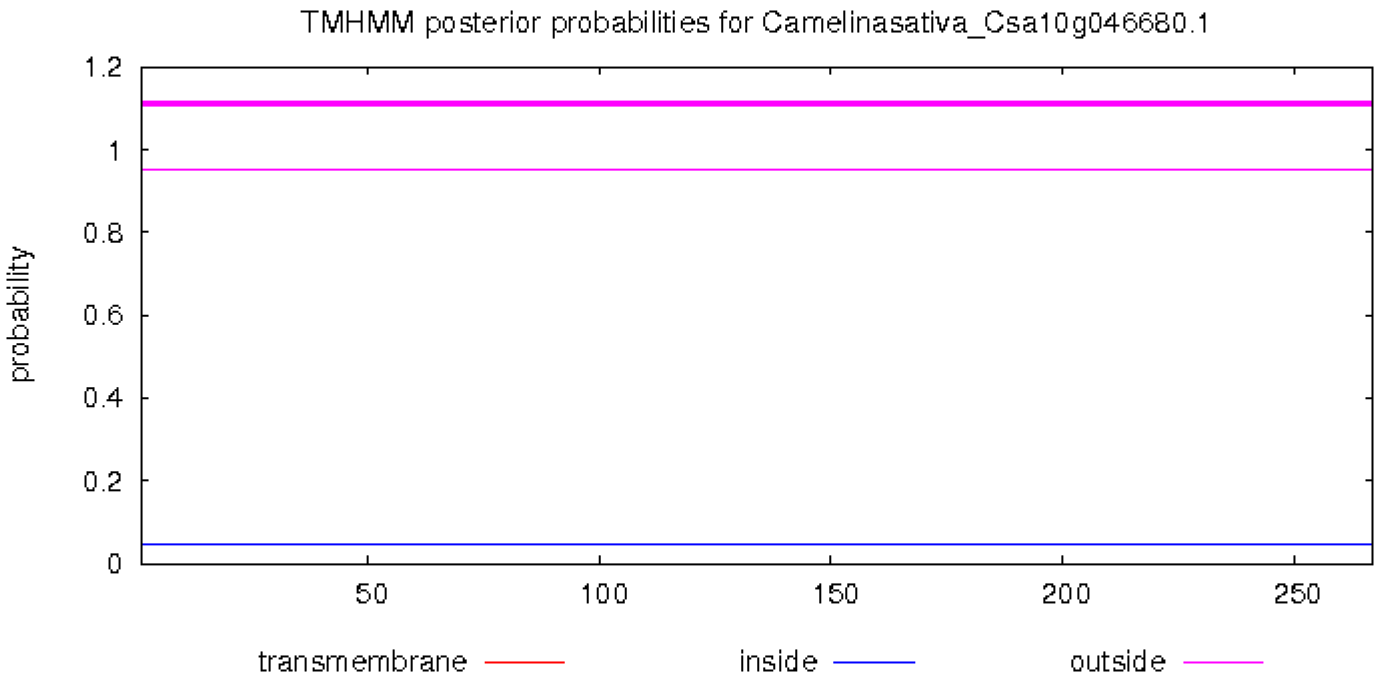

plot in postscript, script for making the plot in gnuplot, data for plot

---

```
# Camelinasativa_Csa10g046940.1 Length: 290
# Camelinasativa_Csa10g046940.1 Number of predicted TMHs:  0
# Camelinasativa_Csa10g046940.1 Exp number of AAs in TMHs: 0.00093
# Camelinasativa_Csa10g046940.1 Exp number, first 60 AAs:  0.00089
# Camelinasativa_Csa10g046940.1 Total prob of N-in:        0.08738
Camelinasativa_Csa10g046940.1	TMHMM2.0	outside	     1   290
```

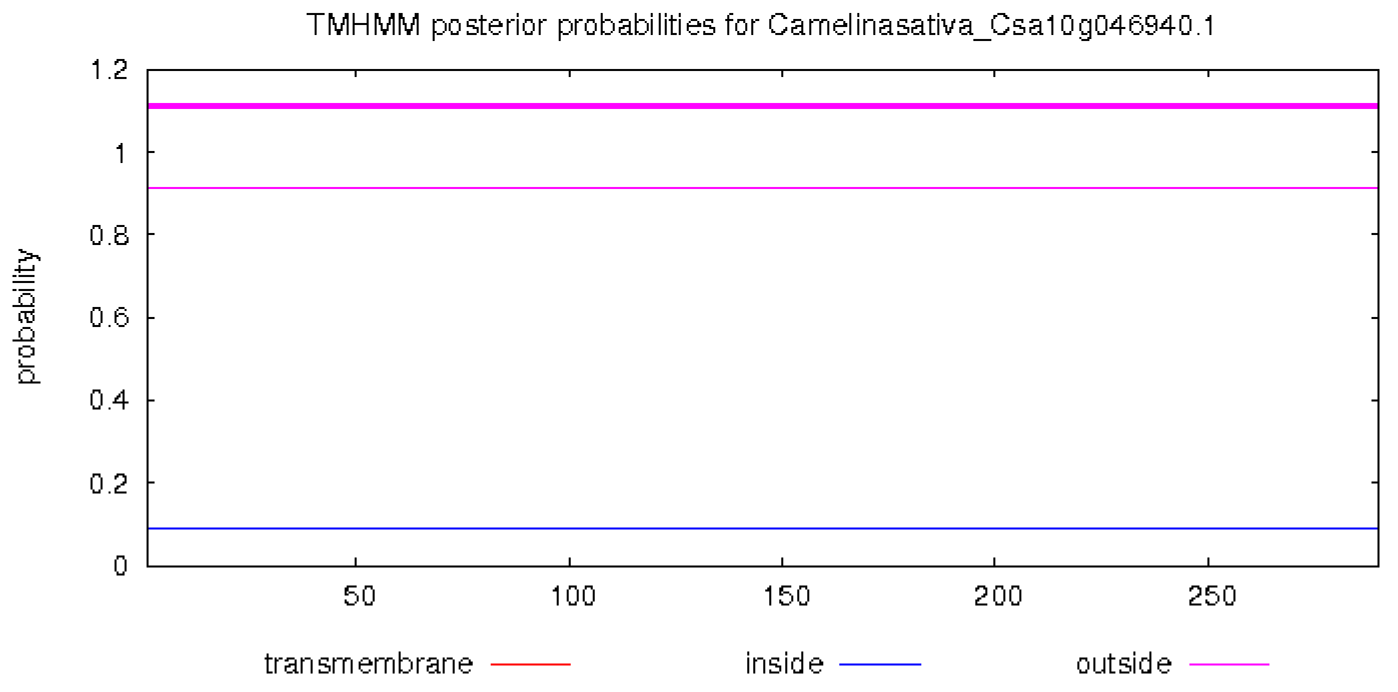

plot in postscript, script for making the plot in gnuplot, data for plot

---

```
# Camelinasativa_Csa10g049630.1 Length: 175
# Camelinasativa_Csa10g049630.1 Number of predicted TMHs:  0
# Camelinasativa_Csa10g049630.1 Exp number of AAs in TMHs: 0.028
# Camelinasativa_Csa10g049630.1 Exp number, first 60 AAs:  0.028
# Camelinasativa_Csa10g049630.1 Total prob of N-in:        0.04328
Camelinasativa_Csa10g049630.1	TMHMM2.0	outside	     1   175
```

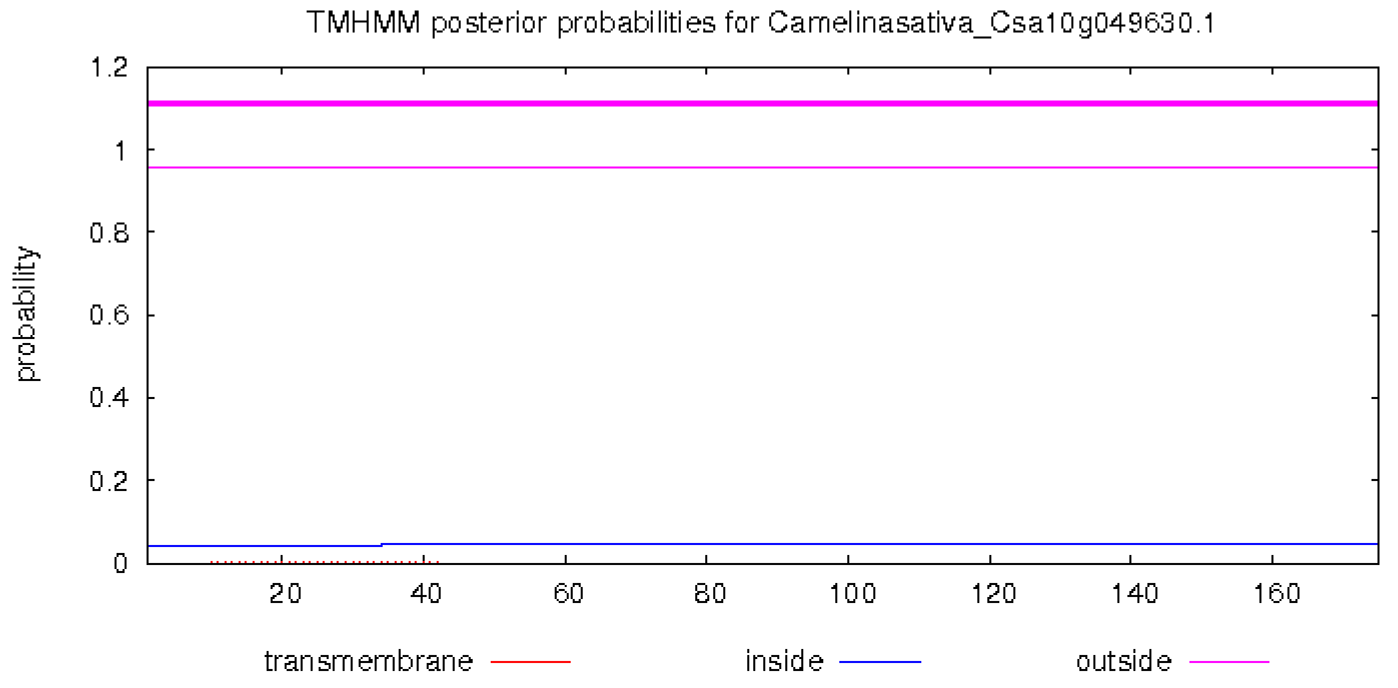

plot in postscript, script for making the plot in gnuplot, data for plot

---

```
# Camelinasativa_Csa11g005520.1 Length: 376
# Camelinasativa_Csa11g005520.1 Number of predicted TMHs:  0
# Camelinasativa_Csa11g005520.1 Exp number of AAs in TMHs: 0
# Camelinasativa_Csa11g005520.1 Exp number, first 60 AAs:  0
# Camelinasativa_Csa11g005520.1 Total prob of N-in:        0.01338
Camelinasativa_Csa11g005520.1   TMHMM2.0        outside      1   376
```

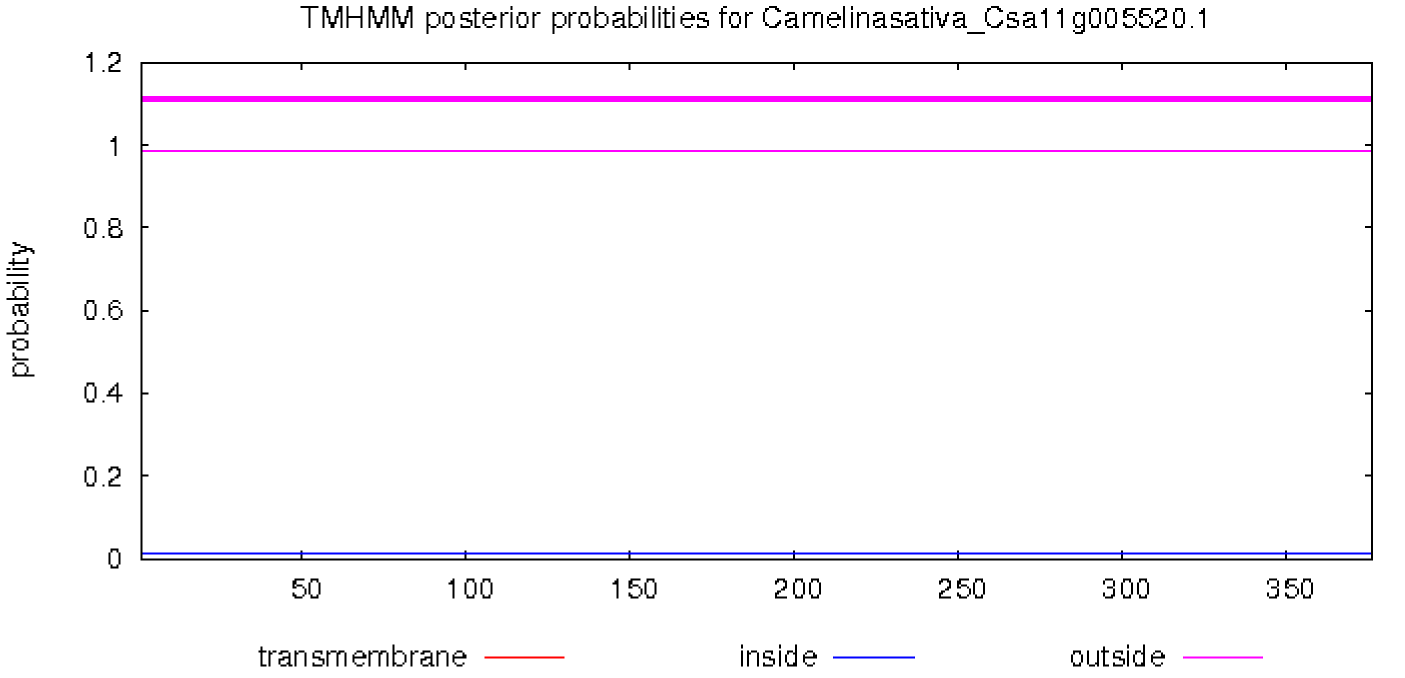

plot in postscript, script for making the plot in gnuplot, data for plot

---

```
# Camelinasativa_Csa11g006210.1 Length: 504
# Camelinasativa_Csa11g006210.1 Number of predicted TMHs:  1
# Camelinasativa_Csa11g006210.1 Exp number of AAs in TMHs: 19.38408
# Camelinasativa_Csa11g006210.1 Exp number, first 60 AAs:  0
# Camelinasativa_Csa11g006210.1 Total prob of N-in:        0.62911
Camelinasativa_Csa11g006210.1   TMHMM2.0        inside       1   478
Camelinasativa_Csa11g006210.1   TMHMM2.0        TMhelix    479   498
Camelinasativa_Csa11g006210.1   TMHMM2.0        outside    499   504
```

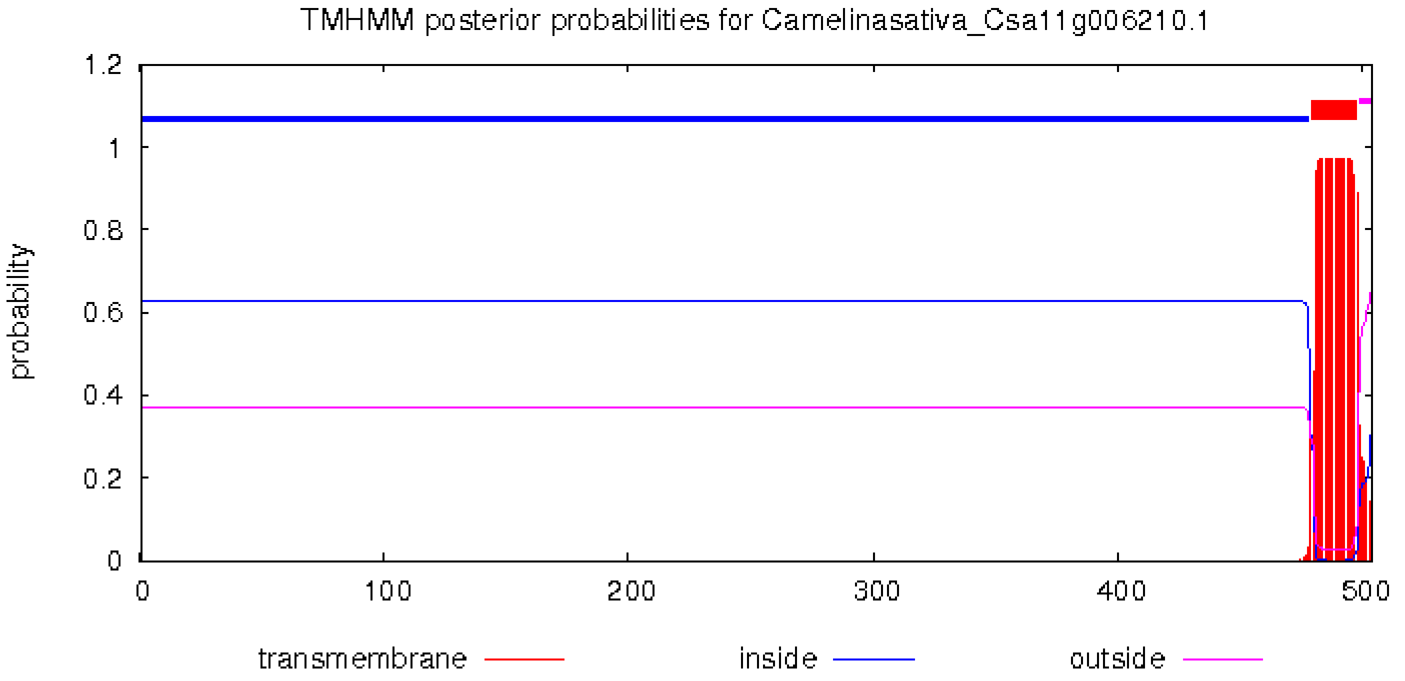

plot in postscript, script for making the plot in gnuplot, data for plot

```
# Camelinasativa_Csa11g014290.1 Length: 495
# Camelinasativa_Csa11g014290.1 Number of predicted TMHs:  0
# Camelinasativa_Csa11g014290.1 Exp number of AAs in TMHs: 6.93779
# Camelinasativa_Csa11g014290.1 Exp number, first 60 AAs:  0
# Camelinasativa_Csa11g014290.1 Total prob of N-in:        0.28819
Camelinasativa_Csa11g014290.1	TMHMM2.0	outside	     1   495
```

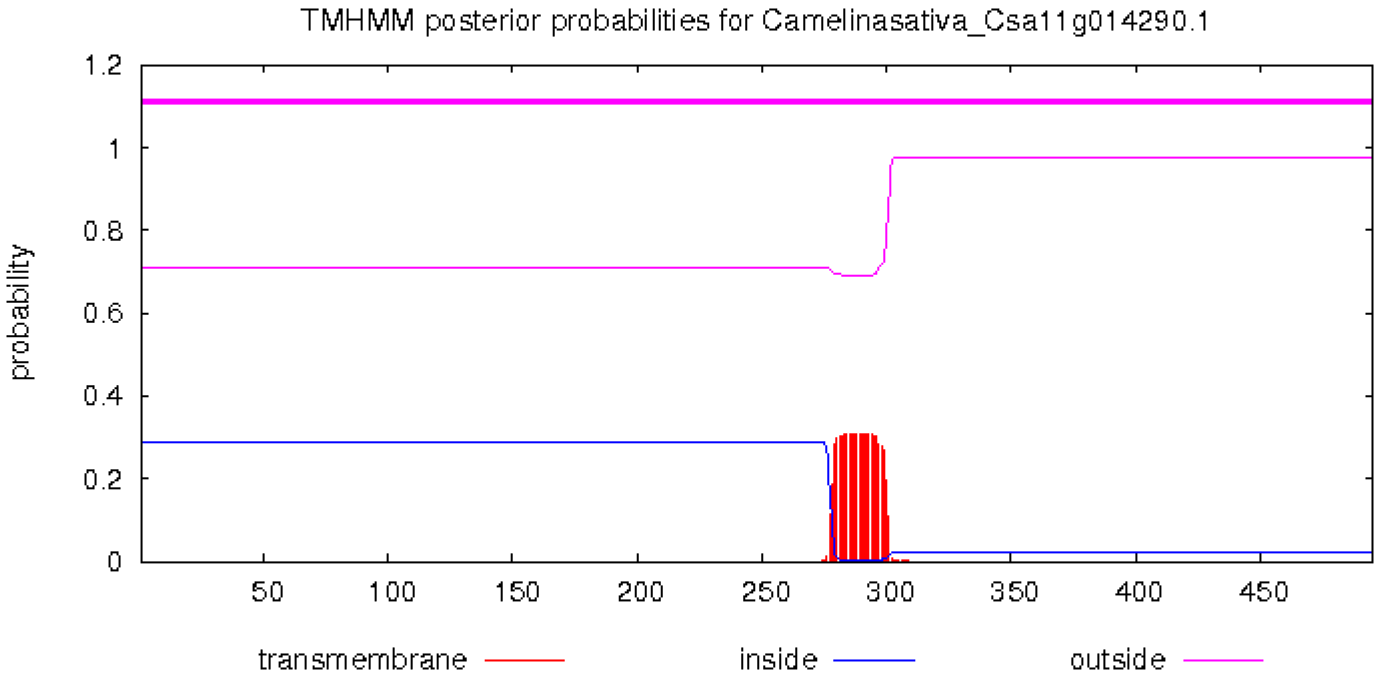

plot in postscript, script for making the plot in gnuplot, data for plot

```
# Camelinasativa_Csa11g015230.1 Length: 304
# Camelinasativa_Csa11g015230.1 Number of predicted TMHs:  0
# Camelinasativa_Csa11g015230.1 Exp number of AAs in TMHs: 0
# Camelinasativa_Csa11g015230.1 Exp number, first 60 AAs:  0
# Camelinasativa_Csa11g015230.1 Total prob of N-in:        0.01021
Camelinasativa_Csa11g015230.1	TMHMM2.0	outside	     1   304
```

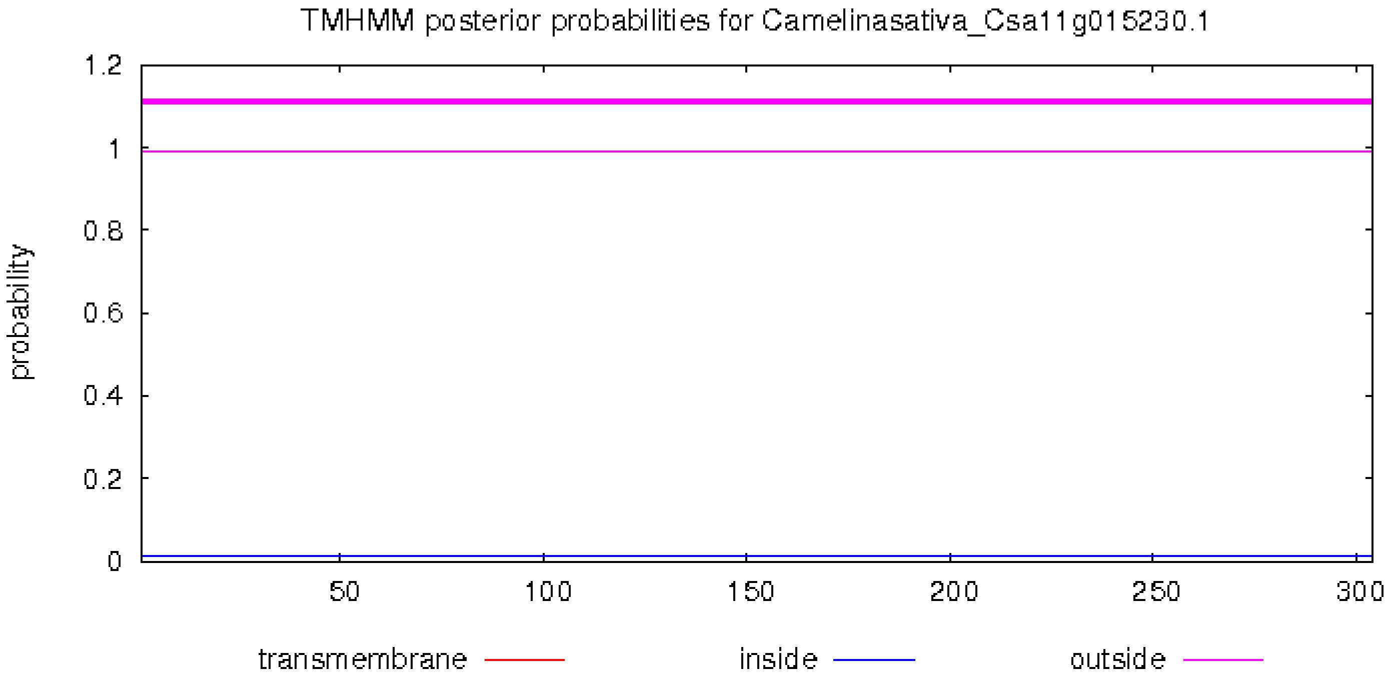

plot in postscript, script for making the plot in gnuplot, data for plot

```
# Camelinasativa_Csa11g015260.1 Length: 306
# Camelinasativa_Csa11g015260.1 Number of predicted TMHs:  0
# Camelinasativa_Csa11g015260.1 Exp number of AAs in TMHs: 0.00106
# Camelinasativa_Csa11g015260.1 Exp number, first 60 AAs:  0
# Camelinasativa_Csa11g015260.1 Total prob of N-in:        0.09756
Camelinasativa_Csa11g015260.1   TMHMM2.0        outside      1   306
```

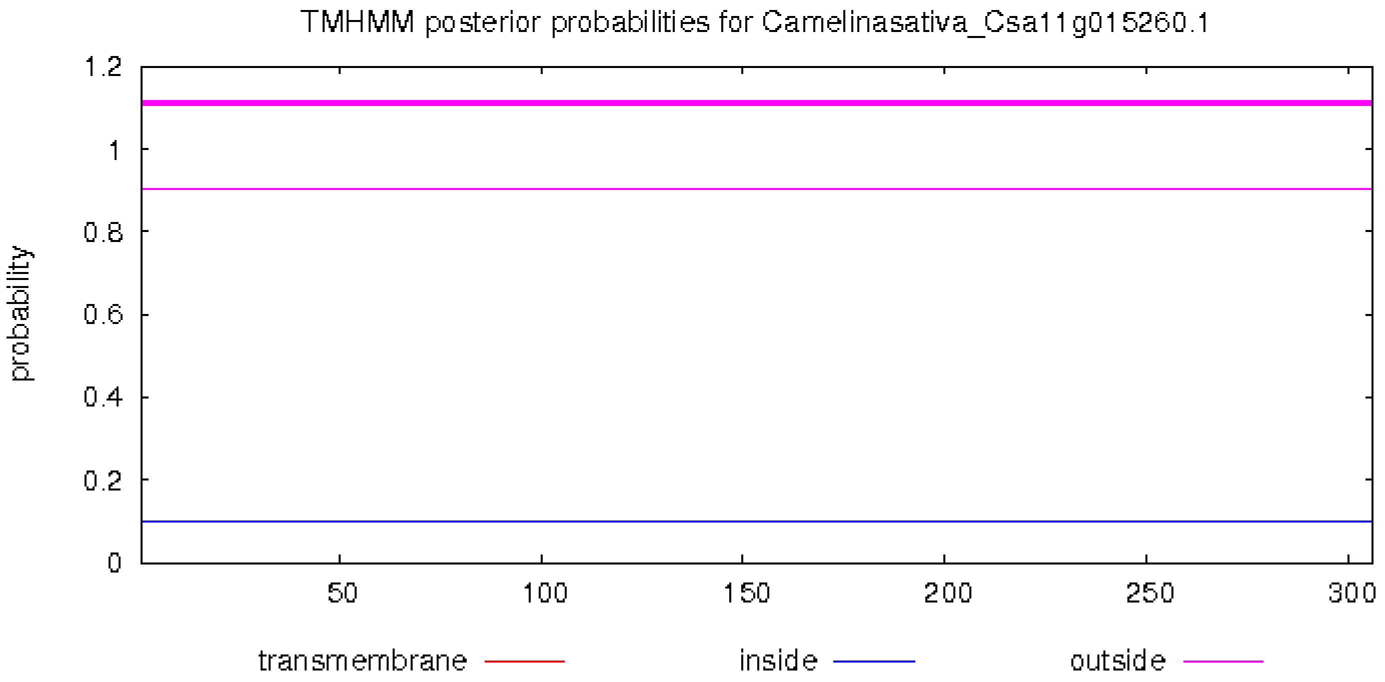

plot in postscript, script for making the plot in gnuplot, data for plot

---

```
# Camelinasativa_Csa11g016750.1 Length: 301
# Camelinasativa_Csa11g016750.1 Number of predicted TMHs:  0
# Camelinasativa_Csa11g016750.1 Exp number of AAs in TMHs: 0.01091
# Camelinasativa_Csa11g016750.1 Exp number, first 60 AAs:  0.00039
# Camelinasativa_Csa11g016750.1 Total prob of N-in:        0.02126
Camelinasativa_Csa11g016750.1   TMHMM2.0        outside      1   301
```

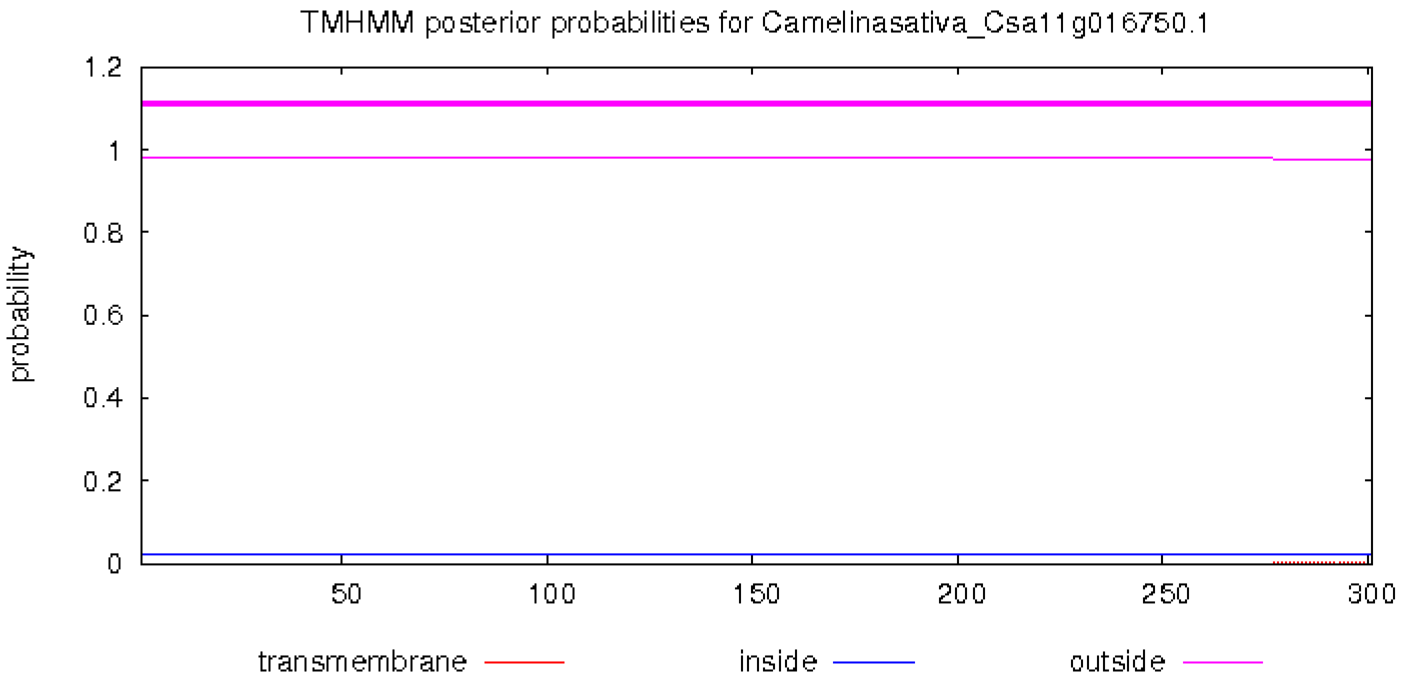

plot in postscript, script for making the plot in gnuplot, data for plot

---

```
# Camelinasativa_Csa11g055650.1 Length: 290
# Camelinasativa_Csa11g055650.1 Number of predicted TMHs:  0
# Camelinasativa_Csa11g055650.1 Exp number of AAs in TMHs: 0.00093
# Camelinasativa_Csa11g055650.1 Exp number, first 60 AAs:  0.00089
# Camelinasativa_Csa11g055650.1 Total prob of N-in:        0.08738
Camelinasativa_Csa11g055650.1   TMHMM2.0        outside      1   290
```

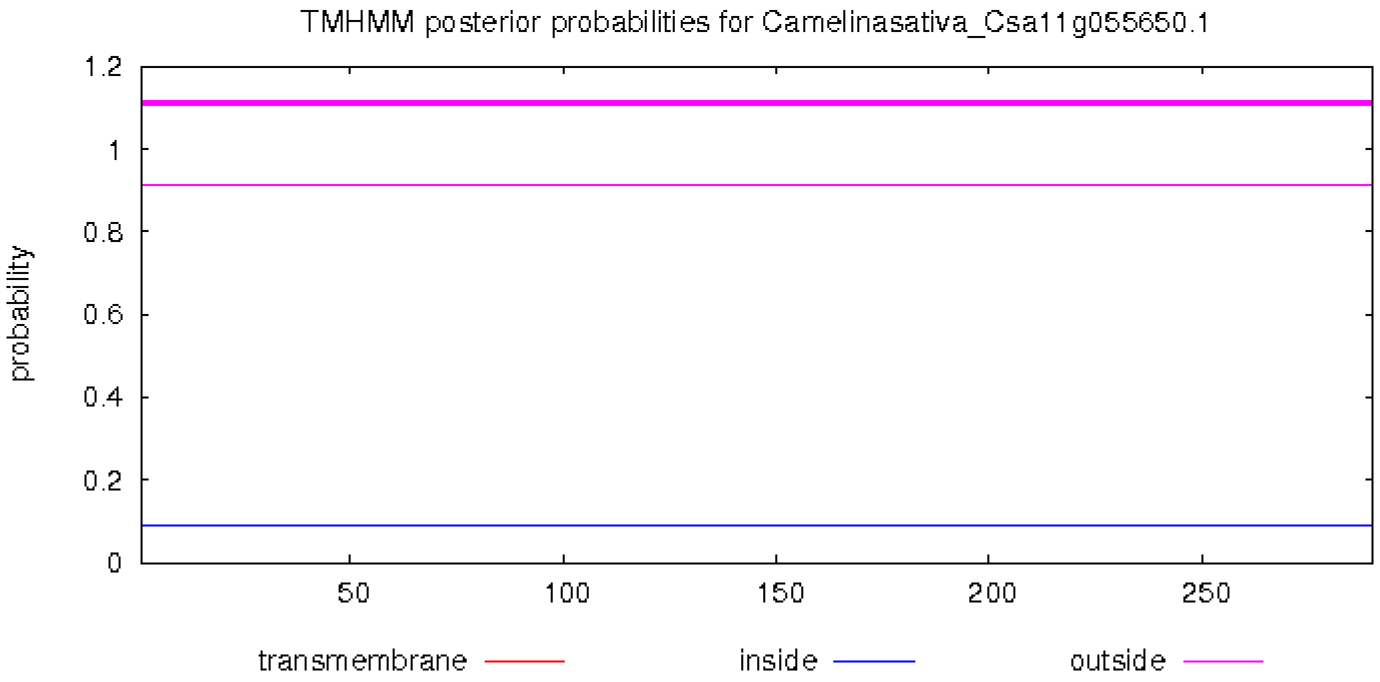

plot in postscript, script for making the plot in gnuplot, data for plot

---

```
# Camelinasativa_Csa11g055880.1 Length: 335
# Camelinasativa_Csa11g055880.1 Number of predicted TMHs:  0
# Camelinasativa_Csa11g055880.1 Exp number of AAs in TMHs: 0.00124
# Camelinasativa_Csa11g055880.1 Exp number, first 60 AAs:  0
# Camelinasativa_Csa11g055880.1 Total prob of N-in:        0.02392
Camelinasativa_Csa11g055880.1   TMHMM2.0        outside      1   335
```

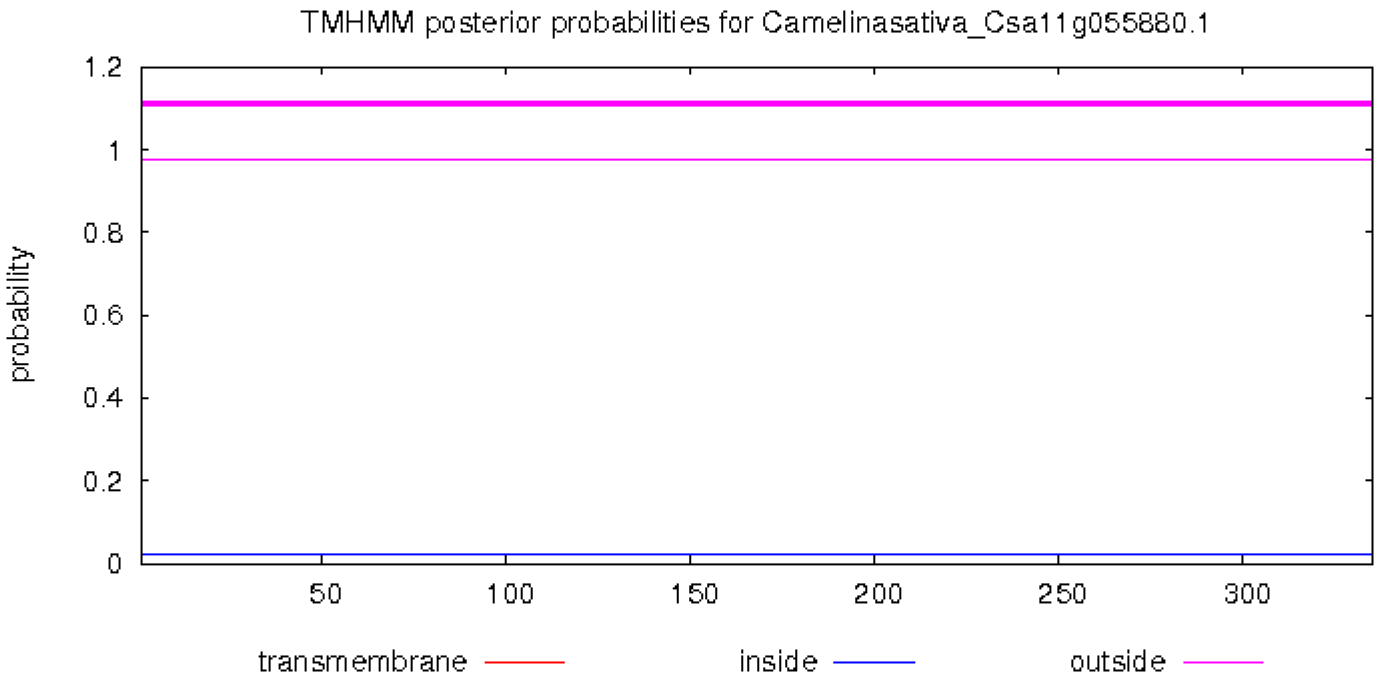

plot in postscript, script for making the plot in gnuplot, data for plot

---

```
# Camelinasativa_Csa11g059640.1 Length: 169
# Camelinasativa_Csa11g059640.1 Number of predicted TMHs:  0
# Camelinasativa_Csa11g059640.1 Exp number of AAs in TMHs: 0.02382
# Camelinasativa_Csa11g059640.1 Exp number, first 60 AAs:  0.02372
# Camelinasativa_Csa11g059640.1 Total prob of N-in:        0.04682
Camelinasativa_Csa11g059640.1	TMHMM2.0	outside	     1   169
```

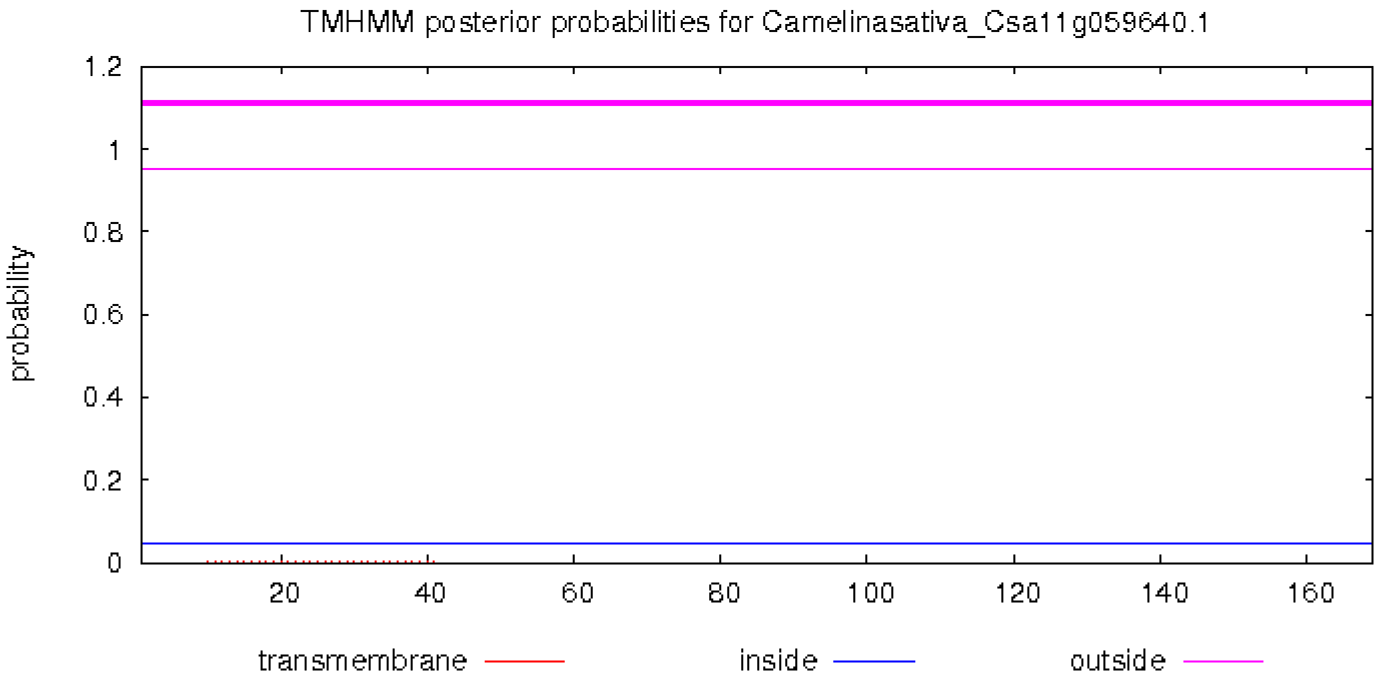

plot in postscript, script for making the plot in gnuplot, data for plot

---

```
# Camelinasativa_Csa11g065390.1 Length: 290
# Camelinasativa_Csa11g065390.1 Number of predicted TMHs:  0
# Camelinasativa_Csa11g065390.1 Exp number of AAs in TMHs: 0.00016
# Camelinasativa_Csa11g065390.1 Exp number, first 60 AAs:  0
# Camelinasativa_Csa11g065390.1 Total prob of N-in:        0.00220
Camelinasativa_Csa11g065390.1	TMHMM2.0	outside	     1   290
```

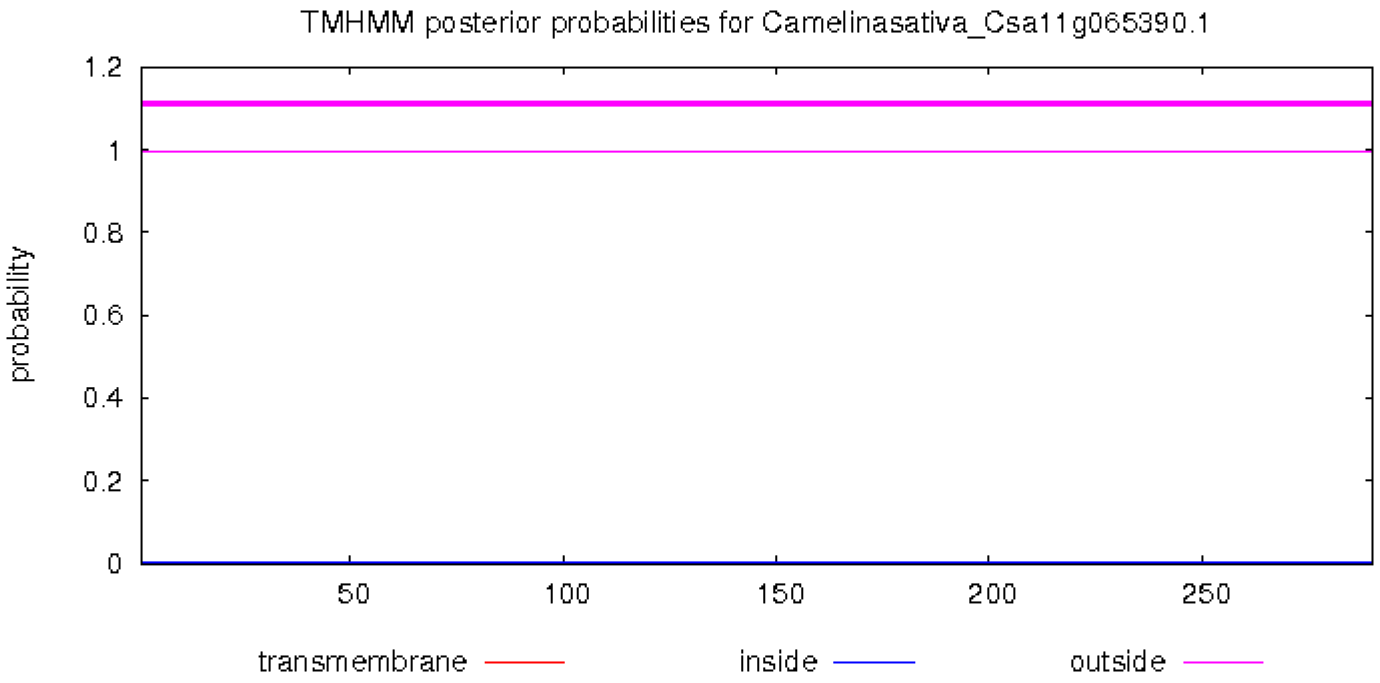

plot in postscript, script for making the plot in gnuplot, data for plot

---

```
# Camelinasativa_Csa11g082140.1 Length: 193
# Camelinasativa_Csa11g082140.1 Number of predicted TMHs:  0
# Camelinasativa_Csa11g082140.1 Exp number of AAs in TMHs: 0.00109
# Camelinasativa_Csa11g082140.1 Exp number, first 60 AAs:  0
# Camelinasativa_Csa11g082140.1 Total prob of N-in:        0.12749
Camelinasativa_Csa11g082140.1   TMHMM2.0        outside      1   193
```

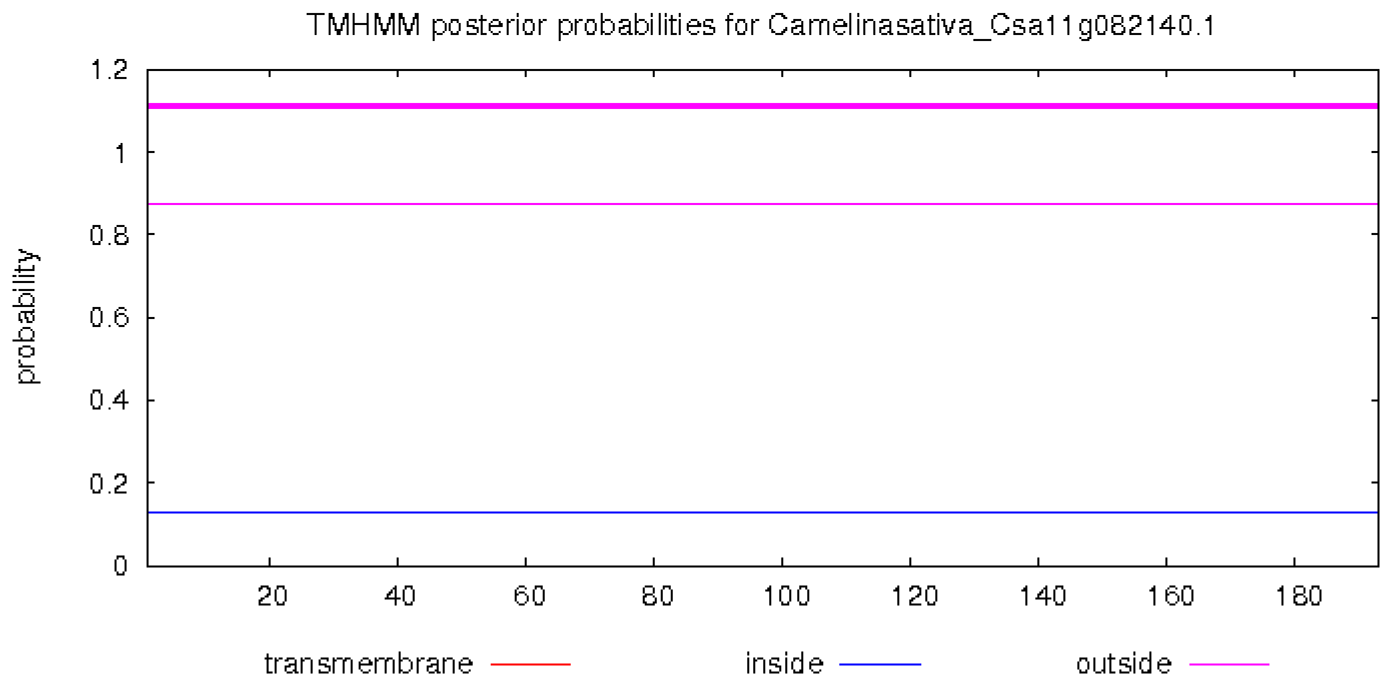

plot in postscript, script for making the plot in gnuplot, data for plot

---

```
# Camelinasativa_Csa11g085880.1 Length: 374
# Camelinasativa_Csa11g085880.1 Number of predicted TMHs:  0
# Camelinasativa_Csa11g085880.1 Exp number of AAs in TMHs: 0.00478
# Camelinasativa_Csa11g085880.1 Exp number, first 60 AAs:  0
# Camelinasativa_Csa11g085880.1 Total prob of N-in:        0.01613
Camelinasativa_Csa11g085880.1   TMHMM2.0        outside      1   374
```

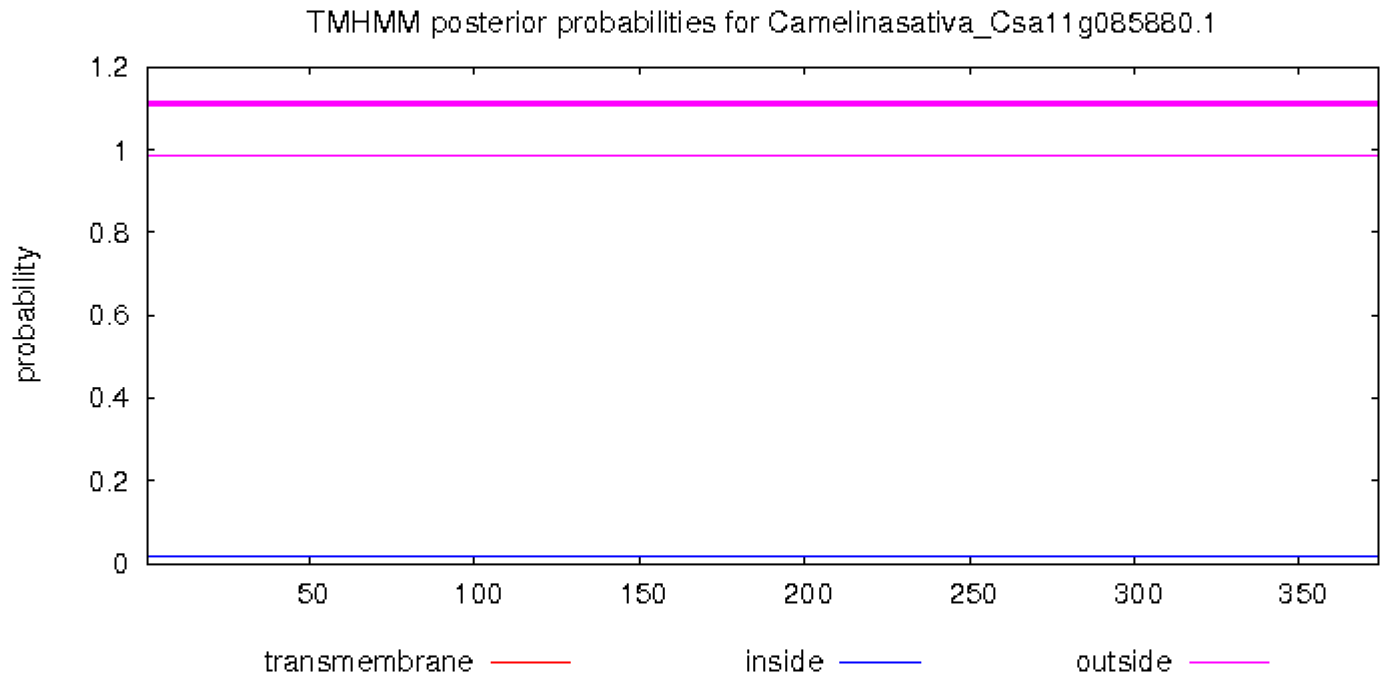

plot in postscript, script for making the plot in gnuplot, data for plot

---

```
# Camelinasativa_Csa11g090050.1 Length: 403
# Camelinasativa_Csa11g090050.1 Number of predicted TMHs:  0
# Camelinasativa_Csa11g090050.1 Exp number of AAs in TMHs: 0.00064
# Camelinasativa_Csa11g090050.1 Exp number, first 60 AAs:  0
# Camelinasativa_Csa11g090050.1 Total prob of N-in:        0.02266
Camelinasativa_Csa11g090050.1	TMHMM2.0	outside	     1   403
```

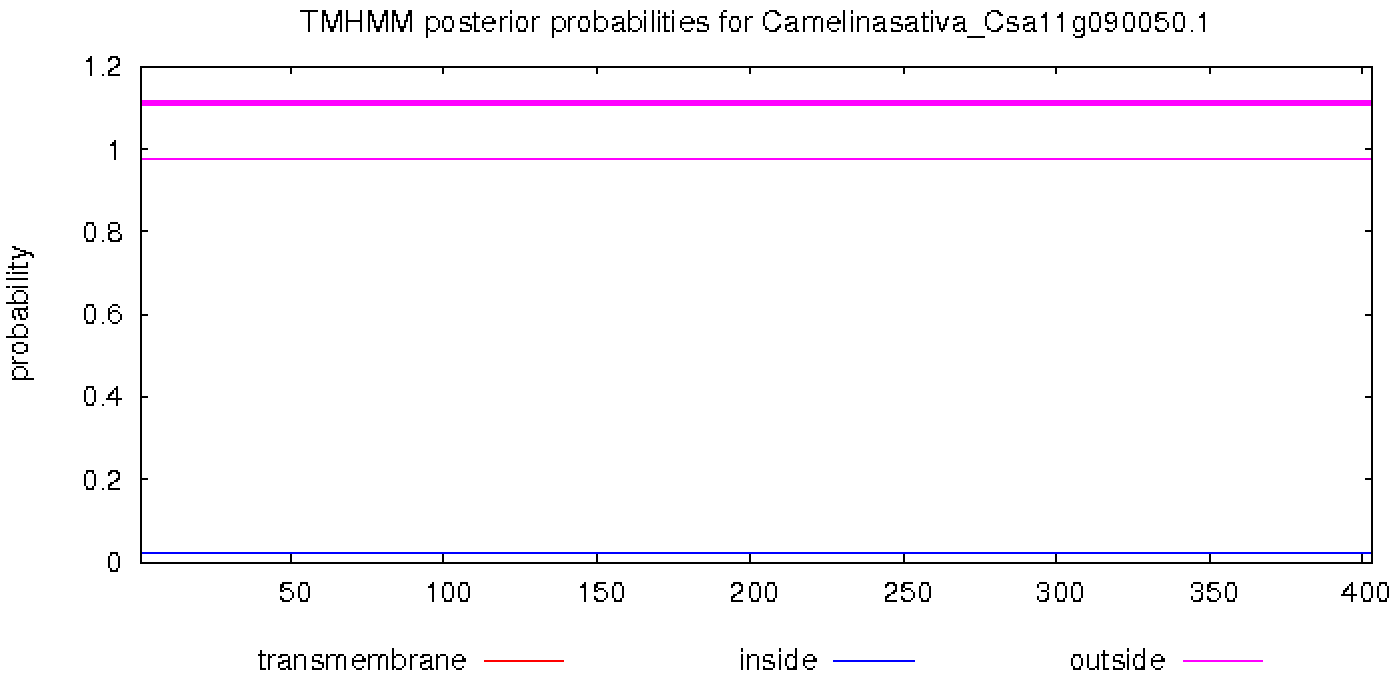

plot in postscript, script for making the plot in gnuplot, data for plot

---

```
# Camelinasativa_Csa11g092840.1 Length: 289
# Camelinasativa_Csa11g092840.1 Number of predicted TMHs:  0
# Camelinasativa_Csa11g092840.1 Exp number of AAs in TMHs: 0
# Camelinasativa_Csa11g092840.1 Exp number, first 60 AAs:  0
# Camelinasativa_Csa11g092840.1 Total prob of N-in:        0.08157
Camelinasativa_Csa11g092840.1	TMHMM2.0	outside	     1   289
```

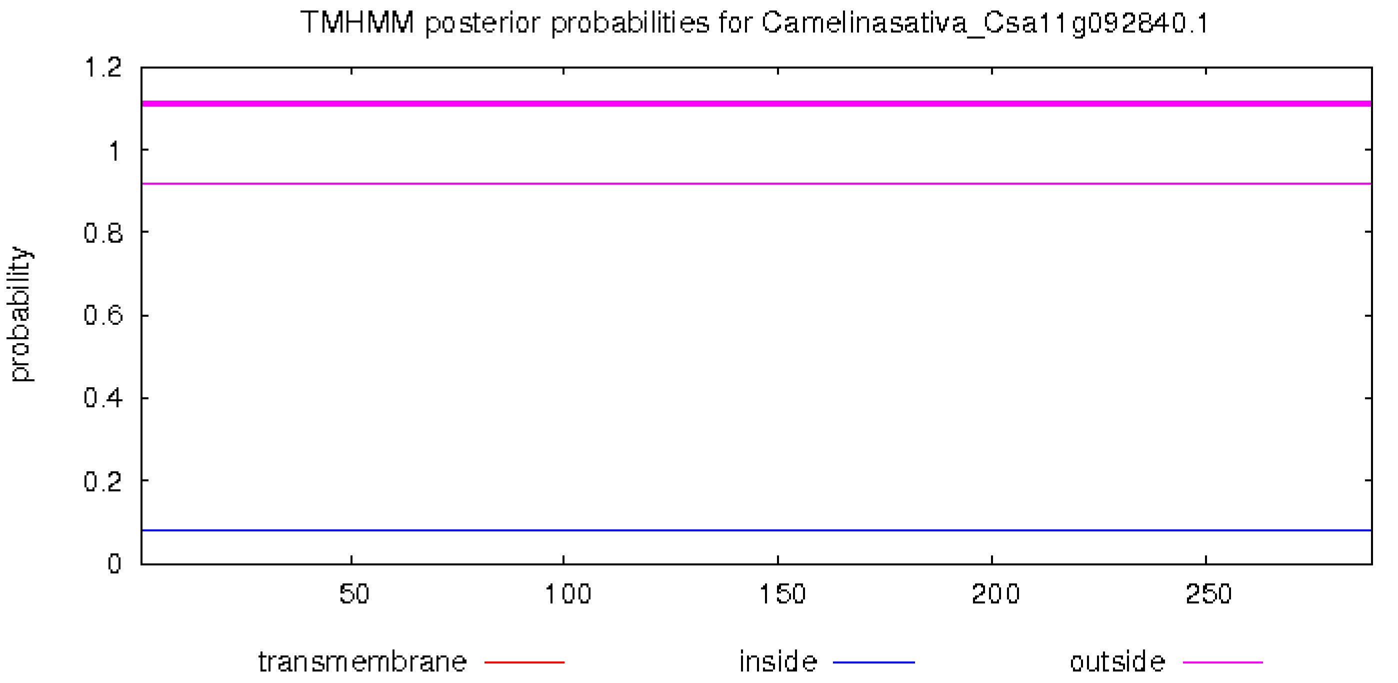

plot in postscript, script for making the plot in gnuplot, data for plot

---

```
# Camelinasativa_Csa11g093080.1 Length: 274
# Camelinasativa_Csa11g093080.1 Number of predicted TMHs:  0
# Camelinasativa_Csa11g093080.1 Exp number of AAs in TMHs: 0.00014
# Camelinasativa_Csa11g093080.1 Exp number, first 60 AAs:  0
# Camelinasativa_Csa11g093080.1 Total prob of N-in:        0.09724
Camelinasativa_Csa11g093080.1	TMHMM2.0	outside	     1   274
```

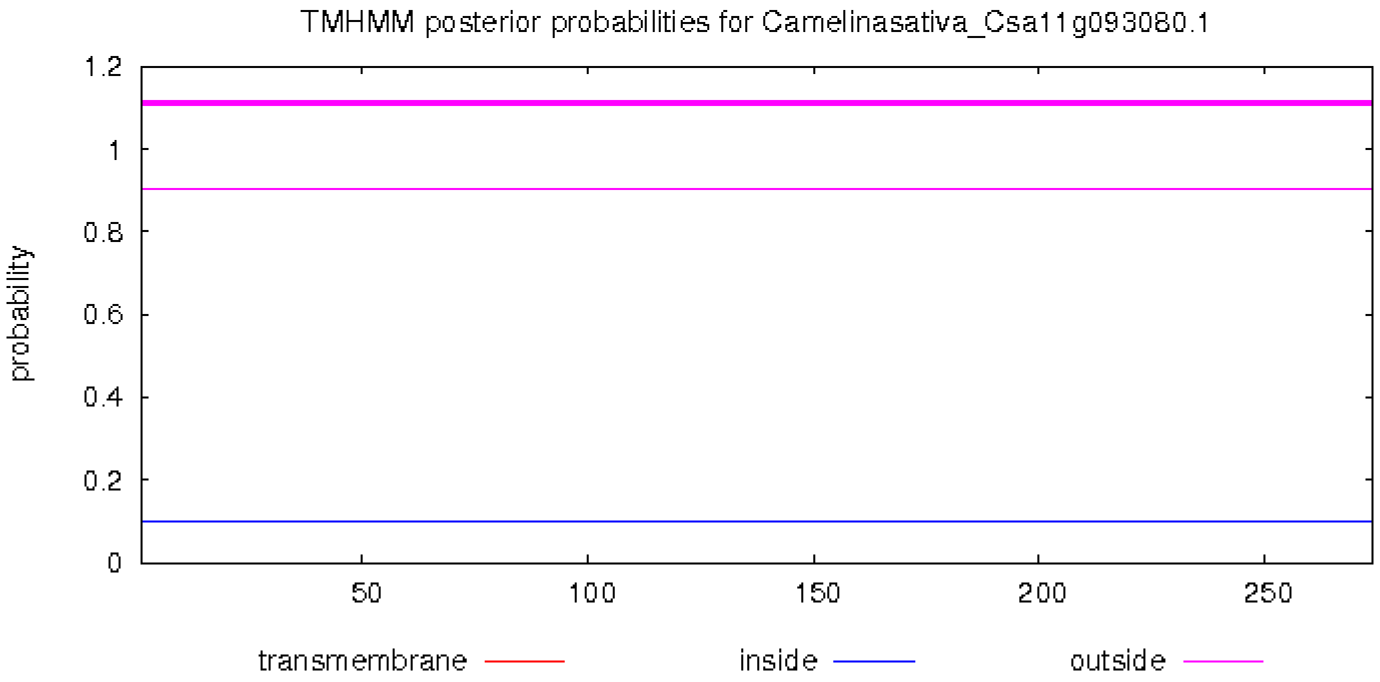

plot in postscript, script for making the plot in gnuplot, data for plot

---

```
# Camelinasativa_Csa11g097230.1 Length: 338
# Camelinasativa_Csa11g097230.1 Number of predicted TMHs:  0
# Camelinasativa_Csa11g097230.1 Exp number of AAs in TMHs: 0.00035
# Camelinasativa_Csa11g097230.1 Exp number, first 60 AAs:  0
# Camelinasativa_Csa11g097230.1 Total prob of N-in:        0.01207
Camelinasativa_Csa11g097230.1	TMHMM2.0	outside	     1   338
```

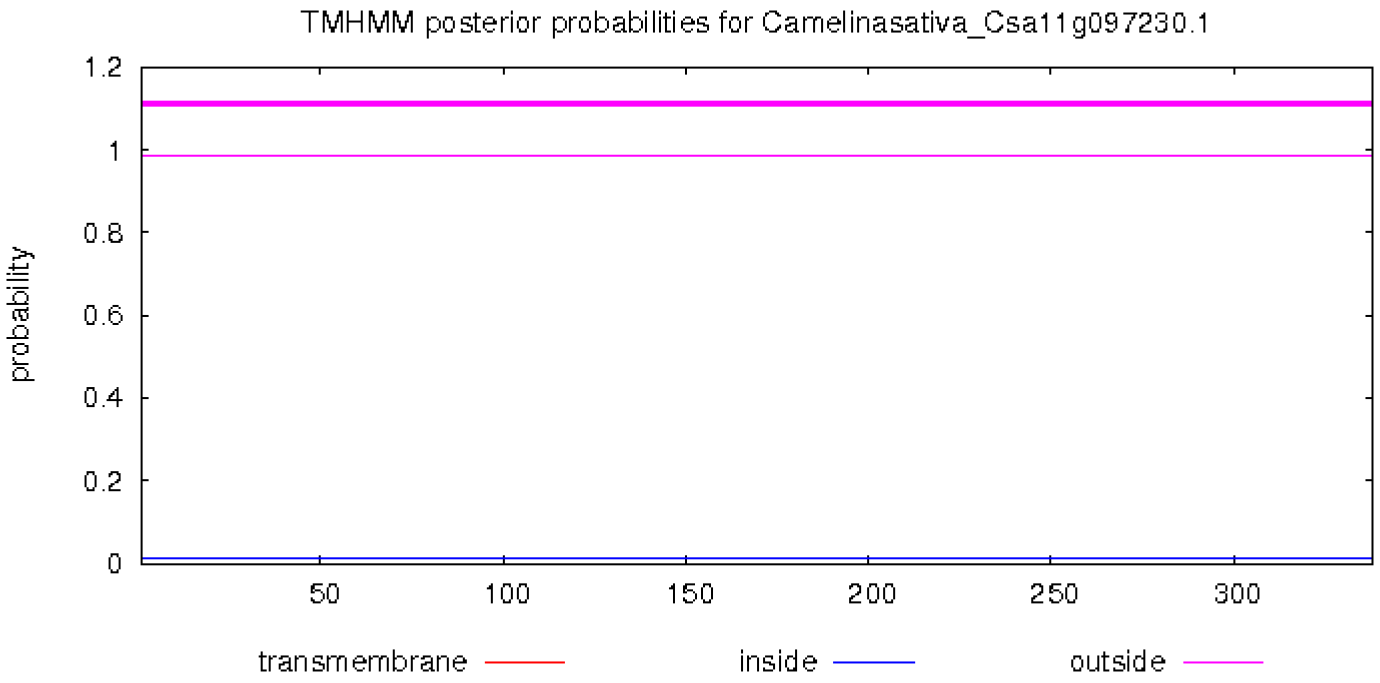

plot in postscript, script for making the plot in gnuplot, data for plot

---

```
# Camelinasativa_Csa11g099250.1 Length: 344
# Camelinasativa_Csa11g099250.1 Number of predicted TMHs:  0
# Camelinasativa_Csa11g099250.1 Exp number of AAs in TMHs: 0.00156
# Camelinasativa_Csa11g099250.1 Exp number, first 60 AAs:  0
# Camelinasativa_Csa11g099250.1 Total prob of N-in:        0.00201
Camelinasativa_Csa11g099250.1   TMHMM2.0        outside      1   344
```

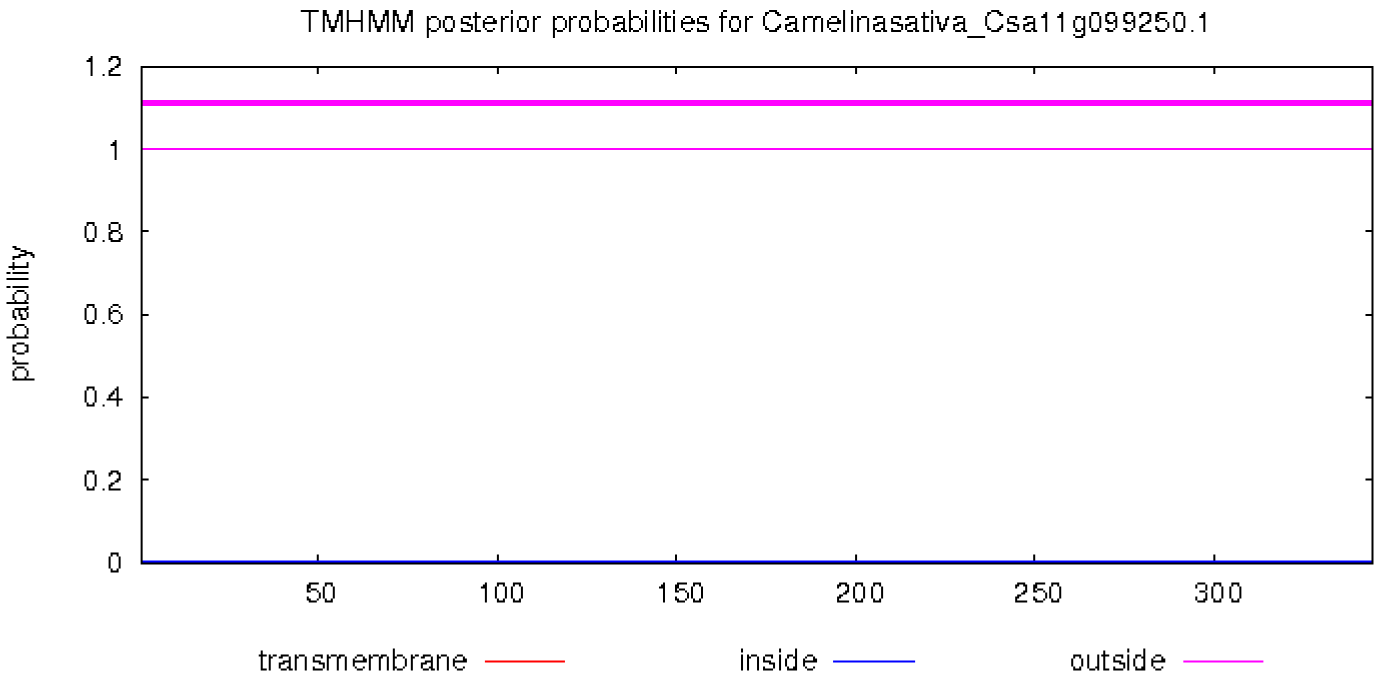

plot in postscript, script for making the plot in gnuplot, data for plot

---

```
# Camelinasativa_Csa11g100860.1 Length: 276
# Camelinasativa_Csa11g100860.1 Number of predicted TMHs:  0
# Camelinasativa_Csa11g100860.1 Exp number of AAs in TMHs: 0.00019
# Camelinasativa_Csa11g100860.1 Exp number, first 60 AAs:  0
# Camelinasativa_Csa11g100860.1 Total prob of N-in:        0.01191
Camelinasativa_Csa11g100860.1   TMHMM2.0        outside      1   276
```

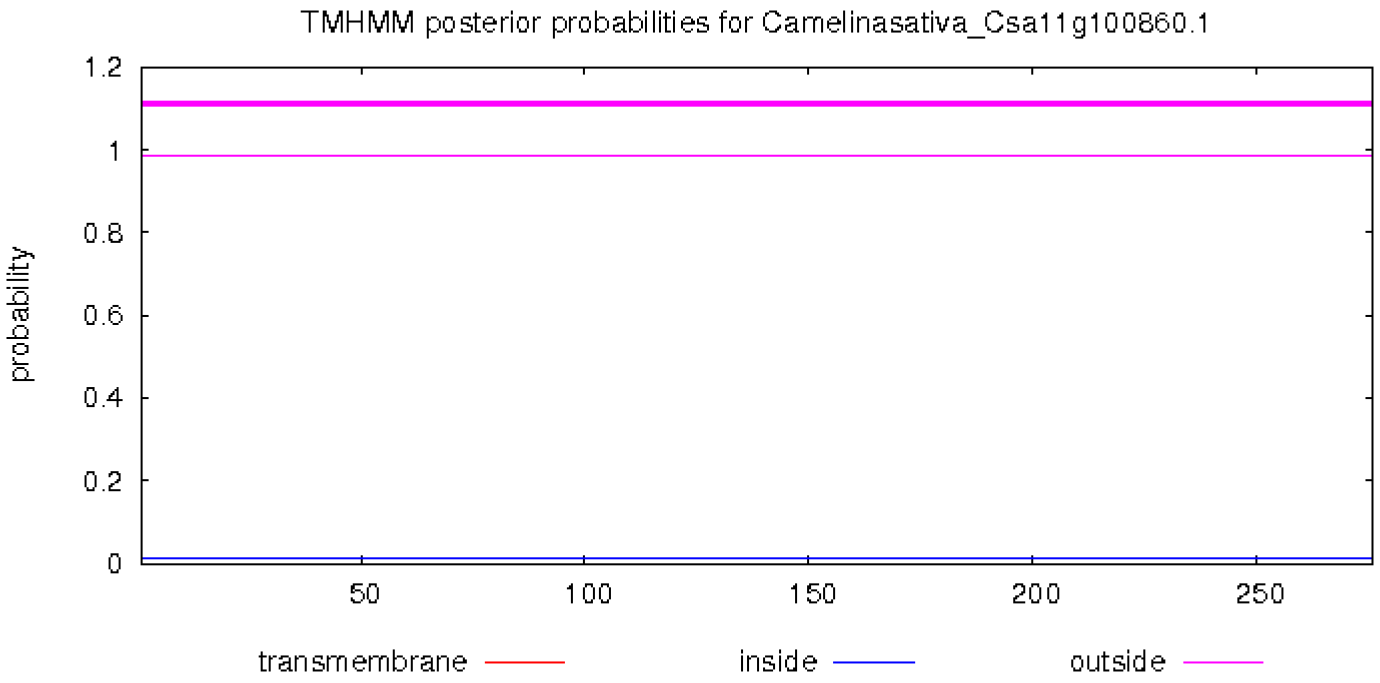

plot in postscript, script for making the plot in gnuplot, data for plot

---

```
# Camelinasativa_Csa11g101160.1 Length: 361
# Camelinasativa_Csa11g101160.1 Number of predicted TMHs:  0
# Camelinasativa_Csa11g101160.1 Exp number of AAs in TMHs: 0.00035
# Camelinasativa_Csa11g101160.1 Exp number, first 60 AAs:  0.00018
# Camelinasativa_Csa11g101160.1 Total prob of N-in:        0.00559
Camelinasativa_Csa11g101160.1   TMHMM2.0        outside      1   361
```

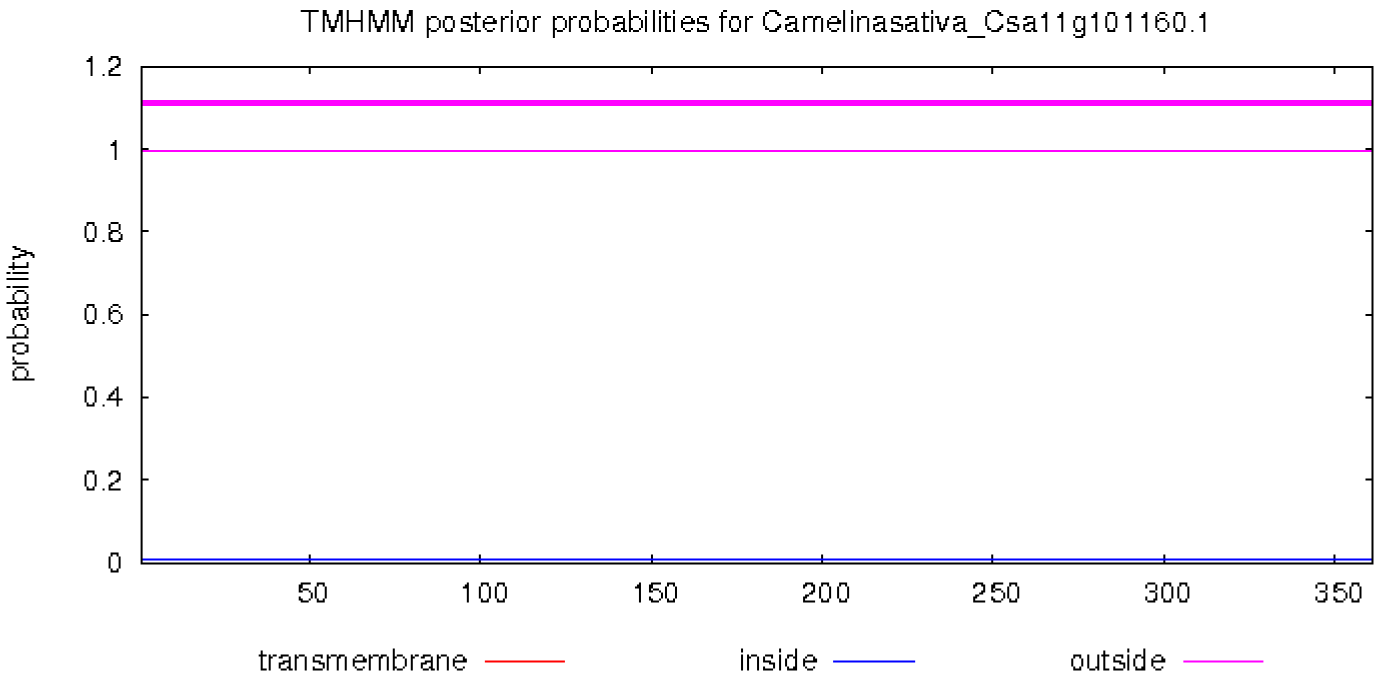

plot in postscript, script for making the plot in gnuplot, data for plot

---

```
# Camelinasativa_Csa11g101660.1 Length: 188
# Camelinasativa_Csa11g101660.1 Number of predicted TMHs:  0
# Camelinasativa_Csa11g101660.1 Exp number of AAs in TMHs: 0
# Camelinasativa_Csa11g101660.1 Exp number, first 60 AAs:  0
# Camelinasativa_Csa11g101660.1 Total prob of N-in:        0.00724
Camelinasativa_Csa11g101660.1   TMHMM2.0        outside      1   188
```

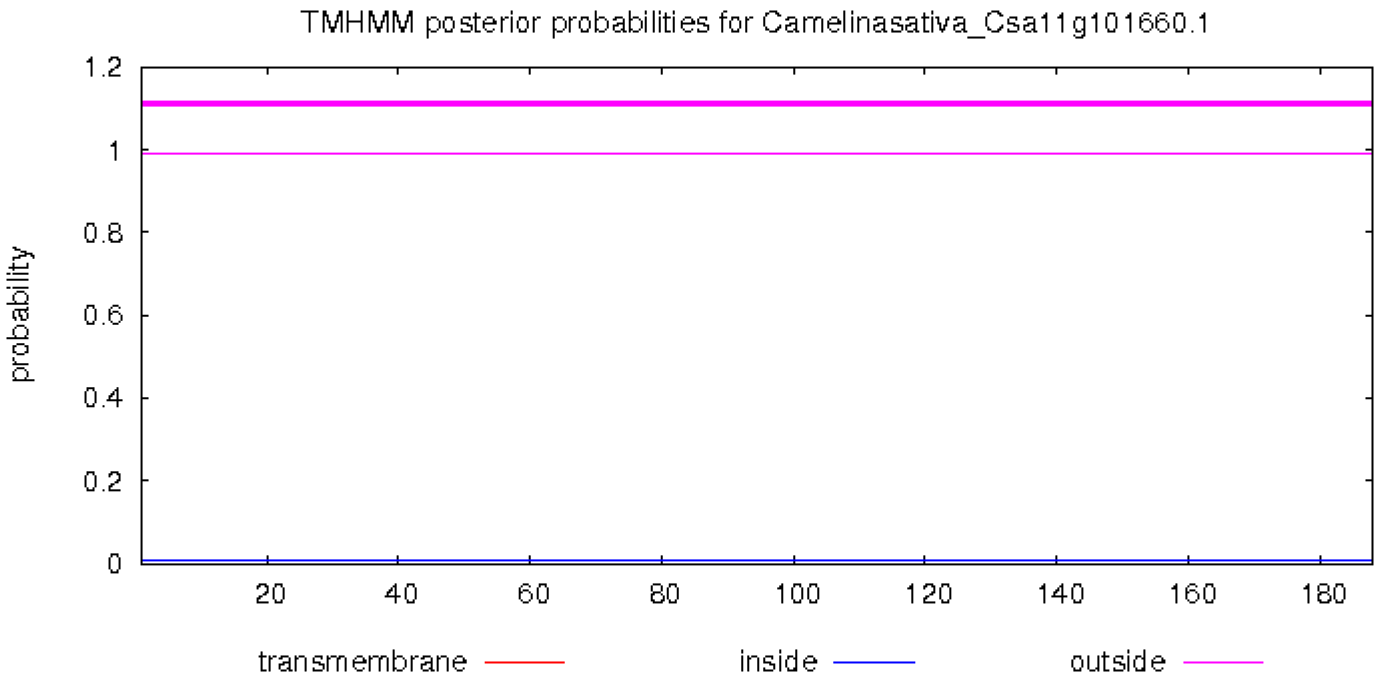

plot in postscript, script for making the plot in gnuplot, data for plot

---

```
# Camelinasativa_Csa11g104660.1 Length: 289
# Camelinasativa_Csa11g104660.1 Number of predicted TMHs:  0
# Camelinasativa_Csa11g104660.1 Exp number of AAs in TMHs: 0.00149
# Camelinasativa_Csa11g104660.1 Exp number, first 60 AAs:  0
# Camelinasativa_Csa11g104660.1 Total prob of N-in:        0.04830
Camelinasativa_Csa11g104660.1   TMHMM2.0        outside      1   289
```

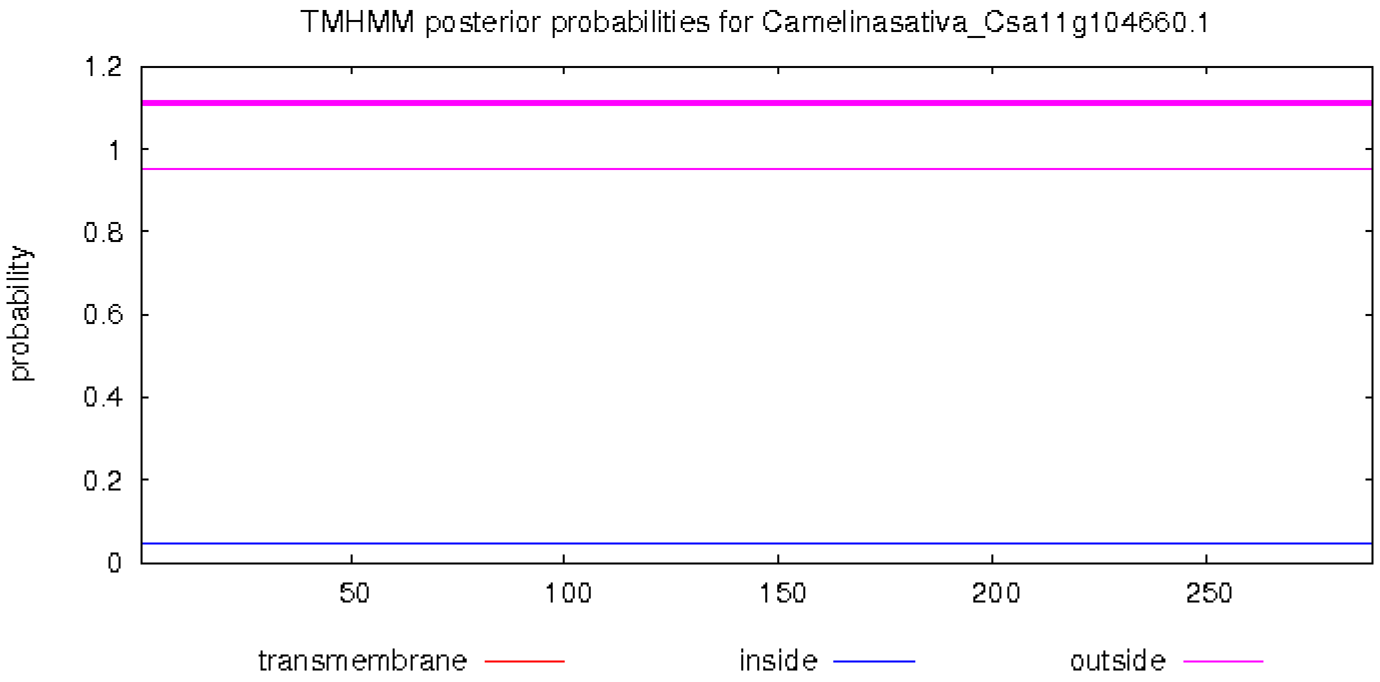

plot in postscript, script for making the plot in gnuplot, data for plot

---

```
# Camelinasativa_Csa12g006210.1 Length: 376
# Camelinasativa_Csa12g006210.1 Number of predicted TMHs:  0
# Camelinasativa_Csa12g006210.1 Exp number of AAs in TMHs: 0
# Camelinasativa_Csa12g006210.1 Exp number, first 60 AAs:  0
# Camelinasativa_Csa12g006210.1 Total prob of N-in:        0.01338
Camelinasativa_Csa12g006210.1   TMHMM2.0        outside      1   376
```

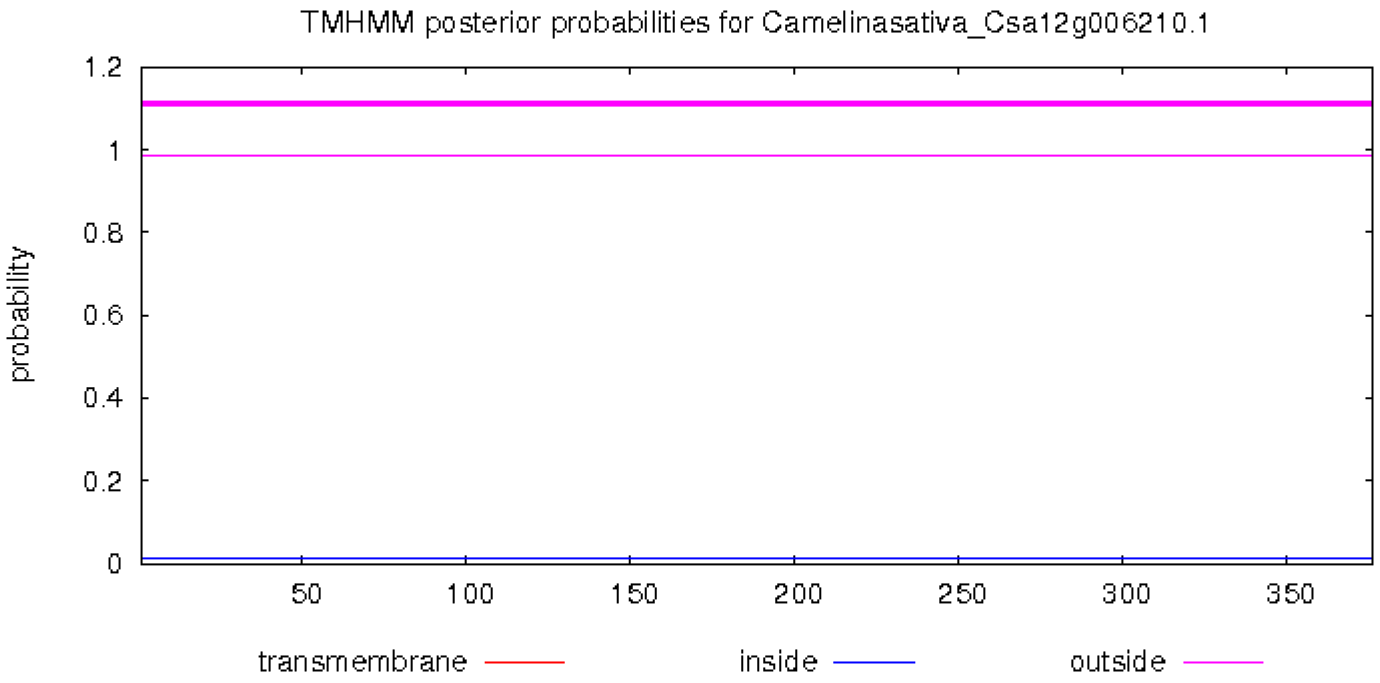

plot in postscript, script for making the plot in gnuplot, data for plot

---

```
# Camelinasativa_Csa12g007970.1 Length: 496
# Camelinasativa_Csa12g007970.1 Number of predicted TMHs:  0
# Camelinasativa_Csa12g007970.1 Exp number of AAs in TMHs: 0
# Camelinasativa_Csa12g007970.1 Exp number, first 60 AAs:  0
# Camelinasativa_Csa12g007970.1 Total prob of N-in:        0.00074
Camelinasativa_Csa12g007970.1	TMHMM2.0	outside	     1   496
```

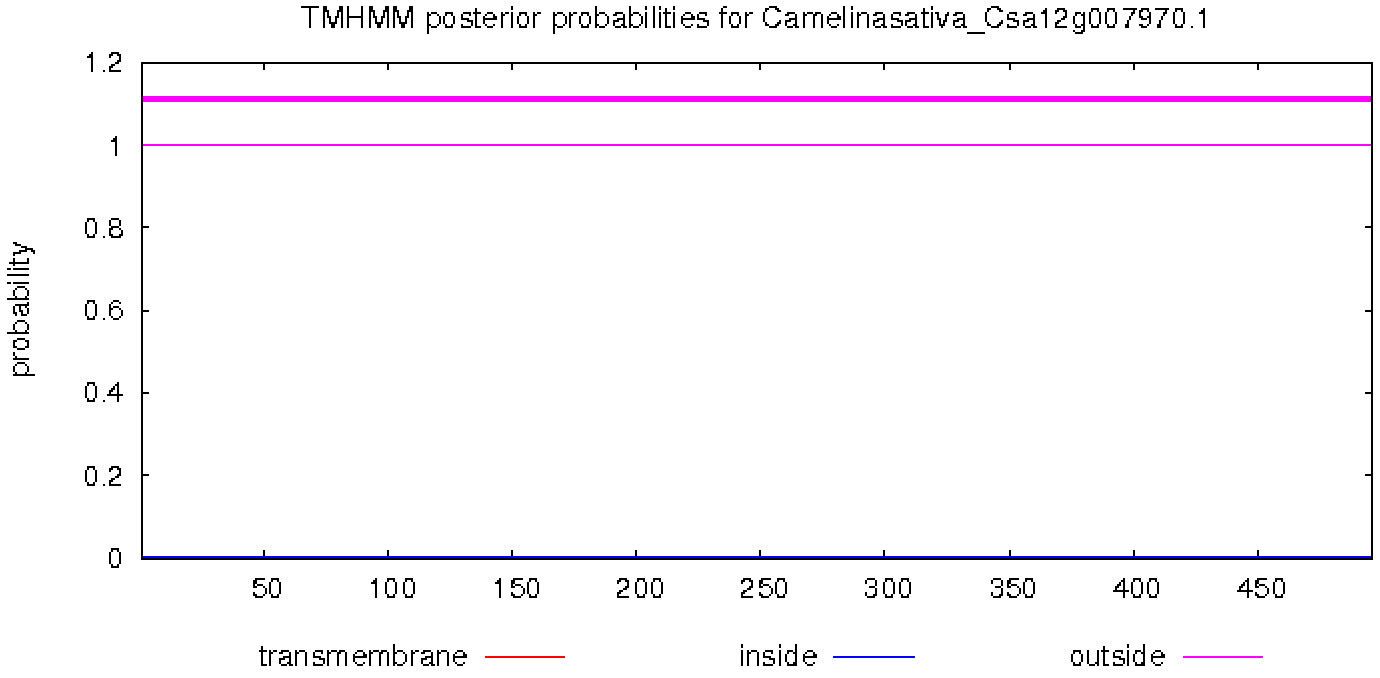

plot in postscript, script for making the plot in gnuplot, data for plot

---

```
# Camelinasativa_Csa12g019250.1 Length: 496
# Camelinasativa_Csa12g019250.1 Number of predicted TMHs:  0
# Camelinasativa_Csa12g019250.1 Exp number of AAs in TMHs: 4.26602
# Camelinasativa_Csa12g019250.1 Exp number, first 60 AAs:  0
# Camelinasativa_Csa12g019250.1 Total prob of N-in:        0.16670
Camelinasativa_Csa12g019250.1	TMHMM2.0	outside	     1   496
```

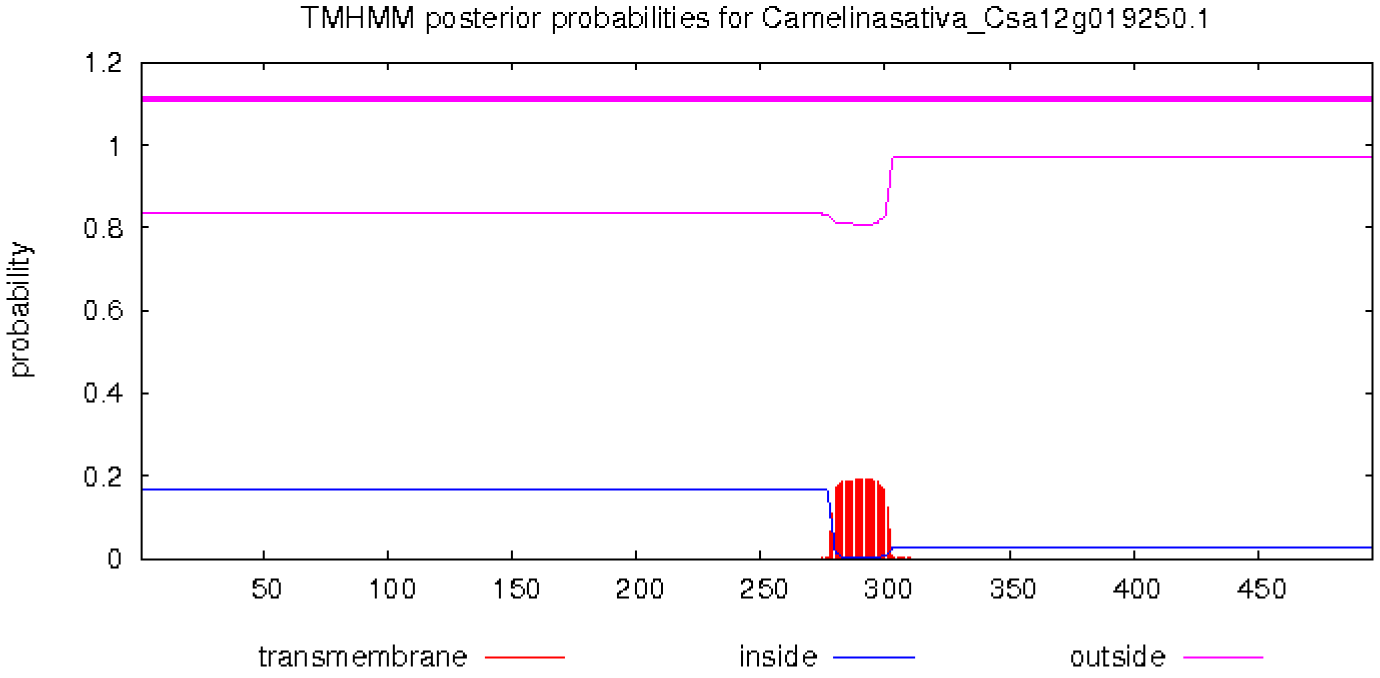

plot in postscript, script for making the plot in gnuplot, data for plot

---

```
# Camelinasativa_Csa12g021980.1 Length: 352
# Camelinasativa_Csa12g021980.1 Number of predicted TMHs:  0
# Camelinasativa_Csa12g021980.1 Exp number of AAs in TMHs: 0
# Camelinasativa_Csa12g021980.1 Exp number, first 60 AAs:  0
# Camelinasativa_Csa12g021980.1 Total prob of N-in:        0.00561
Camelinasativa_Csa12g021980.1   TMHMM2.0        outside      1   352
```

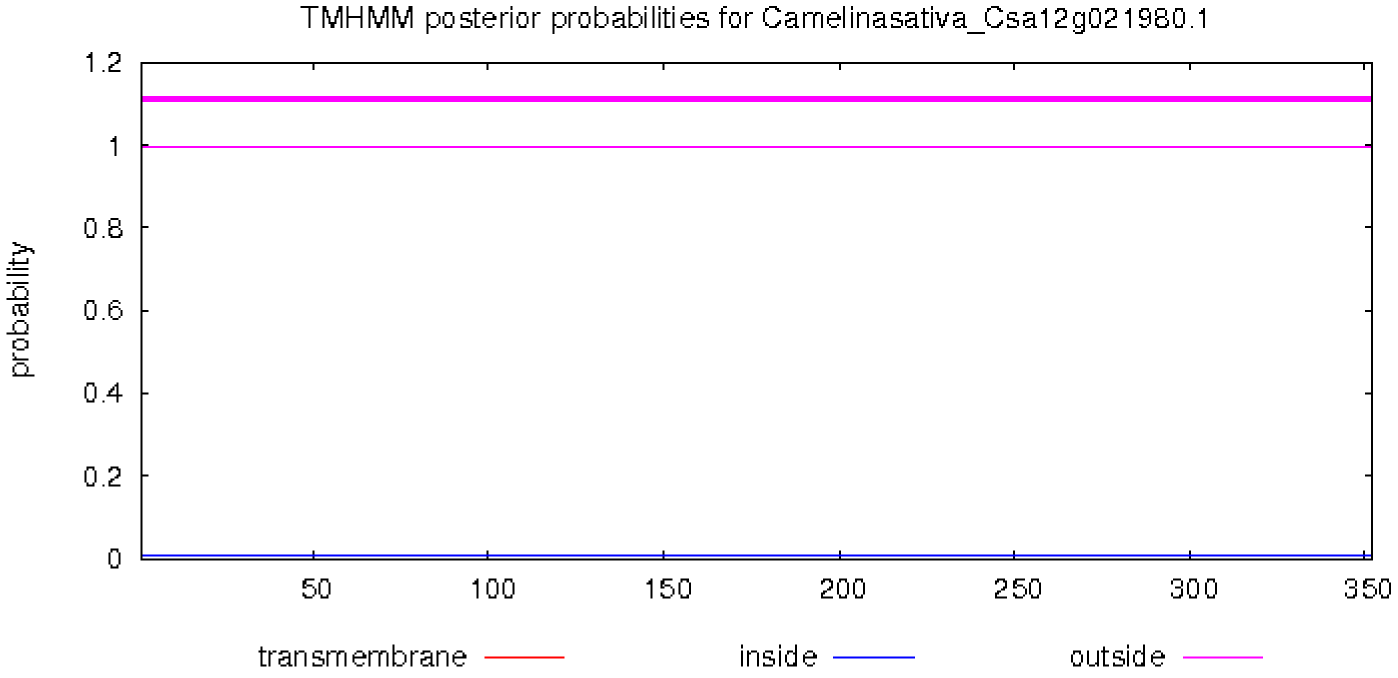

plot in postscript, script for making the plot in gnuplot, data for plot

```
# Camelinasativa_Csa12g022020.1 Length: 306
# Camelinasativa_Csa12g022020.1 Number of predicted TMHs:  0
# Camelinasativa_Csa12g022020.1 Exp number of AAs in TMHs: 0.00171
# Camelinasativa_Csa12g022020.1 Exp number, first 60 AAs:  0
# Camelinasativa_Csa12g022020.1 Total prob of N-in:        0.09778
Camelinasativa_Csa12g022020.1   TMHMM2.0        outside      1   306
```

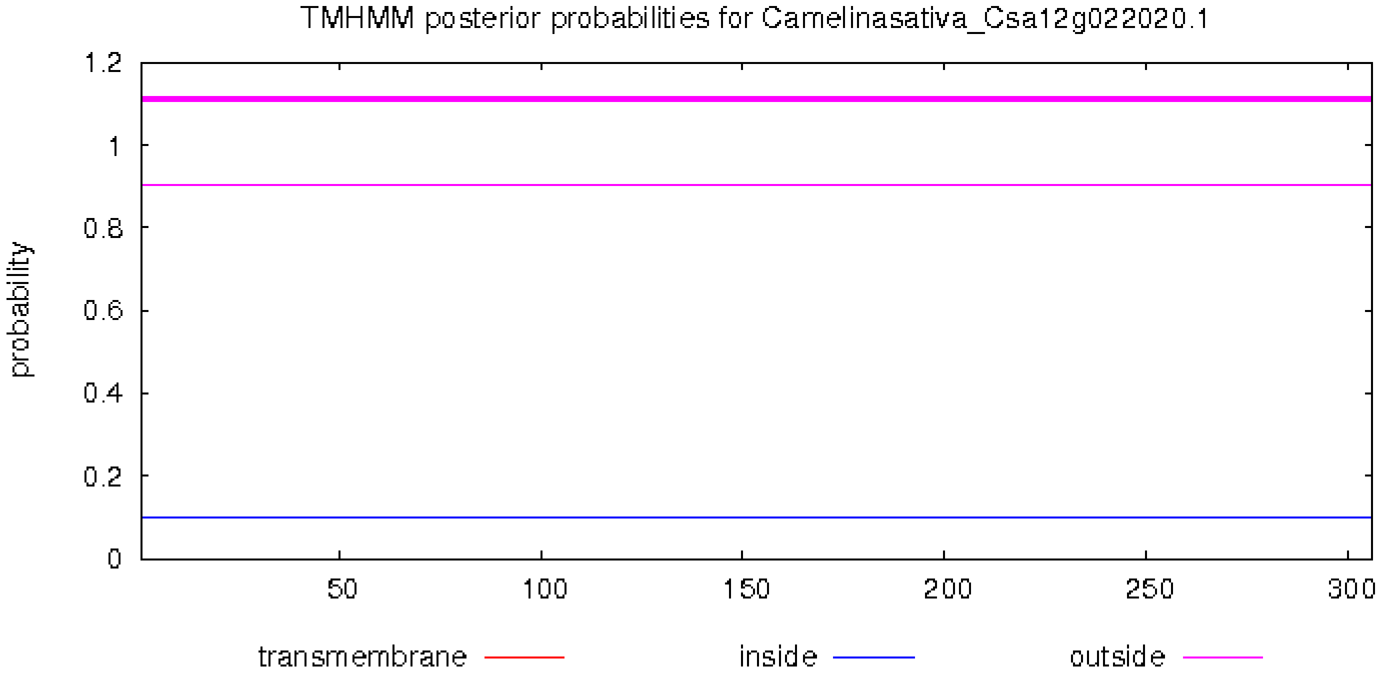

plot in postscript, script for making the plot in gnuplot, data for plot

```
# Camelinasativa_Csa12g023460.1 Length: 240
# Camelinasativa_Csa12g023460.1 Number of predicted TMHs:  0
# Camelinasativa_Csa12g023460.1 Exp number of AAs in TMHs: 0.00292
# Camelinasativa_Csa12g023460.1 Exp number, first 60 AAs:  6e-05
# Camelinasativa_Csa12g023460.1 Total prob of N-in:        0.04368
Camelinasativa_Csa12g023460.1   TMHMM2.0        outside      1   240
```

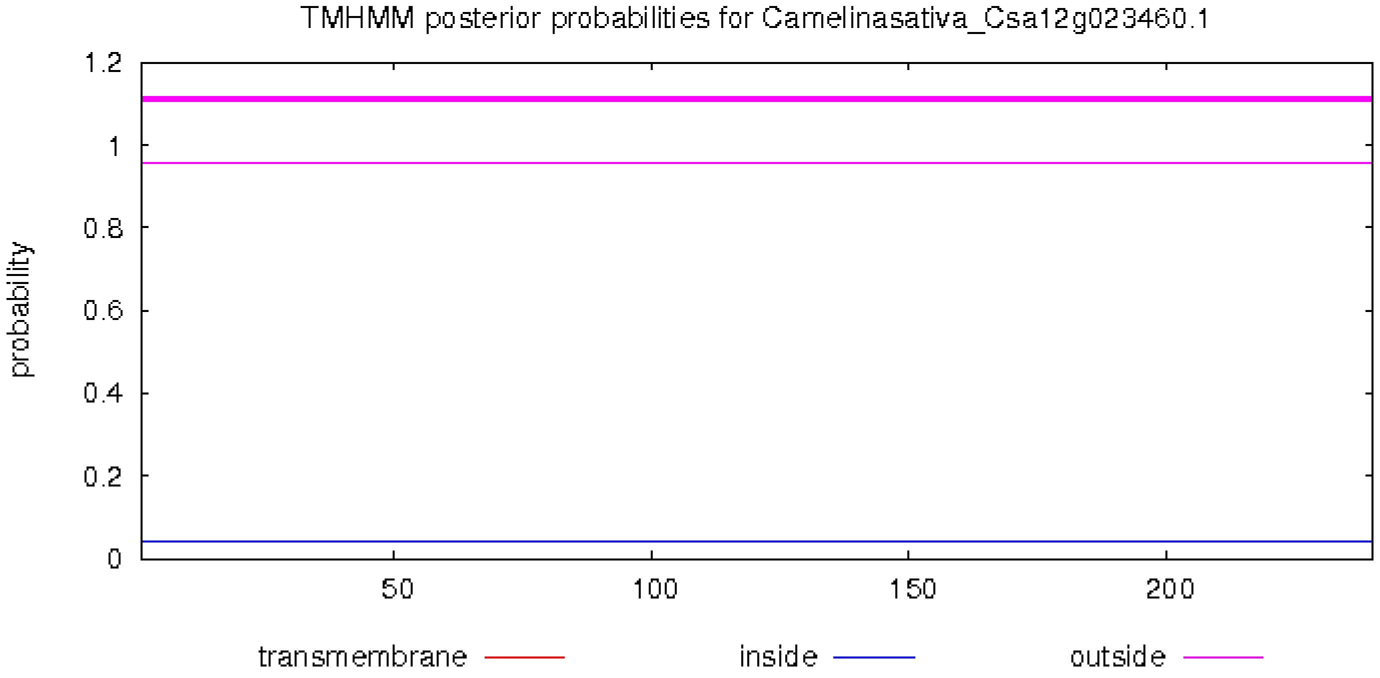

plot in postscript, script for making the plot in gnuplot, data for plot

---

```
# Camelinasativa_Csa12g045480.1 Length: 363
# Camelinasativa_Csa12g045480.1 Number of predicted TMHs:  0
# Camelinasativa_Csa12g045480.1 Exp number of AAs in TMHs: 0
# Camelinasativa_Csa12g045480.1 Exp number, first 60 AAs:  0
# Camelinasativa_Csa12g045480.1 Total prob of N-in:        0.00067
Camelinasativa_Csa12g045480.1   TMHMM2.0        outside      1   363
```

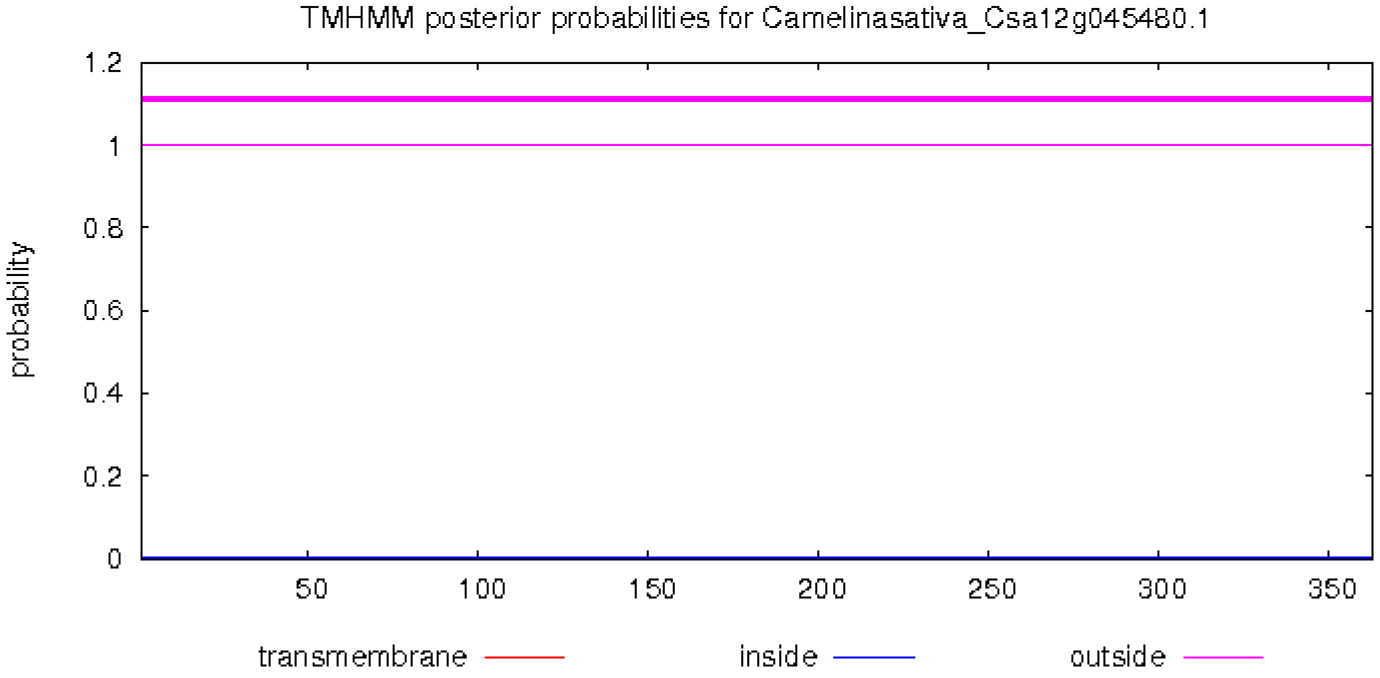

plot in postscript, script for making the plot in gnuplot, data for plot

---

```
# Camelinasativa_Csa12g077490.1 Length: 115
# Camelinasativa_Csa12g077490.1 Number of predicted TMHs:  0
# Camelinasativa_Csa12g077490.1 Exp number of AAs in TMHs: 0.84508
# Camelinasativa_Csa12g077490.1 Exp number, first 60 AAs:  0.0504
# Camelinasativa_Csa12g077490.1 Total prob of N-in:        0.18570
Camelinasativa_Csa12g077490.1   TMHMM2.0        outside      1   115
```

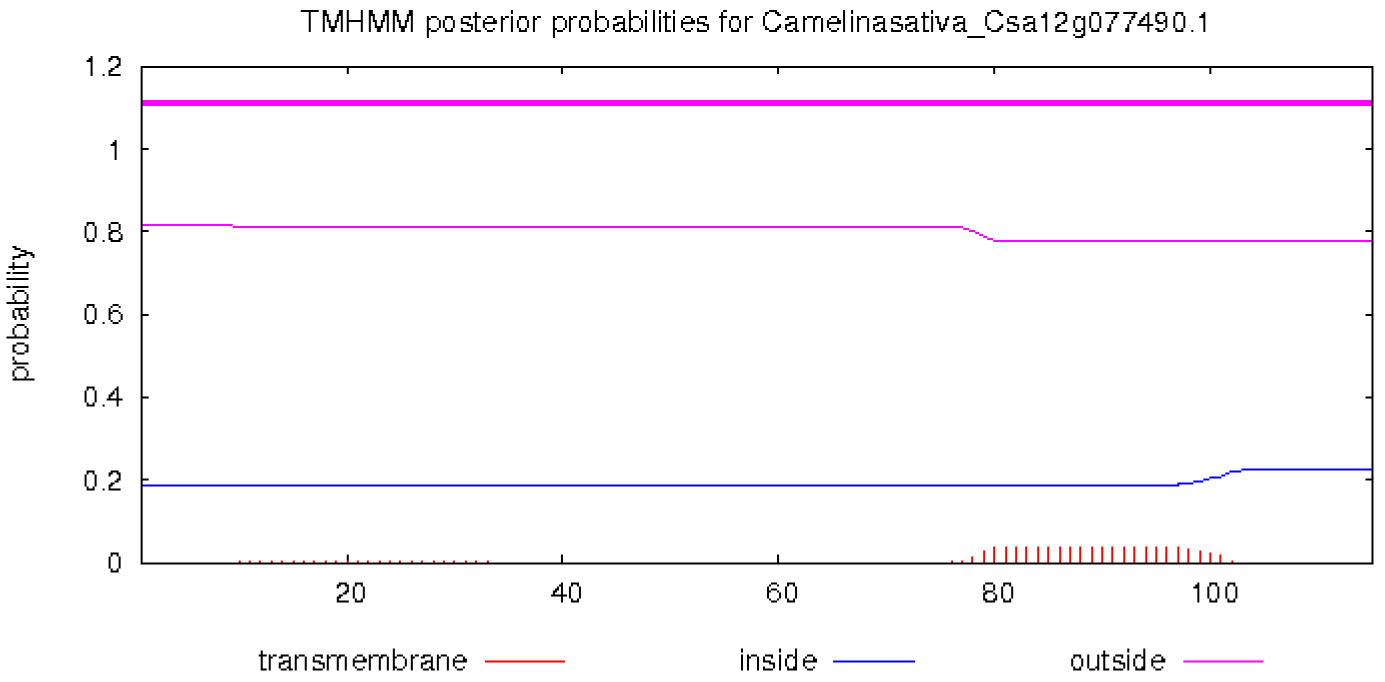

plot in postscript, script for making the plot in gnuplot, data for plot

```
# Camelinasativa_Csa12g081560.1 Length: 363
# Camelinasativa_Csa12g081560.1 Number of predicted TMHs:  0
# Camelinasativa_Csa12g081560.1 Exp number of AAs in TMHs: 0.00182
# Camelinasativa_Csa12g081560.1 Exp number, first 60 AAs:  0
# Camelinasativa_Csa12g081560.1 Total prob of N-in:        0.01700
Camelinasativa_Csa12g081560.1   TMHMM2.0        outside      1   363
```

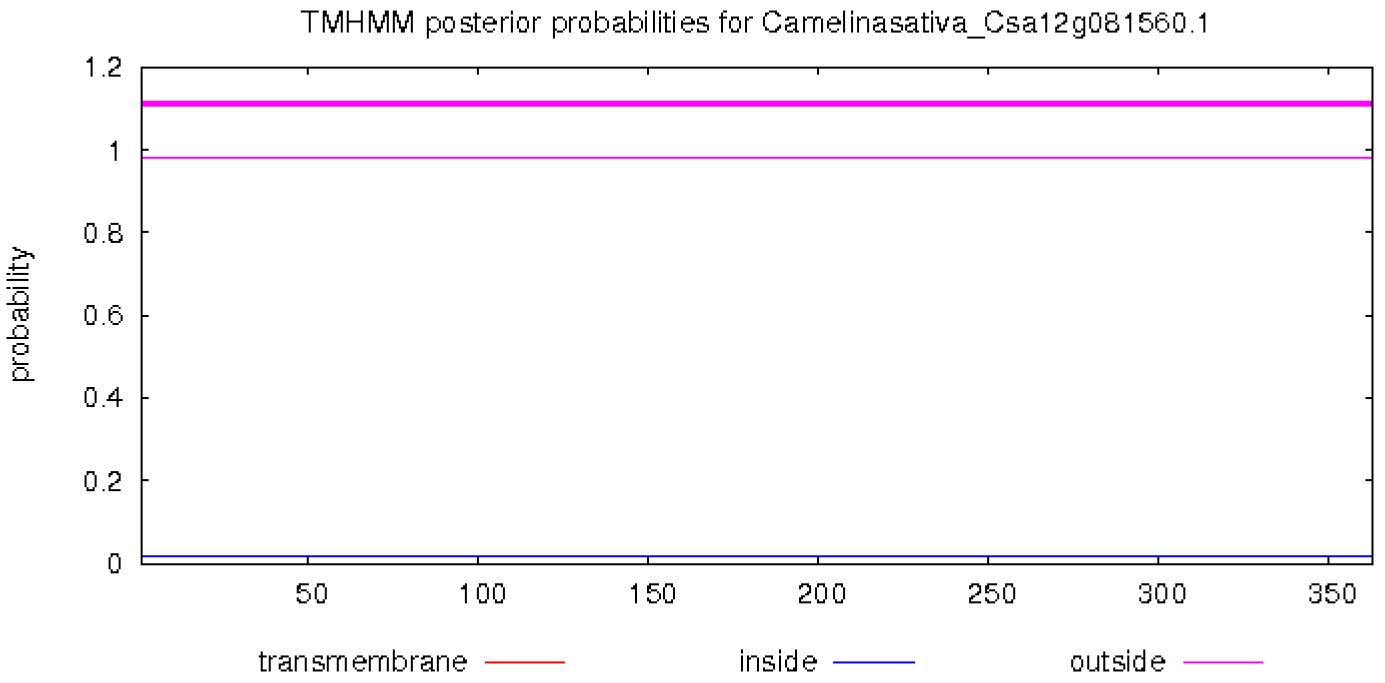

plot in postscript, script for making the plot in gnuplot, data for plot

```
# Camelinasativa_Csa12g081820.1 Length: 476
# Camelinasativa_Csa12g081820.1 Number of predicted TMHs:  0
# Camelinasativa_Csa12g081820.1 Exp number of AAs in TMHs: 0.03694
# Camelinasativa_Csa12g081820.1 Exp number, first 60 AAs:  0
# Camelinasativa_Csa12g081820.1 Total prob of N-in:        0.00287
Camelinasativa_Csa12g081820.1   TMHMM2.0        outside      1   476
```

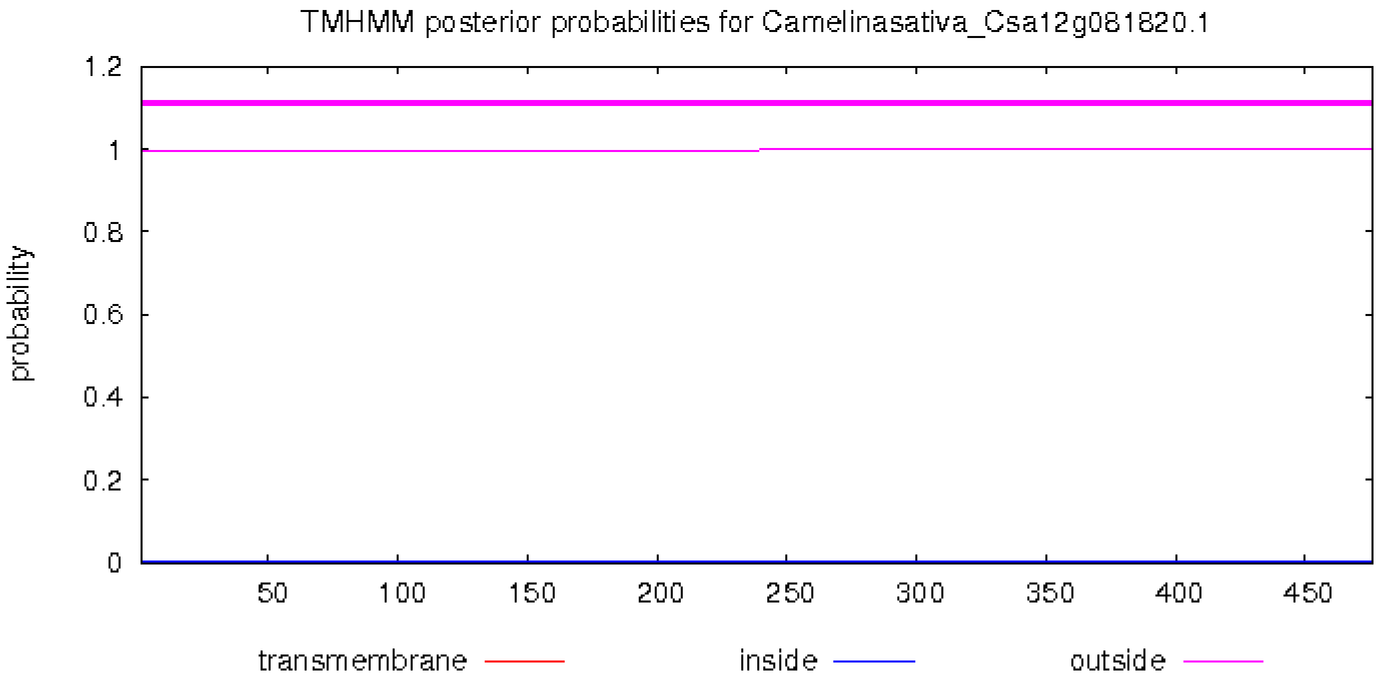

plot in postscript, script for making the plot in gnuplot, data for plot

```
# Camelinasativa_Csa13g006400.1 Length: 386
# Camelinasativa_Csa13g006400.1 Number of predicted TMHs:  0
# Camelinasativa_Csa13g006400.1 Exp number of AAs in TMHs: 0.01244
# Camelinasativa_Csa13g006400.1 Exp number, first 60 AAs:  0.00516
# Camelinasativa_Csa13g006400.1 Total prob of N-in:        0.02709
Camelinasativa_Csa13g006400.1   TMHMM2.0        outside      1   386
```

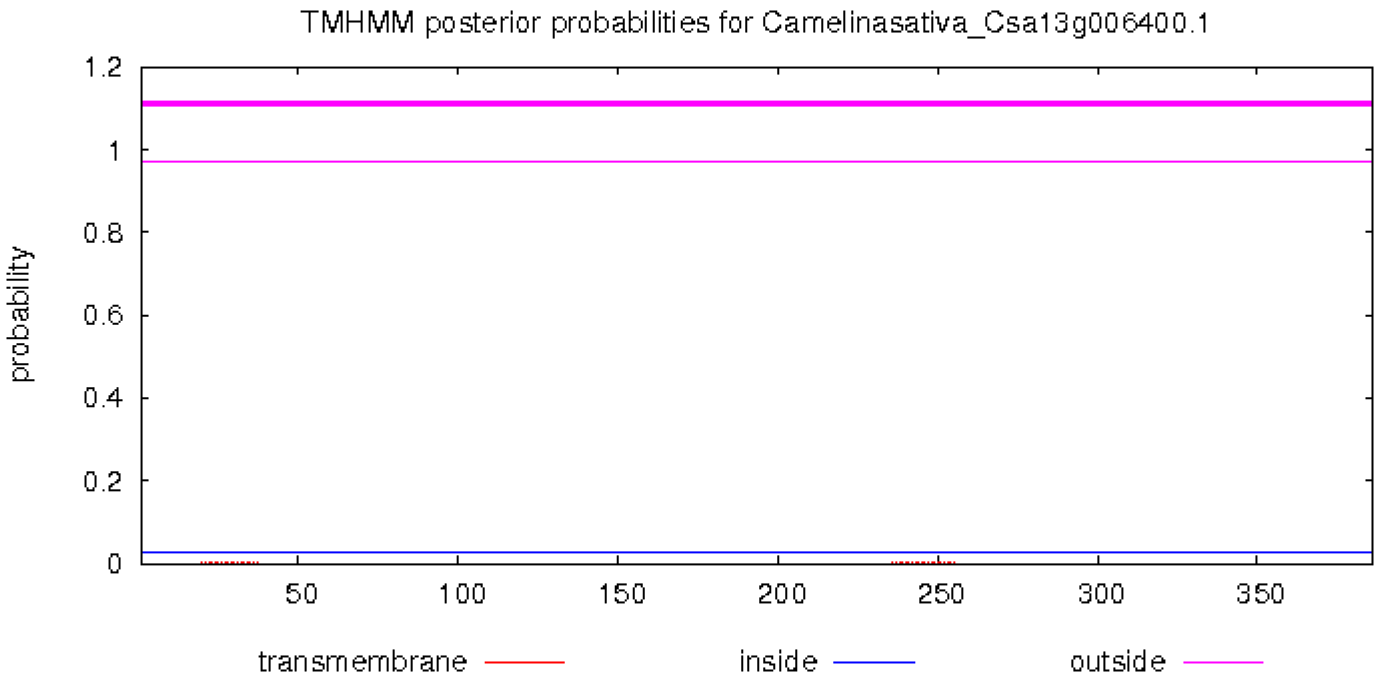

plot in postscript, script for making the plot in gnuplot, data for plot

```
# Camelinasativa_Csa13g006410.1 Length: 562
# Camelinasativa_Csa13g006410.1 Number of predicted TMHs:  1
# Camelinasativa_Csa13g006410.1 Exp number of AAs in TMHs: 21.14118
# Camelinasativa_Csa13g006410.1 Exp number, first 60 AAs:  0
# Camelinasativa_Csa13g006410.1 Total prob of N-in:        0.04469
Camelinasativa_Csa13g006410.1   TMHMM2.0        outside      1   538
Camelinasativa_Csa13g006410.1   TMHMM2.0        TMhelix    539   561
Camelinasativa_Csa13g006410.1   TMHMM2.0        inside     562   562
```

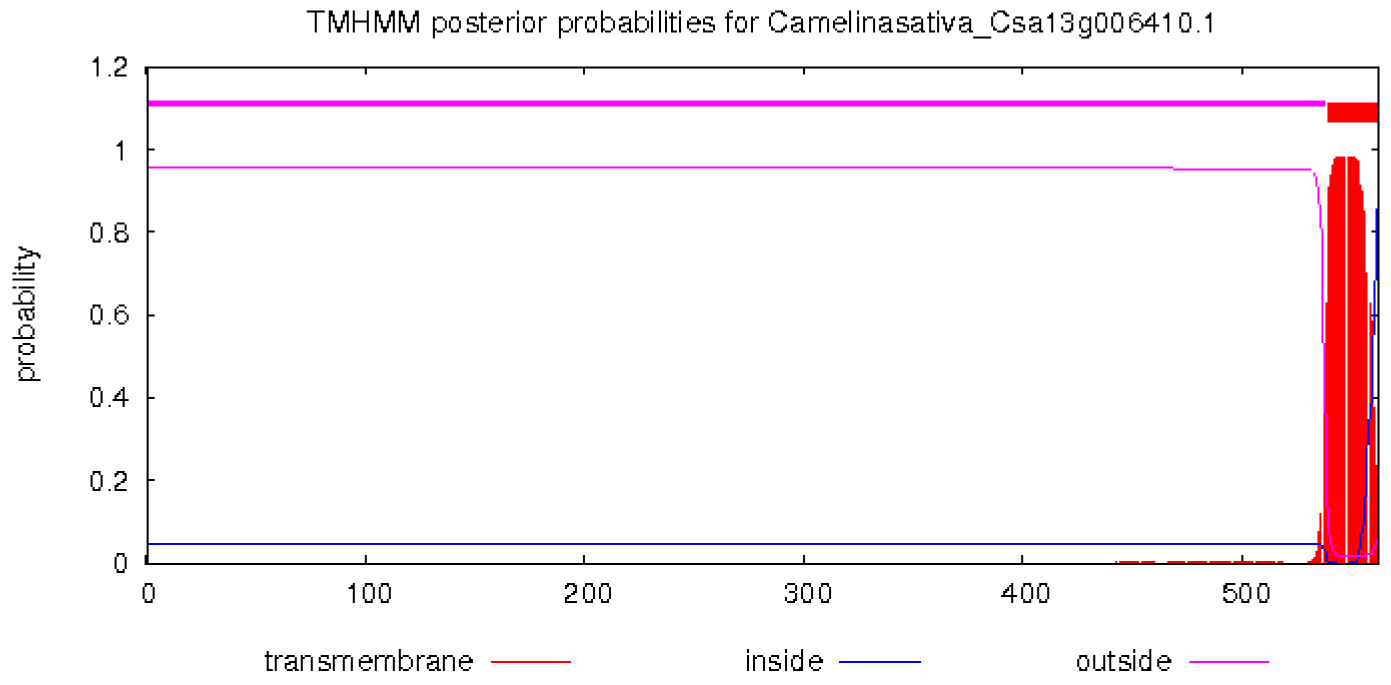

plot in postscript, script for making the plot in gnuplot, data for plot

---

```
# Camelinasativa_Csa13g009710.1 Length: 336
# Camelinasativa_Csa13g009710.1 Number of predicted TMHs:  0
# Camelinasativa_Csa13g009710.1 Exp number of AAs in TMHs: 0.00021
# Camelinasativa_Csa13g009710.1 Exp number, first 60 AAs:  0
# Camelinasativa_Csa13g009710.1 Total prob of N-in:        0.00461
Camelinasativa_Csa13g009710.1   TMHMM2.0        outside      1   336
```

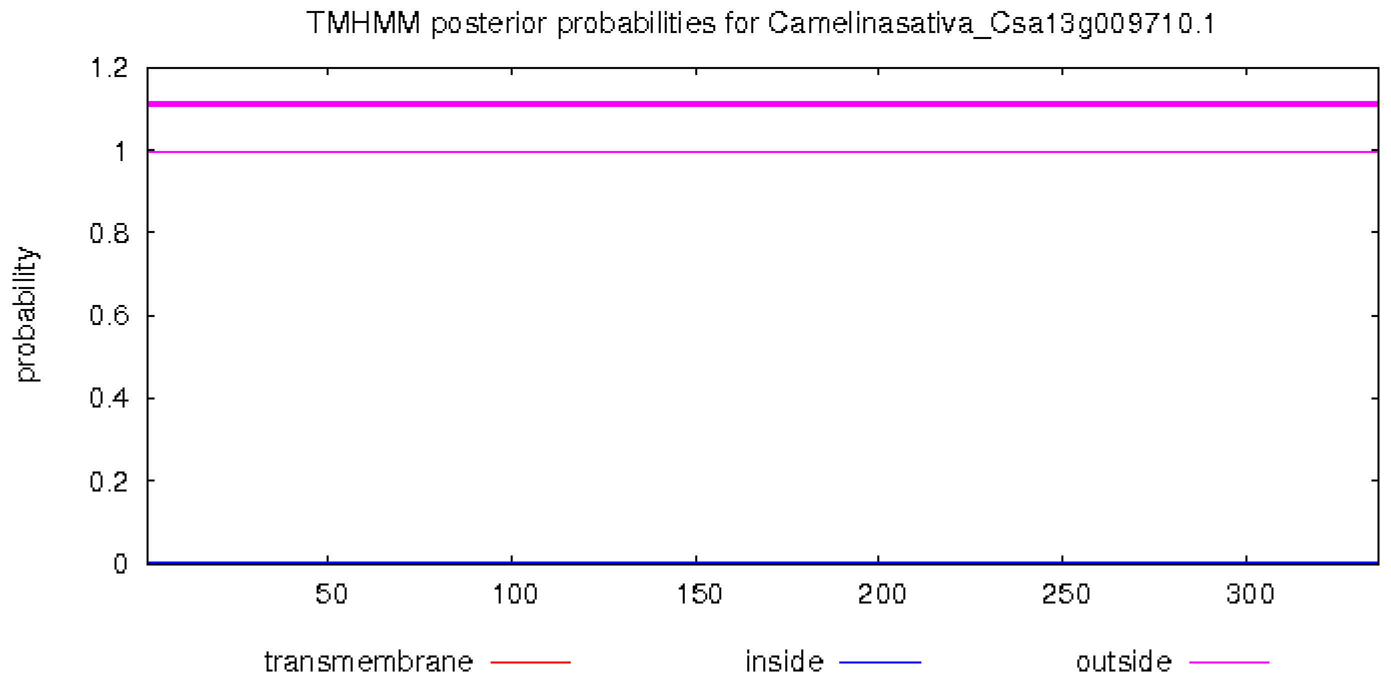

plot in postscript, script for making the plot in gnuplot, data for plot

```
# Camelinasativa_Csa13g010890.1 Length: 230
# Camelinasativa_Csa13g010890.1 Number of predicted TMHs:  0
# Camelinasativa_Csa13g010890.1 Exp number of AAs in TMHs: 0.00098
# Camelinasativa_Csa13g010890.1 Exp number, first 60 AAs:  0.00032
# Camelinasativa_Csa13g010890.1 Total prob of N-in:        0.05281
Camelinasativa_Csa13g010890.1	TMHMM2.0	outside	     1   230
```

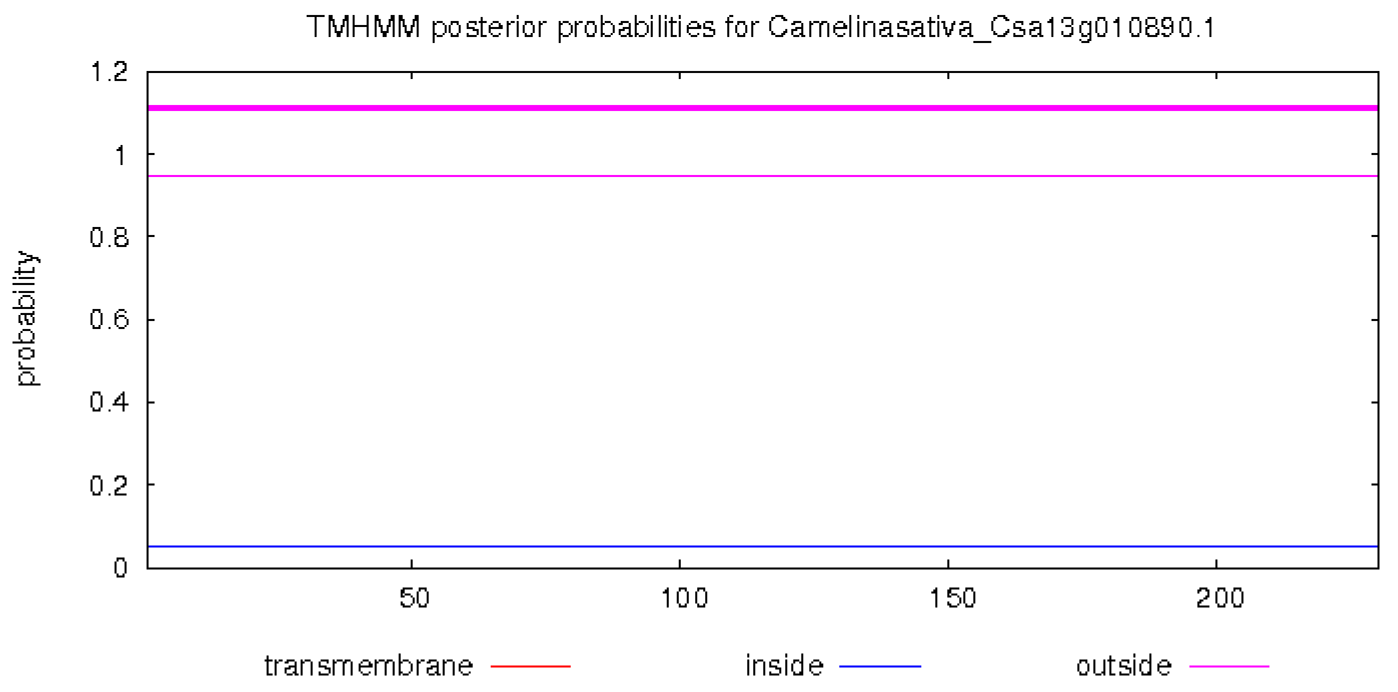

plot in postscript, script for making the plot in gnuplot, data for plot

```
# Camelinasativa_Csa13g011010.1 Length: 399
# Camelinasativa_Csa13g011010.1 Number of predicted TMHs:  0
# Camelinasativa_Csa13g011010.1 Exp number of AAs in TMHs: 0.00018
# Camelinasativa_Csa13g011010.1 Exp number, first 60 AAs:  0.00018
# Camelinasativa_Csa13g011010.1 Total prob of N-in:        0.00355
Camelinasativa_Csa13g011010.1	TMHMM2.0	outside	     1   399
```

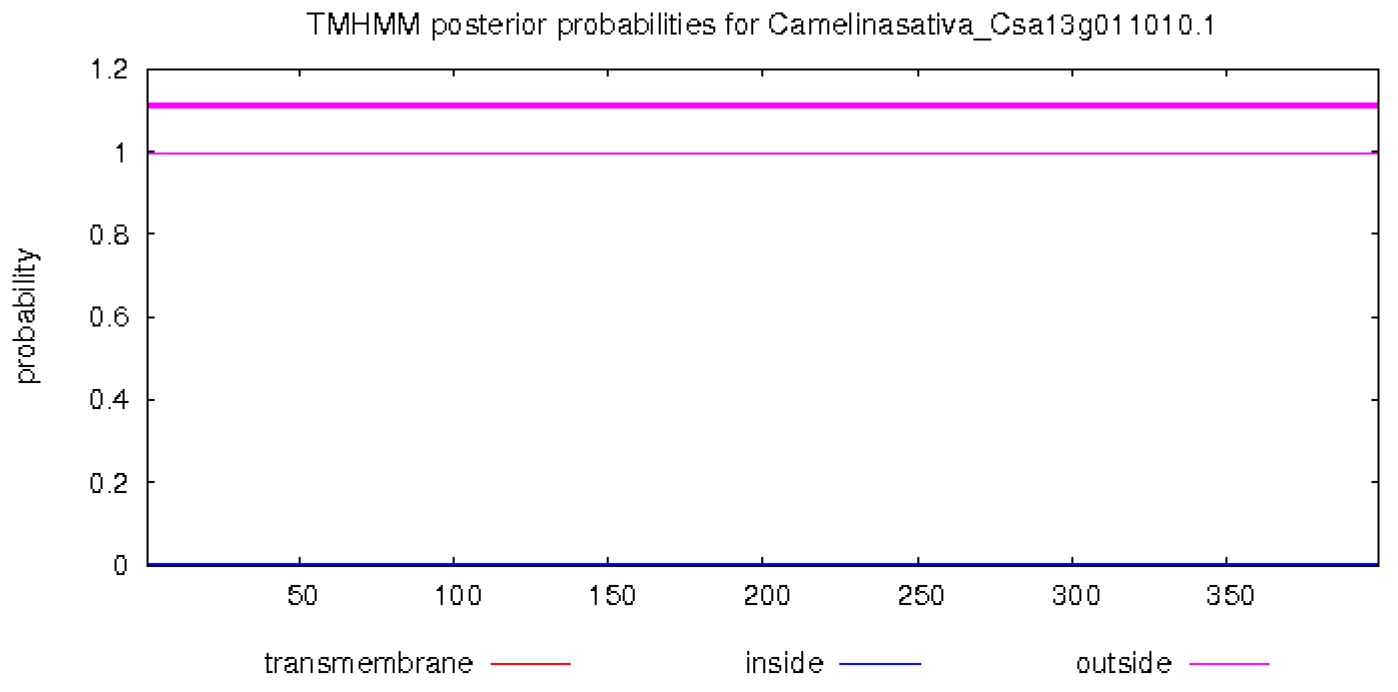

plot in postscript, script for making the plot in gnuplot, data for plot

```
# Camelinasativa_Csa13g015790.1 Length: 257
# Camelinasativa_Csa13g015790.1 Number of predicted TMHs:  0
# Camelinasativa_Csa13g015790.1 Exp number of AAs in TMHs: 0.00036
# Camelinasativa_Csa13g015790.1 Exp number, first 60 AAs:  0
# Camelinasativa_Csa13g015790.1 Total prob of N-in:        0.10509
Camelinasativa_Csa13g015790.1   TMHMM2.0        outside      1   257
```

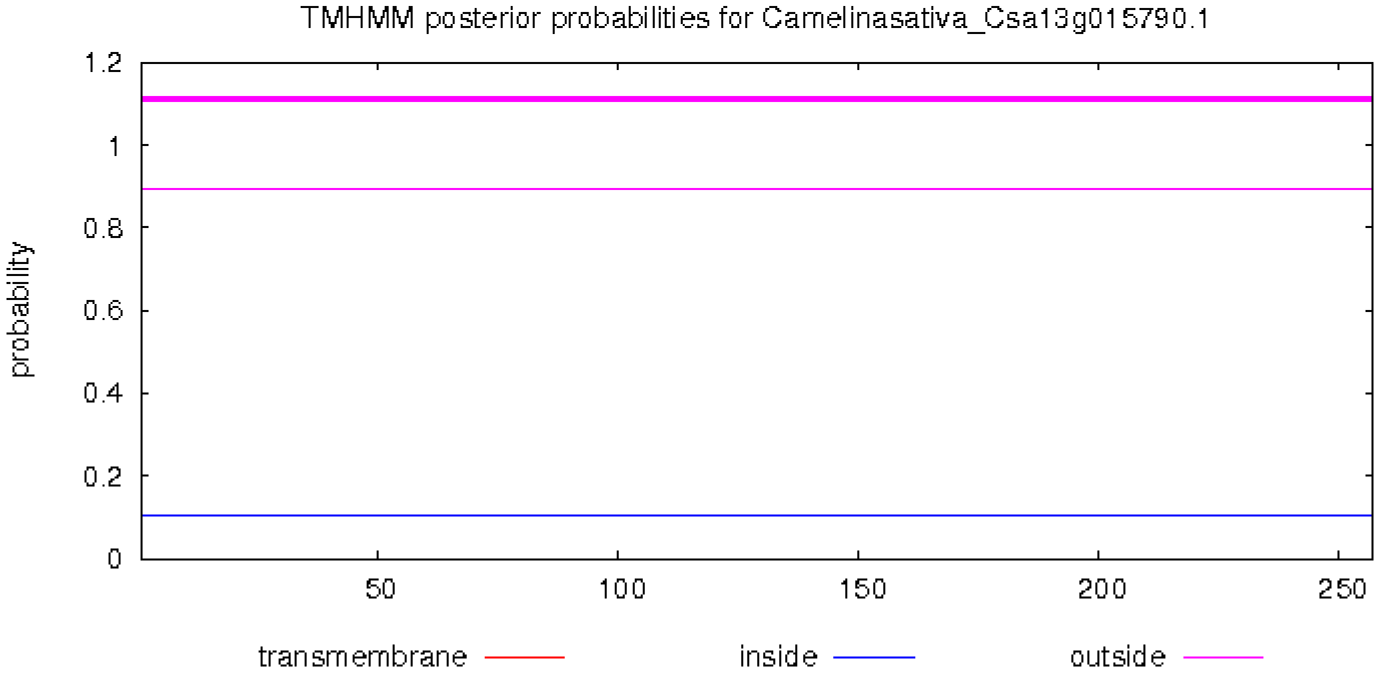

plot in postscript, script for making the plot in gnuplot, data for plot

```
# Camelinasativa_Csa13g016770.1 Length: 206
# Camelinasativa_Csa13g016770.1 Number of predicted TMHs:  0
# Camelinasativa_Csa13g016770.1 Exp number of AAs in TMHs: 1.07011
# Camelinasativa_Csa13g016770.1 Exp number, first 60 AAs:  0.69505
# Camelinasativa_Csa13g016770.1 Total prob of N-in:        0.20125
Camelinasativa_Csa13g016770.1   TMHMM2.0        outside      1   206
```

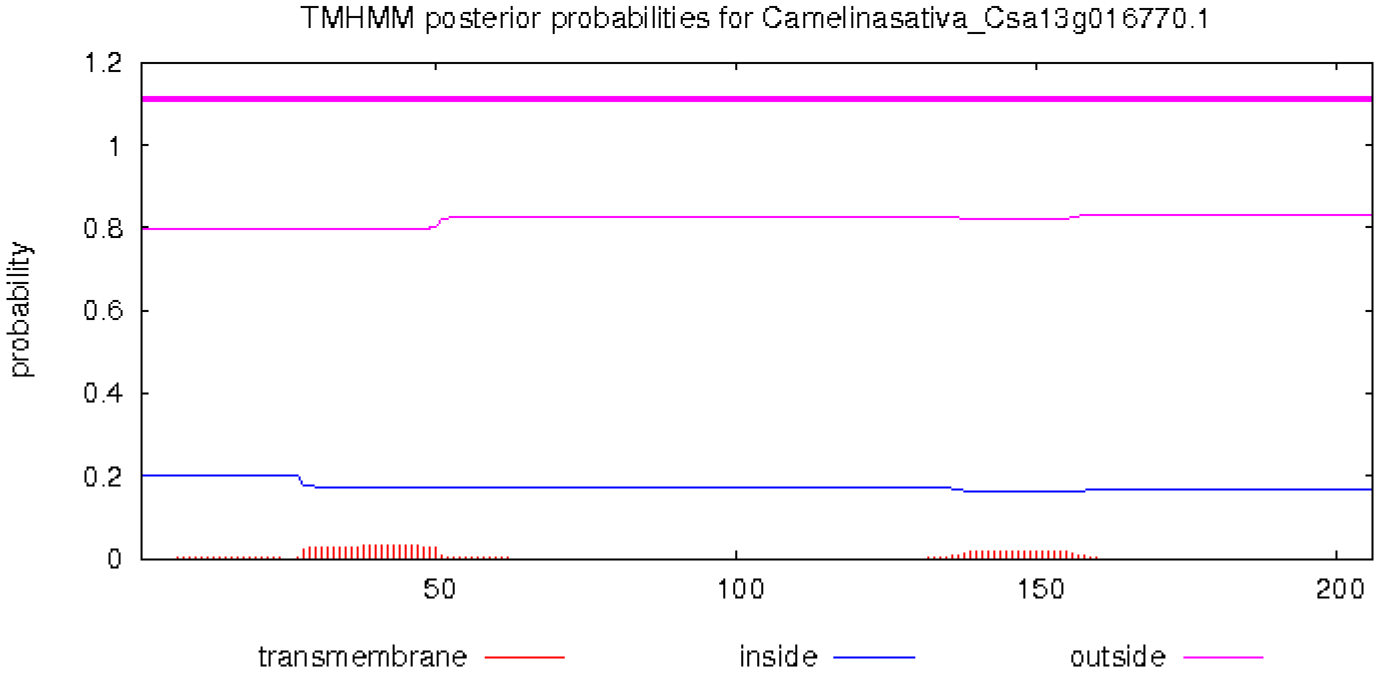

plot in postscript, script for making the plot in gnuplot, data for plot

```
# Camelinasativa_Csa13g017310.1 Length: 357
# Camelinasativa_Csa13g017310.1 Number of predicted TMHs:  0
# Camelinasativa_Csa13g017310.1 Exp number of AAs in TMHs: 0.0002
# Camelinasativa_Csa13g017310.1 Exp number, first 60 AAs:  0
# Camelinasativa_Csa13g017310.1 Total prob of N-in:        0.00931
Camelinasativa_Csa13g017310.1   TMHMM2.0        outside      1   357
```

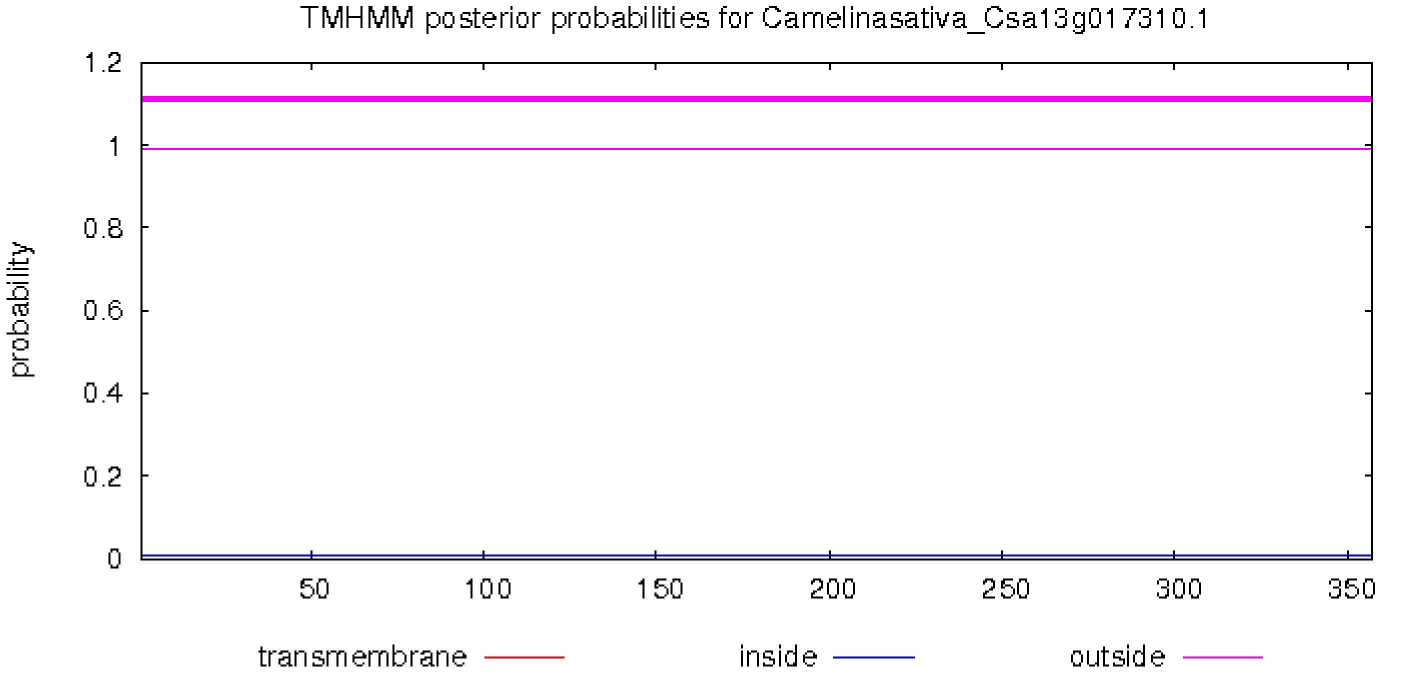

plot in postscript, script for making the plot in gnuplot, data for plot

---

```
# Camelinasativa_Csa13g020020.1 Length: 502
# Camelinasativa_Csa13g020020.1 Number of predicted TMHs:  0
# Camelinasativa_Csa13g020020.1 Exp number of AAs in TMHs: 0.00036
# Camelinasativa_Csa13g020020.1 Exp number, first 60 AAs:  0
# Camelinasativa_Csa13g020020.1 Total prob of N-in:        0.00013
Camelinasativa_Csa13g020020.1   TMHMM2.0        outside      1   502
```

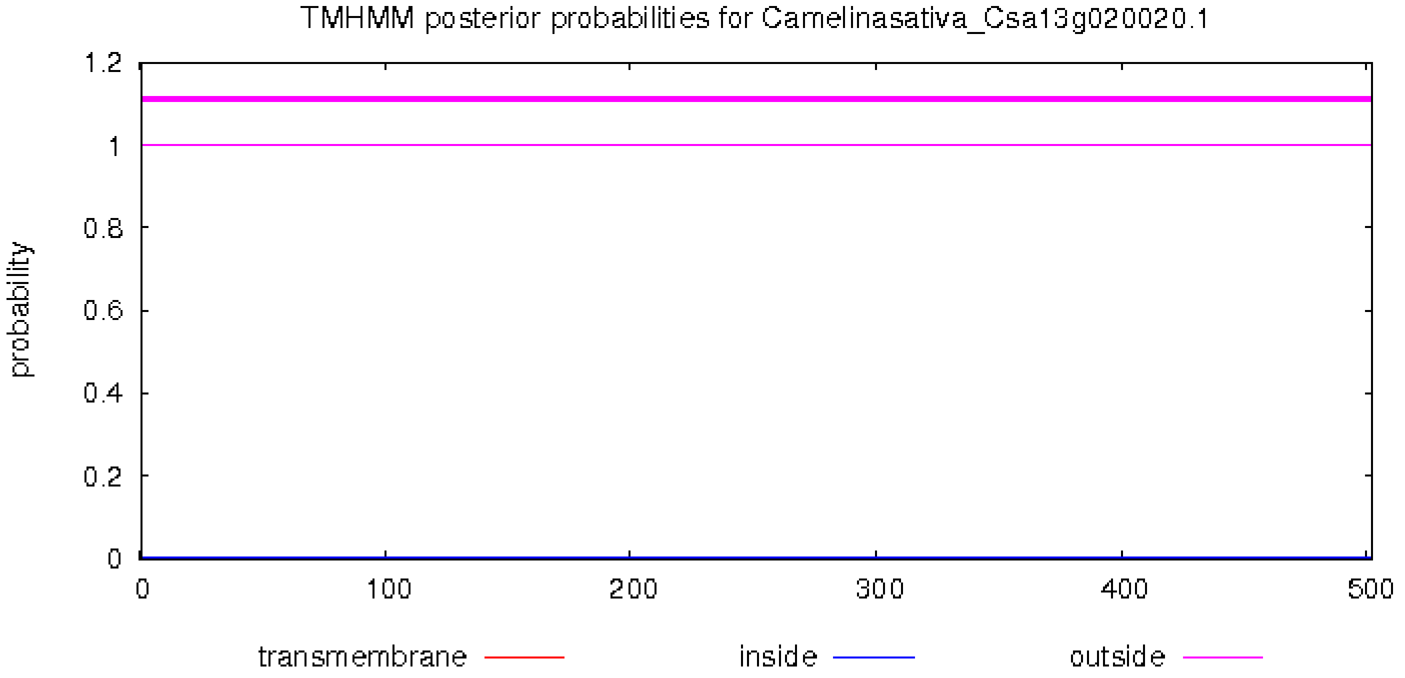

plot in postscript, script for making the plot in gnuplot, data for plot

---

```
# Camelinasativa_Csa13g020920.1 Length: 160
# Camelinasativa_Csa13g020920.1 Number of predicted TMHs:  0
# Camelinasativa_Csa13g020920.1 Exp number of AAs in TMHs: 0.00384
# Camelinasativa_Csa13g020920.1 Exp number, first 60 AAs:  0
# Camelinasativa_Csa13g020920.1 Total prob of N-in:        0.32402
Camelinasativa_Csa13g020920.1   TMHMM2.0        outside      1   160
```

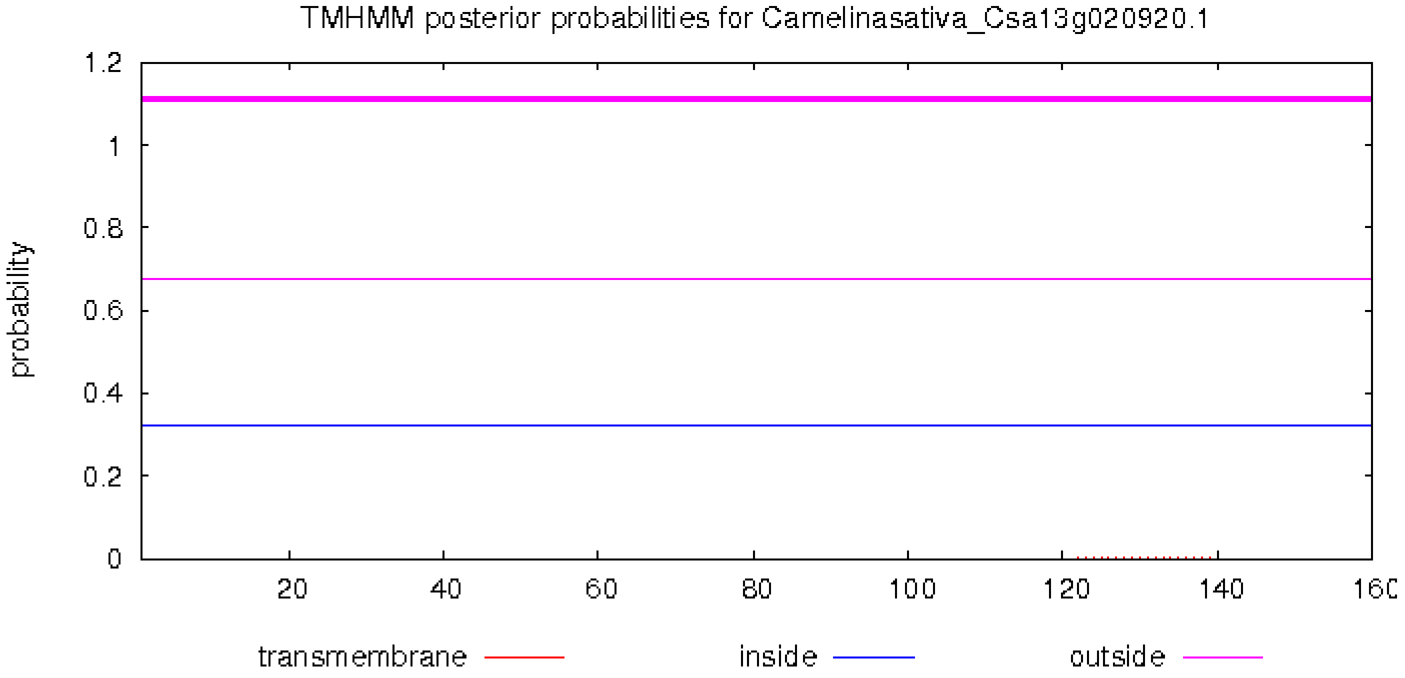

plot in postscript, script for making the plot in gnuplot, data for plot

---

```
# Camelinasativa_Csa13g020970.1 Length: 168
# Camelinasativa_Csa13g020970.1 Number of predicted TMHs:  0
# Camelinasativa_Csa13g020970.1 Exp number of AAs in TMHs: 0.01019
# Camelinasativa_Csa13g020970.1 Exp number, first 60 AAs:  0
# Camelinasativa_Csa13g020970.1 Total prob of N-in:        0.32564
Camelinasativa_Csa13g020970.1   TMHMM2.0        outside      1   168
```

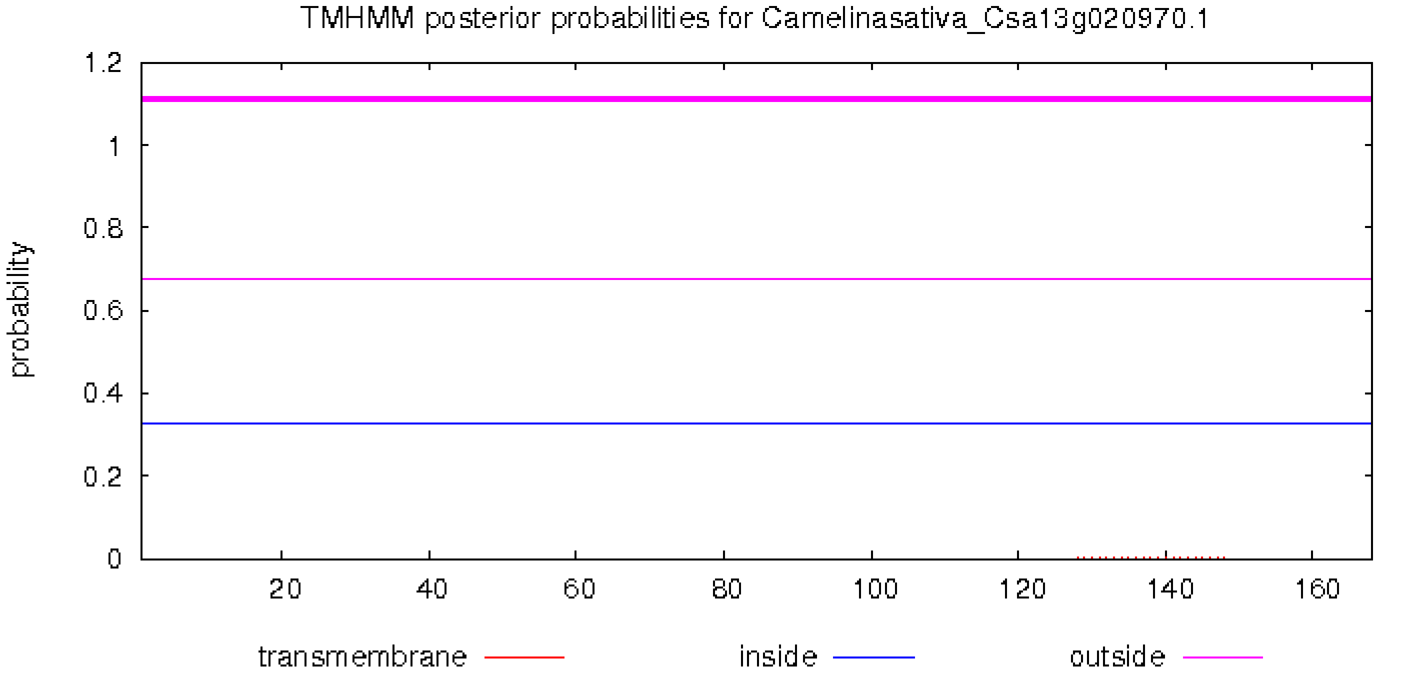

plot in postscript, script for making the plot in gnuplot, data for plot

---

```
# Camelinasativa_Csa13g021170.1 Length: 336
# Camelinasativa_Csa13g021170.1 Number of predicted TMHs:  0
# Camelinasativa_Csa13g021170.1 Exp number of AAs in TMHs: 0.00057
# Camelinasativa_Csa13g021170.1 Exp number, first 60 AAs:  0.00021
# Camelinasativa_Csa13g021170.1 Total prob of N-in:        0.00842
Camelinasativa_Csa13g021170.1	TMHMM2.0	outside	     1   336
```

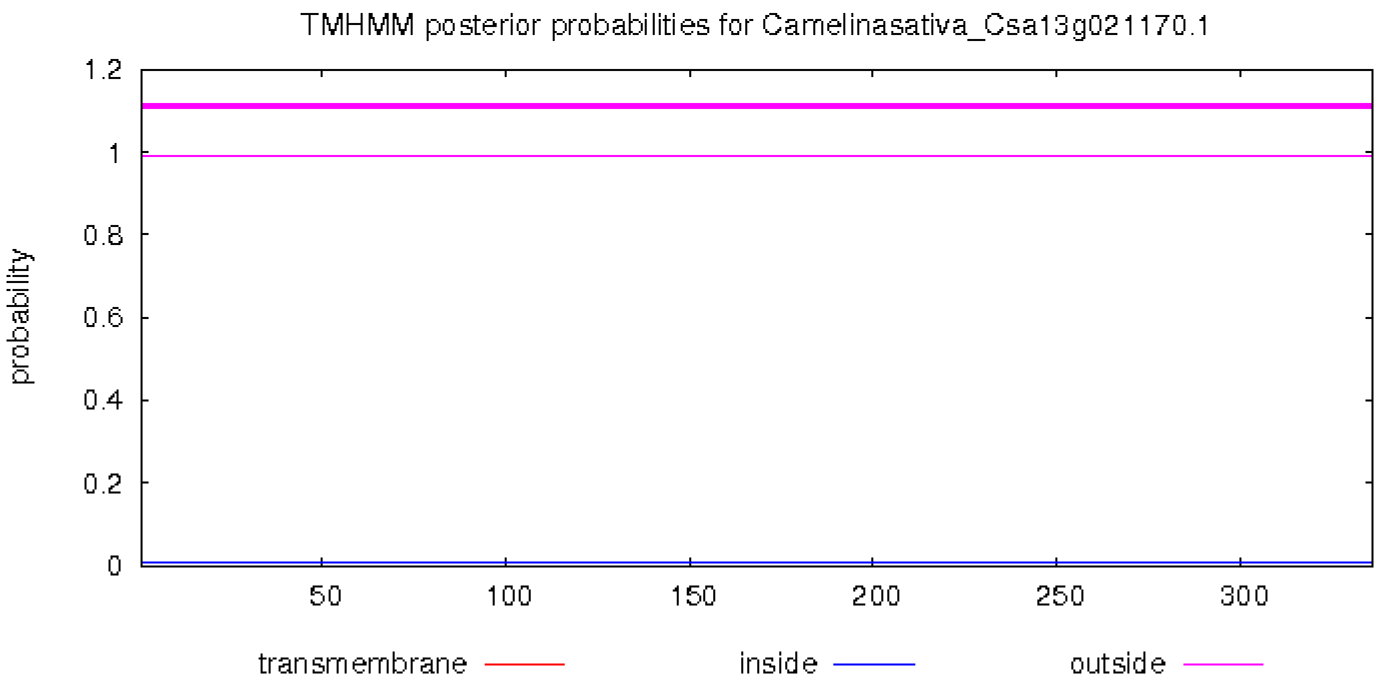

plot in postscript, script for making the plot in gnuplot, data for plot

---

```
# Camelinasativa_Csa13g021200.1 Length: 151
# Camelinasativa_Csa13g021200.1 Number of predicted TMHs:  0
# Camelinasativa_Csa13g021200.1 Exp number of AAs in TMHs: 0.00115
# Camelinasativa_Csa13g021200.1 Exp number, first 60 AAs:  0
# Camelinasativa_Csa13g021200.1 Total prob of N-in:        0.23564
Camelinasativa_Csa13g021200.1	TMHMM2.0	outside	     1   151
```

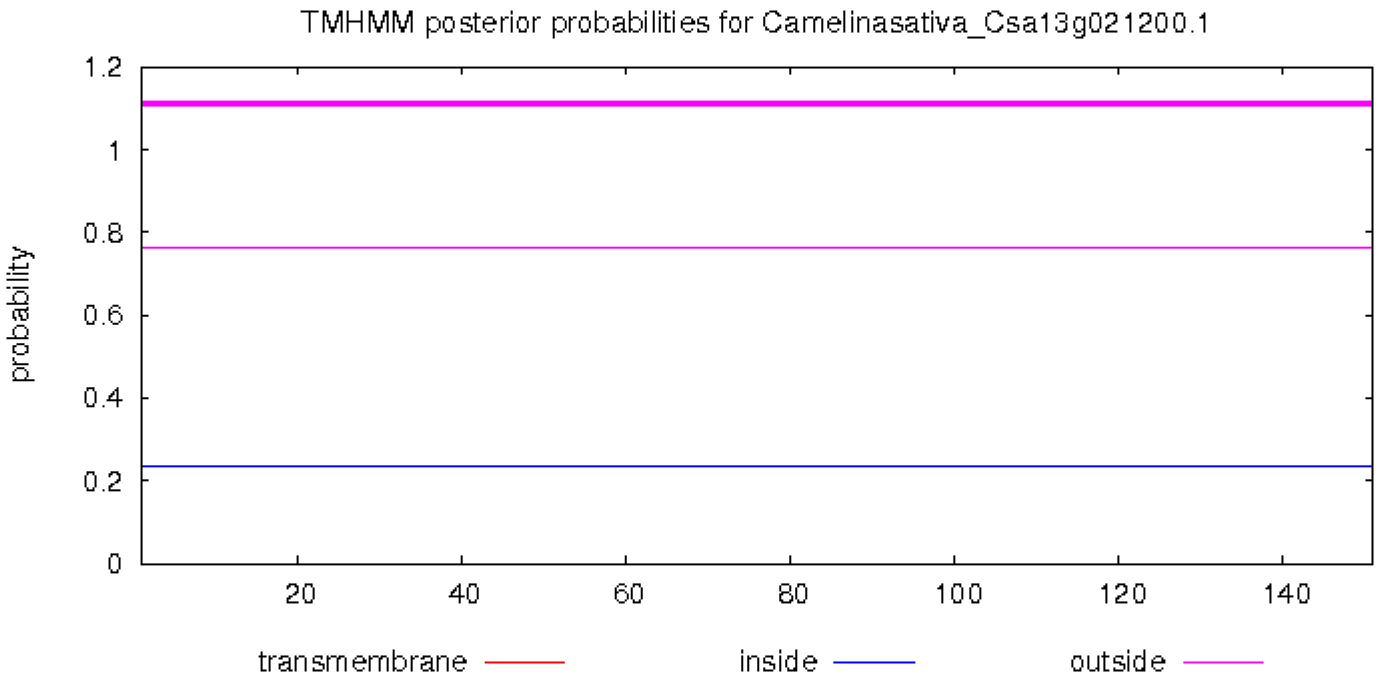

plot in postscript, script for making the plot in gnuplot, data for plot

---

```
# Camelinasativa_Csa13g025730.1 Length: 341
# Camelinasativa_Csa13g025730.1 Number of predicted TMHs:  0
# Camelinasativa_Csa13g025730.1 Exp number of AAs in TMHs: 8.93808
# Camelinasativa_Csa13g025730.1 Exp number, first 60 AAs:  0.01396
# Camelinasativa_Csa13g025730.1 Total prob of N-in:        0.05518
Camelinasativa_Csa13g025730.1	TMHMM2.0	outside	     1   341
```

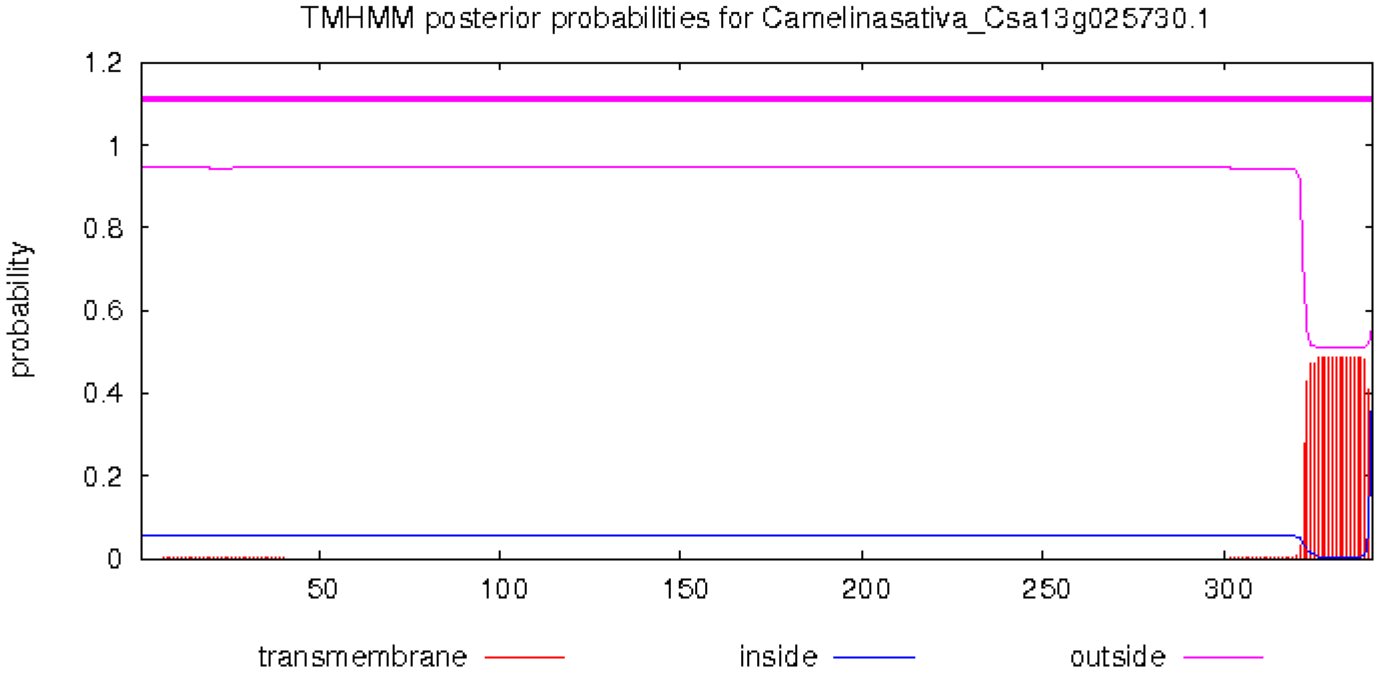

plot in postscript, script for making the plot in gnuplot, data for plot

```
# Camelinasativa_Csa13g025850.1 Length: 238
# Camelinasativa_Csa13g025850.1 Number of predicted TMHs:  0
# Camelinasativa_Csa13g025850.1 Exp number of AAs in TMHs: 0
# Camelinasativa_Csa13g025850.1 Exp number, first 60 AAs:  0
# Camelinasativa_Csa13g025850.1 Total prob of N-in:        0.04644
Camelinasativa_Csa13g025850.1	TMHMM2.0	outside	     1   238
```

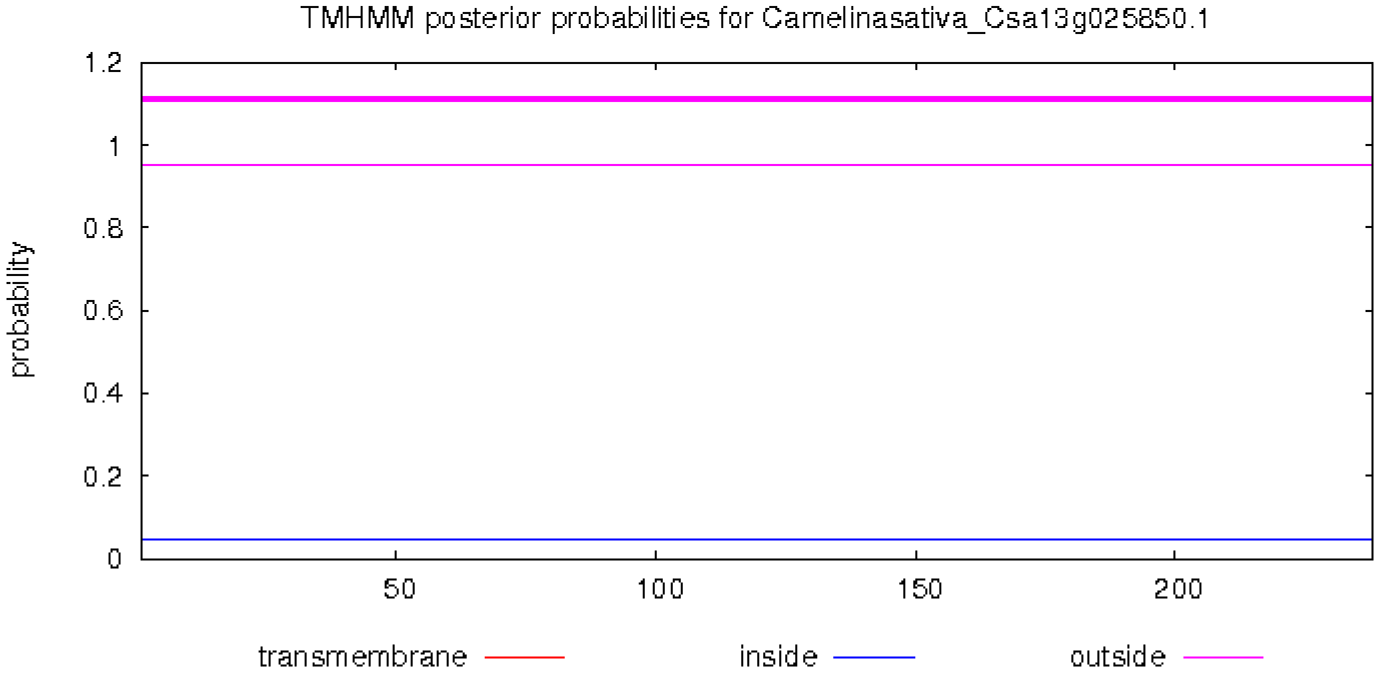

plot in postscript, script for making the plot in gnuplot, data for plot

```
# Camelinasativa_Csa13g030320.1 Length: 251
# Camelinasativa_Csa13g030320.1 Number of predicted TMHs:  0
# Camelinasativa_Csa13g030320.1 Exp number of AAs in TMHs: 0
# Camelinasativa_Csa13g030320.1 Exp number, first 60 AAs:  0
# Camelinasativa_Csa13g030320.1 Total prob of N-in:        0.00543
Camelinasativa_Csa13g030320.1	TMHMM2.0	outside	     1   251
```

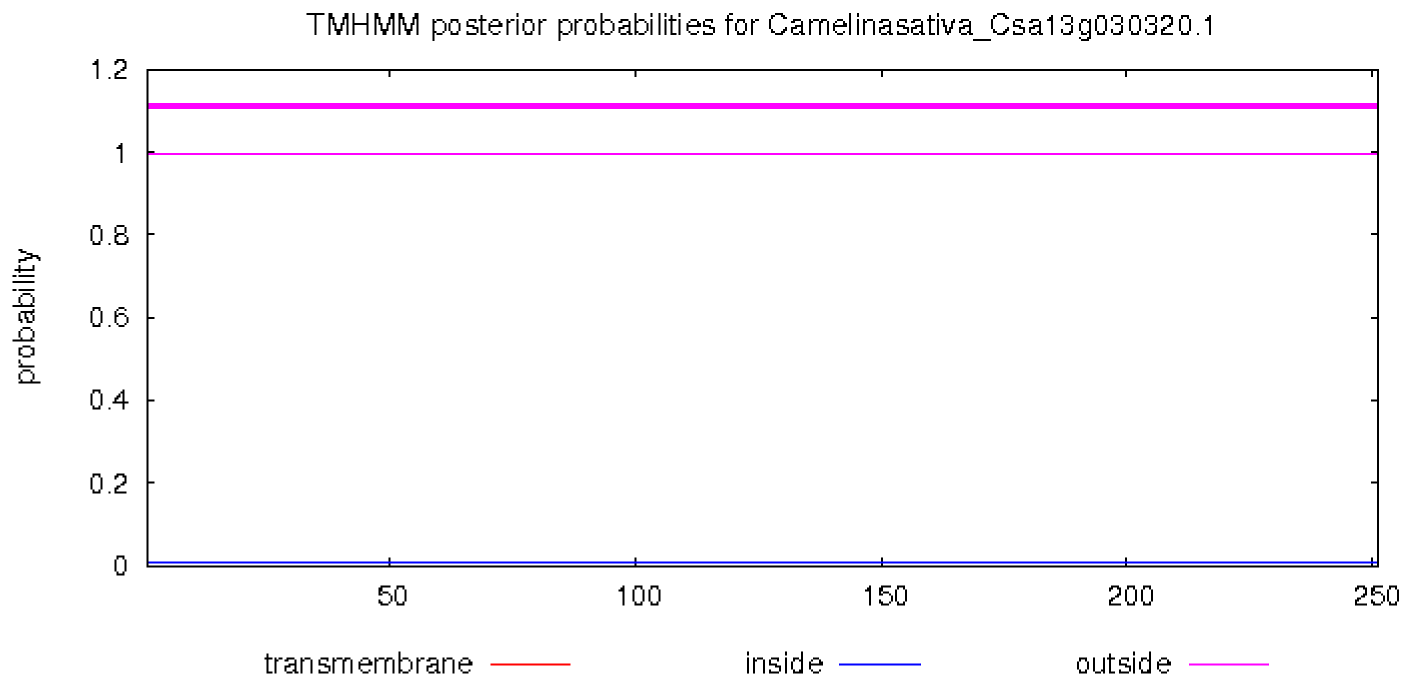

plot in postscript, script for making the plot in gnuplot, data for plot

```
# Camelinasativa_Csa13g047490.1 Length: 340
# Camelinasativa_Csa13g047490.1 Number of predicted TMHs:  0
# Camelinasativa_Csa13g047490.1 Exp number of AAs in TMHs: 0
# Camelinasativa_Csa13g047490.1 Exp number, first 60 AAs:  0
# Camelinasativa_Csa13g047490.1 Total prob of N-in:        0.00442
Camelinasativa_Csa13g047490.1	TMHMM2.0	outside	     1   340
```

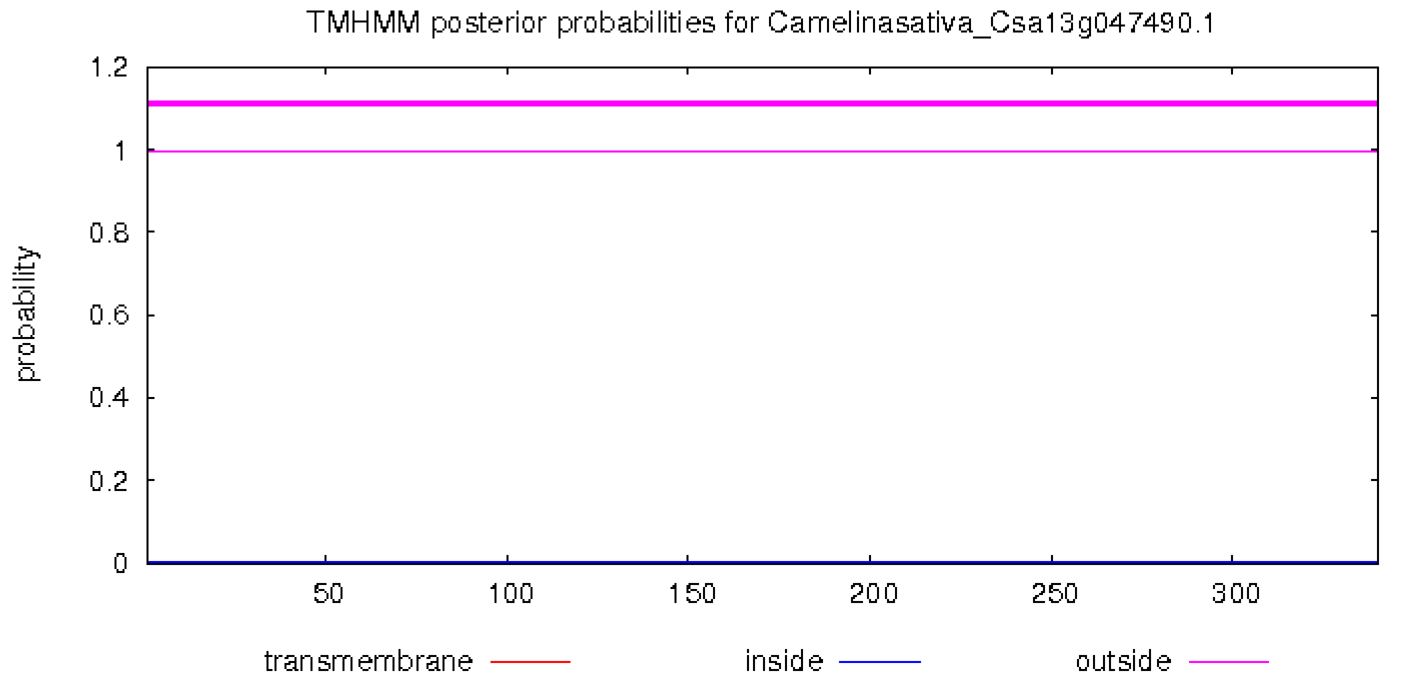

plot in postscript, script for making the plot in gnuplot, data for plot

```
# Camelinasativa_Csa13g055570.1 Length: 1030
# Camelinasativa_Csa13g055570.1 Number of predicted TMHs:  0
# Camelinasativa_Csa13g055570.1 Exp number of AAs in TMHs: 0.00345
# Camelinasativa_Csa13g055570.1 Exp number, first 60 AAs:  0.0002
# Camelinasativa_Csa13g055570.1 Total prob of N-in:        0.00005
Camelinasativa_Csa13g055570.1   TMHMM2.0        outside      1  1030
```

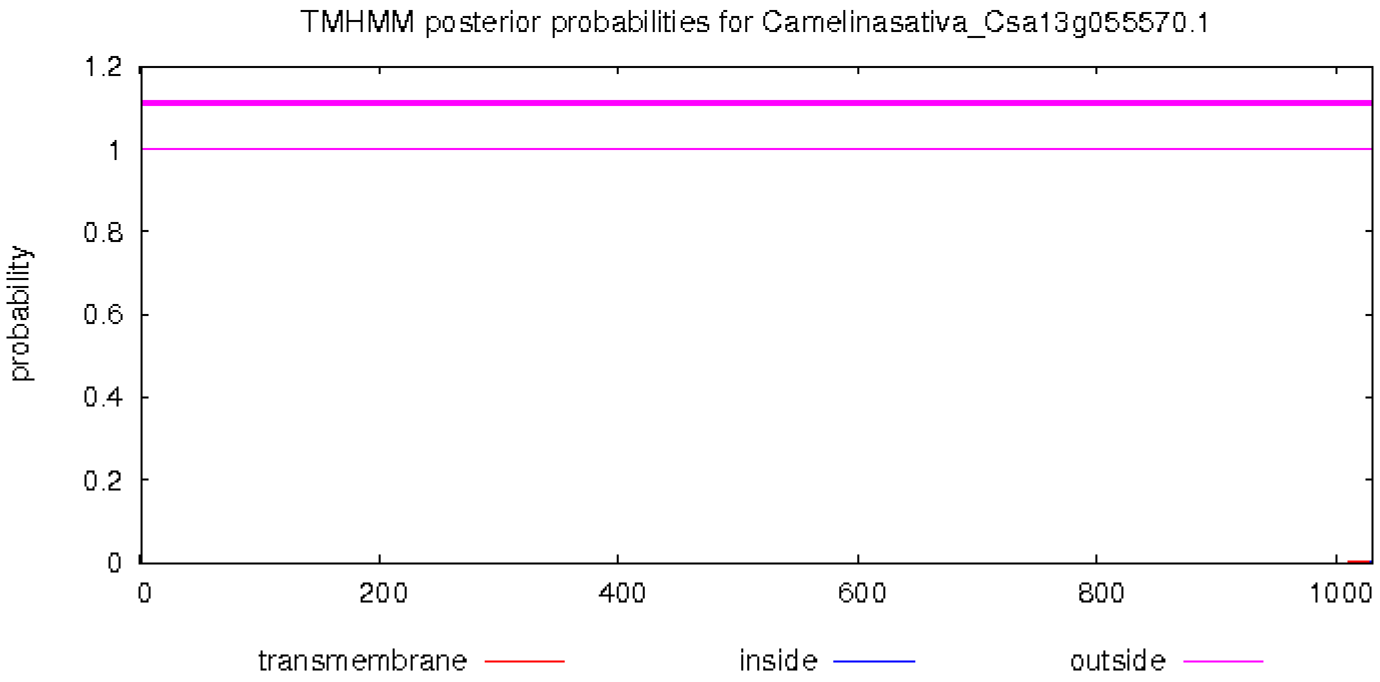

plot in postscript, script for making the plot in gnuplot, data for plot

```
# Camelinasativa_Csa13g055730.1 Length: 437
# Camelinasativa_Csa13g055730.1 Number of predicted TMHs:  1
# Camelinasativa_Csa13g055730.1 Exp number of AAs in TMHs: 22.7156
# Camelinasativa_Csa13g055730.1 Exp number, first 60 AAs:  0.00194
# Camelinasativa_Csa13g055730.1 Total prob of N-in:        0.12490
Camelinasativa_Csa13g055730.1   TMHMM2.0        outside      1   408
Camelinasativa_Csa13g055730.1   TMHMM2.0        TMhelix    409   431
Camelinasativa_Csa13g055730.1   TMHMM2.0        inside     432   437
```

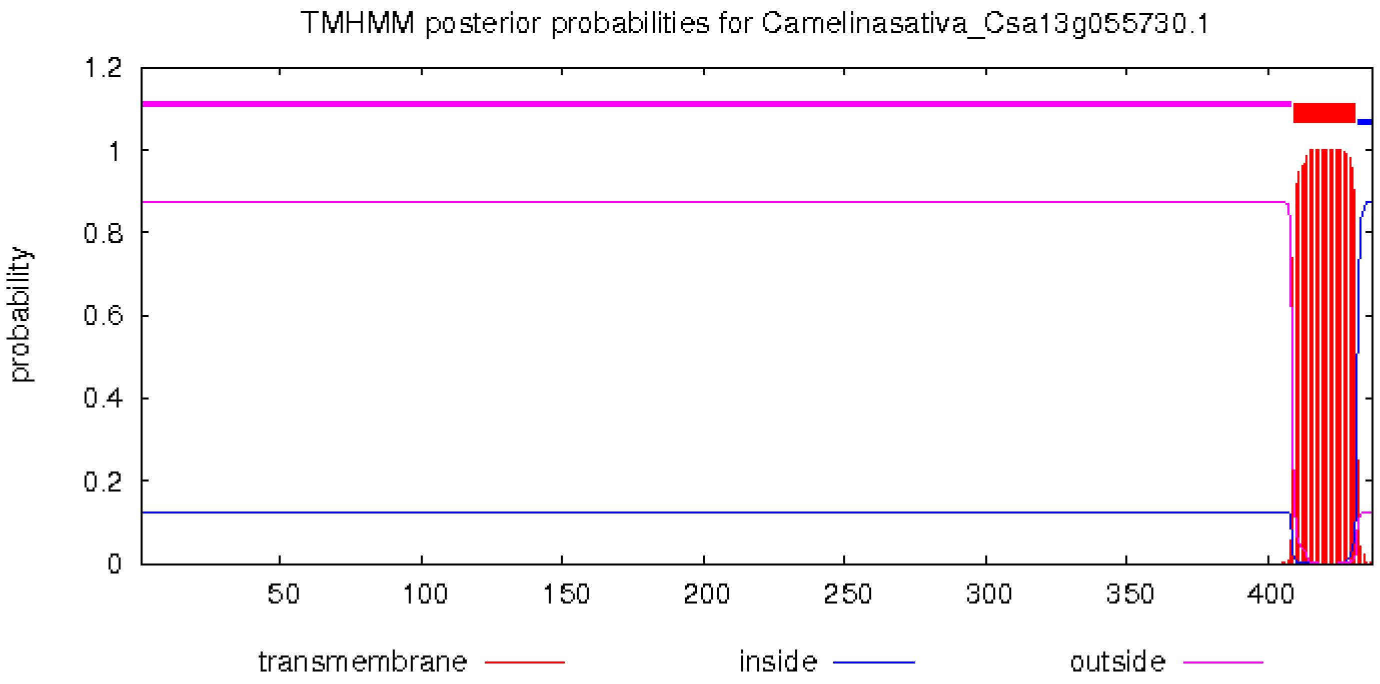

plot in postscript, script for making the plot in gnuplot, data for plot

```
# Camelinasativa_Csa14g002180.1 Length: 823
# Camelinasativa_Csa14g002180.1 Number of predicted TMHs:  1
# Camelinasativa_Csa14g002180.1 Exp number of AAs in TMHs: 22.40945
# Camelinasativa_Csa14g002180.1 Exp number, first 60 AAs:  0
# Camelinasativa_Csa14g002180.1 Total prob of N-in:        0.00303
Camelinasativa_Csa14g002180.1   TMHMM2.0        outside      1   799
Camelinasativa_Csa14g002180.1   TMHMM2.0        TMhelix    800   822
Camelinasativa_Csa14g002180.1   TMHMM2.0        inside     823   823
```

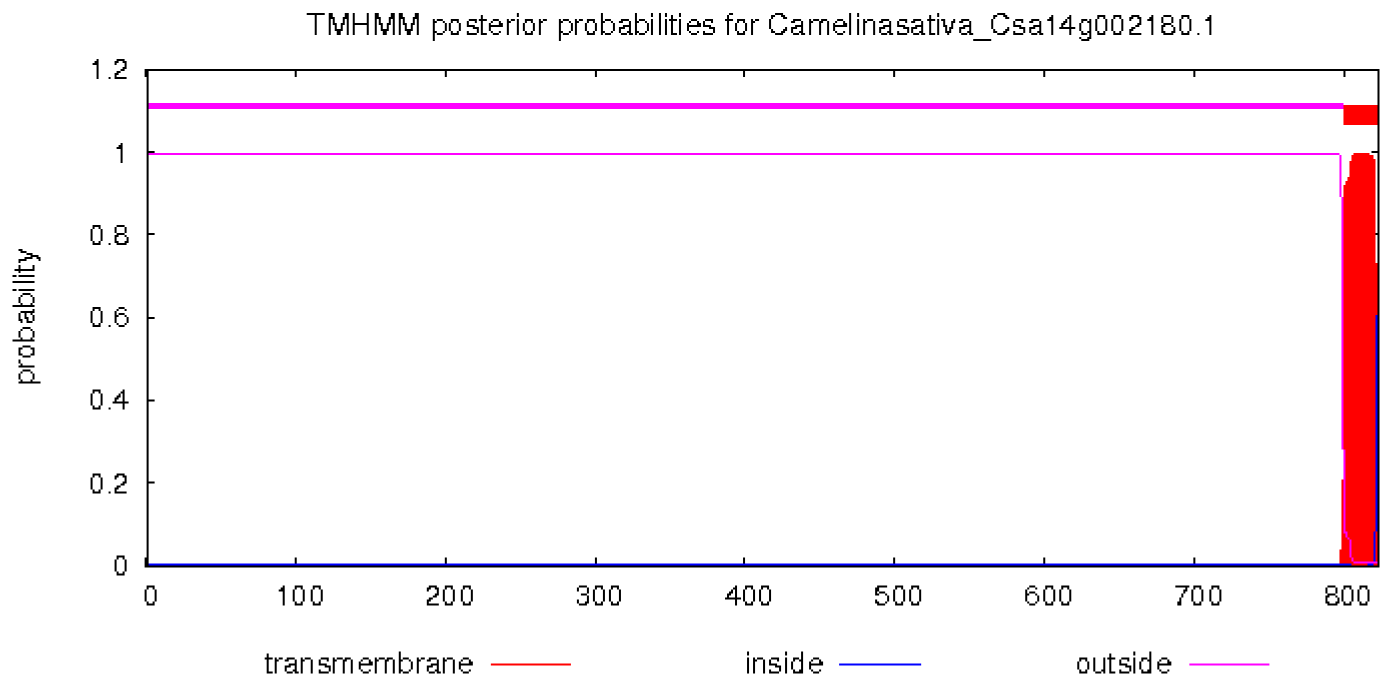

plot in postscript, script for making the plot in gnuplot, data for plot

```
# Camelinasativa_Csa14g002190.1 Length: 384
# Camelinasativa_Csa14g002190.1 Number of predicted TMHs:  0
# Camelinasativa_Csa14g002190.1 Exp number of AAs in TMHs: 0.00036
# Camelinasativa_Csa14g002190.1 Exp number, first 60 AAs:  0
# Camelinasativa_Csa14g002190.1 Total prob of N-in:        0.00342
Camelinasativa_Csa14g002190.1   TMHMM2.0        outside      1   384
```

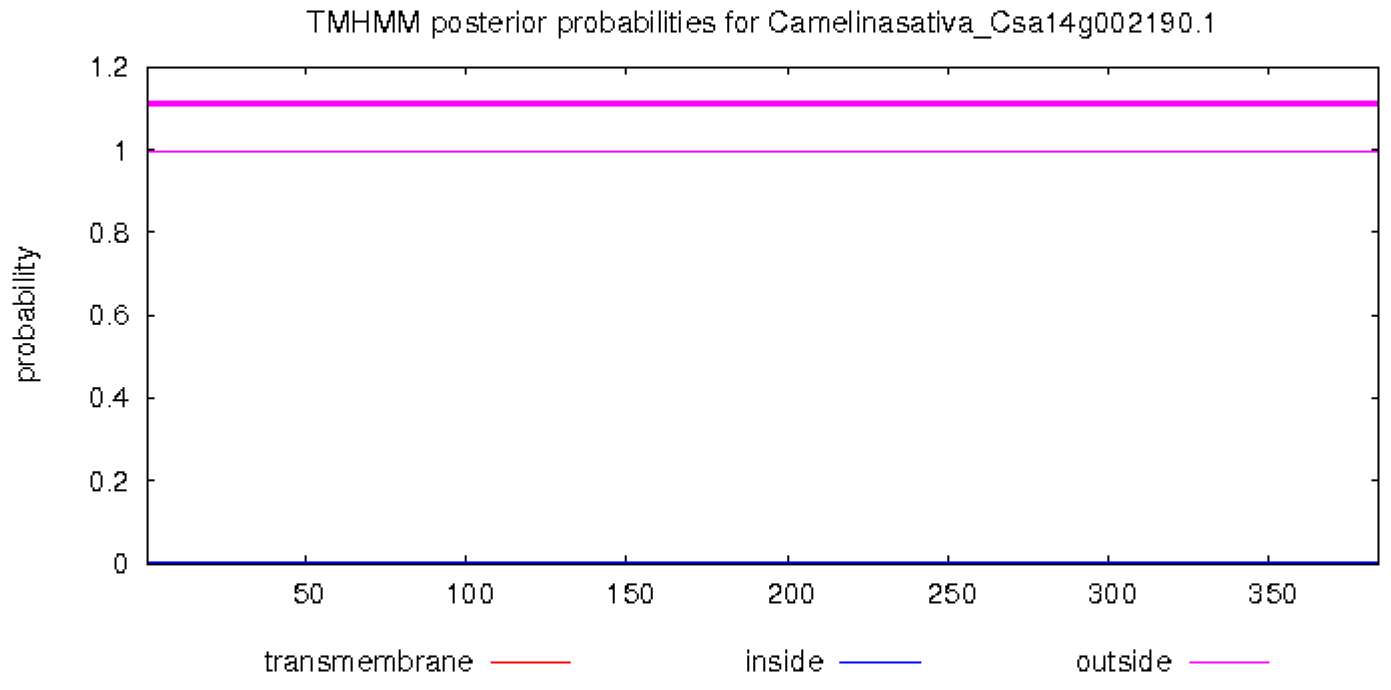

plot in postscript, script for making the plot in gnuplot, data for plot

---

```
# Camelinasativa_Csa14g002230.1 Length: 225
# Camelinasativa_Csa14g002230.1 Number of predicted TMHs:  0
# Camelinasativa_Csa14g002230.1 Exp number of AAs in TMHs: 0.0798499999999999999
# Camelinasativa_Csa14g002230.1 Exp number, first 60 AAs:  0.02359
# Camelinasativa_Csa14g002230.1 Total prob of N-in:        0.05741
Camelinasativa_Csa14g002230.1   TMHMM2.0        outside      1   225
```

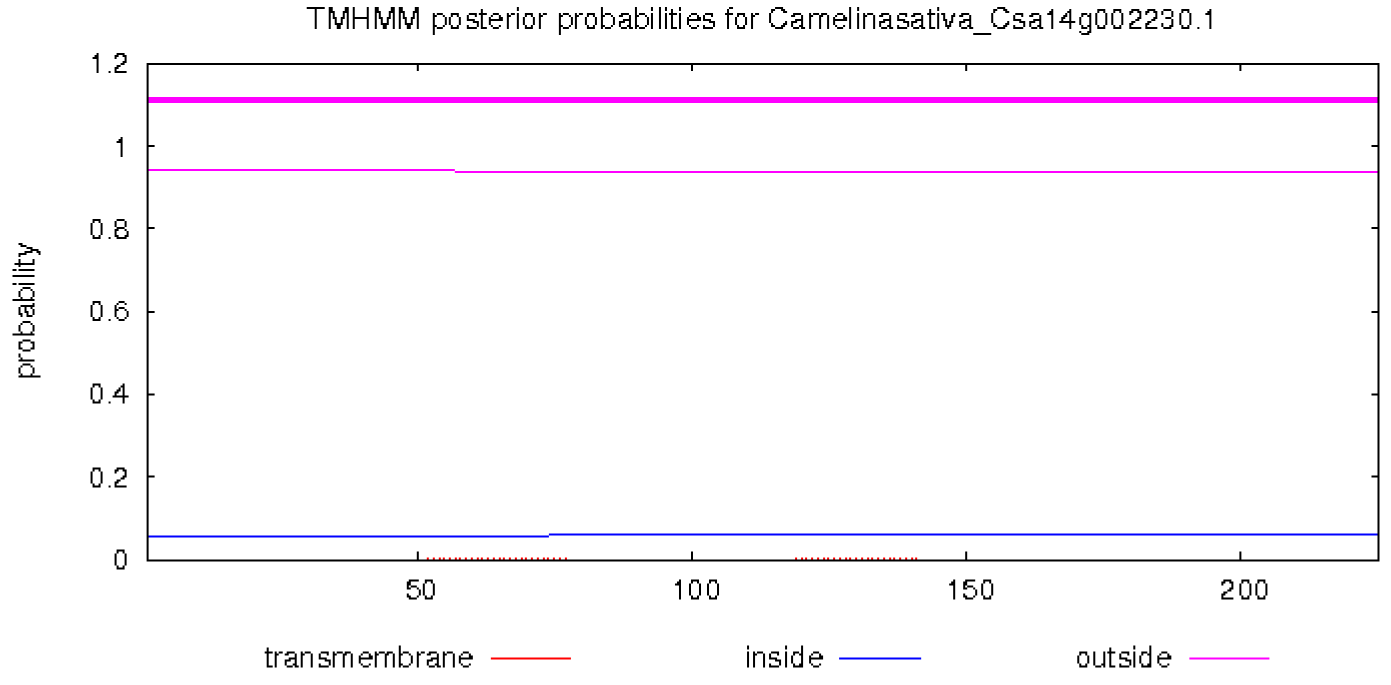

plot in postscript, script for making the plot in gnuplot, data for plot

---

```
# Camelinasativa_Csa14g002590.1 Length: 586
# Camelinasativa_Csa14g002590.1 Number of predicted TMHs:  0
# Camelinasativa_Csa14g002590.1 Exp number of AAs in TMHs: 0.0007
# Camelinasativa_Csa14g002590.1 Exp number, first 60 AAs:  0
# Camelinasativa_Csa14g002590.1 Total prob of N-in:        0.00187
Camelinasativa_Csa14g002590.1   TMHMM2.0        outside      1   586
```

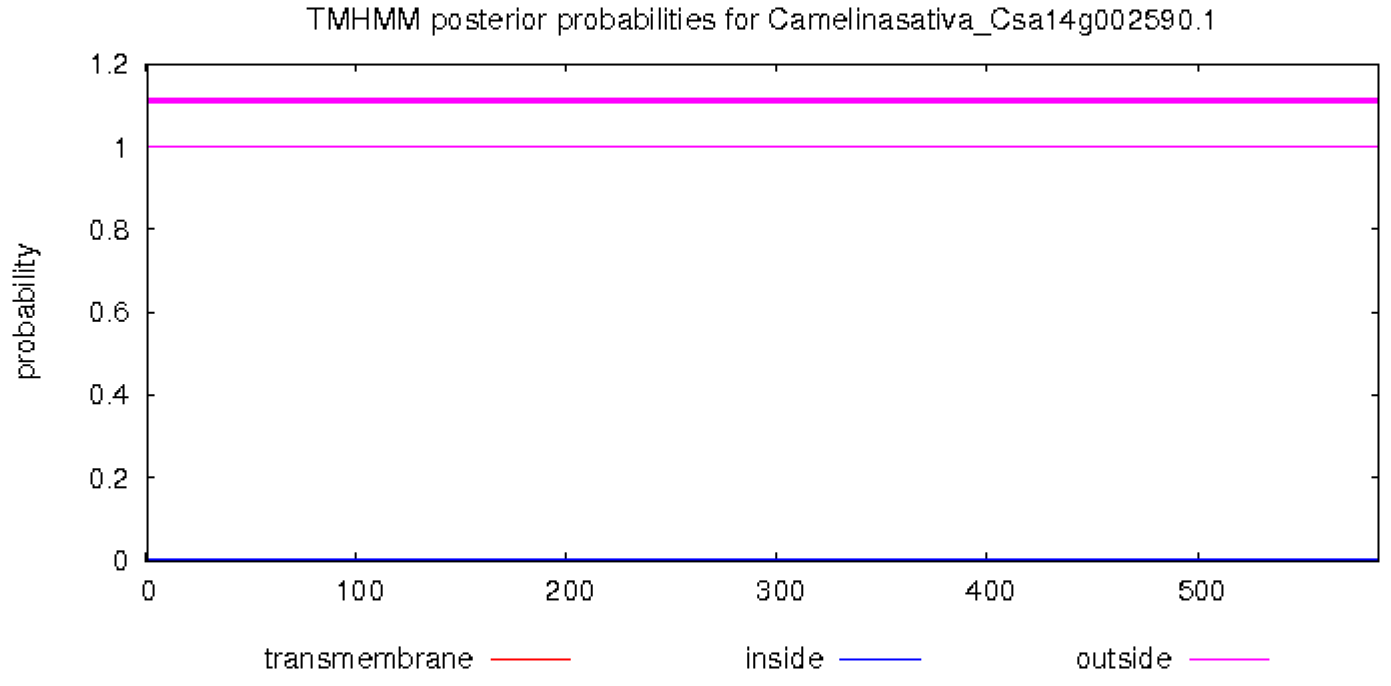

plot in postscript, script for making the plot in gnuplot, data for plot

---

```
# Camelinasativa_Csa14g004560.1 Length: 519
# Camelinasativa_Csa14g004560.1 Number of predicted TMHs:  0
# Camelinasativa_Csa14g004560.1 Exp number of AAs in TMHs: 0.00228
# Camelinasativa_Csa14g004560.1 Exp number, first 60 AAs:  0.00228
# Camelinasativa_Csa14g004560.1 Total prob of N-in:        0.00110
Camelinasativa_Csa14g004560.1	TMHMM2.0	outside	     1   519
```

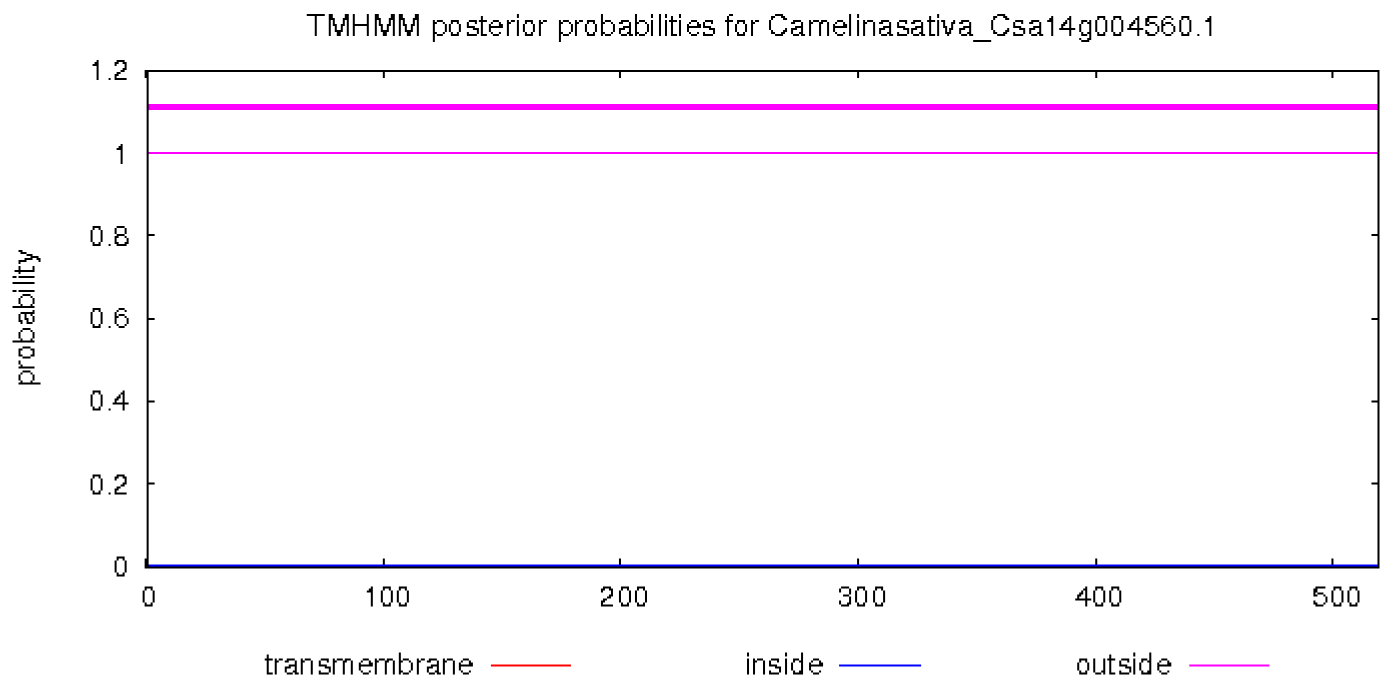

plot in postscript, script for making the plot in gnuplot, data for plot

---

```
# Camelinasativa_Csa14g015200.1 Length: 584
# Camelinasativa_Csa14g015200.1 Number of predicted TMHs:  0
# Camelinasativa_Csa14g015200.1 Exp number of AAs in TMHs: 0.00015
# Camelinasativa_Csa14g015200.1 Exp number, first 60 AAs:  0
# Camelinasativa_Csa14g015200.1 Total prob of N-in:        0.00016
Camelinasativa_Csa14g015200.1	TMHMM2.0	outside	     1   584
```

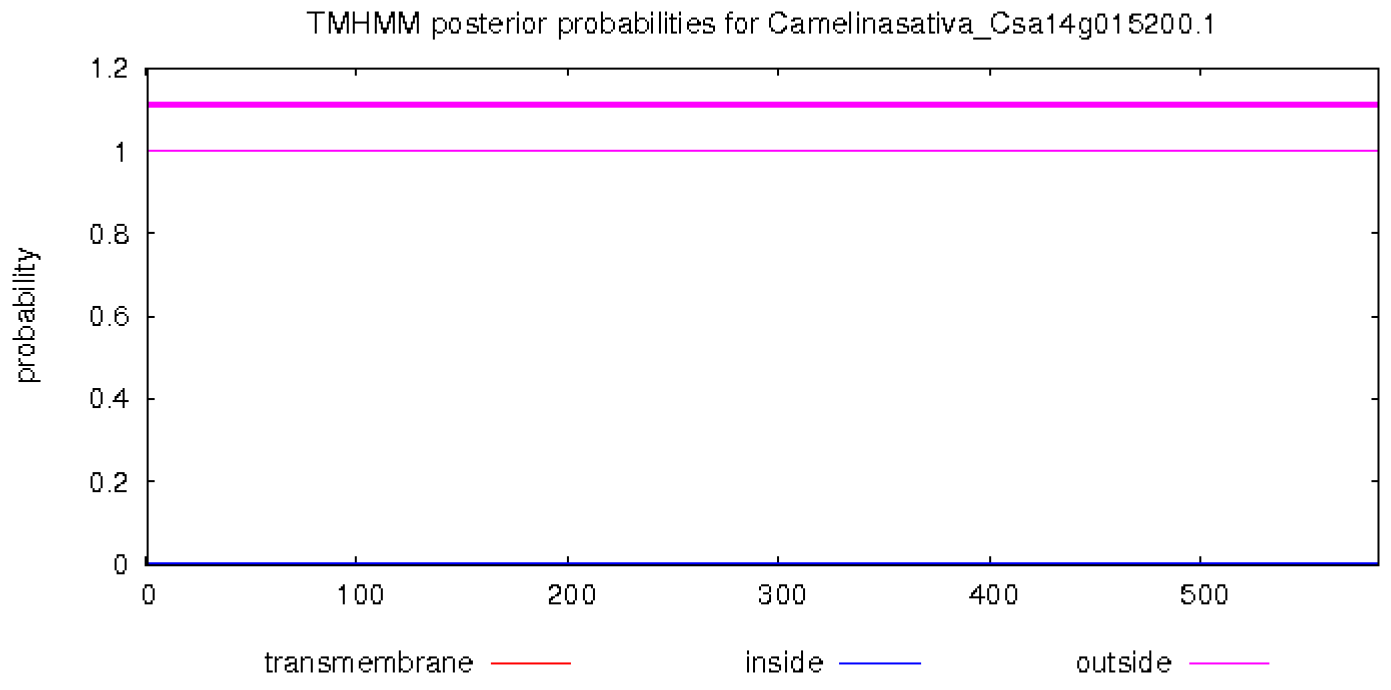

plot in postscript, script for making the plot in gnuplot, data for plot

```
# Camelinasativa_Csa14g031790.1 Length: 187
# Camelinasativa_Csa14g031790.1 Number of predicted TMHs:  0
# Camelinasativa_Csa14g031790.1 Exp number of AAs in TMHs: 0.0002
# Camelinasativa_Csa14g031790.1 Exp number, first 60 AAs:  0.0002
# Camelinasativa_Csa14g031790.1 Total prob of N-in:        0.21647
Camelinasativa_Csa14g031790.1   TMHMM2.0        outside      1   187
```

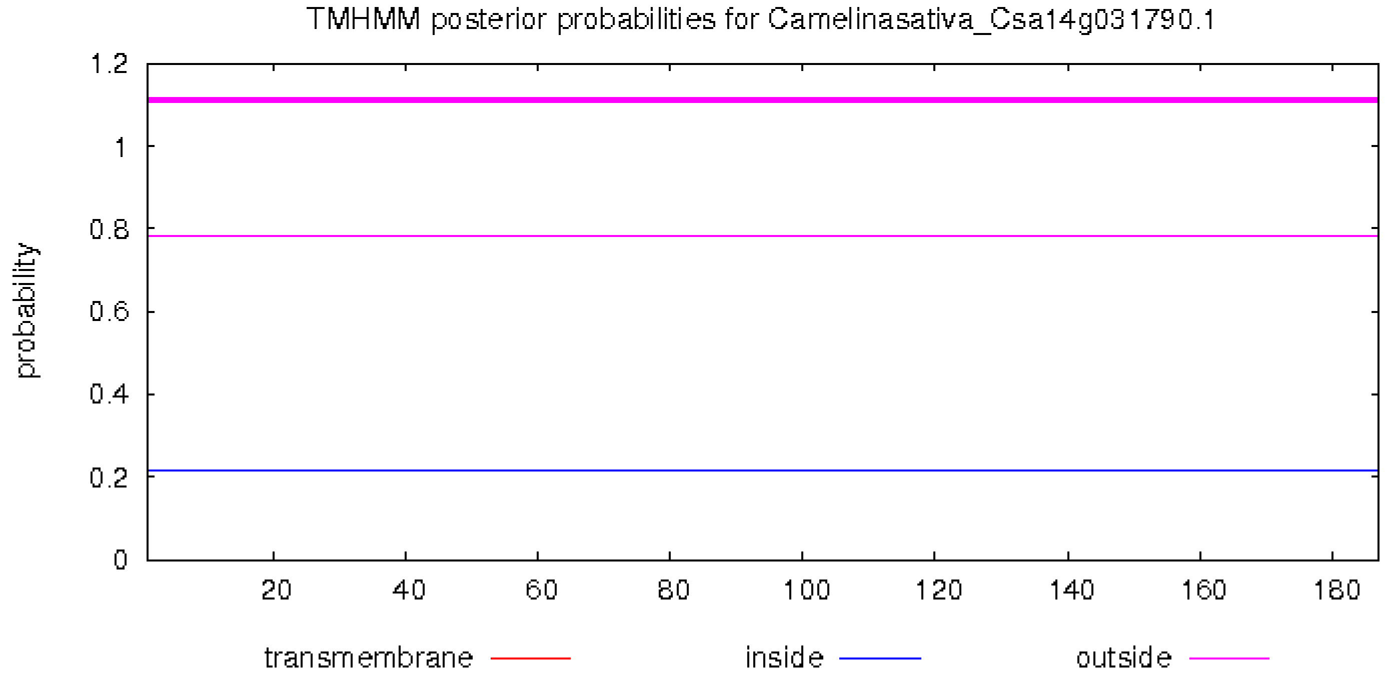

plot in postscript, script for making the plot in gnuplot, data for plot

```
# Camelinasativa_Csa14g032780.1 Length: 459
# Camelinasativa_Csa14g032780.1 Number of predicted TMHs:  0
# Camelinasativa_Csa14g032780.1 Exp number of AAs in TMHs: 0.0002
# Camelinasativa_Csa14g032780.1 Exp number, first 60 AAs:  0
# Camelinasativa_Csa14g032780.1 Total prob of N-in:        0.00266
Camelinasativa_Csa14g032780.1   TMHMM2.0        outside      1   459
```

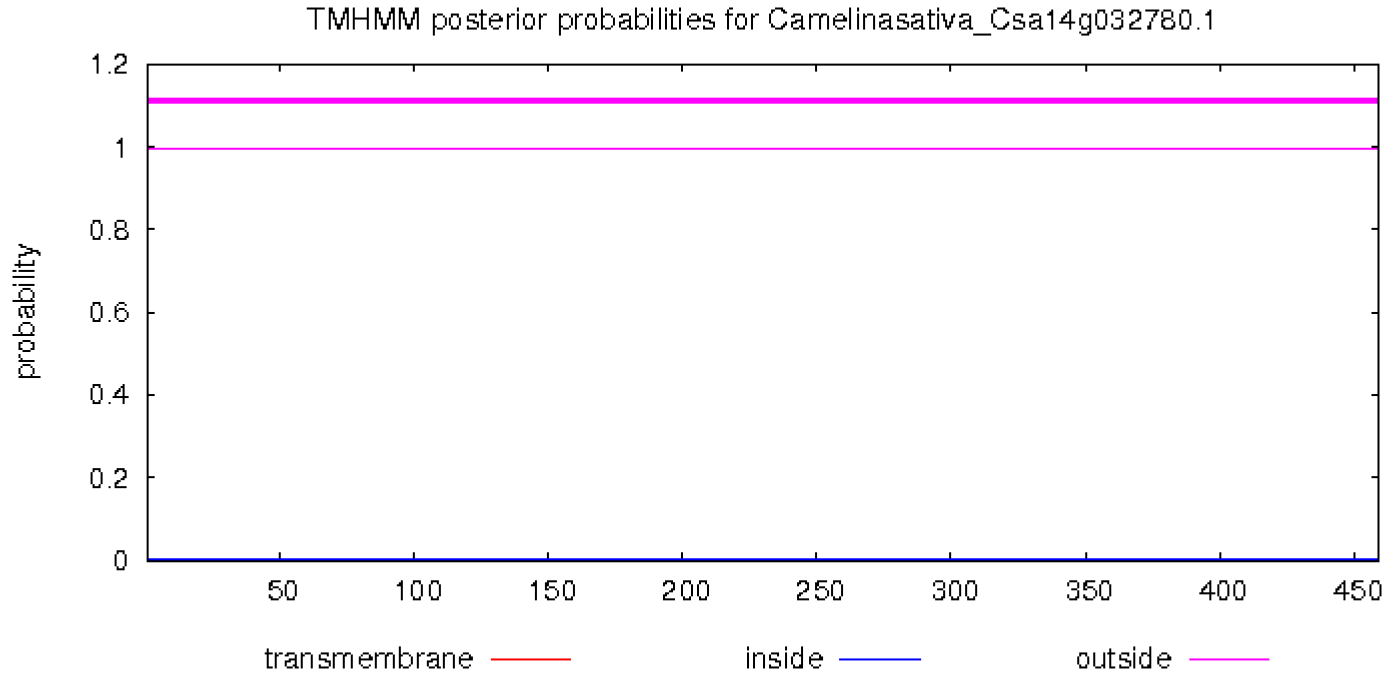

plot in postscript, script for making the plot in gnuplot, data for plot

```
# Camelinasativa_Csa14g036150.1 Length: 133
# Camelinasativa_Csa14g036150.1 Number of predicted TMHs:  0
# Camelinasativa_Csa14g036150.1 Exp number of AAs in TMHs: 0.00038
# Camelinasativa_Csa14g036150.1 Exp number, first 60 AAs:  0.00034
# Camelinasativa_Csa14g036150.1 Total prob of N-in:        0.23273
Camelinasativa_Csa14g036150.1	TMHMM2.0	outside	     1   133
```

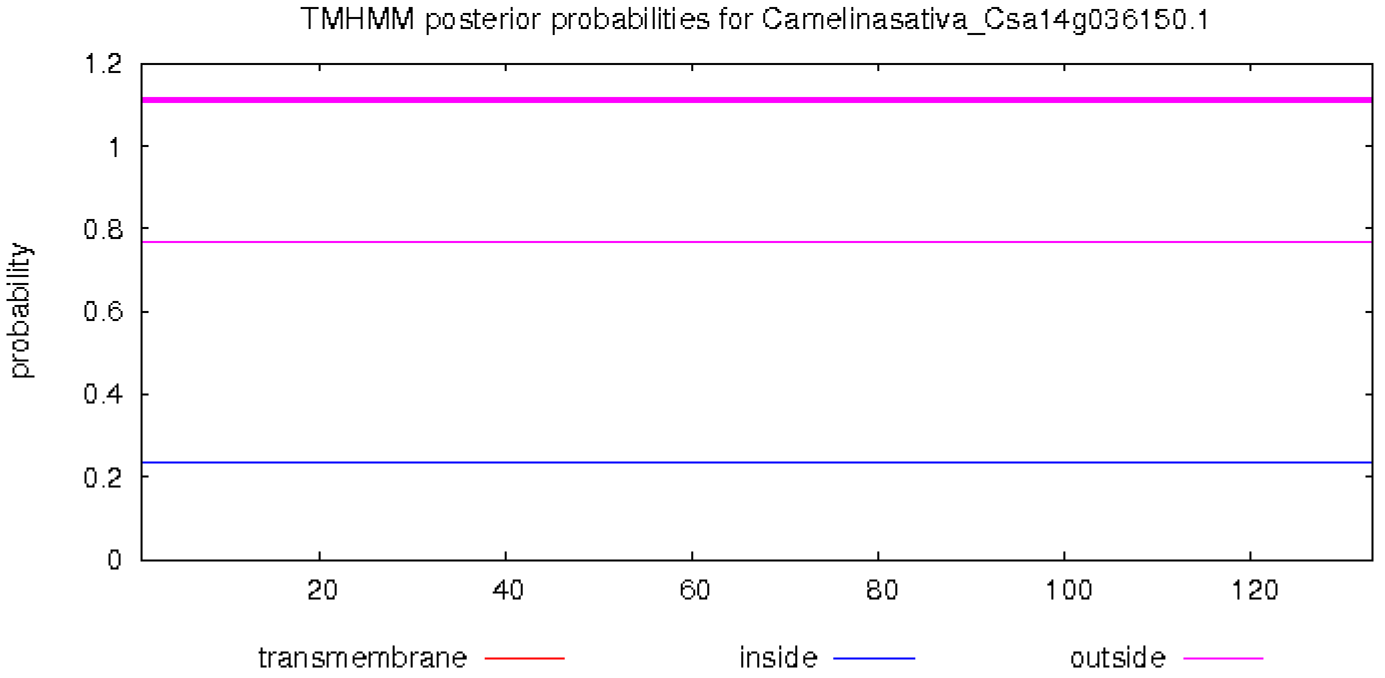

plot in postscript, script for making the plot in gnuplot, data for plot

```
# Camelinasativa_Csa14g042520.1 Length: 145
# Camelinasativa_Csa14g042520.1 Number of predicted TMHs:  0
# Camelinasativa_Csa14g042520.1 Exp number of AAs in TMHs: 0
# Camelinasativa_Csa14g042520.1 Exp number, first 60 AAs:  0
# Camelinasativa_Csa14g042520.1 Total prob of N-in:        0.01272
Camelinasativa_Csa14g042520.1	TMHMM2.0	outside	     1   145
```

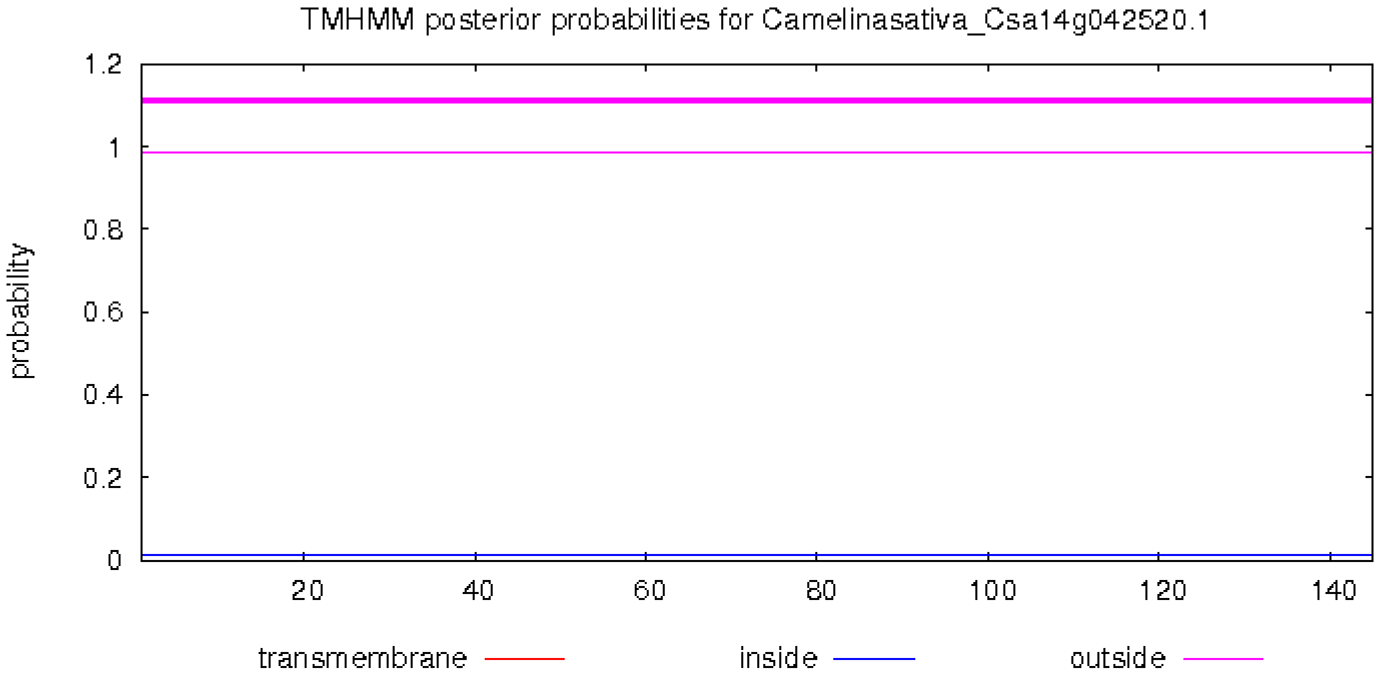

plot in postscript, script for making the plot in gnuplot, data for plot

```
# Camelinasativa_Csa14g042630.1 Length: 560
# Camelinasativa_Csa14g042630.1 Number of predicted TMHs:  2
# Camelinasativa_Csa14g042630.1 Exp number of AAs in TMHs: 43.62347
# Camelinasativa_Csa14g042630.1 Exp number, first 60 AAs:  21.10921
# Camelinasativa_Csa14g042630.1 Total prob of N-in:        0.08356
# Camelinasativa_Csa14g042630.1 POSSIBLE N-term signal sequence
Camelinasativa_Csa14g042630.1   TMHMM2.0        outside      1     9
Camelinasativa_Csa14g042630.1   TMHMM2.0        TMhelix     10    29
Camelinasativa_Csa14g042630.1   TMHMM2.0        inside      30   530
Camelinasativa_Csa14g042630.1   TMHMM2.0        TMhelix    531   553
Camelinasativa_Csa14g042630.1   TMHMM2.0        outside    554   560
```

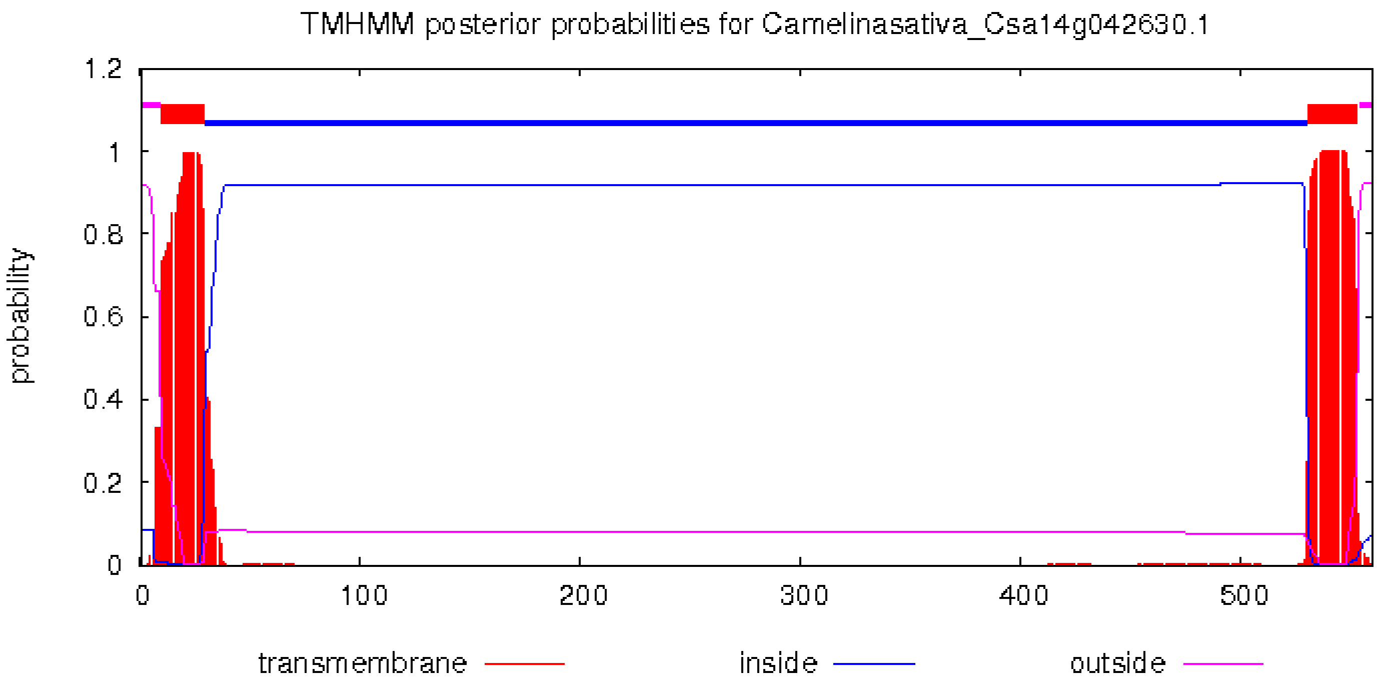

plot in postscript, script for making the plot in gnuplot, data for plot

```
# Camelinasativa_Csa14g042710.1 Length: 362
# Camelinasativa_Csa14g042710.1 Number of predicted TMHs:  0
# Camelinasativa_Csa14g042710.1 Exp number of AAs in TMHs: 0
# Camelinasativa_Csa14g042710.1 Exp number, first 60 AAs:  0
# Camelinasativa_Csa14g042710.1 Total prob of N-in:        0.01802
Camelinasativa_Csa14g042710.1   TMHMM2.0        outside      1   362
```

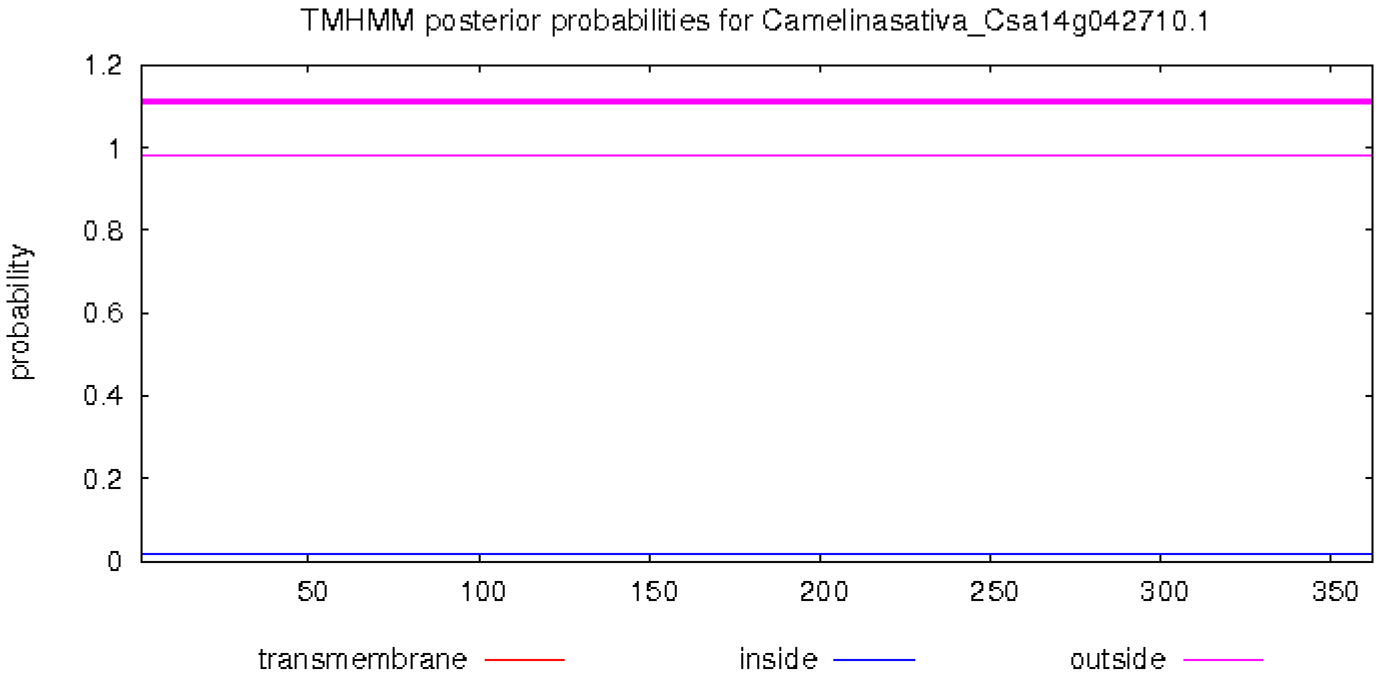

plot in postscript, script for making the plot in gnuplot, data for plot

```
# Camelinasativa_Csa14g043230.1 Length: 308
# Camelinasativa_Csa14g043230.1 Number of predicted TMHs:  0
# Camelinasativa_Csa14g043230.1 Exp number of AAs in TMHs: 0
# Camelinasativa_Csa14g043230.1 Exp number, first 60 AAs:  0
# Camelinasativa_Csa14g043230.1 Total prob of N-in:        0.00478
Camelinasativa_Csa14g043230.1	TMHMM2.0	outside	     1   308
```

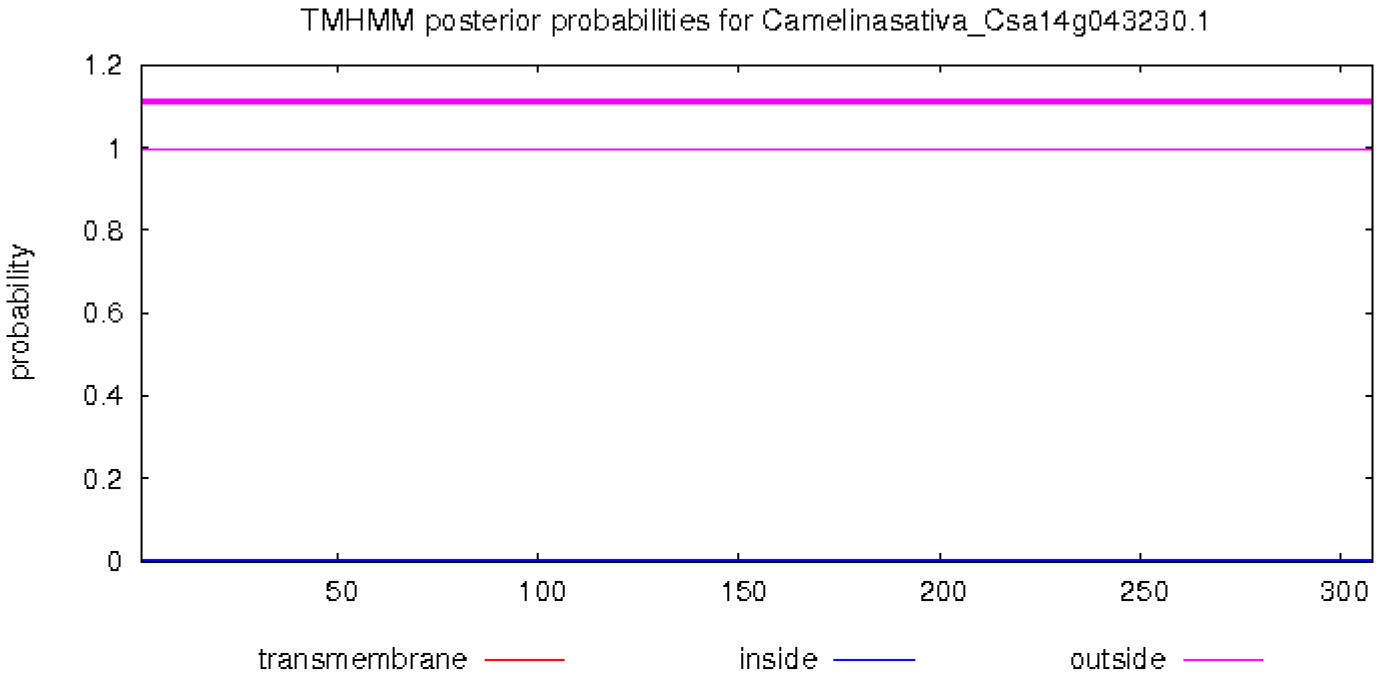

plot in postscript, script for making the plot in gnuplot, data for plot

```
# Camelinasativa_Csa14g044030.1 Length: 1397
# Camelinasativa_Csa14g044030.1 Number of predicted TMHs:  2
# Camelinasativa_Csa14g044030.1 Exp number of AAs in TMHs: 61.25804
# Camelinasativa_Csa14g044030.1 Exp number, first 60 AAs:  0.00249
# Camelinasativa_Csa14g044030.1 Total prob of N-in:        0.31724
Camelinasativa_Csa14g044030.1	TMHMM2.0	inside	     1   578
Camelinasativa_Csa14g044030.1	TMHMM2.0	TMhelix	   579   601
```

```
Camelinasativa_Csa14g044030.1   TMHMM2.0        outside    602  1367
Camelinasativa_Csa14g044030.1   TMHMM2.0        TMhelix   1368  1390
Camelinasativa_Csa14g044030.1   TMHMM2.0        inside    1391  1397
```

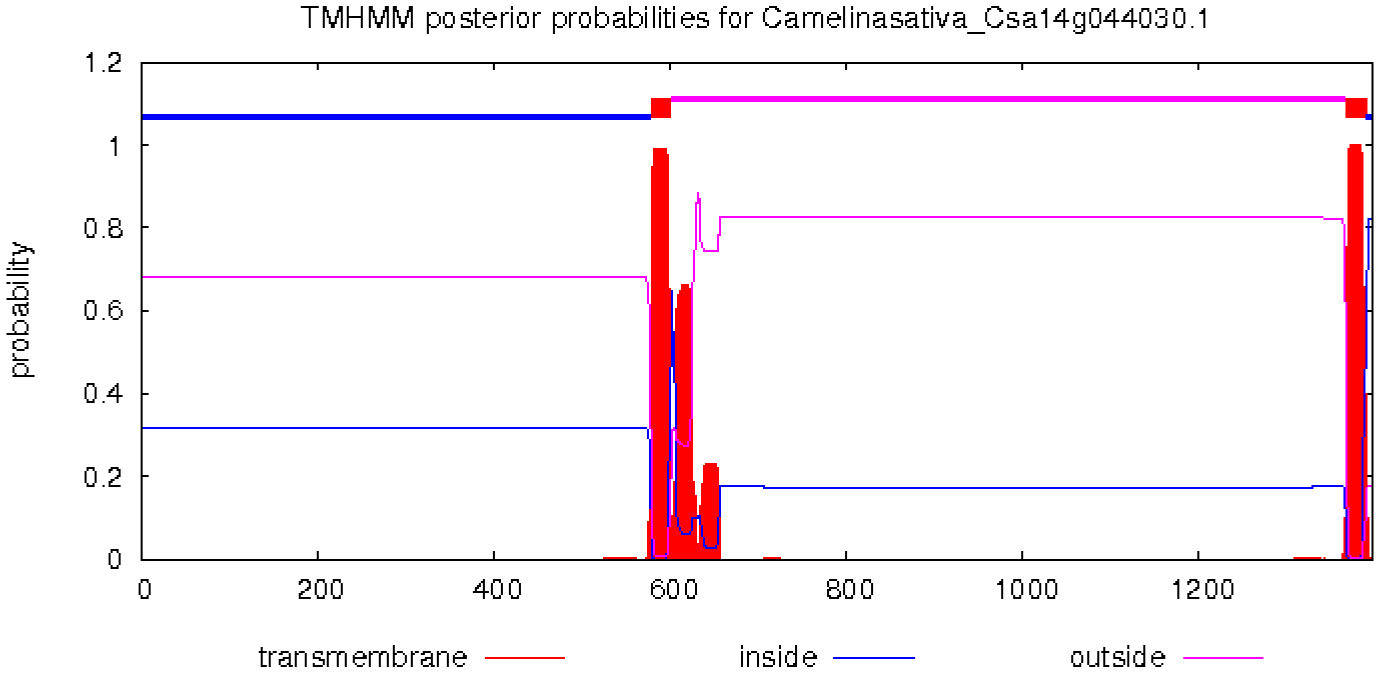

plot in postscript, script for making the plot in gnuplot, data for plot

---

```
# Camelinasativa_Csa14g063200.1 Length: 309
# Camelinasativa_Csa14g063200.1 Number of predicted TMHs:  0
# Camelinasativa_Csa14g063200.1 Exp number of AAs in TMHs: 0.00126
# Camelinasativa_Csa14g063200.1 Exp number, first 60 AAs:  0
# Camelinasativa_Csa14g063200.1 Total prob of N-in:        0.00819
Camelinasativa_Csa14g063200.1   TMHMM2.0        outside      1   309
```

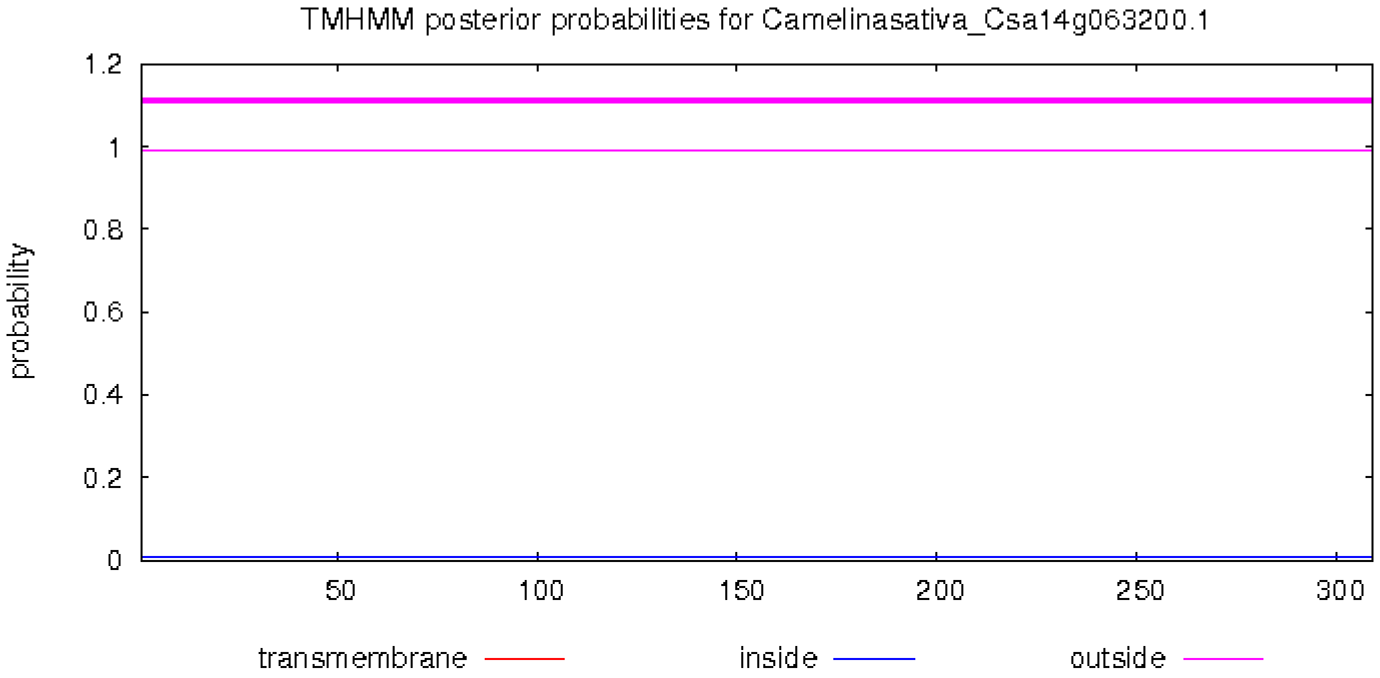

plot in postscript, script for making the plot in gnuplot, data for plot

---

```
# Camelinasativa_Csa14g063210.1 Length: 306
# Camelinasativa_Csa14g063210.1 Number of predicted TMHs:  0
# Camelinasativa_Csa14g063210.1 Exp number of AAs in TMHs: 0.00171
```

```
# Camelinasativa_Csa14g063210.1 Exp number, first 60 AAs:  0.00023
# Camelinasativa_Csa14g063210.1 Total prob of N-in:        0.01521
Camelinasativa_Csa14g063210.1   TMHMM2.0        outside      1   306
```

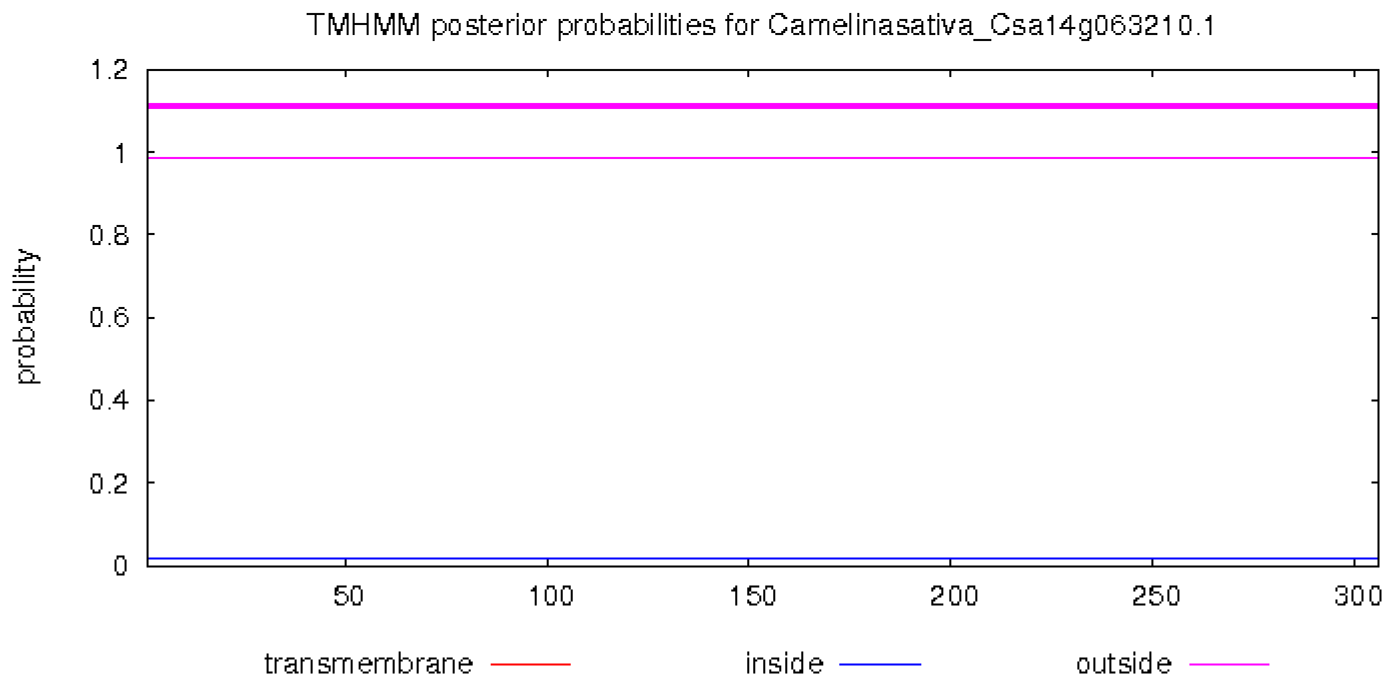

plot in postscript, script for making the plot in gnuplot, data for plot

```
# Camelinasativa_Csa14g067910.1 Length: 323
# Camelinasativa_Csa14g067910.1 Number of predicted TMHs:  0
# Camelinasativa_Csa14g067910.1 Exp number of AAs in TMHs: 0.00079
# Camelinasativa_Csa14g067910.1 Exp number, first 60 AAs:  0
# Camelinasativa_Csa14g067910.1 Total prob of N-in:        0.02372
Camelinasativa_Csa14g067910.1   TMHMM2.0        outside      1   323
```

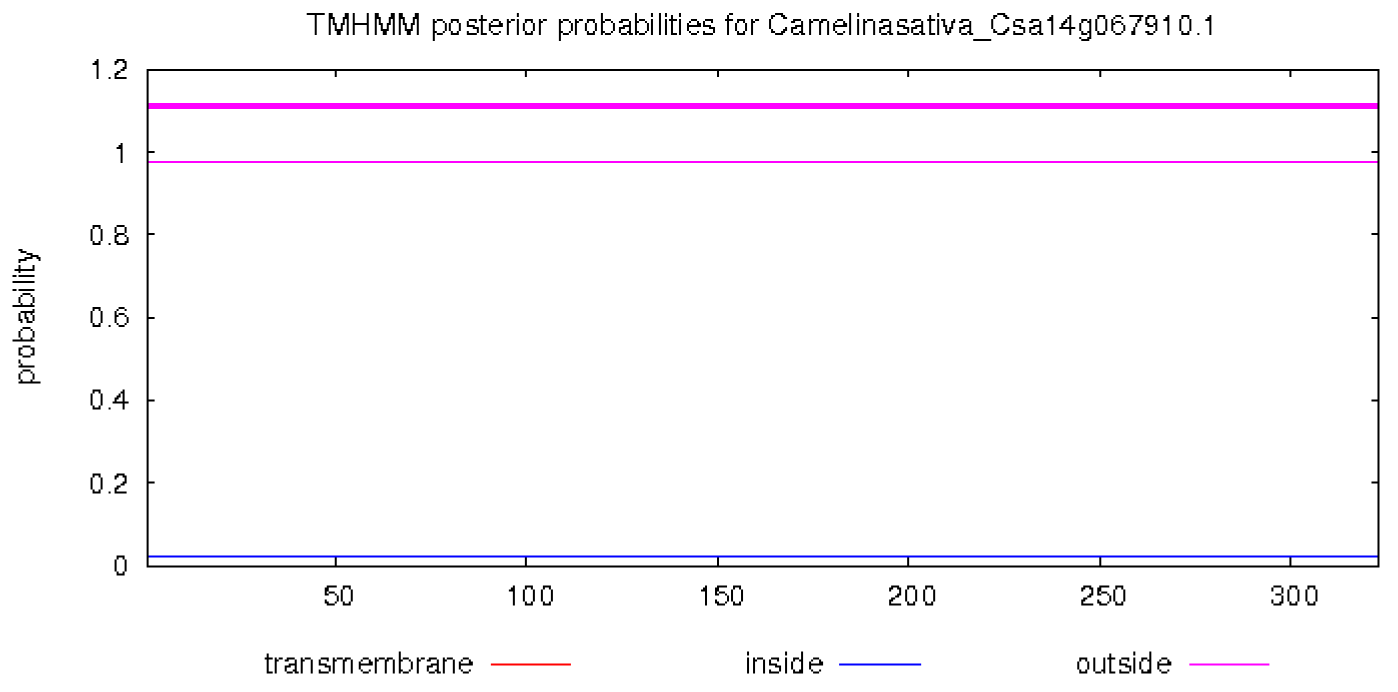

plot in postscript, script for making the plot in gnuplot, data for plot

```
# Camelinasativa_Csa14g069120.1 Length: 304
# Camelinasativa_Csa14g069120.1 Number of predicted TMHs:  0
# Camelinasativa_Csa14g069120.1 Exp number of AAs in TMHs: 0
```

```
# Camelinasativa_Csa14g069120.1 Exp number, first 60 AAs:  0
# Camelinasativa_Csa14g069120.1 Total prob of N-in:        0.00117
Camelinasativa_Csa14g069120.1   TMHMM2.0        outside      1   304
```

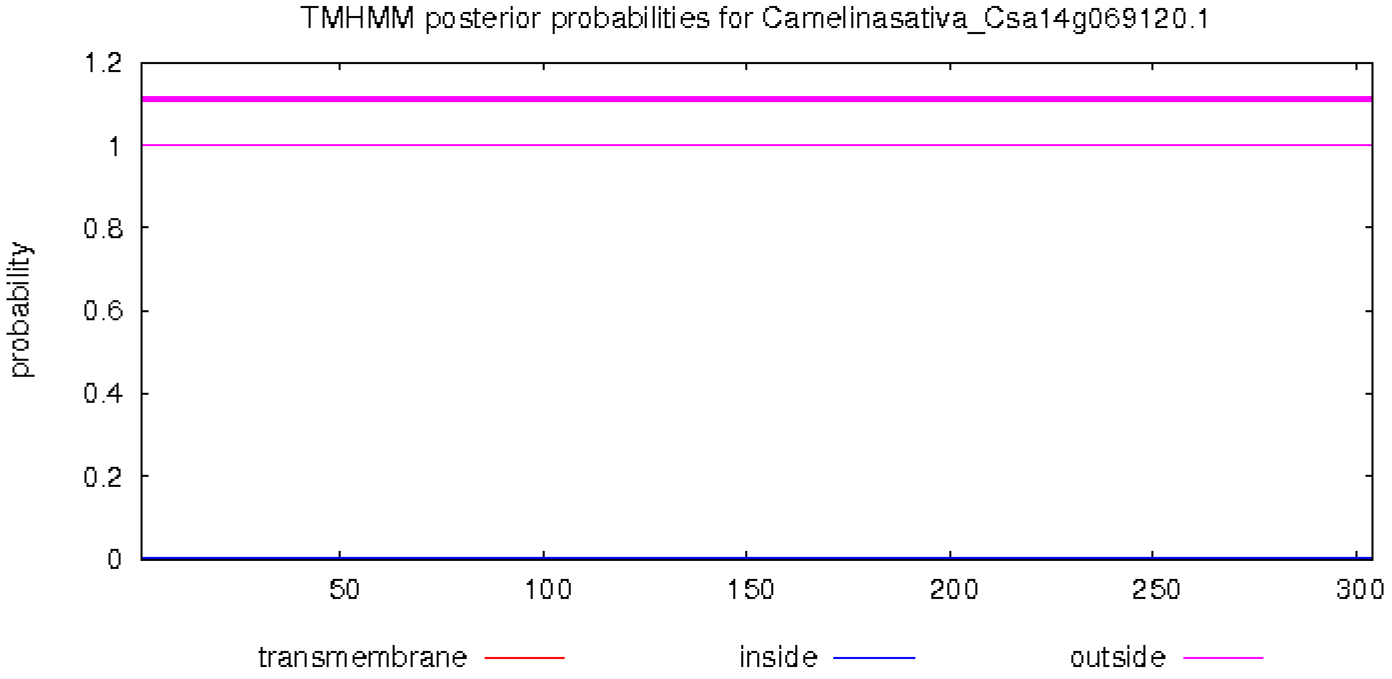

plot in postscript, script for making the plot in gnuplot, data for plot

---

```
# Camelinasativa_Csa15g002950.1 Length: 476
# Camelinasativa_Csa15g002950.1 Number of predicted TMHs:  0
# Camelinasativa_Csa15g002950.1 Exp number of AAs in TMHs: 0.00018
# Camelinasativa_Csa15g002950.1 Exp number, first 60 AAs:  0
# Camelinasativa_Csa15g002950.1 Total prob of N-in:        0.00017
Camelinasativa_Csa15g002950.1   TMHMM2.0        outside      1   476
```

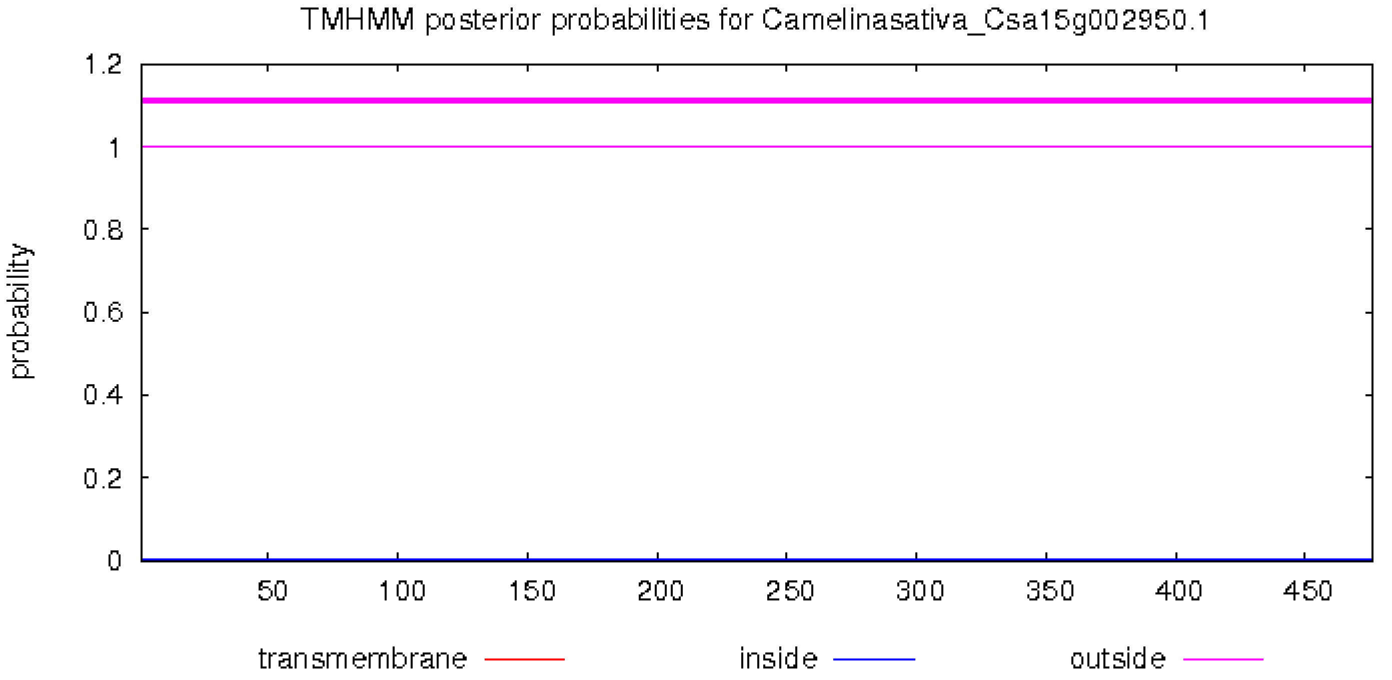

plot in postscript, script for making the plot in gnuplot, data for plot

---

```
# Camelinasativa_Csa15g003940.1 Length: 500
# Camelinasativa_Csa15g003940.1 Number of predicted TMHs:  0
# Camelinasativa_Csa15g003940.1 Exp number of AAs in TMHs: 0.00034
```

```
# Camelinasativa_Csa15g003940.1 Exp number, first 60 AAs:  0.00016
# Camelinasativa_Csa15g003940.1 Total prob of N-in:        0.00155
Camelinasativa_Csa15g003940.1   TMHMM2.0        outside      1   500
```

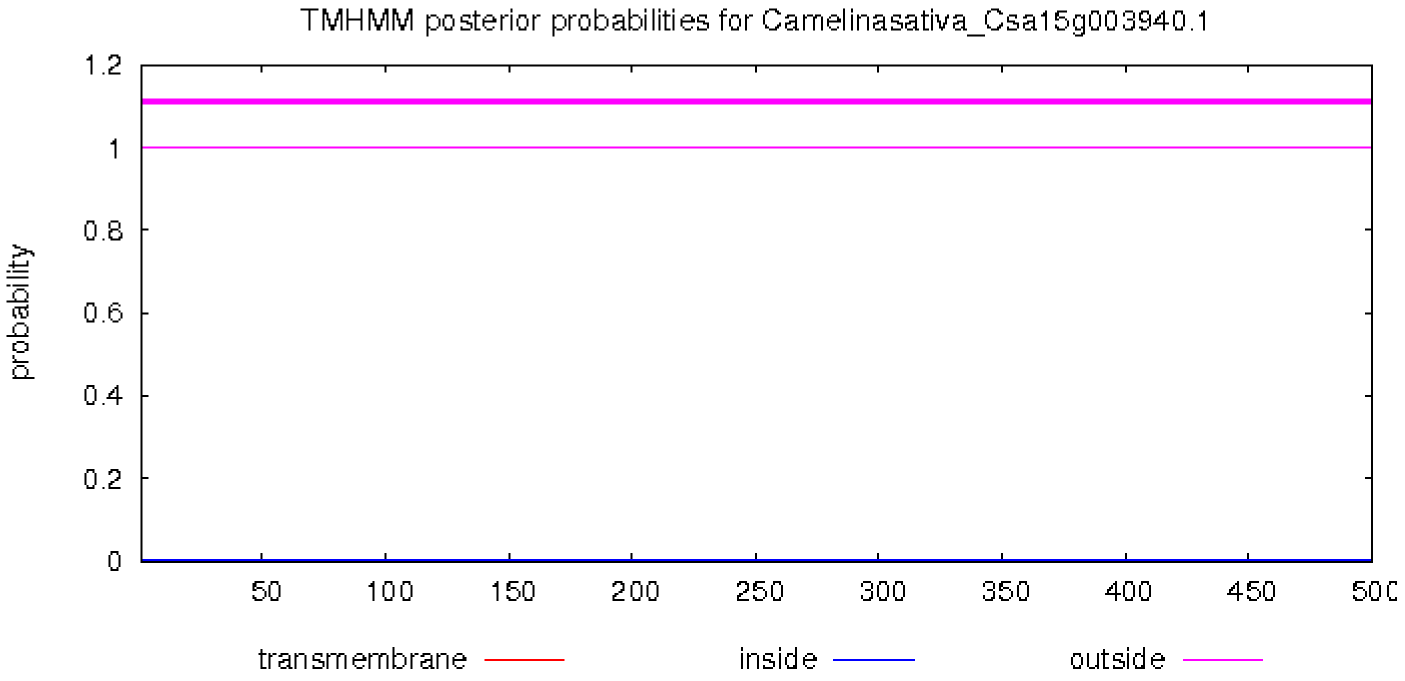

plot in postscript, script for making the plot in gnuplot, data for plot

---

```
# Camelinasativa_Csa15g003950.1 Length: 356
# Camelinasativa_Csa15g003950.1 Number of predicted TMHs:  0
# Camelinasativa_Csa15g003950.1 Exp number of AAs in TMHs: 0
# Camelinasativa_Csa15g003950.1 Exp number, first 60 AAs:  0
# Camelinasativa_Csa15g003950.1 Total prob of N-in:        0.01178
Camelinasativa_Csa15g003950.1   TMHMM2.0        outside      1   356
```

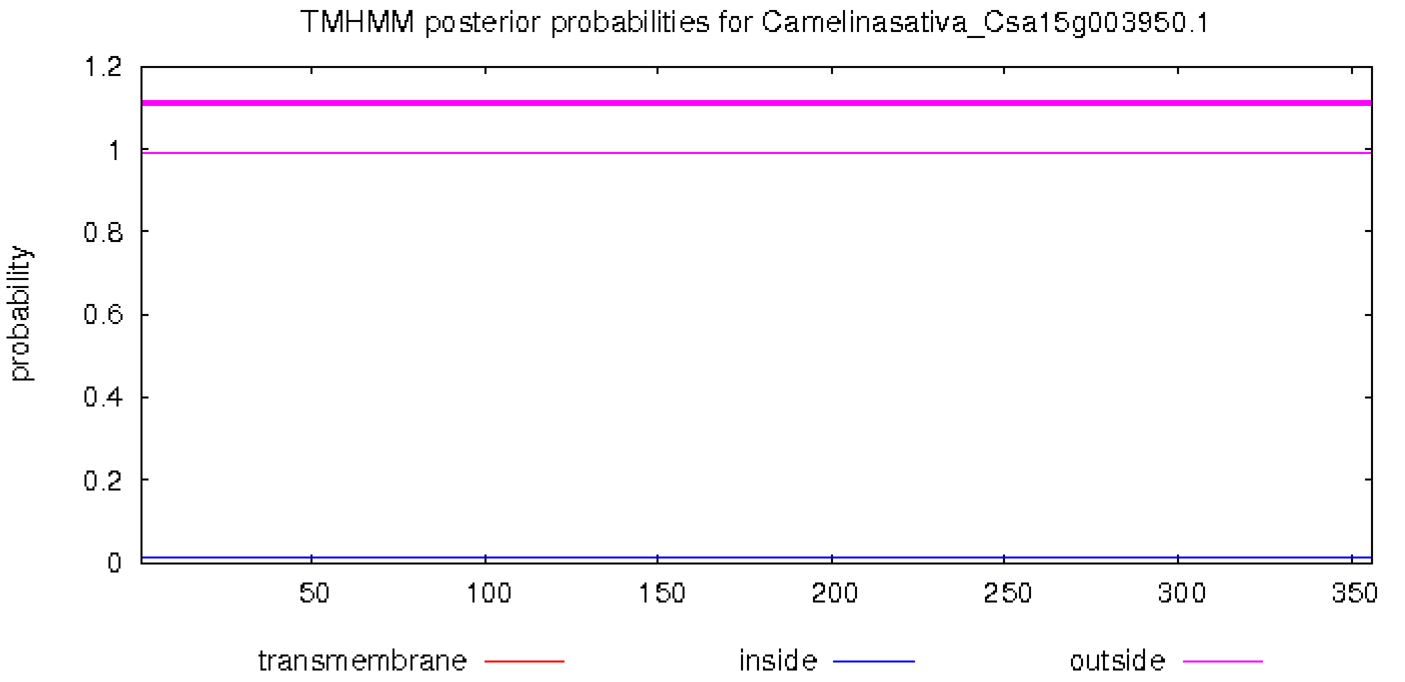

plot in postscript, script for making the plot in gnuplot, data for plot

---

```
# Camelinasativa_Csa15g013970.1 Length: 451
# Camelinasativa_Csa15g013970.1 Number of predicted TMHs:  0
# Camelinasativa_Csa15g013970.1 Exp number of AAs in TMHs: 0.05374
```

```
# Camelinasativa_Csa15g013970.1 Exp number, first 60 AAs:  0.01599
# Camelinasativa_Csa15g013970.1 Total prob of N-in:        0.00331
Camelinasativa_Csa15g013970.1   TMHMM2.0        outside      1   451
```

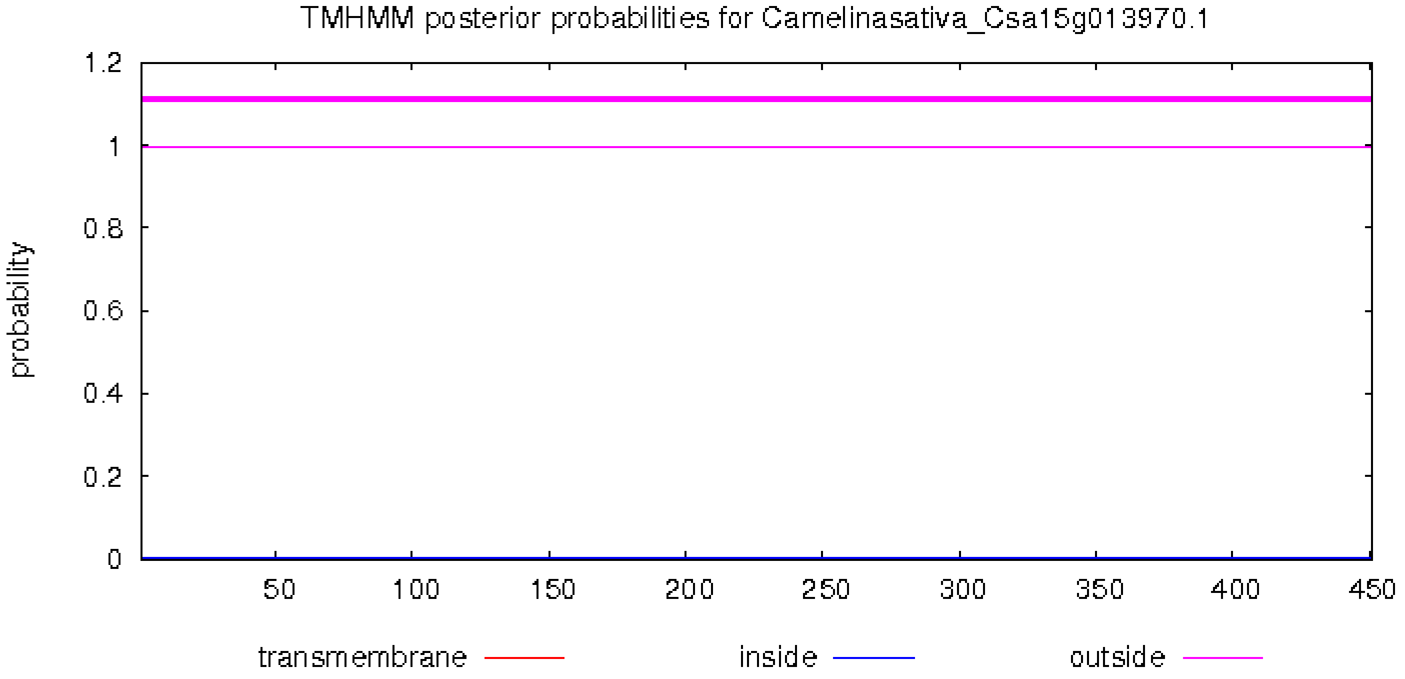

plot in postscript, script for making the plot in gnuplot, data for plot

---

```
# Camelinasativa_Csa15g013990.1 Length: 462
# Camelinasativa_Csa15g013990.1 Number of predicted TMHs:  0
# Camelinasativa_Csa15g013990.1 Exp number of AAs in TMHs: 0.02
# Camelinasativa_Csa15g013990.1 Exp number, first 60 AAs:  0.00086
# Camelinasativa_Csa15g013990.1 Total prob of N-in:        0.00538
Camelinasativa_Csa15g013990.1   TMHMM2.0        outside      1   462
```

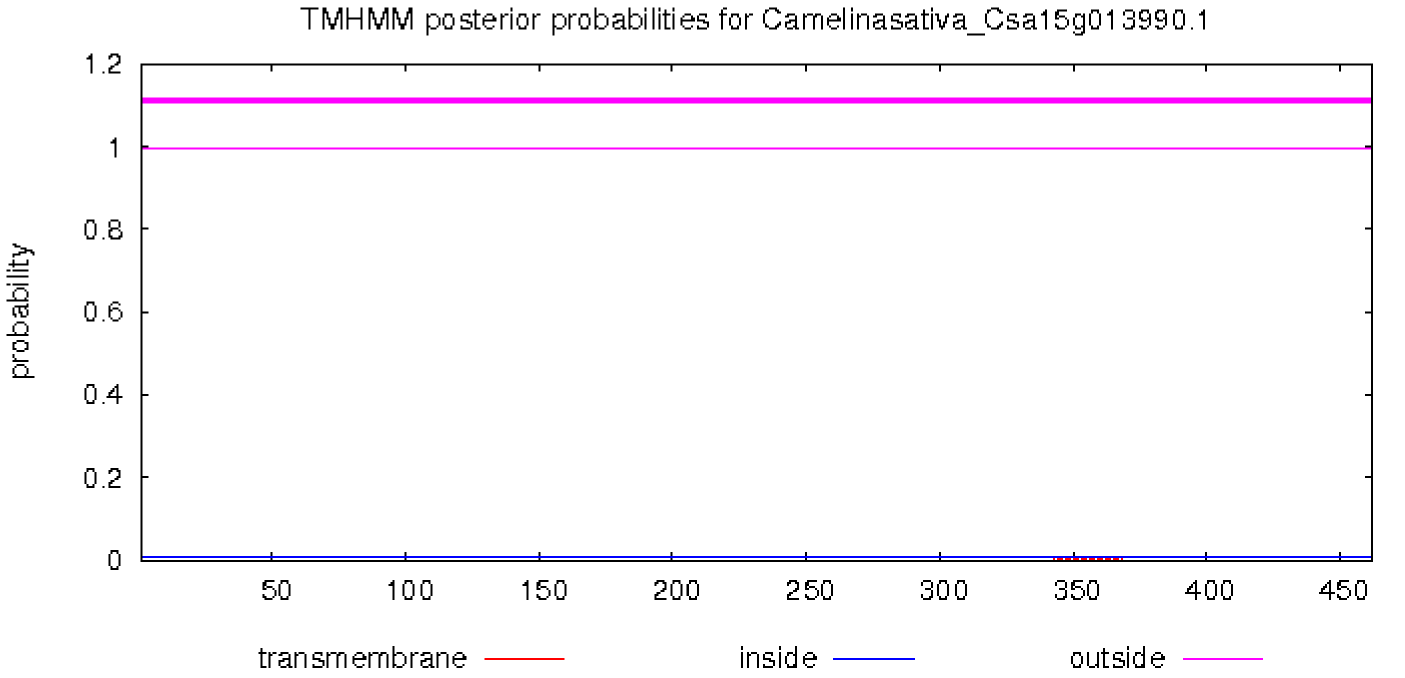

plot in postscript, script for making the plot in gnuplot, data for plot

---

```
# Camelinasativa_Csa15g014000.1 Length: 498
# Camelinasativa_Csa15g014000.1 Number of predicted TMHs:  0
# Camelinasativa_Csa15g014000.1 Exp number of AAs in TMHs: 0.00672
```

```
# Camelinasativa_Csa15g014000.1 Exp number, first 60 AAs:  0
# Camelinasativa_Csa15g014000.1 Total prob of N-in:        0.00073
Camelinasativa_Csa15g014000.1   TMHMM2.0        outside      1   498
```

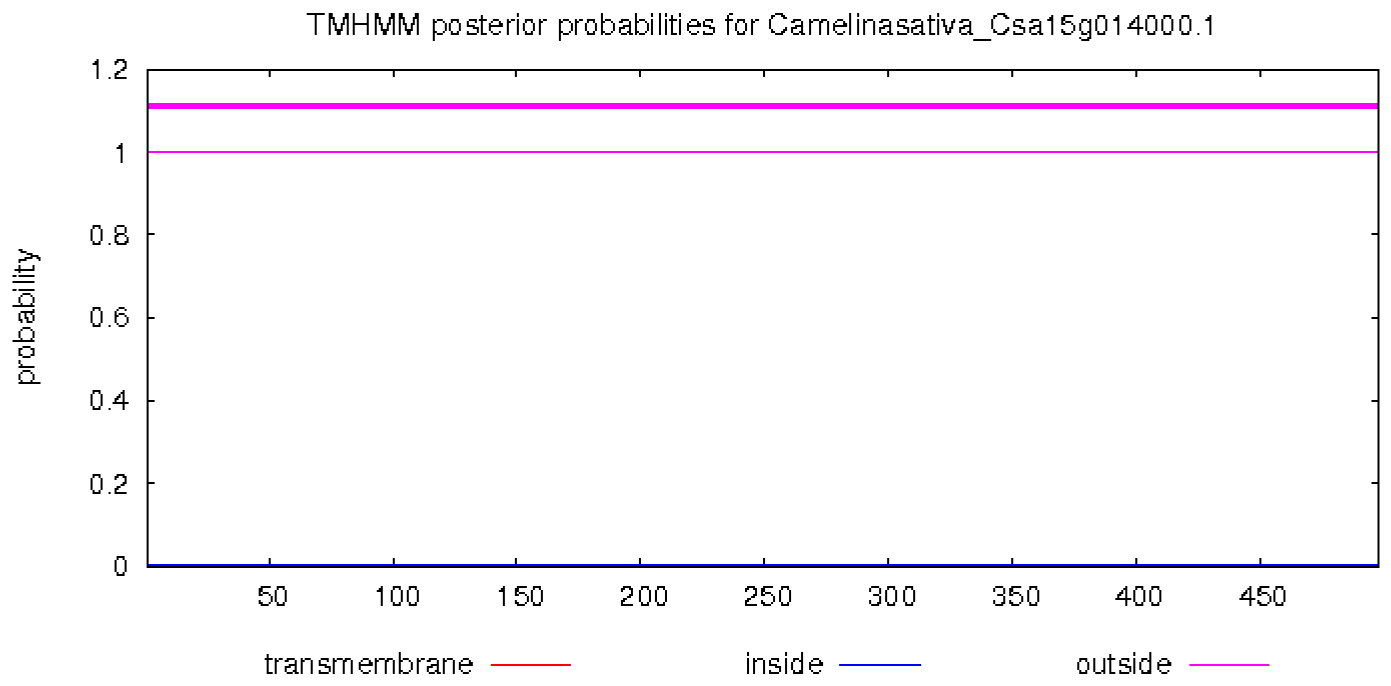

plot in postscript, script for making the plot in gnuplot, data for plot

---

```
# Camelinasativa_Csa15g016850.1 Length: 298
# Camelinasativa_Csa15g016850.1 Number of predicted TMHs:  0
# Camelinasativa_Csa15g016850.1 Exp number of AAs in TMHs: 0.00057
# Camelinasativa_Csa15g016850.1 Exp number, first 60 AAs:  0
# Camelinasativa_Csa15g016850.1 Total prob of N-in:        0.04003
Camelinasativa_Csa15g016850.1   TMHMM2.0        outside      1   298
```

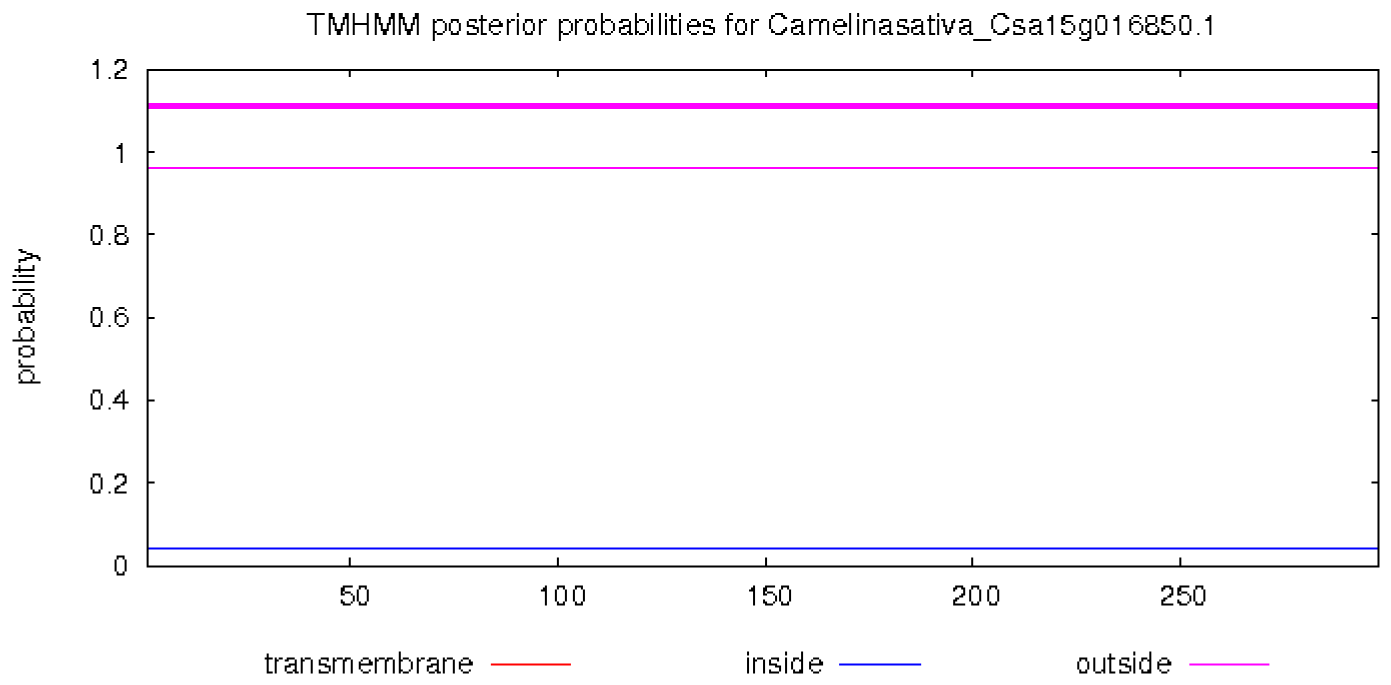

plot in postscript, script for making the plot in gnuplot, data for plot

---

```
# Camelinasativa_Csa15g016960.1 Length: 283
# Camelinasativa_Csa15g016960.1 Number of predicted TMHs:  0
# Camelinasativa_Csa15g016960.1 Exp number of AAs in TMHs: 0.00074
```

```
# Camelinasativa_Csa15g016960.1 Exp number, first 60 AAs:  0
# Camelinasativa_Csa15g016960.1 Total prob of N-in:        0.18179
Camelinasativa_Csa15g016960.1   TMHMM2.0        outside      1   283
```

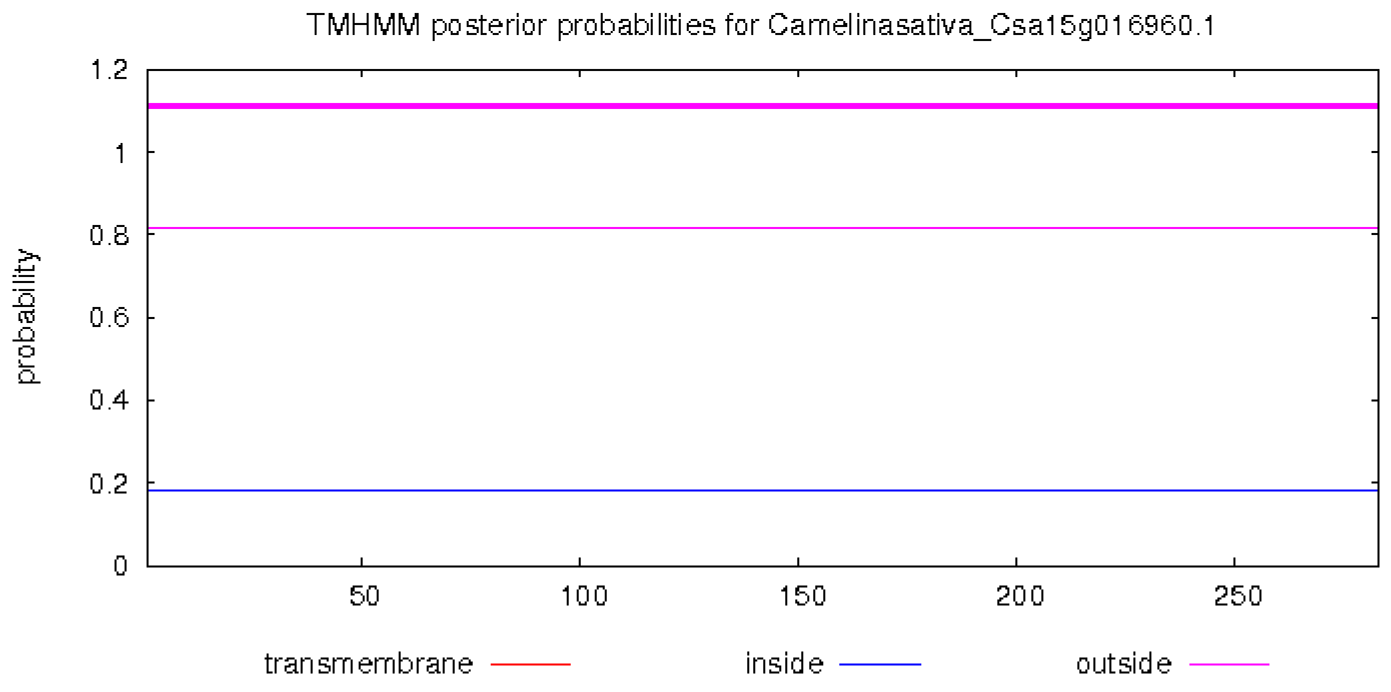

plot in postscript, script for making the plot in gnuplot, data for plot

---

```
# Camelinasativa_Csa15g019650.1 Length: 319
# Camelinasativa_Csa15g019650.1 Number of predicted TMHs:  0
# Camelinasativa_Csa15g019650.1 Exp number of AAs in TMHs: 0.27265
# Camelinasativa_Csa15g019650.1 Exp number, first 60 AAs:  0.00267
# Camelinasativa_Csa15g019650.1 Total prob of N-in:        0.00247
Camelinasativa_Csa15g019650.1   TMHMM2.0        outside      1   319
```

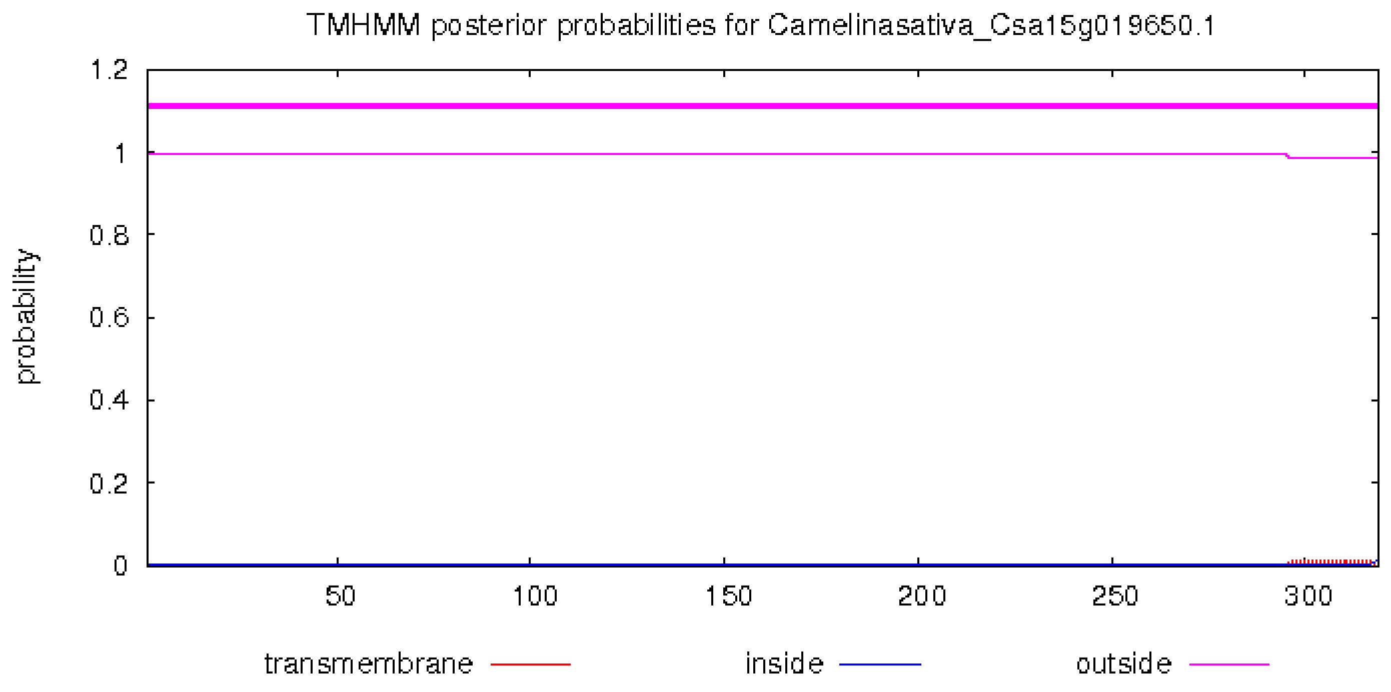

plot in postscript, script for making the plot in gnuplot, data for plot

---

```
# Camelinasativa_Csa15g020040.1 Length: 319
# Camelinasativa_Csa15g020040.1 Number of predicted TMHs:  0
# Camelinasativa_Csa15g020040.1 Exp number of AAs in TMHs: 0
```

```
# Camelinasativa_Csa15g020040.1 Exp number, first 60 AAs:  0
# Camelinasativa_Csa15g020040.1 Total prob of N-in:        0.00235
Camelinasativa_Csa15g020040.1   TMHMM2.0        outside      1   319
```

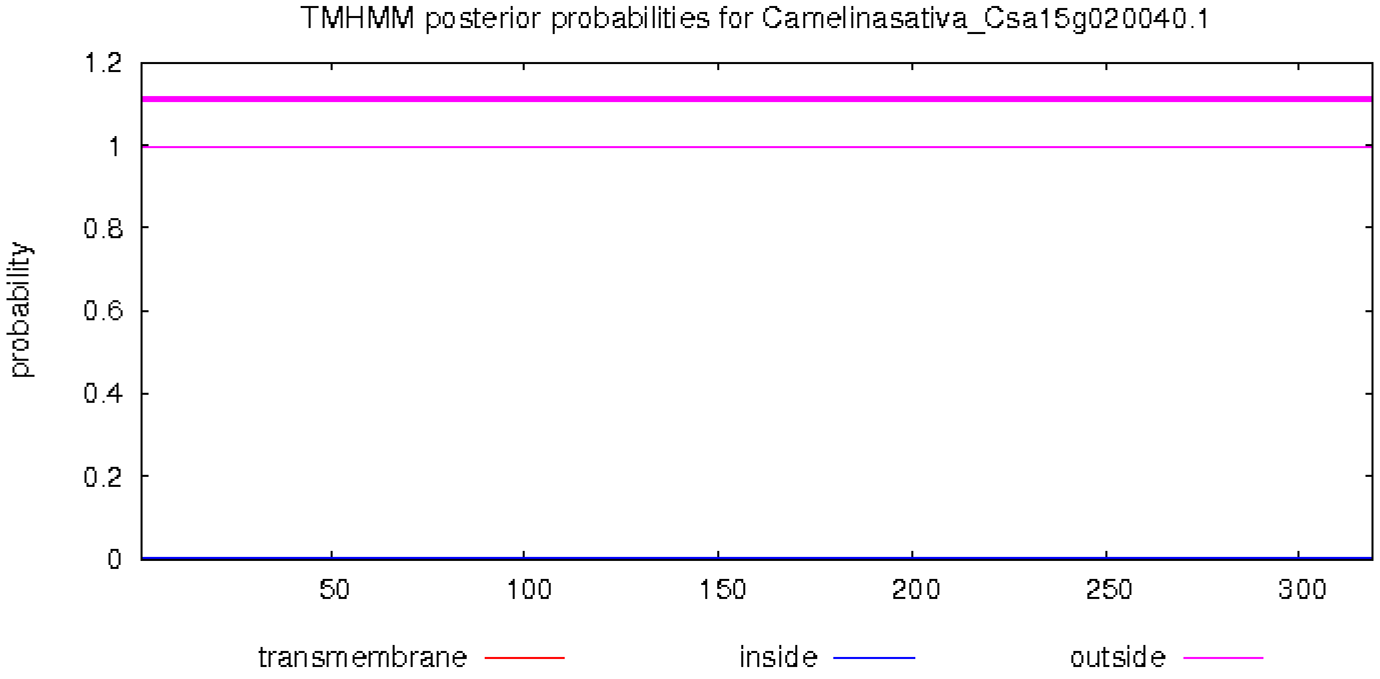

plot in postscript, script for making the plot in gnuplot, data for plot

```
# Camelinasativa_Csa15g020050.1 Length: 369
# Camelinasativa_Csa15g020050.1 Number of predicted TMHs:  0
# Camelinasativa_Csa15g020050.1 Exp number of AAs in TMHs: 0.00223
# Camelinasativa_Csa15g020050.1 Exp number, first 60 AAs:  0.00017
# Camelinasativa_Csa15g020050.1 Total prob of N-in:        0.00382
Camelinasativa_Csa15g020050.1   TMHMM2.0        outside      1   369
```

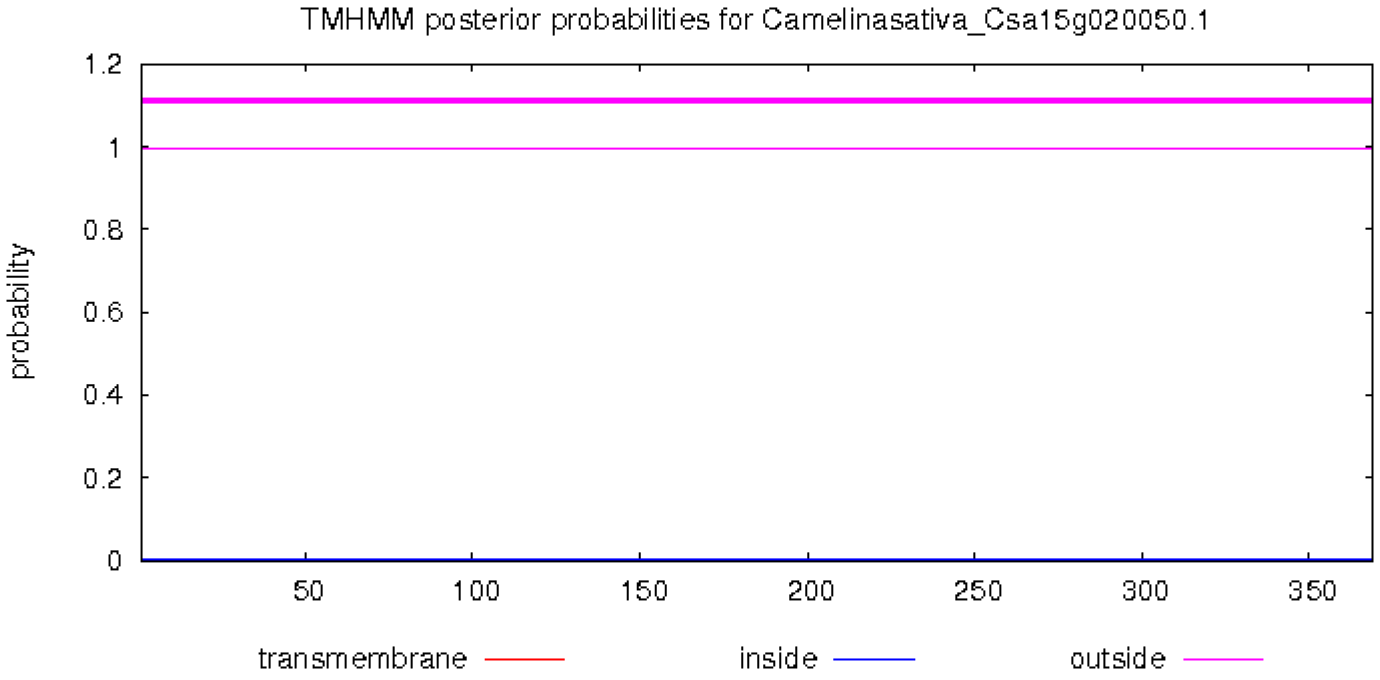

plot in postscript, script for making the plot in gnuplot, data for plot

```
# Camelinasativa_Csa15g023980.1 Length: 247
# Camelinasativa_Csa15g023980.1 Number of predicted TMHs:  0
# Camelinasativa_Csa15g023980.1 Exp number of AAs in TMHs: 0
```

```
# Camelinasativa_Csa15g023980.1 Exp number, first 60 AAs:  0
# Camelinasativa_Csa15g023980.1 Total prob of N-in:        0.00220
Camelinasativa_Csa15g023980.1   TMHMM2.0        outside      1   247
```

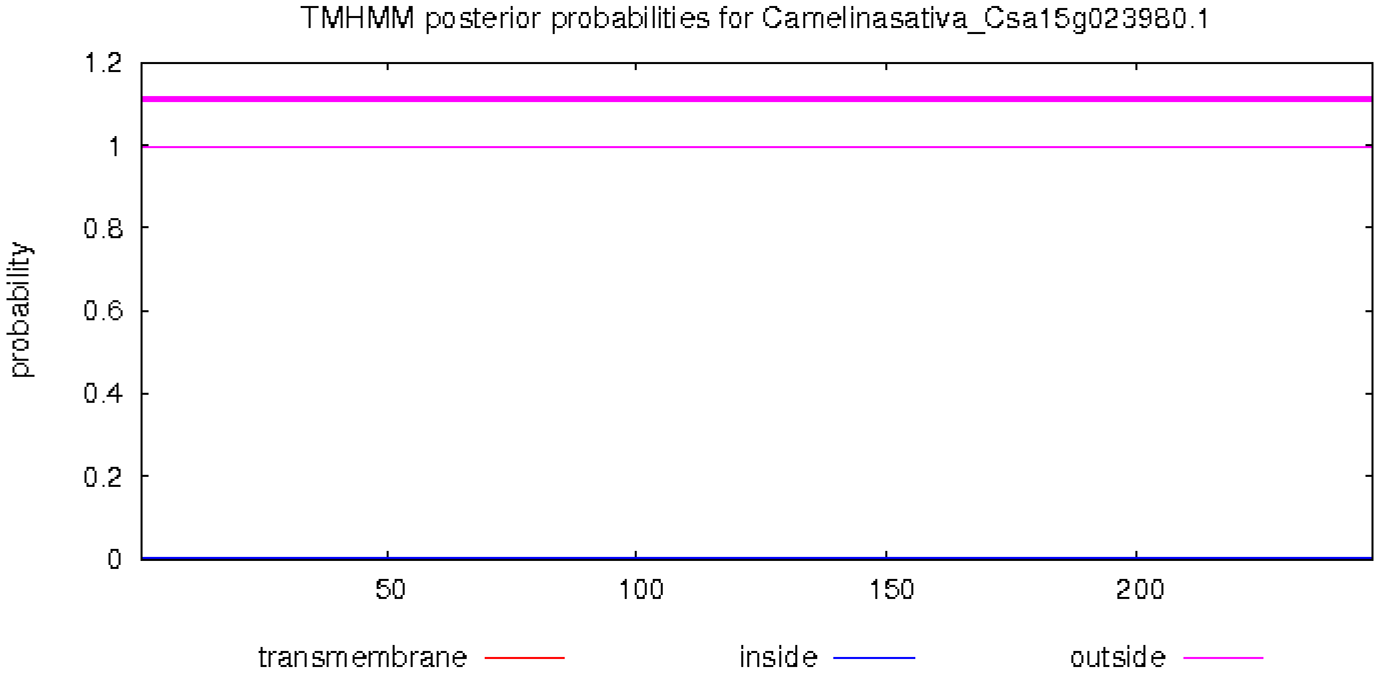

plot in postscript, script for making the plot in gnuplot, data for plot

---

```
# Camelinasativa_Csa15g024970.1 Length: 216
# Camelinasativa_Csa15g024970.1 Number of predicted TMHs:  0
# Camelinasativa_Csa15g024970.1 Exp number of AAs in TMHs: 0.00127
# Camelinasativa_Csa15g024970.1 Exp number, first 60 AAs:  0
# Camelinasativa_Csa15g024970.1 Total prob of N-in:        0.03607
Camelinasativa_Csa15g024970.1   TMHMM2.0        outside      1   216
```

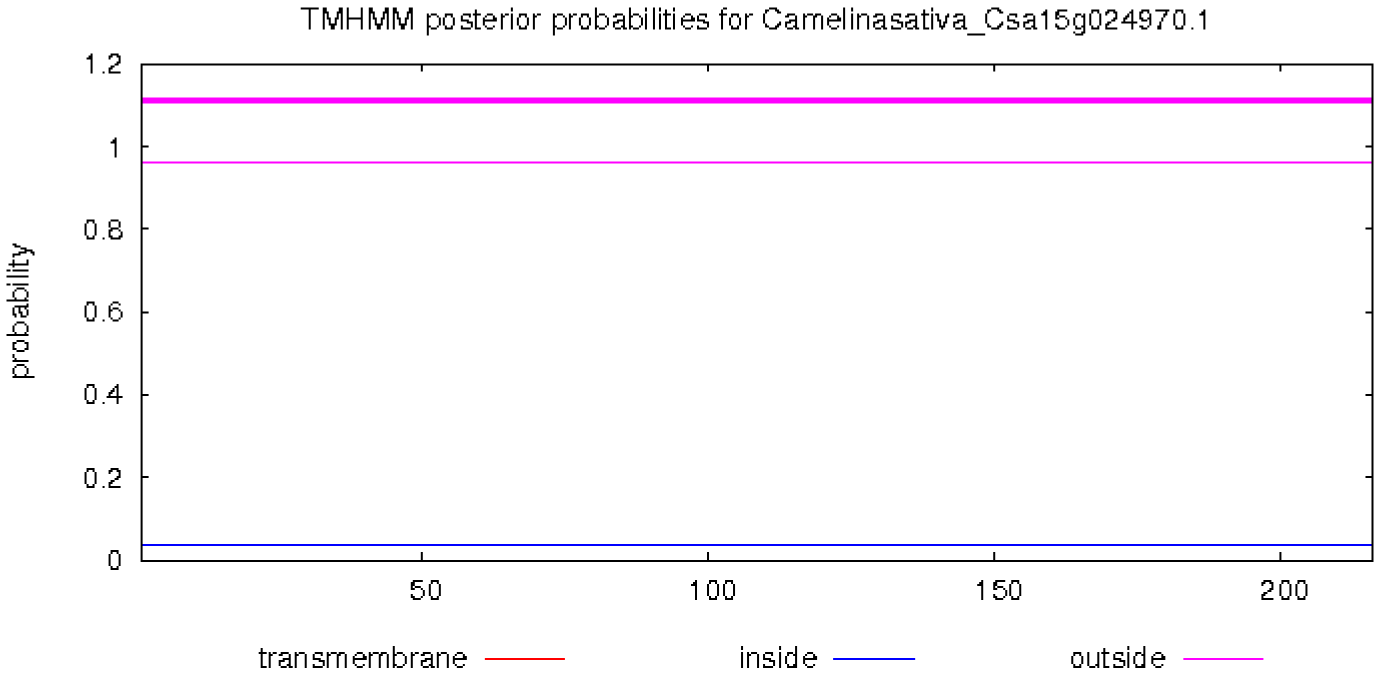

plot in postscript, script for making the plot in gnuplot, data for plot

---

```
# Camelinasativa_Csa15g071340.1 Length: 179
# Camelinasativa_Csa15g071340.1 Number of predicted TMHs:  0
# Camelinasativa_Csa15g071340.1 Exp number of AAs in TMHs: 0.00051
```

```
# Camelinasativa_Csa15g071340.1 Exp number, first 60 AAs:  0.00026
# Camelinasativa_Csa15g071340.1 Total prob of N-in:        0.26784
Camelinasativa_Csa15g071340.1   TMHMM2.0        outside      1   179
```

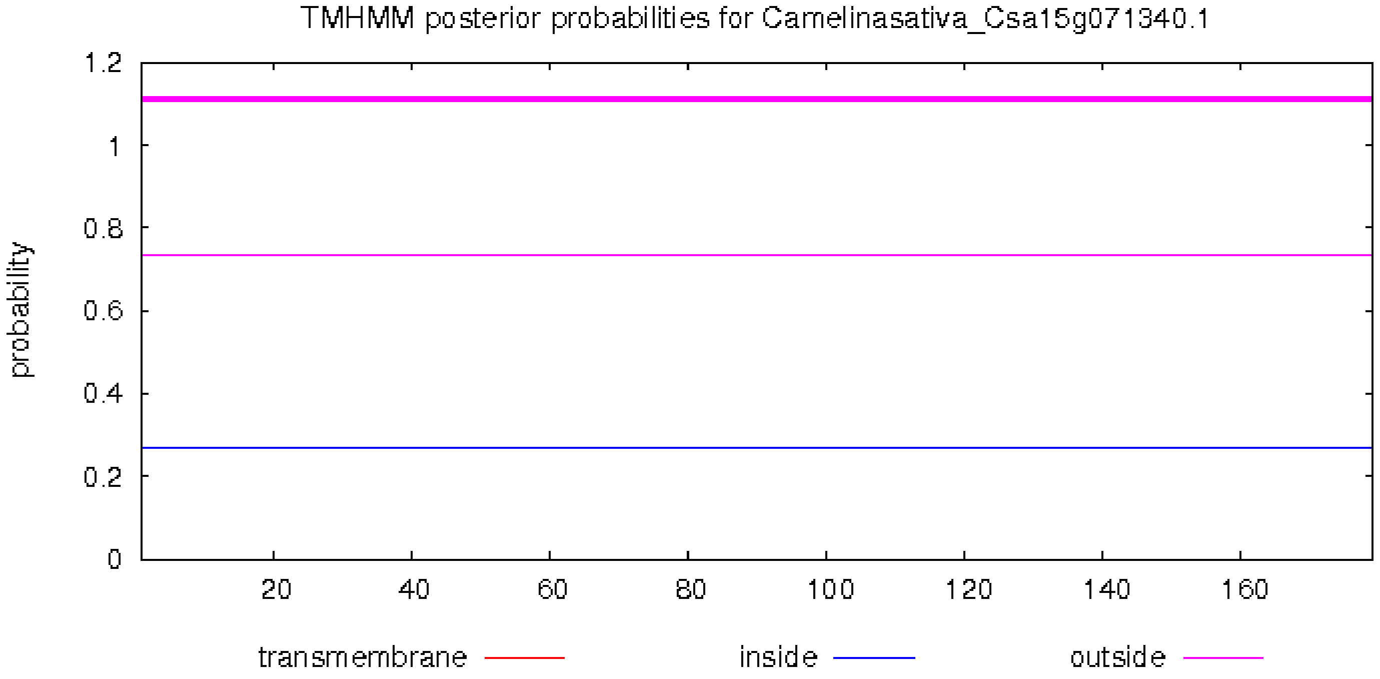

plot in postscript, script for making the plot in gnuplot, data for plot

---

```
# Camelinasativa_Csa15g074090.1 Length: 309
# Camelinasativa_Csa15g074090.1 Number of predicted TMHs:  0
# Camelinasativa_Csa15g074090.1 Exp number of AAs in TMHs: 2.06504
# Camelinasativa_Csa15g074090.1 Exp number, first 60 AAs:  0.00526
# Camelinasativa_Csa15g074090.1 Total prob of N-in:        0.07439
Camelinasativa_Csa15g074090.1   TMHMM2.0        outside      1   309
```

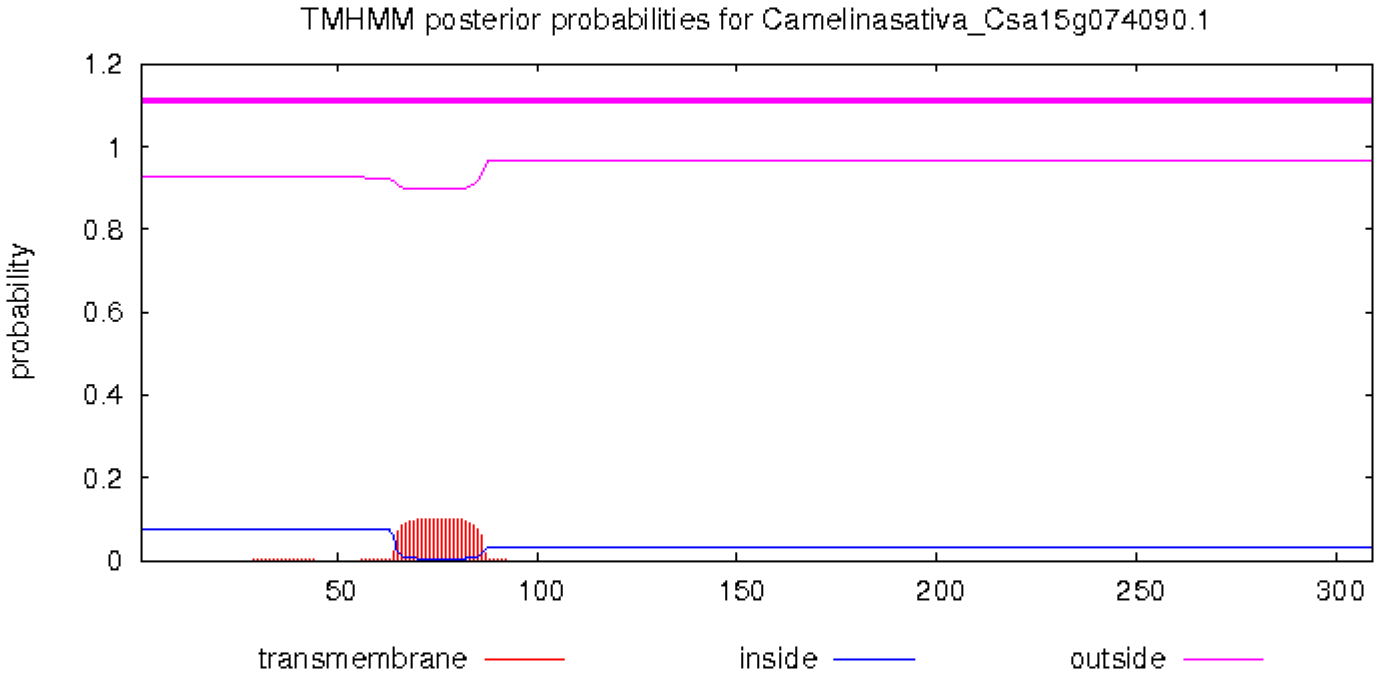

plot in postscript, script for making the plot in gnuplot, data for plot

---

```
# Camelinasativa_Csa16729s010.1 Length: 70
# Camelinasativa_Csa16729s010.1 Number of predicted TMHs:  0
# Camelinasativa_Csa16729s010.1 Exp number of AAs in TMHs: 0
```

```
# Camelinasativa_Csa16729s010.1 Exp number, first 60 AAs:  0
# Camelinasativa_Csa16729s010.1 Total prob of N-in:        0.15407
Camelinasativa_Csa16729s010.1   TMHMM2.0        outside      1    70
```

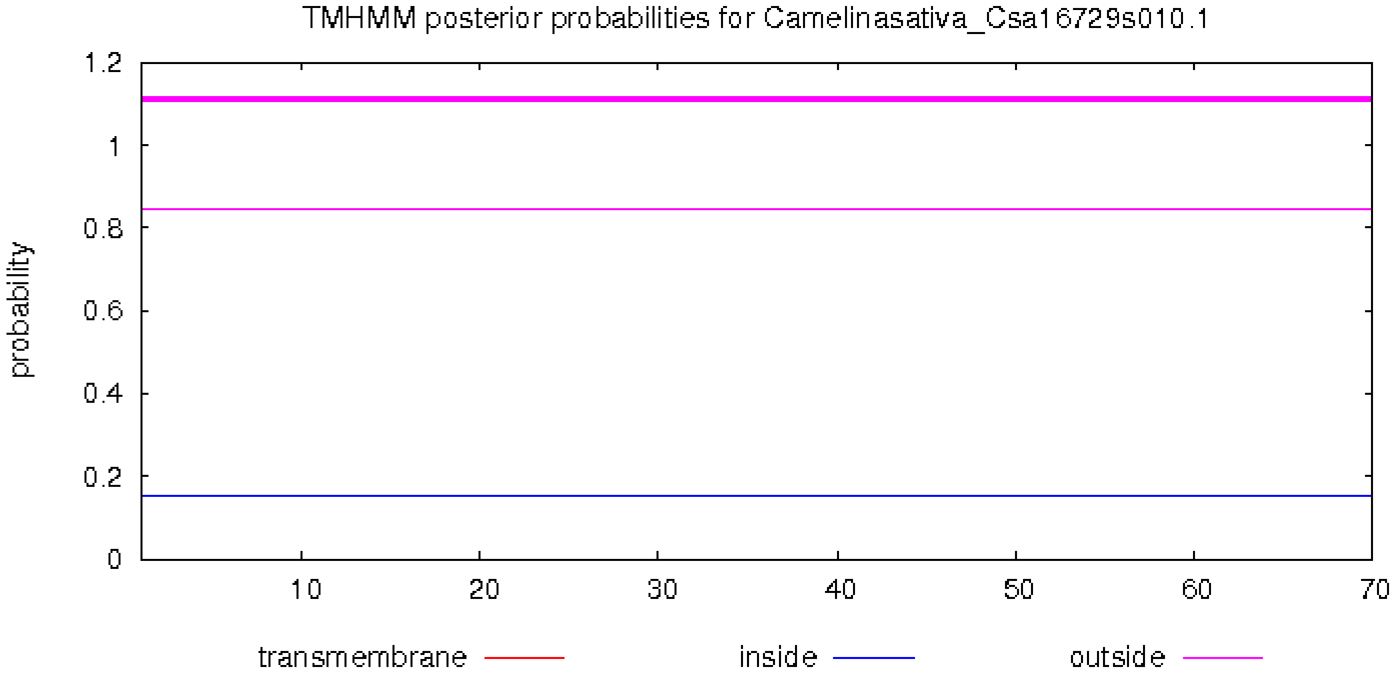

plot in postscript, script for making the plot in gnuplot, data for plot

---

```
# Camelinasativa_Csa16g002750.1 Length: 336
# Camelinasativa_Csa16g002750.1 Number of predicted TMHs:  0
# Camelinasativa_Csa16g002750.1 Exp number of AAs in TMHs: 0
# Camelinasativa_Csa16g002750.1 Exp number, first 60 AAs:  0
# Camelinasativa_Csa16g002750.1 Total prob of N-in:        0.02969
Camelinasativa_Csa16g002750.1   TMHMM2.0        outside      1   336
```

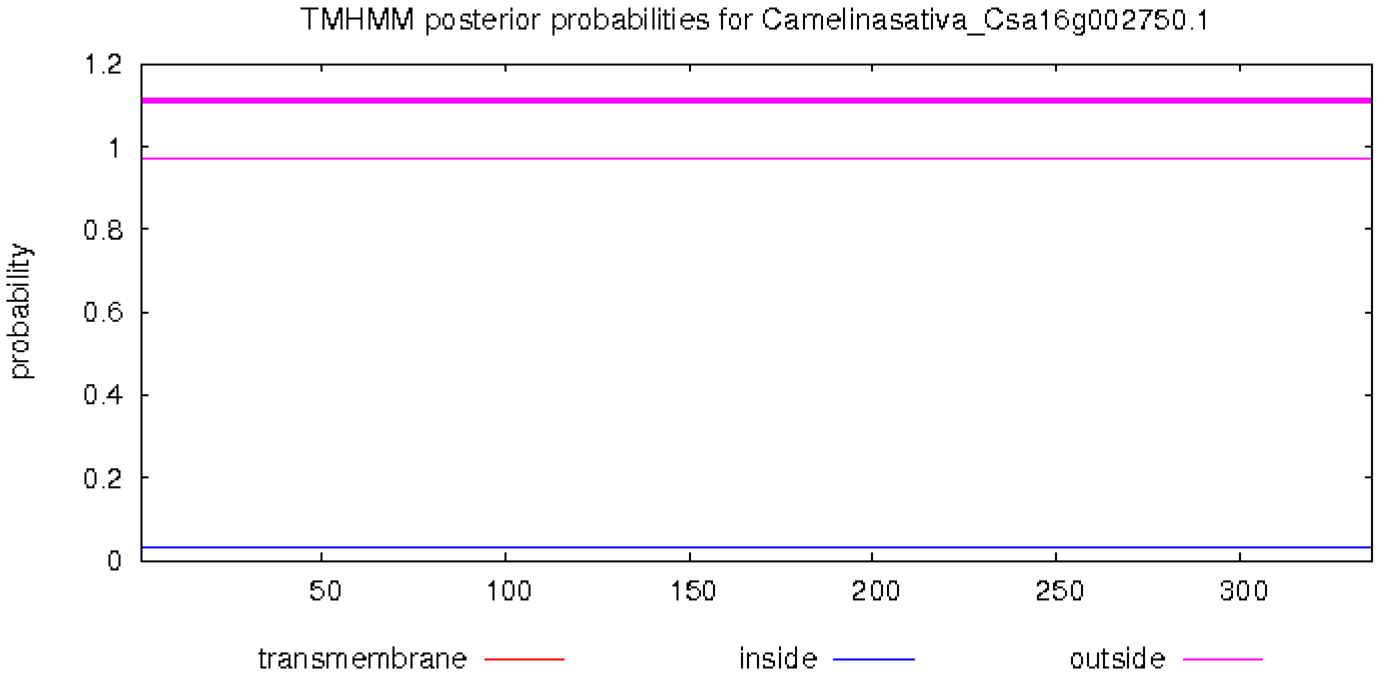

plot in postscript, script for making the plot in gnuplot, data for plot

---

```
# Camelinasativa_Csa16g007760.1 Length: 220
# Camelinasativa_Csa16g007760.1 Number of predicted TMHs:  0
# Camelinasativa_Csa16g007760.1 Exp number of AAs in TMHs: 0.00015
```

```
# Camelinasativa_Csa16g007760.1 Exp number, first 60 AAs:  0
# Camelinasativa_Csa16g007760.1 Total prob of N-in:        0.44044
Camelinasativa_Csa16g007760.1	TMHMM2.0	outside	     1   220
```

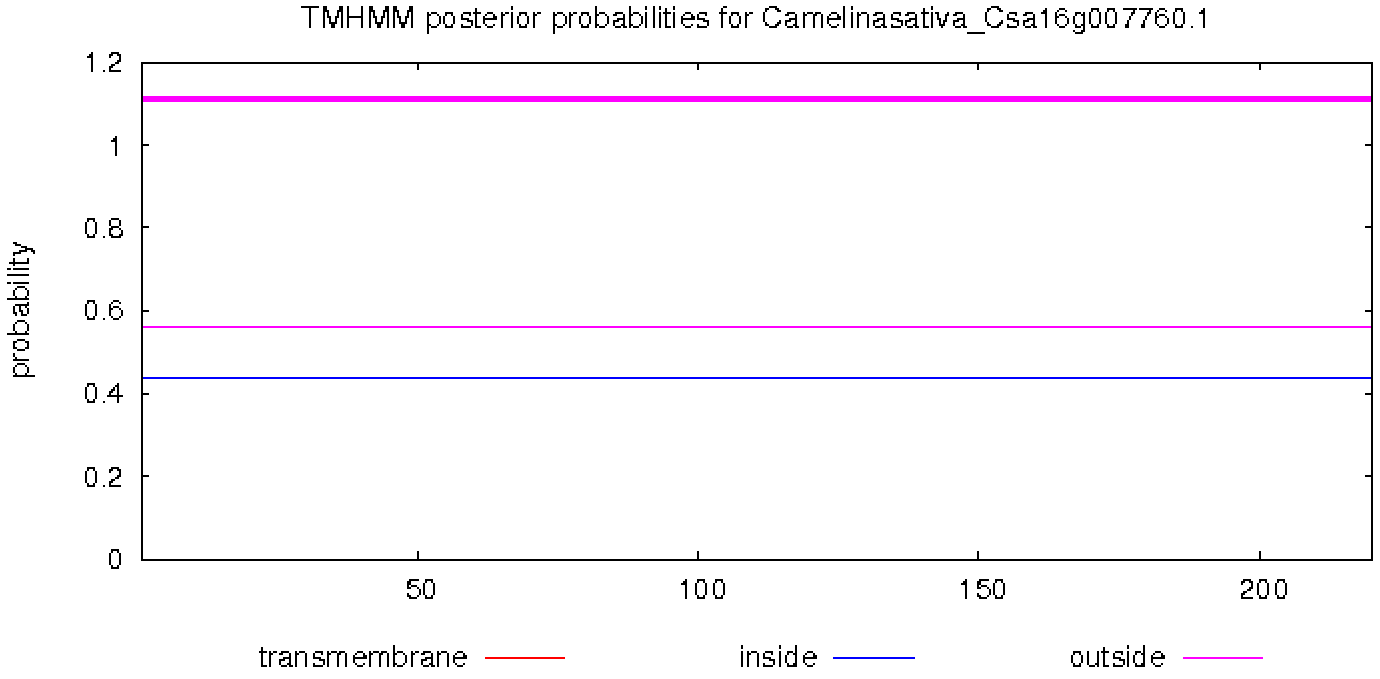

plot in postscript, script for making the plot in gnuplot, data for plot

---

```
# Camelinasativa_Csa16g023250.1 Length: 623
# Camelinasativa_Csa16g023250.1 Number of predicted TMHs:  0
# Camelinasativa_Csa16g023250.1 Exp number of AAs in TMHs: 14.3557
# Camelinasativa_Csa16g023250.1 Exp number, first 60 AAs:  0
# Camelinasativa_Csa16g023250.1 Total prob of N-in:        0.00012
Camelinasativa_Csa16g023250.1	TMHMM2.0	outside	     1   623
```

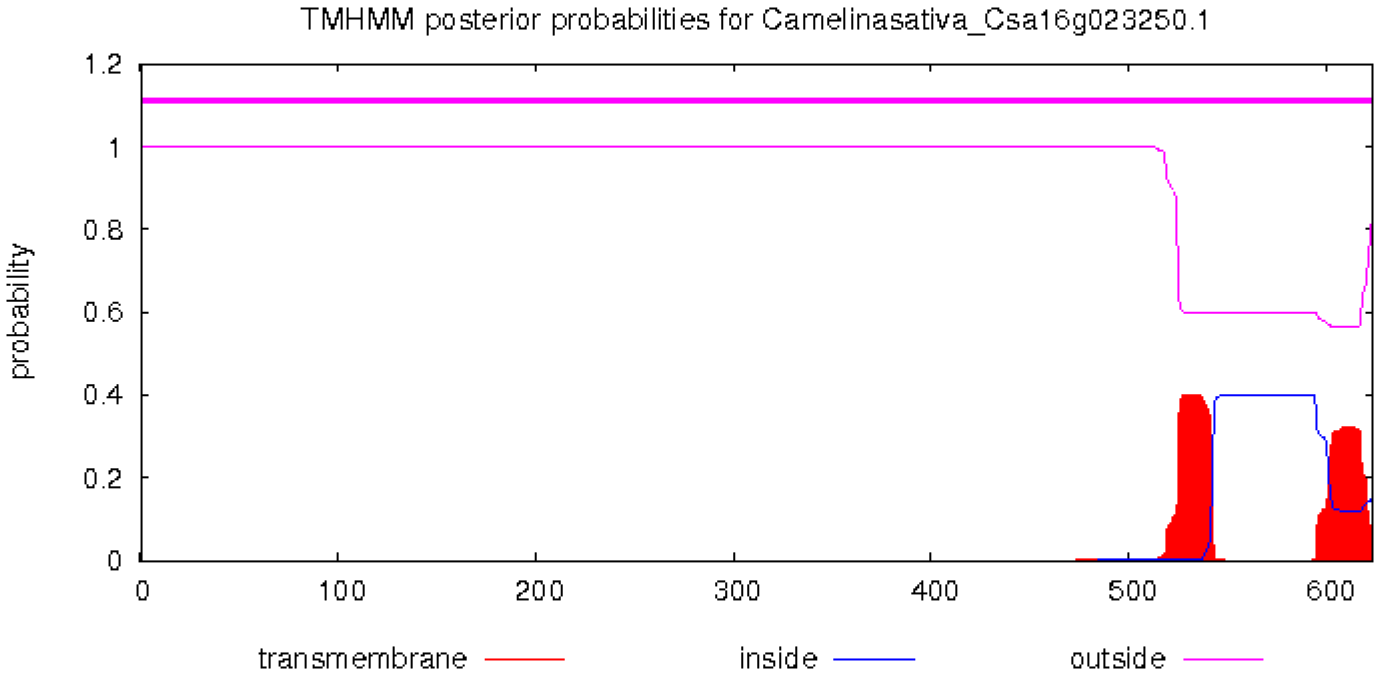

plot in postscript, script for making the plot in gnuplot, data for plot

---

```
# Camelinasativa_Csa16g030850.1 Length: 272
# Camelinasativa_Csa16g030850.1 Number of predicted TMHs:  0
# Camelinasativa_Csa16g030850.1 Exp number of AAs in TMHs: 0.00058
```

```
# Camelinasativa_Csa16g030850.1 Exp number, first 60 AAs:  0
# Camelinasativa_Csa16g030850.1 Total prob of N-in:        0.01822
Camelinasativa_Csa16g030850.1   TMHMM2.0        outside      1   272
```

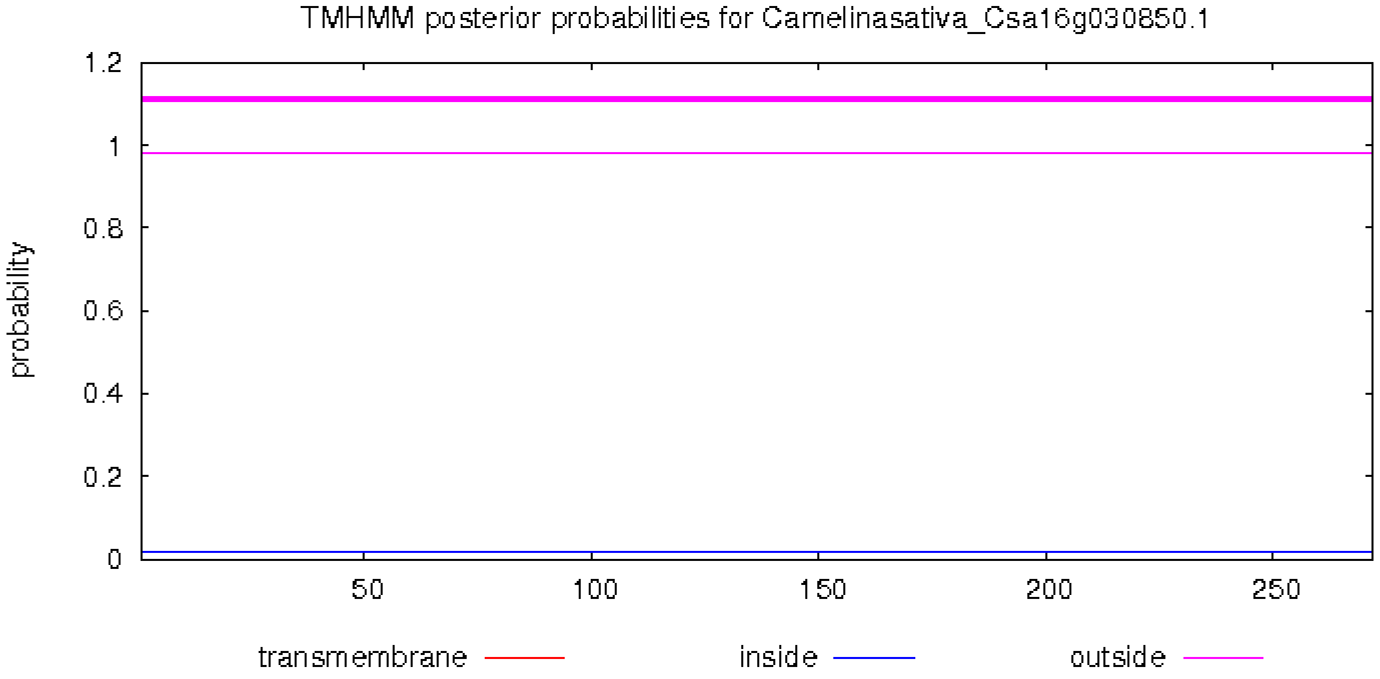

plot in postscript, script for making the plot in gnuplot, data for plot

---

```
# Camelinasativa_Csa16g031130.1 Length: 393
# Camelinasativa_Csa16g031130.1 Number of predicted TMHs:  1
# Camelinasativa_Csa16g031130.1 Exp number of AAs in TMHs: 20.675
# Camelinasativa_Csa16g031130.1 Exp number, first 60 AAs:  0
# Camelinasativa_Csa16g031130.1 Total prob of N-in:        0.18806
Camelinasativa_Csa16g031130.1   TMHMM2.0        outside      1   369
Camelinasativa_Csa16g031130.1   TMHMM2.0        TMhelix    370   392
Camelinasativa_Csa16g031130.1   TMHMM2.0        inside     393   393
```

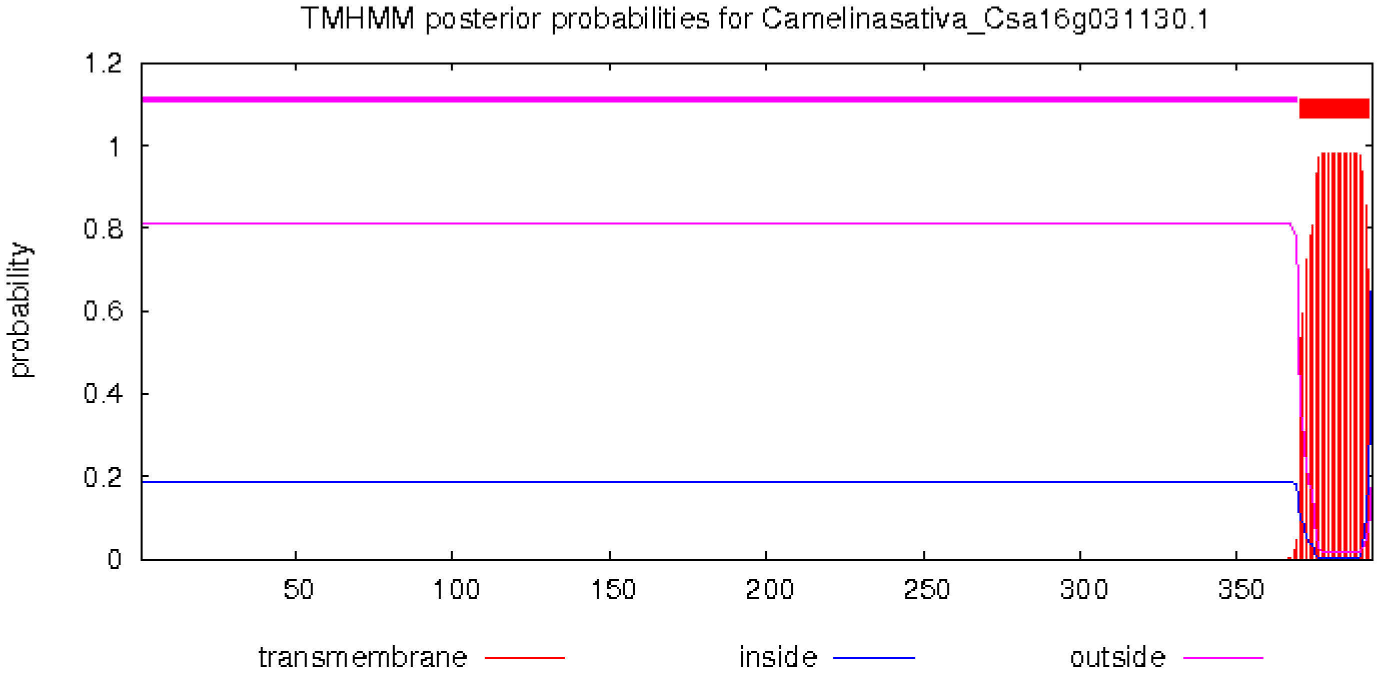

plot in postscript, script for making the plot in gnuplot, data for plot

---

```
# Camelinasativa_Csa16g034580.1 Length: 326
# Camelinasativa_Csa16g034580.1 Number of predicted TMHs:  0
# Camelinasativa_Csa16g034580.1 Exp number of AAs in TMHs: 0
# Camelinasativa_Csa16g034580.1 Exp number, first 60 AAs:  0
# Camelinasativa_Csa16g034580.1 Total prob of N-in:        0.01705
Camelinasativa_Csa16g034580.1   TMHMM2.0        outside      1   326
```

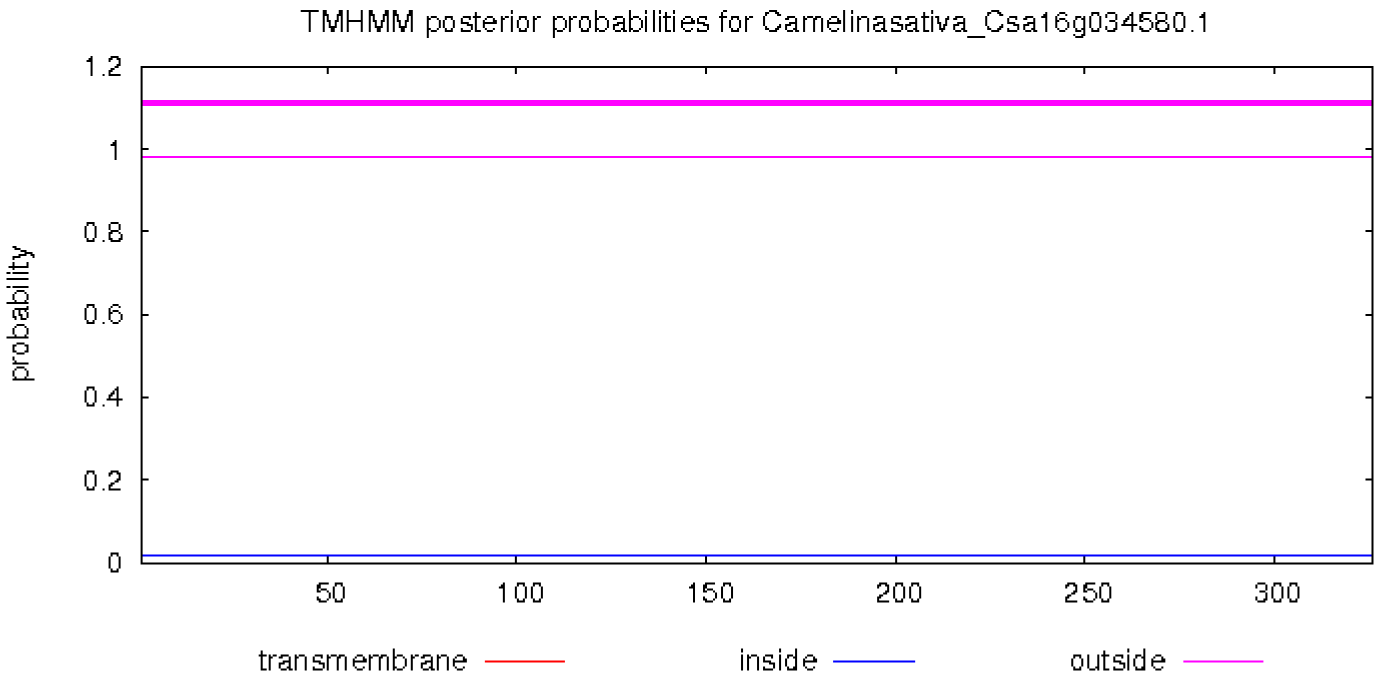

plot in postscript, script for making the plot in gnuplot, data for plot

---

```
# Camelinasativa_Csa16g040560.1 Length: 493
# Camelinasativa_Csa16g040560.1 Number of predicted TMHs:  0
# Camelinasativa_Csa16g040560.1 Exp number of AAs in TMHs: 0.00632
# Camelinasativa_Csa16g040560.1 Exp number, first 60 AAs:  0.00477
# Camelinasativa_Csa16g040560.1 Total prob of N-in:        0.00090
Camelinasativa_Csa16g040560.1   TMHMM2.0        outside      1   493
```

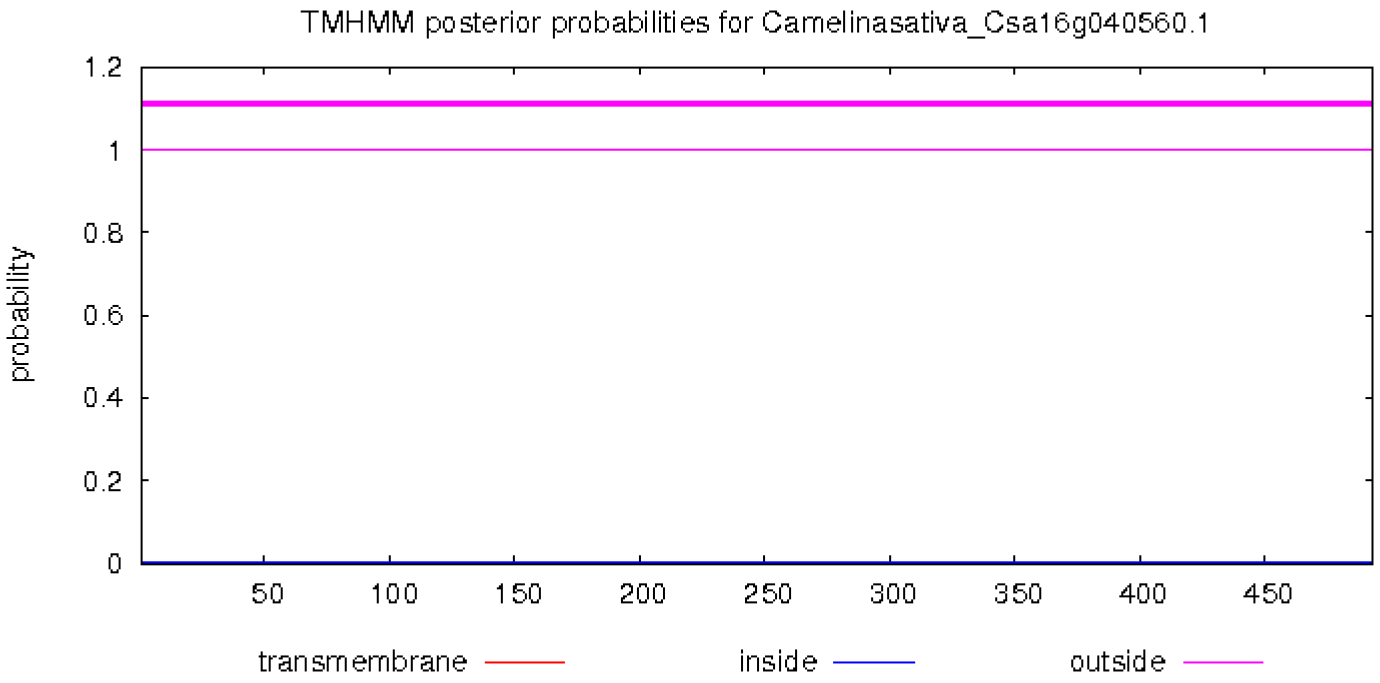

plot in postscript, script for making the plot in gnuplot, data for plot

---

```
# Camelinasativa_Csa16g041630.1 Length: 250
# Camelinasativa_Csa16g041630.1 Number of predicted TMHs:  0
# Camelinasativa_Csa16g041630.1 Exp number of AAs in TMHs: 0.00021
# Camelinasativa_Csa16g041630.1 Exp number, first 60 AAs:  0
# Camelinasativa_Csa16g041630.1 Total prob of N-in:        0.02270
Camelinasativa_Csa16g041630.1	TMHMM2.0	outside	     1   250
```

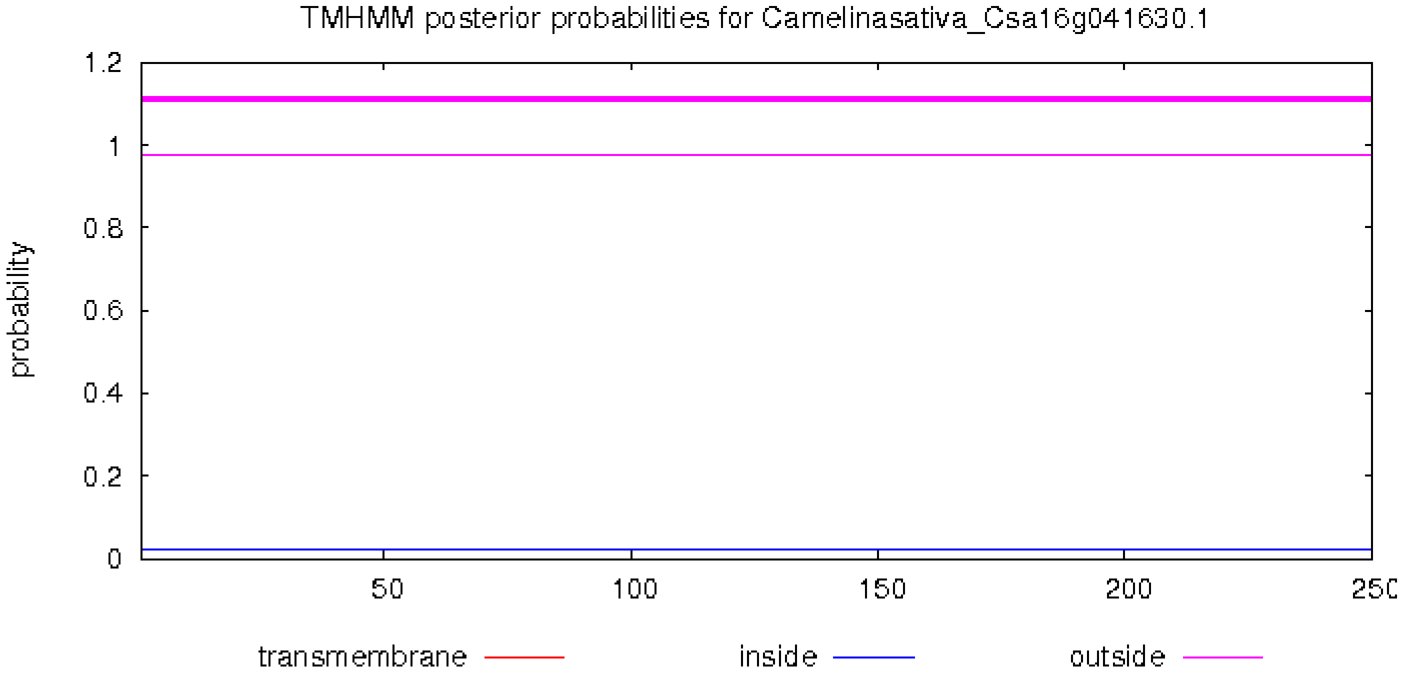

plot in postscript, script for making the plot in gnuplot, data for plot

---

```
# Camelinasativa_Csa16g046090.1 Length: 317
# Camelinasativa_Csa16g046090.1 Number of predicted TMHs:  0
# Camelinasativa_Csa16g046090.1 Exp number of AAs in TMHs: 0.02891
# Camelinasativa_Csa16g046090.1 Exp number, first 60 AAs:  0
# Camelinasativa_Csa16g046090.1 Total prob of N-in:        0.07369
Camelinasativa_Csa16g046090.1	TMHMM2.0	outside	     1   317
```

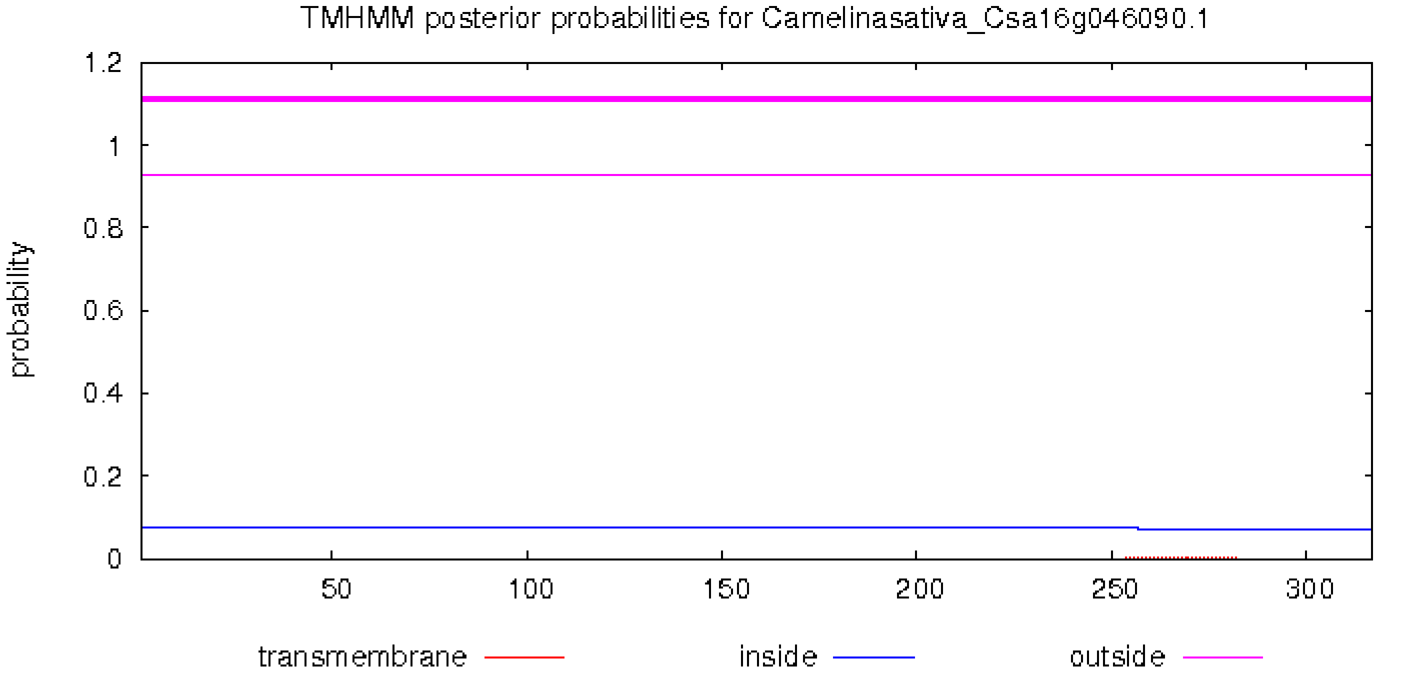

plot in postscript, script for making the plot in gnuplot, data for plot

---

```
# Camelinasativa_Csa16g049230.1 Length: 376
# Camelinasativa_Csa16g049230.1 Number of predicted TMHs:  0
# Camelinasativa_Csa16g049230.1 Exp number of AAs in TMHs: 0.00689
# Camelinasativa_Csa16g049230.1 Exp number, first 60 AAs:  0.00096
# Camelinasativa_Csa16g049230.1 Total prob of N-in:        0.00721
Camelinasativa_Csa16g049230.1   TMHMM2.0        outside      1   376
```

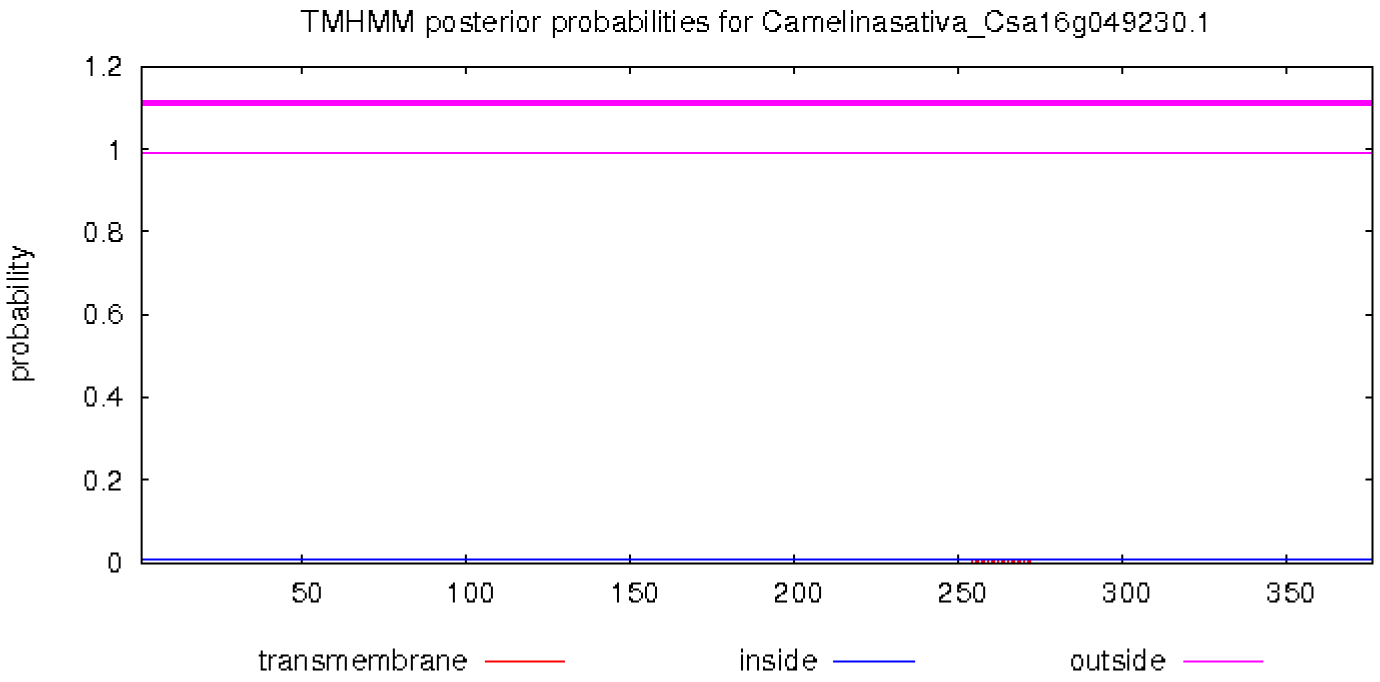

plot in postscript, script for making the plot in gnuplot, data for plot

---

```
# Camelinasativa_Csa16g051500.1 Length: 339
# Camelinasativa_Csa16g051500.1 Number of predicted TMHs:  1
# Camelinasativa_Csa16g051500.1 Exp number of AAs in TMHs: 20.84967
# Camelinasativa_Csa16g051500.1 Exp number, first 60 AAs:  0.11432
# Camelinasativa_Csa16g051500.1 Total prob of N-in:        0.60918
Camelinasativa_Csa16g051500.1   TMHMM2.0        outside      1   315
Camelinasativa_Csa16g051500.1   TMHMM2.0        TMhelix    316   335
Camelinasativa_Csa16g051500.1   TMHMM2.0        inside     336   339
```

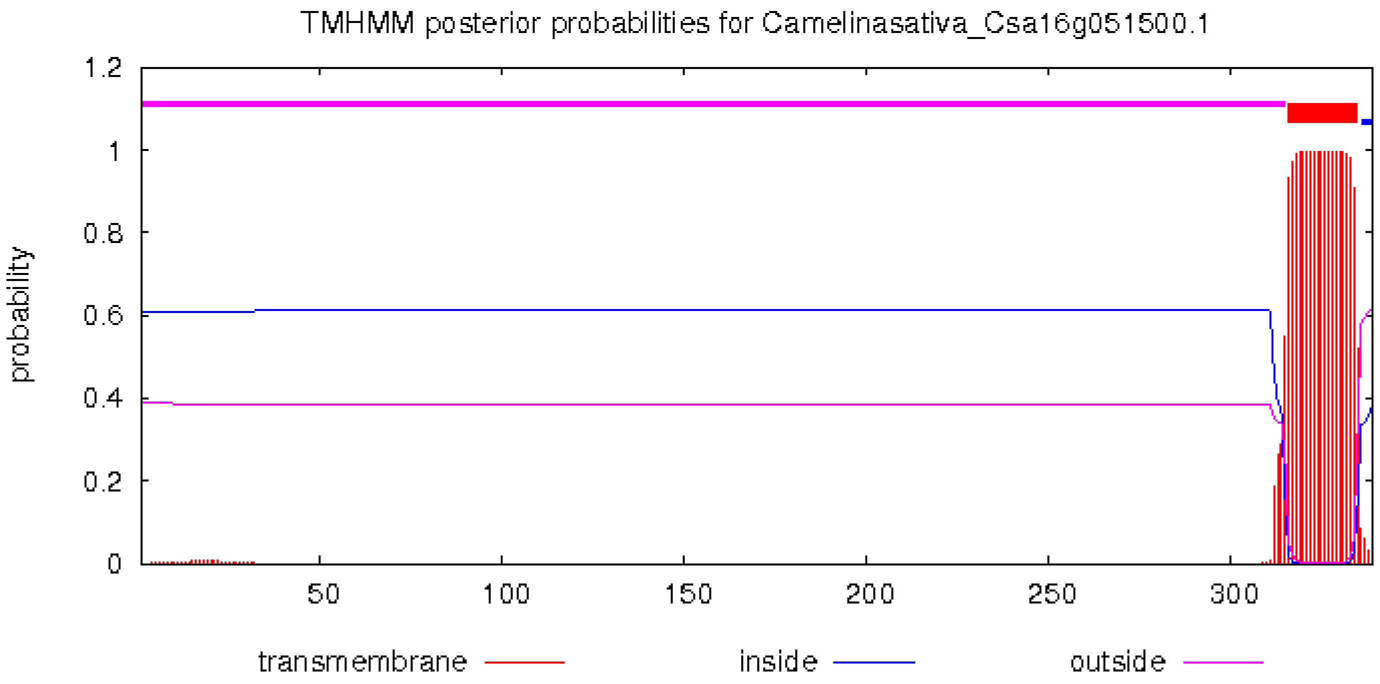

plot in postscript, script for making the plot in gnuplot, data for plot

```
# Camelinasativa_Csa16g052230.1 Length: 180
# Camelinasativa_Csa16g052230.1 Number of predicted TMHs:  0
# Camelinasativa_Csa16g052230.1 Exp number of AAs in TMHs: 0.11876
# Camelinasativa_Csa16g052230.1 Exp number, first 60 AAs:  0.06841
# Camelinasativa_Csa16g052230.1 Total prob of N-in:        0.33330
Camelinasativa_Csa16g052230.1   TMHMM2.0        outside      1   180
```

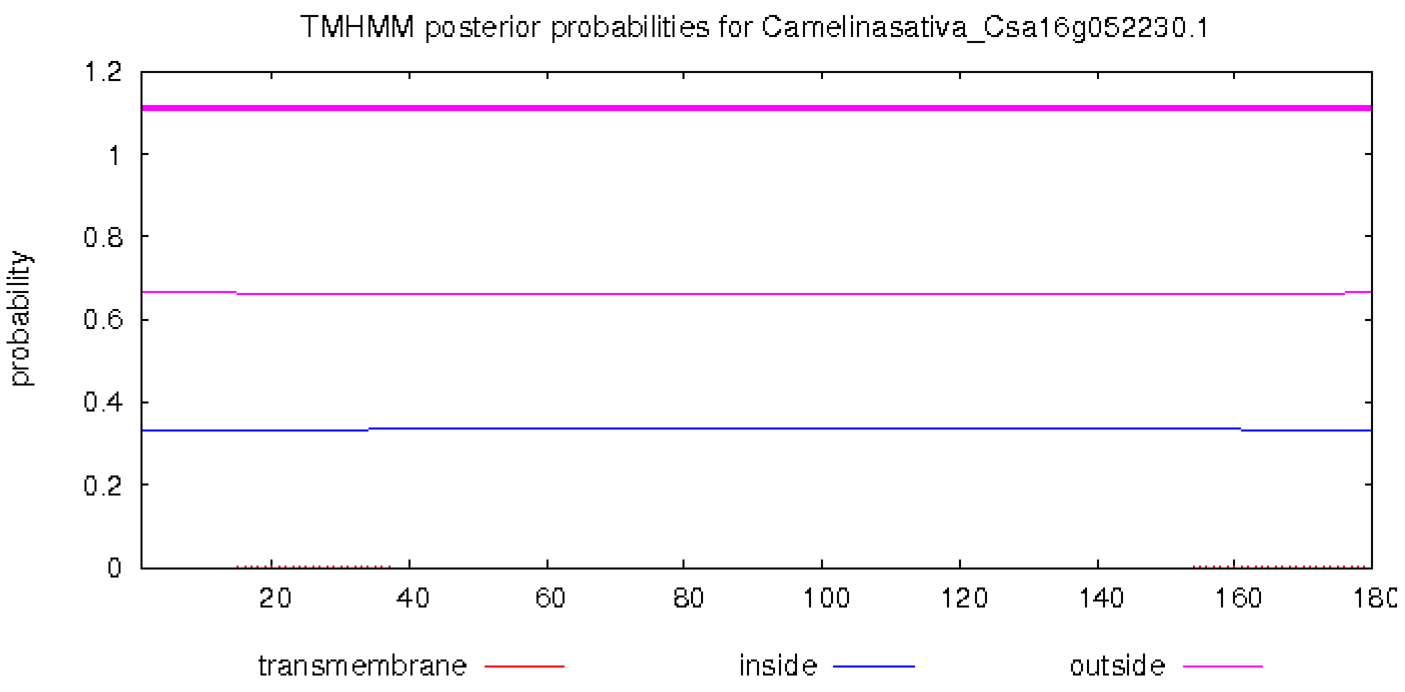

plot in postscript, script for making the plot in gnuplot, data for plot

```
# Camelinasativa_Csa16g052260.1 Length: 784
# Camelinasativa_Csa16g052260.1 Number of predicted TMHs:  0
# Camelinasativa_Csa16g052260.1 Exp number of AAs in TMHs: 0.03755
# Camelinasativa_Csa16g052260.1 Exp number, first 60 AAs:  0.0027
# Camelinasativa_Csa16g052260.1 Total prob of N-in:        0.00184
Camelinasativa_Csa16g052260.1   TMHMM2.0        outside      1   784
```

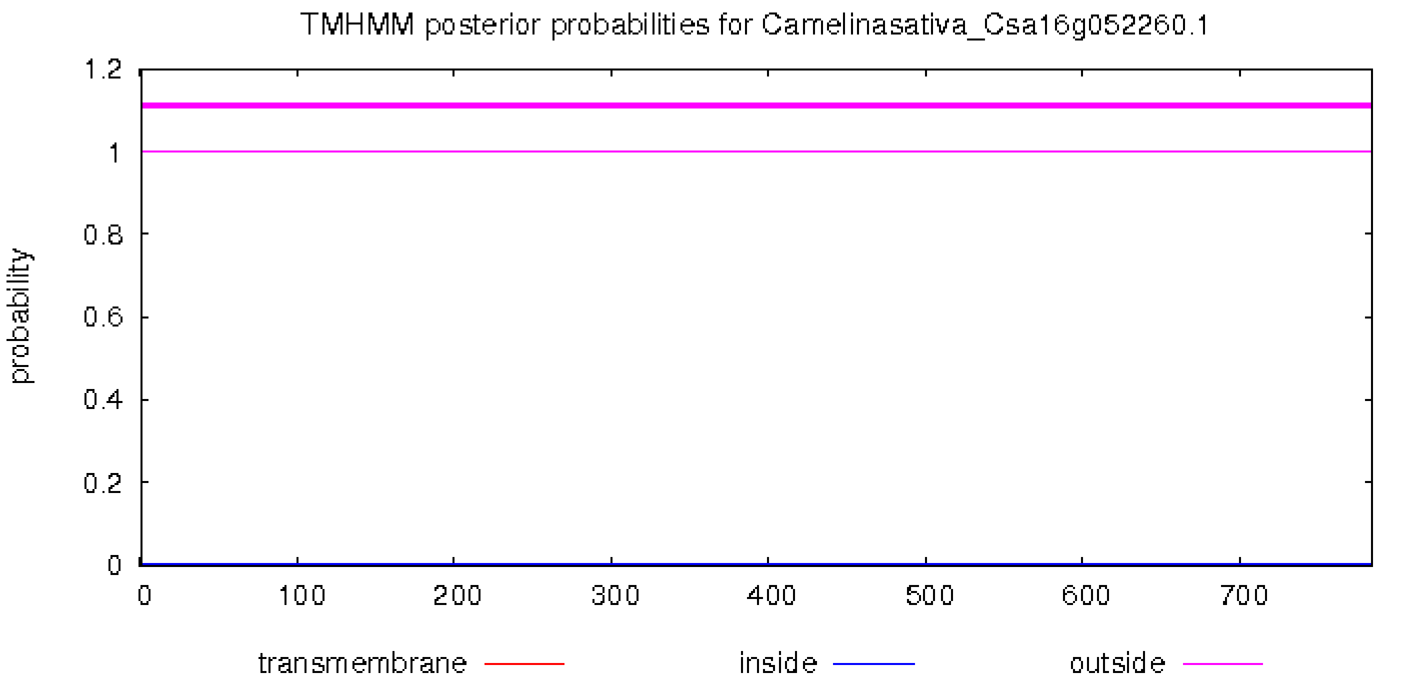

plot in postscript, script for making the plot in gnuplot, data for plot

```
# Camelinasativa_Csa16g053010.1 Length: 328
# Camelinasativa_Csa16g053010.1 Number of predicted TMHs:  0
# Camelinasativa_Csa16g053010.1 Exp number of AAs in TMHs: 0.00046
# Camelinasativa_Csa16g053010.1 Exp number, first 60 AAs:  0.00016
# Camelinasativa_Csa16g053010.1 Total prob of N-in:        0.01073
Camelinasativa_Csa16g053010.1	TMHMM2.0	outside	     1   328
```

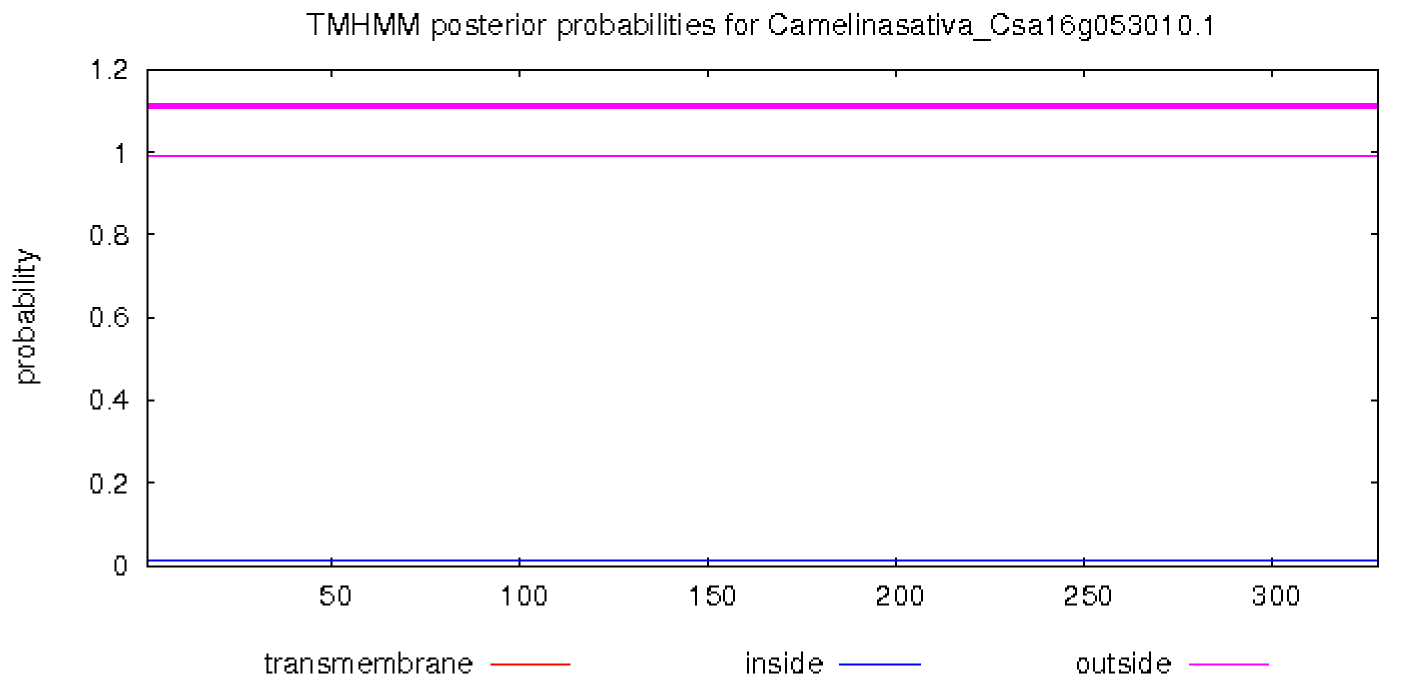

plot in postscript, script for making the plot in gnuplot, data for plot

---

```
# Camelinasativa_Csa16g055400.1 Length: 401
# Camelinasativa_Csa16g055400.1 Number of predicted TMHs:  0
# Camelinasativa_Csa16g055400.1 Exp number of AAs in TMHs: 0.00018
# Camelinasativa_Csa16g055400.1 Exp number, first 60 AAs:  0
# Camelinasativa_Csa16g055400.1 Total prob of N-in:        0.00271
Camelinasativa_Csa16g055400.1	TMHMM2.0	outside	     1   401
```

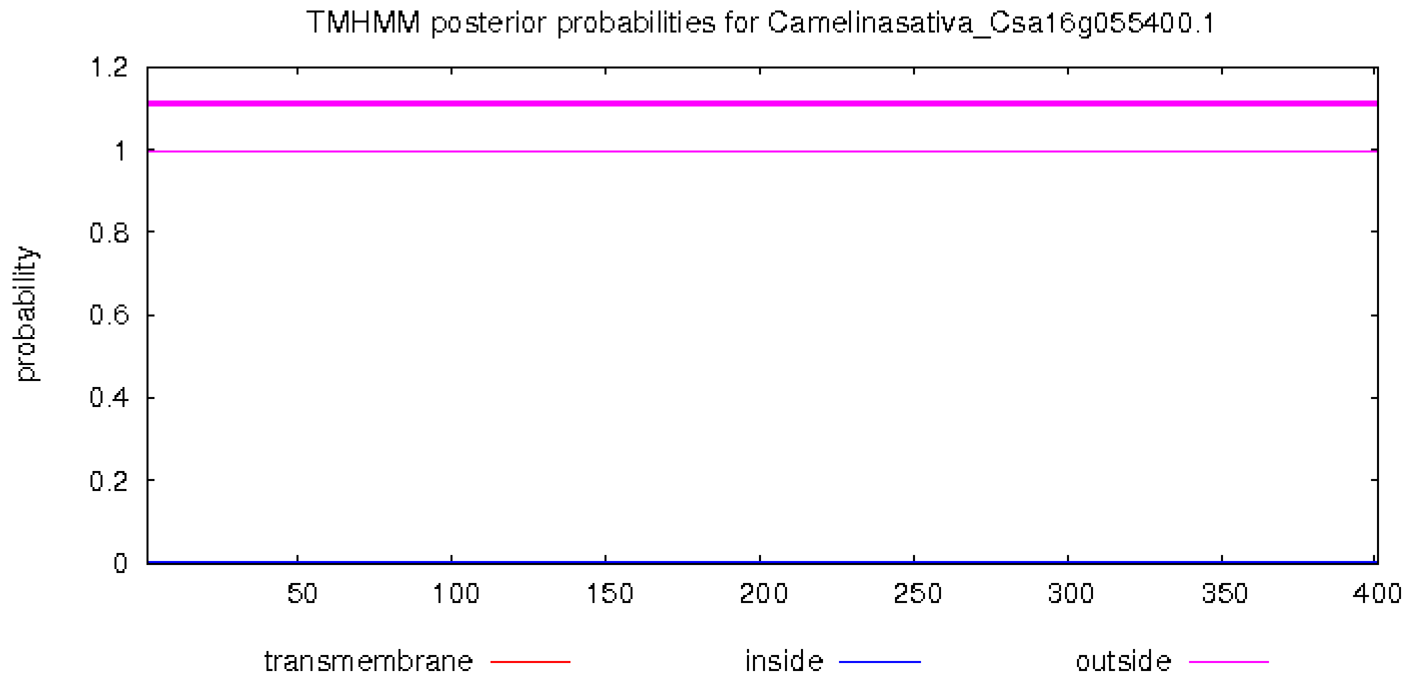

plot in postscript, script for making the plot in gnuplot, data for plot

---

```
# Camelinasativa_Csa17g001000.1 Length: 389
# Camelinasativa_Csa17g001000.1 Number of predicted TMHs:  0
# Camelinasativa_Csa17g001000.1 Exp number of AAs in TMHs: 0
# Camelinasativa_Csa17g001000.1 Exp number, first 60 AAs:  0
# Camelinasativa_Csa17g001000.1 Total prob of N-in:        0.00515
Camelinasativa_Csa17g001000.1   TMHMM2.0        outside      1   389
```

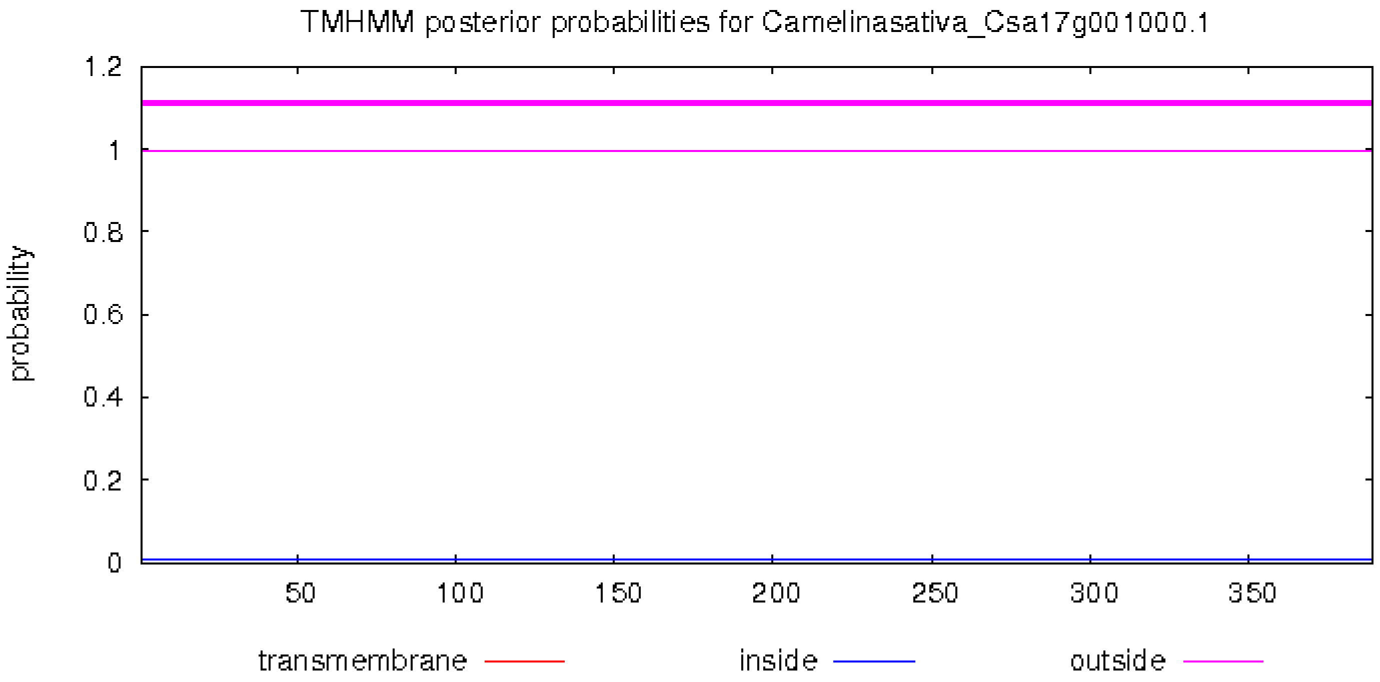

plot in postscript, script for making the plot in gnuplot, data for plot

```
# Camelinasativa_Csa17g001010.1 Length: 351
# Camelinasativa_Csa17g001010.1 Number of predicted TMHs:  0
# Camelinasativa_Csa17g001010.1 Exp number of AAs in TMHs: 0.03902
# Camelinasativa_Csa17g001010.1 Exp number, first 60 AAs:  0.01515
# Camelinasativa_Csa17g001010.1 Total prob of N-in:        0.00820
Camelinasativa_Csa17g001010.1   TMHMM2.0        outside      1   351
```

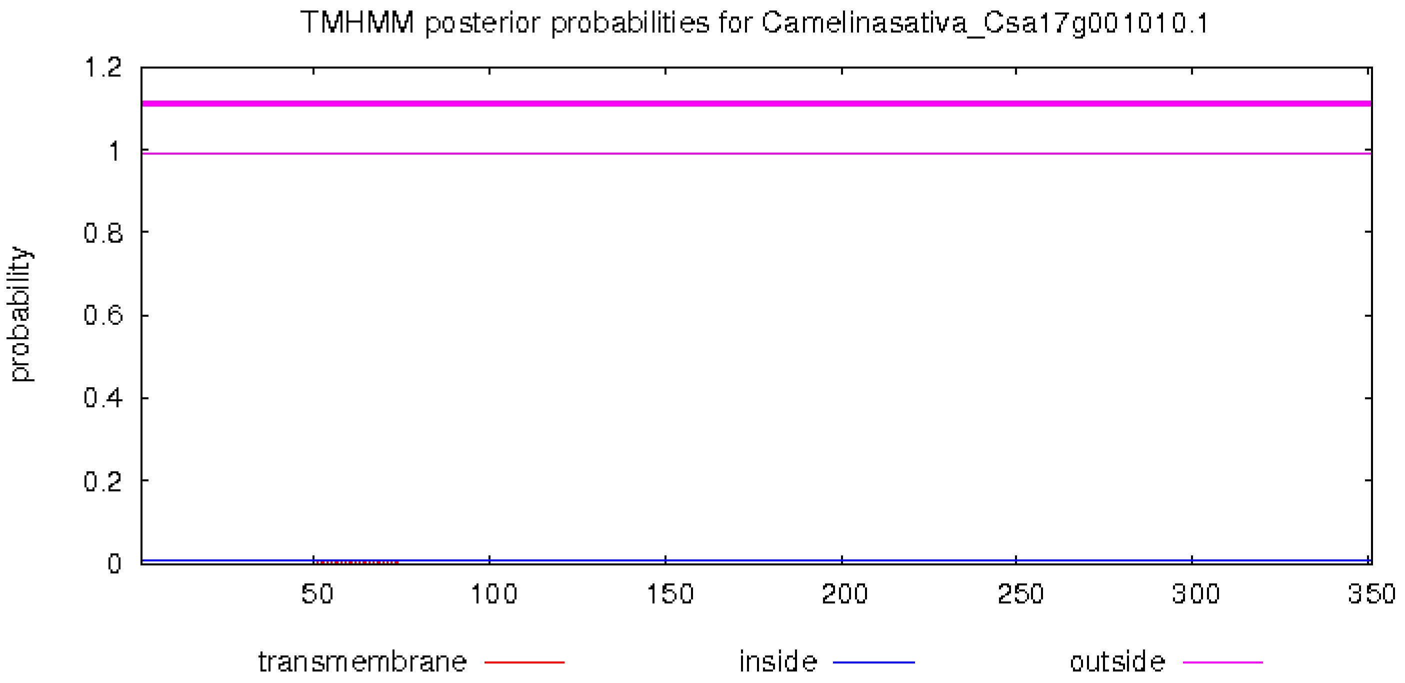

plot in postscript, script for making the plot in gnuplot, data for plot

```
# Camelinasativa_Csa17g001330.1 Length: 691
# Camelinasativa_Csa17g001330.1 Number of predicted TMHs:  0
# Camelinasativa_Csa17g001330.1 Exp number of AAs in TMHs: 0.00199
# Camelinasativa_Csa17g001330.1 Exp number, first 60 AAs:  0
# Camelinasativa_Csa17g001330.1 Total prob of N-in:        0.00051
Camelinasativa_Csa17g001330.1   TMHMM2.0        outside      1   691
```

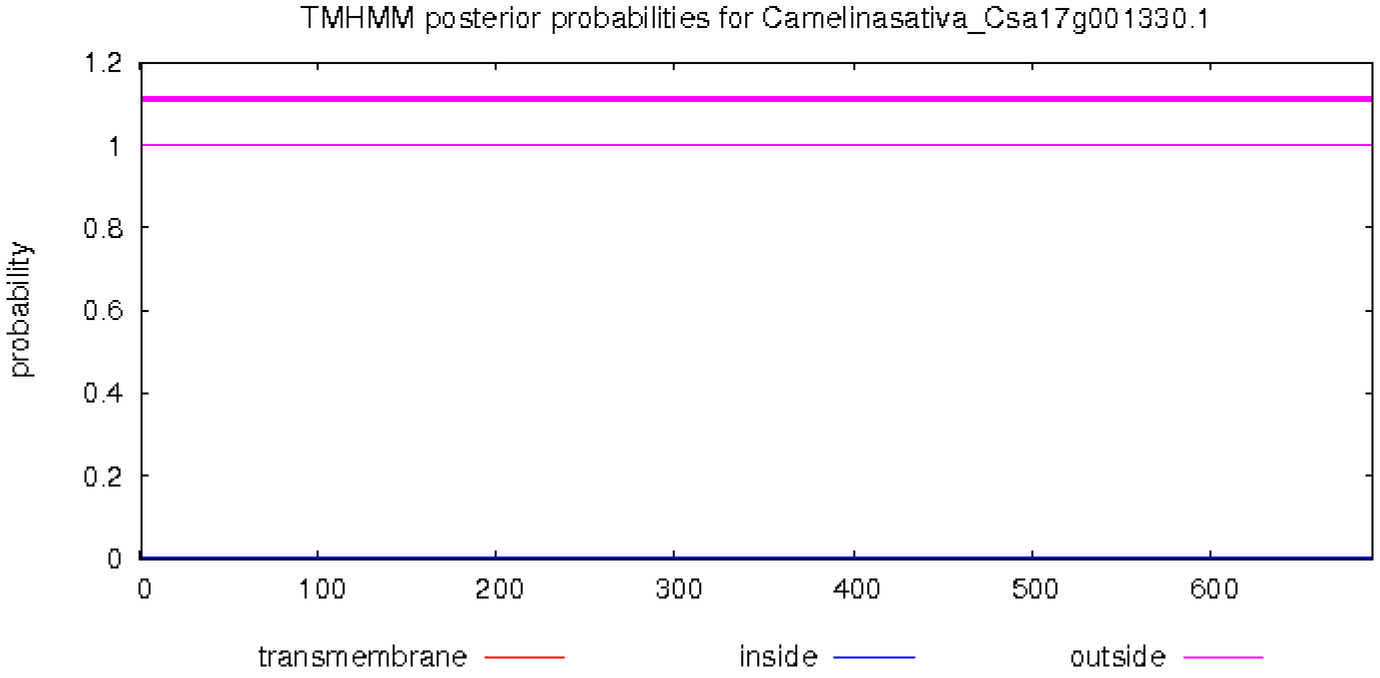

plot in postscript, script for making the plot in gnuplot, data for plot

---

```
# Camelinasativa_Csa17g001470.1 Length: 357
# Camelinasativa_Csa17g001470.1 Number of predicted TMHs:  0
# Camelinasativa_Csa17g001470.1 Exp number of AAs in TMHs: 0
# Camelinasativa_Csa17g001470.1 Exp number, first 60 AAs:  0
# Camelinasativa_Csa17g001470.1 Total prob of N-in:        0.01763
Camelinasativa_Csa17g001470.1   TMHMM2.0        outside      1   357
```

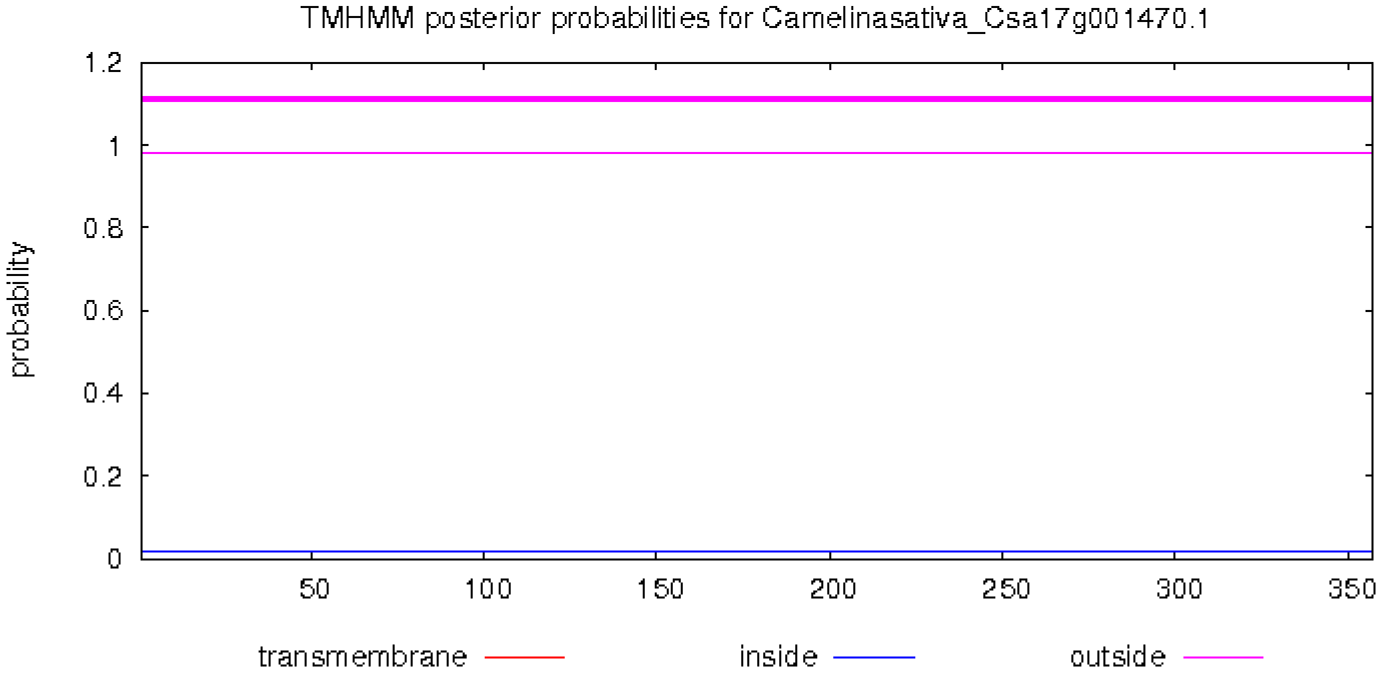

plot in postscript, script for making the plot in gnuplot, data for plot

---

```
# Camelinasativa_Csa17g001480.1 Length: 1045
# Camelinasativa_Csa17g001480.1 Number of predicted TMHs:  1
# Camelinasativa_Csa17g001480.1 Exp number of AAs in TMHs: 36.09227
# Camelinasativa_Csa17g001480.1 Exp number, first 60 AAs:  1e-05
# Camelinasativa_Csa17g001480.1 Total prob of N-in:        0.12256
Camelinasativa_Csa17g001480.1   TMHMM2.0        outside      1   805
Camelinasativa_Csa17g001480.1   TMHMM2.0        TMhelix    806   828
Camelinasativa_Csa17g001480.1   TMHMM2.0        inside     829  1045
```

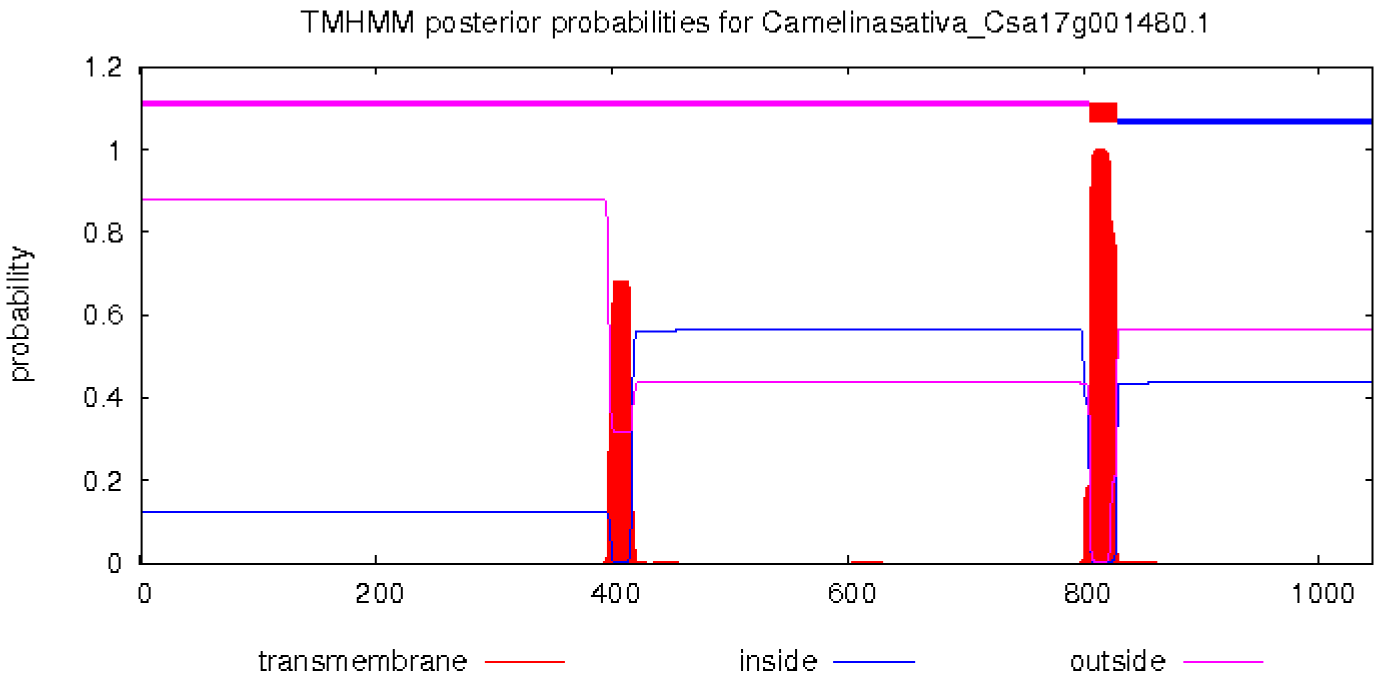

plot in postscript, script for making the plot in gnuplot, data for plot

---

```
# Camelinasativa_Csa17g002230.1 Length: 292
# Camelinasativa_Csa17g002230.1 Number of predicted TMHs:  0
# Camelinasativa_Csa17g002230.1 Exp number of AAs in TMHs: 0.00187
# Camelinasativa_Csa17g002230.1 Exp number, first 60 AAs:  0.00055
# Camelinasativa_Csa17g002230.1 Total prob of N-in:        0.00297
Camelinasativa_Csa17g002230.1   TMHMM2.0        outside      1   292
```

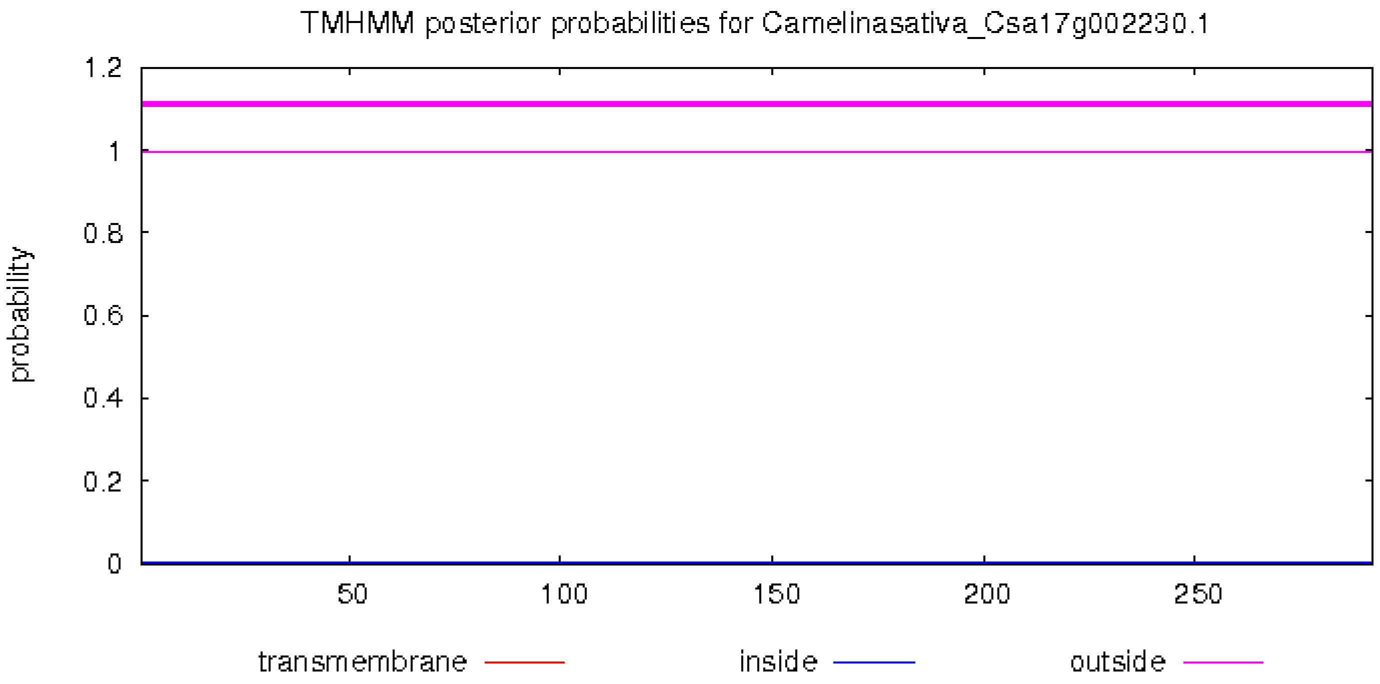

plot in postscript, script for making the plot in gnuplot, data for plot

```
# Camelinasativa_Csa17g005560.1 Length: 242
# Camelinasativa_Csa17g005560.1 Number of predicted TMHs:  0
# Camelinasativa_Csa17g005560.1 Exp number of AAs in TMHs: 0.00131
# Camelinasativa_Csa17g005560.1 Exp number, first 60 AAs:  0.00131
# Camelinasativa_Csa17g005560.1 Total prob of N-in:        0.02555
Camelinasativa_Csa17g005560.1	TMHMM2.0	outside	     1   242
```

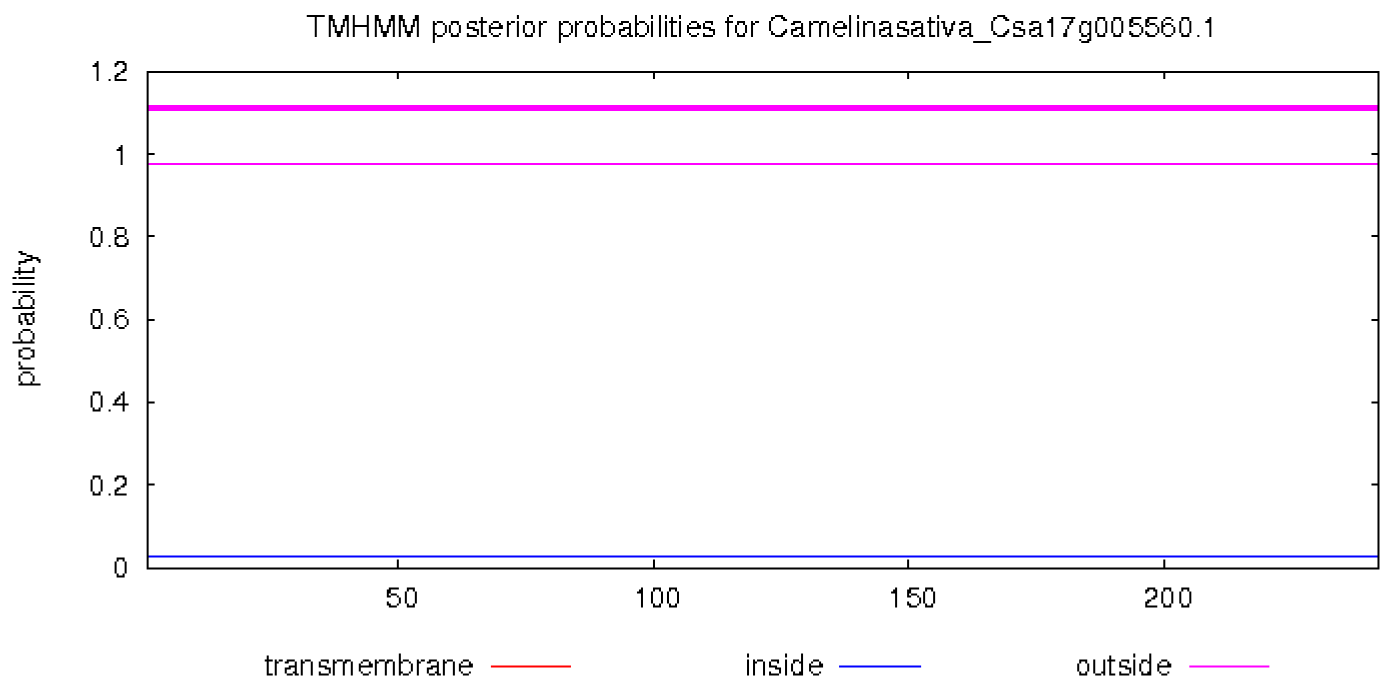

plot in postscript, script for making the plot in gnuplot, data for plot

```
# Camelinasativa_Csa17g016480.1 Length: 342
# Camelinasativa_Csa17g016480.1 Number of predicted TMHs:  0
# Camelinasativa_Csa17g016480.1 Exp number of AAs in TMHs: 0.00018
# Camelinasativa_Csa17g016480.1 Exp number, first 60 AAs:  0
# Camelinasativa_Csa17g016480.1 Total prob of N-in:        0.00322
Camelinasativa_Csa17g016480.1	TMHMM2.0	outside	     1   342
```

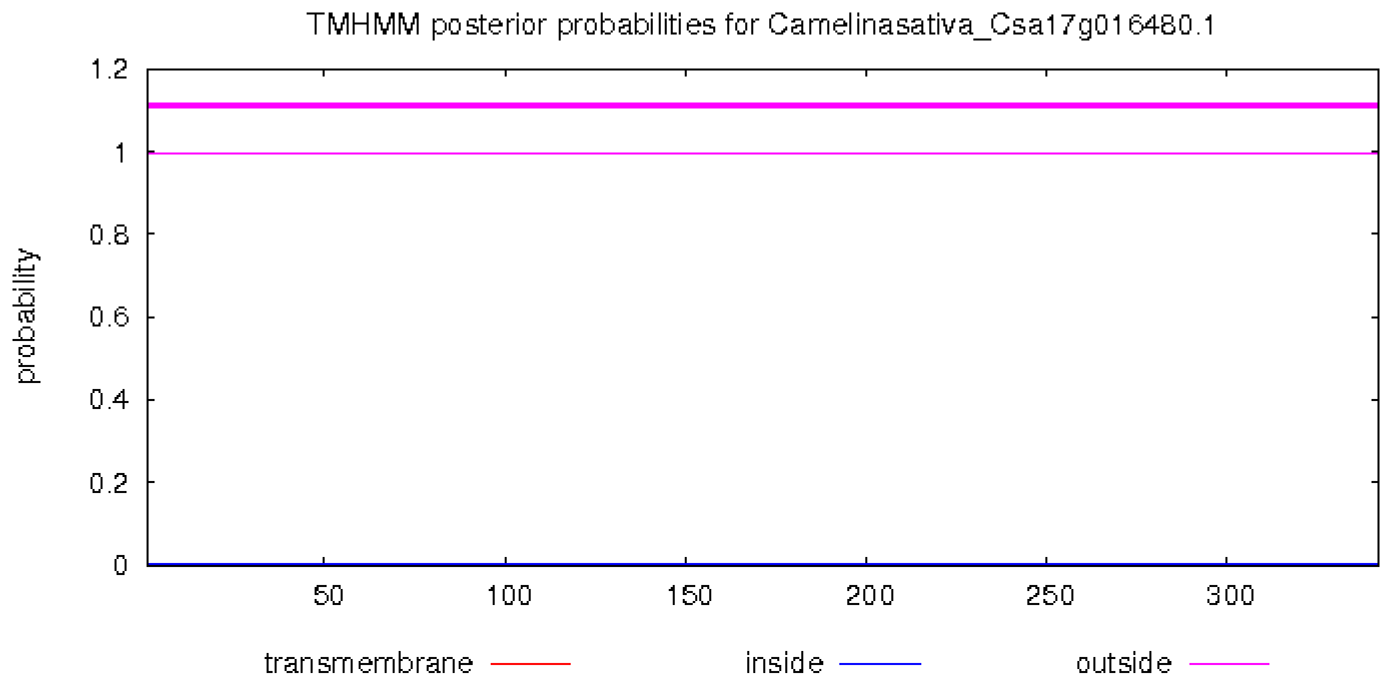

plot in postscript, script for making the plot in gnuplot, data for plot

```
# Camelinasativa_Csa17g033960.1 Length: 530
# Camelinasativa_Csa17g033960.1 Number of predicted TMHs:  0
# Camelinasativa_Csa17g033960.1 Exp number of AAs in TMHs: 0.00058
# Camelinasativa_Csa17g033960.1 Exp number, first 60 AAs:  0
# Camelinasativa_Csa17g033960.1 Total prob of N-in:        0.00287
Camelinasativa_Csa17g033960.1   TMHMM2.0        outside      1   530
```

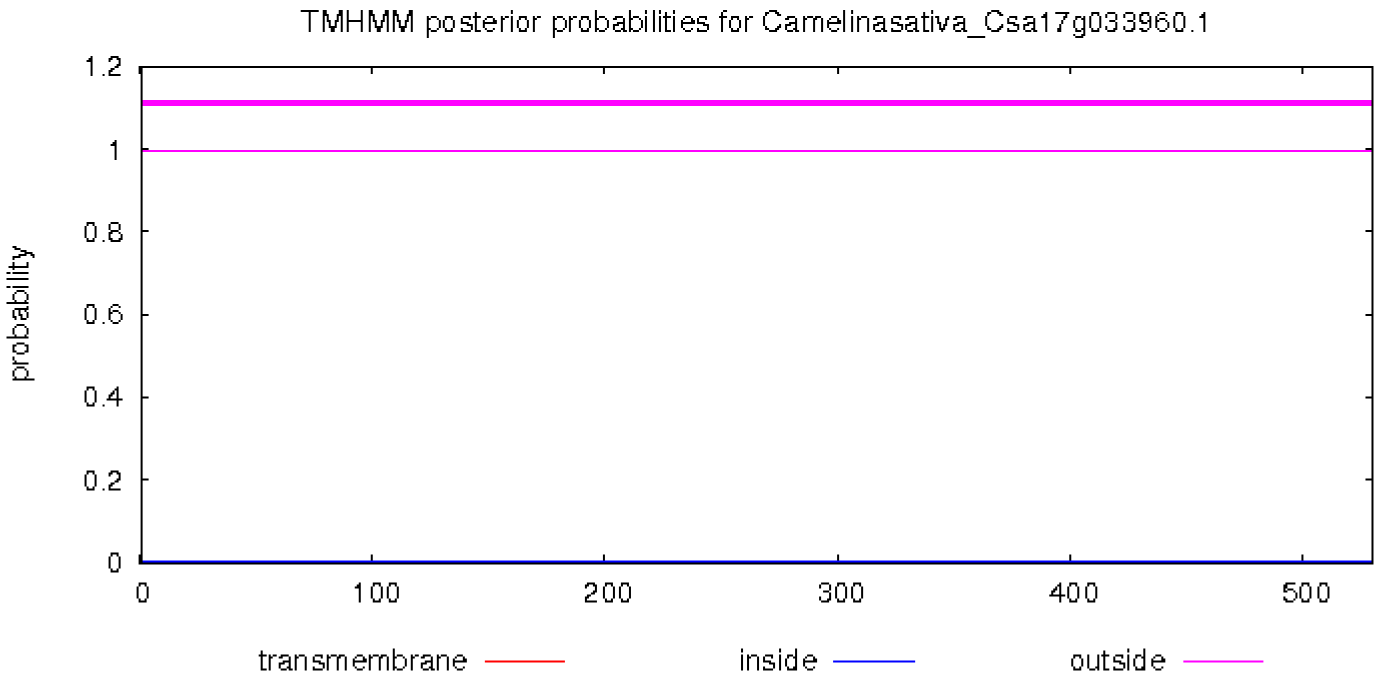

plot in postscript, script for making the plot in gnuplot, data for plot

---

```
# Camelinasativa_Csa17g034890.1 Length: 455
# Camelinasativa_Csa17g034890.1 Number of predicted TMHs:  0
# Camelinasativa_Csa17g034890.1 Exp number of AAs in TMHs: 0.0002
# Camelinasativa_Csa17g034890.1 Exp number, first 60 AAs:  0
# Camelinasativa_Csa17g034890.1 Total prob of N-in:        0.00279
Camelinasativa_Csa17g034890.1   TMHMM2.0        outside      1   455
```

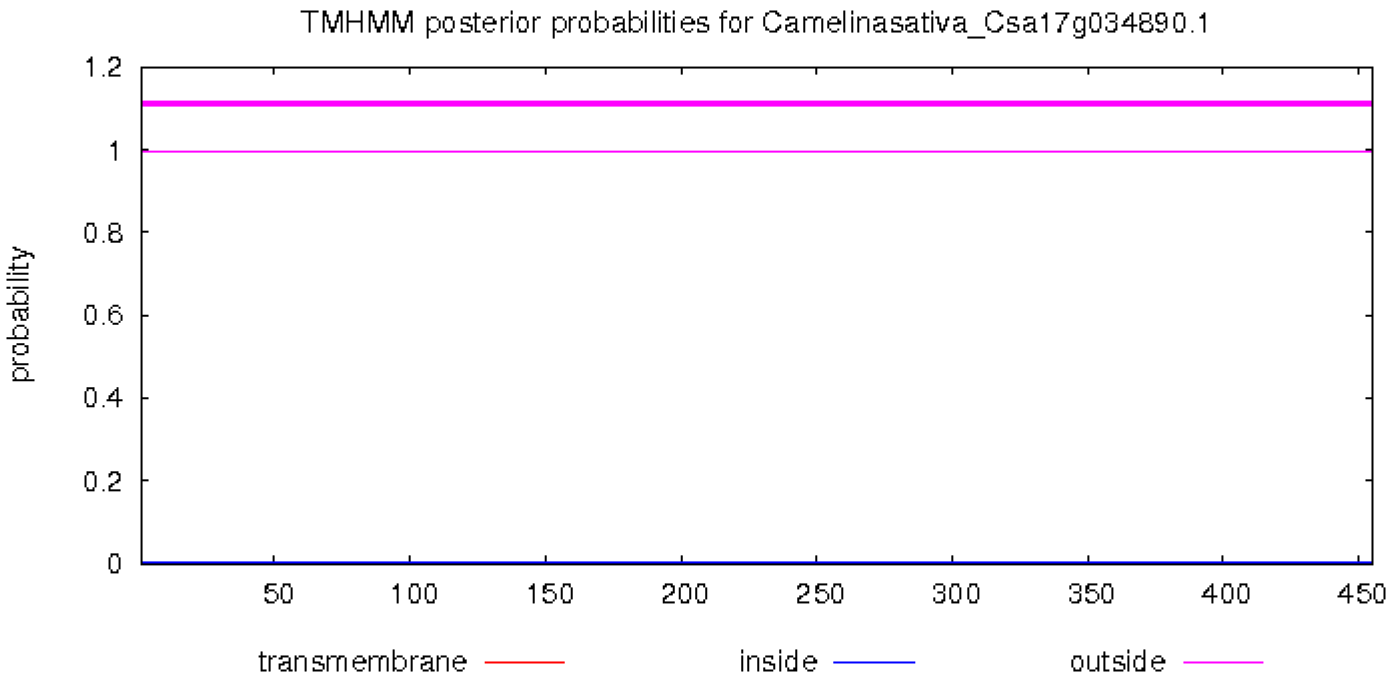

plot in postscript, script for making the plot in gnuplot, data for plot

---

```
# Camelinasativa_Csa17g039350.1 Length: 253
# Camelinasativa_Csa17g039350.1 Number of predicted TMHs:  0
# Camelinasativa_Csa17g039350.1 Exp number of AAs in TMHs: 0.00036
# Camelinasativa_Csa17g039350.1 Exp number, first 60 AAs:  0
# Camelinasativa_Csa17g039350.1 Total prob of N-in:        0.09449
Camelinasativa_Csa17g039350.1	TMHMM2.0	outside	     1   253
```

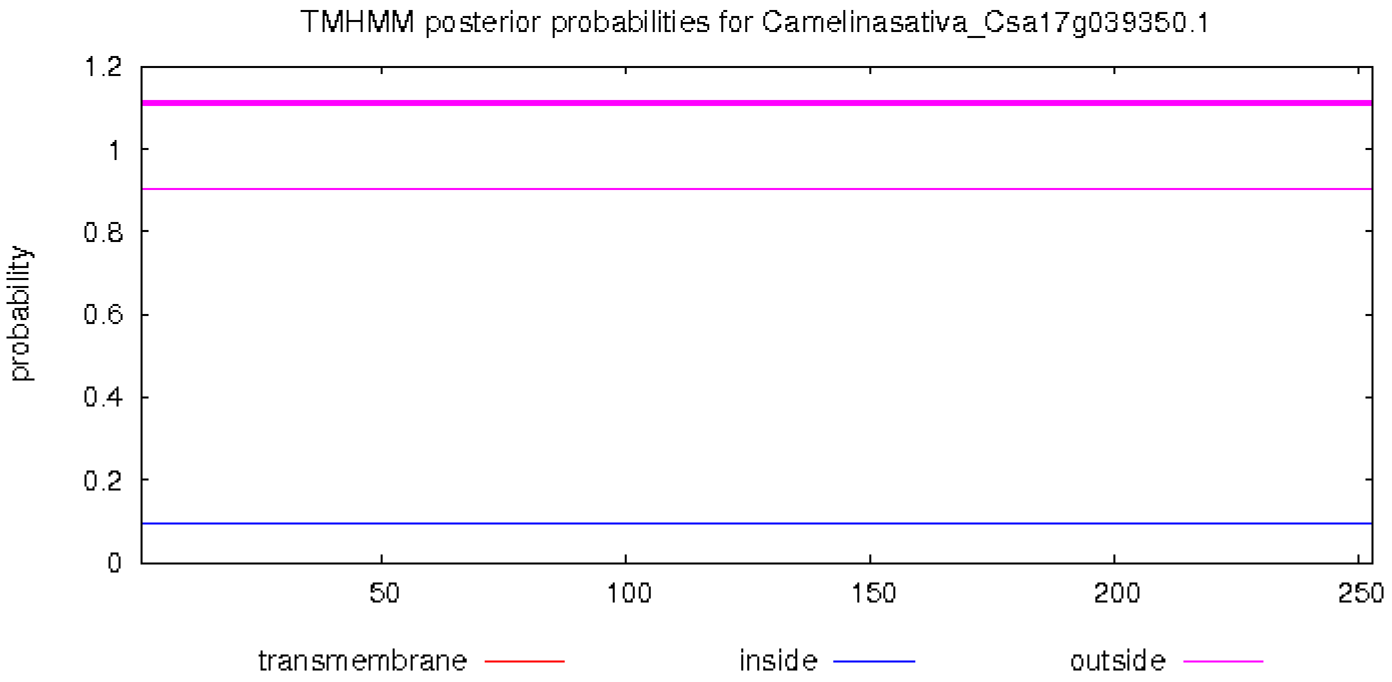

plot in postscript, script for making the plot in gnuplot, data for plot

---

```
# Camelinasativa_Csa17g054170.1 Length: 362
# Camelinasativa_Csa17g054170.1 Number of predicted TMHs:  0
# Camelinasativa_Csa17g054170.1 Exp number of AAs in TMHs: 0
# Camelinasativa_Csa17g054170.1 Exp number, first 60 AAs:  0
# Camelinasativa_Csa17g054170.1 Total prob of N-in:        0.01802
Camelinasativa_Csa17g054170.1	TMHMM2.0	outside	     1   362
```

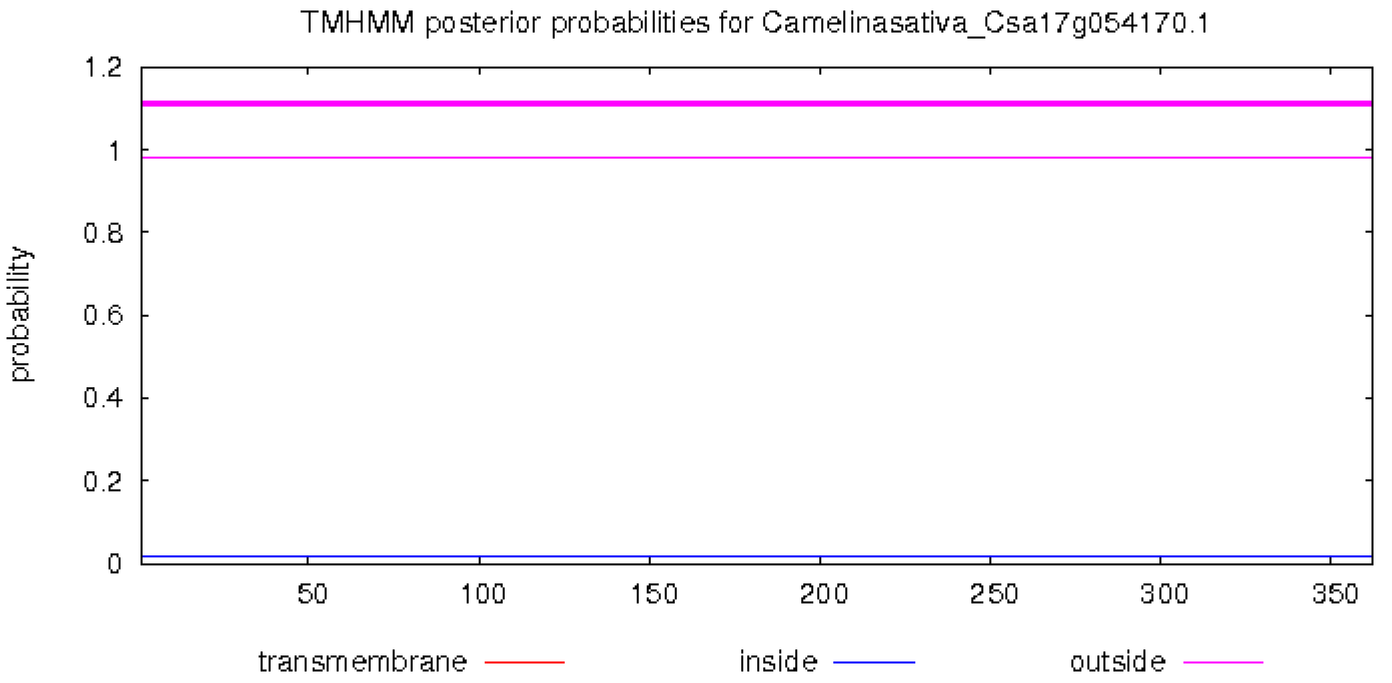

plot in postscript, script for making the plot in gnuplot, data for plot

---

```
# Camelinasativa_Csa17g054250.1 Length: 519
# Camelinasativa_Csa17g054250.1 Number of predicted TMHs:  1
# Camelinasativa_Csa17g054250.1 Exp number of AAs in TMHs: 23.00023
# Camelinasativa_Csa17g054250.1 Exp number, first 60 AAs:  0.03148
# Camelinasativa_Csa17g054250.1 Total prob of N-in:        0.78218
Camelinasativa_Csa17g054250.1   TMHMM2.0        inside       1   489
Camelinasativa_Csa17g054250.1   TMHMM2.0        TMhelix    490   512
Camelinasativa_Csa17g054250.1   TMHMM2.0        outside    513   519
```

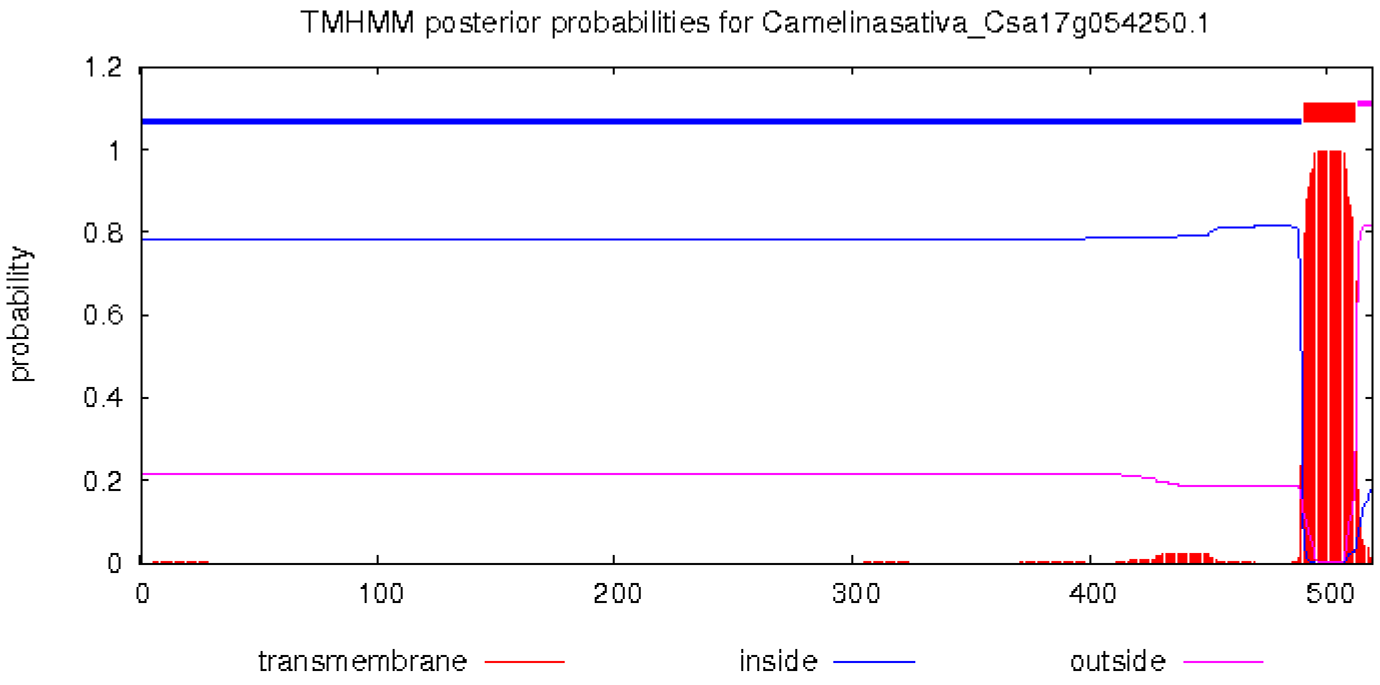

plot in postscript, script for making the plot in gnuplot, data for plot

---

```
# Camelinasativa_Csa17g055470.1 Length: 660
# Camelinasativa_Csa17g055470.1 Number of predicted TMHs:  1
# Camelinasativa_Csa17g055470.1 Exp number of AAs in TMHs: 21.81138
# Camelinasativa_Csa17g055470.1 Exp number, first 60 AAs:  0
# Camelinasativa_Csa17g055470.1 Total prob of N-in:        0.00168
Camelinasativa_Csa17g055470.1   TMHMM2.0        outside      1   614
Camelinasativa_Csa17g055470.1   TMHMM2.0        TMhelix    615   637
Camelinasativa_Csa17g055470.1   TMHMM2.0        inside     638   660
```

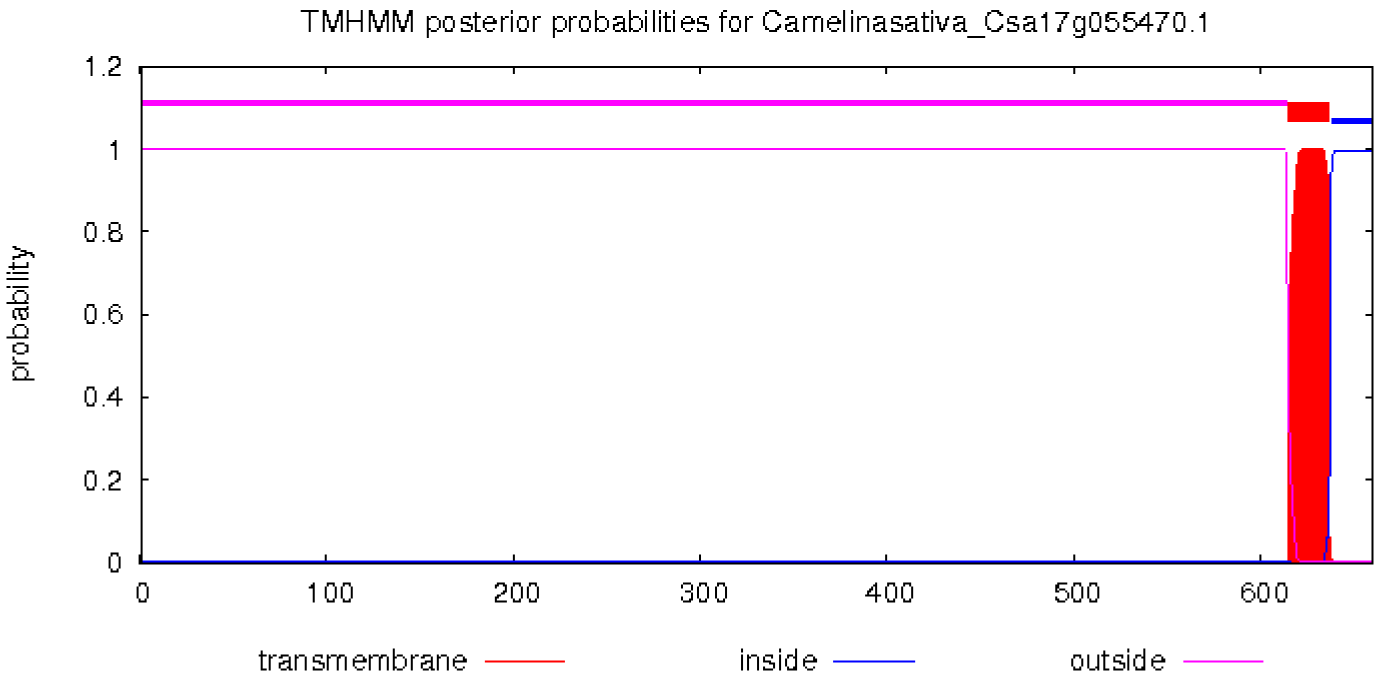

plot in postscript, script for making the plot in gnuplot, data for plot

```
# Camelinasativa_Csa17g055620.1 Length: 306
# Camelinasativa_Csa17g055620.1 Number of predicted TMHs:  0
# Camelinasativa_Csa17g055620.1 Exp number of AAs in TMHs: 0
# Camelinasativa_Csa17g055620.1 Exp number, first 60 AAs:  0
# Camelinasativa_Csa17g055620.1 Total prob of N-in:        0.00490
Camelinasativa_Csa17g055620.1   TMHMM2.0        outside      1   306
```

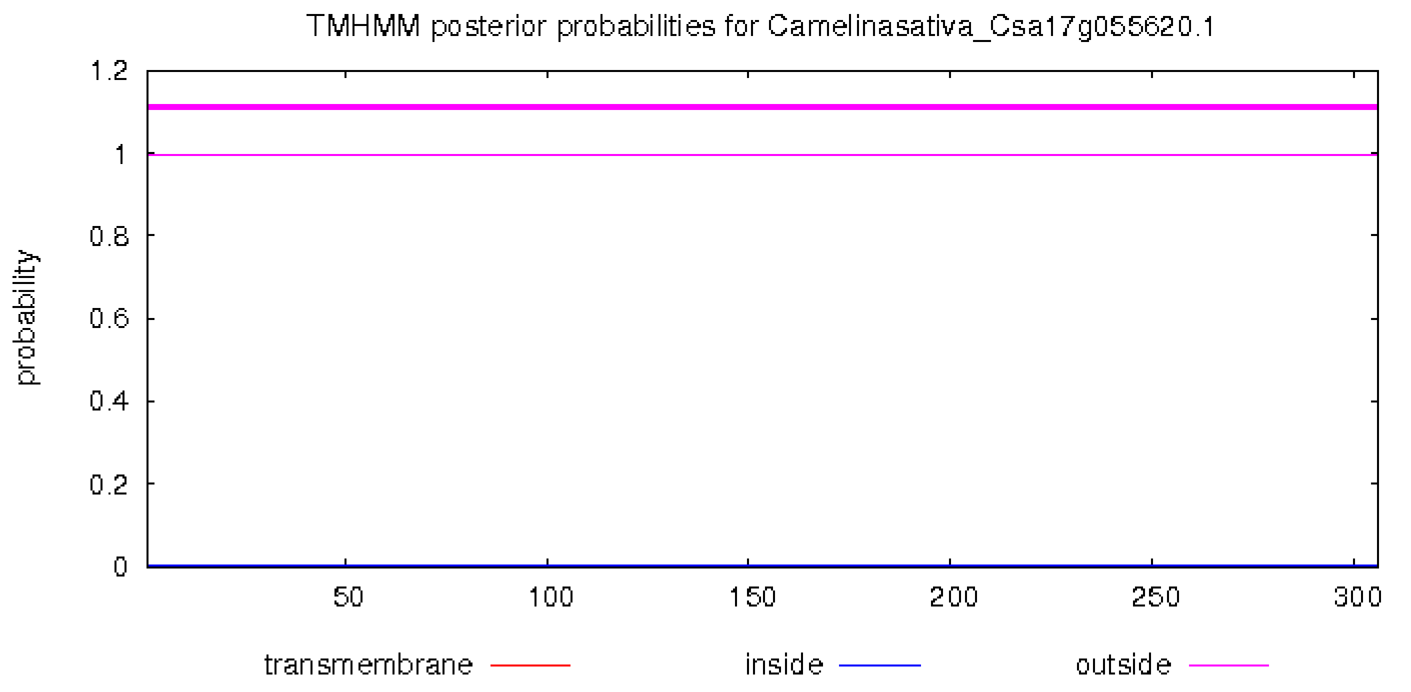

plot in postscript, script for making the plot in gnuplot, data for plot

```
# Camelinasativa_Csa17g057040.1 Length: 200
# Camelinasativa_Csa17g057040.1 Number of predicted TMHs:  0
# Camelinasativa_Csa17g057040.1 Exp number of AAs in TMHs: 0.00514
# Camelinasativa_Csa17g057040.1 Exp number, first 60 AAs:  0.00106
# Camelinasativa_Csa17g057040.1 Total prob of N-in:        0.07111
Camelinasativa_Csa17g057040.1   TMHMM2.0        outside      1   200
```

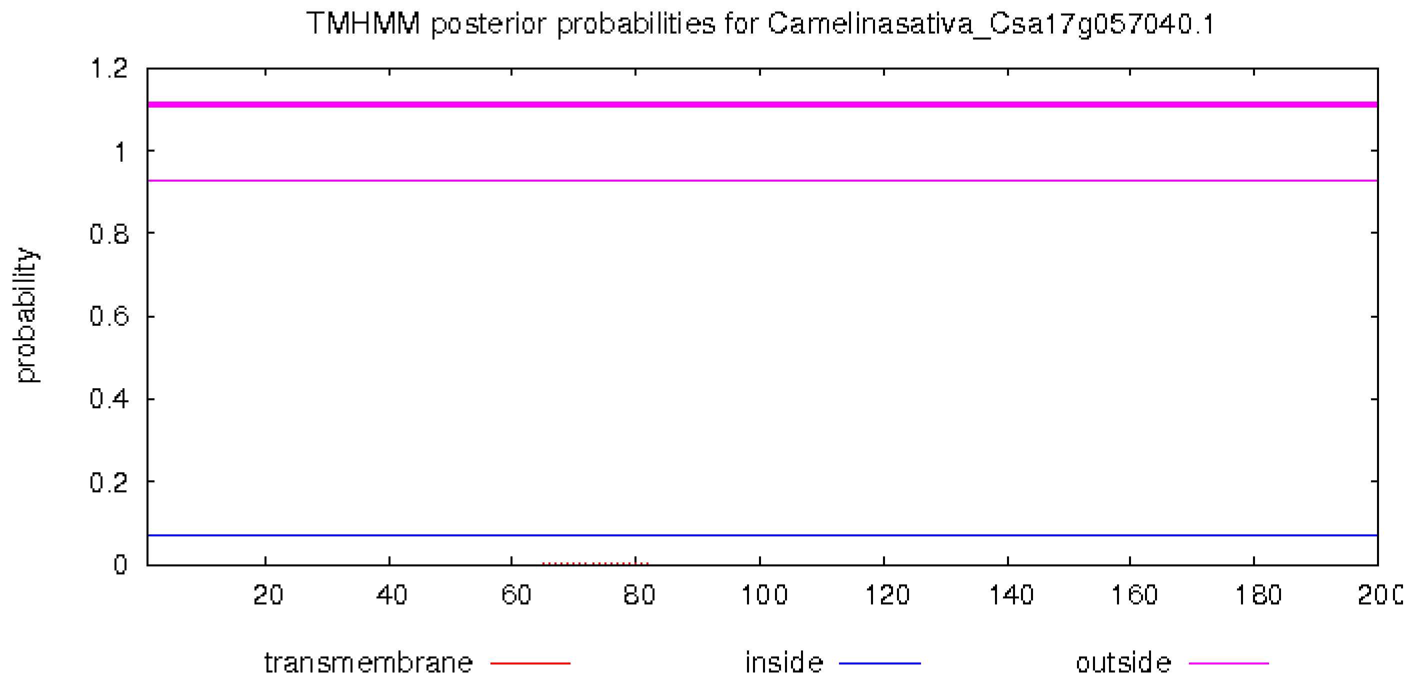

plot in postscript, script for making the plot in gnuplot, data for plot

```
# Camelinasativa_Csa17g059460.1 Length: 553
# Camelinasativa_Csa17g059460.1 Number of predicted TMHs:  1
# Camelinasativa_Csa17g059460.1 Exp number of AAs in TMHs: 23.5825
# Camelinasativa_Csa17g059460.1 Exp number, first 60 AAs:  0.00412
# Camelinasativa_Csa17g059460.1 Total prob of N-in:        0.43820
Camelinasativa_Csa17g059460.1   TMHMM2.0        outside      1   523
Camelinasativa_Csa17g059460.1   TMHMM2.0        TMhelix    524   546
Camelinasativa_Csa17g059460.1   TMHMM2.0        inside     547   553
```

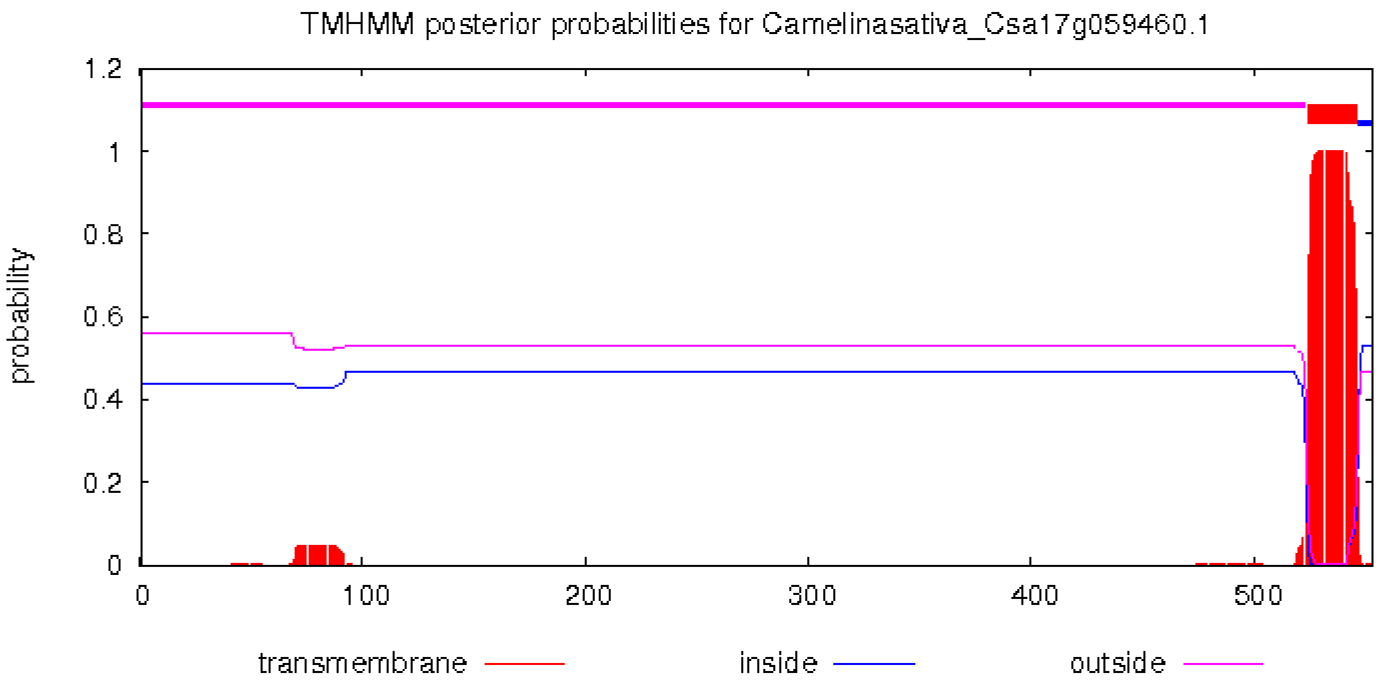

plot in postscript, script for making the plot in gnuplot, data for plot

```
# Camelinasativa_Csa17g059480.1 Length: 545
# Camelinasativa_Csa17g059480.1 Number of predicted TMHs:  1
# Camelinasativa_Csa17g059480.1 Exp number of AAs in TMHs: 21.33283
# Camelinasativa_Csa17g059480.1 Exp number, first 60 AAs:  0.00554
# Camelinasativa_Csa17g059480.1 Total prob of N-in:        0.09163
Camelinasativa_Csa17g059480.1   TMHMM2.0        outside      1   515
Camelinasativa_Csa17g059480.1   TMHMM2.0        TMhelix    516   538
Camelinasativa_Csa17g059480.1   TMHMM2.0        inside     539   545
```

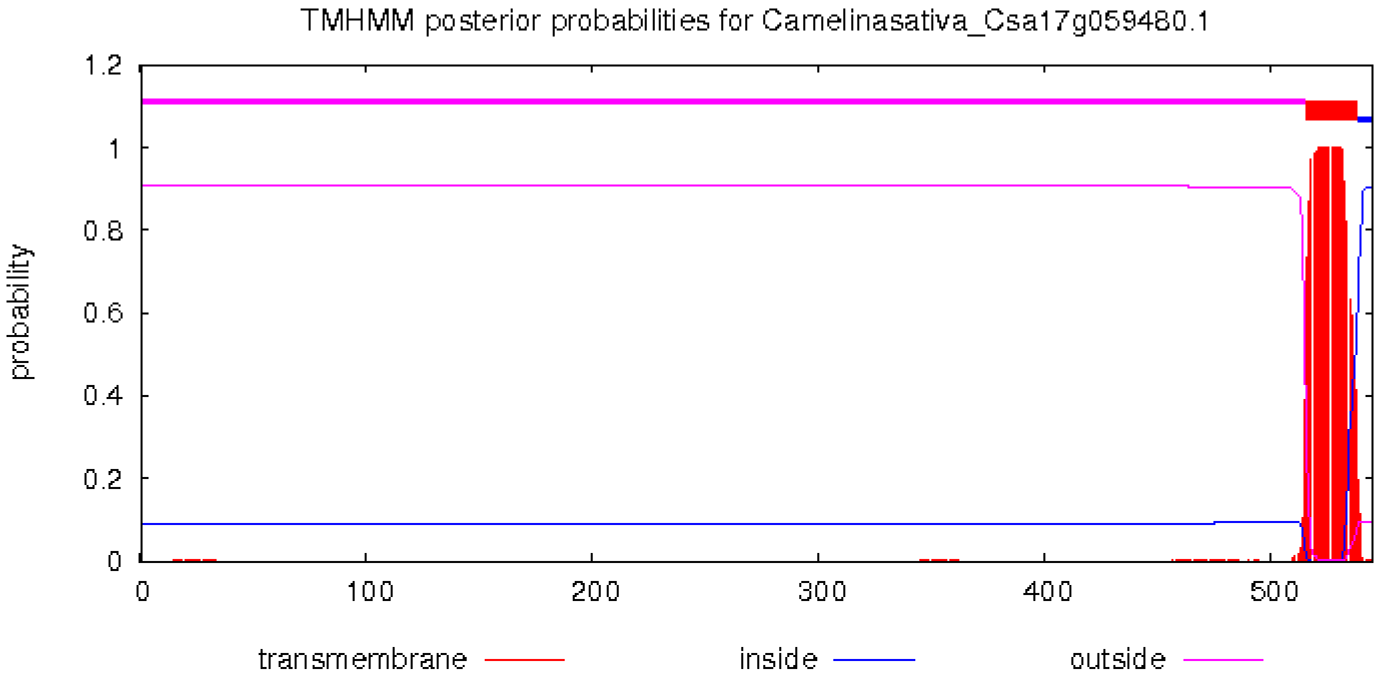

plot in postscript, script for making the plot in gnuplot, data for plot

---

```
# Camelinasativa_Csa17g062260.1 Length: 158
# Camelinasativa_Csa17g062260.1 Number of predicted TMHs:  0
# Camelinasativa_Csa17g062260.1 Exp number of AAs in TMHs: 0.01202
# Camelinasativa_Csa17g062260.1 Exp number, first 60 AAs:  0
# Camelinasativa_Csa17g062260.1 Total prob of N-in:        0.44104
Camelinasativa_Csa17g062260.1   TMHMM2.0        outside      1   158
```

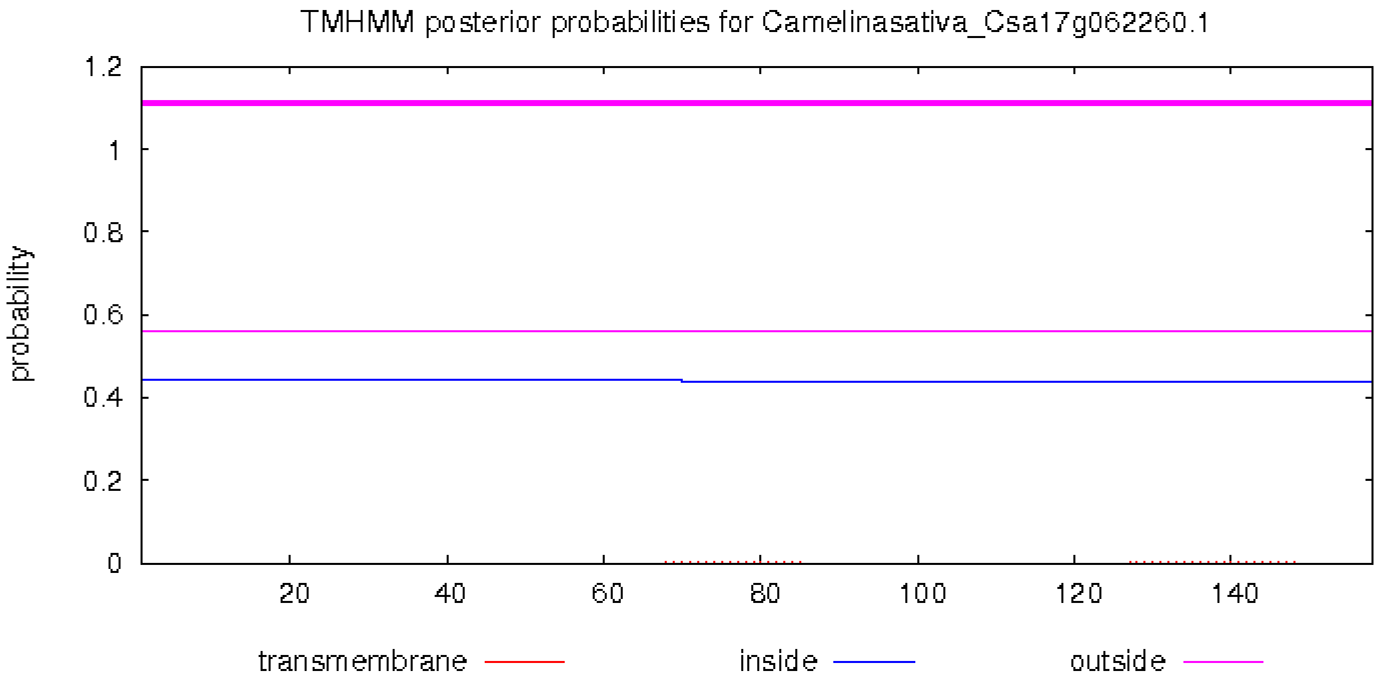

plot in postscript, script for making the plot in gnuplot, data for plot

---

```
# Camelinasativa_Csa17g093060.1 Length: 354
# Camelinasativa_Csa17g093060.1 Number of predicted TMHs:  0
# Camelinasativa_Csa17g093060.1 Exp number of AAs in TMHs: 0.01201
# Camelinasativa_Csa17g093060.1 Exp number, first 60 AAs:  0.01153
# Camelinasativa_Csa17g093060.1 Total prob of N-in:        0.00183
Camelinasativa_Csa17g093060.1   TMHMM2.0        outside      1   354
```

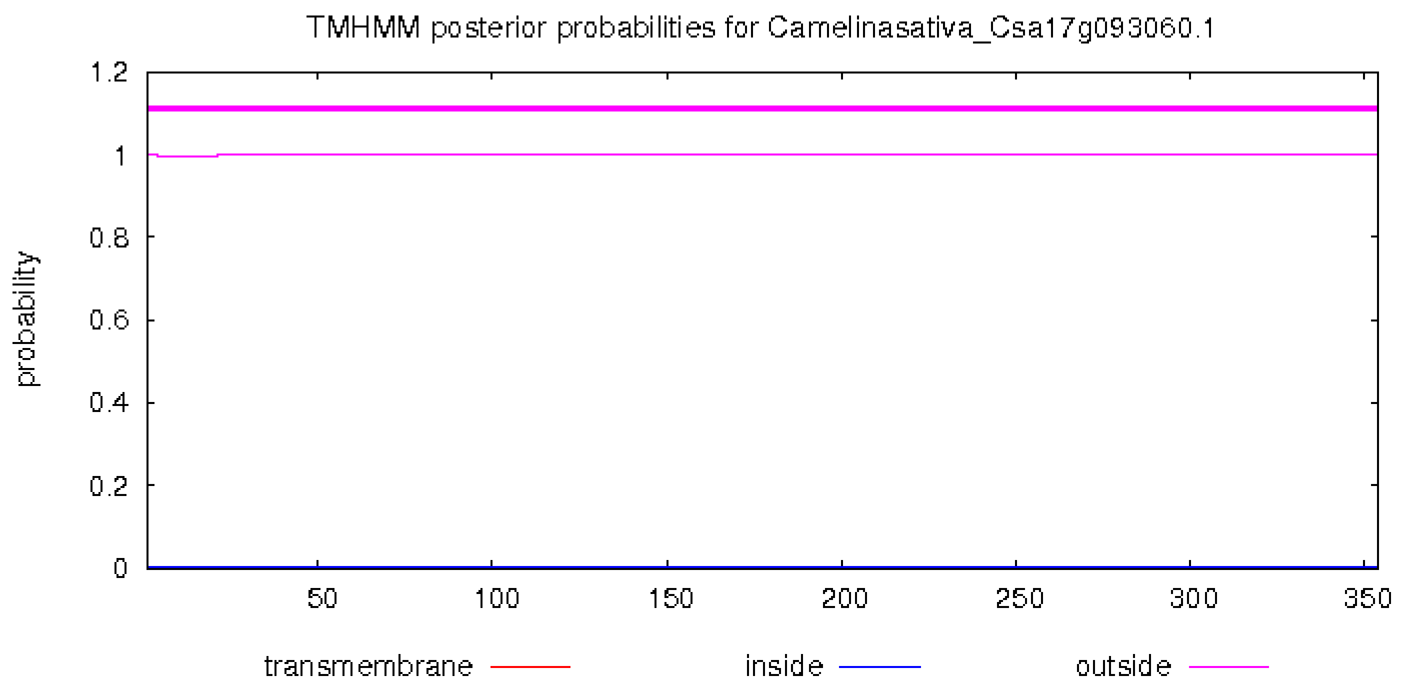

plot in postscript, script for making the plot in gnuplot, data for plot

```
# Camelinasativa_Csa17g093080.1 Length: 307
# Camelinasativa_Csa17g093080.1 Number of predicted TMHs:  0
# Camelinasativa_Csa17g093080.1 Exp number of AAs in TMHs: 0.00591
# Camelinasativa_Csa17g093080.1 Exp number, first 60 AAs:  0.00023
# Camelinasativa_Csa17g093080.1 Total prob of N-in:        0.01512
Camelinasativa_Csa17g093080.1   TMHMM2.0        outside      1   307
```

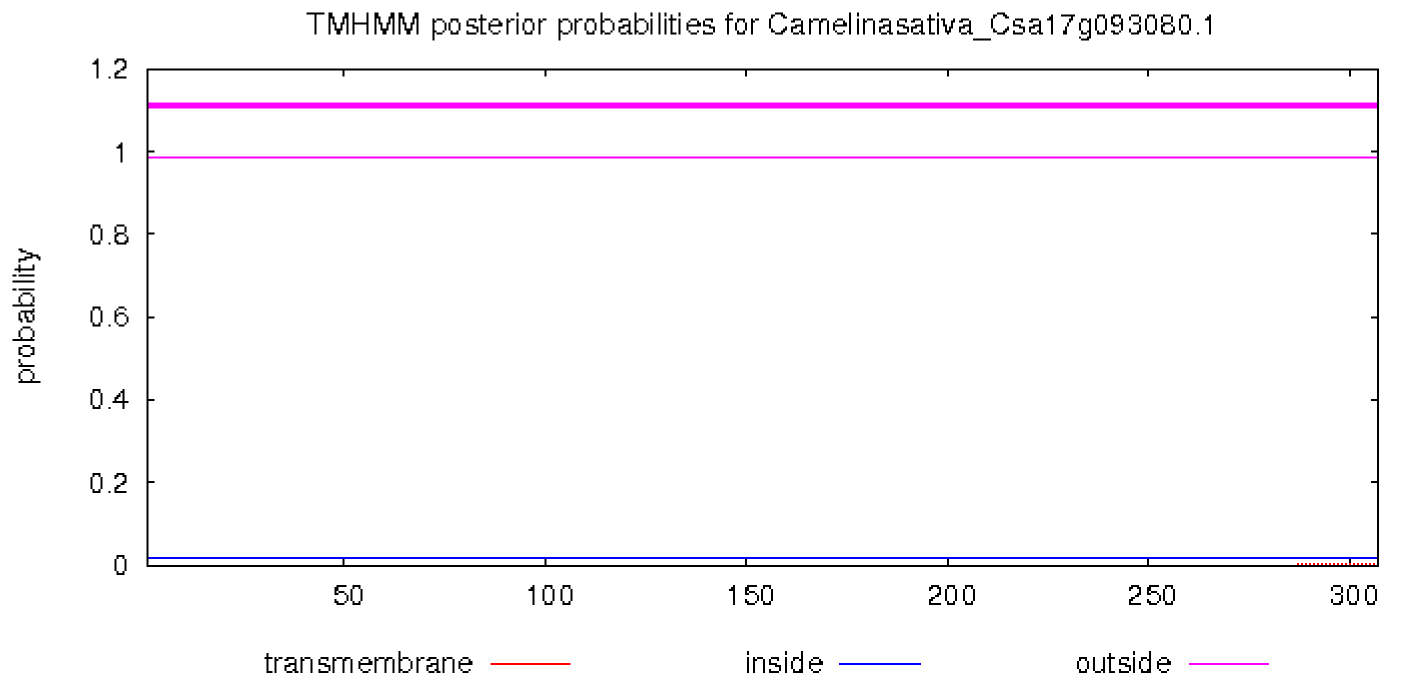

plot in postscript, script for making the plot in gnuplot, data for plot

```
# Camelinasativa_Csa17g095950.1 Length: 323
# Camelinasativa_Csa17g095950.1 Number of predicted TMHs:  0
# Camelinasativa_Csa17g095950.1 Exp number of AAs in TMHs: 0.00039
# Camelinasativa_Csa17g095950.1 Exp number, first 60 AAs:  0
# Camelinasativa_Csa17g095950.1 Total prob of N-in:        0.02389
Camelinasativa_Csa17g095950.1   TMHMM2.0        outside      1   323
```

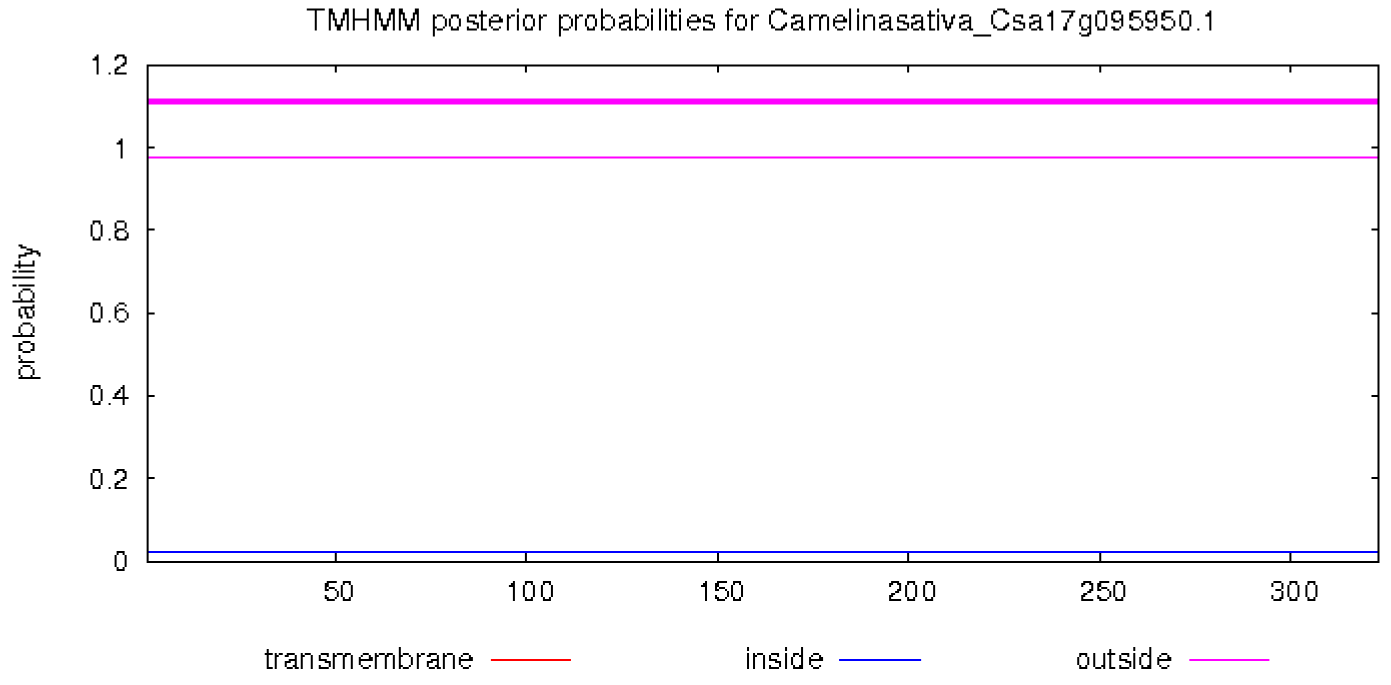

plot in postscript, script for making the plot in gnuplot, data for plot

```
# Camelinasativa_Csa17g097090.1 Length: 532
# Camelinasativa_Csa17g097090.1 Number of predicted TMHs:  0
# Camelinasativa_Csa17g097090.1 Exp number of AAs in TMHs: 0
# Camelinasativa_Csa17g097090.1 Exp number, first 60 AAs:  0
# Camelinasativa_Csa17g097090.1 Total prob of N-in:        0.00008
Camelinasativa_Csa17g097090.1   TMHMM2.0        outside      1   532
```

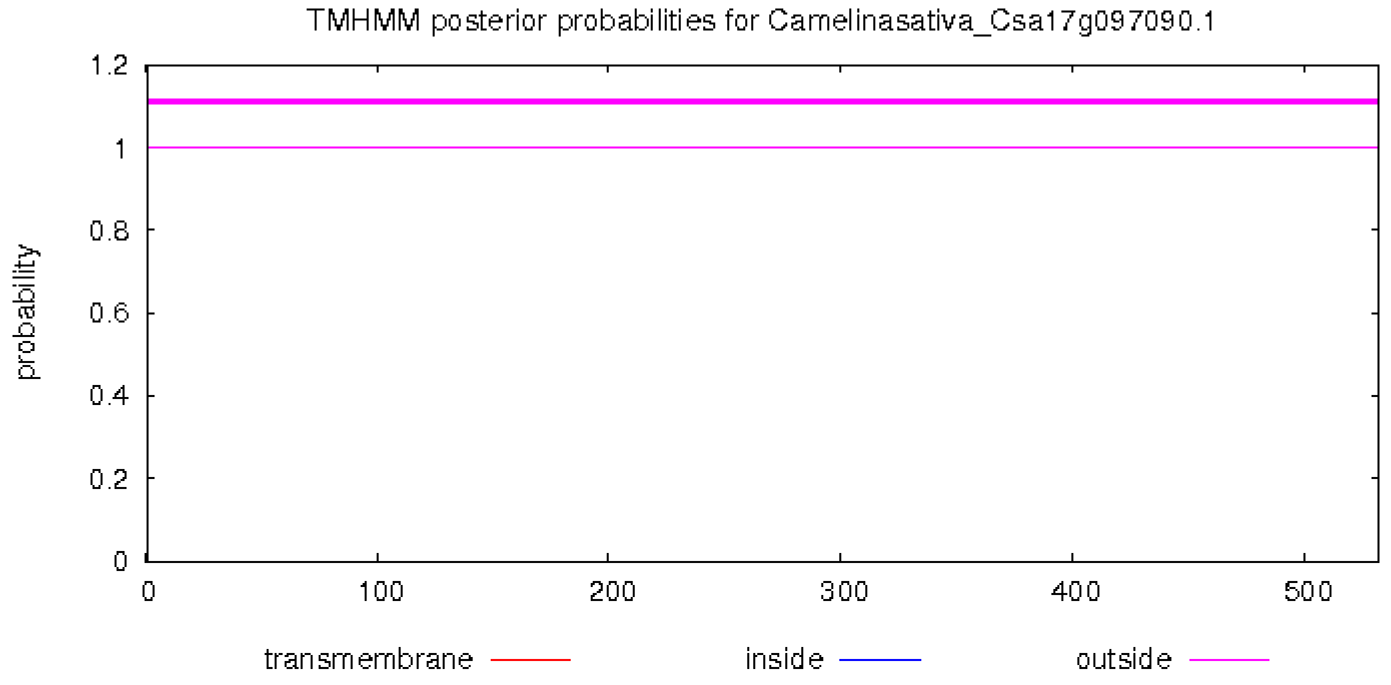

plot in postscript, script for making the plot in gnuplot, data for plot

```
# Camelinasativa_Csa18g003450.1 Length: 293
# Camelinasativa_Csa18g003450.1 Number of predicted TMHs:  0
# Camelinasativa_Csa18g003450.1 Exp number of AAs in TMHs: 0
# Camelinasativa_Csa18g003450.1 Exp number, first 60 AAs:  0
# Camelinasativa_Csa18g003450.1 Total prob of N-in:        0.00212
Camelinasativa_Csa18g003450.1   TMHMM2.0        outside      1   293
```

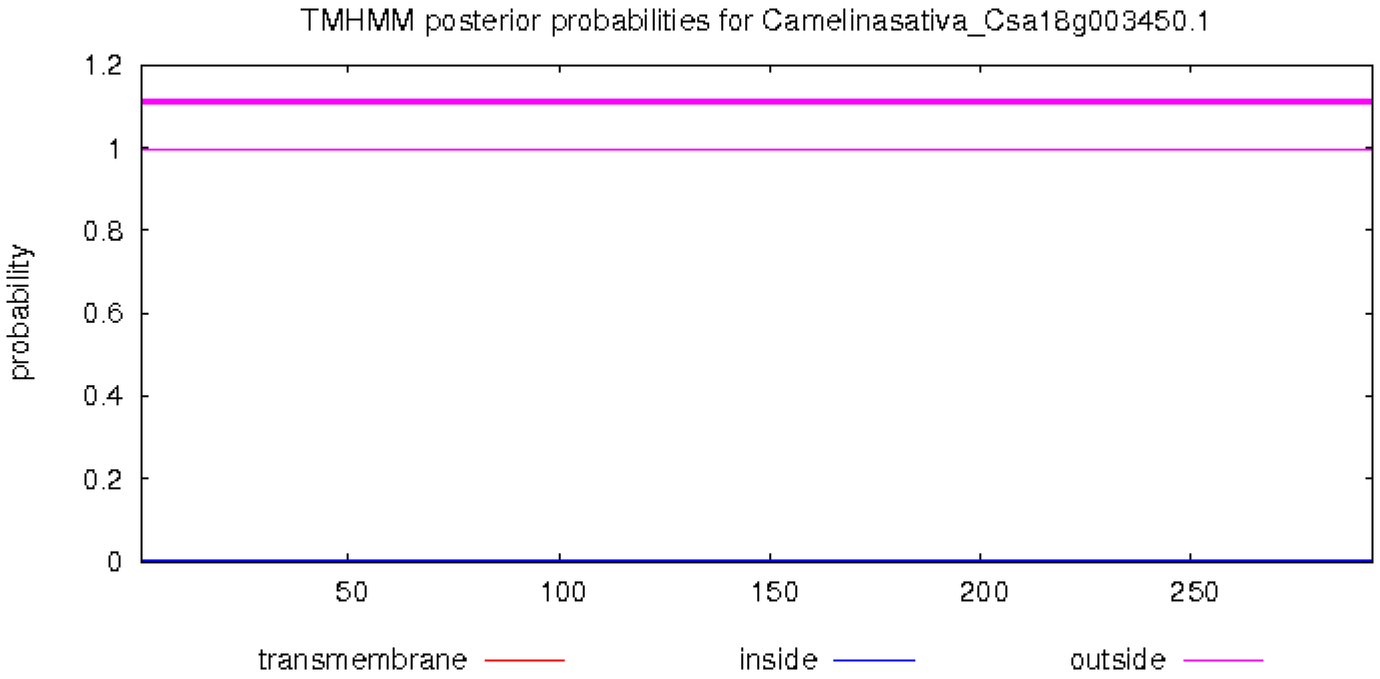

plot in postscript, script for making the plot in gnuplot, data for plot

---

```
# Camelinasativa_Csa18g021500.1 Length: 193
# Camelinasativa_Csa18g021500.1 Number of predicted TMHs:  0
# Camelinasativa_Csa18g021500.1 Exp number of AAs in TMHs: 0.00129
# Camelinasativa_Csa18g021500.1 Exp number, first 60 AAs:  0
# Camelinasativa_Csa18g021500.1 Total prob of N-in:        0.16512
Camelinasativa_Csa18g021500.1   TMHMM2.0        outside      1   193
```

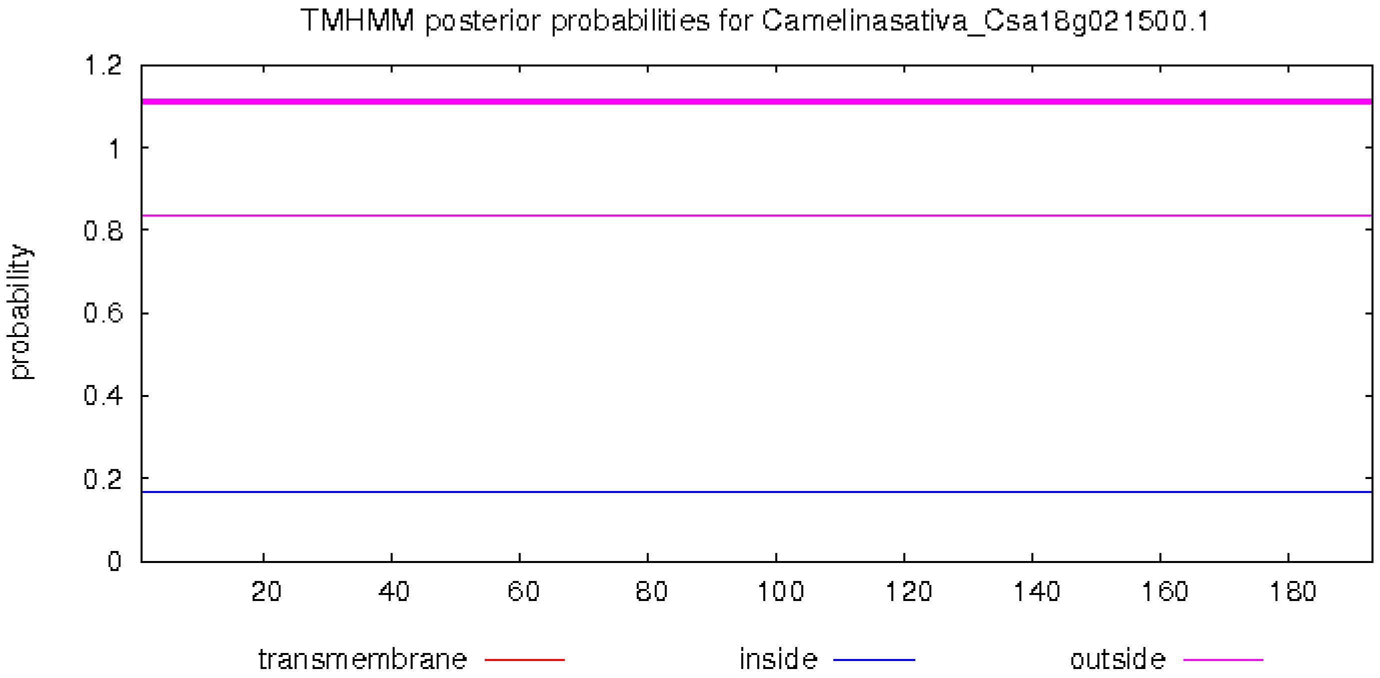

plot in postscript, script for making the plot in gnuplot, data for plot

---

```
# Camelinasativa_Csa18g025220.1 Length: 373
# Camelinasativa_Csa18g025220.1 Number of predicted TMHs:  0
# Camelinasativa_Csa18g025220.1 Exp number of AAs in TMHs: 0.00424
# Camelinasativa_Csa18g025220.1 Exp number, first 60 AAs:  0
# Camelinasativa_Csa18g025220.1 Total prob of N-in:        0.01632
Camelinasativa_Csa18g025220.1   TMHMM2.0        outside      1   373
```

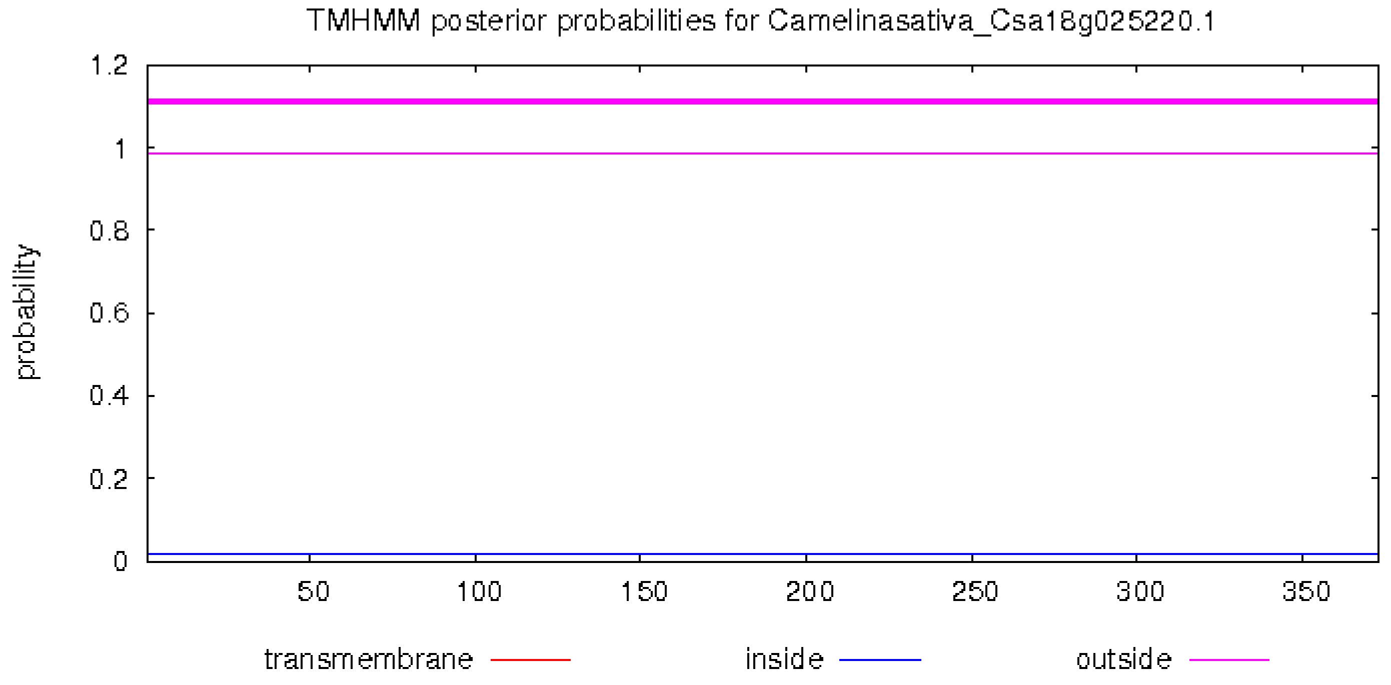

plot in postscript, script for making the plot in gnuplot, data for plot

---

```
# Camelinasativa_Csa18g029530.1 Length: 404
# Camelinasativa_Csa18g029530.1 Number of predicted TMHs:  0
# Camelinasativa_Csa18g029530.1 Exp number of AAs in TMHs: 0.00013
# Camelinasativa_Csa18g029530.1 Exp number, first 60 AAs:  0
# Camelinasativa_Csa18g029530.1 Total prob of N-in:        0.01014
Camelinasativa_Csa18g029530.1   TMHMM2.0        outside      1   404
```

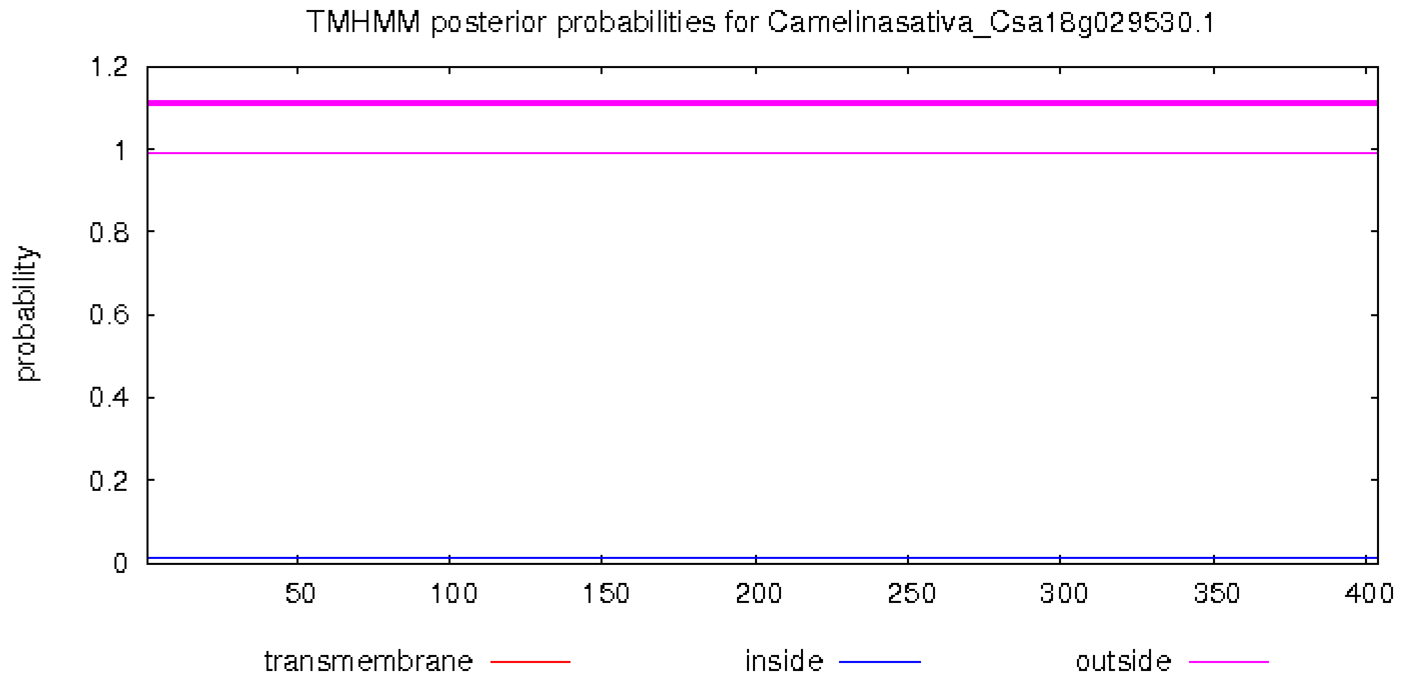

plot in postscript, script for making the plot in gnuplot, data for plot

---

```
# Camelinasativa_Csa18g032320.1 Length: 288
# Camelinasativa_Csa18g032320.1 Number of predicted TMHs:  0
# Camelinasativa_Csa18g032320.1 Exp number of AAs in TMHs: 0.0005
# Camelinasativa_Csa18g032320.1 Exp number, first 60 AAs:  0.0005
# Camelinasativa_Csa18g032320.1 Total prob of N-in:        0.03772
Camelinasativa_Csa18g032320.1   TMHMM2.0        outside      1   288
```

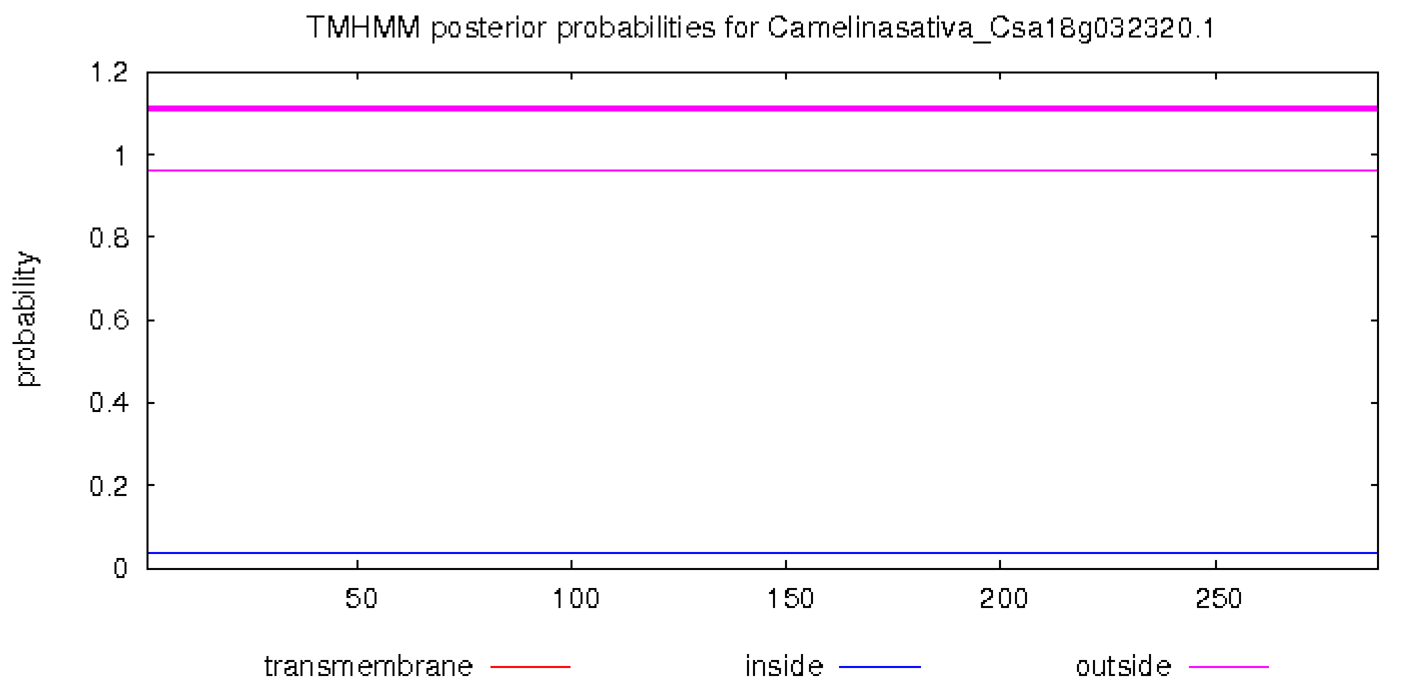

plot in postscript, script for making the plot in gnuplot, data for plot

---

```
# Camelinasativa_Csa18g032550.1 Length: 302
# Camelinasativa_Csa18g032550.1 Number of predicted TMHs:  0
# Camelinasativa_Csa18g032550.1 Exp number of AAs in TMHs: 0
# Camelinasativa_Csa18g032550.1 Exp number, first 60 AAs:  0
# Camelinasativa_Csa18g032550.1 Total prob of N-in:        0.06970
Camelinasativa_Csa18g032550.1   TMHMM2.0        outside      1   302
```

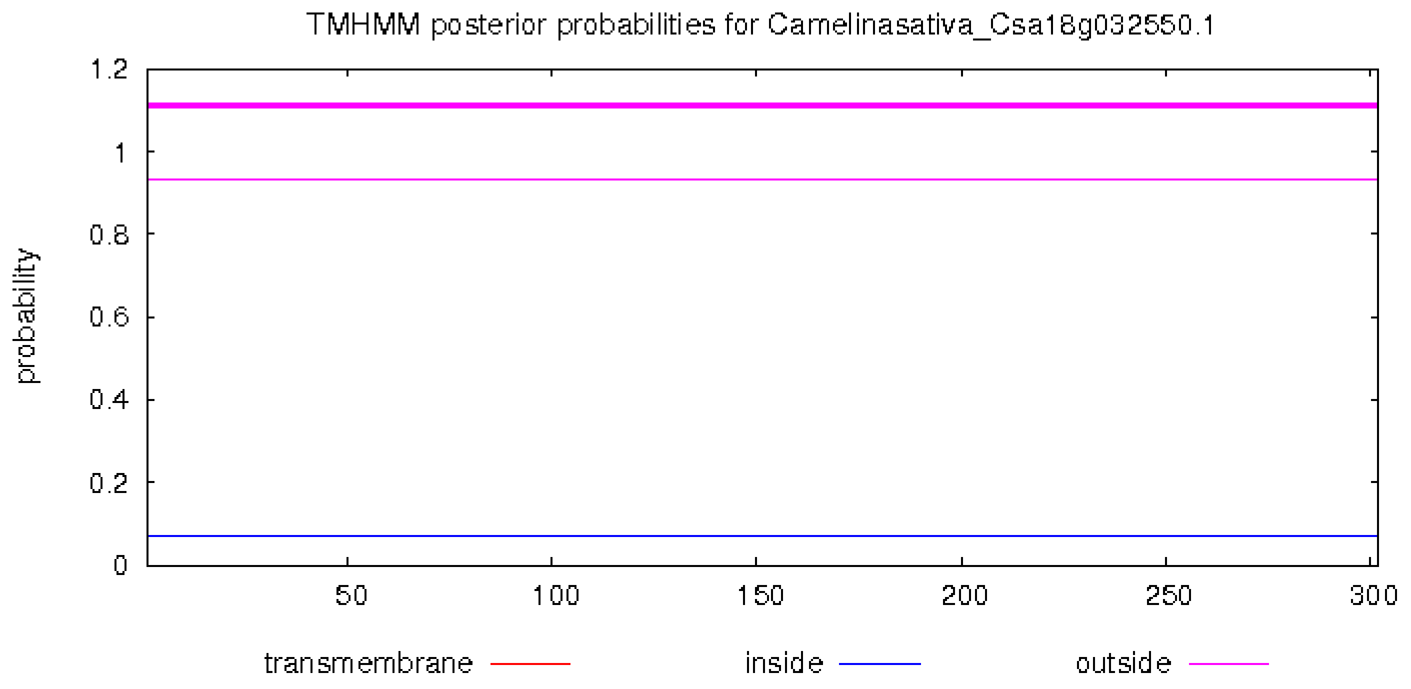

plot in postscript, script for making the plot in gnuplot, data for plot

---

```
# Camelinasativa_Csa18g034760.1 Length: 338
# Camelinasativa_Csa18g034760.1 Number of predicted TMHs:  0
# Camelinasativa_Csa18g034760.1 Exp number of AAs in TMHs: 0.00038
# Camelinasativa_Csa18g034760.1 Exp number, first 60 AAs:  0
# Camelinasativa_Csa18g034760.1 Total prob of N-in:        0.01207
Camelinasativa_Csa18g034760.1   TMHMM2.0        outside      1   338
```

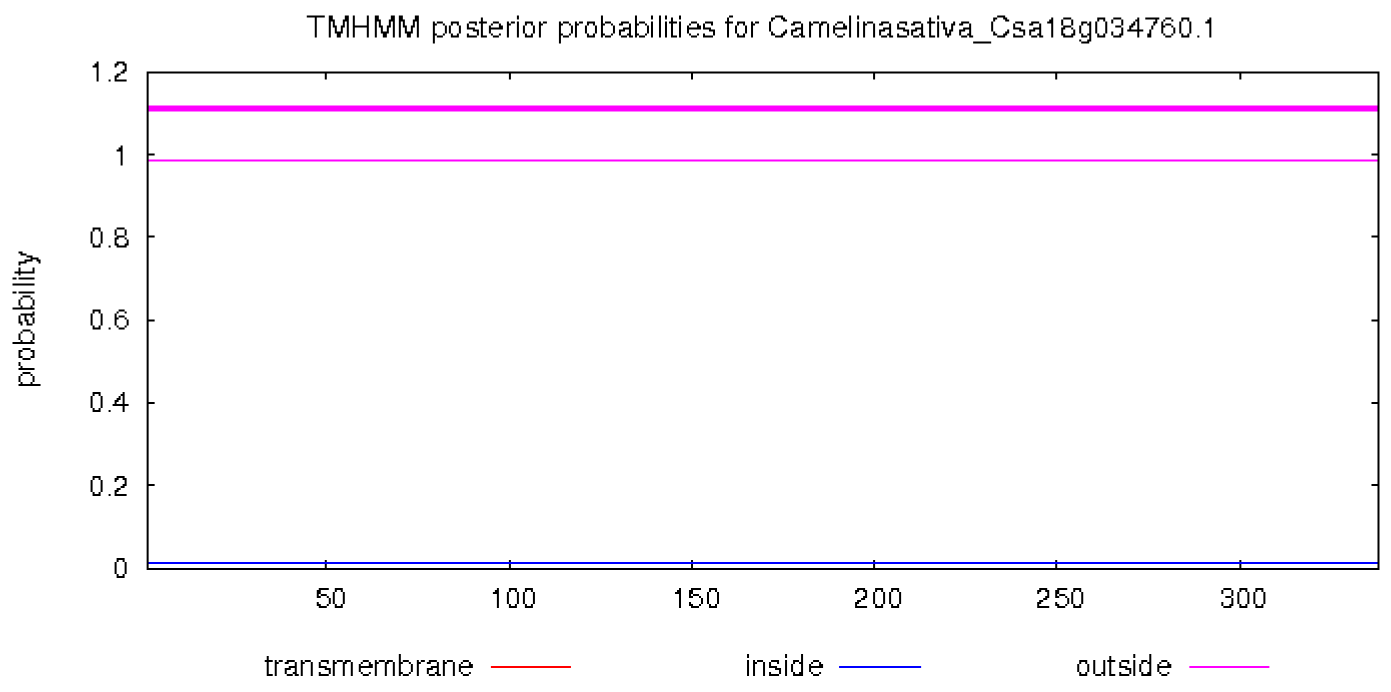

plot in postscript, script for making the plot in gnuplot, data for plot

---

```
# Camelinasativa_Csa18g035850.1 Length: 342
# Camelinasativa_Csa18g035850.1 Number of predicted TMHs:  0
# Camelinasativa_Csa18g035850.1 Exp number of AAs in TMHs: 0.00117
# Camelinasativa_Csa18g035850.1 Exp number, first 60 AAs:  0
# Camelinasativa_Csa18g035850.1 Total prob of N-in:        0.00206
Camelinasativa_Csa18g035850.1	TMHMM2.0	outside	     1   342
```

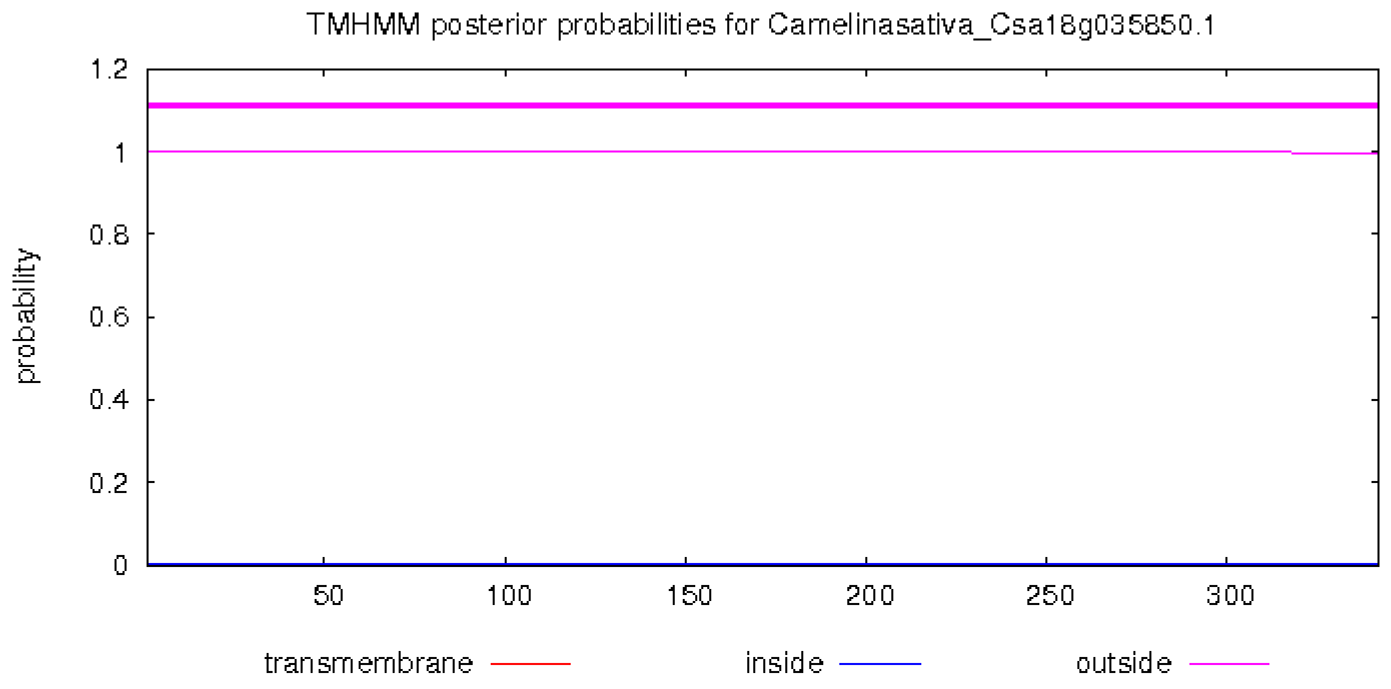

plot in postscript, script for making the plot in gnuplot, data for plot

---

```
# Camelinasativa_Csa18g037380.1 Length: 281
# Camelinasativa_Csa18g037380.1 Number of predicted TMHs:  0
# Camelinasativa_Csa18g037380.1 Exp number of AAs in TMHs: 0.00019
# Camelinasativa_Csa18g037380.1 Exp number, first 60 AAs:  0
# Camelinasativa_Csa18g037380.1 Total prob of N-in:        0.01108
Camelinasativa_Csa18g037380.1	TMHMM2.0	outside	     1   281
```

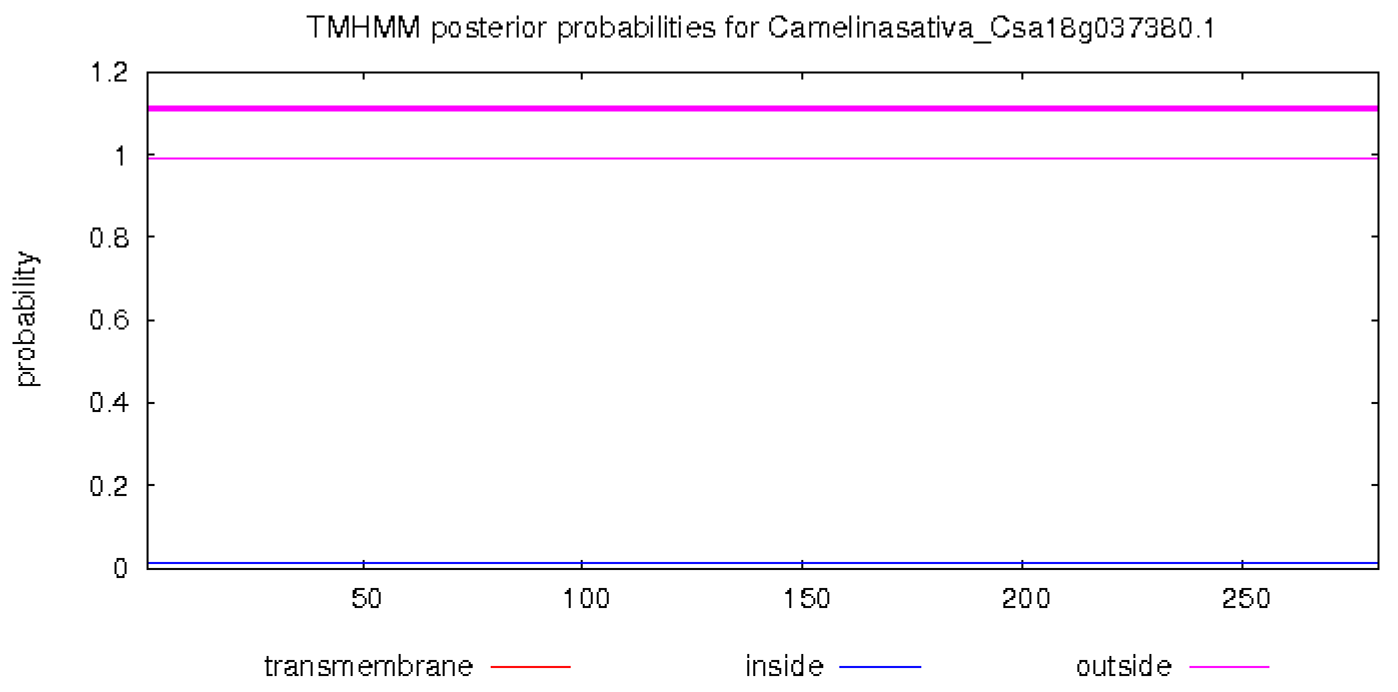

plot in postscript, script for making the plot in gnuplot, data for plot

---

```
# Camelinasativa_Csa18g037670.1 Length: 304
# Camelinasativa_Csa18g037670.1 Number of predicted TMHs:  0
# Camelinasativa_Csa18g037670.1 Exp number of AAs in TMHs: 0.00092
# Camelinasativa_Csa18g037670.1 Exp number, first 60 AAs:  0.00073
# Camelinasativa_Csa18g037670.1 Total prob of N-in:        0.02097
Camelinasativa_Csa18g037670.1	TMHMM2.0	outside	     1   304
```

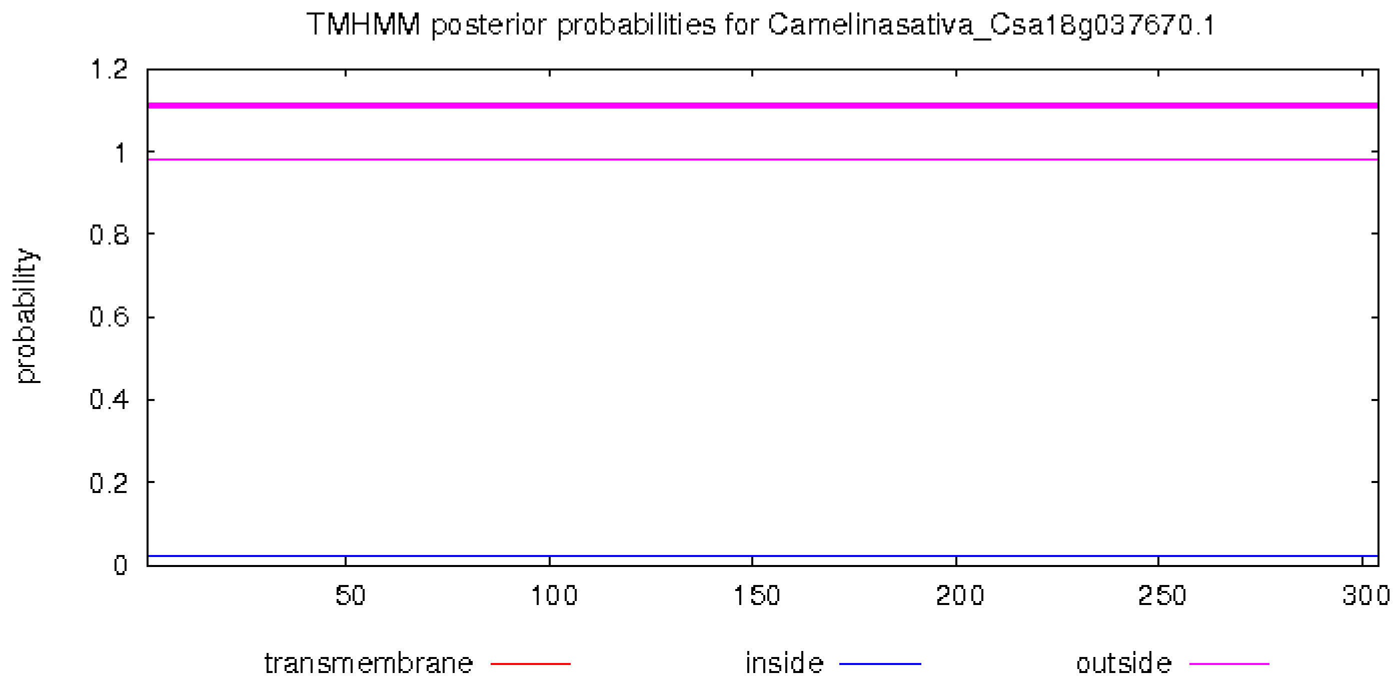

plot in postscript, script for making the plot in gnuplot, data for plot

---

```
# Camelinasativa_Csa18g038180.1 Length: 188
# Camelinasativa_Csa18g038180.1 Number of predicted TMHs:  0
# Camelinasativa_Csa18g038180.1 Exp number of AAs in TMHs: 0
# Camelinasativa_Csa18g038180.1 Exp number, first 60 AAs:  0
# Camelinasativa_Csa18g038180.1 Total prob of N-in:        0.00724
Camelinasativa_Csa18g038180.1	TMHMM2.0	outside	     1   188
```

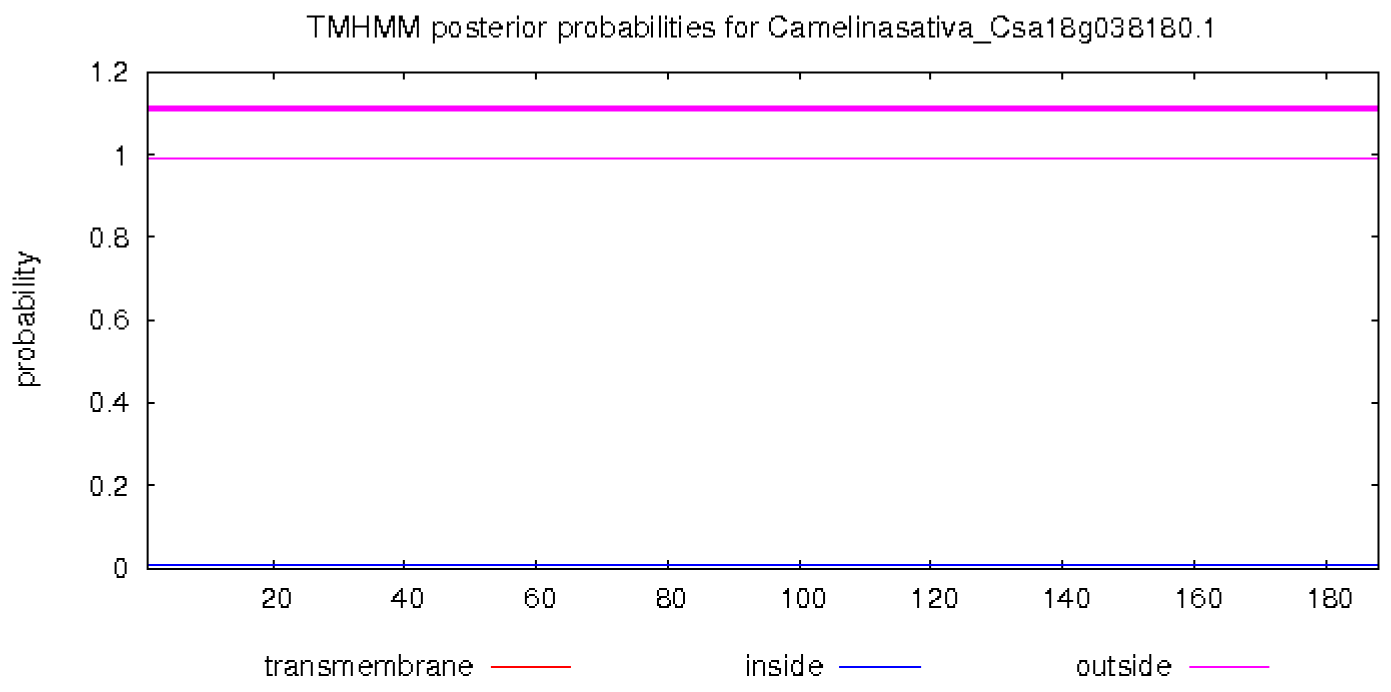

plot in postscript, script for making the plot in gnuplot, data for plot

---

```
# Camelinasativa_Csa18g042250.1 Length: 297
# Camelinasativa_Csa18g042250.1 Number of predicted TMHs:  0
# Camelinasativa_Csa18g042250.1 Exp number of AAs in TMHs: 0.00034
# Camelinasativa_Csa18g042250.1 Exp number, first 60 AAs:  0
# Camelinasativa_Csa18g042250.1 Total prob of N-in:        0.04387
Camelinasativa_Csa18g042250.1   TMHMM2.0        outside      1   297
```

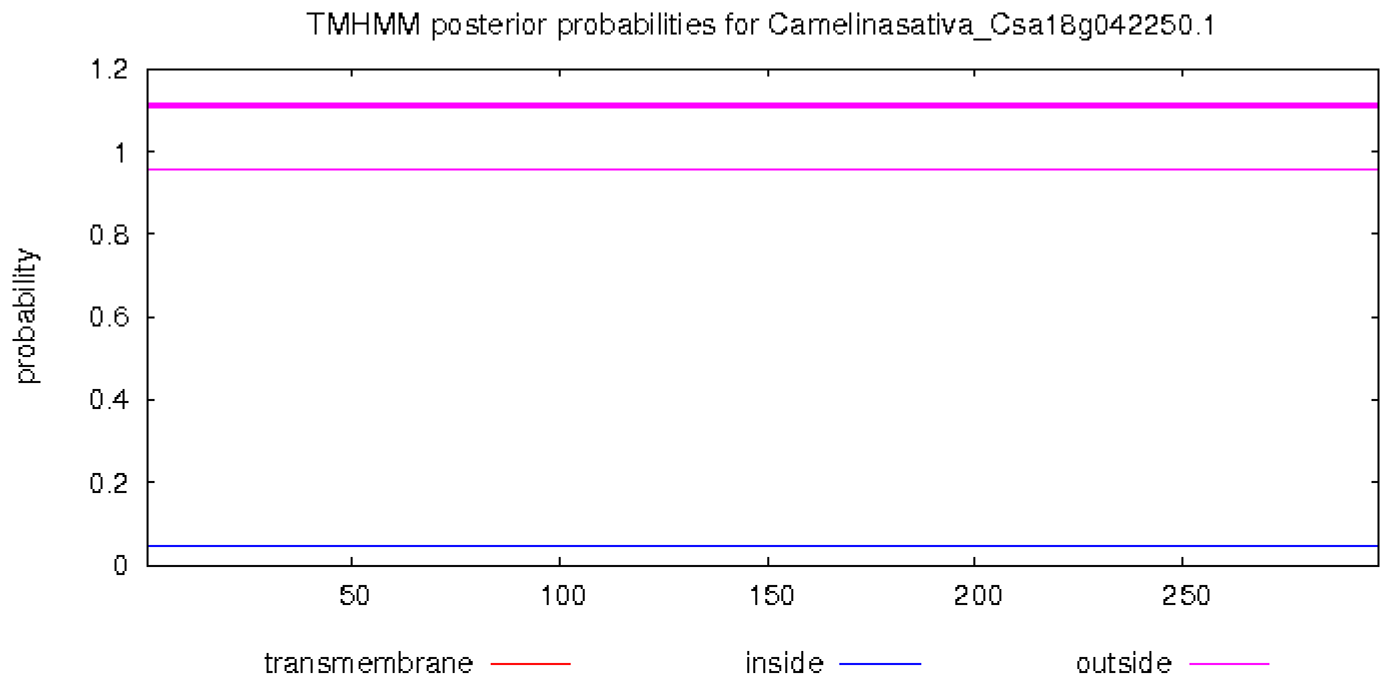

plot in postscript, script for making the plot in gnuplot, data for plot

---

```
# Camelinasativa_Csa19g001330.1 Length: 362
# Camelinasativa_Csa19g001330.1 Number of predicted TMHs:  0
# Camelinasativa_Csa19g001330.1 Exp number of AAs in TMHs: 0.00018
# Camelinasativa_Csa19g001330.1 Exp number, first 60 AAs:  0
# Camelinasativa_Csa19g001330.1 Total prob of N-in:        0.02867
Camelinasativa_Csa19g001330.1   TMHMM2.0        outside      1   362
```

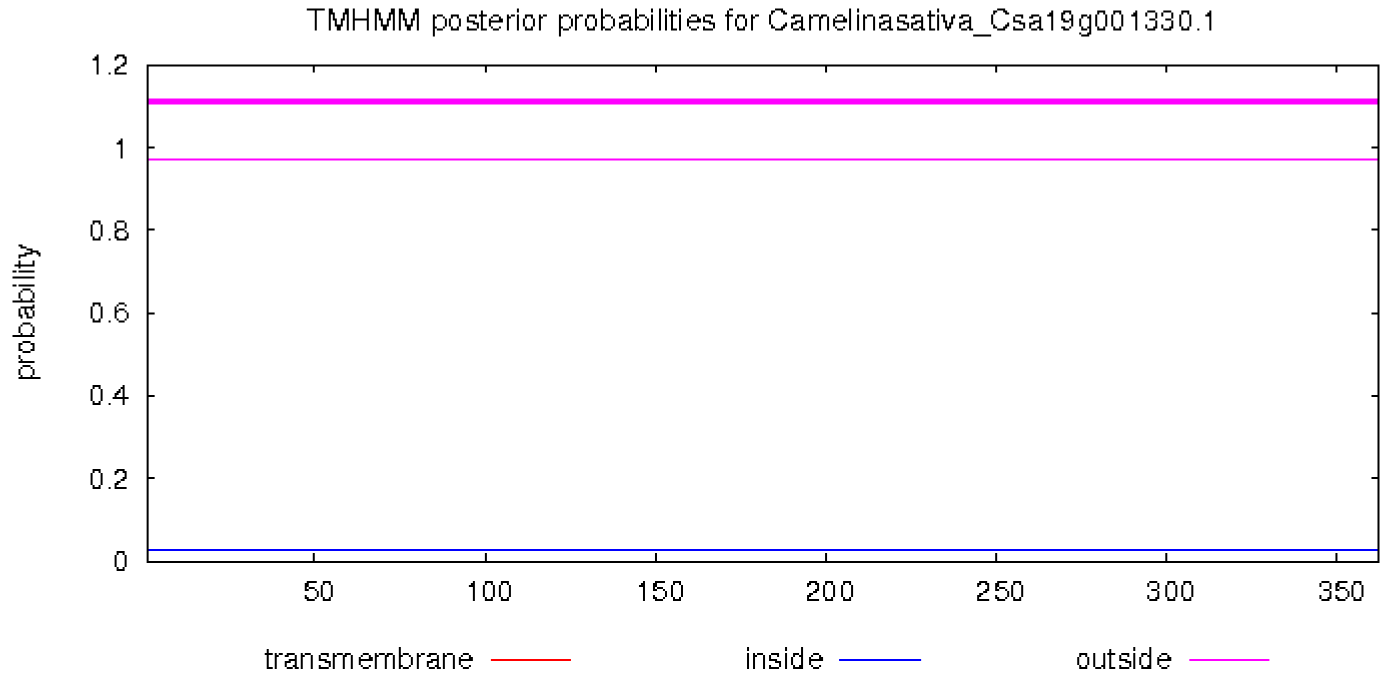

plot in postscript, script for making the plot in gnuplot, data for plot

---

```
# Camelinasativa_Csa19g005320.1 Length: 477
# Camelinasativa_Csa19g005320.1 Number of predicted TMHs:  0
# Camelinasativa_Csa19g005320.1 Exp number of AAs in TMHs: 0.00018
# Camelinasativa_Csa19g005320.1 Exp number, first 60 AAs:  0
# Camelinasativa_Csa19g005320.1 Total prob of N-in:        0.00017
Camelinasativa_Csa19g005320.1	TMHMM2.0	outside	     1   477
```

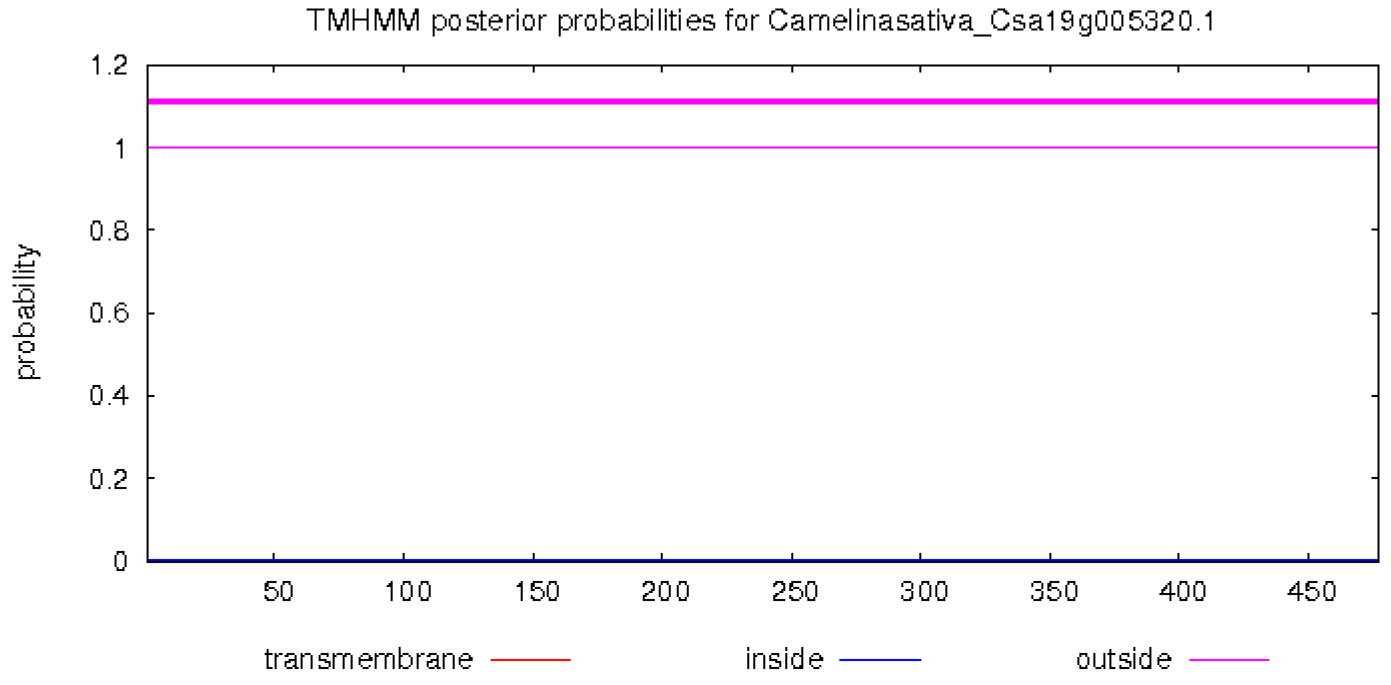

plot in postscript, script for making the plot in gnuplot, data for plot

---

```
# Camelinasativa_Csa19g006270.1 Length: 340
# Camelinasativa_Csa19g006270.1 Number of predicted TMHs:  0
# Camelinasativa_Csa19g006270.1 Exp number of AAs in TMHs: 0.00034
# Camelinasativa_Csa19g006270.1 Exp number, first 60 AAs:  0.00016
# Camelinasativa_Csa19g006270.1 Total prob of N-in:        0.01116
Camelinasativa_Csa19g006270.1	TMHMM2.0	outside	     1   340
```

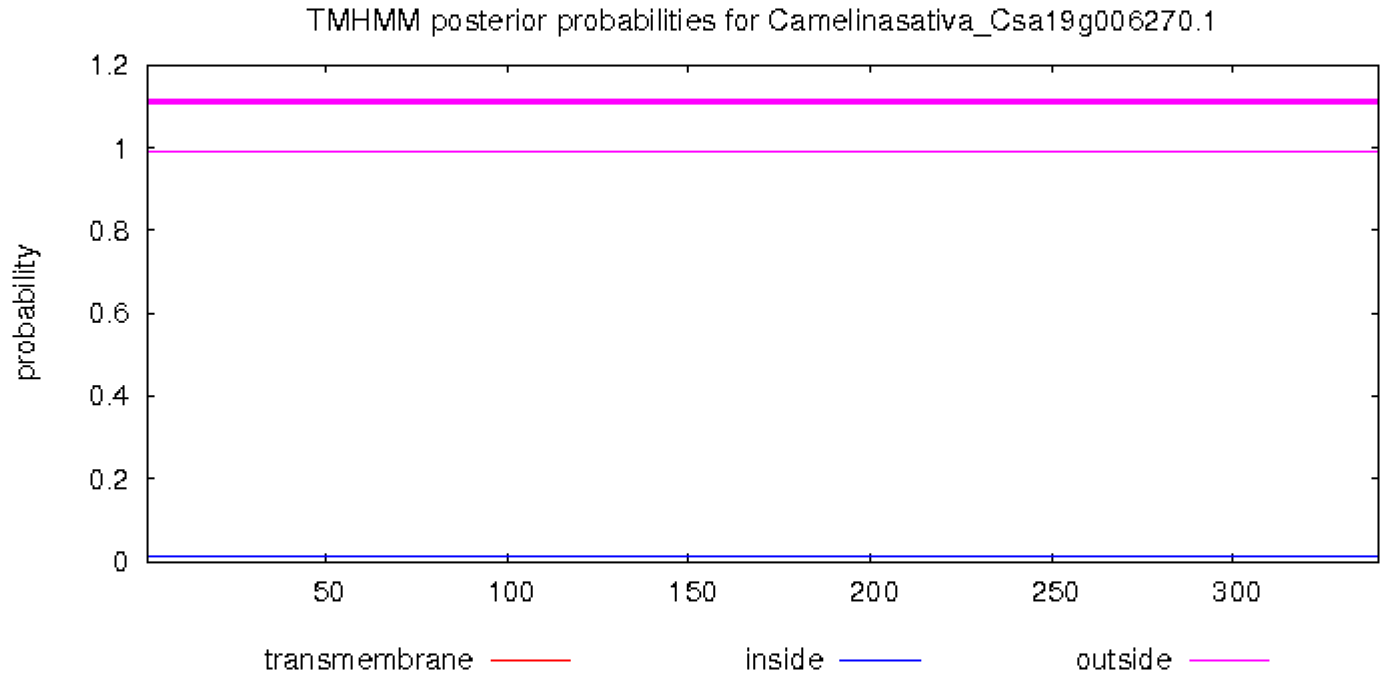

plot in postscript, script for making the plot in gnuplot, data for plot

---

```
# Camelinasativa_Csa19g006280.1 Length: 399
# Camelinasativa_Csa19g006280.1 Number of predicted TMHs:  0
# Camelinasativa_Csa19g006280.1 Exp number of AAs in TMHs: 0.00388
# Camelinasativa_Csa19g006280.1 Exp number, first 60 AAs:  0.00388
# Camelinasativa_Csa19g006280.1 Total prob of N-in:        0.00054
Camelinasativa_Csa19g006280.1   TMHMM2.0        outside      1   399
```

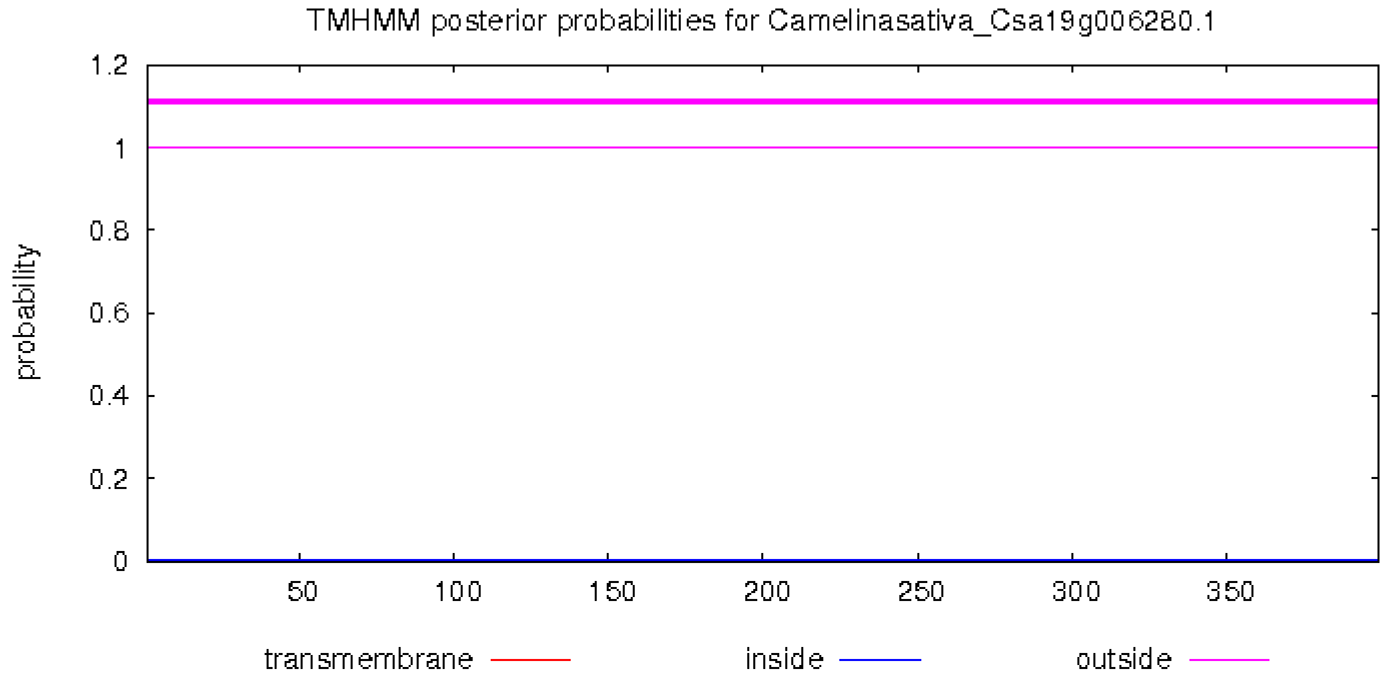

plot in postscript, script for making the plot in gnuplot, data for plot

---

```
# Camelinasativa_Csa19g014110.1 Length: 450
# Camelinasativa_Csa19g014110.1 Number of predicted TMHs:  0
# Camelinasativa_Csa19g014110.1 Exp number of AAs in TMHs: 0.0422
# Camelinasativa_Csa19g014110.1 Exp number, first 60 AAs:  0.01599
# Camelinasativa_Csa19g014110.1 Total prob of N-in:        0.00333
Camelinasativa_Csa19g014110.1   TMHMM2.0        outside      1   450
```

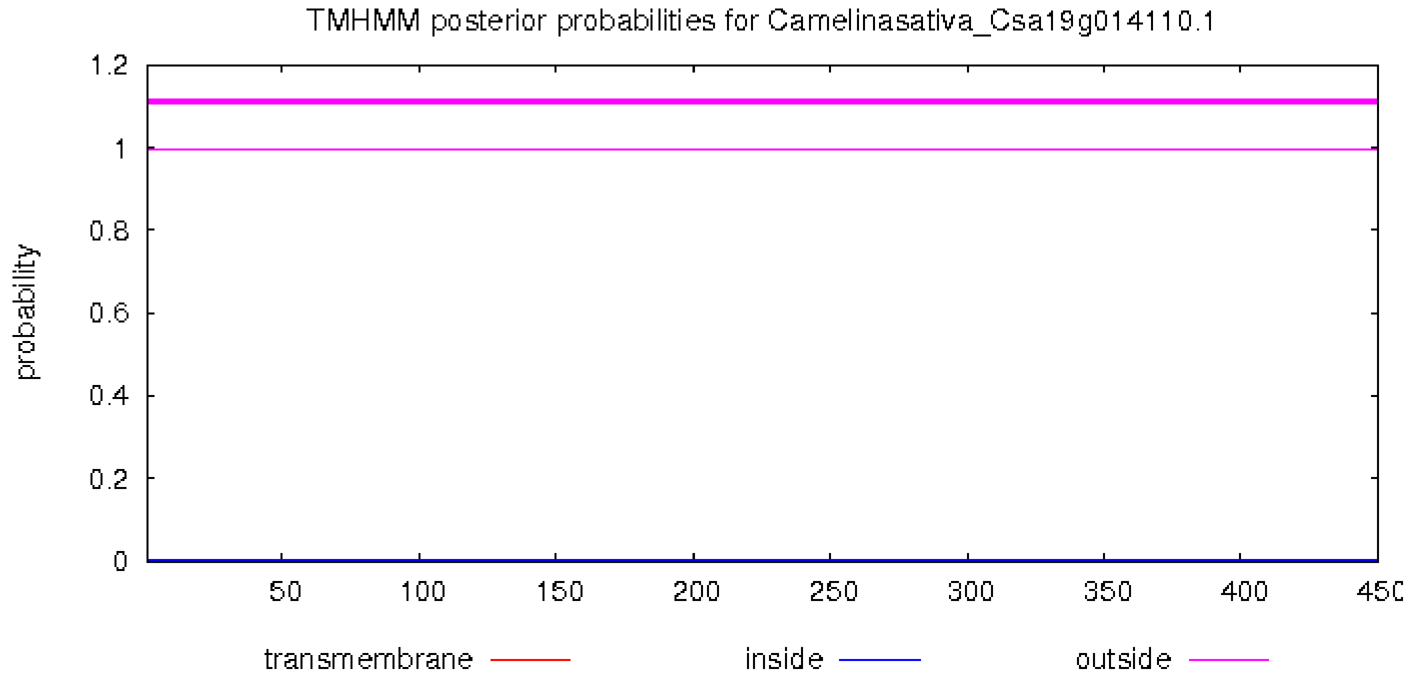

plot in postscript, script for making the plot in gnuplot, data for plot

---

```
# Camelinasativa_Csa19g014120.1 Length: 461
# Camelinasativa_Csa19g014120.1 Number of predicted TMHs:  0
# Camelinasativa_Csa19g014120.1 Exp number of AAs in TMHs: 0.02195
# Camelinasativa_Csa19g014120.1 Exp number, first 60 AAs:  0.00134
# Camelinasativa_Csa19g014120.1 Total prob of N-in:        0.00547
Camelinasativa_Csa19g014120.1	TMHMM2.0	outside	     1   461
```

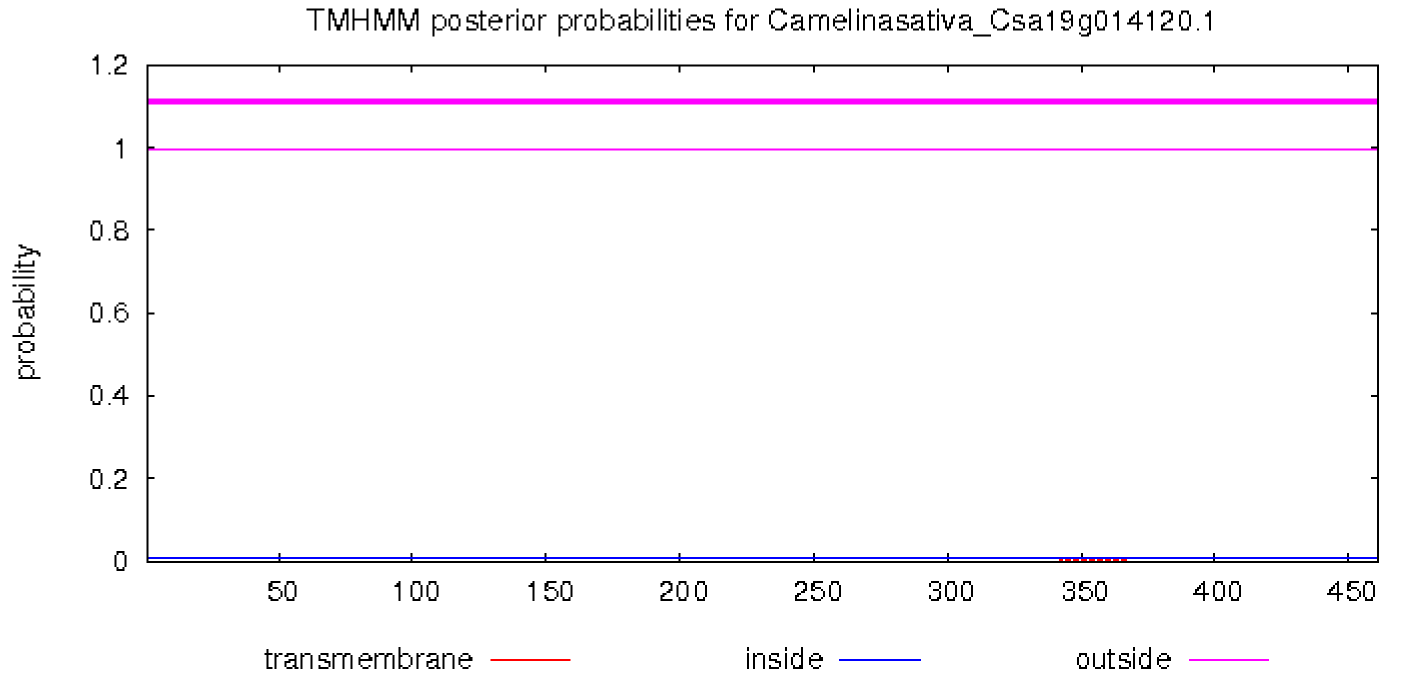

plot in postscript, script for making the plot in gnuplot, data for plot

---

```
# Camelinasativa_Csa19g014130.1 Length: 746
# Camelinasativa_Csa19g014130.1 Number of predicted TMHs:  1
# Camelinasativa_Csa19g014130.1 Exp number of AAs in TMHs: 21.11211
# Camelinasativa_Csa19g014130.1 Exp number, first 60 AAs:  0
# Camelinasativa_Csa19g014130.1 Total prob of N-in:        0.00021
Camelinasativa_Csa19g014130.1	TMHMM2.0	outside	     1   720
```

```
Camelinasativa_Csa19g014130.1   TMHMM2.0        TMhelix    721   743
Camelinasativa_Csa19g014130.1   TMHMM2.0        inside     744   746
```

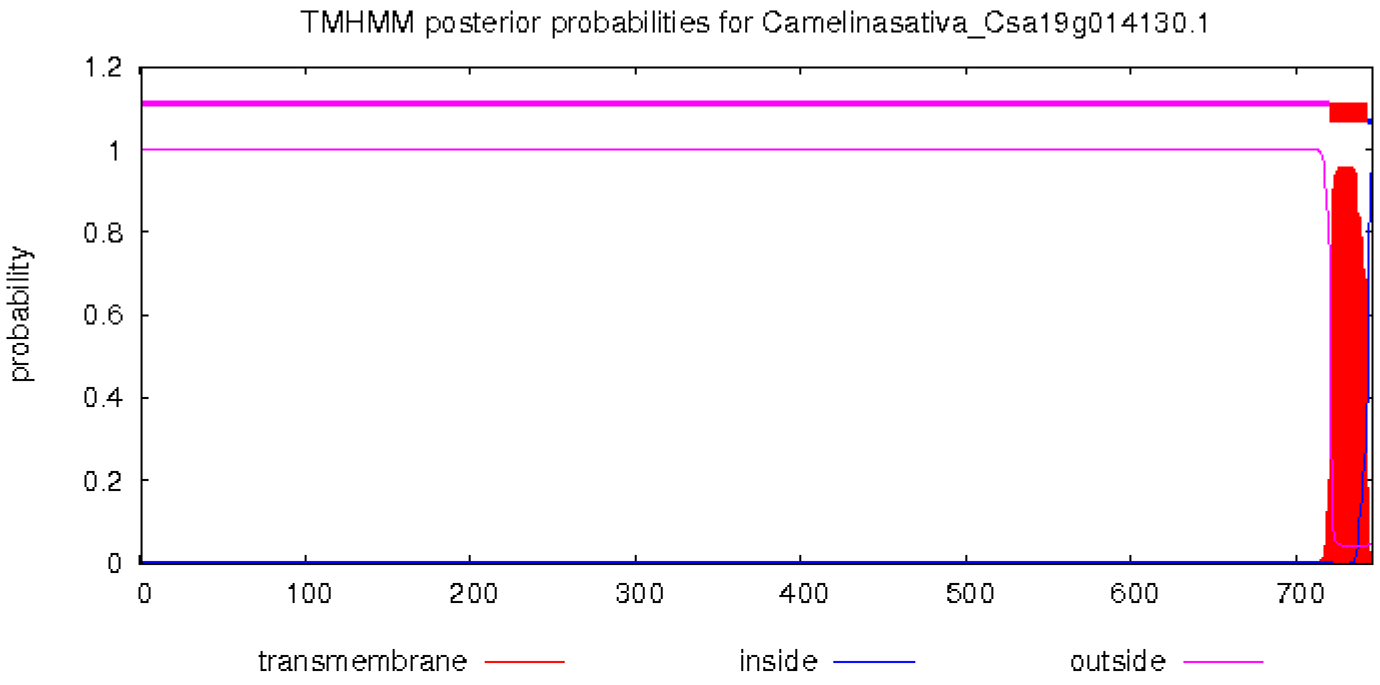

plot in postscript, script for making the plot in gnuplot, data for plot

---

```
# Camelinasativa_Csa19g017110.1 Length: 302
# Camelinasativa_Csa19g017110.1 Number of predicted TMHs:  0
# Camelinasativa_Csa19g017110.1 Exp number of AAs in TMHs: 0.00056
# Camelinasativa_Csa19g017110.1 Exp number, first 60 AAs:  0
# Camelinasativa_Csa19g017110.1 Total prob of N-in:        0.03816
Camelinasativa_Csa19g017110.1   TMHMM2.0        outside      1   302
```

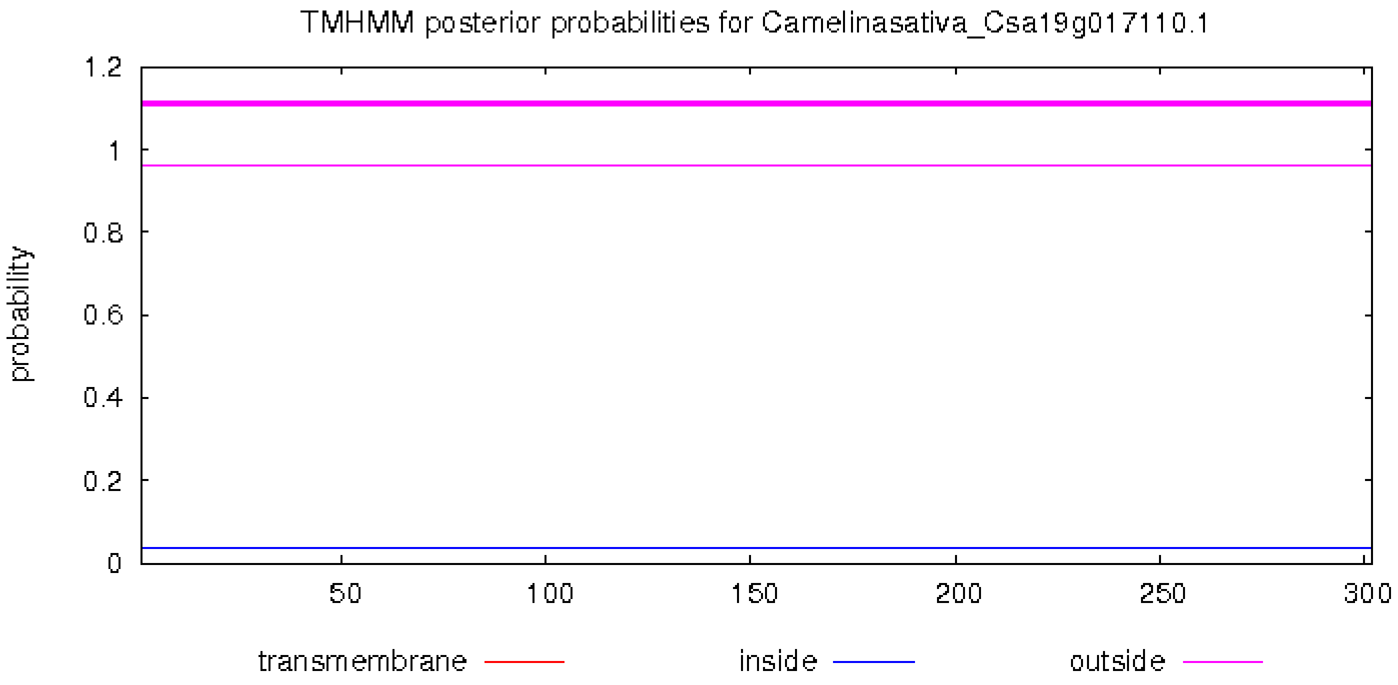

plot in postscript, script for making the plot in gnuplot, data for plot

---

```
# Camelinasativa_Csa19g017210.1 Length: 281
# Camelinasativa_Csa19g017210.1 Number of predicted TMHs:  0
# Camelinasativa_Csa19g017210.1 Exp number of AAs in TMHs: 0.00085
# Camelinasativa_Csa19g017210.1 Exp number, first 60 AAs:  0
```

```
# Camelinasativa_Csa19g017210.1 Total prob of N-in:        0.09682
Camelinasativa_Csa19g017210.1   TMHMM2.0        outside      1   281
```

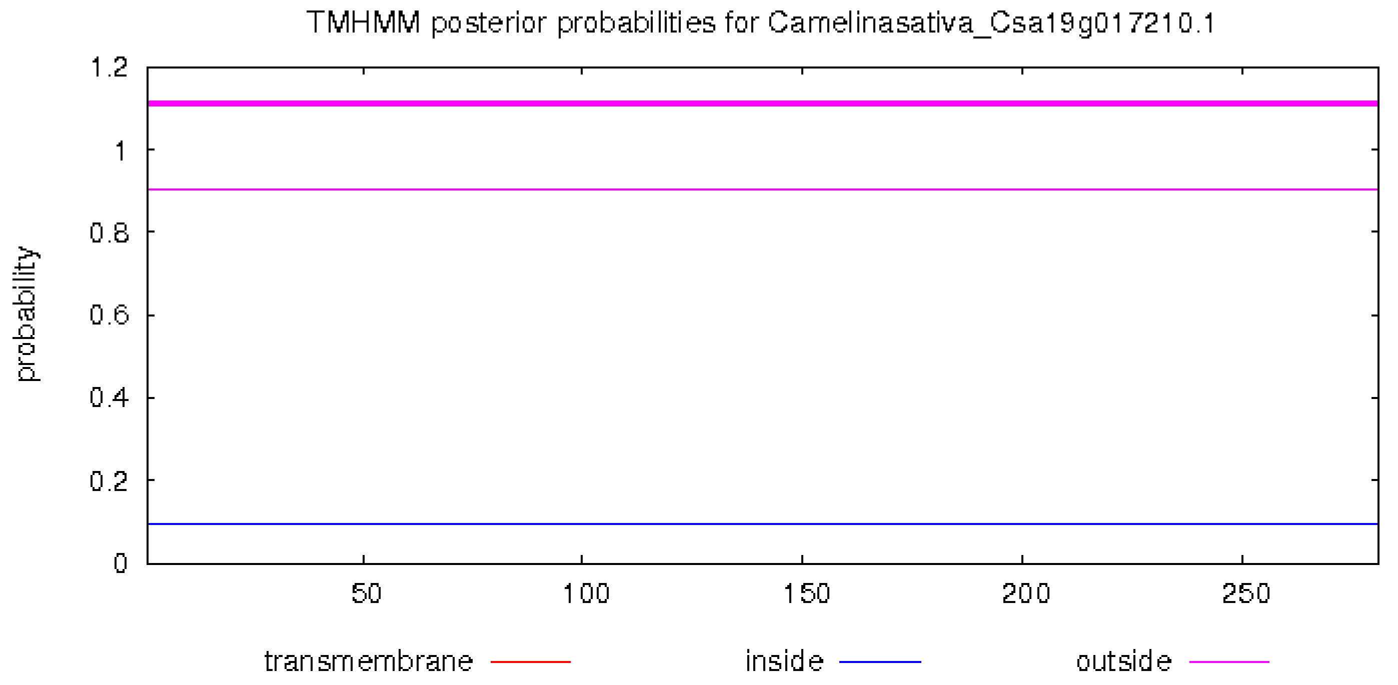

plot in postscript, script for making the plot in gnuplot, data for plot

---

```
# Camelinasativa_Csa19g021860.1 Length: 307
# Camelinasativa_Csa19g021860.1 Number of predicted TMHs:  0
# Camelinasativa_Csa19g021860.1 Exp number of AAs in TMHs: 0.17378
# Camelinasativa_Csa19g021860.1 Exp number, first 60 AAs:  0.00017
# Camelinasativa_Csa19g021860.1 Total prob of N-in:        0.01304
Camelinasativa_Csa19g021860.1   TMHMM2.0        outside      1   307
```

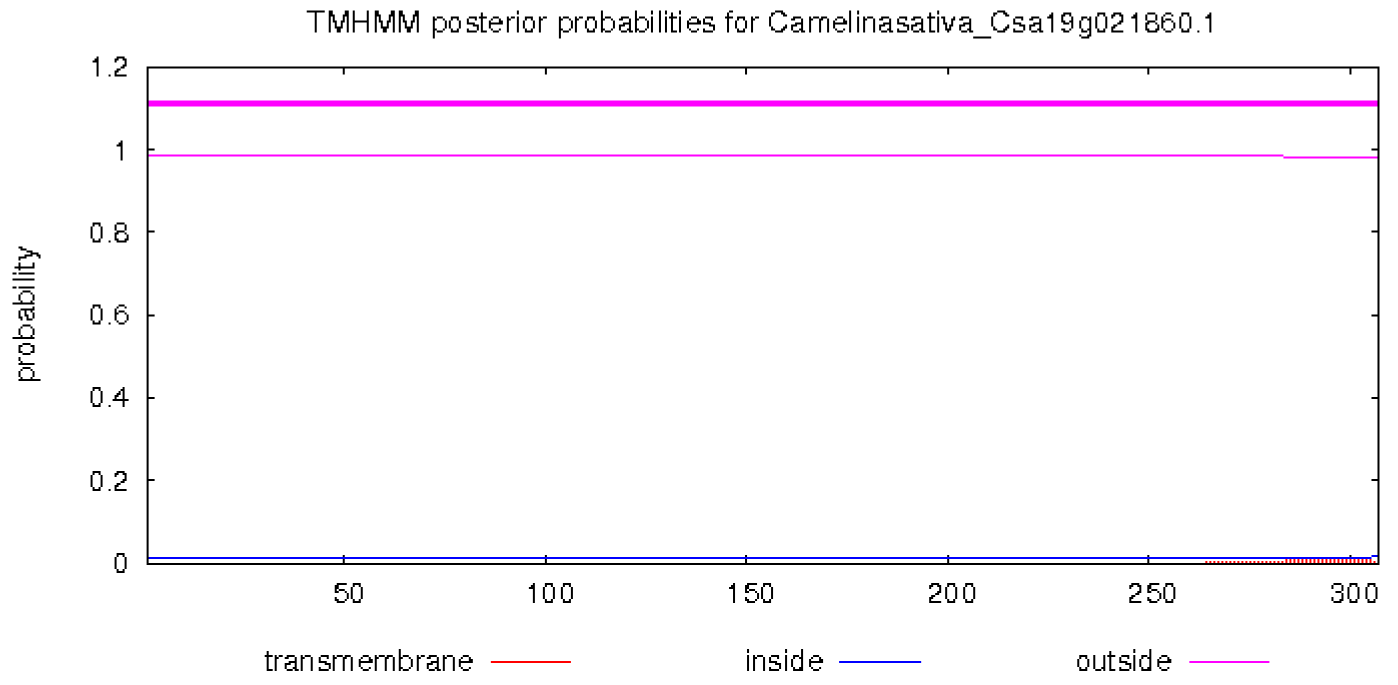

plot in postscript, script for making the plot in gnuplot, data for plot

---

```
# Camelinasativa_Csa19g022270.1 Length: 319
# Camelinasativa_Csa19g022270.1 Number of predicted TMHs:  0
# Camelinasativa_Csa19g022270.1 Exp number of AAs in TMHs: 0
# Camelinasativa_Csa19g022270.1 Exp number, first 60 AAs:  0
```

```
# Camelinasativa_Csa19g022270.1 Total prob of N-in:        0.00235
Camelinasativa_Csa19g022270.1   TMHMM2.0        outside      1   319
```

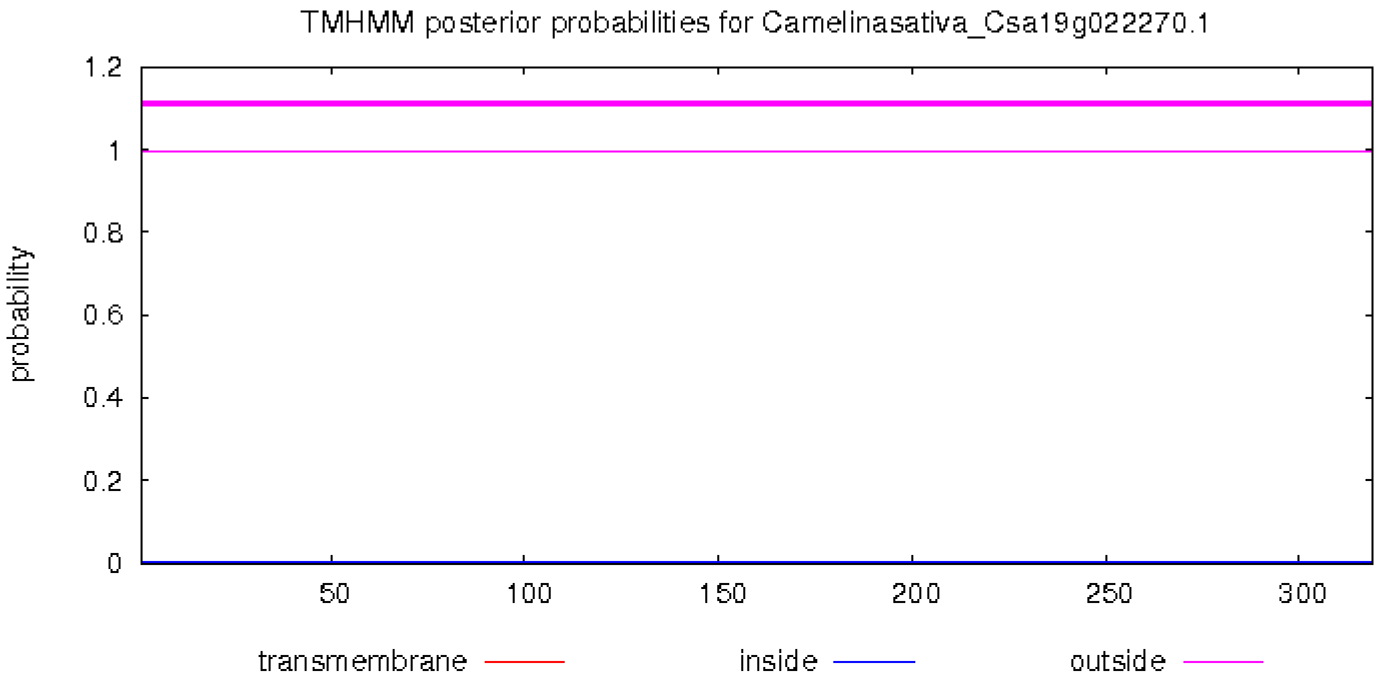

plot in postscript, script for making the plot in gnuplot, data for plot

```
# Camelinasativa_Csa19g022280.1 Length: 299
# Camelinasativa_Csa19g022280.1 Number of predicted TMHs:  0
# Camelinasativa_Csa19g022280.1 Exp number of AAs in TMHs: 0.00147
# Camelinasativa_Csa19g022280.1 Exp number, first 60 AAs:  0
# Camelinasativa_Csa19g022280.1 Total prob of N-in:        0.02023
Camelinasativa_Csa19g022280.1   TMHMM2.0        outside      1   299
```

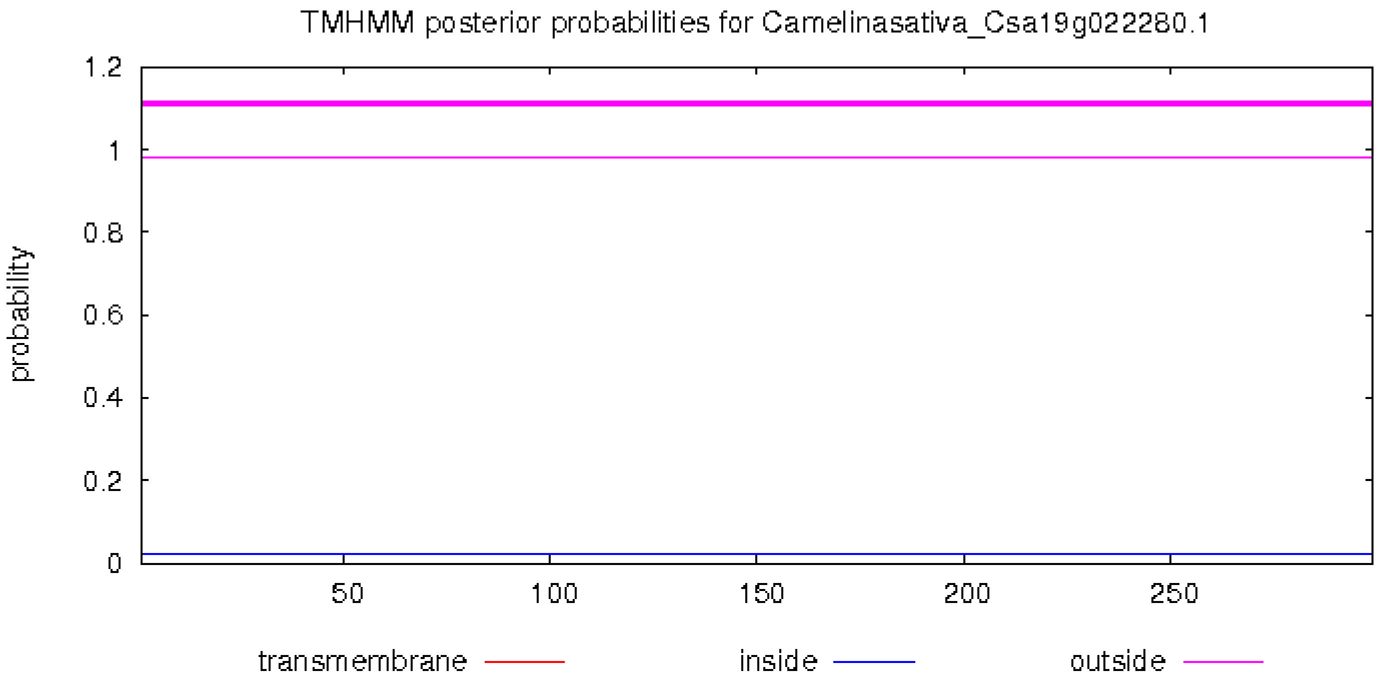

plot in postscript, script for making the plot in gnuplot, data for plot

```
# Camelinasativa_Csa19g024880.1 Length: 247
# Camelinasativa_Csa19g024880.1 Number of predicted TMHs:  0
# Camelinasativa_Csa19g024880.1 Exp number of AAs in TMHs: 0
# Camelinasativa_Csa19g024880.1 Exp number, first 60 AAs:  0
```

```
# Camelinasativa_Csa19g024880.1 Total prob of N-in:        0.00220
Camelinasativa_Csa19g024880.1	TMHMM2.0	outside	     1   247
```

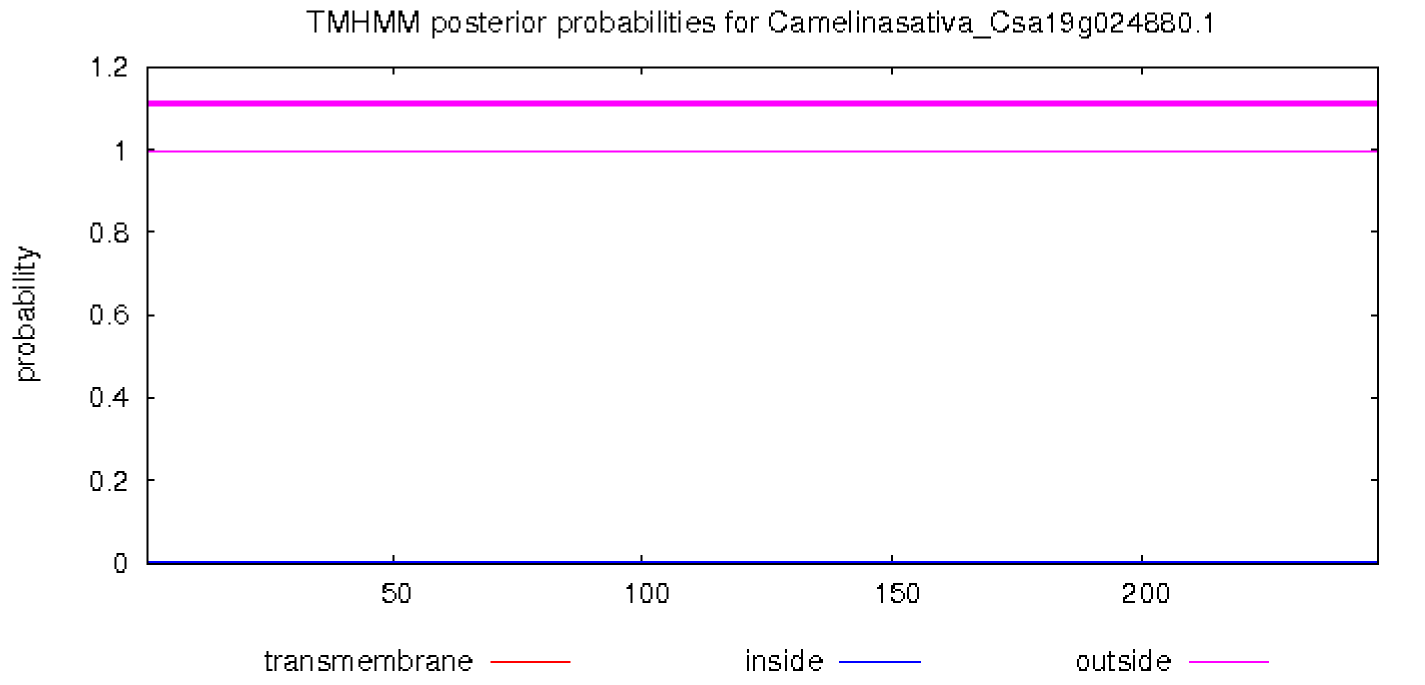

plot in postscript, script for making the plot in gnuplot, data for plot

---

```
# Camelinasativa_Csa19g025790.1 Length: 351
# Camelinasativa_Csa19g025790.1 Number of predicted TMHs:  0
# Camelinasativa_Csa19g025790.1 Exp number of AAs in TMHs: 0.05905
# Camelinasativa_Csa19g025790.1 Exp number, first 60 AAs:  0
# Camelinasativa_Csa19g025790.1 Total prob of N-in:        0.00444
Camelinasativa_Csa19g025790.1	TMHMM2.0	outside	     1   351
```

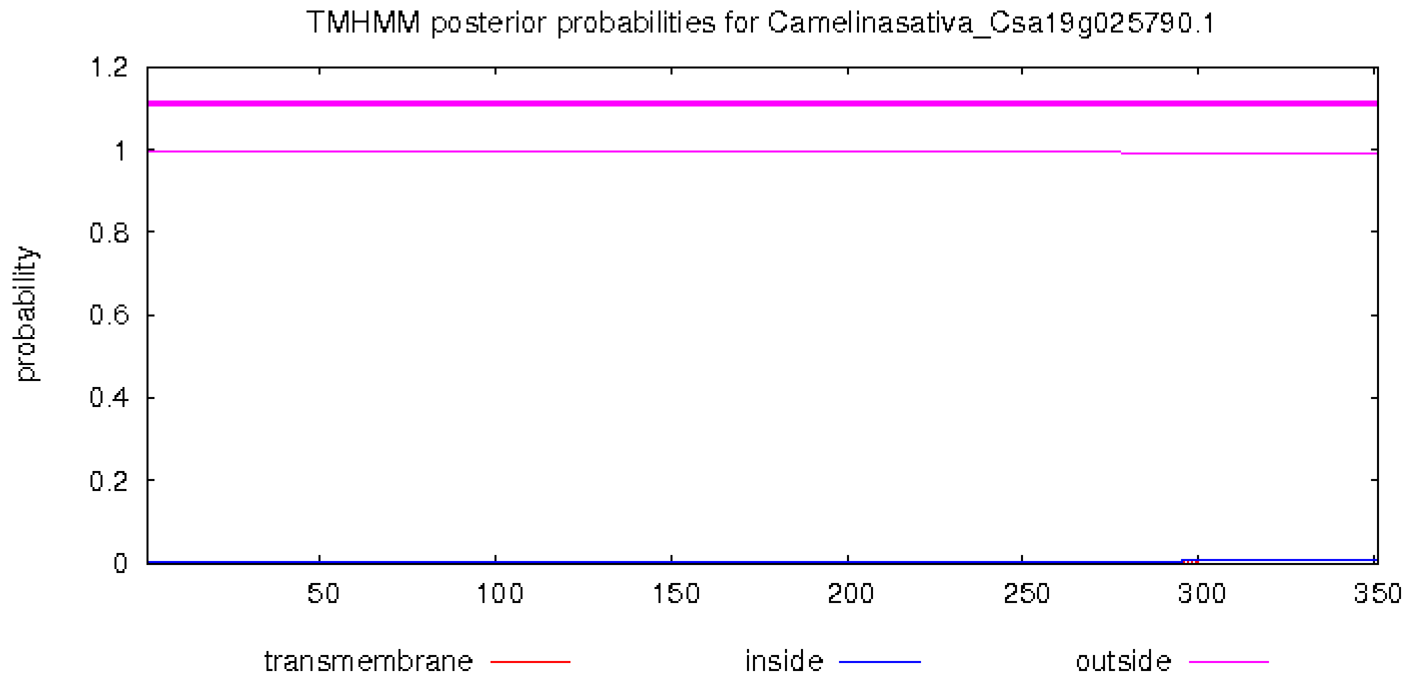

plot in postscript, script for making the plot in gnuplot, data for plot

---

```
# Camelinasativa_Csa19g046490.1 Length: 260
# Camelinasativa_Csa19g046490.1 Number of predicted TMHs:  0
# Camelinasativa_Csa19g046490.1 Exp number of AAs in TMHs: 0.00016
# Camelinasativa_Csa19g046490.1 Exp number, first 60 AAs:  0.00016
```

```
# Camelinasativa_Csa19g046490.1 Total prob of N-in:        0.00909
Camelinasativa_Csa19g046490.1   TMHMM2.0        outside      1   260
```

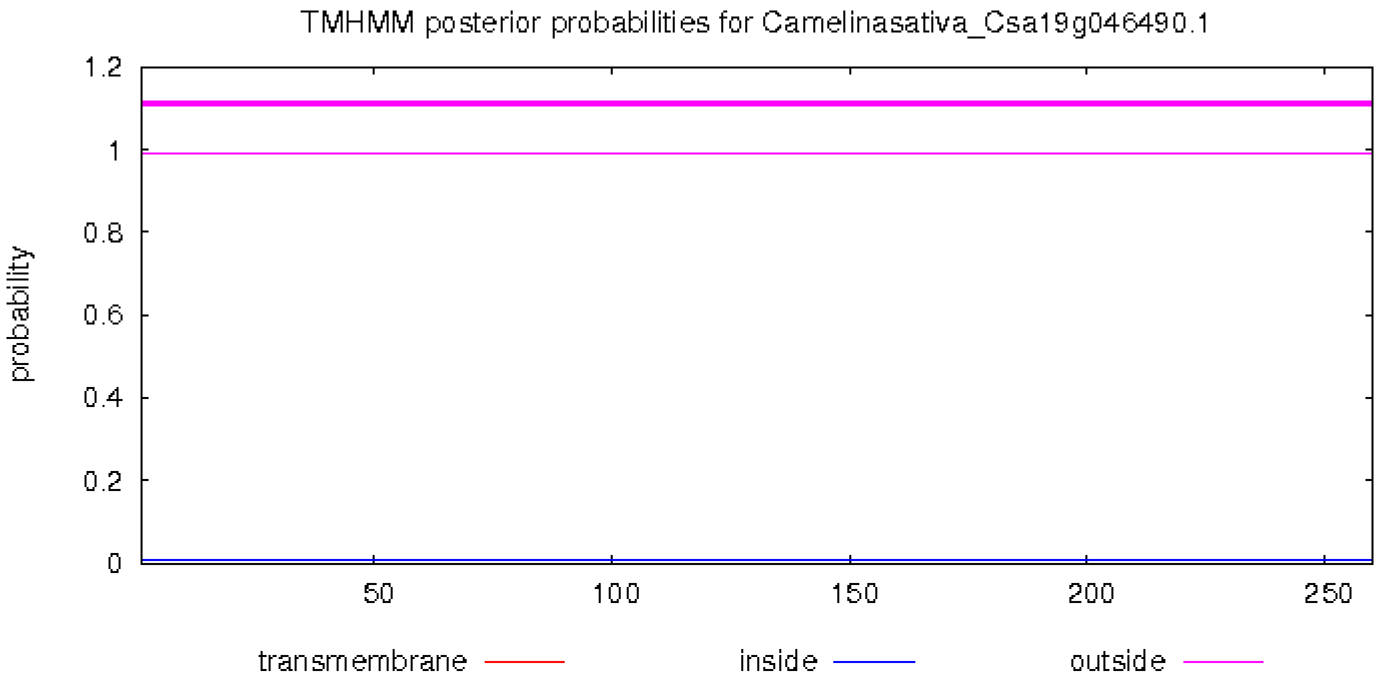

plot in postscript, script for making the plot in gnuplot, data for plot

---

```
# Camelinasativa_Csa19g053810.1 Length: 282
# Camelinasativa_Csa19g053810.1 Number of predicted TMHs:  0
# Camelinasativa_Csa19g053810.1 Exp number of AAs in TMHs: 0.00083
# Camelinasativa_Csa19g053810.1 Exp number, first 60 AAs:  0
# Camelinasativa_Csa19g053810.1 Total prob of N-in:        0.00633
Camelinasativa_Csa19g053810.1   TMHMM2.0        outside      1   282
```

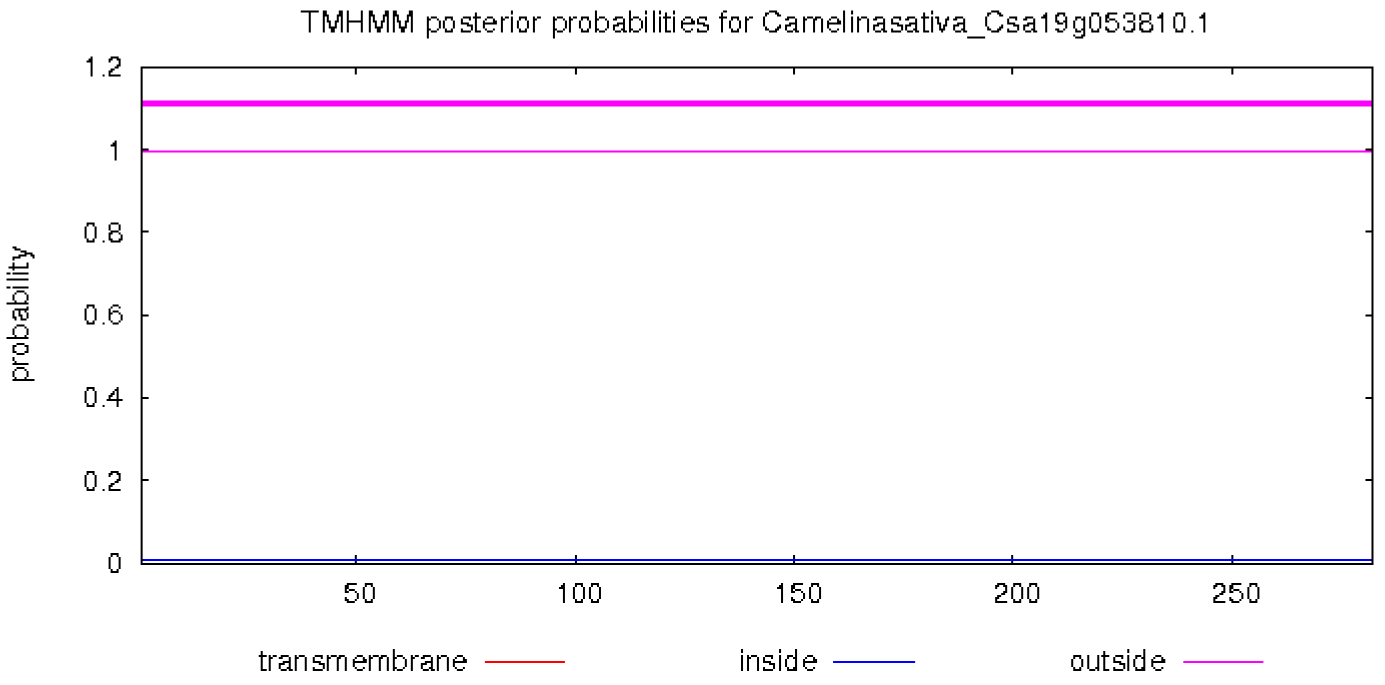

plot in postscript, script for making the plot in gnuplot, data for plot

---

```
# Camelinasativa_Csa20g005600.1 Length: 398
# Camelinasativa_Csa20g005600.1 Number of predicted TMHs:  0
# Camelinasativa_Csa20g005600.1 Exp number of AAs in TMHs: 0.00569
# Camelinasativa_Csa20g005600.1 Exp number, first 60 AAs:  0.00515
```

```
# Camelinasativa_Csa20g005600.1 Total prob of N-in:        0.02351
Camelinasativa_Csa20g005600.1   TMHMM2.0        outside      1   398
```

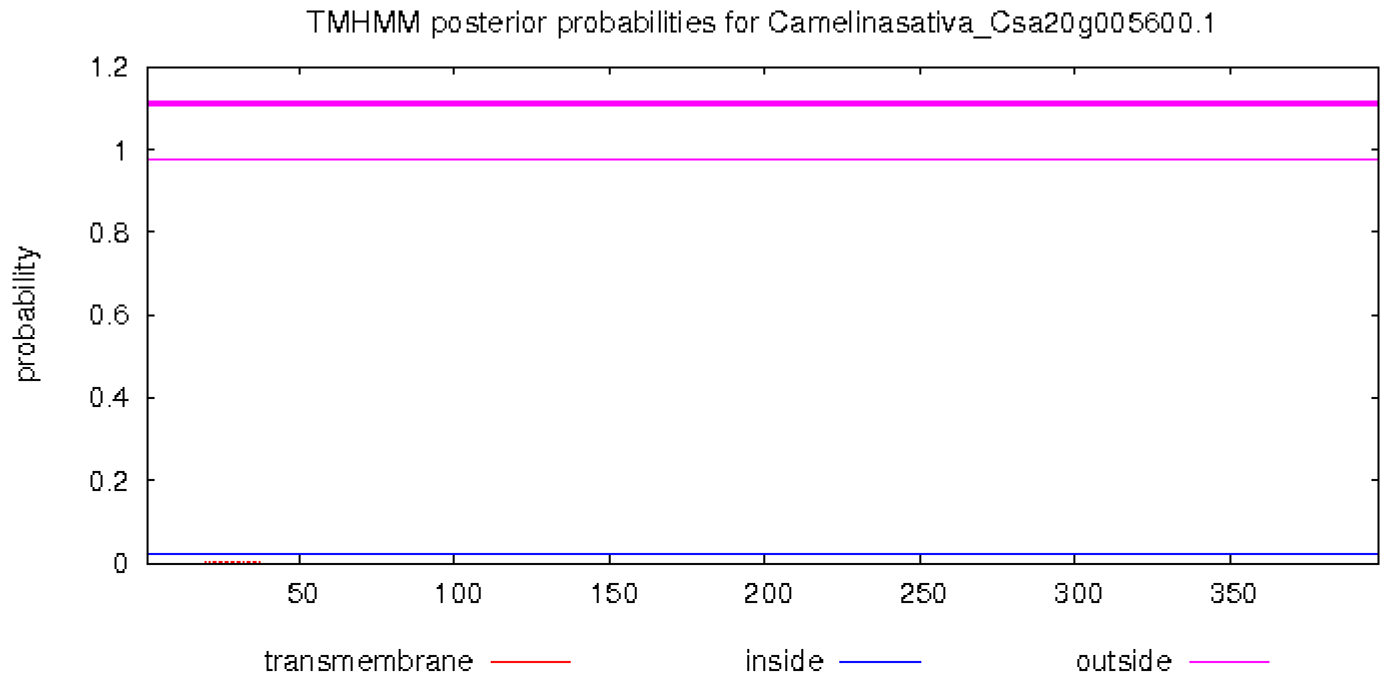

plot in postscript, script for making the plot in gnuplot, data for plot

---

```
# Camelinasativa_Csa20g005610.1 Length: 657
# Camelinasativa_Csa20g005610.1 Number of predicted TMHs:  1
# Camelinasativa_Csa20g005610.1 Exp number of AAs in TMHs: 20.05079
# Camelinasativa_Csa20g005610.1 Exp number, first 60 AAs:  0
# Camelinasativa_Csa20g005610.1 Total prob of N-in:        0.01420
Camelinasativa_Csa20g005610.1   TMHMM2.0        outside      1   631
Camelinasativa_Csa20g005610.1   TMHMM2.0        TMhelix    632   654
Camelinasativa_Csa20g005610.1   TMHMM2.0        inside     655   657
```

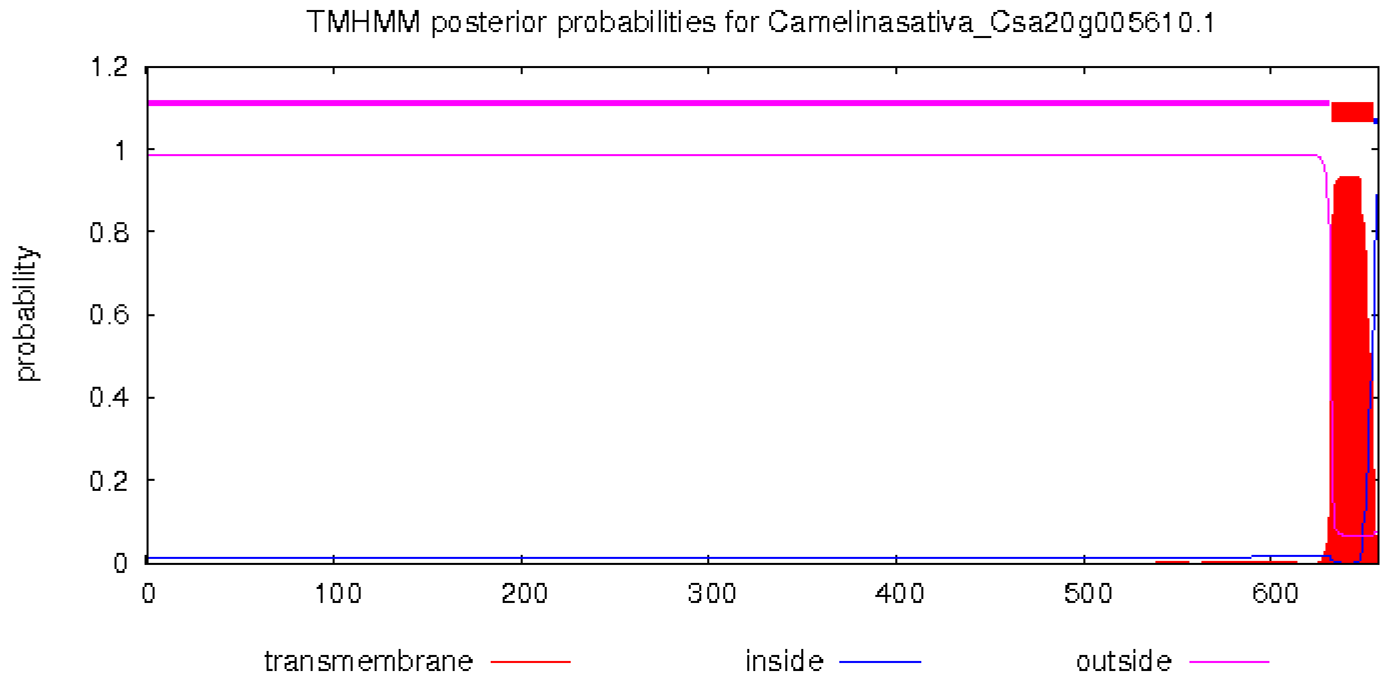

plot in postscript, script for making the plot in gnuplot, data for plot

---

```
# Camelinasativa_Csa20g010110.1 Length: 330
# Camelinasativa_Csa20g010110.1 Number of predicted TMHs:  0
```

```
# Camelinasativa_Csa20g010110.1 Exp number of AAs in TMHs: 0.00022
# Camelinasativa_Csa20g010110.1 Exp number, first 60 AAs:  0
# Camelinasativa_Csa20g010110.1 Total prob of N-in:        0.00495
Camelinasativa_Csa20g010110.1	TMHMM2.0	outside	     1   330
```

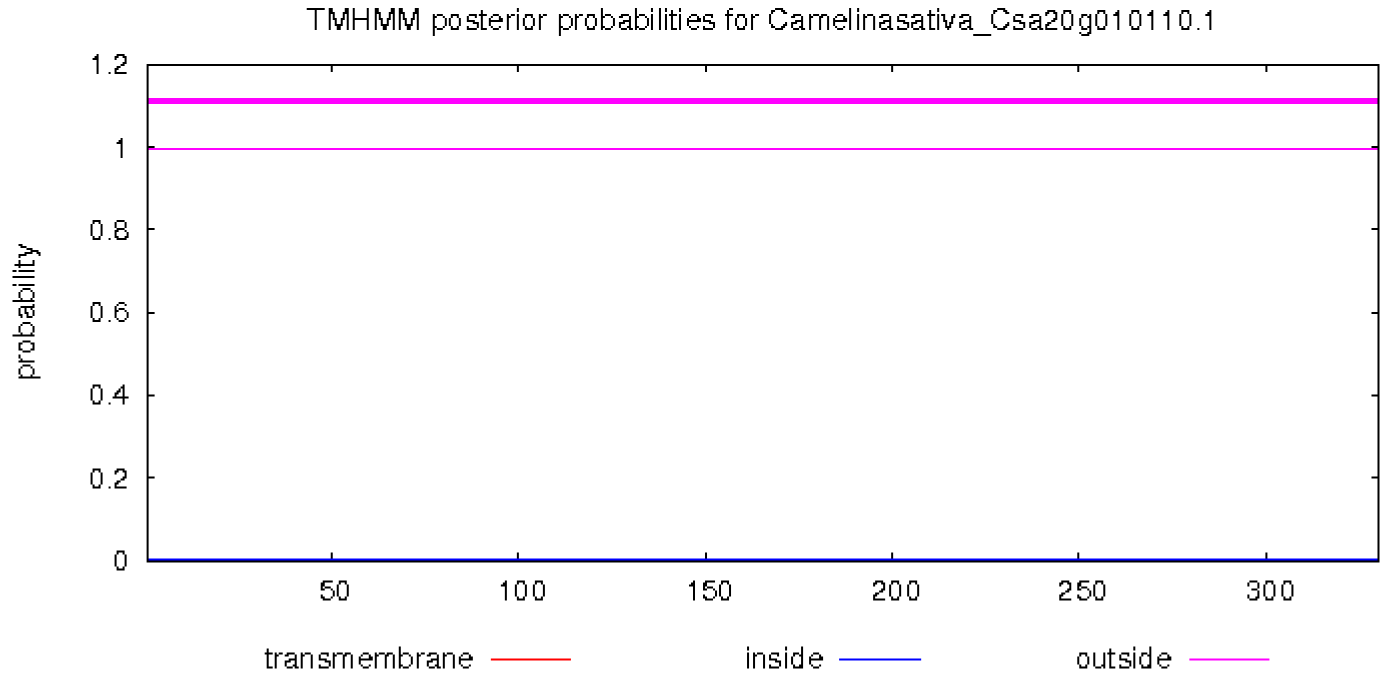

plot in postscript, script for making the plot in gnuplot, data for plot

---

```
# Camelinasativa_Csa20g012360.1 Length: 284
# Camelinasativa_Csa20g012360.1 Number of predicted TMHs:  0
# Camelinasativa_Csa20g012360.1 Exp number of AAs in TMHs: 0.00019
# Camelinasativa_Csa20g012360.1 Exp number, first 60 AAs:  0
# Camelinasativa_Csa20g012360.1 Total prob of N-in:        0.01019
Camelinasativa_Csa20g012360.1	TMHMM2.0	outside	     1   284
```

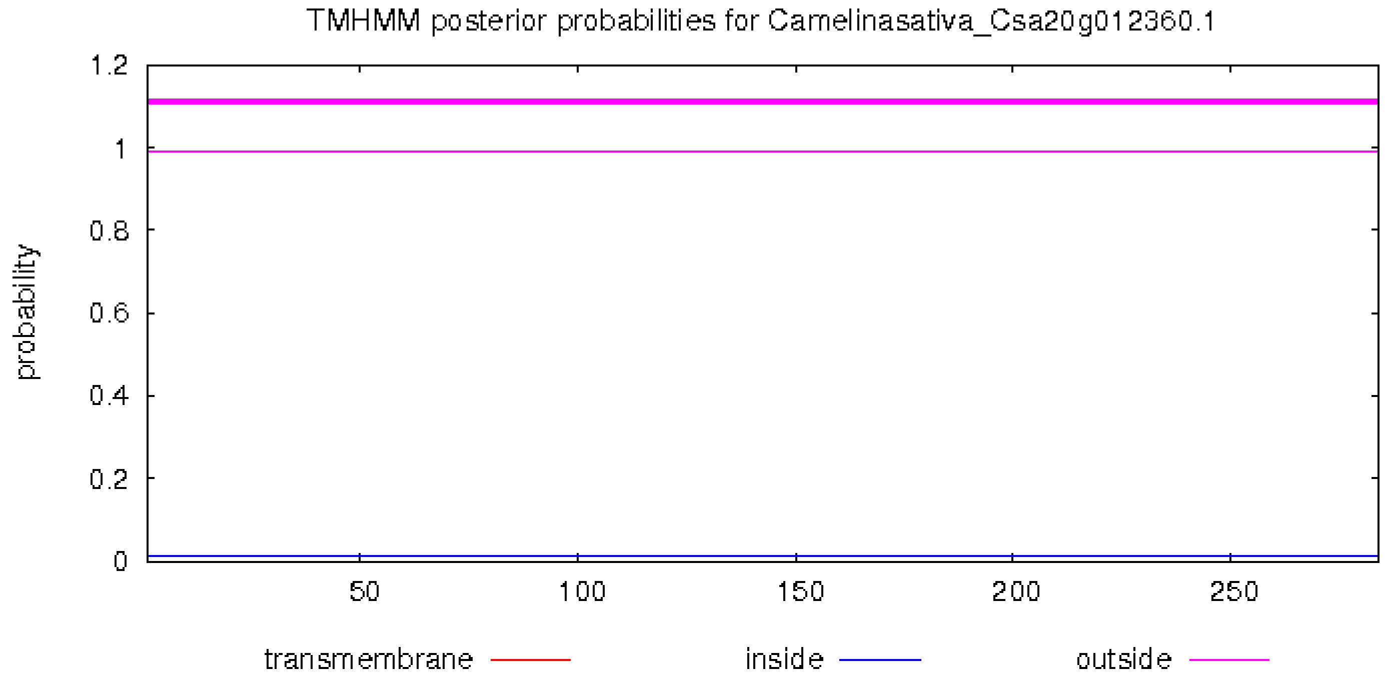

plot in postscript, script for making the plot in gnuplot, data for plot

---

```
# Camelinasativa_Csa20g012490.1 Length: 425
# Camelinasativa_Csa20g012490.1 Number of predicted TMHs:  0
```

```
# Camelinasativa_Csa20g012490.1 Exp number of AAs in TMHs: 2.0571
# Camelinasativa_Csa20g012490.1 Exp number, first 60 AAs:  0.00959
# Camelinasativa_Csa20g012490.1 Total prob of N-in:        0.09179
Camelinasativa_Csa20g012490.1	TMHMM2.0	outside	     1   425
```

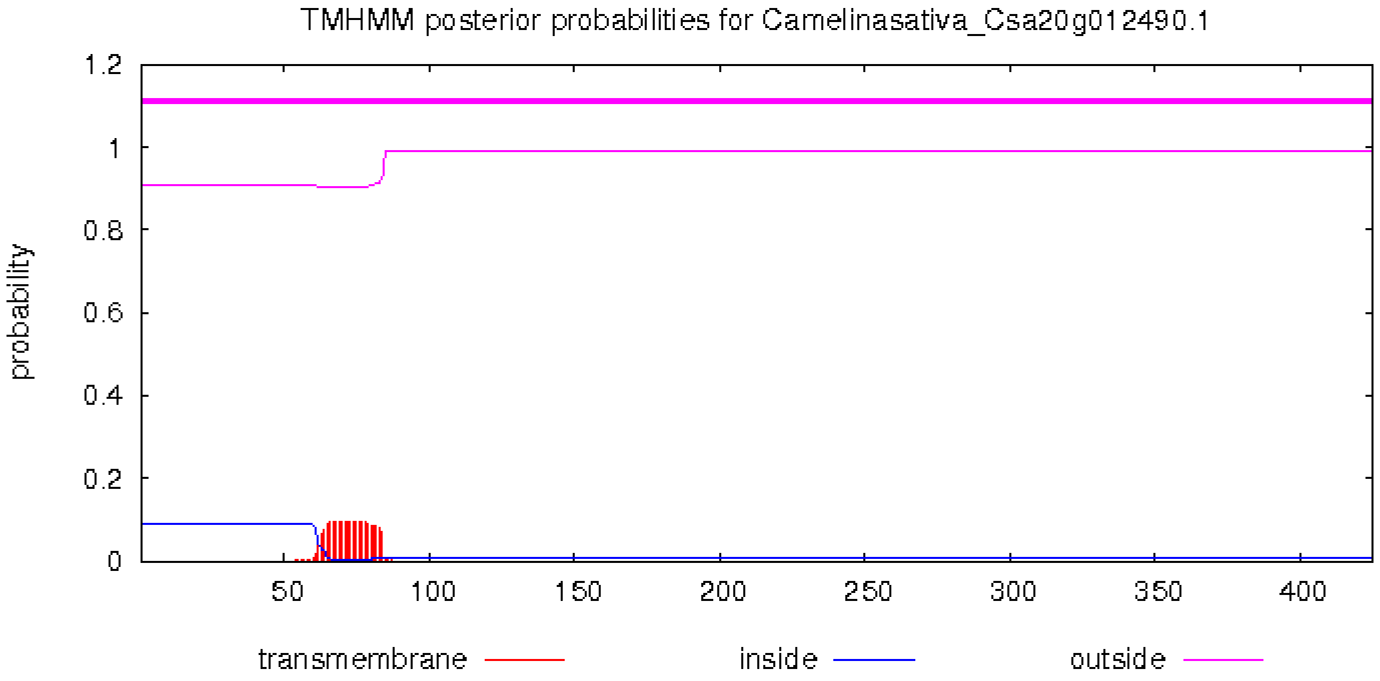

plot in postscript, script for making the plot in gnuplot, data for plot

---

```
# Camelinasativa_Csa20g018530.1 Length: 260
# Camelinasativa_Csa20g018530.1 Number of predicted TMHs:  0
# Camelinasativa_Csa20g018530.1 Exp number of AAs in TMHs: 0.00034
# Camelinasativa_Csa20g018530.1 Exp number, first 60 AAs:  0
# Camelinasativa_Csa20g018530.1 Total prob of N-in:        0.10162
Camelinasativa_Csa20g018530.1	TMHMM2.0	outside	     1   260
```

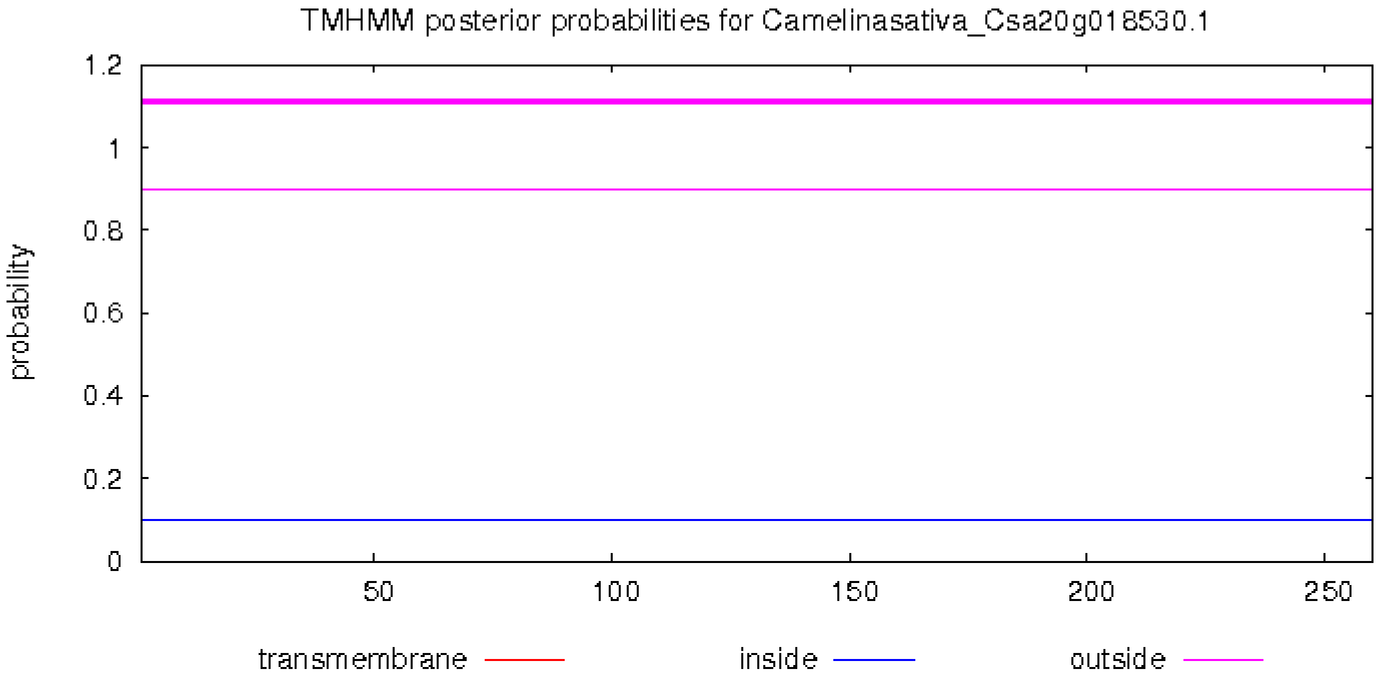

plot in postscript, script for making the plot in gnuplot, data for plot

---

```
# Camelinasativa_Csa20g020460.1 Length: 233
# Camelinasativa_Csa20g020460.1 Number of predicted TMHs:  0
```

```
# Camelinasativa_Csa20g020460.1 Exp number of AAs in TMHs: 0.24142
# Camelinasativa_Csa20g020460.1 Exp number, first 60 AAs:  0.01311
# Camelinasativa_Csa20g020460.1 Total prob of N-in:        0.01417
Camelinasativa_Csa20g020460.1   TMHMM2.0        outside      1   233
```

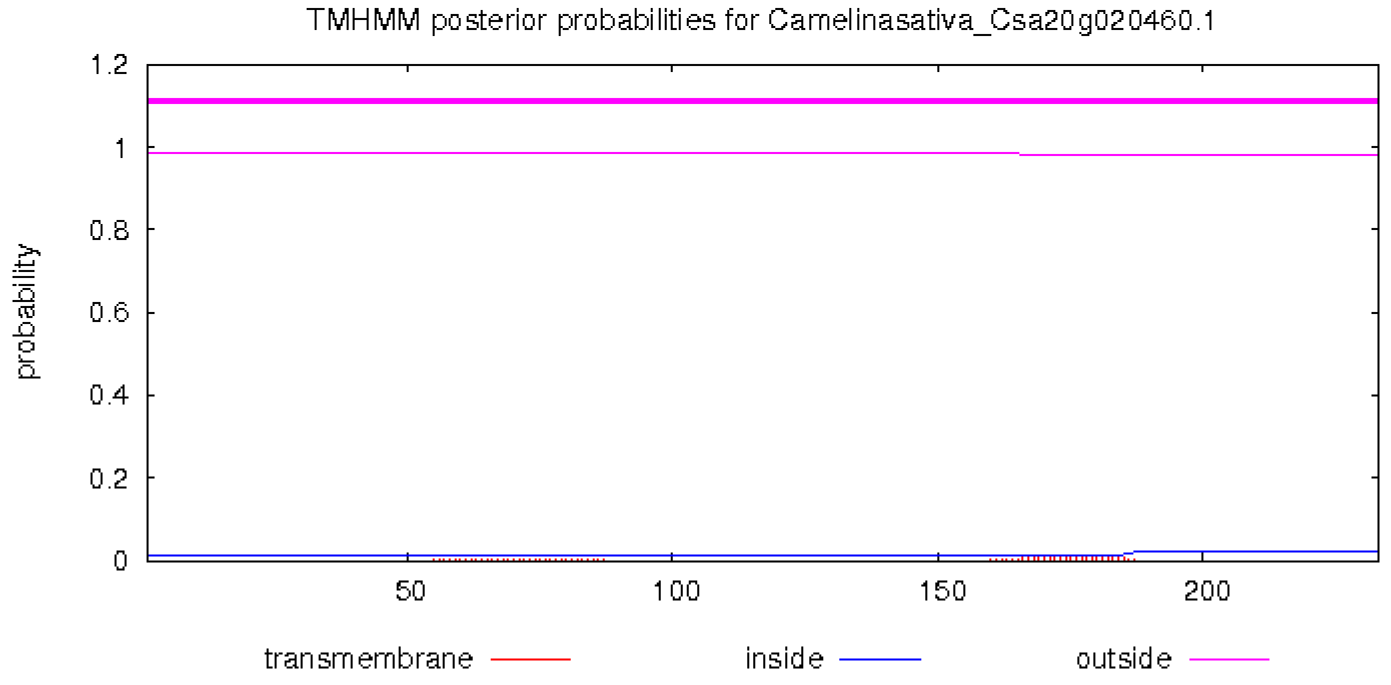

plot in postscript, script for making the plot in gnuplot, data for plot

---

```
# Camelinasativa_Csa20g021030.1 Length: 342
# Camelinasativa_Csa20g021030.1 Number of predicted TMHs:  0
# Camelinasativa_Csa20g021030.1 Exp number of AAs in TMHs: 0
# Camelinasativa_Csa20g021030.1 Exp number, first 60 AAs:  0
# Camelinasativa_Csa20g021030.1 Total prob of N-in:        0.01113
Camelinasativa_Csa20g021030.1   TMHMM2.0        outside      1   342
```

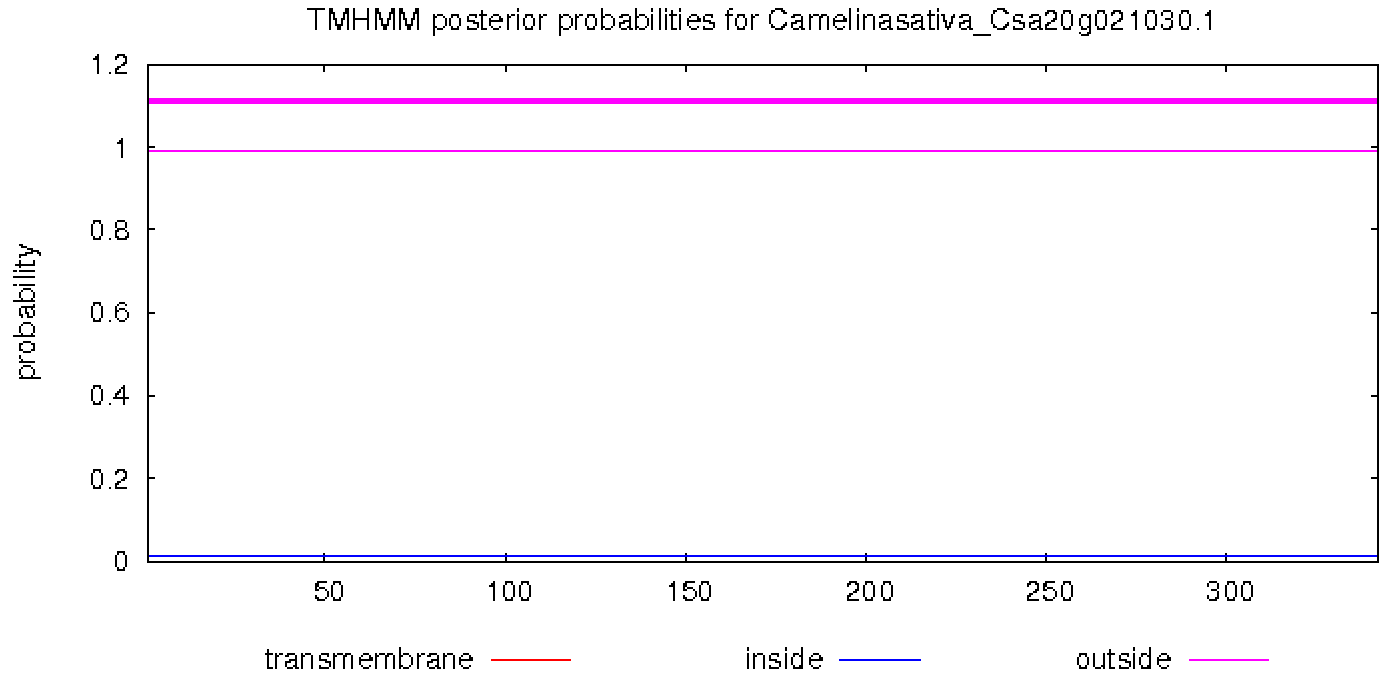

plot in postscript, script for making the plot in gnuplot, data for plot

---

```
# Camelinasativa_Csa20g023680.1 Length: 319
# Camelinasativa_Csa20g023680.1 Number of predicted TMHs:  0
```

```
# Camelinasativa_Csa20g023680.1 Exp number of AAs in TMHs: 0.01257
# Camelinasativa_Csa20g023680.1 Exp number, first 60 AAs:  0.00209
# Camelinasativa_Csa20g023680.1 Total prob of N-in:        0.02010
Camelinasativa_Csa20g023680.1   TMHMM2.0        outside      1   319
```

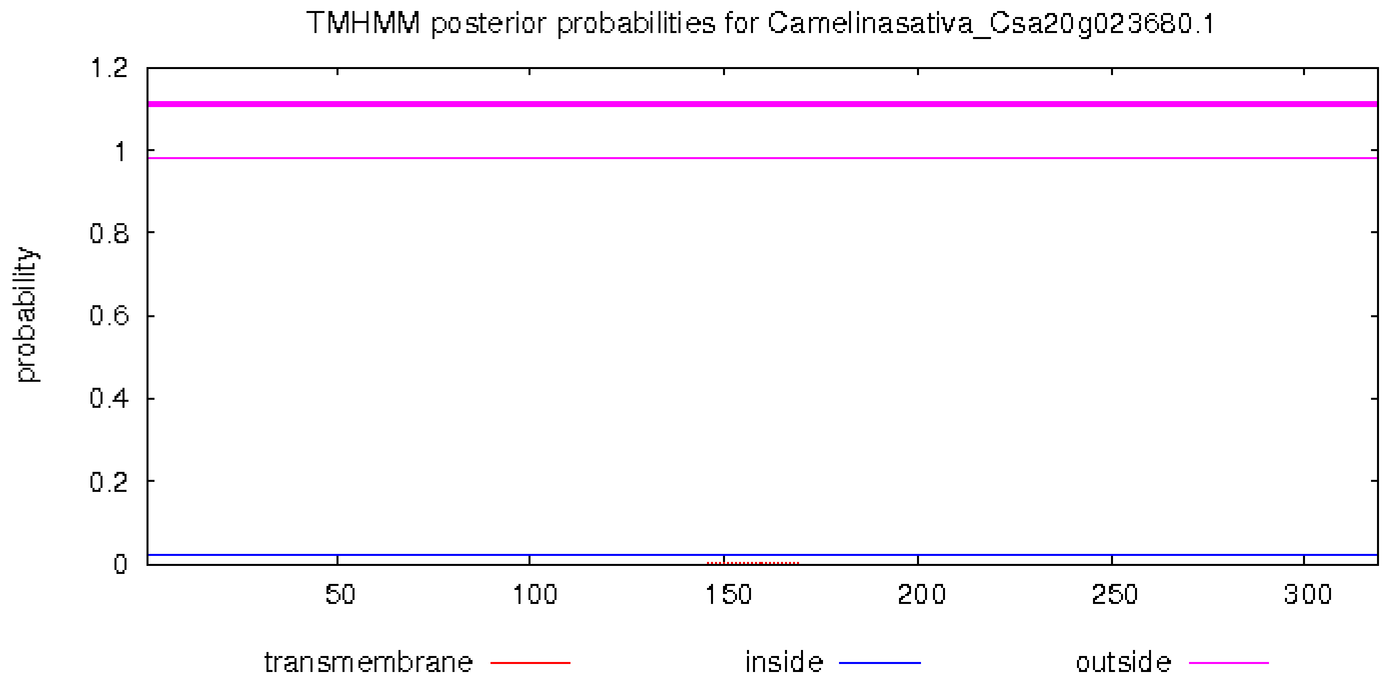

plot in postscript, script for making the plot in gnuplot, data for plot

---

```
# Camelinasativa_Csa20g025340.1 Length: 499
# Camelinasativa_Csa20g025340.1 Number of predicted TMHs:  0
# Camelinasativa_Csa20g025340.1 Exp number of AAs in TMHs: 0.00036
# Camelinasativa_Csa20g025340.1 Exp number, first 60 AAs:  0
# Camelinasativa_Csa20g025340.1 Total prob of N-in:        0.00014
Camelinasativa_Csa20g025340.1   TMHMM2.0        outside      1   499
```

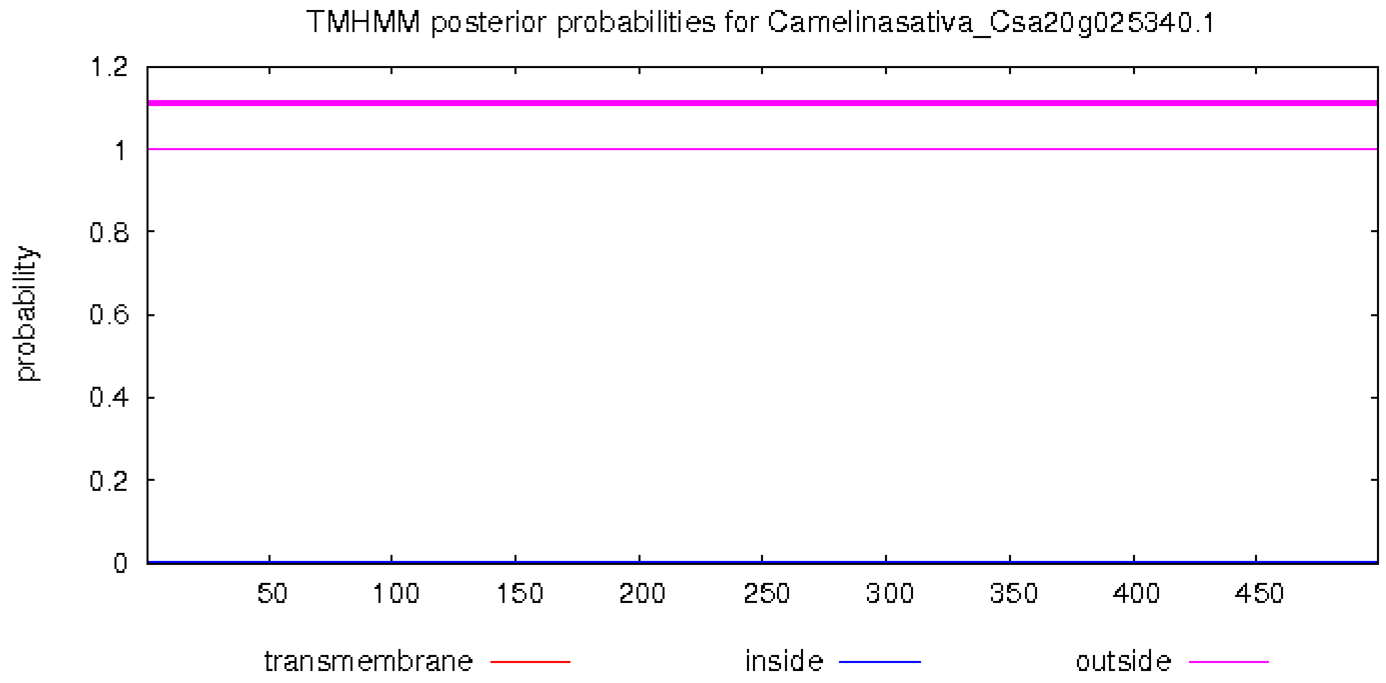

plot in postscript, script for making the plot in gnuplot, data for plot

---

```
# Camelinasativa_Csa20g026310.1 Length: 157
# Camelinasativa_Csa20g026310.1 Number of predicted TMHs:  0
```

```
# Camelinasativa_Csa20g026310.1 Exp number of AAs in TMHs: 0.00298
# Camelinasativa_Csa20g026310.1 Exp number, first 60 AAs:  0.00018
# Camelinasativa_Csa20g026310.1 Total prob of N-in:        0.33305
Camelinasativa_Csa20g026310.1   TMHMM2.0        outside      1   157
```

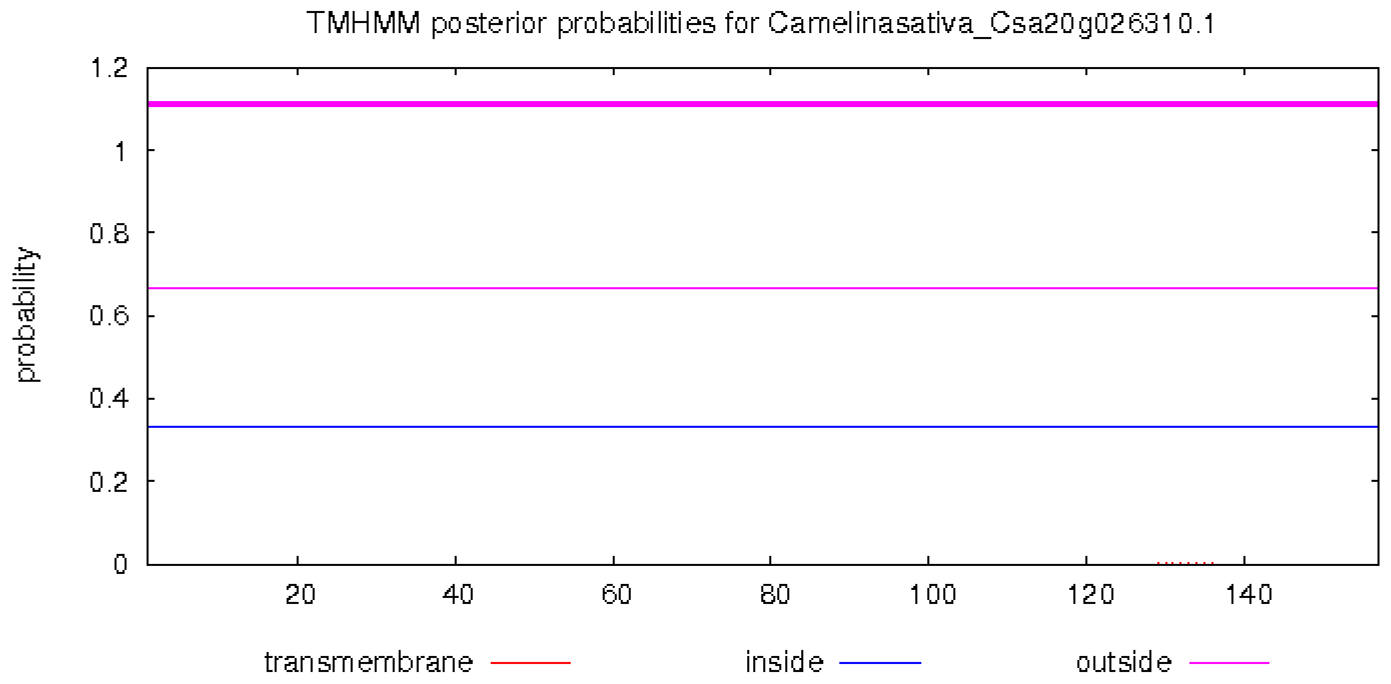

plot in postscript, script for making the plot in gnuplot, data for plot

---

```
# Camelinasativa_Csa20g026350.1 Length: 159
# Camelinasativa_Csa20g026350.1 Number of predicted TMHs:  0
# Camelinasativa_Csa20g026350.1 Exp number of AAs in TMHs: 0.00416
# Camelinasativa_Csa20g026350.1 Exp number, first 60 AAs:  0
# Camelinasativa_Csa20g026350.1 Total prob of N-in:        0.32762
Camelinasativa_Csa20g026350.1   TMHMM2.0        outside      1   159
```

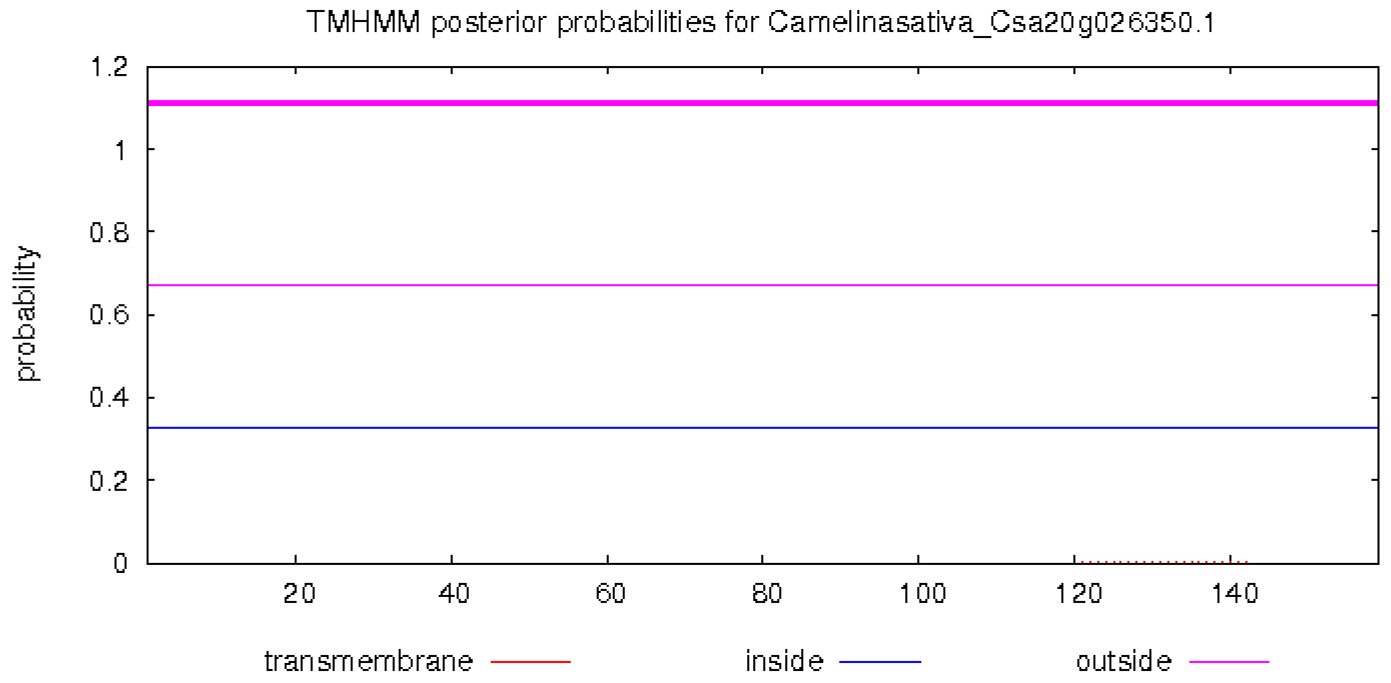

plot in postscript, script for making the plot in gnuplot, data for plot

---

```
# Camelinasativa_Csa20g026400.1 Length: 157
# Camelinasativa_Csa20g026400.1 Number of predicted TMHs:  0
```

```
# Camelinasativa_Csa20g026400.1 Exp number of AAs in TMHs: 0.00421
# Camelinasativa_Csa20g026400.1 Exp number, first 60 AAs:  0.00018
# Camelinasativa_Csa20g026400.1 Total prob of N-in:        0.35943
Camelinasativa_Csa20g026400.1   TMHMM2.0        outside      1   157
```

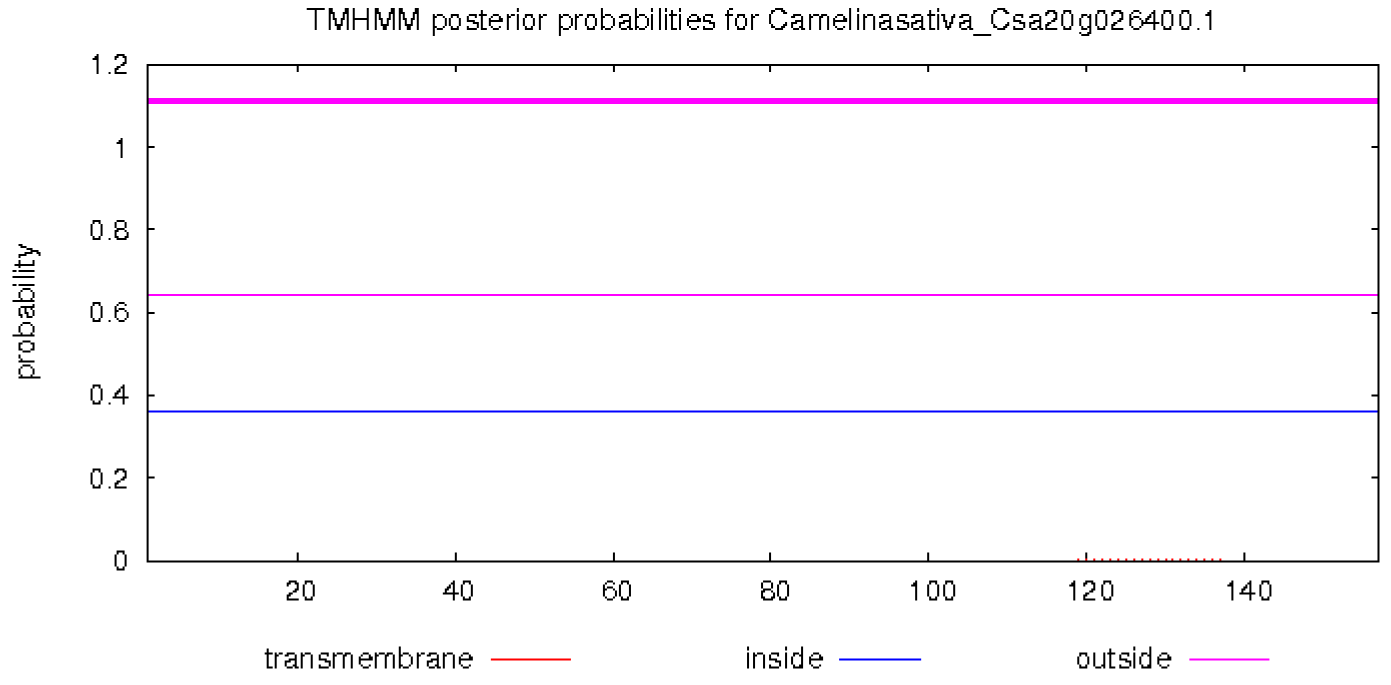

plot in postscript, script for making the plot in gnuplot, data for plot

---

```
# Camelinasativa_Csa20g026650.1 Length: 217
# Camelinasativa_Csa20g026650.1 Number of predicted TMHs:  0
# Camelinasativa_Csa20g026650.1 Exp number of AAs in TMHs: 0
# Camelinasativa_Csa20g026650.1 Exp number, first 60 AAs:  0
# Camelinasativa_Csa20g026650.1 Total prob of N-in:        0.12107
Camelinasativa_Csa20g026650.1   TMHMM2.0        outside      1   217
```

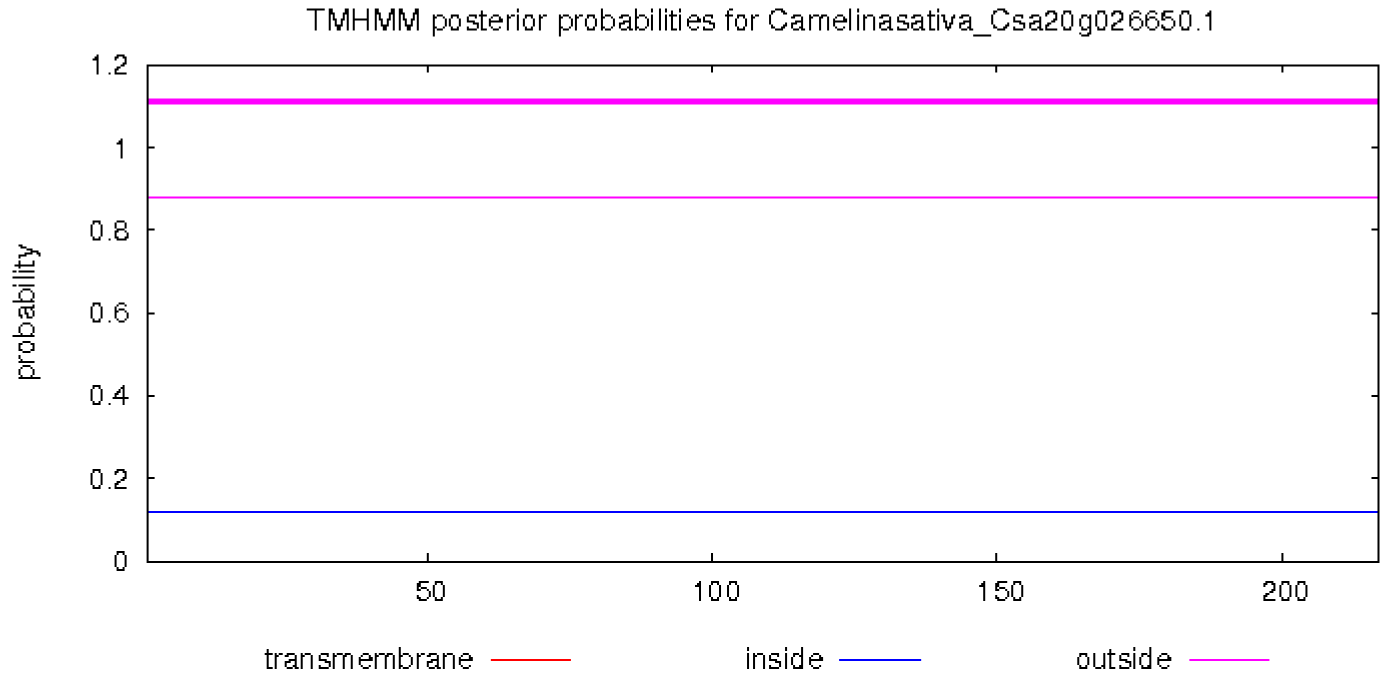

plot in postscript, script for making the plot in gnuplot, data for plot

---

```
# Camelinasativa_Csa20g026680.1 Length: 146
# Camelinasativa_Csa20g026680.1 Number of predicted TMHs:  0
```

```
# Camelinasativa_Csa20g026680.1 Exp number of AAs in TMHs: 0.00173
# Camelinasativa_Csa20g026680.1 Exp number, first 60 AAs:  0
# Camelinasativa_Csa20g026680.1 Total prob of N-in:        0.21829
Camelinasativa_Csa20g026680.1   TMHMM2.0        outside      1   146
```

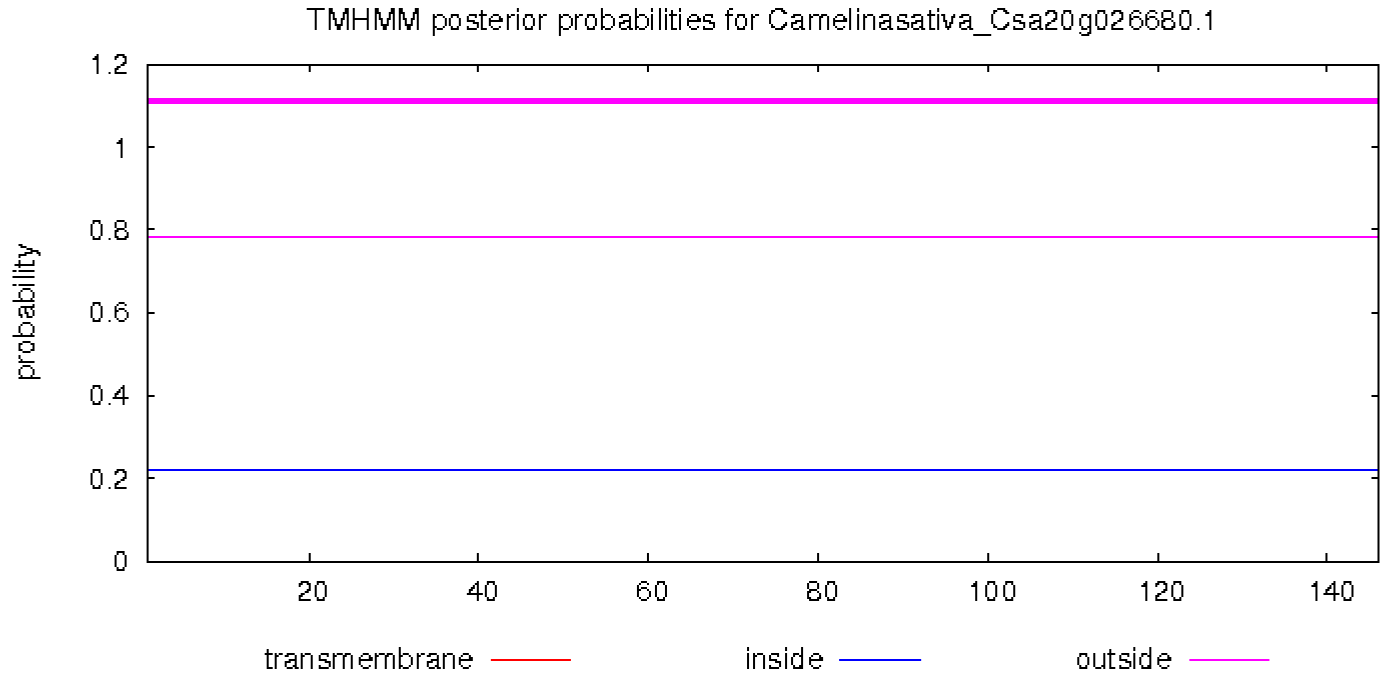

plot in postscript, script for making the plot in gnuplot, data for plot

---

```
# Camelinasativa_Csa20g031750.1 Length: 332
# Camelinasativa_Csa20g031750.1 Number of predicted TMHs:  0
# Camelinasativa_Csa20g031750.1 Exp number of AAs in TMHs: 0.00017
# Camelinasativa_Csa20g031750.1 Exp number, first 60 AAs:  0
# Camelinasativa_Csa20g031750.1 Total prob of N-in:        0.06399
Camelinasativa_Csa20g031750.1   TMHMM2.0        outside      1   332
```

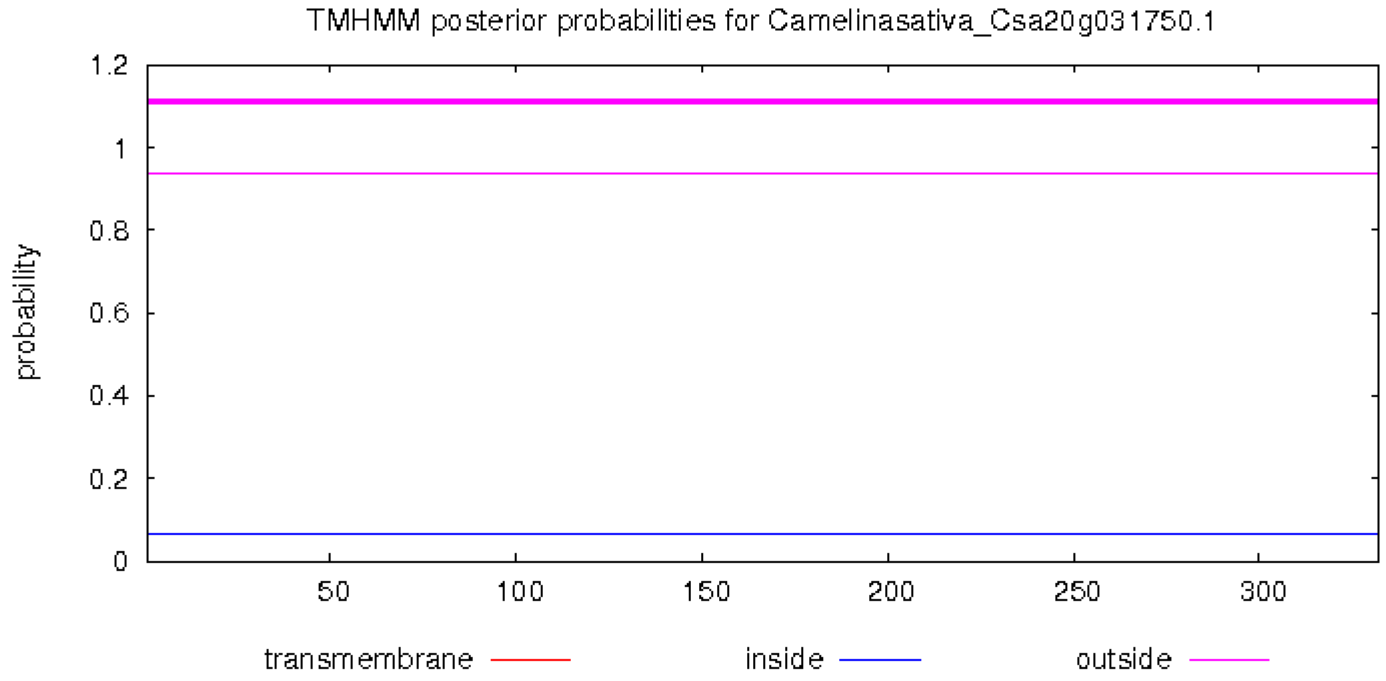

plot in postscript, script for making the plot in gnuplot, data for plot

---

```
# Camelinasativa_Csa20g031760.1 Length: 416
# Camelinasativa_Csa20g031760.1 Number of predicted TMHs:  0
```

```
# Camelinasativa_Csa20g031760.1 Exp number of AAs in TMHs: 0.00263
# Camelinasativa_Csa20g031760.1 Exp number, first 60 AAs:  0
# Camelinasativa_Csa20g031760.1 Total prob of N-in:        0.02728
Camelinasativa_Csa20g031760.1   TMHMM2.0        outside      1   416
```

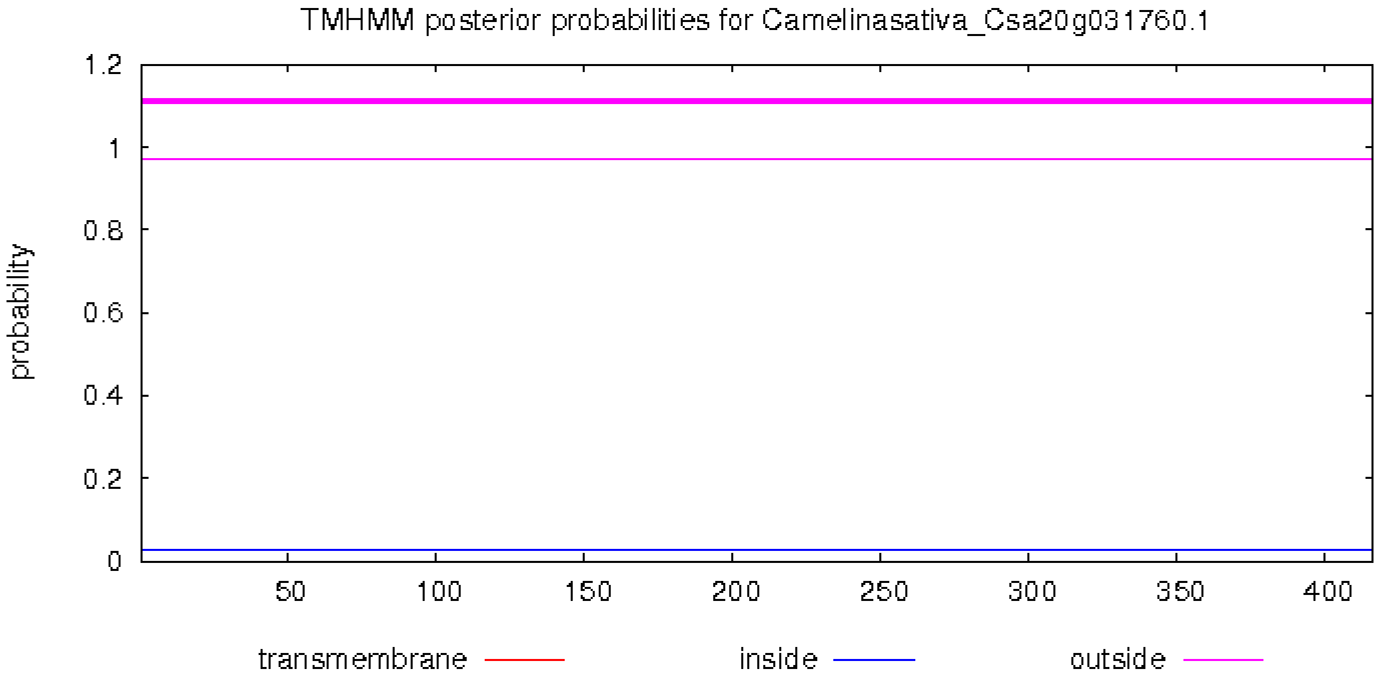

plot in postscript, script for making the plot in gnuplot, data for plot

---

```
# Camelinasativa_Csa20g035850.1 Length: 340
# Camelinasativa_Csa20g035850.1 Number of predicted TMHs:  0
# Camelinasativa_Csa20g035850.1 Exp number of AAs in TMHs: 8.19207
# Camelinasativa_Csa20g035850.1 Exp number, first 60 AAs:  0.00764
# Camelinasativa_Csa20g035850.1 Total prob of N-in:        0.08336
Camelinasativa_Csa20g035850.1   TMHMM2.0        outside      1   340
```

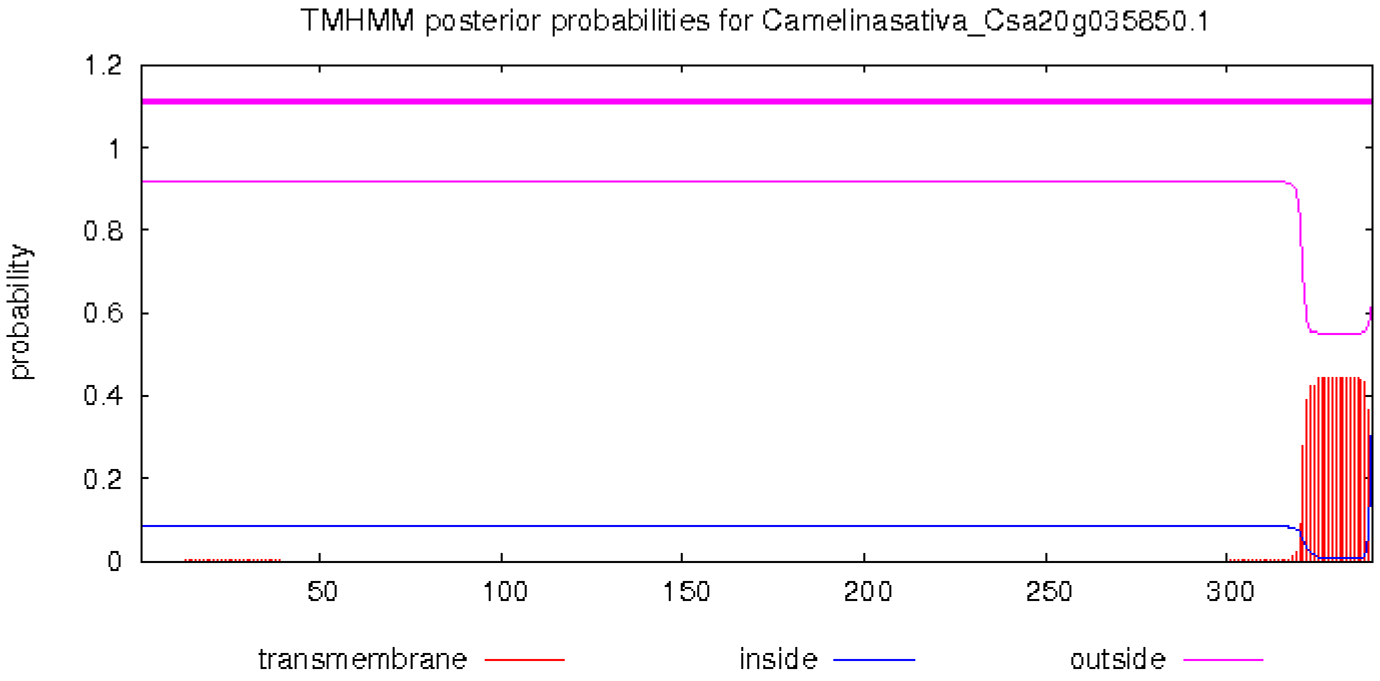

plot in postscript, script for making the plot in gnuplot, data for plot

---

```
# Camelinasativa_Csa20g036010.1 Length: 258
# Camelinasativa_Csa20g036010.1 Number of predicted TMHs:  0
```

```
# Camelinasativa_Csa20g036010.1 Exp number of AAs in TMHs: 0.01261
# Camelinasativa_Csa20g036010.1 Exp number, first 60 AAs:  0.00571
# Camelinasativa_Csa20g036010.1 Total prob of N-in:        0.04968
Camelinasativa_Csa20g036010.1   TMHMM2.0        outside      1   258
```

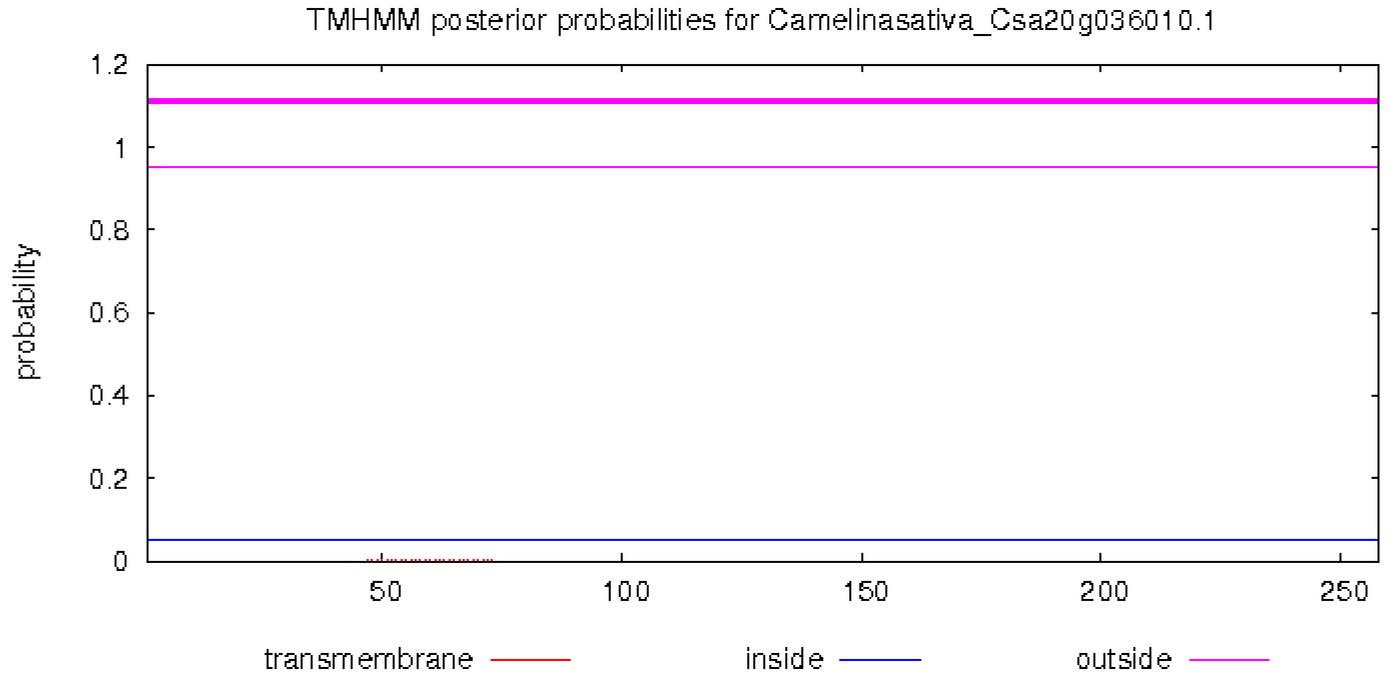

plot in postscript, script for making the plot in gnuplot, data for plot

---

```
# Camelinasativa_Csa20g042750.1 Length: 466
# Camelinasativa_Csa20g042750.1 Number of predicted TMHs:  1
# Camelinasativa_Csa20g042750.1 Exp number of AAs in TMHs: 18.24342
# Camelinasativa_Csa20g042750.1 Exp number, first 60 AAs:  0
# Camelinasativa_Csa20g042750.1 Total prob of N-in:        0.00355
Camelinasativa_Csa20g042750.1   TMHMM2.0        outside      1   445
Camelinasativa_Csa20g042750.1   TMHMM2.0        TMhelix    446   465
Camelinasativa_Csa20g042750.1   TMHMM2.0        inside     466   466
```

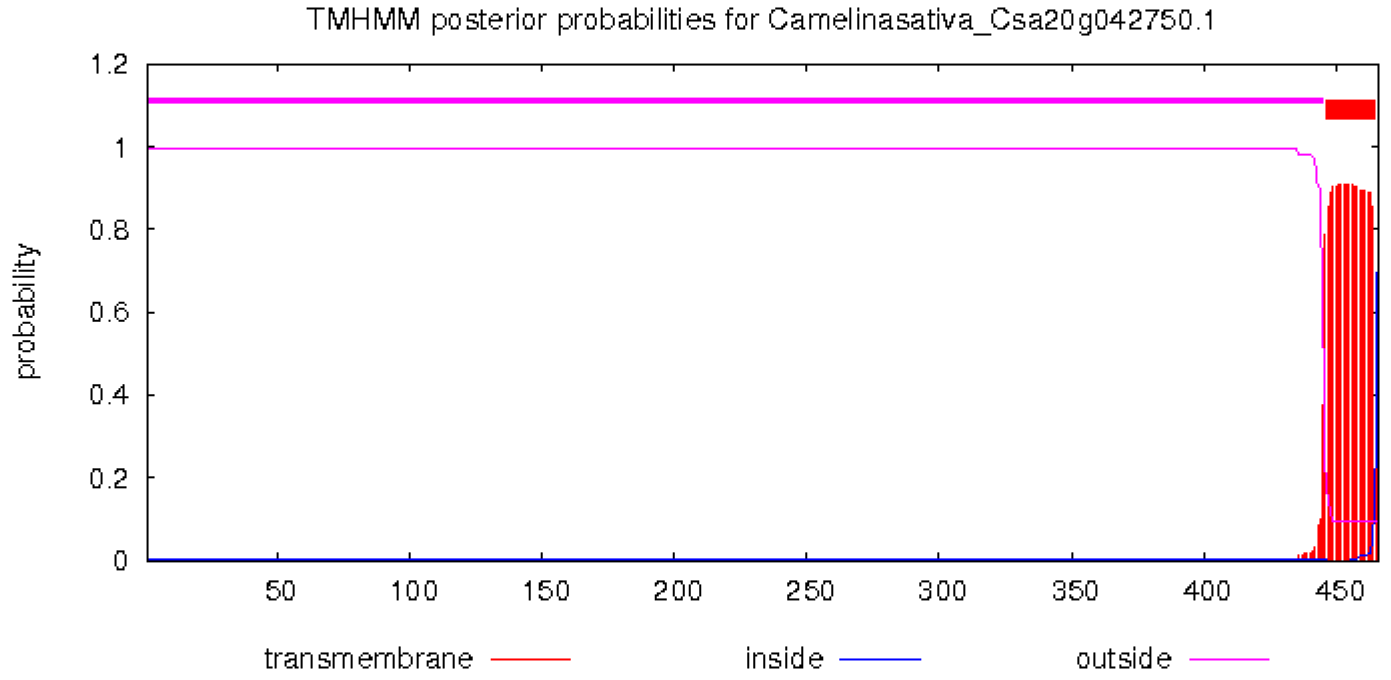

plot in postscript, script for making the plot in gnuplot, data for plot

---

```
# Camelinasativa_Csa20g057380.1 Length: 113
# Camelinasativa_Csa20g057380.1 Number of predicted TMHs:  1
# Camelinasativa_Csa20g057380.1 Exp number of AAs in TMHs: 21.24289
# Camelinasativa_Csa20g057380.1 Exp number, first 60 AAs:  21.23619
# Camelinasativa_Csa20g057380.1 Total prob of N-in:        0.98180
# Camelinasativa_Csa20g057380.1 POSSIBLE N-term signal sequence
Camelinasativa_Csa20g057380.1	TMHMM2.0	inside	     1    20
Camelinasativa_Csa20g057380.1	TMHMM2.0	TMhelix	    21    43
Camelinasativa_Csa20g057380.1	TMHMM2.0	outside	    44   113
```

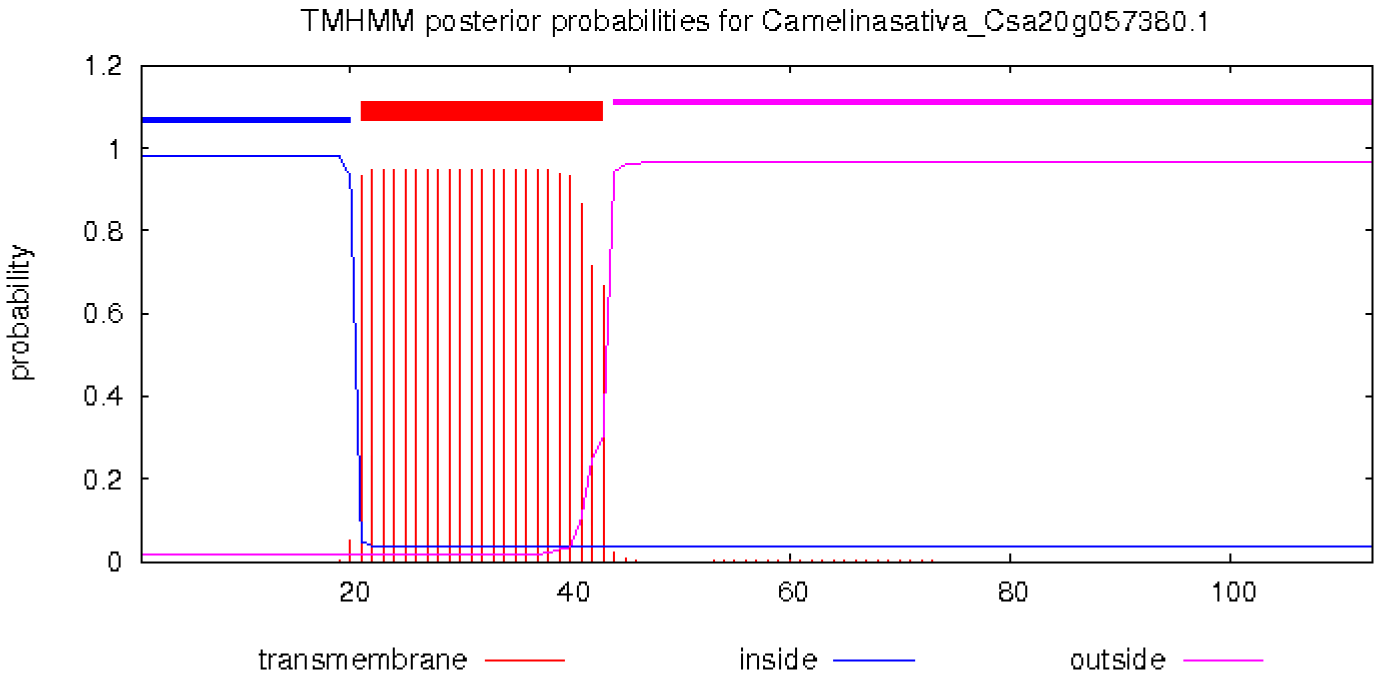

plot in postscript, script for making the plot in gnuplot, data for plot

---

```
# Camelinasativa_Csa20g080730.1 Length: 292
# Camelinasativa_Csa20g080730.1 Number of predicted TMHs:  0
# Camelinasativa_Csa20g080730.1 Exp number of AAs in TMHs: 0.00016
# Camelinasativa_Csa20g080730.1 Exp number, first 60 AAs:  0
# Camelinasativa_Csa20g080730.1 Total prob of N-in:        0.00215
Camelinasativa_Csa20g080730.1	TMHMM2.0	outside	     1   292
```

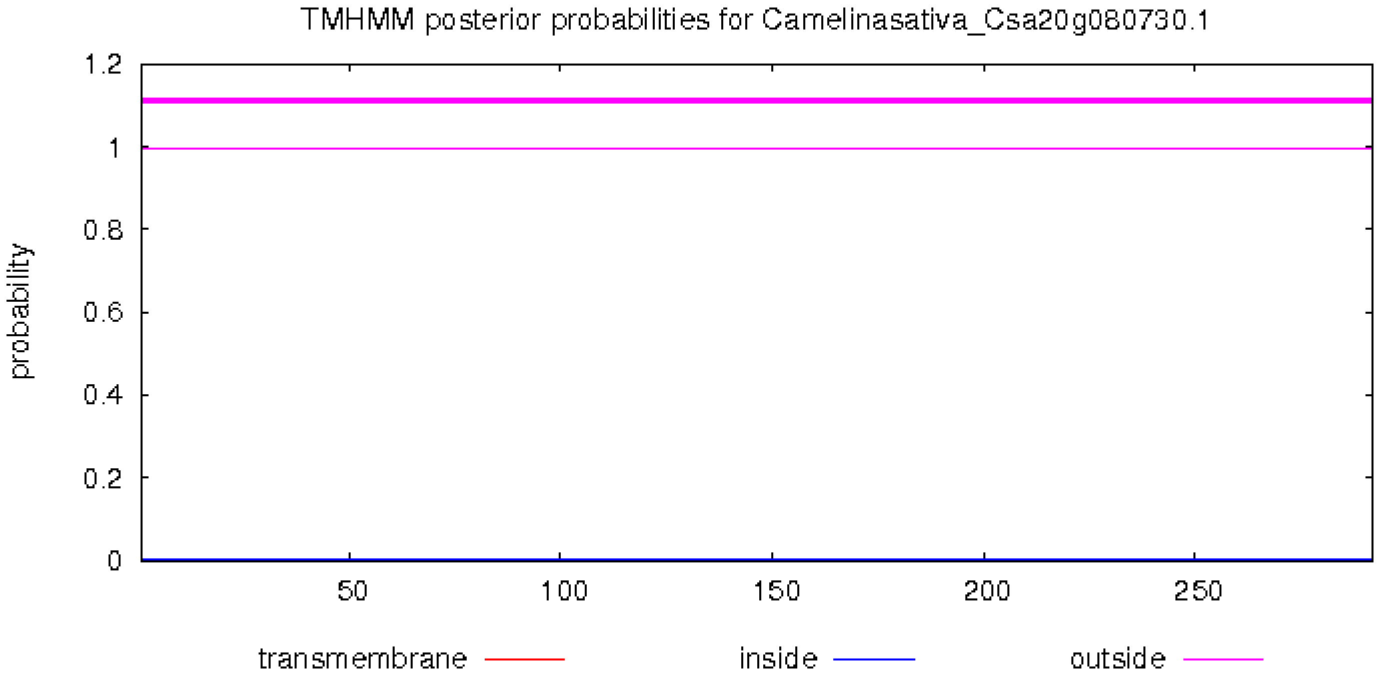

plot in postscript, script for making the plot in gnuplot, data for plot

```
# Camelinasativa_Csa26370s010.1 Length: 79
# Camelinasativa_Csa26370s010.1 Number of predicted TMHs:  0
# Camelinasativa_Csa26370s010.1 Exp number of AAs in TMHs: 0
# Camelinasativa_Csa26370s010.1 Exp number, first 60 AAs:  0
# Camelinasativa_Csa26370s010.1 Total prob of N-in:        0.69791
Camelinasativa_Csa26370s010.1	TMHMM2.0	inside	     1    79
```

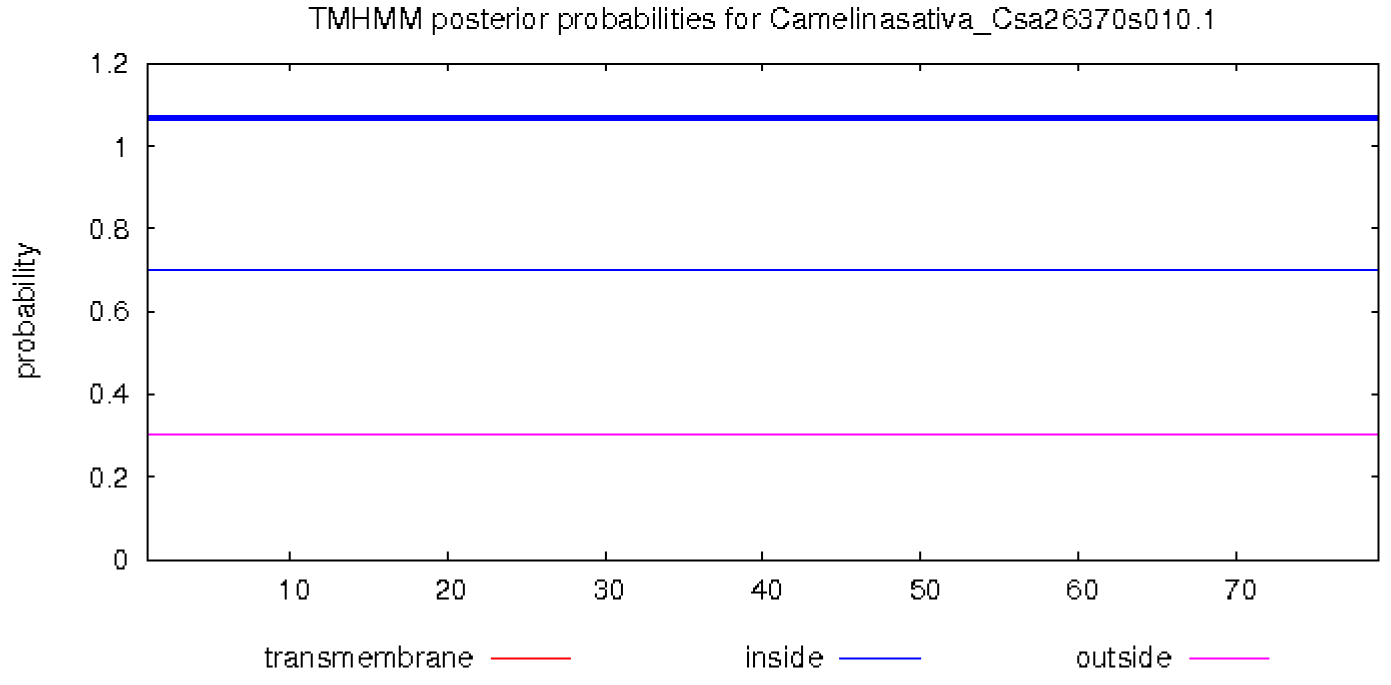

plot in postscript, script for making the plot in gnuplot, data for plot

```
# Camelinasativa_Csa33192s010.1 Length: 66
# Camelinasativa_Csa33192s010.1 Number of predicted TMHs:  0
# Camelinasativa_Csa33192s010.1 Exp number of AAs in TMHs: 0.0013
# Camelinasativa_Csa33192s010.1 Exp number, first 60 AAs:  0.00129
# Camelinasativa_Csa33192s010.1 Total prob of N-in:        0.39485
Camelinasativa_Csa33192s010.1	TMHMM2.0	outside	     1    66
```

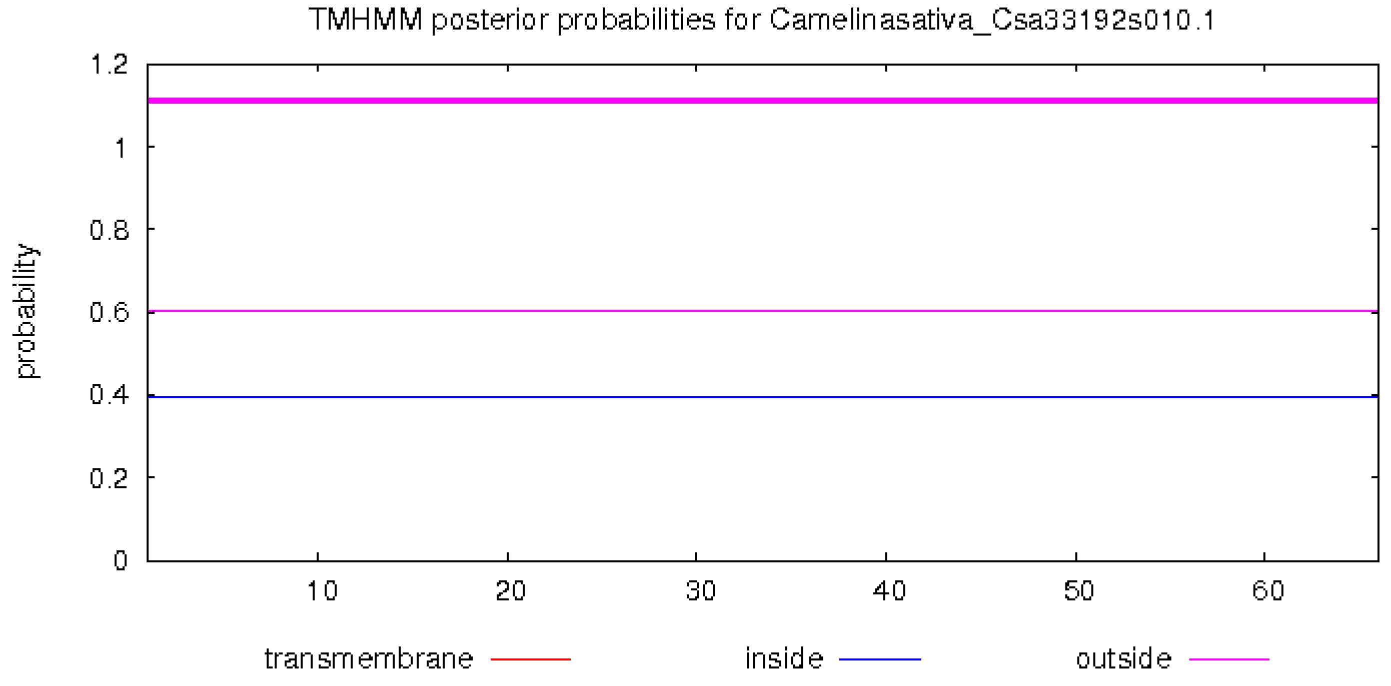

plot in postscript, script for making the plot in gnuplot, data for plot

---

```
# Camelinasativa_Csa34253s010.1 Length: 64
# Camelinasativa_Csa34253s010.1 Number of predicted TMHs:  0
# Camelinasativa_Csa34253s010.1 Exp number of AAs in TMHs: 0
# Camelinasativa_Csa34253s010.1 Exp number, first 60 AAs:  0
# Camelinasativa_Csa34253s010.1 Total prob of N-in:        0.33421
Camelinasativa_Csa34253s010.1   TMHMM2.0        outside      1    64
```

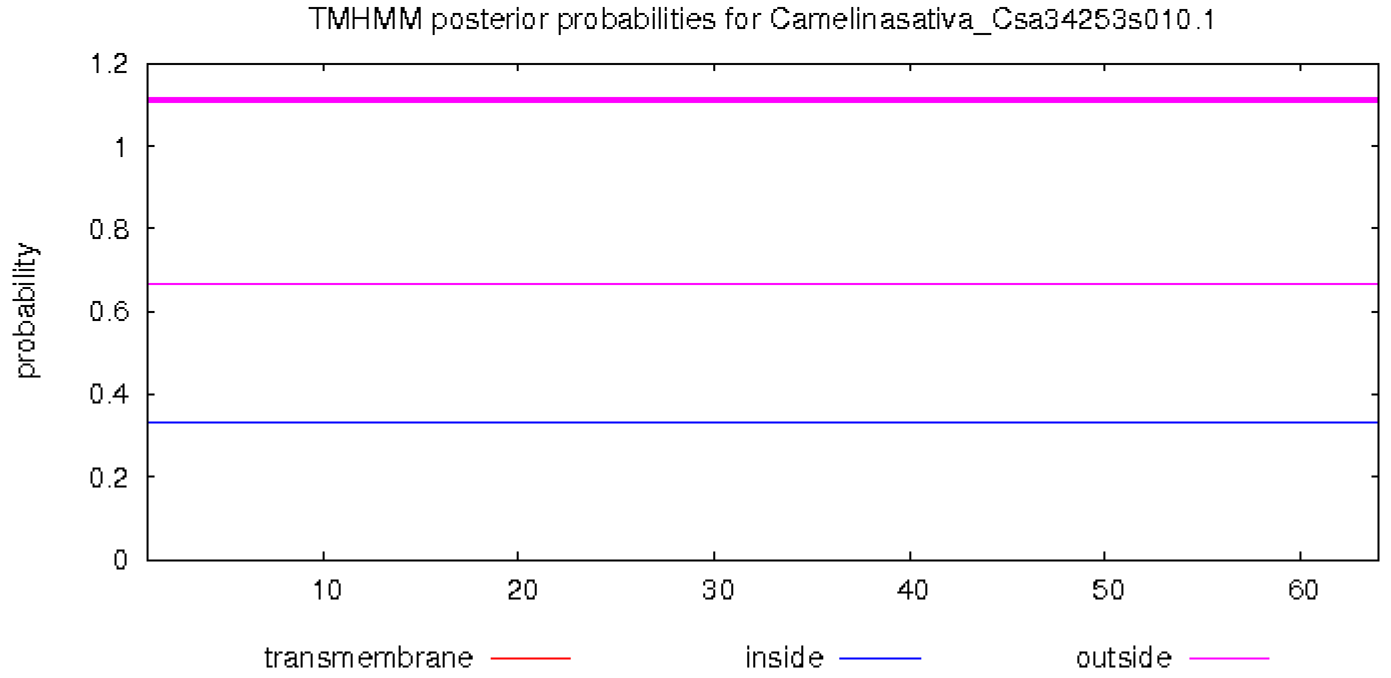

plot in postscript, script for making the plot in gnuplot, data for plot

---

```
# Camelinasativa_Csa34414s010.1 Length: 52
# Camelinasativa_Csa34414s010.1 Number of predicted TMHs:  0
# Camelinasativa_Csa34414s010.1 Exp number of AAs in TMHs: 0.01101
# Camelinasativa_Csa34414s010.1 Exp number, first 60 AAs:  0.01101
# Camelinasativa_Csa34414s010.1 Total prob of N-in:        0.03555
Camelinasativa_Csa34414s010.1   TMHMM2.0        outside      1    52
```

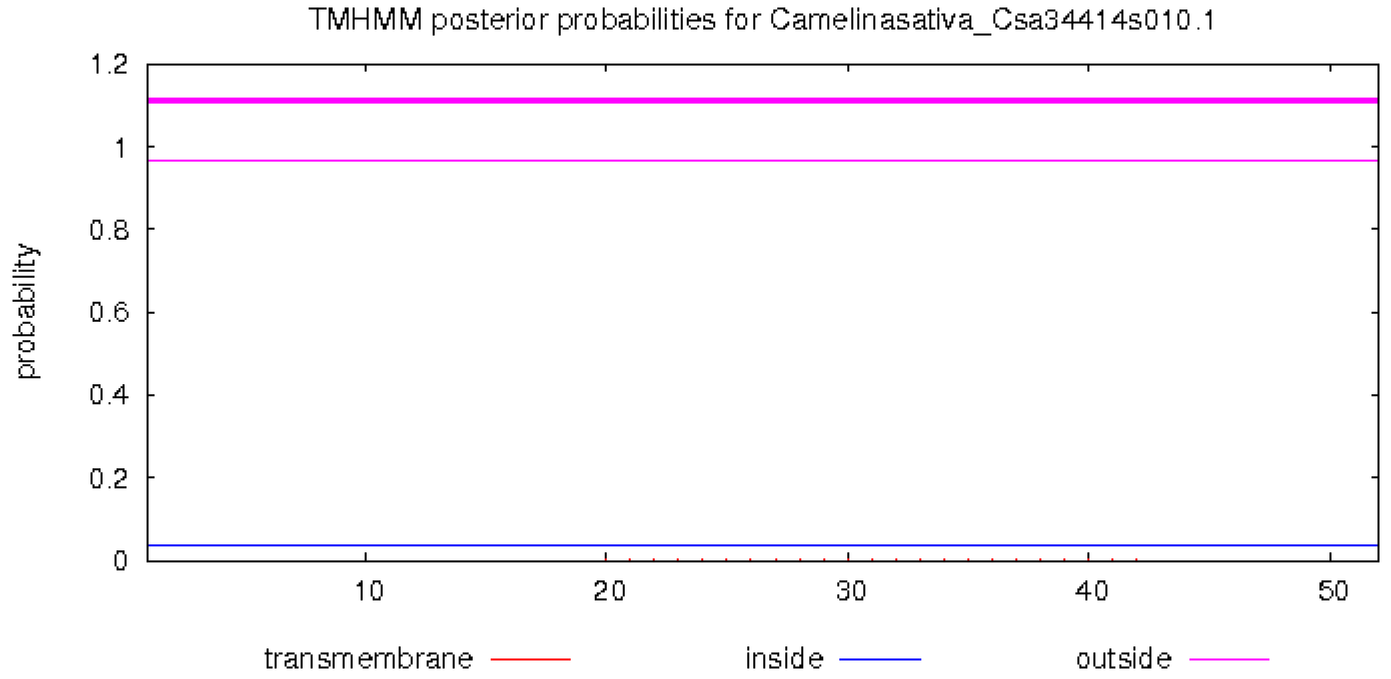

plot in postscript, script for making the plot in gnuplot, data for plot

---

```
# Cannabissativa_PK00339.1 Length: 326
# Cannabissativa_PK00339.1 Number of predicted TMHs:  0
# Cannabissativa_PK00339.1 Exp number of AAs in TMHs: 0.00018
# Cannabissativa_PK00339.1 Exp number, first 60 AAs:  0
# Cannabissativa_PK00339.1 Total prob of N-in:        0.00106
Cannabissativa_PK00339.1        TMHMM2.0        outside      1   326
```

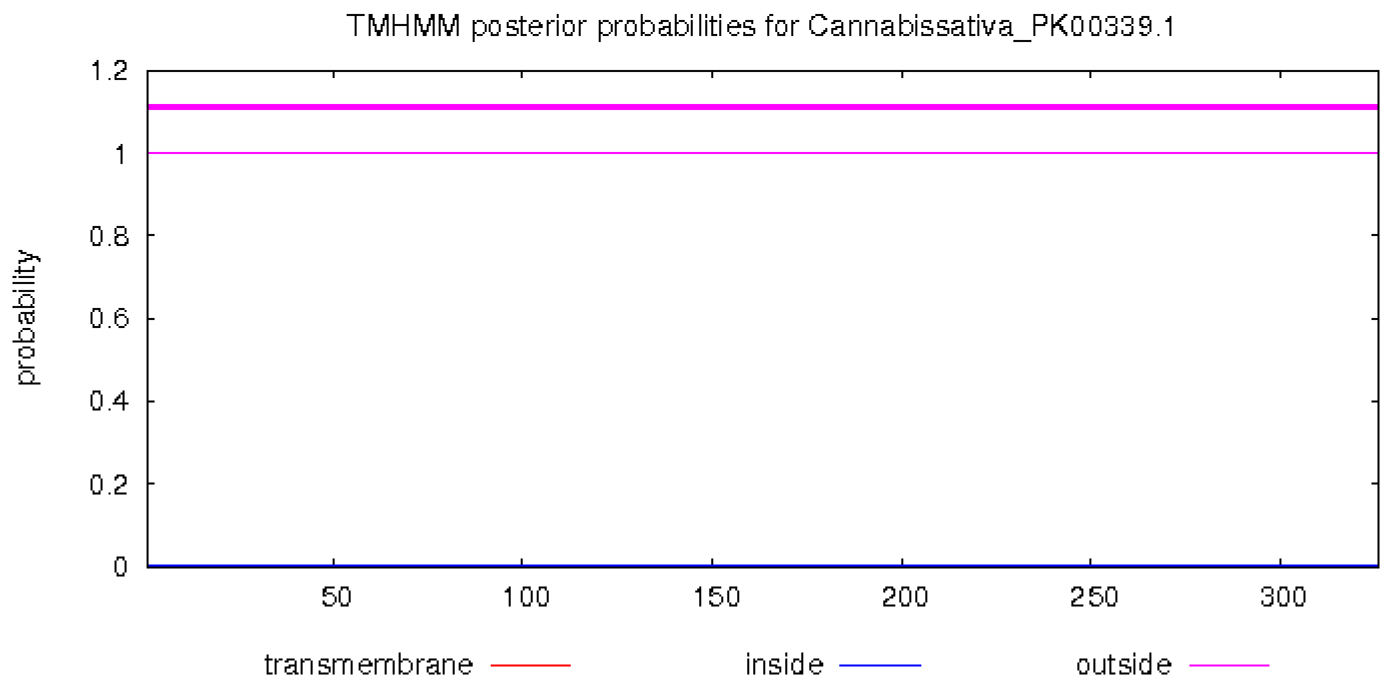

plot in postscript, script for making the plot in gnuplot, data for plot

---

```
# Cannabissativa_PK00547.1 Length: 241
# Cannabissativa_PK00547.1 Number of predicted TMHs:  0
# Cannabissativa_PK00547.1 Exp number of AAs in TMHs: 0.00109
# Cannabissativa_PK00547.1 Exp number, first 60 AAs:  0.00034
# Cannabissativa_PK00547.1 Total prob of N-in:        0.04636
Cannabissativa_PK00547.1        TMHMM2.0        outside      1   241
```

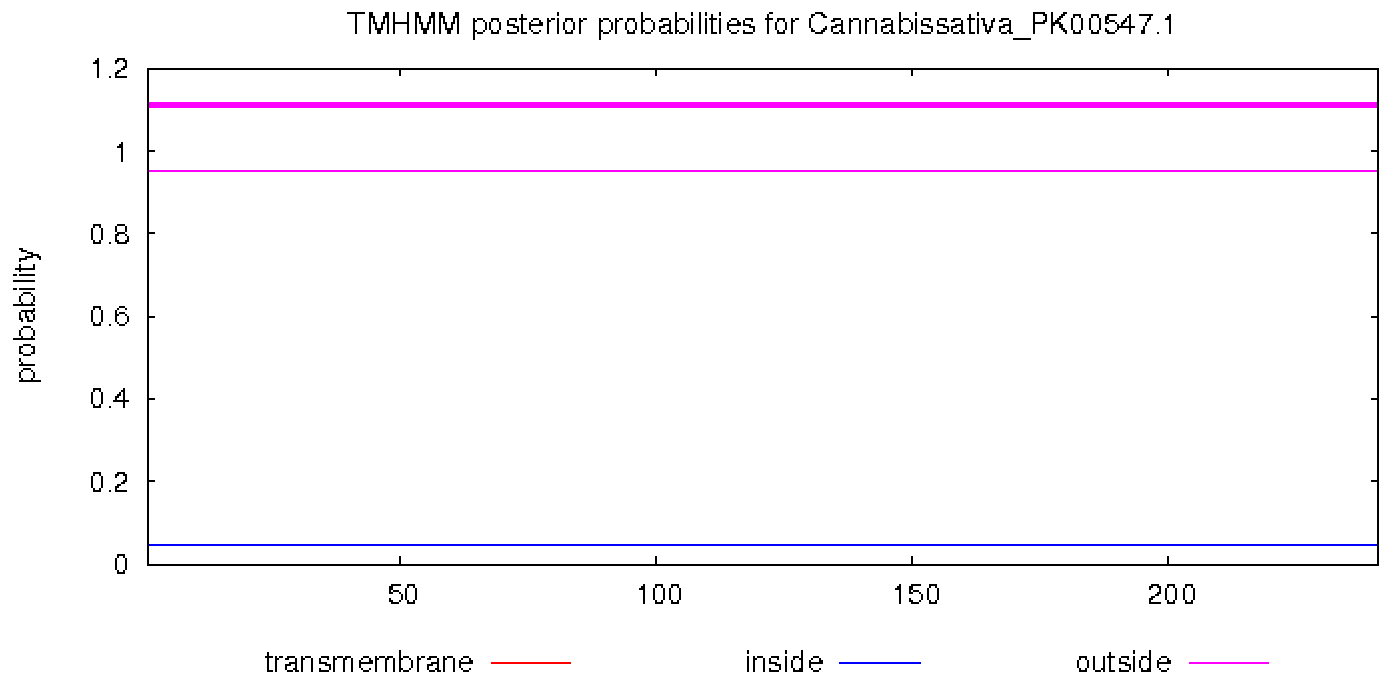

plot in postscript, script for making the plot in gnuplot, data for plot

```
# Cannabissativa_PK01070.1 Length: 84
# Cannabissativa_PK01070.1 Number of predicted TMHs:  0
# Cannabissativa_PK01070.1 Exp number of AAs in TMHs: 0
# Cannabissativa_PK01070.1 Exp number, first 60 AAs:  0
# Cannabissativa_PK01070.1 Total prob of N-in:        0.28725
Cannabissativa_PK01070.1        TMHMM2.0        outside      1    84
```

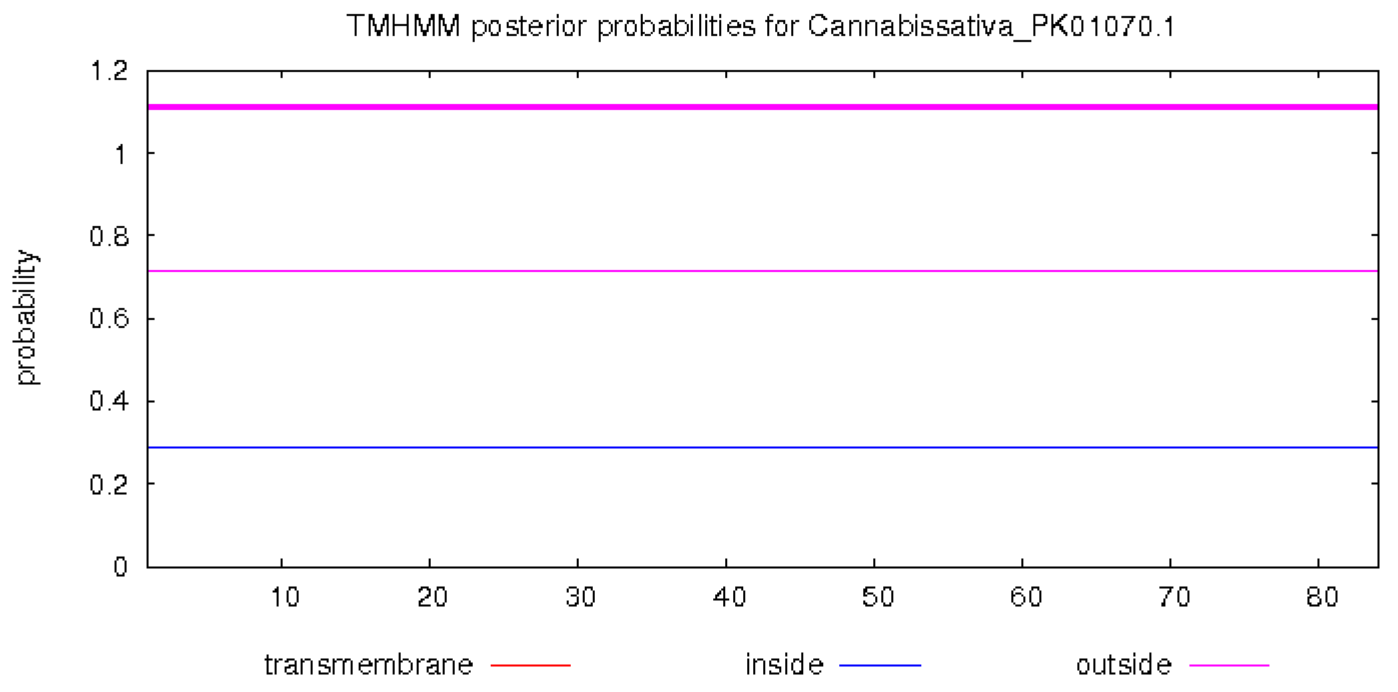

plot in postscript, script for making the plot in gnuplot, data for plot

```
# Cannabissativa_PK01104.1 Length: 254
# Cannabissativa_PK01104.1 Number of predicted TMHs:  0
# Cannabissativa_PK01104.1 Exp number of AAs in TMHs: 0.01914
# Cannabissativa_PK01104.1 Exp number, first 60 AAs:  0
# Cannabissativa_PK01104.1 Total prob of N-in:        0.04157
Cannabissativa_PK01104.1        TMHMM2.0        outside      1   254
```

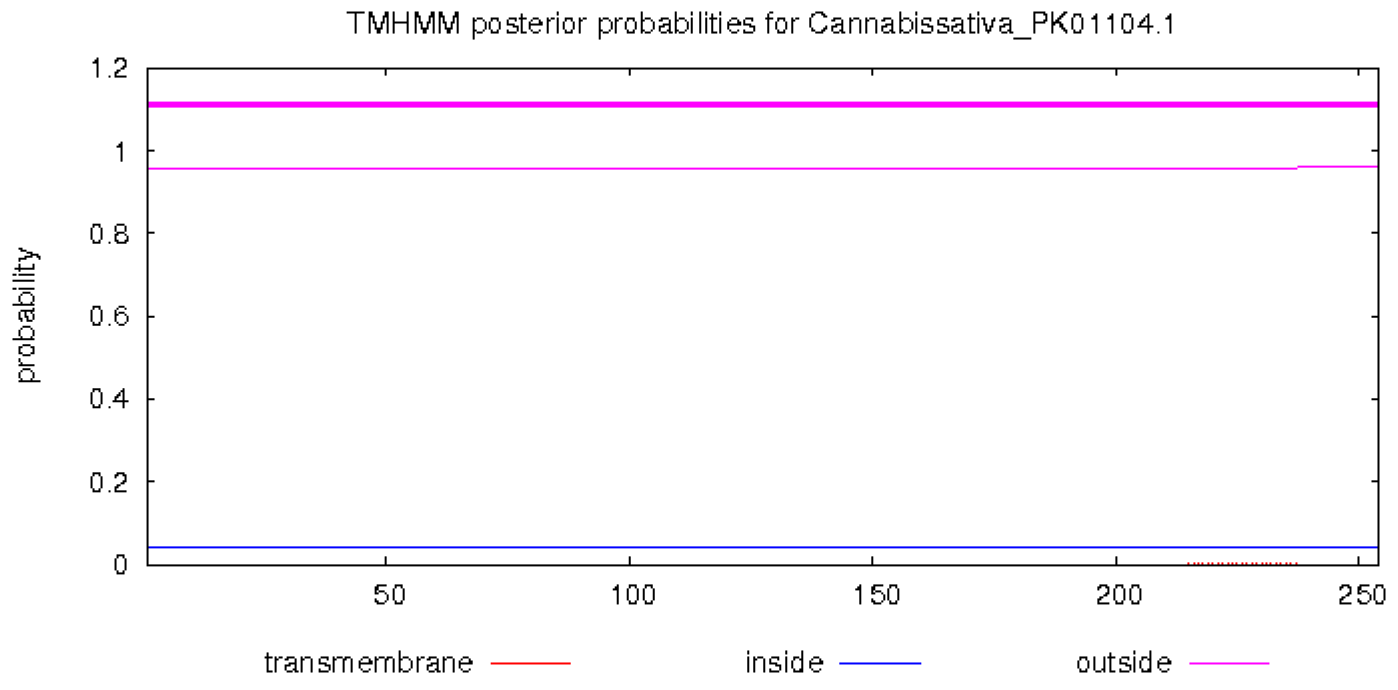

plot in postscript, script for making the plot in gnuplot, data for plot

```
# Cannabissativa_PK02019.1 Length: 860
# Cannabissativa_PK02019.1 Number of predicted TMHs:  1
# Cannabissativa_PK02019.1 Exp number of AAs in TMHs: 22.59023
# Cannabissativa_PK02019.1 Exp number, first 60 AAs:  0
# Cannabissativa_PK02019.1 Total prob of N-in:        0.00010
Cannabissativa_PK02019.1	TMHMM2.0	outside	     1   827
Cannabissativa_PK02019.1	TMHMM2.0	TMhelix	   828   850
Cannabissativa_PK02019.1	TMHMM2.0	inside	   851   860
```

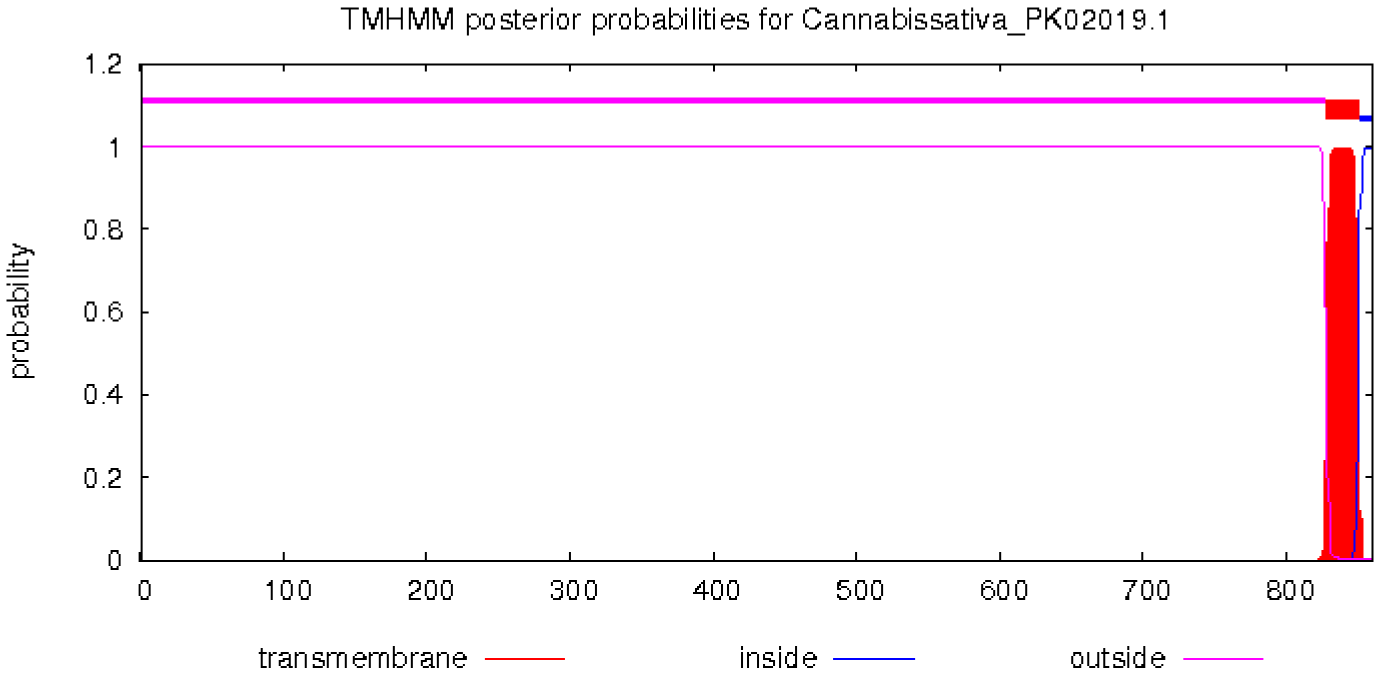

plot in postscript, script for making the plot in gnuplot, data for plot

```
# Cannabissativa_PK02366.1 Length: 376
# Cannabissativa_PK02366.1 Number of predicted TMHs:  0
# Cannabissativa_PK02366.1 Exp number of AAs in TMHs: 0.03871
# Cannabissativa_PK02366.1 Exp number, first 60 AAs:  0.00014
# Cannabissativa_PK02366.1 Total prob of N-in:        0.03641
Cannabissativa_PK02366.1	TMHMM2.0	outside	     1   376
```

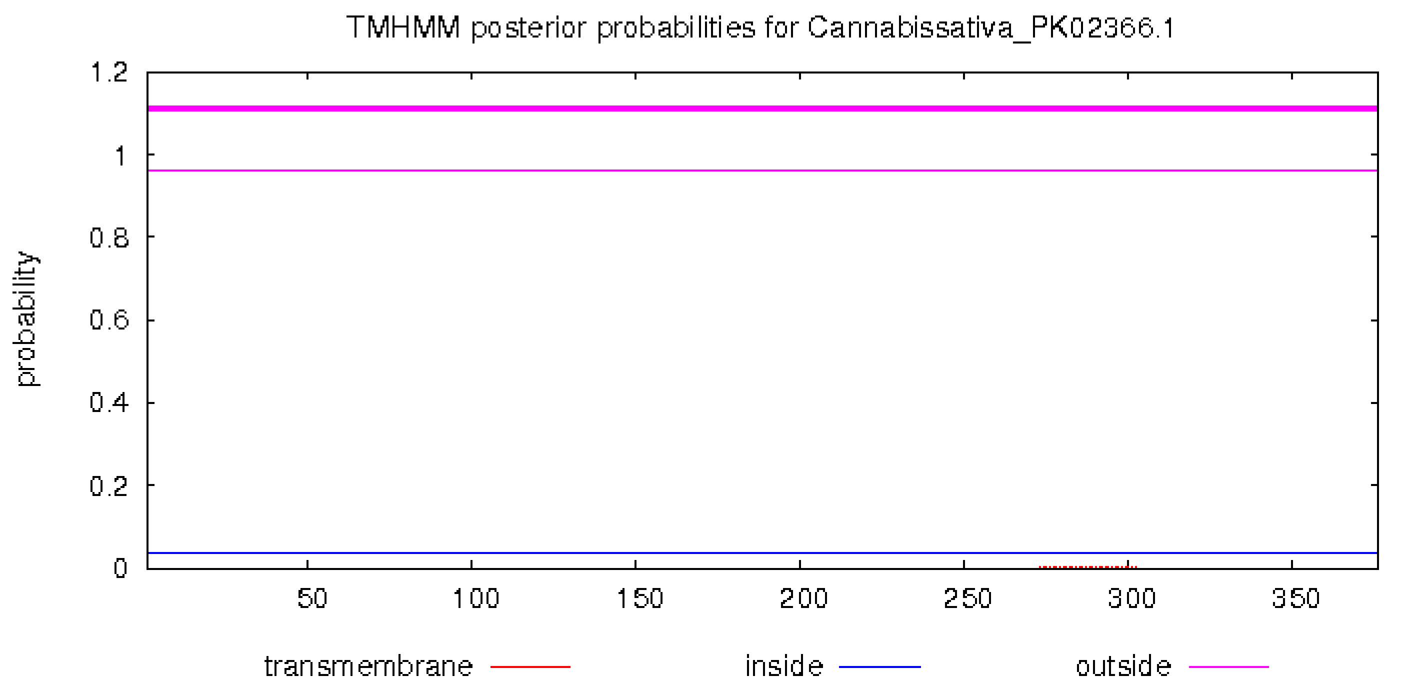

plot in postscript, script for making the plot in gnuplot, data for plot

---

```
# Cannabissativa_PK02804.1 Length: 350
# Cannabissativa_PK02804.1 Number of predicted TMHs:  0
# Cannabissativa_PK02804.1 Exp number of AAs in TMHs: 0
# Cannabissativa_PK02804.1 Exp number, first 60 AAs:  0
# Cannabissativa_PK02804.1 Total prob of N-in:        0.00283
Cannabissativa_PK02804.1        TMHMM2.0        outside      1   350
```

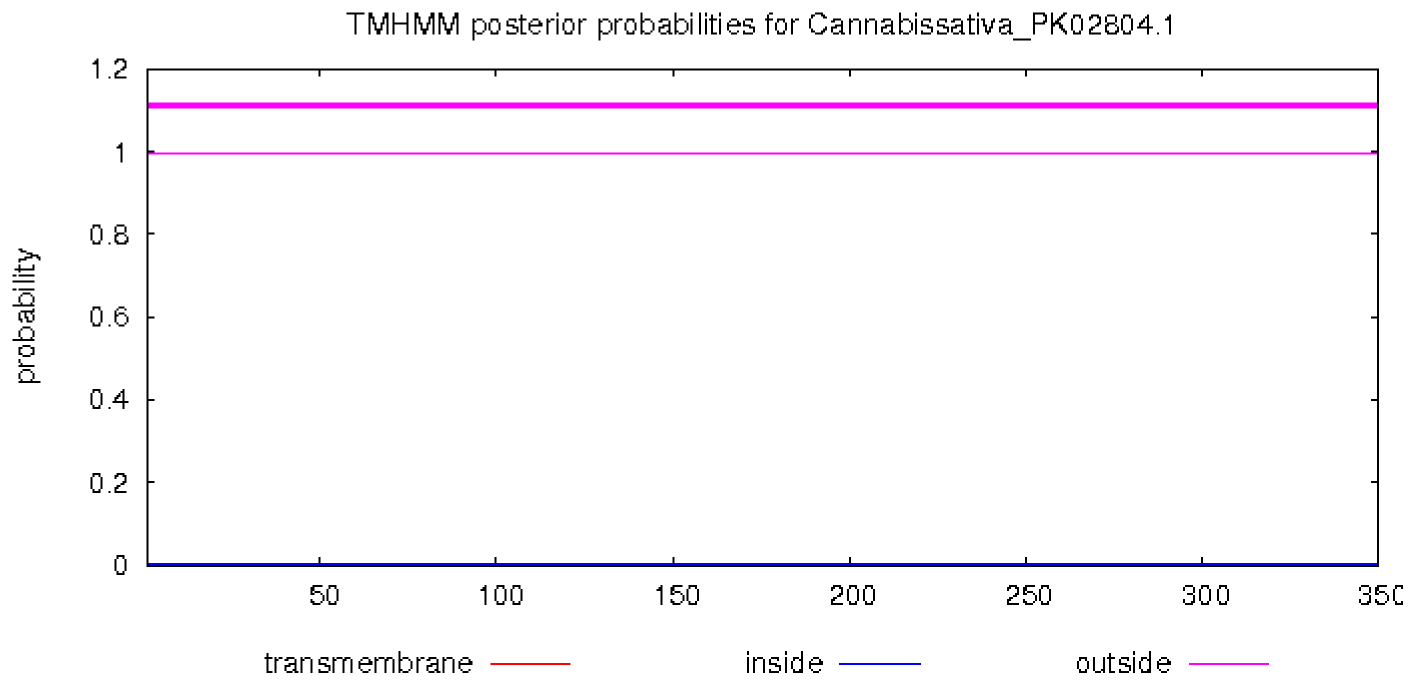

plot in postscript, script for making the plot in gnuplot, data for plot

---

```
# Cannabissativa_PK03341.1 Length: 268
# Cannabissativa_PK03341.1 Number of predicted TMHs:  0
# Cannabissativa_PK03341.1 Exp number of AAs in TMHs: 0
# Cannabissativa_PK03341.1 Exp number, first 60 AAs:  0
# Cannabissativa_PK03341.1 Total prob of N-in:        0.00158
Cannabissativa_PK03341.1        TMHMM2.0        outside      1   268
```

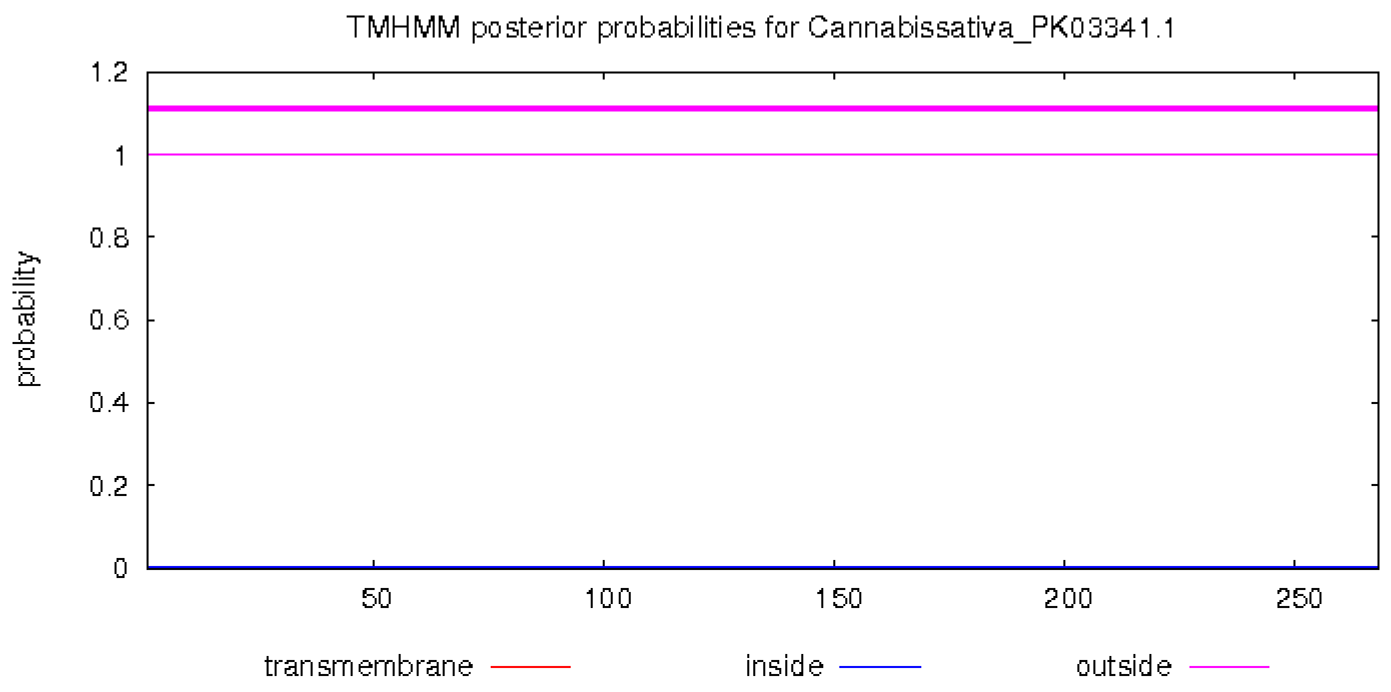

plot in postscript, script for making the plot in gnuplot, data for plot

---

```
# Cannabissativa_PK04961.1 Length: 420
# Cannabissativa_PK04961.1 Number of predicted TMHs:  0
# Cannabissativa_PK04961.1 Exp number of AAs in TMHs: 0.00121
# Cannabissativa_PK04961.1 Exp number, first 60 AAs:  0
# Cannabissativa_PK04961.1 Total prob of N-in:        0.00367
Cannabissativa_PK04961.1        TMHMM2.0        outside      1   420
```

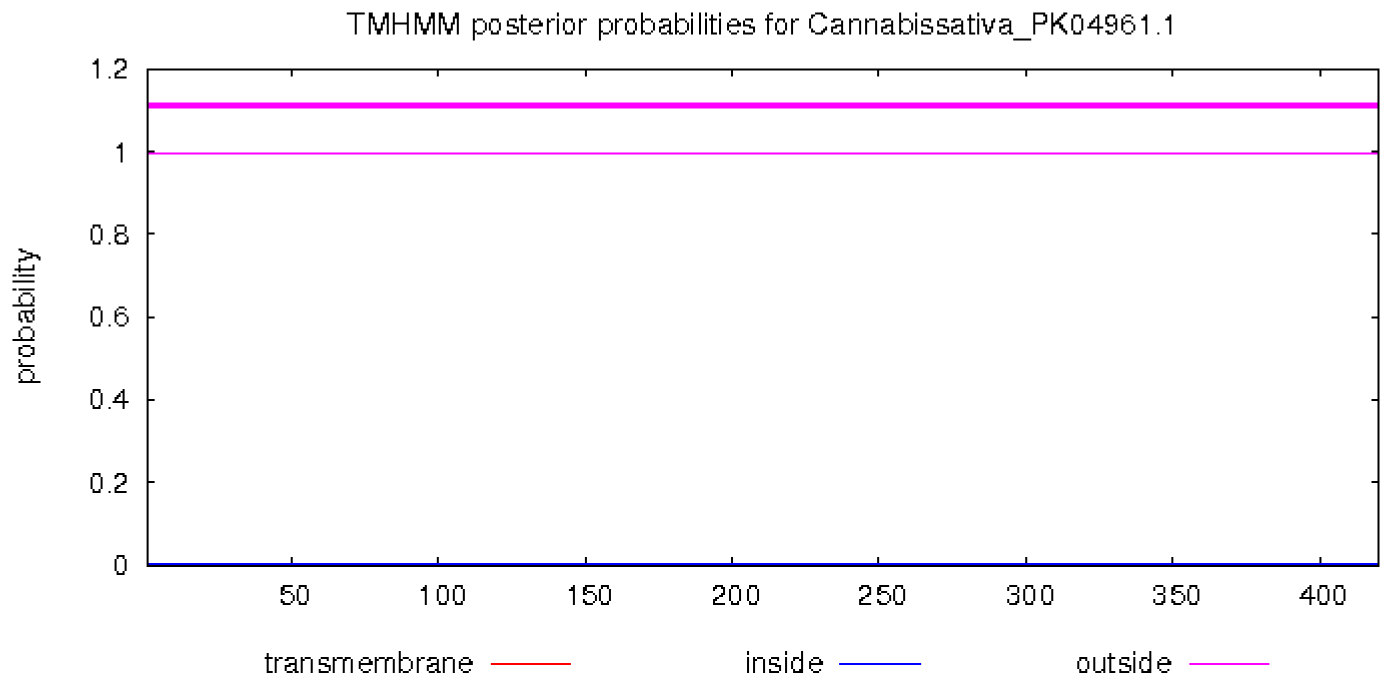

plot in postscript, script for making the plot in gnuplot, data for plot

---

```
# Cannabissativa_PK05686.1 Length: 292
# Cannabissativa_PK05686.1 Number of predicted TMHs:  0
# Cannabissativa_PK05686.1 Exp number of AAs in TMHs: 0.05762
# Cannabissativa_PK05686.1 Exp number, first 60 AAs:  0.05739
# Cannabissativa_PK05686.1 Total prob of N-in:        0.02503
Cannabissativa_PK05686.1        TMHMM2.0        outside      1   292
```

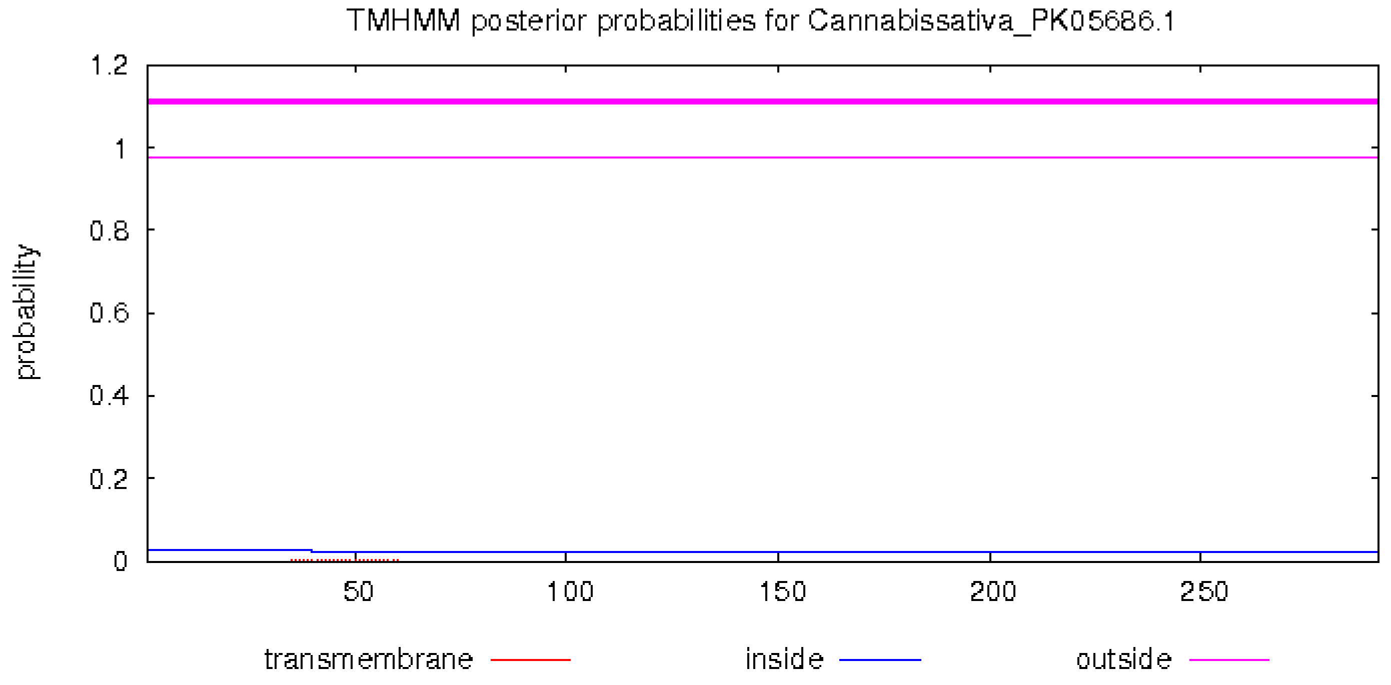

plot in postscript, script for making the plot in gnuplot, data for plot

---

```
# Cannabissativa_PK06287.1 Length: 290
# Cannabissativa_PK06287.1 Number of predicted TMHs:  0
# Cannabissativa_PK06287.1 Exp number of AAs in TMHs: 0.00033
# Cannabissativa_PK06287.1 Exp number, first 60 AAs:  0.00016
# Cannabissativa_PK06287.1 Total prob of N-in:        0.01590
Cannabissativa_PK06287.1        TMHMM2.0        outside      1   290
```

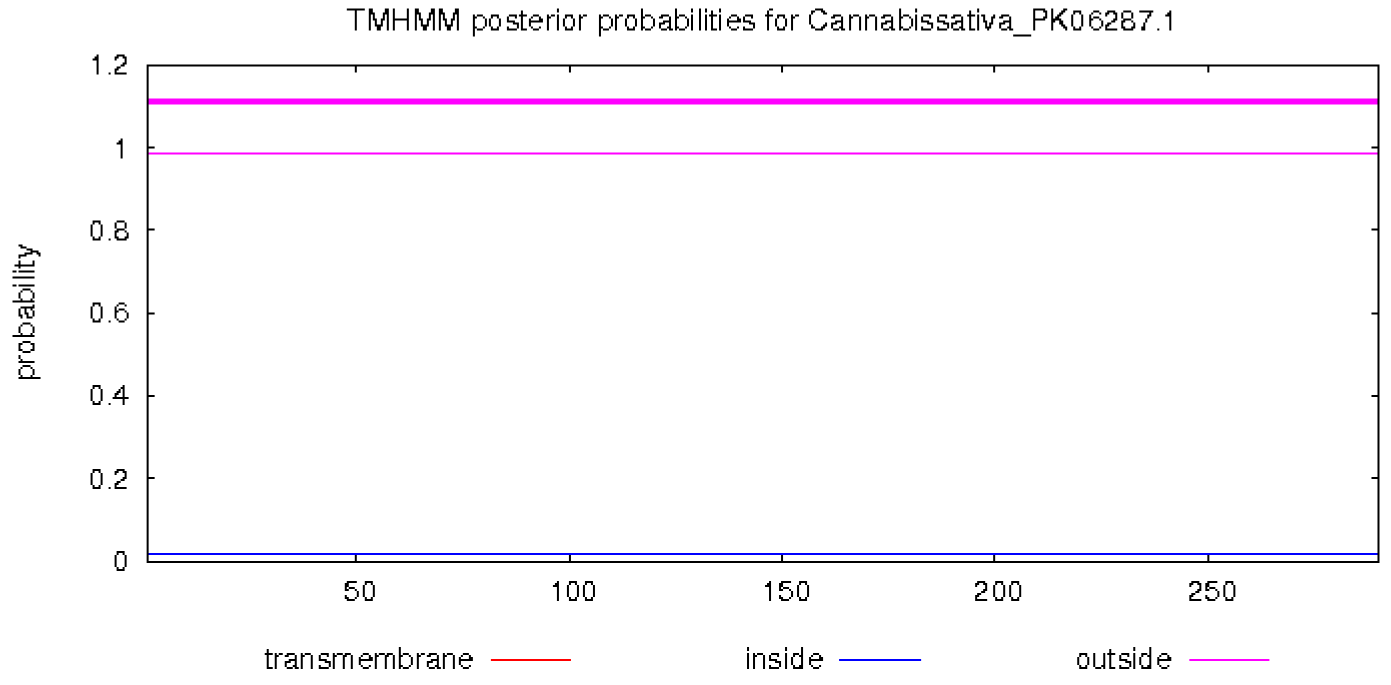

plot in postscript, script for making the plot in gnuplot, data for plot

---

```
# Cannabissativa_PK06477.1 Length: 498
# Cannabissativa_PK06477.1 Number of predicted TMHs:  0
# Cannabissativa_PK06477.1 Exp number of AAs in TMHs: 0.00086
# Cannabissativa_PK06477.1 Exp number, first 60 AAs:  0.00034
# Cannabissativa_PK06477.1 Total prob of N-in:        0.00193
Cannabissativa_PK06477.1        TMHMM2.0        outside      1   498
```

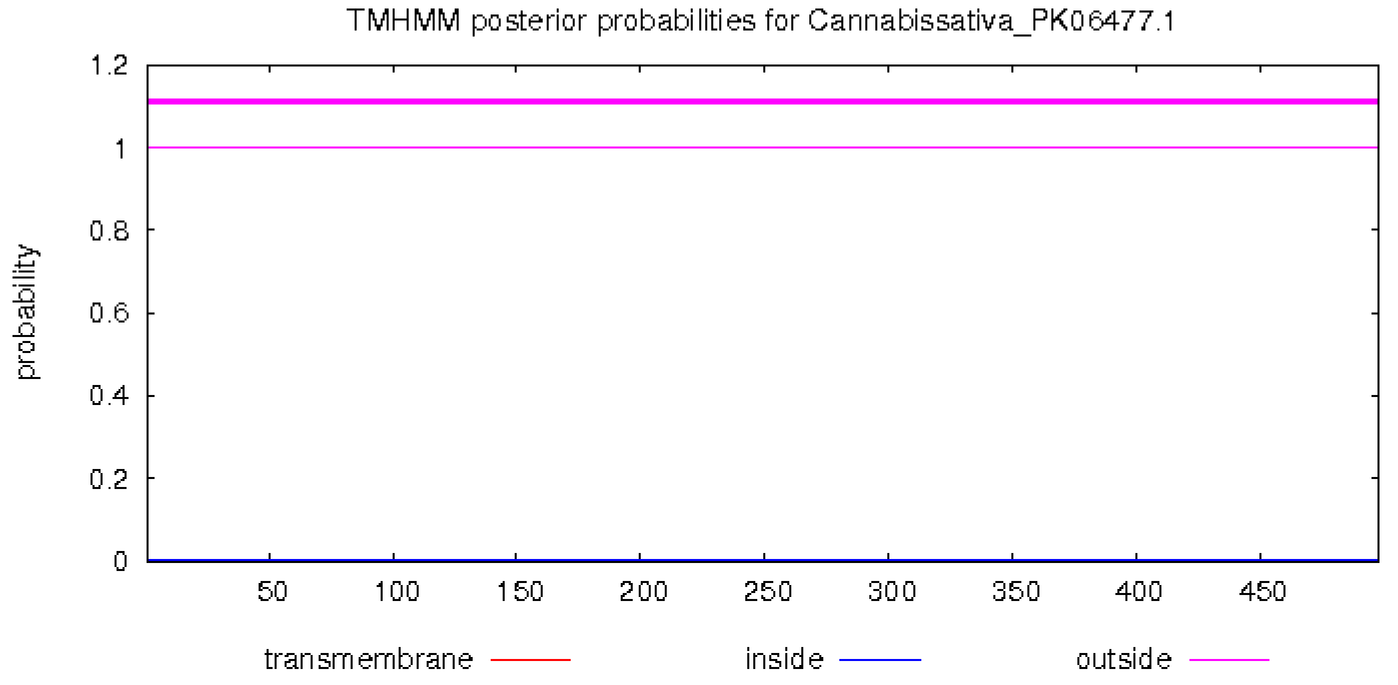

plot in postscript, script for making the plot in gnuplot, data for plot

```
# Cannabissativa_PK06766.1 Length: 405
# Cannabissativa_PK06766.1 Number of predicted TMHs:  0
# Cannabissativa_PK06766.1 Exp number of AAs in TMHs: 0.0098
# Cannabissativa_PK06766.1 Exp number, first 60 AAs:  0.0098
# Cannabissativa_PK06766.1 Total prob of N-in:        0.00331
Cannabissativa_PK06766.1        TMHMM2.0        outside      1   405
```

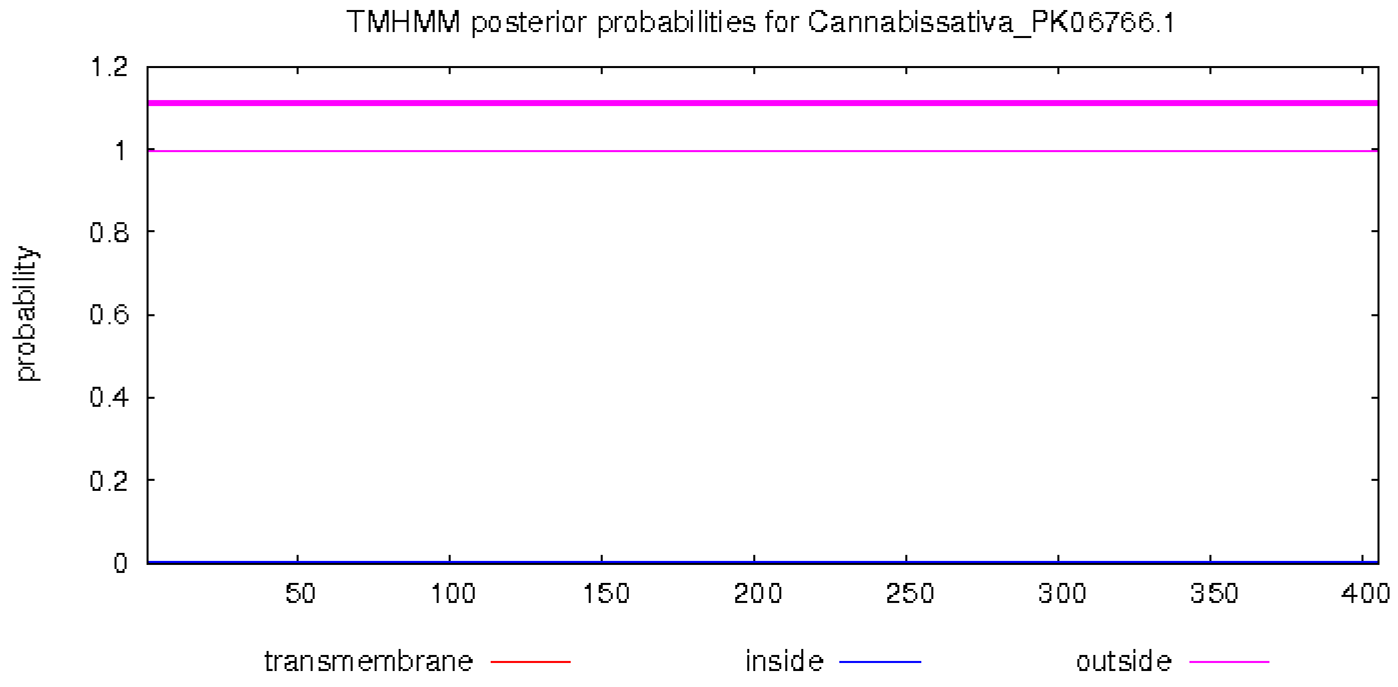

plot in postscript, script for making the plot in gnuplot, data for plot

```
# Cannabissativa_PK07042.1 Length: 344
# Cannabissativa_PK07042.1 Number of predicted TMHs:  0
# Cannabissativa_PK07042.1 Exp number of AAs in TMHs: 0.00651
# Cannabissativa_PK07042.1 Exp number, first 60 AAs:  0.00637
# Cannabissativa_PK07042.1 Total prob of N-in:        0.01657
Cannabissativa_PK07042.1        TMHMM2.0        outside      1   344
```

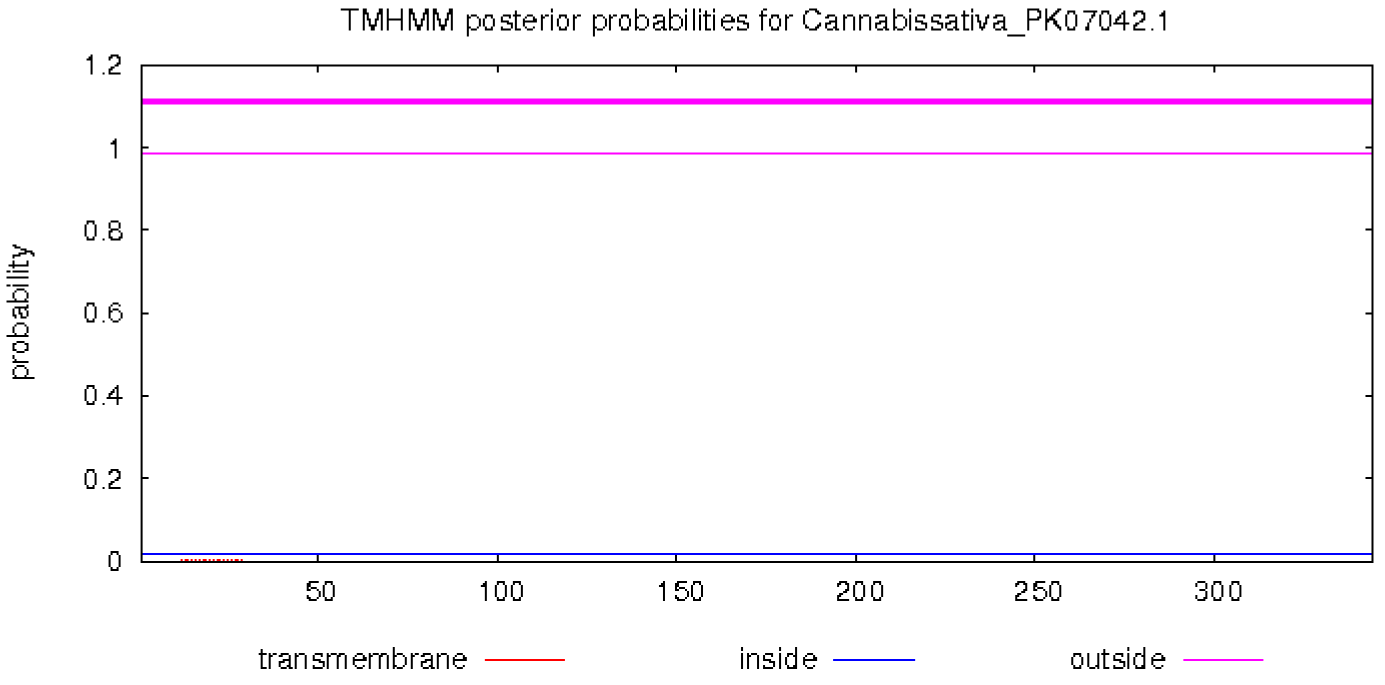

plot in postscript, script for making the plot in gnuplot, data for plot

---

```
# Cannabissativa_PK08180.1 Length: 205
# Cannabissativa_PK08180.1 Number of predicted TMHs:  0
# Cannabissativa_PK08180.1 Exp number of AAs in TMHs: 0
# Cannabissativa_PK08180.1 Exp number, first 60 AAs:  0
# Cannabissativa_PK08180.1 Total prob of N-in:        0.04645
Cannabissativa_PK08180.1        TMHMM2.0        outside      1   205
```

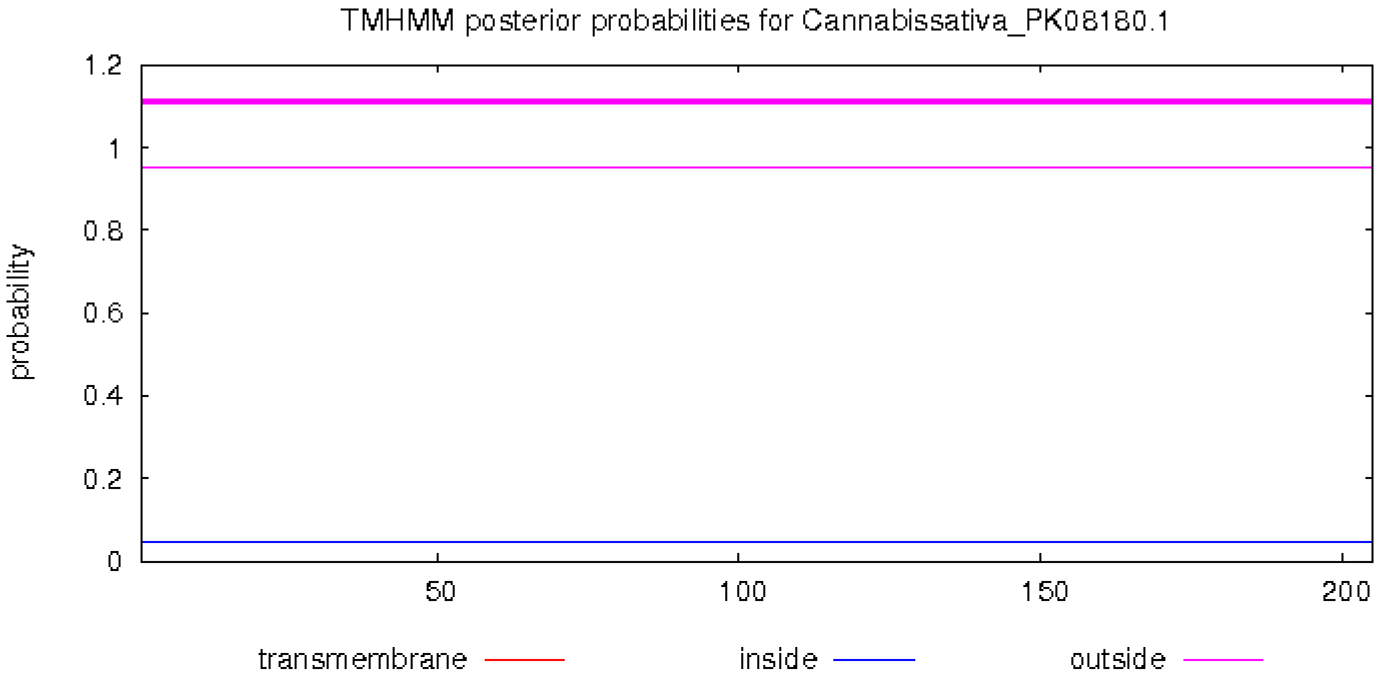

plot in postscript, script for making the plot in gnuplot, data for plot

---

```
# Cannabissativa_PK08629.1 Length: 393
# Cannabissativa_PK08629.1 Number of predicted TMHs:  0
# Cannabissativa_PK08629.1 Exp number of AAs in TMHs: 0.00139
# Cannabissativa_PK08629.1 Exp number, first 60 AAs:  0.00054
# Cannabissativa_PK08629.1 Total prob of N-in:        0.00650
Cannabissativa_PK08629.1        TMHMM2.0        outside      1   393
```

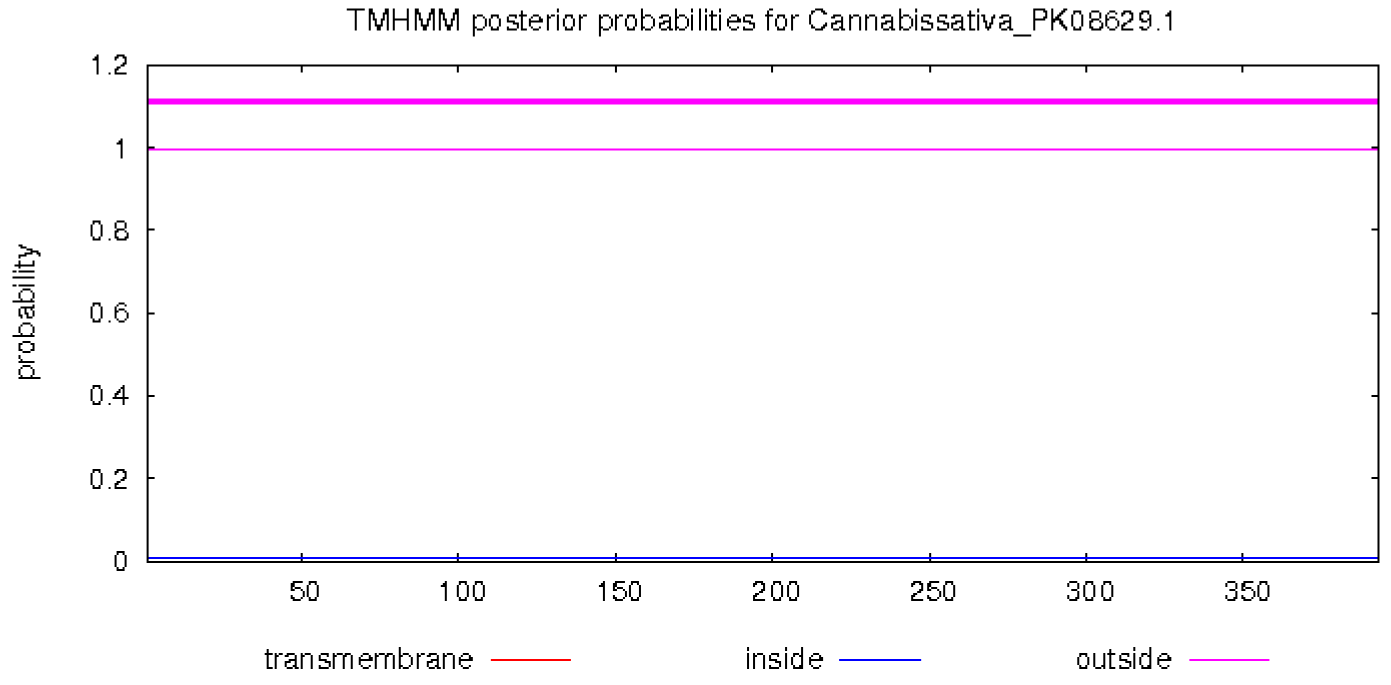

plot in postscript, script for making the plot in gnuplot, data for plot

---

```
# Cannabissativa_PK10271.1 Length: 374
# Cannabissativa_PK10271.1 Number of predicted TMHs:  0
# Cannabissativa_PK10271.1 Exp number of AAs in TMHs: 0
# Cannabissativa_PK10271.1 Exp number, first 60 AAs:  0
# Cannabissativa_PK10271.1 Total prob of N-in:        0.01271
Cannabissativa_PK10271.1        TMHMM2.0        outside      1   374
```

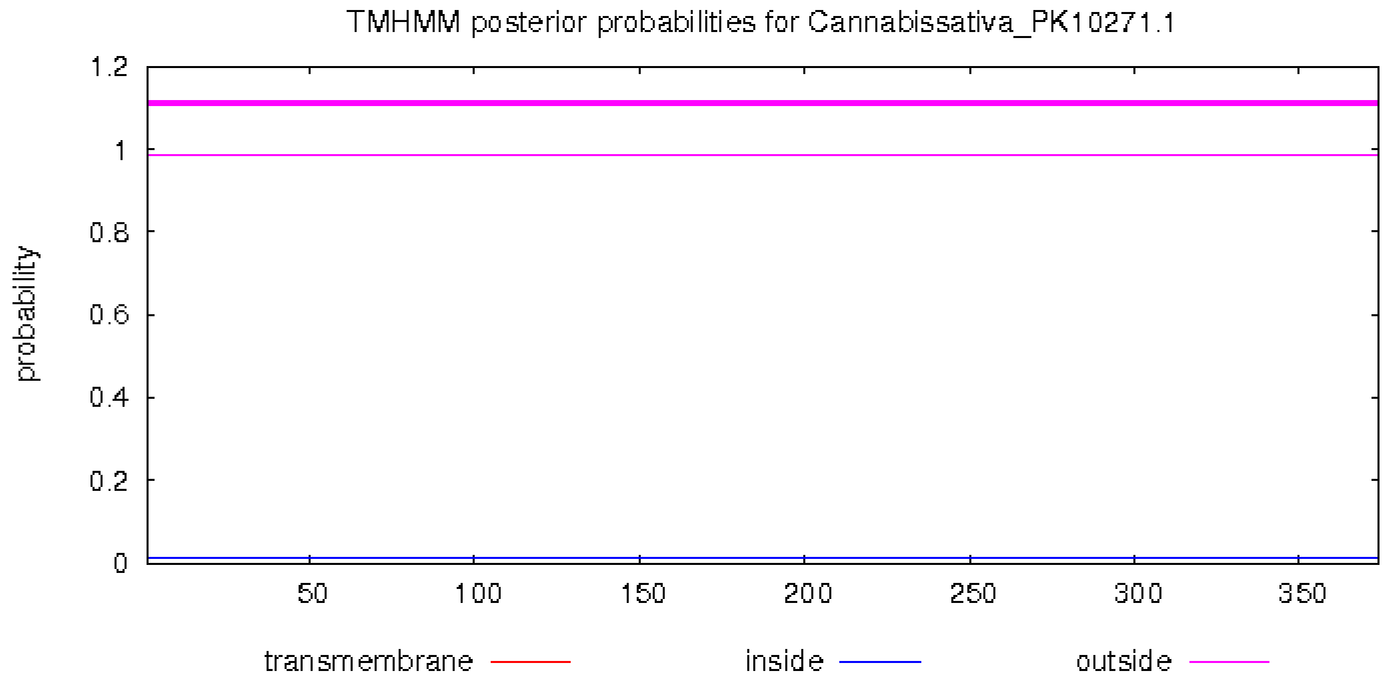

plot in postscript, script for making the plot in gnuplot, data for plot

---

```
# Cannabissativa_PK11173.1 Length: 405
# Cannabissativa_PK11173.1 Number of predicted TMHs:  0
# Cannabissativa_PK11173.1 Exp number of AAs in TMHs: 0
# Cannabissativa_PK11173.1 Exp number, first 60 AAs:  0
# Cannabissativa_PK11173.1 Total prob of N-in:        0.00083
Cannabissativa_PK11173.1        TMHMM2.0        outside      1   405
```

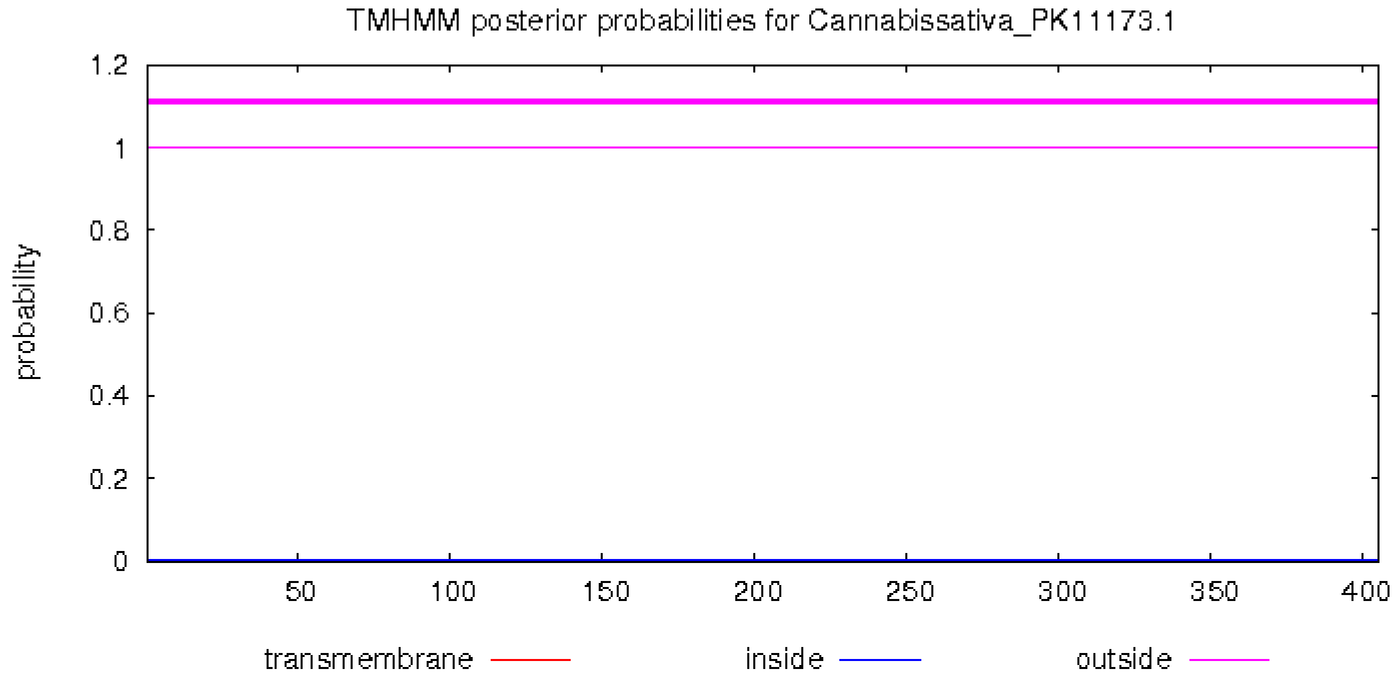

plot in postscript, script for making the plot in gnuplot, data for plot

---

```
# Cannabissativa_PK11281.7 Length: 54
# Cannabissativa_PK11281.7 Number of predicted TMHs:  0
# Cannabissativa_PK11281.7 Exp number of AAs in TMHs: 0.0003
# Cannabissativa_PK11281.7 Exp number, first 60 AAs:  0.0003
# Cannabissativa_PK11281.7 Total prob of N-in:        0.11875
Cannabissativa_PK11281.7        TMHMM2.0        outside      1    54
```

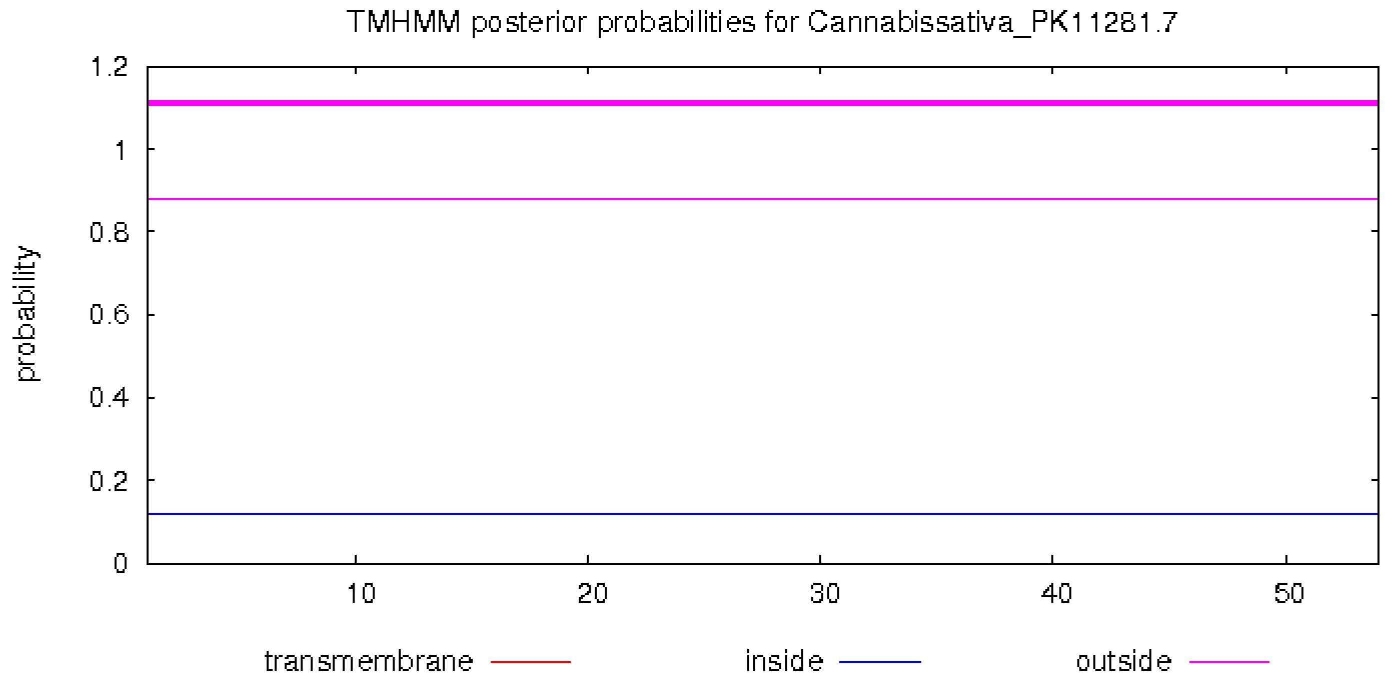

plot in postscript, script for making the plot in gnuplot, data for plot

---

```
# Cannabissativa_PK11467.1 Length: 571
# Cannabissativa_PK11467.1 Number of predicted TMHs:  0
# Cannabissativa_PK11467.1 Exp number of AAs in TMHs: 0.45704
# Cannabissativa_PK11467.1 Exp number, first 60 AAs:  0
# Cannabissativa_PK11467.1 Total prob of N-in:        0.00147
Cannabissativa_PK11467.1        TMHMM2.0        outside      1   571
```

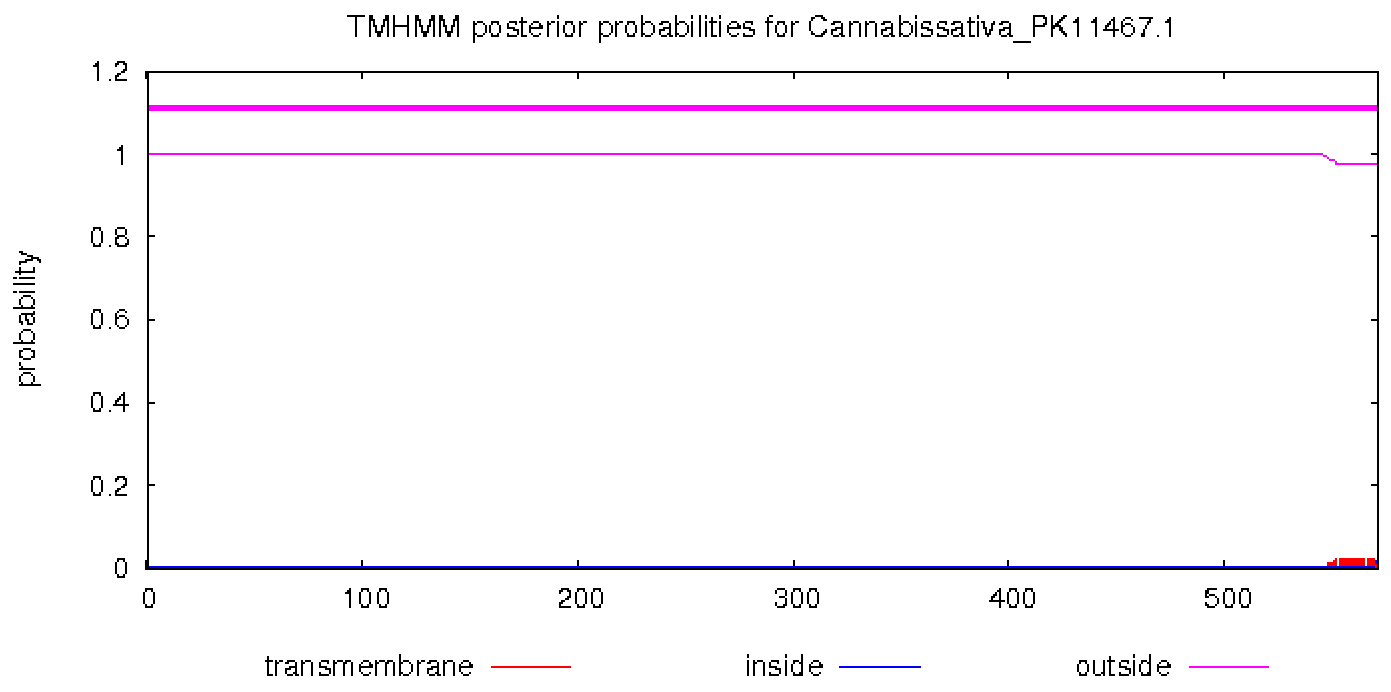

plot in postscript, script for making the plot in gnuplot, data for plot

---

```
# Cannabissativa_PK11990.1 Length: 360
# Cannabissativa_PK11990.1 Number of predicted TMHs:  0
# Cannabissativa_PK11990.1 Exp number of AAs in TMHs: 0.04782
# Cannabissativa_PK11990.1 Exp number, first 60 AAs:  0
# Cannabissativa_PK11990.1 Total prob of N-in:        0.05796
Cannabissativa_PK11990.1        TMHMM2.0        outside      1   360
```

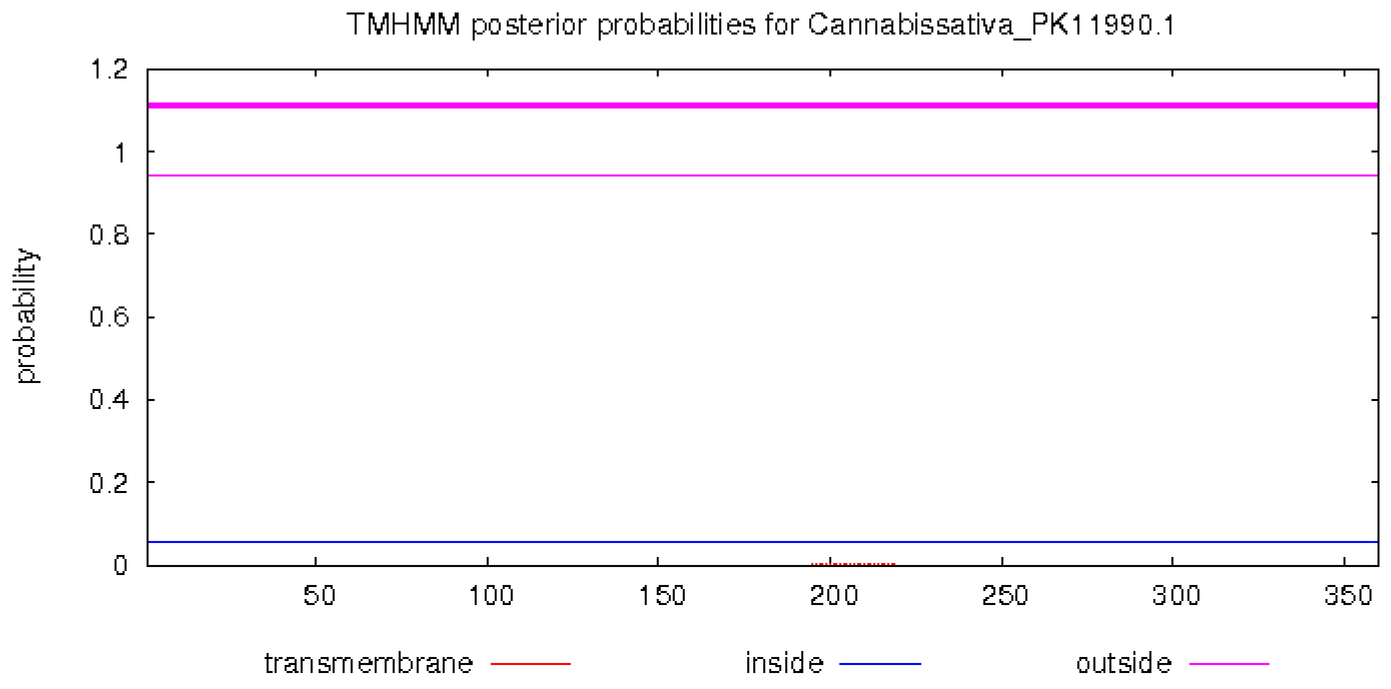

plot in postscript, script for making the plot in gnuplot, data for plot

---

```
# Cannabissativa_PK12461.1 Length: 267
# Cannabissativa_PK12461.1 Number of predicted TMHs:  0
# Cannabissativa_PK12461.1 Exp number of AAs in TMHs: 0.00208
# Cannabissativa_PK12461.1 Exp number, first 60 AAs:  0
# Cannabissativa_PK12461.1 Total prob of N-in:        0.03785
Cannabissativa_PK12461.1        TMHMM2.0        outside      1   267
```

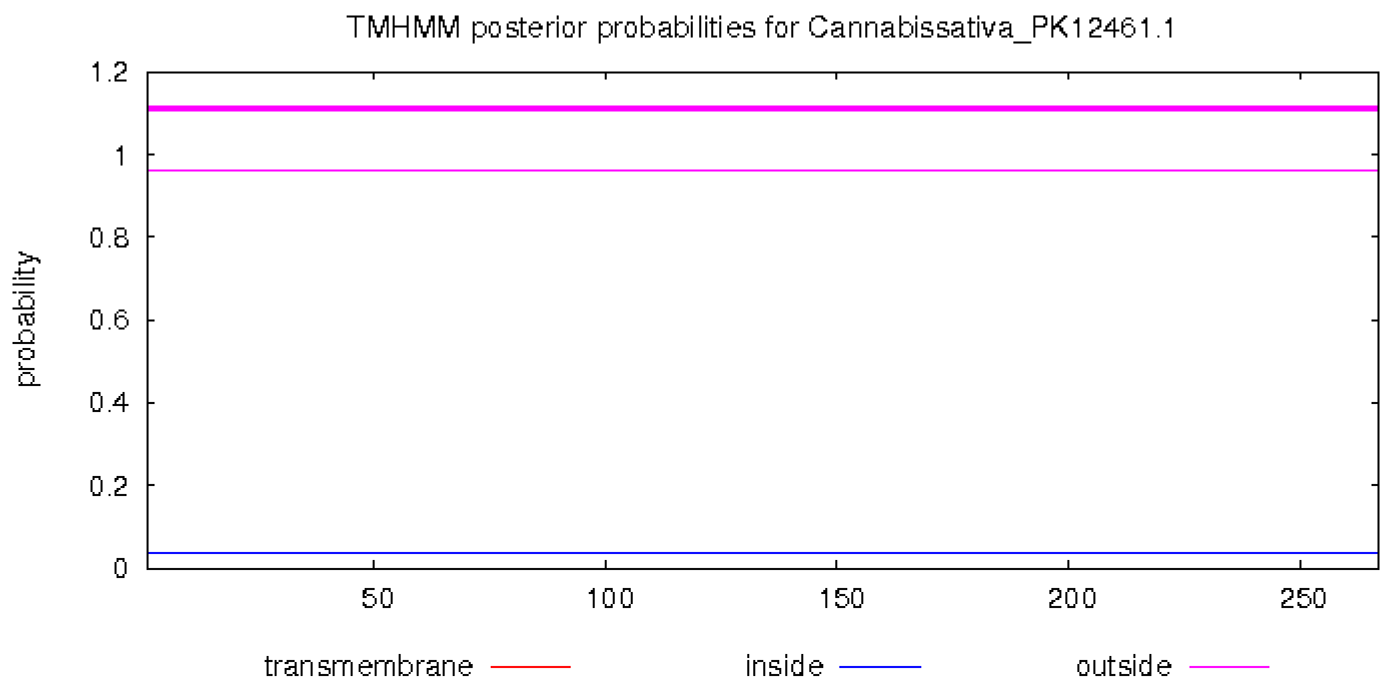

plot in postscript, script for making the plot in gnuplot, data for plot

---

```
# Cannabissativa_PK12901.1 Length: 204
# Cannabissativa_PK12901.1 Number of predicted TMHs:  0
# Cannabissativa_PK12901.1 Exp number of AAs in TMHs: 0.00039
# Cannabissativa_PK12901.1 Exp number, first 60 AAs:  0.0001
# Cannabissativa_PK12901.1 Total prob of N-in:        0.41983
Cannabissativa_PK12901.1        TMHMM2.0        outside      1   204
```

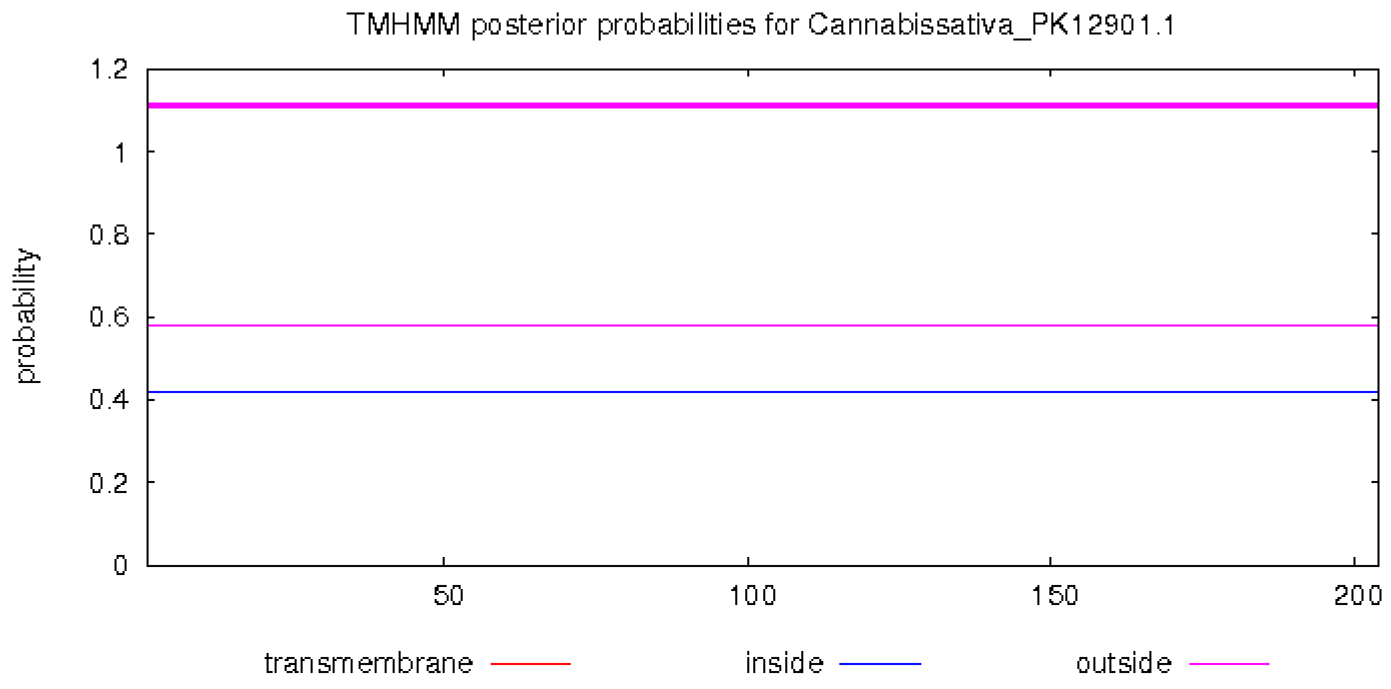

plot in postscript, script for making the plot in gnuplot, data for plot

---

```
# Cannabissativa_PK13251.1 Length: 249
# Cannabissativa_PK13251.1 Number of predicted TMHs:  0
# Cannabissativa_PK13251.1 Exp number of AAs in TMHs: 0.00153
# Cannabissativa_PK13251.1 Exp number, first 60 AAs:  0.00098
# Cannabissativa_PK13251.1 Total prob of N-in:        0.12185
Cannabissativa_PK13251.1        TMHMM2.0        outside      1   249
```

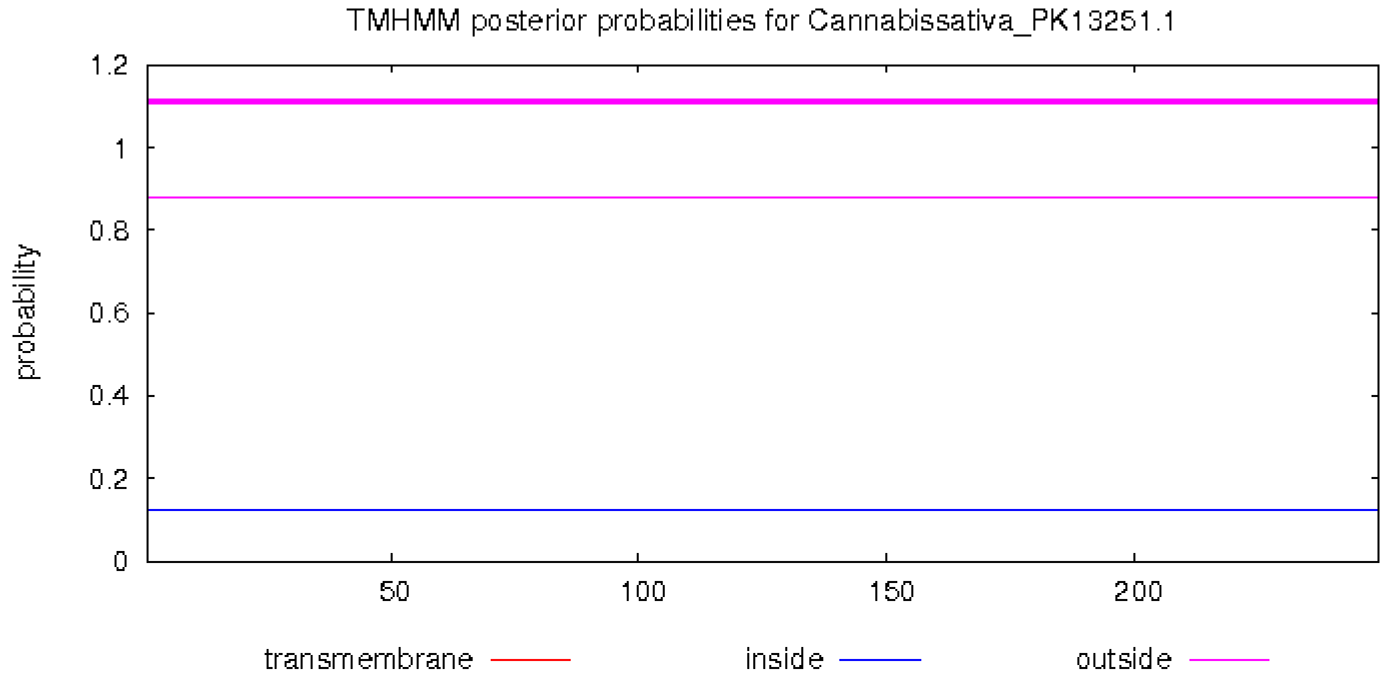

plot in postscript, script for making the plot in gnuplot, data for plot

---

```
# Cannabissativa_PK13860.1 Length: 366
# Cannabissativa_PK13860.1 Number of predicted TMHs:  0
# Cannabissativa_PK13860.1 Exp number of AAs in TMHs: 0.00274
# Cannabissativa_PK13860.1 Exp number, first 60 AAs:  0
# Cannabissativa_PK13860.1 Total prob of N-in:        0.01618
Cannabissativa_PK13860.1        TMHMM2.0        outside      1   366
```

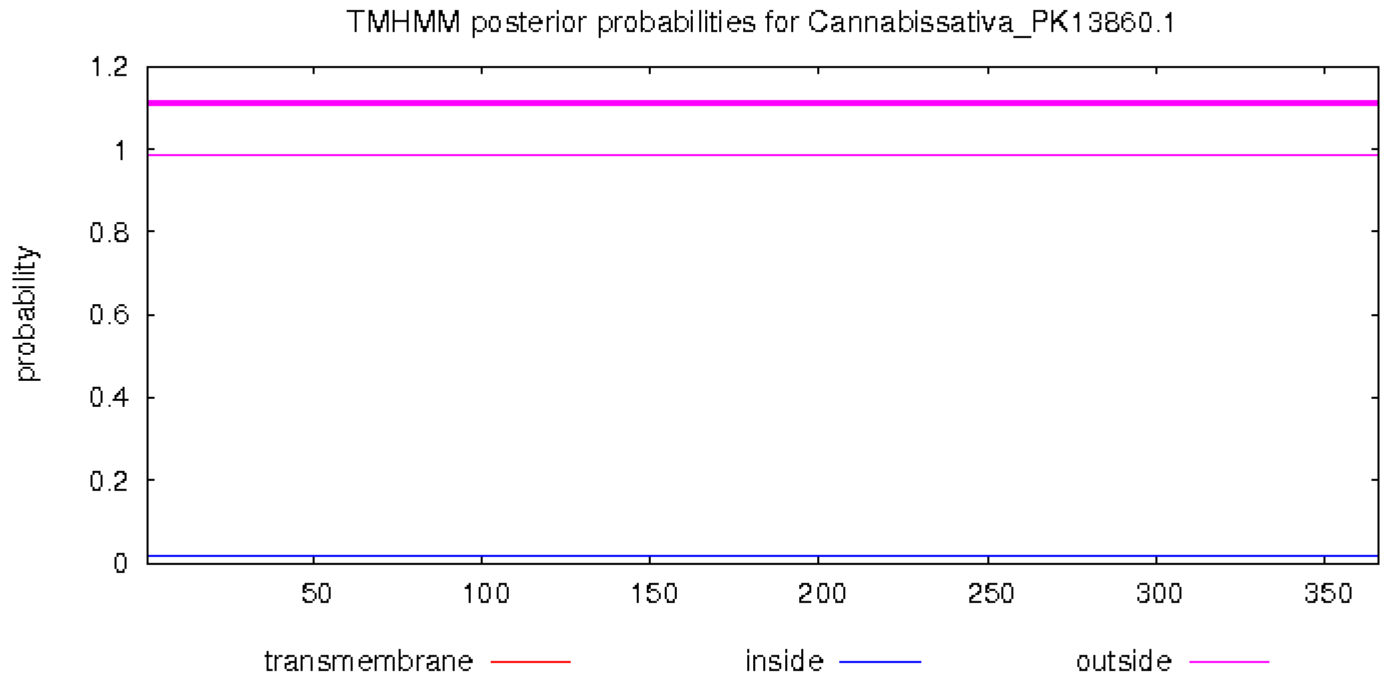

plot in postscript, script for making the plot in gnuplot, data for plot

---

```
# Cannabissativa_PK13964.1 Length: 356
# Cannabissativa_PK13964.1 Number of predicted TMHs:  0
# Cannabissativa_PK13964.1 Exp number of AAs in TMHs: 0.00588
# Cannabissativa_PK13964.1 Exp number, first 60 AAs:  0.00552
# Cannabissativa_PK13964.1 Total prob of N-in:        0.00570
Cannabissativa_PK13964.1        TMHMM2.0        outside      1   356
```

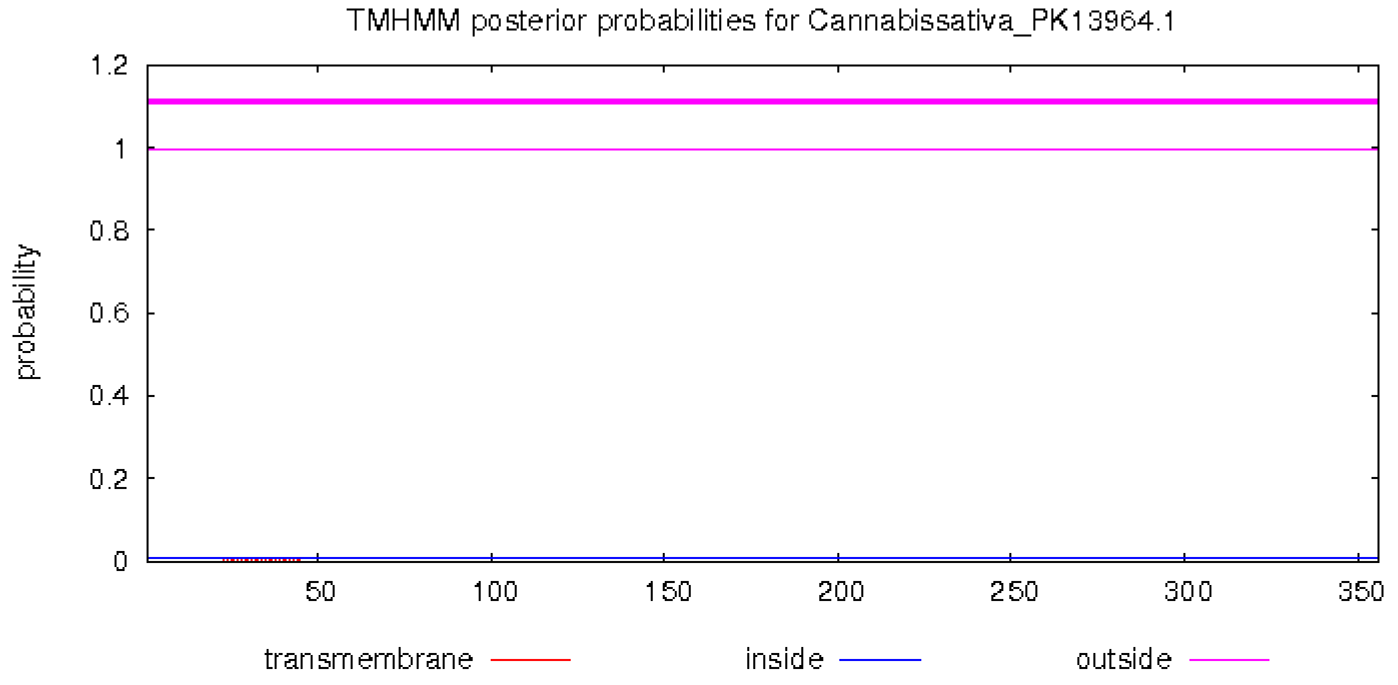

plot in postscript, script for making the plot in gnuplot, data for plot

---

```
# Cannabissativa_PK15363.1 Length: 512
# Cannabissativa_PK15363.1 Number of predicted TMHs:  1
# Cannabissativa_PK15363.1 Exp number of AAs in TMHs: 19.0526
# Cannabissativa_PK15363.1 Exp number, first 60 AAs:  0
# Cannabissativa_PK15363.1 Total prob of N-in:        0.00558
Cannabissativa_PK15363.1	TMHMM2.0	outside	     1   488
Cannabissativa_PK15363.1	TMHMM2.0	TMhelix	   489   511
Cannabissativa_PK15363.1	TMHMM2.0	inside	   512   512
```

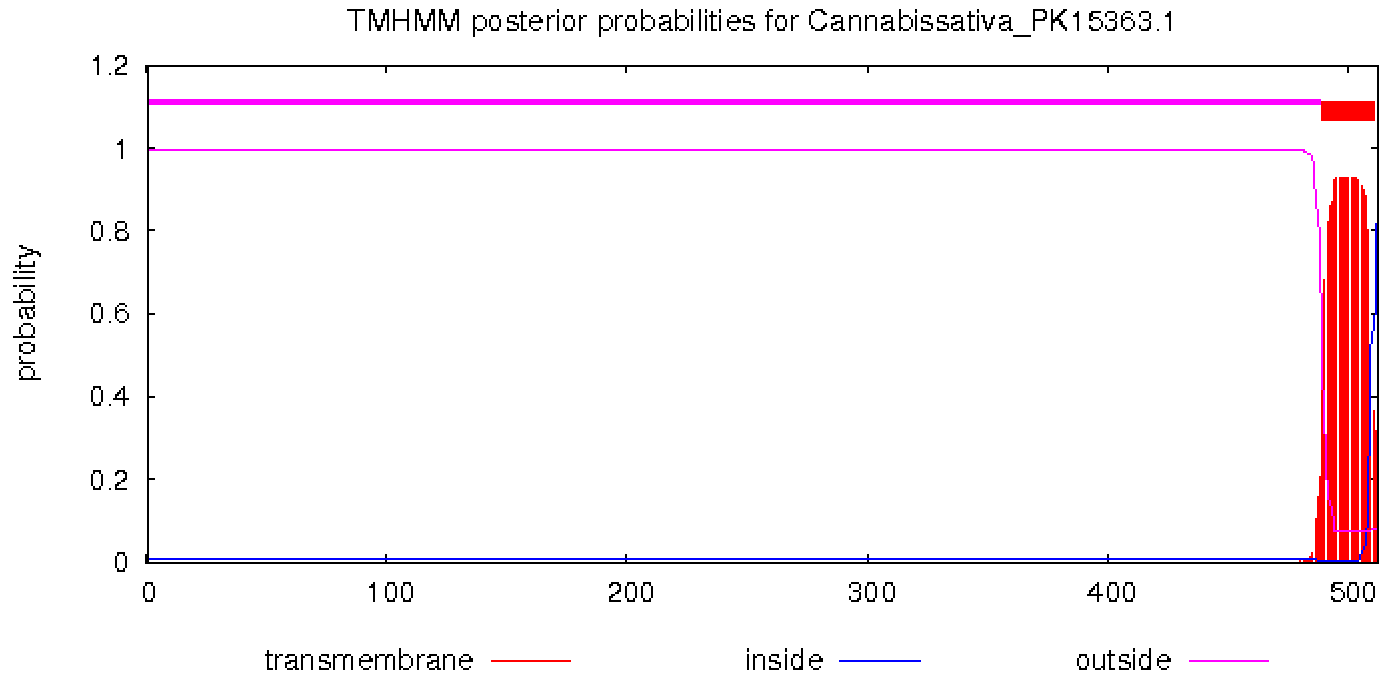

plot in postscript, script for making the plot in gnuplot, data for plot

---

```
# Cannabissativa_PK17141.1 Length: 376
# Cannabissativa_PK17141.1 Number of predicted TMHs:  0
# Cannabissativa_PK17141.1 Exp number of AAs in TMHs: 0.00301
# Cannabissativa_PK17141.1 Exp number, first 60 AAs:  0
```

```
# Cannabissativa_PK17141.1 Total prob of N-in:        0.01729
Cannabissativa_PK17141.1        TMHMM2.0        outside      1   376
```

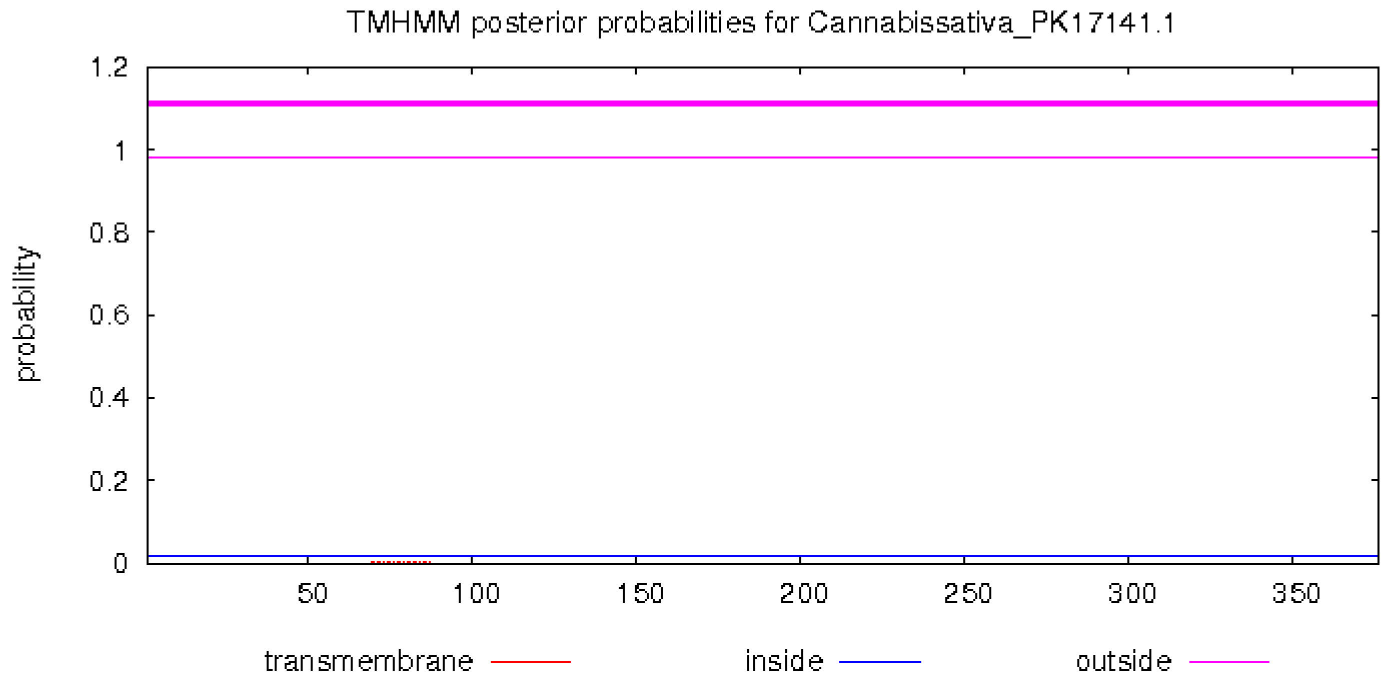

plot in postscript, script for making the plot in gnuplot, data for plot

---

```
# Cannabissativa_PK17295.1 Length: 91
# Cannabissativa_PK17295.1 Number of predicted TMHs:  0
# Cannabissativa_PK17295.1 Exp number of AAs in TMHs: 0.00018
# Cannabissativa_PK17295.1 Exp number, first 60 AAs:  0.00018
# Cannabissativa_PK17295.1 Total prob of N-in:        0.46536
Cannabissativa_PK17295.1        TMHMM2.0        outside      1    91
```

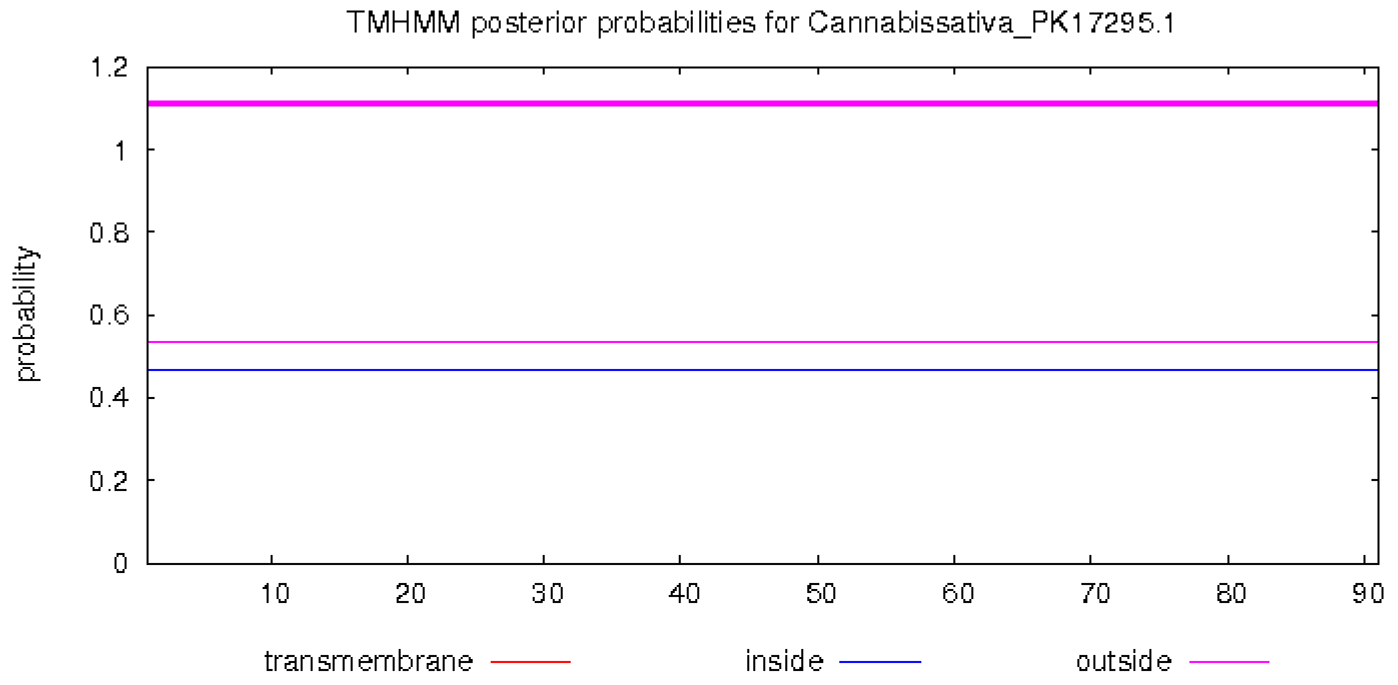

plot in postscript, script for making the plot in gnuplot, data for plot

---

```
# Cannabissativa_PK18158.1 Length: 226
# Cannabissativa_PK18158.1 Number of predicted TMHs:  0
# Cannabissativa_PK18158.1 Exp number of AAs in TMHs: 0.00031
# Cannabissativa_PK18158.1 Exp number, first 60 AAs:  0
```

```
# Cannabissativa_PK18158.1 Total prob of N-in:        0.21453
Cannabissativa_PK18158.1	TMHMM2.0	outside	     1   226
```

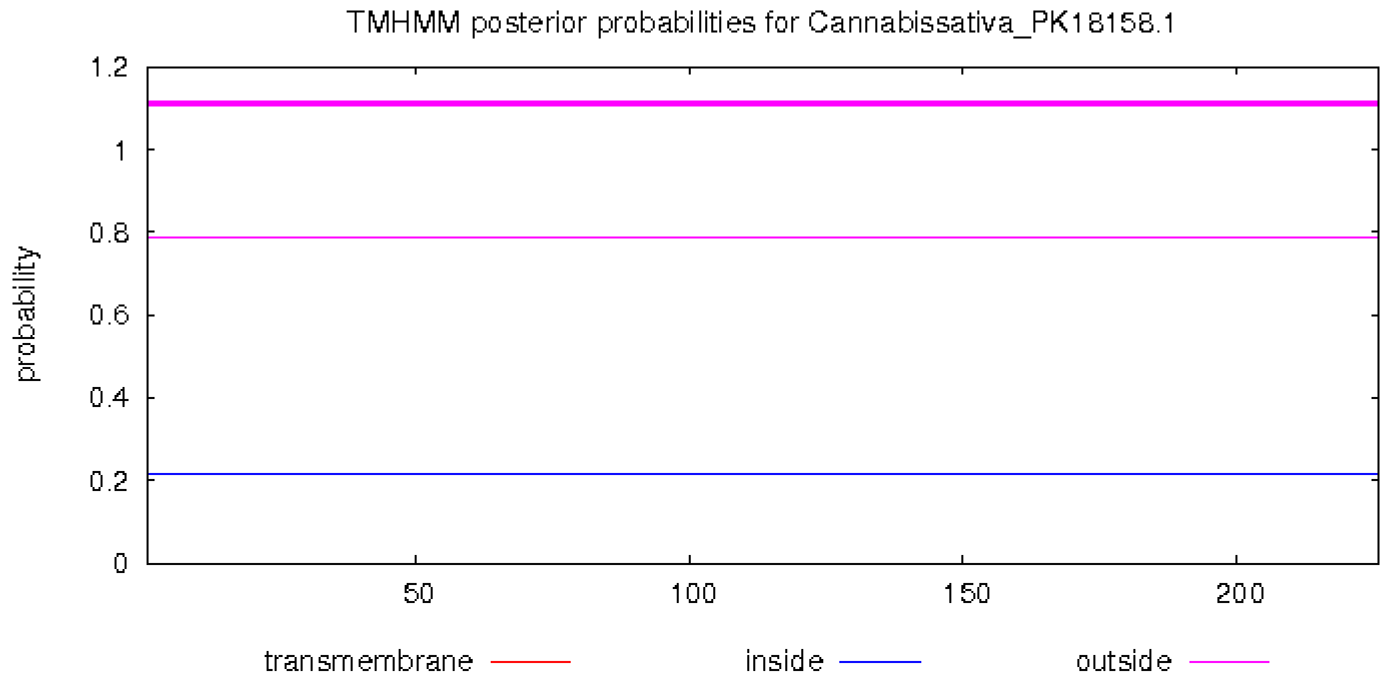

plot in postscript, script for making the plot in gnuplot, data for plot

---

```
# Cannabissativa_PK18821.1 Length: 207
# Cannabissativa_PK18821.1 Number of predicted TMHs:  0
# Cannabissativa_PK18821.1 Exp number of AAs in TMHs: 5e-05
# Cannabissativa_PK18821.1 Exp number, first 60 AAs:  0
# Cannabissativa_PK18821.1 Total prob of N-in:        0.03924
Cannabissativa_PK18821.1	TMHMM2.0	outside	     1   207
```

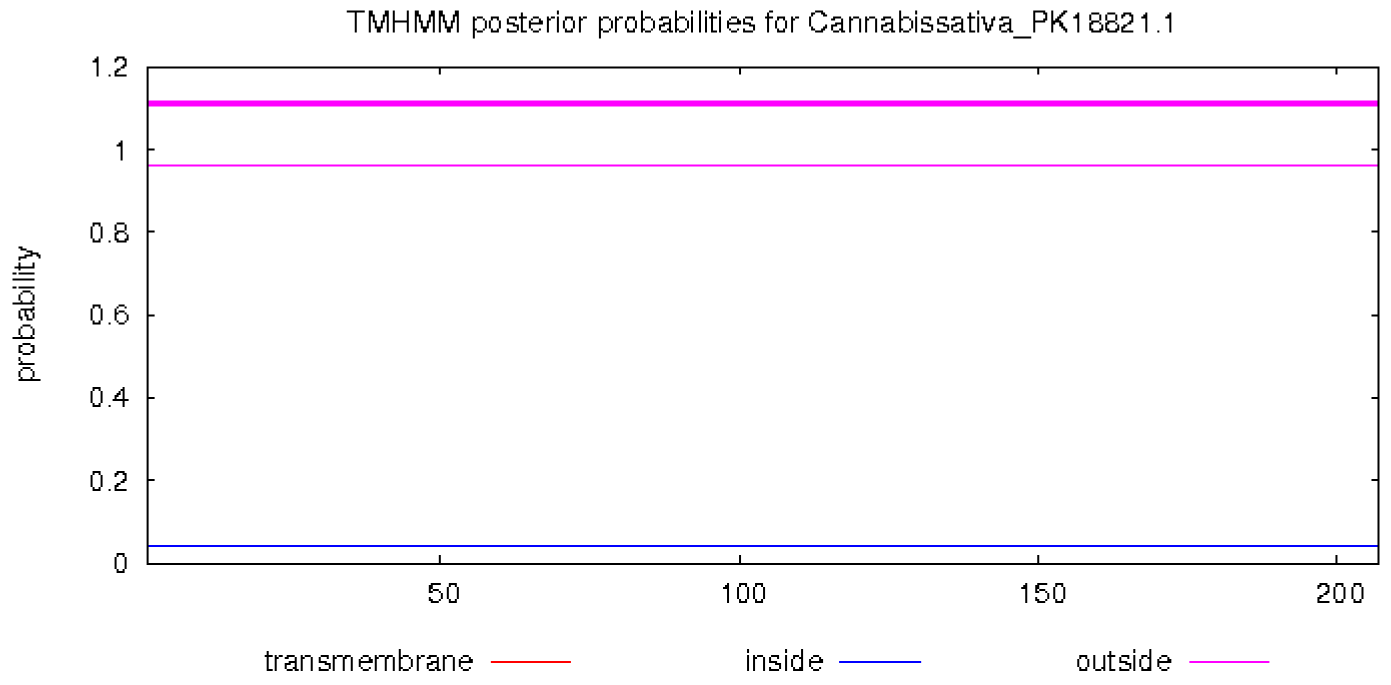

plot in postscript, script for making the plot in gnuplot, data for plot

---

```
# Cannabissativa_PK19051.1 Length: 285
# Cannabissativa_PK19051.1 Number of predicted TMHs:  0
# Cannabissativa_PK19051.1 Exp number of AAs in TMHs: 0.00524
# Cannabissativa_PK19051.1 Exp number, first 60 AAs:  0
```

```
# Cannabissativa_PK19051.1 Total prob of N-in:        0.01423
Cannabissativa_PK19051.1        TMHMM2.0        outside      1   285
```

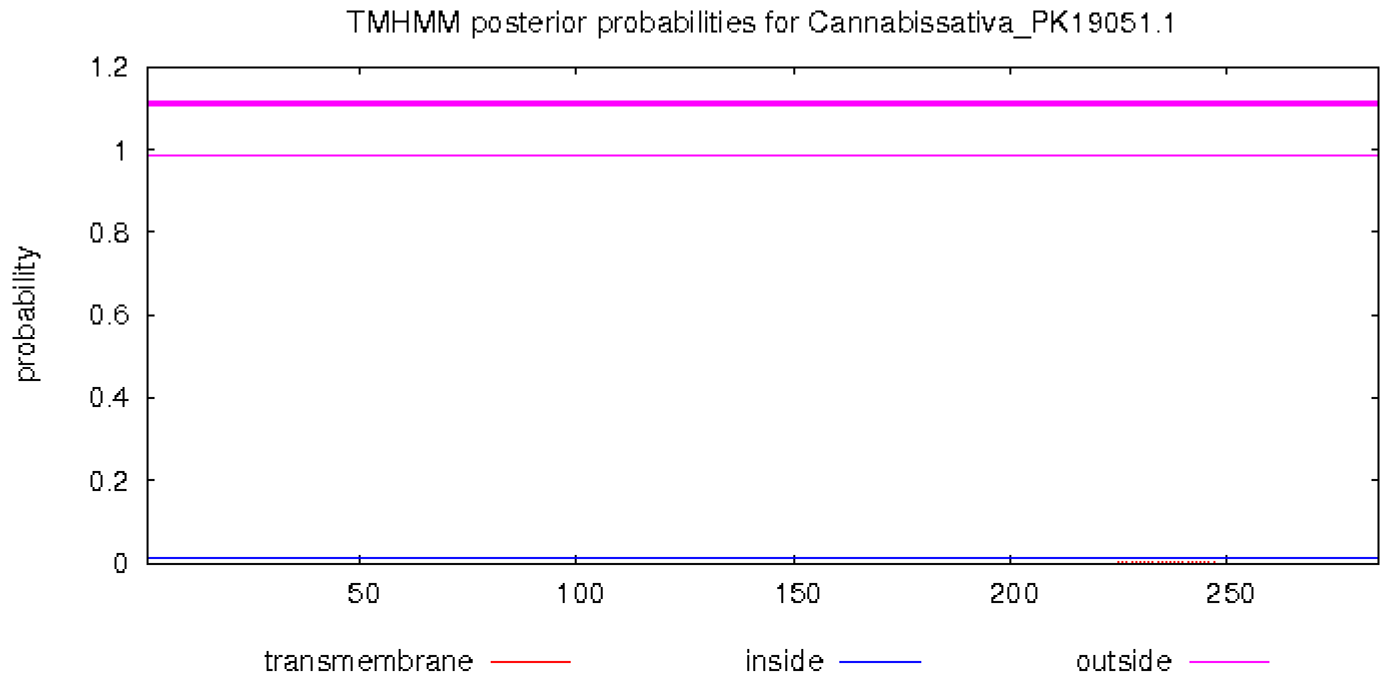

plot in postscript, script for making the plot in gnuplot, data for plot

```
# Cannabissativa_PK19059.1 Length: 164
# Cannabissativa_PK19059.1 Number of predicted TMHs:  0
# Cannabissativa_PK19059.1 Exp number of AAs in TMHs: 0.0009
# Cannabissativa_PK19059.1 Exp number, first 60 AAs:  0.00087
# Cannabissativa_PK19059.1 Total prob of N-in:        0.15786
Cannabissativa_PK19059.1        TMHMM2.0        outside      1   164
```

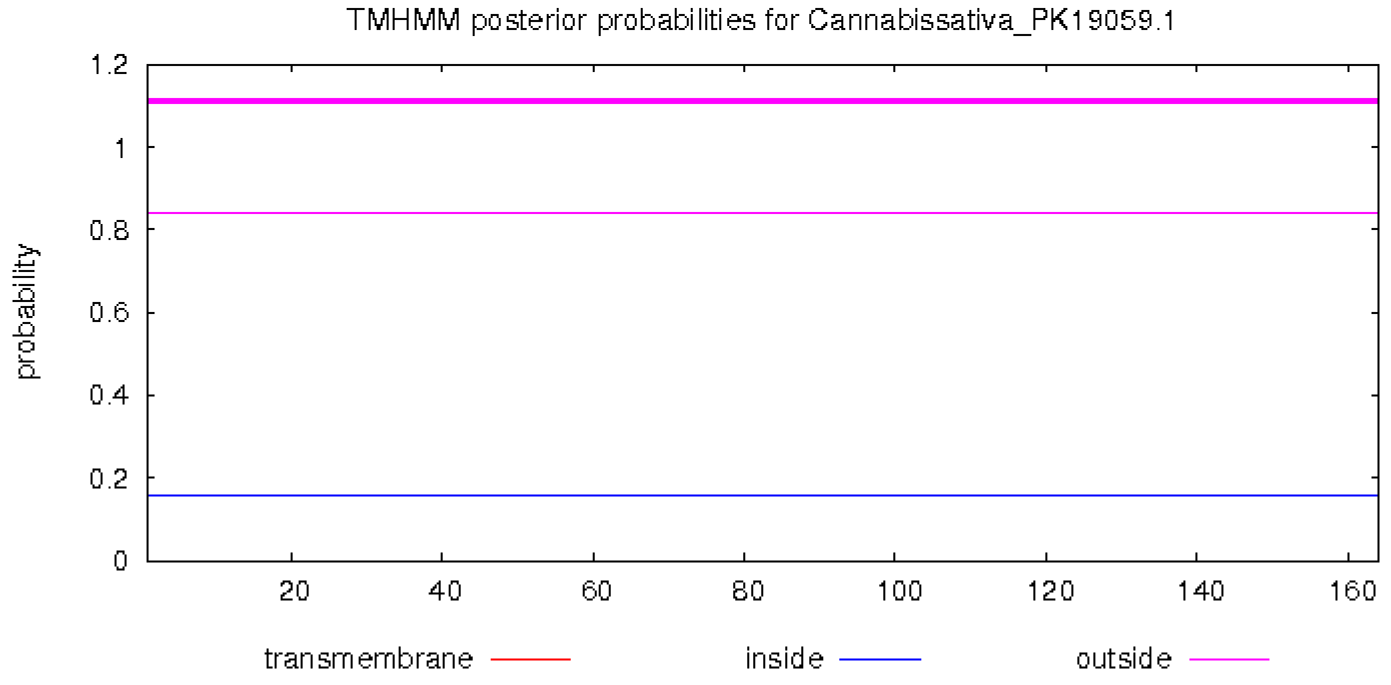

plot in postscript, script for making the plot in gnuplot, data for plot

```
# Cannabissativa_PK19195.1 Length: 275
# Cannabissativa_PK19195.1 Number of predicted TMHs:  0
# Cannabissativa_PK19195.1 Exp number of AAs in TMHs: 0.00366
# Cannabissativa_PK19195.1 Exp number, first 60 AAs:  0.00238
```

```
# Cannabissativa_PK19195.1 Total prob of N-in:        0.01100
Cannabissativa_PK19195.1        TMHMM2.0        outside      1   275
```

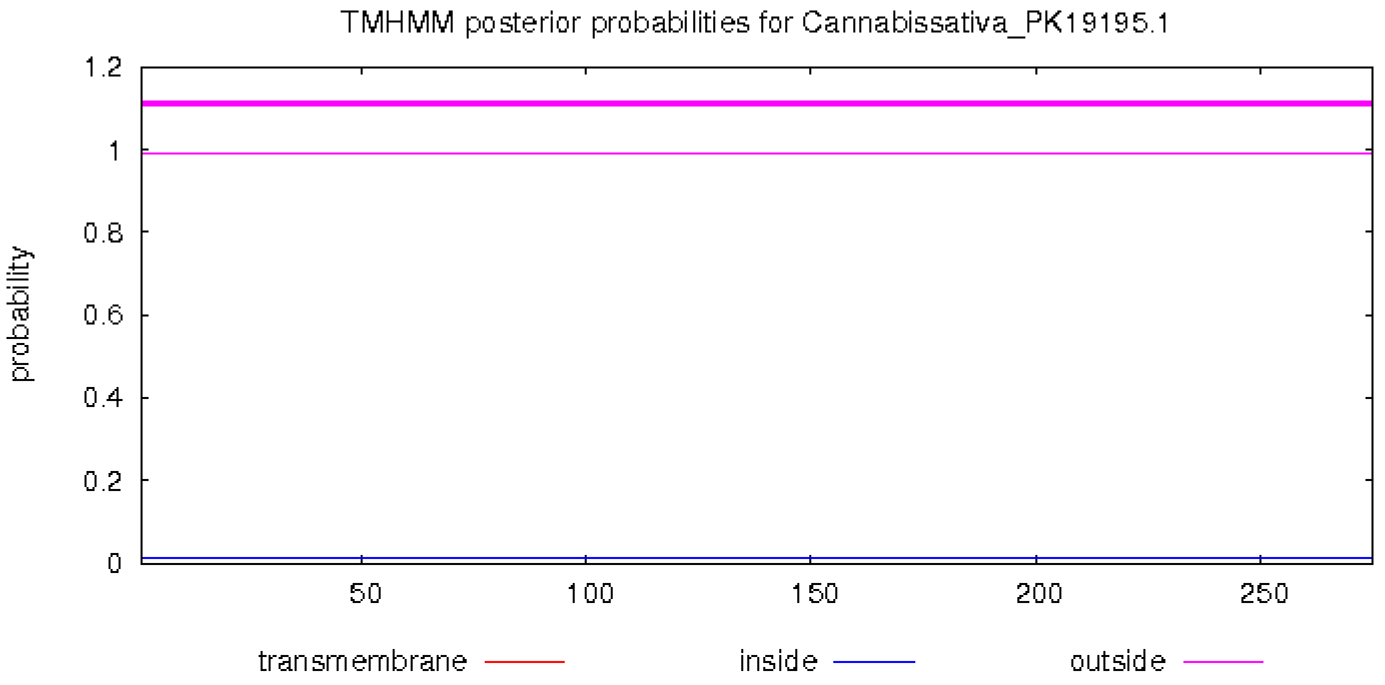

plot in postscript, script for making the plot in gnuplot, data for plot

```
# Cannabissativa_PK19314.1 Length: 729
# Cannabissativa_PK19314.1 Number of predicted TMHs:  0
# Cannabissativa_PK19314.1 Exp number of AAs in TMHs: 27.13438
# Cannabissativa_PK19314.1 Exp number, first 60 AAs:  0
# Cannabissativa_PK19314.1 Total prob of N-in:        0.00011
Cannabissativa_PK19314.1        TMHMM2.0        outside      1   729
```

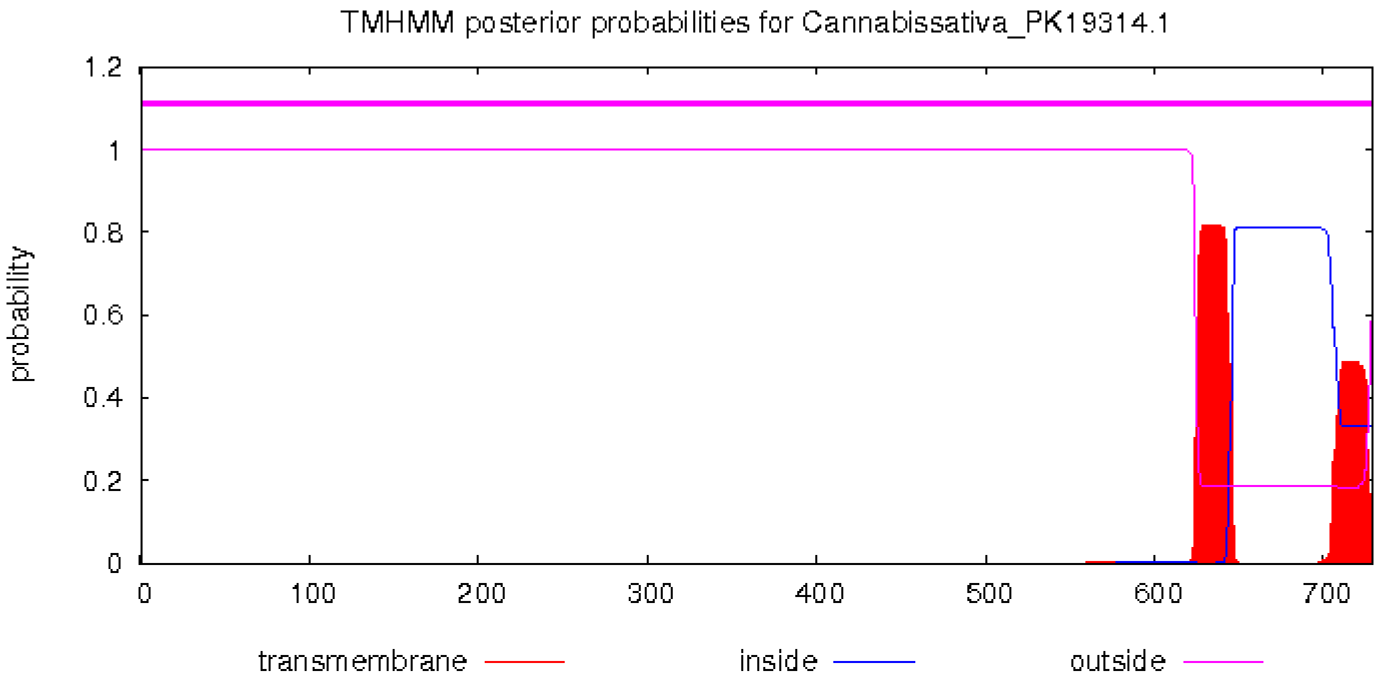

plot in postscript, script for making the plot in gnuplot, data for plot

```
# Cannabissativa_PK20135.1 Length: 352
# Cannabissativa_PK20135.1 Number of predicted TMHs:  0
# Cannabissativa_PK20135.1 Exp number of AAs in TMHs: 0.00023
# Cannabissativa_PK20135.1 Exp number, first 60 AAs:  0
```

```
# Cannabissativa_PK20135.1 Total prob of N-in:        0.00214
Cannabissativa_PK20135.1        TMHMM2.0        outside      1   352
```

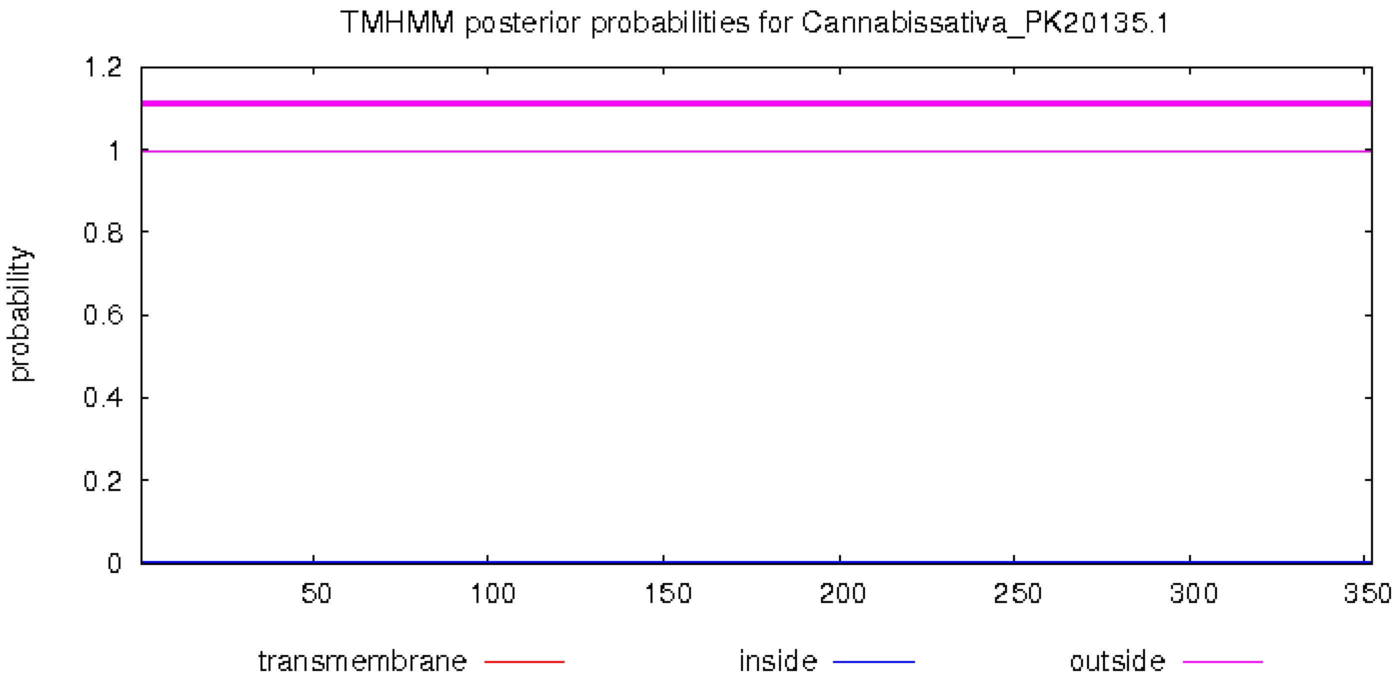

plot in postscript, script for making the plot in gnuplot, data for plot

---

```
# Cannabissativa_PK20262.1 Length: 193
# Cannabissativa_PK20262.1 Number of predicted TMHs:  0
# Cannabissativa_PK20262.1 Exp number of AAs in TMHs: 0.00251
# Cannabissativa_PK20262.1 Exp number, first 60 AAs:  0.00102
# Cannabissativa_PK20262.1 Total prob of N-in:        0.41445
Cannabissativa_PK20262.1        TMHMM2.0        outside      1   193
```

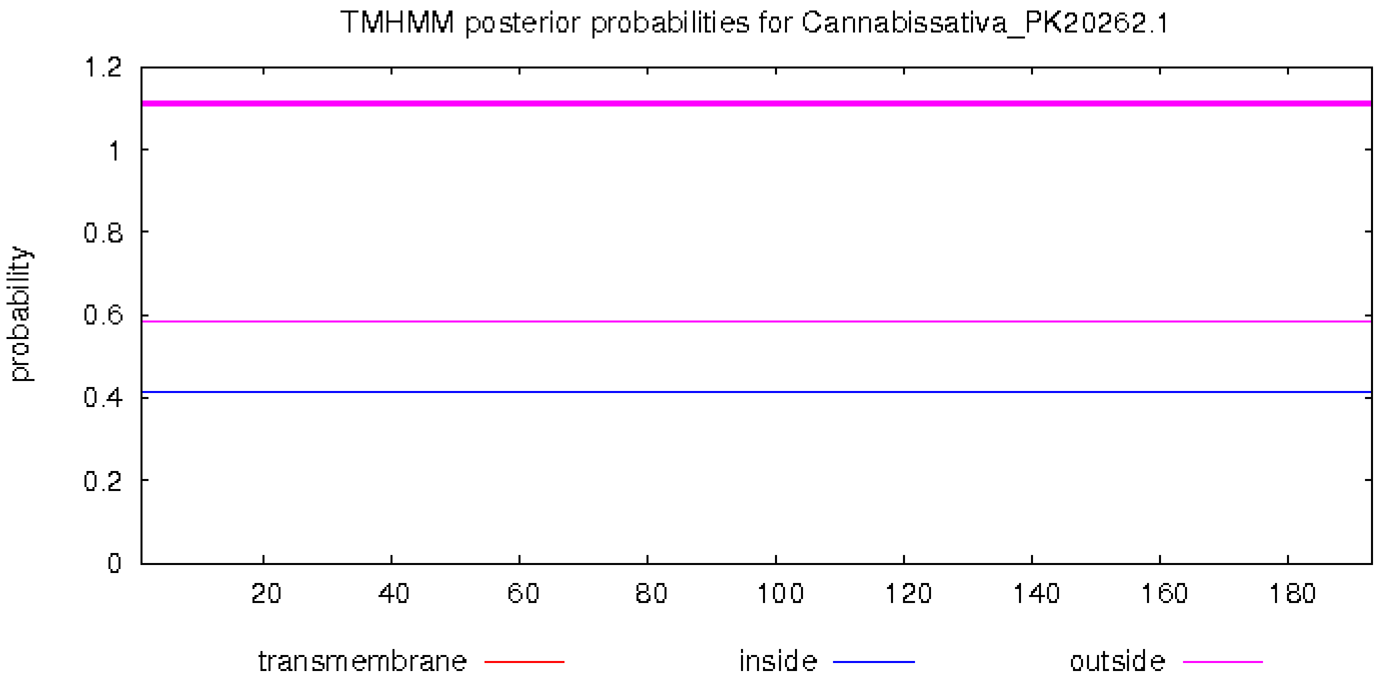

plot in postscript, script for making the plot in gnuplot, data for plot

---

```
# Cannabissativa_PK22182.1 Length: 483
# Cannabissativa_PK22182.1 Number of predicted TMHs:  0
# Cannabissativa_PK22182.1 Exp number of AAs in TMHs: 0
# Cannabissativa_PK22182.1 Exp number, first 60 AAs:  0
```

```
# Cannabissativa_PK22182.1 Total prob of N-in:        0.00264
Cannabissativa_PK22182.1        TMHMM2.0        outside      1   483
```

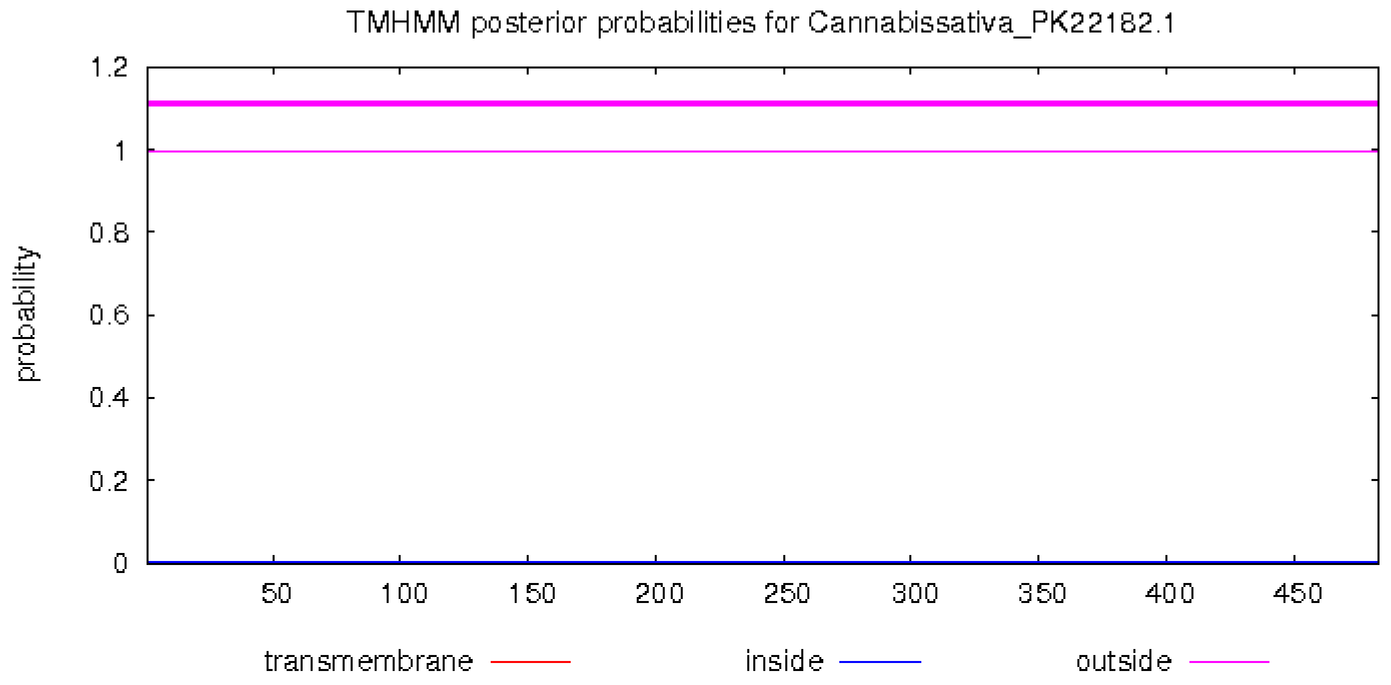

plot in postscript, script for making the plot in gnuplot, data for plot

---

```
# Cannabissativa_PK22221.1 Length: 74
# Cannabissativa_PK22221.1 Number of predicted TMHs:  0
# Cannabissativa_PK22221.1 Exp number of AAs in TMHs: 0
# Cannabissativa_PK22221.1 Exp number, first 60 AAs:  0
# Cannabissativa_PK22221.1 Total prob of N-in:        0.16181
Cannabissativa_PK22221.1        TMHMM2.0        outside      1    74
```

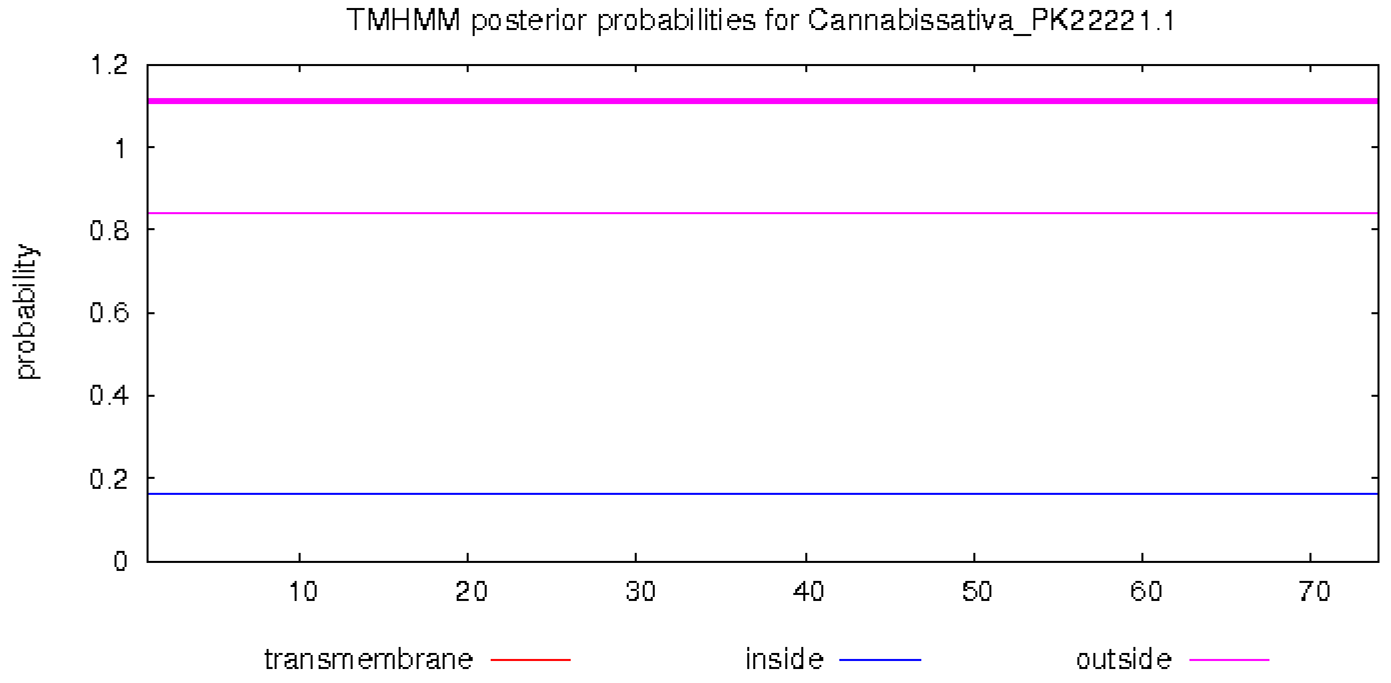

plot in postscript, script for making the plot in gnuplot, data for plot

---

```
# Cannabissativa_PK22704.1 Length: 126
# Cannabissativa_PK22704.1 Number of predicted TMHs:  0
# Cannabissativa_PK22704.1 Exp number of AAs in TMHs: 9e-05
# Cannabissativa_PK22704.1 Exp number, first 60 AAs:  9e-05
```

```
# Cannabissativa_PK22704.1 Total prob of N-in:        0.17190
Cannabissativa_PK22704.1        TMHMM2.0        outside      1   126
```

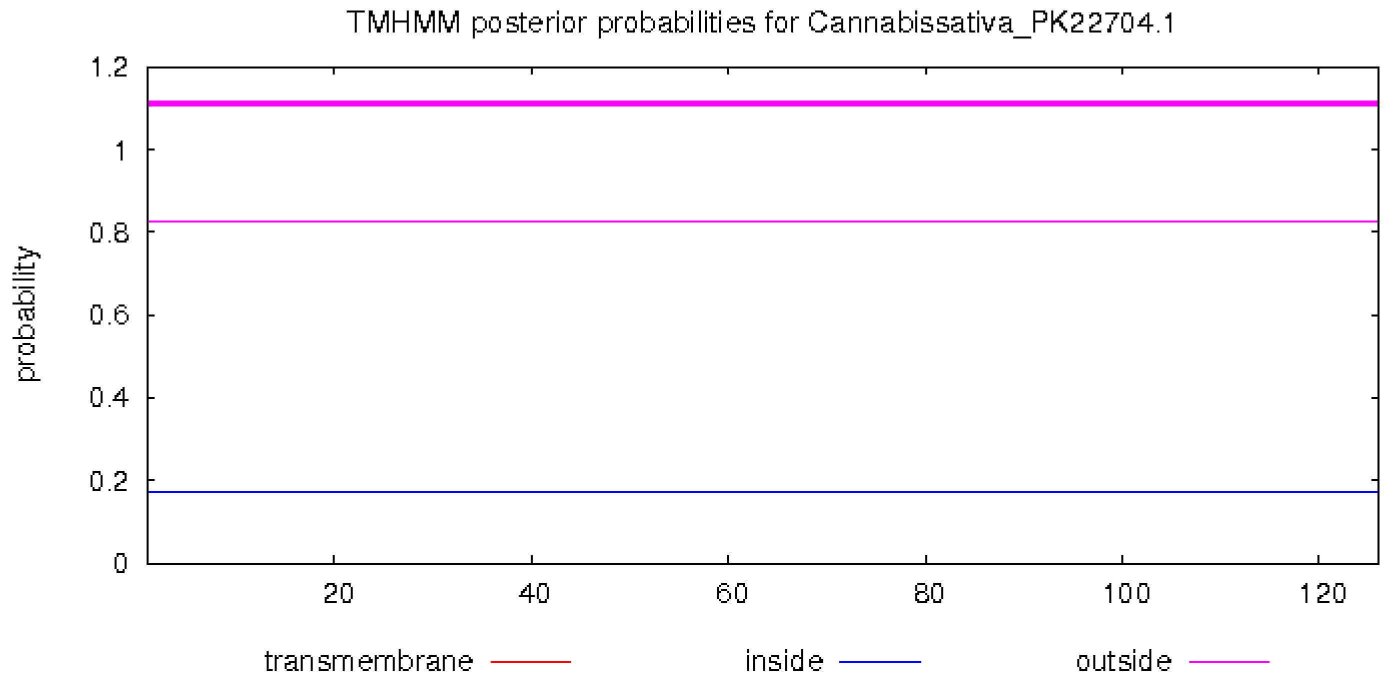

plot in postscript, script for making the plot in gnuplot, data for plot

---

```
# Cannabissativa_PK23050.1 Length: 161
# Cannabissativa_PK23050.1 Number of predicted TMHs:  0
# Cannabissativa_PK23050.1 Exp number of AAs in TMHs: 0.0025
# Cannabissativa_PK23050.1 Exp number, first 60 AAs:  0
# Cannabissativa_PK23050.1 Total prob of N-in:        0.07195
Cannabissativa_PK23050.1        TMHMM2.0        outside      1   161
```

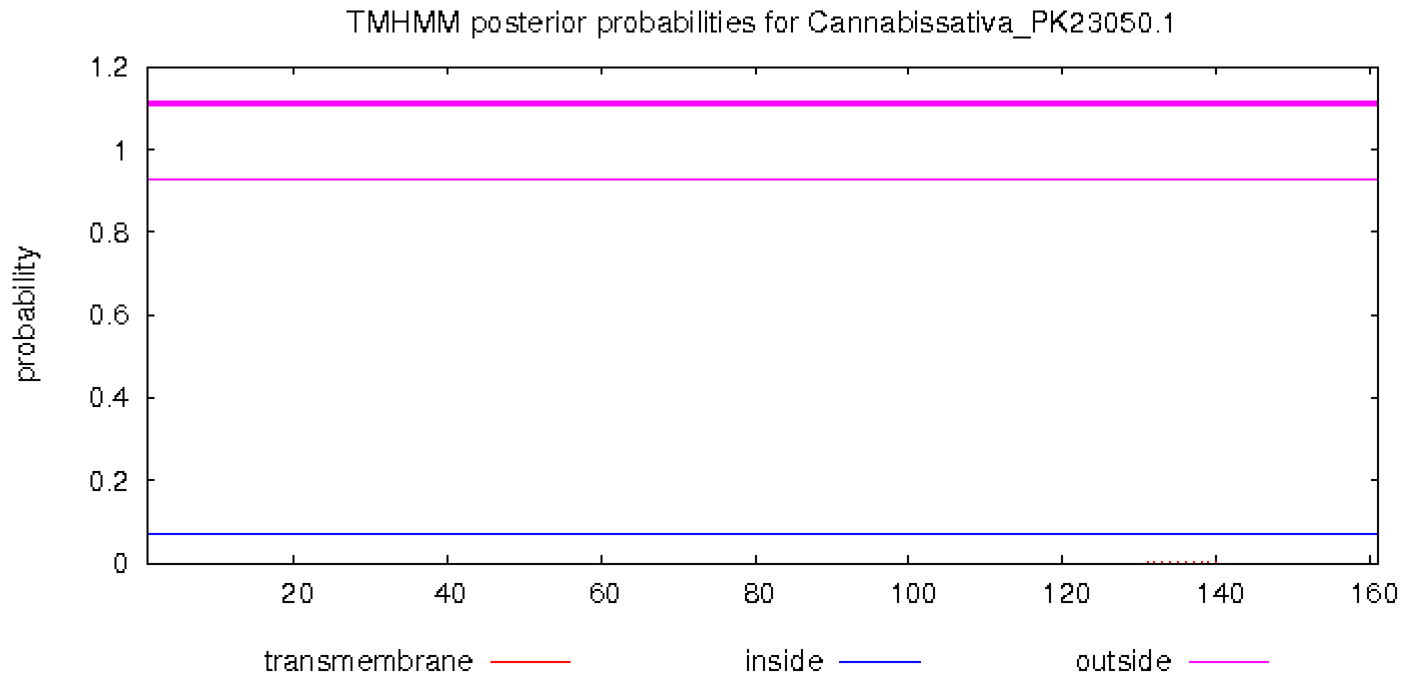

plot in postscript, script for making the plot in gnuplot, data for plot

---

```
# Cannabissativa_PK23416.1 Length: 364
# Cannabissativa_PK23416.1 Number of predicted TMHs:  0
# Cannabissativa_PK23416.1 Exp number of AAs in TMHs: 0.00213
# Cannabissativa_PK23416.1 Exp number, first 60 AAs:  0
```

```
# Cannabissativa_PK23416.1 Total prob of N-in:        0.00342
Cannabissativa_PK23416.1        TMHMM2.0        outside      1   364
```

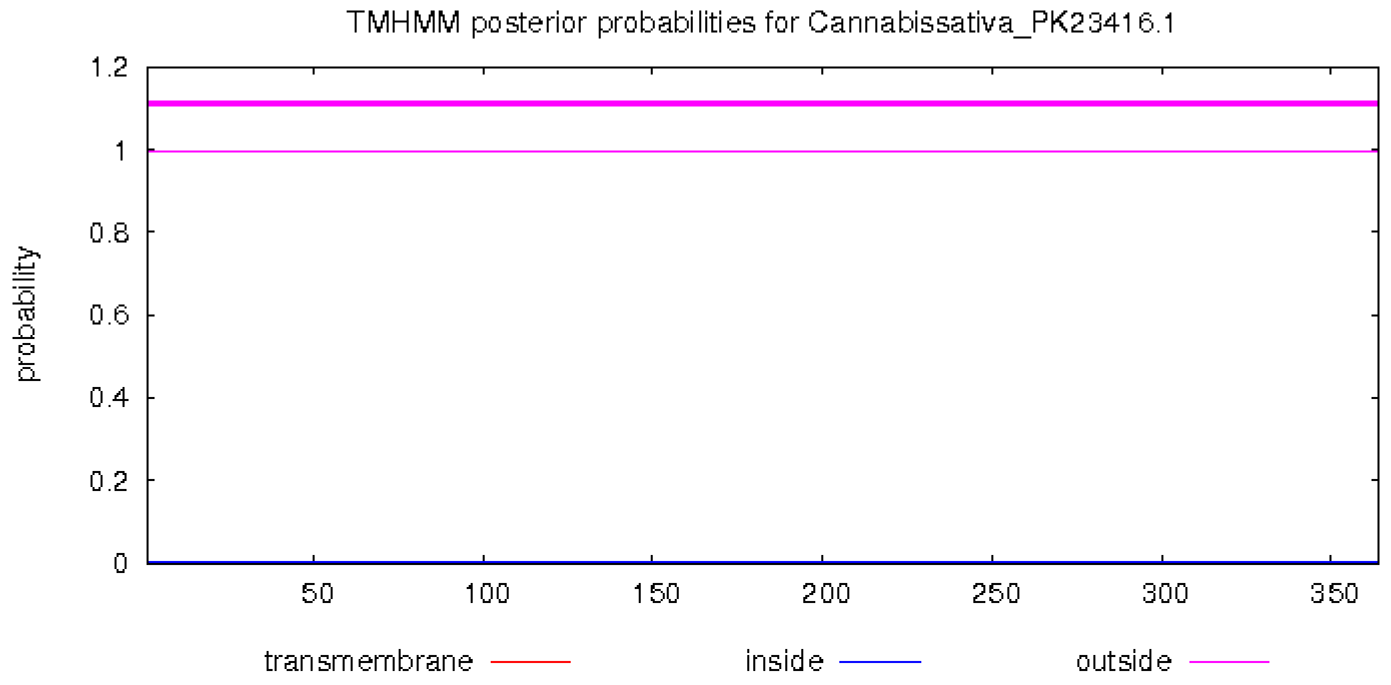

plot in postscript, script for making the plot in gnuplot, data for plot

---

```
# Cannabissativa_PK23481.1 Length: 395
# Cannabissativa_PK23481.1 Number of predicted TMHs:  0
# Cannabissativa_PK23481.1 Exp number of AAs in TMHs: 0
# Cannabissativa_PK23481.1 Exp number, first 60 AAs:  0
# Cannabissativa_PK23481.1 Total prob of N-in:        0.00104
Cannabissativa_PK23481.1        TMHMM2.0        outside      1   395
```

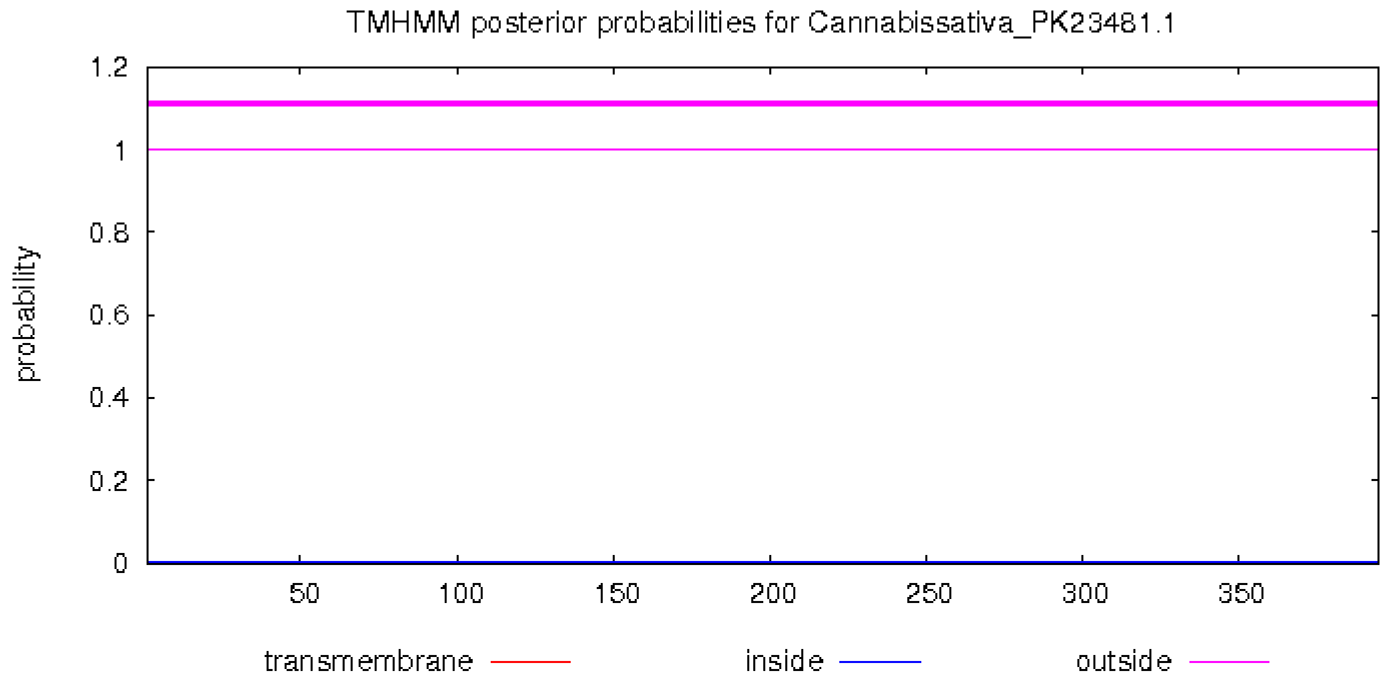

plot in postscript, script for making the plot in gnuplot, data for plot

---

```
# Cannabissativa_PK23488.1 Length: 121
# Cannabissativa_PK23488.1 Number of predicted TMHs:  0
# Cannabissativa_PK23488.1 Exp number of AAs in TMHs: 0.0006
# Cannabissativa_PK23488.1 Exp number, first 60 AAs:  0.0006
```

```
# Cannabissativa_PK23488.1 Total prob of N-in:        0.33970
Cannabissativa_PK23488.1        TMHMM2.0        outside      1   121
```

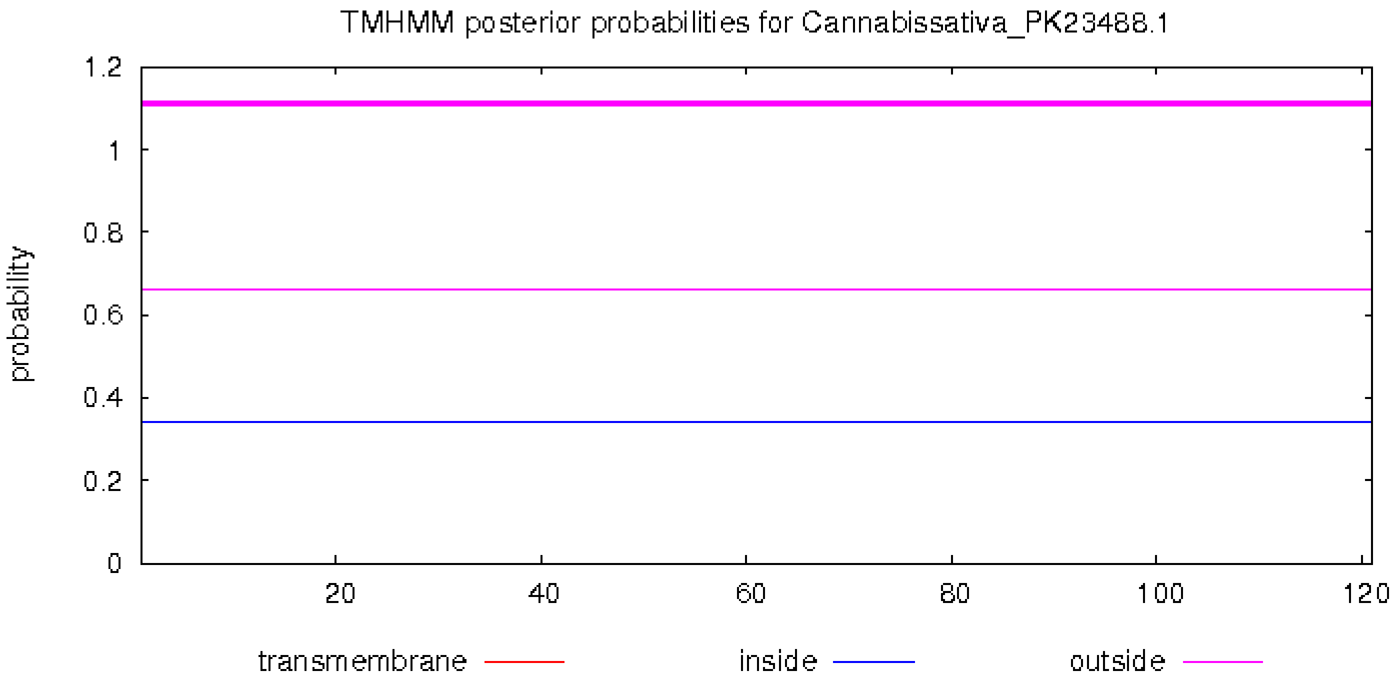

plot in postscript, script for making the plot in gnuplot, data for plot

---

```
# Cannabissativa_PK24240.1 Length: 275
# Cannabissativa_PK24240.1 Number of predicted TMHs:  0
# Cannabissativa_PK24240.1 Exp number of AAs in TMHs: 0.00273
# Cannabissativa_PK24240.1 Exp number, first 60 AAs:  0
# Cannabissativa_PK24240.1 Total prob of N-in:        0.05158
Cannabissativa_PK24240.1        TMHMM2.0        outside      1   275
```

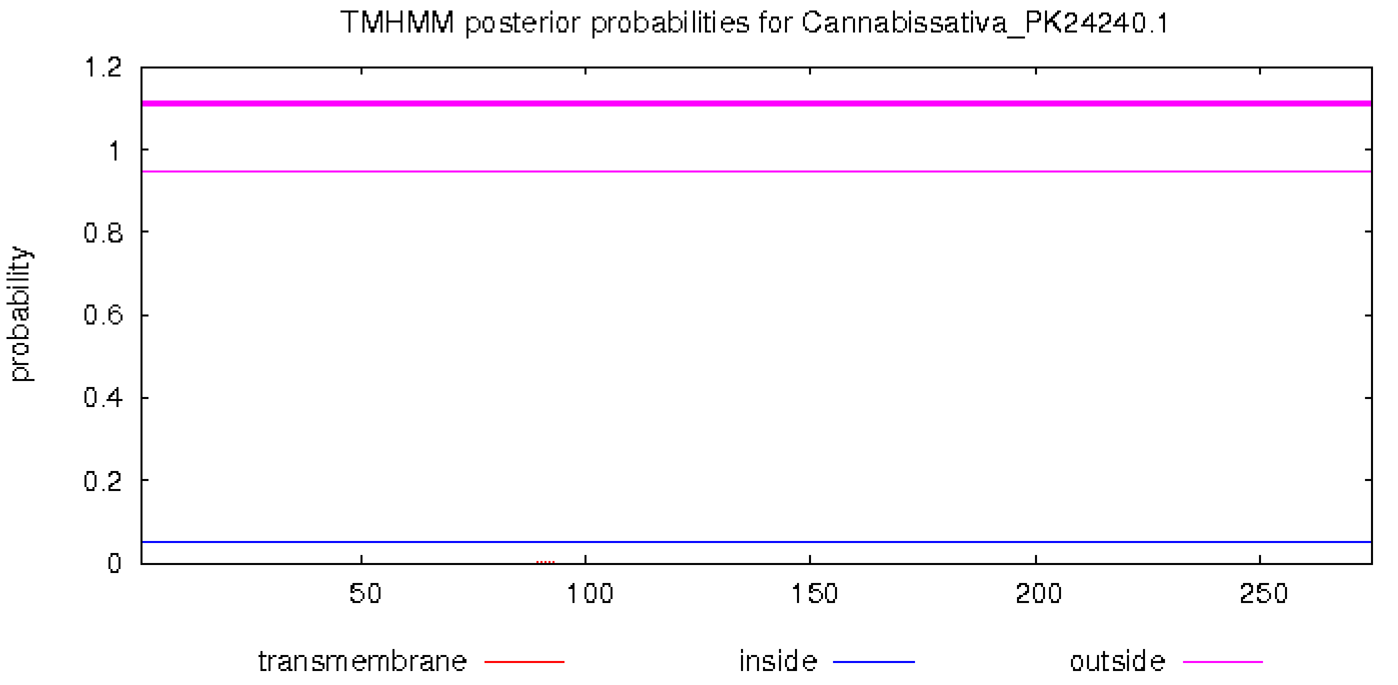

plot in postscript, script for making the plot in gnuplot, data for plot

---

```
# Cannabissativa_PK24620.1 Length: 161
# Cannabissativa_PK24620.1 Number of predicted TMHs:  0
# Cannabissativa_PK24620.1 Exp number of AAs in TMHs: 0.00796
# Cannabissativa_PK24620.1 Exp number, first 60 AAs:  0.0009
```

```
# Cannabissativa_PK24620.1 Total prob of N-in:        0.04792
Cannabissativa_PK24620.1        TMHMM2.0        outside      1   161
```

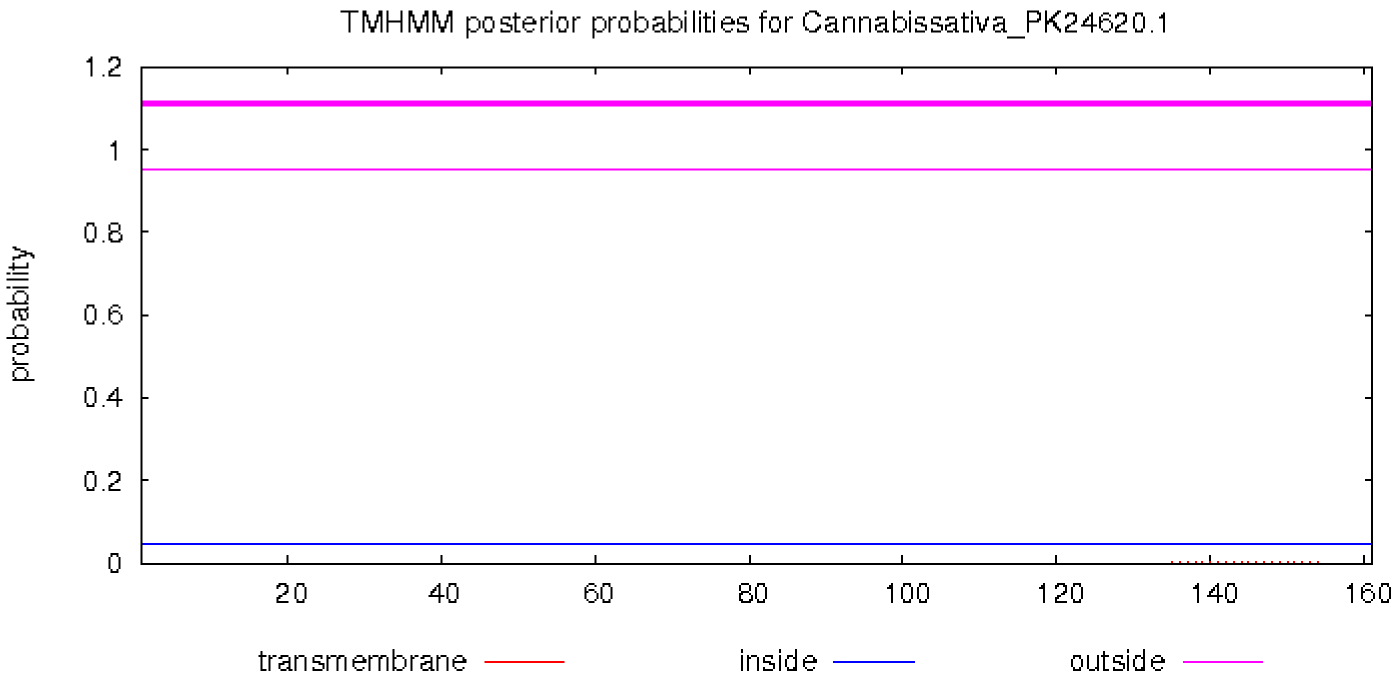

plot in postscript, script for making the plot in gnuplot, data for plot

---

```
# Cannabissativa_PK25119.1 Length: 284
# Cannabissativa_PK25119.1 Number of predicted TMHs:  0
# Cannabissativa_PK25119.1 Exp number of AAs in TMHs: 0.00018
# Cannabissativa_PK25119.1 Exp number, first 60 AAs:  0
# Cannabissativa_PK25119.1 Total prob of N-in:        0.01428
Cannabissativa_PK25119.1        TMHMM2.0        outside      1   284
```

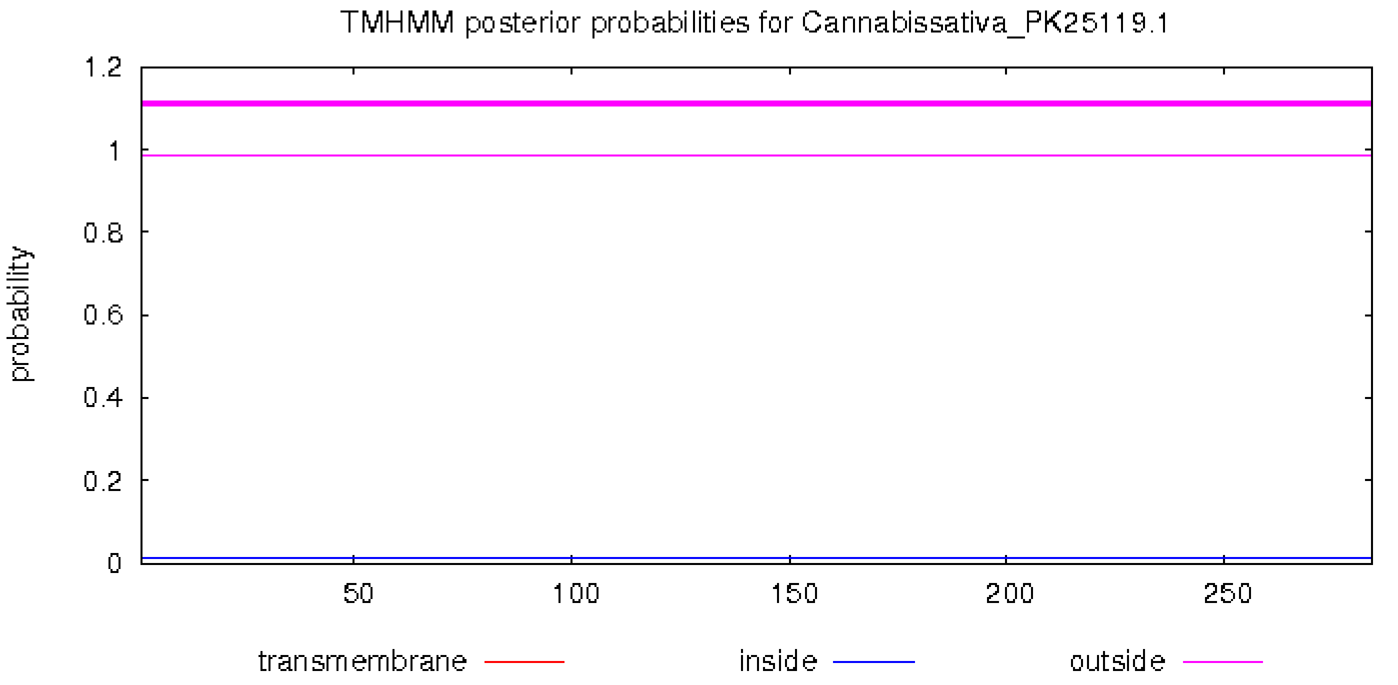

plot in postscript, script for making the plot in gnuplot, data for plot

---

```
# Cannabissativa_PK25206.1 Length: 287
# Cannabissativa_PK25206.1 Number of predicted TMHs:  0
# Cannabissativa_PK25206.1 Exp number of AAs in TMHs: 0.0002
# Cannabissativa_PK25206.1 Exp number, first 60 AAs:  0.0002
```

```
# Cannabissativa_PK25206.1 Total prob of N-in:        0.00250
Cannabissativa_PK25206.1        TMHMM2.0        outside      1   287
```

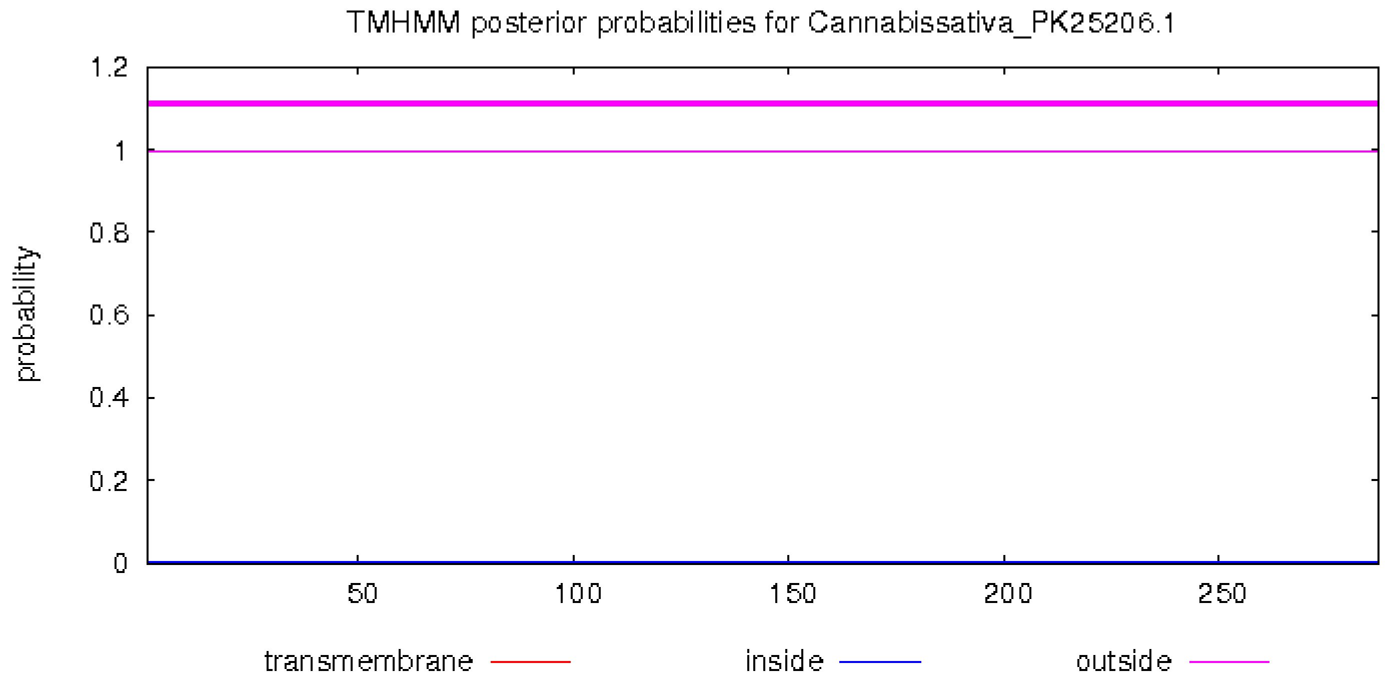

plot in postscript, script for making the plot in gnuplot, data for plot

---

```
# Cannabissativa_PK25436.1 Length: 217
# Cannabissativa_PK25436.1 Number of predicted TMHs:  0
# Cannabissativa_PK25436.1 Exp number of AAs in TMHs: 0.01125
# Cannabissativa_PK25436.1 Exp number, first 60 AAs:  0.01022
# Cannabissativa_PK25436.1 Total prob of N-in:        0.05147
Cannabissativa_PK25436.1        TMHMM2.0        outside      1   217
```

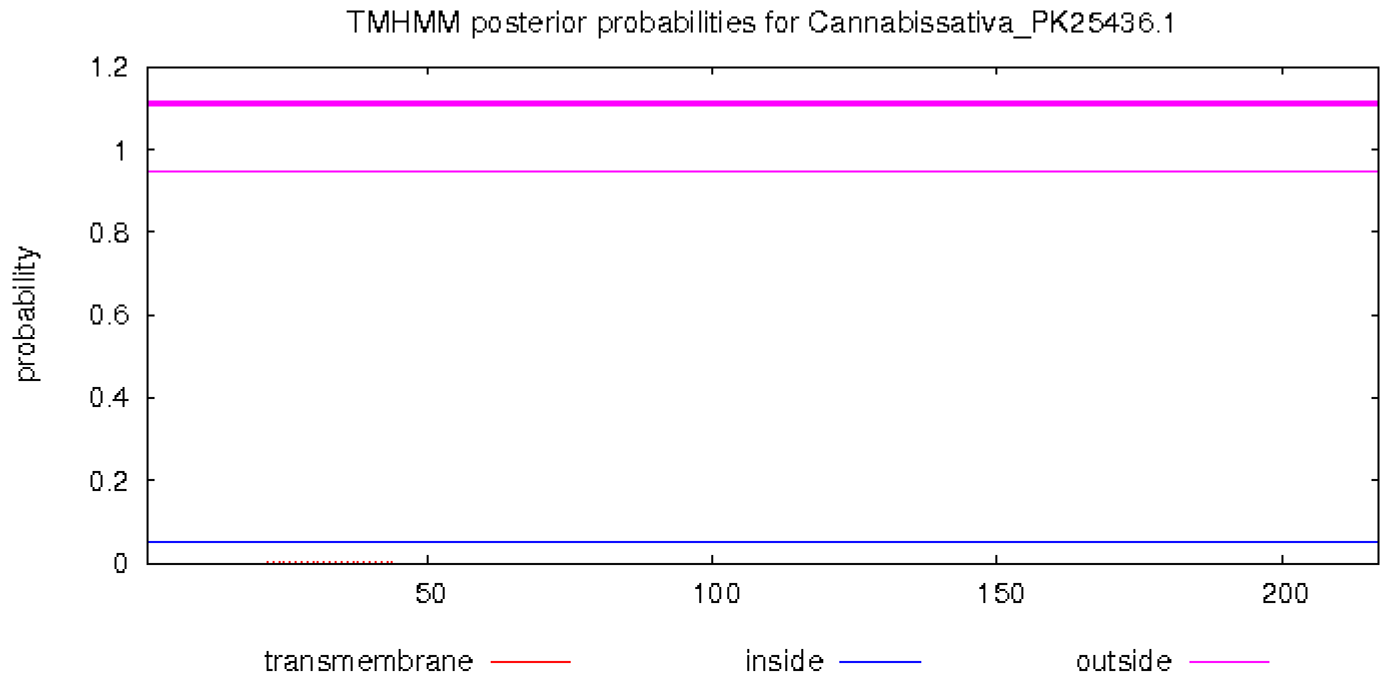

plot in postscript, script for making the plot in gnuplot, data for plot

---

```
# Cannabissativa_PK25812.1 Length: 474
# Cannabissativa_PK25812.1 Number of predicted TMHs:  0
# Cannabissativa_PK25812.1 Exp number of AAs in TMHs: 0
# Cannabissativa_PK25812.1 Exp number, first 60 AAs:  0
```

```
# Cannabissativa_PK25812.1 Total prob of N-in:        0.00079
Cannabissativa_PK25812.1        TMHMM2.0        outside      1   474
```

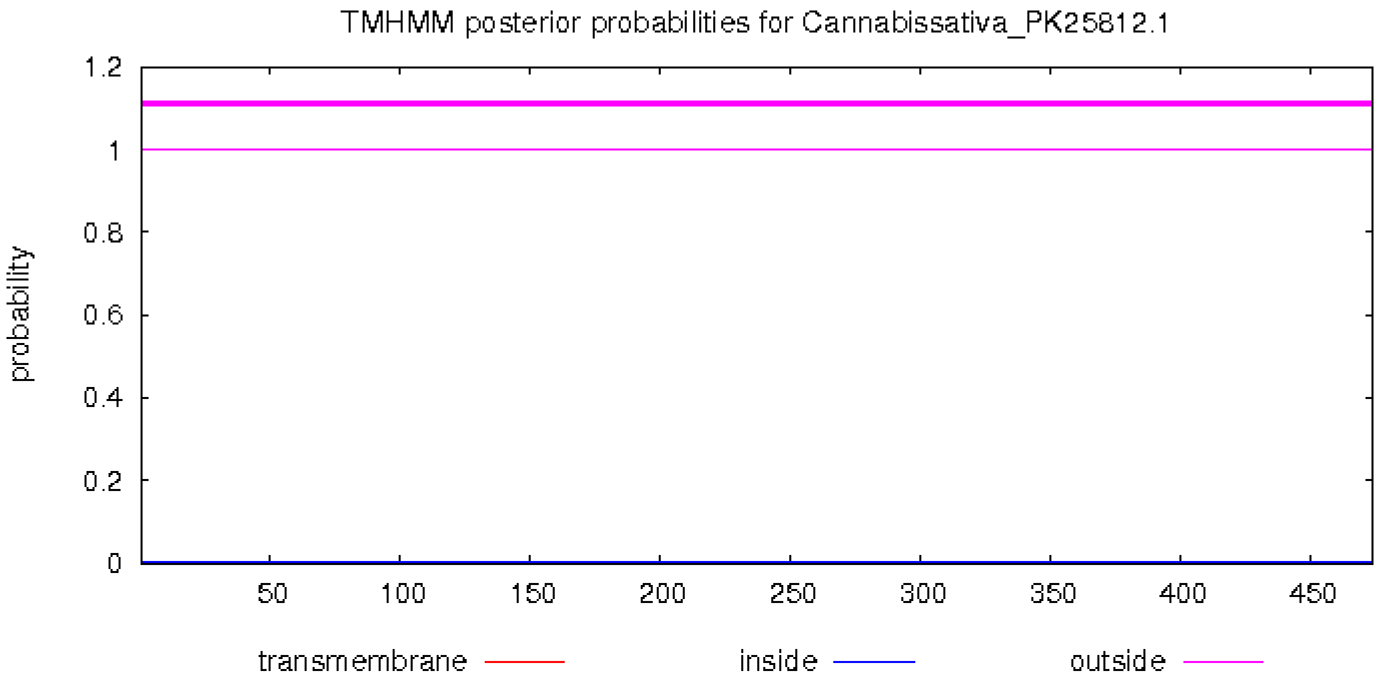

plot in postscript, script for making the plot in gnuplot, data for plot

---

```
# Cannabissativa_PK26157.1 Length: 295
# Cannabissativa_PK26157.1 Number of predicted TMHs:  0
# Cannabissativa_PK26157.1 Exp number of AAs in TMHs: 0
# Cannabissativa_PK26157.1 Exp number, first 60 AAs:  0
# Cannabissativa_PK26157.1 Total prob of N-in:        0.00712
Cannabissativa_PK26157.1        TMHMM2.0        outside      1   295
```

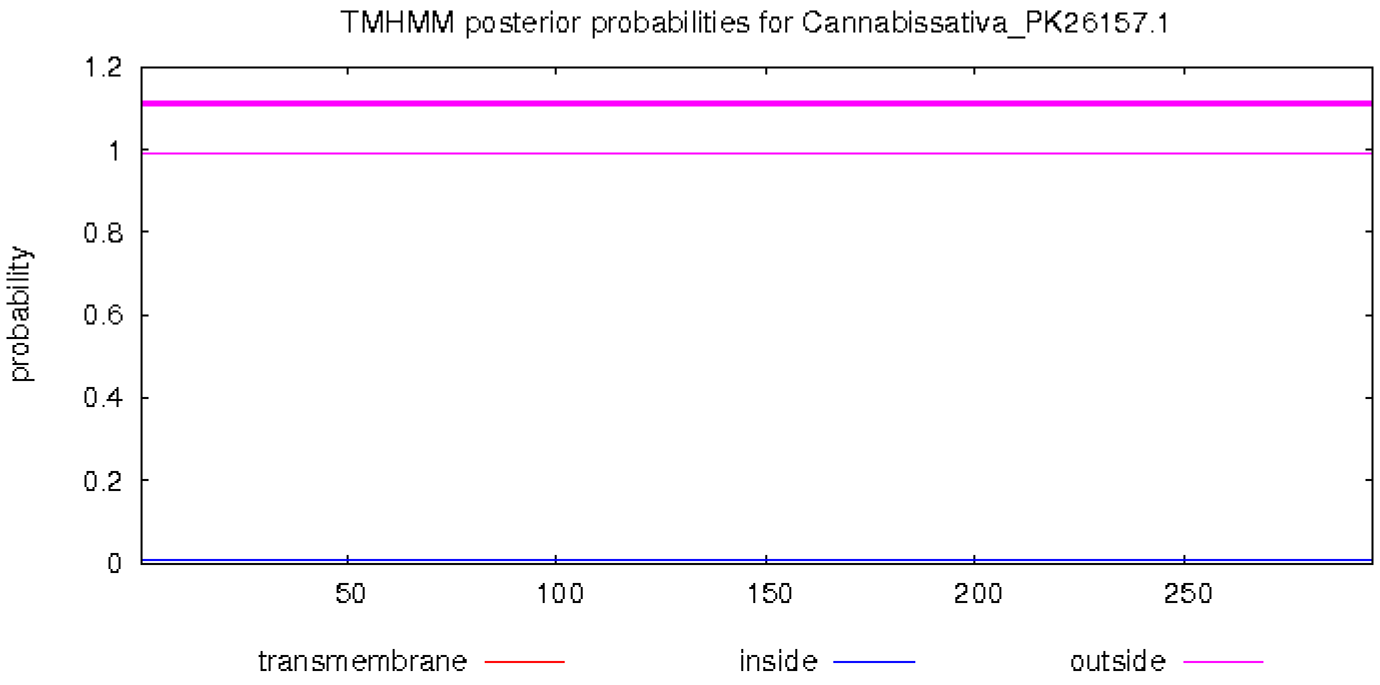

plot in postscript, script for making the plot in gnuplot, data for plot

---

```
# Cannabissativa_PK26618.1 Length: 196
# Cannabissativa_PK26618.1 Number of predicted TMHs:  0
# Cannabissativa_PK26618.1 Exp number of AAs in TMHs: 0.00019
# Cannabissativa_PK26618.1 Exp number, first 60 AAs:  0
```

```
# Cannabissativa_PK26618.1 Total prob of N-in:        0.00545
Cannabissativa_PK26618.1        TMHMM2.0        outside      1   196
```

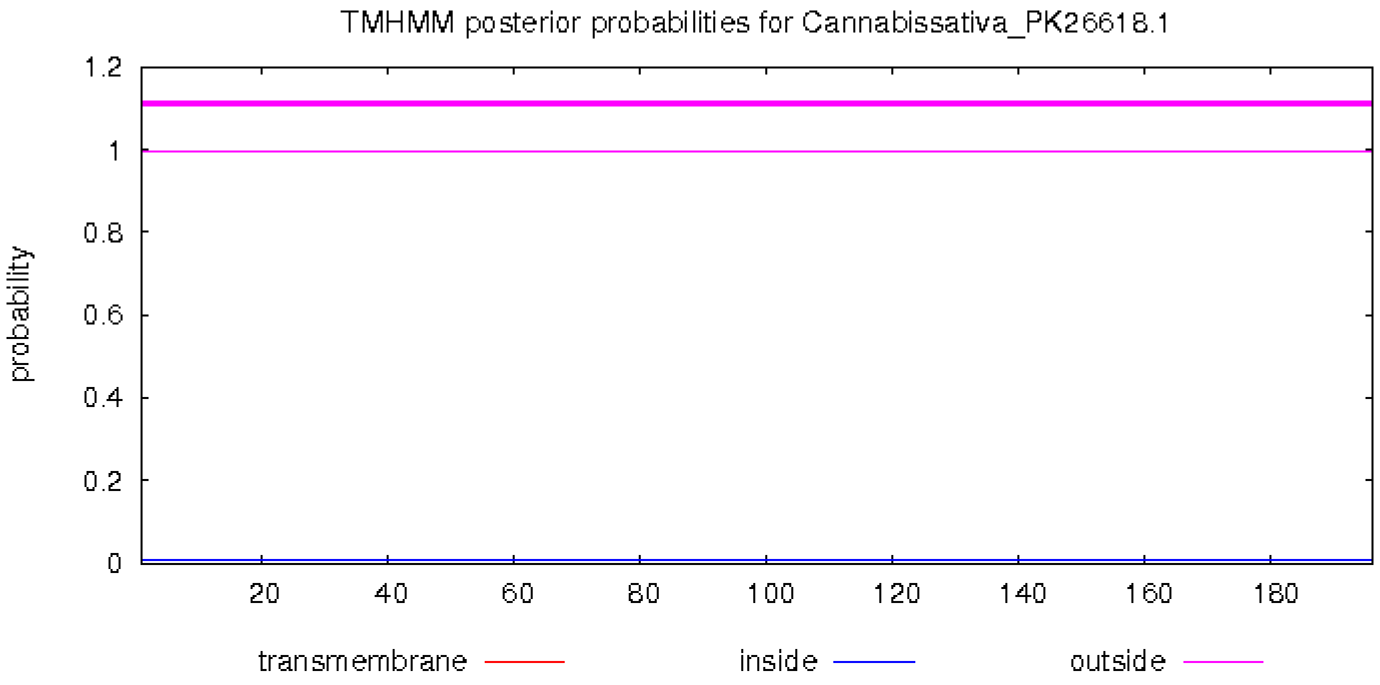

plot in postscript, script for making the plot in gnuplot, data for plot

---

```
# Cannabissativa_PK26741.1 Length: 342
# Cannabissativa_PK26741.1 Number of predicted TMHs:  0
# Cannabissativa_PK26741.1 Exp number of AAs in TMHs: 0.00039
# Cannabissativa_PK26741.1 Exp number, first 60 AAs:  0
# Cannabissativa_PK26741.1 Total prob of N-in:        0.03756
Cannabissativa_PK26741.1        TMHMM2.0        outside      1   342
```

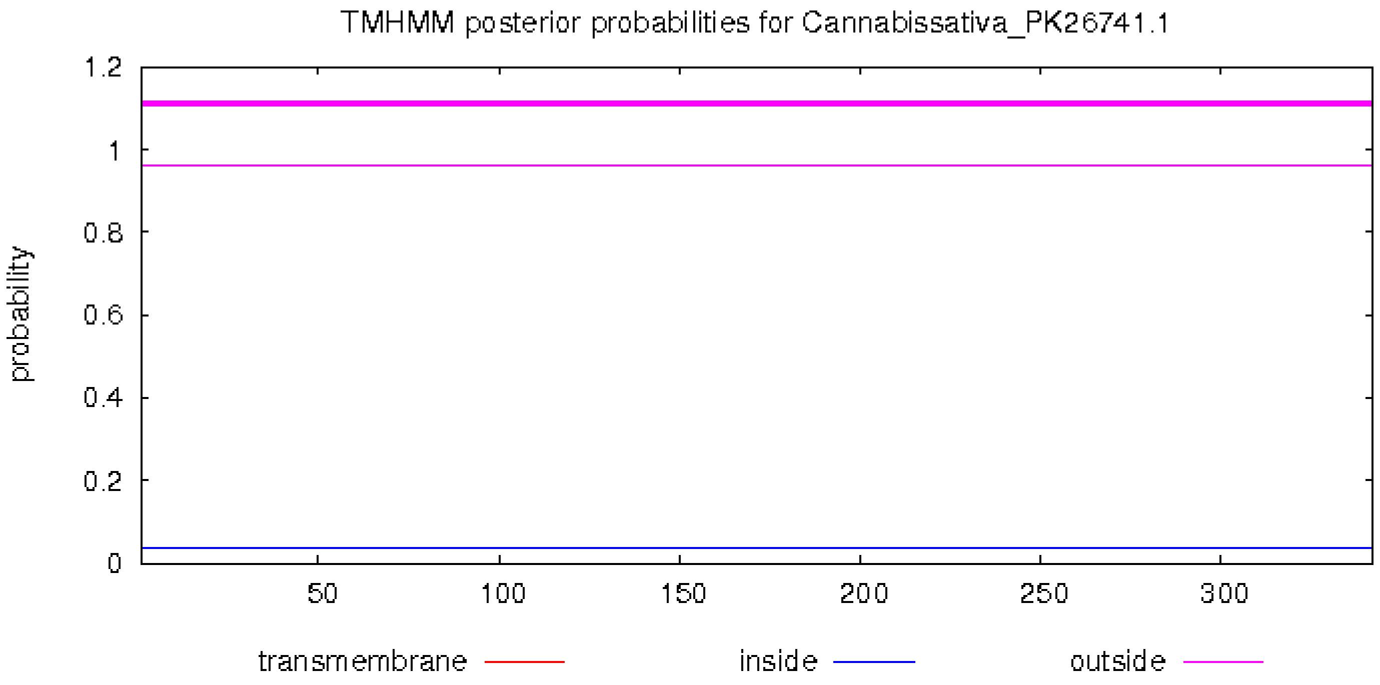

plot in postscript, script for making the plot in gnuplot, data for plot

---

```
# Cannabissativa_PK27085.1 Length: 295
# Cannabissativa_PK27085.1 Number of predicted TMHs:  0
# Cannabissativa_PK27085.1 Exp number of AAs in TMHs: 0.00392
# Cannabissativa_PK27085.1 Exp number, first 60 AAs:  0
```

```
# Cannabissativa_PK27085.1 Total prob of N-in:        0.06528
Cannabissativa_PK27085.1        TMHMM2.0        outside      1   295
```

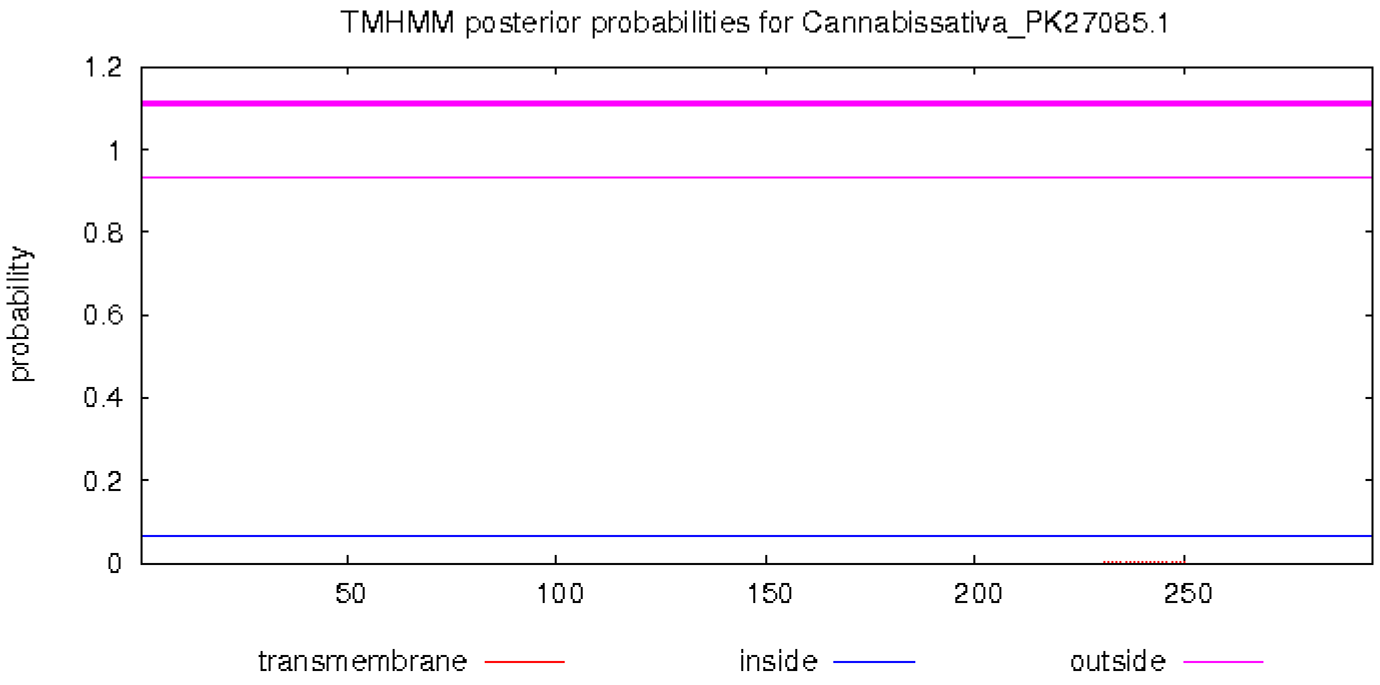

plot in postscript, script for making the plot in gnuplot, data for plot

```
# Cannabissativa_PK27925.1 Length: 310
# Cannabissativa_PK27925.1 Number of predicted TMHs:  0
# Cannabissativa_PK27925.1 Exp number of AAs in TMHs: 0.75749
# Cannabissativa_PK27925.1 Exp number, first 60 AAs:  0
# Cannabissativa_PK27925.1 Total prob of N-in:        0.08472
Cannabissativa_PK27925.1        TMHMM2.0        outside      1   310
```

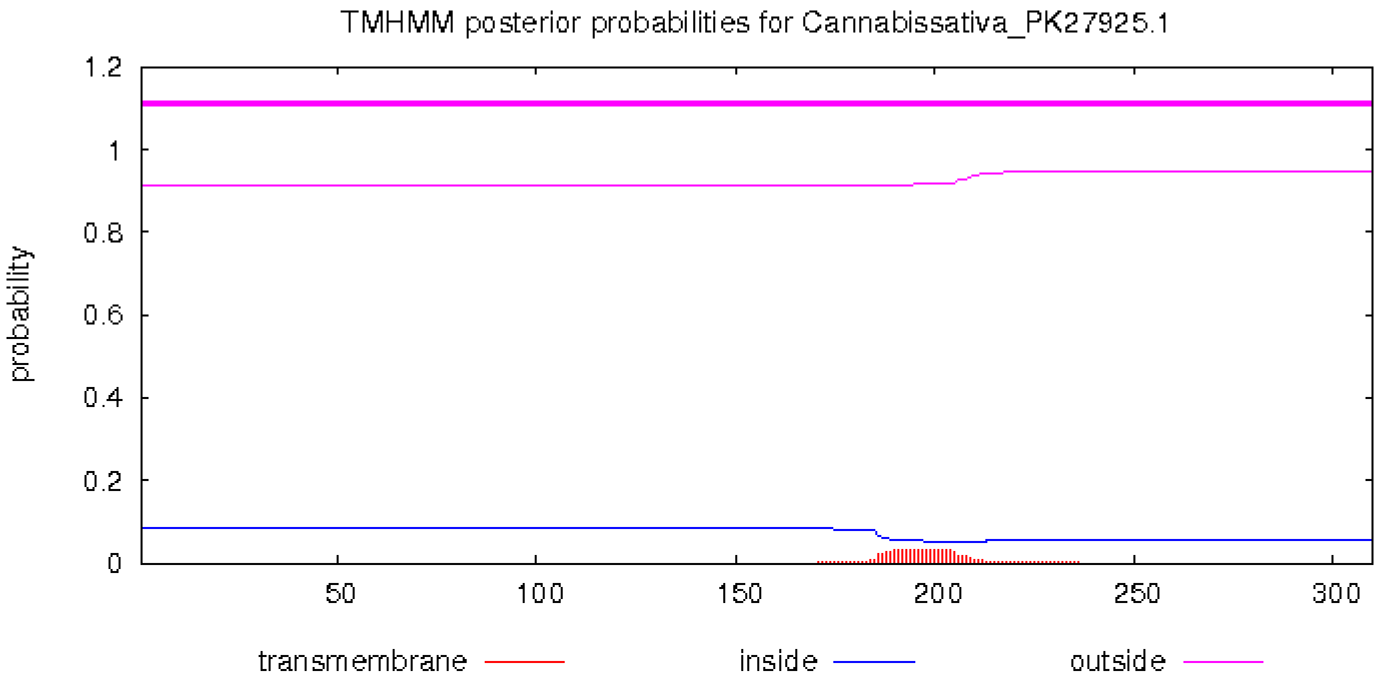

plot in postscript, script for making the plot in gnuplot, data for plot

```
# Cannabissativa_PK28347.1 Length: 269
# Cannabissativa_PK28347.1 Number of predicted TMHs:  0
# Cannabissativa_PK28347.1 Exp number of AAs in TMHs: 0.00148
# Cannabissativa_PK28347.1 Exp number, first 60 AAs:  0
```

```
# Cannabissativa_PK28347.1 Total prob of N-in:        0.09631
Cannabissativa_PK28347.1        TMHMM2.0        outside      1   269
```

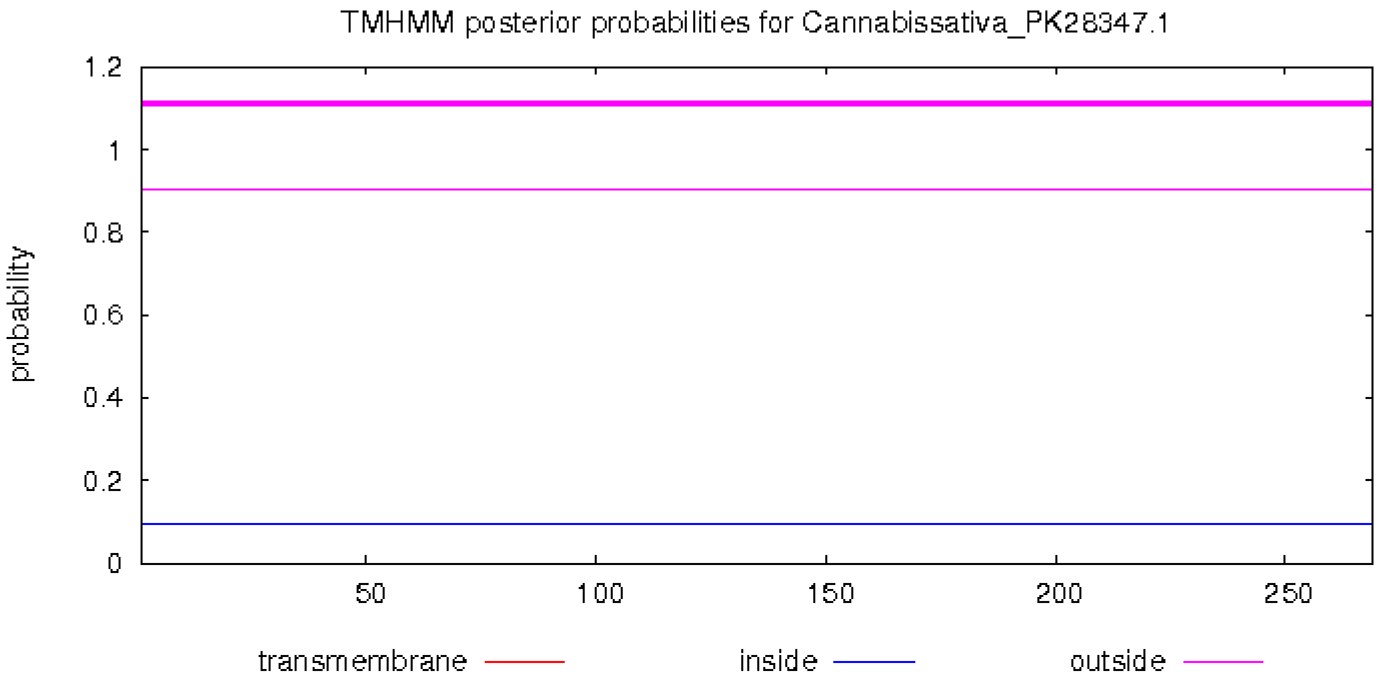

plot in postscript, script for making the plot in gnuplot, data for plot

```
# Cannabissativa_PK28510.1 Length: 407
# Cannabissativa_PK28510.1 Number of predicted TMHs:  0
# Cannabissativa_PK28510.1 Exp number of AAs in TMHs: 0.00023
# Cannabissativa_PK28510.1 Exp number, first 60 AAs:  0
# Cannabissativa_PK28510.1 Total prob of N-in:        0.00154
Cannabissativa_PK28510.1        TMHMM2.0        outside      1   407
```

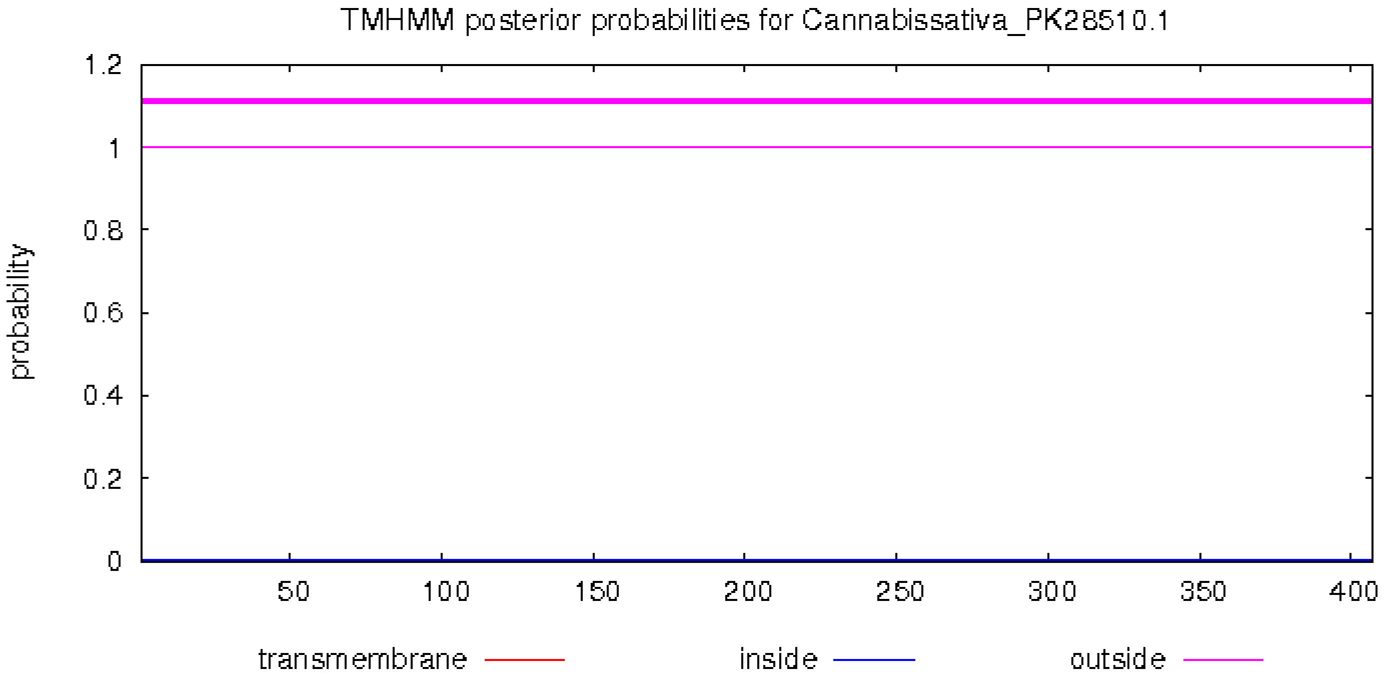

plot in postscript, script for making the plot in gnuplot, data for plot

```
# Cannabissativa_PK29014.1 Length: 231
# Cannabissativa_PK29014.1 Number of predicted TMHs:  0
# Cannabissativa_PK29014.1 Exp number of AAs in TMHs: 0.00037
# Cannabissativa_PK29014.1 Exp number, first 60 AAs:  0.00018
```

```
# Cannabissativa_PK29014.1 Total prob of N-in:        0.04555
Cannabissativa_PK29014.1        TMHMM2.0        outside      1   231
```

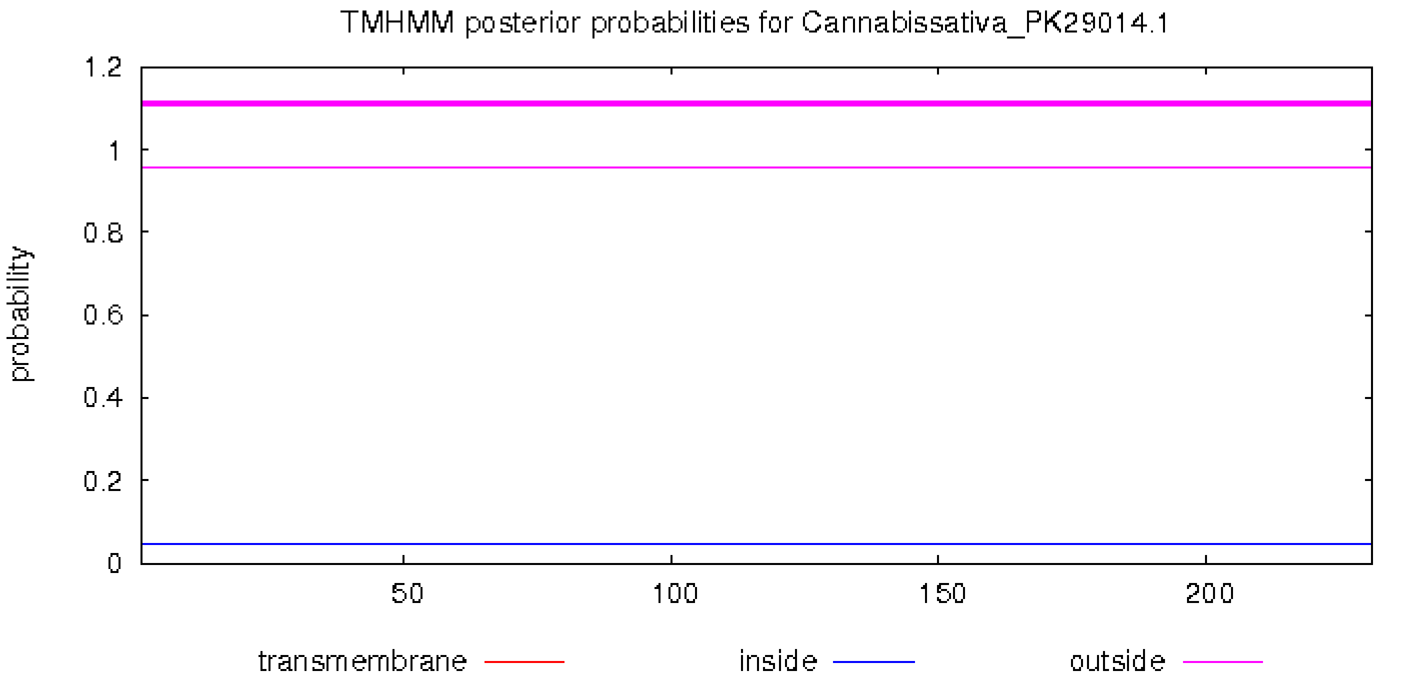

plot in postscript, script for making the plot in gnuplot, data for plot

---

```
# Capsellagrandiflora_Cagra.0007s0060 Length: 300
# Capsellagrandiflora_Cagra.0007s0060 Number of predicted TMHs:  0
# Capsellagrandiflora_Cagra.0007s0060 Exp number of AAs in TMHs: 0.02066
# Capsellagrandiflora_Cagra.0007s0060 Exp number, first 60 AAs:  0.00018
# Capsellagrandiflora_Cagra.0007s0060 Total prob of N-in:        0.01433
Capsellagrandiflora_Cagra.0007s0060     TMHMM2.0        outside      1   300
```

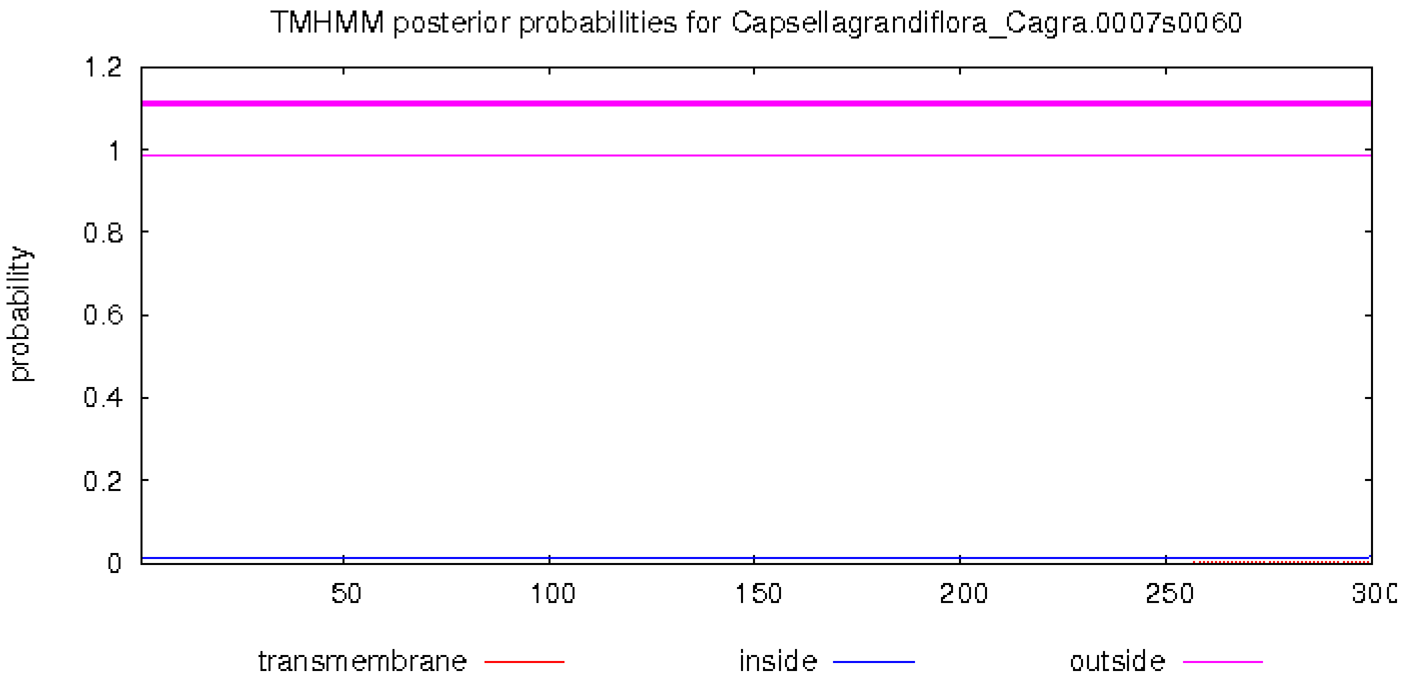

plot in postscript, script for making the plot in gnuplot, data for plot

---

```
# Capsellagrandiflora_Cagra.0007s0097 Length: 318
# Capsellagrandiflora_Cagra.0007s0097 Number of predicted TMHs:  0
# Capsellagrandiflora_Cagra.0007s0097 Exp number of AAs in TMHs: 0
# Capsellagrandiflora_Cagra.0007s0097 Exp number, first 60 AAs:  0
```

```
# Capsellagrandiflora_Cagra.0007s0097 Total prob of N-in:        0.00238
Capsellagrandiflora_Cagra.0007s0097     TMHMM2.0        outside      1   318
```

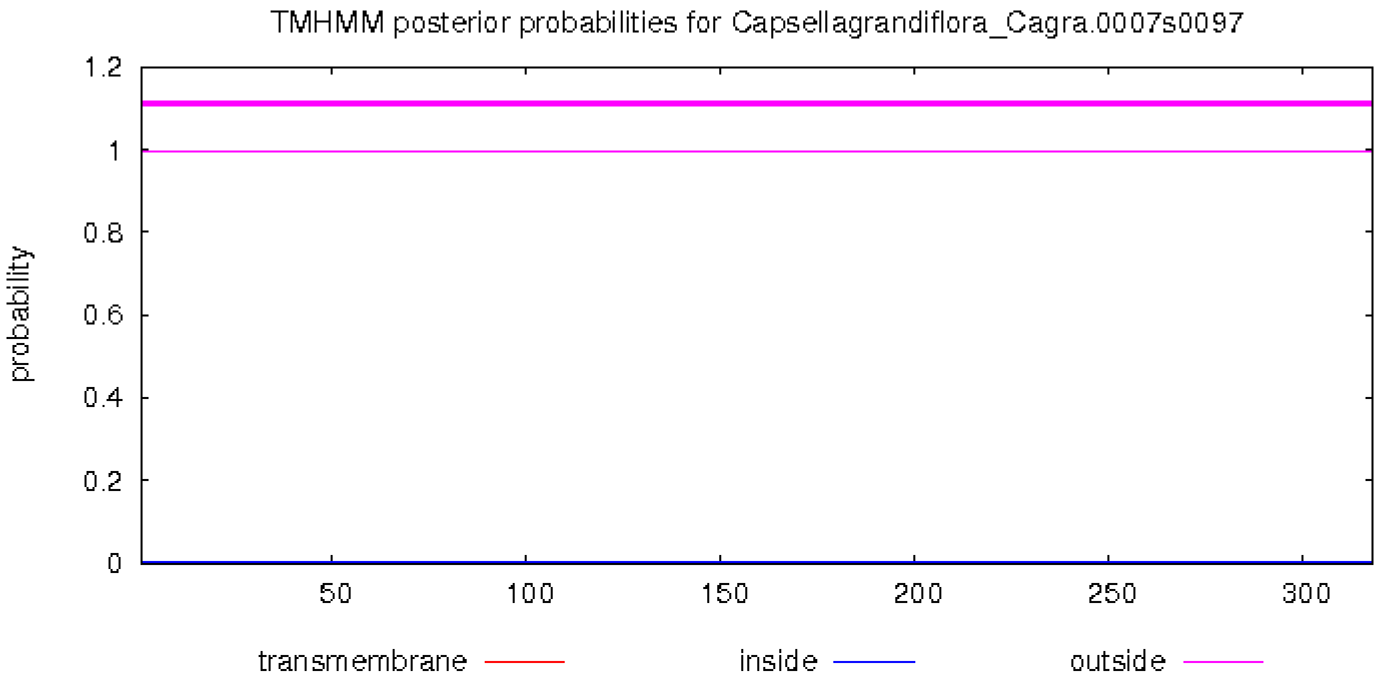

plot in postscript, script for making the plot in gnuplot, data for plot

---

```
# Capsellagrandiflora_Cagra.0050s0024 Length: 358
# Capsellagrandiflora_Cagra.0050s0024 Number of predicted TMHs:  0
# Capsellagrandiflora_Cagra.0050s0024 Exp number of AAs in TMHs: 0.00018
# Capsellagrandiflora_Cagra.0050s0024 Exp number, first 60 AAs:  0
# Capsellagrandiflora_Cagra.0050s0024 Total prob of N-in:        0.01313
Capsellagrandiflora_Cagra.0050s0024     TMHMM2.0        outside      1   358
```

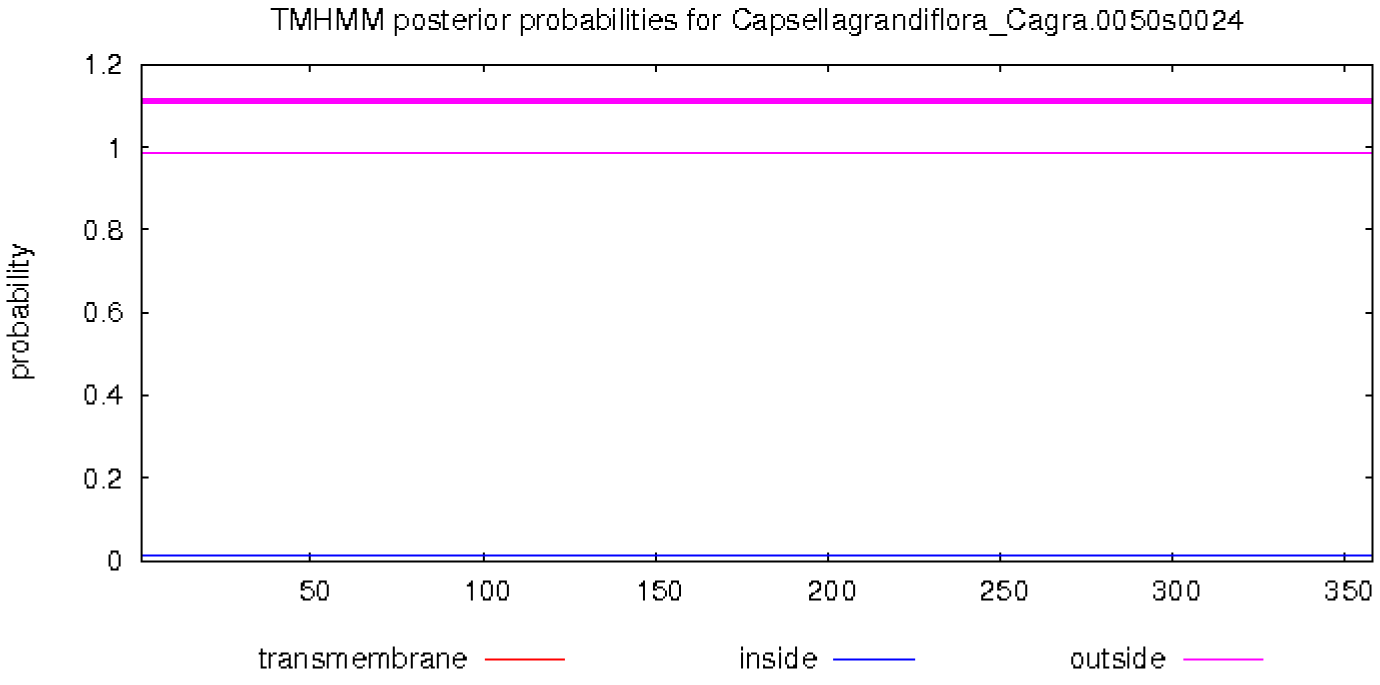

plot in postscript, script for making the plot in gnuplot, data for plot

---

```
# Capsellagrandiflora_Cagra.0096s0087 Length: 255
# Capsellagrandiflora_Cagra.0096s0087 Number of predicted TMHs:  0
# Capsellagrandiflora_Cagra.0096s0087 Exp number of AAs in TMHs: 5e-05
# Capsellagrandiflora_Cagra.0096s0087 Exp number, first 60 AAs:  0
```

```
# Capsellagrandiflora_Cagra.0096s0087 Total prob of N-in:        0.03865
Capsellagrandiflora_Cagra.0096s0087     TMHMM2.0        outside      1   255
```

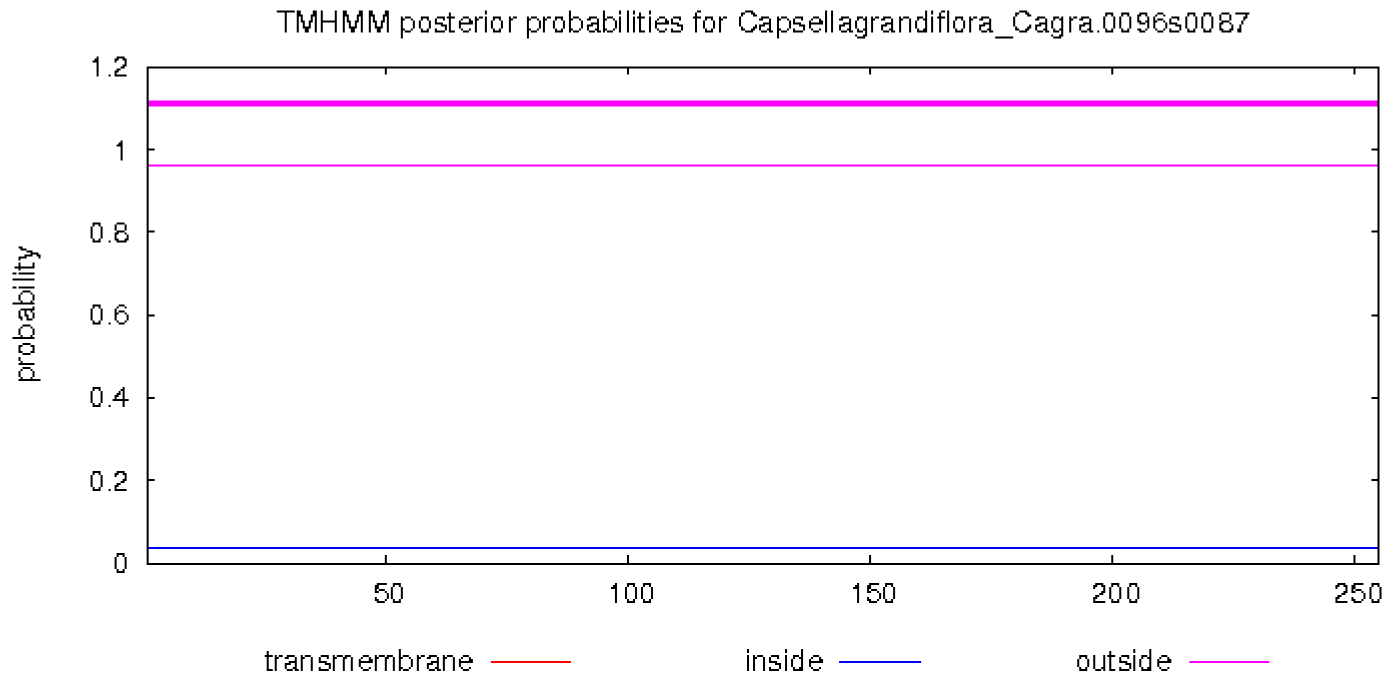

plot in postscript, script for making the plot in gnuplot, data for plot

---

```
# Capsellagrandiflora_Cagra.0202s0012 Length: 278
# Capsellagrandiflora_Cagra.0202s0012 Number of predicted TMHs:  0
# Capsellagrandiflora_Cagra.0202s0012 Exp number of AAs in TMHs: 0.00746
# Capsellagrandiflora_Cagra.0202s0012 Exp number, first 60 AAs:  0
# Capsellagrandiflora_Cagra.0202s0012 Total prob of N-in:        0.02054
Capsellagrandiflora_Cagra.0202s0012     TMHMM2.0        outside      1   278
```

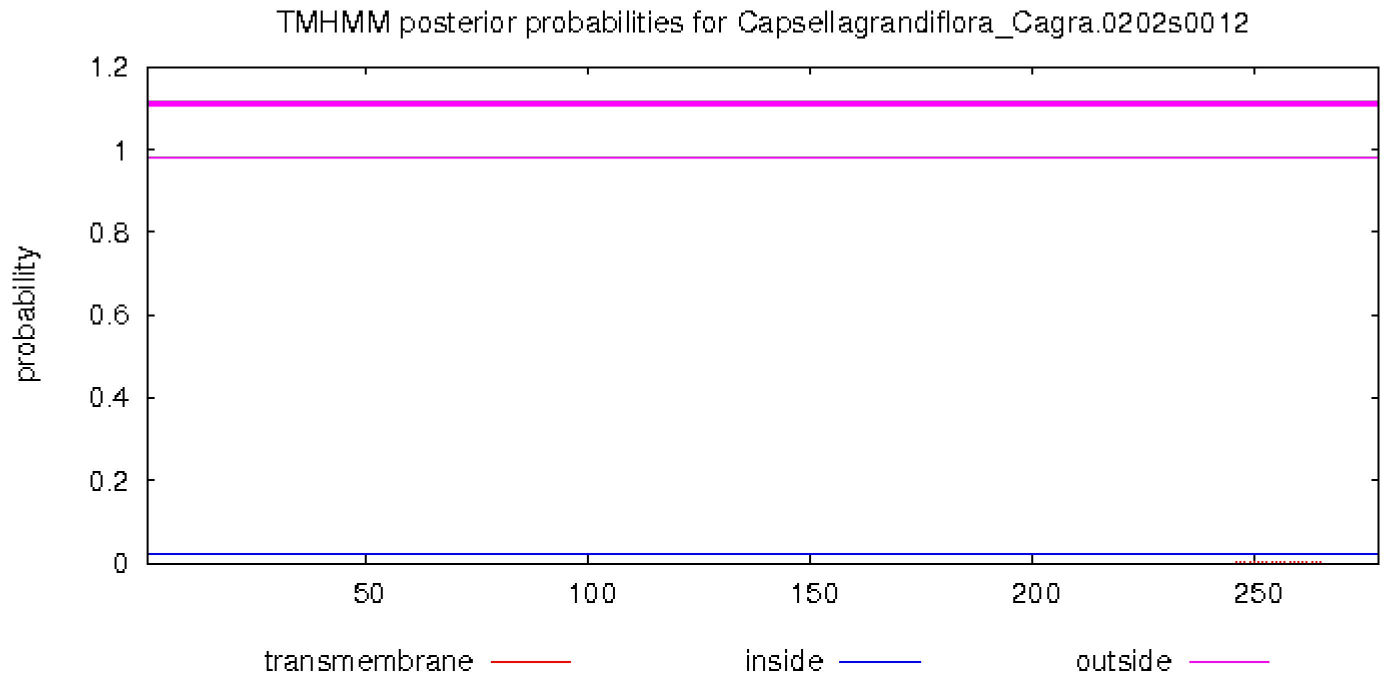

plot in postscript, script for making the plot in gnuplot, data for plot

---

```
# Capsellagrandiflora_Cagra.0248s0059 Length: 270
# Capsellagrandiflora_Cagra.0248s0059 Number of predicted TMHs:  0
# Capsellagrandiflora_Cagra.0248s0059 Exp number of AAs in TMHs: 0.00019
# Capsellagrandiflora_Cagra.0248s0059 Exp number, first 60 AAs:  0
```

```
# Capsellagrandiflora_Cagra.0248s0059 Total prob of N-in:        0.01308
Capsellagrandiflora_Cagra.0248s0059     TMHMM2.0        outside      1   270
```

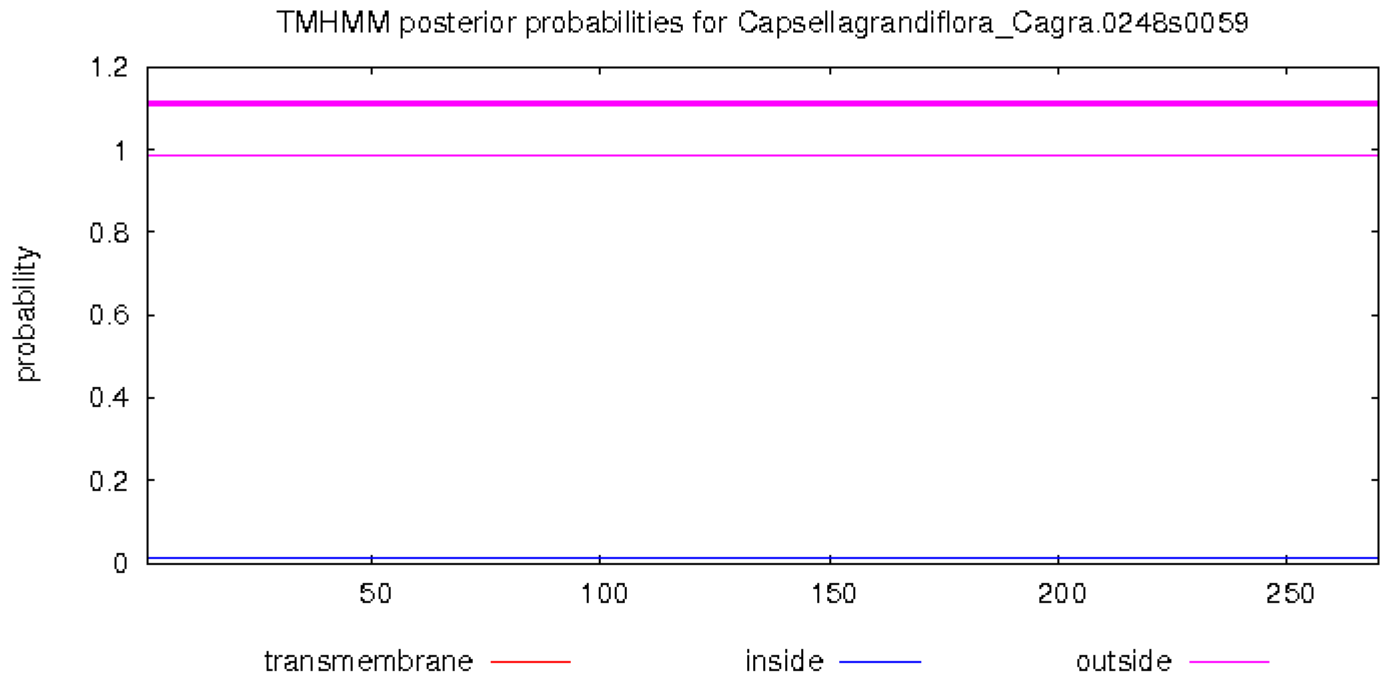

plot in postscript, script for making the plot in gnuplot, data for plot

---

```
# Capsellagrandiflora_Cagra.0248s0087 Length: 364
# Capsellagrandiflora_Cagra.0248s0087 Number of predicted TMHs:  0
# Capsellagrandiflora_Cagra.0248s0087 Exp number of AAs in TMHs: 0.00024
# Capsellagrandiflora_Cagra.0248s0087 Exp number, first 60 AAs:  0.00018
# Capsellagrandiflora_Cagra.0248s0087 Total prob of N-in:        0.00410
Capsellagrandiflora_Cagra.0248s0087     TMHMM2.0        outside      1   364
```

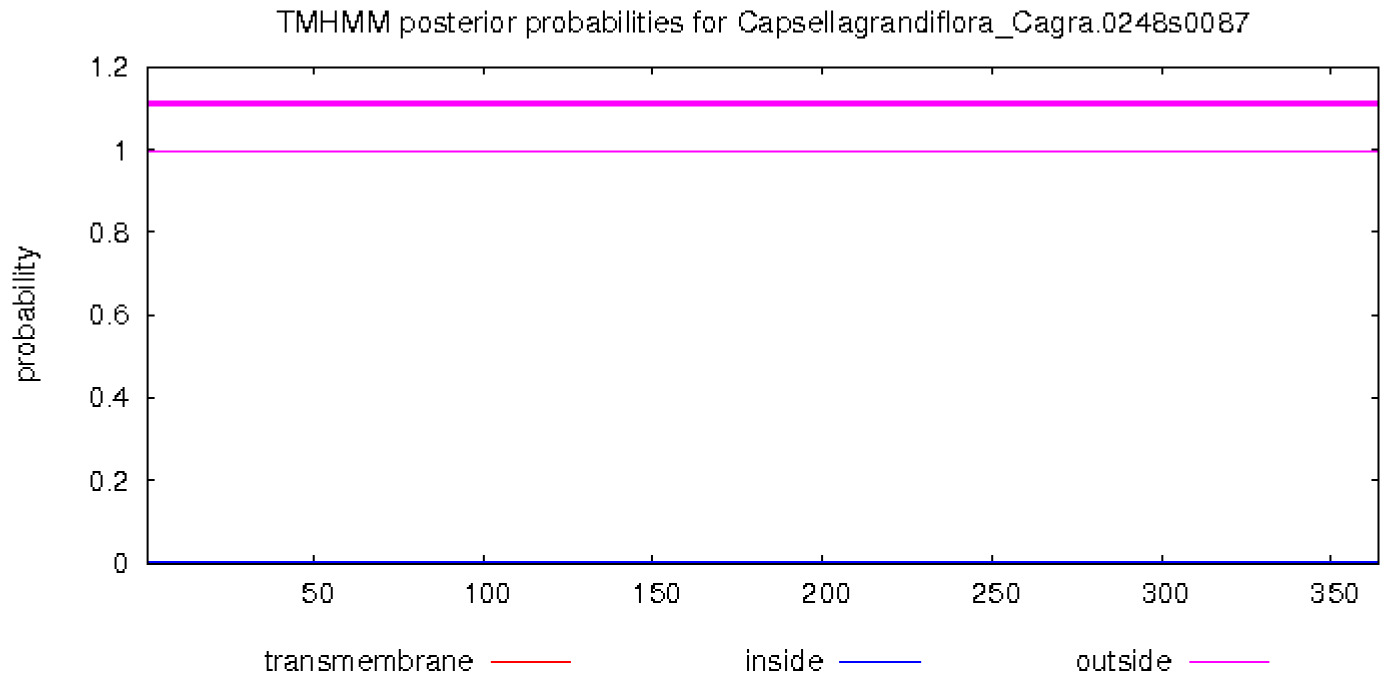

plot in postscript, script for making the plot in gnuplot, data for plot

---

```
# Capsellagrandiflora_Cagra.0248s0135 Length: 187
# Capsellagrandiflora_Cagra.0248s0135 Number of predicted TMHs:  0
# Capsellagrandiflora_Cagra.0248s0135 Exp number of AAs in TMHs: 0
# Capsellagrandiflora_Cagra.0248s0135 Exp number, first 60 AAs:  0
```

```
# Capsellagrandiflora_Cagra.0248s0135 Total prob of N-in:        0.00733
Capsellagrandiflora_Cagra.0248s0135     TMHMM2.0        outside      1   187
```

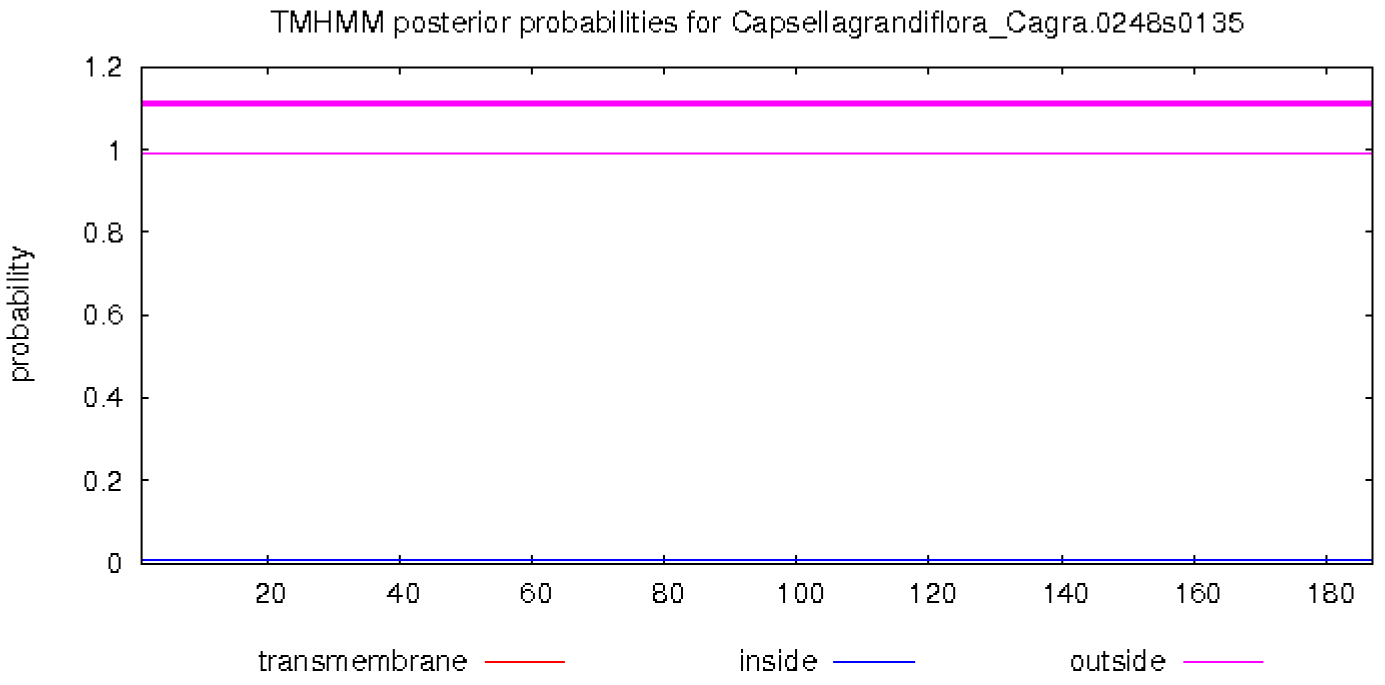

plot in postscript, script for making the plot in gnuplot, data for plot

---

```
# Capsellagrandiflora_Cagra.0301s0034 Length: 270
# Capsellagrandiflora_Cagra.0301s0034 Number of predicted TMHs:  0
# Capsellagrandiflora_Cagra.0301s0034 Exp number of AAs in TMHs: 0.15367
# Capsellagrandiflora_Cagra.0301s0034 Exp number, first 60 AAs:  0
# Capsellagrandiflora_Cagra.0301s0034 Total prob of N-in:        0.23369
Capsellagrandiflora_Cagra.0301s0034     TMHMM2.0        outside      1   270
```

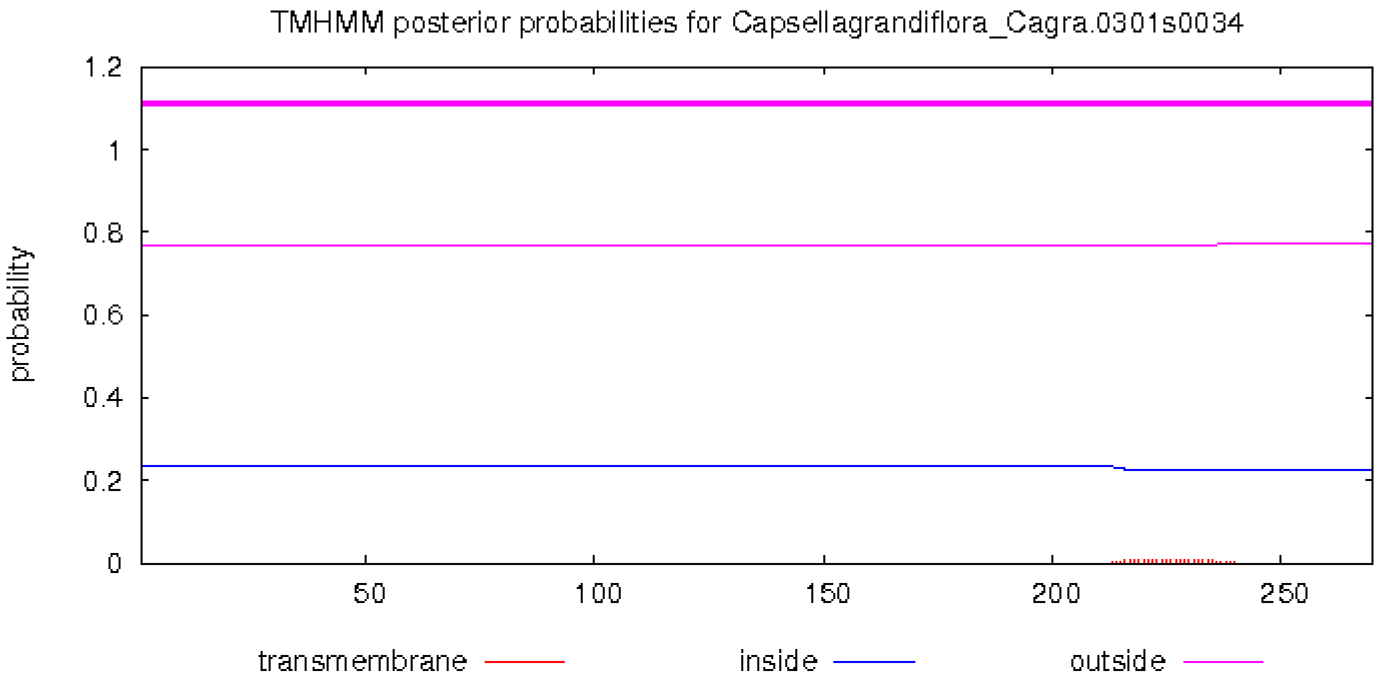

plot in postscript, script for making the plot in gnuplot, data for plot

---

```
# Capsellagrandiflora_Cagra.0334s0001 Length: 403
# Capsellagrandiflora_Cagra.0334s0001 Number of predicted TMHs:  1
# Capsellagrandiflora_Cagra.0334s0001 Exp number of AAs in TMHs: 22.26377
# Capsellagrandiflora_Cagra.0334s0001 Exp number, first 60 AAs:  0.00151
```

```
# Capsellagrandiflora_Cagra.0334s0001 Total prob of N-in:        0.10397
Capsellagrandiflora_Cagra.0334s0001     TMHMM2.0        outside      1   379
Capsellagrandiflora_Cagra.0334s0001     TMHMM2.0        TMhelix    380   402
Capsellagrandiflora_Cagra.0334s0001     TMHMM2.0        inside     403   403
```

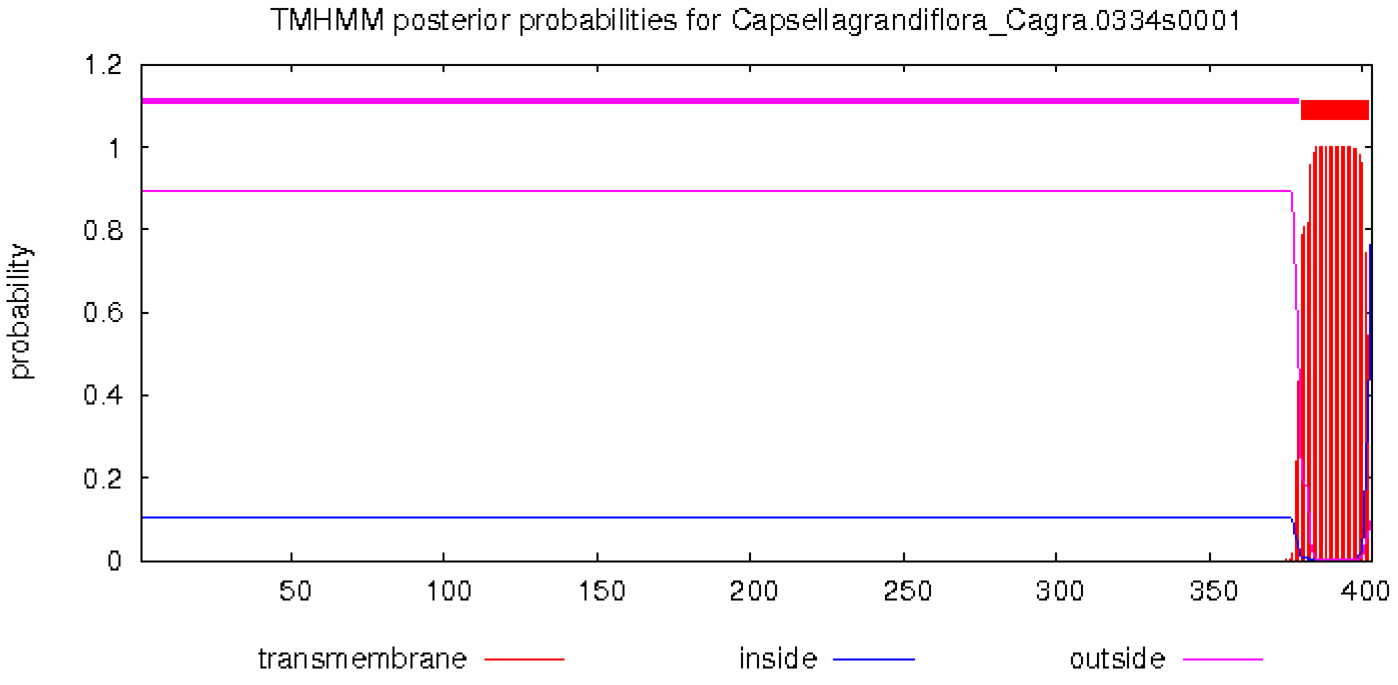

plot in postscript, script for making the plot in gnuplot, data for plot

---

```
# Capsellagrandiflora_Cagra.0483s0052 Length: 322
# Capsellagrandiflora_Cagra.0483s0052 Number of predicted TMHs:  0
# Capsellagrandiflora_Cagra.0483s0052 Exp number of AAs in TMHs: 0
# Capsellagrandiflora_Cagra.0483s0052 Exp number, first 60 AAs:  0
# Capsellagrandiflora_Cagra.0483s0052 Total prob of N-in:        0.03193
Capsellagrandiflora_Cagra.0483s0052     TMHMM2.0        outside      1   322
```

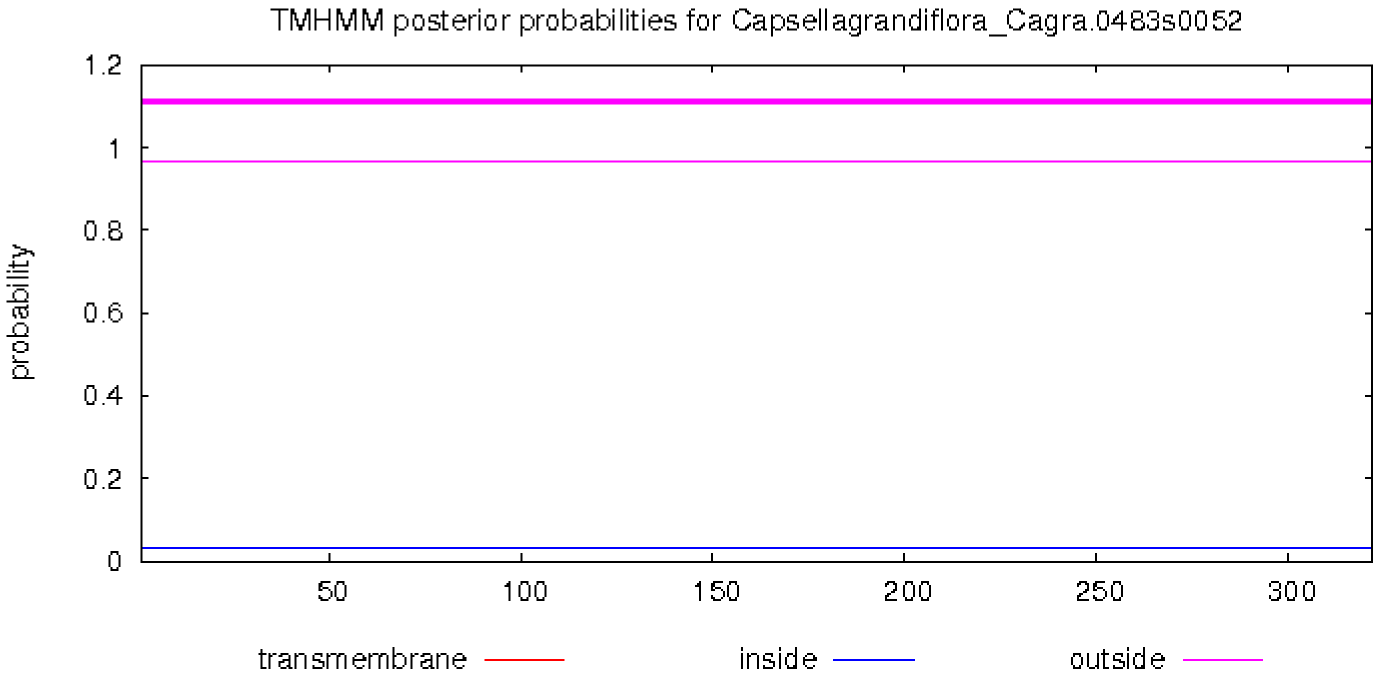

plot in postscript, script for making the plot in gnuplot, data for plot

---

```
# Capsellagrandiflora_Cagra.0534s0046 Length: 417
# Capsellagrandiflora_Cagra.0534s0046 Number of predicted TMHs:  0
```

```
# Capsellagrandiflora_Cagra.0534s0046 Exp number of AAs in TMHs: 0.0517
# Capsellagrandiflora_Cagra.0534s0046 Exp number, first 60 AAs:  0
# Capsellagrandiflora_Cagra.0534s0046 Total prob of N-in:        0.00998
Capsellagrandiflora_Cagra.0534s0046     TMHMM2.0        outside      1   417
```

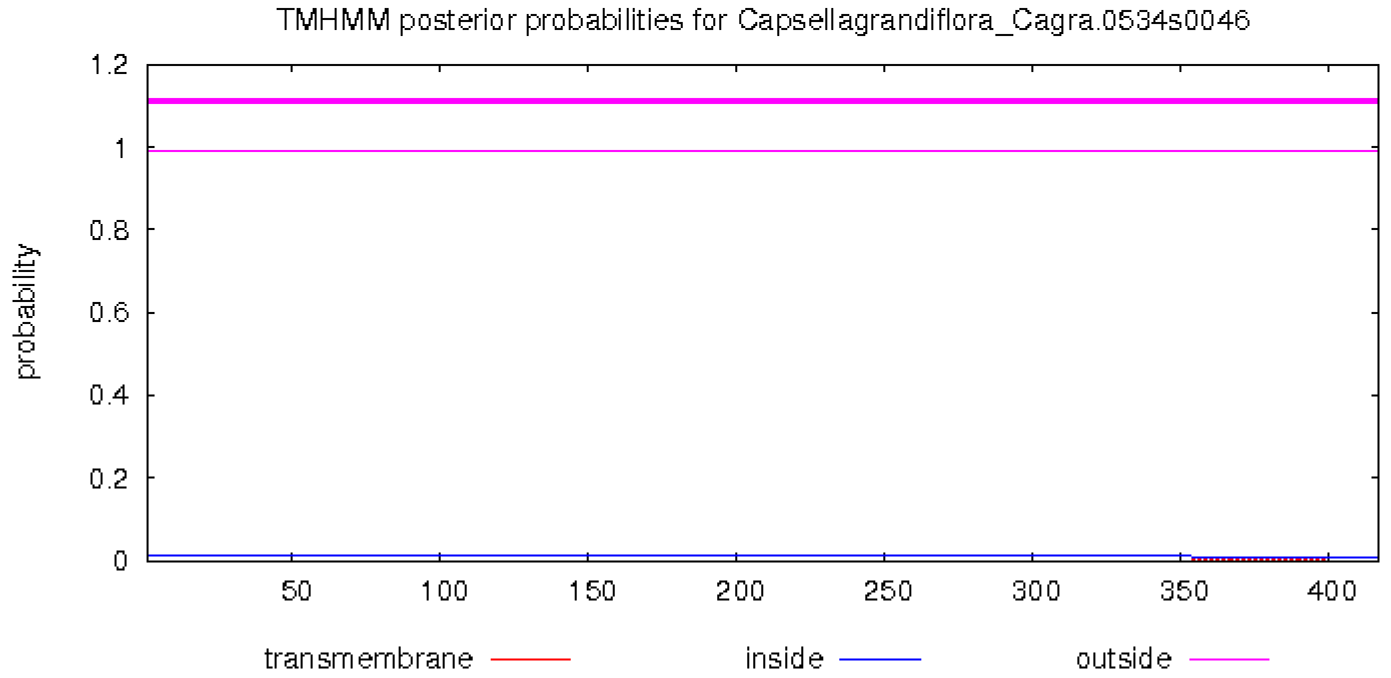

plot in postscript, script for making the plot in gnuplot, data for plot

---

```
# Capsellagrandiflora_Cagra.0542s0037 Length: 333
# Capsellagrandiflora_Cagra.0542s0037 Number of predicted TMHs:  0
# Capsellagrandiflora_Cagra.0542s0037 Exp number of AAs in TMHs: 0.00014
# Capsellagrandiflora_Cagra.0542s0037 Exp number, first 60 AAs:  0
# Capsellagrandiflora_Cagra.0542s0037 Total prob of N-in:        0.01284
Capsellagrandiflora_Cagra.0542s0037     TMHMM2.0        outside      1   333
```

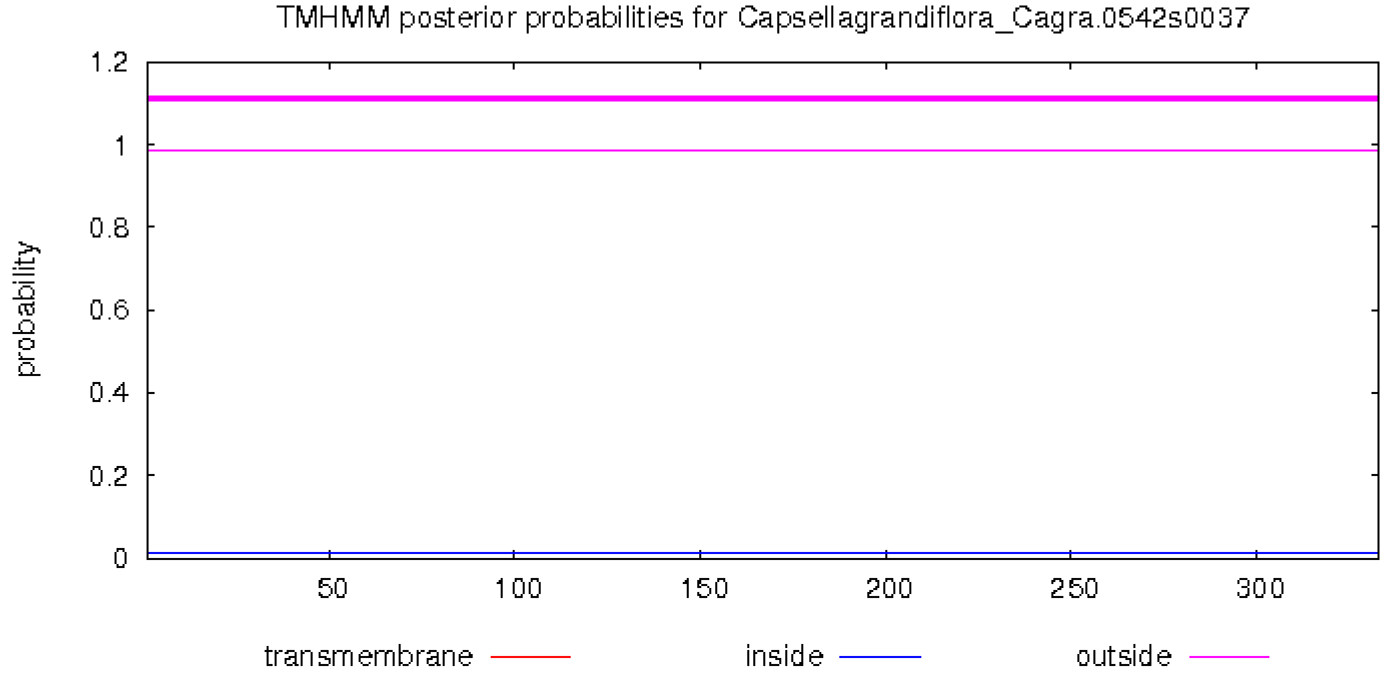

plot in postscript, script for making the plot in gnuplot, data for plot

---

```
# Capsellagrandiflora_Cagra.0568s0135 Length: 344
# Capsellagrandiflora_Cagra.0568s0135 Number of predicted TMHs:  0
```

```
# Capsellagrandiflora_Cagra.0568s0135 Exp number of AAs in TMHs: 0.00018
# Capsellagrandiflora_Cagra.0568s0135 Exp number, first 60 AAs:  0
# Capsellagrandiflora_Cagra.0568s0135 Total prob of N-in:        0.00314
Capsellagrandiflora_Cagra.0568s0135     TMHMM2.0        outside      1   344
```

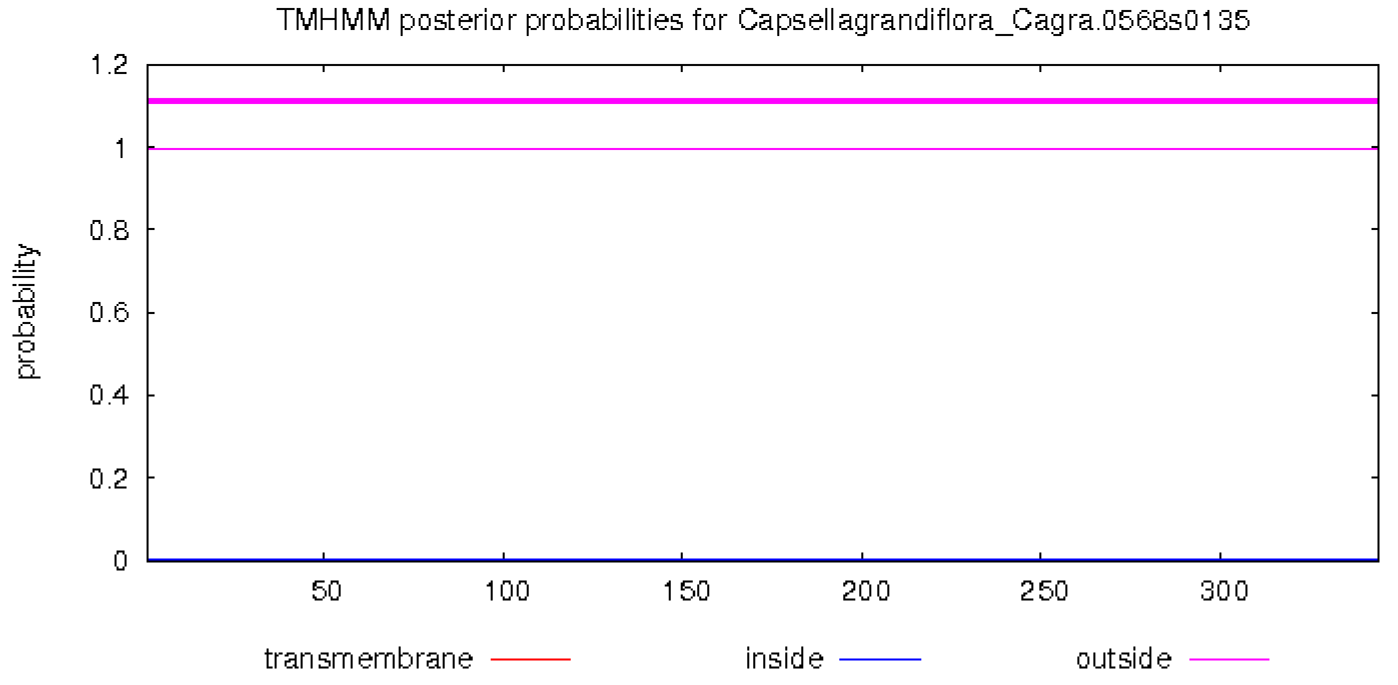

plot in postscript, script for making the plot in gnuplot, data for plot

---

```
# Capsellagrandiflora_Cagra.0569s0030 Length: 291
# Capsellagrandiflora_Cagra.0569s0030 Number of predicted TMHs:  0
# Capsellagrandiflora_Cagra.0569s0030 Exp number of AAs in TMHs: 0
# Capsellagrandiflora_Cagra.0569s0030 Exp number, first 60 AAs:  0
# Capsellagrandiflora_Cagra.0569s0030 Total prob of N-in:        0.00215
Capsellagrandiflora_Cagra.0569s0030     TMHMM2.0        outside      1   291
```

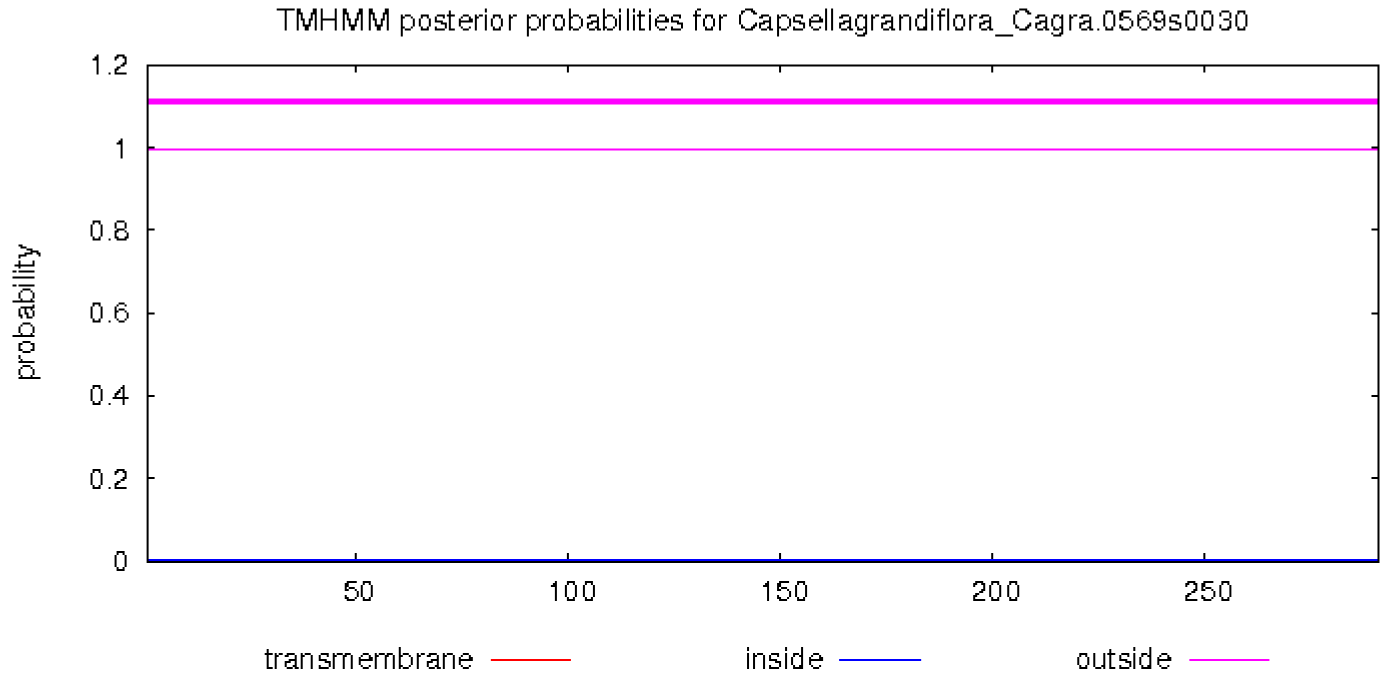

plot in postscript, script for making the plot in gnuplot, data for plot

---

```
# Capsellagrandiflora_Cagra.0570s0027 Length: 301
# Capsellagrandiflora_Cagra.0570s0027 Number of predicted TMHs:  0
```

```
# Capsellagrandiflora_Cagra.0570s0027 Exp number of AAs in TMHs: 0.00224
# Capsellagrandiflora_Cagra.0570s0027 Exp number, first 60 AAs:  0.00039
# Capsellagrandiflora_Cagra.0570s0027 Total prob of N-in:        0.02095
Capsellagrandiflora_Cagra.0570s0027     TMHMM2.0        outside      1   301
```

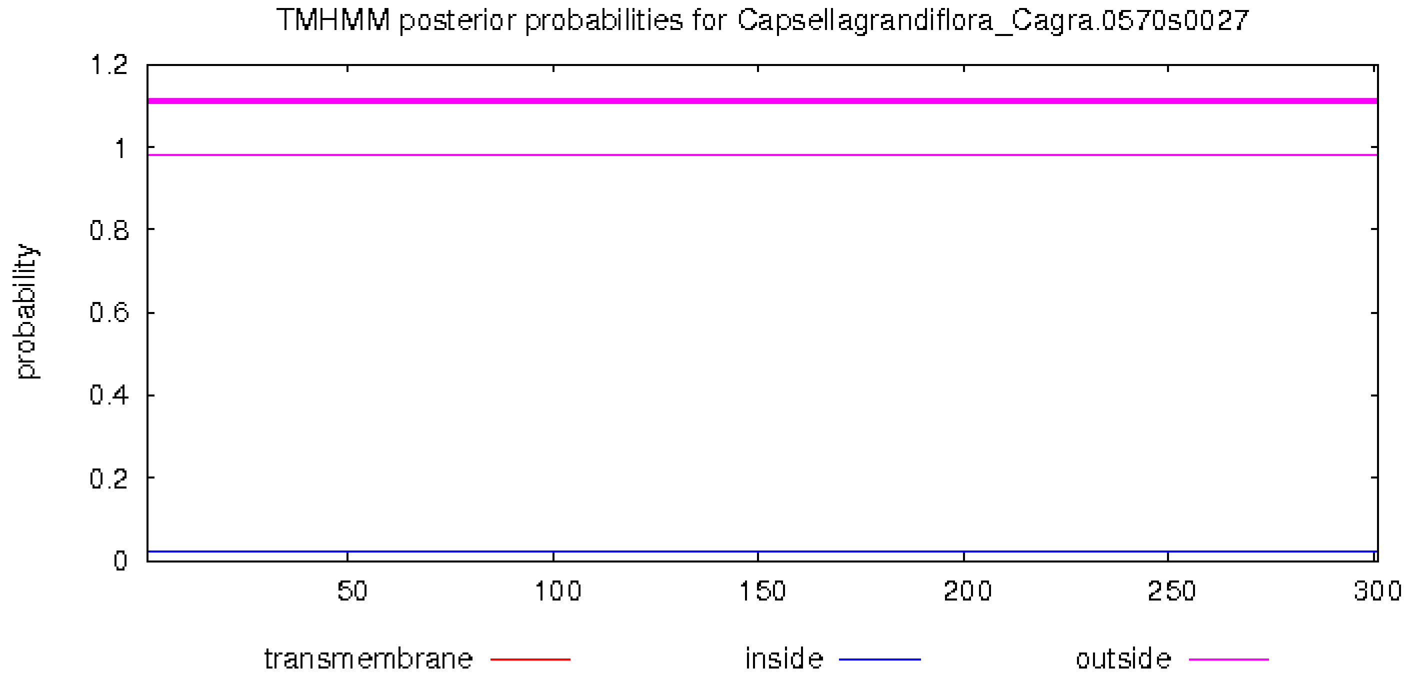

plot in postscript, script for making the plot in gnuplot, data for plot

---

```
# Capsellagrandiflora_Cagra.0596s0010 Length: 257
# Capsellagrandiflora_Cagra.0596s0010 Number of predicted TMHs:  0
# Capsellagrandiflora_Cagra.0596s0010 Exp number of AAs in TMHs: 0.0002
# Capsellagrandiflora_Cagra.0596s0010 Exp number, first 60 AAs:  0
# Capsellagrandiflora_Cagra.0596s0010 Total prob of N-in:        0.10047
Capsellagrandiflora_Cagra.0596s0010     TMHMM2.0        outside      1   257
```

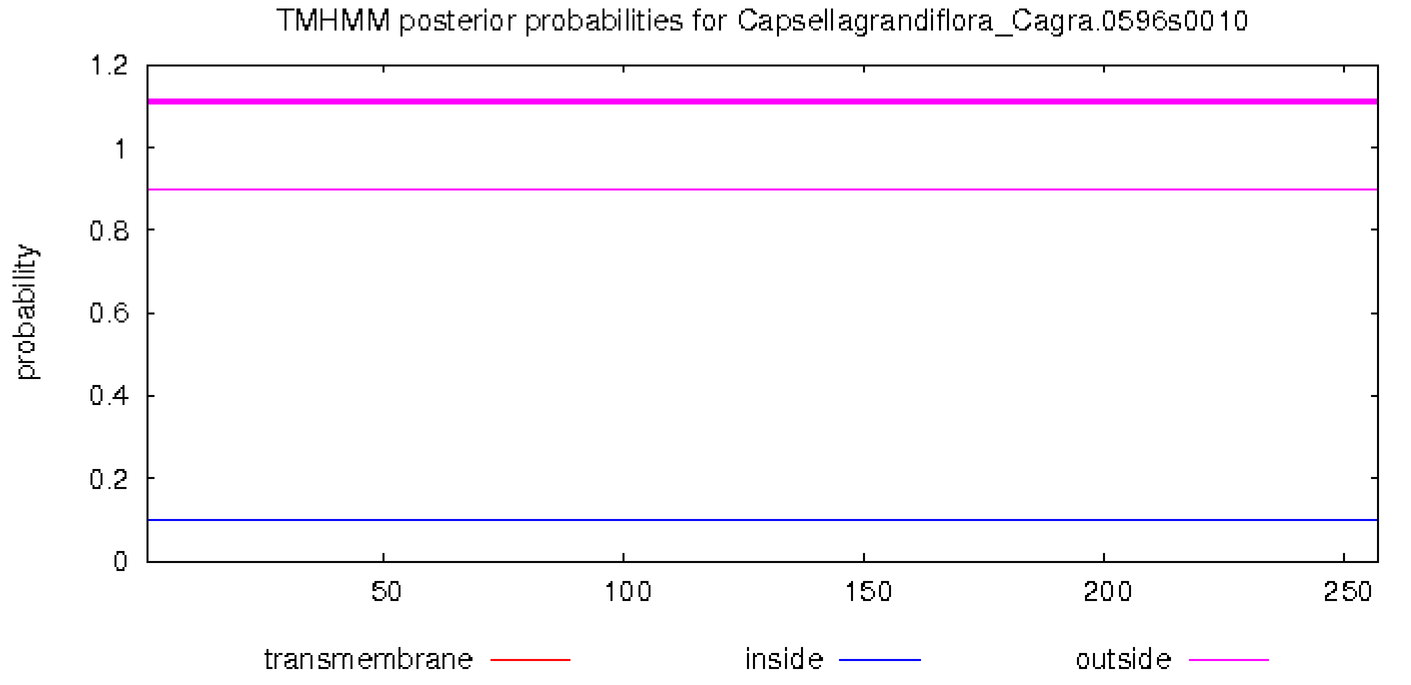

plot in postscript, script for making the plot in gnuplot, data for plot

---

```
# Capsellagrandiflora_Cagra.0799s0114 Length: 340
# Capsellagrandiflora_Cagra.0799s0114 Number of predicted TMHs:  0
```

```
# Capsellagrandiflora_Cagra.0799s0114 Exp number of AAs in TMHs: 0.02168
# Capsellagrandiflora_Cagra.0799s0114 Exp number, first 60 AAs:  0.0166
# Capsellagrandiflora_Cagra.0799s0114 Total prob of N-in:        0.00493
Capsellagrandiflora_Cagra.0799s0114     TMHMM2.0        outside      1   340
```

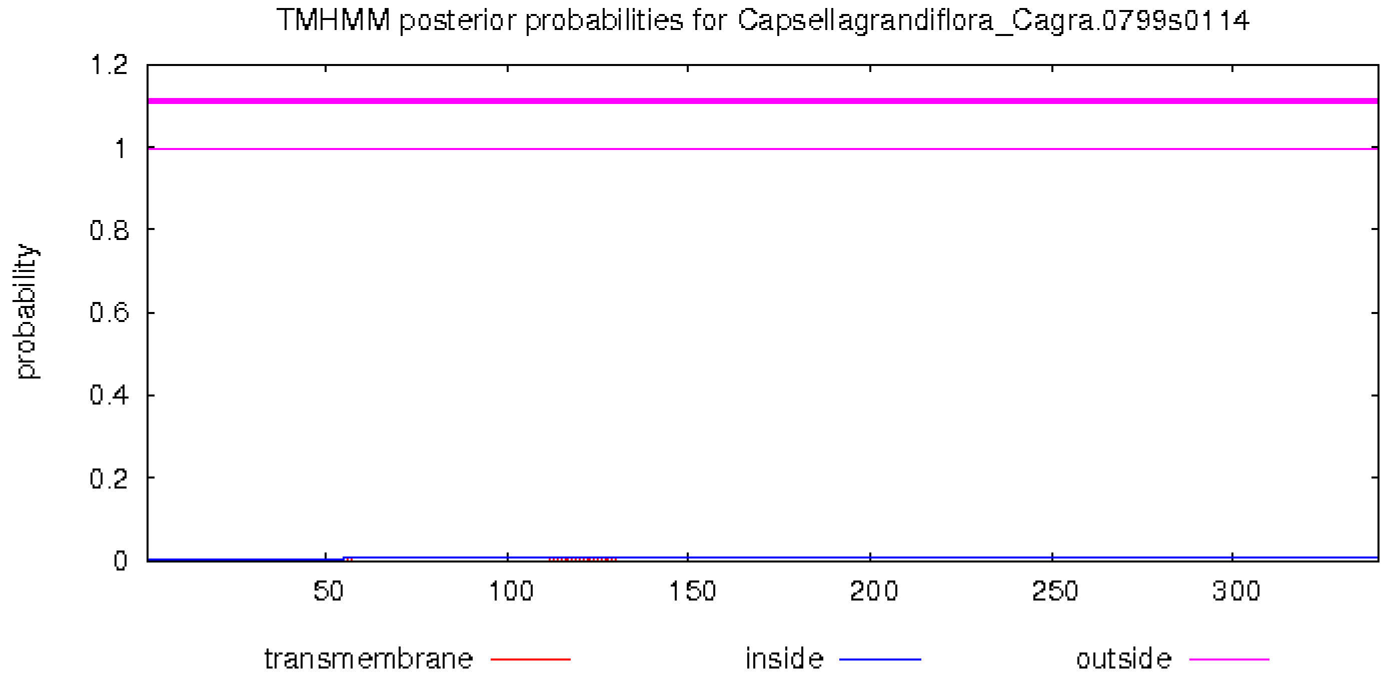

plot in postscript, script for making the plot in gnuplot, data for plot

---

```
# Capsellagrandiflora_Cagra.0805s0005 Length: 280
# Capsellagrandiflora_Cagra.0805s0005 Number of predicted TMHs:  0
# Capsellagrandiflora_Cagra.0805s0005 Exp number of AAs in TMHs: 0.00119
# Capsellagrandiflora_Cagra.0805s0005 Exp number, first 60 AAs:  0
# Capsellagrandiflora_Cagra.0805s0005 Total prob of N-in:        0.00648
Capsellagrandiflora_Cagra.0805s0005     TMHMM2.0        outside      1   280
```

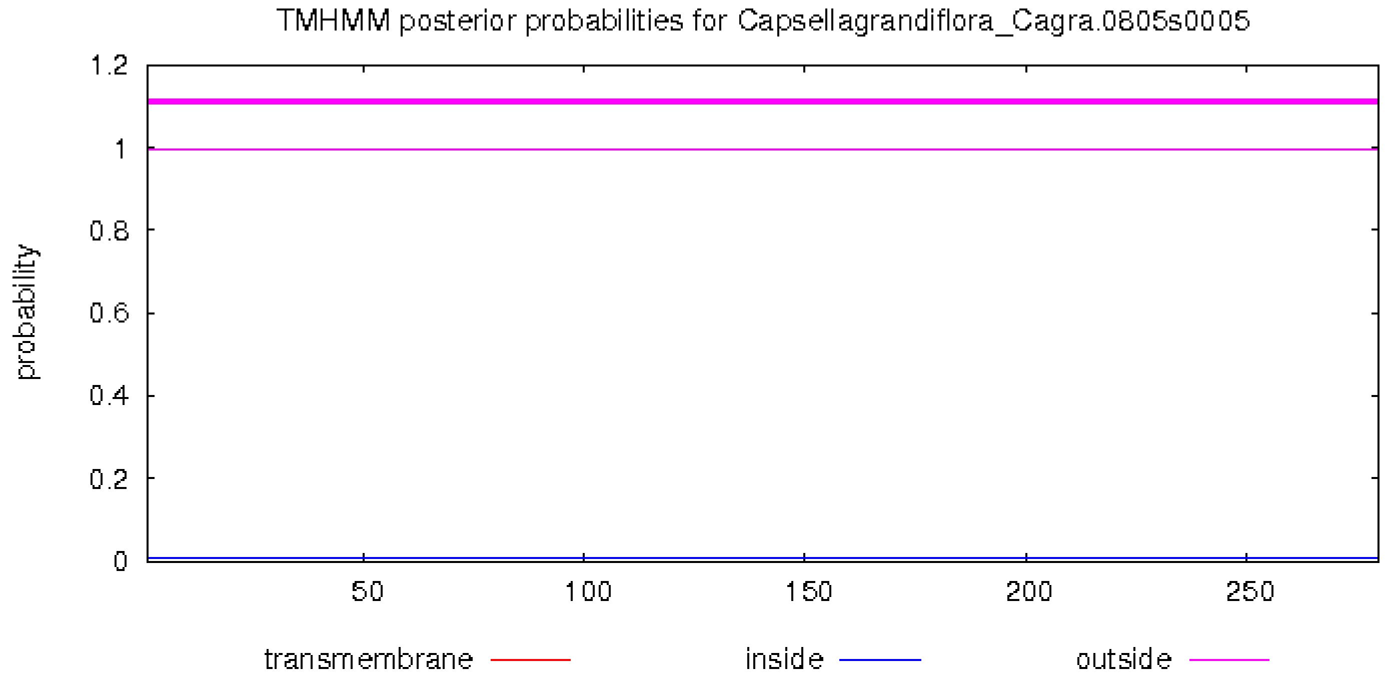

plot in postscript, script for making the plot in gnuplot, data for plot

---

```
# Capsellagrandiflora_Cagra.0834s0027 Length: 559
# Capsellagrandiflora_Cagra.0834s0027 Number of predicted TMHs:  1
```

```
# Capsellagrandiflora_Cagra.0834s0027 Exp number of AAs in TMHs: 22.02649
# Capsellagrandiflora_Cagra.0834s0027 Exp number, first 60 AAs:  0
# Capsellagrandiflora_Cagra.0834s0027 Total prob of N-in:        0.00139
Capsellagrandiflora_Cagra.0834s0027     TMHMM2.0        outside      1   534
Capsellagrandiflora_Cagra.0834s0027     TMHMM2.0        TMhelix    535   557
Capsellagrandiflora_Cagra.0834s0027     TMHMM2.0        inside     558   559
```

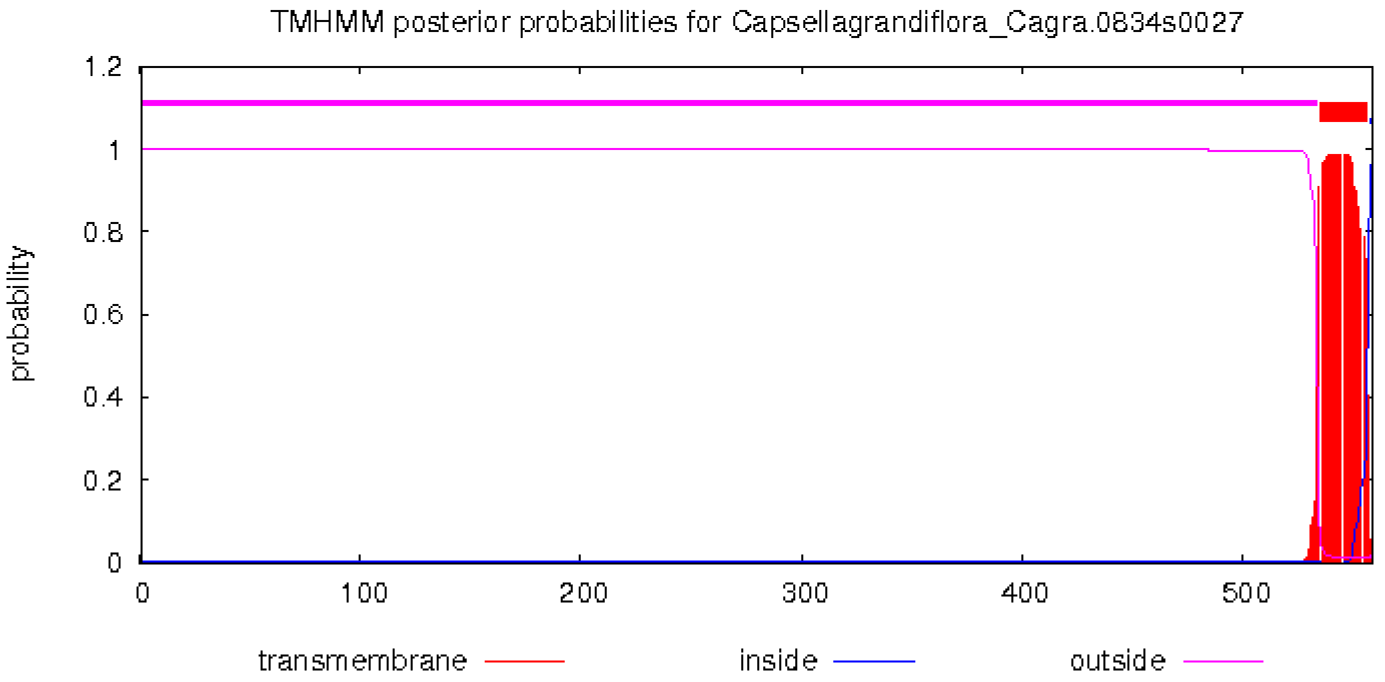

plot in postscript, script for making the plot in gnuplot, data for plot

---

```
# Capsellagrandiflora_Cagra.0834s0028 Length: 448
# Capsellagrandiflora_Cagra.0834s0028 Number of predicted TMHs:  0
# Capsellagrandiflora_Cagra.0834s0028 Exp number of AAs in TMHs: 0.43759
# Capsellagrandiflora_Cagra.0834s0028 Exp number, first 60 AAs:  0.42456
# Capsellagrandiflora_Cagra.0834s0028 Total prob of N-in:        0.02544
Capsellagrandiflora_Cagra.0834s0028     TMHMM2.0        outside      1   448
```

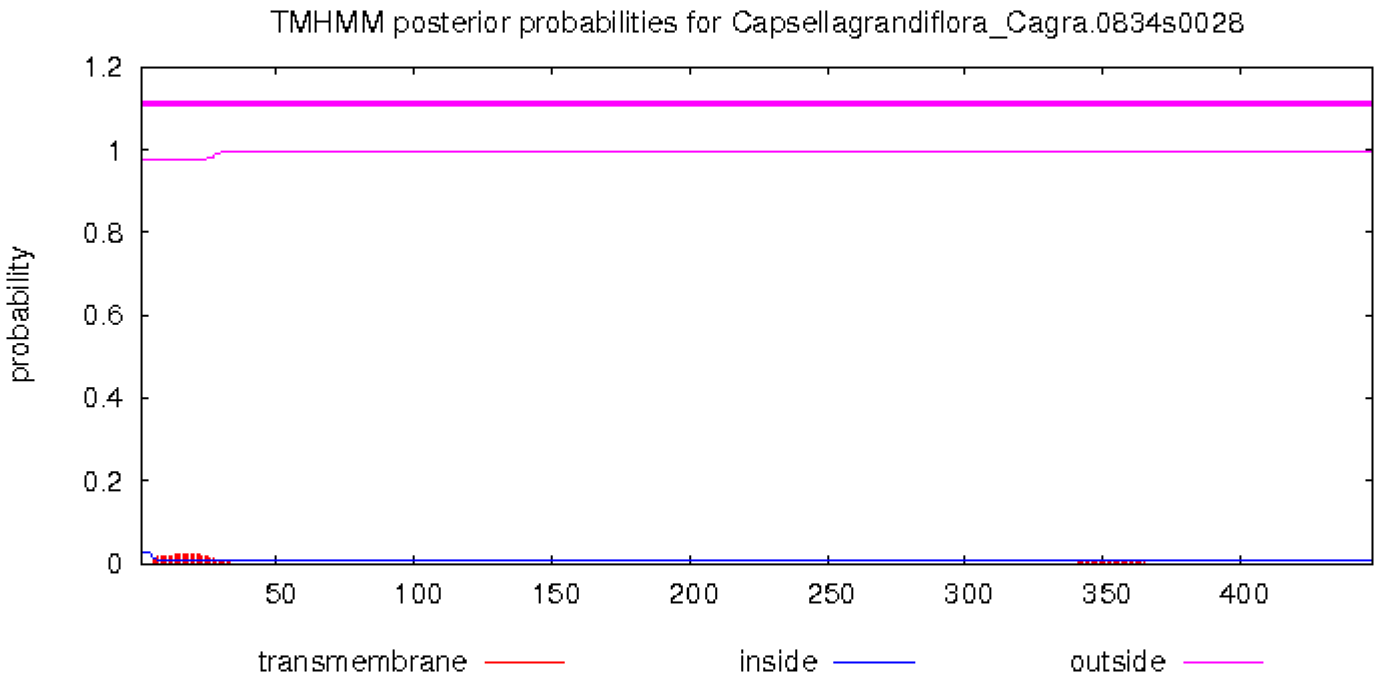

plot in postscript, script for making the plot in gnuplot, data for plot

---

```
# Capsellagrandiflora_Cagra.0834s0029 Length: 448
# Capsellagrandiflora_Cagra.0834s0029 Number of predicted TMHs:  0
# Capsellagrandiflora_Cagra.0834s0029 Exp number of AAs in TMHs: 0.06105
# Capsellagrandiflora_Cagra.0834s0029 Exp number, first 60 AAs:  0.0531
# Capsellagrandiflora_Cagra.0834s0029 Total prob of N-in:        0.00681
Capsellagrandiflora_Cagra.0834s0029     TMHMM2.0        outside      1   448
```

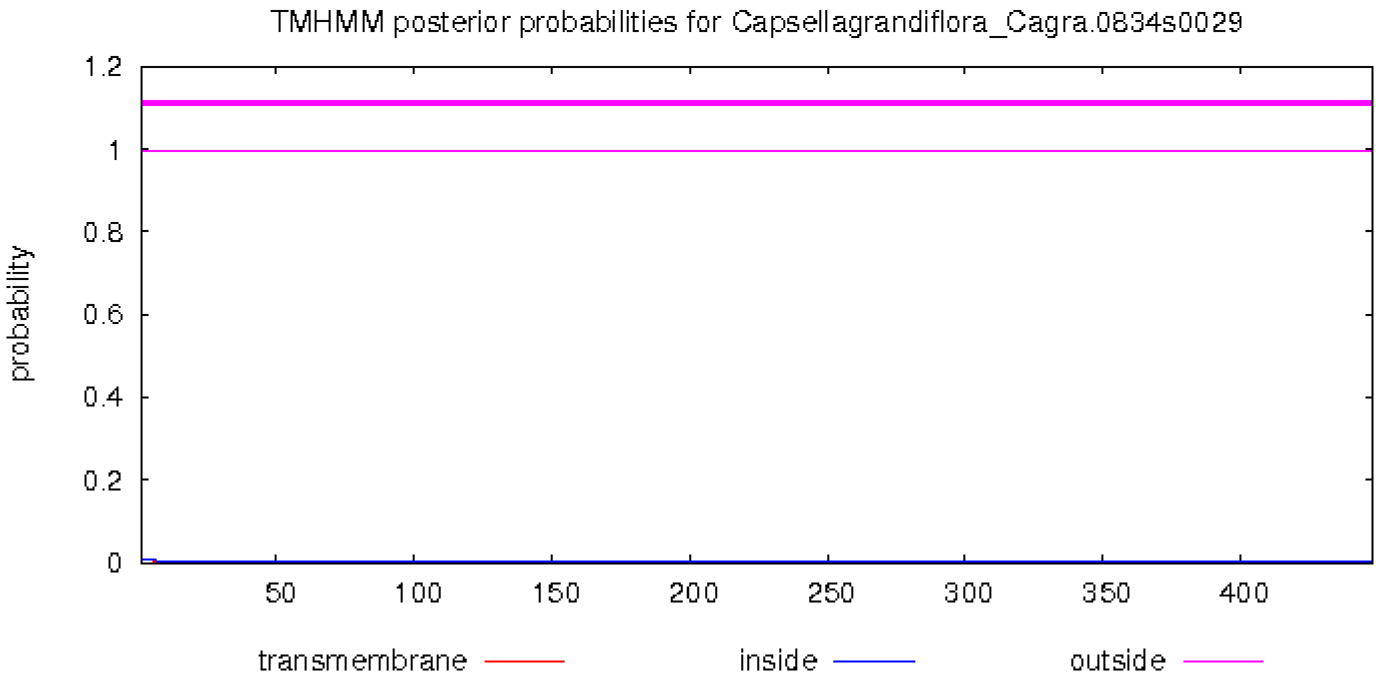

plot in postscript, script for making the plot in gnuplot, data for plot

---

```
# Capsellagrandiflora_Cagra.0846s0004 Length: 340
# Capsellagrandiflora_Cagra.0846s0004 Number of predicted TMHs:  0
# Capsellagrandiflora_Cagra.0846s0004 Exp number of AAs in TMHs: 12.49629
# Capsellagrandiflora_Cagra.0846s0004 Exp number, first 60 AAs:  0.01379
# Capsellagrandiflora_Cagra.0846s0004 Total prob of N-in:        0.14157
Capsellagrandiflora_Cagra.0846s0004     TMHMM2.0        outside      1   340
```

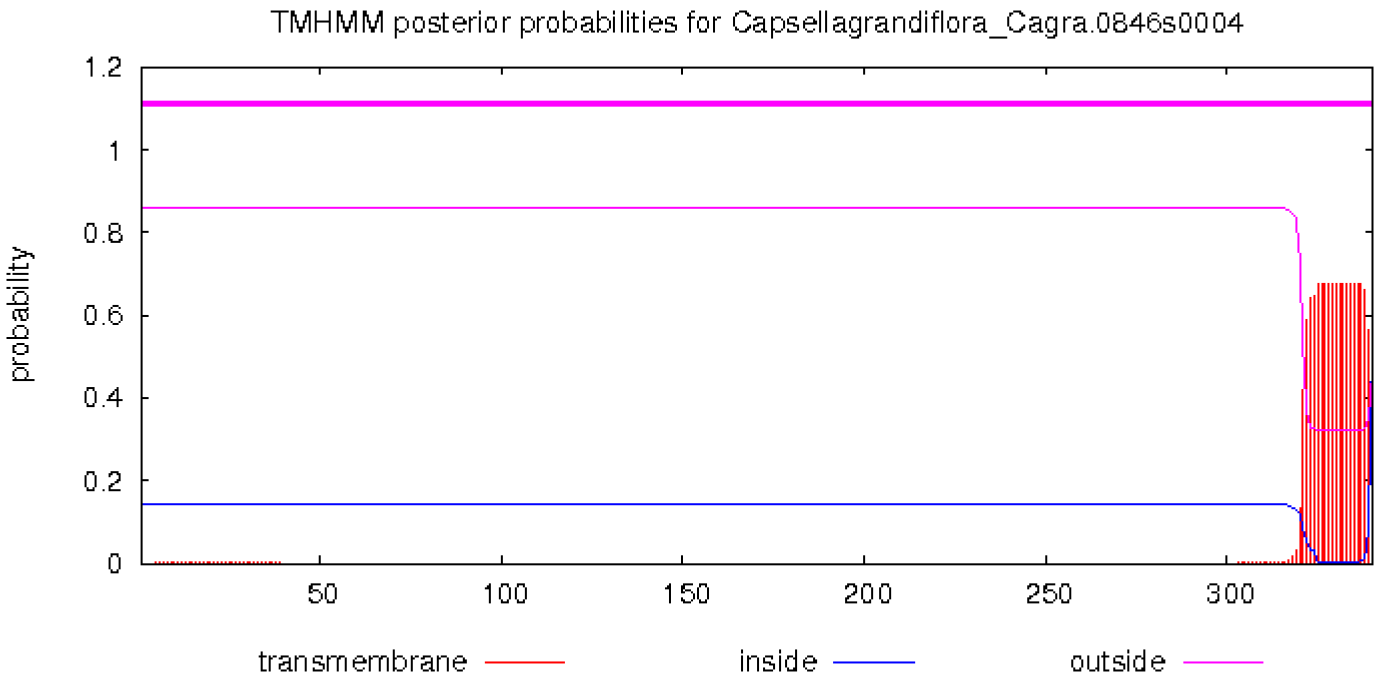

plot in postscript, script for making the plot in gnuplot, data for plot

---

```
# Capsellagrandiflora_Cagra.0846s0014 Length: 238
# Capsellagrandiflora_Cagra.0846s0014 Number of predicted TMHs:  0
# Capsellagrandiflora_Cagra.0846s0014 Exp number of AAs in TMHs: 0.00061
# Capsellagrandiflora_Cagra.0846s0014 Exp number, first 60 AAs:  0
# Capsellagrandiflora_Cagra.0846s0014 Total prob of N-in:        0.04670
Capsellagrandiflora_Cagra.0846s0014     TMHMM2.0        outside      1   238
```

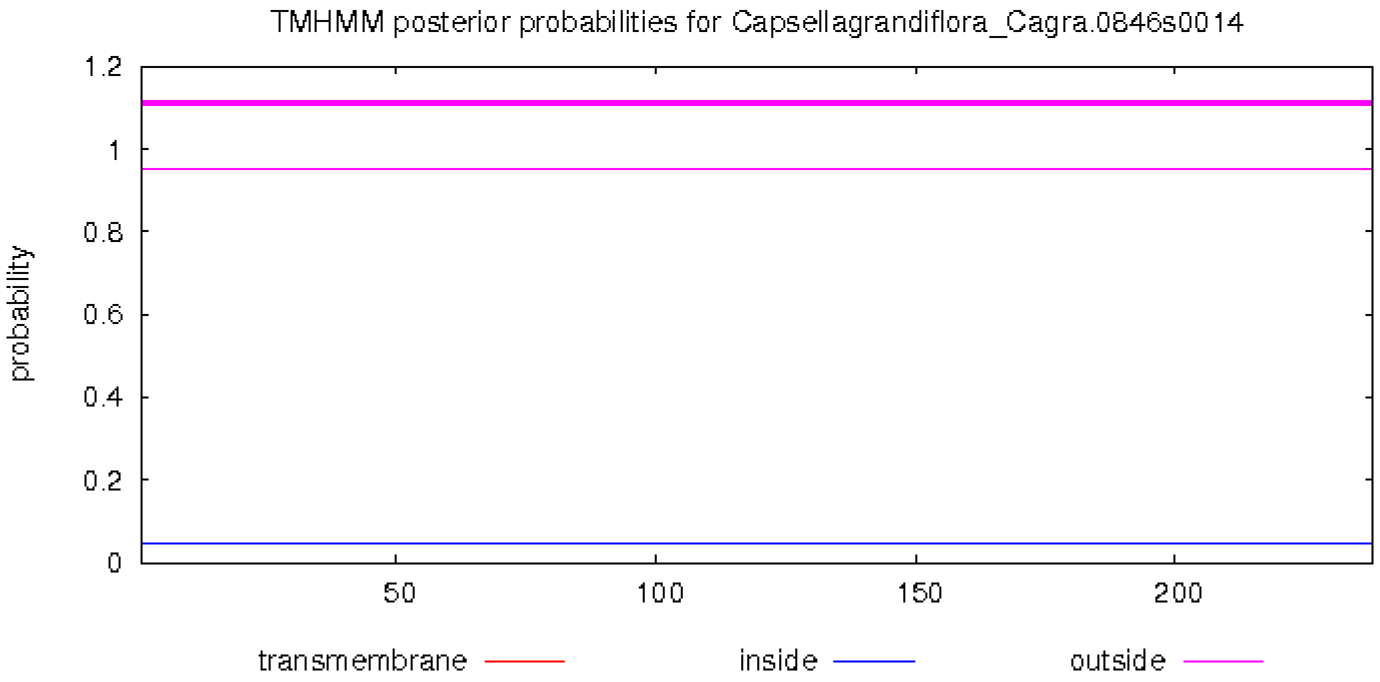

plot in postscript, script for making the plot in gnuplot, data for plot

```
# Capsellagrandiflora_Cagra.0887s0008 Length: 271
# Capsellagrandiflora_Cagra.0887s0008 Number of predicted TMHs:  0
# Capsellagrandiflora_Cagra.0887s0008 Exp number of AAs in TMHs: 0.00245
# Capsellagrandiflora_Cagra.0887s0008 Exp number, first 60 AAs:  0
# Capsellagrandiflora_Cagra.0887s0008 Total prob of N-in:        0.03831
Capsellagrandiflora_Cagra.0887s0008     TMHMM2.0        outside      1   271
```

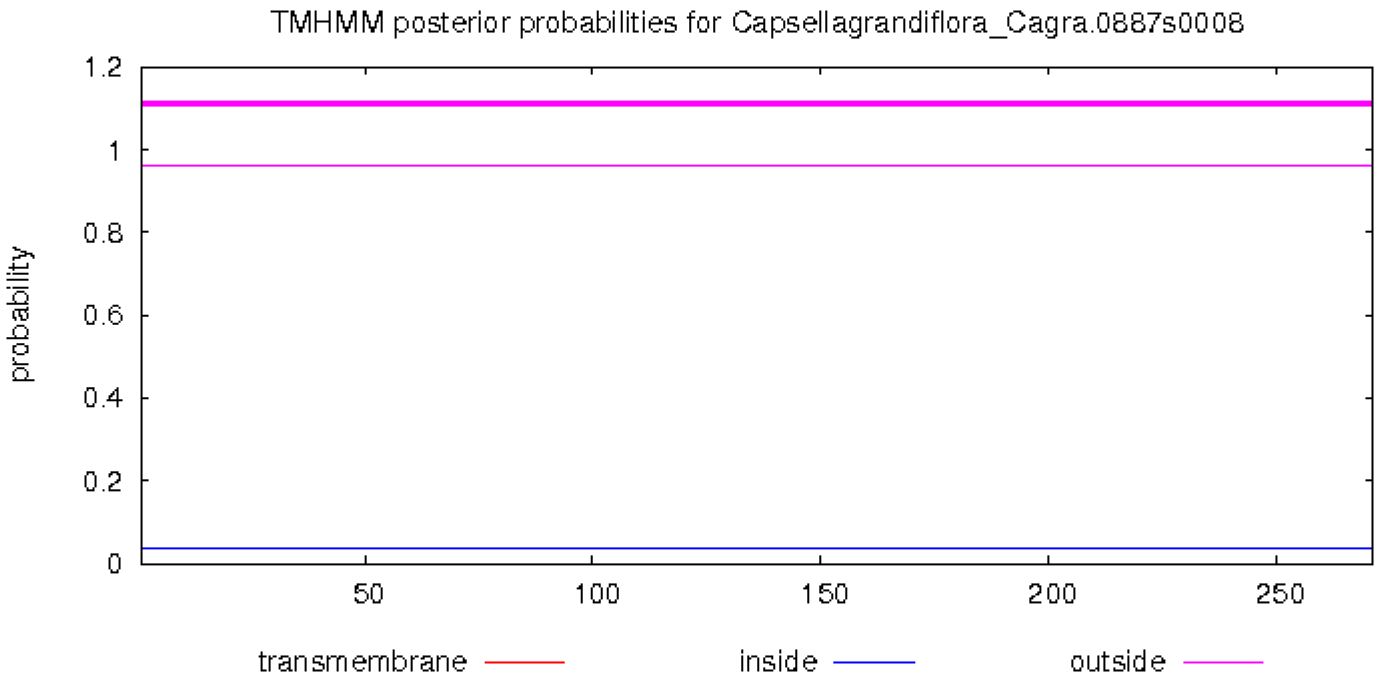

plot in postscript, script for making the plot in gnuplot, data for plot

```
# Capsellagrandiflora_Cagra.0897s0030 Length: 391
# Capsellagrandiflora_Cagra.0897s0030 Number of predicted TMHs:  0
# Capsellagrandiflora_Cagra.0897s0030 Exp number of AAs in TMHs: 0.00023
# Capsellagrandiflora_Cagra.0897s0030 Exp number, first 60 AAs:  0.00023
# Capsellagrandiflora_Cagra.0897s0030 Total prob of N-in:        0.01018
Capsellagrandiflora_Cagra.0897s0030     TMHMM2.0        outside      1   391
```

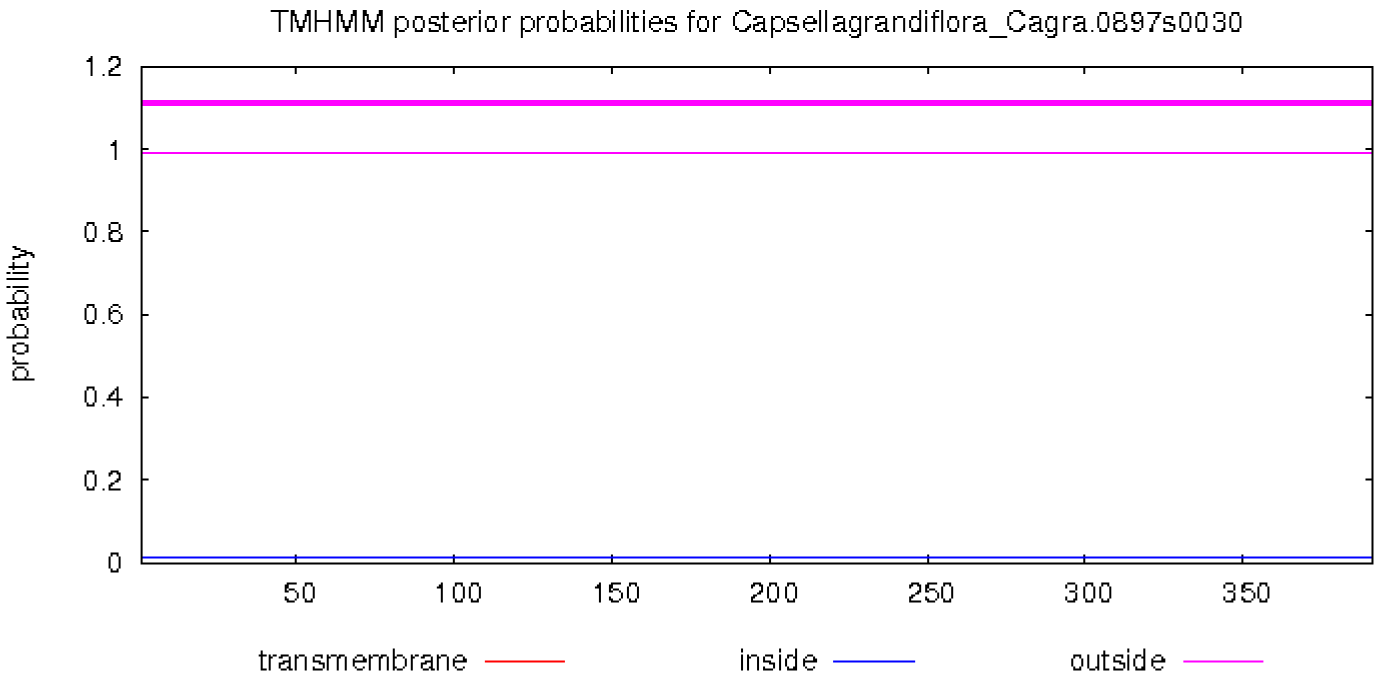

plot in postscript, script for making the plot in gnuplot, data for plot

```
# Capsellagrandiflora_Cagra.0906s0001 Length: 149
# Capsellagrandiflora_Cagra.0906s0001 Number of predicted TMHs:  0
# Capsellagrandiflora_Cagra.0906s0001 Exp number of AAs in TMHs: 0.00092
# Capsellagrandiflora_Cagra.0906s0001 Exp number, first 60 AAs:  0
# Capsellagrandiflora_Cagra.0906s0001 Total prob of N-in:        0.35571
Capsellagrandiflora_Cagra.0906s0001     TMHMM2.0        outside      1   149
```

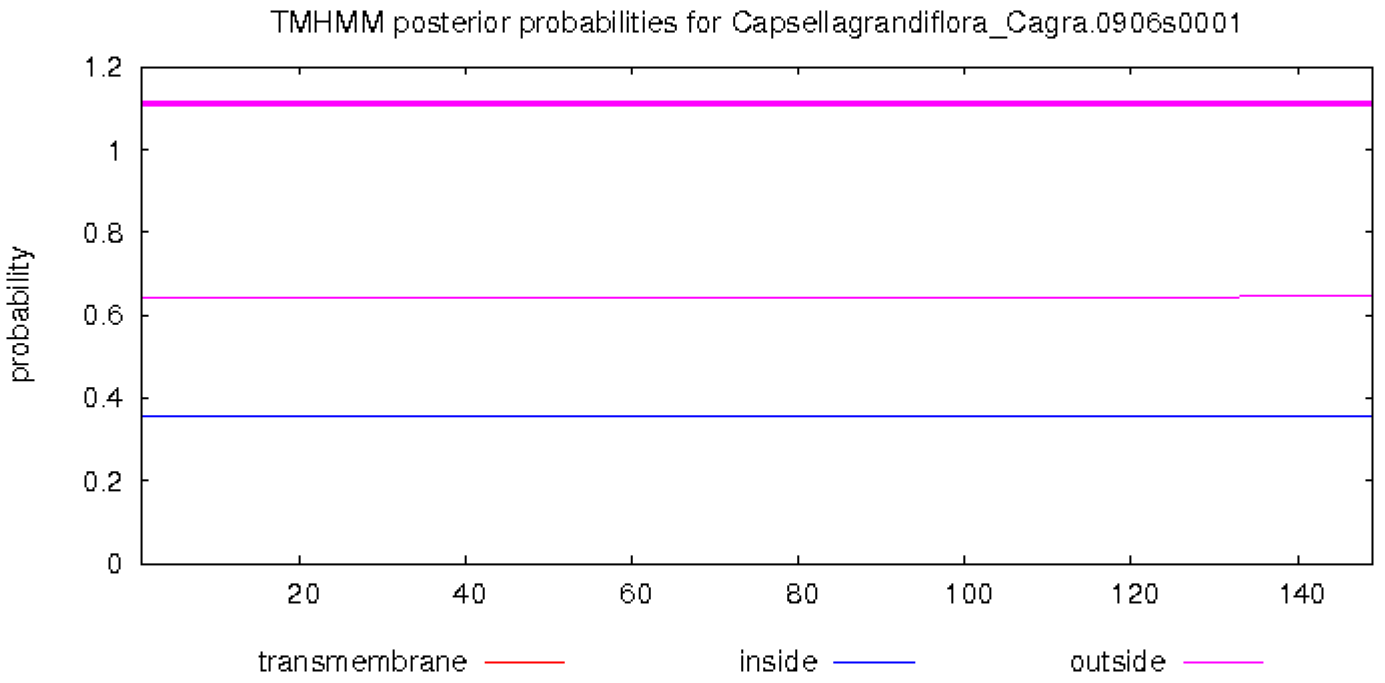

plot in postscript, script for making the plot in gnuplot, data for plot

```
# Capsellagrandiflora_Cagra.0906s0022 Length: 337
# Capsellagrandiflora_Cagra.0906s0022 Number of predicted TMHs:  0
# Capsellagrandiflora_Cagra.0906s0022 Exp number of AAs in TMHs: 0.00032
# Capsellagrandiflora_Cagra.0906s0022 Exp number, first 60 AAs:  0
# Capsellagrandiflora_Cagra.0906s0022 Total prob of N-in:        0.00864
Capsellagrandiflora_Cagra.0906s0022     TMHMM2.0        outside      1   337
```

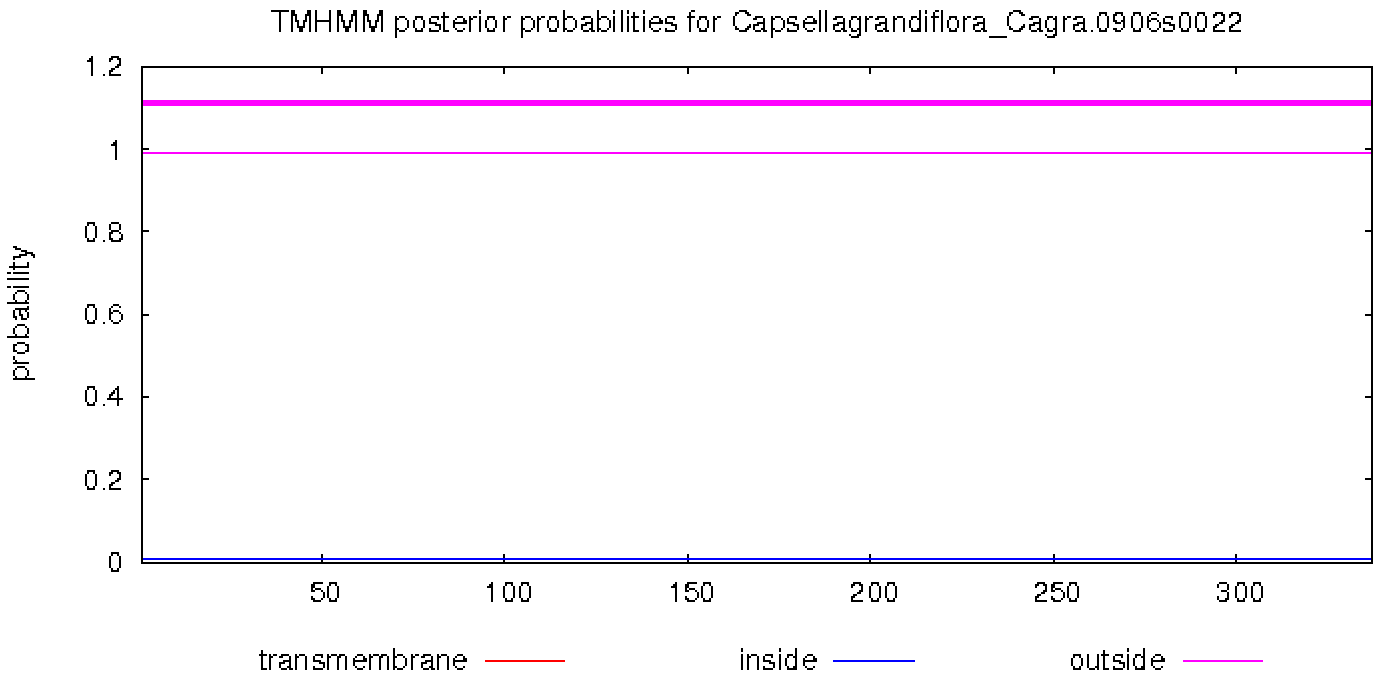

plot in postscript, script for making the plot in gnuplot, data for plot

```
# Capsellagrandiflora_Cagra.0925s0069 Length: 320
# Capsellagrandiflora_Cagra.0925s0069 Number of predicted TMHs:  0
# Capsellagrandiflora_Cagra.0925s0069 Exp number of AAs in TMHs: 0.01067
# Capsellagrandiflora_Cagra.0925s0069 Exp number, first 60 AAs:  0
# Capsellagrandiflora_Cagra.0925s0069 Total prob of N-in:        0.03557
Capsellagrandiflora_Cagra.0925s0069     TMHMM2.0        outside      1   320
```

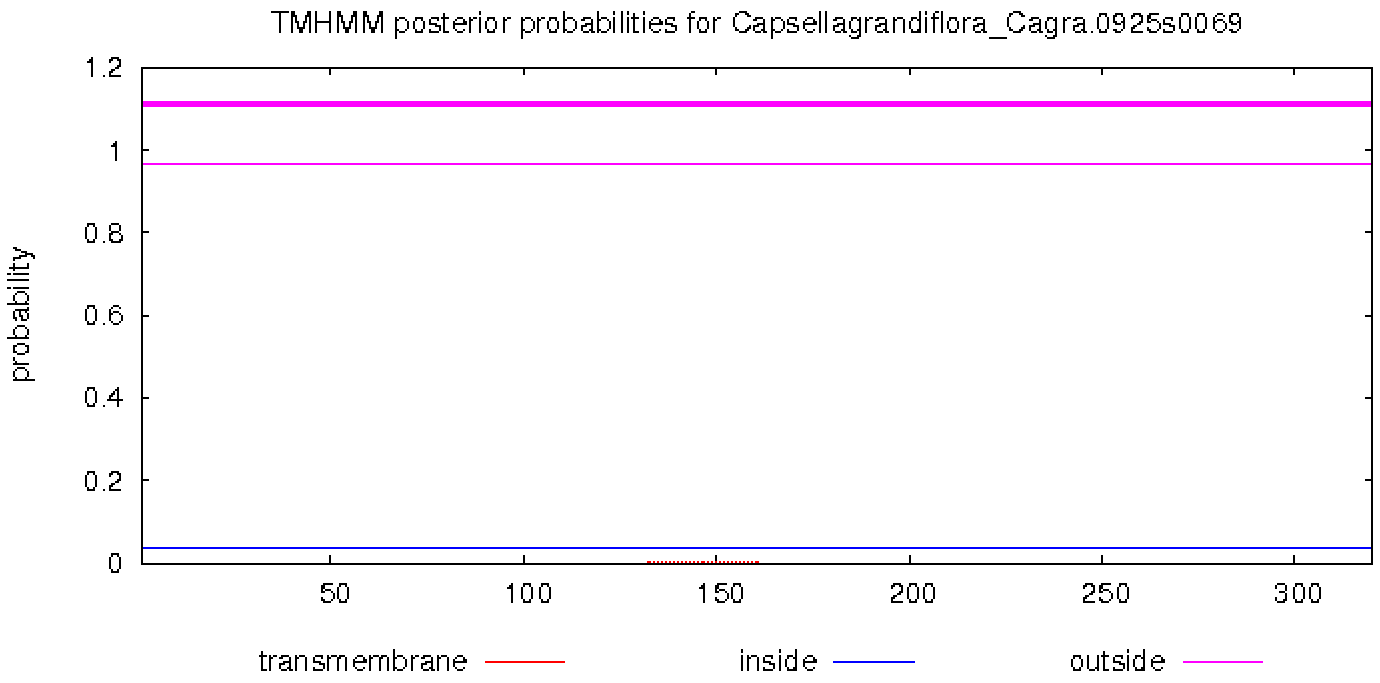

plot in postscript, script for making the plot in gnuplot, data for plot

```
# Capsellagrandiflora_Cagra.0946s0024 Length: 324
# Capsellagrandiflora_Cagra.0946s0024 Number of predicted TMHs:  0
# Capsellagrandiflora_Cagra.0946s0024 Exp number of AAs in TMHs: 0.00085
# Capsellagrandiflora_Cagra.0946s0024 Exp number, first 60 AAs:  0
# Capsellagrandiflora_Cagra.0946s0024 Total prob of N-in:        0.00101
Capsellagrandiflora_Cagra.0946s0024     TMHMM2.0        outside      1   324
```

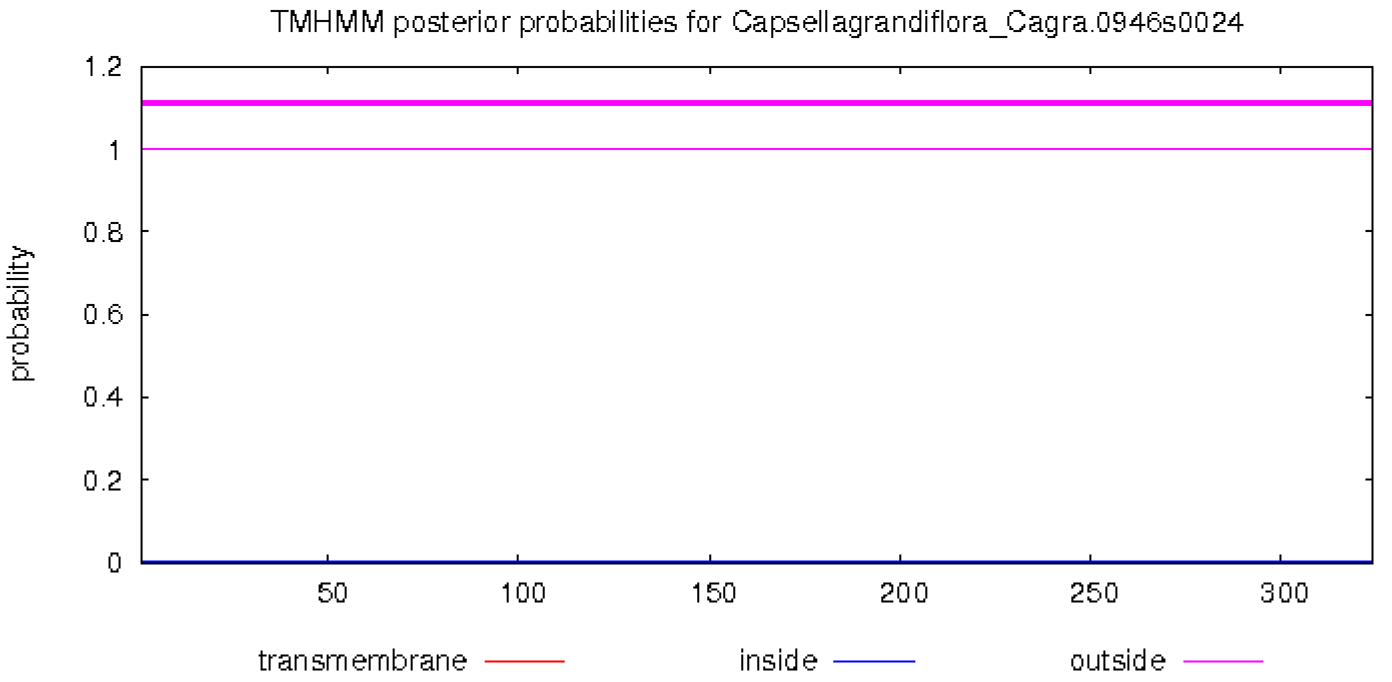

plot in postscript, script for making the plot in gnuplot, data for plot

---

```
# Capsellagrandiflora_Cagra.0946s0025 Length: 314
# Capsellagrandiflora_Cagra.0946s0025 Number of predicted TMHs:  0
# Capsellagrandiflora_Cagra.0946s0025 Exp number of AAs in TMHs: 0.00036
# Capsellagrandiflora_Cagra.0946s0025 Exp number, first 60 AAs:  0
# Capsellagrandiflora_Cagra.0946s0025 Total prob of N-in:        0.00616
Capsellagrandiflora_Cagra.0946s0025     TMHMM2.0        outside      1   314
```

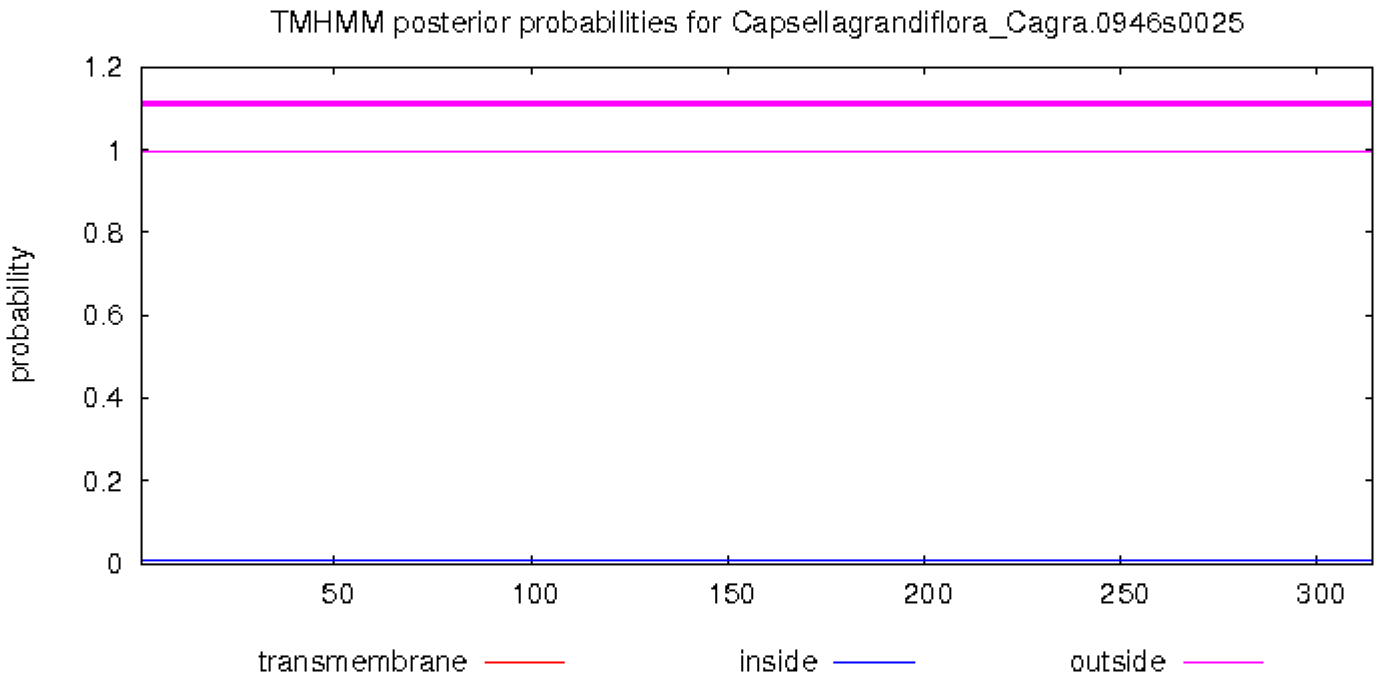

plot in postscript, script for making the plot in gnuplot, data for plot

---

```
# Capsellagrandiflora_Cagra.10427s0040 Length: 289
# Capsellagrandiflora_Cagra.10427s0040 Number of predicted TMHs:  0
# Capsellagrandiflora_Cagra.10427s0040 Exp number of AAs in TMHs: 0.00037
# Capsellagrandiflora_Cagra.10427s0040 Exp number, first 60 AAs:  0
# Capsellagrandiflora_Cagra.10427s0040 Total prob of N-in:        0.02907
Capsellagrandiflora_Cagra.10427s0040	TMHMM2.0	outside	     1   289
```

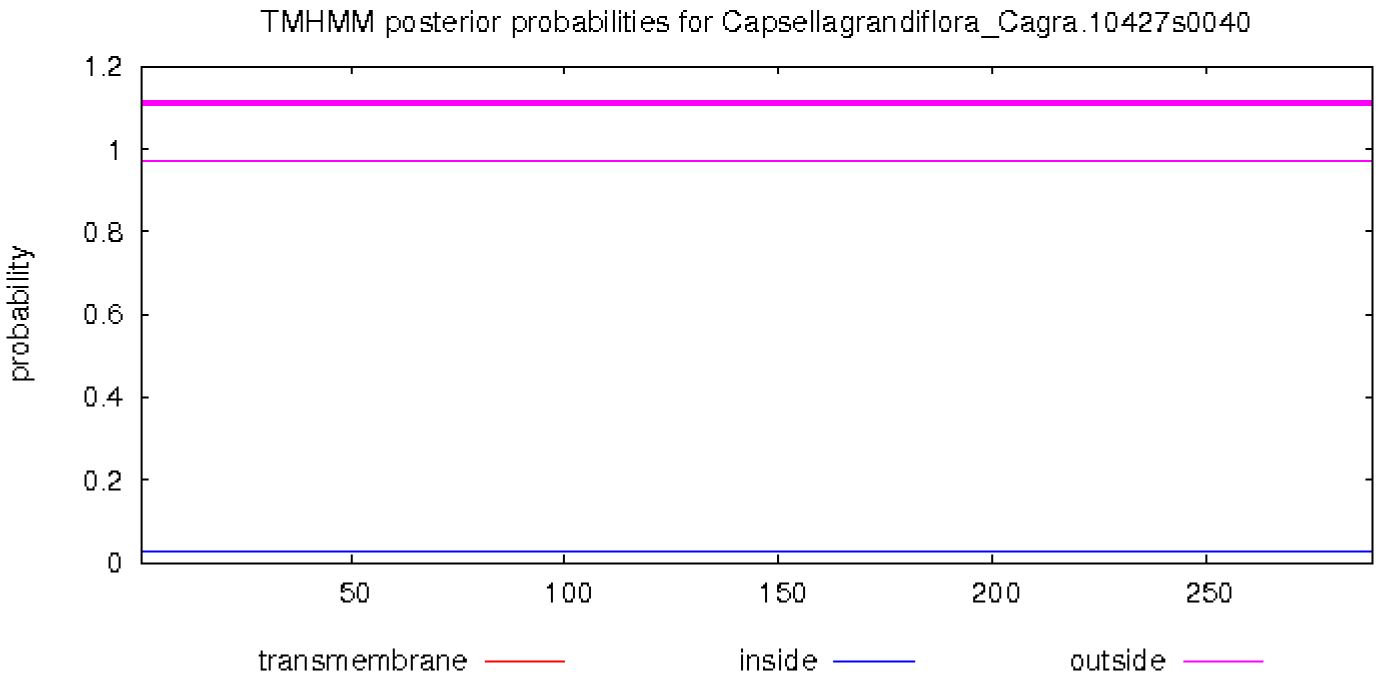

plot in postscript, script for making the plot in gnuplot, data for plot

```
# Capsellagrandiflora_Cagra.11446s0003 Length: 623
# Capsellagrandiflora_Cagra.11446s0003 Number of predicted TMHs:  0
# Capsellagrandiflora_Cagra.11446s0003 Exp number of AAs in TMHs: 6.75224
# Capsellagrandiflora_Cagra.11446s0003 Exp number, first 60 AAs:  0
# Capsellagrandiflora_Cagra.11446s0003 Total prob of N-in:        0.00016
Capsellagrandiflora_Cagra.11446s0003	TMHMM2.0	outside	     1   623
```

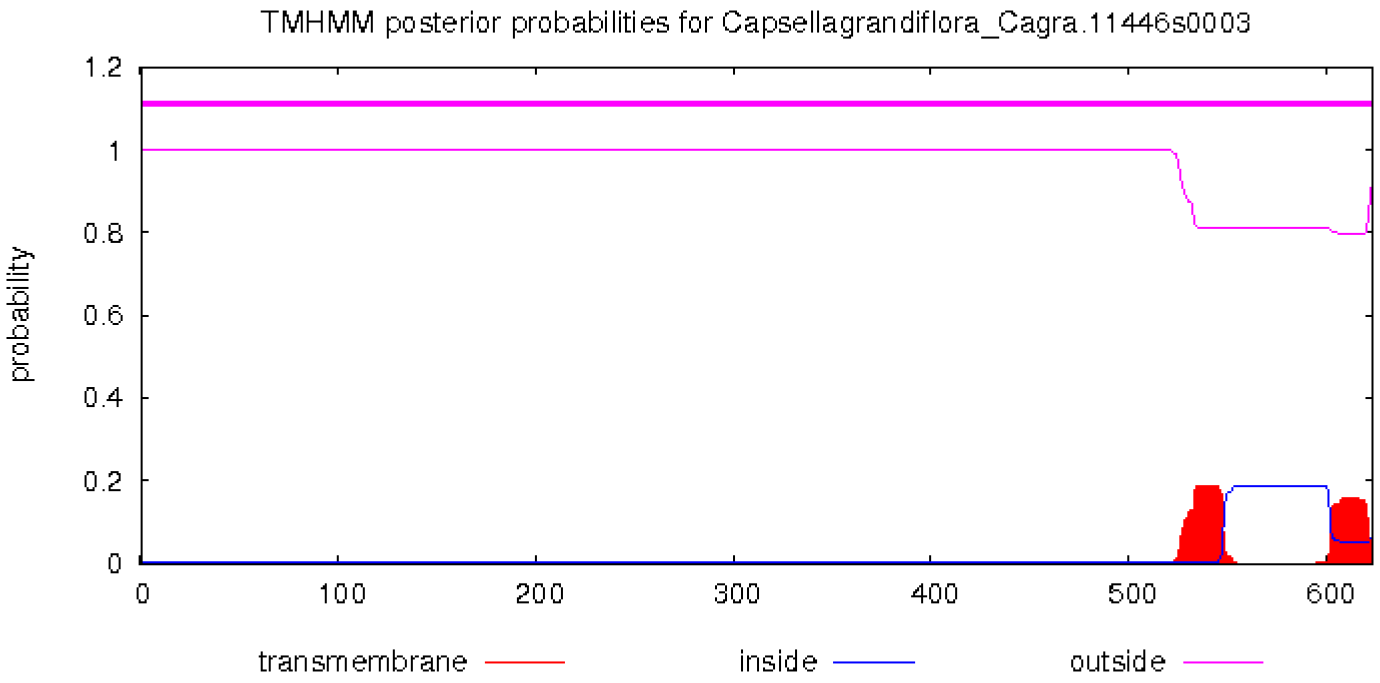

plot in postscript, script for making the plot in gnuplot, data for plot

```
# Capsellagrandiflora_Cagra.1150s0013 Length: 418
# Capsellagrandiflora_Cagra.1150s0013 Number of predicted TMHs:  0
# Capsellagrandiflora_Cagra.1150s0013 Exp number of AAs in TMHs: 0.00034
# Capsellagrandiflora_Cagra.1150s0013 Exp number, first 60 AAs:  0
# Capsellagrandiflora_Cagra.1150s0013 Total prob of N-in:        0.00957
Capsellagrandiflora_Cagra.1150s0013     TMHMM2.0        outside      1   418
```

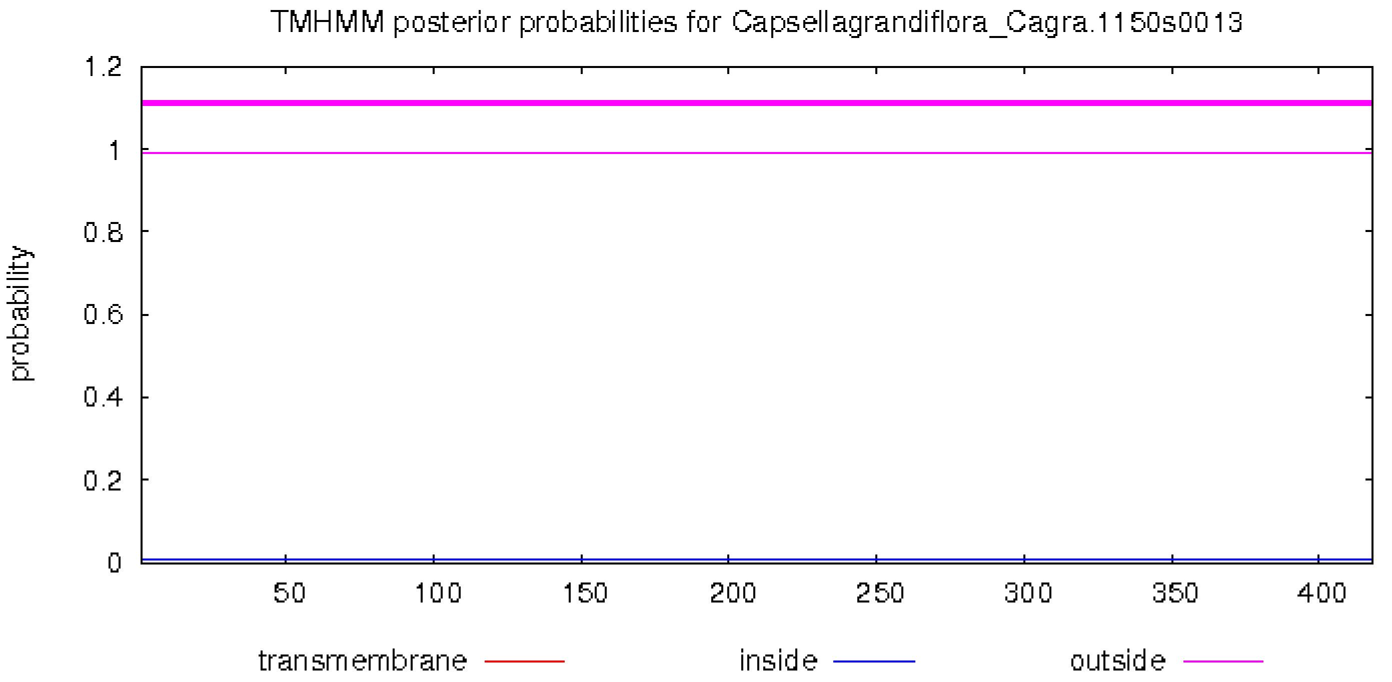

plot in postscript, script for making the plot in gnuplot, data for plot

---

```
# Capsellagrandiflora_Cagra.11546s0003 Length: 457
# Capsellagrandiflora_Cagra.11546s0003 Number of predicted TMHs:  0
# Capsellagrandiflora_Cagra.11546s0003 Exp number of AAs in TMHs: 0.0002
# Capsellagrandiflora_Cagra.11546s0003 Exp number, first 60 AAs:  0
# Capsellagrandiflora_Cagra.11546s0003 Total prob of N-in:        0.00272
Capsellagrandiflora_Cagra.11546s0003    TMHMM2.0        outside      1   457
```

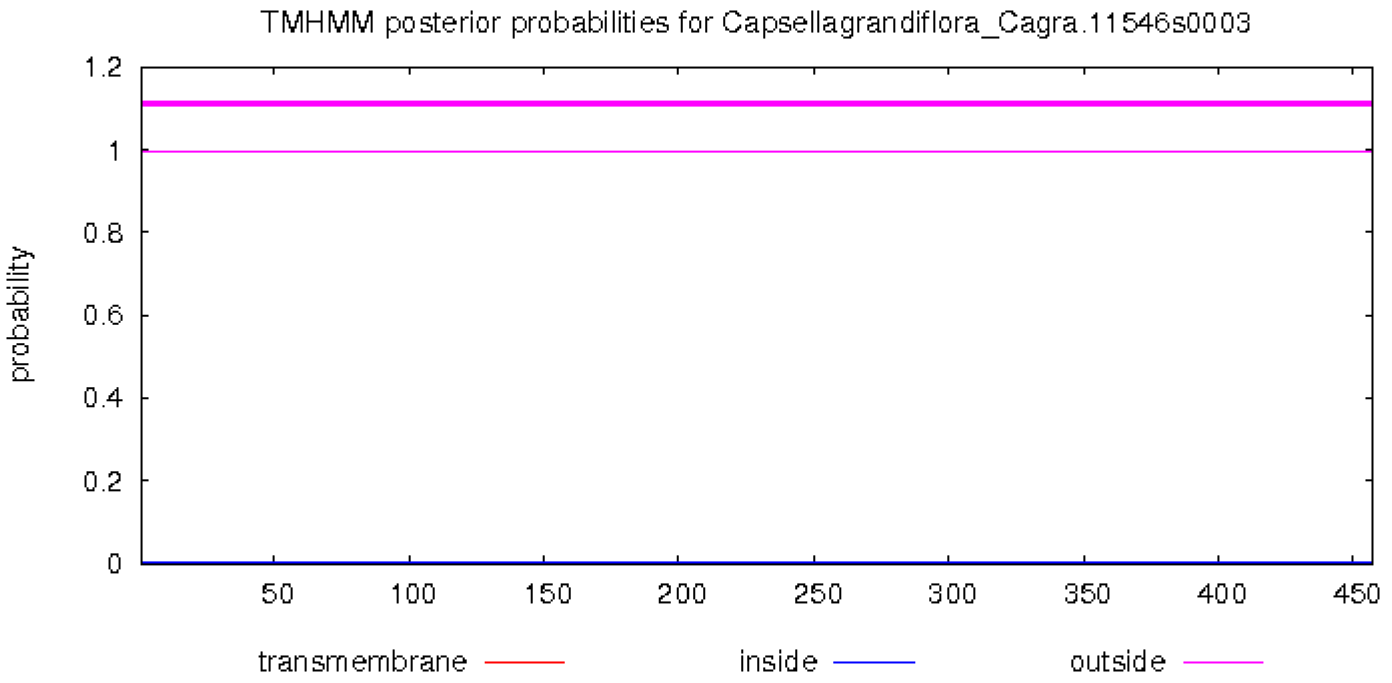

plot in postscript, script for making the plot in gnuplot, data for plot

---

```
# Capsellagrandiflora_Cagra.1225s0038 Length: 352
# Capsellagrandiflora_Cagra.1225s0038 Number of predicted TMHs:  0
# Capsellagrandiflora_Cagra.1225s0038 Exp number of AAs in TMHs: 0
# Capsellagrandiflora_Cagra.1225s0038 Exp number, first 60 AAs:  0
# Capsellagrandiflora_Cagra.1225s0038 Total prob of N-in:        0.00567
Capsellagrandiflora_Cagra.1225s0038     TMHMM2.0        outside      1   352
```

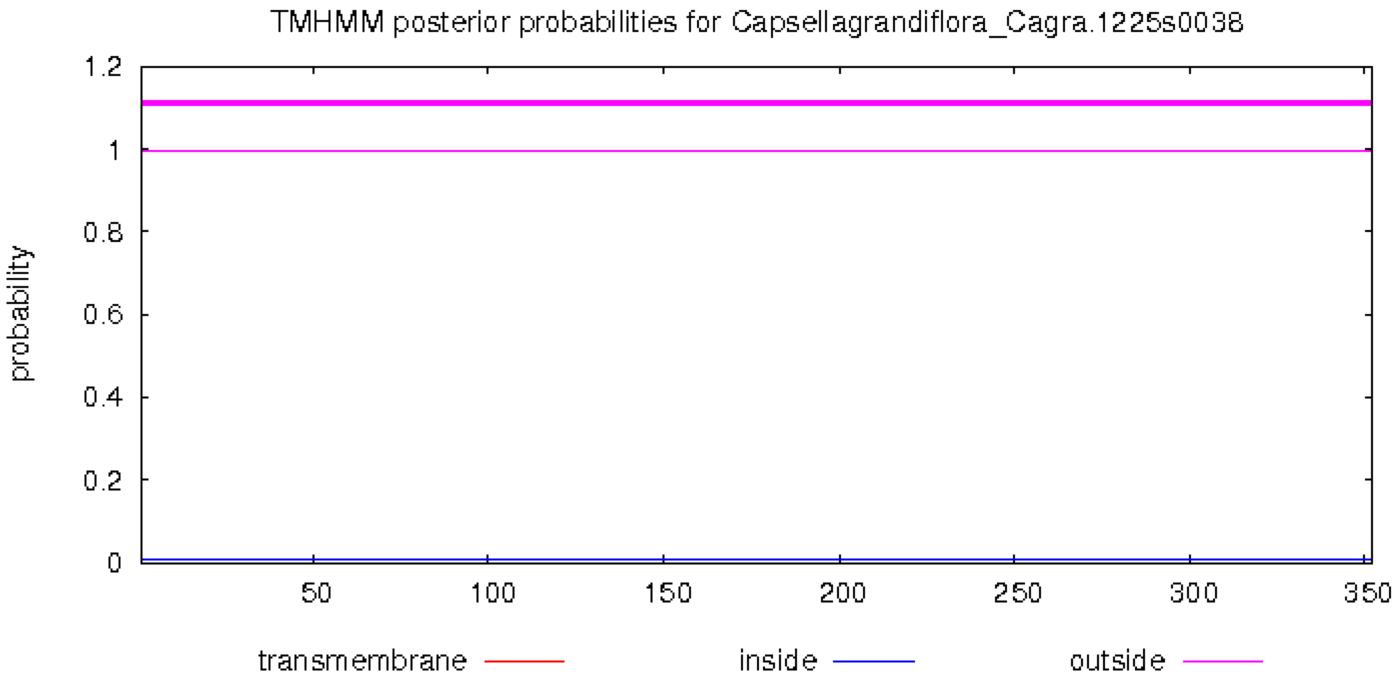

plot in postscript, script for making the plot in gnuplot, data for plot

```
# Capsellagrandiflora_Cagra.12529s0001 Length: 565
# Capsellagrandiflora_Cagra.12529s0001 Number of predicted TMHs:  1
# Capsellagrandiflora_Cagra.12529s0001 Exp number of AAs in TMHs: 23.02766
# Capsellagrandiflora_Cagra.12529s0001 Exp number, first 60 AAs:  0.00466
# Capsellagrandiflora_Cagra.12529s0001 Total prob of N-in:        0.00401
Capsellagrandiflora_Cagra.12529s0001    TMHMM2.0        outside      1   538
Capsellagrandiflora_Cagra.12529s0001    TMHMM2.0        TMhelix    539   561
Capsellagrandiflora_Cagra.12529s0001    TMHMM2.0        inside     562   565
```

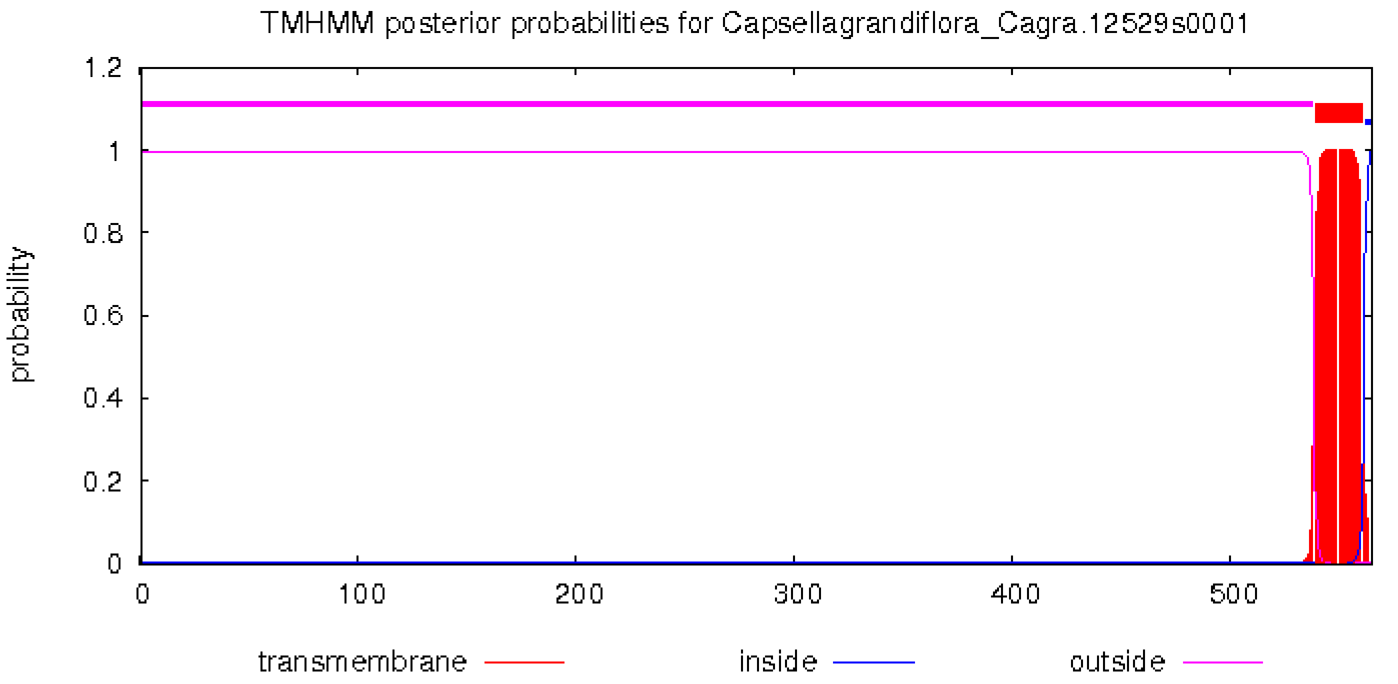

plot in postscript, script for making the plot in gnuplot, data for plot

```
# Capsellagrandiflora_Cagra.12686s0005 Length: 341
# Capsellagrandiflora_Cagra.12686s0005 Number of predicted TMHs:  0
# Capsellagrandiflora_Cagra.12686s0005 Exp number of AAs in TMHs: 0
# Capsellagrandiflora_Cagra.12686s0005 Exp number, first 60 AAs:  0
# Capsellagrandiflora_Cagra.12686s0005 Total prob of N-in:        0.00436
Capsellagrandiflora_Cagra.12686s0005    TMHMM2.0        outside      1   341
```

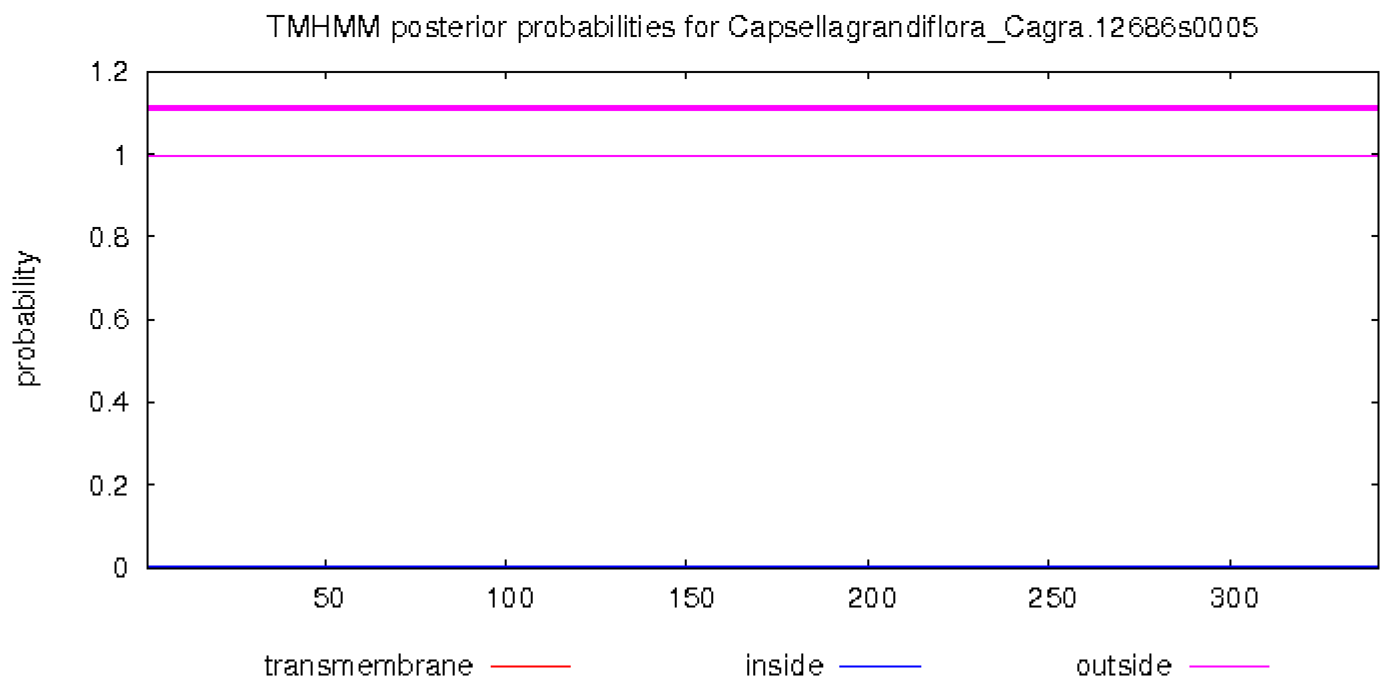

plot in postscript, script for making the plot in gnuplot, data for plot

```
# Capsellagrandiflora_Cagra.1301s0009 Length: 285
# Capsellagrandiflora_Cagra.1301s0009 Number of predicted TMHs:  0
# Capsellagrandiflora_Cagra.1301s0009 Exp number of AAs in TMHs: 0.00031
# Capsellagrandiflora_Cagra.1301s0009 Exp number, first 60 AAs:  0
# Capsellagrandiflora_Cagra.1301s0009 Total prob of N-in:        0.03036
Capsellagrandiflora_Cagra.1301s0009     TMHMM2.0        outside      1   285
```

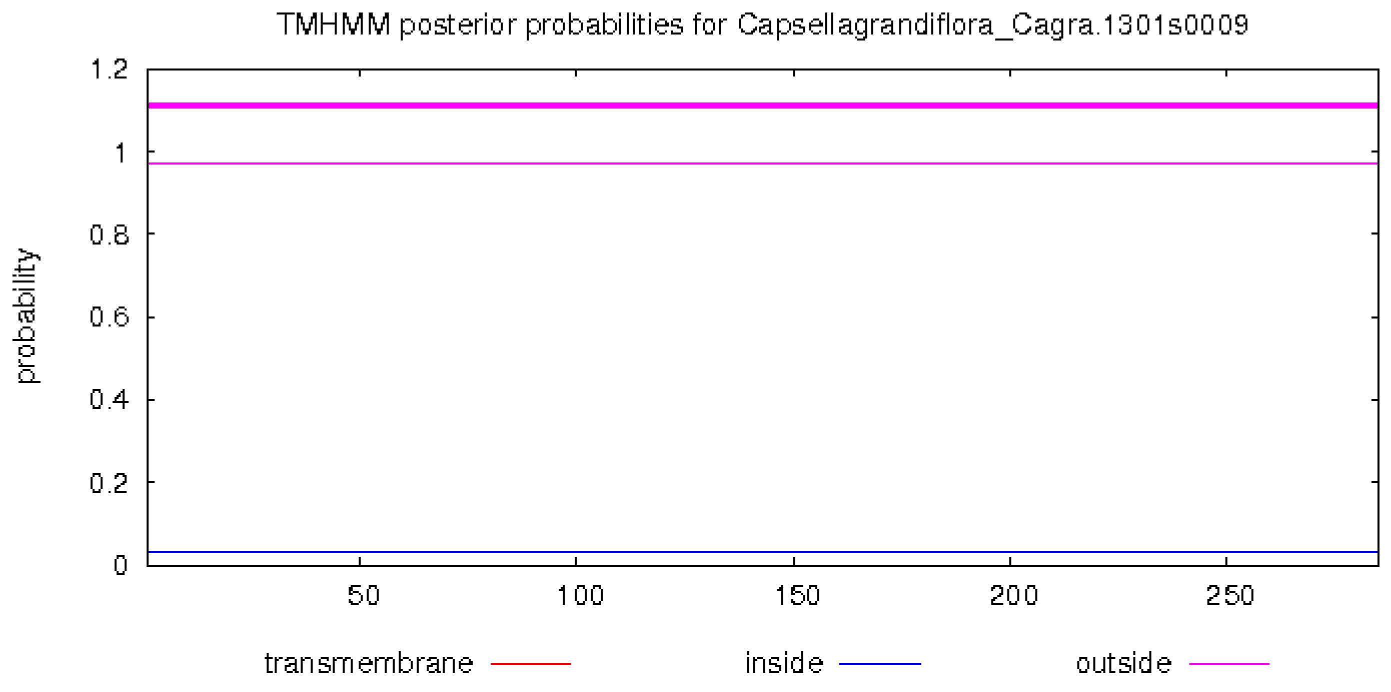

plot in postscript, script for making the plot in gnuplot, data for plot

```
# Capsellagrandiflora_Cagra.1301s0010 Length: 320
# Capsellagrandiflora_Cagra.1301s0010 Number of predicted TMHs:  0
# Capsellagrandiflora_Cagra.1301s0010 Exp number of AAs in TMHs: 0.00272
# Capsellagrandiflora_Cagra.1301s0010 Exp number, first 60 AAs:  0
# Capsellagrandiflora_Cagra.1301s0010 Total prob of N-in:        0.00968
Capsellagrandiflora_Cagra.1301s0010     TMHMM2.0        outside      1   320
```

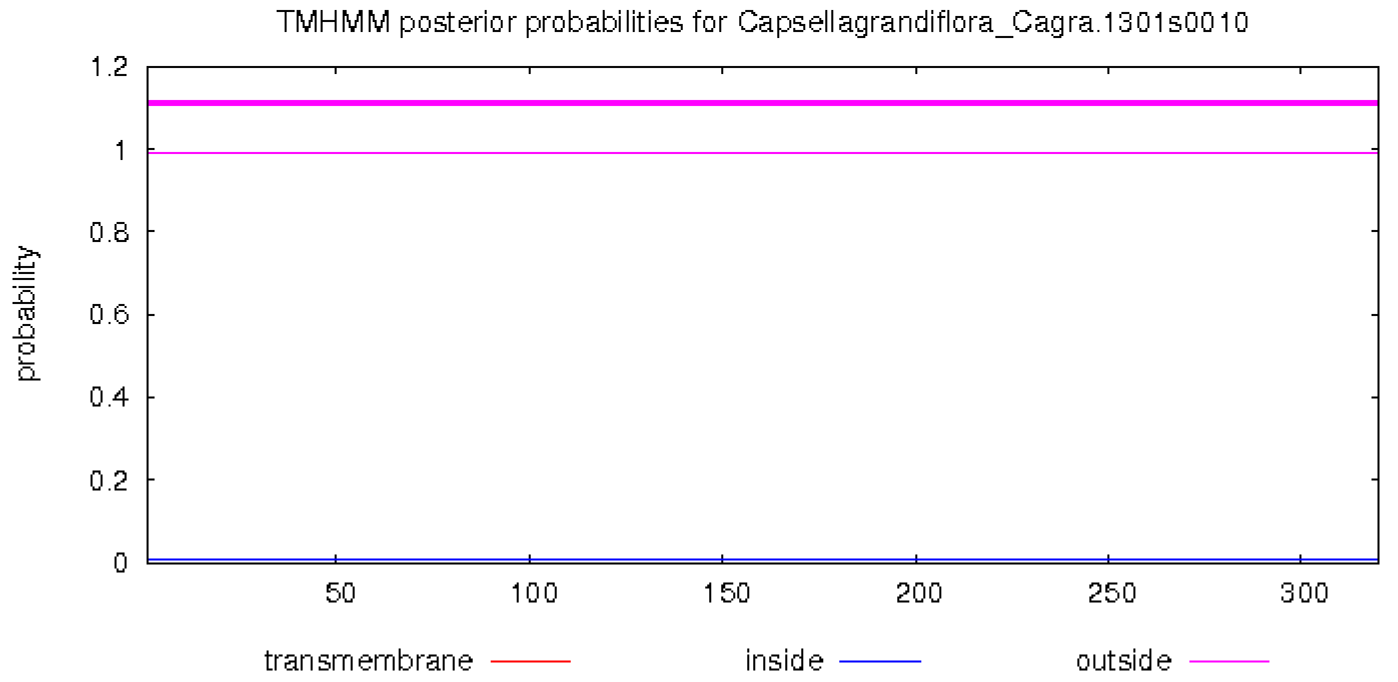

plot in postscript, script for making the plot in gnuplot, data for plot

```
# Capsellagrandiflora_Cagra.1310s0016 Length: 304
# Capsellagrandiflora_Cagra.1310s0016 Number of predicted TMHs:  0
# Capsellagrandiflora_Cagra.1310s0016 Exp number of AAs in TMHs: 0
# Capsellagrandiflora_Cagra.1310s0016 Exp number, first 60 AAs:  0
# Capsellagrandiflora_Cagra.1310s0016 Total prob of N-in:        0.00200
Capsellagrandiflora_Cagra.1310s0016     TMHMM2.0        outside      1   304
```

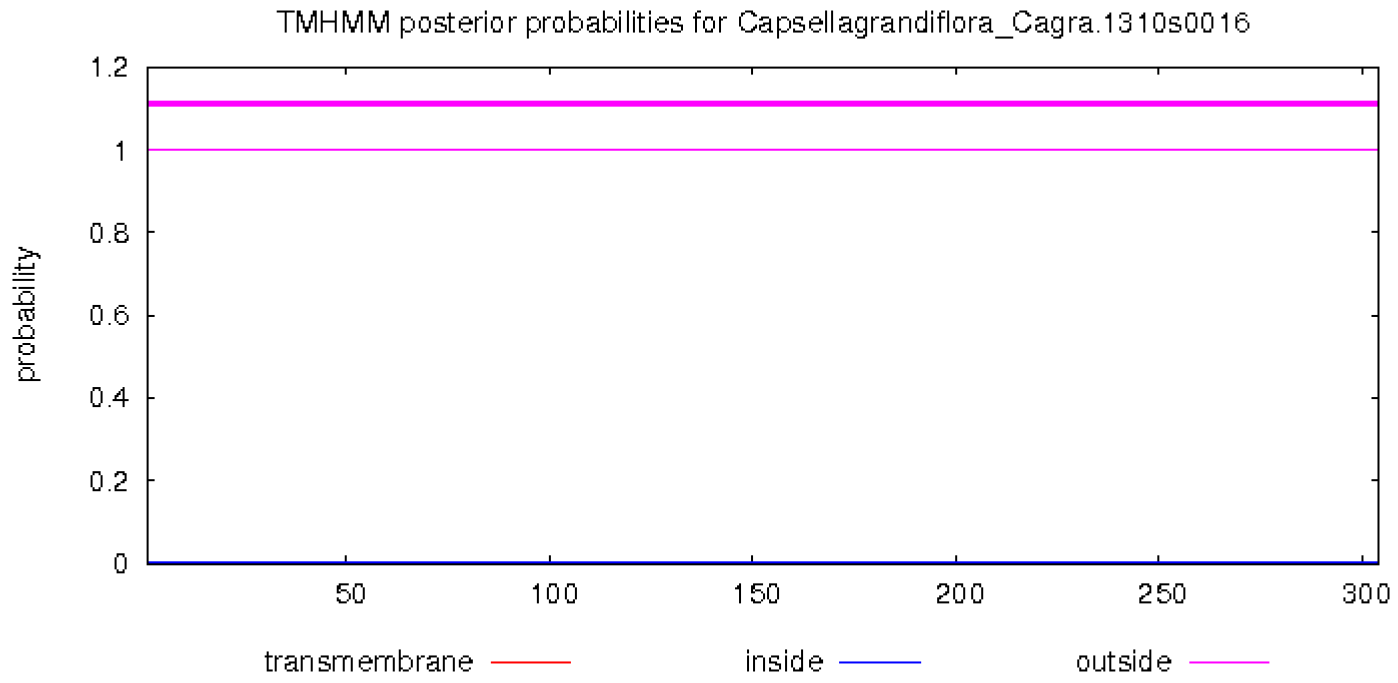

plot in postscript, script for making the plot in gnuplot, data for plot

```
# Capsellagrandiflora_Cagra.1365s0094 Length: 283
# Capsellagrandiflora_Cagra.1365s0094 Number of predicted TMHs:  0
# Capsellagrandiflora_Cagra.1365s0094 Exp number of AAs in TMHs: 8e-05
# Capsellagrandiflora_Cagra.1365s0094 Exp number, first 60 AAs:  0
# Capsellagrandiflora_Cagra.1365s0094 Total prob of N-in:        0.00921
Capsellagrandiflora_Cagra.1365s0094     TMHMM2.0        outside      1   283
```

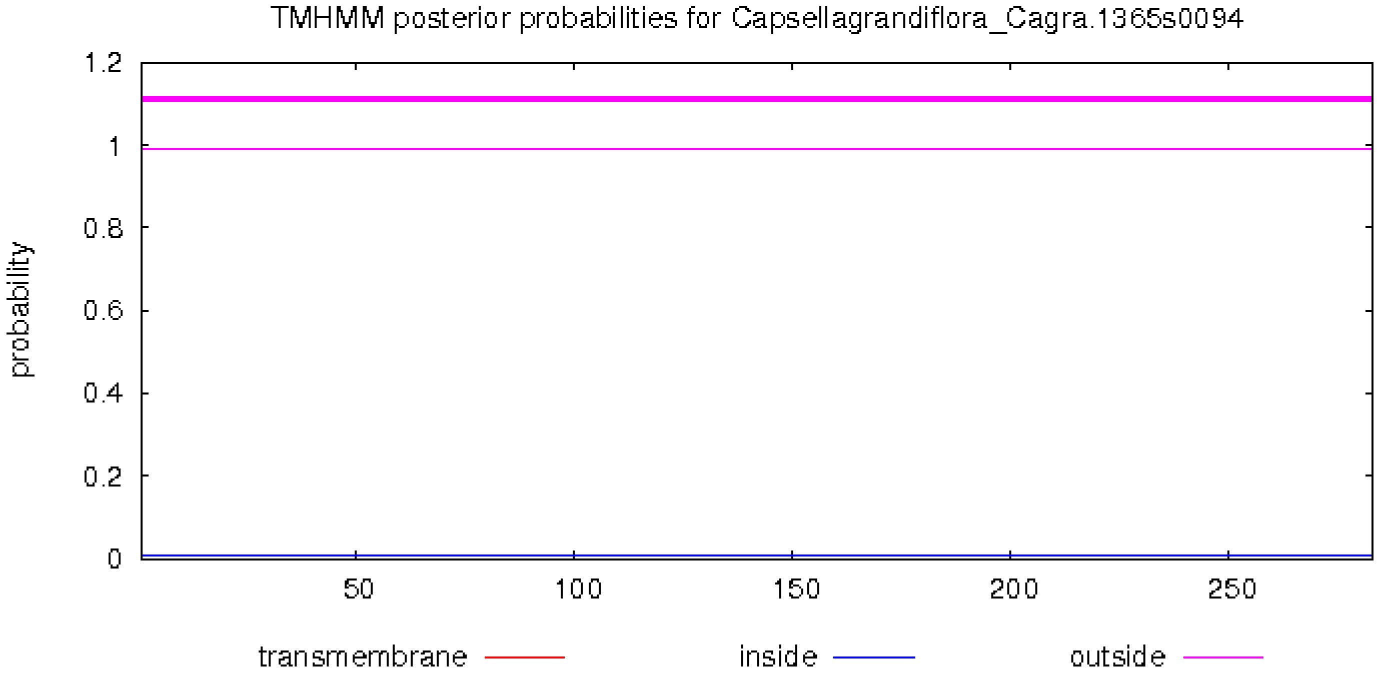

plot in postscript, script for making the plot in gnuplot, data for plot

---

```
# Capsellagrandiflora_Cagra.1365s0106 Length: 393
# Capsellagrandiflora_Cagra.1365s0106 Number of predicted TMHs:  0
# Capsellagrandiflora_Cagra.1365s0106 Exp number of AAs in TMHs: 0.00071
# Capsellagrandiflora_Cagra.1365s0106 Exp number, first 60 AAs:  0.00018
# Capsellagrandiflora_Cagra.1365s0106 Total prob of N-in:        0.00275
Capsellagrandiflora_Cagra.1365s0106     TMHMM2.0        outside      1   393
```

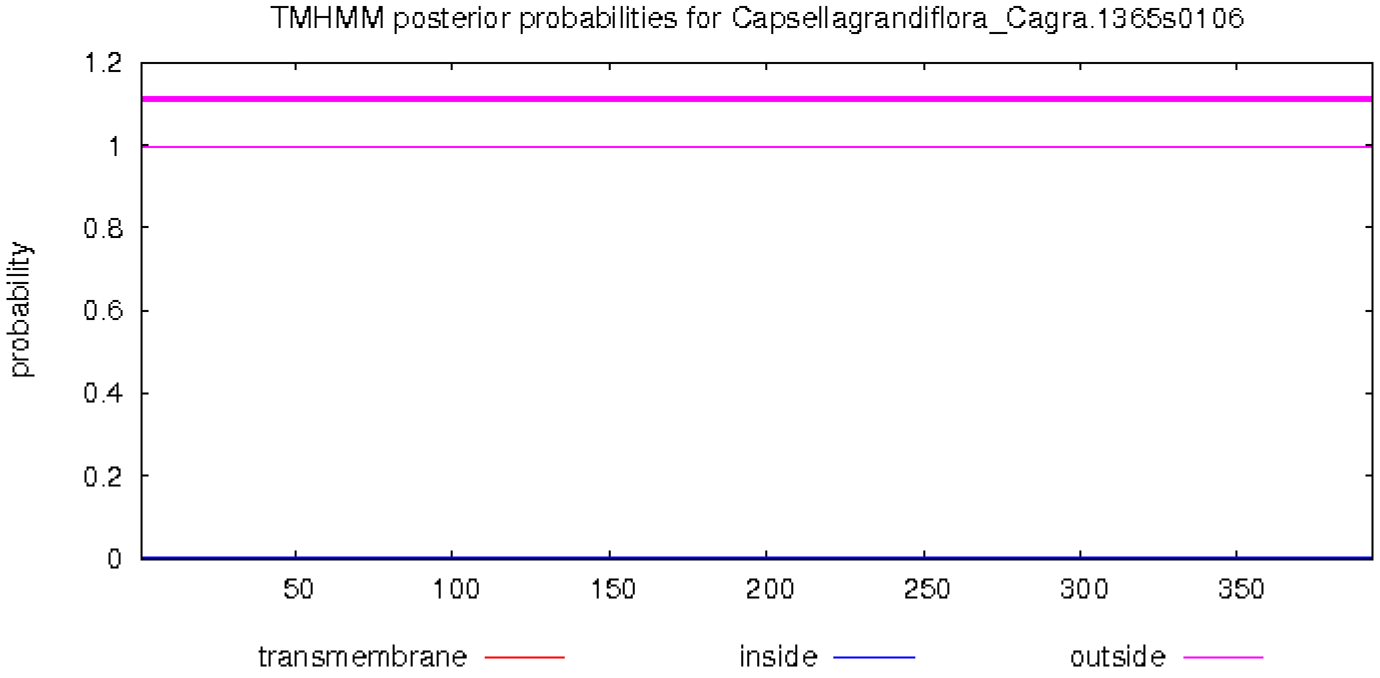

plot in postscript, script for making the plot in gnuplot, data for plot

---

```
# Capsellagrandiflora_Cagra.1378s0012 Length: 234
# Capsellagrandiflora_Cagra.1378s0012 Number of predicted TMHs:  0
# Capsellagrandiflora_Cagra.1378s0012 Exp number of AAs in TMHs: 0
# Capsellagrandiflora_Cagra.1378s0012 Exp number, first 60 AAs:  0
# Capsellagrandiflora_Cagra.1378s0012 Total prob of N-in:        0.02018
Capsellagrandiflora_Cagra.1378s0012     TMHMM2.0        outside      1   234
```

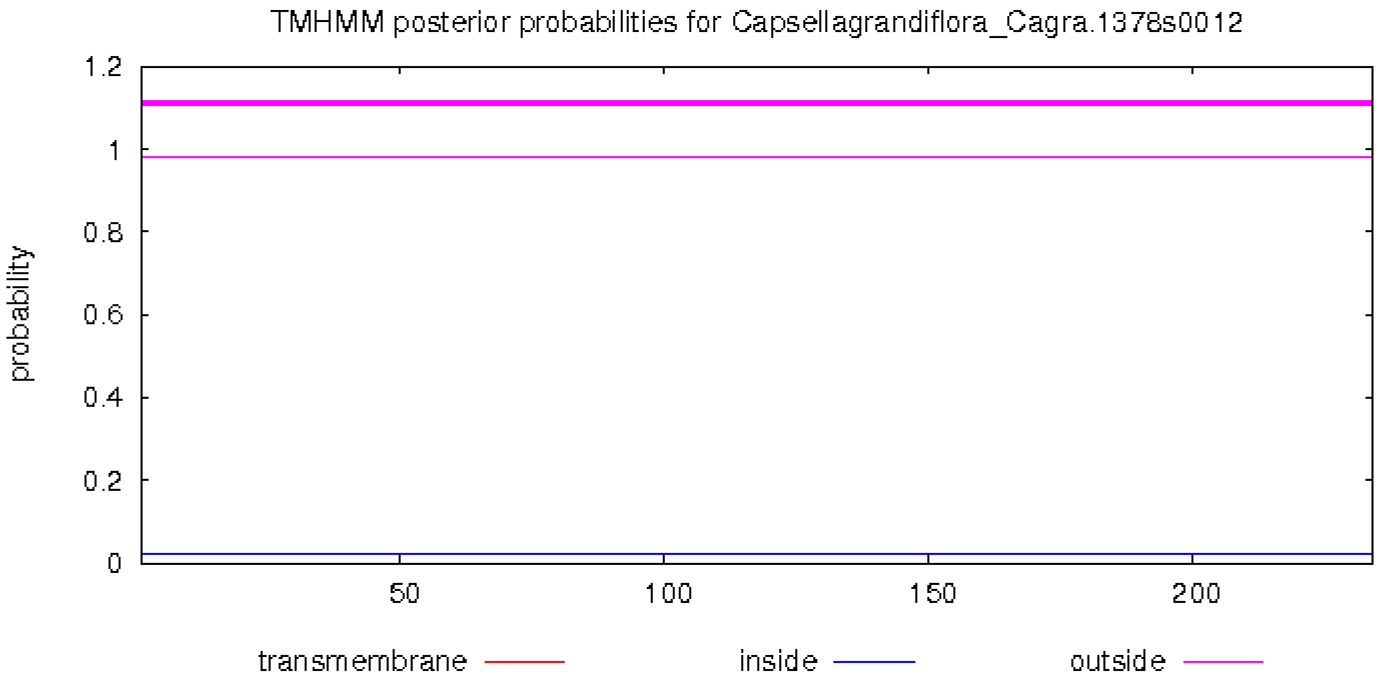

plot in postscript, script for making the plot in gnuplot, data for plot

---

```
# Capsellagrandiflora_Cagra.1443s0001 Length: 656
# Capsellagrandiflora_Cagra.1443s0001 Number of predicted TMHs:  1
# Capsellagrandiflora_Cagra.1443s0001 Exp number of AAs in TMHs: 19.90365
# Capsellagrandiflora_Cagra.1443s0001 Exp number, first 60 AAs:  0
# Capsellagrandiflora_Cagra.1443s0001 Total prob of N-in:        0.00757
Capsellagrandiflora_Cagra.1443s0001     TMHMM2.0        outside      1   613
Capsellagrandiflora_Cagra.1443s0001     TMHMM2.0        TMhelix    614   633
Capsellagrandiflora_Cagra.1443s0001     TMHMM2.0        inside     634   656
```

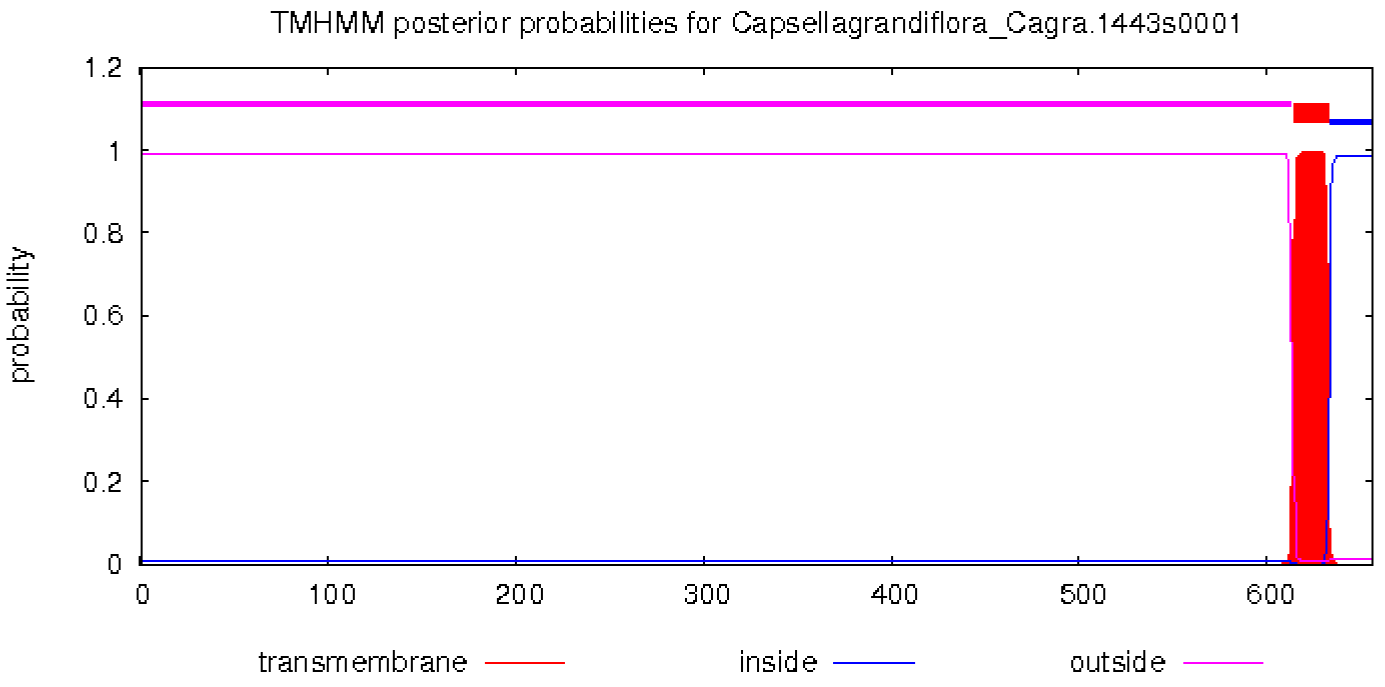

plot in postscript, script for making the plot in gnuplot, data for plot

```
# Capsellagrandiflora_Cagra.1443s0015 Length: 532
# Capsellagrandiflora_Cagra.1443s0015 Number of predicted TMHs:  1
# Capsellagrandiflora_Cagra.1443s0015 Exp number of AAs in TMHs: 25.50261
# Capsellagrandiflora_Cagra.1443s0015 Exp number, first 60 AAs:  0.07768
# Capsellagrandiflora_Cagra.1443s0015 Total prob of N-in:        0.63909
Capsellagrandiflora_Cagra.1443s0015     TMHMM2.0        inside       1   502
Capsellagrandiflora_Cagra.1443s0015     TMHMM2.0        TMhelix    503   525
Capsellagrandiflora_Cagra.1443s0015     TMHMM2.0        outside    526   532
```

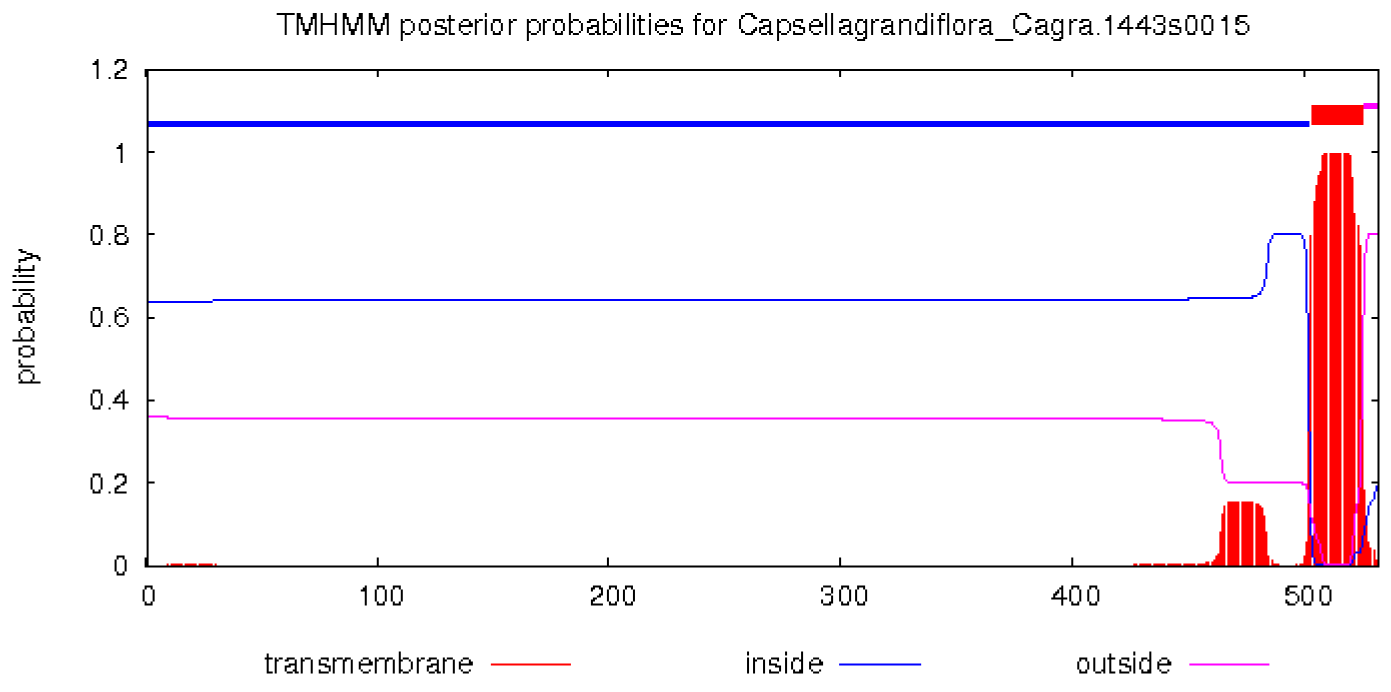

plot in postscript, script for making the plot in gnuplot, data for plot

```
# Capsellagrandiflora_Cagra.14513s0001 Length: 367
# Capsellagrandiflora_Cagra.14513s0001 Number of predicted TMHs:  0
# Capsellagrandiflora_Cagra.14513s0001 Exp number of AAs in TMHs: 0
# Capsellagrandiflora_Cagra.14513s0001 Exp number, first 60 AAs:  0
# Capsellagrandiflora_Cagra.14513s0001 Total prob of N-in:        0.01693
Capsellagrandiflora_Cagra.14513s0001    TMHMM2.0        outside      1   367
```

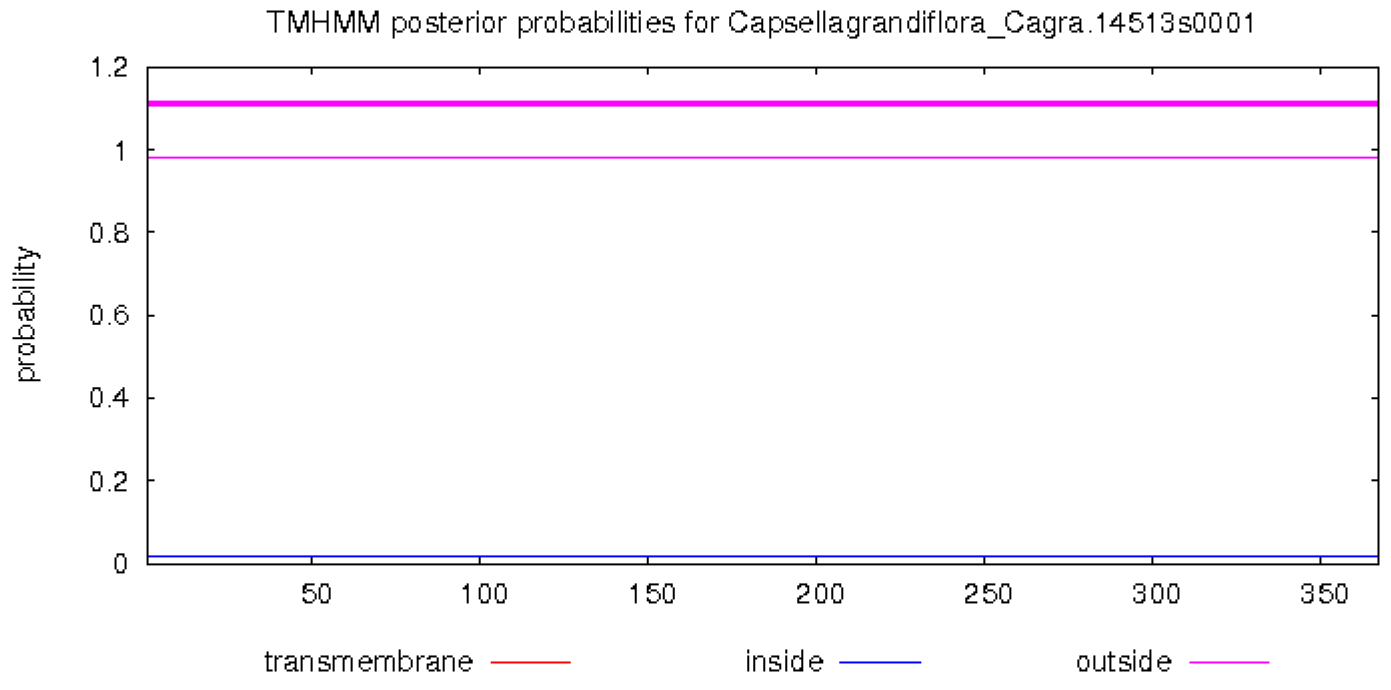

plot in postscript, script for making the plot in gnuplot, data for plot

```
# Capsellagrandiflora_Cagra.1670s0031 Length: 411
# Capsellagrandiflora_Cagra.1670s0031 Number of predicted TMHs:  0
# Capsellagrandiflora_Cagra.1670s0031 Exp number of AAs in TMHs: 0.00018
# Capsellagrandiflora_Cagra.1670s0031 Exp number, first 60 AAs:  0
# Capsellagrandiflora_Cagra.1670s0031 Total prob of N-in:        0.00164
Capsellagrandiflora_Cagra.1670s0031     TMHMM2.0        outside      1   411
```

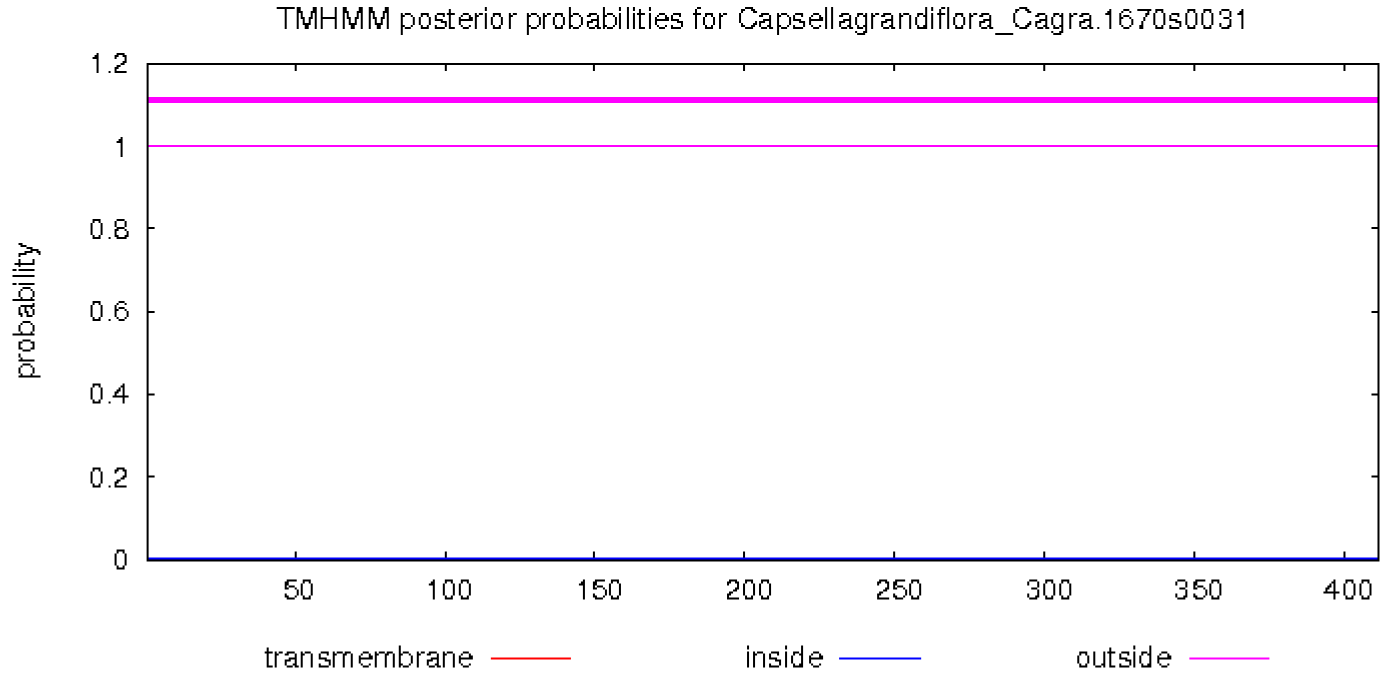

plot in postscript, script for making the plot in gnuplot, data for plot

```
# Capsellagrandiflora_Cagra.1681s0009 Length: 311
# Capsellagrandiflora_Cagra.1681s0009 Number of predicted TMHs:  0
# Capsellagrandiflora_Cagra.1681s0009 Exp number of AAs in TMHs: 0.04294
# Capsellagrandiflora_Cagra.1681s0009 Exp number, first 60 AAs:  0
# Capsellagrandiflora_Cagra.1681s0009 Total prob of N-in:        0.00503
Capsellagrandiflora_Cagra.1681s0009     TMHMM2.0        outside      1   311
```

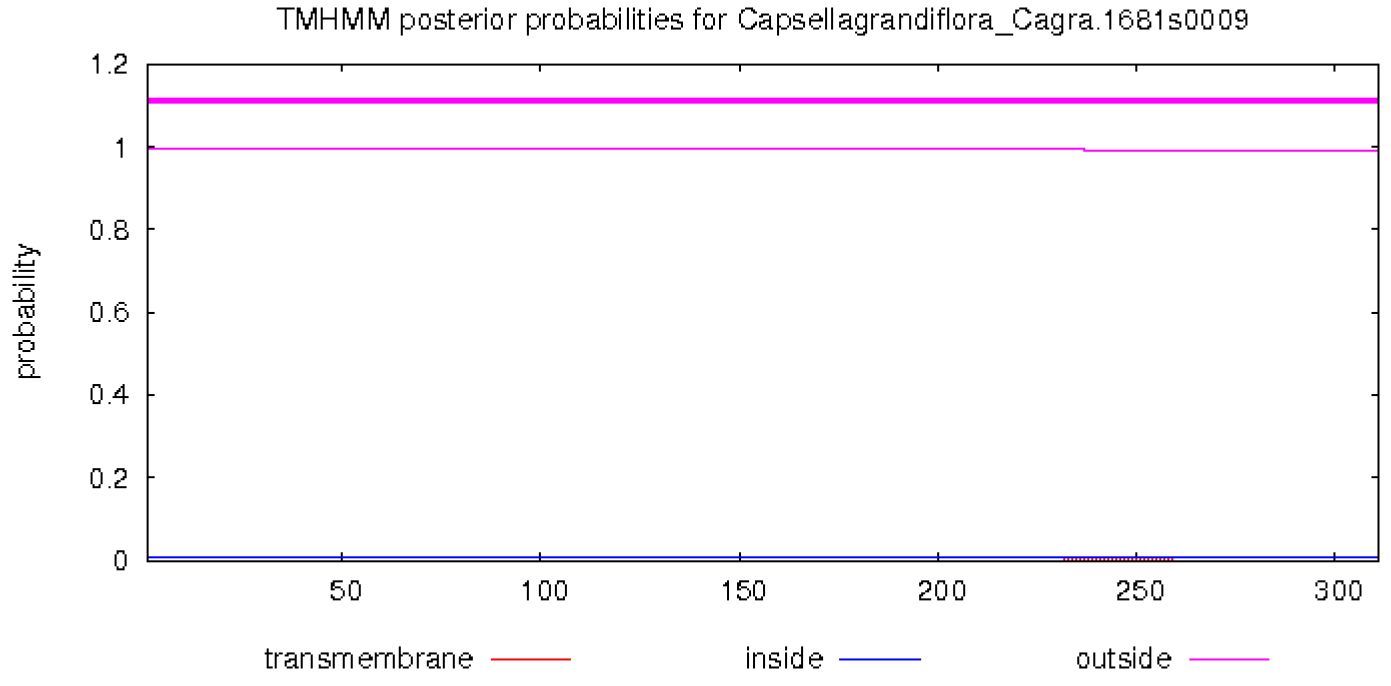

plot in postscript, script for making the plot in gnuplot, data for plot

```
# Capsellagrandiflora_Cagra.1725s0032 Length: 374
# Capsellagrandiflora_Cagra.1725s0032 Number of predicted TMHs:  0
# Capsellagrandiflora_Cagra.1725s0032 Exp number of AAs in TMHs: 0.00501
# Capsellagrandiflora_Cagra.1725s0032 Exp number, first 60 AAs:  0.00096
# Capsellagrandiflora_Cagra.1725s0032 Total prob of N-in:        0.00739
Capsellagrandiflora_Cagra.1725s0032     TMHMM2.0        outside      1   374
```

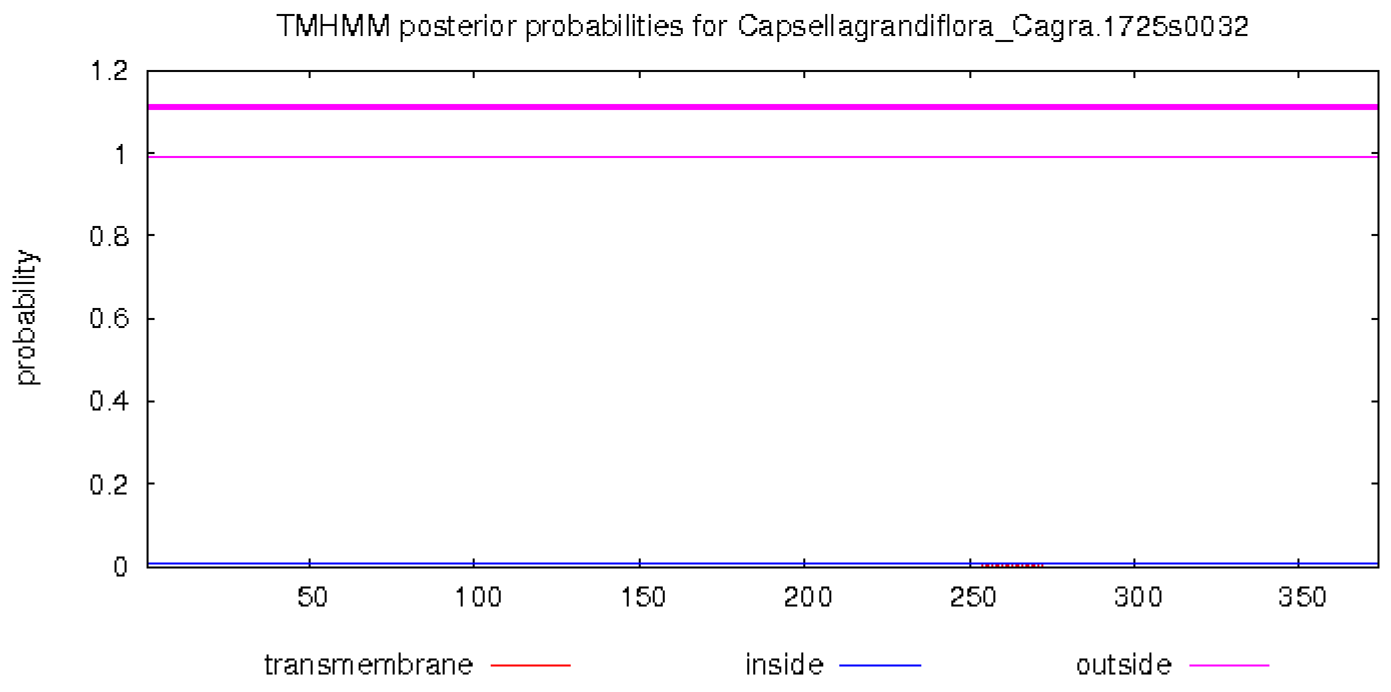

plot in postscript, script for making the plot in gnuplot, data for plot

```
# Capsellagrandiflora_Cagra.1822s0012 Length: 309
# Capsellagrandiflora_Cagra.1822s0012 Number of predicted TMHs:  0
# Capsellagrandiflora_Cagra.1822s0012 Exp number of AAs in TMHs: 0.00021
# Capsellagrandiflora_Cagra.1822s0012 Exp number, first 60 AAs:  0
# Capsellagrandiflora_Cagra.1822s0012 Total prob of N-in:        0.03961
Capsellagrandiflora_Cagra.1822s0012     TMHMM2.0        outside      1   309
```

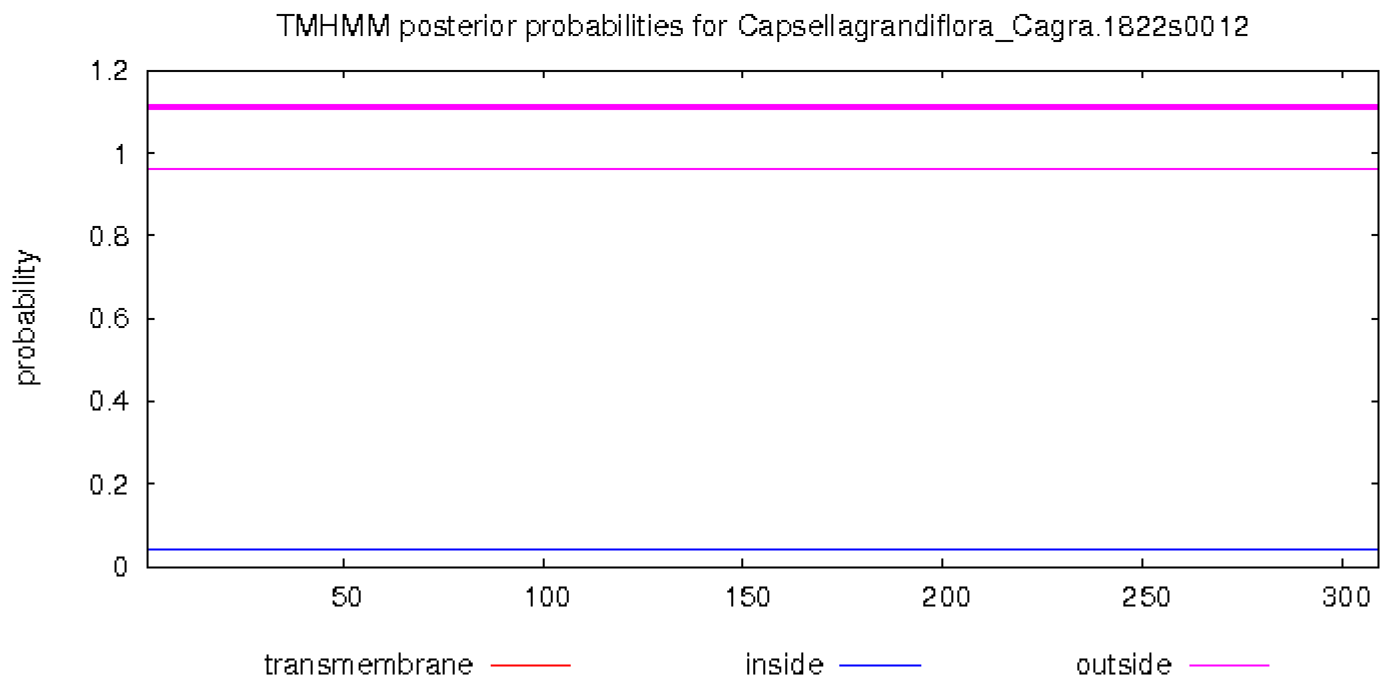

plot in postscript, script for making the plot in gnuplot, data for plot

```
# Capsellagrandiflora_Cagra.1838s0033 Length: 460
# Capsellagrandiflora_Cagra.1838s0033 Number of predicted TMHs:  1
# Capsellagrandiflora_Cagra.1838s0033 Exp number of AAs in TMHs: 17.98455
# Capsellagrandiflora_Cagra.1838s0033 Exp number, first 60 AAs:  0
# Capsellagrandiflora_Cagra.1838s0033 Total prob of N-in:        0.12243
Capsellagrandiflora_Cagra.1838s0033     TMHMM2.0        outside      1   429
Capsellagrandiflora_Cagra.1838s0033     TMHMM2.0        TMhelix    430   452
Capsellagrandiflora_Cagra.1838s0033     TMHMM2.0        inside     453   460
```

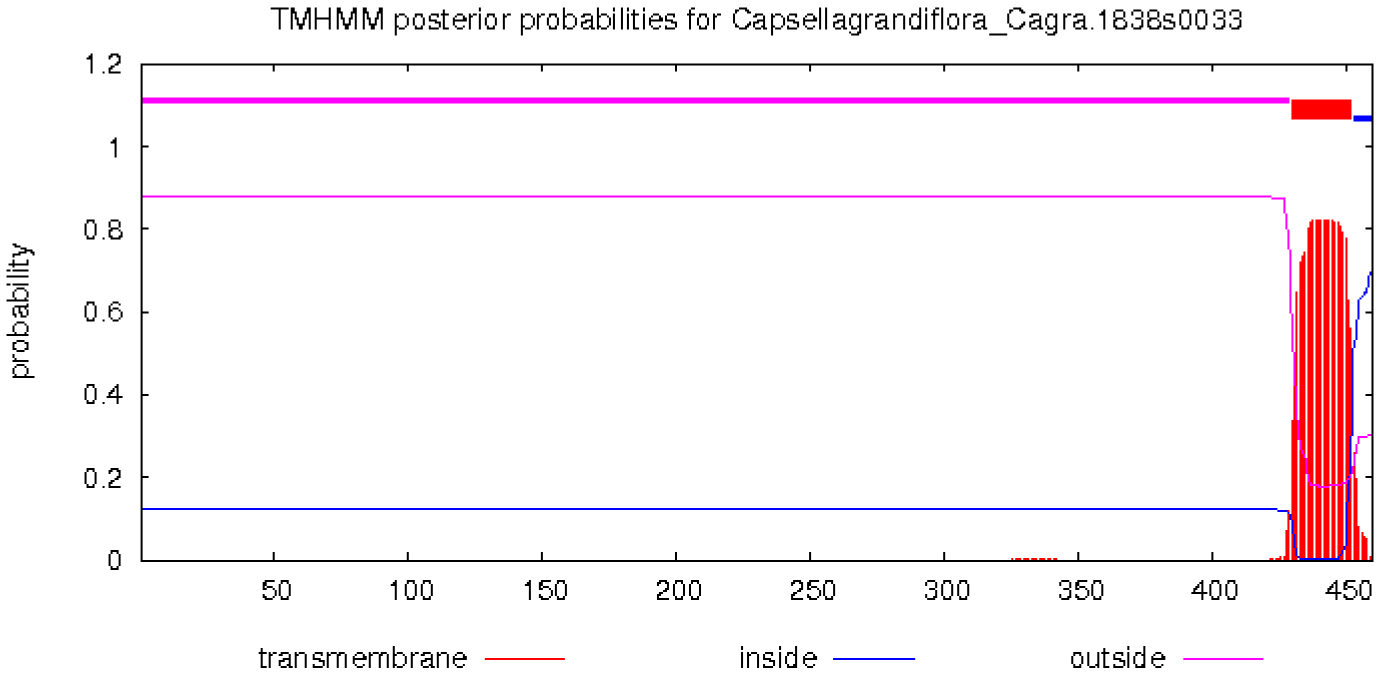

plot in postscript, script for making the plot in gnuplot, data for plot

```
# Capsellagrandiflora_Cagra.1968s0008 Length: 164
# Capsellagrandiflora_Cagra.1968s0008 Number of predicted TMHs:  0
# Capsellagrandiflora_Cagra.1968s0008 Exp number of AAs in TMHs: 0.00206
# Capsellagrandiflora_Cagra.1968s0008 Exp number, first 60 AAs:  0
# Capsellagrandiflora_Cagra.1968s0008 Total prob of N-in:        0.05847
Capsellagrandiflora_Cagra.1968s0008     TMHMM2.0        outside      1   164
```

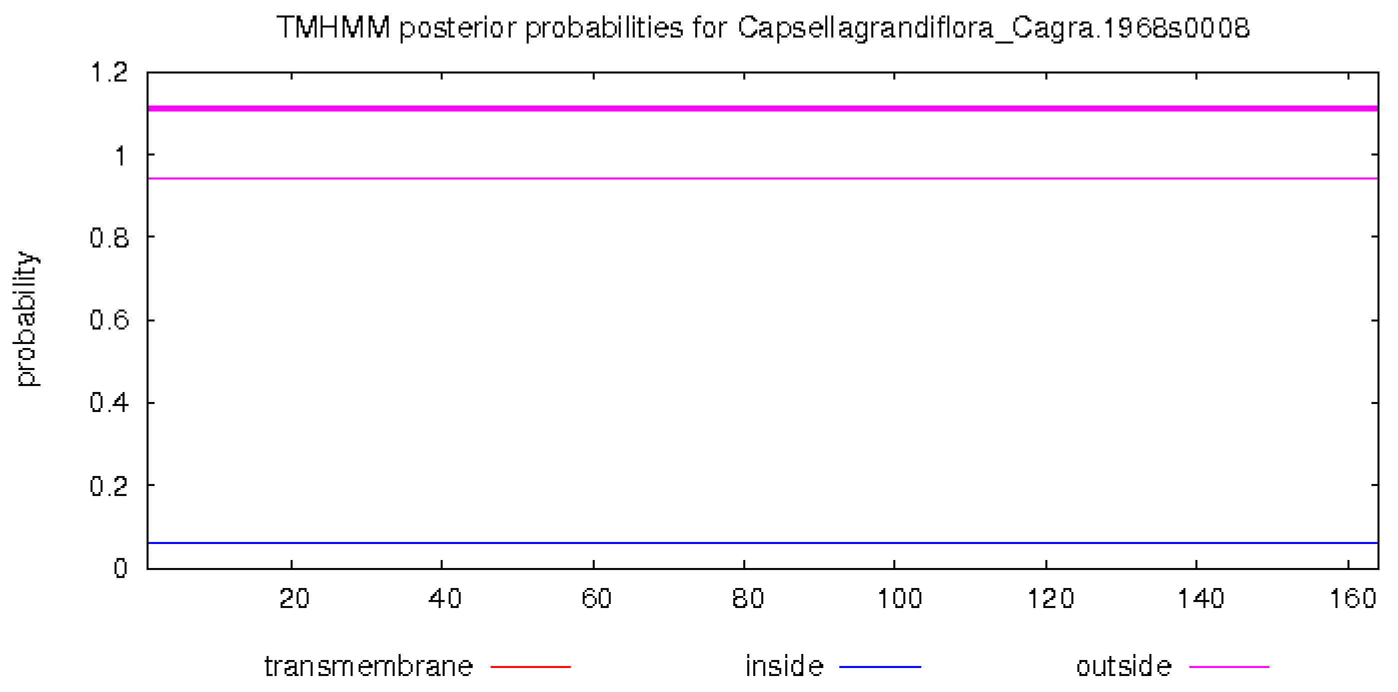

plot in postscript, script for making the plot in gnuplot, data for plot

---

```
# Capsellagrandiflora_Cagra.1968s0009 Length: 381
# Capsellagrandiflora_Cagra.1968s0009 Number of predicted TMHs:  0
# Capsellagrandiflora_Cagra.1968s0009 Exp number of AAs in TMHs: 9e-05
# Capsellagrandiflora_Cagra.1968s0009 Exp number, first 60 AAs:  0
# Capsellagrandiflora_Cagra.1968s0009 Total prob of N-in:        0.00248
Capsellagrandiflora_Cagra.1968s0009     TMHMM2.0        outside      1   381
```

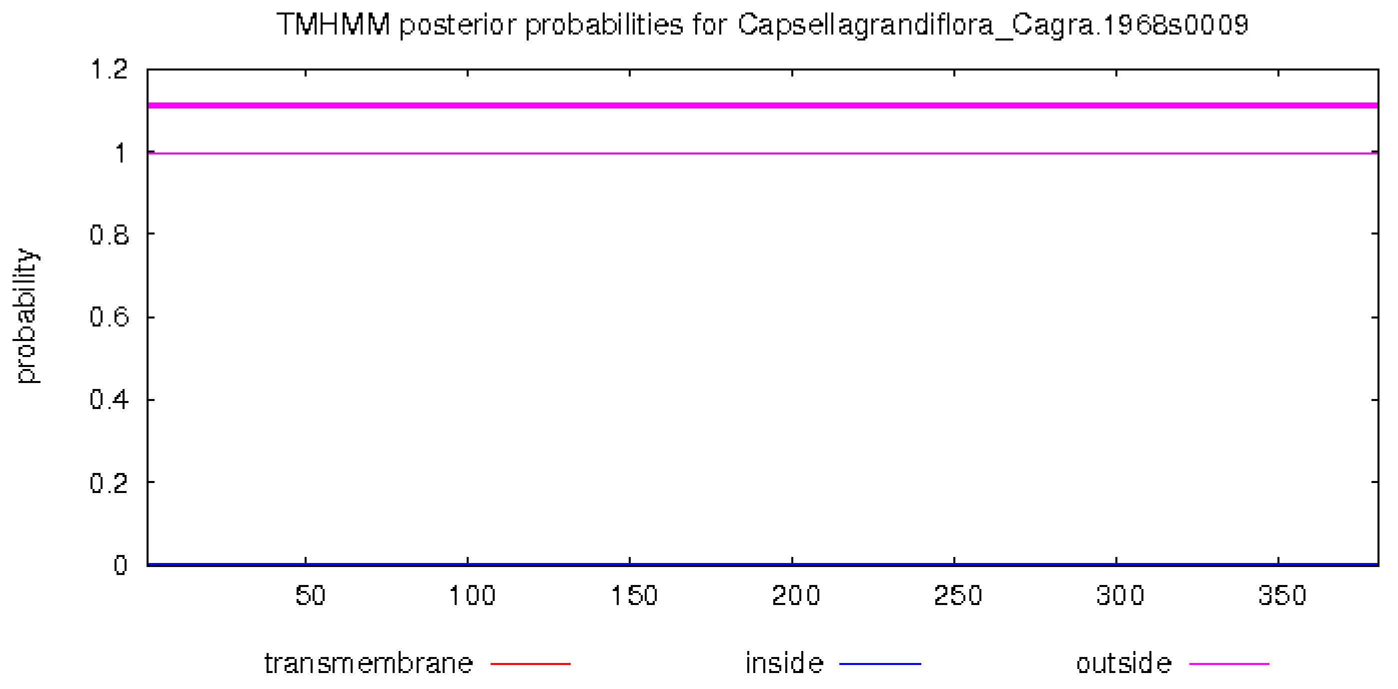

plot in postscript, script for making the plot in gnuplot, data for plot

---

```
# Capsellagrandiflora_Cagra.1968s0040 Length: 326
# Capsellagrandiflora_Cagra.1968s0040 Number of predicted TMHs:  0
# Capsellagrandiflora_Cagra.1968s0040 Exp number of AAs in TMHs: 0.06451
# Capsellagrandiflora_Cagra.1968s0040 Exp number, first 60 AAs:  0.01323
# Capsellagrandiflora_Cagra.1968s0040 Total prob of N-in:        0.00900
Capsellagrandiflora_Cagra.1968s0040     TMHMM2.0        outside      1   326
```

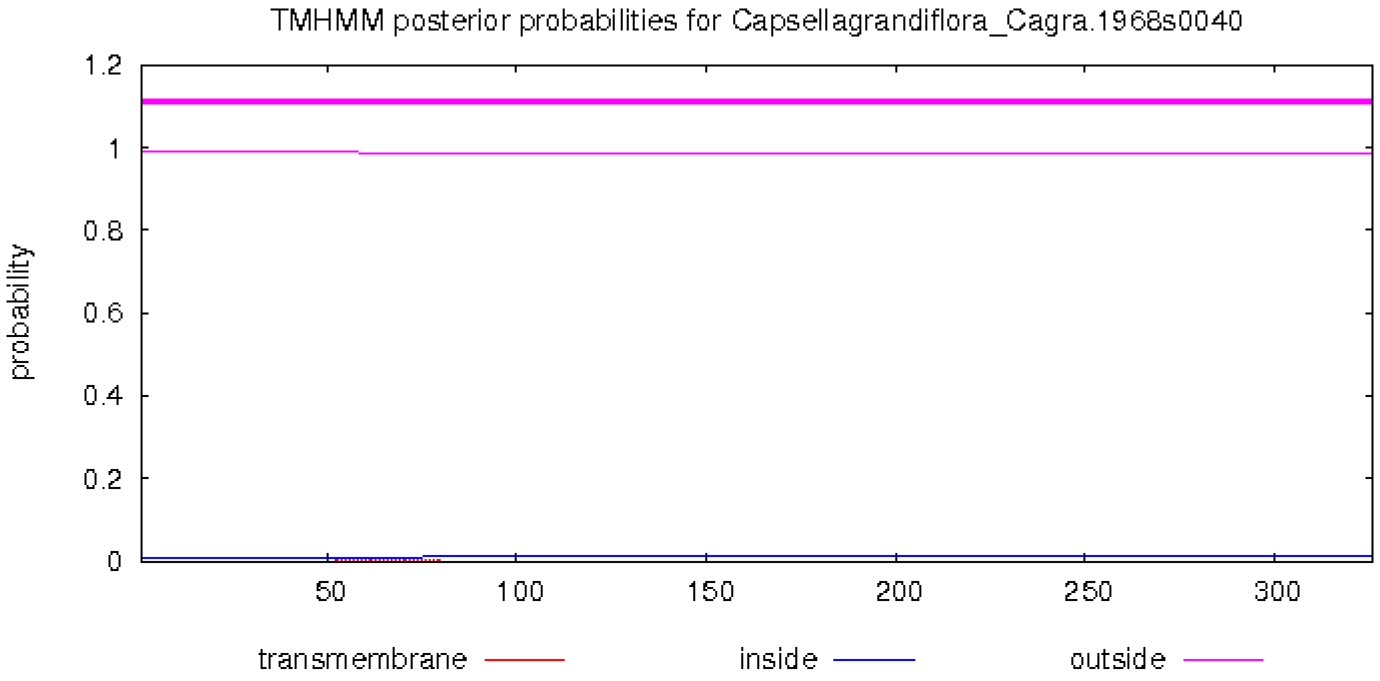

plot in postscript, script for making the plot in gnuplot, data for plot

---

```
# Capsellagrandiflora_Cagra.1968s0041 Length: 288
# Capsellagrandiflora_Cagra.1968s0041 Number of predicted TMHs:  0
# Capsellagrandiflora_Cagra.1968s0041 Exp number of AAs in TMHs: 0.02666
# Capsellagrandiflora_Cagra.1968s0041 Exp number, first 60 AAs:  0
# Capsellagrandiflora_Cagra.1968s0041 Total prob of N-in:        0.02072
Capsellagrandiflora_Cagra.1968s0041     TMHMM2.0        outside      1   288
```

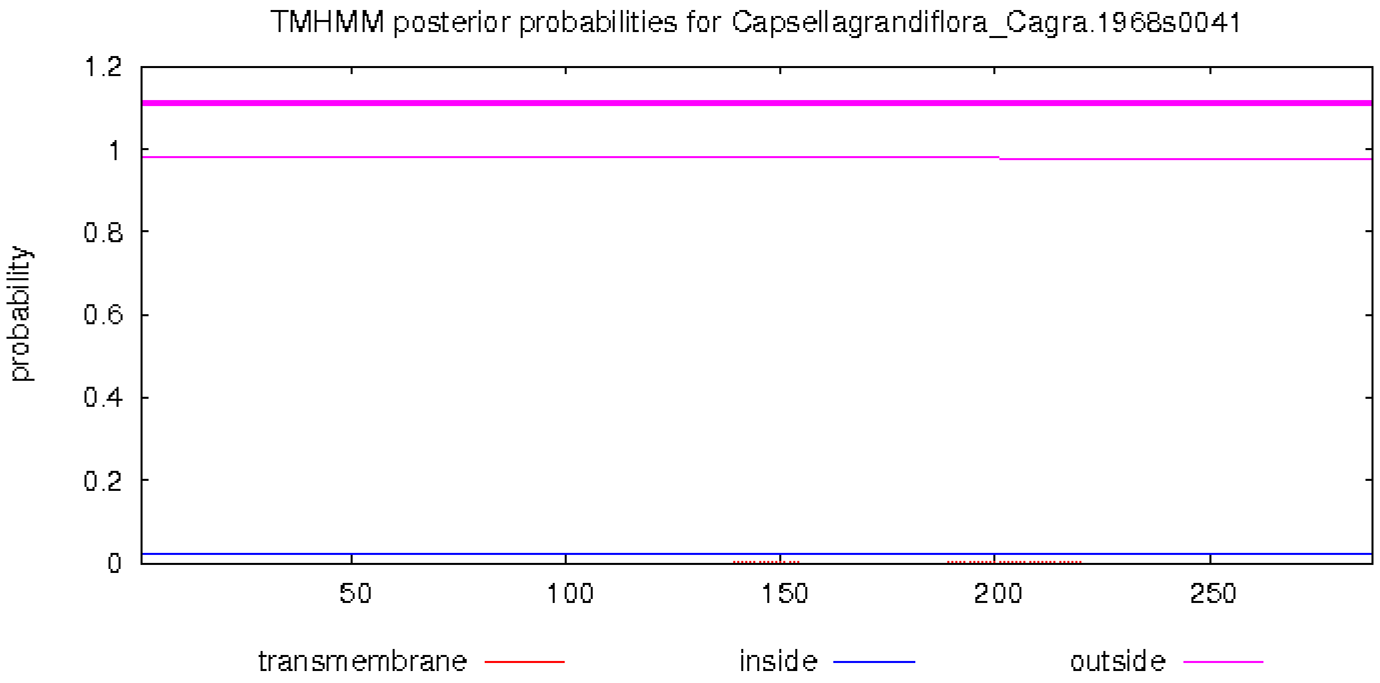

plot in postscript, script for making the plot in gnuplot, data for plot

---

```
# Capsellagrandiflora_Cagra.1968s0043 Length: 76
# Capsellagrandiflora_Cagra.1968s0043 Number of predicted TMHs:  1
# Capsellagrandiflora_Cagra.1968s0043 Exp number of AAs in TMHs: 15.36495
# Capsellagrandiflora_Cagra.1968s0043 Exp number, first 60 AAs:  15.36495
# Capsellagrandiflora_Cagra.1968s0043 Total prob of N-in:        0.03212
# Capsellagrandiflora_Cagra.1968s0043 POSSIBLE N-term signal sequence
Capsellagrandiflora_Cagra.1968s0043     TMHMM2.0        outside      1    27
```

```
Capsellagrandiflora_Cagra.1968s0043     TMHMM2.0        TMhelix     28    45
Capsellagrandiflora_Cagra.1968s0043     TMHMM2.0        inside      46    76
```

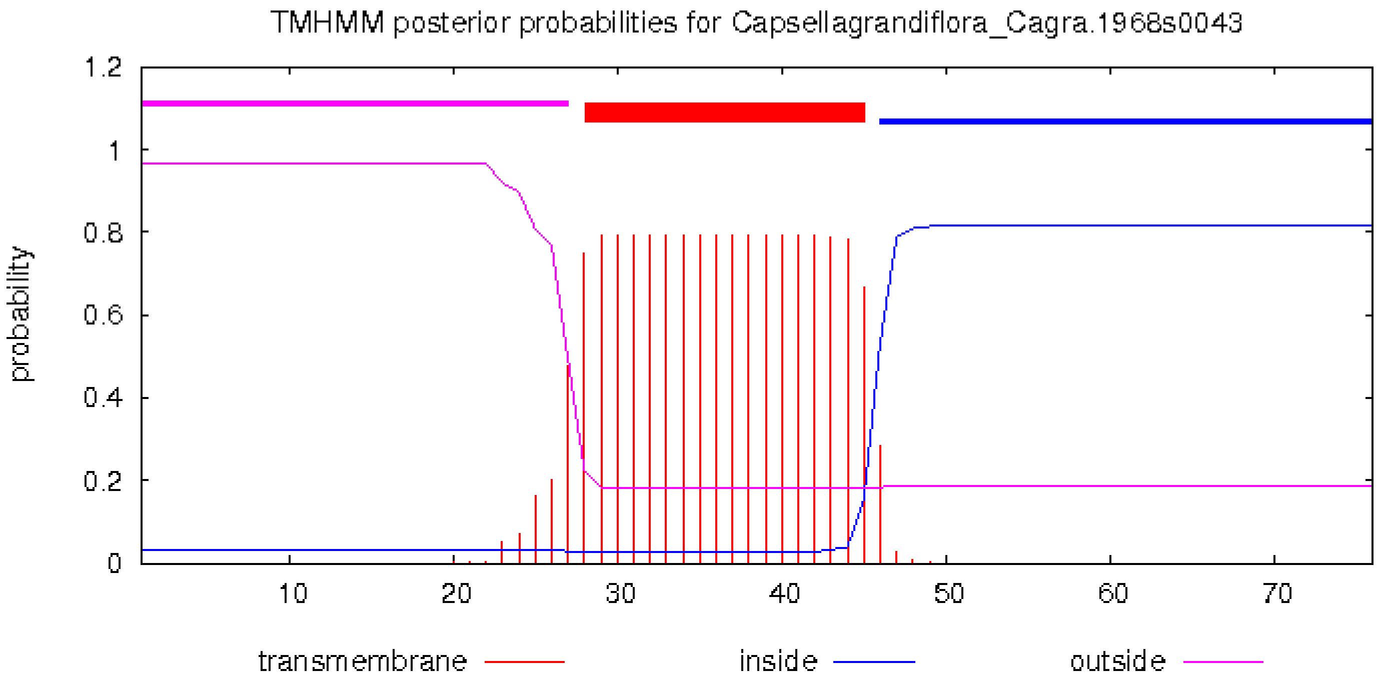

plot in postscript, script for making the plot in gnuplot, data for plot

---

```
# Capsellagrandiflora_Cagra.1968s0044 Length: 229
# Capsellagrandiflora_Cagra.1968s0044 Number of predicted TMHs:  0
# Capsellagrandiflora_Cagra.1968s0044 Exp number of AAs in TMHs: 0.00015
# Capsellagrandiflora_Cagra.1968s0044 Exp number, first 60 AAs:  0
# Capsellagrandiflora_Cagra.1968s0044 Total prob of N-in:        0.05274
Capsellagrandiflora_Cagra.1968s0044     TMHMM2.0        outside      1   229
```

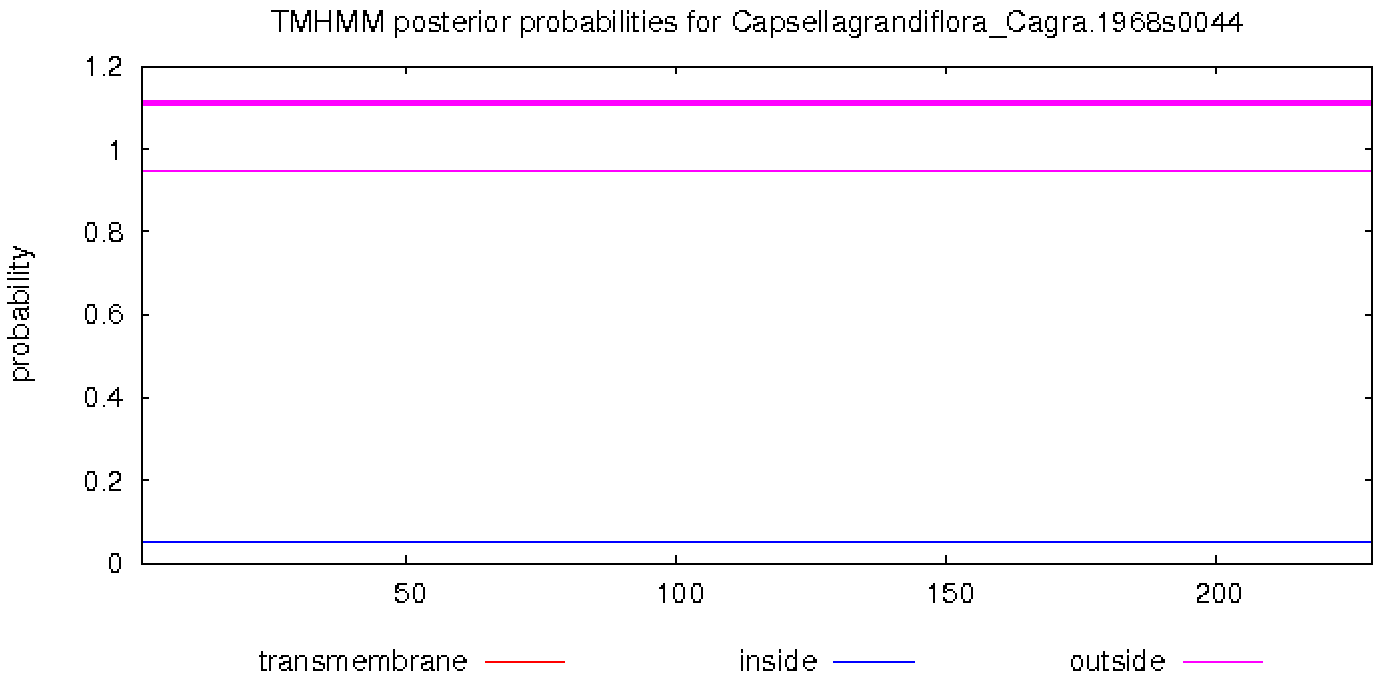

plot in postscript, script for making the plot in gnuplot, data for plot

---

```
# Capsellagrandiflora_Cagra.1968s0045 Length: 469
# Capsellagrandiflora_Cagra.1968s0045 Number of predicted TMHs:  1
# Capsellagrandiflora_Cagra.1968s0045 Exp number of AAs in TMHs: 17.87065
# Capsellagrandiflora_Cagra.1968s0045 Exp number, first 60 AAs:  0.00391
```

```
# Capsellagrandiflora_Cagra.1968s0045 Total prob of N-in:        0.32014
Capsellagrandiflora_Cagra.1968s0045     TMHMM2.0        outside      1   450
Capsellagrandiflora_Cagra.1968s0045     TMHMM2.0        TMhelix    451   468
Capsellagrandiflora_Cagra.1968s0045     TMHMM2.0        inside     469   469
```

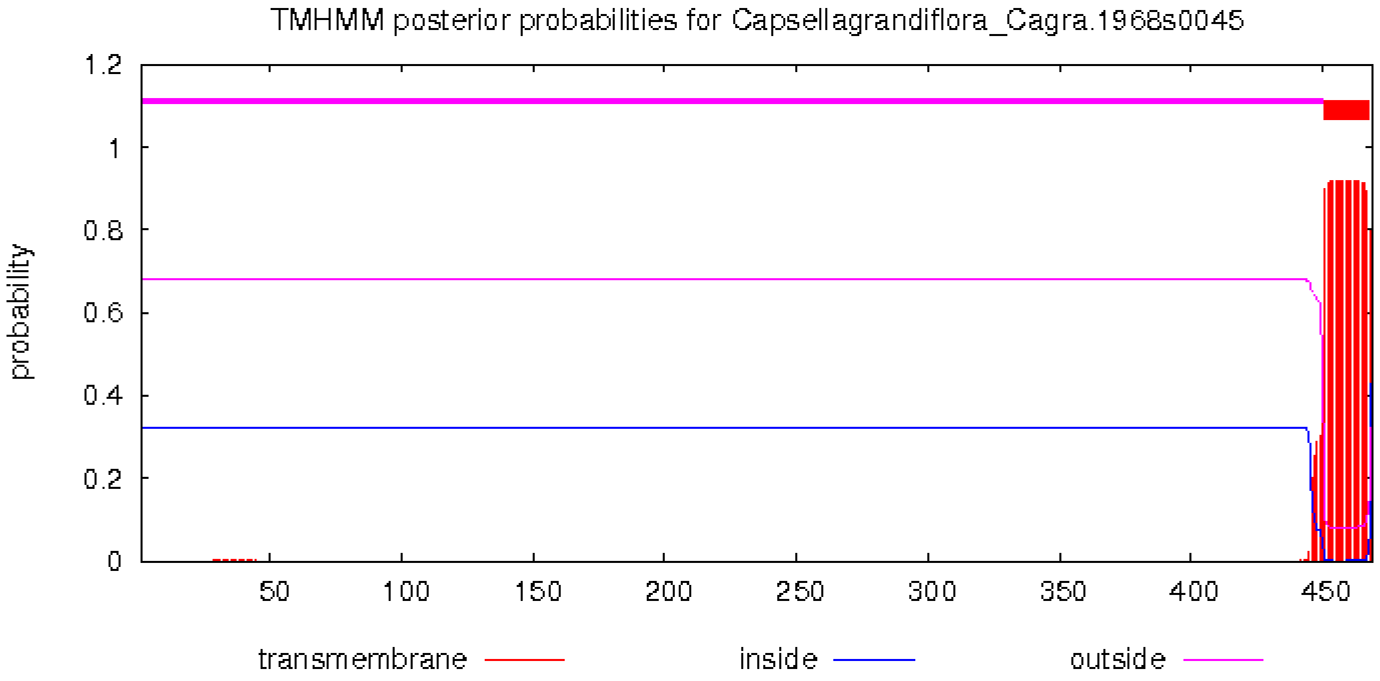

plot in postscript, script for making the plot in gnuplot, data for plot

---

```
# Capsellagrandiflora_Cagra.1968s0046 Length: 308
# Capsellagrandiflora_Cagra.1968s0046 Number of predicted TMHs:  1
# Capsellagrandiflora_Cagra.1968s0046 Exp number of AAs in TMHs: 23.24652
# Capsellagrandiflora_Cagra.1968s0046 Exp number, first 60 AAs:  1.93621
# Capsellagrandiflora_Cagra.1968s0046 Total prob of N-in:        0.84354
Capsellagrandiflora_Cagra.1968s0046     TMHMM2.0        inside       1   284
Capsellagrandiflora_Cagra.1968s0046     TMHMM2.0        TMhelix    285   307
Capsellagrandiflora_Cagra.1968s0046     TMHMM2.0        outside    308   308
```

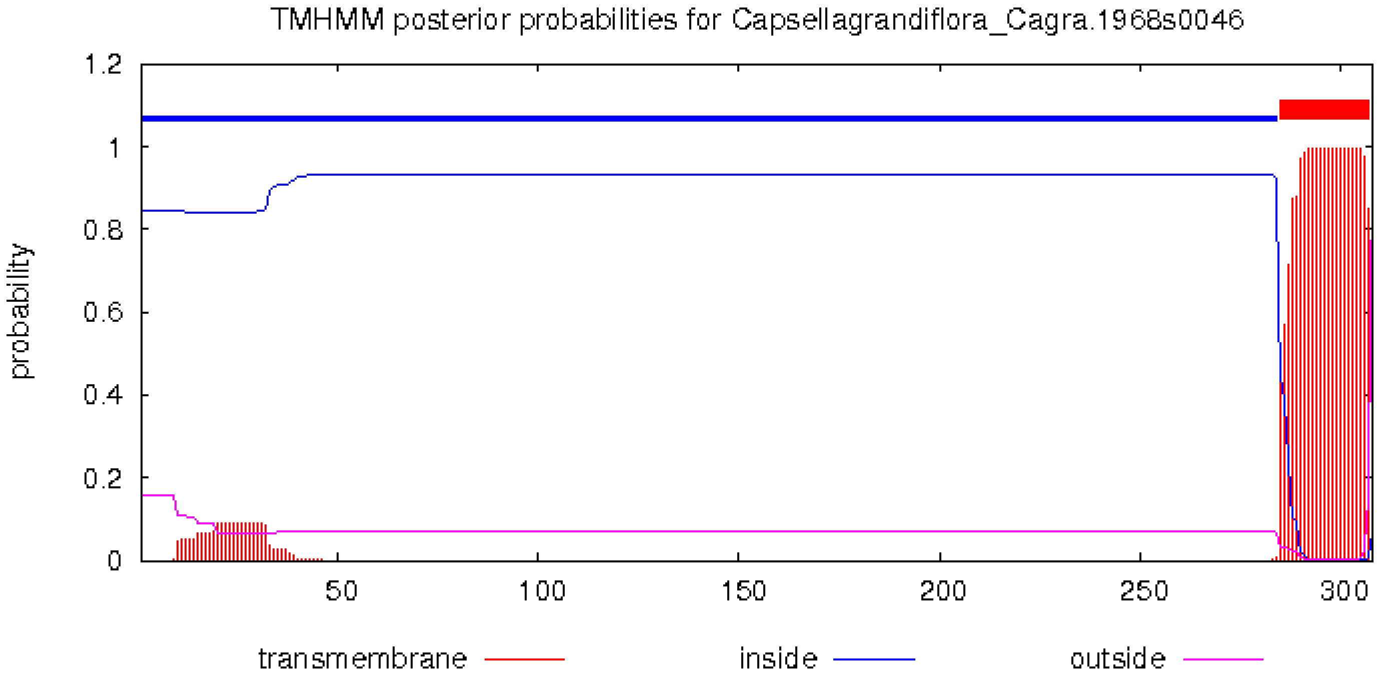

plot in postscript, script for making the plot in gnuplot, data for plot

---

```
# Capsellagrandiflora_Cagra.1968s0112 Length: 286
# Capsellagrandiflora_Cagra.1968s0112 Number of predicted TMHs:  0
# Capsellagrandiflora_Cagra.1968s0112 Exp number of AAs in TMHs: 0.00127
# Capsellagrandiflora_Cagra.1968s0112 Exp number, first 60 AAs:  0.00017
# Capsellagrandiflora_Cagra.1968s0112 Total prob of N-in:        0.00254
Capsellagrandiflora_Cagra.1968s0112     TMHMM2.0        outside      1   286
```

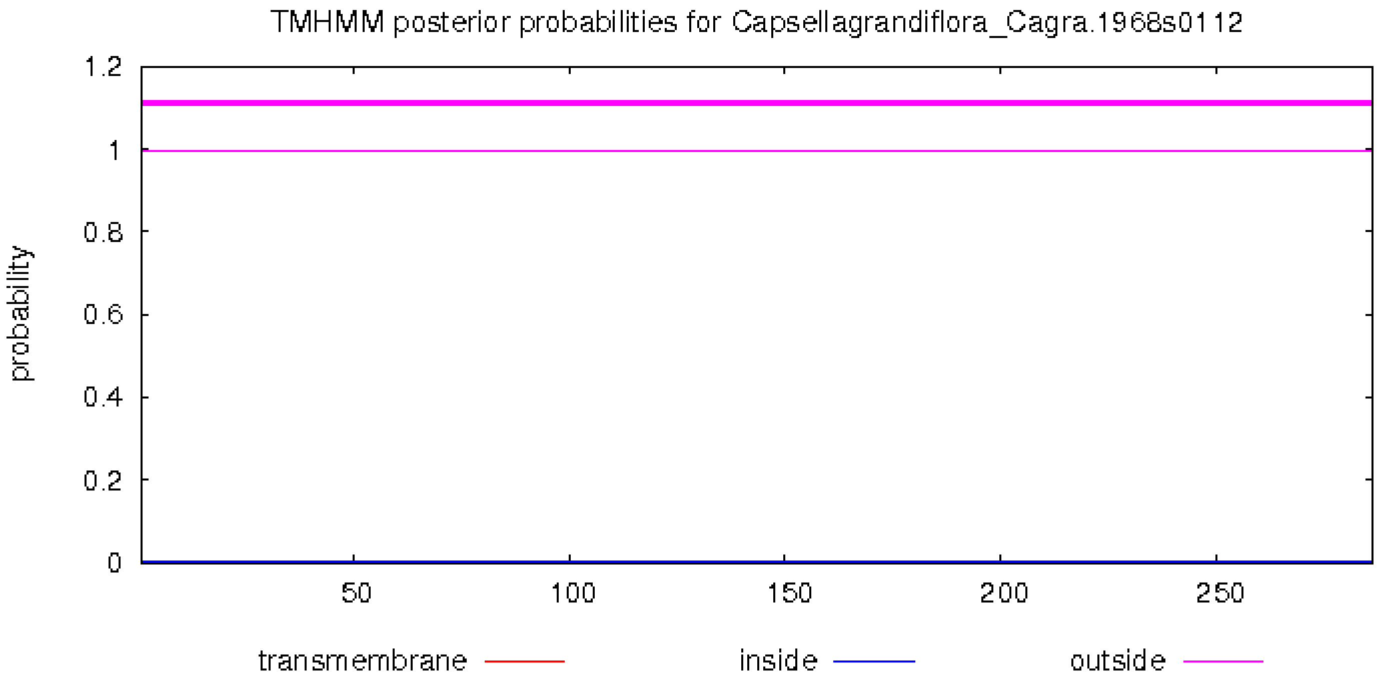

plot in postscript, script for making the plot in gnuplot, data for plot

---

```
# Capsellagrandiflora_Cagra.2026s0001 Length: 367
# Capsellagrandiflora_Cagra.2026s0001 Number of predicted TMHs:  0
# Capsellagrandiflora_Cagra.2026s0001 Exp number of AAs in TMHs: 0
# Capsellagrandiflora_Cagra.2026s0001 Exp number, first 60 AAs:  0
# Capsellagrandiflora_Cagra.2026s0001 Total prob of N-in:        0.01029
Capsellagrandiflora_Cagra.2026s0001     TMHMM2.0        outside      1   367
```

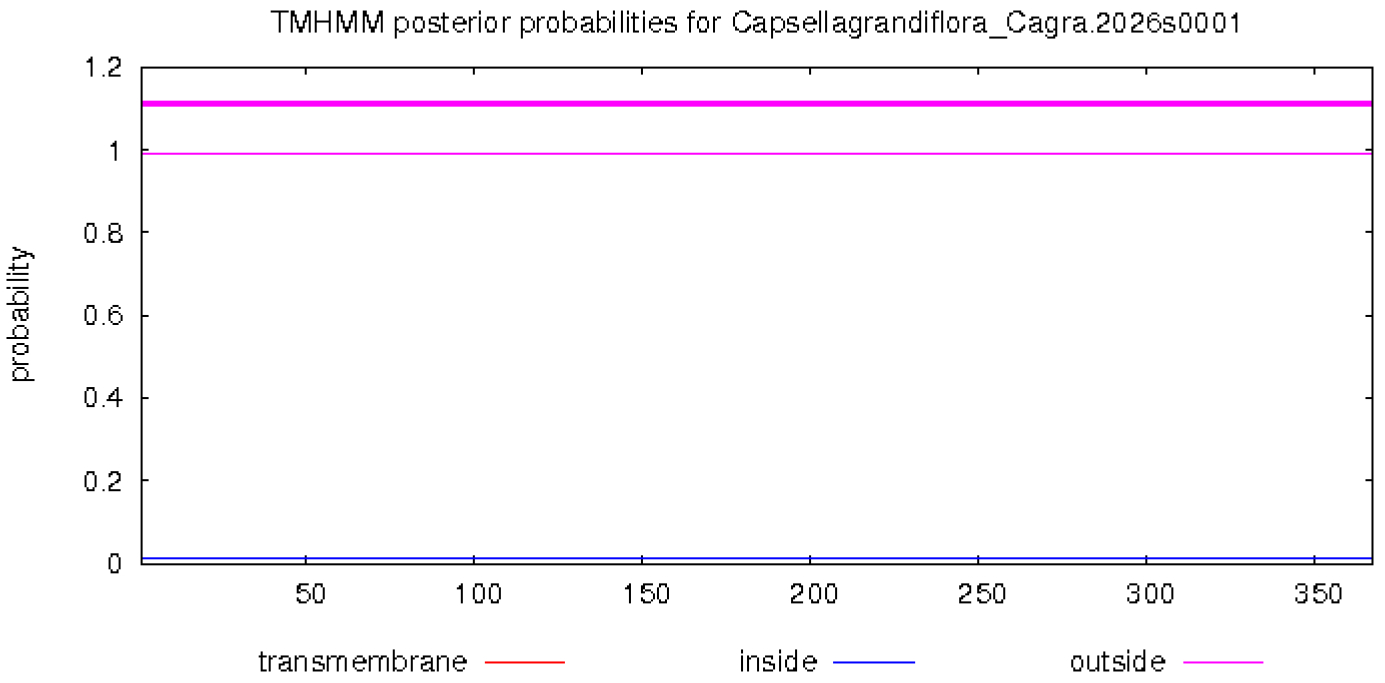

plot in postscript, script for making the plot in gnuplot, data for plot

---

```
# Capsellagrandiflora_Cagra.2026s0002 Length: 339
# Capsellagrandiflora_Cagra.2026s0002 Number of predicted TMHs:  0
# Capsellagrandiflora_Cagra.2026s0002 Exp number of AAs in TMHs: 0.000980000000000000001
# Capsellagrandiflora_Cagra.2026s0002 Exp number, first 60 AAs:  0.00017
# Capsellagrandiflora_Cagra.2026s0002 Total prob of N-in:        0.01066
Capsellagrandiflora_Cagra.2026s0002     TMHMM2.0        outside      1   339
```

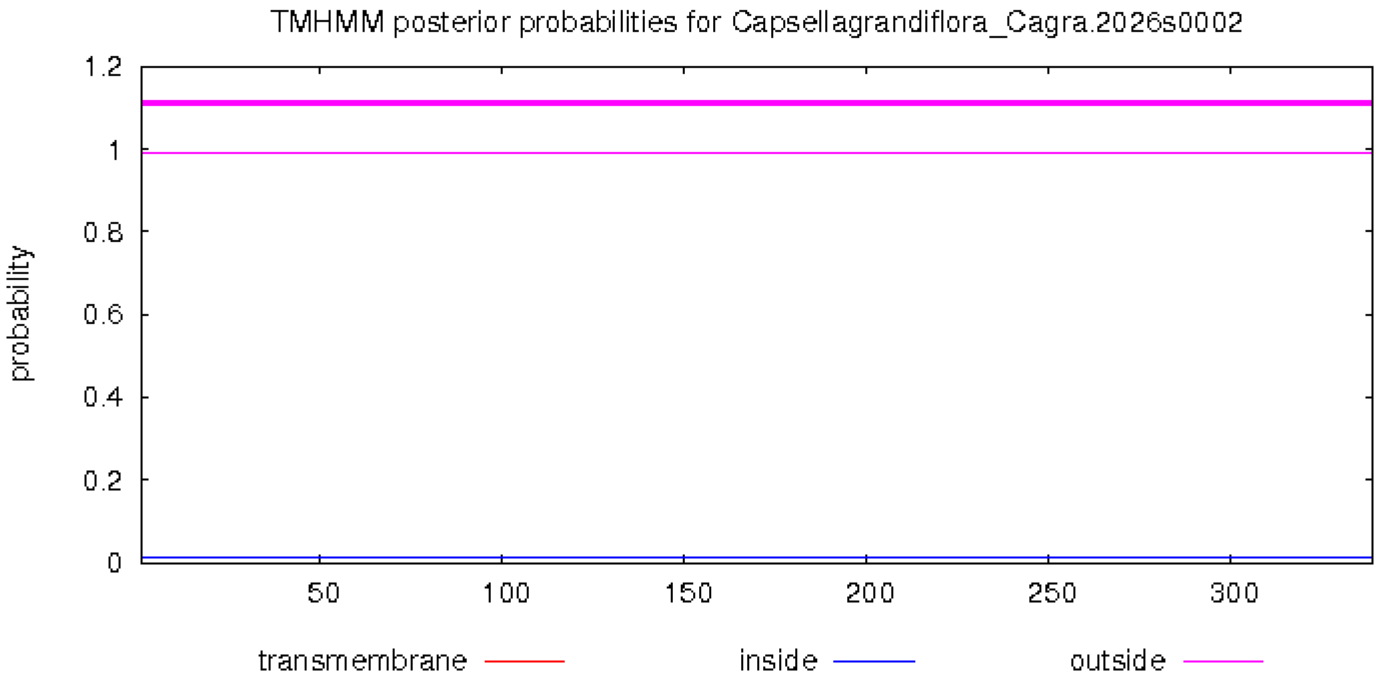

plot in postscript, script for making the plot in gnuplot, data for plot

---

```
# Capsellagrandiflora_Cagra.2098s0066 Length: 480
# Capsellagrandiflora_Cagra.2098s0066 Number of predicted TMHs:  0
# Capsellagrandiflora_Cagra.2098s0066 Exp number of AAs in TMHs: 0.00018
# Capsellagrandiflora_Cagra.2098s0066 Exp number, first 60 AAs:  0
# Capsellagrandiflora_Cagra.2098s0066 Total prob of N-in:        0.00016
Capsellagrandiflora_Cagra.2098s0066     TMHMM2.0        outside      1   480
```

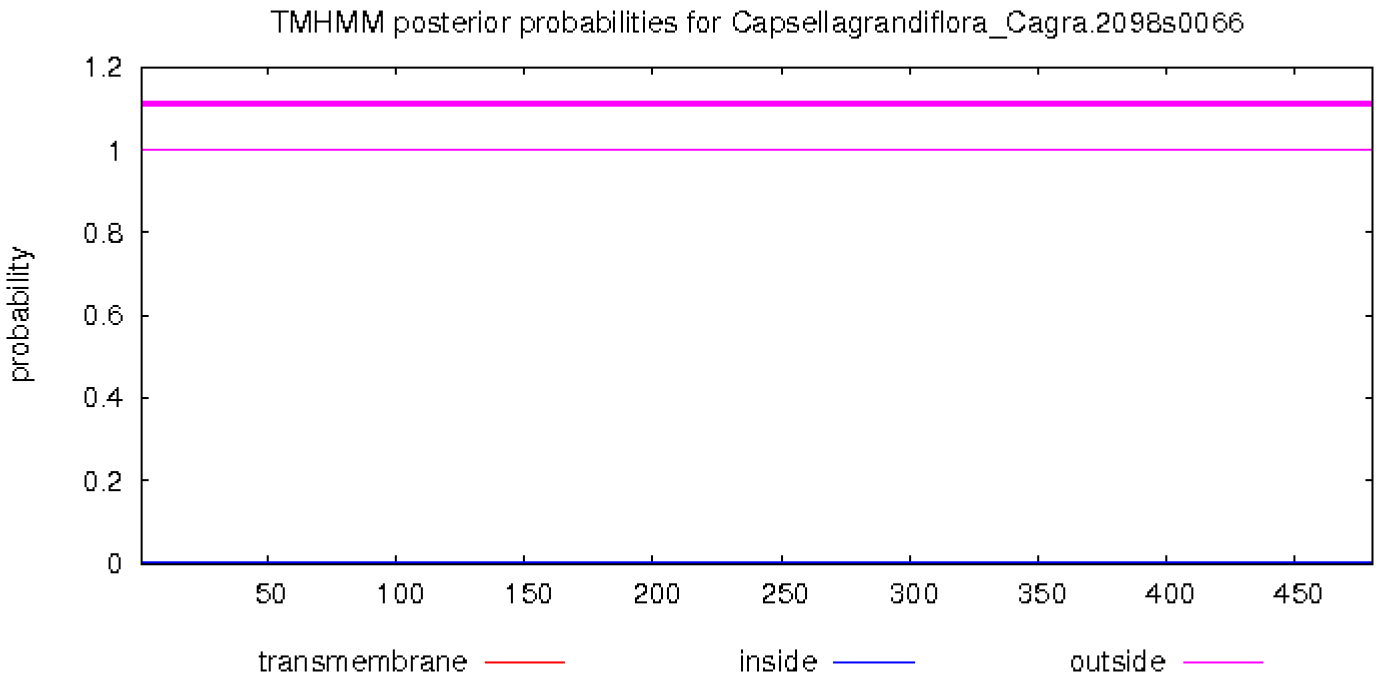

plot in postscript, script for making the plot in gnuplot, data for plot

---

```
# Capsellagrandiflora_Cagra.21346s0001 Length: 466
# Capsellagrandiflora_Cagra.21346s0001 Number of predicted TMHs:  0
# Capsellagrandiflora_Cagra.21346s0001 Exp number of AAs in TMHs: 9.80062
# Capsellagrandiflora_Cagra.21346s0001 Exp number, first 60 AAs:  0.00509
# Capsellagrandiflora_Cagra.21346s0001 Total prob of N-in:        0.01049
Capsellagrandiflora_Cagra.21346s0001	TMHMM2.0	outside	     1   466
```

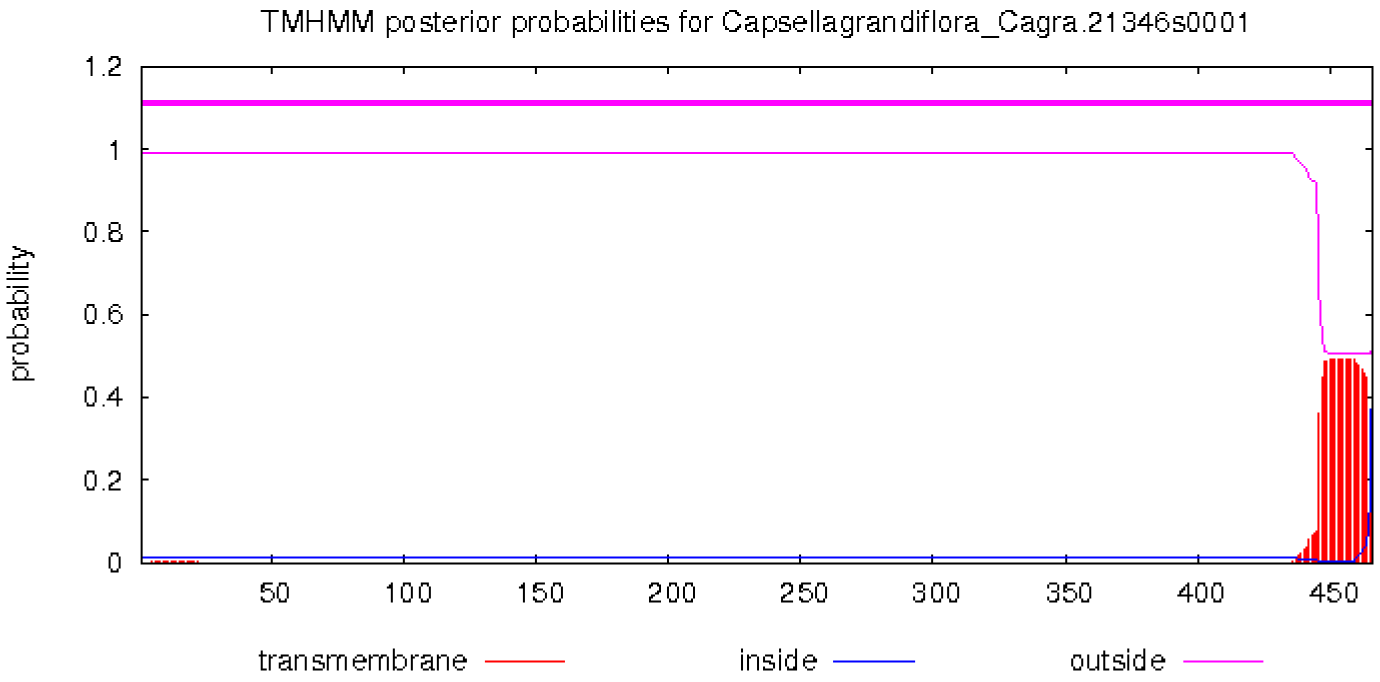

plot in postscript, script for making the plot in gnuplot, data for plot

```
# Capsellagrandiflora_Cagra.2192s0006 Length: 245
# Capsellagrandiflora_Cagra.2192s0006 Number of predicted TMHs:  0
# Capsellagrandiflora_Cagra.2192s0006 Exp number of AAs in TMHs: 0.16945
# Capsellagrandiflora_Cagra.2192s0006 Exp number, first 60 AAs:  0.16848
# Capsellagrandiflora_Cagra.2192s0006 Total prob of N-in:        0.14495
Capsellagrandiflora_Cagra.2192s0006	TMHMM2.0	outside	     1   245
```

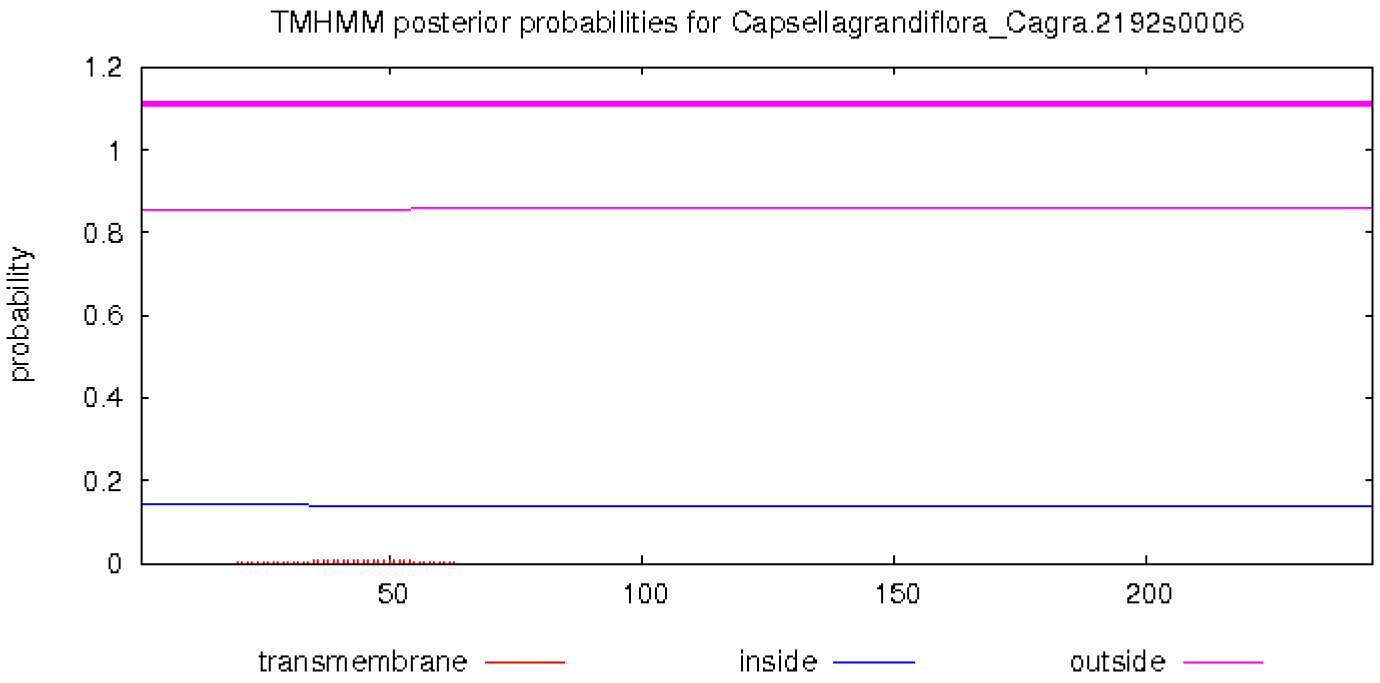

plot in postscript, script for making the plot in gnuplot, data for plot

```
# Capsellagrandiflora_Cagra.2204s0002 Length: 327
# Capsellagrandiflora_Cagra.2204s0002 Number of predicted TMHs:  0
# Capsellagrandiflora_Cagra.2204s0002 Exp number of AAs in TMHs: 0.0002
# Capsellagrandiflora_Cagra.2204s0002 Exp number, first 60 AAs:  0
# Capsellagrandiflora_Cagra.2204s0002 Total prob of N-in:        0.11180
Capsellagrandiflora_Cagra.2204s0002     TMHMM2.0        outside      1   327
```

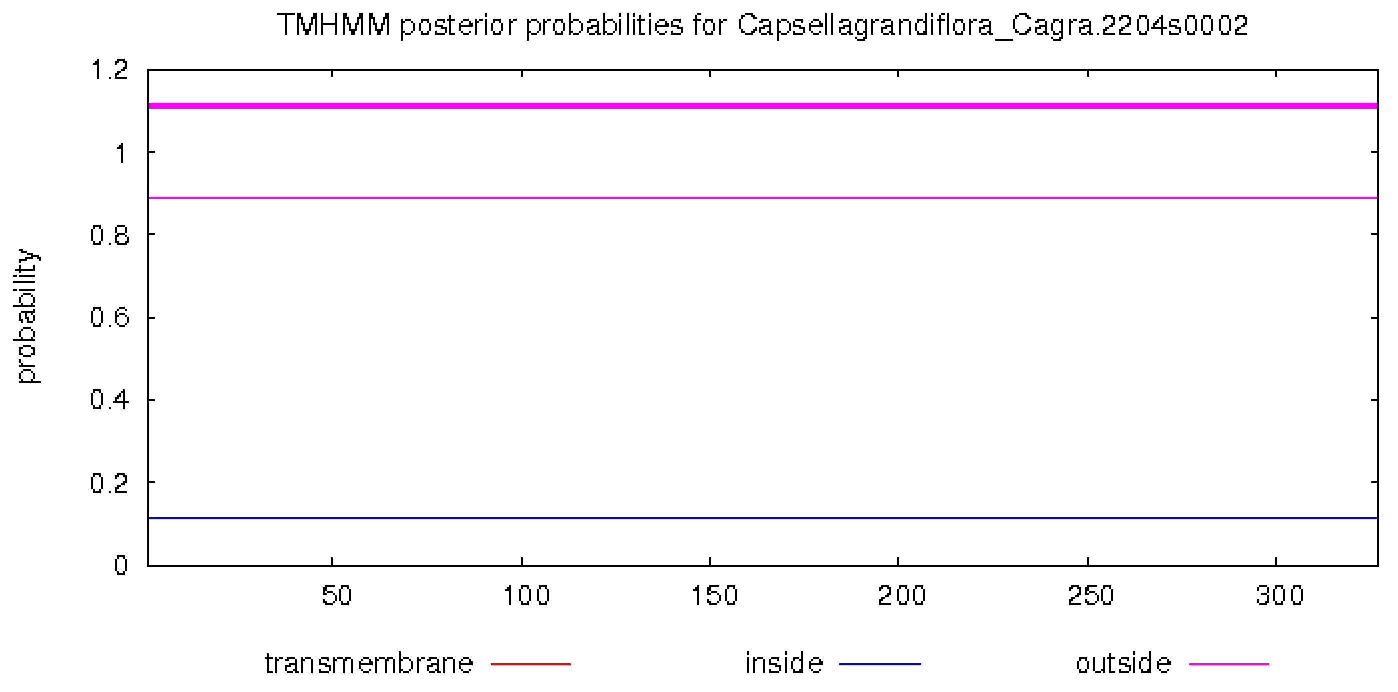

plot in postscript, script for making the plot in gnuplot, data for plot

---

```
# Capsellagrandiflora_Cagra.2213s0001 Length: 165
# Capsellagrandiflora_Cagra.2213s0001 Number of predicted TMHs:  0
# Capsellagrandiflora_Cagra.2213s0001 Exp number of AAs in TMHs: 0.00039
# Capsellagrandiflora_Cagra.2213s0001 Exp number, first 60 AAs:  0.00039
# Capsellagrandiflora_Cagra.2213s0001 Total prob of N-in:        0.11096
Capsellagrandiflora_Cagra.2213s0001     TMHMM2.0        outside      1   165
```

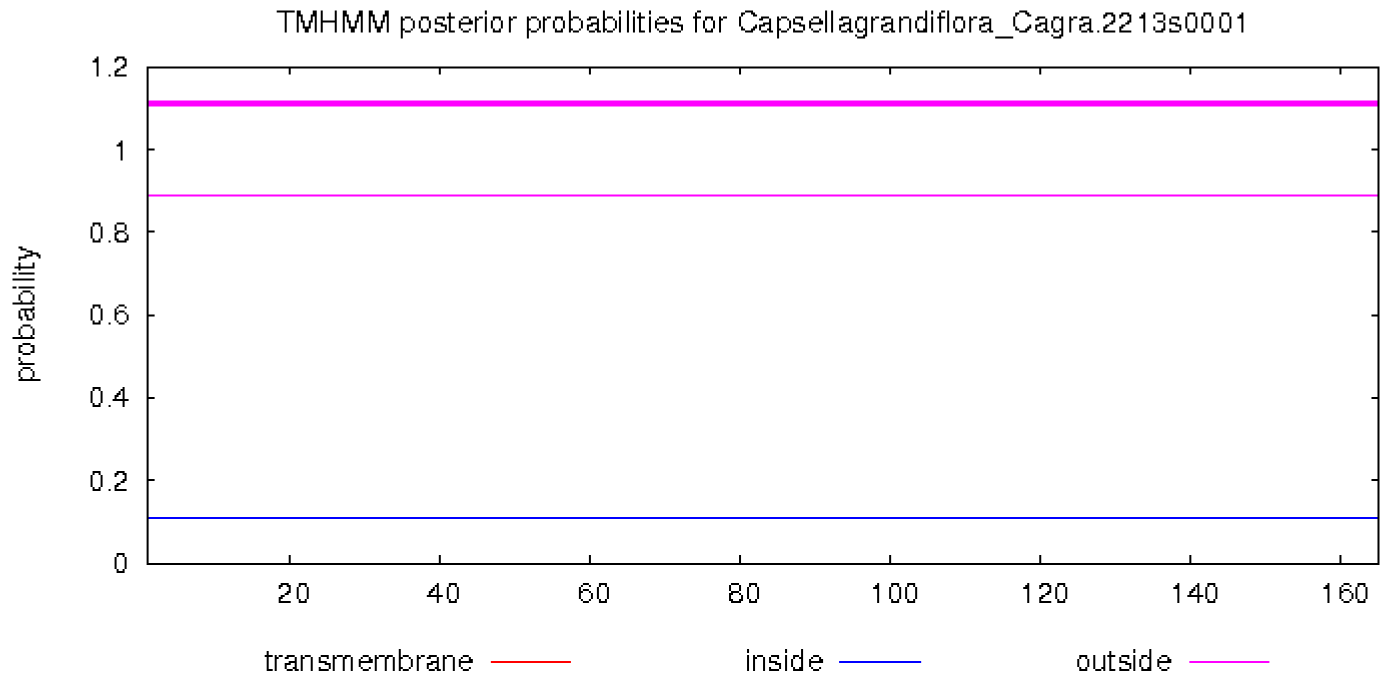

plot in postscript, script for making the plot in gnuplot, data for plot

---

```
# Capsellagrandiflora_Cagra.2350s0100 Length: 526
# Capsellagrandiflora_Cagra.2350s0100 Number of predicted TMHs:  1
# Capsellagrandiflora_Cagra.2350s0100 Exp number of AAs in TMHs: 21.64315
# Capsellagrandiflora_Cagra.2350s0100 Exp number, first 60 AAs:  0
# Capsellagrandiflora_Cagra.2350s0100 Total prob of N-in:        0.13836
Capsellagrandiflora_Cagra.2350s0100     TMHMM2.0        outside      1   502
Capsellagrandiflora_Cagra.2350s0100     TMHMM2.0        TMhelix    503   525
Capsellagrandiflora_Cagra.2350s0100     TMHMM2.0        inside     526   526
```

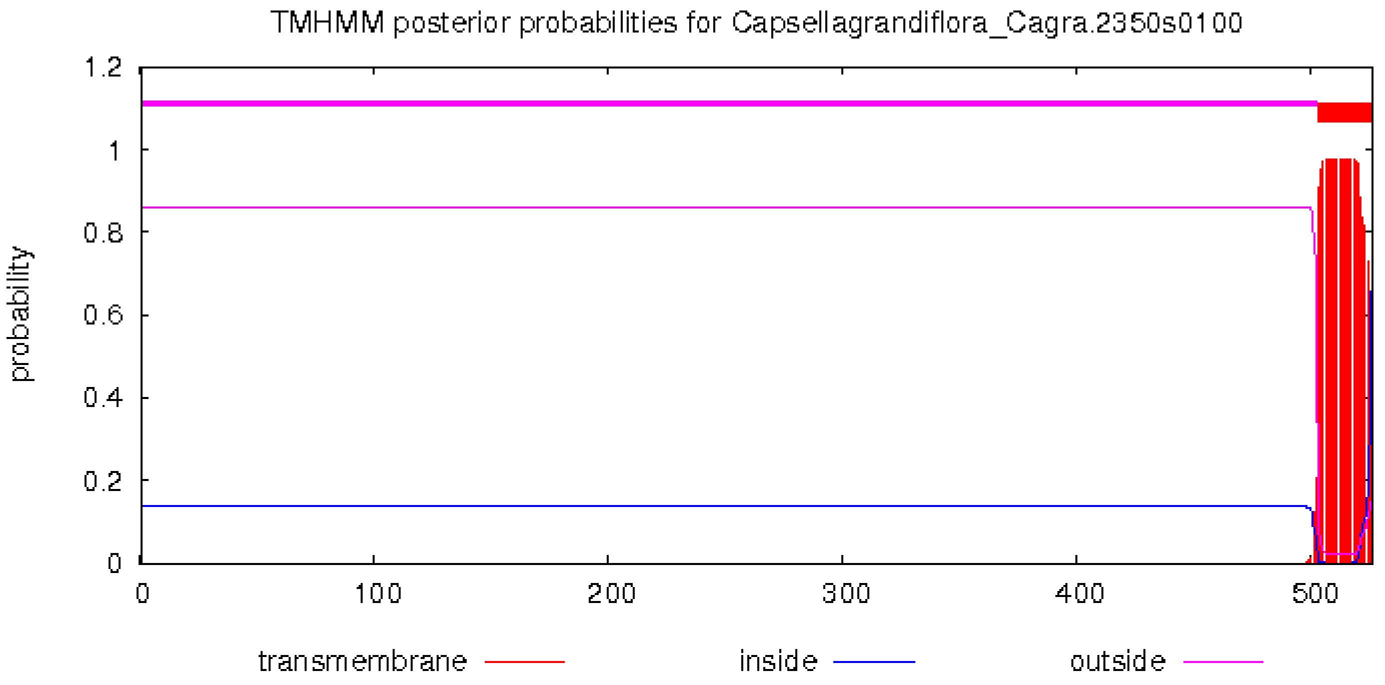

plot in postscript, script for making the plot in gnuplot, data for plot

---

```
# Capsellagrandiflora_Cagra.27691s0001 Length: 208
# Capsellagrandiflora_Cagra.27691s0001 Number of predicted TMHs:  0
# Capsellagrandiflora_Cagra.27691s0001 Exp number of AAs in TMHs: 0.14436
# Capsellagrandiflora_Cagra.27691s0001 Exp number, first 60 AAs:  0.00036
# Capsellagrandiflora_Cagra.27691s0001 Total prob of N-in:        0.30835
Capsellagrandiflora_Cagra.27691s0001    TMHMM2.0        outside      1   208
```

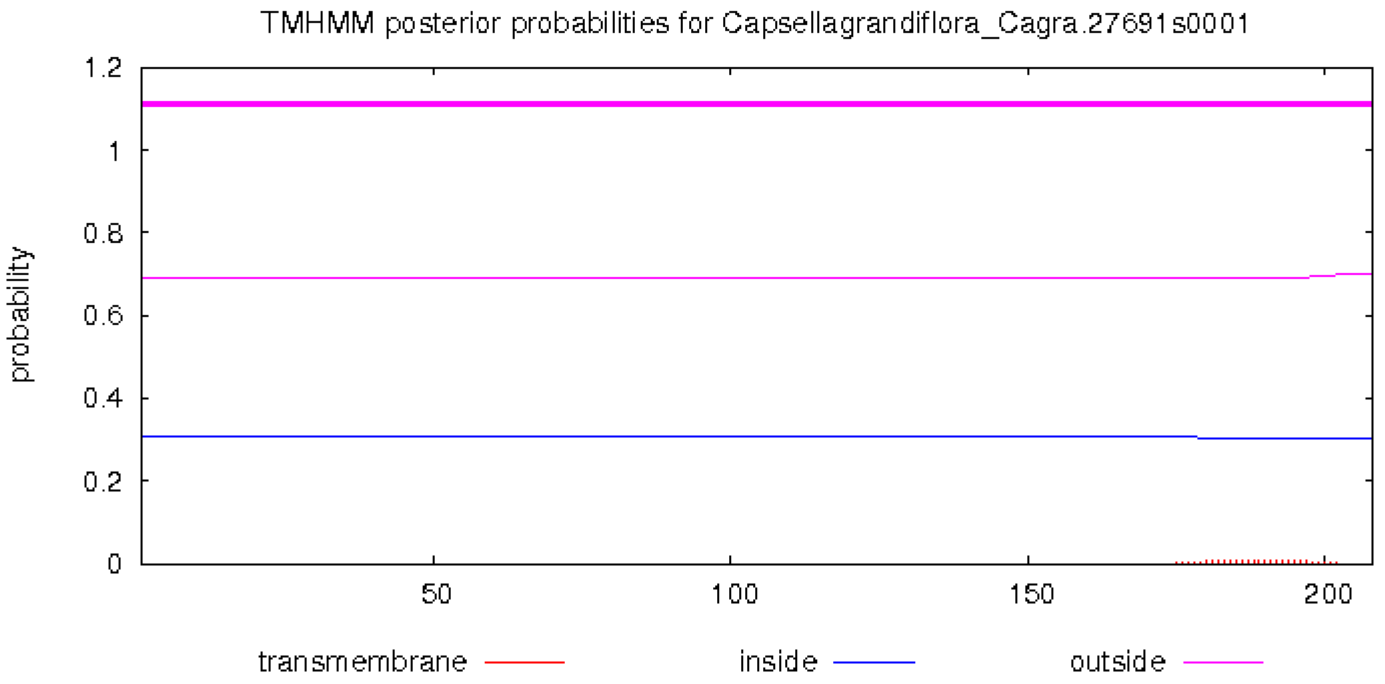

plot in postscript, script for making the plot in gnuplot, data for plot

```
# Capsellagrandiflora_Cagra.27949s0001 Length: 345
# Capsellagrandiflora_Cagra.27949s0001 Number of predicted TMHs:  0
# Capsellagrandiflora_Cagra.27949s0001 Exp number of AAs in TMHs: 0.00313
# Capsellagrandiflora_Cagra.27949s0001 Exp number, first 60 AAs:  0.00134
# Capsellagrandiflora_Cagra.27949s0001 Total prob of N-in:        0.00359
Capsellagrandiflora_Cagra.27949s0001	TMHMM2.0	outside	     1   345
```

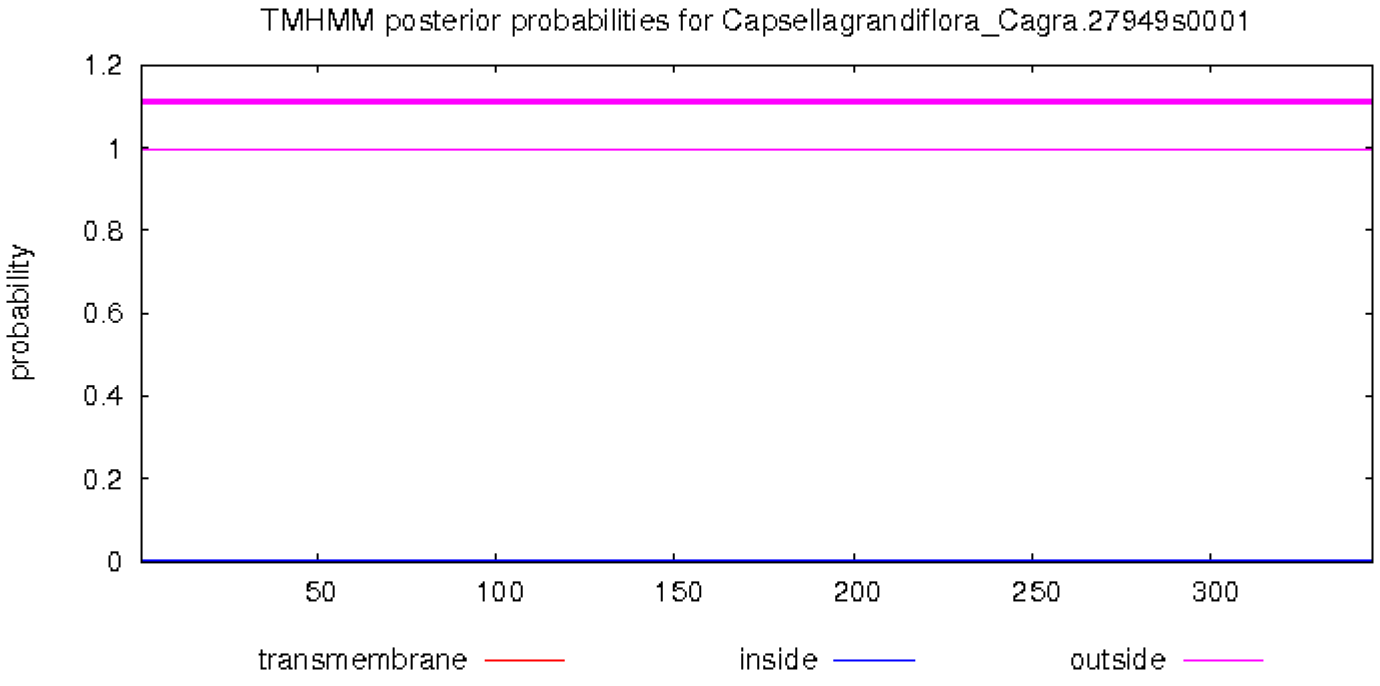

plot in postscript, script for making the plot in gnuplot, data for plot

```
# Capsellagrandiflora_Cagra.2925s0018 Length: 346
# Capsellagrandiflora_Cagra.2925s0018 Number of predicted TMHs:  0
# Capsellagrandiflora_Cagra.2925s0018 Exp number of AAs in TMHs: 0.00038
# Capsellagrandiflora_Cagra.2925s0018 Exp number, first 60 AAs:  0
# Capsellagrandiflora_Cagra.2925s0018 Total prob of N-in:        0.00723
Capsellagrandiflora_Cagra.2925s0018	TMHMM2.0	outside	     1   346
```

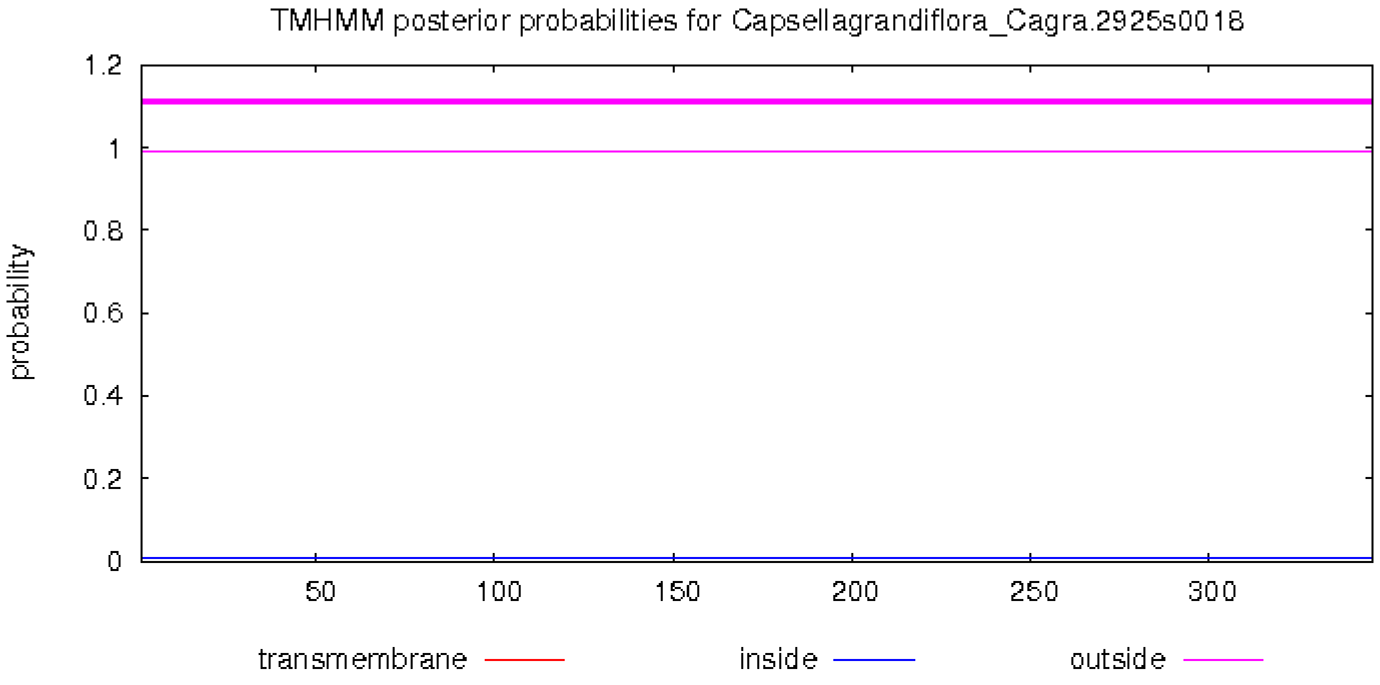

plot in postscript, script for making the plot in gnuplot, data for plot

```
# Capsellagrandiflora_Cagra.3125s0012 Length: 340
# Capsellagrandiflora_Cagra.3125s0012 Number of predicted TMHs:  1
# Capsellagrandiflora_Cagra.3125s0012 Exp number of AAs in TMHs: 21.40323
# Capsellagrandiflora_Cagra.3125s0012 Exp number, first 60 AAs:  0.19516
# Capsellagrandiflora_Cagra.3125s0012 Total prob of N-in:        0.55277
Capsellagrandiflora_Cagra.3125s0012     TMHMM2.0        outside      1   315
Capsellagrandiflora_Cagra.3125s0012     TMHMM2.0        TMhelix    316   335
Capsellagrandiflora_Cagra.3125s0012     TMHMM2.0        inside     336   340
```

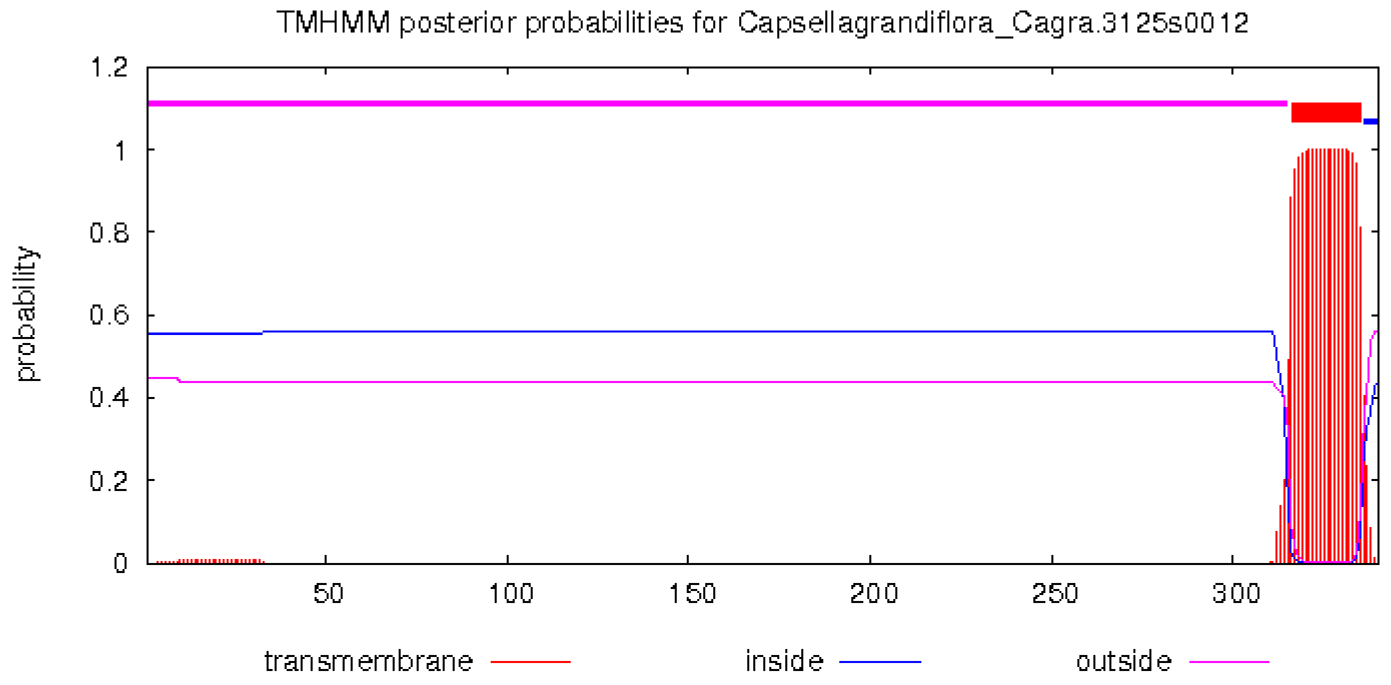

plot in postscript, script for making the plot in gnuplot, data for plot

---

```
# Capsellagrandiflora_Cagra.3356s0092 Length: 225
# Capsellagrandiflora_Cagra.3356s0092 Number of predicted TMHs:  0
# Capsellagrandiflora_Cagra.3356s0092 Exp number of AAs in TMHs: 0.00018
# Capsellagrandiflora_Cagra.3356s0092 Exp number, first 60 AAs:  0
# Capsellagrandiflora_Cagra.3356s0092 Total prob of N-in:        0.01152
Capsellagrandiflora_Cagra.3356s0092     TMHMM2.0        outside      1   225
```

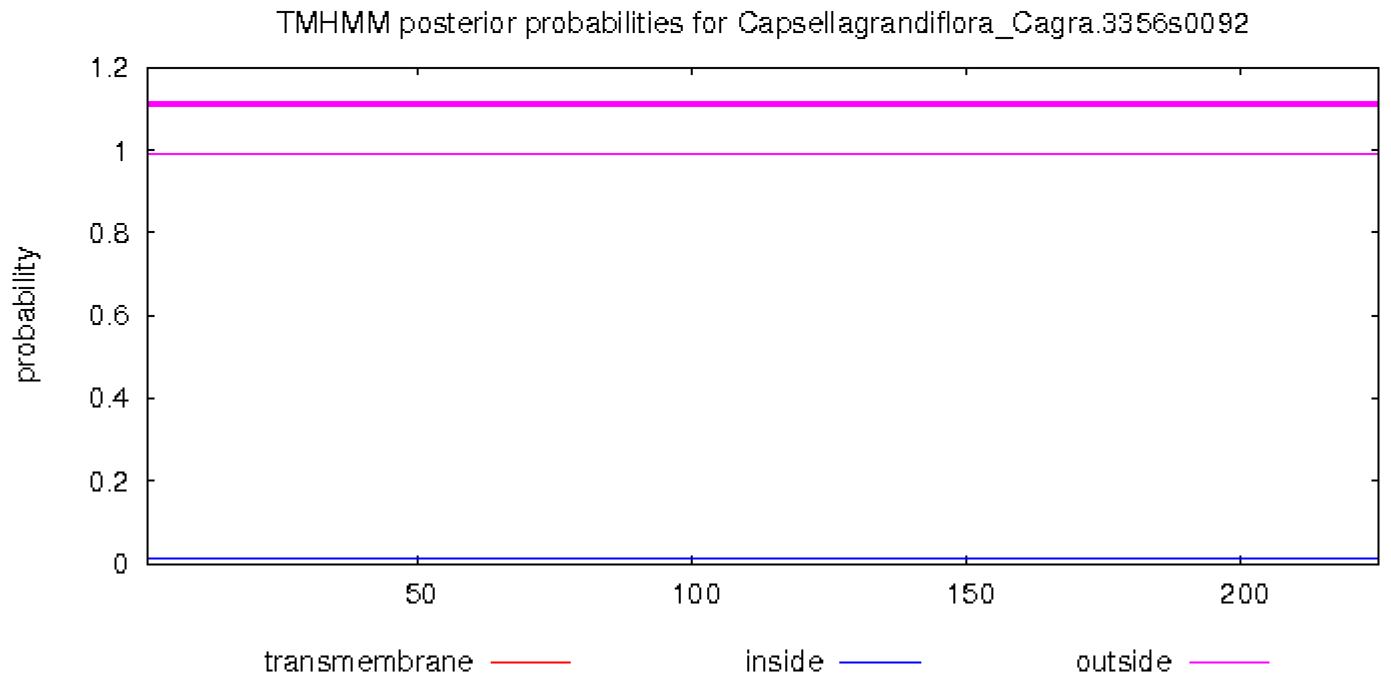

plot in postscript, script for making the plot in gnuplot, data for plot

```
# Capsellagrandiflora_Cagra.3375s0004 Length: 551
# Capsellagrandiflora_Cagra.3375s0004 Number of predicted TMHs:  1
# Capsellagrandiflora_Cagra.3375s0004 Exp number of AAs in TMHs: 21.37083
# Capsellagrandiflora_Cagra.3375s0004 Exp number, first 60 AAs:  0.00363
# Capsellagrandiflora_Cagra.3375s0004 Total prob of N-in:        0.05876
Capsellagrandiflora_Cagra.3375s0004     TMHMM2.0        outside      1   521
Capsellagrandiflora_Cagra.3375s0004     TMHMM2.0        TMhelix    522   544
Capsellagrandiflora_Cagra.3375s0004     TMHMM2.0        inside     545   551
```

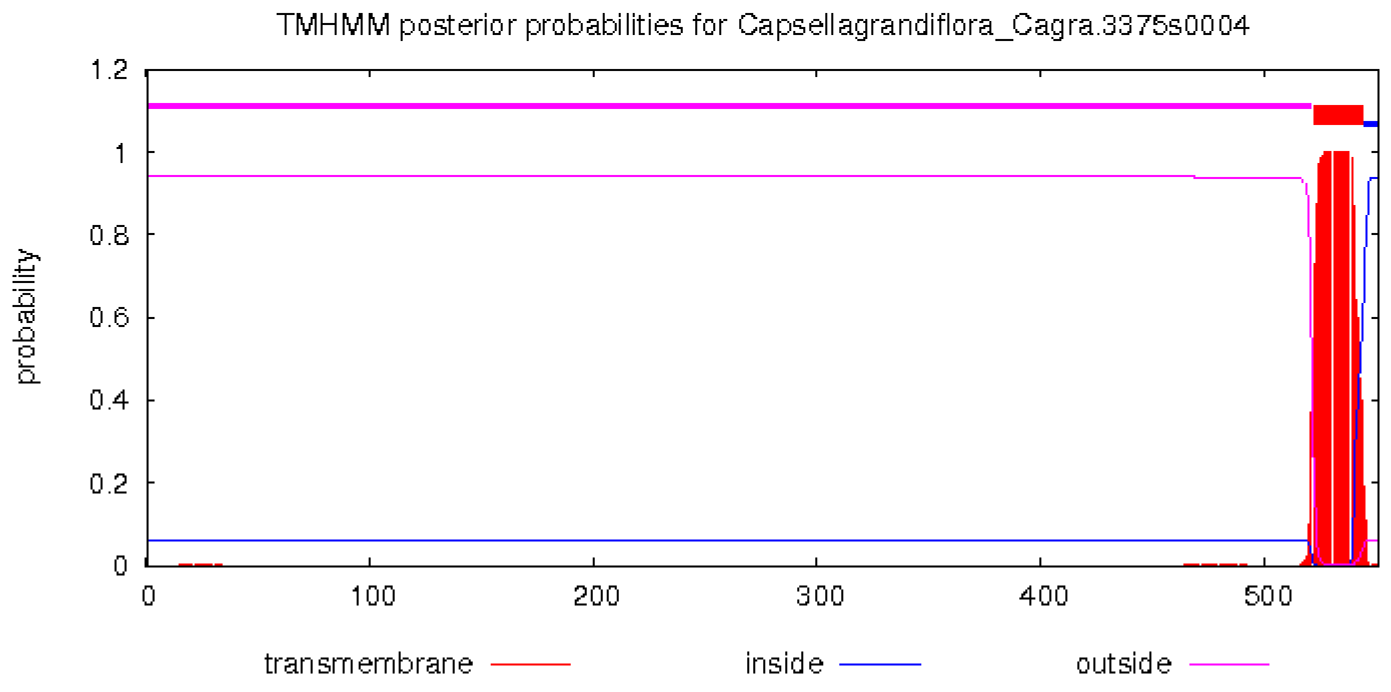

plot in postscript, script for making the plot in gnuplot, data for plot

```
# Capsellagrandiflora_Cagra.3375s0006 Length: 572
# Capsellagrandiflora_Cagra.3375s0006 Number of predicted TMHs:  1
# Capsellagrandiflora_Cagra.3375s0006 Exp number of AAs in TMHs: 22.3584
# Capsellagrandiflora_Cagra.3375s0006 Exp number, first 60 AAs:  0.00239
# Capsellagrandiflora_Cagra.3375s0006 Total prob of N-in:        0.58723
Capsellagrandiflora_Cagra.3375s0006     TMHMM2.0        outside      1   541
Capsellagrandiflora_Cagra.3375s0006     TMHMM2.0        TMhelix    542   564
Capsellagrandiflora_Cagra.3375s0006     TMHMM2.0        inside     565   572
```

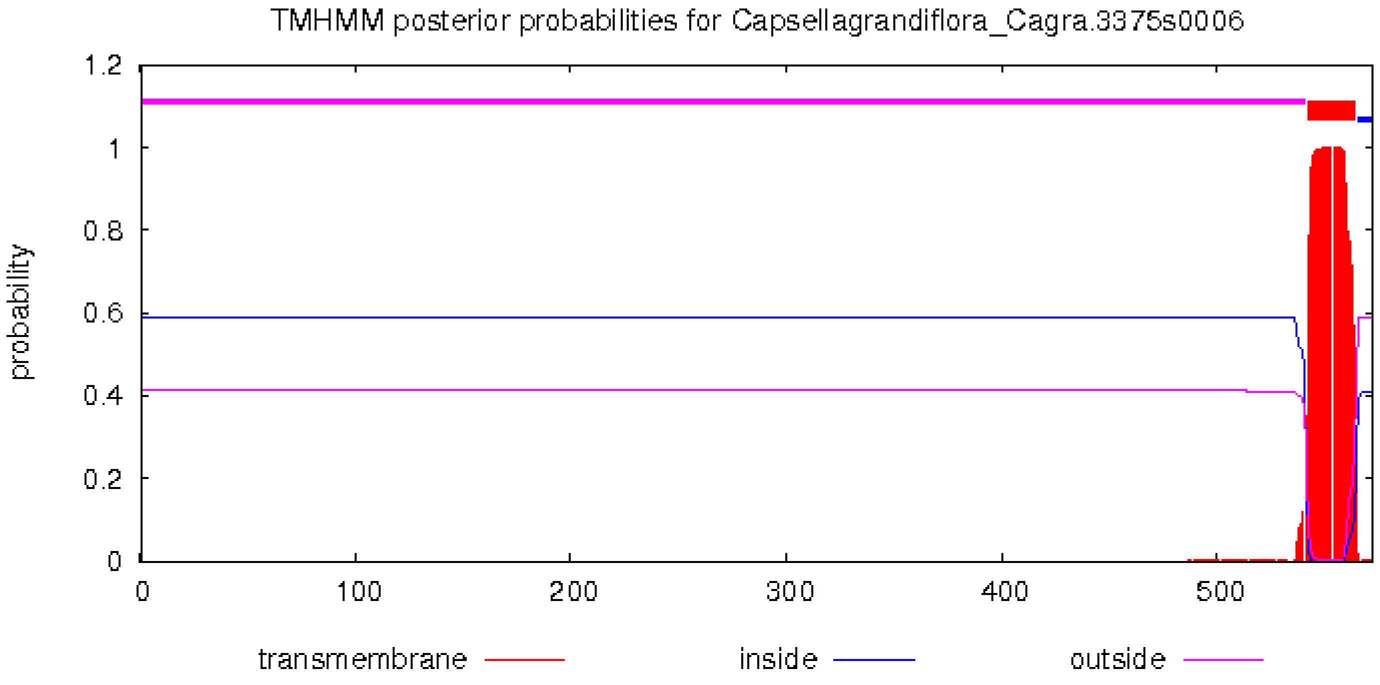

plot in postscript, script for making the plot in gnuplot, data for plot

```
# Capsellagrandiflora_Cagra.3545s0001 Length: 189
# Capsellagrandiflora_Cagra.3545s0001 Number of predicted TMHs:  0
# Capsellagrandiflora_Cagra.3545s0001 Exp number of AAs in TMHs: 0.06944
# Capsellagrandiflora_Cagra.3545s0001 Exp number, first 60 AAs:  0
# Capsellagrandiflora_Cagra.3545s0001 Total prob of N-in:        0.24332
Capsellagrandiflora_Cagra.3545s0001     TMHMM2.0        outside      1   189
```

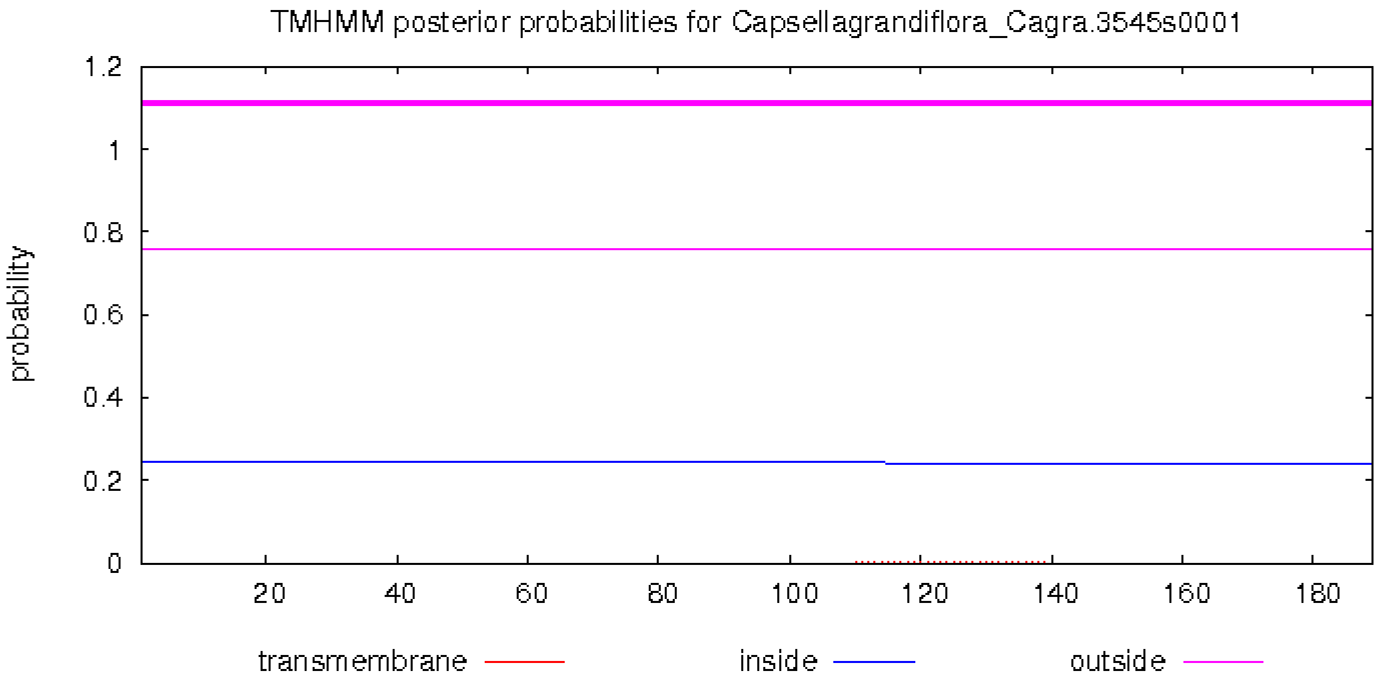

plot in postscript, script for making the plot in gnuplot, data for plot

```
# Capsellagrandiflora_Cagra.4003s0027 Length: 349
# Capsellagrandiflora_Cagra.4003s0027 Number of predicted TMHs:  0
# Capsellagrandiflora_Cagra.4003s0027 Exp number of AAs in TMHs: 0.00087
# Capsellagrandiflora_Cagra.4003s0027 Exp number, first 60 AAs:  0
# Capsellagrandiflora_Cagra.4003s0027 Total prob of N-in:        0.00194
Capsellagrandiflora_Cagra.4003s0027     TMHMM2.0        outside      1   349
```

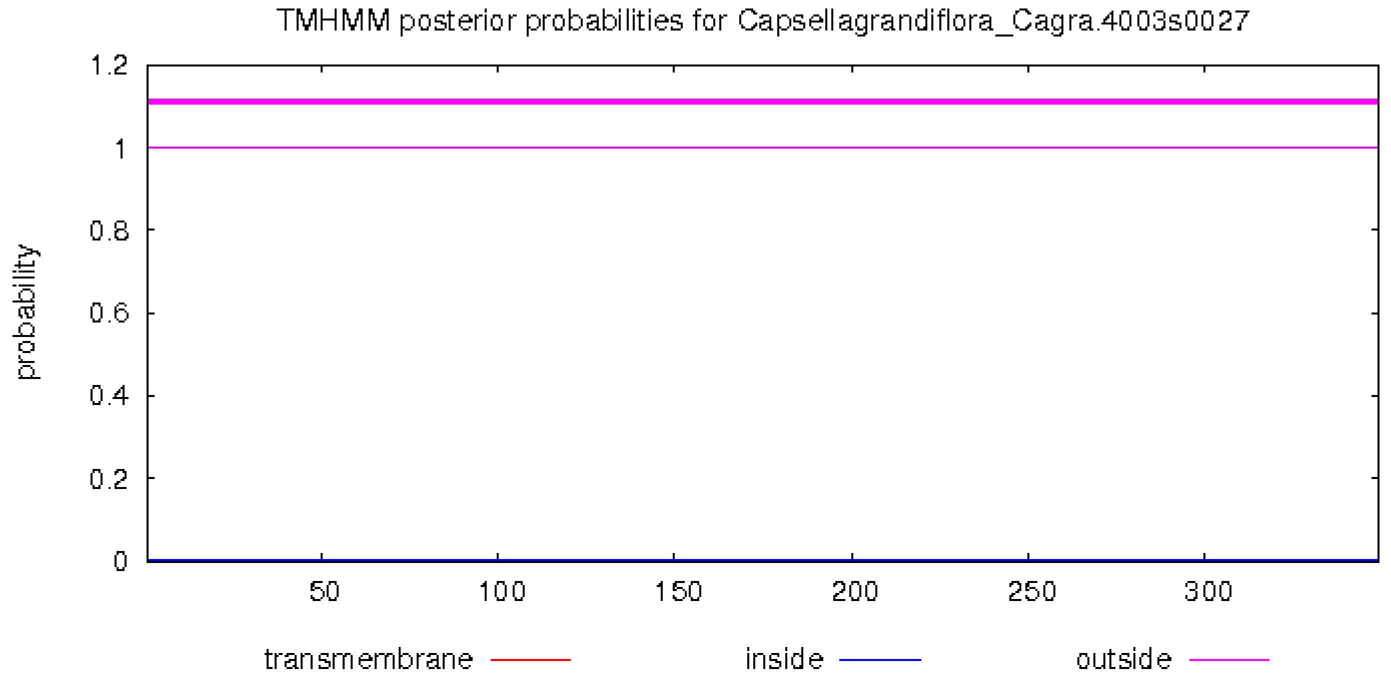

plot in postscript, script for making the plot in gnuplot, data for plot

---

```
# Capsellagrandiflora_Cagra.4006s0034 Length: 327
# Capsellagrandiflora_Cagra.4006s0034 Number of predicted TMHs:  0
# Capsellagrandiflora_Cagra.4006s0034 Exp number of AAs in TMHs: 0
# Capsellagrandiflora_Cagra.4006s0034 Exp number, first 60 AAs:  0
# Capsellagrandiflora_Cagra.4006s0034 Total prob of N-in:        0.01684
Capsellagrandiflora_Cagra.4006s0034     TMHMM2.0        outside      1   327
```

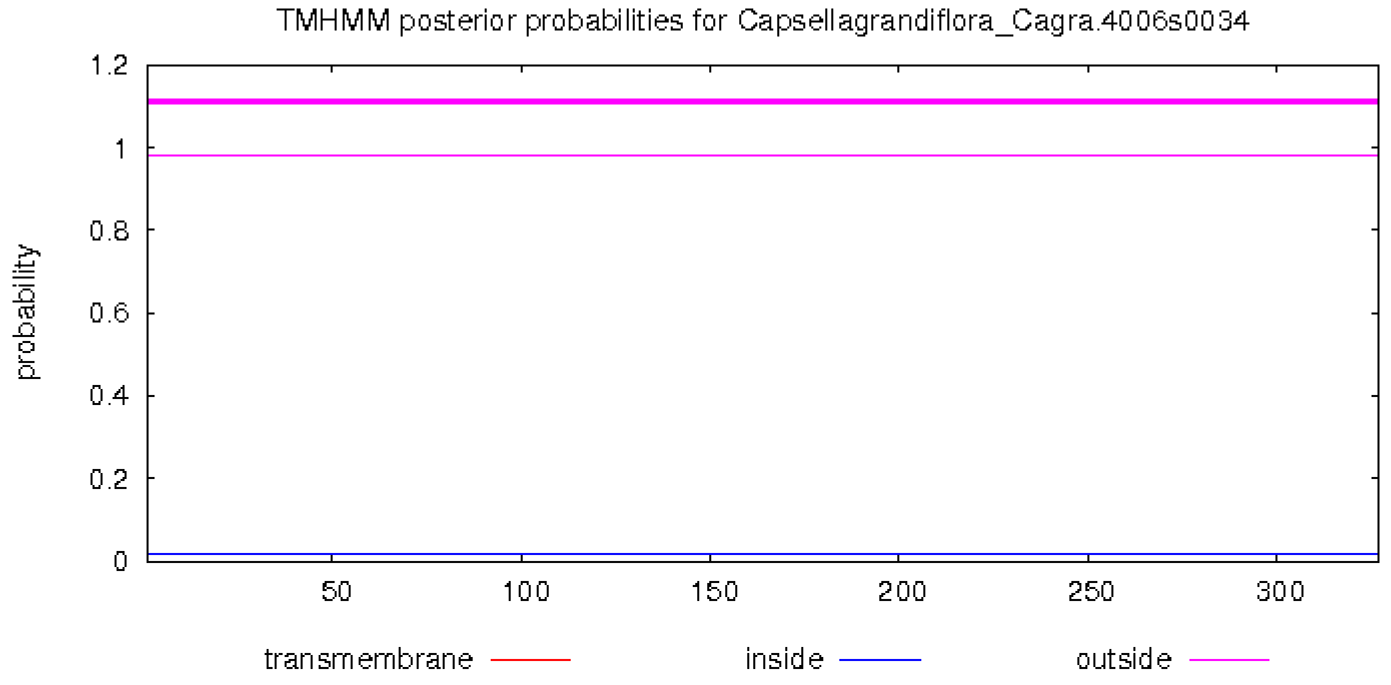

plot in postscript, script for making the plot in gnuplot, data for plot

---

```
# Capsellagrandiflora_Cagra.4091s0015 Length: 318
# Capsellagrandiflora_Cagra.4091s0015 Number of predicted TMHs:  0
# Capsellagrandiflora_Cagra.4091s0015 Exp number of AAs in TMHs: 0.01776
# Capsellagrandiflora_Cagra.4091s0015 Exp number, first 60 AAs:  0
# Capsellagrandiflora_Cagra.4091s0015 Total prob of N-in:        0.07240
Capsellagrandiflora_Cagra.4091s0015     TMHMM2.0        outside      1   318
```

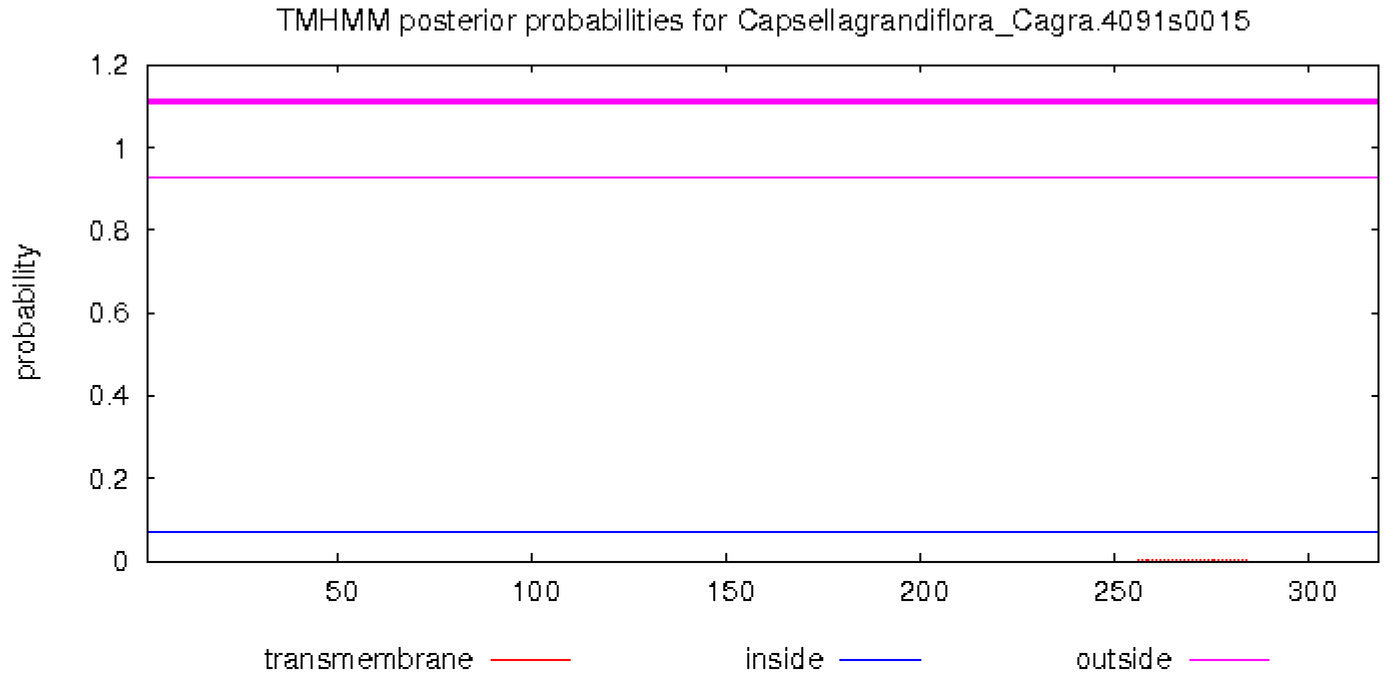

plot in postscript, script for making the plot in gnuplot, data for plot

```
# Capsellagrandiflora_Cagra.4344s0001 Length: 274
# Capsellagrandiflora_Cagra.4344s0001 Number of predicted TMHs:  1
# Capsellagrandiflora_Cagra.4344s0001 Exp number of AAs in TMHs: 18.27345
# Capsellagrandiflora_Cagra.4344s0001 Exp number, first 60 AAs:  0.00014
# Capsellagrandiflora_Cagra.4344s0001 Total prob of N-in:        0.48072
Capsellagrandiflora_Cagra.4344s0001     TMHMM2.0        outside      1   255
Capsellagrandiflora_Cagra.4344s0001     TMHMM2.0        TMhelix    256   273
Capsellagrandiflora_Cagra.4344s0001     TMHMM2.0        inside     274   274
```

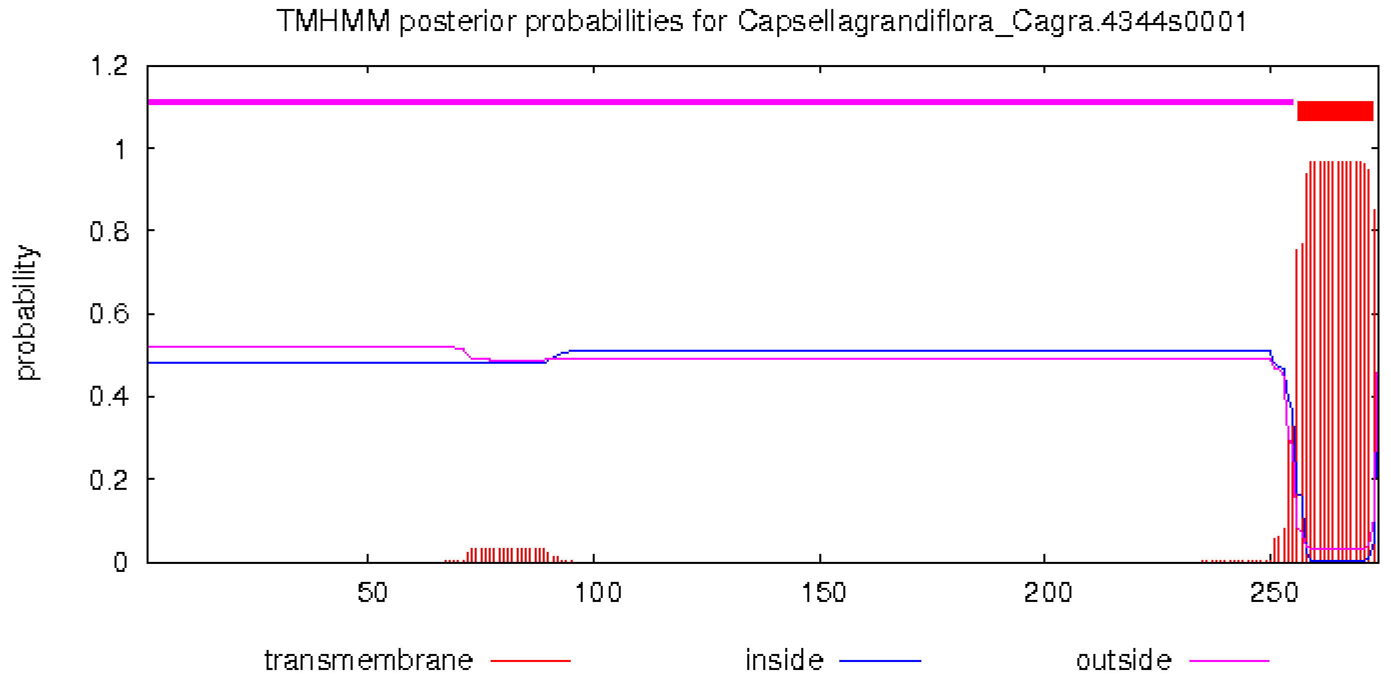

plot in postscript, script for making the plot in gnuplot, data for plot

```
# Capsellagrandiflora_Cagra.4622s0005 Length: 376
# Capsellagrandiflora_Cagra.4622s0005 Number of predicted TMHs:  0
# Capsellagrandiflora_Cagra.4622s0005 Exp number of AAs in TMHs: 0.01121
# Capsellagrandiflora_Cagra.4622s0005 Exp number, first 60 AAs:  0
```

```
# Capsellagrandiflora_Cagra.4622s0005 Total prob of N-in:        0.01682
Capsellagrandiflora_Cagra.4622s0005     TMHMM2.0        outside      1   376
```

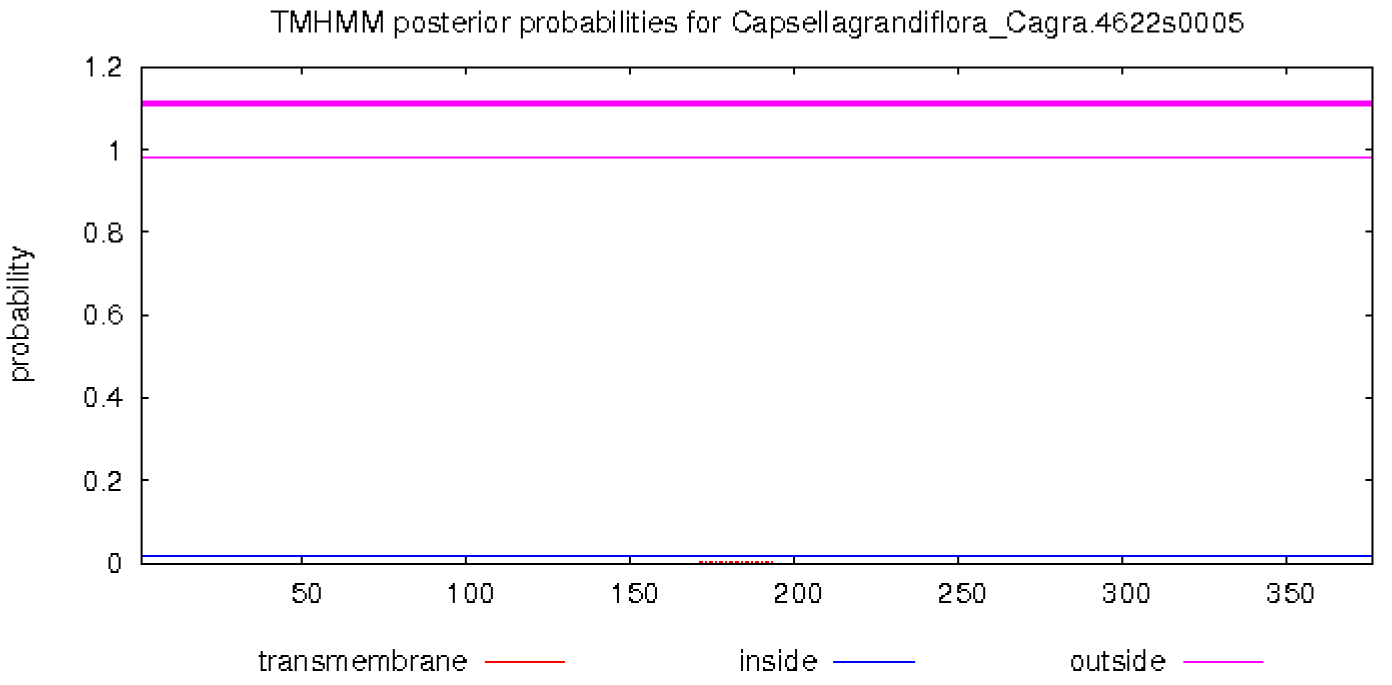

plot in postscript, script for making the plot in gnuplot, data for plot

---

```
# Capsellagrandiflora_Cagra.4734s0006 Length: 376
# Capsellagrandiflora_Cagra.4734s0006 Number of predicted TMHs:  0
# Capsellagrandiflora_Cagra.4734s0006 Exp number of AAs in TMHs: 0
# Capsellagrandiflora_Cagra.4734s0006 Exp number, first 60 AAs:  0
# Capsellagrandiflora_Cagra.4734s0006 Total prob of N-in:        0.01248
Capsellagrandiflora_Cagra.4734s0006     TMHMM2.0        outside      1   376
```

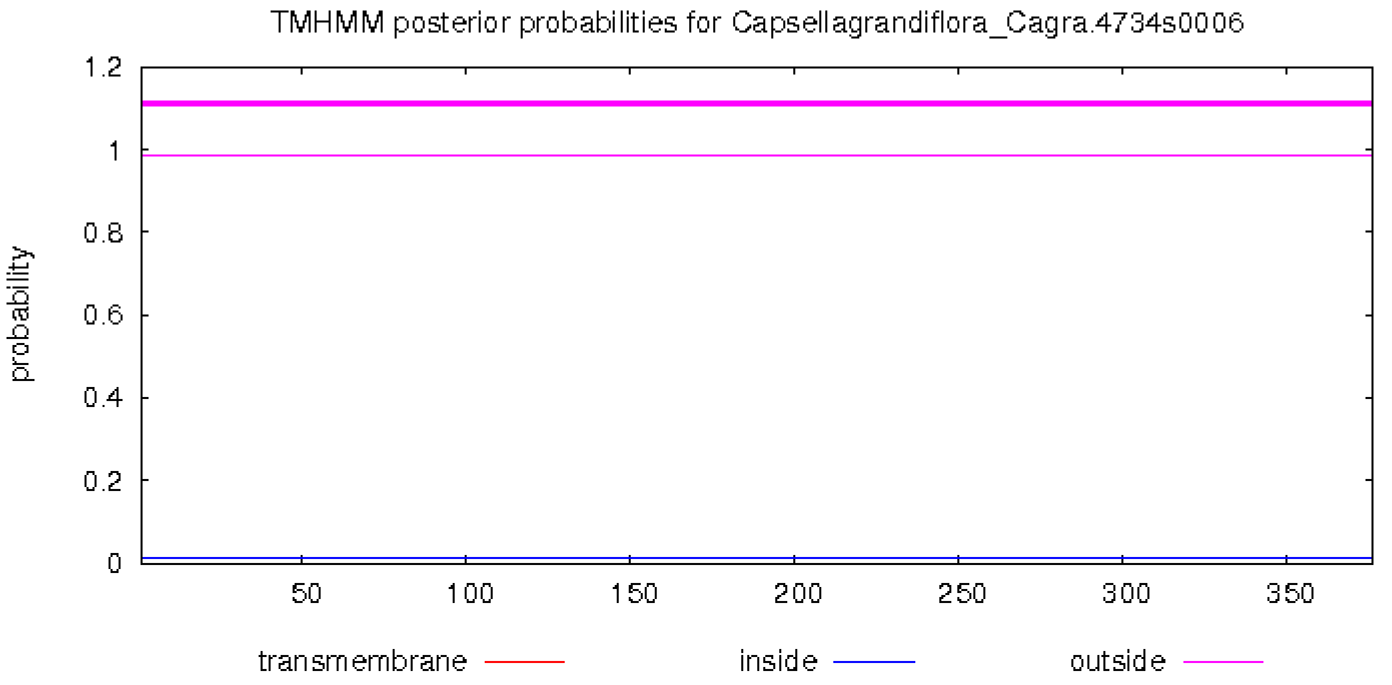

plot in postscript, script for making the plot in gnuplot, data for plot

---

```
# Capsellagrandiflora_Cagra.4849s0008 Length: 491
# Capsellagrandiflora_Cagra.4849s0008 Number of predicted TMHs:  0
# Capsellagrandiflora_Cagra.4849s0008 Exp number of AAs in TMHs: 0.00019
# Capsellagrandiflora_Cagra.4849s0008 Exp number, first 60 AAs:  0
```

```
# Capsellagrandiflora_Cagra.4849s0008 Total prob of N-in:        0.00015
Capsellagrandiflora_Cagra.4849s0008     TMHMM2.0        outside      1   491
```

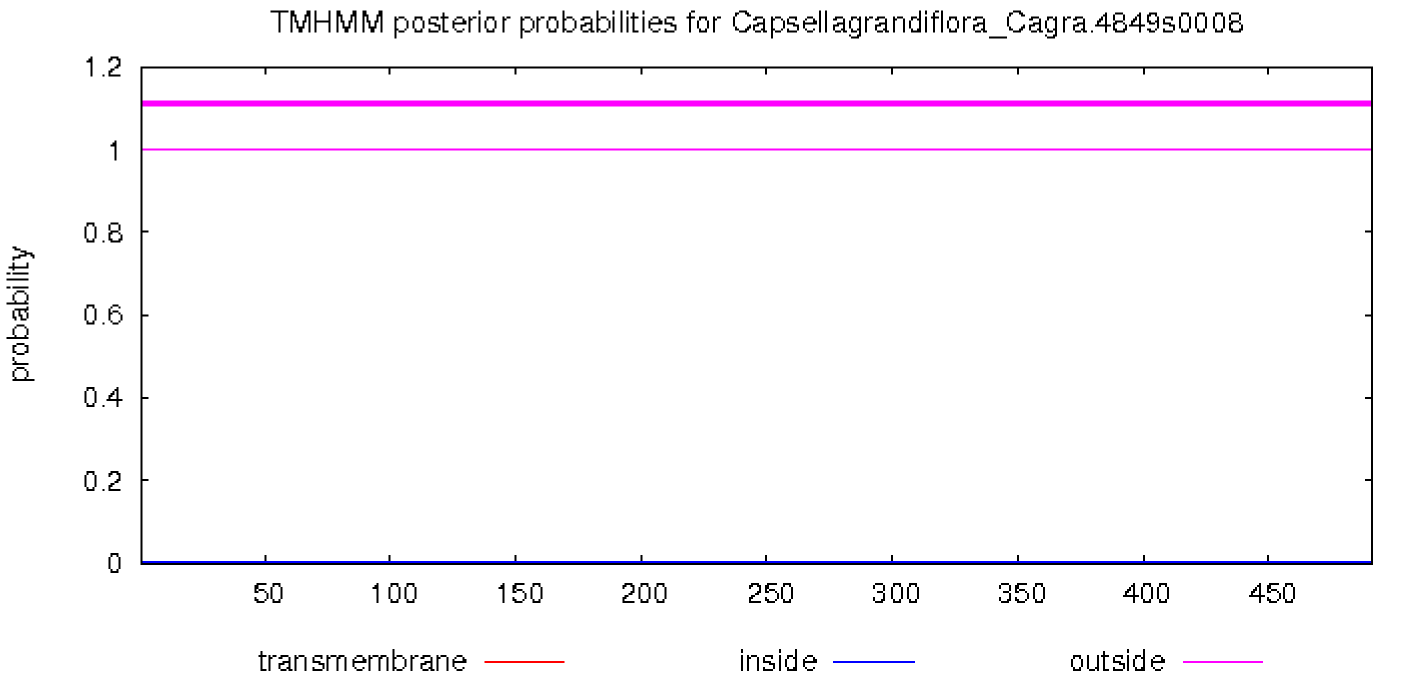

plot in postscript, script for making the plot in gnuplot, data for plot

---

```
# Capsellagrandiflora_Cagra.5641s0055 Length: 301
# Capsellagrandiflora_Cagra.5641s0055 Number of predicted TMHs:  0
# Capsellagrandiflora_Cagra.5641s0055 Exp number of AAs in TMHs: 0.00069
# Capsellagrandiflora_Cagra.5641s0055 Exp number, first 60 AAs:  0
# Capsellagrandiflora_Cagra.5641s0055 Total prob of N-in:        0.03862
Capsellagrandiflora_Cagra.5641s0055     TMHMM2.0        outside      1   301
```

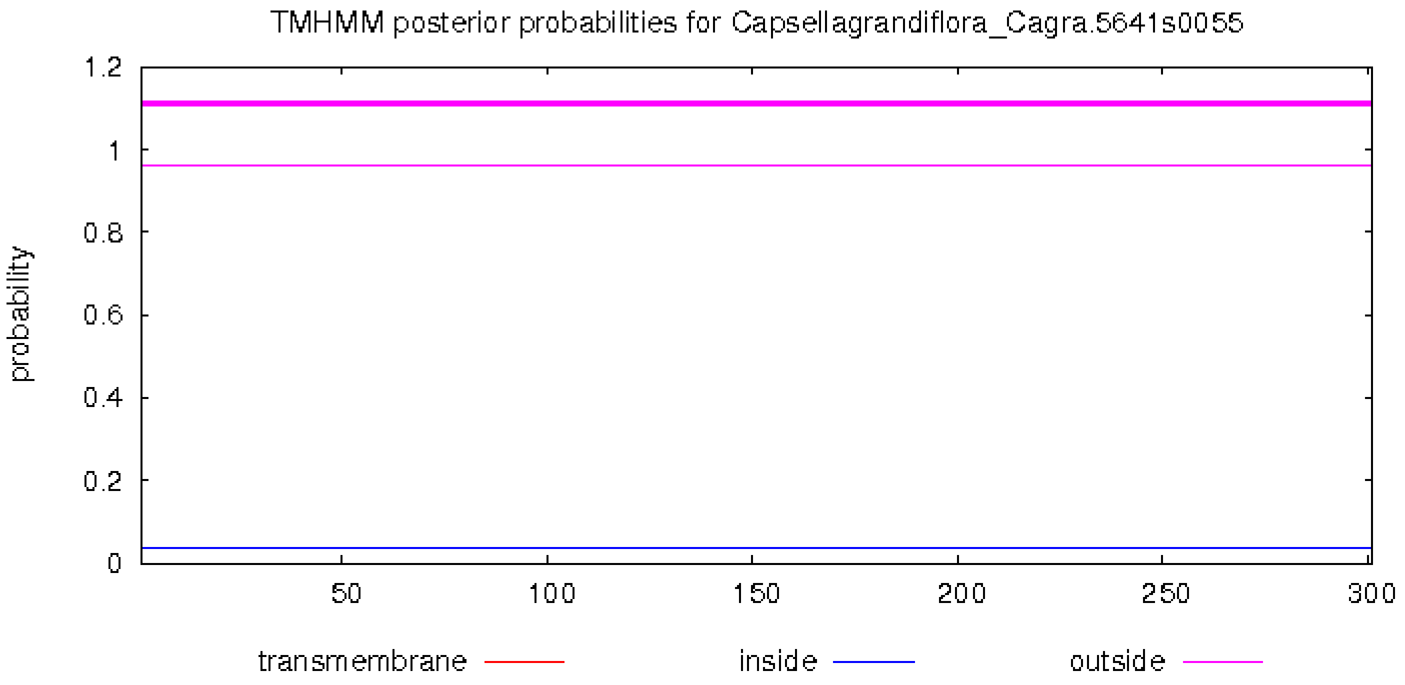

plot in postscript, script for making the plot in gnuplot, data for plot

---

```
# Capsellagrandiflora_Cagra.5641s0060 Length: 248
# Capsellagrandiflora_Cagra.5641s0060 Number of predicted TMHs:  0
# Capsellagrandiflora_Cagra.5641s0060 Exp number of AAs in TMHs: 0
# Capsellagrandiflora_Cagra.5641s0060 Exp number, first 60 AAs:  0
```

```
# Capsellagrandiflora_Cagra.5641s0060 Total prob of N-in:        0.08977
Capsellagrandiflora_Cagra.5641s0060     TMHMM2.0        outside      1   248
```

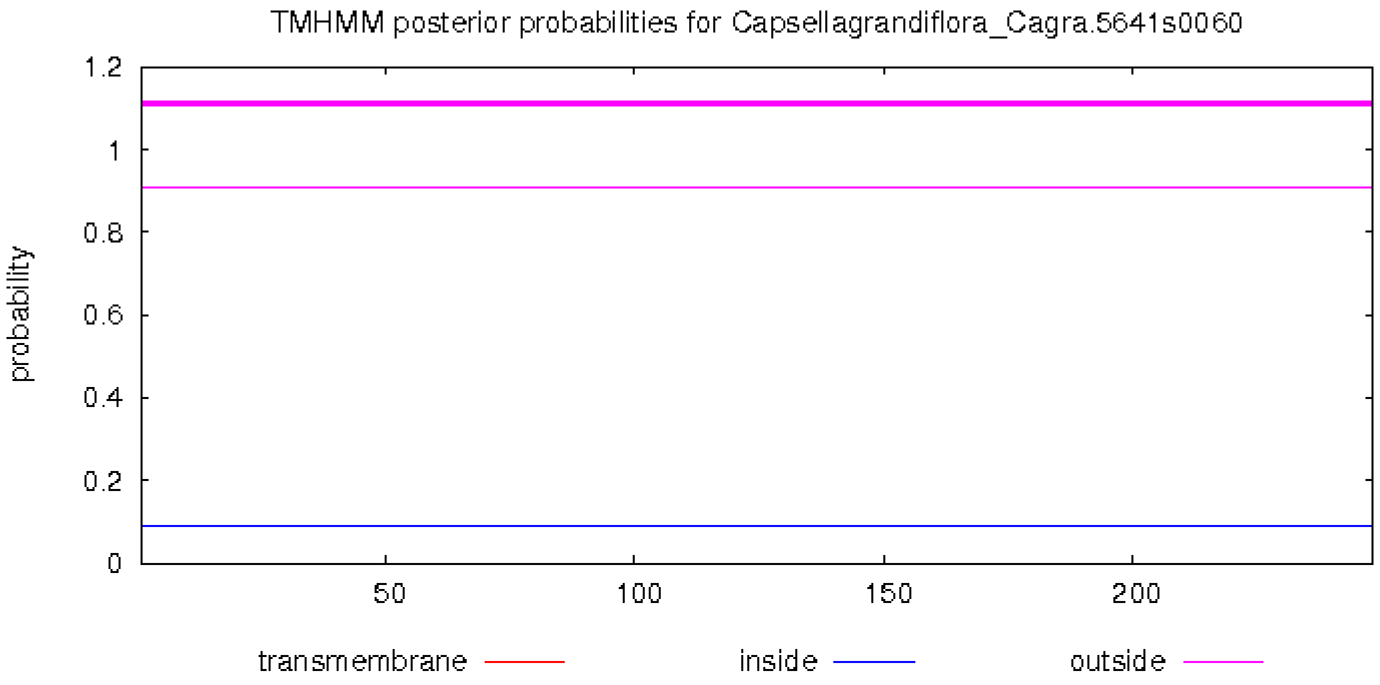

plot in postscript, script for making the plot in gnuplot, data for plot

---

```
# Capsellagrandiflora_Cagra.5905s0003 Length: 343
# Capsellagrandiflora_Cagra.5905s0003 Number of predicted TMHs:  0
# Capsellagrandiflora_Cagra.5905s0003 Exp number of AAs in TMHs: 0.00144
# Capsellagrandiflora_Cagra.5905s0003 Exp number, first 60 AAs:  0
# Capsellagrandiflora_Cagra.5905s0003 Total prob of N-in:        0.00783
Capsellagrandiflora_Cagra.5905s0003     TMHMM2.0        outside      1   343
```

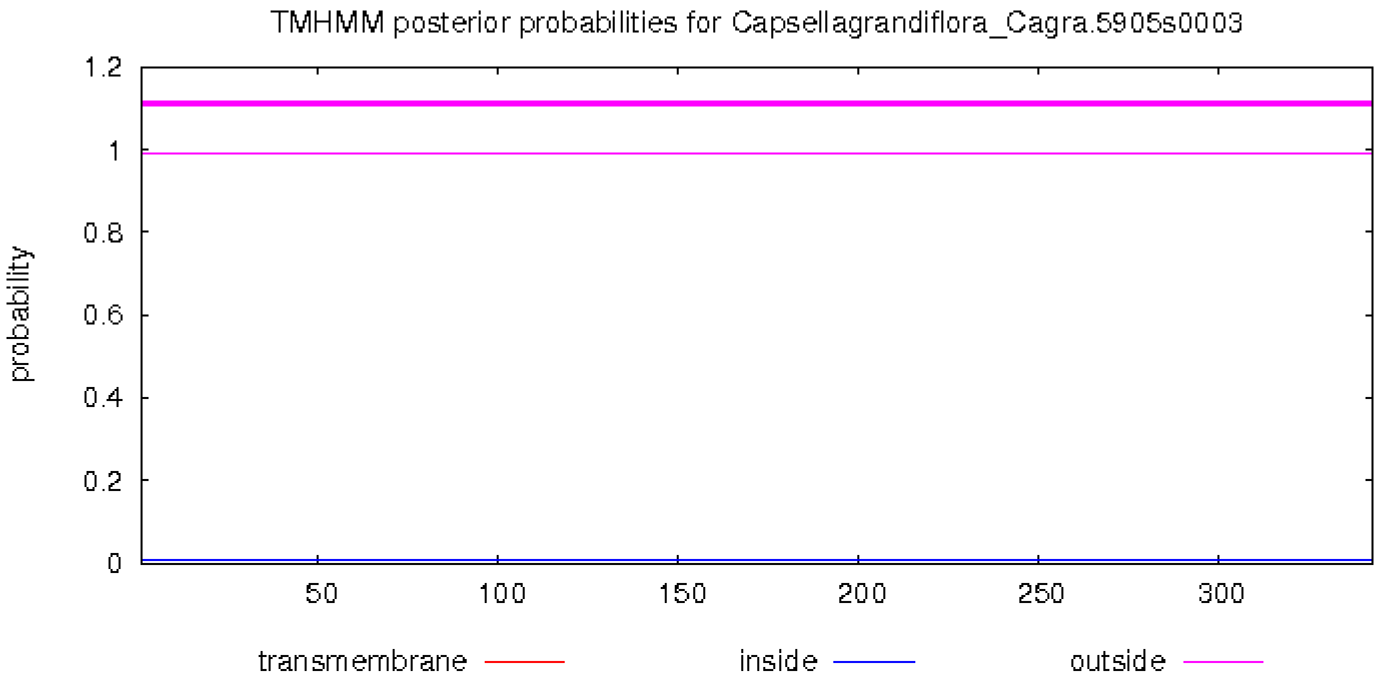

plot in postscript, script for making the plot in gnuplot, data for plot

---

```
# Capsellagrandiflora_Cagra.6133s0030 Length: 313
# Capsellagrandiflora_Cagra.6133s0030 Number of predicted TMHs:  0
# Capsellagrandiflora_Cagra.6133s0030 Exp number of AAs in TMHs: 0
# Capsellagrandiflora_Cagra.6133s0030 Exp number, first 60 AAs:  0
```

```
# Capsellagrandiflora_Cagra.6133s0030 Total prob of N-in:        0.00449
Capsellagrandiflora_Cagra.6133s0030     TMHMM2.0        outside      1   313
```

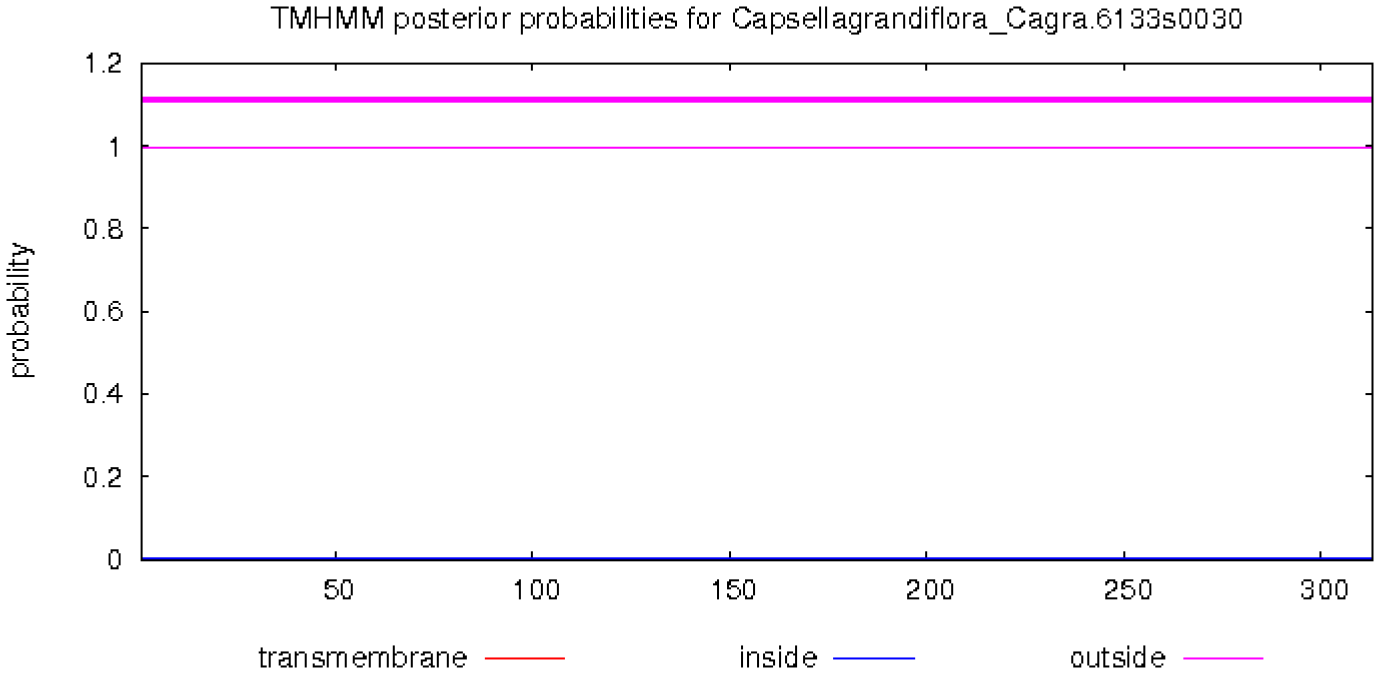

plot in postscript, script for making the plot in gnuplot, data for plot

---

```
# Capsellagrandiflora_Cagra.6372s0006 Length: 323
# Capsellagrandiflora_Cagra.6372s0006 Number of predicted TMHs:  0
# Capsellagrandiflora_Cagra.6372s0006 Exp number of AAs in TMHs: 0.00031
# Capsellagrandiflora_Cagra.6372s0006 Exp number, first 60 AAs:  0
# Capsellagrandiflora_Cagra.6372s0006 Total prob of N-in:        0.02489
Capsellagrandiflora_Cagra.6372s0006     TMHMM2.0        outside      1   323
```

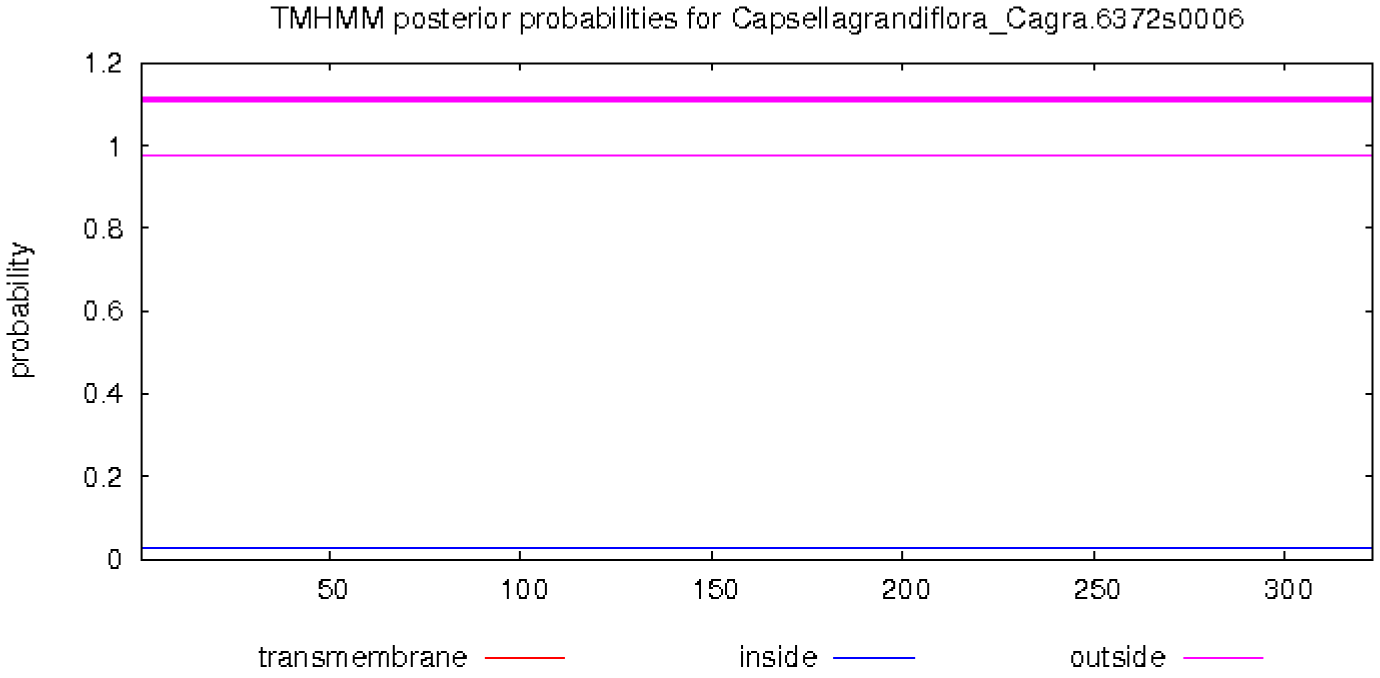

plot in postscript, script for making the plot in gnuplot, data for plot

---

```
# Capsellagrandiflora_Cagra.6981s0001 Length: 279
# Capsellagrandiflora_Cagra.6981s0001 Number of predicted TMHs:  0
# Capsellagrandiflora_Cagra.6981s0001 Exp number of AAs in TMHs: 0.00023
# Capsellagrandiflora_Cagra.6981s0001 Exp number, first 60 AAs:  0.00023
```

```
# Capsellagrandiflora_Cagra.6981s0001 Total prob of N-in:        0.03049
Capsellagrandiflora_Cagra.6981s0001     TMHMM2.0        outside      1   279
```

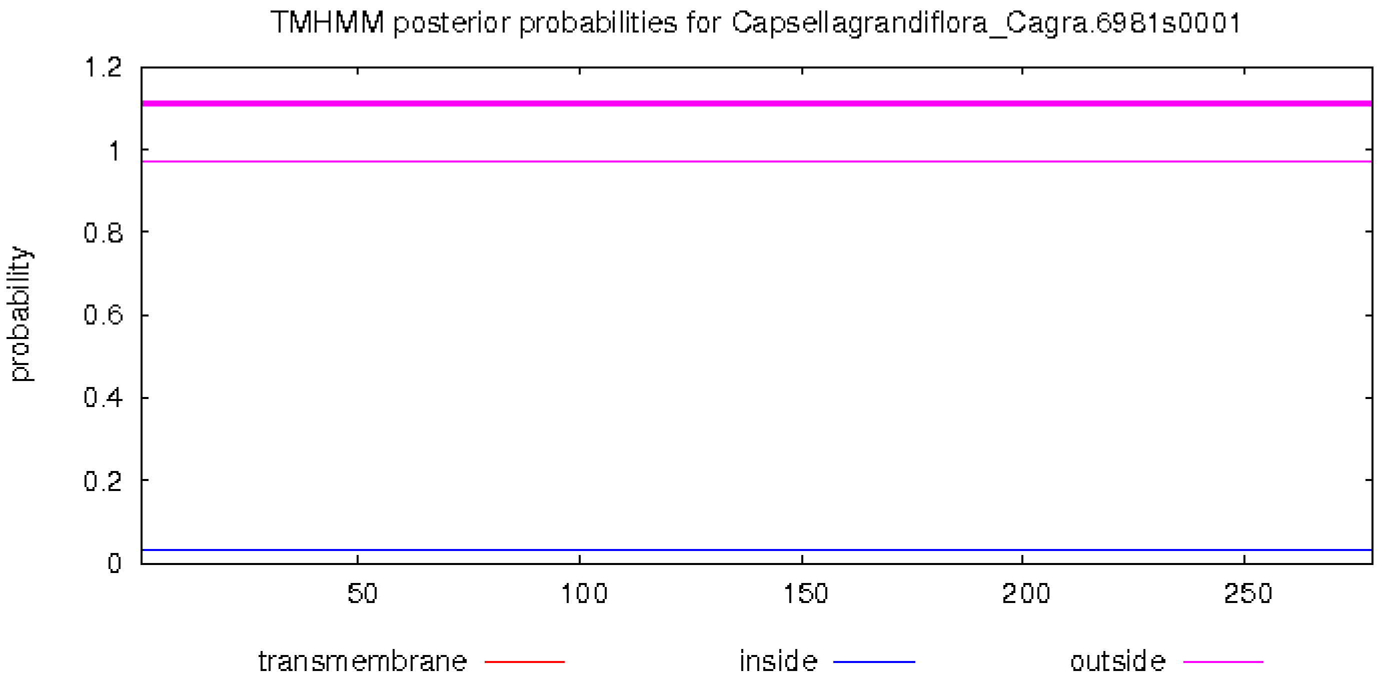

plot in postscript, script for making the plot in gnuplot, data for plot

---

```
# Capsellagrandiflora_Cagra.7393s0016 Length: 194
# Capsellagrandiflora_Cagra.7393s0016 Number of predicted TMHs:  0
# Capsellagrandiflora_Cagra.7393s0016 Exp number of AAs in TMHs: 0.00137
# Capsellagrandiflora_Cagra.7393s0016 Exp number, first 60 AAs:  0
# Capsellagrandiflora_Cagra.7393s0016 Total prob of N-in:        0.12612
Capsellagrandiflora_Cagra.7393s0016     TMHMM2.0        outside      1   194
```

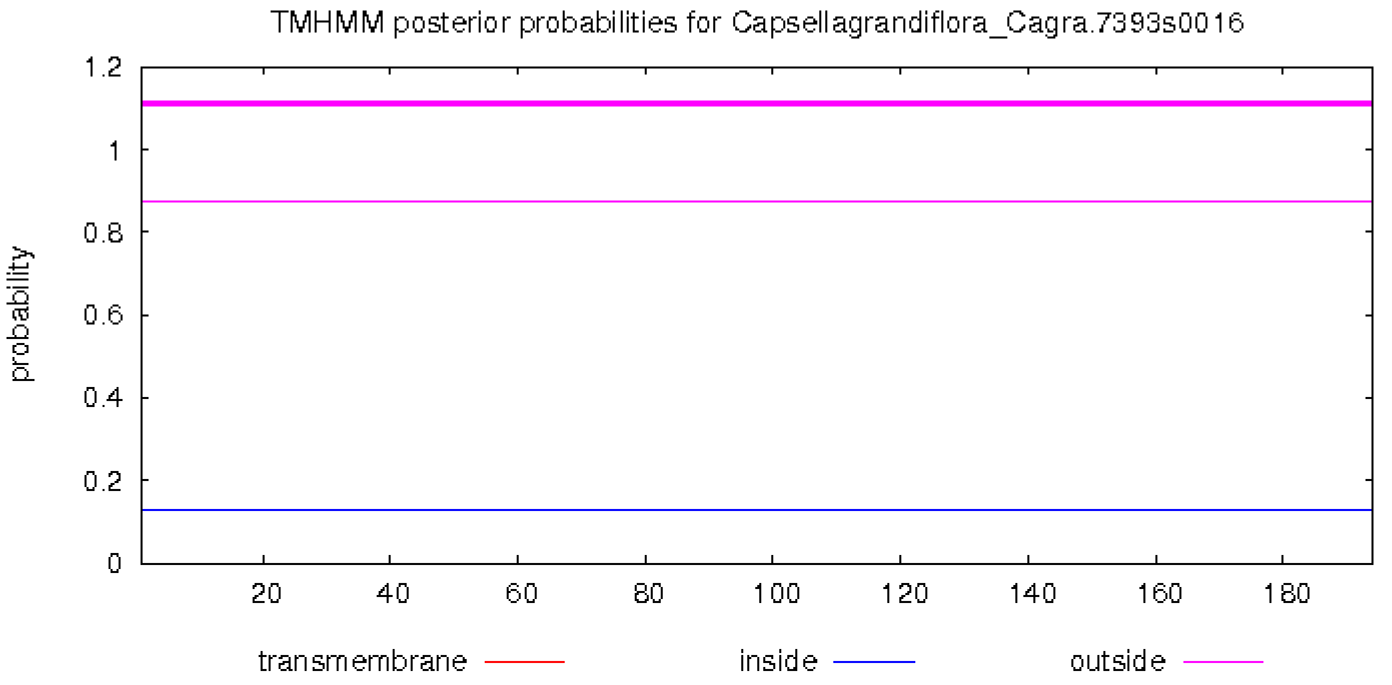

plot in postscript, script for making the plot in gnuplot, data for plot

---

```
# Capsellagrandiflora_Cagra.7865s0013 Length: 258
# Capsellagrandiflora_Cagra.7865s0013 Number of predicted TMHs:  0
# Capsellagrandiflora_Cagra.7865s0013 Exp number of AAs in TMHs: 0.00042
# Capsellagrandiflora_Cagra.7865s0013 Exp number, first 60 AAs:  0
```

```
# Capsellagrandiflora_Cagra.7865s0013 Total prob of N-in:        0.00330
Capsellagrandiflora_Cagra.7865s0013     TMHMM2.0        outside      1   258
```

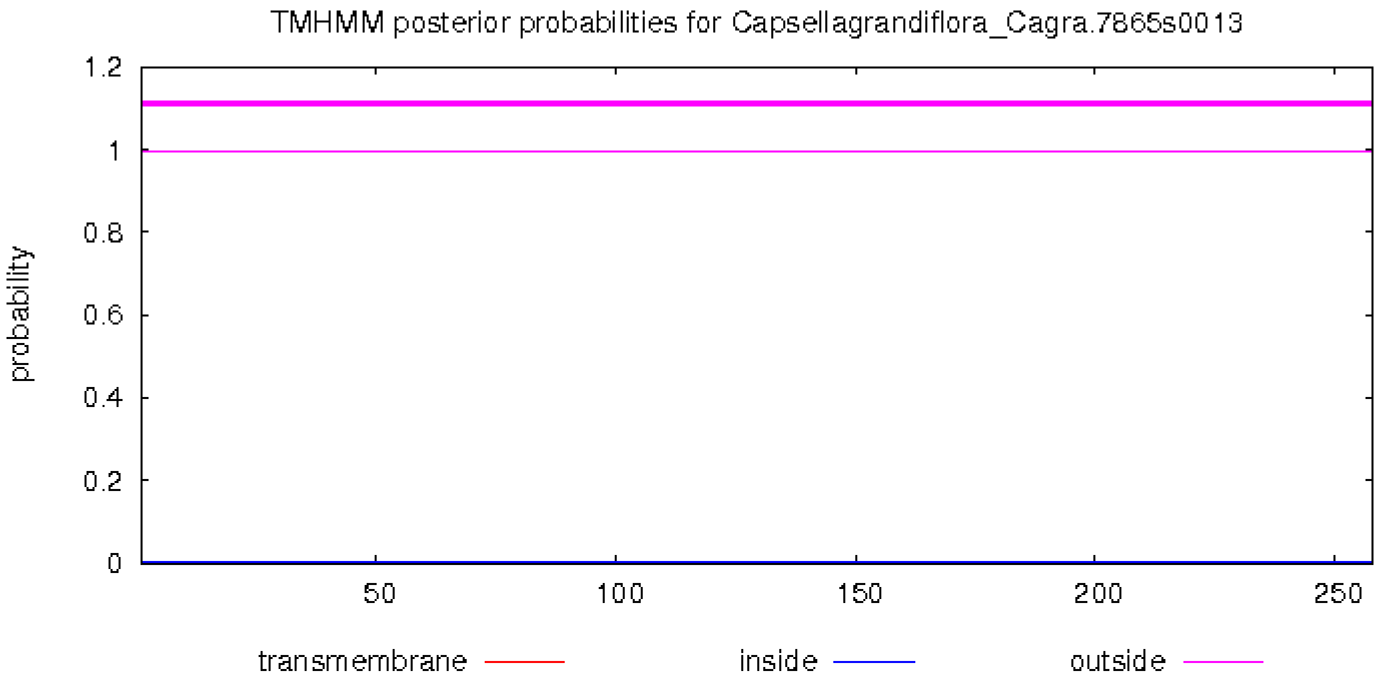

plot in postscript, script for making the plot in gnuplot, data for plot

---

```
# Capsellagrandiflora_Cagra.8367s0003 Length: 378
# Capsellagrandiflora_Cagra.8367s0003 Number of predicted TMHs:  0
# Capsellagrandiflora_Cagra.8367s0003 Exp number of AAs in TMHs: 0
# Capsellagrandiflora_Cagra.8367s0003 Exp number, first 60 AAs:  0
# Capsellagrandiflora_Cagra.8367s0003 Total prob of N-in:        0.00137
Capsellagrandiflora_Cagra.8367s0003     TMHMM2.0        outside      1   378
```

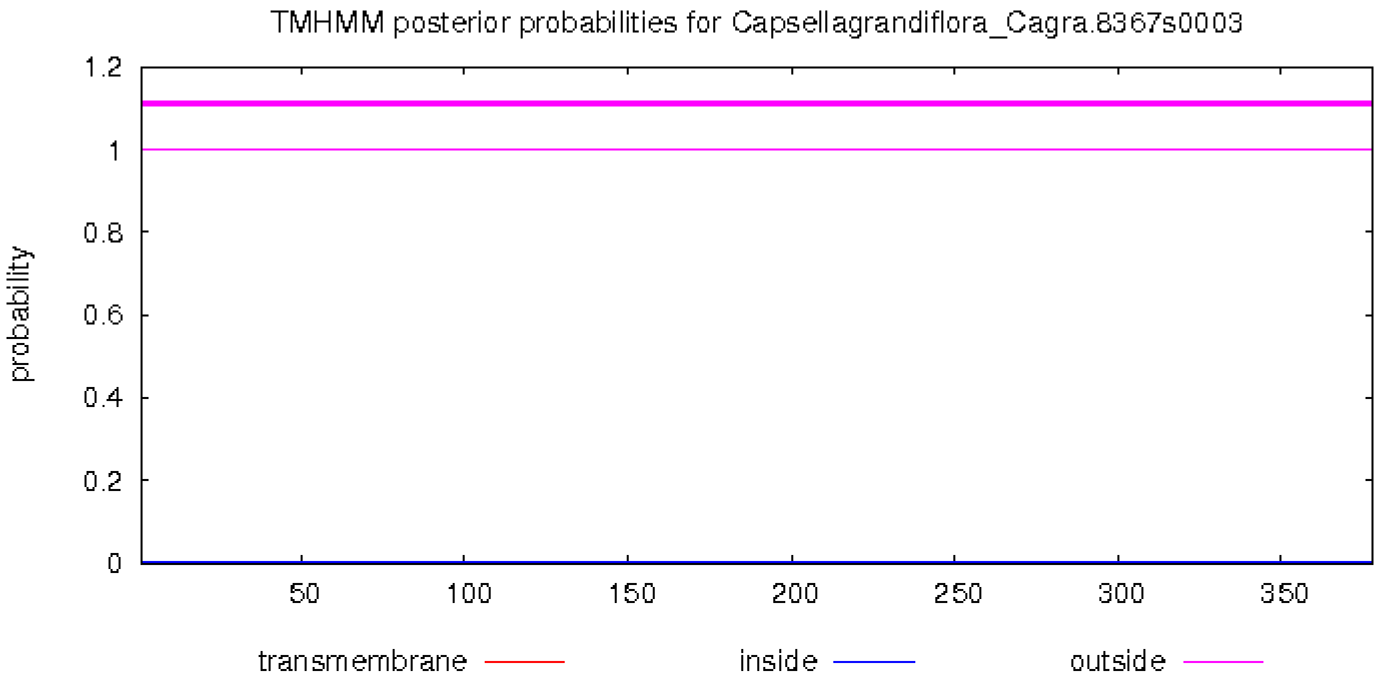

plot in postscript, script for making the plot in gnuplot, data for plot

---

```
# Capsellagrandiflora_Cagra.9217s0020 Length: 404
# Capsellagrandiflora_Cagra.9217s0020 Number of predicted TMHs:  0
# Capsellagrandiflora_Cagra.9217s0020 Exp number of AAs in TMHs: 0.03496
# Capsellagrandiflora_Cagra.9217s0020 Exp number, first 60 AAs:  0.01504
```

```
# Capsellagrandiflora_Cagra.9217s0020 Total prob of N-in:        0.02591
Capsellagrandiflora_Cagra.9217s0020     TMHMM2.0        outside      1   404
```

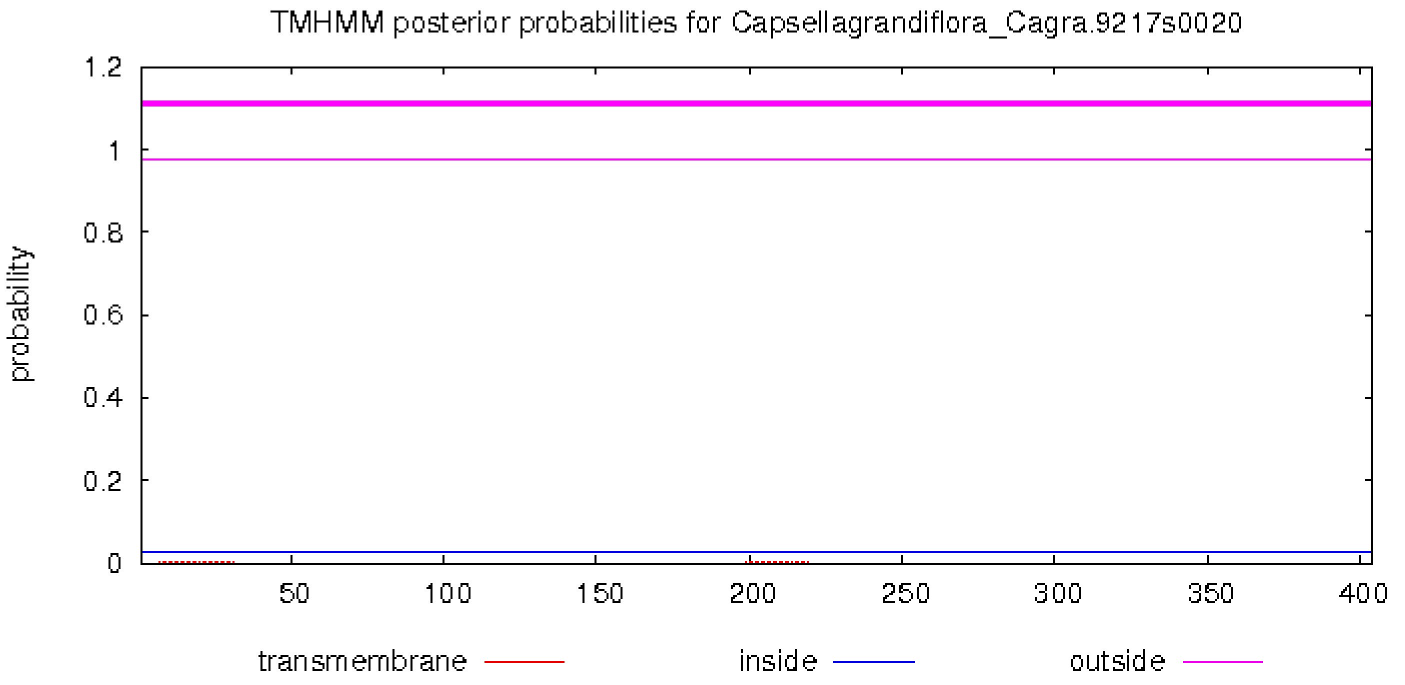

plot in postscript, script for making the plot in gnuplot, data for plot

```
# Capsellagrandiflora_Cagra.9217s0021 Length: 569
# Capsellagrandiflora_Cagra.9217s0021 Number of predicted TMHs:  1
# Capsellagrandiflora_Cagra.9217s0021 Exp number of AAs in TMHs: 21.52252
# Capsellagrandiflora_Cagra.9217s0021 Exp number, first 60 AAs:  0
# Capsellagrandiflora_Cagra.9217s0021 Total prob of N-in:        0.01889
Capsellagrandiflora_Cagra.9217s0021     TMHMM2.0        outside      1   543
Capsellagrandiflora_Cagra.9217s0021     TMHMM2.0        TMhelix    544   566
Capsellagrandiflora_Cagra.9217s0021     TMHMM2.0        inside     567   569
```

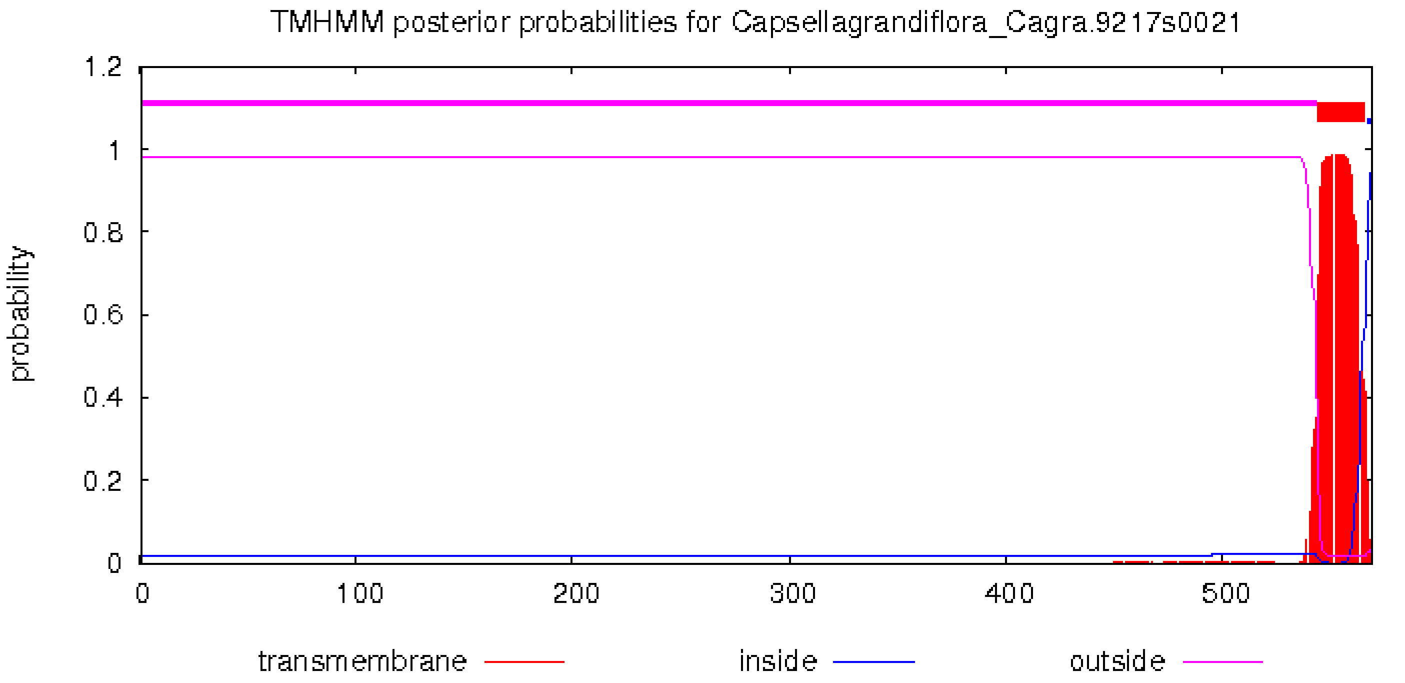

plot in postscript, script for making the plot in gnuplot, data for plot

```
# Capsellarubella_Carubv10000489m Length: 614
# Capsellarubella_Carubv10000489m Number of predicted TMHs:  1
```

```
# Capsellarubella_Carubv10000489m Exp number of AAs in TMHs: 22.25262
# Capsellarubella_Carubv10000489m Exp number, first 60 AAs:  0.05833
# Capsellarubella_Carubv10000489m Total prob of N-in:        0.01049
Capsellarubella_Carubv10000489m TMHMM2.0        outside      1   592
Capsellarubella_Carubv10000489m TMHMM2.0        TMhelix    593   612
Capsellarubella_Carubv10000489m TMHMM2.0        inside     613   614
```

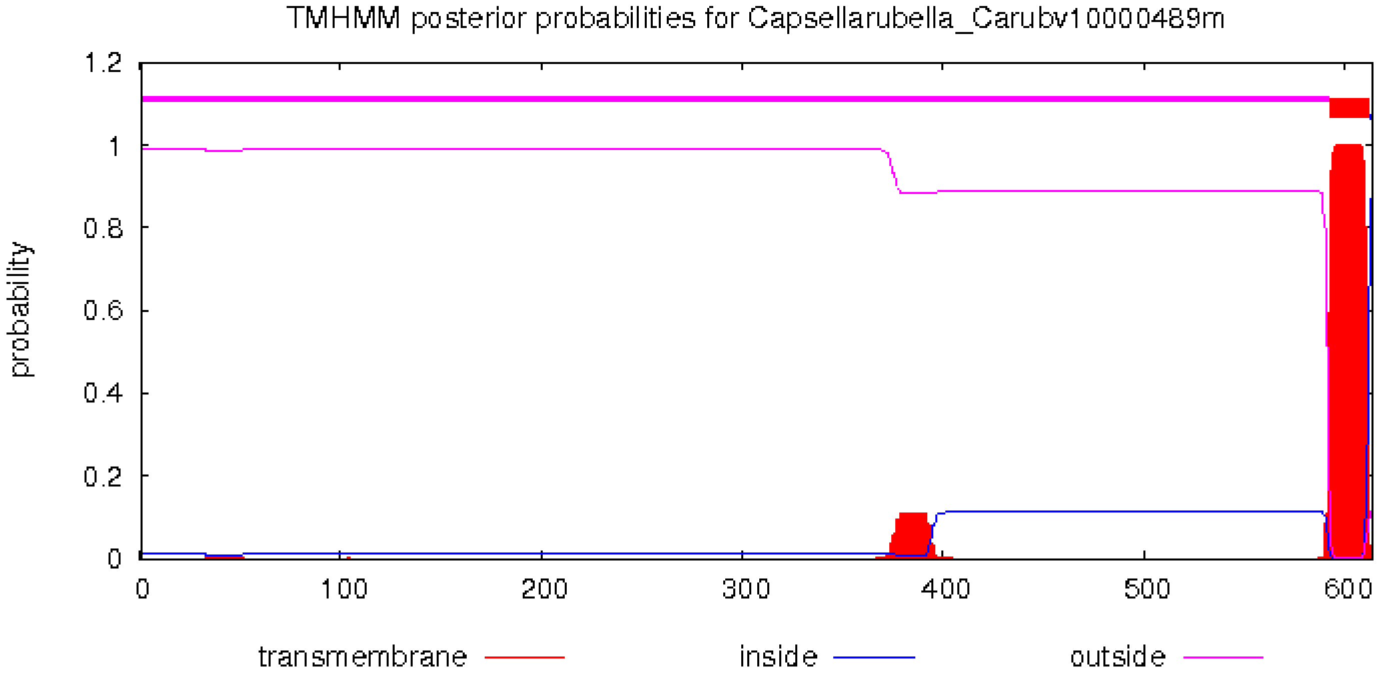

plot in postscript, script for making the plot in gnuplot, data for plot

---

```
# Capsellarubella_Carubv10000500m Length: 611
# Capsellarubella_Carubv10000500m Number of predicted TMHs:  1
# Capsellarubella_Carubv10000500m Exp number of AAs in TMHs: 20.1595
# Capsellarubella_Carubv10000500m Exp number, first 60 AAs:  0.0493
# Capsellarubella_Carubv10000500m Total prob of N-in:        0.00814
Capsellarubella_Carubv10000500m TMHMM2.0        outside      1   589
Capsellarubella_Carubv10000500m TMHMM2.0        TMhelix    590   609
Capsellarubella_Carubv10000500m TMHMM2.0        inside     610   611
```

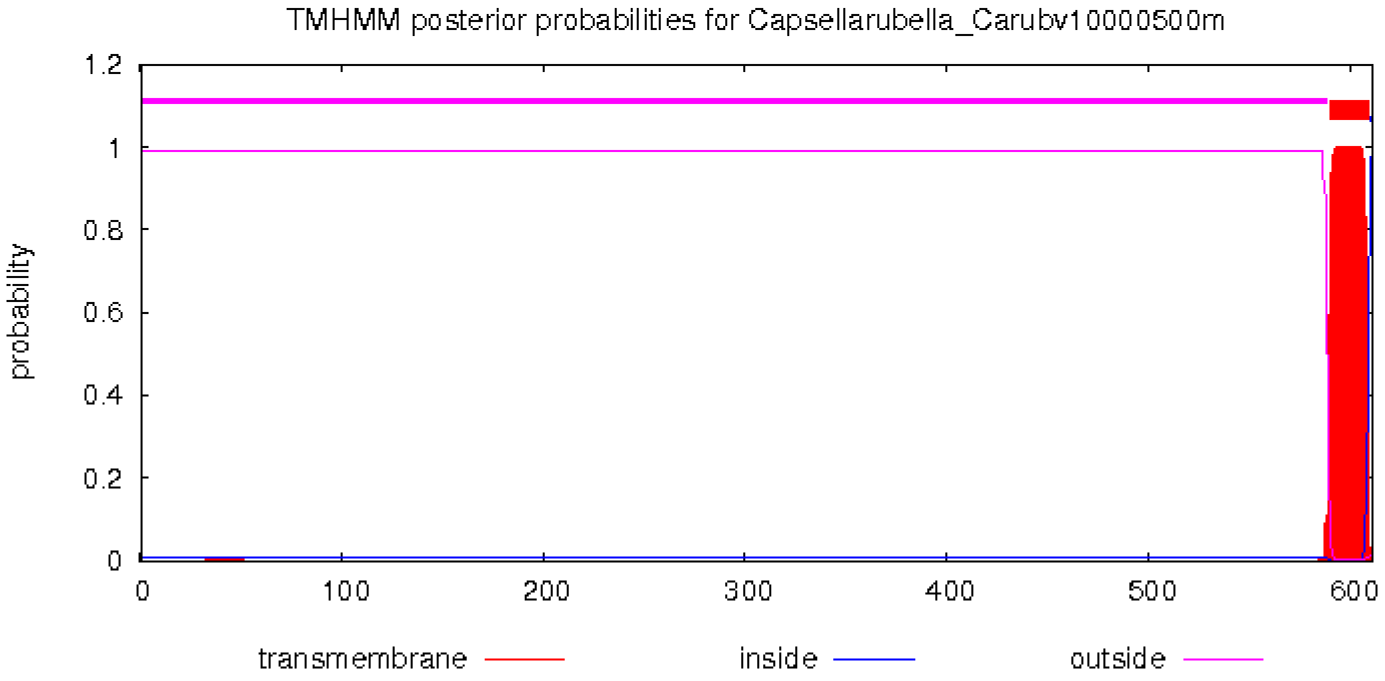

plot in postscript, script for making the plot in gnuplot, data for plot

```
# Capsellarubella_Carubv10000892m Length: 466
# Capsellarubella_Carubv10000892m Number of predicted TMHs:  0
# Capsellarubella_Carubv10000892m Exp number of AAs in TMHs: 13.10461
# Capsellarubella_Carubv10000892m Exp number, first 60 AAs:  0.00522
# Capsellarubella_Carubv10000892m Total prob of N-in:        0.01325
Capsellarubella_Carubv10000892m TMHMM2.0        outside      1   466
```

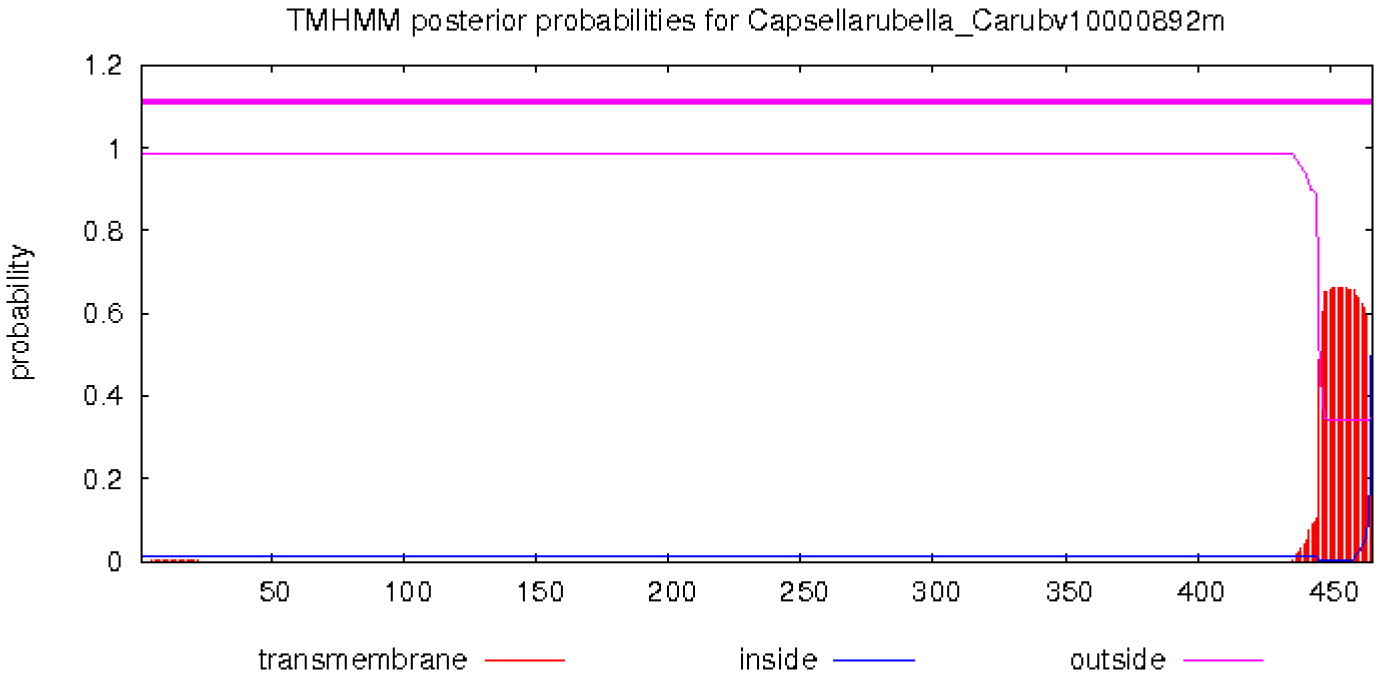

plot in postscript, script for making the plot in gnuplot, data for plot

```
# Capsellarubella_Carubv10001072m Length: 414
# Capsellarubella_Carubv10001072m Number of predicted TMHs:  0
# Capsellarubella_Carubv10001072m Exp number of AAs in TMHs: 0.00497
# Capsellarubella_Carubv10001072m Exp number, first 60 AAs:  0.00492
# Capsellarubella_Carubv10001072m Total prob of N-in:        0.00602
Capsellarubella_Carubv10001072m TMHMM2.0        outside      1   414
```

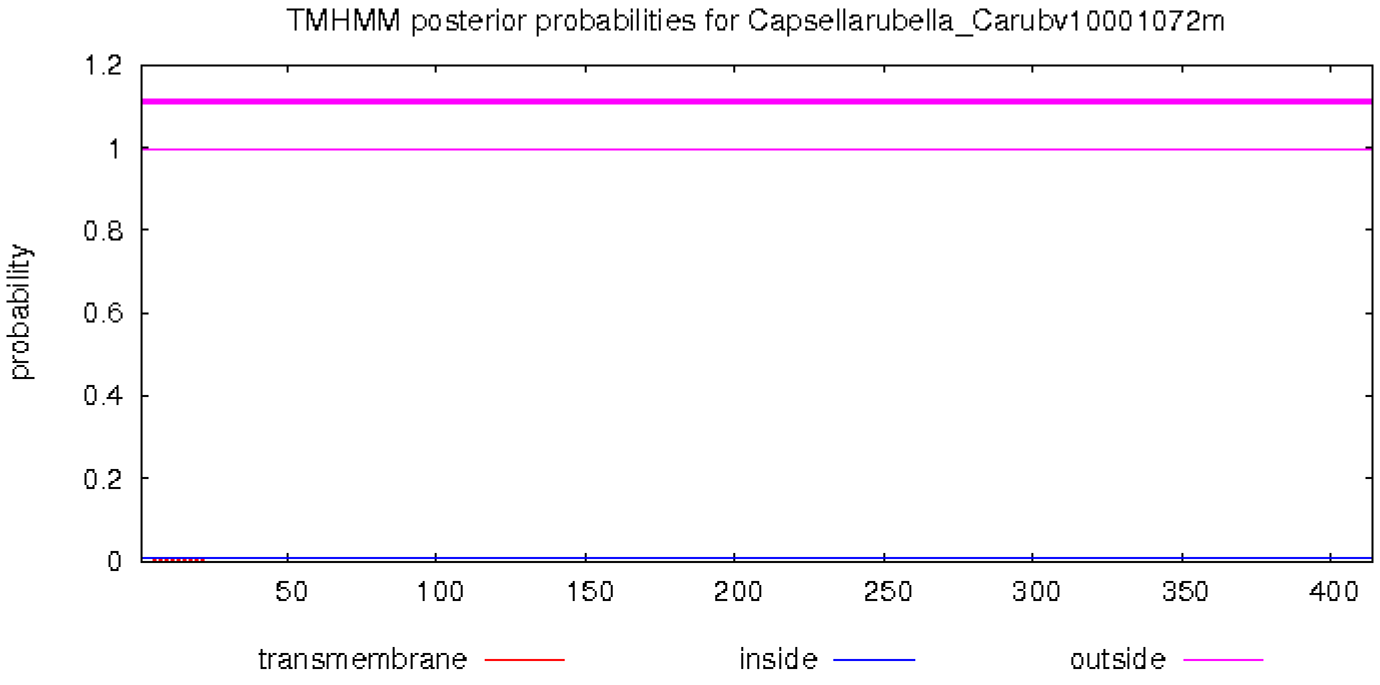

plot in postscript, script for making the plot in gnuplot, data for plot

```
# Capsellarubella_Carubv10001139m Length: 393
# Capsellarubella_Carubv10001139m Number of predicted TMHs:  0
# Capsellarubella_Carubv10001139m Exp number of AAs in TMHs: 0.00082
# Capsellarubella_Carubv10001139m Exp number, first 60 AAs:  0.00018
# Capsellarubella_Carubv10001139m Total prob of N-in:        0.00276
Capsellarubella_Carubv10001139m TMHMM2.0        outside      1   393
```

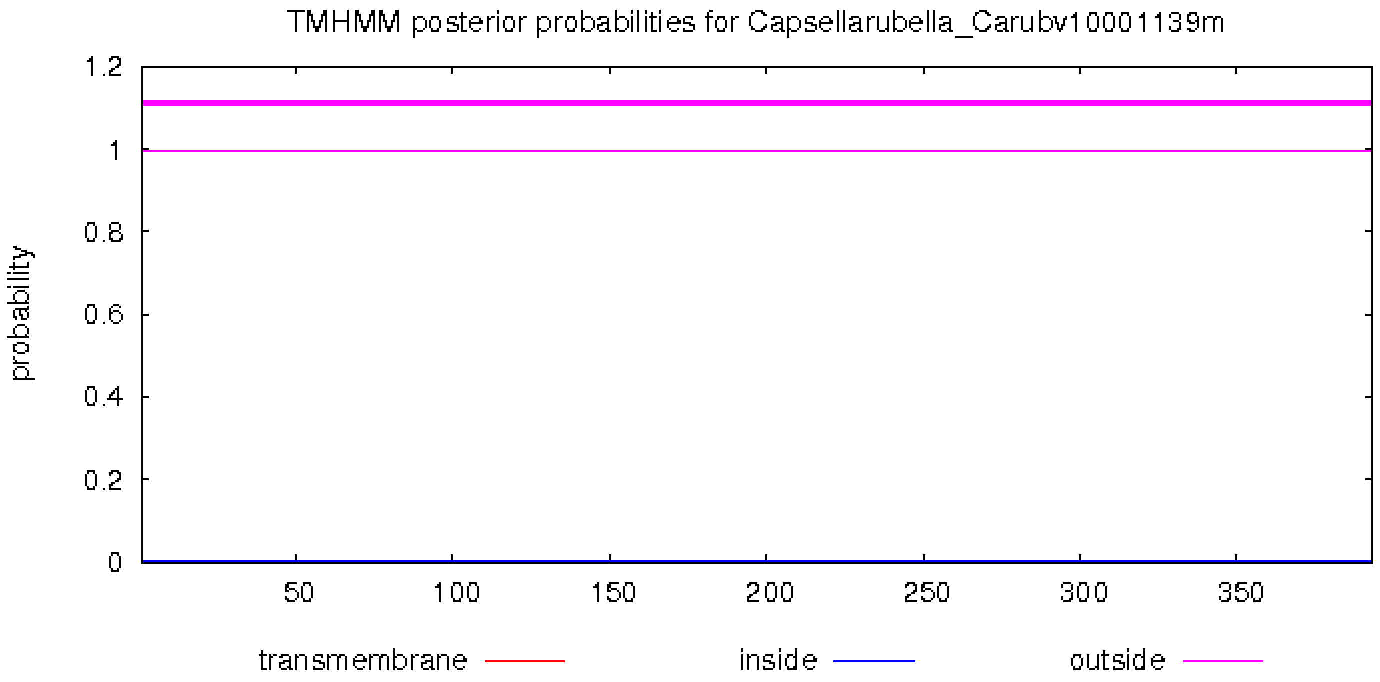

plot in postscript, script for making the plot in gnuplot, data for plot

```
# Capsellarubella_Carubv10001357m Length: 340
# Capsellarubella_Carubv10001357m Number of predicted TMHs:  0
# Capsellarubella_Carubv10001357m Exp number of AAs in TMHs: 12.4951
# Capsellarubella_Carubv10001357m Exp number, first 60 AAs:  0.01379
# Capsellarubella_Carubv10001357m Total prob of N-in:        0.14157
Capsellarubella_Carubv10001357m TMHMM2.0        outside      1   340
```

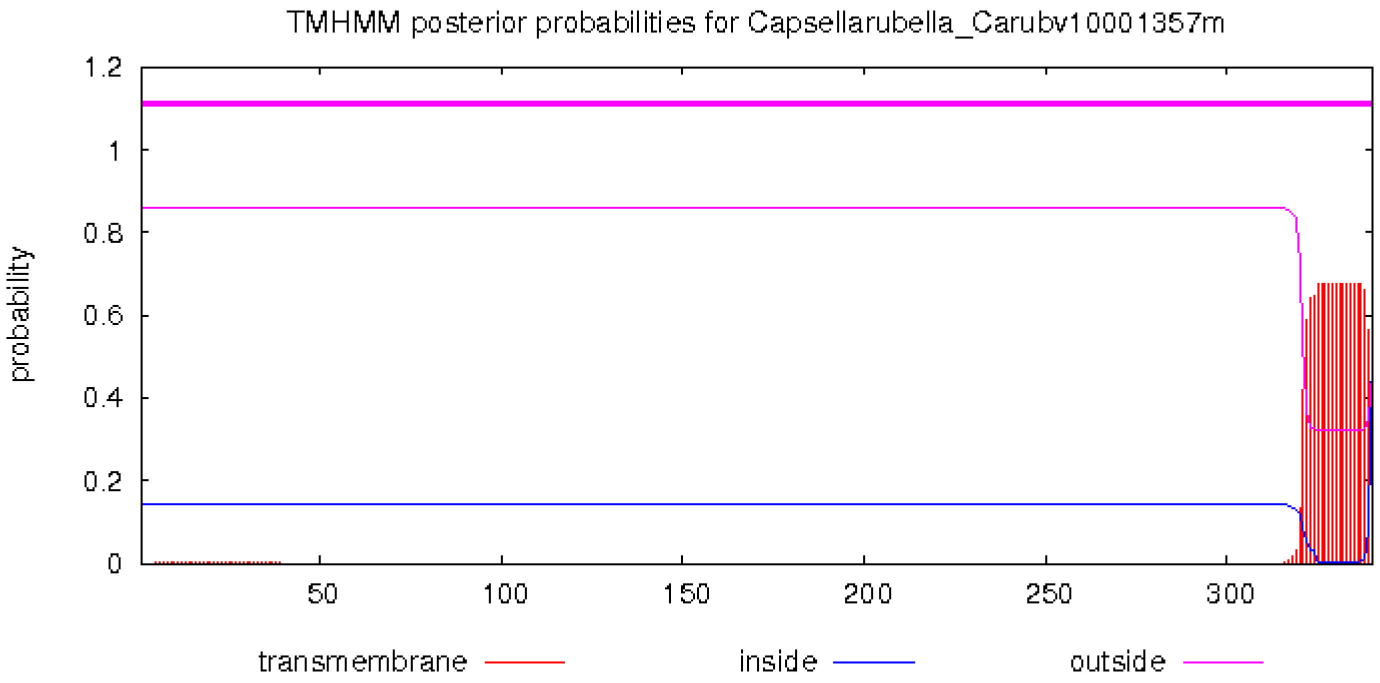

plot in postscript, script for making the plot in gnuplot, data for plot

```
# Capsellarubella_Carubv10001372m Length: 337
# Capsellarubella_Carubv10001372m Number of predicted TMHs:  0
# Capsellarubella_Carubv10001372m Exp number of AAs in TMHs: 0.00022
# Capsellarubella_Carubv10001372m Exp number, first 60 AAs:  0
# Capsellarubella_Carubv10001372m Total prob of N-in:        0.00808
Capsellarubella_Carubv10001372m TMHMM2.0        outside      1   337
```

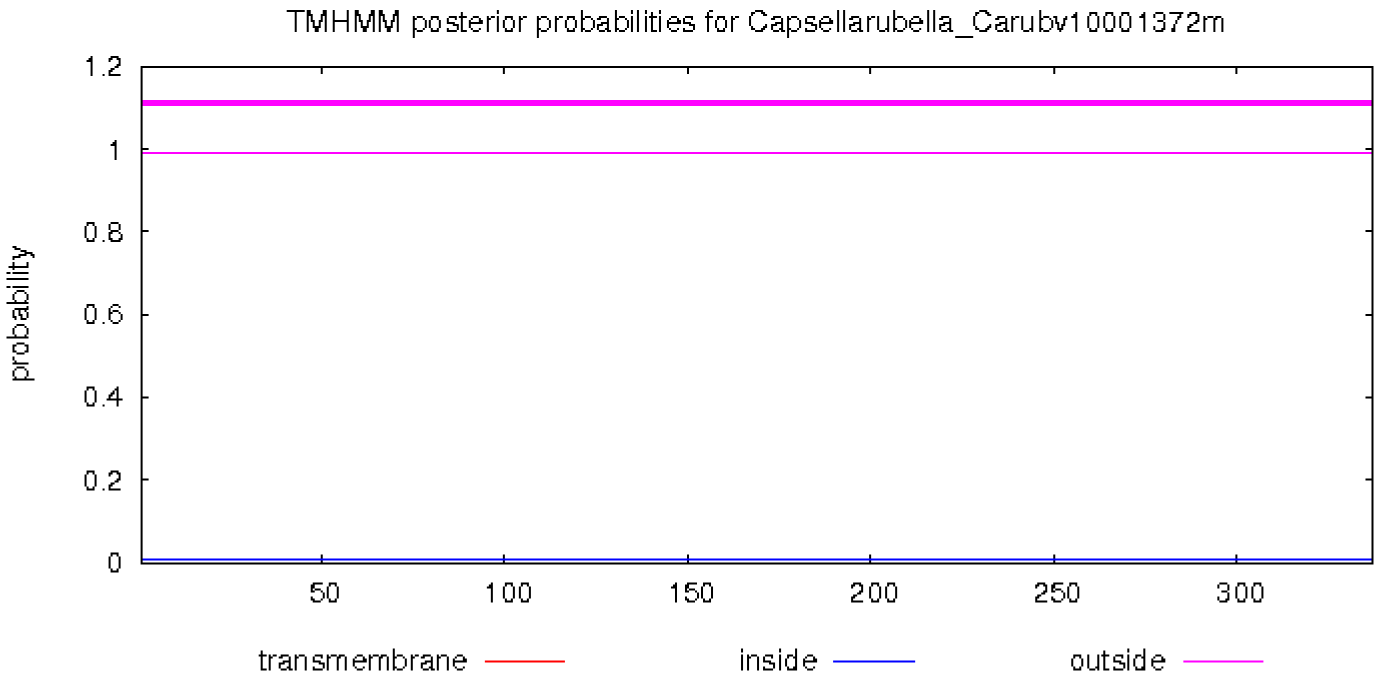

plot in postscript, script for making the plot in gnuplot, data for plot

```
# Capsellarubella_Carubv10001373m Length: 337
# Capsellarubella_Carubv10001373m Number of predicted TMHs:  0
# Capsellarubella_Carubv10001373m Exp number of AAs in TMHs: 0.00032
# Capsellarubella_Carubv10001373m Exp number, first 60 AAs:  0
# Capsellarubella_Carubv10001373m Total prob of N-in:        0.00864
Capsellarubella_Carubv10001373m TMHMM2.0        outside      1   337
```

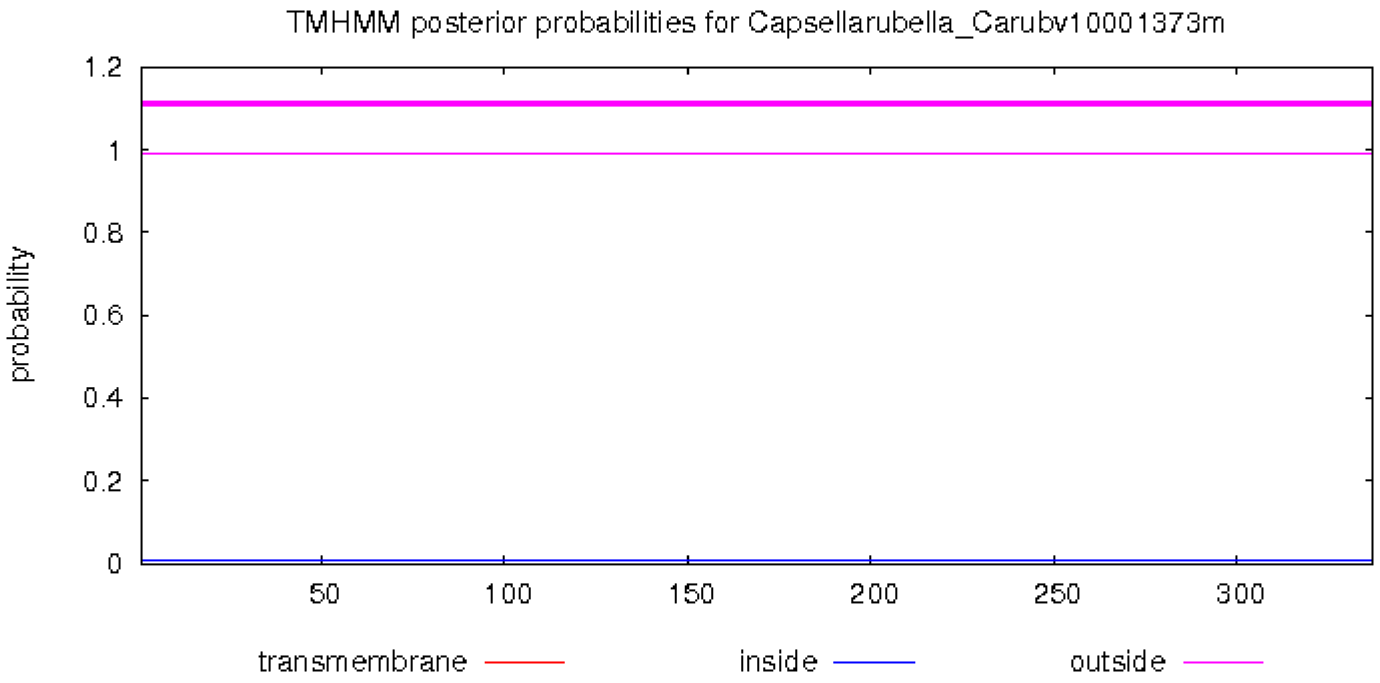

plot in postscript, script for making the plot in gnuplot, data for plot

```
# Capsellarubella_Carubv10001688m Length: 274
# Capsellarubella_Carubv10001688m Number of predicted TMHs:  0
# Capsellarubella_Carubv10001688m Exp number of AAs in TMHs: 13.19669
# Capsellarubella_Carubv10001688m Exp number, first 60 AAs:  11.21964
# Capsellarubella_Carubv10001688m Total prob of N-in:        0.54930
# Capsellarubella_Carubv10001688m POSSIBLE N-term signal sequence
Capsellarubella_Carubv10001688m TMHMM2.0        outside      1   274
```

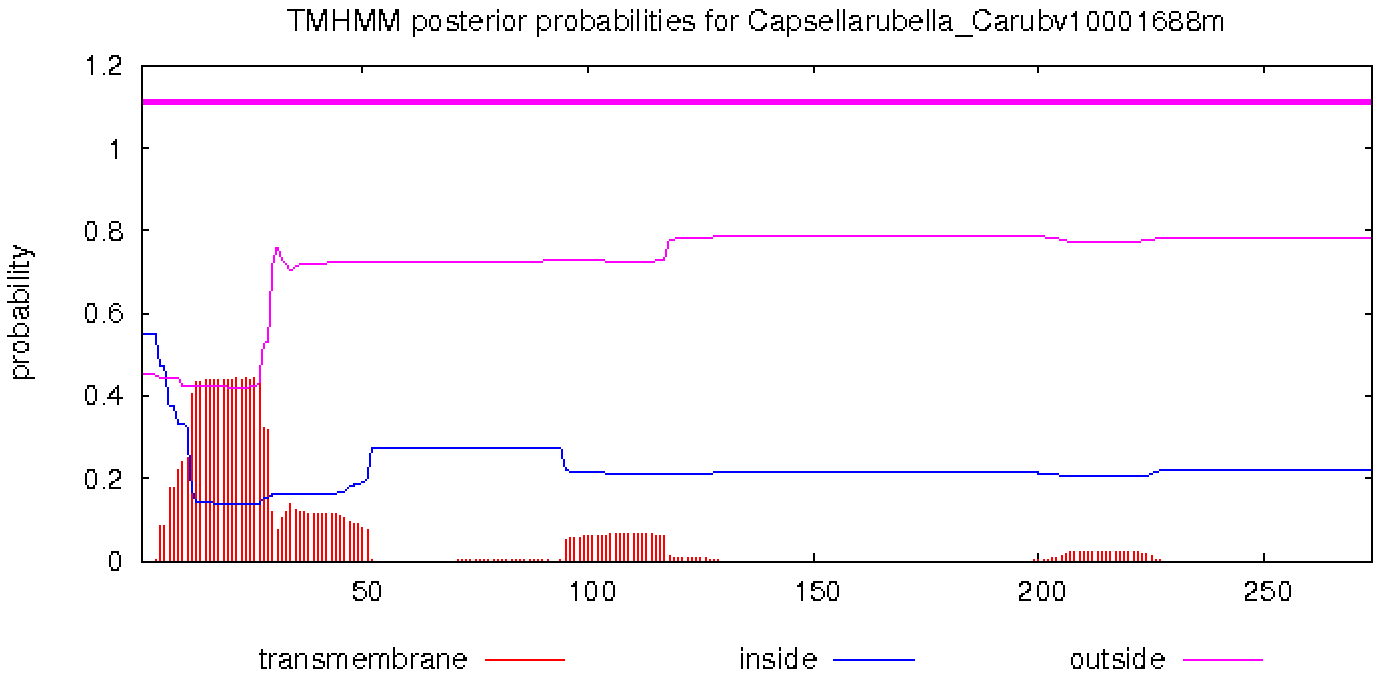

plot in postscript, script for making the plot in gnuplot, data for plot

---

```
# Capsellarubella_Carubv10001788m Length: 253
# Capsellarubella_Carubv10001788m Number of predicted TMHs:  0
# Capsellarubella_Carubv10001788m Exp number of AAs in TMHs: 0.00193
# Capsellarubella_Carubv10001788m Exp number, first 60 AAs:  0.0013
# Capsellarubella_Carubv10001788m Total prob of N-in:        0.05251
Capsellarubella_Carubv10001788m TMHMM2.0        outside      1   253
```

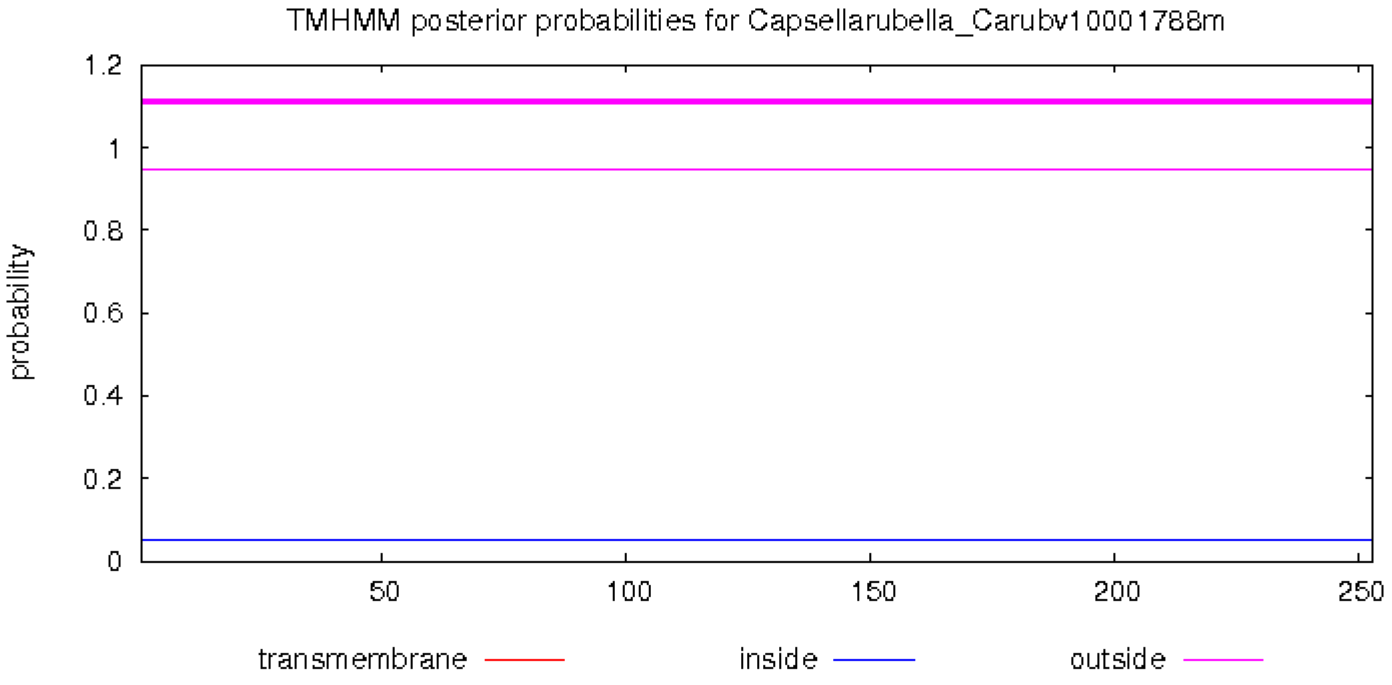

plot in postscript, script for making the plot in gnuplot, data for plot

---

```
# Capsellarubella_Carubv10002548m Length: 257
# Capsellarubella_Carubv10002548m Number of predicted TMHs:  0
# Capsellarubella_Carubv10002548m Exp number of AAs in TMHs: 0.0002
# Capsellarubella_Carubv10002548m Exp number, first 60 AAs:  0
# Capsellarubella_Carubv10002548m Total prob of N-in:        0.10047
Capsellarubella_Carubv10002548m TMHMM2.0        outside      1   257
```

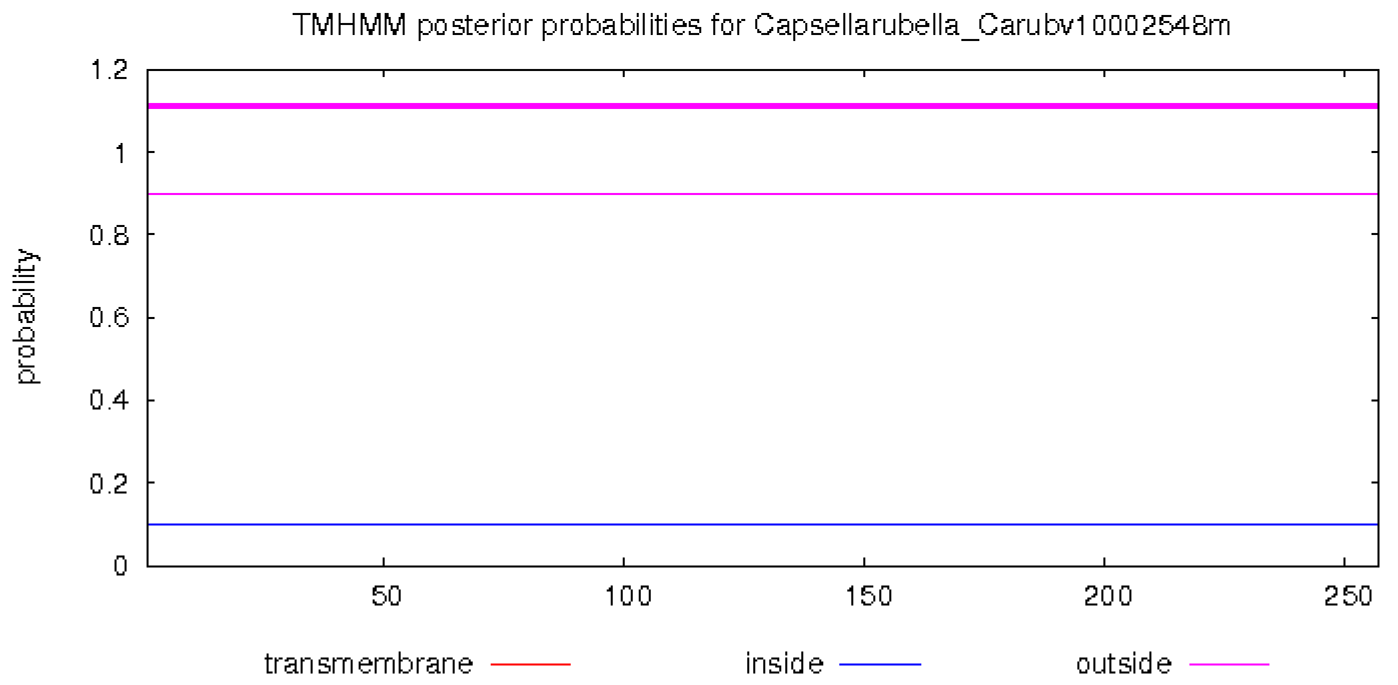

plot in postscript, script for making the plot in gnuplot, data for plot

---

```
# Capsellarubella_Carubv10002665m Length: 362
# Capsellarubella_Carubv10002665m Number of predicted TMHs:  0
# Capsellarubella_Carubv10002665m Exp number of AAs in TMHs: 0
# Capsellarubella_Carubv10002665m Exp number, first 60 AAs:  0
# Capsellarubella_Carubv10002665m Total prob of N-in:        0.01713
Capsellarubella_Carubv10002665m TMHMM2.0        outside      1   362
```

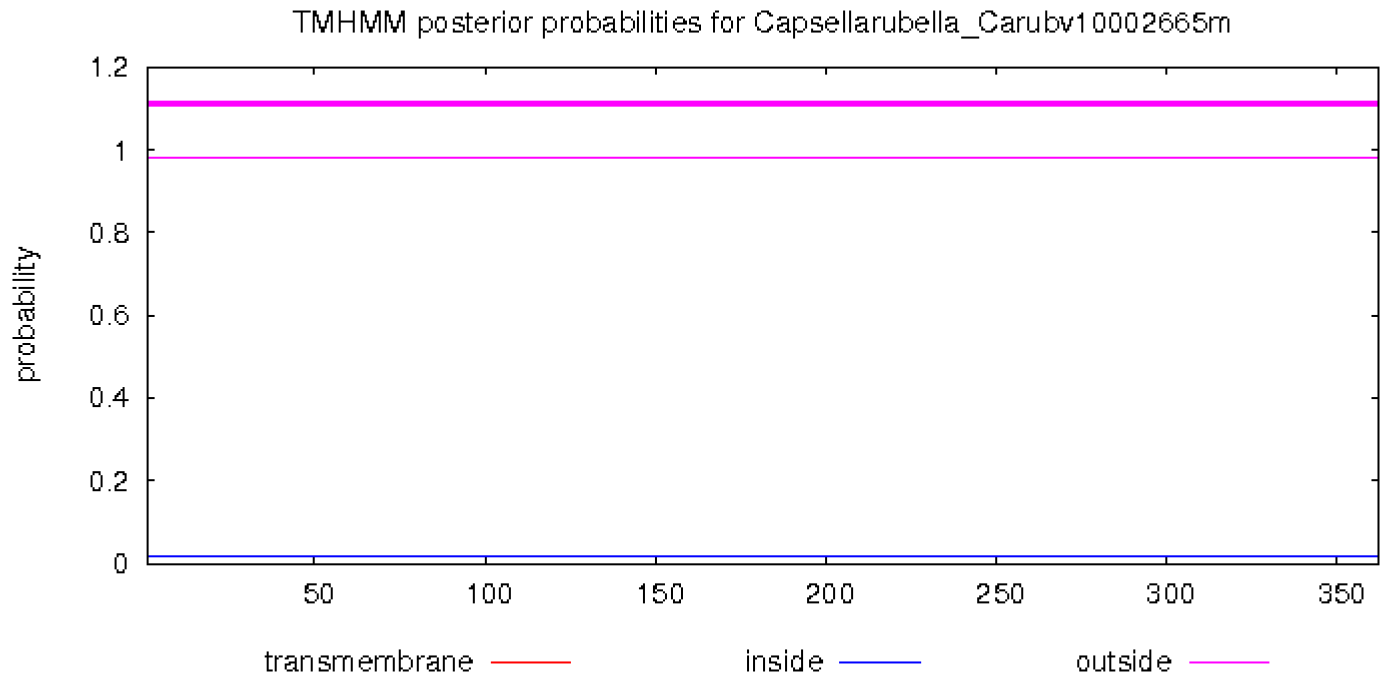

plot in postscript, script for making the plot in gnuplot, data for plot

---

```
# Capsellarubella_Carubv10002791m Length: 143
# Capsellarubella_Carubv10002791m Number of predicted TMHs:  0
# Capsellarubella_Carubv10002791m Exp number of AAs in TMHs: 0
# Capsellarubella_Carubv10002791m Exp number, first 60 AAs:  0
# Capsellarubella_Carubv10002791m Total prob of N-in:        0.52578
Capsellarubella_Carubv10002791m TMHMM2.0        inside       1   143
```

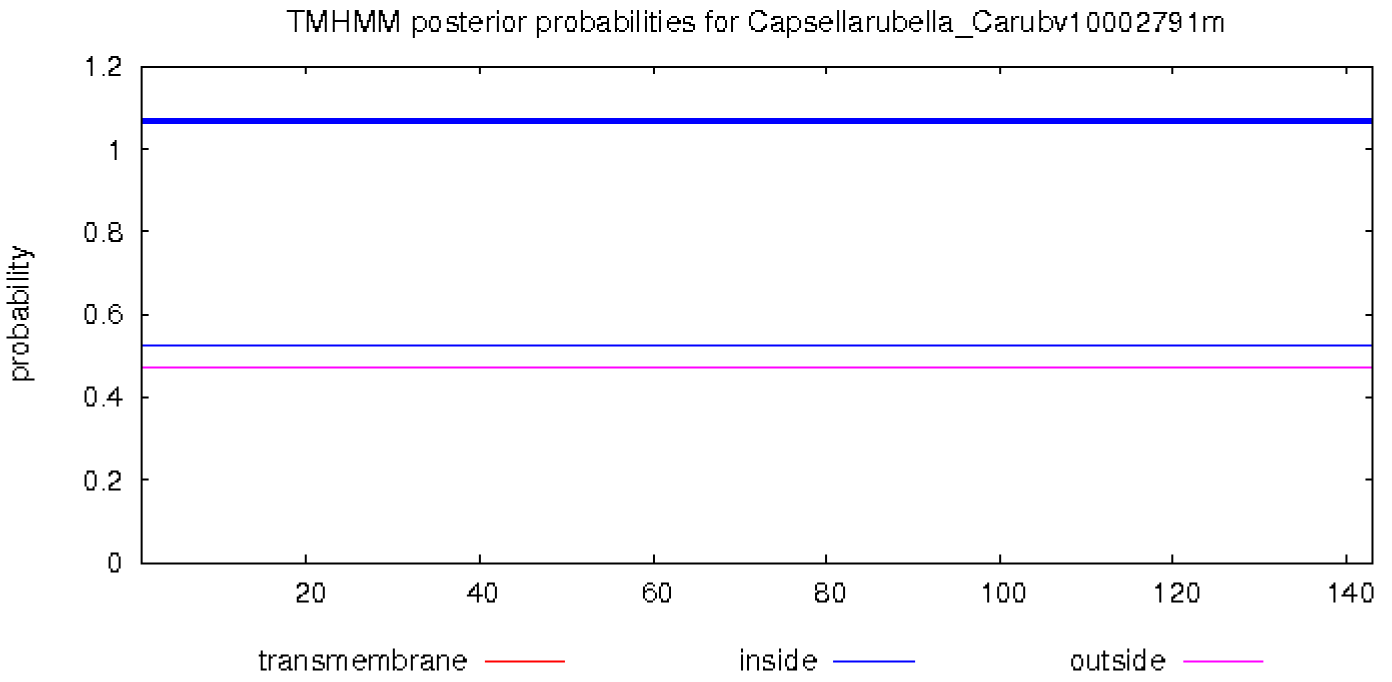

plot in postscript, script for making the plot in gnuplot, data for plot

---

```
# Capsellarubella_Carubv10003054m Length: 202
# Capsellarubella_Carubv10003054m Number of predicted TMHs:  0
# Capsellarubella_Carubv10003054m Exp number of AAs in TMHs: 0.00217
# Capsellarubella_Carubv10003054m Exp number, first 60 AAs:  0.00209
# Capsellarubella_Carubv10003054m Total prob of N-in:        0.02489
Capsellarubella_Carubv10003054m TMHMM2.0        outside      1   202
```

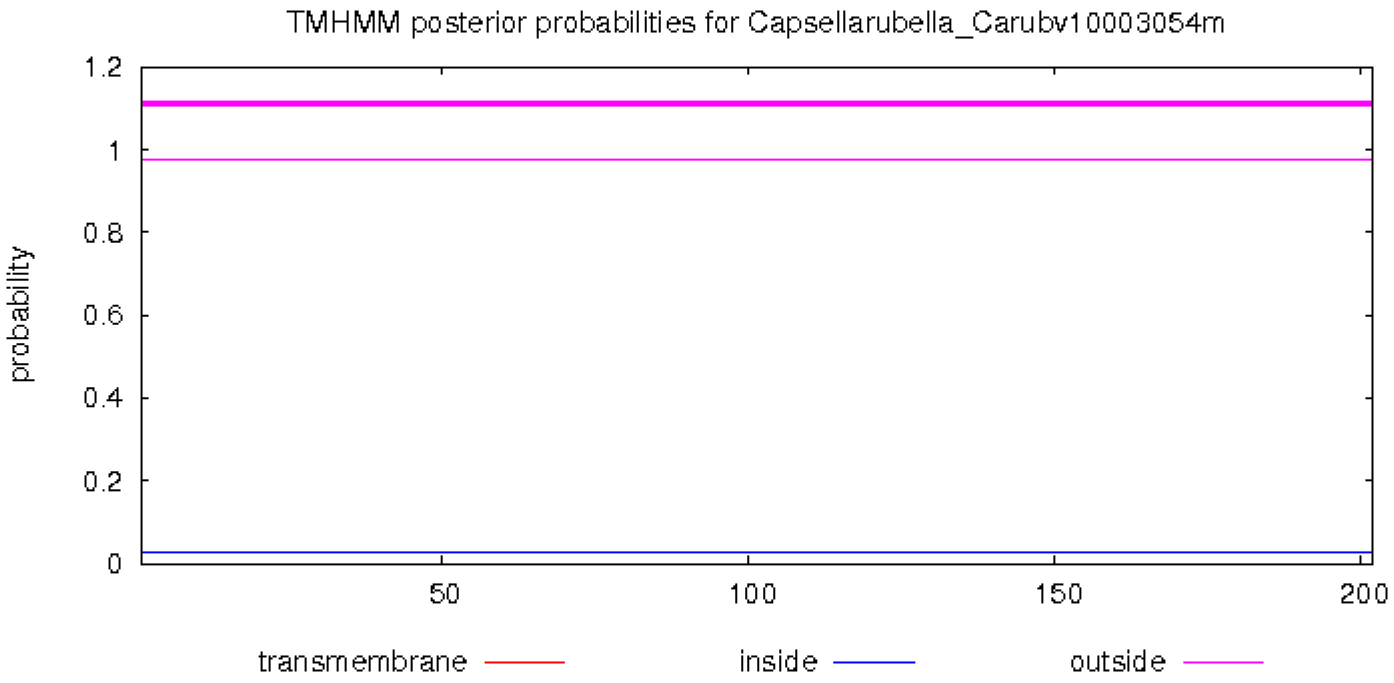

plot in postscript, script for making the plot in gnuplot, data for plot

---

```
# Capsellarubella_Carubv10003254m Length: 284
# Capsellarubella_Carubv10003254m Number of predicted TMHs:  0
# Capsellarubella_Carubv10003254m Exp number of AAs in TMHs: 6e-05
# Capsellarubella_Carubv10003254m Exp number, first 60 AAs:  0
# Capsellarubella_Carubv10003254m Total prob of N-in:        0.00896
Capsellarubella_Carubv10003254m TMHMM2.0        outside      1   284
```

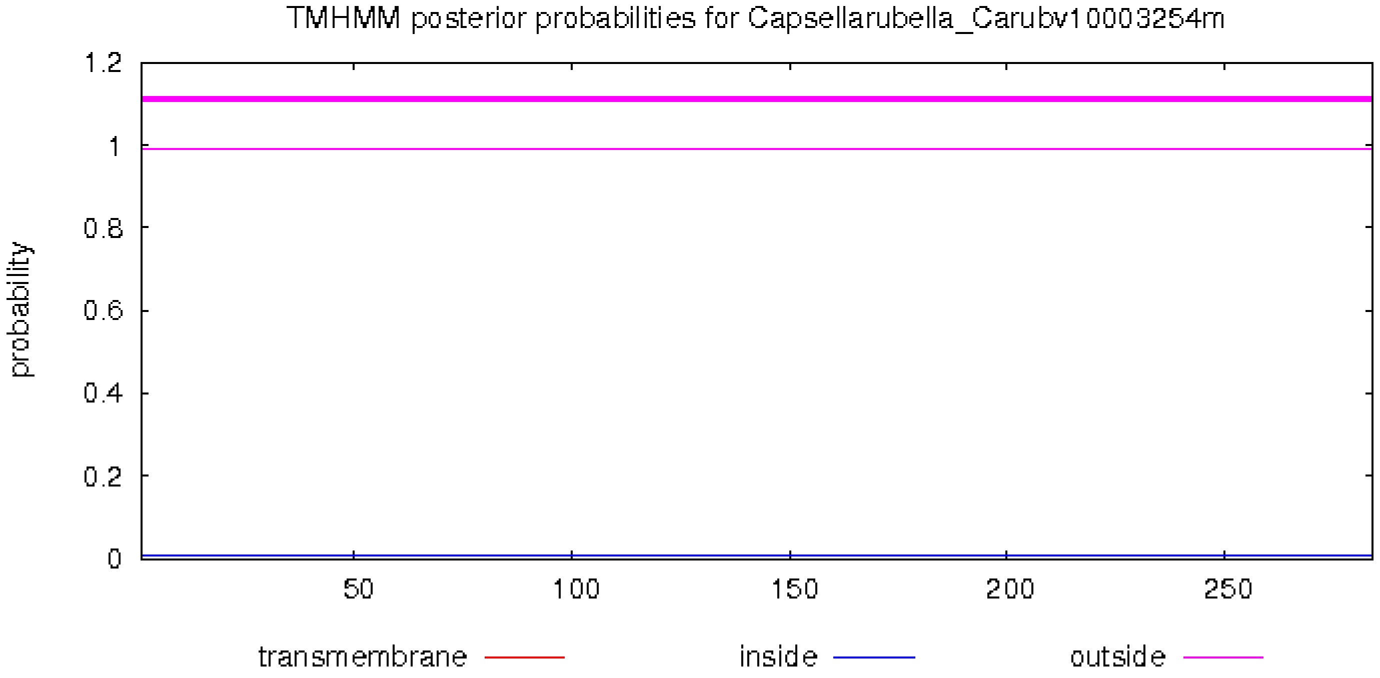

plot in postscript, script for making the plot in gnuplot, data for plot

```
# Capsellarubella_Carubv10003260m Length: 435
# Capsellarubella_Carubv10003260m Number of predicted TMHs:  1
# Capsellarubella_Carubv10003260m Exp number of AAs in TMHs: 22.27085
# Capsellarubella_Carubv10003260m Exp number, first 60 AAs:  0.00048
# Capsellarubella_Carubv10003260m Total prob of N-in:        0.12174
Capsellarubella_Carubv10003260m TMHMM2.0        outside      1   411
Capsellarubella_Carubv10003260m TMHMM2.0        TMhelix    412   434
Capsellarubella_Carubv10003260m TMHMM2.0        inside     435   435
```

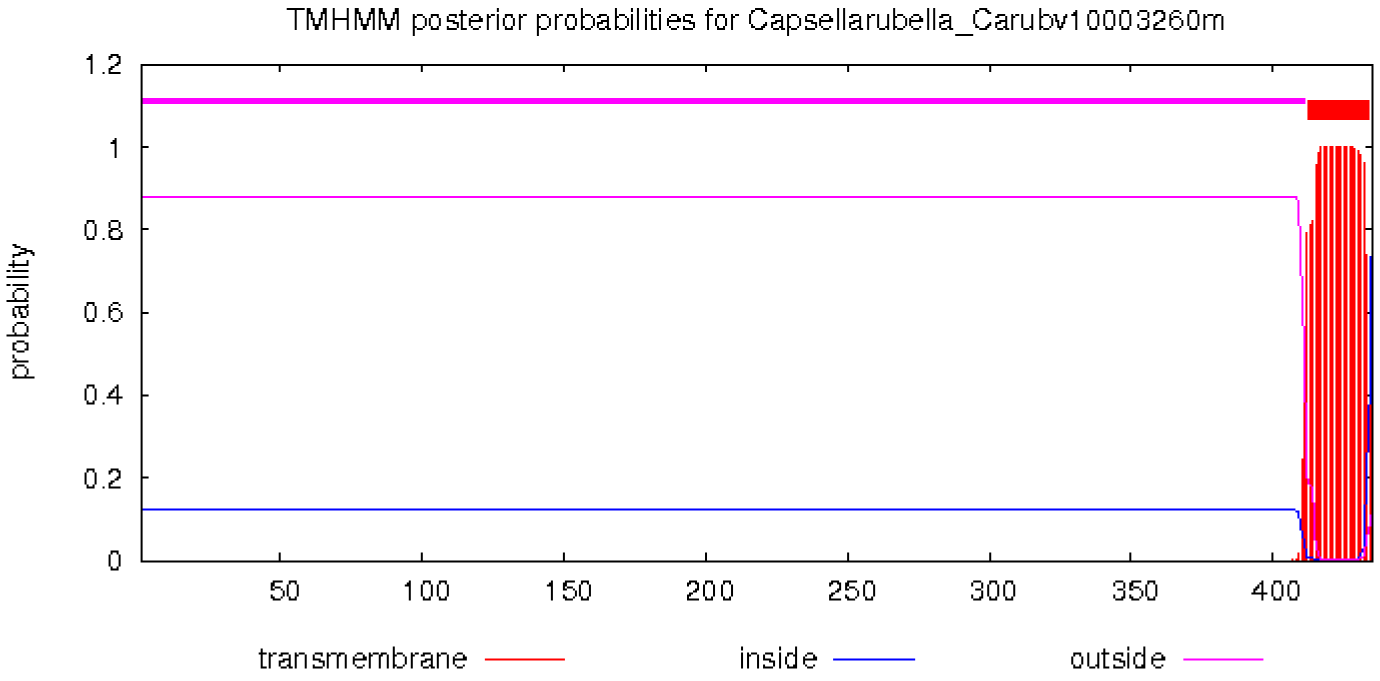

plot in postscript, script for making the plot in gnuplot, data for plot

```
# Capsellarubella_Carubv10003671m Length: 145
# Capsellarubella_Carubv10003671m Number of predicted TMHs:  0
# Capsellarubella_Carubv10003671m Exp number of AAs in TMHs: 0.01611
# Capsellarubella_Carubv10003671m Exp number, first 60 AAs:  0.01249
# Capsellarubella_Carubv10003671m Total prob of N-in:        0.10001
Capsellarubella_Carubv10003671m TMHMM2.0        outside      1   145
```

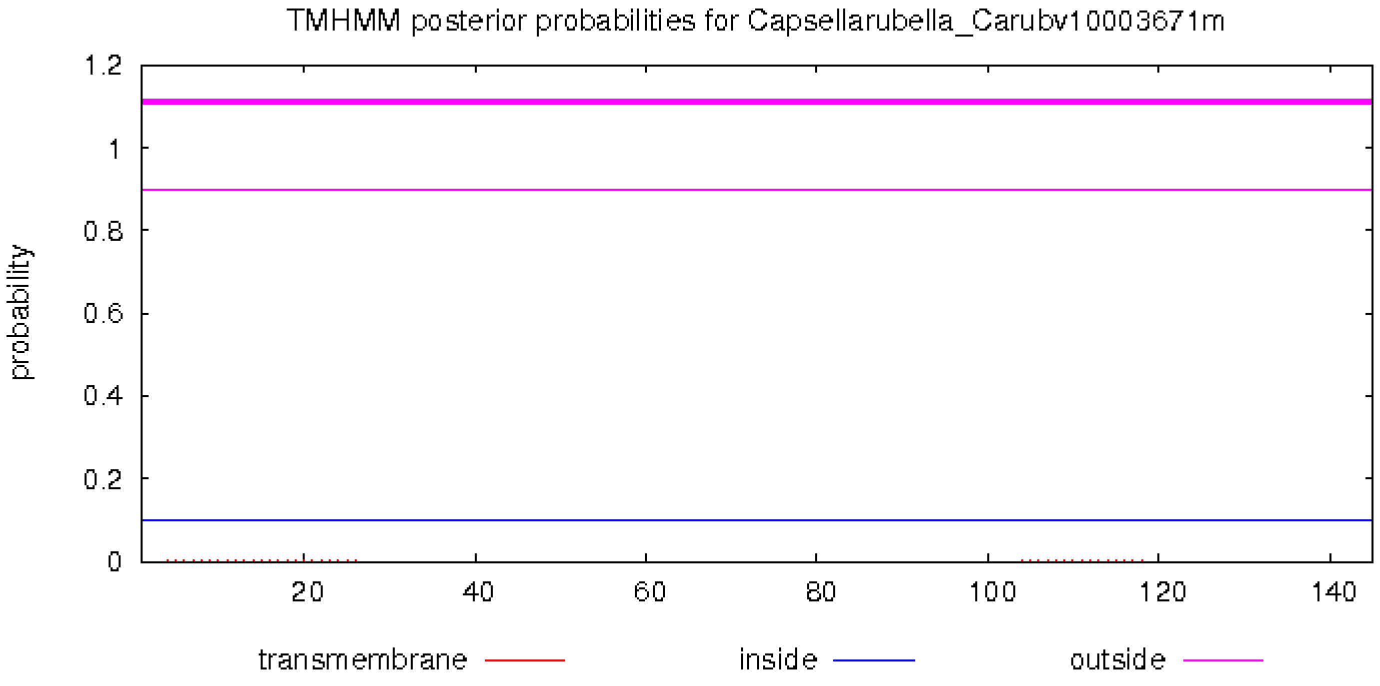

plot in postscript, script for making the plot in gnuplot, data for plot

```
# Capsellarubella_Carubv10003768m Length: 623
# Capsellarubella_Carubv10003768m Number of predicted TMHs:  2
# Capsellarubella_Carubv10003768m Exp number of AAs in TMHs: 45.27191
# Capsellarubella_Carubv10003768m Exp number, first 60 AAs:  0.23588
# Capsellarubella_Carubv10003768m Total prob of N-in:        0.18708
Capsellarubella_Carubv10003768m TMHMM2.0        outside      1   105
Capsellarubella_Carubv10003768m TMHMM2.0        TMhelix    106   128
Capsellarubella_Carubv10003768m TMHMM2.0        inside     129   599
Capsellarubella_Carubv10003768m TMHMM2.0        TMhelix    600   622
Capsellarubella_Carubv10003768m TMHMM2.0        outside    623   623
```

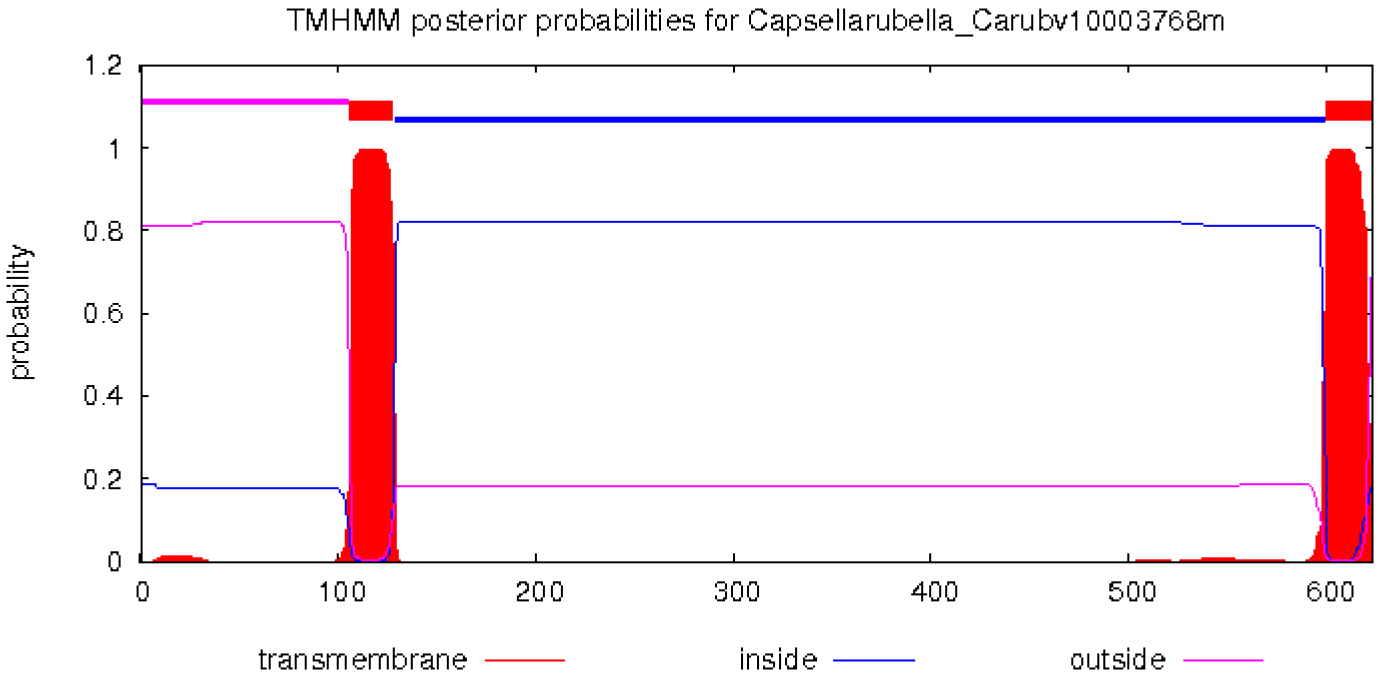

plot in postscript, script for making the plot in gnuplot, data for plot

---

```
# Capsellarubella_Carubv10003817m Length: 242
# Capsellarubella_Carubv10003817m Number of predicted TMHs:  0
# Capsellarubella_Carubv10003817m Exp number of AAs in TMHs: 0
# Capsellarubella_Carubv10003817m Exp number, first 60 AAs:  0
# Capsellarubella_Carubv10003817m Total prob of N-in:        0.05224
Capsellarubella_Carubv10003817m TMHMM2.0        outside      1   242
```

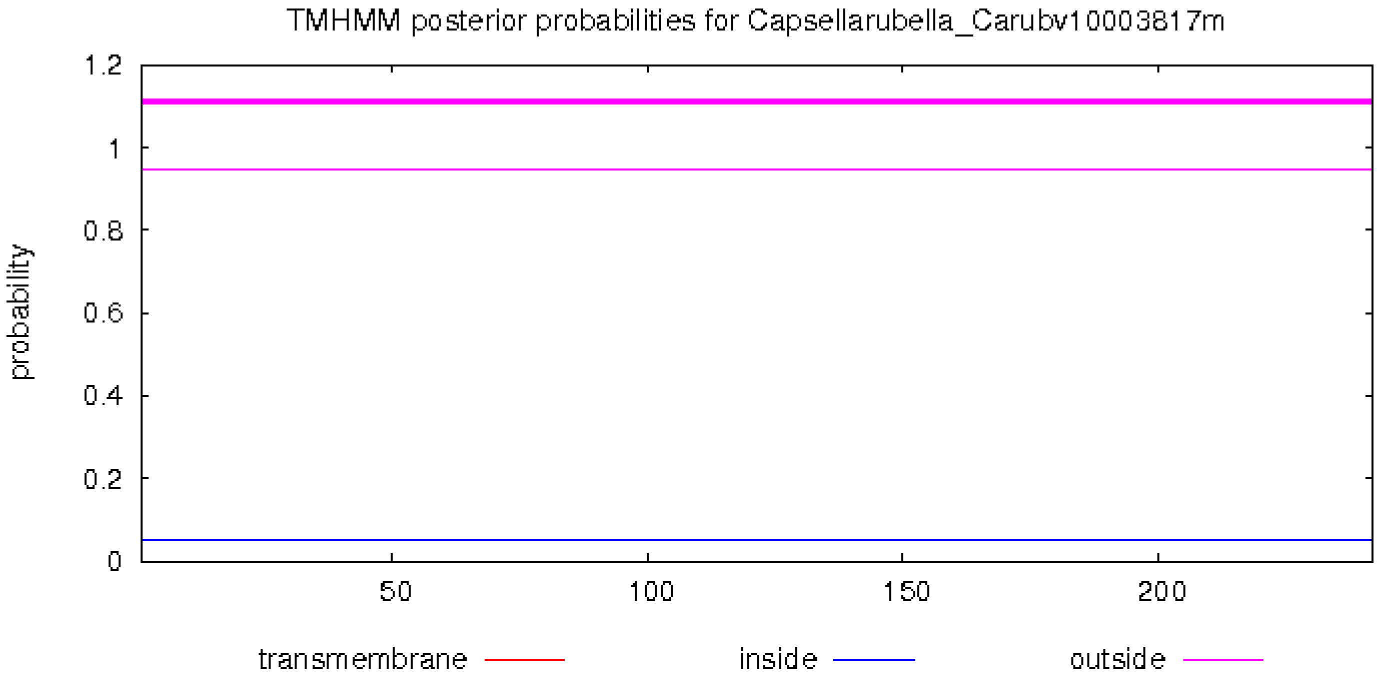

plot in postscript, script for making the plot in gnuplot, data for plot

---

```
# Capsellarubella_Carubv10003869m Length: 489
# Capsellarubella_Carubv10003869m Number of predicted TMHs:  0
# Capsellarubella_Carubv10003869m Exp number of AAs in TMHs: 0.00019
# Capsellarubella_Carubv10003869m Exp number, first 60 AAs:  0
# Capsellarubella_Carubv10003869m Total prob of N-in:        0.00015
Capsellarubella_Carubv10003869m TMHMM2.0        outside      1   489
```

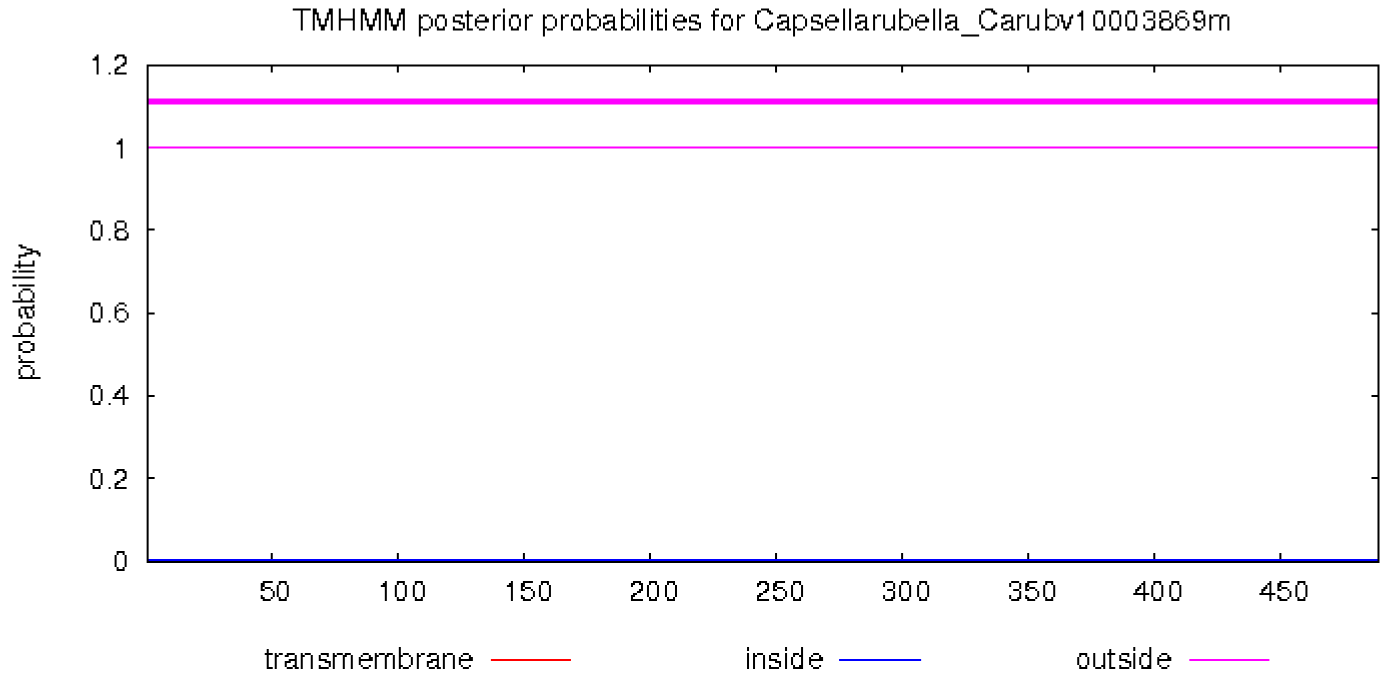

plot in postscript, script for making the plot in gnuplot, data for plot

---

```
# Capsellarubella_Carubv10003879m Length: 341
# Capsellarubella_Carubv10003879m Number of predicted TMHs:  0
# Capsellarubella_Carubv10003879m Exp number of AAs in TMHs: 0
# Capsellarubella_Carubv10003879m Exp number, first 60 AAs:  0
# Capsellarubella_Carubv10003879m Total prob of N-in:        0.00436
Capsellarubella_Carubv10003879m TMHMM2.0        outside      1   341
```

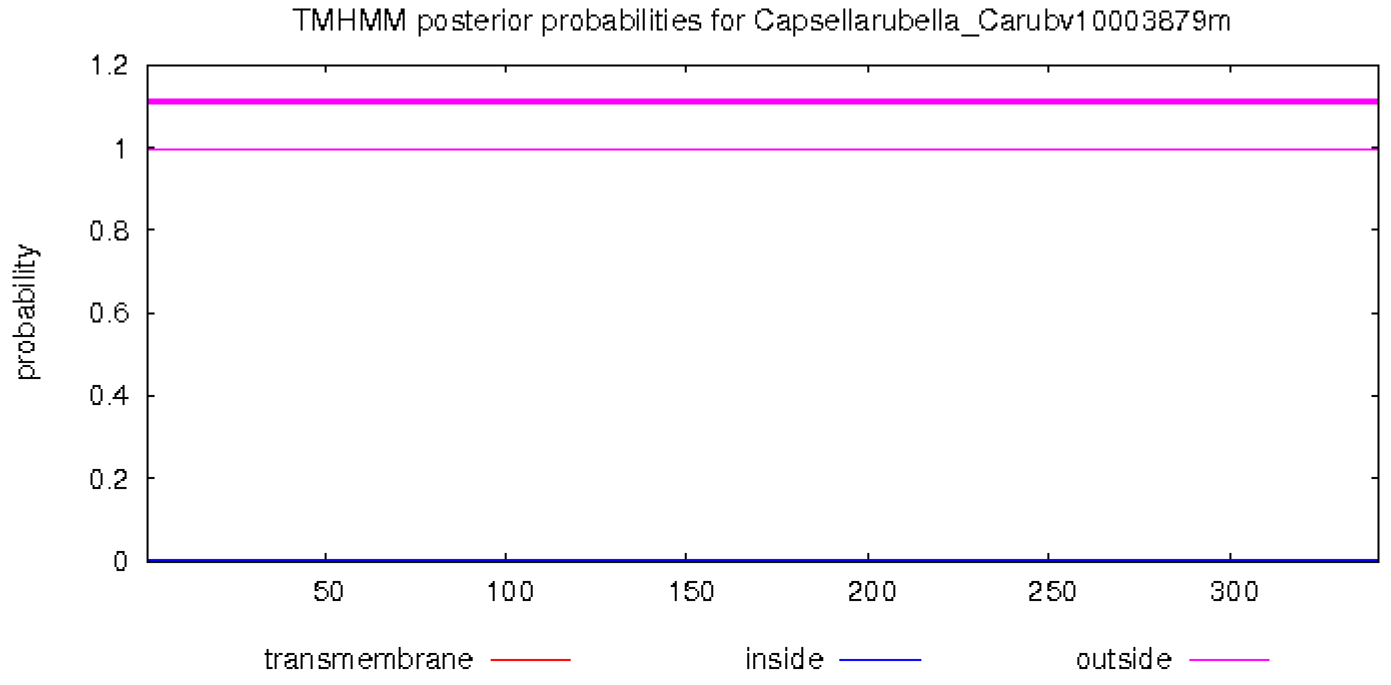

plot in postscript, script for making the plot in gnuplot, data for plot

---

```
# Capsellarubella_Carubv10004602m Length: 515
# Capsellarubella_Carubv10004602m Number of predicted TMHs:  0
# Capsellarubella_Carubv10004602m Exp number of AAs in TMHs: 6.95184
# Capsellarubella_Carubv10004602m Exp number, first 60 AAs:  0
# Capsellarubella_Carubv10004602m Total prob of N-in:        0.29193
Capsellarubella_Carubv10004602m TMHMM2.0        outside      1   515
```

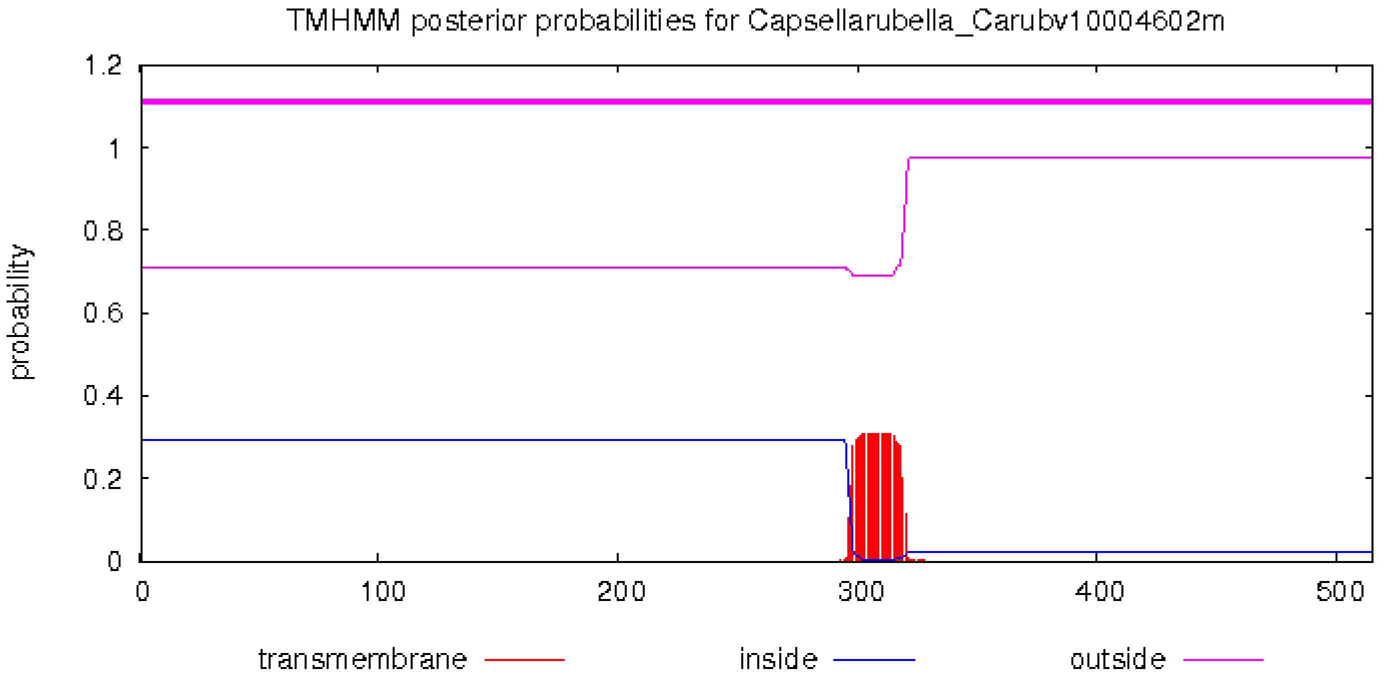

plot in postscript, script for making the plot in gnuplot, data for plot

---

```
# Capsellarubella_Carubv10004997m Length: 392
# Capsellarubella_Carubv10004997m Number of predicted TMHs:  0
# Capsellarubella_Carubv10004997m Exp number of AAs in TMHs: 0
# Capsellarubella_Carubv10004997m Exp number, first 60 AAs:  0
# Capsellarubella_Carubv10004997m Total prob of N-in:        0.00906
Capsellarubella_Carubv10004997m TMHMM2.0        outside      1   392
```

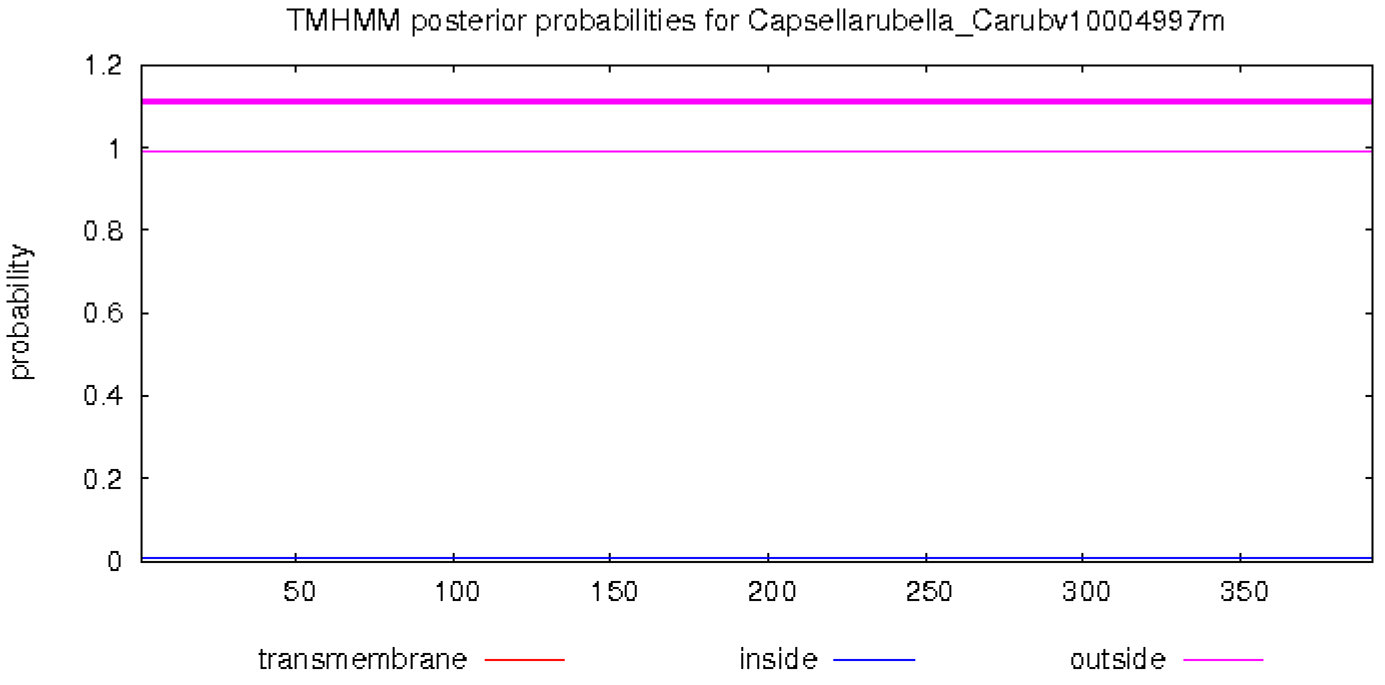

plot in postscript, script for making the plot in gnuplot, data for plot

---

```
# Capsellarubella_Carubv10005139m Length: 353
# Capsellarubella_Carubv10005139m Number of predicted TMHs:  0
# Capsellarubella_Carubv10005139m Exp number of AAs in TMHs: 0
# Capsellarubella_Carubv10005139m Exp number, first 60 AAs:  0
# Capsellarubella_Carubv10005139m Total prob of N-in:        0.00560
Capsellarubella_Carubv10005139m TMHMM2.0        outside      1   353
```

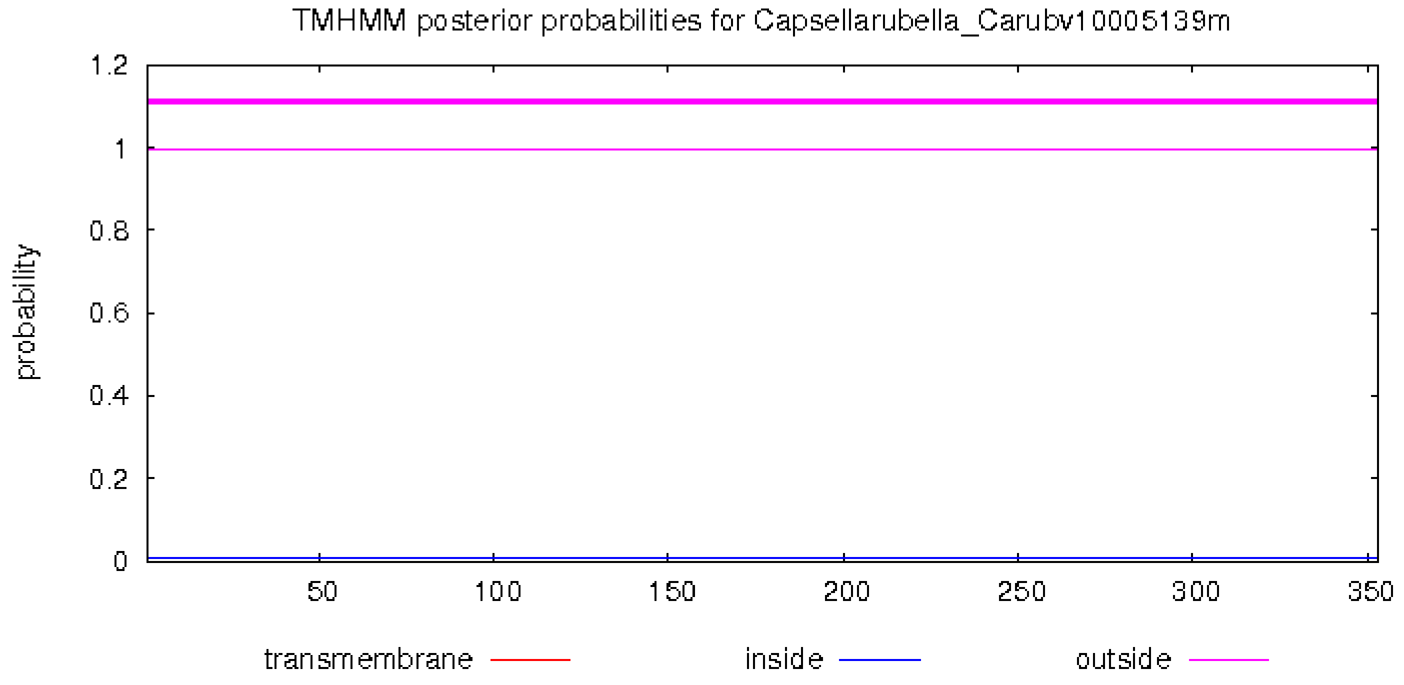

plot in postscript, script for making the plot in gnuplot, data for plot

---

```
# Capsellarubella_Carubv10005201m Length: 336
# Capsellarubella_Carubv10005201m Number of predicted TMHs:  0
# Capsellarubella_Carubv10005201m Exp number of AAs in TMHs: 0.48755
# Capsellarubella_Carubv10005201m Exp number, first 60 AAs:  0.48755
# Capsellarubella_Carubv10005201m Total prob of N-in:        0.02907
Capsellarubella_Carubv10005201m TMHMM2.0        outside      1   336
```

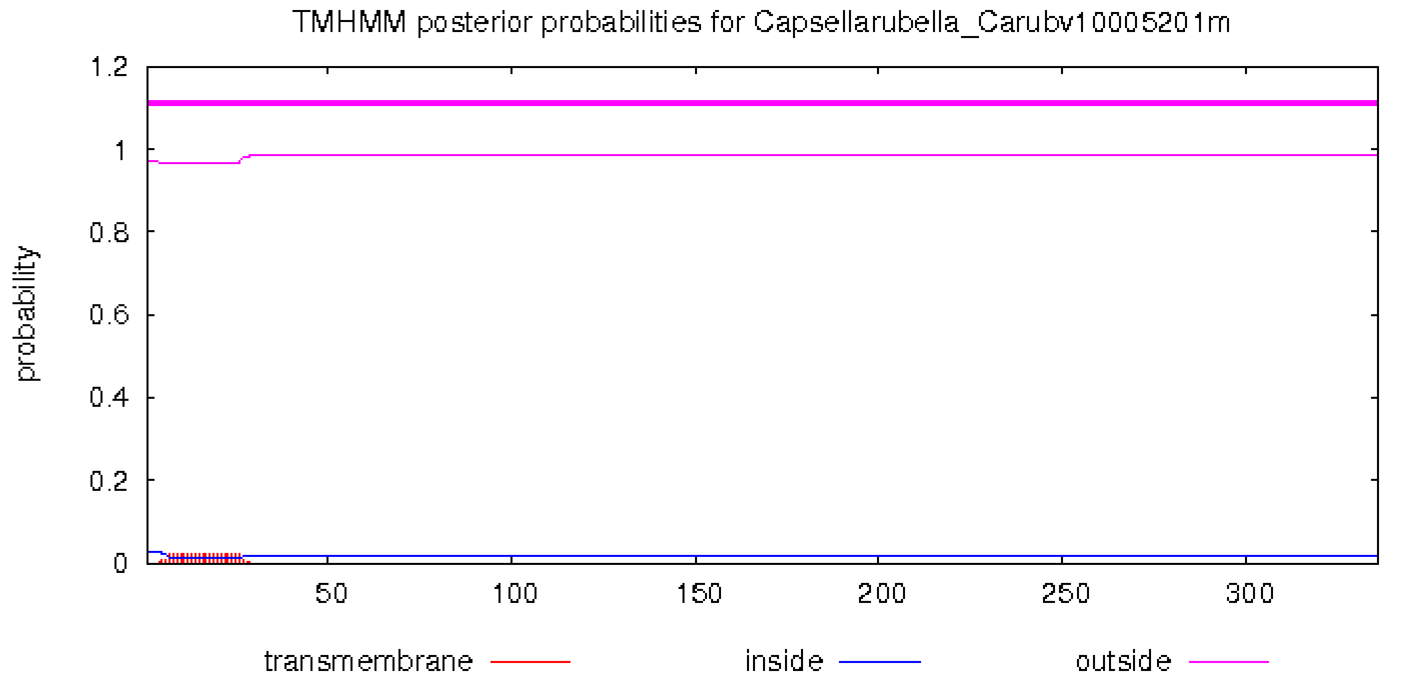

plot in postscript, script for making the plot in gnuplot, data for plot

---

```
# Capsellarubella_Carubv10005324m Length: 306
# Capsellarubella_Carubv10005324m Number of predicted TMHs:  0
# Capsellarubella_Carubv10005324m Exp number of AAs in TMHs: 0.00179
# Capsellarubella_Carubv10005324m Exp number, first 60 AAs:  0
# Capsellarubella_Carubv10005324m Total prob of N-in:        0.13586
Capsellarubella_Carubv10005324m TMHMM2.0        outside      1   306
```

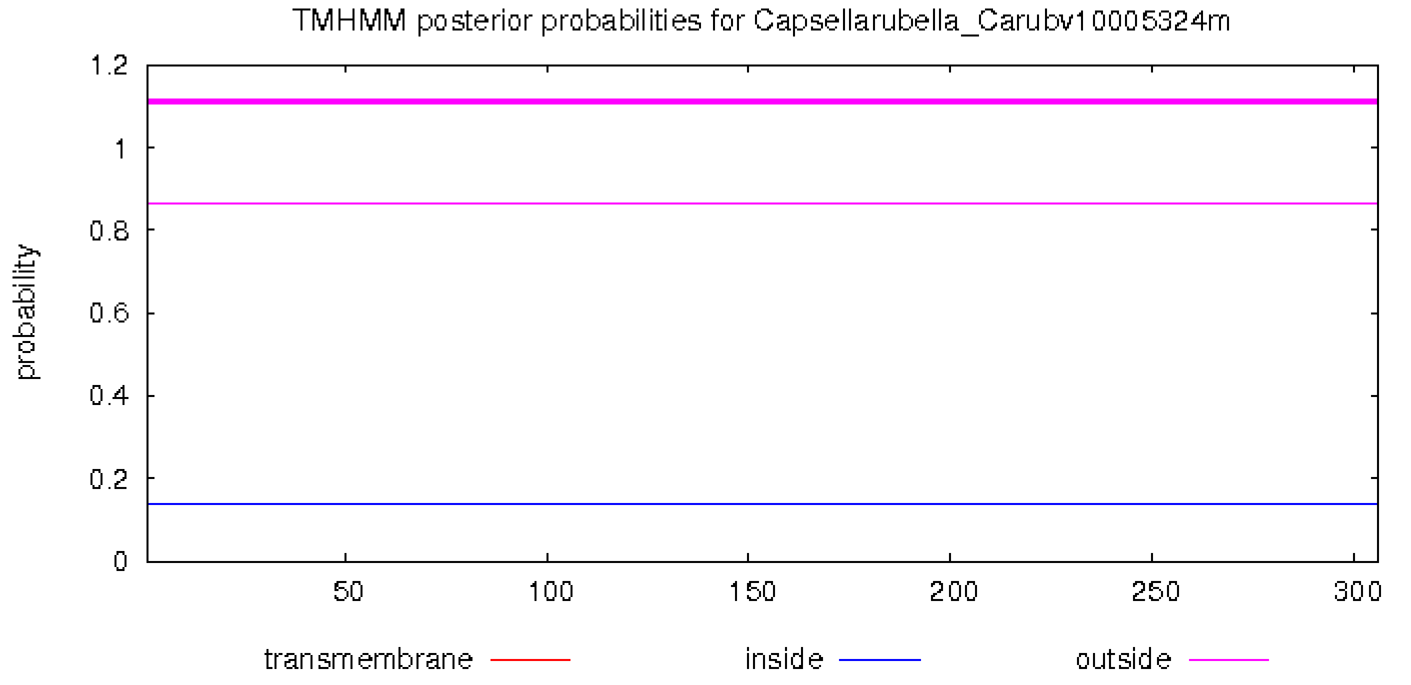

plot in postscript, script for making the plot in gnuplot, data for plot

---

```
# Capsellarubella_Carubv10005353m Length: 302
# Capsellarubella_Carubv10005353m Number of predicted TMHs:  0
# Capsellarubella_Carubv10005353m Exp number of AAs in TMHs: 0.0023
# Capsellarubella_Carubv10005353m Exp number, first 60 AAs:  0.00039
# Capsellarubella_Carubv10005353m Total prob of N-in:        0.02071
Capsellarubella_Carubv10005353m TMHMM2.0        outside      1   302
```

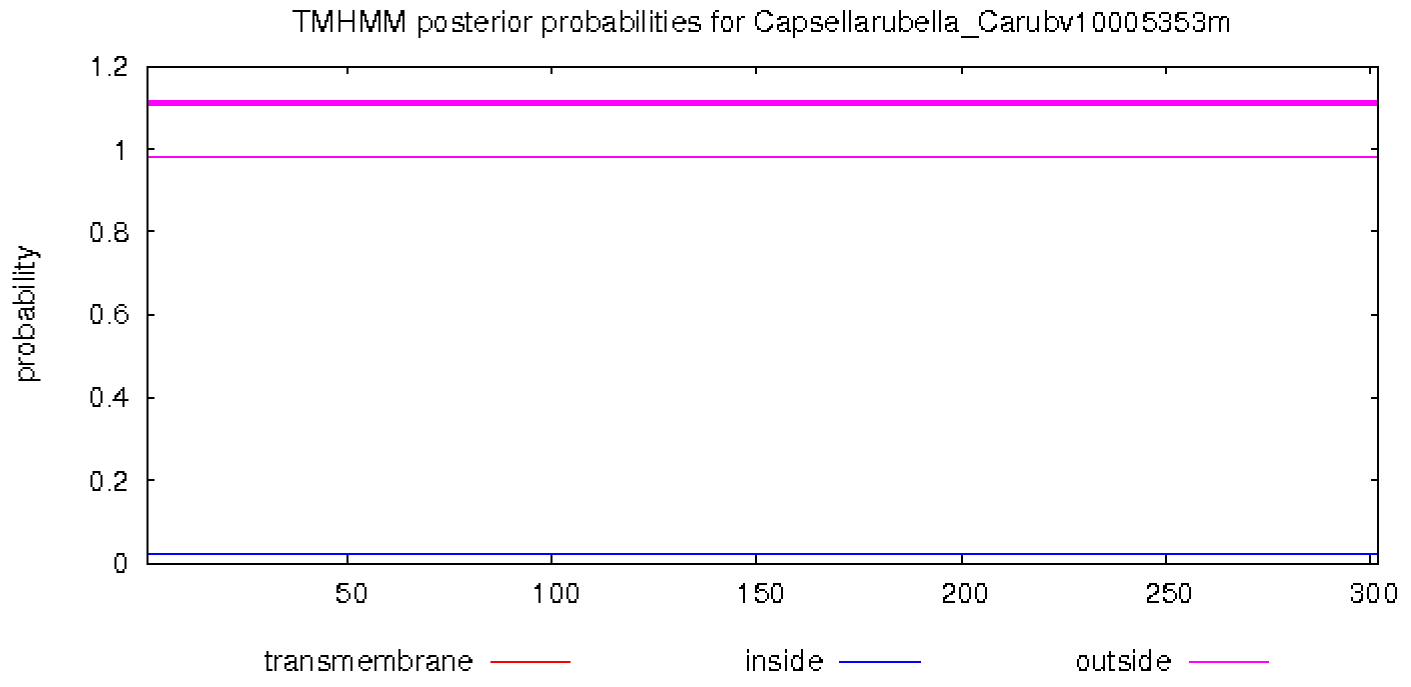

plot in postscript, script for making the plot in gnuplot, data for plot

---

```
# Capsellarubella_Carubv10005574m Length: 251
# Capsellarubella_Carubv10005574m Number of predicted TMHs:  1
# Capsellarubella_Carubv10005574m Exp number of AAs in TMHs: 19.63484
# Capsellarubella_Carubv10005574m Exp number, first 60 AAs:  19.63446
# Capsellarubella_Carubv10005574m Total prob of N-in:        0.43794
# Capsellarubella_Carubv10005574m POSSIBLE N-term signal sequence
Capsellarubella_Carubv10005574m TMHMM2.0        outside      1     9
```

```
Capsellarubella_Carubv10005574m TMHMM2.0        TMhelix     10    29
Capsellarubella_Carubv10005574m TMHMM2.0        inside      30   251
```

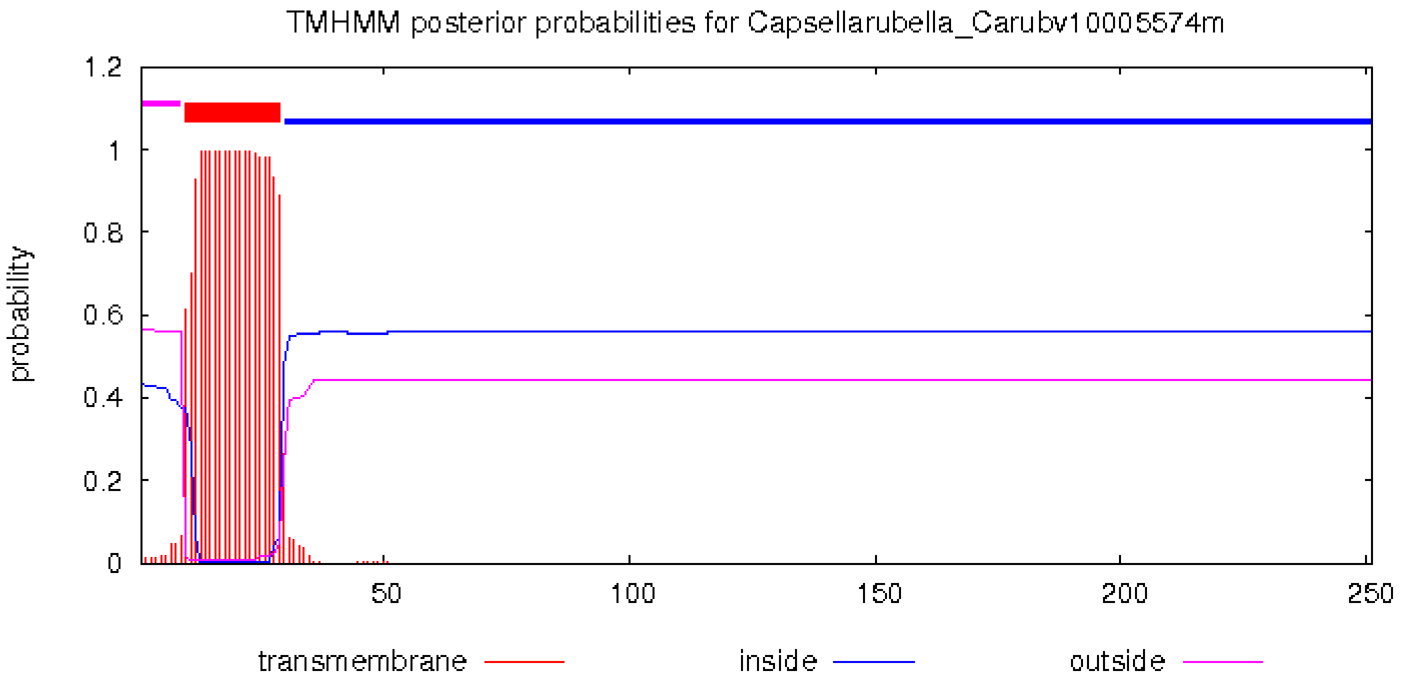

plot in postscript, script for making the plot in gnuplot, data for plot

---

```
# Capsellarubella_Carubv10006906m Length: 342
# Capsellarubella_Carubv10006906m Number of predicted TMHs:  0
# Capsellarubella_Carubv10006906m Exp number of AAs in TMHs: 0.00057
# Capsellarubella_Carubv10006906m Exp number, first 60 AAs:  0
# Capsellarubella_Carubv10006906m Total prob of N-in:        0.00792
Capsellarubella_Carubv10006906m TMHMM2.0        outside      1   342
```

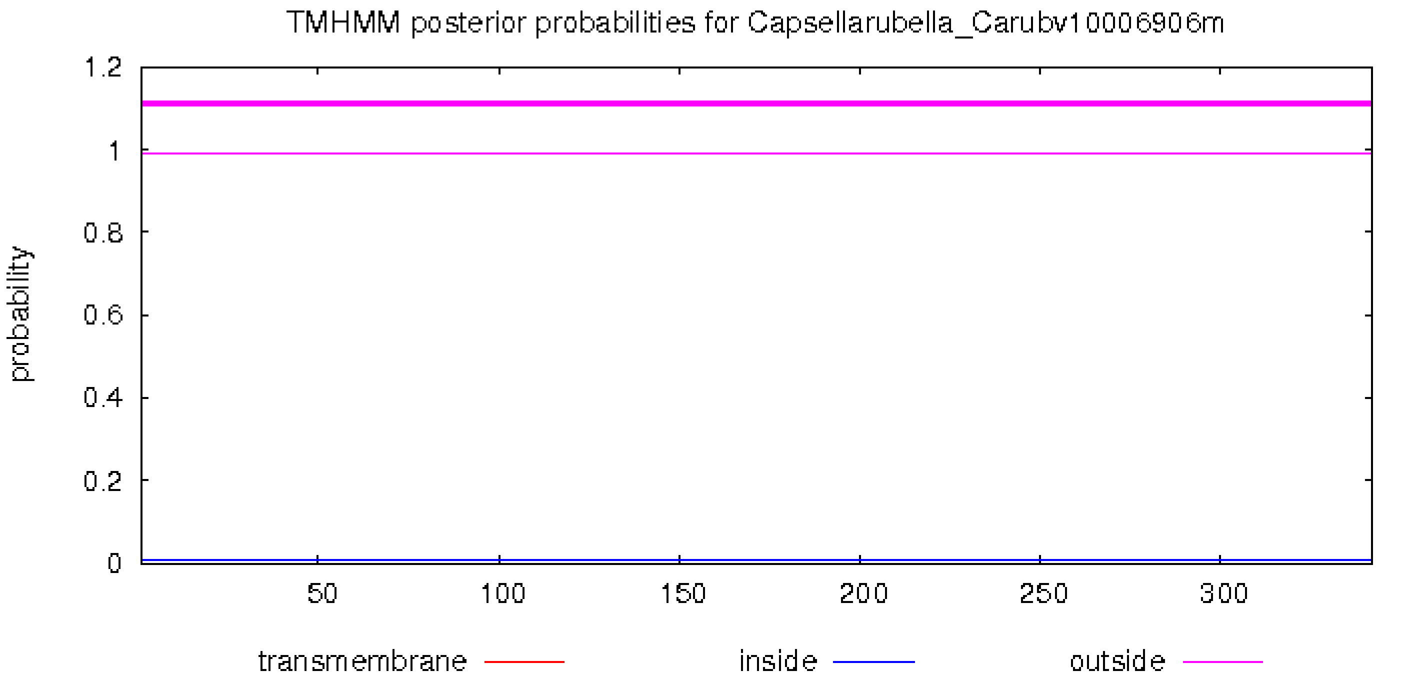

plot in postscript, script for making the plot in gnuplot, data for plot

---

```
# Capsellarubella_Carubv10007390m Length: 262
# Capsellarubella_Carubv10007390m Number of predicted TMHs:  0
# Capsellarubella_Carubv10007390m Exp number of AAs in TMHs: 0
# Capsellarubella_Carubv10007390m Exp number, first 60 AAs:  0
```

```
# Capsellarubella_Carubv10007390m Total prob of N-in:        0.00229
Capsellarubella_Carubv10007390m TMHMM2.0        outside      1   262
```

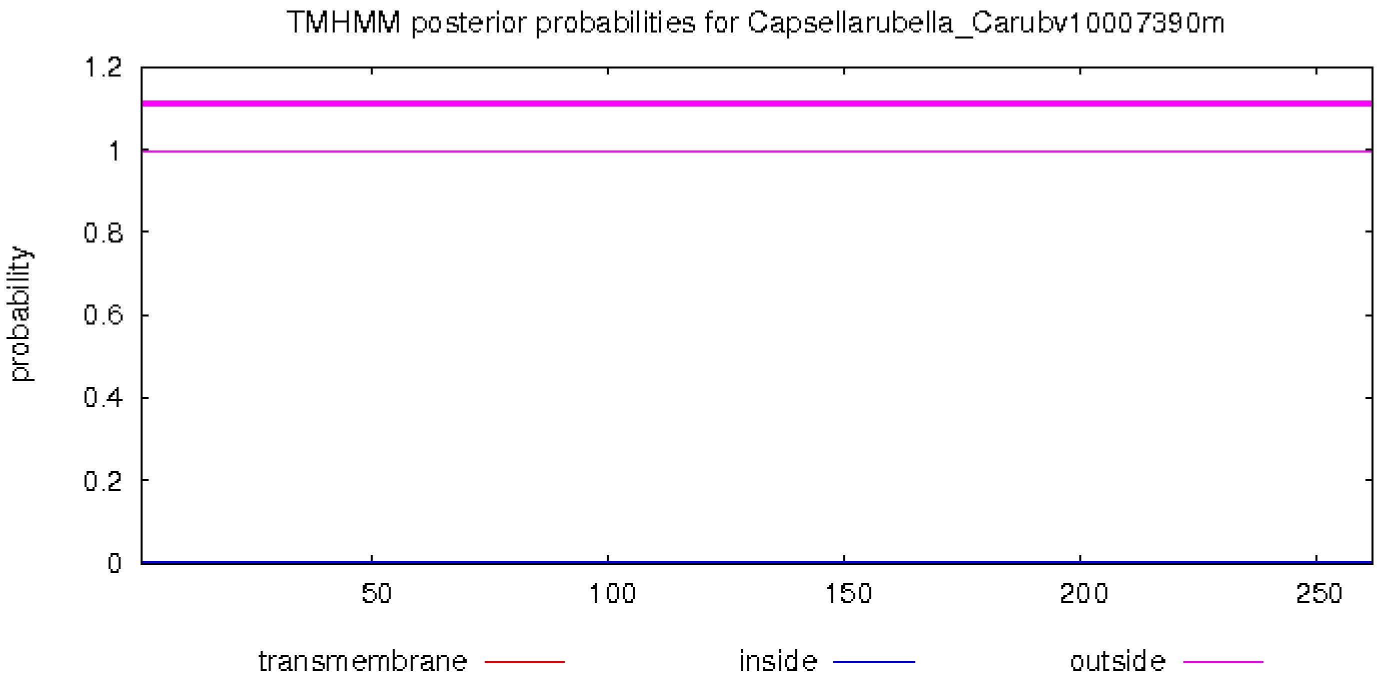

plot in postscript, script for making the plot in gnuplot, data for plot

---

```
# Capsellarubella_Carubv10007613m Length: 526
# Capsellarubella_Carubv10007613m Number of predicted TMHs:  1
# Capsellarubella_Carubv10007613m Exp number of AAs in TMHs: 21.575
# Capsellarubella_Carubv10007613m Exp number, first 60 AAs:  0
# Capsellarubella_Carubv10007613m Total prob of N-in:        0.12692
Capsellarubella_Carubv10007613m TMHMM2.0        outside      1   502
Capsellarubella_Carubv10007613m TMHMM2.0        TMhelix    503   525
Capsellarubella_Carubv10007613m TMHMM2.0        inside     526   526
```

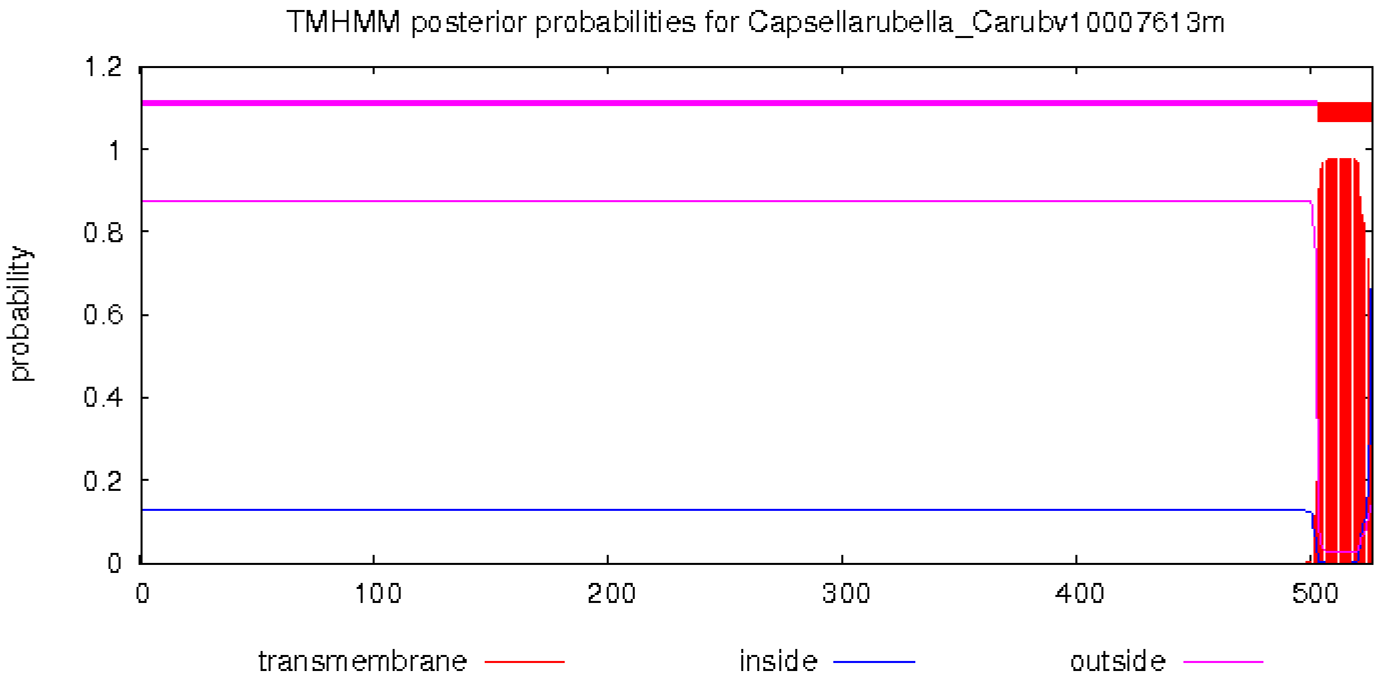

plot in postscript, script for making the plot in gnuplot, data for plot

---

```
# Capsellarubella_Carubv10008035m Length: 354
# Capsellarubella_Carubv10008035m Number of predicted TMHs:  0
```

```
# Capsellarubella_Carubv10008035m Exp number of AAs in TMHs: 0.00853
# Capsellarubella_Carubv10008035m Exp number, first 60 AAs:  0
# Capsellarubella_Carubv10008035m Total prob of N-in:        0.00593
Capsellarubella_Carubv10008035m TMHMM2.0        outside      1   354
```

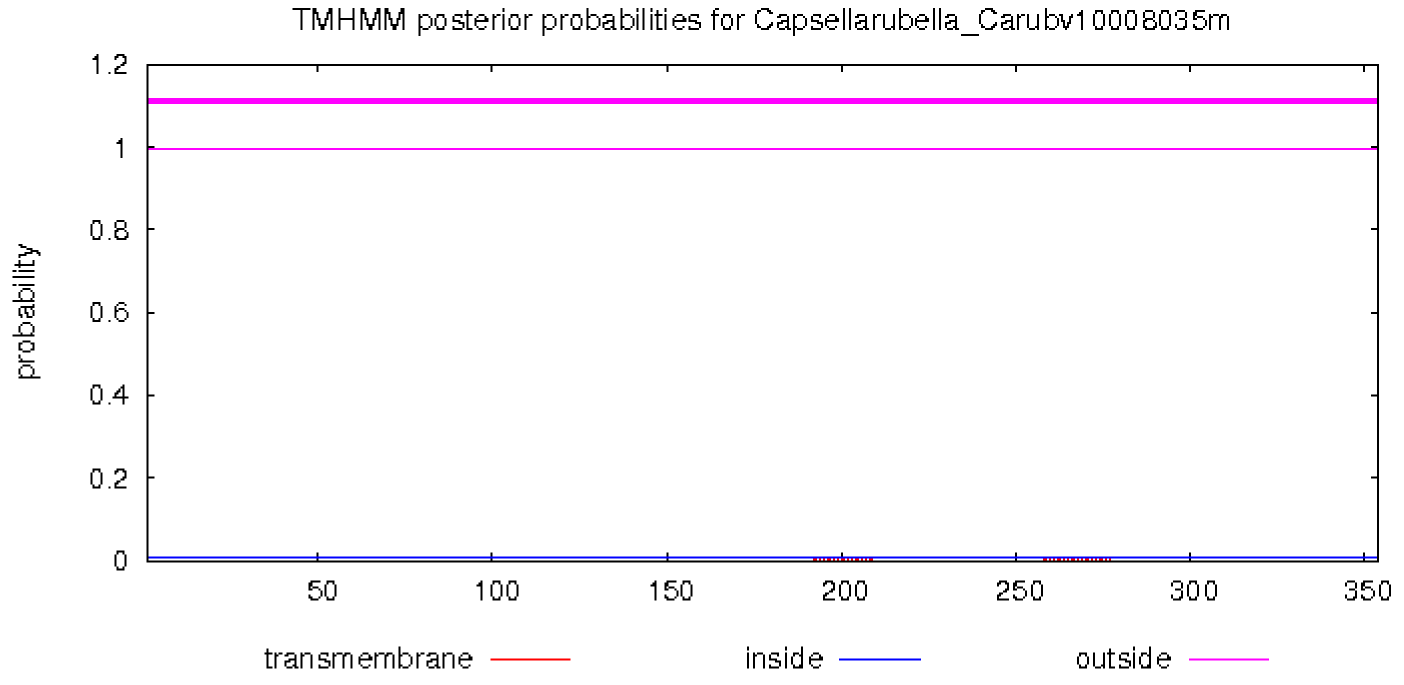

plot in postscript, script for making the plot in gnuplot, data for plot

---

```
# Capsellarubella_Carubv10008036m Length: 320
# Capsellarubella_Carubv10008036m Number of predicted TMHs:  0
# Capsellarubella_Carubv10008036m Exp number of AAs in TMHs: 0.06187
# Capsellarubella_Carubv10008036m Exp number, first 60 AAs:  0
# Capsellarubella_Carubv10008036m Total prob of N-in:        0.01063
Capsellarubella_Carubv10008036m TMHMM2.0        outside      1   320
```

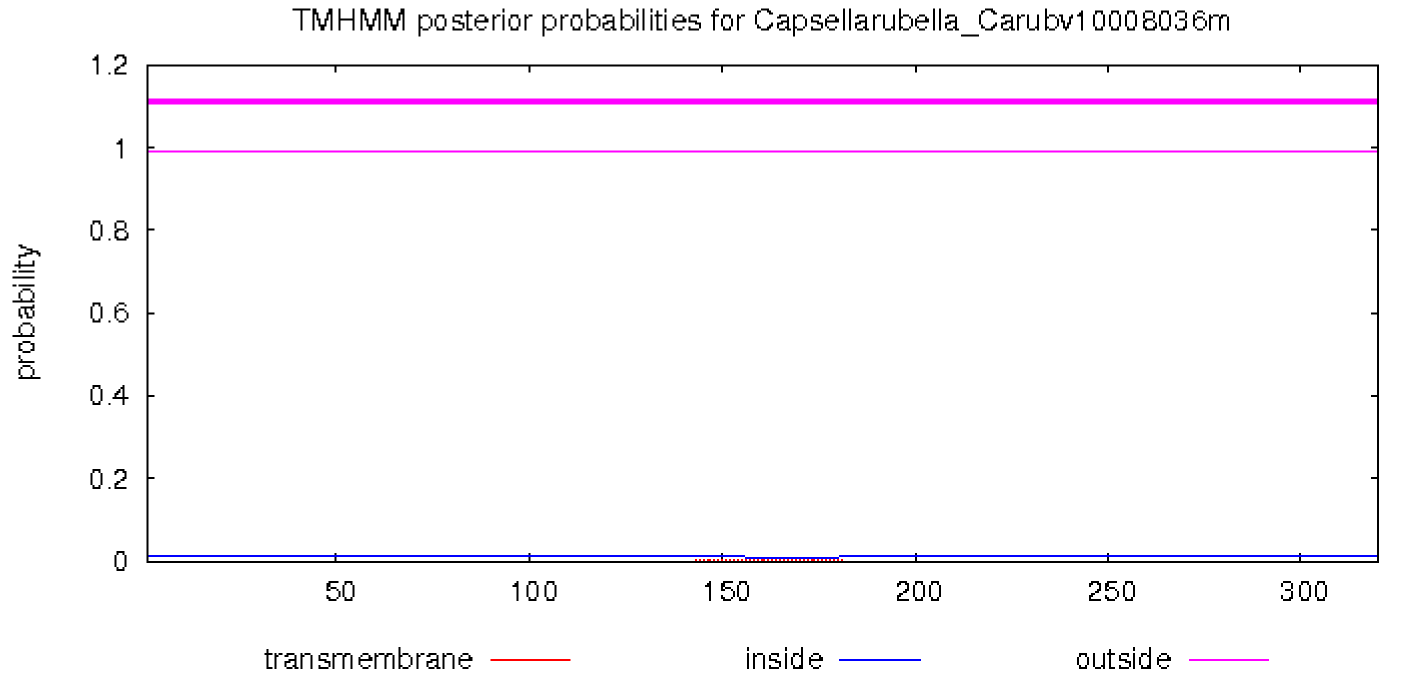

plot in postscript, script for making the plot in gnuplot, data for plot

---

```
# Capsellarubella_Carubv10008529m Length: 658
# Capsellarubella_Carubv10008529m Number of predicted TMHs:  1
```

```
# Capsellarubella_Carubv10008529m Exp number of AAs in TMHs: 19.89856
# Capsellarubella_Carubv10008529m Exp number, first 60 AAs:  0
# Capsellarubella_Carubv10008529m Total prob of N-in:        0.00695
Capsellarubella_Carubv10008529m TMHMM2.0        outside      1   615
Capsellarubella_Carubv10008529m TMHMM2.0        TMhelix    616   635
Capsellarubella_Carubv10008529m TMHMM2.0        inside     636   658
```

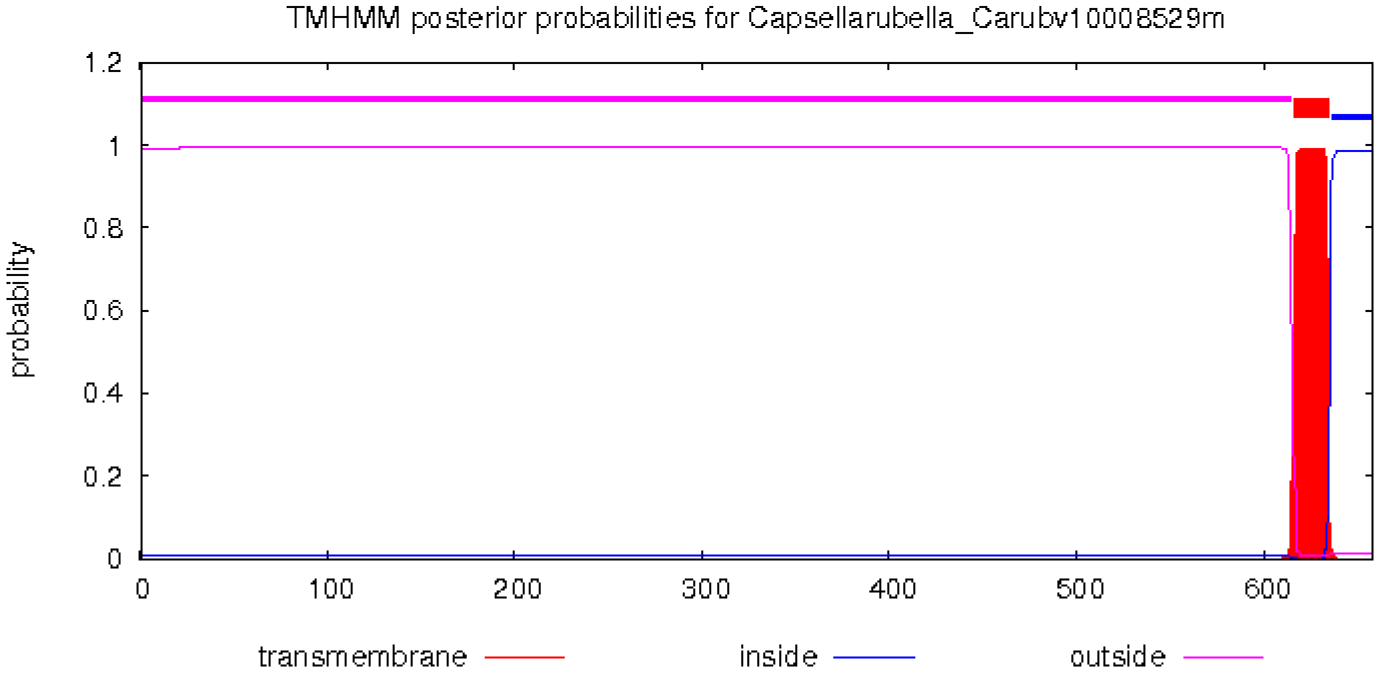

plot in postscript, script for making the plot in gnuplot, data for plot

---

```
# Capsellarubella_Carubv10008531m Length: 657
# Capsellarubella_Carubv10008531m Number of predicted TMHs:  1
# Capsellarubella_Carubv10008531m Exp number of AAs in TMHs: 19.89863
# Capsellarubella_Carubv10008531m Exp number, first 60 AAs:  0
# Capsellarubella_Carubv10008531m Total prob of N-in:        0.00703
Capsellarubella_Carubv10008531m TMHMM2.0        outside      1   614
Capsellarubella_Carubv10008531m TMHMM2.0        TMhelix    615   634
Capsellarubella_Carubv10008531m TMHMM2.0        inside     635   657
```

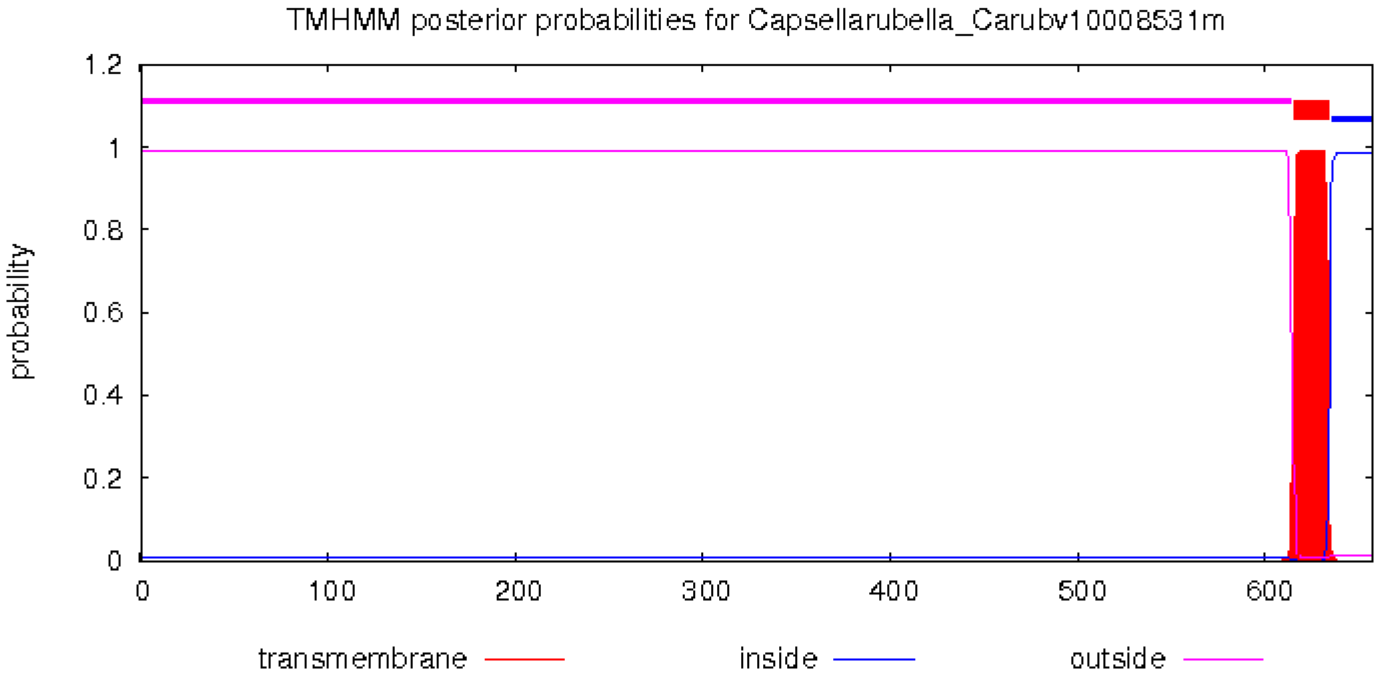

plot in postscript, script for making the plot in gnuplot, data for plot

```
# Capsellarubella_Carubv10008533m Length: 655
# Capsellarubella_Carubv10008533m Number of predicted TMHs:  1
# Capsellarubella_Carubv10008533m Exp number of AAs in TMHs: 19.89877
# Capsellarubella_Carubv10008533m Exp number, first 60 AAs:  0
# Capsellarubella_Carubv10008533m Total prob of N-in:        0.00721
Capsellarubella_Carubv10008533m TMHMM2.0        outside      1   612
Capsellarubella_Carubv10008533m TMHMM2.0        TMhelix    613   632
Capsellarubella_Carubv10008533m TMHMM2.0        inside     633   655
```

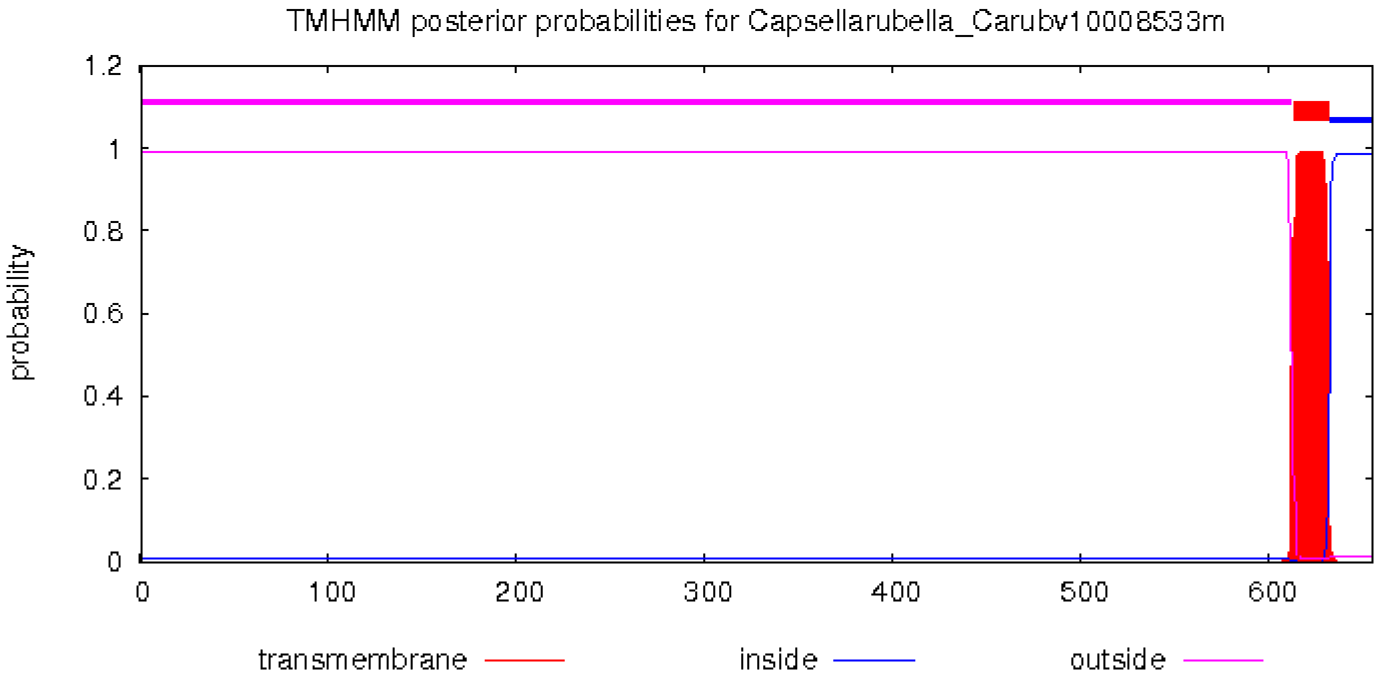

plot in postscript, script for making the plot in gnuplot, data for plot

```
# Capsellarubella_Carubv10008706m Length: 572
# Capsellarubella_Carubv10008706m Number of predicted TMHs:  1
# Capsellarubella_Carubv10008706m Exp number of AAs in TMHs: 22.56683
# Capsellarubella_Carubv10008706m Exp number, first 60 AAs:  0.00241
# Capsellarubella_Carubv10008706m Total prob of N-in:        0.57453
Capsellarubella_Carubv10008706m TMHMM2.0        outside      1   541
Capsellarubella_Carubv10008706m TMHMM2.0        TMhelix    542   564
Capsellarubella_Carubv10008706m TMHMM2.0        inside     565   572
```

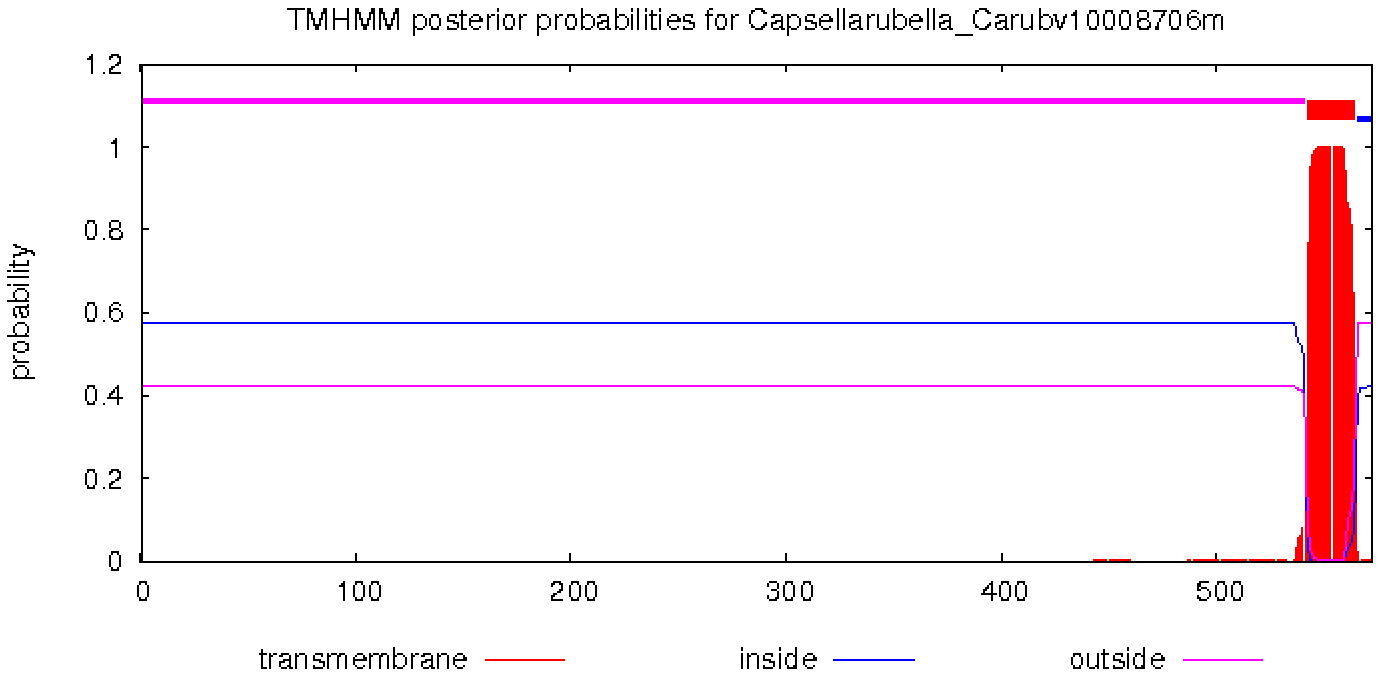

plot in postscript, script for making the plot in gnuplot, data for plot

---

```
# Capsellarubella_Carubv10008761m Length: 551
# Capsellarubella_Carubv10008761m Number of predicted TMHs:  1
# Capsellarubella_Carubv10008761m Exp number of AAs in TMHs: 21.37073
# Capsellarubella_Carubv10008761m Exp number, first 60 AAs:  0.00363
# Capsellarubella_Carubv10008761m Total prob of N-in:        0.05876
Capsellarubella_Carubv10008761m TMHMM2.0        outside      1   521
Capsellarubella_Carubv10008761m TMHMM2.0        TMhelix    522   544
Capsellarubella_Carubv10008761m TMHMM2.0        inside     545   551
```

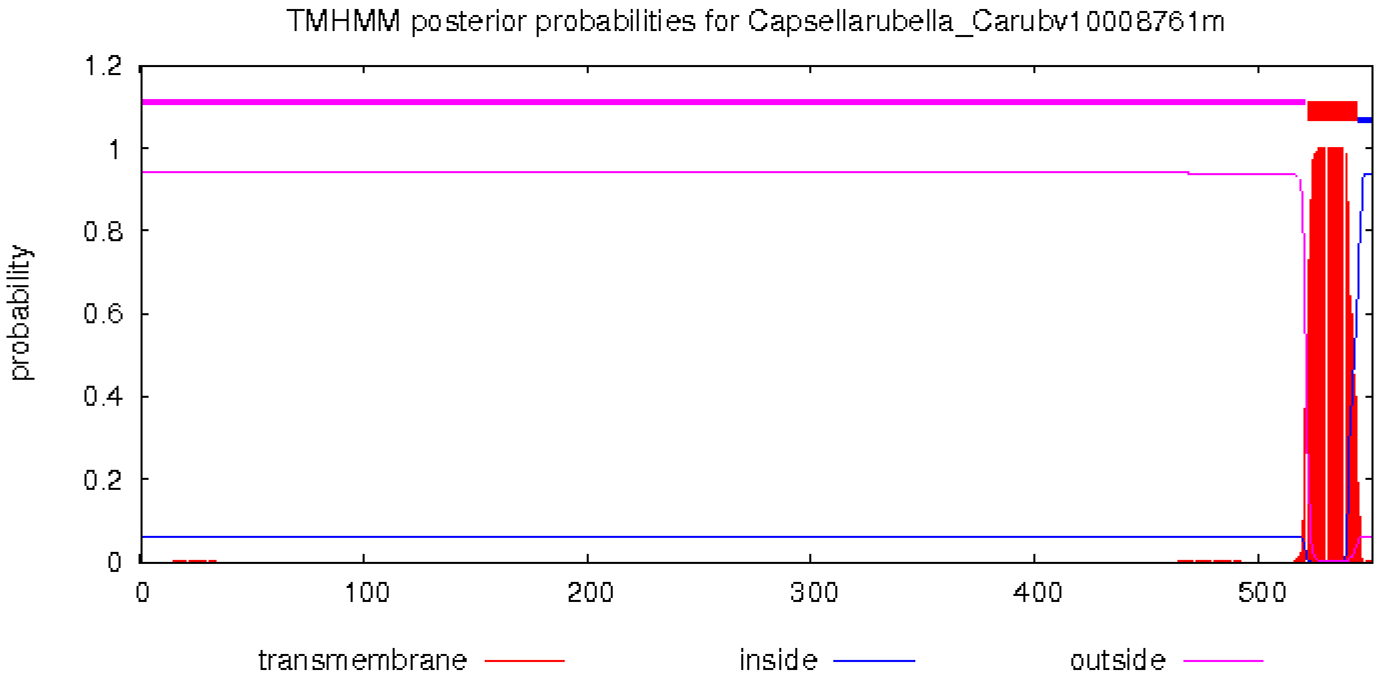

plot in postscript, script for making the plot in gnuplot, data for plot

---

```
# Capsellarubella_Carubv10008817m Length: 532
# Capsellarubella_Carubv10008817m Number of predicted TMHs:  1
# Capsellarubella_Carubv10008817m Exp number of AAs in TMHs: 23.52646
# Capsellarubella_Carubv10008817m Exp number, first 60 AAs:  0.08817
# Capsellarubella_Carubv10008817m Total prob of N-in:        0.72587
```

```
Capsellarubella_Carubv10008817m TMHMM2.0        inside       1   502
Capsellarubella_Carubv10008817m TMHMM2.0        TMhelix    503   525
Capsellarubella_Carubv10008817m TMHMM2.0        outside    526   532
```

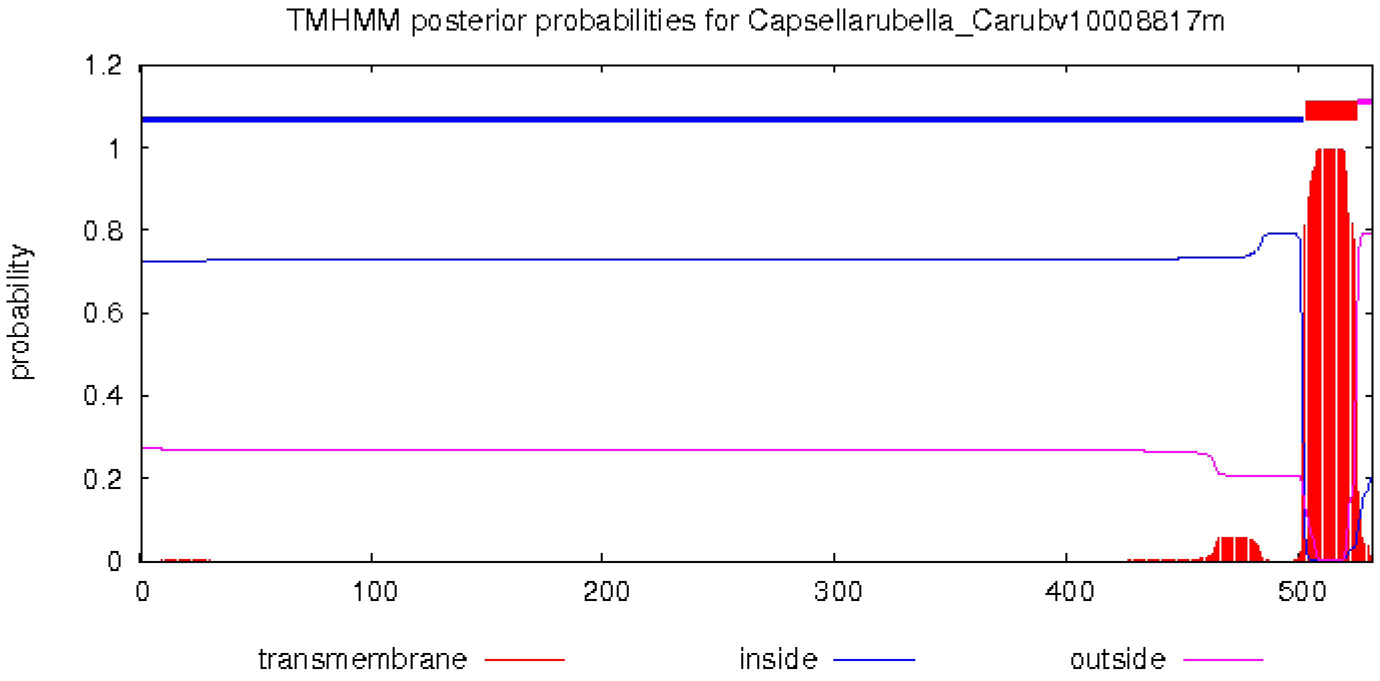

plot in postscript, script for making the plot in gnuplot, data for plot

---

```
# Capsellarubella_Carubv10008886m Length: 511
# Capsellarubella_Carubv10008886m Number of predicted TMHs:  1
# Capsellarubella_Carubv10008886m Exp number of AAs in TMHs: 22.57
# Capsellarubella_Carubv10008886m Exp number, first 60 AAs:  0
# Capsellarubella_Carubv10008886m Total prob of N-in:        0.71390
Capsellarubella_Carubv10008886m TMHMM2.0        inside       1   480
Capsellarubella_Carubv10008886m TMHMM2.0        TMhelix    481   503
Capsellarubella_Carubv10008886m TMHMM2.0        outside    504   511
```

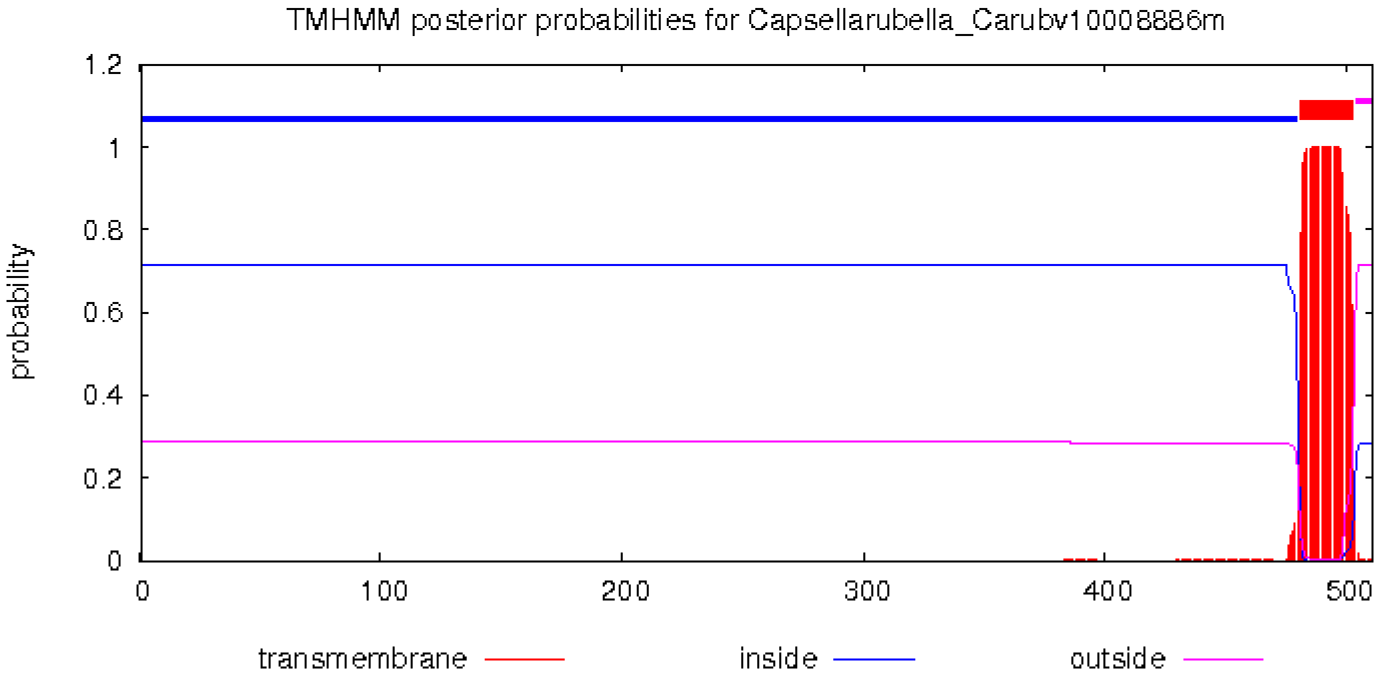

plot in postscript, script for making the plot in gnuplot, data for plot

---

```
# Capsellarubella_Carubv10009049m Length: 469
# Capsellarubella_Carubv10009049m Number of predicted TMHs:  1
# Capsellarubella_Carubv10009049m Exp number of AAs in TMHs: 17.89615
# Capsellarubella_Carubv10009049m Exp number, first 60 AAs:  0.00391
# Capsellarubella_Carubv10009049m Total prob of N-in:        0.32049
Capsellarubella_Carubv10009049m TMHMM2.0        outside      1   450
Capsellarubella_Carubv10009049m TMHMM2.0        TMhelix    451   468
Capsellarubella_Carubv10009049m TMHMM2.0        inside     469   469
```

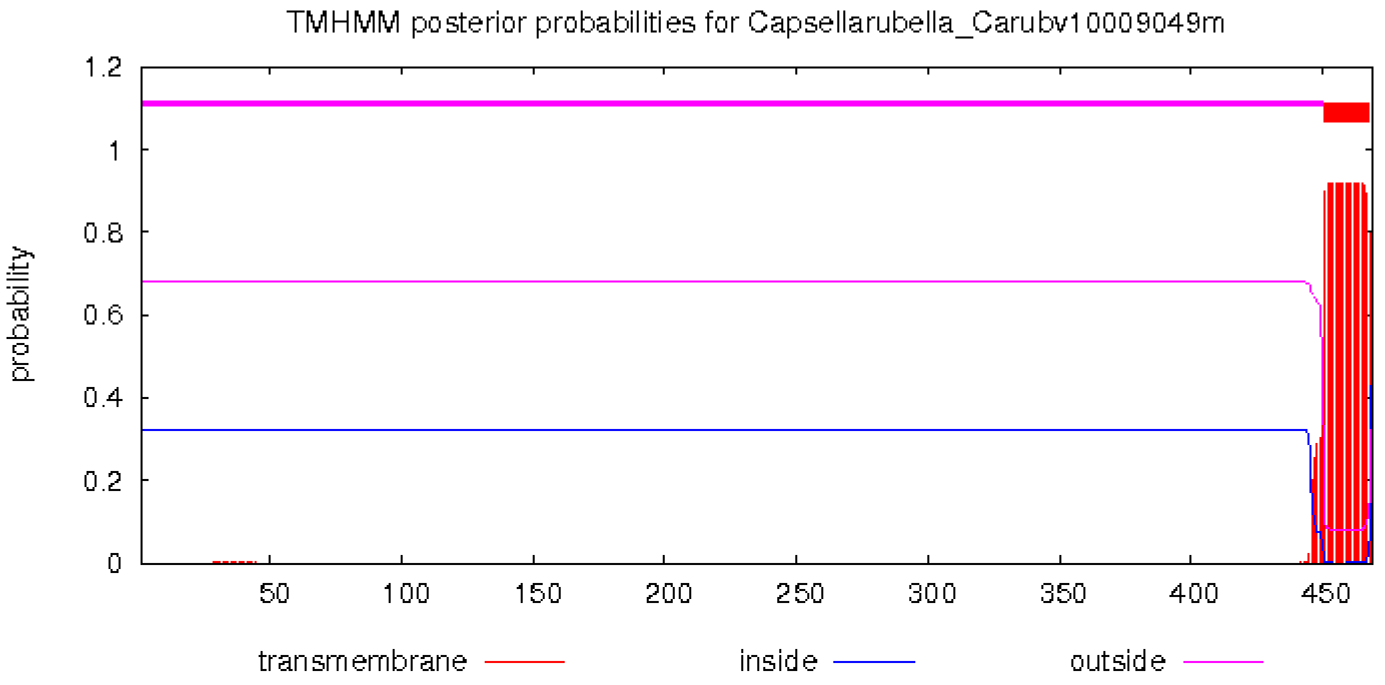

plot in postscript, script for making the plot in gnuplot, data for plot

---

```
# Capsellarubella_Carubv10009087m Length: 457
# Capsellarubella_Carubv10009087m Number of predicted TMHs:  0
# Capsellarubella_Carubv10009087m Exp number of AAs in TMHs: 0.0002
# Capsellarubella_Carubv10009087m Exp number, first 60 AAs:  0
# Capsellarubella_Carubv10009087m Total prob of N-in:        0.00272
Capsellarubella_Carubv10009087m TMHMM2.0        outside      1   457
```

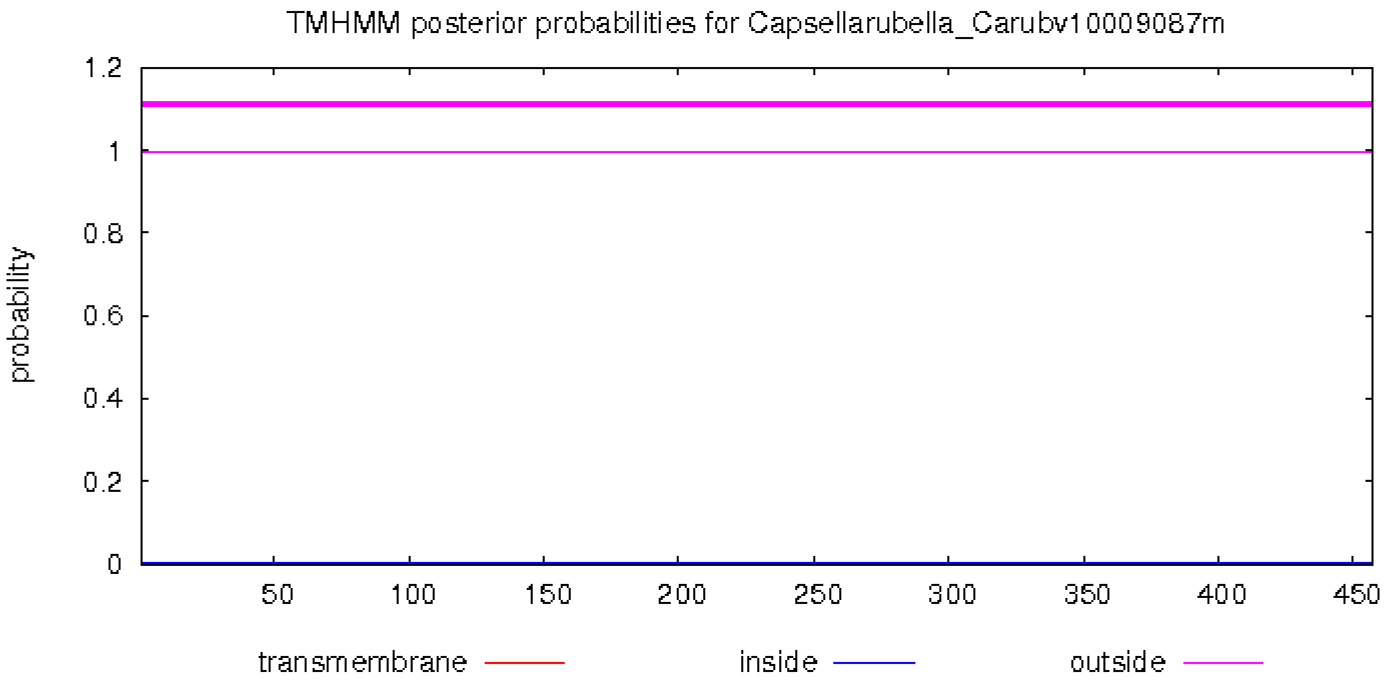

plot in postscript, script for making the plot in gnuplot, data for plot

```
# Capsellarubella_Carubv10009366m Length: 395
# Capsellarubella_Carubv10009366m Number of predicted TMHs:  0
# Capsellarubella_Carubv10009366m Exp number of AAs in TMHs: 0.00018
# Capsellarubella_Carubv10009366m Exp number, first 60 AAs:  0
# Capsellarubella_Carubv10009366m Total prob of N-in:        0.00167
Capsellarubella_Carubv10009366m TMHMM2.0        outside      1   395
```

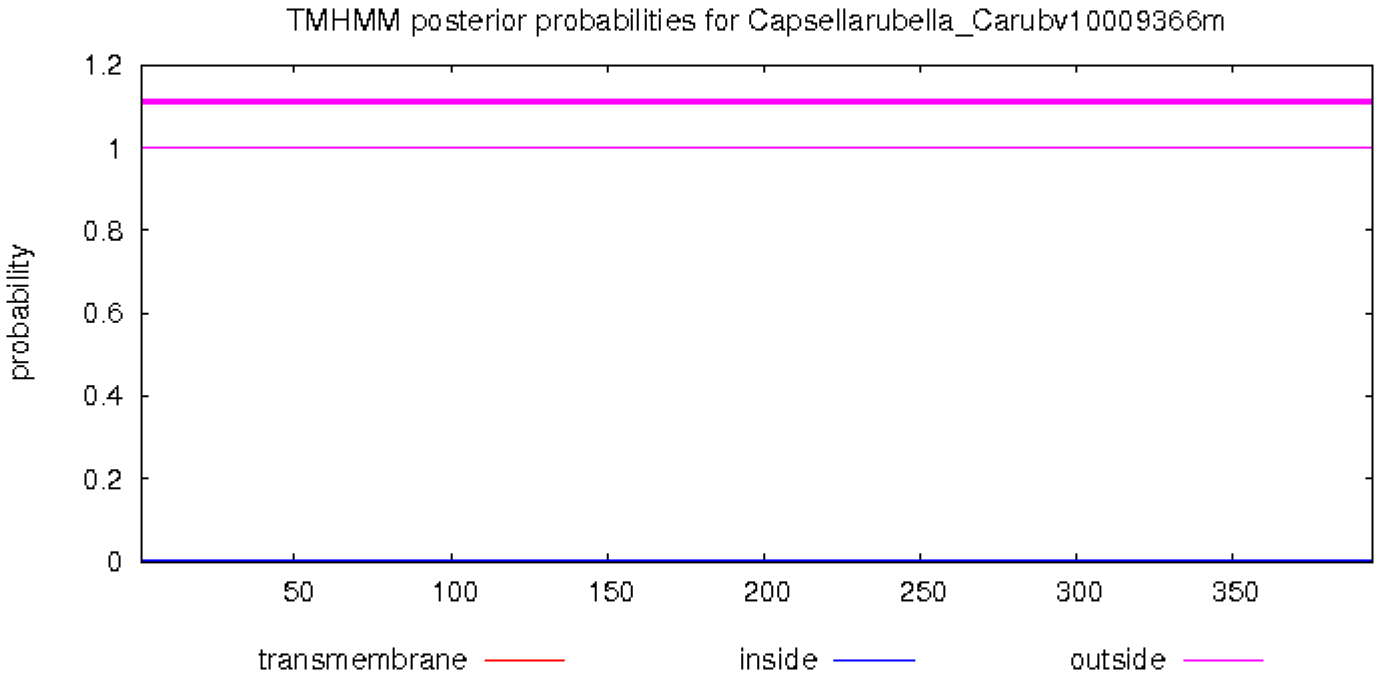

plot in postscript, script for making the plot in gnuplot, data for plot

```
# Capsellarubella_Carubv10009390m Length: 391
# Capsellarubella_Carubv10009390m Number of predicted TMHs:  0
# Capsellarubella_Carubv10009390m Exp number of AAs in TMHs: 0.00376
# Capsellarubella_Carubv10009390m Exp number, first 60 AAs:  0.00018
# Capsellarubella_Carubv10009390m Total prob of N-in:        0.02026
Capsellarubella_Carubv10009390m TMHMM2.0        outside      1   391
```

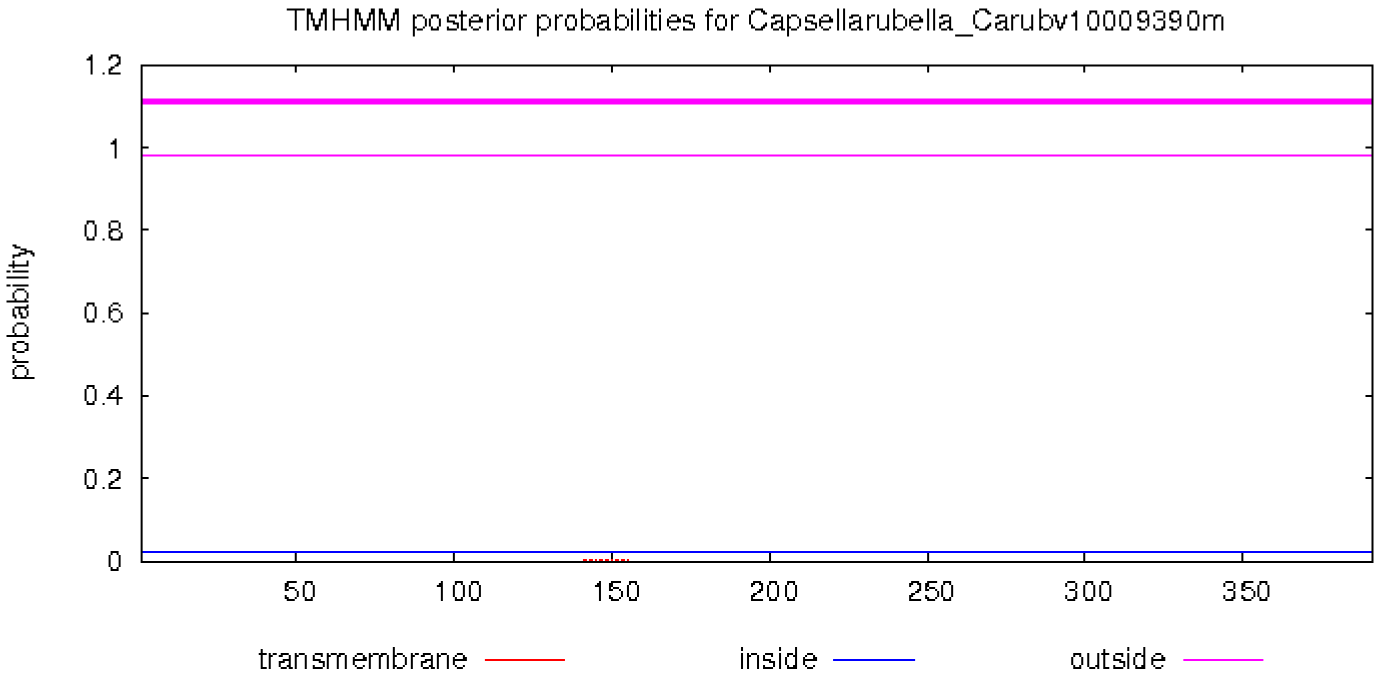

plot in postscript, script for making the plot in gnuplot, data for plot

```
# Capsellarubella_Carubv10009451m Length: 379
# Capsellarubella_Carubv10009451m Number of predicted TMHs:  1
# Capsellarubella_Carubv10009451m Exp number of AAs in TMHs: 20.69954
# Capsellarubella_Carubv10009451m Exp number, first 60 AAs:  0.00033
# Capsellarubella_Carubv10009451m Total prob of N-in:        0.45205
Capsellarubella_Carubv10009451m TMHMM2.0        outside      1   355
Capsellarubella_Carubv10009451m TMHMM2.0        TMhelix    356   378
Capsellarubella_Carubv10009451m TMHMM2.0        inside     379   379
```

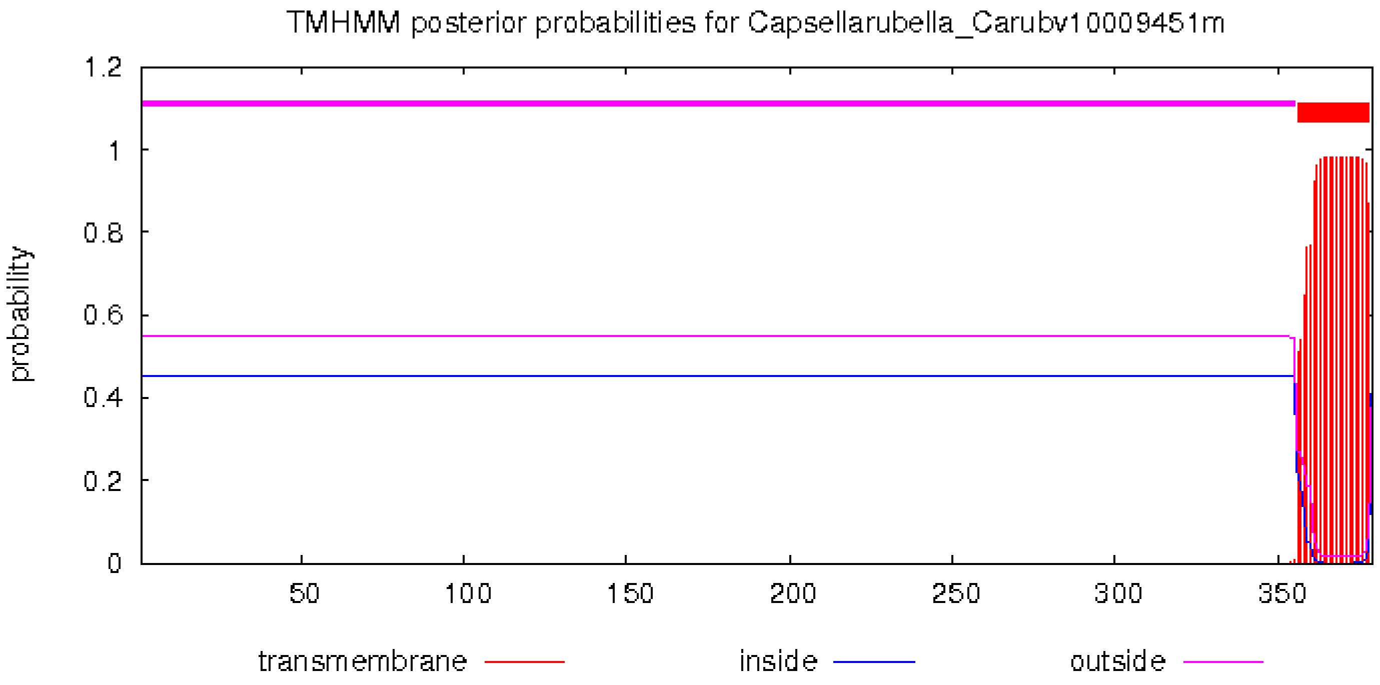

plot in postscript, script for making the plot in gnuplot, data for plot

---

```
# Capsellarubella_Carubv10009524m Length: 363
# Capsellarubella_Carubv10009524m Number of predicted TMHs:  0
# Capsellarubella_Carubv10009524m Exp number of AAs in TMHs: 0.00974
# Capsellarubella_Carubv10009524m Exp number, first 60 AAs:  0.00212
# Capsellarubella_Carubv10009524m Total prob of N-in:        0.04523
Capsellarubella_Carubv10009524m TMHMM2.0        outside      1   363
```

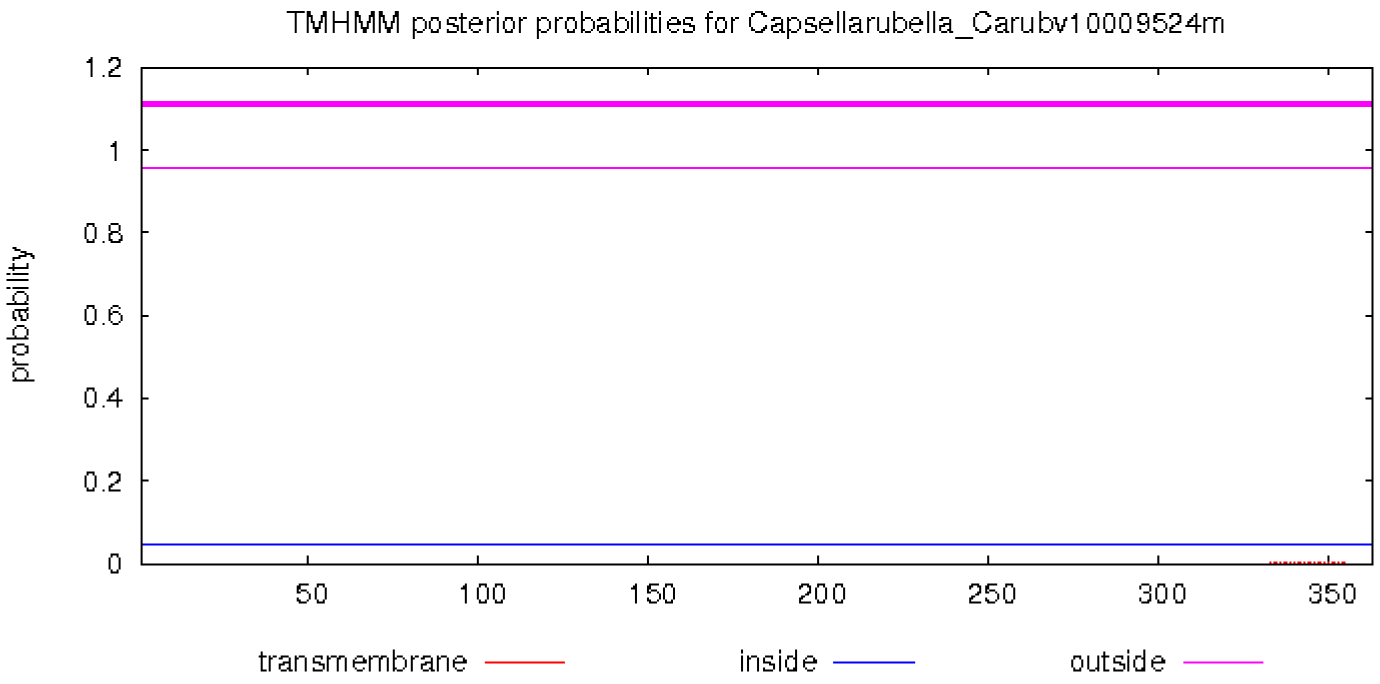

plot in postscript, script for making the plot in gnuplot, data for plot

```
# Capsellarubella_Carubv10009638m Length: 342
# Capsellarubella_Carubv10009638m Number of predicted TMHs:  0
# Capsellarubella_Carubv10009638m Exp number of AAs in TMHs: 0.0002
# Capsellarubella_Carubv10009638m Exp number, first 60 AAs:  0
# Capsellarubella_Carubv10009638m Total prob of N-in:        0.02114
Capsellarubella_Carubv10009638m TMHMM2.0        outside      1   342
```

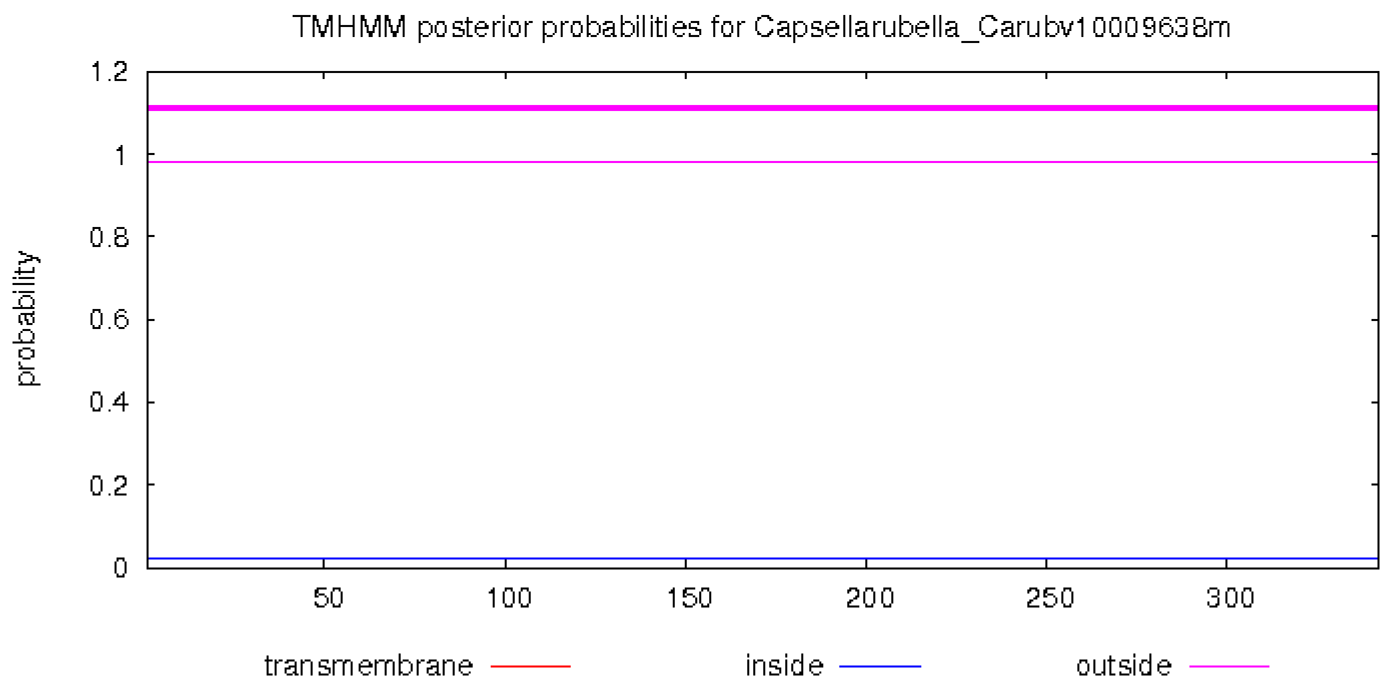

plot in postscript, script for making the plot in gnuplot, data for plot

```
# Capsellarubella_Carubv10009640m Length: 342
# Capsellarubella_Carubv10009640m Number of predicted TMHs:  0
# Capsellarubella_Carubv10009640m Exp number of AAs in TMHs: 0.00018
# Capsellarubella_Carubv10009640m Exp number, first 60 AAs:  0
# Capsellarubella_Carubv10009640m Total prob of N-in:        0.00322
Capsellarubella_Carubv10009640m TMHMM2.0        outside      1   342
```

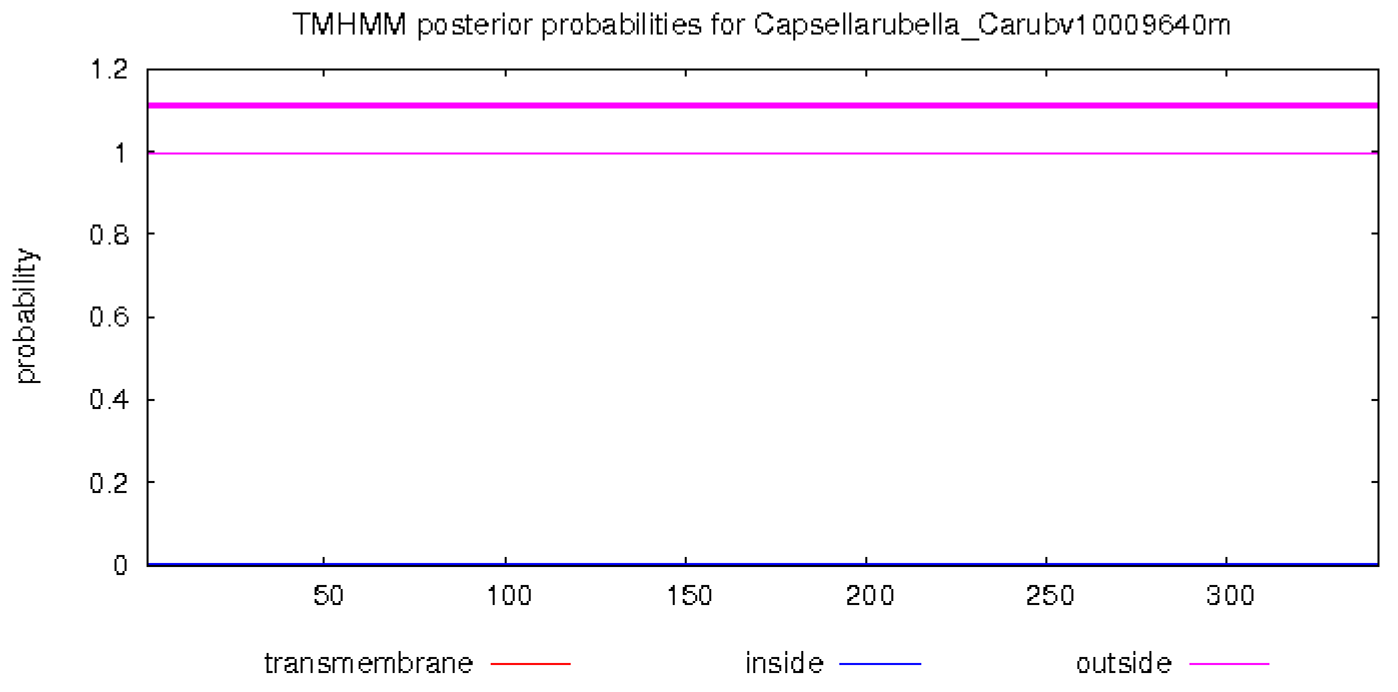

plot in postscript, script for making the plot in gnuplot, data for plot

```
# Capsellarubella_Carubv10009727m Length: 326
# Capsellarubella_Carubv10009727m Number of predicted TMHs:  0
# Capsellarubella_Carubv10009727m Exp number of AAs in TMHs: 0.06333
# Capsellarubella_Carubv10009727m Exp number, first 60 AAs:  0.01297
# Capsellarubella_Carubv10009727m Total prob of N-in:        0.00882
Capsellarubella_Carubv10009727m TMHMM2.0        outside      1   326
```

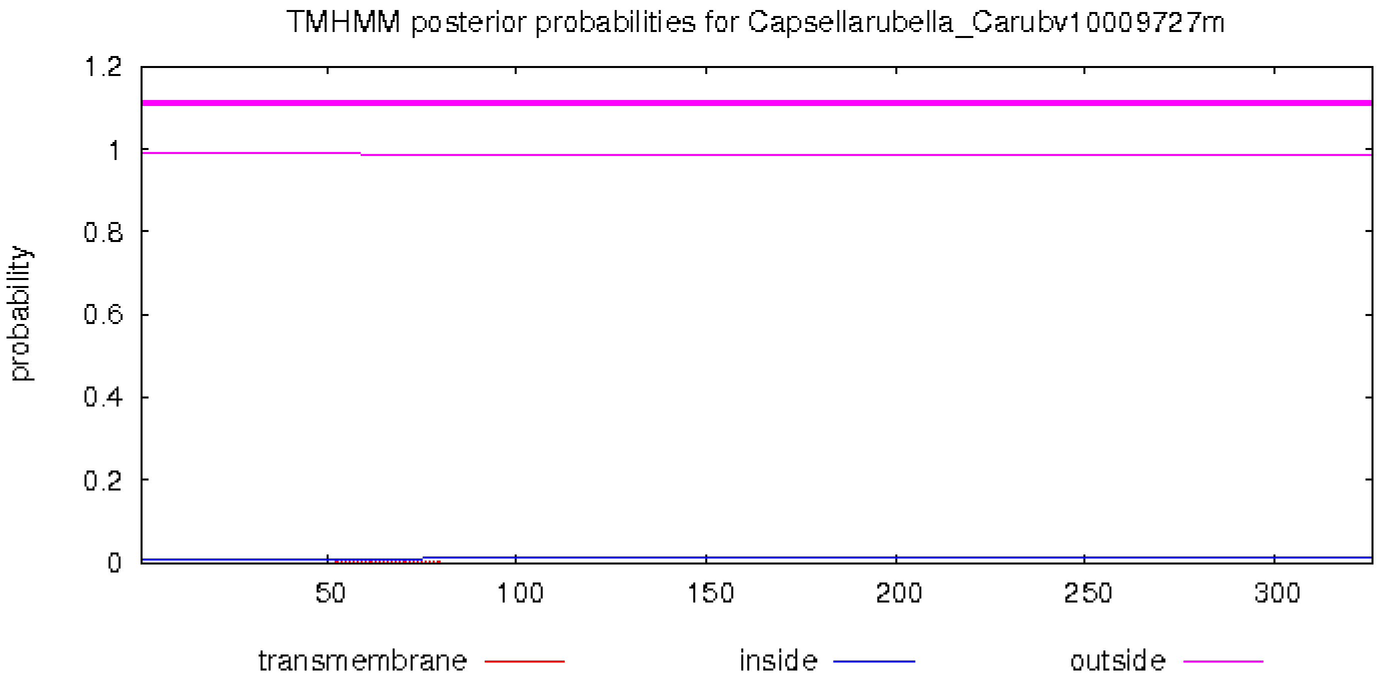

plot in postscript, script for making the plot in gnuplot, data for plot

---

```
# Capsellarubella_Carubv10009816m Length: 311
# Capsellarubella_Carubv10009816m Number of predicted TMHs:  0
# Capsellarubella_Carubv10009816m Exp number of AAs in TMHs: 0.00036
# Capsellarubella_Carubv10009816m Exp number, first 60 AAs:  0
# Capsellarubella_Carubv10009816m Total prob of N-in:        0.00640
Capsellarubella_Carubv10009816m TMHMM2.0        outside      1   311
```

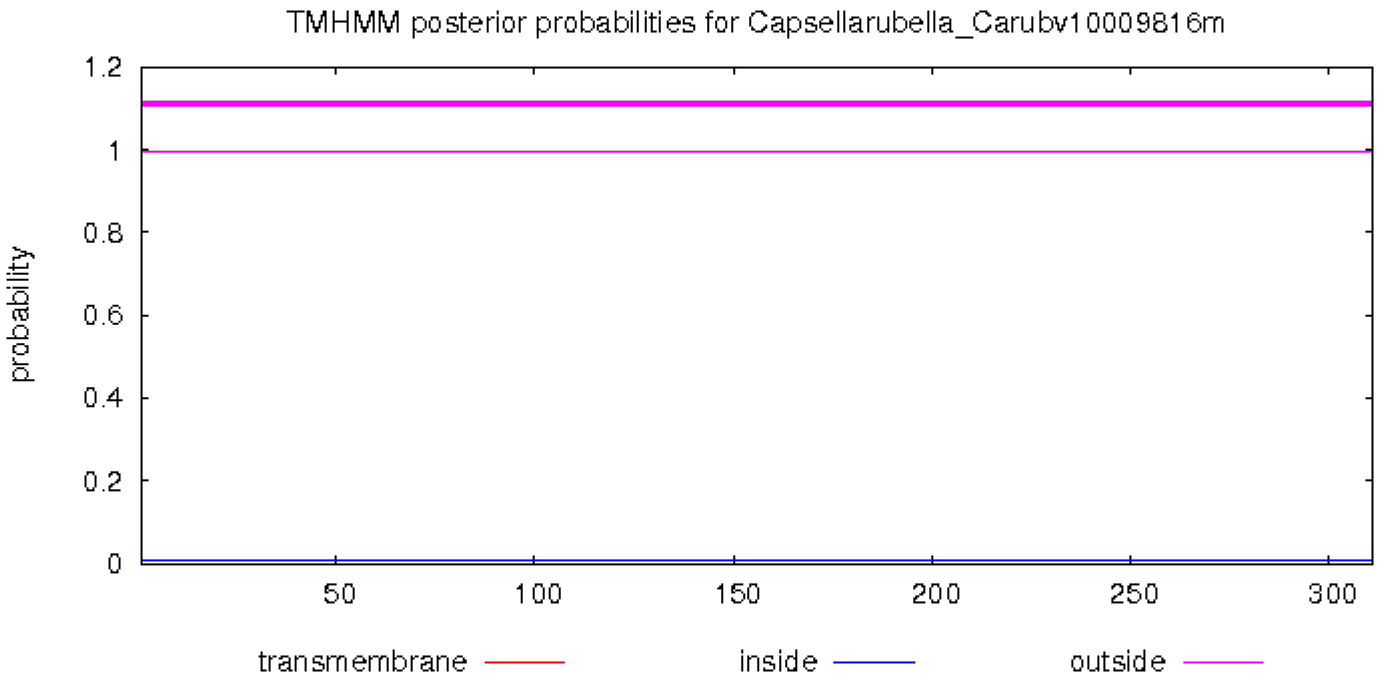

plot in postscript, script for making the plot in gnuplot, data for plot

---

```
# Capsellarubella_Carubv10009870m Length: 301
# Capsellarubella_Carubv10009870m Number of predicted TMHs:  0
# Capsellarubella_Carubv10009870m Exp number of AAs in TMHs: 0.00016
# Capsellarubella_Carubv10009870m Exp number, first 60 AAs:  0
# Capsellarubella_Carubv10009870m Total prob of N-in:        0.00617
Capsellarubella_Carubv10009870m TMHMM2.0        outside      1   301
```

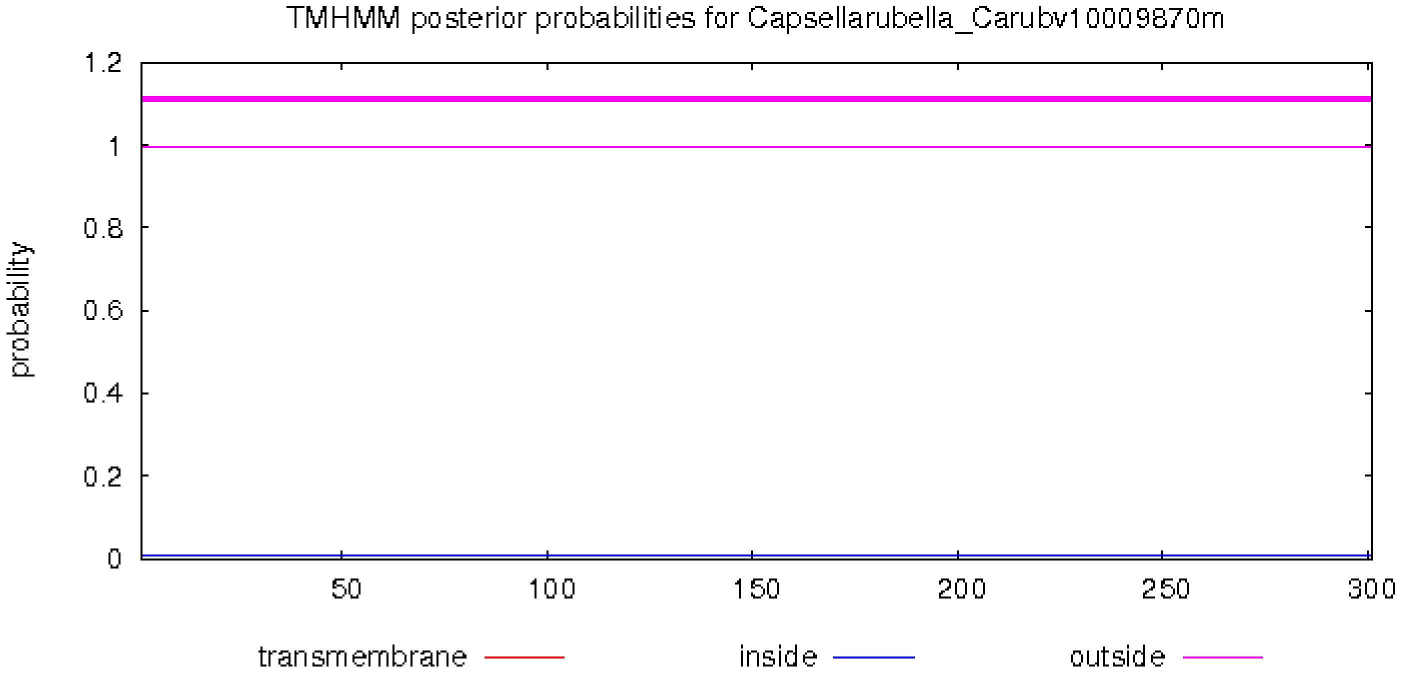

plot in postscript, script for making the plot in gnuplot, data for plot

---

```
# Capsellarubella_Carubv10009926m Length: 286
# Capsellarubella_Carubv10009926m Number of predicted TMHs:  0
# Capsellarubella_Carubv10009926m Exp number of AAs in TMHs: 0.00127
# Capsellarubella_Carubv10009926m Exp number, first 60 AAs:  0.00017
# Capsellarubella_Carubv10009926m Total prob of N-in:        0.00254
Capsellarubella_Carubv10009926m TMHMM2.0        outside      1   286
```

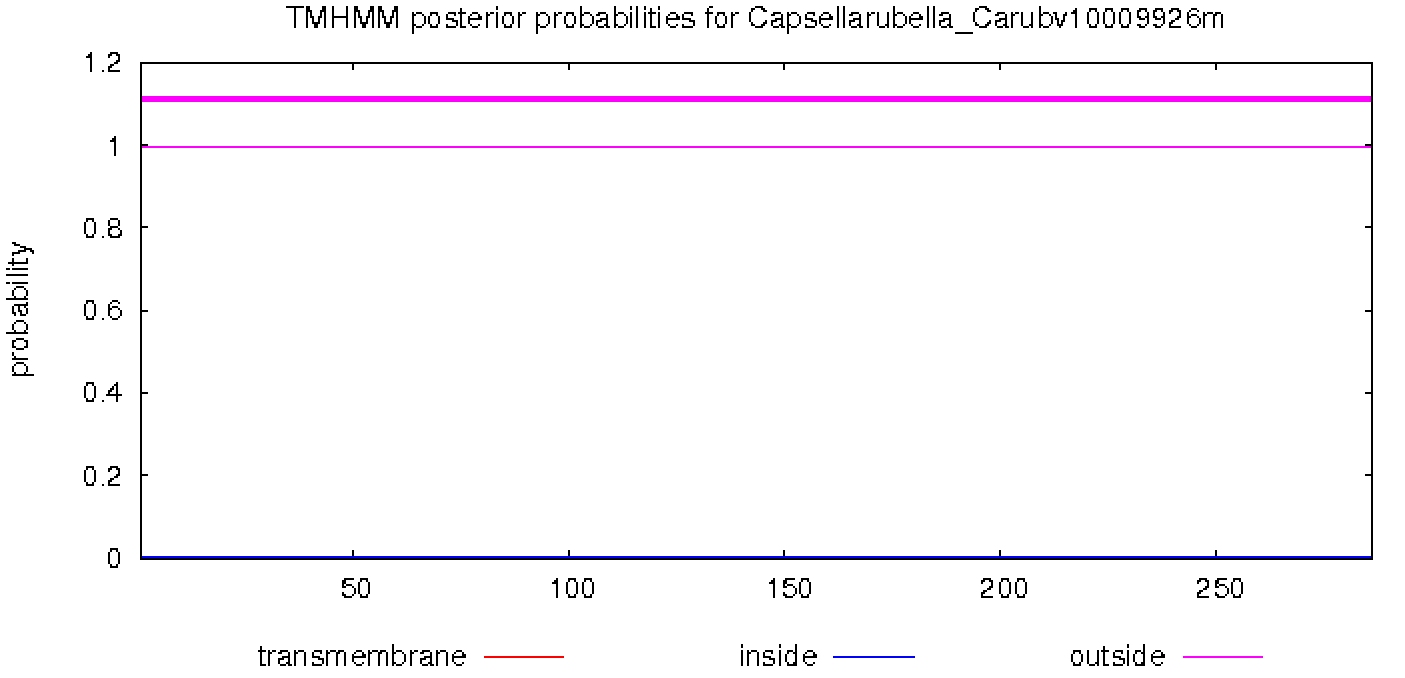

plot in postscript, script for making the plot in gnuplot, data for plot

---

```
# Capsellarubella_Carubv10010920m Length: 313
# Capsellarubella_Carubv10010920m Number of predicted TMHs:  0
# Capsellarubella_Carubv10010920m Exp number of AAs in TMHs: 0
# Capsellarubella_Carubv10010920m Exp number, first 60 AAs:  0
# Capsellarubella_Carubv10010920m Total prob of N-in:        0.00449
Capsellarubella_Carubv10010920m TMHMM2.0        outside      1   313
```

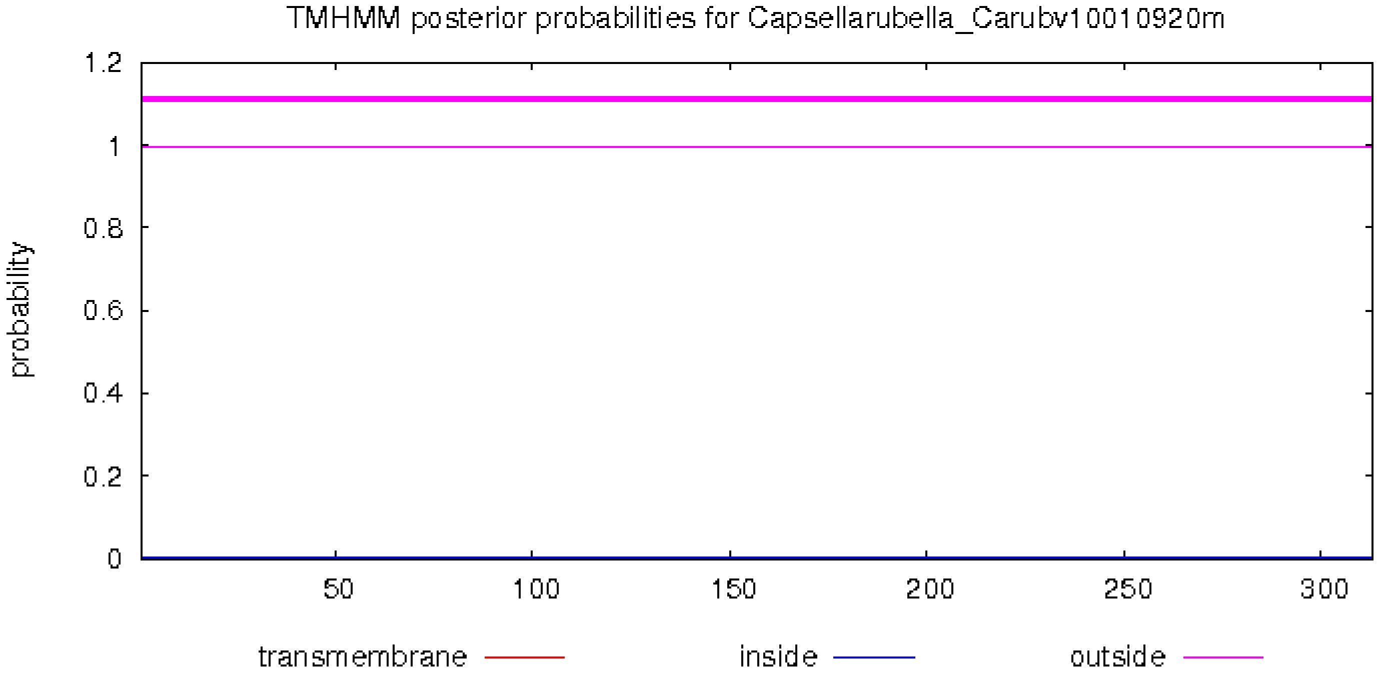

plot in postscript, script for making the plot in gnuplot, data for plot

---

```
# Capsellarubella_Carubv10011081m Length: 323
# Capsellarubella_Carubv10011081m Number of predicted TMHs:  0
# Capsellarubella_Carubv10011081m Exp number of AAs in TMHs: 0.00031
# Capsellarubella_Carubv10011081m Exp number, first 60 AAs:  0
# Capsellarubella_Carubv10011081m Total prob of N-in:        0.02489
Capsellarubella_Carubv10011081m TMHMM2.0        outside      1   323
```

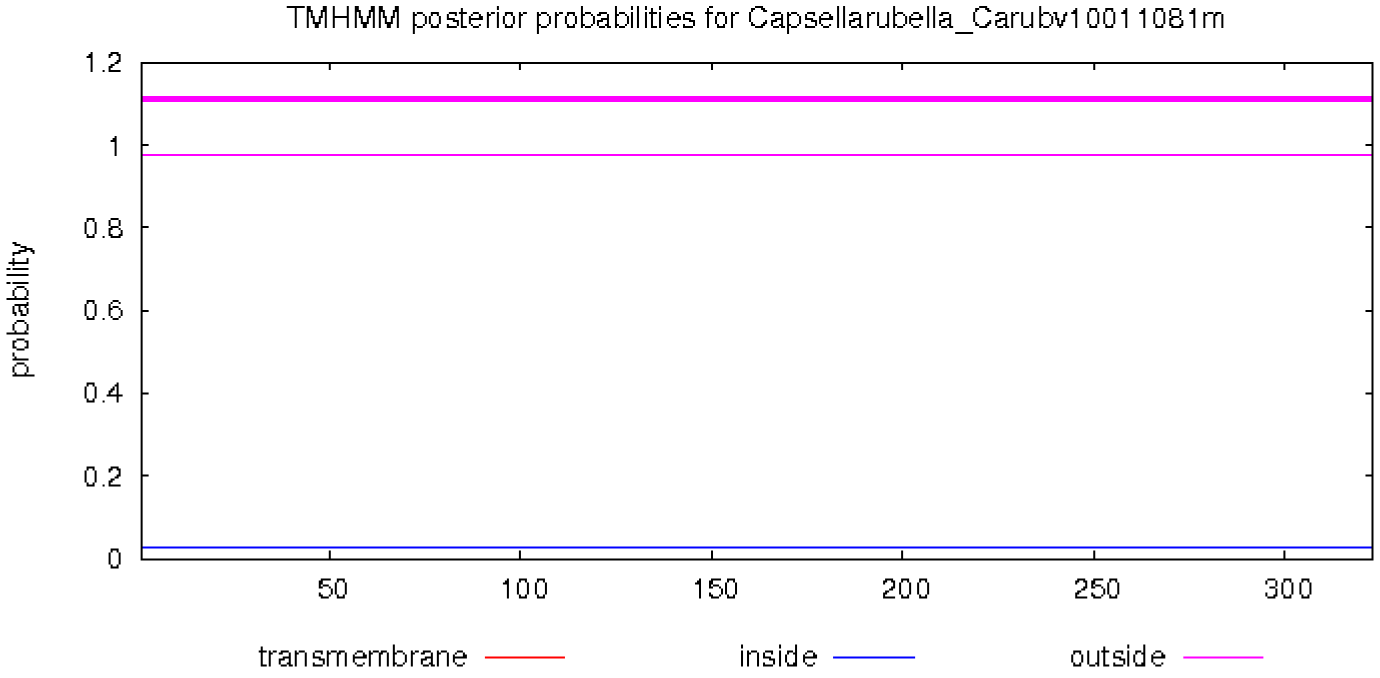

plot in postscript, script for making the plot in gnuplot, data for plot

---

```
# Capsellarubella_Carubv10011108m Length: 262
# Capsellarubella_Carubv10011108m Number of predicted TMHs:  0
# Capsellarubella_Carubv10011108m Exp number of AAs in TMHs: 0.00043
# Capsellarubella_Carubv10011108m Exp number, first 60 AAs:  0
# Capsellarubella_Carubv10011108m Total prob of N-in:        0.00406
Capsellarubella_Carubv10011108m TMHMM2.0        outside      1   262
```

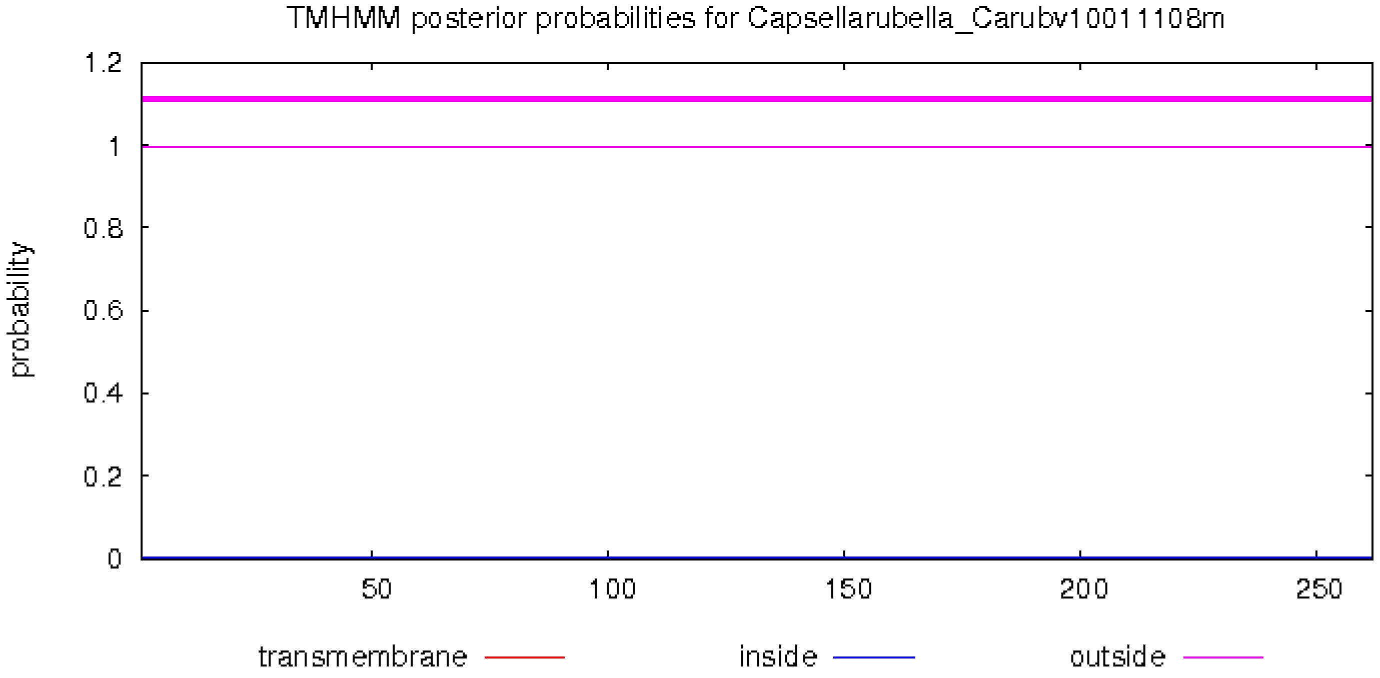

plot in postscript, script for making the plot in gnuplot, data for plot

---

```
# Capsellarubella_Carubv10011253m Length: 452
# Capsellarubella_Carubv10011253m Number of predicted TMHs:  1
# Capsellarubella_Carubv10011253m Exp number of AAs in TMHs: 17.04601
# Capsellarubella_Carubv10011253m Exp number, first 60 AAs:  17.04541
# Capsellarubella_Carubv10011253m Total prob of N-in:        0.82547
# Capsellarubella_Carubv10011253m POSSIBLE N-term signal sequence
Capsellarubella_Carubv10011253m TMHMM2.0        inside       1     6
Capsellarubella_Carubv10011253m TMHMM2.0        TMhelix      7    29
Capsellarubella_Carubv10011253m TMHMM2.0        outside     30   452
```

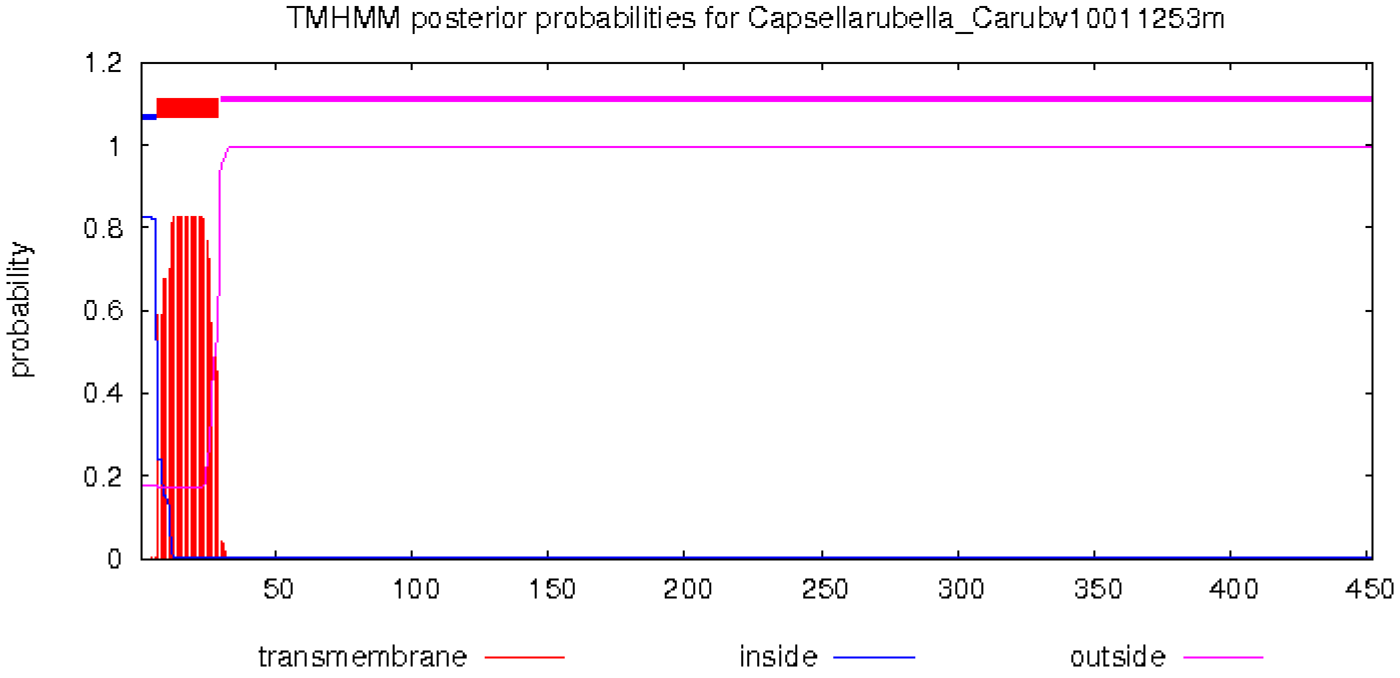

plot in postscript, script for making the plot in gnuplot, data for plot

---

```
# Capsellarubella_Carubv10011623m Length: 251
# Capsellarubella_Carubv10011623m Number of predicted TMHs:  0
# Capsellarubella_Carubv10011623m Exp number of AAs in TMHs: 0.00854
# Capsellarubella_Carubv10011623m Exp number, first 60 AAs:  0
# Capsellarubella_Carubv10011623m Total prob of N-in:        0.03905
Capsellarubella_Carubv10011623m TMHMM2.0        outside      1   251
```

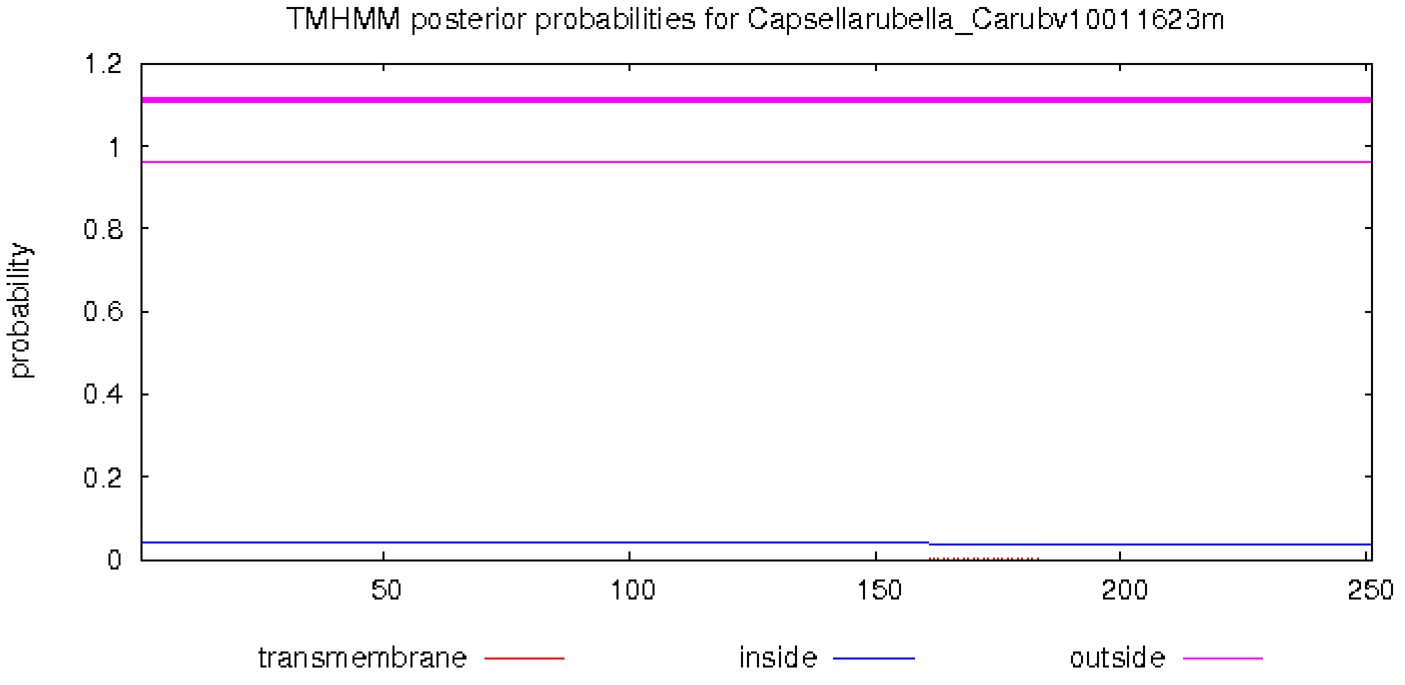

plot in postscript, script for making the plot in gnuplot, data for plot

---

```
# Capsellarubella_Carubv10011900m Length: 368
# Capsellarubella_Carubv10011900m Number of predicted TMHs:  0
# Capsellarubella_Carubv10011900m Exp number of AAs in TMHs: 0.0002
# Capsellarubella_Carubv10011900m Exp number, first 60 AAs:  0
# Capsellarubella_Carubv10011900m Total prob of N-in:        0.02373
Capsellarubella_Carubv10011900m TMHMM2.0        outside      1   368
```

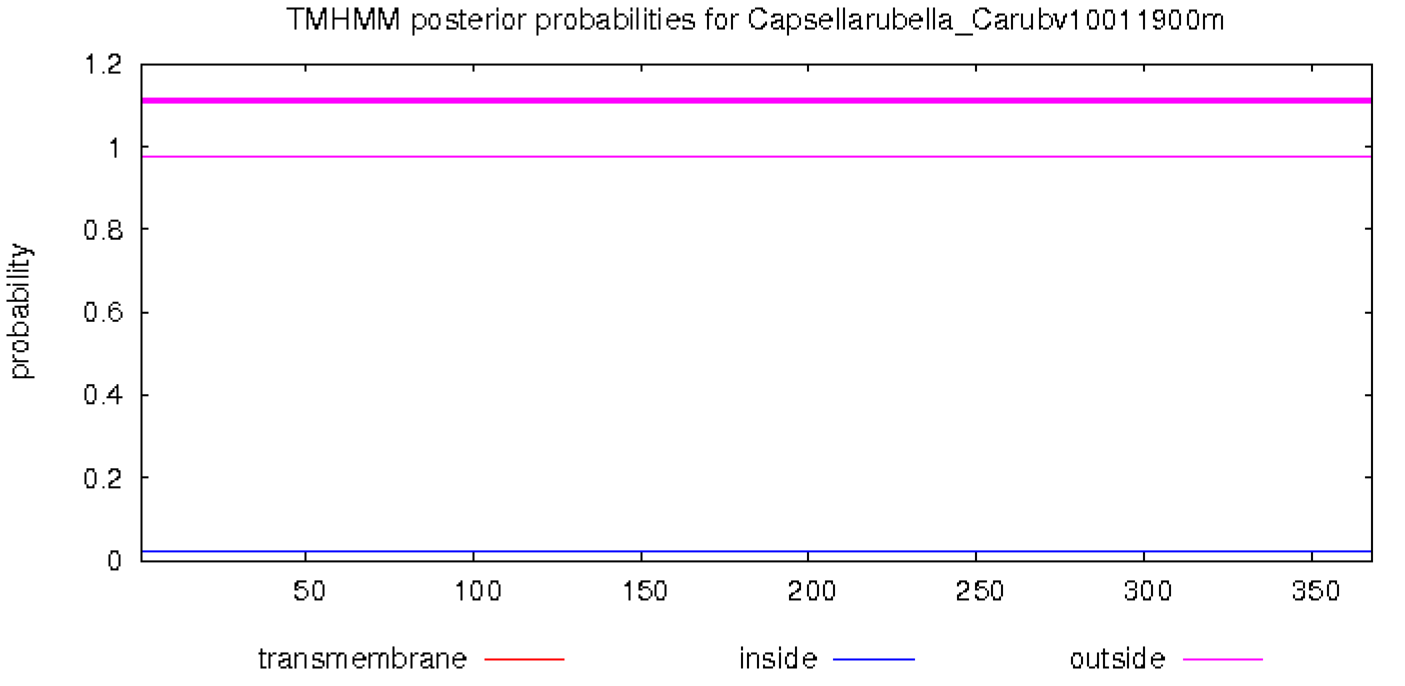

plot in postscript, script for making the plot in gnuplot, data for plot

```
# Capsellarubella_Carubv10011902m Length: 166
# Capsellarubella_Carubv10011902m Number of predicted TMHs:  0
# Capsellarubella_Carubv10011902m Exp number of AAs in TMHs: 0.00644
# Capsellarubella_Carubv10011902m Exp number, first 60 AAs:  1e-05
# Capsellarubella_Carubv10011902m Total prob of N-in:        0.27079
Capsellarubella_Carubv10011902m TMHMM2.0        outside      1   166
```

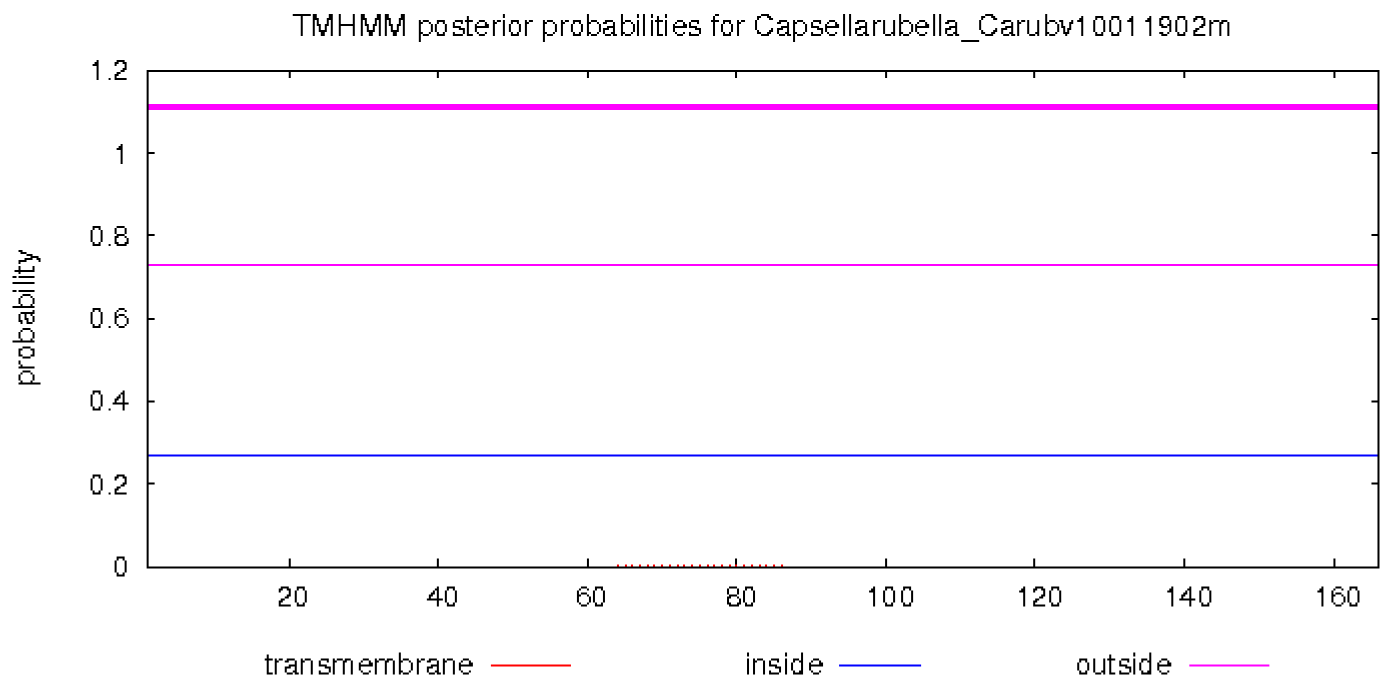

plot in postscript, script for making the plot in gnuplot, data for plot

```
# Capsellarubella_Carubv10012163m Length: 304
# Capsellarubella_Carubv10012163m Number of predicted TMHs:  0
# Capsellarubella_Carubv10012163m Exp number of AAs in TMHs: 0
# Capsellarubella_Carubv10012163m Exp number, first 60 AAs:  0
# Capsellarubella_Carubv10012163m Total prob of N-in:        0.00138
Capsellarubella_Carubv10012163m TMHMM2.0        outside      1   304
```

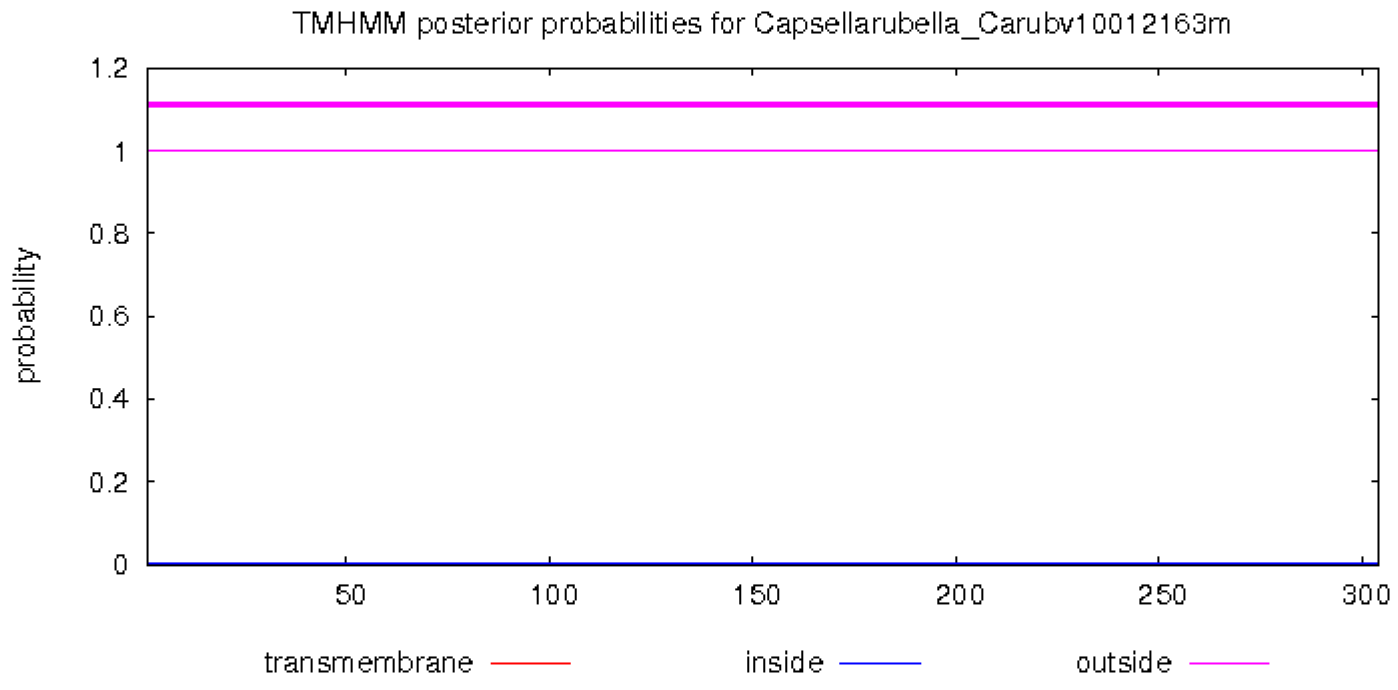

plot in postscript, script for making the plot in gnuplot, data for plot

```
# Capsellarubella_Carubv10012206m Length: 447
# Capsellarubella_Carubv10012206m Number of predicted TMHs:  0
# Capsellarubella_Carubv10012206m Exp number of AAs in TMHs: 0.00031
# Capsellarubella_Carubv10012206m Exp number, first 60 AAs:  0.00031
# Capsellarubella_Carubv10012206m Total prob of N-in:        0.00333
Capsellarubella_Carubv10012206m TMHMM2.0        outside      1   447
```

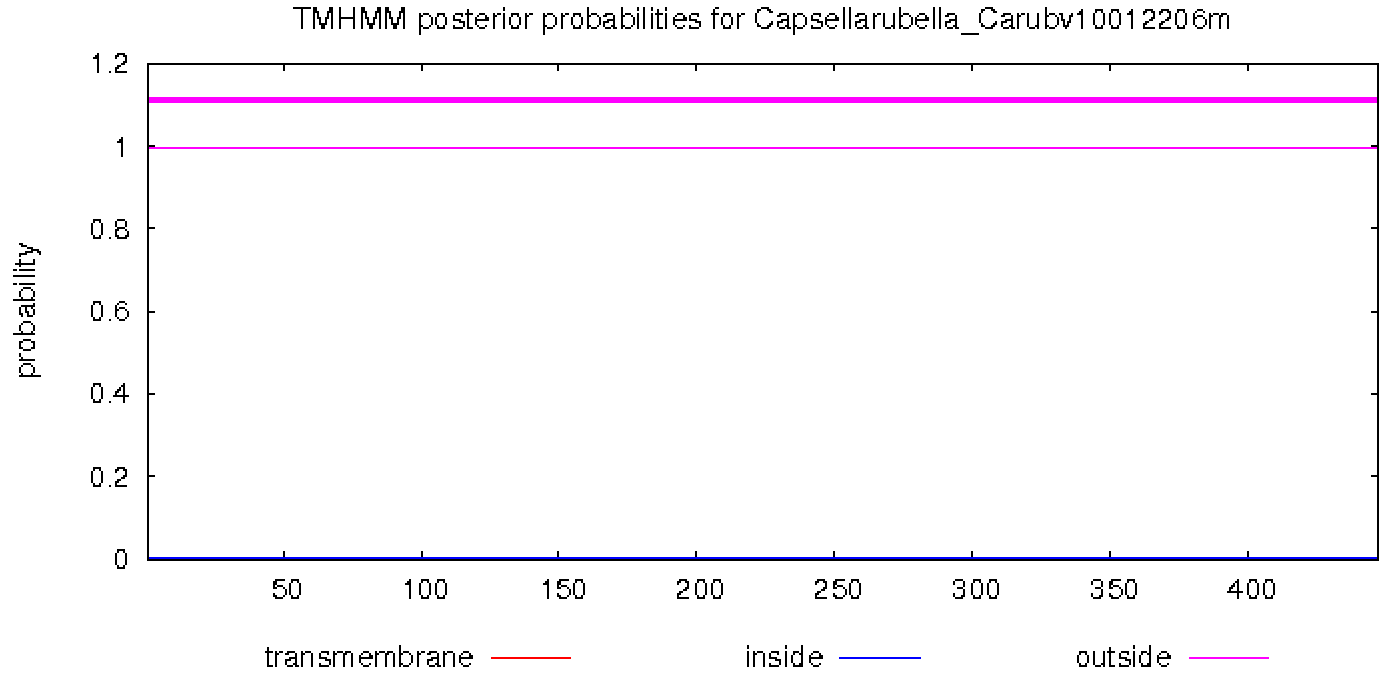

plot in postscript, script for making the plot in gnuplot, data for plot

```
# Capsellarubella_Carubv10013370m Length: 558
# Capsellarubella_Carubv10013370m Number of predicted TMHs:  1
# Capsellarubella_Carubv10013370m Exp number of AAs in TMHs: 22.01674
# Capsellarubella_Carubv10013370m Exp number, first 60 AAs:  0
# Capsellarubella_Carubv10013370m Total prob of N-in:        0.00136
Capsellarubella_Carubv10013370m TMHMM2.0        outside      1   533
Capsellarubella_Carubv10013370m TMHMM2.0        TMhelix    534   556
Capsellarubella_Carubv10013370m TMHMM2.0        inside     557   558
```

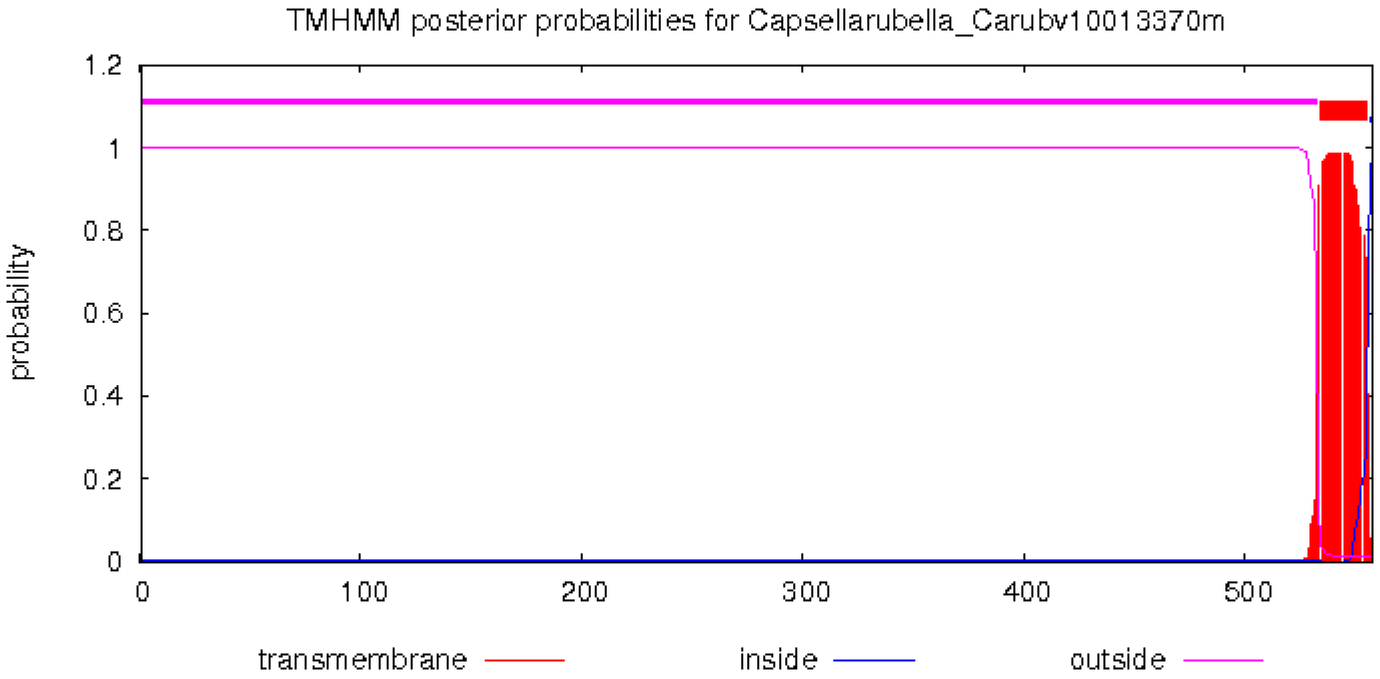

plot in postscript, script for making the plot in gnuplot, data for plot

---

```
# Capsellarubella_Carubv10013564m Length: 484
# Capsellarubella_Carubv10013564m Number of predicted TMHs:  0
# Capsellarubella_Carubv10013564m Exp number of AAs in TMHs: 0.07647
# Capsellarubella_Carubv10013564m Exp number, first 60 AAs:  0.04638
# Capsellarubella_Carubv10013564m Total prob of N-in:        0.00501
Capsellarubella_Carubv10013564m TMHMM2.0        outside      1   484
```

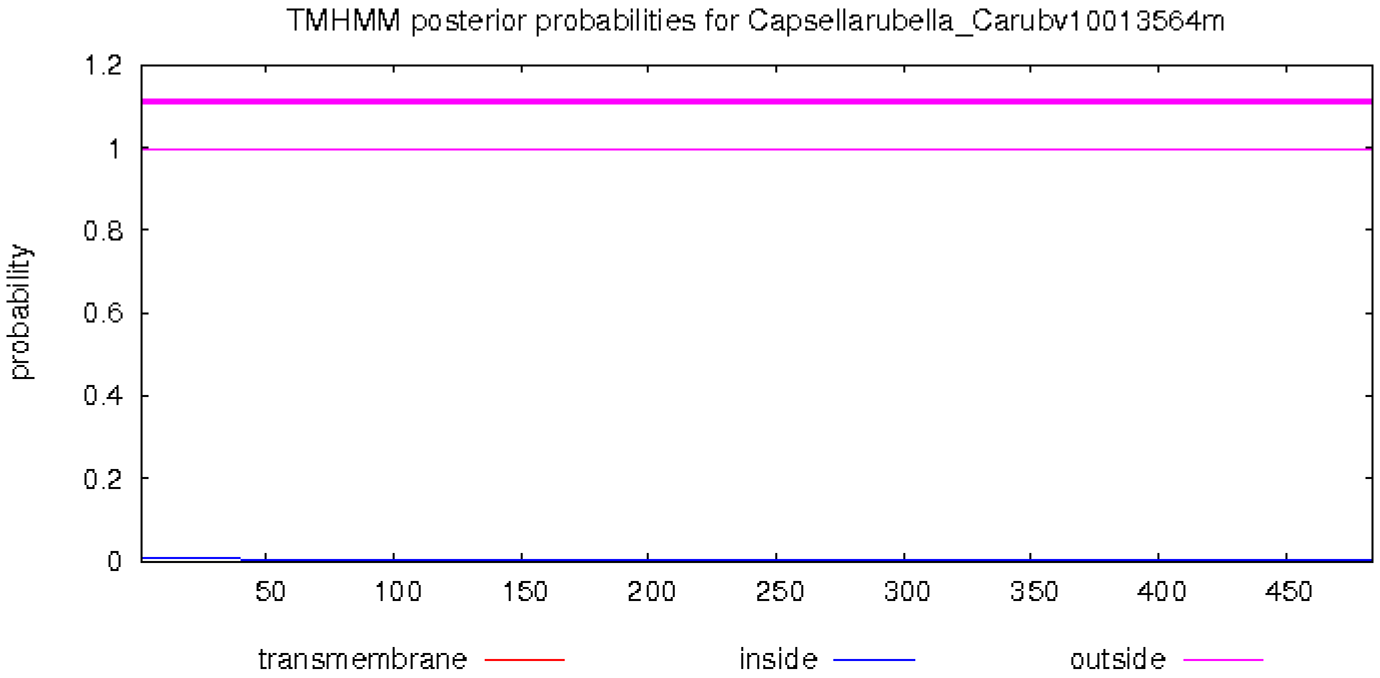

plot in postscript, script for making the plot in gnuplot, data for plot

---

```
# Capsellarubella_Carubv10013584m Length: 480
# Capsellarubella_Carubv10013584m Number of predicted TMHs:  0
# Capsellarubella_Carubv10013584m Exp number of AAs in TMHs: 0.00018
# Capsellarubella_Carubv10013584m Exp number, first 60 AAs:  0
# Capsellarubella_Carubv10013584m Total prob of N-in:        0.00016
Capsellarubella_Carubv10013584m TMHMM2.0        outside      1   480
```

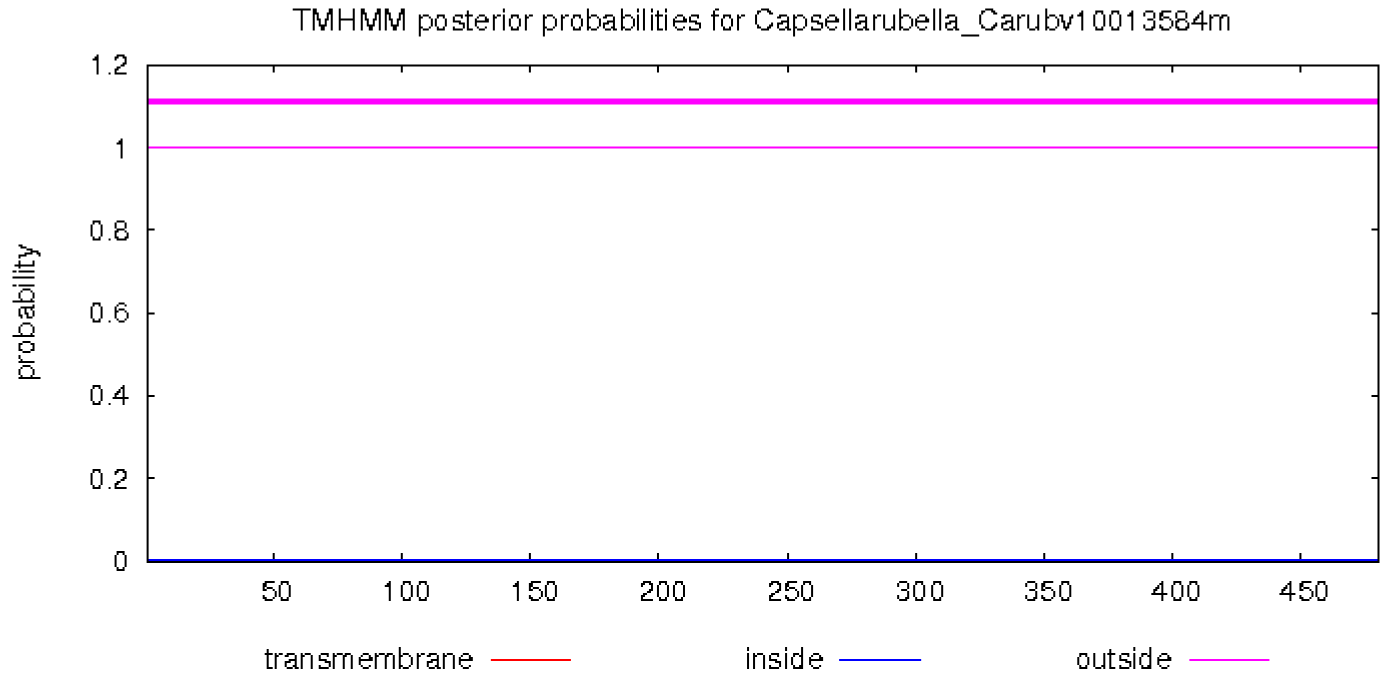

plot in postscript, script for making the plot in gnuplot, data for plot

---

```
# Capsellarubella_Carubv10013694m Length: 447
# Capsellarubella_Carubv10013694m Number of predicted TMHs:  0
# Capsellarubella_Carubv10013694m Exp number of AAs in TMHs: 0.06106
# Capsellarubella_Carubv10013694m Exp number, first 60 AAs:  0.0531
# Capsellarubella_Carubv10013694m Total prob of N-in:        0.00687
Capsellarubella_Carubv10013694m TMHMM2.0        outside      1   447
```

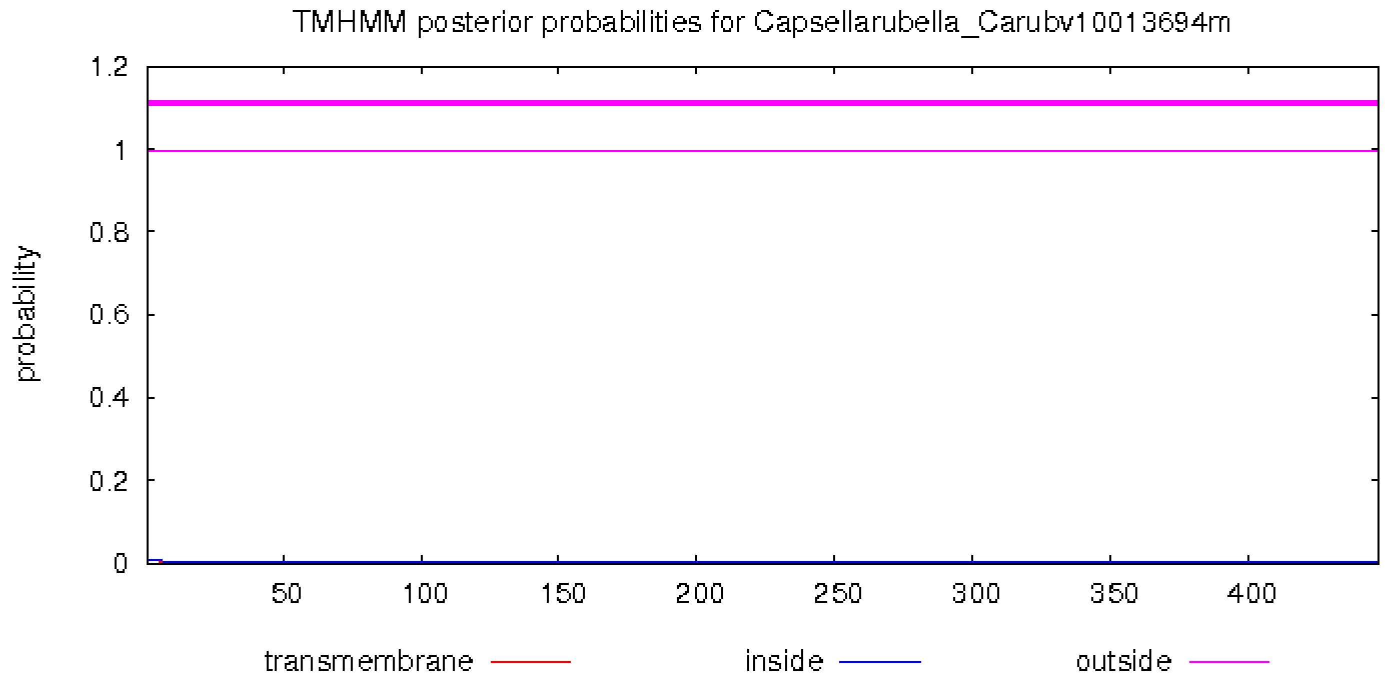

plot in postscript, script for making the plot in gnuplot, data for plot

---

```
# Capsellarubella_Carubv10014000m Length: 367
# Capsellarubella_Carubv10014000m Number of predicted TMHs:  0
# Capsellarubella_Carubv10014000m Exp number of AAs in TMHs: 0
# Capsellarubella_Carubv10014000m Exp number, first 60 AAs:  0
# Capsellarubella_Carubv10014000m Total prob of N-in:        0.01057
Capsellarubella_Carubv10014000m TMHMM2.0        outside      1   367
```

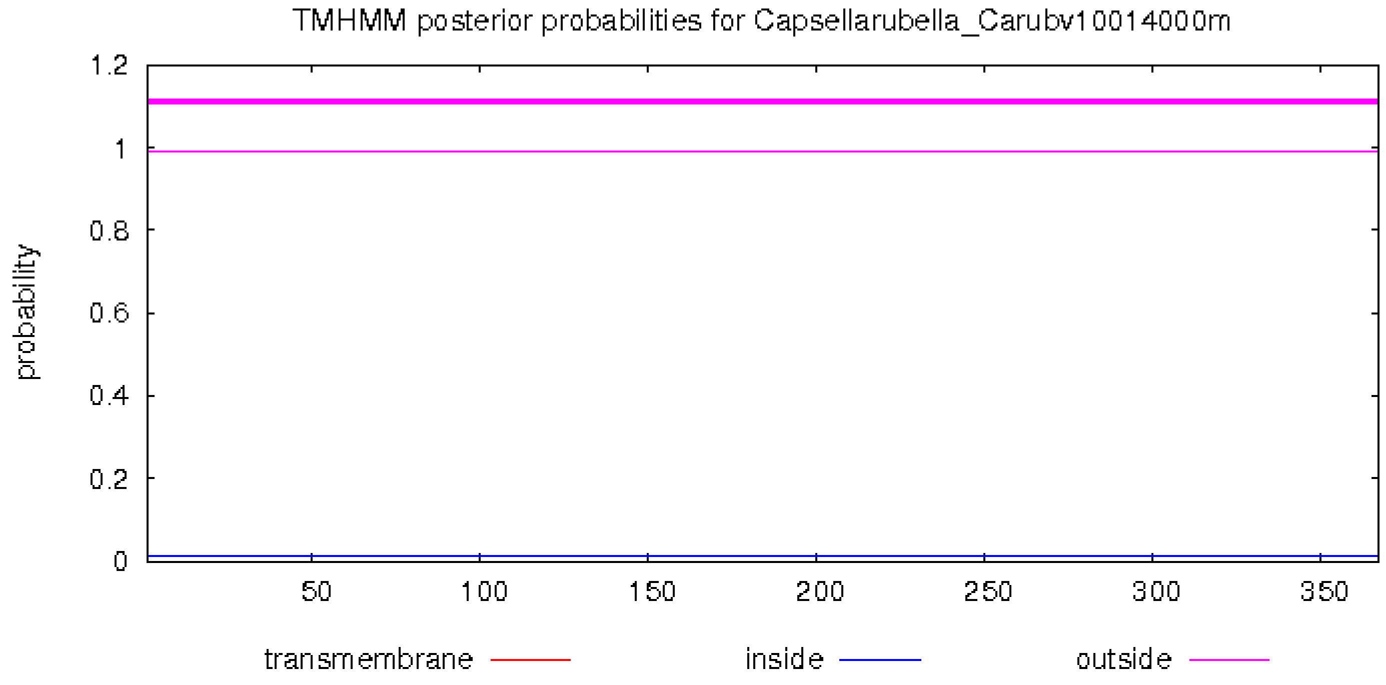

plot in postscript, script for making the plot in gnuplot, data for plot

---

```
# Capsellarubella_Carubv10014004m Length: 366
# Capsellarubella_Carubv10014004m Number of predicted TMHs:  0
# Capsellarubella_Carubv10014004m Exp number of AAs in TMHs: 0.00018
# Capsellarubella_Carubv10014004m Exp number, first 60 AAs:  0
# Capsellarubella_Carubv10014004m Total prob of N-in:        0.02583
Capsellarubella_Carubv10014004m TMHMM2.0        outside      1   366
```

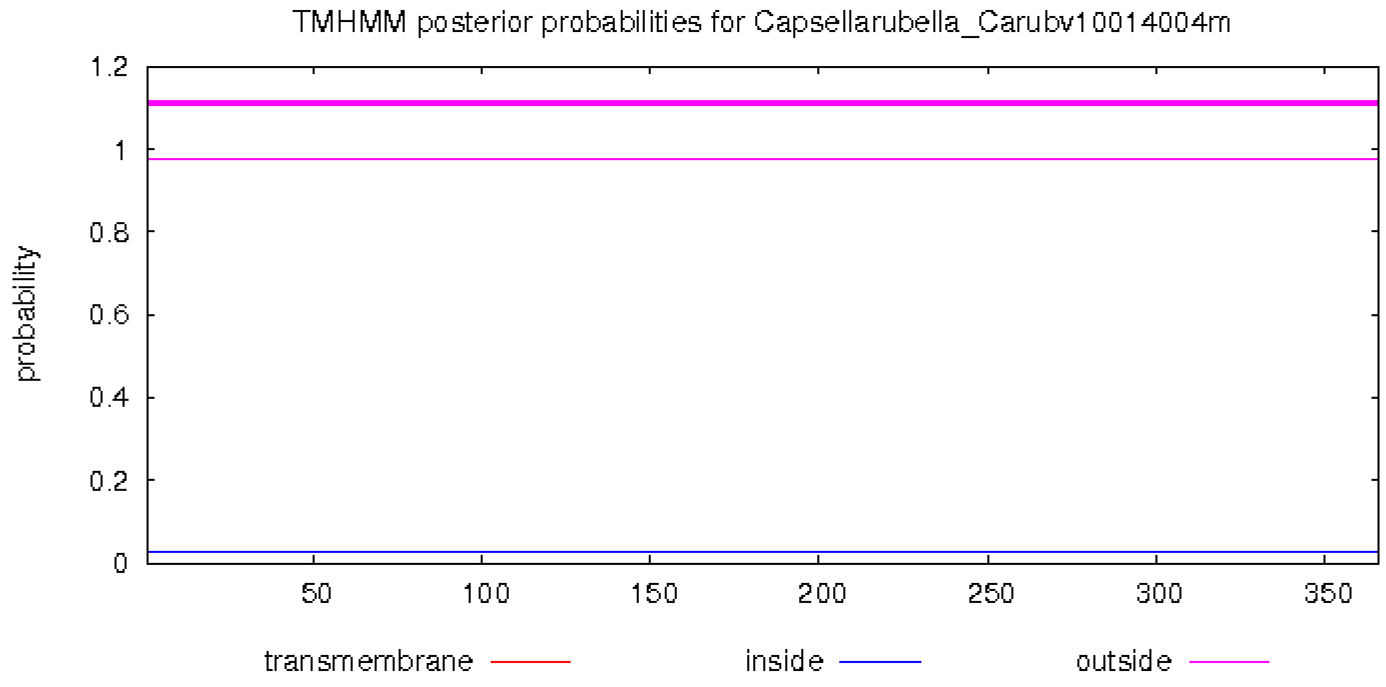

plot in postscript, script for making the plot in gnuplot, data for plot

---

```
# Capsellarubella_Carubv10014008m Length: 366
# Capsellarubella_Carubv10014008m Number of predicted TMHs:  0
# Capsellarubella_Carubv10014008m Exp number of AAs in TMHs: 0.00189
# Capsellarubella_Carubv10014008m Exp number, first 60 AAs:  0.00017
# Capsellarubella_Carubv10014008m Total prob of N-in:        0.00396
Capsellarubella_Carubv10014008m TMHMM2.0        outside      1   366
```

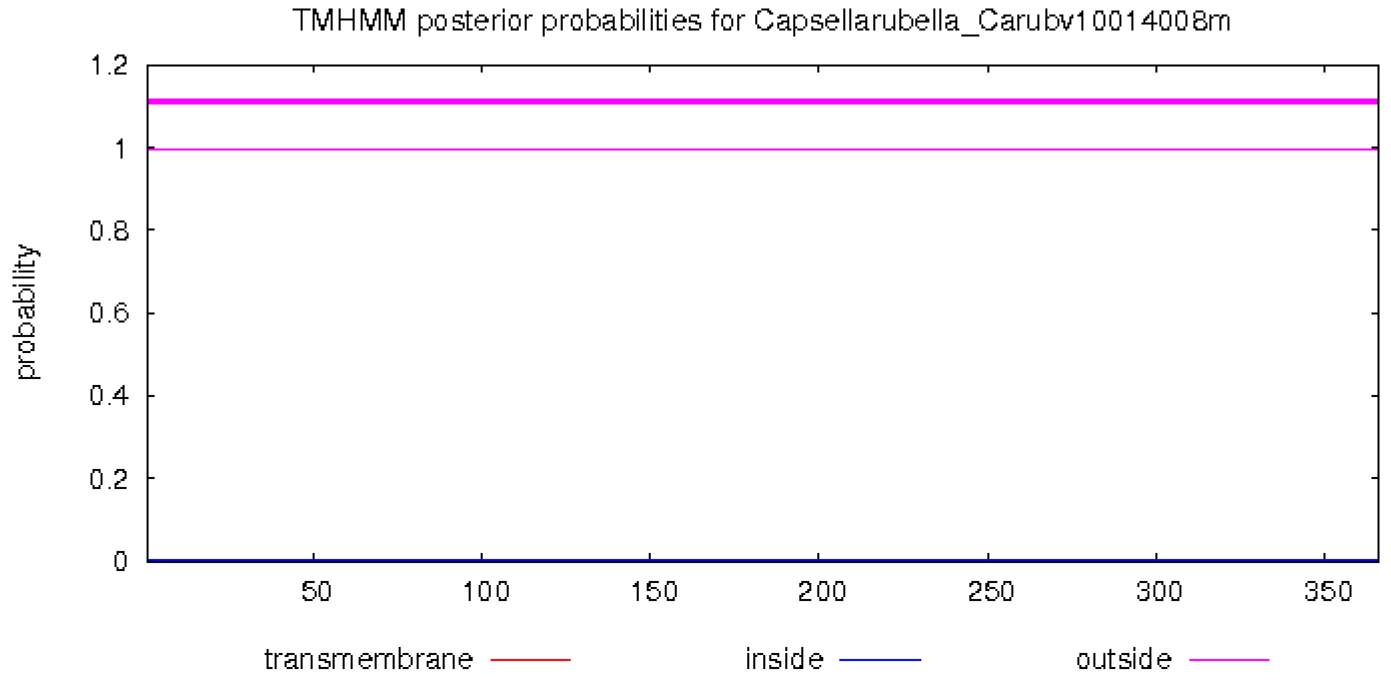

plot in postscript, script for making the plot in gnuplot, data for plot

---

```
# Capsellarubella_Carubv10014118m Length: 339
# Capsellarubella_Carubv10014118m Number of predicted TMHs:  0
# Capsellarubella_Carubv10014118m Exp number of AAs in TMHs: 0.000950000000000000001
# Capsellarubella_Carubv10014118m Exp number, first 60 AAs:  0.00017
# Capsellarubella_Carubv10014118m Total prob of N-in:        0.01066
Capsellarubella_Carubv10014118m TMHMM2.0        outside      1   339
```

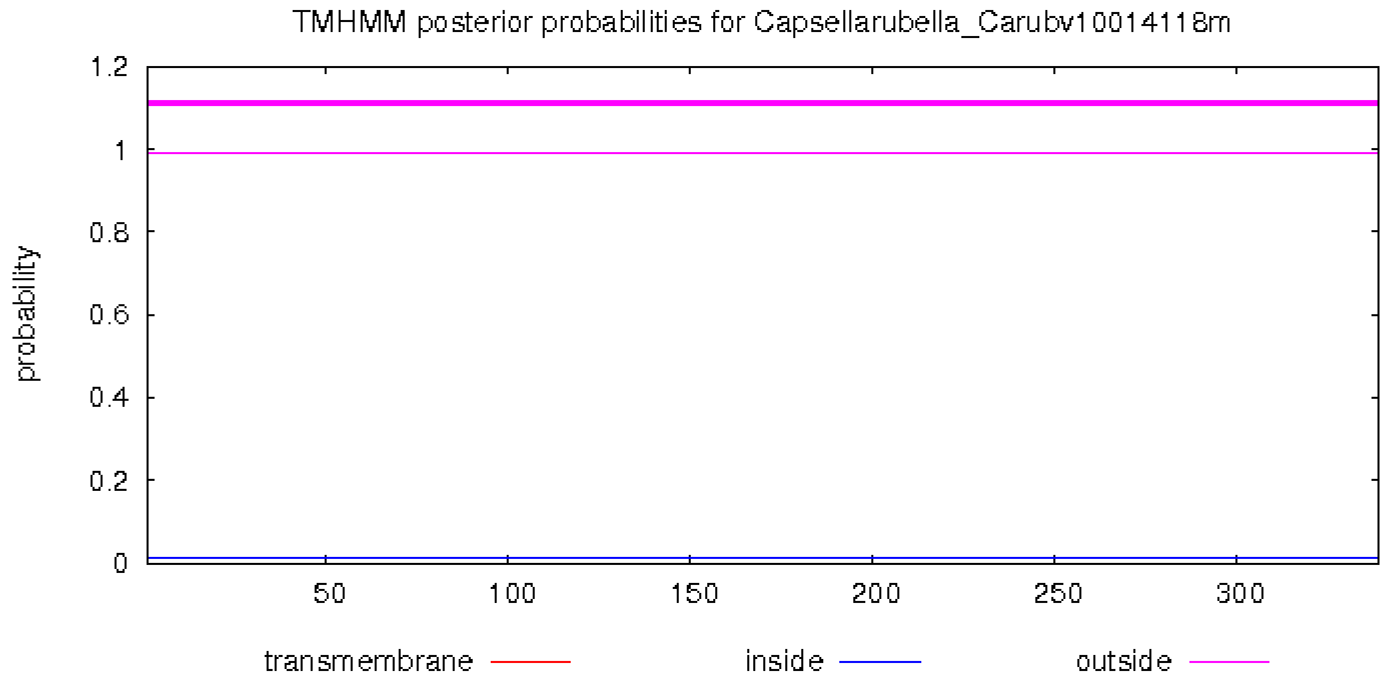

plot in postscript, script for making the plot in gnuplot, data for plot

---

```
# Capsellarubella_Carubv10014211m Length: 318
# Capsellarubella_Carubv10014211m Number of predicted TMHs:  0
# Capsellarubella_Carubv10014211m Exp number of AAs in TMHs: 0
# Capsellarubella_Carubv10014211m Exp number, first 60 AAs:  0
# Capsellarubella_Carubv10014211m Total prob of N-in:        0.00238
Capsellarubella_Carubv10014211m TMHMM2.0        outside      1   318
```

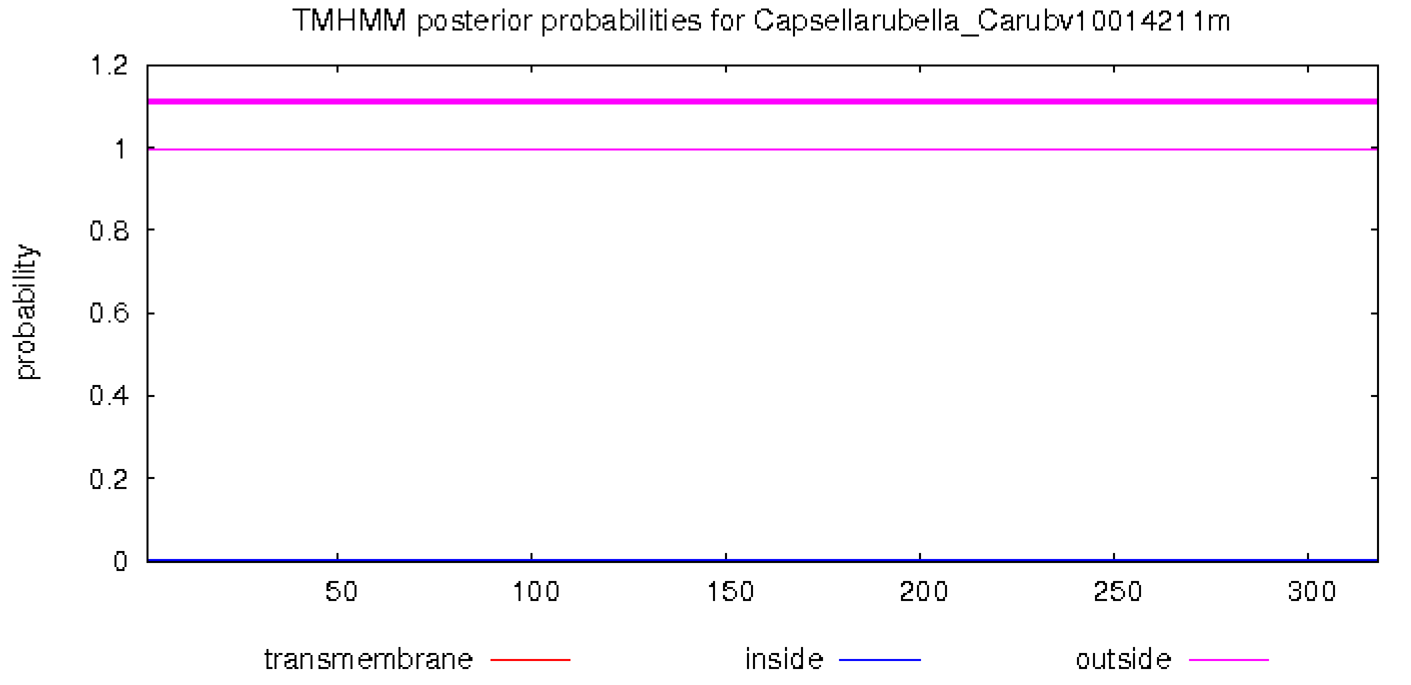

plot in postscript, script for making the plot in gnuplot, data for plot

---

```
# Capsellarubella_Carubv10014270m Length: 301
# Capsellarubella_Carubv10014270m Number of predicted TMHs:  0
# Capsellarubella_Carubv10014270m Exp number of AAs in TMHs: 0.00069
# Capsellarubella_Carubv10014270m Exp number, first 60 AAs:  0
# Capsellarubella_Carubv10014270m Total prob of N-in:        0.03862
Capsellarubella_Carubv10014270m TMHMM2.0        outside      1   301
```

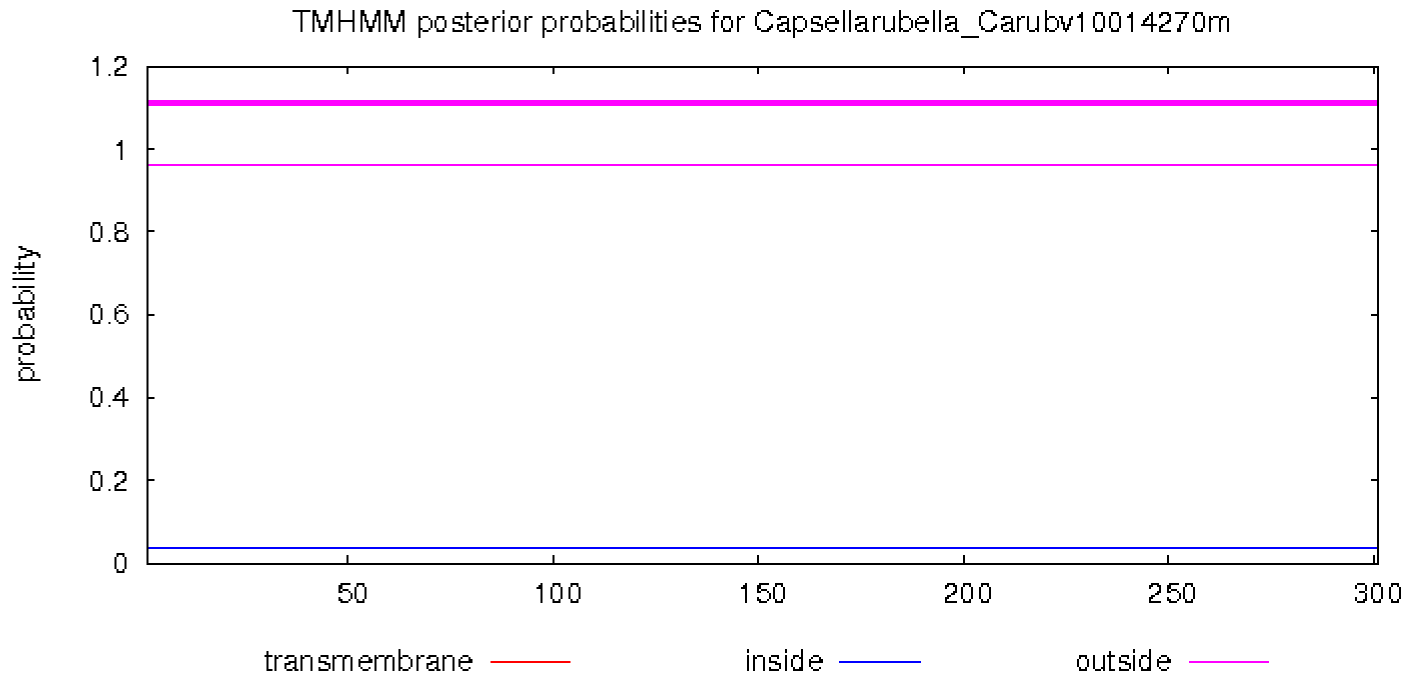

plot in postscript, script for making the plot in gnuplot, data for plot

---

```
# Capsellarubella_Carubv10014272m Length: 301
# Capsellarubella_Carubv10014272m Number of predicted TMHs:  0
# Capsellarubella_Carubv10014272m Exp number of AAs in TMHs: 0.00088
# Capsellarubella_Carubv10014272m Exp number, first 60 AAs:  0
# Capsellarubella_Carubv10014272m Total prob of N-in:        0.03862
Capsellarubella_Carubv10014272m TMHMM2.0        outside      1   301
```

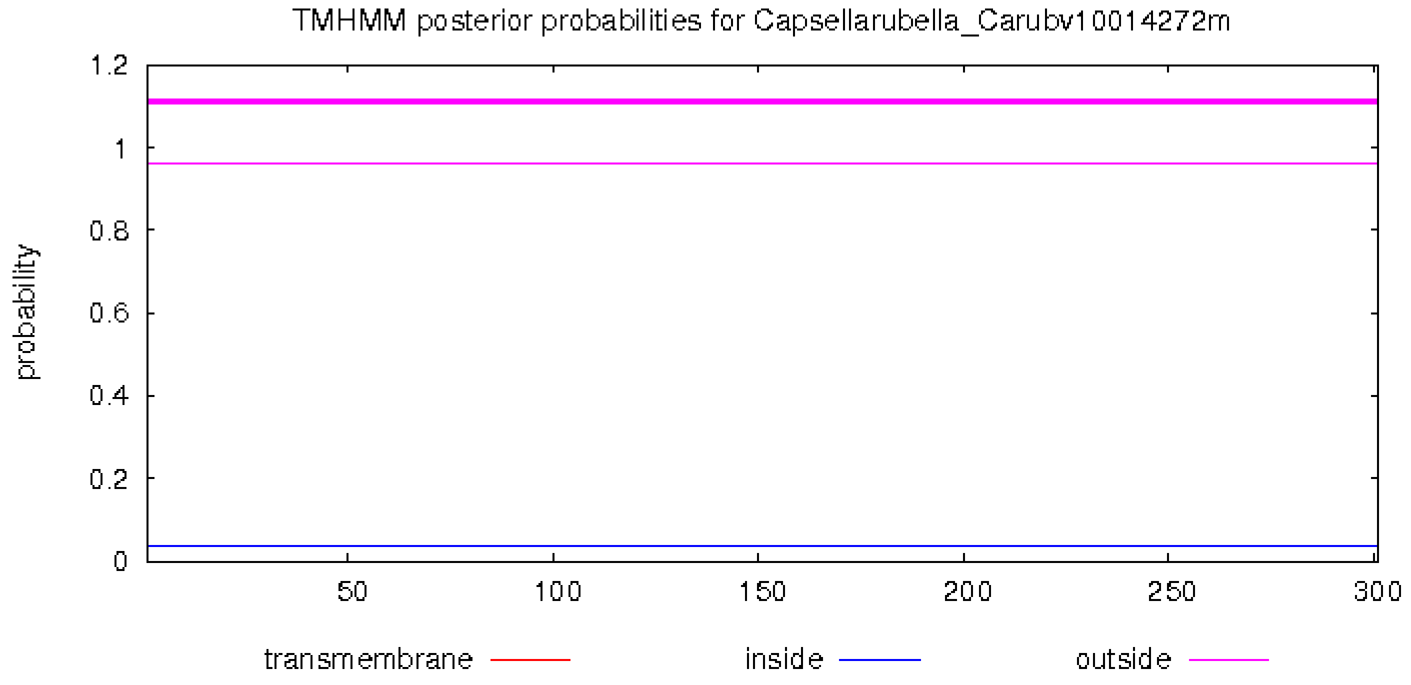

plot in postscript, script for making the plot in gnuplot, data for plot

---

```
# Capsellarubella_Carubv10014276m Length: 300
# Capsellarubella_Carubv10014276m Number of predicted TMHs:  0
# Capsellarubella_Carubv10014276m Exp number of AAs in TMHs: 0.02066
# Capsellarubella_Carubv10014276m Exp number, first 60 AAs:  0.00018
# Capsellarubella_Carubv10014276m Total prob of N-in:        0.01433
Capsellarubella_Carubv10014276m TMHMM2.0        outside      1   300
```

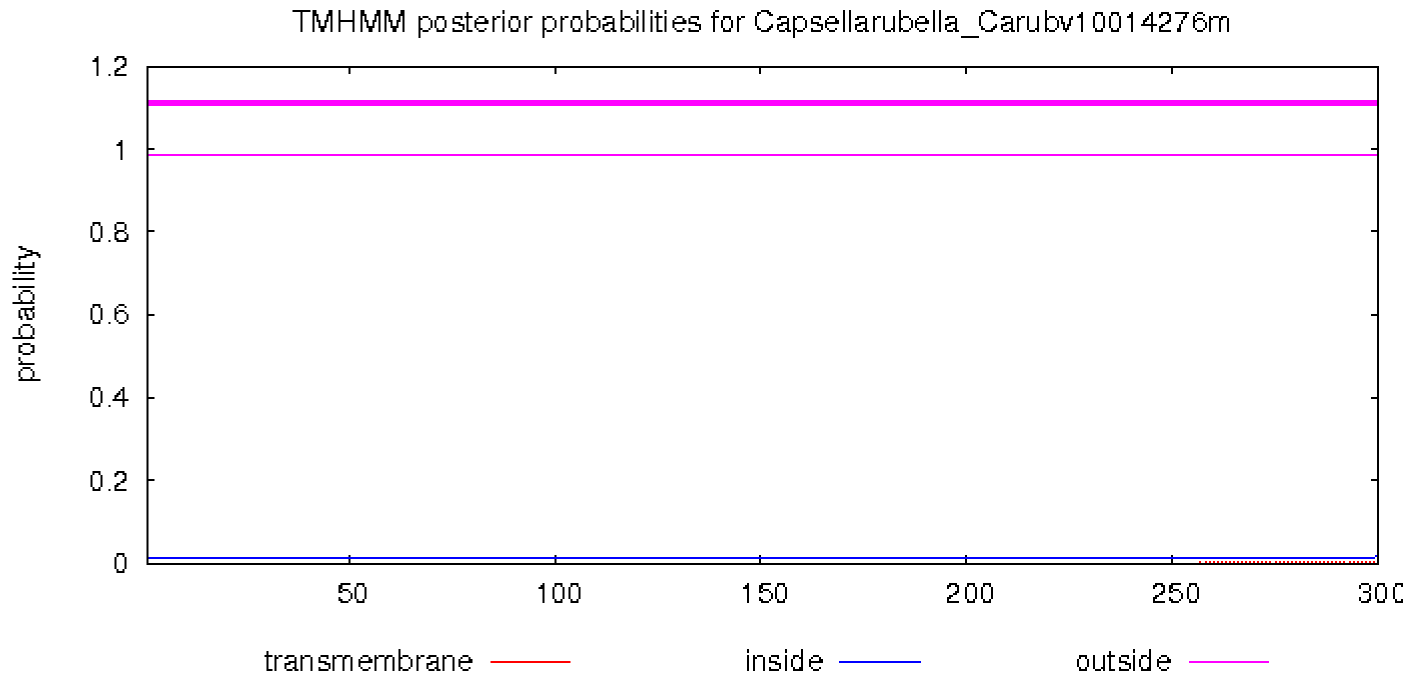

plot in postscript, script for making the plot in gnuplot, data for plot

---

```
# Capsellarubella_Carubv10014350m Length: 280
# Capsellarubella_Carubv10014350m Number of predicted TMHs:  0
# Capsellarubella_Carubv10014350m Exp number of AAs in TMHs: 0.00119
# Capsellarubella_Carubv10014350m Exp number, first 60 AAs:  0
# Capsellarubella_Carubv10014350m Total prob of N-in:        0.00648
Capsellarubella_Carubv10014350m TMHMM2.0        outside      1   280
```

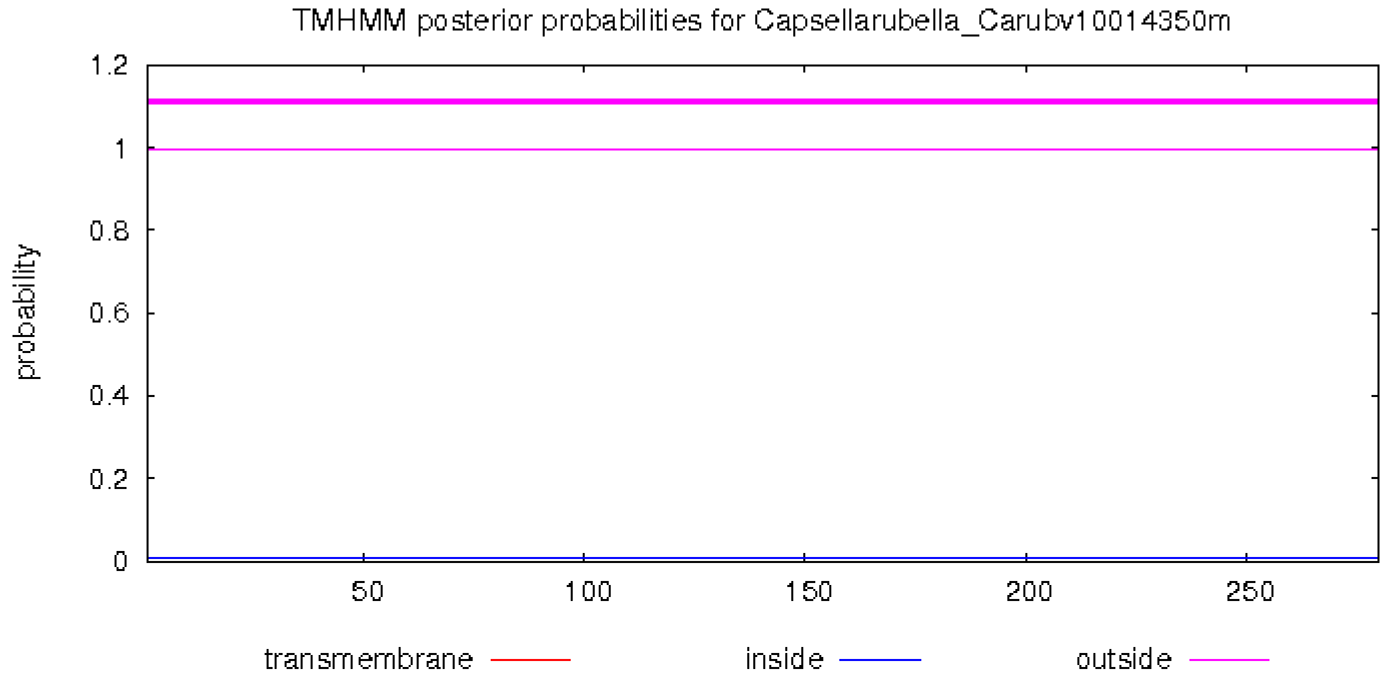

plot in postscript, script for making the plot in gnuplot, data for plot

```
# Capsellarubella_Carubv10014426m Length: 261
# Capsellarubella_Carubv10014426m Number of predicted TMHs:  0
# Capsellarubella_Carubv10014426m Exp number of AAs in TMHs: 0.70855
# Capsellarubella_Carubv10014426m Exp number, first 60 AAs:  0.70855
# Capsellarubella_Carubv10014426m Total prob of N-in:        0.10363
Capsellarubella_Carubv10014426m TMHMM2.0        outside      1   261
```

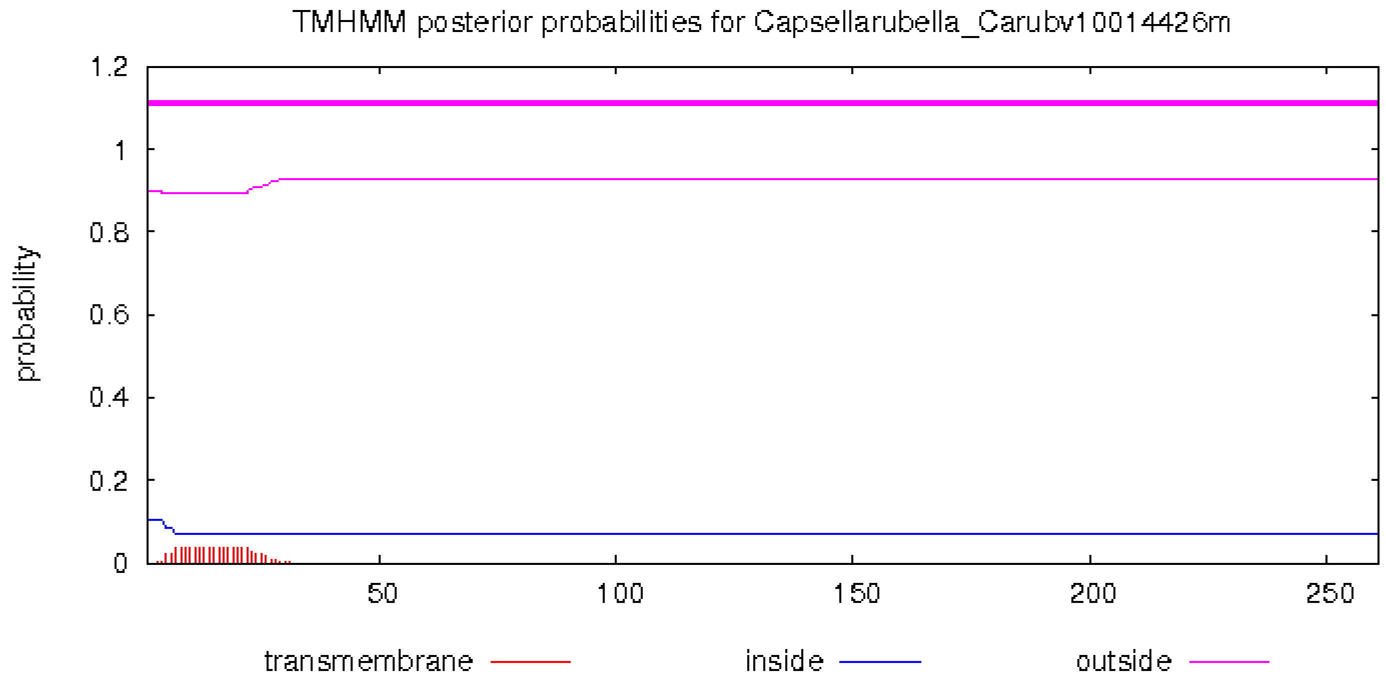

plot in postscript, script for making the plot in gnuplot, data for plot

```
# Capsellarubella_Carubv10015289m Length: 388
# Capsellarubella_Carubv10015289m Number of predicted TMHs:  0
# Capsellarubella_Carubv10015289m Exp number of AAs in TMHs: 0.00423
# Capsellarubella_Carubv10015289m Exp number, first 60 AAs:  0
# Capsellarubella_Carubv10015289m Total prob of N-in:        0.05662
Capsellarubella_Carubv10015289m TMHMM2.0        outside      1   388
```

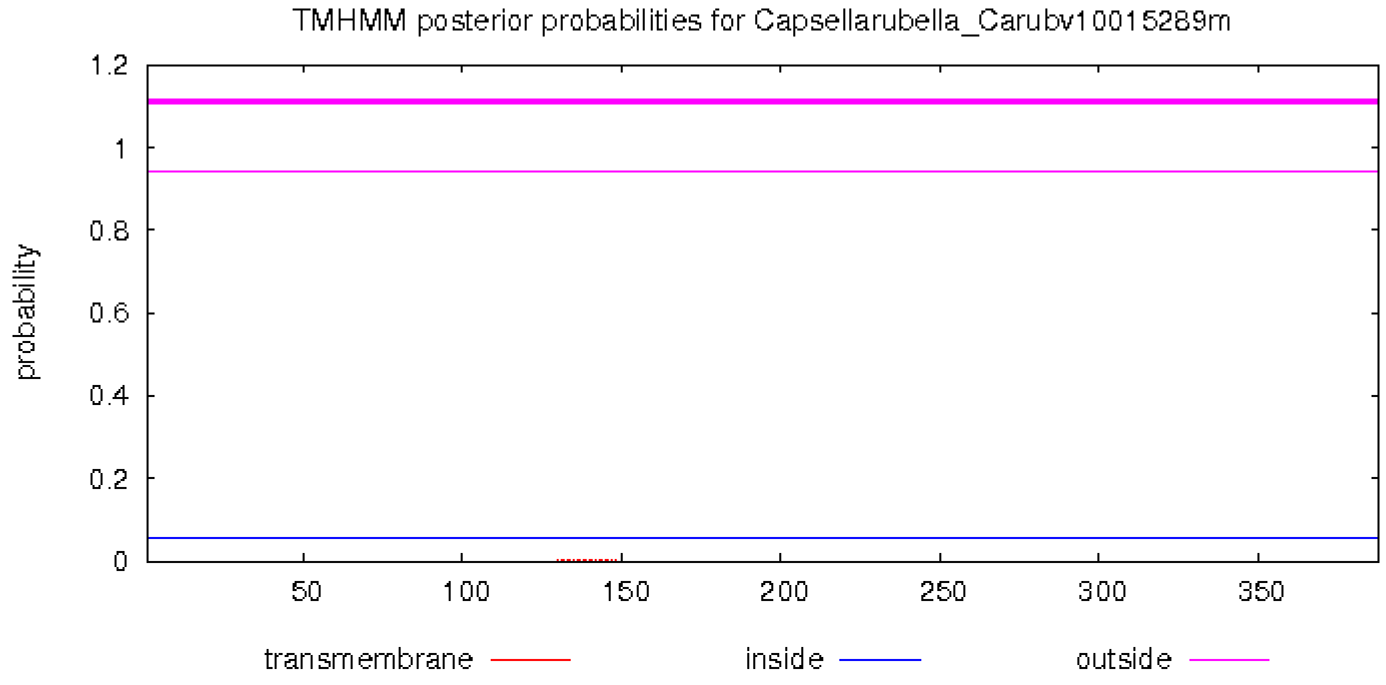

plot in postscript, script for making the plot in gnuplot, data for plot

---

```
# Capsellarubella_Carubv10015990m Length: 311
# Capsellarubella_Carubv10015990m Number of predicted TMHs:  0
# Capsellarubella_Carubv10015990m Exp number of AAs in TMHs: 0.04077
# Capsellarubella_Carubv10015990m Exp number, first 60 AAs:  0
# Capsellarubella_Carubv10015990m Total prob of N-in:        0.00405
Capsellarubella_Carubv10015990m TMHMM2.0        outside      1   311
```

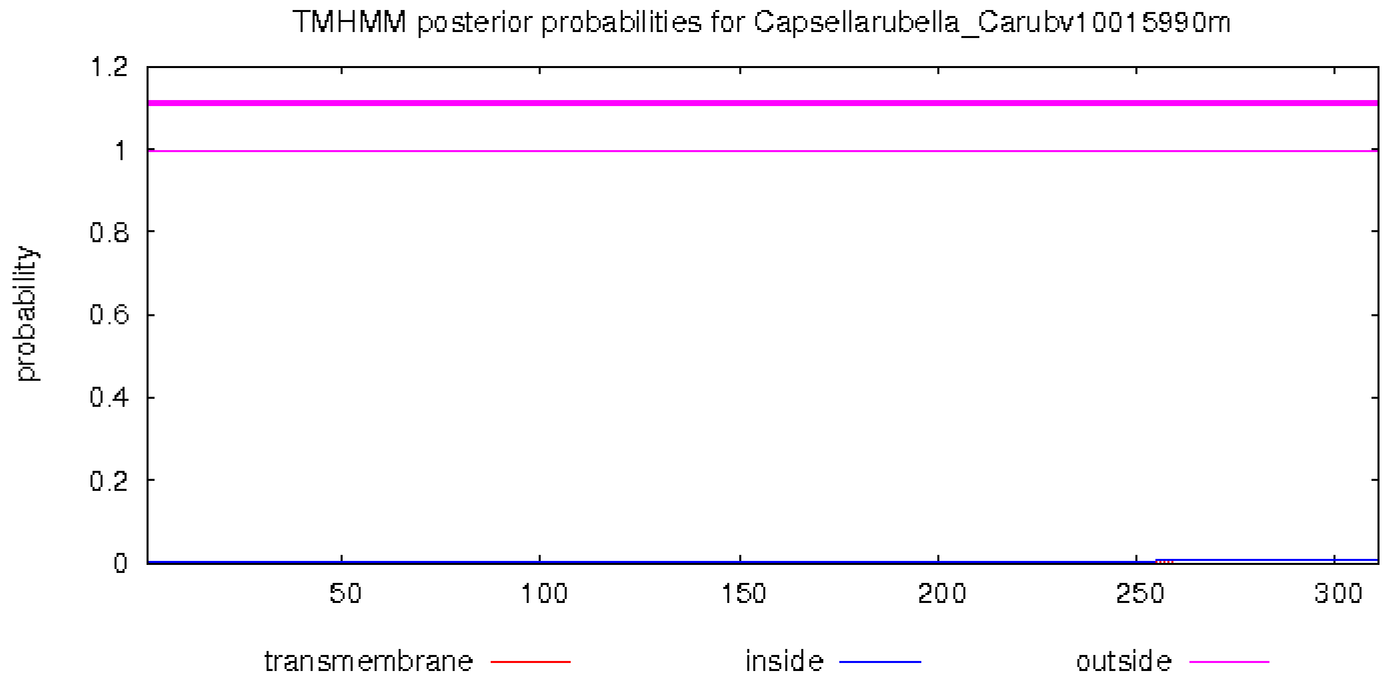

plot in postscript, script for making the plot in gnuplot, data for plot

---

```
# Capsellarubella_Carubv10017199m Length: 460
# Capsellarubella_Carubv10017199m Number of predicted TMHs:  0
# Capsellarubella_Carubv10017199m Exp number of AAs in TMHs: 15.34508
# Capsellarubella_Carubv10017199m Exp number, first 60 AAs:  0
# Capsellarubella_Carubv10017199m Total prob of N-in:        0.09669
Capsellarubella_Carubv10017199m TMHMM2.0        outside      1   460
```

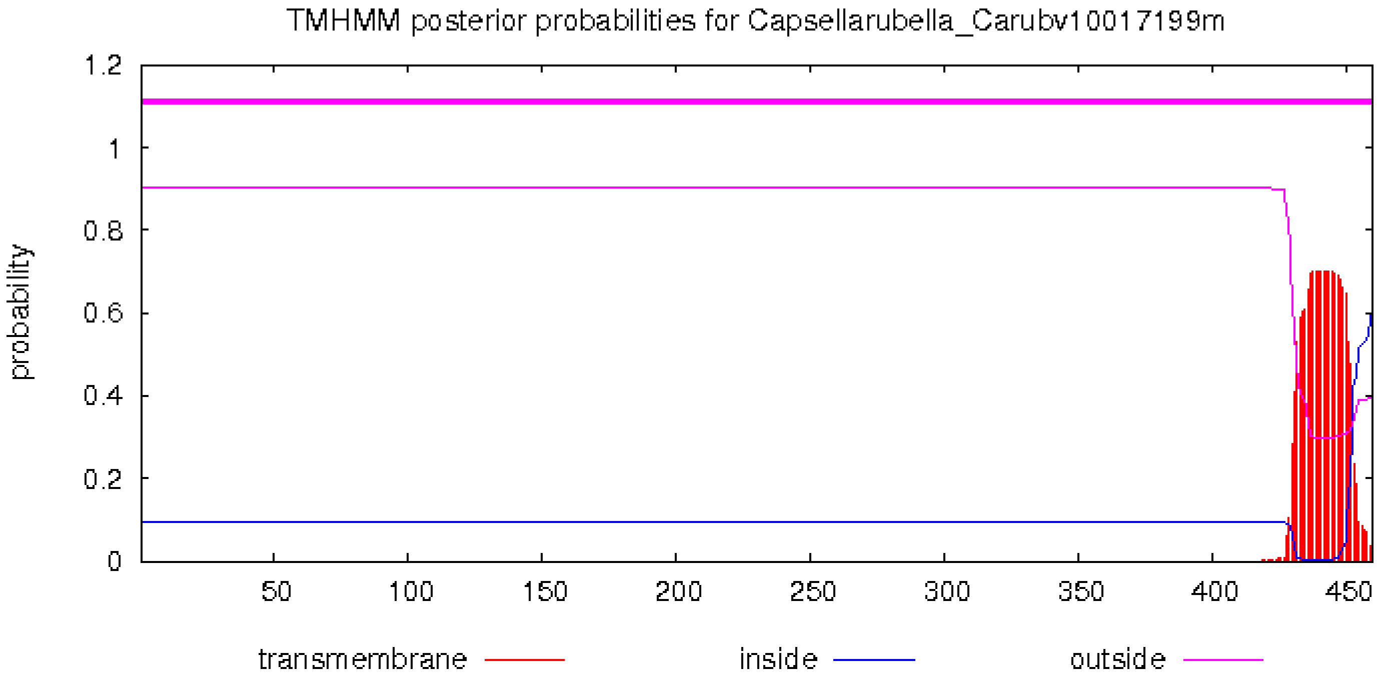

plot in postscript, script for making the plot in gnuplot, data for plot

---

```
# Capsellarubella_Carubv10017334m Length: 418
# Capsellarubella_Carubv10017334m Number of predicted TMHs:  0
# Capsellarubella_Carubv10017334m Exp number of AAs in TMHs: 0.0885
# Capsellarubella_Carubv10017334m Exp number, first 60 AAs:  0
# Capsellarubella_Carubv10017334m Total prob of N-in:        0.01121
Capsellarubella_Carubv10017334m TMHMM2.0        outside      1   418
```

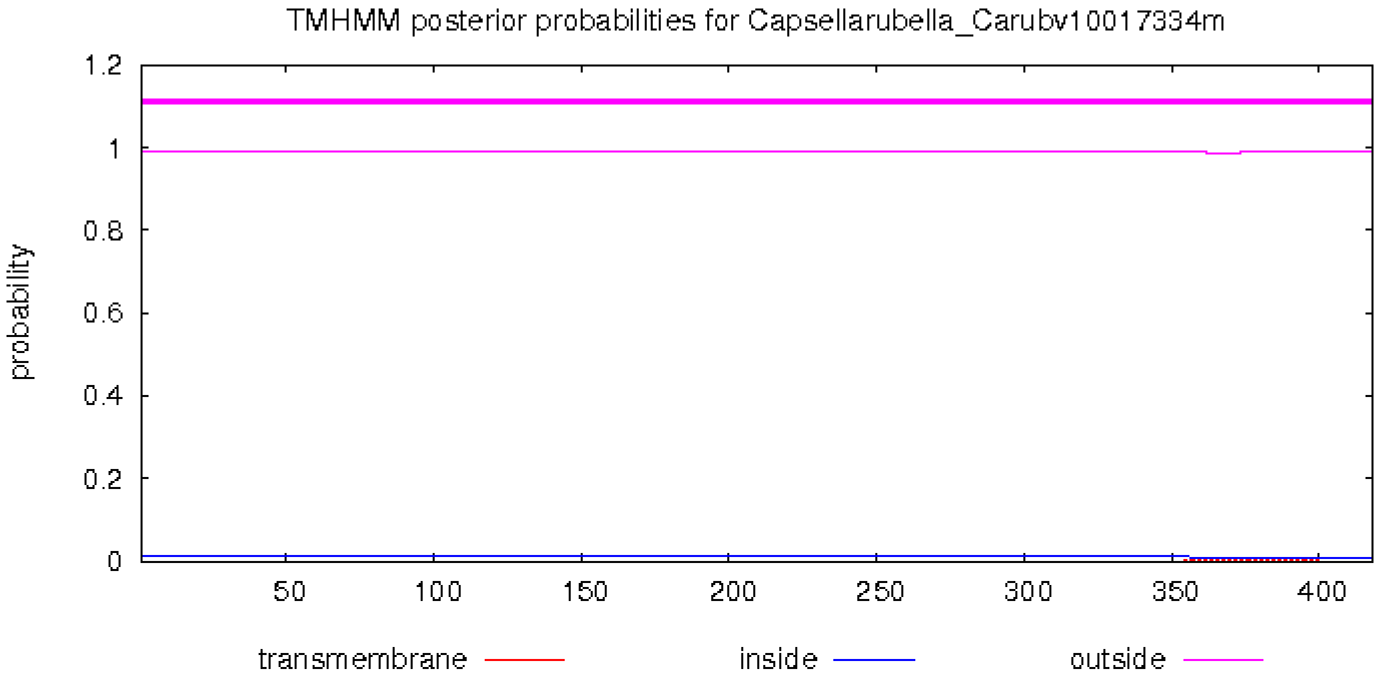

plot in postscript, script for making the plot in gnuplot, data for plot

---

```
# Capsellarubella_Carubv10017737m Length: 296
# Capsellarubella_Carubv10017737m Number of predicted TMHs:  0
# Capsellarubella_Carubv10017737m Exp number of AAs in TMHs: 0.00684
# Capsellarubella_Carubv10017737m Exp number, first 60 AAs:  0.00039
# Capsellarubella_Carubv10017737m Total prob of N-in:        0.05429
Capsellarubella_Carubv10017737m TMHMM2.0        outside      1   296
```

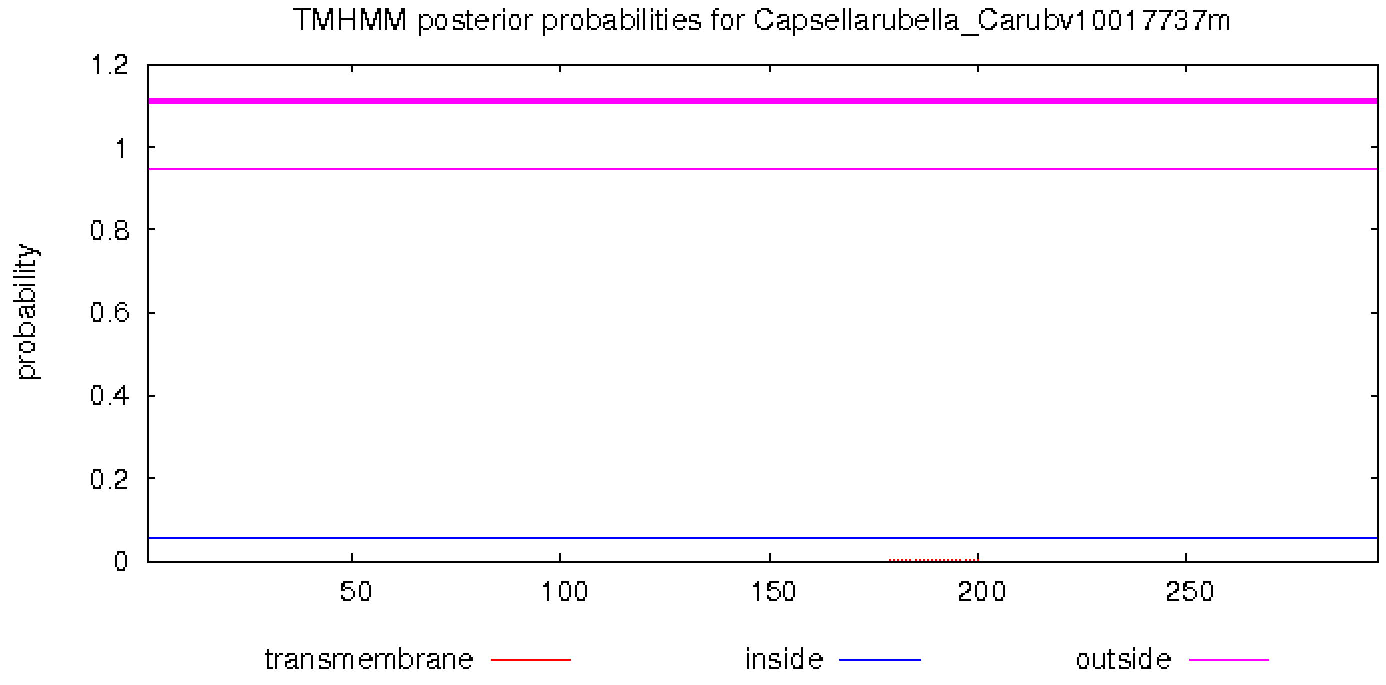

plot in postscript, script for making the plot in gnuplot, data for plot

---

```
# Capsellarubella_Carubv10017870m Length: 258
# Capsellarubella_Carubv10017870m Number of predicted TMHs:  0
# Capsellarubella_Carubv10017870m Exp number of AAs in TMHs: 0.17073
# Capsellarubella_Carubv10017870m Exp number, first 60 AAs:  0.16976
# Capsellarubella_Carubv10017870m Total prob of N-in:        0.12697
Capsellarubella_Carubv10017870m TMHMM2.0        outside      1   258
```

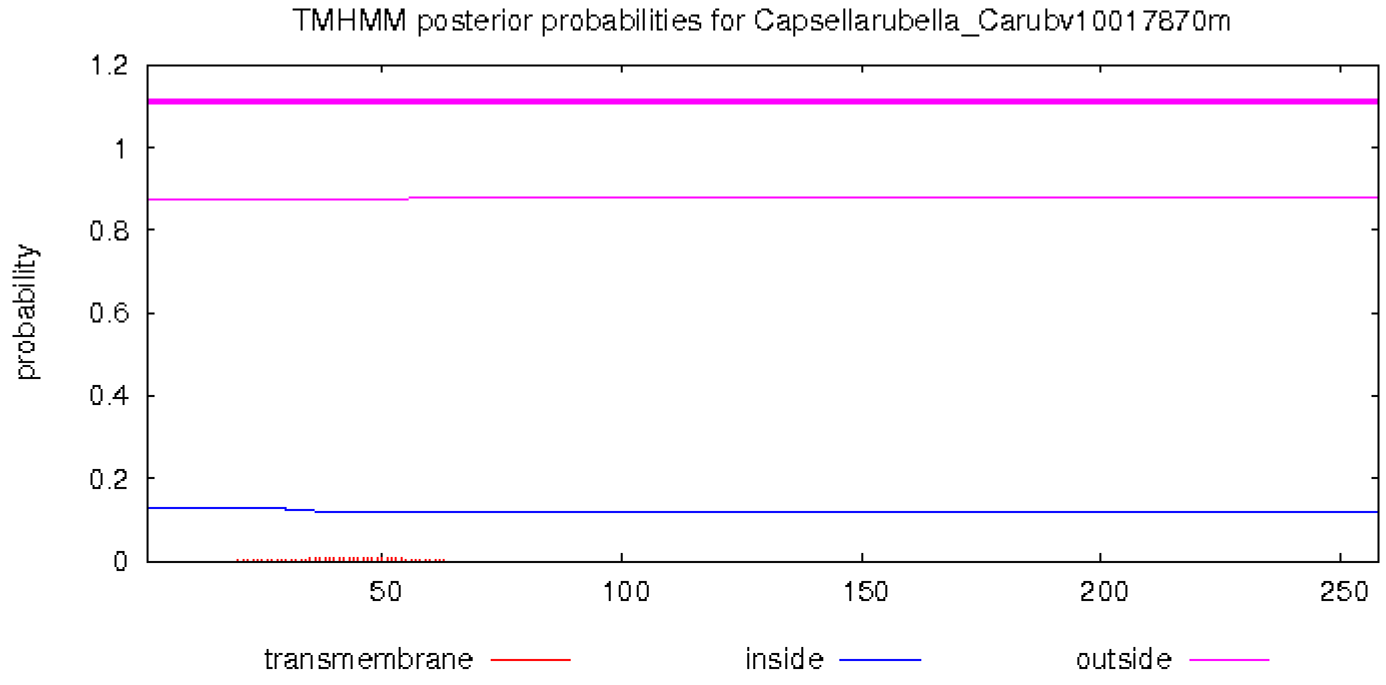

plot in postscript, script for making the plot in gnuplot, data for plot

---

```
# Capsellarubella_Carubv10017972m Length: 228
# Capsellarubella_Carubv10017972m Number of predicted TMHs:  0
# Capsellarubella_Carubv10017972m Exp number of AAs in TMHs: 0.01355
# Capsellarubella_Carubv10017972m Exp number, first 60 AAs:  0
# Capsellarubella_Carubv10017972m Total prob of N-in:        0.11627
Capsellarubella_Carubv10017972m TMHMM2.0        outside      1   228
```

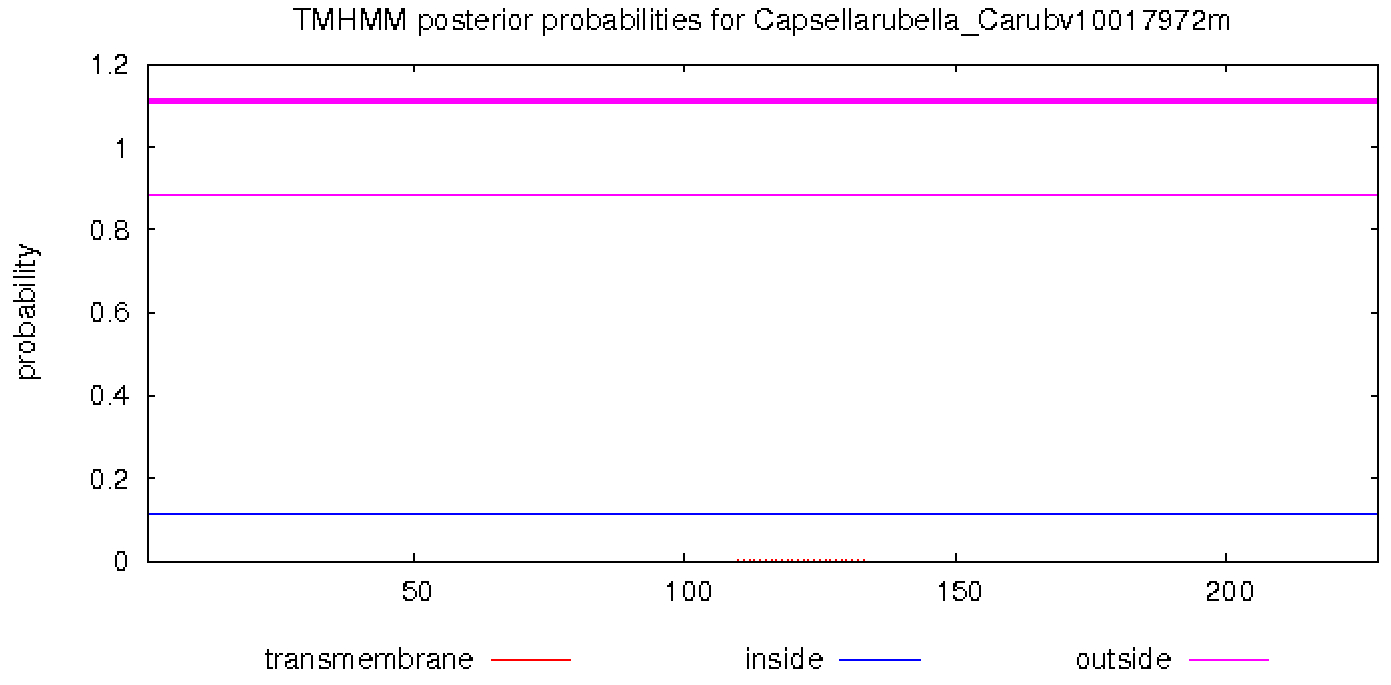

plot in postscript, script for making the plot in gnuplot, data for plot

```
# Capsellarubella_Carubv10018723m Length: 156
# Capsellarubella_Carubv10018723m Number of predicted TMHs:  0
# Capsellarubella_Carubv10018723m Exp number of AAs in TMHs: 0.00174
# Capsellarubella_Carubv10018723m Exp number, first 60 AAs:  0
# Capsellarubella_Carubv10018723m Total prob of N-in:        0.24886
Capsellarubella_Carubv10018723m TMHMM2.0        outside      1   156
```

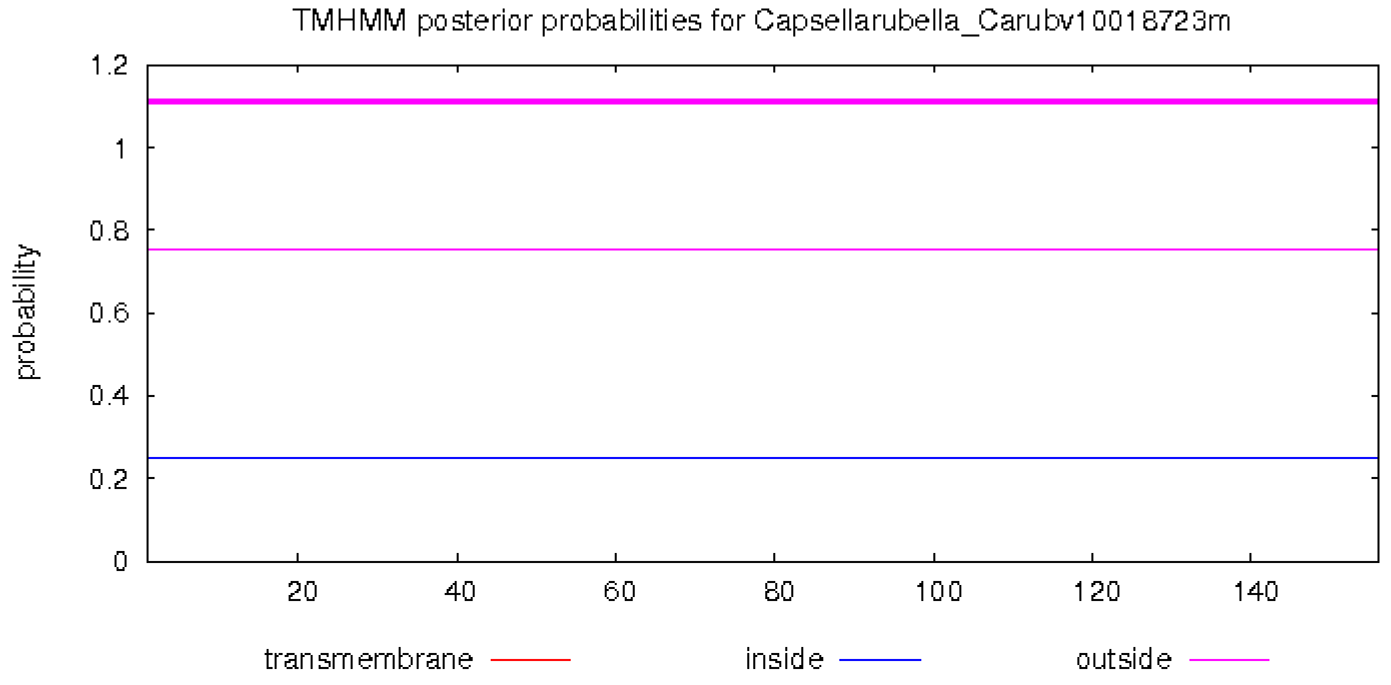

plot in postscript, script for making the plot in gnuplot, data for plot

```
# Capsellarubella_Carubv10019084m Length: 156
# Capsellarubella_Carubv10019084m Number of predicted TMHs:  0
# Capsellarubella_Carubv10019084m Exp number of AAs in TMHs: 0.00082
# Capsellarubella_Carubv10019084m Exp number, first 60 AAs:  0
# Capsellarubella_Carubv10019084m Total prob of N-in:        0.28231
Capsellarubella_Carubv10019084m TMHMM2.0        outside      1   156
```

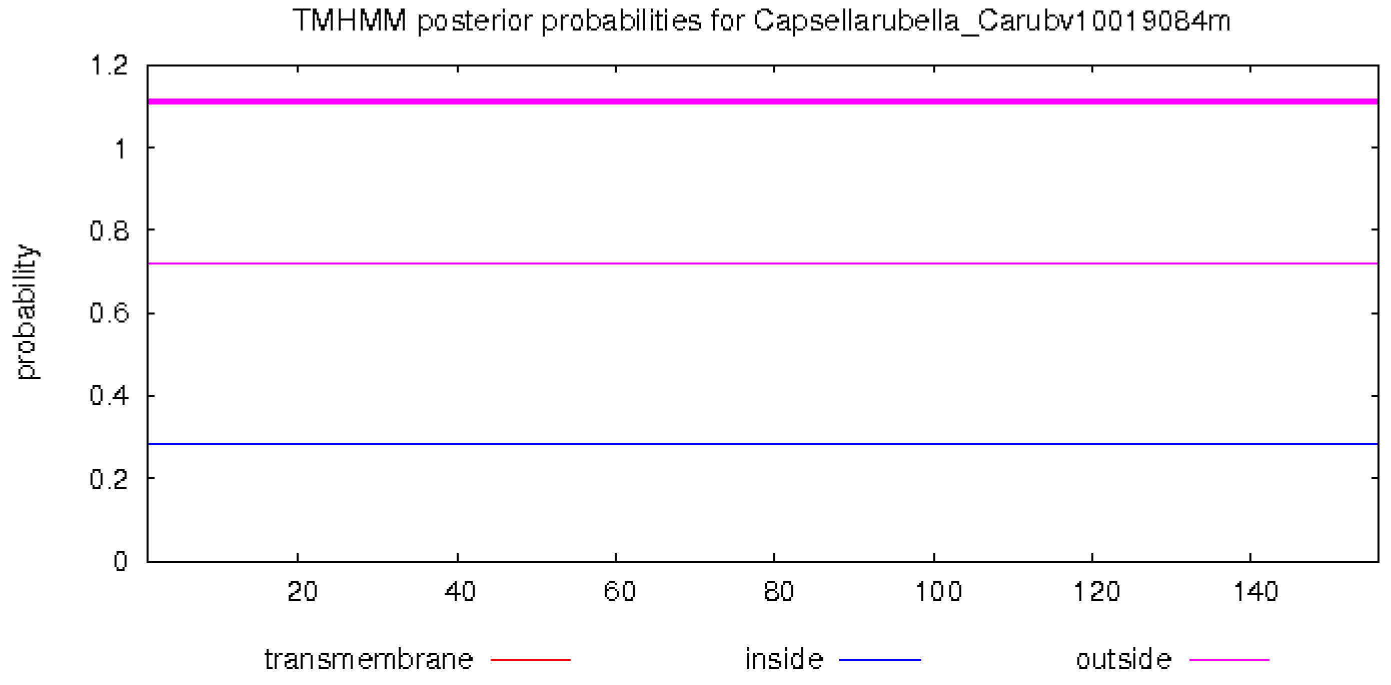

plot in postscript, script for making the plot in gnuplot, data for plot

---

```
# Capsellarubella_Carubv10019187m Length: 334
# Capsellarubella_Carubv10019187m Number of predicted TMHs:  1
# Capsellarubella_Carubv10019187m Exp number of AAs in TMHs: 17.89405
# Capsellarubella_Carubv10019187m Exp number, first 60 AAs:  0.05392
# Capsellarubella_Carubv10019187m Total prob of N-in:        0.14029
Capsellarubella_Carubv10019187m TMHMM2.0        outside      1   315
Capsellarubella_Carubv10019187m TMHMM2.0        TMhelix    316   333
Capsellarubella_Carubv10019187m TMHMM2.0        inside     334   334
```

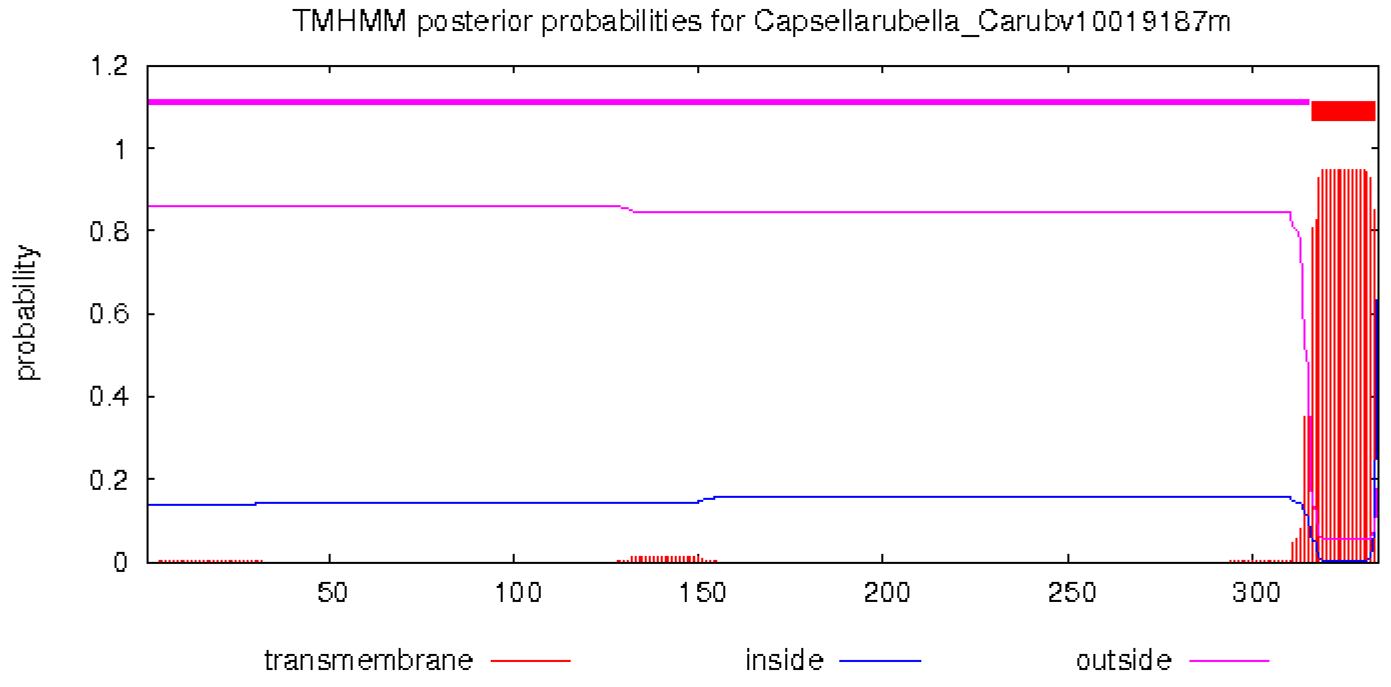

plot in postscript, script for making the plot in gnuplot, data for plot

---

```
# Capsellarubella_Carubv10019223m Length: 322
# Capsellarubella_Carubv10019223m Number of predicted TMHs:  0
# Capsellarubella_Carubv10019223m Exp number of AAs in TMHs: 0
# Capsellarubella_Carubv10019223m Exp number, first 60 AAs:  0
```

```
# Capsellarubella_Carubv10019223m Total prob of N-in:        0.03193
Capsellarubella_Carubv10019223m TMHMM2.0        outside      1   322
```

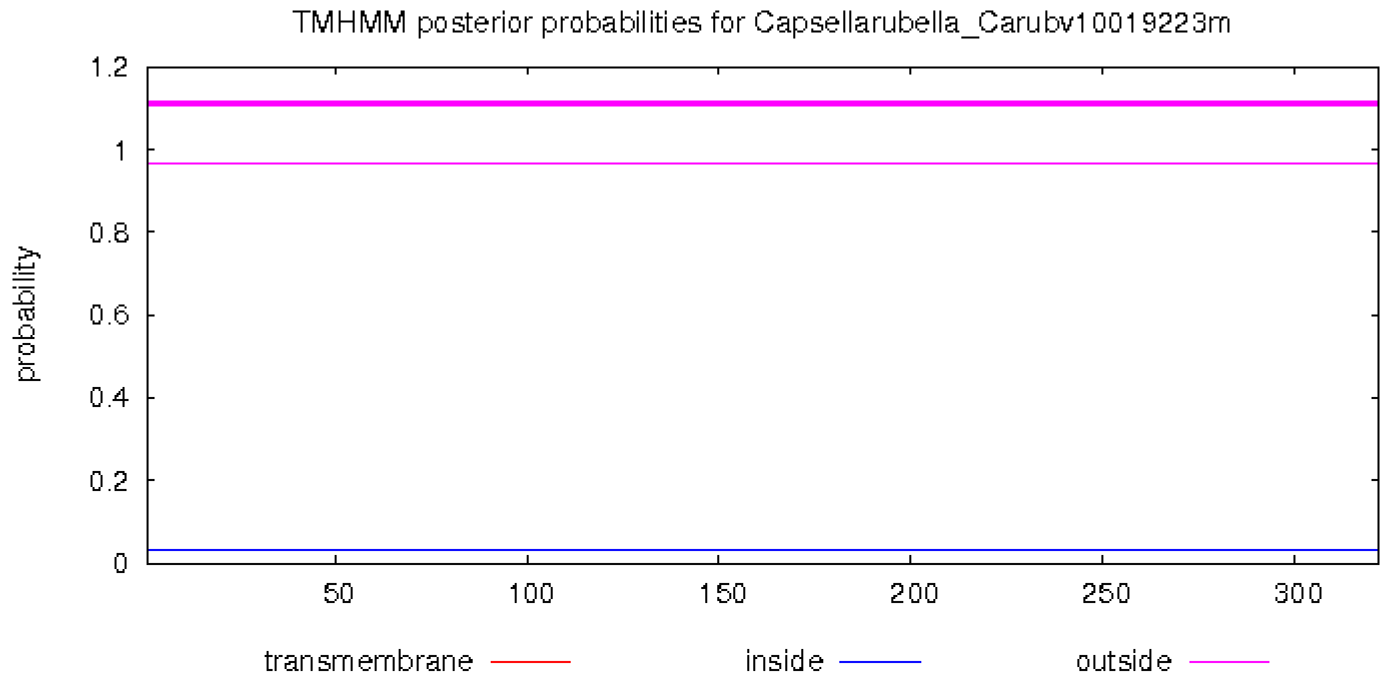

plot in postscript, script for making the plot in gnuplot, data for plot

---

```
# Capsellarubella_Carubv10019452m Length: 520
# Capsellarubella_Carubv10019452m Number of predicted TMHs:  0
# Capsellarubella_Carubv10019452m Exp number of AAs in TMHs: 0
# Capsellarubella_Carubv10019452m Exp number, first 60 AAs:  0
# Capsellarubella_Carubv10019452m Total prob of N-in:        0.00104
Capsellarubella_Carubv10019452m TMHMM2.0        outside      1   520
```

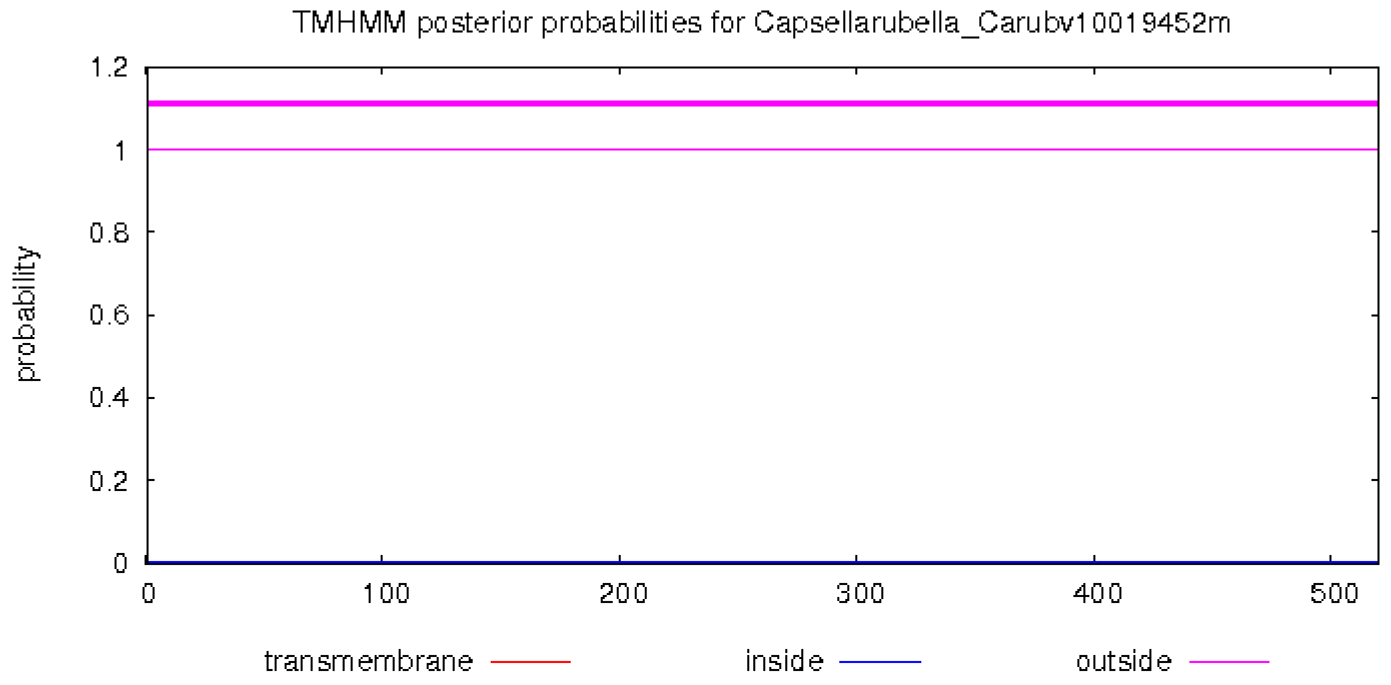

plot in postscript, script for making the plot in gnuplot, data for plot

---

```
# Capsellarubella_Carubv10020504m Length: 368
# Capsellarubella_Carubv10020504m Number of predicted TMHs:  0
# Capsellarubella_Carubv10020504m Exp number of AAs in TMHs: 0.0052
# Capsellarubella_Carubv10020504m Exp number, first 60 AAs:  0.00096
```

```
# Capsellarubella_Carubv10020504m Total prob of N-in:        0.00796
Capsellarubella_Carubv10020504m TMHMM2.0        outside      1   368
```

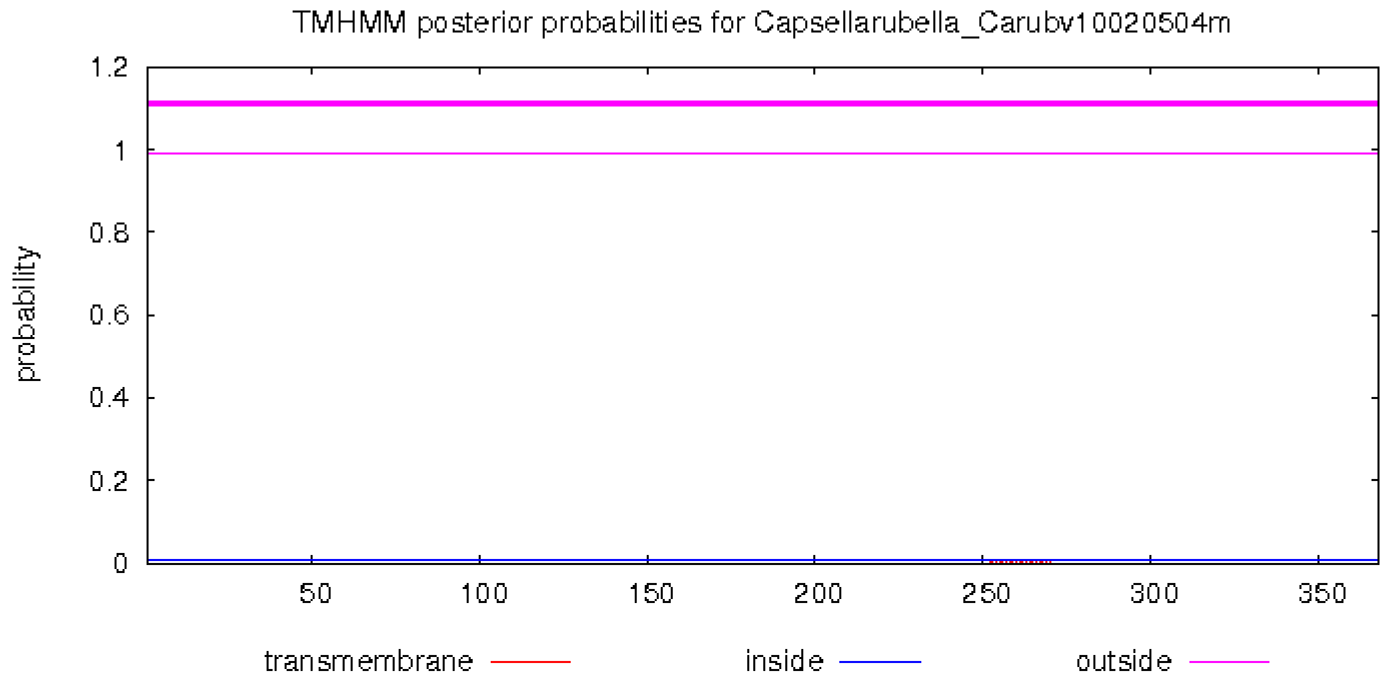

plot in postscript, script for making the plot in gnuplot, data for plot

---

```
# Capsellarubella_Carubv10020645m Length: 327
# Capsellarubella_Carubv10020645m Number of predicted TMHs:  0
# Capsellarubella_Carubv10020645m Exp number of AAs in TMHs: 0
# Capsellarubella_Carubv10020645m Exp number, first 60 AAs:  0
# Capsellarubella_Carubv10020645m Total prob of N-in:        0.01684
Capsellarubella_Carubv10020645m TMHMM2.0        outside      1   327
```

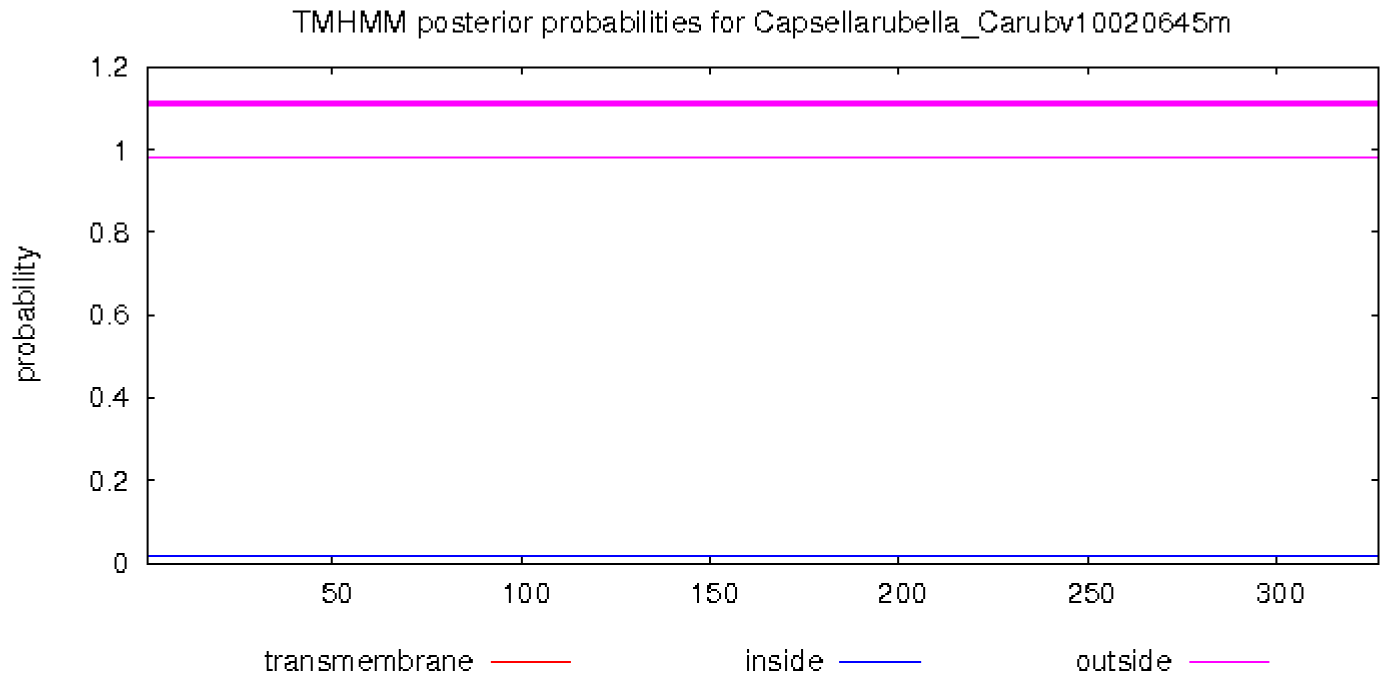

plot in postscript, script for making the plot in gnuplot, data for plot

---

```
# Capsellarubella_Carubv10020776m Length: 276
# Capsellarubella_Carubv10020776m Number of predicted TMHs:  0
# Capsellarubella_Carubv10020776m Exp number of AAs in TMHs: 0.00069
# Capsellarubella_Carubv10020776m Exp number, first 60 AAs:  0
```

```
# Capsellarubella_Carubv10020776m Total prob of N-in:        0.03601
Capsellarubella_Carubv10020776m TMHMM2.0        outside      1   276
```

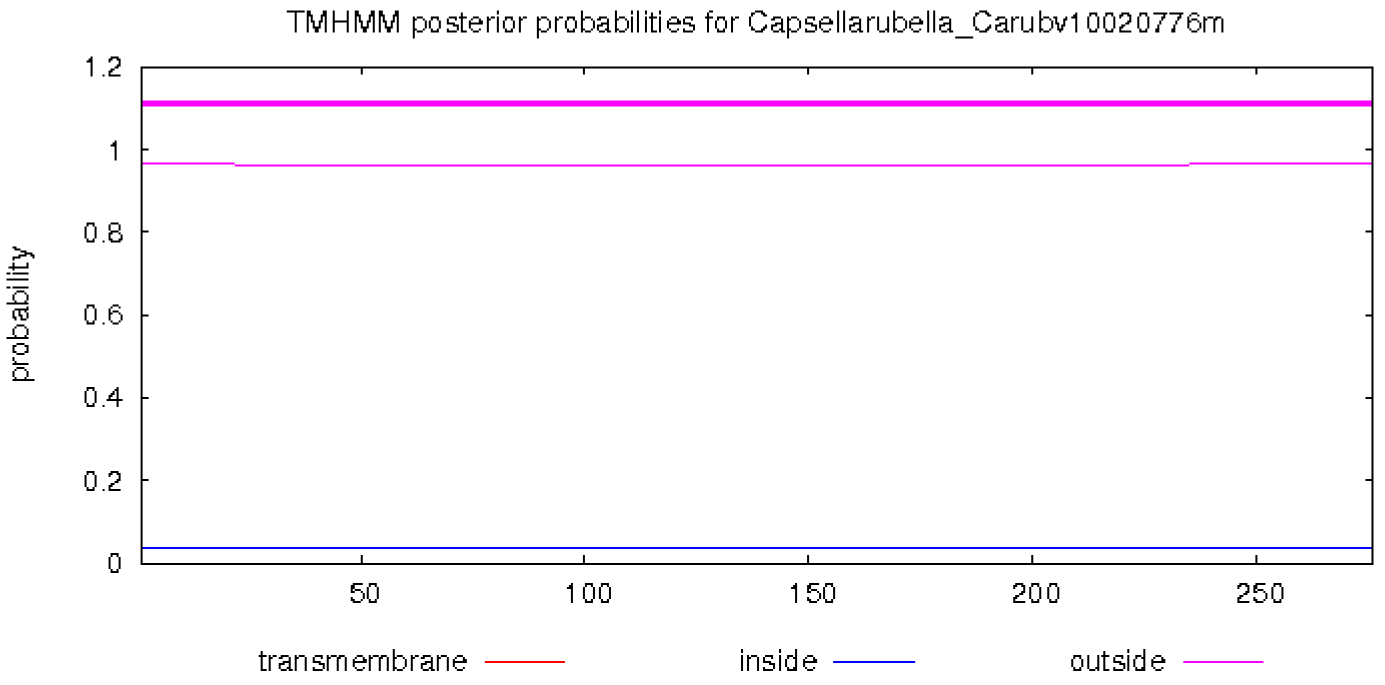

plot in postscript, script for making the plot in gnuplot, data for plot

---

```
# Capsellarubella_Carubv10020834m Length: 255
# Capsellarubella_Carubv10020834m Number of predicted TMHs:  0
# Capsellarubella_Carubv10020834m Exp number of AAs in TMHs: 5e-05
# Capsellarubella_Carubv10020834m Exp number, first 60 AAs:  0
# Capsellarubella_Carubv10020834m Total prob of N-in:        0.03865
Capsellarubella_Carubv10020834m TMHMM2.0        outside      1   255
```

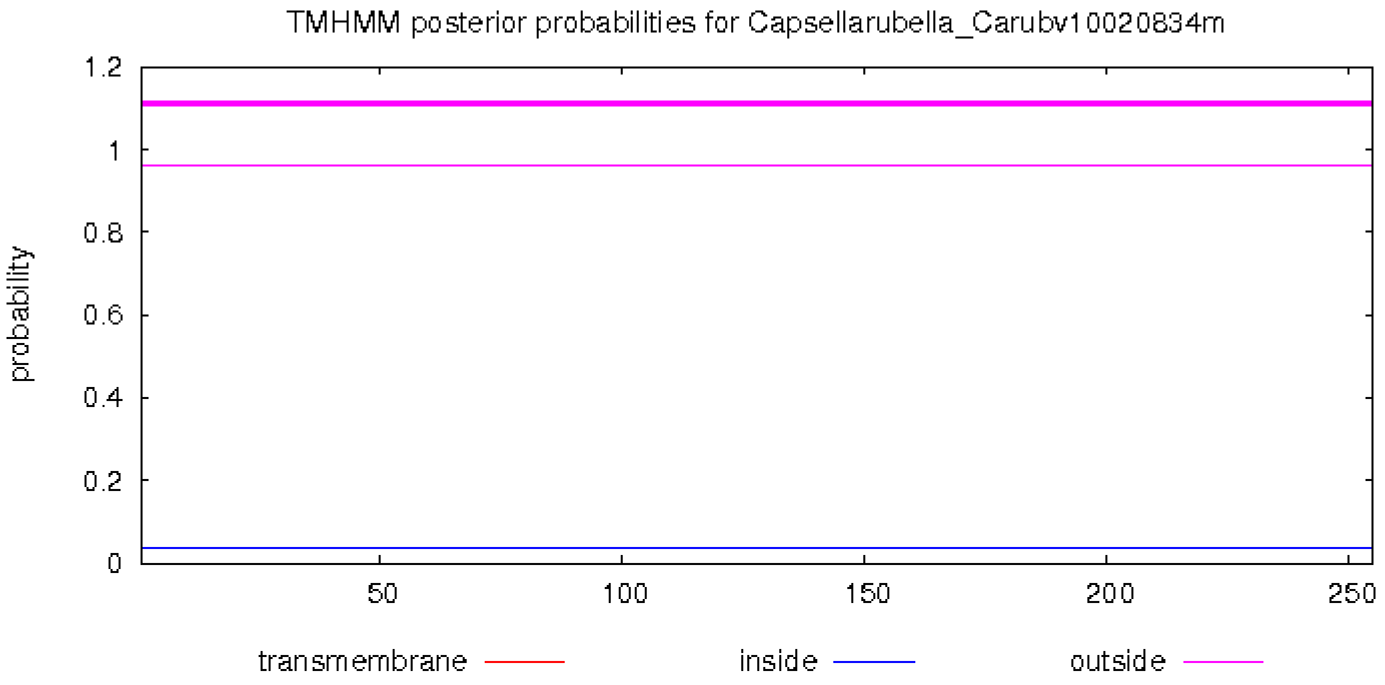

plot in postscript, script for making the plot in gnuplot, data for plot

---

```
# Capsellarubella_Carubv10021319m Length: 335
# Capsellarubella_Carubv10021319m Number of predicted TMHs:  0
# Capsellarubella_Carubv10021319m Exp number of AAs in TMHs: 0.00522
# Capsellarubella_Carubv10021319m Exp number, first 60 AAs:  0
```

```
# Capsellarubella_Carubv10021319m Total prob of N-in:        0.04225
Capsellarubella_Carubv10021319m TMHMM2.0        outside      1   335
```

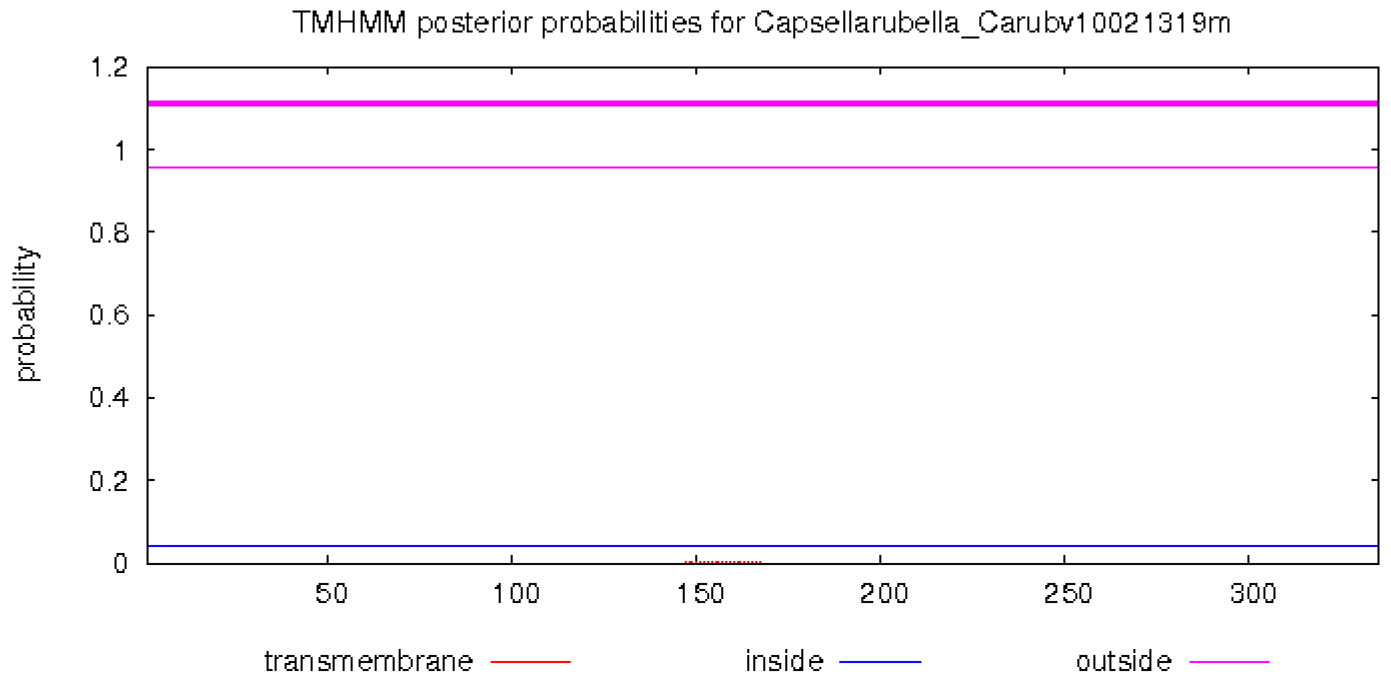

plot in postscript, script for making the plot in gnuplot, data for plot

---

```
# Capsellarubella_Carubv10021371m Length: 324
# Capsellarubella_Carubv10021371m Number of predicted TMHs:  0
# Capsellarubella_Carubv10021371m Exp number of AAs in TMHs: 0.00035
# Capsellarubella_Carubv10021371m Exp number, first 60 AAs:  0.00016
# Capsellarubella_Carubv10021371m Total prob of N-in:        0.01182
Capsellarubella_Carubv10021371m TMHMM2.0        outside      1   324
```

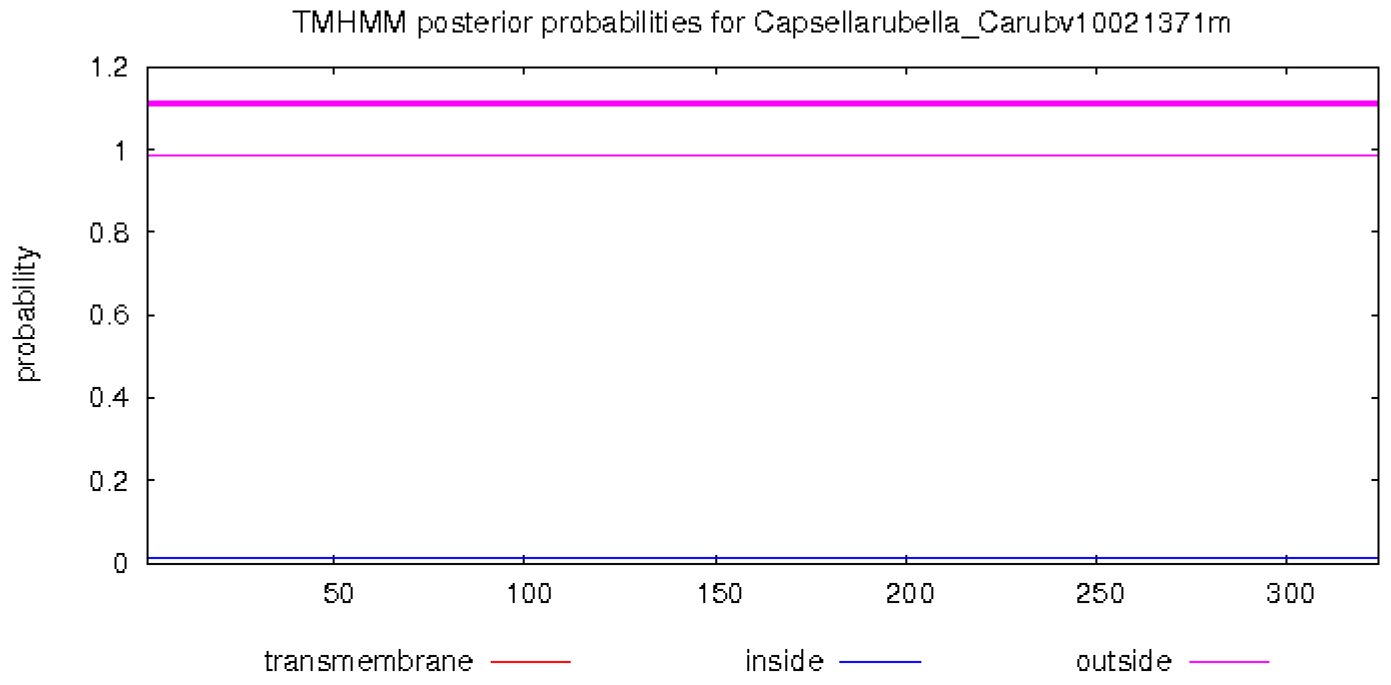

plot in postscript, script for making the plot in gnuplot, data for plot

---

```
# Capsellarubella_Carubv10021514m Length: 387
# Capsellarubella_Carubv10021514m Number of predicted TMHs:  0
# Capsellarubella_Carubv10021514m Exp number of AAs in TMHs: 0.00017
# Capsellarubella_Carubv10021514m Exp number, first 60 AAs:  0
```

```
# Capsellarubella_Carubv10021514m Total prob of N-in:        0.01013
Capsellarubella_Carubv10021514m TMHMM2.0        outside      1   387
```

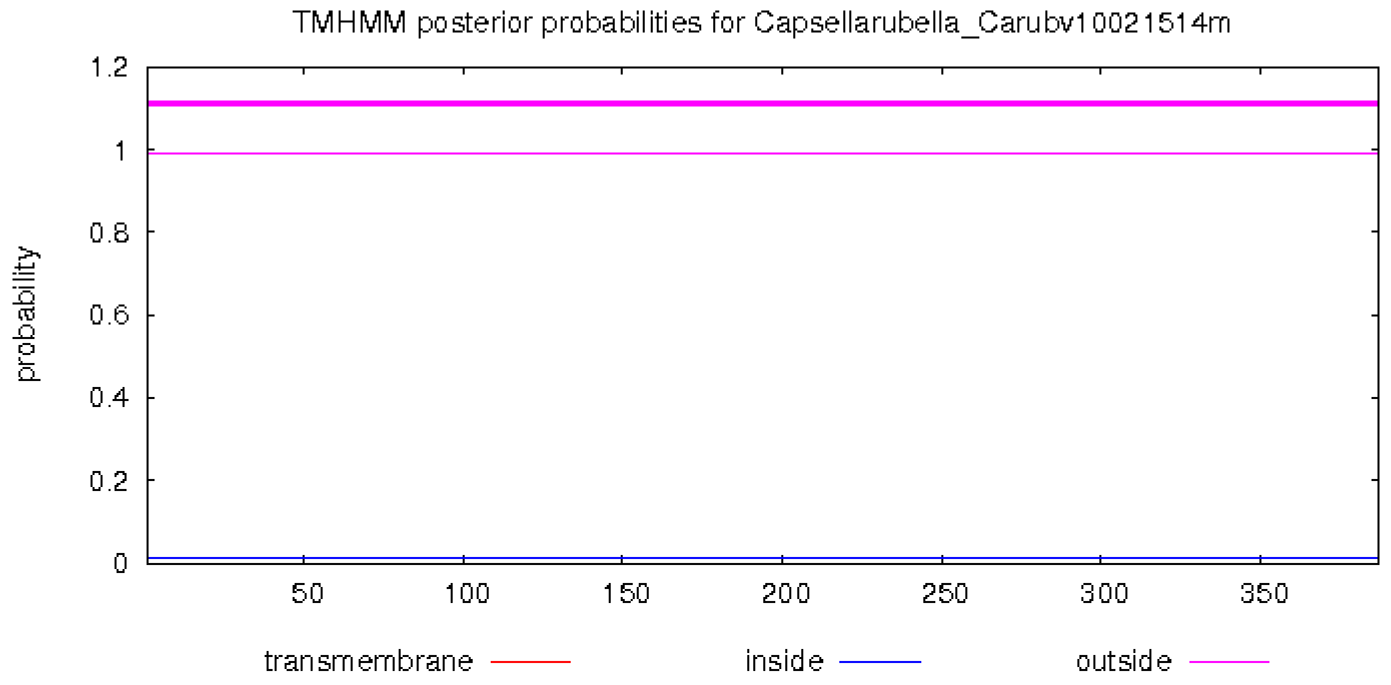

plot in postscript, script for making the plot in gnuplot, data for plot

```
# Capsellarubella_Carubv10021788m Length: 627
# Capsellarubella_Carubv10021788m Number of predicted TMHs:  0
# Capsellarubella_Carubv10021788m Exp number of AAs in TMHs: 9.48287
# Capsellarubella_Carubv10021788m Exp number, first 60 AAs:  0
# Capsellarubella_Carubv10021788m Total prob of N-in:        0.00020
Capsellarubella_Carubv10021788m TMHMM2.0        outside      1   627
```

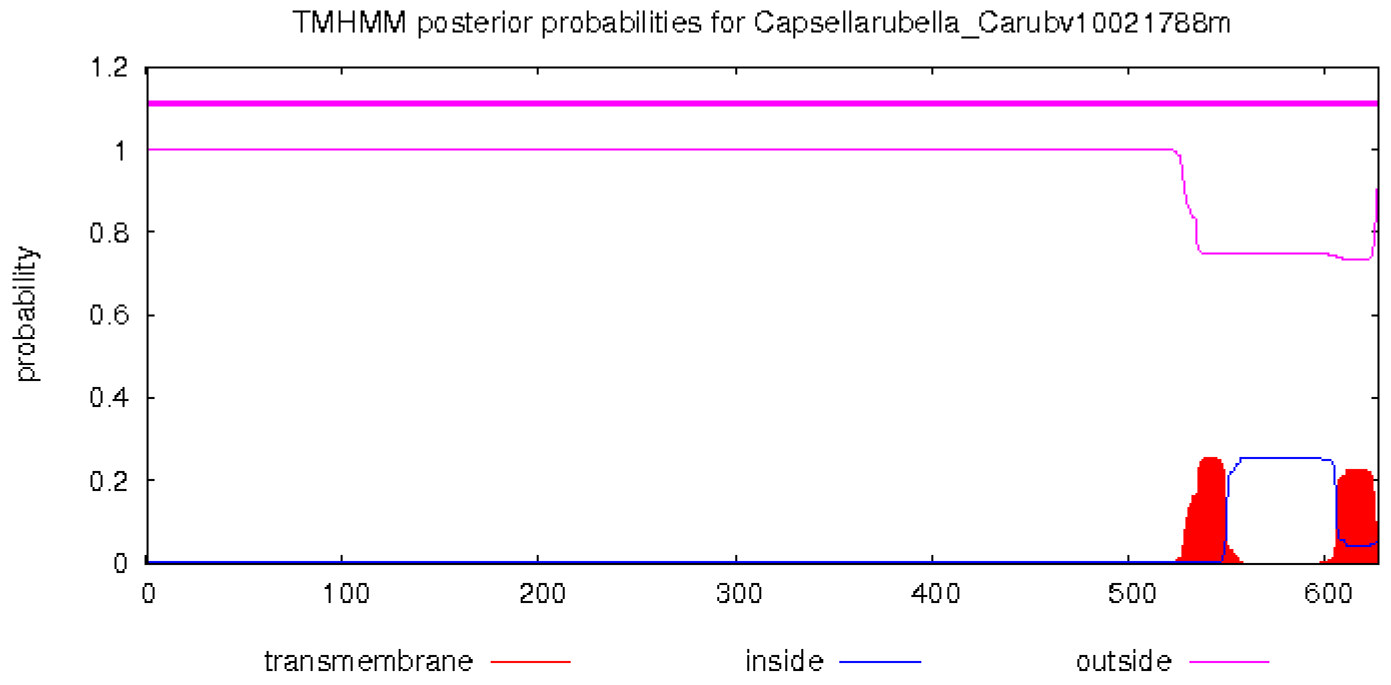

plot in postscript, script for making the plot in gnuplot, data for plot

```
# Capsellarubella_Carubv10021818m Length: 320
# Capsellarubella_Carubv10021818m Number of predicted TMHs:  0
# Capsellarubella_Carubv10021818m Exp number of AAs in TMHs: 0.01107
# Capsellarubella_Carubv10021818m Exp number, first 60 AAs:  0.00019
```

```
# Capsellarubella_Carubv10021818m Total prob of N-in:        0.01097
Capsellarubella_Carubv10021818m TMHMM2.0        outside      1   320
```

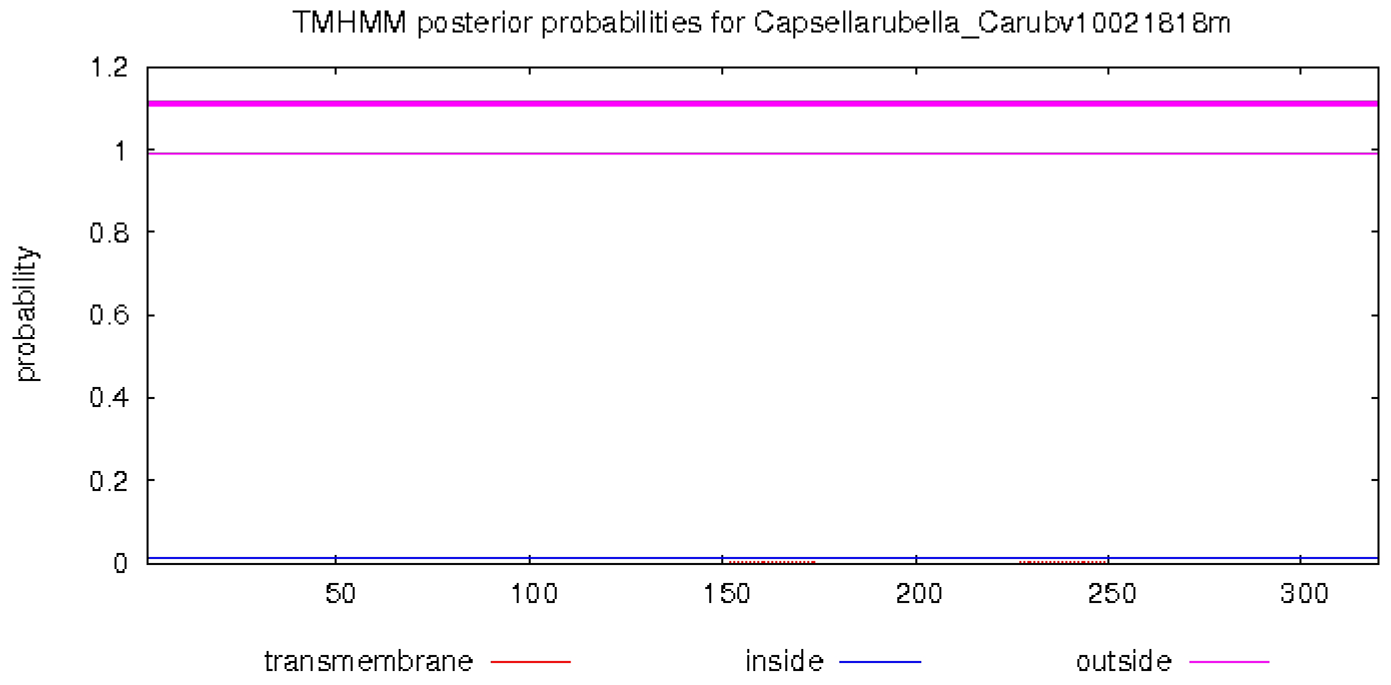

plot in postscript, script for making the plot in gnuplot, data for plot

---

```
# Capsellarubella_Carubv10022010m Length: 173
# Capsellarubella_Carubv10022010m Number of predicted TMHs:  0
# Capsellarubella_Carubv10022010m Exp number of AAs in TMHs: 0.01034
# Capsellarubella_Carubv10022010m Exp number, first 60 AAs:  0.0057
# Capsellarubella_Carubv10022010m Total prob of N-in:        0.09685
Capsellarubella_Carubv10022010m TMHMM2.0        outside      1   173
```

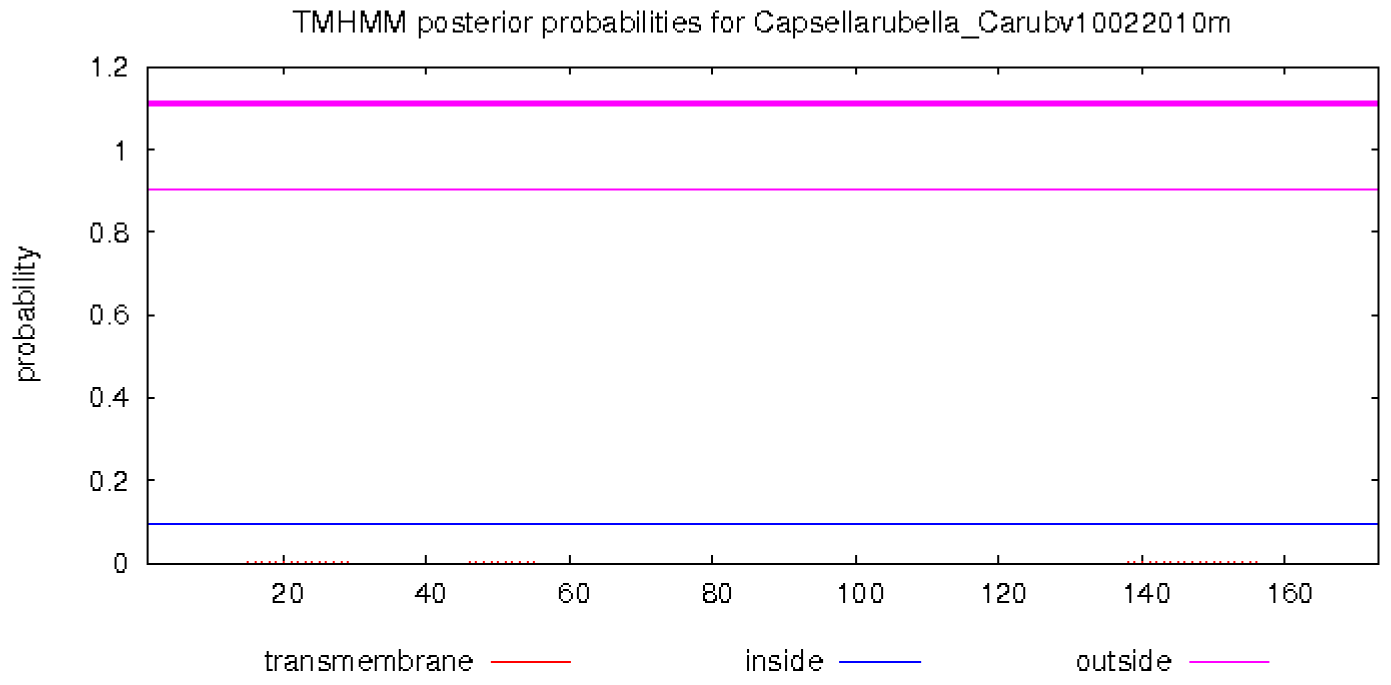

plot in postscript, script for making the plot in gnuplot, data for plot

---

```
# Capsellarubella_Carubv10022061m Length: 341
# Capsellarubella_Carubv10022061m Number of predicted TMHs:  0
# Capsellarubella_Carubv10022061m Exp number of AAs in TMHs: 0.02139
# Capsellarubella_Carubv10022061m Exp number, first 60 AAs:  0.01646
```

```
# Capsellarubella_Carubv10022061m Total prob of N-in:        0.00487
Capsellarubella_Carubv10022061m TMHMM2.0        outside      1   341
```

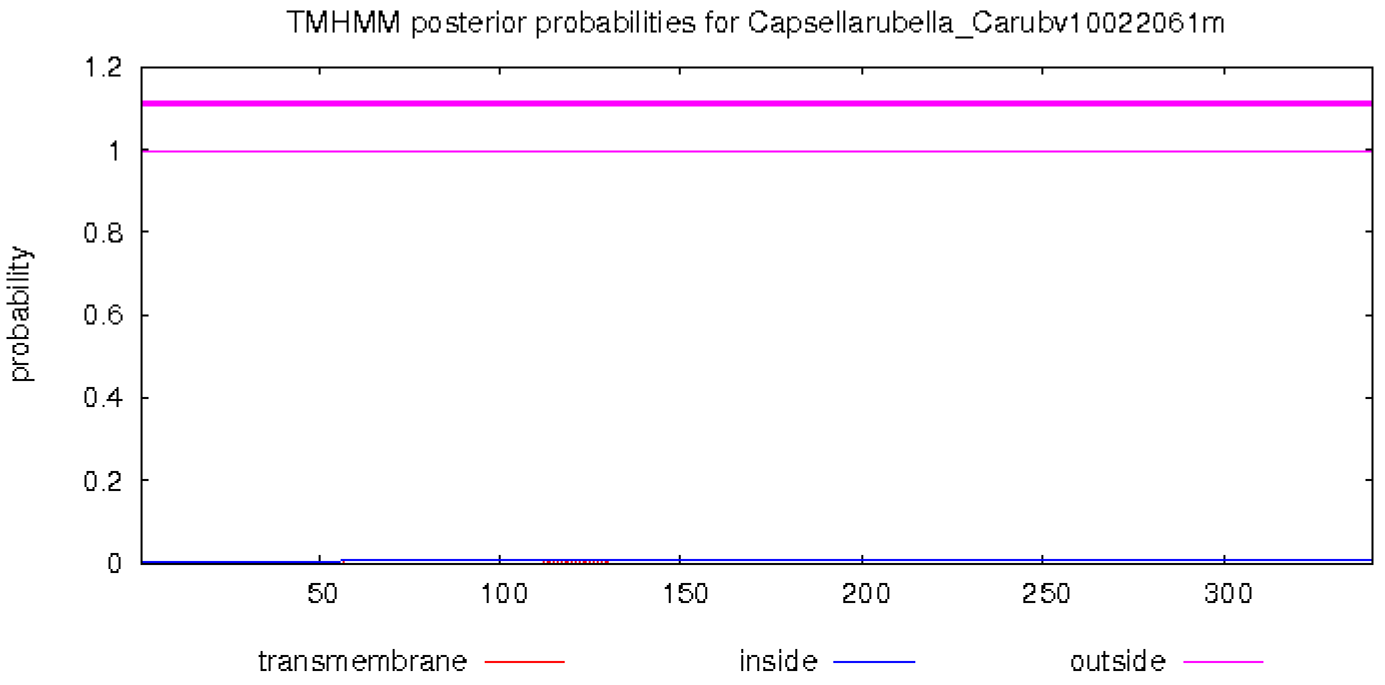

plot in postscript, script for making the plot in gnuplot, data for plot

---

```
# Capsellarubella_Carubv10022099m Length: 325
# Capsellarubella_Carubv10022099m Number of predicted TMHs:  0
# Capsellarubella_Carubv10022099m Exp number of AAs in TMHs: 0.00146
# Capsellarubella_Carubv10022099m Exp number, first 60 AAs:  0
# Capsellarubella_Carubv10022099m Total prob of N-in:        0.00843
Capsellarubella_Carubv10022099m TMHMM2.0        outside      1   325
```

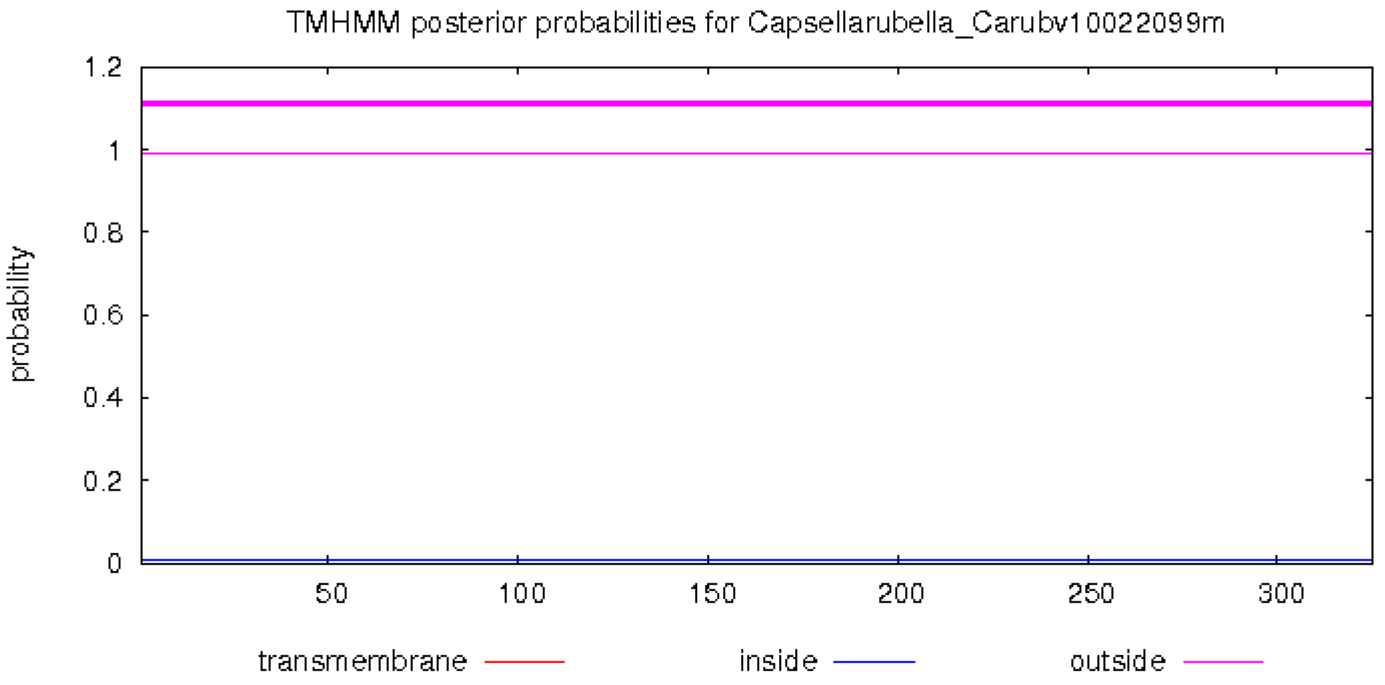

plot in postscript, script for making the plot in gnuplot, data for plot

---

```
# Capsellarubella_Carubv10022302m Length: 437
# Capsellarubella_Carubv10022302m Number of predicted TMHs:  0
# Capsellarubella_Carubv10022302m Exp number of AAs in TMHs: 0
# Capsellarubella_Carubv10022302m Exp number, first 60 AAs:  0
```

```
# Capsellarubella_Carubv10022302m Total prob of N-in:        0.00249
Capsellarubella_Carubv10022302m TMHMM2.0        outside      1   437
```

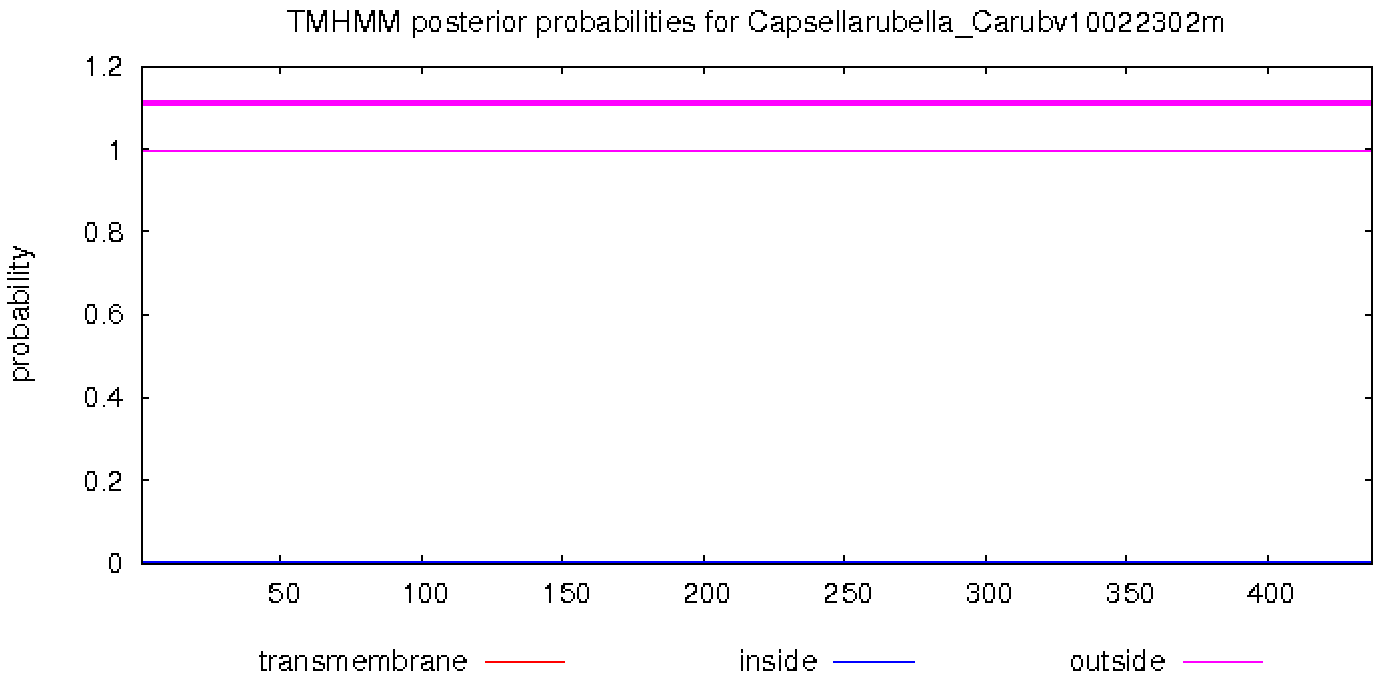

plot in postscript, script for making the plot in gnuplot, data for plot

---

```
# Capsellarubella_Carubv10022351m Length: 407
# Capsellarubella_Carubv10022351m Number of predicted TMHs:  0
# Capsellarubella_Carubv10022351m Exp number of AAs in TMHs: 0.00018
# Capsellarubella_Carubv10022351m Exp number, first 60 AAs:  0
# Capsellarubella_Carubv10022351m Total prob of N-in:        0.00173
Capsellarubella_Carubv10022351m TMHMM2.0        outside      1   407
```

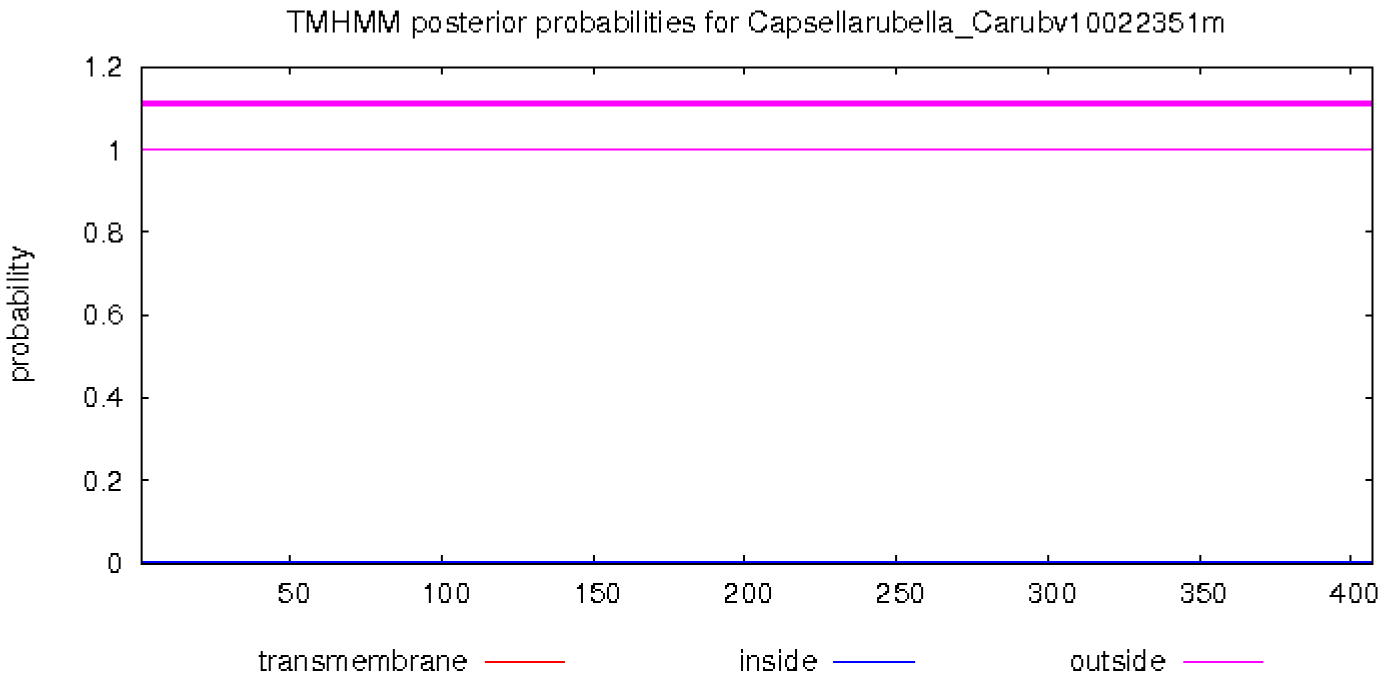

plot in postscript, script for making the plot in gnuplot, data for plot

---

```
# Capsellarubella_Carubv10022440m Length: 414
# Capsellarubella_Carubv10022440m Number of predicted TMHs:  0
# Capsellarubella_Carubv10022440m Exp number of AAs in TMHs: 0.00018
# Capsellarubella_Carubv10022440m Exp number, first 60 AAs:  0.00018
```

```
# Capsellarubella_Carubv10022440m Total prob of N-in:        0.02171
Capsellarubella_Carubv10022440m TMHMM2.0        outside      1   414
```

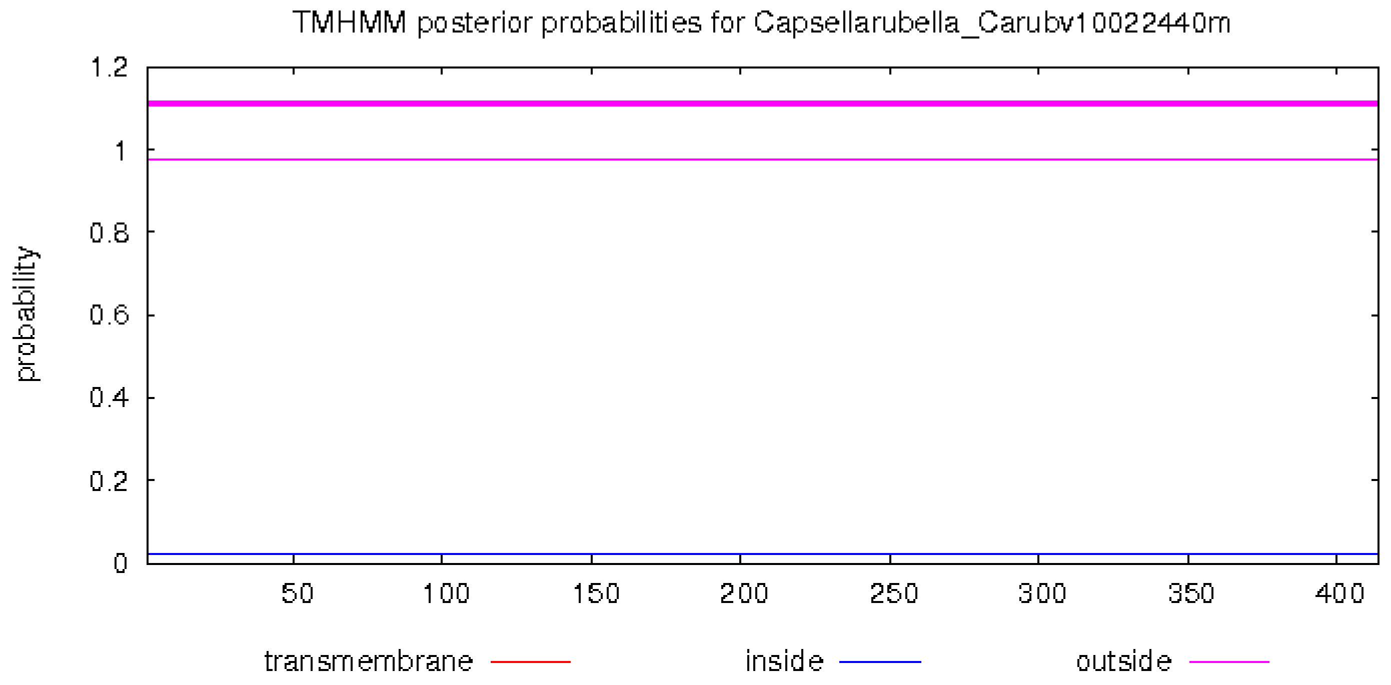

plot in postscript, script for making the plot in gnuplot, data for plot

---

```
# Capsellarubella_Carubv10023503m Length: 358
# Capsellarubella_Carubv10023503m Number of predicted TMHs:  0
# Capsellarubella_Carubv10023503m Exp number of AAs in TMHs: 0
# Capsellarubella_Carubv10023503m Exp number, first 60 AAs:  0
# Capsellarubella_Carubv10023503m Total prob of N-in:        0.01313
Capsellarubella_Carubv10023503m TMHMM2.0        outside      1   358
```

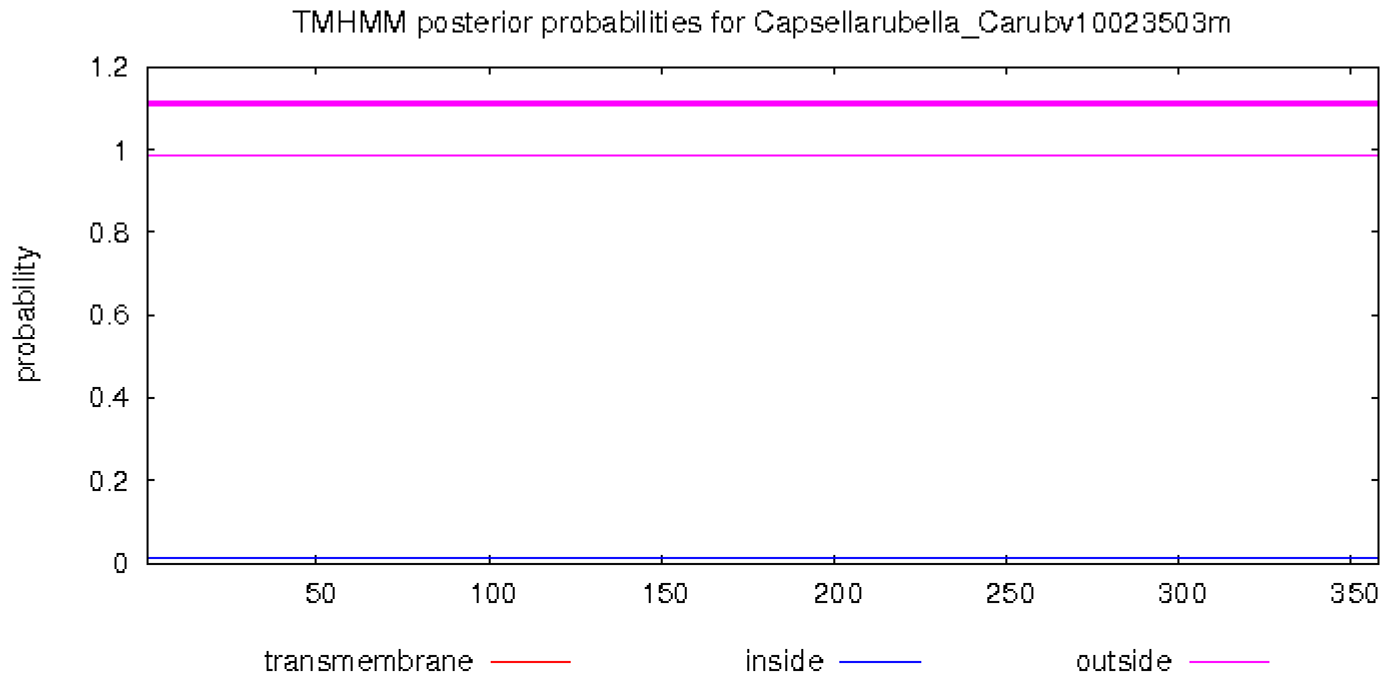

plot in postscript, script for making the plot in gnuplot, data for plot

---

```
# Capsellarubella_Carubv10023568m Length: 340
# Capsellarubella_Carubv10023568m Number of predicted TMHs:  1
# Capsellarubella_Carubv10023568m Exp number of AAs in TMHs: 21.40323
# Capsellarubella_Carubv10023568m Exp number, first 60 AAs:  0.19516
```

```
# Capsellarubella_Carubv10023568m Total prob of N-in:        0.55277
Capsellarubella_Carubv10023568m TMHMM2.0        outside      1   315
Capsellarubella_Carubv10023568m TMHMM2.0        TMhelix    316   335
Capsellarubella_Carubv10023568m TMHMM2.0        inside     336   340
```

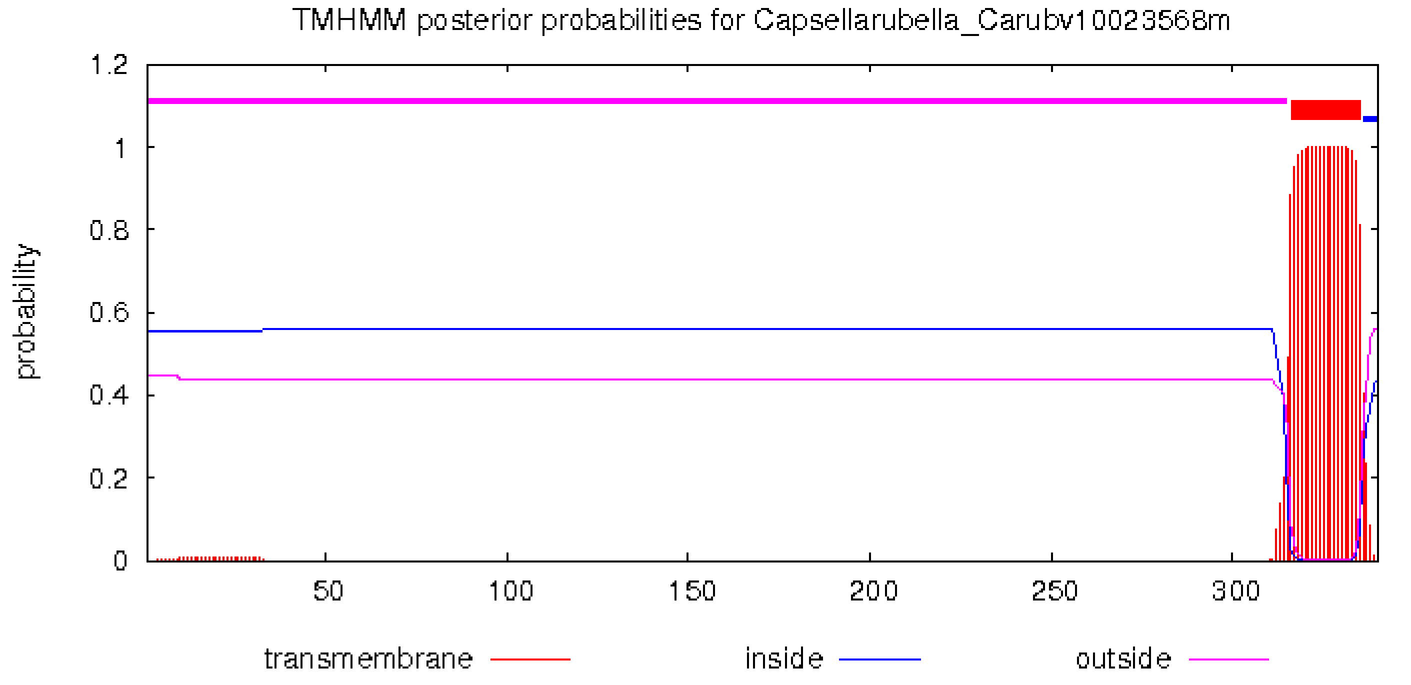

plot in postscript, script for making the plot in gnuplot, data for plot

---

```
# Capsellarubella_Carubv10023730m Length: 300
# Capsellarubella_Carubv10023730m Number of predicted TMHs:  0
# Capsellarubella_Carubv10023730m Exp number of AAs in TMHs: 0.00107
# Capsellarubella_Carubv10023730m Exp number, first 60 AAs:  0
# Capsellarubella_Carubv10023730m Total prob of N-in:        0.03849
Capsellarubella_Carubv10023730m TMHMM2.0        outside      1   300
```

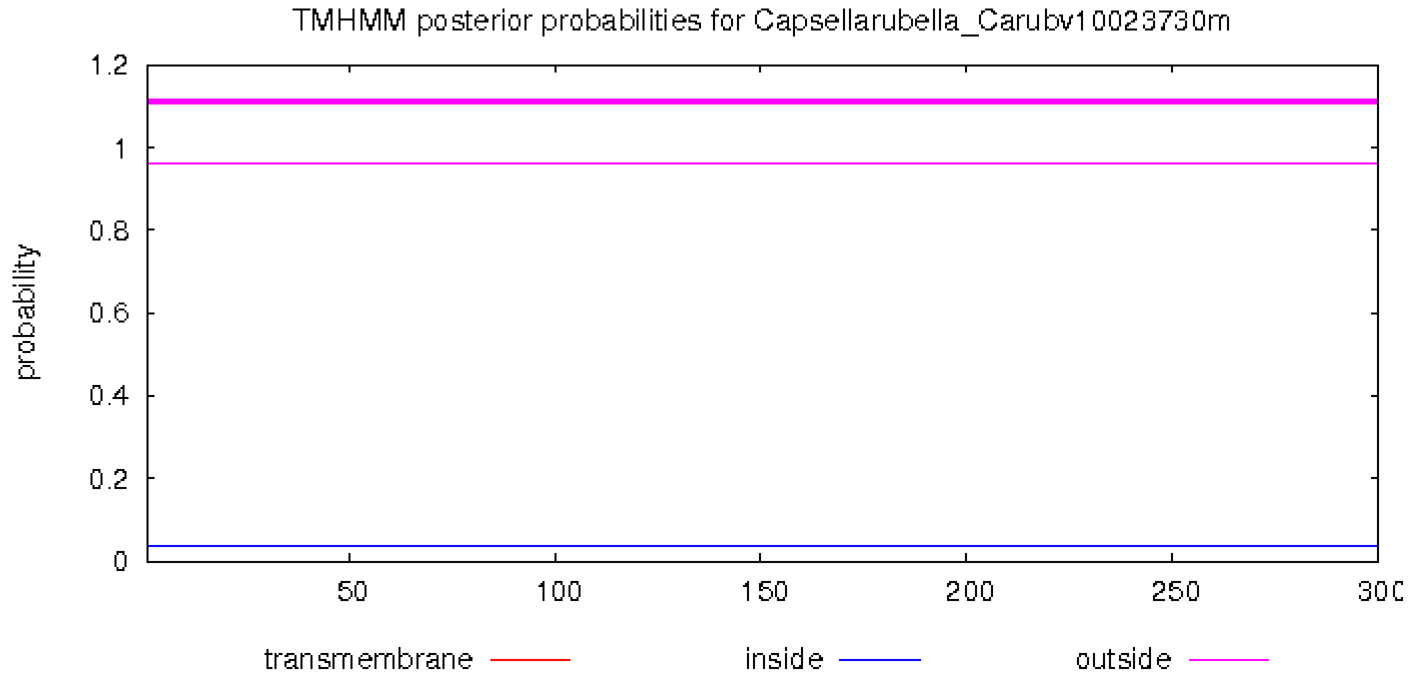

plot in postscript, script for making the plot in gnuplot, data for plot

---

```
# Capsellarubella_Carubv10023833m Length: 273
# Capsellarubella_Carubv10023833m Number of predicted TMHs:  0
```

```
# Capsellarubella_Carubv10023833m Exp number of AAs in TMHs: 0.18739
# Capsellarubella_Carubv10023833m Exp number, first 60 AAs:  0
# Capsellarubella_Carubv10023833m Total prob of N-in:        0.22843
Capsellarubella_Carubv10023833m TMHMM2.0        outside      1   273
```

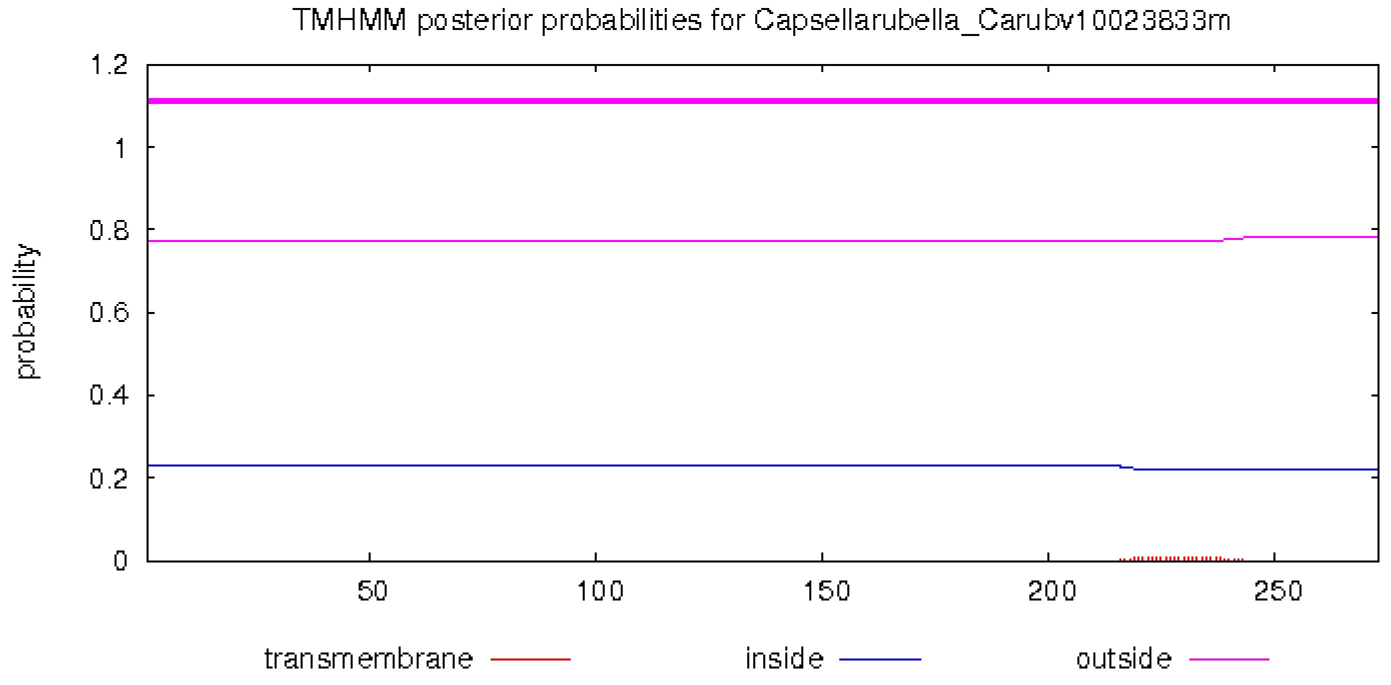

plot in postscript, script for making the plot in gnuplot, data for plot

```
# Capsellarubella_Carubv10025508m Length: 318
# Capsellarubella_Carubv10025508m Number of predicted TMHs:  0
# Capsellarubella_Carubv10025508m Exp number of AAs in TMHs: 0.01776
# Capsellarubella_Carubv10025508m Exp number, first 60 AAs:  0
# Capsellarubella_Carubv10025508m Total prob of N-in:        0.07240
Capsellarubella_Carubv10025508m TMHMM2.0        outside      1   318
```

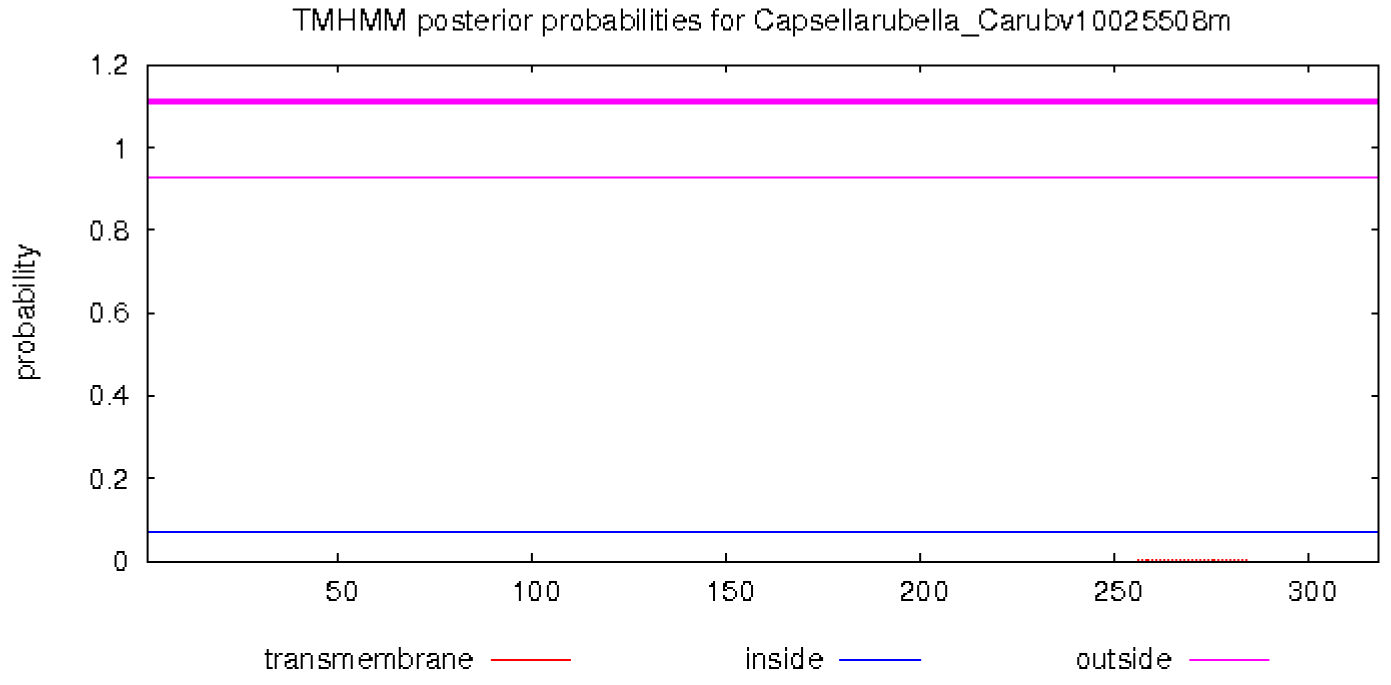

plot in postscript, script for making the plot in gnuplot, data for plot

```
# Capsellarubella_Carubv10026643m Length: 364
# Capsellarubella_Carubv10026643m Number of predicted TMHs:  0
```

```
# Capsellarubella_Carubv10026643m Exp number of AAs in TMHs: 0.00018
# Capsellarubella_Carubv10026643m Exp number, first 60 AAs:  0.00018
# Capsellarubella_Carubv10026643m Total prob of N-in:        0.00409
Capsellarubella_Carubv10026643m TMHMM2.0        outside      1   364
```

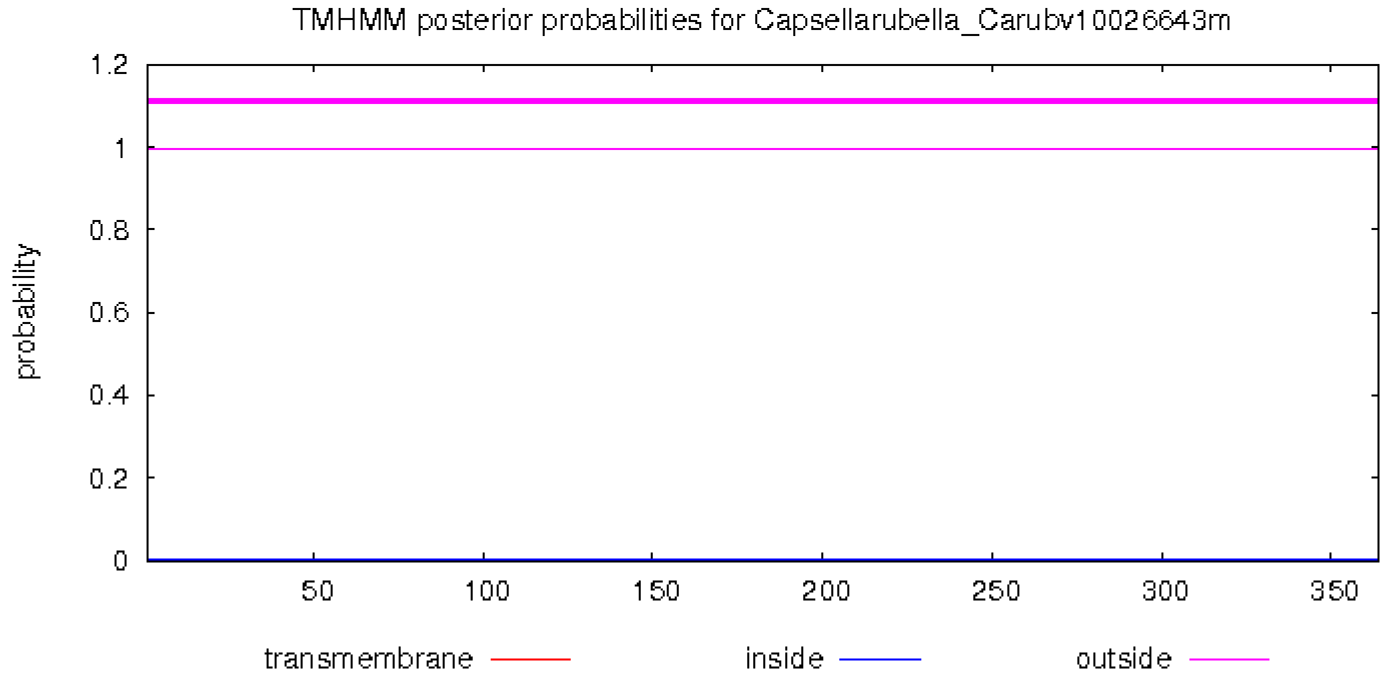

plot in postscript, script for making the plot in gnuplot, data for plot

---

```
# Capsellarubella_Carubv10026697m Length: 349
# Capsellarubella_Carubv10026697m Number of predicted TMHs:  0
# Capsellarubella_Carubv10026697m Exp number of AAs in TMHs: 0.00087
# Capsellarubella_Carubv10026697m Exp number, first 60 AAs:  0
# Capsellarubella_Carubv10026697m Total prob of N-in:        0.00194
Capsellarubella_Carubv10026697m TMHMM2.0        outside      1   349
```

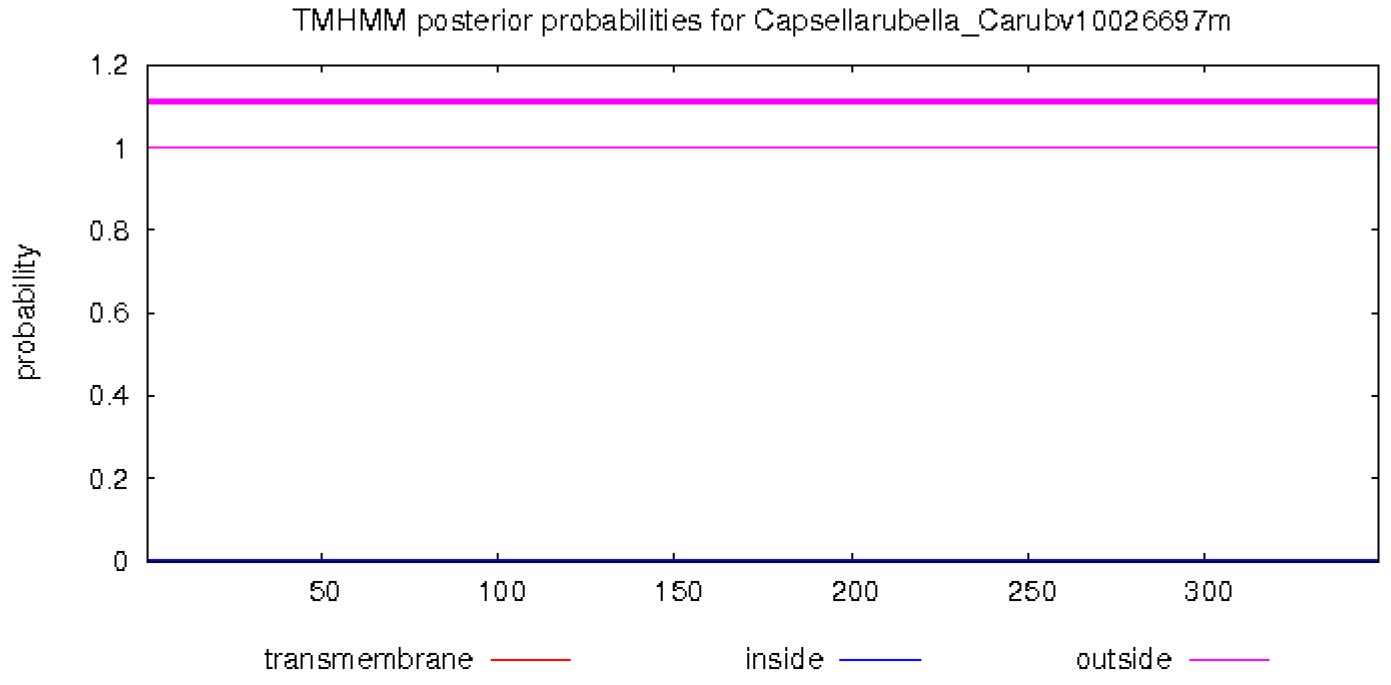

plot in postscript, script for making the plot in gnuplot, data for plot

---

```
# Capsellarubella_Carubv10026752m Length: 333
# Capsellarubella_Carubv10026752m Number of predicted TMHs:  0
```

```
# Capsellarubella_Carubv10026752m Exp number of AAs in TMHs: 0.00014
# Capsellarubella_Carubv10026752m Exp number, first 60 AAs:  0
# Capsellarubella_Carubv10026752m Total prob of N-in:        0.01284
Capsellarubella_Carubv10026752m TMHMM2.0        outside      1   333
```

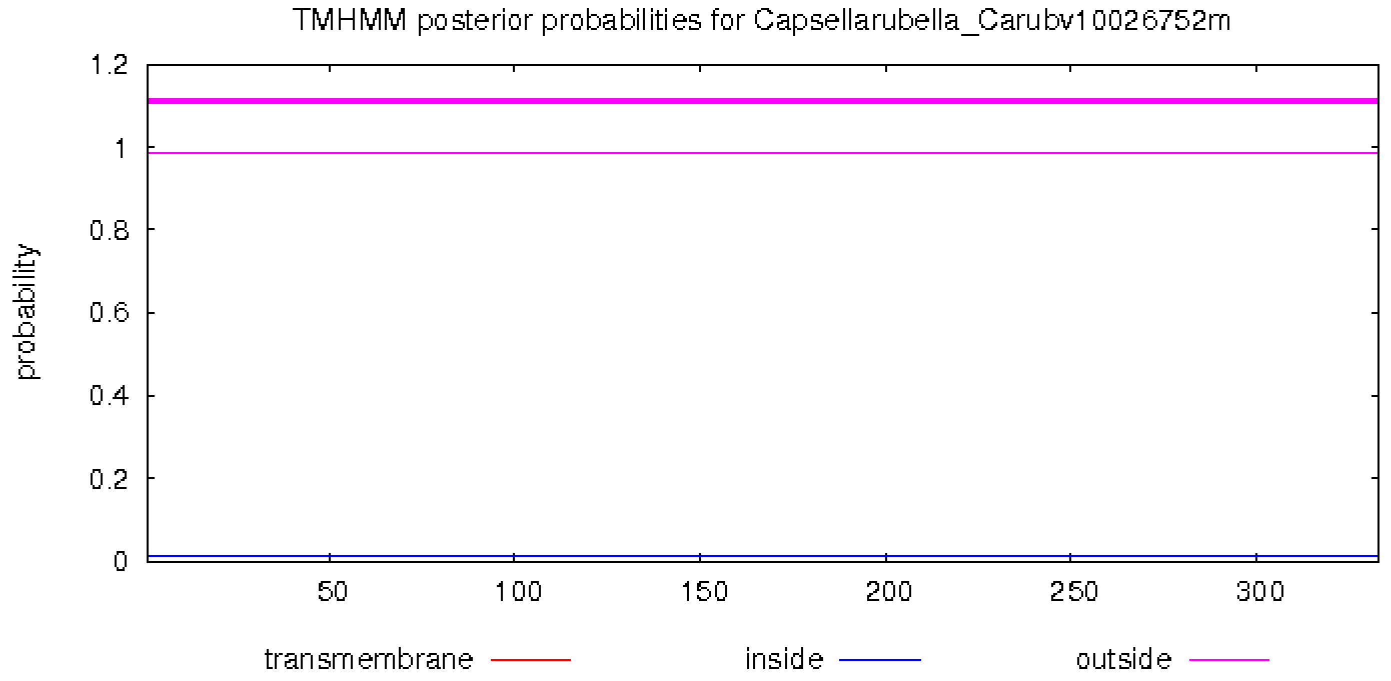

plot in postscript, script for making the plot in gnuplot, data for plot

```
# Capsellarubella_Carubv10026898m Length: 289
# Capsellarubella_Carubv10026898m Number of predicted TMHs:  0
# Capsellarubella_Carubv10026898m Exp number of AAs in TMHs: 0.00037
# Capsellarubella_Carubv10026898m Exp number, first 60 AAs:  0
# Capsellarubella_Carubv10026898m Total prob of N-in:        0.02907
Capsellarubella_Carubv10026898m TMHMM2.0        outside      1   289
```

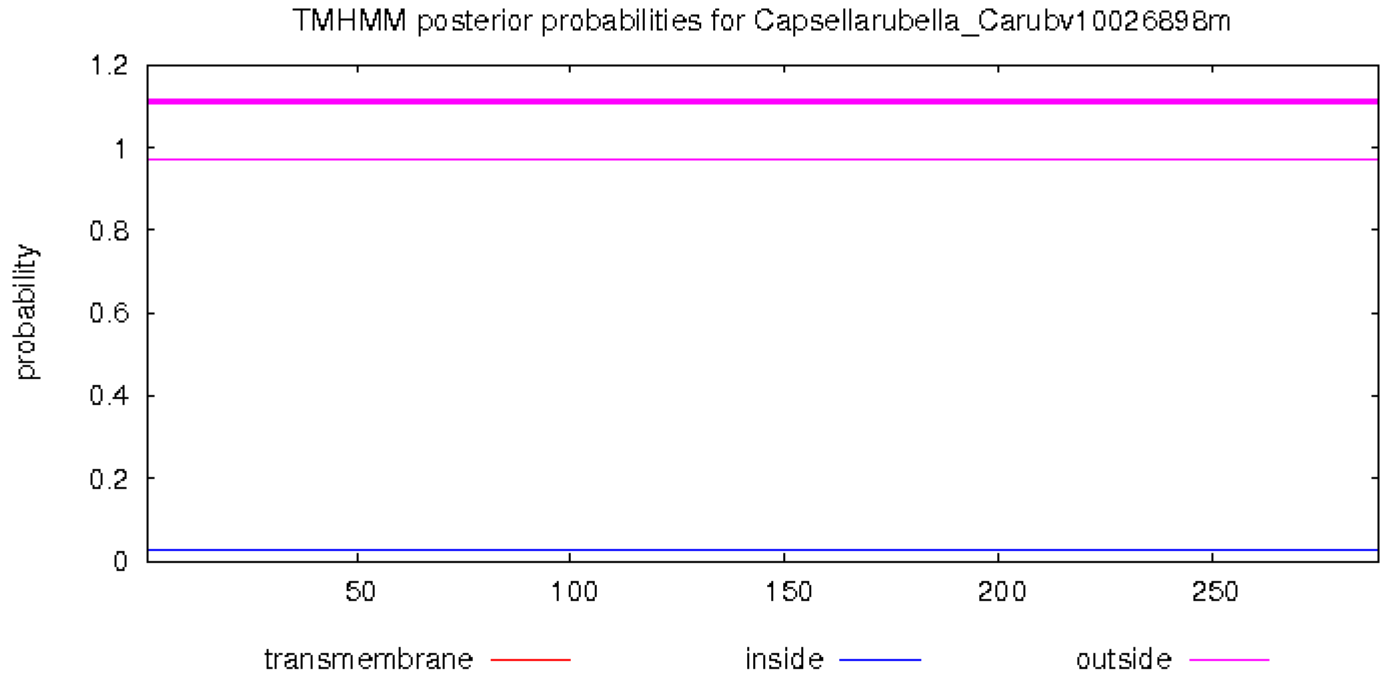

plot in postscript, script for making the plot in gnuplot, data for plot

```
# Capsellarubella_Carubv10026953m Length: 270
# Capsellarubella_Carubv10026953m Number of predicted TMHs:  0
```

```
# Capsellarubella_Carubv10026953m Exp number of AAs in TMHs: 0.00019
# Capsellarubella_Carubv10026953m Exp number, first 60 AAs:  0
# Capsellarubella_Carubv10026953m Total prob of N-in:        0.01308
Capsellarubella_Carubv10026953m TMHMM2.0        outside      1   270
```

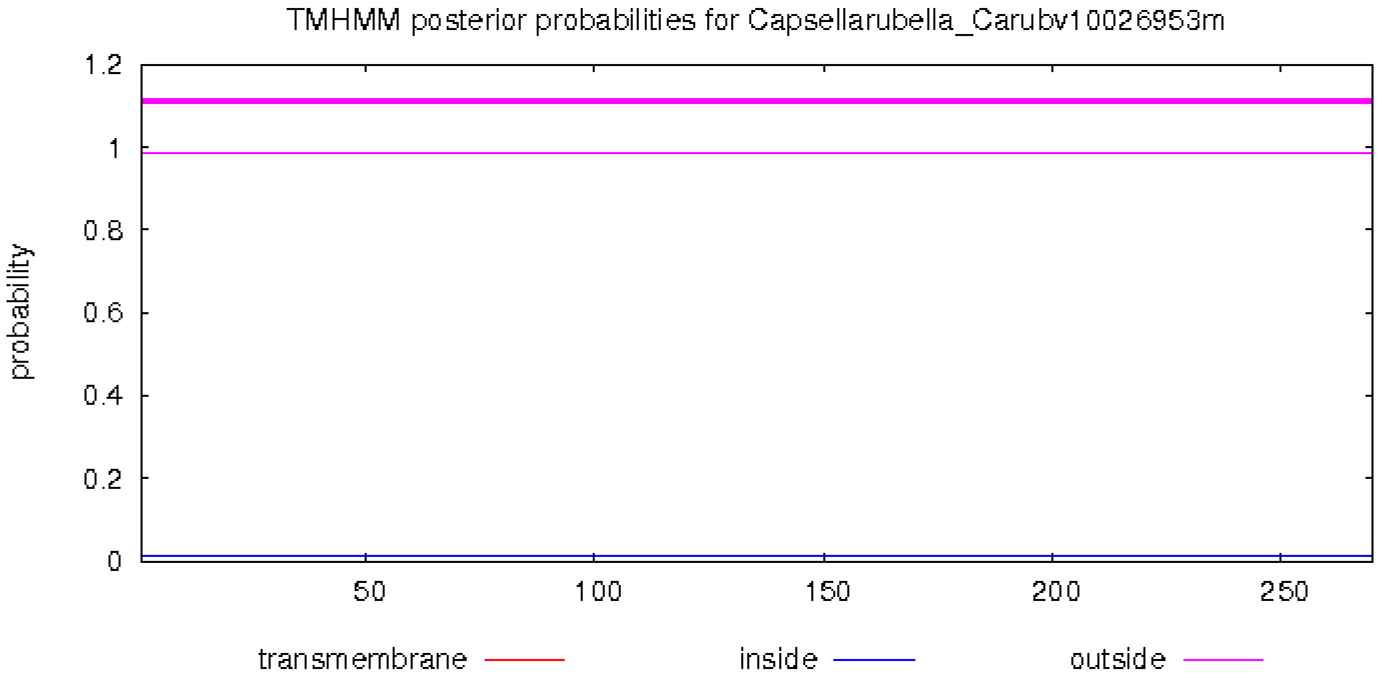

plot in postscript, script for making the plot in gnuplot, data for plot

---

```
# Capsellarubella_Carubv10027180m Length: 194
# Capsellarubella_Carubv10027180m Number of predicted TMHs:  0
# Capsellarubella_Carubv10027180m Exp number of AAs in TMHs: 0.00137
# Capsellarubella_Carubv10027180m Exp number, first 60 AAs:  0
# Capsellarubella_Carubv10027180m Total prob of N-in:        0.12612
Capsellarubella_Carubv10027180m TMHMM2.0        outside      1   194
```

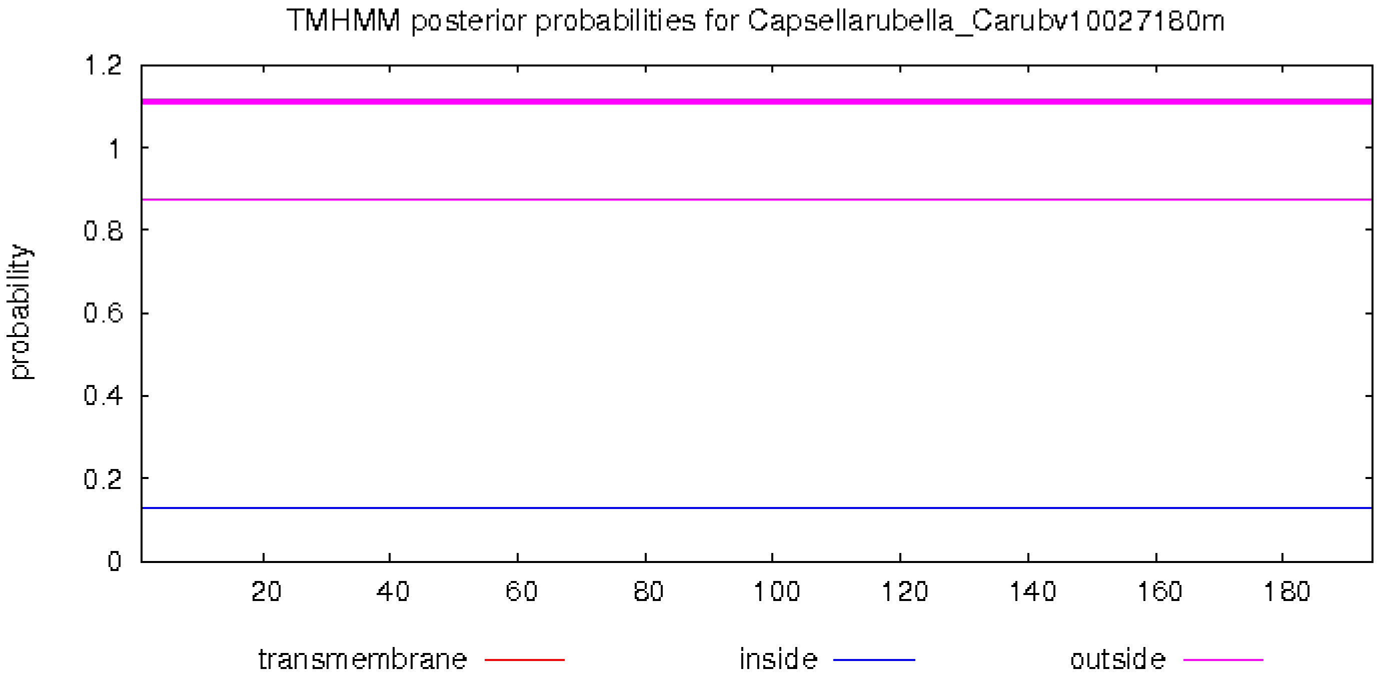

plot in postscript, script for making the plot in gnuplot, data for plot

---

```
# Capsellarubella_Carubv10027198m Length: 187
# Capsellarubella_Carubv10027198m Number of predicted TMHs:  0
```

```
# Capsellarubella_Carubv10027198m Exp number of AAs in TMHs: 0
# Capsellarubella_Carubv10027198m Exp number, first 60 AAs:  0
# Capsellarubella_Carubv10027198m Total prob of N-in:        0.00733
Capsellarubella_Carubv10027198m TMHMM2.0        outside      1   187
```

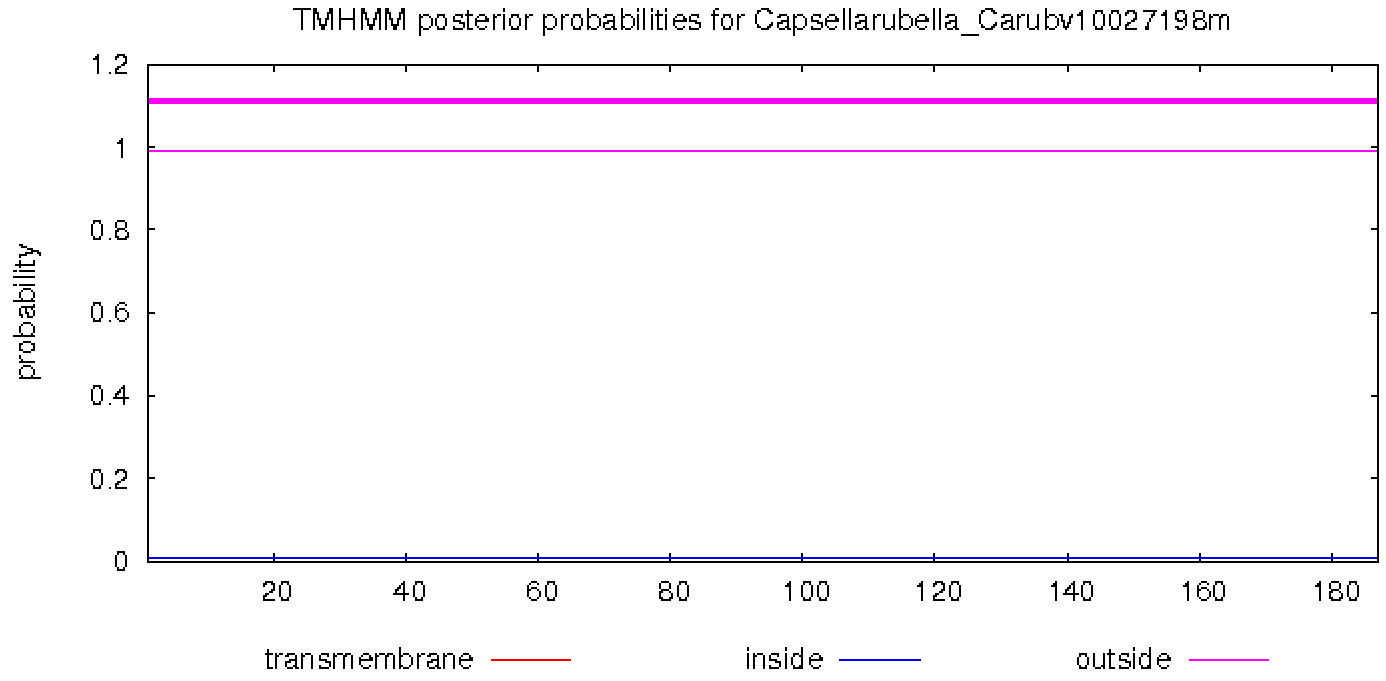

plot in postscript, script for making the plot in gnuplot, data for plot

---

```
# Capsellarubella_Carubv10027934m Length: 378
# Capsellarubella_Carubv10027934m Number of predicted TMHs:  0
# Capsellarubella_Carubv10027934m Exp number of AAs in TMHs: 0
# Capsellarubella_Carubv10027934m Exp number, first 60 AAs:  0
# Capsellarubella_Carubv10027934m Total prob of N-in:        0.02801
Capsellarubella_Carubv10027934m TMHMM2.0        outside      1   378
```

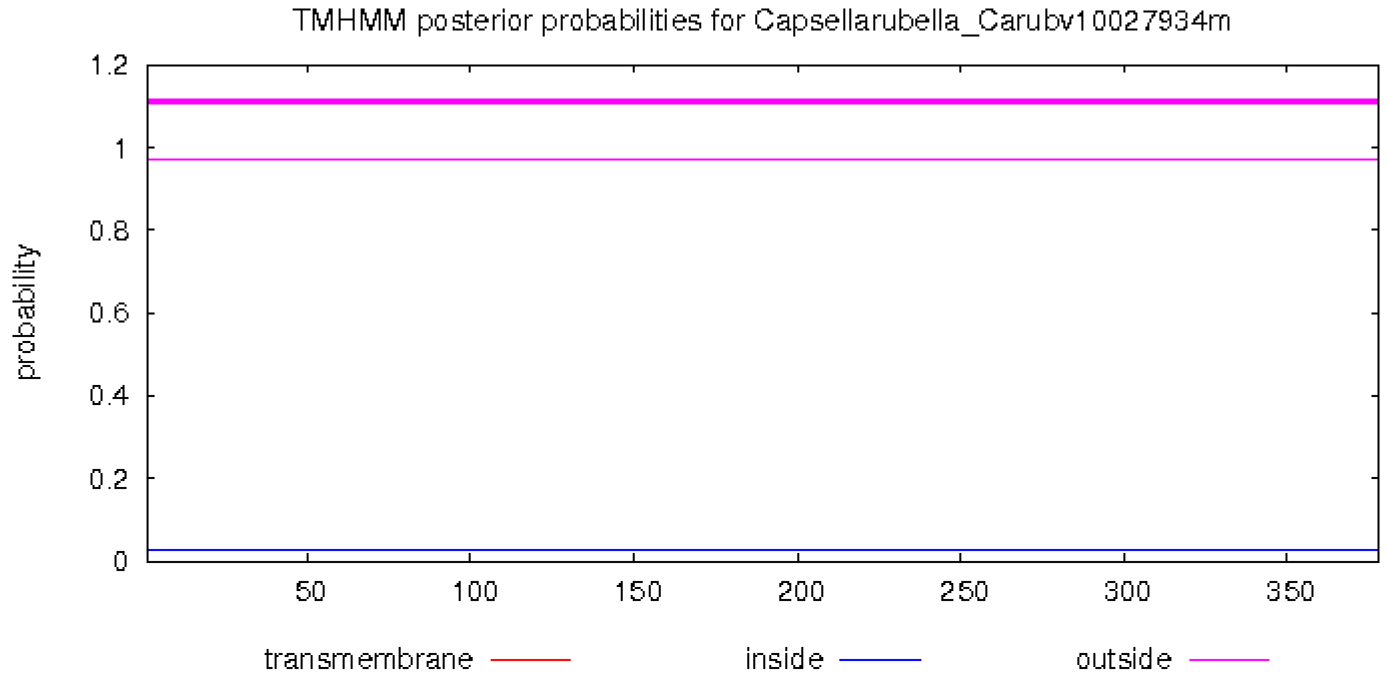

plot in postscript, script for making the plot in gnuplot, data for plot

---

```
# Capsellarubella_Carubv10028131m Length: 385
# Capsellarubella_Carubv10028131m Number of predicted TMHs:  0
```

```
# Capsellarubella_Carubv10028131m Exp number of AAs in TMHs: 0.01474
# Capsellarubella_Carubv10028131m Exp number, first 60 AAs:  0
# Capsellarubella_Carubv10028131m Total prob of N-in:        0.04081
Capsellarubella_Carubv10028131m TMHMM2.0        outside      1   385
```

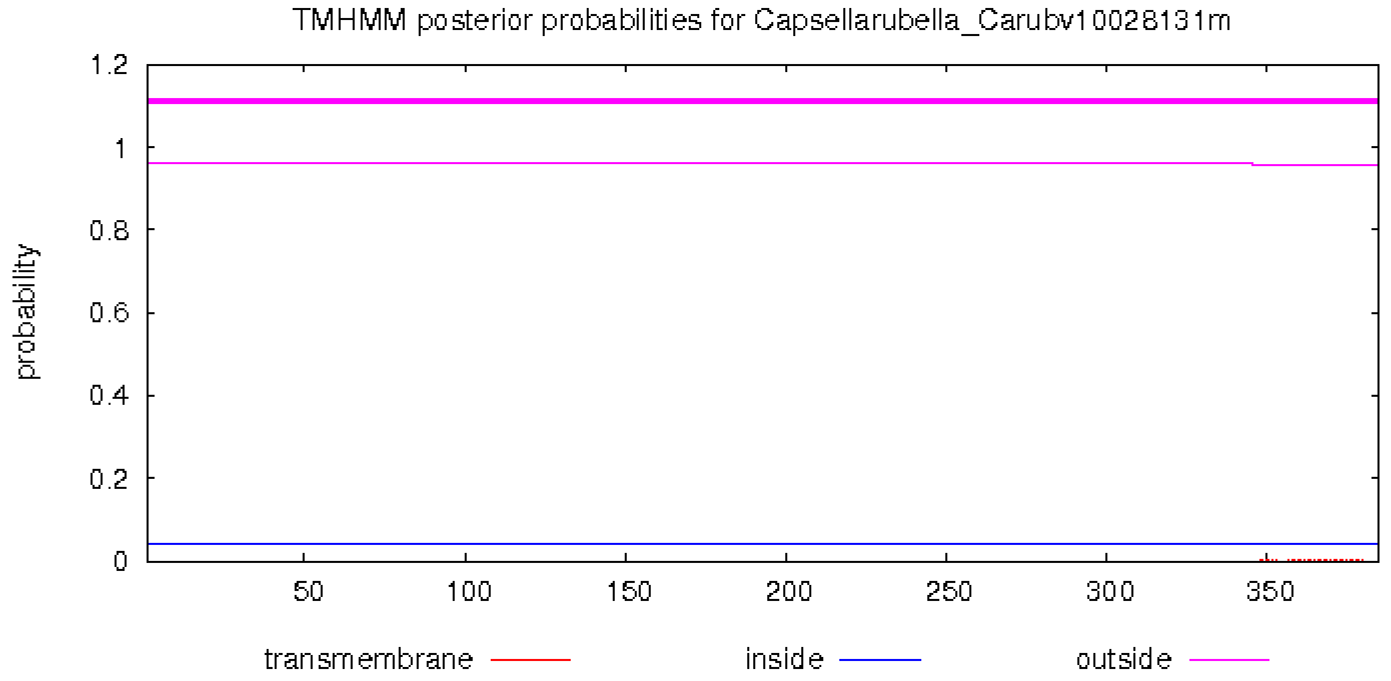

plot in postscript, script for making the plot in gnuplot, data for plot

---

```
# Capsellarubella_Carubv10028284m Length: 291
# Capsellarubella_Carubv10028284m Number of predicted TMHs:  0
# Capsellarubella_Carubv10028284m Exp number of AAs in TMHs: 0
# Capsellarubella_Carubv10028284m Exp number, first 60 AAs:  0
# Capsellarubella_Carubv10028284m Total prob of N-in:        0.00216
Capsellarubella_Carubv10028284m TMHMM2.0        outside      1   291
```

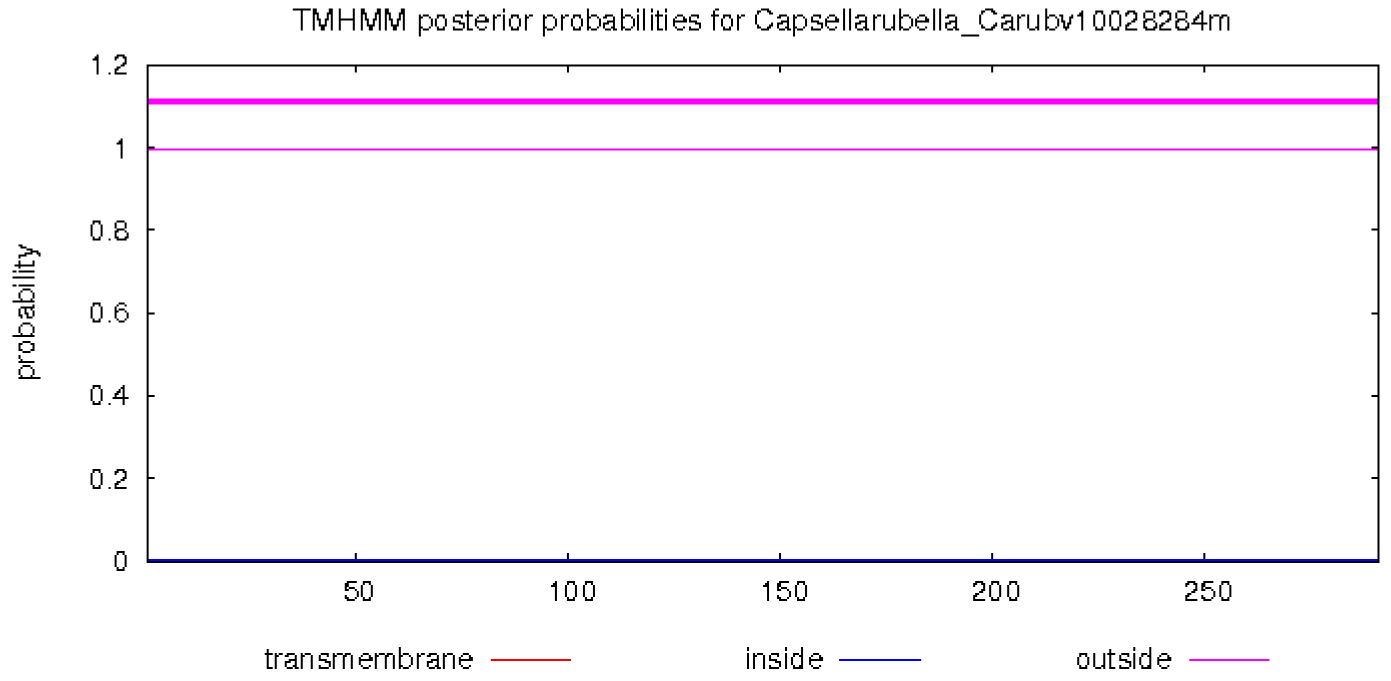

plot in postscript, script for making the plot in gnuplot, data for plot

---

```
# Capsellarubella_Carubv10028330m Length: 376
# Capsellarubella_Carubv10028330m Number of predicted TMHs:  0
```

```
# Capsellarubella_Carubv10028330m Exp number of AAs in TMHs: 0.01123
# Capsellarubella_Carubv10028330m Exp number, first 60 AAs:  0
# Capsellarubella_Carubv10028330m Total prob of N-in:        0.01682
Capsellarubella_Carubv10028330m TMHMM2.0        outside      1   376
```

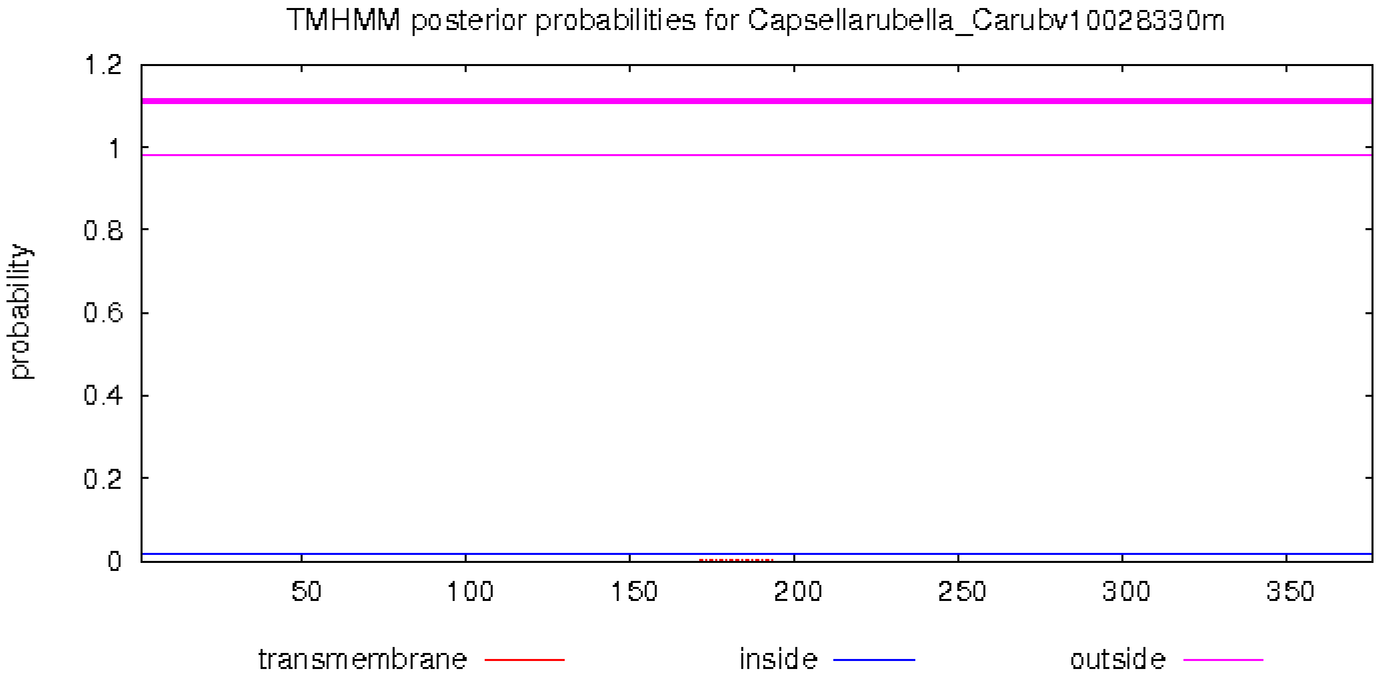

plot in postscript, script for making the plot in gnuplot, data for plot

---

```
# Capsellarubella_Carubv10028442m Length: 151
# Capsellarubella_Carubv10028442m Number of predicted TMHs:  0
# Capsellarubella_Carubv10028442m Exp number of AAs in TMHs: 0
# Capsellarubella_Carubv10028442m Exp number, first 60 AAs:  0
# Capsellarubella_Carubv10028442m Total prob of N-in:        0.09348
Capsellarubella_Carubv10028442m TMHMM2.0        outside      1   151
```

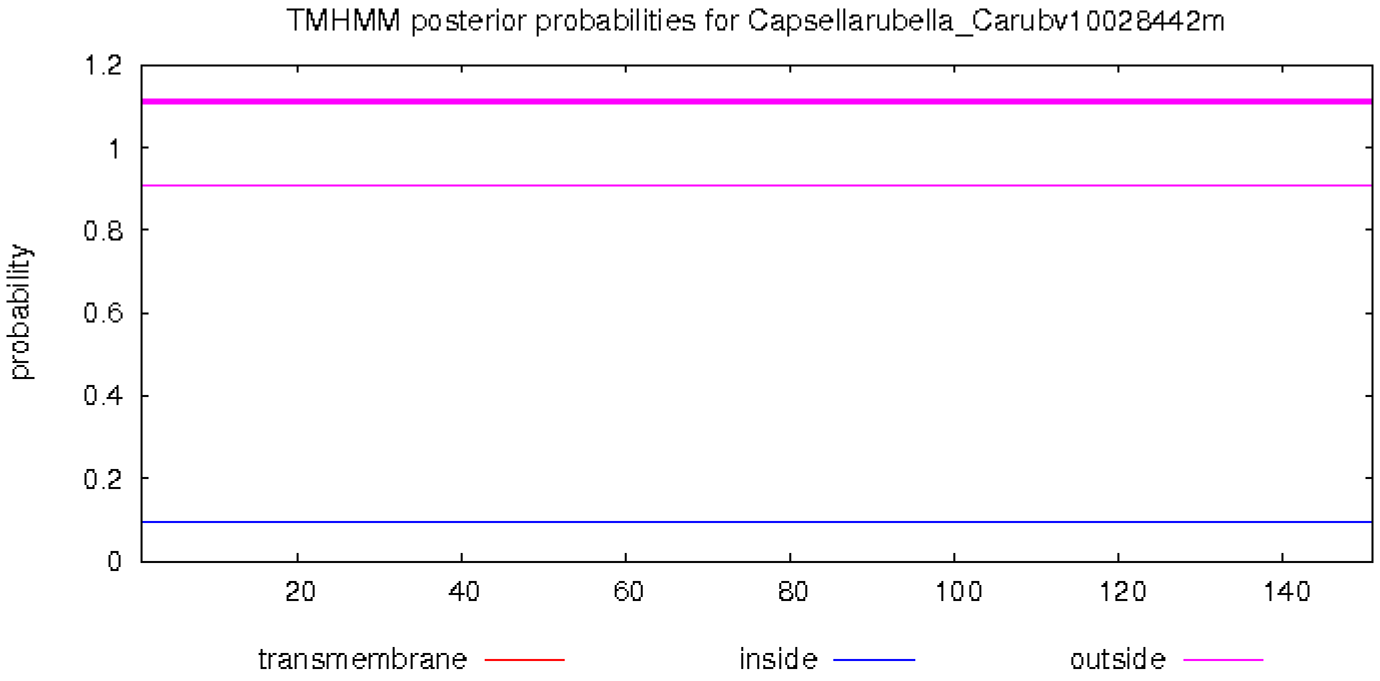

plot in postscript, script for making the plot in gnuplot, data for plot

---

```
# Capsicumannuum_CA00g03050 Length: 290
# Capsicumannuum_CA00g03050 Number of predicted TMHs:  0
```

```
# Capsicumannuum_CA00g03050 Exp number of AAs in TMHs: 0.00016
# Capsicumannuum_CA00g03050 Exp number, first 60 AAs:  0.00016
# Capsicumannuum_CA00g03050 Total prob of N-in:        0.00813
Capsicumannuum_CA00g03050	TMHMM2.0	outside	     1   290
```

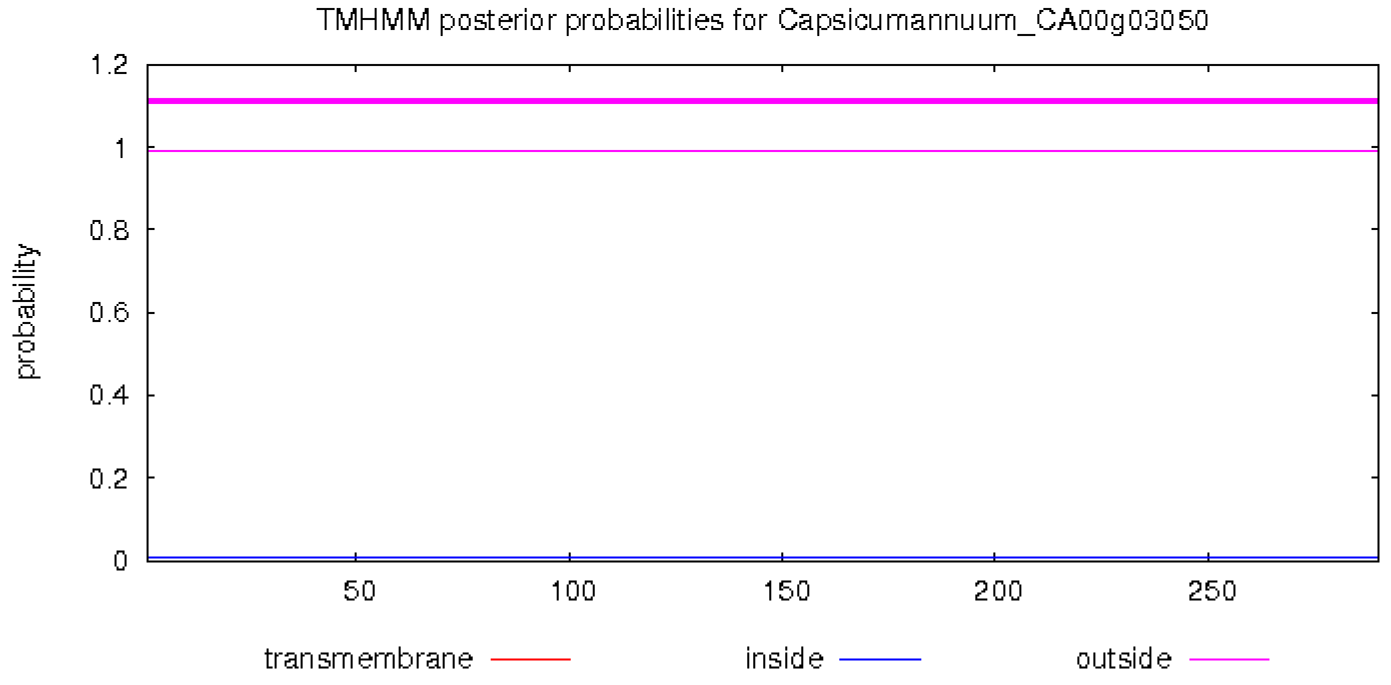

plot in postscript, script for making the plot in gnuplot, data for plot

---

```
# Capsicumannuum_CA00g46270 Length: 290
# Capsicumannuum_CA00g46270 Number of predicted TMHs:  0
# Capsicumannuum_CA00g46270 Exp number of AAs in TMHs: 0.00132
# Capsicumannuum_CA00g46270 Exp number, first 60 AAs:  0
# Capsicumannuum_CA00g46270 Total prob of N-in:        0.01255
Capsicumannuum_CA00g46270	TMHMM2.0	outside	     1   290
```

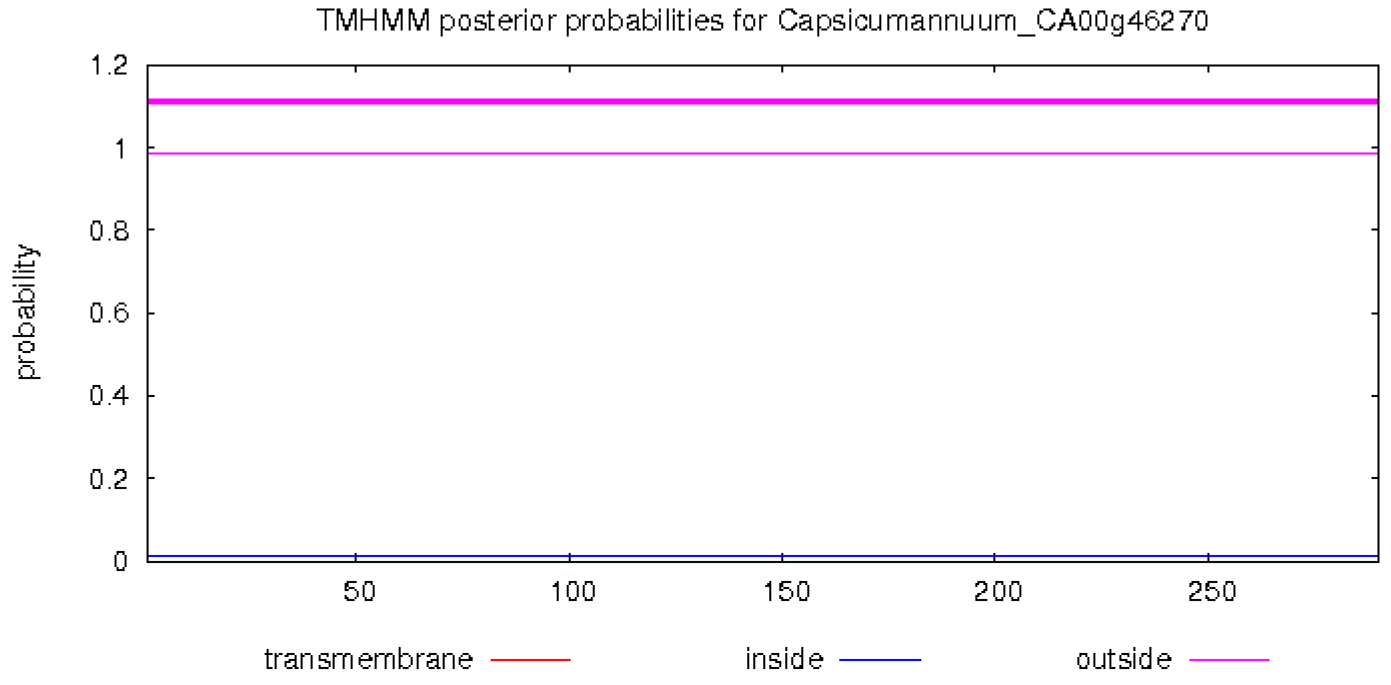

plot in postscript, script for making the plot in gnuplot, data for plot

---

```
# Capsicumannuum_CA00g54690 Length: 248
# Capsicumannuum_CA00g54690 Number of predicted TMHs:  0
```

```
# Capsicumannuum_CA00g54690 Exp number of AAs in TMHs: 0.00048
# Capsicumannuum_CA00g54690 Exp number, first 60 AAs:  0.00017
# Capsicumannuum_CA00g54690 Total prob of N-in:        0.18346
Capsicumannuum_CA00g54690       TMHMM2.0        outside      1   248
```

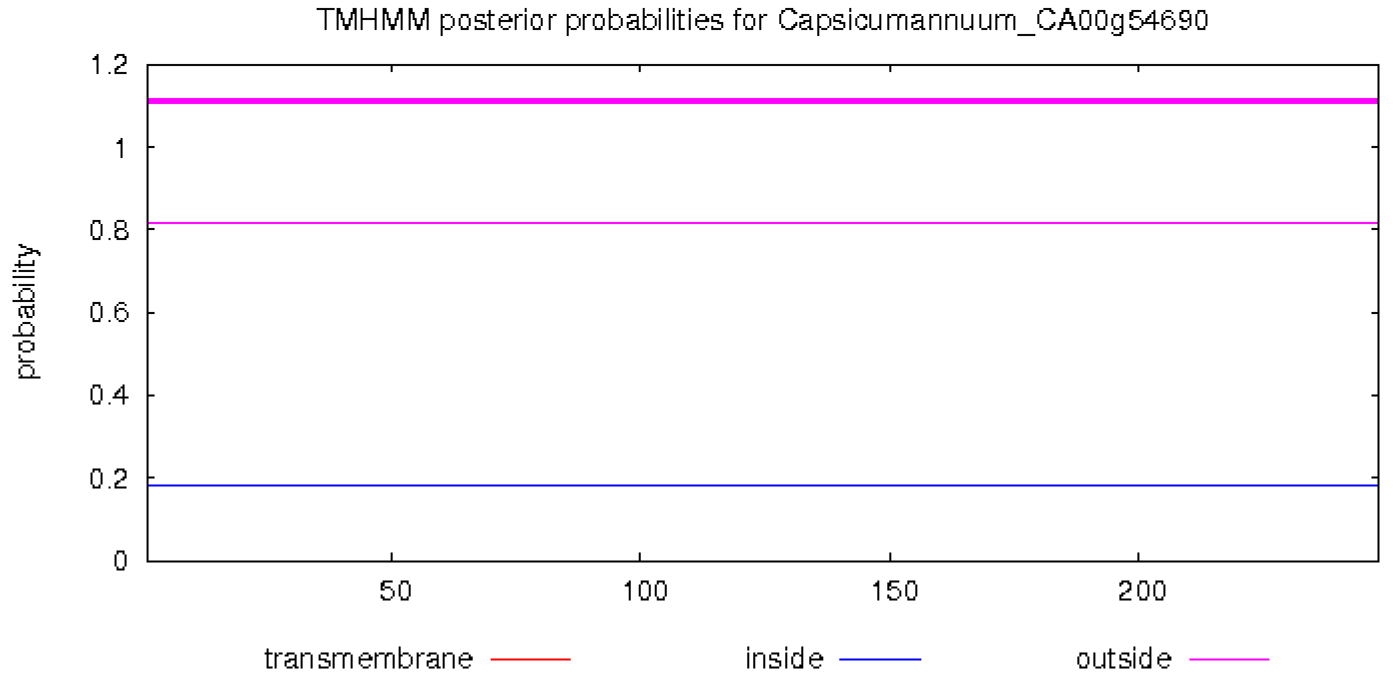

plot in postscript, script for making the plot in gnuplot, data for plot

---

```
# Capsicumannuum_CA00g66680 Length: 178
# Capsicumannuum_CA00g66680 Number of predicted TMHs:  0
# Capsicumannuum_CA00g66680 Exp number of AAs in TMHs: 0.00016
# Capsicumannuum_CA00g66680 Exp number, first 60 AAs:  0.00016
# Capsicumannuum_CA00g66680 Total prob of N-in:        0.13467
Capsicumannuum_CA00g66680       TMHMM2.0        outside      1   178
```

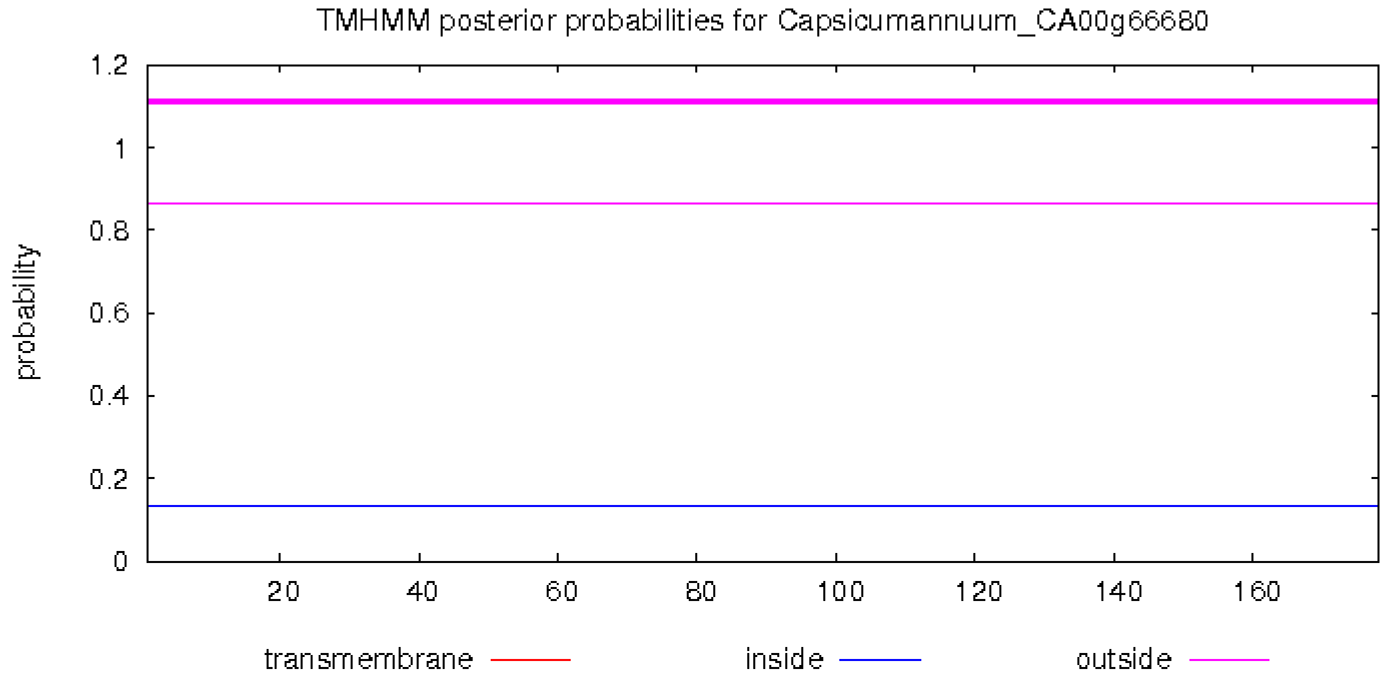

plot in postscript, script for making the plot in gnuplot, data for plot

---

```
# Capsicumannuum_CA00g67620 Length: 190
# Capsicumannuum_CA00g67620 Number of predicted TMHs:  0
```

```
# Capsicumannuum_CA00g67620 Exp number of AAs in TMHs: 0.00013
# Capsicumannuum_CA00g67620 Exp number, first 60 AAs:  0
# Capsicumannuum_CA00g67620 Total prob of N-in:        0.06289
Capsicumannuum_CA00g67620       TMHMM2.0        outside      1   190
```

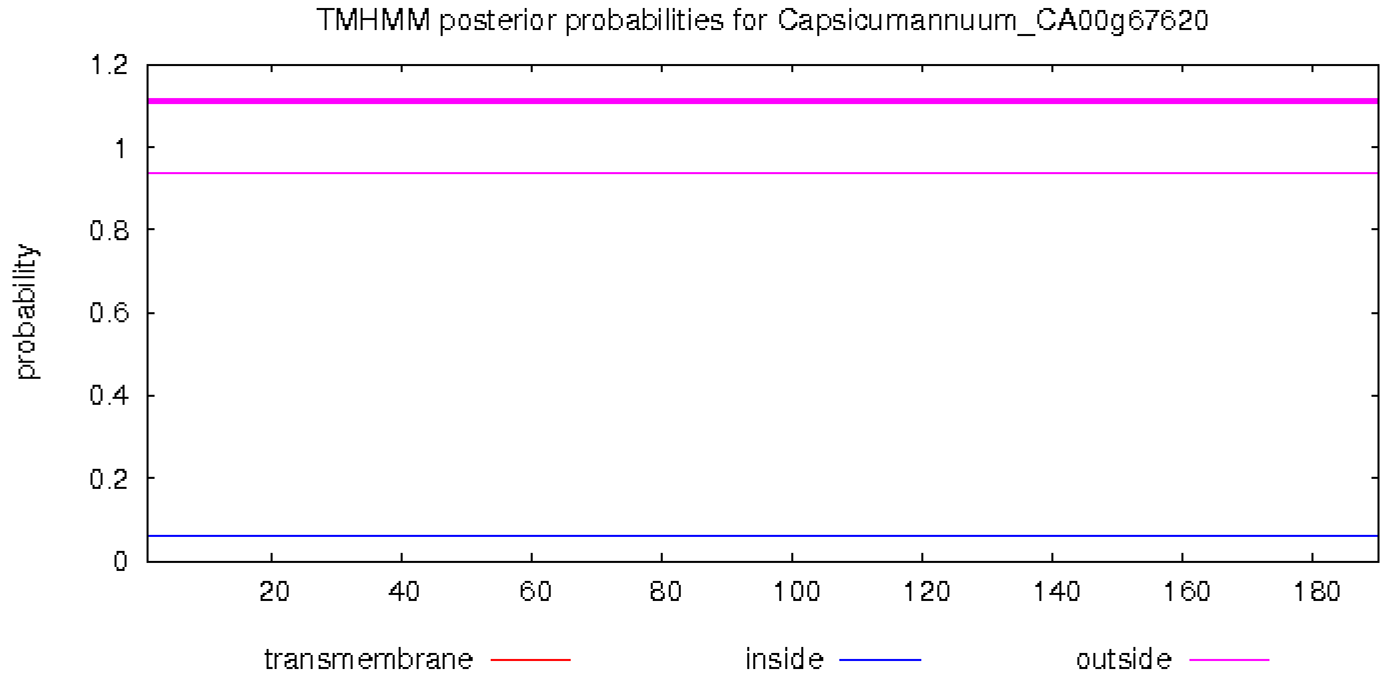

plot in postscript, script for making the plot in gnuplot, data for plot

---

```
# Capsicumannuum_CA00g73220 Length: 178
# Capsicumannuum_CA00g73220 Number of predicted TMHs:  0
# Capsicumannuum_CA00g73220 Exp number of AAs in TMHs: 0.00017
# Capsicumannuum_CA00g73220 Exp number, first 60 AAs:  5e-05
# Capsicumannuum_CA00g73220 Total prob of N-in:        0.03878
Capsicumannuum_CA00g73220       TMHMM2.0        outside      1   178
```

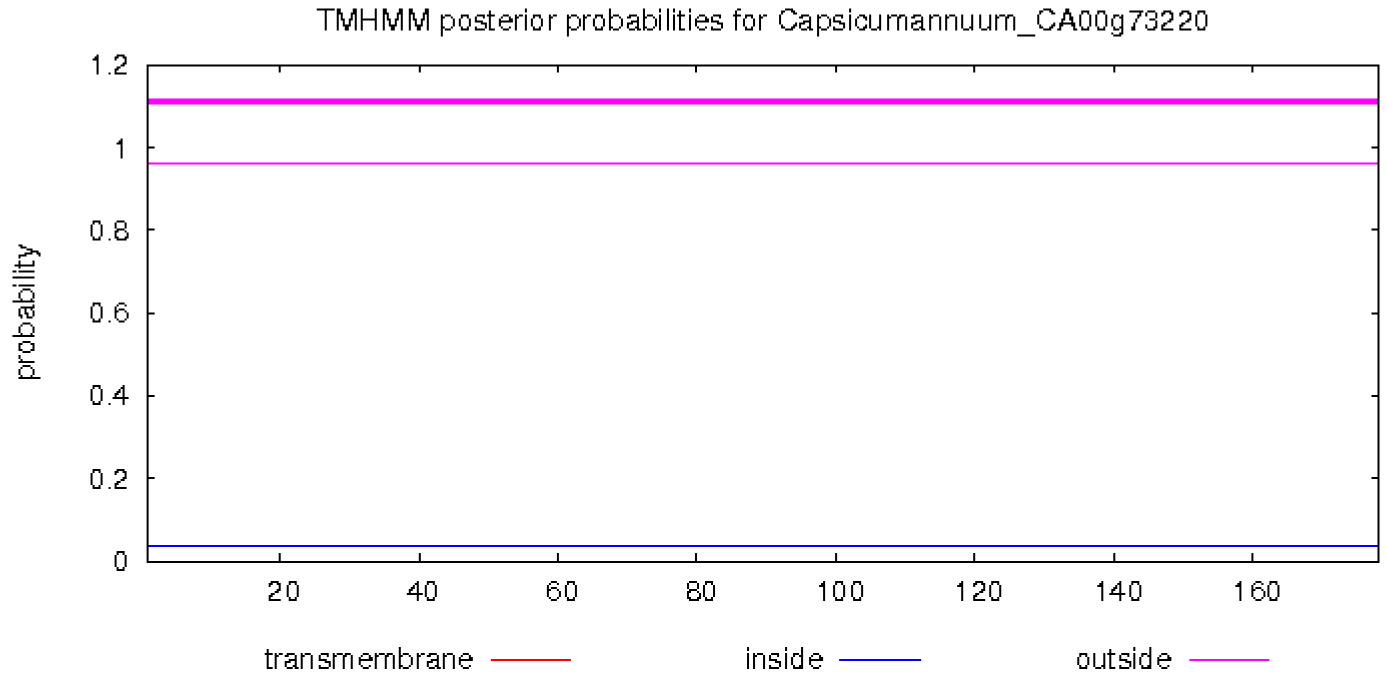

plot in postscript, script for making the plot in gnuplot, data for plot

---

```
# Capsicumannuum_CA00g77790 Length: 334
# Capsicumannuum_CA00g77790 Number of predicted TMHs:  0
```

```
# Capsicumannuum_CA00g77790 Exp number of AAs in TMHs: 0.00104
# Capsicumannuum_CA00g77790 Exp number, first 60 AAs:  0
# Capsicumannuum_CA00g77790 Total prob of N-in:        0.00168
Capsicumannuum_CA00g77790	TMHMM2.0	outside	     1   334
```

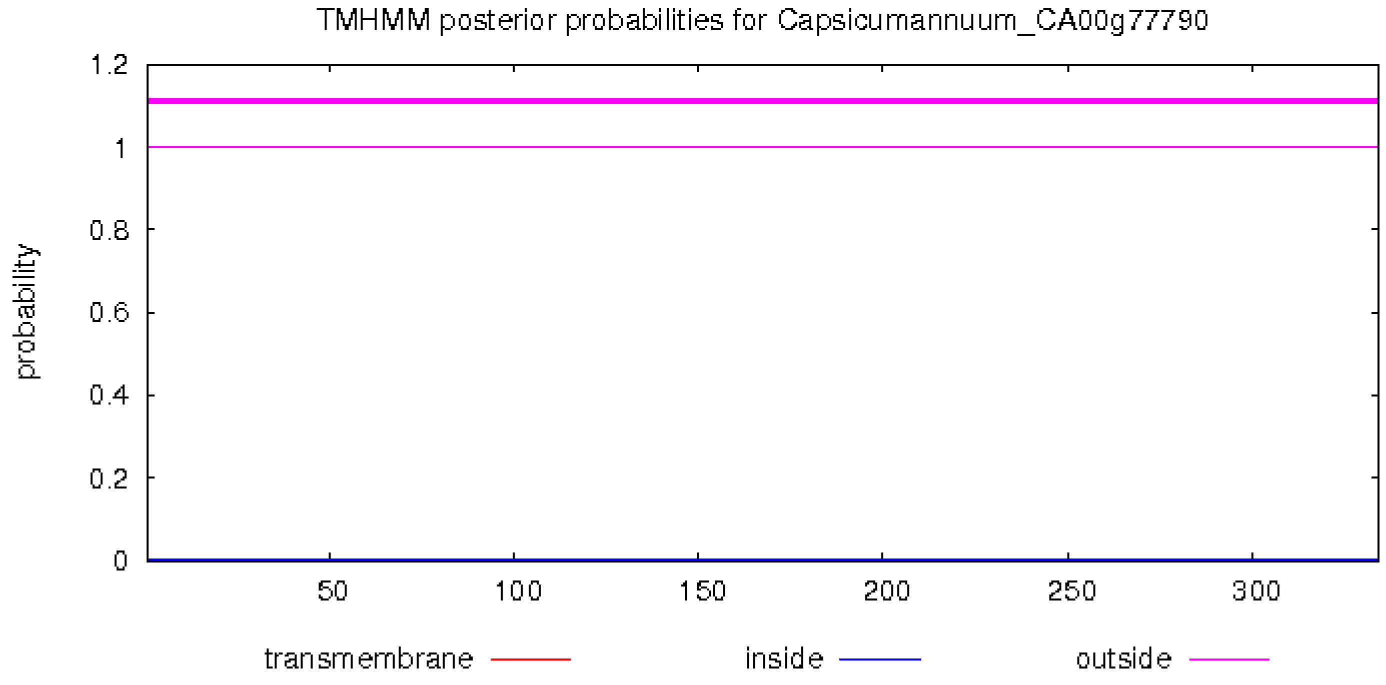

plot in postscript, script for making the plot in gnuplot, data for plot

---

```
# Capsicumannuum_CA00g84200 Length: 292
# Capsicumannuum_CA00g84200 Number of predicted TMHs:  0
# Capsicumannuum_CA00g84200 Exp number of AAs in TMHs: 0.00021
# Capsicumannuum_CA00g84200 Exp number, first 60 AAs:  0
# Capsicumannuum_CA00g84200 Total prob of N-in:        0.04849
Capsicumannuum_CA00g84200	TMHMM2.0	outside	     1   292
```

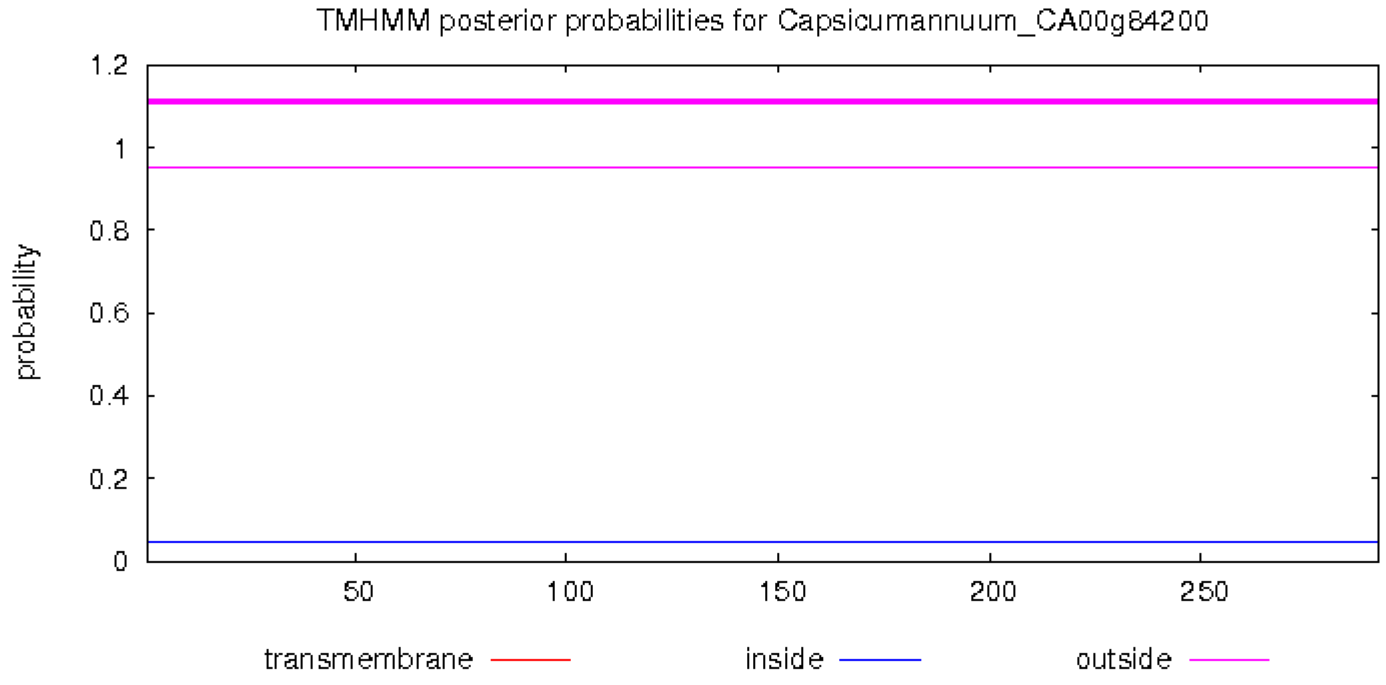

plot in postscript, script for making the plot in gnuplot, data for plot

---

```
# Capsicumannuum_CA00g86400 Length: 382
# Capsicumannuum_CA00g86400 Number of predicted TMHs:  0
```

```
# Capsicumannuum_CA00g86400 Exp number of AAs in TMHs: 0.00032
# Capsicumannuum_CA00g86400 Exp number, first 60 AAs:  0
# Capsicumannuum_CA00g86400 Total prob of N-in:        0.00663
Capsicumannuum_CA00g86400       TMHMM2.0        outside      1   382
```

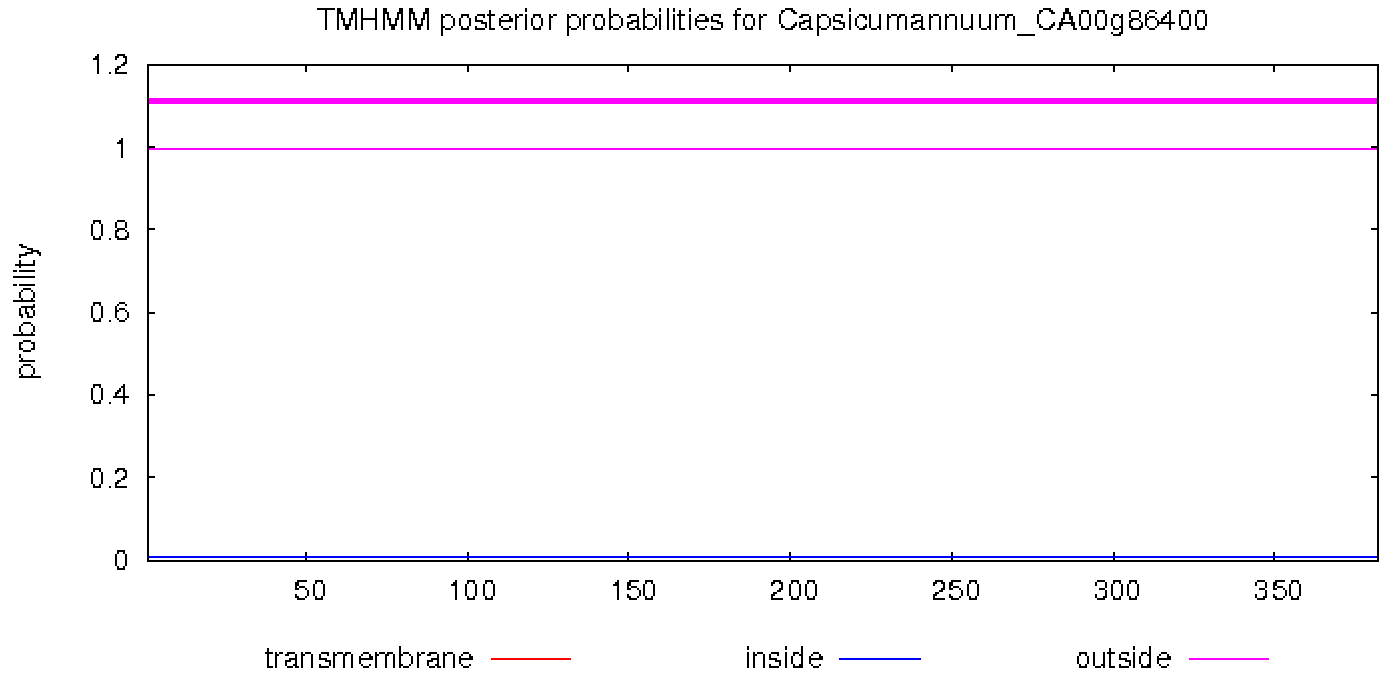

plot in postscript, script for making the plot in gnuplot, data for plot

---

```
# Capsicumannuum_CA00g86410 Length: 590
# Capsicumannuum_CA00g86410 Number of predicted TMHs:  1
# Capsicumannuum_CA00g86410 Exp number of AAs in TMHs: 22.40302
# Capsicumannuum_CA00g86410 Exp number, first 60 AAs:  0
# Capsicumannuum_CA00g86410 Total prob of N-in:        0.00494
Capsicumannuum_CA00g86410       TMHMM2.0        outside      1   555
Capsicumannuum_CA00g86410       TMHMM2.0        TMhelix    556   578
Capsicumannuum_CA00g86410       TMHMM2.0        inside     579   590
```

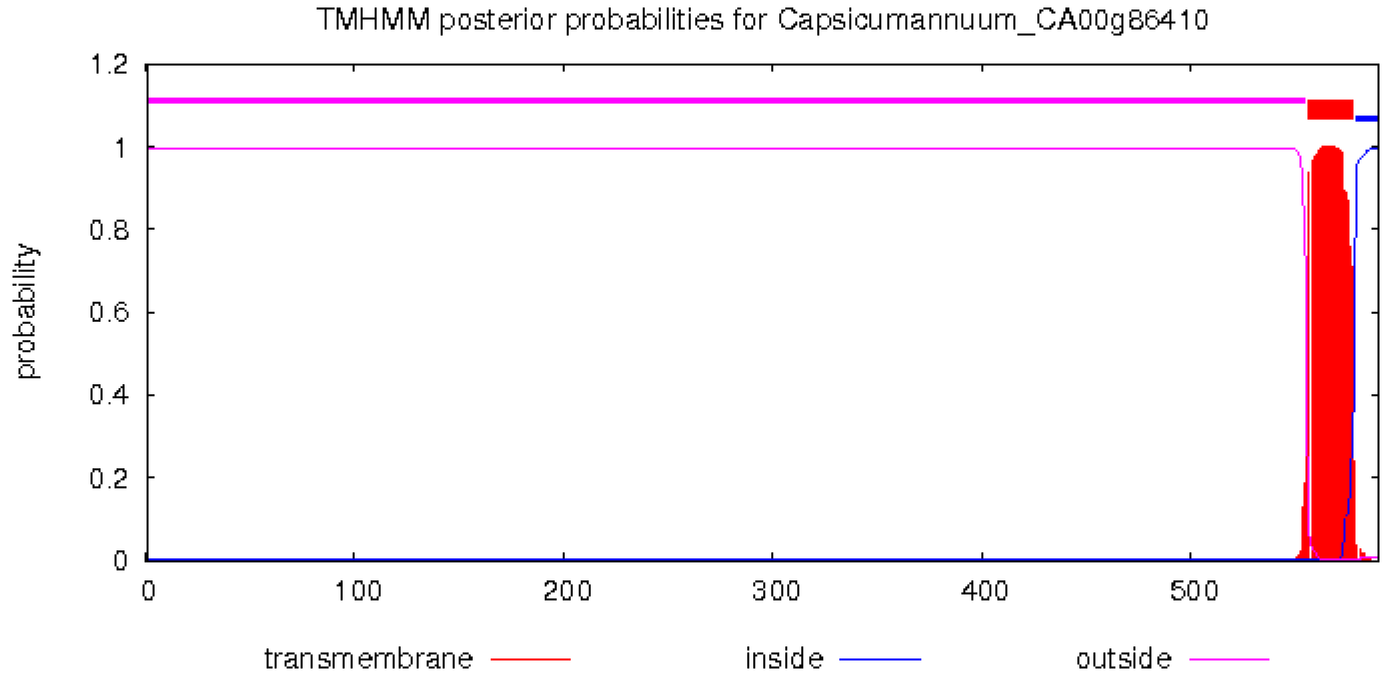

plot in postscript, script for making the plot in gnuplot, data for plot

---

```
# Capsicumannuum_CA00g89750 Length: 171
# Capsicumannuum_CA00g89750 Number of predicted TMHs:  0
# Capsicumannuum_CA00g89750 Exp number of AAs in TMHs: 0.00148
# Capsicumannuum_CA00g89750 Exp number, first 60 AAs:  0.00016
# Capsicumannuum_CA00g89750 Total prob of N-in:        0.02736
Capsicumannuum_CA00g89750	TMHMM2.0	outside	     1   171
```

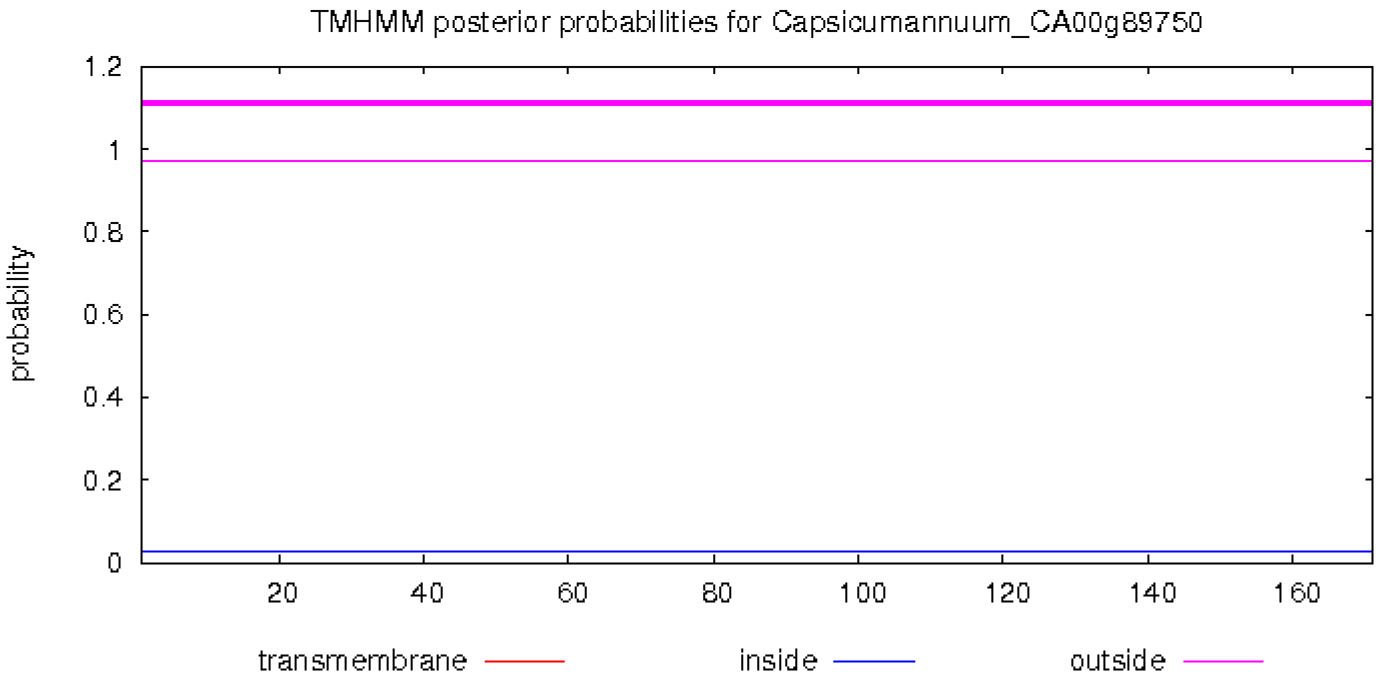

plot in postscript, script for making the plot in gnuplot, data for plot

---

```
# Capsicumannuum_CA00g89790 Length: 348
# Capsicumannuum_CA00g89790 Number of predicted TMHs:  0
# Capsicumannuum_CA00g89790 Exp number of AAs in TMHs: 0
# Capsicumannuum_CA00g89790 Exp number, first 60 AAs:  0
# Capsicumannuum_CA00g89790 Total prob of N-in:        0.01095
Capsicumannuum_CA00g89790	TMHMM2.0	outside	     1   348
```

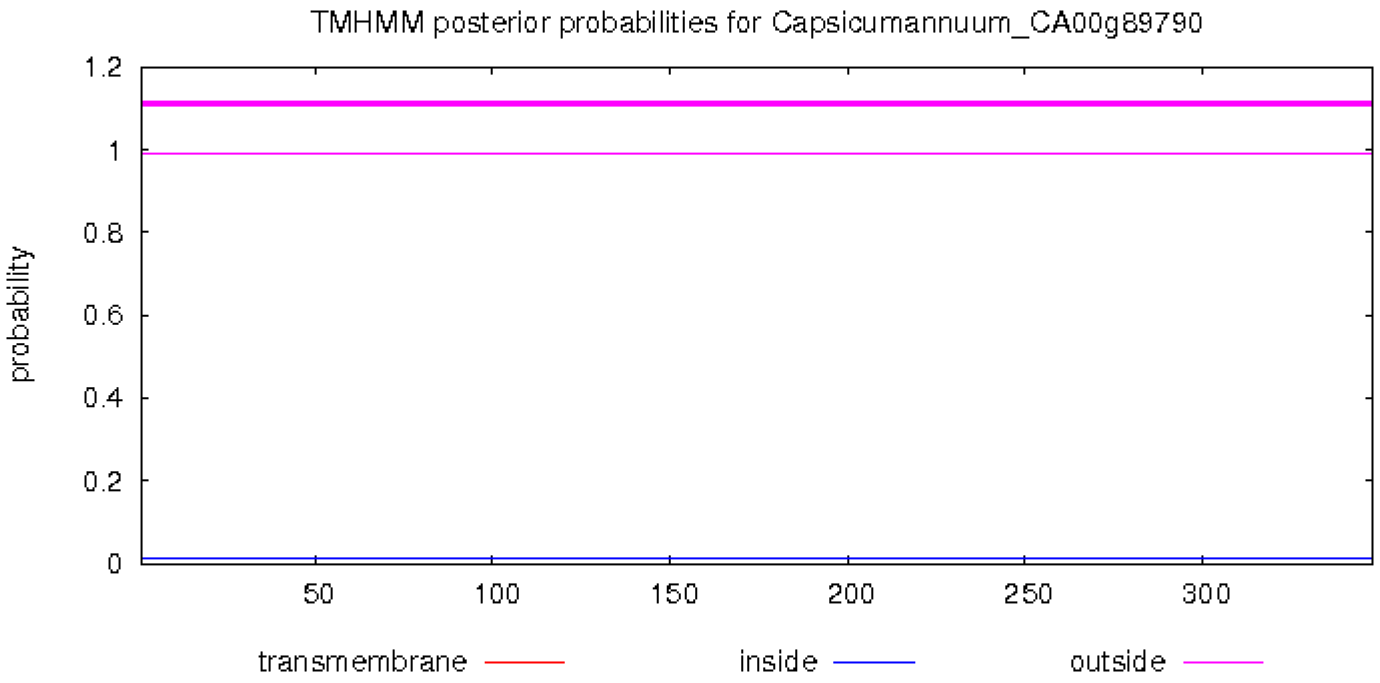

plot in postscript, script for making the plot in gnuplot, data for plot

---

```
# Capsicumannuum_CA01g04740 Length: 336
# Capsicumannuum_CA01g04740 Number of predicted TMHs:  0
# Capsicumannuum_CA01g04740 Exp number of AAs in TMHs: 0
# Capsicumannuum_CA01g04740 Exp number, first 60 AAs:  0
# Capsicumannuum_CA01g04740 Total prob of N-in:        0.00498
Capsicumannuum_CA01g04740	TMHMM2.0	outside	     1   336
```

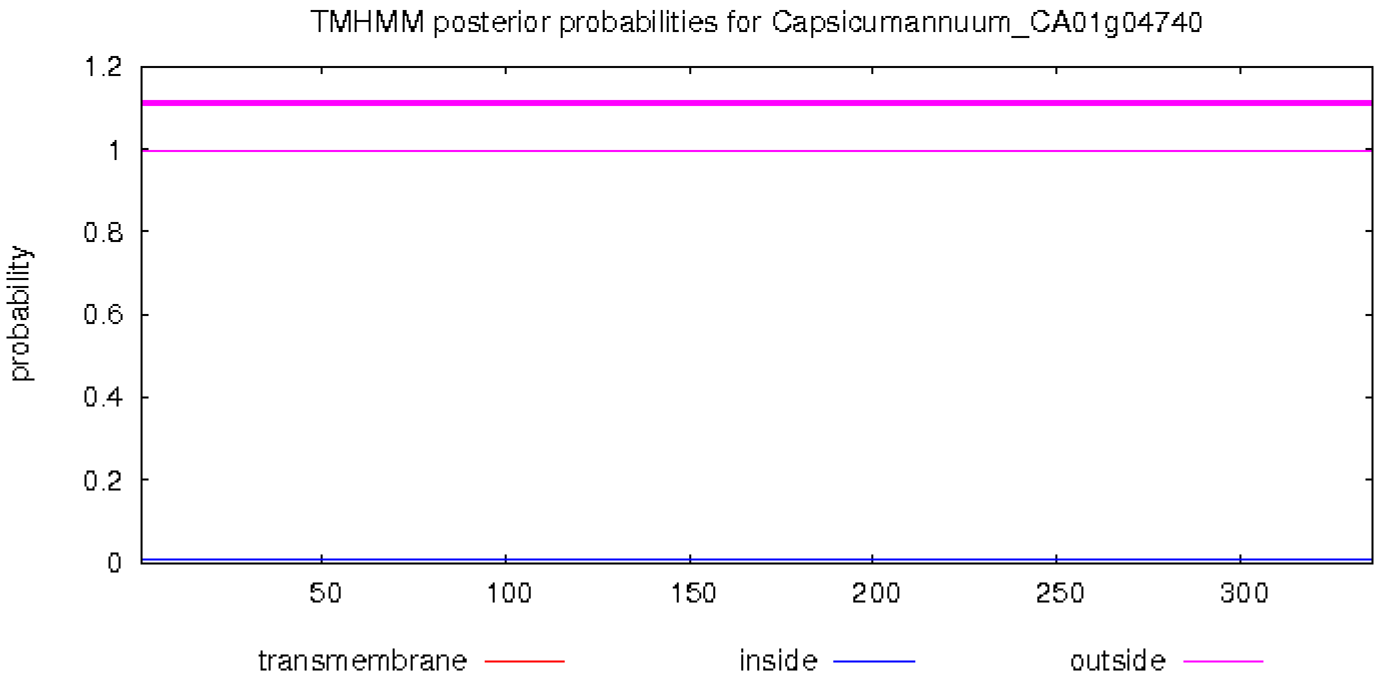

plot in postscript, script for making the plot in gnuplot, data for plot

---

```
# Capsicumannuum_CA01g07550 Length: 418
# Capsicumannuum_CA01g07550 Number of predicted TMHs:  0
# Capsicumannuum_CA01g07550 Exp number of AAs in TMHs: 0.00019
# Capsicumannuum_CA01g07550 Exp number, first 60 AAs:  0.00019
# Capsicumannuum_CA01g07550 Total prob of N-in:        0.00418
Capsicumannuum_CA01g07550	TMHMM2.0	outside	     1   418
```

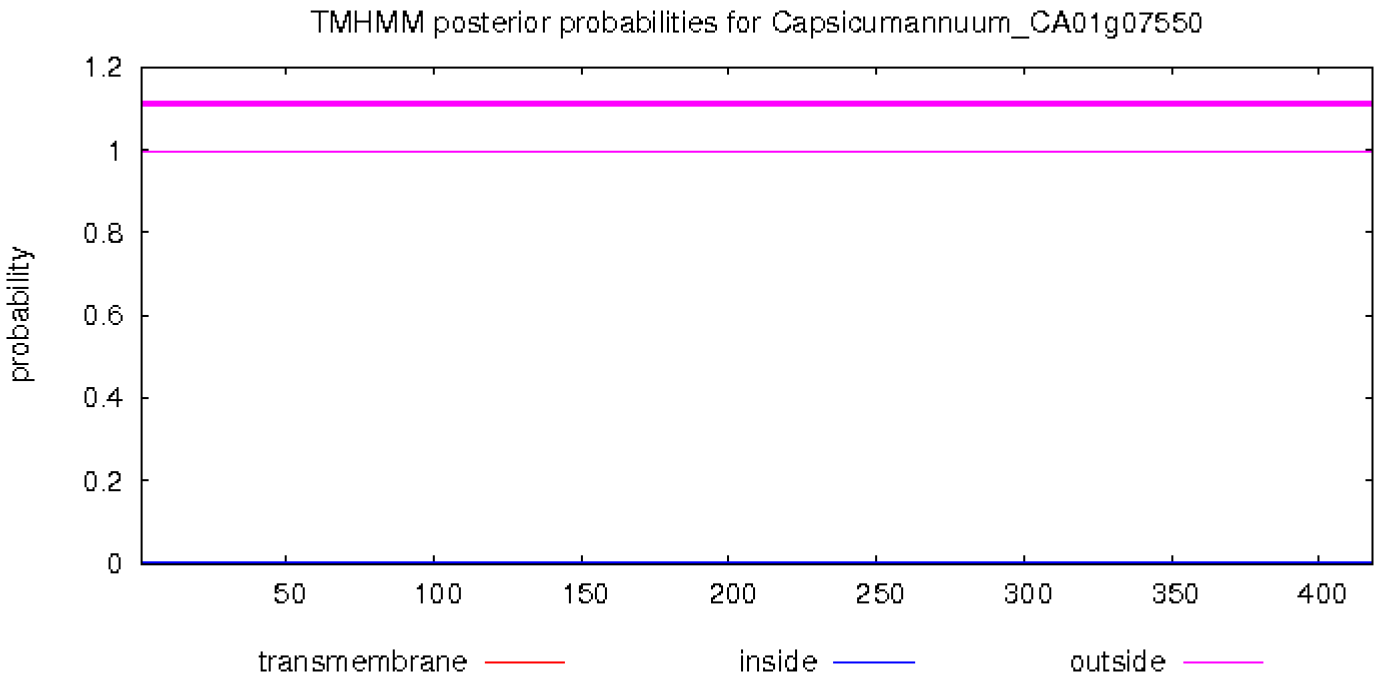

plot in postscript, script for making the plot in gnuplot, data for plot

---

```
# Capsicumannuum_CA01g09650 Length: 311
# Capsicumannuum_CA01g09650 Number of predicted TMHs:  0
# Capsicumannuum_CA01g09650 Exp number of AAs in TMHs: 0.00581
# Capsicumannuum_CA01g09650 Exp number, first 60 AAs:  0
# Capsicumannuum_CA01g09650 Total prob of N-in:        0.01422
Capsicumannuum_CA01g09650	TMHMM2.0	outside	     1   311
```

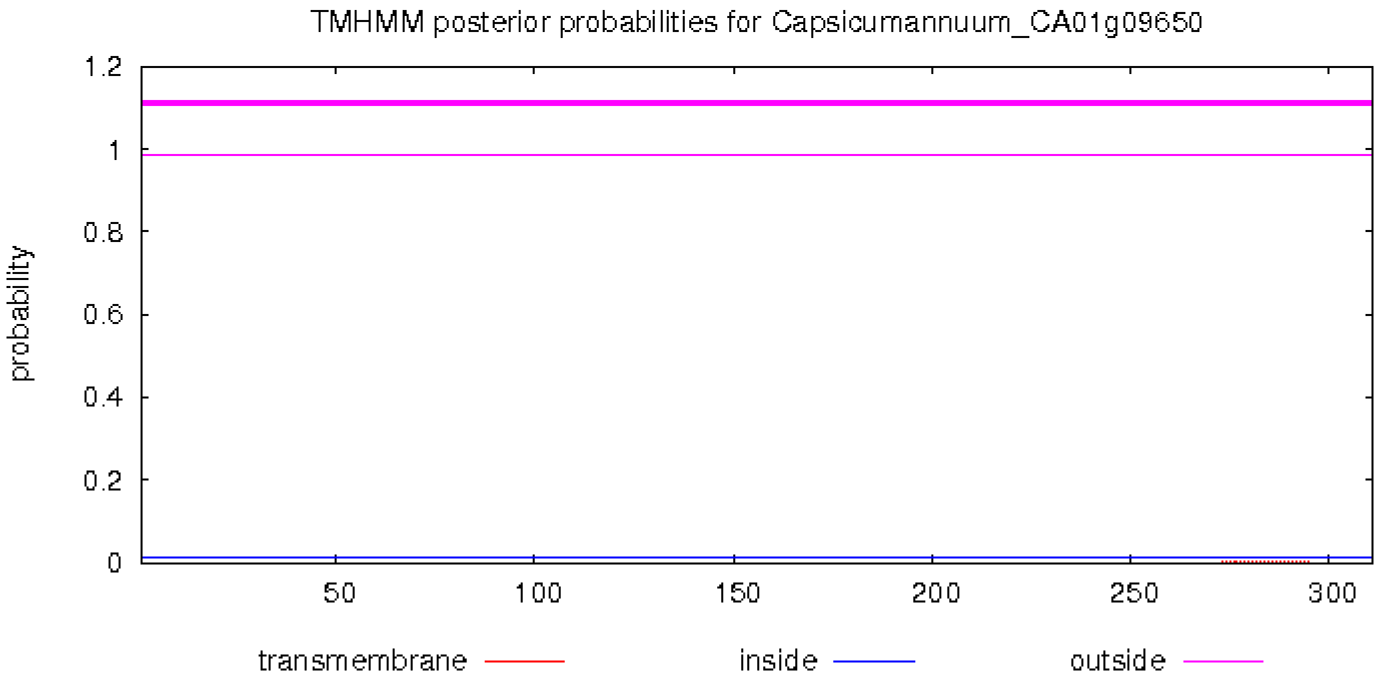

plot in postscript, script for making the plot in gnuplot, data for plot

```
# Capsicumannuum_CA01g11960 Length: 276
# Capsicumannuum_CA01g11960 Number of predicted TMHs:  0
# Capsicumannuum_CA01g11960 Exp number of AAs in TMHs: 0.15047
# Capsicumannuum_CA01g11960 Exp number, first 60 AAs:  0.15047
# Capsicumannuum_CA01g11960 Total prob of N-in:        0.01506
Capsicumannuum_CA01g11960	TMHMM2.0	outside	     1   276
```

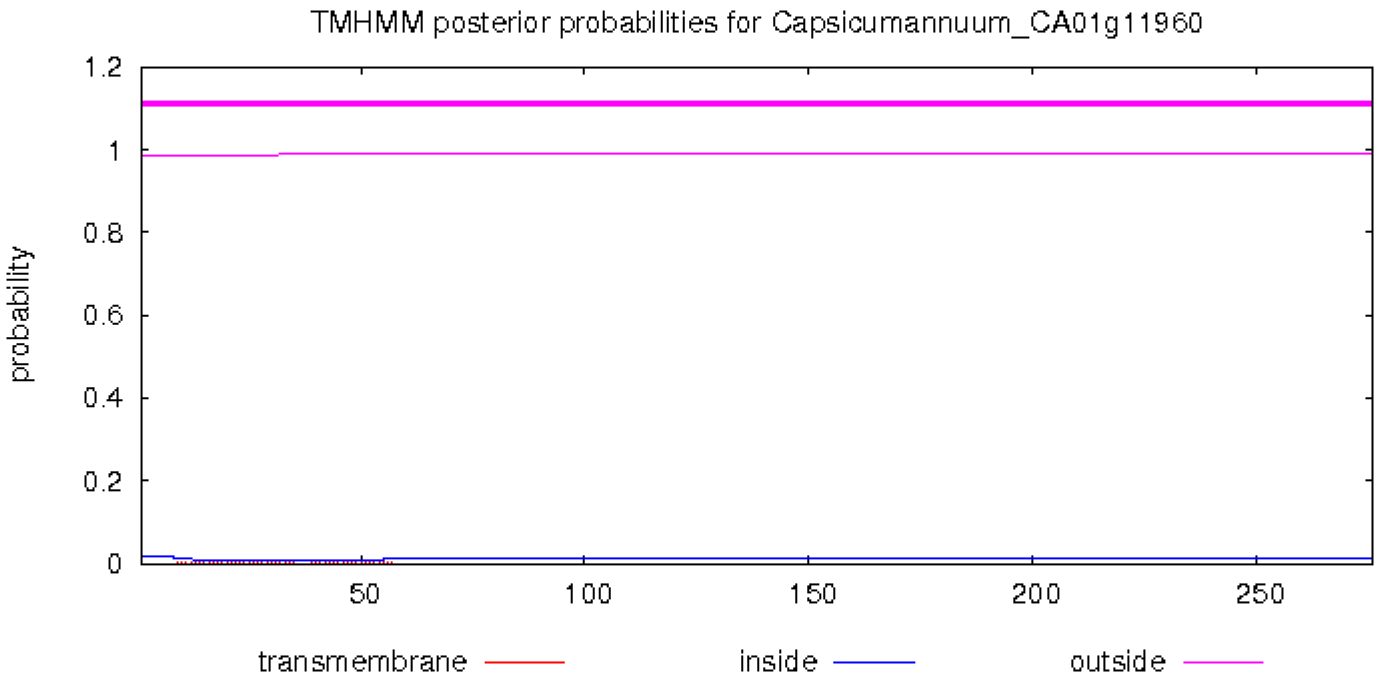

plot in postscript, script for making the plot in gnuplot, data for plot

```
# Capsicumannuum_CA01g17160 Length: 229
# Capsicumannuum_CA01g17160 Number of predicted TMHs:  0
# Capsicumannuum_CA01g17160 Exp number of AAs in TMHs: 1.52382
# Capsicumannuum_CA01g17160 Exp number, first 60 AAs:  0
# Capsicumannuum_CA01g17160 Total prob of N-in:        0.04166
Capsicumannuum_CA01g17160       TMHMM2.0        outside      1   229
```

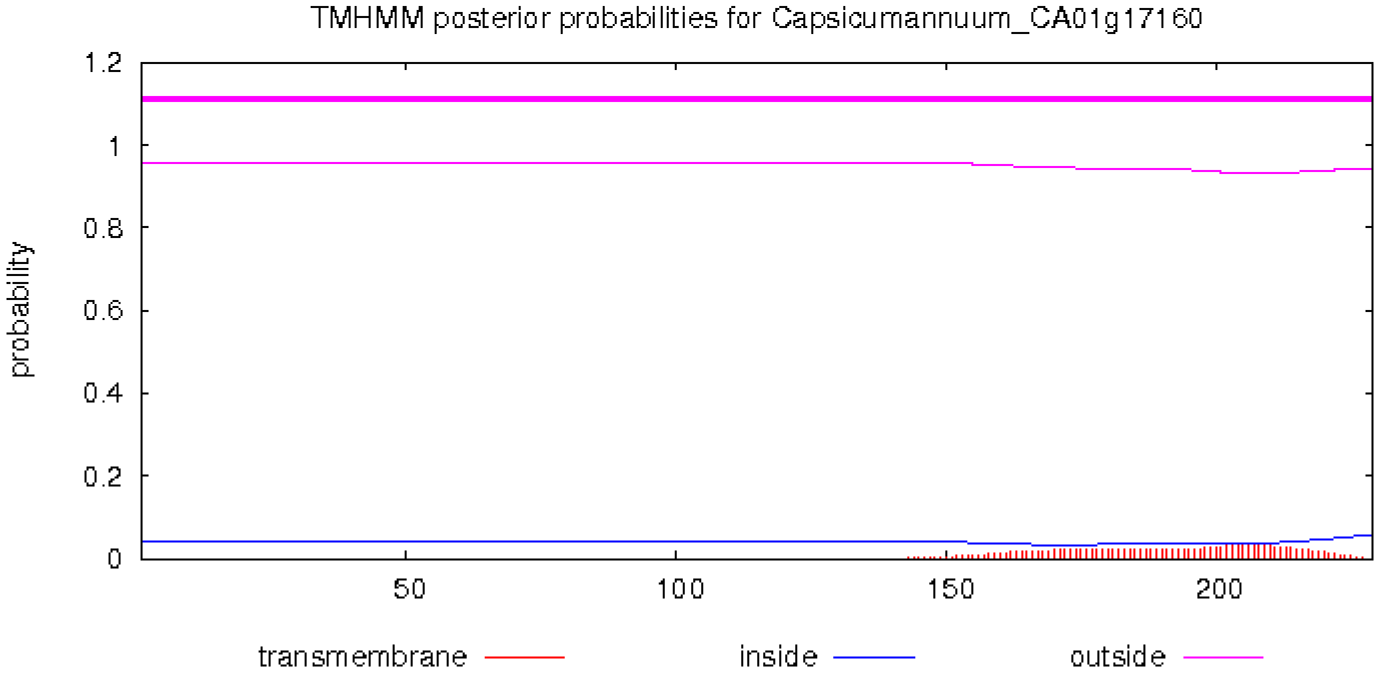

plot in postscript, script for making the plot in gnuplot, data for plot

---

```
# Capsicumannuum_CA01g26660 Length: 151
# Capsicumannuum_CA01g26660 Number of predicted TMHs:  0
# Capsicumannuum_CA01g26660 Exp number of AAs in TMHs: 0.04367
# Capsicumannuum_CA01g26660 Exp number, first 60 AAs:  0.00021
# Capsicumannuum_CA01g26660 Total prob of N-in:        0.33029
Capsicumannuum_CA01g26660       TMHMM2.0        outside      1   151
```

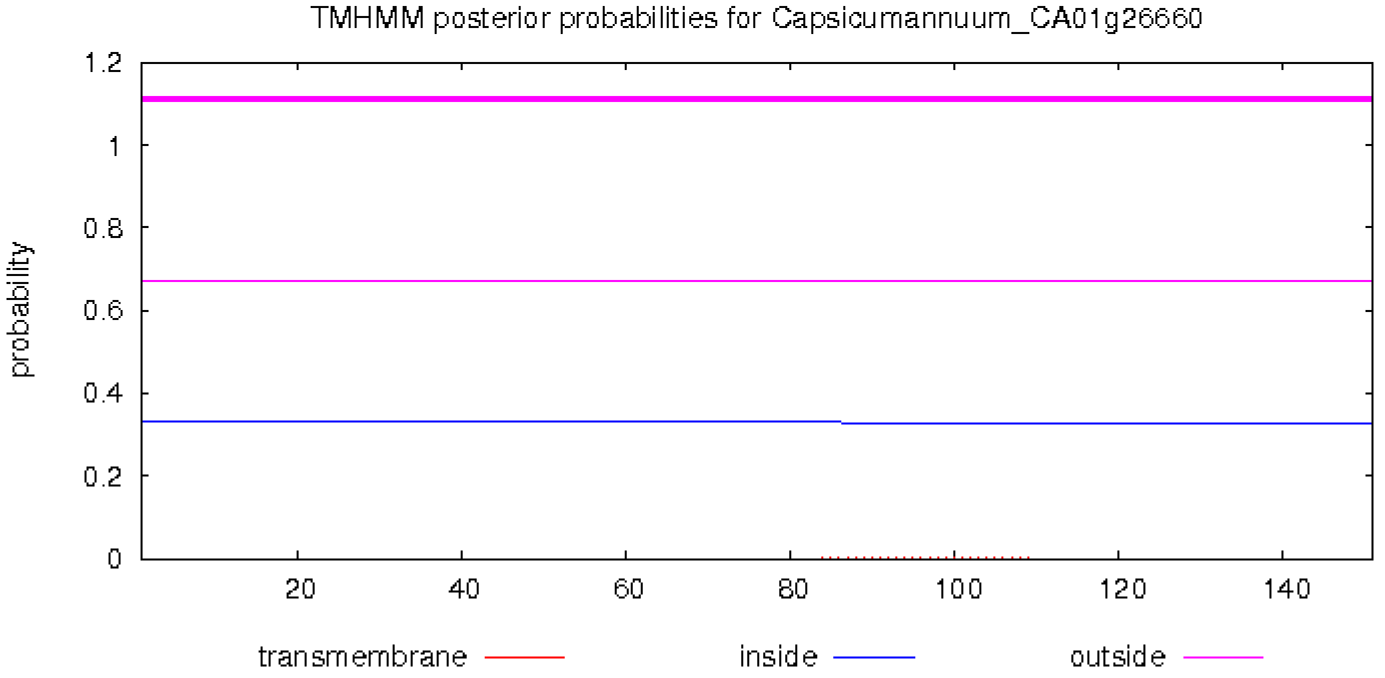

plot in postscript, script for making the plot in gnuplot, data for plot

---

```
# Capsicumannuum_CA01g30550 Length: 353
# Capsicumannuum_CA01g30550 Number of predicted TMHs:  0
# Capsicumannuum_CA01g30550 Exp number of AAs in TMHs: 0.00023
# Capsicumannuum_CA01g30550 Exp number, first 60 AAs:  0
# Capsicumannuum_CA01g30550 Total prob of N-in:        0.02616
Capsicumannuum_CA01g30550	TMHMM2.0	outside	     1   353
```

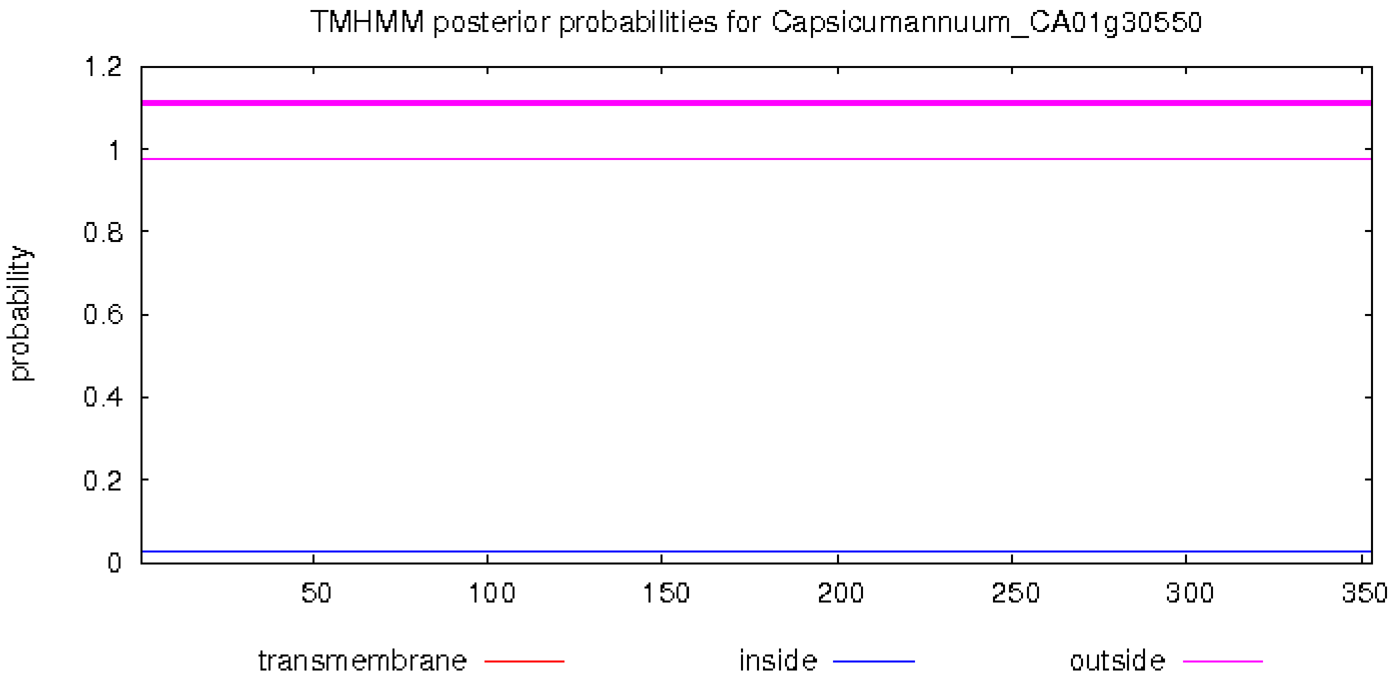

plot in postscript, script for making the plot in gnuplot, data for plot

---

```
# Capsicumannuum_CA01g34750 Length: 313
# Capsicumannuum_CA01g34750 Number of predicted TMHs:  0
# Capsicumannuum_CA01g34750 Exp number of AAs in TMHs: 0.00055
# Capsicumannuum_CA01g34750 Exp number, first 60 AAs:  0
# Capsicumannuum_CA01g34750 Total prob of N-in:        0.03313
Capsicumannuum_CA01g34750	TMHMM2.0	outside	     1   313
```

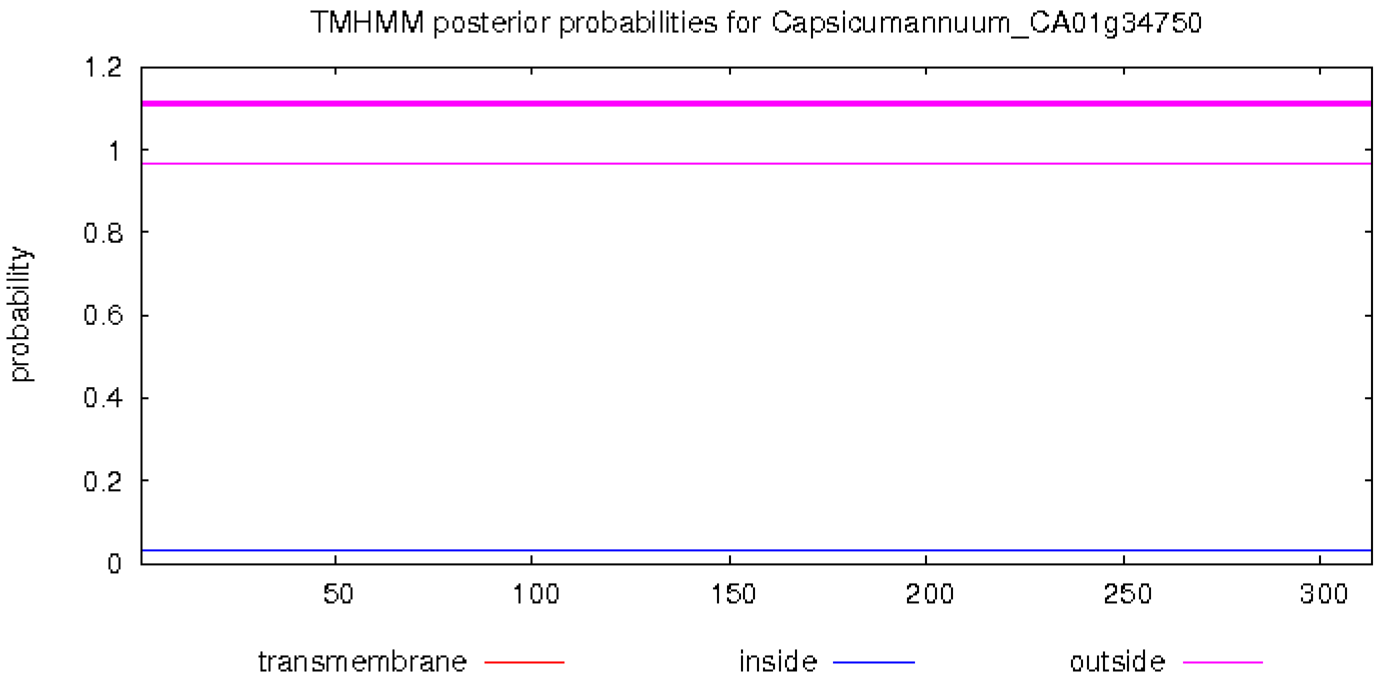

plot in postscript, script for making the plot in gnuplot, data for plot

---

```
# Capsicumannuum_CA02g03060 Length: 181
# Capsicumannuum_CA02g03060 Number of predicted TMHs:  0
# Capsicumannuum_CA02g03060 Exp number of AAs in TMHs: 0.0021
# Capsicumannuum_CA02g03060 Exp number, first 60 AAs:  0
# Capsicumannuum_CA02g03060 Total prob of N-in:        0.16062
Capsicumannuum_CA02g03060       TMHMM2.0        outside      1   181
```

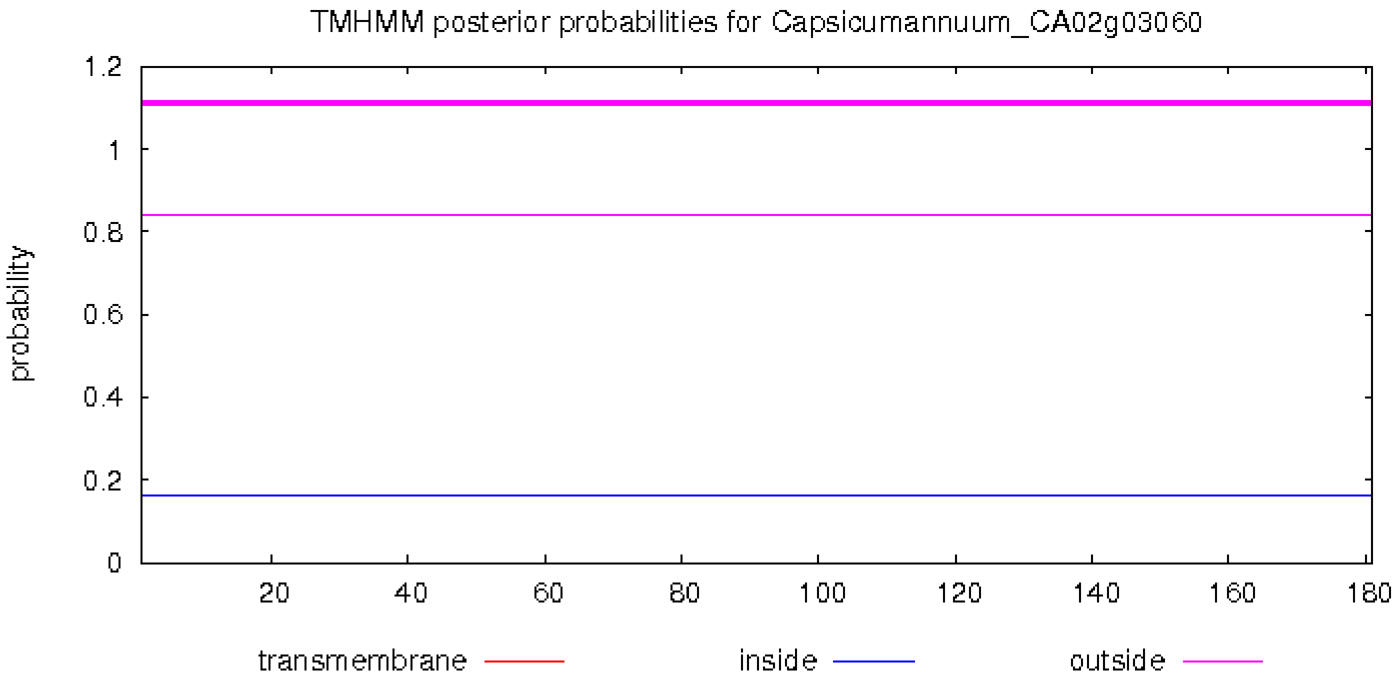

plot in postscript, script for making the plot in gnuplot, data for plot

---

```
# Capsicumannuum_CA02g04820 Length: 322
# Capsicumannuum_CA02g04820 Number of predicted TMHs:  0
# Capsicumannuum_CA02g04820 Exp number of AAs in TMHs: 0.00019
# Capsicumannuum_CA02g04820 Exp number, first 60 AAs:  0
# Capsicumannuum_CA02g04820 Total prob of N-in:        0.03113
Capsicumannuum_CA02g04820       TMHMM2.0        outside      1   322
```

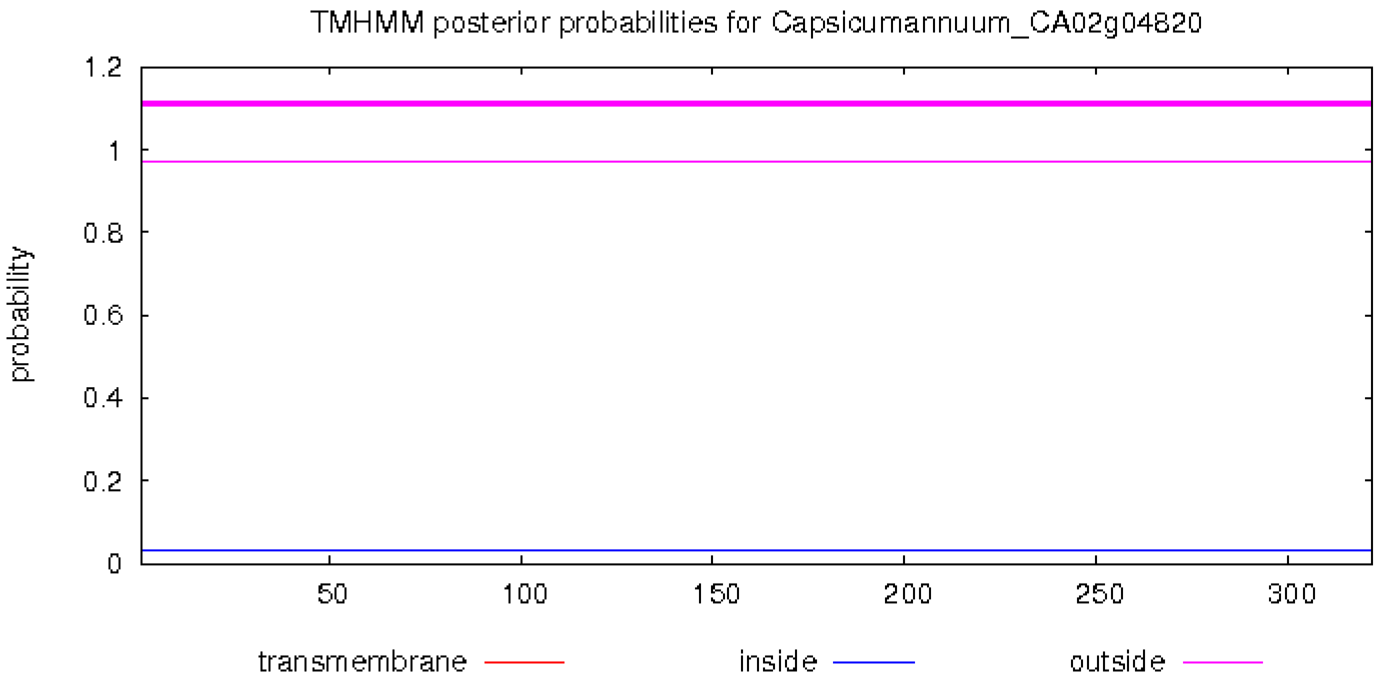

plot in postscript, script for making the plot in gnuplot, data for plot

---

```
# Capsicumannuum_CA02g05470 Length: 116
# Capsicumannuum_CA02g05470 Number of predicted TMHs:  0
# Capsicumannuum_CA02g05470 Exp number of AAs in TMHs: 0.23212
# Capsicumannuum_CA02g05470 Exp number, first 60 AAs:  0.22097
# Capsicumannuum_CA02g05470 Total prob of N-in:        0.06695
Capsicumannuum_CA02g05470       TMHMM2.0        outside      1   116
```

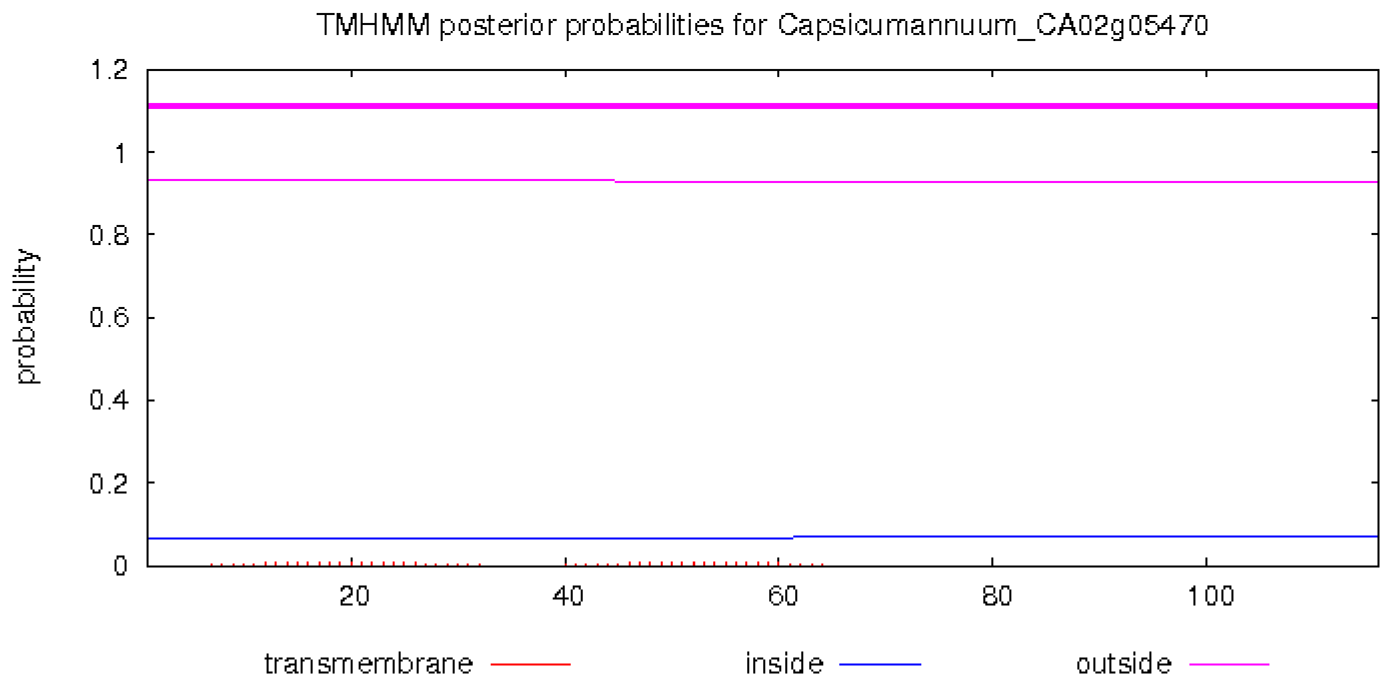

plot in postscript, script for making the plot in gnuplot, data for plot

---

```
# Capsicumannuum_CA02g06450 Length: 577
# Capsicumannuum_CA02g06450 Number of predicted TMHs:  0
# Capsicumannuum_CA02g06450 Exp number of AAs in TMHs: 2.26291
# Capsicumannuum_CA02g06450 Exp number, first 60 AAs:  0
# Capsicumannuum_CA02g06450 Total prob of N-in:        0.00080
Capsicumannuum_CA02g06450       TMHMM2.0        outside      1   577
```

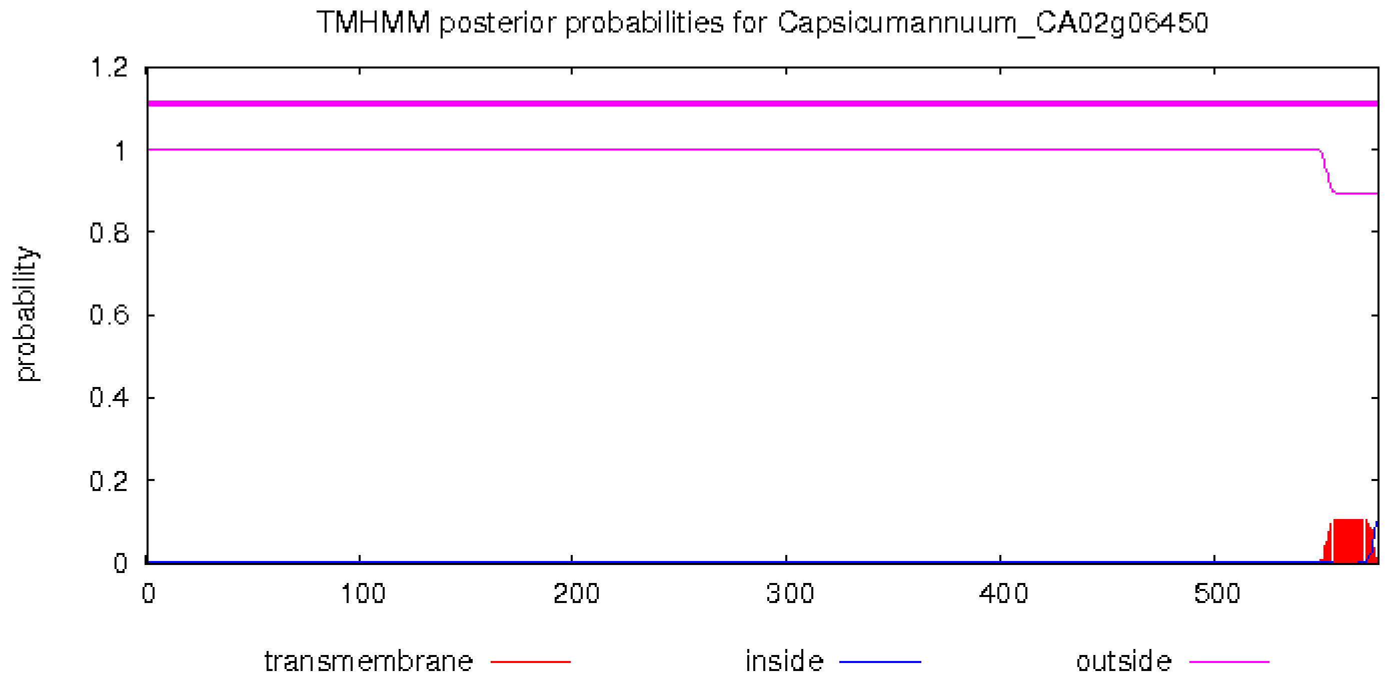

plot in postscript, script for making the plot in gnuplot, data for plot

---

```
# Capsicumannuum_CA02g15520 Length: 290
# Capsicumannuum_CA02g15520 Number of predicted TMHs:  0
# Capsicumannuum_CA02g15520 Exp number of AAs in TMHs: 0.02344
# Capsicumannuum_CA02g15520 Exp number, first 60 AAs:  0.00022
# Capsicumannuum_CA02g15520 Total prob of N-in:        0.09772
Capsicumannuum_CA02g15520       TMHMM2.0        outside      1   290
```

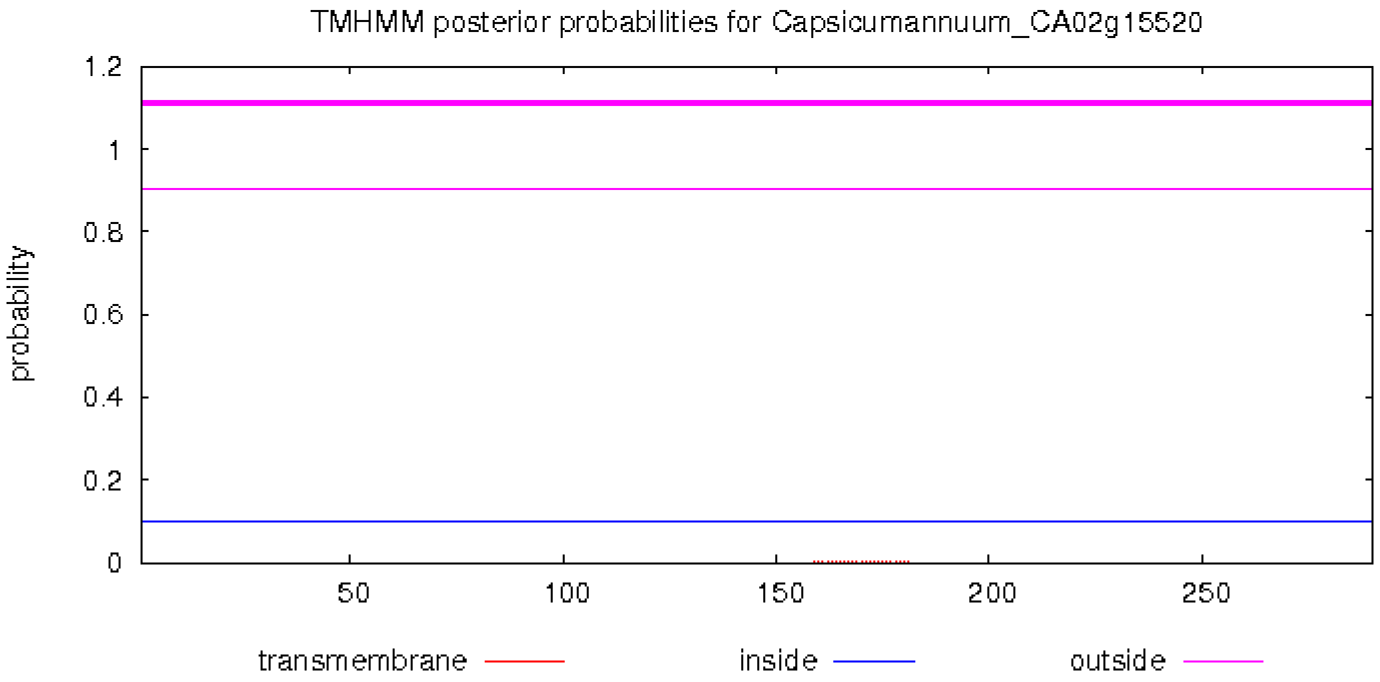

plot in postscript, script for making the plot in gnuplot, data for plot

```
# Capsicumannuum_CA02g17910 Length: 246
# Capsicumannuum_CA02g17910 Number of predicted TMHs:  0
# Capsicumannuum_CA02g17910 Exp number of AAs in TMHs: 0.00074
# Capsicumannuum_CA02g17910 Exp number, first 60 AAs:  0.00074
# Capsicumannuum_CA02g17910 Total prob of N-in:        0.21967
Capsicumannuum_CA02g17910       TMHMM2.0        outside      1   246
```

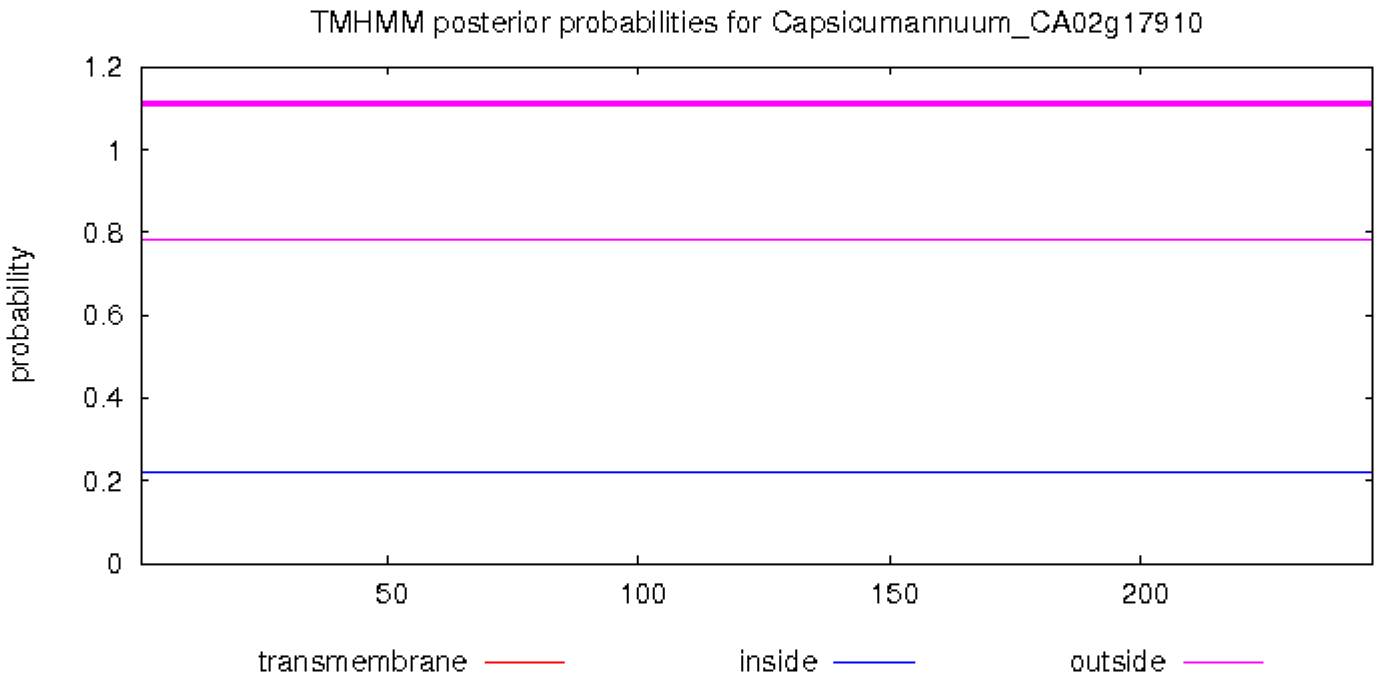

plot in postscript, script for making the plot in gnuplot, data for plot

```
# Capsicumannuum_CA02g20290 Length: 632
# Capsicumannuum_CA02g20290 Number of predicted TMHs:  0
# Capsicumannuum_CA02g20290 Exp number of AAs in TMHs: 4.81942
# Capsicumannuum_CA02g20290 Exp number, first 60 AAs:  0
# Capsicumannuum_CA02g20290 Total prob of N-in:        0.00046
Capsicumannuum_CA02g20290       TMHMM2.0        outside      1   632
```

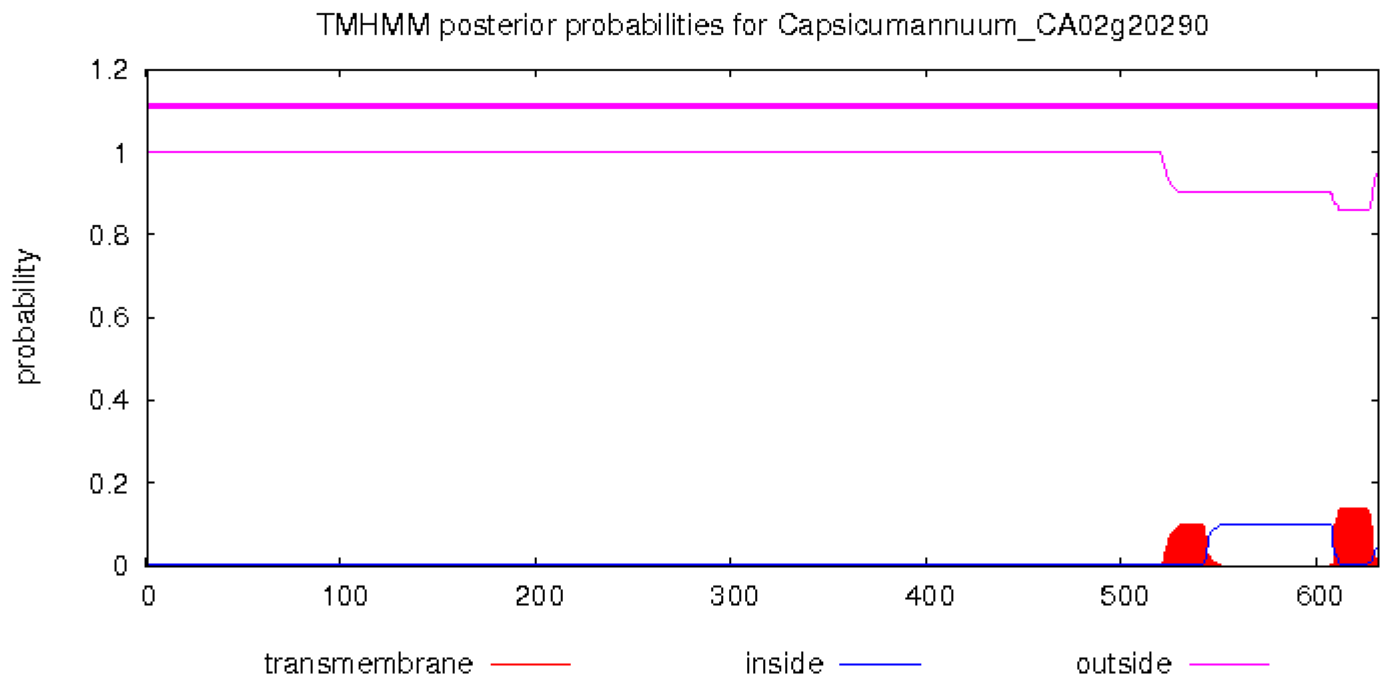

plot in postscript, script for making the plot in gnuplot, data for plot

---

```
# Capsicumannuum_CA02g22730 Length: 218
# Capsicumannuum_CA02g22730 Number of predicted TMHs:  1
# Capsicumannuum_CA02g22730 Exp number of AAs in TMHs: 18.68468
# Capsicumannuum_CA02g22730 Exp number, first 60 AAs:  0.00111
# Capsicumannuum_CA02g22730 Total prob of N-in:        0.63743
Capsicumannuum_CA02g22730       TMHMM2.0        inside       1   193
Capsicumannuum_CA02g22730       TMHMM2.0        TMhelix    194   216
Capsicumannuum_CA02g22730       TMHMM2.0        outside    217   218
```

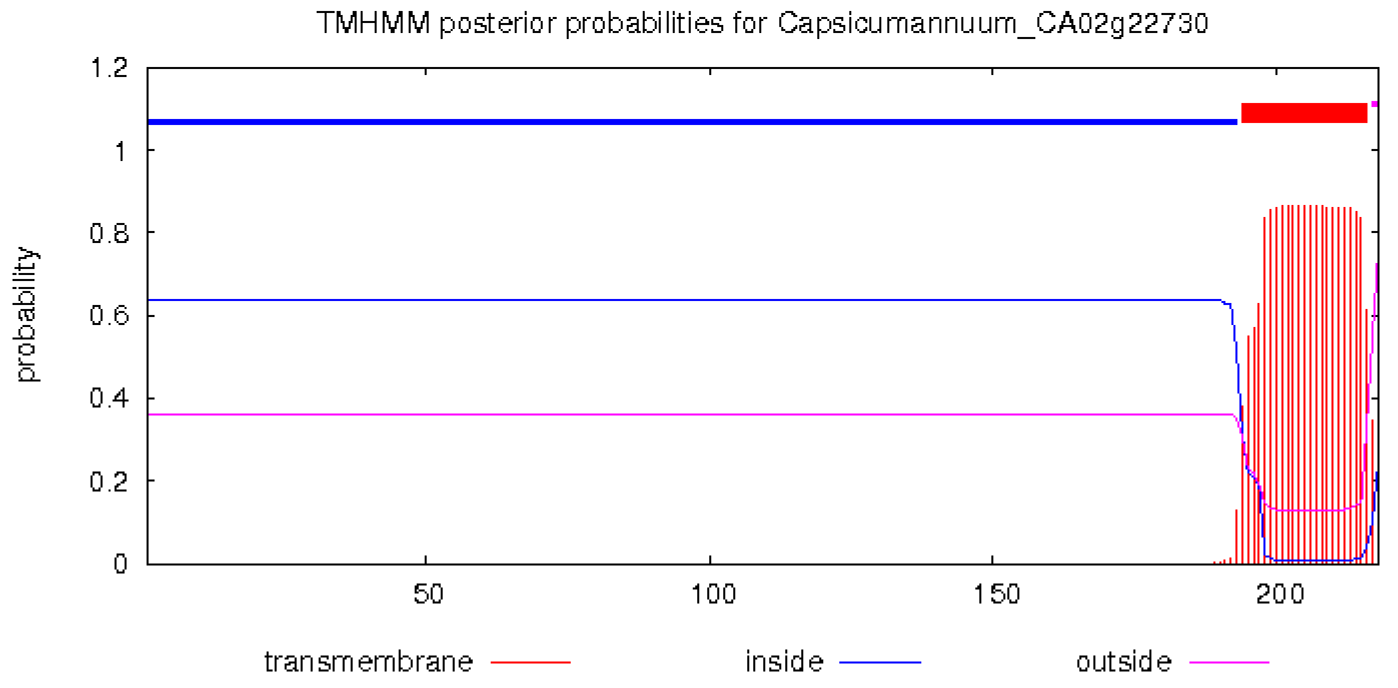

plot in postscript, script for making the plot in gnuplot, data for plot

```
# Capsicumannuum_CA02g28070 Length: 326
# Capsicumannuum_CA02g28070 Number of predicted TMHs:  0
# Capsicumannuum_CA02g28070 Exp number of AAs in TMHs: 0
# Capsicumannuum_CA02g28070 Exp number, first 60 AAs:  0
# Capsicumannuum_CA02g28070 Total prob of N-in:        0.00920
Capsicumannuum_CA02g28070       TMHMM2.0        outside      1   326
```

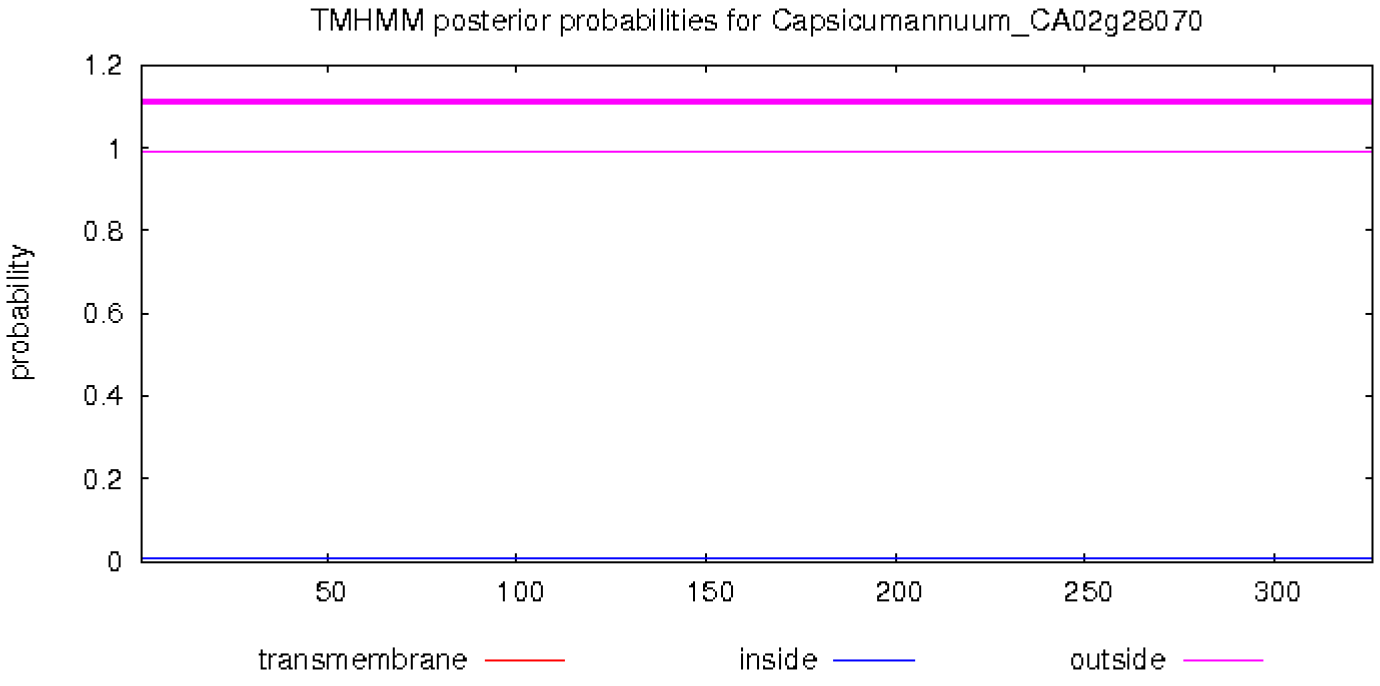

plot in postscript, script for making the plot in gnuplot, data for plot

```
# Capsicumannuum_CA02g28290 Length: 221
# Capsicumannuum_CA02g28290 Number of predicted TMHs:  1
# Capsicumannuum_CA02g28290 Exp number of AAs in TMHs: 20.40126
# Capsicumannuum_CA02g28290 Exp number, first 60 AAs:  0.00087
# Capsicumannuum_CA02g28290 Total prob of N-in:        0.29756
Capsicumannuum_CA02g28290       TMHMM2.0        outside      1    69
Capsicumannuum_CA02g28290       TMHMM2.0        TMhelix     70    92
Capsicumannuum_CA02g28290       TMHMM2.0        inside      93   221
```

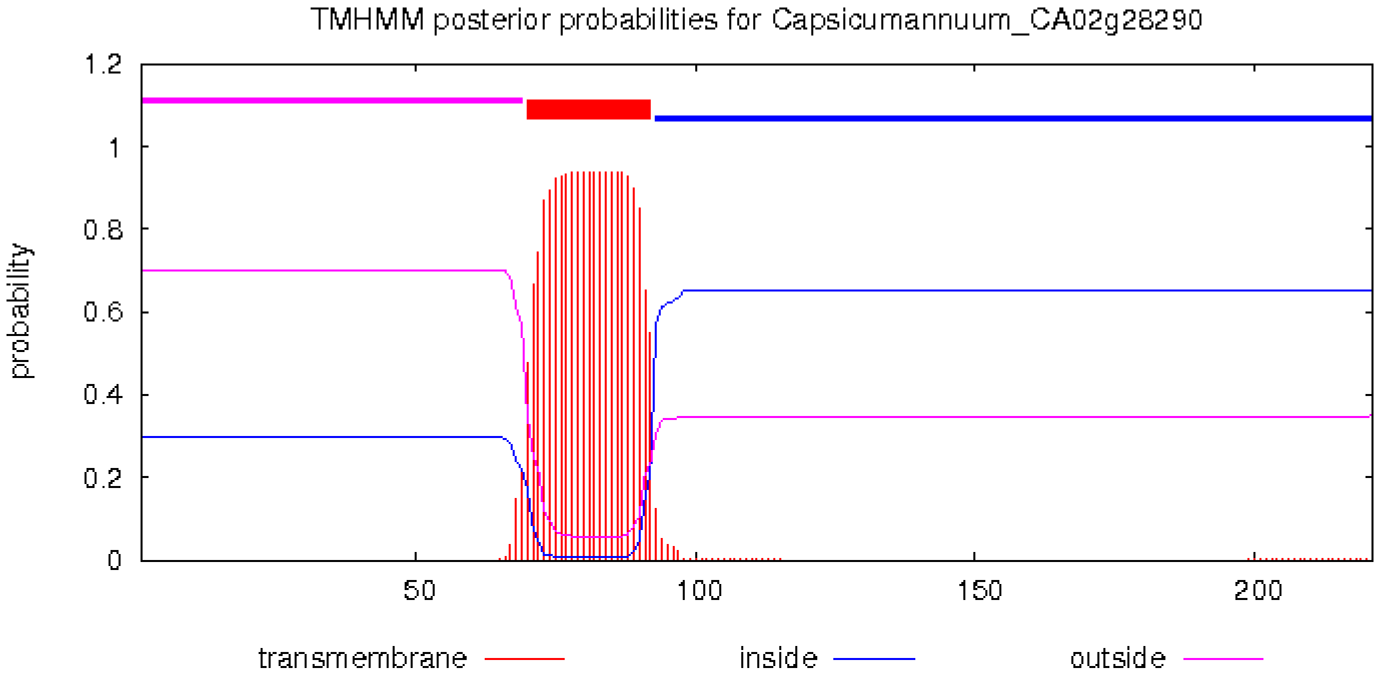

plot in postscript, script for making the plot in gnuplot, data for plot

```
# Capsicumannuum_CA02g30090 Length: 387
# Capsicumannuum_CA02g30090 Number of predicted TMHs:  0
# Capsicumannuum_CA02g30090 Exp number of AAs in TMHs: 0
# Capsicumannuum_CA02g30090 Exp number, first 60 AAs:  0
# Capsicumannuum_CA02g30090 Total prob of N-in:        0.00829
Capsicumannuum_CA02g30090       TMHMM2.0        outside      1   387
```

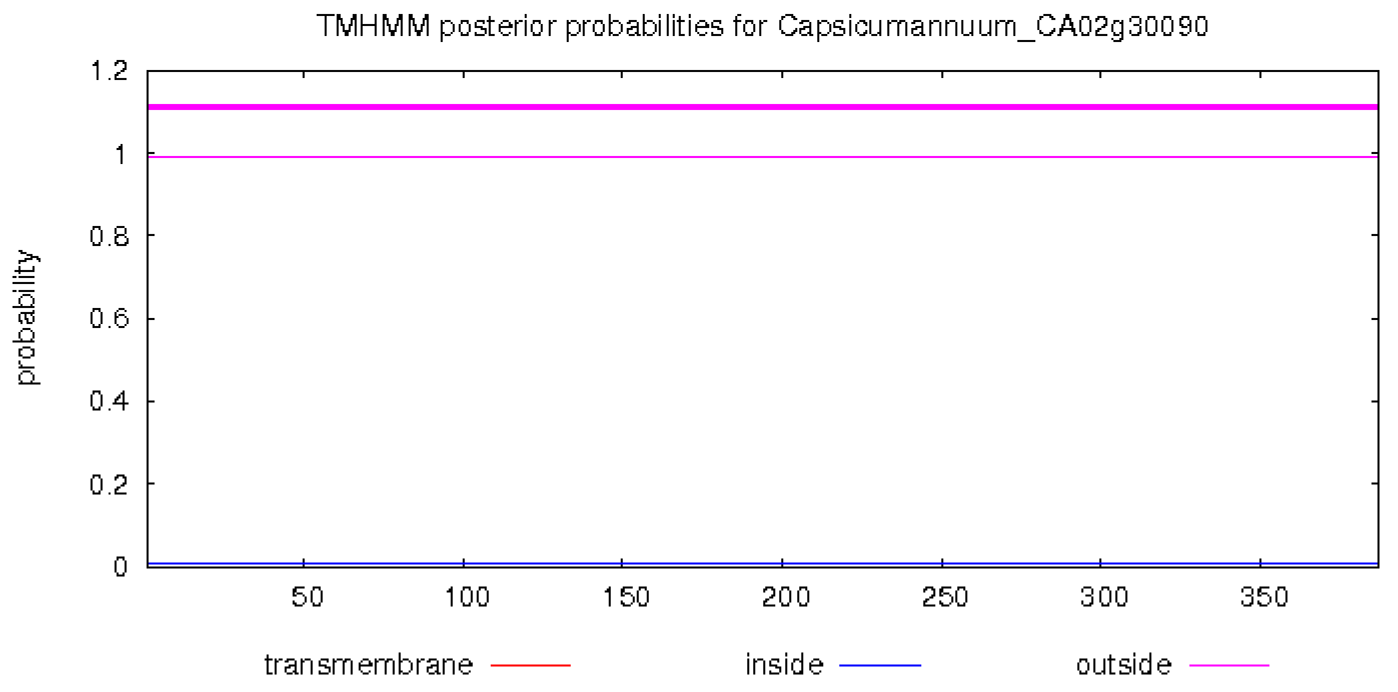

plot in postscript, script for making the plot in gnuplot, data for plot

```
# Capsicumannuum_CA03g14470 Length: 344
# Capsicumannuum_CA03g14470 Number of predicted TMHs:  0
# Capsicumannuum_CA03g14470 Exp number of AAs in TMHs: 0.01057
# Capsicumannuum_CA03g14470 Exp number, first 60 AAs:  0
# Capsicumannuum_CA03g14470 Total prob of N-in:        0.02378
Capsicumannuum_CA03g14470       TMHMM2.0        outside      1   344
```

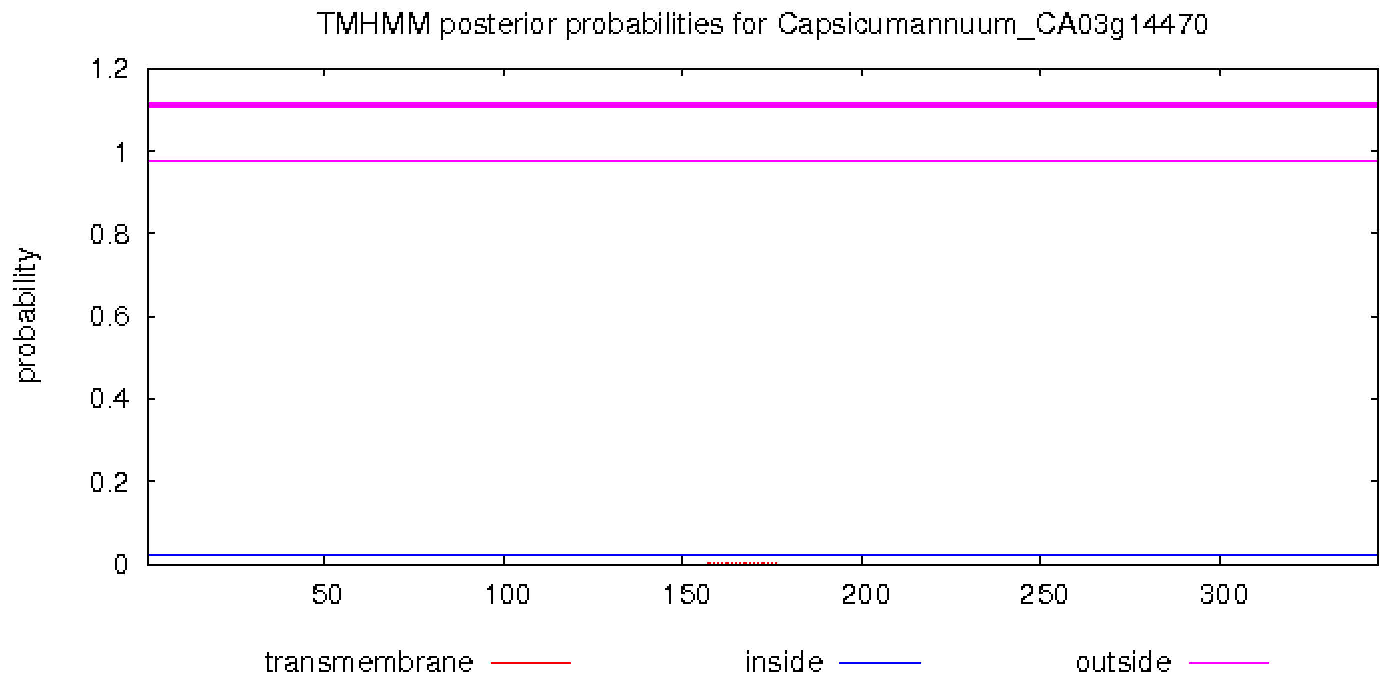

plot in postscript, script for making the plot in gnuplot, data for plot

---

```
# Capsicumannuum_CA03g15410 Length: 170
# Capsicumannuum_CA03g15410 Number of predicted TMHs:  0
# Capsicumannuum_CA03g15410 Exp number of AAs in TMHs: 0.00609
# Capsicumannuum_CA03g15410 Exp number, first 60 AAs:  0
# Capsicumannuum_CA03g15410 Total prob of N-in:        0.02907
Capsicumannuum_CA03g15410       TMHMM2.0        outside      1   170
```

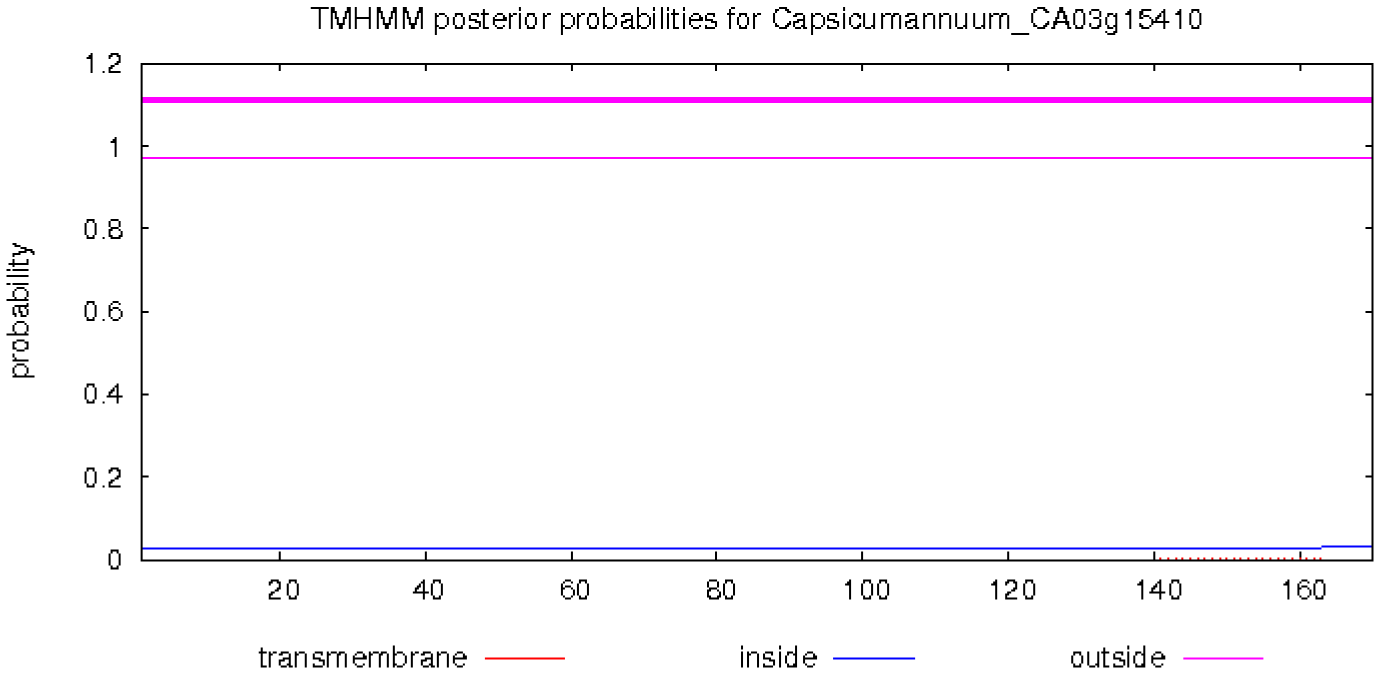

plot in postscript, script for making the plot in gnuplot, data for plot

---

```
# Capsicumannuum_CA03g21000 Length: 633
# Capsicumannuum_CA03g21000 Number of predicted TMHs:  1
# Capsicumannuum_CA03g21000 Exp number of AAs in TMHs: 21.73715
# Capsicumannuum_CA03g21000 Exp number, first 60 AAs:  0
# Capsicumannuum_CA03g21000 Total prob of N-in:        0.00059
Capsicumannuum_CA03g21000       TMHMM2.0        outside      1   606
Capsicumannuum_CA03g21000       TMHMM2.0        TMhelix    607   629
Capsicumannuum_CA03g21000       TMHMM2.0        inside     630   633
```

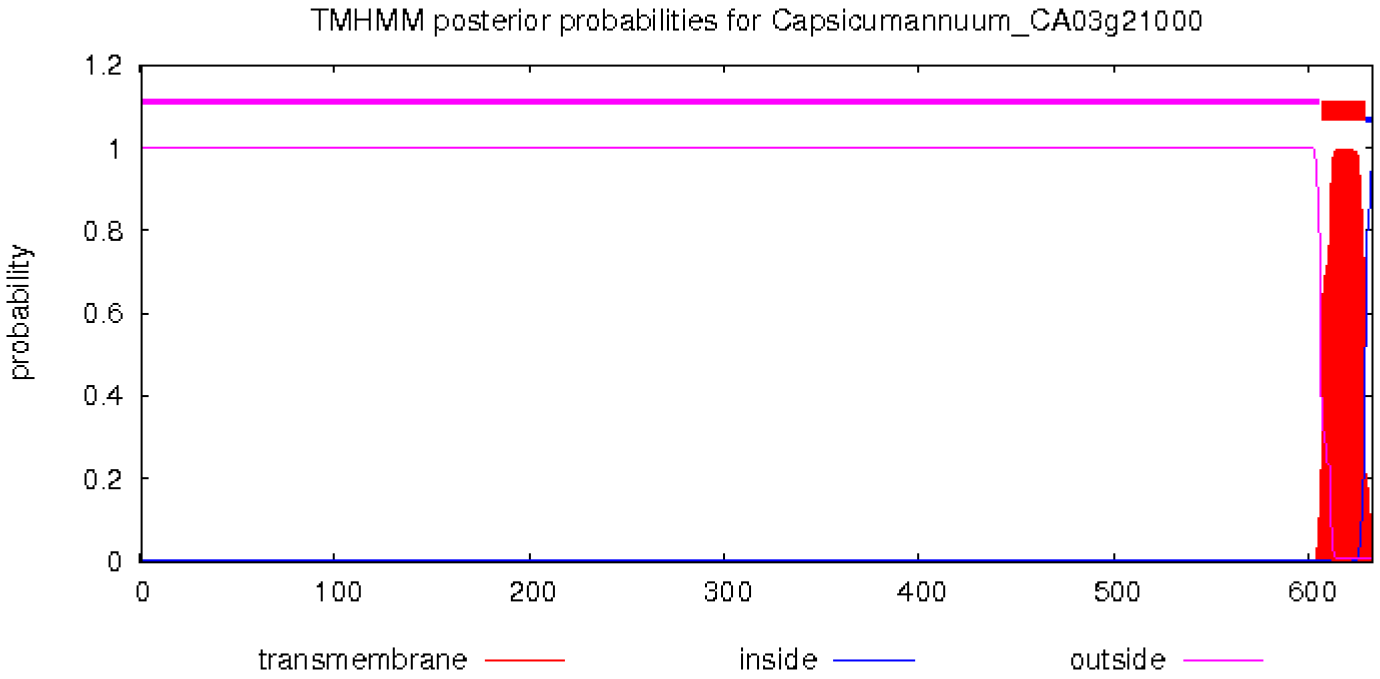

plot in postscript, script for making the plot in gnuplot, data for plot

---

```
# Capsicumannuum_CA03g22350 Length: 262
# Capsicumannuum_CA03g22350 Number of predicted TMHs:  0
# Capsicumannuum_CA03g22350 Exp number of AAs in TMHs: 0.00244
# Capsicumannuum_CA03g22350 Exp number, first 60 AAs:  0.00041
# Capsicumannuum_CA03g22350 Total prob of N-in:        0.03893
Capsicumannuum_CA03g22350       TMHMM2.0        outside      1   262
```

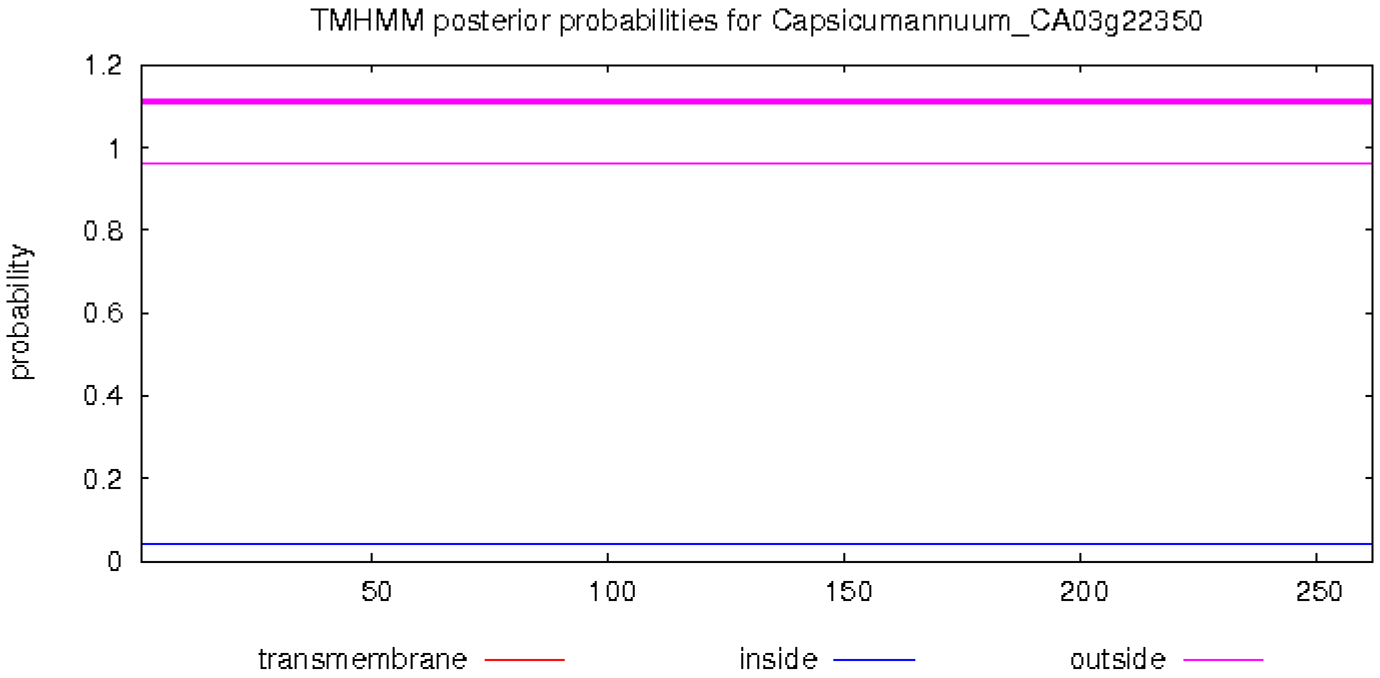

plot in postscript, script for making the plot in gnuplot, data for plot

---

```
# Capsicumannuum_CA03g23090 Length: 150
# Capsicumannuum_CA03g23090 Number of predicted TMHs:  0
# Capsicumannuum_CA03g23090 Exp number of AAs in TMHs: 0
# Capsicumannuum_CA03g23090 Exp number, first 60 AAs:  0
# Capsicumannuum_CA03g23090 Total prob of N-in:        0.00763
Capsicumannuum_CA03g23090       TMHMM2.0        outside      1   150
```

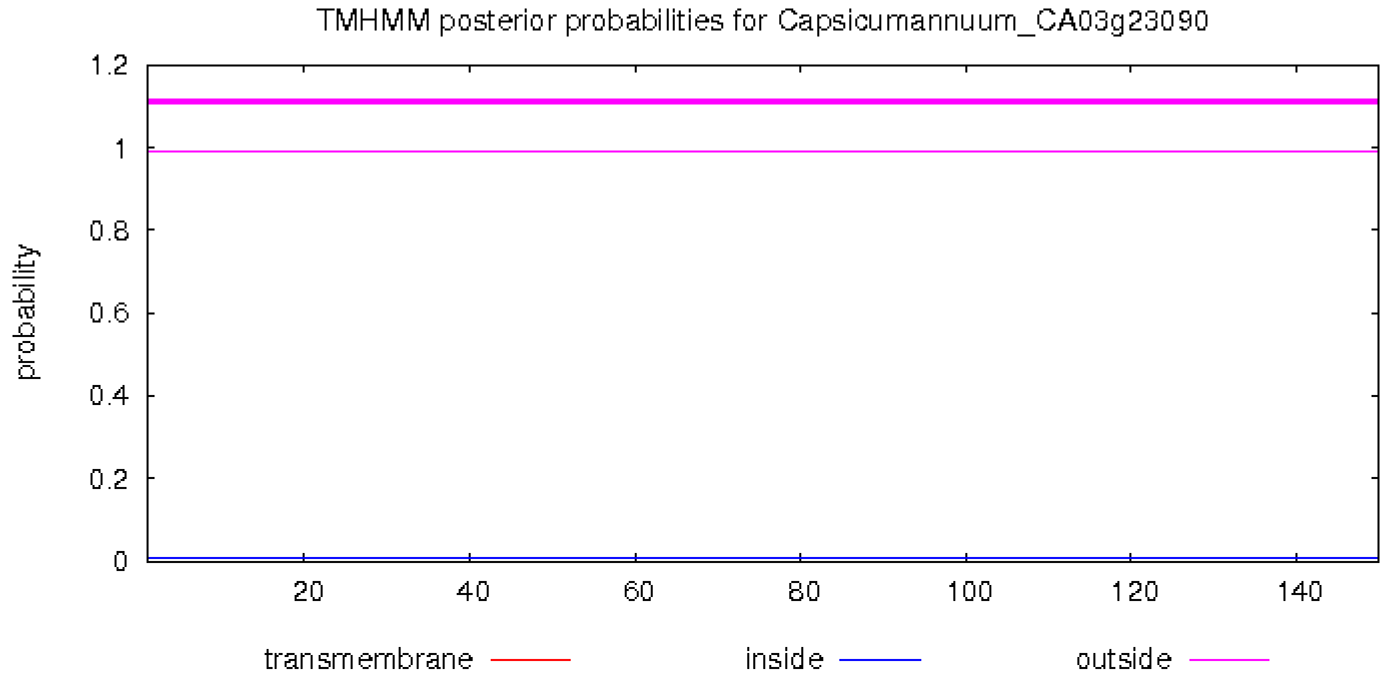

plot in postscript, script for making the plot in gnuplot, data for plot

---

```
# Capsicumannuum_CA03g27130 Length: 245
# Capsicumannuum_CA03g27130 Number of predicted TMHs:  0
# Capsicumannuum_CA03g27130 Exp number of AAs in TMHs: 0.00013
# Capsicumannuum_CA03g27130 Exp number, first 60 AAs:  0.00013
# Capsicumannuum_CA03g27130 Total prob of N-in:        0.02953
Capsicumannuum_CA03g27130	TMHMM2.0	outside	     1   245
```

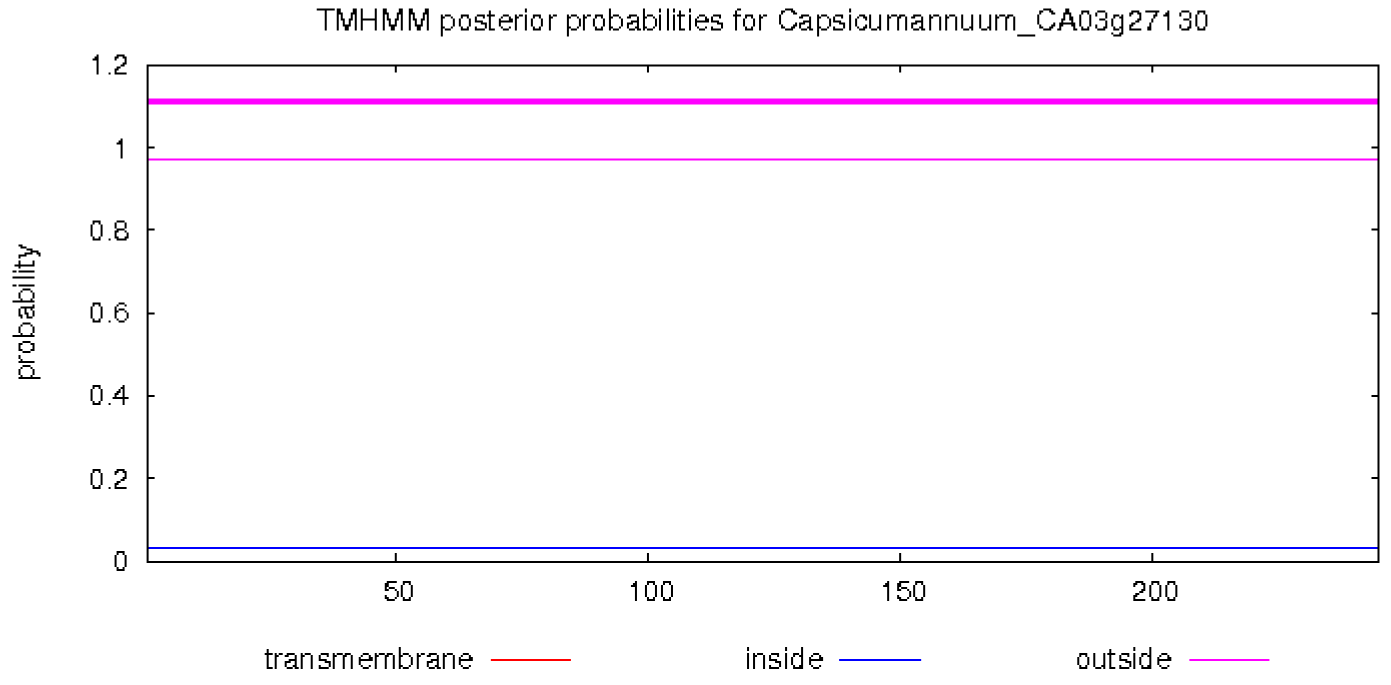

plot in postscript, script for making the plot in gnuplot, data for plot

---

```
# Capsicumannuum_CA03g27300 Length: 284
# Capsicumannuum_CA03g27300 Number of predicted TMHs:  0
# Capsicumannuum_CA03g27300 Exp number of AAs in TMHs: 0
# Capsicumannuum_CA03g27300 Exp number, first 60 AAs:  0
# Capsicumannuum_CA03g27300 Total prob of N-in:        0.01868
Capsicumannuum_CA03g27300	TMHMM2.0	outside	     1   284
```

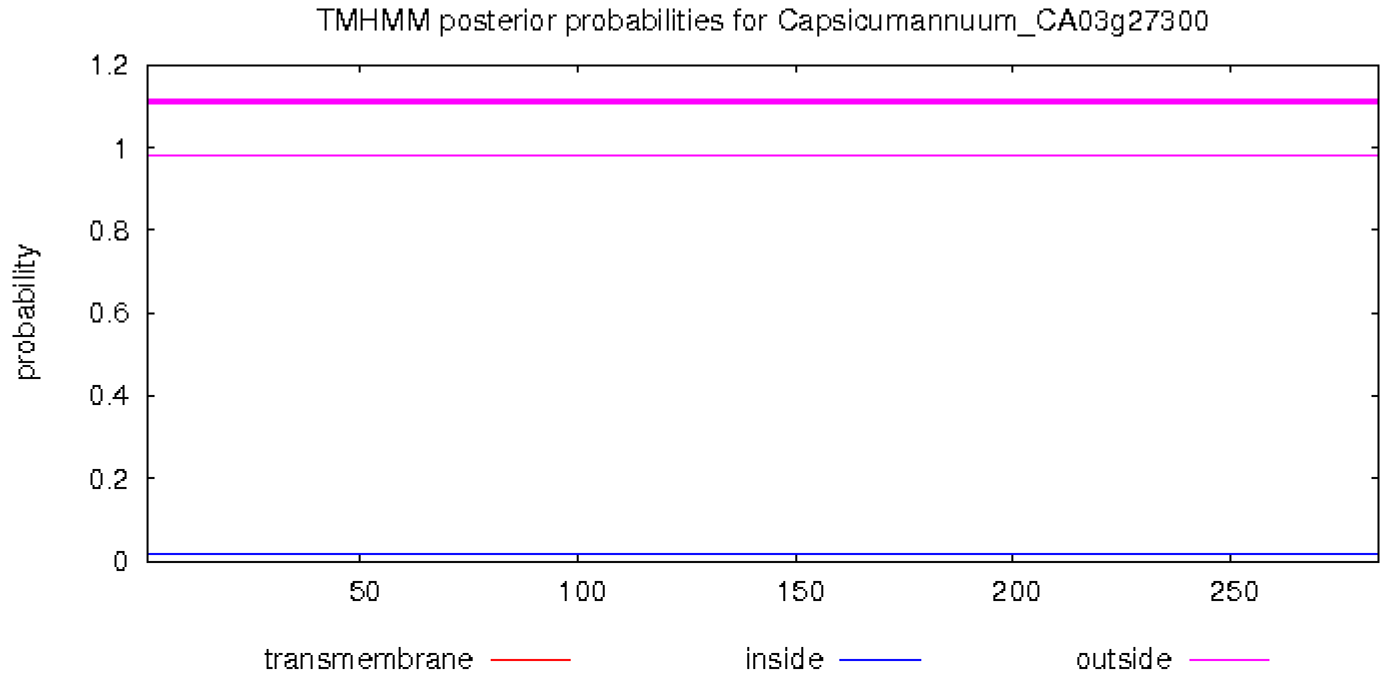

plot in postscript, script for making the plot in gnuplot, data for plot

```
# Capsicumannuum_CA03g28100 Length: 236
# Capsicumannuum_CA03g28100 Number of predicted TMHs:  0
# Capsicumannuum_CA03g28100 Exp number of AAs in TMHs: 0.00014
# Capsicumannuum_CA03g28100 Exp number, first 60 AAs:  0
# Capsicumannuum_CA03g28100 Total prob of N-in:        0.04285
Capsicumannuum_CA03g28100       TMHMM2.0        outside      1   236
```

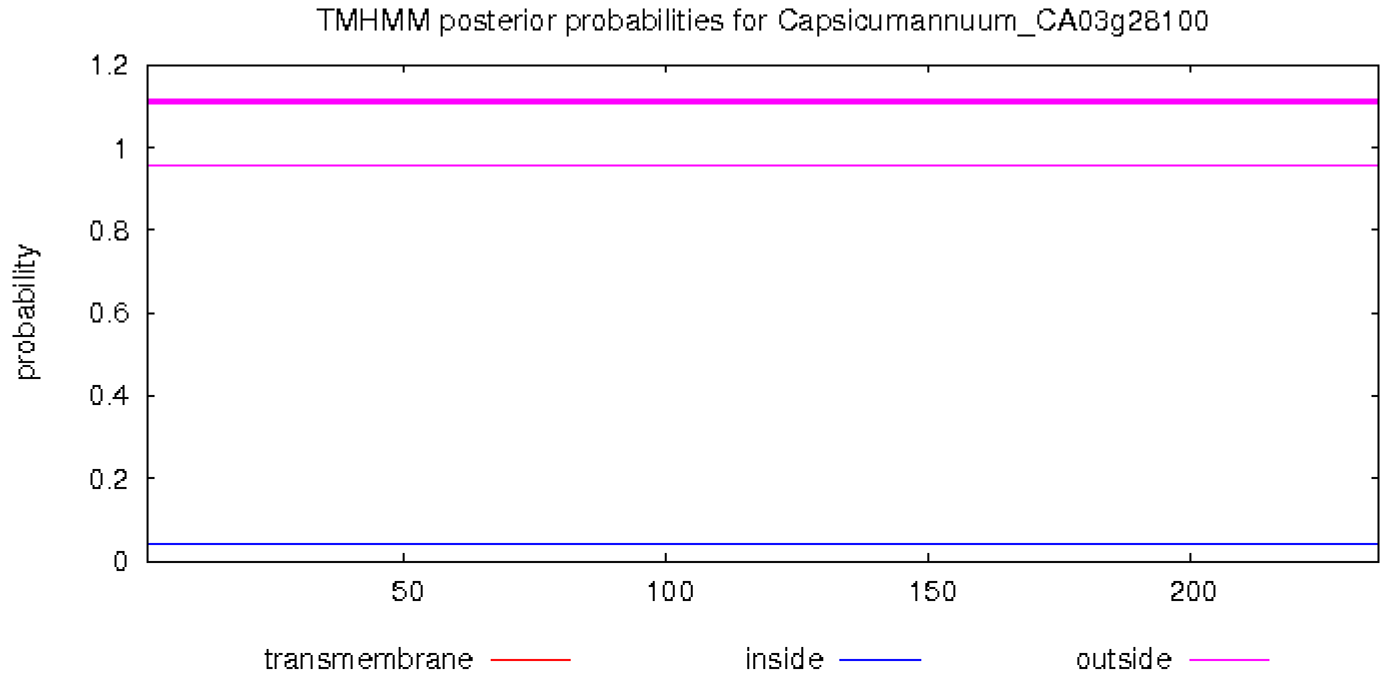

plot in postscript, script for making the plot in gnuplot, data for plot

```
# Capsicumannuum_CA04g14740 Length: 513
# Capsicumannuum_CA04g14740 Number of predicted TMHs:  0
# Capsicumannuum_CA04g14740 Exp number of AAs in TMHs: 0
# Capsicumannuum_CA04g14740 Exp number, first 60 AAs:  0
# Capsicumannuum_CA04g14740 Total prob of N-in:        0.00055
Capsicumannuum_CA04g14740       TMHMM2.0        outside      1   513
```

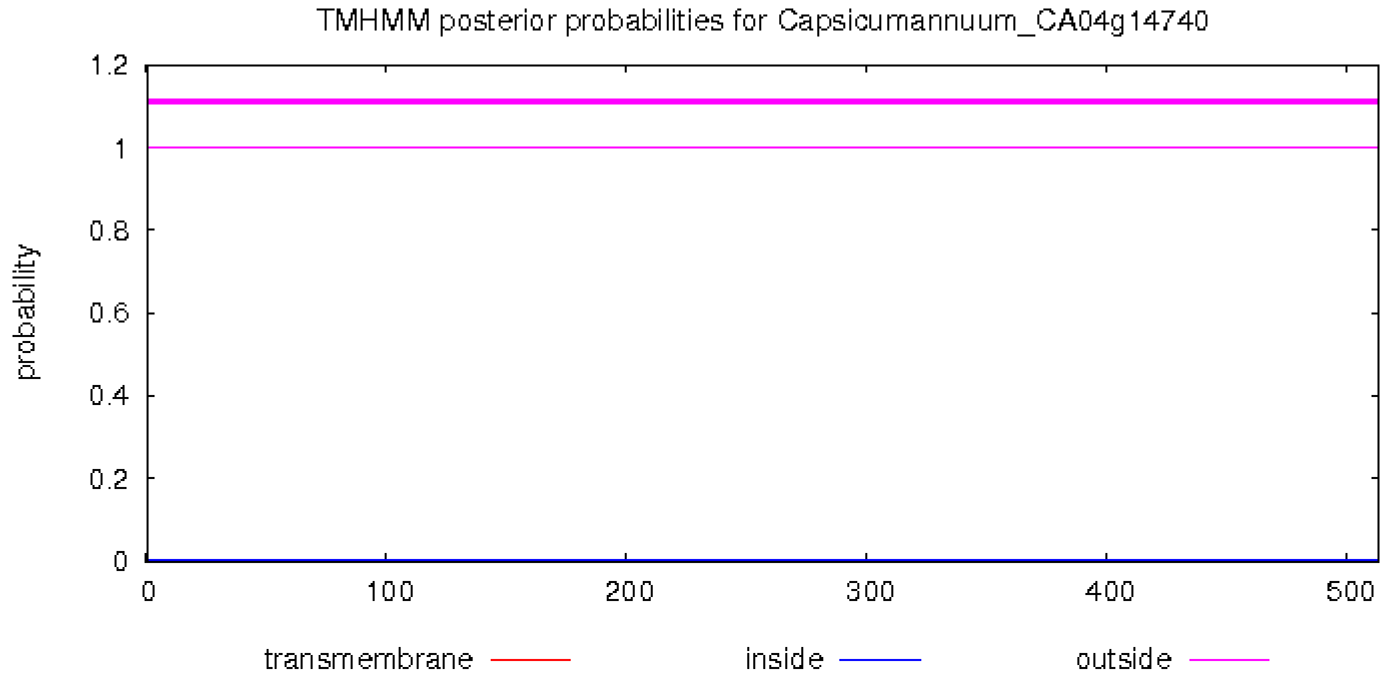

plot in postscript, script for making the plot in gnuplot, data for plot

---

```
# Capsicumannuum_CA04g20070 Length: 371
# Capsicumannuum_CA04g20070 Number of predicted TMHs:  0
# Capsicumannuum_CA04g20070 Exp number of AAs in TMHs: 0.00042
# Capsicumannuum_CA04g20070 Exp number, first 60 AAs:  0
# Capsicumannuum_CA04g20070 Total prob of N-in:        0.00102
Capsicumannuum_CA04g20070       TMHMM2.0        outside      1   371
```

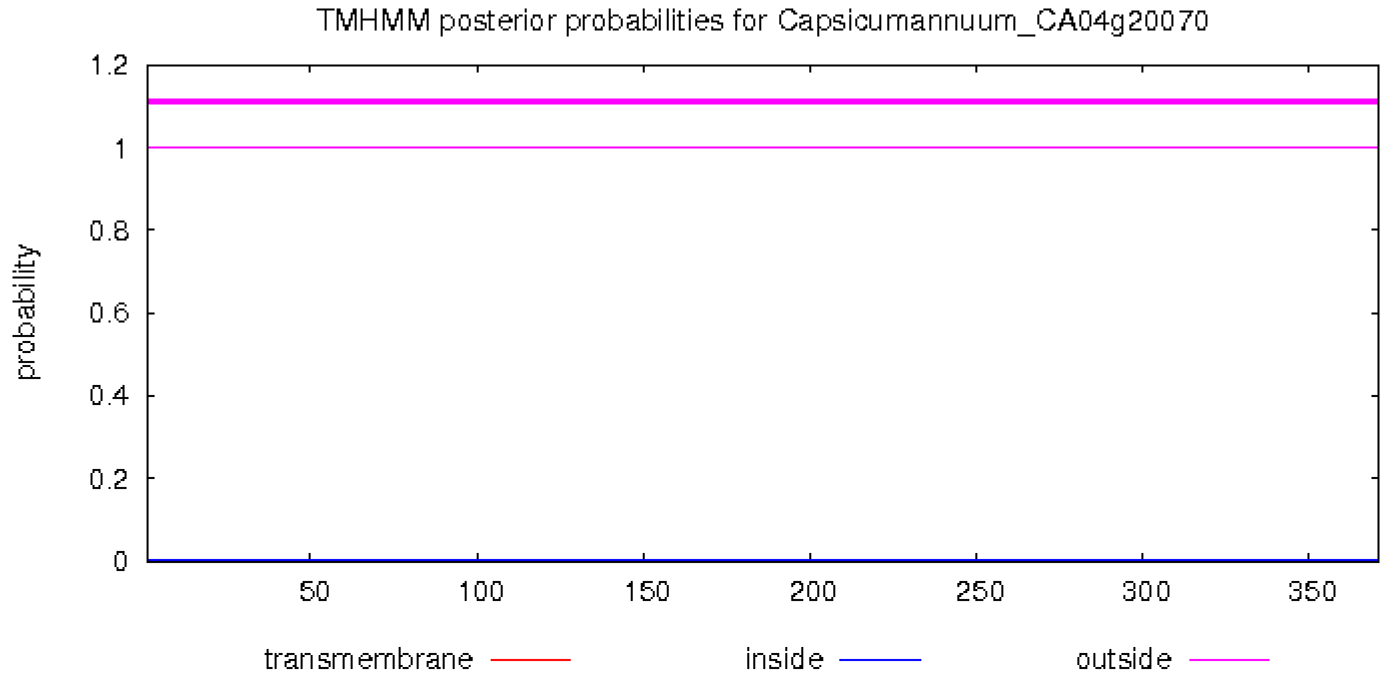

plot in postscript, script for making the plot in gnuplot, data for plot

---

```
# Capsicumannuum_CA04g23340 Length: 390
# Capsicumannuum_CA04g23340 Number of predicted TMHs:  0
# Capsicumannuum_CA04g23340 Exp number of AAs in TMHs: 0.00779
# Capsicumannuum_CA04g23340 Exp number, first 60 AAs:  0.00697
# Capsicumannuum_CA04g23340 Total prob of N-in:        0.00309
Capsicumannuum_CA04g23340       TMHMM2.0        outside      1   390
```

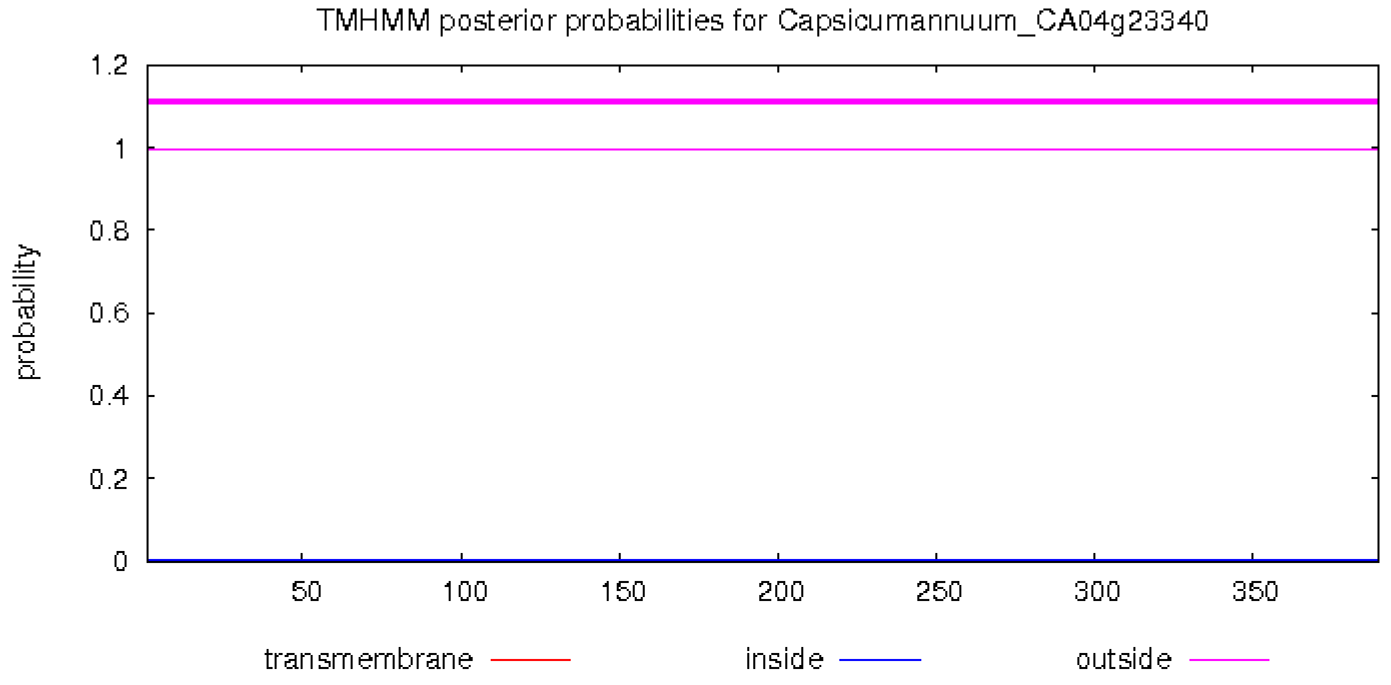

plot in postscript, script for making the plot in gnuplot, data for plot

---

```
# Capsicumannuum_CA05g04410 Length: 313
# Capsicumannuum_CA05g04410 Number of predicted TMHs:  0
# Capsicumannuum_CA05g04410 Exp number of AAs in TMHs: 0.00056
# Capsicumannuum_CA05g04410 Exp number, first 60 AAs:  0.00037
# Capsicumannuum_CA05g04410 Total prob of N-in:        0.04353
Capsicumannuum_CA05g04410       TMHMM2.0        outside      1   313
```

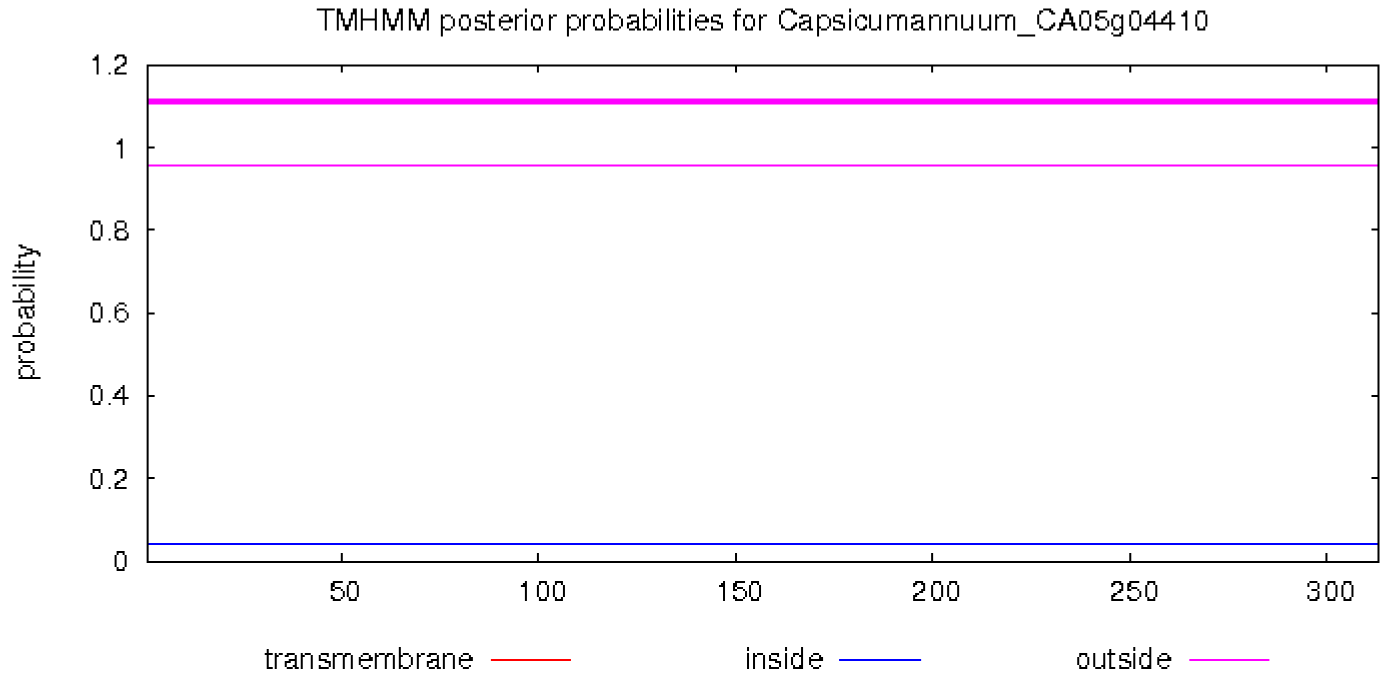

plot in postscript, script for making the plot in gnuplot, data for plot

---

```
# Capsicumannuum_CA05g09500 Length: 313
# Capsicumannuum_CA05g09500 Number of predicted TMHs:  0
# Capsicumannuum_CA05g09500 Exp number of AAs in TMHs: 0.01485
# Capsicumannuum_CA05g09500 Exp number, first 60 AAs:  0
# Capsicumannuum_CA05g09500 Total prob of N-in:        0.02490
Capsicumannuum_CA05g09500       TMHMM2.0        outside      1   313
```

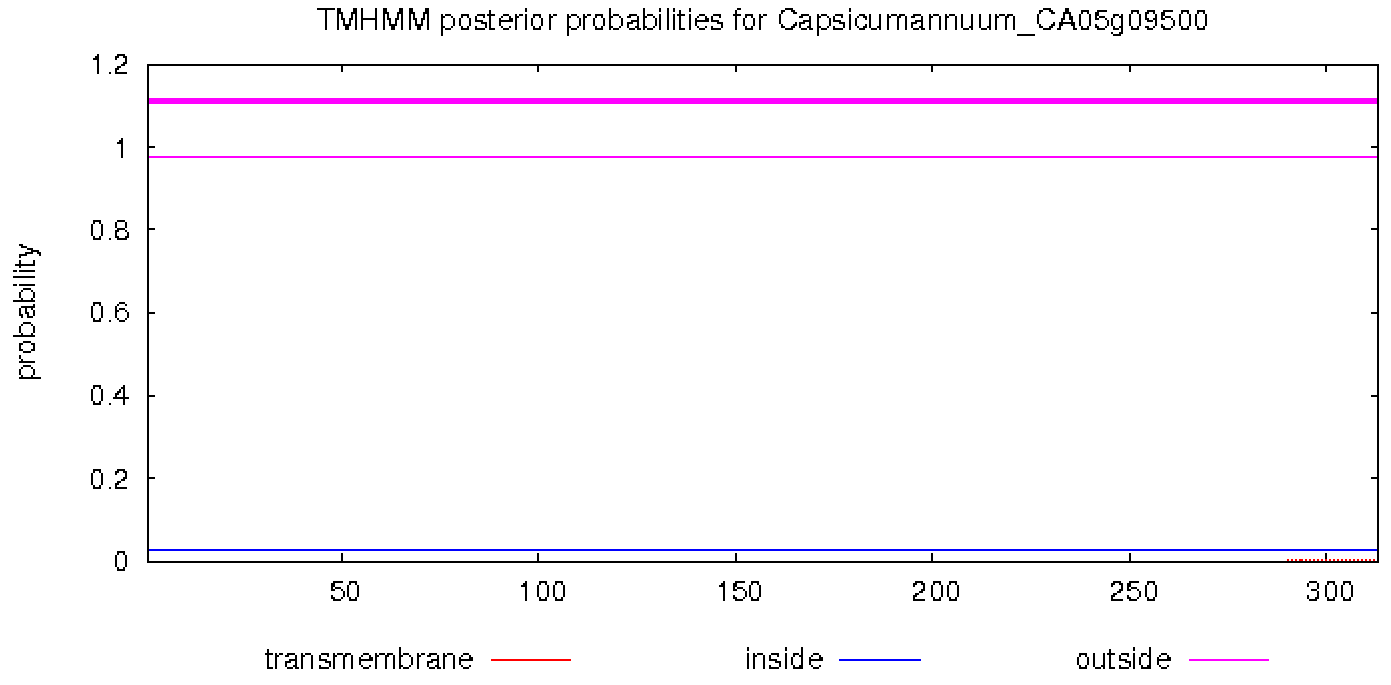

plot in postscript, script for making the plot in gnuplot, data for plot

```
# Capsicumannuum_CA05g09600 Length: 103
# Capsicumannuum_CA05g09600 Number of predicted TMHs:  0
# Capsicumannuum_CA05g09600 Exp number of AAs in TMHs: 0.07073
# Capsicumannuum_CA05g09600 Exp number, first 60 AAs:  0.00021
# Capsicumannuum_CA05g09600 Total prob of N-in:        0.19492
Capsicumannuum_CA05g09600       TMHMM2.0        outside      1   103
```

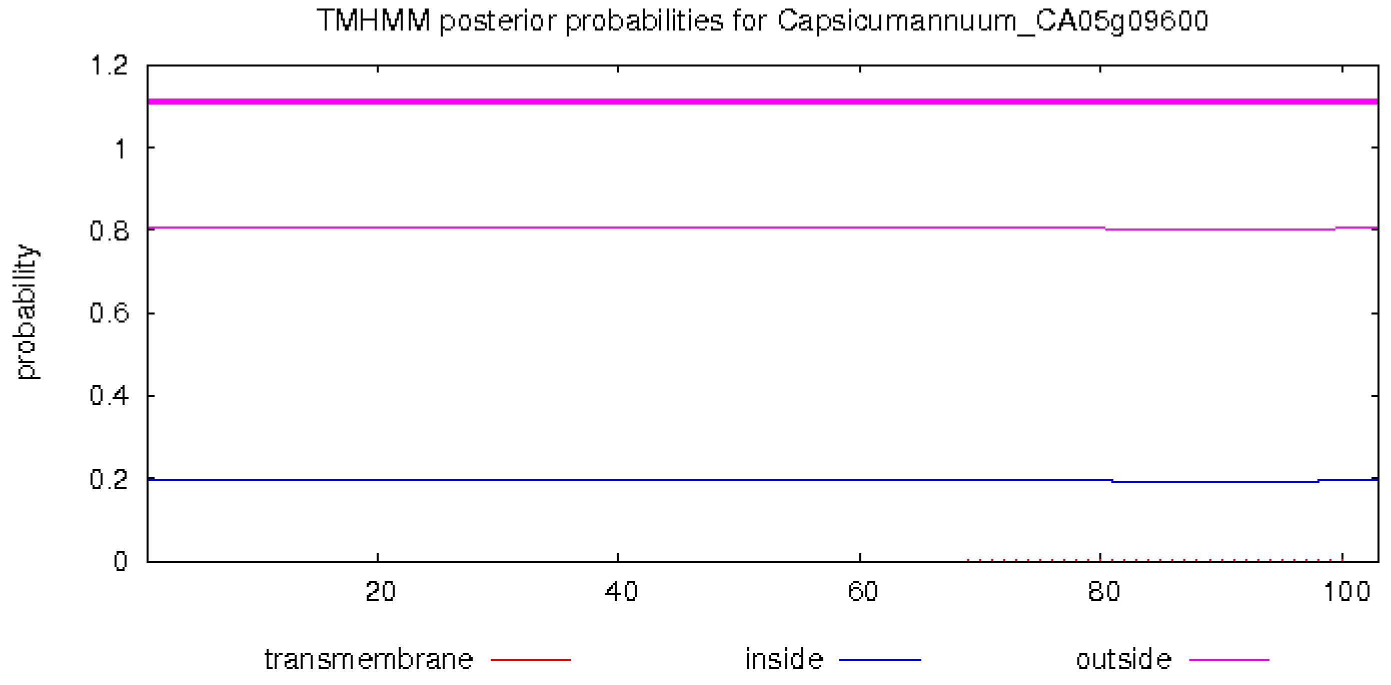

plot in postscript, script for making the plot in gnuplot, data for plot

```
# Capsicumannuum_CA06g01680 Length: 290
# Capsicumannuum_CA06g01680 Number of predicted TMHs:  0
# Capsicumannuum_CA06g01680 Exp number of AAs in TMHs: 0
# Capsicumannuum_CA06g01680 Exp number, first 60 AAs:  0
# Capsicumannuum_CA06g01680 Total prob of N-in:        0.01097
Capsicumannuum_CA06g01680       TMHMM2.0        outside      1   290
```

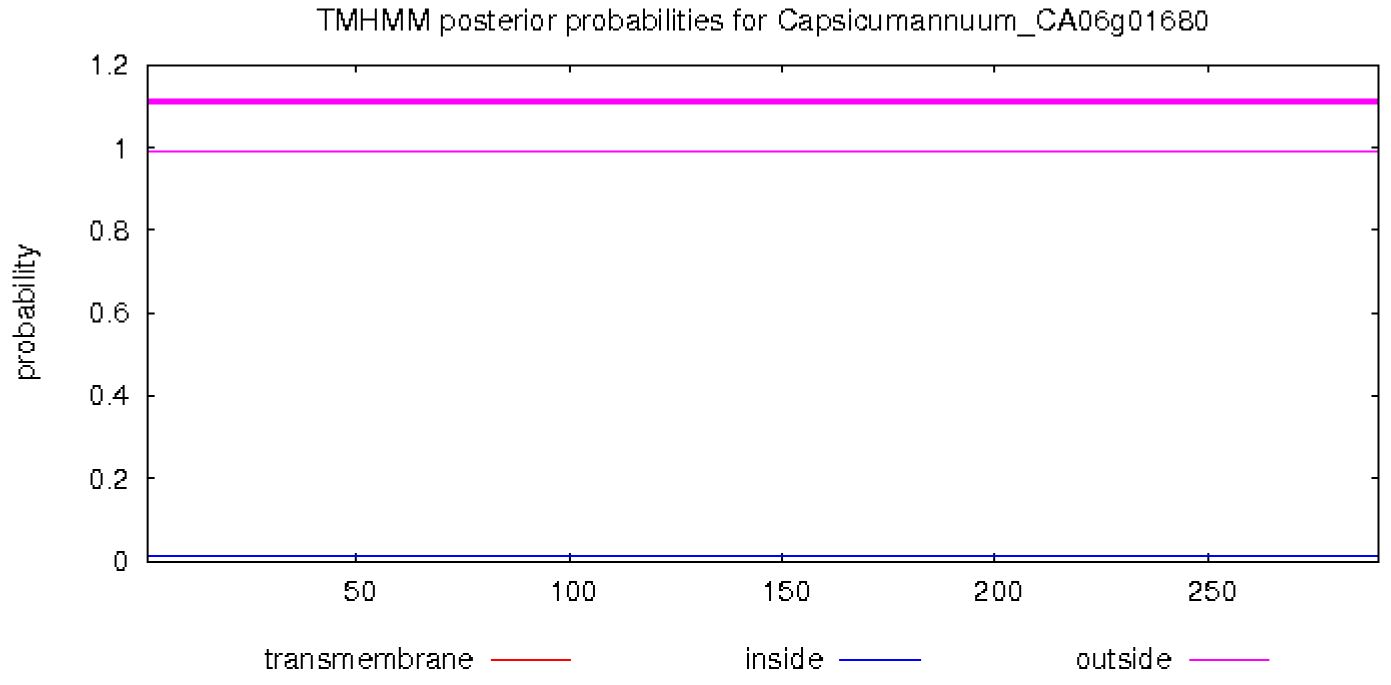

plot in postscript, script for making the plot in gnuplot, data for plot

```
# Capsicumannuum_CA06g02260 Length: 178
# Capsicumannuum_CA06g02260 Number of predicted TMHs:  0
# Capsicumannuum_CA06g02260 Exp number of AAs in TMHs: 0.0002
# Capsicumannuum_CA06g02260 Exp number, first 60 AAs:  7e-05
# Capsicumannuum_CA06g02260 Total prob of N-in:        0.05501
Capsicumannuum_CA06g02260       TMHMM2.0        outside      1   178
```

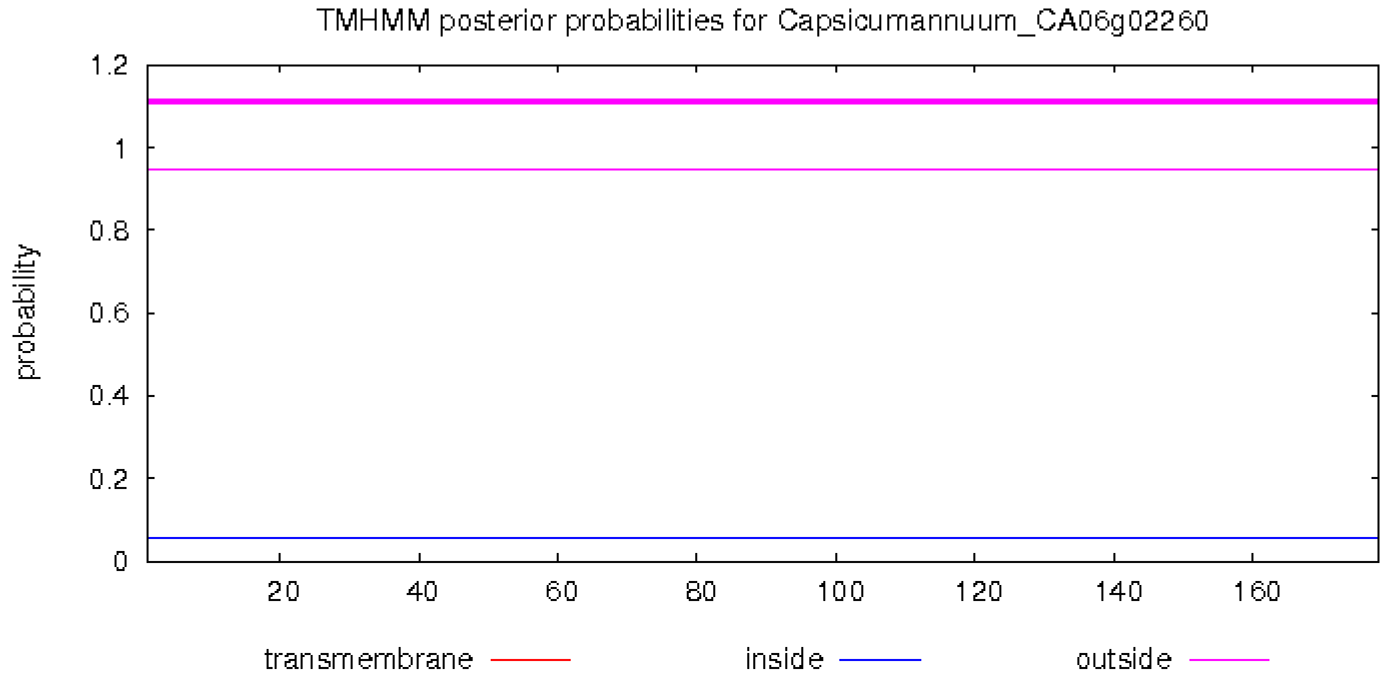

plot in postscript, script for making the plot in gnuplot, data for plot

```
# Capsicumannuum_CA06g06410 Length: 347
# Capsicumannuum_CA06g06410 Number of predicted TMHs:  0
# Capsicumannuum_CA06g06410 Exp number of AAs in TMHs: 0
# Capsicumannuum_CA06g06410 Exp number, first 60 AAs:  0
# Capsicumannuum_CA06g06410 Total prob of N-in:        0.00299
Capsicumannuum_CA06g06410       TMHMM2.0        outside      1   347
```

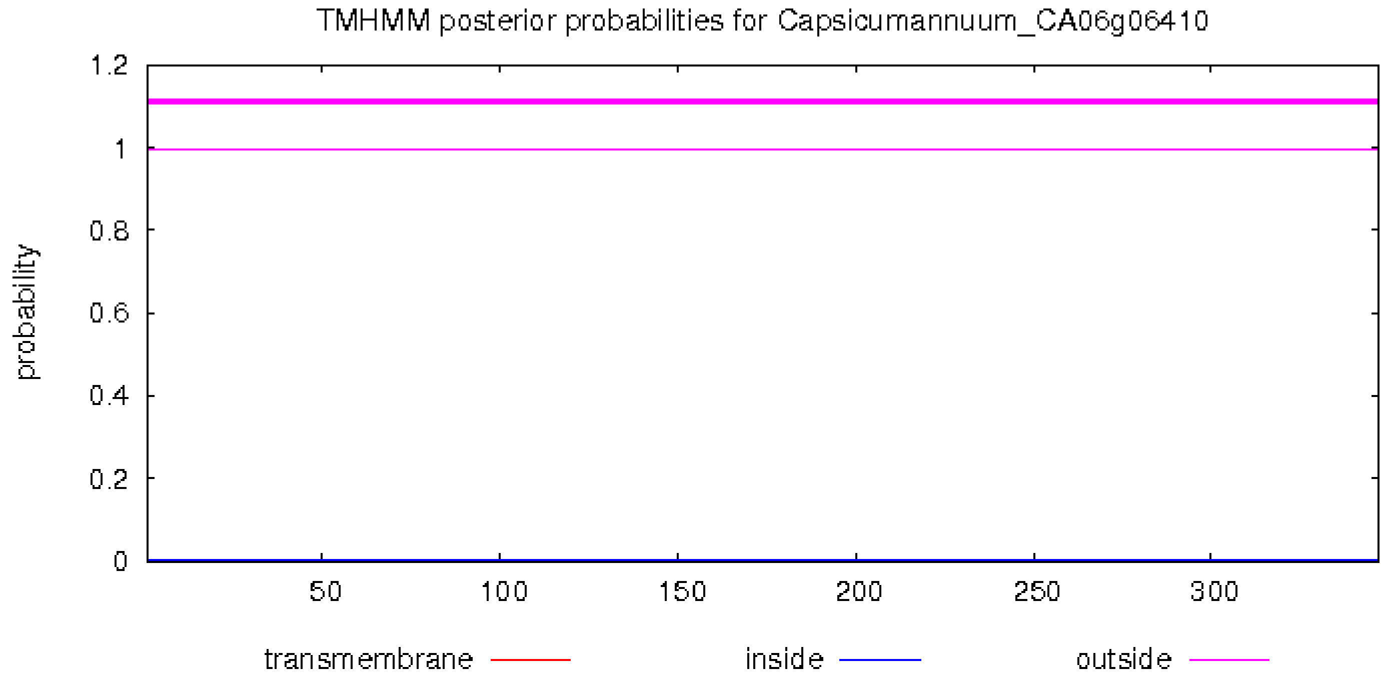

plot in postscript, script for making the plot in gnuplot, data for plot

---

```
# Capsicumannuum_CA06g11310 Length: 294
# Capsicumannuum_CA06g11310 Number of predicted TMHs:  0
# Capsicumannuum_CA06g11310 Exp number of AAs in TMHs: 0.00039
# Capsicumannuum_CA06g11310 Exp number, first 60 AAs:  0.00023
# Capsicumannuum_CA06g11310 Total prob of N-in:        0.00526
Capsicumannuum_CA06g11310	TMHMM2.0	outside	     1   294
```

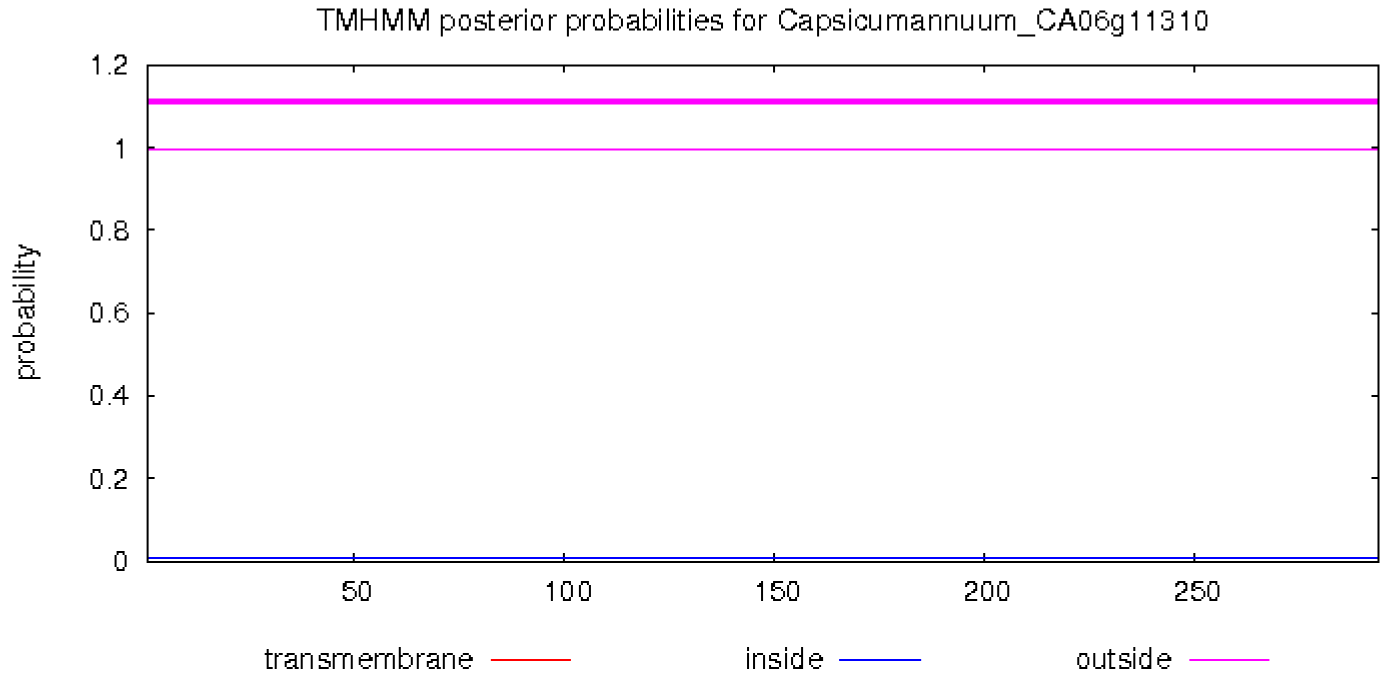

plot in postscript, script for making the plot in gnuplot, data for plot

---

```
# Capsicumannuum_CA06g13110 Length: 342
# Capsicumannuum_CA06g13110 Number of predicted TMHs:  0
# Capsicumannuum_CA06g13110 Exp number of AAs in TMHs: 0.00109
# Capsicumannuum_CA06g13110 Exp number, first 60 AAs:  0
# Capsicumannuum_CA06g13110 Total prob of N-in:        0.00078
Capsicumannuum_CA06g13110	TMHMM2.0	outside	     1   342
```

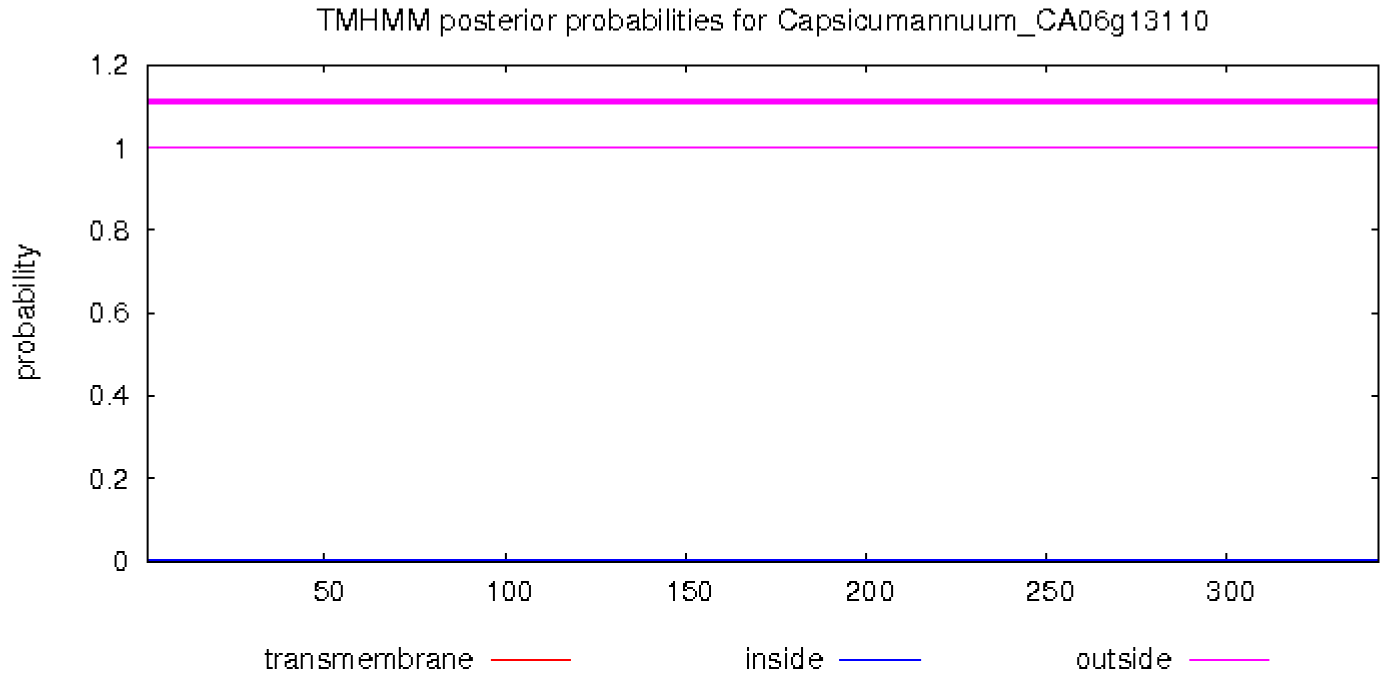

plot in postscript, script for making the plot in gnuplot, data for plot

---

```
# Capsicumannuum_CA06g14050 Length: 322
# Capsicumannuum_CA06g14050 Number of predicted TMHs:  0
# Capsicumannuum_CA06g14050 Exp number of AAs in TMHs: 0
# Capsicumannuum_CA06g14050 Exp number, first 60 AAs:  0
# Capsicumannuum_CA06g14050 Total prob of N-in:        0.01294
Capsicumannuum_CA06g14050       TMHMM2.0        outside      1   322
```

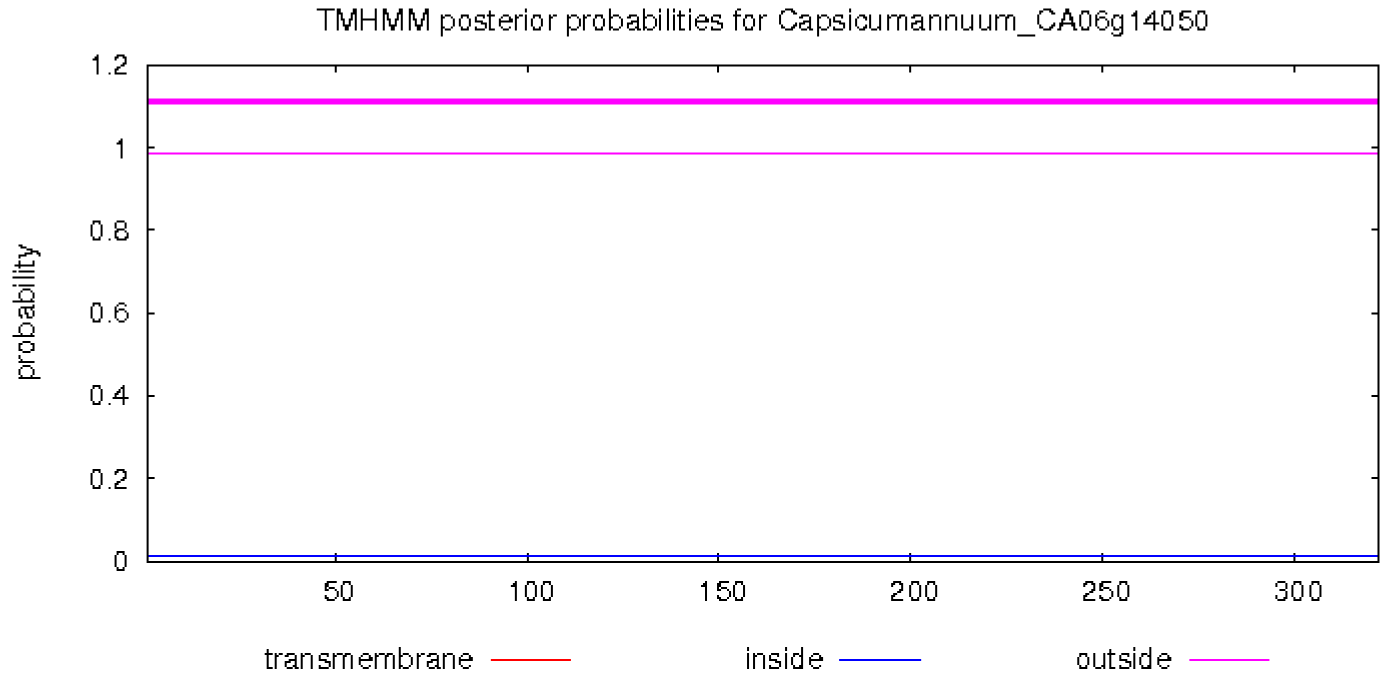

plot in postscript, script for making the plot in gnuplot, data for plot

---

```
# Capsicumannuum_CA06g14960 Length: 341
# Capsicumannuum_CA06g14960 Number of predicted TMHs:  0
# Capsicumannuum_CA06g14960 Exp number of AAs in TMHs: 0.00027
# Capsicumannuum_CA06g14960 Exp number, first 60 AAs:  0.00027
# Capsicumannuum_CA06g14960 Total prob of N-in:        0.01532
Capsicumannuum_CA06g14960       TMHMM2.0        outside      1   341
```

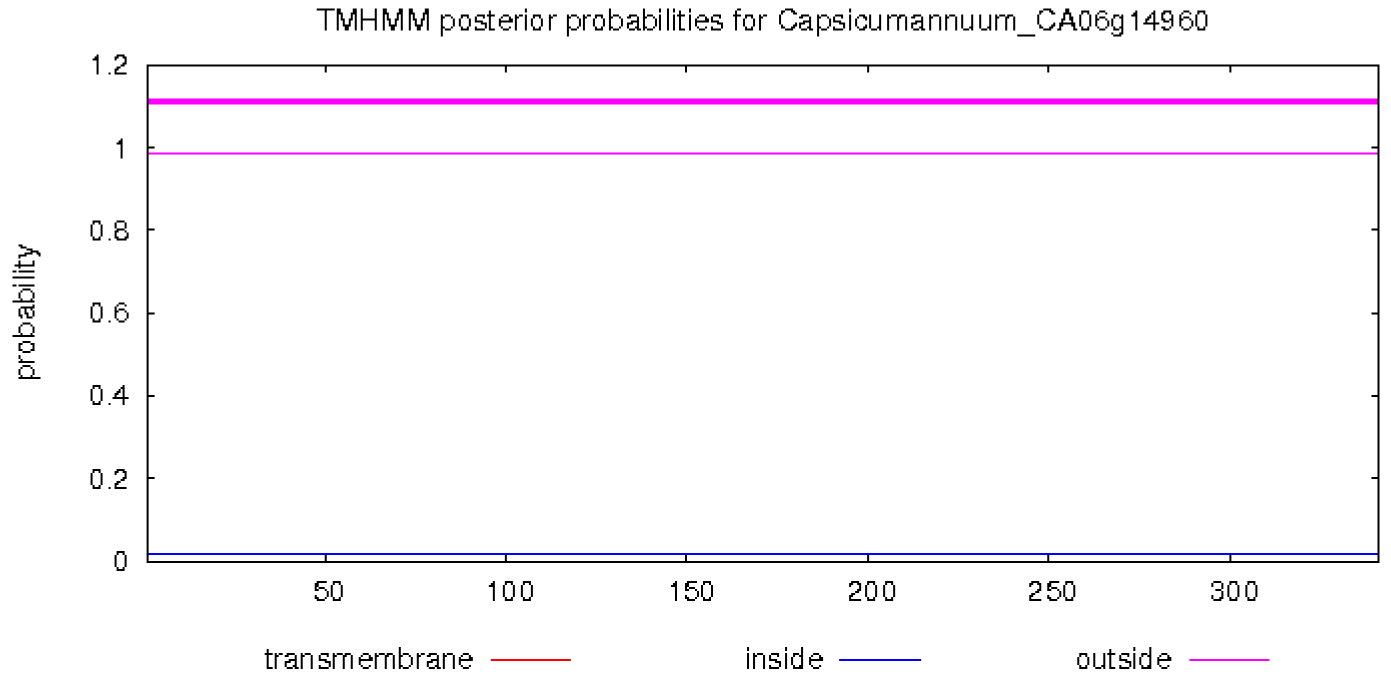

plot in postscript, script for making the plot in gnuplot, data for plot

---

```
# Capsicumannuum_CA06g15350 Length: 534
# Capsicumannuum_CA06g15350 Number of predicted TMHs:  0
# Capsicumannuum_CA06g15350 Exp number of AAs in TMHs: 0.00039
# Capsicumannuum_CA06g15350 Exp number, first 60 AAs:  0.00039
# Capsicumannuum_CA06g15350 Total prob of N-in:        0.00063
Capsicumannuum_CA06g15350	TMHMM2.0	outside	     1   534
```

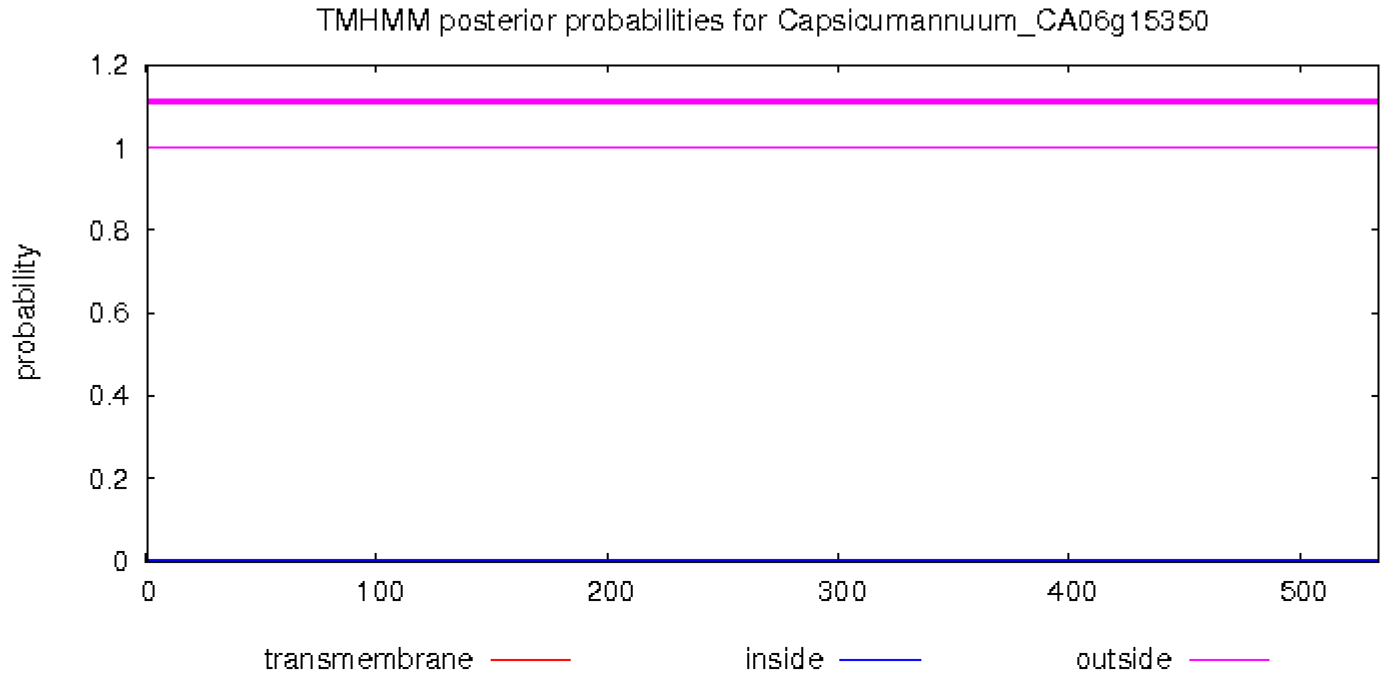

plot in postscript, script for making the plot in gnuplot, data for plot

---

```
# Capsicumannuum_CA06g17290 Length: 379
# Capsicumannuum_CA06g17290 Number of predicted TMHs:  0
# Capsicumannuum_CA06g17290 Exp number of AAs in TMHs: 0.00131
# Capsicumannuum_CA06g17290 Exp number, first 60 AAs:  0
# Capsicumannuum_CA06g17290 Total prob of N-in:        0.01018
Capsicumannuum_CA06g17290	TMHMM2.0	outside	     1   379
```

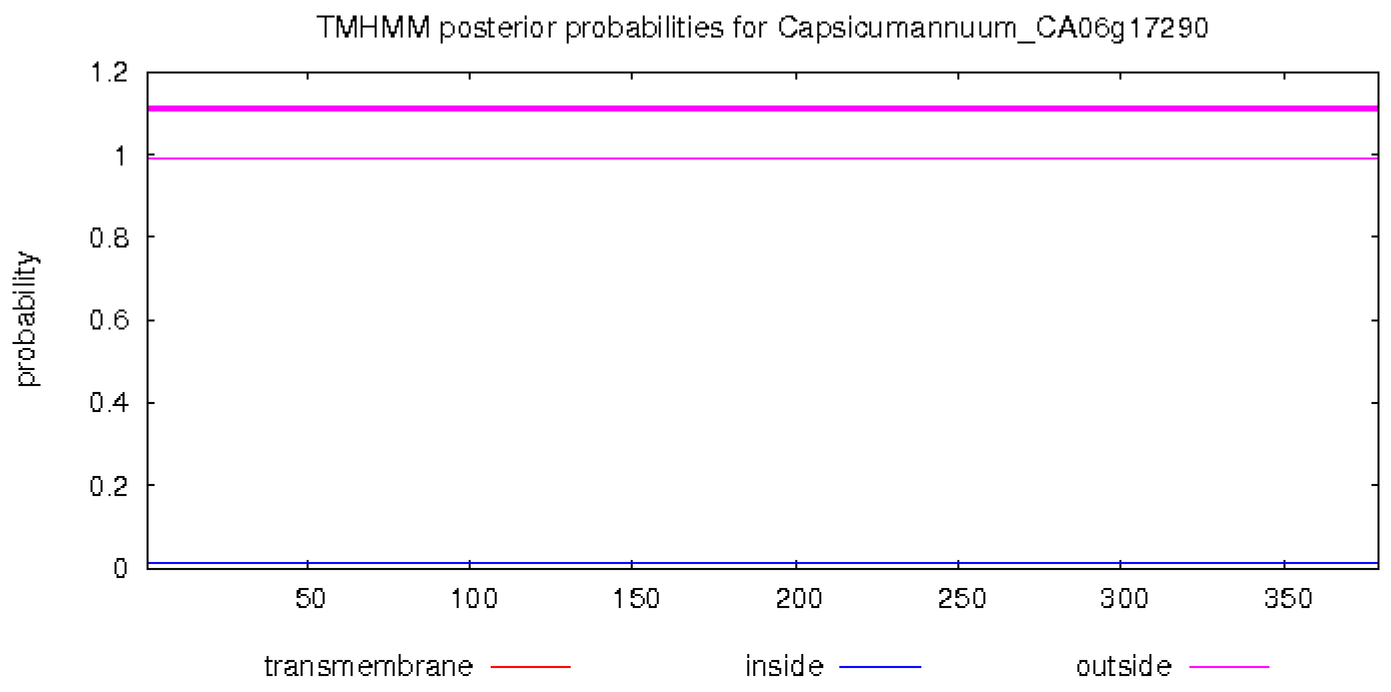

plot in postscript, script for making the plot in gnuplot, data for plot

```
# Capsicumannuum_CA06g17870 Length: 317
# Capsicumannuum_CA06g17870 Number of predicted TMHs:  0
# Capsicumannuum_CA06g17870 Exp number of AAs in TMHs: 0.02111
# Capsicumannuum_CA06g17870 Exp number, first 60 AAs:  0
# Capsicumannuum_CA06g17870 Total prob of N-in:        0.00383
Capsicumannuum_CA06g17870       TMHMM2.0        outside      1   317
```

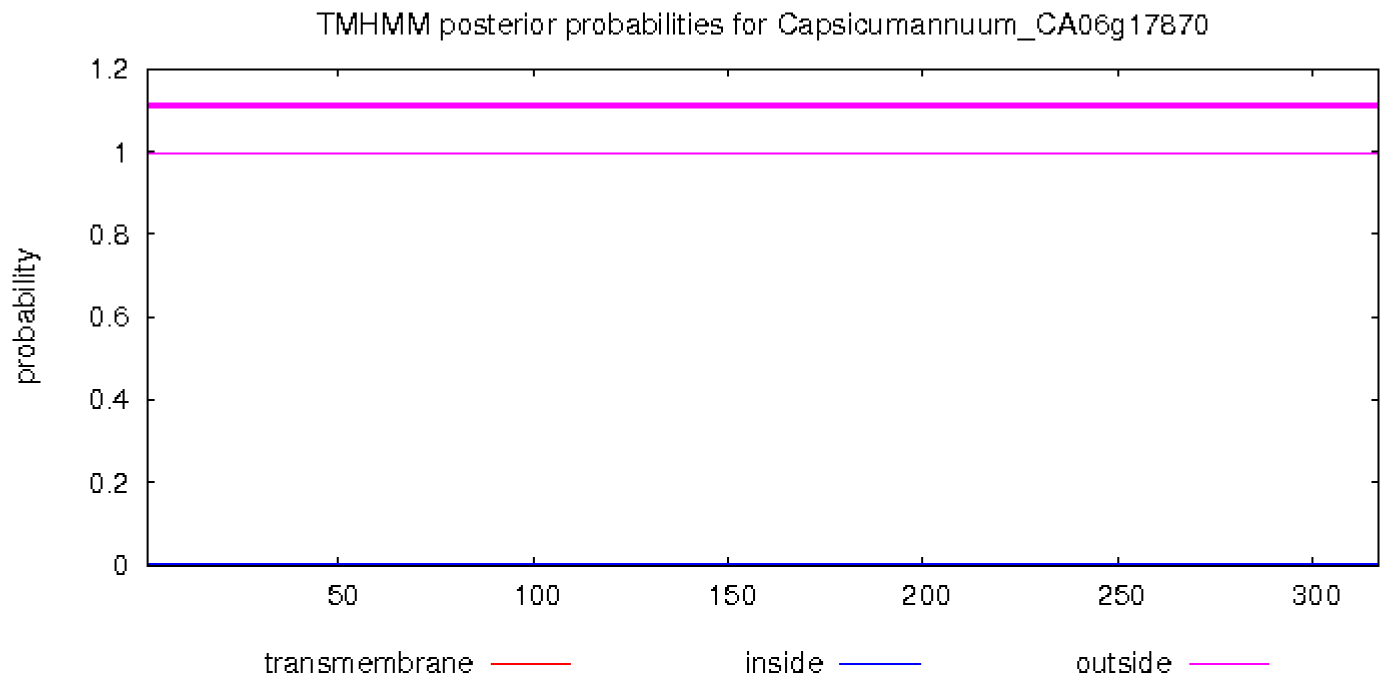

plot in postscript, script for making the plot in gnuplot, data for plot

```
# Capsicumannuum_CA06g18770 Length: 377
# Capsicumannuum_CA06g18770 Number of predicted TMHs:  0
# Capsicumannuum_CA06g18770 Exp number of AAs in TMHs: 0.0263
# Capsicumannuum_CA06g18770 Exp number, first 60 AAs:  0
# Capsicumannuum_CA06g18770 Total prob of N-in:        0.01173
Capsicumannuum_CA06g18770       TMHMM2.0        outside      1   377
```

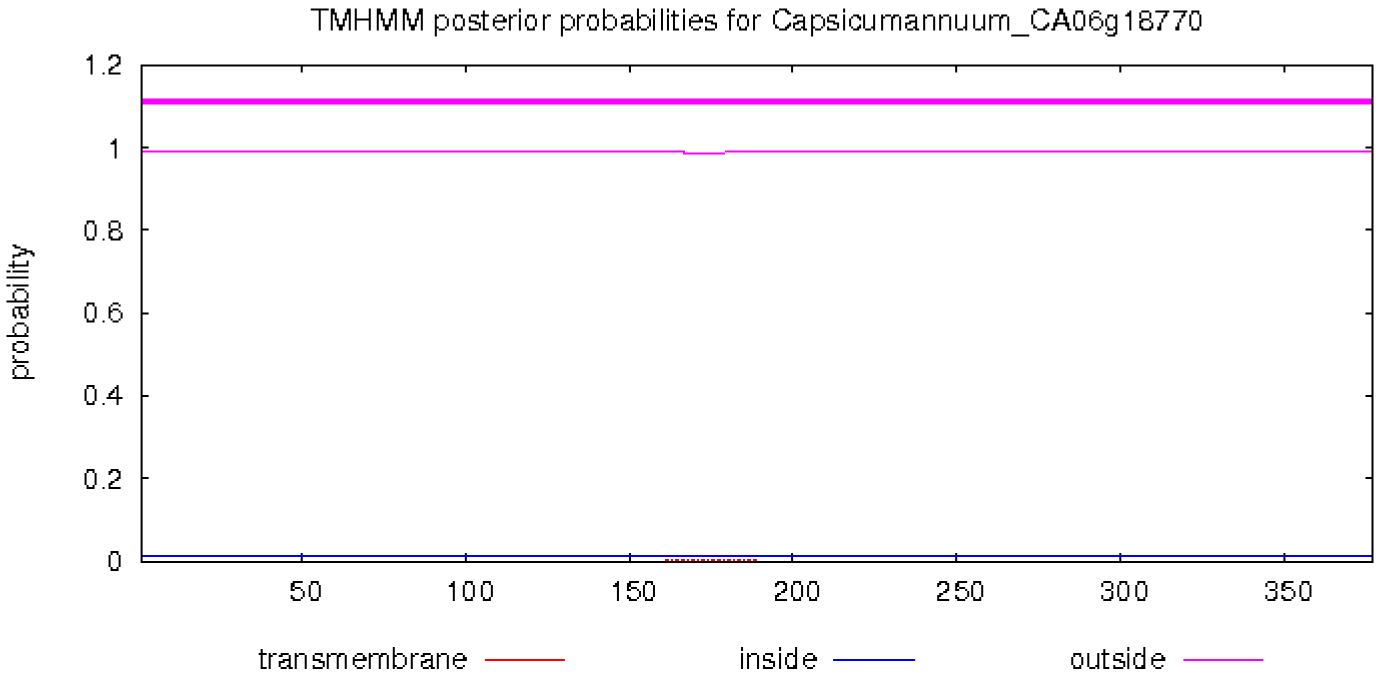

plot in postscript, script for making the plot in gnuplot, data for plot

---

```
# Capsicumannuum_CA06g21590 Length: 637
# Capsicumannuum_CA06g21590 Number of predicted TMHs:  1
# Capsicumannuum_CA06g21590 Exp number of AAs in TMHs: 22.21663
# Capsicumannuum_CA06g21590 Exp number, first 60 AAs:  0.00026
# Capsicumannuum_CA06g21590 Total prob of N-in:        0.00027
Capsicumannuum_CA06g21590	TMHMM2.0	outside	     1   611
Capsicumannuum_CA06g21590	TMHMM2.0	TMhelix	   612   634
Capsicumannuum_CA06g21590	TMHMM2.0	inside	   635   637
```

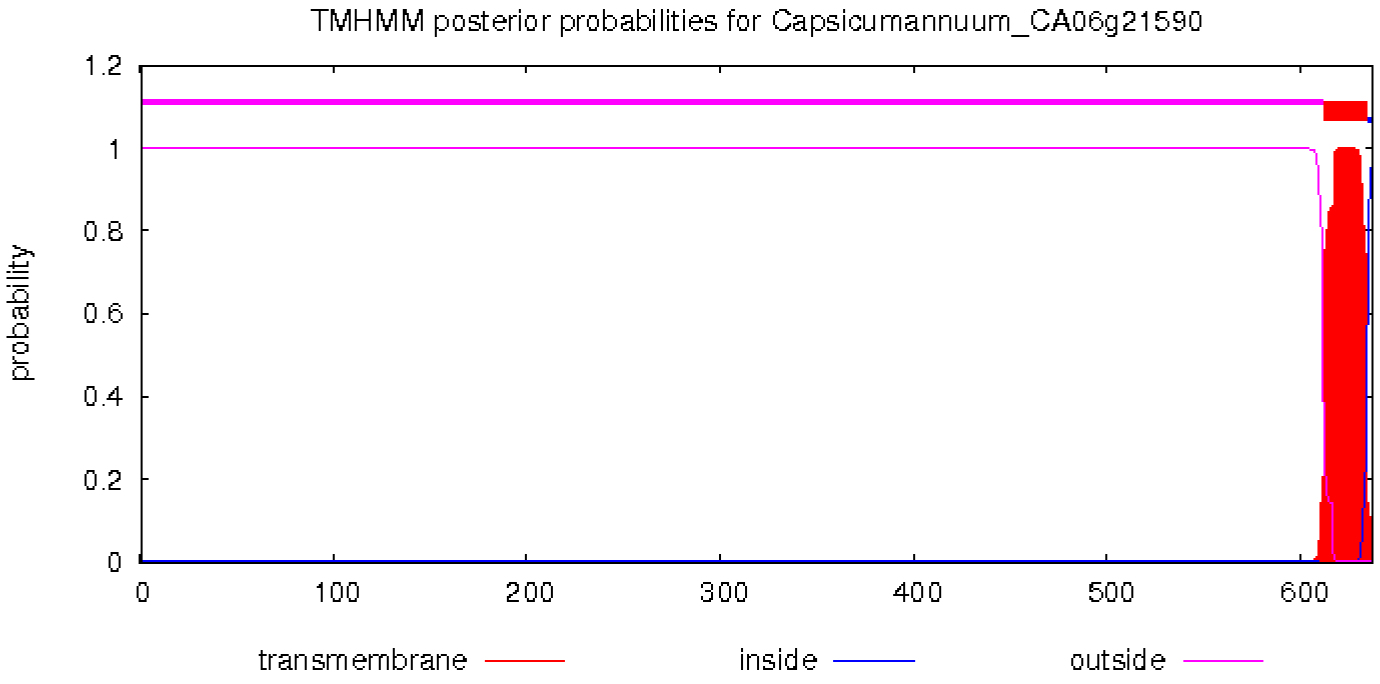

plot in postscript, script for making the plot in gnuplot, data for plot

---

```
# Capsicumannuum_CA06g22780 Length: 432
# Capsicumannuum_CA06g22780 Number of predicted TMHs:  1
# Capsicumannuum_CA06g22780 Exp number of AAs in TMHs: 21.99548
# Capsicumannuum_CA06g22780 Exp number, first 60 AAs:  0.03615
# Capsicumannuum_CA06g22780 Total prob of N-in:        0.01533
```

```
Capsicumannuum_CA06g22780       TMHMM2.0        outside      1   392
Capsicumannuum_CA06g22780       TMHMM2.0        TMhelix    393   415
Capsicumannuum_CA06g22780       TMHMM2.0        inside     416   432
```

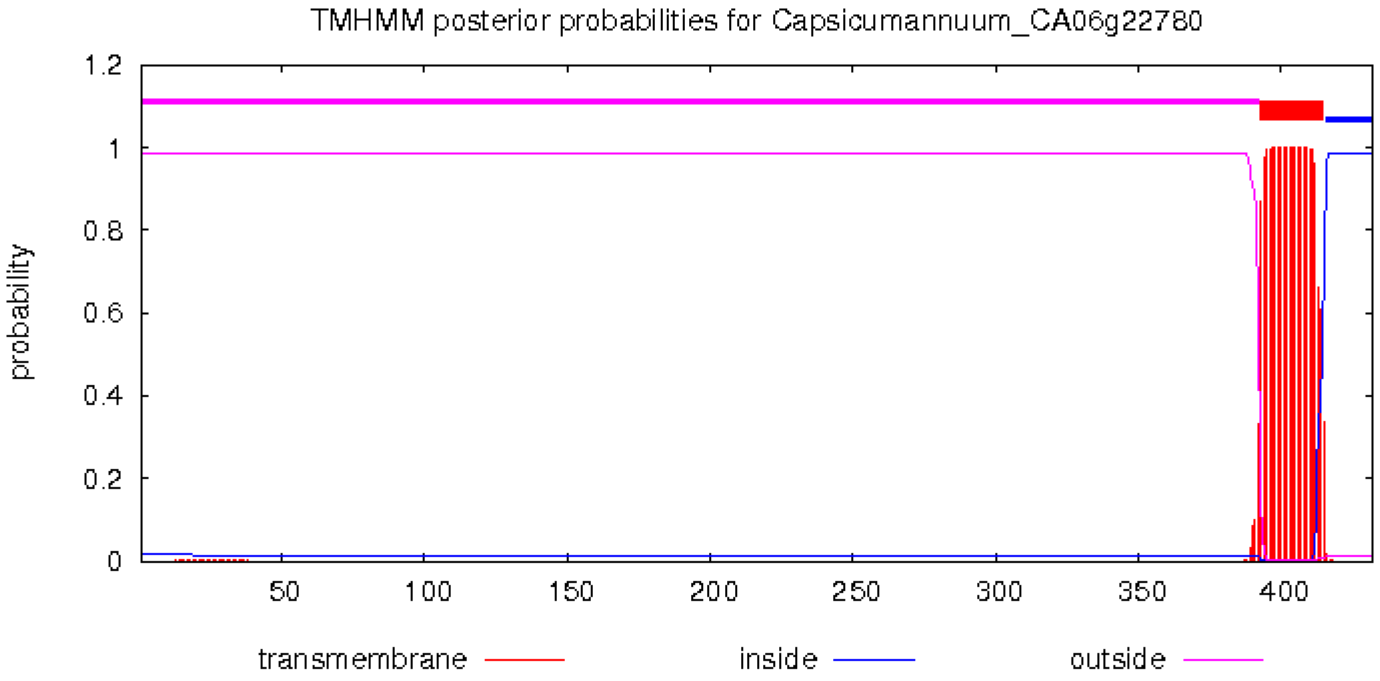

plot in postscript, script for making the plot in gnuplot, data for plot

---

```
# Capsicumannuum_CA06g25420 Length: 467
# Capsicumannuum_CA06g25420 Number of predicted TMHs:  0
# Capsicumannuum_CA06g25420 Exp number of AAs in TMHs: 0.00034
# Capsicumannuum_CA06g25420 Exp number, first 60 AAs:  0.00034
# Capsicumannuum_CA06g25420 Total prob of N-in:        0.00475
Capsicumannuum_CA06g25420       TMHMM2.0        outside      1   467
```

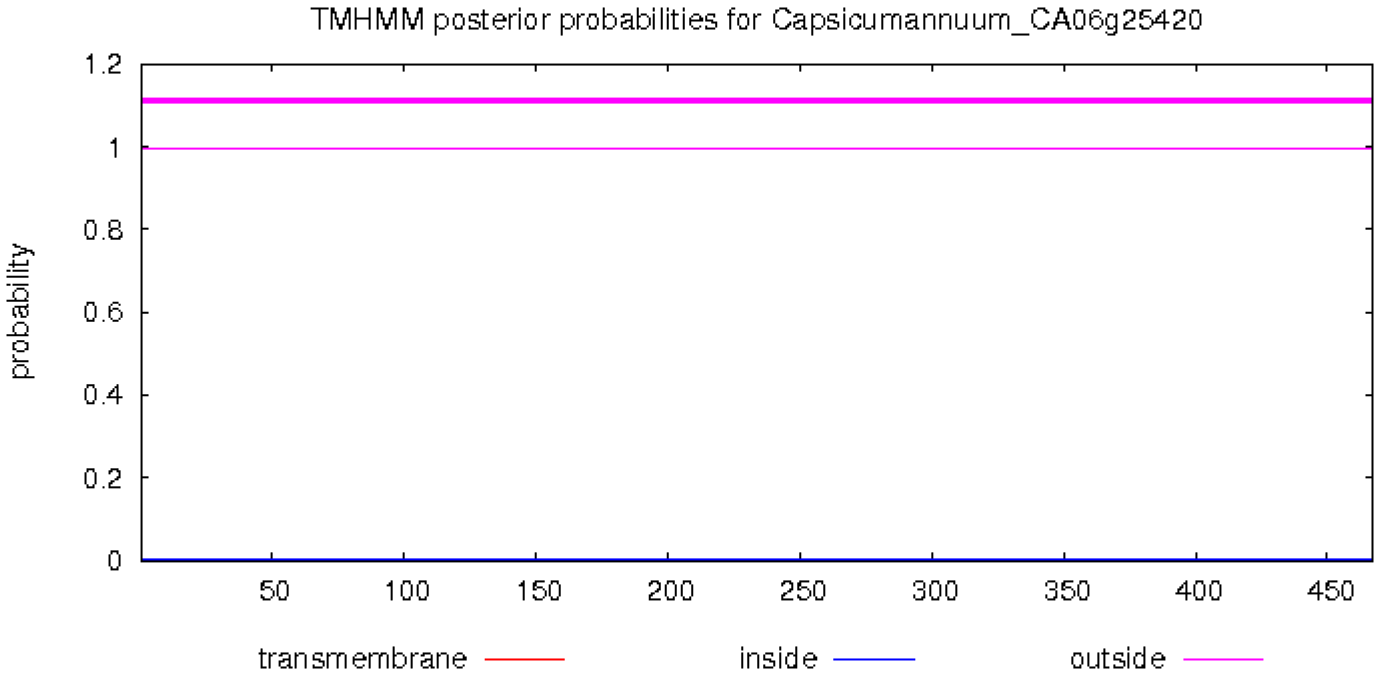

plot in postscript, script for making the plot in gnuplot, data for plot

---

```
# Capsicumannuum_CA06g27940 Length: 287
# Capsicumannuum_CA06g27940 Number of predicted TMHs:  0
# Capsicumannuum_CA06g27940 Exp number of AAs in TMHs: 0
```

```
# Capsicumannuum_CA06g27940 Exp number, first 60 AAs:  0
# Capsicumannuum_CA06g27940 Total prob of N-in:        0.01620
Capsicumannuum_CA06g27940       TMHMM2.0        outside      1   287
```

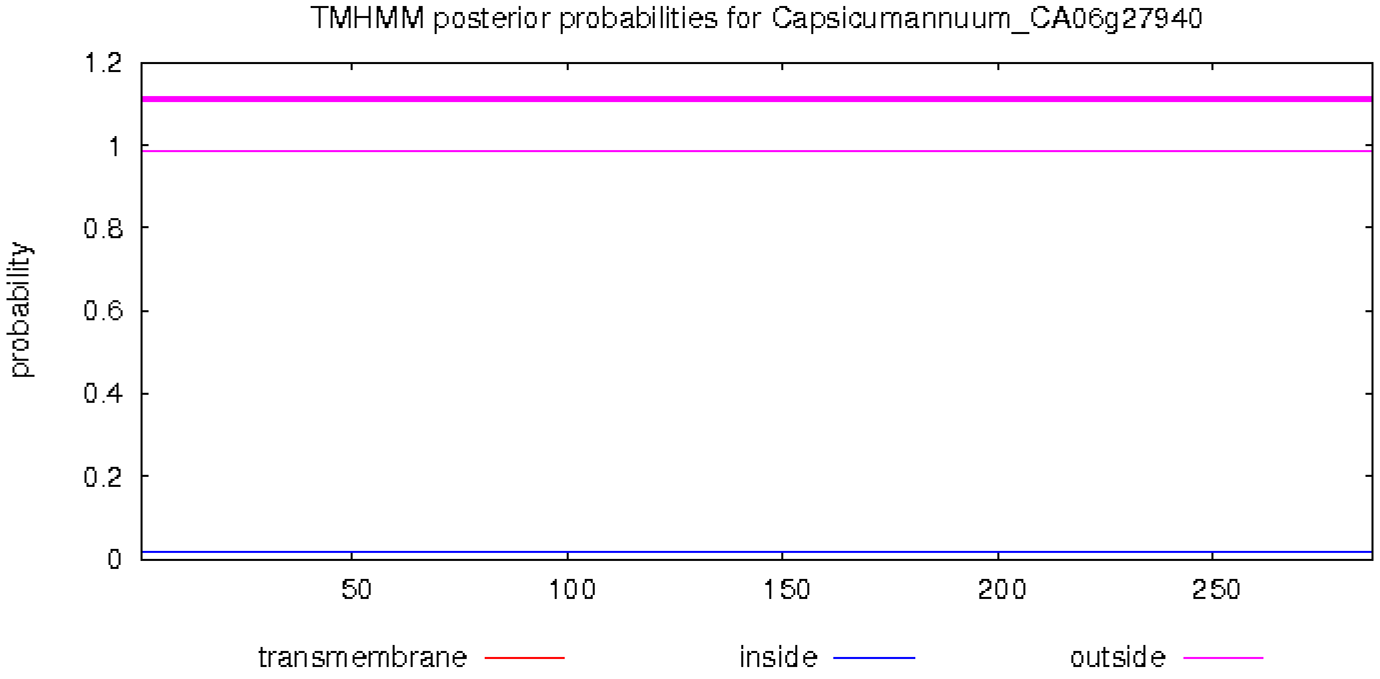

plot in postscript, script for making the plot in gnuplot, data for plot

---

```
# Capsicumannuum_CA07g00500 Length: 415
# Capsicumannuum_CA07g00500 Number of predicted TMHs:  0
# Capsicumannuum_CA07g00500 Exp number of AAs in TMHs: 0.00294
# Capsicumannuum_CA07g00500 Exp number, first 60 AAs:  0
# Capsicumannuum_CA07g00500 Total prob of N-in:        0.00910
Capsicumannuum_CA07g00500       TMHMM2.0        outside      1   415
```

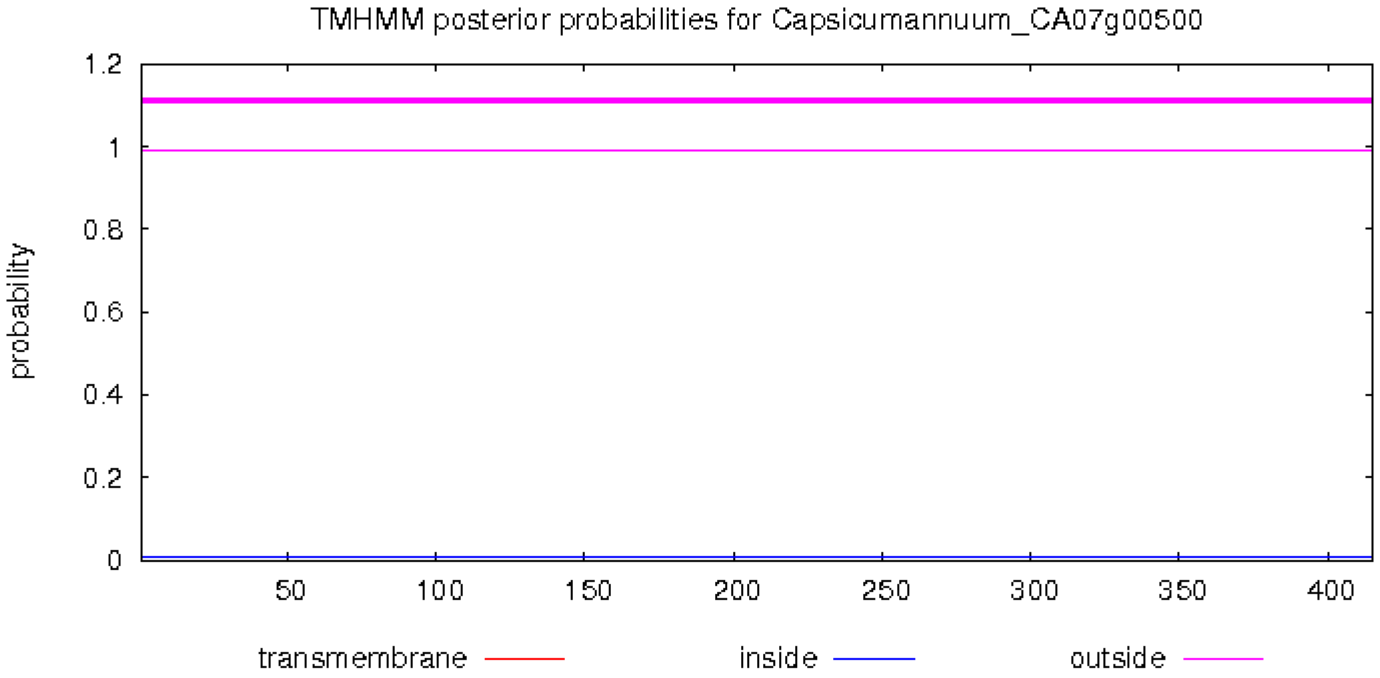

plot in postscript, script for making the plot in gnuplot, data for plot

---

```
# Capsicumannuum_CA07g02120 Length: 117
# Capsicumannuum_CA07g02120 Number of predicted TMHs:  0
# Capsicumannuum_CA07g02120 Exp number of AAs in TMHs: 0.00015
```

```
# Capsicumannuum_CA07g02120 Exp number, first 60 AAs:  0.00015
# Capsicumannuum_CA07g02120 Total prob of N-in:        0.03283
Capsicumannuum_CA07g02120       TMHMM2.0        outside      1   117
```

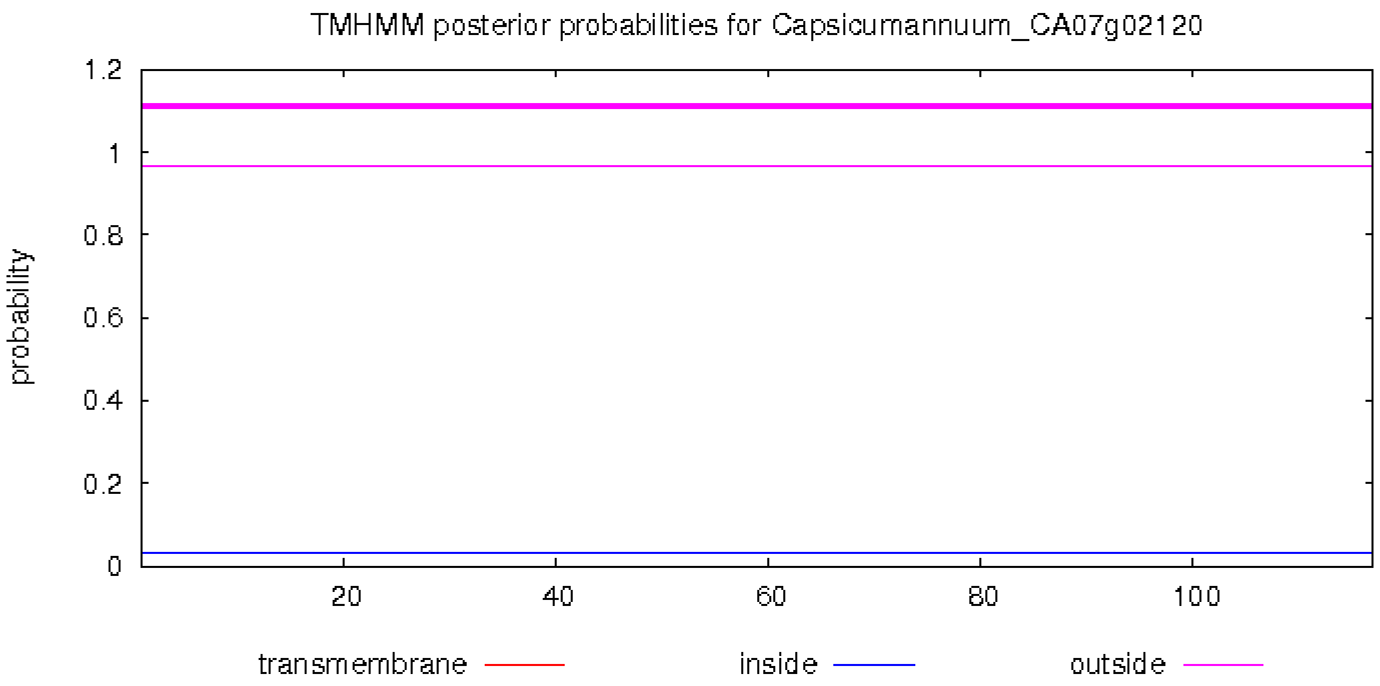

plot in postscript, script for making the plot in gnuplot, data for plot

---

```
# Capsicumannuum_CA07g03900 Length: 137
# Capsicumannuum_CA07g03900 Number of predicted TMHs:  0
# Capsicumannuum_CA07g03900 Exp number of AAs in TMHs: 0.01936
# Capsicumannuum_CA07g03900 Exp number, first 60 AAs:  0.01936
# Capsicumannuum_CA07g03900 Total prob of N-in:        0.07134
Capsicumannuum_CA07g03900       TMHMM2.0        outside      1   137
```

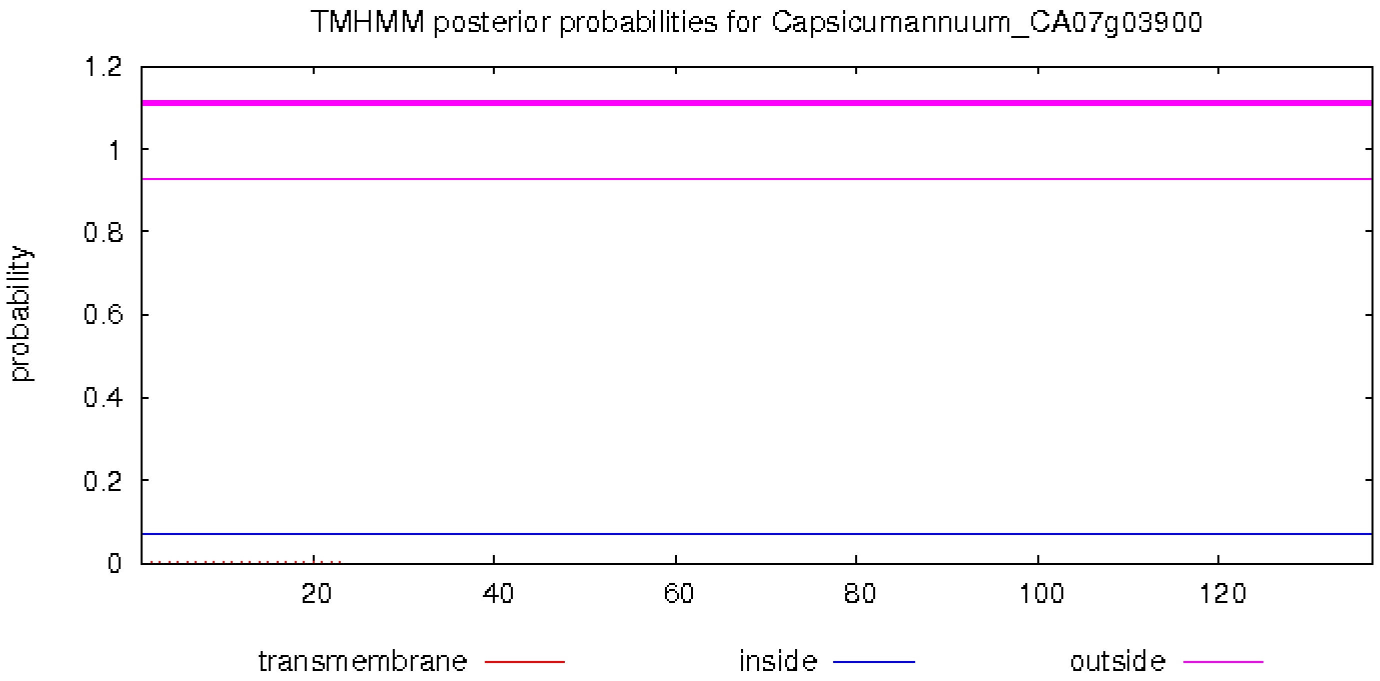

plot in postscript, script for making the plot in gnuplot, data for plot

---

```
# Capsicumannuum_CA07g16790 Length: 154
# Capsicumannuum_CA07g16790 Number of predicted TMHs:  0
# Capsicumannuum_CA07g16790 Exp number of AAs in TMHs: 0.35451
```

```
# Capsicumannuum_CA07g16790 Exp number, first 60 AAs:  0.35451
# Capsicumannuum_CA07g16790 Total prob of N-in:        0.28981
Capsicumannuum_CA07g16790       TMHMM2.0        outside      1   154
```

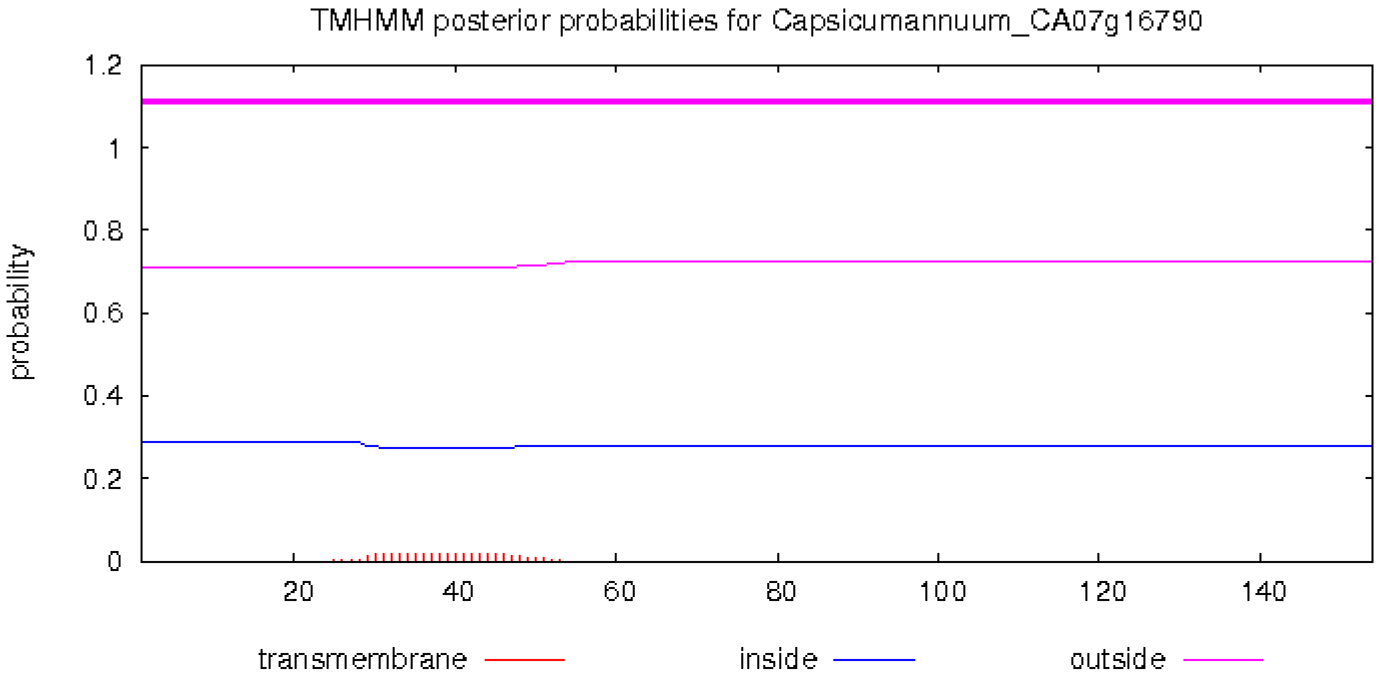

plot in postscript, script for making the plot in gnuplot, data for plot

---

```
# Capsicumannuum_CA07g17460 Length: 263
# Capsicumannuum_CA07g17460 Number of predicted TMHs:  0
# Capsicumannuum_CA07g17460 Exp number of AAs in TMHs: 0.00777
# Capsicumannuum_CA07g17460 Exp number, first 60 AAs:  0.00176
# Capsicumannuum_CA07g17460 Total prob of N-in:        0.01859
Capsicumannuum_CA07g17460       TMHMM2.0        outside      1   263
```

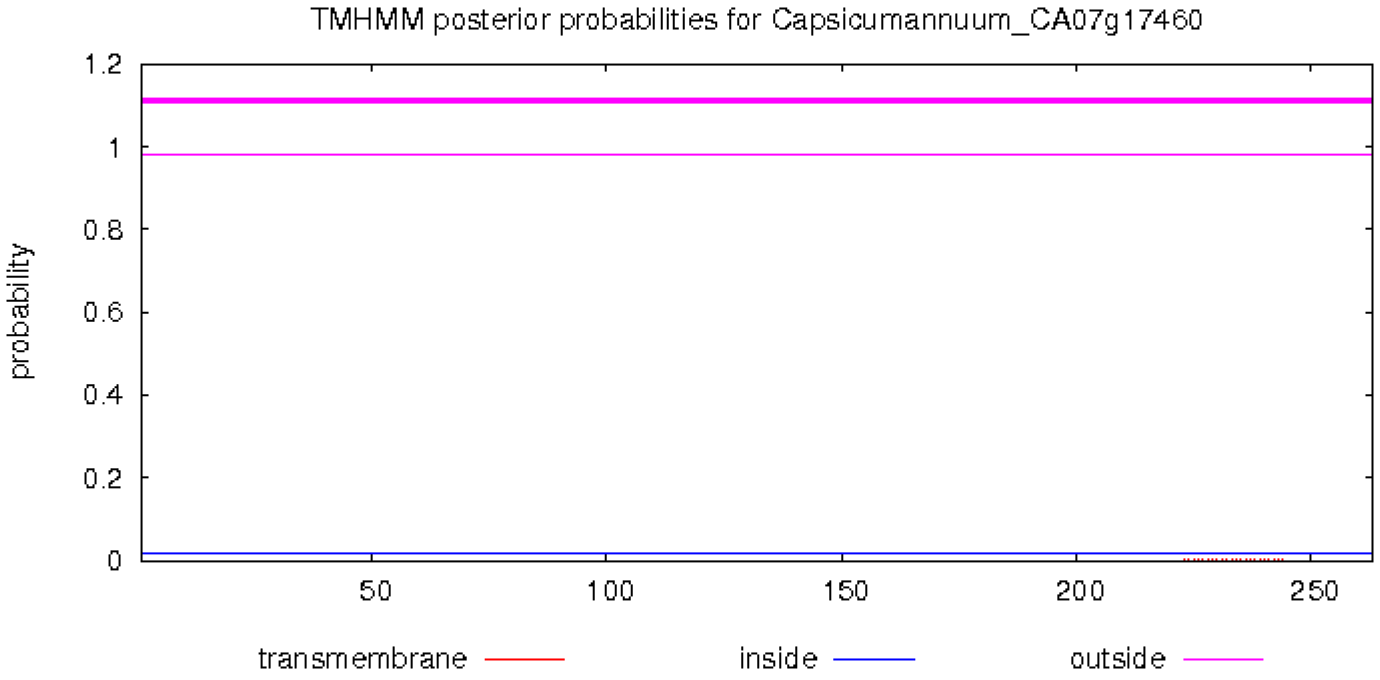

plot in postscript, script for making the plot in gnuplot, data for plot

---

```
# Capsicumannuum_CA07g18020 Length: 456
# Capsicumannuum_CA07g18020 Number of predicted TMHs:  0
# Capsicumannuum_CA07g18020 Exp number of AAs in TMHs: 8.00385
```

```
# Capsicumannuum_CA07g18020 Exp number, first 60 AAs:  0
# Capsicumannuum_CA07g18020 Total prob of N-in:        0.00821
Capsicumannuum_CA07g18020       TMHMM2.0        outside      1   456
```

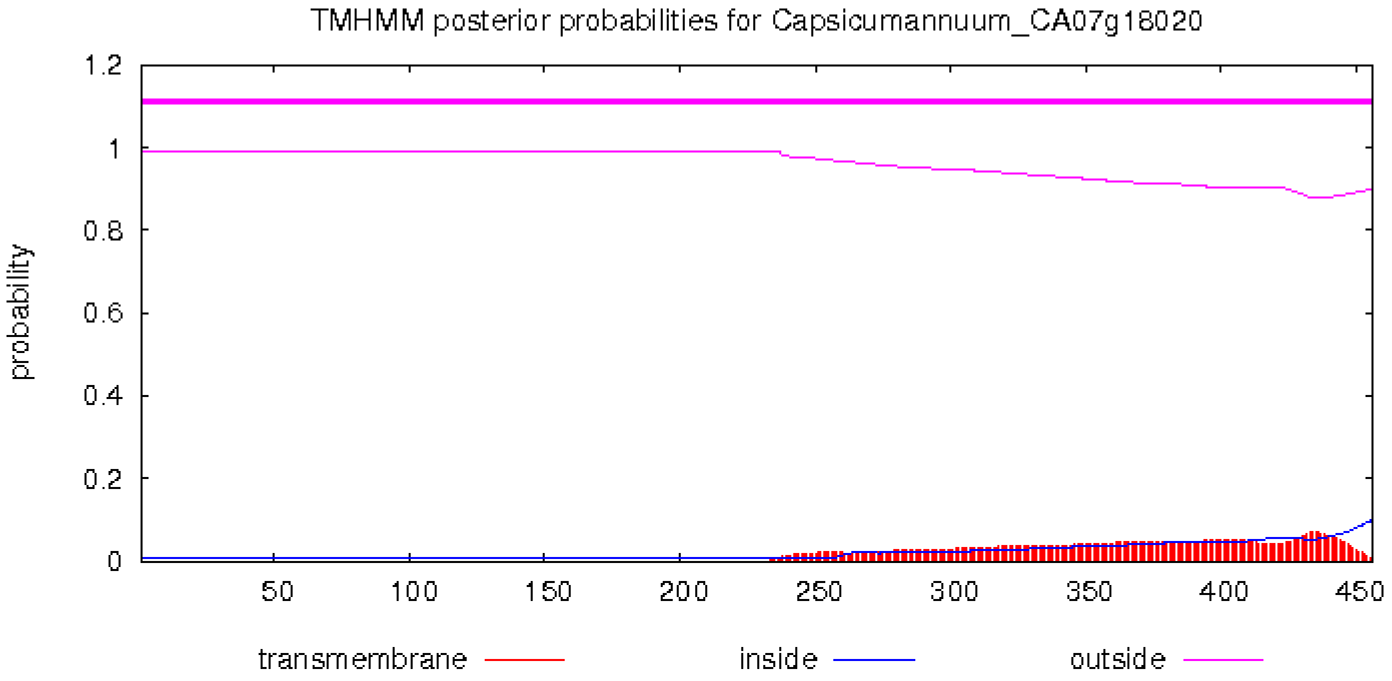

plot in postscript, script for making the plot in gnuplot, data for plot

---

```
# Capsicumannuum_CA07g18030 Length: 313
# Capsicumannuum_CA07g18030 Number of predicted TMHs:  3
# Capsicumannuum_CA07g18030 Exp number of AAs in TMHs: 56.78252
# Capsicumannuum_CA07g18030 Exp number, first 60 AAs:  0.27165
# Capsicumannuum_CA07g18030 Total prob of N-in:        0.13124
Capsicumannuum_CA07g18030       TMHMM2.0        outside      1    60
Capsicumannuum_CA07g18030       TMHMM2.0        TMhelix     61    78
Capsicumannuum_CA07g18030       TMHMM2.0        inside      79    84
Capsicumannuum_CA07g18030       TMHMM2.0        TMhelix     85   107
Capsicumannuum_CA07g18030       TMHMM2.0        outside    108   110
Capsicumannuum_CA07g18030       TMHMM2.0        TMhelix    111   133
Capsicumannuum_CA07g18030       TMHMM2.0        inside     134   313
```

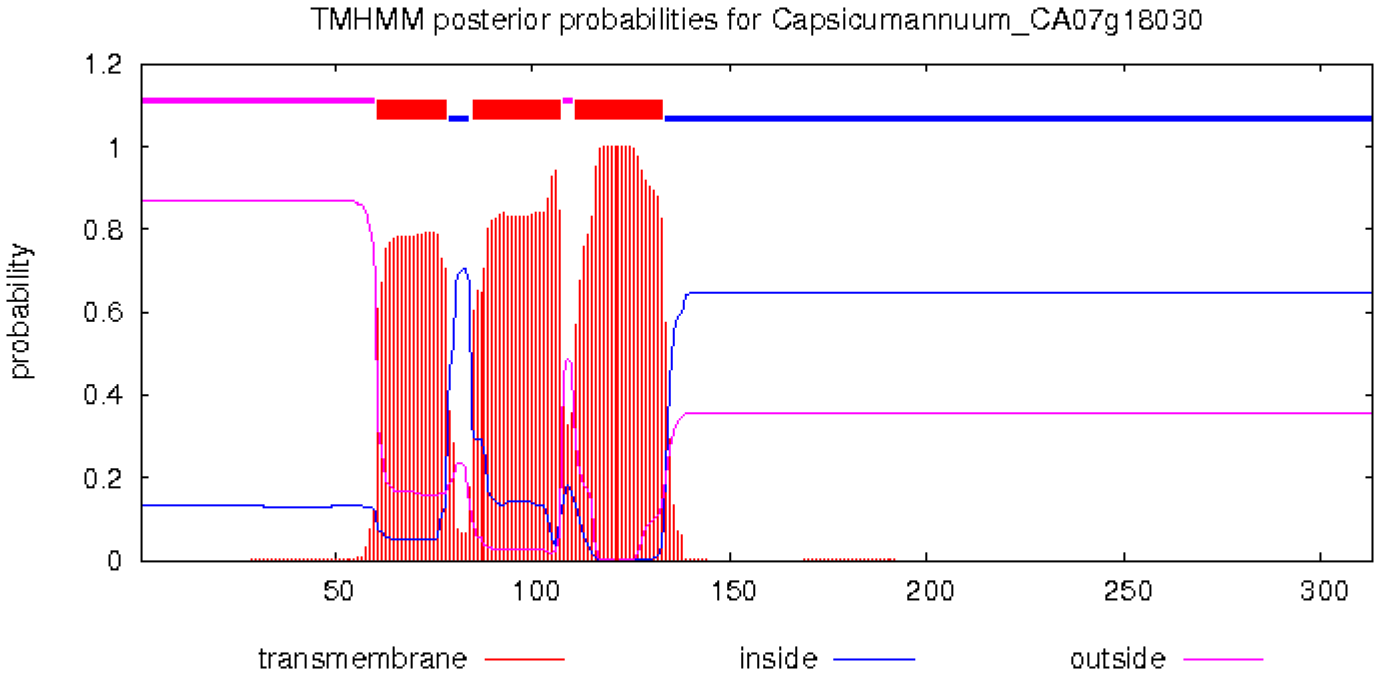

plot in postscript, script for making the plot in gnuplot, data for plot

```
# Capsicumannuum_CA07g21320 Length: 330
# Capsicumannuum_CA07g21320 Number of predicted TMHs:  0
# Capsicumannuum_CA07g21320 Exp number of AAs in TMHs: 0.00147
# Capsicumannuum_CA07g21320 Exp number, first 60 AAs:  0
# Capsicumannuum_CA07g21320 Total prob of N-in:        0.02551
Capsicumannuum_CA07g21320	TMHMM2.0	outside	     1   330
```

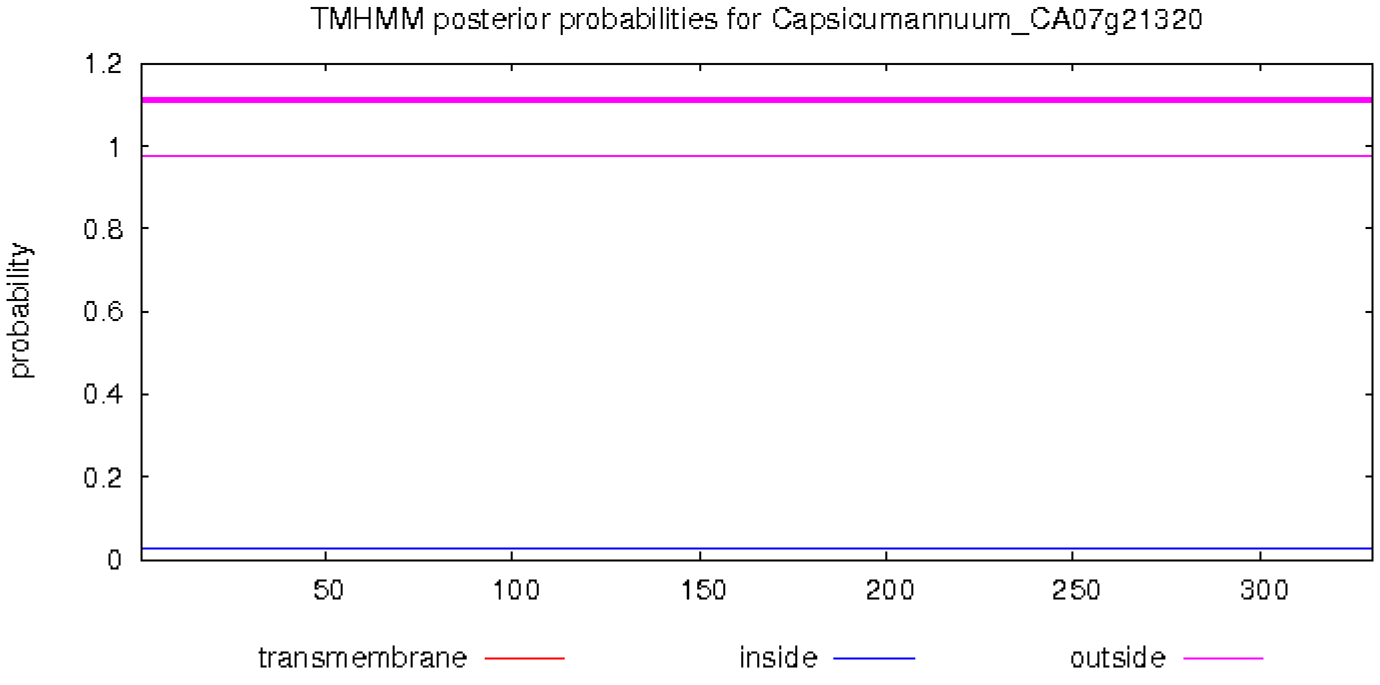

plot in postscript, script for making the plot in gnuplot, data for plot

```
# Capsicumannuum_CA07g21470 Length: 328
# Capsicumannuum_CA07g21470 Number of predicted TMHs:  0
# Capsicumannuum_CA07g21470 Exp number of AAs in TMHs: 1.07476
# Capsicumannuum_CA07g21470 Exp number, first 60 AAs:  0
# Capsicumannuum_CA07g21470 Total prob of N-in:        0.01675
Capsicumannuum_CA07g21470	TMHMM2.0	outside	     1   328
```

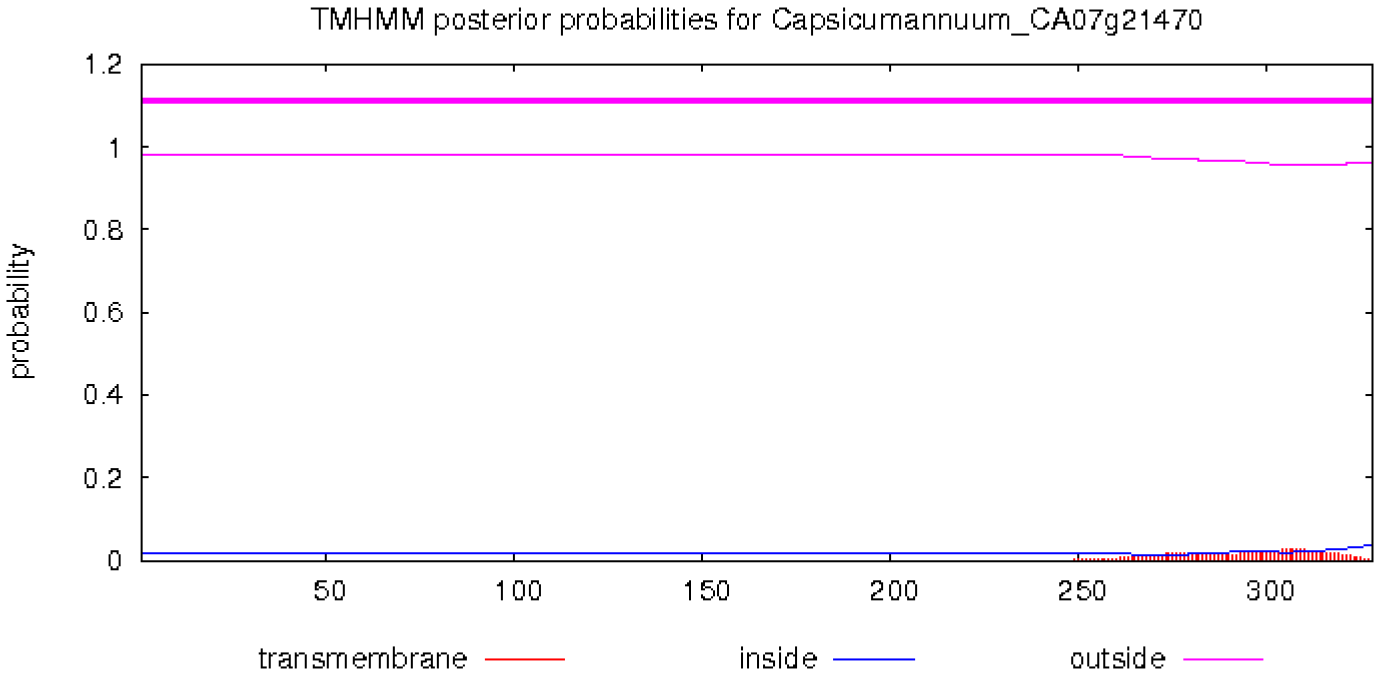

plot in postscript, script for making the plot in gnuplot, data for plot

```
# Capsicumannuum_CA08g06780 Length: 242
# Capsicumannuum_CA08g06780 Number of predicted TMHs:  0
# Capsicumannuum_CA08g06780 Exp number of AAs in TMHs: 0.0074
# Capsicumannuum_CA08g06780 Exp number, first 60 AAs:  0.00663
# Capsicumannuum_CA08g06780 Total prob of N-in:        0.17026
Capsicumannuum_CA08g06780	TMHMM2.0	outside	     1   242
```

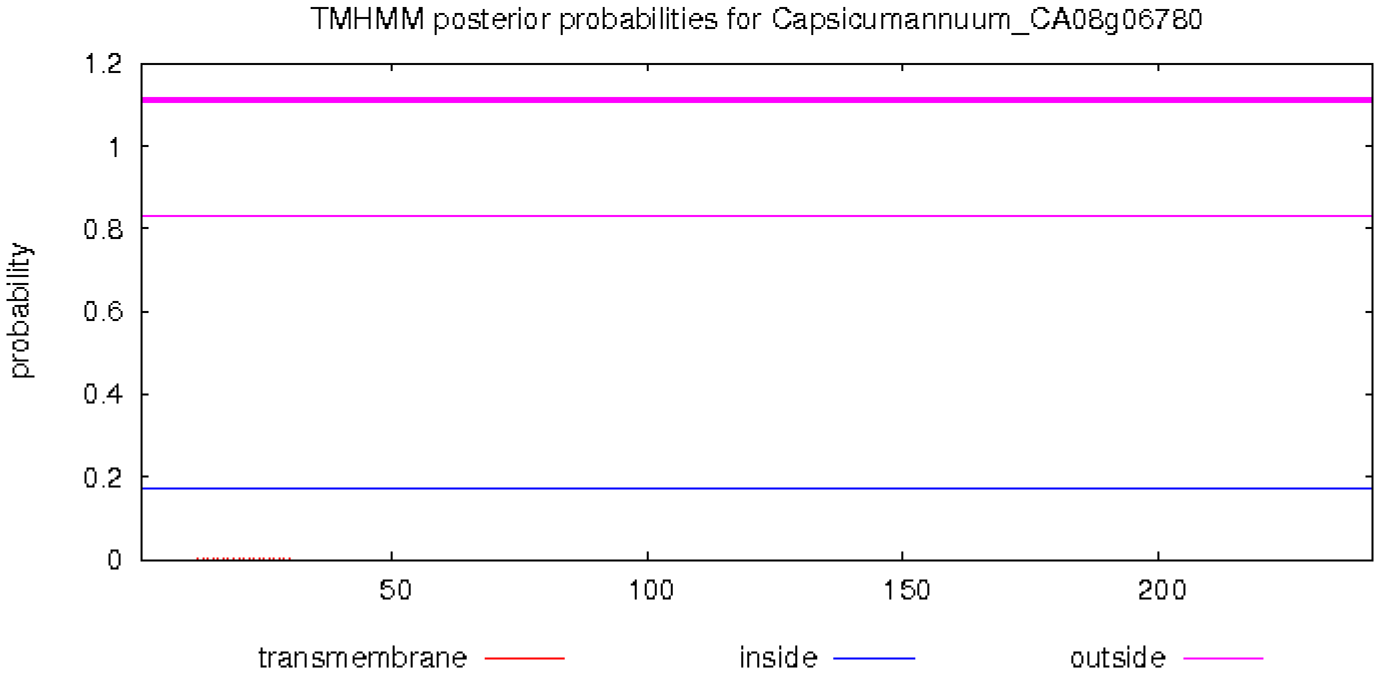

plot in postscript, script for making the plot in gnuplot, data for plot

```
# Capsicumannuum_CA08g06790 Length: 219
# Capsicumannuum_CA08g06790 Number of predicted TMHs:  0
# Capsicumannuum_CA08g06790 Exp number of AAs in TMHs: 0.00734
# Capsicumannuum_CA08g06790 Exp number, first 60 AAs:  0.0072
# Capsicumannuum_CA08g06790 Total prob of N-in:        0.21495
Capsicumannuum_CA08g06790	TMHMM2.0	outside	     1   219
```

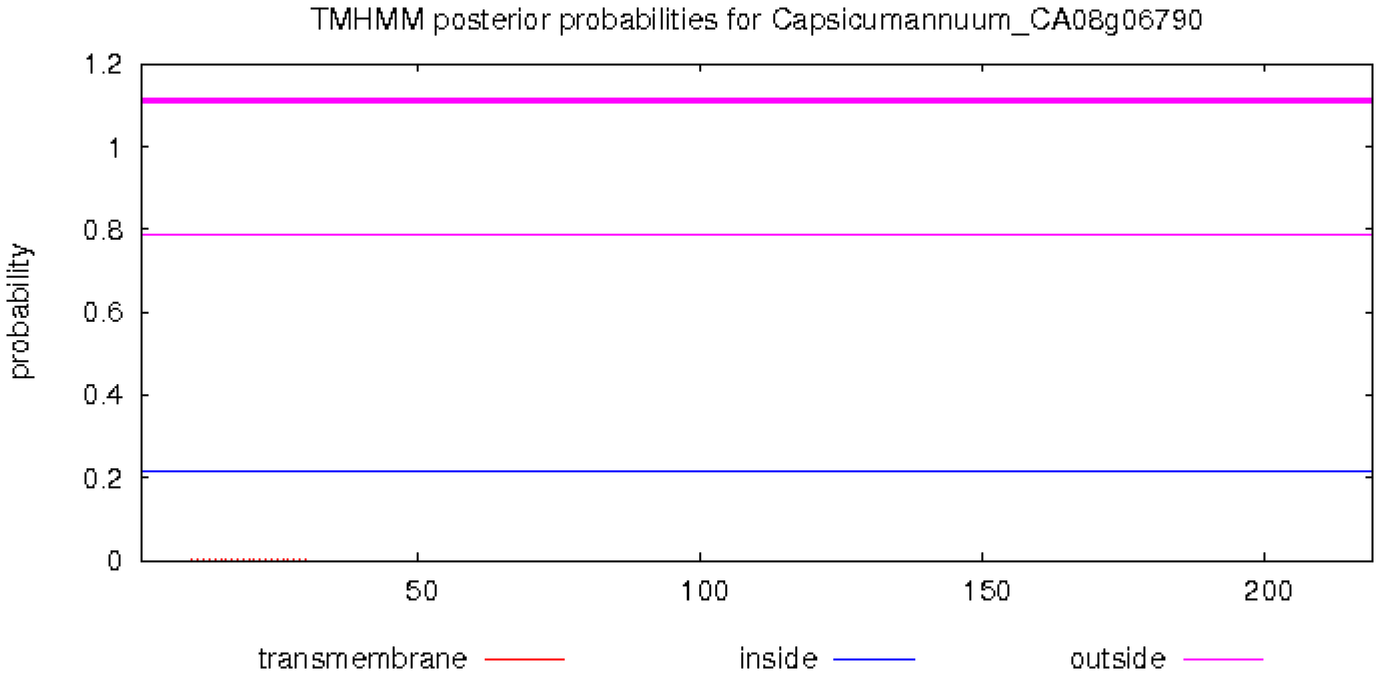

plot in postscript, script for making the plot in gnuplot, data for plot

```
# Capsicumannuum_CA08g13660 Length: 194
# Capsicumannuum_CA08g13660 Number of predicted TMHs:  0
# Capsicumannuum_CA08g13660 Exp number of AAs in TMHs: 0.00264
# Capsicumannuum_CA08g13660 Exp number, first 60 AAs:  0
# Capsicumannuum_CA08g13660 Total prob of N-in:        0.07912
Capsicumannuum_CA08g13660	TMHMM2.0	outside	     1   194
```

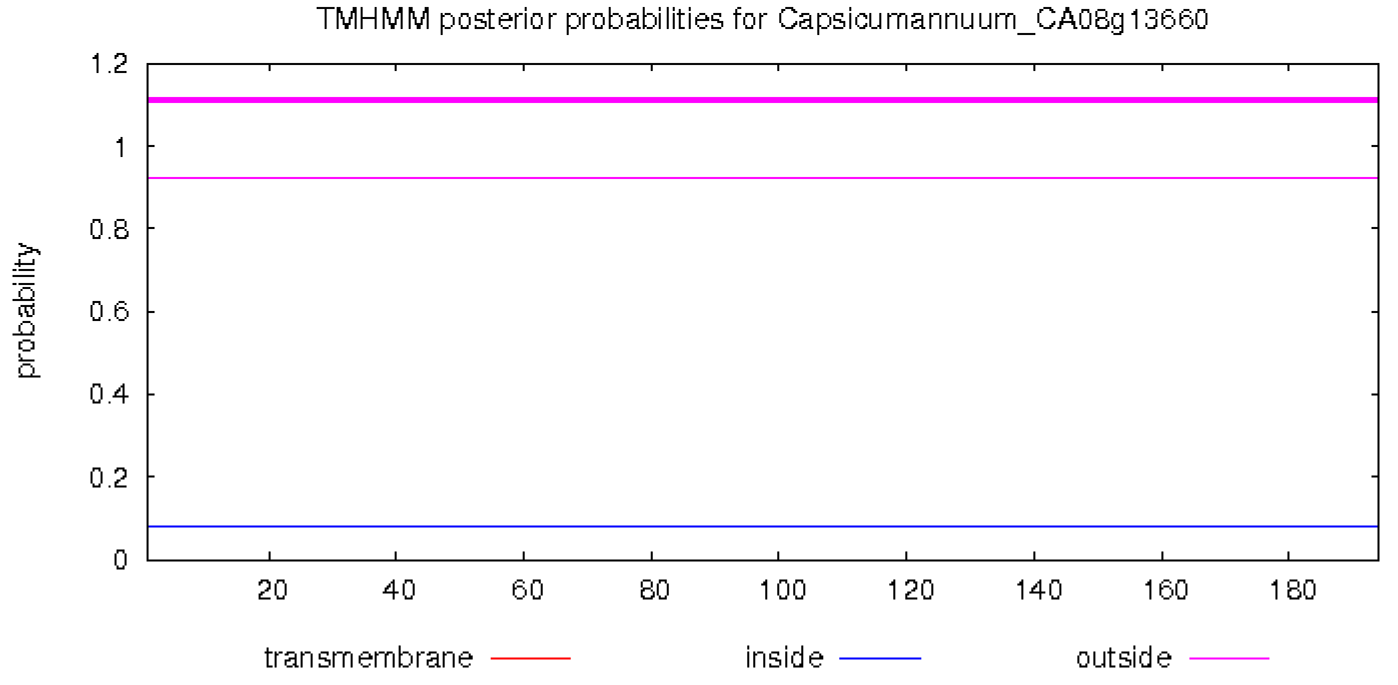

plot in postscript, script for making the plot in gnuplot, data for plot

```
# Capsicumannuum_CA08g15460 Length: 195
# Capsicumannuum_CA08g15460 Number of predicted TMHs:  0
# Capsicumannuum_CA08g15460 Exp number of AAs in TMHs: 0
# Capsicumannuum_CA08g15460 Exp number, first 60 AAs:  0
# Capsicumannuum_CA08g15460 Total prob of N-in:        0.02568
Capsicumannuum_CA08g15460	TMHMM2.0	outside	     1   195
```

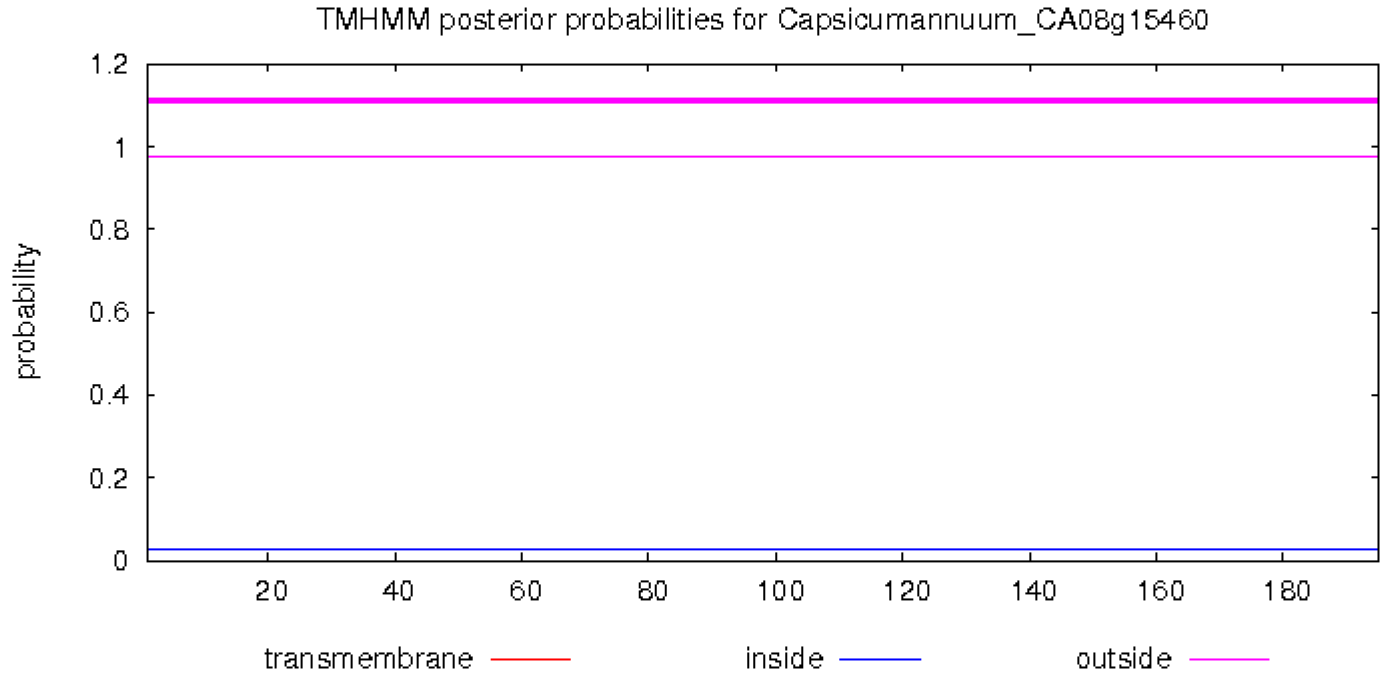

plot in postscript, script for making the plot in gnuplot, data for plot

```
# Capsicumannuum_CA09g00160 Length: 161
# Capsicumannuum_CA09g00160 Number of predicted TMHs:  0
# Capsicumannuum_CA09g00160 Exp number of AAs in TMHs: 0.00935
# Capsicumannuum_CA09g00160 Exp number, first 60 AAs:  0.00059
# Capsicumannuum_CA09g00160 Total prob of N-in:        0.36391
Capsicumannuum_CA09g00160	TMHMM2.0	outside	     1   161
```

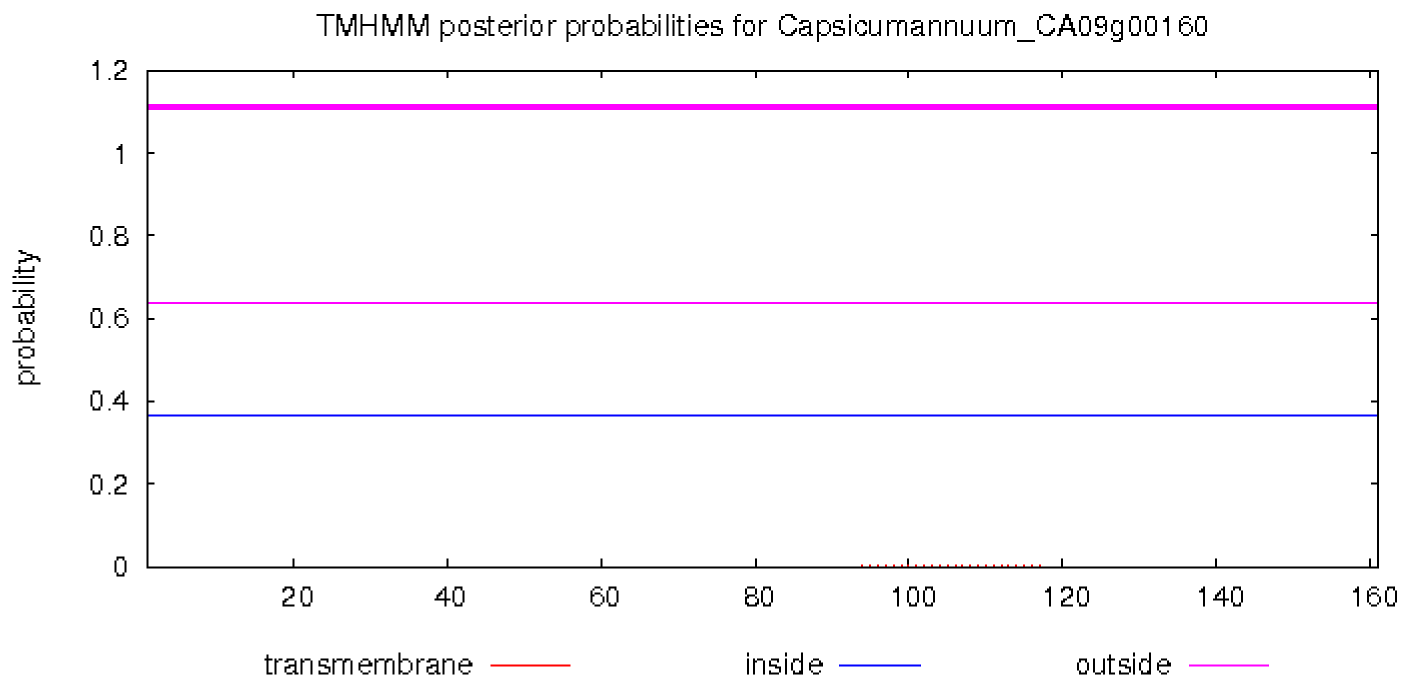

plot in postscript, script for making the plot in gnuplot, data for plot

```
# Capsicumannuum_CA09g12970 Length: 350
# Capsicumannuum_CA09g12970 Number of predicted TMHs:  0
# Capsicumannuum_CA09g12970 Exp number of AAs in TMHs: 0.00027
# Capsicumannuum_CA09g12970 Exp number, first 60 AAs:  0
# Capsicumannuum_CA09g12970 Total prob of N-in:        0.00469
Capsicumannuum_CA09g12970	TMHMM2.0	outside	     1   350
```

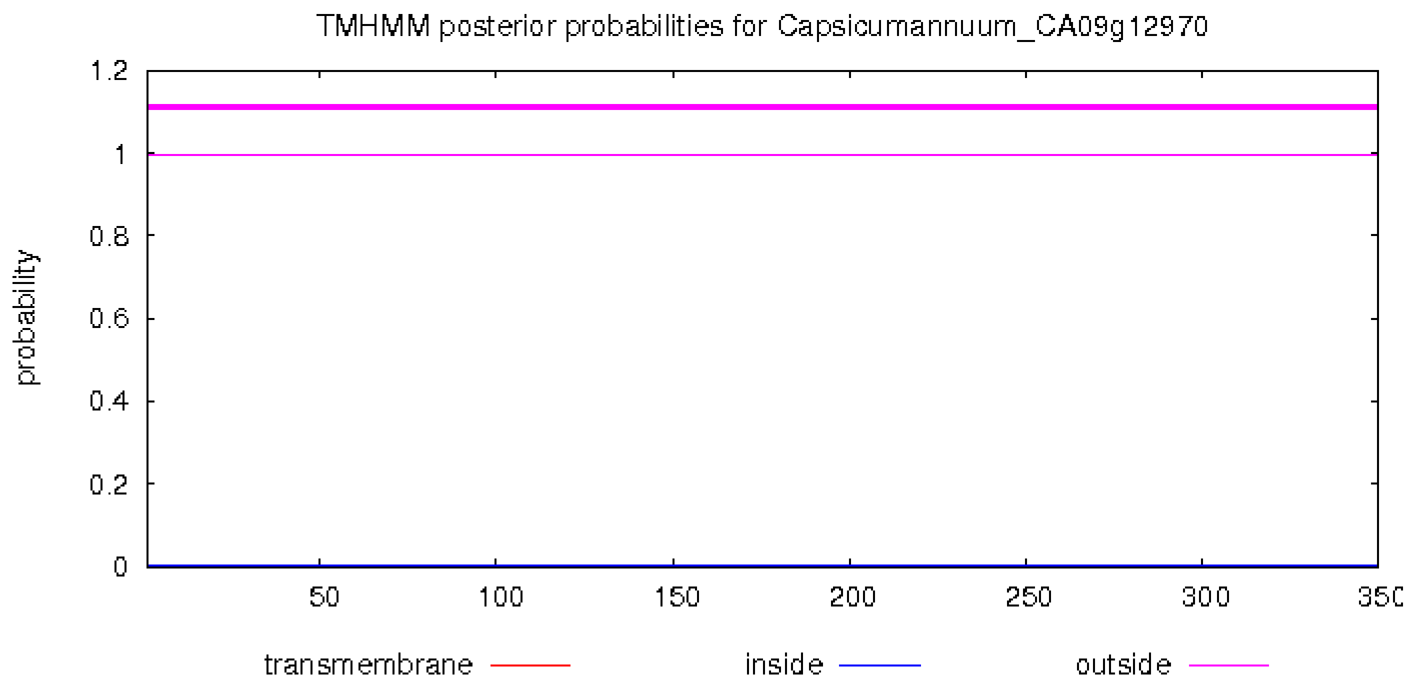

plot in postscript, script for making the plot in gnuplot, data for plot

---

```
# Capsicumannuum_CA09g18600 Length: 175
# Capsicumannuum_CA09g18600 Number of predicted TMHs:  0
# Capsicumannuum_CA09g18600 Exp number of AAs in TMHs: 0
# Capsicumannuum_CA09g18600 Exp number, first 60 AAs:  0
# Capsicumannuum_CA09g18600 Total prob of N-in:        0.12870
Capsicumannuum_CA09g18600       TMHMM2.0        outside      1   175
```

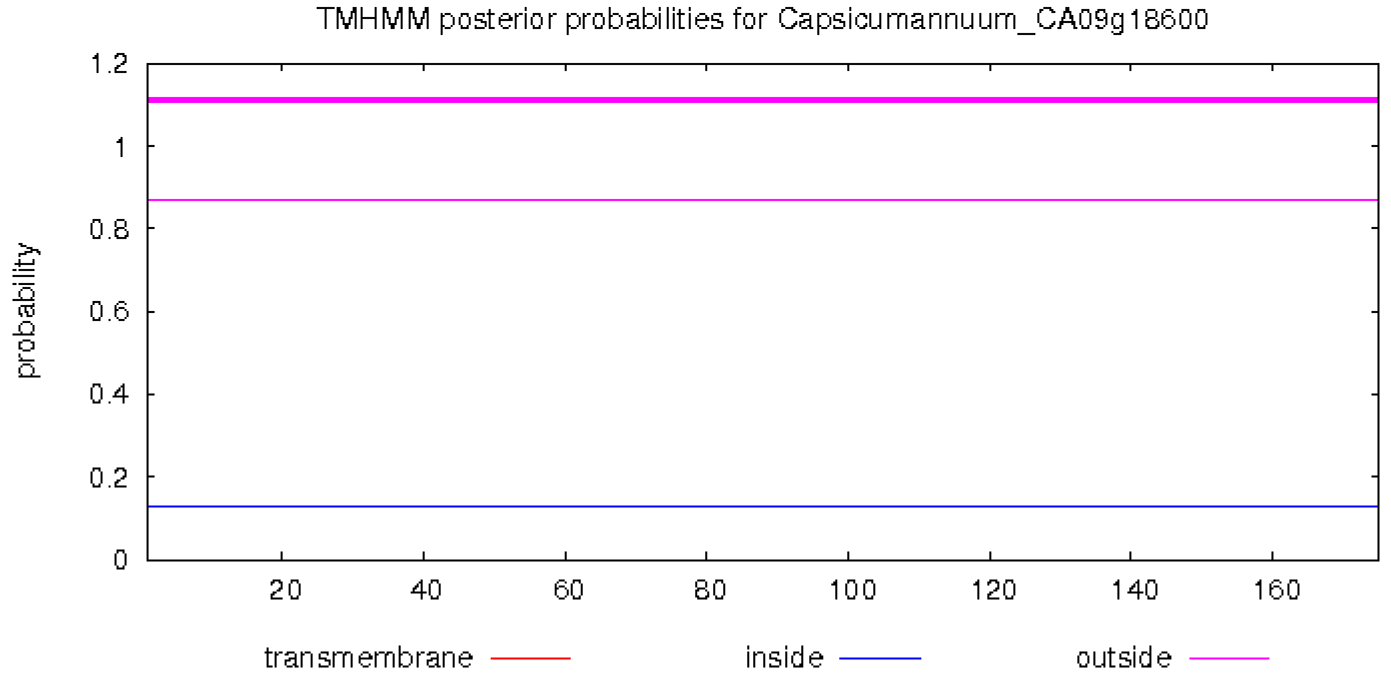

plot in postscript, script for making the plot in gnuplot, data for plot

---

```
# Capsicumannuum_CA10g00460 Length: 292
# Capsicumannuum_CA10g00460 Number of predicted TMHs:  1
# Capsicumannuum_CA10g00460 Exp number of AAs in TMHs: 19.88636
# Capsicumannuum_CA10g00460 Exp number, first 60 AAs:  0.00259
# Capsicumannuum_CA10g00460 Total prob of N-in:        0.35204
Capsicumannuum_CA10g00460       TMHMM2.0        outside      1    97
Capsicumannuum_CA10g00460       TMHMM2.0        TMhelix     98   120
Capsicumannuum_CA10g00460       TMHMM2.0        inside     121   292
```

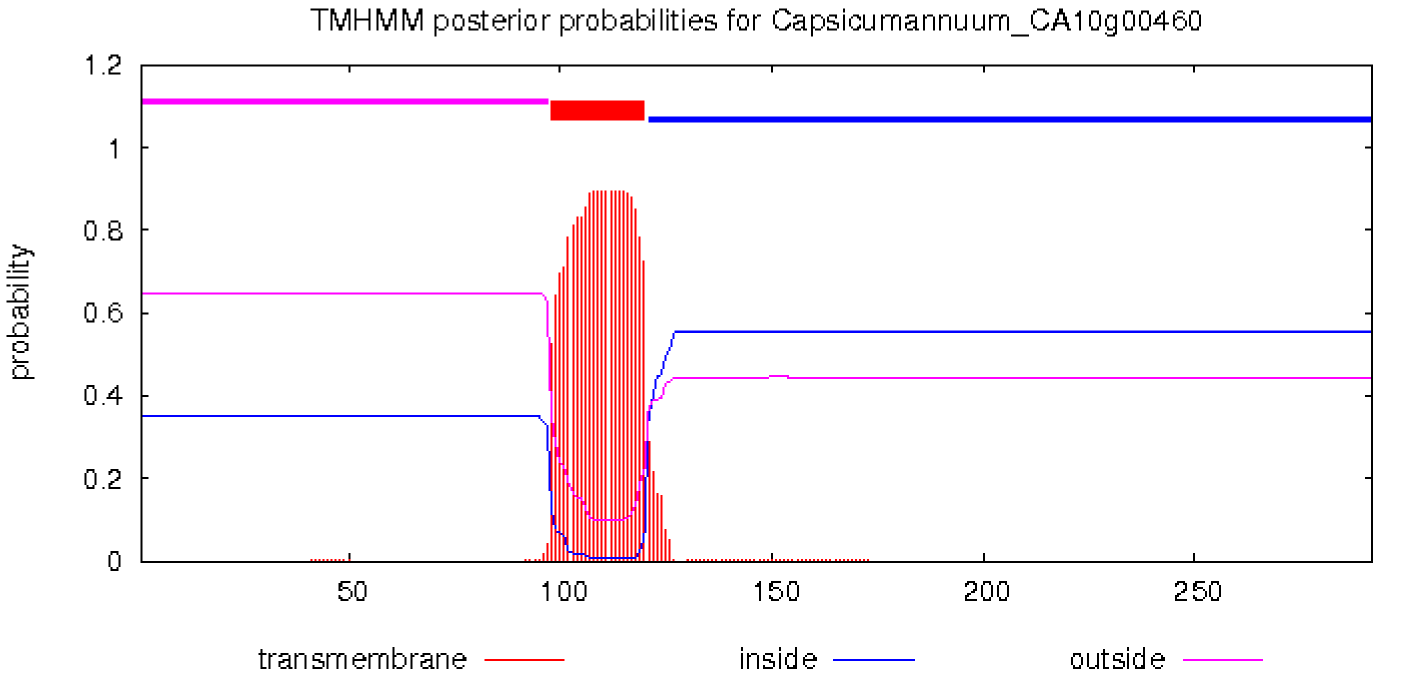

plot in postscript, script for making the plot in gnuplot, data for plot

```
# Capsicumannuum_CA10g09910 Length: 160
# Capsicumannuum_CA10g09910 Number of predicted TMHs:  0
# Capsicumannuum_CA10g09910 Exp number of AAs in TMHs: 0.17732
# Capsicumannuum_CA10g09910 Exp number, first 60 AAs:  0.00031
# Capsicumannuum_CA10g09910 Total prob of N-in:        0.03962
Capsicumannuum_CA10g09910       TMHMM2.0        outside      1   160
```

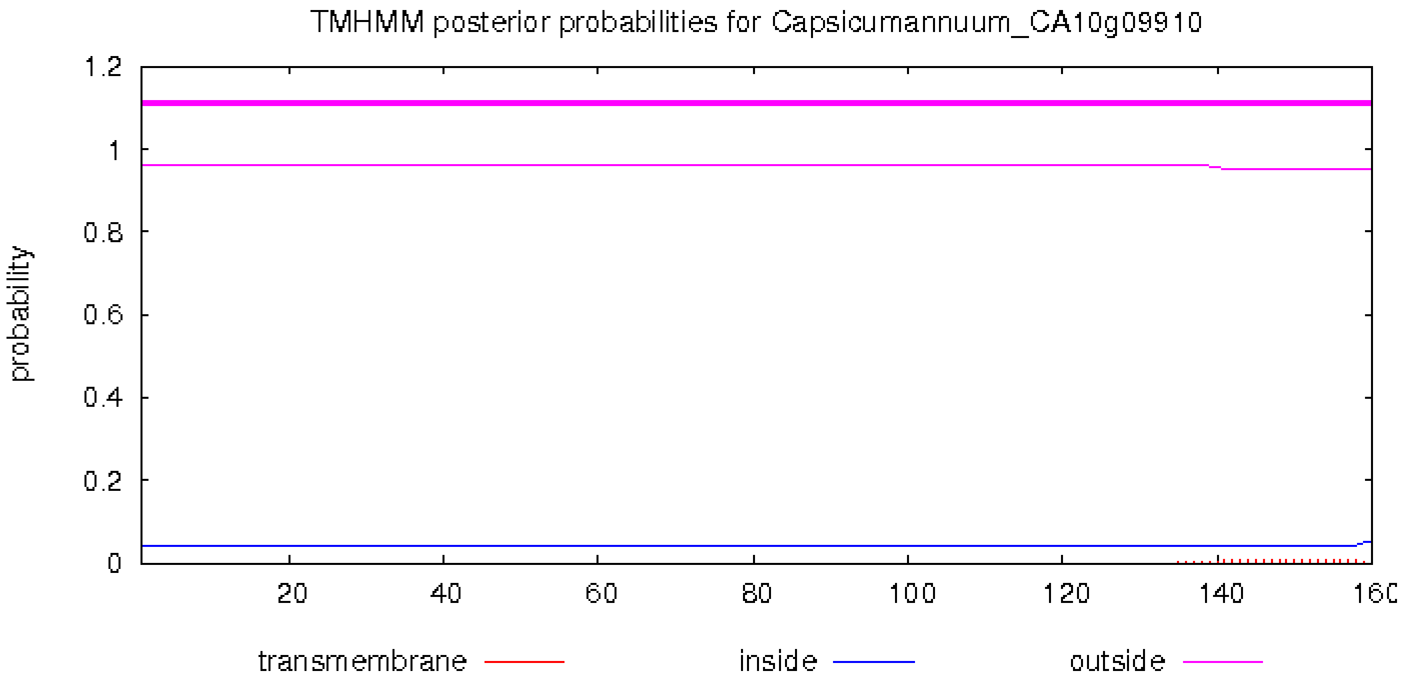

plot in postscript, script for making the plot in gnuplot, data for plot

```
# Capsicumannuum_CA10g13700 Length: 167
# Capsicumannuum_CA10g13700 Number of predicted TMHs:  0
# Capsicumannuum_CA10g13700 Exp number of AAs in TMHs: 0.01763
# Capsicumannuum_CA10g13700 Exp number, first 60 AAs:  0.01763
# Capsicumannuum_CA10g13700 Total prob of N-in:        0.35175
Capsicumannuum_CA10g13700       TMHMM2.0        outside      1   167
```

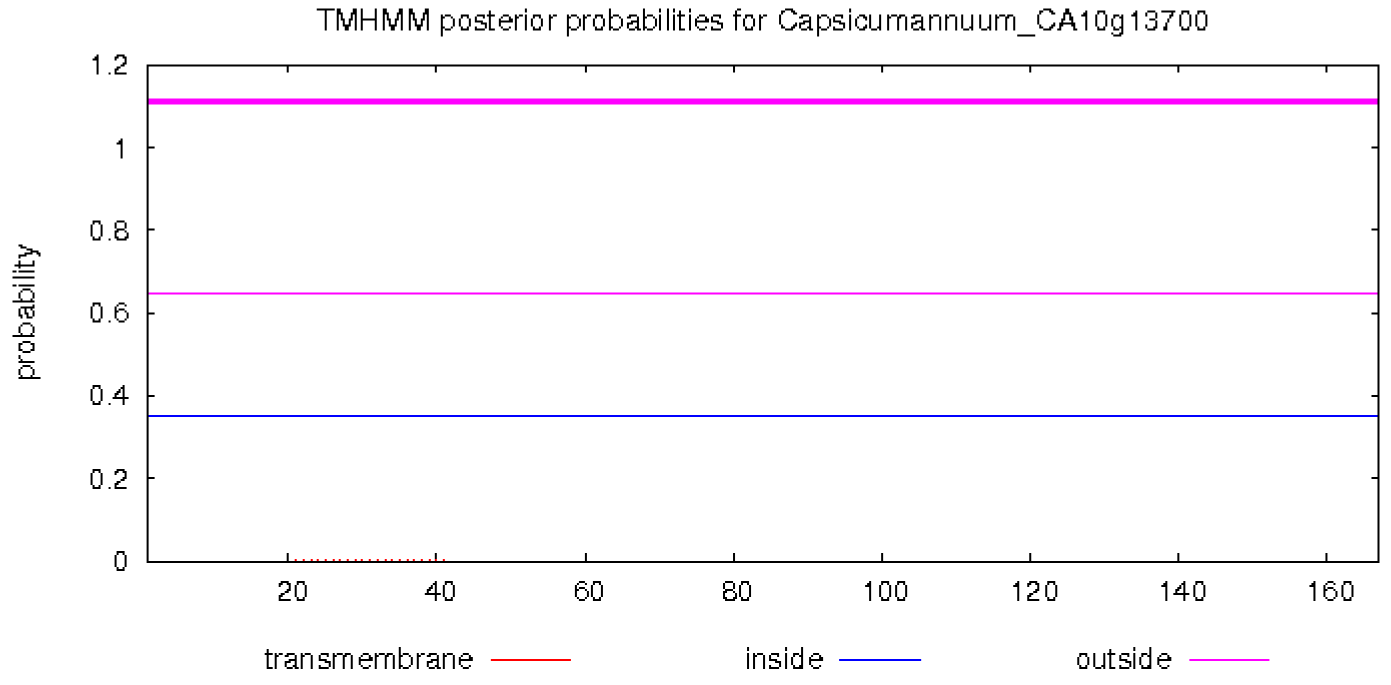

plot in postscript, script for making the plot in gnuplot, data for plot

---

```
# Capsicumannuum_CA10g13720 Length: 177
# Capsicumannuum_CA10g13720 Number of predicted TMHs:  0
# Capsicumannuum_CA10g13720 Exp number of AAs in TMHs: 0
# Capsicumannuum_CA10g13720 Exp number, first 60 AAs:  0
# Capsicumannuum_CA10g13720 Total prob of N-in:        0.13280
Capsicumannuum_CA10g13720       TMHMM2.0        outside      1   177
```

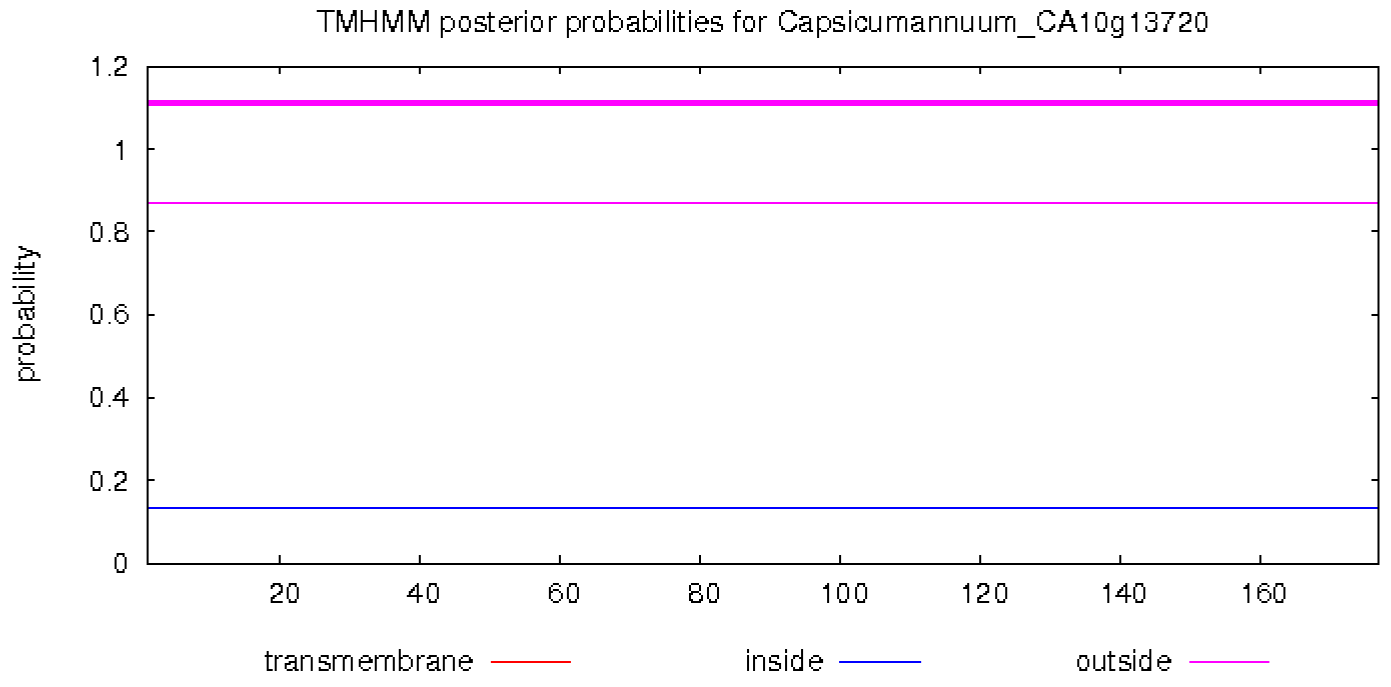

plot in postscript, script for making the plot in gnuplot, data for plot

---

```
# Capsicumannuum_CA10g20530 Length: 160
# Capsicumannuum_CA10g20530 Number of predicted TMHs:  0
# Capsicumannuum_CA10g20530 Exp number of AAs in TMHs: 0
# Capsicumannuum_CA10g20530 Exp number, first 60 AAs:  0
# Capsicumannuum_CA10g20530 Total prob of N-in:        0.01596
Capsicumannuum_CA10g20530       TMHMM2.0        outside      1   160
```

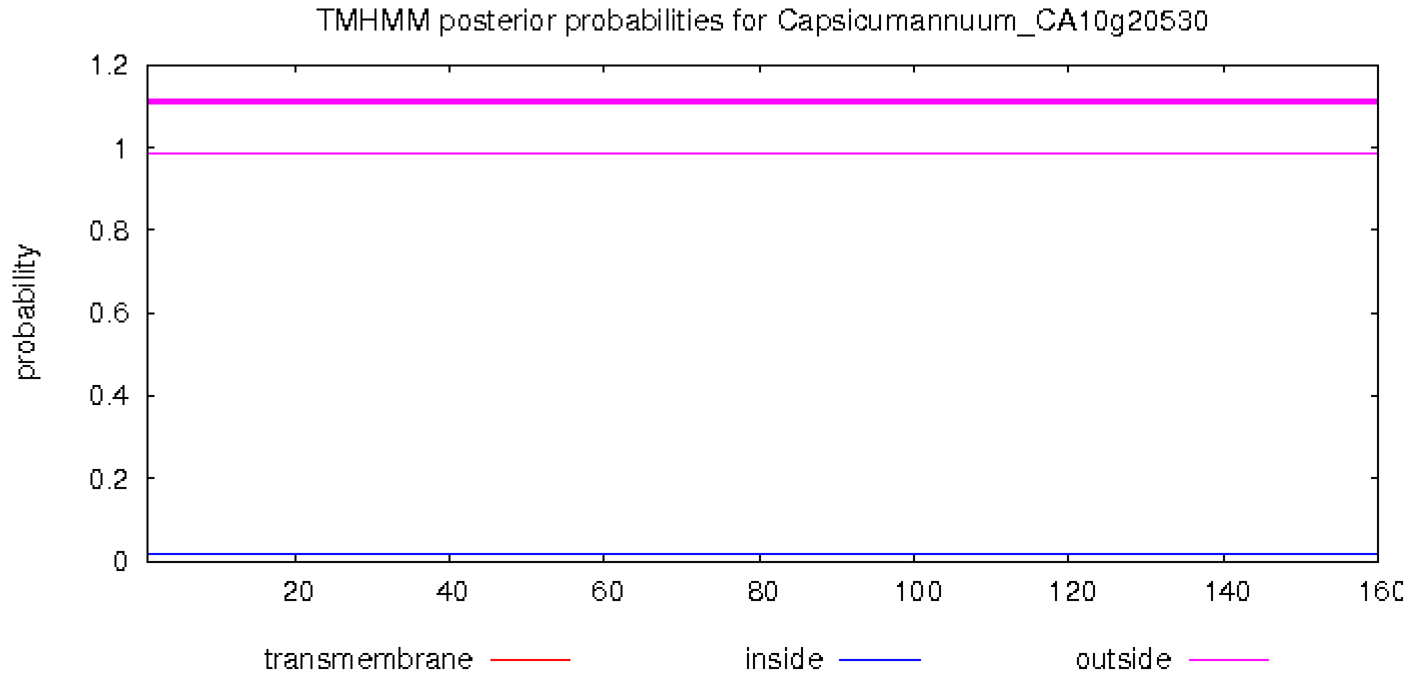

plot in postscript, script for making the plot in gnuplot, data for plot

---

```
# Capsicumannuum_CA10g20640 Length: 362
# Capsicumannuum_CA10g20640 Number of predicted TMHs:  0
# Capsicumannuum_CA10g20640 Exp number of AAs in TMHs: 0.0003
# Capsicumannuum_CA10g20640 Exp number, first 60 AAs:  0
# Capsicumannuum_CA10g20640 Total prob of N-in:        0.01575
Capsicumannuum_CA10g20640       TMHMM2.0        outside      1   362
```

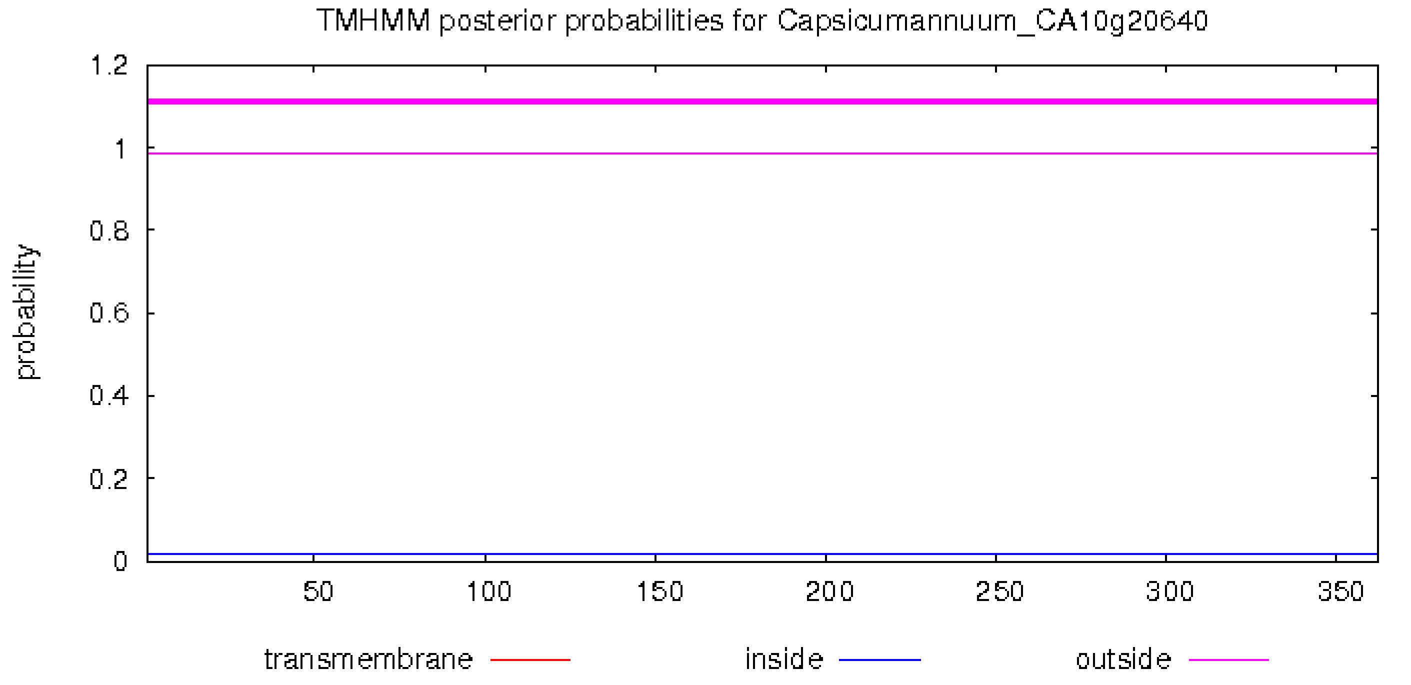

plot in postscript, script for making the plot in gnuplot, data for plot

---

```
# Capsicumannuum_CA11g01550 Length: 329
# Capsicumannuum_CA11g01550 Number of predicted TMHs:  0
# Capsicumannuum_CA11g01550 Exp number of AAs in TMHs: 8.82277
# Capsicumannuum_CA11g01550 Exp number, first 60 AAs:  1.07332
# Capsicumannuum_CA11g01550 Total prob of N-in:        0.05518
Capsicumannuum_CA11g01550       TMHMM2.0        outside      1   329
```

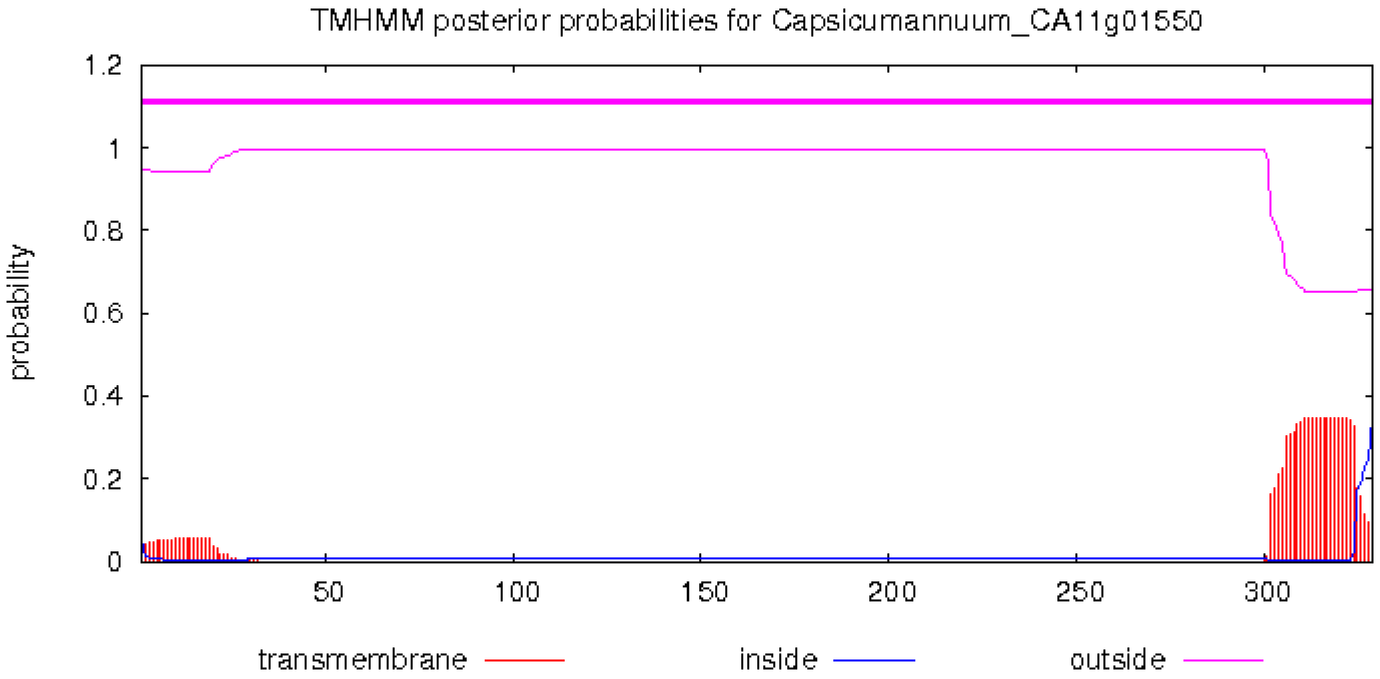

plot in postscript, script for making the plot in gnuplot, data for plot

---

```
# Capsicumannuum_CA11g04440 Length: 294
# Capsicumannuum_CA11g04440 Number of predicted TMHs:  0
# Capsicumannuum_CA11g04440 Exp number of AAs in TMHs: 0.00029
# Capsicumannuum_CA11g04440 Exp number, first 60 AAs:  0
# Capsicumannuum_CA11g04440 Total prob of N-in:        0.00990
Capsicumannuum_CA11g04440       TMHMM2.0        outside      1   294
```

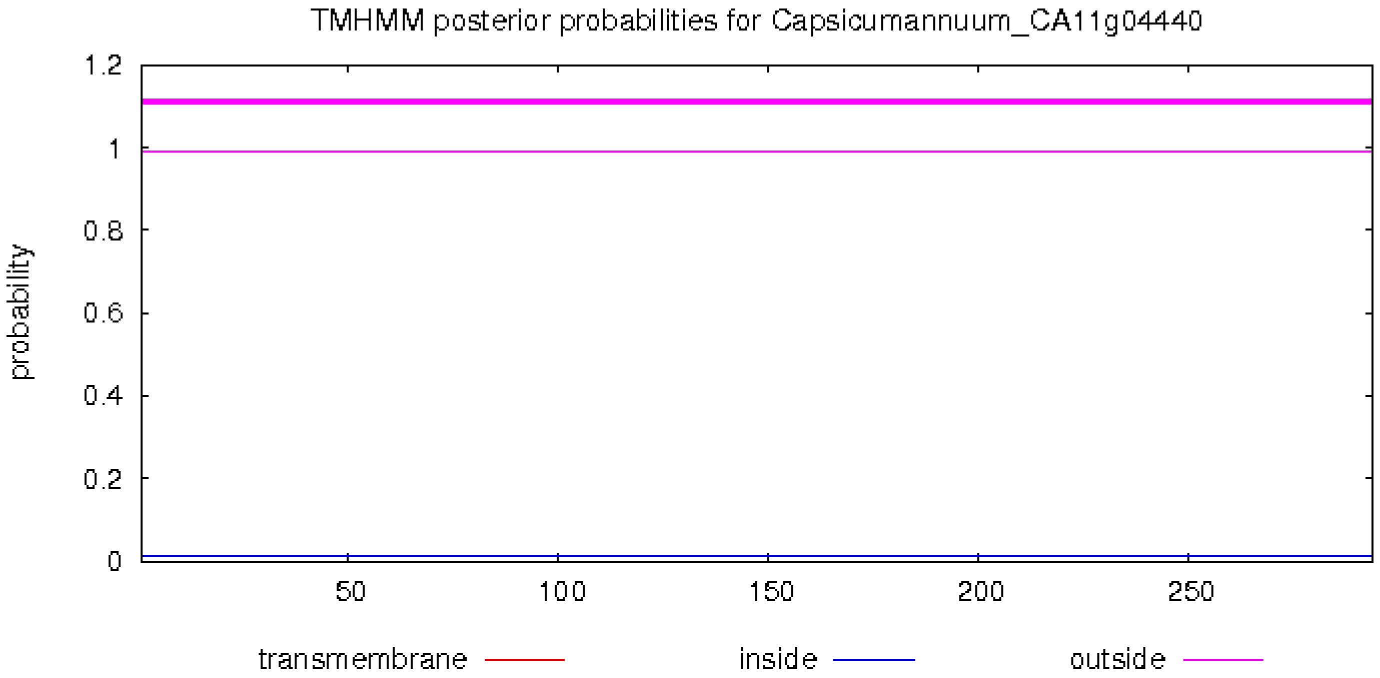

plot in postscript, script for making the plot in gnuplot, data for plot

---

```
# Capsicumannuum_CA11g04580 Length: 409
# Capsicumannuum_CA11g04580 Number of predicted TMHs:  0
# Capsicumannuum_CA11g04580 Exp number of AAs in TMHs: 0.00944
# Capsicumannuum_CA11g04580 Exp number, first 60 AAs:  0
# Capsicumannuum_CA11g04580 Total prob of N-in:        0.03730
Capsicumannuum_CA11g04580       TMHMM2.0        outside      1   409
```

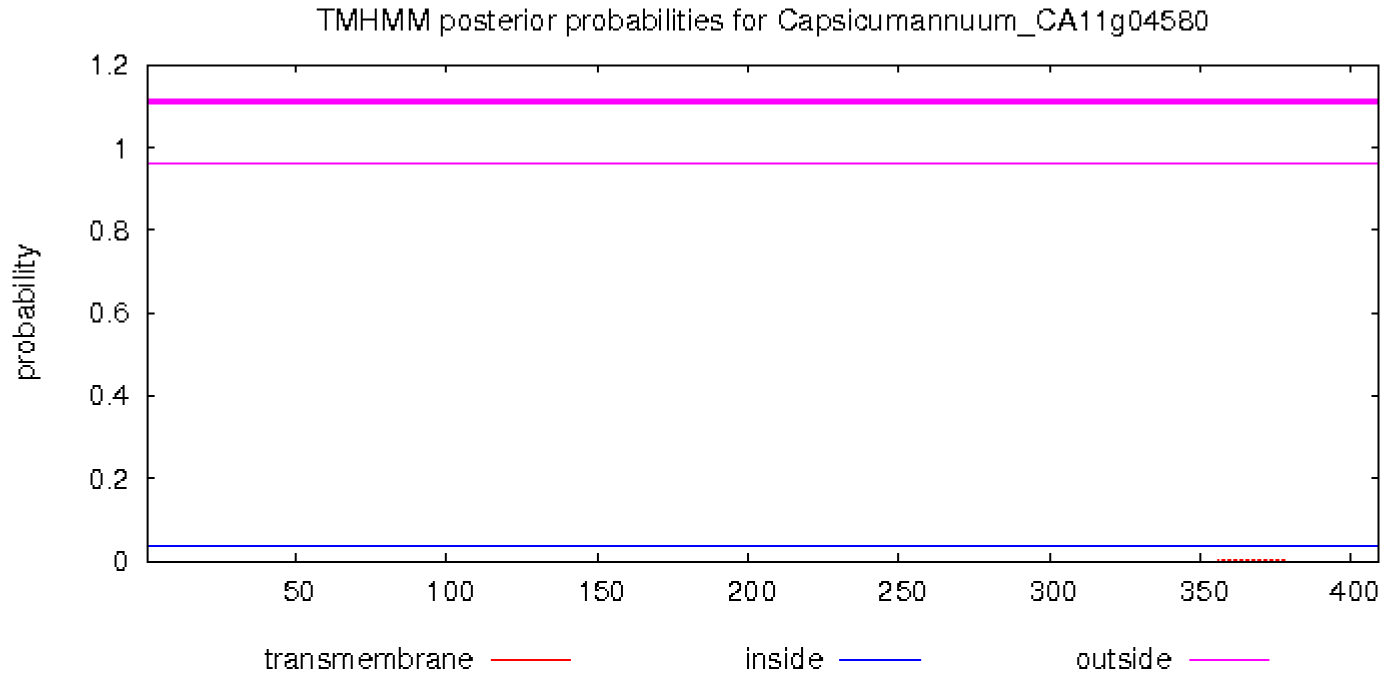

plot in postscript, script for making the plot in gnuplot, data for plot

---

```
# Capsicumannuum_CA11g08290 Length: 341
# Capsicumannuum_CA11g08290 Number of predicted TMHs:  0
# Capsicumannuum_CA11g08290 Exp number of AAs in TMHs: 0.00016
# Capsicumannuum_CA11g08290 Exp number, first 60 AAs:  0
# Capsicumannuum_CA11g08290 Total prob of N-in:        0.01241
Capsicumannuum_CA11g08290       TMHMM2.0        outside      1   341
```

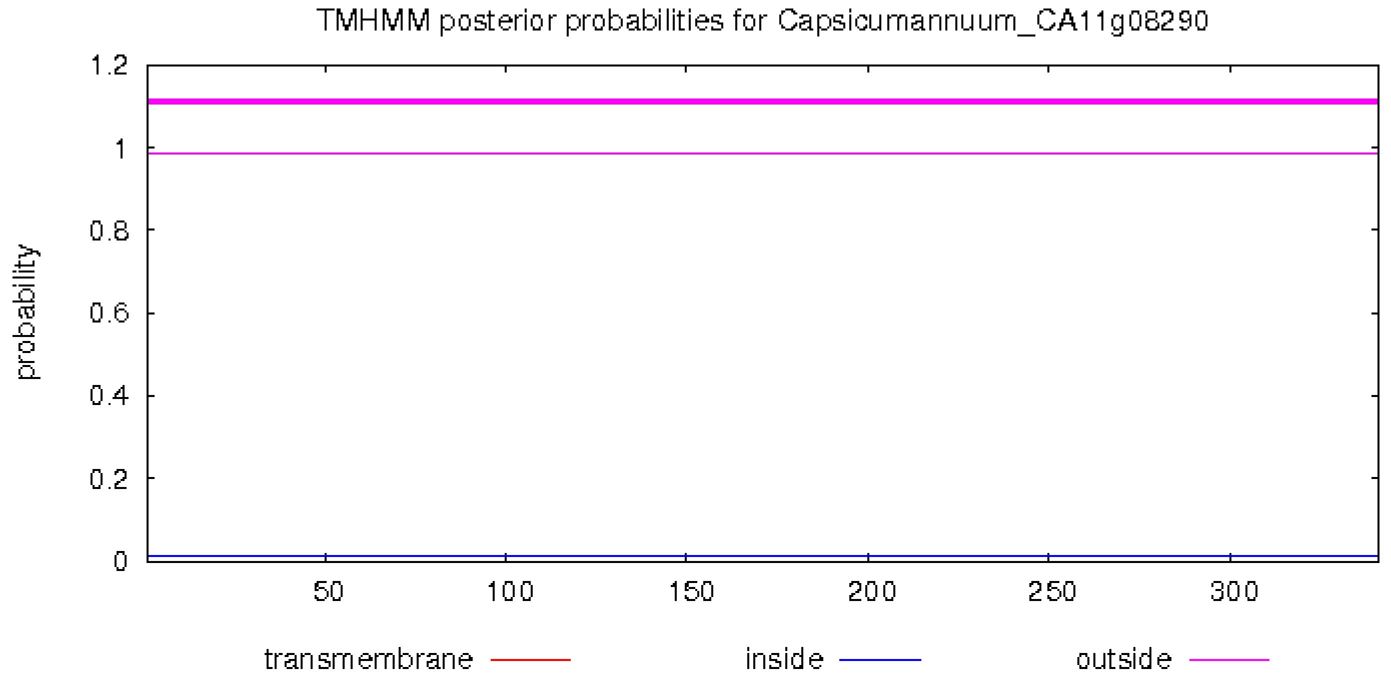

plot in postscript, script for making the plot in gnuplot, data for plot

---

```
# Capsicumannuum_CA11g12260 Length: 368
# Capsicumannuum_CA11g12260 Number of predicted TMHs:  0
# Capsicumannuum_CA11g12260 Exp number of AAs in TMHs: 0.00078
# Capsicumannuum_CA11g12260 Exp number, first 60 AAs:  0
# Capsicumannuum_CA11g12260 Total prob of N-in:        0.01286
Capsicumannuum_CA11g12260       TMHMM2.0        outside      1   368
```

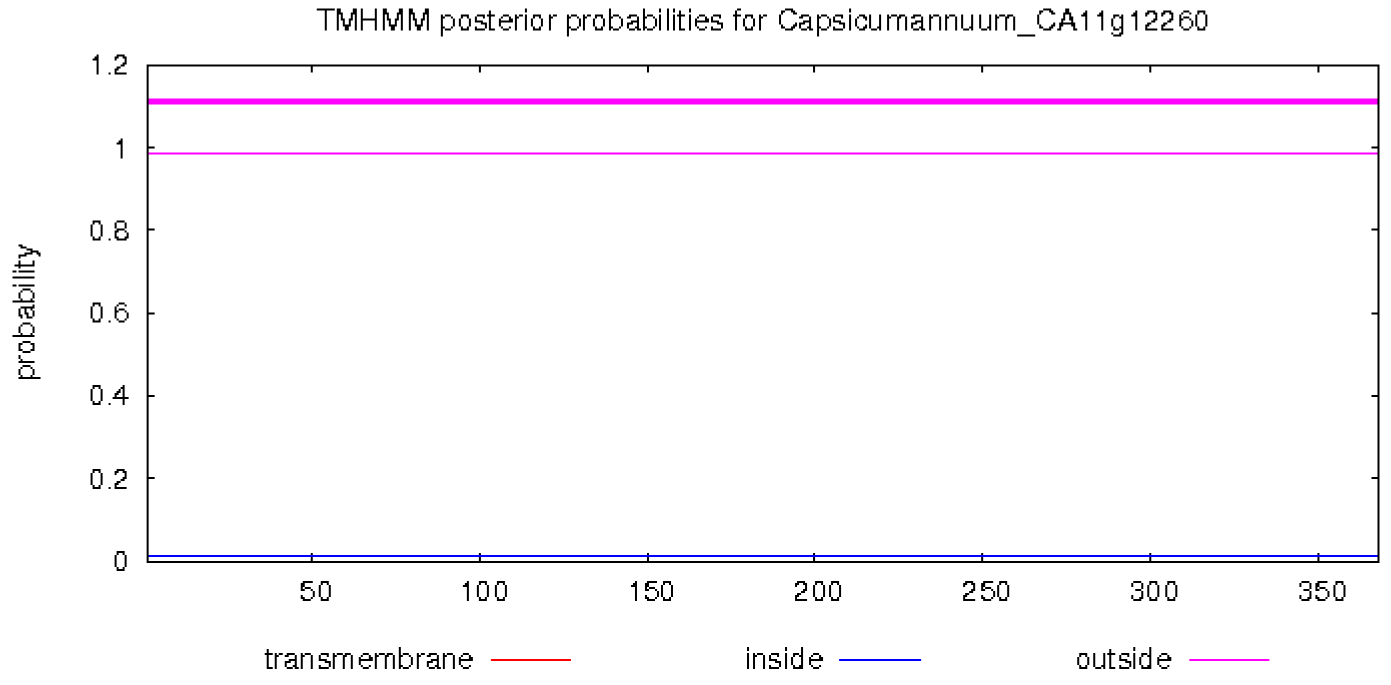

plot in postscript, script for making the plot in gnuplot, data for plot

---

```
# Capsicumannuum_CA11g16490 Length: 414
# Capsicumannuum_CA11g16490 Number of predicted TMHs:  1
# Capsicumannuum_CA11g16490 Exp number of AAs in TMHs: 21.45564
# Capsicumannuum_CA11g16490 Exp number, first 60 AAs:  0.01173
# Capsicumannuum_CA11g16490 Total prob of N-in:        0.01625
Capsicumannuum_CA11g16490       TMHMM2.0        outside      1   381
Capsicumannuum_CA11g16490       TMHMM2.0        TMhelix    382   404
Capsicumannuum_CA11g16490       TMHMM2.0        inside     405   414
```

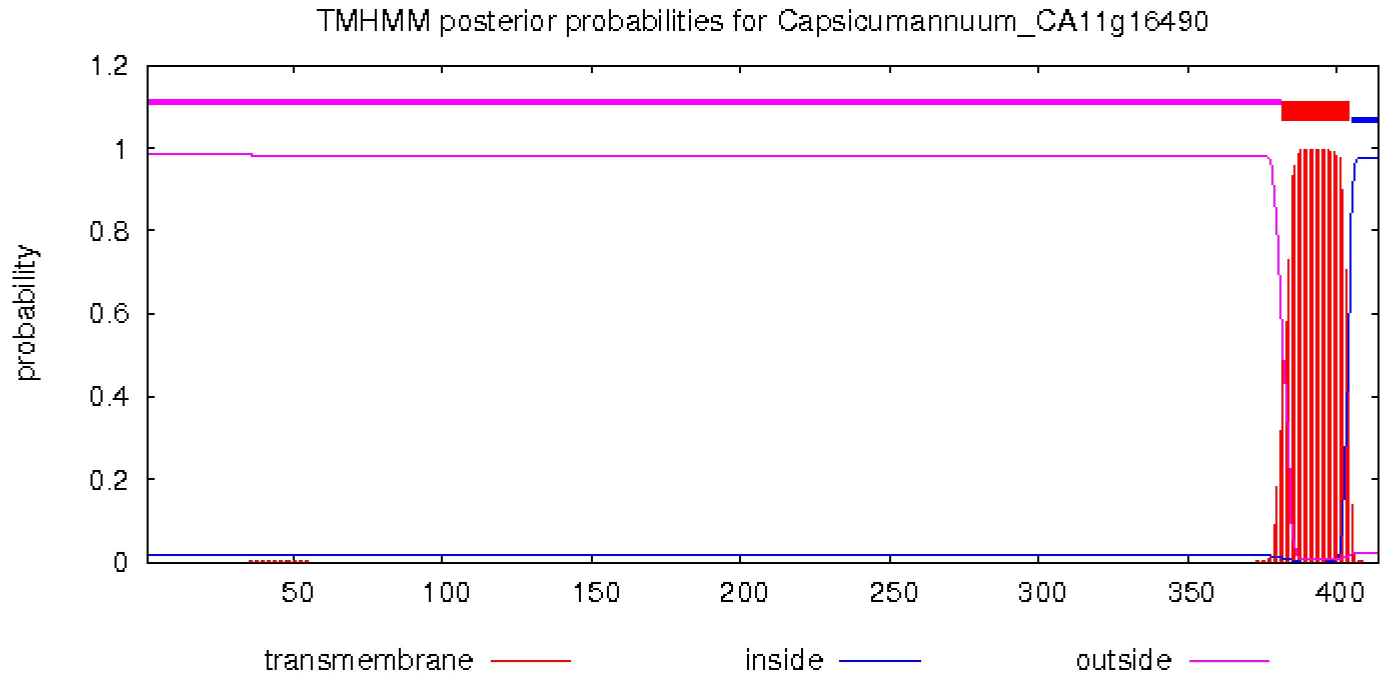

plot in postscript, script for making the plot in gnuplot, data for plot

---

```
# Capsicumannuum_CA12g00080 Length: 195
# Capsicumannuum_CA12g00080 Number of predicted TMHs:  0
# Capsicumannuum_CA12g00080 Exp number of AAs in TMHs: 0.00054
# Capsicumannuum_CA12g00080 Exp number, first 60 AAs:  0.00026
```

```
# Capsicumannuum_CA12g00080 Total prob of N-in:        0.00378
Capsicumannuum_CA12g00080       TMHMM2.0        outside      1   195
```

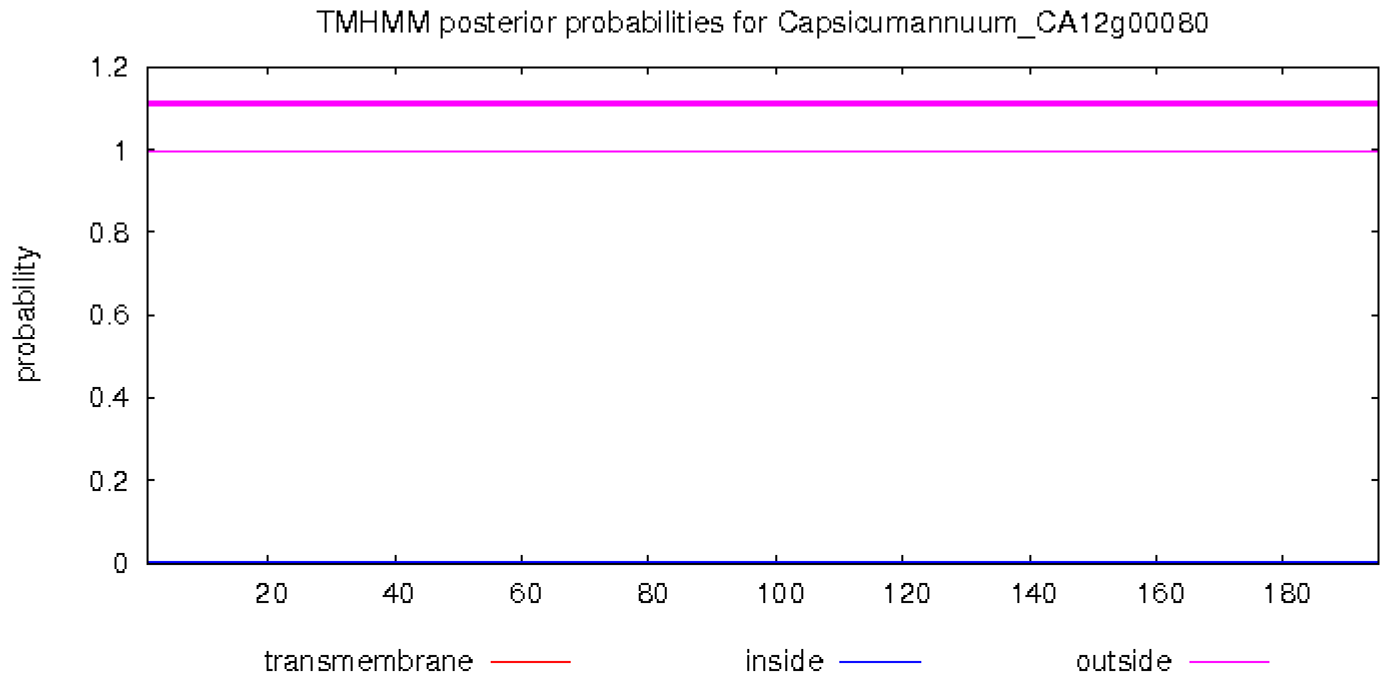

plot in postscript, script for making the plot in gnuplot, data for plot

---

```
# Capsicumannuum_CA12g04950 Length: 382
# Capsicumannuum_CA12g04950 Number of predicted TMHs:  0
# Capsicumannuum_CA12g04950 Exp number of AAs in TMHs: 0
# Capsicumannuum_CA12g04950 Exp number, first 60 AAs:  0
# Capsicumannuum_CA12g04950 Total prob of N-in:        0.00071
Capsicumannuum_CA12g04950       TMHMM2.0        outside      1   382
```

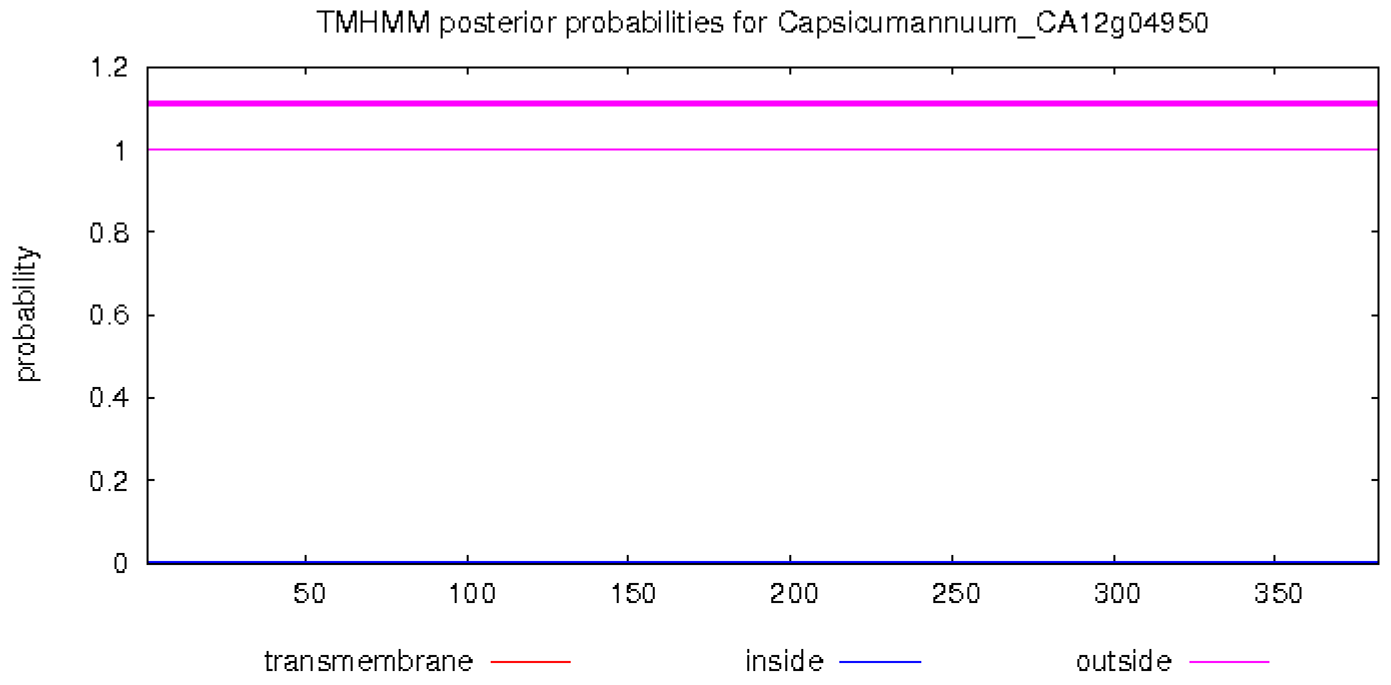

plot in postscript, script for making the plot in gnuplot, data for plot

---

```
# Capsicumannuum_CA12g04970 Length: 529
# Capsicumannuum_CA12g04970 Number of predicted TMHs:  1
# Capsicumannuum_CA12g04970 Exp number of AAs in TMHs: 20.63241
# Capsicumannuum_CA12g04970 Exp number, first 60 AAs:  0
```

```
# Capsicumannuum_CA12g04970 Total prob of N-in:        0.02506
Capsicumannuum_CA12g04970       TMHMM2.0        outside      1   504
Capsicumannuum_CA12g04970       TMHMM2.0        TMhelix    505   527
Capsicumannuum_CA12g04970       TMHMM2.0        inside     528   529
```

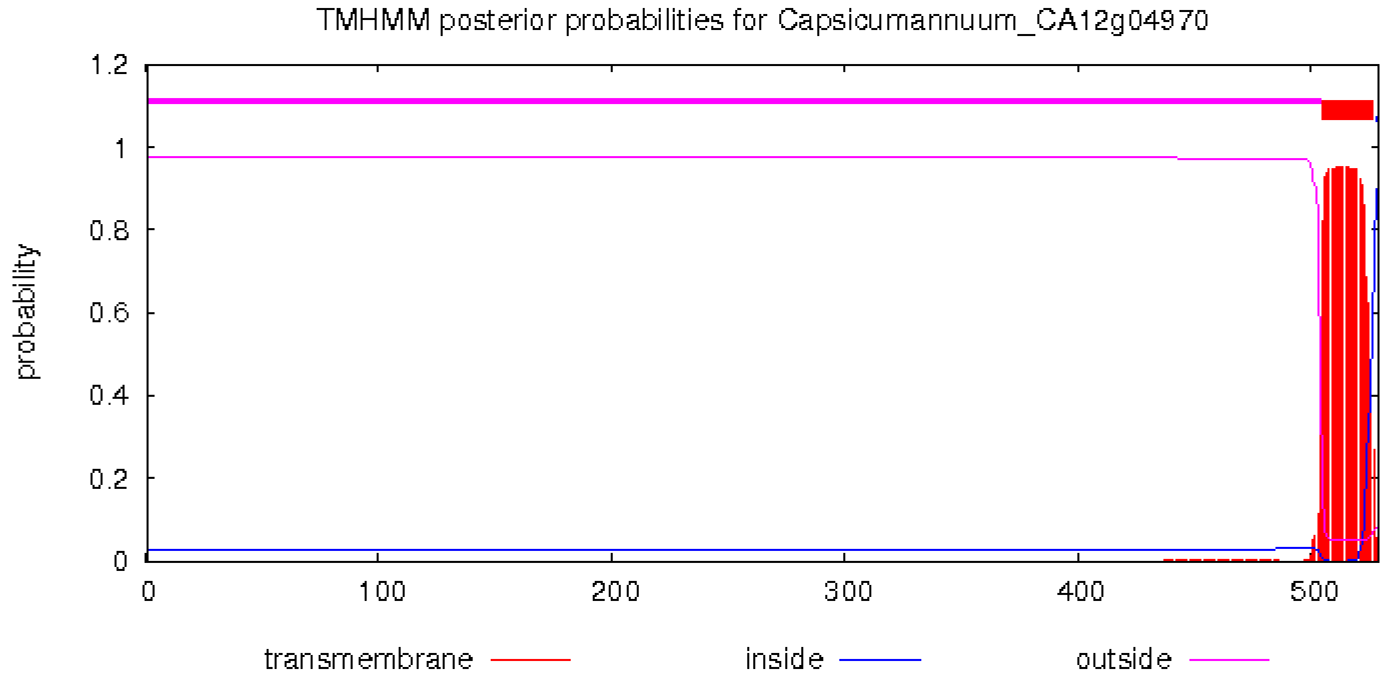

plot in postscript, script for making the plot in gnuplot, data for plot

---

```
# Capsicumannuum_CA12g05890 Length: 154
# Capsicumannuum_CA12g05890 Number of predicted TMHs:  0
# Capsicumannuum_CA12g05890 Exp number of AAs in TMHs: 0.00115
# Capsicumannuum_CA12g05890 Exp number, first 60 AAs:  0.00115
# Capsicumannuum_CA12g05890 Total prob of N-in:        0.14447
Capsicumannuum_CA12g05890       TMHMM2.0        outside      1   154
```

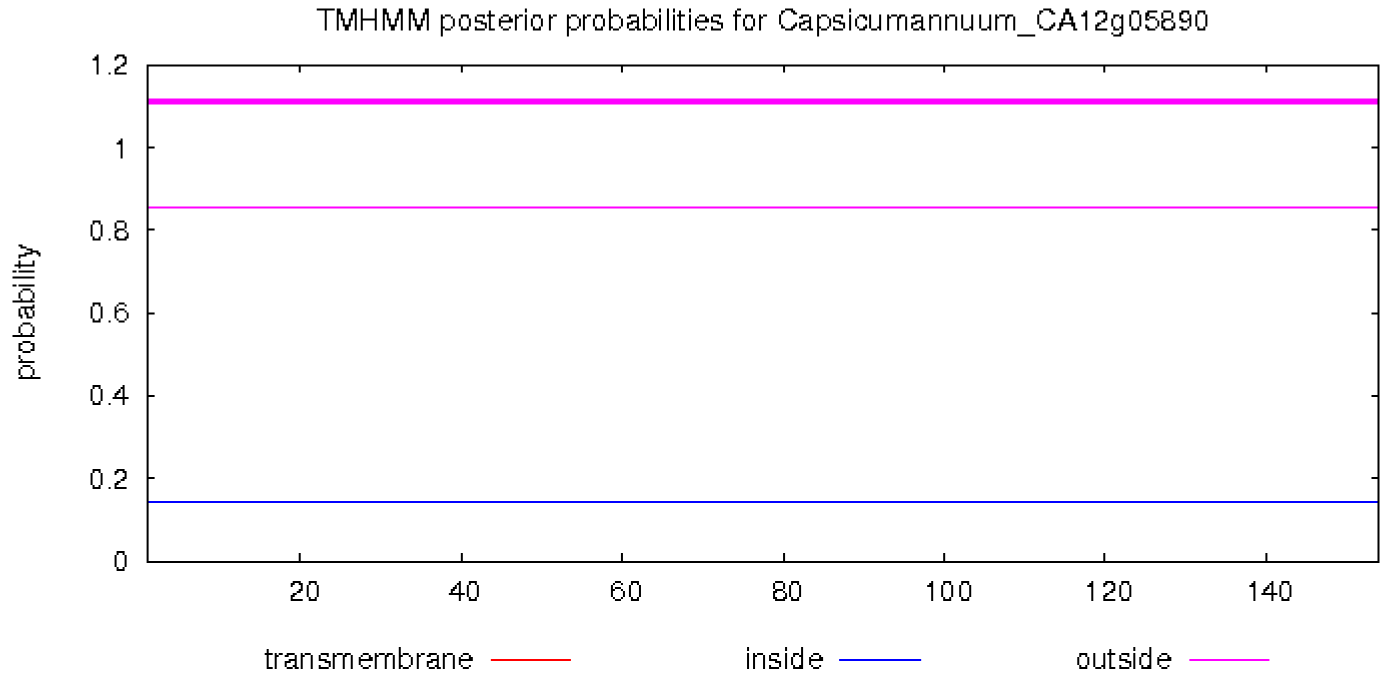

plot in postscript, script for making the plot in gnuplot, data for plot

---

```
# Capsicumannuum_CA12g05910 Length: 269
# Capsicumannuum_CA12g05910 Number of predicted TMHs:  0
```

```
# Capsicumannuum_CA12g05910 Exp number of AAs in TMHs: 0
# Capsicumannuum_CA12g05910 Exp number, first 60 AAs:  0
# Capsicumannuum_CA12g05910 Total prob of N-in:        0.00702
Capsicumannuum_CA12g05910       TMHMM2.0        outside      1   269
```

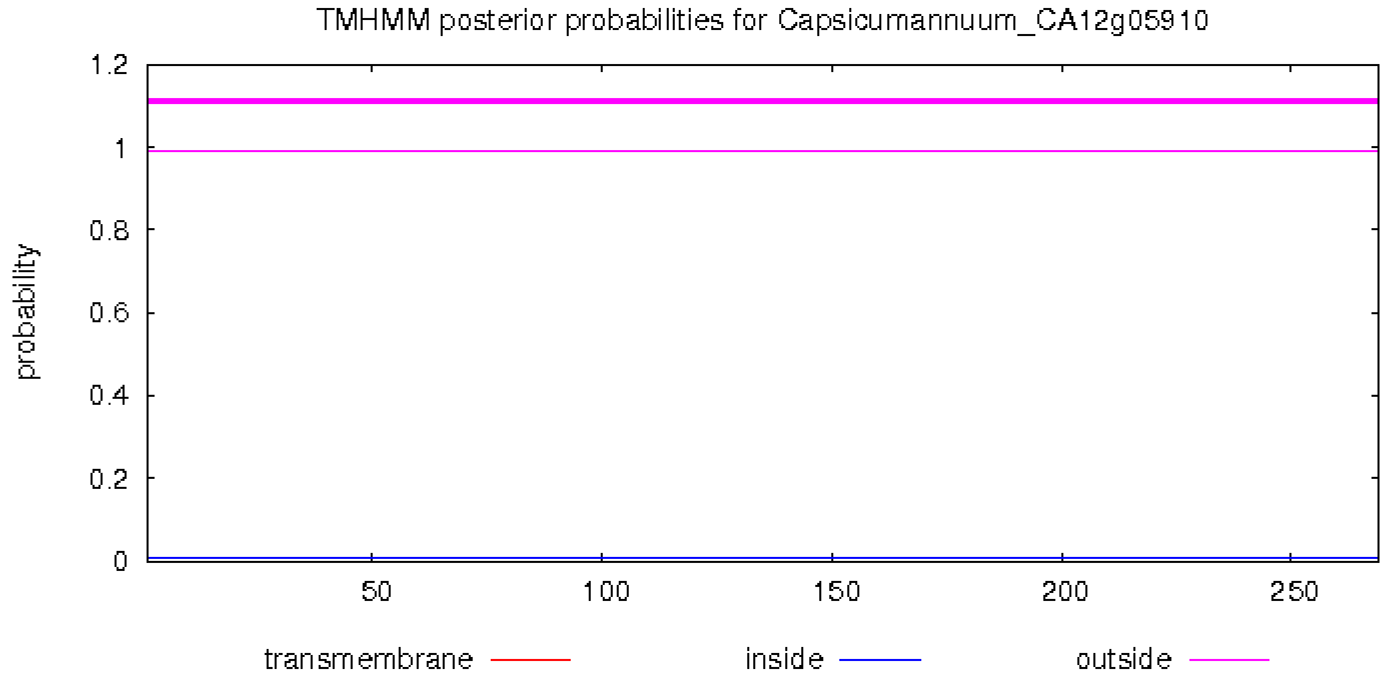

[plot](#) in postscript, [script](#) for making the plot in gnuplot, [data](#) for plot

---

```
# Capsicumannuum_CA12g06210 Length: 224
# Capsicumannuum_CA12g06210 Number of predicted TMHs:  0
# Capsicumannuum_CA12g06210 Exp number of AAs in TMHs: 0
# Capsicumannuum_CA12g06210 Exp number, first 60 AAs:  0
# Capsicumannuum_CA12g06210 Total prob of N-in:        0.00728
Capsicumannuum_CA12g06210       TMHMM2.0        outside      1   224
```

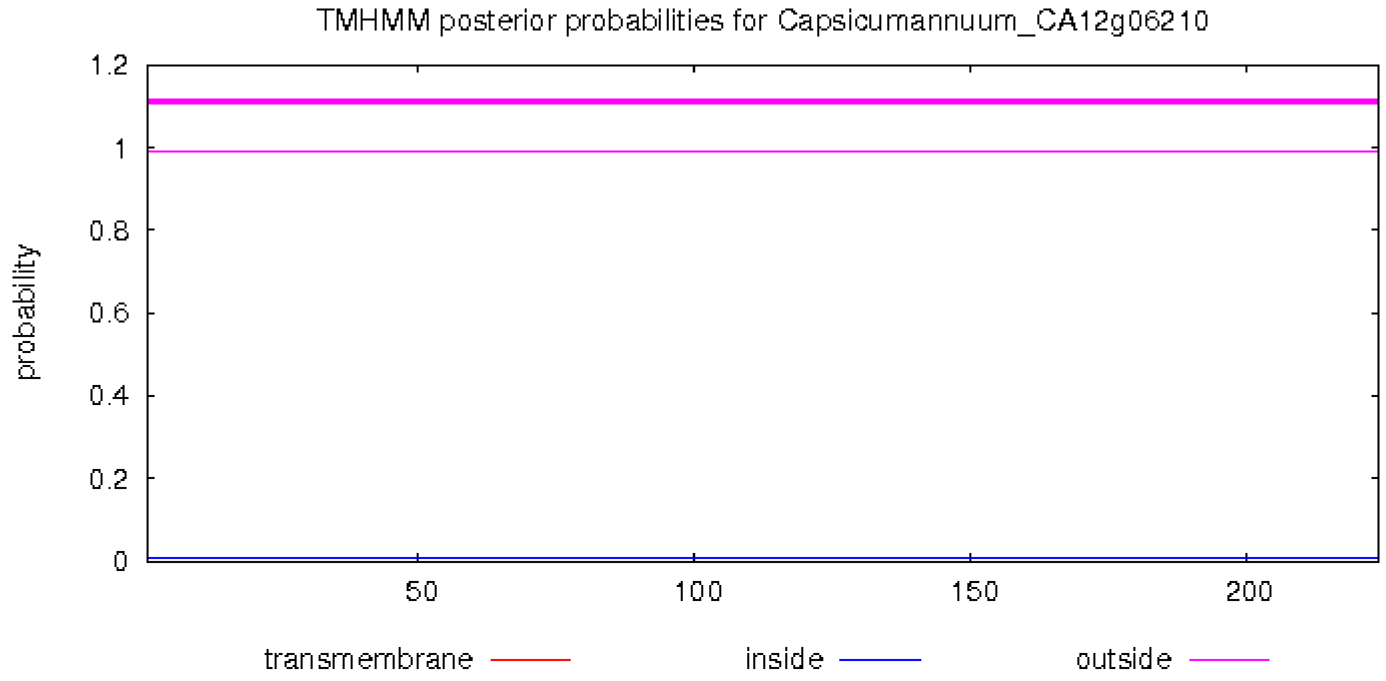

[plot](#) in postscript, [script](#) for making the plot in gnuplot, [data](#) for plot

---

```
# Capsicumannuum_CA12g07920 Length: 584
# Capsicumannuum_CA12g07920 Number of predicted TMHs:  0
```

```
# Capsicumannuum_CA12g07920 Exp number of AAs in TMHs: 15.31256
# Capsicumannuum_CA12g07920 Exp number, first 60 AAs:  0.00112
# Capsicumannuum_CA12g07920 Total prob of N-in:        0.00923
Capsicumannuum_CA12g07920       TMHMM2.0        outside      1   584
```

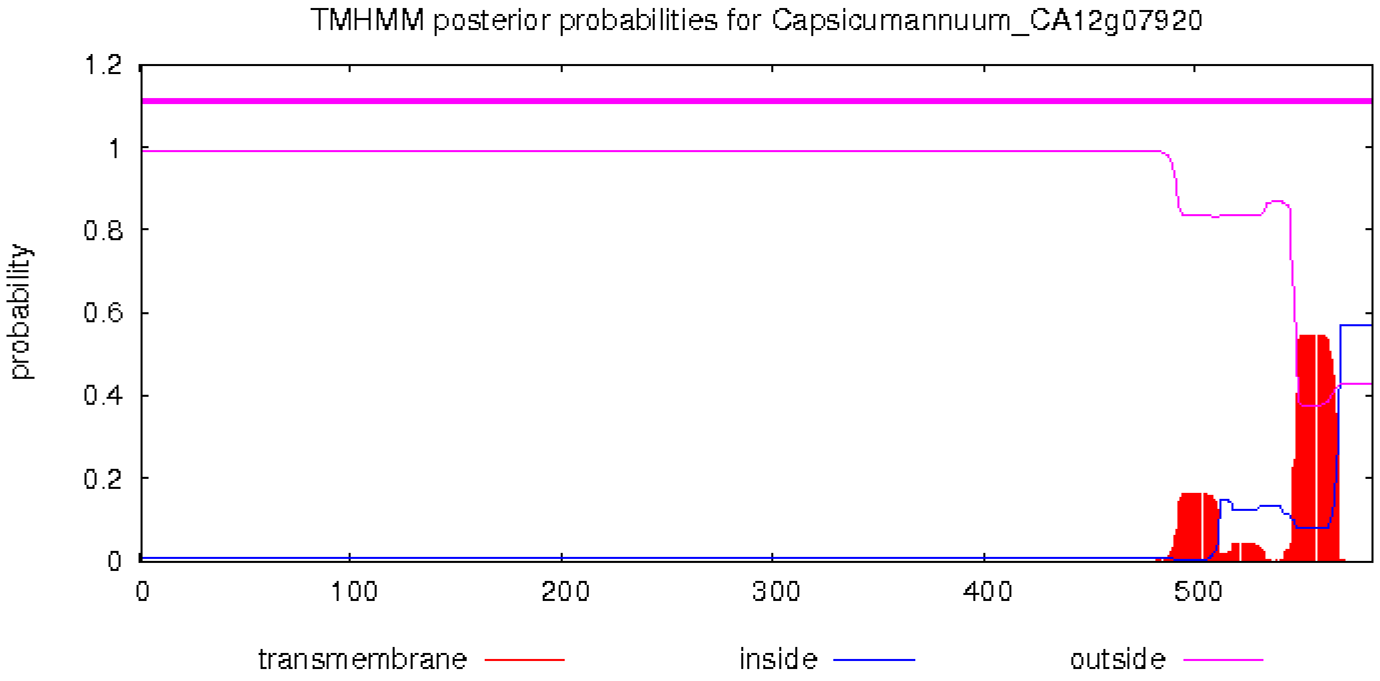

plot in postscript, script for making the plot in gnuplot, data for plot

---

```
# Capsicumannuum_CA12g13470 Length: 333
# Capsicumannuum_CA12g13470 Number of predicted TMHs:  0
# Capsicumannuum_CA12g13470 Exp number of AAs in TMHs: 0.0003
# Capsicumannuum_CA12g13470 Exp number, first 60 AAs:  9e-05
# Capsicumannuum_CA12g13470 Total prob of N-in:        0.01311
Capsicumannuum_CA12g13470       TMHMM2.0        outside      1   333
```

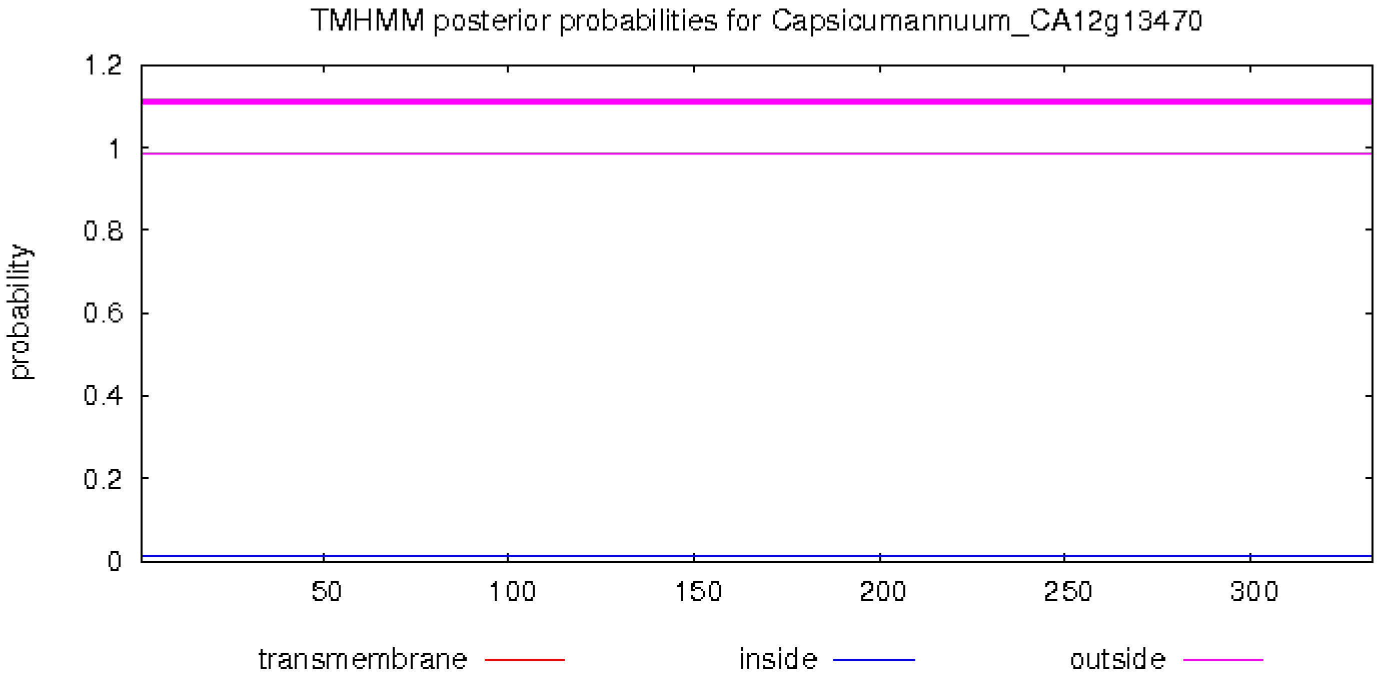

plot in postscript, script for making the plot in gnuplot, data for plot

---

```
# Caricapapaya_evm.model.supercontig_10.25 Length: 293
# Caricapapaya_evm.model.supercontig_10.25 Number of predicted TMHs:  0
```

```
# Caricapapaya_evm.model.supercontig_10.25 Exp number of AAs in TMHs: 0.00061
# Caricapapaya_evm.model.supercontig_10.25 Exp number, first 60 AAs:  0
# Caricapapaya_evm.model.supercontig_10.25 Total prob of N-in:        0.03054
Caricapapaya_evm.model.supercontig_10.25	TMHMM2.0	outside	     1   293
```

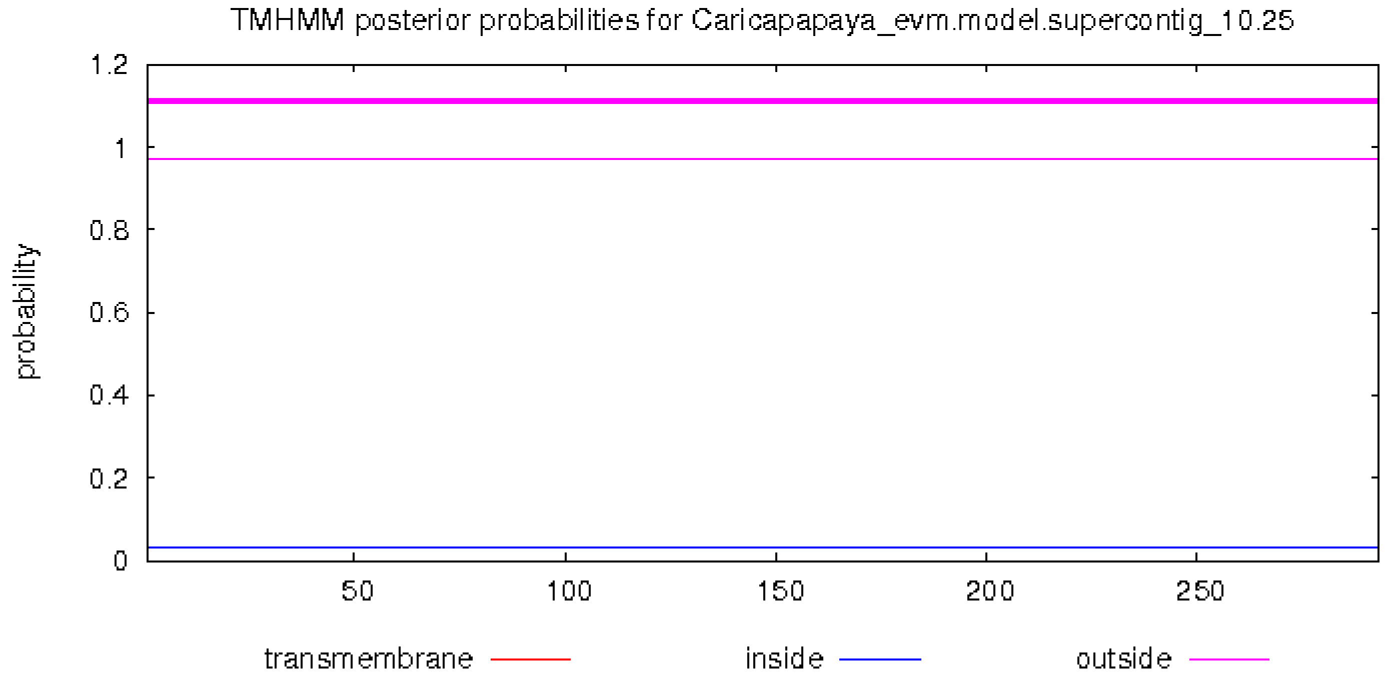

plot in postscript, script for making the plot in gnuplot, data for plot

---

```
# Caricapapaya_evm.model.supercontig_104.98 Length: 259
# Caricapapaya_evm.model.supercontig_104.98 Number of predicted TMHs:  0
# Caricapapaya_evm.model.supercontig_104.98 Exp number of AAs in TMHs: 0.02456
# Caricapapaya_evm.model.supercontig_104.98 Exp number, first 60 AAs:  0
# Caricapapaya_evm.model.supercontig_104.98 Total prob of N-in:        0.00750
Caricapapaya_evm.model.supercontig_104.98	TMHMM2.0	outside	     1   259
```

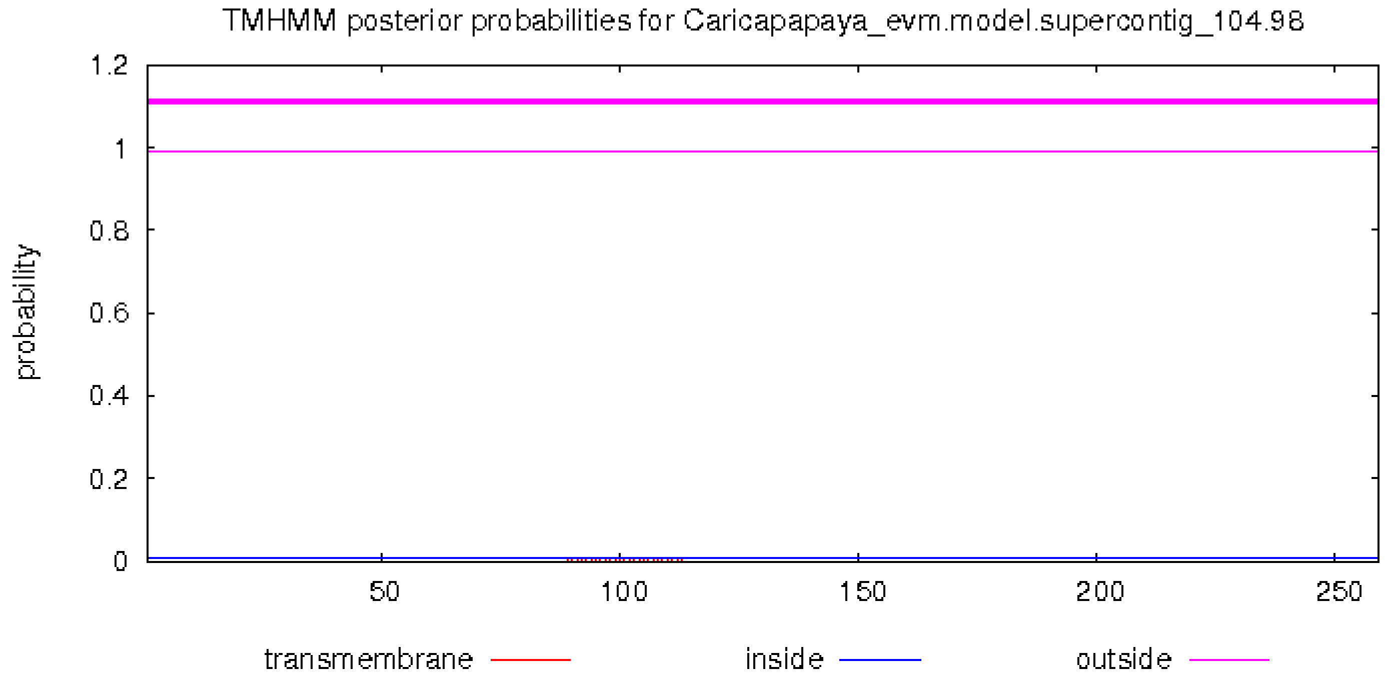

plot in postscript, script for making the plot in gnuplot, data for plot

---

```
# Caricapapaya_evm.model.supercontig_106.80 Length: 209
# Caricapapaya_evm.model.supercontig_106.80 Number of predicted TMHs:  0
```

```
# Caricapapaya_evm.model.supercontig_106.80 Exp number of AAs in TMHs: 1.20184
# Caricapapaya_evm.model.supercontig_106.80 Exp number, first 60 AAs:  0.01442
# Caricapapaya_evm.model.supercontig_106.80 Total prob of N-in:        0.08894
Caricapapaya_evm.model.supercontig_106.80       TMHMM2.0        outside      1   209
```

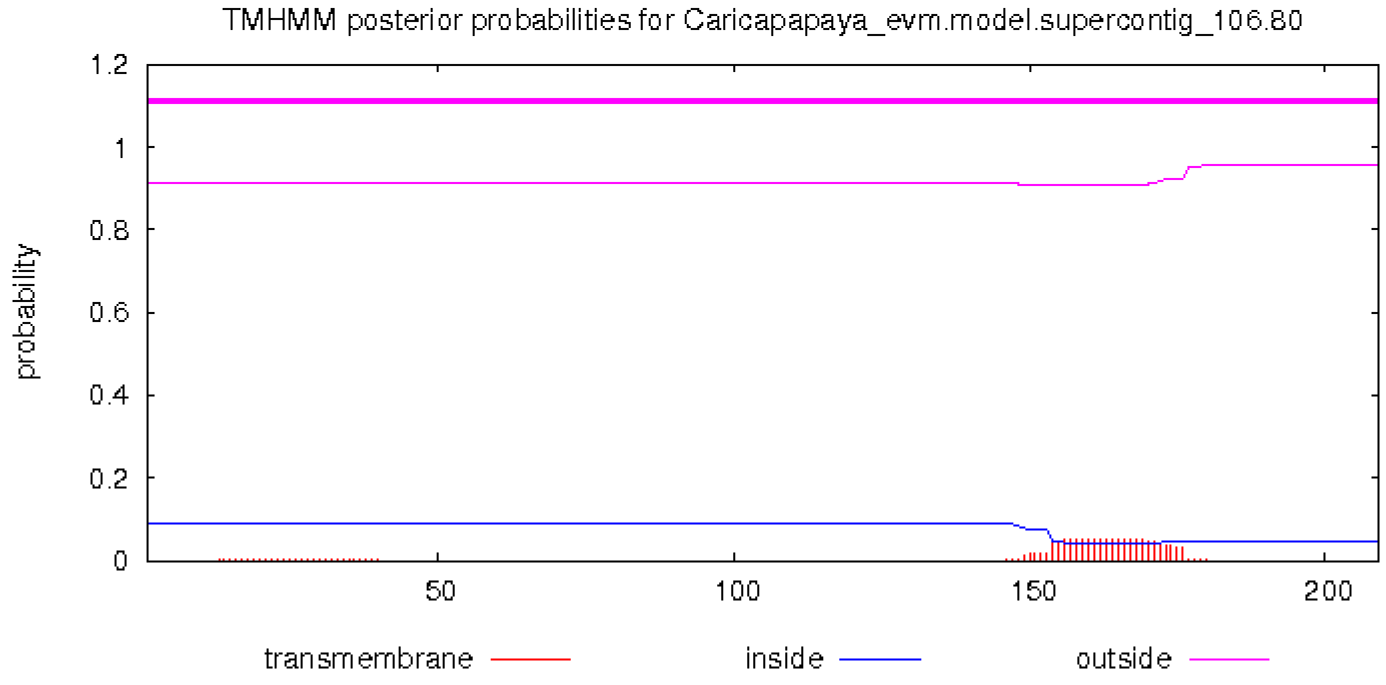

plot in postscript, script for making the plot in gnuplot, data for plot

---

```
# Caricapapaya_evm.model.supercontig_1.108 Length: 313
# Caricapapaya_evm.model.supercontig_1.108 Number of predicted TMHs:  0
# Caricapapaya_evm.model.supercontig_1.108 Exp number of AAs in TMHs: 0.01845
# Caricapapaya_evm.model.supercontig_1.108 Exp number, first 60 AAs:  0
# Caricapapaya_evm.model.supercontig_1.108 Total prob of N-in:        0.04144
Caricapapaya_evm.model.supercontig_1.108        TMHMM2.0        outside      1   313
```

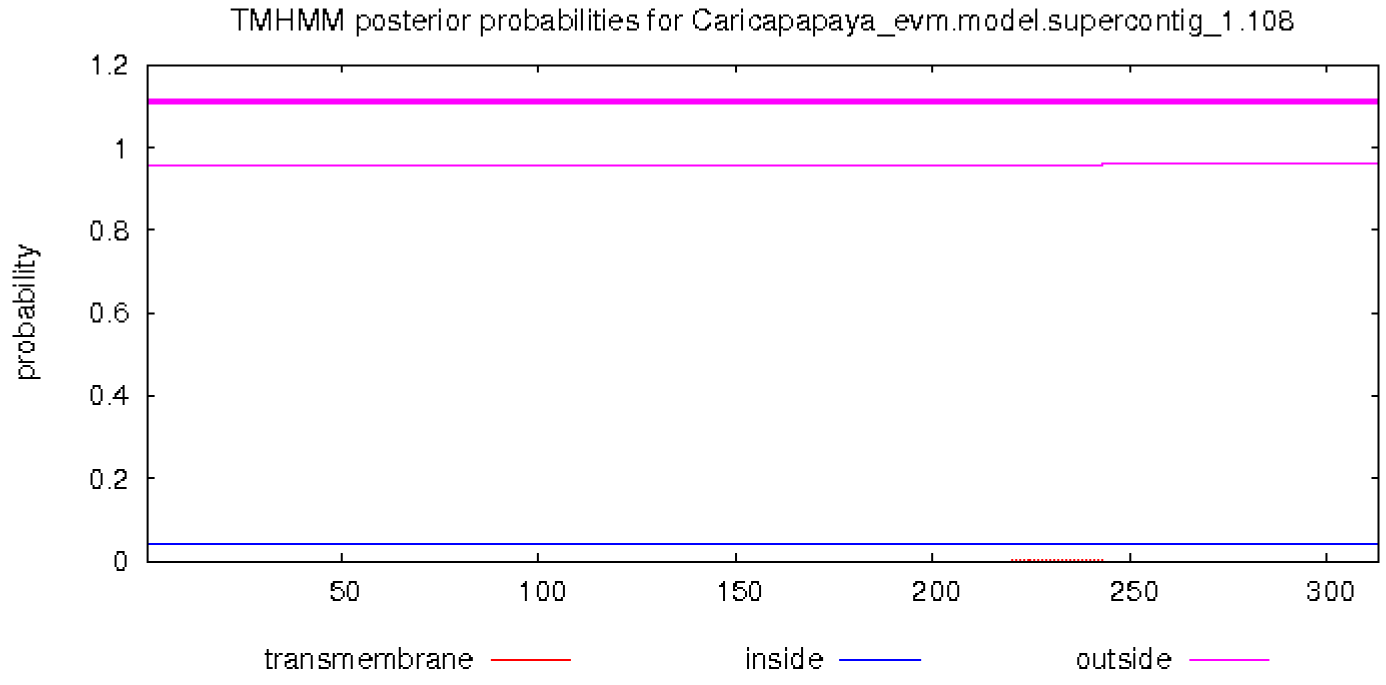

plot in postscript, script for making the plot in gnuplot, data for plot

---

```
# Caricapapaya_evm.model.supercontig_111.22 Length: 340
# Caricapapaya_evm.model.supercontig_111.22 Number of predicted TMHs:  0
```

```
# Caricapapaya_evm.model.supercontig_111.22 Exp number of AAs in TMHs: 0.00087
# Caricapapaya_evm.model.supercontig_111.22 Exp number, first 60 AAs:  0
# Caricapapaya_evm.model.supercontig_111.22 Total prob of N-in:        0.03422
Caricapapaya_evm.model.supercontig_111.22       TMHMM2.0        outside      1   340
```

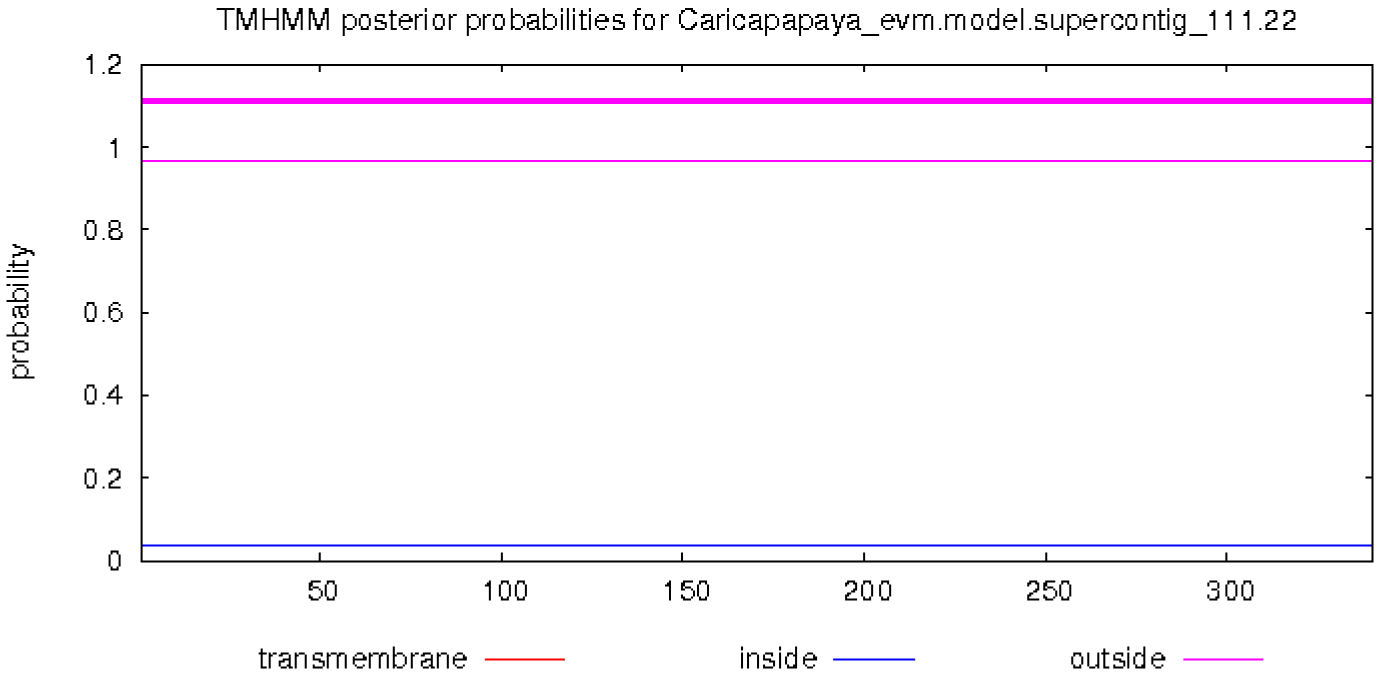

plot in postscript, script for making the plot in gnuplot, data for plot

---

```
# Caricapapaya_evm.model.supercontig_113.19 Length: 382
# Caricapapaya_evm.model.supercontig_113.19 Number of predicted TMHs:  0
# Caricapapaya_evm.model.supercontig_113.19 Exp number of AAs in TMHs: 0.94994
# Caricapapaya_evm.model.supercontig_113.19 Exp number, first 60 AAs:  0.54425
# Caricapapaya_evm.model.supercontig_113.19 Total prob of N-in:        0.05870
Caricapapaya_evm.model.supercontig_113.19       TMHMM2.0        outside      1   382
```

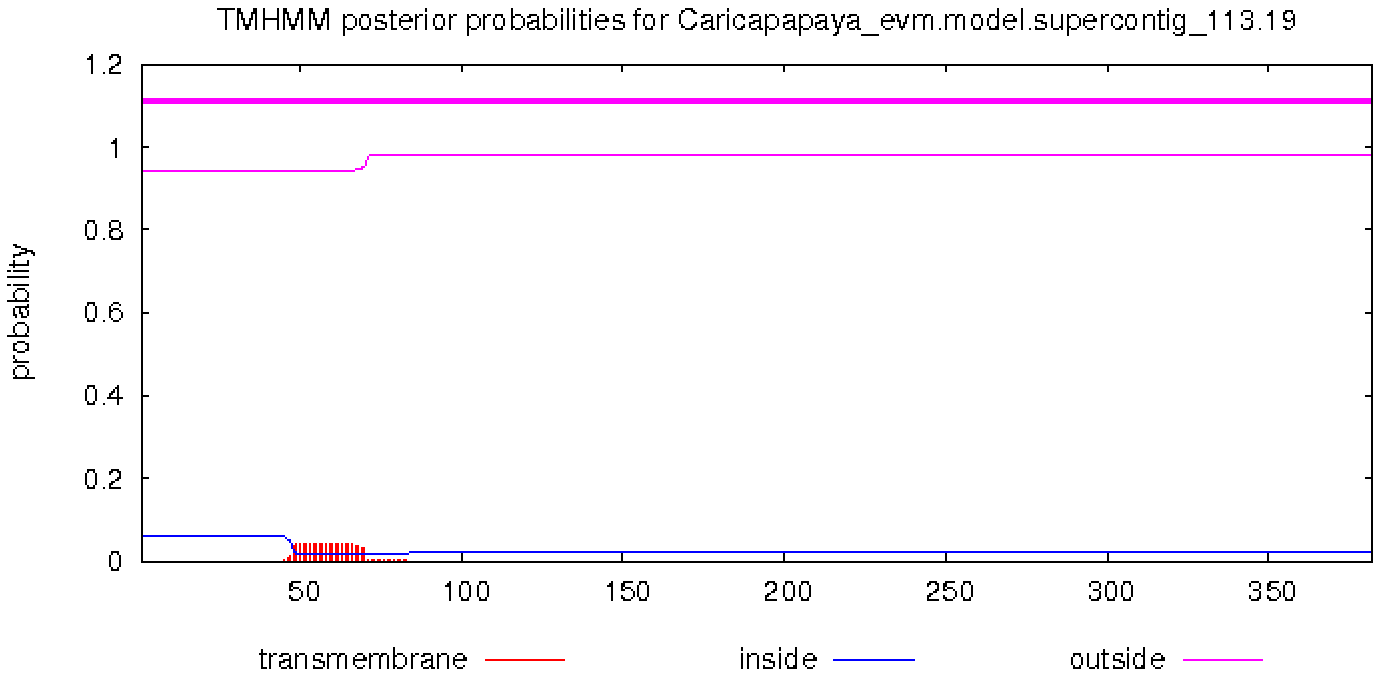

plot in postscript, script for making the plot in gnuplot, data for plot

---

```
# Caricapapaya_evm.model.supercontig_113.36 Length: 290
# Caricapapaya_evm.model.supercontig_113.36 Number of predicted TMHs:  0
```

```
# Caricapapaya_evm.model.supercontig_113.36 Exp number of AAs in TMHs: 1.30884
# Caricapapaya_evm.model.supercontig_113.36 Exp number, first 60 AAs:  1.30472
# Caricapapaya_evm.model.supercontig_113.36 Total prob of N-in:        0.07539
Caricapapaya_evm.model.supercontig_113.36       TMHMM2.0        outside      1   290
```

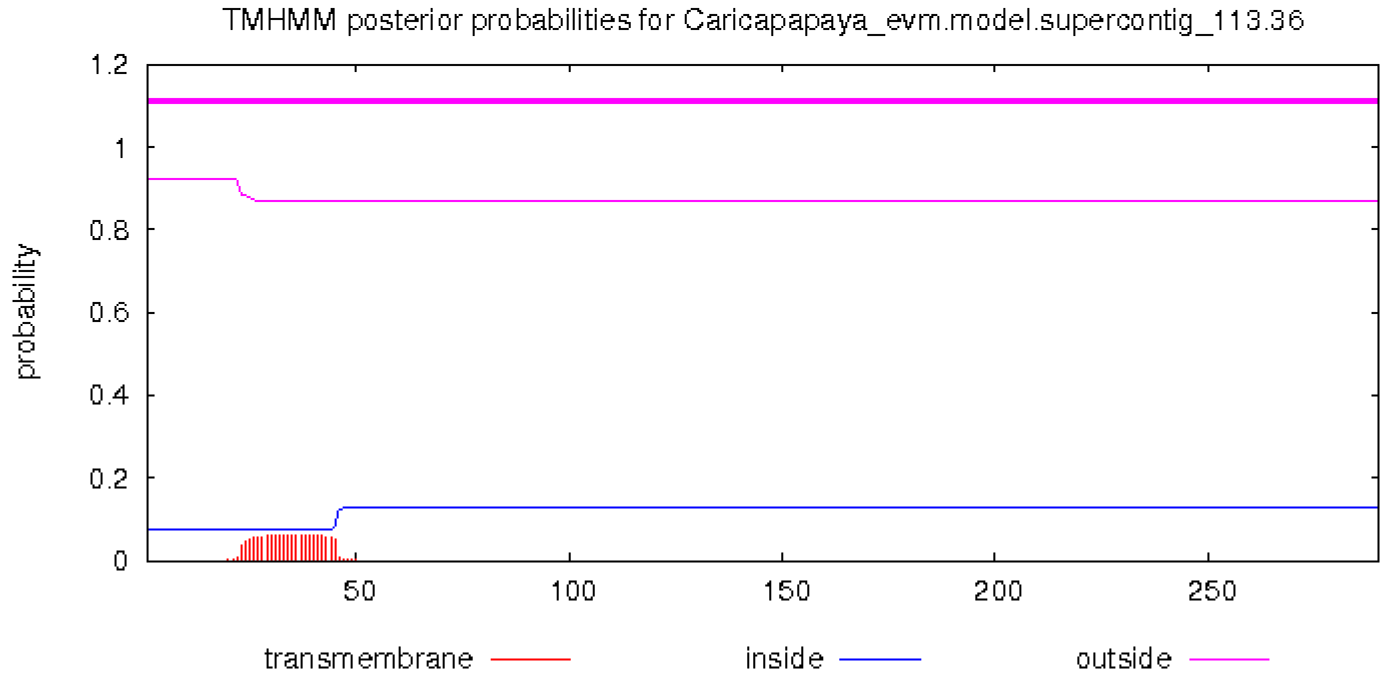

plot in postscript, script for making the plot in gnuplot, data for plot

---

```
# Caricapapaya_evm.model.supercontig_1.141 Length: 407
# Caricapapaya_evm.model.supercontig_1.141 Number of predicted TMHs:  0
# Caricapapaya_evm.model.supercontig_1.141 Exp number of AAs in TMHs: 0.00347
# Caricapapaya_evm.model.supercontig_1.141 Exp number, first 60 AAs:  0
# Caricapapaya_evm.model.supercontig_1.141 Total prob of N-in:        0.00422
Caricapapaya_evm.model.supercontig_1.141        TMHMM2.0        outside      1   407
```

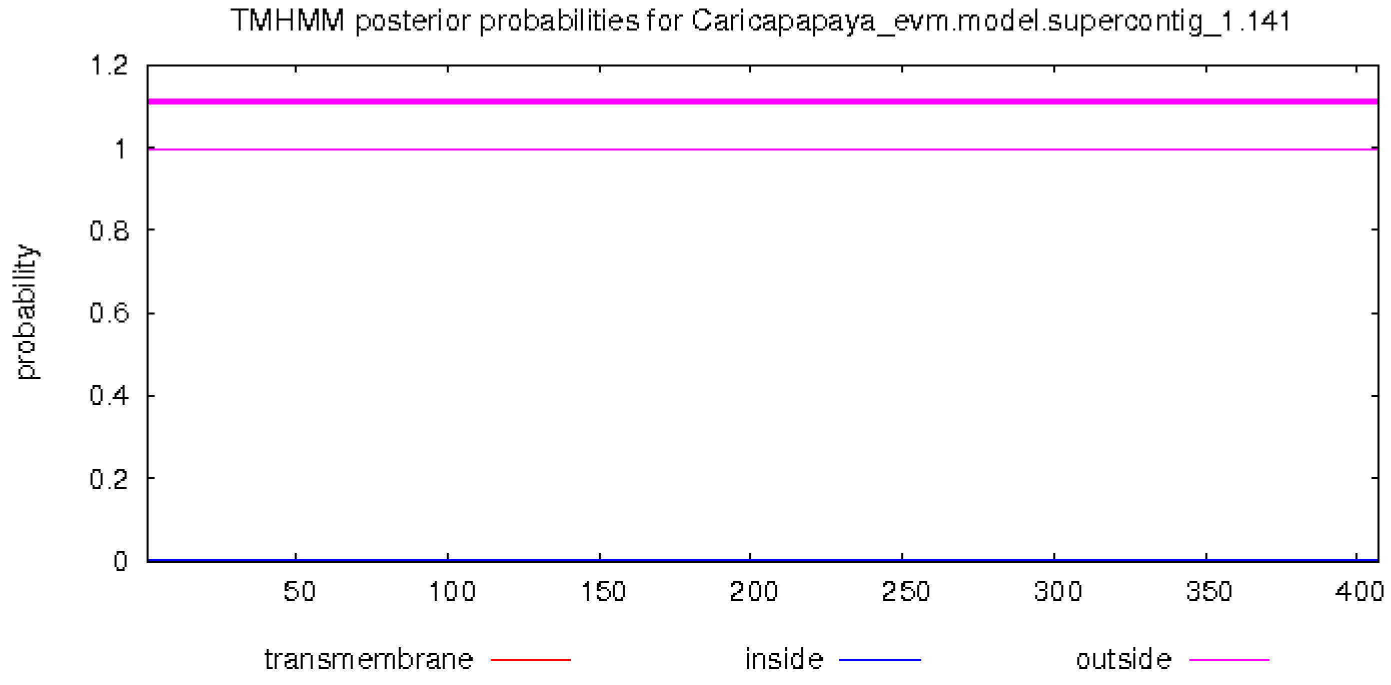

plot in postscript, script for making the plot in gnuplot, data for plot

---

```
# Caricapapaya_evm.model.supercontig_11.52 Length: 409
# Caricapapaya_evm.model.supercontig_11.52 Number of predicted TMHs:  0
```

```
# Caricapapaya_evm.model.supercontig_11.52 Exp number of AAs in TMHs: 0.00106
# Caricapapaya_evm.model.supercontig_11.52 Exp number, first 60 AAs:  0.00015
# Caricapapaya_evm.model.supercontig_11.52 Total prob of N-in:        0.01886
Caricapapaya_evm.model.supercontig_11.52        TMHMM2.0        outside      1   409
```

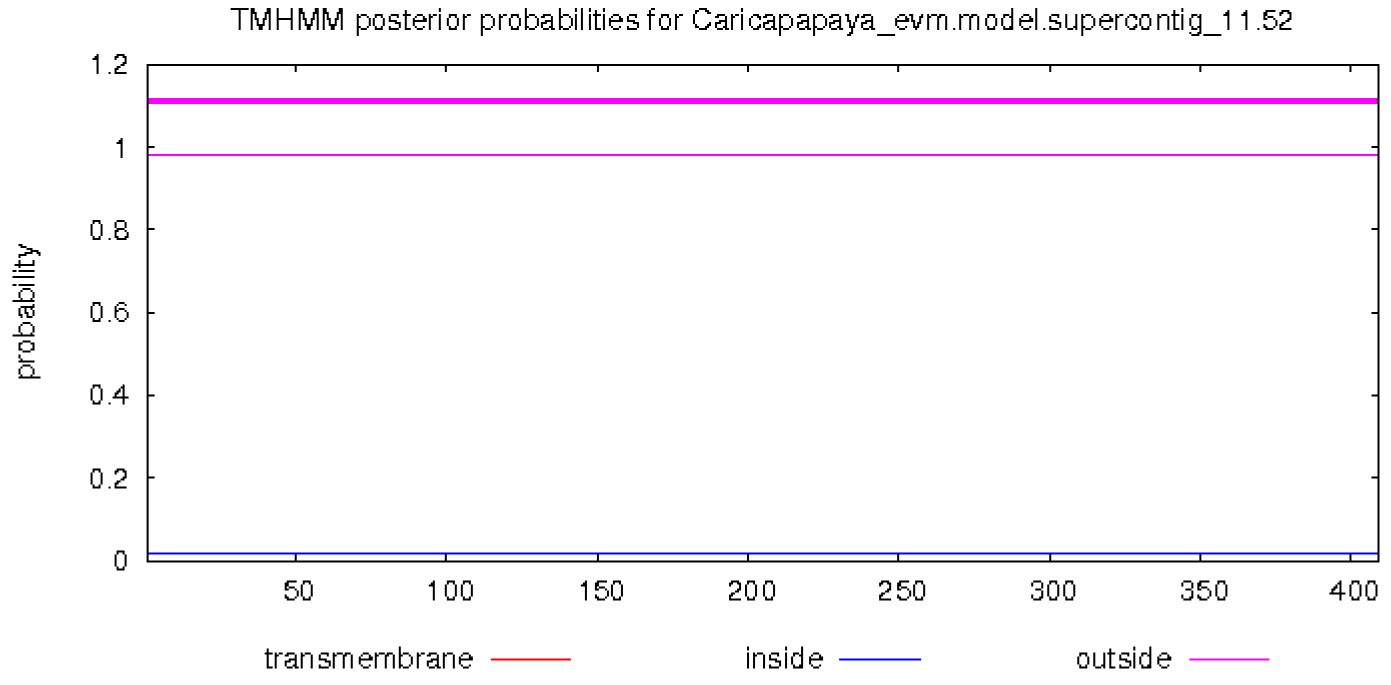

plot in postscript, script for making the plot in gnuplot, data for plot

---

```
# Caricapapaya_evm.model.supercontig_118.26 Length: 315
# Caricapapaya_evm.model.supercontig_118.26 Number of predicted TMHs:  0
# Caricapapaya_evm.model.supercontig_118.26 Exp number of AAs in TMHs: 0
# Caricapapaya_evm.model.supercontig_118.26 Exp number, first 60 AAs:  0
# Caricapapaya_evm.model.supercontig_118.26 Total prob of N-in:        0.01179
Caricapapaya_evm.model.supercontig_118.26       TMHMM2.0        outside      1   315
```

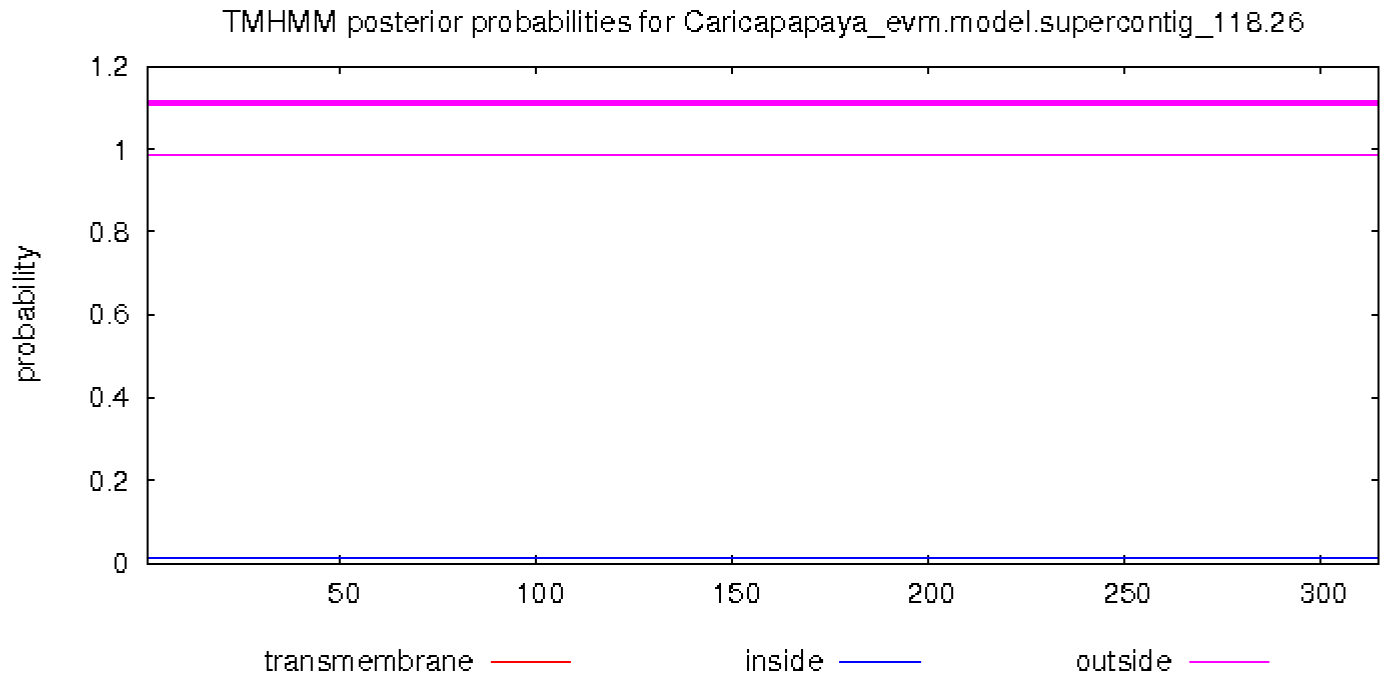

plot in postscript, script for making the plot in gnuplot, data for plot

---

```
# Caricapapaya_evm.model.supercontig_12.156 Length: 417
# Caricapapaya_evm.model.supercontig_12.156 Number of predicted TMHs:  0
```

```
# Caricapapaya_evm.model.supercontig_12.156 Exp number of AAs in TMHs: 0.00312
# Caricapapaya_evm.model.supercontig_12.156 Exp number, first 60 AAs:  0
# Caricapapaya_evm.model.supercontig_12.156 Total prob of N-in:        0.00282
Caricapapaya_evm.model.supercontig_12.156	TMHMM2.0	outside	     1   417
```

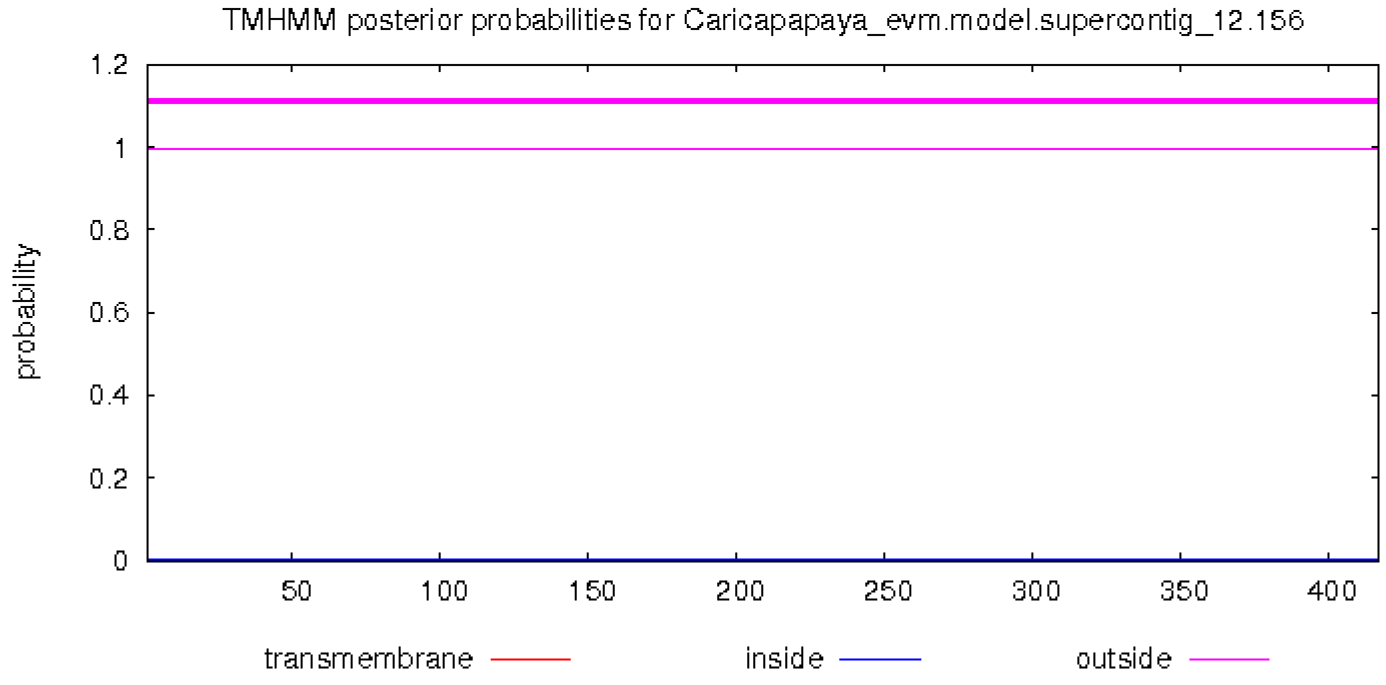

plot in postscript, script for making the plot in gnuplot, data for plot

---

```
# Caricapapaya_evm.model.supercontig_128.20 Length: 396
# Caricapapaya_evm.model.supercontig_128.20 Number of predicted TMHs:  0
# Caricapapaya_evm.model.supercontig_128.20 Exp number of AAs in TMHs: 0.00071
# Caricapapaya_evm.model.supercontig_128.20 Exp number, first 60 AAs:  0
# Caricapapaya_evm.model.supercontig_128.20 Total prob of N-in:        0.00981
Caricapapaya_evm.model.supercontig_128.20	TMHMM2.0	outside	     1   396
```

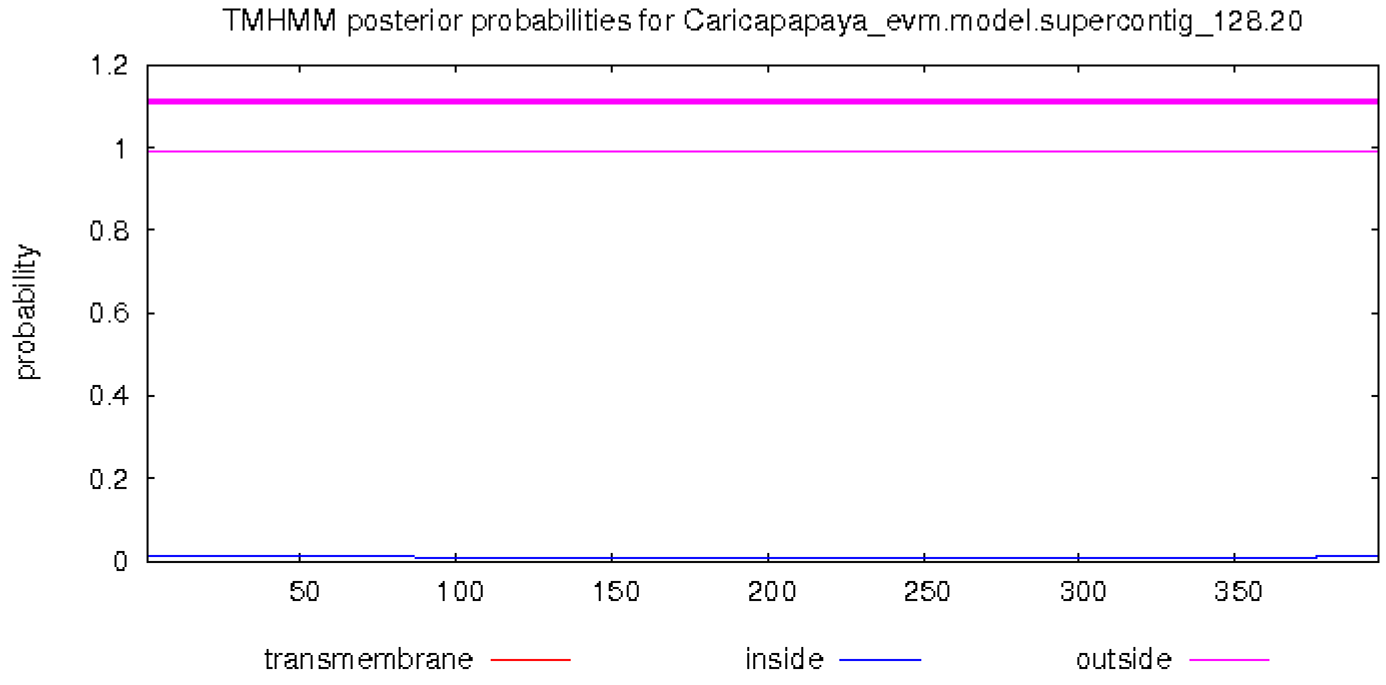

plot in postscript, script for making the plot in gnuplot, data for plot

---

```
# Caricapapaya_evm.model.supercontig_131.88 Length: 364
# Caricapapaya_evm.model.supercontig_131.88 Number of predicted TMHs:  0
```

```
# Caricapapaya_evm.model.supercontig_131.88 Exp number of AAs in TMHs: 0.01114
# Caricapapaya_evm.model.supercontig_131.88 Exp number, first 60 AAs:  0
# Caricapapaya_evm.model.supercontig_131.88 Total prob of N-in:        0.01375
Caricapapaya_evm.model.supercontig_131.88       TMHMM2.0        outside      1   364
```

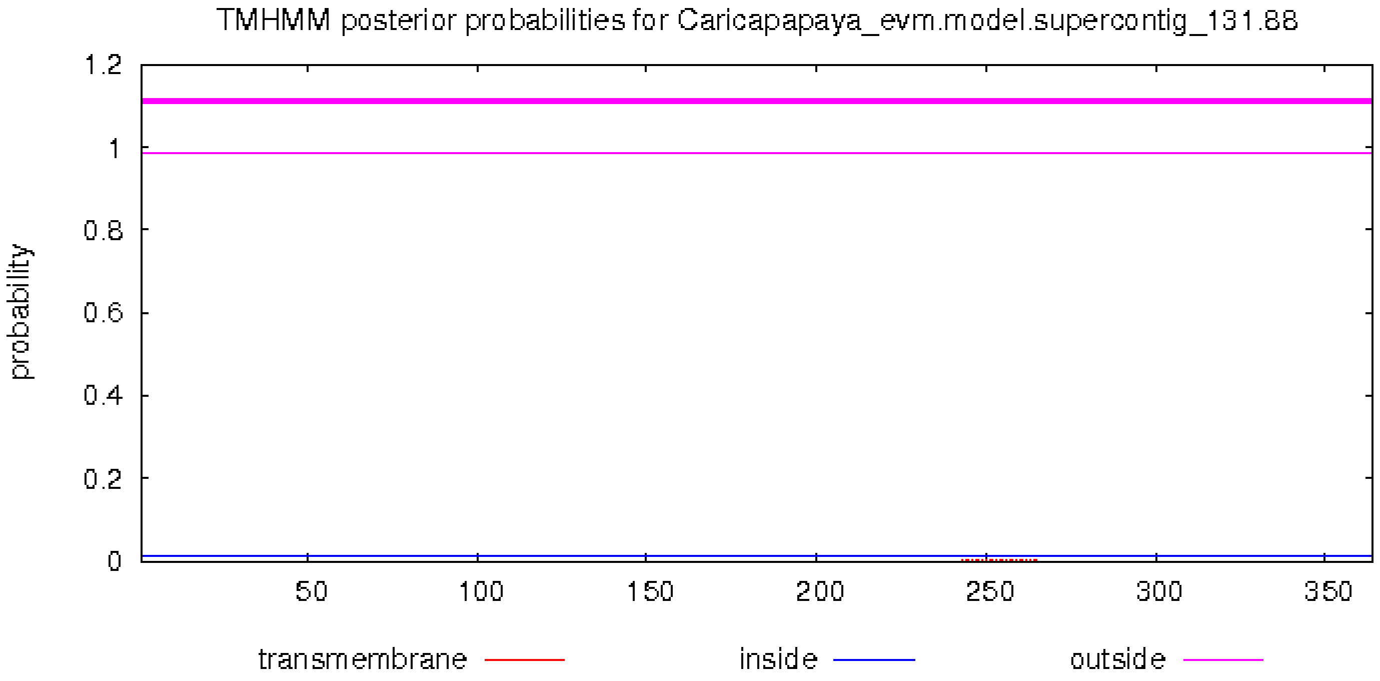

plot in postscript, script for making the plot in gnuplot, data for plot

---

```
# Caricapapaya_evm.model.supercontig_14.101 Length: 346
# Caricapapaya_evm.model.supercontig_14.101 Number of predicted TMHs:  0
# Caricapapaya_evm.model.supercontig_14.101 Exp number of AAs in TMHs: 0
# Caricapapaya_evm.model.supercontig_14.101 Exp number, first 60 AAs:  0
# Caricapapaya_evm.model.supercontig_14.101 Total prob of N-in:        0.05545
Caricapapaya_evm.model.supercontig_14.101       TMHMM2.0        outside      1   346
```

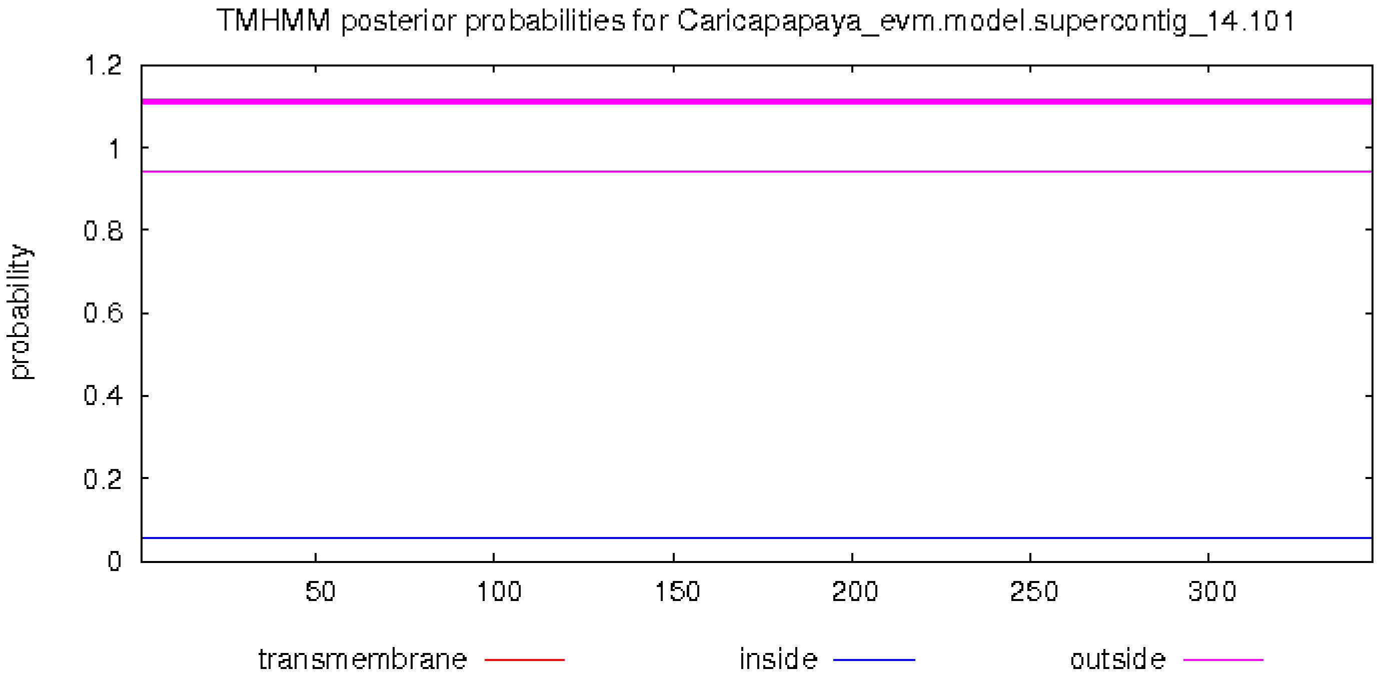

plot in postscript, script for making the plot in gnuplot, data for plot

---

```
# Caricapapaya_evm.model.supercontig_14.2 Length: 342
# Caricapapaya_evm.model.supercontig_14.2 Number of predicted TMHs:  0
```

```
# Caricapapaya_evm.model.supercontig_14.2 Exp number of AAs in TMHs: 0.06462
# Caricapapaya_evm.model.supercontig_14.2 Exp number, first 60 AAs:  0.00036
# Caricapapaya_evm.model.supercontig_14.2 Total prob of N-in:        0.04820
Caricapapaya_evm.model.supercontig_14.2 TMHMM2.0        outside      1   342
```

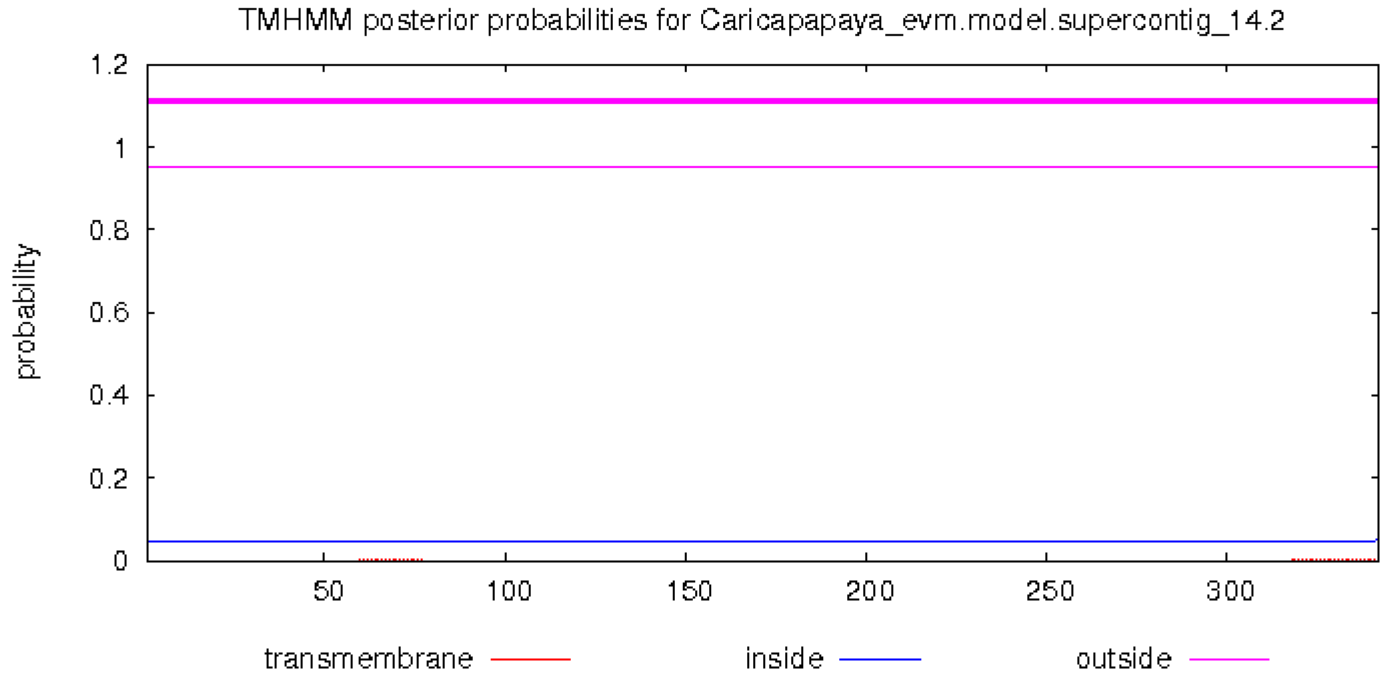

plot in postscript, script for making the plot in gnuplot, data for plot

---

```
# Caricapapaya_evm.model.supercontig_14.213 Length: 197
# Caricapapaya_evm.model.supercontig_14.213 Number of predicted TMHs:  0
# Caricapapaya_evm.model.supercontig_14.213 Exp number of AAs in TMHs: 0.06069
# Caricapapaya_evm.model.supercontig_14.213 Exp number, first 60 AAs:  0.05913
# Caricapapaya_evm.model.supercontig_14.213 Total prob of N-in:        0.14572
Caricapapaya_evm.model.supercontig_14.213       TMHMM2.0        outside      1   197
```

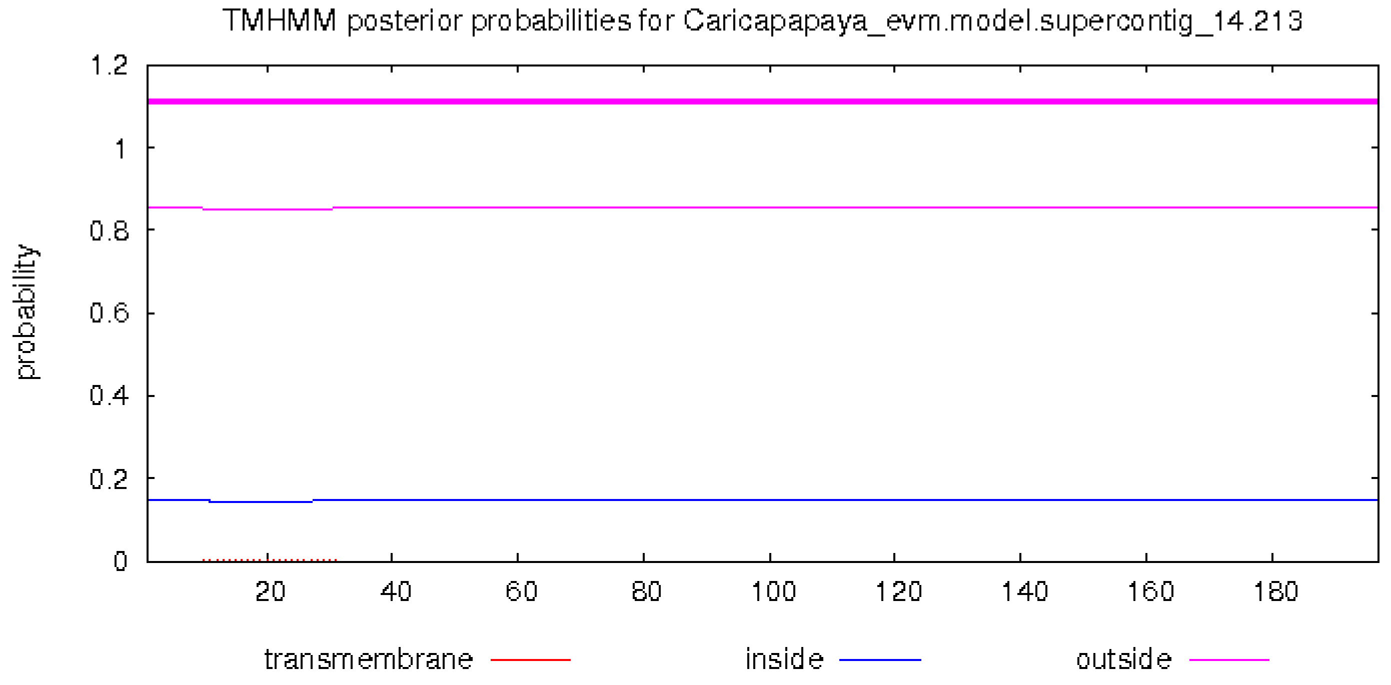

plot in postscript, script for making the plot in gnuplot, data for plot

---

```
# Caricapapaya_evm.model.supercontig_1.48 Length: 181
# Caricapapaya_evm.model.supercontig_1.48 Number of predicted TMHs:  0
```

```
# Caricapapaya_evm.model.supercontig_1.48 Exp number of AAs in TMHs: 0.00469
# Caricapapaya_evm.model.supercontig_1.48 Exp number, first 60 AAs:  0.00018
# Caricapapaya_evm.model.supercontig_1.48 Total prob of N-in:        0.06531
Caricapapaya_evm.model.supercontig_1.48 TMHMM2.0        outside      1   181
```

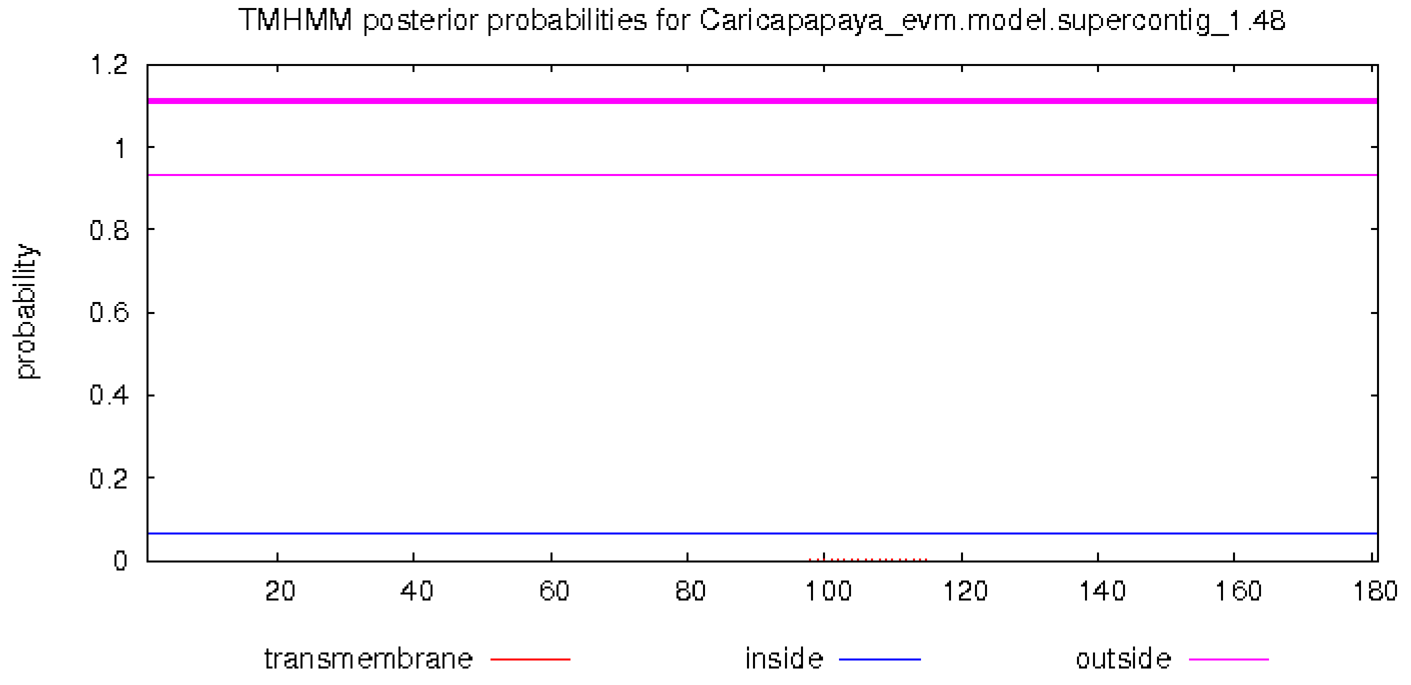

plot in postscript, script for making the plot in gnuplot, data for plot

---

```
# Caricapapaya_evm.model.supercontig_165.12 Length: 307
# Caricapapaya_evm.model.supercontig_165.12 Number of predicted TMHs:  0
# Caricapapaya_evm.model.supercontig_165.12 Exp number of AAs in TMHs: 0.00012
# Caricapapaya_evm.model.supercontig_165.12 Exp number, first 60 AAs:  0
# Caricapapaya_evm.model.supercontig_165.12 Total prob of N-in:        0.01064
Caricapapaya_evm.model.supercontig_165.12       TMHMM2.0        outside      1   307
```

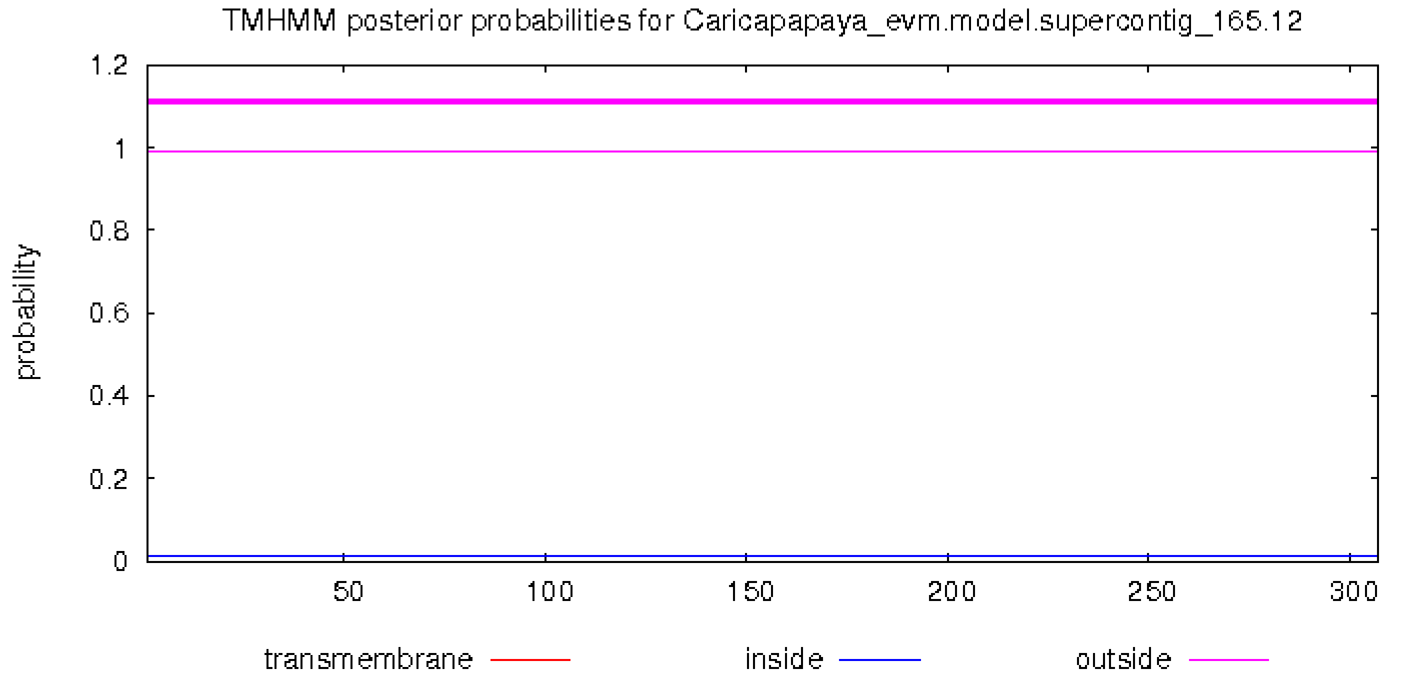

plot in postscript, script for making the plot in gnuplot, data for plot

---

```
# Caricapapaya_evm.model.supercontig_17.29 Length: 177
# Caricapapaya_evm.model.supercontig_17.29 Number of predicted TMHs:  0
```

```
# Caricapapaya_evm.model.supercontig_17.29 Exp number of AAs in TMHs: 0.01162
# Caricapapaya_evm.model.supercontig_17.29 Exp number, first 60 AAs:  0.00031
# Caricapapaya_evm.model.supercontig_17.29 Total prob of N-in:        0.21332
Caricapapaya_evm.model.supercontig_17.29	TMHMM2.0	outside	     1   177
```

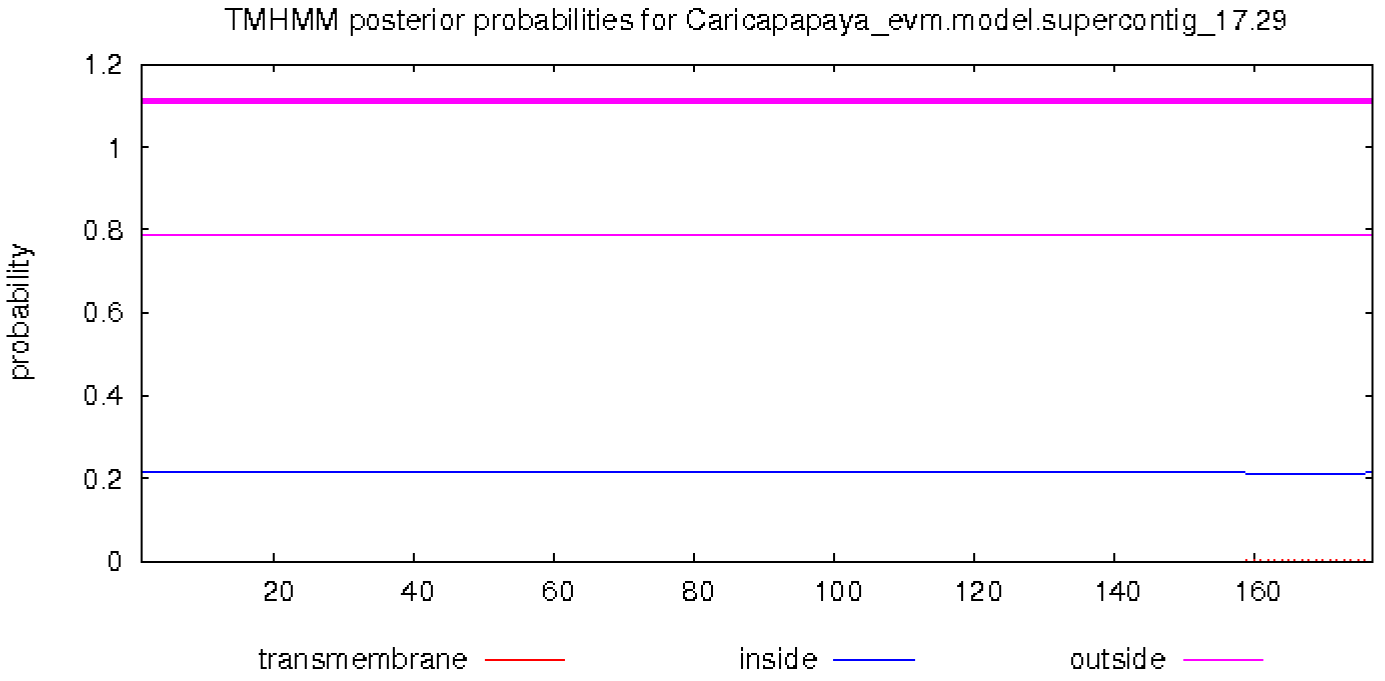

plot in postscript, script for making the plot in gnuplot, data for plot

---

```
# Caricapapaya_evm.model.supercontig_178.32 Length: 491
# Caricapapaya_evm.model.supercontig_178.32 Number of predicted TMHs:  2
# Caricapapaya_evm.model.supercontig_178.32 Exp number of AAs in TMHs: 35.71895
# Caricapapaya_evm.model.supercontig_178.32 Exp number, first 60 AAs:  0
# Caricapapaya_evm.model.supercontig_178.32 Total prob of N-in:        0.00585
Caricapapaya_evm.model.supercontig_178.32	TMHMM2.0	outside	     1   388
Caricapapaya_evm.model.supercontig_178.32	TMHMM2.0	TMhelix	   389   411
Caricapapaya_evm.model.supercontig_178.32	TMHMM2.0	inside	   412   470
Caricapapaya_evm.model.supercontig_178.32	TMHMM2.0	TMhelix	   471   488
Caricapapaya_evm.model.supercontig_178.32	TMHMM2.0	outside	   489   491
```

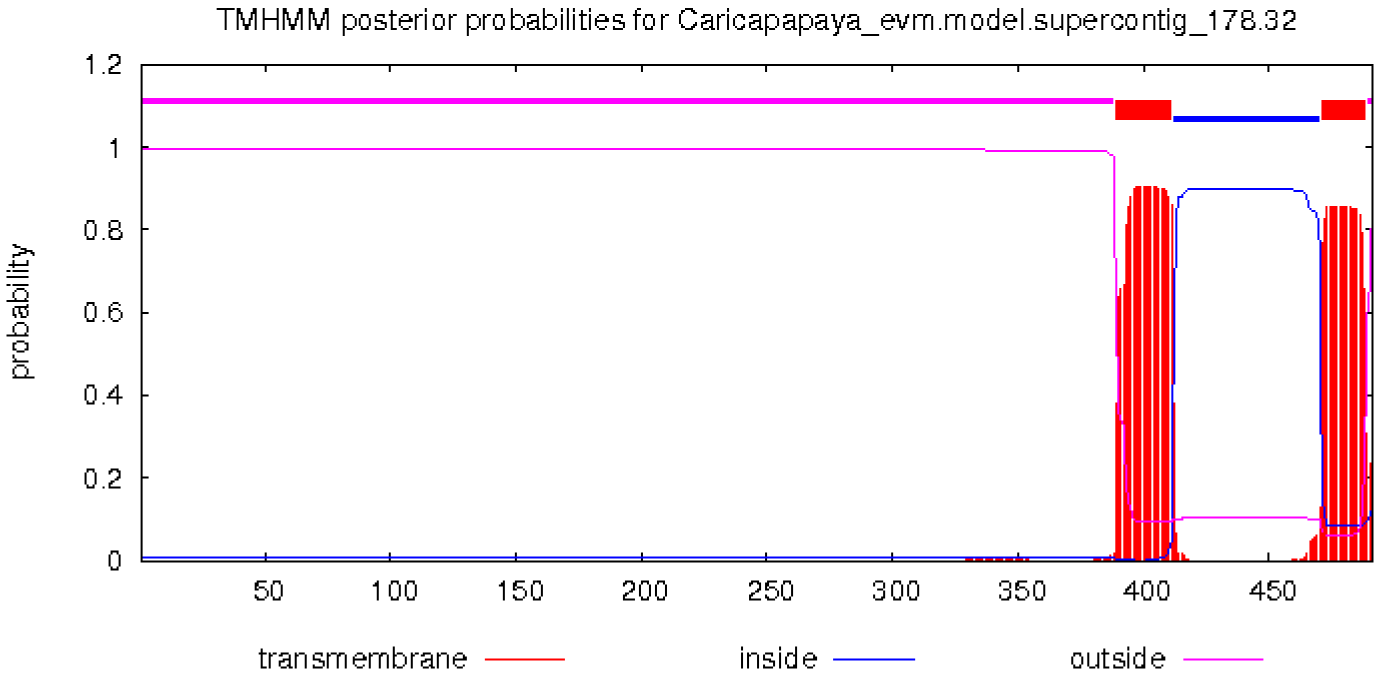

plot in postscript, script for making the plot in gnuplot, data for plot

```
# Caricapapaya_evm.model.supercontig_192.19 Length: 259
# Caricapapaya_evm.model.supercontig_192.19 Number of predicted TMHs:  0
# Caricapapaya_evm.model.supercontig_192.19 Exp number of AAs in TMHs: 0.08532
# Caricapapaya_evm.model.supercontig_192.19 Exp number, first 60 AAs:  0.04313
# Caricapapaya_evm.model.supercontig_192.19 Total prob of N-in:        0.06118
Caricapapaya_evm.model.supercontig_192.19	TMHMM2.0	outside	     1   259
```

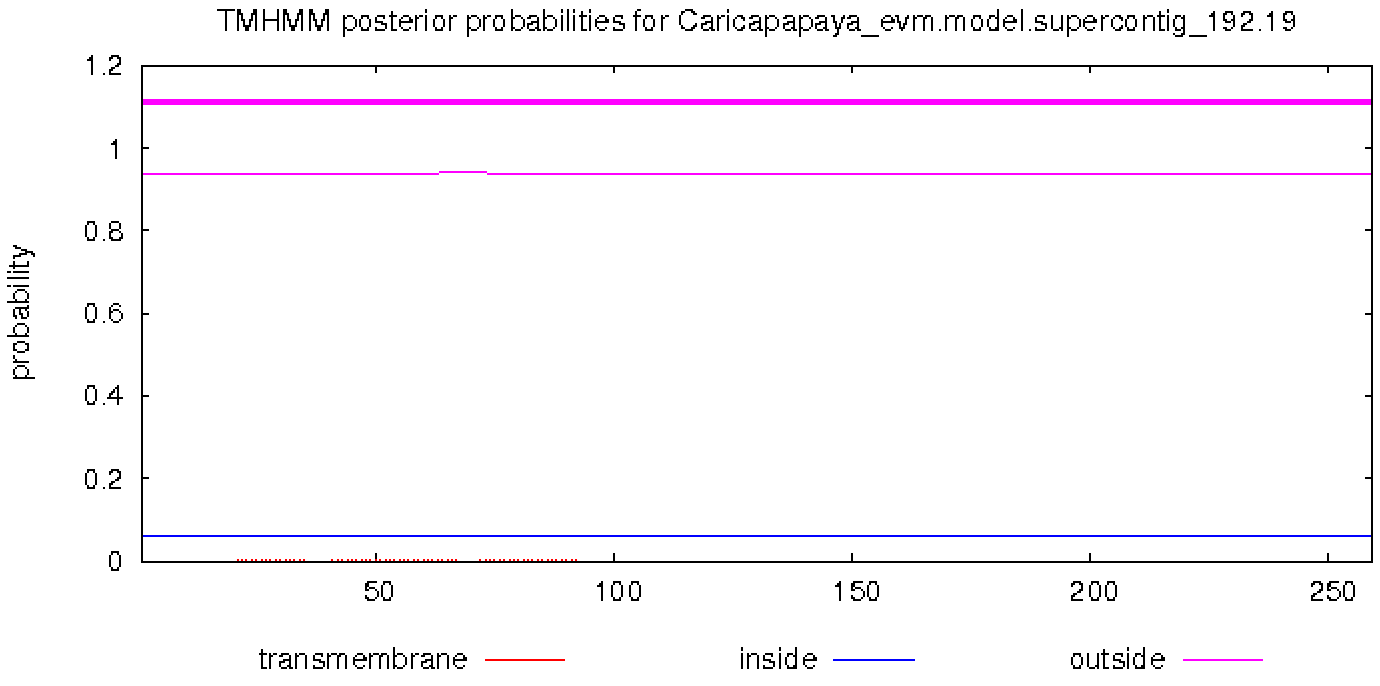

plot in postscript, script for making the plot in gnuplot, data for plot

```
# Caricapapaya_evm.model.supercontig_195.14 Length: 245
# Caricapapaya_evm.model.supercontig_195.14 Number of predicted TMHs:  0
# Caricapapaya_evm.model.supercontig_195.14 Exp number of AAs in TMHs: 0.00033
# Caricapapaya_evm.model.supercontig_195.14 Exp number, first 60 AAs:  0
# Caricapapaya_evm.model.supercontig_195.14 Total prob of N-in:        0.00851
Caricapapaya_evm.model.supercontig_195.14	TMHMM2.0	outside	     1   245
```

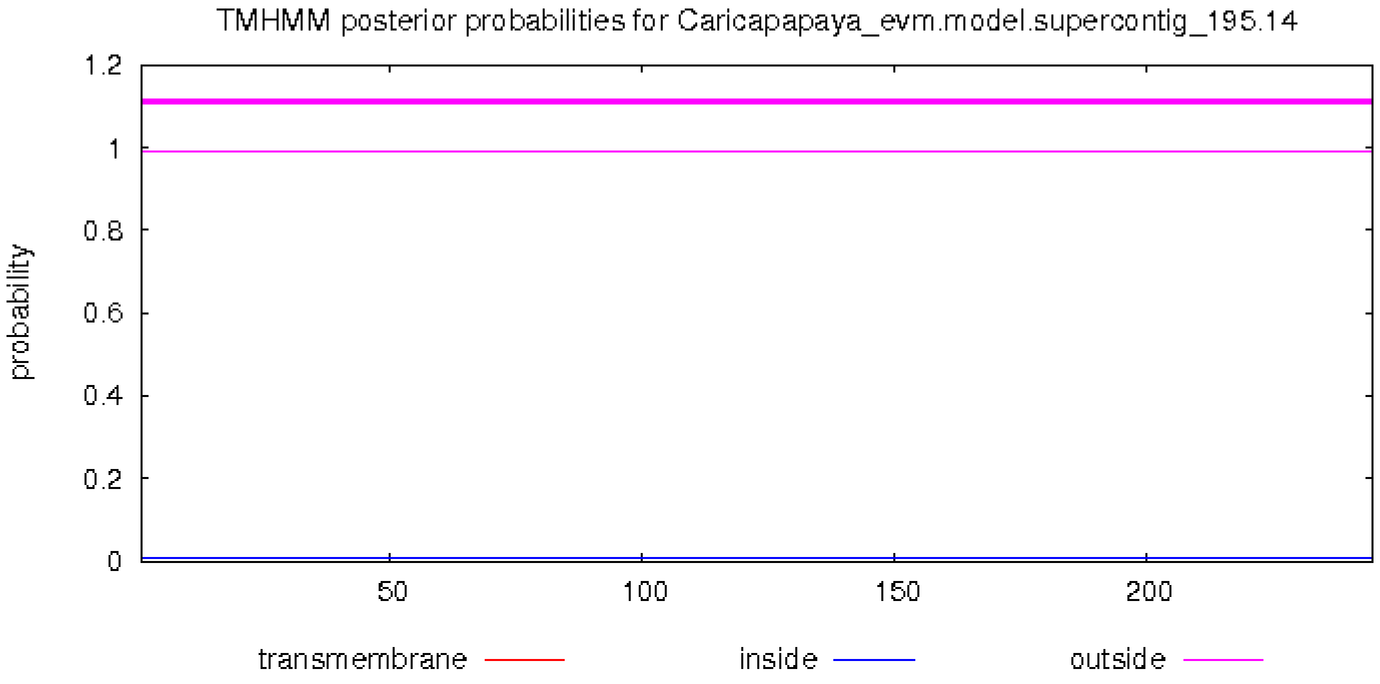

plot in postscript, script for making the plot in gnuplot, data for plot

```
# Caricapapaya_evm.model.supercontig_195.35 Length: 435
# Caricapapaya_evm.model.supercontig_195.35 Number of predicted TMHs:  1
# Caricapapaya_evm.model.supercontig_195.35 Exp number of AAs in TMHs: 19.79794
# Caricapapaya_evm.model.supercontig_195.35 Exp number, first 60 AAs:  0.00916
# Caricapapaya_evm.model.supercontig_195.35 Total prob of N-in:        0.09229
Caricapapaya_evm.model.supercontig_195.35	TMHMM2.0	outside	     1   400
Caricapapaya_evm.model.supercontig_195.35	TMHMM2.0	TMhelix	   401   420
Caricapapaya_evm.model.supercontig_195.35	TMHMM2.0	inside	   421   435
```

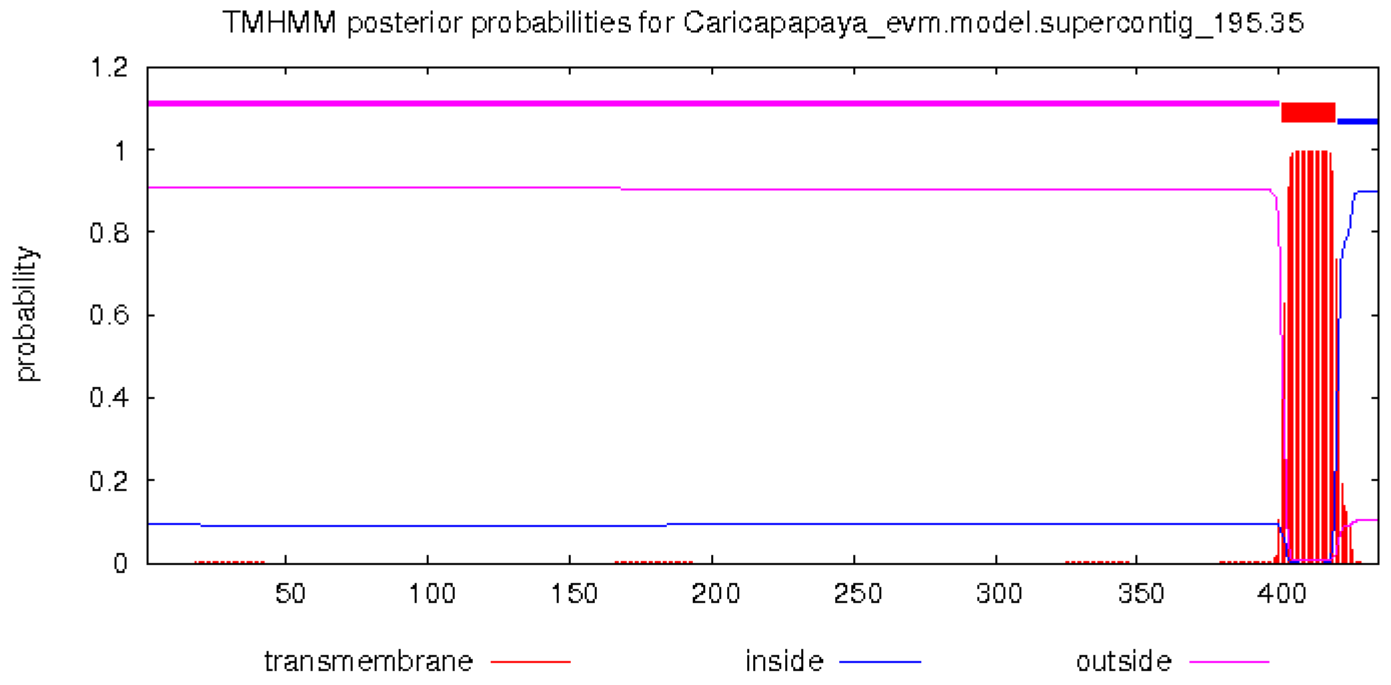

plot in postscript, script for making the plot in gnuplot, data for plot

---

```
# Caricapapaya_evm.model.supercontig_200.10 Length: 163
# Caricapapaya_evm.model.supercontig_200.10 Number of predicted TMHs:  0
# Caricapapaya_evm.model.supercontig_200.10 Exp number of AAs in TMHs: 0.00128
# Caricapapaya_evm.model.supercontig_200.10 Exp number, first 60 AAs:  0.00128
# Caricapapaya_evm.model.supercontig_200.10 Total prob of N-in:        0.12079
Caricapapaya_evm.model.supercontig_200.10	TMHMM2.0	outside	     1   163
```

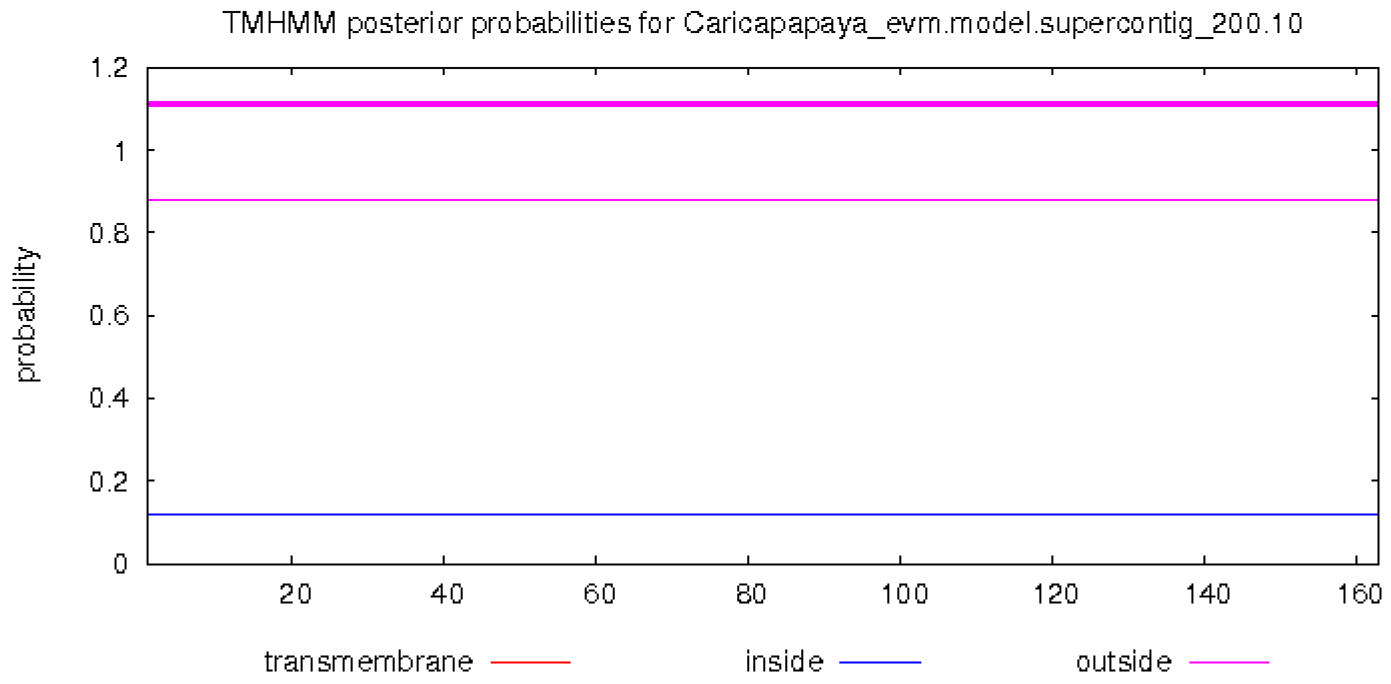

plot in postscript, script for making the plot in gnuplot, data for plot

```
# Caricapapaya_evm.model.supercontig_202.7 Length: 141
# Caricapapaya_evm.model.supercontig_202.7 Number of predicted TMHs:  0
# Caricapapaya_evm.model.supercontig_202.7 Exp number of AAs in TMHs: 0.0018
# Caricapapaya_evm.model.supercontig_202.7 Exp number, first 60 AAs:  0.00141
# Caricapapaya_evm.model.supercontig_202.7 Total prob of N-in:        0.56500
Caricapapaya_evm.model.supercontig_202.7	TMHMM2.0	inside	     1   141
```

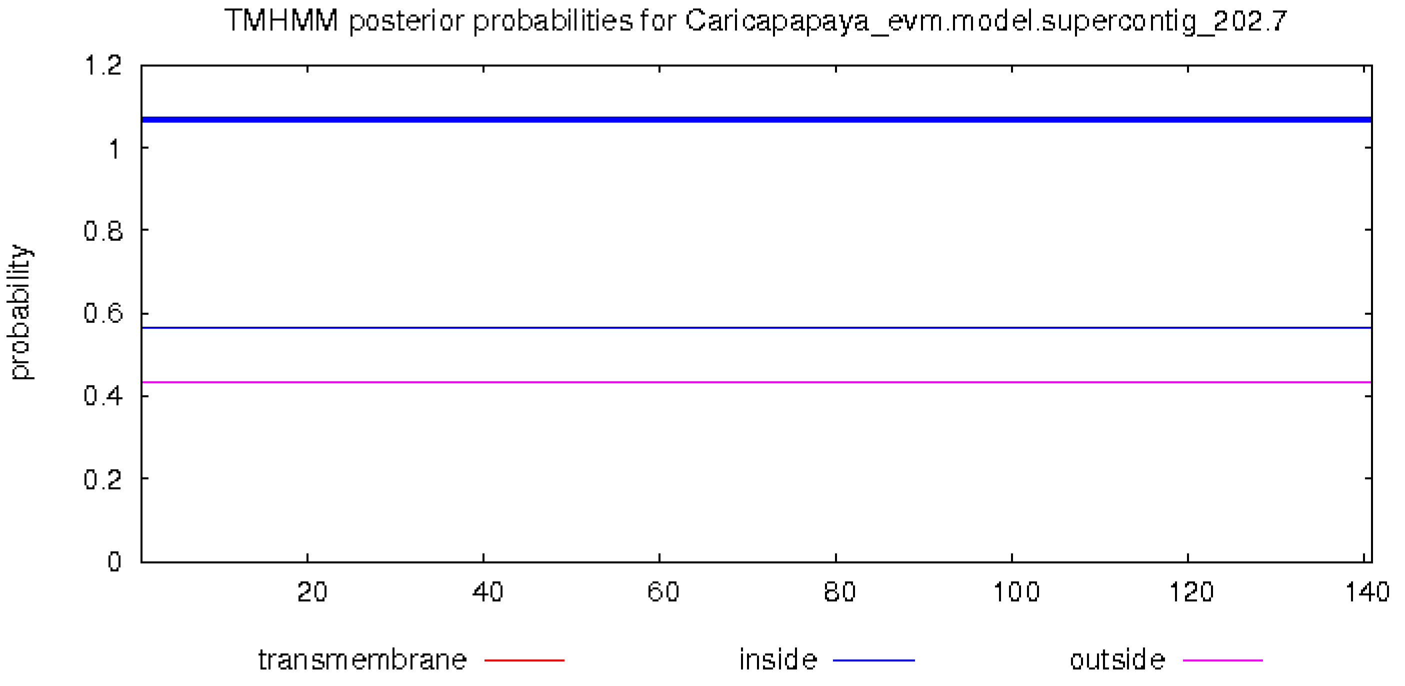

plot in postscript, script for making the plot in gnuplot, data for plot

```
# Caricapapaya_evm.model.supercontig_21.173 Length: 100
# Caricapapaya_evm.model.supercontig_21.173 Number of predicted TMHs:  0
# Caricapapaya_evm.model.supercontig_21.173 Exp number of AAs in TMHs: 0
# Caricapapaya_evm.model.supercontig_21.173 Exp number, first 60 AAs:  0
# Caricapapaya_evm.model.supercontig_21.173 Total prob of N-in:        0.52191
Caricapapaya_evm.model.supercontig_21.173	TMHMM2.0	inside	     1   100
```

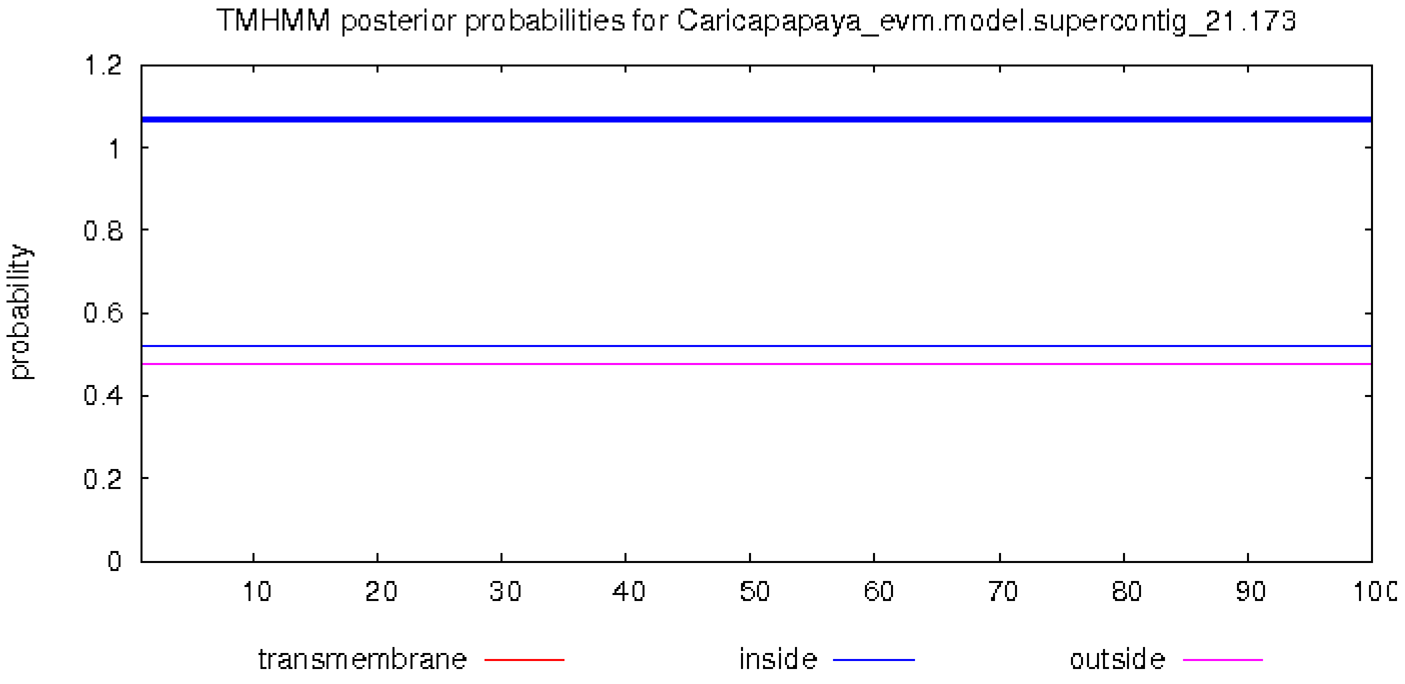

plot in postscript, script for making the plot in gnuplot, data for plot

```
# Caricapapaya_evm.model.supercontig_244.7 Length: 334
# Caricapapaya_evm.model.supercontig_244.7 Number of predicted TMHs:  0
# Caricapapaya_evm.model.supercontig_244.7 Exp number of AAs in TMHs: 0.03195
# Caricapapaya_evm.model.supercontig_244.7 Exp number, first 60 AAs:  0.00061
# Caricapapaya_evm.model.supercontig_244.7 Total prob of N-in:        0.03785
Caricapapaya_evm.model.supercontig_244.7        TMHMM2.0        outside      1   334
```

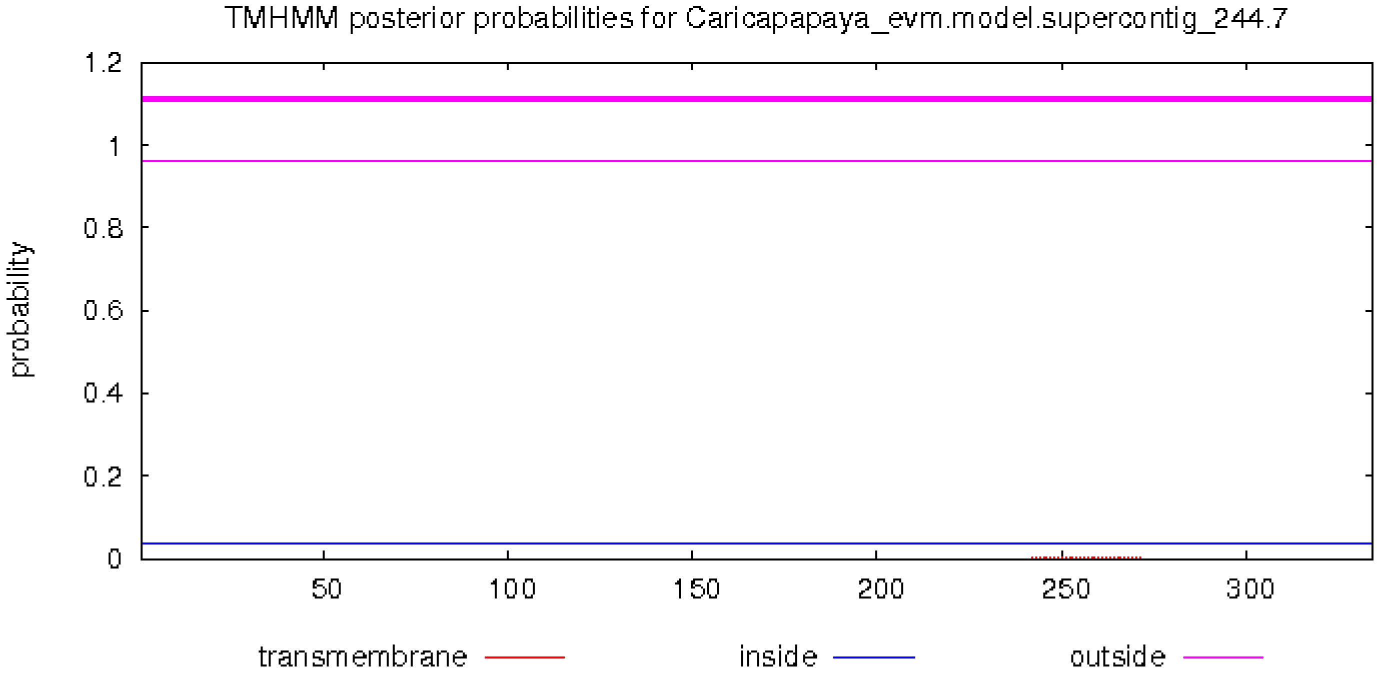

plot in postscript, script for making the plot in gnuplot, data for plot

---

```
# Caricapapaya_evm.model.supercontig_2586.1 Length: 328
# Caricapapaya_evm.model.supercontig_2586.1 Number of predicted TMHs:  0
# Caricapapaya_evm.model.supercontig_2586.1 Exp number of AAs in TMHs: 0.00754
# Caricapapaya_evm.model.supercontig_2586.1 Exp number, first 60 AAs:  0.00033
# Caricapapaya_evm.model.supercontig_2586.1 Total prob of N-in:        0.12438
Caricapapaya_evm.model.supercontig_2586.1       TMHMM2.0        outside      1   328
```

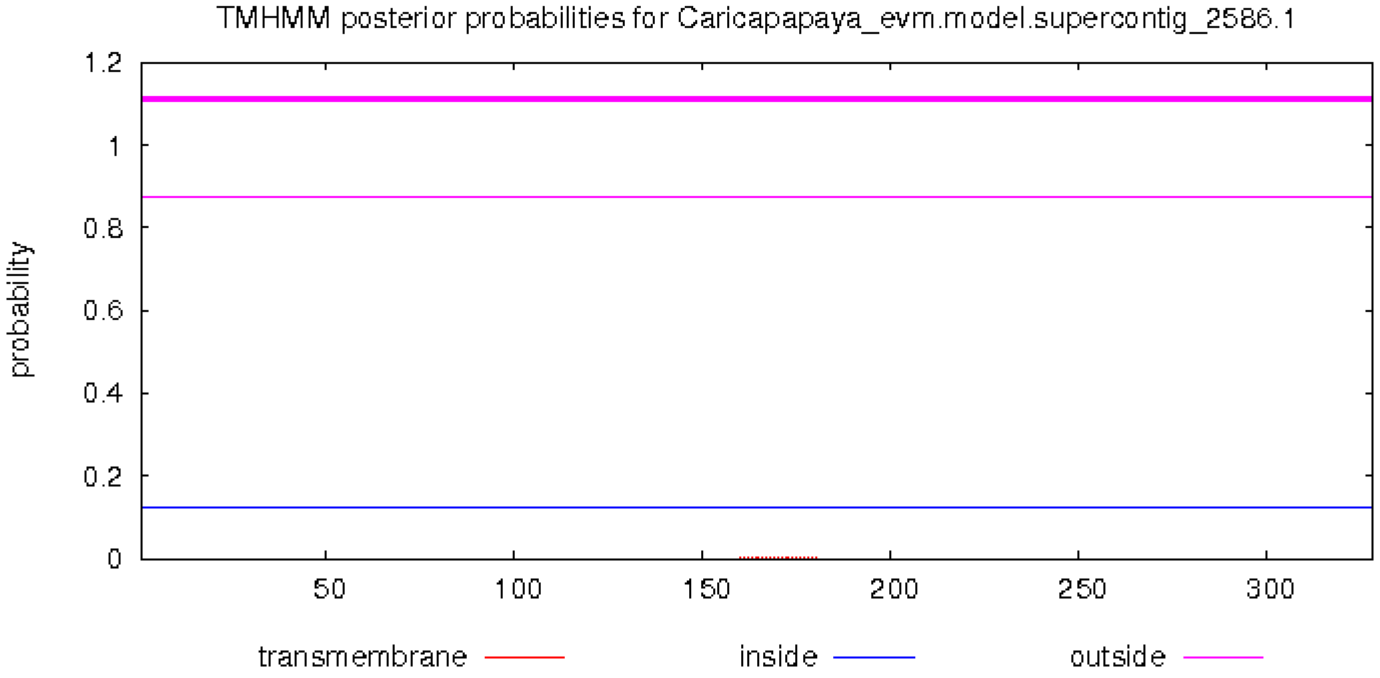

plot in postscript, script for making the plot in gnuplot, data for plot

---

```
# Caricapapaya_evm.model.supercontig_26.135 Length: 482
# Caricapapaya_evm.model.supercontig_26.135 Number of predicted TMHs:  0
# Caricapapaya_evm.model.supercontig_26.135 Exp number of AAs in TMHs: 0
# Caricapapaya_evm.model.supercontig_26.135 Exp number, first 60 AAs:  0
# Caricapapaya_evm.model.supercontig_26.135 Total prob of N-in:        0.00212
Caricapapaya_evm.model.supercontig_26.135	TMHMM2.0	outside	     1   482
```

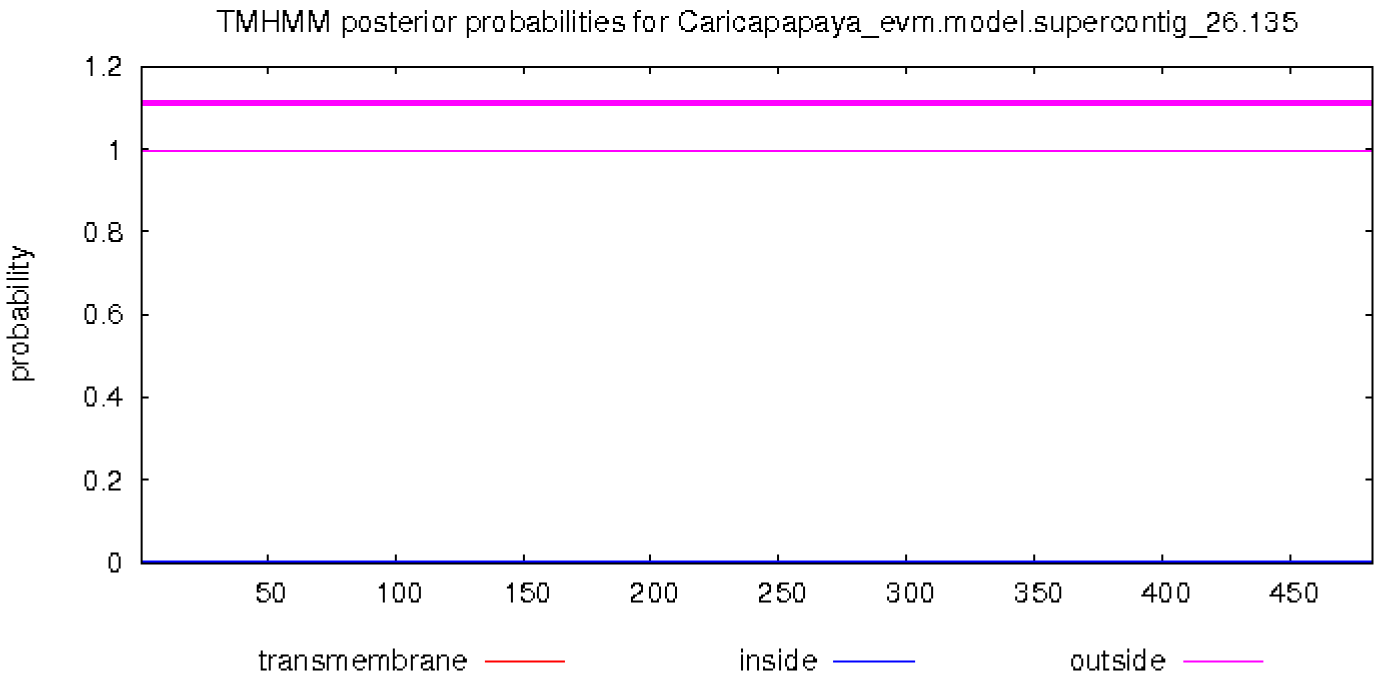

plot in postscript, script for making the plot in gnuplot, data for plot

---

```
# Caricapapaya_evm.model.supercontig_27.81 Length: 385
# Caricapapaya_evm.model.supercontig_27.81 Number of predicted TMHs:  0
# Caricapapaya_evm.model.supercontig_27.81 Exp number of AAs in TMHs: 0.00035
# Caricapapaya_evm.model.supercontig_27.81 Exp number, first 60 AAs:  0.00017
# Caricapapaya_evm.model.supercontig_27.81 Total prob of N-in:        0.00630
Caricapapaya_evm.model.supercontig_27.81	TMHMM2.0	outside	     1   385
```

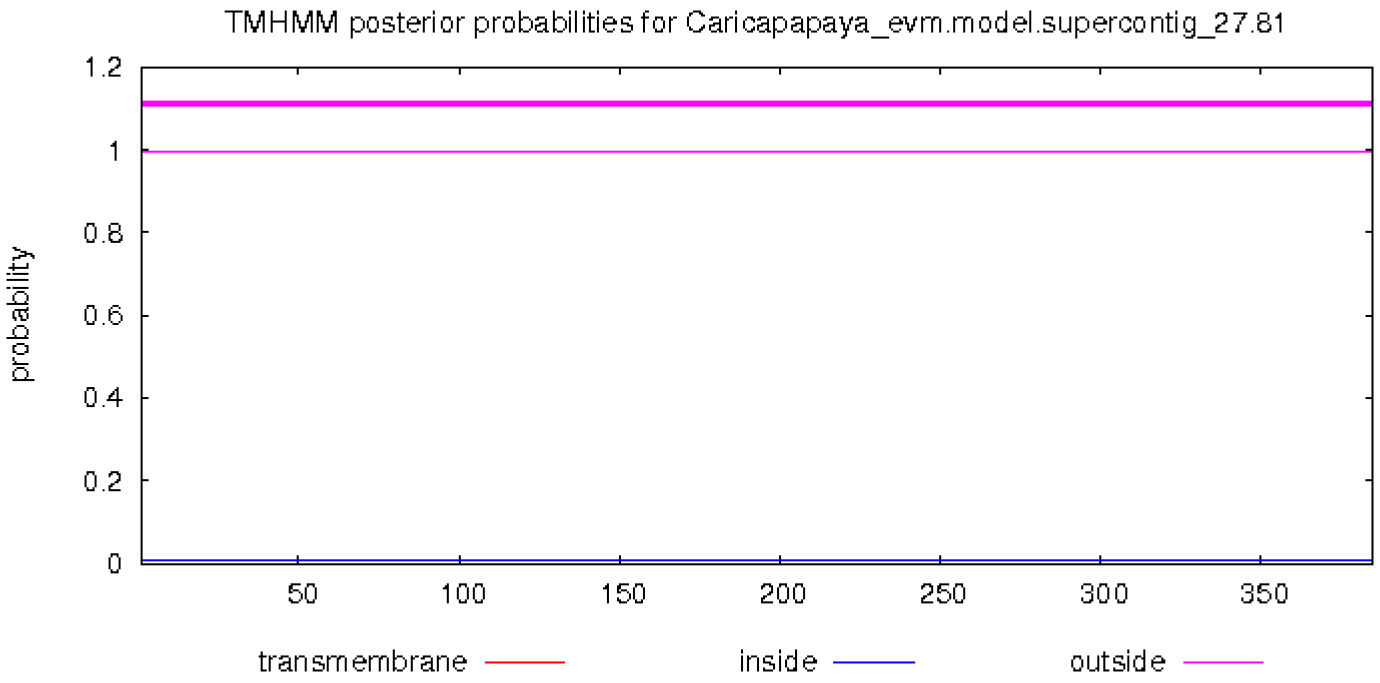

plot in postscript, script for making the plot in gnuplot, data for plot

---

```
# Caricapapaya_evm.model.supercontig_286.12 Length: 360
# Caricapapaya_evm.model.supercontig_286.12 Number of predicted TMHs:  0
# Caricapapaya_evm.model.supercontig_286.12 Exp number of AAs in TMHs: 0.02176
# Caricapapaya_evm.model.supercontig_286.12 Exp number, first 60 AAs:  0.01732
# Caricapapaya_evm.model.supercontig_286.12 Total prob of N-in:        0.00656
Caricapapaya_evm.model.supercontig_286.12	TMHMM2.0	outside	     1   360
```

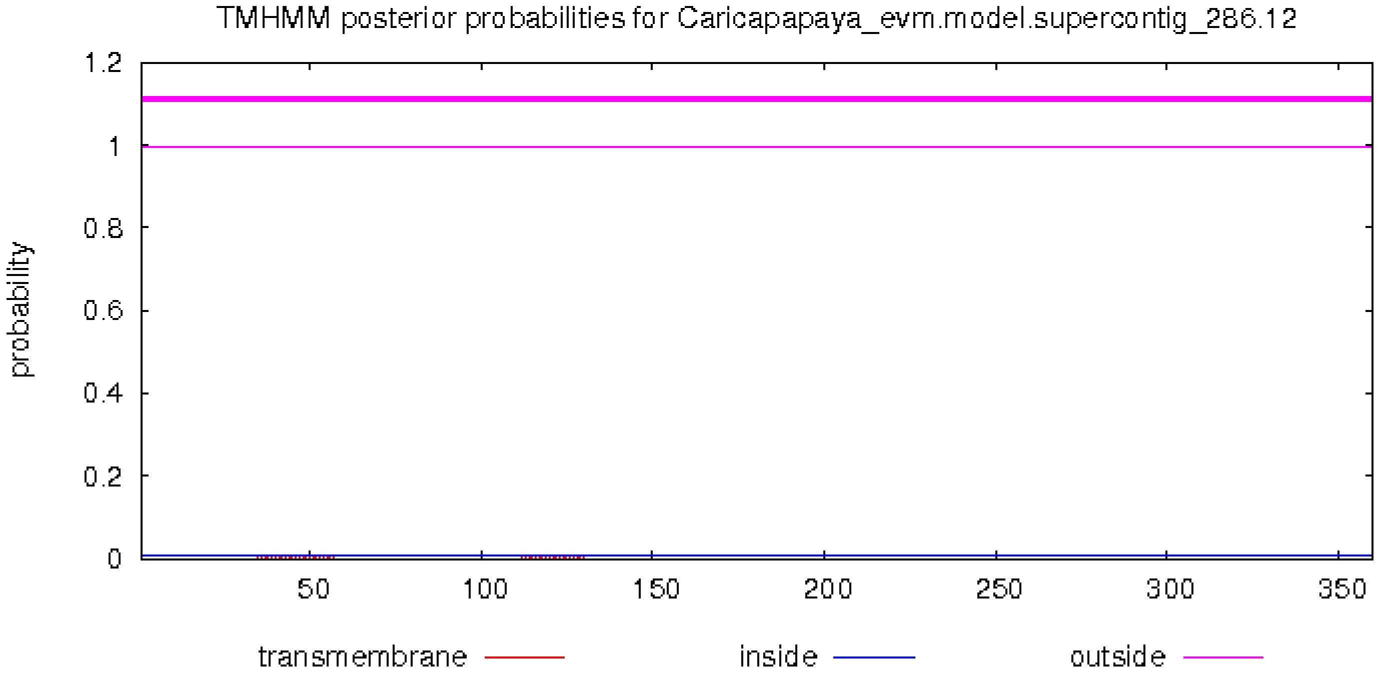

plot in postscript, script for making the plot in gnuplot, data for plot

---

```
# Caricapapaya_evm.model.supercontig_29.118 Length: 307
# Caricapapaya_evm.model.supercontig_29.118 Number of predicted TMHs:  0
# Caricapapaya_evm.model.supercontig_29.118 Exp number of AAs in TMHs: 0
# Caricapapaya_evm.model.supercontig_29.118 Exp number, first 60 AAs:  0
# Caricapapaya_evm.model.supercontig_29.118 Total prob of N-in:        0.00570
Caricapapaya_evm.model.supercontig_29.118	TMHMM2.0	outside	     1   307
```

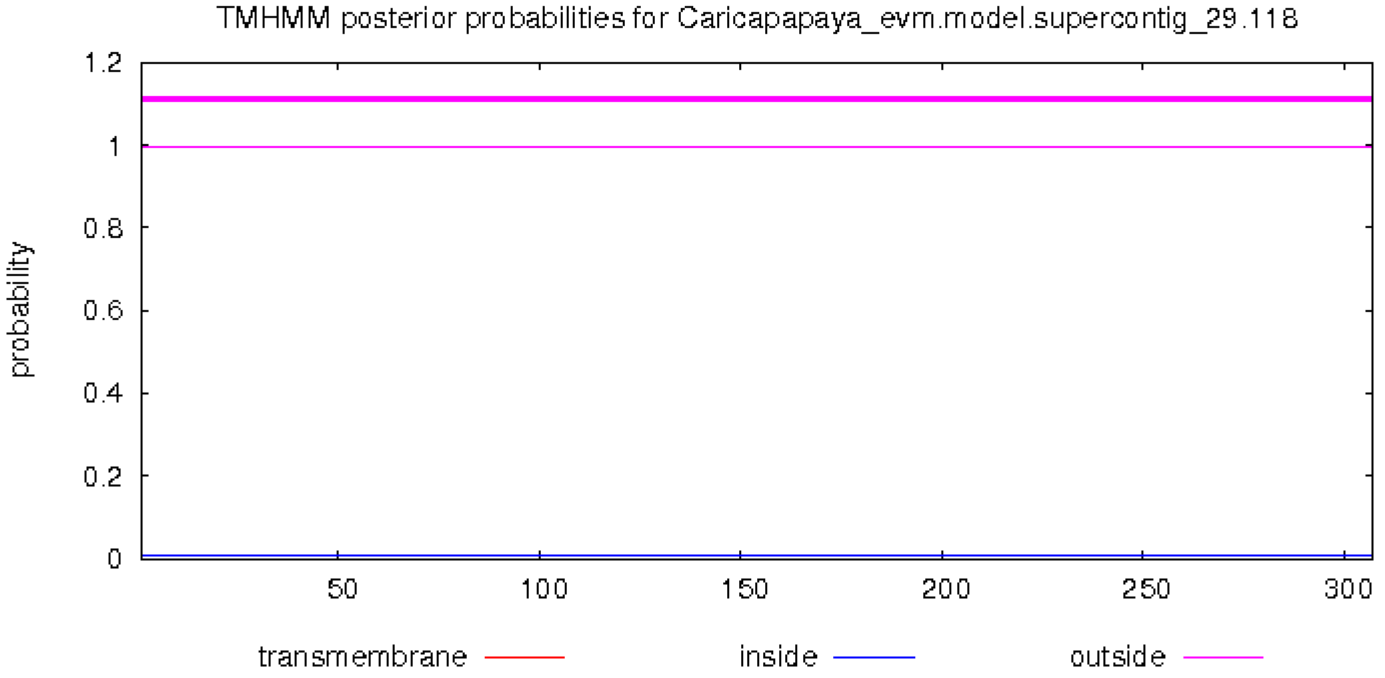

plot in postscript, script for making the plot in gnuplot, data for plot

---

```
# Caricapapaya_evm.model.supercontig_3.170 Length: 608
# Caricapapaya_evm.model.supercontig_3.170 Number of predicted TMHs:  1
# Caricapapaya_evm.model.supercontig_3.170 Exp number of AAs in TMHs: 21.11441
# Caricapapaya_evm.model.supercontig_3.170 Exp number, first 60 AAs:  0
# Caricapapaya_evm.model.supercontig_3.170 Total prob of N-in:        0.00494
Caricapapaya_evm.model.supercontig_3.170	TMHMM2.0	outside	     1   581
Caricapapaya_evm.model.supercontig_3.170	TMHMM2.0	TMhelix	   582   604
Caricapapaya_evm.model.supercontig_3.170	TMHMM2.0	inside	   605   608
```

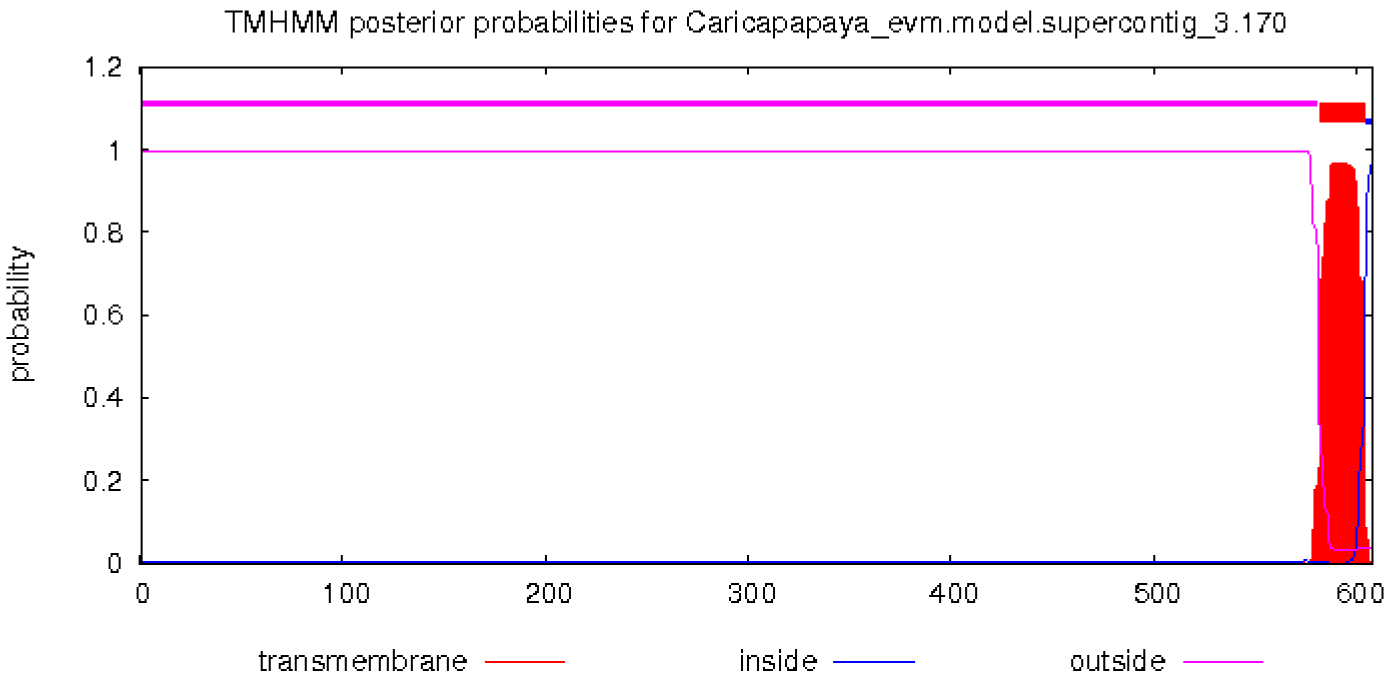

plot in postscript, script for making the plot in gnuplot, data for plot

---

```
# Caricapapaya_evm.model.supercontig_320.2 Length: 287
# Caricapapaya_evm.model.supercontig_320.2 Number of predicted TMHs:  0
# Caricapapaya_evm.model.supercontig_320.2 Exp number of AAs in TMHs: 0.00346
# Caricapapaya_evm.model.supercontig_320.2 Exp number, first 60 AAs:  0.00058
# Caricapapaya_evm.model.supercontig_320.2 Total prob of N-in:        0.01469
Caricapapaya_evm.model.supercontig_320.2	TMHMM2.0	outside	     1   287
```

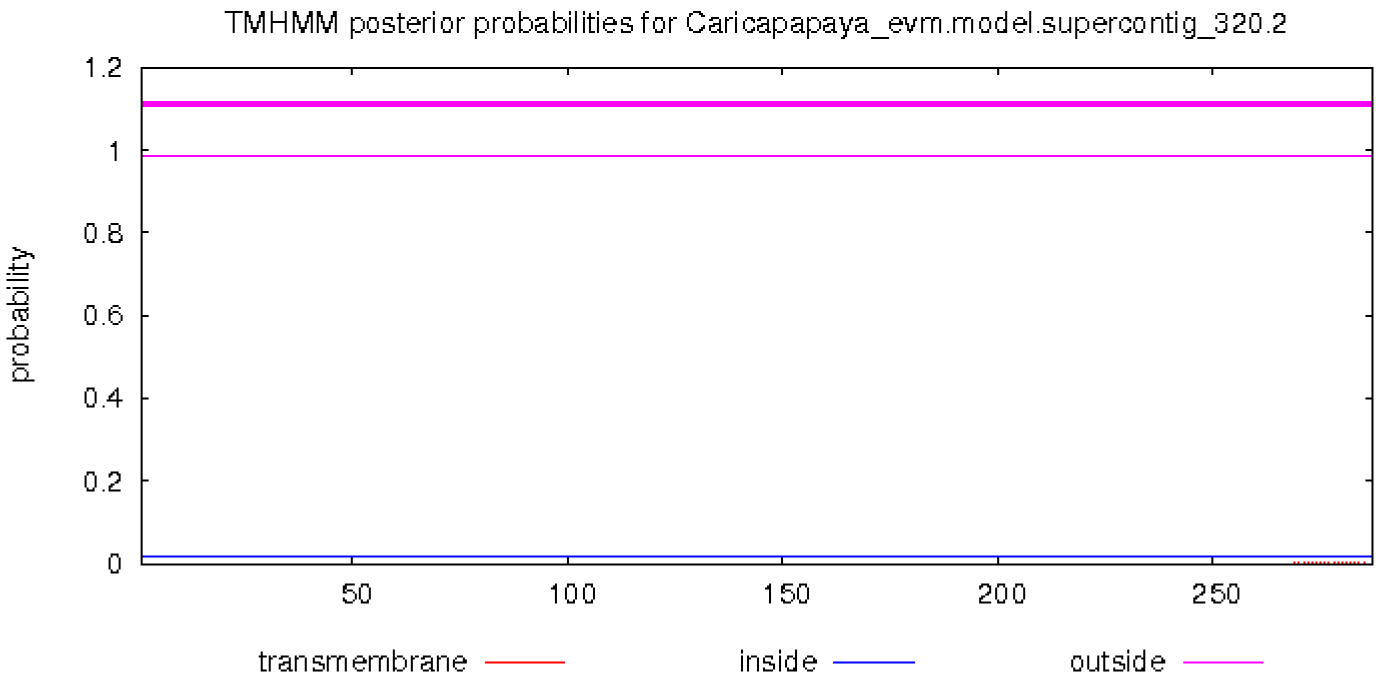

plot in postscript, script for making the plot in gnuplot, data for plot

```
# Caricapapaya_evm.model.supercontig_3.311 Length: 346
# Caricapapaya_evm.model.supercontig_3.311 Number of predicted TMHs:  0
# Caricapapaya_evm.model.supercontig_3.311 Exp number of AAs in TMHs: 0
# Caricapapaya_evm.model.supercontig_3.311 Exp number, first 60 AAs:  0
# Caricapapaya_evm.model.supercontig_3.311 Total prob of N-in:        0.01283
Caricapapaya_evm.model.supercontig_3.311        TMHMM2.0        outside      1   346
```

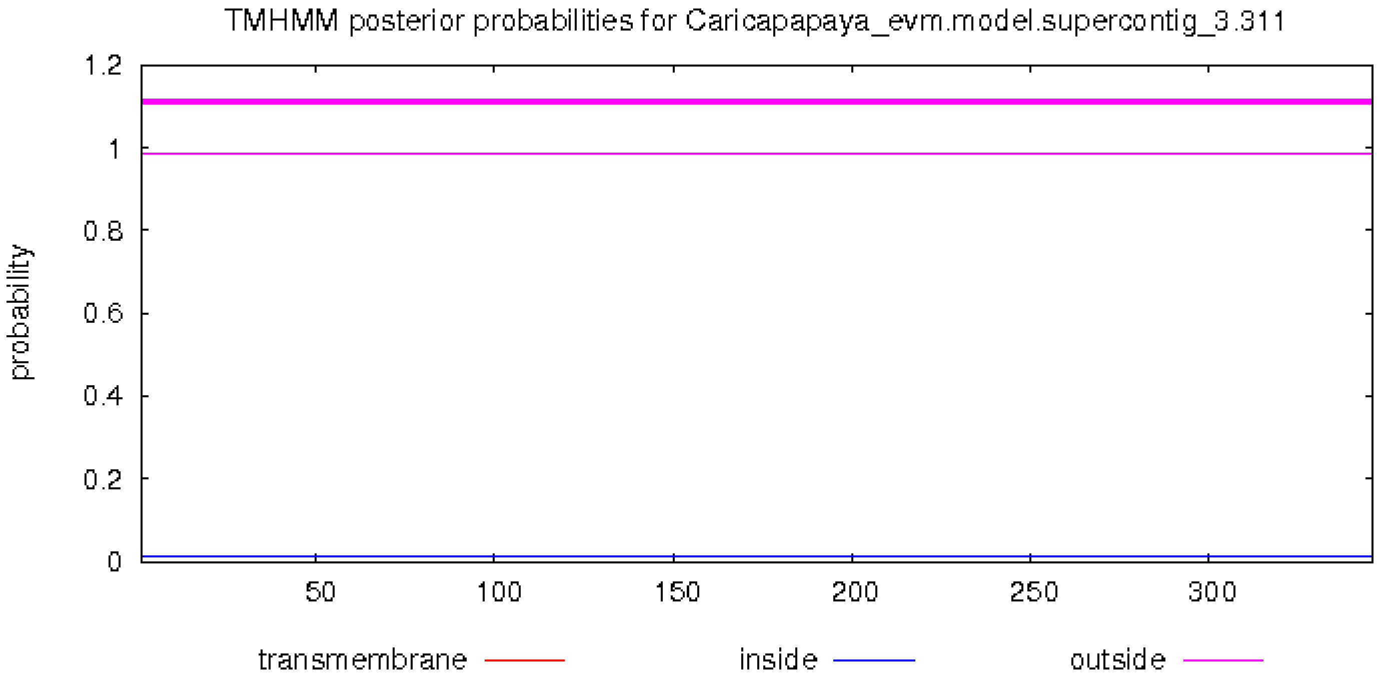

plot in postscript, script for making the plot in gnuplot, data for plot

```
# Caricapapaya_evm.model.supercontig_33.206 Length: 237
# Caricapapaya_evm.model.supercontig_33.206 Number of predicted TMHs:  0
# Caricapapaya_evm.model.supercontig_33.206 Exp number of AAs in TMHs: 0.00089
# Caricapapaya_evm.model.supercontig_33.206 Exp number, first 60 AAs:  7e-05
# Caricapapaya_evm.model.supercontig_33.206 Total prob of N-in:        0.01182
Caricapapaya_evm.model.supercontig_33.206       TMHMM2.0        outside      1   237
```

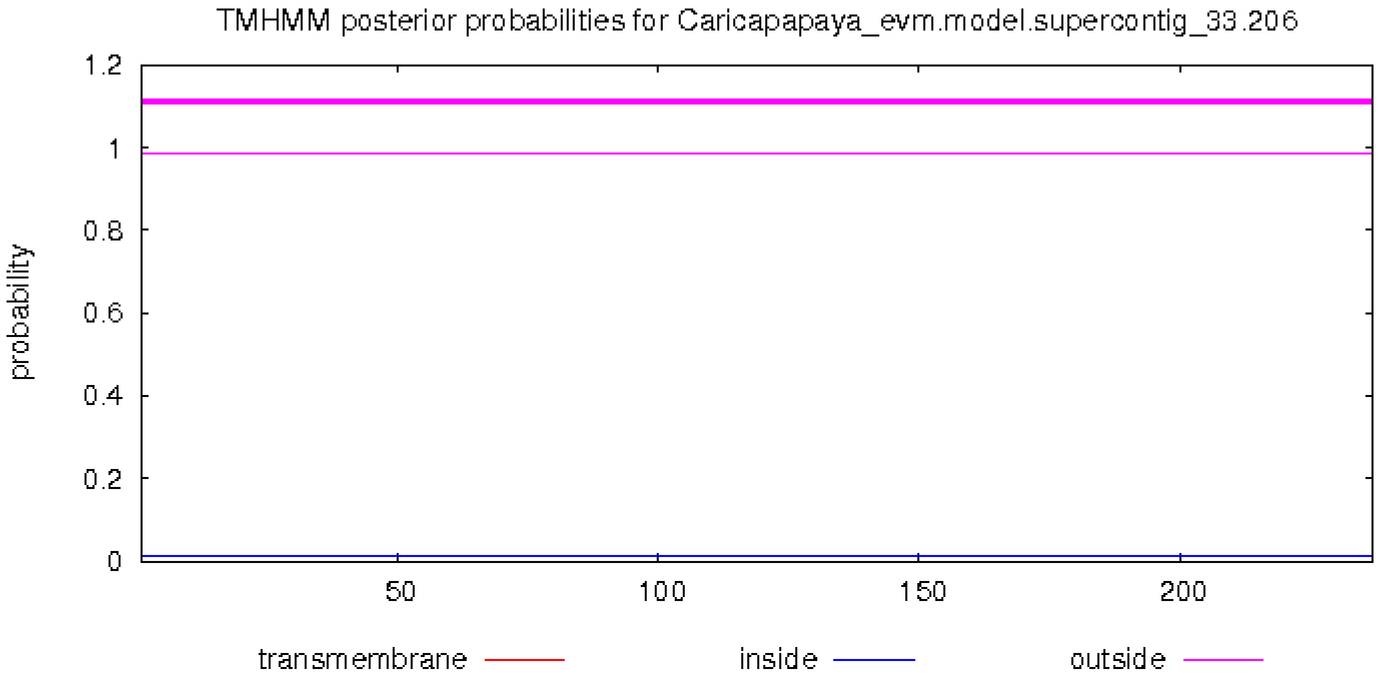

plot in postscript, script for making the plot in gnuplot, data for plot

```
# Caricapapaya_evm.model.supercontig_33.207 Length: 250
# Caricapapaya_evm.model.supercontig_33.207 Number of predicted TMHs:  0
# Caricapapaya_evm.model.supercontig_33.207 Exp number of AAs in TMHs: 0.00118
# Caricapapaya_evm.model.supercontig_33.207 Exp number, first 60 AAs:  0.001
# Caricapapaya_evm.model.supercontig_33.207 Total prob of N-in:        0.02460
Caricapapaya_evm.model.supercontig_33.207	TMHMM2.0	outside	     1   250
```

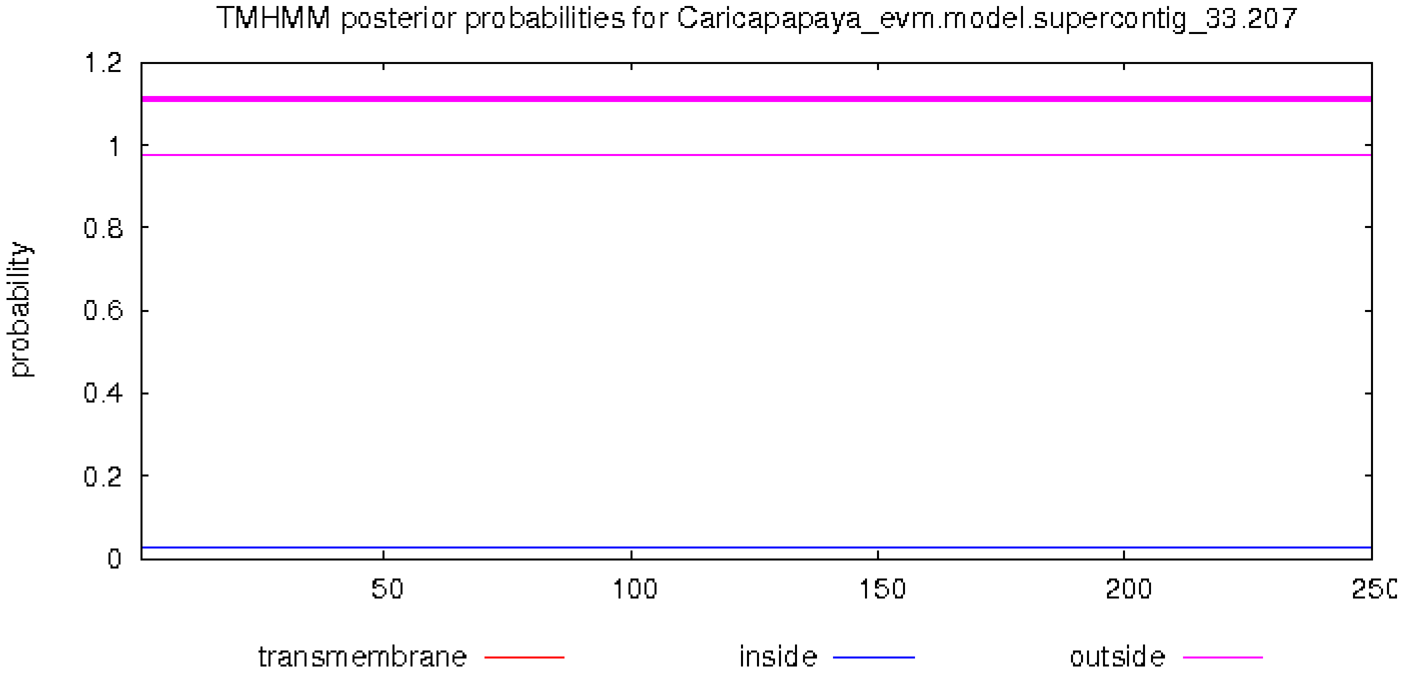

plot in postscript, script for making the plot in gnuplot, data for plot

---

```
# Caricapapaya_evm.model.supercontig_3.509 Length: 337
# Caricapapaya_evm.model.supercontig_3.509 Number of predicted TMHs:  0
# Caricapapaya_evm.model.supercontig_3.509 Exp number of AAs in TMHs: 0.19431
# Caricapapaya_evm.model.supercontig_3.509 Exp number, first 60 AAs:  0
# Caricapapaya_evm.model.supercontig_3.509 Total prob of N-in:        0.00356
Caricapapaya_evm.model.supercontig_3.509	TMHMM2.0	outside	     1   337
```

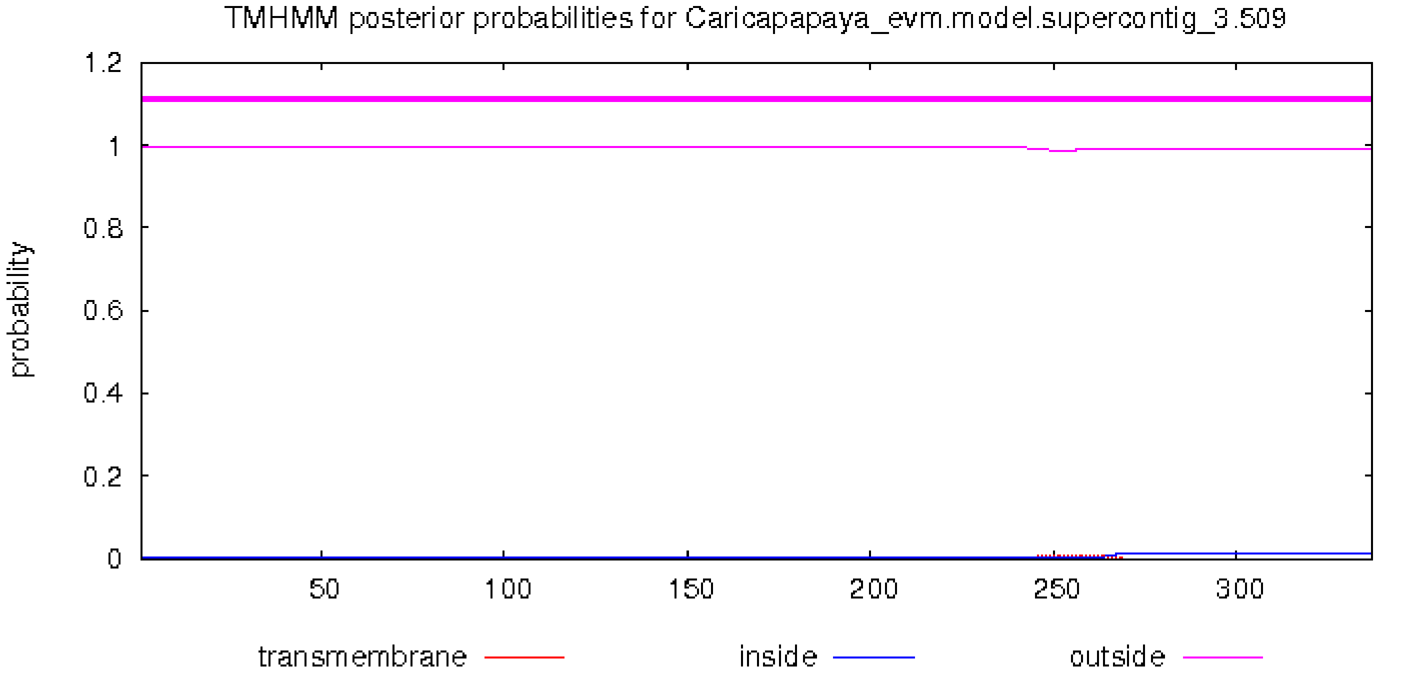

plot in postscript, script for making the plot in gnuplot, data for plot

---

```
# Caricapapaya_evm.model.supercontig_37.112 Length: 631
# Caricapapaya_evm.model.supercontig_37.112 Number of predicted TMHs:  1
# Caricapapaya_evm.model.supercontig_37.112 Exp number of AAs in TMHs: 22.34026
# Caricapapaya_evm.model.supercontig_37.112 Exp number, first 60 AAs:  0
# Caricapapaya_evm.model.supercontig_37.112 Total prob of N-in:        0.00056
Caricapapaya_evm.model.supercontig_37.112       TMHMM2.0        outside      1   604
Caricapapaya_evm.model.supercontig_37.112       TMHMM2.0        TMhelix    605   627
Caricapapaya_evm.model.supercontig_37.112       TMHMM2.0        inside     628   631
```

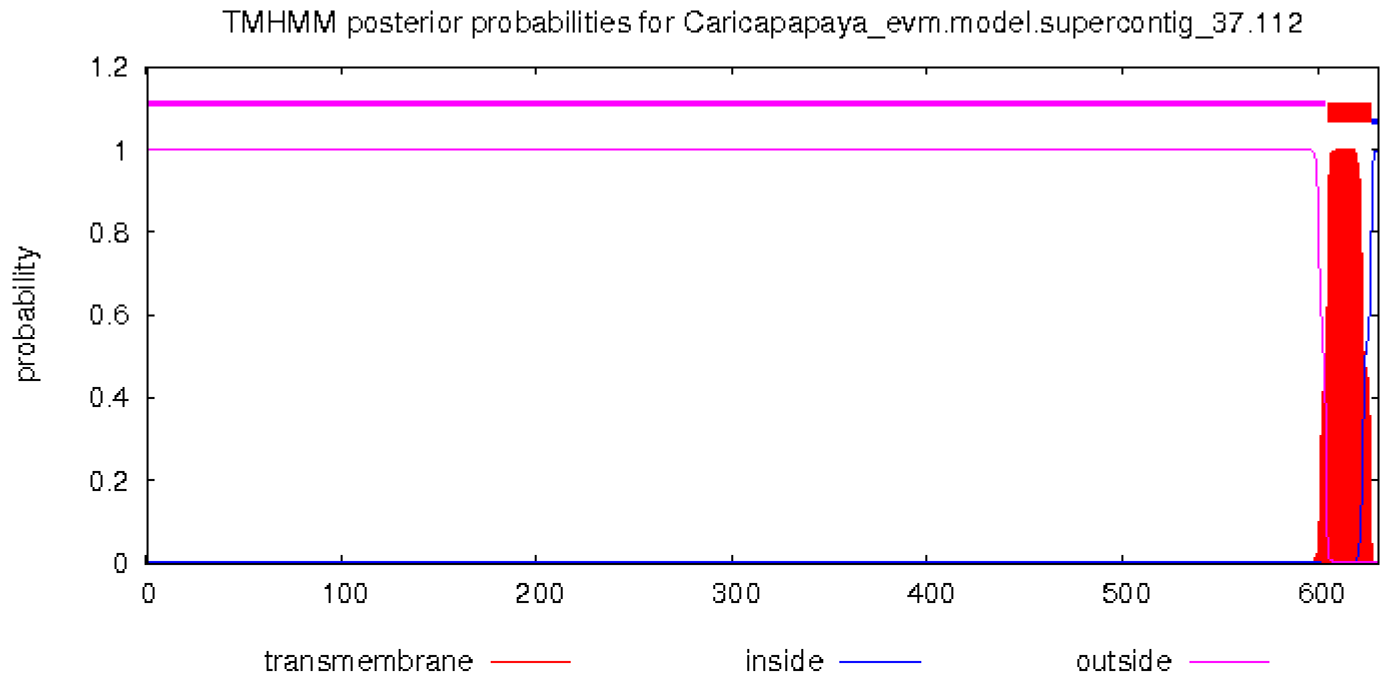

plot in postscript, script for making the plot in gnuplot, data for plot

---

```
# Caricapapaya_evm.model.supercontig_37.28 Length: 276
# Caricapapaya_evm.model.supercontig_37.28 Number of predicted TMHs:  0
# Caricapapaya_evm.model.supercontig_37.28 Exp number of AAs in TMHs: 0
# Caricapapaya_evm.model.supercontig_37.28 Exp number, first 60 AAs:  0
# Caricapapaya_evm.model.supercontig_37.28 Total prob of N-in:        0.00504
Caricapapaya_evm.model.supercontig_37.28        TMHMM2.0        outside      1   276
```

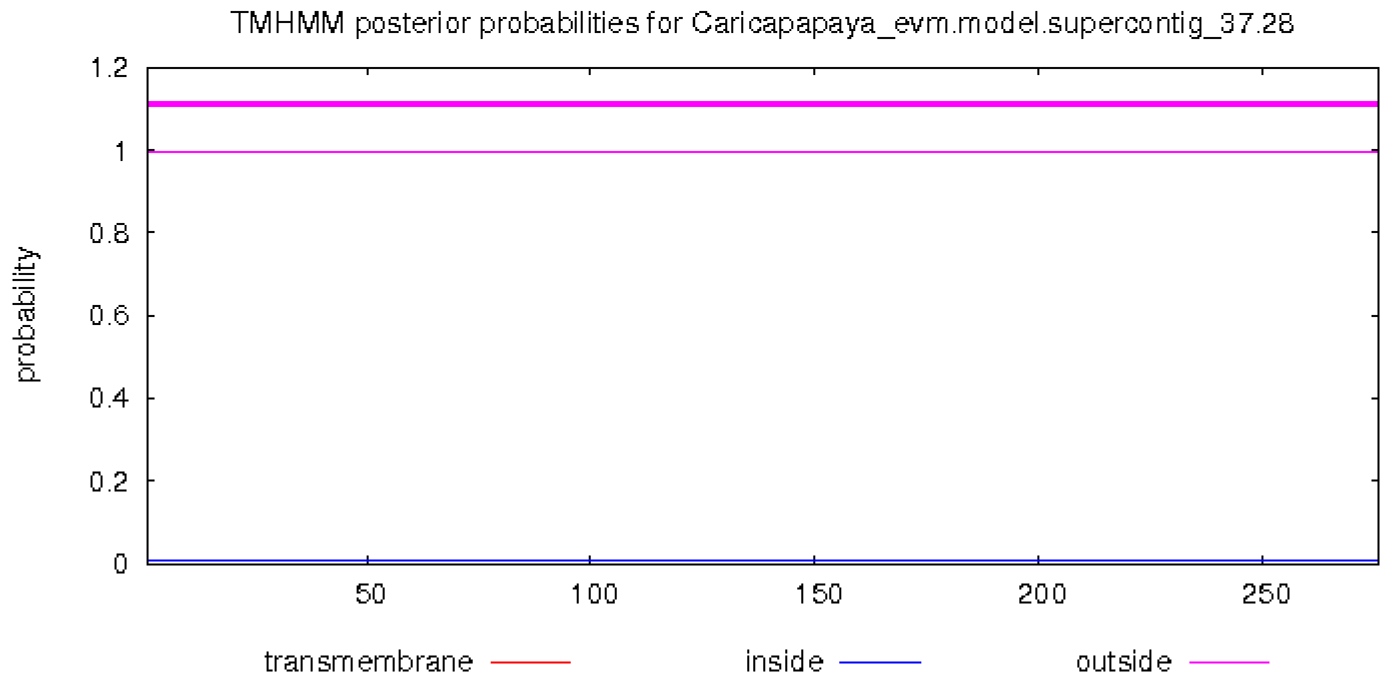

plot in postscript, script for making the plot in gnuplot, data for plot

```
# Caricapapaya_evm.model.supercontig_37.89 Length: 437
# Caricapapaya_evm.model.supercontig_37.89 Number of predicted TMHs:  0
# Caricapapaya_evm.model.supercontig_37.89 Exp number of AAs in TMHs: 0
# Caricapapaya_evm.model.supercontig_37.89 Exp number, first 60 AAs:  0
# Caricapapaya_evm.model.supercontig_37.89 Total prob of N-in:        0.00248
Caricapapaya_evm.model.supercontig_37.89	TMHMM2.0	outside	     1   437
```

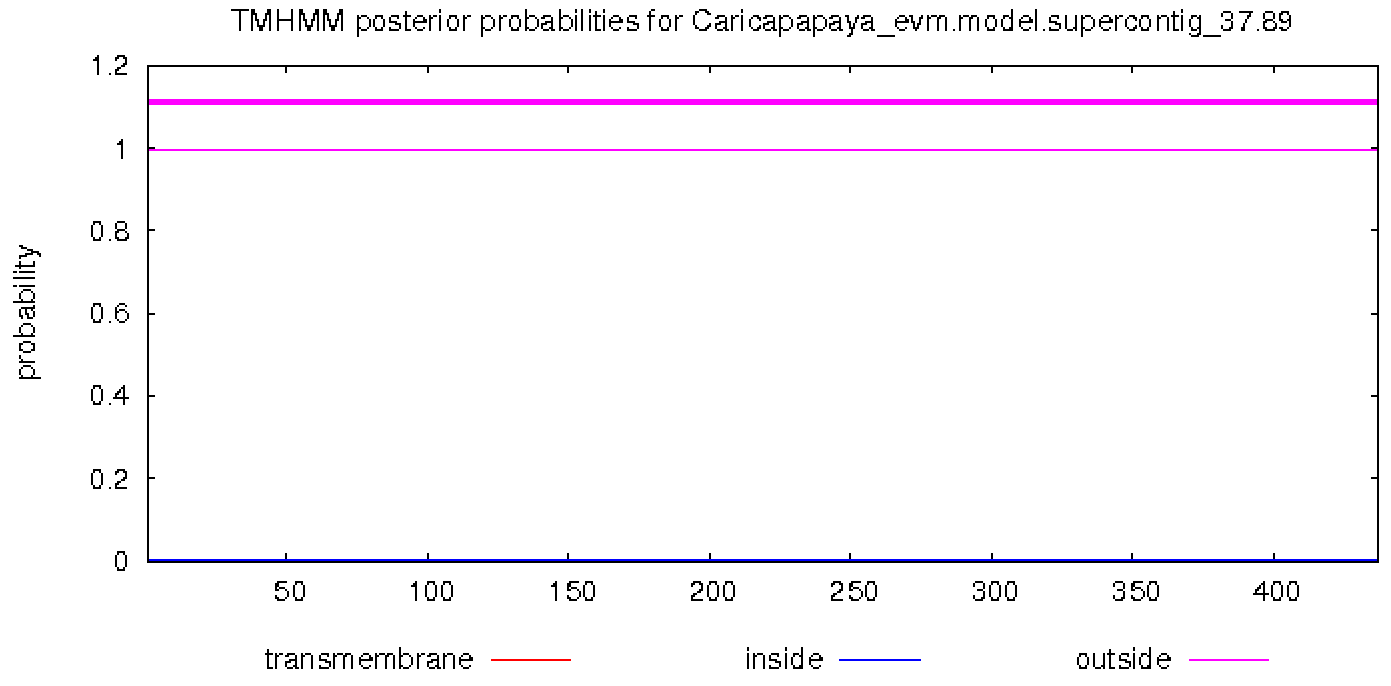

plot in postscript, script for making the plot in gnuplot, data for plot

```
# Caricapapaya_evm.model.supercontig_38.43 Length: 368
# Caricapapaya_evm.model.supercontig_38.43 Number of predicted TMHs:  0
# Caricapapaya_evm.model.supercontig_38.43 Exp number of AAs in TMHs: 0
# Caricapapaya_evm.model.supercontig_38.43 Exp number, first 60 AAs:  0
# Caricapapaya_evm.model.supercontig_38.43 Total prob of N-in:        0.00085
Caricapapaya_evm.model.supercontig_38.43	TMHMM2.0	outside	     1   368
```

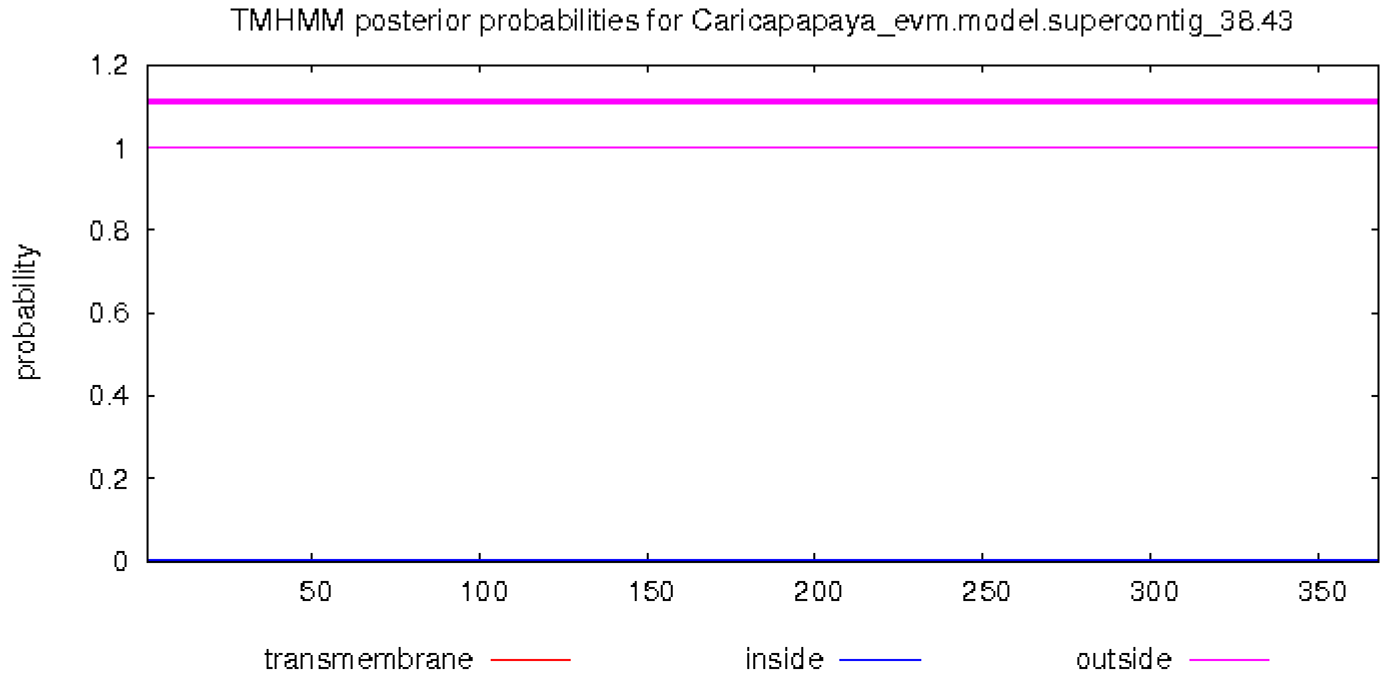

plot in postscript, script for making the plot in gnuplot, data for plot

```
# Caricapapaya_evm.model.supercontig_4056.1 Length: 154
# Caricapapaya_evm.model.supercontig_4056.1 Number of predicted TMHs:  0
# Caricapapaya_evm.model.supercontig_4056.1 Exp number of AAs in TMHs: 0.00826
# Caricapapaya_evm.model.supercontig_4056.1 Exp number, first 60 AAs:  0.00765
# Caricapapaya_evm.model.supercontig_4056.1 Total prob of N-in:        0.16864
Caricapapaya_evm.model.supercontig_4056.1	TMHMM2.0	outside	     1   154
```

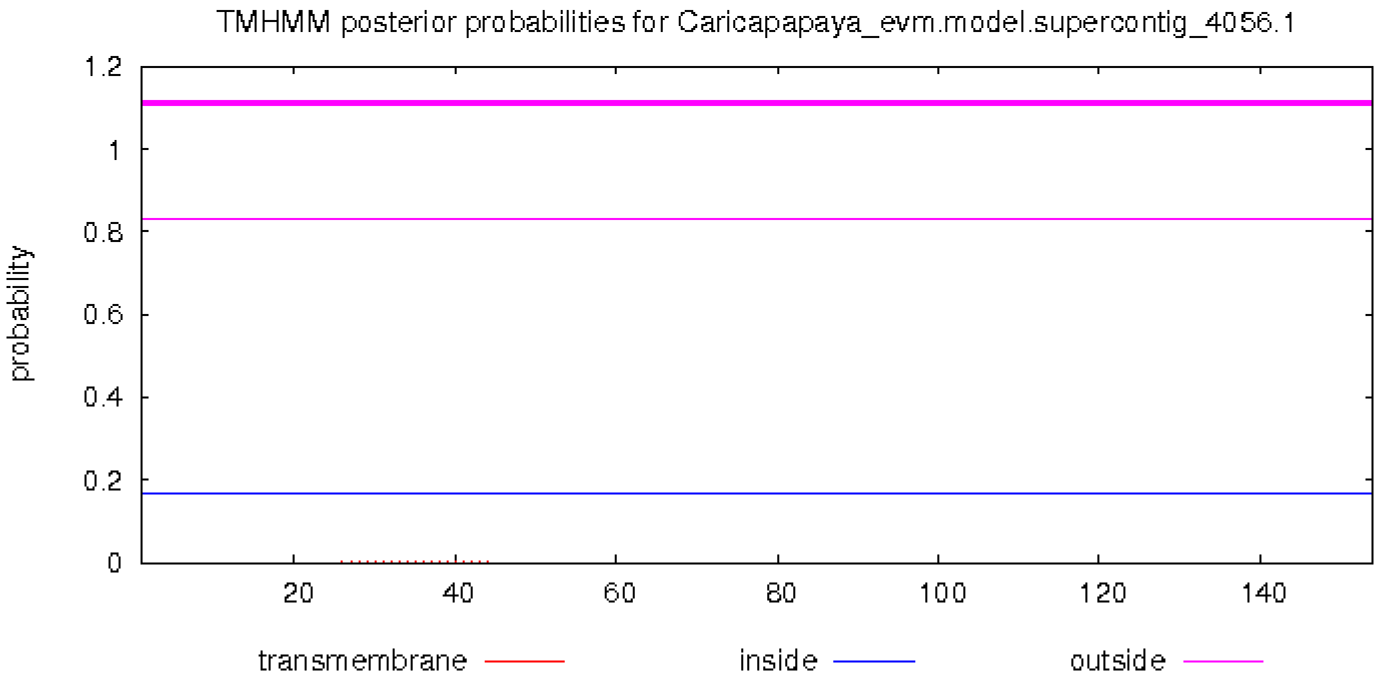

plot in postscript, script for making the plot in gnuplot, data for plot

---

```
# Caricapapaya_evm.model.supercontig_4.138 Length: 227
# Caricapapaya_evm.model.supercontig_4.138 Number of predicted TMHs:  0
# Caricapapaya_evm.model.supercontig_4.138 Exp number of AAs in TMHs: 0.00288
# Caricapapaya_evm.model.supercontig_4.138 Exp number, first 60 AAs:  0.00281
# Caricapapaya_evm.model.supercontig_4.138 Total prob of N-in:        0.06139
Caricapapaya_evm.model.supercontig_4.138	TMHMM2.0	outside	     1   227
```

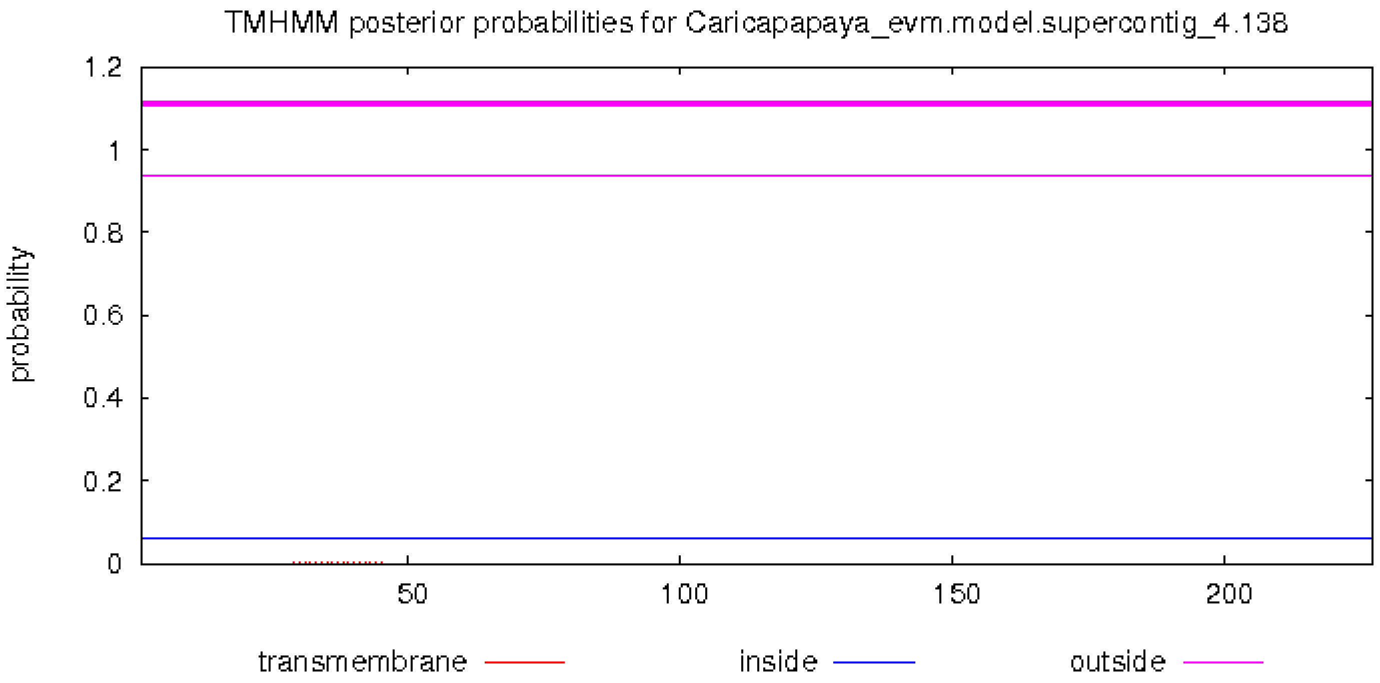

plot in postscript, script for making the plot in gnuplot, data for plot

---

```
# Caricapapaya_evm.model.supercontig_42.100 Length: 85
# Caricapapaya_evm.model.supercontig_42.100 Number of predicted TMHs:  0
# Caricapapaya_evm.model.supercontig_42.100 Exp number of AAs in TMHs: 0.04075
# Caricapapaya_evm.model.supercontig_42.100 Exp number, first 60 AAs:  0.02039
# Caricapapaya_evm.model.supercontig_42.100 Total prob of N-in:        0.52092
Caricapapaya_evm.model.supercontig_42.100       TMHMM2.0        inside       1    85
```

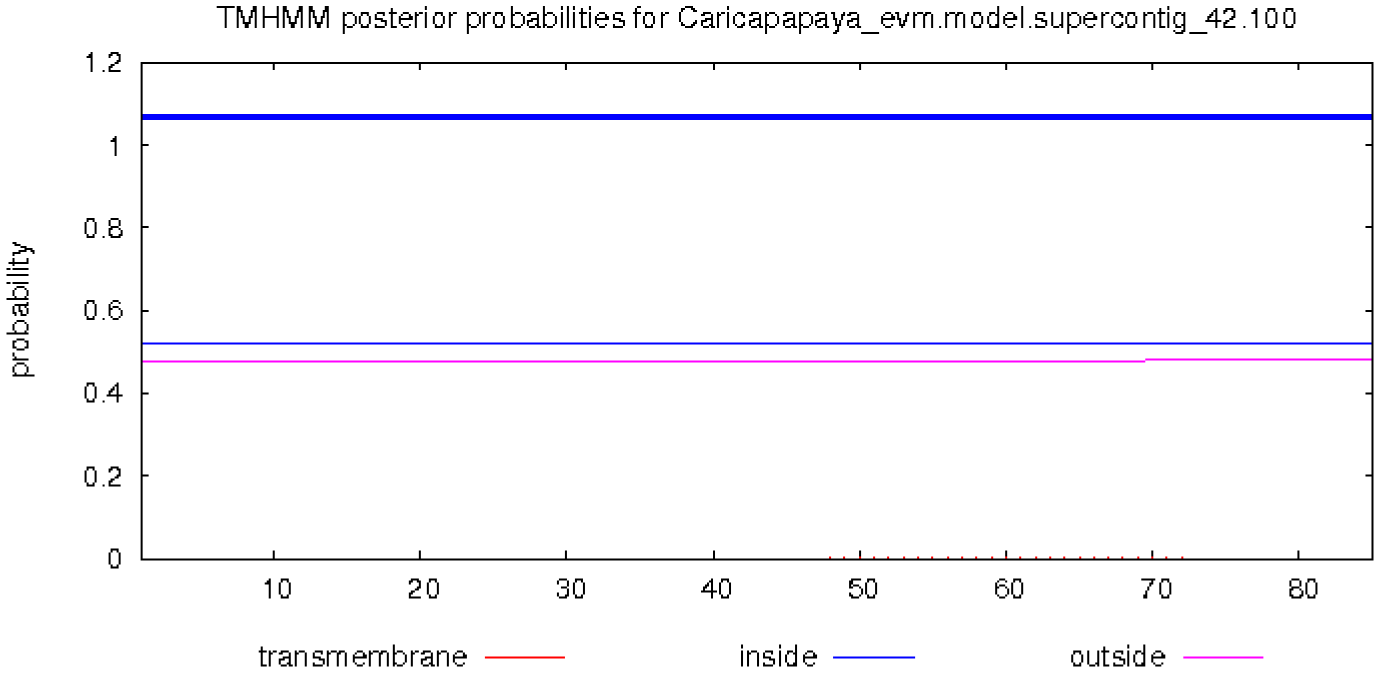

plot in postscript, script for making the plot in gnuplot, data for plot

---

```
# Caricapapaya_evm.model.supercontig_42.145 Length: 376
# Caricapapaya_evm.model.supercontig_42.145 Number of predicted TMHs:  0
# Caricapapaya_evm.model.supercontig_42.145 Exp number of AAs in TMHs: 0
# Caricapapaya_evm.model.supercontig_42.145 Exp number, first 60 AAs:  0
# Caricapapaya_evm.model.supercontig_42.145 Total prob of N-in:        0.00185
Caricapapaya_evm.model.supercontig_42.145       TMHMM2.0        outside      1   376
```

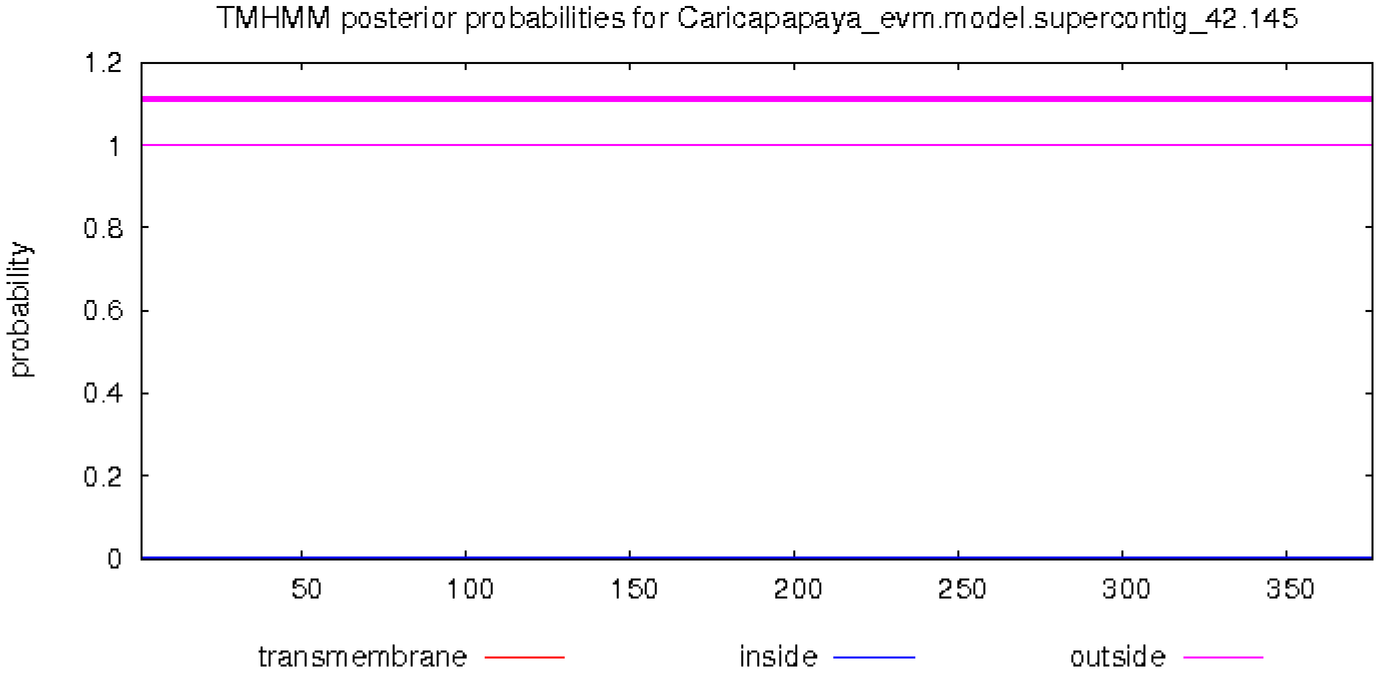

plot in postscript, script for making the plot in gnuplot, data for plot

---

```
# Caricapapaya_evm.model.supercontig_42.167 Length: 202
# Caricapapaya_evm.model.supercontig_42.167 Number of predicted TMHs:  0
# Caricapapaya_evm.model.supercontig_42.167 Exp number of AAs in TMHs: 0.0014
# Caricapapaya_evm.model.supercontig_42.167 Exp number, first 60 AAs:  0.0001
# Caricapapaya_evm.model.supercontig_42.167 Total prob of N-in:        0.01393
Caricapapaya_evm.model.supercontig_42.167       TMHMM2.0        outside      1   202
```

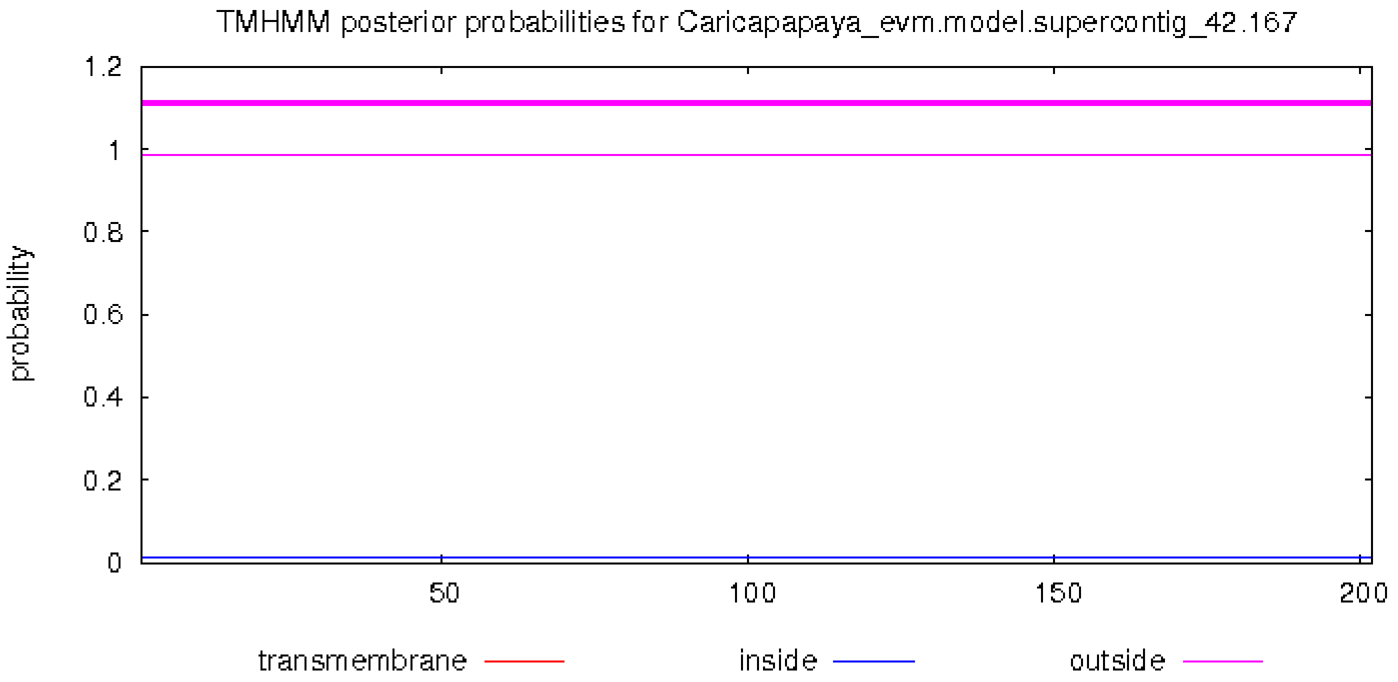

plot in postscript, script for making the plot in gnuplot, data for plot

---

```
# Caricapapaya_evm.model.supercontig_435.1 Length: 198
# Caricapapaya_evm.model.supercontig_435.1 Number of predicted TMHs:  0
# Caricapapaya_evm.model.supercontig_435.1 Exp number of AAs in TMHs: 0.00037
# Caricapapaya_evm.model.supercontig_435.1 Exp number, first 60 AAs:  0
# Caricapapaya_evm.model.supercontig_435.1 Total prob of N-in:        0.03172
Caricapapaya_evm.model.supercontig_435.1        TMHMM2.0        outside      1   198
```

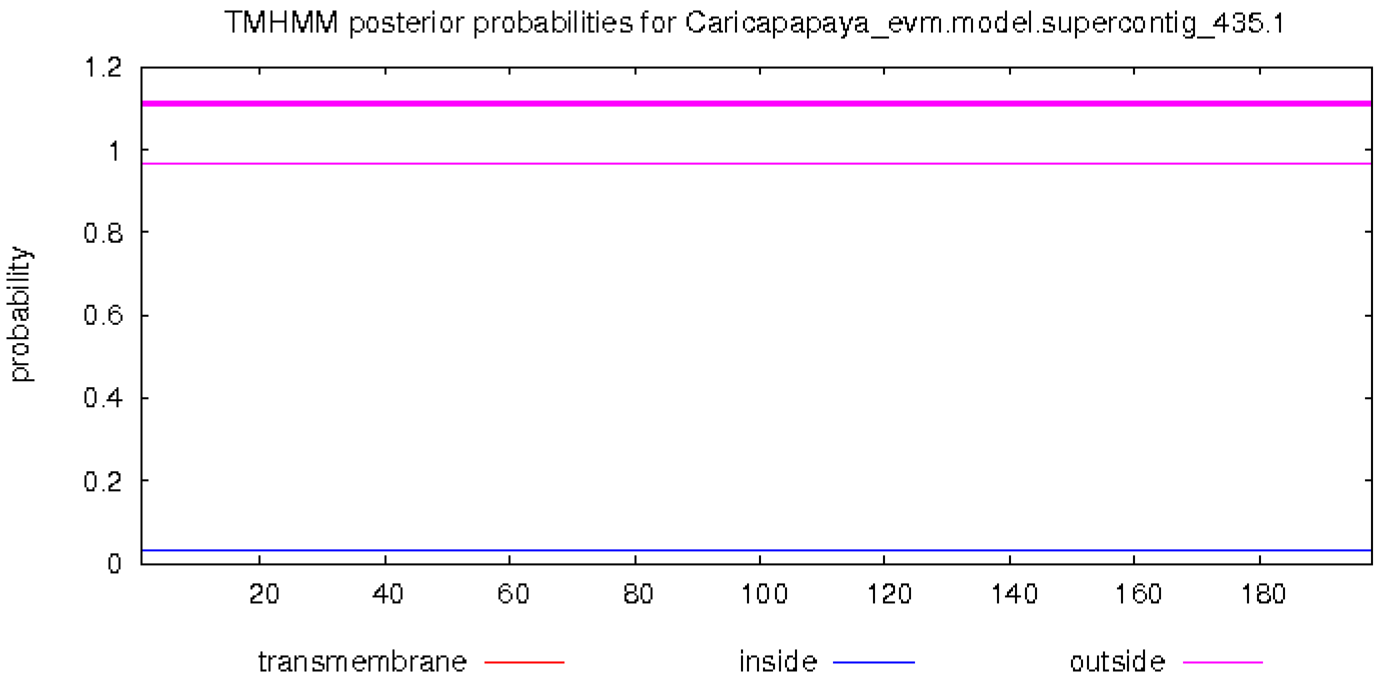

plot in postscript, script for making the plot in gnuplot, data for plot

---

```
# Caricapapaya_evm.model.supercontig_53.116 Length: 448
# Caricapapaya_evm.model.supercontig_53.116 Number of predicted TMHs:  0
# Caricapapaya_evm.model.supercontig_53.116 Exp number of AAs in TMHs: 0.00085
# Caricapapaya_evm.model.supercontig_53.116 Exp number, first 60 AAs:  0.00085
# Caricapapaya_evm.model.supercontig_53.116 Total prob of N-in:        0.00624
Caricapapaya_evm.model.supercontig_53.116	TMHMM2.0	outside	     1   448
```

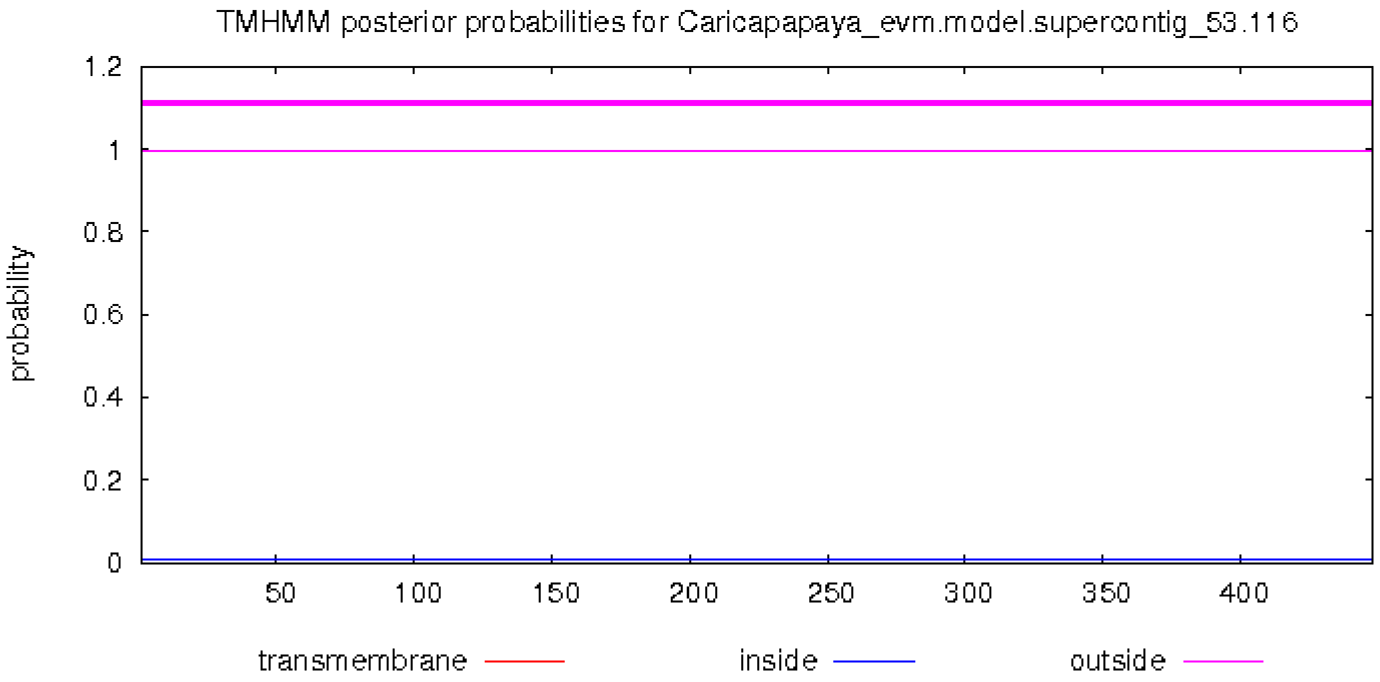

plot in postscript, script for making the plot in gnuplot, data for plot

---

```
# Caricapapaya_evm.model.supercontig_53.164 Length: 134
# Caricapapaya_evm.model.supercontig_53.164 Number of predicted TMHs:  0
# Caricapapaya_evm.model.supercontig_53.164 Exp number of AAs in TMHs: 0.00546
# Caricapapaya_evm.model.supercontig_53.164 Exp number, first 60 AAs:  0.00546
# Caricapapaya_evm.model.supercontig_53.164 Total prob of N-in:        0.09707
Caricapapaya_evm.model.supercontig_53.164	TMHMM2.0	outside	     1   134
```

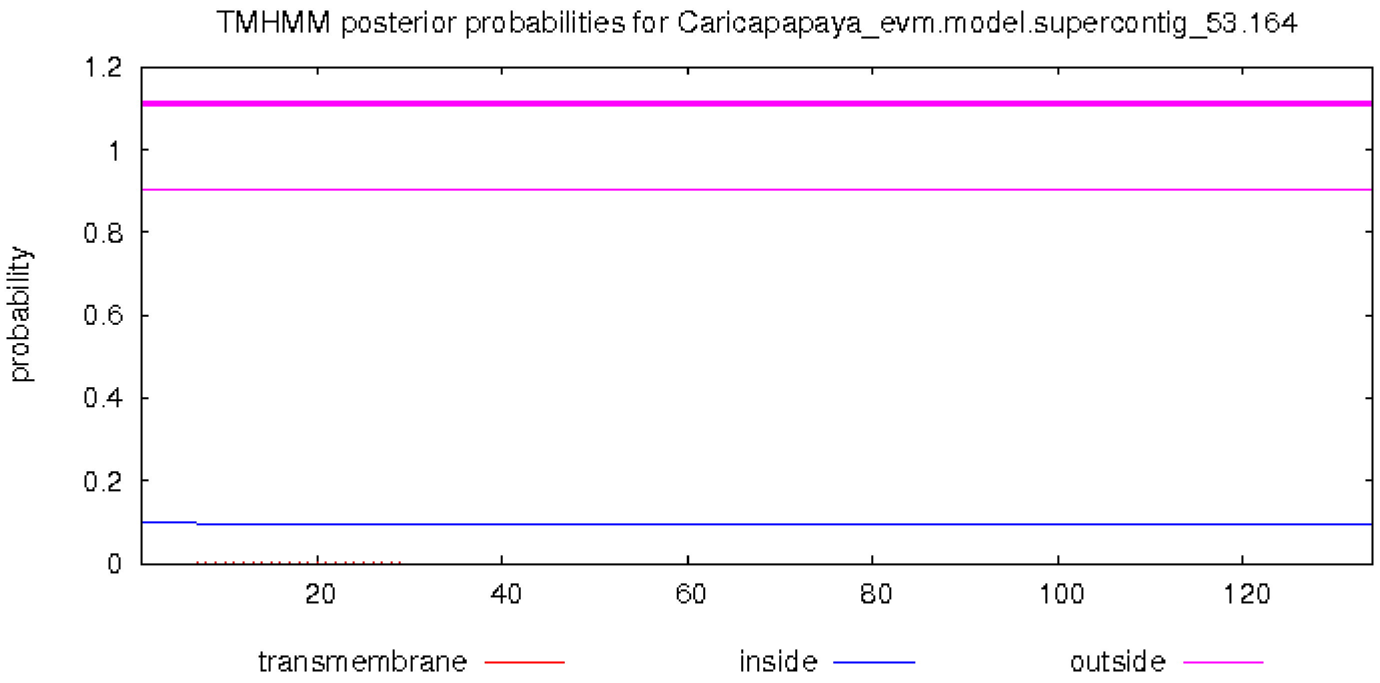

plot in postscript, script for making the plot in gnuplot, data for plot

---

```
# Caricapapaya_evm.model.supercontig_566.2 Length: 281
# Caricapapaya_evm.model.supercontig_566.2 Number of predicted TMHs:  0
# Caricapapaya_evm.model.supercontig_566.2 Exp number of AAs in TMHs: 0.00168
# Caricapapaya_evm.model.supercontig_566.2 Exp number, first 60 AAs:  0
# Caricapapaya_evm.model.supercontig_566.2 Total prob of N-in:        0.04959
Caricapapaya_evm.model.supercontig_566.2	TMHMM2.0	outside	     1   281
```

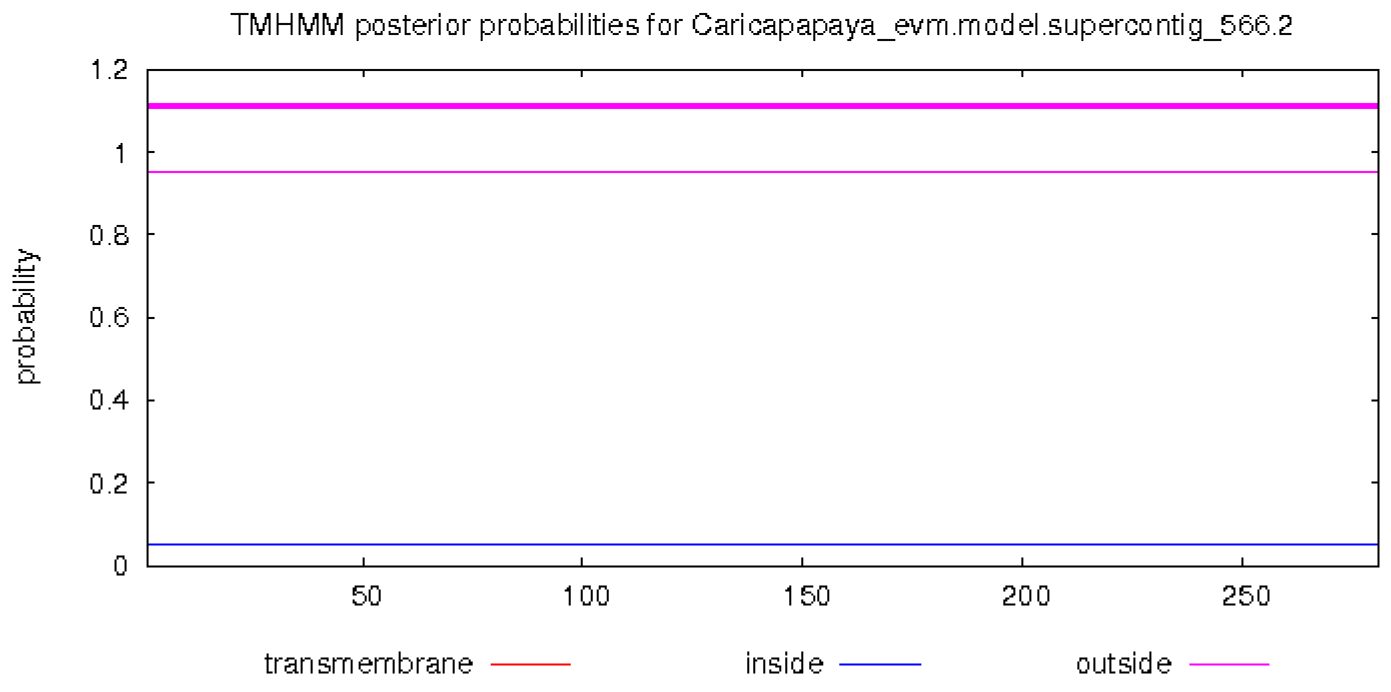

plot in postscript, script for making the plot in gnuplot, data for plot

---

```
# Caricapapaya_evm.model.supercontig_566.3 Length: 93
# Caricapapaya_evm.model.supercontig_566.3 Number of predicted TMHs:  0
# Caricapapaya_evm.model.supercontig_566.3 Exp number of AAs in TMHs: 0.00368
# Caricapapaya_evm.model.supercontig_566.3 Exp number, first 60 AAs:  0
# Caricapapaya_evm.model.supercontig_566.3 Total prob of N-in:        0.40268
Caricapapaya_evm.model.supercontig_566.3	TMHMM2.0	outside	     1    93
```

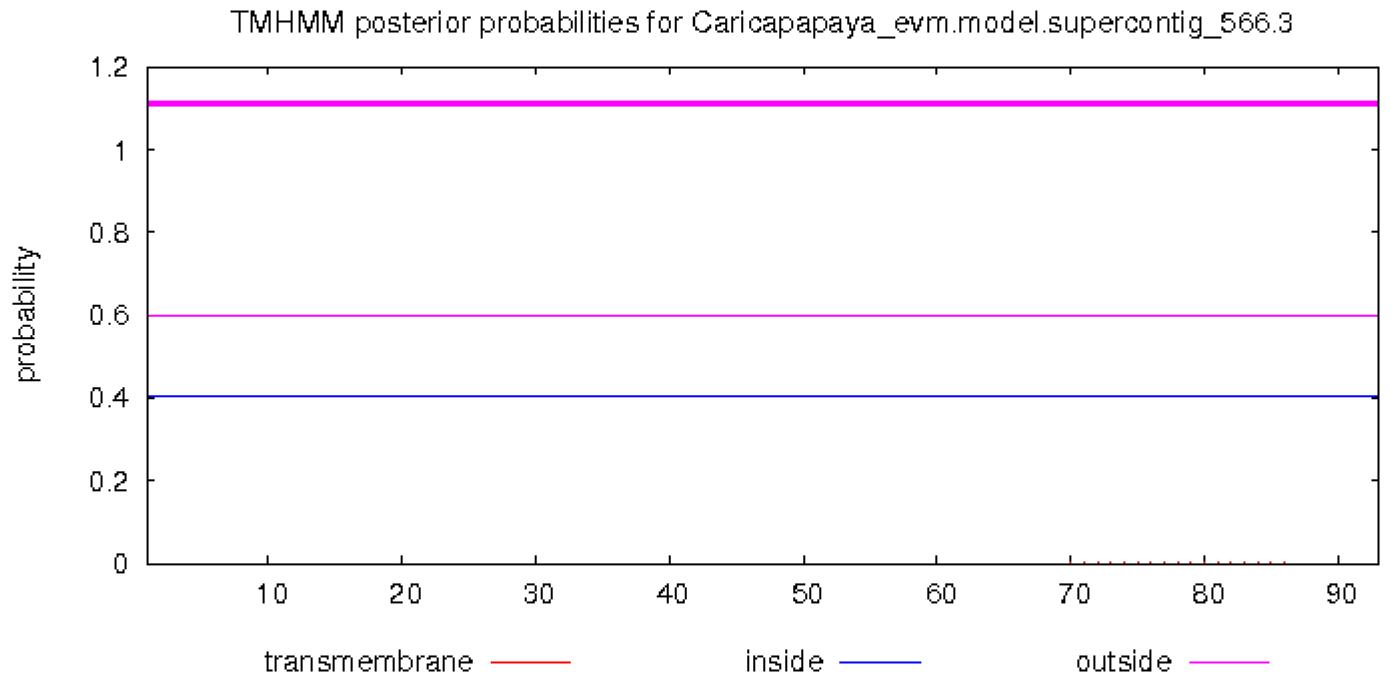

plot in postscript, script for making the plot in gnuplot, data for plot

---

```
# Caricapapaya_evm.model.supercontig_57.70 Length: 505
# Caricapapaya_evm.model.supercontig_57.70 Number of predicted TMHs:  0
# Caricapapaya_evm.model.supercontig_57.70 Exp number of AAs in TMHs: 0.00036
# Caricapapaya_evm.model.supercontig_57.70 Exp number, first 60 AAs:  0.00036
# Caricapapaya_evm.model.supercontig_57.70 Total prob of N-in:        0.00220
Caricapapaya_evm.model.supercontig_57.70	TMHMM2.0	outside	     1   505
```

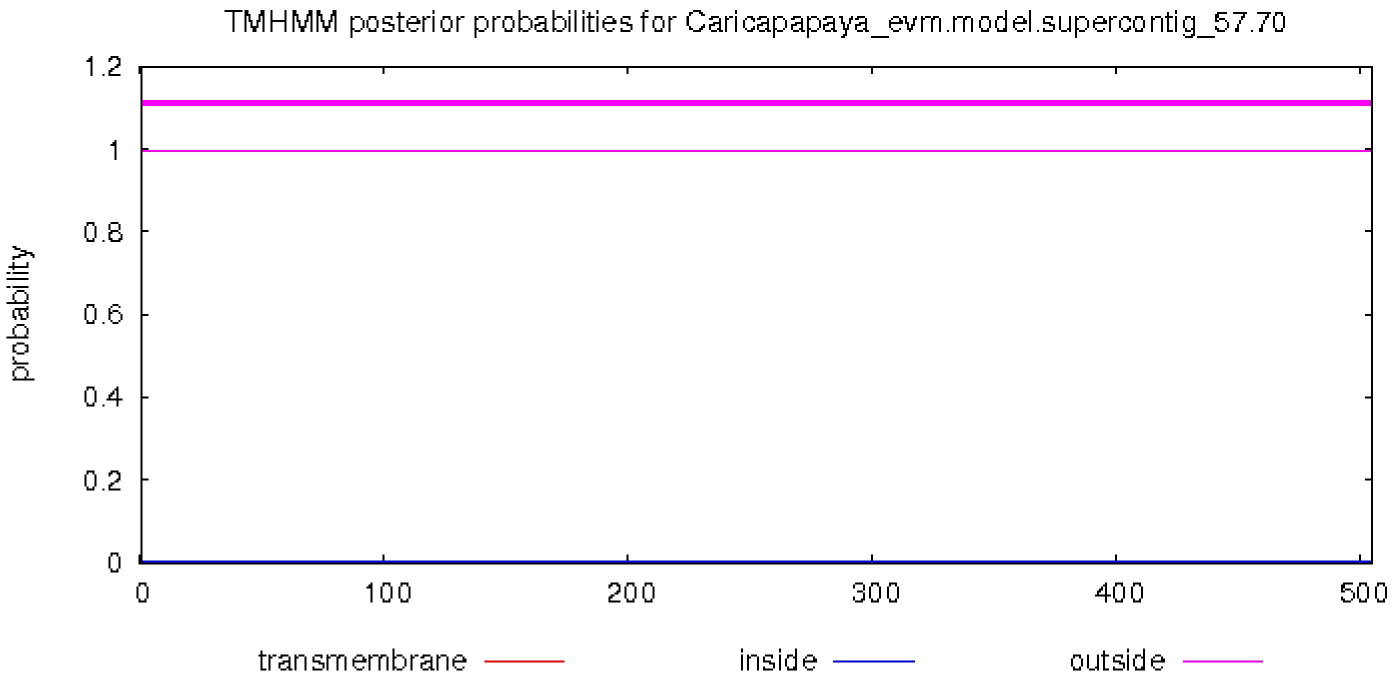

plot in postscript, script for making the plot in gnuplot, data for plot

---

```
# Caricapapaya_evm.model.supercontig_594.1 Length: 373
# Caricapapaya_evm.model.supercontig_594.1 Number of predicted TMHs:  0
# Caricapapaya_evm.model.supercontig_594.1 Exp number of AAs in TMHs: 0.00255
# Caricapapaya_evm.model.supercontig_594.1 Exp number, first 60 AAs:  0.00139
# Caricapapaya_evm.model.supercontig_594.1 Total prob of N-in:        0.03873
Caricapapaya_evm.model.supercontig_594.1	TMHMM2.0	outside	     1   373
```

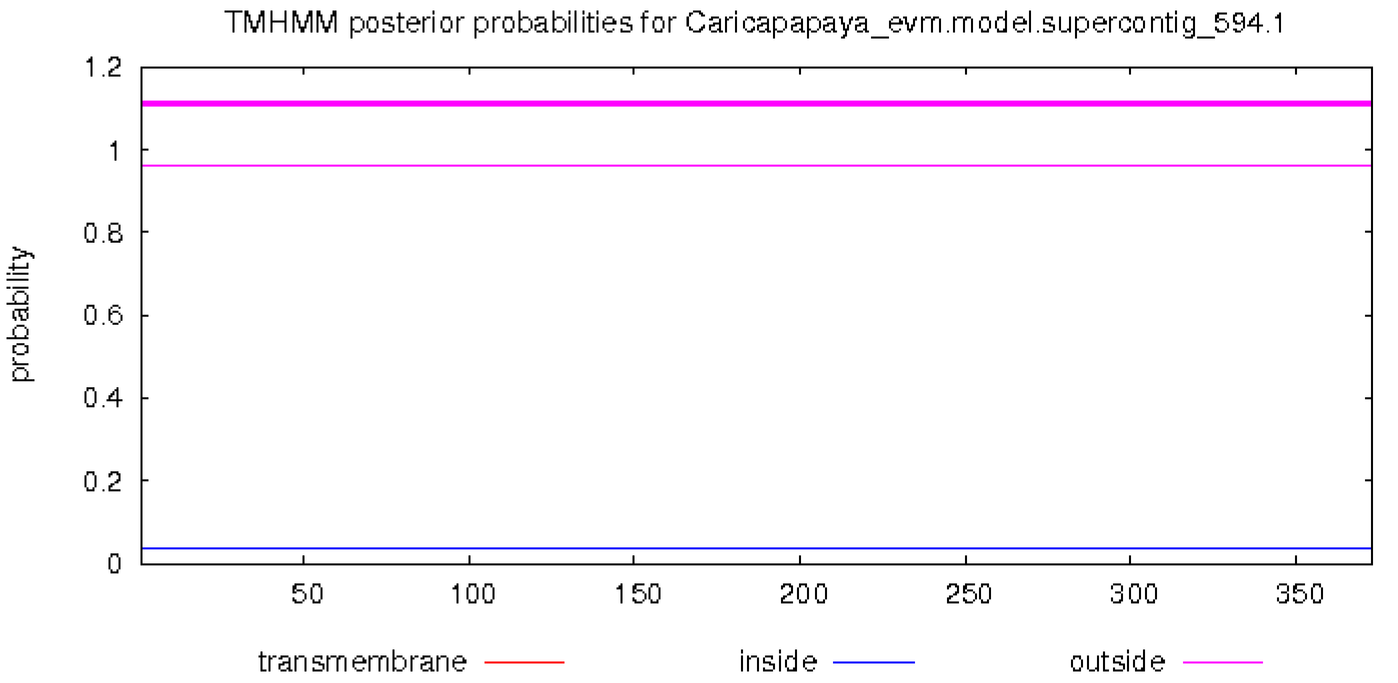

plot in postscript, script for making the plot in gnuplot, data for plot

---

```
# Caricapapaya_evm.model.supercontig_6.226 Length: 158
# Caricapapaya_evm.model.supercontig_6.226 Number of predicted TMHs:  0
# Caricapapaya_evm.model.supercontig_6.226 Exp number of AAs in TMHs: 0.00016
# Caricapapaya_evm.model.supercontig_6.226 Exp number, first 60 AAs:  0
# Caricapapaya_evm.model.supercontig_6.226 Total prob of N-in:        0.06603
Caricapapaya_evm.model.supercontig_6.226	TMHMM2.0	outside	     1   158
```

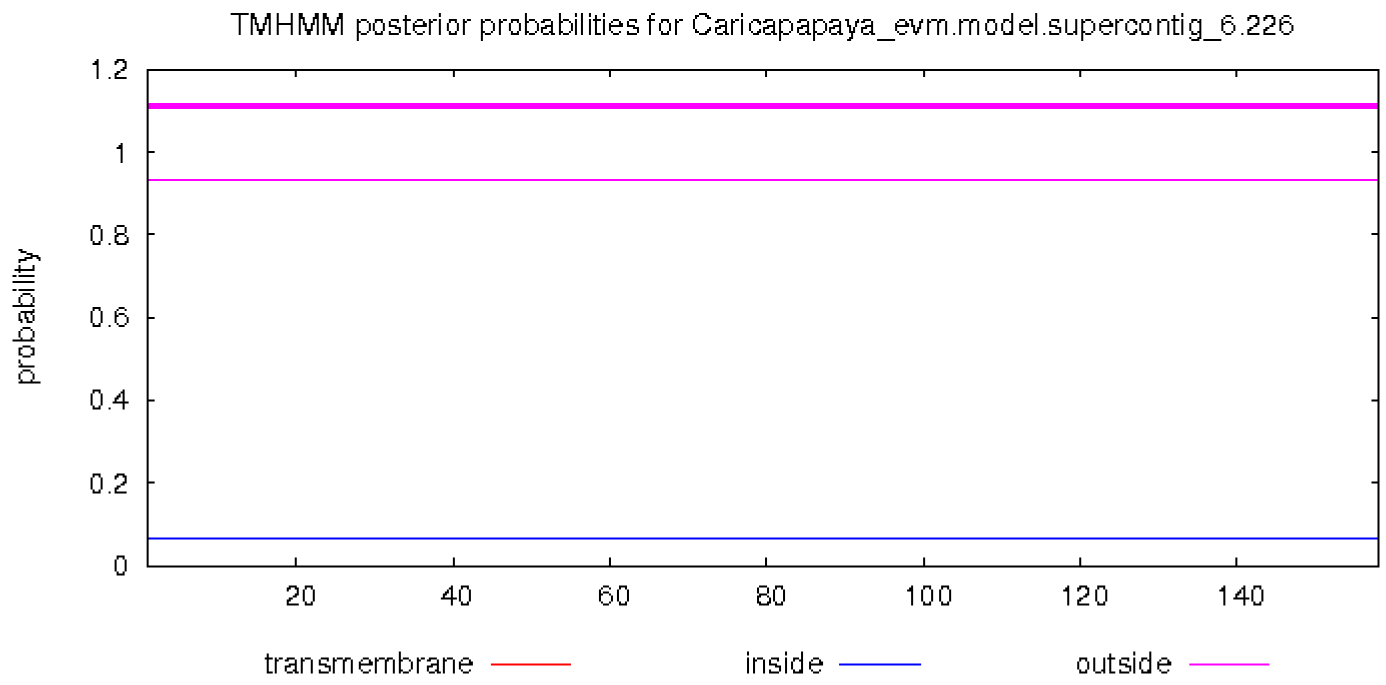

plot in postscript, script for making the plot in gnuplot, data for plot

```
# Caricapapaya_evm.model.supercontig_63.29 Length: 174
# Caricapapaya_evm.model.supercontig_63.29 Number of predicted TMHs:  0
# Caricapapaya_evm.model.supercontig_63.29 Exp number of AAs in TMHs: 0.05634
# Caricapapaya_evm.model.supercontig_63.29 Exp number, first 60 AAs:  0.00036
# Caricapapaya_evm.model.supercontig_63.29 Total prob of N-in:        0.02869
Caricapapaya_evm.model.supercontig_63.29	TMHMM2.0	outside	     1   174
```

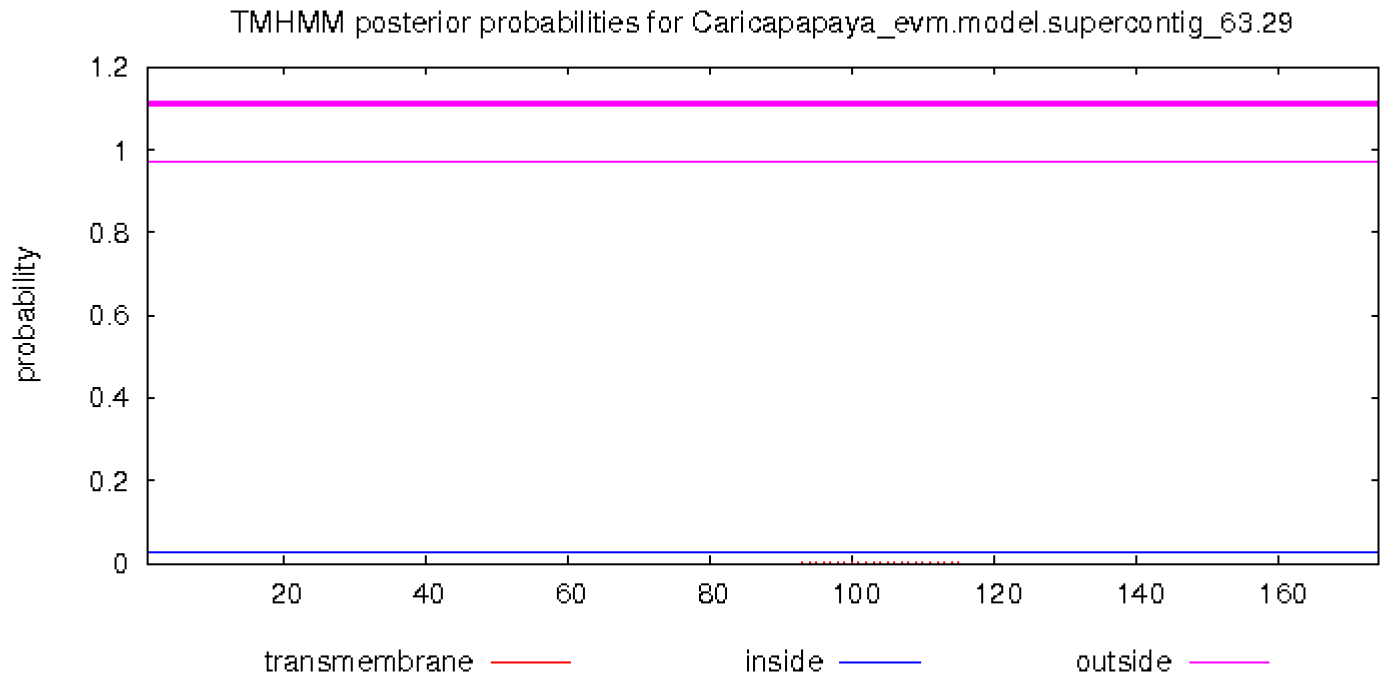

plot in postscript, script for making the plot in gnuplot, data for plot

```
# Caricapapaya_evm.model.supercontig_64.115 Length: 301
# Caricapapaya_evm.model.supercontig_64.115 Number of predicted TMHs:  0
# Caricapapaya_evm.model.supercontig_64.115 Exp number of AAs in TMHs: 0.00065
# Caricapapaya_evm.model.supercontig_64.115 Exp number, first 60 AAs:  0
# Caricapapaya_evm.model.supercontig_64.115 Total prob of N-in:        0.01535
Caricapapaya_evm.model.supercontig_64.115	TMHMM2.0	outside	     1   301
```

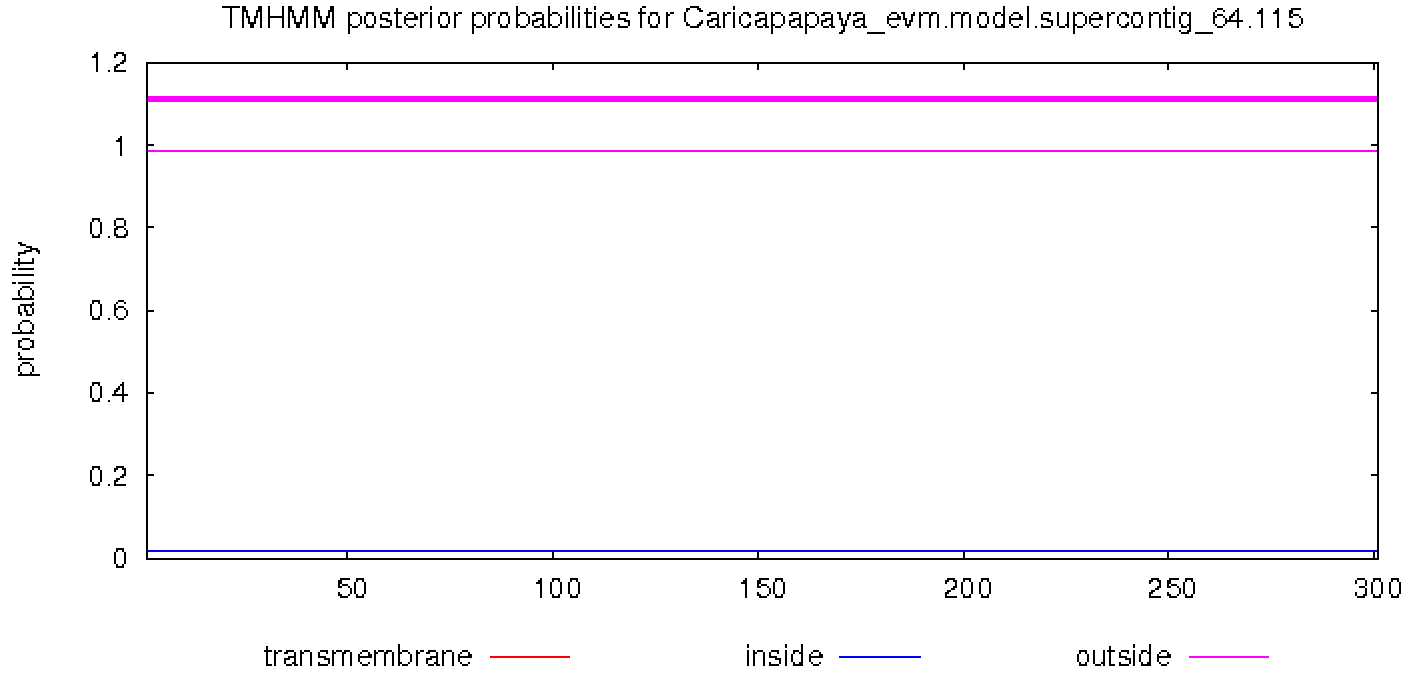

plot in postscript, script for making the plot in gnuplot, data for plot

---

```
# Caricapapaya_evm.model.supercontig_64.53 Length: 299
# Caricapapaya_evm.model.supercontig_64.53 Number of predicted TMHs:  0
# Caricapapaya_evm.model.supercontig_64.53 Exp number of AAs in TMHs: 0.00726
# Caricapapaya_evm.model.supercontig_64.53 Exp number, first 60 AAs:  0.00151
# Caricapapaya_evm.model.supercontig_64.53 Total prob of N-in:        0.01793
Caricapapaya_evm.model.supercontig_64.53	TMHMM2.0	outside	     1   299
```

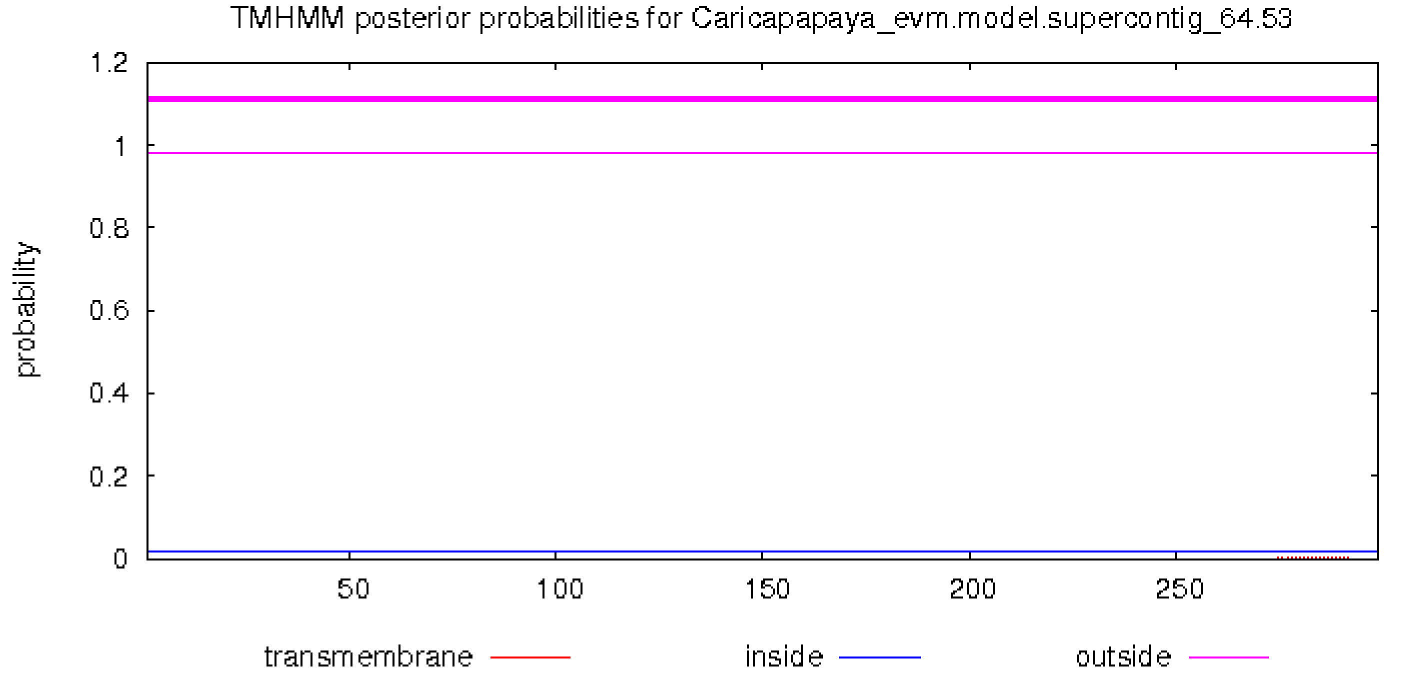

plot in postscript, script for making the plot in gnuplot, data for plot

---

```
# Caricapapaya_evm.model.supercontig_67.60 Length: 296
# Caricapapaya_evm.model.supercontig_67.60 Number of predicted TMHs:  0
# Caricapapaya_evm.model.supercontig_67.60 Exp number of AAs in TMHs: 0.0009
# Caricapapaya_evm.model.supercontig_67.60 Exp number, first 60 AAs:  0
# Caricapapaya_evm.model.supercontig_67.60 Total prob of N-in:        0.10111
Caricapapaya_evm.model.supercontig_67.60        TMHMM2.0        outside      1   296
```

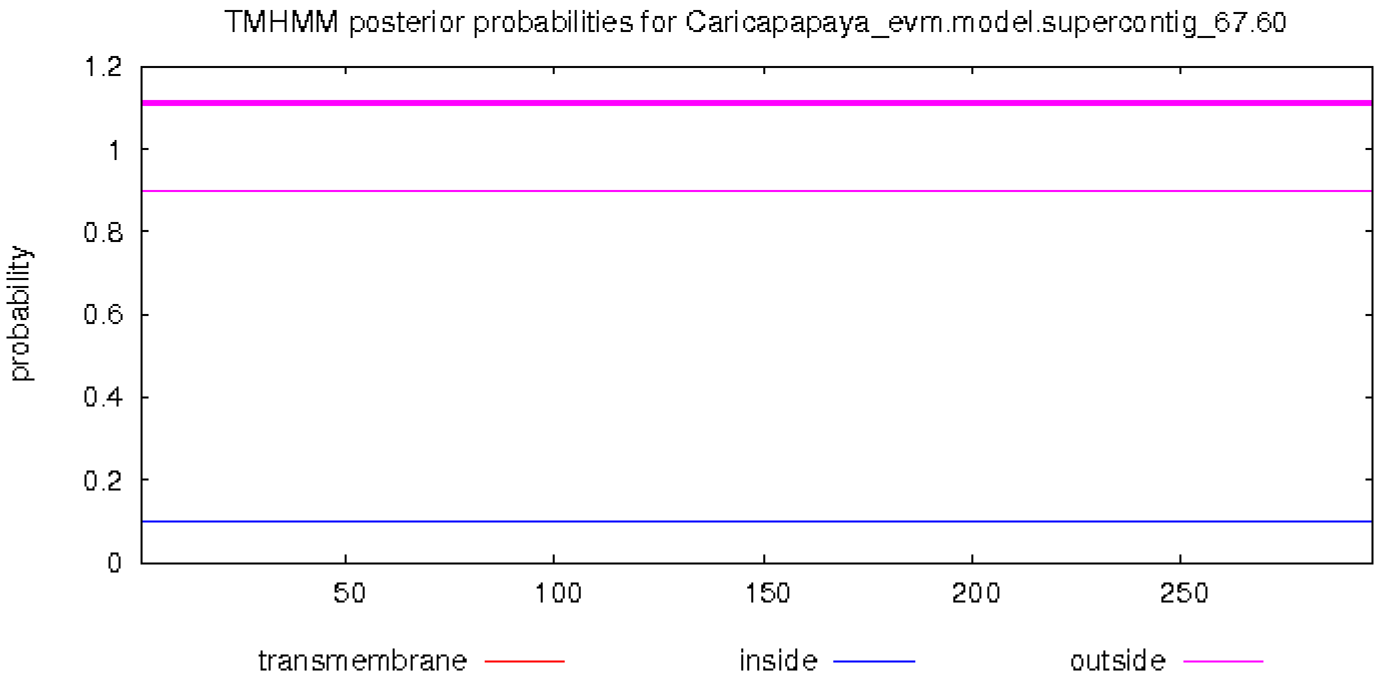

plot in postscript, script for making the plot in gnuplot, data for plot

---

```
# Caricapapaya_evm.model.supercontig_70.104 Length: 230
# Caricapapaya_evm.model.supercontig_70.104 Number of predicted TMHs:  0
# Caricapapaya_evm.model.supercontig_70.104 Exp number of AAs in TMHs: 0.15929
# Caricapapaya_evm.model.supercontig_70.104 Exp number, first 60 AAs:  0.12431
# Caricapapaya_evm.model.supercontig_70.104 Total prob of N-in:        0.04512
Caricapapaya_evm.model.supercontig_70.104       TMHMM2.0        outside      1   230
```

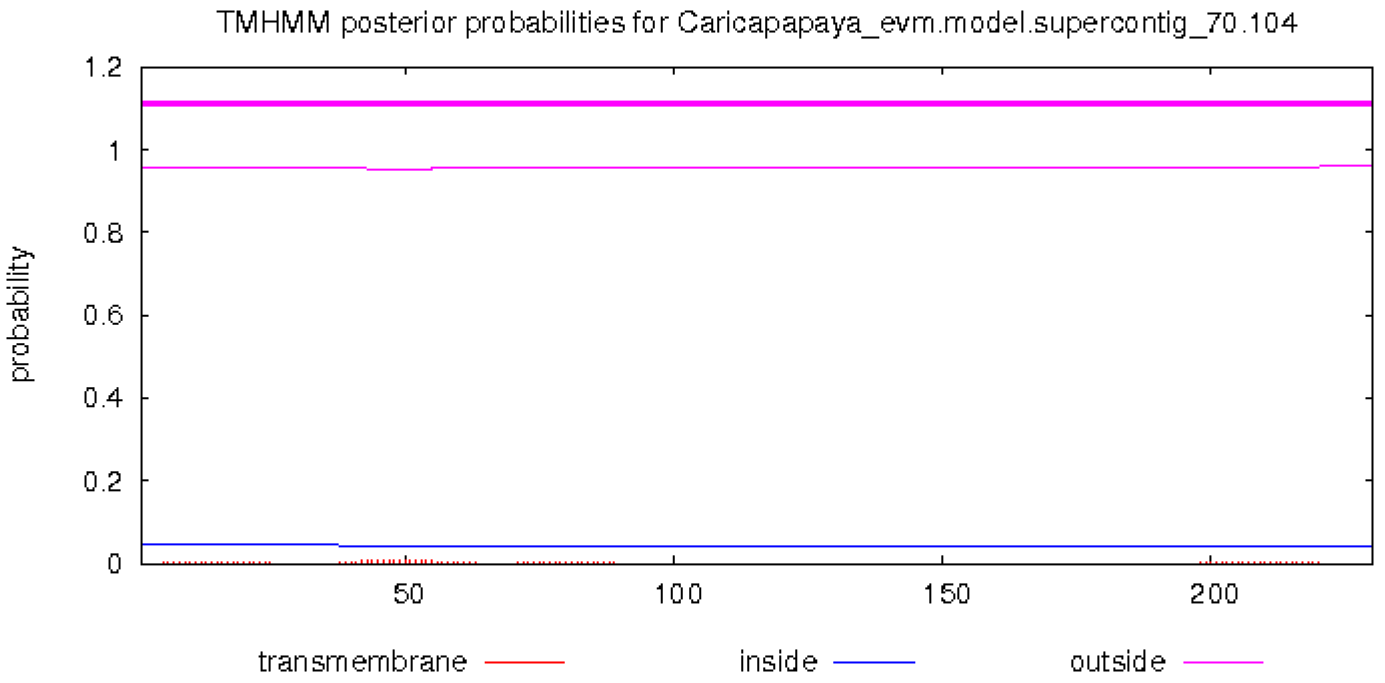

plot in postscript, script for making the plot in gnuplot, data for plot

---

```
# Caricapapaya_evm.model.supercontig_78.40 Length: 449
# Caricapapaya_evm.model.supercontig_78.40 Number of predicted TMHs:  0
# Caricapapaya_evm.model.supercontig_78.40 Exp number of AAs in TMHs: 0
# Caricapapaya_evm.model.supercontig_78.40 Exp number, first 60 AAs:  0
# Caricapapaya_evm.model.supercontig_78.40 Total prob of N-in:        0.00524
Caricapapaya_evm.model.supercontig_78.40	TMHMM2.0	outside	     1   449
```

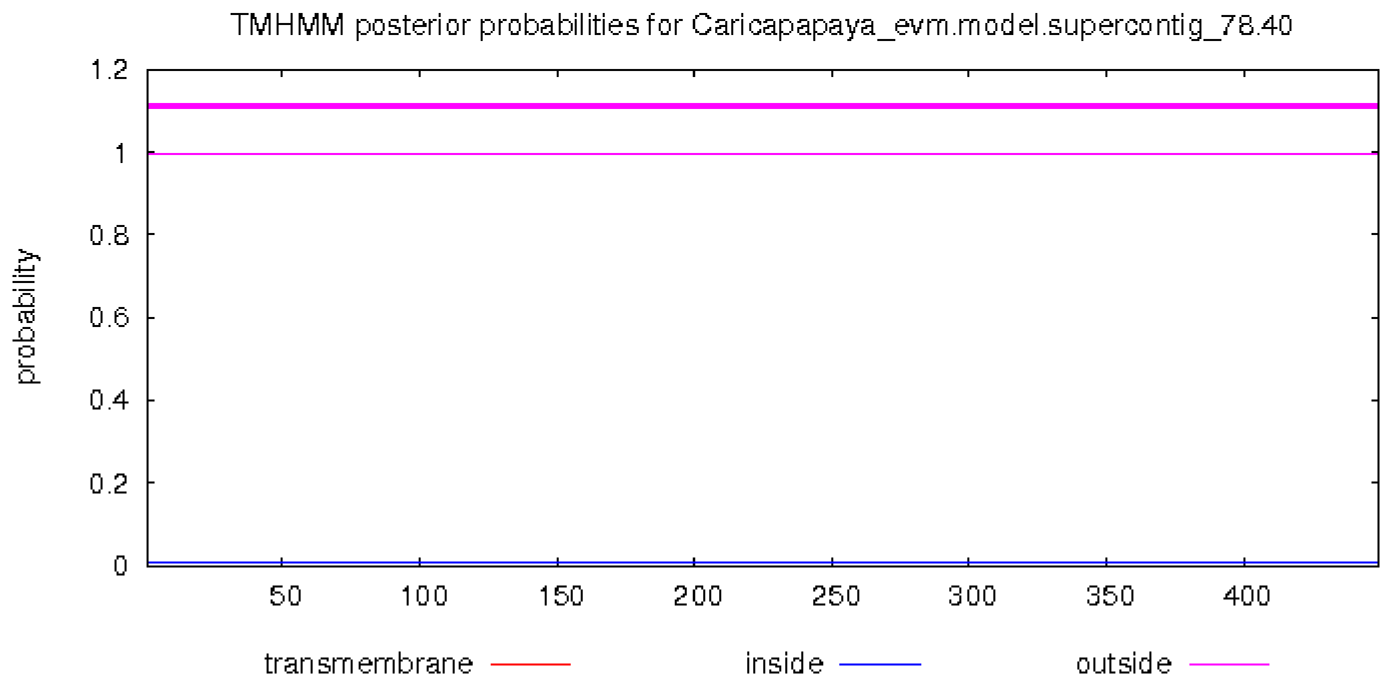

plot in postscript, script for making the plot in gnuplot, data for plot

```
# Caricapapaya_evm.model.supercontig_80.14 Length: 512
# Caricapapaya_evm.model.supercontig_80.14 Number of predicted TMHs:  0
# Caricapapaya_evm.model.supercontig_80.14 Exp number of AAs in TMHs: 0.25774
# Caricapapaya_evm.model.supercontig_80.14 Exp number, first 60 AAs:  0
# Caricapapaya_evm.model.supercontig_80.14 Total prob of N-in:        0.00323
Caricapapaya_evm.model.supercontig_80.14	TMHMM2.0	outside	     1   512
```

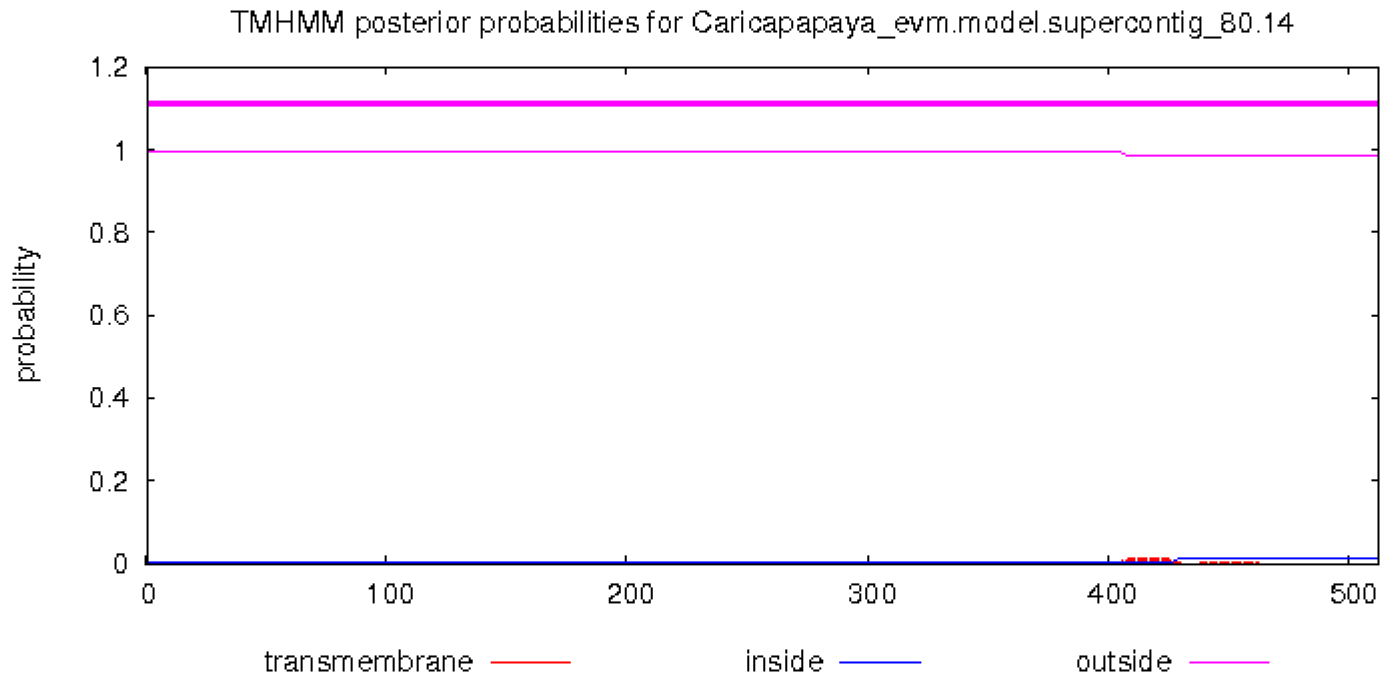

plot in postscript, script for making the plot in gnuplot, data for plot

```
# Caricapapaya_evm.model.supercontig_80.18 Length: 267
# Caricapapaya_evm.model.supercontig_80.18 Number of predicted TMHs:  0
# Caricapapaya_evm.model.supercontig_80.18 Exp number of AAs in TMHs: 0.01288
# Caricapapaya_evm.model.supercontig_80.18 Exp number, first 60 AAs:  0.00016
# Caricapapaya_evm.model.supercontig_80.18 Total prob of N-in:        0.01768
Caricapapaya_evm.model.supercontig_80.18	TMHMM2.0	outside	     1   267
```

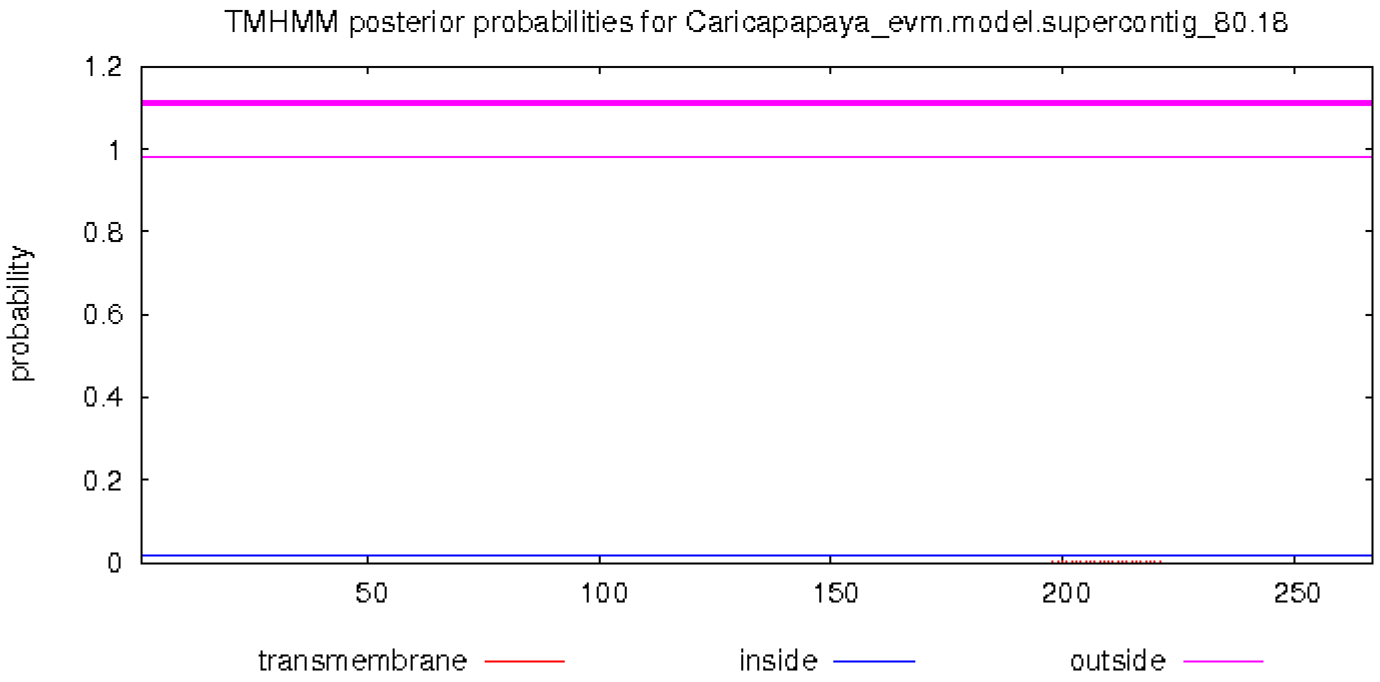

plot in postscript, script for making the plot in gnuplot, data for plot

---

```
# Caricapapaya_evm.model.supercontig_80.19 Length: 393
# Caricapapaya_evm.model.supercontig_80.19 Number of predicted TMHs:  0
# Caricapapaya_evm.model.supercontig_80.19 Exp number of AAs in TMHs: 0.05144
# Caricapapaya_evm.model.supercontig_80.19 Exp number, first 60 AAs:  0
# Caricapapaya_evm.model.supercontig_80.19 Total prob of N-in:        0.00229
Caricapapaya_evm.model.supercontig_80.19	TMHMM2.0	outside	     1   393
```

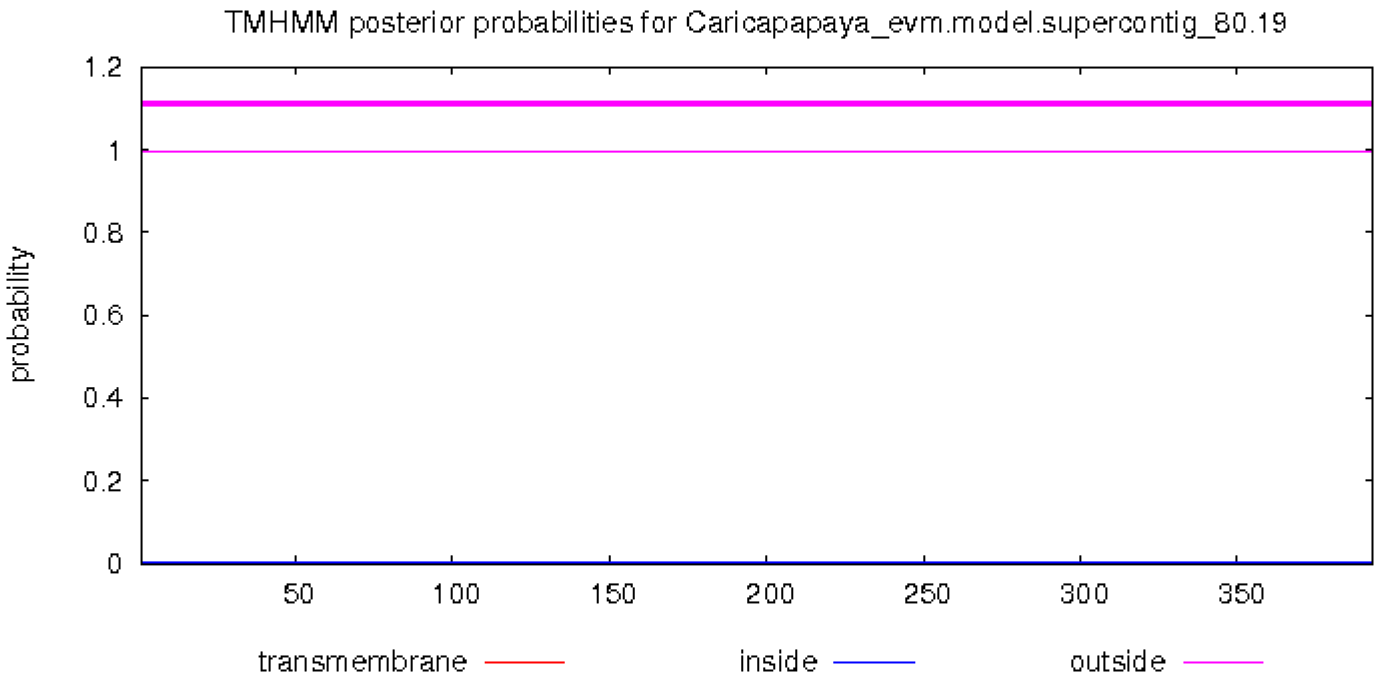

plot in postscript, script for making the plot in gnuplot, data for plot

---

```
# Caricapapaya_evm.model.supercontig_80.20 Length: 365
# Caricapapaya_evm.model.supercontig_80.20 Number of predicted TMHs:  0
# Caricapapaya_evm.model.supercontig_80.20 Exp number of AAs in TMHs: 0.00782
# Caricapapaya_evm.model.supercontig_80.20 Exp number, first 60 AAs:  0
# Caricapapaya_evm.model.supercontig_80.20 Total prob of N-in:        0.00614
Caricapapaya_evm.model.supercontig_80.20        TMHMM2.0        outside      1   365
```

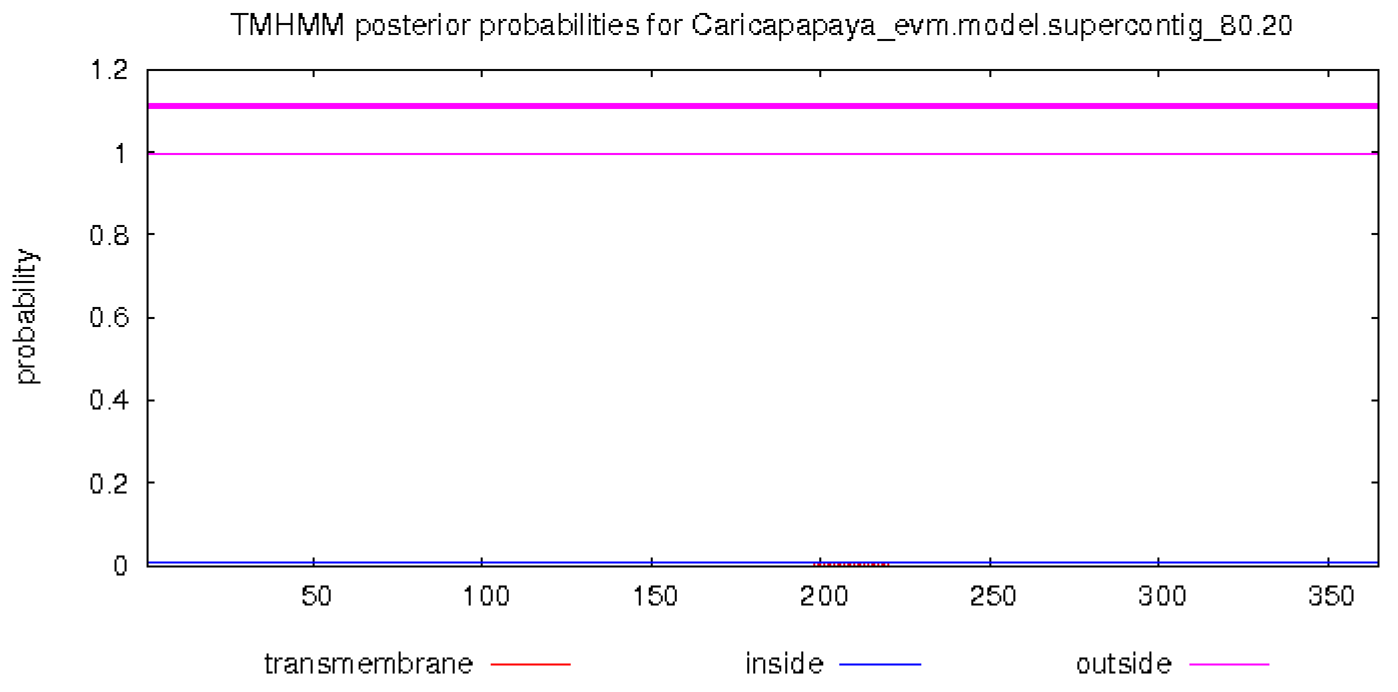

plot in postscript, script for making the plot in gnuplot, data for plot

---

```
# Caricapapaya_evm.model.supercontig_80.21 Length: 322
# Caricapapaya_evm.model.supercontig_80.21 Number of predicted TMHs:  0
# Caricapapaya_evm.model.supercontig_80.21 Exp number of AAs in TMHs: 0.00428
# Caricapapaya_evm.model.supercontig_80.21 Exp number, first 60 AAs:  0
# Caricapapaya_evm.model.supercontig_80.21 Total prob of N-in:        0.00539
Caricapapaya_evm.model.supercontig_80.21        TMHMM2.0        outside      1   322
```

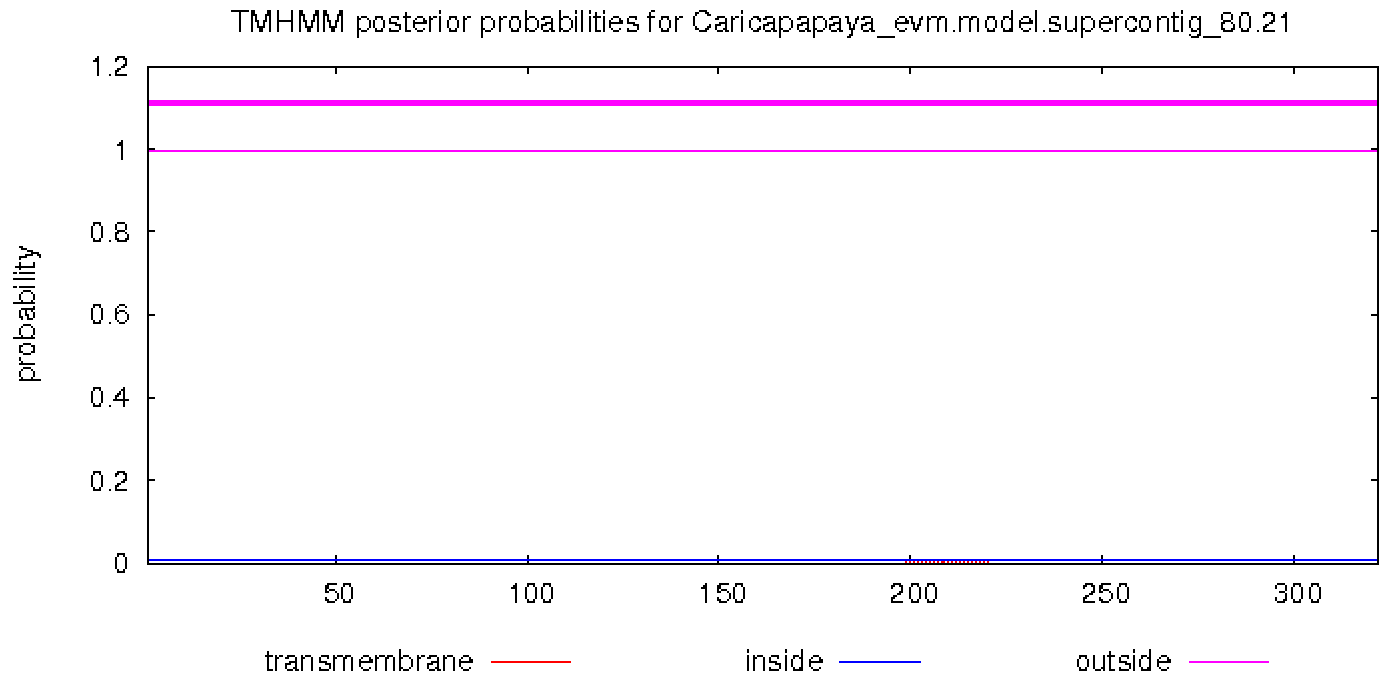

plot in postscript, script for making the plot in gnuplot, data for plot

---

```
# Caricapapaya_evm.model.supercontig_80.23 Length: 440
# Caricapapaya_evm.model.supercontig_80.23 Number of predicted TMHs:  0
# Caricapapaya_evm.model.supercontig_80.23 Exp number of AAs in TMHs: 0.00329
# Caricapapaya_evm.model.supercontig_80.23 Exp number, first 60 AAs:  0
# Caricapapaya_evm.model.supercontig_80.23 Total prob of N-in:        0.00223
Caricapapaya_evm.model.supercontig_80.23        TMHMM2.0        outside      1   440
```

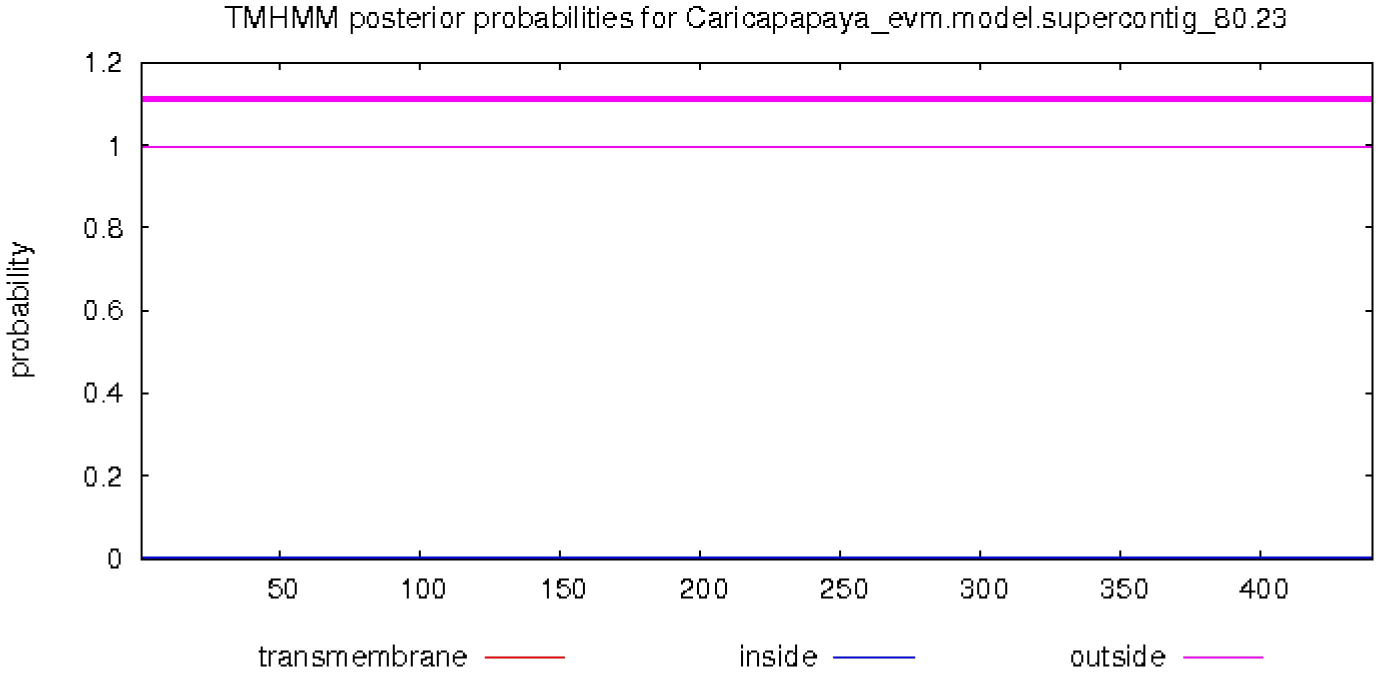

plot in postscript, script for making the plot in gnuplot, data for plot

---

```
# Caricapapaya_evm.model.supercontig_80.24 Length: 393
# Caricapapaya_evm.model.supercontig_80.24 Number of predicted TMHs:  0
# Caricapapaya_evm.model.supercontig_80.24 Exp number of AAs in TMHs: 0.05144
# Caricapapaya_evm.model.supercontig_80.24 Exp number, first 60 AAs:  0
# Caricapapaya_evm.model.supercontig_80.24 Total prob of N-in:        0.00229
Caricapapaya_evm.model.supercontig_80.24        TMHMM2.0        outside      1   393
```

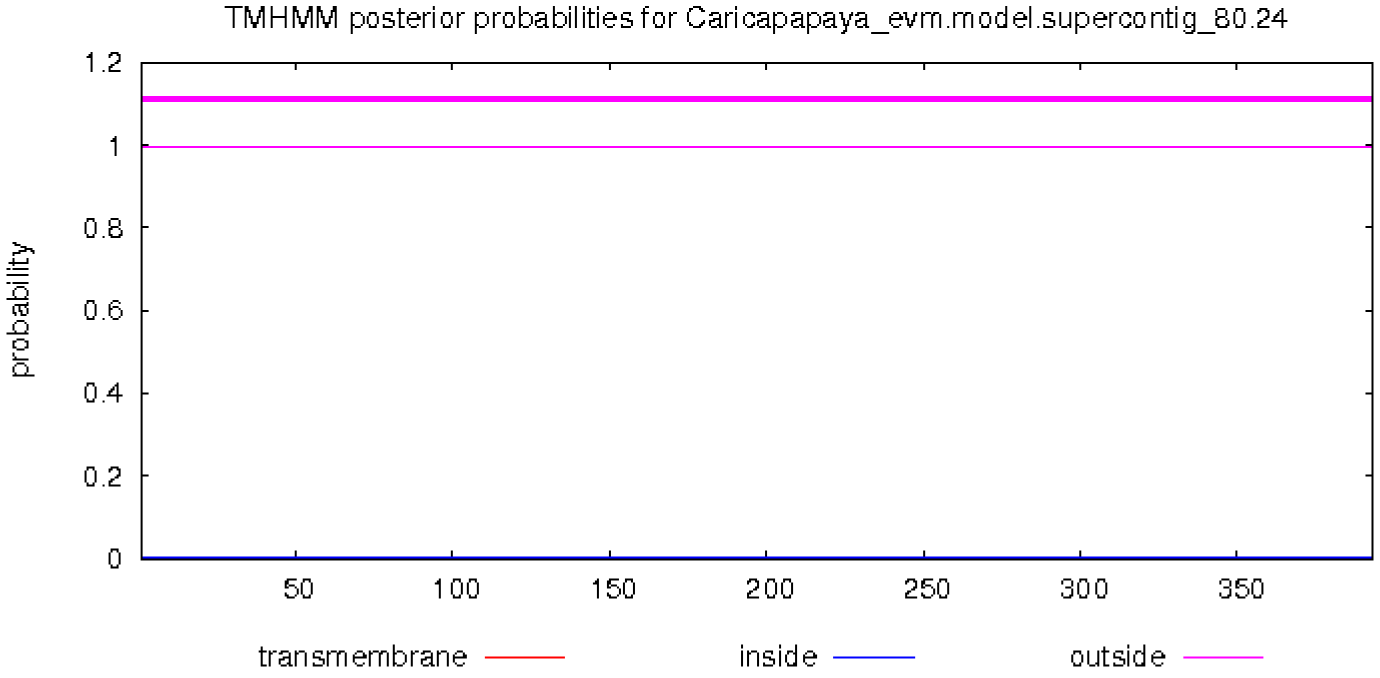

plot in postscript, script for making the plot in gnuplot, data for plot

---

```
# Caricapapaya_evm.model.supercontig_80.93 Length: 339
# Caricapapaya_evm.model.supercontig_80.93 Number of predicted TMHs:  0
# Caricapapaya_evm.model.supercontig_80.93 Exp number of AAs in TMHs: 0.00042
# Caricapapaya_evm.model.supercontig_80.93 Exp number, first 60 AAs:  0
# Caricapapaya_evm.model.supercontig_80.93 Total prob of N-in:        0.00945
Caricapapaya_evm.model.supercontig_80.93        TMHMM2.0        outside      1   339
```

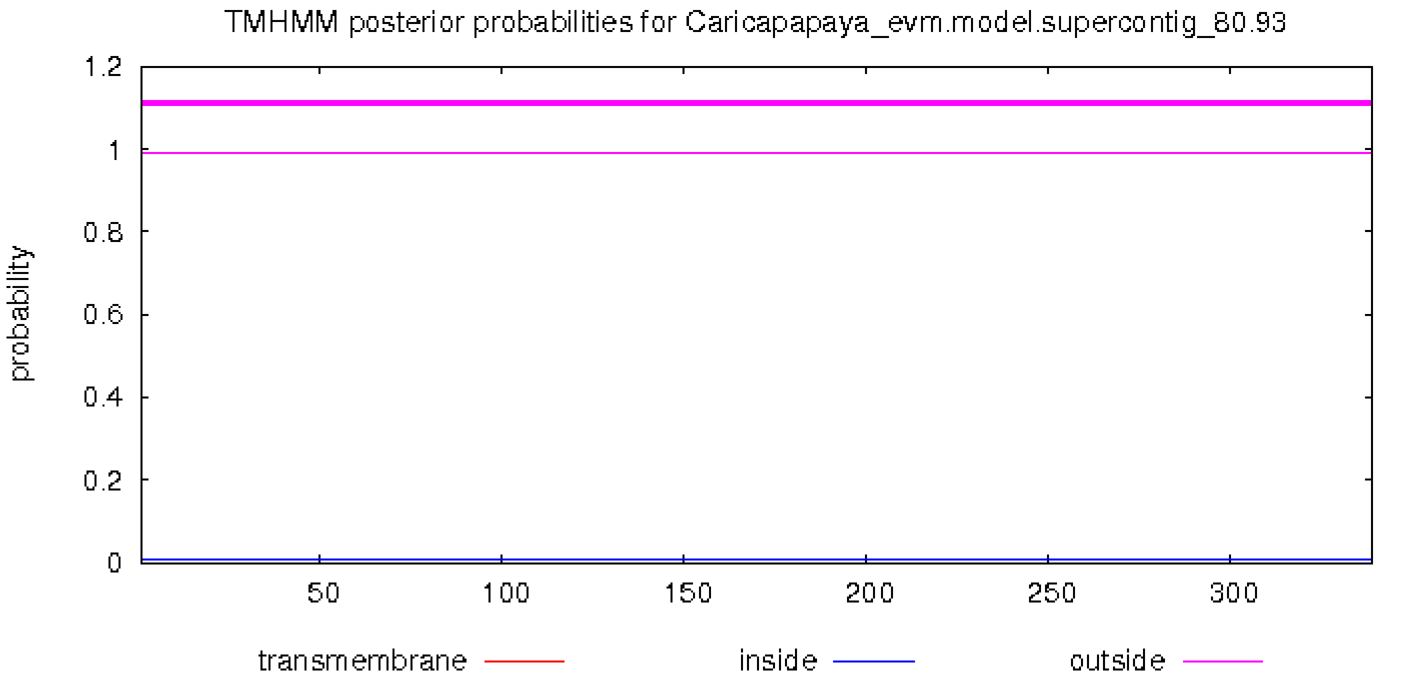

plot in postscript, script for making the plot in gnuplot, data for plot

```
# Caricapapaya_evm.model.supercontig_80.94 Length: 352
# Caricapapaya_evm.model.supercontig_80.94 Number of predicted TMHs:  0
# Caricapapaya_evm.model.supercontig_80.94 Exp number of AAs in TMHs: 0.00085
# Caricapapaya_evm.model.supercontig_80.94 Exp number, first 60 AAs:  0.00017
# Caricapapaya_evm.model.supercontig_80.94 Total prob of N-in:        0.01033
Caricapapaya_evm.model.supercontig_80.94        TMHMM2.0        outside      1   352
```

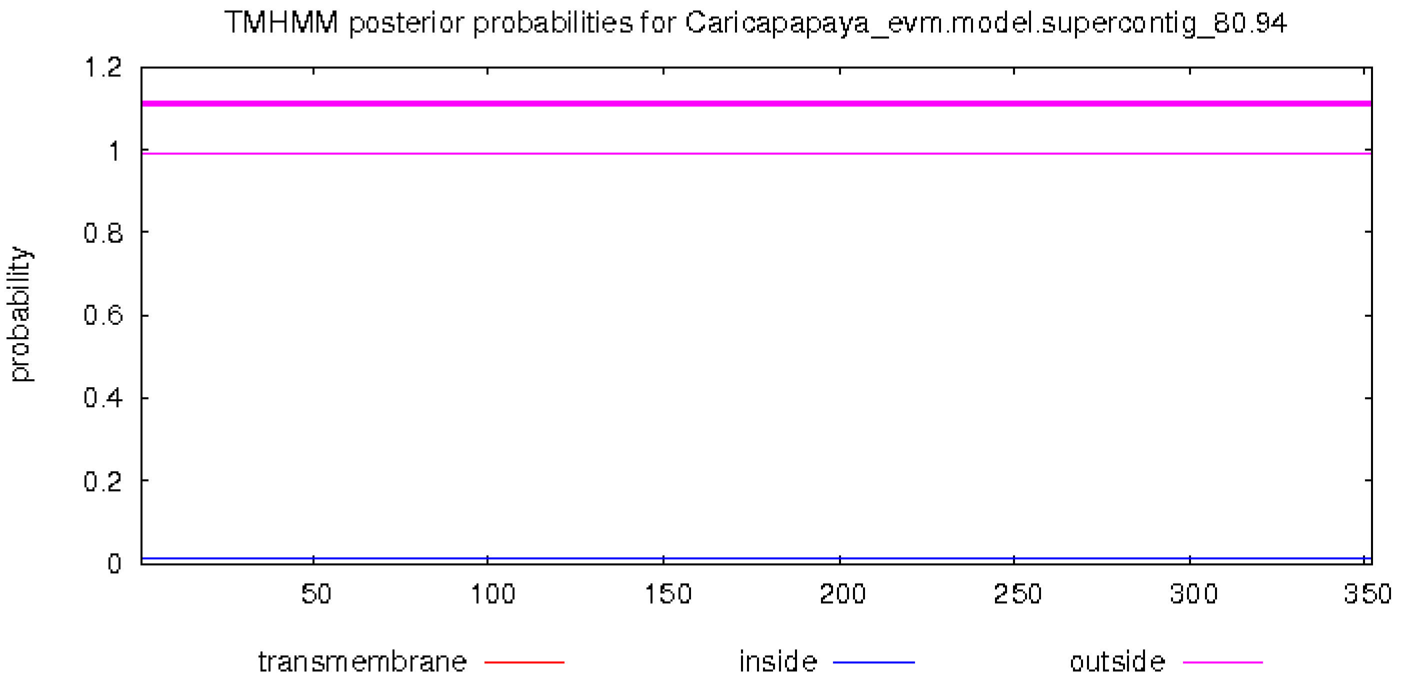

plot in postscript, script for making the plot in gnuplot, data for plot

```
# Caricapapaya_evm.model.supercontig_83.99 Length: 313
# Caricapapaya_evm.model.supercontig_83.99 Number of predicted TMHs:  0
# Caricapapaya_evm.model.supercontig_83.99 Exp number of AAs in TMHs: 0.00061
# Caricapapaya_evm.model.supercontig_83.99 Exp number, first 60 AAs:  0.00021
# Caricapapaya_evm.model.supercontig_83.99 Total prob of N-in:        0.04829
Caricapapaya_evm.model.supercontig_83.99        TMHMM2.0        outside      1   313
```

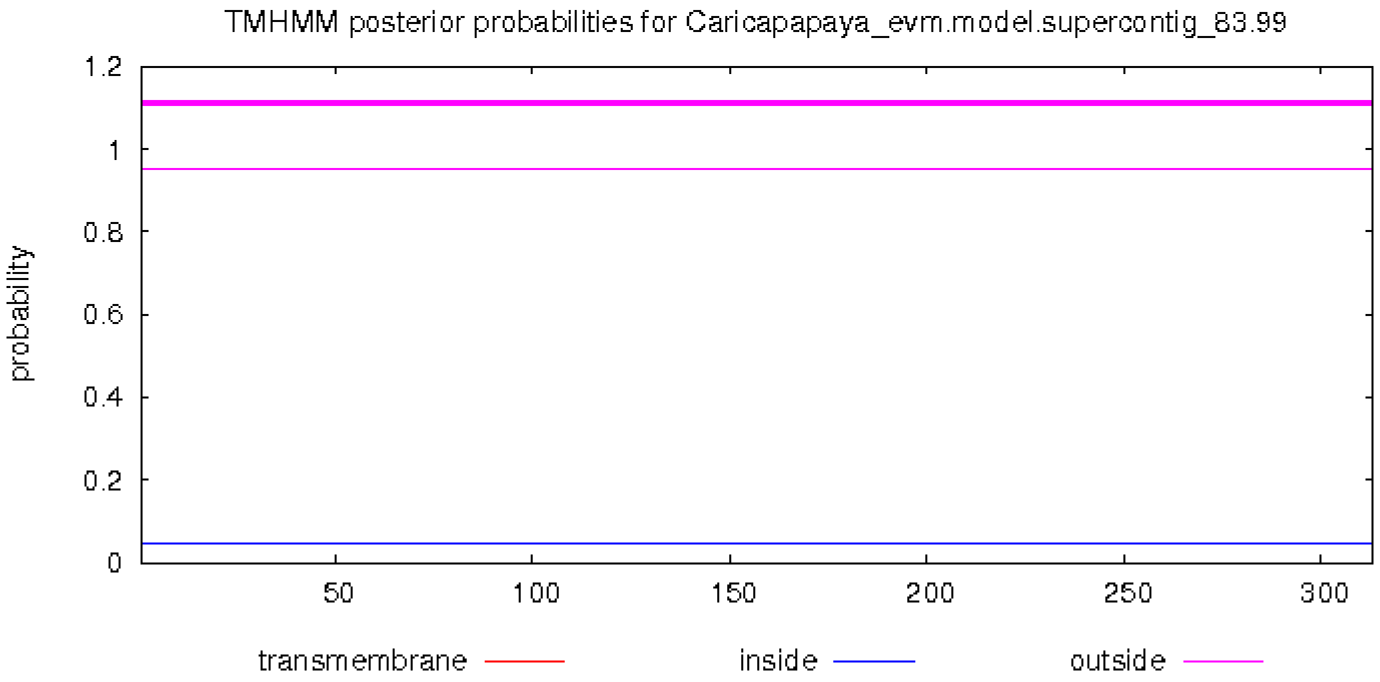

plot in postscript, script for making the plot in gnuplot, data for plot

---

```
# Caricapapaya_evm.model.supercontig_84.41 Length: 549
# Caricapapaya_evm.model.supercontig_84.41 Number of predicted TMHs:  0
# Caricapapaya_evm.model.supercontig_84.41 Exp number of AAs in TMHs: 0
# Caricapapaya_evm.model.supercontig_84.41 Exp number, first 60 AAs:  0
# Caricapapaya_evm.model.supercontig_84.41 Total prob of N-in:        0.00022
Caricapapaya_evm.model.supercontig_84.41        TMHMM2.0        outside      1   549
```

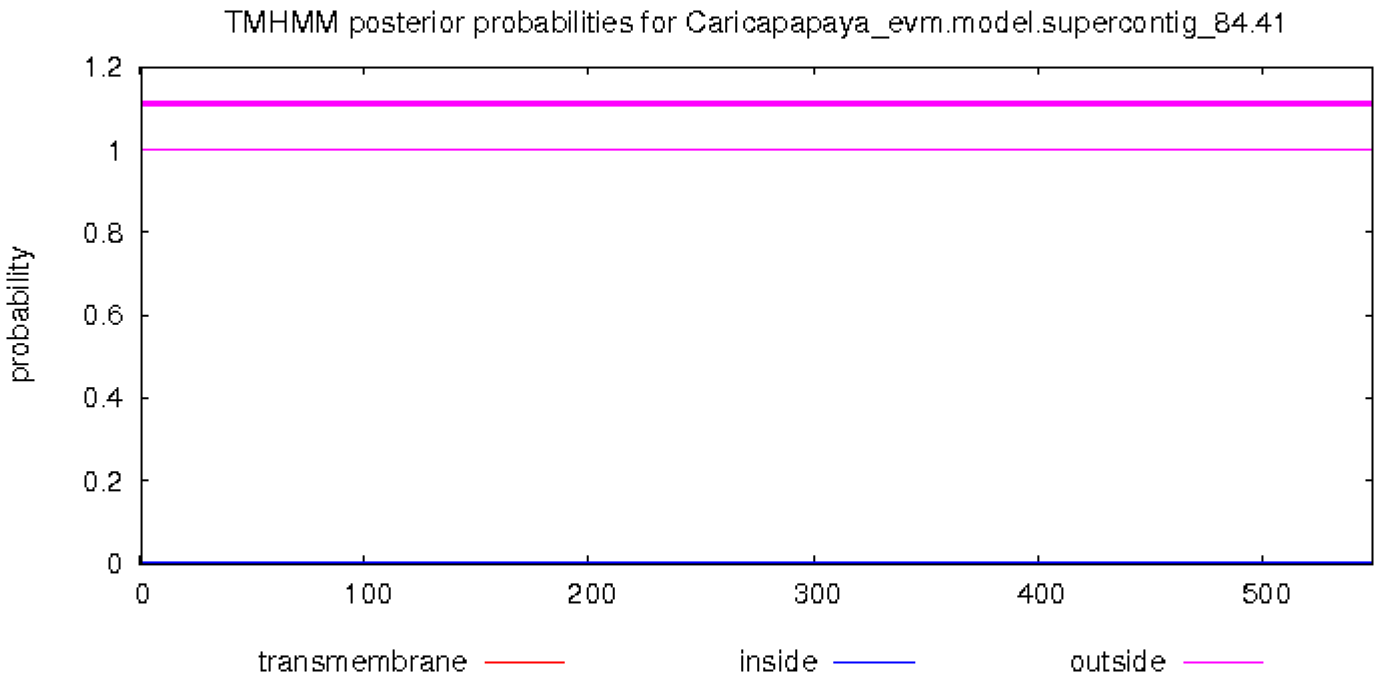

plot in postscript, script for making the plot in gnuplot, data for plot

---

```
# Caricapapaya_evm.model.supercontig_90.52 Length: 309
# Caricapapaya_evm.model.supercontig_90.52 Number of predicted TMHs:  0
# Caricapapaya_evm.model.supercontig_90.52 Exp number of AAs in TMHs: 0.00023
# Caricapapaya_evm.model.supercontig_90.52 Exp number, first 60 AAs:  0
# Caricapapaya_evm.model.supercontig_90.52 Total prob of N-in:        0.00291
Caricapapaya_evm.model.supercontig_90.52	TMHMM2.0	outside	     1   309
```

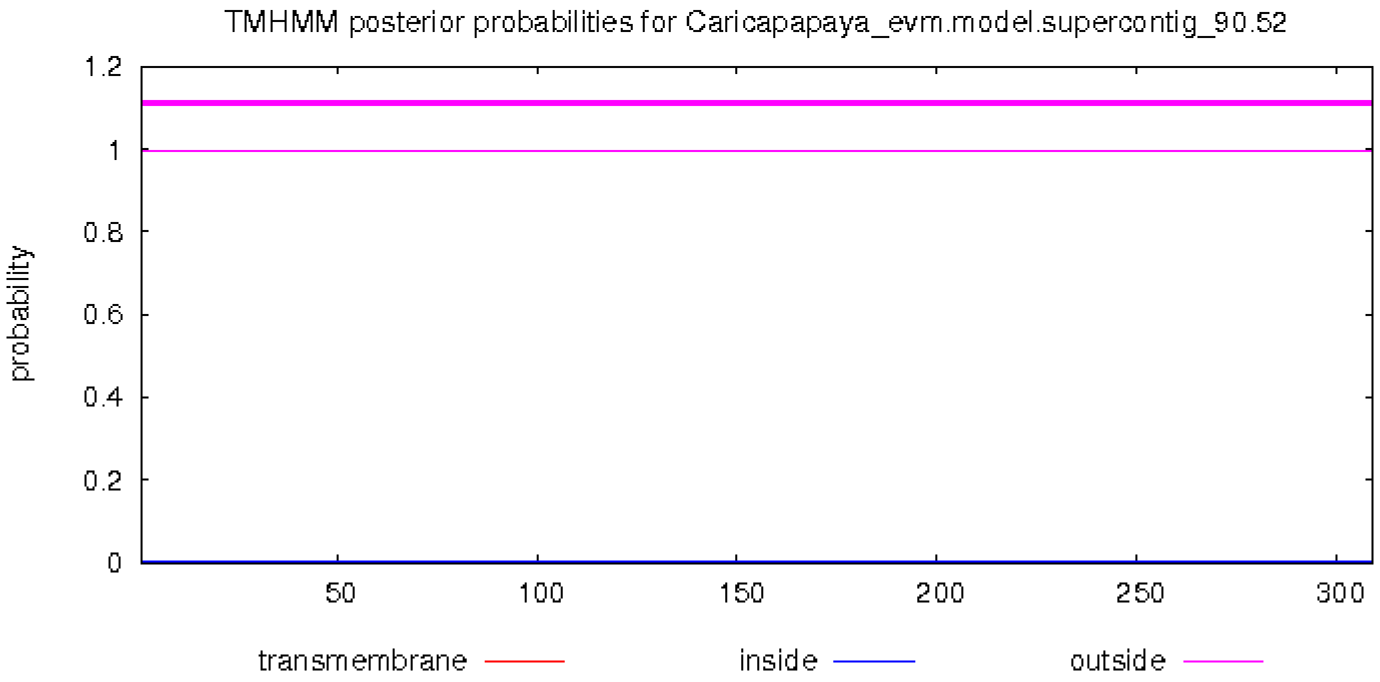

plot in postscript, script for making the plot in gnuplot, data for plot

---

```
# Caricapapaya_evm.model.supercontig_94.23 Length: 179
# Caricapapaya_evm.model.supercontig_94.23 Number of predicted TMHs:  0
# Caricapapaya_evm.model.supercontig_94.23 Exp number of AAs in TMHs: 0.30647
# Caricapapaya_evm.model.supercontig_94.23 Exp number, first 60 AAs:  0.00677
# Caricapapaya_evm.model.supercontig_94.23 Total prob of N-in:        0.38674
Caricapapaya_evm.model.supercontig_94.23	TMHMM2.0	outside	     1   179
```

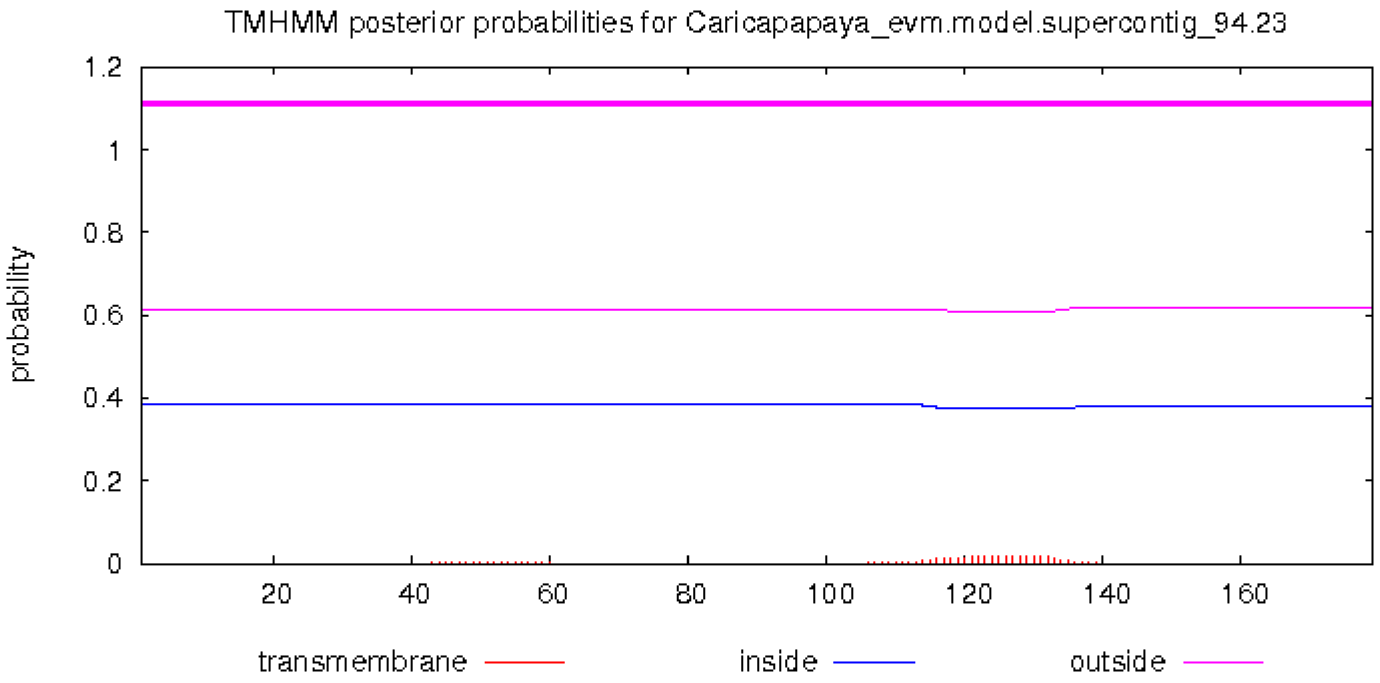

plot in postscript, script for making the plot in gnuplot, data for plot

---

```
# Caricapapaya_evm.model.supercontig_94.54 Length: 403
# Caricapapaya_evm.model.supercontig_94.54 Number of predicted TMHs:  0
# Caricapapaya_evm.model.supercontig_94.54 Exp number of AAs in TMHs: 0.00065
# Caricapapaya_evm.model.supercontig_94.54 Exp number, first 60 AAs:  0
# Caricapapaya_evm.model.supercontig_94.54 Total prob of N-in:        0.00683
Caricapapaya_evm.model.supercontig_94.54	TMHMM2.0	outside	     1   403
```

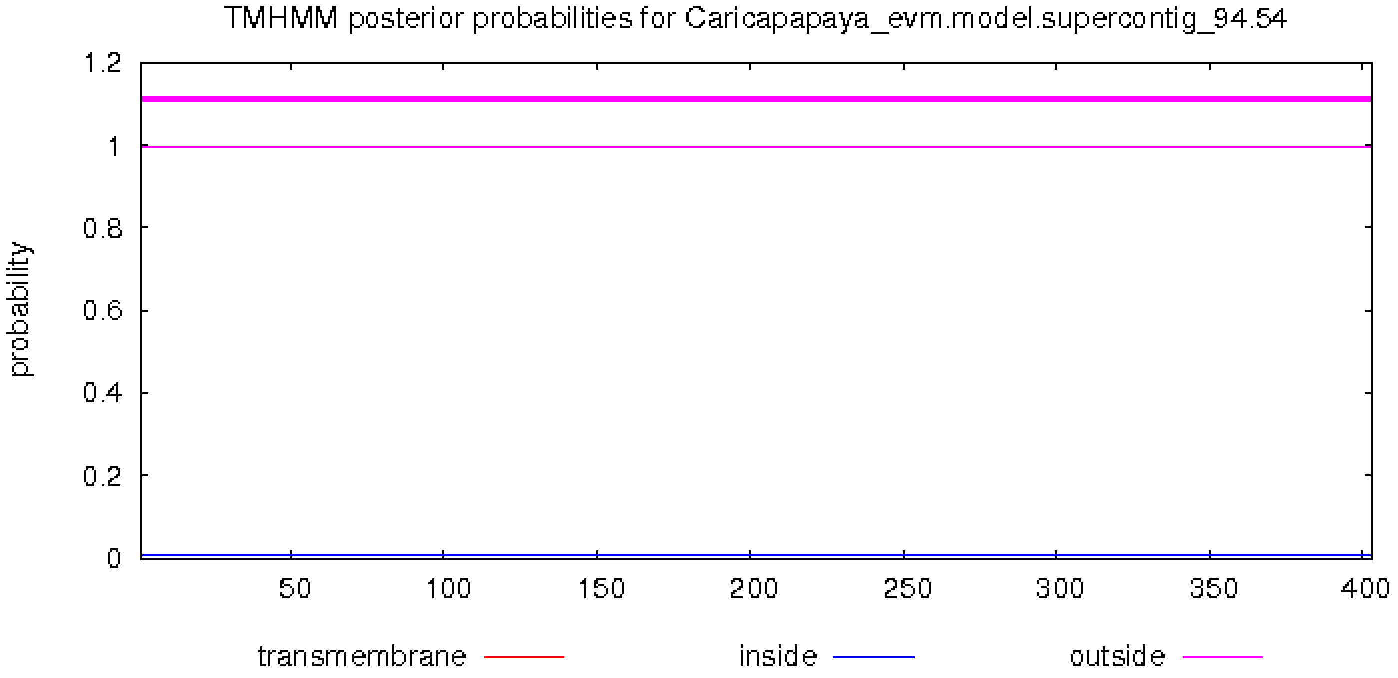

plot in postscript, script for making the plot in gnuplot, data for plot

---

```
# Caricapapaya_evm.model.supercontig_99.63 Length: 209
# Caricapapaya_evm.model.supercontig_99.63 Number of predicted TMHs:  0
# Caricapapaya_evm.model.supercontig_99.63 Exp number of AAs in TMHs: 0.00012
# Caricapapaya_evm.model.supercontig_99.63 Exp number, first 60 AAs:  0
# Caricapapaya_evm.model.supercontig_99.63 Total prob of N-in:        0.07863
Caricapapaya_evm.model.supercontig_99.63	TMHMM2.0	outside	     1   209
```

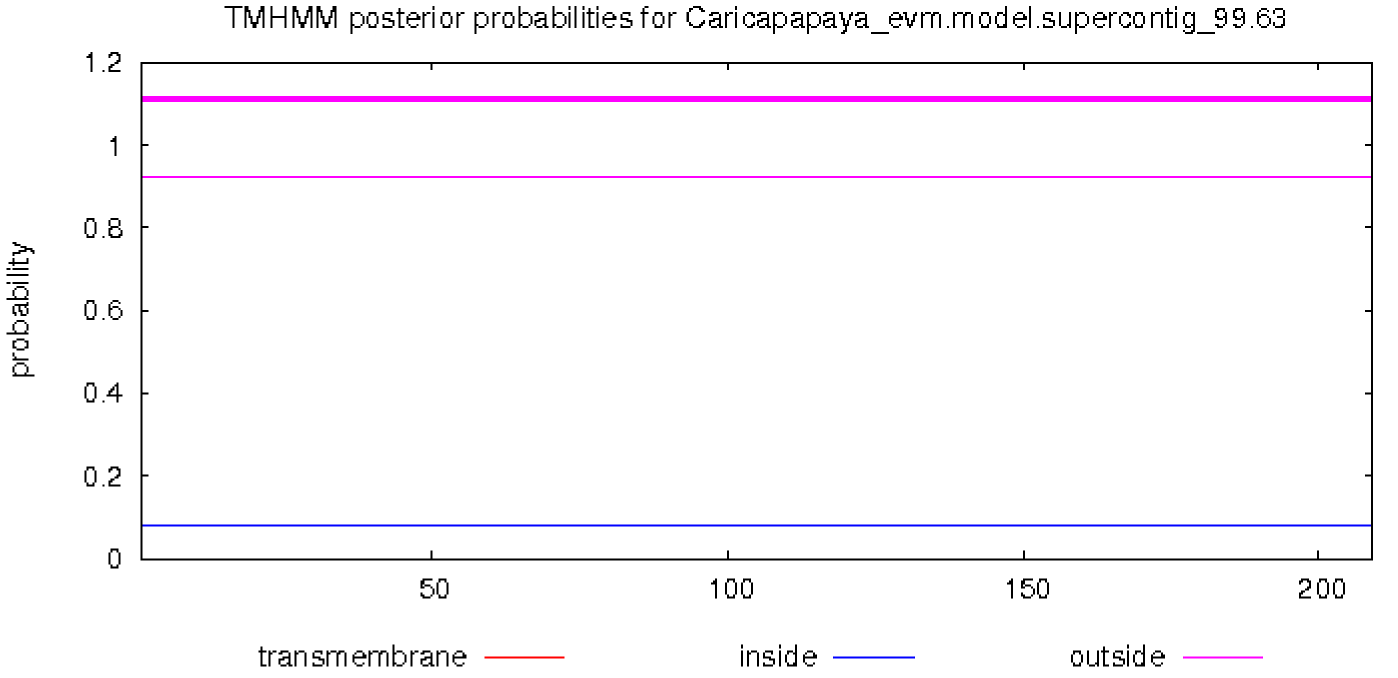

plot in postscript, script for making the plot in gnuplot, data for plot

---

```
# Caricapapaya_evm.TU.contig_29561.1 Length: 247
# Caricapapaya_evm.TU.contig_29561.1 Number of predicted TMHs:  0
# Caricapapaya_evm.TU.contig_29561.1 Exp number of AAs in TMHs: 0
# Caricapapaya_evm.TU.contig_29561.1 Exp number, first 60 AAs:  0
# Caricapapaya_evm.TU.contig_29561.1 Total prob of N-in:        0.00224
Caricapapaya_evm.TU.contig_29561.1      TMHMM2.0        outside      1   247
```

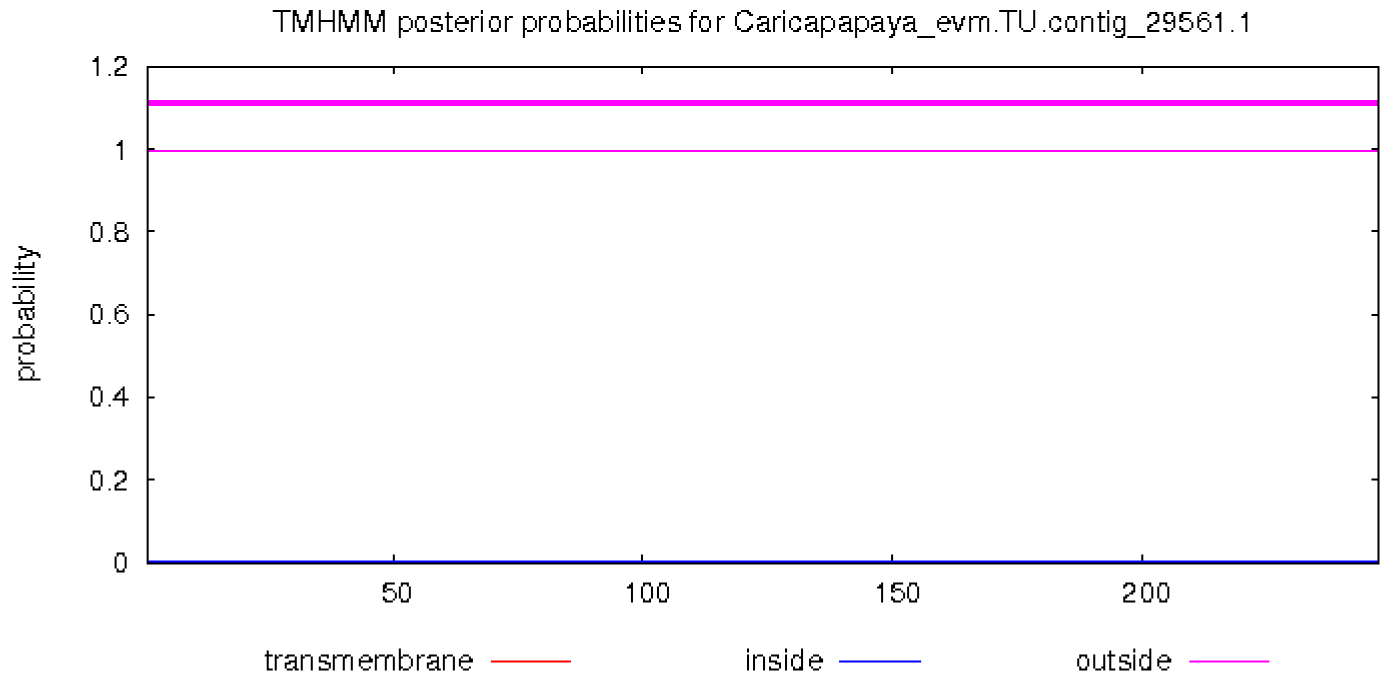

plot in postscript, script for making the plot in gnuplot, data for plot

```
# Caricapapaya_evm.TU.contig_30558.1 Length: 329
# Caricapapaya_evm.TU.contig_30558.1 Number of predicted TMHs:  0
# Caricapapaya_evm.TU.contig_30558.1 Exp number of AAs in TMHs: 0
# Caricapapaya_evm.TU.contig_30558.1 Exp number, first 60 AAs:  0
# Caricapapaya_evm.TU.contig_30558.1 Total prob of N-in:        0.00302
Caricapapaya_evm.TU.contig_30558.1      TMHMM2.0        outside      1   329
```

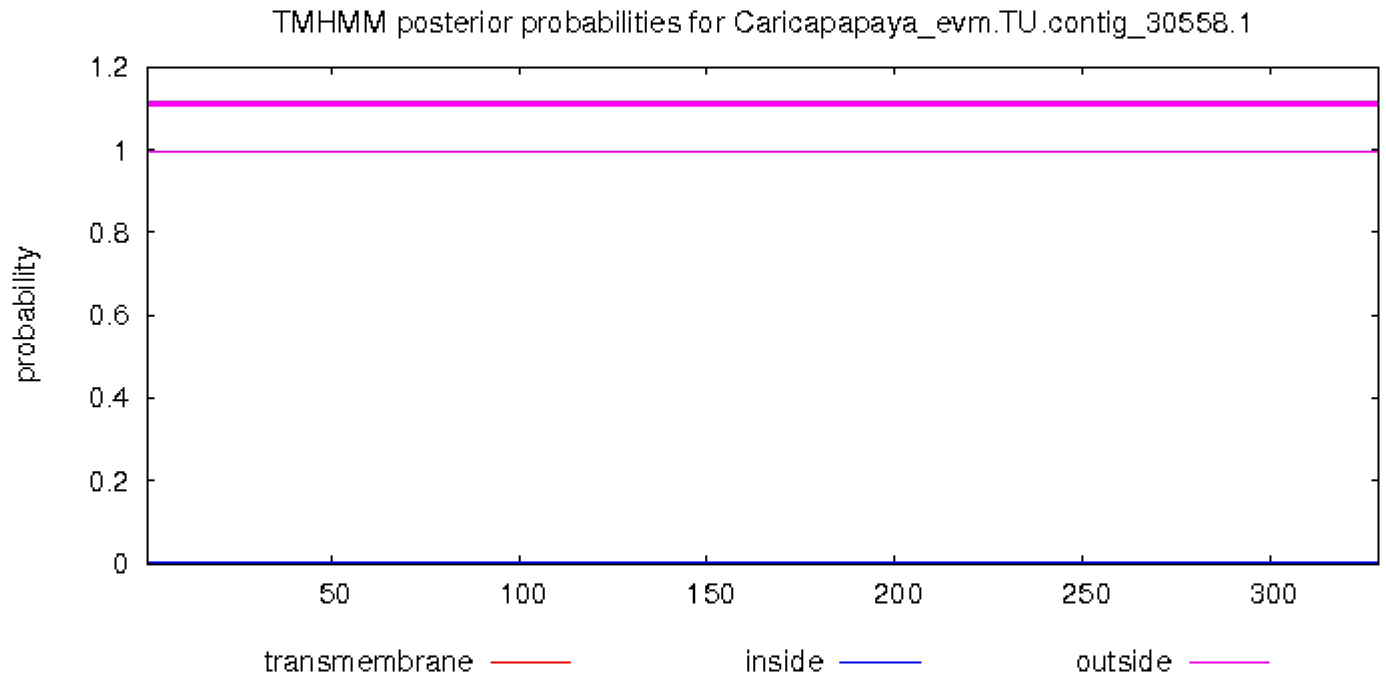

plot in postscript, script for making the plot in gnuplot, data for plot

```
# Caricapapaya_evm.TU.contig_33298.1 Length: 327
# Caricapapaya_evm.TU.contig_33298.1 Number of predicted TMHs:  0
# Caricapapaya_evm.TU.contig_33298.1 Exp number of AAs in TMHs: 0.00016
# Caricapapaya_evm.TU.contig_33298.1 Exp number, first 60 AAs:  0.00016
# Caricapapaya_evm.TU.contig_33298.1 Total prob of N-in:        0.00474
Caricapapaya_evm.TU.contig_33298.1      TMHMM2.0        outside      1   327
```

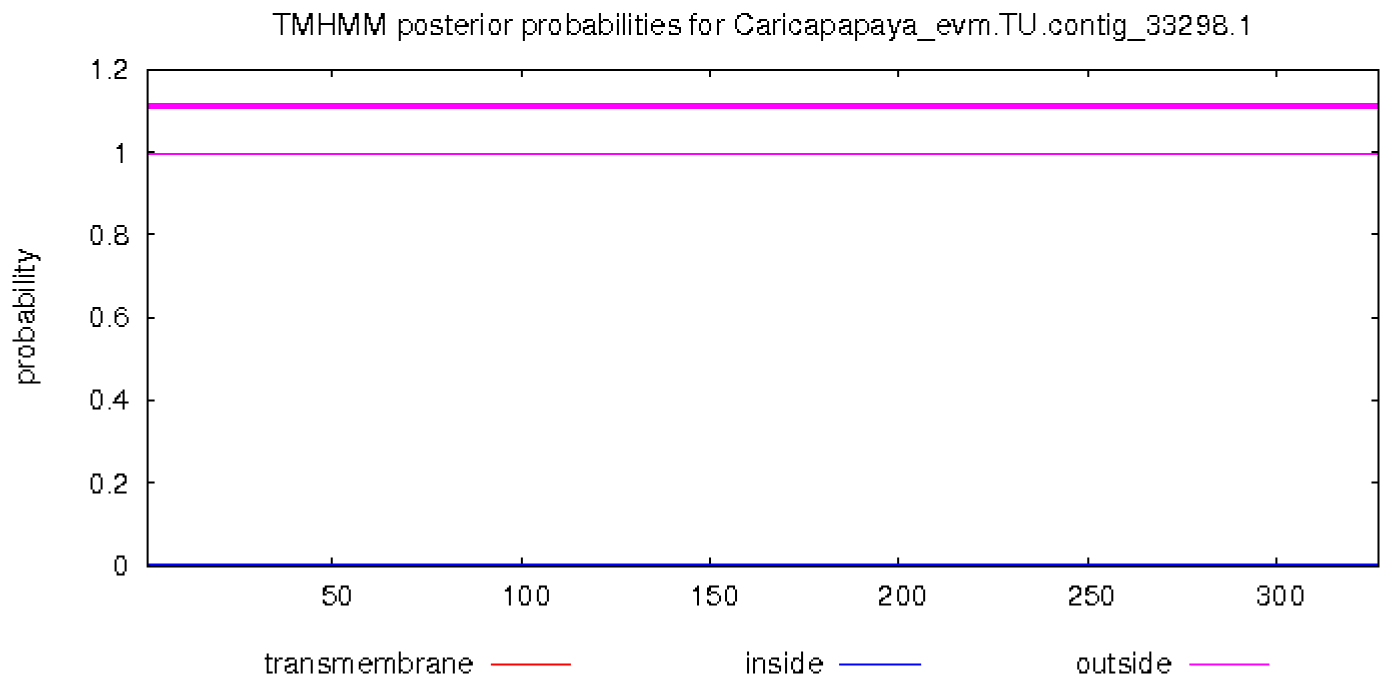

plot in postscript, script for making the plot in gnuplot, data for plot

```
# Caricapapaya_evm.TU.contig_37010.1 Length: 214
# Caricapapaya_evm.TU.contig_37010.1 Number of predicted TMHs:  0
# Caricapapaya_evm.TU.contig_37010.1 Exp number of AAs in TMHs: 0.00161
# Caricapapaya_evm.TU.contig_37010.1 Exp number, first 60 AAs:  0.00143
# Caricapapaya_evm.TU.contig_37010.1 Total prob of N-in:        0.03716
Caricapapaya_evm.TU.contig_37010.1      TMHMM2.0        outside      1   214
```

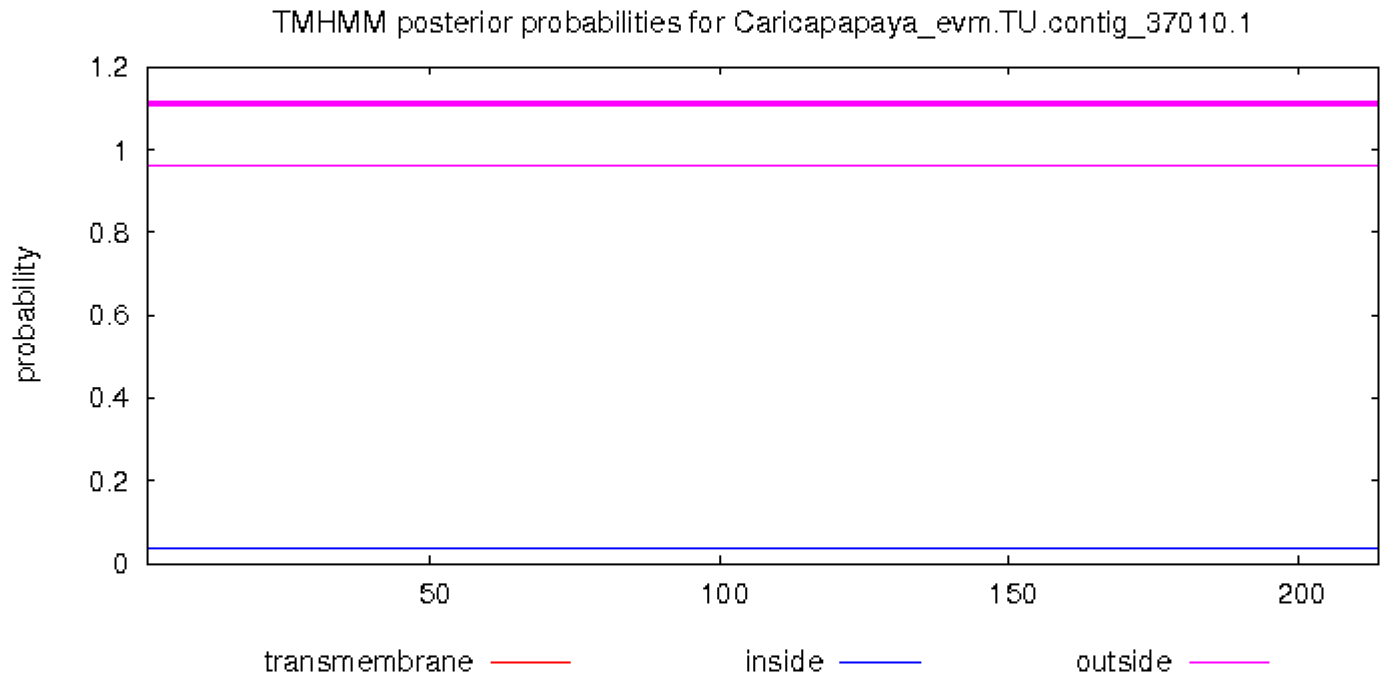

plot in postscript, script for making the plot in gnuplot, data for plot

```
# Caricapapaya_evm.TU.contig_38280.1 Length: 313
# Caricapapaya_evm.TU.contig_38280.1 Number of predicted TMHs:  0
# Caricapapaya_evm.TU.contig_38280.1 Exp number of AAs in TMHs: 0.01086
# Caricapapaya_evm.TU.contig_38280.1 Exp number, first 60 AAs:  0
# Caricapapaya_evm.TU.contig_38280.1 Total prob of N-in:        0.00867
Caricapapaya_evm.TU.contig_38280.1      TMHMM2.0        outside      1   313
```

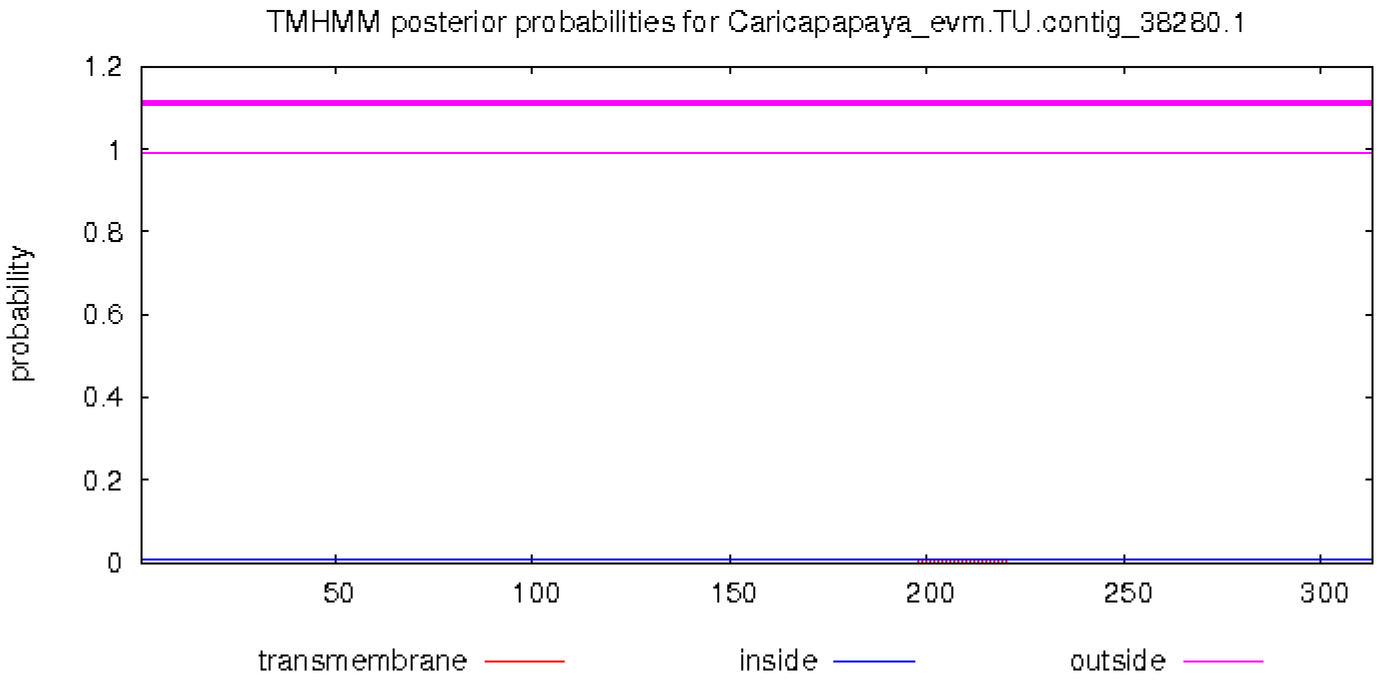

plot in postscript, script for making the plot in gnuplot, data for plot

---

```
# Caricapapaya_evm.TU.contig_45261.1 Length: 124
# Caricapapaya_evm.TU.contig_45261.1 Number of predicted TMHs:  0
# Caricapapaya_evm.TU.contig_45261.1 Exp number of AAs in TMHs: 0.00036
# Caricapapaya_evm.TU.contig_45261.1 Exp number, first 60 AAs:  0.00032
# Caricapapaya_evm.TU.contig_45261.1 Total prob of N-in:        0.10597
Caricapapaya_evm.TU.contig_45261.1      TMHMM2.0        outside      1   124
```

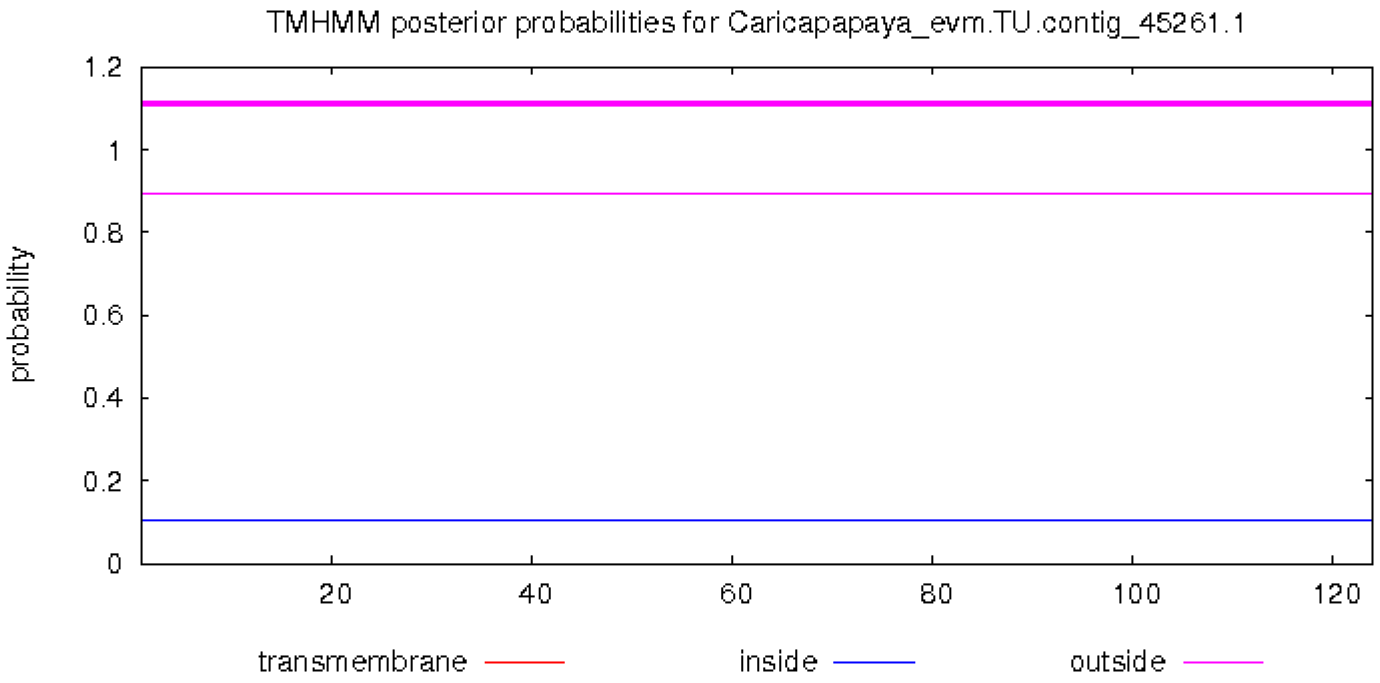

plot in postscript, script for making the plot in gnuplot, data for plot

---

```
# Castaneamollissima_scaffold00033-abinit-gene-2.0 Length: 404
# Castaneamollissima_scaffold00033-abinit-gene-2.0 Number of predicted TMHs:  0
# Castaneamollissima_scaffold00033-abinit-gene-2.0 Exp number of AAs in TMHs: 0
# Castaneamollissima_scaffold00033-abinit-gene-2.0 Exp number, first 60 AAs:  0
# Castaneamollissima_scaffold00033-abinit-gene-2.0 Total prob of N-in:        0.01644
Castaneamollissima_scaffold00033-abinit-gene-2.0        TMHMM2.0        outside      1   404
```

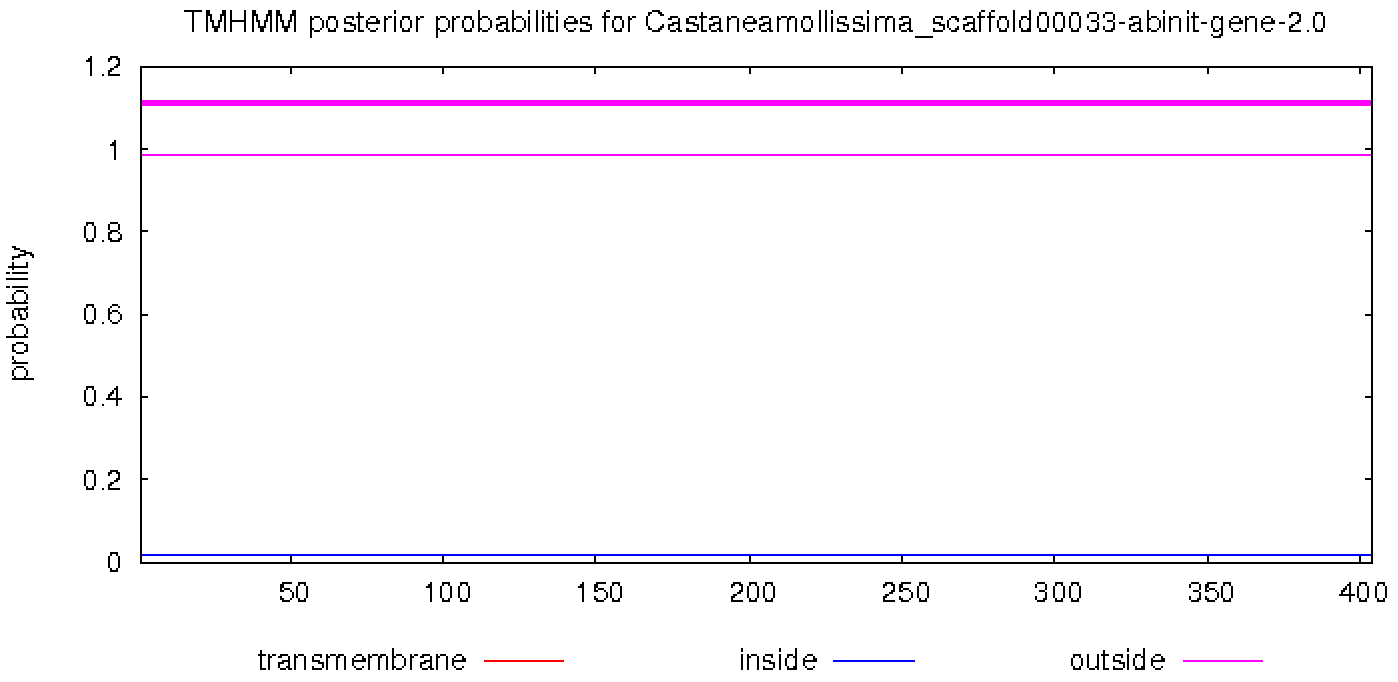

plot in postscript, script for making the plot in gnuplot, data for plot

---

```
# Castaneamollissima_scaffold00498-abinit-gene-0.4d00498 Length: 168
# Castaneamollissima_scaffold00498-abinit-gene-0.4d00498 Number of predicted TMHs:  0
# Castaneamollissima_scaffold00498-abinit-gene-0.4d00498 Exp number of AAs in TMHs: 0.00013
# Castaneamollissima_scaffold00498-abinit-gene-0.4d00498 Exp number, first 60 AAs:  0
# Castaneamollissima_scaffold00498-abinit-gene-0.4d00498 Total prob of N-in:        0.14577
Castaneamollissima_scaffold00498-abinit-gene-0.4d00498  TMHMM2.0        outside      1   168
```

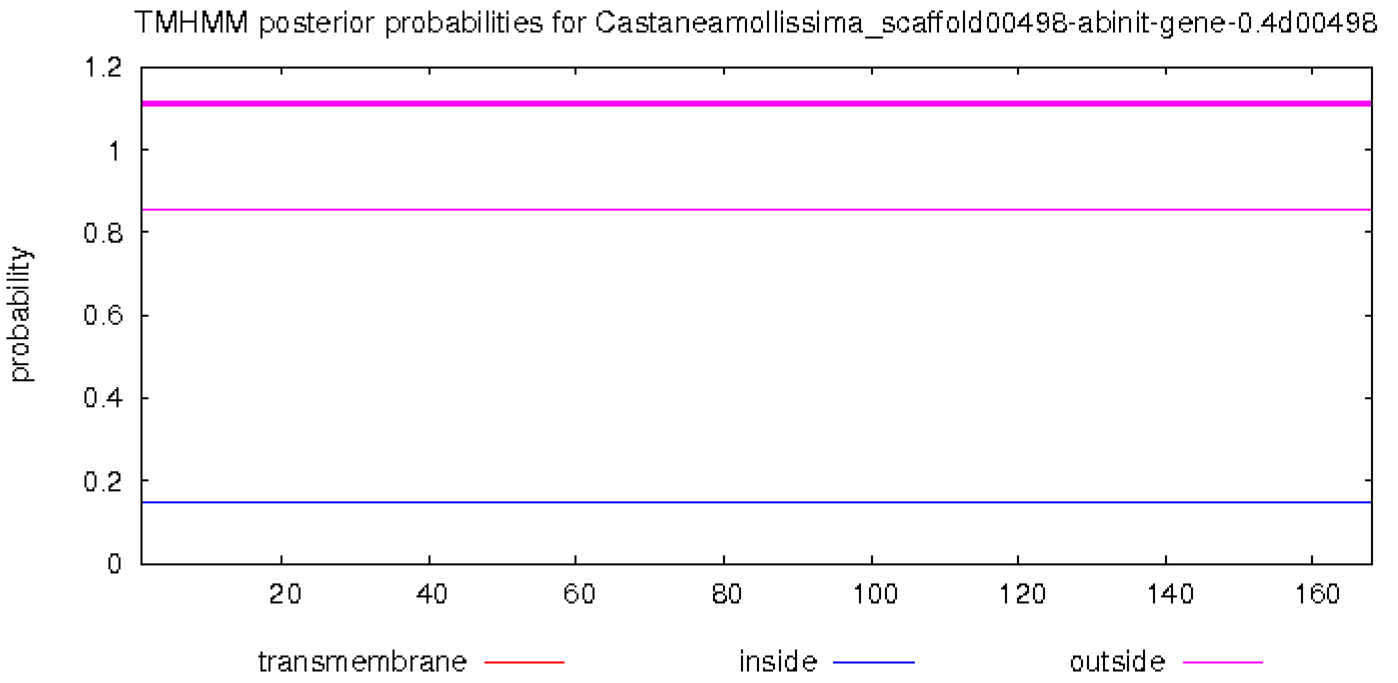

plot in postscript, script for making the plot in gnuplot, data for plot

---

```
# Castaneamollissima_scaffold00534-abinit-gene-0.10d00534 Length: 267
# Castaneamollissima_scaffold00534-abinit-gene-0.10d00534 Number of predicted TMHs:  0
# Castaneamollissima_scaffold00534-abinit-gene-0.10d00534 Exp number of AAs in TMHs: 0
# Castaneamollissima_scaffold00534-abinit-gene-0.10d00534 Exp number, first 60 AAs:  0
# Castaneamollissima_scaffold00534-abinit-gene-0.10d00534 Total prob of N-in:        0.01693
Castaneamollissima_scaffold00534-abinit-gene-0.10d00534 TMHMM2.0        outside      1   267
```

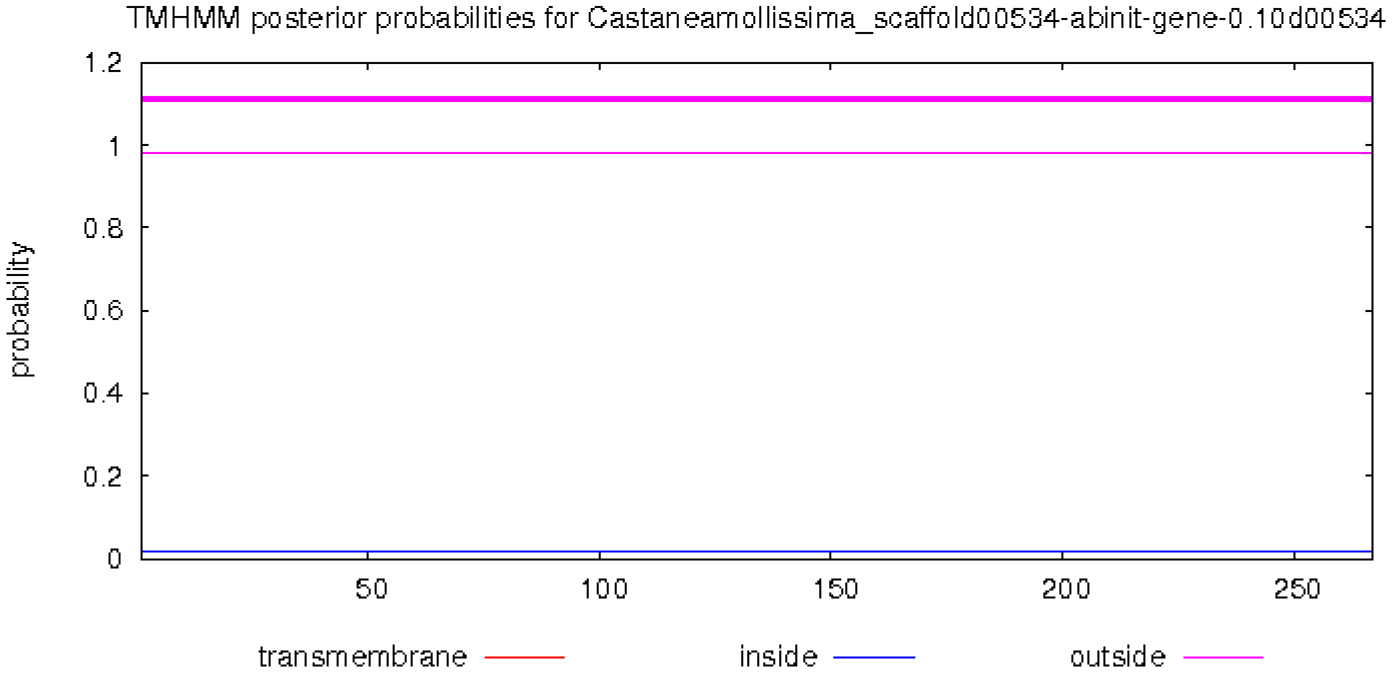

plot in postscript, script for making the plot in gnuplot, data for plot

---

```
# Castaneamollissima_scaffold00906-abinit-gene-0.7d00906 Length: 567
# Castaneamollissima_scaffold00906-abinit-gene-0.7d00906 Number of predicted TMHs:  1
# Castaneamollissima_scaffold00906-abinit-gene-0.7d00906 Exp number of AAs in TMHs: 21.89696
# Castaneamollissima_scaffold00906-abinit-gene-0.7d00906 Exp number, first 60 AAs:  0.00036
# Castaneamollissima_scaffold00906-abinit-gene-0.7d00906 Total prob of N-in:        0.02821
Castaneamollissima_scaffold00906-abinit-gene-0.7d00906  TMHMM2.0        outside      1   538
Castaneamollissima_scaffold00906-abinit-gene-0.7d00906  TMHMM2.0        TMhelix    539   561
Castaneamollissima_scaffold00906-abinit-gene-0.7d00906  TMHMM2.0        inside     562   567
```

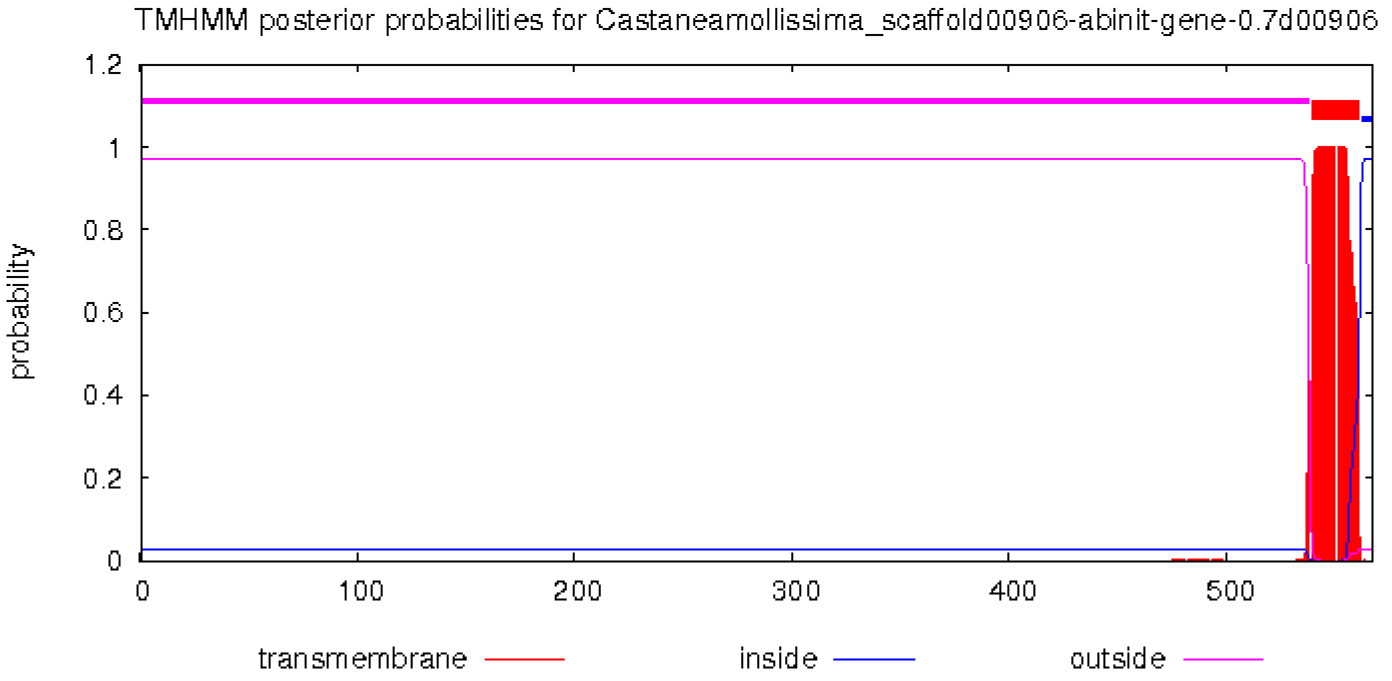

plot in postscript, script for making the plot in gnuplot, data for plot

```
# Castaneamollissima_scaffold01826-abinit-gene-0.0d01826 Length: 278
# Castaneamollissima_scaffold01826-abinit-gene-0.0d01826 Number of predicted TMHs:  0
# Castaneamollissima_scaffold01826-abinit-gene-0.0d01826 Exp number of AAs in TMHs: 0.000900000000000000001
# Castaneamollissima_scaffold01826-abinit-gene-0.0d01826 Exp number, first 60 AAs:  0
# Castaneamollissima_scaffold01826-abinit-gene-0.0d01826 Total prob of N-in:        0.14743
Castaneamollissima_scaffold01826-abinit-gene-0.0d01826  TMHMM2.0        outside      1   278
```

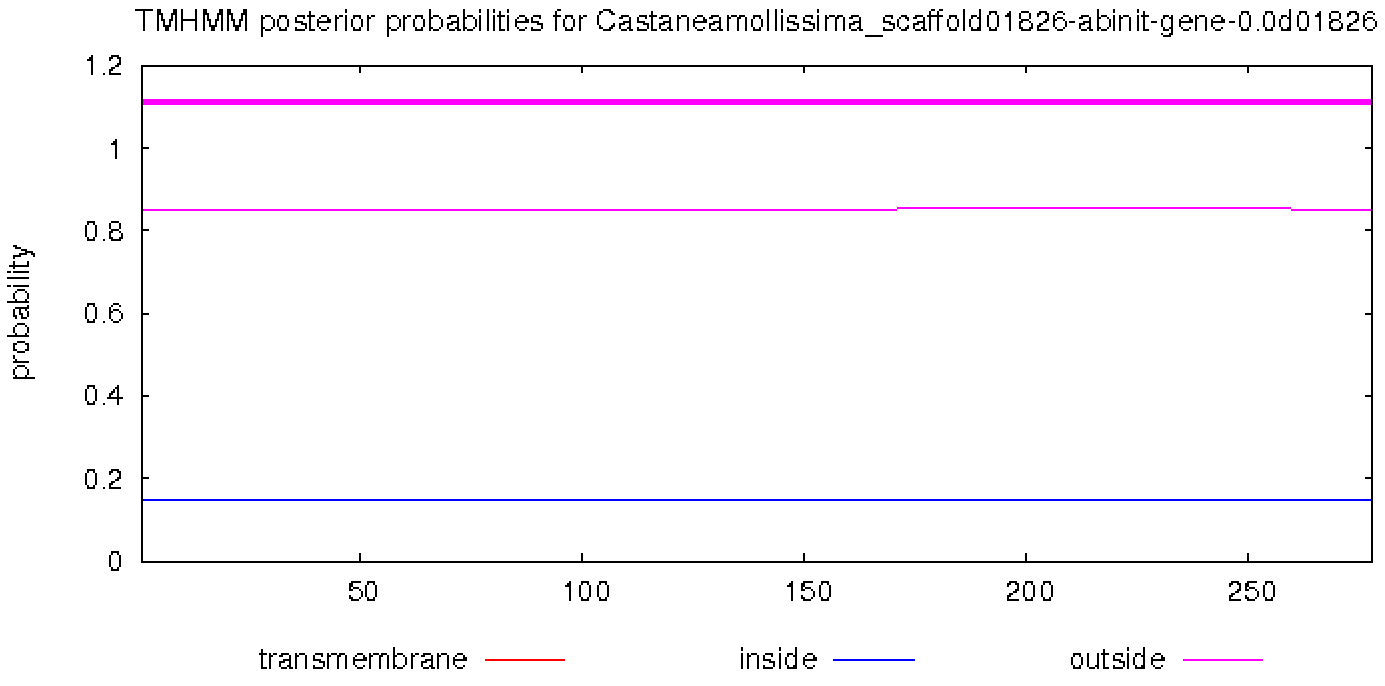

plot in postscript, script for making the plot in gnuplot, data for plot

```
# Castaneamollissima_scaffold02790-abinit-gene-0.8d02790 Length: 290
# Castaneamollissima_scaffold02790-abinit-gene-0.8d02790 Number of predicted TMHs:  0
# Castaneamollissima_scaffold02790-abinit-gene-0.8d02790 Exp number of AAs in TMHs: 0.00015
# Castaneamollissima_scaffold02790-abinit-gene-0.8d02790 Exp number, first 60 AAs:  0
# Castaneamollissima_scaffold02790-abinit-gene-0.8d02790 Total prob of N-in:        0.00525
Castaneamollissima_scaffold02790-abinit-gene-0.8d02790  TMHMM2.0        outside      1   290
```

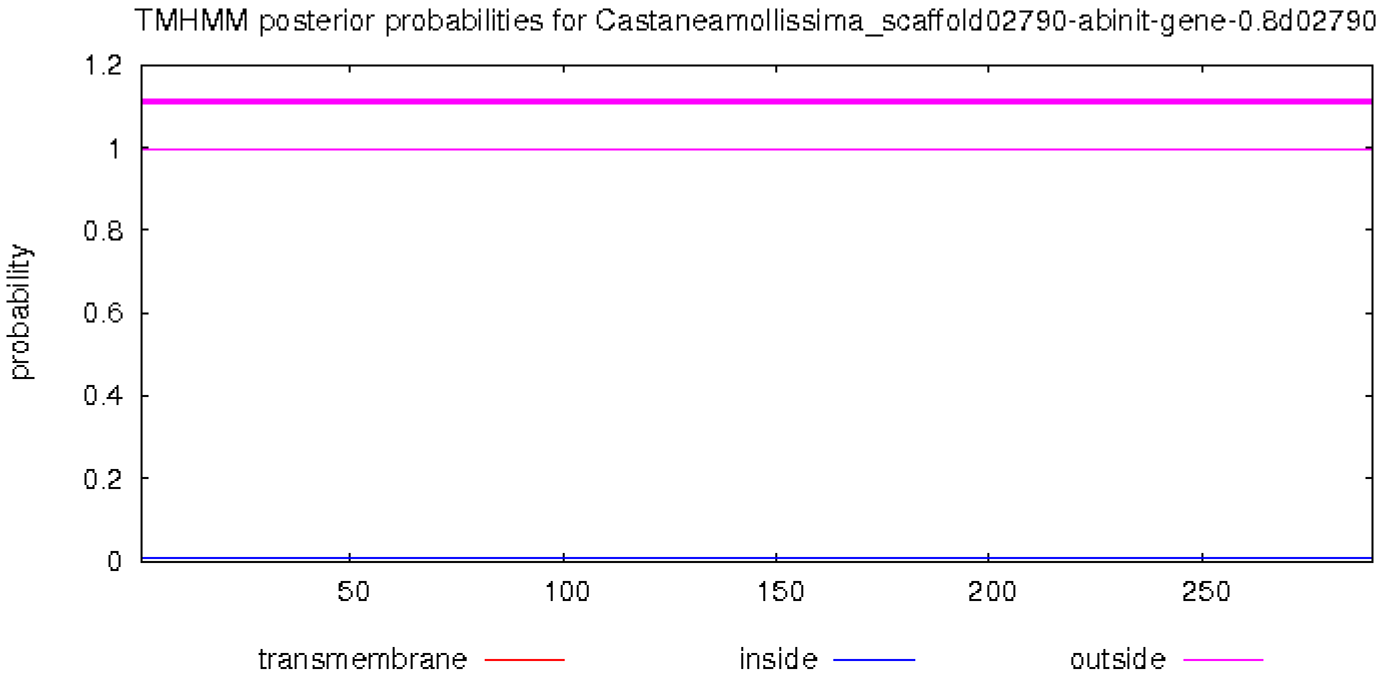

plot in postscript, script for making the plot in gnuplot, data for plot

```
# Castaneamollissima_scaffold02867-abinit-gene-0.1d02867 Length: 364
# Castaneamollissima_scaffold02867-abinit-gene-0.1d02867 Number of predicted TMHs:  0
# Castaneamollissima_scaffold02867-abinit-gene-0.1d02867 Exp number of AAs in TMHs: 0.00113
# Castaneamollissima_scaffold02867-abinit-gene-0.1d02867 Exp number, first 60 AAs:  0
# Castaneamollissima_scaffold02867-abinit-gene-0.1d02867 Total prob of N-in:        0.02651
Castaneamollissima_scaffold02867-abinit-gene-0.1d02867  TMHMM2.0        outside      1   364
```

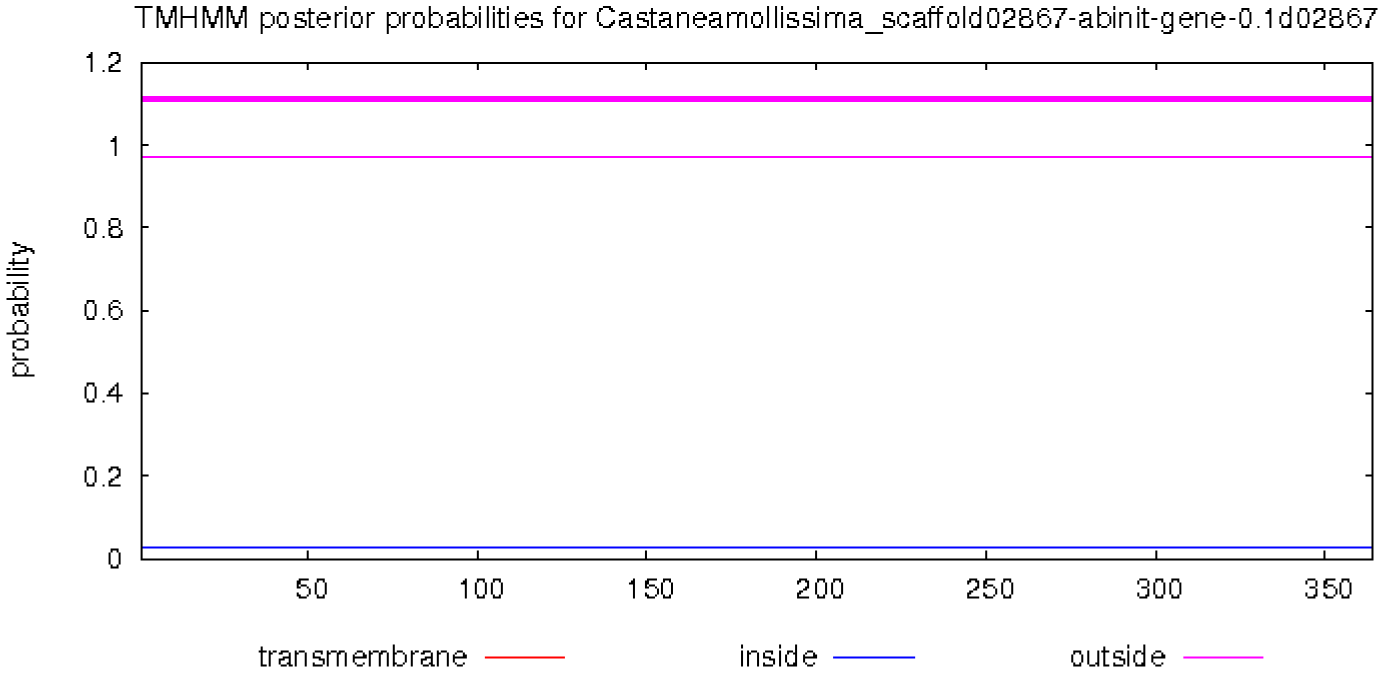

plot in postscript, script for making the plot in gnuplot, data for plot

---

```
# Castaneamollissima_scaffold03666-abinit-gene-0.5d03666 Length: 560
# Castaneamollissima_scaffold03666-abinit-gene-0.5d03666 Number of predicted TMHs:  0
# Castaneamollissima_scaffold03666-abinit-gene-0.5d03666 Exp number of AAs in TMHs: 0.07431
# Castaneamollissima_scaffold03666-abinit-gene-0.5d03666 Exp number, first 60 AAs:  0.07374
# Castaneamollissima_scaffold03666-abinit-gene-0.5d03666 Total prob of N-in:        0.00480
Castaneamollissima_scaffold03666-abinit-gene-0.5d03666  TMHMM2.0        outside      1   560
```

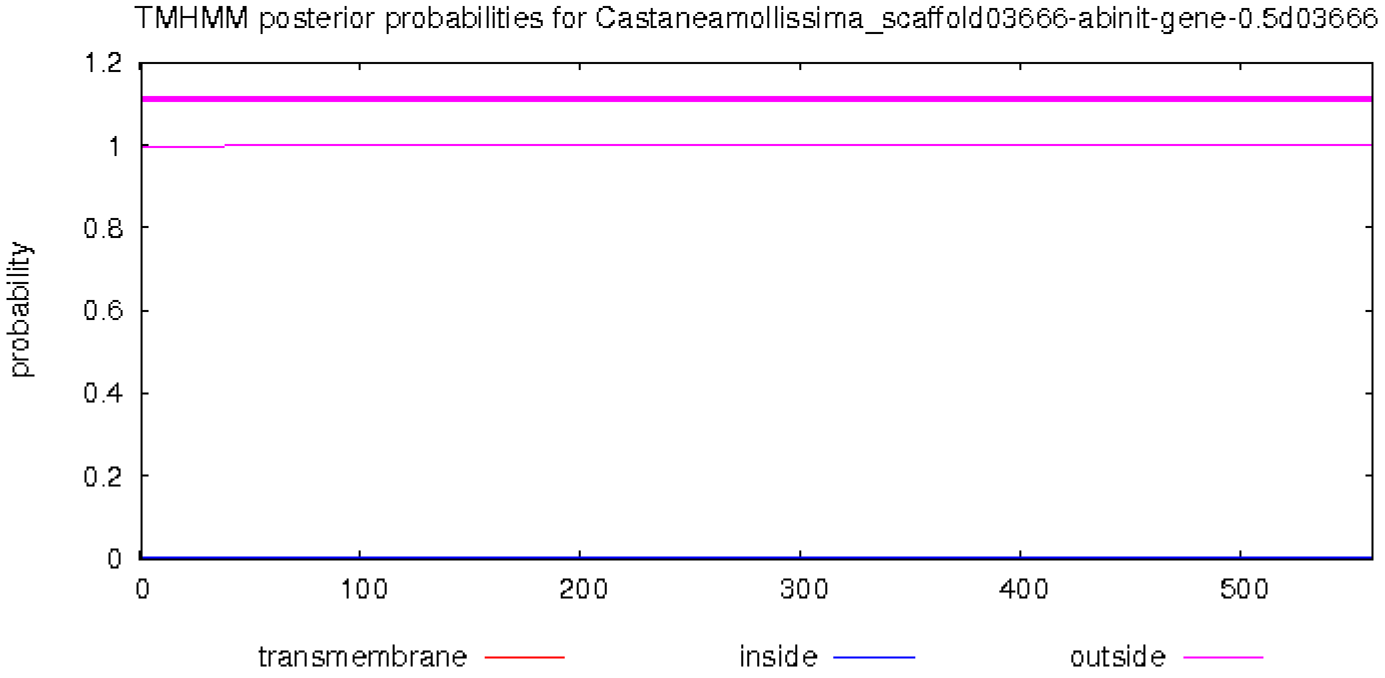

plot in postscript, script for making the plot in gnuplot, data for plot

---

```
# Castaneamollissima_scaffold04550-abinit-gene-0.6d04550 Length: 724
# Castaneamollissima_scaffold04550-abinit-gene-0.6d04550 Number of predicted TMHs:  0
# Castaneamollissima_scaffold04550-abinit-gene-0.6d04550 Exp number of AAs in TMHs: 0.00926
# Castaneamollissima_scaffold04550-abinit-gene-0.6d04550 Exp number, first 60 AAs:  0.00209
# Castaneamollissima_scaffold04550-abinit-gene-0.6d04550 Total prob of N-in:        0.00062
Castaneamollissima_scaffold04550-abinit-gene-0.6d04550  TMHMM2.0        outside      1   724
```

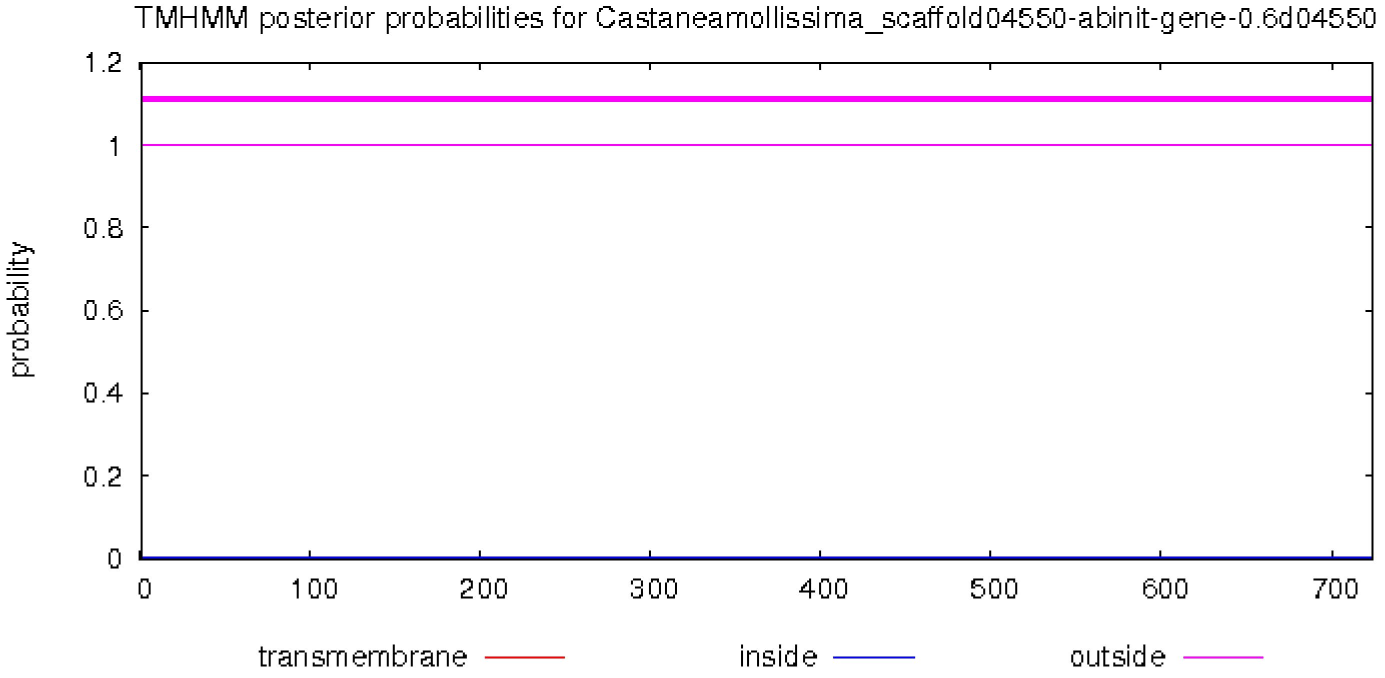

plot in postscript, script for making the plot in gnuplot, data for plot

---

```
# Castaneamollissima_scaffold10669-abinit-gene-0.0d10669 Length: 334
# Castaneamollissima_scaffold10669-abinit-gene-0.0d10669 Number of predicted TMHs:  0
# Castaneamollissima_scaffold10669-abinit-gene-0.0d10669 Exp number of AAs in TMHs: 0.00075
# Castaneamollissima_scaffold10669-abinit-gene-0.0d10669 Exp number, first 60 AAs:  0.0002
# Castaneamollissima_scaffold10669-abinit-gene-0.0d10669 Total prob of N-in:        0.07709
Castaneamollissima_scaffold10669-abinit-gene-0.0d10669  TMHMM2.0        outside      1   334
```

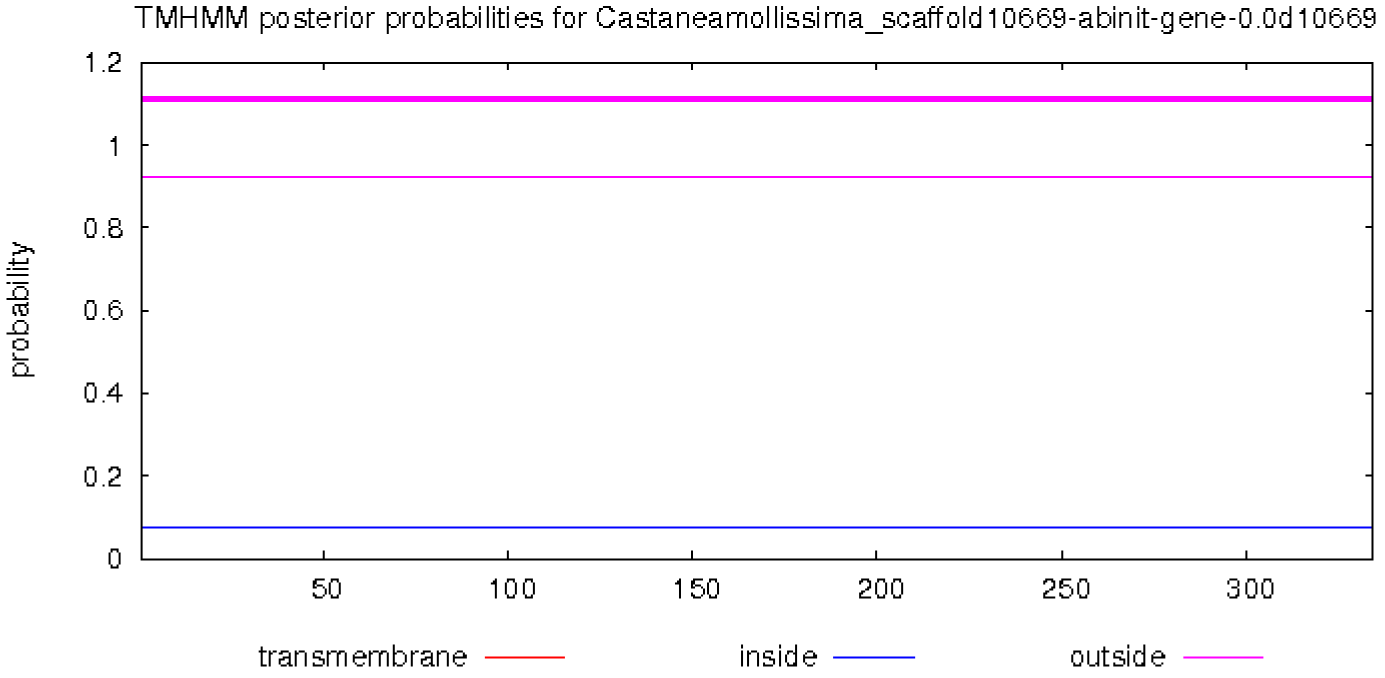

plot in postscript, script for making the plot in gnuplot, data for plot

---

```
# Castaneamollissima_scaffold12148-abinit-gene-0.2d12148 Length: 165
# Castaneamollissima_scaffold12148-abinit-gene-0.2d12148 Number of predicted TMHs:  0
# Castaneamollissima_scaffold12148-abinit-gene-0.2d12148 Exp number of AAs in TMHs: 0.00037
# Castaneamollissima_scaffold12148-abinit-gene-0.2d12148 Exp number, first 60 AAs:  0.00018
# Castaneamollissima_scaffold12148-abinit-gene-0.2d12148 Total prob of N-in:        0.30194
Castaneamollissima_scaffold12148-abinit-gene-0.2d12148  TMHMM2.0        outside      1   165
```

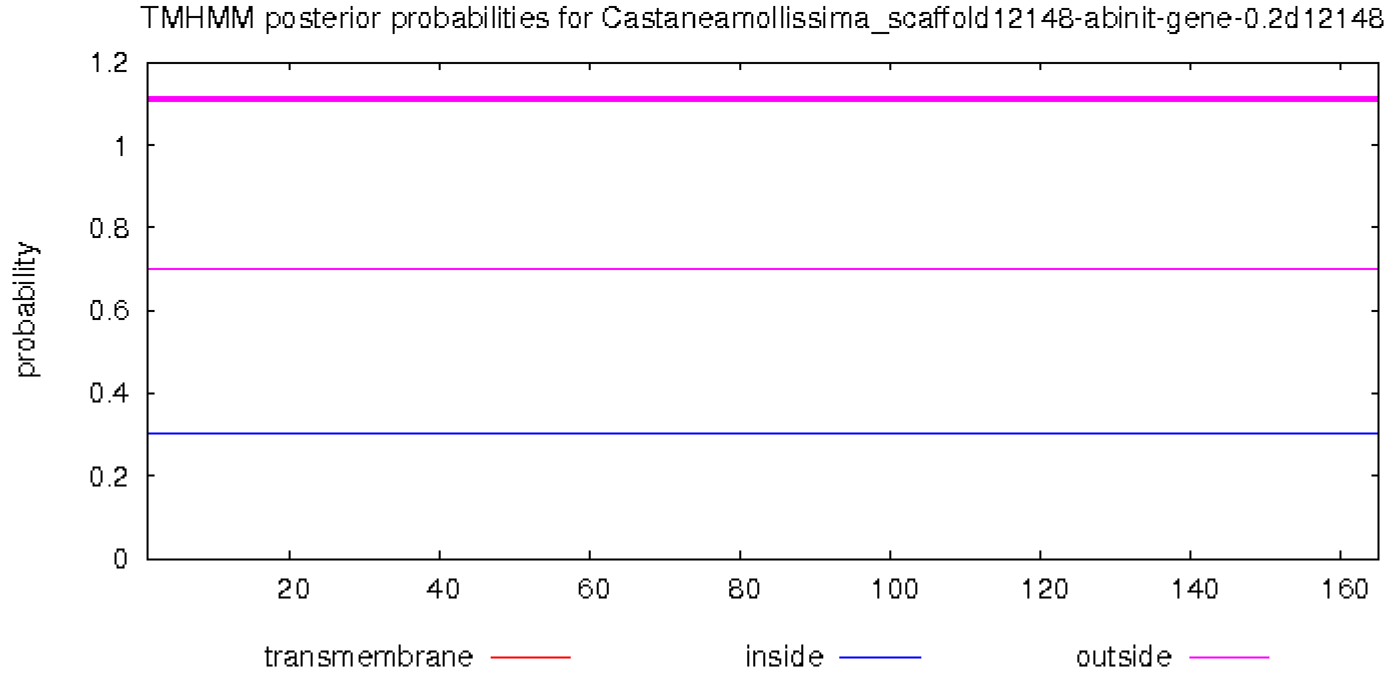

plot in postscript, script for making the plot in gnuplot, data for plot

---

```
# Castaneamollissima_scaffold13692-abinit-gene-0.0d13692 Length: 62
# Castaneamollissima_scaffold13692-abinit-gene-0.0d13692 Number of predicted TMHs:  0
# Castaneamollissima_scaffold13692-abinit-gene-0.0d13692 Exp number of AAs in TMHs: 0.00335
# Castaneamollissima_scaffold13692-abinit-gene-0.0d13692 Exp number, first 60 AAs:  0.00335
# Castaneamollissima_scaffold13692-abinit-gene-0.0d13692 Total prob of N-in:        0.21937
Castaneamollissima_scaffold13692-abinit-gene-0.0d13692  TMHMM2.0        outside      1    62
```

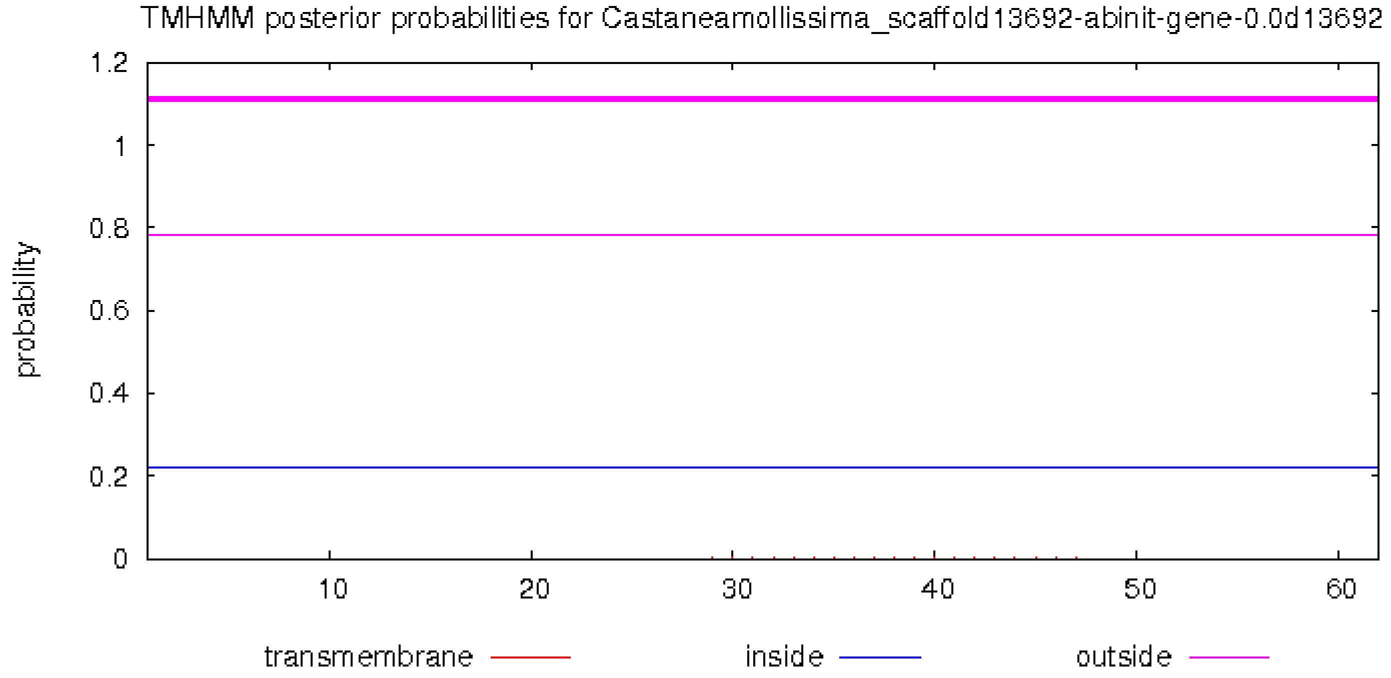

plot in postscript, script for making the plot in gnuplot, data for plot

---

```
# Castaneamollissima_scaffold00002-augustus-gene-1.34 Length: 288
# Castaneamollissima_scaffold00002-augustus-gene-1.34 Number of predicted TMHs:  0
# Castaneamollissima_scaffold00002-augustus-gene-1.34 Exp number of AAs in TMHs: 0.00043
# Castaneamollissima_scaffold00002-augustus-gene-1.34 Exp number, first 60 AAs:  0.00043
# Castaneamollissima_scaffold00002-augustus-gene-1.34 Total prob of N-in:        0.00361
Castaneamollissima_scaffold00002-augustus-gene-1.34	TMHMM2.0	outside	     1   288
```

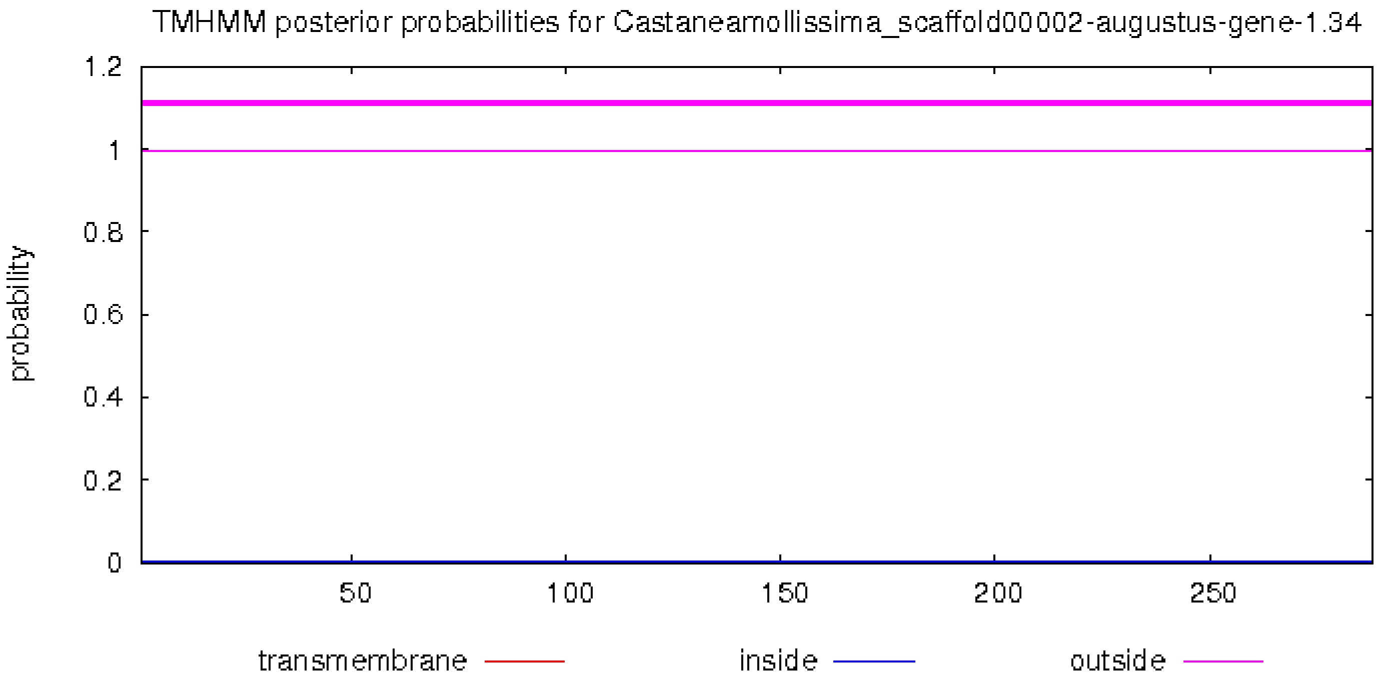

plot in postscript, script for making the plot in gnuplot, data for plot

---

```
# Castaneamollissima_scaffold00005-augustus-gene-0.28 Length: 242
# Castaneamollissima_scaffold00005-augustus-gene-0.28 Number of predicted TMHs:  0
# Castaneamollissima_scaffold00005-augustus-gene-0.28 Exp number of AAs in TMHs: 0.00015
# Castaneamollissima_scaffold00005-augustus-gene-0.28 Exp number, first 60 AAs:  0
# Castaneamollissima_scaffold00005-augustus-gene-0.28 Total prob of N-in:        0.00248
Castaneamollissima_scaffold00005-augustus-gene-0.28	TMHMM2.0	outside	     1   242
```

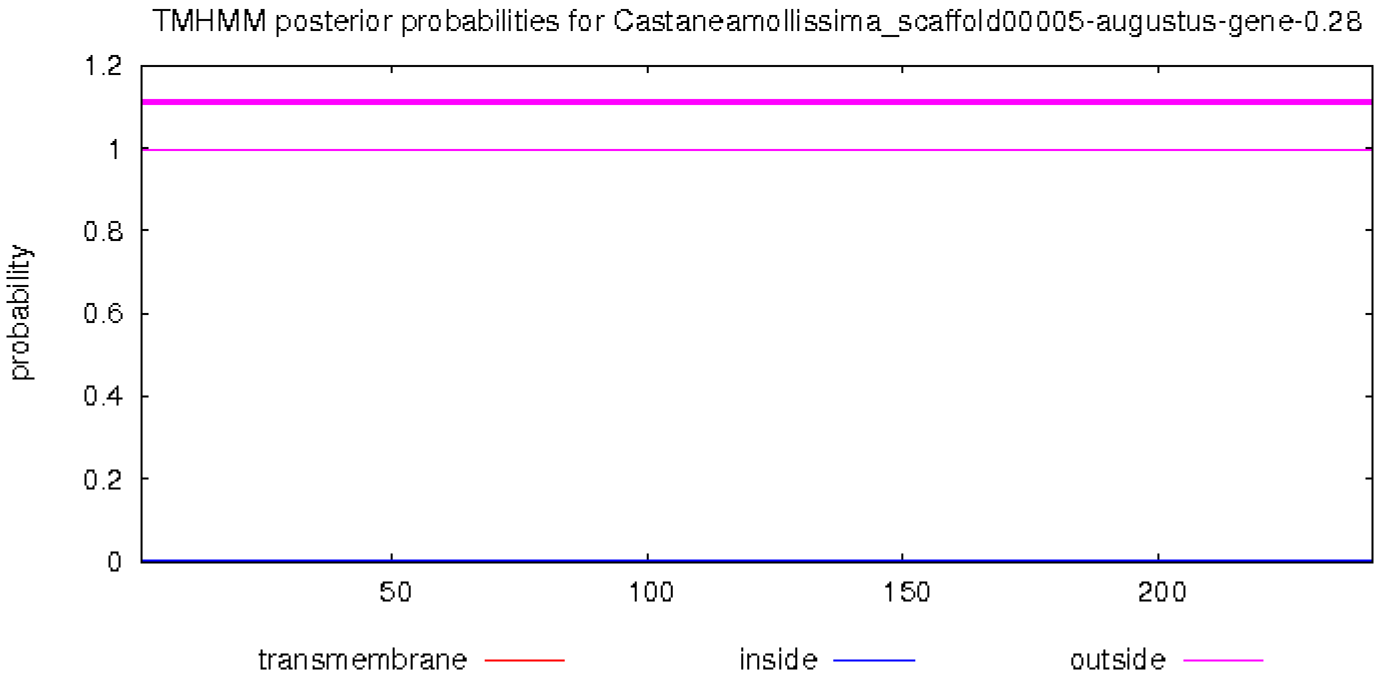

plot in postscript, script for making the plot in gnuplot, data for plot

---

```
# Castaneamollissima_scaffold00018-augustus-gene-0.30 Length: 406
# Castaneamollissima_scaffold00018-augustus-gene-0.30 Number of predicted TMHs:  0
# Castaneamollissima_scaffold00018-augustus-gene-0.30 Exp number of AAs in TMHs: 0
# Castaneamollissima_scaffold00018-augustus-gene-0.30 Exp number, first 60 AAs:  0
# Castaneamollissima_scaffold00018-augustus-gene-0.30 Total prob of N-in:        0.00618
Castaneamollissima_scaffold00018-augustus-gene-0.30     TMHMM2.0        outside      1   406
```

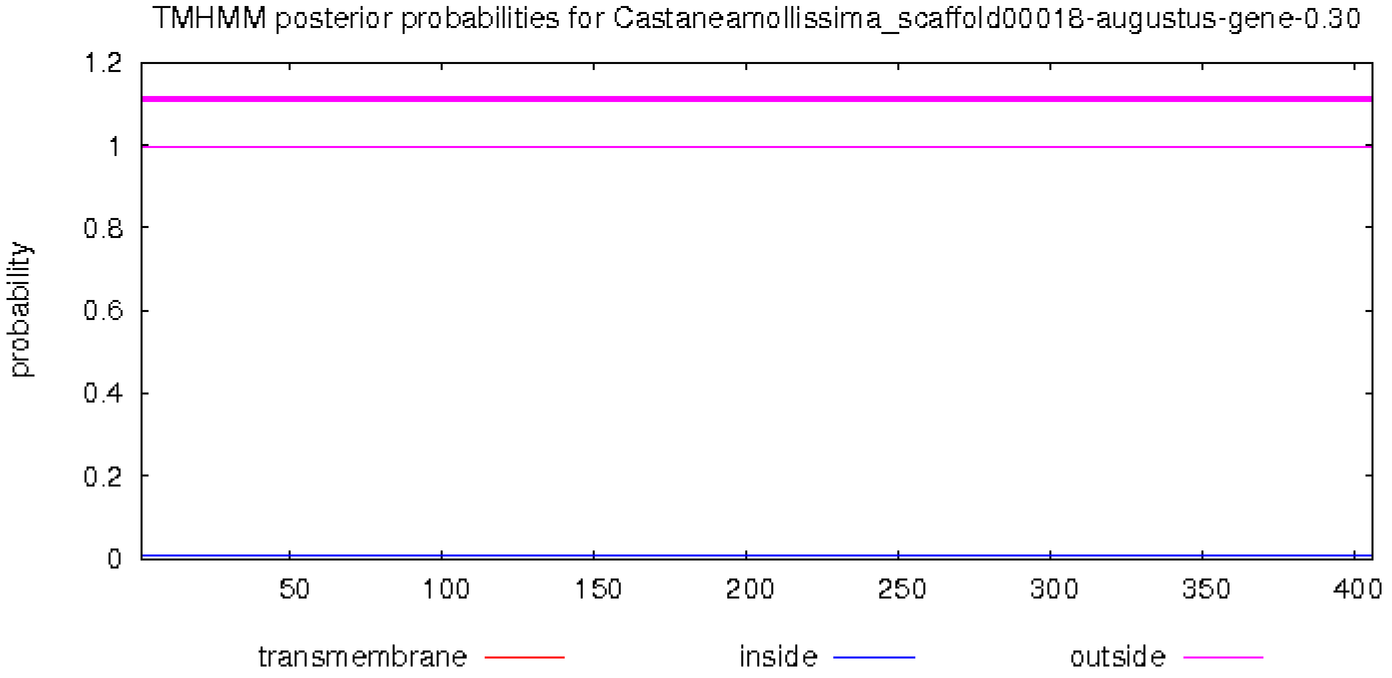

plot in postscript, script for making the plot in gnuplot, data for plot

---

```
# Castaneamollissima_scaffold00057-snap-gene-0.53 Length: 339
# Castaneamollissima_scaffold00057-snap-gene-0.53 Number of predicted TMHs:  0
# Castaneamollissima_scaffold00057-snap-gene-0.53 Exp number of AAs in TMHs: 0.00018
# Castaneamollissima_scaffold00057-snap-gene-0.53 Exp number, first 60 AAs:  0
# Castaneamollissima_scaffold00057-snap-gene-0.53 Total prob of N-in:        0.02941
Castaneamollissima_scaffold00057-snap-gene-0.53 TMHMM2.0        outside      1   339
```

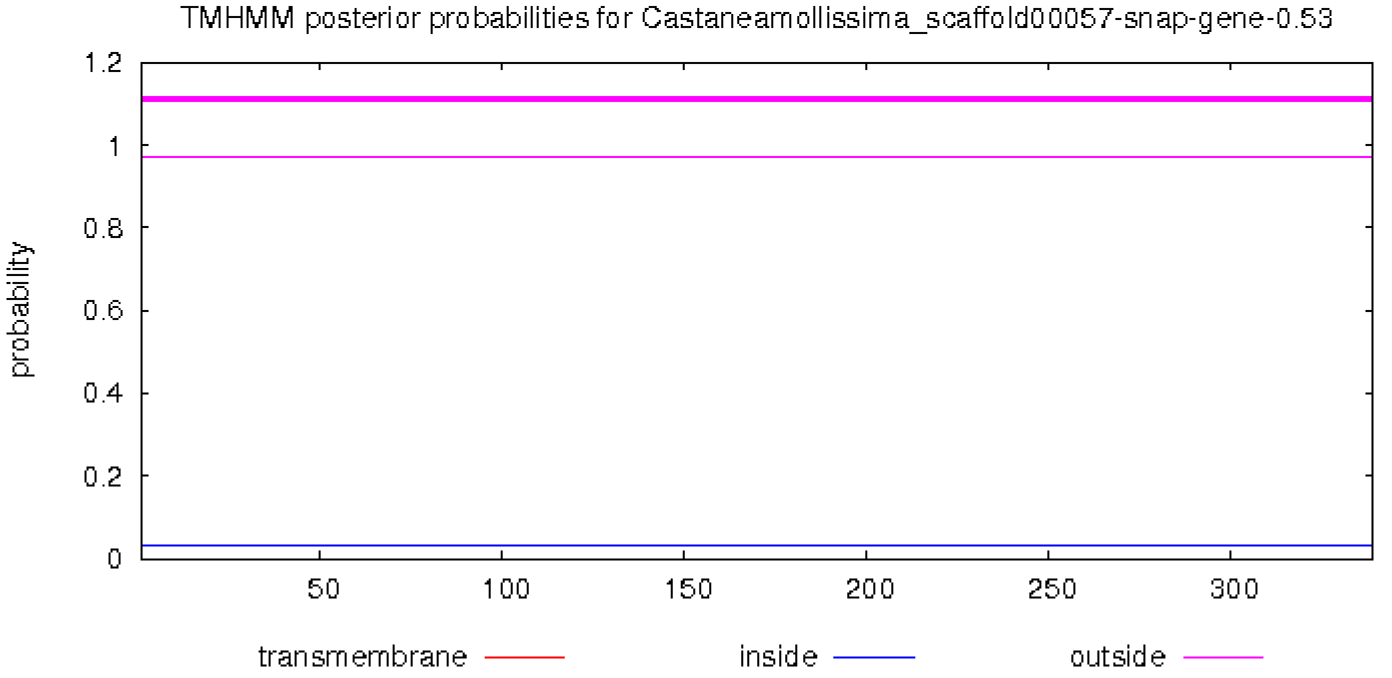

plot in postscript, script for making the plot in gnuplot, data for plot

---

```
# Castaneamollissima_scaffold00345-augustus-gene-0.38 Length: 317
# Castaneamollissima_scaffold00345-augustus-gene-0.38 Number of predicted TMHs:  0
# Castaneamollissima_scaffold00345-augustus-gene-0.38 Exp number of AAs in TMHs: 0.02244
# Castaneamollissima_scaffold00345-augustus-gene-0.38 Exp number, first 60 AAs:  0
# Castaneamollissima_scaffold00345-augustus-gene-0.38 Total prob of N-in:        0.00298
Castaneamollissima_scaffold00345-augustus-gene-0.38     TMHMM2.0        outside      1   317
```

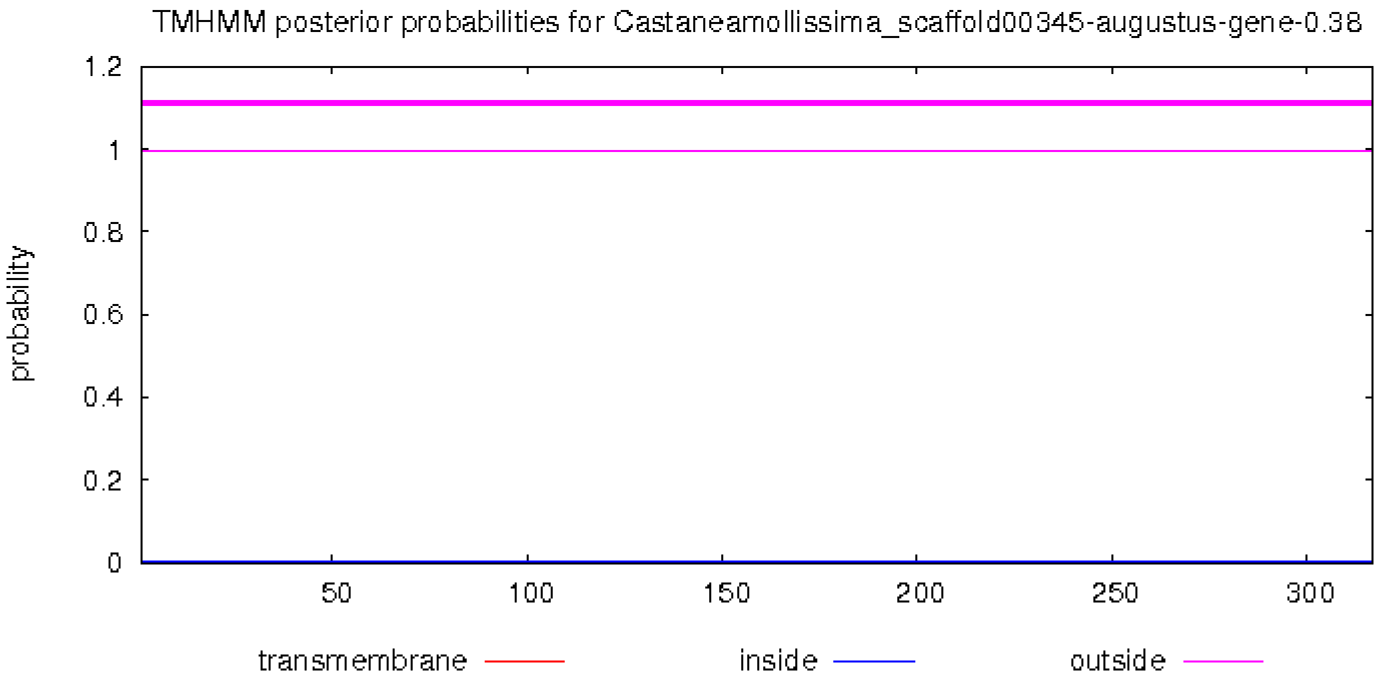

plot in postscript, script for making the plot in gnuplot, data for plot

---

```
# Castaneamollissima_scaffold00745-snap-gene-0.42 Length: 317
# Castaneamollissima_scaffold00745-snap-gene-0.42 Number of predicted TMHs:  0
# Castaneamollissima_scaffold00745-snap-gene-0.42 Exp number of AAs in TMHs: 0
# Castaneamollissima_scaffold00745-snap-gene-0.42 Exp number, first 60 AAs:  0
# Castaneamollissima_scaffold00745-snap-gene-0.42 Total prob of N-in:        0.00532
Castaneamollissima_scaffold00745-snap-gene-0.42 TMHMM2.0        outside      1   317
```

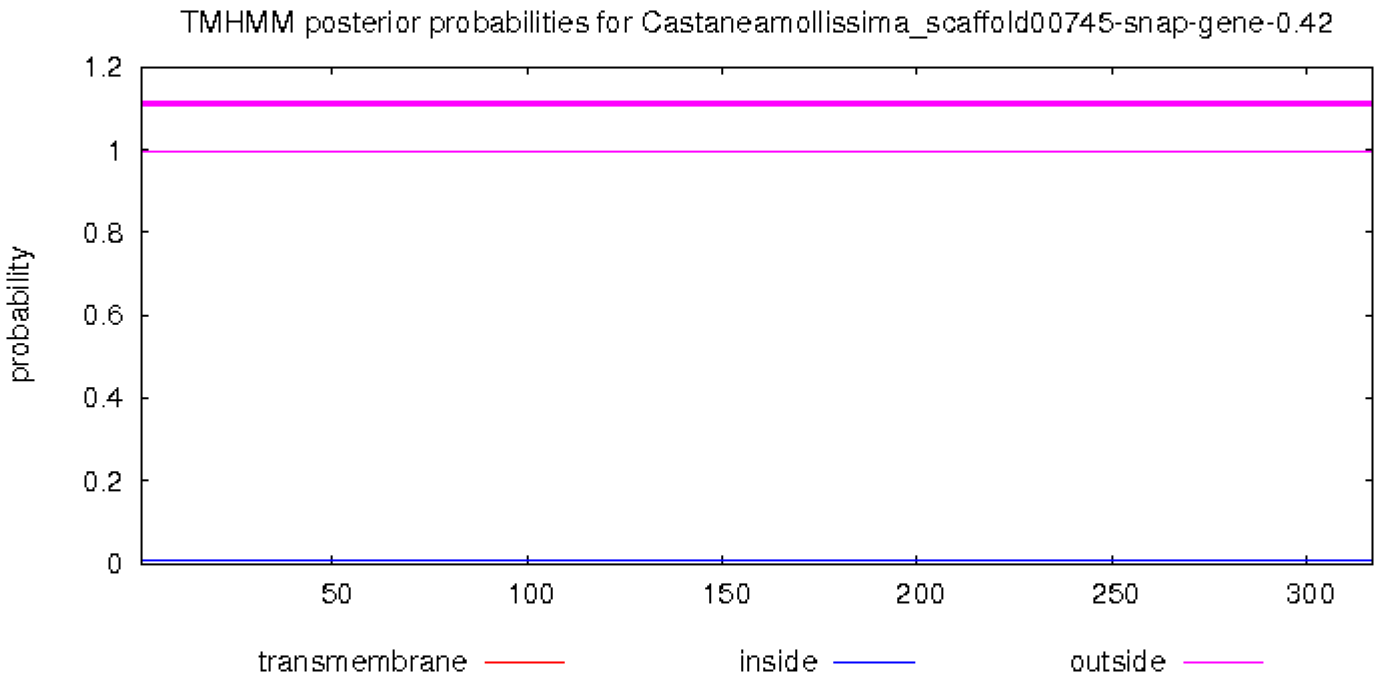

plot in postscript, script for making the plot in gnuplot, data for plot

---

```
# Castaneamollissima_scaffold00830-augustus-gene-0.42 Length: 292
# Castaneamollissima_scaffold00830-augustus-gene-0.42 Number of predicted TMHs:  0
# Castaneamollissima_scaffold00830-augustus-gene-0.42 Exp number of AAs in TMHs: 0.00063
# Castaneamollissima_scaffold00830-augustus-gene-0.42 Exp number, first 60 AAs:  0.00063
# Castaneamollissima_scaffold00830-augustus-gene-0.42 Total prob of N-in:        0.00207
Castaneamollissima_scaffold00830-augustus-gene-0.42     TMHMM2.0        outside      1   292
```

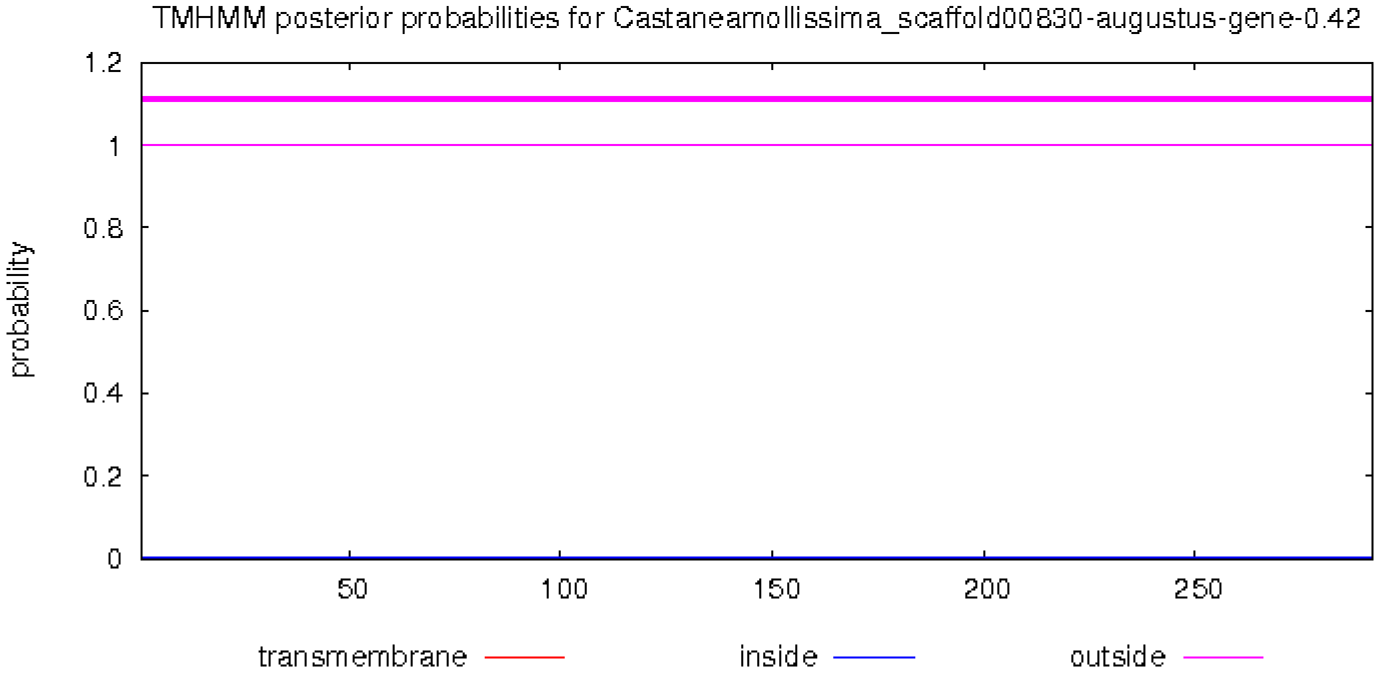

plot in postscript, script for making the plot in gnuplot, data for plot

---

```
# Castaneamollissima_scaffold00860-augustus-gene-0.36 Length: 314
# Castaneamollissima_scaffold00860-augustus-gene-0.36 Number of predicted TMHs:  0
# Castaneamollissima_scaffold00860-augustus-gene-0.36 Exp number of AAs in TMHs: 0.00011
# Castaneamollissima_scaffold00860-augustus-gene-0.36 Exp number, first 60 AAs:  0
# Castaneamollissima_scaffold00860-augustus-gene-0.36 Total prob of N-in:        0.02098
Castaneamollissima_scaffold00860-augustus-gene-0.36     TMHMM2.0        outside      1   314
```

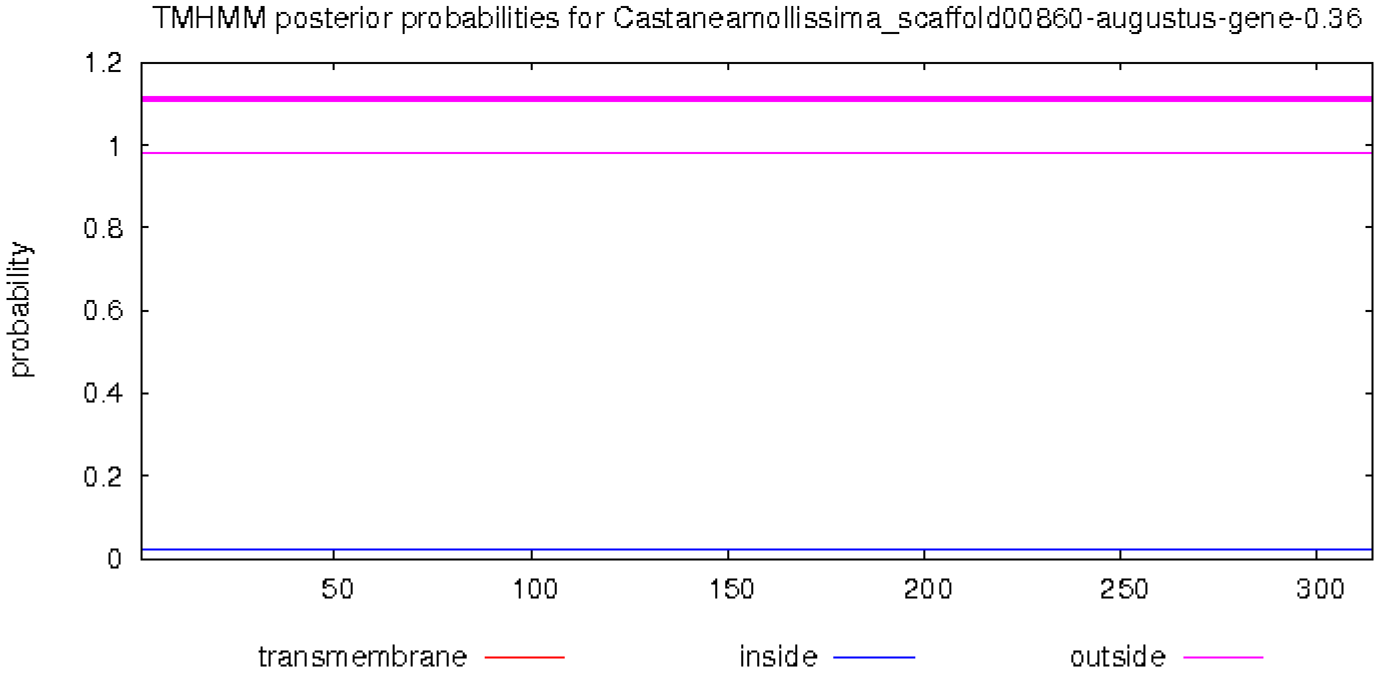

plot in postscript, script for making the plot in gnuplot, data for plot

---

```
# Castaneamollissima_scaffold00873-snap-gene-0.35 Length: 441
# Castaneamollissima_scaffold00873-snap-gene-0.35 Number of predicted TMHs:  0
# Castaneamollissima_scaffold00873-snap-gene-0.35 Exp number of AAs in TMHs: 0
# Castaneamollissima_scaffold00873-snap-gene-0.35 Exp number, first 60 AAs:  0
# Castaneamollissima_scaffold00873-snap-gene-0.35 Total prob of N-in:        0.01229
Castaneamollissima_scaffold00873-snap-gene-0.35 TMHMM2.0        outside      1   441
```

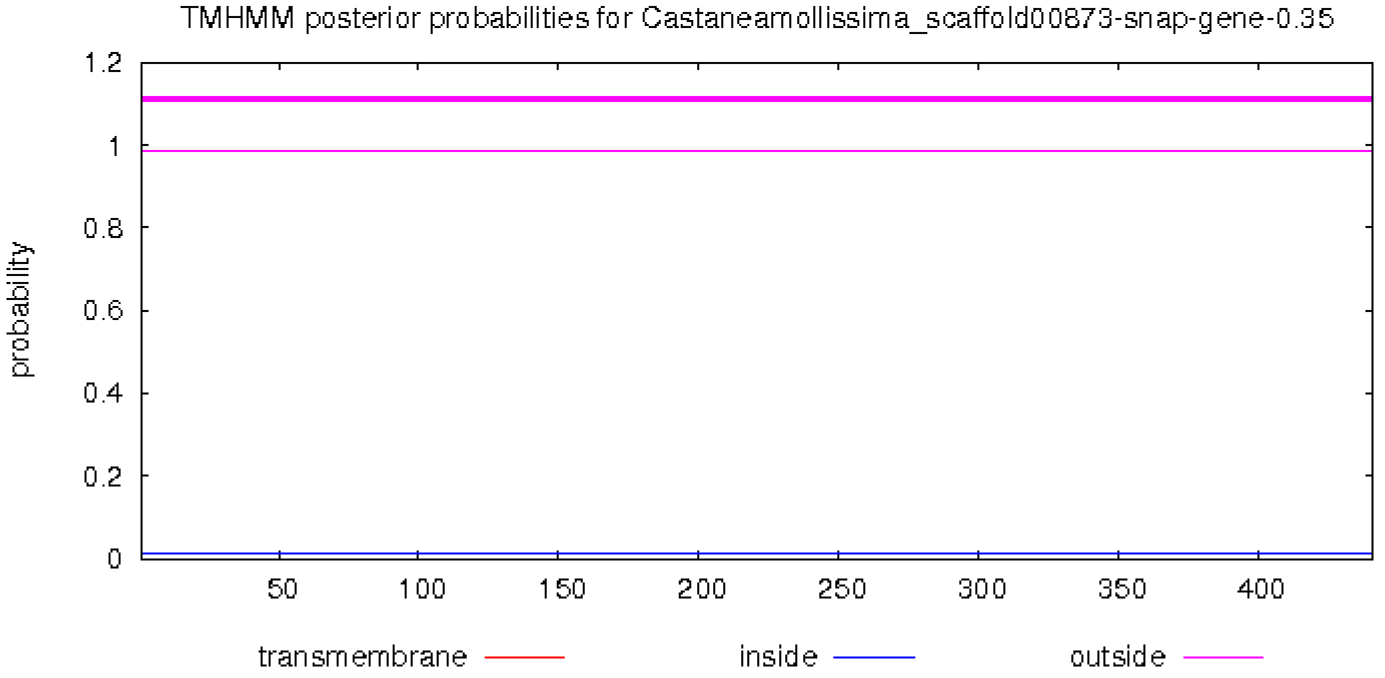

plot in postscript, script for making the plot in gnuplot, data for plot

```
# Castaneamollissima_scaffold00890-augustus-gene-0.33 Length: 355
# Castaneamollissima_scaffold00890-augustus-gene-0.33 Number of predicted TMHs:  0
# Castaneamollissima_scaffold00890-augustus-gene-0.33 Exp number of AAs in TMHs: 0.00053
# Castaneamollissima_scaffold00890-augustus-gene-0.33 Exp number, first 60 AAs:  0.00014
# Castaneamollissima_scaffold00890-augustus-gene-0.33 Total prob of N-in:        0.00471
Castaneamollissima_scaffold00890-augustus-gene-0.33     TMHMM2.0        outside      1   355
```

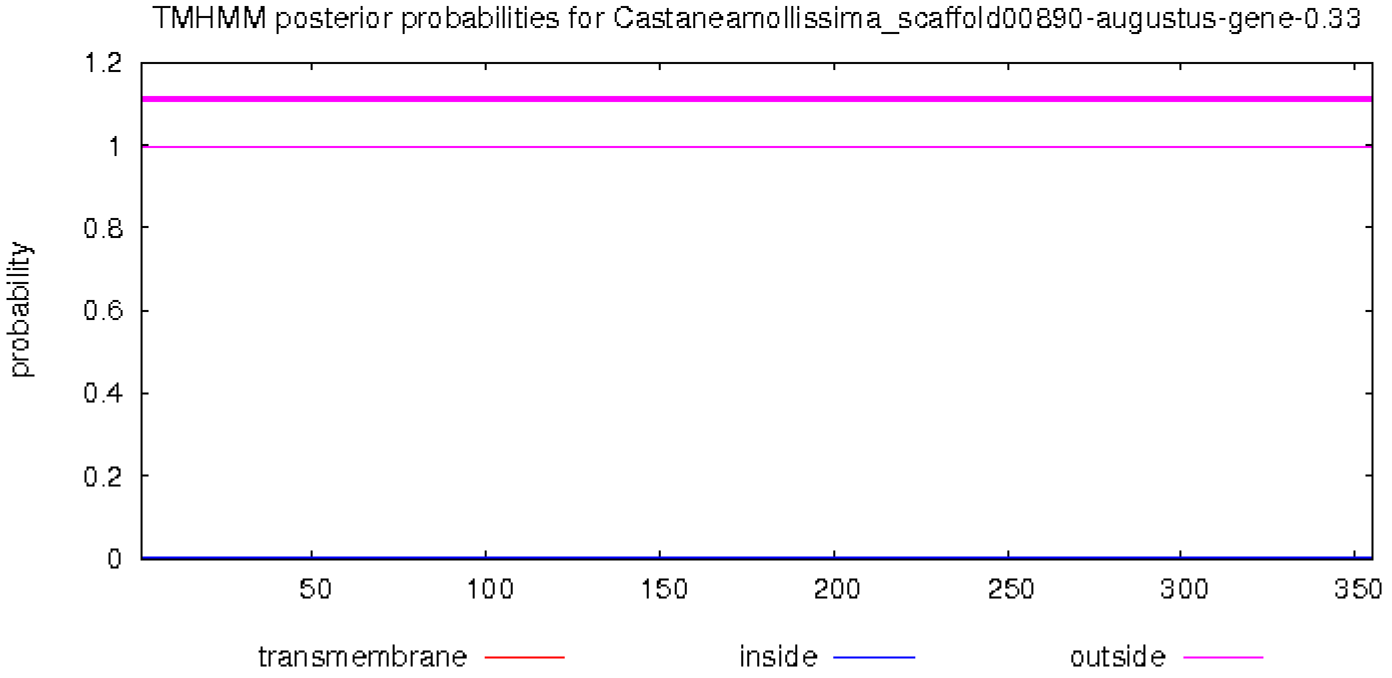

plot in postscript, script for making the plot in gnuplot, data for plot

```
# Castaneamollissima_scaffold00983-snap-gene-0.43 Length: 99
# Castaneamollissima_scaffold00983-snap-gene-0.43 Number of predicted TMHs:  0
# Castaneamollissima_scaffold00983-snap-gene-0.43 Exp number of AAs in TMHs: 0.00178
# Castaneamollissima_scaffold00983-snap-gene-0.43 Exp number, first 60 AAs:  0.00087
# Castaneamollissima_scaffold00983-snap-gene-0.43 Total prob of N-in:        0.54058
Castaneamollissima_scaffold00983-snap-gene-0.43 TMHMM2.0        inside       1    99
```

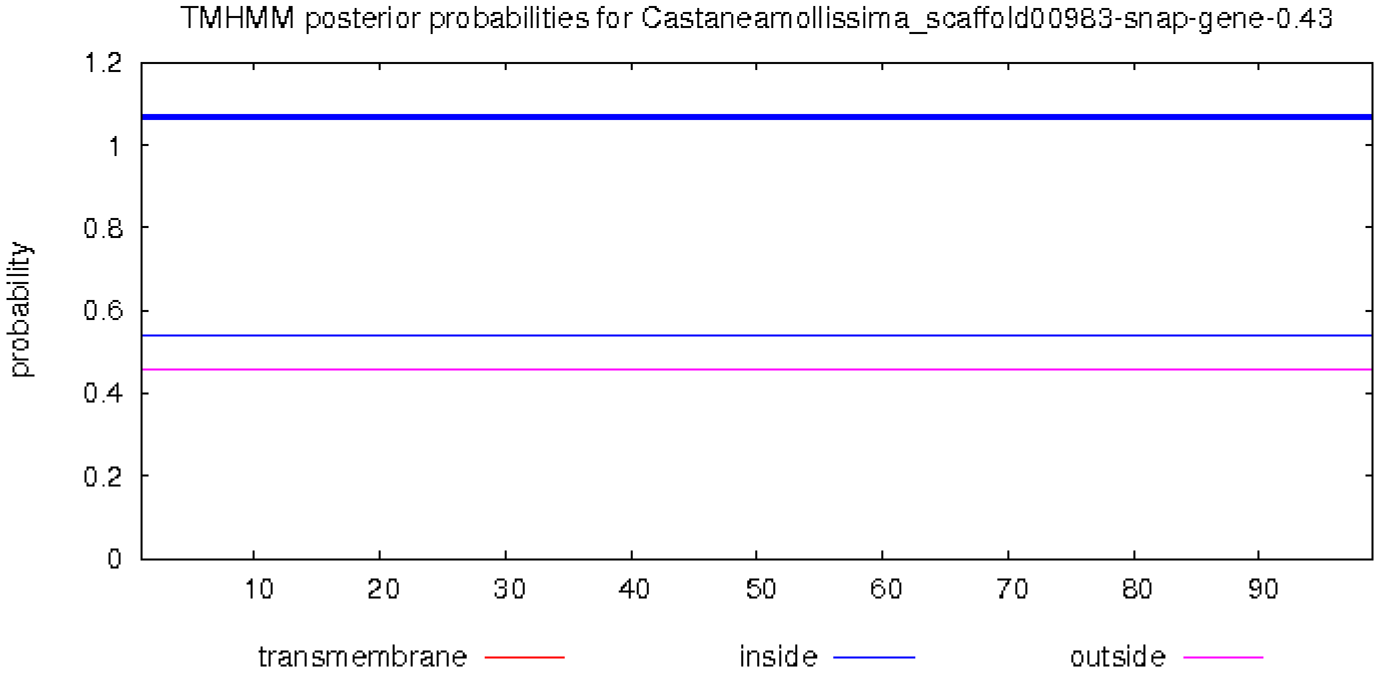

plot in postscript, script for making the plot in gnuplot, data for plot

---

```
# Castaneamollissima_scaffold01061-augustus-gene-0.30 Length: 188
# Castaneamollissima_scaffold01061-augustus-gene-0.30 Number of predicted TMHs:  0
# Castaneamollissima_scaffold01061-augustus-gene-0.30 Exp number of AAs in TMHs: 0.00093
# Castaneamollissima_scaffold01061-augustus-gene-0.30 Exp number, first 60 AAs:  0
# Castaneamollissima_scaffold01061-augustus-gene-0.30 Total prob of N-in:        0.04837
Castaneamollissima_scaffold01061-augustus-gene-0.30     TMHMM2.0        outside      1   188
```

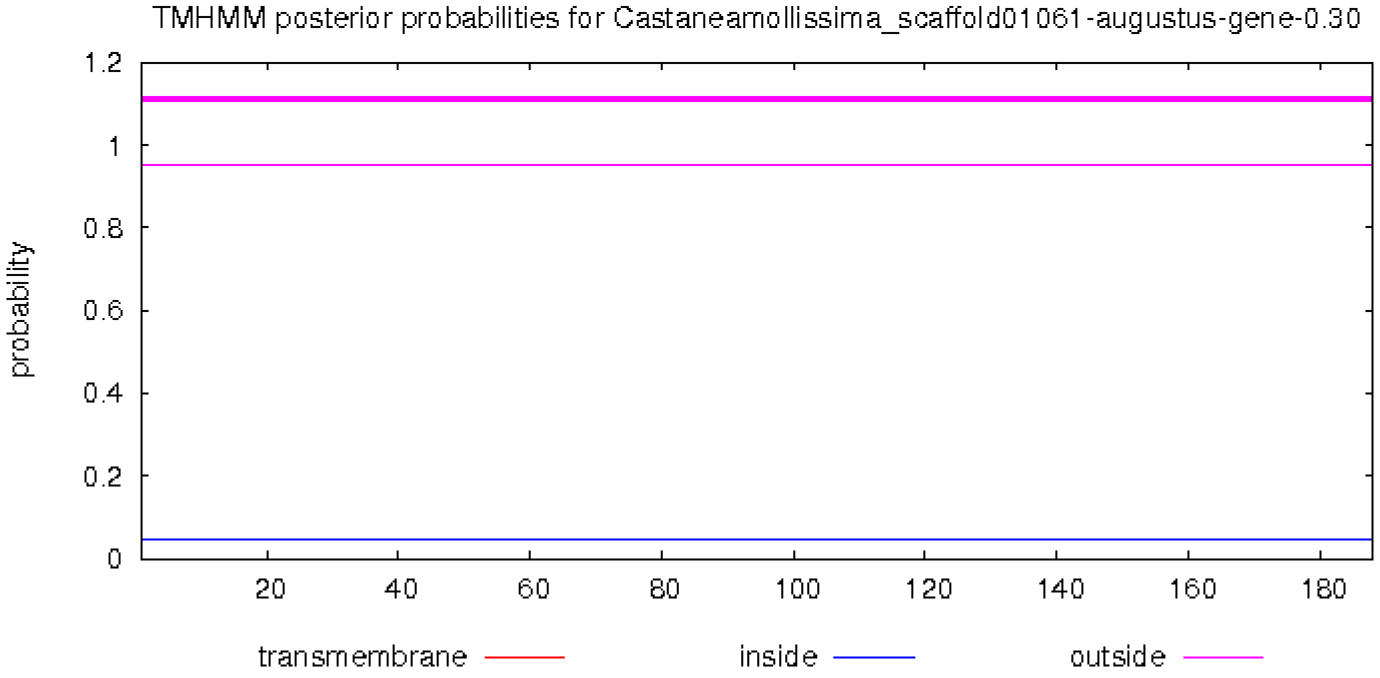

plot in postscript, script for making the plot in gnuplot, data for plot

---

```
# Castaneamollissima_scaffold01636-snap-gene-0.46 Length: 183
# Castaneamollissima_scaffold01636-snap-gene-0.46 Number of predicted TMHs:  0
# Castaneamollissima_scaffold01636-snap-gene-0.46 Exp number of AAs in TMHs: 0.01104
# Castaneamollissima_scaffold01636-snap-gene-0.46 Exp number, first 60 AAs:  0
# Castaneamollissima_scaffold01636-snap-gene-0.46 Total prob of N-in:        0.06942
Castaneamollissima_scaffold01636-snap-gene-0.46 TMHMM2.0        outside      1   183
```

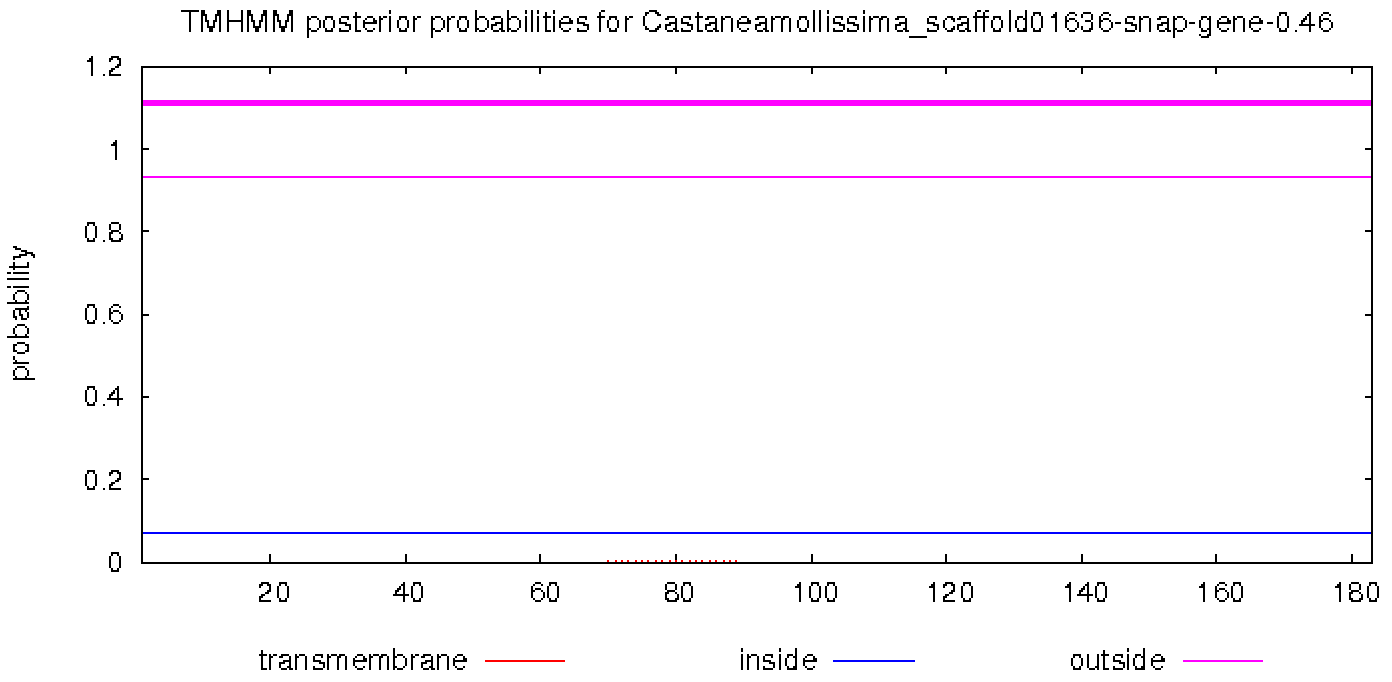

plot in postscript, script for making the plot in gnuplot, data for plot

---

```
# Castaneamollissima_scaffold01655-augustus-gene-0.20 Length: 344
# Castaneamollissima_scaffold01655-augustus-gene-0.20 Number of predicted TMHs:  0
# Castaneamollissima_scaffold01655-augustus-gene-0.20 Exp number of AAs in TMHs: 0.0098
# Castaneamollissima_scaffold01655-augustus-gene-0.20 Exp number, first 60 AAs:  0.0002
# Castaneamollissima_scaffold01655-augustus-gene-0.20 Total prob of N-in:        0.04615
Castaneamollissima_scaffold01655-augustus-gene-0.20     TMHMM2.0        outside      1   344
```

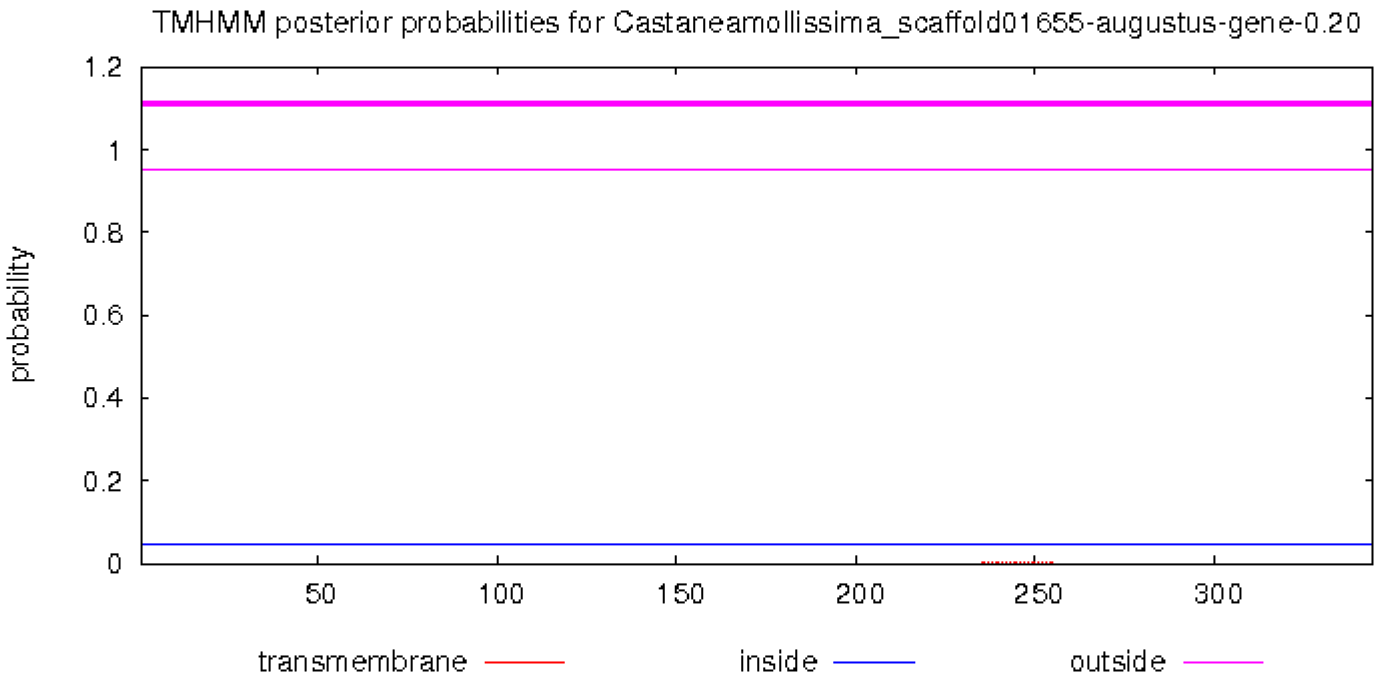

plot in postscript, script for making the plot in gnuplot, data for plot

---

```
# Castaneamollissima_scaffold01684-snap-gene-0.33 Length: 432
# Castaneamollissima_scaffold01684-snap-gene-0.33 Number of predicted TMHs:  1
# Castaneamollissima_scaffold01684-snap-gene-0.33 Exp number of AAs in TMHs: 21.90669
# Castaneamollissima_scaffold01684-snap-gene-0.33 Exp number, first 60 AAs:  1.77003
# Castaneamollissima_scaffold01684-snap-gene-0.33 Total prob of N-in:        0.84603
Castaneamollissima_scaffold01684-snap-gene-0.33 TMHMM2.0        inside       1    73
Castaneamollissima_scaffold01684-snap-gene-0.33 TMHMM2.0        TMhelix     74    96
Castaneamollissima_scaffold01684-snap-gene-0.33 TMHMM2.0        outside     97   432
```

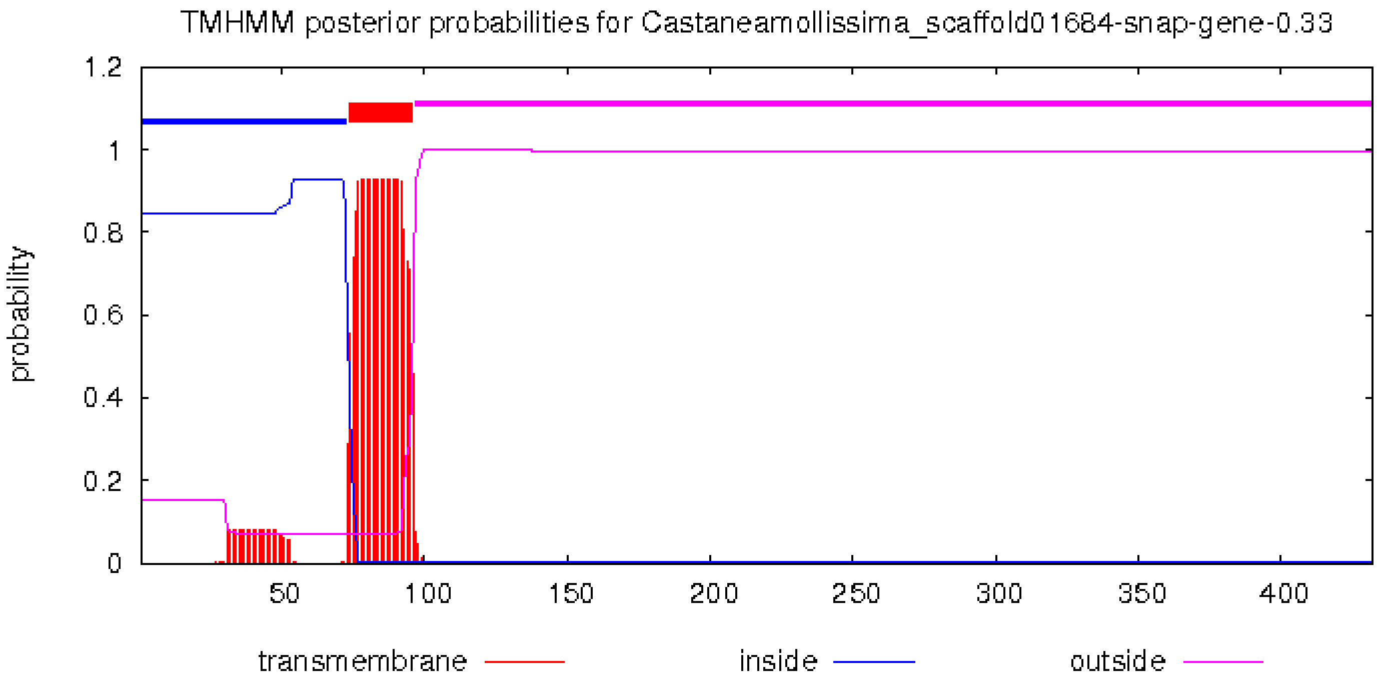

plot in postscript, script for making the plot in gnuplot, data for plot

---

```
# Castaneamollissima_scaffold01789-snap-gene-0.35 Length: 303
# Castaneamollissima_scaffold01789-snap-gene-0.35 Number of predicted TMHs:  0
# Castaneamollissima_scaffold01789-snap-gene-0.35 Exp number of AAs in TMHs: 0.0023
# Castaneamollissima_scaffold01789-snap-gene-0.35 Exp number, first 60 AAs:  0
# Castaneamollissima_scaffold01789-snap-gene-0.35 Total prob of N-in:        0.01874
Castaneamollissima_scaffold01789-snap-gene-0.35 TMHMM2.0        outside      1   303
```

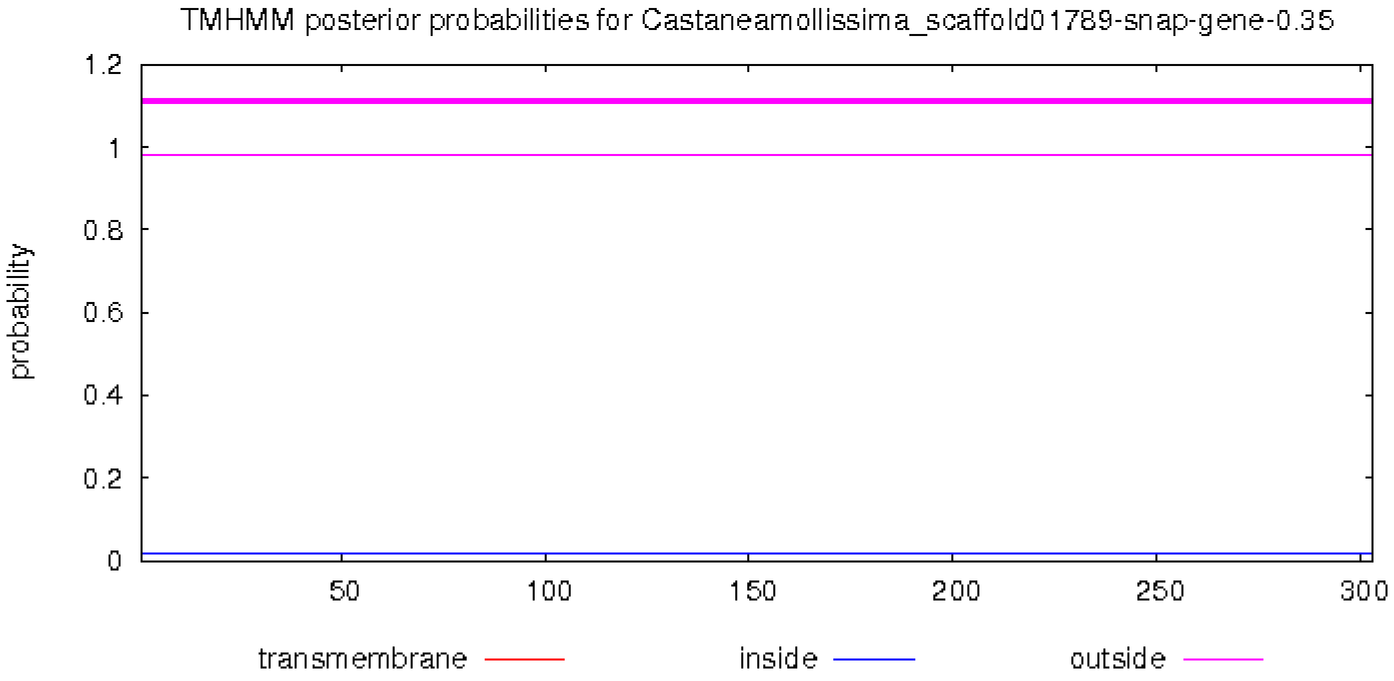

plot in postscript, script for making the plot in gnuplot, data for plot

```
# Castaneamollissima_scaffold02051-augustus-gene-0.10 Length: 291
# Castaneamollissima_scaffold02051-augustus-gene-0.10 Number of predicted TMHs:  0
# Castaneamollissima_scaffold02051-augustus-gene-0.10 Exp number of AAs in TMHs: 0.00091
# Castaneamollissima_scaffold02051-augustus-gene-0.10 Exp number, first 60 AAs:  0.00071
# Castaneamollissima_scaffold02051-augustus-gene-0.10 Total prob of N-in:        0.02127
Castaneamollissima_scaffold02051-augustus-gene-0.10     TMHMM2.0        outside      1   291
```

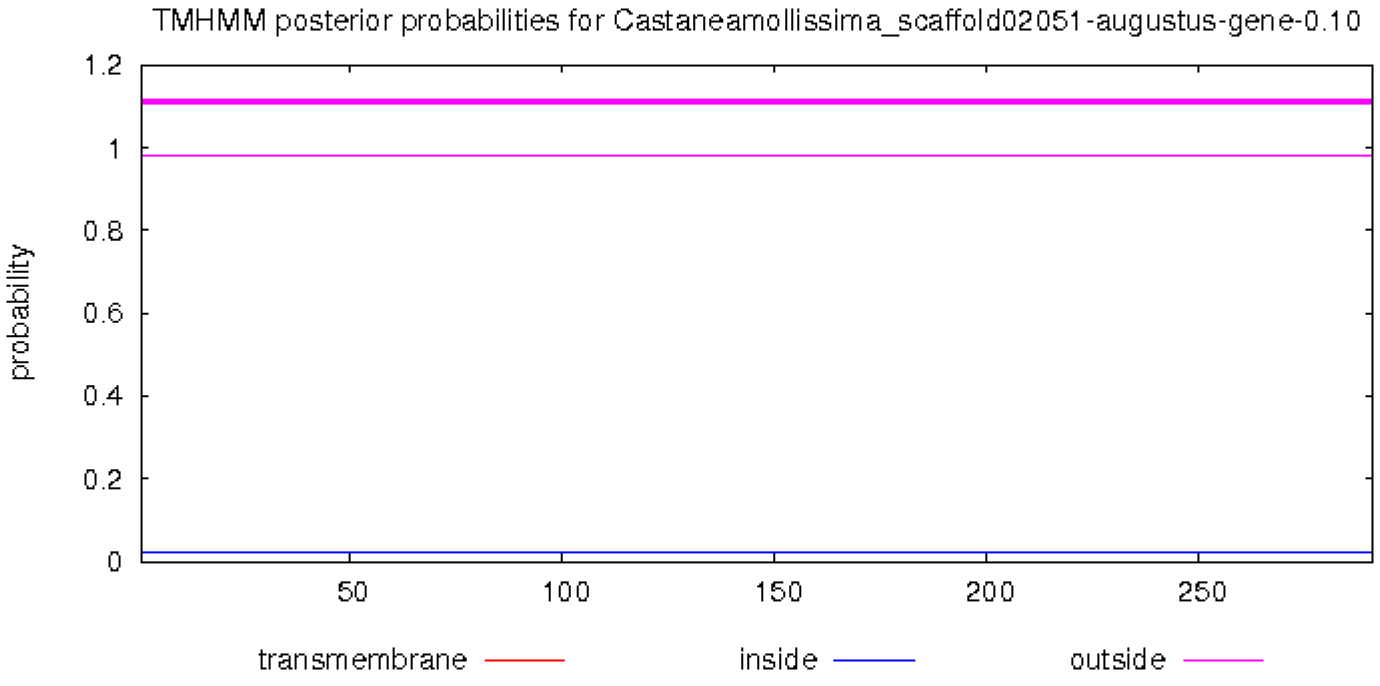

plot in postscript, script for making the plot in gnuplot, data for plot

```
# Castaneamollissima_scaffold02306-augustus-gene-0.21 Length: 346
# Castaneamollissima_scaffold02306-augustus-gene-0.21 Number of predicted TMHs:  0
# Castaneamollissima_scaffold02306-augustus-gene-0.21 Exp number of AAs in TMHs: 0.01205
# Castaneamollissima_scaffold02306-augustus-gene-0.21 Exp number, first 60 AAs:  9e-05
# Castaneamollissima_scaffold02306-augustus-gene-0.21 Total prob of N-in:        0.01471
Castaneamollissima_scaffold02306-augustus-gene-0.21     TMHMM2.0        outside      1   346
```

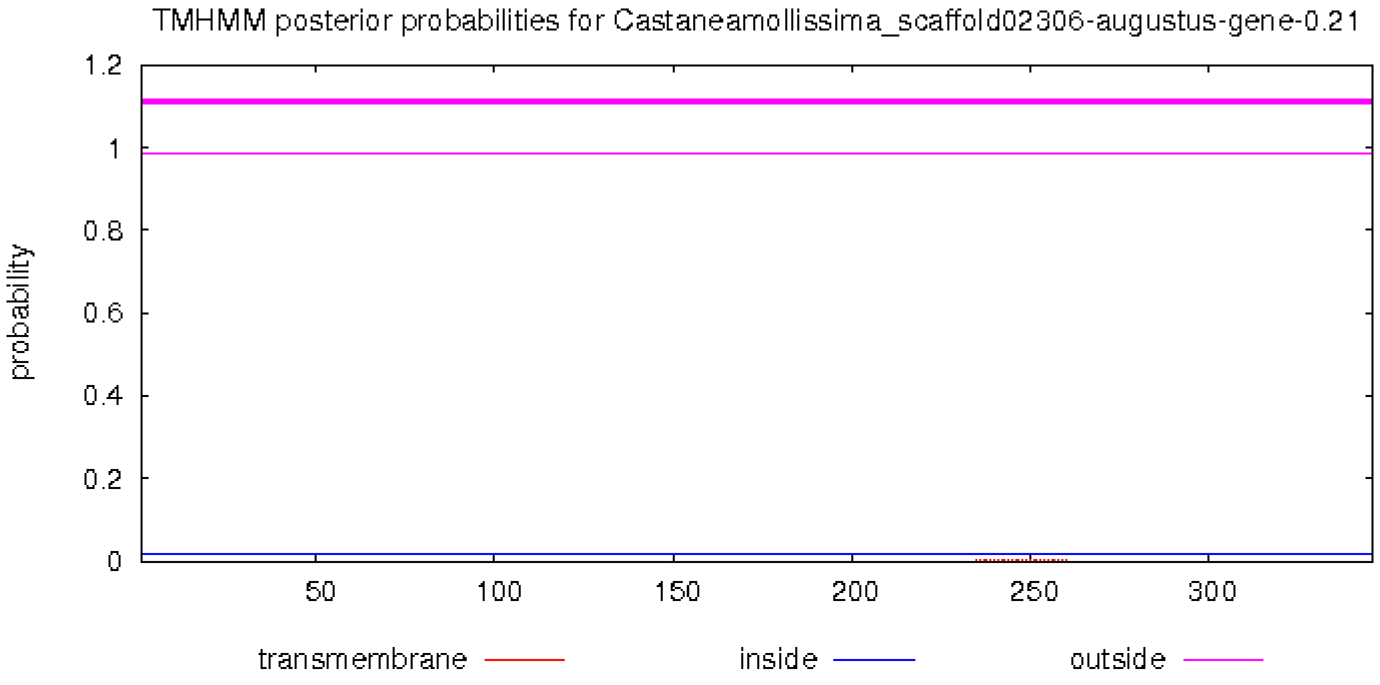

plot in postscript, script for making the plot in gnuplot, data for plot

```
# Castaneamollissima_scaffold02433-augustus-gene-0.7 Length: 332
# Castaneamollissima_scaffold02433-augustus-gene-0.7 Number of predicted TMHs:  0
# Castaneamollissima_scaffold02433-augustus-gene-0.7 Exp number of AAs in TMHs: 0
# Castaneamollissima_scaffold02433-augustus-gene-0.7 Exp number, first 60 AAs:  0
# Castaneamollissima_scaffold02433-augustus-gene-0.7 Total prob of N-in:        0.00331
Castaneamollissima_scaffold02433-augustus-gene-0.7      TMHMM2.0        outside      1   332
```

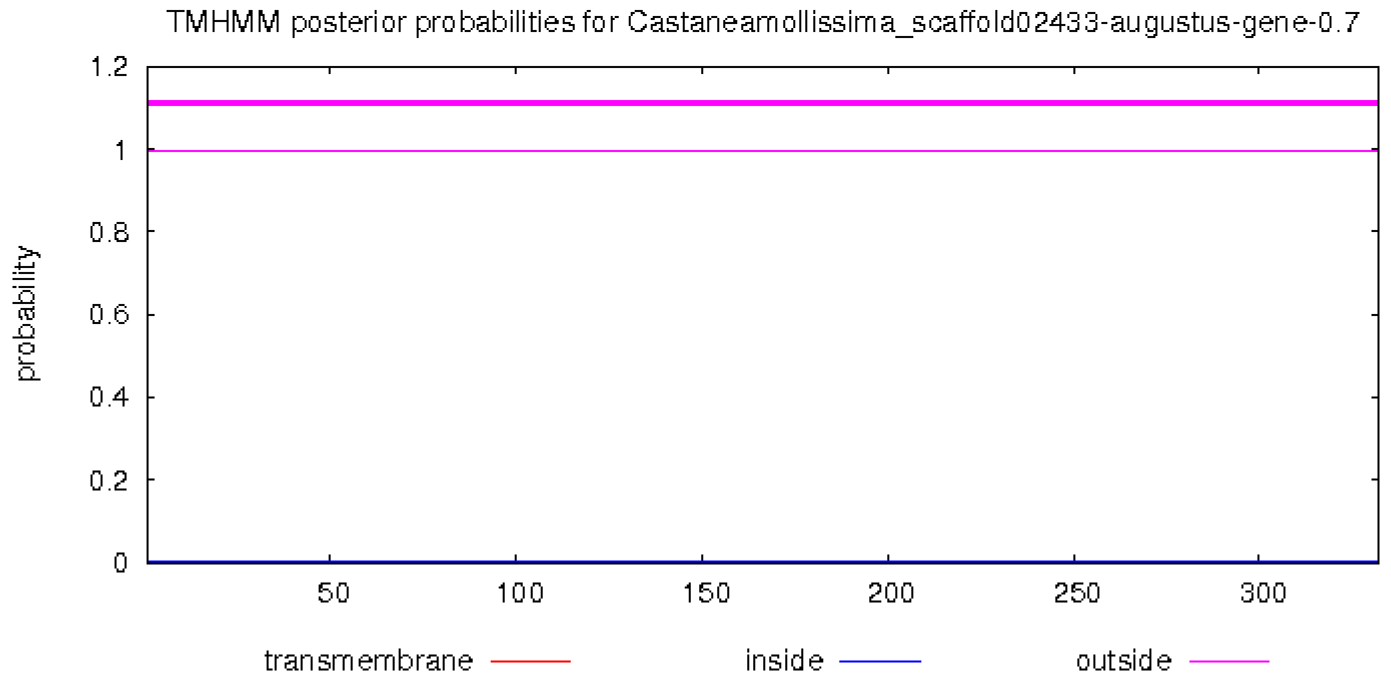

plot in postscript, script for making the plot in gnuplot, data for plot

---

```
# Castaneamollissima_scaffold02656-augustus-gene-0.15 Length: 379
# Castaneamollissima_scaffold02656-augustus-gene-0.15 Number of predicted TMHs:  0
# Castaneamollissima_scaffold02656-augustus-gene-0.15 Exp number of AAs in TMHs: 0.0002
# Castaneamollissima_scaffold02656-augustus-gene-0.15 Exp number, first 60 AAs:  0
# Castaneamollissima_scaffold02656-augustus-gene-0.15 Total prob of N-in:        0.00288
Castaneamollissima_scaffold02656-augustus-gene-0.15     TMHMM2.0        outside      1   379
```

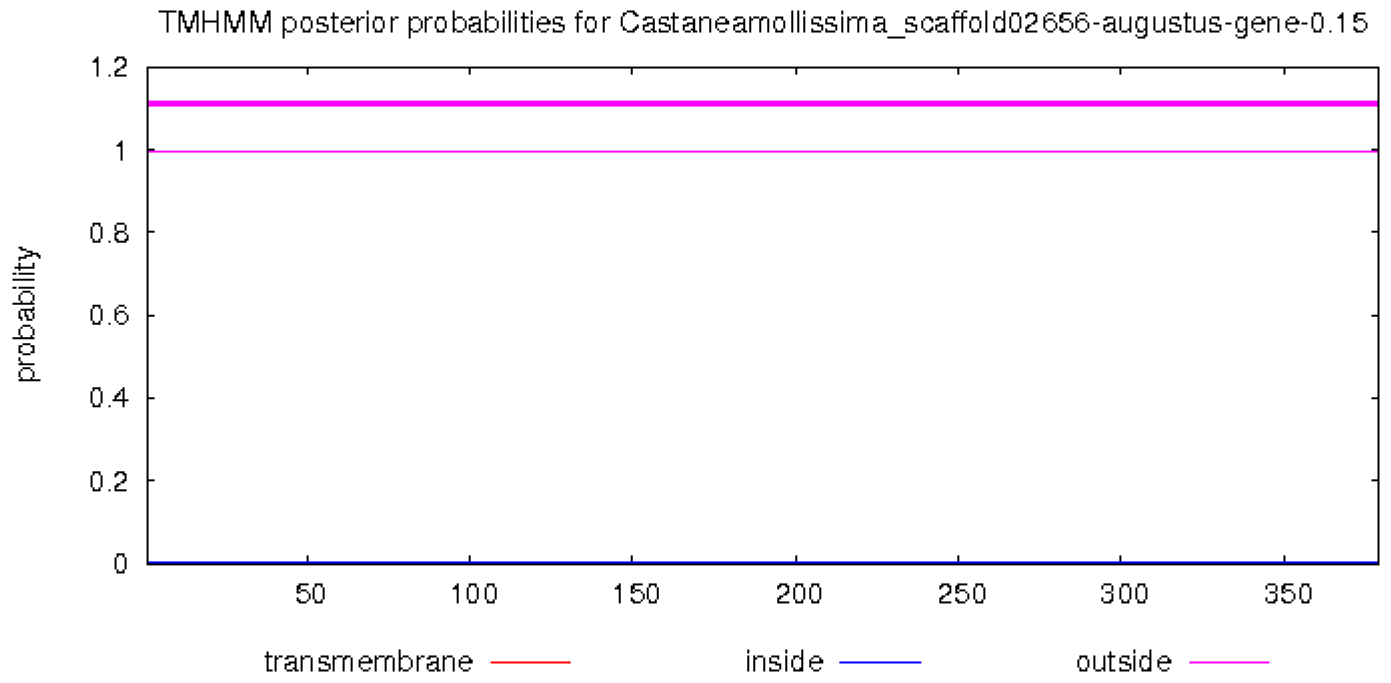

plot in postscript, script for making the plot in gnuplot, data for plot

---

```
# Castaneamollissima_scaffold02751-augustus-gene-0.20 Length: 296
# Castaneamollissima_scaffold02751-augustus-gene-0.20 Number of predicted TMHs:  0
# Castaneamollissima_scaffold02751-augustus-gene-0.20 Exp number of AAs in TMHs: 0.00128
# Castaneamollissima_scaffold02751-augustus-gene-0.20 Exp number, first 60 AAs:  0
# Castaneamollissima_scaffold02751-augustus-gene-0.20 Total prob of N-in:        0.04733
Castaneamollissima_scaffold02751-augustus-gene-0.20     TMHMM2.0        outside      1   296
```

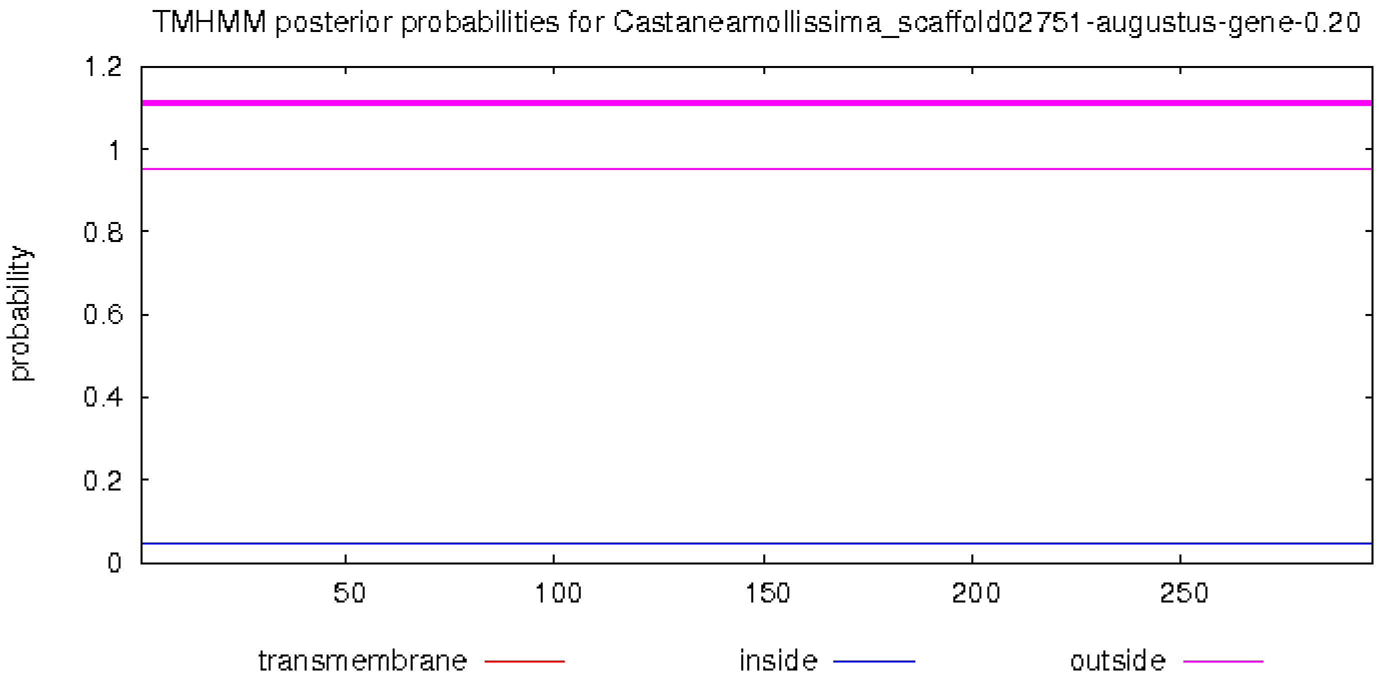

plot in postscript, script for making the plot in gnuplot, data for plot

---

```
# Castaneamollissima_scaffold02784-augustus-gene-0.30 Length: 192
# Castaneamollissima_scaffold02784-augustus-gene-0.30 Number of predicted TMHs:  0
# Castaneamollissima_scaffold02784-augustus-gene-0.30 Exp number of AAs in TMHs: 0.01162
# Castaneamollissima_scaffold02784-augustus-gene-0.30 Exp number, first 60 AAs:  0
# Castaneamollissima_scaffold02784-augustus-gene-0.30 Total prob of N-in:        0.13189
Castaneamollissima_scaffold02784-augustus-gene-0.30     TMHMM2.0        outside      1   192
```

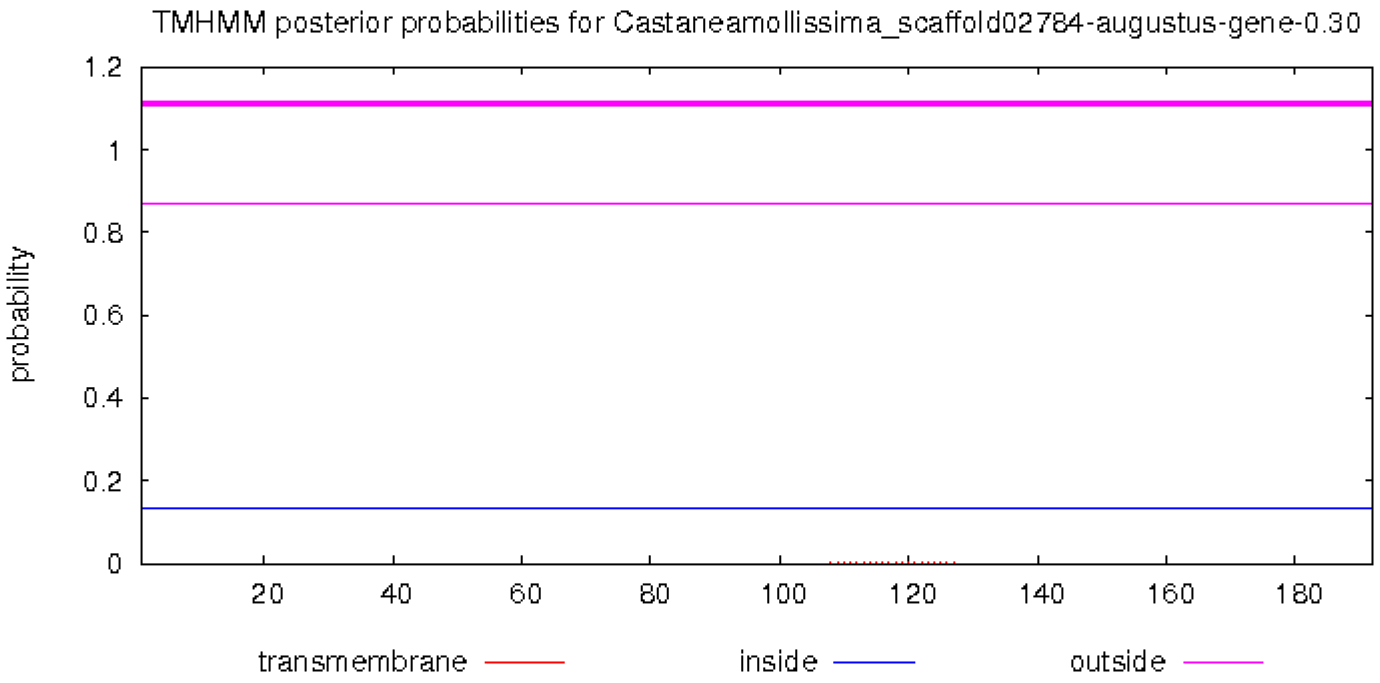

plot in postscript, script for making the plot in gnuplot, data for plot

---

```
# Castaneamollissima_scaffold02784-augustus-gene-0.30 Length: 187
# Castaneamollissima_scaffold02784-augustus-gene-0.30 Number of predicted TMHs:  0
# Castaneamollissima_scaffold02784-augustus-gene-0.30 Exp number of AAs in TMHs: 0.00441
# Castaneamollissima_scaffold02784-augustus-gene-0.30 Exp number, first 60 AAs:  0
# Castaneamollissima_scaffold02784-augustus-gene-0.30 Total prob of N-in:        0.04080
Castaneamollissima_scaffold02784-augustus-gene-0.30     TMHMM2.0        outside      1   187
```

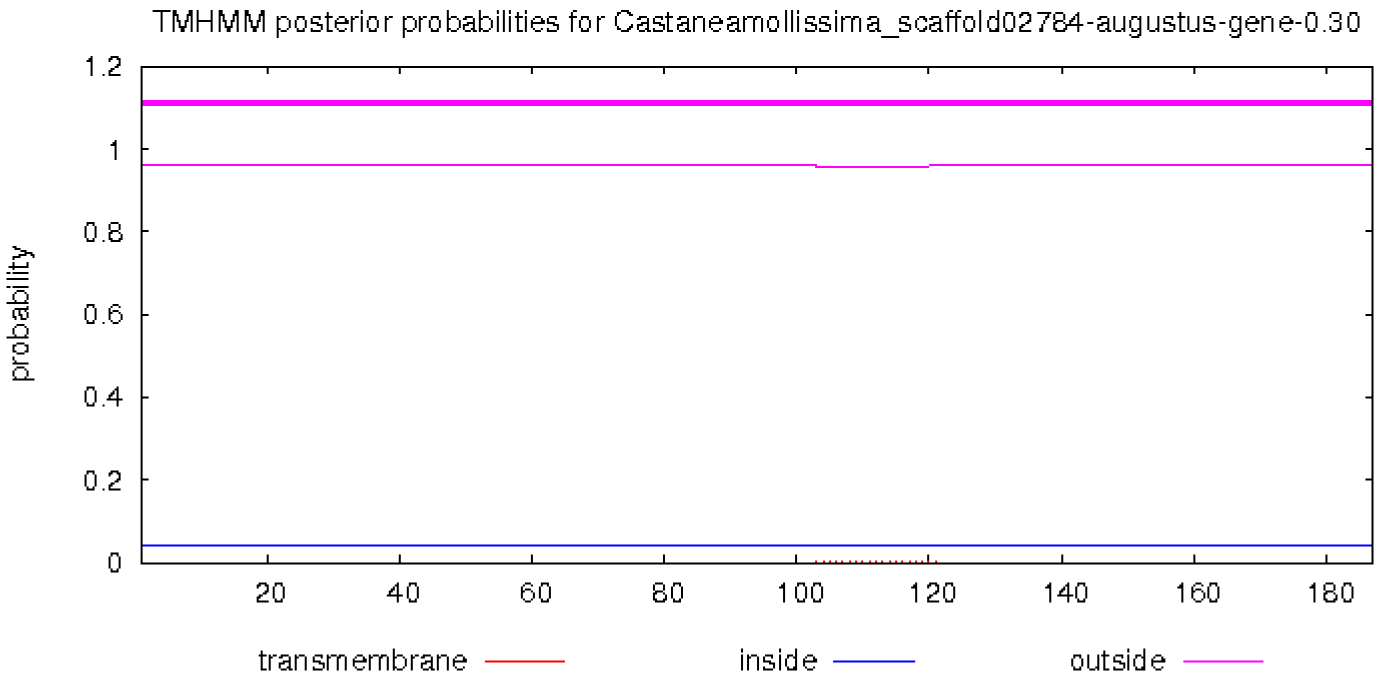

plot in postscript, script for making the plot in gnuplot, data for plot

---

```
# Castaneamollissima_scaffold02799-augustus-gene-0.20 Length: 403
# Castaneamollissima_scaffold02799-augustus-gene-0.20 Number of predicted TMHs:  0
# Castaneamollissima_scaffold02799-augustus-gene-0.20 Exp number of AAs in TMHs: 0.07699
# Castaneamollissima_scaffold02799-augustus-gene-0.20 Exp number, first 60 AAs:  0.07699
# Castaneamollissima_scaffold02799-augustus-gene-0.20 Total prob of N-in:        0.01083
Castaneamollissima_scaffold02799-augustus-gene-0.20     TMHMM2.0        outside      1   403
```

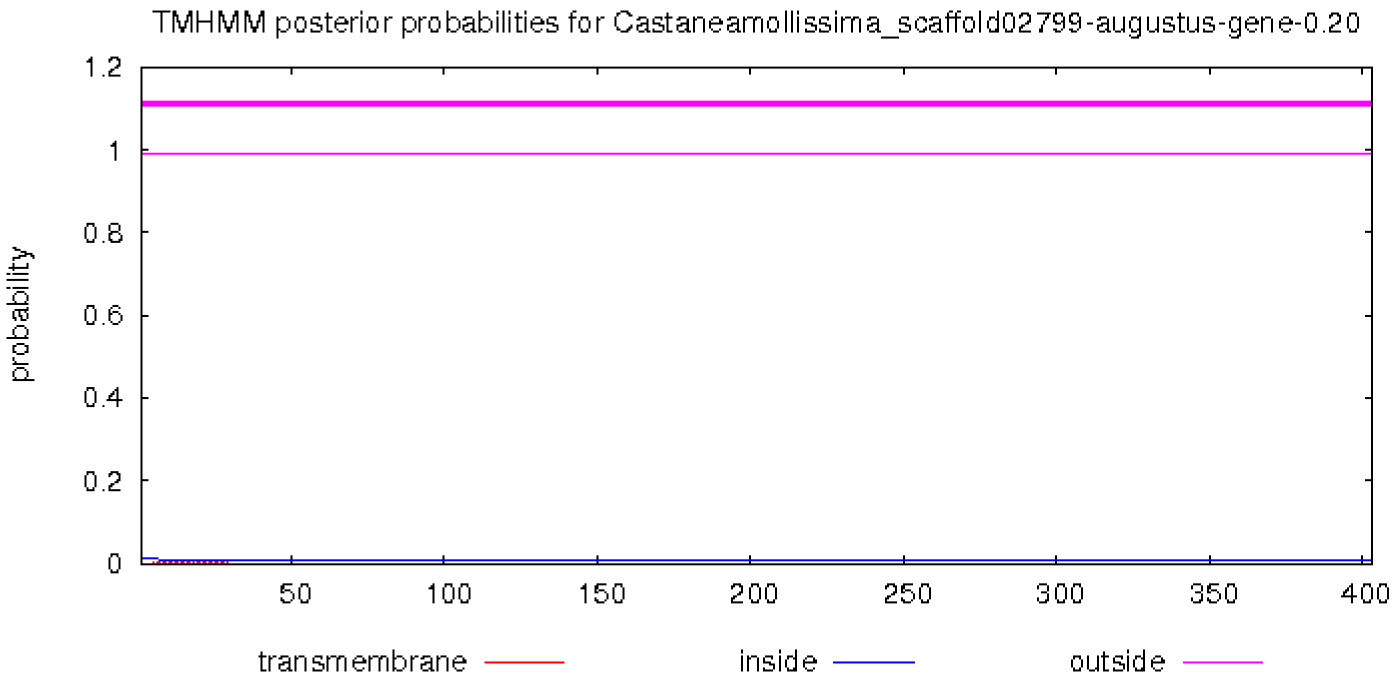

plot in postscript, script for making the plot in gnuplot, data for plot

---

```
# Castaneamollissima_scaffold02824-augustus-gene-0.20 Length: 285
# Castaneamollissima_scaffold02824-augustus-gene-0.20 Number of predicted TMHs:  0
# Castaneamollissima_scaffold02824-augustus-gene-0.20 Exp number of AAs in TMHs: 0.00226
# Castaneamollissima_scaffold02824-augustus-gene-0.20 Exp number, first 60 AAs:  0
# Castaneamollissima_scaffold02824-augustus-gene-0.20 Total prob of N-in:        0.03260
Castaneamollissima_scaffold02824-augustus-gene-0.20     TMHMM2.0        outside      1   285
```

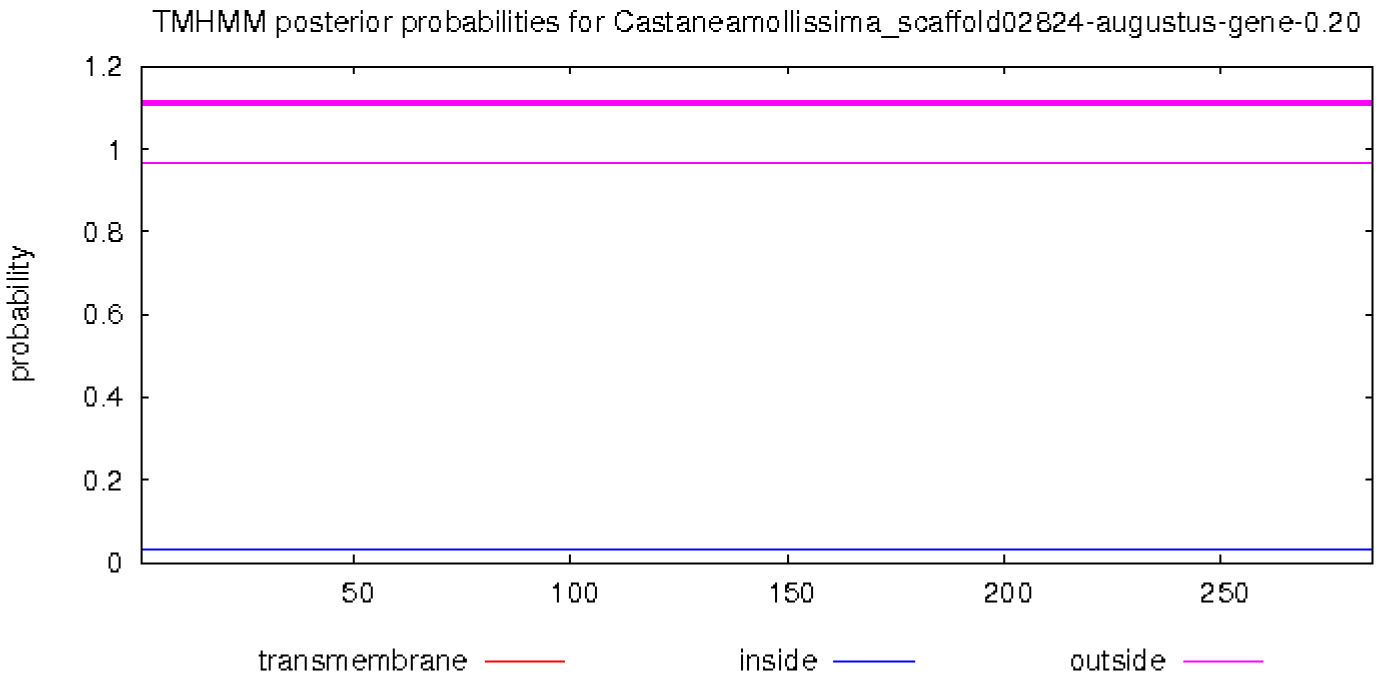

plot in postscript, script for making the plot in gnuplot, data for plot

---

```
# Castaneamollissima_scaffold02829-snap-gene-0.27 Length: 516
# Castaneamollissima_scaffold02829-snap-gene-0.27 Number of predicted TMHs:  0
# Castaneamollissima_scaffold02829-snap-gene-0.27 Exp number of AAs in TMHs: 0.00037
# Castaneamollissima_scaffold02829-snap-gene-0.27 Exp number, first 60 AAs:  0
# Castaneamollissima_scaffold02829-snap-gene-0.27 Total prob of N-in:        0.00156
Castaneamollissima_scaffold02829-snap-gene-0.27 TMHMM2.0        outside      1   516
```

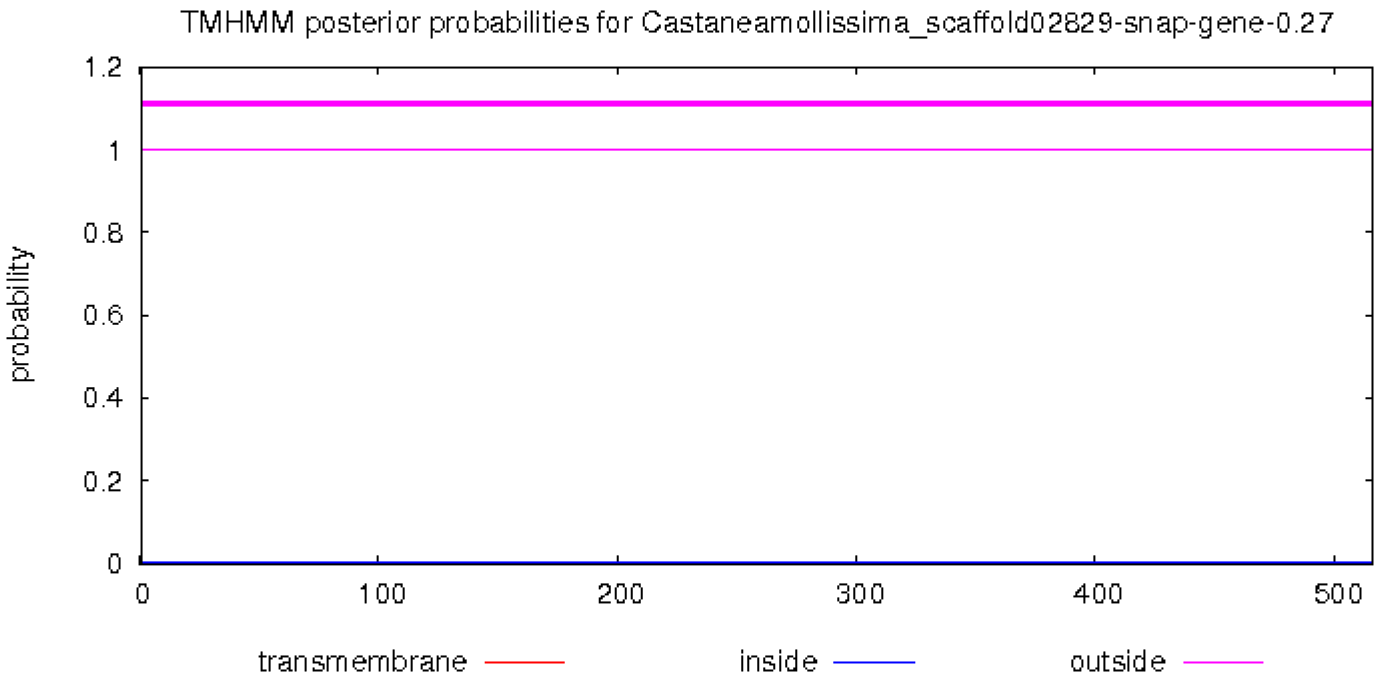

plot in postscript, script for making the plot in gnuplot, data for plot

---

```
# Castaneamollissima_scaffold02959-augustus-gene-0.14 Length: 303
# Castaneamollissima_scaffold02959-augustus-gene-0.14 Number of predicted TMHs:  0
# Castaneamollissima_scaffold02959-augustus-gene-0.14 Exp number of AAs in TMHs: 0.00074
# Castaneamollissima_scaffold02959-augustus-gene-0.14 Exp number, first 60 AAs:  0
# Castaneamollissima_scaffold02959-augustus-gene-0.14 Total prob of N-in:        0.04019
Castaneamollissima_scaffold02959-augustus-gene-0.14	TMHMM2.0	outside	     1   303
```

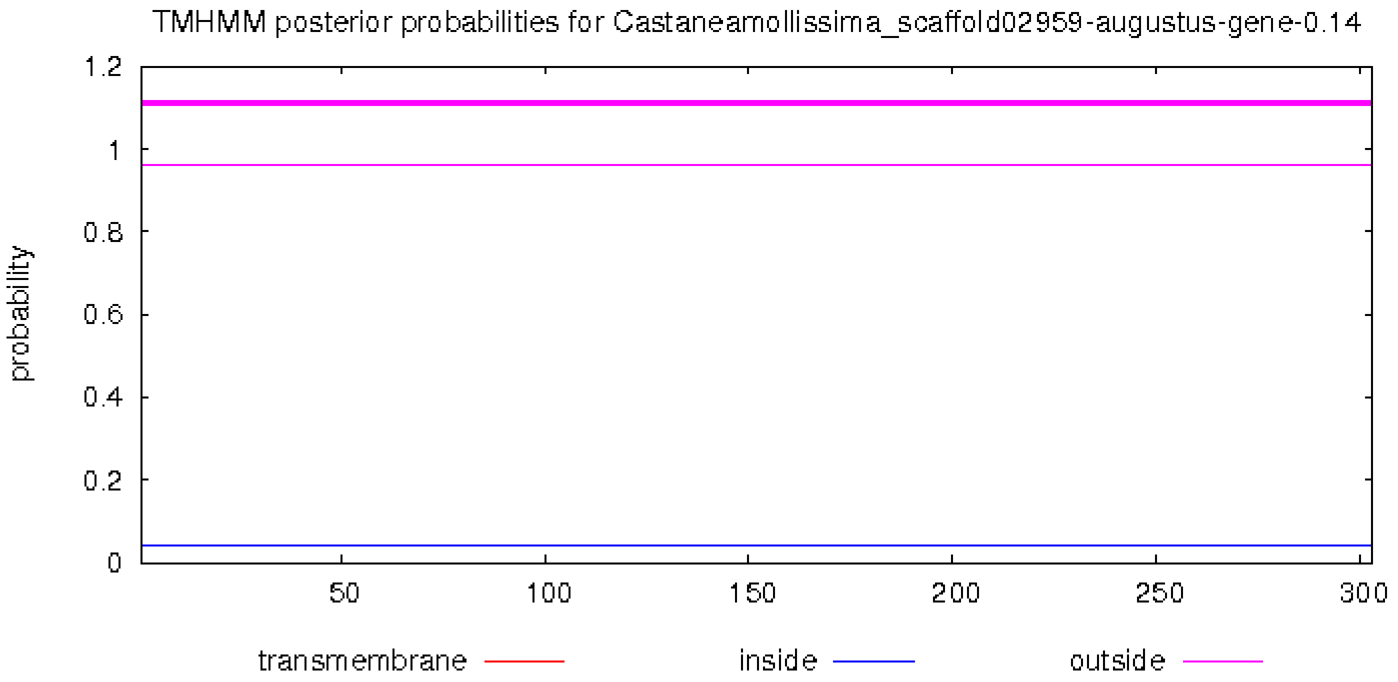

plot in postscript, script for making the plot in gnuplot, data for plot

---

```
# Castaneamollissima_scaffold03016-augustus-gene-0.16 Length: 343
# Castaneamollissima_scaffold03016-augustus-gene-0.16 Number of predicted TMHs:  0
# Castaneamollissima_scaffold03016-augustus-gene-0.16 Exp number of AAs in TMHs: 0
# Castaneamollissima_scaffold03016-augustus-gene-0.16 Exp number, first 60 AAs:  0
# Castaneamollissima_scaffold03016-augustus-gene-0.16 Total prob of N-in:        0.00084
Castaneamollissima_scaffold03016-augustus-gene-0.16	TMHMM2.0	outside	     1   343
```

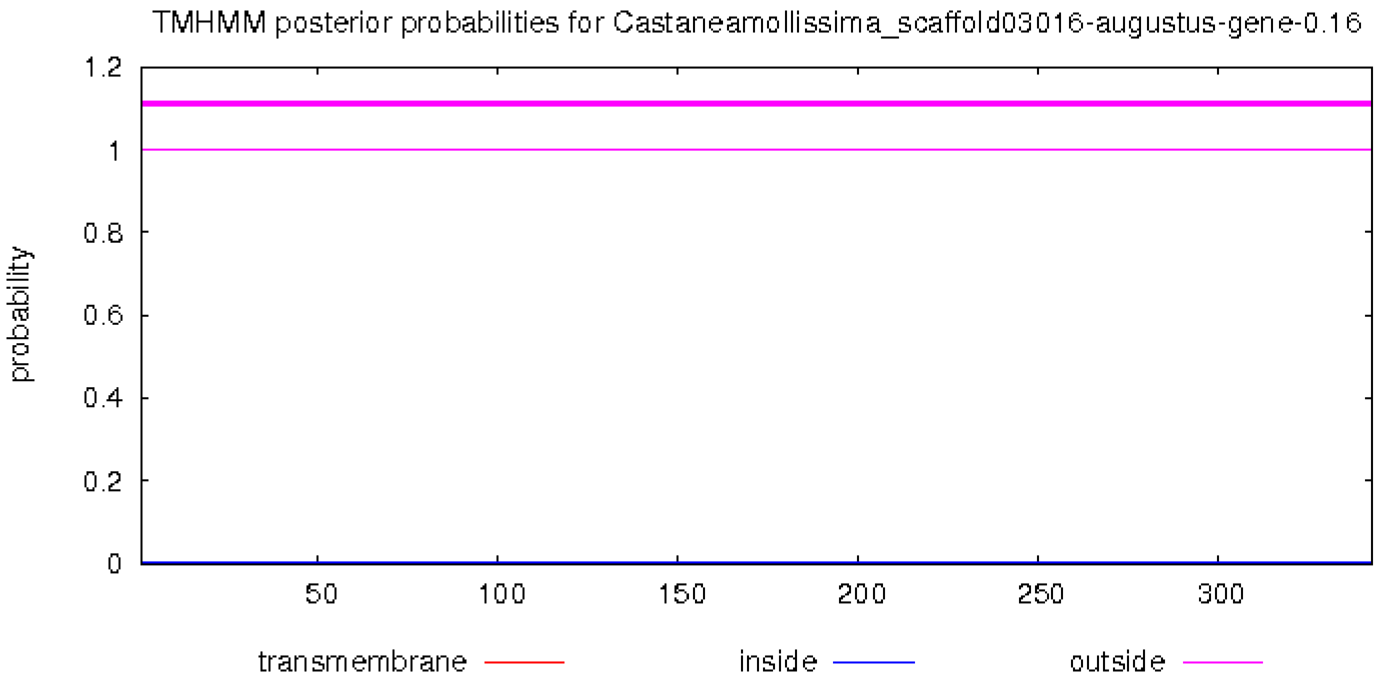

plot in postscript, script for making the plot in gnuplot, data for plot

---

```
# Castaneamollissima_scaffold03032-augustus-gene-0.24 Length: 309
# Castaneamollissima_scaffold03032-augustus-gene-0.24 Number of predicted TMHs:  0
# Castaneamollissima_scaffold03032-augustus-gene-0.24 Exp number of AAs in TMHs: 0.00018
# Castaneamollissima_scaffold03032-augustus-gene-0.24 Exp number, first 60 AAs:  0
# Castaneamollissima_scaffold03032-augustus-gene-0.24 Total prob of N-in:        0.00648
Castaneamollissima_scaffold03032-augustus-gene-0.24	TMHMM2.0	outside	     1   309
```

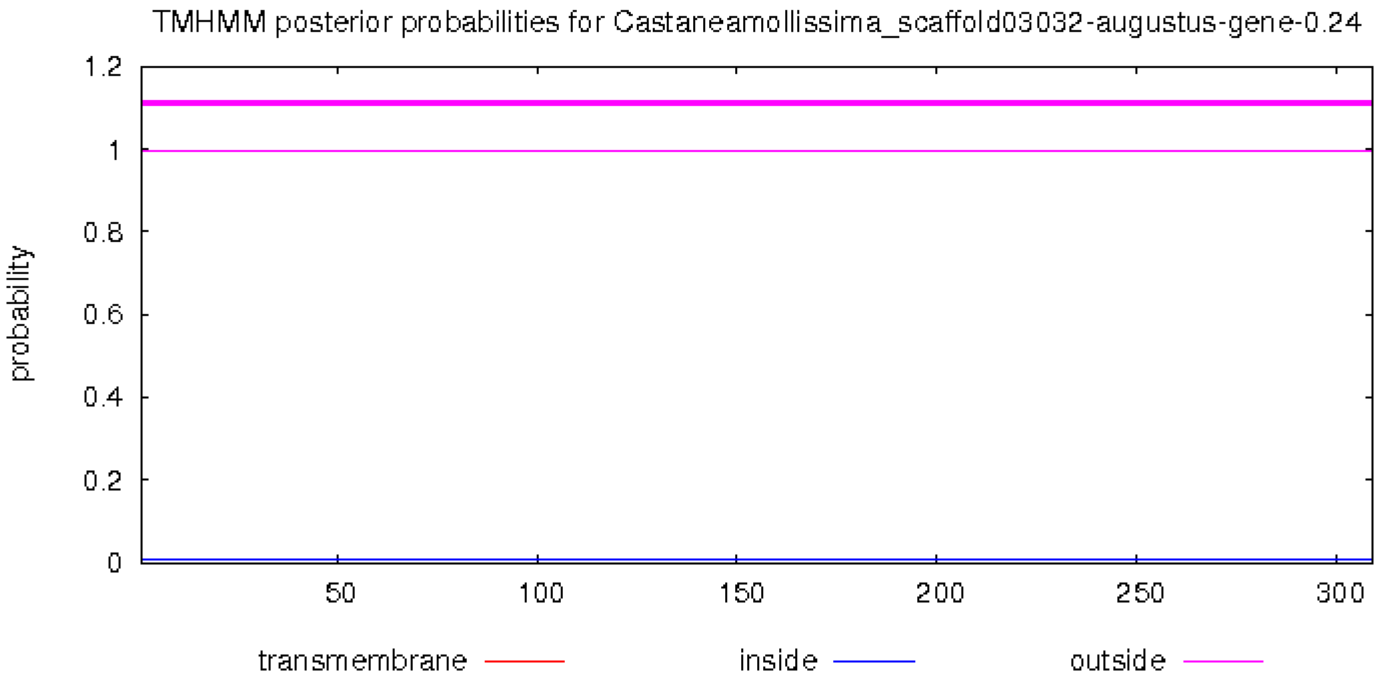

plot in postscript, script for making the plot in gnuplot, data for plot

---

```
# Castaneamollissima_scaffold03547-augustus-gene-0.16 Length: 149
# Castaneamollissima_scaffold03547-augustus-gene-0.16 Number of predicted TMHs:  0
# Castaneamollissima_scaffold03547-augustus-gene-0.16 Exp number of AAs in TMHs: 0.00248
# Castaneamollissima_scaffold03547-augustus-gene-0.16 Exp number, first 60 AAs:  0.00248
# Castaneamollissima_scaffold03547-augustus-gene-0.16 Total prob of N-in:        0.19546
Castaneamollissima_scaffold03547-augustus-gene-0.16	TMHMM2.0	outside	     1   149
```

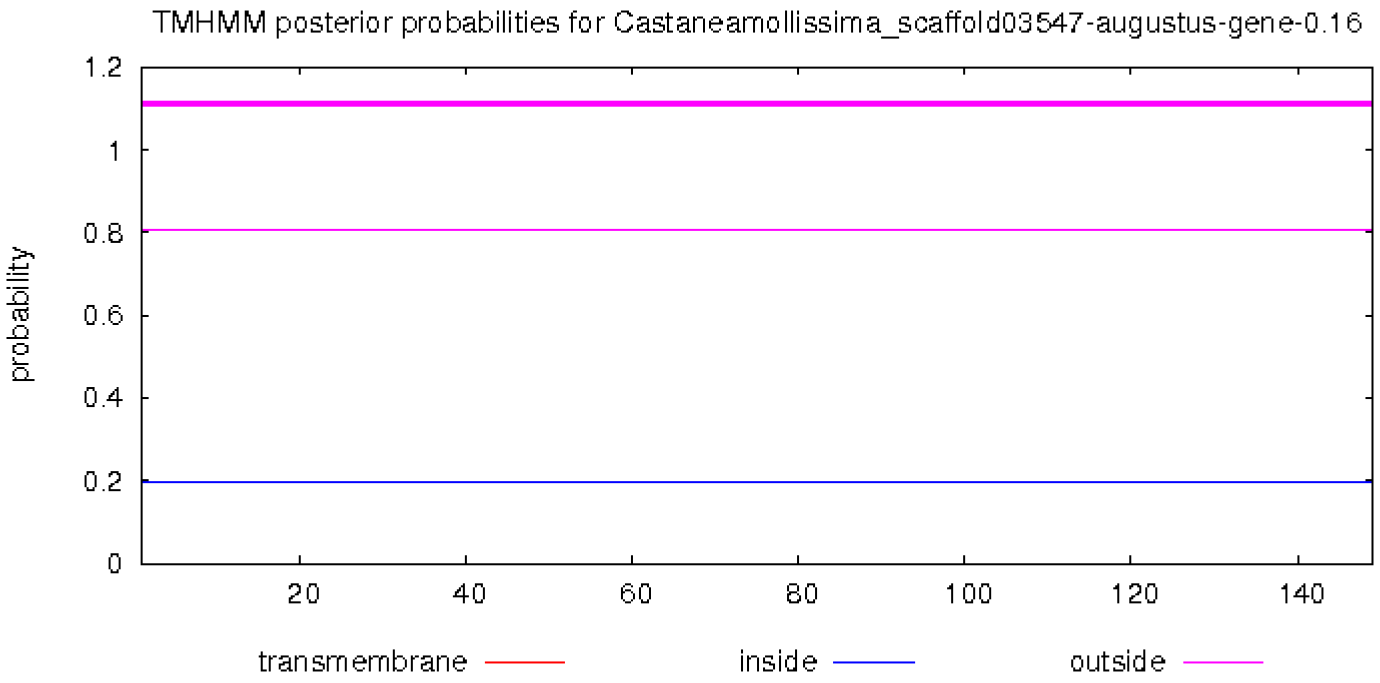

plot in postscript, script for making the plot in gnuplot, data for plot

---

```
# Castaneamollissima_scaffold03703-augustus-gene-0.17 Length: 613
# Castaneamollissima_scaffold03703-augustus-gene-0.17 Number of predicted TMHs:  1
# Castaneamollissima_scaffold03703-augustus-gene-0.17 Exp number of AAs in TMHs: 22.46635
# Castaneamollissima_scaffold03703-augustus-gene-0.17 Exp number, first 60 AAs:  0
# Castaneamollissima_scaffold03703-augustus-gene-0.17 Total prob of N-in:        0.00183
Castaneamollissima_scaffold03703-augustus-gene-0.17     TMHMM2.0        outside      1   588
Castaneamollissima_scaffold03703-augustus-gene-0.17     TMHMM2.0        TMhelix    589   611
Castaneamollissima_scaffold03703-augustus-gene-0.17     TMHMM2.0        inside     612   613
```

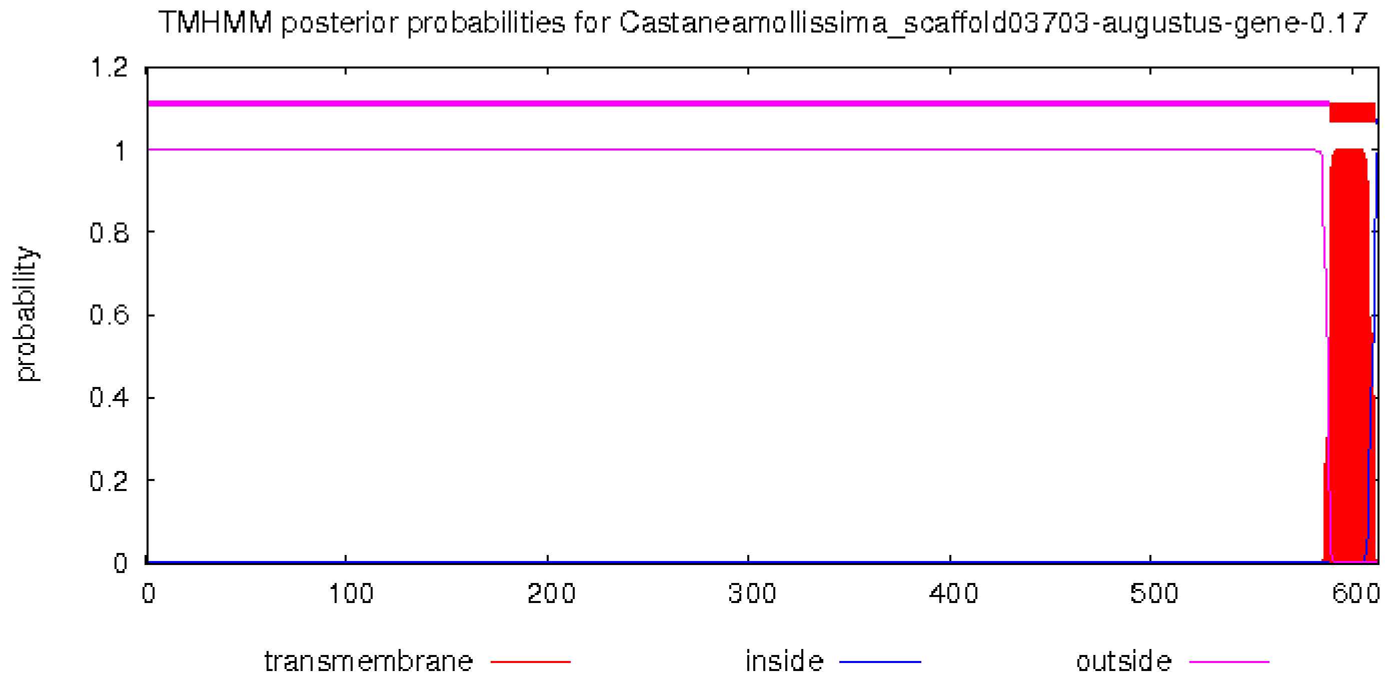

plot in postscript, script for making the plot in gnuplot, data for plot

---

```
# Castaneamollissima_scaffold03973-snap-gene-0.12 Length: 429
# Castaneamollissima_scaffold03973-snap-gene-0.12 Number of predicted TMHs:  0
# Castaneamollissima_scaffold03973-snap-gene-0.12 Exp number of AAs in TMHs: 0
# Castaneamollissima_scaffold03973-snap-gene-0.12 Exp number, first 60 AAs:  0
# Castaneamollissima_scaffold03973-snap-gene-0.12 Total prob of N-in:        0.00400
Castaneamollissima_scaffold03973-snap-gene-0.12 TMHMM2.0        outside      1   429
```

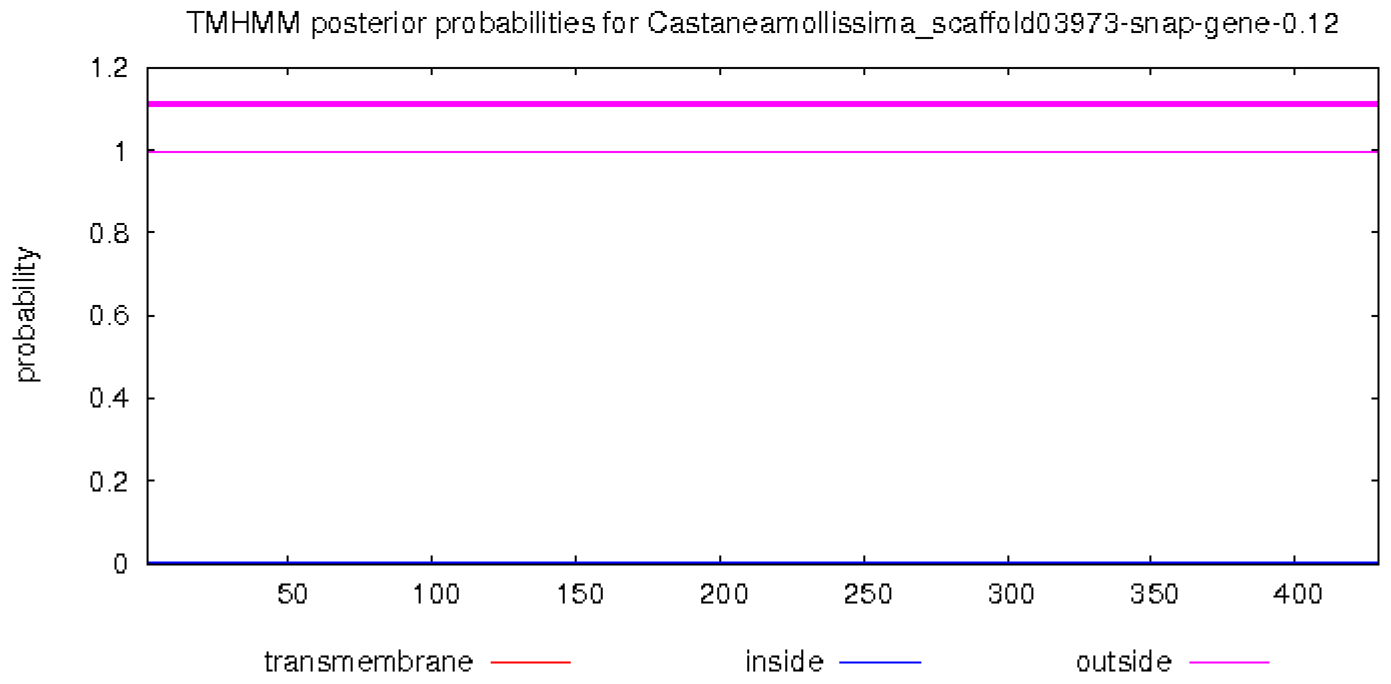

plot in postscript, script for making the plot in gnuplot, data for plot

```
# Castaneamollissima_scaffold04069-snap-gene-0.18 Length: 189
# Castaneamollissima_scaffold04069-snap-gene-0.18 Number of predicted TMHs:  0
# Castaneamollissima_scaffold04069-snap-gene-0.18 Exp number of AAs in TMHs: 0.00283
# Castaneamollissima_scaffold04069-snap-gene-0.18 Exp number, first 60 AAs:  0.00019
# Castaneamollissima_scaffold04069-snap-gene-0.18 Total prob of N-in:        0.01624
Castaneamollissima_scaffold04069-snap-gene-0.18 TMHMM2.0        outside      1   189
```

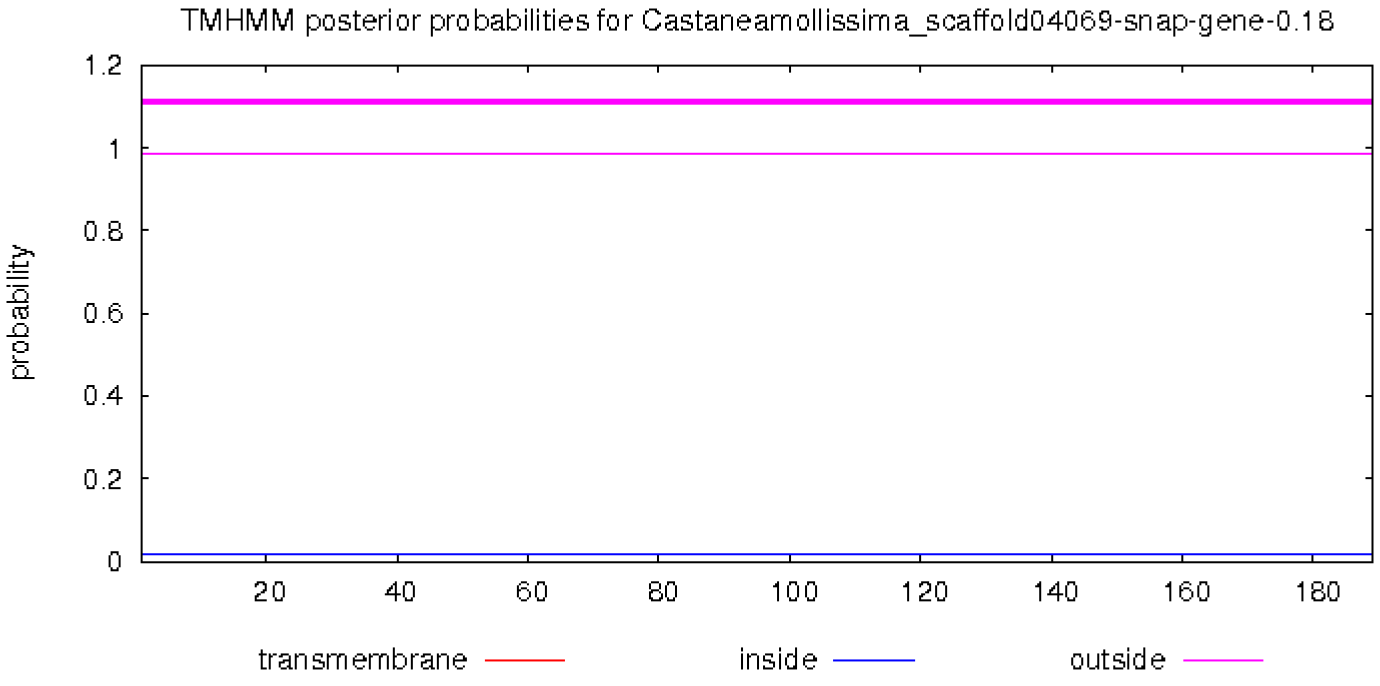

plot in postscript, script for making the plot in gnuplot, data for plot

```
# Castaneamollissima_scaffold04103-augustus-gene-0.16 Length: 325
# Castaneamollissima_scaffold04103-augustus-gene-0.16 Number of predicted TMHs:  0
# Castaneamollissima_scaffold04103-augustus-gene-0.16 Exp number of AAs in TMHs: 0.00031
# Castaneamollissima_scaffold04103-augustus-gene-0.16 Exp number, first 60 AAs:  0
# Castaneamollissima_scaffold04103-augustus-gene-0.16 Total prob of N-in:        0.05206
Castaneamollissima_scaffold04103-augustus-gene-0.16     TMHMM2.0        outside      1   325
```

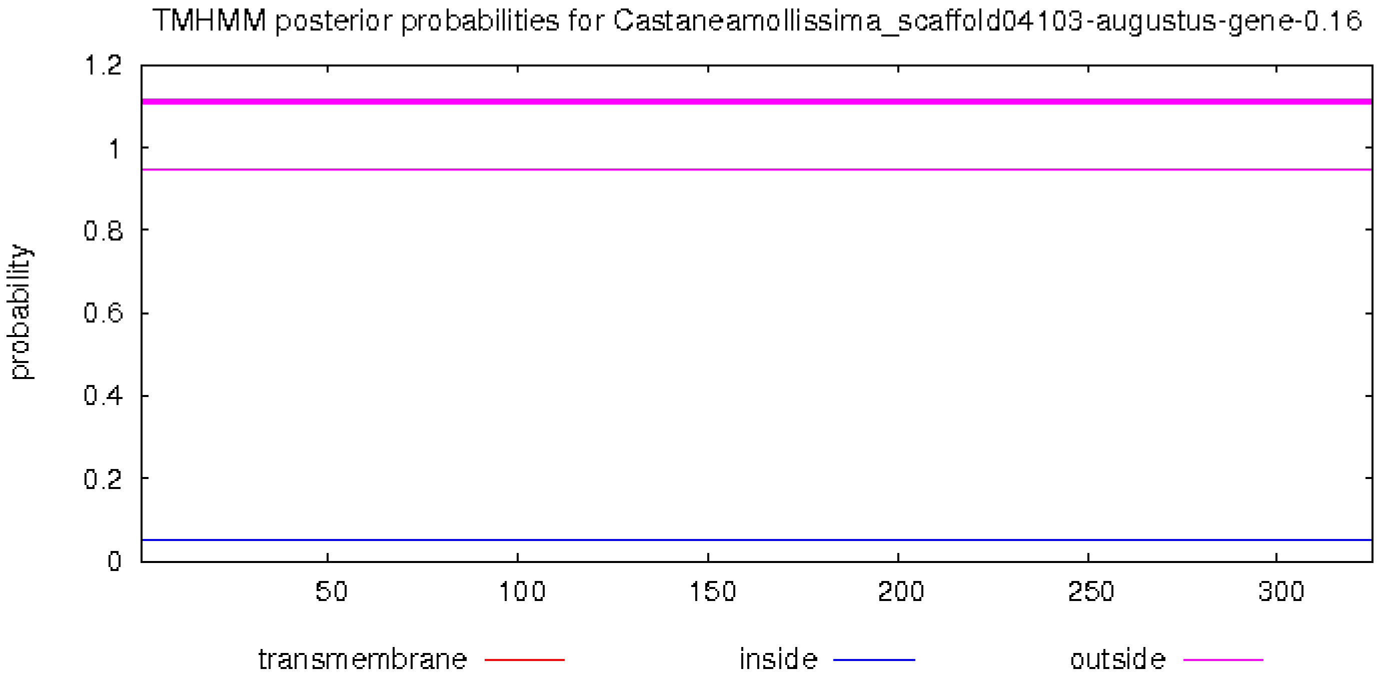

plot in postscript, script for making the plot in gnuplot, data for plot

```
# Castaneamollissima_scaffold04107-augustus-gene-0.9 Length: 391
# Castaneamollissima_scaffold04107-augustus-gene-0.9 Number of predicted TMHs:  0
# Castaneamollissima_scaffold04107-augustus-gene-0.9 Exp number of AAs in TMHs: 0
# Castaneamollissima_scaffold04107-augustus-gene-0.9 Exp number, first 60 AAs:  0
# Castaneamollissima_scaffold04107-augustus-gene-0.9 Total prob of N-in:        0.00626
Castaneamollissima_scaffold04107-augustus-gene-0.9      TMHMM2.0        outside      1   391
```

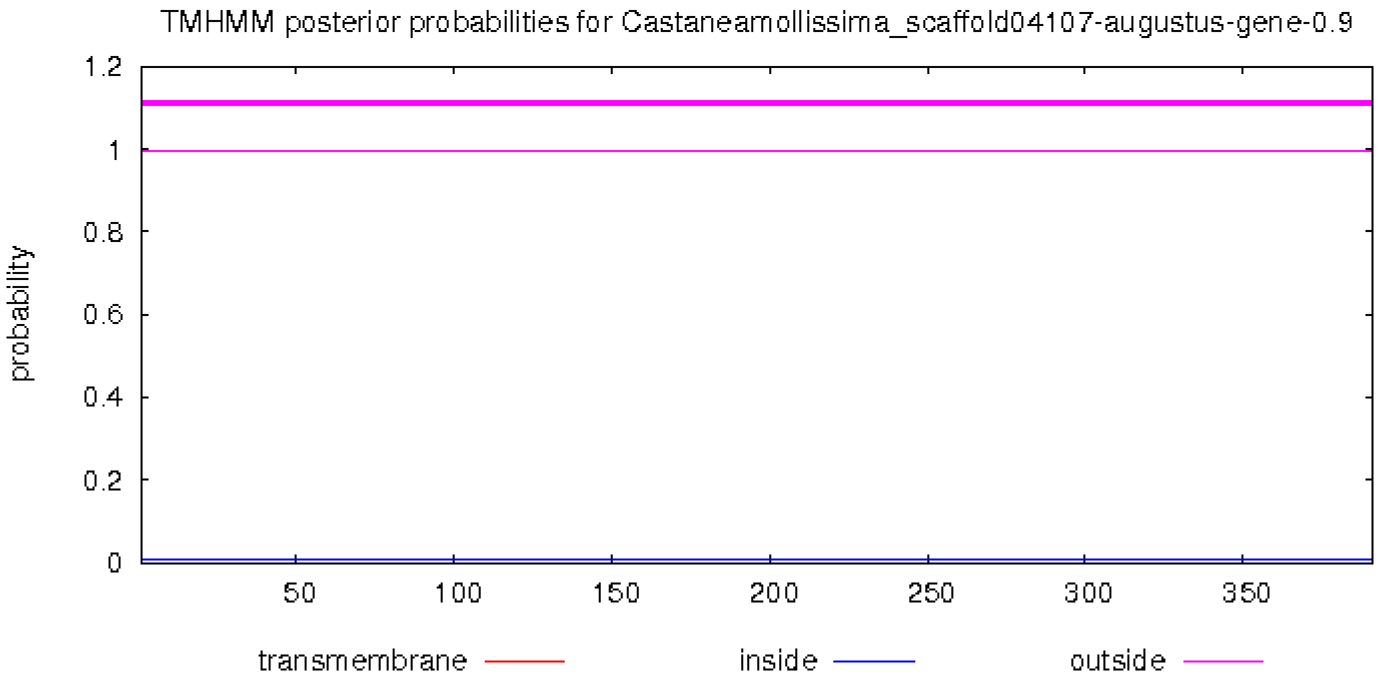

plot in postscript, script for making the plot in gnuplot, data for plot

---

```
# Castaneamollissima_scaffold04312-snap-gene-0.20 Length: 433
# Castaneamollissima_scaffold04312-snap-gene-0.20 Number of predicted TMHs:  0
# Castaneamollissima_scaffold04312-snap-gene-0.20 Exp number of AAs in TMHs: 0
# Castaneamollissima_scaffold04312-snap-gene-0.20 Exp number, first 60 AAs:  0
# Castaneamollissima_scaffold04312-snap-gene-0.20 Total prob of N-in:        0.00105
Castaneamollissima_scaffold04312-snap-gene-0.20 TMHMM2.0        outside      1   433
```

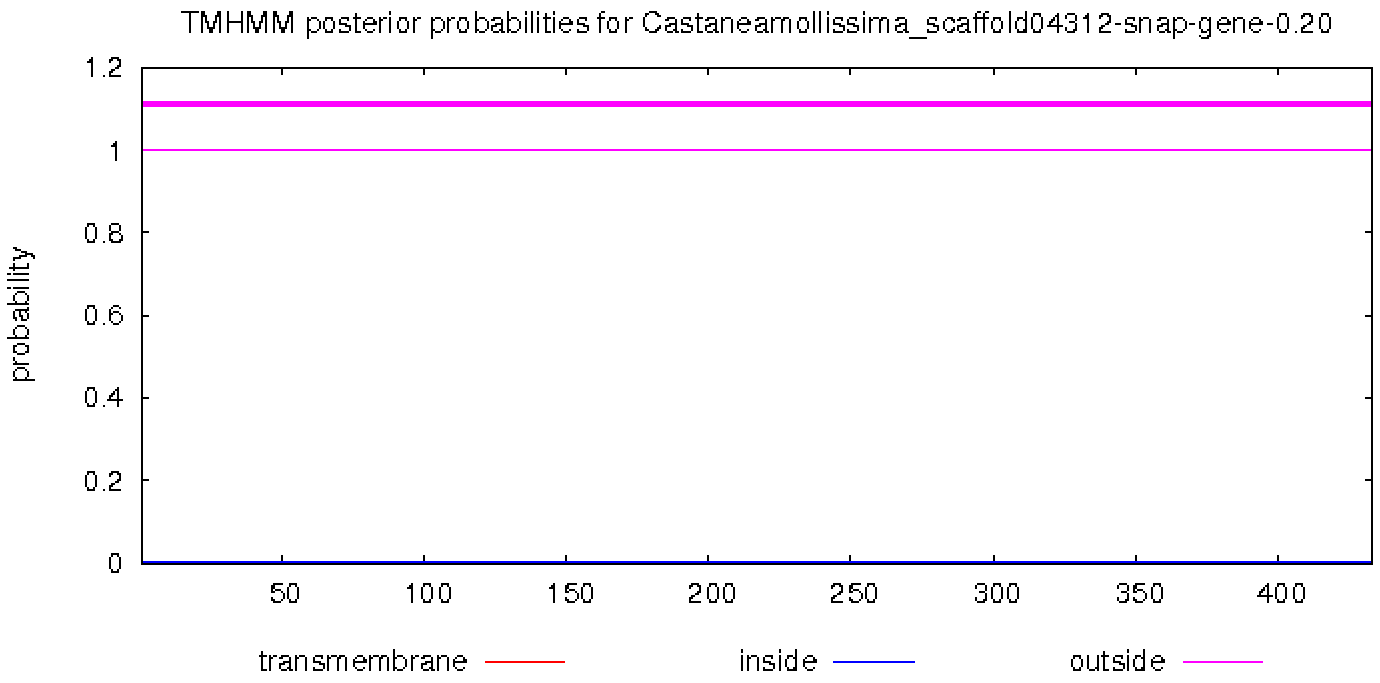

plot in postscript, script for making the plot in gnuplot, data for plot

---

```
# Castaneamollissima_scaffold04312-snap-gene-0.21 Length: 148
# Castaneamollissima_scaffold04312-snap-gene-0.21 Number of predicted TMHs:  0
# Castaneamollissima_scaffold04312-snap-gene-0.21 Exp number of AAs in TMHs: 0
# Castaneamollissima_scaffold04312-snap-gene-0.21 Exp number, first 60 AAs:  0
# Castaneamollissima_scaffold04312-snap-gene-0.21 Total prob of N-in:        0.03123
Castaneamollissima_scaffold04312-snap-gene-0.21 TMHMM2.0        outside      1   148
```

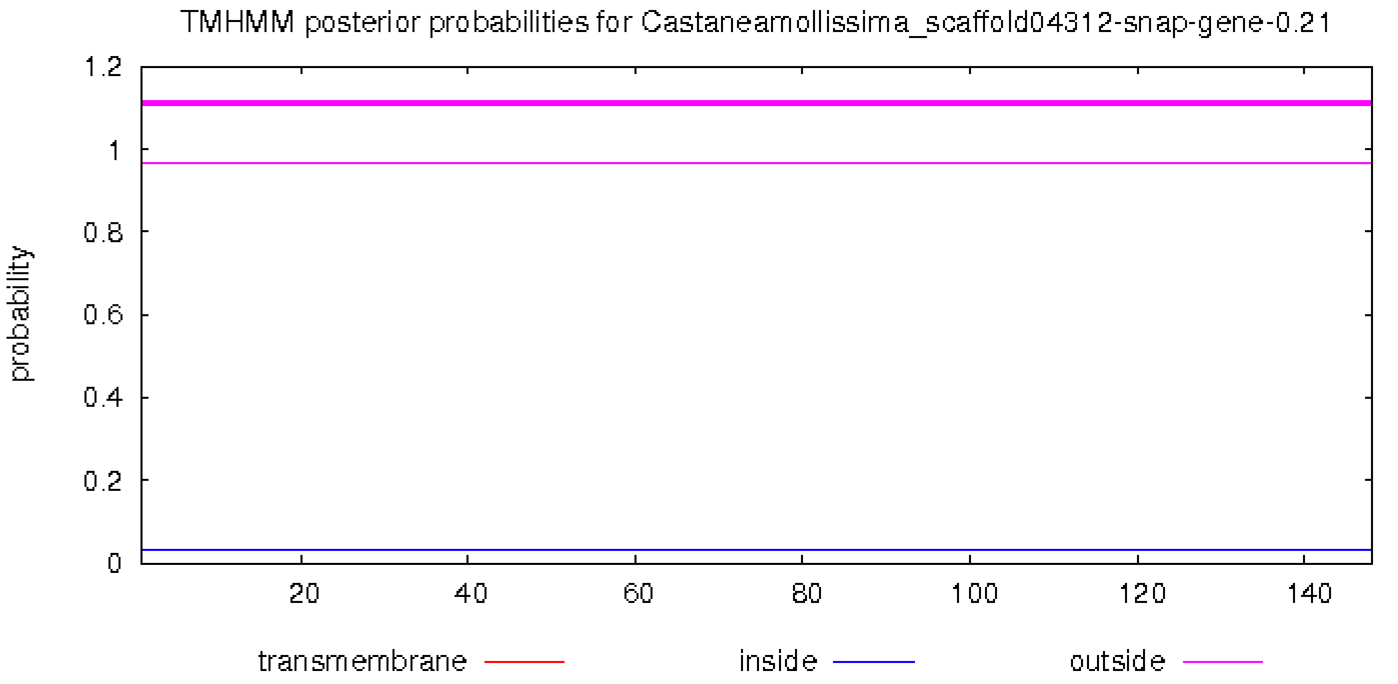

plot in postscript, script for making the plot in gnuplot, data for plot

---

```
# Castaneamollissima_scaffold04399-augustus-gene-0.11 Length: 404
# Castaneamollissima_scaffold04399-augustus-gene-0.11 Number of predicted TMHs:  0
# Castaneamollissima_scaffold04399-augustus-gene-0.11 Exp number of AAs in TMHs: 0
# Castaneamollissima_scaffold04399-augustus-gene-0.11 Exp number, first 60 AAs:  0
# Castaneamollissima_scaffold04399-augustus-gene-0.11 Total prob of N-in:        0.00908
Castaneamollissima_scaffold04399-augustus-gene-0.11     TMHMM2.0        outside      1   404
```

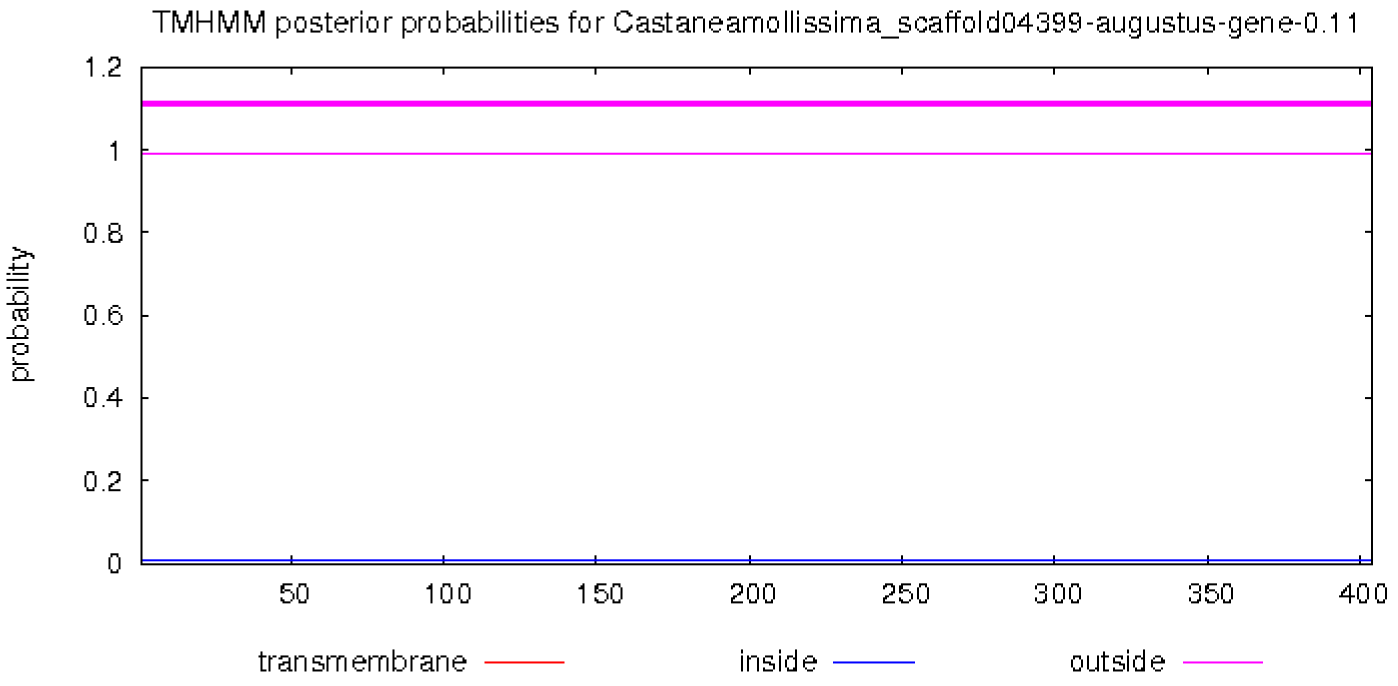

plot in postscript, script for making the plot in gnuplot, data for plot

---

```
# Castaneamollissima_scaffold04399-augustus-gene-0.11 Length: 362
# Castaneamollissima_scaffold04399-augustus-gene-0.11 Number of predicted TMHs:  0
# Castaneamollissima_scaffold04399-augustus-gene-0.11 Exp number of AAs in TMHs: 0.00018
# Castaneamollissima_scaffold04399-augustus-gene-0.11 Exp number, first 60 AAs:  0
# Castaneamollissima_scaffold04399-augustus-gene-0.11 Total prob of N-in:        0.02689
Castaneamollissima_scaffold04399-augustus-gene-0.11     TMHMM2.0        outside      1   362
```

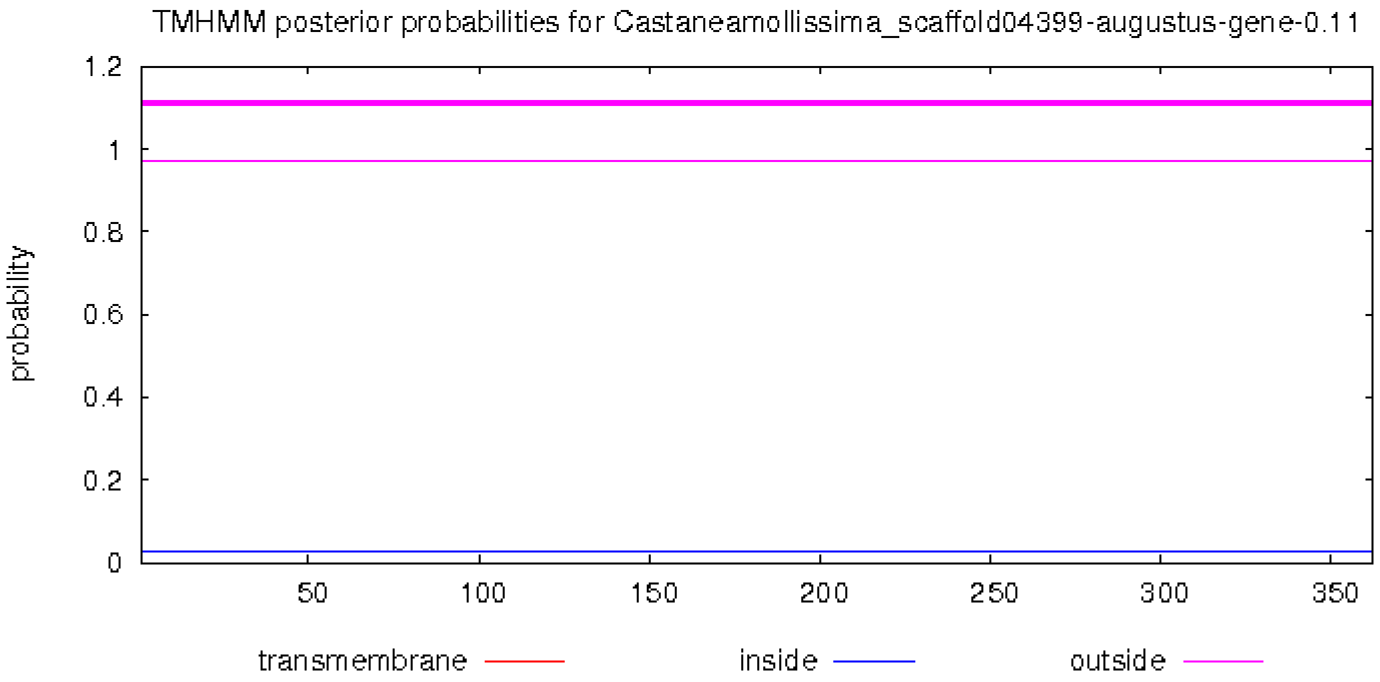

plot in postscript, script for making the plot in gnuplot, data for plot

---

```
# Castaneamollissima_scaffold04550-snap-gene-0.24 Length: 607
# Castaneamollissima_scaffold04550-snap-gene-0.24 Number of predicted TMHs:  0
# Castaneamollissima_scaffold04550-snap-gene-0.24 Exp number of AAs in TMHs: 0.00756
# Castaneamollissima_scaffold04550-snap-gene-0.24 Exp number, first 60 AAs:  0.00727
# Castaneamollissima_scaffold04550-snap-gene-0.24 Total prob of N-in:        0.00178
Castaneamollissima_scaffold04550-snap-gene-0.24 TMHMM2.0        outside      1   607
```

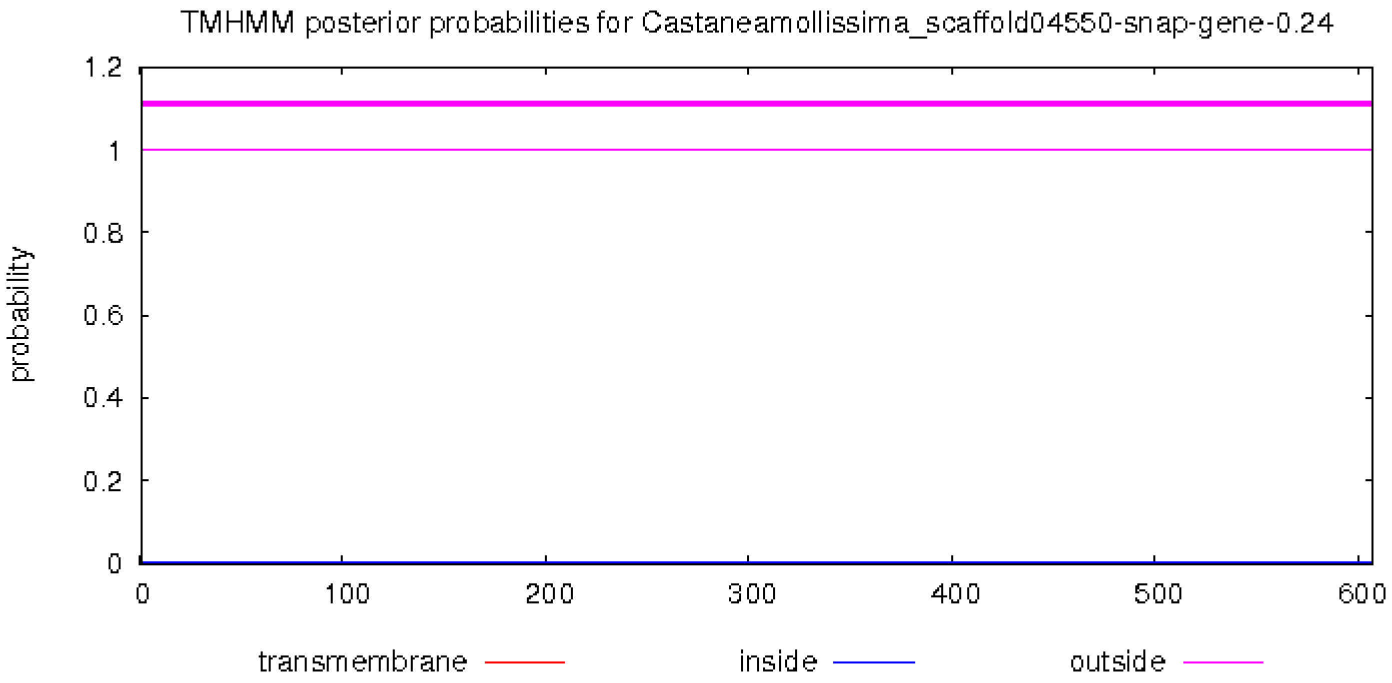

plot in postscript, script for making the plot in gnuplot, data for plot

---

```
# Castaneamollissima_scaffold04550-snap-gene-0.25 Length: 276
# Castaneamollissima_scaffold04550-snap-gene-0.25 Number of predicted TMHs:  0
# Castaneamollissima_scaffold04550-snap-gene-0.25 Exp number of AAs in TMHs: 0.02238
# Castaneamollissima_scaffold04550-snap-gene-0.25 Exp number, first 60 AAs:  0
# Castaneamollissima_scaffold04550-snap-gene-0.25 Total prob of N-in:        0.00945
Castaneamollissima_scaffold04550-snap-gene-0.25 TMHMM2.0        outside      1   276
```

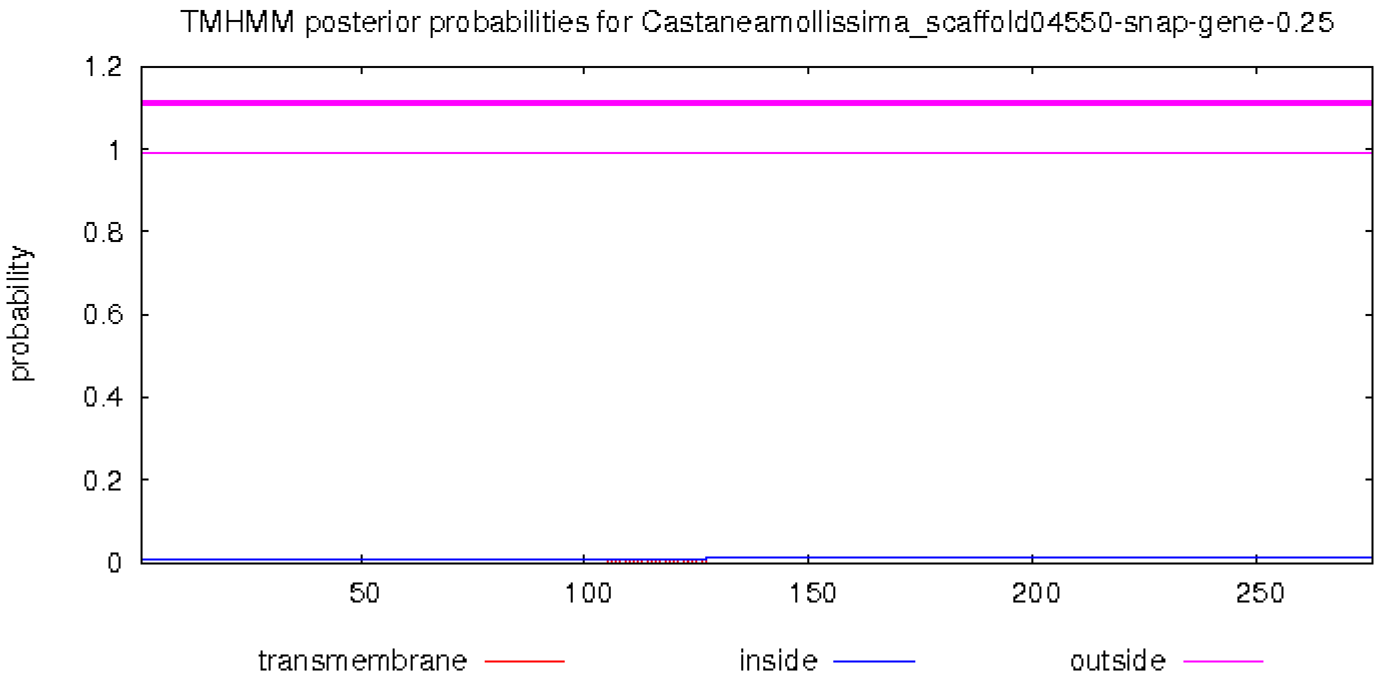

plot in postscript, script for making the plot in gnuplot, data for plot

---

```
# Castaneamollissima_scaffold04583-augustus-gene-0.13 Length: 361
# Castaneamollissima_scaffold04583-augustus-gene-0.13 Number of predicted TMHs:  0
# Castaneamollissima_scaffold04583-augustus-gene-0.13 Exp number of AAs in TMHs: 0
# Castaneamollissima_scaffold04583-augustus-gene-0.13 Exp number, first 60 AAs:  0
# Castaneamollissima_scaffold04583-augustus-gene-0.13 Total prob of N-in:        0.00379
Castaneamollissima_scaffold04583-augustus-gene-0.13     TMHMM2.0        outside      1   361
```

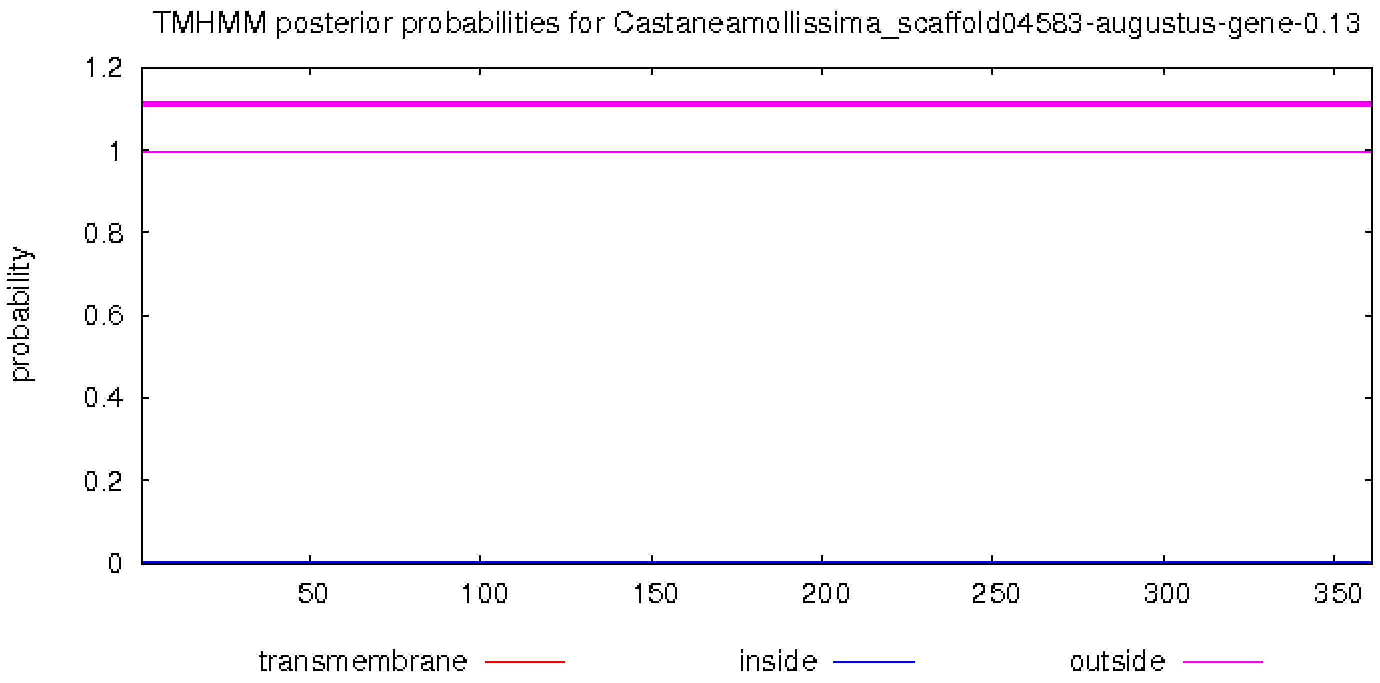

plot in postscript, script for making the plot in gnuplot, data for plot

---

```
# Castaneamollissima_scaffold04981-augustus-gene-0.2 Length: 358
# Castaneamollissima_scaffold04981-augustus-gene-0.2 Number of predicted TMHs:  0
# Castaneamollissima_scaffold04981-augustus-gene-0.2 Exp number of AAs in TMHs: 0.00011
# Castaneamollissima_scaffold04981-augustus-gene-0.2 Exp number, first 60 AAs:  0
# Castaneamollissima_scaffold04981-augustus-gene-0.2 Total prob of N-in:        0.01182
Castaneamollissima_scaffold04981-augustus-gene-0.2	TMHMM2.0	outside	     1   358
```

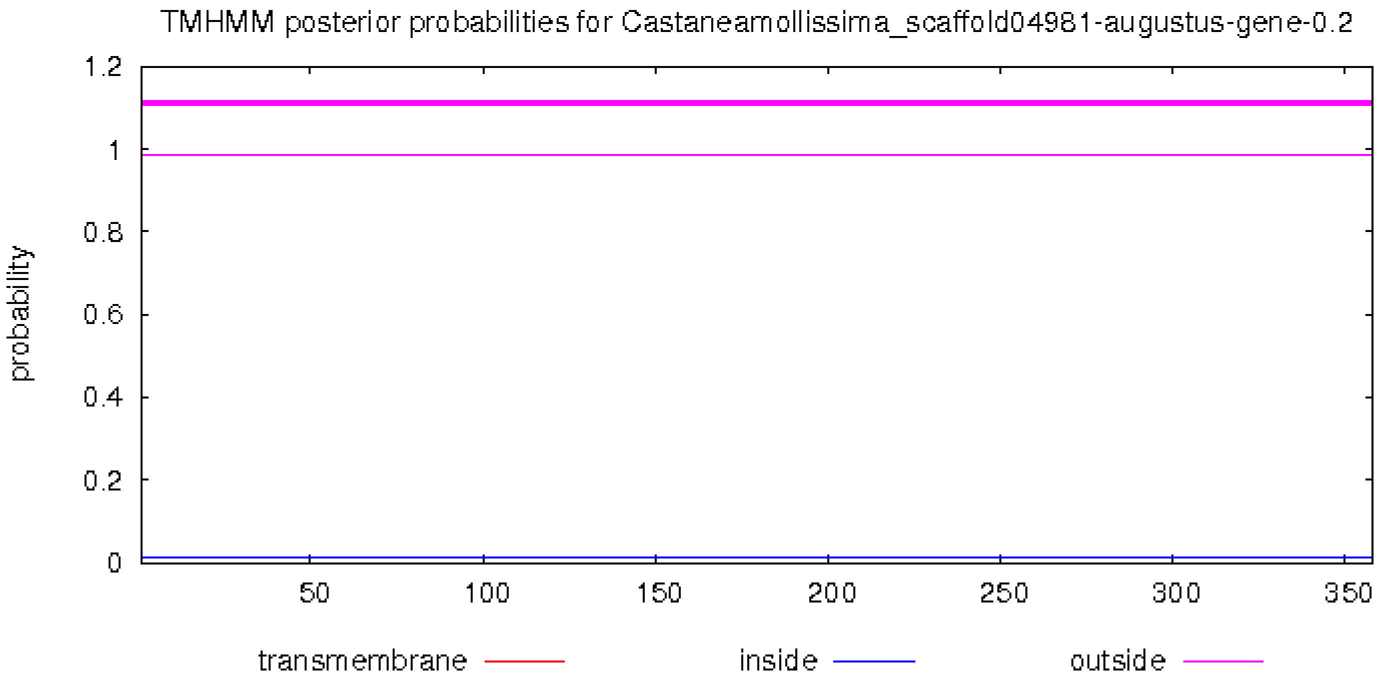

plot in postscript, script for making the plot in gnuplot, data for plot

---

```
# Castaneamollissima_scaffold05403-augustus-gene-0.18 Length: 199
# Castaneamollissima_scaffold05403-augustus-gene-0.18 Number of predicted TMHs:  0
# Castaneamollissima_scaffold05403-augustus-gene-0.18 Exp number of AAs in TMHs: 0.05462
# Castaneamollissima_scaffold05403-augustus-gene-0.18 Exp number, first 60 AAs:  0.00022
# Castaneamollissima_scaffold05403-augustus-gene-0.18 Total prob of N-in:        0.27966
Castaneamollissima_scaffold05403-augustus-gene-0.18	TMHMM2.0	outside	     1   199
```

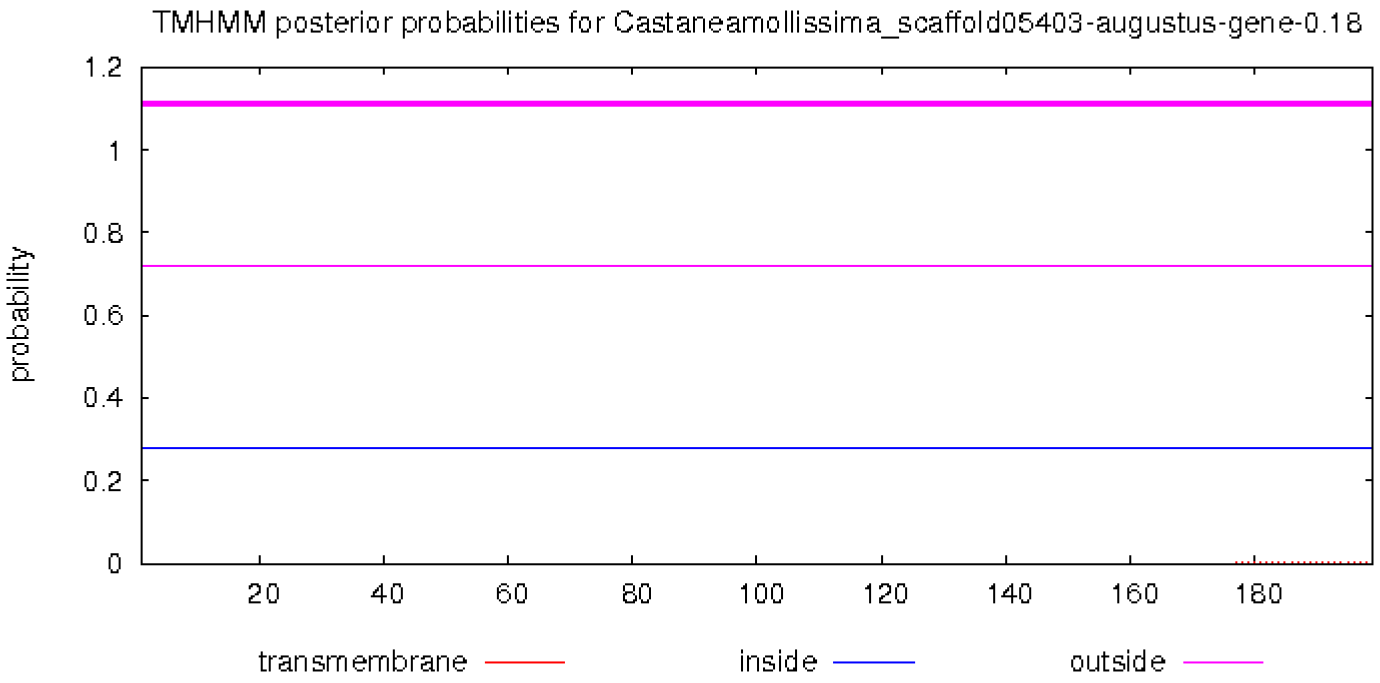

plot in postscript, script for making the plot in gnuplot, data for plot

---

```
# Castaneamollissima_scaffold05469-snap-gene-0.9 Length: 325
# Castaneamollissima_scaffold05469-snap-gene-0.9 Number of predicted TMHs:  0
# Castaneamollissima_scaffold05469-snap-gene-0.9 Exp number of AAs in TMHs: 0.00497
# Castaneamollissima_scaffold05469-snap-gene-0.9 Exp number, first 60 AAs:  0.0002
# Castaneamollissima_scaffold05469-snap-gene-0.9 Total prob of N-in:        0.03203
Castaneamollissima_scaffold05469-snap-gene-0.9  TMHMM2.0        outside      1   325
```

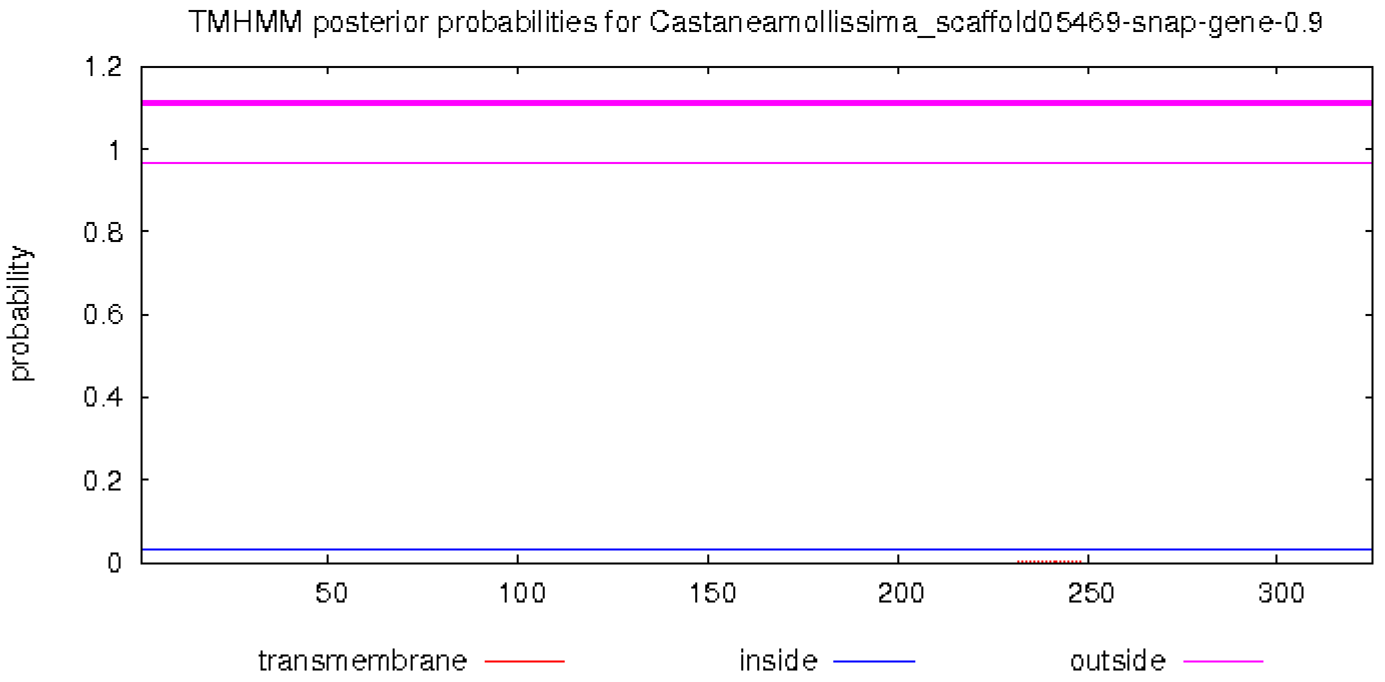

plot in postscript, script for making the plot in gnuplot, data for plot

---

```
# Castaneamollissima_scaffold06450-augustus-gene-0.17 Length: 254
# Castaneamollissima_scaffold06450-augustus-gene-0.17 Number of predicted TMHs:  0
# Castaneamollissima_scaffold06450-augustus-gene-0.17 Exp number of AAs in TMHs: 0
# Castaneamollissima_scaffold06450-augustus-gene-0.17 Exp number, first 60 AAs:  0
# Castaneamollissima_scaffold06450-augustus-gene-0.17 Total prob of N-in:        0.06235
Castaneamollissima_scaffold06450-augustus-gene-0.17     TMHMM2.0        outside      1   254
```

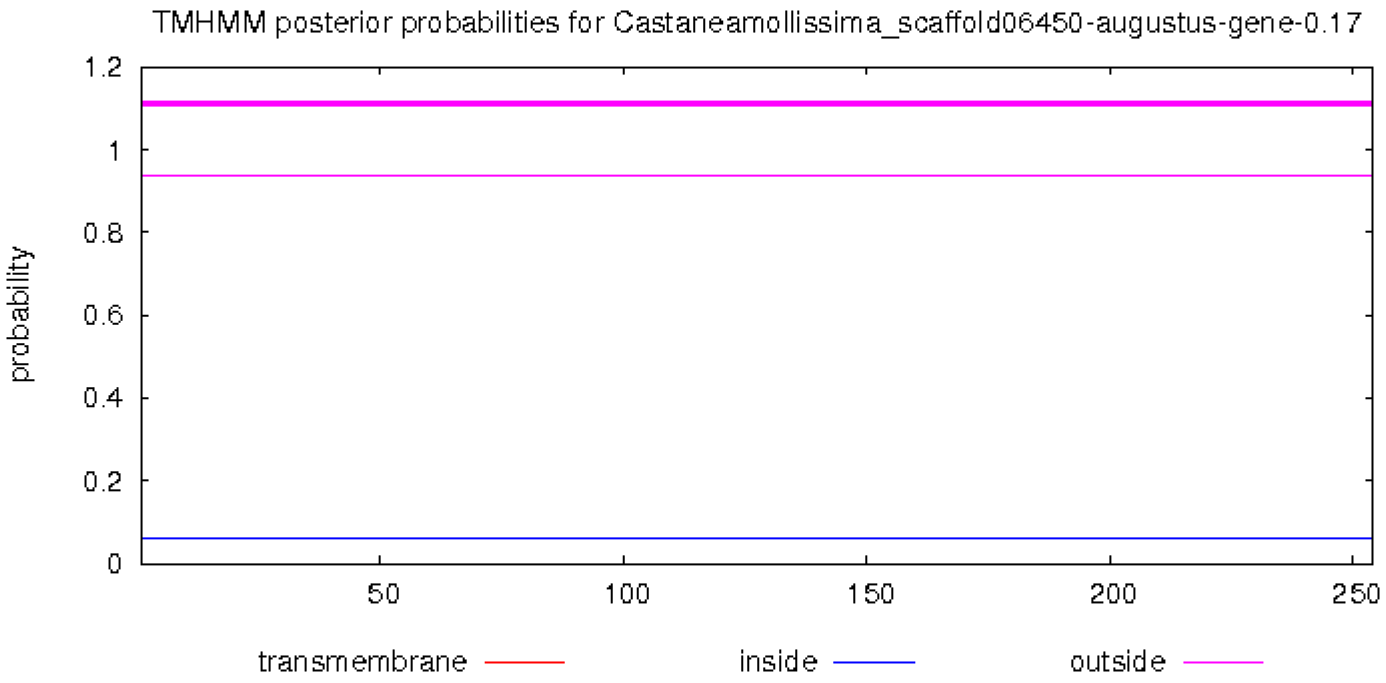

plot in postscript, script for making the plot in gnuplot, data for plot

---

```
# Castaneamollissima_scaffold06646-augustus-gene-0.12 Length: 203
# Castaneamollissima_scaffold06646-augustus-gene-0.12 Number of predicted TMHs:  0
# Castaneamollissima_scaffold06646-augustus-gene-0.12 Exp number of AAs in TMHs: 0.0248
# Castaneamollissima_scaffold06646-augustus-gene-0.12 Exp number, first 60 AAs:  0.00311
# Castaneamollissima_scaffold06646-augustus-gene-0.12 Total prob of N-in:        0.02414
Castaneamollissima_scaffold06646-augustus-gene-0.12     TMHMM2.0        outside      1   203
```

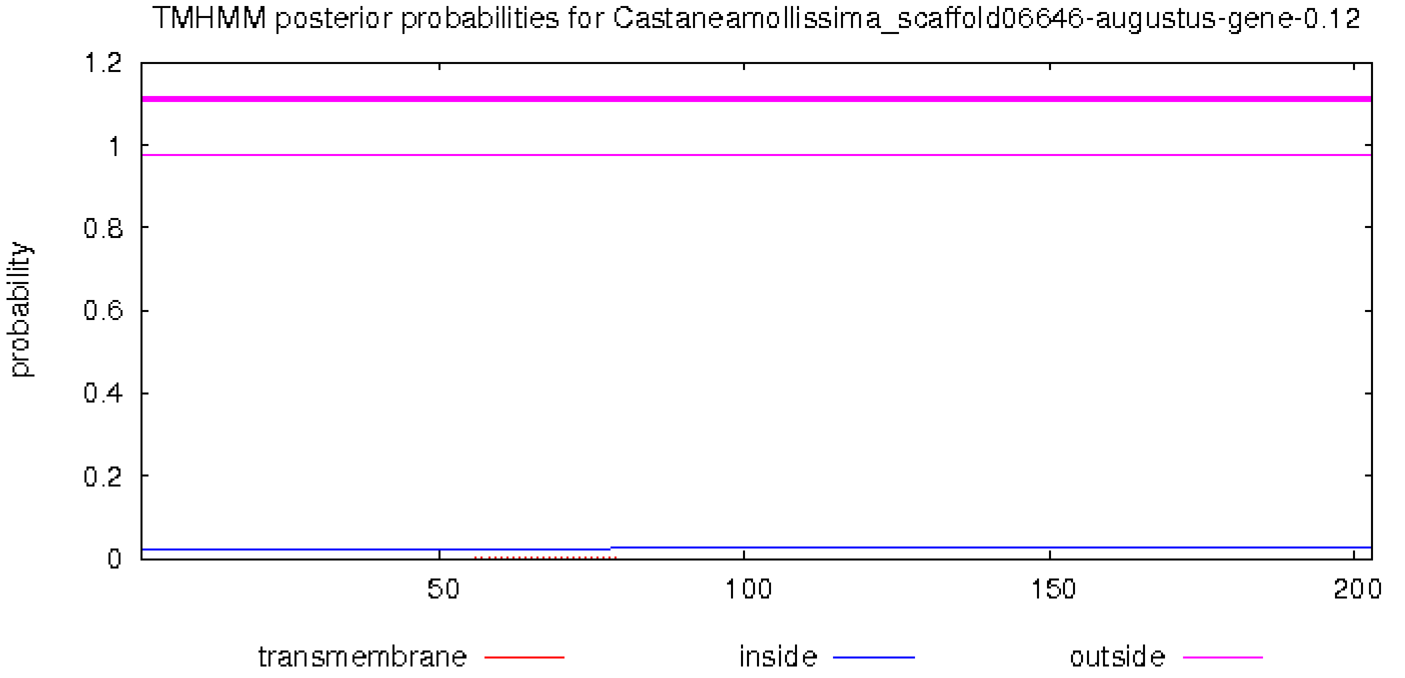

plot in postscript, script for making the plot in gnuplot, data for plot

---

```
# Castaneamollissima_scaffold07067-augustus-gene-0.6 Length: 705
# Castaneamollissima_scaffold07067-augustus-gene-0.6 Number of predicted TMHs:  0
# Castaneamollissima_scaffold07067-augustus-gene-0.6 Exp number of AAs in TMHs: 0.00449
# Castaneamollissima_scaffold07067-augustus-gene-0.6 Exp number, first 60 AAs:  0.00108
# Castaneamollissima_scaffold07067-augustus-gene-0.6 Total prob of N-in:        0.00019
Castaneamollissima_scaffold07067-augustus-gene-0.6      TMHMM2.0        outside      1   705
```

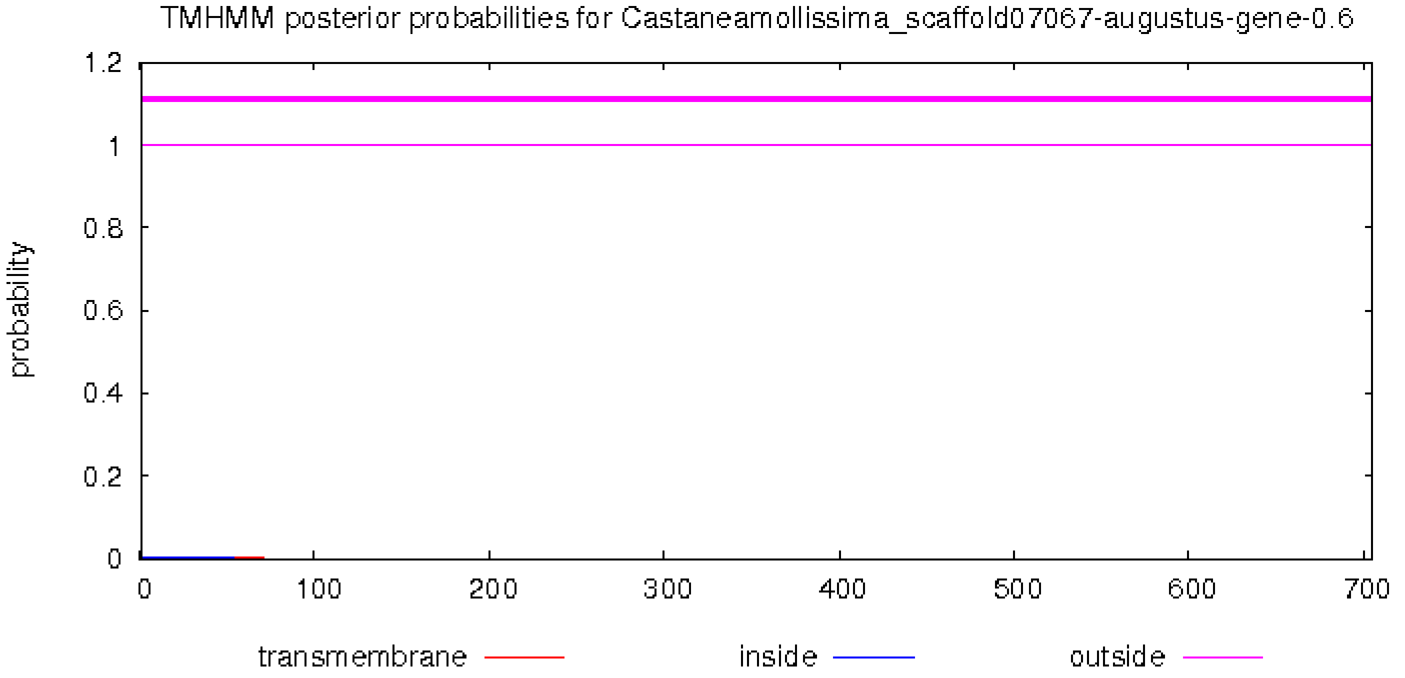

plot in postscript, script for making the plot in gnuplot, data for plot

---

```
# Castaneamollissima_scaffold07067-augustus-gene-0.6 Length: 640
# Castaneamollissima_scaffold07067-augustus-gene-0.6 Number of predicted TMHs:  0
# Castaneamollissima_scaffold07067-augustus-gene-0.6 Exp number of AAs in TMHs: 0.00019
# Castaneamollissima_scaffold07067-augustus-gene-0.6 Exp number, first 60 AAs:  0
# Castaneamollissima_scaffold07067-augustus-gene-0.6 Total prob of N-in:        0.00007
Castaneamollissima_scaffold07067-augustus-gene-0.6	TMHMM2.0	outside	     1   640
```

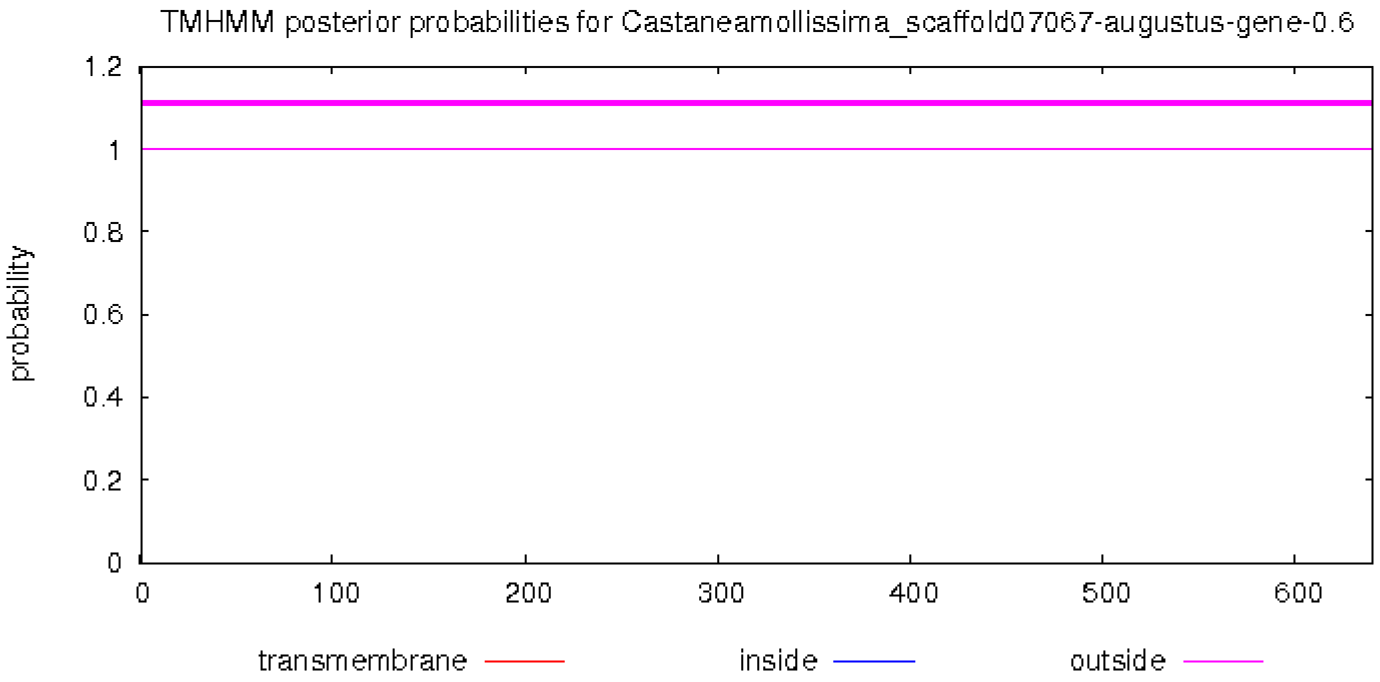

plot in postscript, script for making the plot in gnuplot, data for plot

---

```
# Castaneamollissima_scaffold07067-augustus-gene-0.6 Length: 175
# Castaneamollissima_scaffold07067-augustus-gene-0.6 Number of predicted TMHs:  0
# Castaneamollissima_scaffold07067-augustus-gene-0.6 Exp number of AAs in TMHs: 0.0002
# Castaneamollissima_scaffold07067-augustus-gene-0.6 Exp number, first 60 AAs:  0
# Castaneamollissima_scaffold07067-augustus-gene-0.6 Total prob of N-in:        0.02407
Castaneamollissima_scaffold07067-augustus-gene-0.6	TMHMM2.0	outside	     1   175
```

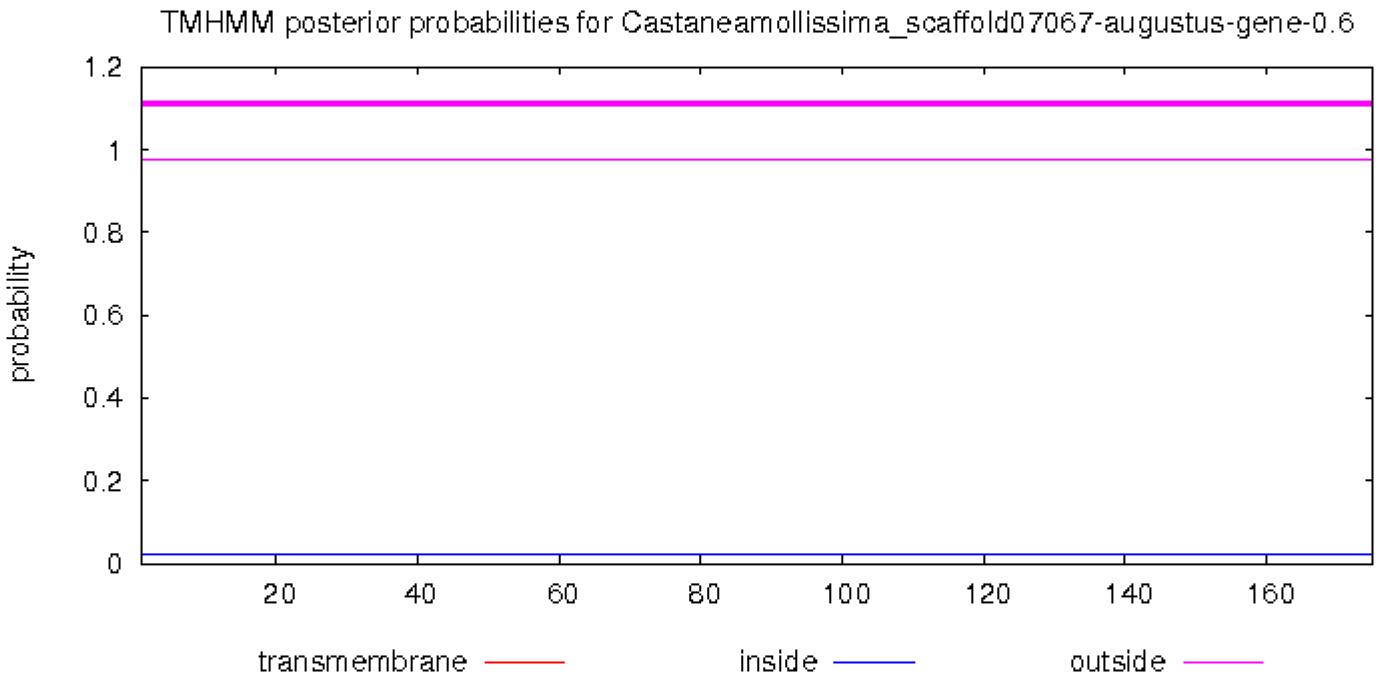

plot in postscript, script for making the plot in gnuplot, data for plot

---

```
# Castaneamollissima_scaffold07459-snap-gene-0.9 Length: 544
# Castaneamollissima_scaffold07459-snap-gene-0.9 Number of predicted TMHs:  1
# Castaneamollissima_scaffold07459-snap-gene-0.9 Exp number of AAs in TMHs: 21.82846
# Castaneamollissima_scaffold07459-snap-gene-0.9 Exp number, first 60 AAs:  0.00033
# Castaneamollissima_scaffold07459-snap-gene-0.9 Total prob of N-in:        0.05004
Castaneamollissima_scaffold07459-snap-gene-0.9  TMHMM2.0        outside      1   519
Castaneamollissima_scaffold07459-snap-gene-0.9  TMHMM2.0        TMhelix    520   542
Castaneamollissima_scaffold07459-snap-gene-0.9  TMHMM2.0        inside     543   544
```

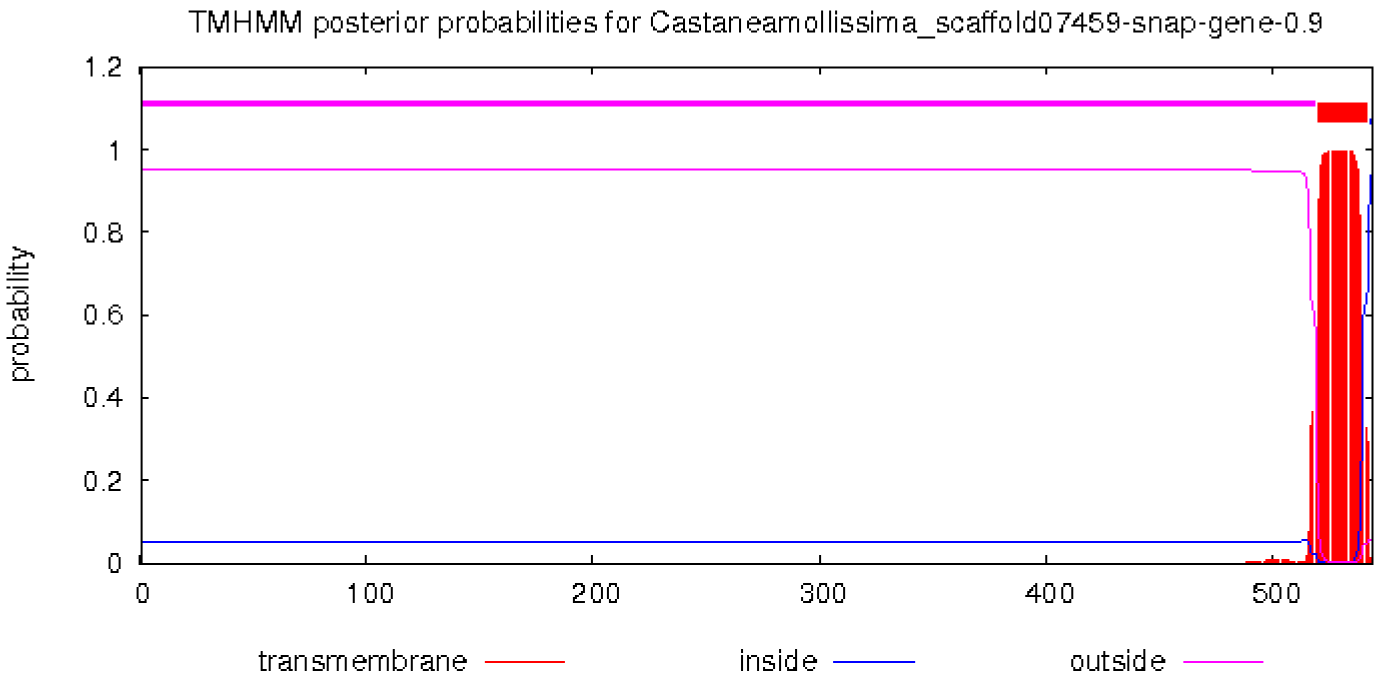

plot in postscript, script for making the plot in gnuplot, data for plot

---

```
# Castaneamollissima_scaffold08043-augustus-gene-0.8 Length: 253
# Castaneamollissima_scaffold08043-augustus-gene-0.8 Number of predicted TMHs:  0
# Castaneamollissima_scaffold08043-augustus-gene-0.8 Exp number of AAs in TMHs: 0.00043
# Castaneamollissima_scaffold08043-augustus-gene-0.8 Exp number, first 60 AAs:  0
# Castaneamollissima_scaffold08043-augustus-gene-0.8 Total prob of N-in:        0.00420
Castaneamollissima_scaffold08043-augustus-gene-0.8      TMHMM2.0        outside      1   253
```

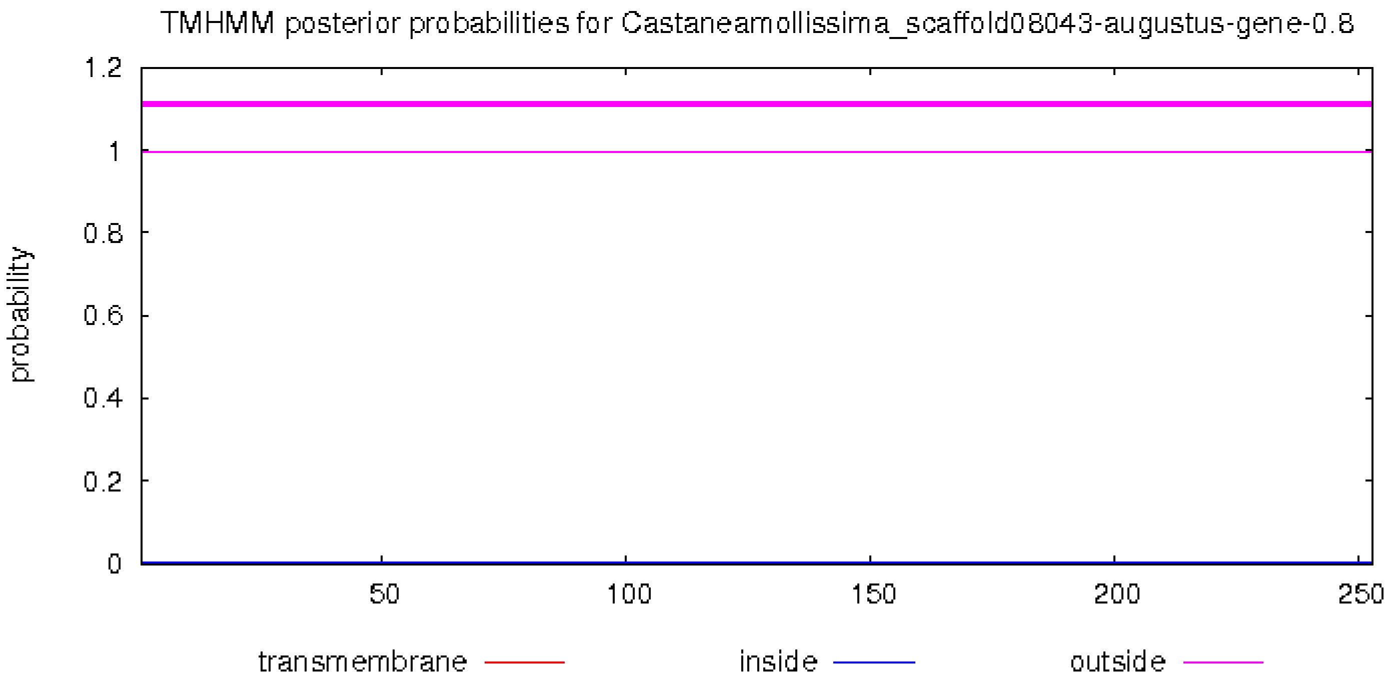

plot in postscript, script for making the plot in gnuplot, data for plot

```
# Castaneamollissima_scaffold08137-snap-gene-0.8 Length: 934
# Castaneamollissima_scaffold08137-snap-gene-0.8 Number of predicted TMHs:  1
# Castaneamollissima_scaffold08137-snap-gene-0.8 Exp number of AAs in TMHs: 20.60784
# Castaneamollissima_scaffold08137-snap-gene-0.8 Exp number, first 60 AAs:  0
# Castaneamollissima_scaffold08137-snap-gene-0.8 Total prob of N-in:        0.00100
Castaneamollissima_scaffold08137-snap-gene-0.8  TMHMM2.0        outside      1   906
Castaneamollissima_scaffold08137-snap-gene-0.8  TMHMM2.0        TMhelix    907   929
Castaneamollissima_scaffold08137-snap-gene-0.8  TMHMM2.0        inside     930   934
```

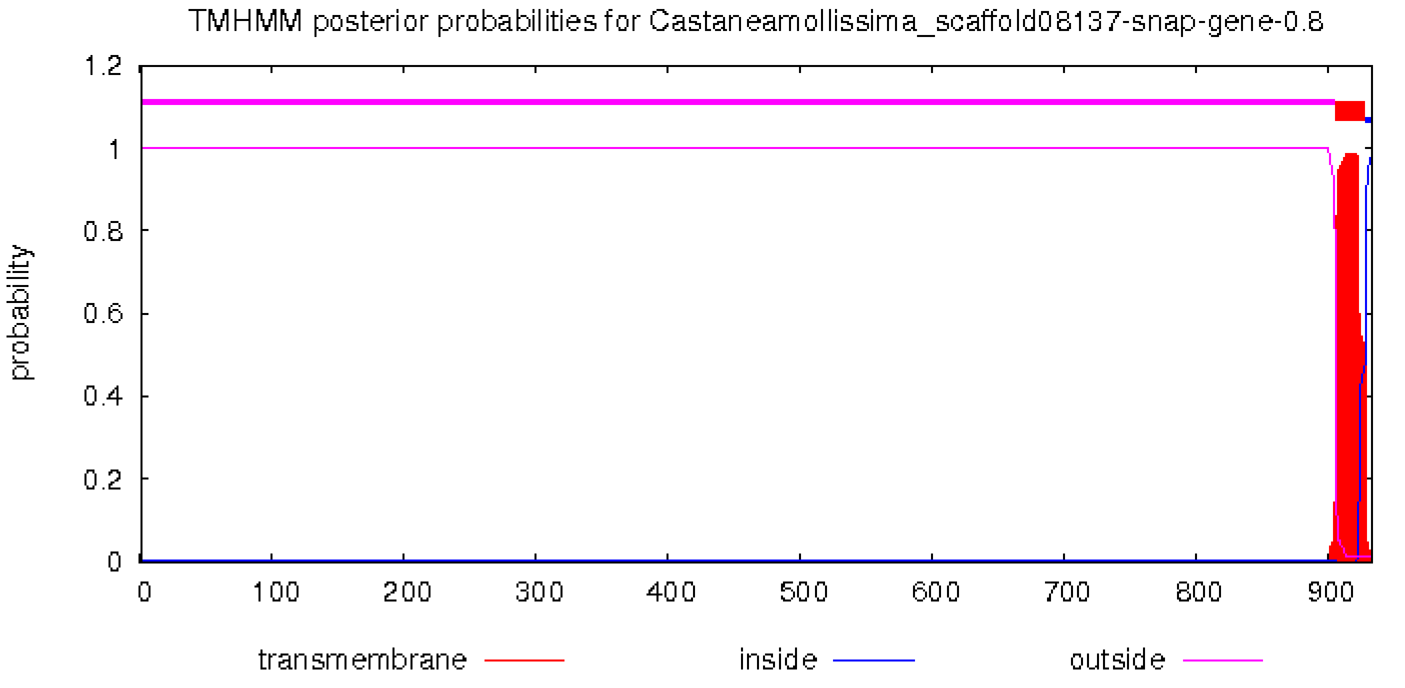

plot in postscript, script for making the plot in gnuplot, data for plot

```
# Castaneamollissima_scaffold08188-augustus-gene-0.7 Length: 160
# Castaneamollissima_scaffold08188-augustus-gene-0.7 Number of predicted TMHs:  0
# Castaneamollissima_scaffold08188-augustus-gene-0.7 Exp number of AAs in TMHs: 0
# Castaneamollissima_scaffold08188-augustus-gene-0.7 Exp number, first 60 AAs:  0
# Castaneamollissima_scaffold08188-augustus-gene-0.7 Total prob of N-in:        0.01867
Castaneamollissima_scaffold08188-augustus-gene-0.7      TMHMM2.0        outside      1   160
```

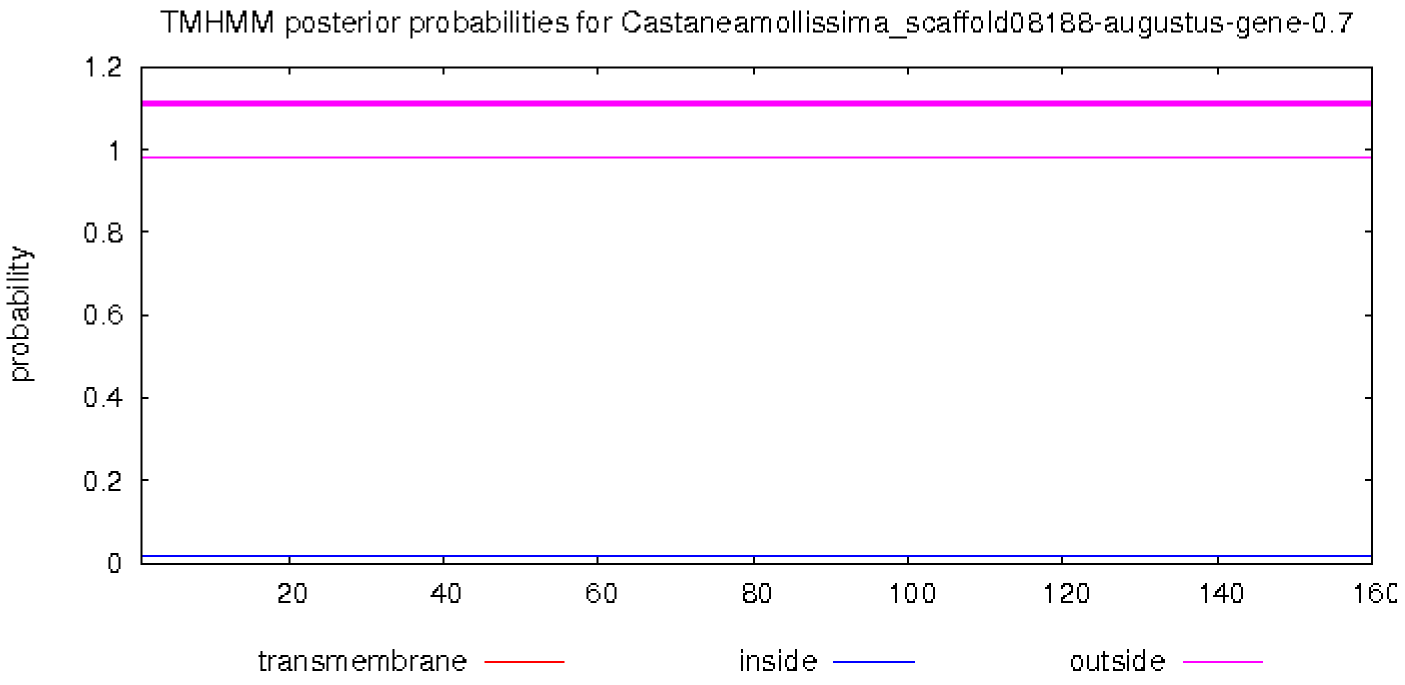

plot in postscript, script for making the plot in gnuplot, data for plot

---

```
# Castaneamollissima_scaffold08294-augustus-gene-0.4 Length: 332
# Castaneamollissima_scaffold08294-augustus-gene-0.4 Number of predicted TMHs:  0
# Castaneamollissima_scaffold08294-augustus-gene-0.4 Exp number of AAs in TMHs: 0.00015
# Castaneamollissima_scaffold08294-augustus-gene-0.4 Exp number, first 60 AAs:  0
# Castaneamollissima_scaffold08294-augustus-gene-0.4 Total prob of N-in:        0.01892
Castaneamollissima_scaffold08294-augustus-gene-0.4	TMHMM2.0	outside	     1   332
```

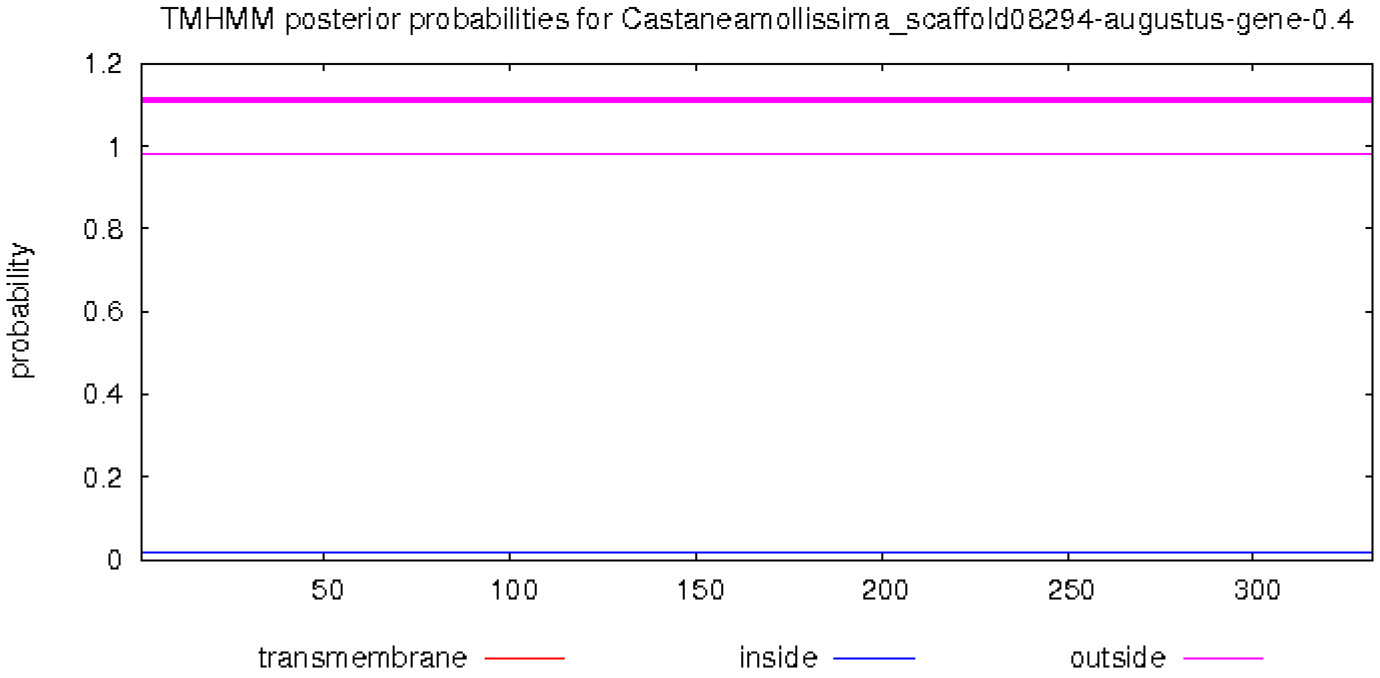

plot in postscript, script for making the plot in gnuplot, data for plot

---

```
# Castaneamollissima_scaffold08297-augustus-gene-0.5 Length: 191
# Castaneamollissima_scaffold08297-augustus-gene-0.5 Number of predicted TMHs:  0
# Castaneamollissima_scaffold08297-augustus-gene-0.5 Exp number of AAs in TMHs: 1.28598
# Castaneamollissima_scaffold08297-augustus-gene-0.5 Exp number, first 60 AAs:  0.13725
# Castaneamollissima_scaffold08297-augustus-gene-0.5 Total prob of N-in:        0.00946
Castaneamollissima_scaffold08297-augustus-gene-0.5	TMHMM2.0	outside	     1   191
```

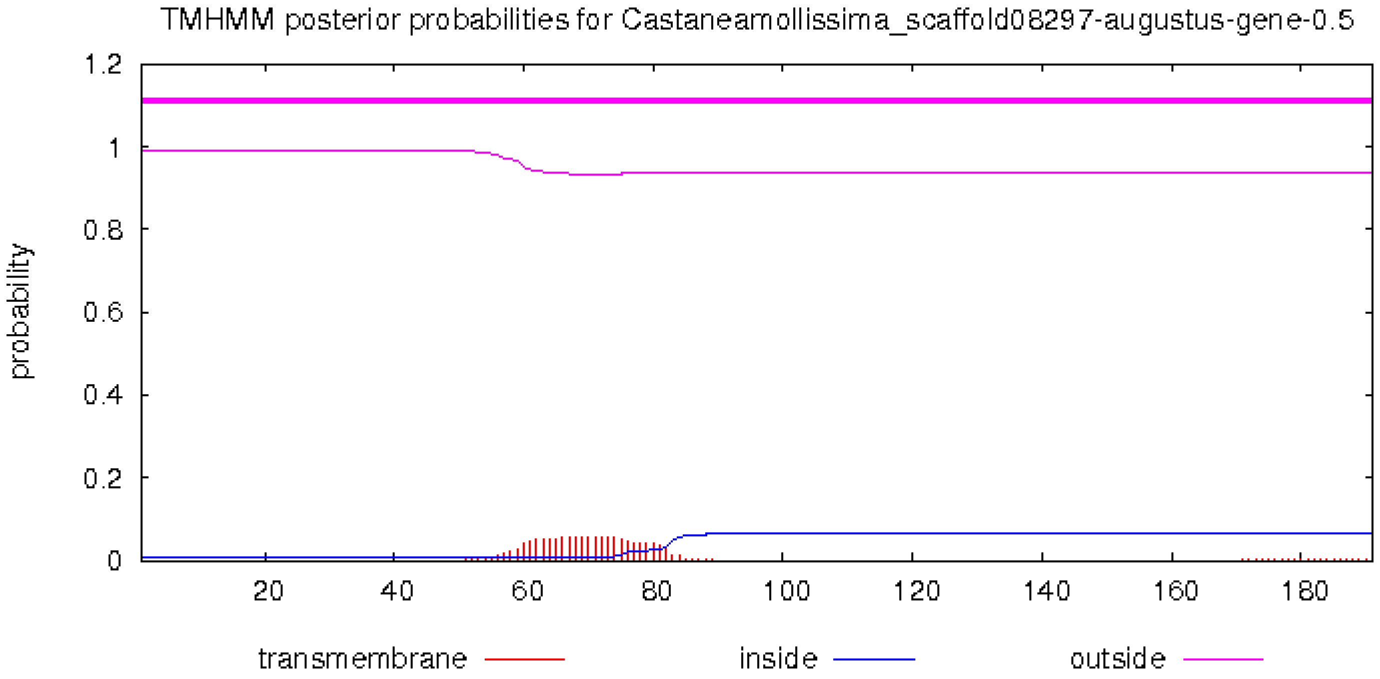

plot in postscript, script for making the plot in gnuplot, data for plot

```
# Castaneamollissima_scaffold08573-augustus-gene-0.8 Length: 531
# Castaneamollissima_scaffold08573-augustus-gene-0.8 Number of predicted TMHs:  0
# Castaneamollissima_scaffold08573-augustus-gene-0.8 Exp number of AAs in TMHs: 0.01521
# Castaneamollissima_scaffold08573-augustus-gene-0.8 Exp number, first 60 AAs:  0.01239
# Castaneamollissima_scaffold08573-augustus-gene-0.8 Total prob of N-in:        0.00068
Castaneamollissima_scaffold08573-augustus-gene-0.8      TMHMM2.0        outside      1   531
```

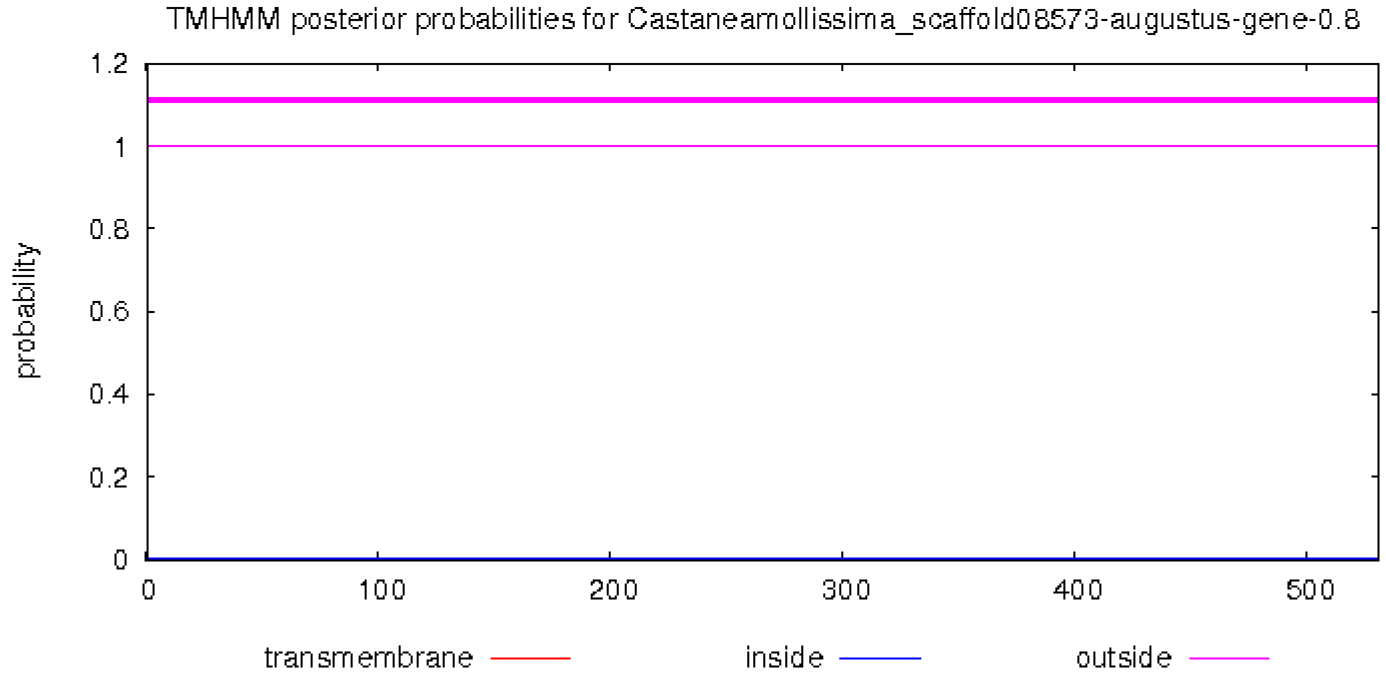

plot in postscript, script for making the plot in gnuplot, data for plot

```
# Castaneamollissima_scaffold09059-snap-gene-0.18 Length: 449
# Castaneamollissima_scaffold09059-snap-gene-0.18 Number of predicted TMHs:  0
# Castaneamollissima_scaffold09059-snap-gene-0.18 Exp number of AAs in TMHs: 0
# Castaneamollissima_scaffold09059-snap-gene-0.18 Exp number, first 60 AAs:  0
# Castaneamollissima_scaffold09059-snap-gene-0.18 Total prob of N-in:        0.00304
Castaneamollissima_scaffold09059-snap-gene-0.18 TMHMM2.0        outside      1   449
```

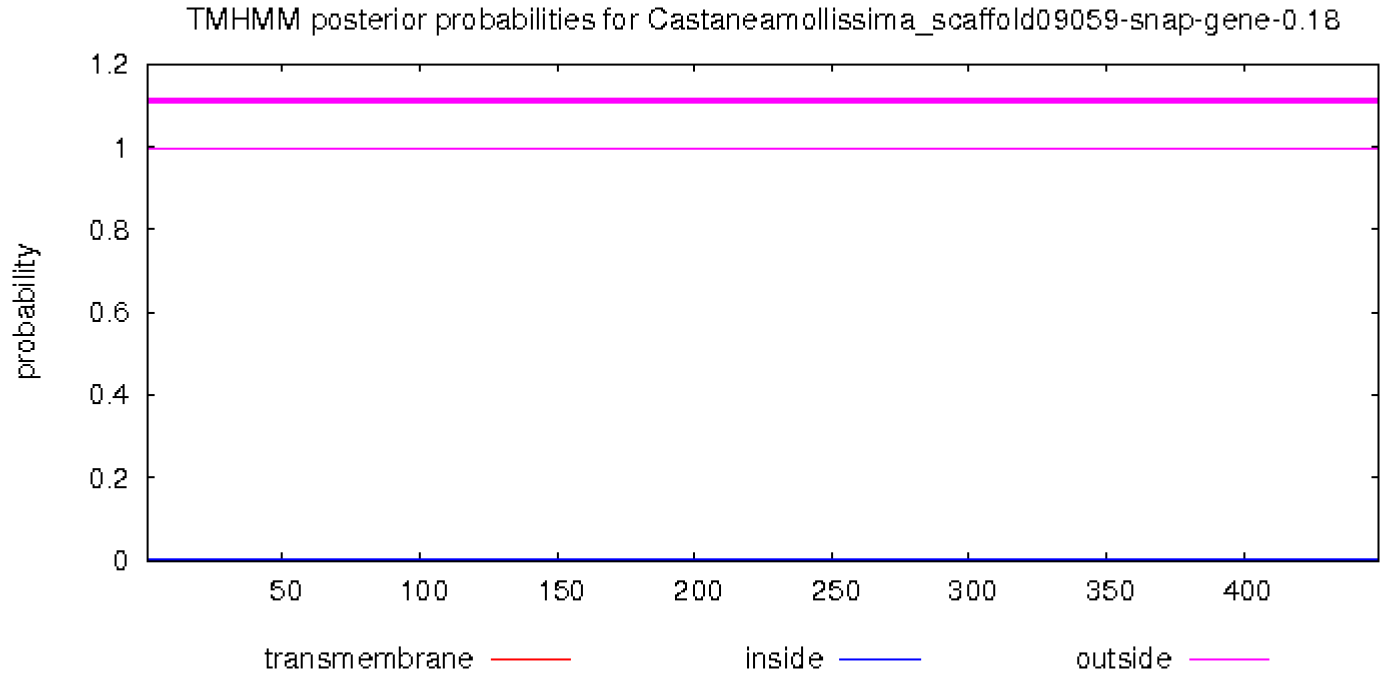

plot in postscript, script for making the plot in gnuplot, data for plot

```
# Castaneamollissima_scaffold09484-snap-gene-0.17 Length: 160
# Castaneamollissima_scaffold09484-snap-gene-0.17 Number of predicted TMHs:  0
# Castaneamollissima_scaffold09484-snap-gene-0.17 Exp number of AAs in TMHs: 0.00111
# Castaneamollissima_scaffold09484-snap-gene-0.17 Exp number, first 60 AAs:  0
# Castaneamollissima_scaffold09484-snap-gene-0.17 Total prob of N-in:        0.15193
Castaneamollissima_scaffold09484-snap-gene-0.17 TMHMM2.0        outside      1   160
```

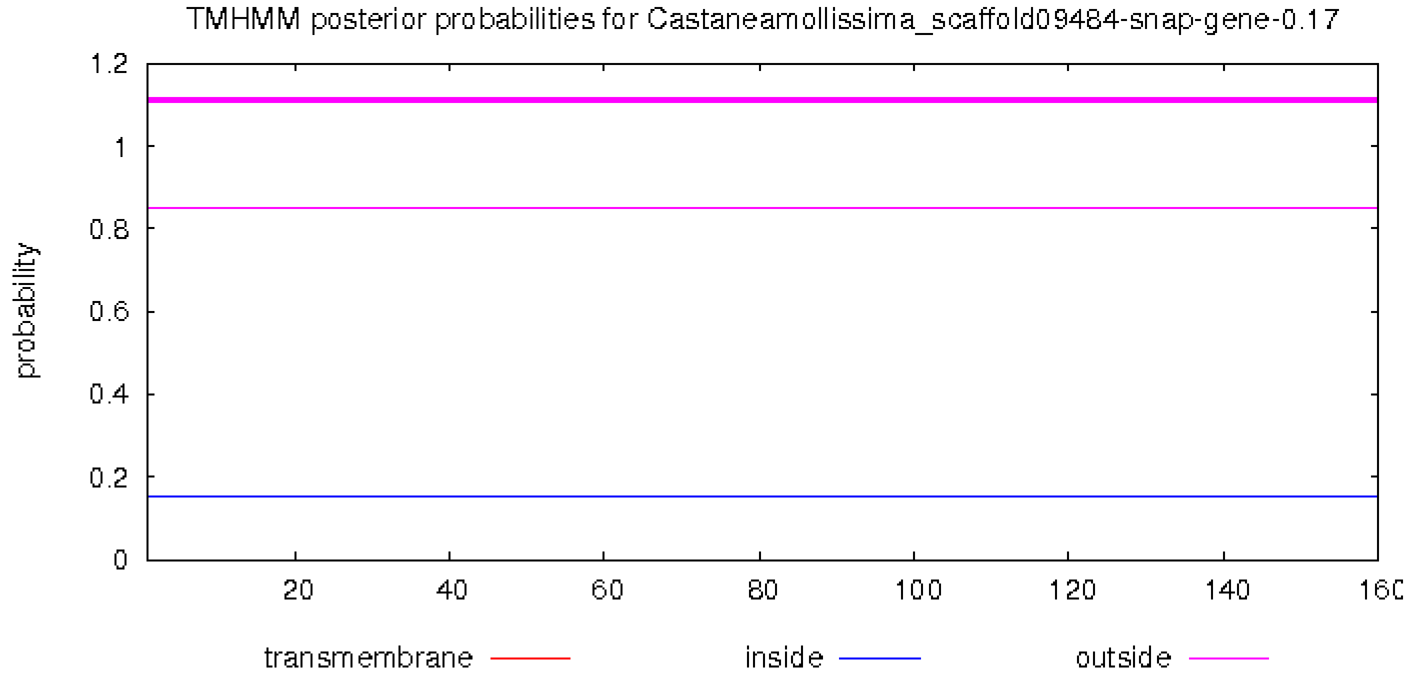

plot in postscript, script for making the plot in gnuplot, data for plot

```
# Castaneamollissima_scaffold09786-augustus-gene-0.7 Length: 345
# Castaneamollissima_scaffold09786-augustus-gene-0.7 Number of predicted TMHs:  0
# Castaneamollissima_scaffold09786-augustus-gene-0.7 Exp number of AAs in TMHs: 0.00018
# Castaneamollissima_scaffold09786-augustus-gene-0.7 Exp number, first 60 AAs:  0
# Castaneamollissima_scaffold09786-augustus-gene-0.7 Total prob of N-in:        0.00075
Castaneamollissima_scaffold09786-augustus-gene-0.7      TMHMM2.0        outside      1   345
```

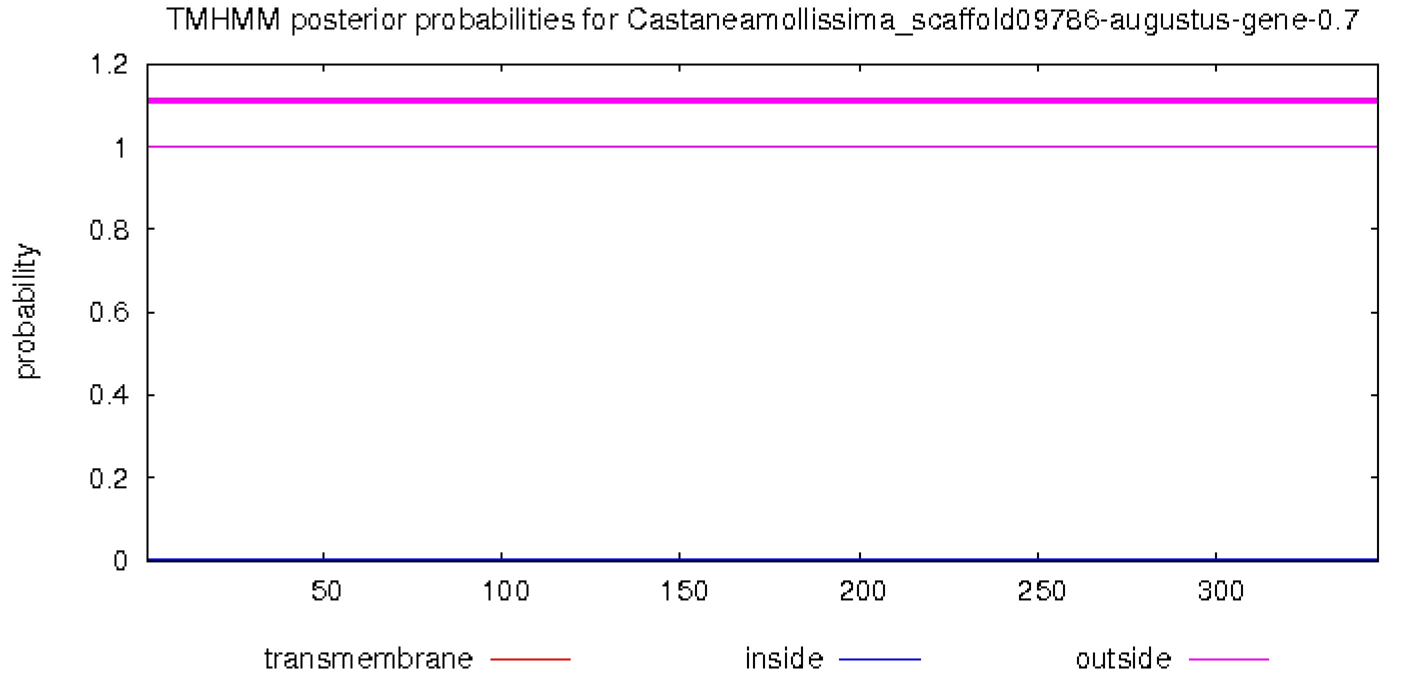

plot in postscript, script for making the plot in gnuplot, data for plot

```
# Castaneamollissima_scaffold09973-snap-gene-0.10 Length: 240
# Castaneamollissima_scaffold09973-snap-gene-0.10 Number of predicted TMHs:  1
# Castaneamollissima_scaffold09973-snap-gene-0.10 Exp number of AAs in TMHs: 20.1491
# Castaneamollissima_scaffold09973-snap-gene-0.10 Exp number, first 60 AAs:  20.14776
# Castaneamollissima_scaffold09973-snap-gene-0.10 Total prob of N-in:        0.92577
# Castaneamollissima_scaffold09973-snap-gene-0.10 POSSIBLE N-term signal sequence
Castaneamollissima_scaffold09973-snap-gene-0.10 TMHMM2.0        inside       1    20
Castaneamollissima_scaffold09973-snap-gene-0.10 TMHMM2.0        TMhelix     21    38
Castaneamollissima_scaffold09973-snap-gene-0.10 TMHMM2.0        outside     39   240
```

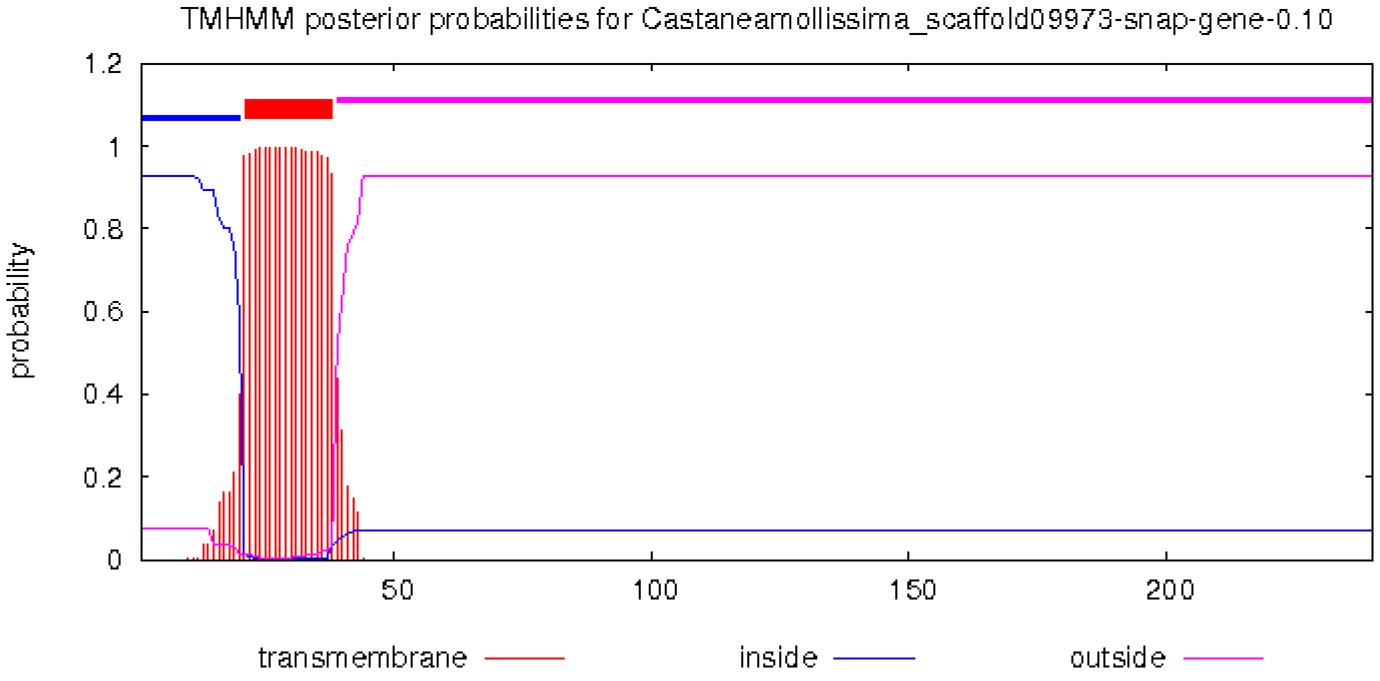

plot in postscript, script for making the plot in gnuplot, data for plot

```
# Castaneamollissima_scaffold09973-snap-gene-0.11 Length: 452
# Castaneamollissima_scaffold09973-snap-gene-0.11 Number of predicted TMHs:  0
# Castaneamollissima_scaffold09973-snap-gene-0.11 Exp number of AAs in TMHs: 0
# Castaneamollissima_scaffold09973-snap-gene-0.11 Exp number, first 60 AAs:  0
# Castaneamollissima_scaffold09973-snap-gene-0.11 Total prob of N-in:        0.00086
Castaneamollissima_scaffold09973-snap-gene-0.11 TMHMM2.0        outside      1   452
```

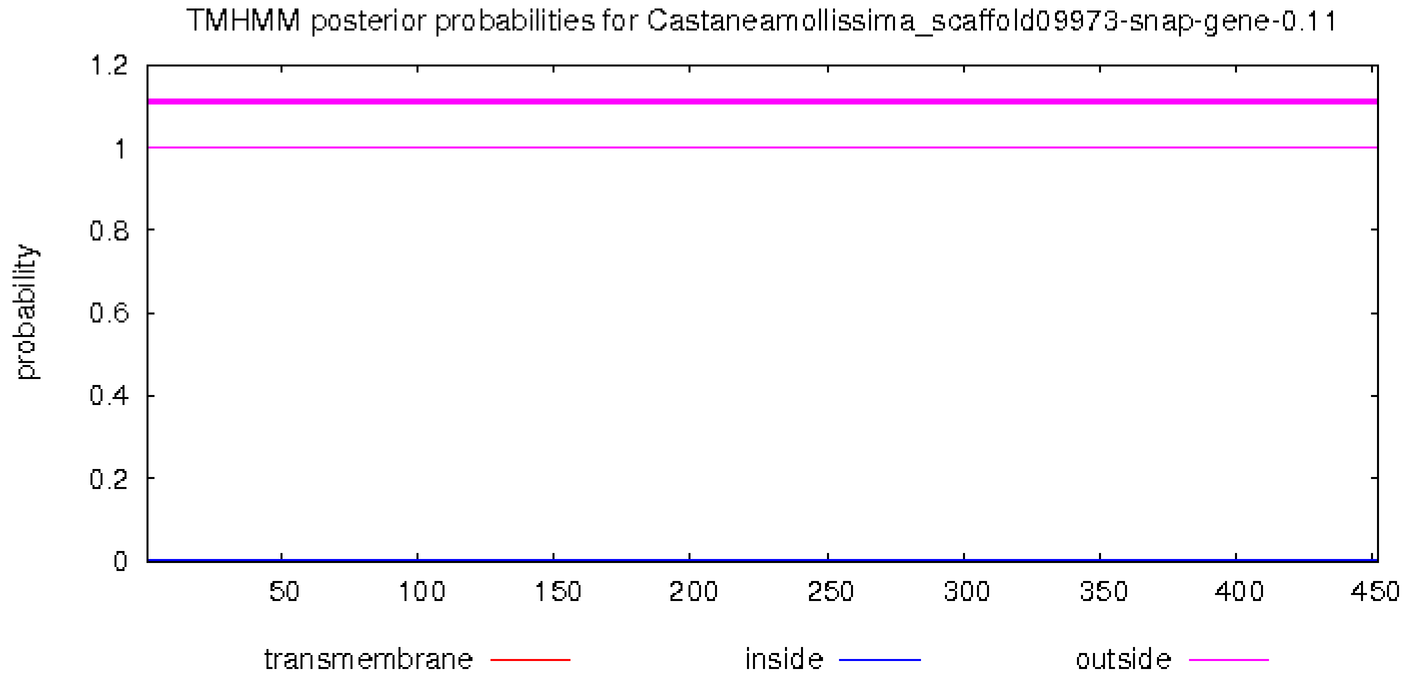

plot in postscript, script for making the plot in gnuplot, data for plot

---

```
# Castaneamollissima_scaffold10692-snap-gene-0.12 Length: 162
# Castaneamollissima_scaffold10692-snap-gene-0.12 Number of predicted TMHs:  0
# Castaneamollissima_scaffold10692-snap-gene-0.12 Exp number of AAs in TMHs: 0
# Castaneamollissima_scaffold10692-snap-gene-0.12 Exp number, first 60 AAs:  0
# Castaneamollissima_scaffold10692-snap-gene-0.12 Total prob of N-in:        0.01760
Castaneamollissima_scaffold10692-snap-gene-0.12 TMHMM2.0        outside      1   162
```

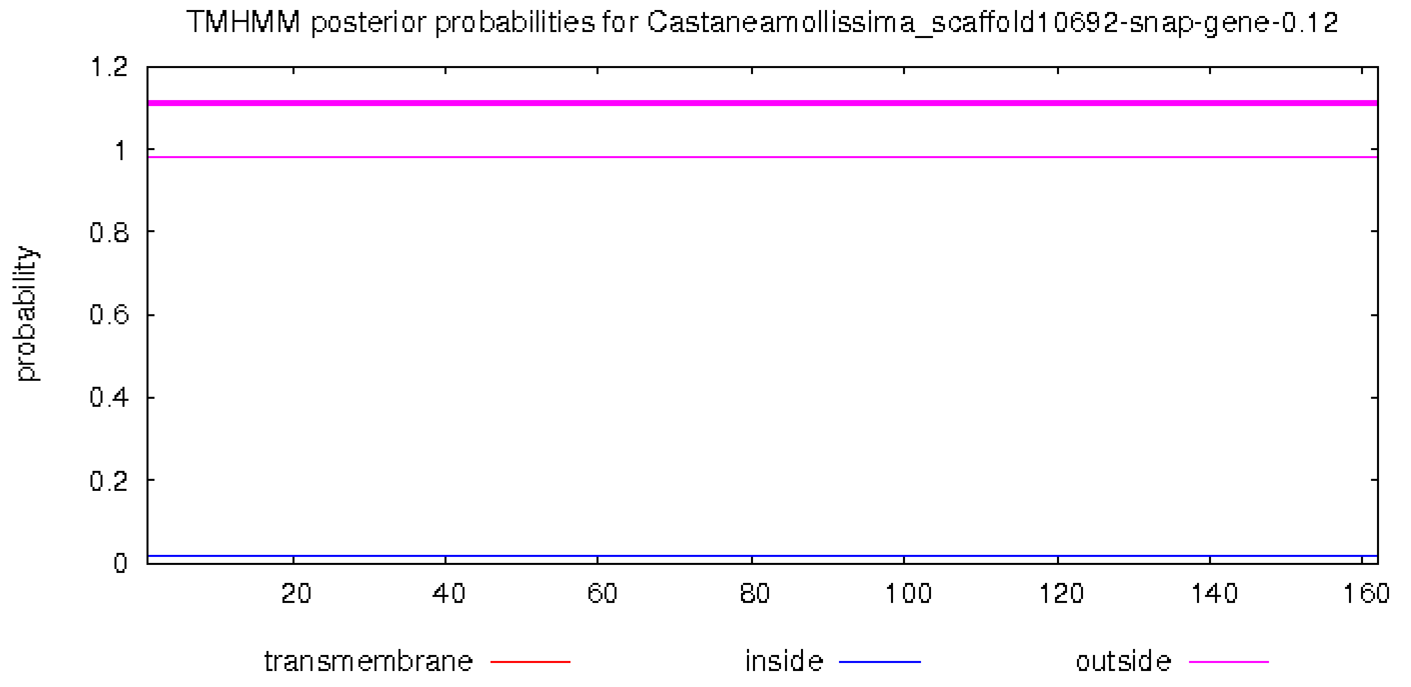

plot in postscript, script for making the plot in gnuplot, data for plot

---

```
# Castaneamollissima_scaffold12065-snap-gene-0.6-mRNA Length: 468
# Castaneamollissima_scaffold12065-snap-gene-0.6-mRNA Number of predicted TMHs:  0
# Castaneamollissima_scaffold12065-snap-gene-0.6-mRNA Exp number of AAs in TMHs: 0.00032
# Castaneamollissima_scaffold12065-snap-gene-0.6-mRNA Exp number, first 60 AAs:  0.00018
# Castaneamollissima_scaffold12065-snap-gene-0.6-mRNA Total prob of N-in:        0.00346
Castaneamollissima_scaffold12065-snap-gene-0.6-mRNA     TMHMM2.0        outside      1   468
```

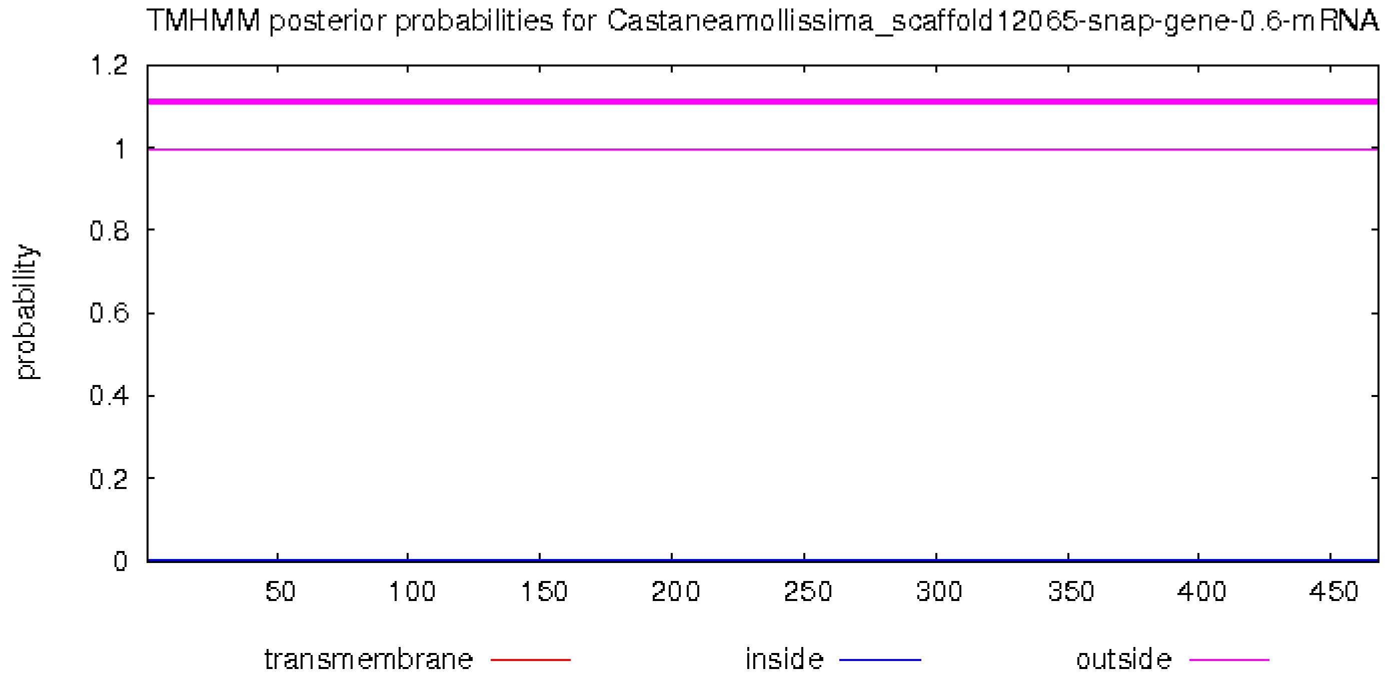

plot in postscript, script for making the plot in gnuplot, data for plot

---

```
# Castaneamollissima_scaffold13586-augustus-gene-0.8 Length: 171
# Castaneamollissima_scaffold13586-augustus-gene-0.8 Number of predicted TMHs:  0
# Castaneamollissima_scaffold13586-augustus-gene-0.8 Exp number of AAs in TMHs: 0
# Castaneamollissima_scaffold13586-augustus-gene-0.8 Exp number, first 60 AAs:  0
# Castaneamollissima_scaffold13586-augustus-gene-0.8 Total prob of N-in:        0.02303
Castaneamollissima_scaffold13586-augustus-gene-0.8      TMHMM2.0        outside      1   171
```

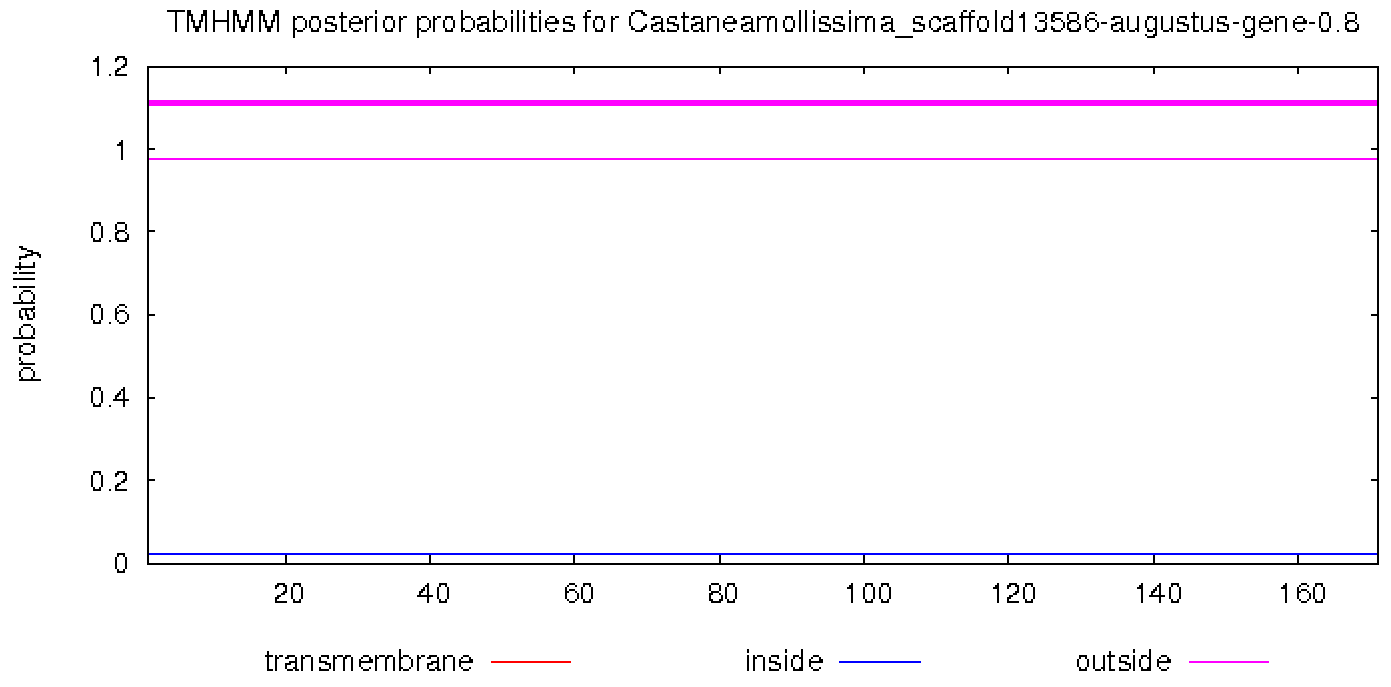

plot in postscript, script for making the plot in gnuplot, data for plot

---

```
# Castaneamollissima_scaffold13838-snap-gene-0.5 Length: 681
# Castaneamollissima_scaffold13838-snap-gene-0.5 Number of predicted TMHs:  0
# Castaneamollissima_scaffold13838-snap-gene-0.5 Exp number of AAs in TMHs: 25.87383
# Castaneamollissima_scaffold13838-snap-gene-0.5 Exp number, first 60 AAs:  0.0001
# Castaneamollissima_scaffold13838-snap-gene-0.5 Total prob of N-in:        0.00154
Castaneamollissima_scaffold13838-snap-gene-0.5  TMHMM2.0        outside      1   681
```

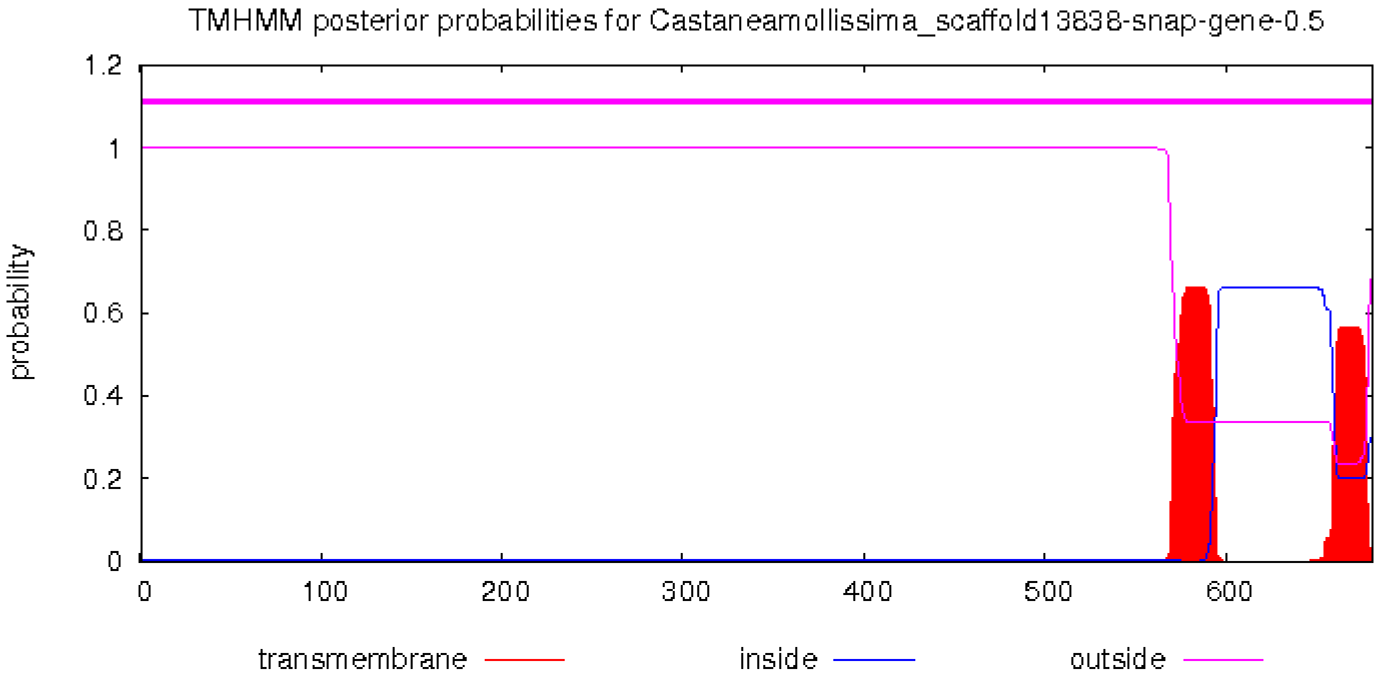

plot in postscript, script for making the plot in gnuplot, data for plot

```
# Castaneamollissima_scaffold14402-augustus-gene-0.4 Length: 243
# Castaneamollissima_scaffold14402-augustus-gene-0.4 Number of predicted TMHs:  0
# Castaneamollissima_scaffold14402-augustus-gene-0.4 Exp number of AAs in TMHs: 0.00034
# Castaneamollissima_scaffold14402-augustus-gene-0.4 Exp number, first 60 AAs:  0
# Castaneamollissima_scaffold14402-augustus-gene-0.4 Total prob of N-in:        0.01214
Castaneamollissima_scaffold14402-augustus-gene-0.4      TMHMM2.0        outside      1   243
```

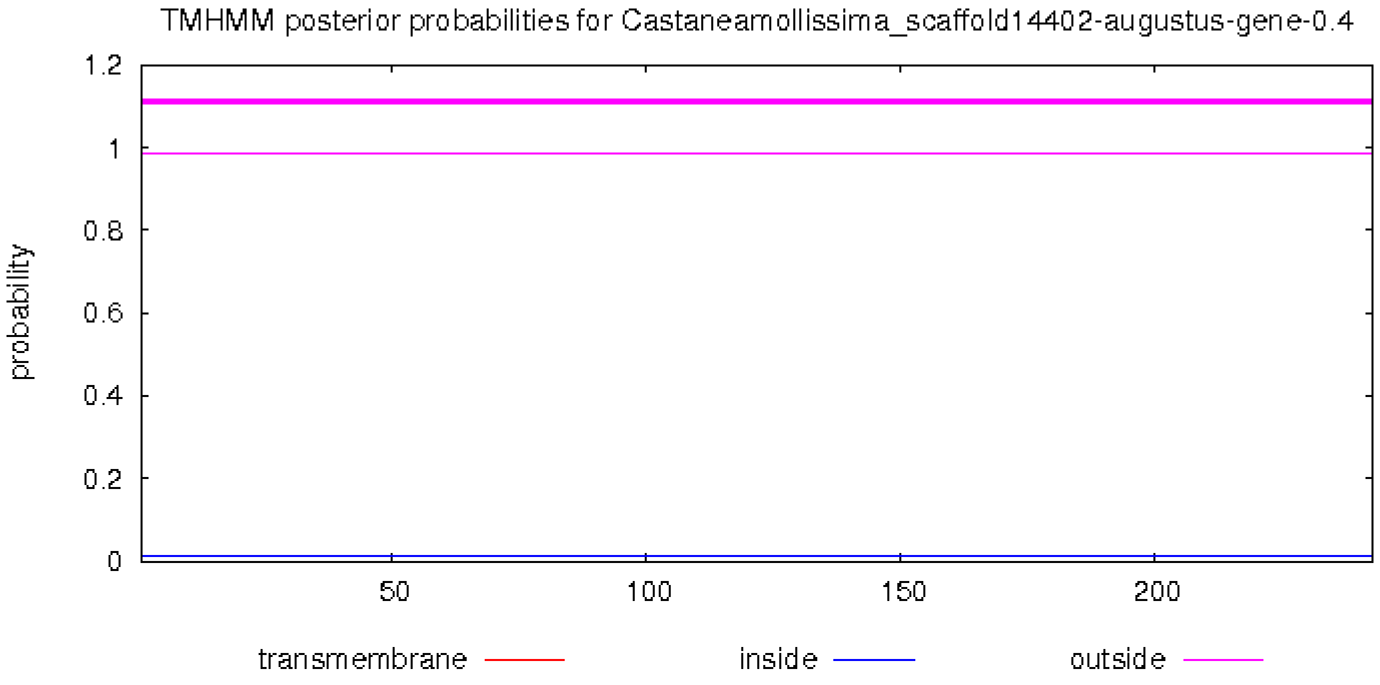

plot in postscript, script for making the plot in gnuplot, data for plot

```
# Castaneamollissima_scaffold14907-augustus-gene-0.3 Length: 607
# Castaneamollissima_scaffold14907-augustus-gene-0.3 Number of predicted TMHs:  0
# Castaneamollissima_scaffold14907-augustus-gene-0.3 Exp number of AAs in TMHs: 0
# Castaneamollissima_scaffold14907-augustus-gene-0.3 Exp number, first 60 AAs:  0
# Castaneamollissima_scaffold14907-augustus-gene-0.3 Total prob of N-in:        0.00030
Castaneamollissima_scaffold14907-augustus-gene-0.3      TMHMM2.0        outside      1   607
```

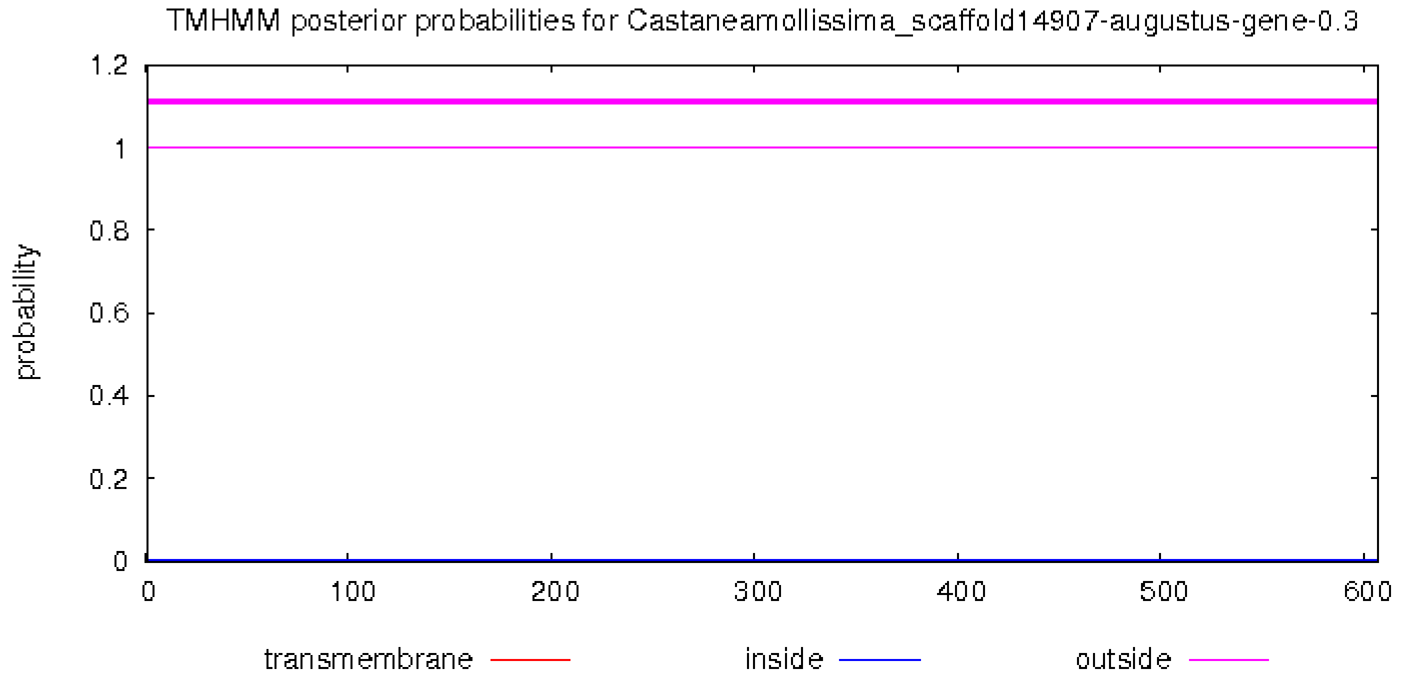

plot in postscript, script for making the plot in gnuplot, data for plot

---

```
# Castaneamollissima_scaffold15610-augustus-gene-0.4 Length: 576
# Castaneamollissima_scaffold15610-augustus-gene-0.4 Number of predicted TMHs:  1
# Castaneamollissima_scaffold15610-augustus-gene-0.4 Exp number of AAs in TMHs: 20.76403
# Castaneamollissima_scaffold15610-augustus-gene-0.4 Exp number, first 60 AAs:  0
# Castaneamollissima_scaffold15610-augustus-gene-0.4 Total prob of N-in:        0.01135
Castaneamollissima_scaffold15610-augustus-gene-0.4      TMHMM2.0        outside      1   552
Castaneamollissima_scaffold15610-augustus-gene-0.4      TMHMM2.0        TMhelix    553   575
Castaneamollissima_scaffold15610-augustus-gene-0.4      TMHMM2.0        inside     576   576
```

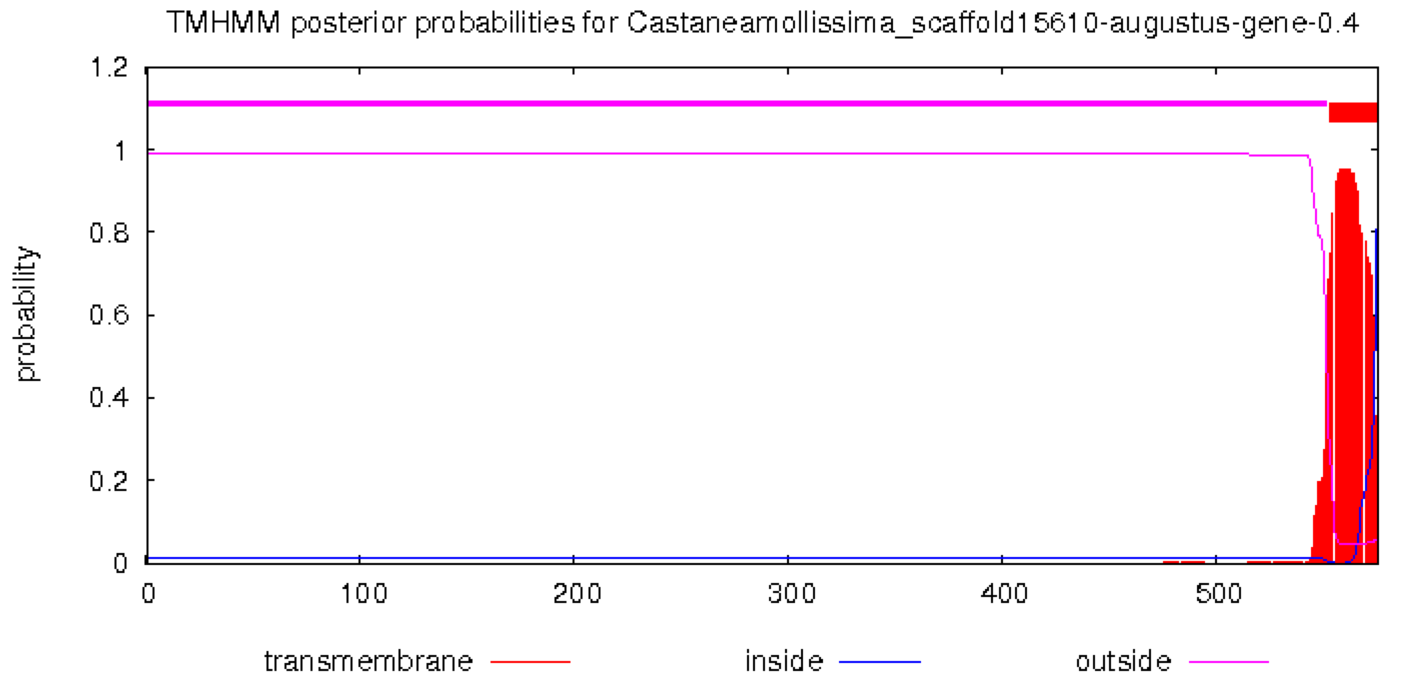

plot in postscript, script for making the plot in gnuplot, data for plot

---

```
# Castaneamollissima_scaffold16081-augustus-gene-0.6 Length: 177
# Castaneamollissima_scaffold16081-augustus-gene-0.6 Number of predicted TMHs:  0
# Castaneamollissima_scaffold16081-augustus-gene-0.6 Exp number of AAs in TMHs: 0.0065
# Castaneamollissima_scaffold16081-augustus-gene-0.6 Exp number, first 60 AAs:  0
```

```
# Castaneamollissima_scaffold16081-augustus-gene-0.6 Total prob of N-in:        0.02223
Castaneamollissima_scaffold16081-augustus-gene-0.6      TMHMM2.0        outside      1   177
```

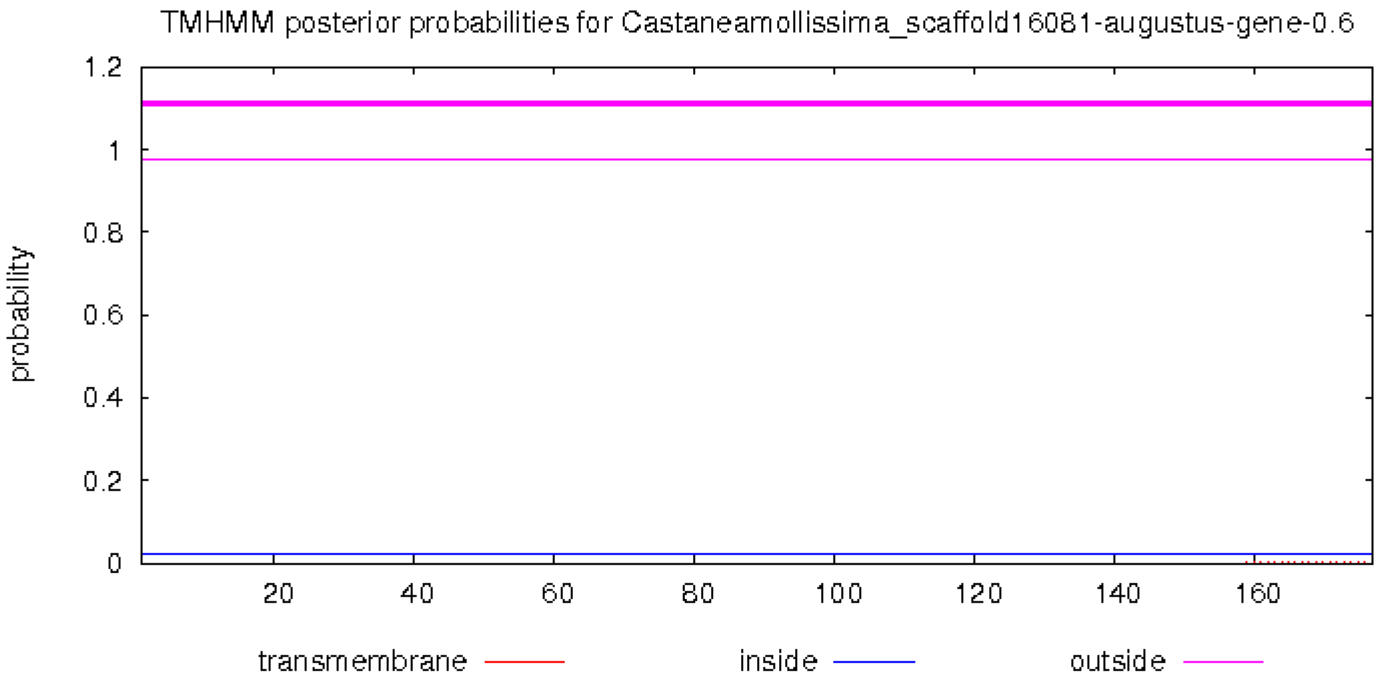

plot in postscript, script for making the plot in gnuplot, data for plot

---

```
# Castaneamollissima_scaffold16464-augustus-gene-0.2 Length: 347
# Castaneamollissima_scaffold16464-augustus-gene-0.2 Number of predicted TMHs:  0
# Castaneamollissima_scaffold16464-augustus-gene-0.2 Exp number of AAs in TMHs: 0.00072
# Castaneamollissima_scaffold16464-augustus-gene-0.2 Exp number, first 60 AAs:  0.00054
# Castaneamollissima_scaffold16464-augustus-gene-0.2 Total prob of N-in:        0.00968
Castaneamollissima_scaffold16464-augustus-gene-0.2      TMHMM2.0        outside      1   347
```

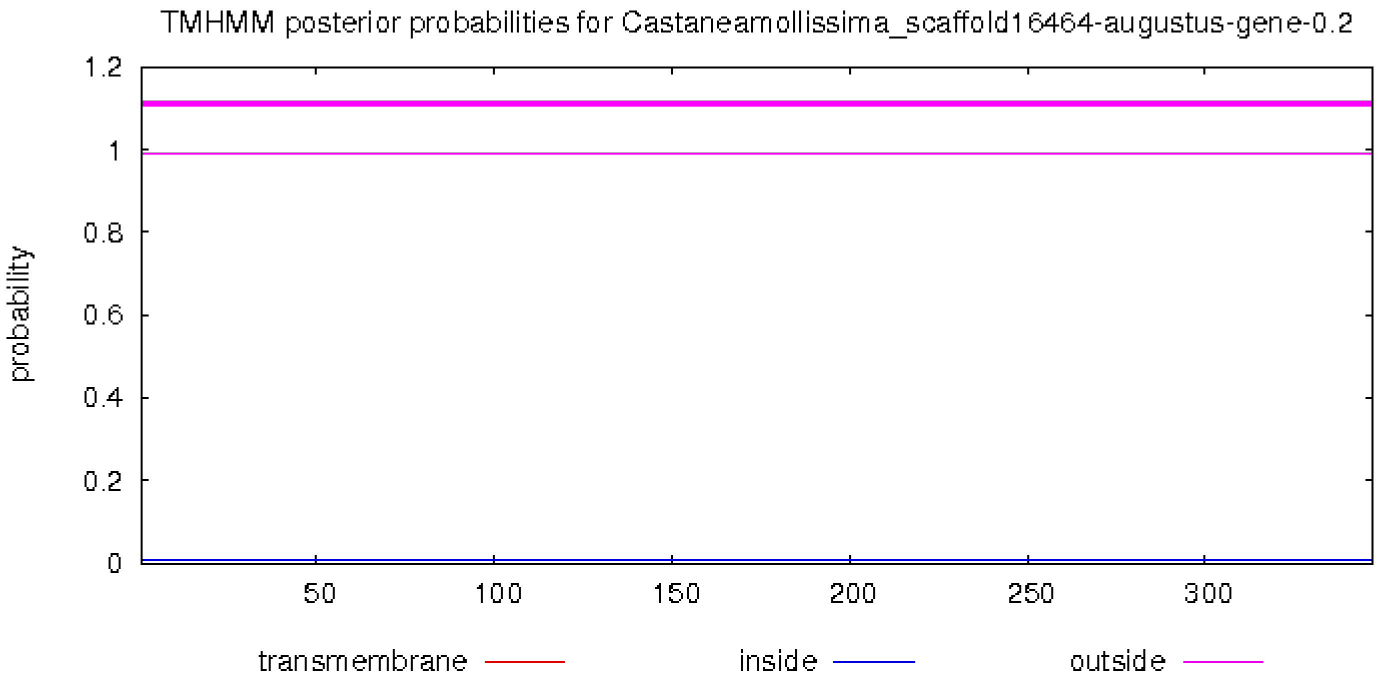

plot in postscript, script for making the plot in gnuplot, data for plot

---

```
# Castaneamollissima_scaffold17927-snap-gene-0.8 Length: 161
# Castaneamollissima_scaffold17927-snap-gene-0.8 Number of predicted TMHs:  0
# Castaneamollissima_scaffold17927-snap-gene-0.8 Exp number of AAs in TMHs: 0.00275
# Castaneamollissima_scaffold17927-snap-gene-0.8 Exp number, first 60 AAs:  0
```

```
# Castaneamollissima_scaffold17927-snap-gene-0.8 Total prob of N-in:        0.00983
Castaneamollissima_scaffold17927-snap-gene-0.8  TMHMM2.0        outside      1   161
```

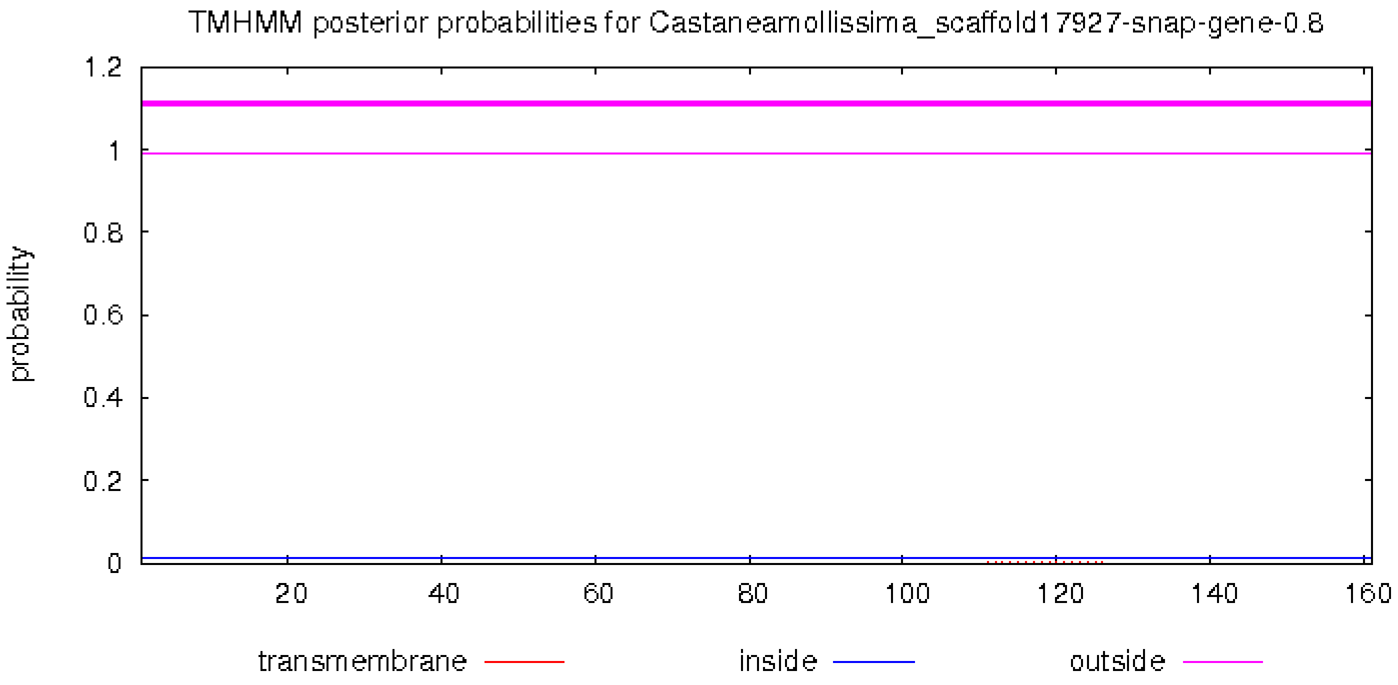

plot in postscript, script for making the plot in gnuplot, data for plot

---

```
# Castaneamollissima_scaffold22384-snap-gene-0.5 Length: 122
# Castaneamollissima_scaffold22384-snap-gene-0.5 Number of predicted TMHs:  0
# Castaneamollissima_scaffold22384-snap-gene-0.5 Exp number of AAs in TMHs: 0
# Castaneamollissima_scaffold22384-snap-gene-0.5 Exp number, first 60 AAs:  0
# Castaneamollissima_scaffold22384-snap-gene-0.5 Total prob of N-in:        0.41893
Castaneamollissima_scaffold22384-snap-gene-0.5  TMHMM2.0        outside      1   122
```

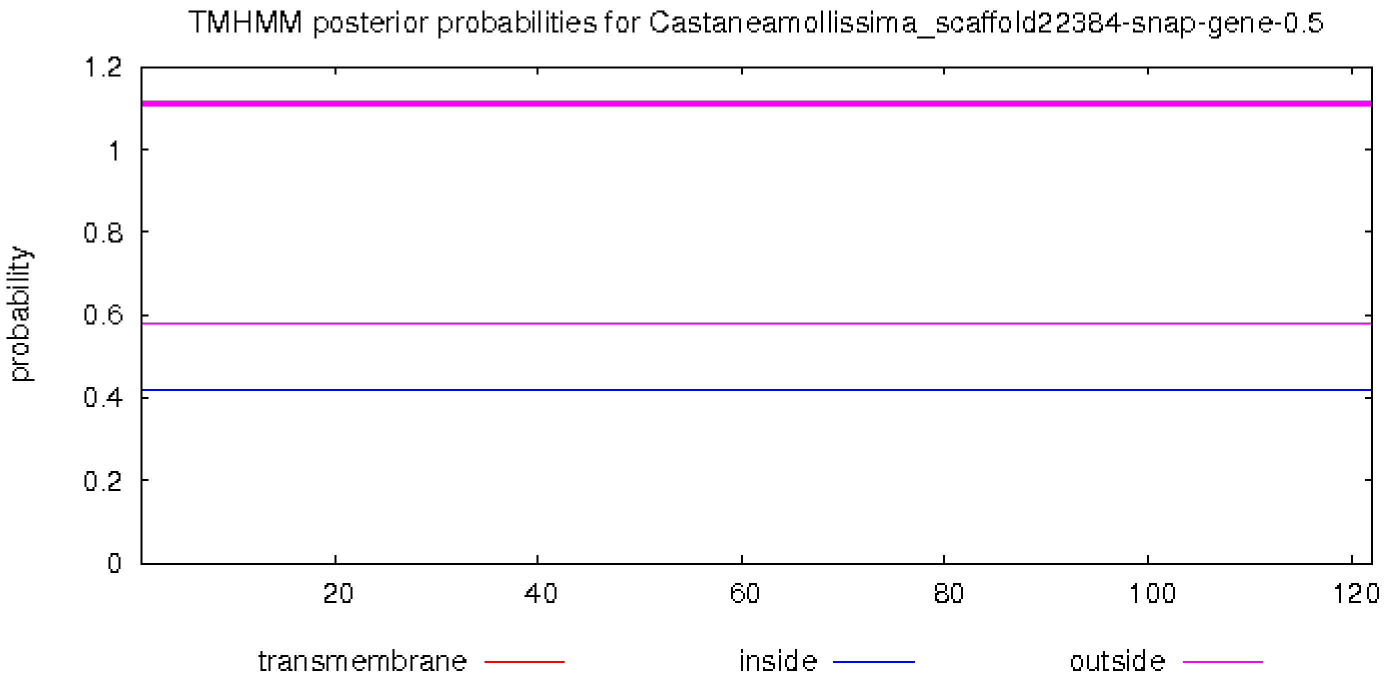

plot in postscript, script for making the plot in gnuplot, data for plot

---

```
# Castaneamollissima_scaffold23714-snap-gene-0.3 Length: 295
# Castaneamollissima_scaffold23714-snap-gene-0.3 Number of predicted TMHs:  0
# Castaneamollissima_scaffold23714-snap-gene-0.3 Exp number of AAs in TMHs: 0.0043
# Castaneamollissima_scaffold23714-snap-gene-0.3 Exp number, first 60 AAs:  0.0043
```

```
# Castaneamollissima_scaffold23714-snap-gene-0.3 Total prob of N-in:        0.04493
Castaneamollissima_scaffold23714-snap-gene-0.3  TMHMM2.0        outside      1   295
```

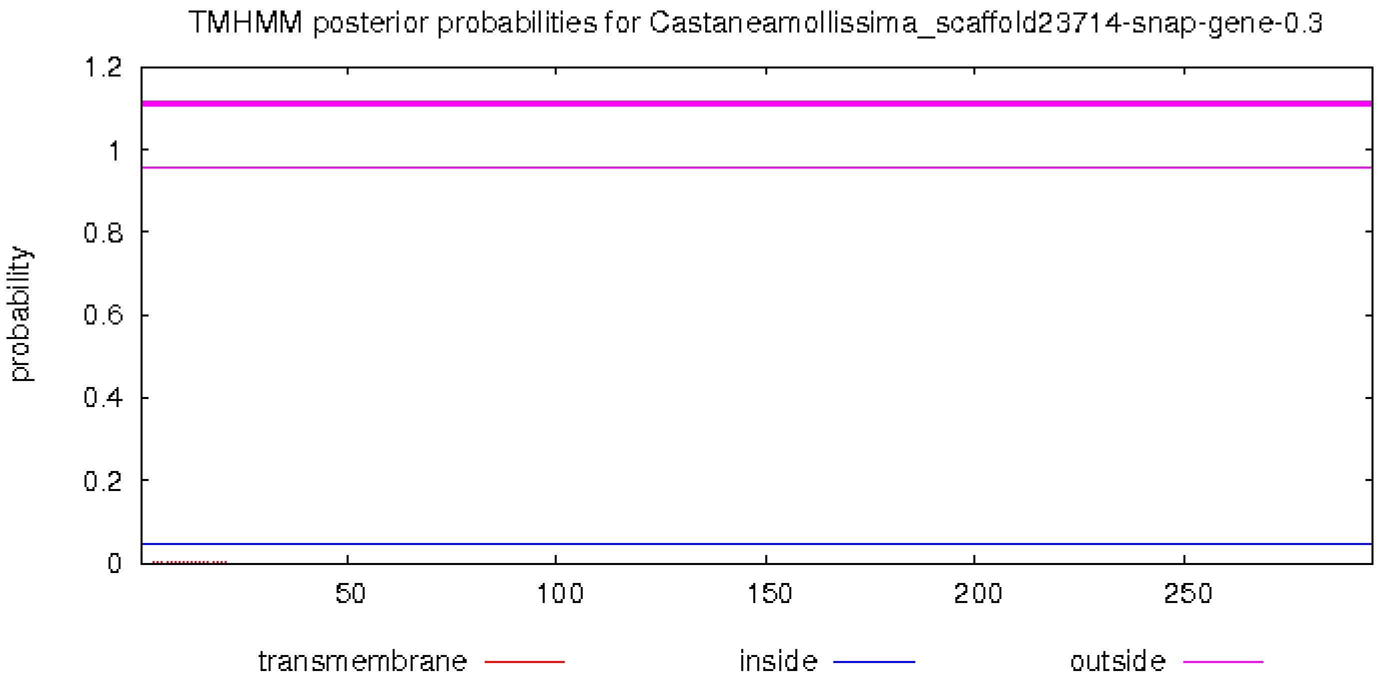

plot in postscript, script for making the plot in gnuplot, data for plot

---

```
# Castaneamollissima_scaffold08137-abinit-gene-0.4 Length: 496
# Castaneamollissima_scaffold08137-abinit-gene-0.4 Number of predicted TMHs:  0
# Castaneamollissima_scaffold08137-abinit-gene-0.4 Exp number of AAs in TMHs: 0.00018
# Castaneamollissima_scaffold08137-abinit-gene-0.4 Exp number, first 60 AAs:  0
# Castaneamollissima_scaffold08137-abinit-gene-0.4 Total prob of N-in:        0.00387
Castaneamollissima_scaffold08137-abinit-gene-0.4        TMHMM2.0        outside      1   496
```

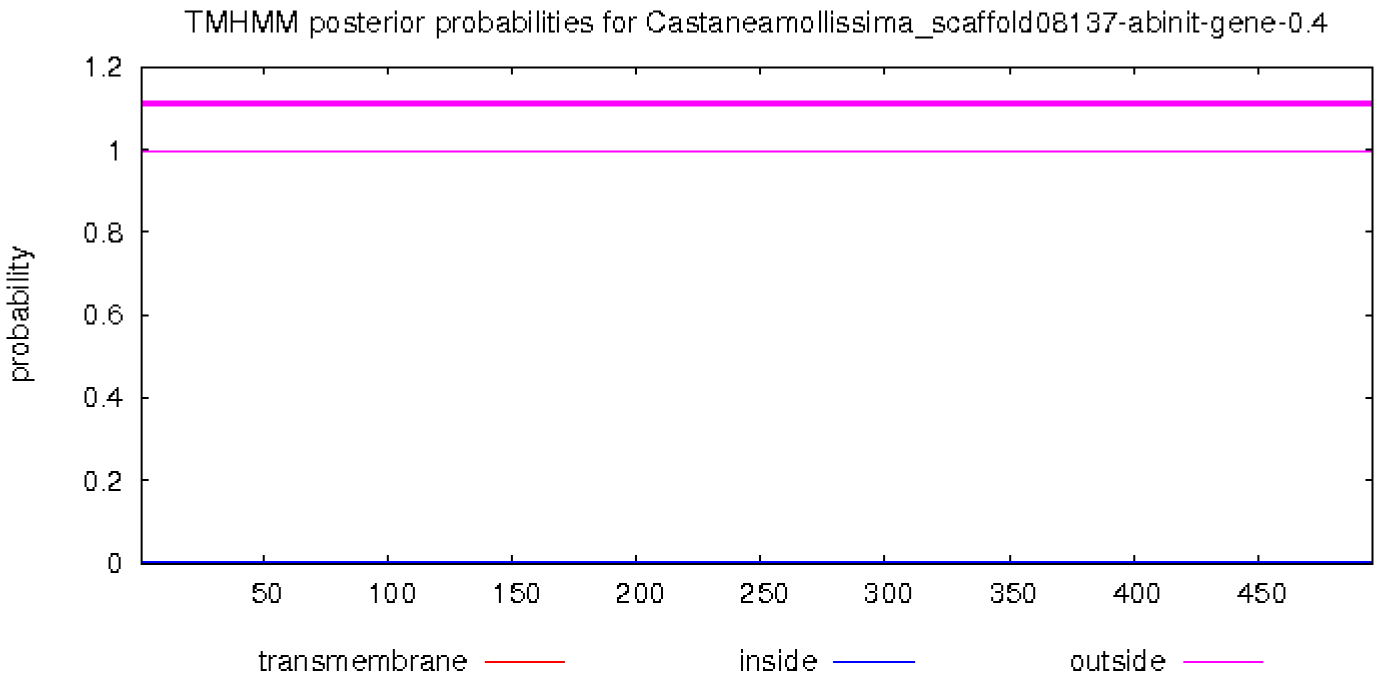

plot in postscript, script for making the plot in gnuplot, data for plot

---

```
# Castaneamollissima_scaffold08253-abinit-gene-0.10 Length: 129
# Castaneamollissima_scaffold08253-abinit-gene-0.10 Number of predicted TMHs:  0
# Castaneamollissima_scaffold08253-abinit-gene-0.10 Exp number of AAs in TMHs: 0.00016
# Castaneamollissima_scaffold08253-abinit-gene-0.10 Exp number, first 60 AAs:  0
```

```
# Castaneamollissima_scaffold08253-abinit-gene-0.10 Total prob of N-in:        0.36210
Castaneamollissima_scaffold08253-abinit-gene-0.10       TMHMM2.0        outside      1   129
```

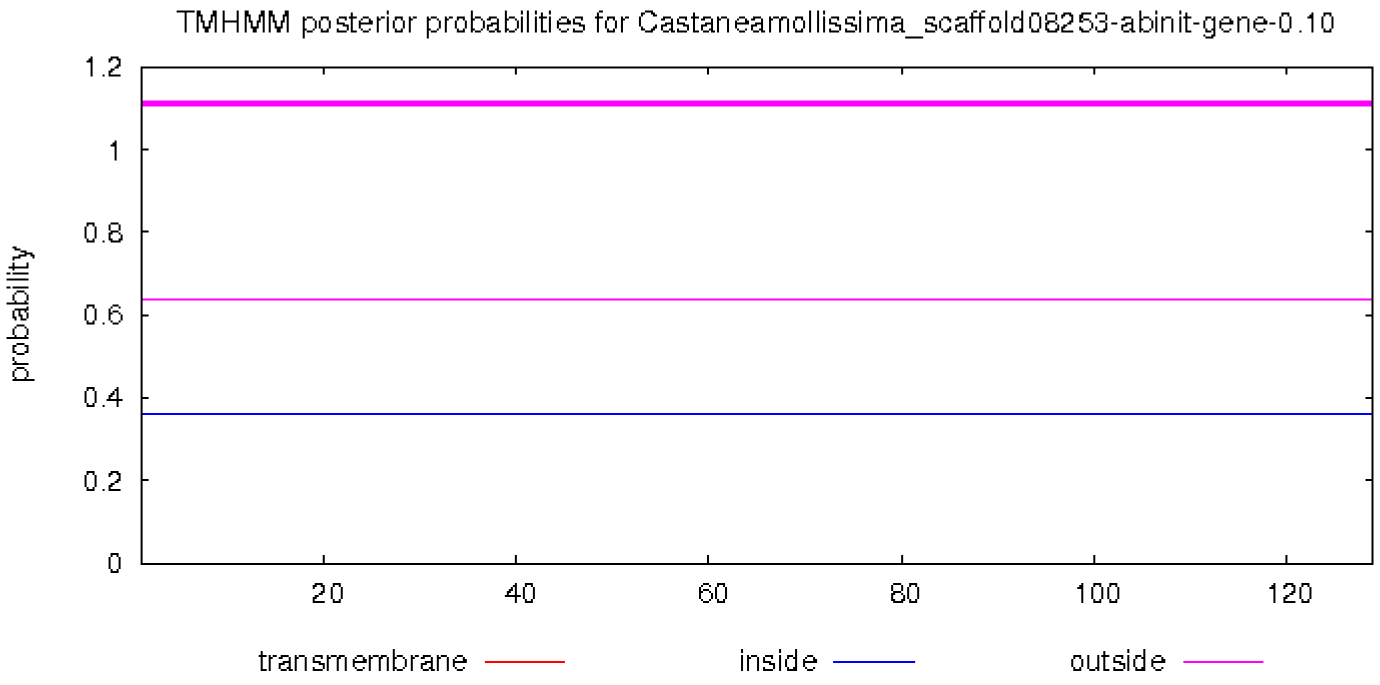

plot in postscript, script for making the plot in gnuplot, data for plot

---

```
# Castaneamollissima_scaffold08322-abinit-gene-0.9 Length: 76
# Castaneamollissima_scaffold08322-abinit-gene-0.9 Number of predicted TMHs:  0
# Castaneamollissima_scaffold08322-abinit-gene-0.9 Exp number of AAs in TMHs: 0.03161
# Castaneamollissima_scaffold08322-abinit-gene-0.9 Exp number, first 60 AAs:  0.03161
# Castaneamollissima_scaffold08322-abinit-gene-0.9 Total prob of N-in:        0.31607
Castaneamollissima_scaffold08322-abinit-gene-0.9        TMHMM2.0        outside      1    76
```

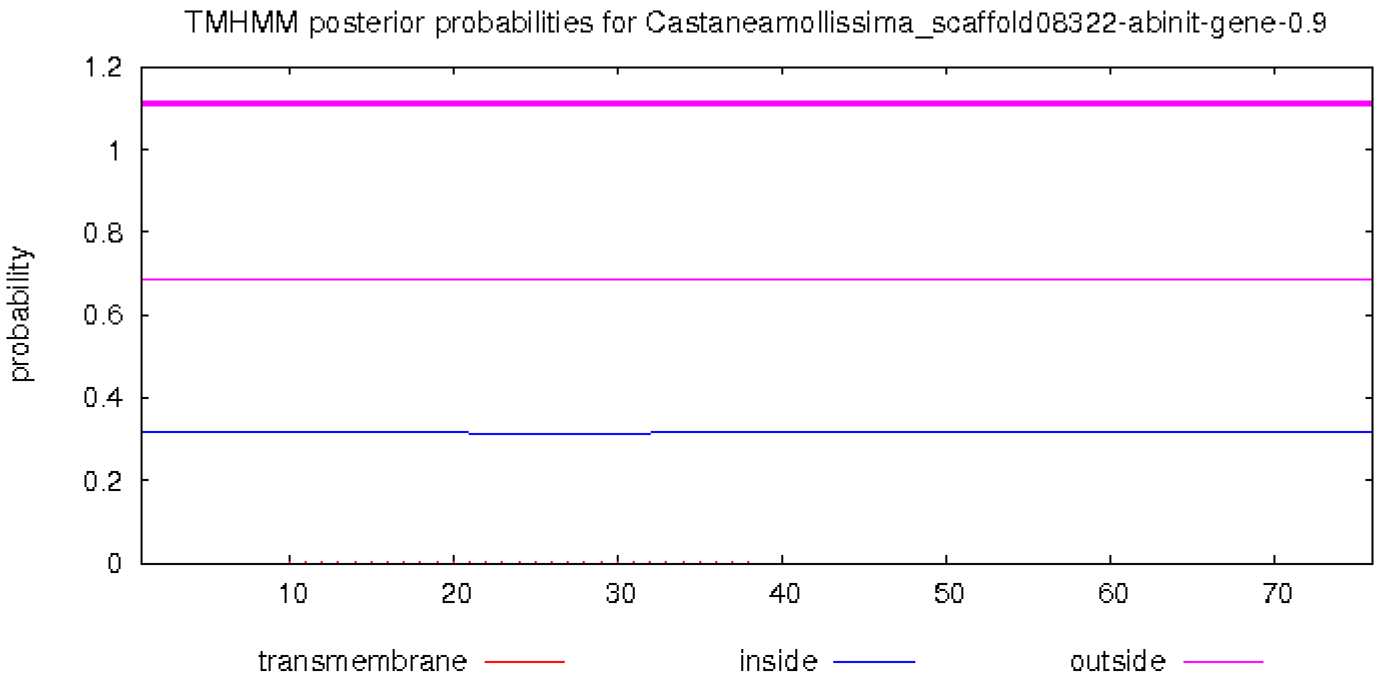

plot in postscript, script for making the plot in gnuplot, data for plot

---

```
# Castaneamollissima_scaffold12800-abinit-gene-0.4 Length: 115
# Castaneamollissima_scaffold12800-abinit-gene-0.4 Number of predicted TMHs:  0
# Castaneamollissima_scaffold12800-abinit-gene-0.4 Exp number of AAs in TMHs: 4e-05
# Castaneamollissima_scaffold12800-abinit-gene-0.4 Exp number, first 60 AAs:  0
```

```
# Castaneamollissima_scaffold12800-abinit-gene-0.4 Total prob of N-in:        0.06178
Castaneamollissima_scaffold12800-abinit-gene-0.4        TMHMM2.0        outside      1   115
```

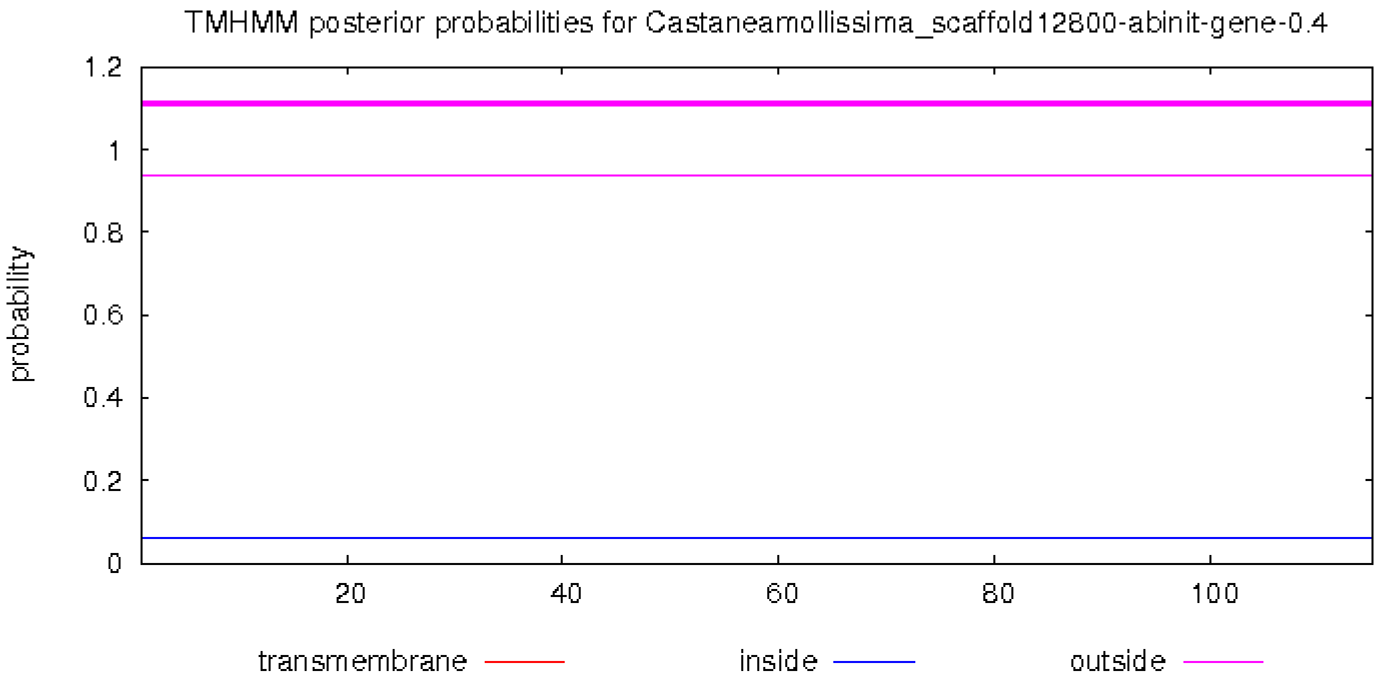

plot in postscript, script for making the plot in gnuplot, data for plot

---

```
# Castaneamollissima_scaffold13705-abinit-gene-0.5 Length: 97
# Castaneamollissima_scaffold13705-abinit-gene-0.5 Number of predicted TMHs:  0
# Castaneamollissima_scaffold13705-abinit-gene-0.5 Exp number of AAs in TMHs: 0.21701
# Castaneamollissima_scaffold13705-abinit-gene-0.5 Exp number, first 60 AAs:  0.03744
# Castaneamollissima_scaffold13705-abinit-gene-0.5 Total prob of N-in:        0.05870
Castaneamollissima_scaffold13705-abinit-gene-0.5        TMHMM2.0        outside      1    97
```

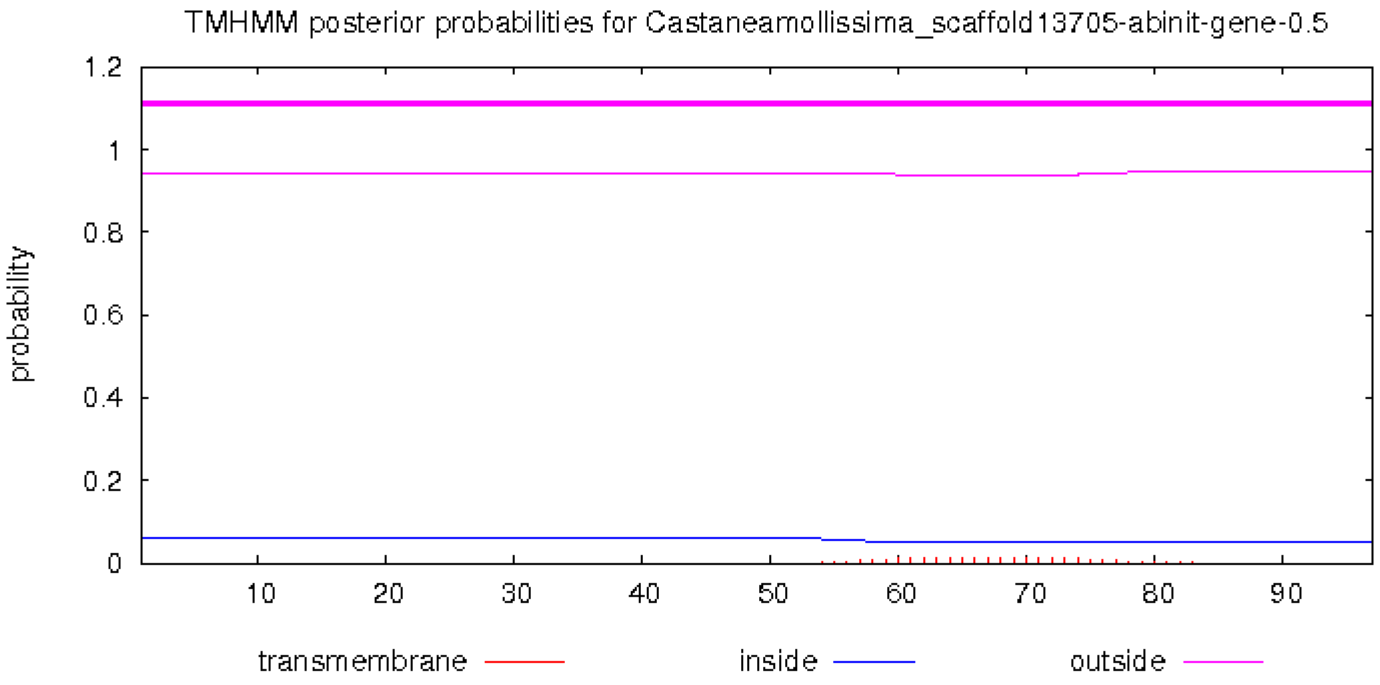

plot in postscript, script for making the plot in gnuplot, data for plot

---

```
# Catharanthusroseus_cra_locus_10450_iso_3 Length: 142
# Catharanthusroseus_cra_locus_10450_iso_3 Number of predicted TMHs:  0
# Catharanthusroseus_cra_locus_10450_iso_3 Exp number of AAs in TMHs: 0.00054
# Catharanthusroseus_cra_locus_10450_iso_3 Exp number, first 60 AAs:  1e-05
```

```
# Catharanthusroseus_cra_locus_10450_iso_3 Total prob of N-in:        0.28952
Catharanthusroseus_cra_locus_10450_iso_3        TMHMM2.0        outside      1   142
```

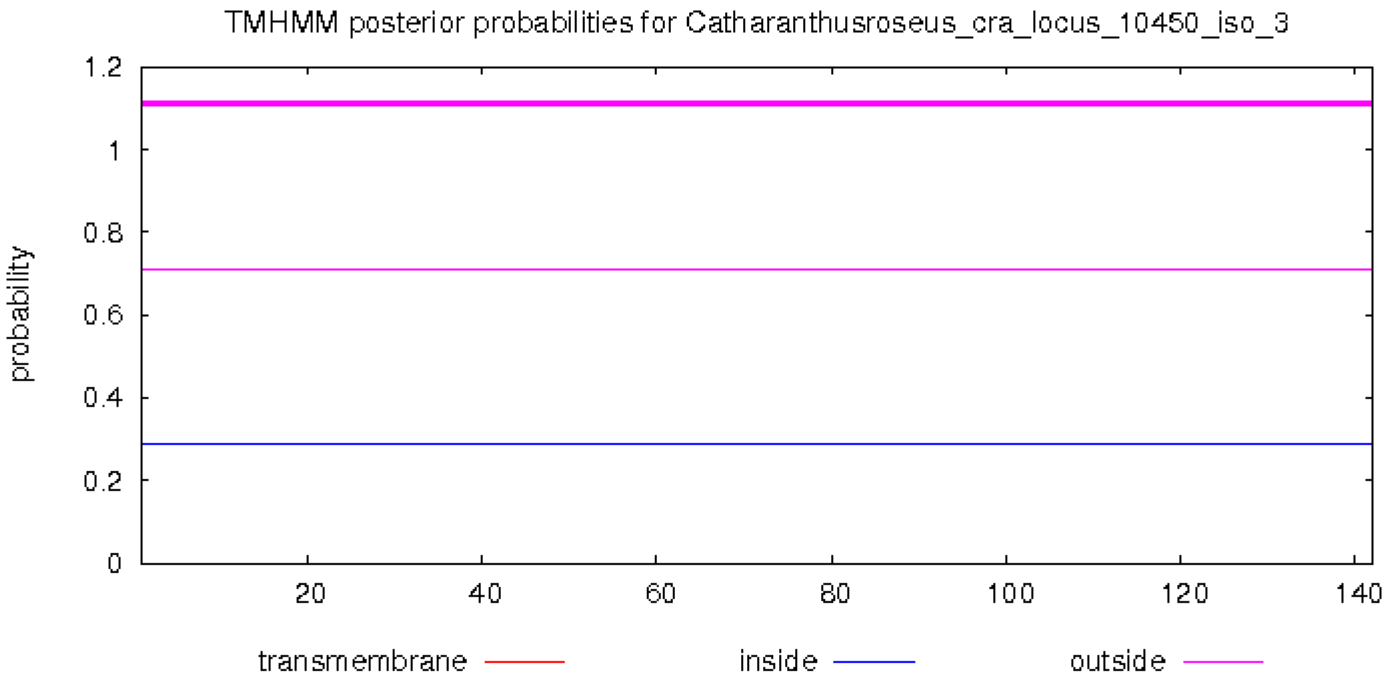

plot in postscript, script for making the plot in gnuplot, data for plot

---

```
# Catharanthusroseus_cra_locus_10450_iso_4 Length: 185
# Catharanthusroseus_cra_locus_10450_iso_4 Number of predicted TMHs:  0
# Catharanthusroseus_cra_locus_10450_iso_4 Exp number of AAs in TMHs: 0.00102
# Catharanthusroseus_cra_locus_10450_iso_4 Exp number, first 60 AAs:  0.00025
# Catharanthusroseus_cra_locus_10450_iso_4 Total prob of N-in:        0.14268
Catharanthusroseus_cra_locus_10450_iso_4        TMHMM2.0        outside      1   185
```

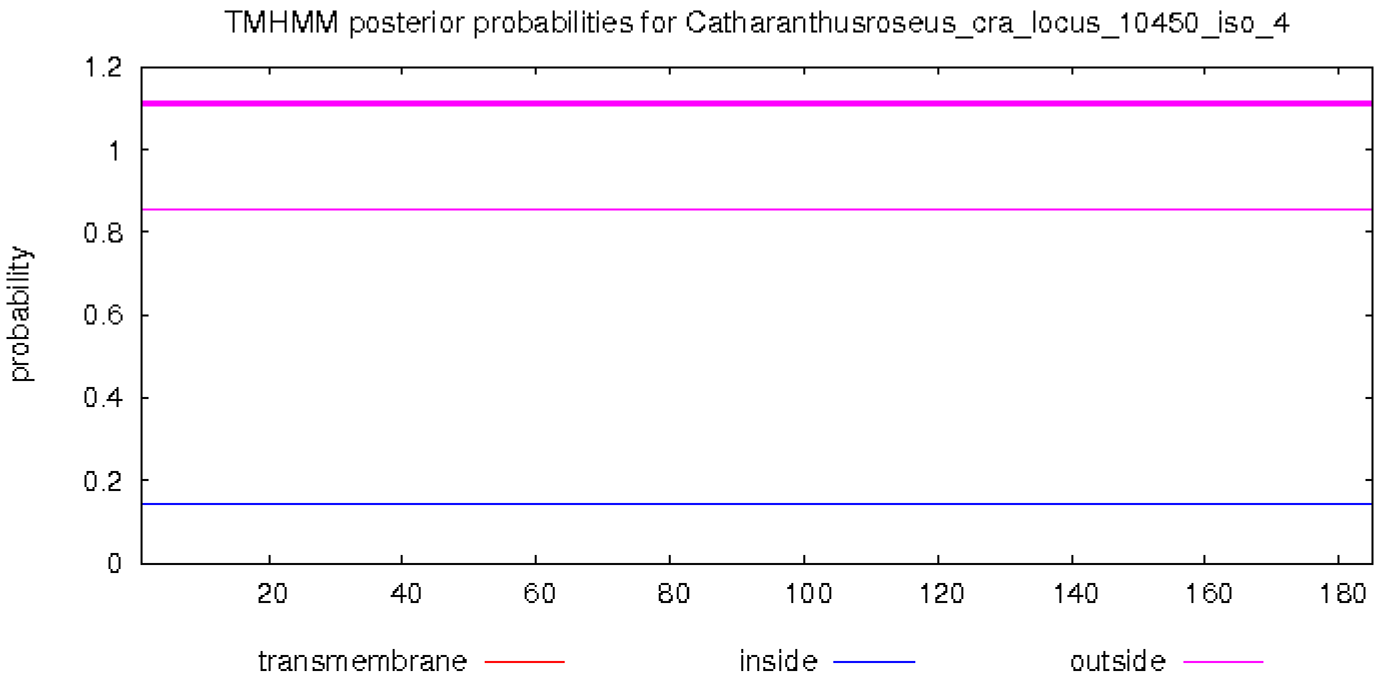

plot in postscript, script for making the plot in gnuplot, data for plot

---

```
# Catharanthusroseus_cra_locus_10904_iso_1 Length: 317
# Catharanthusroseus_cra_locus_10904_iso_1 Number of predicted TMHs:  0
# Catharanthusroseus_cra_locus_10904_iso_1 Exp number of AAs in TMHs: 0.0002
# Catharanthusroseus_cra_locus_10904_iso_1 Exp number, first 60 AAs:  0
```

```
# Catharanthusroseus_cra_locus_10904_iso_1 Total prob of N-in:        0.05837
Catharanthusroseus_cra_locus_10904_iso_1        TMHMM2.0        outside      1   317
```

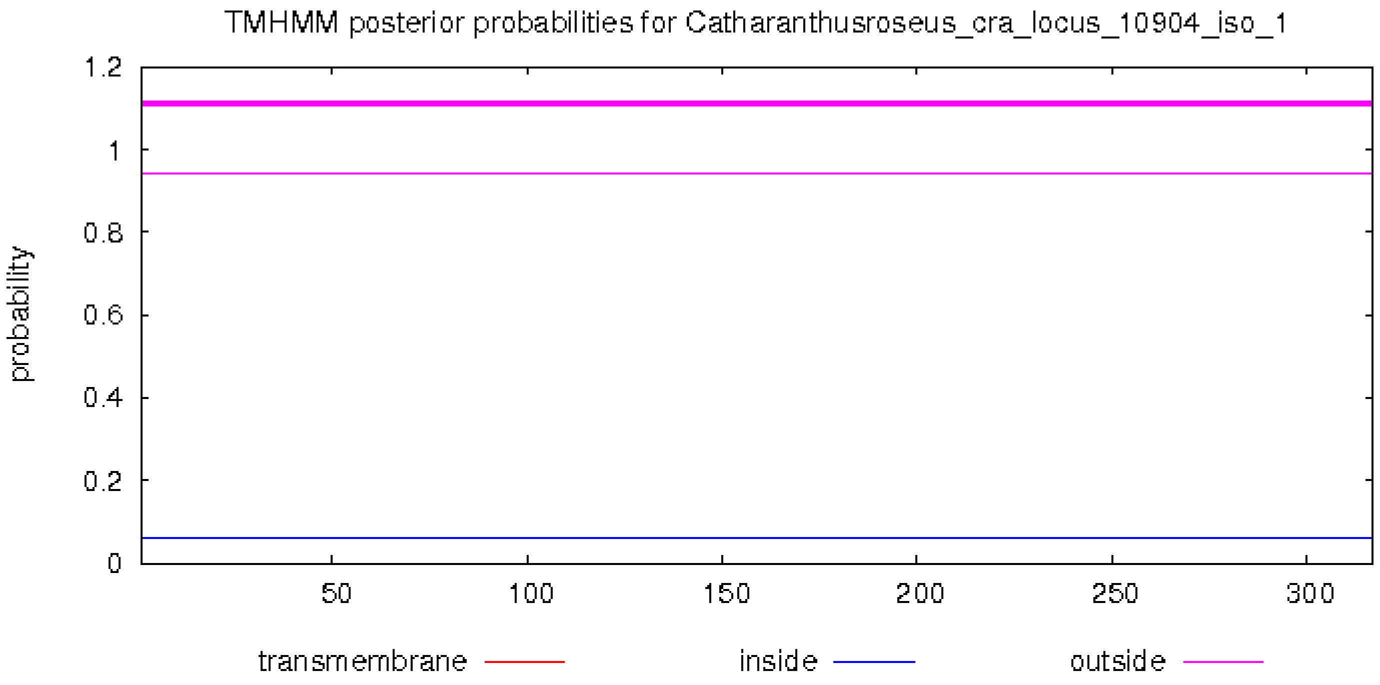

plot in postscript, script for making the plot in gnuplot, data for plot

---

```
# Catharanthusroseus_cra_locus_10904_iso_2 Length: 280
# Catharanthusroseus_cra_locus_10904_iso_2 Number of predicted TMHs:  0
# Catharanthusroseus_cra_locus_10904_iso_2 Exp number of AAs in TMHs: 0.0002
# Catharanthusroseus_cra_locus_10904_iso_2 Exp number, first 60 AAs:  0
# Catharanthusroseus_cra_locus_10904_iso_2 Total prob of N-in:        0.08954
Catharanthusroseus_cra_locus_10904_iso_2        TMHMM2.0        outside      1   280
```

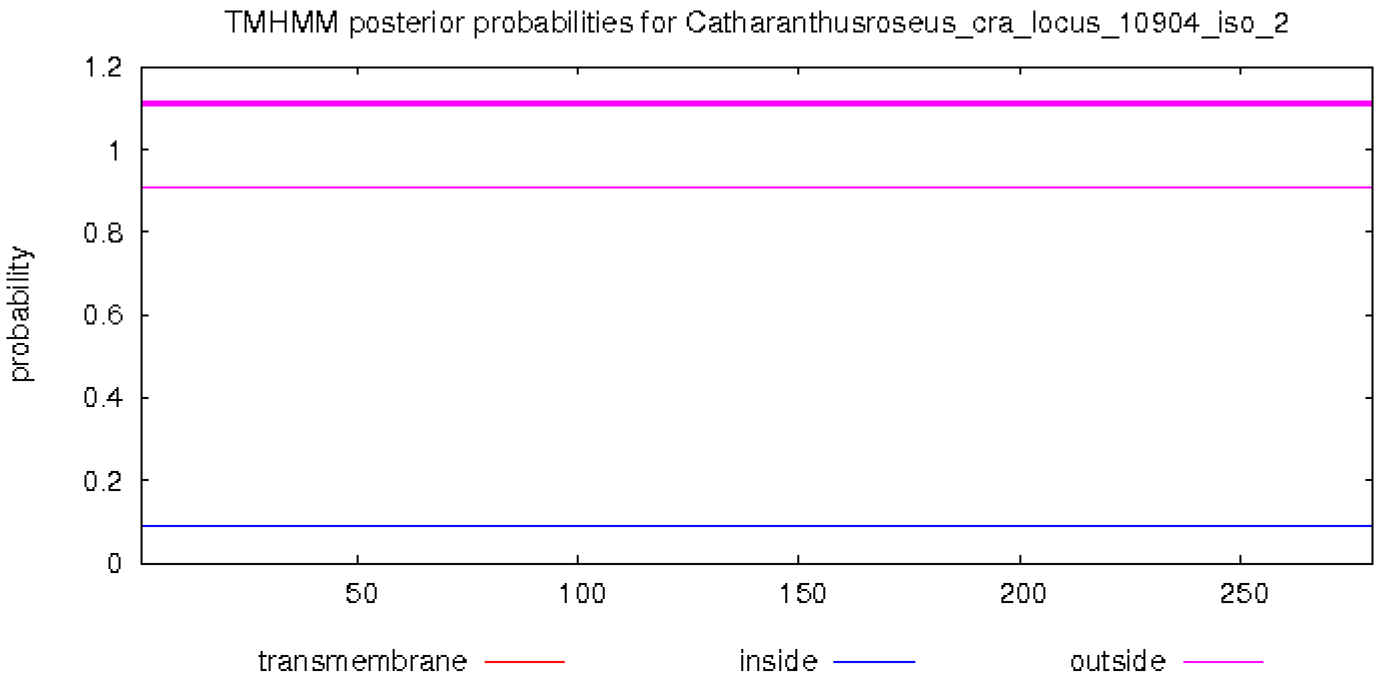

plot in postscript, script for making the plot in gnuplot, data for plot

---

```
# Catharanthusroseus_cra_locus_10952_iso_1 Length: 369
# Catharanthusroseus_cra_locus_10952_iso_1 Number of predicted TMHs:  0
# Catharanthusroseus_cra_locus_10952_iso_1 Exp number of AAs in TMHs: 0.00053
# Catharanthusroseus_cra_locus_10952_iso_1 Exp number, first 60 AAs:  0.00015
```

```
# Catharanthusroseus_cra_locus_10952_iso_1 Total prob of N-in:        0.00925
Catharanthusroseus_cra_locus_10952_iso_1        TMHMM2.0        outside      1   369
```

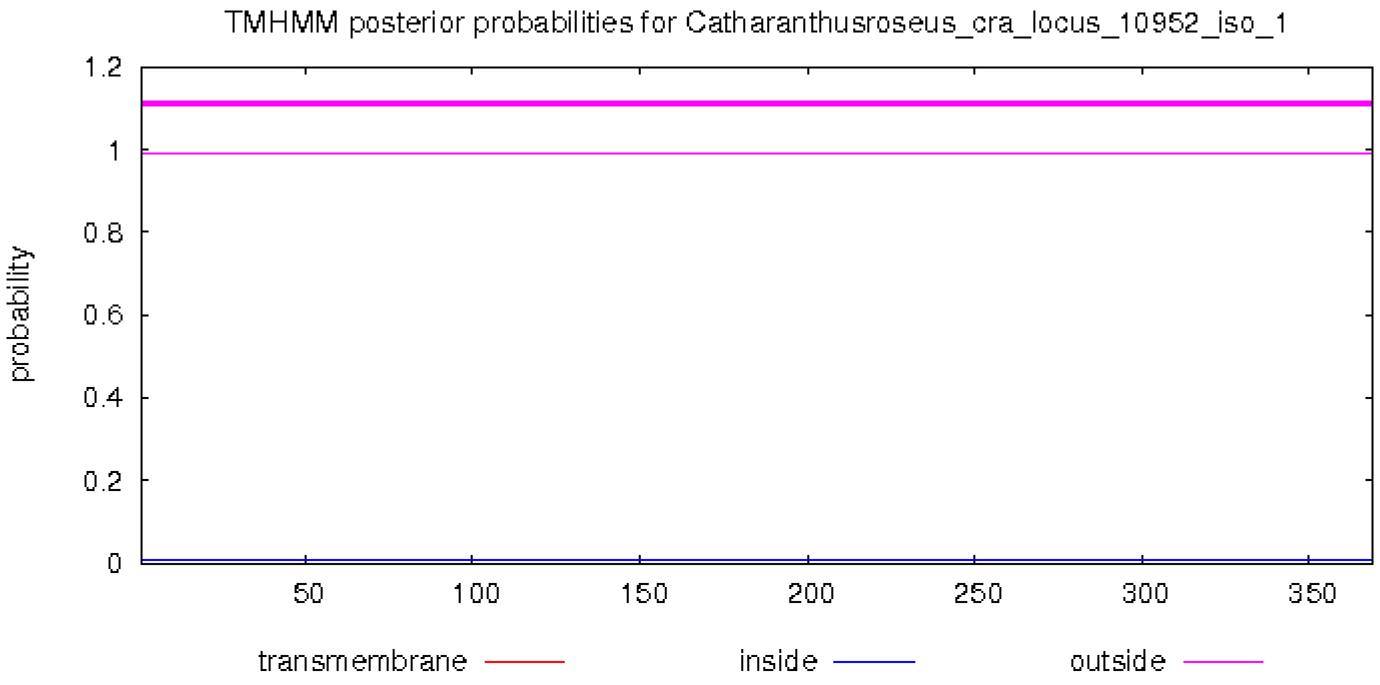

plot in postscript, script for making the plot in gnuplot, data for plot

---

```
# Catharanthusroseus_cra_locus_10952_iso_2 Length: 359
# Catharanthusroseus_cra_locus_10952_iso_2 Number of predicted TMHs:  0
# Catharanthusroseus_cra_locus_10952_iso_2 Exp number of AAs in TMHs: 0.00055
# Catharanthusroseus_cra_locus_10952_iso_2 Exp number, first 60 AAs:  0.00017
# Catharanthusroseus_cra_locus_10952_iso_2 Total prob of N-in:        0.01047
Catharanthusroseus_cra_locus_10952_iso_2        TMHMM2.0        outside      1   359
```

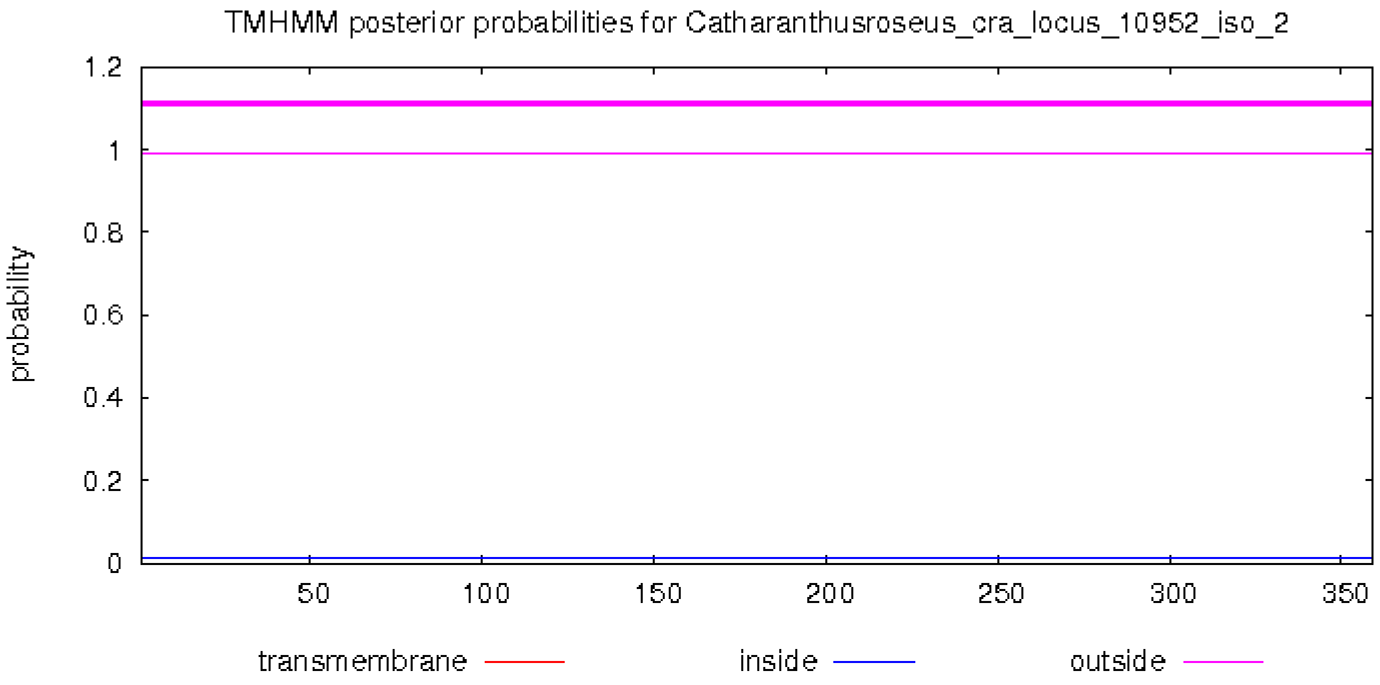

plot in postscript, script for making the plot in gnuplot, data for plot

---

```
# Catharanthusroseus_cra_locus_112403_iso_1 Length: 135
# Catharanthusroseus_cra_locus_112403_iso_1 Number of predicted TMHs:  0
# Catharanthusroseus_cra_locus_112403_iso_1 Exp number of AAs in TMHs: 0.02475
# Catharanthusroseus_cra_locus_112403_iso_1 Exp number, first 60 AAs:  0
```

```
# Catharanthusroseus_cra_locus_112403_iso_1 Total prob of N-in:        0.09057
Catharanthusroseus_cra_locus_112403_iso_1       TMHMM2.0        outside      1   135
```

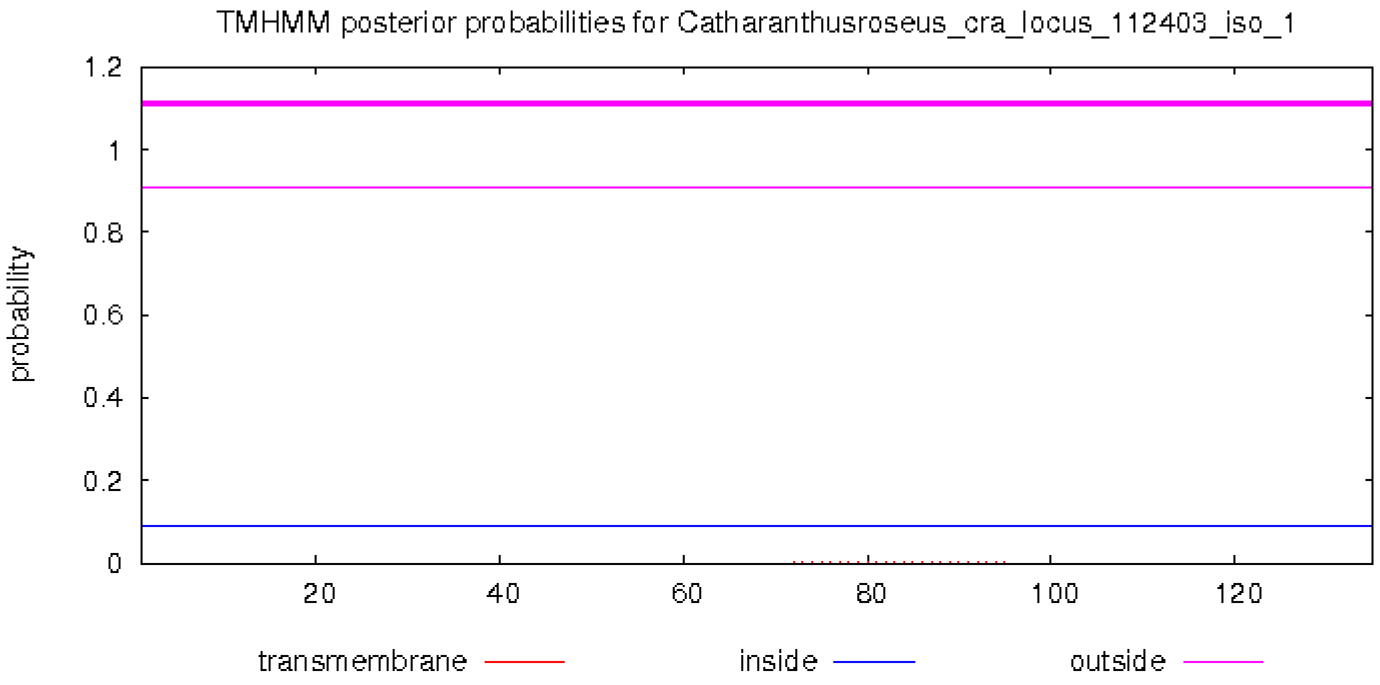

plot in postscript, script for making the plot in gnuplot, data for plot

---

```
# Catharanthusroseus_cra_locus_112403_iso_2 Length: 182
# Catharanthusroseus_cra_locus_112403_iso_2 Number of predicted TMHs:  0
# Catharanthusroseus_cra_locus_112403_iso_2 Exp number of AAs in TMHs: 0.01217
# Catharanthusroseus_cra_locus_112403_iso_2 Exp number, first 60 AAs:  0.0002
# Catharanthusroseus_cra_locus_112403_iso_2 Total prob of N-in:        0.14047
Catharanthusroseus_cra_locus_112403_iso_2       TMHMM2.0        outside      1   182
```

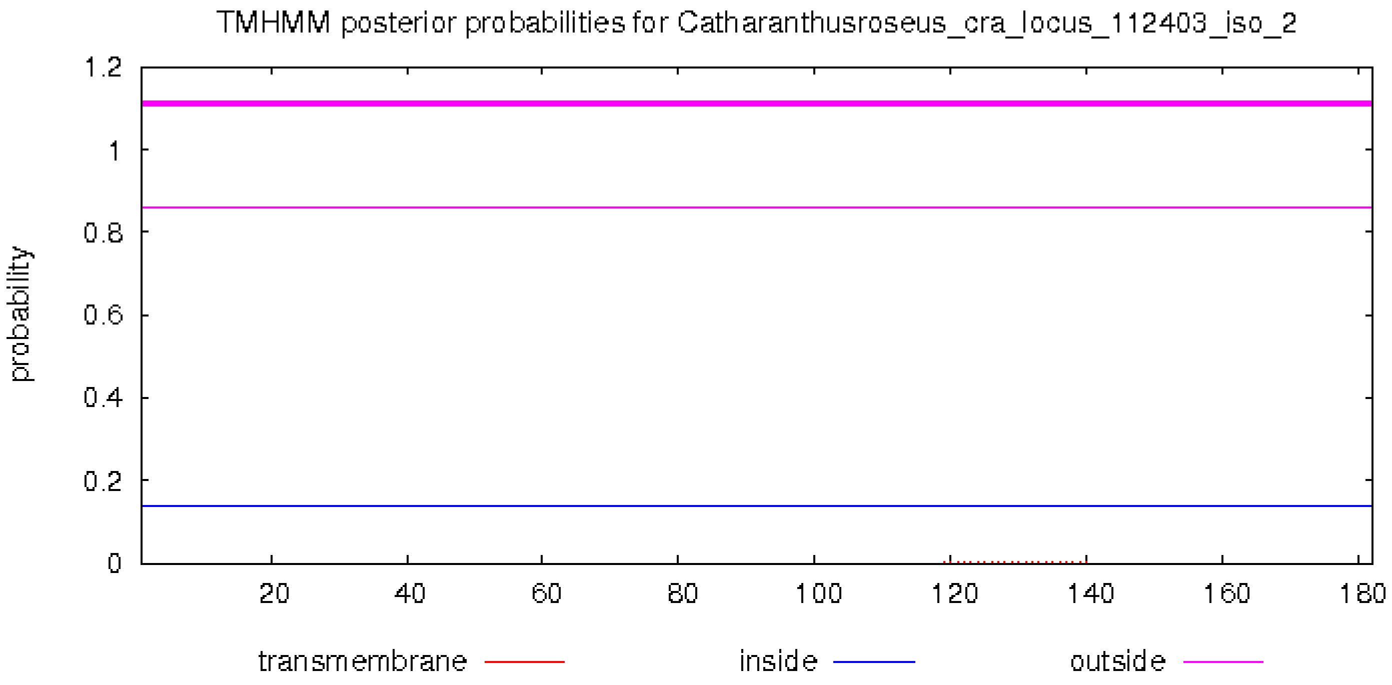

plot in postscript, script for making the plot in gnuplot, data for plot

---

```
# Catharanthusroseus_cra_locus_112403_iso_3 Length: 193
# Catharanthusroseus_cra_locus_112403_iso_3 Number of predicted TMHs:  0
# Catharanthusroseus_cra_locus_112403_iso_3 Exp number of AAs in TMHs: 1.71386
# Catharanthusroseus_cra_locus_112403_iso_3 Exp number, first 60 AAs:  0.0002
```

```
# Catharanthusroseus_cra_locus_112403_iso_3 Total prob of N-in:        0.16600
Catharanthusroseus_cra_locus_112403_iso_3       TMHMM2.0        outside      1   193
```

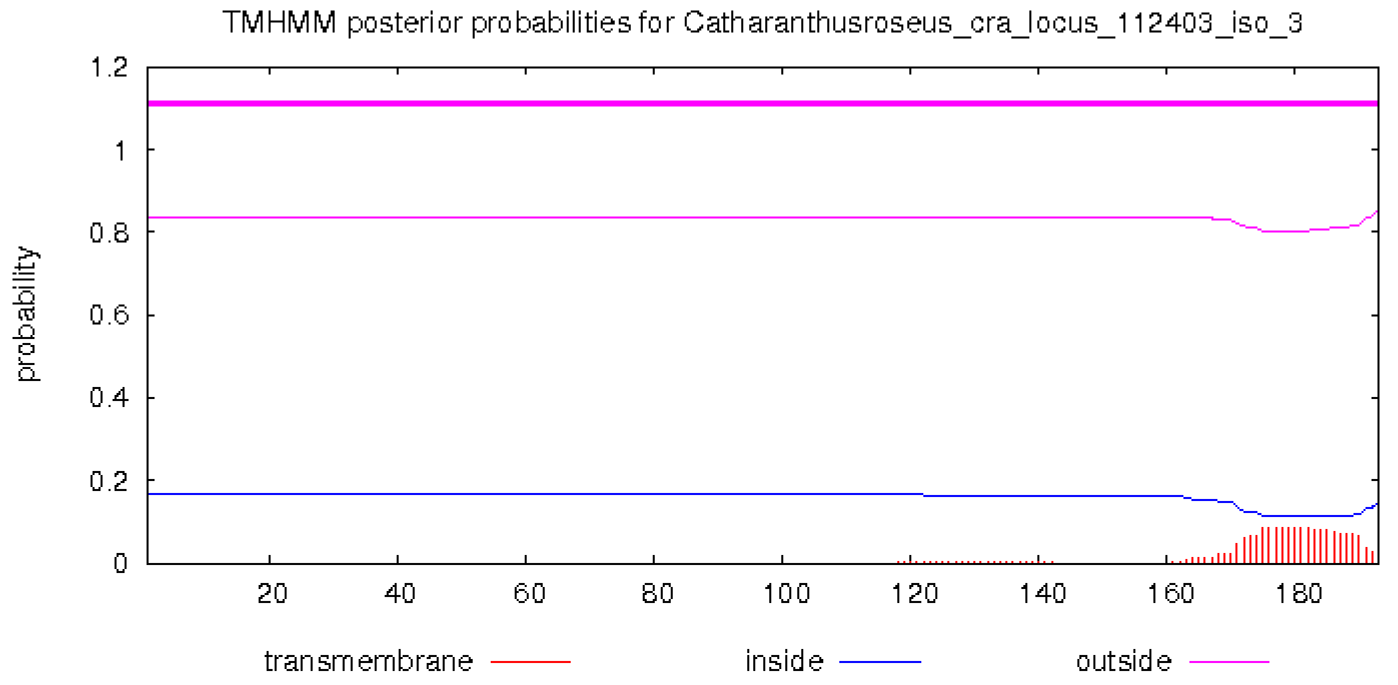

plot in postscript, script for making the plot in gnuplot, data for plot

---

```
# Catharanthusroseus_cra_locus_112403_iso_4 Length: 182
# Catharanthusroseus_cra_locus_112403_iso_4 Number of predicted TMHs:  0
# Catharanthusroseus_cra_locus_112403_iso_4 Exp number of AAs in TMHs: 0.0123
# Catharanthusroseus_cra_locus_112403_iso_4 Exp number, first 60 AAs:  0.0002
# Catharanthusroseus_cra_locus_112403_iso_4 Total prob of N-in:        0.14375
Catharanthusroseus_cra_locus_112403_iso_4       TMHMM2.0        outside      1   182
```

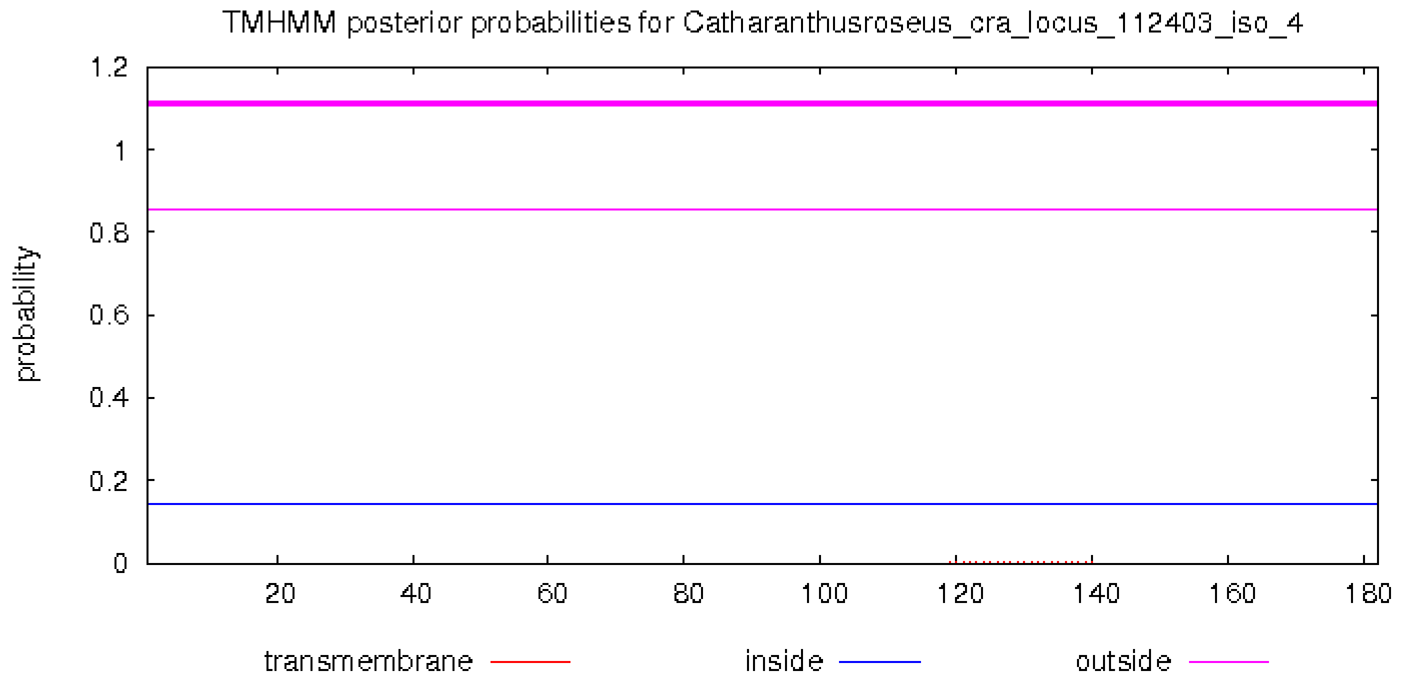

plot in postscript, script for making the plot in gnuplot, data for plot

---

```
# Catharanthusroseus_cra_locus_11431_iso_1 Length: 199
# Catharanthusroseus_cra_locus_11431_iso_1 Number of predicted TMHs:  0
# Catharanthusroseus_cra_locus_11431_iso_1 Exp number of AAs in TMHs: 0.01111
# Catharanthusroseus_cra_locus_11431_iso_1 Exp number, first 60 AAs:  0.00962
```

```
# Catharanthusroseus_cra_locus_11431_iso_1 Total prob of N-in:        0.04932
Catharanthusroseus_cra_locus_11431_iso_1        TMHMM2.0        outside      1   199
```

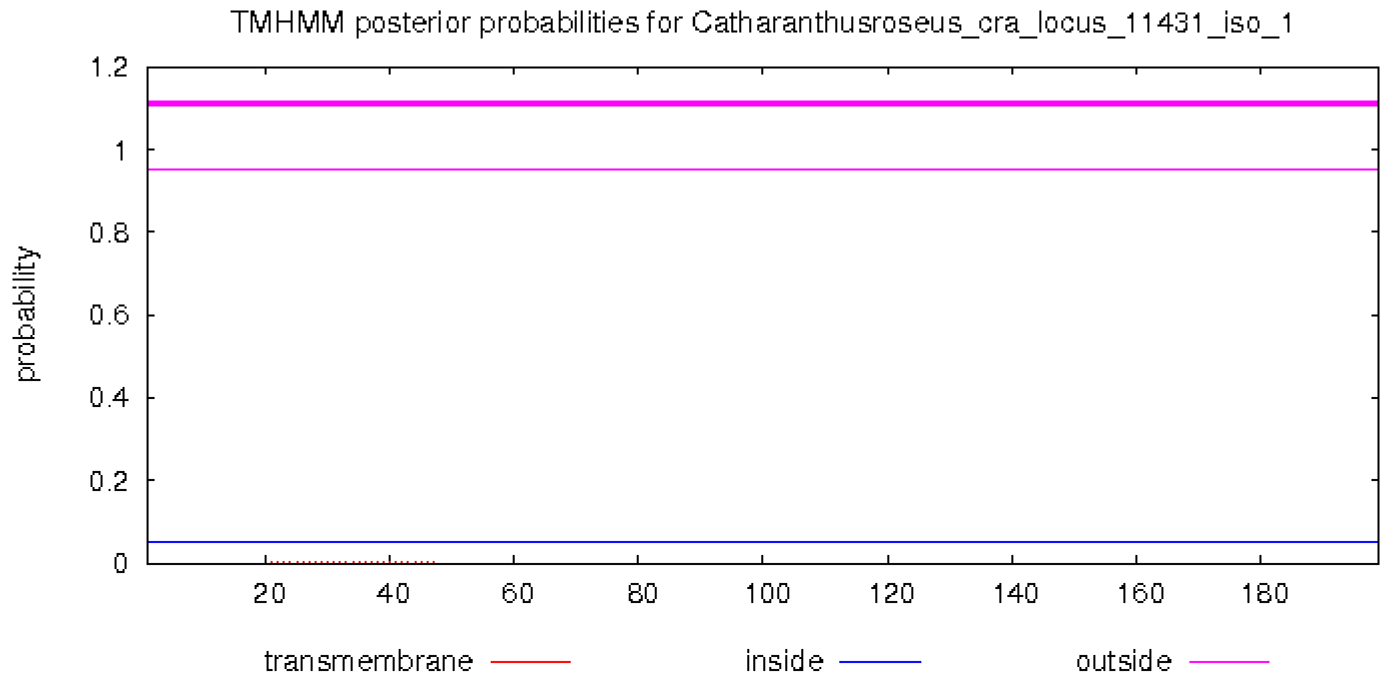

plot in postscript, script for making the plot in gnuplot, data for plot

---

```
# Catharanthusroseus_cra_locus_12026_iso_1 Length: 288
# Catharanthusroseus_cra_locus_12026_iso_1 Number of predicted TMHs:  0
# Catharanthusroseus_cra_locus_12026_iso_1 Exp number of AAs in TMHs: 0.00066
# Catharanthusroseus_cra_locus_12026_iso_1 Exp number, first 60 AAs:  0.00066
# Catharanthusroseus_cra_locus_12026_iso_1 Total prob of N-in:        0.00393
Catharanthusroseus_cra_locus_12026_iso_1        TMHMM2.0        outside      1   288
```

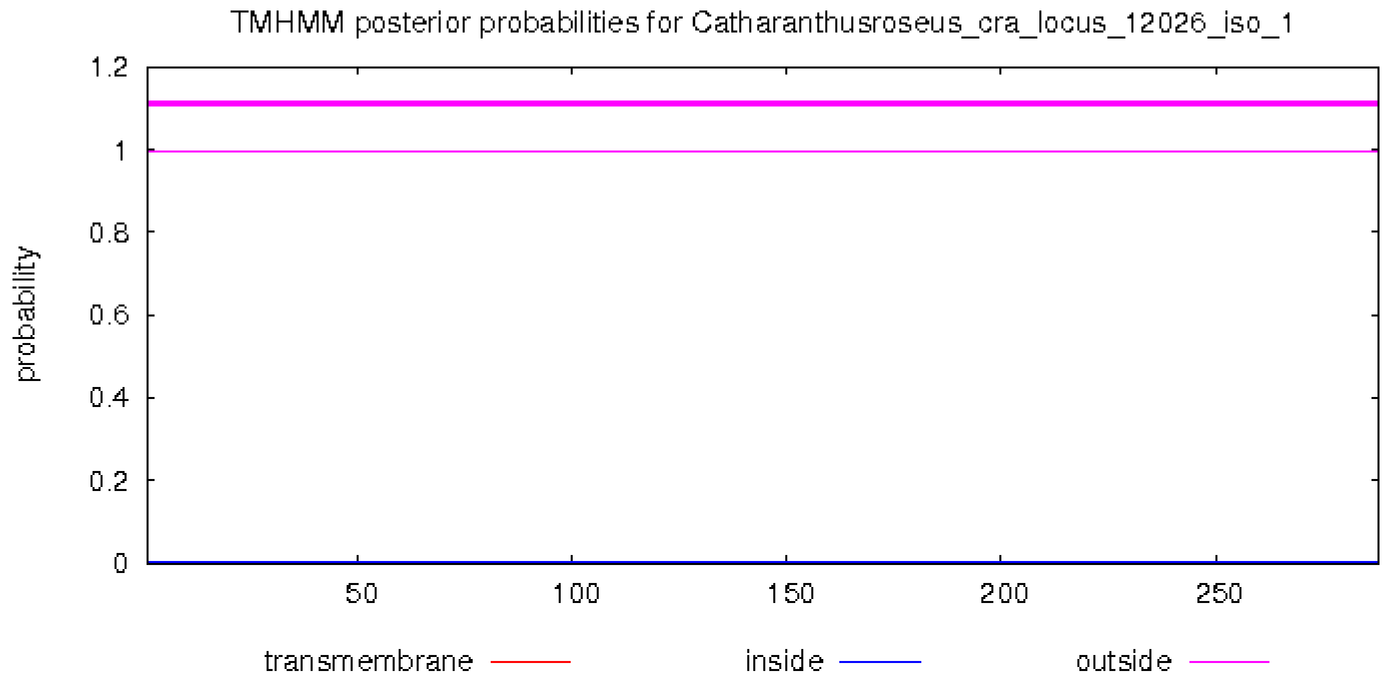

plot in postscript, script for making the plot in gnuplot, data for plot

---

```
# Catharanthusroseus_cra_locus_12026_iso_3 Length: 288
# Catharanthusroseus_cra_locus_12026_iso_3 Number of predicted TMHs:  0
# Catharanthusroseus_cra_locus_12026_iso_3 Exp number of AAs in TMHs: 0.00066
# Catharanthusroseus_cra_locus_12026_iso_3 Exp number, first 60 AAs:  0.00066
```

```
# Catharanthusroseus_cra_locus_12026_iso_3 Total prob of N-in:        0.00393
Catharanthusroseus_cra_locus_12026_iso_3        TMHMM2.0        outside      1   288
```

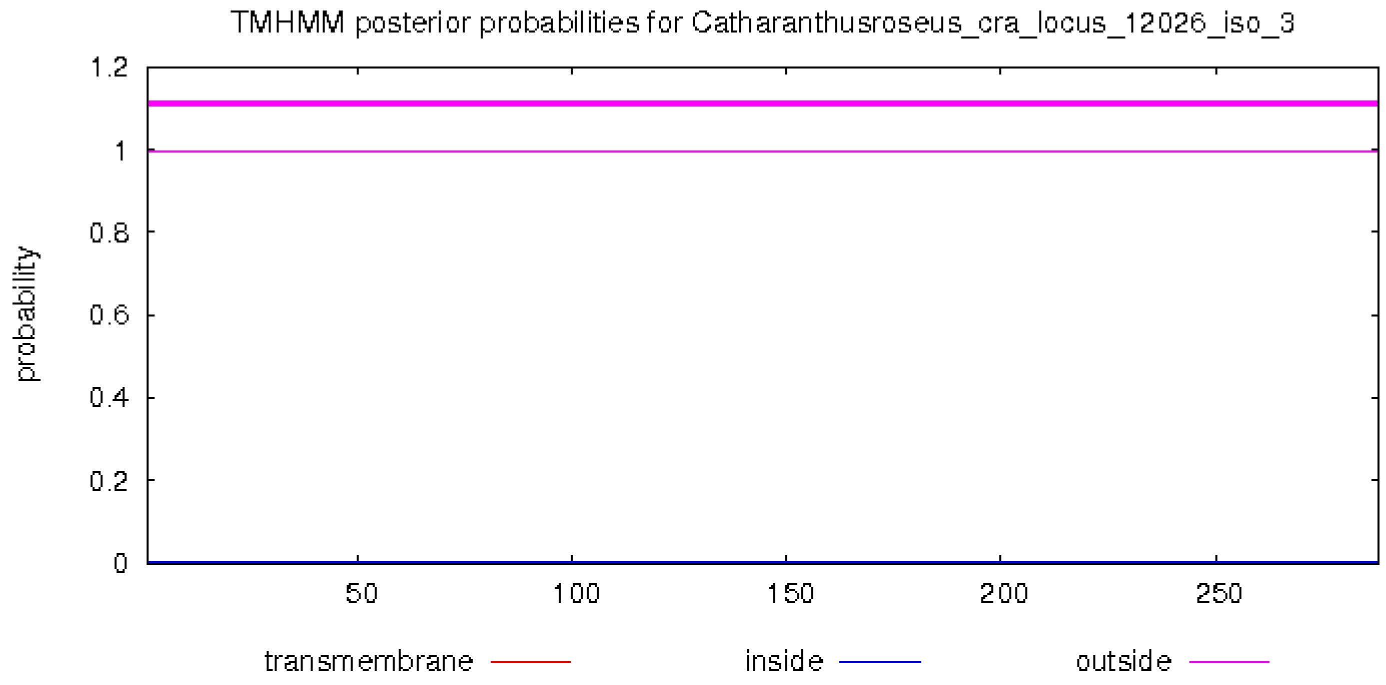

plot in postscript, script for making the plot in gnuplot, data for plot

---

```
# Catharanthusroseus_cra_locus_12026_iso_4 Length: 288
# Catharanthusroseus_cra_locus_12026_iso_4 Number of predicted TMHs:  0
# Catharanthusroseus_cra_locus_12026_iso_4 Exp number of AAs in TMHs: 0.00066
# Catharanthusroseus_cra_locus_12026_iso_4 Exp number, first 60 AAs:  0.00066
# Catharanthusroseus_cra_locus_12026_iso_4 Total prob of N-in:        0.00393
Catharanthusroseus_cra_locus_12026_iso_4        TMHMM2.0        outside      1   288
```

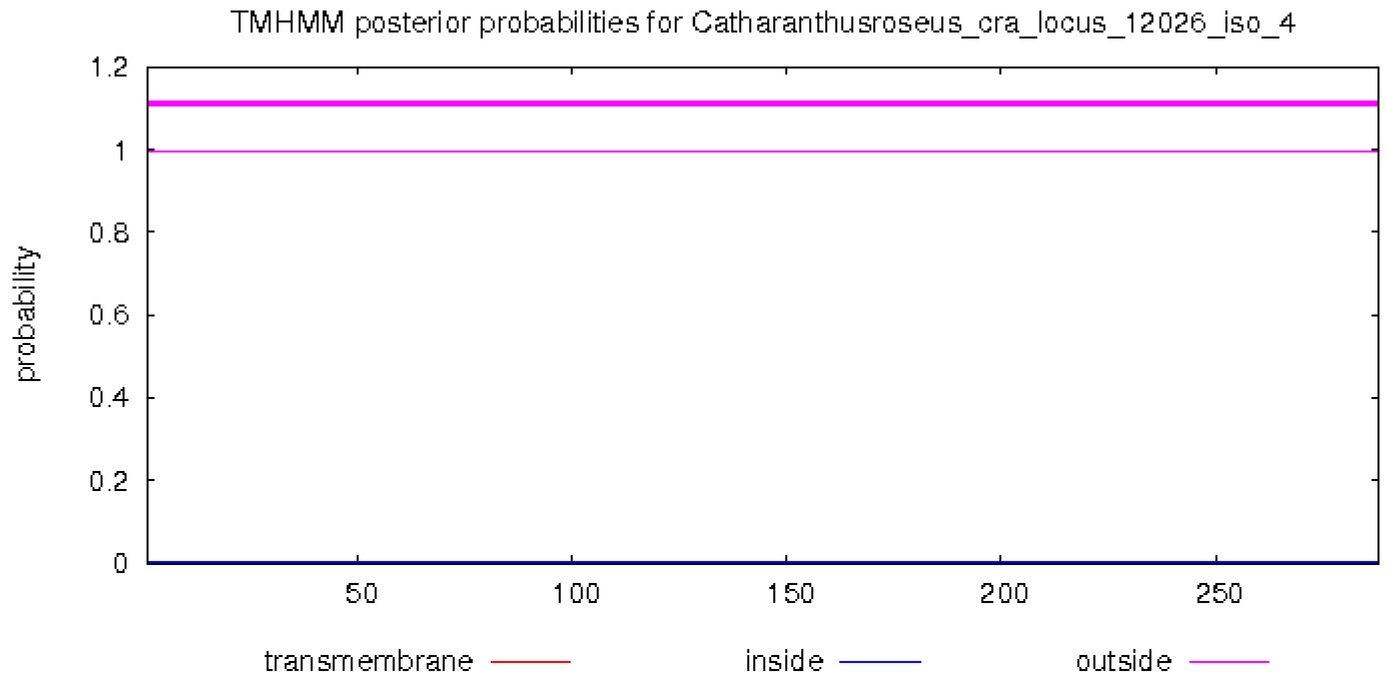

plot in postscript, script for making the plot in gnuplot, data for plot

---

```
# Catharanthusroseus_cra_locus_12026_iso_5 Length: 319
# Catharanthusroseus_cra_locus_12026_iso_5 Number of predicted TMHs:  0
# Catharanthusroseus_cra_locus_12026_iso_5 Exp number of AAs in TMHs: 0.27439
# Catharanthusroseus_cra_locus_12026_iso_5 Exp number, first 60 AAs:  0.10513
```

```
# Catharanthusroseus_cra_locus_12026_iso_5 Total prob of N-in:        0.00449
Catharanthusroseus_cra_locus_12026_iso_5        TMHMM2.0        outside      1   319
```

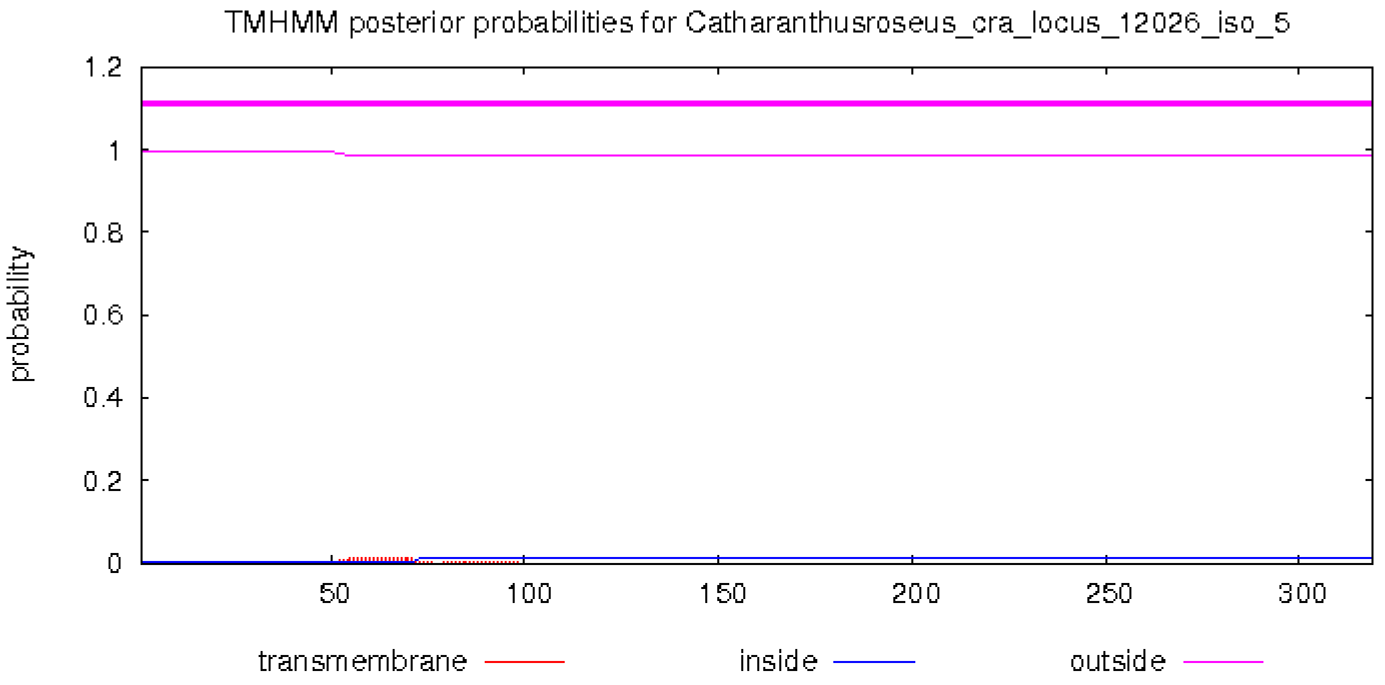

plot in postscript, script for making the plot in gnuplot, data for plot

---

```
# Catharanthusroseus_cra_locus_12026_iso_6 Length: 288
# Catharanthusroseus_cra_locus_12026_iso_6 Number of predicted TMHs:  0
# Catharanthusroseus_cra_locus_12026_iso_6 Exp number of AAs in TMHs: 0.00066
# Catharanthusroseus_cra_locus_12026_iso_6 Exp number, first 60 AAs:  0.00066
# Catharanthusroseus_cra_locus_12026_iso_6 Total prob of N-in:        0.00393
Catharanthusroseus_cra_locus_12026_iso_6        TMHMM2.0        outside      1   288
```

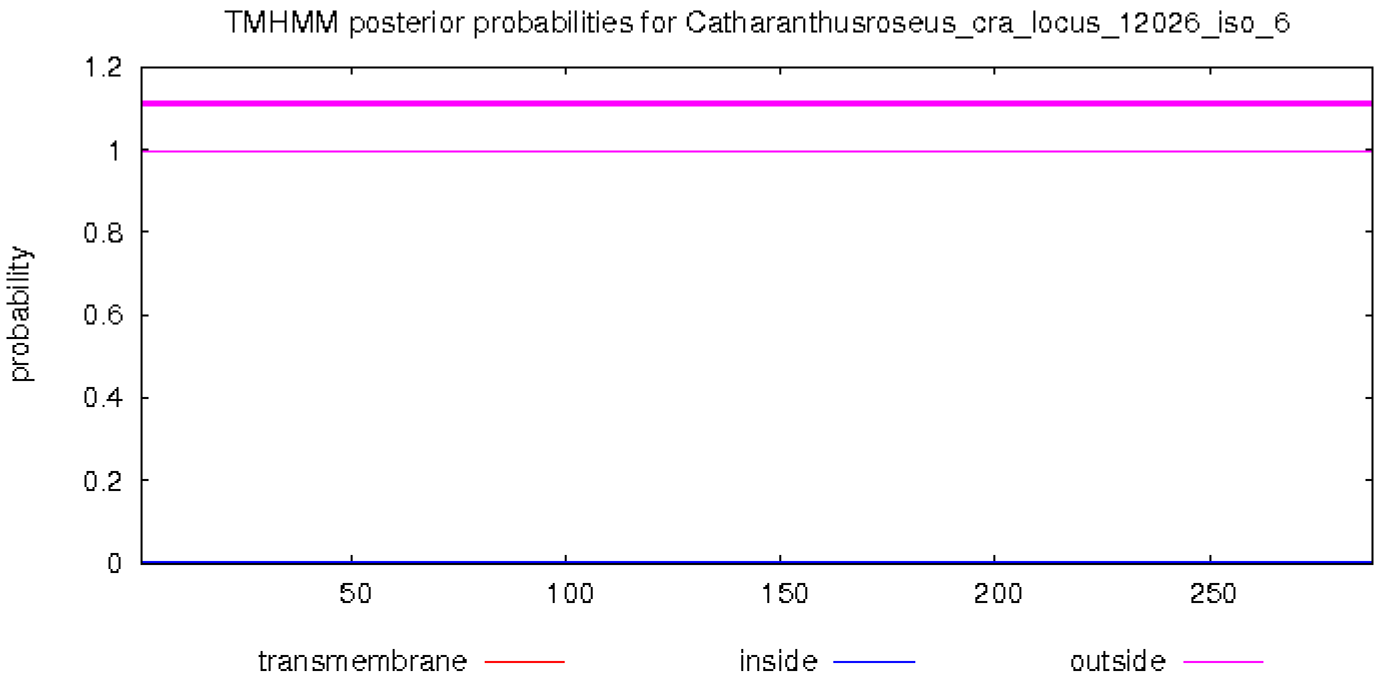

plot in postscript, script for making the plot in gnuplot, data for plot

---

```
# Catharanthusroseus_cra_locus_12235_iso_1 Length: 427
# Catharanthusroseus_cra_locus_12235_iso_1 Number of predicted TMHs:  1
# Catharanthusroseus_cra_locus_12235_iso_1 Exp number of AAs in TMHs: 21.61045
# Catharanthusroseus_cra_locus_12235_iso_1 Exp number, first 60 AAs:  0.00075
```

```
# Catharanthusroseus_cra_locus_12235_iso_1 Total prob of N-in:        0.19206
Catharanthusroseus_cra_locus_12235_iso_1        TMHMM2.0        outside      1   407
Catharanthusroseus_cra_locus_12235_iso_1        TMHMM2.0        TMhelix    408   425
Catharanthusroseus_cra_locus_12235_iso_1        TMHMM2.0        inside     426   427
```

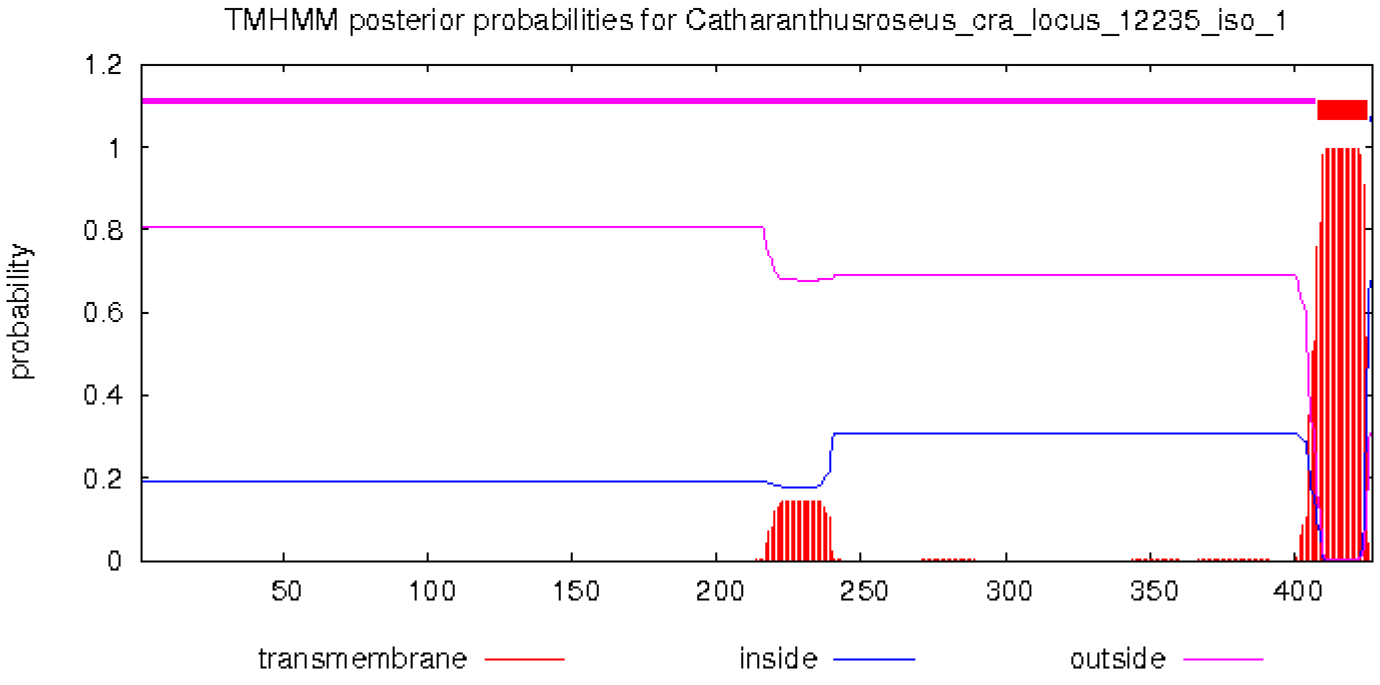

plot in postscript, script for making the plot in gnuplot, data for plot

---

```
# Catharanthusroseus_cra_locus_12235_iso_3 Length: 346
# Catharanthusroseus_cra_locus_12235_iso_3 Number of predicted TMHs:  1
# Catharanthusroseus_cra_locus_12235_iso_3 Exp number of AAs in TMHs: 18.62856
# Catharanthusroseus_cra_locus_12235_iso_3 Exp number, first 60 AAs:  0.00059
# Catharanthusroseus_cra_locus_12235_iso_3 Total prob of N-in:        0.42021
Catharanthusroseus_cra_locus_12235_iso_3        TMHMM2.0        outside      1   326
Catharanthusroseus_cra_locus_12235_iso_3        TMHMM2.0        TMhelix    327   344
Catharanthusroseus_cra_locus_12235_iso_3        TMHMM2.0        inside     345   346
```

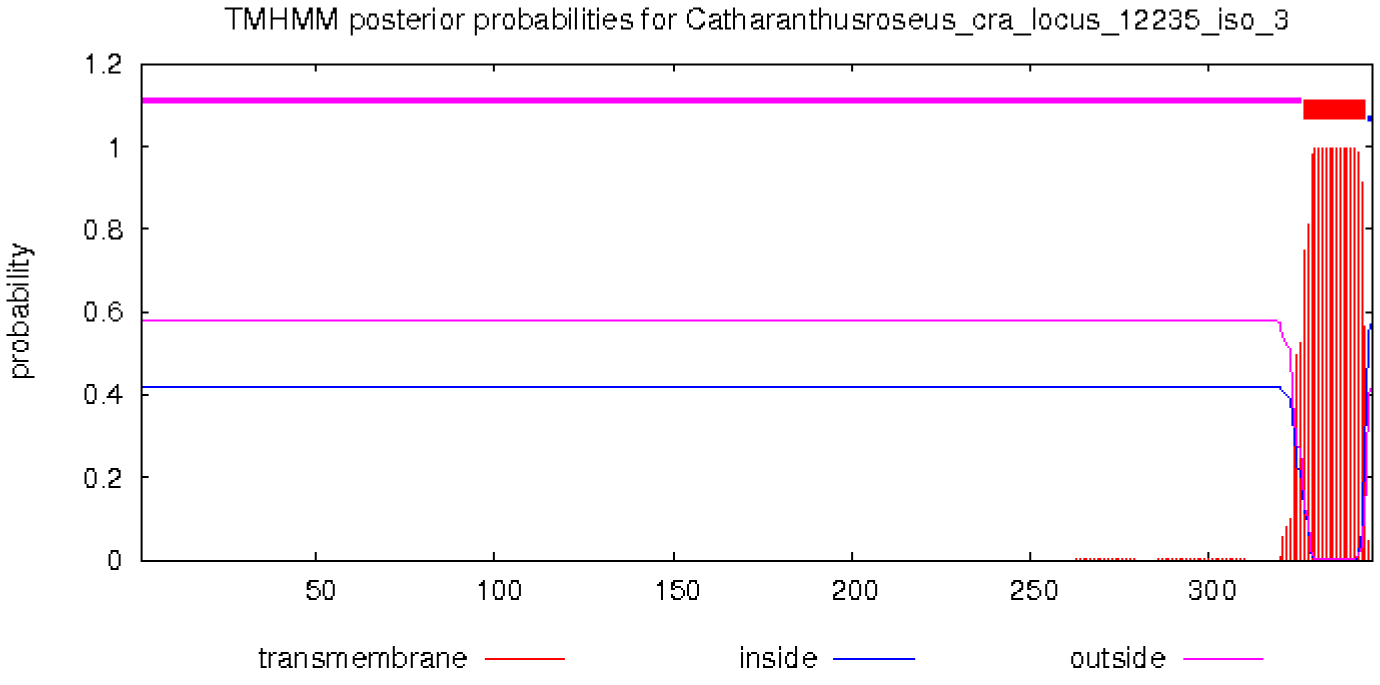

plot in postscript, script for making the plot in gnuplot, data for plot

---

```
# Catharanthusroseus_cra_locus_12235_iso_4 Length: 354
# Catharanthusroseus_cra_locus_12235_iso_4 Number of predicted TMHs:  1
# Catharanthusroseus_cra_locus_12235_iso_4 Exp number of AAs in TMHs: 18.63161
# Catharanthusroseus_cra_locus_12235_iso_4 Exp number, first 60 AAs:  0.00059
# Catharanthusroseus_cra_locus_12235_iso_4 Total prob of N-in:        0.39611
Catharanthusroseus_cra_locus_12235_iso_4	TMHMM2.0	outside	     1   334
Catharanthusroseus_cra_locus_12235_iso_4	TMHMM2.0	TMhelix	   335   352
Catharanthusroseus_cra_locus_12235_iso_4	TMHMM2.0	inside	   353   354
```

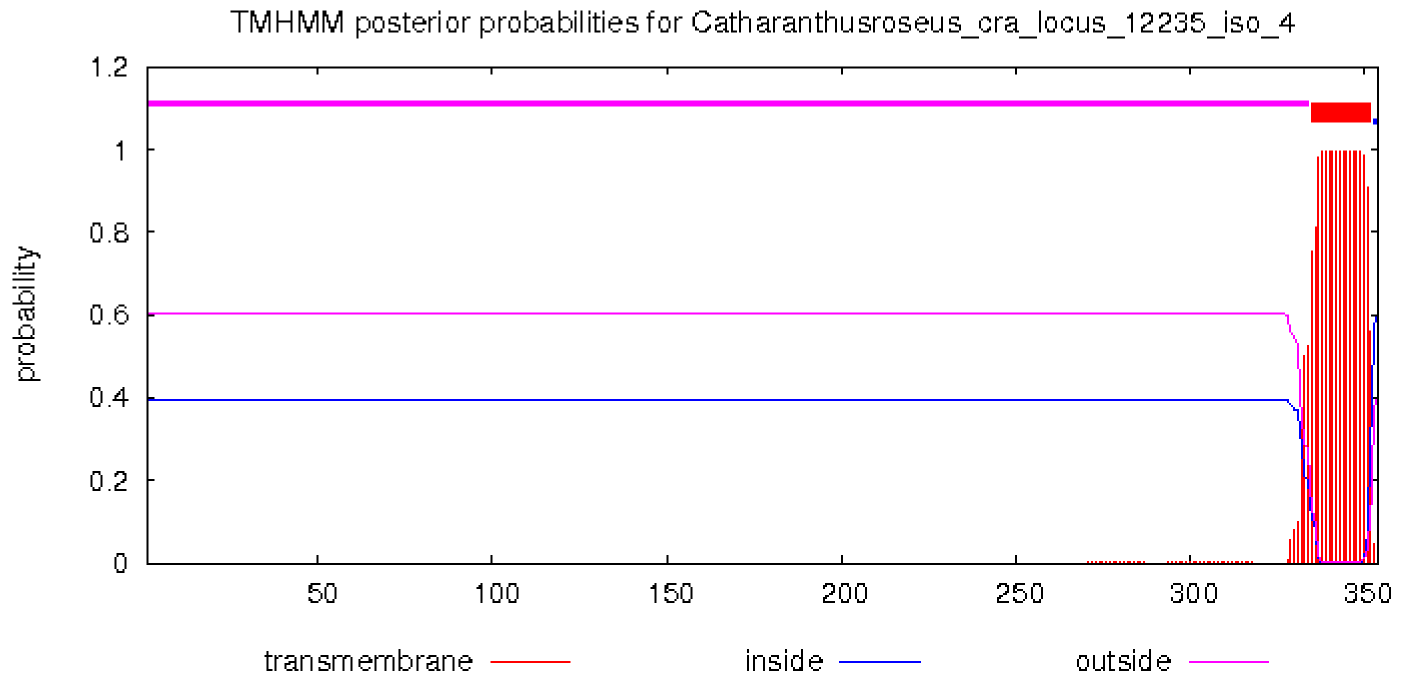

plot in postscript, script for making the plot in gnuplot, data for plot

---

```
# Catharanthusroseus_cra_locus_12712_iso_1 Length: 338
# Catharanthusroseus_cra_locus_12712_iso_1 Number of predicted TMHs:  0
# Catharanthusroseus_cra_locus_12712_iso_1 Exp number of AAs in TMHs: 0.00398
# Catharanthusroseus_cra_locus_12712_iso_1 Exp number, first 60 AAs:  0
# Catharanthusroseus_cra_locus_12712_iso_1 Total prob of N-in:        0.01372
Catharanthusroseus_cra_locus_12712_iso_1	TMHMM2.0	outside	     1   338
```

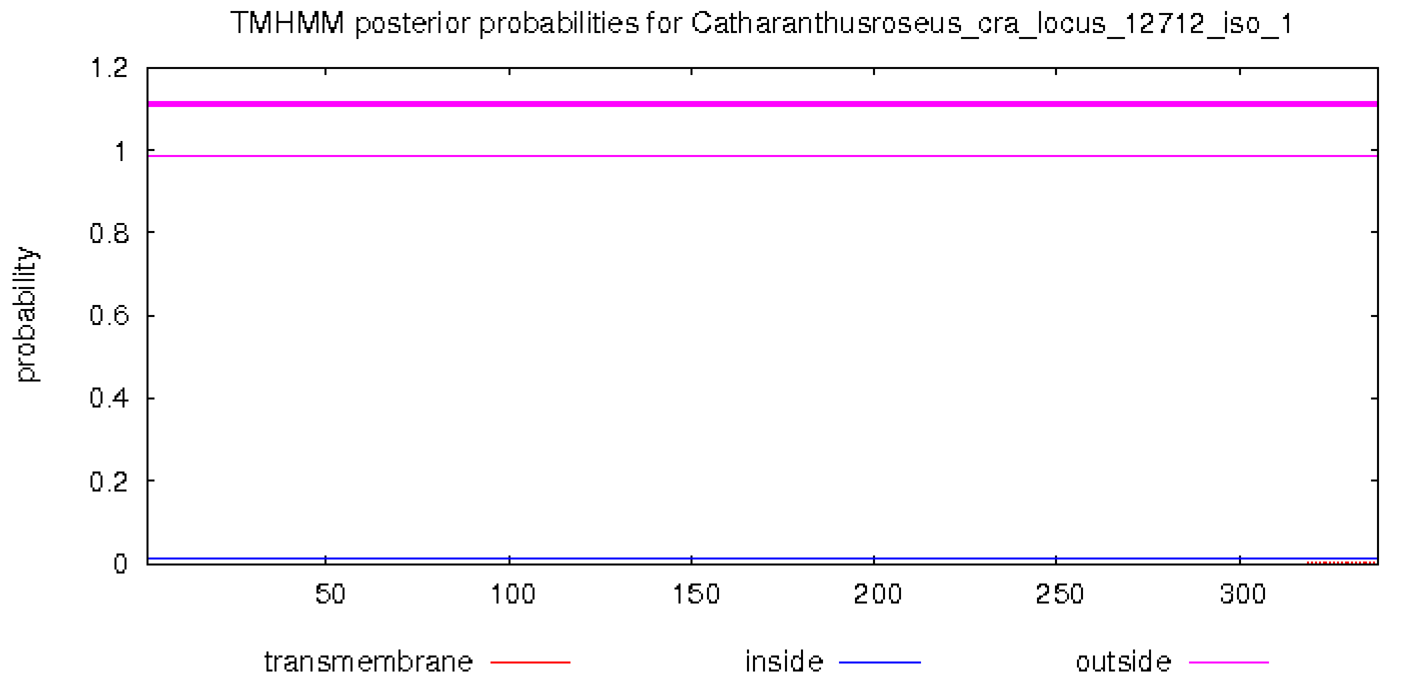

plot in postscript, script for making the plot in gnuplot, data for plot

```
# Catharanthusroseus_cra_locus_12712_iso_2 Length: 100
# Catharanthusroseus_cra_locus_12712_iso_2 Number of predicted TMHs:  0
# Catharanthusroseus_cra_locus_12712_iso_2 Exp number of AAs in TMHs: 0.00037
# Catharanthusroseus_cra_locus_12712_iso_2 Exp number, first 60 AAs:  0
# Catharanthusroseus_cra_locus_12712_iso_2 Total prob of N-in:        0.28462
Catharanthusroseus_cra_locus_12712_iso_2        TMHMM2.0        outside      1   100
```

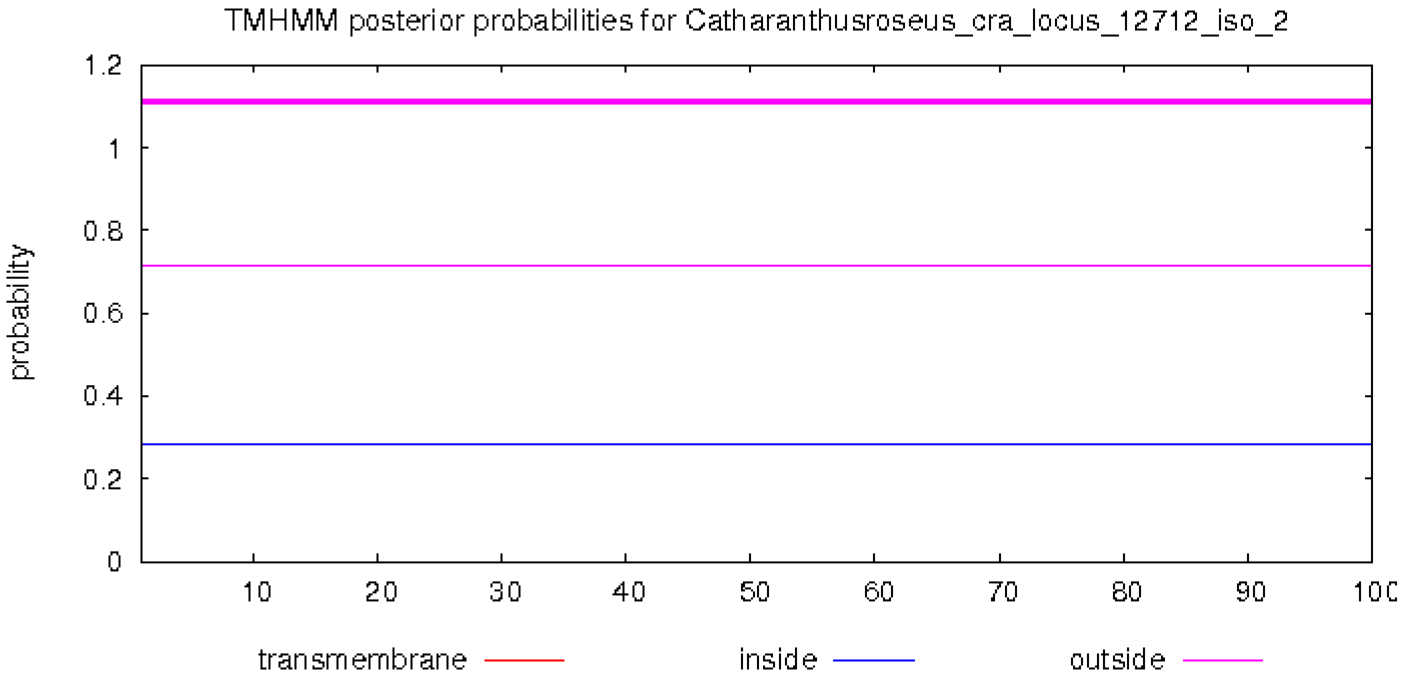

plot in postscript, script for making the plot in gnuplot, data for plot

```
# Catharanthusroseus_cra_locus_12712_iso_3 Length: 339
# Catharanthusroseus_cra_locus_12712_iso_3 Number of predicted TMHs:  0
# Catharanthusroseus_cra_locus_12712_iso_3 Exp number of AAs in TMHs: 0.01043
# Catharanthusroseus_cra_locus_12712_iso_3 Exp number, first 60 AAs:  0
# Catharanthusroseus_cra_locus_12712_iso_3 Total prob of N-in:        0.01367
Catharanthusroseus_cra_locus_12712_iso_3        TMHMM2.0        outside      1   339
```

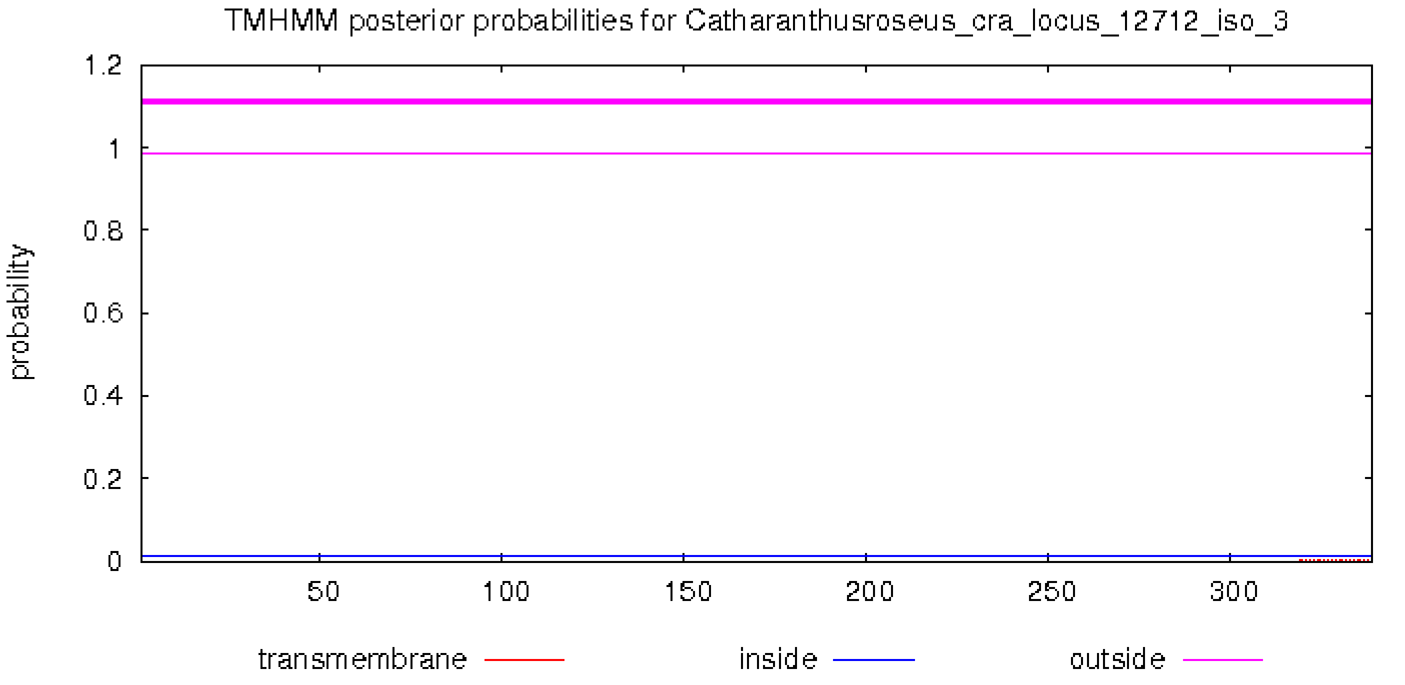

plot in postscript, script for making the plot in gnuplot, data for plot

```
# Catharanthusroseus_cra_locus_1291_iso_2 Length: 562
# Catharanthusroseus_cra_locus_1291_iso_2 Number of predicted TMHs:  0
# Catharanthusroseus_cra_locus_1291_iso_2 Exp number of AAs in TMHs: 0.00182
# Catharanthusroseus_cra_locus_1291_iso_2 Exp number, first 60 AAs:  0.00182
# Catharanthusroseus_cra_locus_1291_iso_2 Total prob of N-in:        0.00037
Catharanthusroseus_cra_locus_1291_iso_2 TMHMM2.0        outside      1   562
```

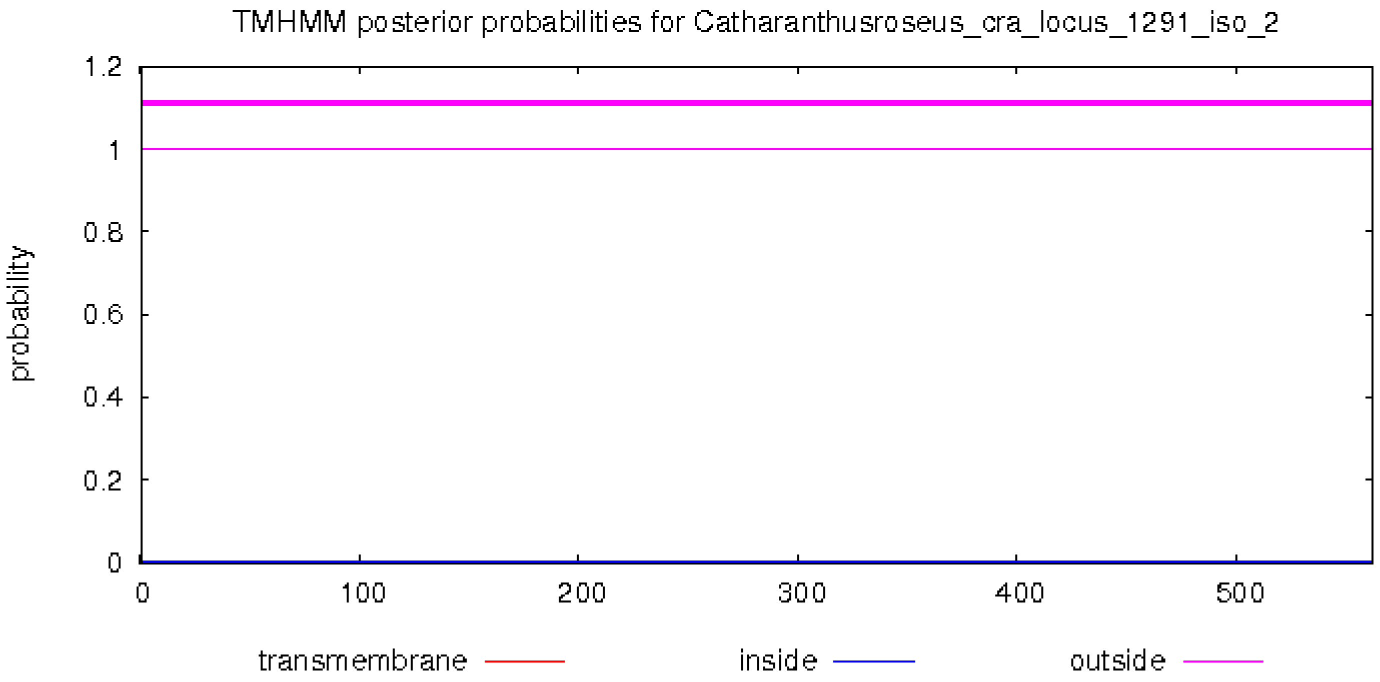

plot in postscript, script for making the plot in gnuplot, data for plot

---

```
# Catharanthusroseus_cra_locus_1291_iso_3 Length: 672
# Catharanthusroseus_cra_locus_1291_iso_3 Number of predicted TMHs:  0
# Catharanthusroseus_cra_locus_1291_iso_3 Exp number of AAs in TMHs: 0.01386
# Catharanthusroseus_cra_locus_1291_iso_3 Exp number, first 60 AAs:  0.00182
# Catharanthusroseus_cra_locus_1291_iso_3 Total prob of N-in:        0.00016
Catharanthusroseus_cra_locus_1291_iso_3 TMHMM2.0        outside      1   672
```

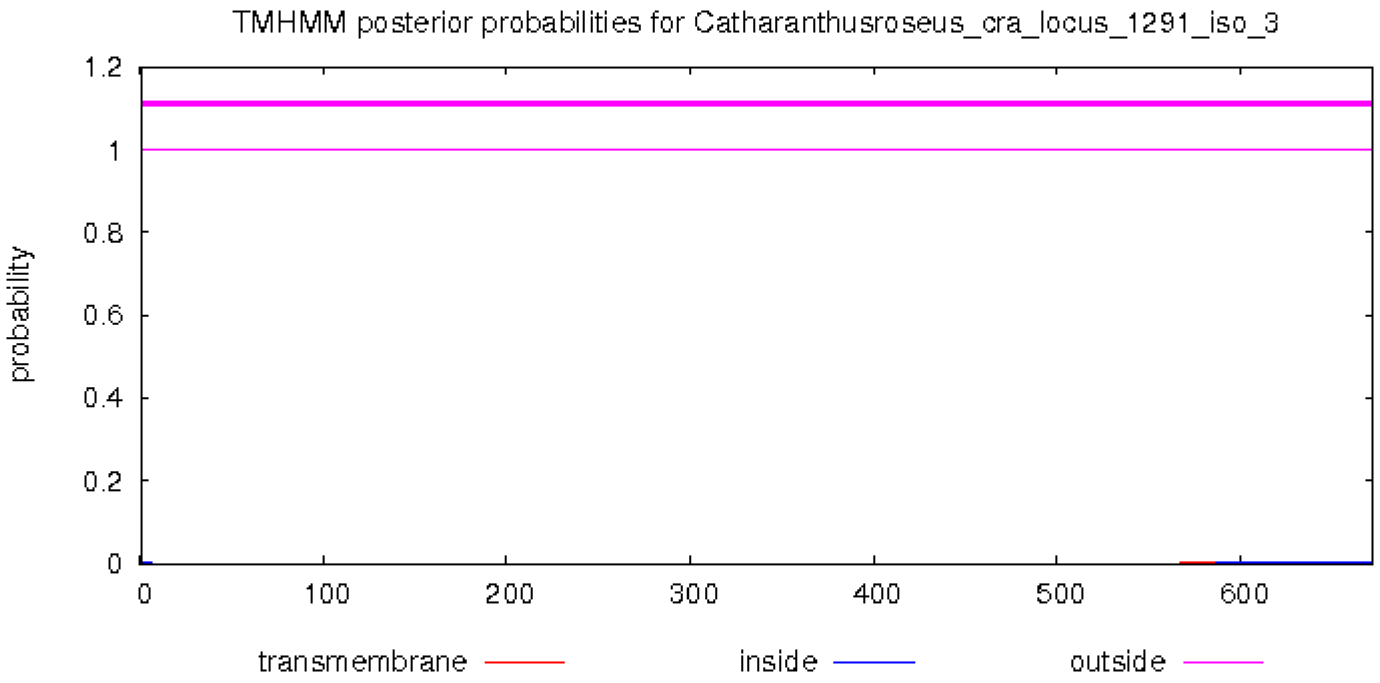

plot in postscript, script for making the plot in gnuplot, data for plot

---

```
# Catharanthusroseus_cra_locus_12960_iso_1 Length: 325
# Catharanthusroseus_cra_locus_12960_iso_1 Number of predicted TMHs:  0
# Catharanthusroseus_cra_locus_12960_iso_1 Exp number of AAs in TMHs: 0.00041
# Catharanthusroseus_cra_locus_12960_iso_1 Exp number, first 60 AAs:  0.00023
# Catharanthusroseus_cra_locus_12960_iso_1 Total prob of N-in:        0.03164
Catharanthusroseus_cra_locus_12960_iso_1        TMHMM2.0        outside      1   325
```

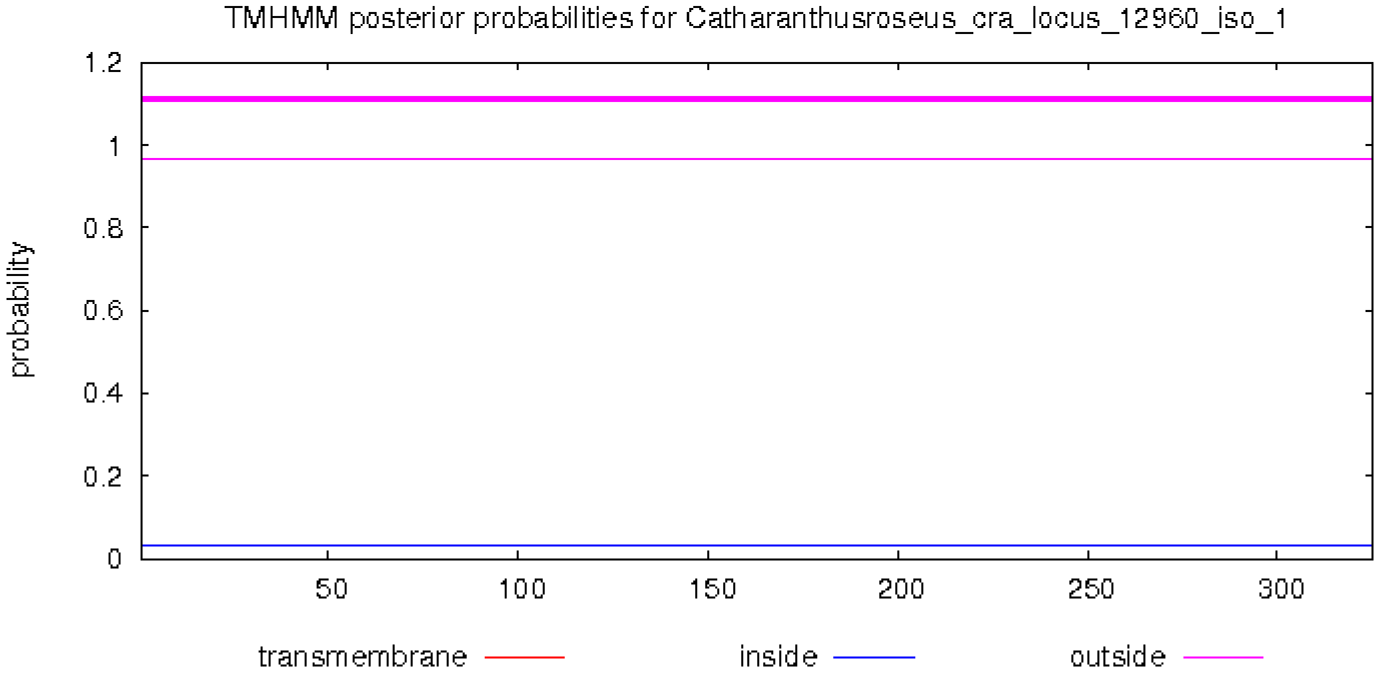

plot in postscript, script for making the plot in gnuplot, data for plot

---

```
# Catharanthusroseus_cra_locus_12983_iso_1 Length: 368
# Catharanthusroseus_cra_locus_12983_iso_1 Number of predicted TMHs:  0
# Catharanthusroseus_cra_locus_12983_iso_1 Exp number of AAs in TMHs: 0
# Catharanthusroseus_cra_locus_12983_iso_1 Exp number, first 60 AAs:  0
# Catharanthusroseus_cra_locus_12983_iso_1 Total prob of N-in:        0.00832
Catharanthusroseus_cra_locus_12983_iso_1        TMHMM2.0        outside      1   368
```

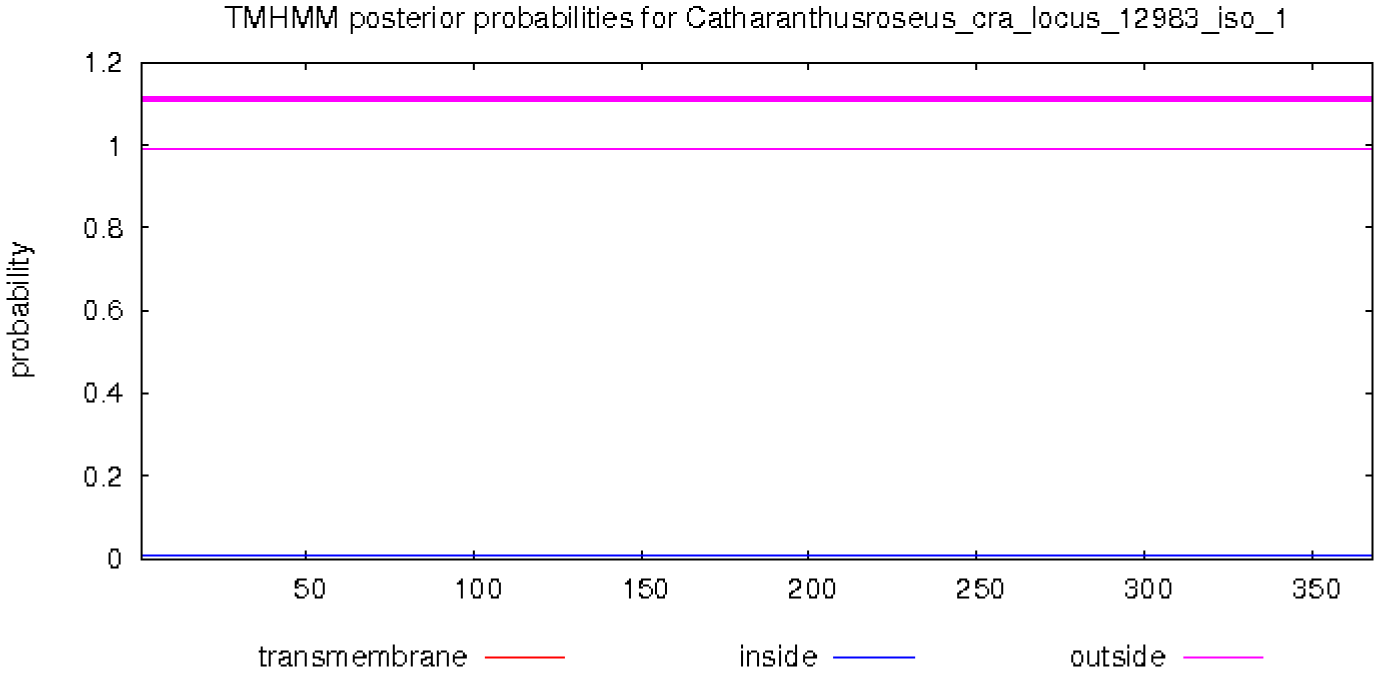

plot in postscript, script for making the plot in gnuplot, data for plot

---

```
# Catharanthusroseus_cra_locus_13006_iso_2 Length: 453
# Catharanthusroseus_cra_locus_13006_iso_2 Number of predicted TMHs:  0
# Catharanthusroseus_cra_locus_13006_iso_2 Exp number of AAs in TMHs: 0.01192
# Catharanthusroseus_cra_locus_13006_iso_2 Exp number, first 60 AAs:  0
# Catharanthusroseus_cra_locus_13006_iso_2 Total prob of N-in:        0.00081
Catharanthusroseus_cra_locus_13006_iso_2	TMHMM2.0	outside	     1   453
```

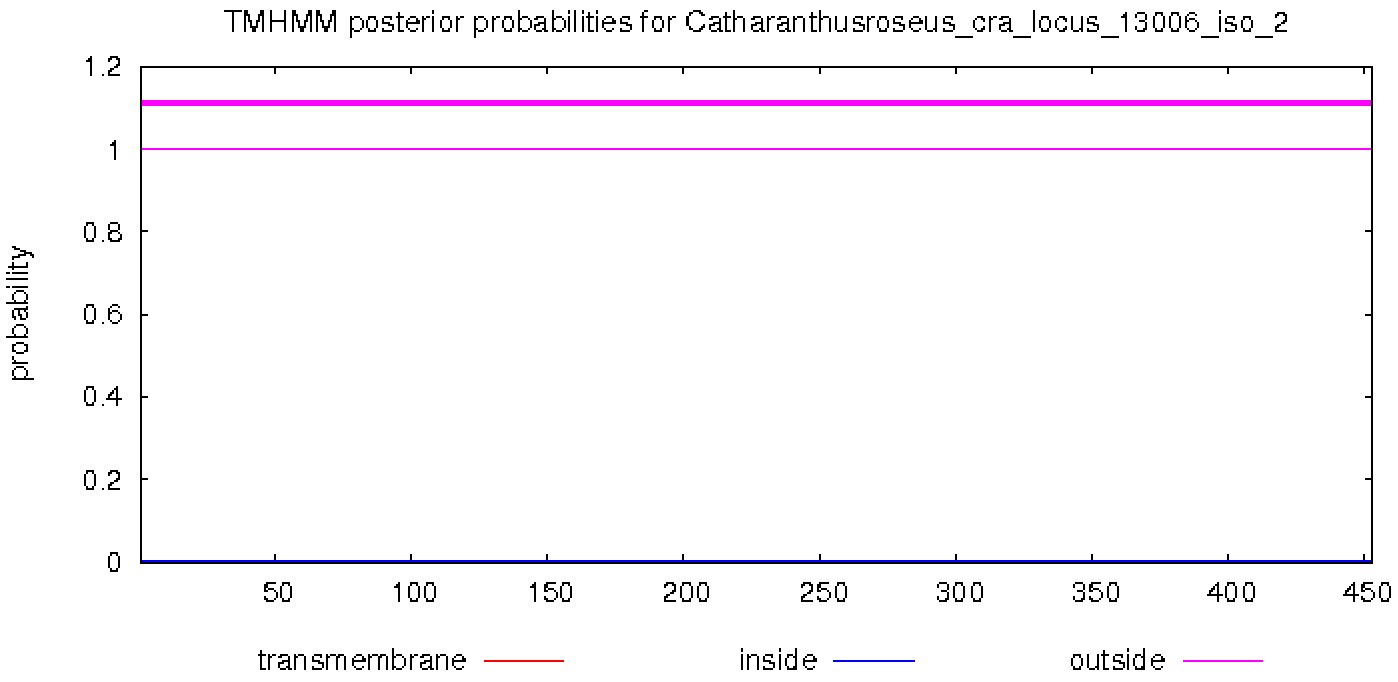

plot in postscript, script for making the plot in gnuplot, data for plot

---

```
# Catharanthusroseus_cra_locus_13006_iso_4 Length: 233
# Catharanthusroseus_cra_locus_13006_iso_4 Number of predicted TMHs:  0
# Catharanthusroseus_cra_locus_13006_iso_4 Exp number of AAs in TMHs: 0
# Catharanthusroseus_cra_locus_13006_iso_4 Exp number, first 60 AAs:  0
# Catharanthusroseus_cra_locus_13006_iso_4 Total prob of N-in:        0.01362
Catharanthusroseus_cra_locus_13006_iso_4	TMHMM2.0	outside	     1   233
```

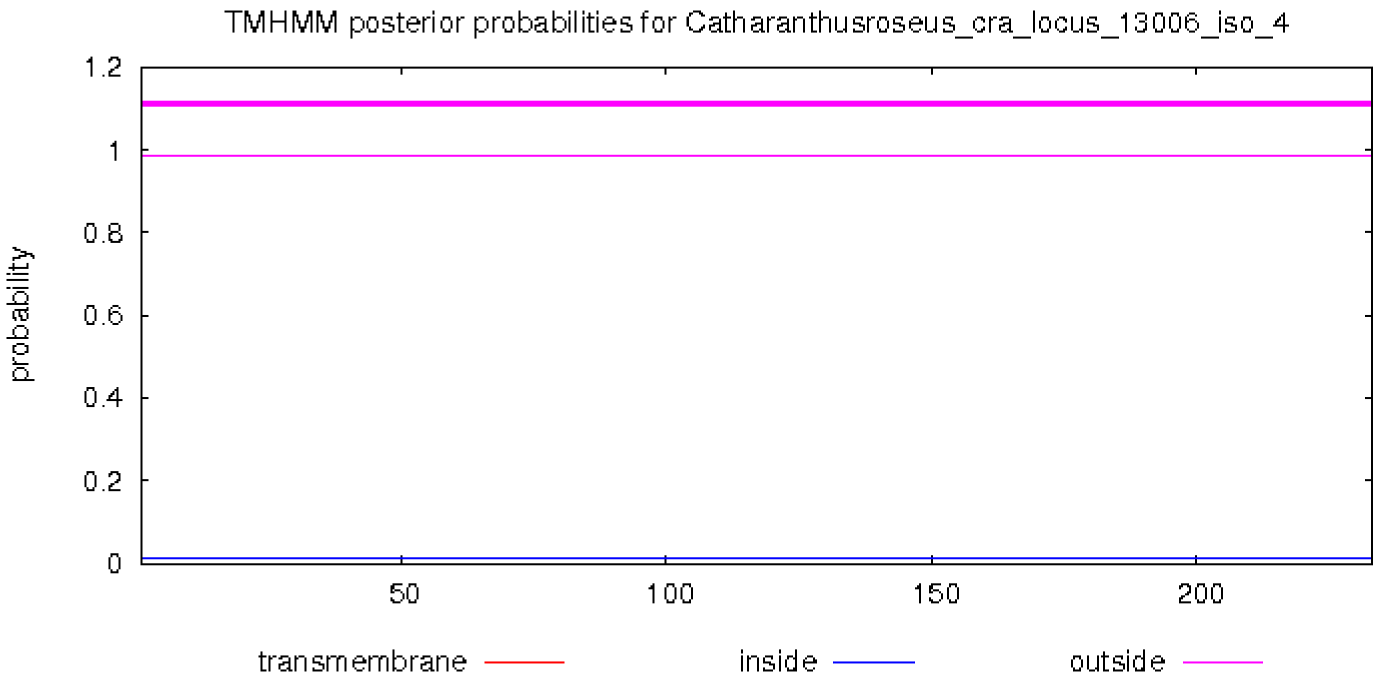

plot in postscript, script for making the plot in gnuplot, data for plot

---

```
# Catharanthusroseus_cra_locus_13006_iso_5 Length: 217
# Catharanthusroseus_cra_locus_13006_iso_5 Number of predicted TMHs:  0
# Catharanthusroseus_cra_locus_13006_iso_5 Exp number of AAs in TMHs: 0.12159
# Catharanthusroseus_cra_locus_13006_iso_5 Exp number, first 60 AAs:  0
# Catharanthusroseus_cra_locus_13006_iso_5 Total prob of N-in:        0.01241
Catharanthusroseus_cra_locus_13006_iso_5	TMHMM2.0	outside	     1   217
```

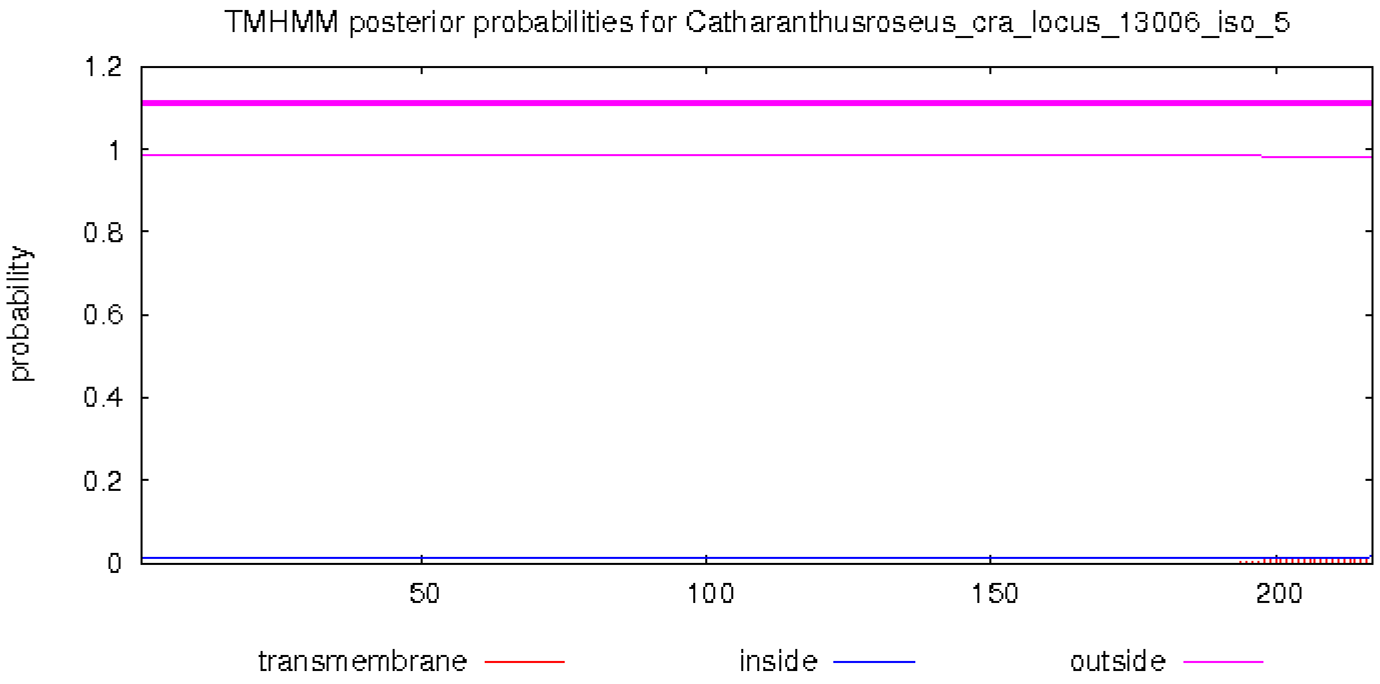

plot in postscript, script for making the plot in gnuplot, data for plot

---

```
# Catharanthusroseus_cra_locus_13006_iso_6 Length: 379
# Catharanthusroseus_cra_locus_13006_iso_6 Number of predicted TMHs:  0
# Catharanthusroseus_cra_locus_13006_iso_6 Exp number of AAs in TMHs: 0
# Catharanthusroseus_cra_locus_13006_iso_6 Exp number, first 60 AAs:  0
# Catharanthusroseus_cra_locus_13006_iso_6 Total prob of N-in:        0.00190
Catharanthusroseus_cra_locus_13006_iso_6	TMHMM2.0	outside	     1   379
```

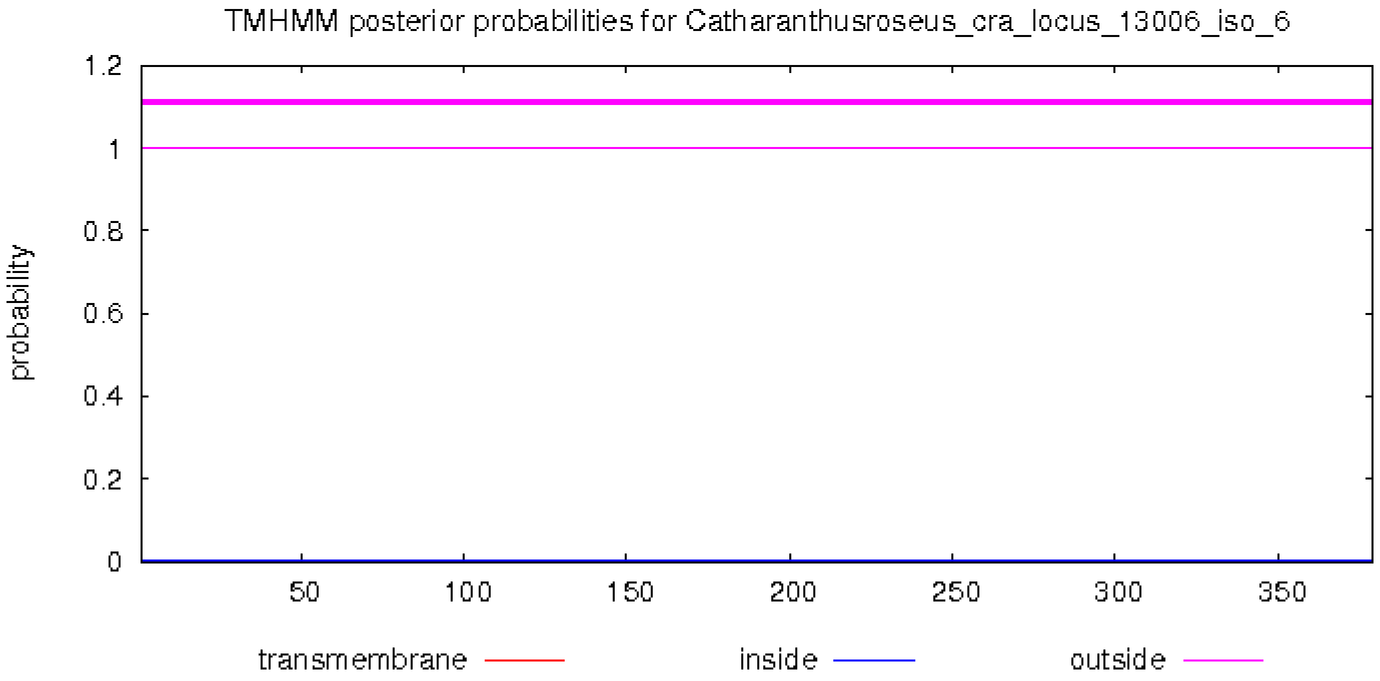

plot in postscript, script for making the plot in gnuplot, data for plot

---

```
# Catharanthusroseus_cra_locus_13047_iso_1 Length: 301
# Catharanthusroseus_cra_locus_13047_iso_1 Number of predicted TMHs:  0
# Catharanthusroseus_cra_locus_13047_iso_1 Exp number of AAs in TMHs: 0.0017
# Catharanthusroseus_cra_locus_13047_iso_1 Exp number, first 60 AAs:  0.00089
# Catharanthusroseus_cra_locus_13047_iso_1 Total prob of N-in:        0.03364
Catharanthusroseus_cra_locus_13047_iso_1        TMHMM2.0        outside      1   301
```

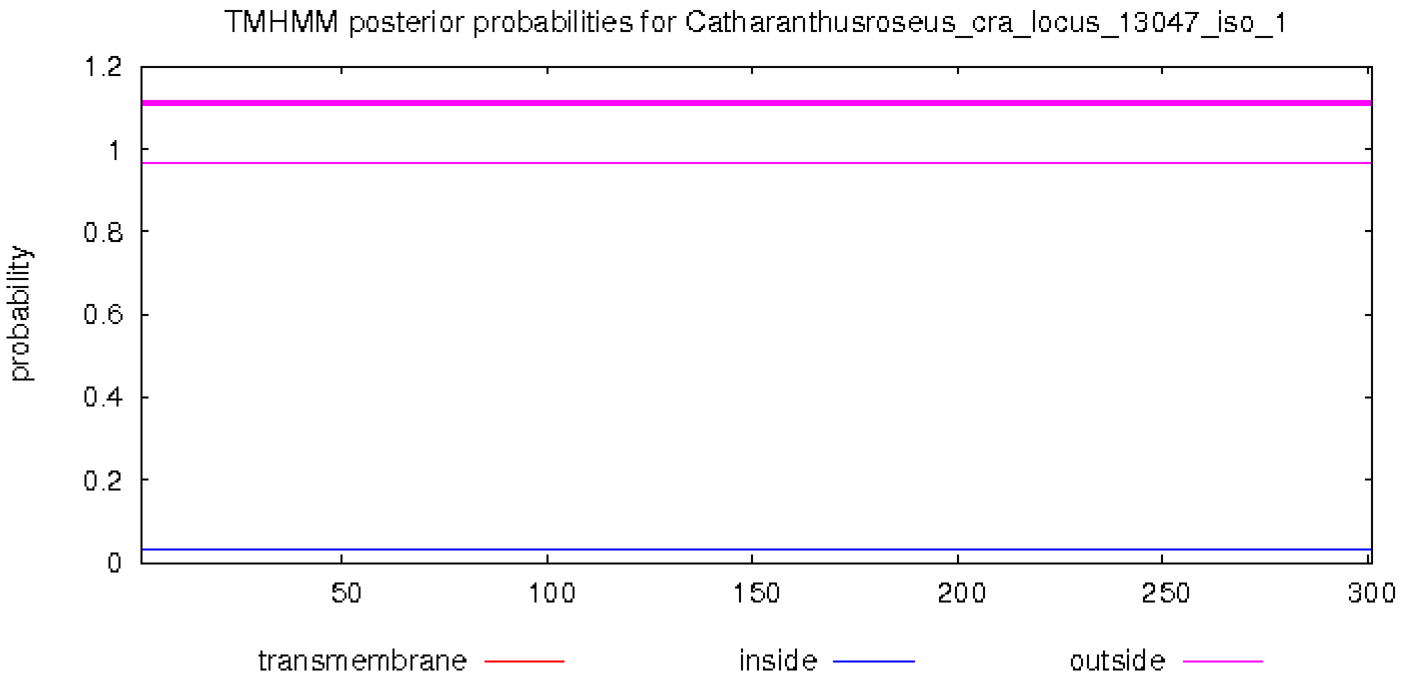

plot in postscript, script for making the plot in gnuplot, data for plot

---

```
# Catharanthusroseus_cra_locus_13047_iso_2 Length: 300
# Catharanthusroseus_cra_locus_13047_iso_2 Number of predicted TMHs:  0
# Catharanthusroseus_cra_locus_13047_iso_2 Exp number of AAs in TMHs: 0.0017
# Catharanthusroseus_cra_locus_13047_iso_2 Exp number, first 60 AAs:  0.00089
# Catharanthusroseus_cra_locus_13047_iso_2 Total prob of N-in:        0.03404
Catharanthusroseus_cra_locus_13047_iso_2        TMHMM2.0        outside      1   300
```

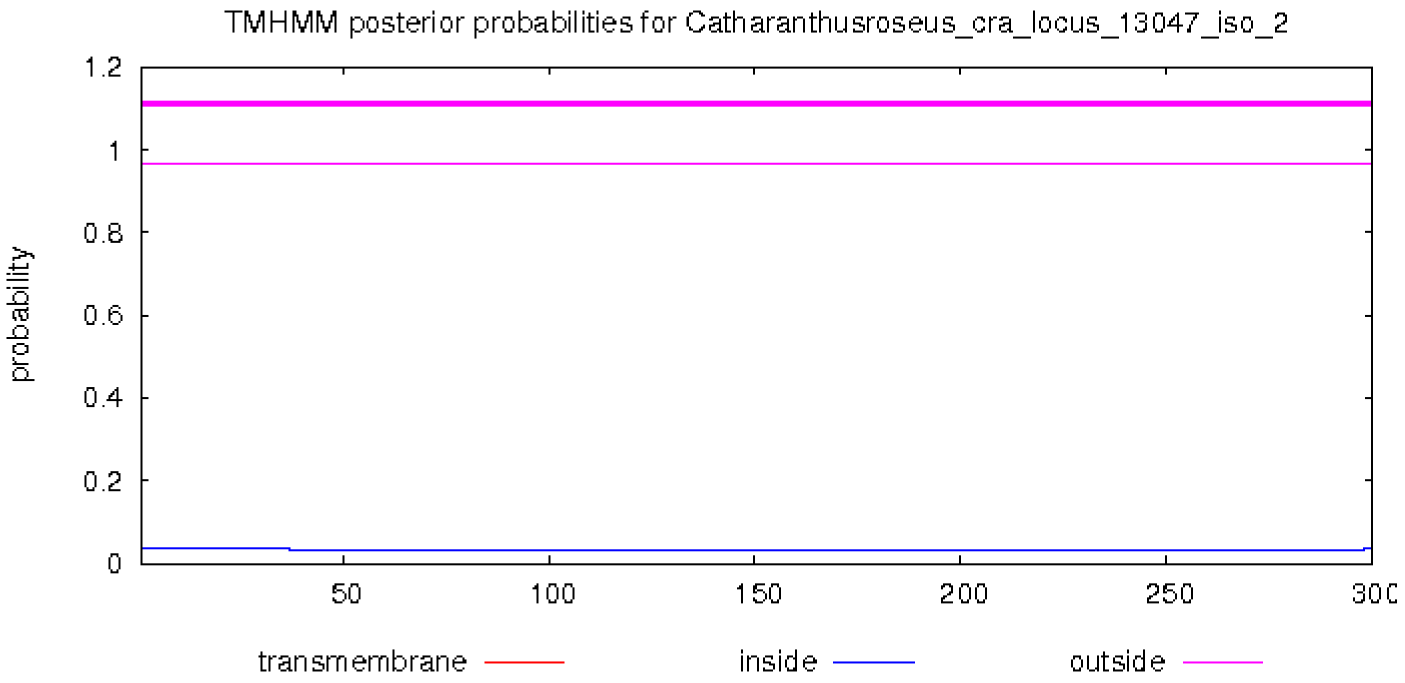

plot in postscript, script for making the plot in gnuplot, data for plot

---

```
# Catharanthusroseus_cra_locus_1368_iso_1 Length: 298
# Catharanthusroseus_cra_locus_1368_iso_1 Number of predicted TMHs:  0
# Catharanthusroseus_cra_locus_1368_iso_1 Exp number of AAs in TMHs: 0.26641
# Catharanthusroseus_cra_locus_1368_iso_1 Exp number, first 60 AAs:  0.26467
# Catharanthusroseus_cra_locus_1368_iso_1 Total prob of N-in:        0.35547
Catharanthusroseus_cra_locus_1368_iso_1 TMHMM2.0        outside      1   298
```

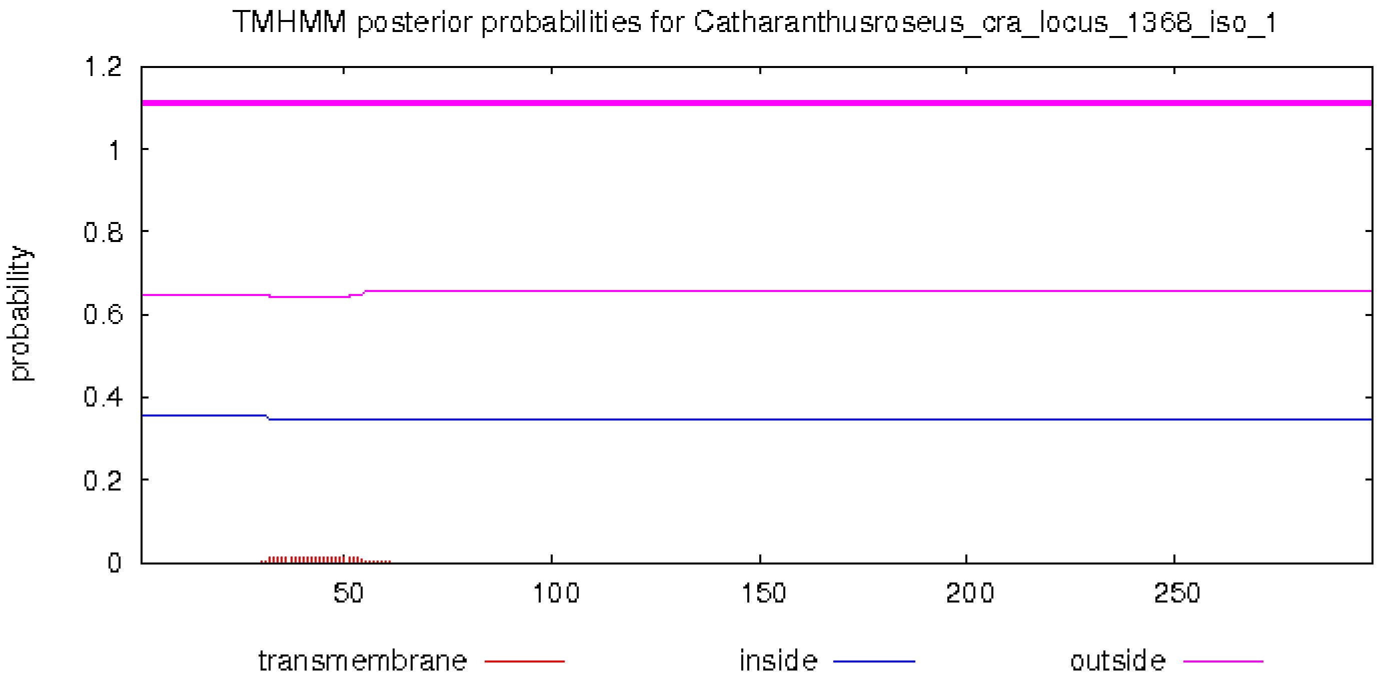

plot in postscript, script for making the plot in gnuplot, data for plot

---

```
# Catharanthusroseus_cra_locus_1368_iso_3 Length: 985
# Catharanthusroseus_cra_locus_1368_iso_3 Number of predicted TMHs:  0
# Catharanthusroseus_cra_locus_1368_iso_3 Exp number of AAs in TMHs: 1.2003
# Catharanthusroseus_cra_locus_1368_iso_3 Exp number, first 60 AAs:  0.00132
# Catharanthusroseus_cra_locus_1368_iso_3 Total prob of N-in:        0.03918
Catharanthusroseus_cra_locus_1368_iso_3 TMHMM2.0        outside      1   985
```

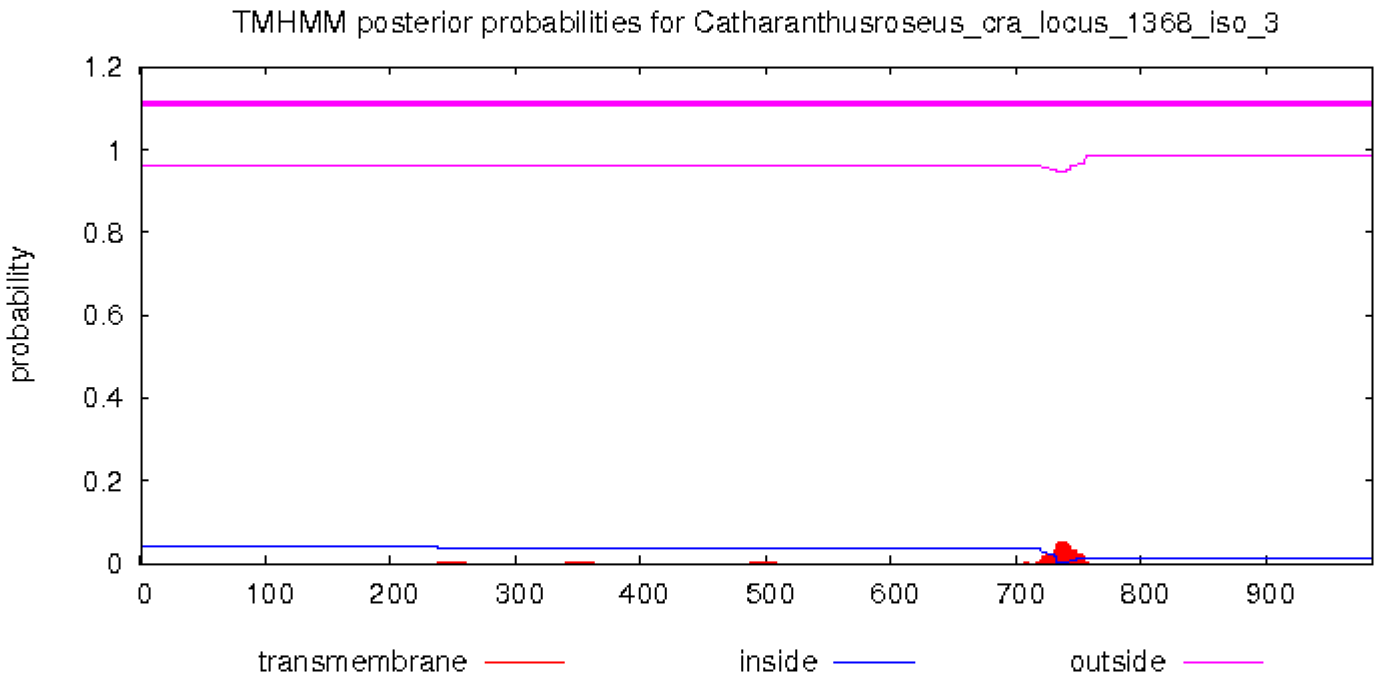

plot in postscript, script for making the plot in gnuplot, data for plot

---

```
# Catharanthusroseus_cra_locus_1368_iso_4 Length: 1123
# Catharanthusroseus_cra_locus_1368_iso_4 Number of predicted TMHs:  0
# Catharanthusroseus_cra_locus_1368_iso_4 Exp number of AAs in TMHs: 0.9542
# Catharanthusroseus_cra_locus_1368_iso_4 Exp number, first 60 AAs:  0.00132
# Catharanthusroseus_cra_locus_1368_iso_4 Total prob of N-in:        0.03937
Catharanthusroseus_cra_locus_1368_iso_4 TMHMM2.0        outside      1  1123
```

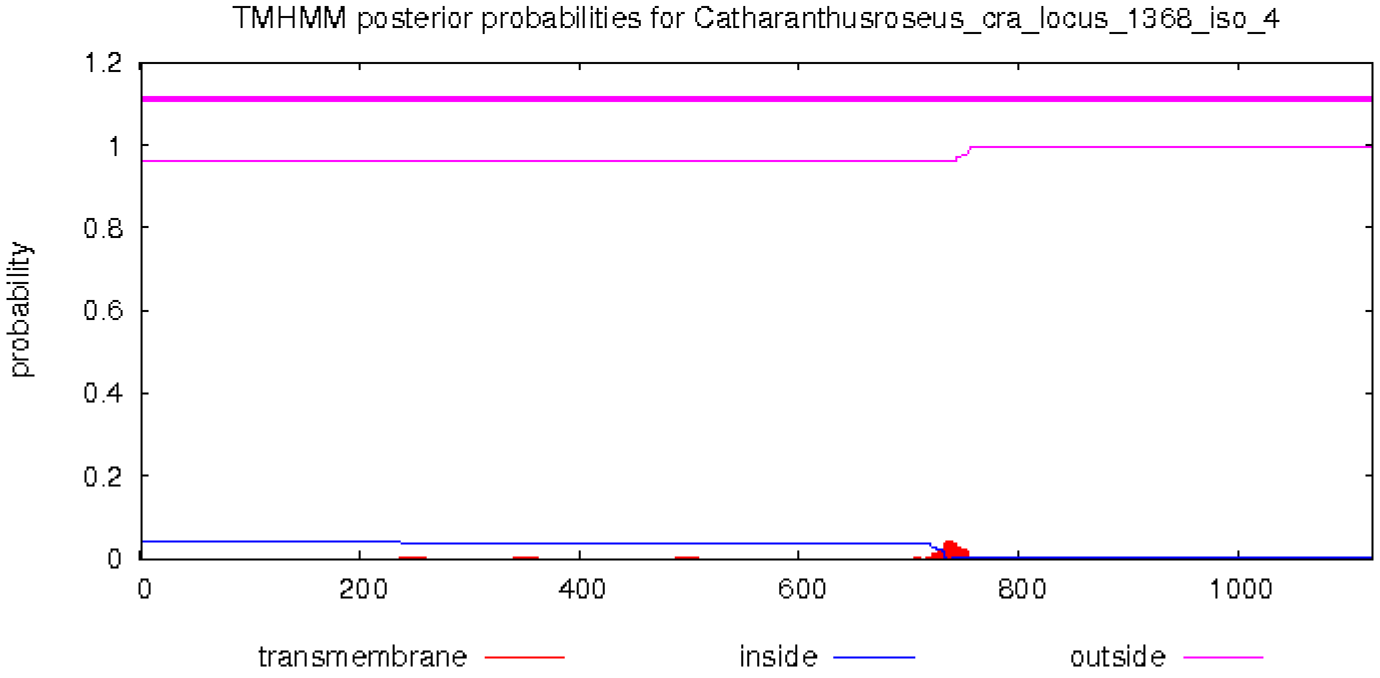

plot in postscript, script for making the plot in gnuplot, data for plot

---

```
# Catharanthusroseus_cra_locus_1368_iso_6 Length: 178
# Catharanthusroseus_cra_locus_1368_iso_6 Number of predicted TMHs:  0
# Catharanthusroseus_cra_locus_1368_iso_6 Exp number of AAs in TMHs: 0.0029
# Catharanthusroseus_cra_locus_1368_iso_6 Exp number, first 60 AAs:  0.0029
# Catharanthusroseus_cra_locus_1368_iso_6 Total prob of N-in:        0.01150
Catharanthusroseus_cra_locus_1368_iso_6 TMHMM2.0        outside      1   178
```

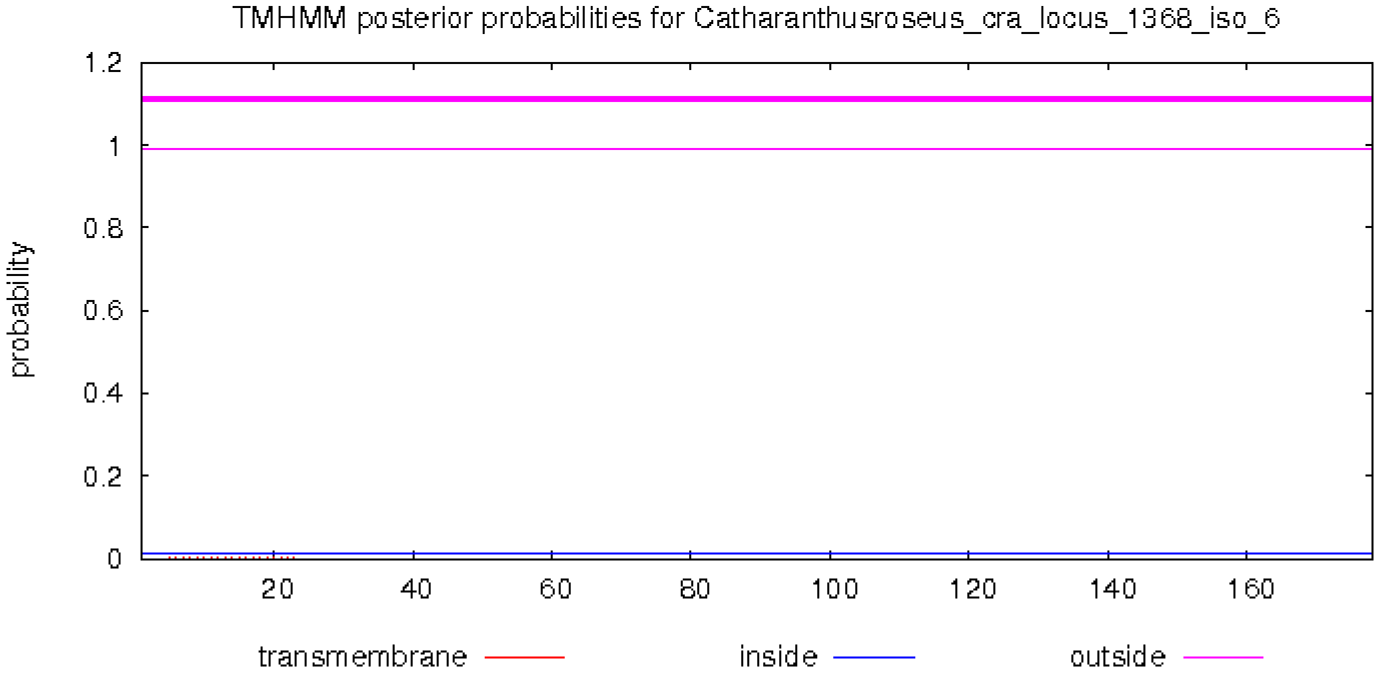

plot in postscript, script for making the plot in gnuplot, data for plot

---

```
# Catharanthusroseus_cra_locus_1368_iso_8 Length: 339
# Catharanthusroseus_cra_locus_1368_iso_8 Number of predicted TMHs:  0
# Catharanthusroseus_cra_locus_1368_iso_8 Exp number of AAs in TMHs: 0.30307
# Catharanthusroseus_cra_locus_1368_iso_8 Exp number, first 60 AAs:  0.30168
# Catharanthusroseus_cra_locus_1368_iso_8 Total prob of N-in:        0.25252
Catharanthusroseus_cra_locus_1368_iso_8 TMHMM2.0        outside      1   339
```

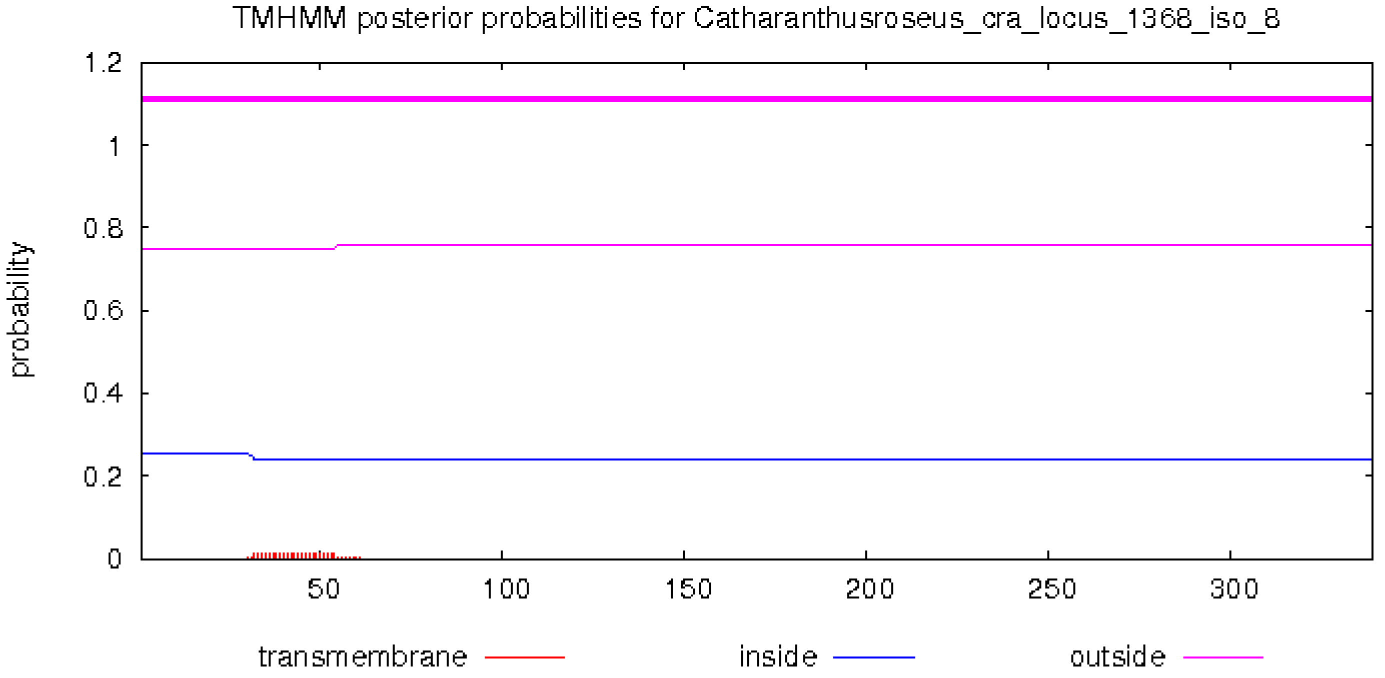

plot in postscript, script for making the plot in gnuplot, data for plot

---

```
# Catharanthusroseus_cra_locus_15344_iso_1 Length: 319
# Catharanthusroseus_cra_locus_15344_iso_1 Number of predicted TMHs:  0
# Catharanthusroseus_cra_locus_15344_iso_1 Exp number of AAs in TMHs: 0.00341
# Catharanthusroseus_cra_locus_15344_iso_1 Exp number, first 60 AAs:  0.00059
# Catharanthusroseus_cra_locus_15344_iso_1 Total prob of N-in:        0.02575
Catharanthusroseus_cra_locus_15344_iso_1        TMHMM2.0        outside      1   319
```

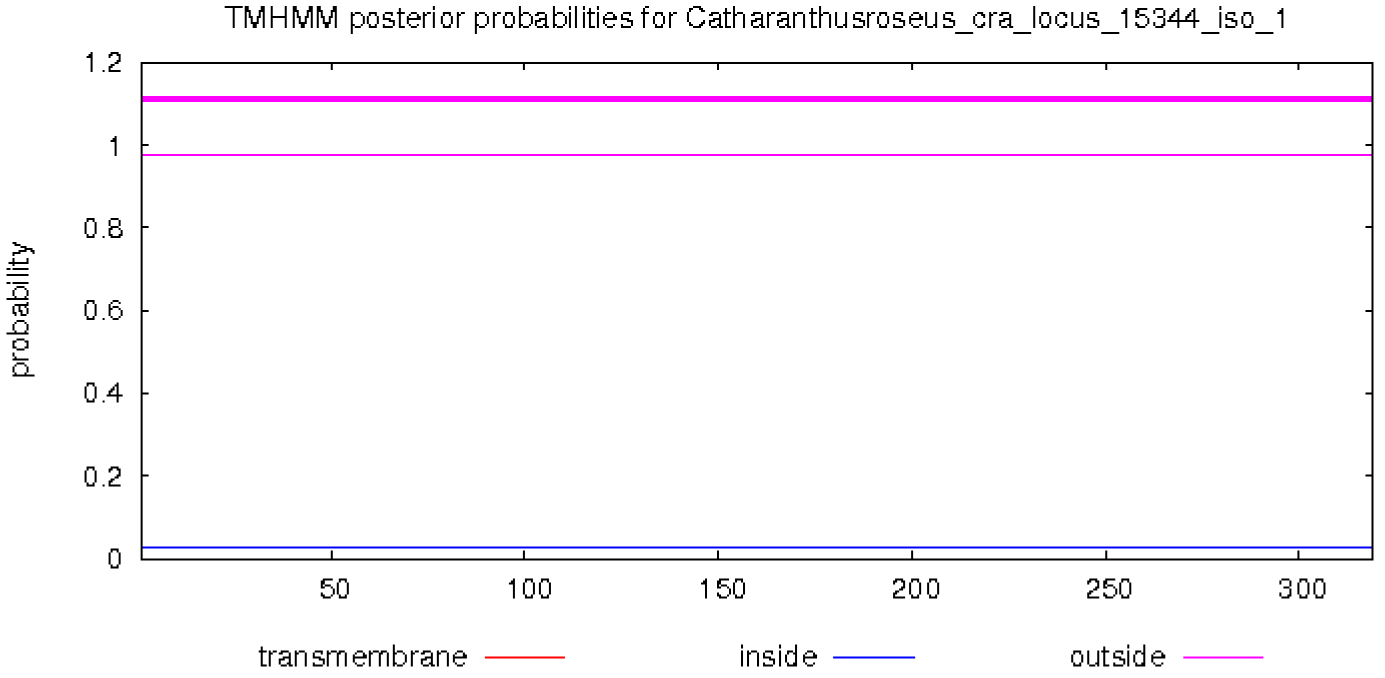

plot in postscript, script for making the plot in gnuplot, data for plot

---

```
# Catharanthusroseus_cra_locus_15356_iso_1 Length: 296
# Catharanthusroseus_cra_locus_15356_iso_1 Number of predicted TMHs:  0
# Catharanthusroseus_cra_locus_15356_iso_1 Exp number of AAs in TMHs: 0
# Catharanthusroseus_cra_locus_15356_iso_1 Exp number, first 60 AAs:  0
# Catharanthusroseus_cra_locus_15356_iso_1 Total prob of N-in:        0.14266
Catharanthusroseus_cra_locus_15356_iso_1	TMHMM2.0	outside	     1   296
```

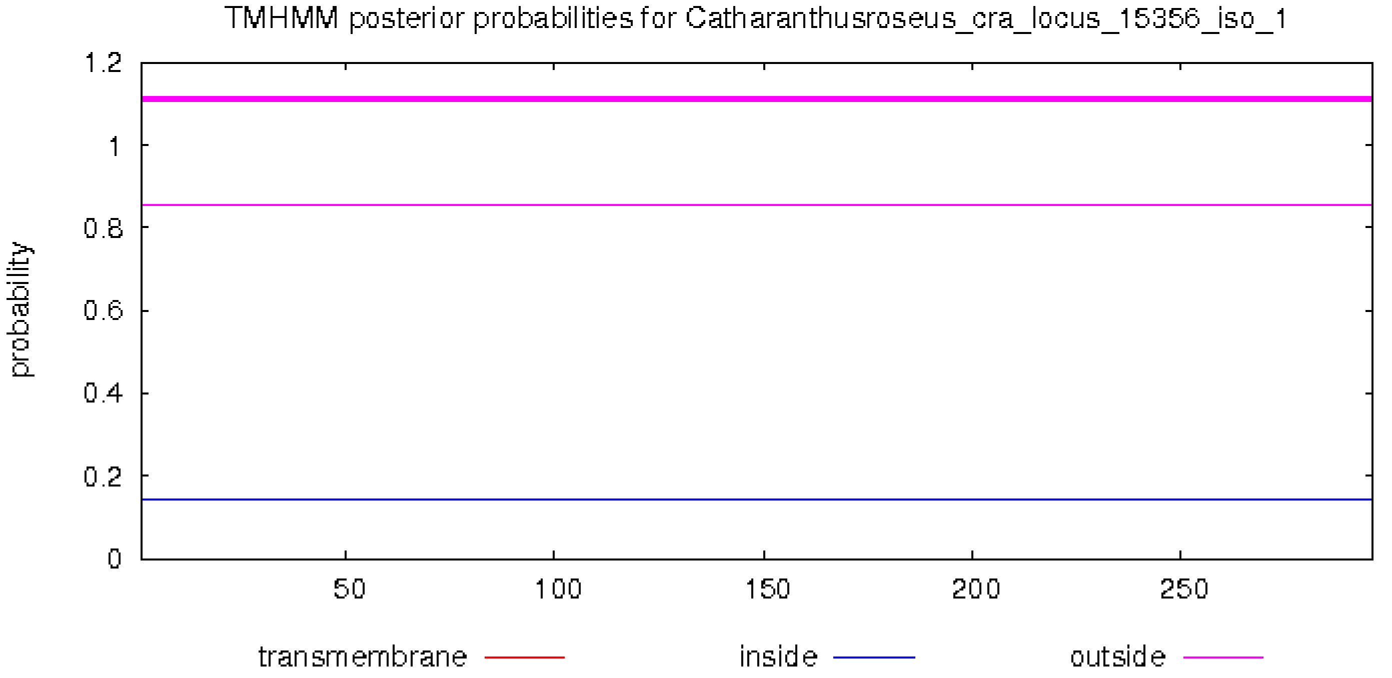

plot in postscript, script for making the plot in gnuplot, data for plot

---

```
# Catharanthusroseus_cra_locus_16332_iso_1 Length: 253
# Catharanthusroseus_cra_locus_16332_iso_1 Number of predicted TMHs:  0
# Catharanthusroseus_cra_locus_16332_iso_1 Exp number of AAs in TMHs: 4e-05
# Catharanthusroseus_cra_locus_16332_iso_1 Exp number, first 60 AAs:  0
# Catharanthusroseus_cra_locus_16332_iso_1 Total prob of N-in:        0.02022
Catharanthusroseus_cra_locus_16332_iso_1	TMHMM2.0	outside	     1   253
```

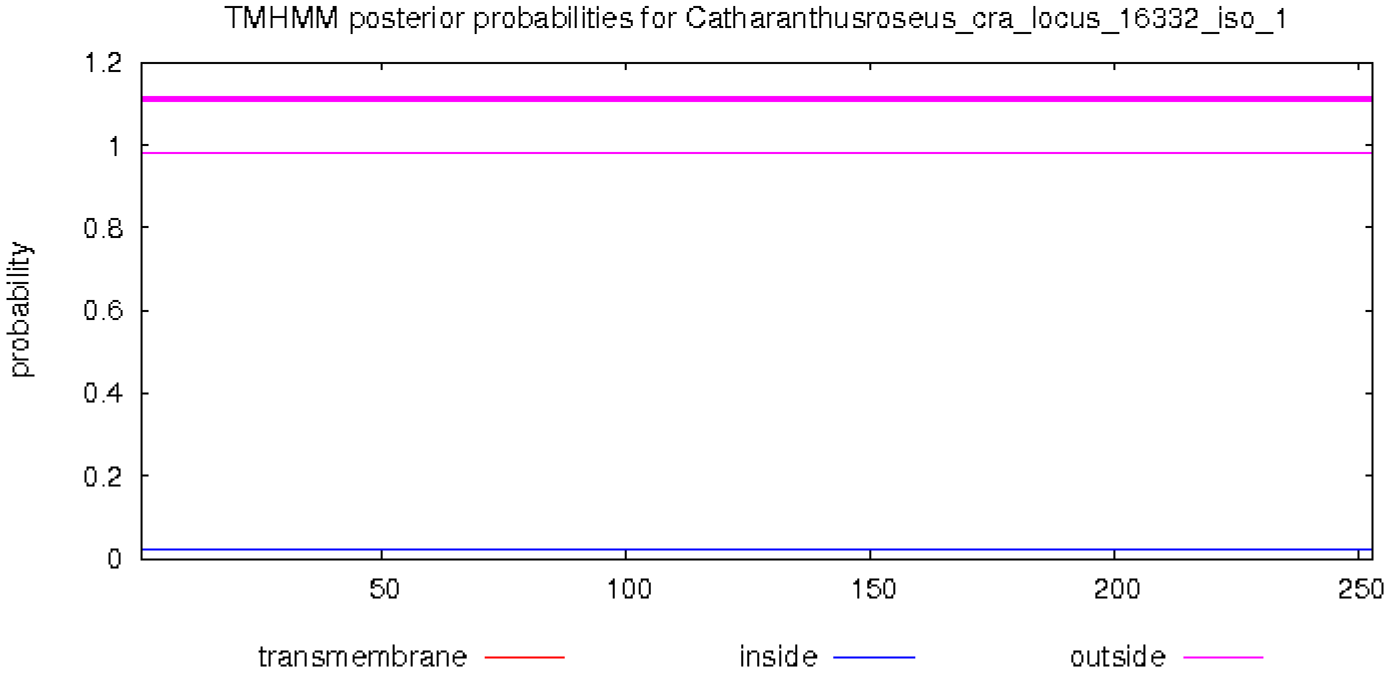

plot in postscript, script for making the plot in gnuplot, data for plot

---

```
# Catharanthusroseus_cra_locus_16332_iso_2 Length: 258
# Catharanthusroseus_cra_locus_16332_iso_2 Number of predicted TMHs:  0
# Catharanthusroseus_cra_locus_16332_iso_2 Exp number of AAs in TMHs: 4e-05
# Catharanthusroseus_cra_locus_16332_iso_2 Exp number, first 60 AAs:  0
# Catharanthusroseus_cra_locus_16332_iso_2 Total prob of N-in:        0.02042
Catharanthusroseus_cra_locus_16332_iso_2	TMHMM2.0	outside	     1   258
```

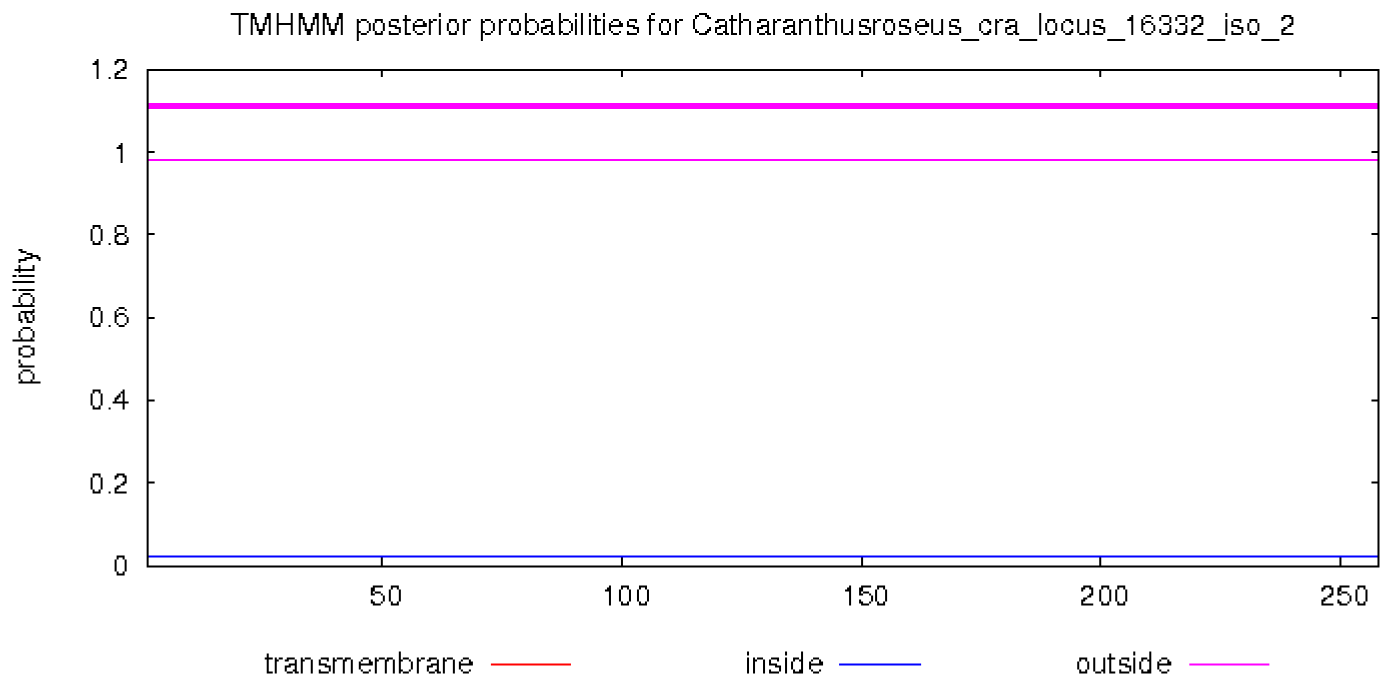

plot in postscript, script for making the plot in gnuplot, data for plot

---

```
# Catharanthusroseus_cra_locus_16332_iso_3 Length: 270
# Catharanthusroseus_cra_locus_16332_iso_3 Number of predicted TMHs:  0
# Catharanthusroseus_cra_locus_16332_iso_3 Exp number of AAs in TMHs: 0.00217
# Catharanthusroseus_cra_locus_16332_iso_3 Exp number, first 60 AAs:  0.00092
# Catharanthusroseus_cra_locus_16332_iso_3 Total prob of N-in:        0.09155
Catharanthusroseus_cra_locus_16332_iso_3	TMHMM2.0	outside	     1   270
```

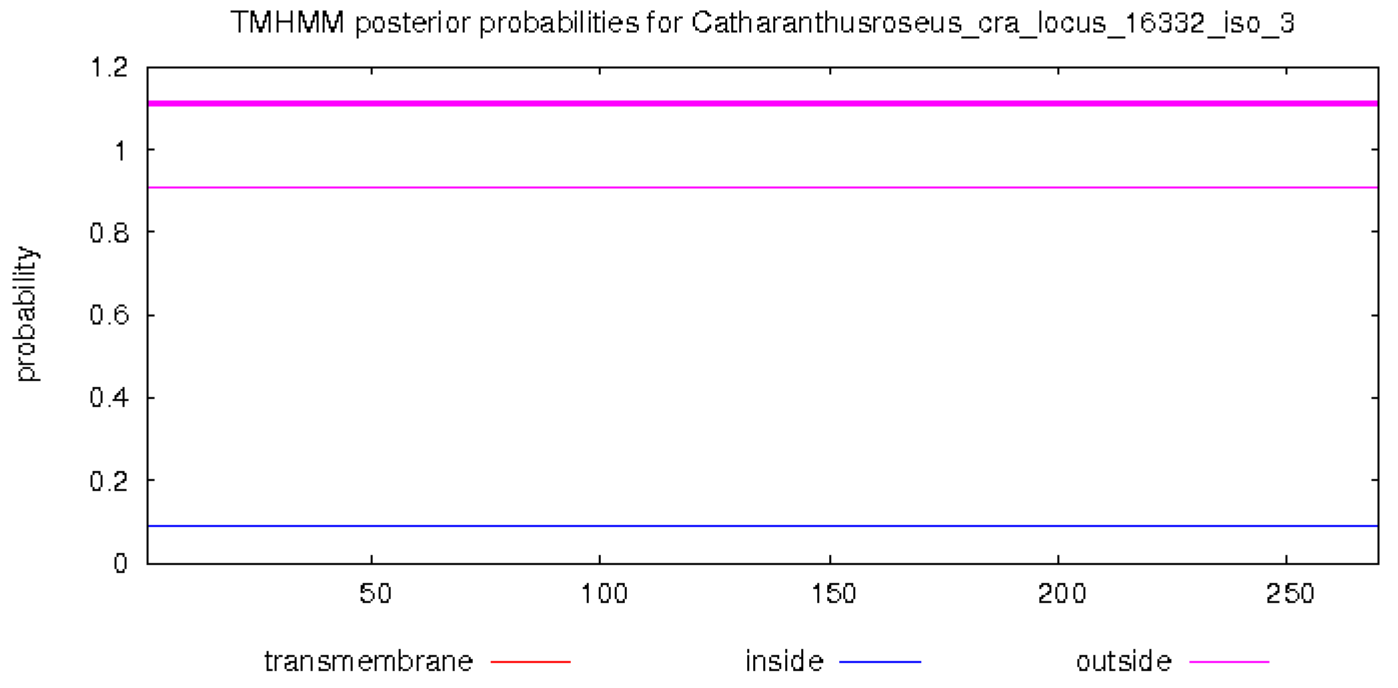

plot in postscript, script for making the plot in gnuplot, data for plot

---

```
# Catharanthusroseus_cra_locus_16332_iso_4 Length: 385
# Catharanthusroseus_cra_locus_16332_iso_4 Number of predicted TMHs:  0
# Catharanthusroseus_cra_locus_16332_iso_4 Exp number of AAs in TMHs: 0.05666
# Catharanthusroseus_cra_locus_16332_iso_4 Exp number, first 60 AAs:  0
# Catharanthusroseus_cra_locus_16332_iso_4 Total prob of N-in:        0.00737
Catharanthusroseus_cra_locus_16332_iso_4	TMHMM2.0	outside	     1   385
```

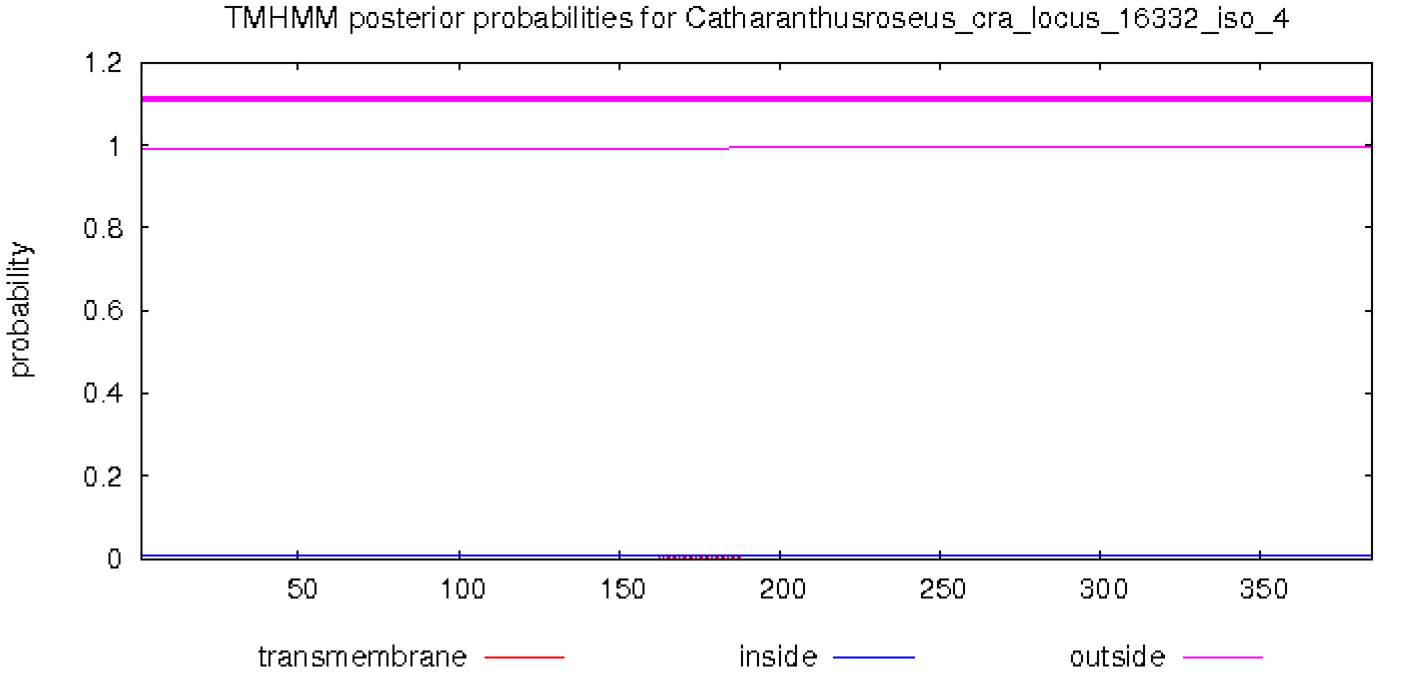

plot in postscript, script for making the plot in gnuplot, data for plot

---

```
# Catharanthusroseus_cra_locus_16332_iso_5 Length: 351
# Catharanthusroseus_cra_locus_16332_iso_5 Number of predicted TMHs:  0
# Catharanthusroseus_cra_locus_16332_iso_5 Exp number of AAs in TMHs: 0.0006
# Catharanthusroseus_cra_locus_16332_iso_5 Exp number, first 60 AAs:  0
# Catharanthusroseus_cra_locus_16332_iso_5 Total prob of N-in:        0.00790
Catharanthusroseus_cra_locus_16332_iso_5	TMHMM2.0	outside	     1   351
```

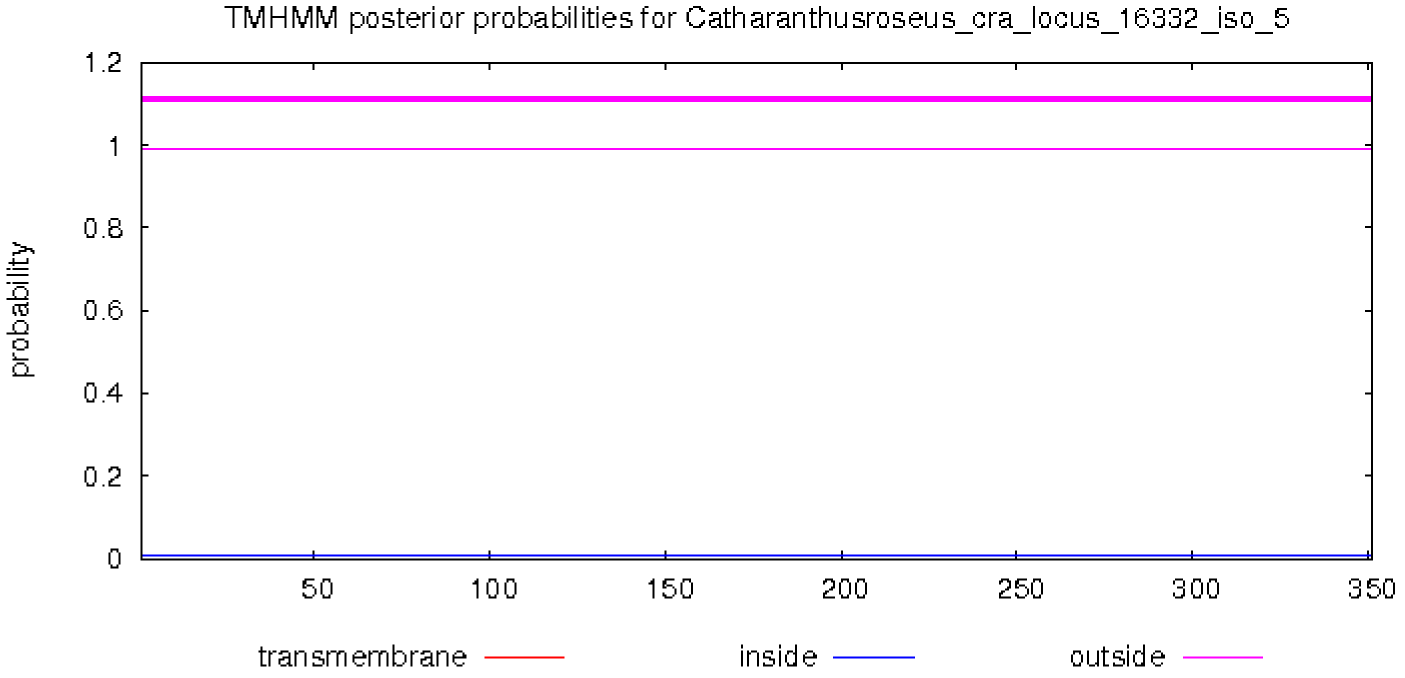

plot in postscript, script for making the plot in gnuplot, data for plot

---

```
# Catharanthusroseus_cra_locus_16366_iso_1 Length: 222
# Catharanthusroseus_cra_locus_16366_iso_1 Number of predicted TMHs:  0
# Catharanthusroseus_cra_locus_16366_iso_1 Exp number of AAs in TMHs: 0.00376
# Catharanthusroseus_cra_locus_16366_iso_1 Exp number, first 60 AAs:  0
# Catharanthusroseus_cra_locus_16366_iso_1 Total prob of N-in:        0.14975
Catharanthusroseus_cra_locus_16366_iso_1	TMHMM2.0	outside	     1   222
```

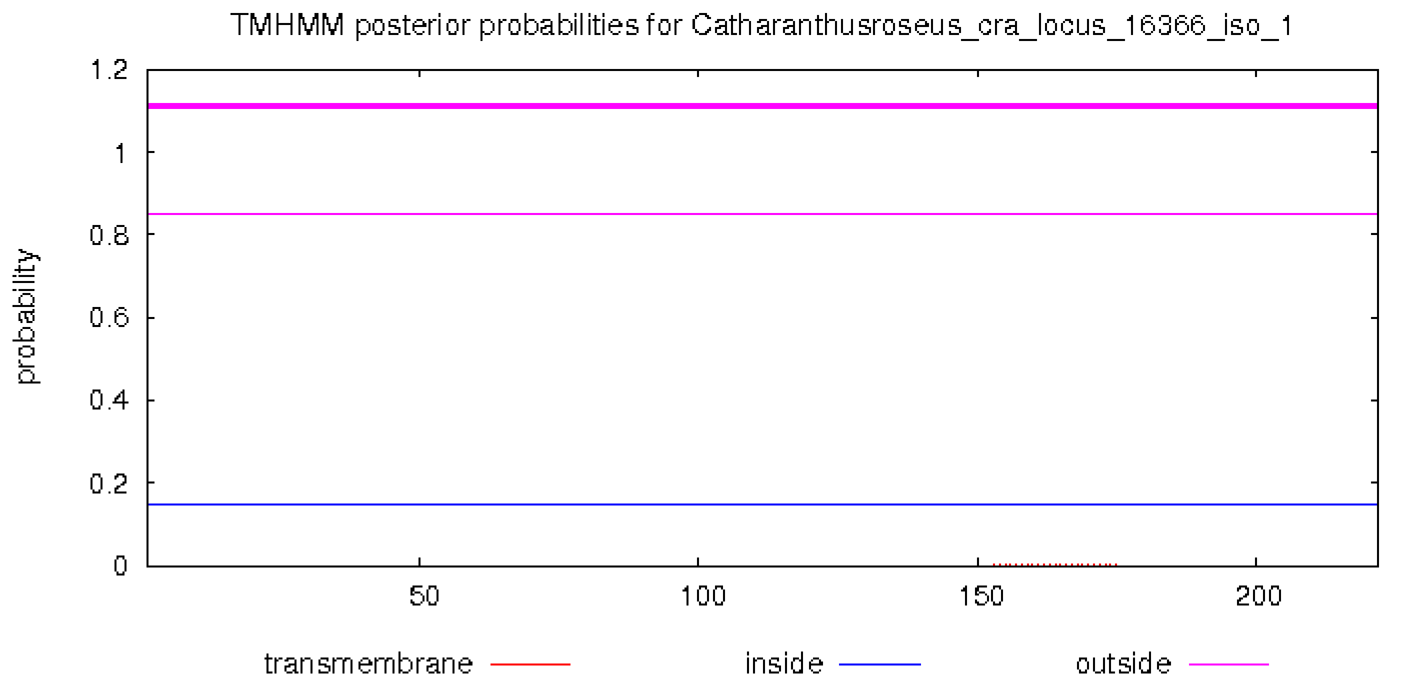

plot in postscript, script for making the plot in gnuplot, data for plot

---

```
# Catharanthusroseus_cra_locus_16366_iso_2 Length: 301
# Catharanthusroseus_cra_locus_16366_iso_2 Number of predicted TMHs:  0
# Catharanthusroseus_cra_locus_16366_iso_2 Exp number of AAs in TMHs: 0.00749
# Catharanthusroseus_cra_locus_16366_iso_2 Exp number, first 60 AAs:  0
# Catharanthusroseus_cra_locus_16366_iso_2 Total prob of N-in:        0.06177
Catharanthusroseus_cra_locus_16366_iso_2	TMHMM2.0	outside	     1   301
```

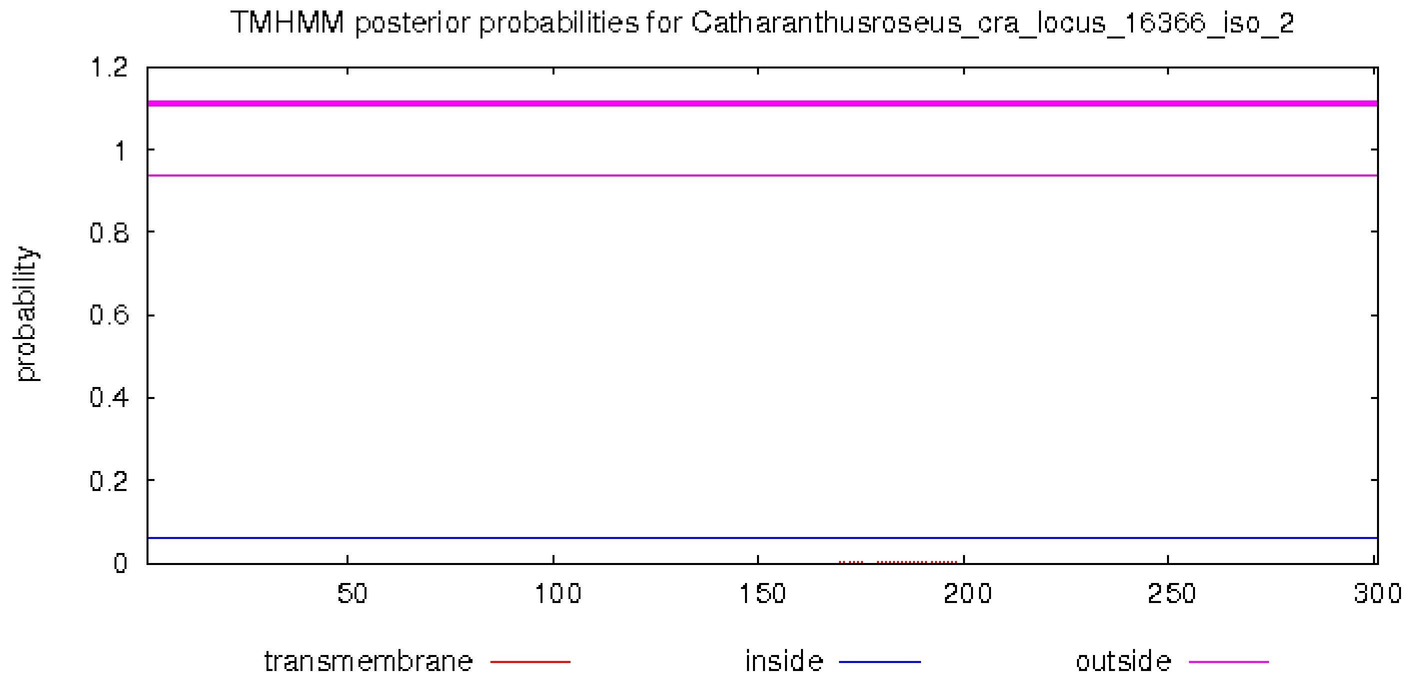

plot in postscript, script for making the plot in gnuplot, data for plot

---

```
# Catharanthusroseus_cra_locus_16366_iso_5 Length: 311
# Catharanthusroseus_cra_locus_16366_iso_5 Number of predicted TMHs:  0
# Catharanthusroseus_cra_locus_16366_iso_5 Exp number of AAs in TMHs: 0.00824
# Catharanthusroseus_cra_locus_16366_iso_5 Exp number, first 60 AAs:  0
# Catharanthusroseus_cra_locus_16366_iso_5 Total prob of N-in:        0.05487
Catharanthusroseus_cra_locus_16366_iso_5        TMHMM2.0        outside      1   311
```

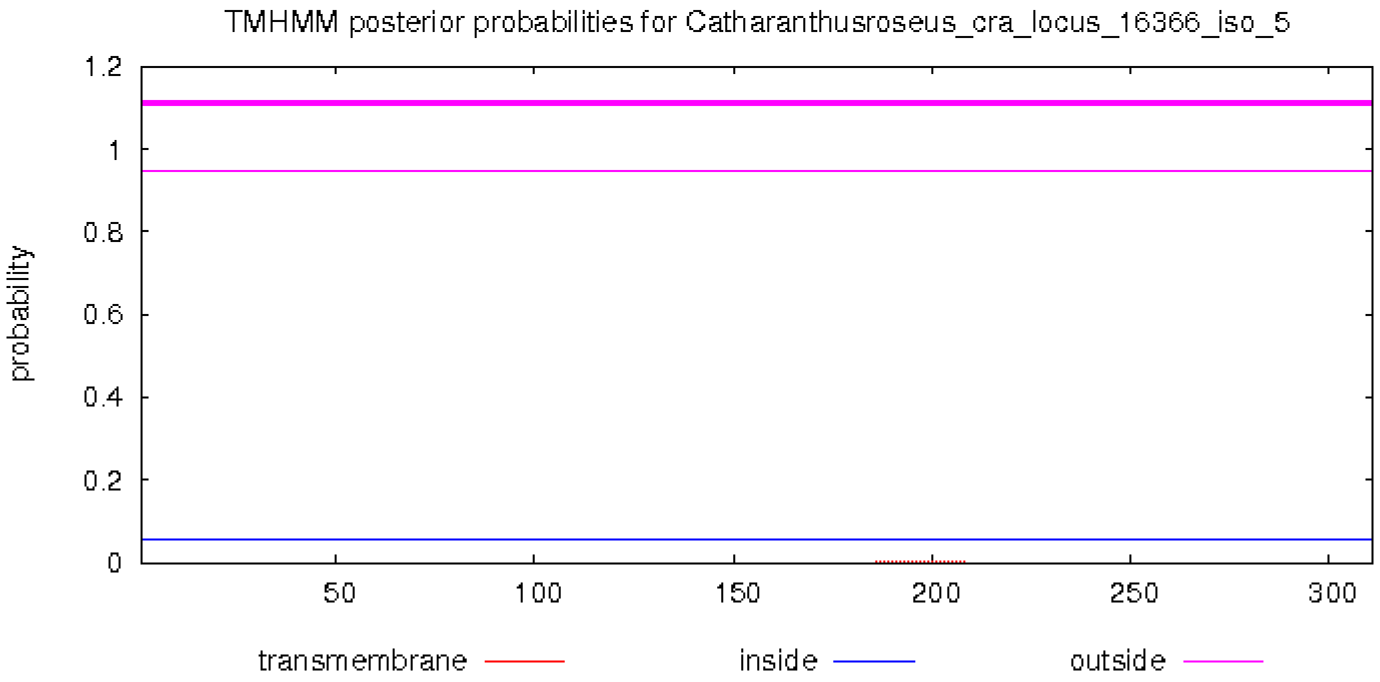

plot in postscript, script for making the plot in gnuplot, data for plot

---

```
# Catharanthusroseus_cra_locus_16413_iso_1 Length: 278
# Catharanthusroseus_cra_locus_16413_iso_1 Number of predicted TMHs:  0
# Catharanthusroseus_cra_locus_16413_iso_1 Exp number of AAs in TMHs: 0
# Catharanthusroseus_cra_locus_16413_iso_1 Exp number, first 60 AAs:  0
# Catharanthusroseus_cra_locus_16413_iso_1 Total prob of N-in:        0.01067
Catharanthusroseus_cra_locus_16413_iso_1        TMHMM2.0        outside      1   278
```

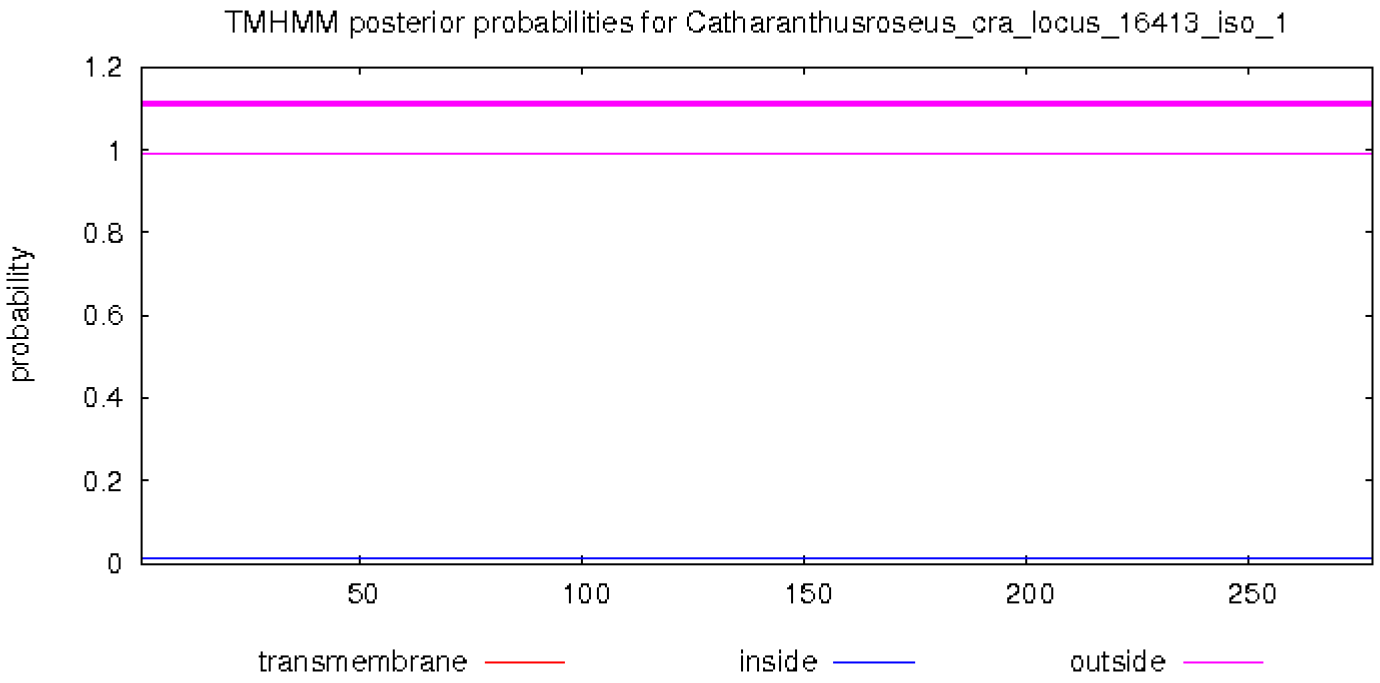

plot in postscript, script for making the plot in gnuplot, data for plot

---

```
# Catharanthusroseus_cra_locus_16413_iso_2 Length: 103
# Catharanthusroseus_cra_locus_16413_iso_2 Number of predicted TMHs:  0
# Catharanthusroseus_cra_locus_16413_iso_2 Exp number of AAs in TMHs: 0.00704
# Catharanthusroseus_cra_locus_16413_iso_2 Exp number, first 60 AAs:  0.00303
# Catharanthusroseus_cra_locus_16413_iso_2 Total prob of N-in:        0.09902
Catharanthusroseus_cra_locus_16413_iso_2	TMHMM2.0	outside	     1   103
```

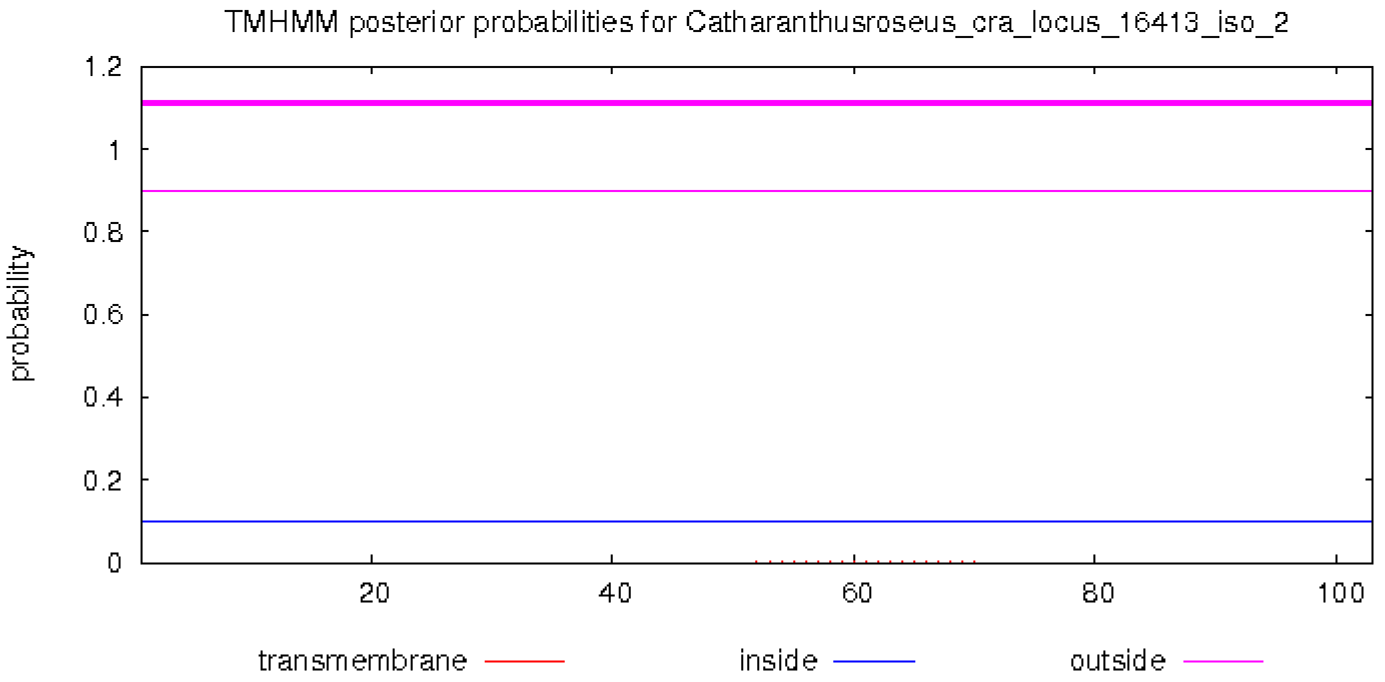

plot in postscript, script for making the plot in gnuplot, data for plot

---

```
# Catharanthusroseus_cra_locus_17418_iso_1 Length: 390
# Catharanthusroseus_cra_locus_17418_iso_1 Number of predicted TMHs:  0
# Catharanthusroseus_cra_locus_17418_iso_1 Exp number of AAs in TMHs: 0
# Catharanthusroseus_cra_locus_17418_iso_1 Exp number, first 60 AAs:  0
# Catharanthusroseus_cra_locus_17418_iso_1 Total prob of N-in:        0.01447
Catharanthusroseus_cra_locus_17418_iso_1	TMHMM2.0	outside	     1   390
```

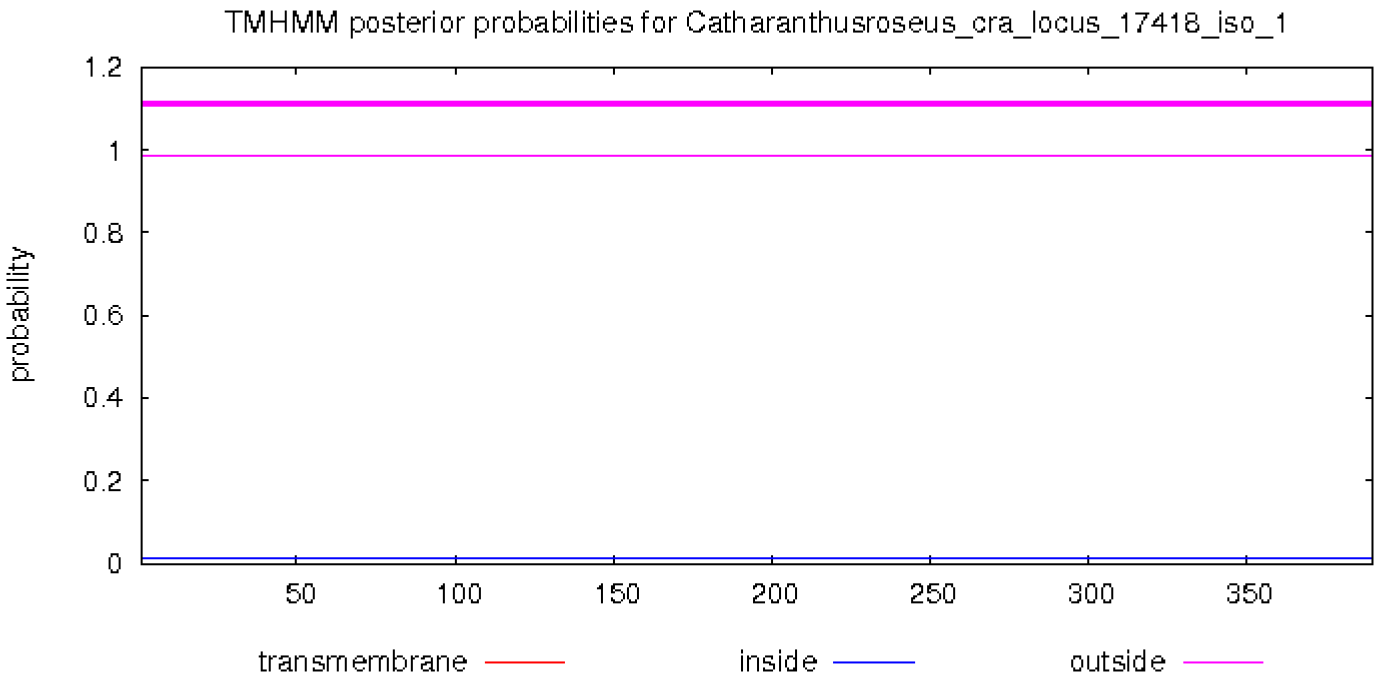

plot in postscript, script for making the plot in gnuplot, data for plot

---

```
# Catharanthusroseus_cra_locus_18519_iso_1 Length: 372
# Catharanthusroseus_cra_locus_18519_iso_1 Number of predicted TMHs:  0
# Catharanthusroseus_cra_locus_18519_iso_1 Exp number of AAs in TMHs: 0.00147
# Catharanthusroseus_cra_locus_18519_iso_1 Exp number, first 60 AAs:  0
# Catharanthusroseus_cra_locus_18519_iso_1 Total prob of N-in:        0.03207
Catharanthusroseus_cra_locus_18519_iso_1        TMHMM2.0        outside      1   372
```

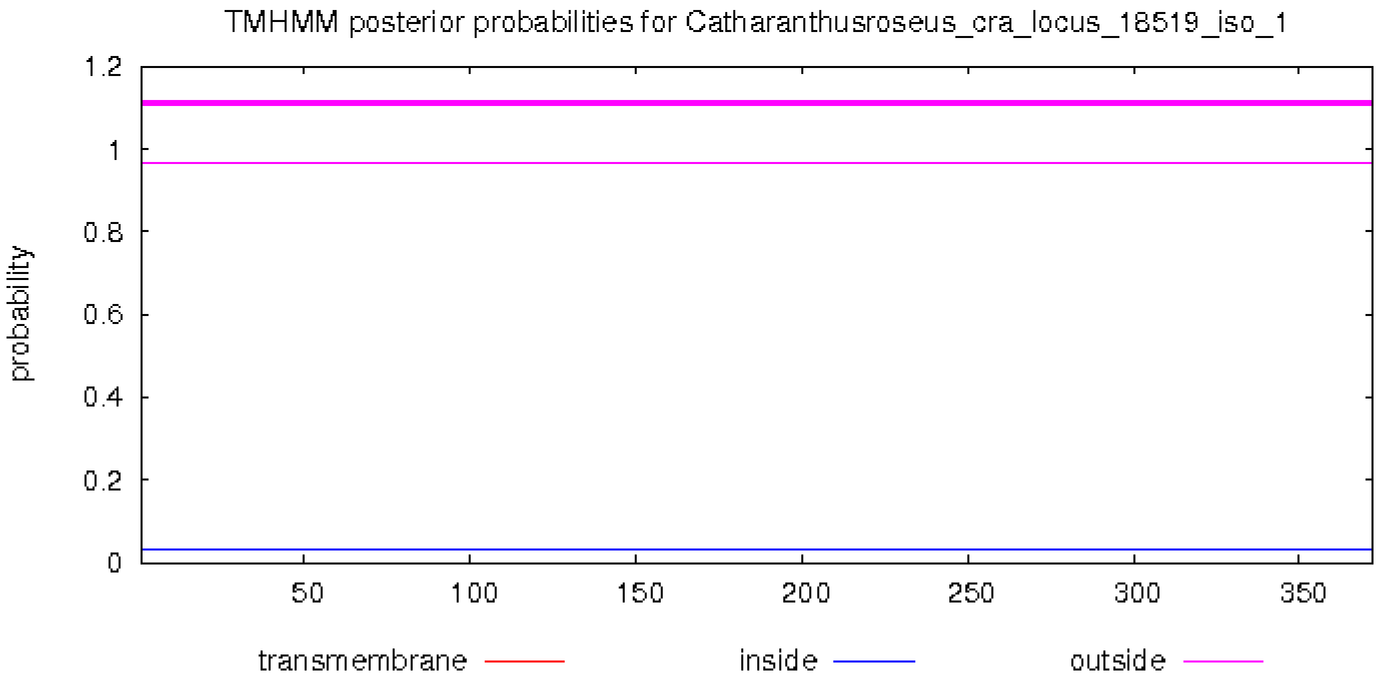

plot in postscript, script for making the plot in gnuplot, data for plot

---

```
# Catharanthusroseus_cra_locus_18775_iso_1 Length: 383
# Catharanthusroseus_cra_locus_18775_iso_1 Number of predicted TMHs:  0
# Catharanthusroseus_cra_locus_18775_iso_1 Exp number of AAs in TMHs: 0.01017
# Catharanthusroseus_cra_locus_18775_iso_1 Exp number, first 60 AAs:  0.00022
# Catharanthusroseus_cra_locus_18775_iso_1 Total prob of N-in:        0.02826
Catharanthusroseus_cra_locus_18775_iso_1        TMHMM2.0        outside      1   383
```

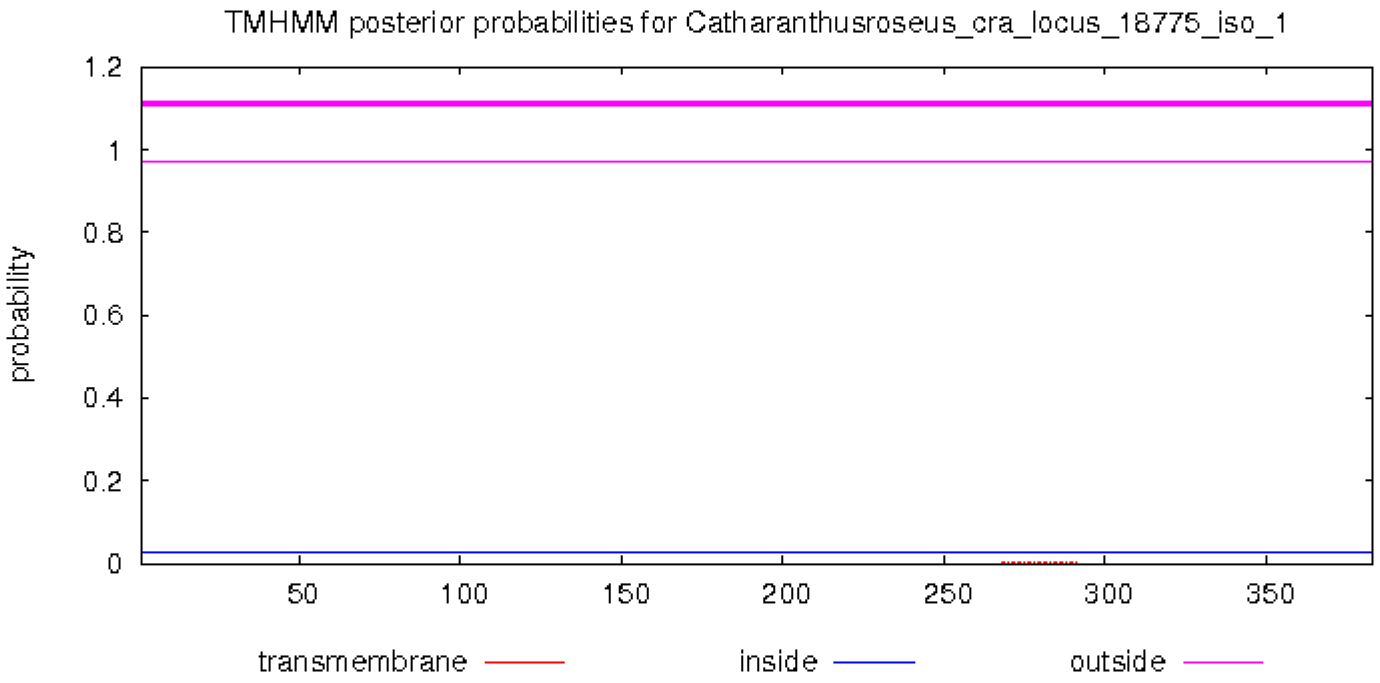

plot in postscript, script for making the plot in gnuplot, data for plot

---

```
# Catharanthusroseus_cra_locus_19122_iso_1 Length: 196
# Catharanthusroseus_cra_locus_19122_iso_1 Number of predicted TMHs:  0
# Catharanthusroseus_cra_locus_19122_iso_1 Exp number of AAs in TMHs: 0.00062
# Catharanthusroseus_cra_locus_19122_iso_1 Exp number, first 60 AAs:  0
# Catharanthusroseus_cra_locus_19122_iso_1 Total prob of N-in:        0.03562
Catharanthusroseus_cra_locus_19122_iso_1	TMHMM2.0	outside	     1   196
```

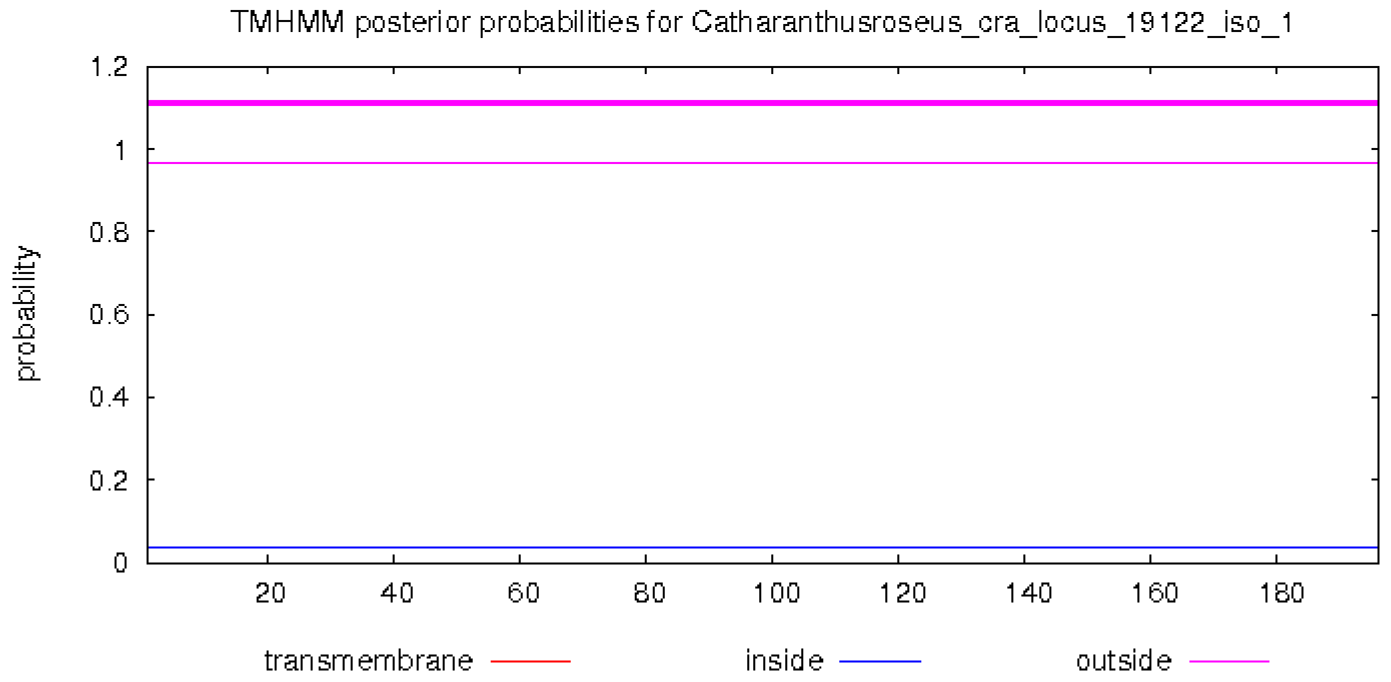

plot in postscript, script for making the plot in gnuplot, data for plot

```
# Catharanthusroseus_cra_locus_19122_iso_2 Length: 196
# Catharanthusroseus_cra_locus_19122_iso_2 Number of predicted TMHs:  0
# Catharanthusroseus_cra_locus_19122_iso_2 Exp number of AAs in TMHs: 0.00062
# Catharanthusroseus_cra_locus_19122_iso_2 Exp number, first 60 AAs:  0
# Catharanthusroseus_cra_locus_19122_iso_2 Total prob of N-in:        0.03562
Catharanthusroseus_cra_locus_19122_iso_2	TMHMM2.0	outside	     1   196
```

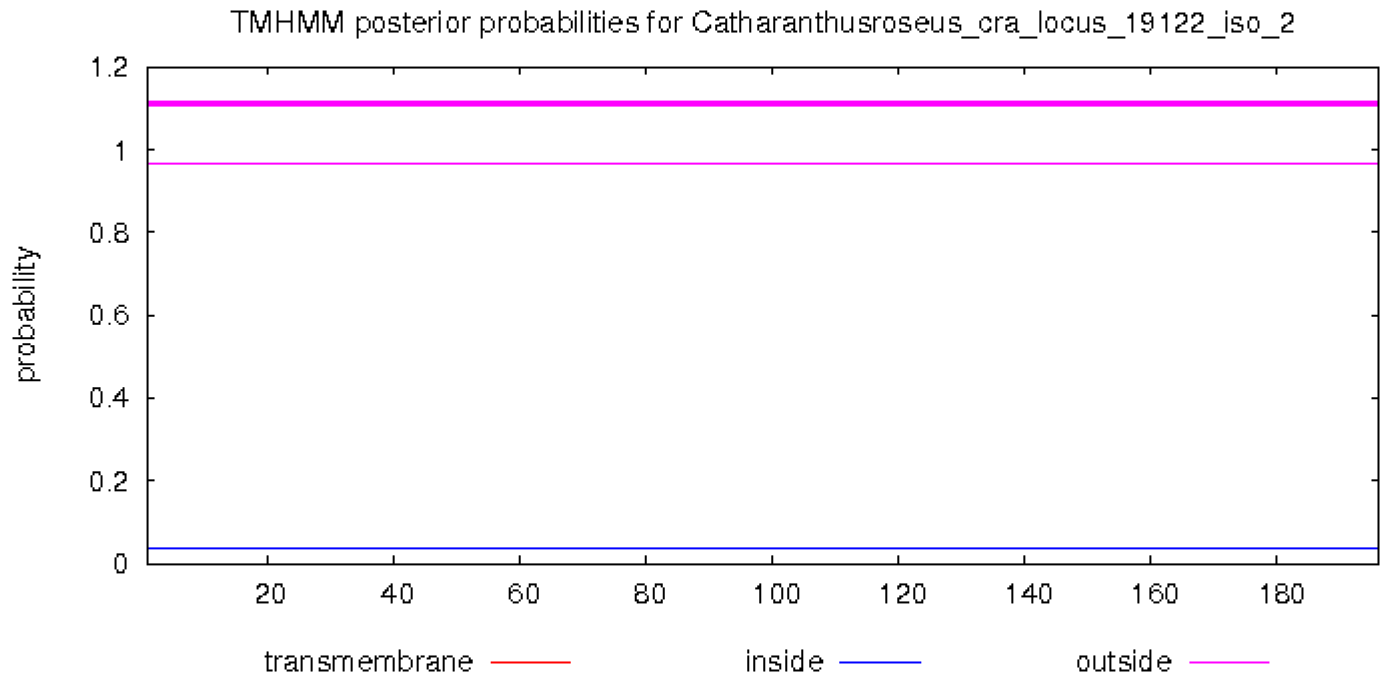

plot in postscript, script for making the plot in gnuplot, data for plot

```
# Catharanthusroseus_cra_locus_19122_iso_3 Length: 196
# Catharanthusroseus_cra_locus_19122_iso_3 Number of predicted TMHs:  0
# Catharanthusroseus_cra_locus_19122_iso_3 Exp number of AAs in TMHs: 0.00062
# Catharanthusroseus_cra_locus_19122_iso_3 Exp number, first 60 AAs:  0
# Catharanthusroseus_cra_locus_19122_iso_3 Total prob of N-in:        0.03562
Catharanthusroseus_cra_locus_19122_iso_3	TMHMM2.0	outside	     1   196
```

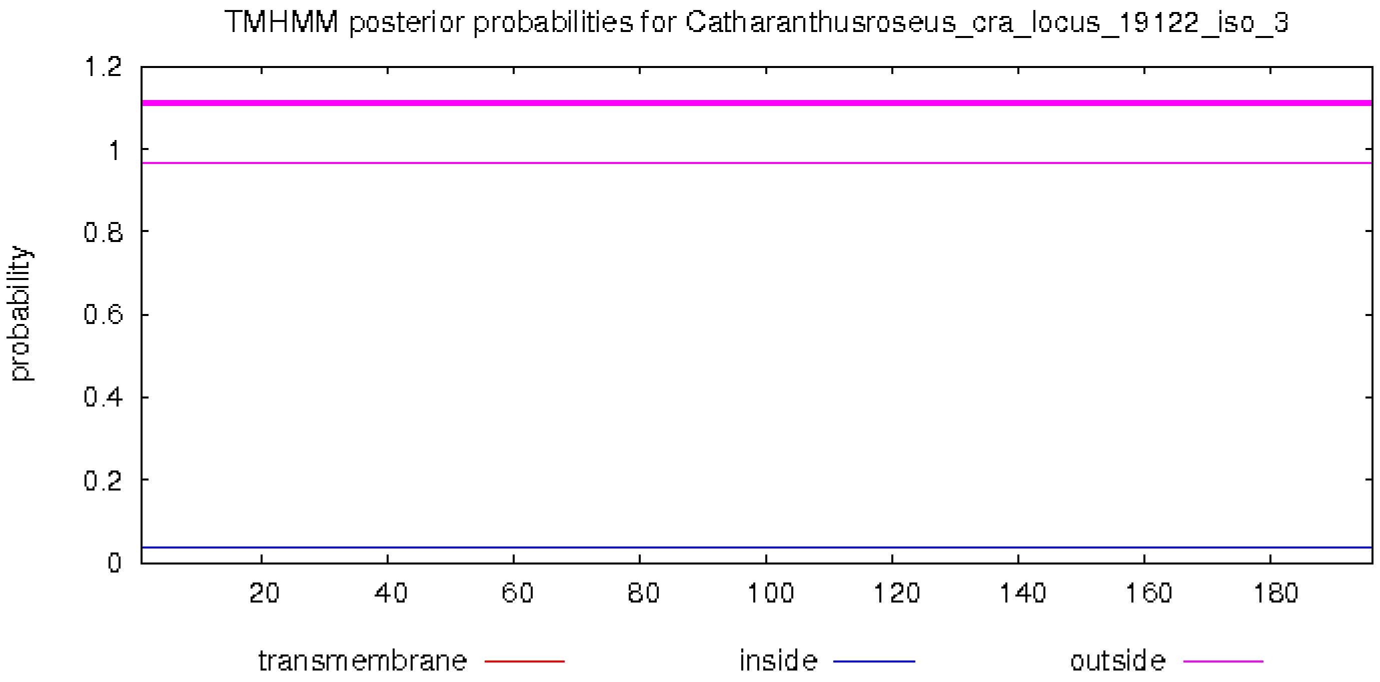

plot in postscript, script for making the plot in gnuplot, data for plot

---

```
# Catharanthusroseus_cra_locus_19122_iso_4 Length: 196
# Catharanthusroseus_cra_locus_19122_iso_4 Number of predicted TMHs:  0
# Catharanthusroseus_cra_locus_19122_iso_4 Exp number of AAs in TMHs: 0.00062
# Catharanthusroseus_cra_locus_19122_iso_4 Exp number, first 60 AAs:  0
# Catharanthusroseus_cra_locus_19122_iso_4 Total prob of N-in:        0.03562
Catharanthusroseus_cra_locus_19122_iso_4	TMHMM2.0	outside	     1   196
```

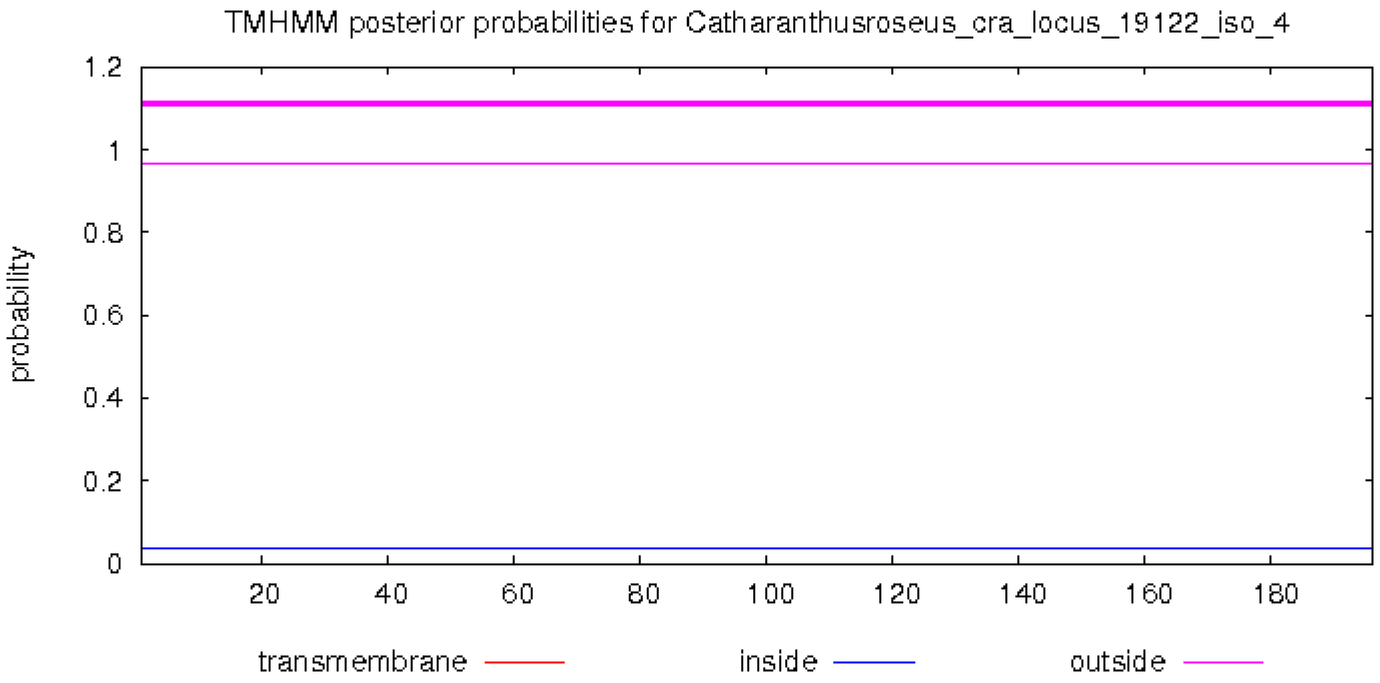

plot in postscript, script for making the plot in gnuplot, data for plot

---

```
# Catharanthusroseus_cra_locus_19122_iso_5 Length: 196
# Catharanthusroseus_cra_locus_19122_iso_5 Number of predicted TMHs:  0
# Catharanthusroseus_cra_locus_19122_iso_5 Exp number of AAs in TMHs: 0.00062
# Catharanthusroseus_cra_locus_19122_iso_5 Exp number, first 60 AAs:  0
# Catharanthusroseus_cra_locus_19122_iso_5 Total prob of N-in:        0.03562
Catharanthusroseus_cra_locus_19122_iso_5	TMHMM2.0	outside	     1   196
```

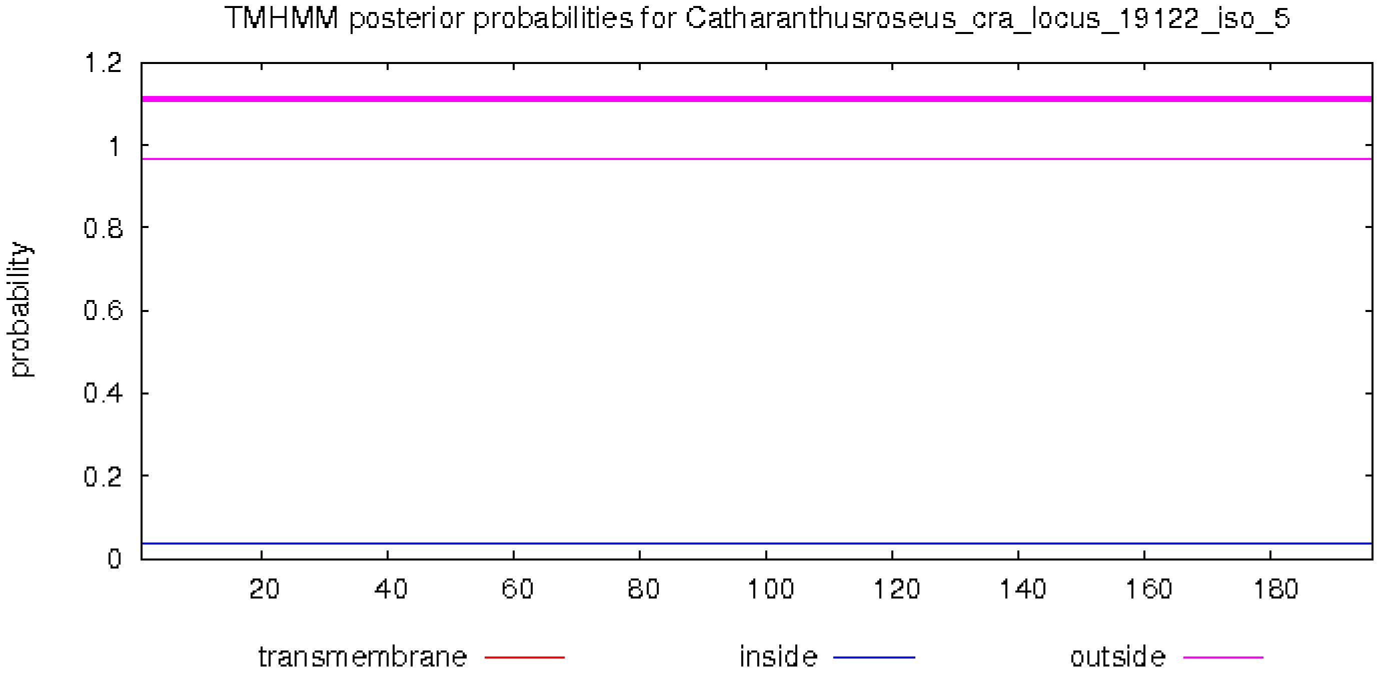

plot in postscript, script for making the plot in gnuplot, data for plot

---

```
# Catharanthusroseus_cra_locus_21223_iso_1 Length: 367
# Catharanthusroseus_cra_locus_21223_iso_1 Number of predicted TMHs:  0
# Catharanthusroseus_cra_locus_21223_iso_1 Exp number of AAs in TMHs: 0.00497
# Catharanthusroseus_cra_locus_21223_iso_1 Exp number, first 60 AAs:  0
# Catharanthusroseus_cra_locus_21223_iso_1 Total prob of N-in:        0.00738
Catharanthusroseus_cra_locus_21223_iso_1	TMHMM2.0	outside	     1   367
```

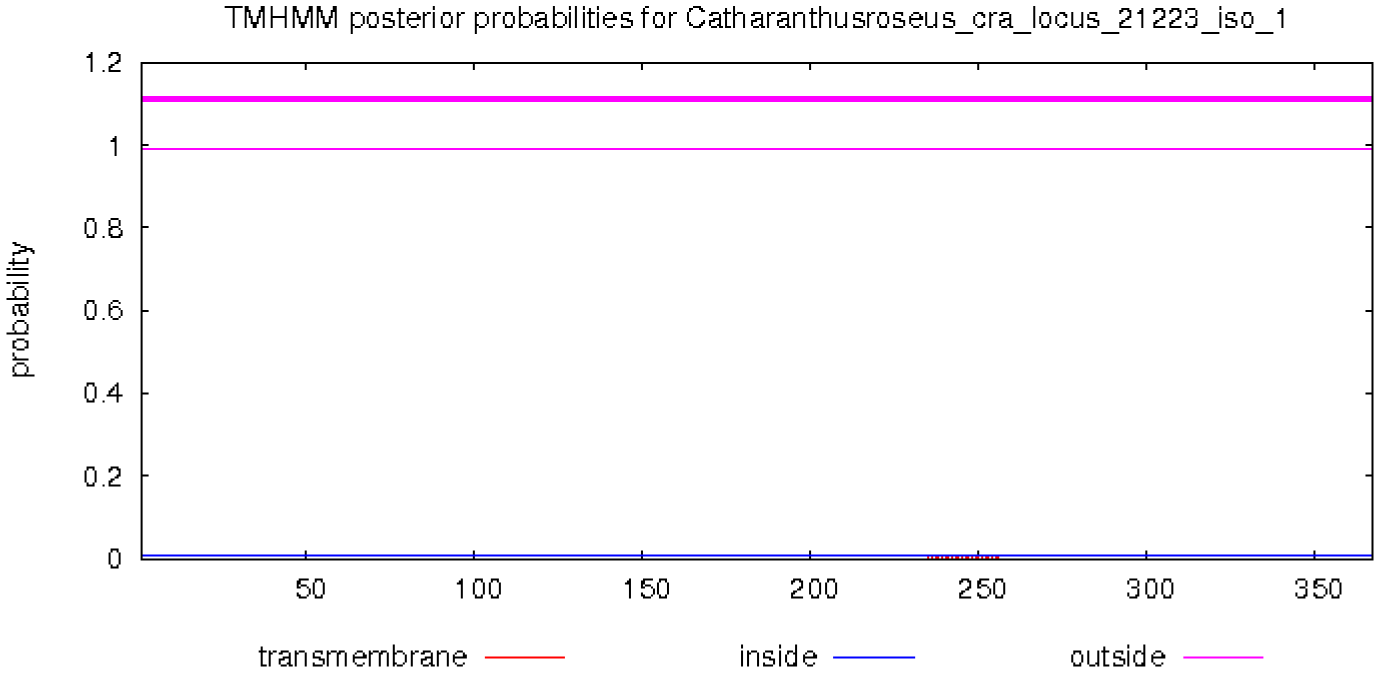

plot in postscript, script for making the plot in gnuplot, data for plot

---

```
# Catharanthusroseus_cra_locus_21223_iso_2 Length: 388
# Catharanthusroseus_cra_locus_21223_iso_2 Number of predicted TMHs:  0
# Catharanthusroseus_cra_locus_21223_iso_2 Exp number of AAs in TMHs: 0.00043
# Catharanthusroseus_cra_locus_21223_iso_2 Exp number, first 60 AAs:  0
# Catharanthusroseus_cra_locus_21223_iso_2 Total prob of N-in:        0.00564
Catharanthusroseus_cra_locus_21223_iso_2        TMHMM2.0        outside      1   388
```

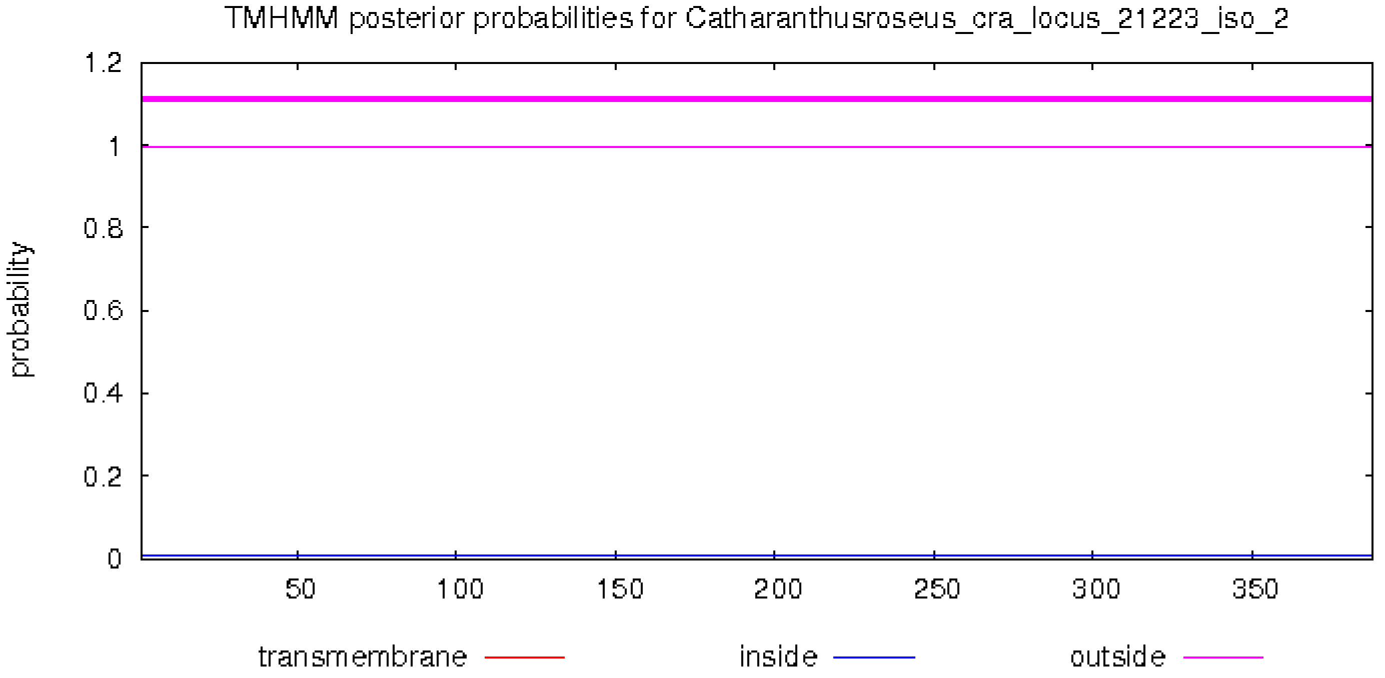

plot in postscript, script for making the plot in gnuplot, data for plot

---

```
# Catharanthusroseus_cra_locus_2128_iso_10 Length: 524
# Catharanthusroseus_cra_locus_2128_iso_10 Number of predicted TMHs:  0
# Catharanthusroseus_cra_locus_2128_iso_10 Exp number of AAs in TMHs: 0.00013
# Catharanthusroseus_cra_locus_2128_iso_10 Exp number, first 60 AAs:  0
# Catharanthusroseus_cra_locus_2128_iso_10 Total prob of N-in:        0.00076
Catharanthusroseus_cra_locus_2128_iso_10        TMHMM2.0        outside      1   524
```

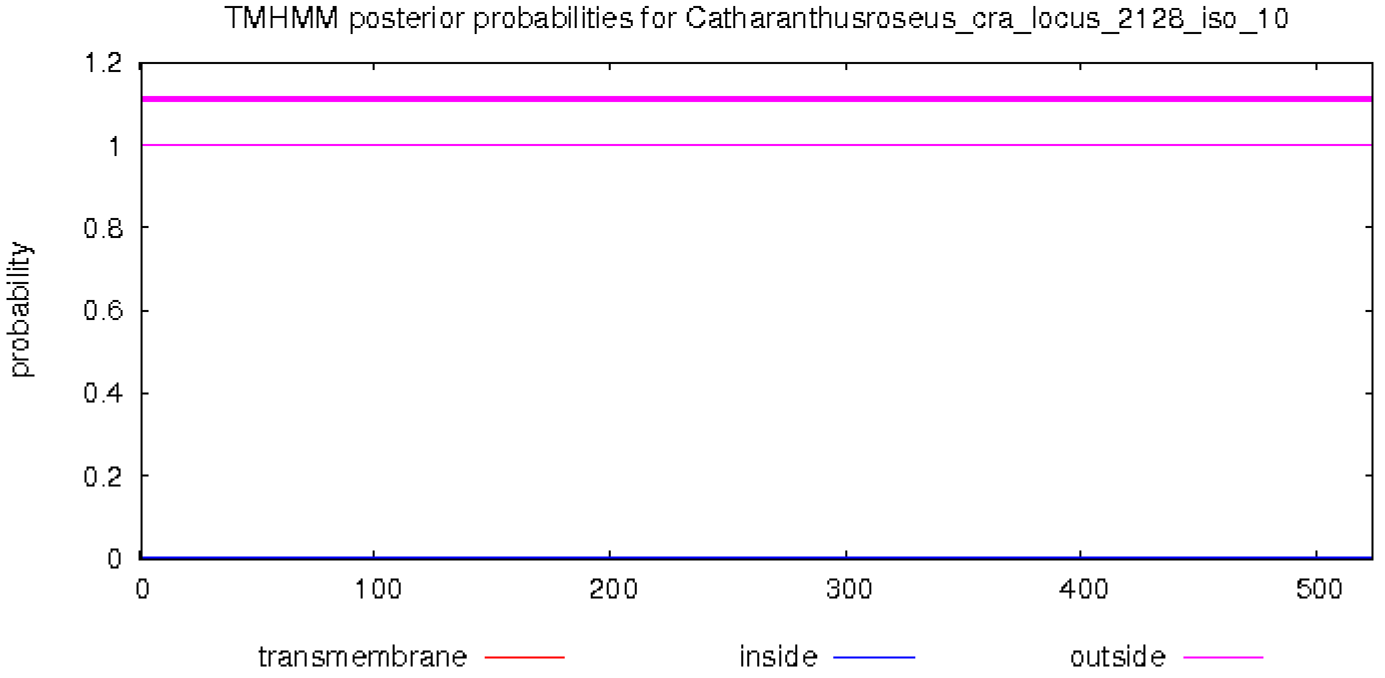

plot in postscript, script for making the plot in gnuplot, data for plot

---

```
# Catharanthusroseus_cra_locus_2128_iso_1 Length: 377
# Catharanthusroseus_cra_locus_2128_iso_1 Number of predicted TMHs:  0
# Catharanthusroseus_cra_locus_2128_iso_1 Exp number of AAs in TMHs: 0.00013
# Catharanthusroseus_cra_locus_2128_iso_1 Exp number, first 60 AAs:  0
# Catharanthusroseus_cra_locus_2128_iso_1 Total prob of N-in:        0.00471
Catharanthusroseus_cra_locus_2128_iso_1 TMHMM2.0        outside      1   377
```

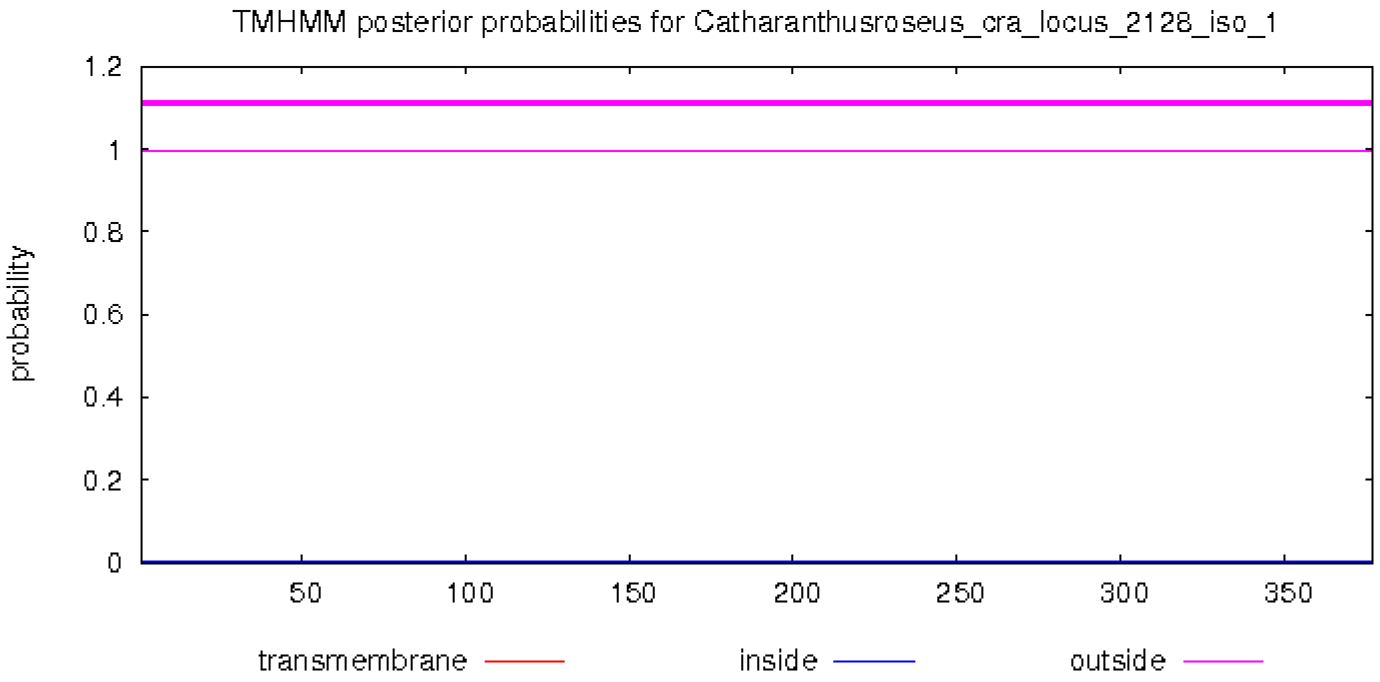

plot in postscript, script for making the plot in gnuplot, data for plot

---

```
# Catharanthusroseus_cra_locus_2128_iso_5 Length: 468
# Catharanthusroseus_cra_locus_2128_iso_5 Number of predicted TMHs:  0
# Catharanthusroseus_cra_locus_2128_iso_5 Exp number of AAs in TMHs: 0.00013
# Catharanthusroseus_cra_locus_2128_iso_5 Exp number, first 60 AAs:  0
# Catharanthusroseus_cra_locus_2128_iso_5 Total prob of N-in:        0.00577
Catharanthusroseus_cra_locus_2128_iso_5 TMHMM2.0        outside      1   468
```

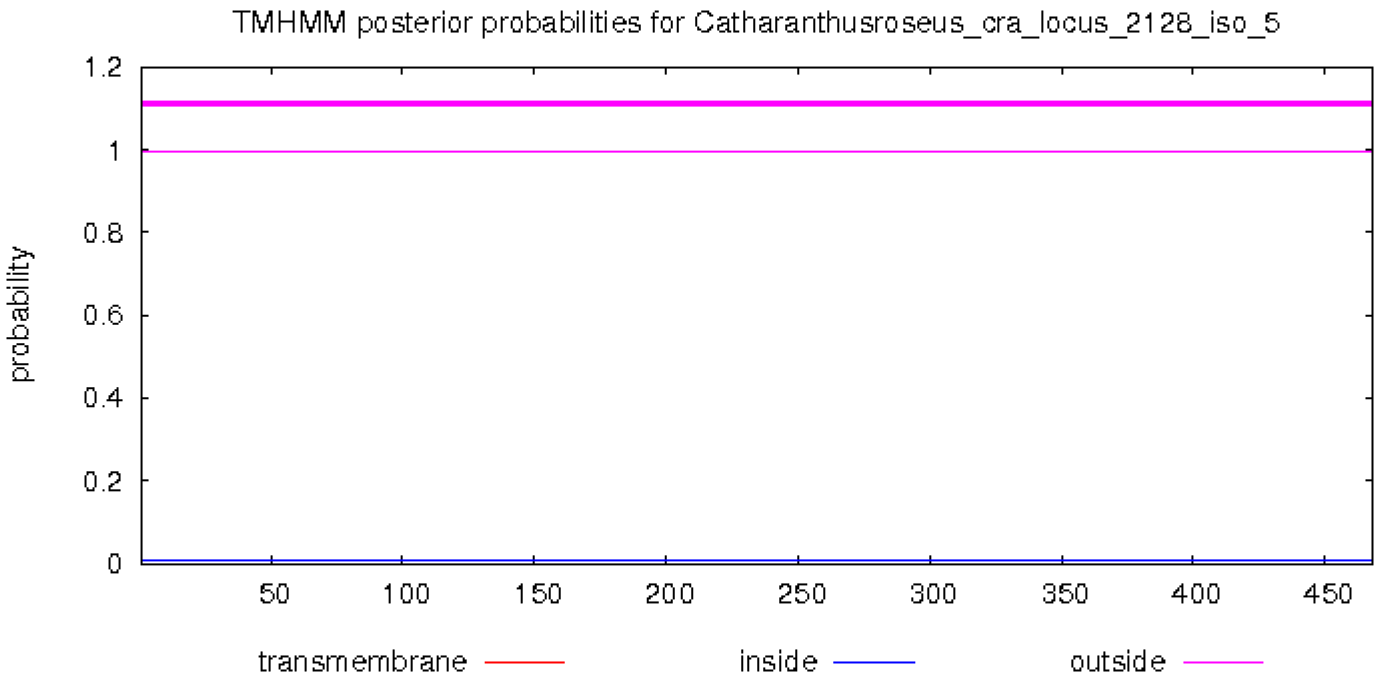

plot in postscript, script for making the plot in gnuplot, data for plot

---

```
# Catharanthusroseus_cra_locus_2128_iso_6 Length: 553
# Catharanthusroseus_cra_locus_2128_iso_6 Number of predicted TMHs:  0
# Catharanthusroseus_cra_locus_2128_iso_6 Exp number of AAs in TMHs: 0.00083
# Catharanthusroseus_cra_locus_2128_iso_6 Exp number, first 60 AAs:  0
# Catharanthusroseus_cra_locus_2128_iso_6 Total prob of N-in:        0.00056
Catharanthusroseus_cra_locus_2128_iso_6 TMHMM2.0        outside      1   553
```

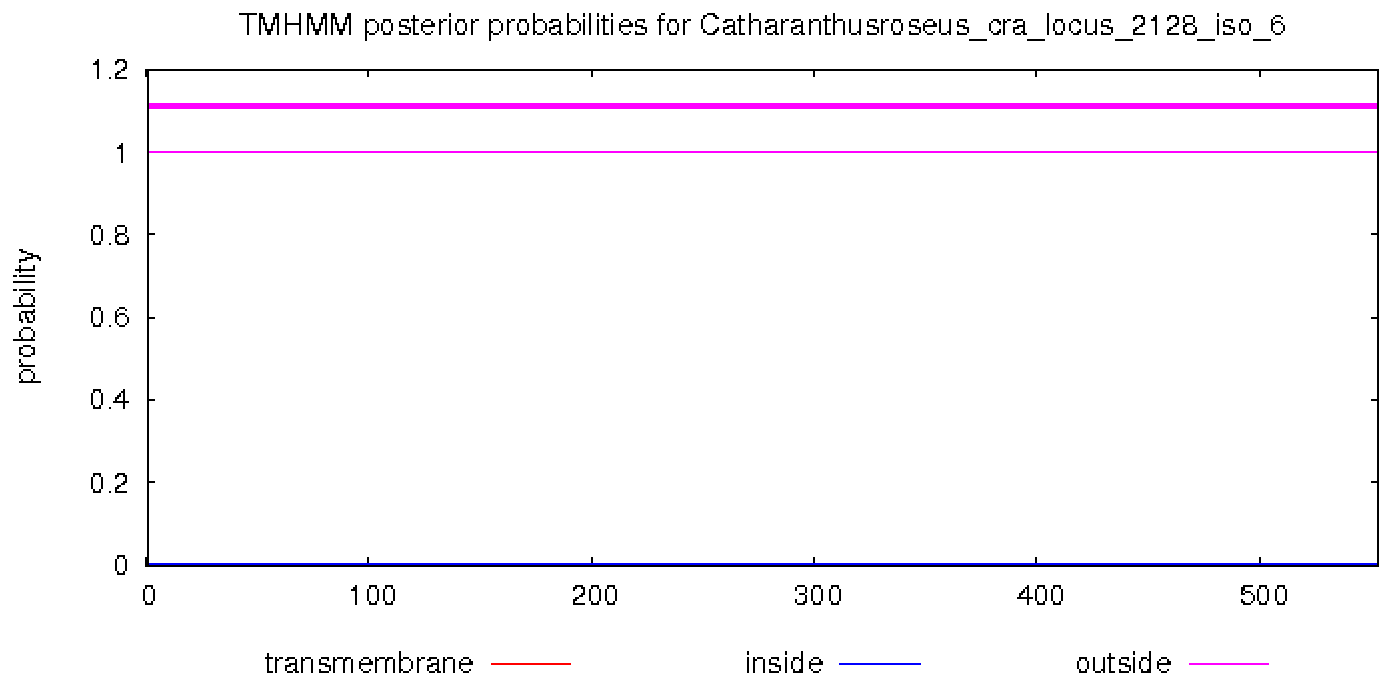

plot in postscript, script for making the plot in gnuplot, data for plot

---

```
# Catharanthusroseus_cra_locus_21743_iso_1 Length: 172
# Catharanthusroseus_cra_locus_21743_iso_1 Number of predicted TMHs:  0
# Catharanthusroseus_cra_locus_21743_iso_1 Exp number of AAs in TMHs: 0
# Catharanthusroseus_cra_locus_21743_iso_1 Exp number, first 60 AAs:  0
# Catharanthusroseus_cra_locus_21743_iso_1 Total prob of N-in:        0.03564
Catharanthusroseus_cra_locus_21743_iso_1        TMHMM2.0        outside      1   172
```

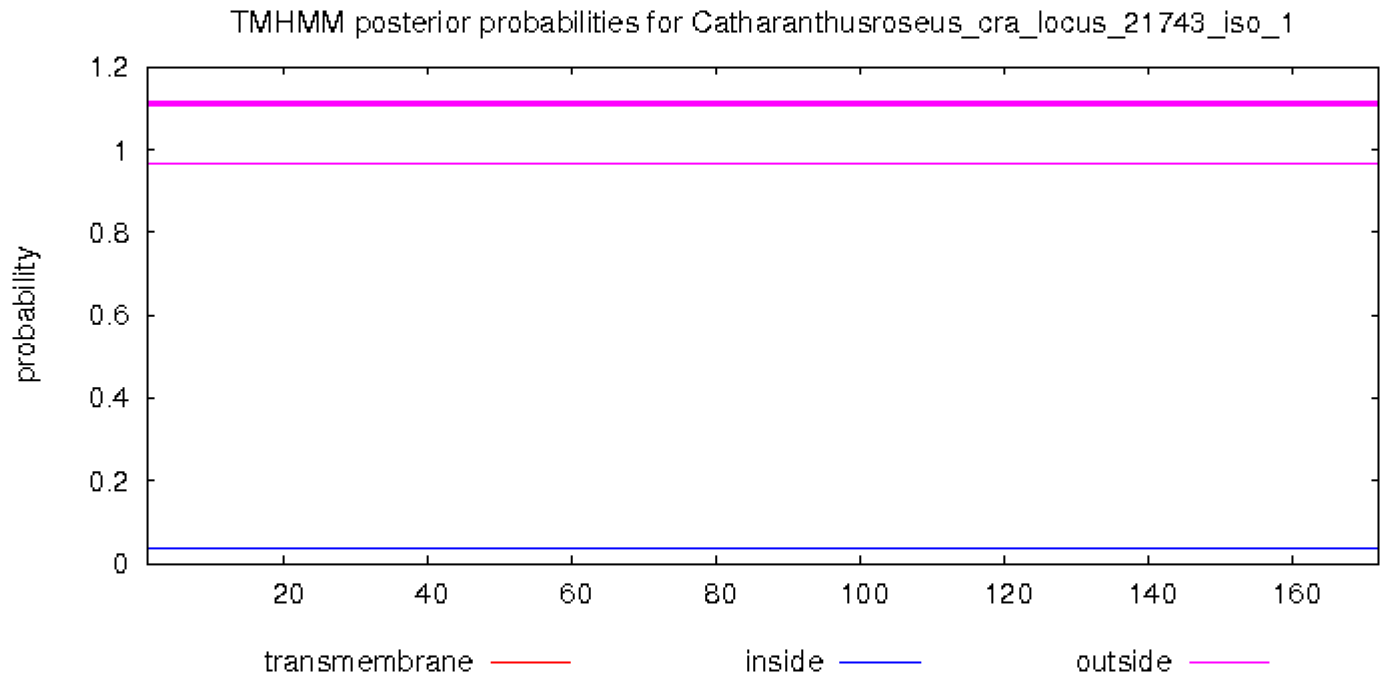

plot in postscript, script for making the plot in gnuplot, data for plot

---

```
# Catharanthusroseus_cra_locus_21743_iso_2 Length: 142
# Catharanthusroseus_cra_locus_21743_iso_2 Number of predicted TMHs:  0
# Catharanthusroseus_cra_locus_21743_iso_2 Exp number of AAs in TMHs: 0
# Catharanthusroseus_cra_locus_21743_iso_2 Exp number, first 60 AAs:  0
# Catharanthusroseus_cra_locus_21743_iso_2 Total prob of N-in:        0.52442
Catharanthusroseus_cra_locus_21743_iso_2        TMHMM2.0        inside       1   142
```

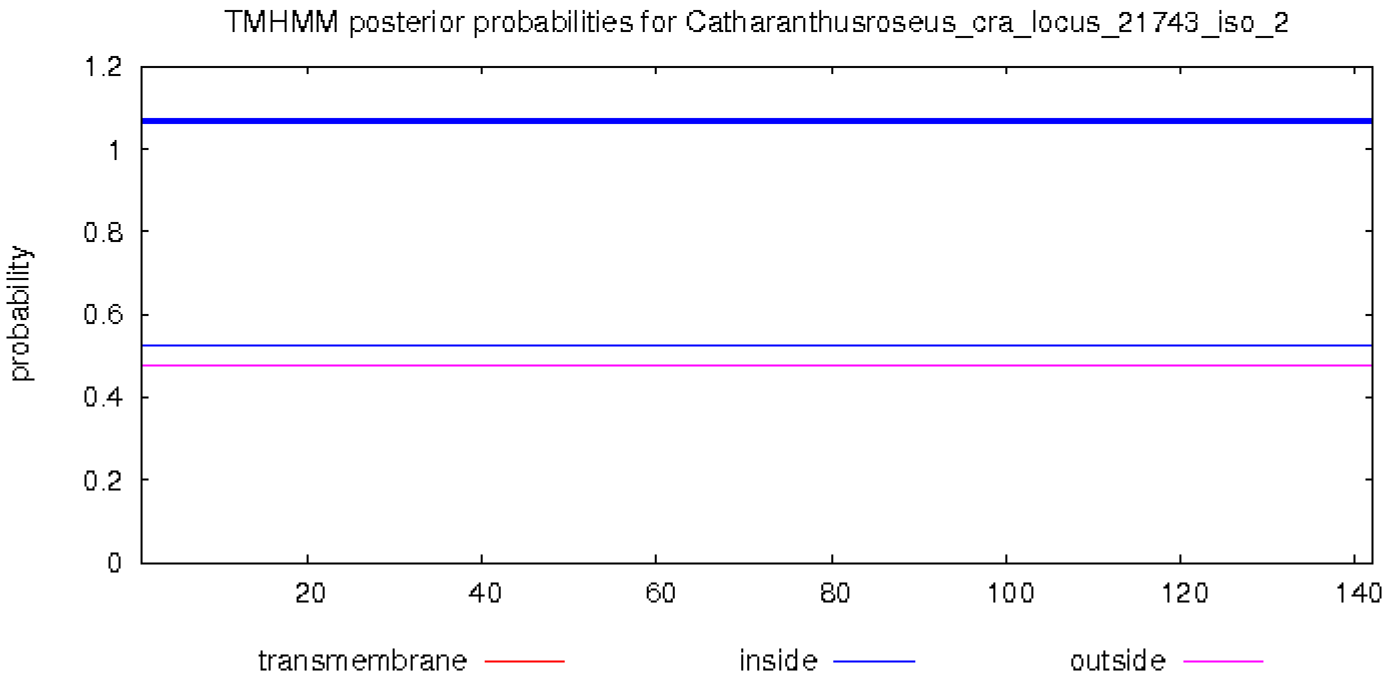

plot in postscript, script for making the plot in gnuplot, data for plot

---

```
# Catharanthusroseus_cra_locus_22400_iso_1 Length: 631
# Catharanthusroseus_cra_locus_22400_iso_1 Number of predicted TMHs:  1
# Catharanthusroseus_cra_locus_22400_iso_1 Exp number of AAs in TMHs: 21.33683
# Catharanthusroseus_cra_locus_22400_iso_1 Exp number, first 60 AAs:  0
# Catharanthusroseus_cra_locus_22400_iso_1 Total prob of N-in:        0.00059
Catharanthusroseus_cra_locus_22400_iso_1        TMHMM2.0        outside      1   604
Catharanthusroseus_cra_locus_22400_iso_1        TMHMM2.0        TMhelix    605   627
Catharanthusroseus_cra_locus_22400_iso_1        TMHMM2.0        inside     628   631
```

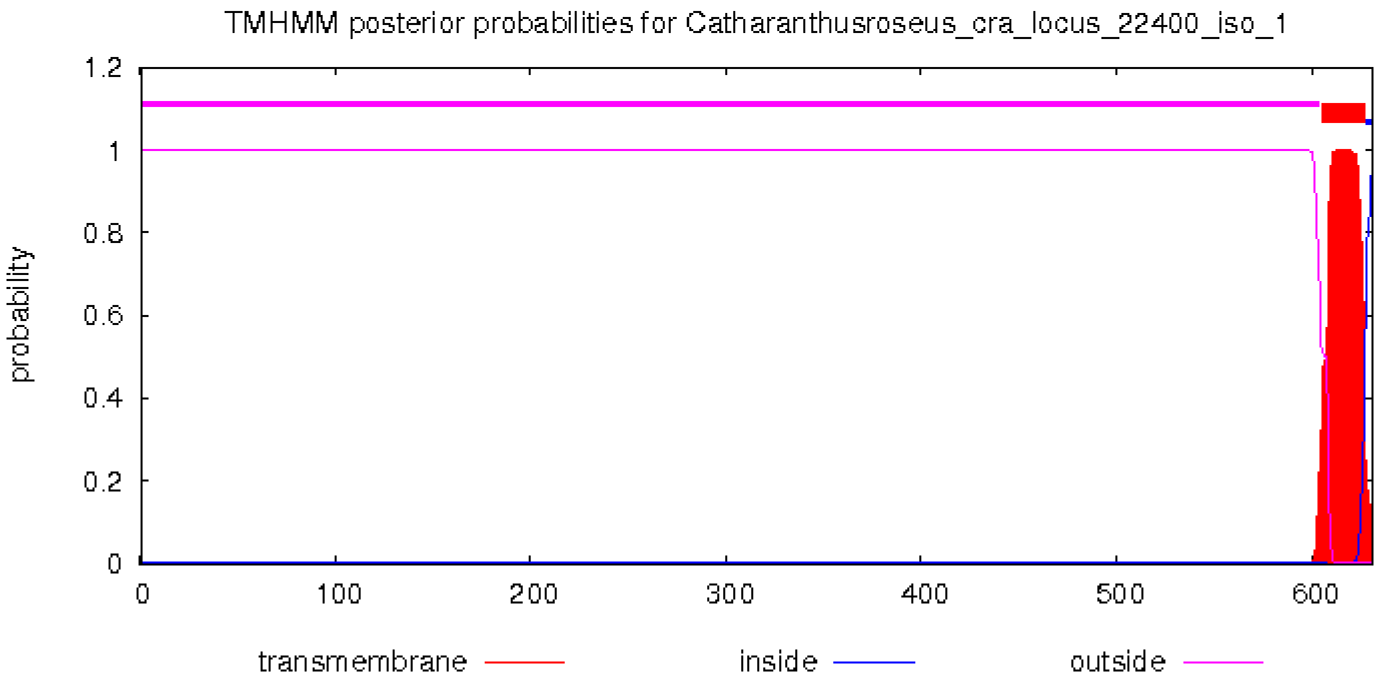

plot in postscript, script for making the plot in gnuplot, data for plot

```
# Catharanthusroseus_cra_locus_22400_iso_2 Length: 631
# Catharanthusroseus_cra_locus_22400_iso_2 Number of predicted TMHs:  1
# Catharanthusroseus_cra_locus_22400_iso_2 Exp number of AAs in TMHs: 21.33683
# Catharanthusroseus_cra_locus_22400_iso_2 Exp number, first 60 AAs:  0
# Catharanthusroseus_cra_locus_22400_iso_2 Total prob of N-in:        0.00059
Catharanthusroseus_cra_locus_22400_iso_2        TMHMM2.0        outside      1   604
Catharanthusroseus_cra_locus_22400_iso_2        TMHMM2.0        TMhelix    605   627
Catharanthusroseus_cra_locus_22400_iso_2        TMHMM2.0        inside     628   631
```

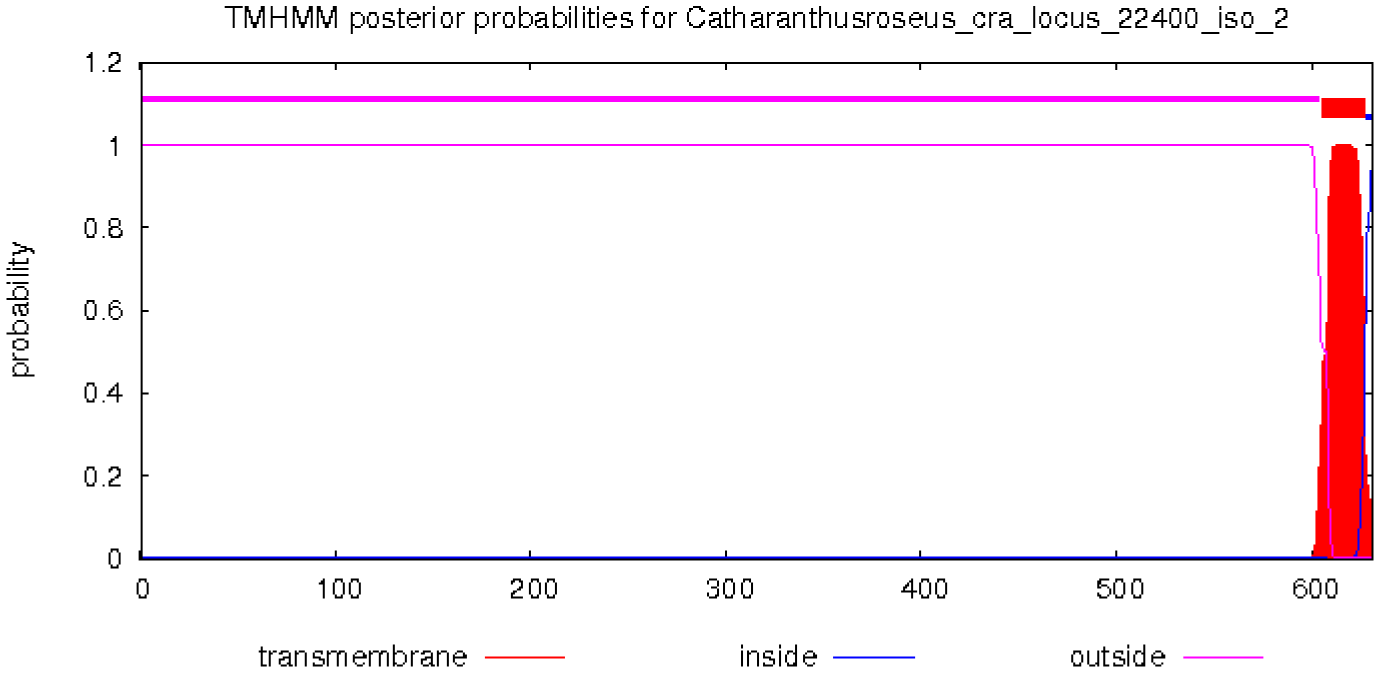

plot in postscript, script for making the plot in gnuplot, data for plot

```
# Catharanthusroseus_cra_locus_22400_iso_3 Length: 631
# Catharanthusroseus_cra_locus_22400_iso_3 Number of predicted TMHs:  1
# Catharanthusroseus_cra_locus_22400_iso_3 Exp number of AAs in TMHs: 21.33683
# Catharanthusroseus_cra_locus_22400_iso_3 Exp number, first 60 AAs:  0
# Catharanthusroseus_cra_locus_22400_iso_3 Total prob of N-in:        0.00059
Catharanthusroseus_cra_locus_22400_iso_3        TMHMM2.0        outside      1   604
Catharanthusroseus_cra_locus_22400_iso_3        TMHMM2.0        TMhelix    605   627
Catharanthusroseus_cra_locus_22400_iso_3        TMHMM2.0        inside     628   631
```

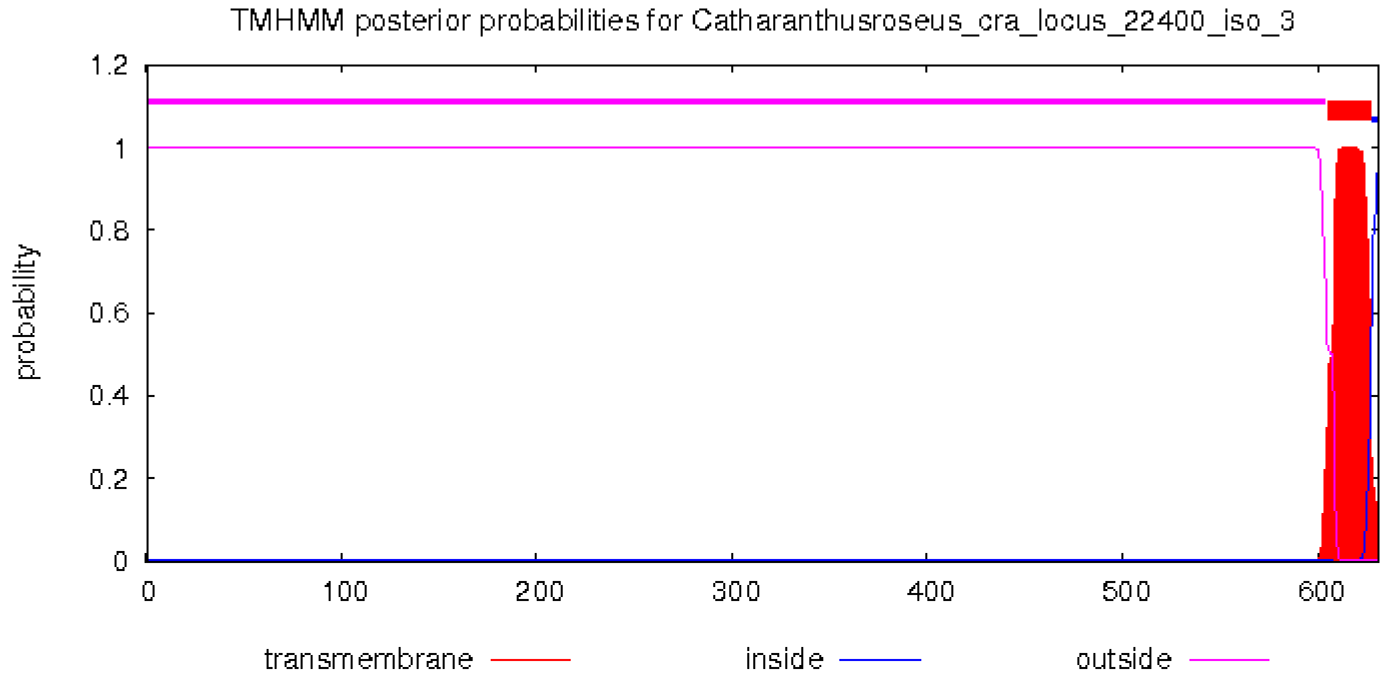

plot in postscript, script for making the plot in gnuplot, data for plot

---

```
# Catharanthusroseus_cra_locus_22400_iso_4 Length: 418
# Catharanthusroseus_cra_locus_22400_iso_4 Number of predicted TMHs:  0
# Catharanthusroseus_cra_locus_22400_iso_4 Exp number of AAs in TMHs: 0.02644
# Catharanthusroseus_cra_locus_22400_iso_4 Exp number, first 60 AAs:  0
# Catharanthusroseus_cra_locus_22400_iso_4 Total prob of N-in:        0.00134
Catharanthusroseus_cra_locus_22400_iso_4        TMHMM2.0        outside      1   418
```

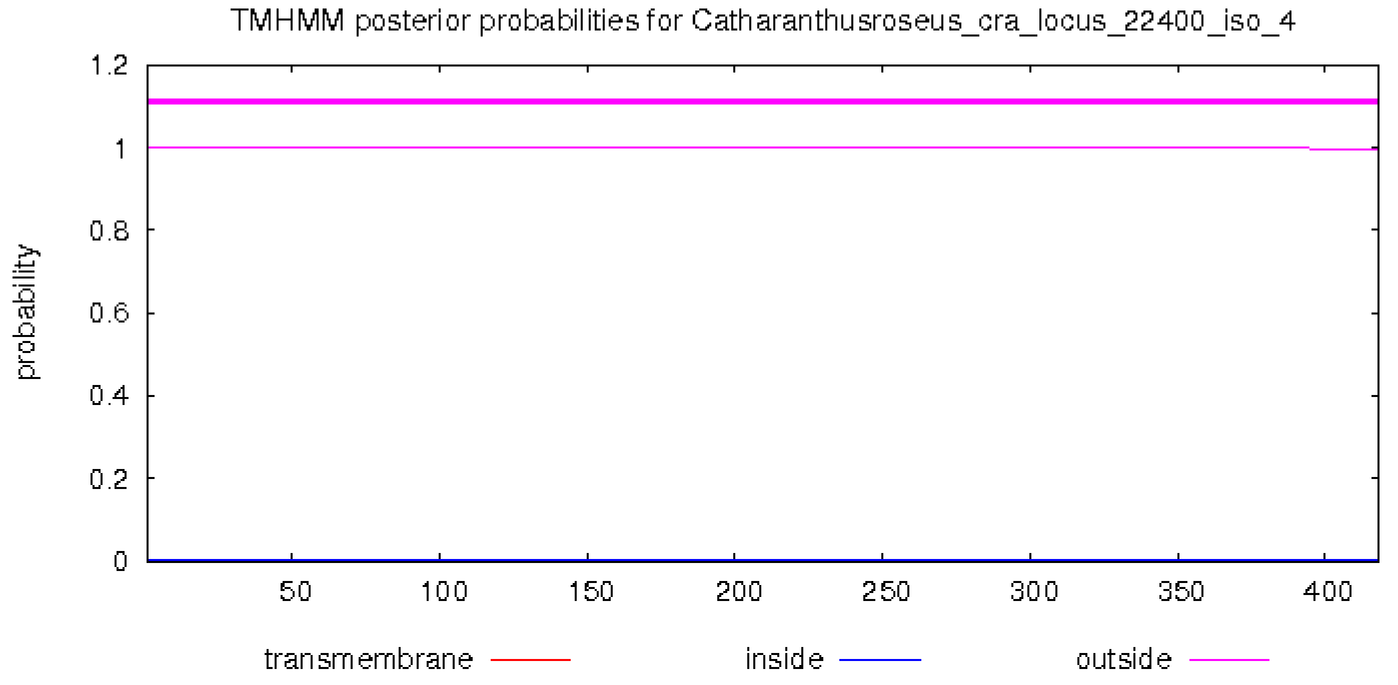

plot in postscript, script for making the plot in gnuplot, data for plot

---

```
# Catharanthusroseus_cra_locus_22400_iso_5 Length: 656
# Catharanthusroseus_cra_locus_22400_iso_5 Number of predicted TMHs:  1
# Catharanthusroseus_cra_locus_22400_iso_5 Exp number of AAs in TMHs: 21.34241
# Catharanthusroseus_cra_locus_22400_iso_5 Exp number, first 60 AAs:  0
# Catharanthusroseus_cra_locus_22400_iso_5 Total prob of N-in:        0.00043
Catharanthusroseus_cra_locus_22400_iso_5        TMHMM2.0        outside      1   629
```

```
Catharanthusroseus_cra_locus_22400_iso_5        TMHMM2.0        TMhelix    630   652
Catharanthusroseus_cra_locus_22400_iso_5        TMHMM2.0        inside     653   656
```

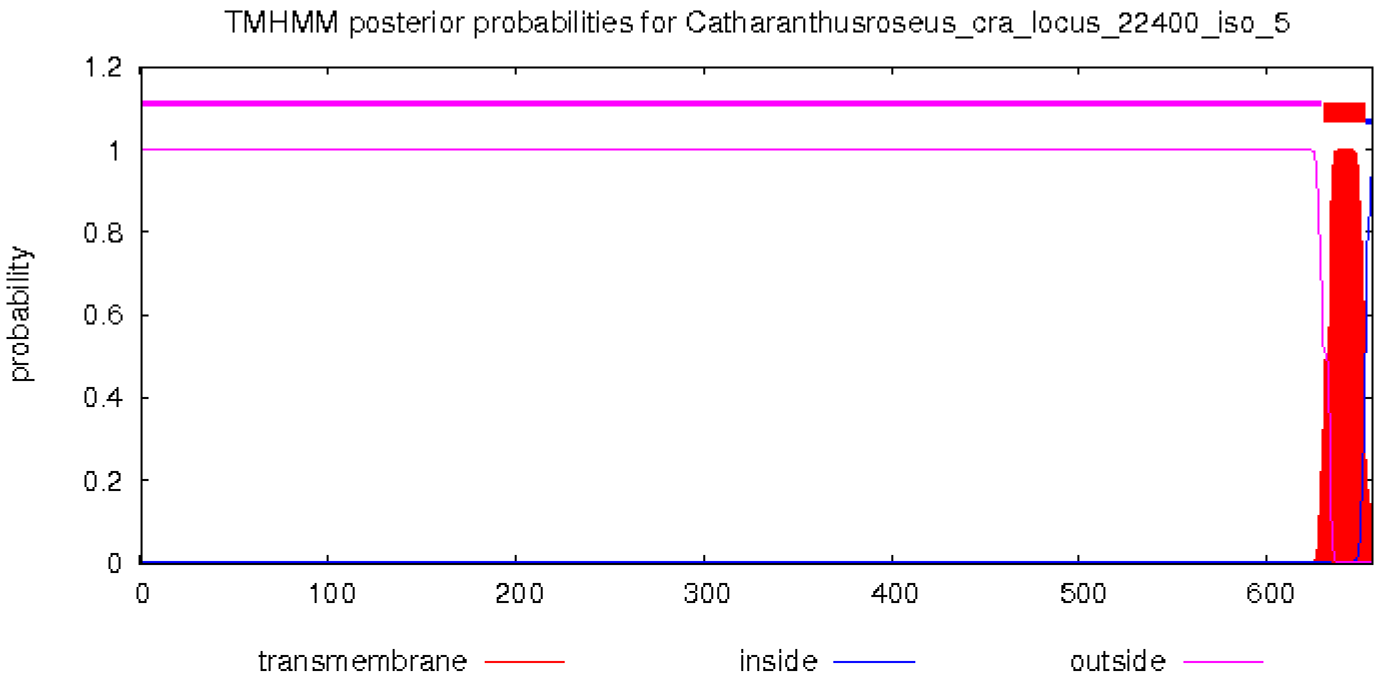

plot in postscript, script for making the plot in gnuplot, data for plot

---

```
# Catharanthusroseus_cra_locus_22400_iso_6 Length: 631
# Catharanthusroseus_cra_locus_22400_iso_6 Number of predicted TMHs:  1
# Catharanthusroseus_cra_locus_22400_iso_6 Exp number of AAs in TMHs: 21.33683
# Catharanthusroseus_cra_locus_22400_iso_6 Exp number, first 60 AAs:  0
# Catharanthusroseus_cra_locus_22400_iso_6 Total prob of N-in:        0.00059
Catharanthusroseus_cra_locus_22400_iso_6        TMHMM2.0        outside      1   604
Catharanthusroseus_cra_locus_22400_iso_6        TMHMM2.0        TMhelix    605   627
Catharanthusroseus_cra_locus_22400_iso_6        TMHMM2.0        inside     628   631
```

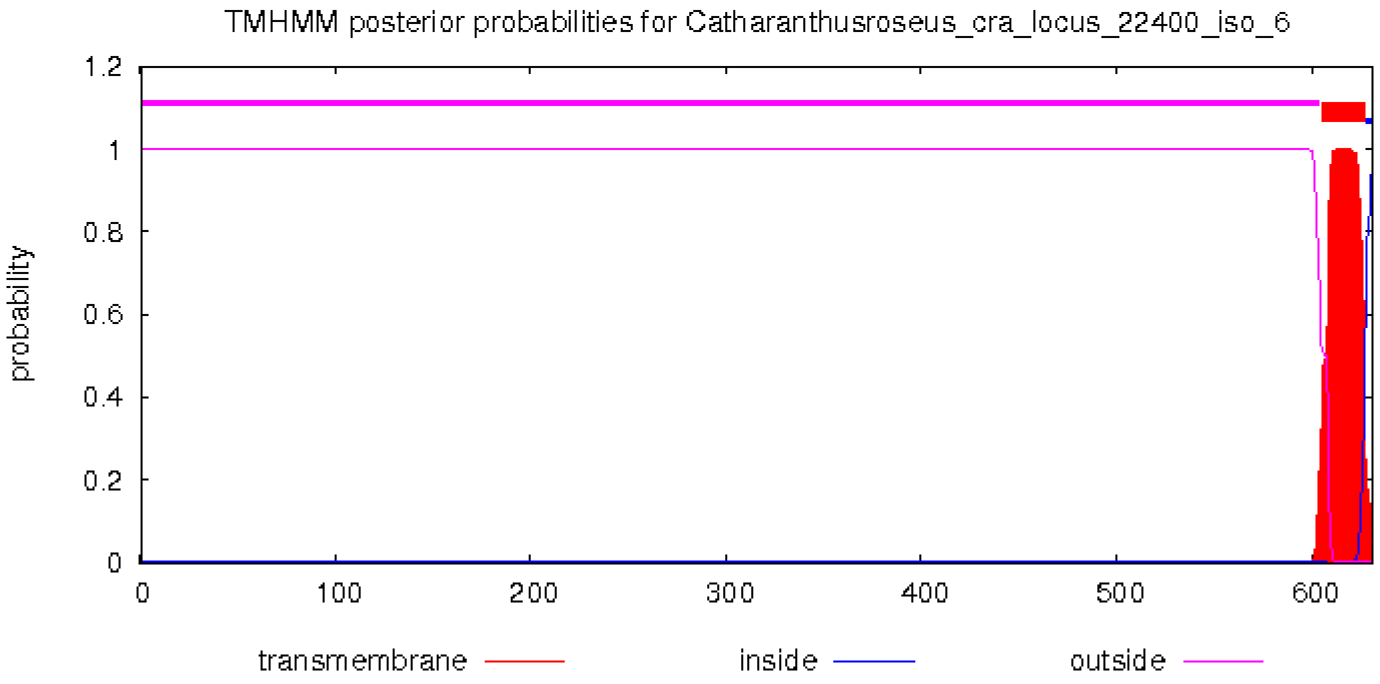

plot in postscript, script for making the plot in gnuplot, data for plot

---

```
# Catharanthusroseus_cra_locus_2287_iso_2 Length: 249
# Catharanthusroseus_cra_locus_2287_iso_2 Number of predicted TMHs:  0
```

```
# Catharanthusroseus_cra_locus_2287_iso_2 Exp number of AAs in TMHs: 0.1843
# Catharanthusroseus_cra_locus_2287_iso_2 Exp number, first 60 AAs:  0.1834
# Catharanthusroseus_cra_locus_2287_iso_2 Total prob of N-in:        0.02734
Catharanthusroseus_cra_locus_2287_iso_2 TMHMM2.0        outside      1   249
```

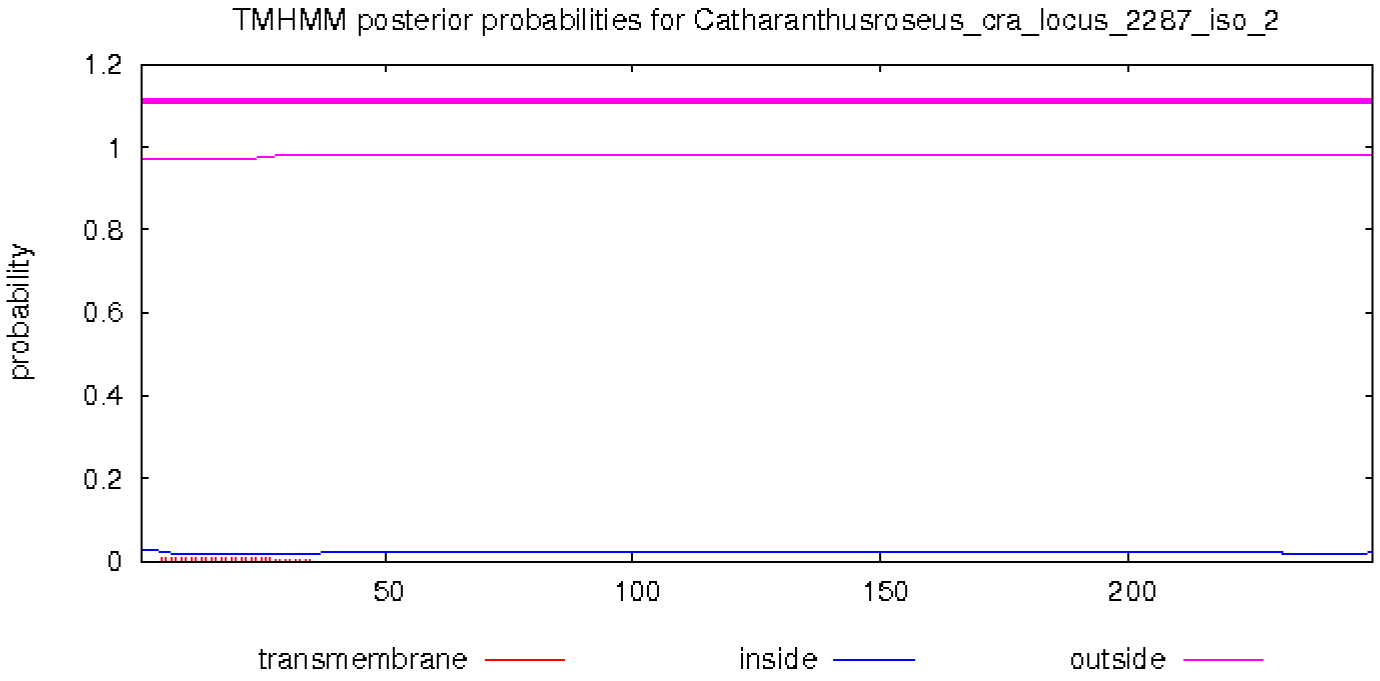

plot in postscript, script for making the plot in gnuplot, data for plot

---

```
# Catharanthusroseus_cra_locus_2287_iso_4 Length: 429
# Catharanthusroseus_cra_locus_2287_iso_4 Number of predicted TMHs:  0
# Catharanthusroseus_cra_locus_2287_iso_4 Exp number of AAs in TMHs: 0.18083
# Catharanthusroseus_cra_locus_2287_iso_4 Exp number, first 60 AAs:  0.18054
# Catharanthusroseus_cra_locus_2287_iso_4 Total prob of N-in:        0.01055
Catharanthusroseus_cra_locus_2287_iso_4 TMHMM2.0        outside      1   429
```

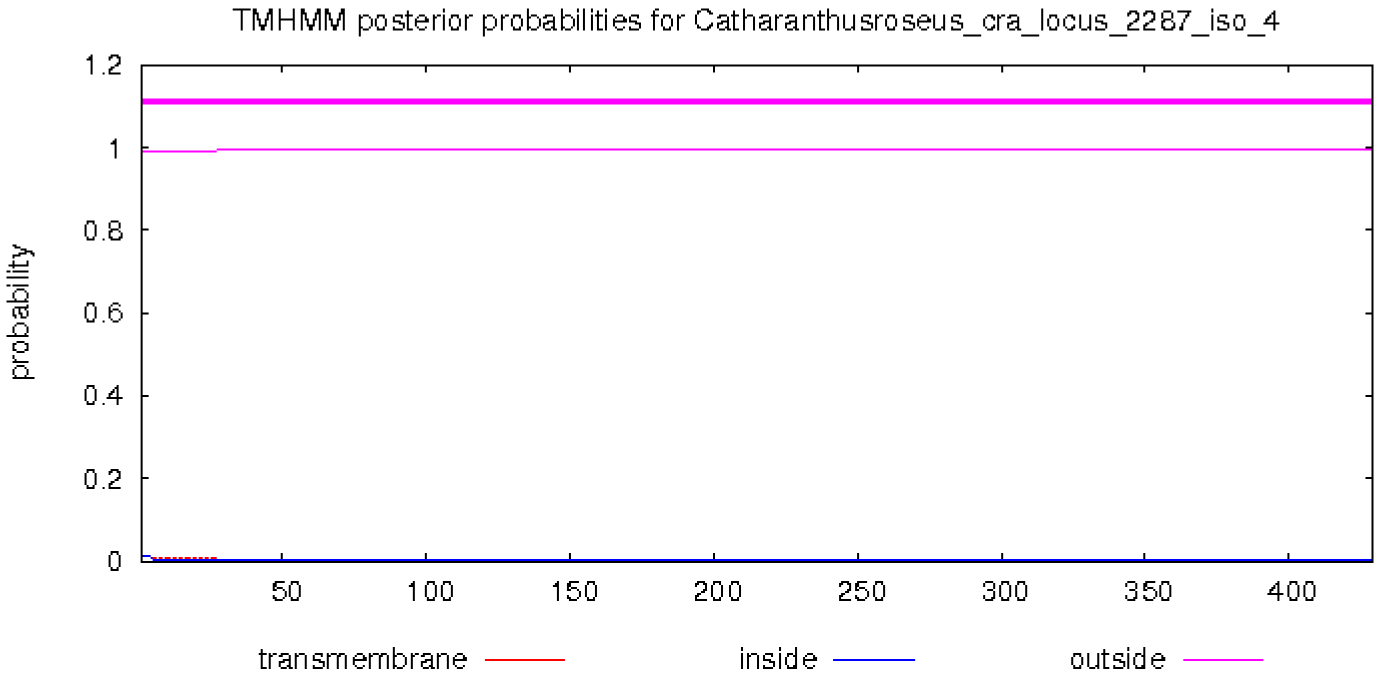

plot in postscript, script for making the plot in gnuplot, data for plot

---

```
# Catharanthusroseus_cra_locus_2287_iso_5 Length: 396
# Catharanthusroseus_cra_locus_2287_iso_5 Number of predicted TMHs:  0
```

```
# Catharanthusroseus_cra_locus_2287_iso_5 Exp number of AAs in TMHs: 0.1808
# Catharanthusroseus_cra_locus_2287_iso_5 Exp number, first 60 AAs:  0.1807
# Catharanthusroseus_cra_locus_2287_iso_5 Total prob of N-in:        0.01169
Catharanthusroseus_cra_locus_2287_iso_5 TMHMM2.0        outside      1   396
```

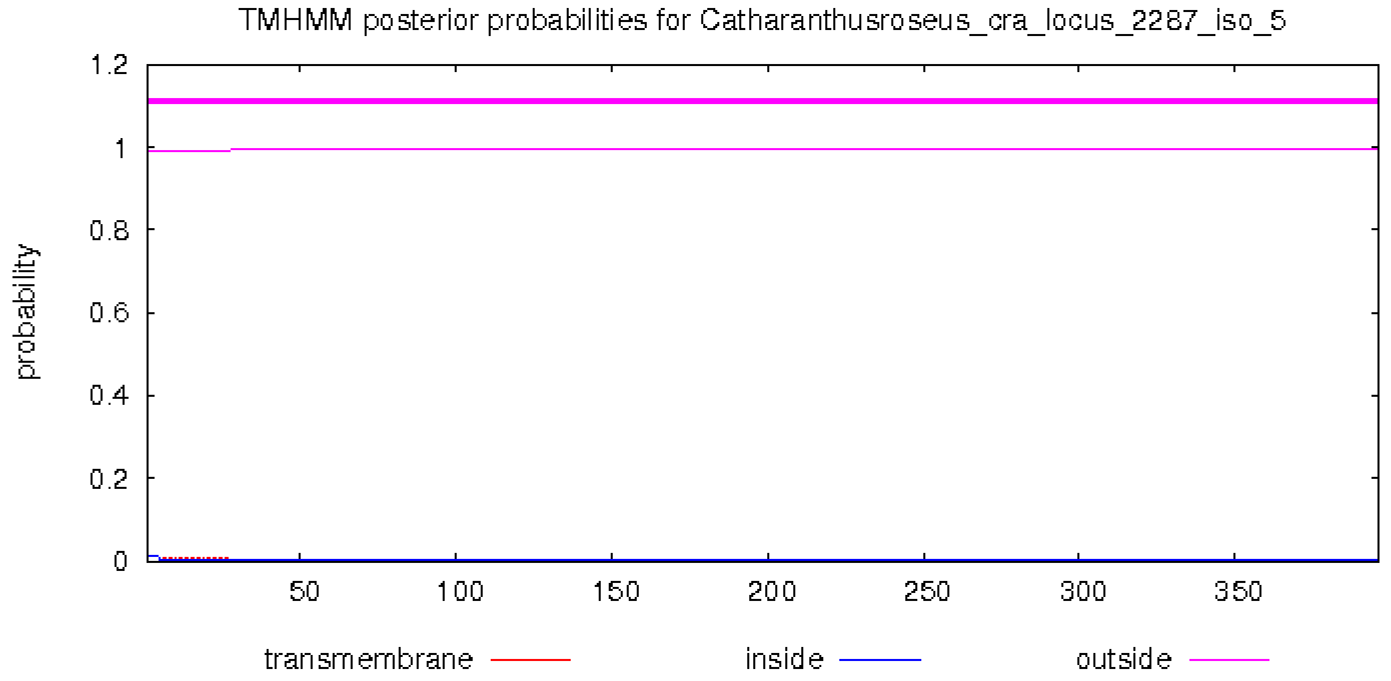

plot in postscript, script for making the plot in gnuplot, data for plot

---

```
# Catharanthusroseus_cra_locus_23250_iso_1 Length: 68
# Catharanthusroseus_cra_locus_23250_iso_1 Number of predicted TMHs:  0
# Catharanthusroseus_cra_locus_23250_iso_1 Exp number of AAs in TMHs: 0
# Catharanthusroseus_cra_locus_23250_iso_1 Exp number, first 60 AAs:  0
# Catharanthusroseus_cra_locus_23250_iso_1 Total prob of N-in:        0.31062
Catharanthusroseus_cra_locus_23250_iso_1        TMHMM2.0        outside      1    68
```

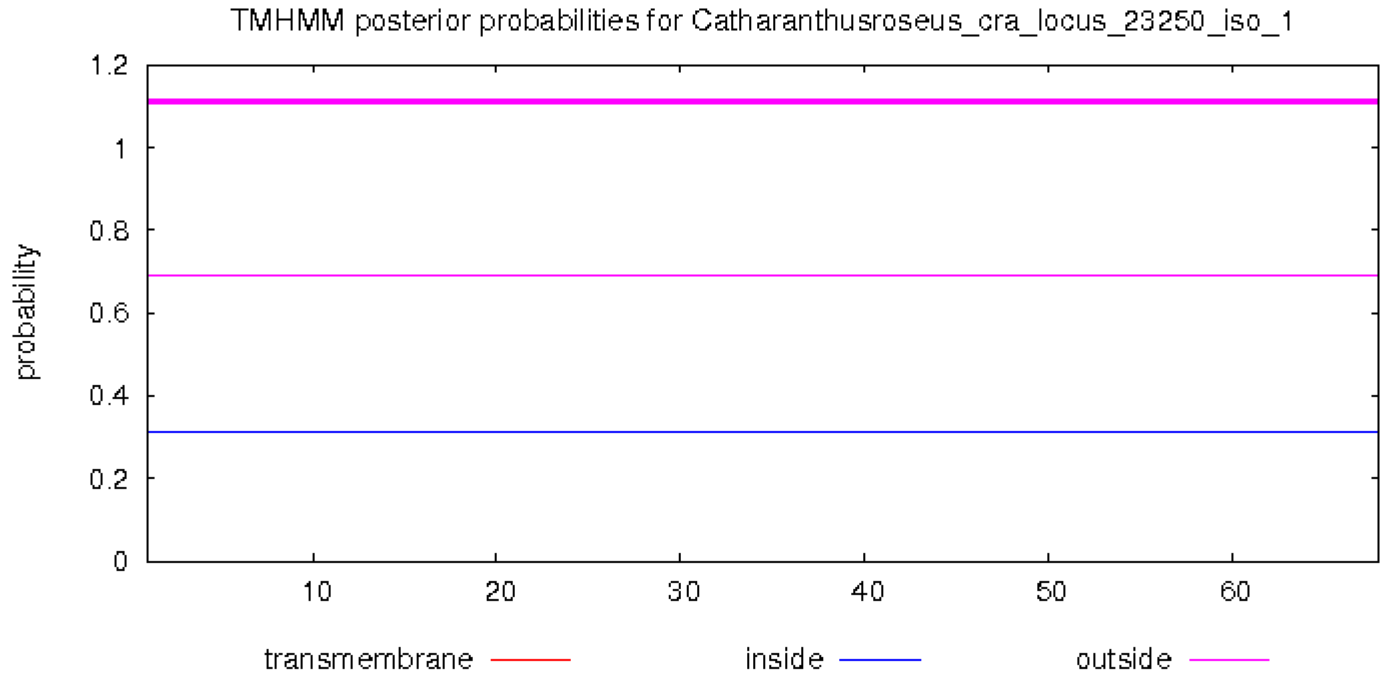

plot in postscript, script for making the plot in gnuplot, data for plot

---

```
# Catharanthusroseus_cra_locus_23250_iso_2 Length: 356
# Catharanthusroseus_cra_locus_23250_iso_2 Number of predicted TMHs:  0
```

```
# Catharanthusroseus_cra_locus_23250_iso_2 Exp number of AAs in TMHs: 0.00011
# Catharanthusroseus_cra_locus_23250_iso_2 Exp number, first 60 AAs:  0
# Catharanthusroseus_cra_locus_23250_iso_2 Total prob of N-in:        0.01610
Catharanthusroseus_cra_locus_23250_iso_2        TMHMM2.0        outside      1   356
```

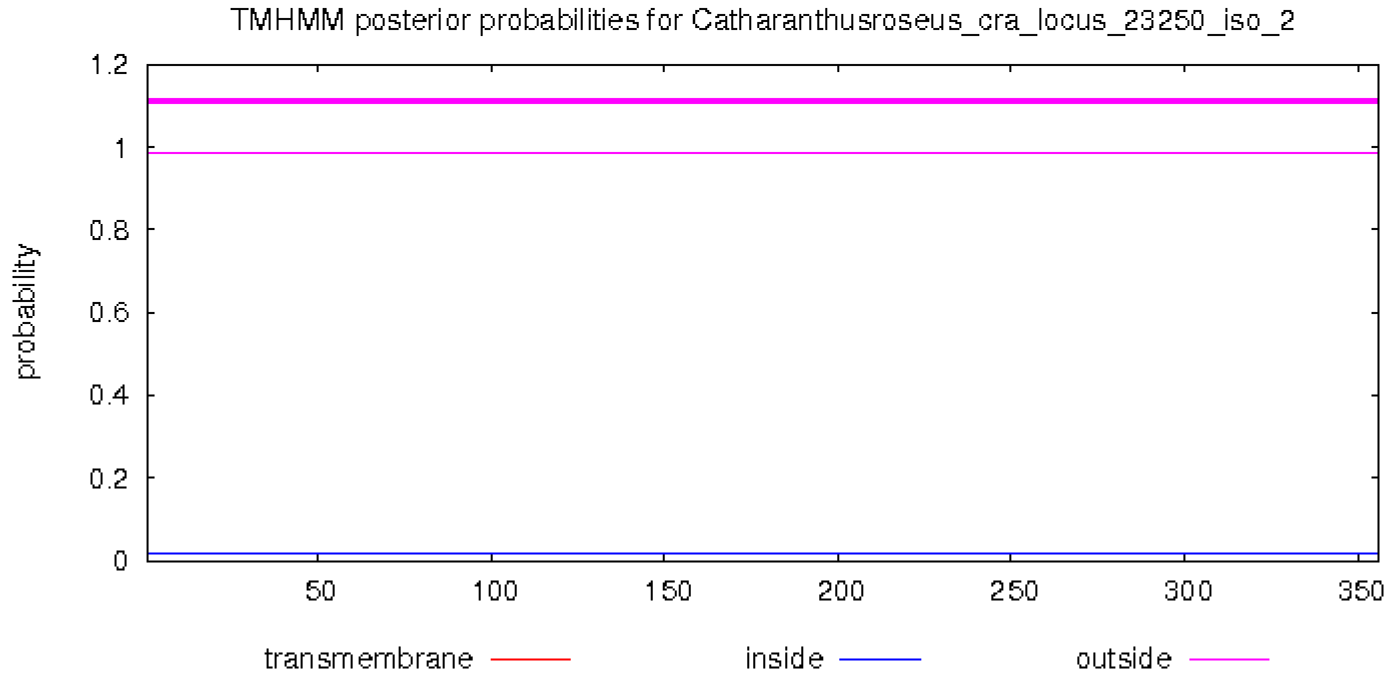

plot in postscript, script for making the plot in gnuplot, data for plot

---

```
# Catharanthusroseus_cra_locus_23496_iso_1 Length: 233
# Catharanthusroseus_cra_locus_23496_iso_1 Number of predicted TMHs:  0
# Catharanthusroseus_cra_locus_23496_iso_1 Exp number of AAs in TMHs: 0.00072
# Catharanthusroseus_cra_locus_23496_iso_1 Exp number, first 60 AAs:  0
# Catharanthusroseus_cra_locus_23496_iso_1 Total prob of N-in:        0.06635
Catharanthusroseus_cra_locus_23496_iso_1        TMHMM2.0        outside      1   233
```

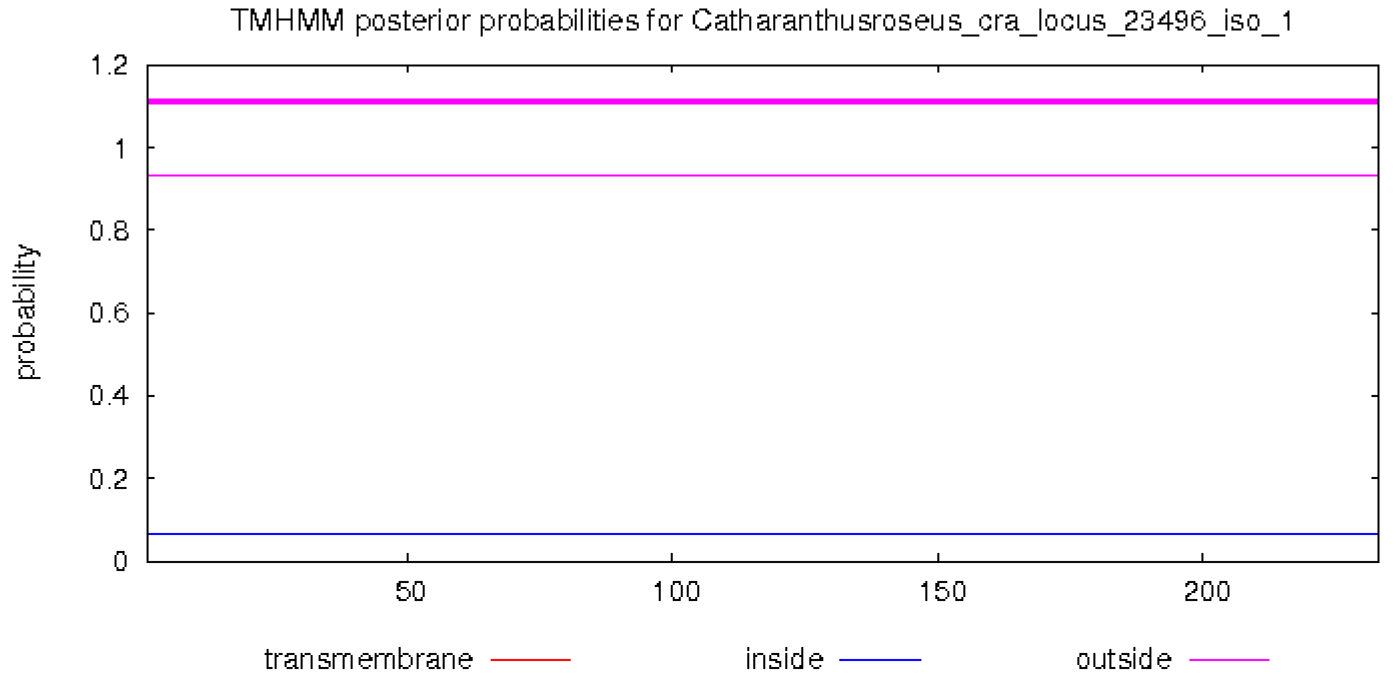

plot in postscript, script for making the plot in gnuplot, data for plot

---

```
# Catharanthusroseus_cra_locus_24682_iso_1 Length: 330
# Catharanthusroseus_cra_locus_24682_iso_1 Number of predicted TMHs:  0
```

```
# Catharanthusroseus_cra_locus_24682_iso_1 Exp number of AAs in TMHs: 0.00301
# Catharanthusroseus_cra_locus_24682_iso_1 Exp number, first 60 AAs:  0
# Catharanthusroseus_cra_locus_24682_iso_1 Total prob of N-in:        0.00091
Catharanthusroseus_cra_locus_24682_iso_1	TMHMM2.0	outside	     1   330
```

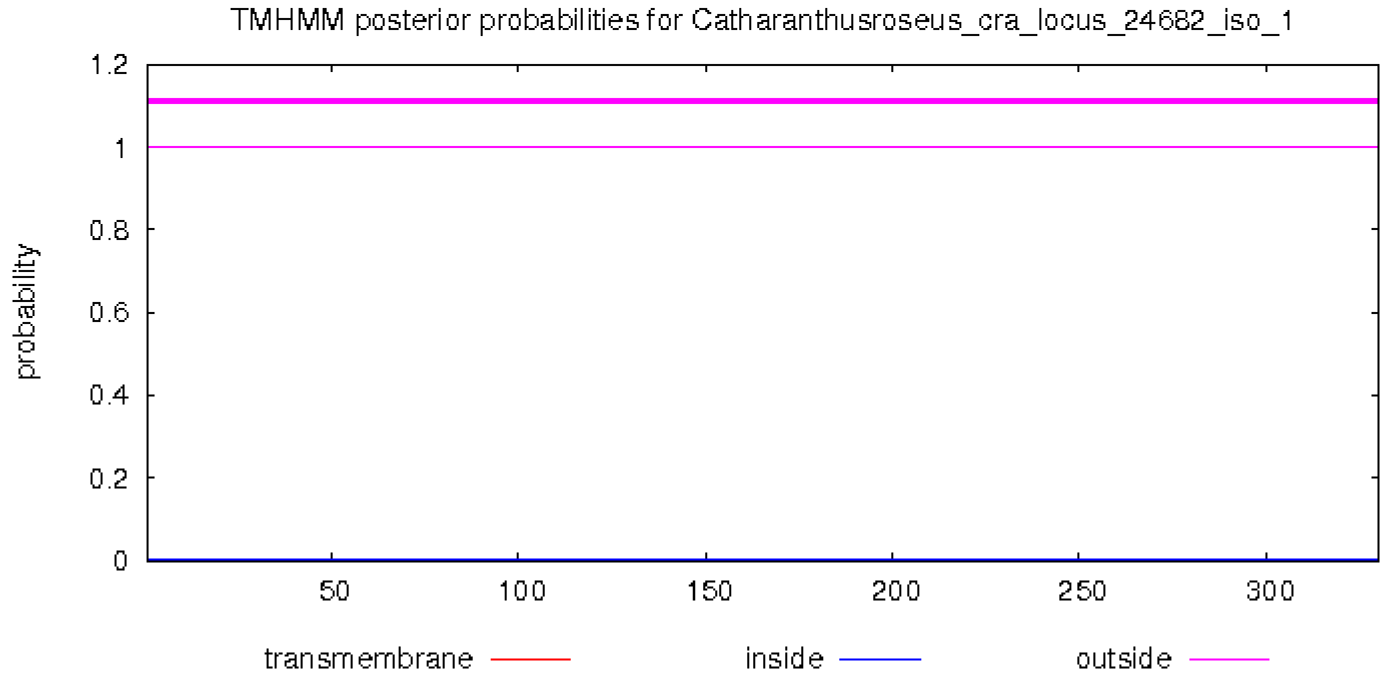

plot in postscript, script for making the plot in gnuplot, data for plot

---

```
# Catharanthusroseus_cra_locus_24682_iso_2 Length: 335
# Catharanthusroseus_cra_locus_24682_iso_2 Number of predicted TMHs:  0
# Catharanthusroseus_cra_locus_24682_iso_2 Exp number of AAs in TMHs: 0.00672
# Catharanthusroseus_cra_locus_24682_iso_2 Exp number, first 60 AAs:  0
# Catharanthusroseus_cra_locus_24682_iso_2 Total prob of N-in:        0.00088
Catharanthusroseus_cra_locus_24682_iso_2	TMHMM2.0	outside	     1   335
```

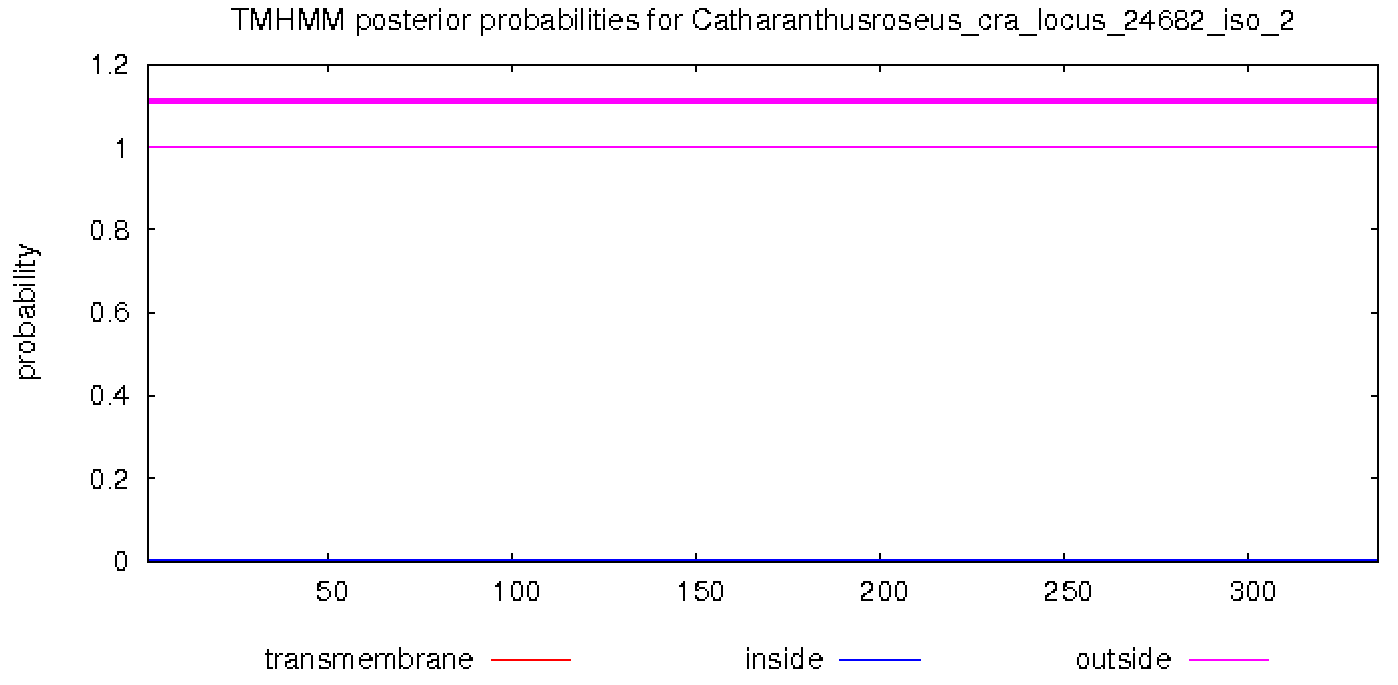

plot in postscript, script for making the plot in gnuplot, data for plot

---

```
# Catharanthusroseus_cra_locus_2614_iso_1 Length: 329
# Catharanthusroseus_cra_locus_2614_iso_1 Number of predicted TMHs:  0
```

```
# Catharanthusroseus_cra_locus_2614_iso_1 Exp number of AAs in TMHs: 0
# Catharanthusroseus_cra_locus_2614_iso_1 Exp number, first 60 AAs:  0
# Catharanthusroseus_cra_locus_2614_iso_1 Total prob of N-in:        0.01794
Catharanthusroseus_cra_locus_2614_iso_1 TMHMM2.0        outside      1   329
```

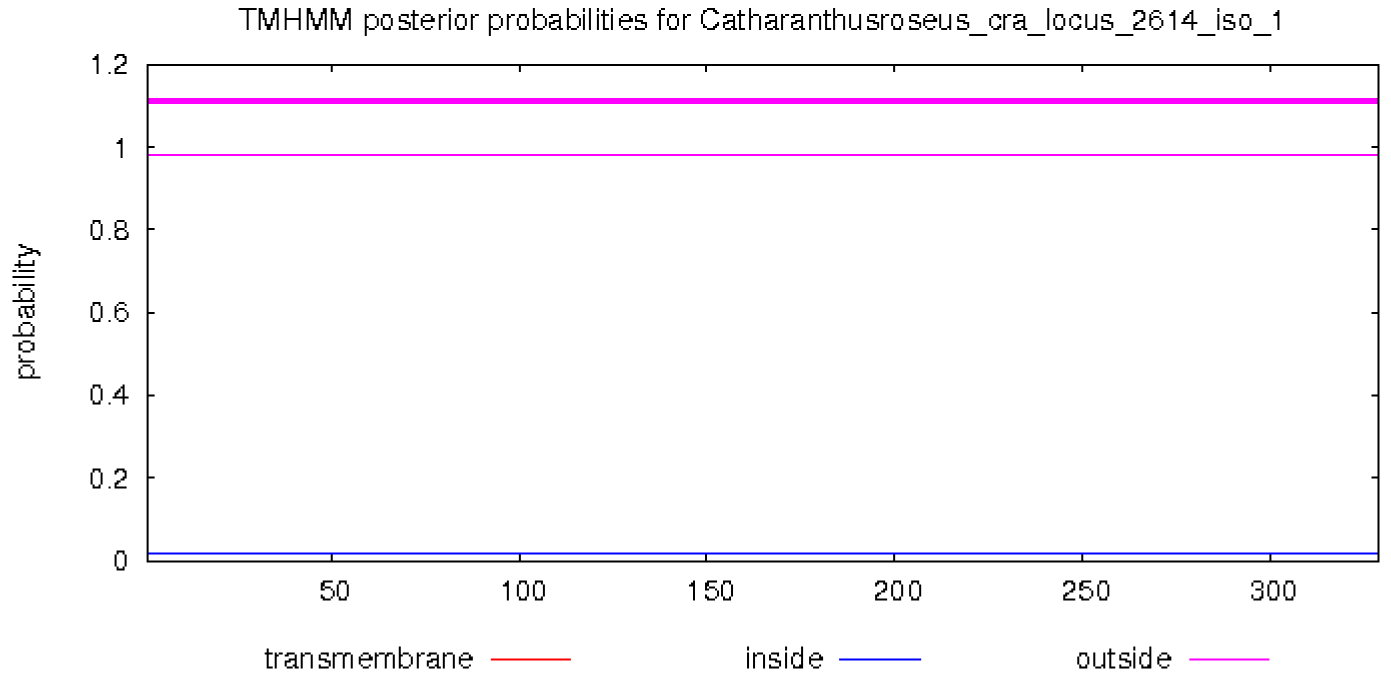

plot in postscript, script for making the plot in gnuplot, data for plot

---

```
# Catharanthusroseus_cra_locus_2614_iso_2 Length: 270
# Catharanthusroseus_cra_locus_2614_iso_2 Number of predicted TMHs:  0
# Catharanthusroseus_cra_locus_2614_iso_2 Exp number of AAs in TMHs: 0
# Catharanthusroseus_cra_locus_2614_iso_2 Exp number, first 60 AAs:  0
# Catharanthusroseus_cra_locus_2614_iso_2 Total prob of N-in:        0.02150
Catharanthusroseus_cra_locus_2614_iso_2 TMHMM2.0        outside      1   270
```

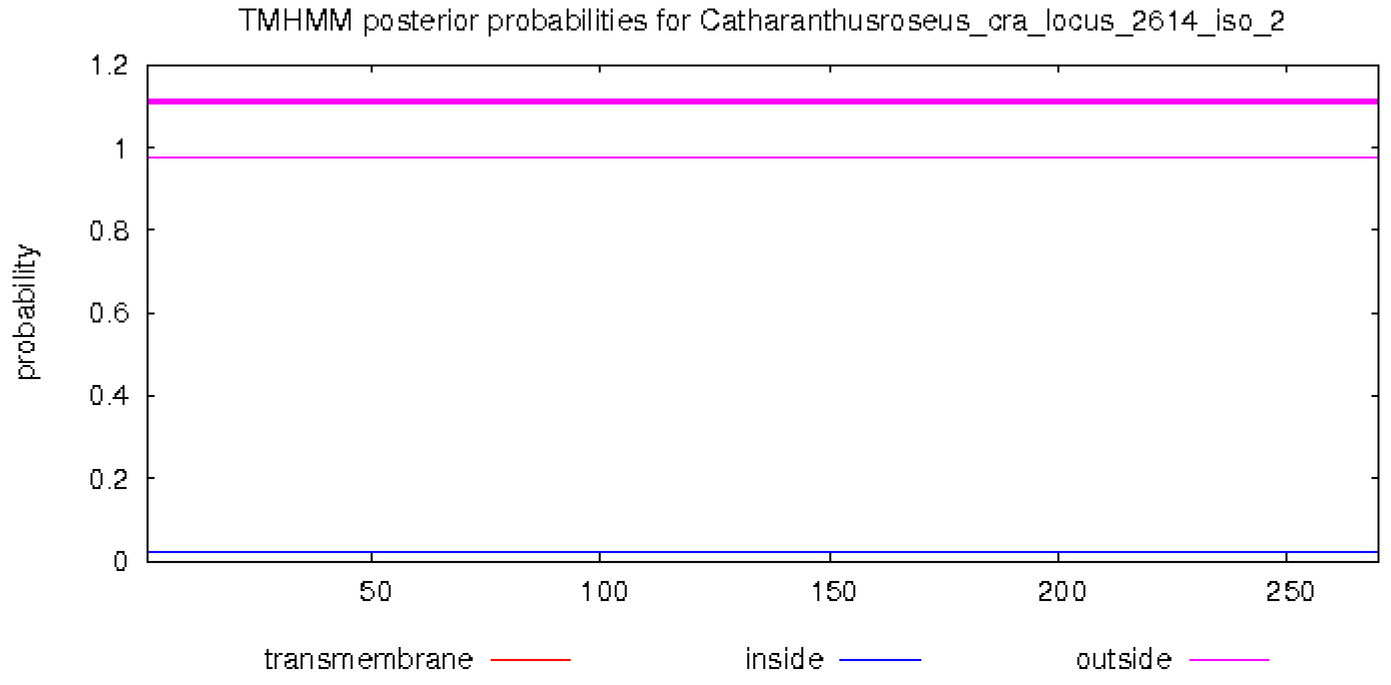

plot in postscript, script for making the plot in gnuplot, data for plot

---

```
# Catharanthusroseus_cra_locus_2614_iso_3 Length: 127
# Catharanthusroseus_cra_locus_2614_iso_3 Number of predicted TMHs:  0
```

```
# Catharanthusroseus_cra_locus_2614_iso_3 Exp number of AAs in TMHs: 0.00081
# Catharanthusroseus_cra_locus_2614_iso_3 Exp number, first 60 AAs:  0.00023
# Catharanthusroseus_cra_locus_2614_iso_3 Total prob of N-in:        0.19301
Catharanthusroseus_cra_locus_2614_iso_3 TMHMM2.0        outside      1   127
```

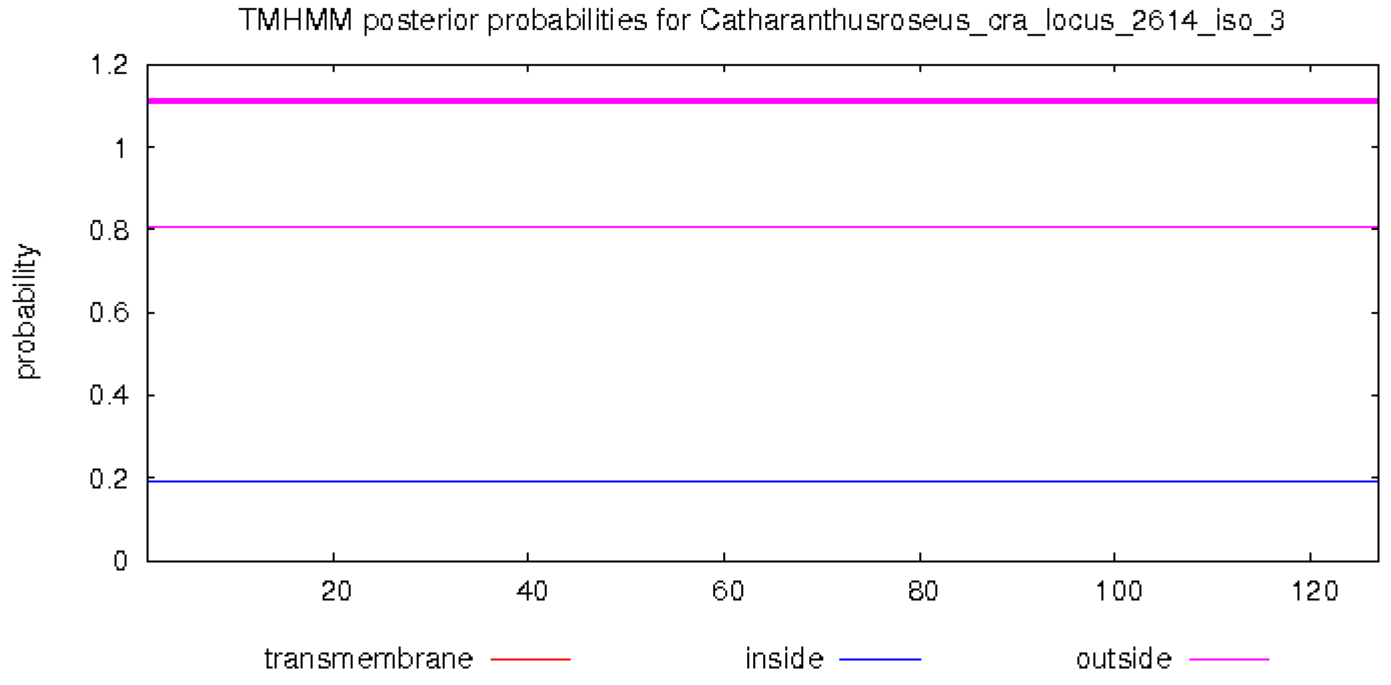

[plot](#) in postscript, [script](#) for making the plot in gnuplot, [data](#) for plot

---

```
# Catharanthusroseus_cra_locus_2614_iso_5 Length: 270
# Catharanthusroseus_cra_locus_2614_iso_5 Number of predicted TMHs:  0
# Catharanthusroseus_cra_locus_2614_iso_5 Exp number of AAs in TMHs: 0
# Catharanthusroseus_cra_locus_2614_iso_5 Exp number, first 60 AAs:  0
# Catharanthusroseus_cra_locus_2614_iso_5 Total prob of N-in:        0.02150
Catharanthusroseus_cra_locus_2614_iso_5 TMHMM2.0        outside      1   270
```

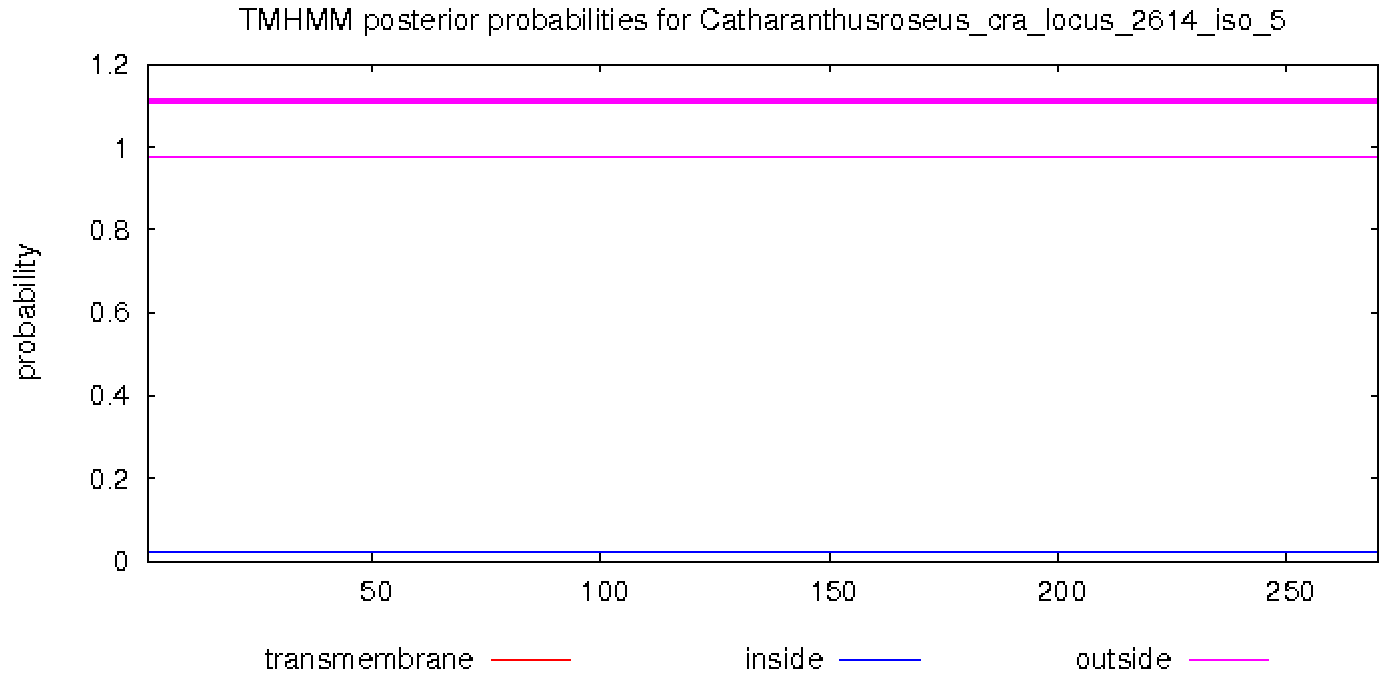

[plot](#) in postscript, [script](#) for making the plot in gnuplot, [data](#) for plot

---

```
# Catharanthusroseus_cra_locus_2614_iso_7 Length: 329
# Catharanthusroseus_cra_locus_2614_iso_7 Number of predicted TMHs:  0
```

```
# Catharanthusroseus_cra_locus_2614_iso_7 Exp number of AAs in TMHs: 0
# Catharanthusroseus_cra_locus_2614_iso_7 Exp number, first 60 AAs:  0
# Catharanthusroseus_cra_locus_2614_iso_7 Total prob of N-in:        0.01794
Catharanthusroseus_cra_locus_2614_iso_7 TMHMM2.0        outside      1   329
```

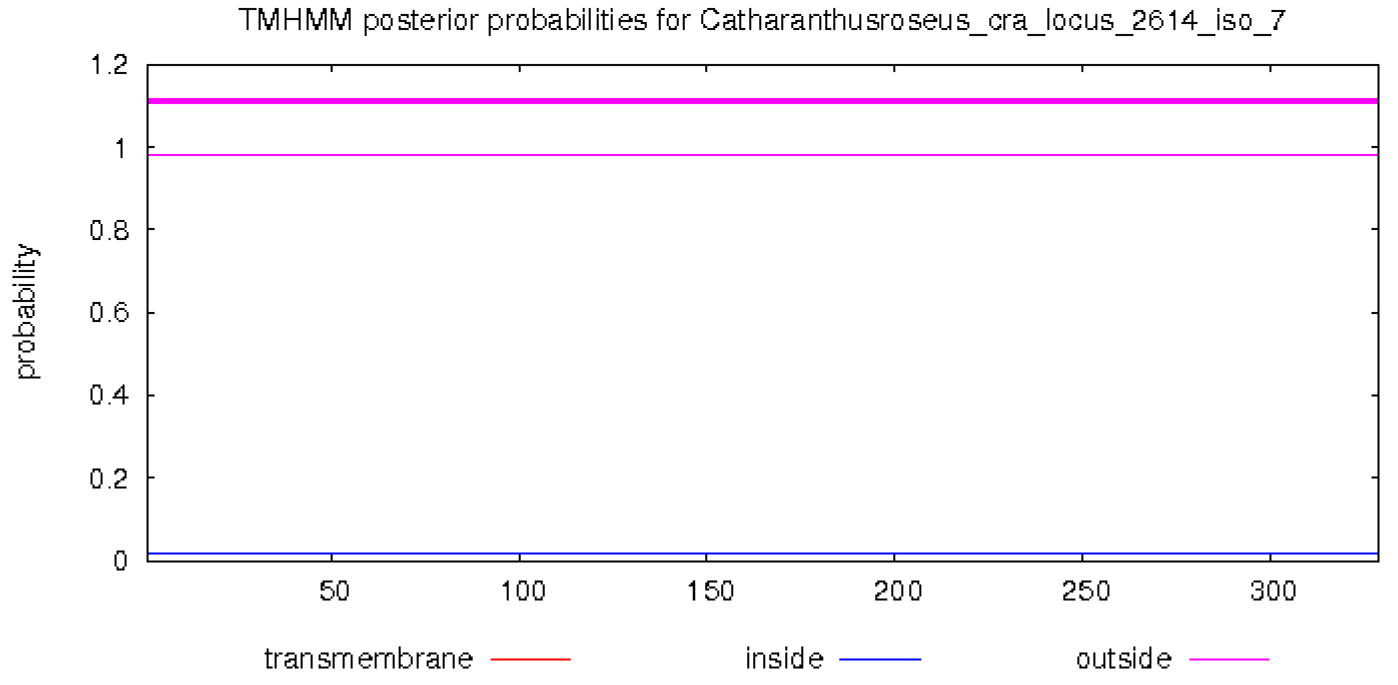

plot in postscript, script for making the plot in gnuplot, data for plot

---

```
# Catharanthusroseus_cra_locus_26911_iso_1 Length: 214
# Catharanthusroseus_cra_locus_26911_iso_1 Number of predicted TMHs:  0
# Catharanthusroseus_cra_locus_26911_iso_1 Exp number of AAs in TMHs: 0.00079
# Catharanthusroseus_cra_locus_26911_iso_1 Exp number, first 60 AAs:  0.0002
# Catharanthusroseus_cra_locus_26911_iso_1 Total prob of N-in:        0.13071
Catharanthusroseus_cra_locus_26911_iso_1        TMHMM2.0        outside      1   214
```

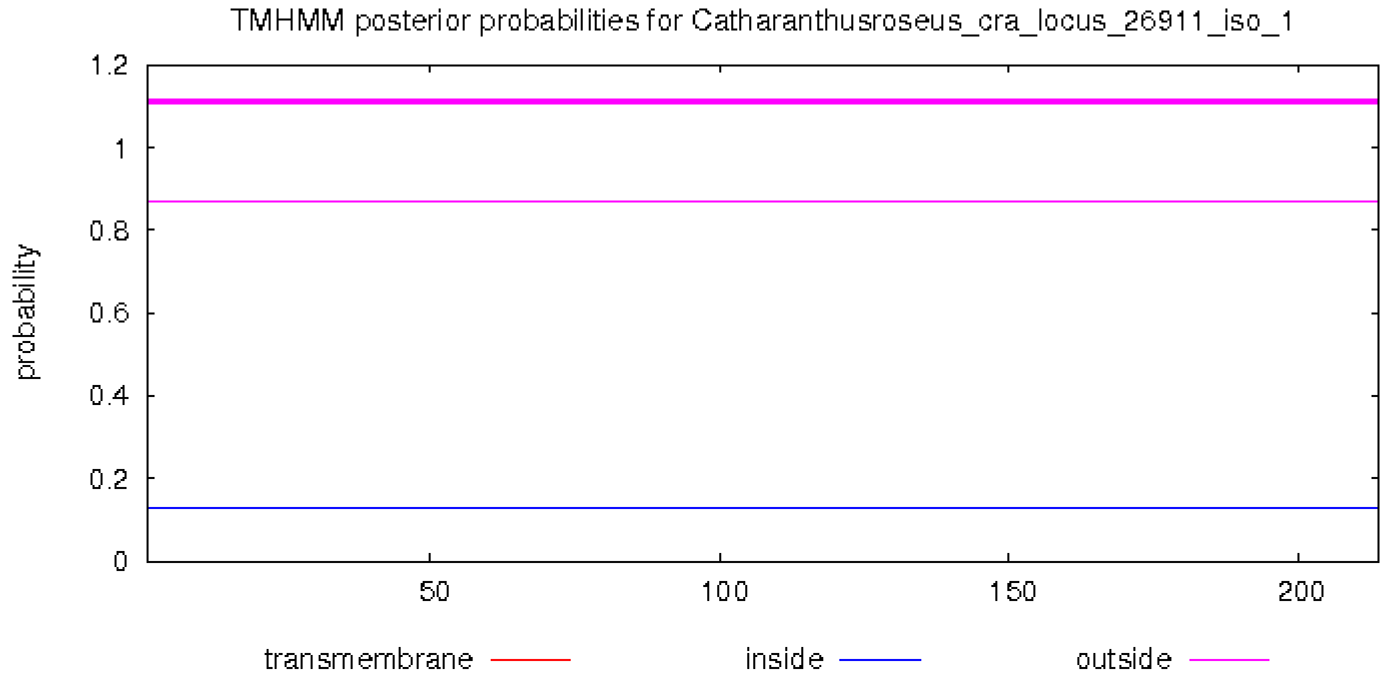

plot in postscript, script for making the plot in gnuplot, data for plot

---

```
# Catharanthusroseus_cra_locus_290_iso_1 Length: 246
# Catharanthusroseus_cra_locus_290_iso_1 Number of predicted TMHs:  0
```

```
# Catharanthusroseus_cra_locus_290_iso_1 Exp number of AAs in TMHs: 0.00056
# Catharanthusroseus_cra_locus_290_iso_1 Exp number, first 60 AAs:  0.0002
# Catharanthusroseus_cra_locus_290_iso_1 Total prob of N-in:        0.06464
Catharanthusroseus_cra_locus_290_iso_1  TMHMM2.0        outside      1   246
```

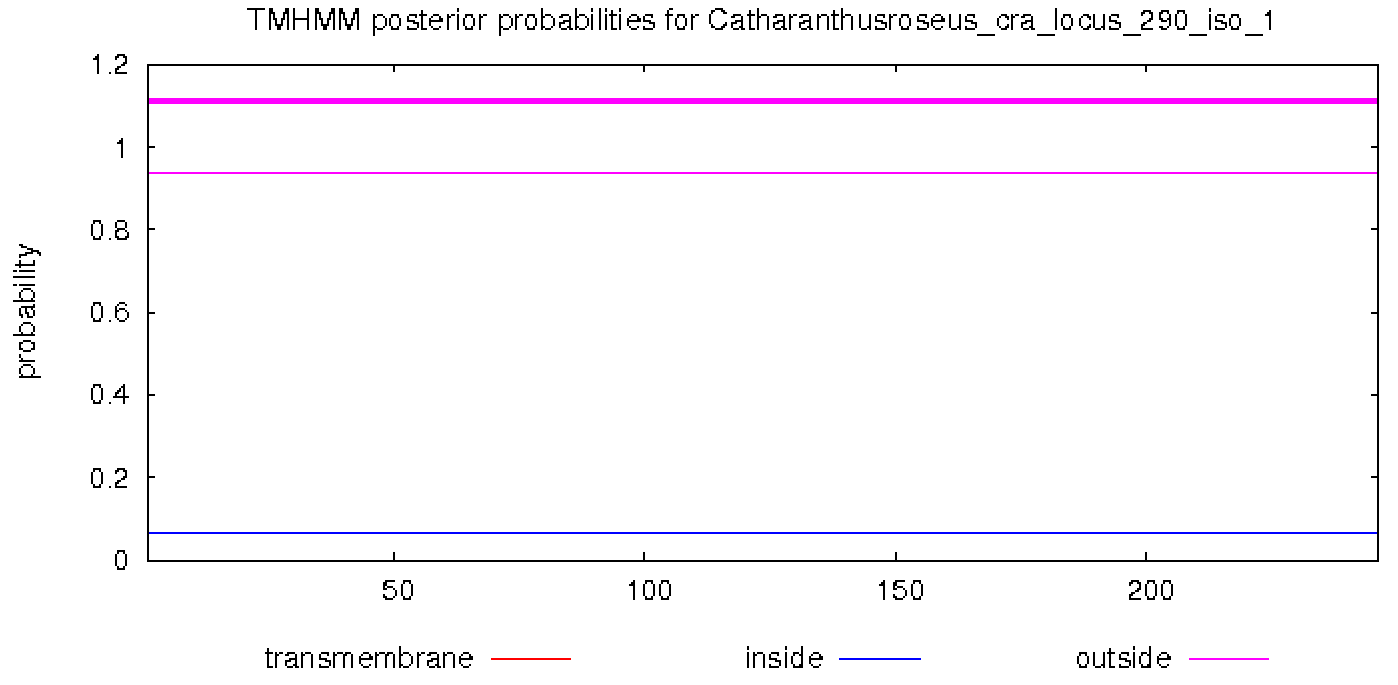

plot in postscript, script for making the plot in gnuplot, data for plot

```
# Catharanthusroseus_cra_locus_290_iso_2 Length: 399
# Catharanthusroseus_cra_locus_290_iso_2 Number of predicted TMHs:  0
# Catharanthusroseus_cra_locus_290_iso_2 Exp number of AAs in TMHs: 0.03428
# Catharanthusroseus_cra_locus_290_iso_2 Exp number, first 60 AAs:  0.0002
# Catharanthusroseus_cra_locus_290_iso_2 Total prob of N-in:        0.00547
Catharanthusroseus_cra_locus_290_iso_2  TMHMM2.0        outside      1   399
```

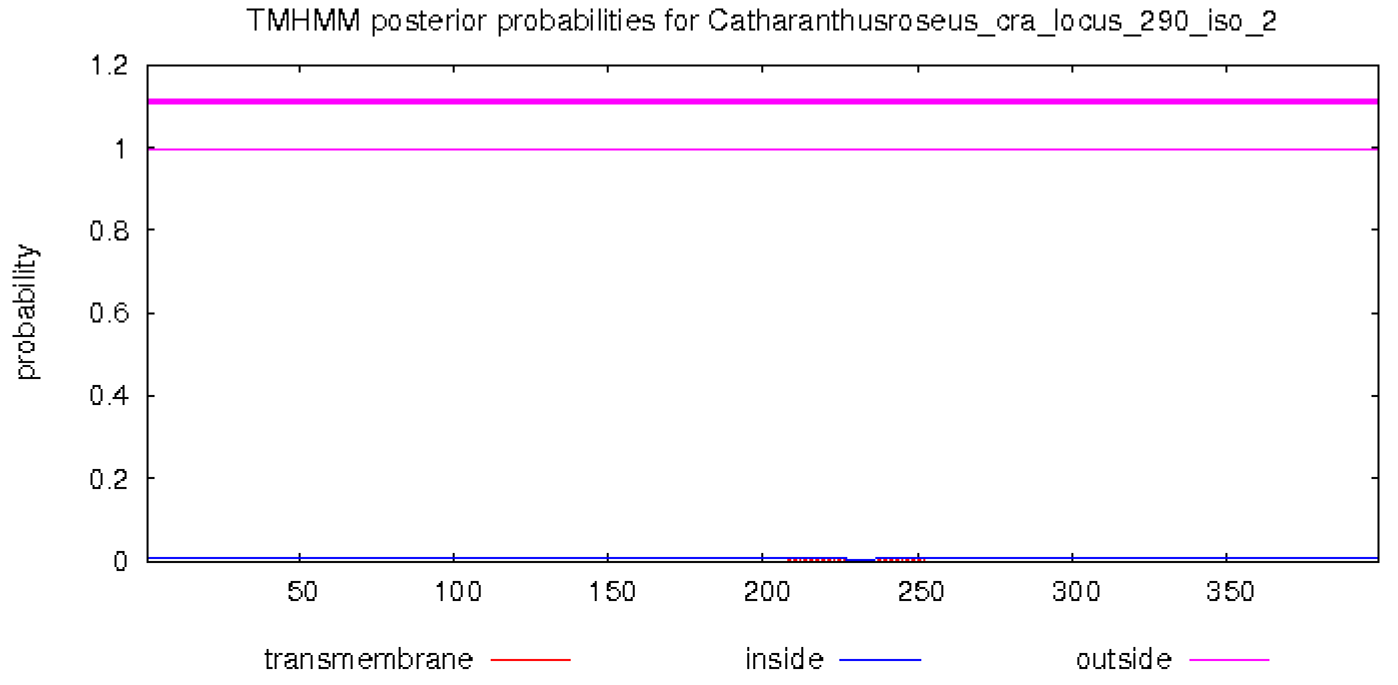

plot in postscript, script for making the plot in gnuplot, data for plot

```
# Catharanthusroseus_cra_locus_290_iso_3 Length: 362
# Catharanthusroseus_cra_locus_290_iso_3 Number of predicted TMHs:  0
```

```
# Catharanthusroseus_cra_locus_290_iso_3 Exp number of AAs in TMHs: 0.00038
# Catharanthusroseus_cra_locus_290_iso_3 Exp number, first 60 AAs:  0.00038
# Catharanthusroseus_cra_locus_290_iso_3 Total prob of N-in:        0.00758
Catharanthusroseus_cra_locus_290_iso_3  TMHMM2.0        outside      1   362
```

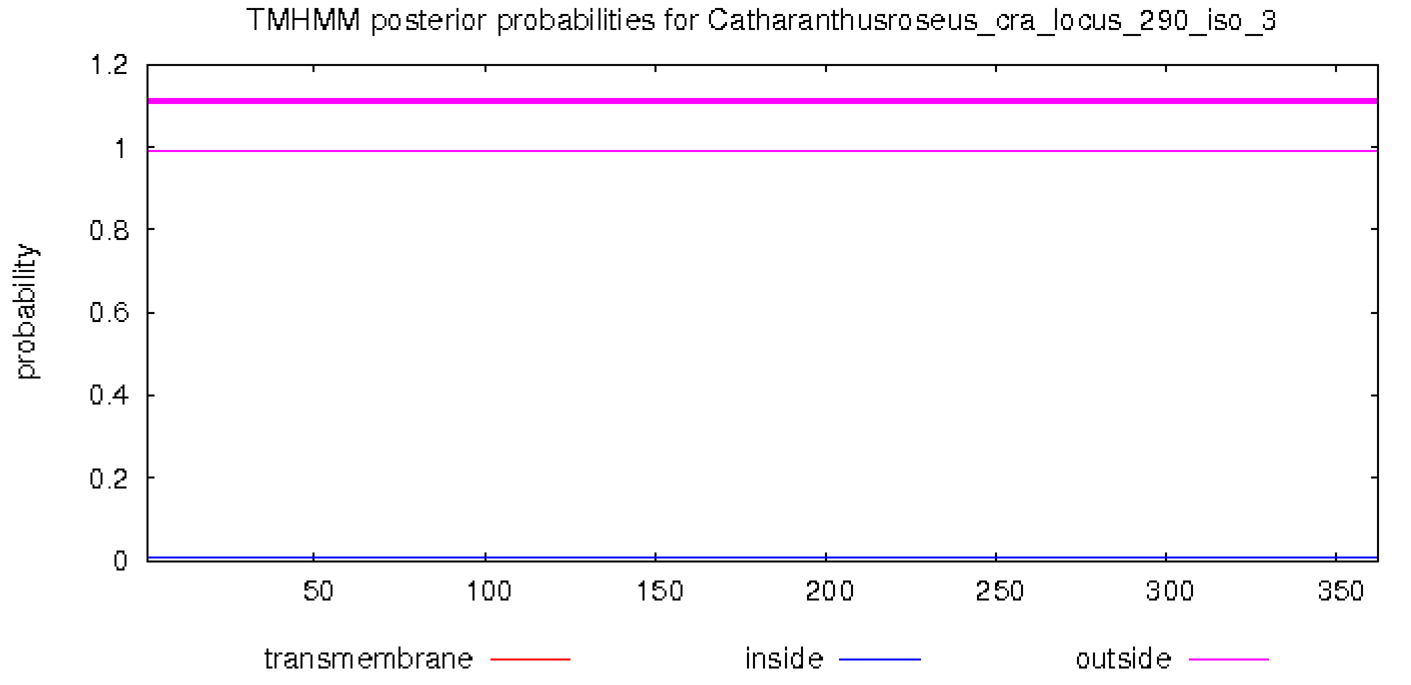

plot in postscript, script for making the plot in gnuplot, data for plot

---

```
# Catharanthusroseus_cra_locus_31747_iso_1 Length: 141
# Catharanthusroseus_cra_locus_31747_iso_1 Number of predicted TMHs:  0
# Catharanthusroseus_cra_locus_31747_iso_1 Exp number of AAs in TMHs: 0
# Catharanthusroseus_cra_locus_31747_iso_1 Exp number, first 60 AAs:  0
# Catharanthusroseus_cra_locus_31747_iso_1 Total prob of N-in:        0.35157
Catharanthusroseus_cra_locus_31747_iso_1        TMHMM2.0        outside      1   141
```

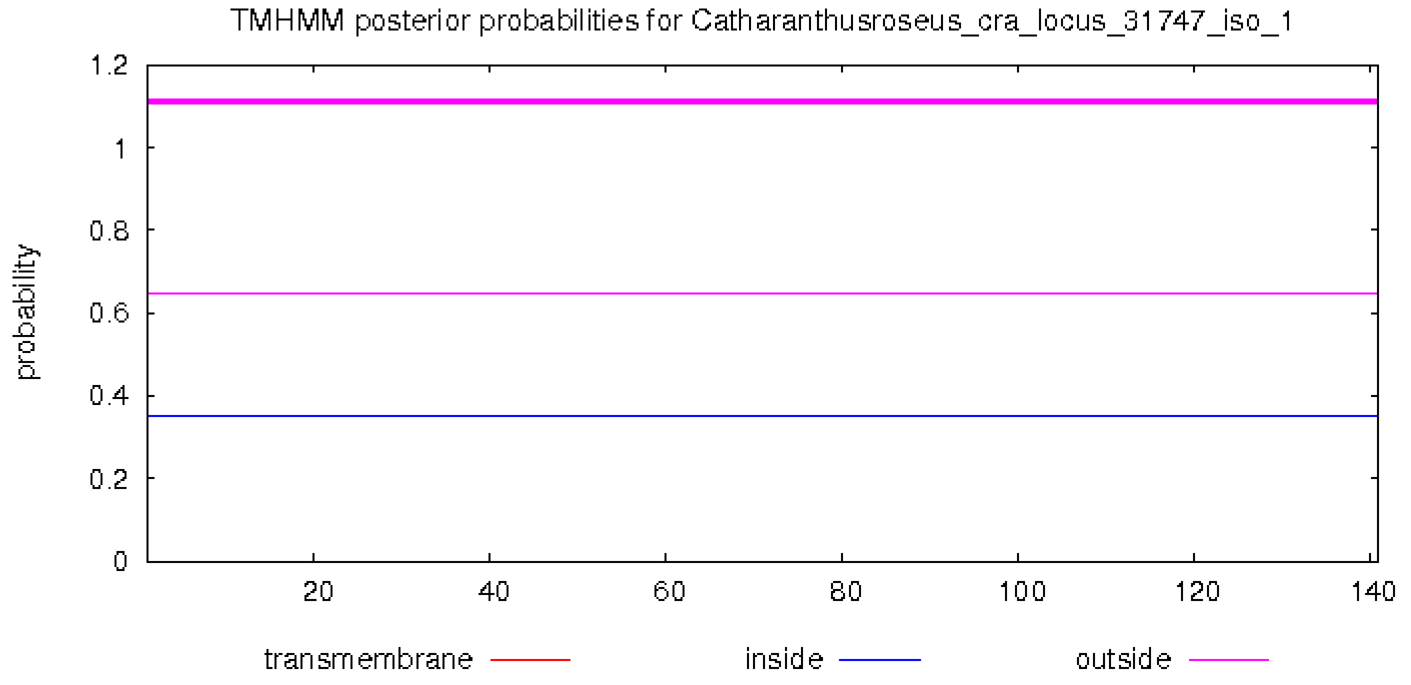

plot in postscript, script for making the plot in gnuplot, data for plot

---

```
# Catharanthusroseus_cra_locus_31747_iso_2 Length: 112
# Catharanthusroseus_cra_locus_31747_iso_2 Number of predicted TMHs:  0
```

```
# Catharanthusroseus_cra_locus_31747_iso_2 Exp number of AAs in TMHs: 0.00084
# Catharanthusroseus_cra_locus_31747_iso_2 Exp number, first 60 AAs:  0
# Catharanthusroseus_cra_locus_31747_iso_2 Total prob of N-in:        0.40813
Catharanthusroseus_cra_locus_31747_iso_2        TMHMM2.0        outside      1   112
```

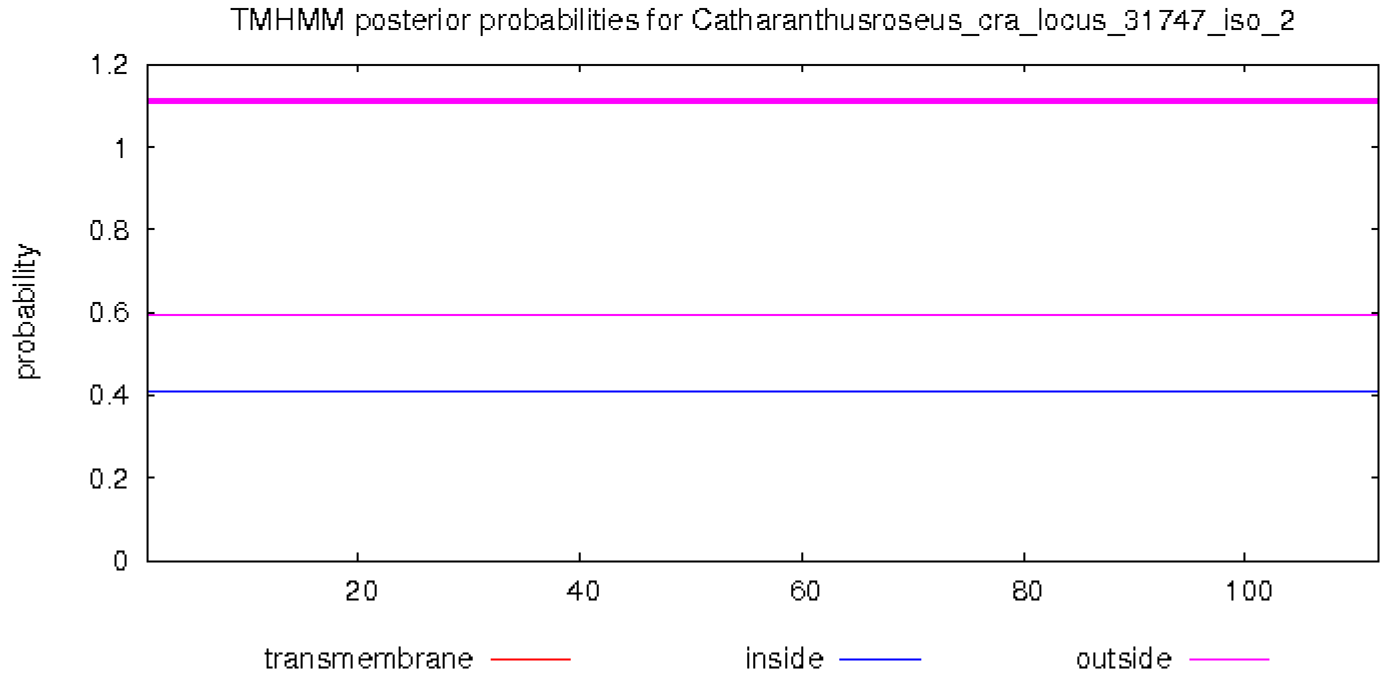

plot in postscript, script for making the plot in gnuplot, data for plot

```
# Catharanthusroseus_cra_locus_31747_iso_3 Length: 127
# Catharanthusroseus_cra_locus_31747_iso_3 Number of predicted TMHs:  0
# Catharanthusroseus_cra_locus_31747_iso_3 Exp number of AAs in TMHs: 0
# Catharanthusroseus_cra_locus_31747_iso_3 Exp number, first 60 AAs:  0
# Catharanthusroseus_cra_locus_31747_iso_3 Total prob of N-in:        0.35953
Catharanthusroseus_cra_locus_31747_iso_3        TMHMM2.0        outside      1   127
```

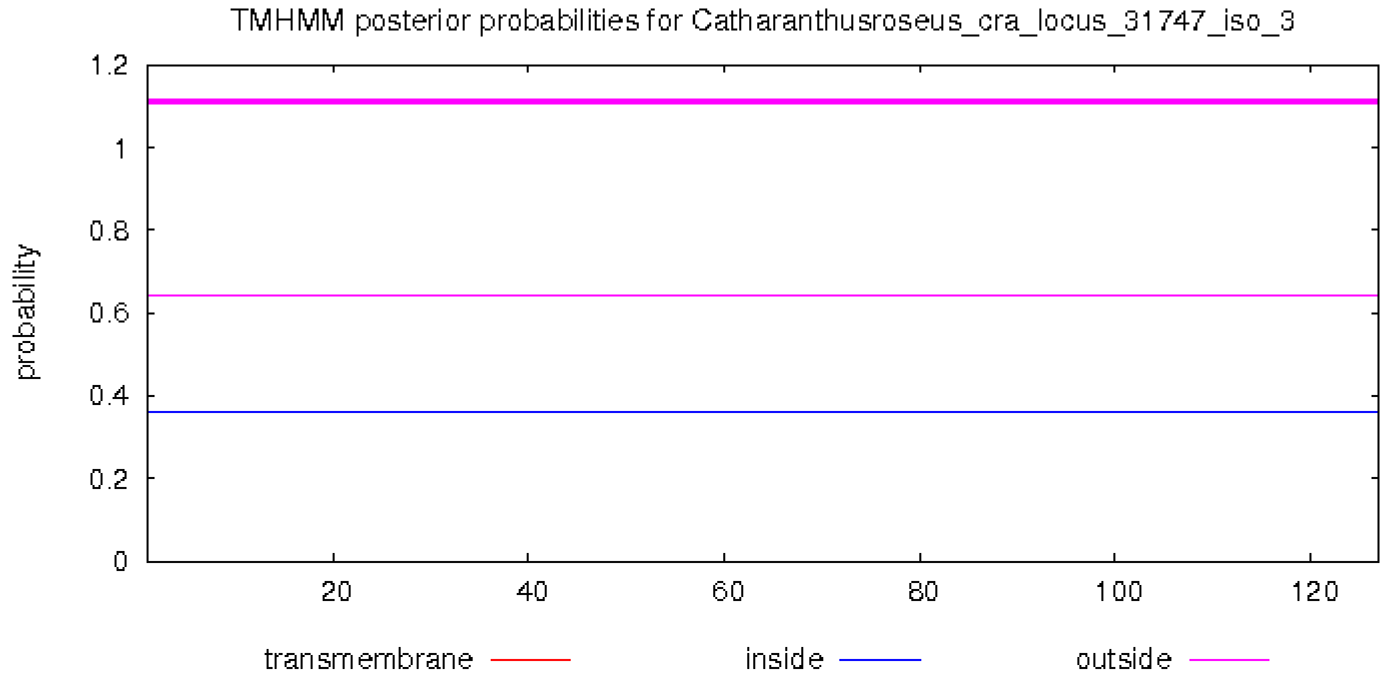

plot in postscript, script for making the plot in gnuplot, data for plot

```
# Catharanthusroseus_cra_locus_35120_iso_1 Length: 171
# Catharanthusroseus_cra_locus_35120_iso_1 Number of predicted TMHs:  0
```

```
# Catharanthusroseus_cra_locus_35120_iso_1 Exp number of AAs in TMHs: 0
# Catharanthusroseus_cra_locus_35120_iso_1 Exp number, first 60 AAs:  0
# Catharanthusroseus_cra_locus_35120_iso_1 Total prob of N-in:        0.05812
Catharanthusroseus_cra_locus_35120_iso_1        TMHMM2.0        outside      1   171
```

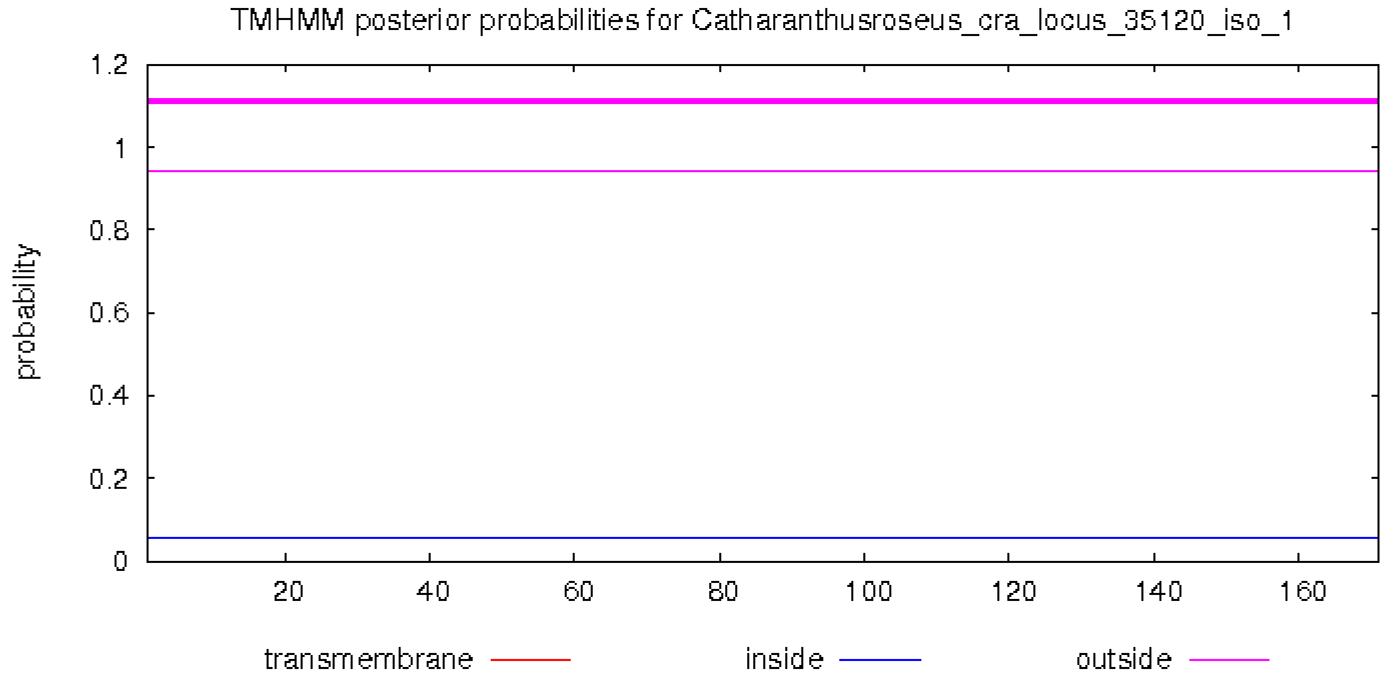

plot in postscript, script for making the plot in gnuplot, data for plot

---

```
# Catharanthusroseus_cra_locus_35121_iso_1 Length: 98
# Catharanthusroseus_cra_locus_35121_iso_1 Number of predicted TMHs:  0
# Catharanthusroseus_cra_locus_35121_iso_1 Exp number of AAs in TMHs: 0
# Catharanthusroseus_cra_locus_35121_iso_1 Exp number, first 60 AAs:  0
# Catharanthusroseus_cra_locus_35121_iso_1 Total prob of N-in:        0.18733
Catharanthusroseus_cra_locus_35121_iso_1        TMHMM2.0        outside      1    98
```

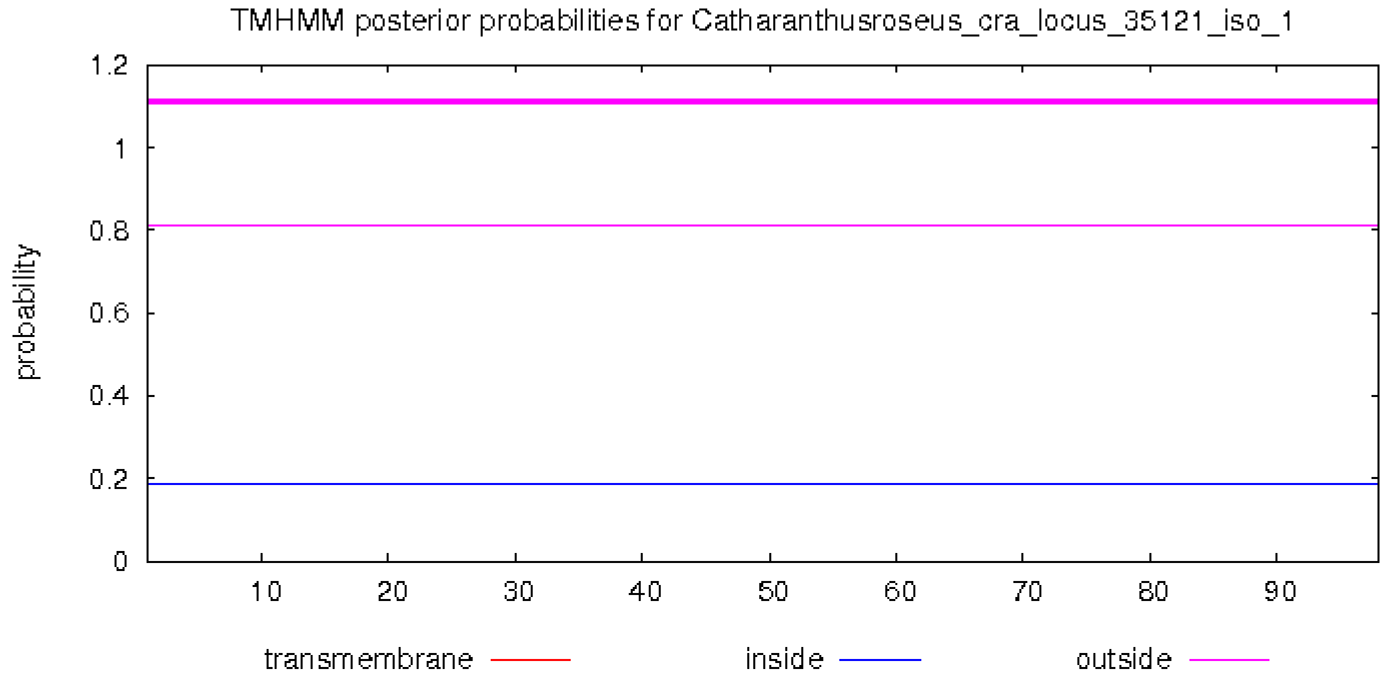

plot in postscript, script for making the plot in gnuplot, data for plot

---

```
# Catharanthusroseus_cra_locus_4016_iso_1 Length: 257
# Catharanthusroseus_cra_locus_4016_iso_1 Number of predicted TMHs:  0
```

```
# Catharanthusroseus_cra_locus_4016_iso_1 Exp number of AAs in TMHs: 0.00036
# Catharanthusroseus_cra_locus_4016_iso_1 Exp number, first 60 AAs:  0
# Catharanthusroseus_cra_locus_4016_iso_1 Total prob of N-in:        0.09945
Catharanthusroseus_cra_locus_4016_iso_1 TMHMM2.0        outside      1   257
```

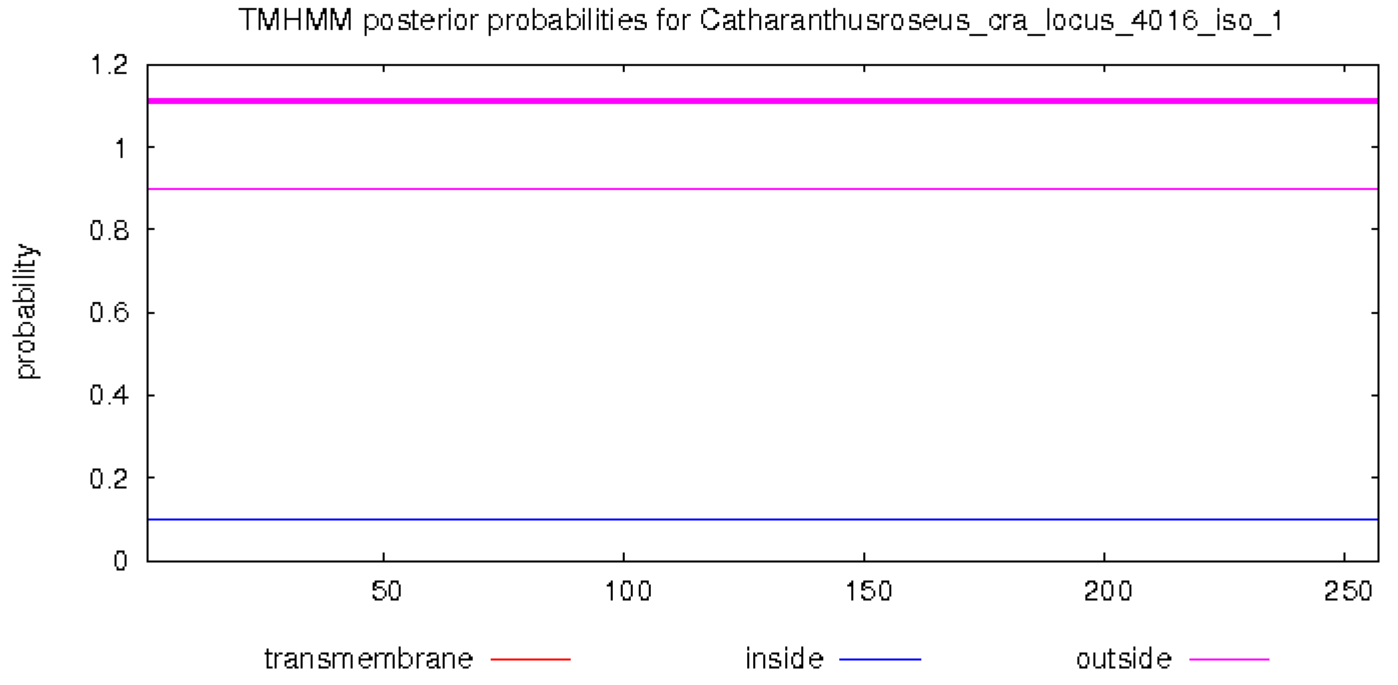

plot in postscript, script for making the plot in gnuplot, data for plot

---

```
# Catharanthusroseus_cra_locus_4016_iso_3 Length: 257
# Catharanthusroseus_cra_locus_4016_iso_3 Number of predicted TMHs:  0
# Catharanthusroseus_cra_locus_4016_iso_3 Exp number of AAs in TMHs: 0.00036
# Catharanthusroseus_cra_locus_4016_iso_3 Exp number, first 60 AAs:  0
# Catharanthusroseus_cra_locus_4016_iso_3 Total prob of N-in:        0.09945
Catharanthusroseus_cra_locus_4016_iso_3 TMHMM2.0        outside      1   257
```

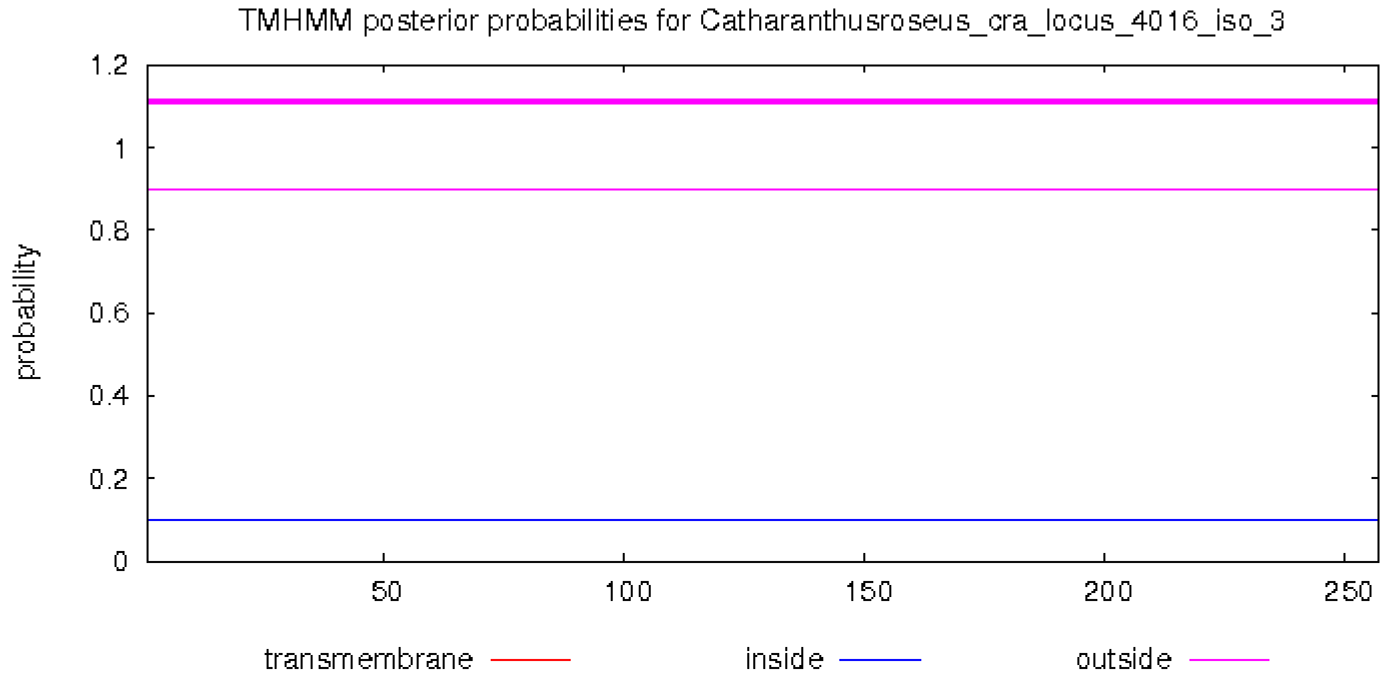

plot in postscript, script for making the plot in gnuplot, data for plot

---

```
# Catharanthusroseus_cra_locus_5081_iso_1 Length: 246
# Catharanthusroseus_cra_locus_5081_iso_1 Number of predicted TMHs:  0
```

```
# Catharanthusroseus_cra_locus_5081_iso_1 Exp number of AAs in TMHs: 0.00103
# Catharanthusroseus_cra_locus_5081_iso_1 Exp number, first 60 AAs:  0
# Catharanthusroseus_cra_locus_5081_iso_1 Total prob of N-in:        0.00343
Catharanthusroseus_cra_locus_5081_iso_1 TMHMM2.0        outside      1   246
```

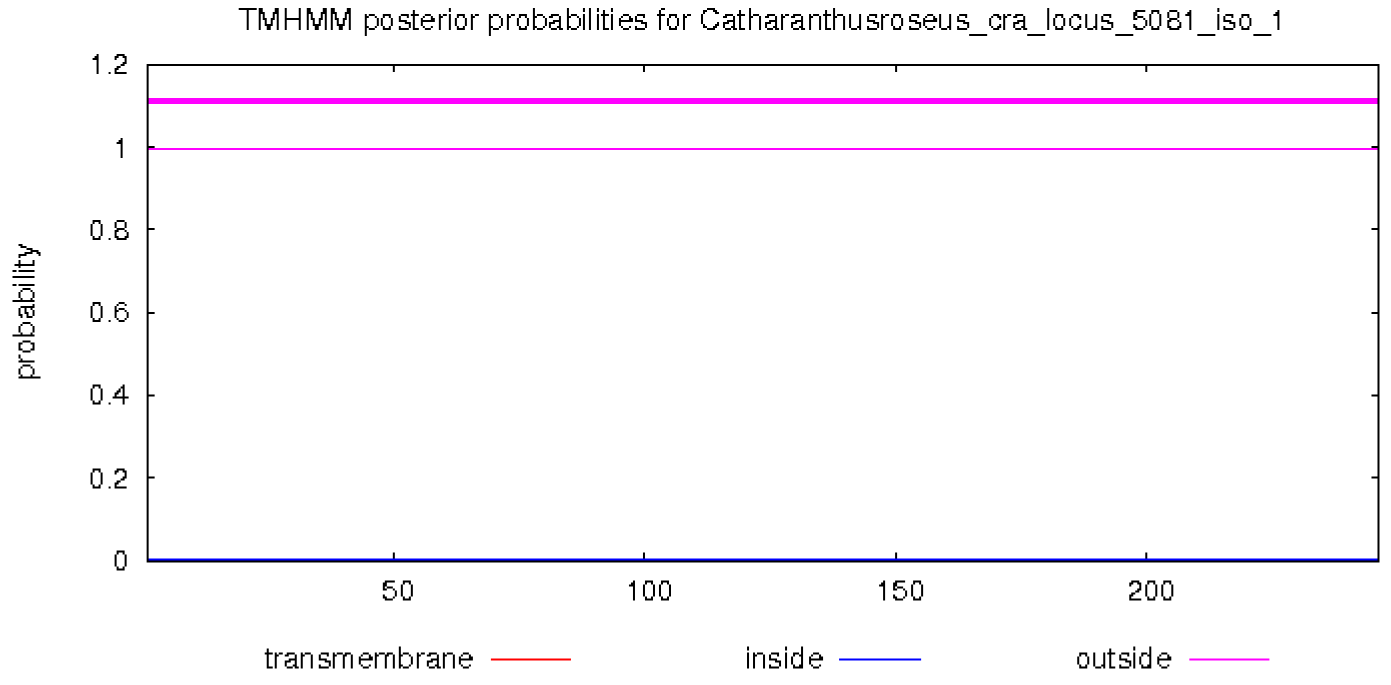

plot in postscript, script for making the plot in gnuplot, data for plot

---

```
# Catharanthusroseus_cra_locus_5081_iso_2 Length: 280
# Catharanthusroseus_cra_locus_5081_iso_2 Number of predicted TMHs:  0
# Catharanthusroseus_cra_locus_5081_iso_2 Exp number of AAs in TMHs: 0.00043
# Catharanthusroseus_cra_locus_5081_iso_2 Exp number, first 60 AAs:  0
# Catharanthusroseus_cra_locus_5081_iso_2 Total prob of N-in:        0.00214
Catharanthusroseus_cra_locus_5081_iso_2 TMHMM2.0        outside      1   280
```

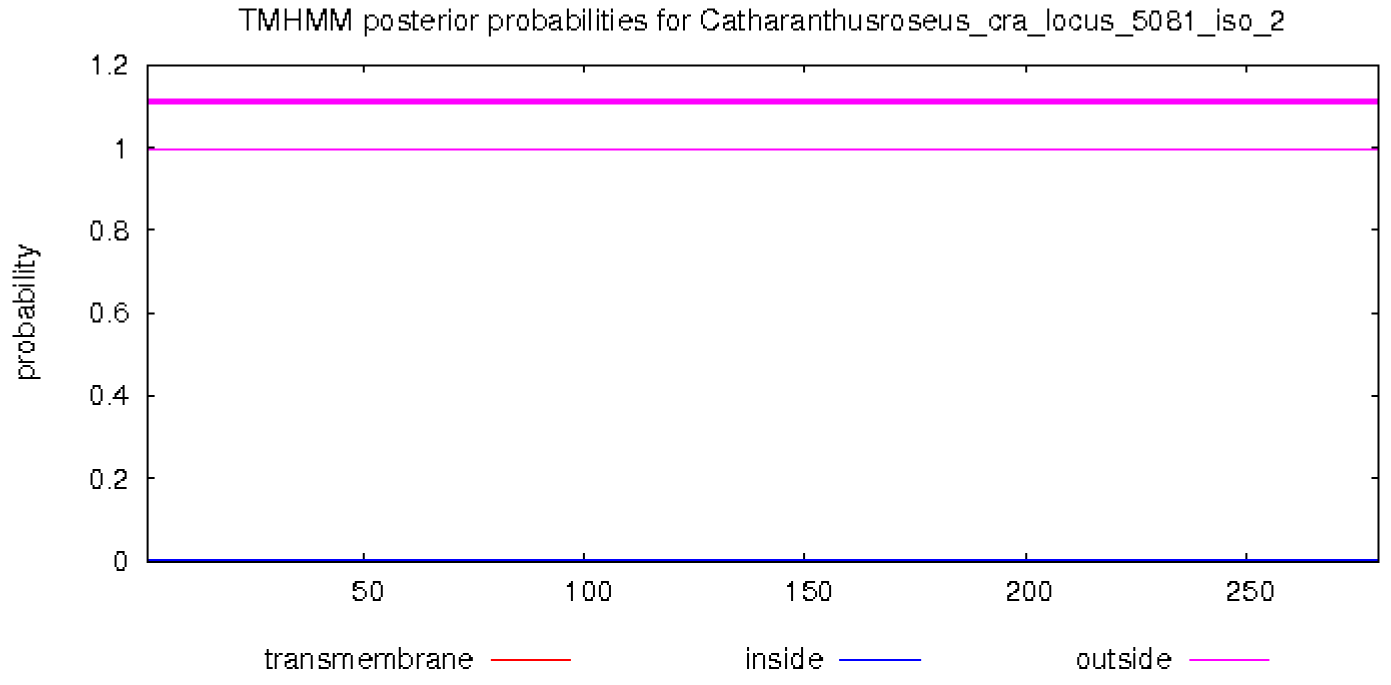

plot in postscript, script for making the plot in gnuplot, data for plot

---

```
# Catharanthusroseus_cra_locus_5081_iso_3 Length: 61
# Catharanthusroseus_cra_locus_5081_iso_3 Number of predicted TMHs:  0
```

```
# Catharanthusroseus_cra_locus_5081_iso_3 Exp number of AAs in TMHs: 0
# Catharanthusroseus_cra_locus_5081_iso_3 Exp number, first 60 AAs:  0
# Catharanthusroseus_cra_locus_5081_iso_3 Total prob of N-in:        0.02977
Catharanthusroseus_cra_locus_5081_iso_3 TMHMM2.0        outside      1    61
```

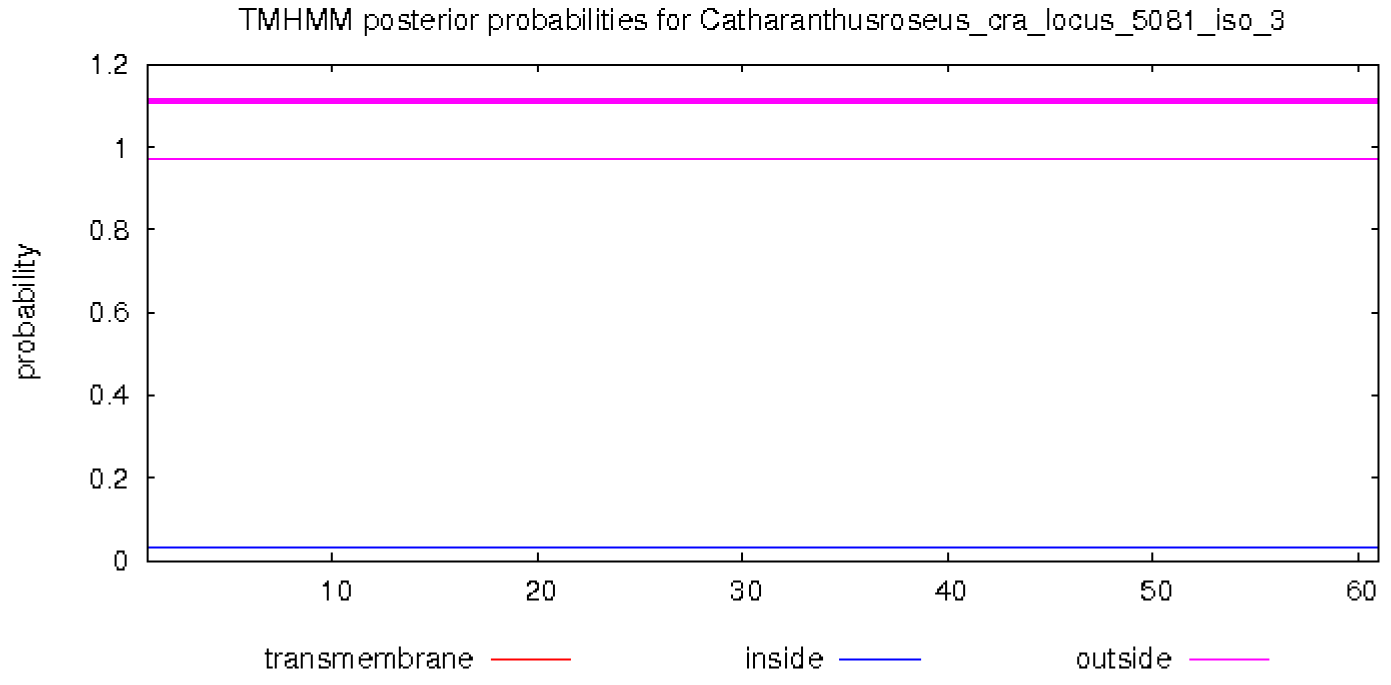

plot in postscript, script for making the plot in gnuplot, data for plot

---

```
# Catharanthusroseus_cra_locus_5081_iso_4 Length: 227
# Catharanthusroseus_cra_locus_5081_iso_4 Number of predicted TMHs:  0
# Catharanthusroseus_cra_locus_5081_iso_4 Exp number of AAs in TMHs: 0.00082
# Catharanthusroseus_cra_locus_5081_iso_4 Exp number, first 60 AAs:  0.00023
# Catharanthusroseus_cra_locus_5081_iso_4 Total prob of N-in:        0.02708
Catharanthusroseus_cra_locus_5081_iso_4 TMHMM2.0        outside      1   227
```

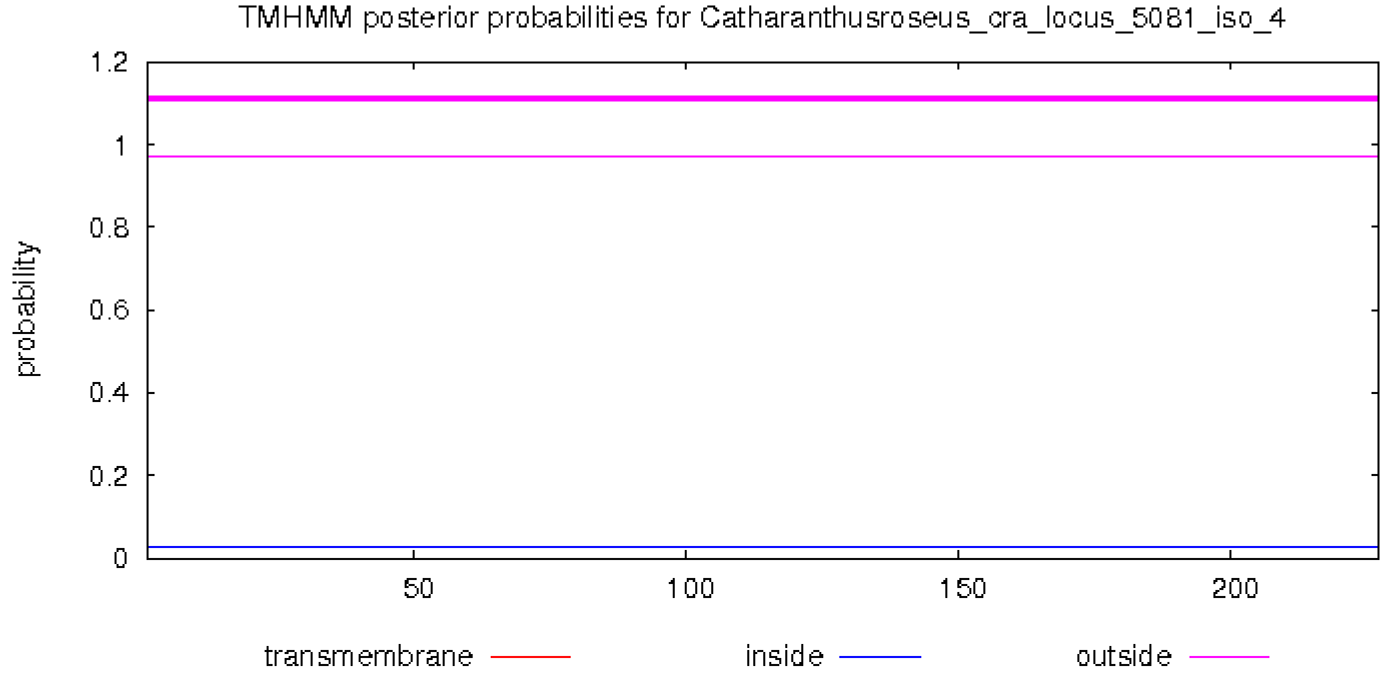

plot in postscript, script for making the plot in gnuplot, data for plot

---

```
# Catharanthusroseus_cra_locus_5081_iso_5 Length: 165
# Catharanthusroseus_cra_locus_5081_iso_5 Number of predicted TMHs:  0
```

```
# Catharanthusroseus_cra_locus_5081_iso_5 Exp number of AAs in TMHs: 0.0001
# Catharanthusroseus_cra_locus_5081_iso_5 Exp number, first 60 AAs:  0
# Catharanthusroseus_cra_locus_5081_iso_5 Total prob of N-in:        0.01047
Catharanthusroseus_cra_locus_5081_iso_5 TMHMM2.0        outside      1   165
```

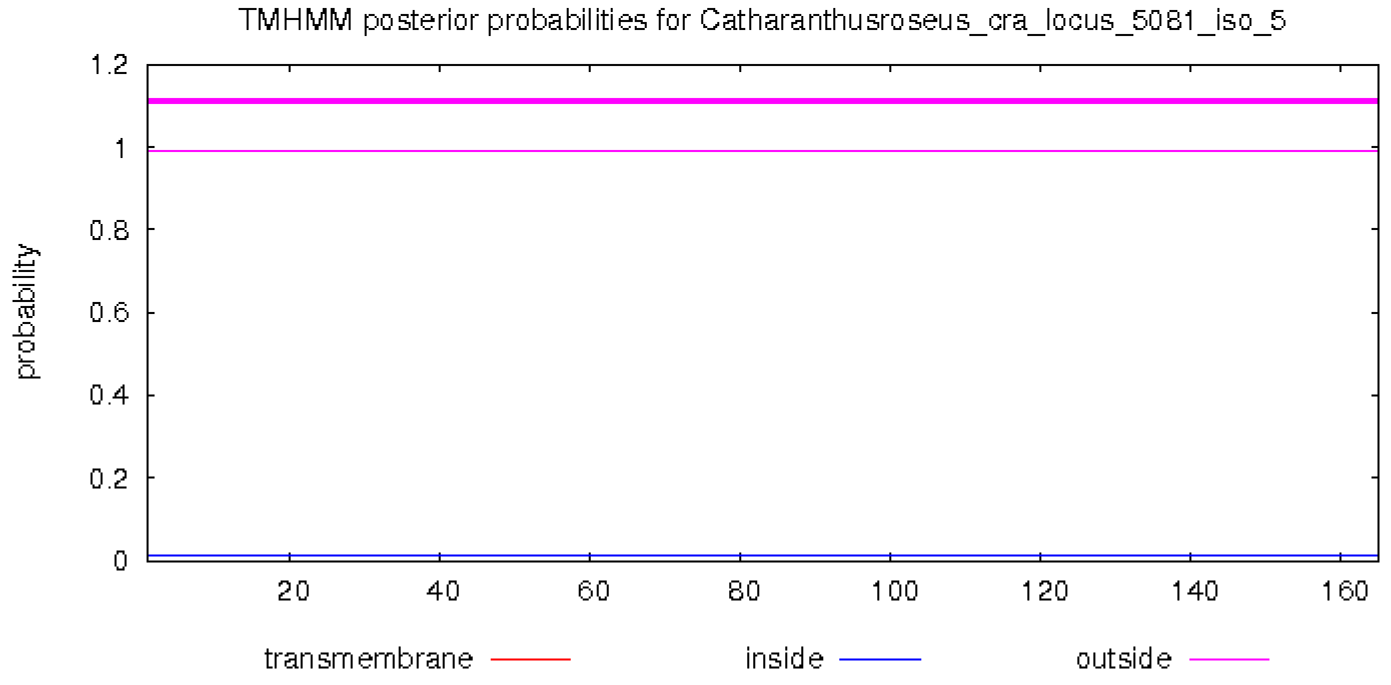

plot in postscript, script for making the plot in gnuplot, data for plot

---

```
# Catharanthusroseus_cra_locus_5081_iso_6 Length: 281
# Catharanthusroseus_cra_locus_5081_iso_6 Number of predicted TMHs:  0
# Catharanthusroseus_cra_locus_5081_iso_6 Exp number of AAs in TMHs: 0.00043
# Catharanthusroseus_cra_locus_5081_iso_6 Exp number, first 60 AAs:  0
# Catharanthusroseus_cra_locus_5081_iso_6 Total prob of N-in:        0.00211
Catharanthusroseus_cra_locus_5081_iso_6 TMHMM2.0        outside      1   281
```

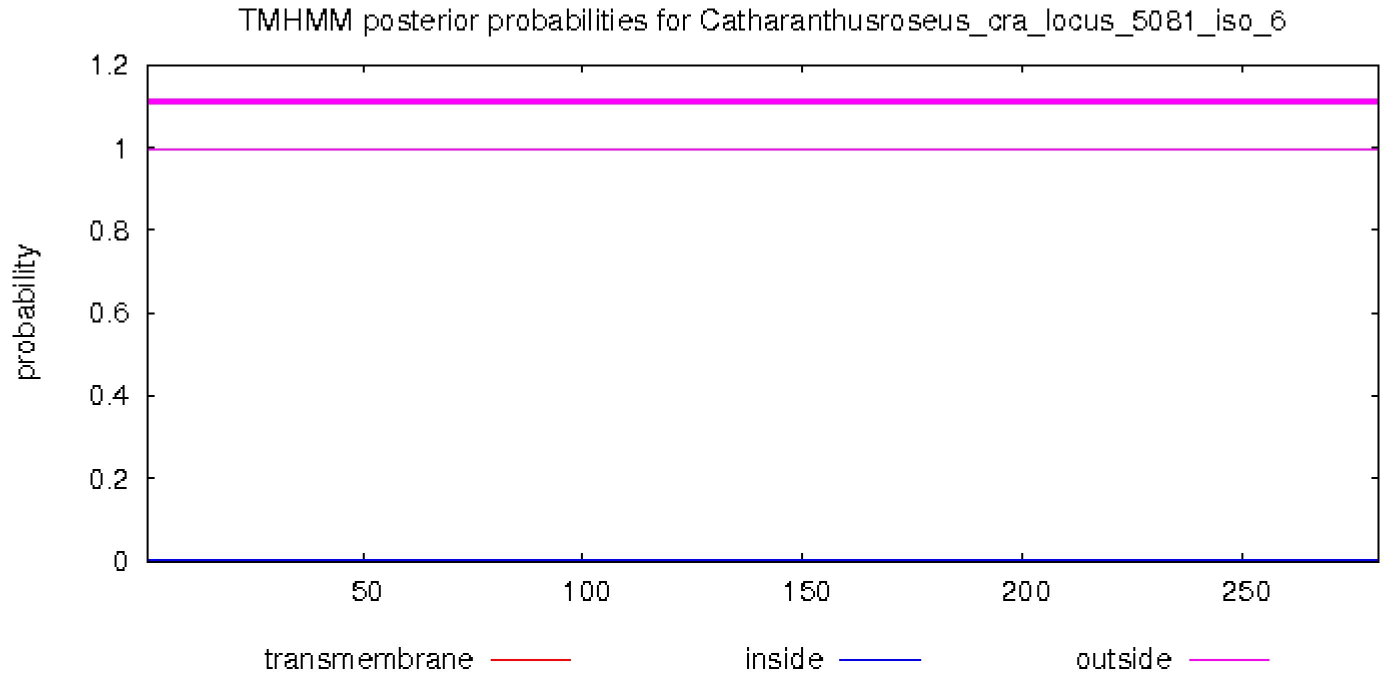

plot in postscript, script for making the plot in gnuplot, data for plot

---

```
# Catharanthusroseus_cra_locus_5366_iso_3 Length: 483
# Catharanthusroseus_cra_locus_5366_iso_3 Number of predicted TMHs:  0
```

```
# Catharanthusroseus_cra_locus_5366_iso_3 Exp number of AAs in TMHs: 0
# Catharanthusroseus_cra_locus_5366_iso_3 Exp number, first 60 AAs:  0
# Catharanthusroseus_cra_locus_5366_iso_3 Total prob of N-in:        0.00807
Catharanthusroseus_cra_locus_5366_iso_3 TMHMM2.0        outside      1   483
```

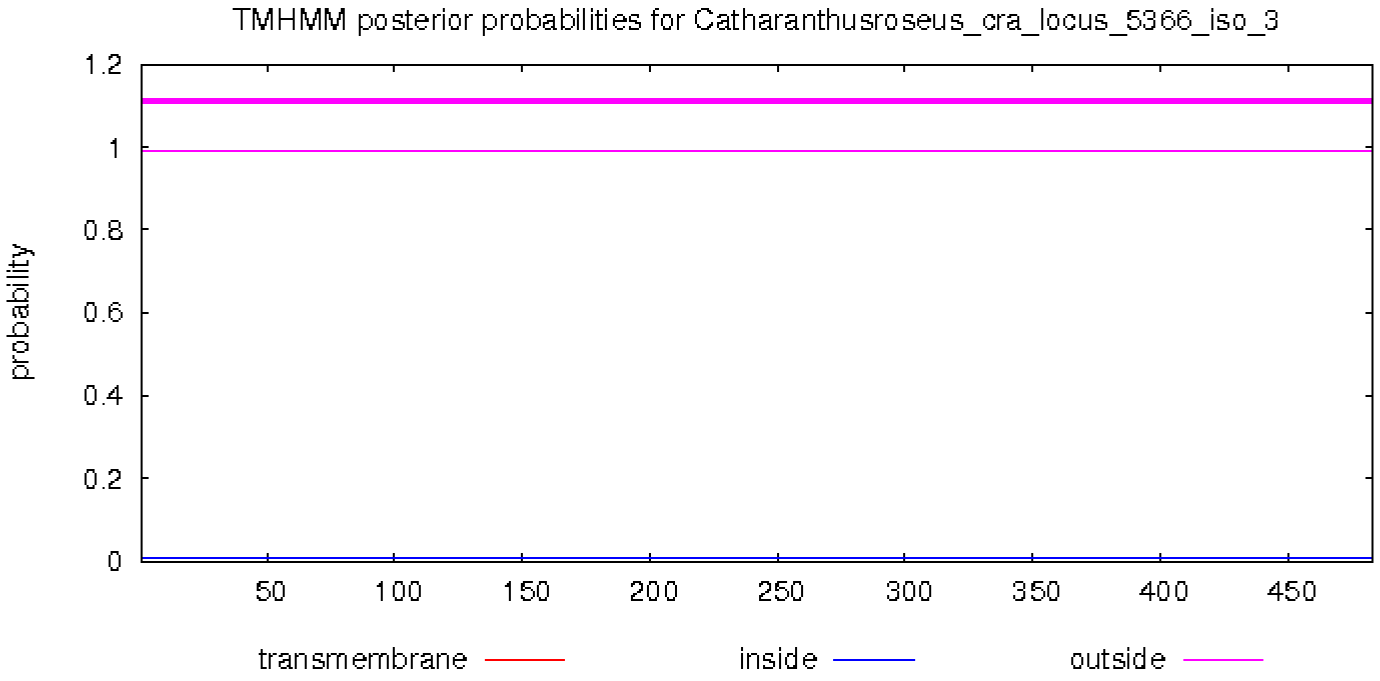

plot in postscript, script for making the plot in gnuplot, data for plot

---

```
# Catharanthusroseus_cra_locus_5366_iso_4 Length: 586
# Catharanthusroseus_cra_locus_5366_iso_4 Number of predicted TMHs:  1
# Catharanthusroseus_cra_locus_5366_iso_4 Exp number of AAs in TMHs: 21.91296
# Catharanthusroseus_cra_locus_5366_iso_4 Exp number, first 60 AAs:  0.00177
# Catharanthusroseus_cra_locus_5366_iso_4 Total prob of N-in:        0.36869
Catharanthusroseus_cra_locus_5366_iso_4 TMHMM2.0        outside      1   559
Catharanthusroseus_cra_locus_5366_iso_4 TMHMM2.0        TMhelix    560   582
Catharanthusroseus_cra_locus_5366_iso_4 TMHMM2.0        inside     583   586
```

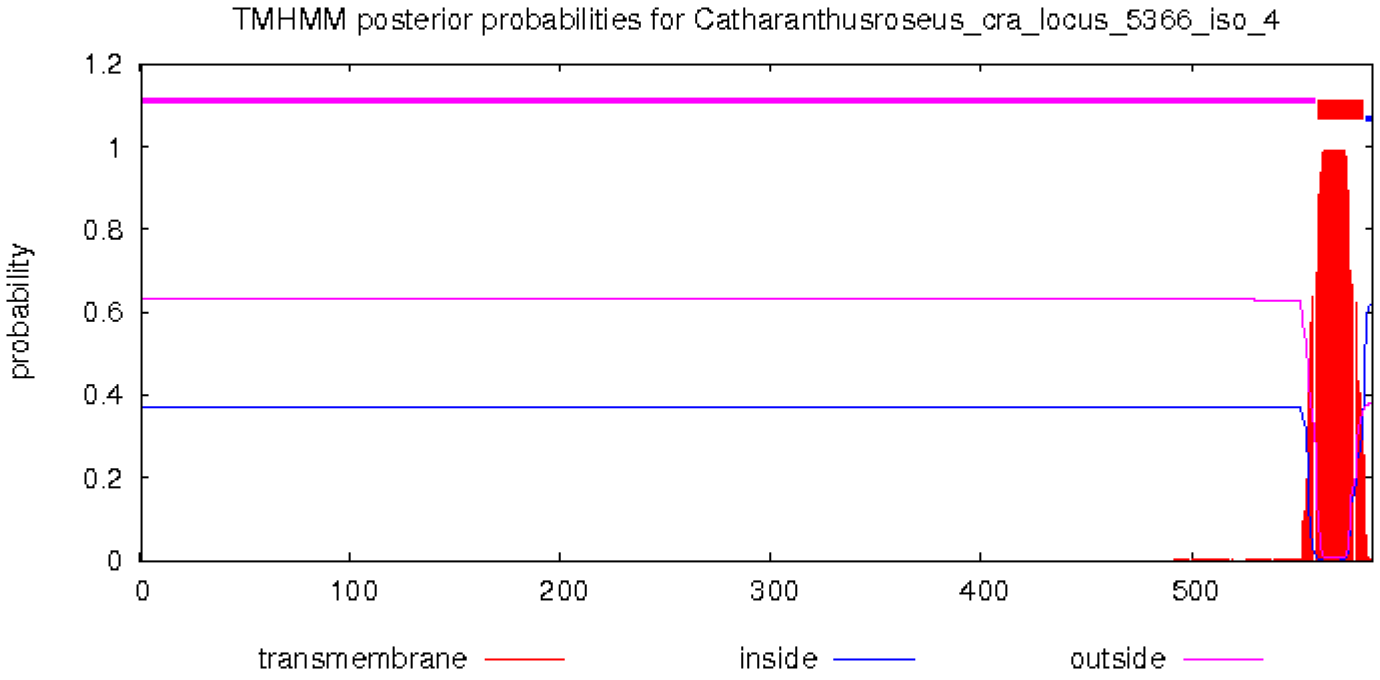

plot in postscript, script for making the plot in gnuplot, data for plot

---

```
# Catharanthusroseus_cra_locus_5600_iso_1 Length: 262
# Catharanthusroseus_cra_locus_5600_iso_1 Number of predicted TMHs:  0
# Catharanthusroseus_cra_locus_5600_iso_1 Exp number of AAs in TMHs: 0.07893
# Catharanthusroseus_cra_locus_5600_iso_1 Exp number, first 60 AAs:  0
# Catharanthusroseus_cra_locus_5600_iso_1 Total prob of N-in:        0.08883
Catharanthusroseus_cra_locus_5600_iso_1 TMHMM2.0        outside      1   262
```

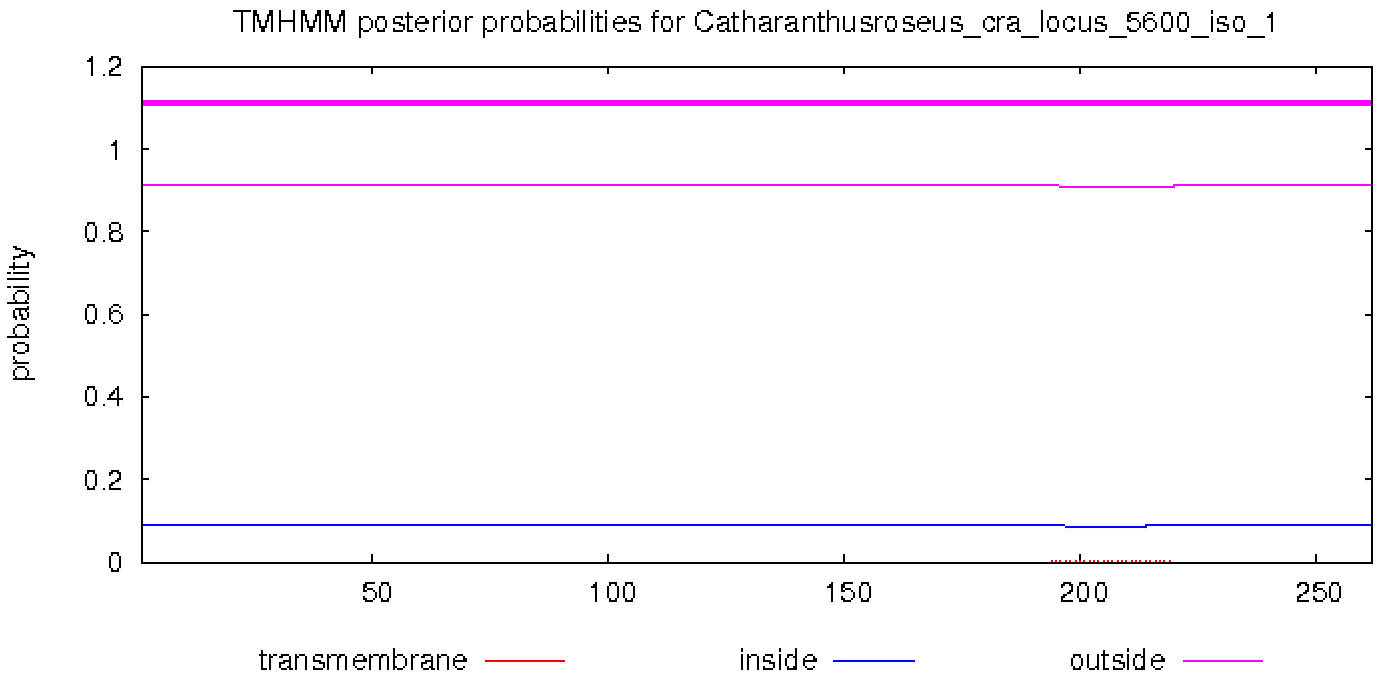

plot in postscript, script for making the plot in gnuplot, data for plot

```
# Catharanthusroseus_cra_locus_5600_iso_2 Length: 242
# Catharanthusroseus_cra_locus_5600_iso_2 Number of predicted TMHs:  0
# Catharanthusroseus_cra_locus_5600_iso_2 Exp number of AAs in TMHs: 0.44595
# Catharanthusroseus_cra_locus_5600_iso_2 Exp number, first 60 AAs:  0.18879
# Catharanthusroseus_cra_locus_5600_iso_2 Total prob of N-in:        0.23070
Catharanthusroseus_cra_locus_5600_iso_2 TMHMM2.0        outside      1   242
```

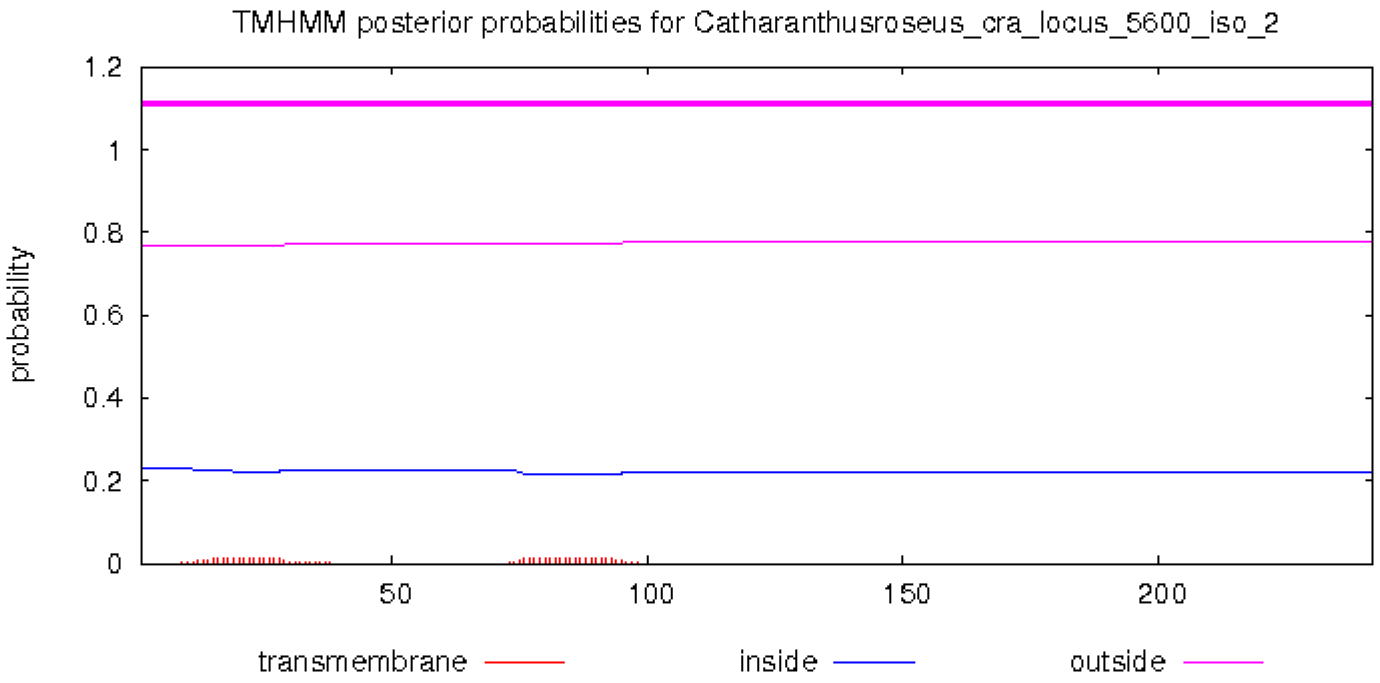

plot in postscript, script for making the plot in gnuplot, data for plot

```
# Catharanthusroseus_cra_locus_5600_iso_3 Length: 331
# Catharanthusroseus_cra_locus_5600_iso_3 Number of predicted TMHs:  0
# Catharanthusroseus_cra_locus_5600_iso_3 Exp number of AAs in TMHs: 0.11498
# Catharanthusroseus_cra_locus_5600_iso_3 Exp number, first 60 AAs:  0
# Catharanthusroseus_cra_locus_5600_iso_3 Total prob of N-in:        0.13463
Catharanthusroseus_cra_locus_5600_iso_3 TMHMM2.0        outside      1   331
```

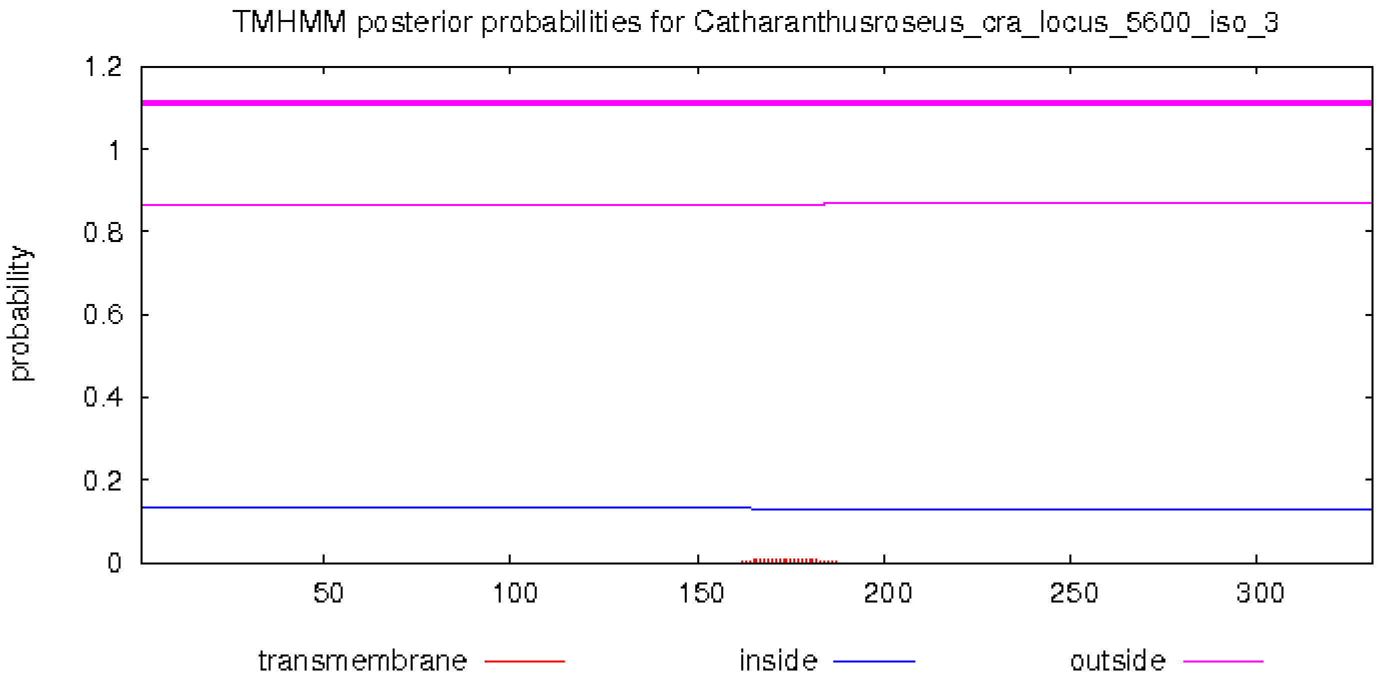

plot in postscript, script for making the plot in gnuplot, data for plot

---

```
# Catharanthusroseus_cra_locus_5600_iso_4 Length: 363
# Catharanthusroseus_cra_locus_5600_iso_4 Number of predicted TMHs:  0
# Catharanthusroseus_cra_locus_5600_iso_4 Exp number of AAs in TMHs: 0.03249
# Catharanthusroseus_cra_locus_5600_iso_4 Exp number, first 60 AAs:  0
# Catharanthusroseus_cra_locus_5600_iso_4 Total prob of N-in:        0.02595
Catharanthusroseus_cra_locus_5600_iso_4 TMHMM2.0        outside      1   363
```

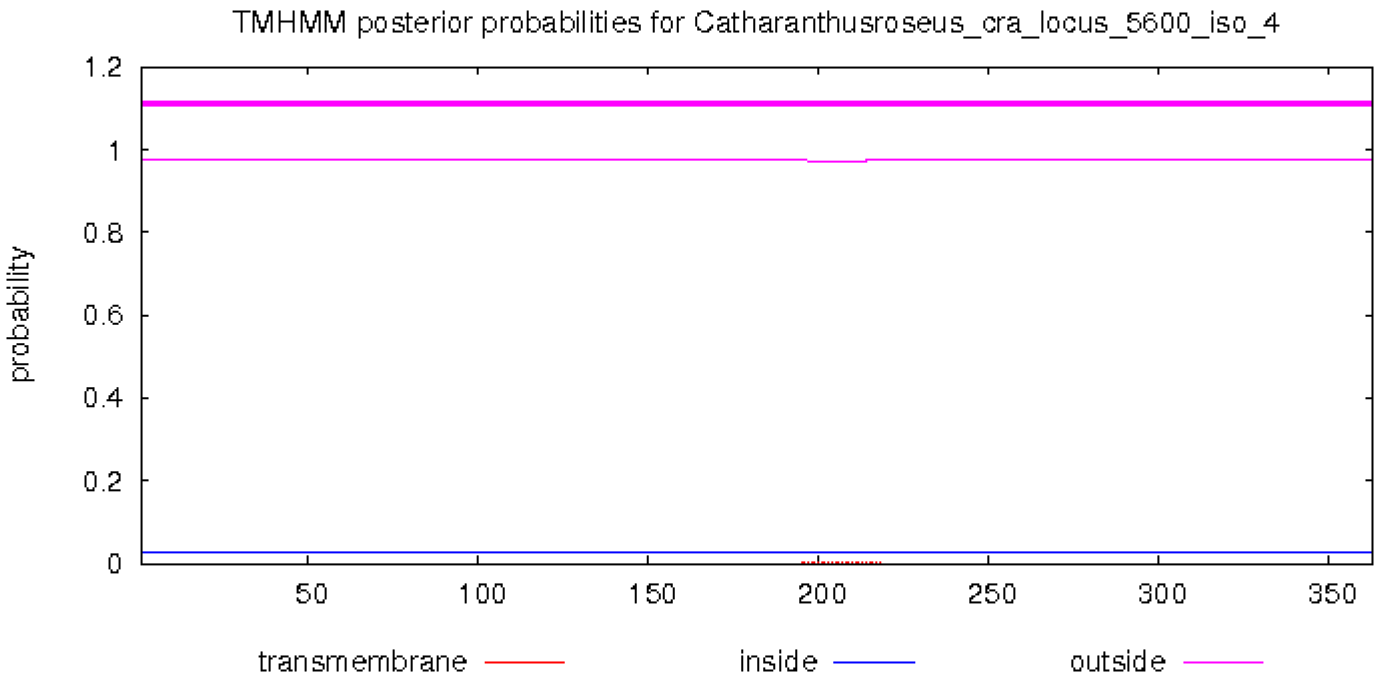

plot in postscript, script for making the plot in gnuplot, data for plot

---

```
# Catharanthusroseus_cra_locus_61901_iso_1 Length: 122
# Catharanthusroseus_cra_locus_61901_iso_1 Number of predicted TMHs:  0
# Catharanthusroseus_cra_locus_61901_iso_1 Exp number of AAs in TMHs: 0.00062
# Catharanthusroseus_cra_locus_61901_iso_1 Exp number, first 60 AAs:  0.00047
# Catharanthusroseus_cra_locus_61901_iso_1 Total prob of N-in:        0.09283
Catharanthusroseus_cra_locus_61901_iso_1        TMHMM2.0        outside      1   122
```

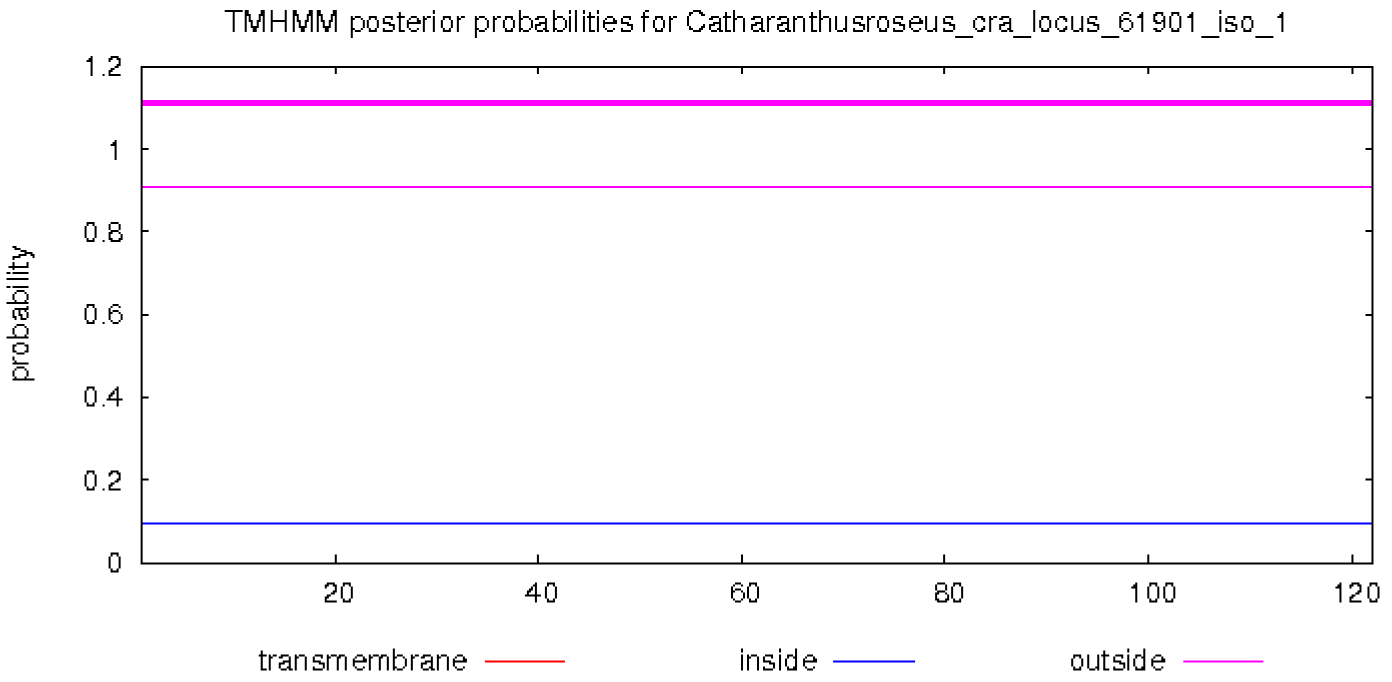

plot in postscript, script for making the plot in gnuplot, data for plot

---

```
# Catharanthusroseus_cra_locus_6334_iso_1 Length: 375
# Catharanthusroseus_cra_locus_6334_iso_1 Number of predicted TMHs:  0
# Catharanthusroseus_cra_locus_6334_iso_1 Exp number of AAs in TMHs: 0
# Catharanthusroseus_cra_locus_6334_iso_1 Exp number, first 60 AAs:  0
# Catharanthusroseus_cra_locus_6334_iso_1 Total prob of N-in:        0.00702
Catharanthusroseus_cra_locus_6334_iso_1 TMHMM2.0        outside      1   375
```

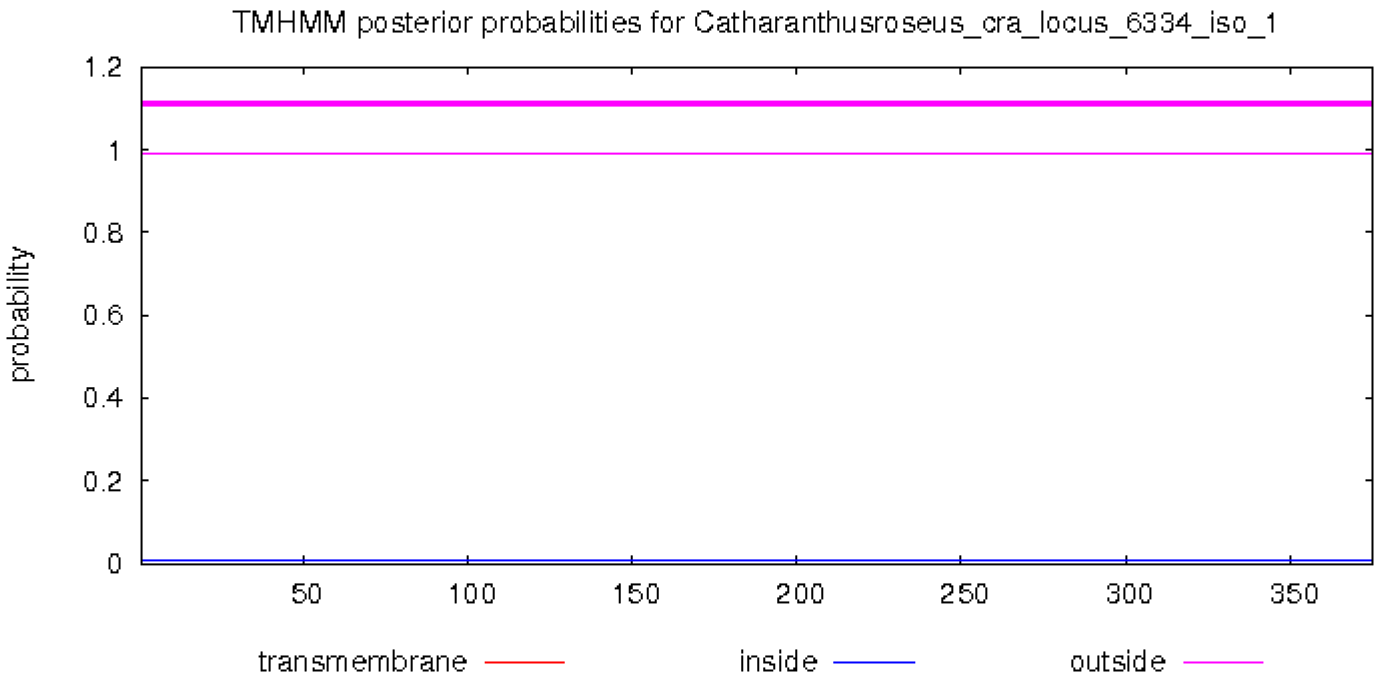

plot in postscript, script for making the plot in gnuplot, data for plot

---

```
# Catharanthusroseus_cra_locus_6334_iso_2 Length: 420
# Catharanthusroseus_cra_locus_6334_iso_2 Number of predicted TMHs:  0
# Catharanthusroseus_cra_locus_6334_iso_2 Exp number of AAs in TMHs: 0
# Catharanthusroseus_cra_locus_6334_iso_2 Exp number, first 60 AAs:  0
# Catharanthusroseus_cra_locus_6334_iso_2 Total prob of N-in:        0.00356
Catharanthusroseus_cra_locus_6334_iso_2 TMHMM2.0        outside      1   420
```

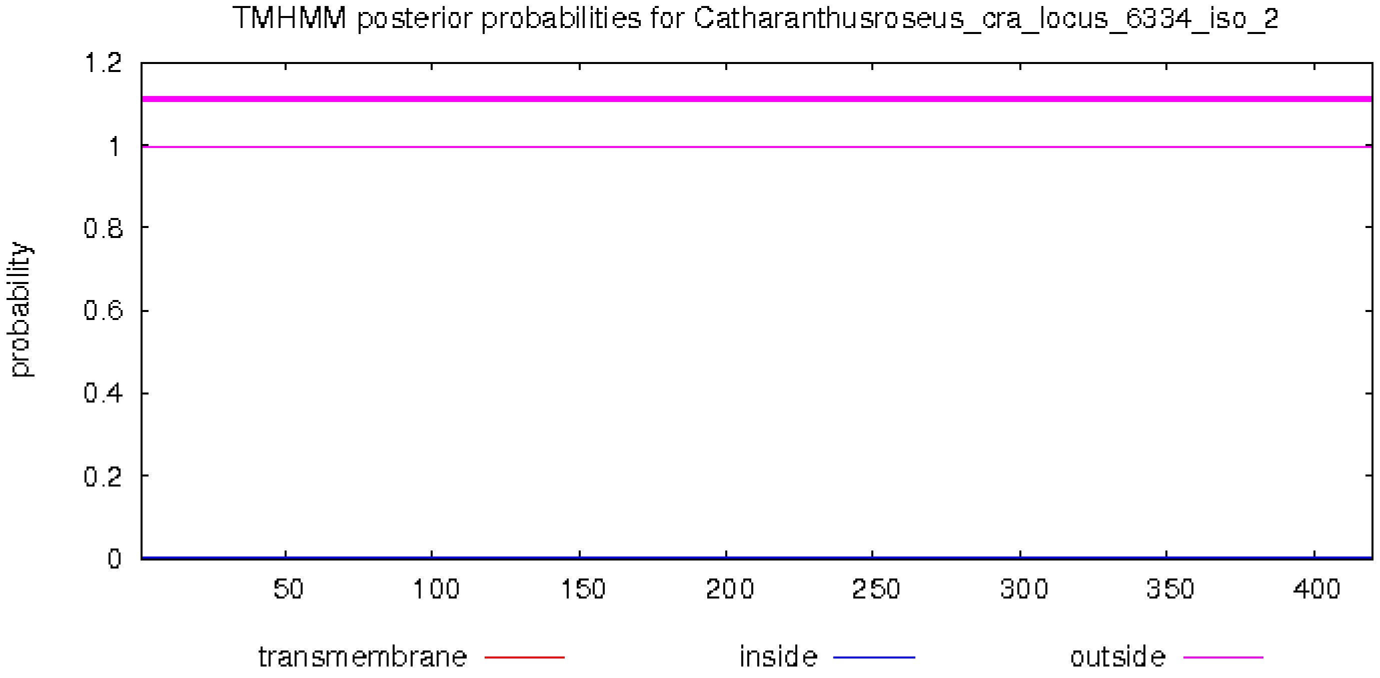

plot in postscript, script for making the plot in gnuplot, data for plot

---

```
# Catharanthusroseus_cra_locus_6334_iso_3 Length: 497
# Catharanthusroseus_cra_locus_6334_iso_3 Number of predicted TMHs:  1
# Catharanthusroseus_cra_locus_6334_iso_3 Exp number of AAs in TMHs: 27.74821
# Catharanthusroseus_cra_locus_6334_iso_3 Exp number, first 60 AAs:  0
# Catharanthusroseus_cra_locus_6334_iso_3 Total prob of N-in:        0.49607
Catharanthusroseus_cra_locus_6334_iso_3 TMHMM2.0        inside       1   308
Catharanthusroseus_cra_locus_6334_iso_3 TMHMM2.0        TMhelix    309   328
Catharanthusroseus_cra_locus_6334_iso_3 TMHMM2.0        outside    329   497
```

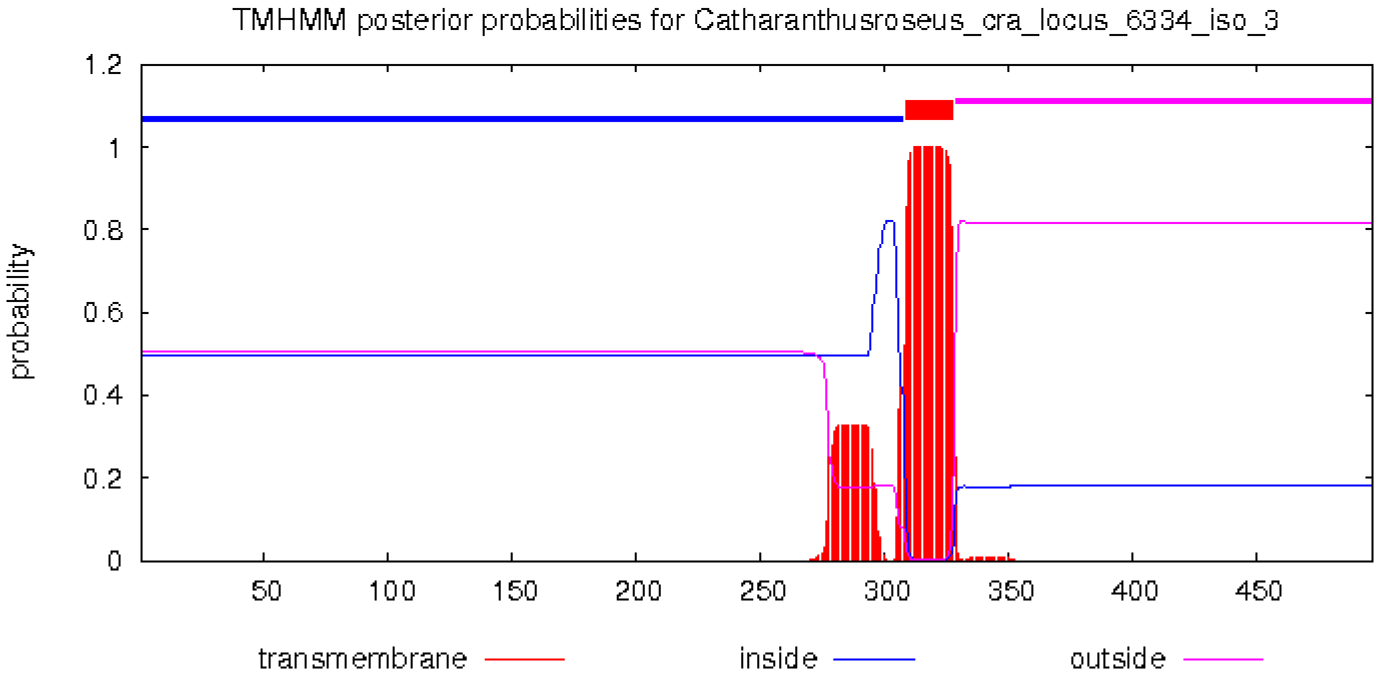

plot in postscript, script for making the plot in gnuplot, data for plot

```
# Catharanthusroseus_cra_locus_6334_iso_4 Length: 422
# Catharanthusroseus_cra_locus_6334_iso_4 Number of predicted TMHs:  0
# Catharanthusroseus_cra_locus_6334_iso_4 Exp number of AAs in TMHs: 0
# Catharanthusroseus_cra_locus_6334_iso_4 Exp number, first 60 AAs:  0
# Catharanthusroseus_cra_locus_6334_iso_4 Total prob of N-in:        0.00392
Catharanthusroseus_cra_locus_6334_iso_4 TMHMM2.0        outside      1   422
```

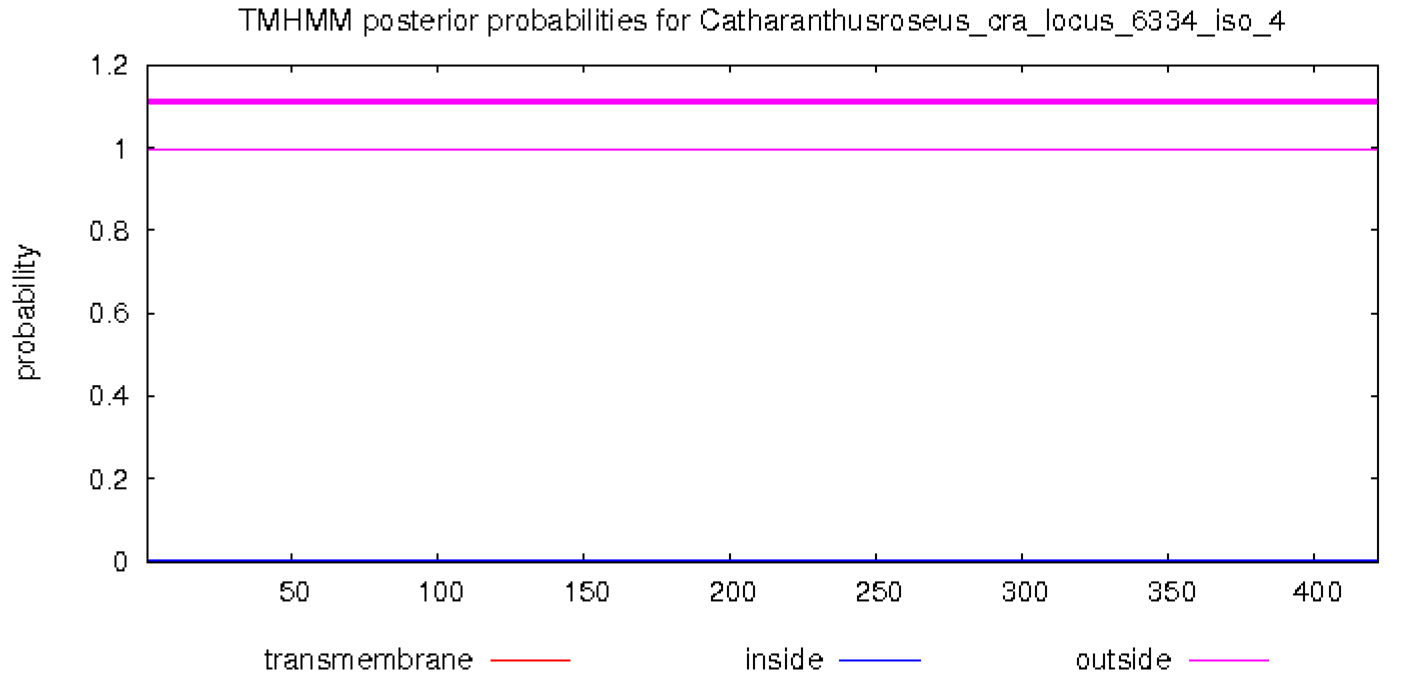

plot in postscript, script for making the plot in gnuplot, data for plot

```
# Catharanthusroseus_cra_locus_63860_iso_1 Length: 120
# Catharanthusroseus_cra_locus_63860_iso_1 Number of predicted TMHs:  0
# Catharanthusroseus_cra_locus_63860_iso_1 Exp number of AAs in TMHs: 0.0001
# Catharanthusroseus_cra_locus_63860_iso_1 Exp number, first 60 AAs:  0.0001
# Catharanthusroseus_cra_locus_63860_iso_1 Total prob of N-in:        0.33591
Catharanthusroseus_cra_locus_63860_iso_1        TMHMM2.0        outside      1   120
```

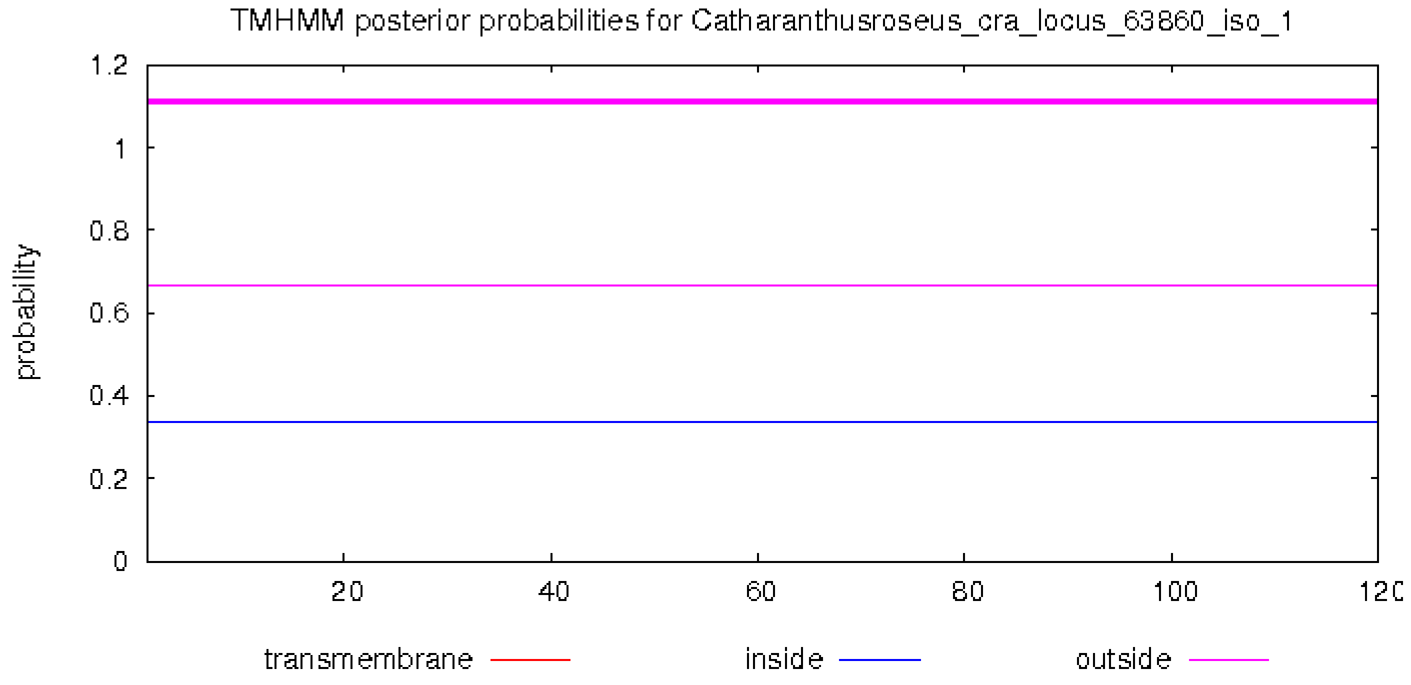

plot in postscript, script for making the plot in gnuplot, data for plot

```
# Catharanthusroseus_cra_locus_6720_iso_3 Length: 158
# Catharanthusroseus_cra_locus_6720_iso_3 Number of predicted TMHs:  0
# Catharanthusroseus_cra_locus_6720_iso_3 Exp number of AAs in TMHs: 0
# Catharanthusroseus_cra_locus_6720_iso_3 Exp number, first 60 AAs:  0
# Catharanthusroseus_cra_locus_6720_iso_3 Total prob of N-in:        0.01089
Catharanthusroseus_cra_locus_6720_iso_3 TMHMM2.0        outside      1   158
```

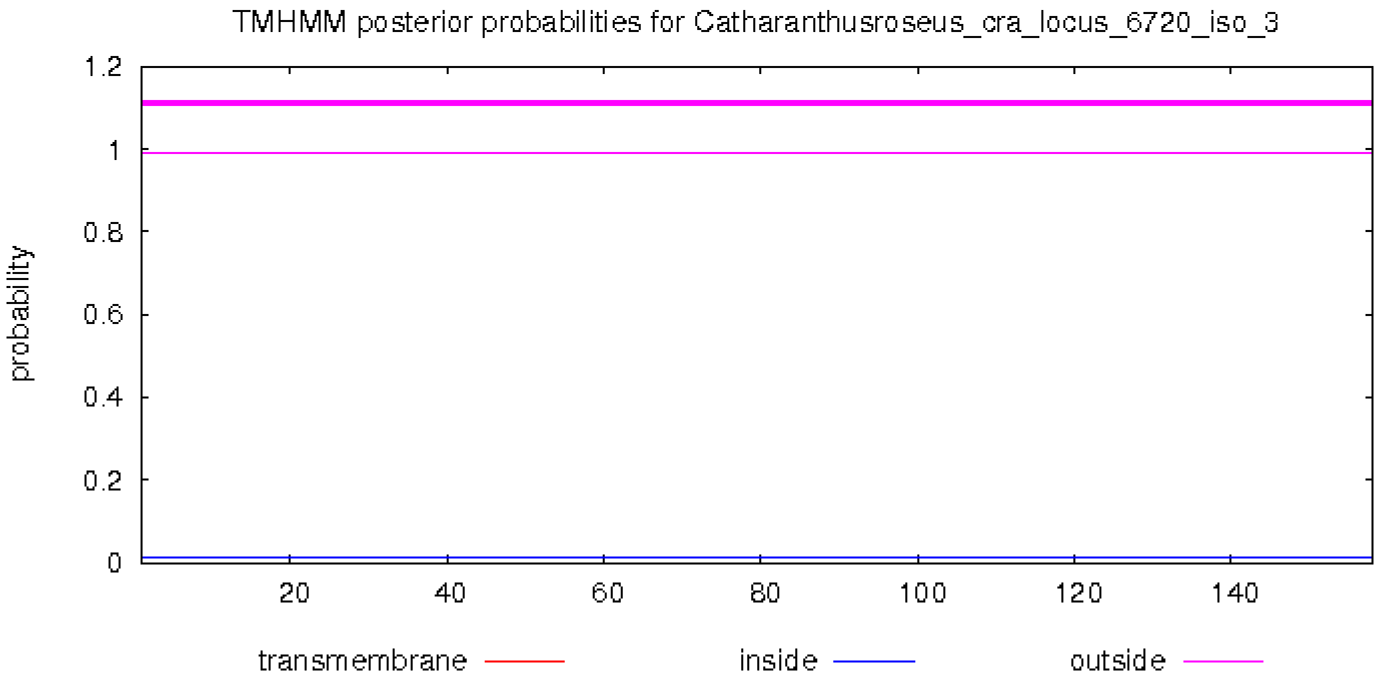

plot in postscript, script for making the plot in gnuplot, data for plot

---

```
# Catharanthusroseus_cra_locus_6720_iso_4 Length: 246
# Catharanthusroseus_cra_locus_6720_iso_4 Number of predicted TMHs:  0
# Catharanthusroseus_cra_locus_6720_iso_4 Exp number of AAs in TMHs: 8.40014
# Catharanthusroseus_cra_locus_6720_iso_4 Exp number, first 60 AAs:  8.40014
# Catharanthusroseus_cra_locus_6720_iso_4 Total prob of N-in:        0.40311
Catharanthusroseus_cra_locus_6720_iso_4 TMHMM2.0        outside      1   246
```

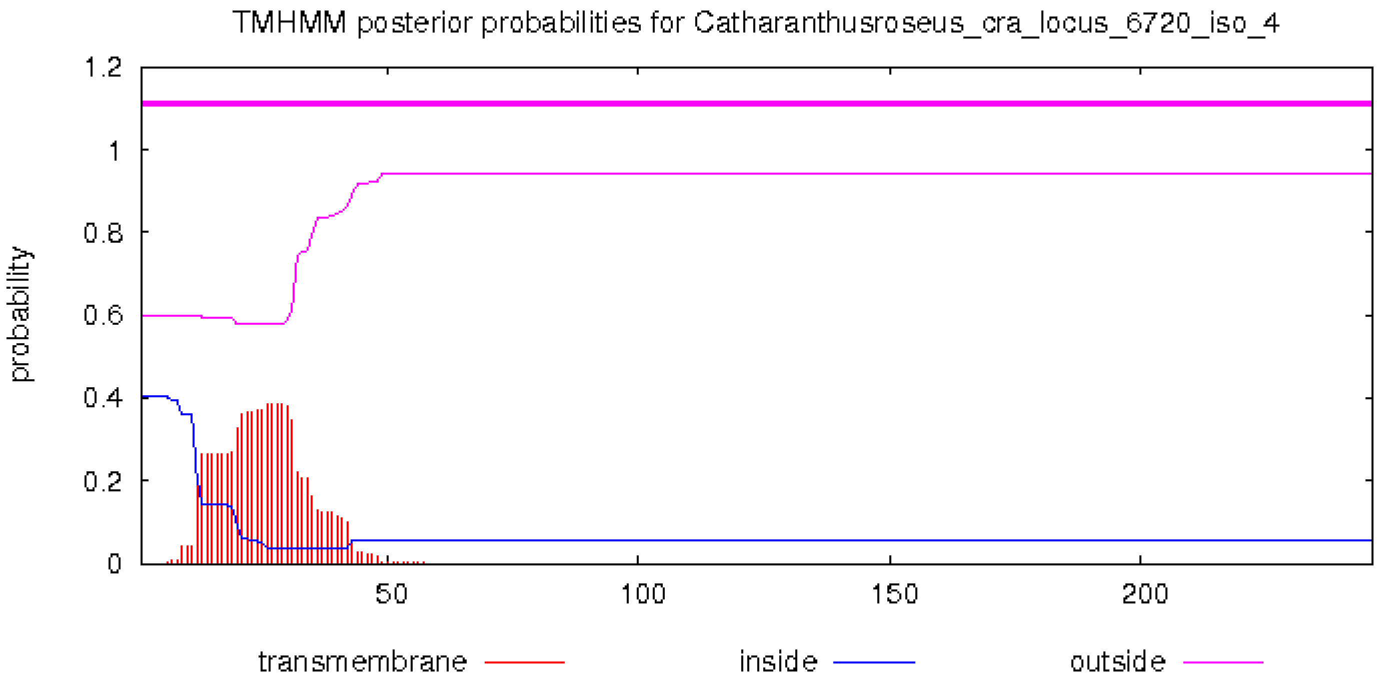

plot in postscript, script for making the plot in gnuplot, data for plot

---

```
# Catharanthusroseus_cra_locus_6720_iso_7 Length: 253
# Catharanthusroseus_cra_locus_6720_iso_7 Number of predicted TMHs:  0
# Catharanthusroseus_cra_locus_6720_iso_7 Exp number of AAs in TMHs: 0
# Catharanthusroseus_cra_locus_6720_iso_7 Exp number, first 60 AAs:  0
# Catharanthusroseus_cra_locus_6720_iso_7 Total prob of N-in:        0.00330
Catharanthusroseus_cra_locus_6720_iso_7 TMHMM2.0        outside      1   253
```

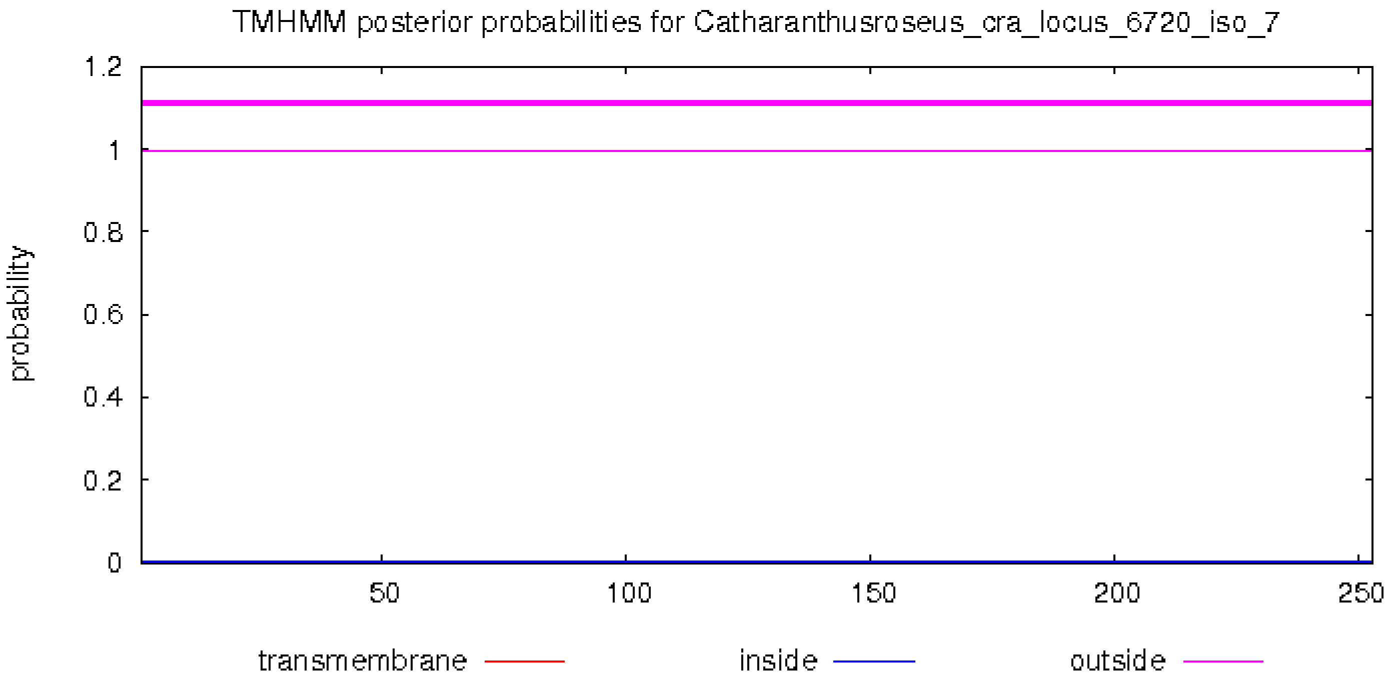

plot in postscript, script for making the plot in gnuplot, data for plot

---

```
# Catharanthusroseus_cra_locus_7294_iso_2 Length: 386
# Catharanthusroseus_cra_locus_7294_iso_2 Number of predicted TMHs:  0
# Catharanthusroseus_cra_locus_7294_iso_2 Exp number of AAs in TMHs: 0.00023
# Catharanthusroseus_cra_locus_7294_iso_2 Exp number, first 60 AAs:  0
# Catharanthusroseus_cra_locus_7294_iso_2 Total prob of N-in:        0.00704
Catharanthusroseus_cra_locus_7294_iso_2 TMHMM2.0        outside      1   386
```

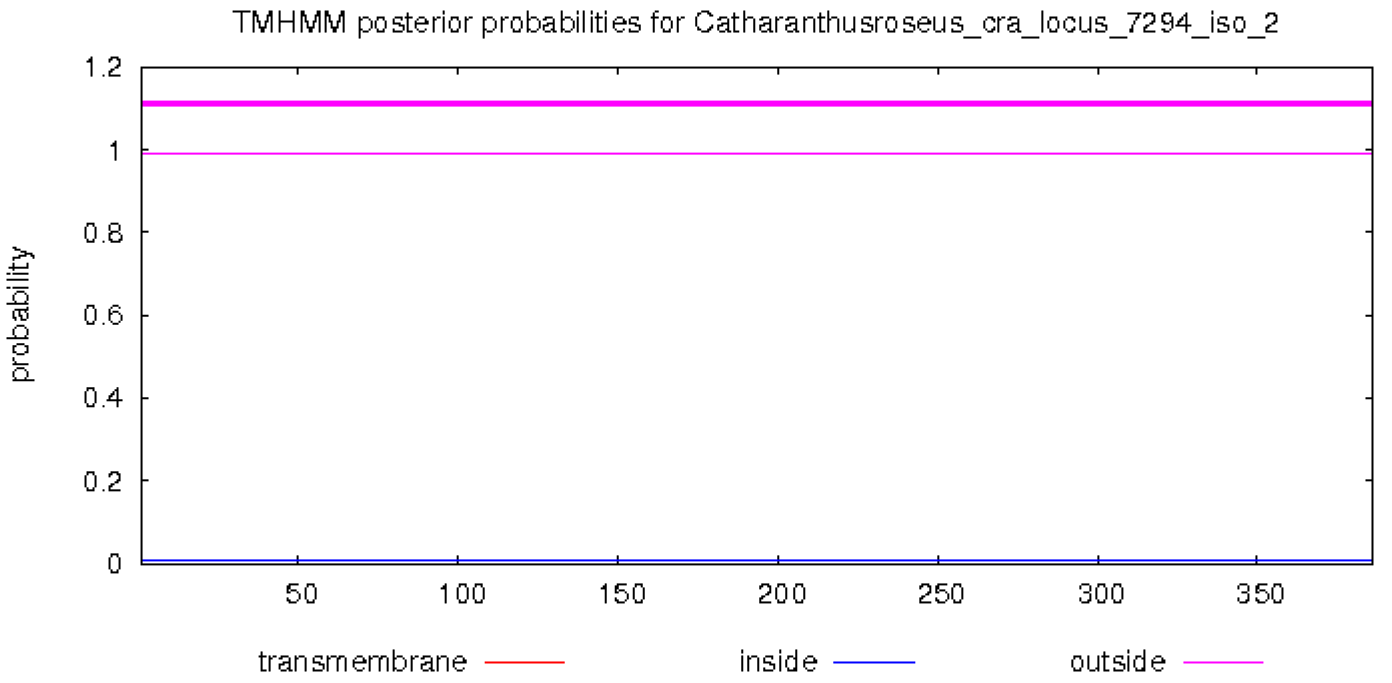

plot in postscript, script for making the plot in gnuplot, data for plot

---

```
# Catharanthusroseus_cra_locus_7294_iso_3 Length: 387
# Catharanthusroseus_cra_locus_7294_iso_3 Number of predicted TMHs:  0
# Catharanthusroseus_cra_locus_7294_iso_3 Exp number of AAs in TMHs: 0.00023
# Catharanthusroseus_cra_locus_7294_iso_3 Exp number, first 60 AAs:  0
# Catharanthusroseus_cra_locus_7294_iso_3 Total prob of N-in:        0.00695
Catharanthusroseus_cra_locus_7294_iso_3 TMHMM2.0        outside      1   387
```

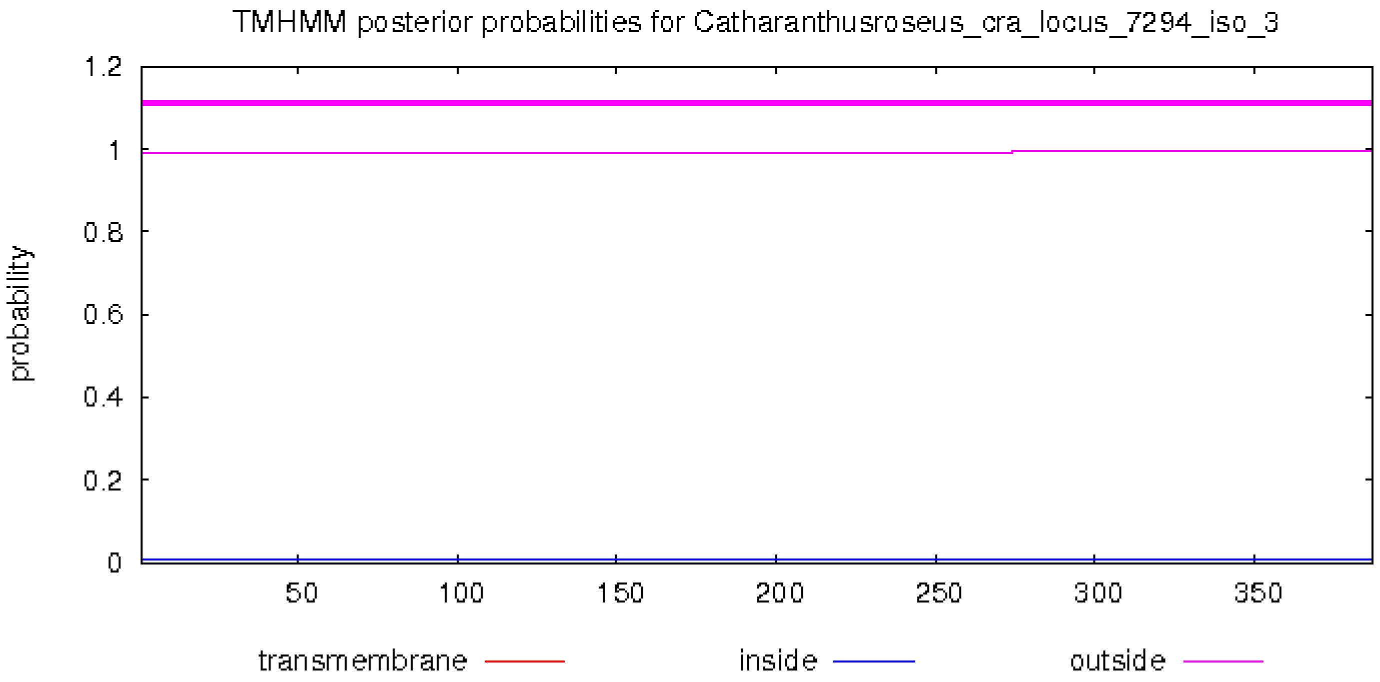

plot in postscript, script for making the plot in gnuplot, data for plot

---

```
# Catharanthusroseus_cra_locus_84210_iso_1 Length: 193
# Catharanthusroseus_cra_locus_84210_iso_1 Number of predicted TMHs:  0
# Catharanthusroseus_cra_locus_84210_iso_1 Exp number of AAs in TMHs: 0.00263
# Catharanthusroseus_cra_locus_84210_iso_1 Exp number, first 60 AAs:  0
# Catharanthusroseus_cra_locus_84210_iso_1 Total prob of N-in:        0.19573
Catharanthusroseus_cra_locus_84210_iso_1        TMHMM2.0        outside      1   193
```

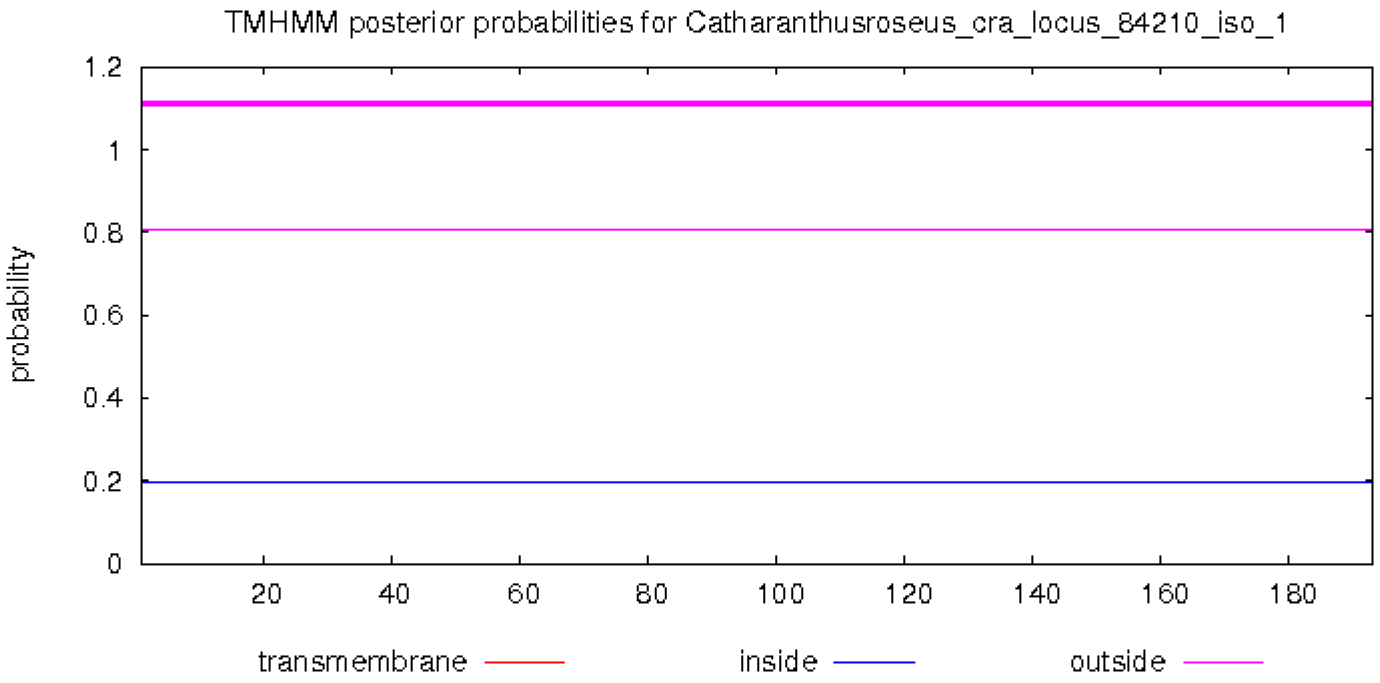

plot in postscript, script for making the plot in gnuplot, data for plot

---

```
# Catharanthusroseus_cra_locus_9229_iso_1 Length: 243
# Catharanthusroseus_cra_locus_9229_iso_1 Number of predicted TMHs:  0
# Catharanthusroseus_cra_locus_9229_iso_1 Exp number of AAs in TMHs: 0.00075
# Catharanthusroseus_cra_locus_9229_iso_1 Exp number, first 60 AAs:  0.00049
# Catharanthusroseus_cra_locus_9229_iso_1 Total prob of N-in:        0.05254
Catharanthusroseus_cra_locus_9229_iso_1 TMHMM2.0        outside      1   243
```

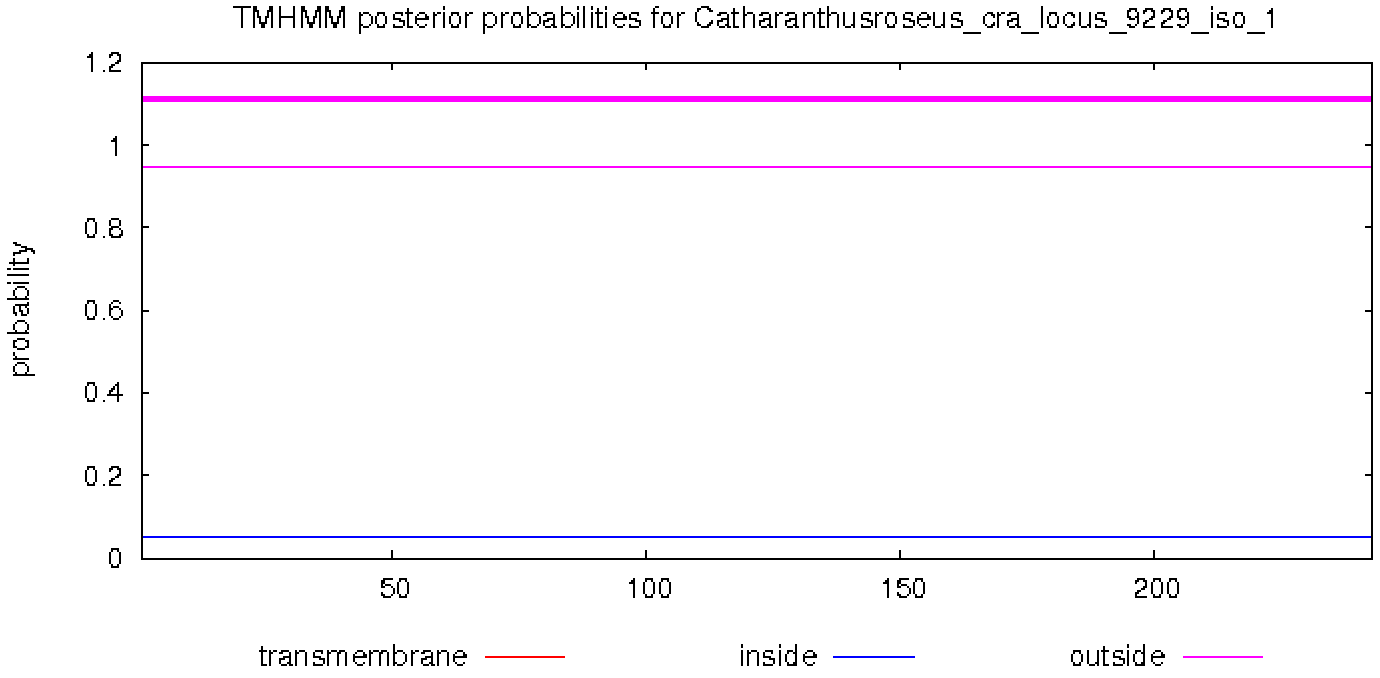

plot in postscript, script for making the plot in gnuplot, data for plot

---

```
# Catharanthusroseus_cra_locus_9524_iso_1 Length: 347
# Catharanthusroseus_cra_locus_9524_iso_1 Number of predicted TMHs:  0
# Catharanthusroseus_cra_locus_9524_iso_1 Exp number of AAs in TMHs: 0.0017
# Catharanthusroseus_cra_locus_9524_iso_1 Exp number, first 60 AAs:  0
# Catharanthusroseus_cra_locus_9524_iso_1 Total prob of N-in:        0.02635
Catharanthusroseus_cra_locus_9524_iso_1 TMHMM2.0        outside      1   347
```

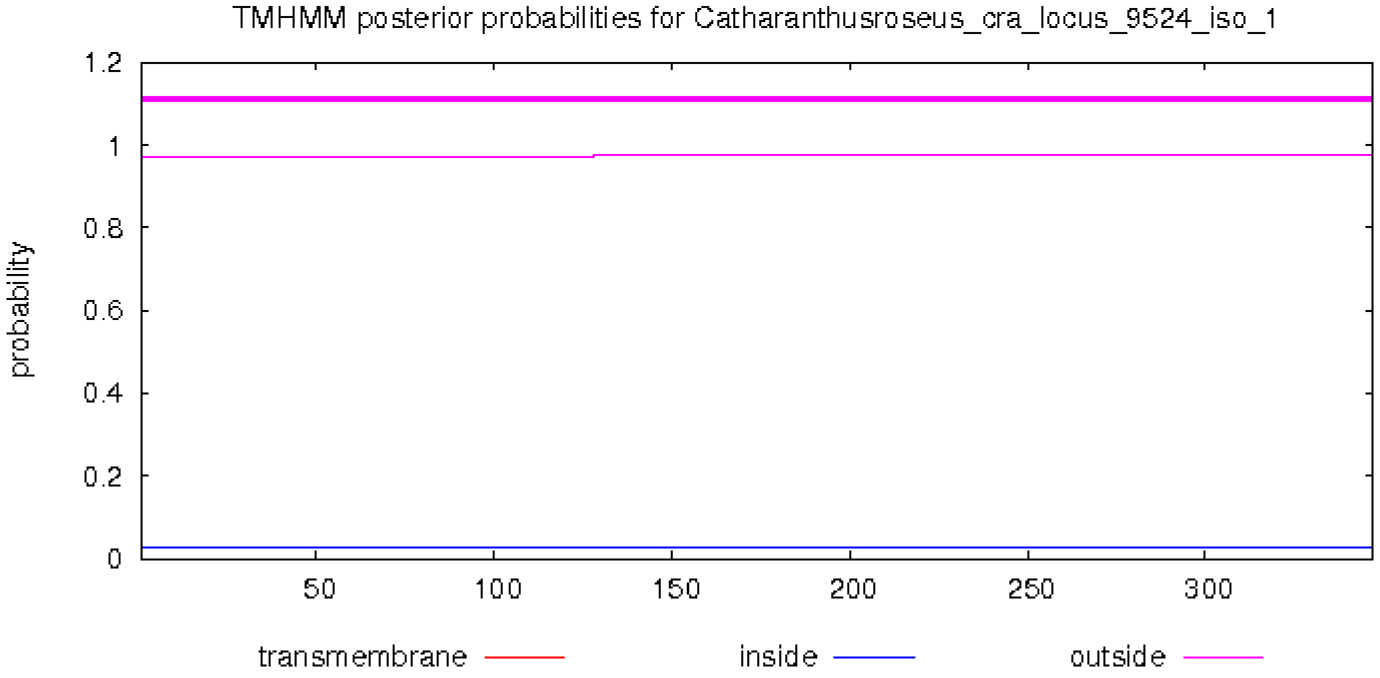

plot in postscript, script for making the plot in gnuplot, data for plot

---

```
# Chenopodiumquinoa_AUR62000492 Length: 327
# Chenopodiumquinoa_AUR62000492 Number of predicted TMHs:  0
# Chenopodiumquinoa_AUR62000492 Exp number of AAs in TMHs: 0.05781
# Chenopodiumquinoa_AUR62000492 Exp number, first 60 AAs:  0
# Chenopodiumquinoa_AUR62000492 Total prob of N-in:        0.01048
Chenopodiumquinoa_AUR62000492	TMHMM2.0	outside	     1   327
```

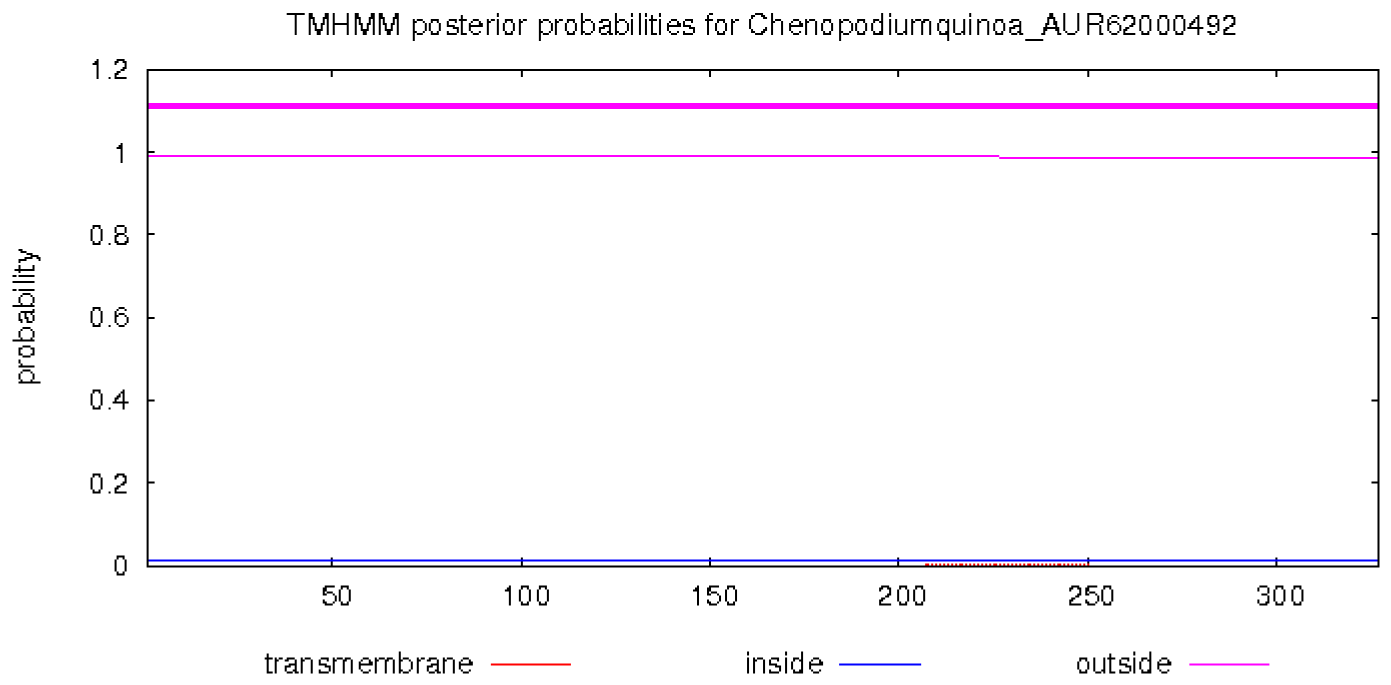

plot in postscript, script for making the plot in gnuplot, data for plot

---

```
# Chenopodiumquinoa_AUR62000493 Length: 329
# Chenopodiumquinoa_AUR62000493 Number of predicted TMHs:  0
# Chenopodiumquinoa_AUR62000493 Exp number of AAs in TMHs: 0.00139
# Chenopodiumquinoa_AUR62000493 Exp number, first 60 AAs:  0
# Chenopodiumquinoa_AUR62000493 Total prob of N-in:        0.00336
Chenopodiumquinoa_AUR62000493	TMHMM2.0	outside	     1   329
```

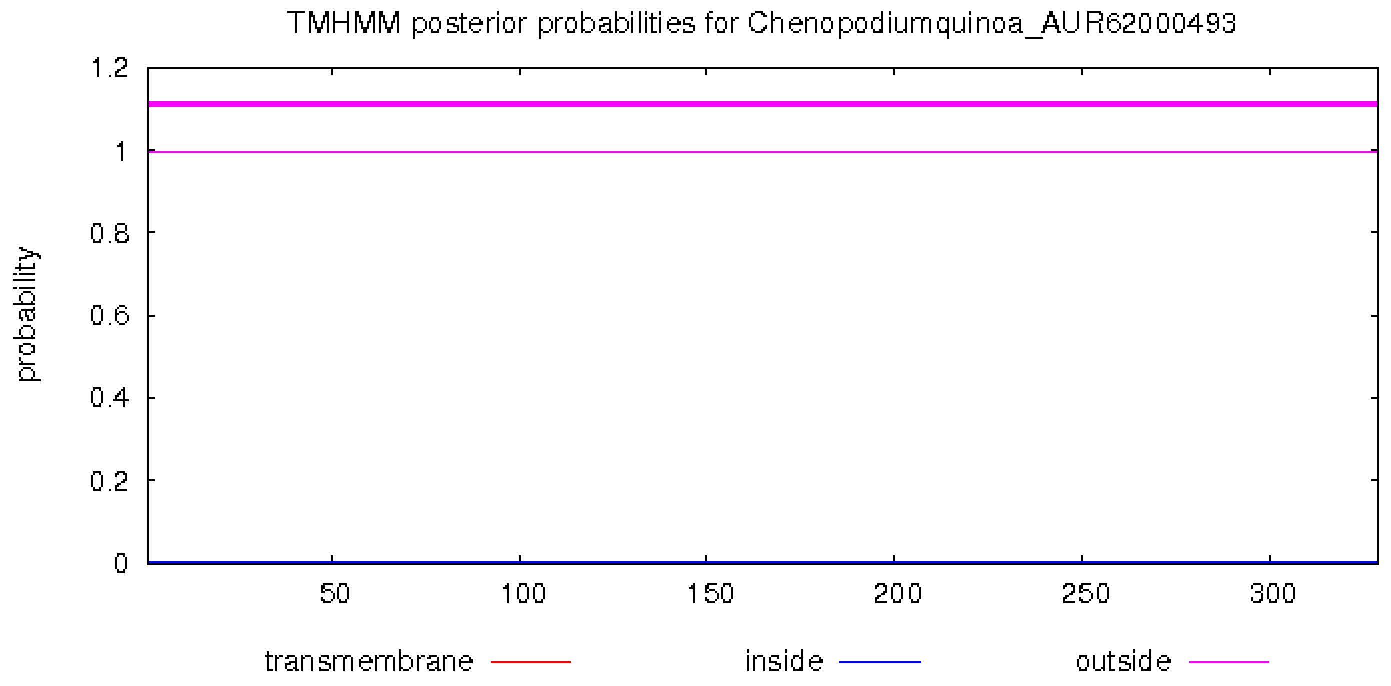

plot in postscript, script for making the plot in gnuplot, data for plot

---

```
# Chenopodiumquinoa_AUR62000536 Length: 248
# Chenopodiumquinoa_AUR62000536 Number of predicted TMHs:  0
# Chenopodiumquinoa_AUR62000536 Exp number of AAs in TMHs: 0.00053
# Chenopodiumquinoa_AUR62000536 Exp number, first 60 AAs:  0.0002
# Chenopodiumquinoa_AUR62000536 Total prob of N-in:        0.05561
Chenopodiumquinoa_AUR62000536   TMHMM2.0        outside      1   248
```

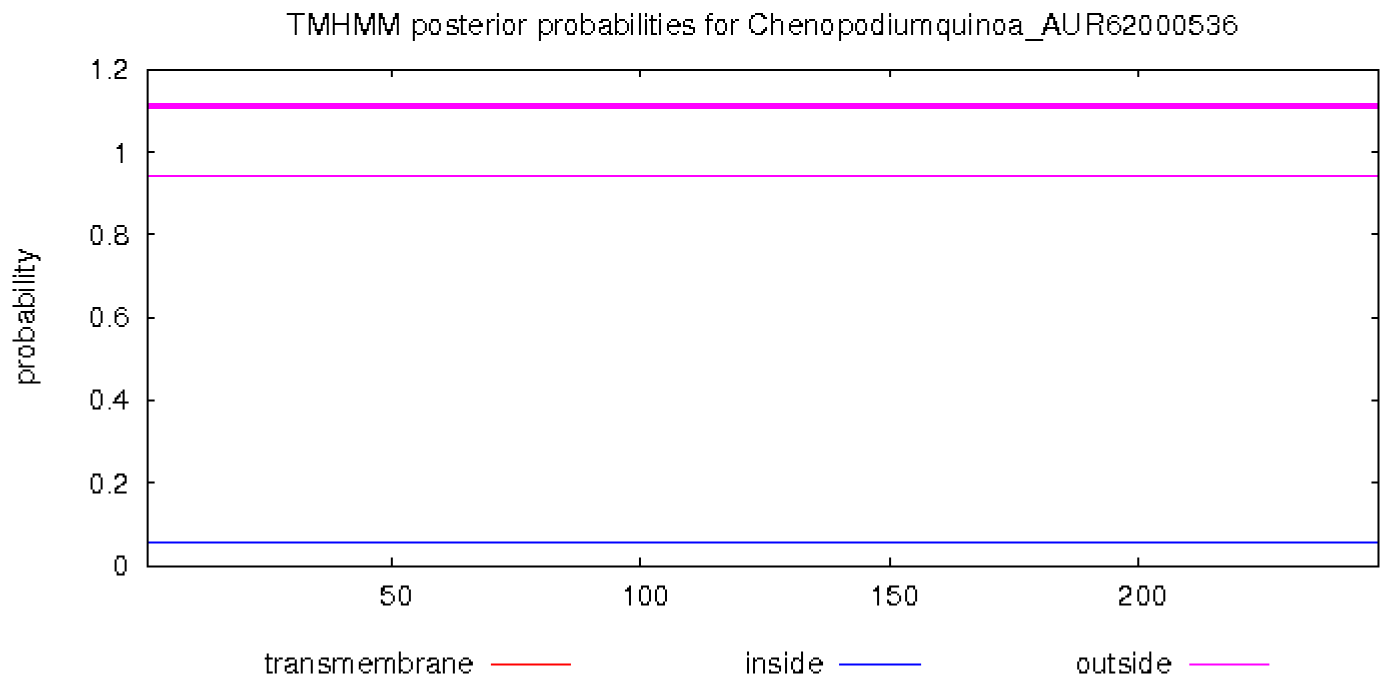

plot in postscript, script for making the plot in gnuplot, data for plot

---

```
# Chenopodiumquinoa_AUR62000898 Length: 418
# Chenopodiumquinoa_AUR62000898 Number of predicted TMHs:  0
# Chenopodiumquinoa_AUR62000898 Exp number of AAs in TMHs: 0.00961
# Chenopodiumquinoa_AUR62000898 Exp number, first 60 AAs:  0.00015
# Chenopodiumquinoa_AUR62000898 Total prob of N-in:        0.02009
Chenopodiumquinoa_AUR62000898   TMHMM2.0        outside      1   418
```

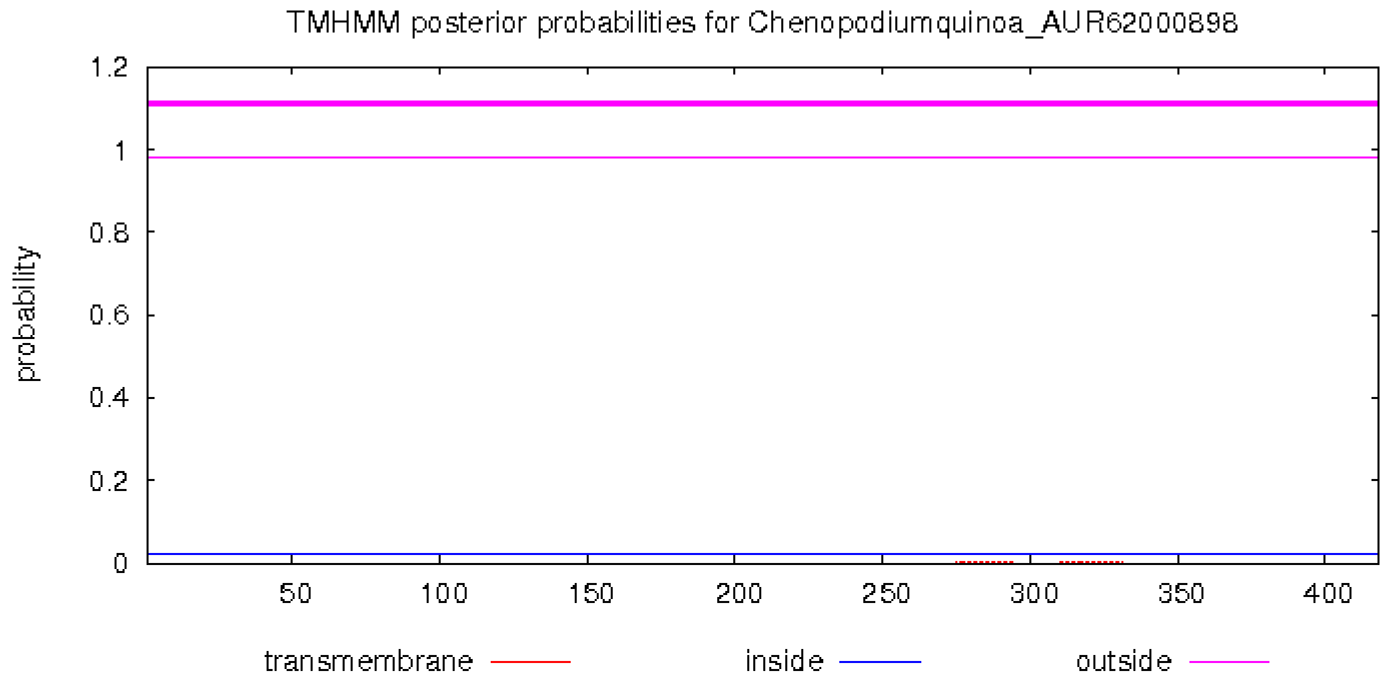

plot in postscript, script for making the plot in gnuplot, data for plot

---

```
# Chenopodiumquinoa_AUR62001365 Length: 307
# Chenopodiumquinoa_AUR62001365 Number of predicted TMHs:  0
# Chenopodiumquinoa_AUR62001365 Exp number of AAs in TMHs: 0.00055
# Chenopodiumquinoa_AUR62001365 Exp number, first 60 AAs:  0
# Chenopodiumquinoa_AUR62001365 Total prob of N-in:        0.02479
Chenopodiumquinoa_AUR62001365   TMHMM2.0        outside      1   307
```

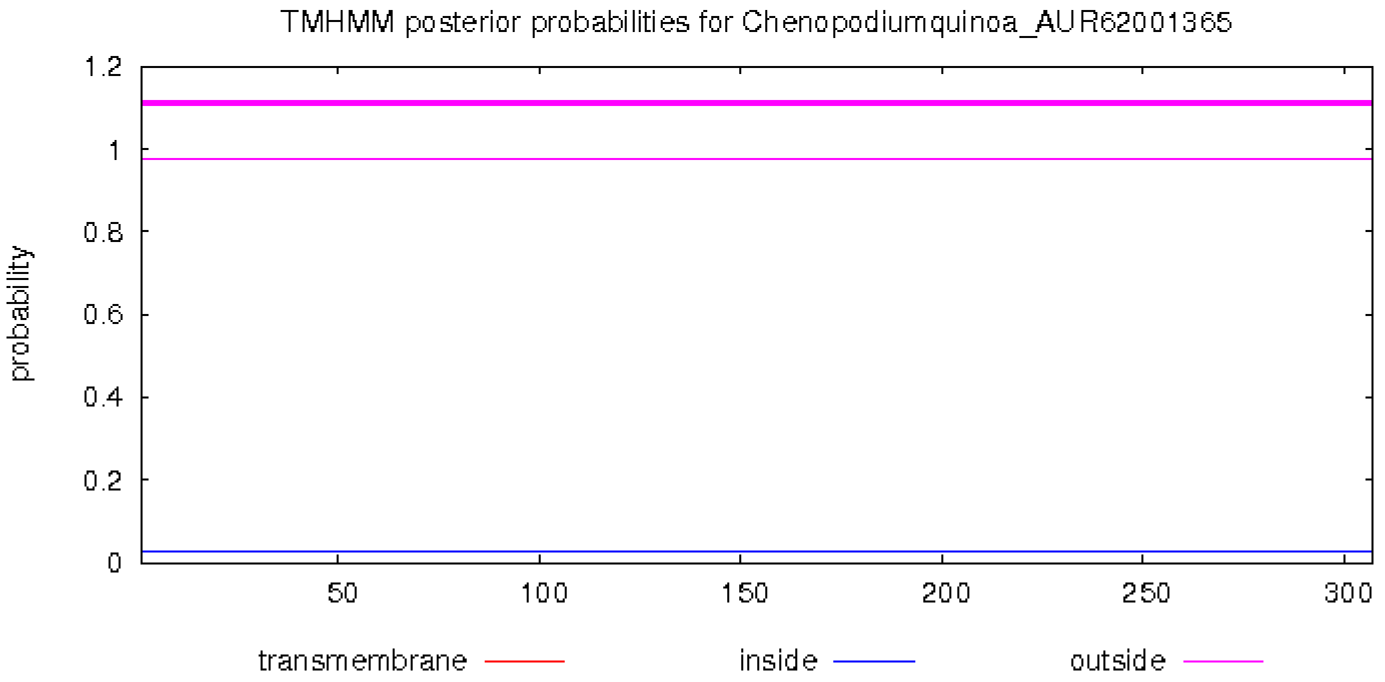

plot in postscript, script for making the plot in gnuplot, data for plot

---

```
# Chenopodiumquinoa_AUR62001522 Length: 268
# Chenopodiumquinoa_AUR62001522 Number of predicted TMHs:  0
# Chenopodiumquinoa_AUR62001522 Exp number of AAs in TMHs: 0.00011
# Chenopodiumquinoa_AUR62001522 Exp number, first 60 AAs:  0
# Chenopodiumquinoa_AUR62001522 Total prob of N-in:        0.00876
Chenopodiumquinoa_AUR62001522   TMHMM2.0        outside      1   268
```

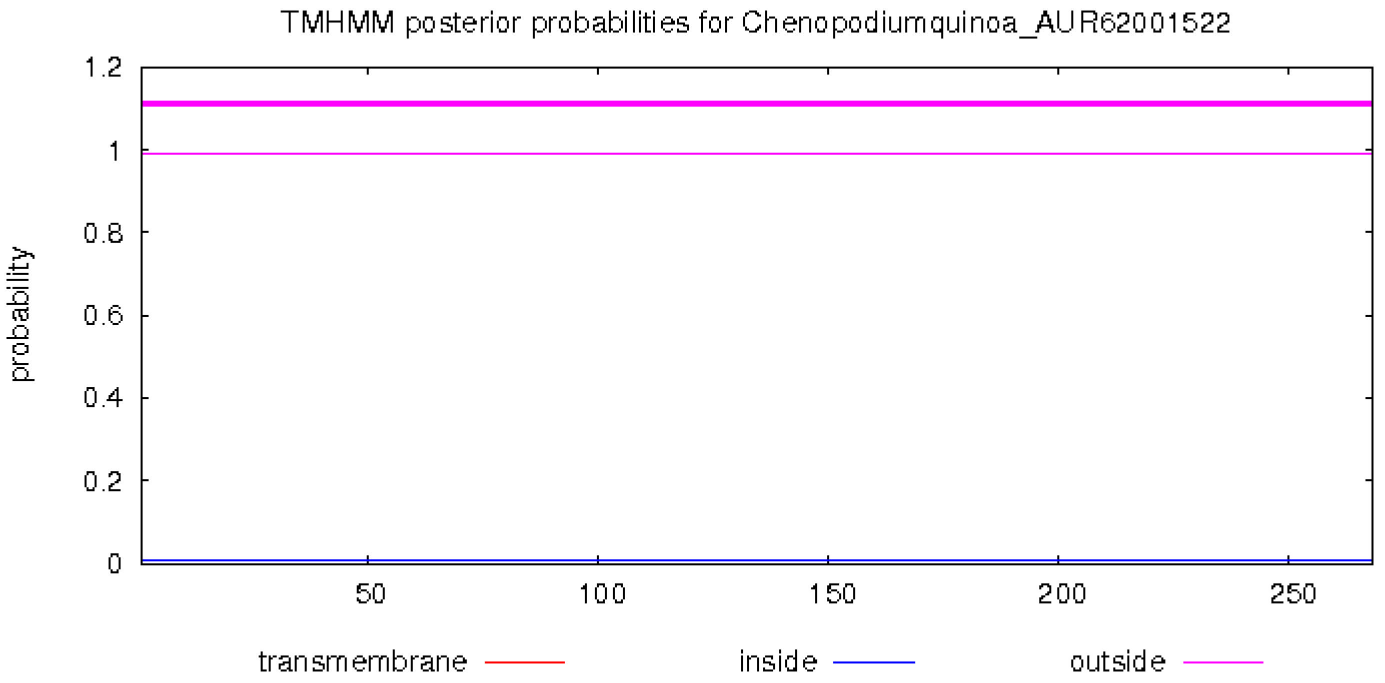

plot in postscript, script for making the plot in gnuplot, data for plot

---

```
# Chenopodiumquinoa_AUR62001531 Length: 330
# Chenopodiumquinoa_AUR62001531 Number of predicted TMHs:  1
# Chenopodiumquinoa_AUR62001531 Exp number of AAs in TMHs: 20.15642
# Chenopodiumquinoa_AUR62001531 Exp number, first 60 AAs:  0
# Chenopodiumquinoa_AUR62001531 Total prob of N-in:        0.99295
Chenopodiumquinoa_AUR62001531   TMHMM2.0        inside       1   305
Chenopodiumquinoa_AUR62001531   TMHMM2.0        TMhelix    306   323
Chenopodiumquinoa_AUR62001531   TMHMM2.0        outside    324   330
```

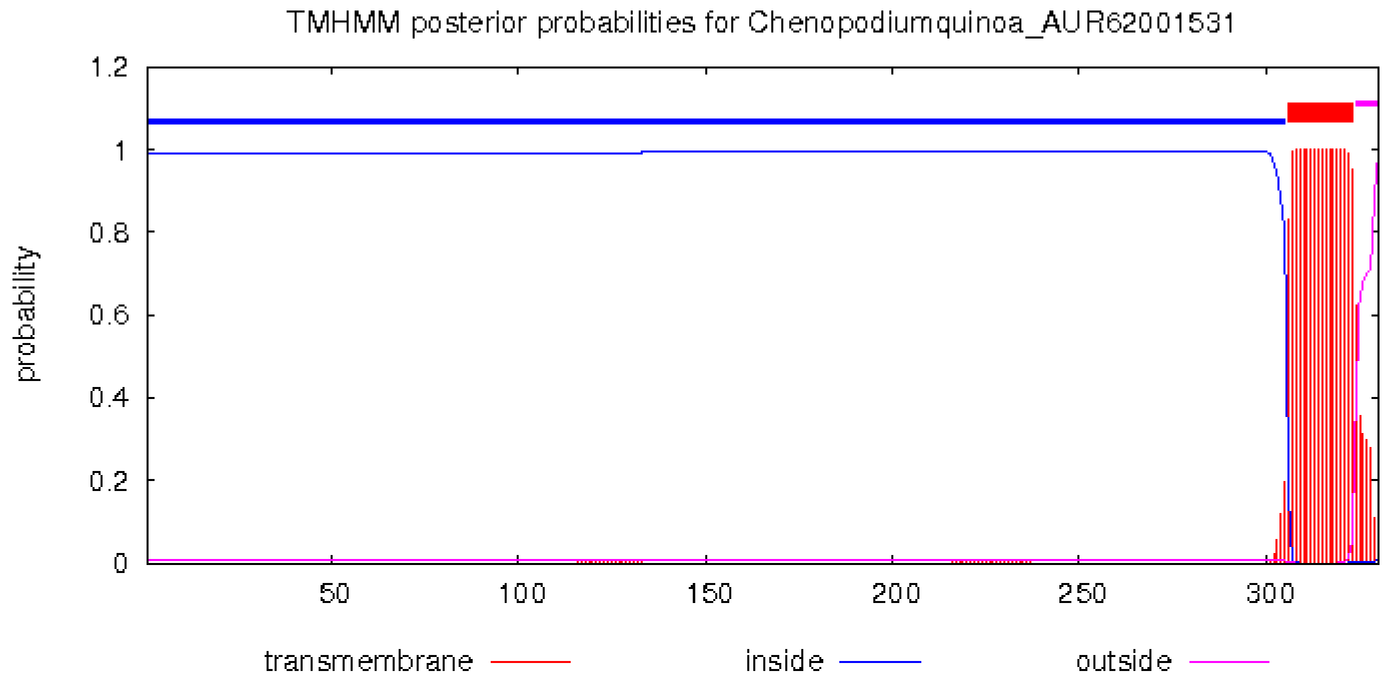

plot in postscript, script for making the plot in gnuplot, data for plot

---

```
# Chenopodiumquinoa_AUR62002191 Length: 249
# Chenopodiumquinoa_AUR62002191 Number of predicted TMHs:  0
# Chenopodiumquinoa_AUR62002191 Exp number of AAs in TMHs: 0.00118
# Chenopodiumquinoa_AUR62002191 Exp number, first 60 AAs:  0
# Chenopodiumquinoa_AUR62002191 Total prob of N-in:        0.03959
Chenopodiumquinoa_AUR62002191   TMHMM2.0        outside      1   249
```

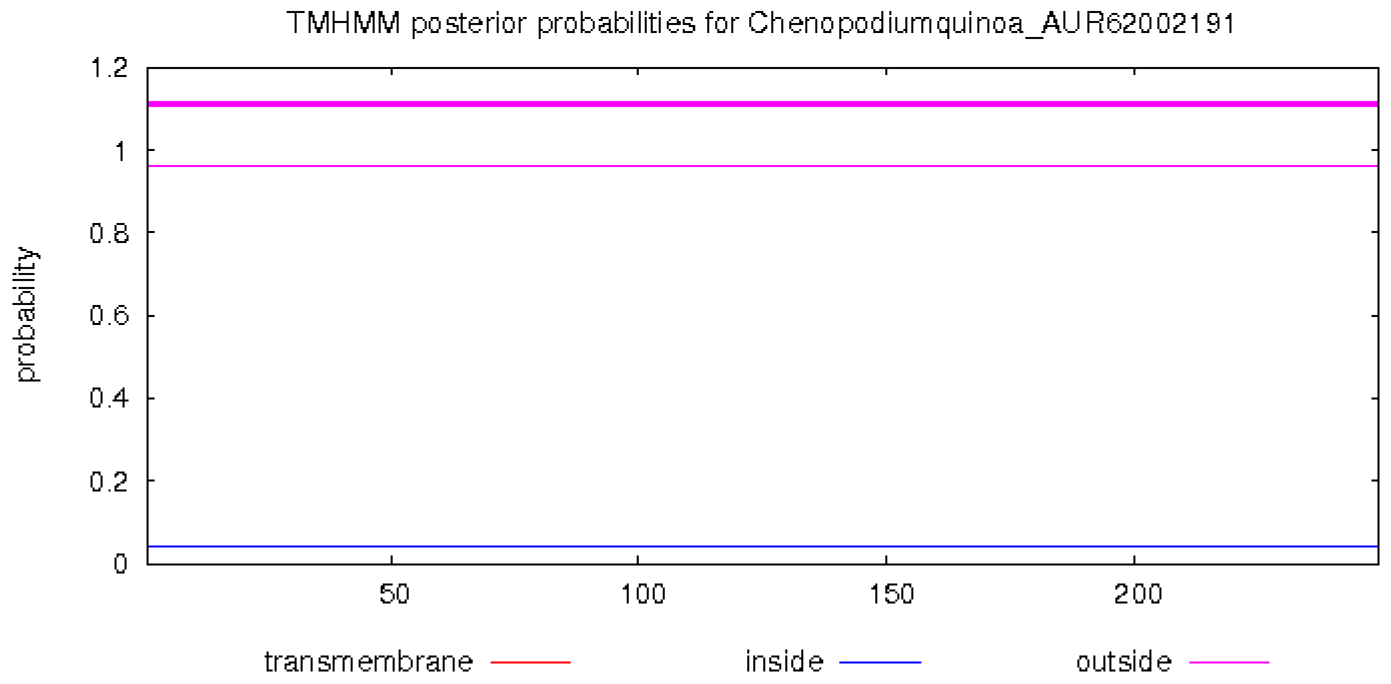

plot in postscript, script for making the plot in gnuplot, data for plot

```
# Chenopodiumquinoa_AUR62003400 Length: 573
# Chenopodiumquinoa_AUR62003400 Number of predicted TMHs:  0
# Chenopodiumquinoa_AUR62003400 Exp number of AAs in TMHs: 0.33327
# Chenopodiumquinoa_AUR62003400 Exp number, first 60 AAs:  0
# Chenopodiumquinoa_AUR62003400 Total prob of N-in:        0.00011
Chenopodiumquinoa_AUR62003400   TMHMM2.0        outside      1   573
```

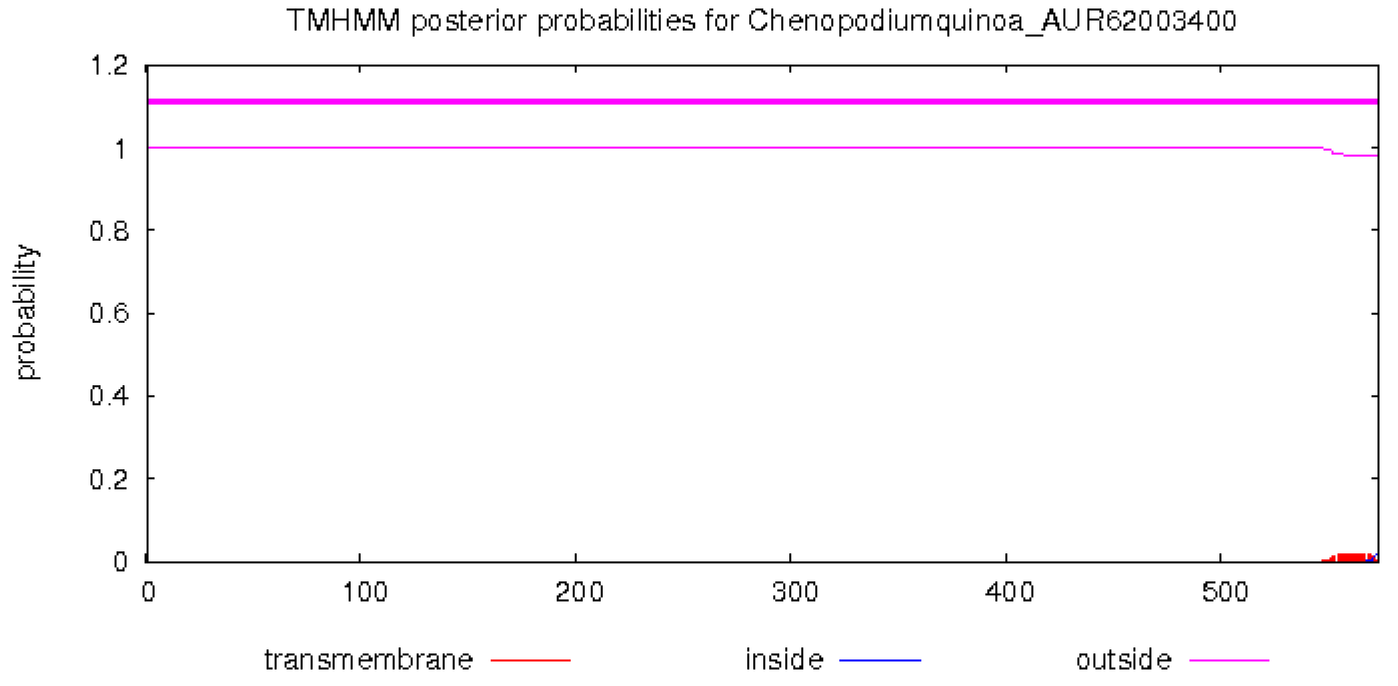

plot in postscript, script for making the plot in gnuplot, data for plot

```
# Chenopodiumquinoa_AUR62003583 Length: 224
# Chenopodiumquinoa_AUR62003583 Number of predicted TMHs:  0
# Chenopodiumquinoa_AUR62003583 Exp number of AAs in TMHs: 0.00074
# Chenopodiumquinoa_AUR62003583 Exp number, first 60 AAs:  0
# Chenopodiumquinoa_AUR62003583 Total prob of N-in:        0.07653
Chenopodiumquinoa_AUR62003583   TMHMM2.0        outside      1   224
```

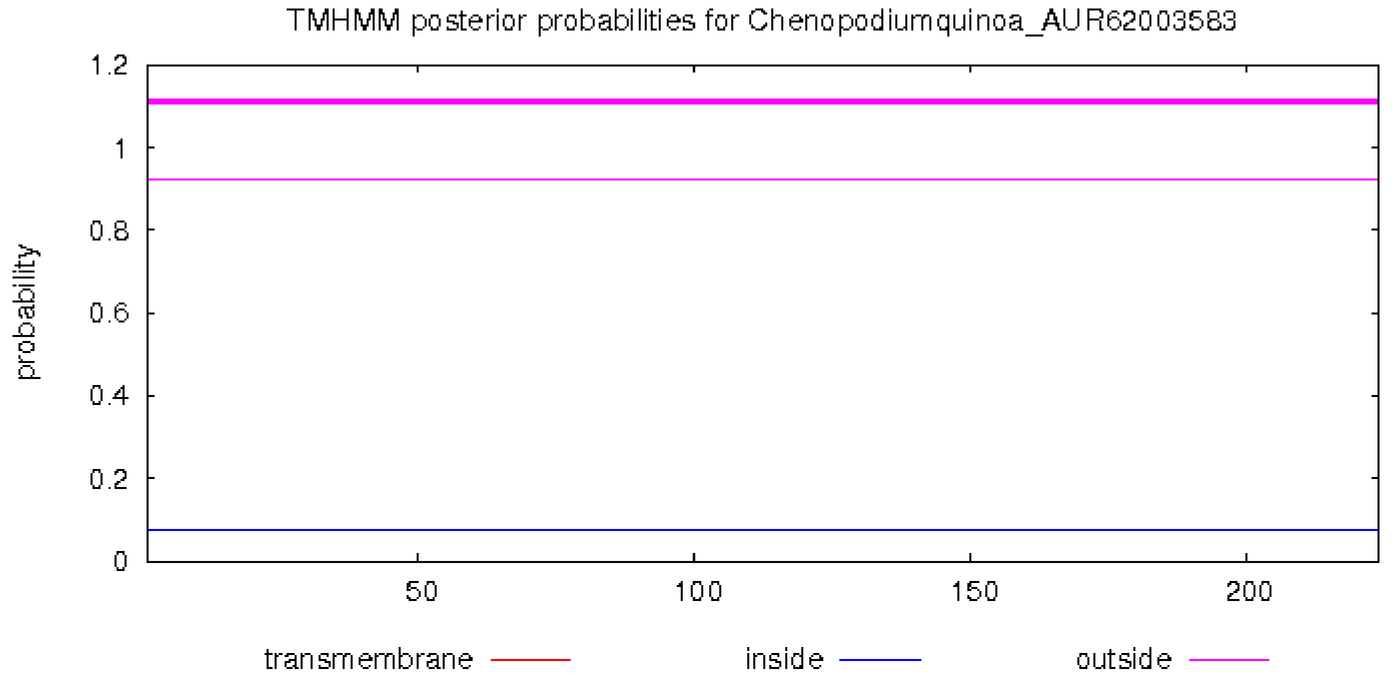

plot in postscript, script for making the plot in gnuplot, data for plot

```
# Chenopodiumquinoa_AUR62004554 Length: 334
# Chenopodiumquinoa_AUR62004554 Number of predicted TMHs:  0
# Chenopodiumquinoa_AUR62004554 Exp number of AAs in TMHs: 0
# Chenopodiumquinoa_AUR62004554 Exp number, first 60 AAs:  0
# Chenopodiumquinoa_AUR62004554 Total prob of N-in:        0.08559
Chenopodiumquinoa_AUR62004554	TMHMM2.0	outside	     1   334
```

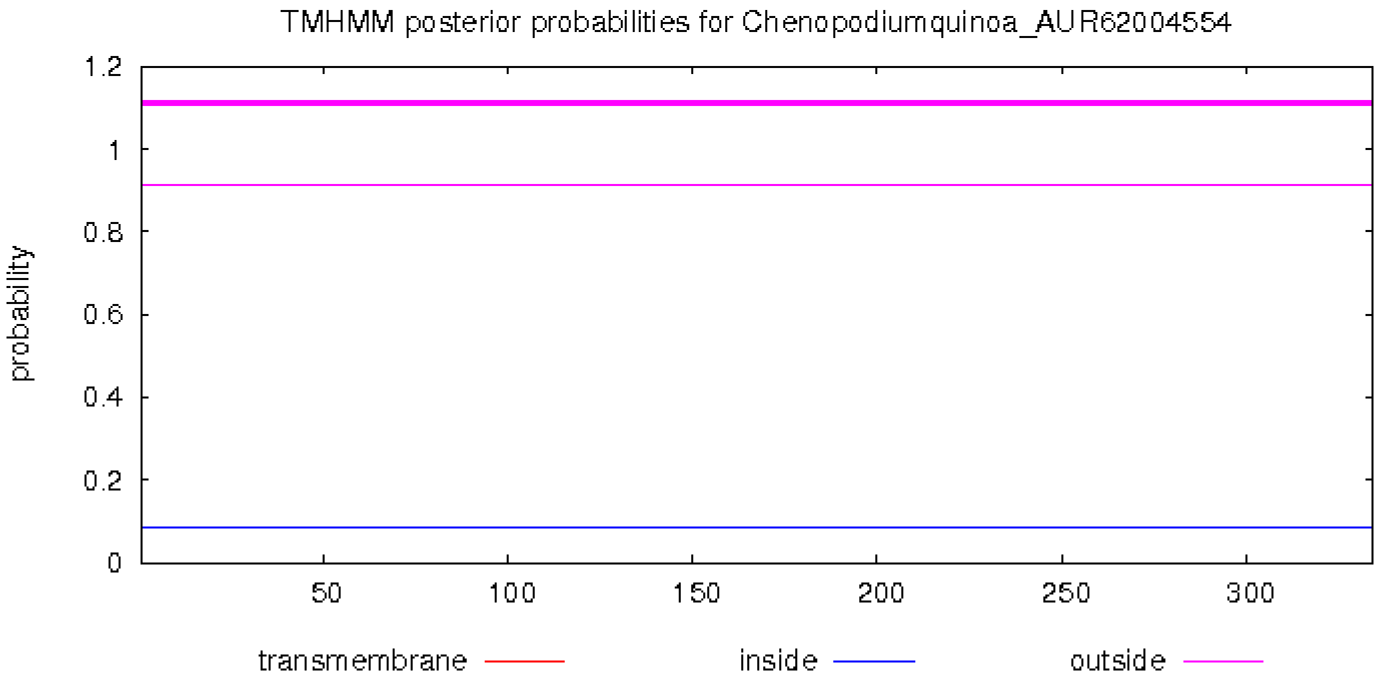

plot in postscript, script for making the plot in gnuplot, data for plot

---

```
# Chenopodiumquinoa_AUR62004648 Length: 334
# Chenopodiumquinoa_AUR62004648 Number of predicted TMHs:  0
# Chenopodiumquinoa_AUR62004648 Exp number of AAs in TMHs: 0
# Chenopodiumquinoa_AUR62004648 Exp number, first 60 AAs:  0
# Chenopodiumquinoa_AUR62004648 Total prob of N-in:        0.00799
Chenopodiumquinoa_AUR62004648	TMHMM2.0	outside	     1   334
```

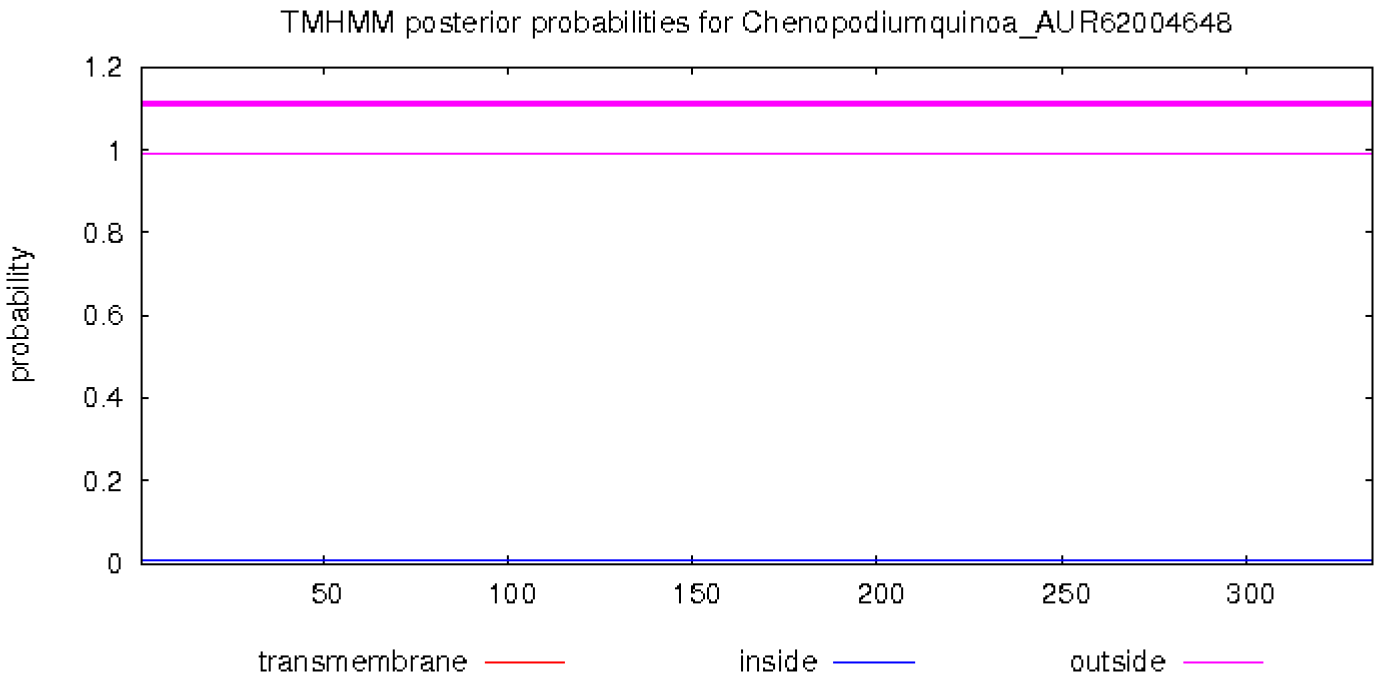

plot in postscript, script for making the plot in gnuplot, data for plot

---

```
# Chenopodiumquinoa_AUR62004773 Length: 140
# Chenopodiumquinoa_AUR62004773 Number of predicted TMHs:  0
# Chenopodiumquinoa_AUR62004773 Exp number of AAs in TMHs: 0
# Chenopodiumquinoa_AUR62004773 Exp number, first 60 AAs:  0
# Chenopodiumquinoa_AUR62004773 Total prob of N-in:        0.33765
Chenopodiumquinoa_AUR62004773   TMHMM2.0        outside      1   140
```

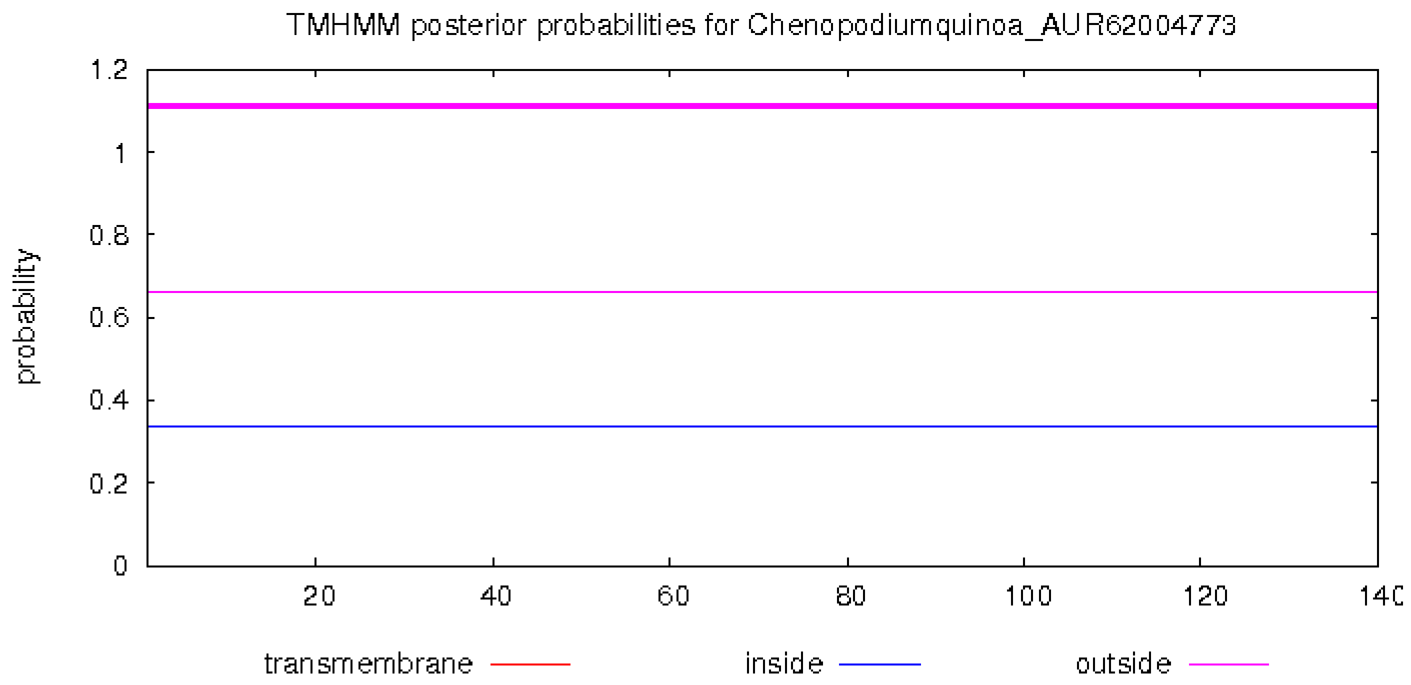

plot in postscript, script for making the plot in gnuplot, data for plot

---

```
# Chenopodiumquinoa_AUR62004778 Length: 576
# Chenopodiumquinoa_AUR62004778 Number of predicted TMHs:  0
# Chenopodiumquinoa_AUR62004778 Exp number of AAs in TMHs: 0.2334
# Chenopodiumquinoa_AUR62004778 Exp number, first 60 AAs:  0
# Chenopodiumquinoa_AUR62004778 Total prob of N-in:        0.00009
Chenopodiumquinoa_AUR62004778   TMHMM2.0        outside      1   576
```

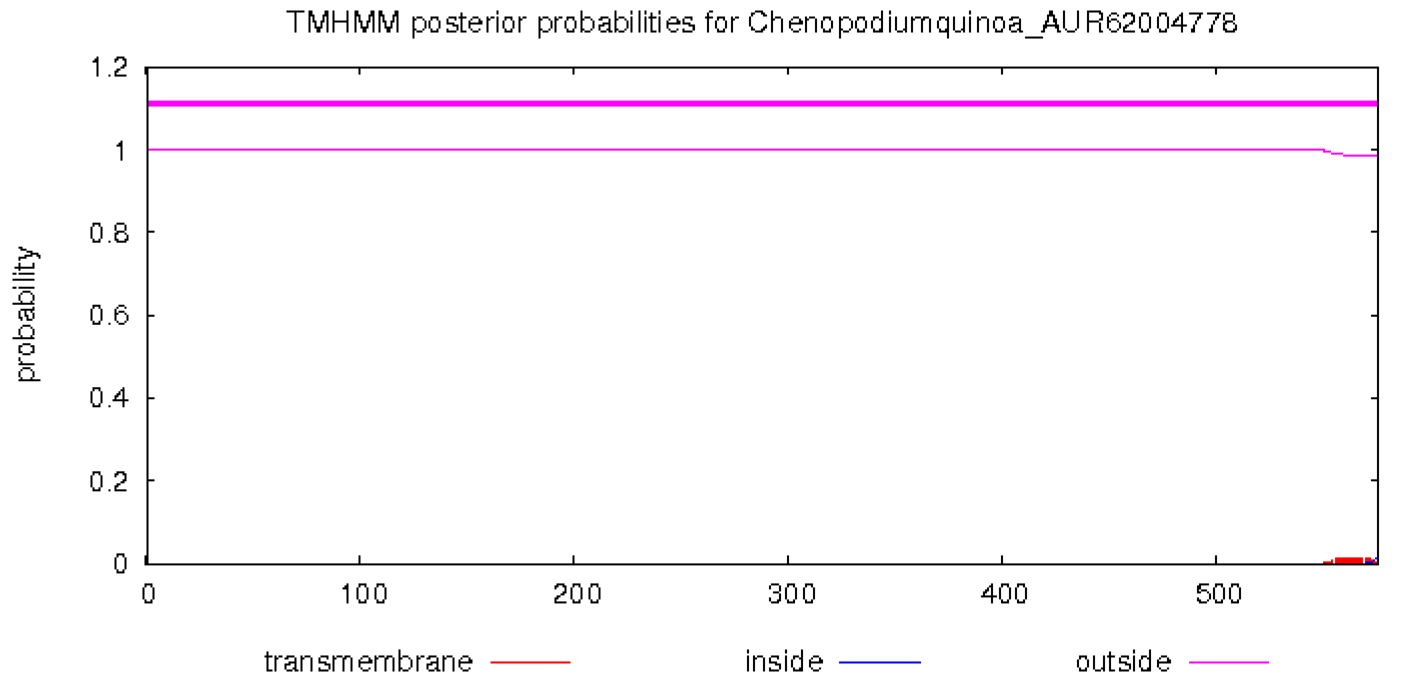

plot in postscript, script for making the plot in gnuplot, data for plot

---

```
# Chenopodiumquinoa_AUR62005686 Length: 256
# Chenopodiumquinoa_AUR62005686 Number of predicted TMHs:  0
# Chenopodiumquinoa_AUR62005686 Exp number of AAs in TMHs: 0.00018
# Chenopodiumquinoa_AUR62005686 Exp number, first 60 AAs:  0
# Chenopodiumquinoa_AUR62005686 Total prob of N-in:        0.08919
Chenopodiumquinoa_AUR62005686   TMHMM2.0        outside      1   256
```

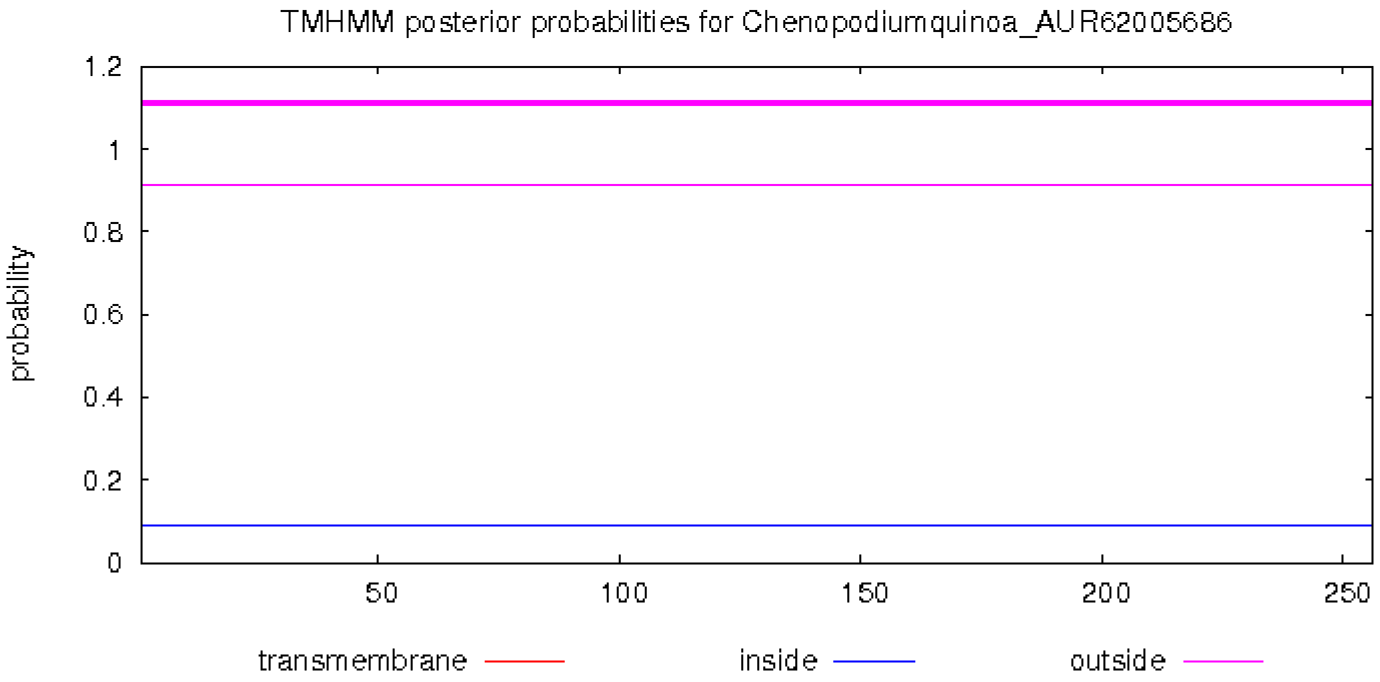

plot in postscript, script for making the plot in gnuplot, data for plot

---

```
# Chenopodiumquinoa_AUR62005772 Length: 446
# Chenopodiumquinoa_AUR62005772 Number of predicted TMHs:  0
# Chenopodiumquinoa_AUR62005772 Exp number of AAs in TMHs: 0.00036
# Chenopodiumquinoa_AUR62005772 Exp number, first 60 AAs:  0
# Chenopodiumquinoa_AUR62005772 Total prob of N-in:        0.01390
Chenopodiumquinoa_AUR62005772   TMHMM2.0        outside      1   446
```

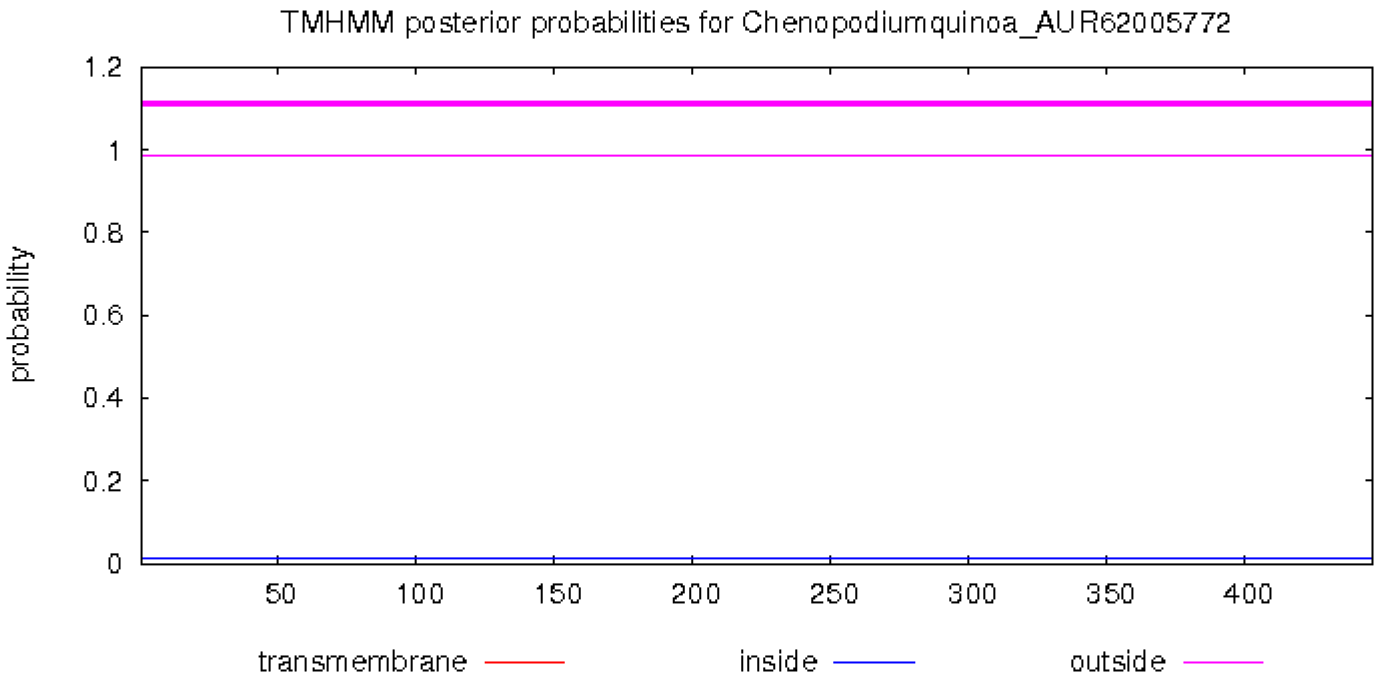

plot in postscript, script for making the plot in gnuplot, data for plot

---

```
# Chenopodiumquinoa_AUR62005821 Length: 288
# Chenopodiumquinoa_AUR62005821 Number of predicted TMHs:  0
# Chenopodiumquinoa_AUR62005821 Exp number of AAs in TMHs: 0.00096
# Chenopodiumquinoa_AUR62005821 Exp number, first 60 AAs:  6e-05
# Chenopodiumquinoa_AUR62005821 Total prob of N-in:        0.01954
Chenopodiumquinoa_AUR62005821   TMHMM2.0        outside      1   288
```

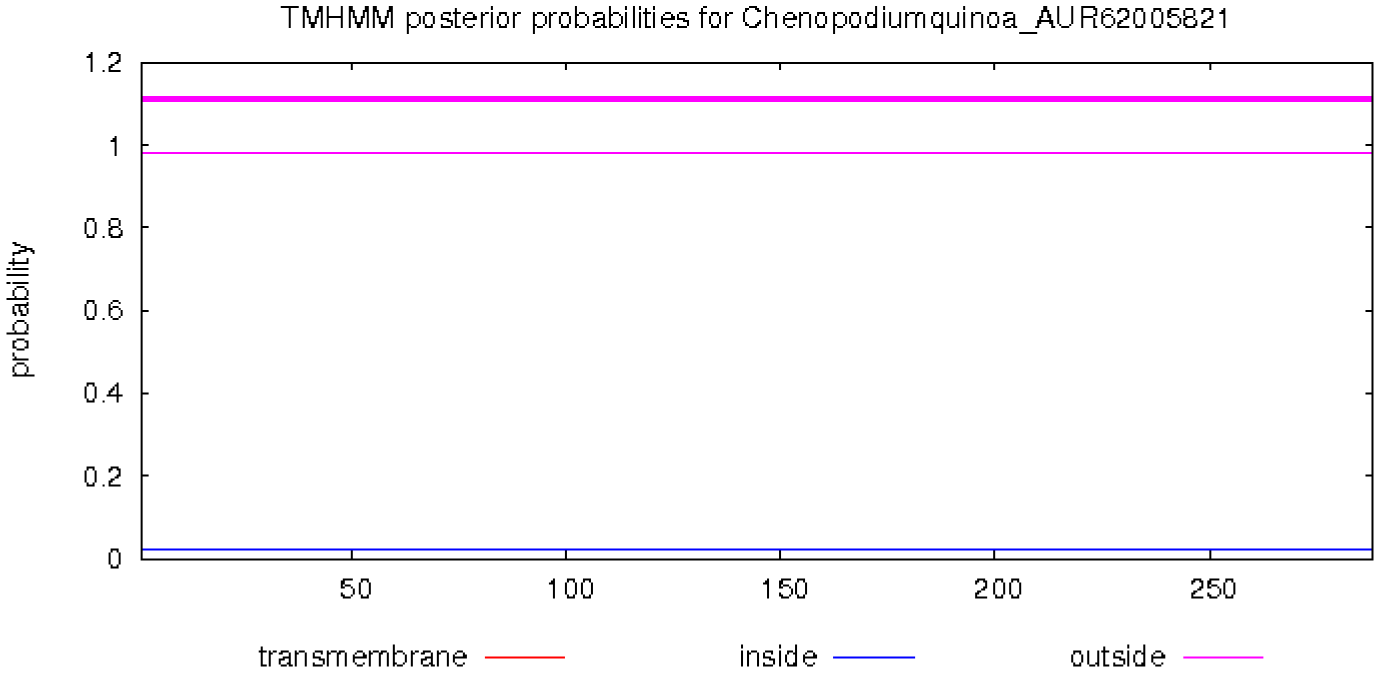

plot in postscript, script for making the plot in gnuplot, data for plot

---

```
# Chenopodiumquinoa_AUR62005966 Length: 631
# Chenopodiumquinoa_AUR62005966 Number of predicted TMHs:  1
# Chenopodiumquinoa_AUR62005966 Exp number of AAs in TMHs: 22.67114
# Chenopodiumquinoa_AUR62005966 Exp number, first 60 AAs:  0
# Chenopodiumquinoa_AUR62005966 Total prob of N-in:        0.00273
Chenopodiumquinoa_AUR62005966   TMHMM2.0        outside      1   599
Chenopodiumquinoa_AUR62005966   TMHMM2.0        TMhelix    600   622
Chenopodiumquinoa_AUR62005966   TMHMM2.0        inside     623   631
```

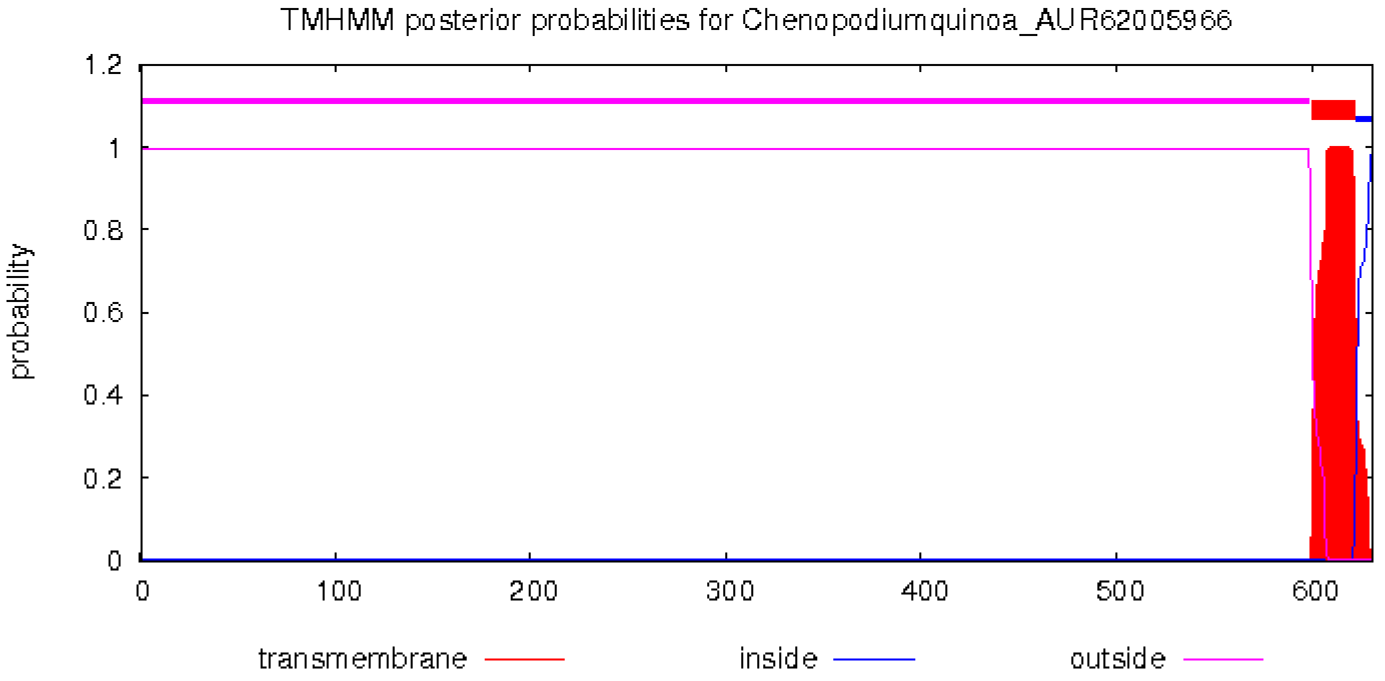

plot in postscript, script for making the plot in gnuplot, data for plot

```
# Chenopodiumquinoa_AUR62006223 Length: 276
# Chenopodiumquinoa_AUR62006223 Number of predicted TMHs:  0
# Chenopodiumquinoa_AUR62006223 Exp number of AAs in TMHs: 0.00046
# Chenopodiumquinoa_AUR62006223 Exp number, first 60 AAs:  0
# Chenopodiumquinoa_AUR62006223 Total prob of N-in:        0.00755
Chenopodiumquinoa_AUR62006223	TMHMM2.0	outside	     1   276
```

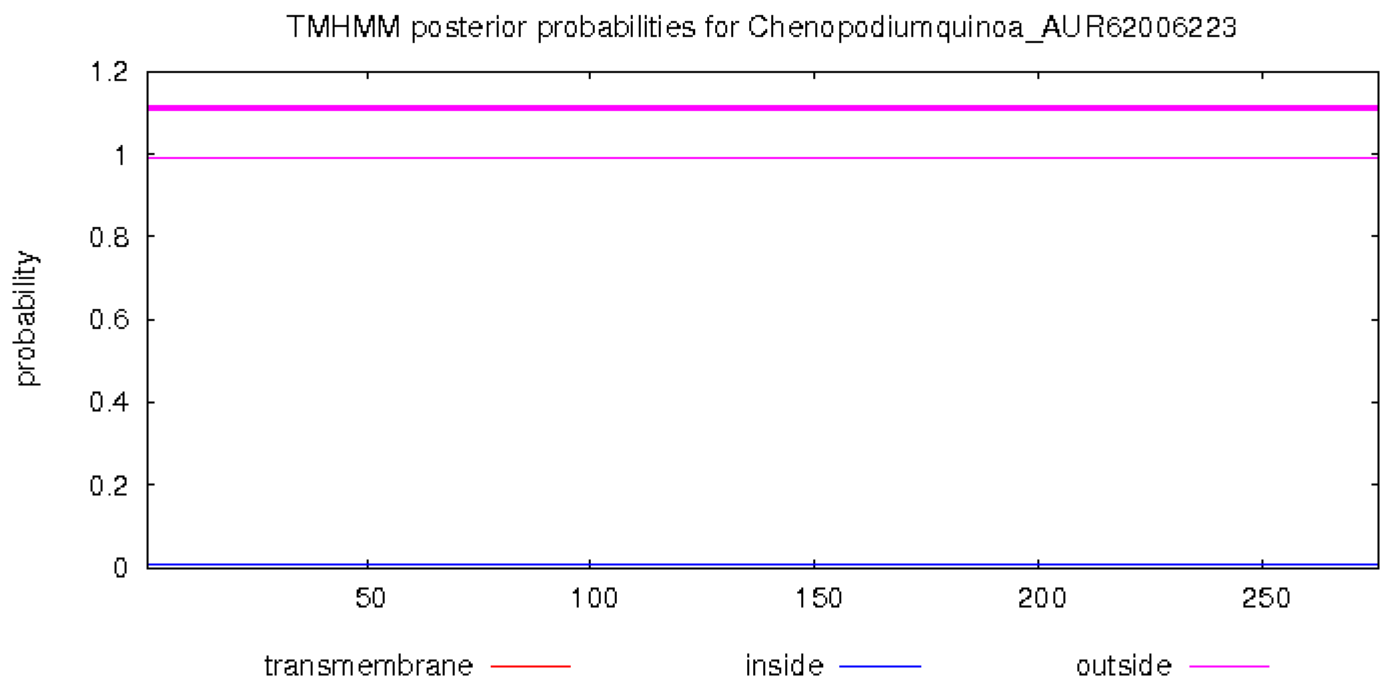

plot in postscript, script for making the plot in gnuplot, data for plot

```
# Chenopodiumquinoa_AUR62006407 Length: 372
# Chenopodiumquinoa_AUR62006407 Number of predicted TMHs:  0
# Chenopodiumquinoa_AUR62006407 Exp number of AAs in TMHs: 0.00826
# Chenopodiumquinoa_AUR62006407 Exp number, first 60 AAs:  0.00044
# Chenopodiumquinoa_AUR62006407 Total prob of N-in:        0.00315
Chenopodiumquinoa_AUR62006407	TMHMM2.0	outside	     1   372
```

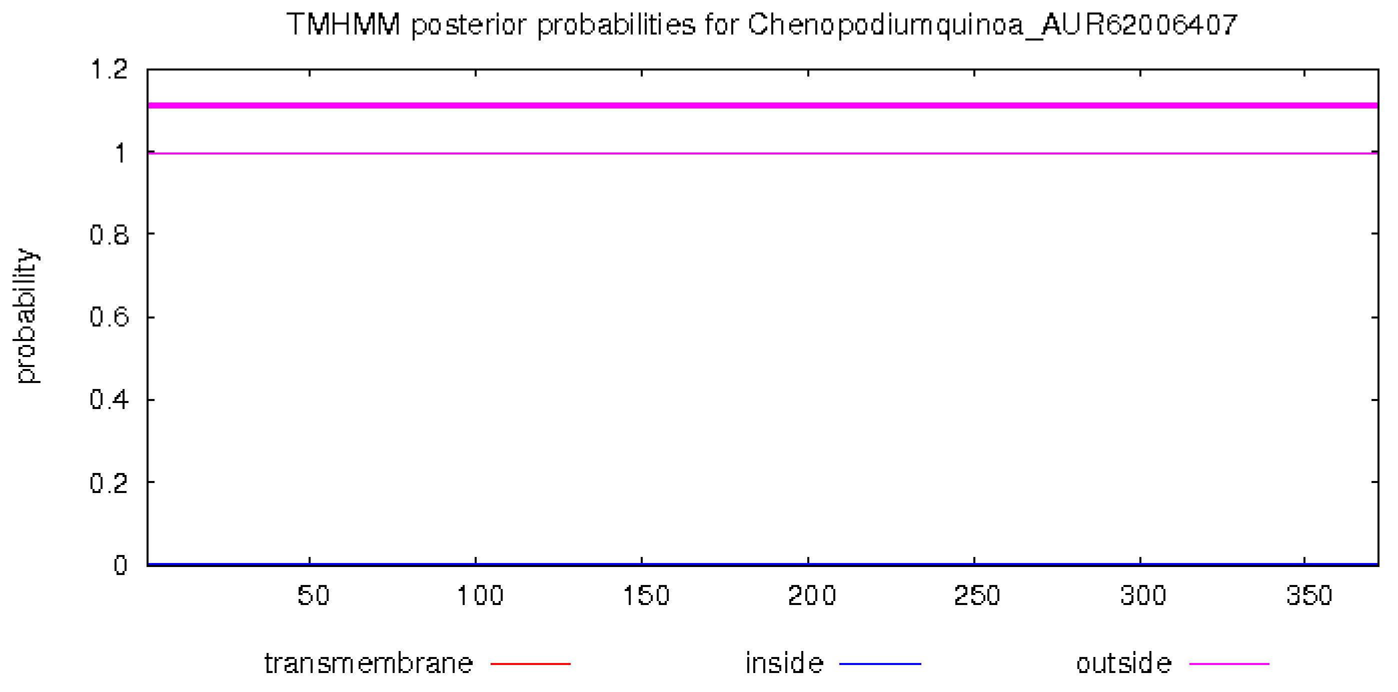

plot in postscript, script for making the plot in gnuplot, data for plot

```
# Chenopodiumquinoa_AUR62006836 Length: 315
# Chenopodiumquinoa_AUR62006836 Number of predicted TMHs:  0
# Chenopodiumquinoa_AUR62006836 Exp number of AAs in TMHs: 0.65749
# Chenopodiumquinoa_AUR62006836 Exp number, first 60 AAs:  0
# Chenopodiumquinoa_AUR62006836 Total prob of N-in:        0.06752
Chenopodiumquinoa_AUR62006836   TMHMM2.0        outside      1   315
```

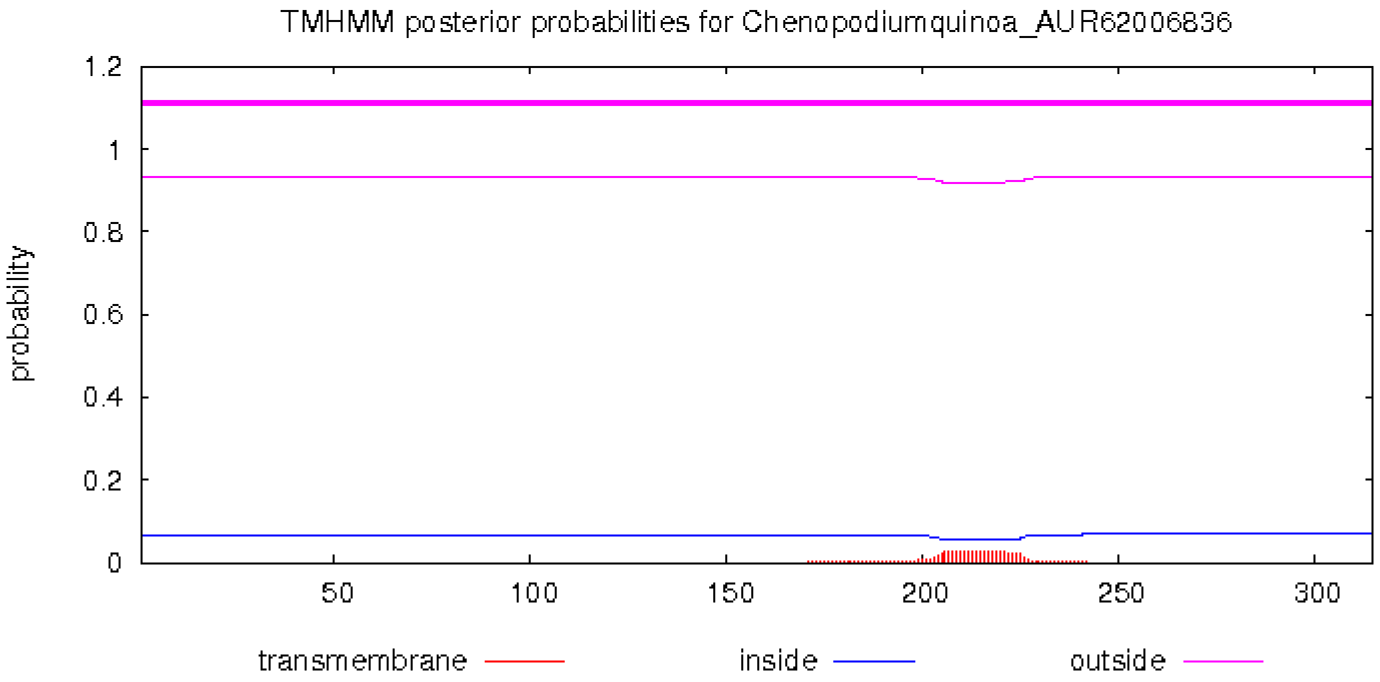

plot in postscript, script for making the plot in gnuplot, data for plot

---

```
# Chenopodiumquinoa_AUR62006837 Length: 319
# Chenopodiumquinoa_AUR62006837 Number of predicted TMHs:  0
# Chenopodiumquinoa_AUR62006837 Exp number of AAs in TMHs: 0.01485
# Chenopodiumquinoa_AUR62006837 Exp number, first 60 AAs:  0
# Chenopodiumquinoa_AUR62006837 Total prob of N-in:        0.00403
Chenopodiumquinoa_AUR62006837   TMHMM2.0        outside      1   319
```

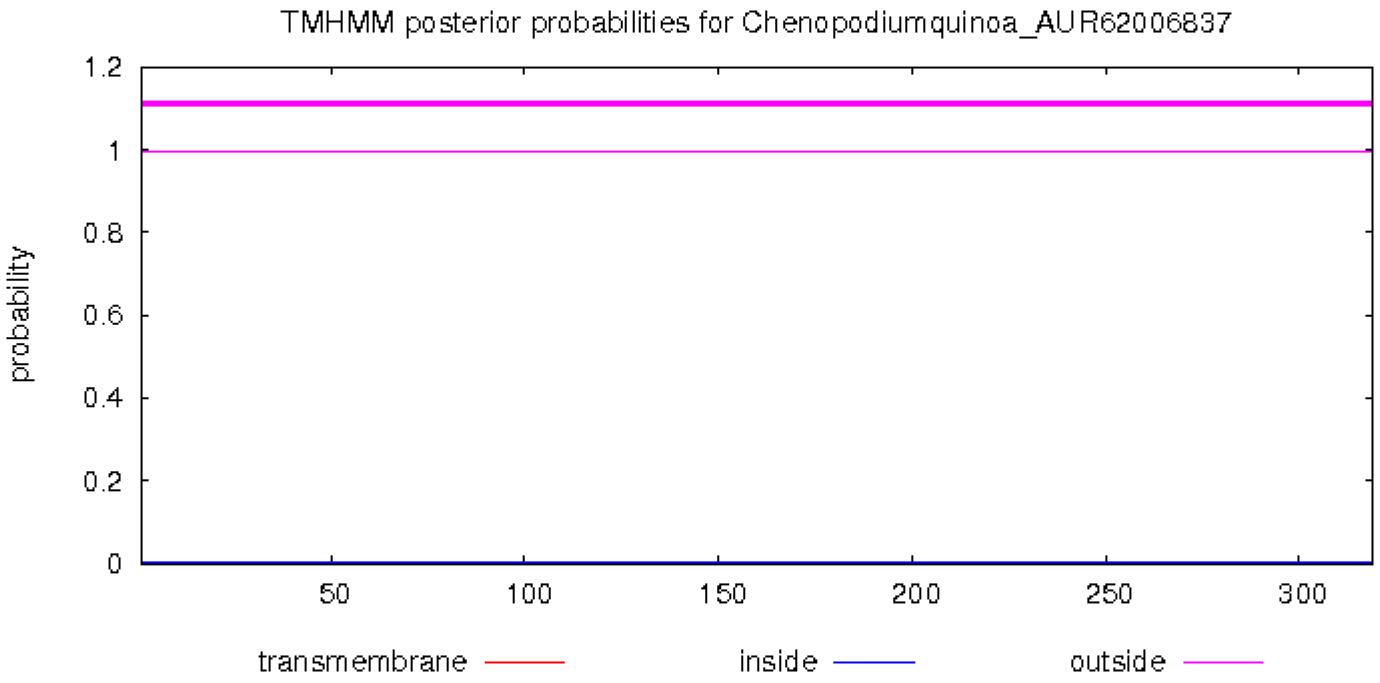

plot in postscript, script for making the plot in gnuplot, data for plot

---

```
# Chenopodiumquinoa_AUR62006885 Length: 329
# Chenopodiumquinoa_AUR62006885 Number of predicted TMHs:  0
# Chenopodiumquinoa_AUR62006885 Exp number of AAs in TMHs: 0.0688999999999999999
# Chenopodiumquinoa_AUR62006885 Exp number, first 60 AAs:  0.06847
# Chenopodiumquinoa_AUR62006885 Total prob of N-in:        0.06921
Chenopodiumquinoa_AUR62006885   TMHMM2.0        outside      1   329
```

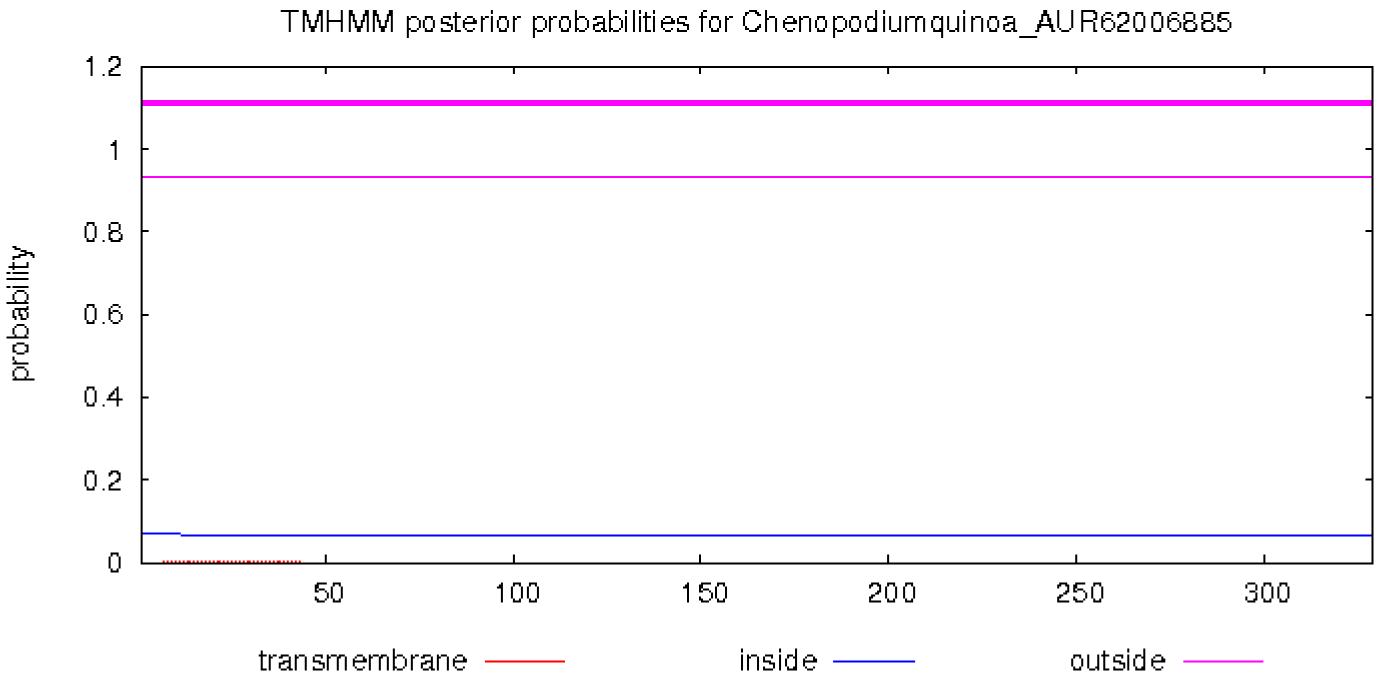

plot in postscript, script for making the plot in gnuplot, data for plot

```
# Chenopodiumquinoa_AUR62007071 Length: 279
# Chenopodiumquinoa_AUR62007071 Number of predicted TMHs:  0
# Chenopodiumquinoa_AUR62007071 Exp number of AAs in TMHs: 0
# Chenopodiumquinoa_AUR62007071 Exp number, first 60 AAs:  0
# Chenopodiumquinoa_AUR62007071 Total prob of N-in:        0.04251
Chenopodiumquinoa_AUR62007071   TMHMM2.0        outside      1   279
```

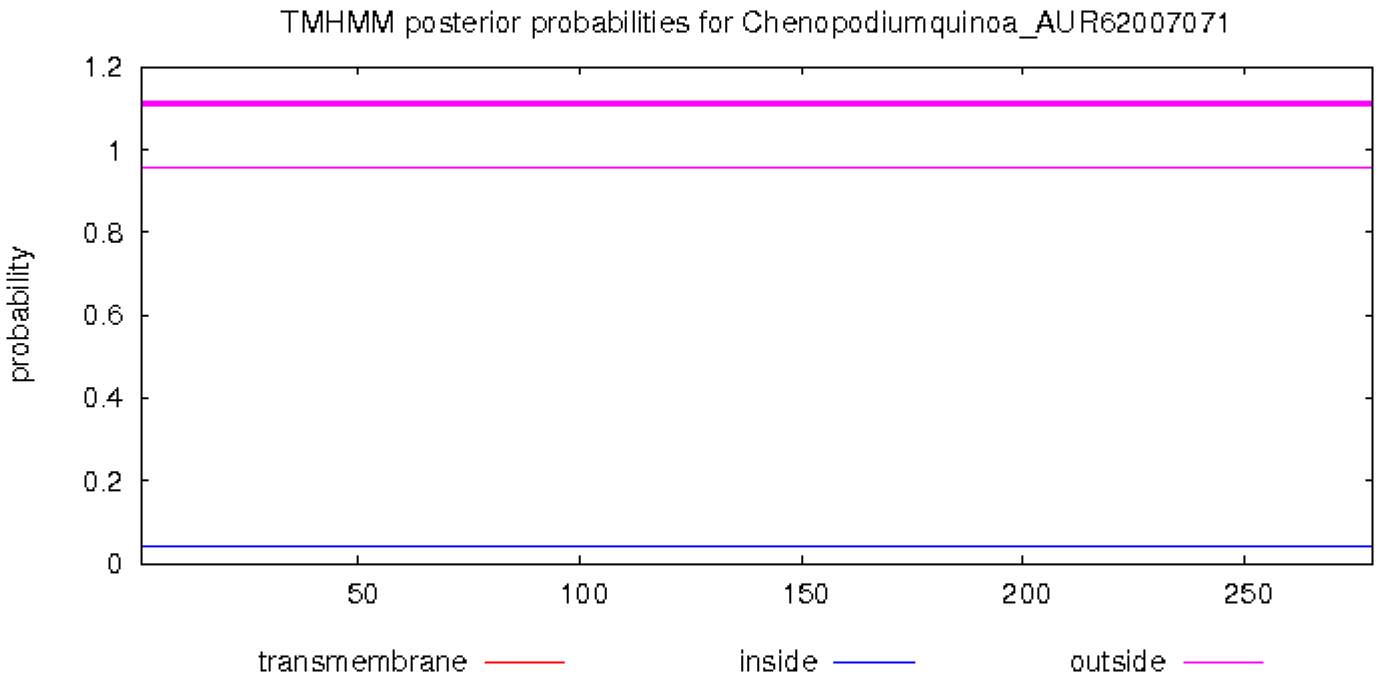

plot in postscript, script for making the plot in gnuplot, data for plot

```
# Chenopodiumquinoa_AUR62007247 Length: 327
# Chenopodiumquinoa_AUR62007247 Number of predicted TMHs:  0
# Chenopodiumquinoa_AUR62007247 Exp number of AAs in TMHs: 0.00022
# Chenopodiumquinoa_AUR62007247 Exp number, first 60 AAs:  0
# Chenopodiumquinoa_AUR62007247 Total prob of N-in:        0.00167
Chenopodiumquinoa_AUR62007247   TMHMM2.0        outside      1   327
```

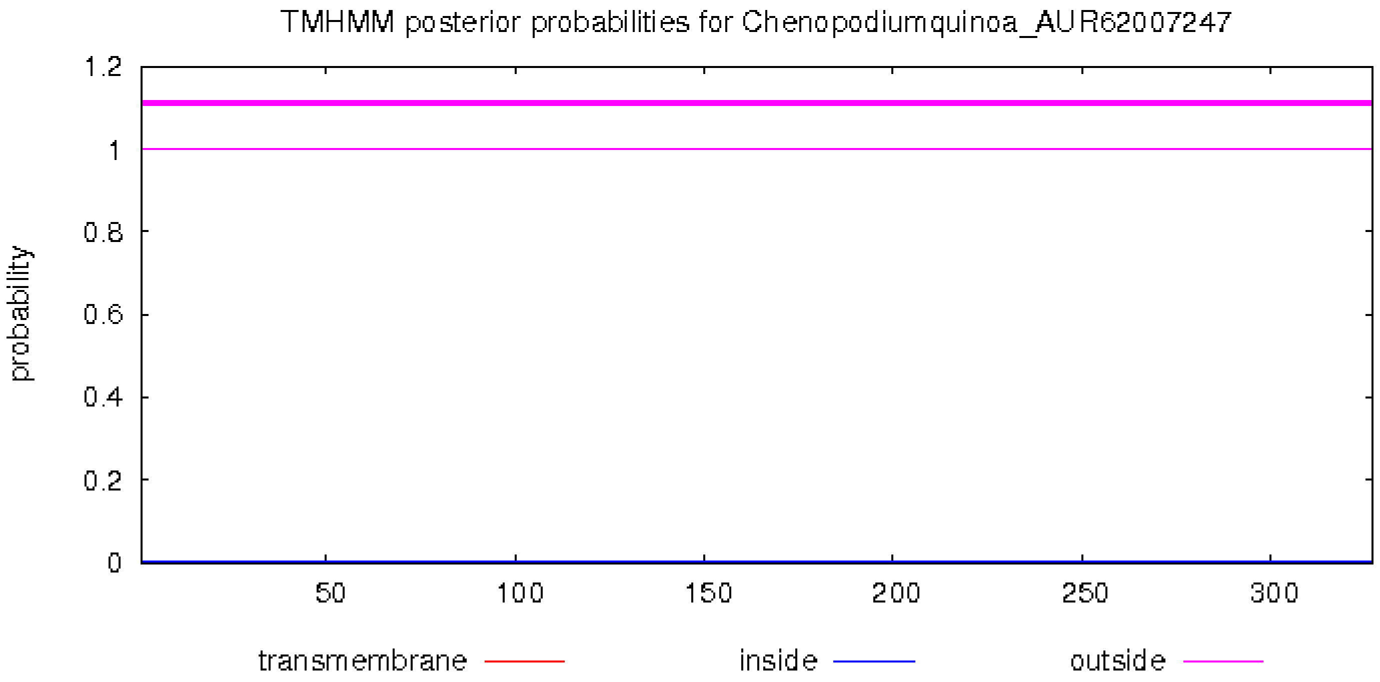

plot in postscript, script for making the plot in gnuplot, data for plot

---

```
# Chenopodiumquinoa_AUR62007469 Length: 370
# Chenopodiumquinoa_AUR62007469 Number of predicted TMHs:  0
# Chenopodiumquinoa_AUR62007469 Exp number of AAs in TMHs: 0
# Chenopodiumquinoa_AUR62007469 Exp number, first 60 AAs:  0
# Chenopodiumquinoa_AUR62007469 Total prob of N-in:        0.00343
Chenopodiumquinoa_AUR62007469   TMHMM2.0        outside      1   370
```

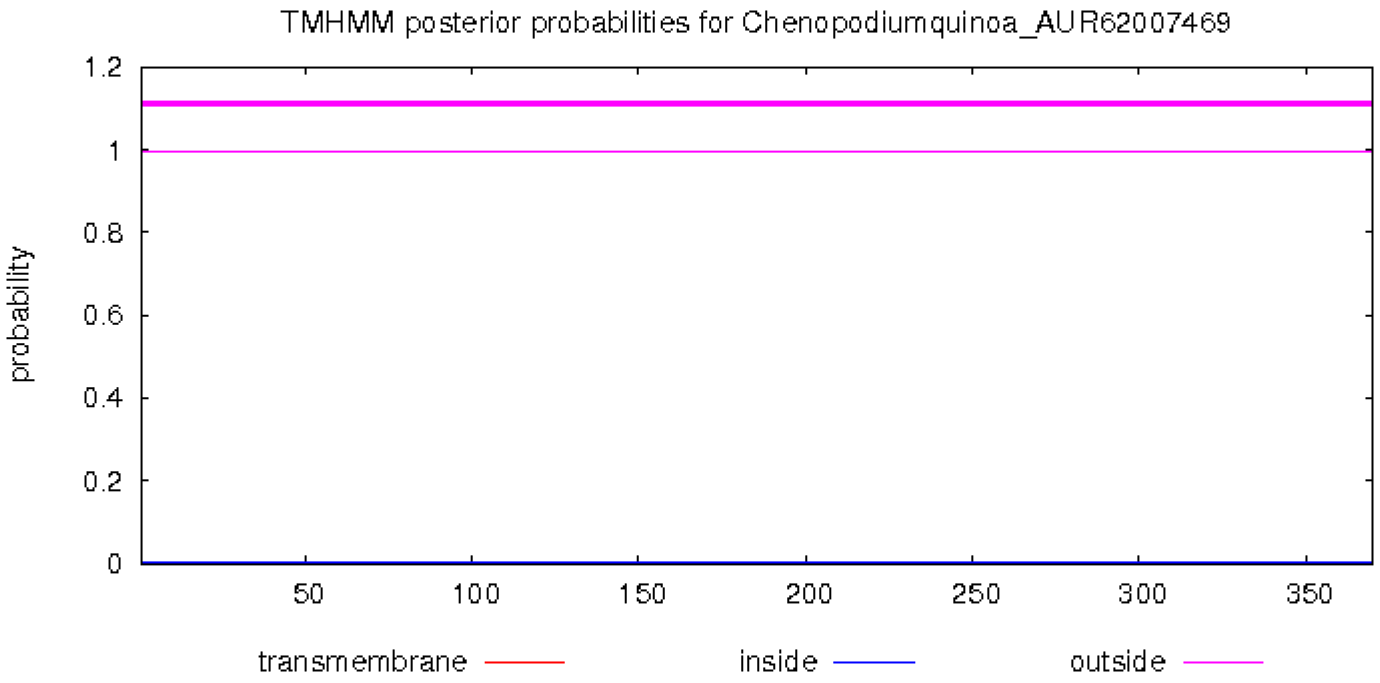

plot in postscript, script for making the plot in gnuplot, data for plot

---

```
# Chenopodiumquinoa_AUR62008438 Length: 364
# Chenopodiumquinoa_AUR62008438 Number of predicted TMHs:  0
# Chenopodiumquinoa_AUR62008438 Exp number of AAs in TMHs: 0
# Chenopodiumquinoa_AUR62008438 Exp number, first 60 AAs:  0
# Chenopodiumquinoa_AUR62008438 Total prob of N-in:        0.00553
Chenopodiumquinoa_AUR62008438   TMHMM2.0        outside      1   364
```

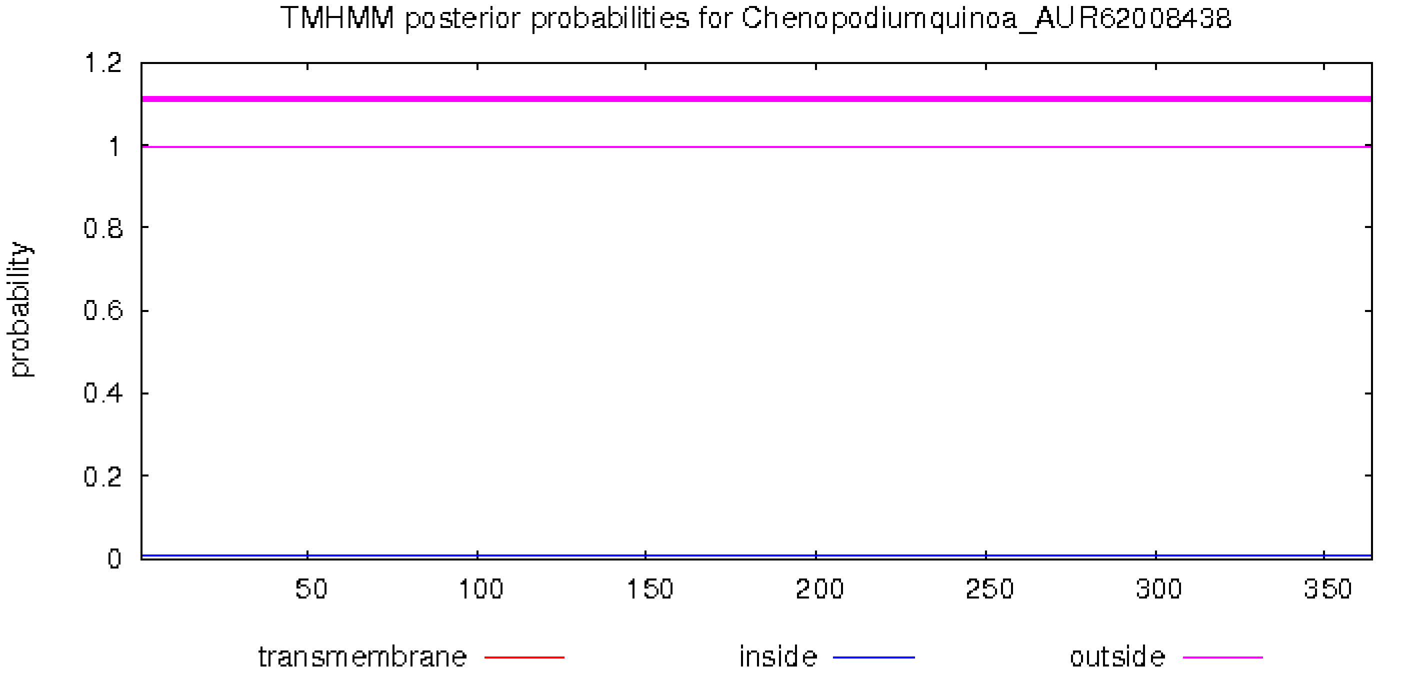

plot in postscript, script for making the plot in gnuplot, data for plot

---

```
# Chenopodiumquinoa_AUR62010080 Length: 352
# Chenopodiumquinoa_AUR62010080 Number of predicted TMHs:  0
# Chenopodiumquinoa_AUR62010080 Exp number of AAs in TMHs: 0.00038
# Chenopodiumquinoa_AUR62010080 Exp number, first 60 AAs:  0
# Chenopodiumquinoa_AUR62010080 Total prob of N-in:        0.00071
Chenopodiumquinoa_AUR62010080   TMHMM2.0        outside      1   352
```

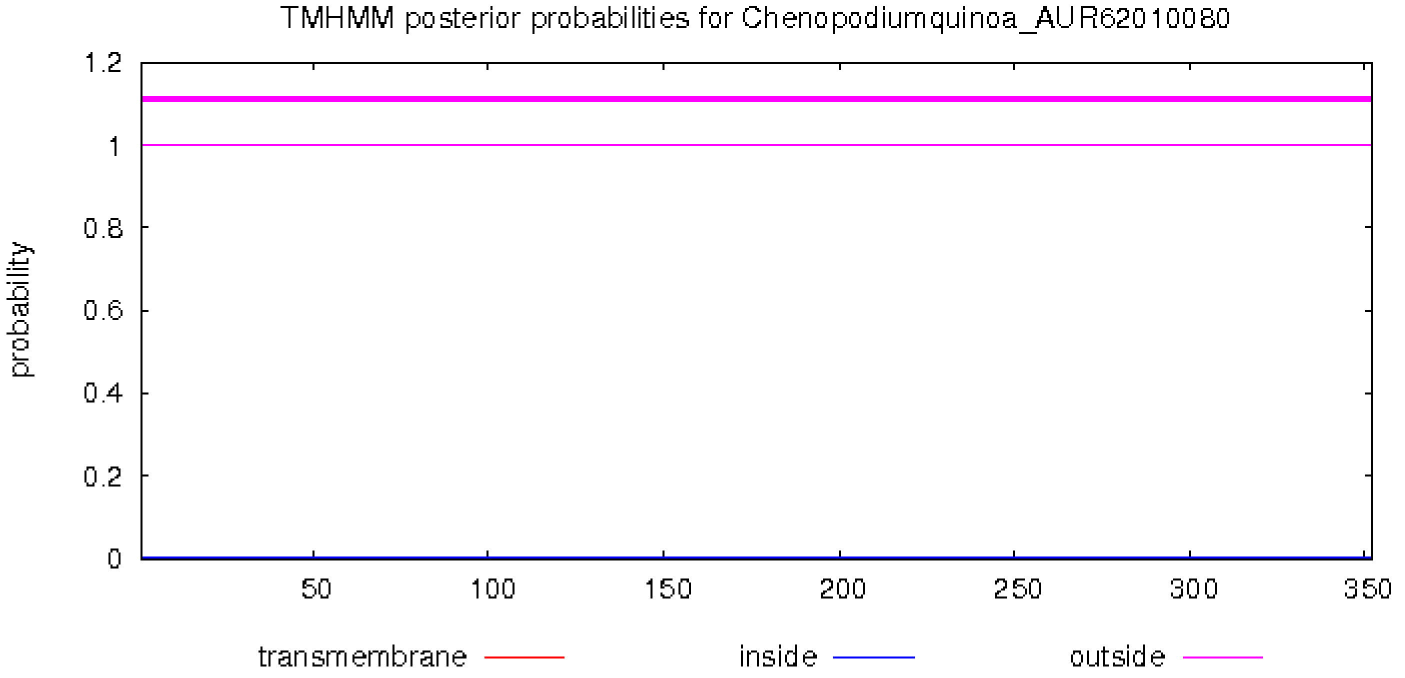

plot in postscript, script for making the plot in gnuplot, data for plot

---

```
# Chenopodiumquinoa_AUR62011638 Length: 414
# Chenopodiumquinoa_AUR62011638 Number of predicted TMHs:  0
# Chenopodiumquinoa_AUR62011638 Exp number of AAs in TMHs: 0.0007
# Chenopodiumquinoa_AUR62011638 Exp number, first 60 AAs:  0
# Chenopodiumquinoa_AUR62011638 Total prob of N-in:        0.00603
Chenopodiumquinoa_AUR62011638	TMHMM2.0	outside	     1   414
```

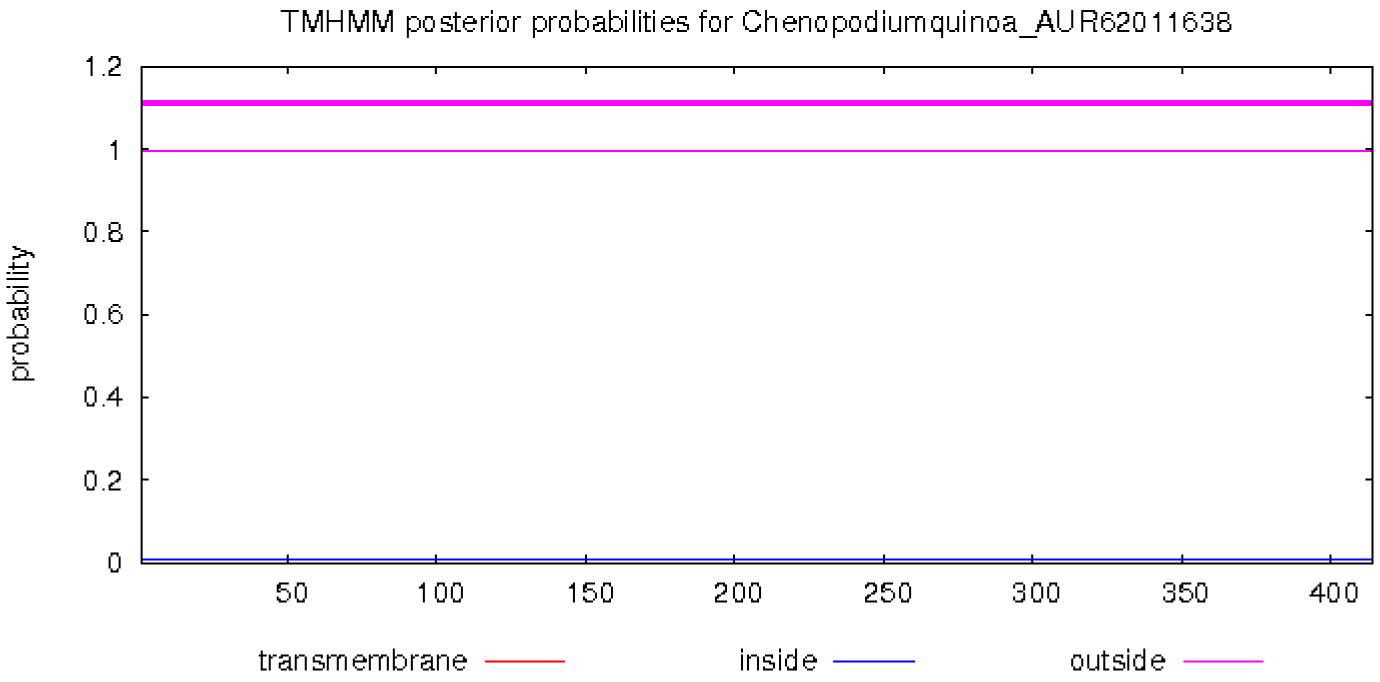

plot in postscript, script for making the plot in gnuplot, data for plot

---

```
# Chenopodiumquinoa_AUR62011853 Length: 145
# Chenopodiumquinoa_AUR62011853 Number of predicted TMHs:  0
# Chenopodiumquinoa_AUR62011853 Exp number of AAs in TMHs: 0.00037
# Chenopodiumquinoa_AUR62011853 Exp number, first 60 AAs:  7e-05
# Chenopodiumquinoa_AUR62011853 Total prob of N-in:        0.07402
Chenopodiumquinoa_AUR62011853	TMHMM2.0	outside	     1   145
```

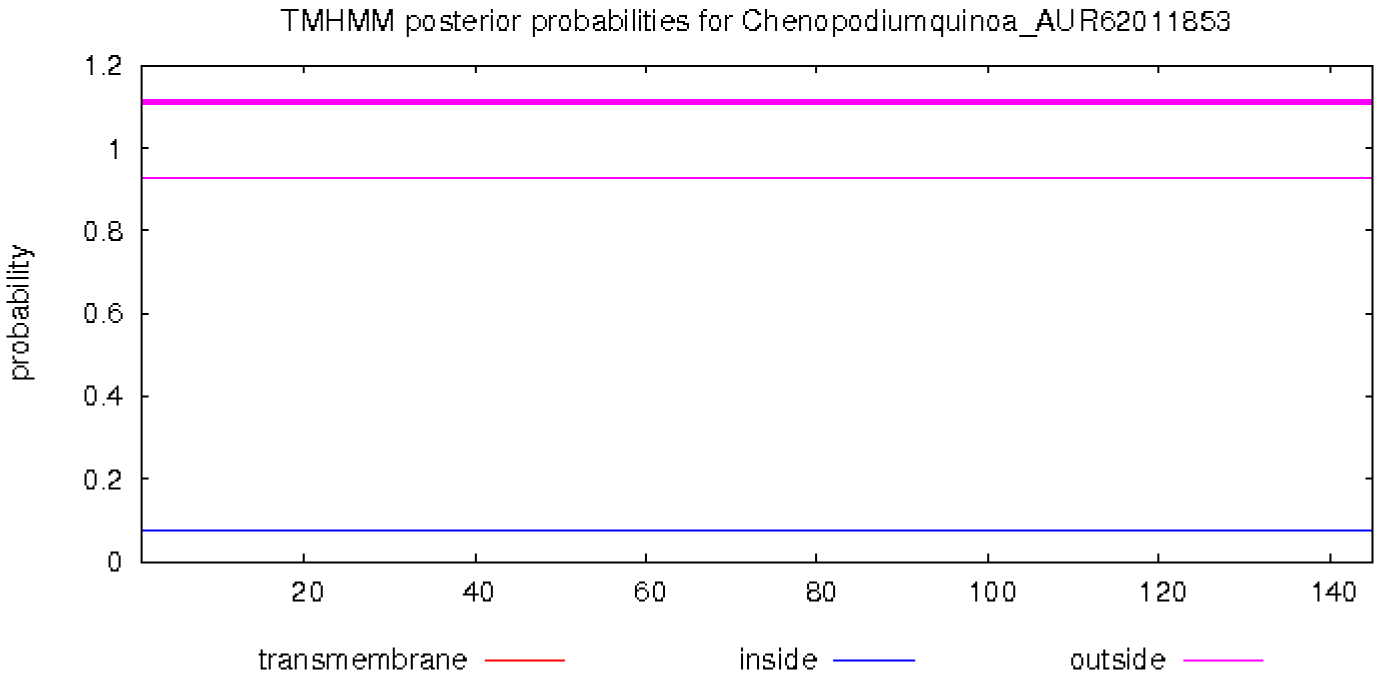

plot in postscript, script for making the plot in gnuplot, data for plot

---

```
# Chenopodiumquinoa_AUR62012948 Length: 146
# Chenopodiumquinoa_AUR62012948 Number of predicted TMHs:  0
# Chenopodiumquinoa_AUR62012948 Exp number of AAs in TMHs: 0.00064
# Chenopodiumquinoa_AUR62012948 Exp number, first 60 AAs:  0.00061
# Chenopodiumquinoa_AUR62012948 Total prob of N-in:        0.15979
Chenopodiumquinoa_AUR62012948   TMHMM2.0        outside      1   146
```

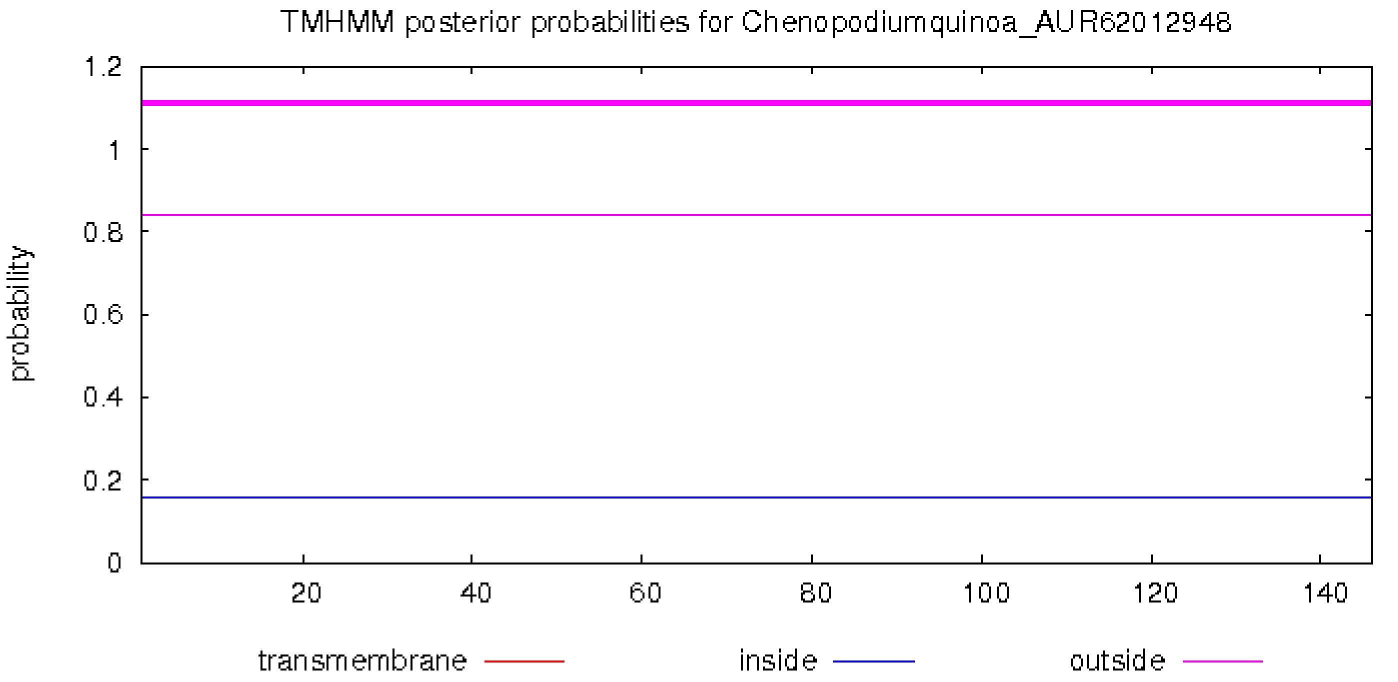

plot in postscript, script for making the plot in gnuplot, data for plot

---

```
# Chenopodiumquinoa_AUR62013074 Length: 352
# Chenopodiumquinoa_AUR62013074 Number of predicted TMHs:  0
# Chenopodiumquinoa_AUR62013074 Exp number of AAs in TMHs: 0.00022
# Chenopodiumquinoa_AUR62013074 Exp number, first 60 AAs:  0
# Chenopodiumquinoa_AUR62013074 Total prob of N-in:        0.00071
Chenopodiumquinoa_AUR62013074   TMHMM2.0        outside      1   352
```

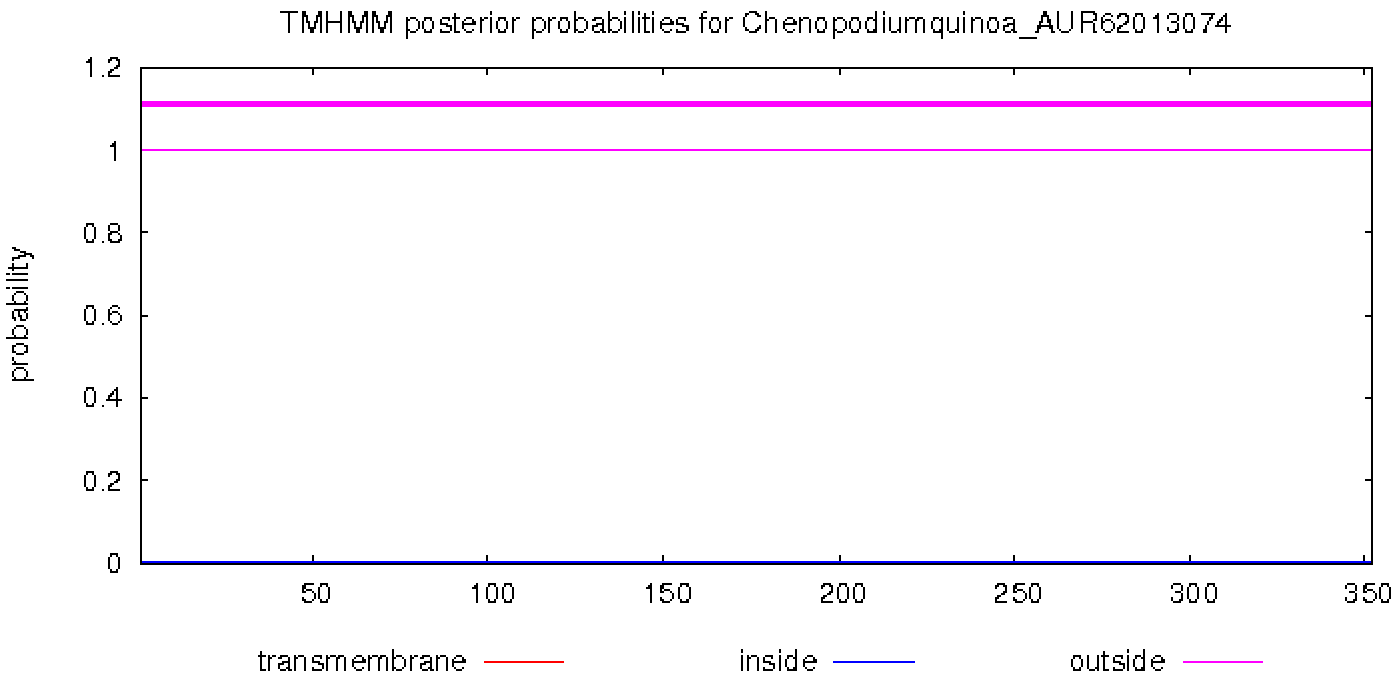

plot in postscript, script for making the plot in gnuplot, data for plot

---

```
# Chenopodiumquinoa_AUR62013541 Length: 346
# Chenopodiumquinoa_AUR62013541 Number of predicted TMHs:  0
# Chenopodiumquinoa_AUR62013541 Exp number of AAs in TMHs: 0.00353
# Chenopodiumquinoa_AUR62013541 Exp number, first 60 AAs:  0
# Chenopodiumquinoa_AUR62013541 Total prob of N-in:        0.00712
Chenopodiumquinoa_AUR62013541   TMHMM2.0        outside      1   346
```

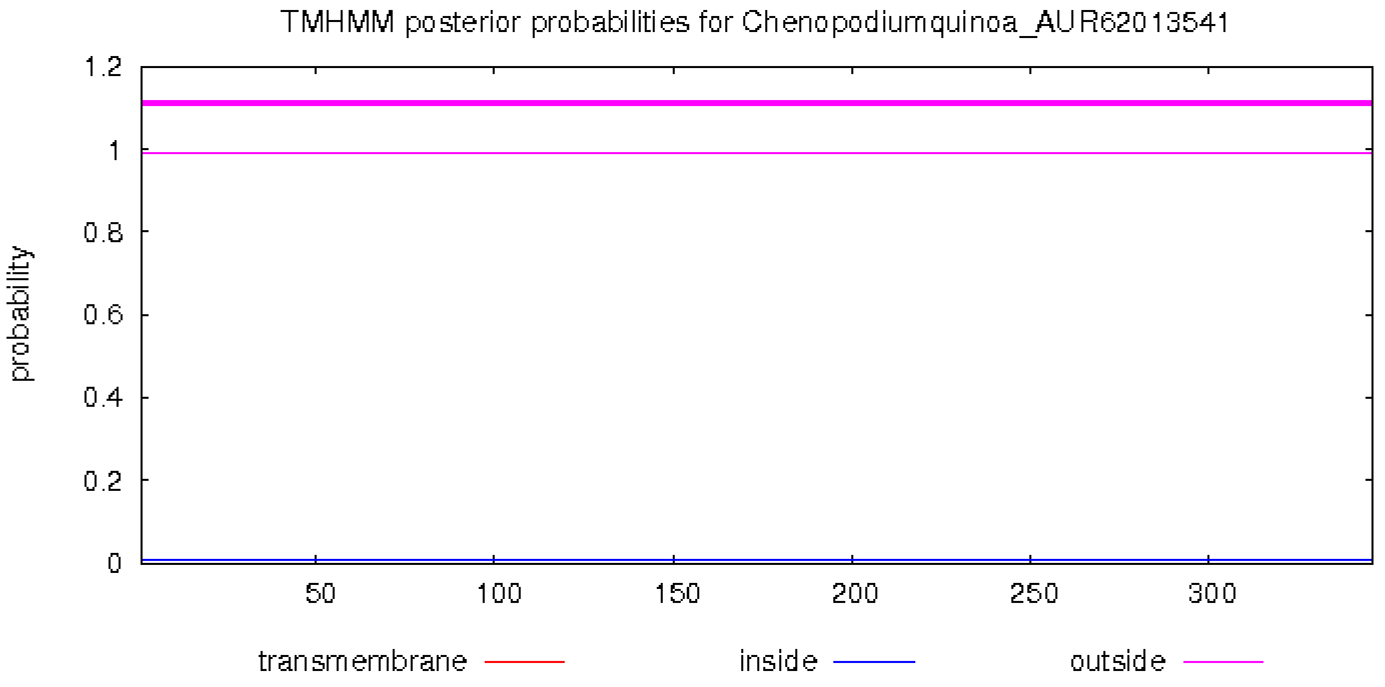

plot in postscript, script for making the plot in gnuplot, data for plot

---

```
# Chenopodiumquinoa_AUR62014768 Length: 343
# Chenopodiumquinoa_AUR62014768 Number of predicted TMHs:  0
# Chenopodiumquinoa_AUR62014768 Exp number of AAs in TMHs: 0.00305
# Chenopodiumquinoa_AUR62014768 Exp number, first 60 AAs:  0.00019
# Chenopodiumquinoa_AUR62014768 Total prob of N-in:        0.02582
Chenopodiumquinoa_AUR62014768   TMHMM2.0        outside      1   343
```

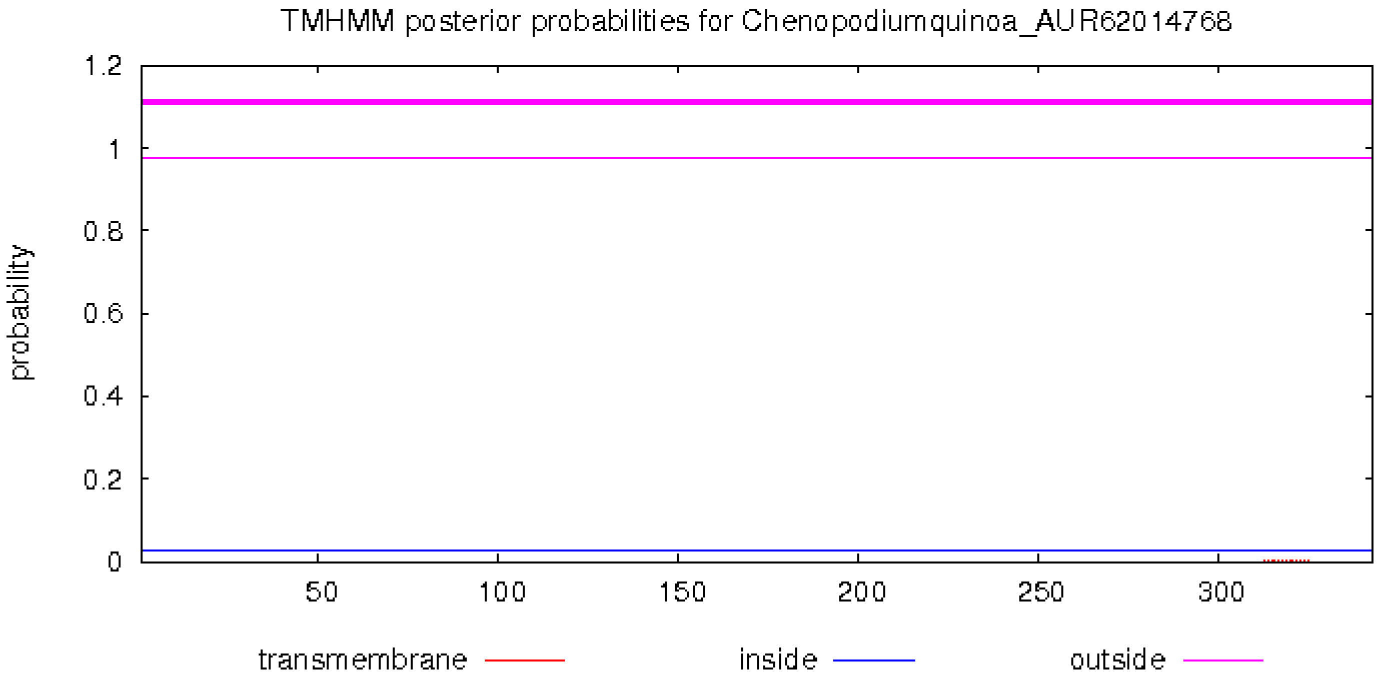

plot in postscript, script for making the plot in gnuplot, data for plot

---

```
# Chenopodiumquinoa_AUR62015520 Length: 248
# Chenopodiumquinoa_AUR62015520 Number of predicted TMHs:  0
# Chenopodiumquinoa_AUR62015520 Exp number of AAs in TMHs: 0.00018
# Chenopodiumquinoa_AUR62015520 Exp number, first 60 AAs:  0
# Chenopodiumquinoa_AUR62015520 Total prob of N-in:        0.04001
Chenopodiumquinoa_AUR62015520   TMHMM2.0        outside      1   248
```

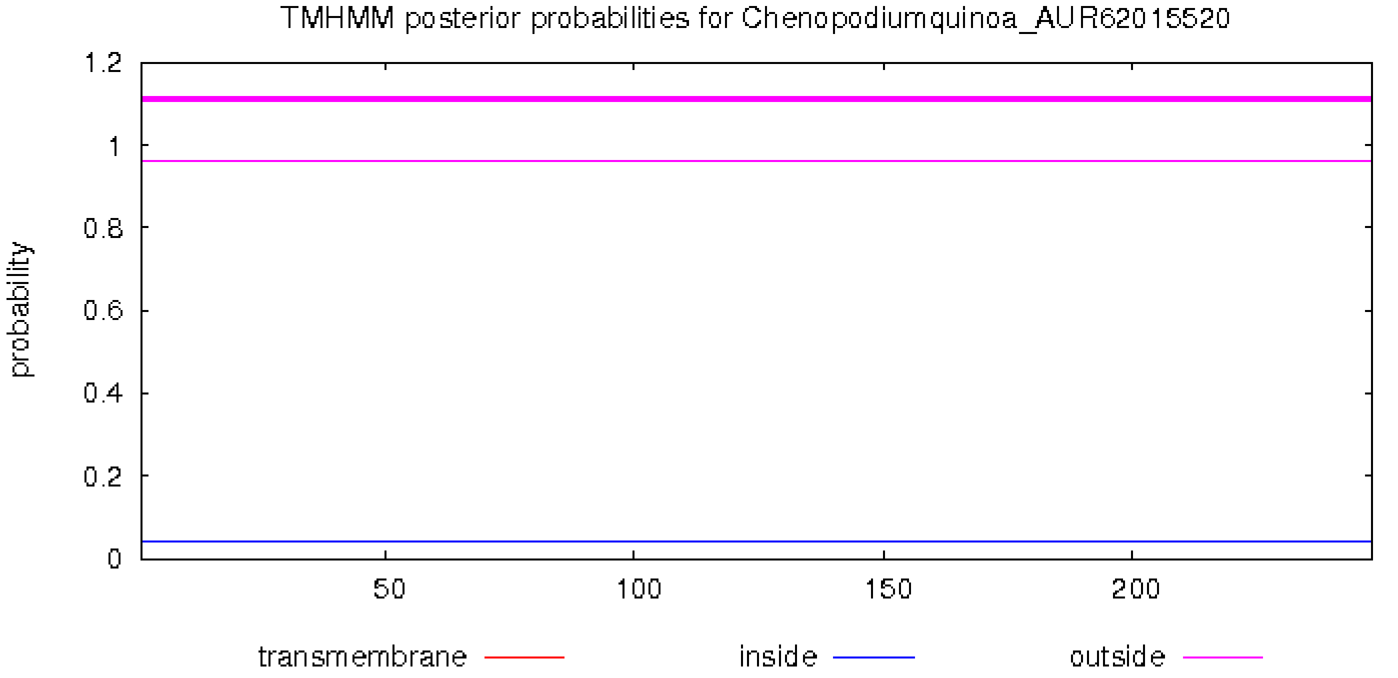

plot in postscript, script for making the plot in gnuplot, data for plot

---

```
# Chenopodiumquinoa_AUR62015629 Length: 302
# Chenopodiumquinoa_AUR62015629 Number of predicted TMHs:  0
# Chenopodiumquinoa_AUR62015629 Exp number of AAs in TMHs: 0.00157
# Chenopodiumquinoa_AUR62015629 Exp number, first 60 AAs:  6e-05
# Chenopodiumquinoa_AUR62015629 Total prob of N-in:        0.00719
Chenopodiumquinoa_AUR62015629   TMHMM2.0        outside      1   302
```

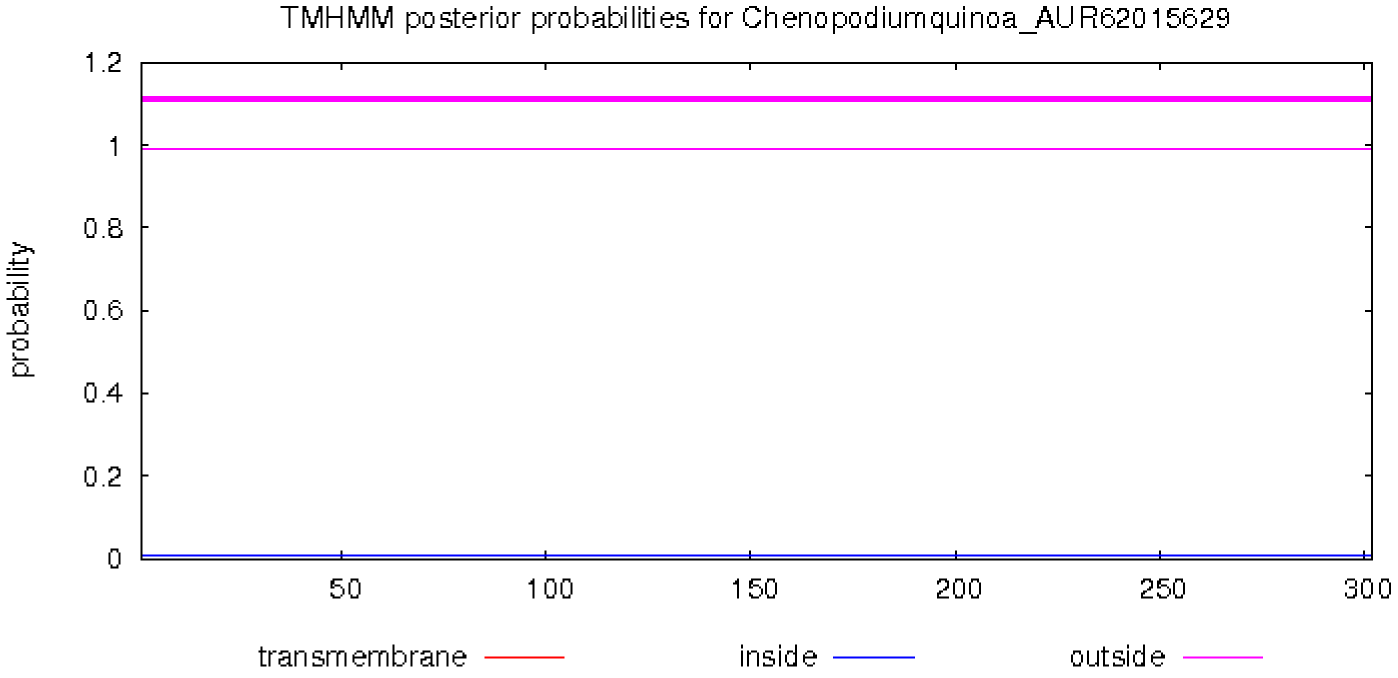

plot in postscript, script for making the plot in gnuplot, data for plot

---

```
# Chenopodiumquinoa_AUR62015630 Length: 278
# Chenopodiumquinoa_AUR62015630 Number of predicted TMHs:  0
# Chenopodiumquinoa_AUR62015630 Exp number of AAs in TMHs: 0.022
# Chenopodiumquinoa_AUR62015630 Exp number, first 60 AAs:  0.0002
# Chenopodiumquinoa_AUR62015630 Total prob of N-in:        0.01867
Chenopodiumquinoa_AUR62015630   TMHMM2.0        outside      1   278
```

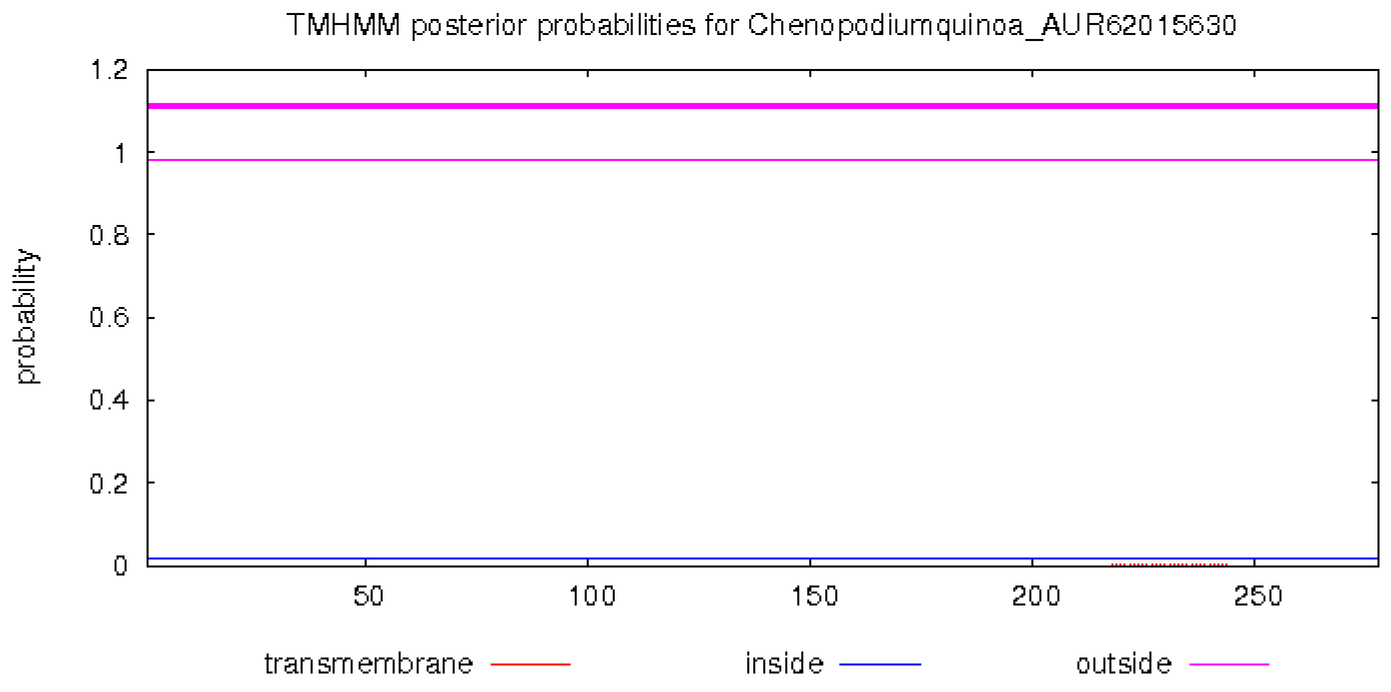

plot in postscript, script for making the plot in gnuplot, data for plot

```
# Chenopodiumquinoa_AUR62016200 Length: 257
# Chenopodiumquinoa_AUR62016200 Number of predicted TMHs:  0
# Chenopodiumquinoa_AUR62016200 Exp number of AAs in TMHs: 0.00399
# Chenopodiumquinoa_AUR62016200 Exp number, first 60 AAs:  0
# Chenopodiumquinoa_AUR62016200 Total prob of N-in:        0.01704
Chenopodiumquinoa_AUR62016200   TMHMM2.0        outside      1   257
```

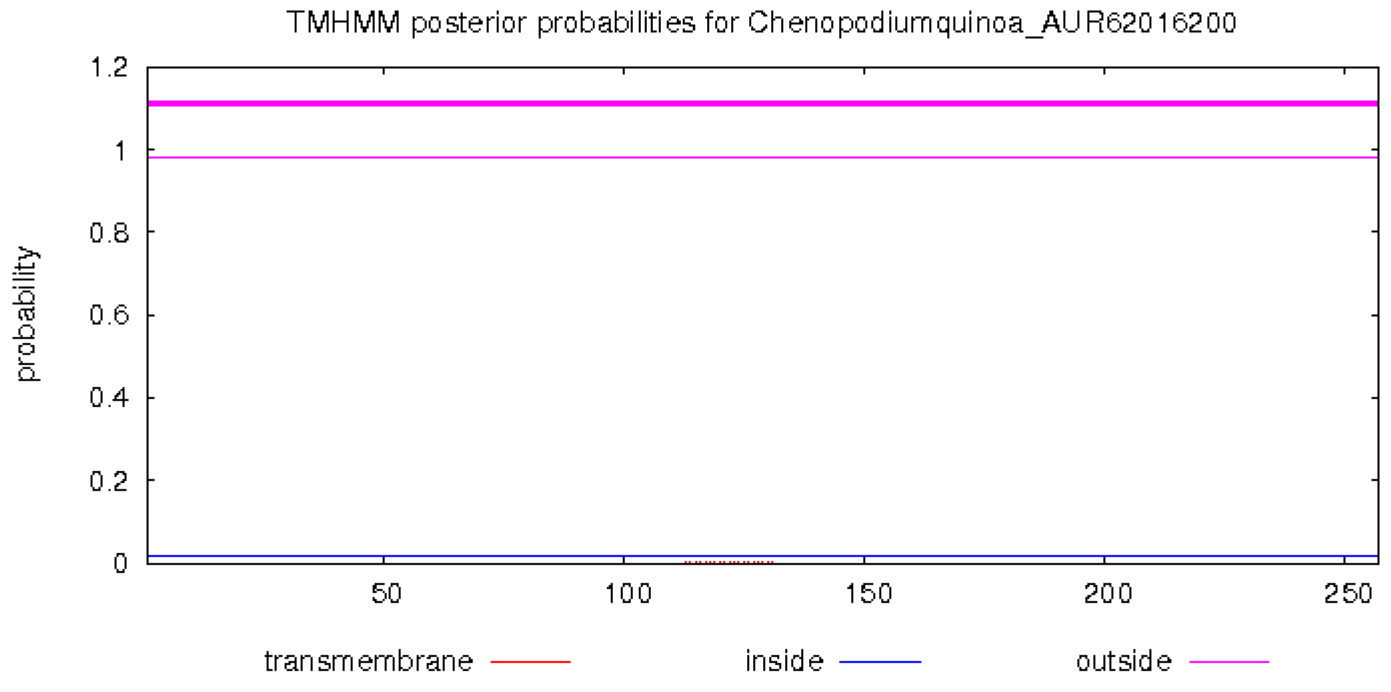

plot in postscript, script for making the plot in gnuplot, data for plot

```
# Chenopodiumquinoa_AUR62016213 Length: 389
# Chenopodiumquinoa_AUR62016213 Number of predicted TMHs:  0
# Chenopodiumquinoa_AUR62016213 Exp number of AAs in TMHs: 0
# Chenopodiumquinoa_AUR62016213 Exp number, first 60 AAs:  0
# Chenopodiumquinoa_AUR62016213 Total prob of N-in:        0.00467
Chenopodiumquinoa_AUR62016213   TMHMM2.0        outside      1   389
```

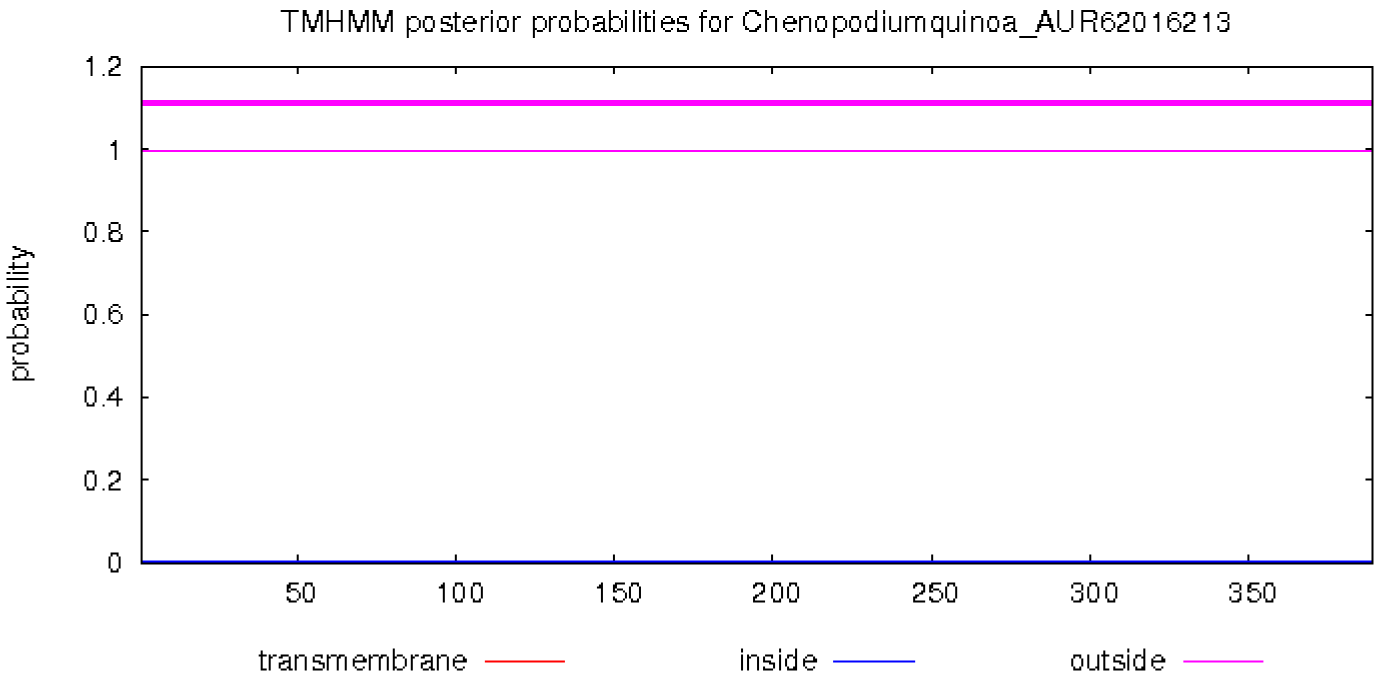

plot in postscript, script for making the plot in gnuplot, data for plot

```
# Chenopodiumquinoa_AUR62016954 Length: 327
# Chenopodiumquinoa_AUR62016954 Number of predicted TMHs:  0
# Chenopodiumquinoa_AUR62016954 Exp number of AAs in TMHs: 0.05627
# Chenopodiumquinoa_AUR62016954 Exp number, first 60 AAs:  0.04329
# Chenopodiumquinoa_AUR62016954 Total prob of N-in:        0.03303
Chenopodiumquinoa_AUR62016954   TMHMM2.0        outside      1   327
```

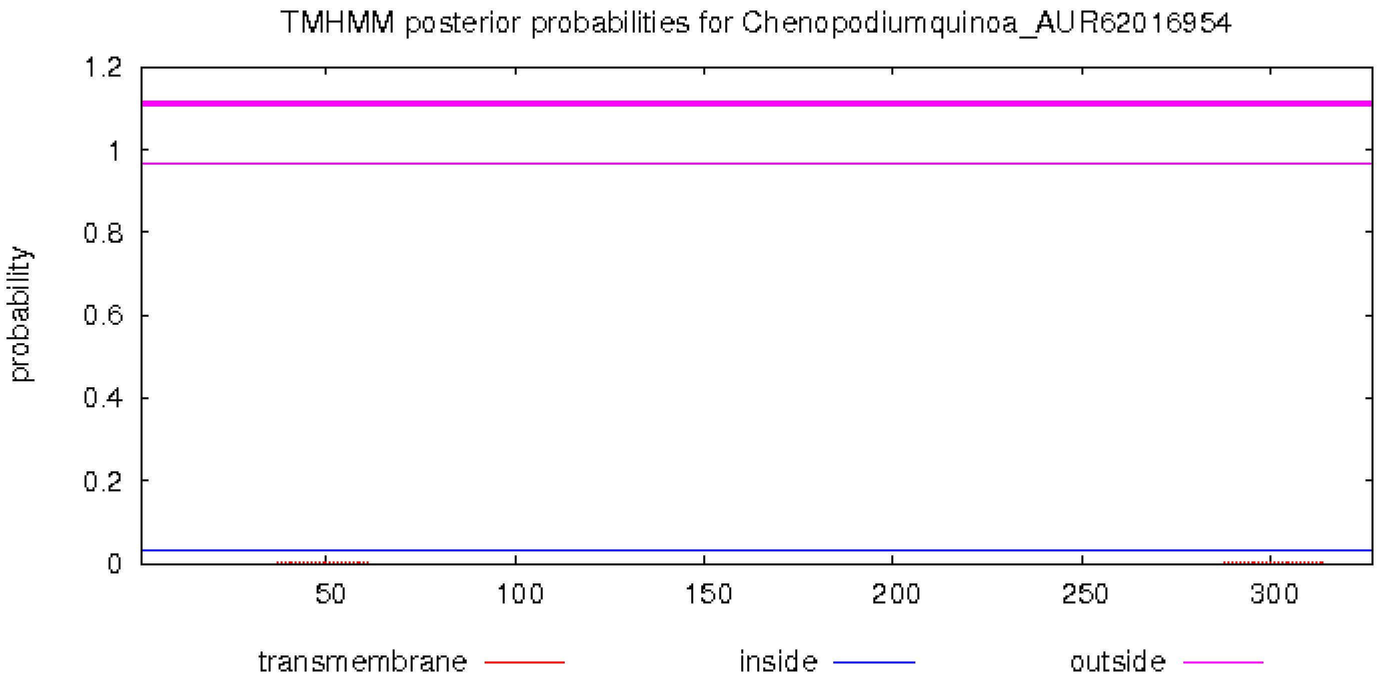

plot in postscript, script for making the plot in gnuplot, data for plot

```
# Chenopodiumquinoa_AUR62017681 Length: 418
# Chenopodiumquinoa_AUR62017681 Number of predicted TMHs:  0
# Chenopodiumquinoa_AUR62017681 Exp number of AAs in TMHs: 0.00583
# Chenopodiumquinoa_AUR62017681 Exp number, first 60 AAs:  0
# Chenopodiumquinoa_AUR62017681 Total prob of N-in:        0.00224
Chenopodiumquinoa_AUR62017681   TMHMM2.0        outside      1   418
```

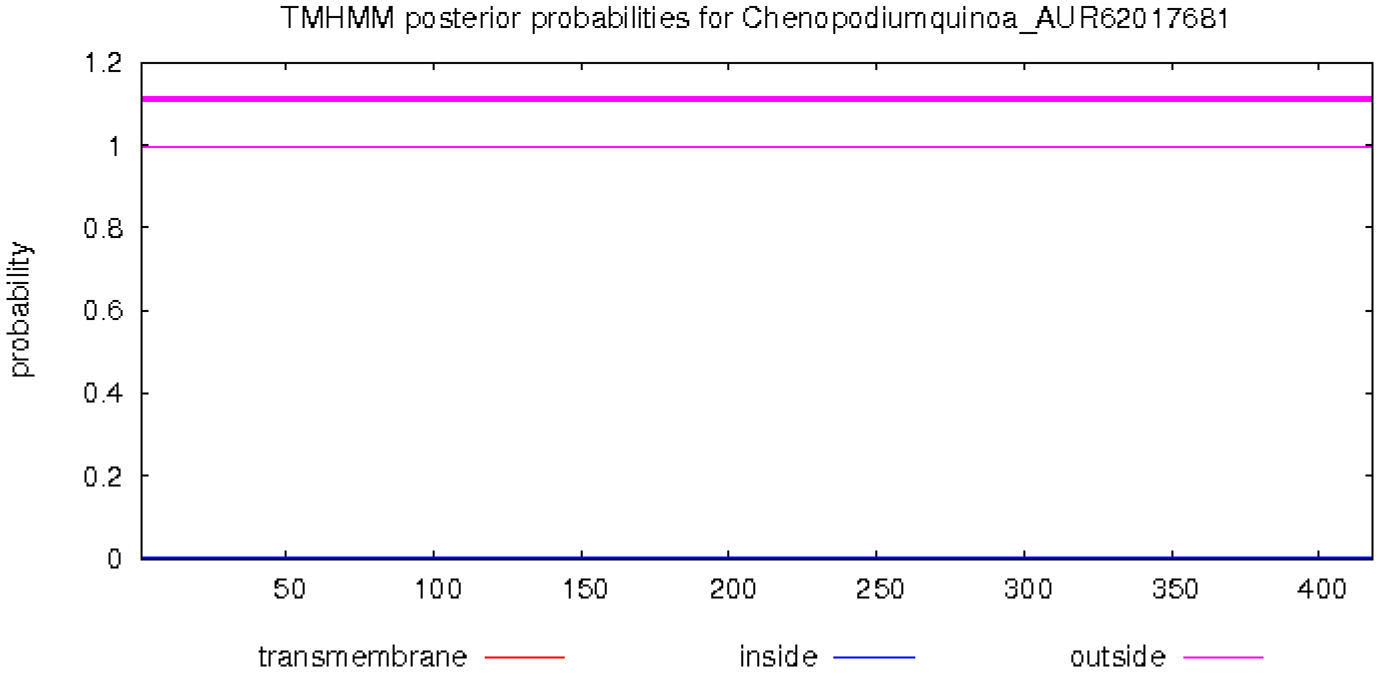

plot in postscript, script for making the plot in gnuplot, data for plot

---

```
# Chenopodiumquinoa_AUR62017896 Length: 224
# Chenopodiumquinoa_AUR62017896 Number of predicted TMHs:  0
# Chenopodiumquinoa_AUR62017896 Exp number of AAs in TMHs: 0.00097
# Chenopodiumquinoa_AUR62017896 Exp number, first 60 AAs:  0
# Chenopodiumquinoa_AUR62017896 Total prob of N-in:        0.07293
Chenopodiumquinoa_AUR62017896   TMHMM2.0        outside      1   224
```

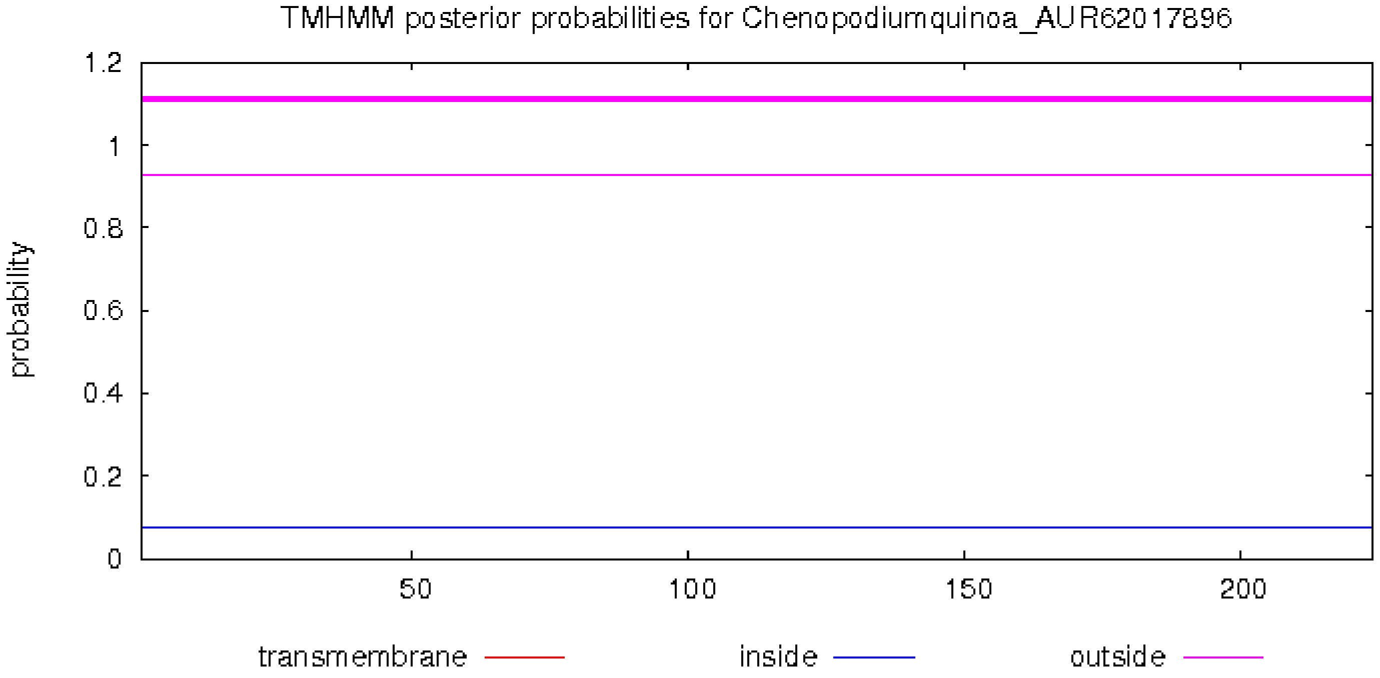

plot in postscript, script for making the plot in gnuplot, data for plot

---

```
# Chenopodiumquinoa_AUR62018102 Length: 333
# Chenopodiumquinoa_AUR62018102 Number of predicted TMHs:  0
# Chenopodiumquinoa_AUR62018102 Exp number of AAs in TMHs: 0.00134
# Chenopodiumquinoa_AUR62018102 Exp number, first 60 AAs:  0
# Chenopodiumquinoa_AUR62018102 Total prob of N-in:        0.04335
Chenopodiumquinoa_AUR62018102   TMHMM2.0        outside      1   333
```

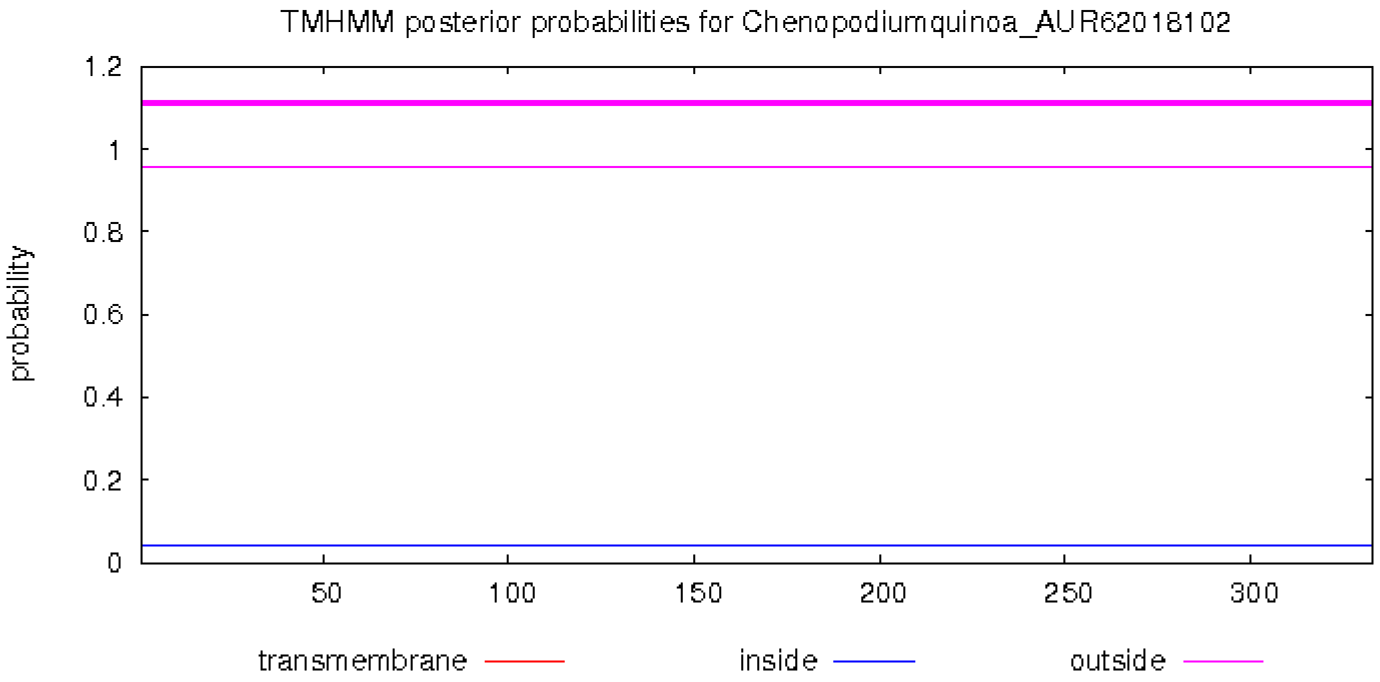

plot in postscript, script for making the plot in gnuplot, data for plot

---

```
# Chenopodiumquinoa_AUR62018752 Length: 325
# Chenopodiumquinoa_AUR62018752 Number of predicted TMHs:  0
# Chenopodiumquinoa_AUR62018752 Exp number of AAs in TMHs: 0.0009
# Chenopodiumquinoa_AUR62018752 Exp number, first 60 AAs:  0
# Chenopodiumquinoa_AUR62018752 Total prob of N-in:        0.00171
Chenopodiumquinoa_AUR62018752   TMHMM2.0        outside      1   325
```

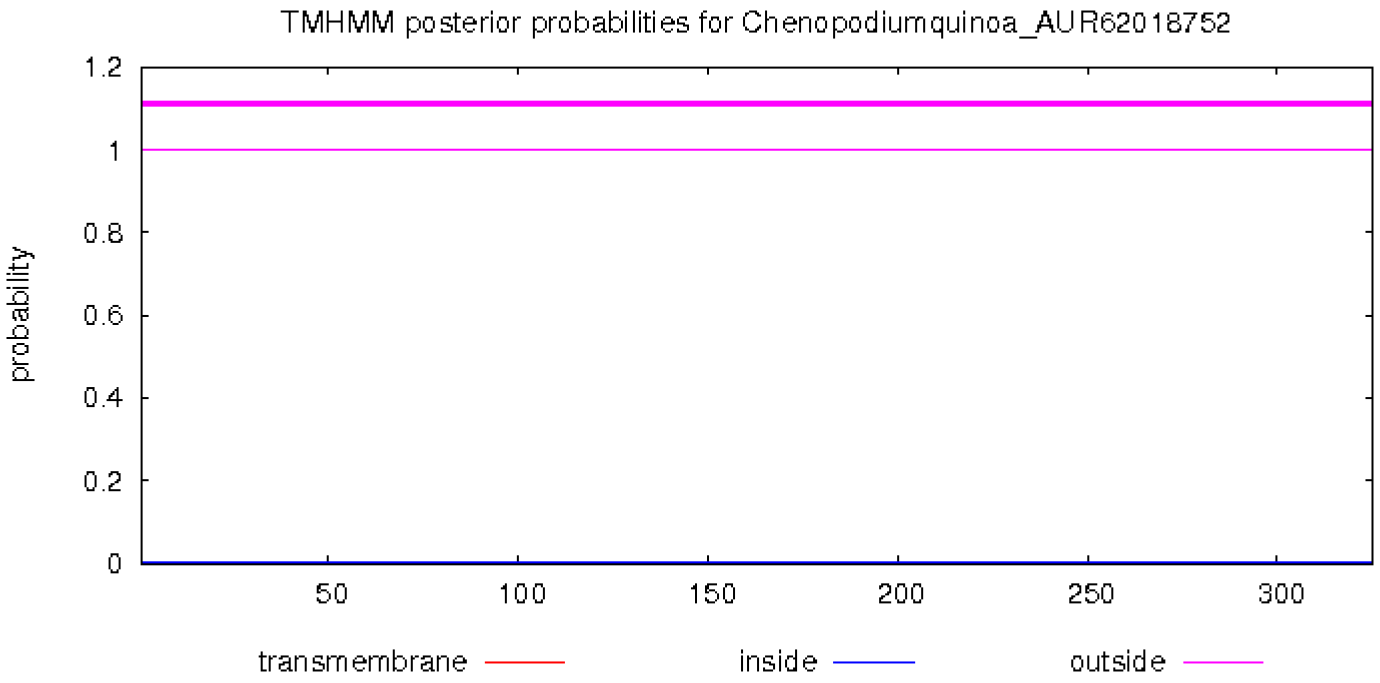

plot in postscript, script for making the plot in gnuplot, data for plot

---

```
# Chenopodiumquinoa_AUR62019477 Length: 400
# Chenopodiumquinoa_AUR62019477 Number of predicted TMHs:  0
# Chenopodiumquinoa_AUR62019477 Exp number of AAs in TMHs: 0.07029
# Chenopodiumquinoa_AUR62019477 Exp number, first 60 AAs:  0
# Chenopodiumquinoa_AUR62019477 Total prob of N-in:        0.00096
Chenopodiumquinoa_AUR62019477   TMHMM2.0        outside      1   400
```

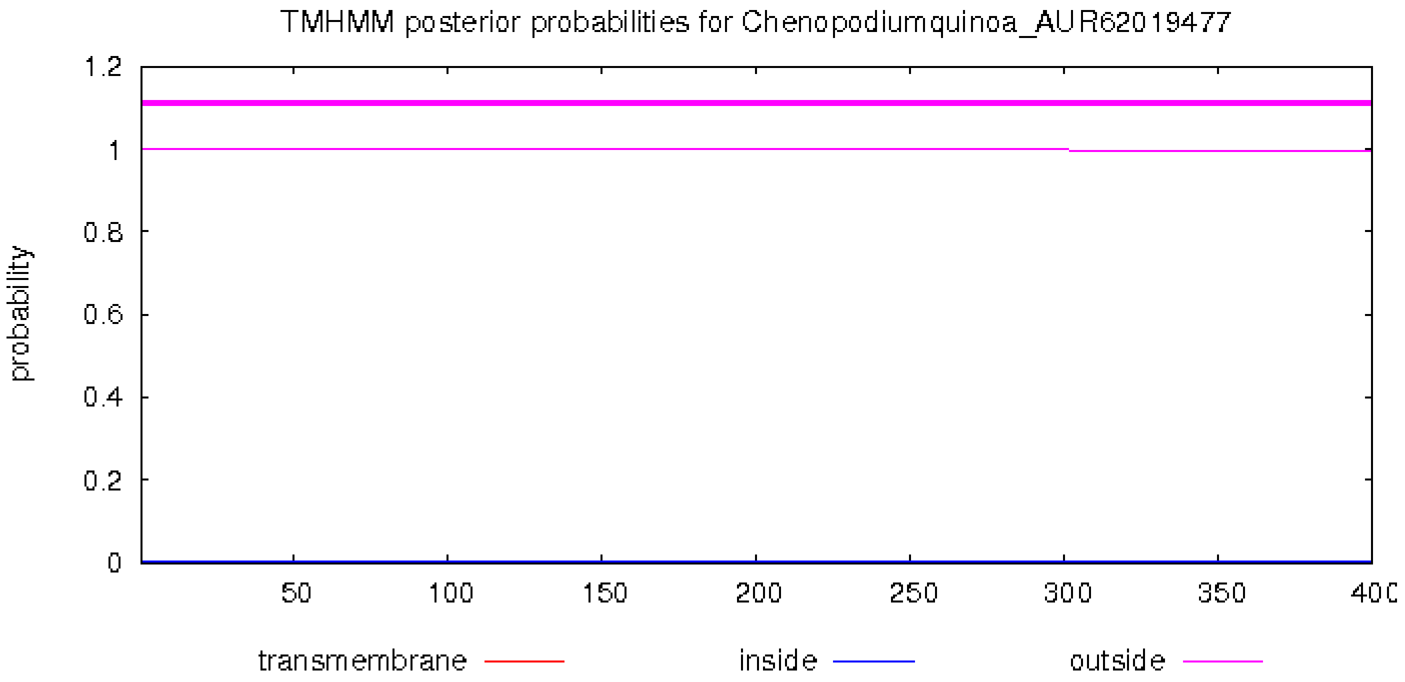

plot in postscript, script for making the plot in gnuplot, data for plot

---

```
# Chenopodiumquinoa_AUR62019936 Length: 341
# Chenopodiumquinoa_AUR62019936 Number of predicted TMHs:  0
# Chenopodiumquinoa_AUR62019936 Exp number of AAs in TMHs: 0
# Chenopodiumquinoa_AUR62019936 Exp number, first 60 AAs:  0
# Chenopodiumquinoa_AUR62019936 Total prob of N-in:        0.01818
Chenopodiumquinoa_AUR62019936   TMHMM2.0        outside      1   341
```

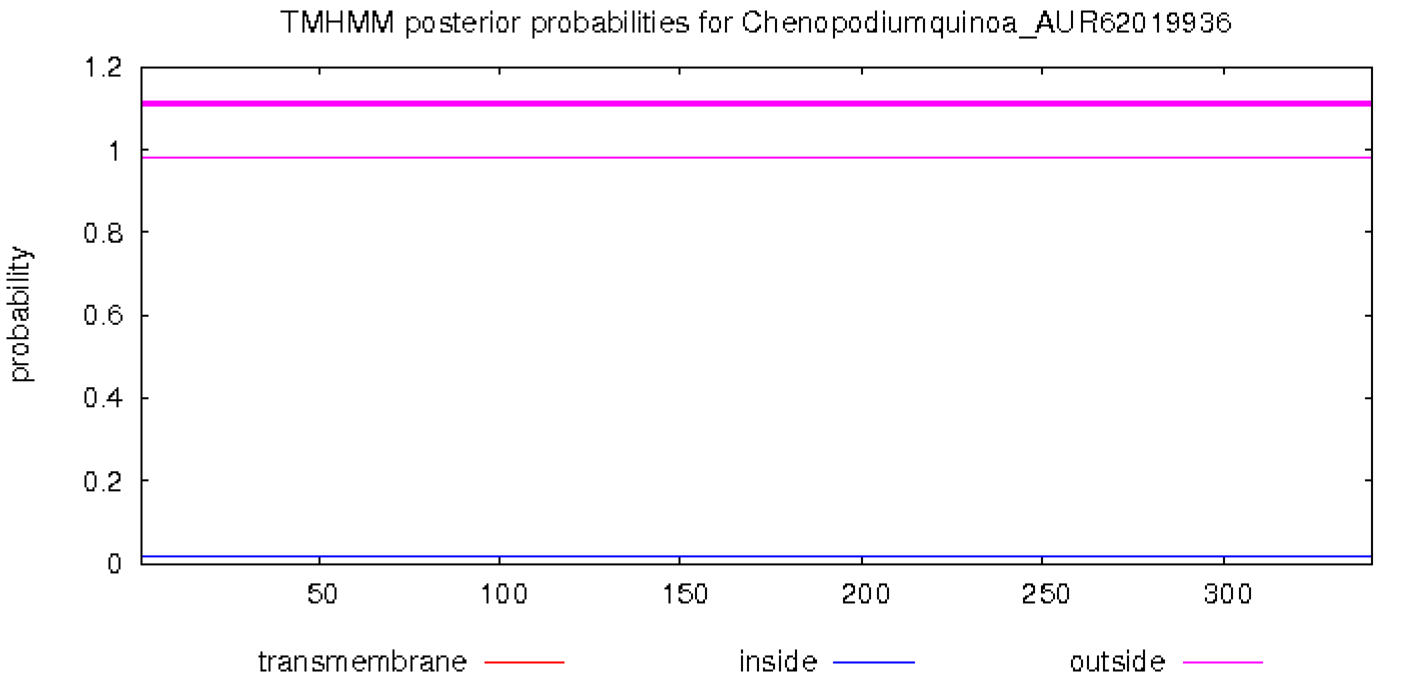

plot in postscript, script for making the plot in gnuplot, data for plot

---

```
# Chenopodiumquinoa_AUR62020251 Length: 430
# Chenopodiumquinoa_AUR62020251 Number of predicted TMHs:  1
# Chenopodiumquinoa_AUR62020251 Exp number of AAs in TMHs: 22.59623
# Chenopodiumquinoa_AUR62020251 Exp number, first 60 AAs:  0.04298
# Chenopodiumquinoa_AUR62020251 Total prob of N-in:        0.72075
Chenopodiumquinoa_AUR62020251   TMHMM2.0        inside       1   405
Chenopodiumquinoa_AUR62020251   TMHMM2.0        TMhelix    406   428
Chenopodiumquinoa_AUR62020251   TMHMM2.0        outside    429   430
```

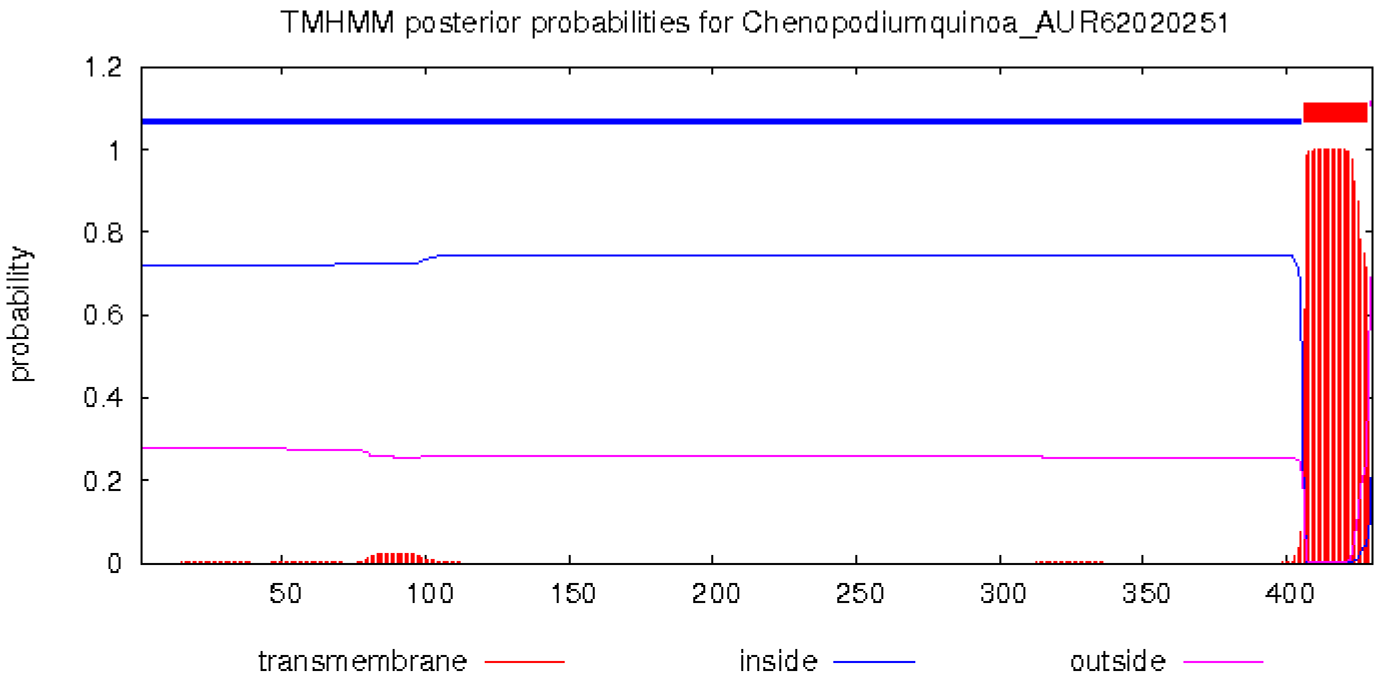

plot in postscript, script for making the plot in gnuplot, data for plot

---

```
# Chenopodiumquinoa_AUR62020261 Length: 258
# Chenopodiumquinoa_AUR62020261 Number of predicted TMHs:  0
# Chenopodiumquinoa_AUR62020261 Exp number of AAs in TMHs: 8e-05
# Chenopodiumquinoa_AUR62020261 Exp number, first 60 AAs:  0
# Chenopodiumquinoa_AUR62020261 Total prob of N-in:        0.00546
Chenopodiumquinoa_AUR62020261   TMHMM2.0        outside      1   258
```

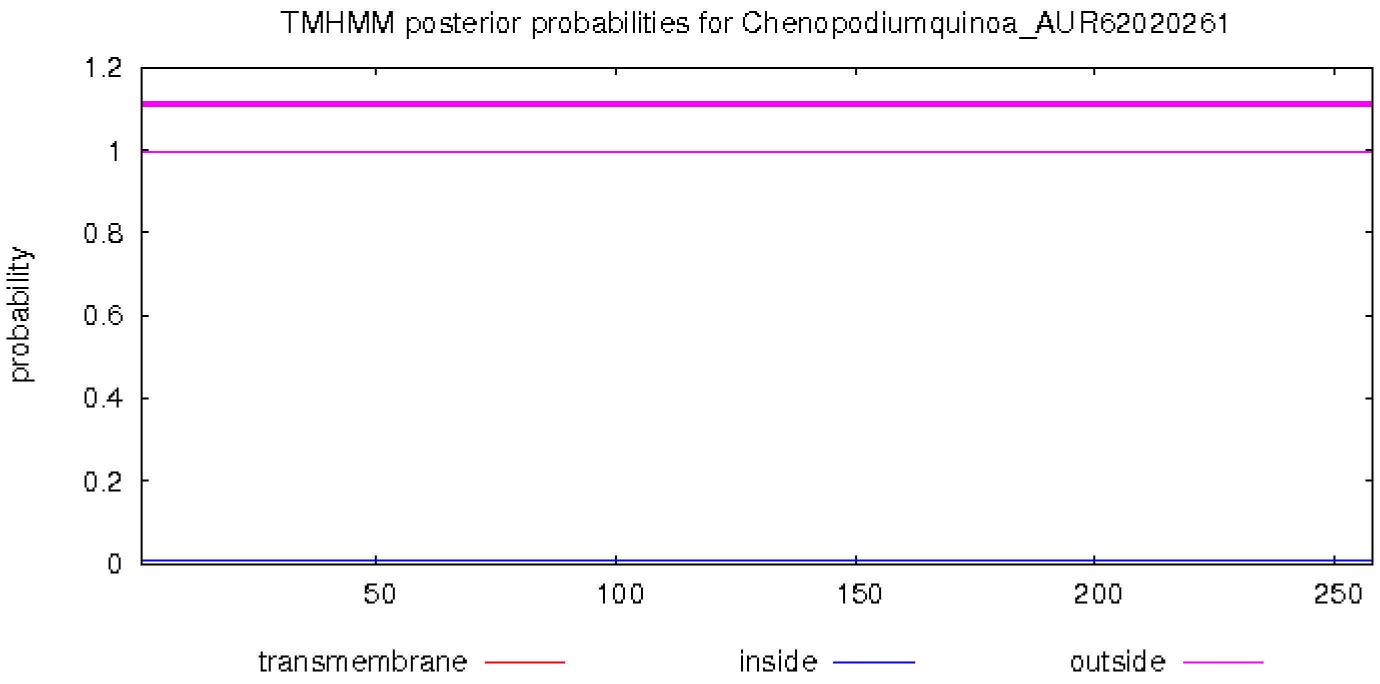

plot in postscript, script for making the plot in gnuplot, data for plot

```
# Chenopodiumquinoa_AUR62020634 Length: 297
# Chenopodiumquinoa_AUR62020634 Number of predicted TMHs:  0
# Chenopodiumquinoa_AUR62020634 Exp number of AAs in TMHs: 0.00214
# Chenopodiumquinoa_AUR62020634 Exp number, first 60 AAs:  0.00037
# Chenopodiumquinoa_AUR62020634 Total prob of N-in:        0.03232
Chenopodiumquinoa_AUR62020634	TMHMM2.0	outside	     1   297
```

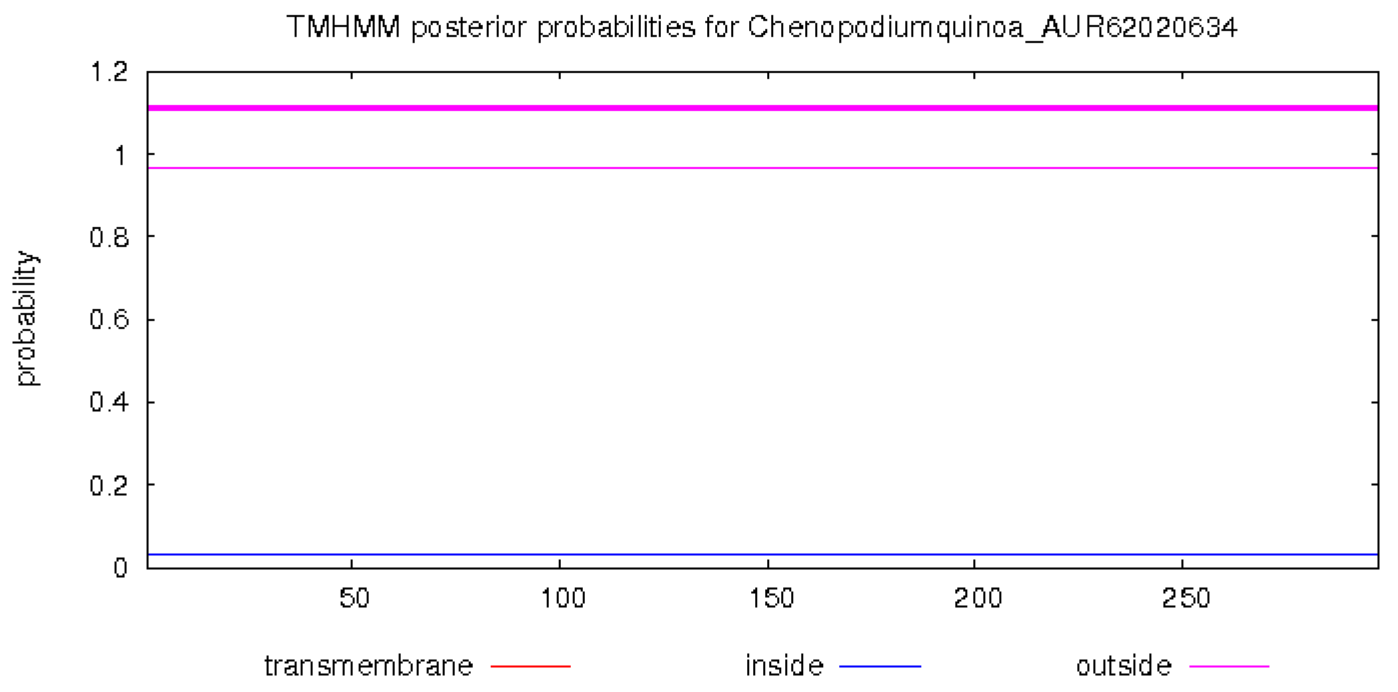

plot in postscript, script for making the plot in gnuplot, data for plot

```
# Chenopodiumquinoa_AUR62021621 Length: 198
# Chenopodiumquinoa_AUR62021621 Number of predicted TMHs:  0
# Chenopodiumquinoa_AUR62021621 Exp number of AAs in TMHs: 2.5001
# Chenopodiumquinoa_AUR62021621 Exp number, first 60 AAs:  0
# Chenopodiumquinoa_AUR62021621 Total prob of N-in:        0.07910
Chenopodiumquinoa_AUR62021621	TMHMM2.0	outside	     1   198
```

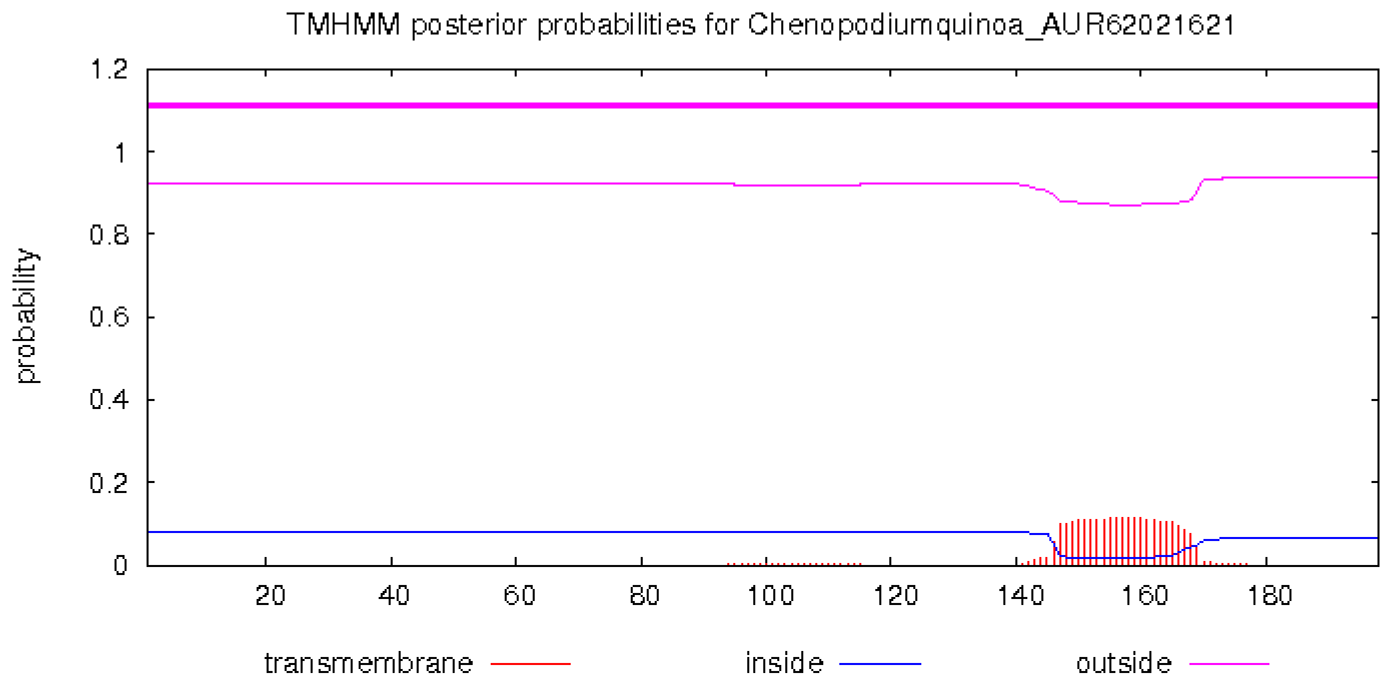

plot in postscript, script for making the plot in gnuplot, data for plot

```
# Chenopodiumquinoa_AUR62021652 Length: 361
# Chenopodiumquinoa_AUR62021652 Number of predicted TMHs:  0
# Chenopodiumquinoa_AUR62021652 Exp number of AAs in TMHs: 0
# Chenopodiumquinoa_AUR62021652 Exp number, first 60 AAs:  0
# Chenopodiumquinoa_AUR62021652 Total prob of N-in:        0.00670
Chenopodiumquinoa_AUR62021652   TMHMM2.0        outside      1   361
```

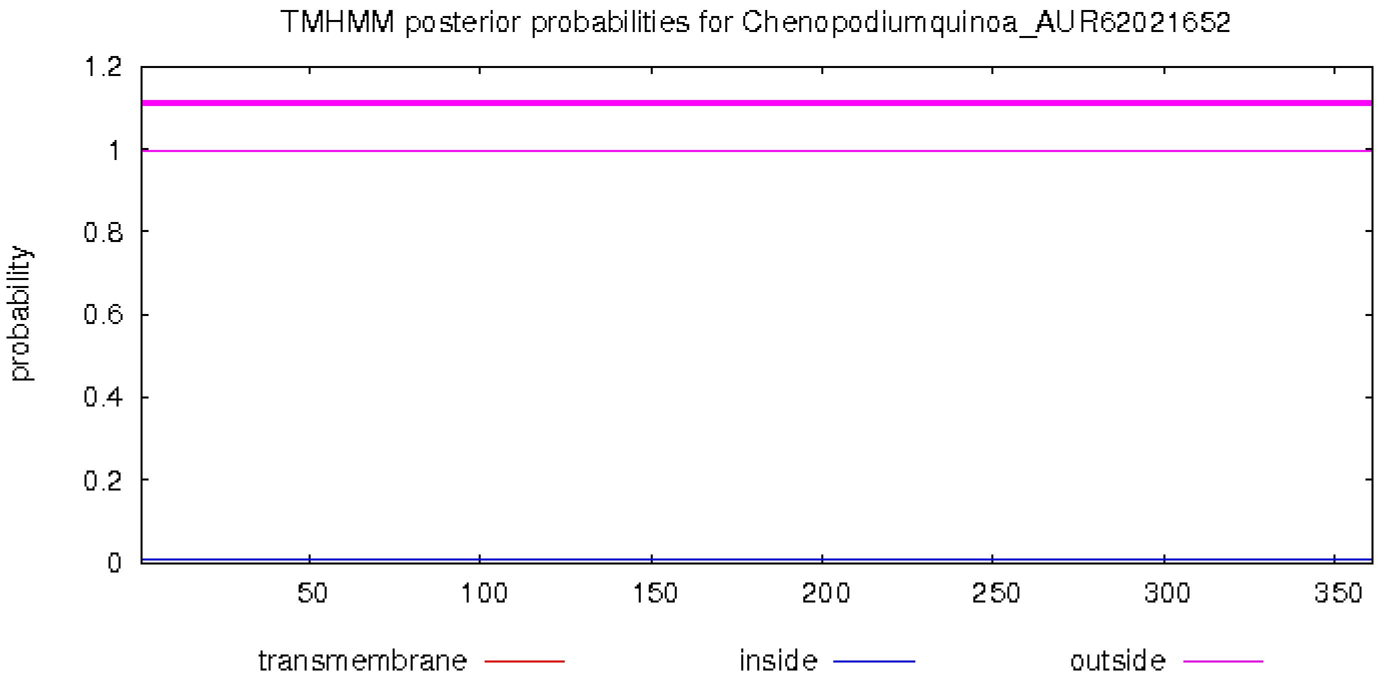

plot in postscript, script for making the plot in gnuplot, data for plot

```
# Chenopodiumquinoa_AUR62022486 Length: 386
# Chenopodiumquinoa_AUR62022486 Number of predicted TMHs:  0
# Chenopodiumquinoa_AUR62022486 Exp number of AAs in TMHs: 0.0225
# Chenopodiumquinoa_AUR62022486 Exp number, first 60 AAs:  0
# Chenopodiumquinoa_AUR62022486 Total prob of N-in:        0.00286
Chenopodiumquinoa_AUR62022486   TMHMM2.0        outside      1   386
```

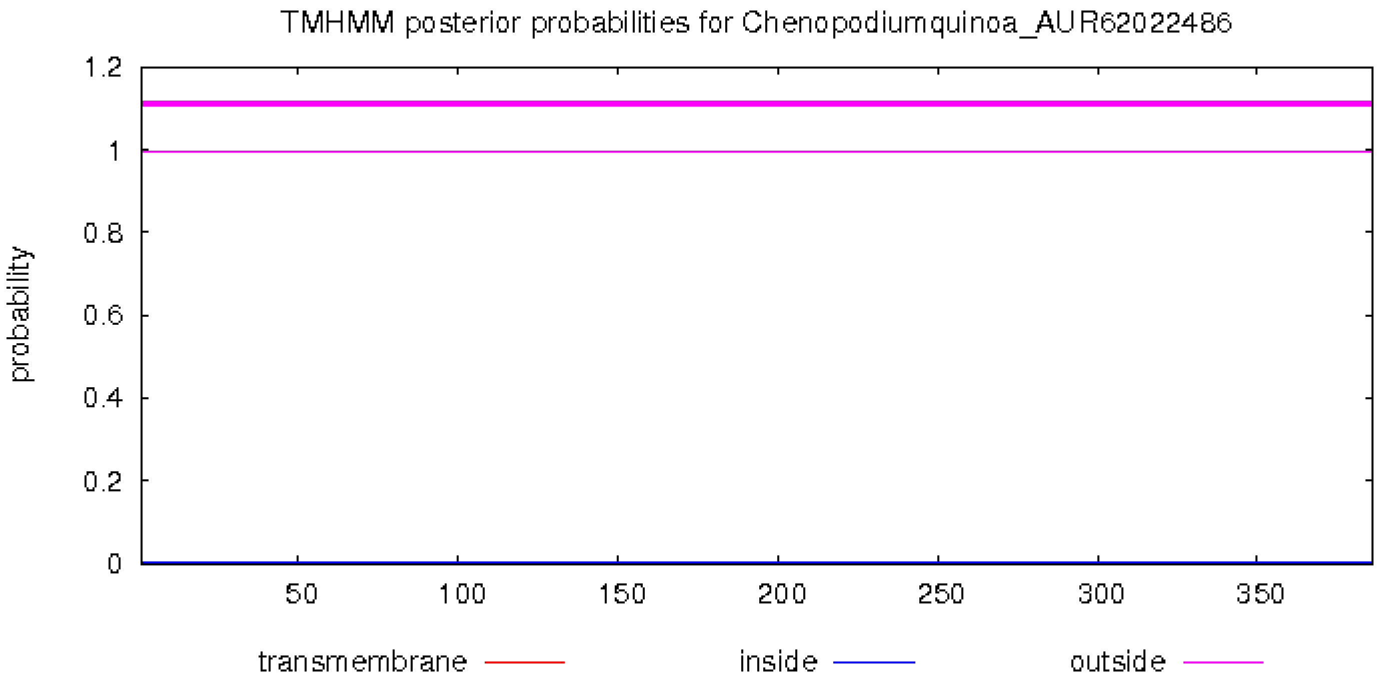

plot in postscript, script for making the plot in gnuplot, data for plot

```
# Chenopodiumquinoa_AUR62022669 Length: 250
# Chenopodiumquinoa_AUR62022669 Number of predicted TMHs:  0
# Chenopodiumquinoa_AUR62022669 Exp number of AAs in TMHs: 0
# Chenopodiumquinoa_AUR62022669 Exp number, first 60 AAs:  0
# Chenopodiumquinoa_AUR62022669 Total prob of N-in:        0.17672
Chenopodiumquinoa_AUR62022669   TMHMM2.0        outside      1   250
```

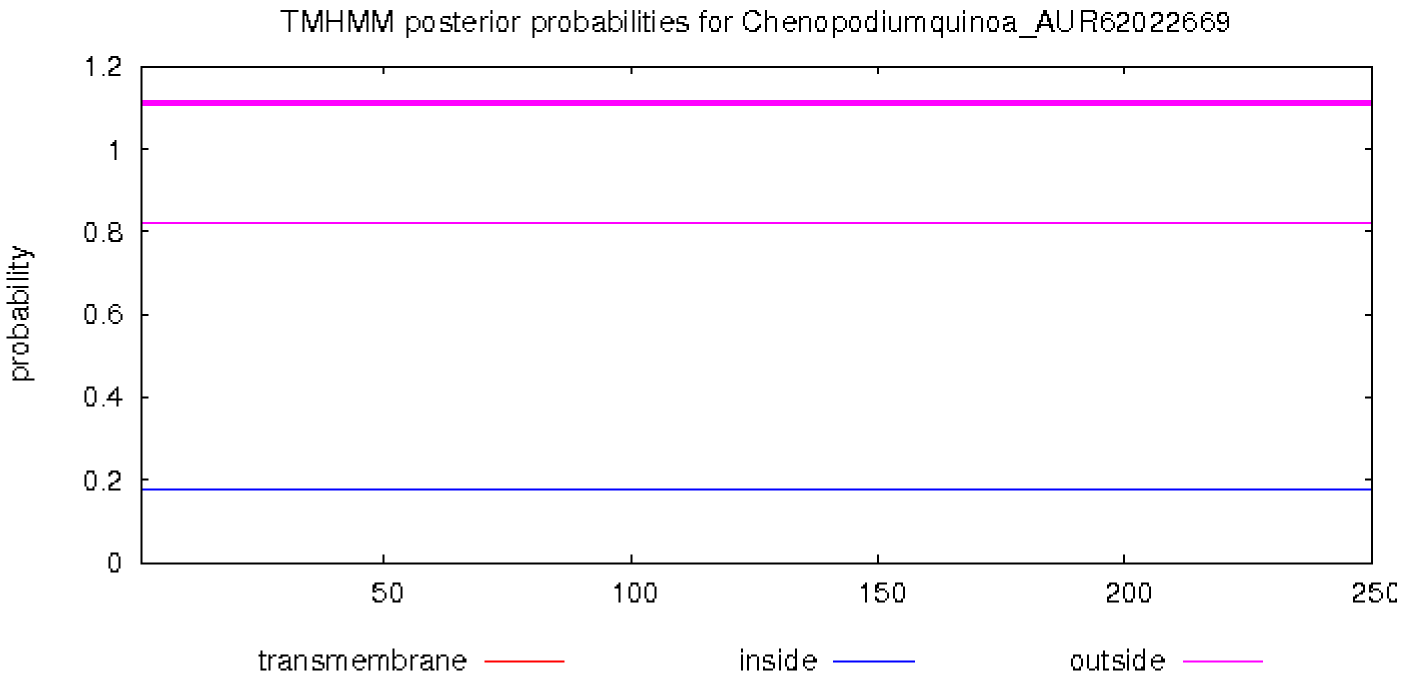

plot in postscript, script for making the plot in gnuplot, data for plot

---

```
# Chenopodiumquinoa_AUR62022690 Length: 571
# Chenopodiumquinoa_AUR62022690 Number of predicted TMHs:  0
# Chenopodiumquinoa_AUR62022690 Exp number of AAs in TMHs: 34.16465
# Chenopodiumquinoa_AUR62022690 Exp number, first 60 AAs:  0
# Chenopodiumquinoa_AUR62022690 Total prob of N-in:        0.20412
Chenopodiumquinoa_AUR62022690   TMHMM2.0        outside      1   571
```

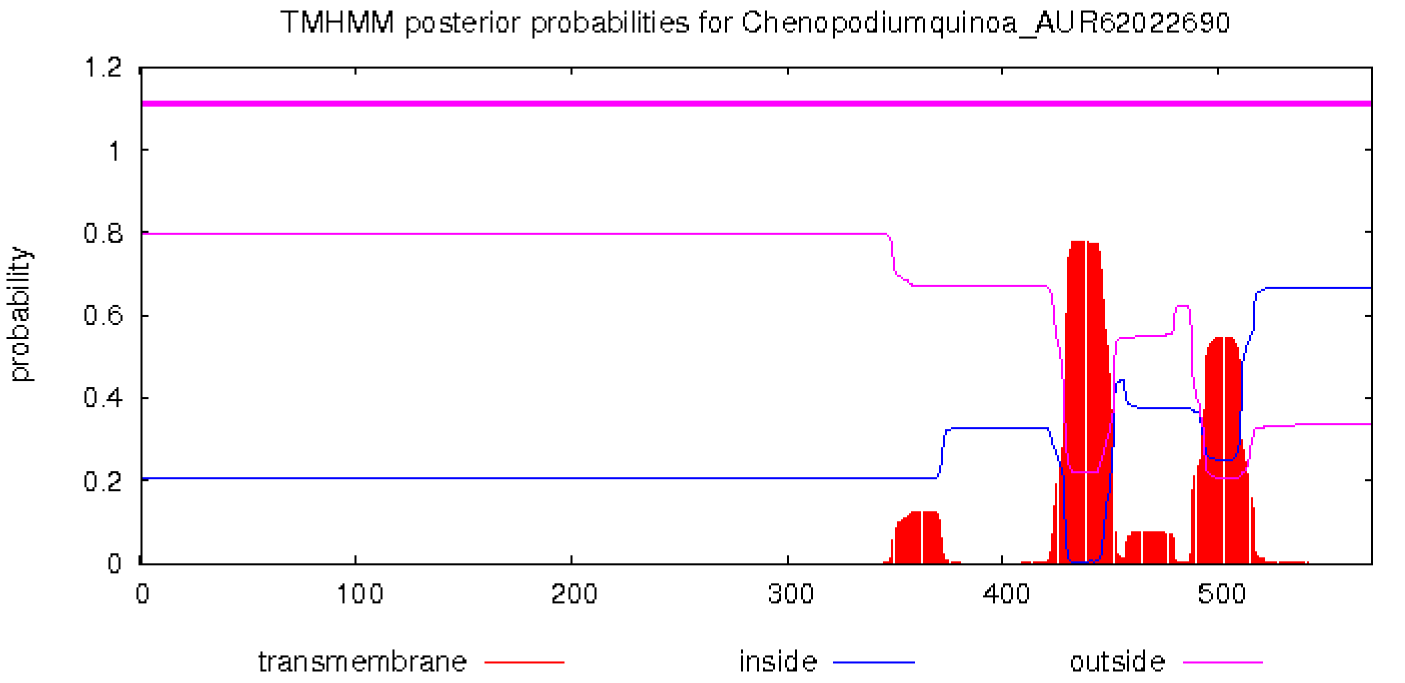

plot in postscript, script for making the plot in gnuplot, data for plot

---

```
# Chenopodiumquinoa_AUR62024101 Length: 248
# Chenopodiumquinoa_AUR62024101 Number of predicted TMHs:  0
# Chenopodiumquinoa_AUR62024101 Exp number of AAs in TMHs: 14.2879
# Chenopodiumquinoa_AUR62024101 Exp number, first 60 AAs:  0.06207
# Chenopodiumquinoa_AUR62024101 Total prob of N-in:        0.41938
Chenopodiumquinoa_AUR62024101   TMHMM2.0        outside      1   248
```

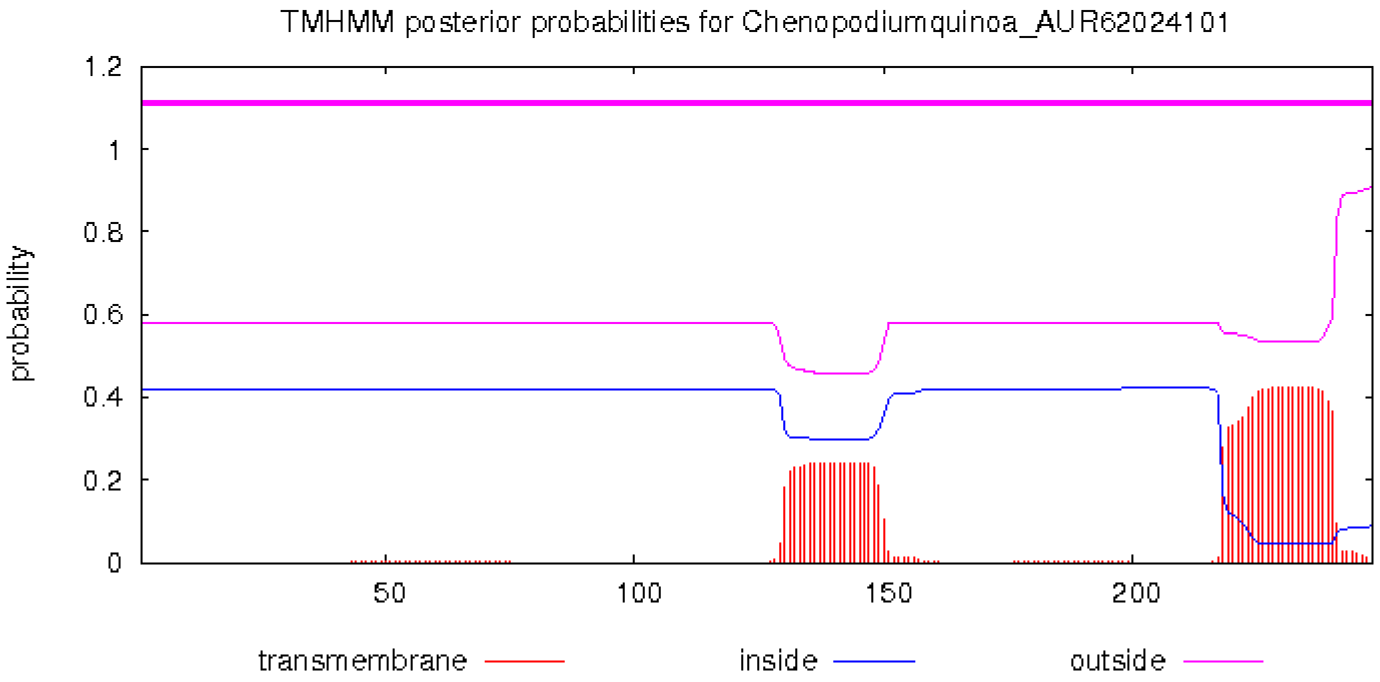

plot in postscript, script for making the plot in gnuplot, data for plot

---

```
# Chenopodiumquinoa_AUR62024698 Length: 149
# Chenopodiumquinoa_AUR62024698 Number of predicted TMHs:  0
# Chenopodiumquinoa_AUR62024698 Exp number of AAs in TMHs: 0.00037
# Chenopodiumquinoa_AUR62024698 Exp number, first 60 AAs:  7e-05
# Chenopodiumquinoa_AUR62024698 Total prob of N-in:        0.07037
Chenopodiumquinoa_AUR62024698   TMHMM2.0        outside      1   149
```

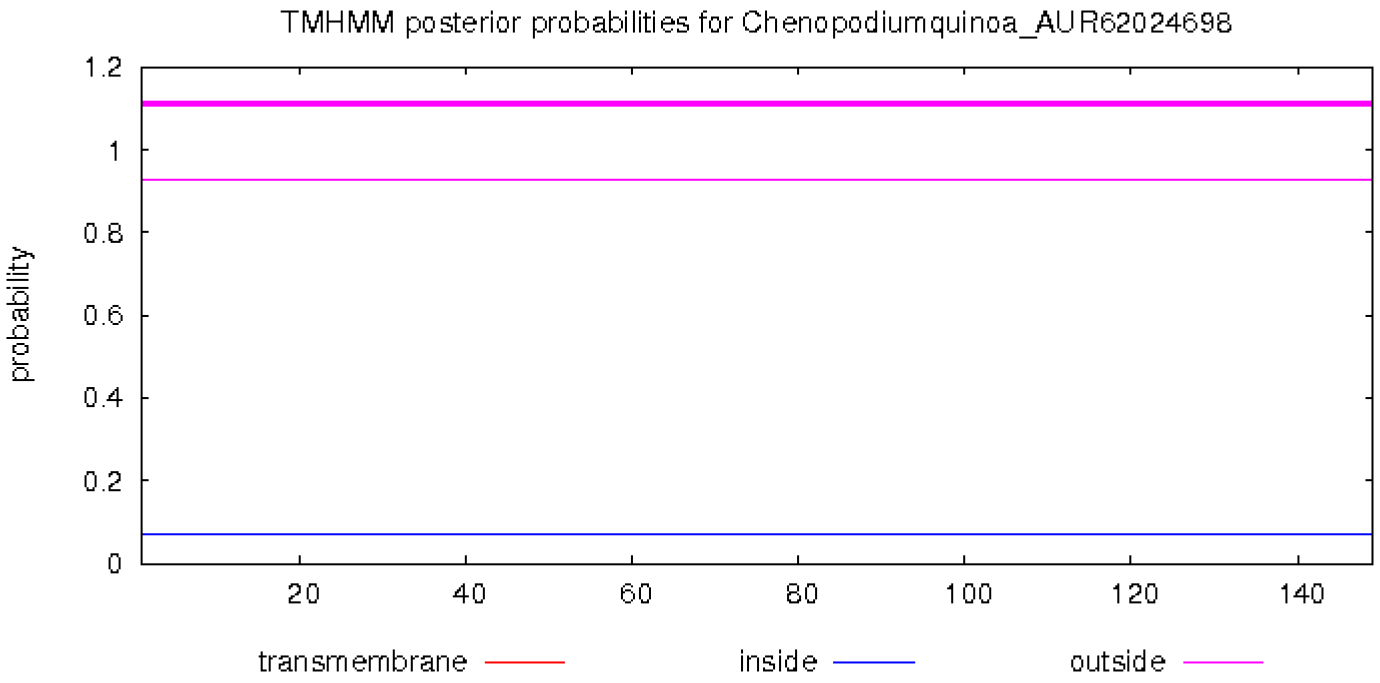

plot in postscript, script for making the plot in gnuplot, data for plot

---

```
# Chenopodiumquinoa_AUR62025580 Length: 342
# Chenopodiumquinoa_AUR62025580 Number of predicted TMHs:  0
# Chenopodiumquinoa_AUR62025580 Exp number of AAs in TMHs: 0.00017
# Chenopodiumquinoa_AUR62025580 Exp number, first 60 AAs:  0.0001
# Chenopodiumquinoa_AUR62025580 Total prob of N-in:        0.02694
Chenopodiumquinoa_AUR62025580   TMHMM2.0        outside      1   342
```

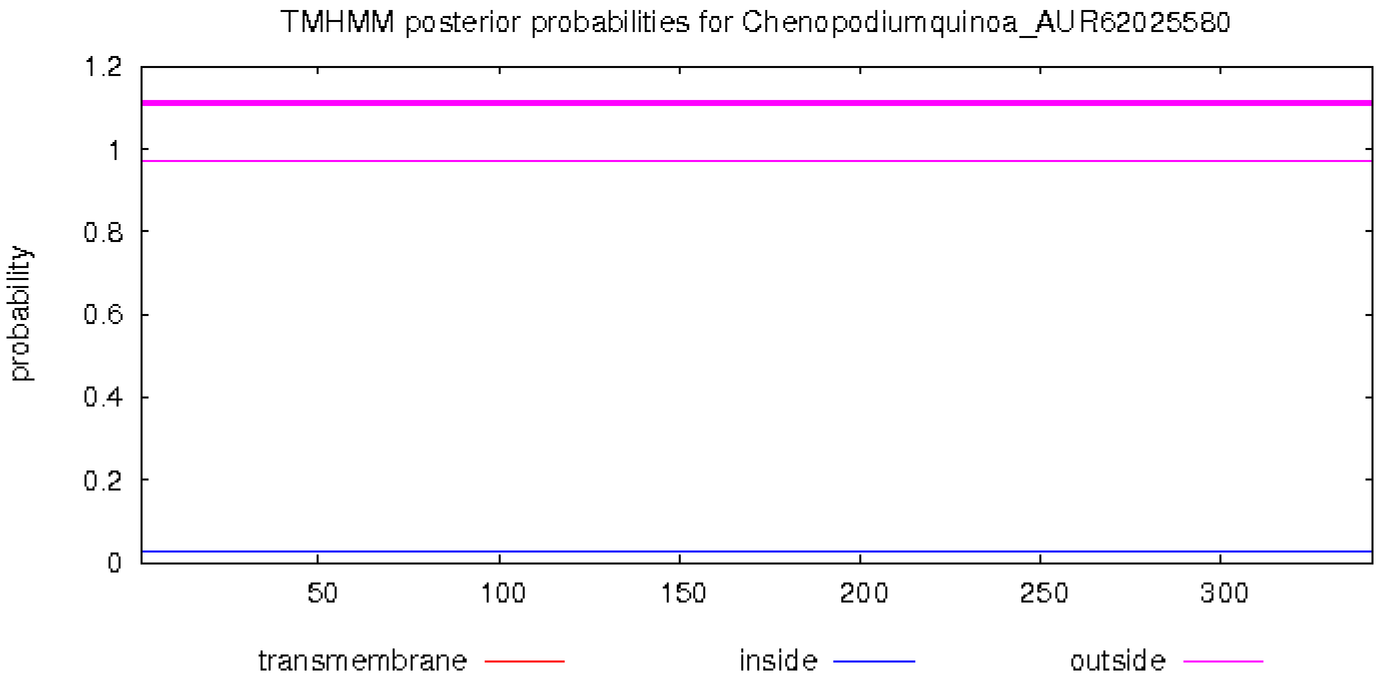

plot in postscript, script for making the plot in gnuplot, data for plot

---

```
# Chenopodiumquinoa_AUR62025636 Length: 354
# Chenopodiumquinoa_AUR62025636 Number of predicted TMHs:  0
# Chenopodiumquinoa_AUR62025636 Exp number of AAs in TMHs: 0
# Chenopodiumquinoa_AUR62025636 Exp number, first 60 AAs:  0
# Chenopodiumquinoa_AUR62025636 Total prob of N-in:        0.01406
Chenopodiumquinoa_AUR62025636   TMHMM2.0        outside      1   354
```

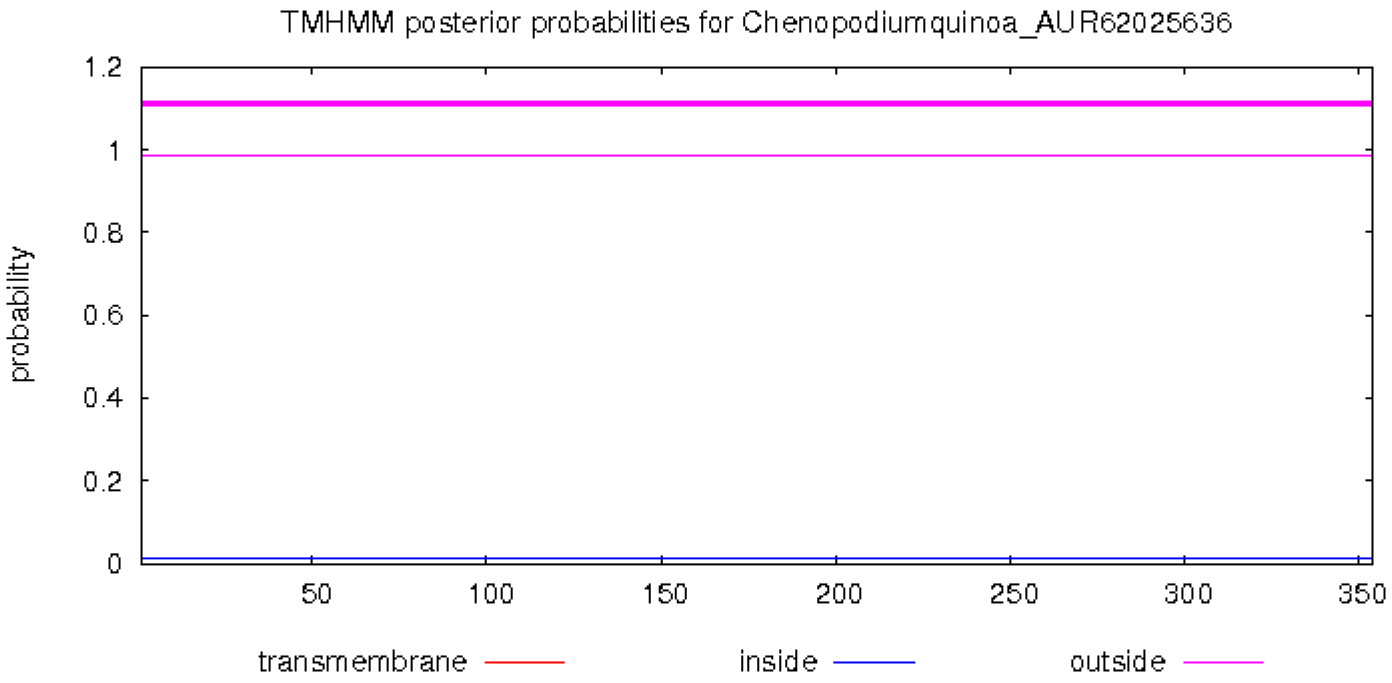

plot in postscript, script for making the plot in gnuplot, data for plot

---

```
# Chenopodiumquinoa_AUR62026741 Length: 283
# Chenopodiumquinoa_AUR62026741 Number of predicted TMHs:  0
# Chenopodiumquinoa_AUR62026741 Exp number of AAs in TMHs: 0.00086
# Chenopodiumquinoa_AUR62026741 Exp number, first 60 AAs:  0
# Chenopodiumquinoa_AUR62026741 Total prob of N-in:        0.02035
Chenopodiumquinoa_AUR62026741   TMHMM2.0        outside      1   283
```

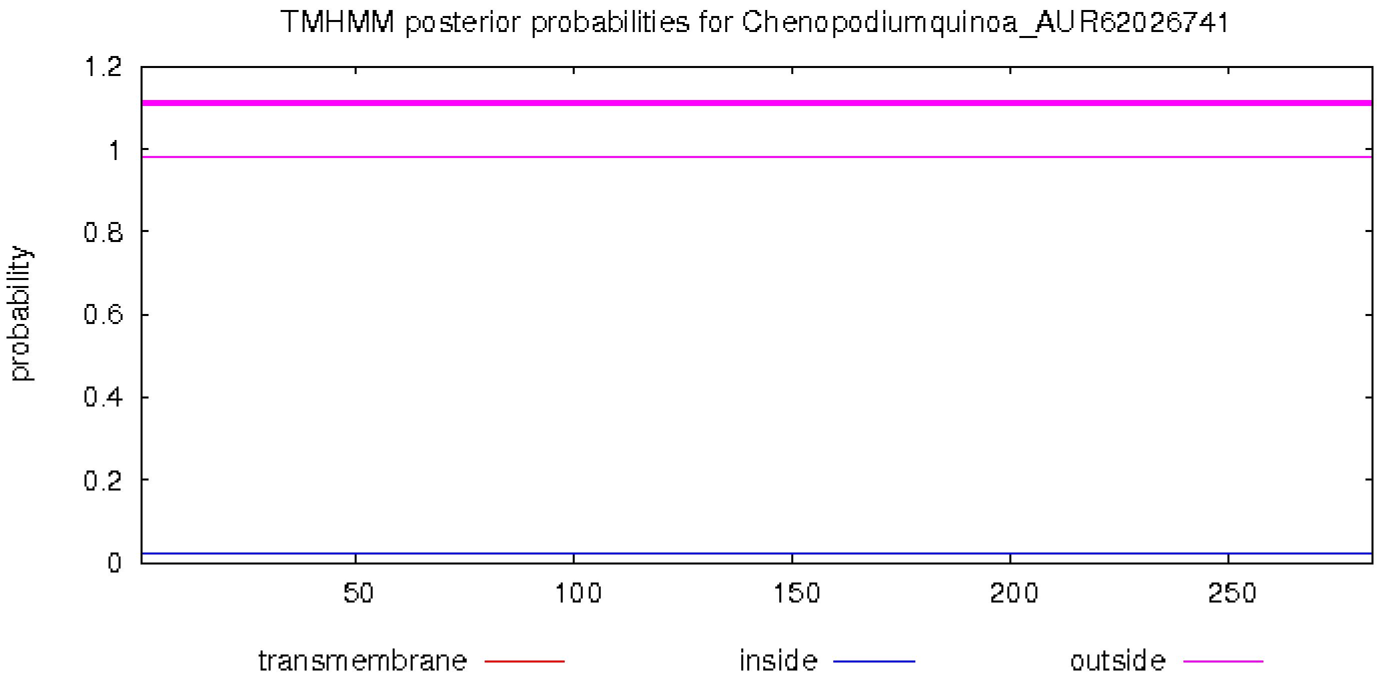

plot in postscript, script for making the plot in gnuplot, data for plot

```
# Chenopodiumquinoa_AUR62026904 Length: 344
# Chenopodiumquinoa_AUR62026904 Number of predicted TMHs:  0
# Chenopodiumquinoa_AUR62026904 Exp number of AAs in TMHs: 0.00066
# Chenopodiumquinoa_AUR62026904 Exp number, first 60 AAs:  0
# Chenopodiumquinoa_AUR62026904 Total prob of N-in:        0.05095
Chenopodiumquinoa_AUR62026904   TMHMM2.0        outside      1   344
```

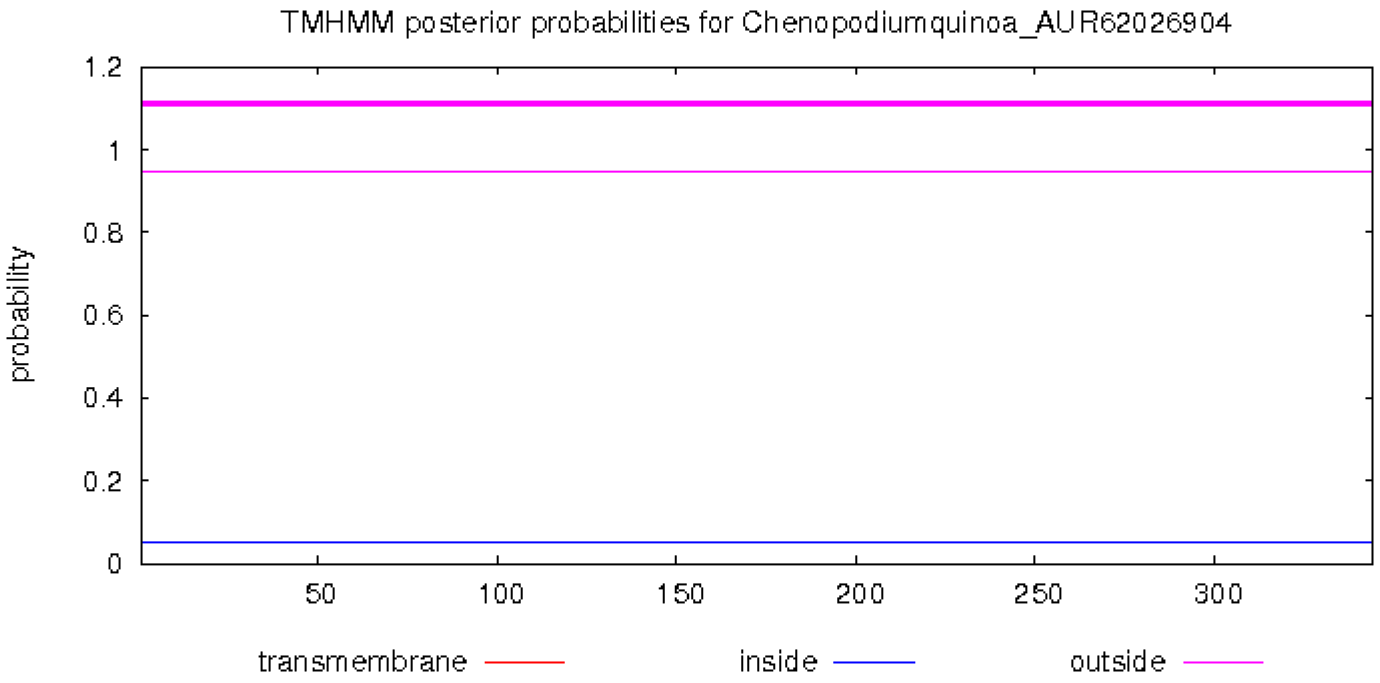

plot in postscript, script for making the plot in gnuplot, data for plot

```
# Chenopodiumquinoa_AUR62027152 Length: 222
# Chenopodiumquinoa_AUR62027152 Number of predicted TMHs:  0
# Chenopodiumquinoa_AUR62027152 Exp number of AAs in TMHs: 0.00101
# Chenopodiumquinoa_AUR62027152 Exp number, first 60 AAs:  0.00057
# Chenopodiumquinoa_AUR62027152 Total prob of N-in:        0.06634
Chenopodiumquinoa_AUR62027152   TMHMM2.0        outside      1   222
```

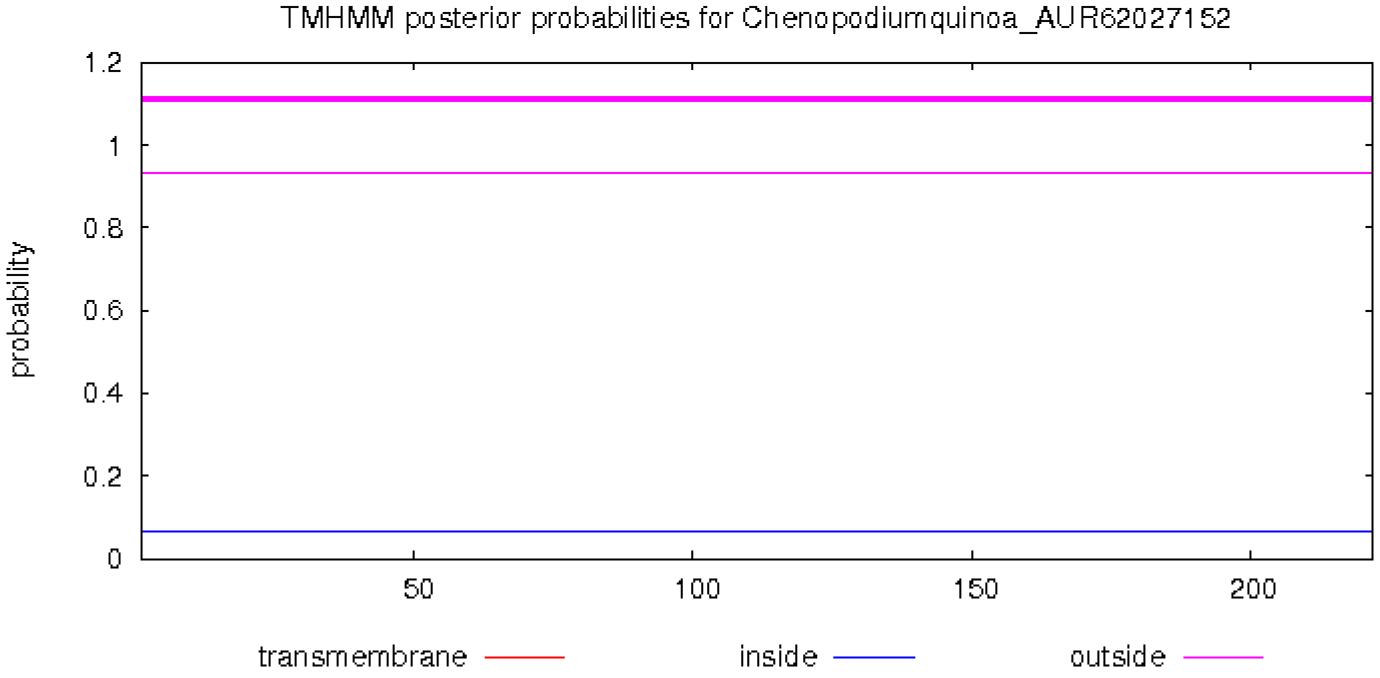

plot in postscript, script for making the plot in gnuplot, data for plot

```
# Chenopodiumquinoa_AUR62027390 Length: 168
# Chenopodiumquinoa_AUR62027390 Number of predicted TMHs:  0
# Chenopodiumquinoa_AUR62027390 Exp number of AAs in TMHs: 0.01739
# Chenopodiumquinoa_AUR62027390 Exp number, first 60 AAs:  8e-05
# Chenopodiumquinoa_AUR62027390 Total prob of N-in:        0.09680
Chenopodiumquinoa_AUR62027390   TMHMM2.0        outside      1   168
```

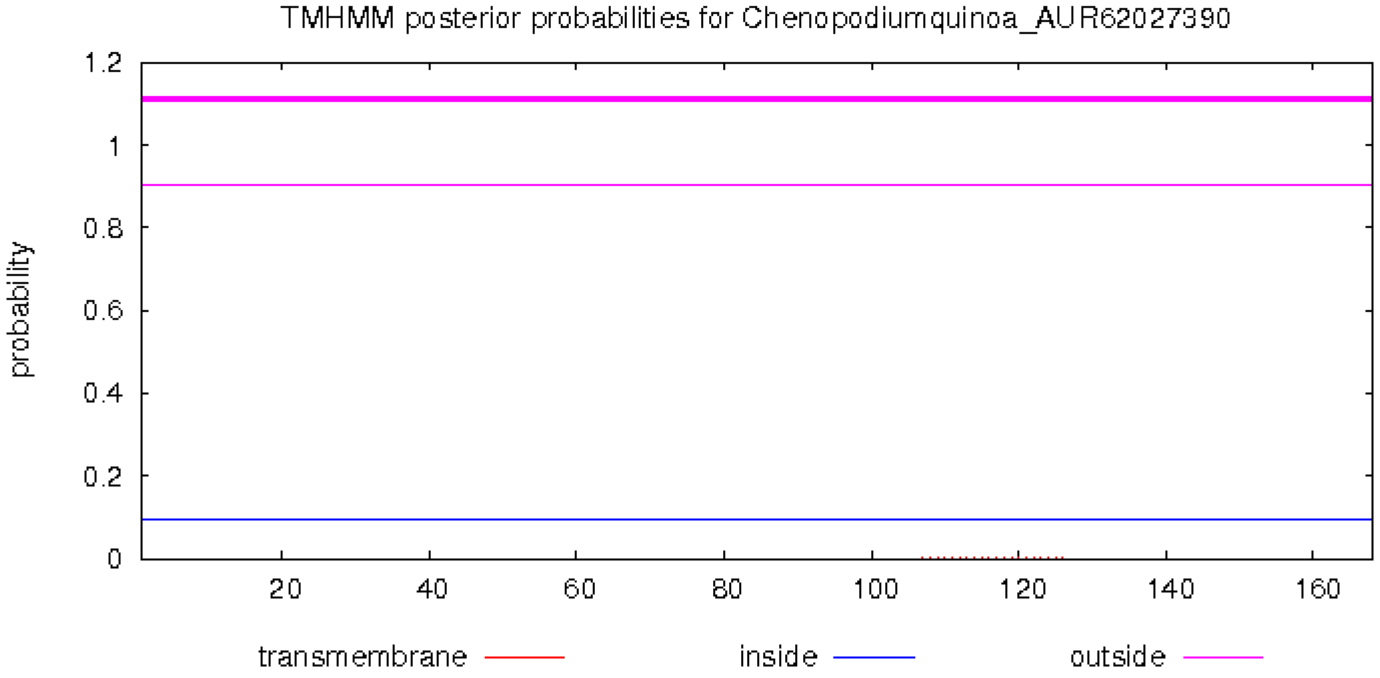

plot in postscript, script for making the plot in gnuplot, data for plot

```
# Chenopodiumquinoa_AUR62028103 Length: 630
# Chenopodiumquinoa_AUR62028103 Number of predicted TMHs:  1
# Chenopodiumquinoa_AUR62028103 Exp number of AAs in TMHs: 22.50828
# Chenopodiumquinoa_AUR62028103 Exp number, first 60 AAs:  0
# Chenopodiumquinoa_AUR62028103 Total prob of N-in:        0.00613
Chenopodiumquinoa_AUR62028103   TMHMM2.0        outside      1   592
Chenopodiumquinoa_AUR62028103   TMHMM2.0        TMhelix    593   615
Chenopodiumquinoa_AUR62028103   TMHMM2.0        inside     616   630
```

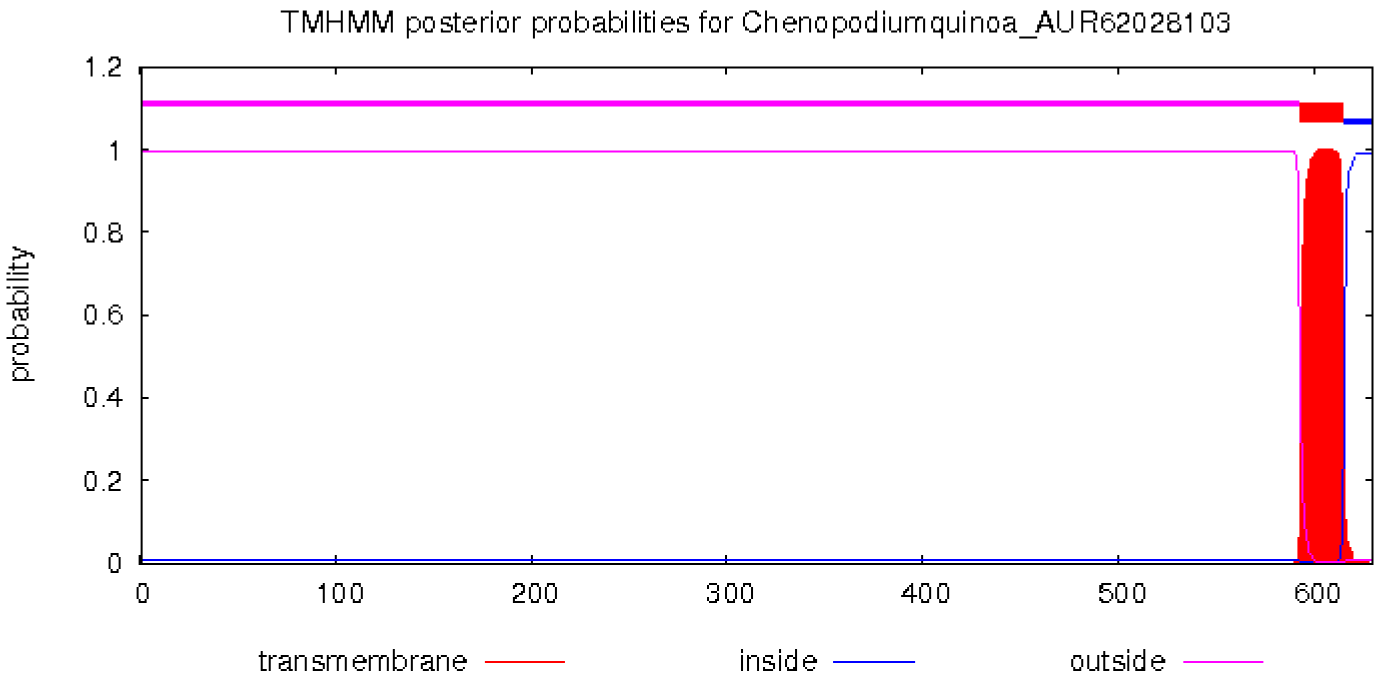

plot in postscript, script for making the plot in gnuplot, data for plot

```
# Chenopodiumquinoa_AUR62028872 Length: 257
# Chenopodiumquinoa_AUR62028872 Number of predicted TMHs:  0
# Chenopodiumquinoa_AUR62028872 Exp number of AAs in TMHs: 0
# Chenopodiumquinoa_AUR62028872 Exp number, first 60 AAs:  0
# Chenopodiumquinoa_AUR62028872 Total prob of N-in:        0.08818
Chenopodiumquinoa_AUR62028872   TMHMM2.0        outside      1   257
```

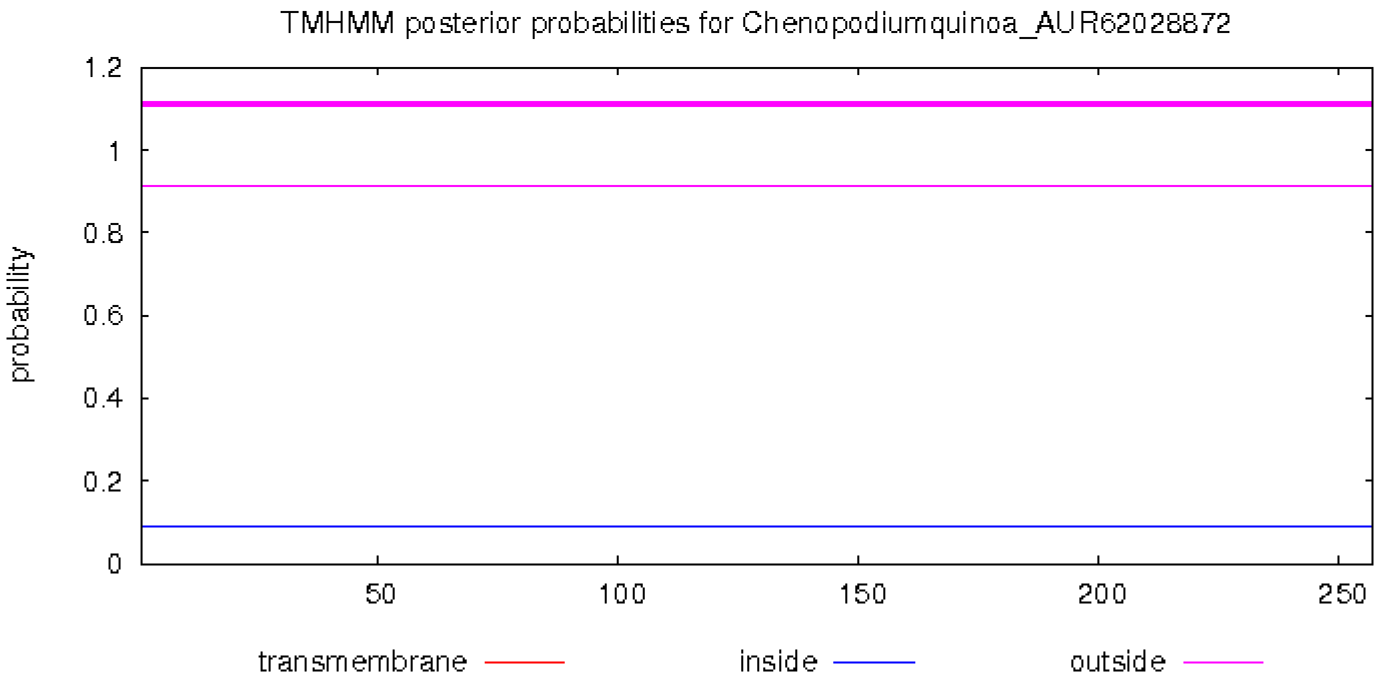

plot in postscript, script for making the plot in gnuplot, data for plot

```
# Chenopodiumquinoa_AUR62029057 Length: 524
# Chenopodiumquinoa_AUR62029057 Number of predicted TMHs:  1
# Chenopodiumquinoa_AUR62029057 Exp number of AAs in TMHs: 22.54631
# Chenopodiumquinoa_AUR62029057 Exp number, first 60 AAs:  0.00017
# Chenopodiumquinoa_AUR62029057 Total prob of N-in:        0.01220
Chenopodiumquinoa_AUR62029057   TMHMM2.0        outside      1   492
Chenopodiumquinoa_AUR62029057   TMHMM2.0        TMhelix    493   515
Chenopodiumquinoa_AUR62029057   TMHMM2.0        inside     516   524
```

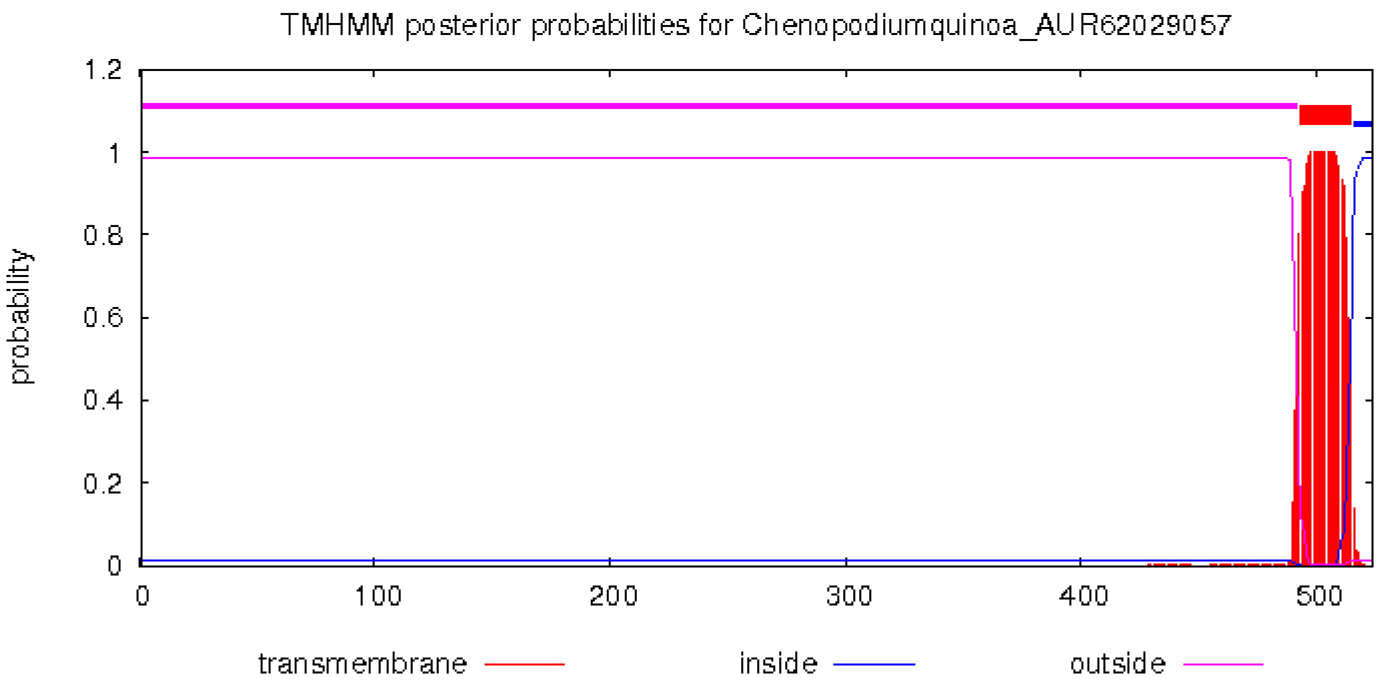

plot in postscript, script for making the plot in gnuplot, data for plot

```
# Chenopodiumquinoa_AUR62029330 Length: 328
# Chenopodiumquinoa_AUR62029330 Number of predicted TMHs:  0
# Chenopodiumquinoa_AUR62029330 Exp number of AAs in TMHs: 0.00285
# Chenopodiumquinoa_AUR62029330 Exp number, first 60 AAs:  0.00021
# Chenopodiumquinoa_AUR62029330 Total prob of N-in:        0.02570
Chenopodiumquinoa_AUR62029330   TMHMM2.0        outside      1   328
```

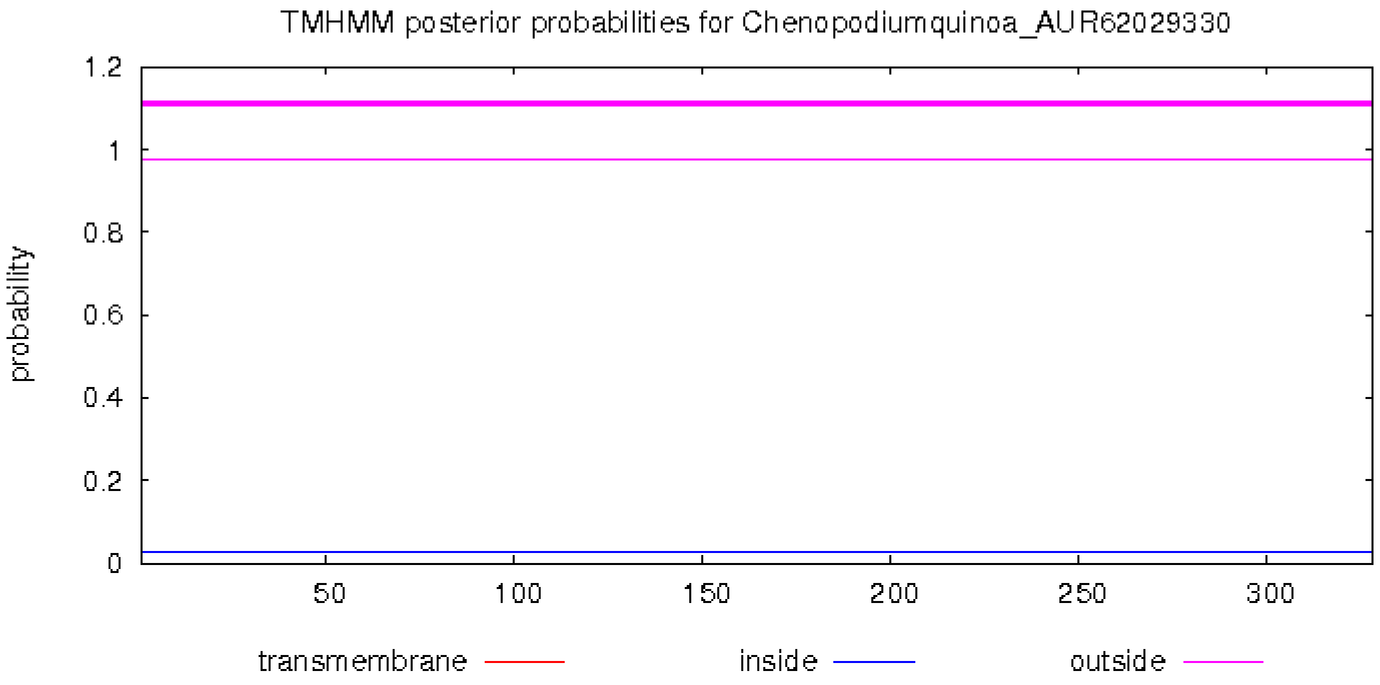

plot in postscript, script for making the plot in gnuplot, data for plot

```
# Chenopodiumquinoa_AUR62029331 Length: 327
# Chenopodiumquinoa_AUR62029331 Number of predicted TMHs:  0
# Chenopodiumquinoa_AUR62029331 Exp number of AAs in TMHs: 0.0015
# Chenopodiumquinoa_AUR62029331 Exp number, first 60 AAs:  0
# Chenopodiumquinoa_AUR62029331 Total prob of N-in:        0.02640
Chenopodiumquinoa_AUR62029331   TMHMM2.0        outside      1   327
```

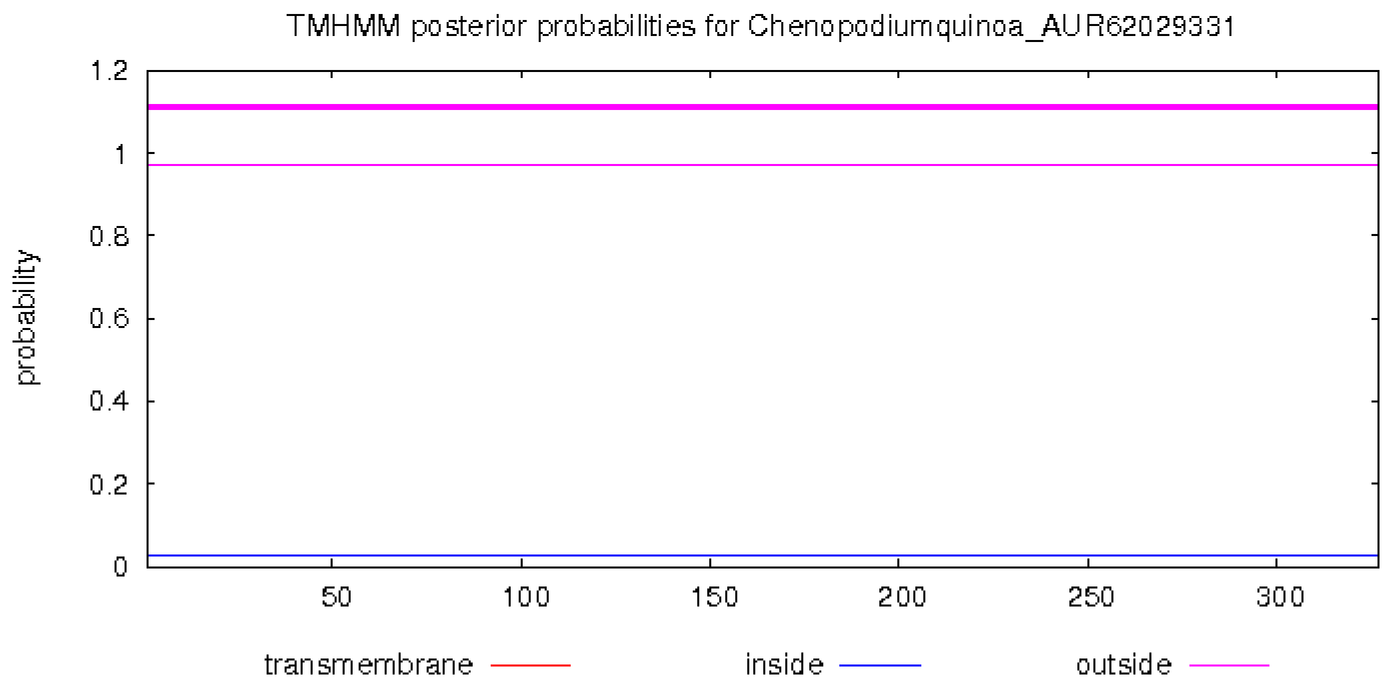

plot in postscript, script for making the plot in gnuplot, data for plot

```
# Chenopodiumquinoa_AUR62029344 Length: 298
# Chenopodiumquinoa_AUR62029344 Number of predicted TMHs:  0
# Chenopodiumquinoa_AUR62029344 Exp number of AAs in TMHs: 0.00614
# Chenopodiumquinoa_AUR62029344 Exp number, first 60 AAs:  0.00023
# Chenopodiumquinoa_AUR62029344 Total prob of N-in:        0.01728
Chenopodiumquinoa_AUR62029344   TMHMM2.0        outside      1   298
```

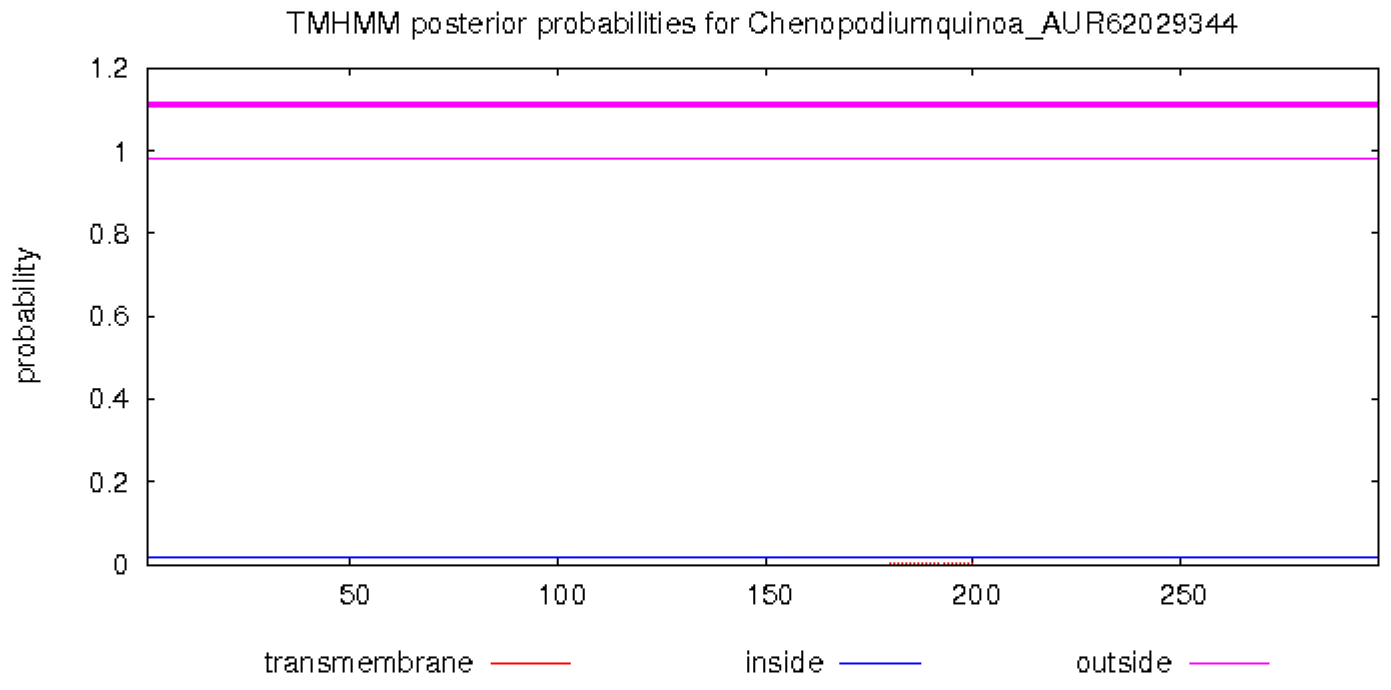

plot in postscript, script for making the plot in gnuplot, data for plot

```
# Chenopodiumquinoa_AUR62029543 Length: 365
# Chenopodiumquinoa_AUR62029543 Number of predicted TMHs:  0
# Chenopodiumquinoa_AUR62029543 Exp number of AAs in TMHs: 0.00441
# Chenopodiumquinoa_AUR62029543 Exp number, first 60 AAs:  0.00441
# Chenopodiumquinoa_AUR62029543 Total prob of N-in:        0.02158
Chenopodiumquinoa_AUR62029543	TMHMM2.0	outside	     1   365
```

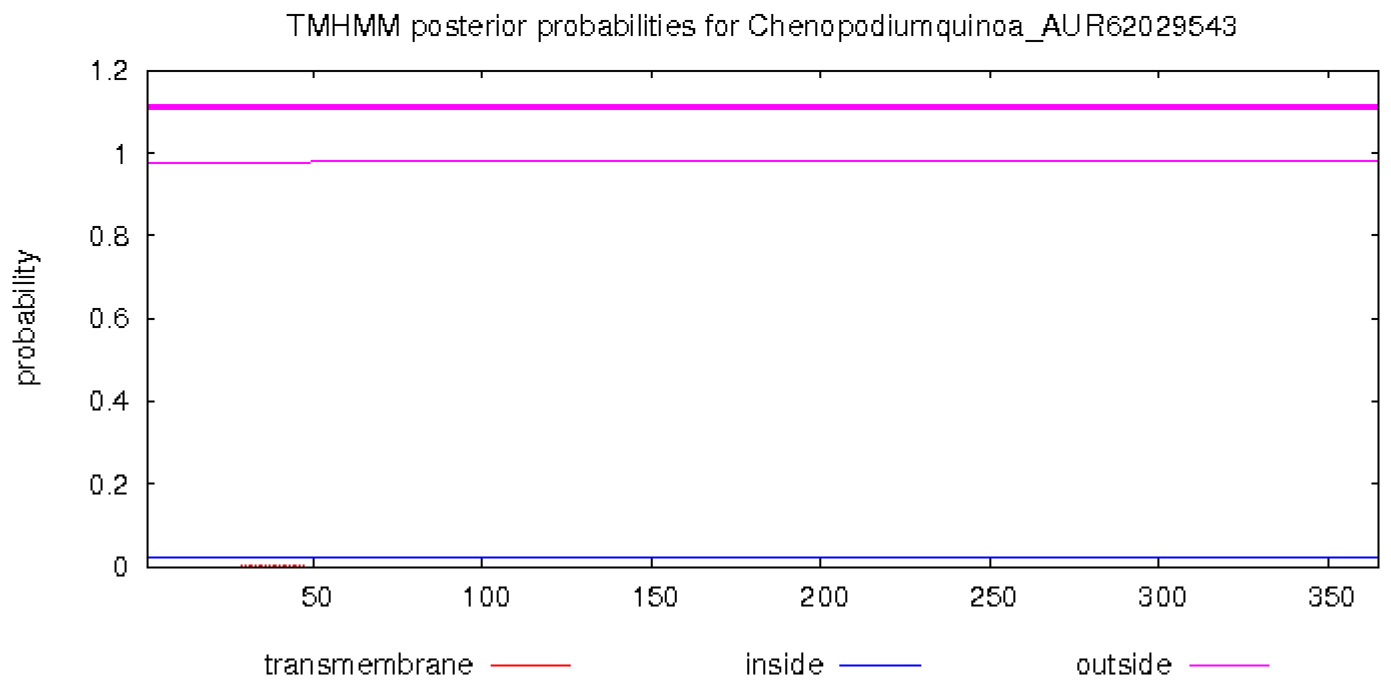

plot in postscript, script for making the plot in gnuplot, data for plot

```
# Chenopodiumquinoa_AUR62029755 Length: 368
# Chenopodiumquinoa_AUR62029755 Number of predicted TMHs:  0
# Chenopodiumquinoa_AUR62029755 Exp number of AAs in TMHs: 0.00499
# Chenopodiumquinoa_AUR62029755 Exp number, first 60 AAs:  0.00029
# Chenopodiumquinoa_AUR62029755 Total prob of N-in:        0.00330
Chenopodiumquinoa_AUR62029755	TMHMM2.0	outside	     1   368
```

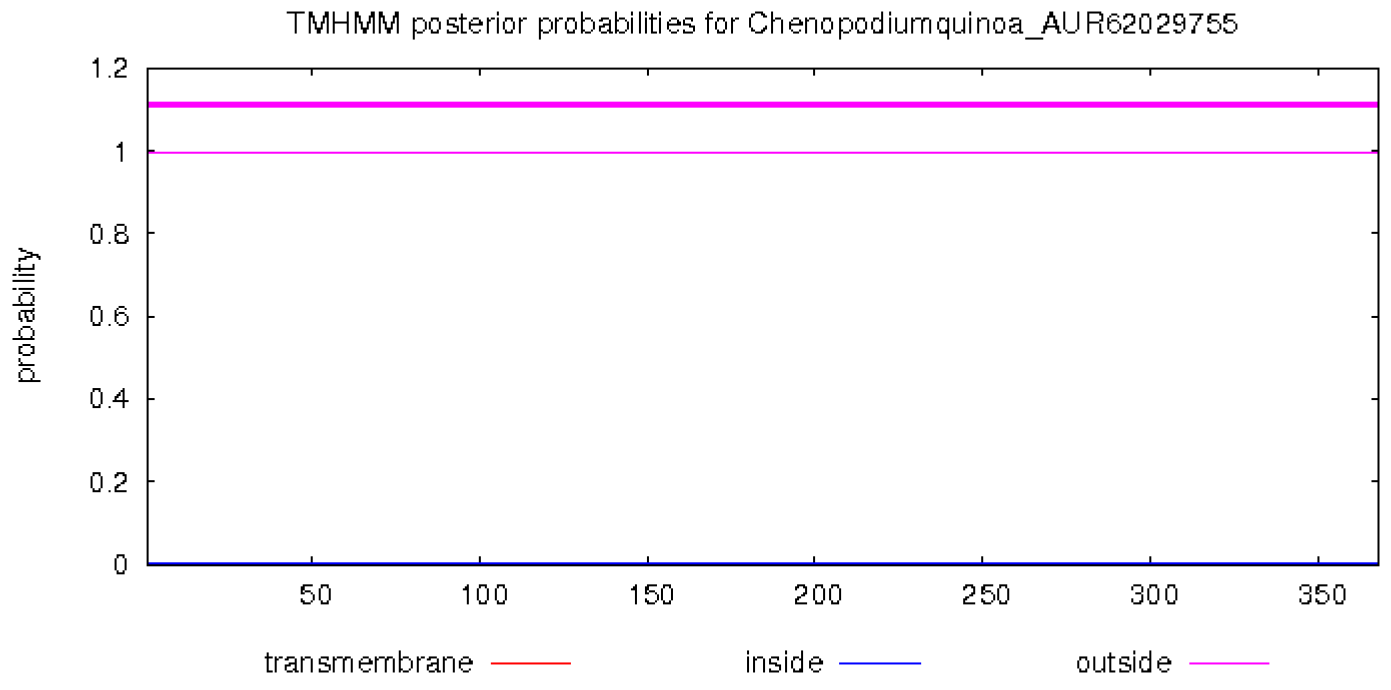

plot in postscript, script for making the plot in gnuplot, data for plot

---

```
# Chenopodiumquinoa_AUR62031305 Length: 158
# Chenopodiumquinoa_AUR62031305 Number of predicted TMHs:  0
# Chenopodiumquinoa_AUR62031305 Exp number of AAs in TMHs: 0.03497
# Chenopodiumquinoa_AUR62031305 Exp number, first 60 AAs:  0.00012
# Chenopodiumquinoa_AUR62031305 Total prob of N-in:        0.15225
Chenopodiumquinoa_AUR62031305   TMHMM2.0        outside      1   158
```

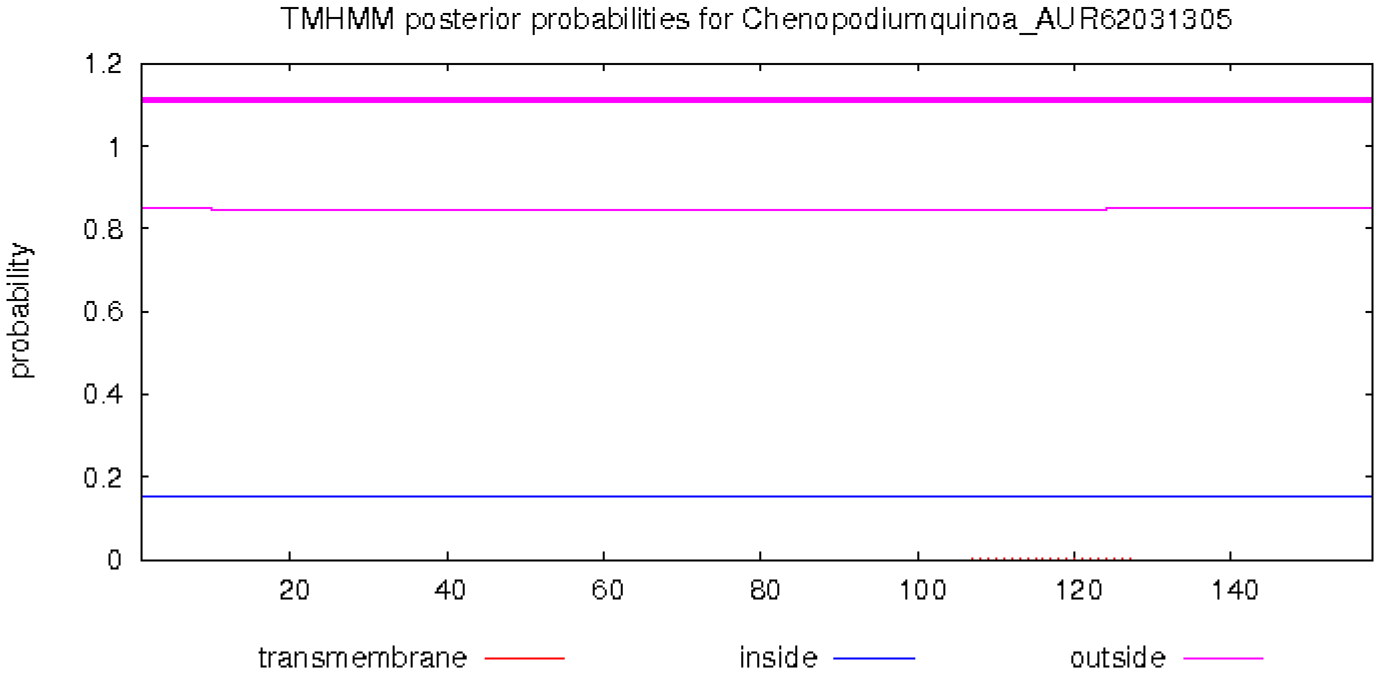

plot in postscript, script for making the plot in gnuplot, data for plot

---

```
# Chenopodiumquinoa_AUR62032488 Length: 516
# Chenopodiumquinoa_AUR62032488 Number of predicted TMHs:  1
# Chenopodiumquinoa_AUR62032488 Exp number of AAs in TMHs: 20.83958
# Chenopodiumquinoa_AUR62032488 Exp number, first 60 AAs:  0.00031
# Chenopodiumquinoa_AUR62032488 Total prob of N-in:        0.10319
Chenopodiumquinoa_AUR62032488   TMHMM2.0        outside      1   491
Chenopodiumquinoa_AUR62032488   TMHMM2.0        TMhelix    492   514
Chenopodiumquinoa_AUR62032488   TMHMM2.0        inside     515   516
```

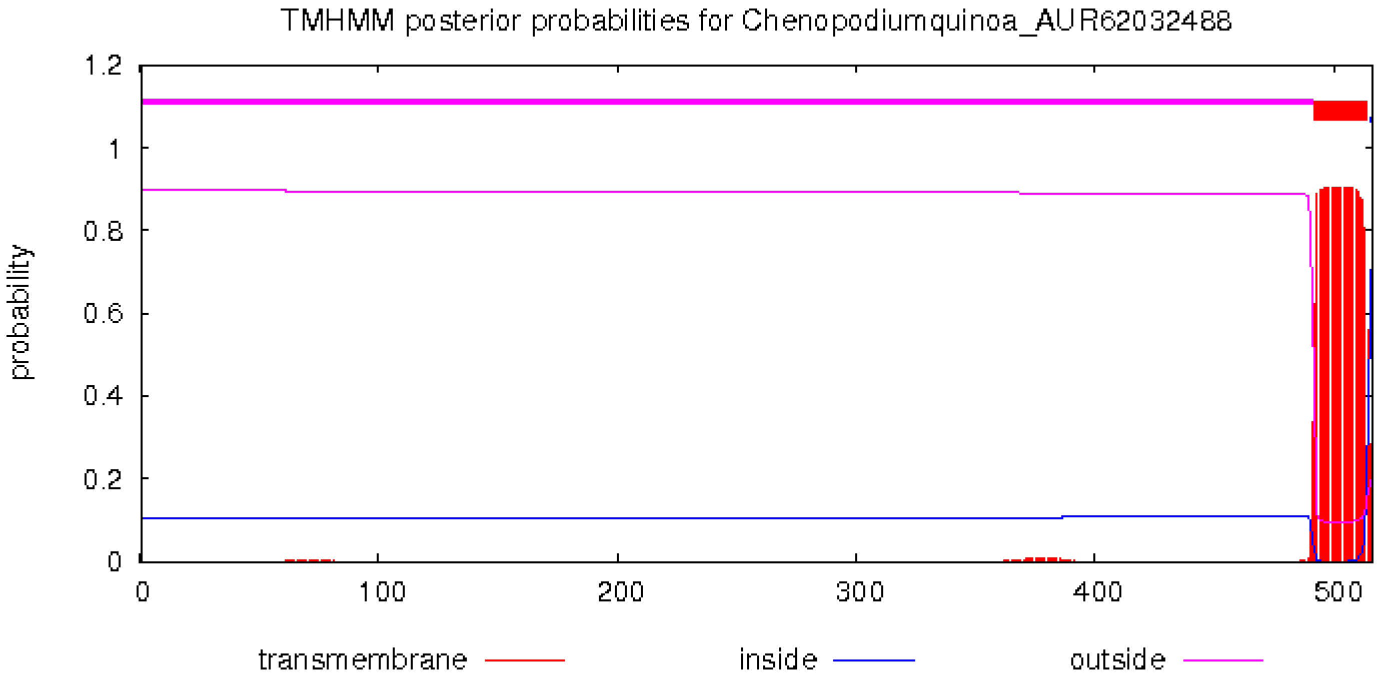

plot in postscript, script for making the plot in gnuplot, data for plot

---

```
# Chenopodiumquinoa_AUR62032816 Length: 272
# Chenopodiumquinoa_AUR62032816 Number of predicted TMHs:  0
# Chenopodiumquinoa_AUR62032816 Exp number of AAs in TMHs: 0.00246
# Chenopodiumquinoa_AUR62032816 Exp number, first 60 AAs:  0
# Chenopodiumquinoa_AUR62032816 Total prob of N-in:        0.07248
Chenopodiumquinoa_AUR62032816	TMHMM2.0	outside	     1   272
```

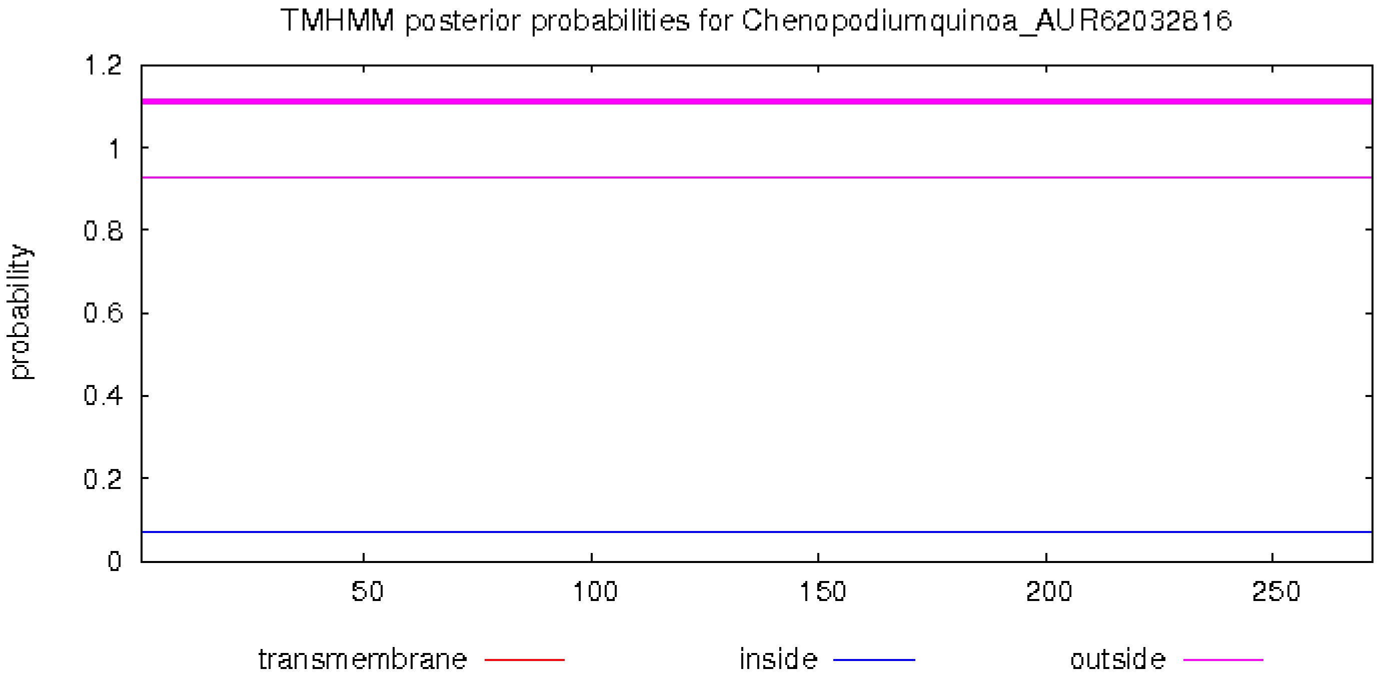

plot in postscript, script for making the plot in gnuplot, data for plot

---

```
# Chenopodiumquinoa_AUR62033952 Length: 530
# Chenopodiumquinoa_AUR62033952 Number of predicted TMHs:  1
# Chenopodiumquinoa_AUR62033952 Exp number of AAs in TMHs: 22.55472
# Chenopodiumquinoa_AUR62033952 Exp number, first 60 AAs:  0
# Chenopodiumquinoa_AUR62033952 Total prob of N-in:        0.01451
Chenopodiumquinoa_AUR62033952	TMHMM2.0	outside	     1   498
```

```
Chenopodiumquinoa_AUR62033952   TMHMM2.0        TMhelix    499   521
Chenopodiumquinoa_AUR62033952   TMHMM2.0        inside     522   530
```

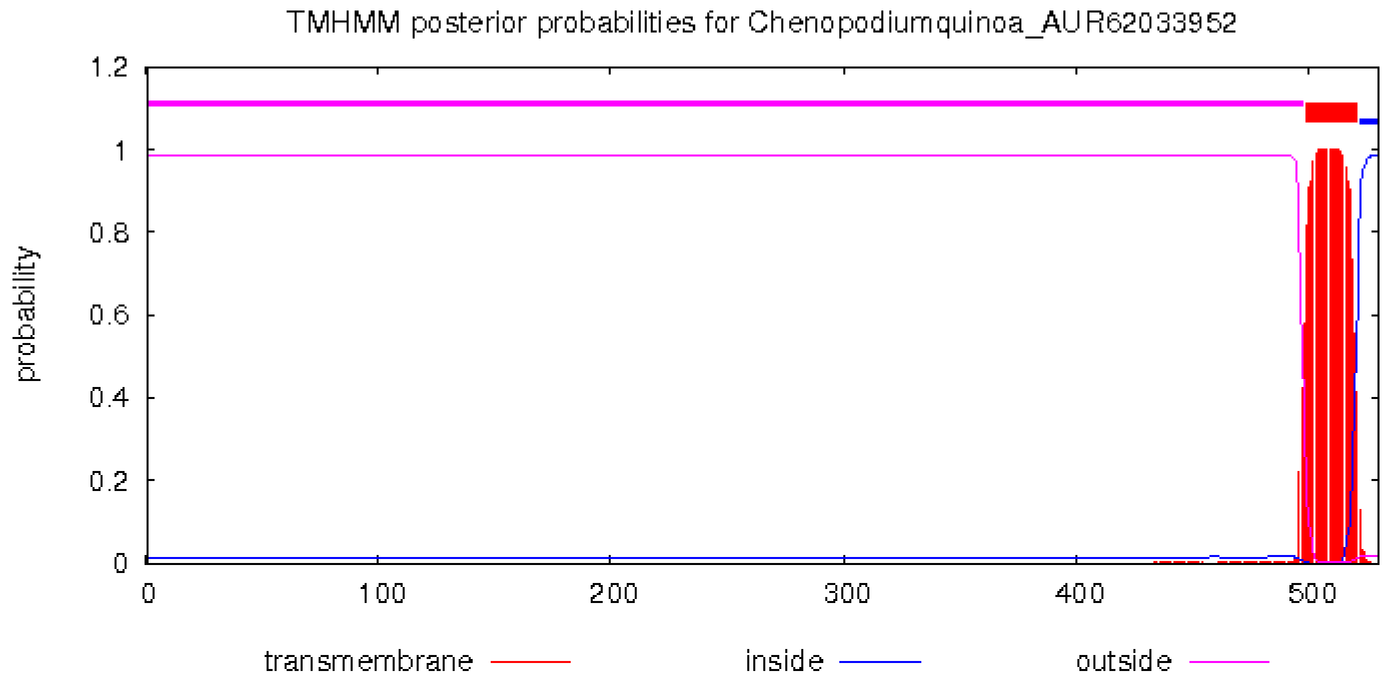

plot in postscript, script for making the plot in gnuplot, data for plot

---

```
# Chenopodiumquinoa_AUR62034400 Length: 385
# Chenopodiumquinoa_AUR62034400 Number of predicted TMHs:  0
# Chenopodiumquinoa_AUR62034400 Exp number of AAs in TMHs: 0.0046
# Chenopodiumquinoa_AUR62034400 Exp number, first 60 AAs:  0
# Chenopodiumquinoa_AUR62034400 Total prob of N-in:        0.02722
Chenopodiumquinoa_AUR62034400   TMHMM2.0        outside      1   385
```

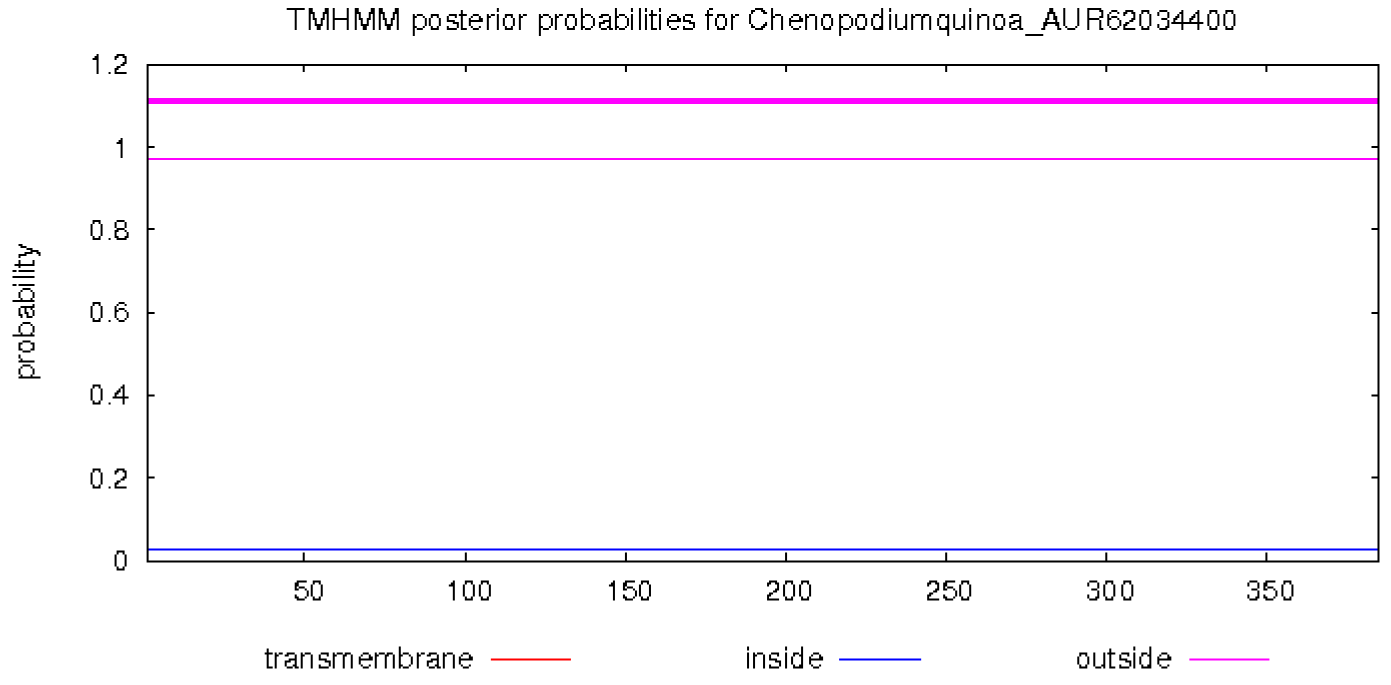

plot in postscript, script for making the plot in gnuplot, data for plot

---

```
# Chenopodiumquinoa_AUR62034435 Length: 341
# Chenopodiumquinoa_AUR62034435 Number of predicted TMHs:  0
# Chenopodiumquinoa_AUR62034435 Exp number of AAs in TMHs: 0.0023
# Chenopodiumquinoa_AUR62034435 Exp number, first 60 AAs:  0
```

```
# Chenopodiumquinoa_AUR62034435 Total prob of N-in:        0.03945
Chenopodiumquinoa_AUR62034435   TMHMM2.0        outside      1   341
```

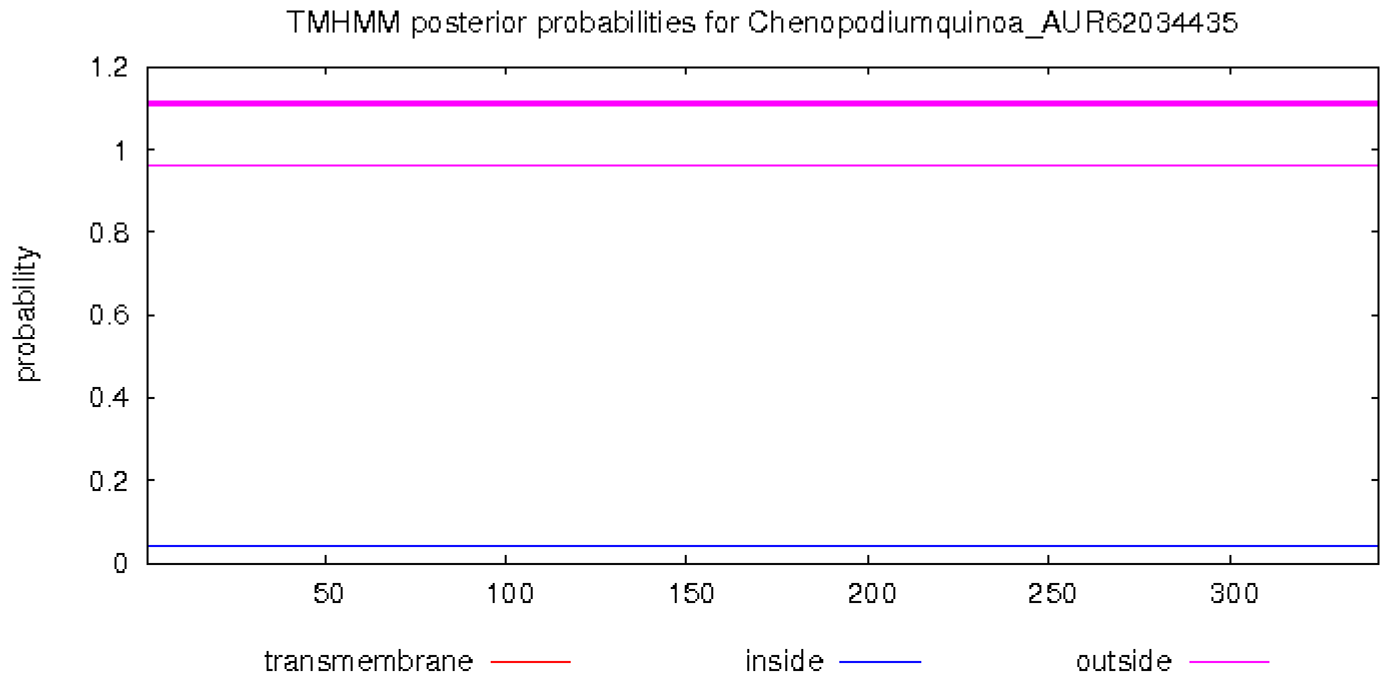

plot in postscript, script for making the plot in gnuplot, data for plot

---

```
# Chenopodiumquinoa_AUR62034813 Length: 228
# Chenopodiumquinoa_AUR62034813 Number of predicted TMHs:  0
# Chenopodiumquinoa_AUR62034813 Exp number of AAs in TMHs: 0.00357
# Chenopodiumquinoa_AUR62034813 Exp number, first 60 AAs:  0.00258
# Chenopodiumquinoa_AUR62034813 Total prob of N-in:        0.12316
Chenopodiumquinoa_AUR62034813   TMHMM2.0        outside      1   228
```

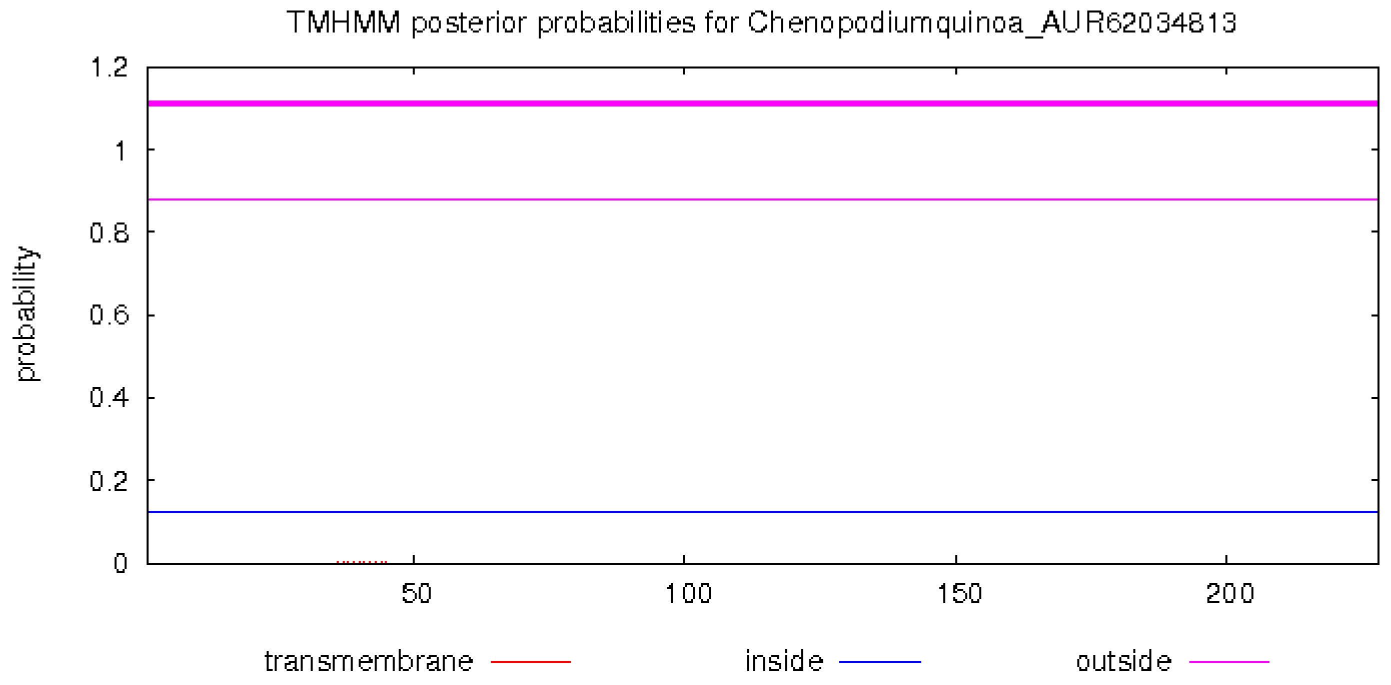

plot in postscript, script for making the plot in gnuplot, data for plot

---

```
# Chenopodiumquinoa_AUR62035165 Length: 101
# Chenopodiumquinoa_AUR62035165 Number of predicted TMHs:  0
# Chenopodiumquinoa_AUR62035165 Exp number of AAs in TMHs: 9.30101
# Chenopodiumquinoa_AUR62035165 Exp number, first 60 AAs:  9.17575
```

```
# Chenopodiumquinoa_AUR62035165 Total prob of N-in:        0.36584
Chenopodiumquinoa_AUR62035165   TMHMM2.0        outside      1   101
```

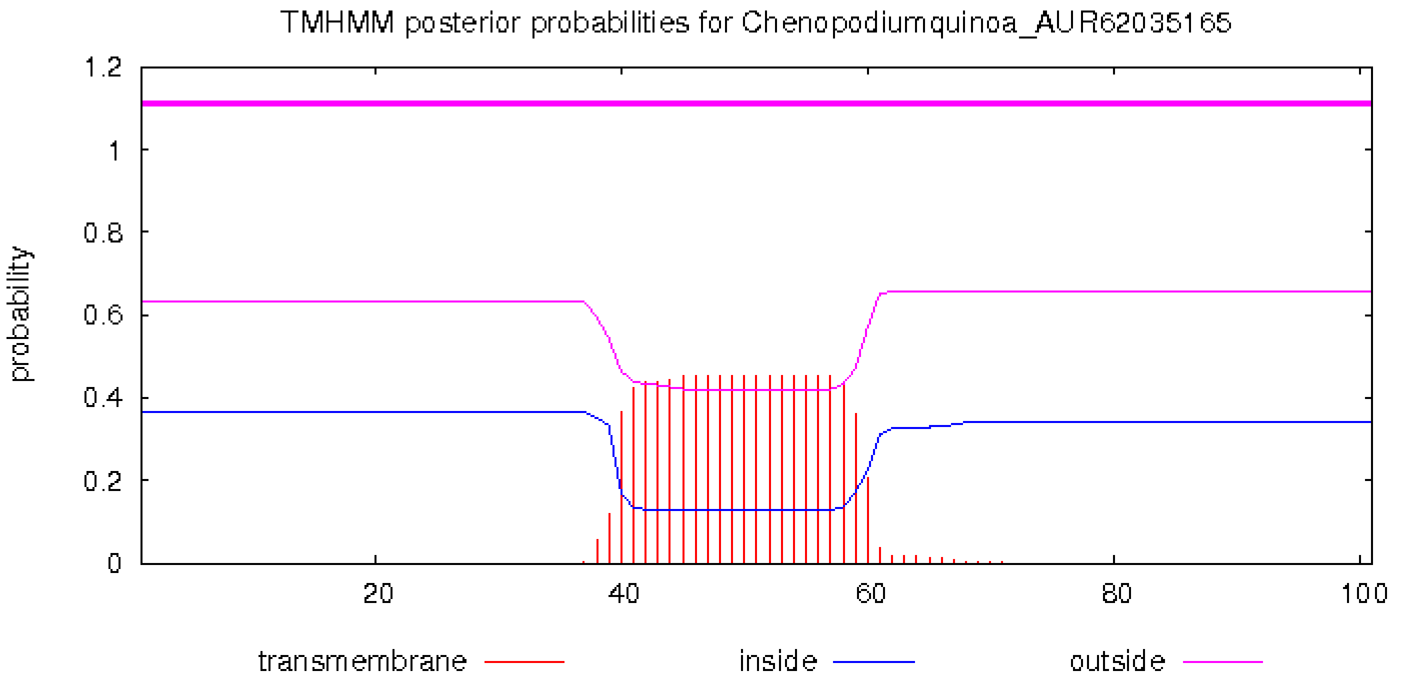

plot in postscript, script for making the plot in gnuplot, data for plot

---

```
# Chenopodiumquinoa_AUR62035514 Length: 153
# Chenopodiumquinoa_AUR62035514 Number of predicted TMHs:  0
# Chenopodiumquinoa_AUR62035514 Exp number of AAs in TMHs: 0.00099
# Chenopodiumquinoa_AUR62035514 Exp number, first 60 AAs:  0
# Chenopodiumquinoa_AUR62035514 Total prob of N-in:        0.14046
Chenopodiumquinoa_AUR62035514   TMHMM2.0        outside      1   153
```

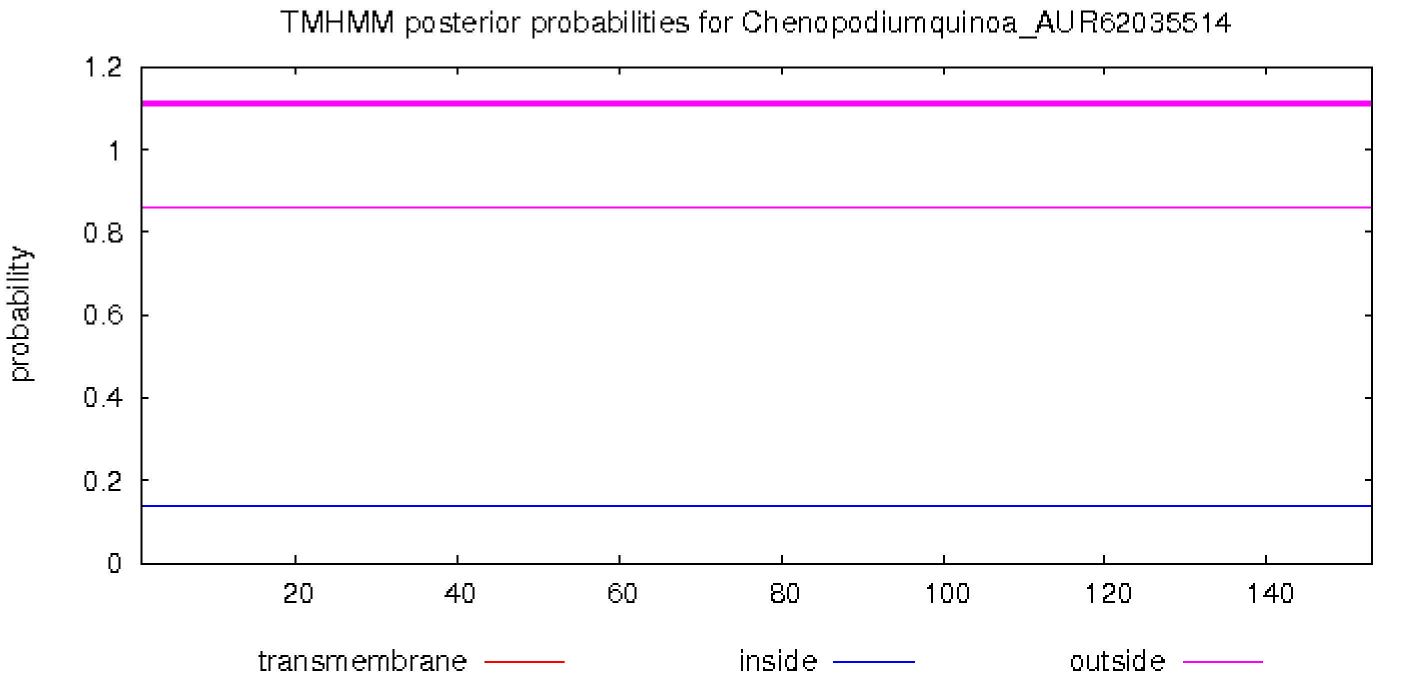

plot in postscript, script for making the plot in gnuplot, data for plot

---

```
# Chenopodiumquinoa_AUR62036158 Length: 324
# Chenopodiumquinoa_AUR62036158 Number of predicted TMHs:  0
# Chenopodiumquinoa_AUR62036158 Exp number of AAs in TMHs: 0.19575
# Chenopodiumquinoa_AUR62036158 Exp number, first 60 AAs:  0
```

```
# Chenopodiumquinoa_AUR62036158 Total prob of N-in:        0.00222
Chenopodiumquinoa_AUR62036158   TMHMM2.0        outside      1   324
```

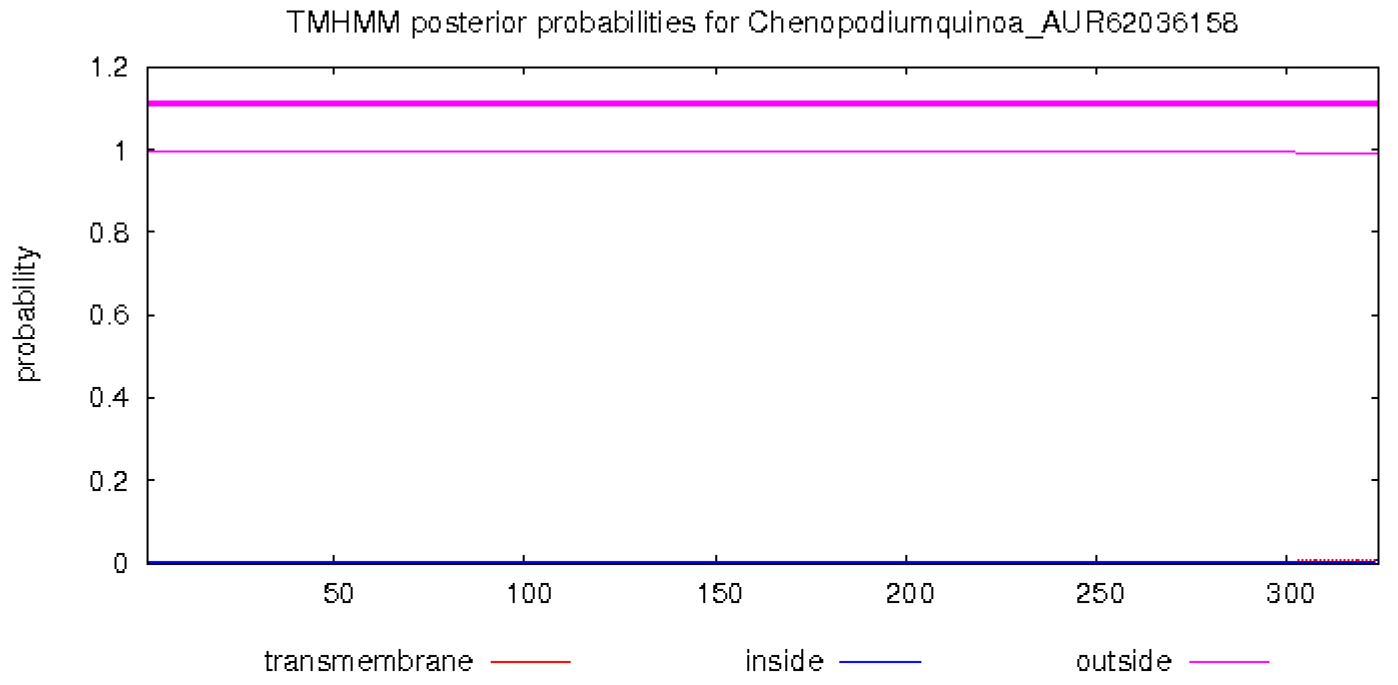

plot in postscript, script for making the plot in gnuplot, data for plot

---

```
# Chenopodiumquinoa_AUR62036626 Length: 279
# Chenopodiumquinoa_AUR62036626 Number of predicted TMHs:  0
# Chenopodiumquinoa_AUR62036626 Exp number of AAs in TMHs: 0.06223
# Chenopodiumquinoa_AUR62036626 Exp number, first 60 AAs:  0
# Chenopodiumquinoa_AUR62036626 Total prob of N-in:        0.00707
Chenopodiumquinoa_AUR62036626   TMHMM2.0        outside      1   279
```

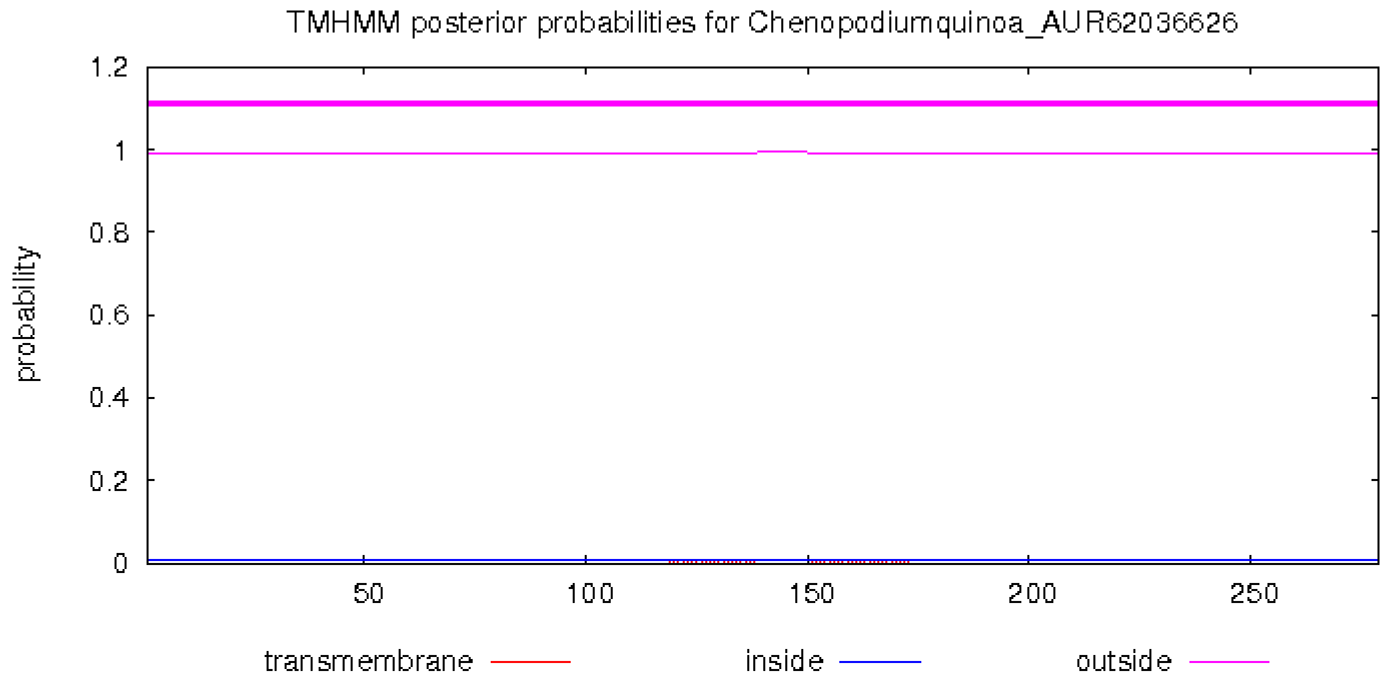

plot in postscript, script for making the plot in gnuplot, data for plot

---

```
# Chenopodiumquinoa_AUR62036991 Length: 151
# Chenopodiumquinoa_AUR62036991 Number of predicted TMHs:  0
# Chenopodiumquinoa_AUR62036991 Exp number of AAs in TMHs: 0.01635
# Chenopodiumquinoa_AUR62036991 Exp number, first 60 AAs:  0
```

```
# Chenopodiumquinoa_AUR62036991 Total prob of N-in:        0.03030
Chenopodiumquinoa_AUR62036991   TMHMM2.0        outside      1   151
```

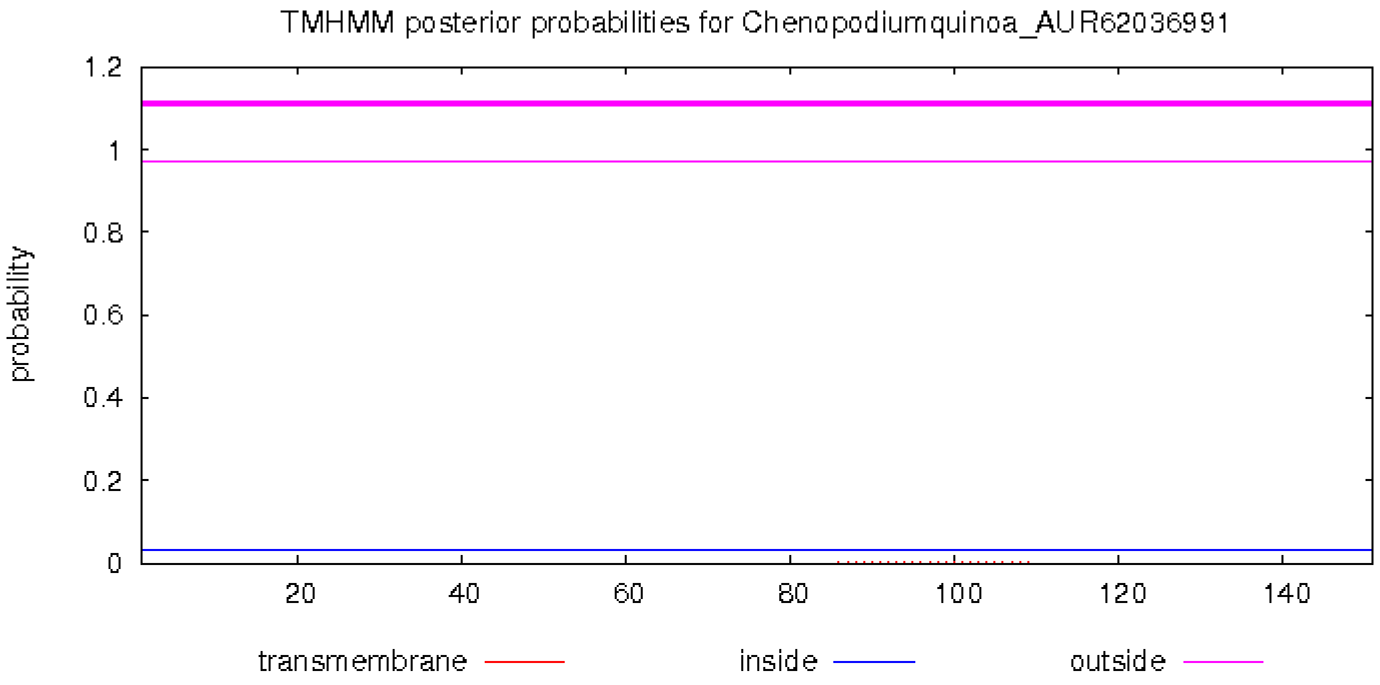

plot in postscript, script for making the plot in gnuplot, data for plot

---

```
# Chenopodiumquinoa_AUR62037478 Length: 241
# Chenopodiumquinoa_AUR62037478 Number of predicted TMHs:  0
# Chenopodiumquinoa_AUR62037478 Exp number of AAs in TMHs: 0
# Chenopodiumquinoa_AUR62037478 Exp number, first 60 AAs:  0
# Chenopodiumquinoa_AUR62037478 Total prob of N-in:        0.00330
Chenopodiumquinoa_AUR62037478   TMHMM2.0        outside      1   241
```

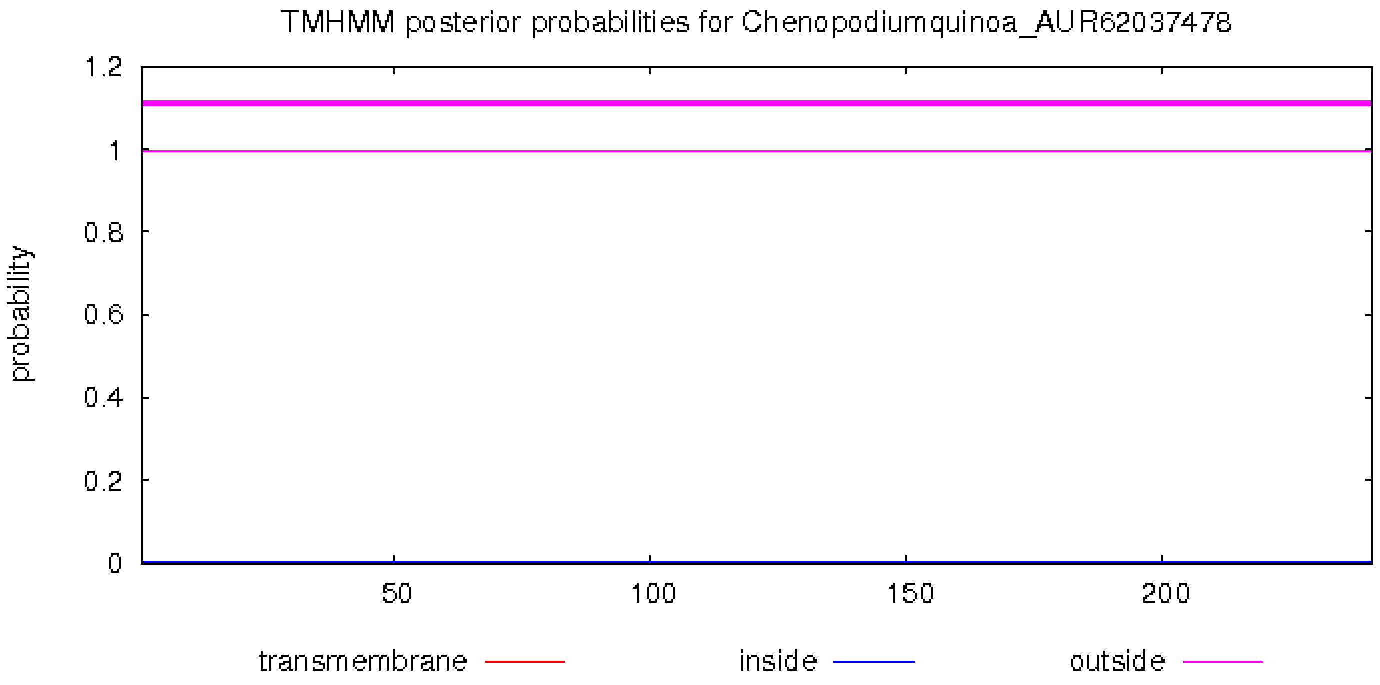

plot in postscript, script for making the plot in gnuplot, data for plot

---

```
# Chenopodiumquinoa_AUR62038748 Length: 294
# Chenopodiumquinoa_AUR62038748 Number of predicted TMHs:  0
# Chenopodiumquinoa_AUR62038748 Exp number of AAs in TMHs: 0.00299
# Chenopodiumquinoa_AUR62038748 Exp number, first 60 AAs:  0.00037
```

```
# Chenopodiumquinoa_AUR62038748 Total prob of N-in:        0.03355
Chenopodiumquinoa_AUR62038748   TMHMM2.0        outside      1   294
```

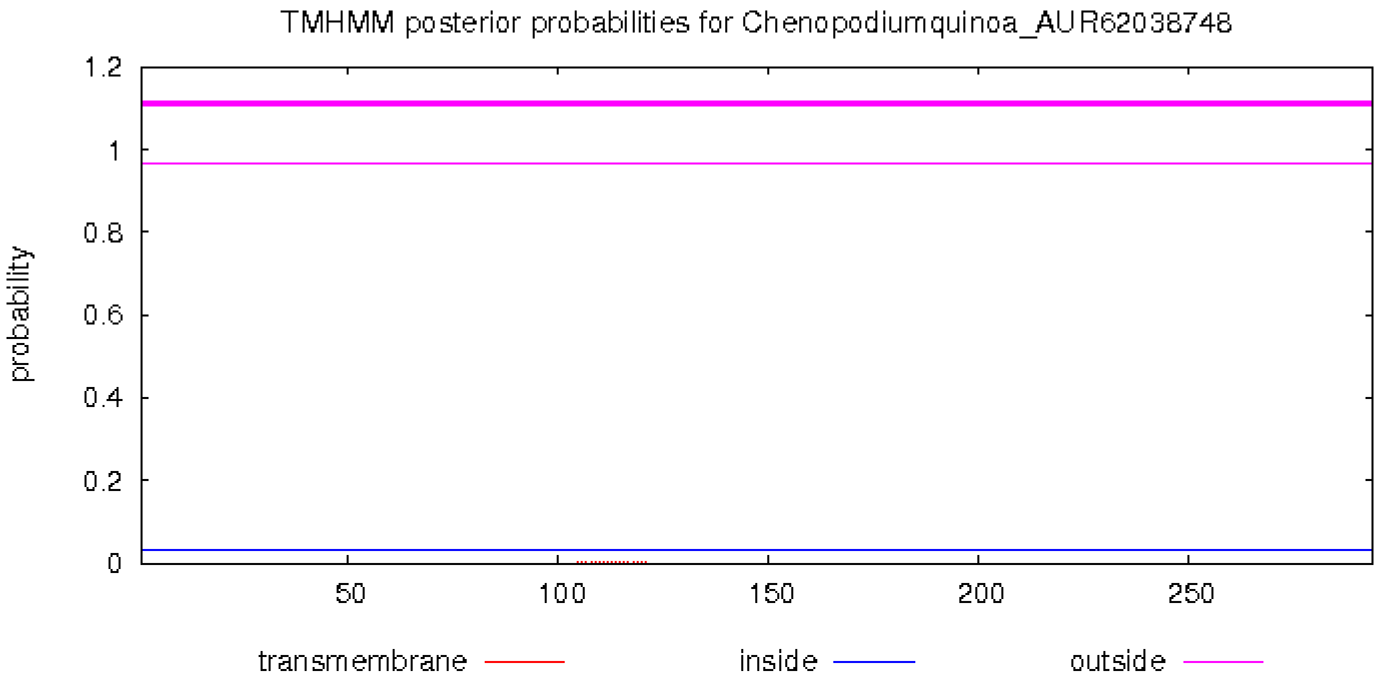

plot in postscript, script for making the plot in gnuplot, data for plot

---

```
# Chenopodiumquinoa_AUR62039815 Length: 385
# Chenopodiumquinoa_AUR62039815 Number of predicted TMHs:  0
# Chenopodiumquinoa_AUR62039815 Exp number of AAs in TMHs: 0.00517
# Chenopodiumquinoa_AUR62039815 Exp number, first 60 AAs:  0.00016
# Chenopodiumquinoa_AUR62039815 Total prob of N-in:        0.02720
Chenopodiumquinoa_AUR62039815   TMHMM2.0        outside      1   385
```

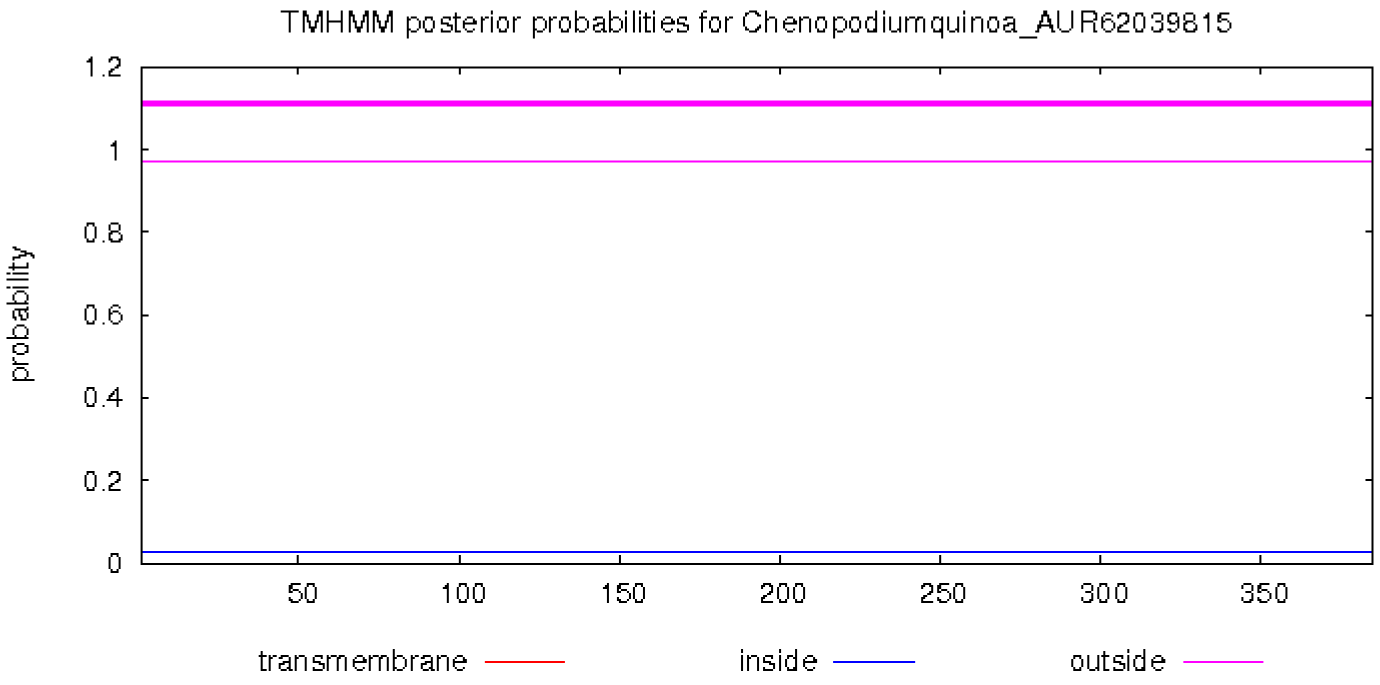

plot in postscript, script for making the plot in gnuplot, data for plot

---

```
# Chenopodiumquinoa_AUR62039927 Length: 370
# Chenopodiumquinoa_AUR62039927 Number of predicted TMHs:  0
# Chenopodiumquinoa_AUR62039927 Exp number of AAs in TMHs: 0
# Chenopodiumquinoa_AUR62039927 Exp number, first 60 AAs:  0
```

```
# Chenopodiumquinoa_AUR62039927 Total prob of N-in:        0.00343
Chenopodiumquinoa_AUR62039927   TMHMM2.0        outside      1   370
```

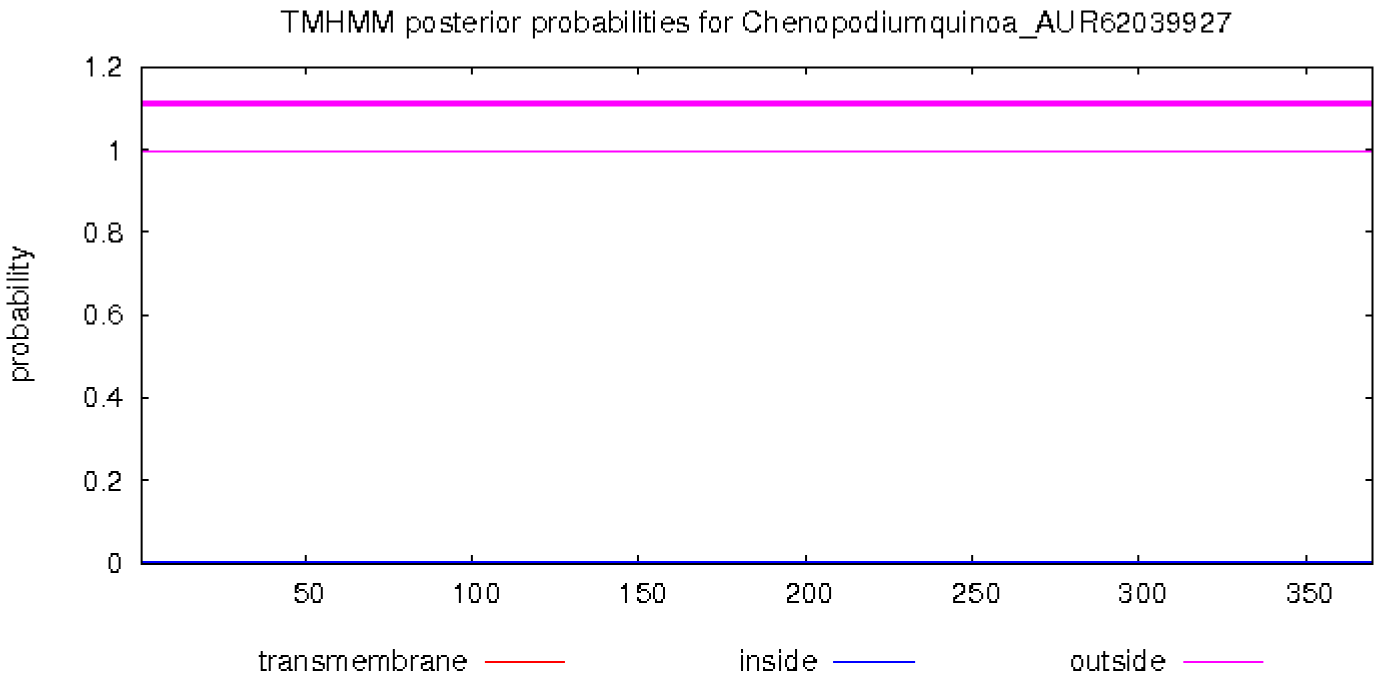

plot in postscript, script for making the plot in gnuplot, data for plot

---

```
# Chenopodiumquinoa_AUR62040120 Length: 390
# Chenopodiumquinoa_AUR62040120 Number of predicted TMHs:  0
# Chenopodiumquinoa_AUR62040120 Exp number of AAs in TMHs: 0.00646
# Chenopodiumquinoa_AUR62040120 Exp number, first 60 AAs:  0.0004
# Chenopodiumquinoa_AUR62040120 Total prob of N-in:        0.00166
Chenopodiumquinoa_AUR62040120   TMHMM2.0        outside      1   390
```

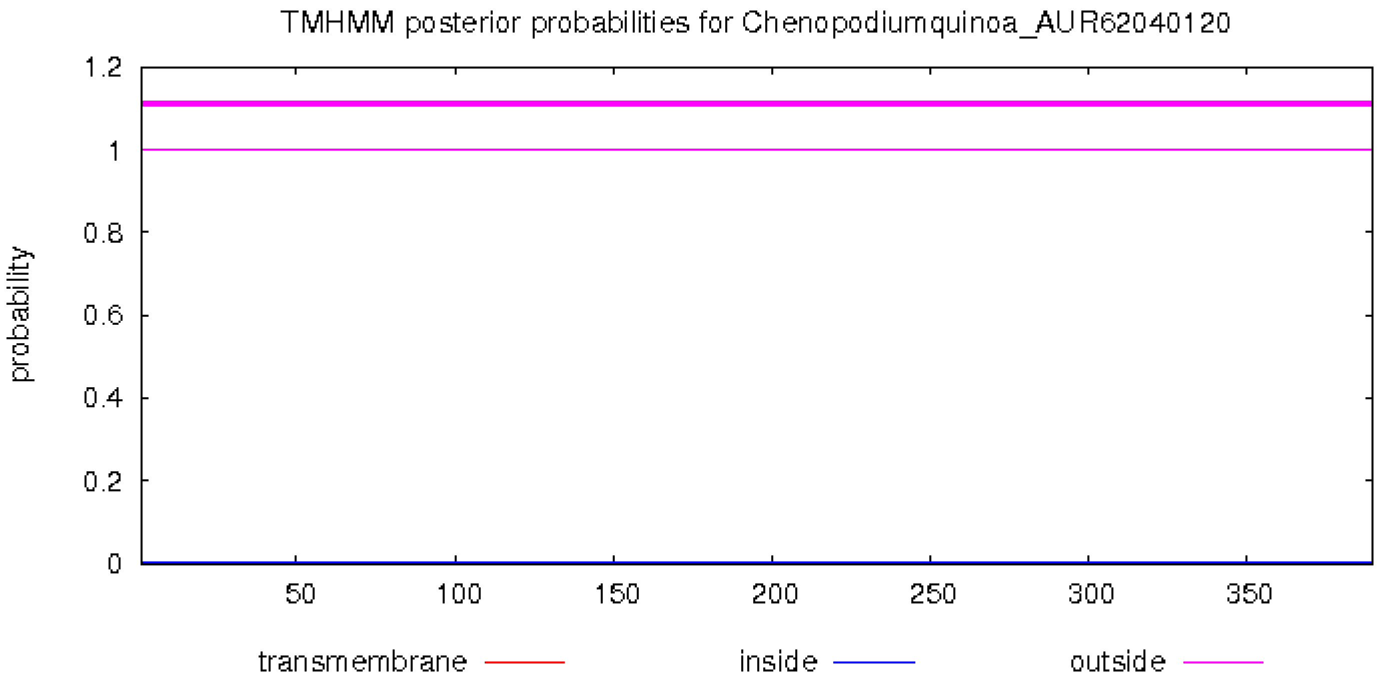

plot in postscript, script for making the plot in gnuplot, data for plot

---

```
# Chenopodiumquinoa_AUR62040889 Length: 213
# Chenopodiumquinoa_AUR62040889 Number of predicted TMHs:  0
# Chenopodiumquinoa_AUR62040889 Exp number of AAs in TMHs: 0.0176
# Chenopodiumquinoa_AUR62040889 Exp number, first 60 AAs:  0
```

```
# Chenopodiumquinoa_AUR62040889 Total prob of N-in:        0.08899
Chenopodiumquinoa_AUR62040889   TMHMM2.0        outside      1   213
```

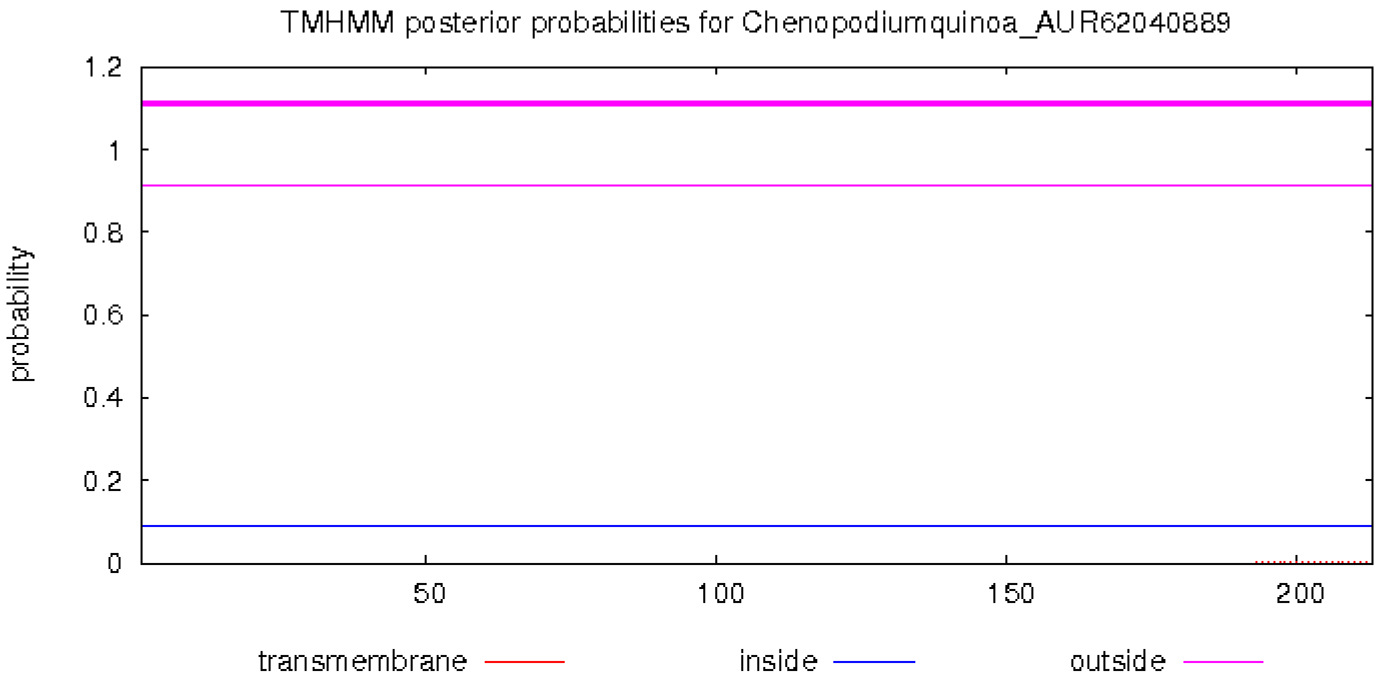

plot in postscript, script for making the plot in gnuplot, data for plot

---

```
# Chenopodiumquinoa_AUR62041525 Length: 550
# Chenopodiumquinoa_AUR62041525 Number of predicted TMHs:  1
# Chenopodiumquinoa_AUR62041525 Exp number of AAs in TMHs: 22.70282
# Chenopodiumquinoa_AUR62041525 Exp number, first 60 AAs:  0
# Chenopodiumquinoa_AUR62041525 Total prob of N-in:        0.01171
Chenopodiumquinoa_AUR62041525   TMHMM2.0        outside      1   525
Chenopodiumquinoa_AUR62041525   TMHMM2.0        TMhelix    526   548
Chenopodiumquinoa_AUR62041525   TMHMM2.0        inside     549   550
```

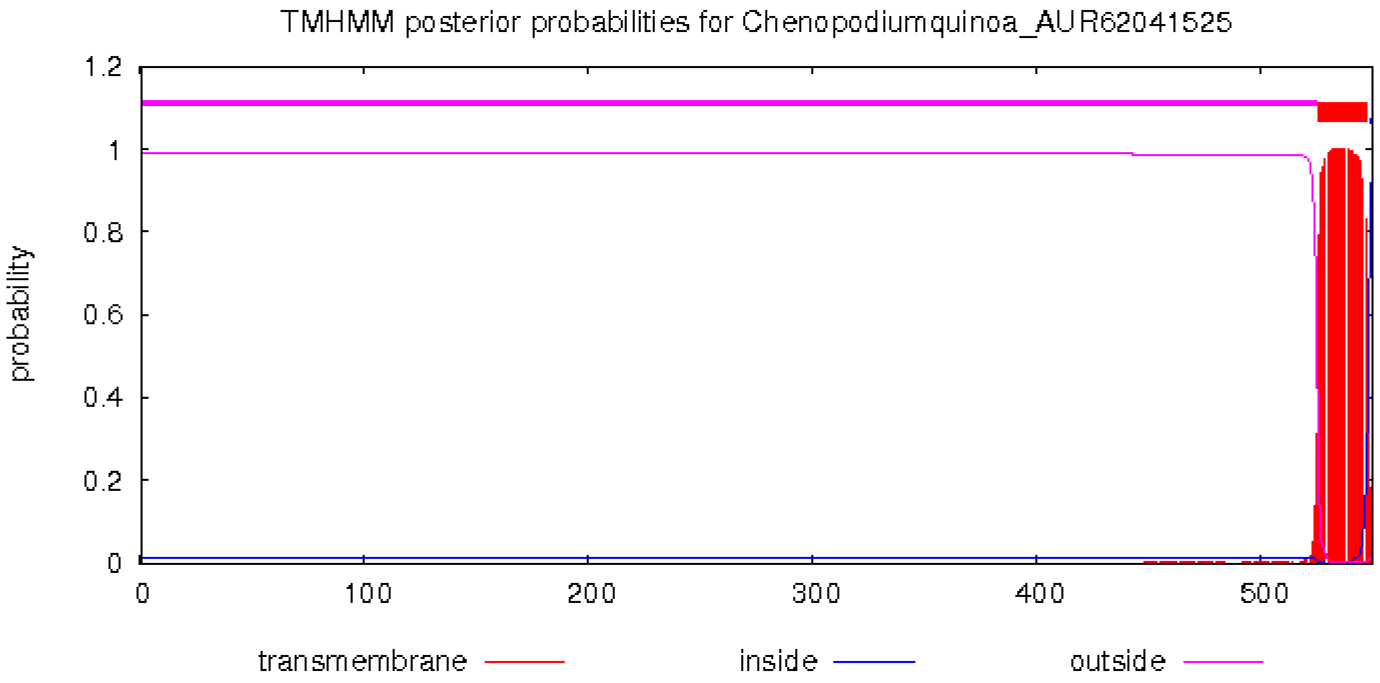

plot in postscript, script for making the plot in gnuplot, data for plot

---

```
# Chenopodiumquinoa_AUR62041539 Length: 556
# Chenopodiumquinoa_AUR62041539 Number of predicted TMHs:  1
```

```
# Chenopodiumquinoa_AUR62041539 Exp number of AAs in TMHs: 22.66141
# Chenopodiumquinoa_AUR62041539 Exp number, first 60 AAs:  0.00023
# Chenopodiumquinoa_AUR62041539 Total prob of N-in:        0.01779
Chenopodiumquinoa_AUR62041539   TMHMM2.0        outside      1   531
Chenopodiumquinoa_AUR62041539   TMHMM2.0        TMhelix    532   554
Chenopodiumquinoa_AUR62041539   TMHMM2.0        inside     555   556
```

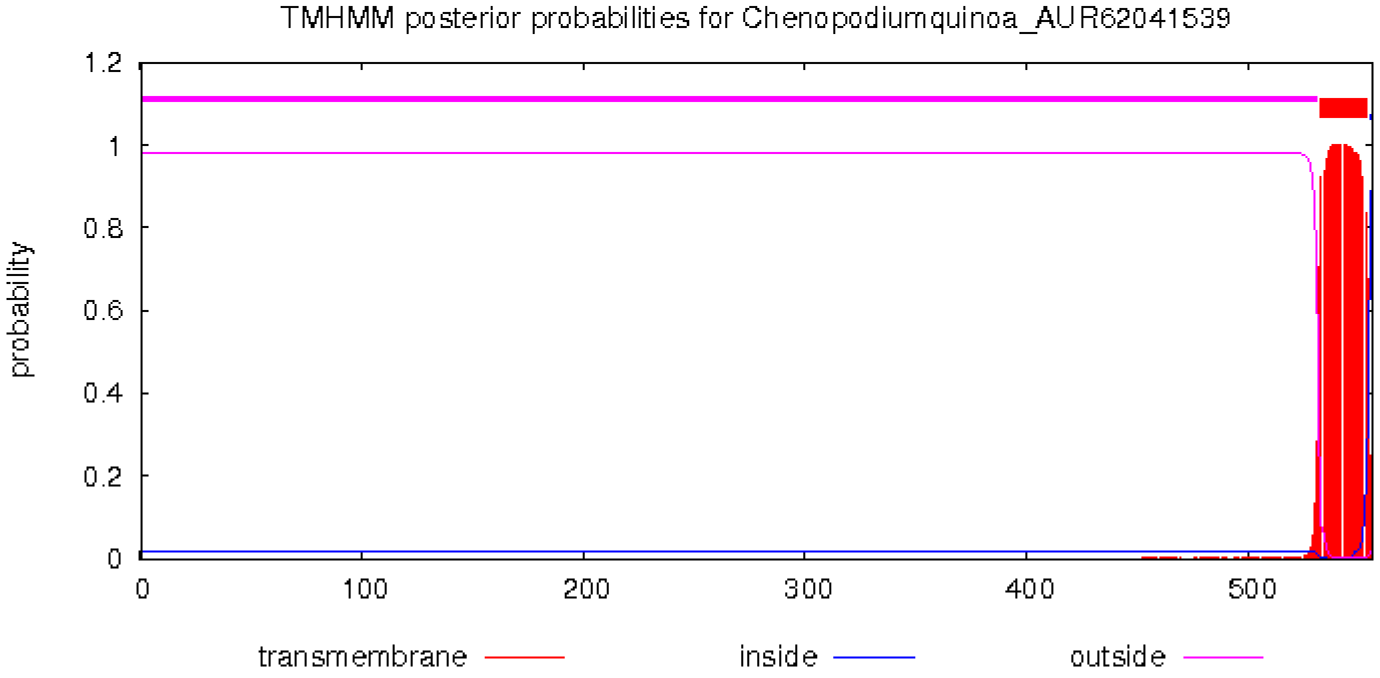

plot in postscript, script for making the plot in gnuplot, data for plot

```
# Chenopodiumquinoa_AUR62043235 Length: 265
# Chenopodiumquinoa_AUR62043235 Number of predicted TMHs:  0
# Chenopodiumquinoa_AUR62043235 Exp number of AAs in TMHs: 0.00042
# Chenopodiumquinoa_AUR62043235 Exp number, first 60 AAs:  0
# Chenopodiumquinoa_AUR62043235 Total prob of N-in:        0.01427
Chenopodiumquinoa_AUR62043235   TMHMM2.0        outside      1   265
```

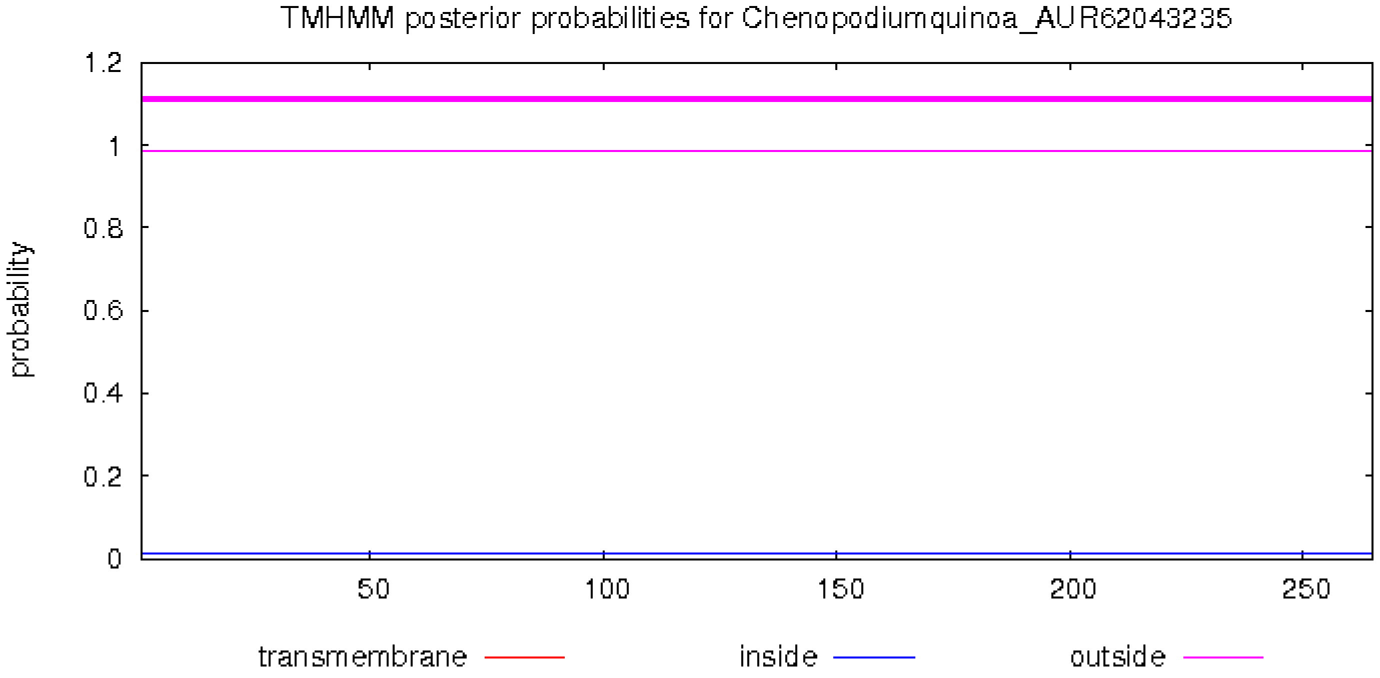

plot in postscript, script for making the plot in gnuplot, data for plot

```
# Chenopodiumquinoa_AUR62043491 Length: 393
# Chenopodiumquinoa_AUR62043491 Number of predicted TMHs:  0
# Chenopodiumquinoa_AUR62043491 Exp number of AAs in TMHs: 0
# Chenopodiumquinoa_AUR62043491 Exp number, first 60 AAs:  0
# Chenopodiumquinoa_AUR62043491 Total prob of N-in:        0.00033
Chenopodiumquinoa_AUR62043491   TMHMM2.0        outside      1   393
```

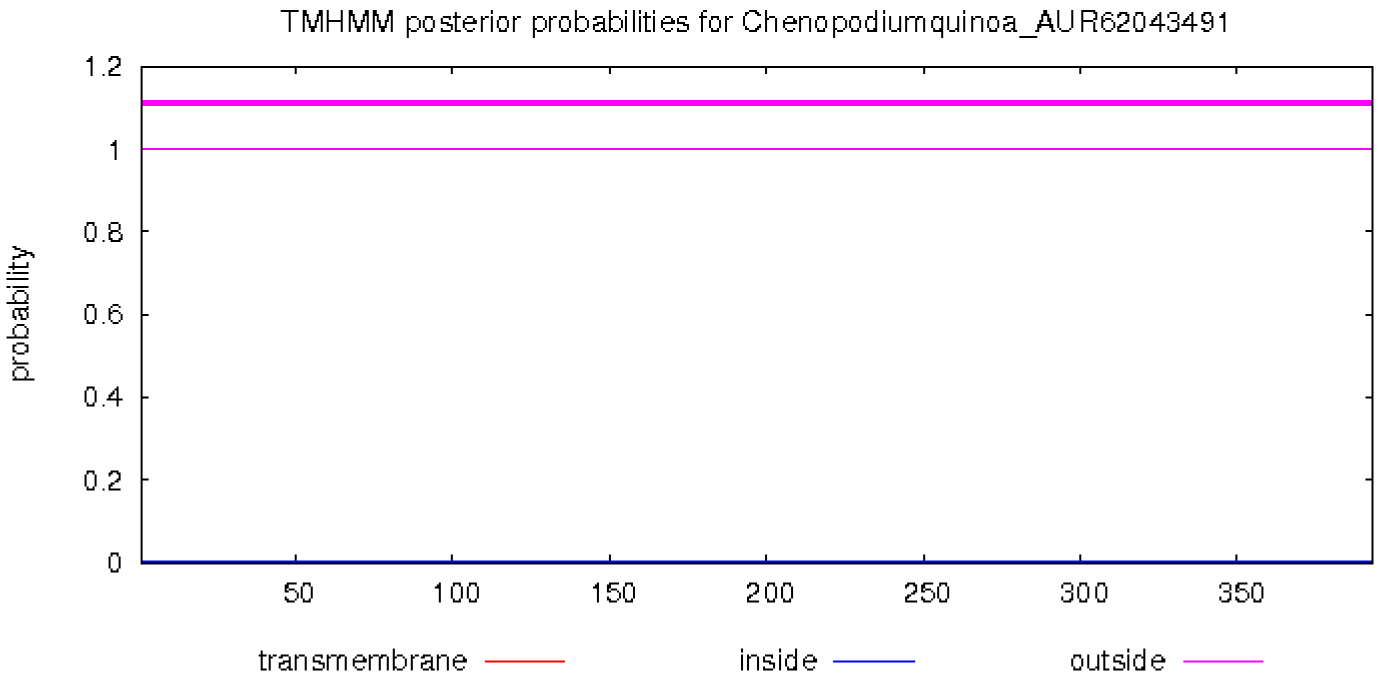

plot in postscript, script for making the plot in gnuplot, data for plot

---

```
# Chenopodiumquinoa_AUR62043497 Length: 155
# Chenopodiumquinoa_AUR62043497 Number of predicted TMHs:  0
# Chenopodiumquinoa_AUR62043497 Exp number of AAs in TMHs: 0.00112
# Chenopodiumquinoa_AUR62043497 Exp number, first 60 AAs:  0
# Chenopodiumquinoa_AUR62043497 Total prob of N-in:        0.13392
Chenopodiumquinoa_AUR62043497   TMHMM2.0        outside      1   155
```

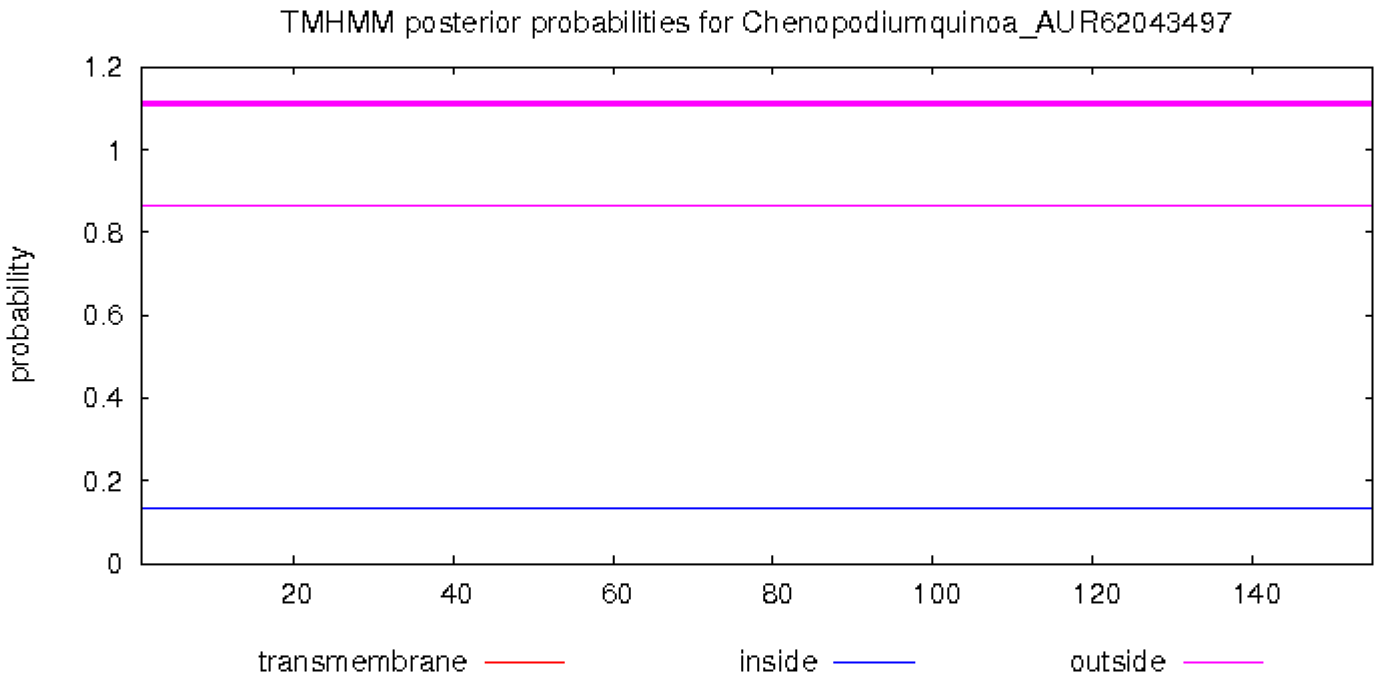

plot in postscript, script for making the plot in gnuplot, data for plot

---

```
# Chenopodiumquinoa_AUR62043713 Length: 265
# Chenopodiumquinoa_AUR62043713 Number of predicted TMHs:  0
# Chenopodiumquinoa_AUR62043713 Exp number of AAs in TMHs: 0.00042
# Chenopodiumquinoa_AUR62043713 Exp number, first 60 AAs:  0
# Chenopodiumquinoa_AUR62043713 Total prob of N-in:        0.01427
Chenopodiumquinoa_AUR62043713   TMHMM2.0        outside      1   265
```

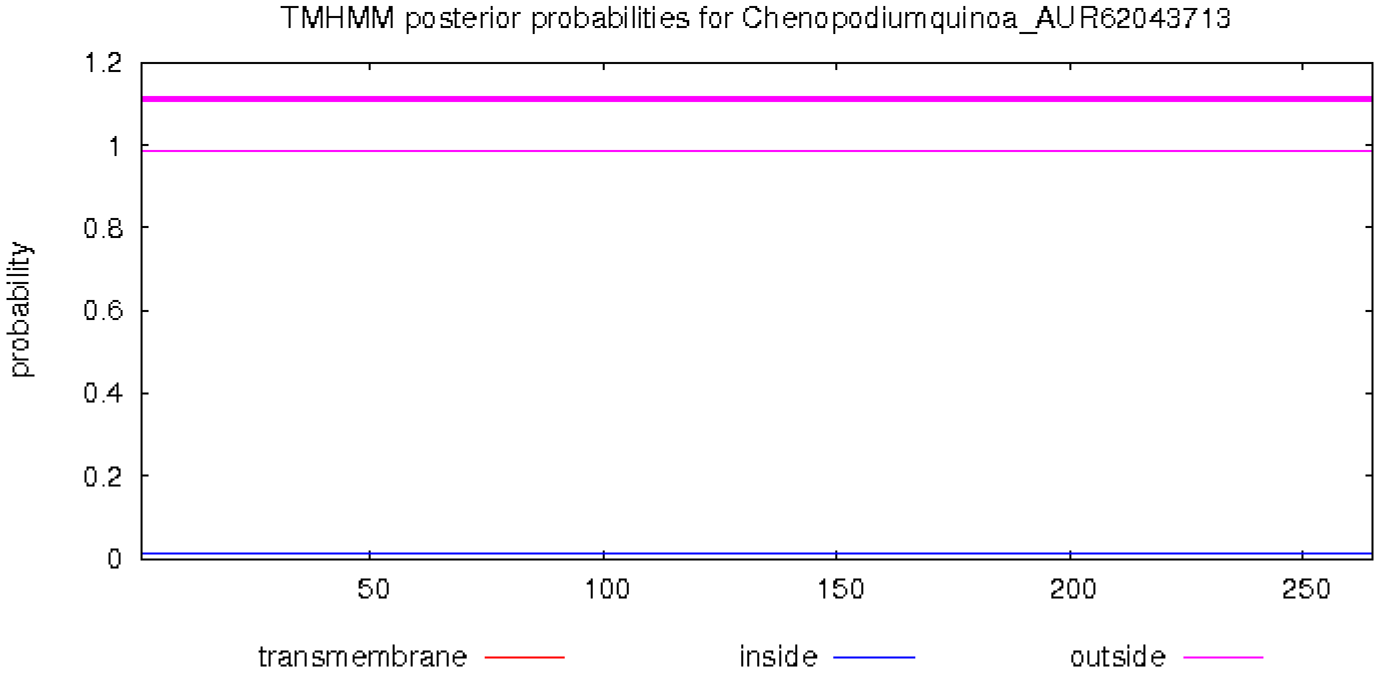

plot in postscript, script for making the plot in gnuplot, data for plot

---

```
# Chenopodiumquinoa_AUR62043741 Length: 355
# Chenopodiumquinoa_AUR62043741 Number of predicted TMHs:  0
# Chenopodiumquinoa_AUR62043741 Exp number of AAs in TMHs: 0.0006
# Chenopodiumquinoa_AUR62043741 Exp number, first 60 AAs:  0.00042
# Chenopodiumquinoa_AUR62043741 Total prob of N-in:        0.01100
Chenopodiumquinoa_AUR62043741   TMHMM2.0        outside      1   355
```

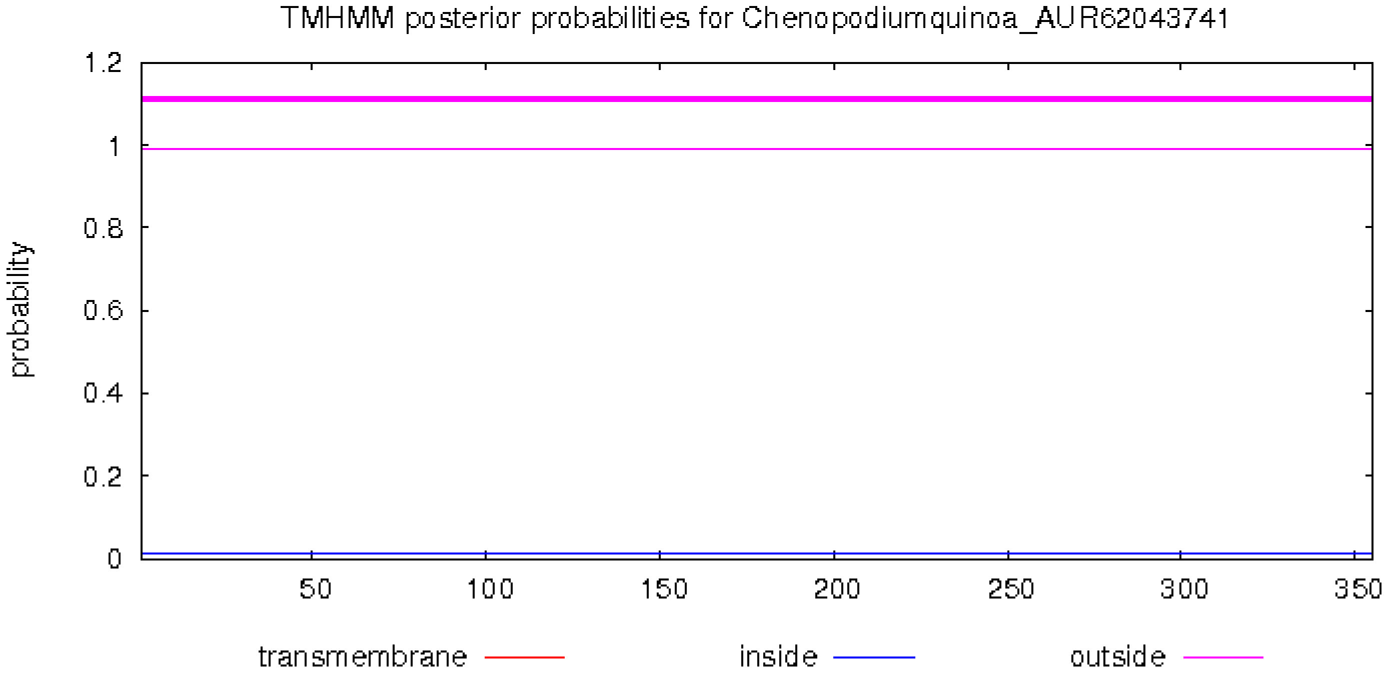

plot in postscript, script for making the plot in gnuplot, data for plot

---

```
# Chenopodiumquinoa_AUR62044373 Length: 454
# Chenopodiumquinoa_AUR62044373 Number of predicted TMHs:  0
# Chenopodiumquinoa_AUR62044373 Exp number of AAs in TMHs: 0
# Chenopodiumquinoa_AUR62044373 Exp number, first 60 AAs:  0
# Chenopodiumquinoa_AUR62044373 Total prob of N-in:        0.00779
Chenopodiumquinoa_AUR62044373   TMHMM2.0        outside      1   454
```

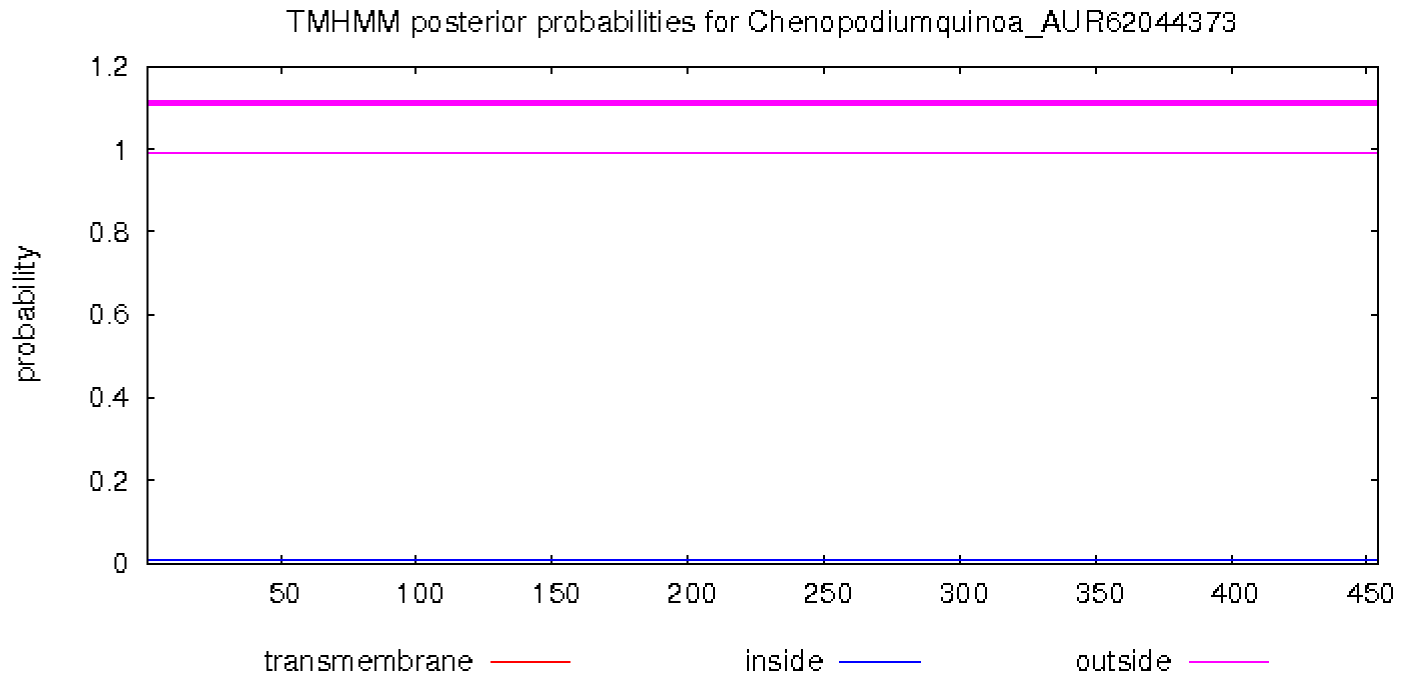

plot in postscript, script for making the plot in gnuplot, data for plot

---

```
# Cicerarietinum_NP_001266054.1 Length: 191
# Cicerarietinum_NP_001266054.1 Number of predicted TMHs:  0
# Cicerarietinum_NP_001266054.1 Exp number of AAs in TMHs: 0.00749
# Cicerarietinum_NP_001266054.1 Exp number, first 60 AAs:  0.00023
# Cicerarietinum_NP_001266054.1 Total prob of N-in:        0.02711
Cicerarietinum_NP_001266054.1   TMHMM2.0        outside      1   191
```

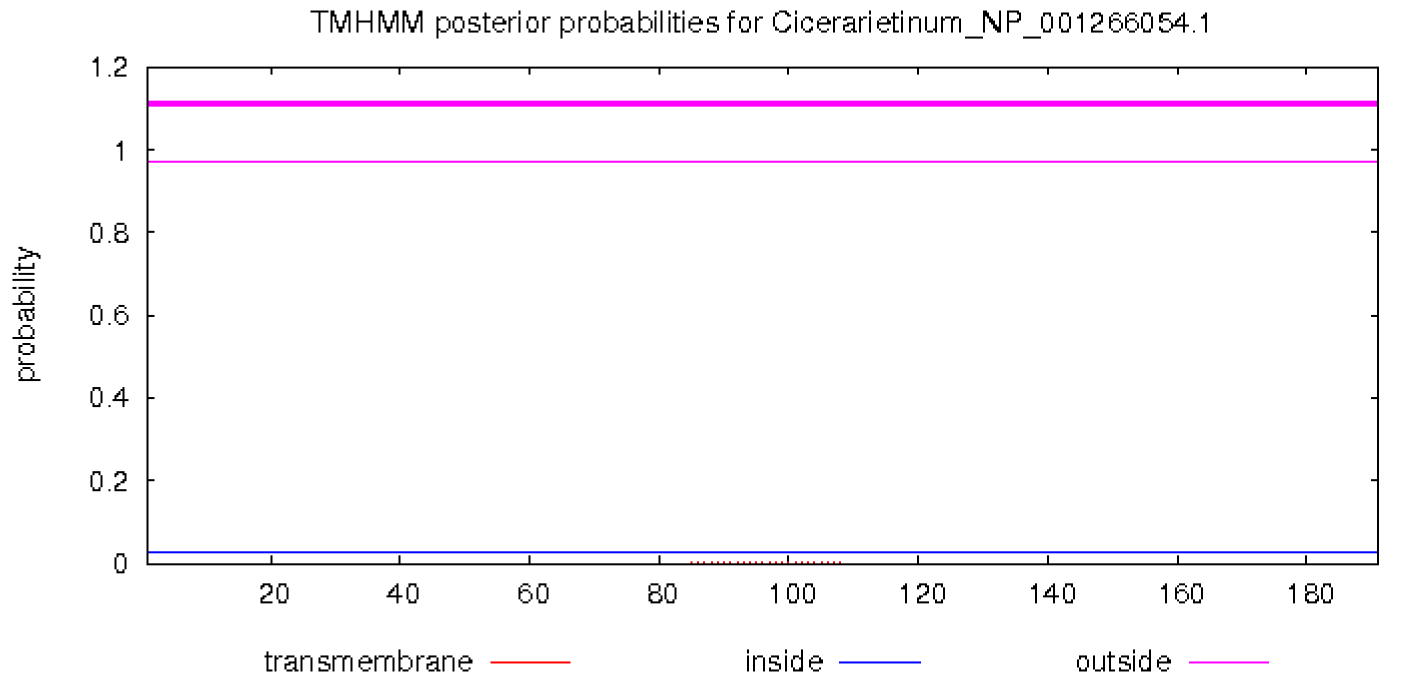

plot in postscript, script for making the plot in gnuplot, data for plot

---

```
# Ciceraríetinum_NP_001266090.1 Length: 239
# Ciceraríetinum_NP_001266090.1 Number of predicted TMHs:  0
# Ciceraríetinum_NP_001266090.1 Exp number of AAs in TMHs: 0.03807
# Ciceraríetinum_NP_001266090.1 Exp number, first 60 AAs:  0.00072
# Ciceraríetinum_NP_001266090.1 Total prob of N-in:        0.14375
Ciceraríetinum_NP_001266090.1   TMHMM2.0        outside      1   239
```

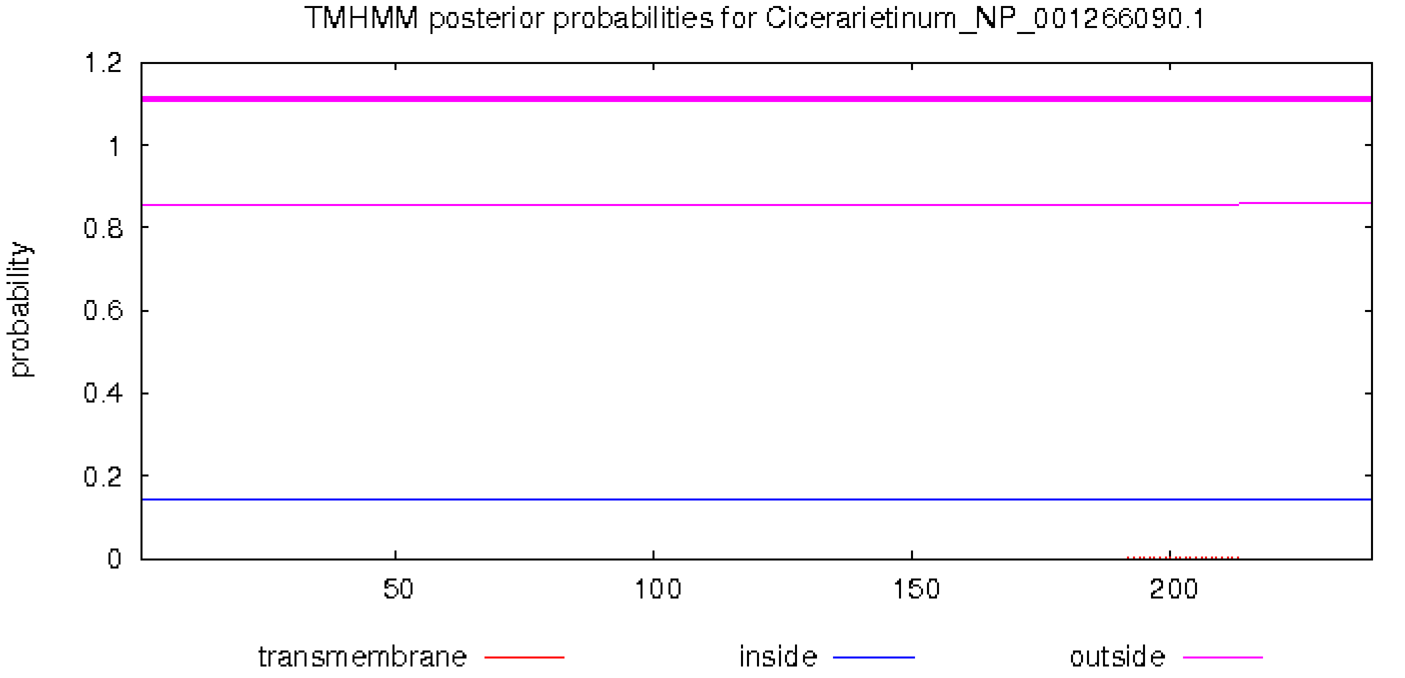

plot in postscript, script for making the plot in gnuplot, data for plot

```
# Ciceraríetinum_XP_004485547.1 Length: 201
# Ciceraríetinum_XP_004485547.1 Number of predicted TMHs:  0
# Ciceraríetinum_XP_004485547.1 Exp number of AAs in TMHs: 0.00188
# Ciceraríetinum_XP_004485547.1 Exp number, first 60 AAs:  0.00036
# Ciceraríetinum_XP_004485547.1 Total prob of N-in:        0.26599
Ciceraríetinum_XP_004485547.1   TMHMM2.0        outside      1   201
```

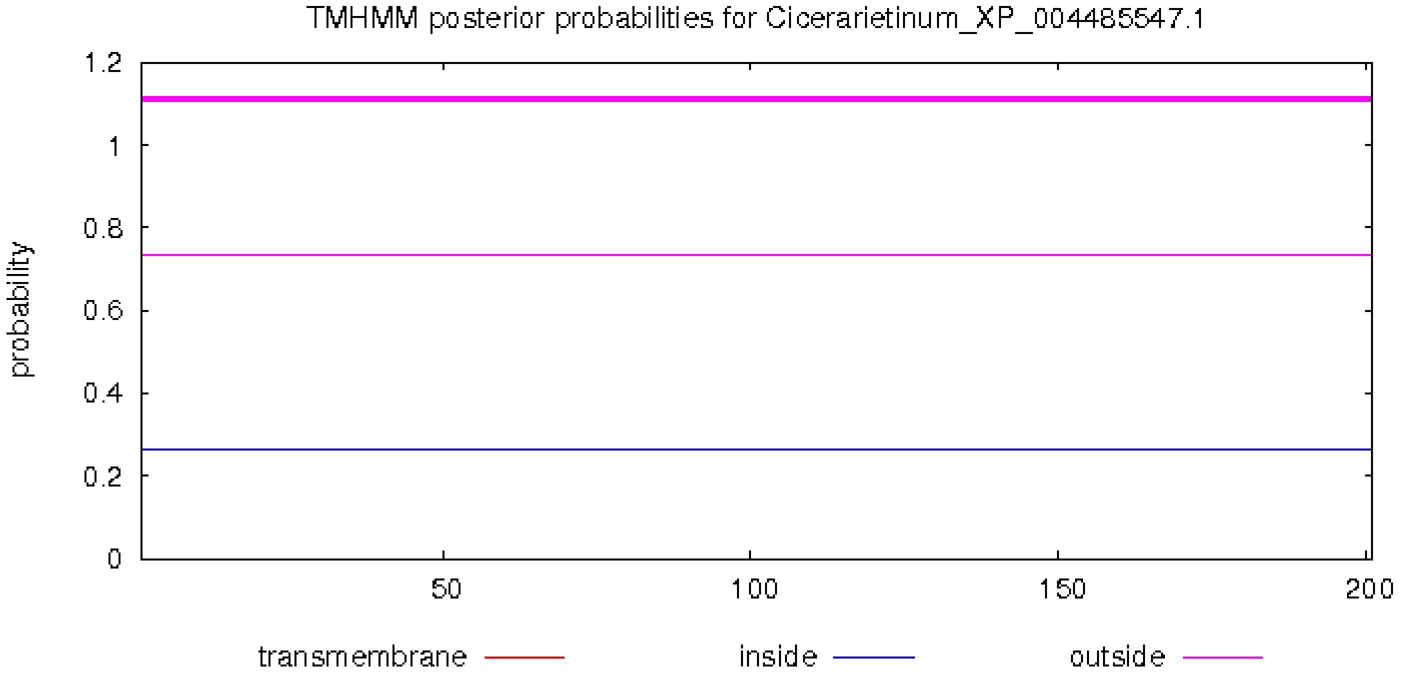

plot in postscript, script for making the plot in gnuplot, data for plot

```
# Cicerarietinum_XP_004487133.1 Length: 339
# Cicerarietinum_XP_004487133.1 Number of predicted TMHs:  0
# Cicerarietinum_XP_004487133.1 Exp number of AAs in TMHs: 0.01152
# Cicerarietinum_XP_004487133.1 Exp number, first 60 AAs:  0.0002
# Cicerarietinum_XP_004487133.1 Total prob of N-in:        0.02798
Cicerarietinum_XP_004487133.1   TMHMM2.0        outside      1   339
```

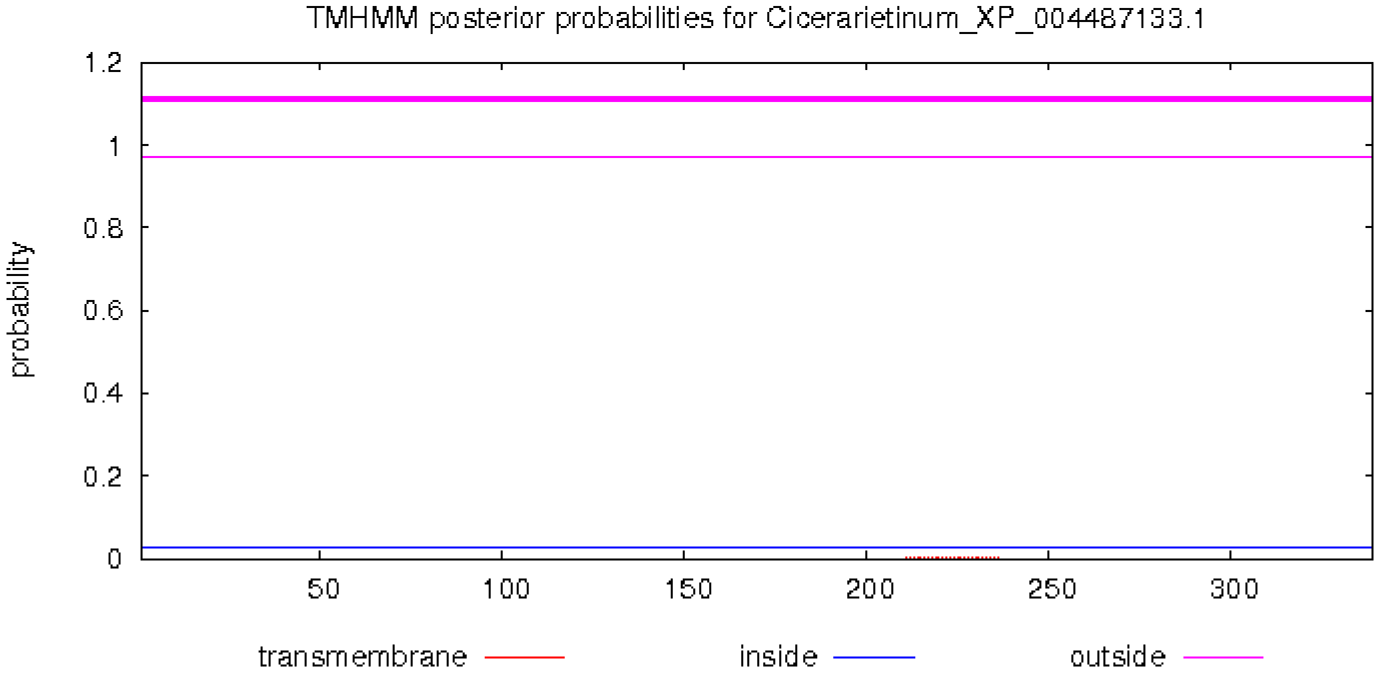

plot in postscript, script for making the plot in gnuplot, data for plot

```
# Cicerarietinum_XP_004487194.1 Length: 286
# Cicerarietinum_XP_004487194.1 Number of predicted TMHs:  0
# Cicerarietinum_XP_004487194.1 Exp number of AAs in TMHs: 0.00039
# Cicerarietinum_XP_004487194.1 Exp number, first 60 AAs:  0
# Cicerarietinum_XP_004487194.1 Total prob of N-in:        0.07485
Cicerarietinum_XP_004487194.1   TMHMM2.0        outside      1   286
```

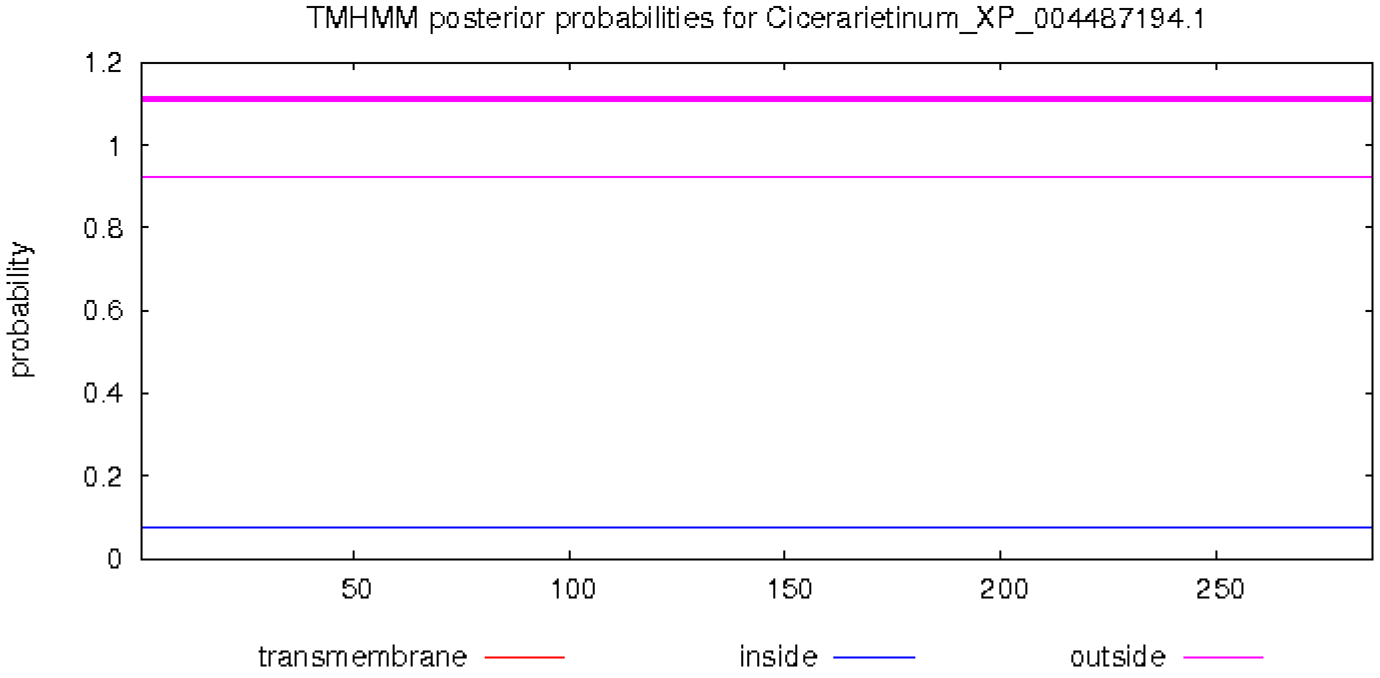

plot in postscript, script for making the plot in gnuplot, data for plot

```
# Cicerarietinum_XP_004487678.1 Length: 356
# Cicerarietinum_XP_004487678.1 Number of predicted TMHs:  0
# Cicerarietinum_XP_004487678.1 Exp number of AAs in TMHs: 0.000860000000000000001
# Cicerarietinum_XP_004487678.1 Exp number, first 60 AAs:  0.00057
# Cicerarietinum_XP_004487678.1 Total prob of N-in:        0.01379
Cicerarietinum_XP_004487678.1   TMHMM2.0        outside      1   356
```

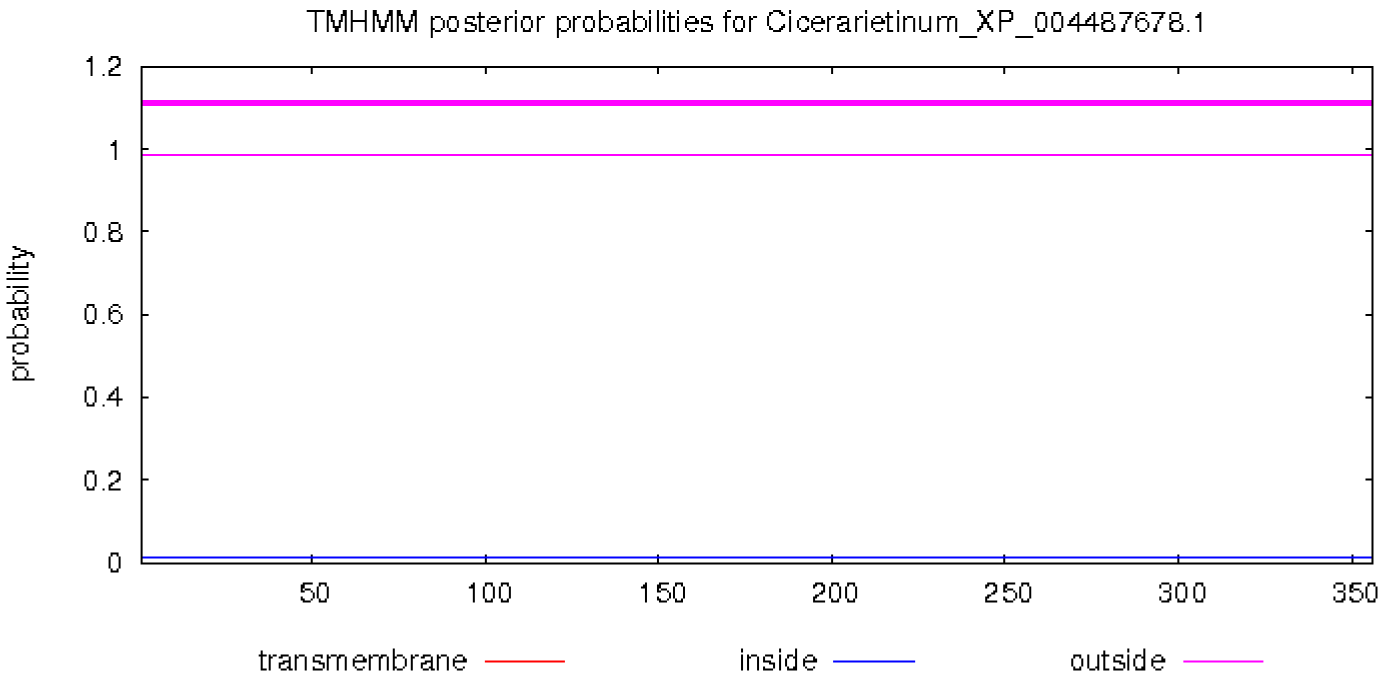

plot in postscript, script for making the plot in gnuplot, data for plot

---

```
# Cicerarietinum_XP_004487679.1 Length: 339
# Cicerarietinum_XP_004487679.1 Number of predicted TMHs:  0
# Cicerarietinum_XP_004487679.1 Exp number of AAs in TMHs: 0.00055
# Cicerarietinum_XP_004487679.1 Exp number, first 60 AAs:  0.00023
# Cicerarietinum_XP_004487679.1 Total prob of N-in:        0.00856
Cicerarietinum_XP_004487679.1   TMHMM2.0        outside      1   339
```

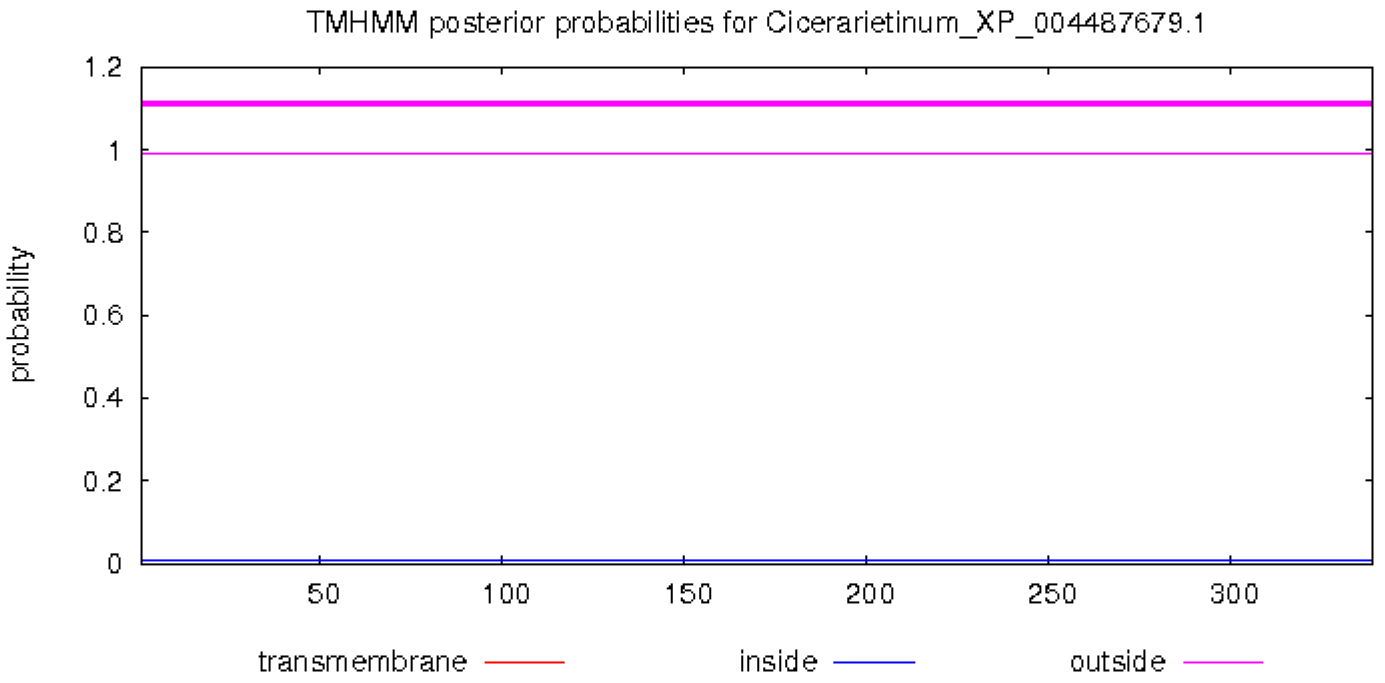

plot in postscript, script for making the plot in gnuplot, data for plot

---

```
# Cicerarietinum_XP_004488556.1 Length: 346
# Cicerarietinum_XP_004488556.1 Number of predicted TMHs:  0
# Cicerarietinum_XP_004488556.1 Exp number of AAs in TMHs: 8e-05
# Cicerarietinum_XP_004488556.1 Exp number, first 60 AAs:  0
# Cicerarietinum_XP_004488556.1 Total prob of N-in:        0.00128
Cicerarietinum_XP_004488556.1   TMHMM2.0        outside      1   346
```

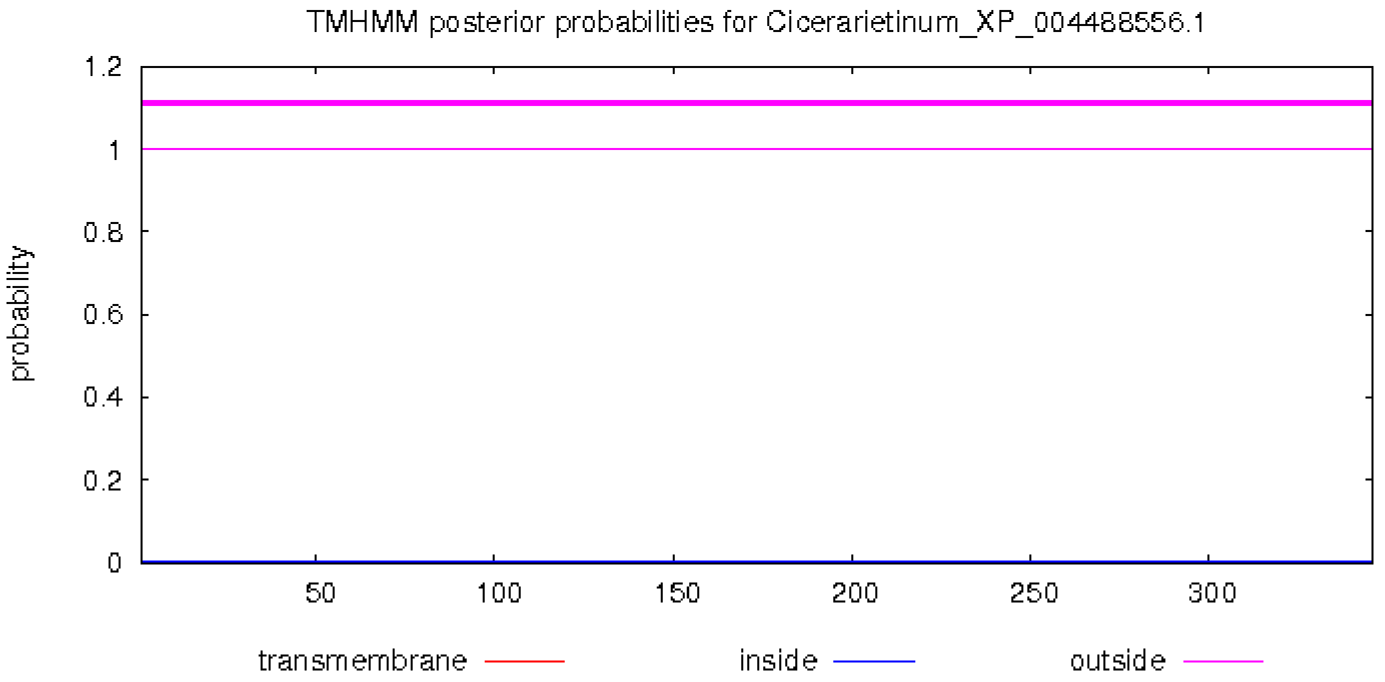

plot in postscript, script for making the plot in gnuplot, data for plot

```
# Cicerarietinum_XP_004488594.1 Length: 339
# Cicerarietinum_XP_004488594.1 Number of predicted TMHs:  0
# Cicerarietinum_XP_004488594.1 Exp number of AAs in TMHs: 0
# Cicerarietinum_XP_004488594.1 Exp number, first 60 AAs:  0
# Cicerarietinum_XP_004488594.1 Total prob of N-in:        0.01424
Cicerarietinum_XP_004488594.1   TMHMM2.0        outside      1   339
```

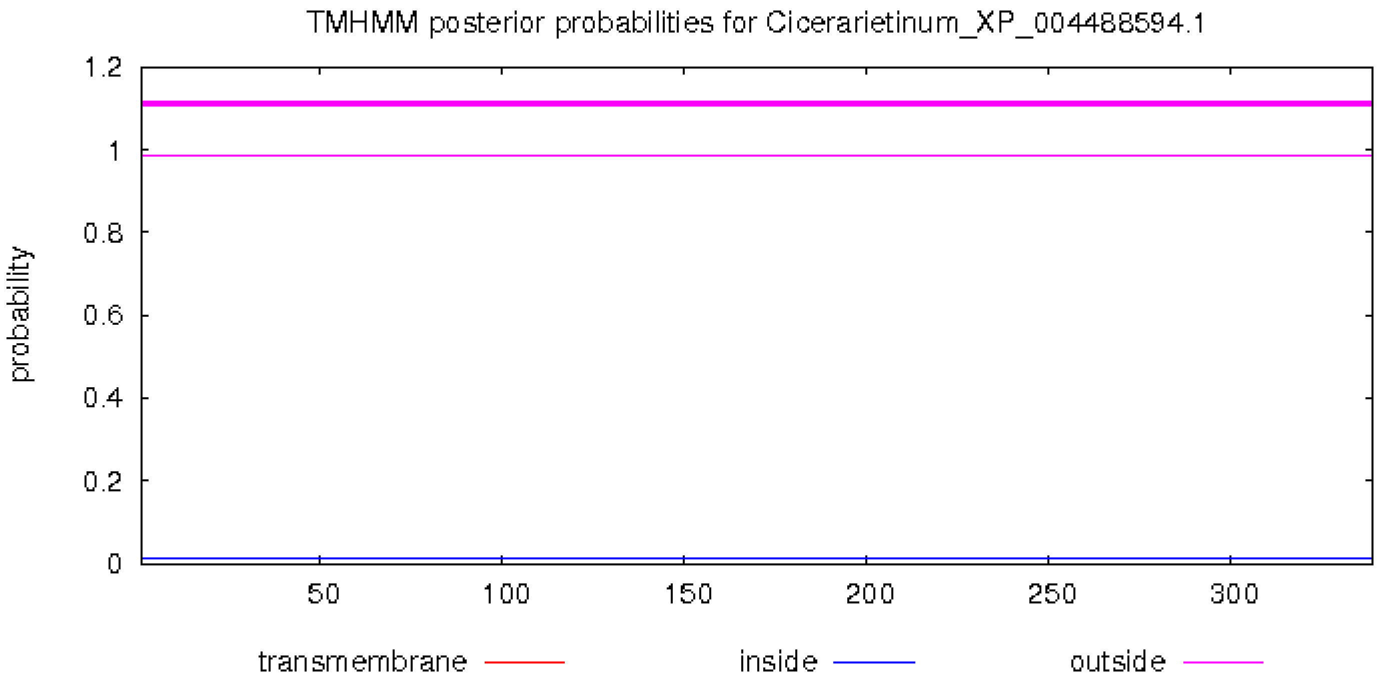

plot in postscript, script for making the plot in gnuplot, data for plot

```
# Cicerarietinum_XP_004488746.1 Length: 386
# Cicerarietinum_XP_004488746.1 Number of predicted TMHs:  0
# Cicerarietinum_XP_004488746.1 Exp number of AAs in TMHs: 0.00133
# Cicerarietinum_XP_004488746.1 Exp number, first 60 AAs:  4e-05
# Cicerarietinum_XP_004488746.1 Total prob of N-in:        0.01986
Cicerarietinum_XP_004488746.1   TMHMM2.0        outside      1   386
```

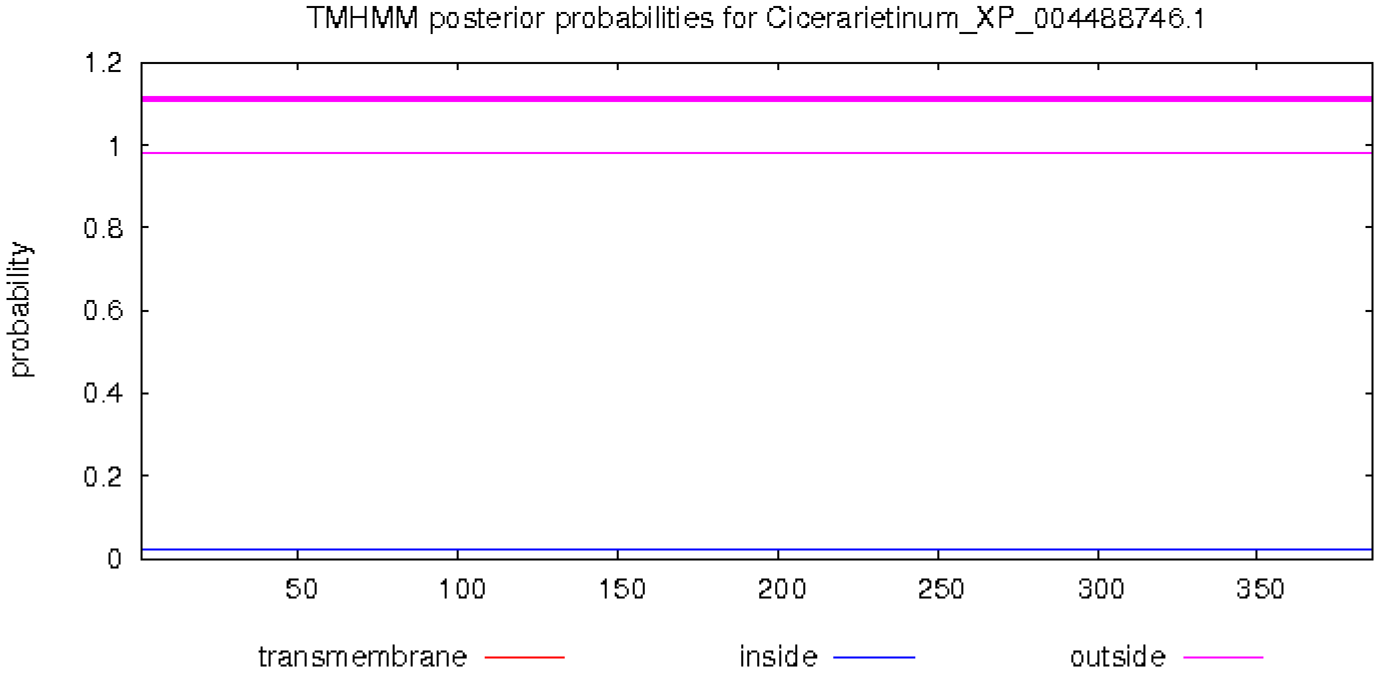

plot in postscript, script for making the plot in gnuplot, data for plot

---

```
# Cicerarietinum_XP_004488808.1 Length: 308
# Cicerarietinum_XP_004488808.1 Number of predicted TMHs:  0
# Cicerarietinum_XP_004488808.1 Exp number of AAs in TMHs: 0.00053
# Cicerarietinum_XP_004488808.1 Exp number, first 60 AAs:  0
# Cicerarietinum_XP_004488808.1 Total prob of N-in:        0.03394
Cicerarietinum_XP_004488808.1   TMHMM2.0        outside      1   308
```

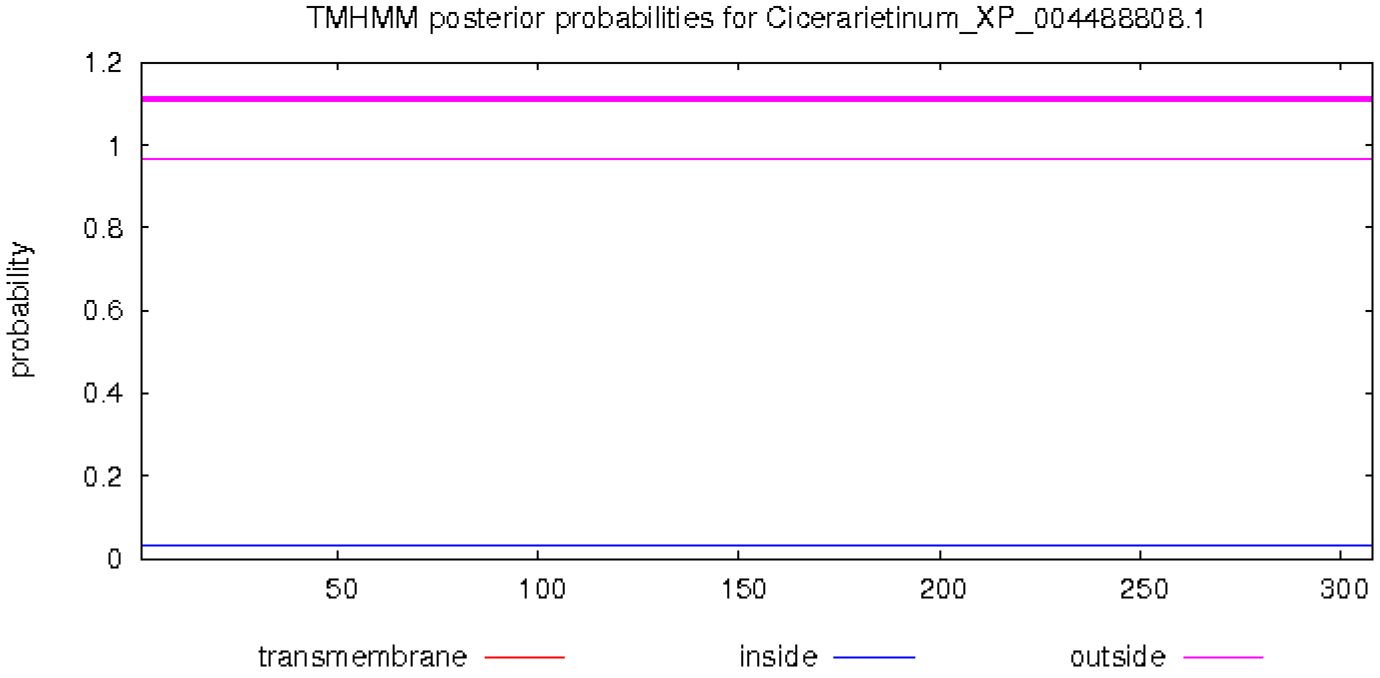

plot in postscript, script for making the plot in gnuplot, data for plot

---

```
# Cicerarietinum_XP_004488843.1 Length: 314
# Cicerarietinum_XP_004488843.1 Number of predicted TMHs:  0
# Cicerarietinum_XP_004488843.1 Exp number of AAs in TMHs: 0.00055
# Cicerarietinum_XP_004488843.1 Exp number, first 60 AAs:  0
# Cicerarietinum_XP_004488843.1 Total prob of N-in:        0.04061
Cicerarietinum_XP_004488843.1	TMHMM2.0	outside	     1   314
```

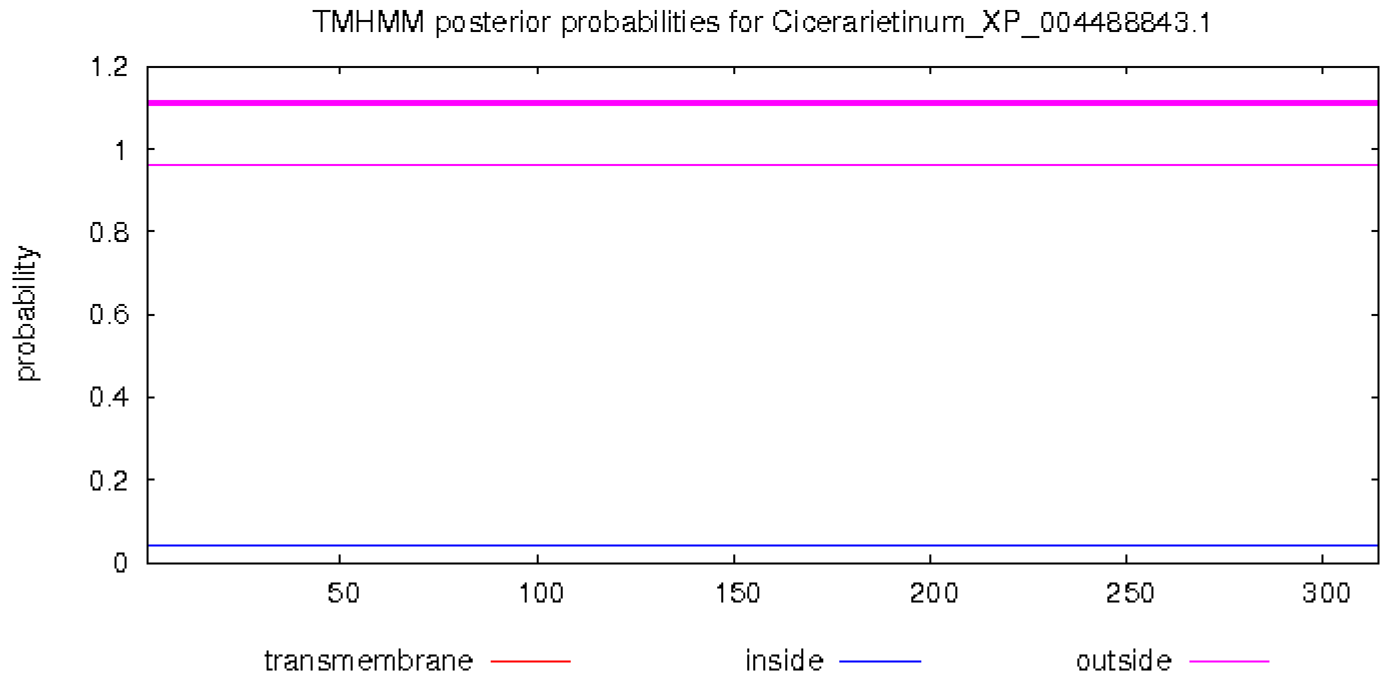

plot in postscript, script for making the plot in gnuplot, data for plot

---

```
# Cicerarietinum_XP_004489410.1 Length: 347
# Cicerarietinum_XP_004489410.1 Number of predicted TMHs:  0
# Cicerarietinum_XP_004489410.1 Exp number of AAs in TMHs: 0.00211
# Cicerarietinum_XP_004489410.1 Exp number, first 60 AAs:  0.00211
# Cicerarietinum_XP_004489410.1 Total prob of N-in:        0.06214
Cicerarietinum_XP_004489410.1	TMHMM2.0	outside	     1   347
```

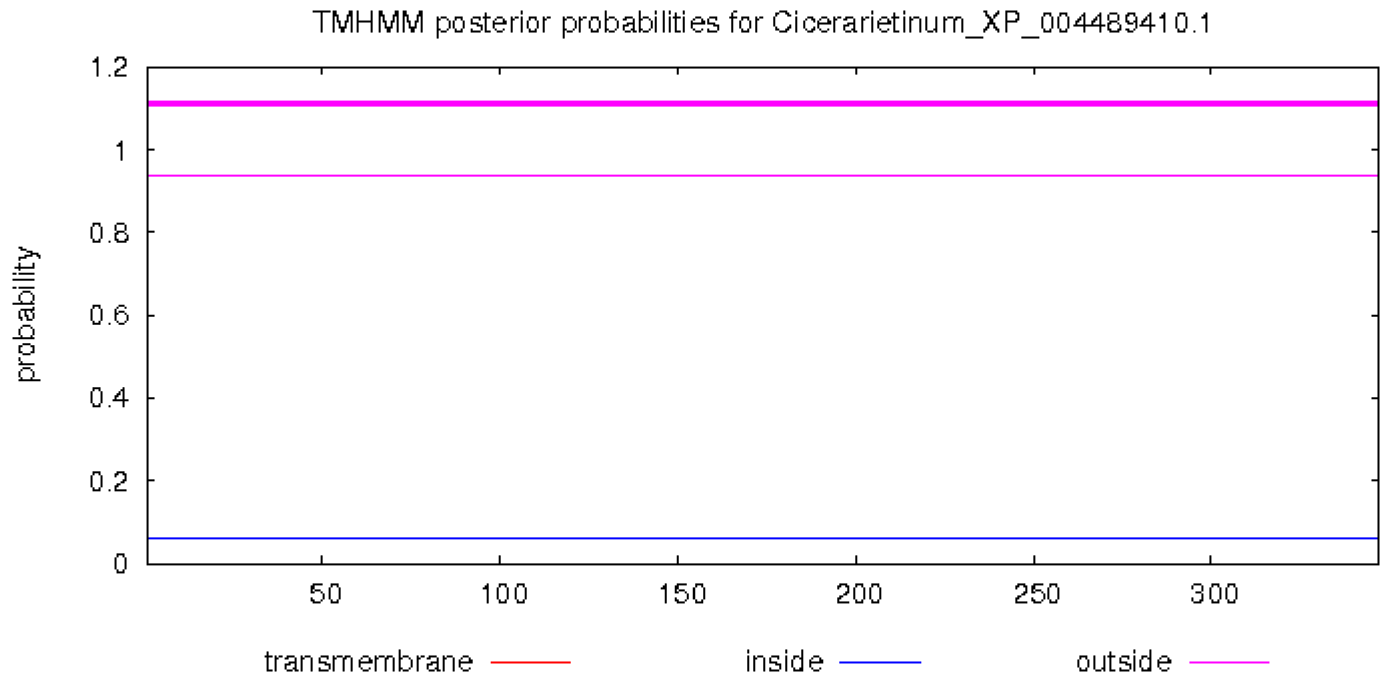

plot in postscript, script for making the plot in gnuplot, data for plot

---

```
# Cicerarietinum_XP_004489492.1 Length: 390
# Cicerarietinum_XP_004489492.1 Number of predicted TMHs:  0
# Cicerarietinum_XP_004489492.1 Exp number of AAs in TMHs: 0
# Cicerarietinum_XP_004489492.1 Exp number, first 60 AAs:  0
# Cicerarietinum_XP_004489492.1 Total prob of N-in:        0.03758
Cicerarietinum_XP_004489492.1   TMHMM2.0        outside      1   390
```

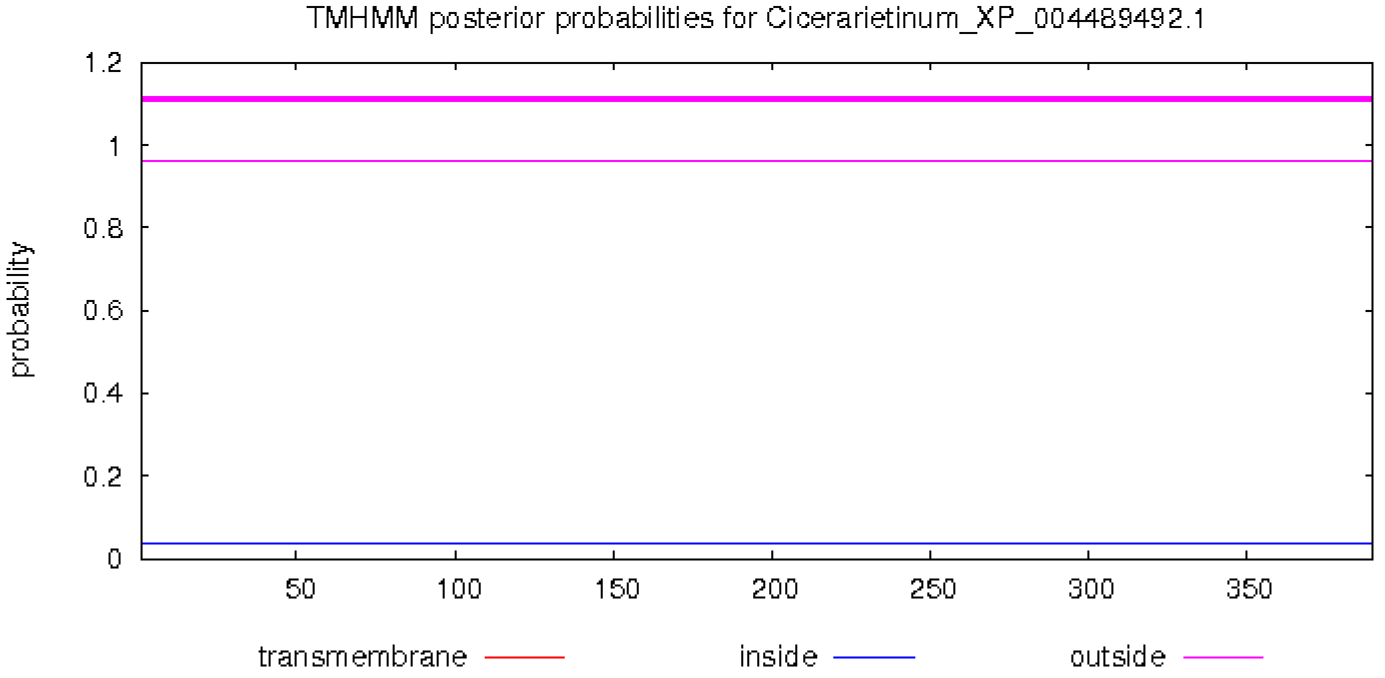

plot in postscript, script for making the plot in gnuplot, data for plot

---

```
# Cicerarietinum_XP_004489720.1 Length: 365
# Cicerarietinum_XP_004489720.1 Number of predicted TMHs:  0
# Cicerarietinum_XP_004489720.1 Exp number of AAs in TMHs: 0.00043
# Cicerarietinum_XP_004489720.1 Exp number, first 60 AAs:  0.00043
# Cicerarietinum_XP_004489720.1 Total prob of N-in:        0.00751
Cicerarietinum_XP_004489720.1   TMHMM2.0        outside      1   365
```

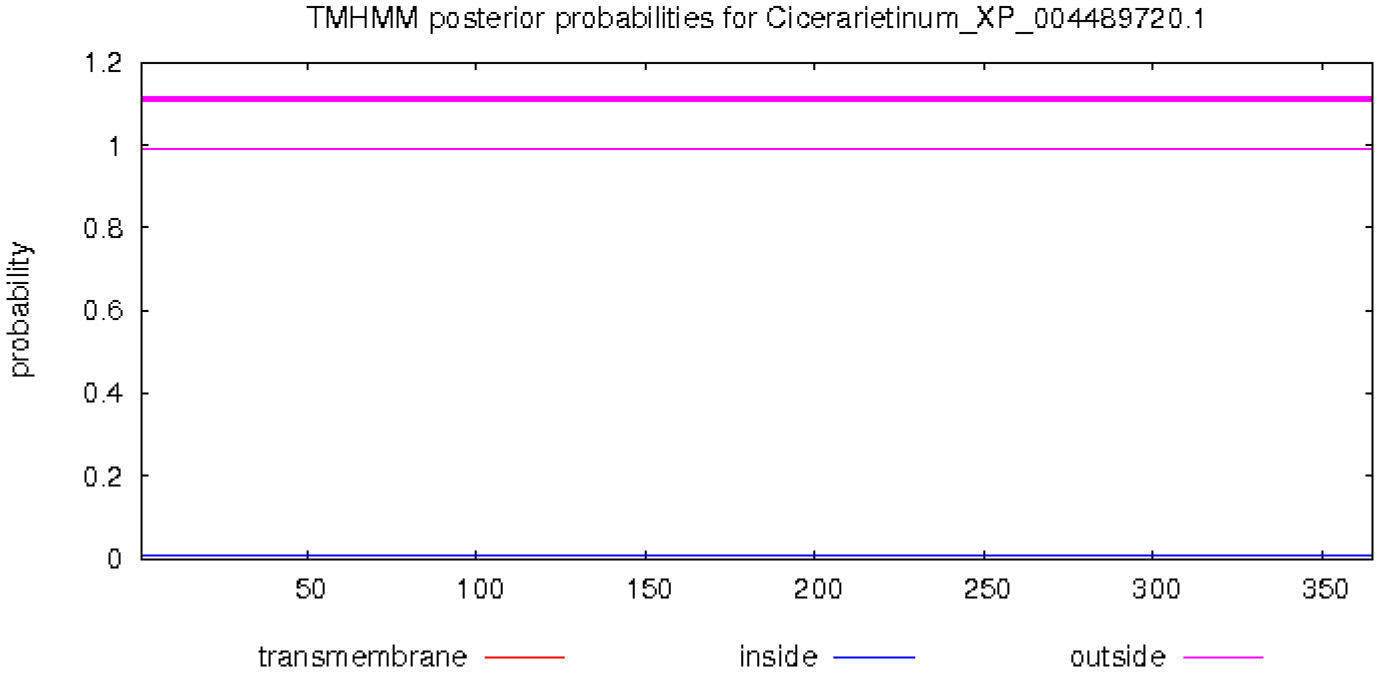

plot in postscript, script for making the plot in gnuplot, data for plot

---

```
# Cicerarietinum_XP_004490046.1 Length: 406
# Cicerarietinum_XP_004490046.1 Number of predicted TMHs:  0
# Cicerarietinum_XP_004490046.1 Exp number of AAs in TMHs: 0.00051
# Cicerarietinum_XP_004490046.1 Exp number, first 60 AAs:  0
# Cicerarietinum_XP_004490046.1 Total prob of N-in:        0.01155
Cicerarietinum_XP_004490046.1   TMHMM2.0        outside      1   406
```

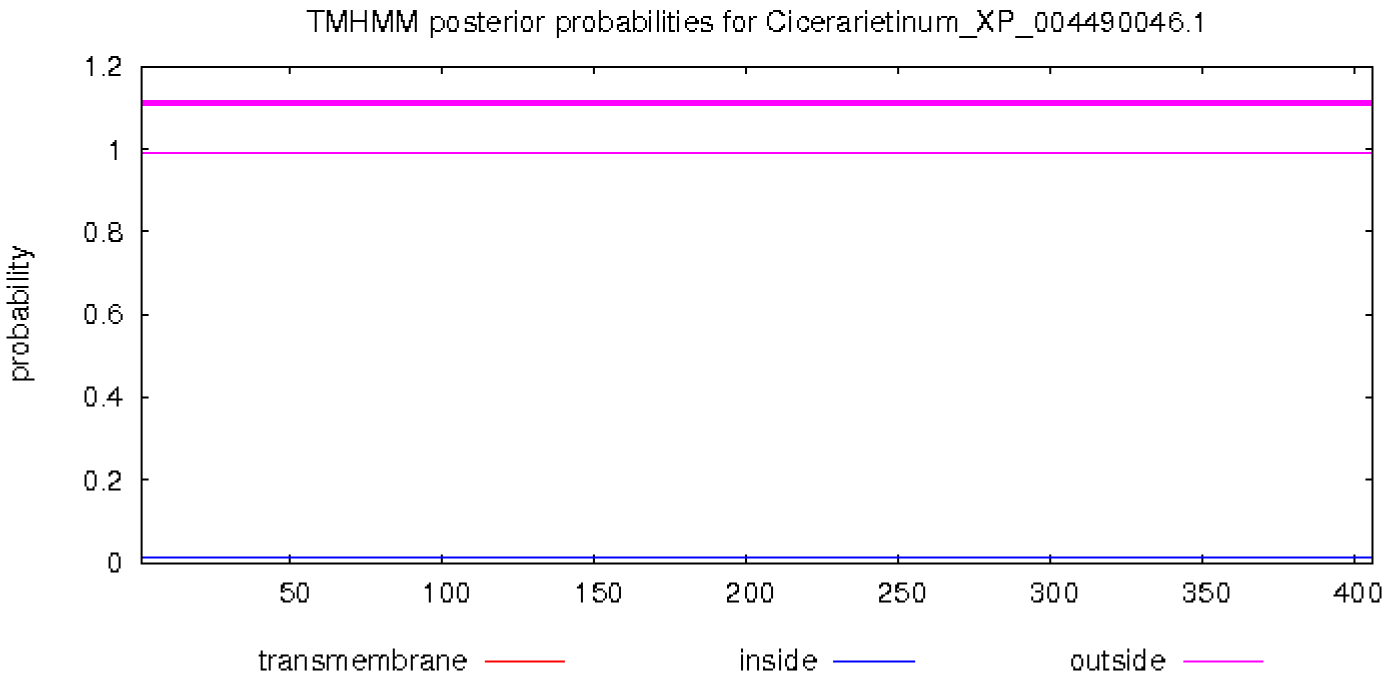

plot in postscript, script for making the plot in gnuplot, data for plot

---

```
# Cicerarietinum_XP_004490093.1 Length: 285
# Cicerarietinum_XP_004490093.1 Number of predicted TMHs:  0
# Cicerarietinum_XP_004490093.1 Exp number of AAs in TMHs: 0.00059
# Cicerarietinum_XP_004490093.1 Exp number, first 60 AAs:  0.00041
# Cicerarietinum_XP_004490093.1 Total prob of N-in:        0.02541
Cicerarietinum_XP_004490093.1   TMHMM2.0        outside      1   285
```

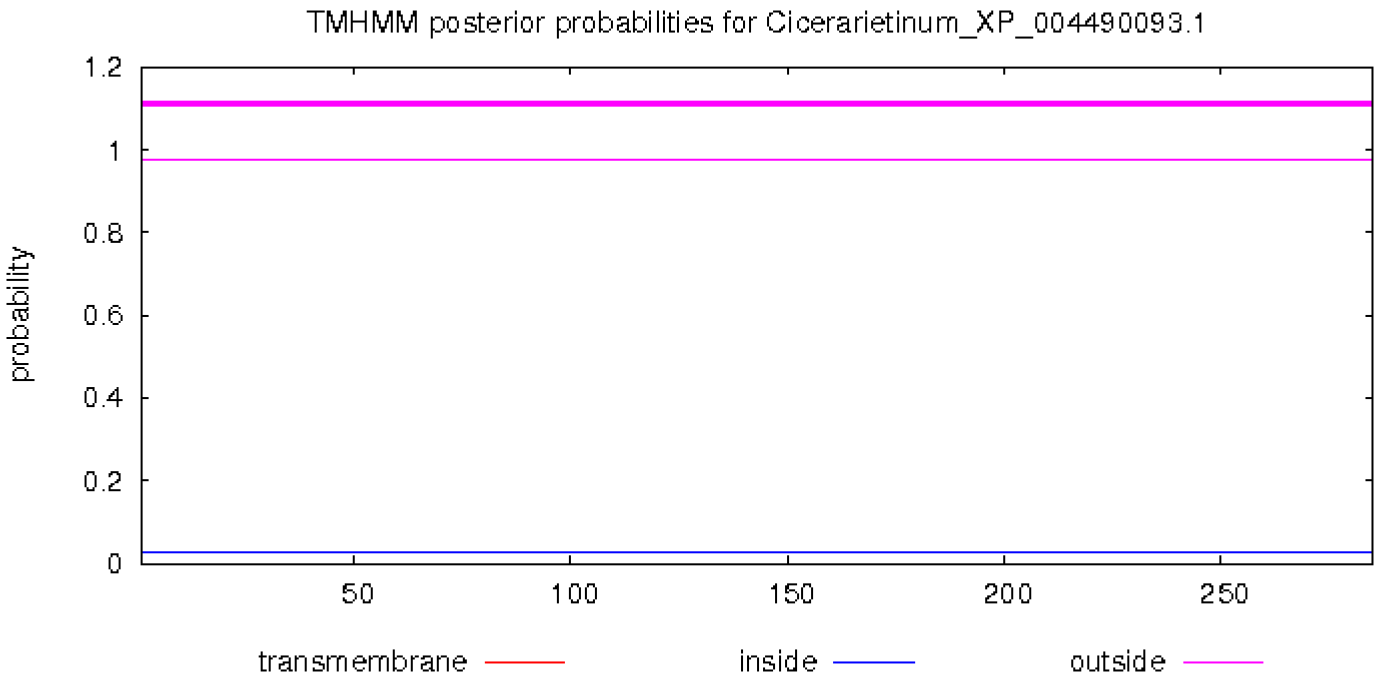

plot in postscript, script for making the plot in gnuplot, data for plot

---

```
# Cicerarietinum_XP_004490175.1 Length: 370
# Cicerarietinum_XP_004490175.1 Number of predicted TMHs:  0
# Cicerarietinum_XP_004490175.1 Exp number of AAs in TMHs: 0
# Cicerarietinum_XP_004490175.1 Exp number, first 60 AAs:  0
# Cicerarietinum_XP_004490175.1 Total prob of N-in:        0.01094
Cicerarietinum_XP_004490175.1   TMHMM2.0        outside      1   370
```

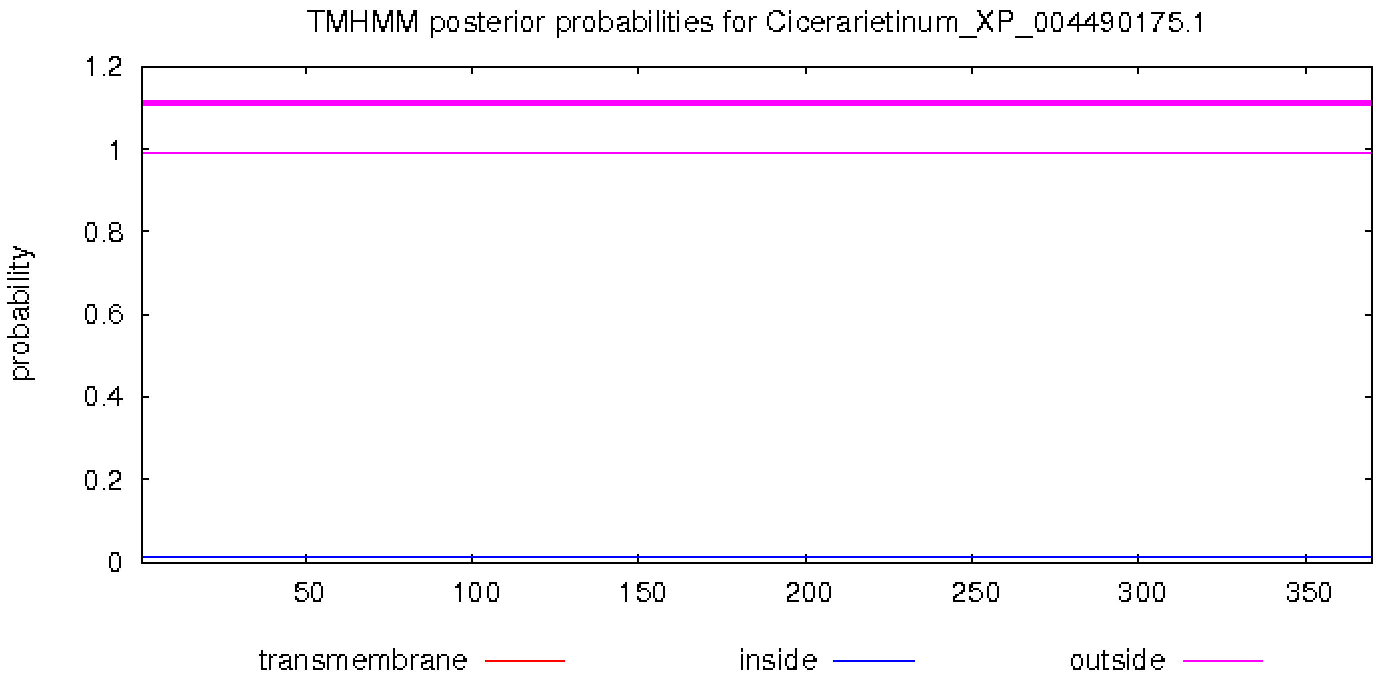

plot in postscript, script for making the plot in gnuplot, data for plot

```
# Cicerarietinum_XP_004490275.1 Length: 426
# Cicerarietinum_XP_004490275.1 Number of predicted TMHs:  0
# Cicerarietinum_XP_004490275.1 Exp number of AAs in TMHs: 0.0006
# Cicerarietinum_XP_004490275.1 Exp number, first 60 AAs:  0
# Cicerarietinum_XP_004490275.1 Total prob of N-in:        0.00293
Cicerarietinum_XP_004490275.1   TMHMM2.0        outside      1   426
```

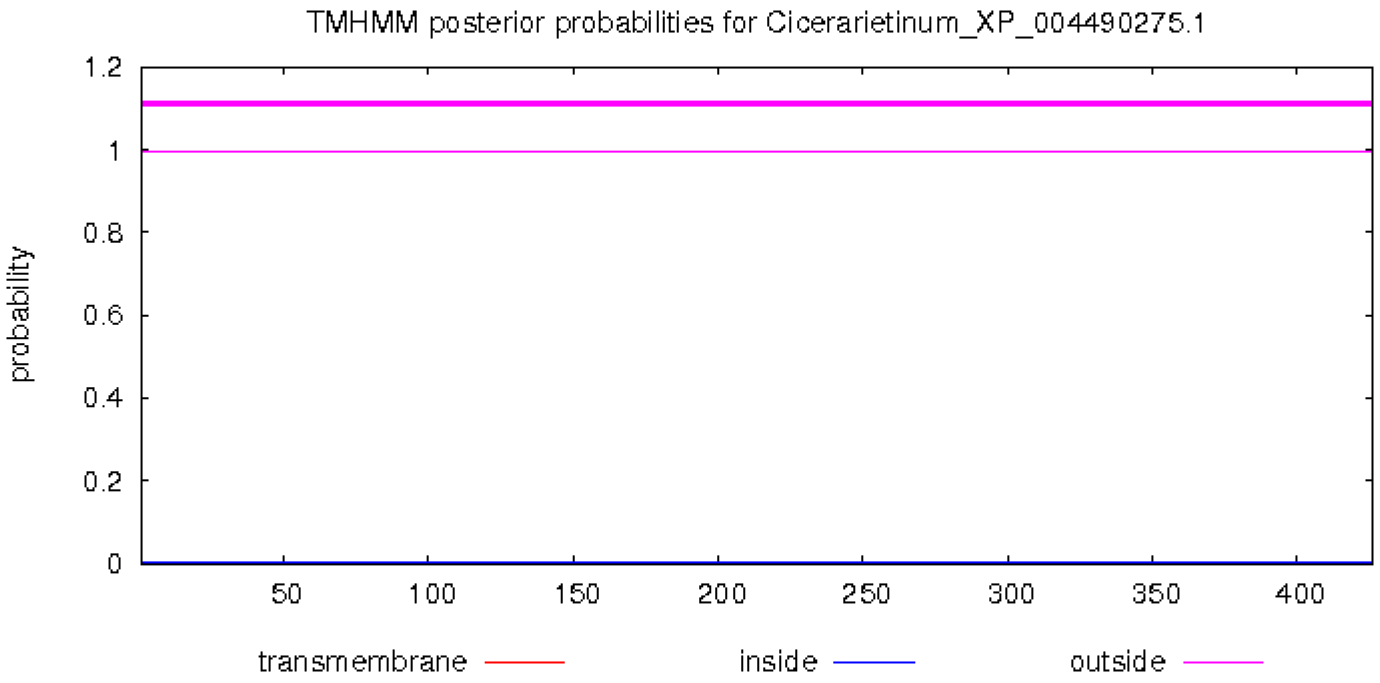

plot in postscript, script for making the plot in gnuplot, data for plot

```
# Cicerarietinum_XP_004490776.1 Length: 624
# Cicerarietinum_XP_004490776.1 Number of predicted TMHs:  2
# Cicerarietinum_XP_004490776.1 Exp number of AAs in TMHs: 38.70603
# Cicerarietinum_XP_004490776.1 Exp number, first 60 AAs:  0
# Cicerarietinum_XP_004490776.1 Total prob of N-in:        0.00016
Cicerarietinum_XP_004490776.1   TMHMM2.0        outside      1   534
Cicerarietinum_XP_004490776.1   TMHMM2.0        TMhelix    535   552
Cicerarietinum_XP_004490776.1   TMHMM2.0        inside     553   599
Cicerarietinum_XP_004490776.1   TMHMM2.0        TMhelix    600   622
Cicerarietinum_XP_004490776.1   TMHMM2.0        outside    623   624
```

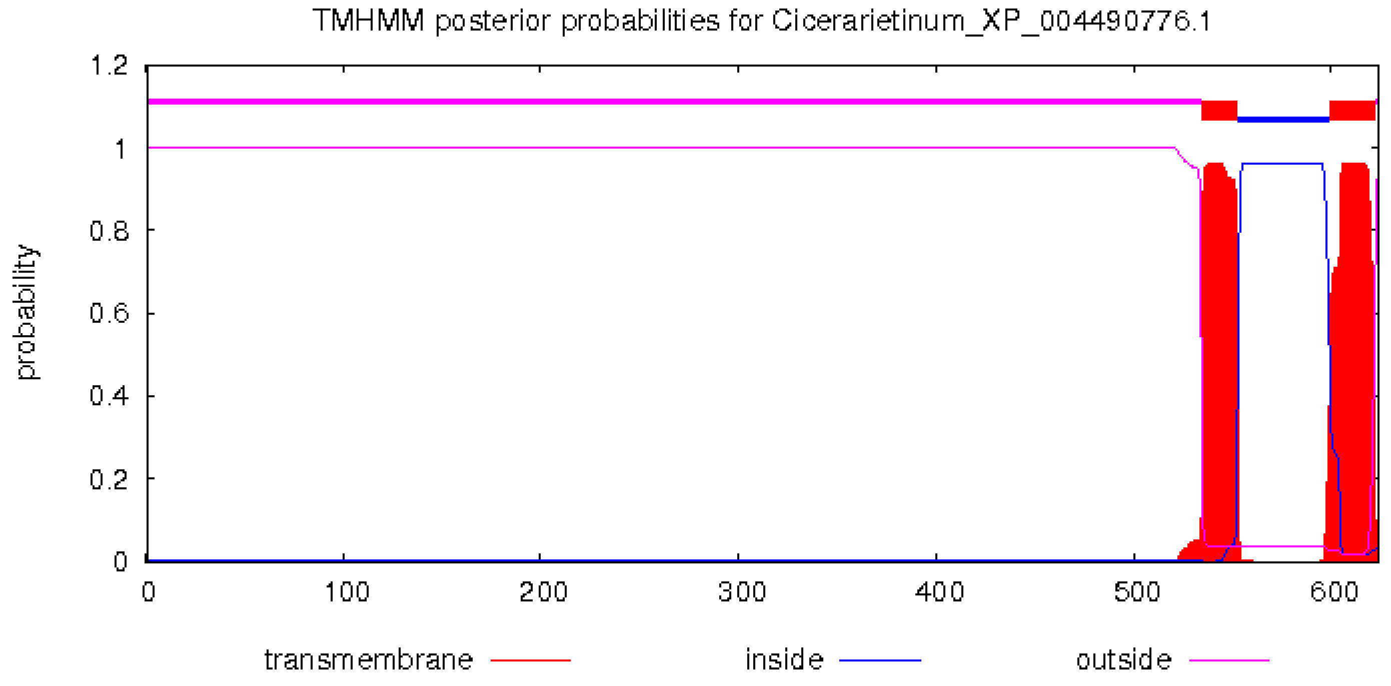

plot in postscript, script for making the plot in gnuplot, data for plot

---

```
# Cicerarietinum_XP_004491313.1 Length: 298
# Cicerarietinum_XP_004491313.1 Number of predicted TMHs:  0
# Cicerarietinum_XP_004491313.1 Exp number of AAs in TMHs: 0
# Cicerarietinum_XP_004491313.1 Exp number, first 60 AAs:  0
# Cicerarietinum_XP_004491313.1 Total prob of N-in:        0.05967
Cicerarietinum_XP_004491313.1   TMHMM2.0        outside      1   298
```

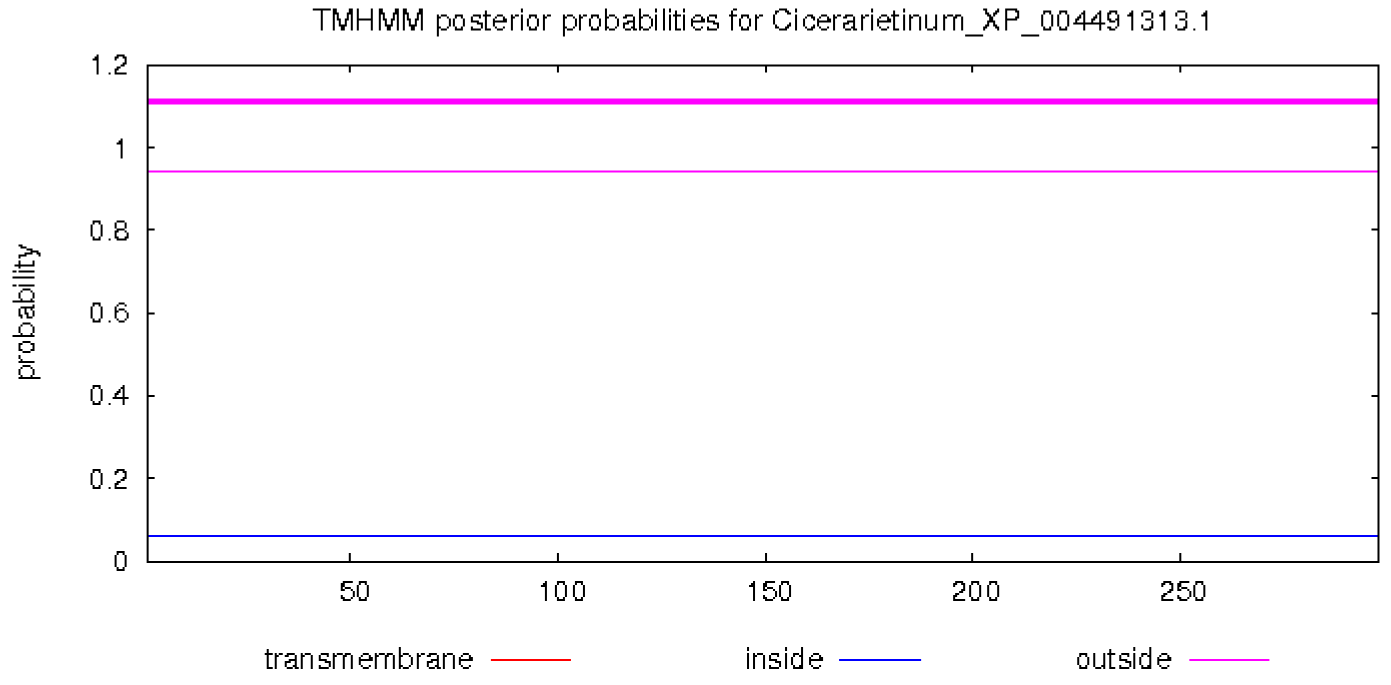

plot in postscript, script for making the plot in gnuplot, data for plot

```
# Cicerarietinum_XP_004491830.1 Length: 363
# Cicerarietinum_XP_004491830.1 Number of predicted TMHs:  0
# Cicerarietinum_XP_004491830.1 Exp number of AAs in TMHs: 0
# Cicerarietinum_XP_004491830.1 Exp number, first 60 AAs:  0
# Cicerarietinum_XP_004491830.1 Total prob of N-in:        0.00527
Cicerarietinum_XP_004491830.1   TMHMM2.0        outside      1   363
```

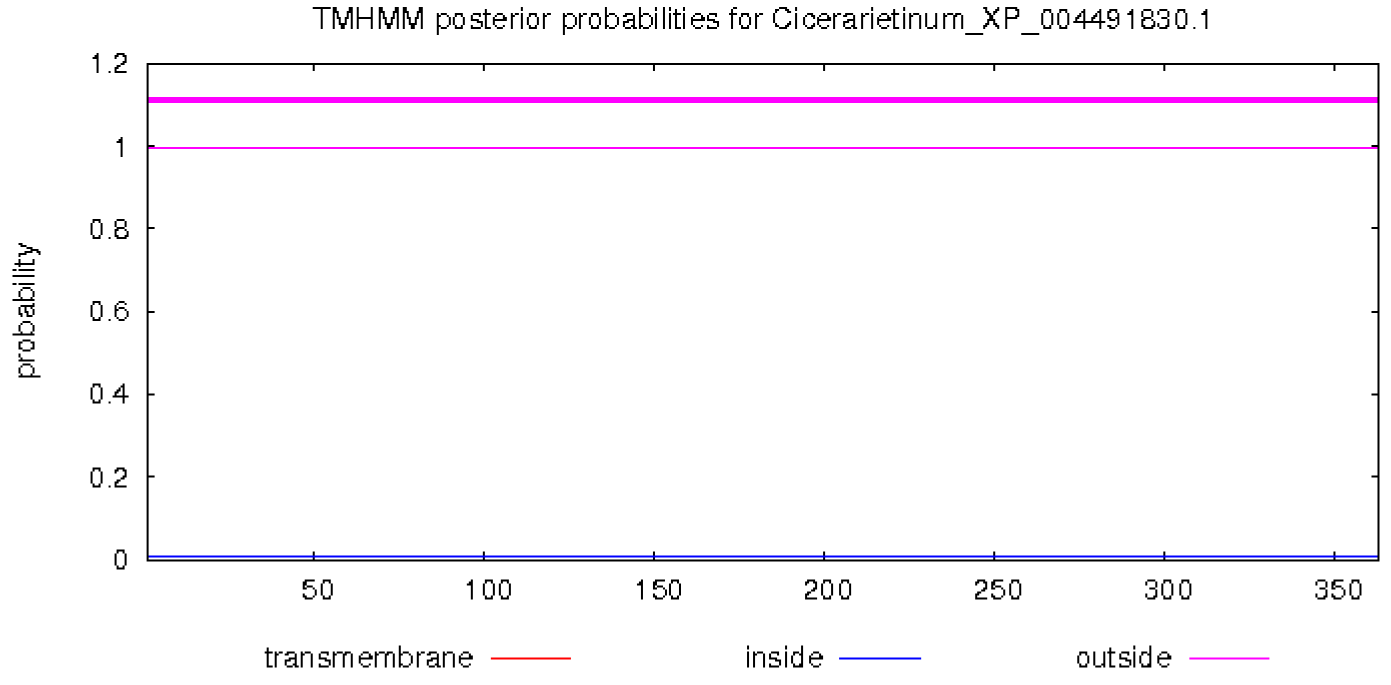

plot in postscript, script for making the plot in gnuplot, data for plot

```
# Cicerarietinum_XP_004491831.1 Length: 363
# Cicerarietinum_XP_004491831.1 Number of predicted TMHs:  0
# Cicerarietinum_XP_004491831.1 Exp number of AAs in TMHs: 0
# Cicerarietinum_XP_004491831.1 Exp number, first 60 AAs:  0
# Cicerarietinum_XP_004491831.1 Total prob of N-in:        0.00527
Cicerarietinum_XP_004491831.1   TMHMM2.0        outside      1   363
```

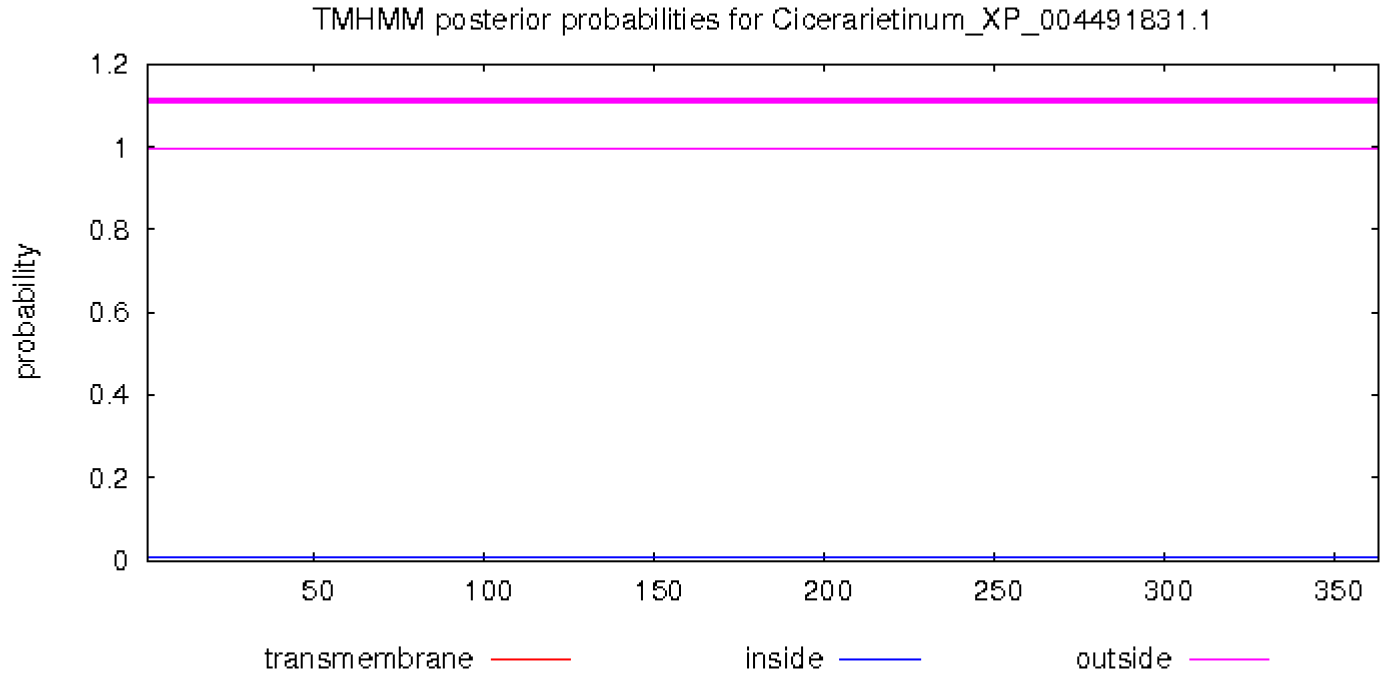

plot in postscript, script for making the plot in gnuplot, data for plot

```
# Cicerarietinum_XP_004492996.1 Length: 197
# Cicerarietinum_XP_004492996.1 Number of predicted TMHs:  0
# Cicerarietinum_XP_004492996.1 Exp number of AAs in TMHs: 0.10058
# Cicerarietinum_XP_004492996.1 Exp number, first 60 AAs:  0.00122
# Cicerarietinum_XP_004492996.1 Total prob of N-in:        0.01191
Cicerarietinum_XP_004492996.1	TMHMM2.0	outside	     1   197
```

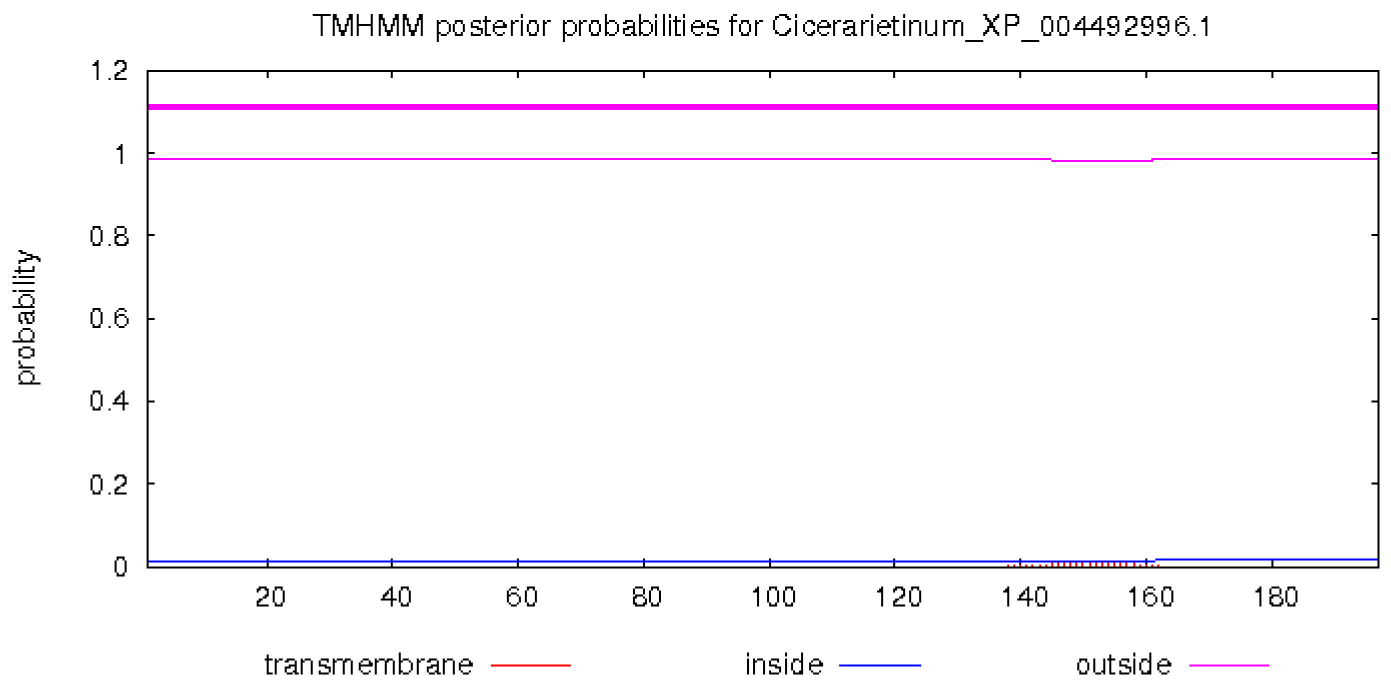

plot in postscript, script for making the plot in gnuplot, data for plot

```
# Cicerarietinum_XP_004493136.1 Length: 335
# Cicerarietinum_XP_004493136.1 Number of predicted TMHs:  0
# Cicerarietinum_XP_004493136.1 Exp number of AAs in TMHs: 0.00085
# Cicerarietinum_XP_004493136.1 Exp number, first 60 AAs:  0.00085
# Cicerarietinum_XP_004493136.1 Total prob of N-in:        0.00368
Cicerarietinum_XP_004493136.1	TMHMM2.0	outside	     1   335
```

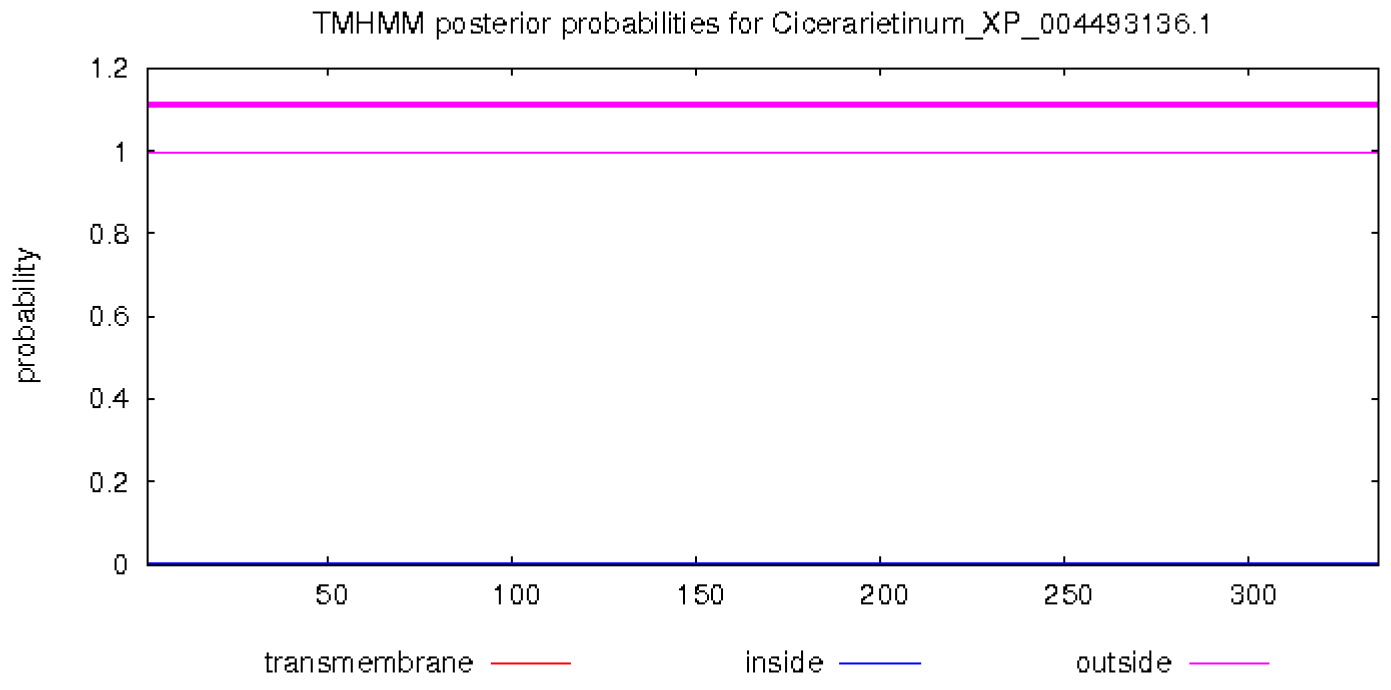

plot in postscript, script for making the plot in gnuplot, data for plot

```
# Cicerarietinum_XP_004493138.1 Length: 388
# Cicerarietinum_XP_004493138.1 Number of predicted TMHs:  0
# Cicerarietinum_XP_004493138.1 Exp number of AAs in TMHs: 0
# Cicerarietinum_XP_004493138.1 Exp number, first 60 AAs:  0
# Cicerarietinum_XP_004493138.1 Total prob of N-in:        0.00584
Cicerarietinum_XP_004493138.1   TMHMM2.0        outside      1   388
```

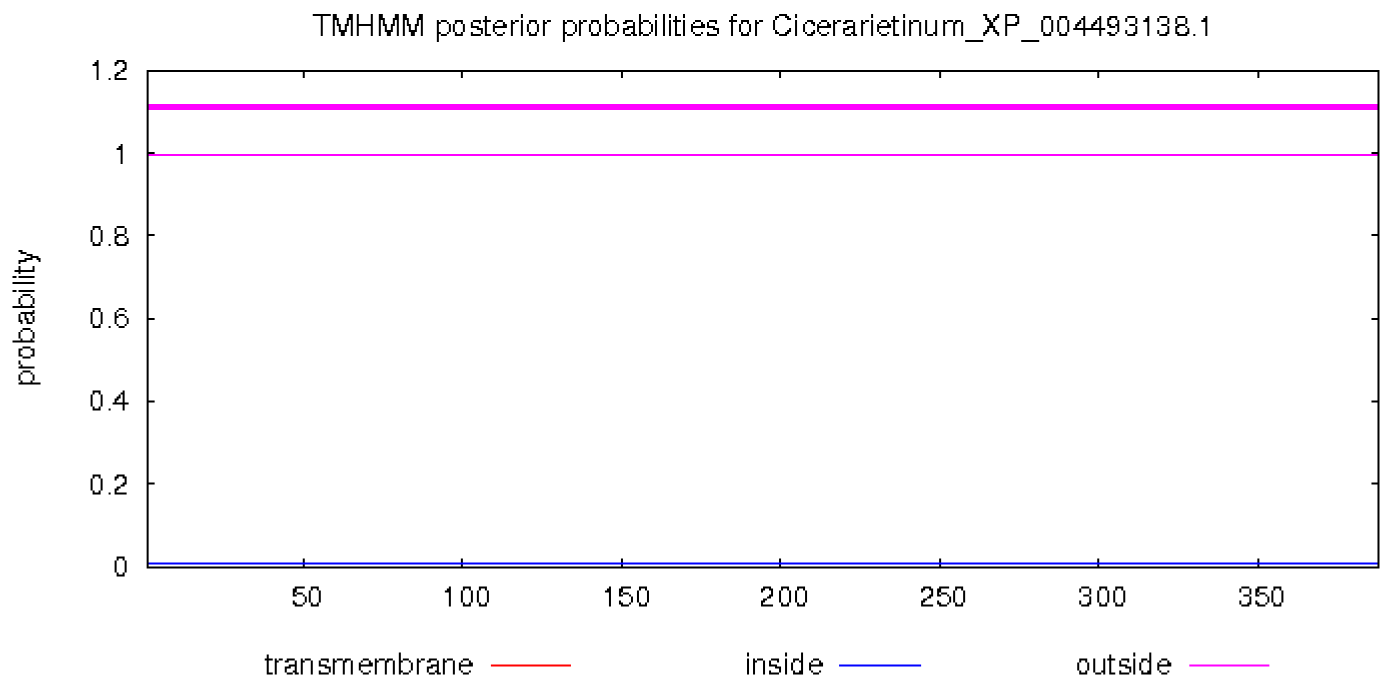

plot in postscript, script for making the plot in gnuplot, data for plot

```
# Cicerarietinum_XP_004493788.1 Length: 291
# Cicerarietinum_XP_004493788.1 Number of predicted TMHs:  0
# Cicerarietinum_XP_004493788.1 Exp number of AAs in TMHs: 0
# Cicerarietinum_XP_004493788.1 Exp number, first 60 AAs:  0
# Cicerarietinum_XP_004493788.1 Total prob of N-in:        0.00891
Cicerarietinum_XP_004493788.1   TMHMM2.0        outside      1   291
```

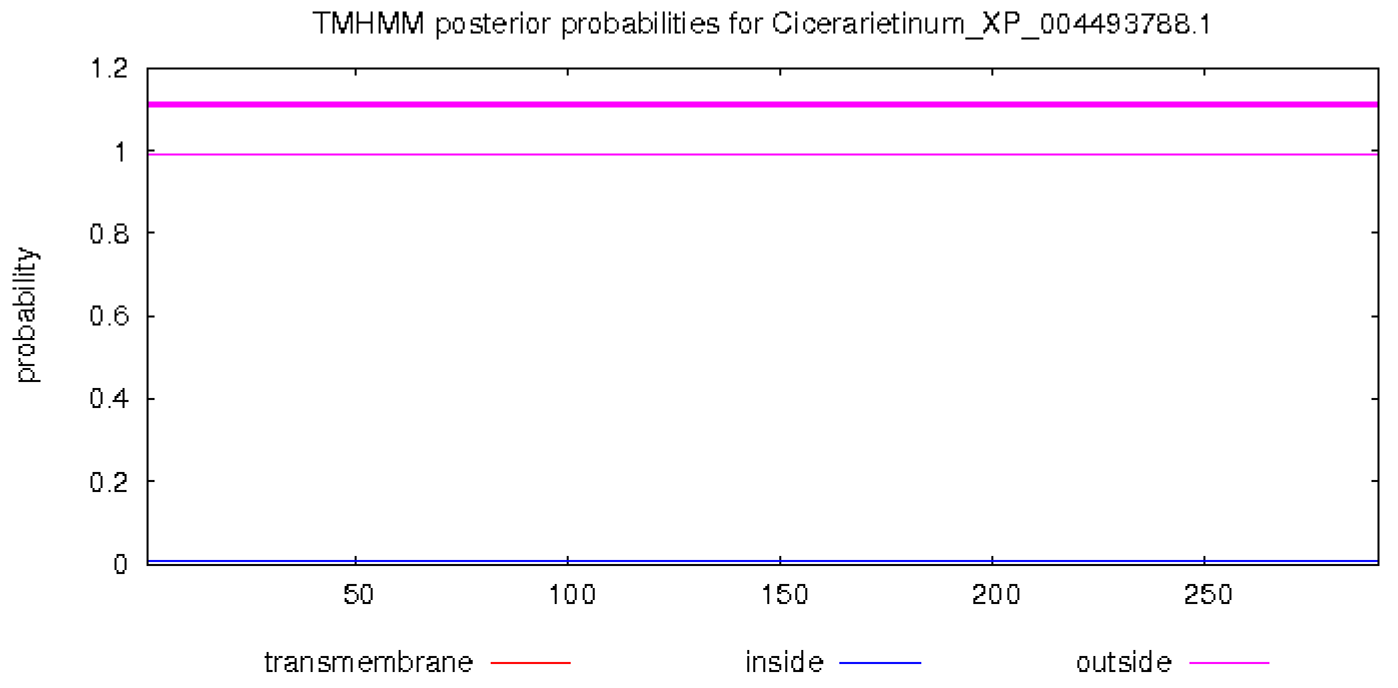

plot in postscript, script for making the plot in gnuplot, data for plot

---

```
# Cicerarietinum_XP_004493961.2 Length: 365
# Cicerarietinum_XP_004493961.2 Number of predicted TMHs:  0
# Cicerarietinum_XP_004493961.2 Exp number of AAs in TMHs: 0.834750000000000001
# Cicerarietinum_XP_004493961.2 Exp number, first 60 AAs:  0.83089
# Cicerarietinum_XP_004493961.2 Total prob of N-in:        0.08940
Cicerarietinum_XP_004493961.2	TMHMM2.0	outside	     1   365
```

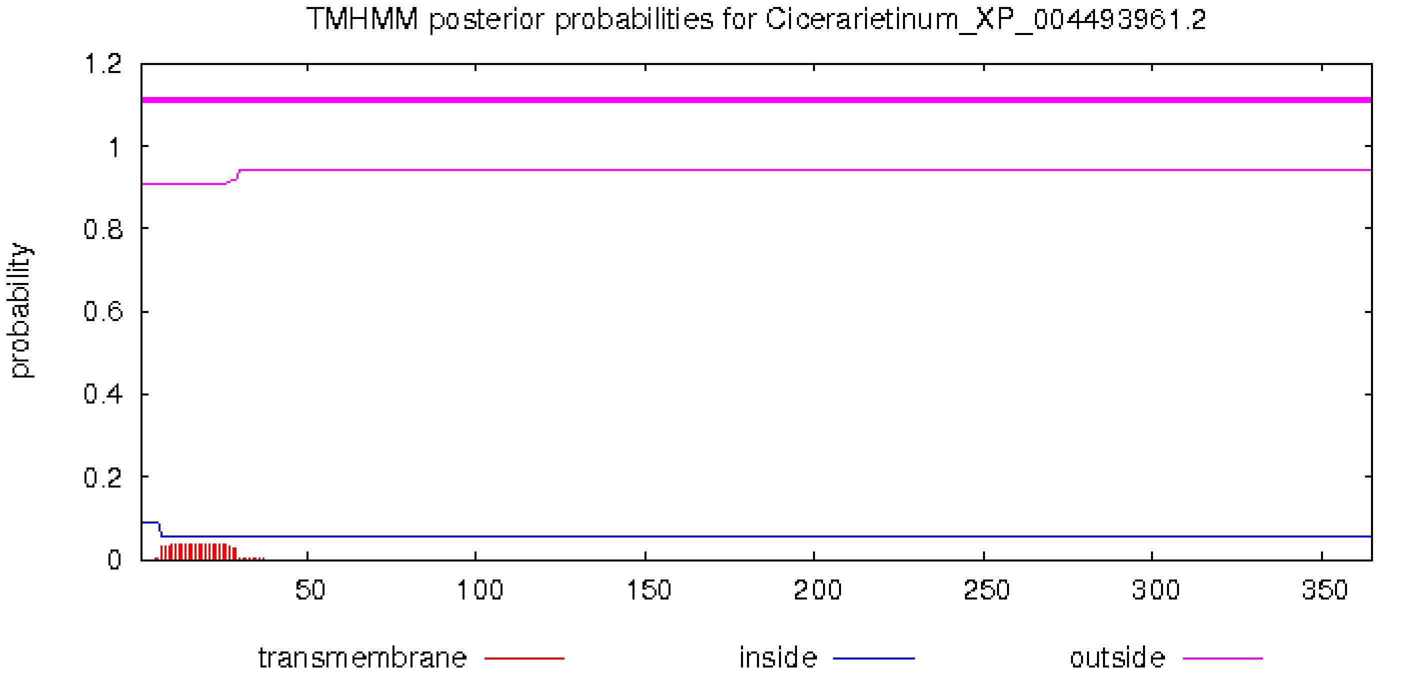

plot in postscript, script for making the plot in gnuplot, data for plot

---

```
# Cicerarietinum_XP_004494211.1 Length: 250
# Cicerarietinum_XP_004494211.1 Number of predicted TMHs:  0
# Cicerarietinum_XP_004494211.1 Exp number of AAs in TMHs: 0.00022
# Cicerarietinum_XP_004494211.1 Exp number, first 60 AAs:  0
# Cicerarietinum_XP_004494211.1 Total prob of N-in:        0.01841
Cicerarietinum_XP_004494211.1	TMHMM2.0	outside	     1   250
```

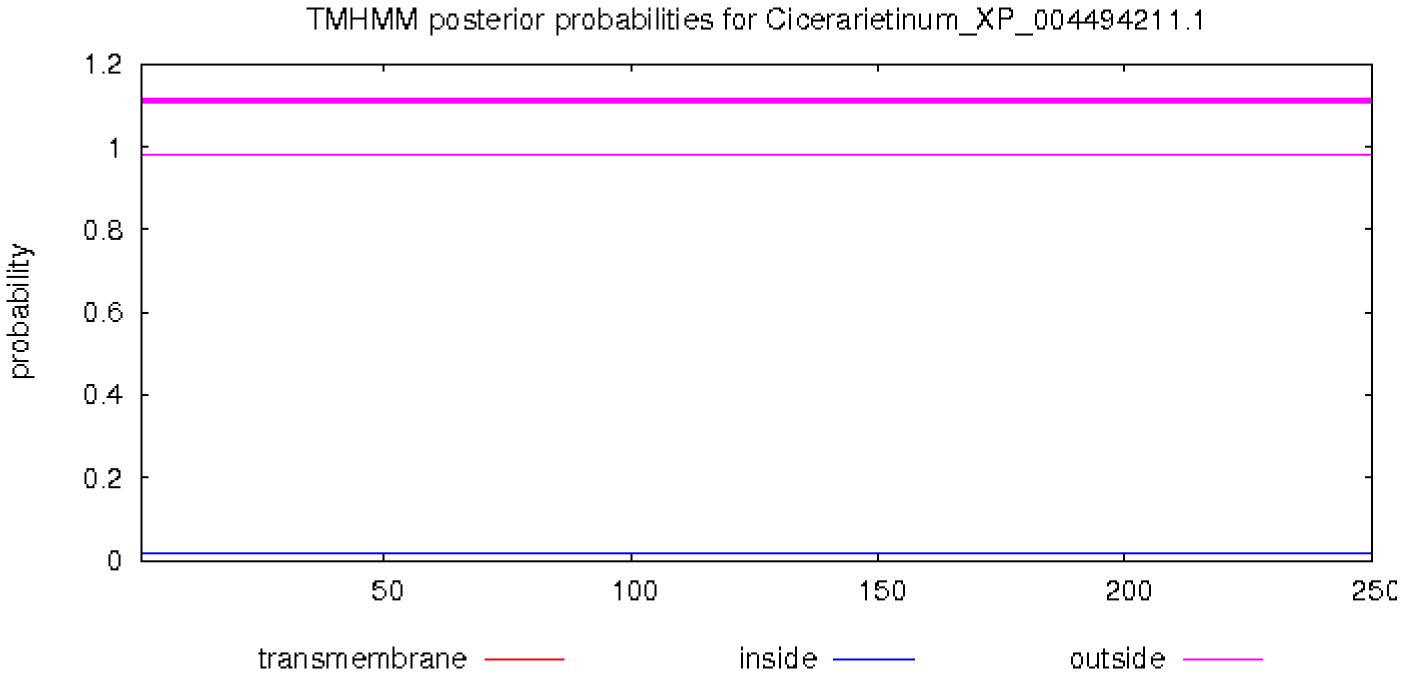

plot in postscript, script for making the plot in gnuplot, data for plot

---

```
# Cicerarietinum_XP_004496131.1 Length: 462
# Cicerarietinum_XP_004496131.1 Number of predicted TMHs:  0
# Cicerarietinum_XP_004496131.1 Exp number of AAs in TMHs: 0.00884
# Cicerarietinum_XP_004496131.1 Exp number, first 60 AAs:  0.0006
# Cicerarietinum_XP_004496131.1 Total prob of N-in:        0.00144
Cicerarietinum_XP_004496131.1   TMHMM2.0        outside      1   462
```

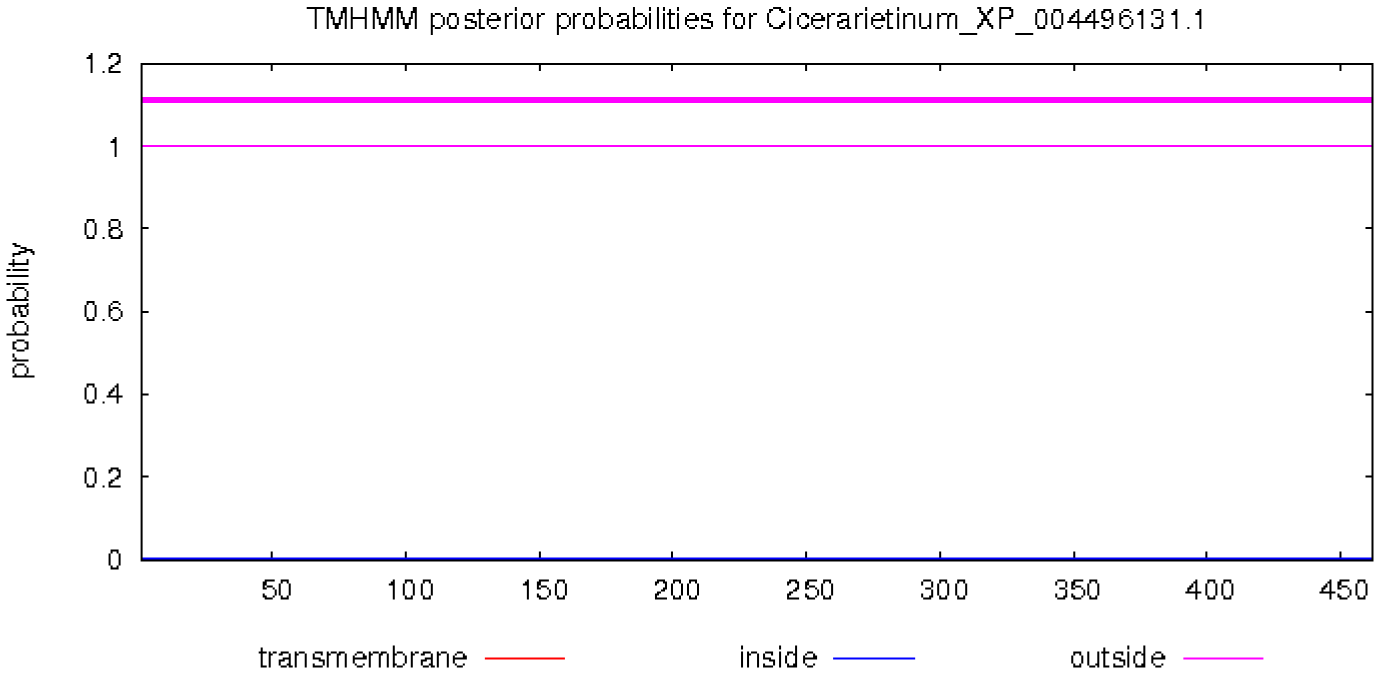

plot in postscript, script for making the plot in gnuplot, data for plot

---

```
# Cicerarietinum_XP_004496133.1 Length: 578
# Cicerarietinum_XP_004496133.1 Number of predicted TMHs:  1
# Cicerarietinum_XP_004496133.1 Exp number of AAs in TMHs: 20.90626
# Cicerarietinum_XP_004496133.1 Exp number, first 60 AAs:  0.0002
# Cicerarietinum_XP_004496133.1 Total prob of N-in:        0.00384
Cicerarietinum_XP_004496133.1   TMHMM2.0        outside      1   552
Cicerarietinum_XP_004496133.1   TMHMM2.0        TMhelix    553   575
Cicerarietinum_XP_004496133.1   TMHMM2.0        inside     576   578
```

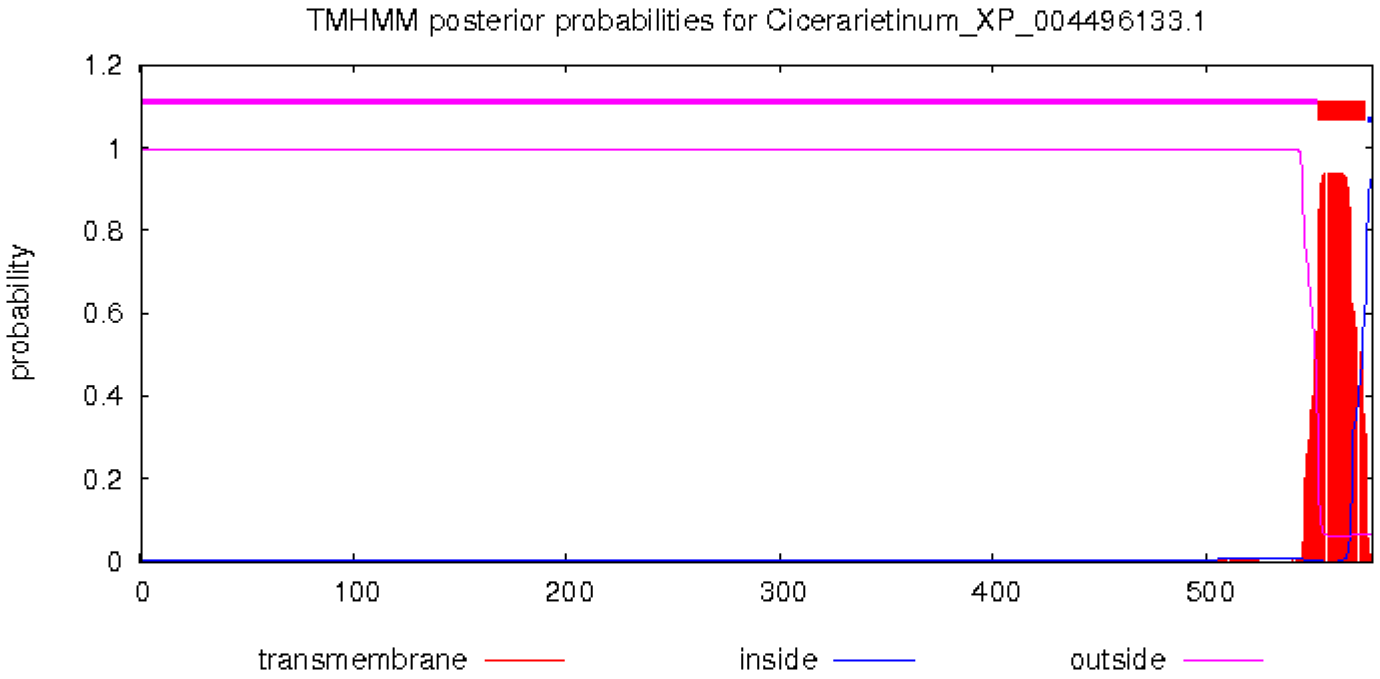

plot in postscript, script for making the plot in gnuplot, data for plot

---

```
# Cicerarietinum_XP_004496134.1 Length: 577
# Cicerarietinum_XP_004496134.1 Number of predicted TMHs:  1
# Cicerarietinum_XP_004496134.1 Exp number of AAs in TMHs: 20.90645
# Cicerarietinum_XP_004496134.1 Exp number, first 60 AAs:  0.0002
# Cicerarietinum_XP_004496134.1 Total prob of N-in:        0.00389
Cicerarietinum_XP_004496134.1   TMHMM2.0        outside      1   551
Cicerarietinum_XP_004496134.1   TMHMM2.0        TMhelix    552   574
Cicerarietinum_XP_004496134.1   TMHMM2.0        inside     575   577
```

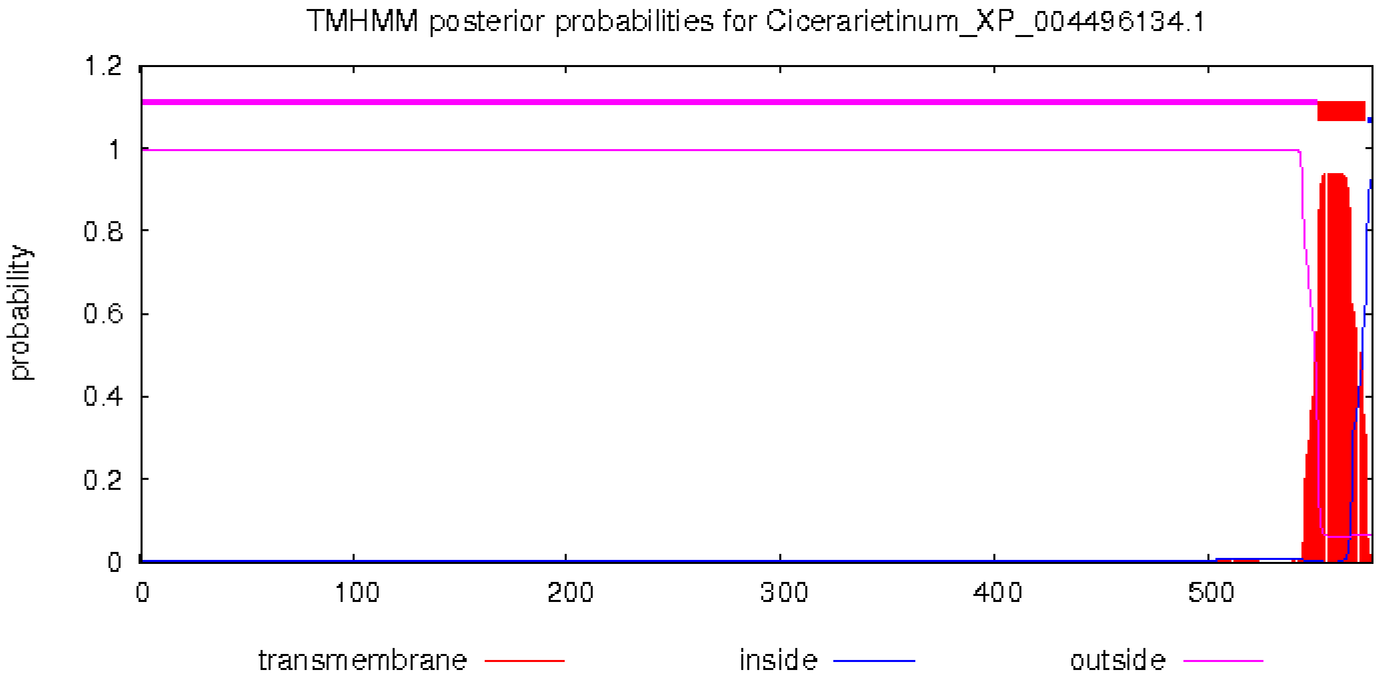

plot in postscript, script for making the plot in gnuplot, data for plot

---

```
# Cicerarietinum_XP_004496367.1 Length: 316
# Cicerarietinum_XP_004496367.1 Number of predicted TMHs:  0
# Cicerarietinum_XP_004496367.1 Exp number of AAs in TMHs: 0
# Cicerarietinum_XP_004496367.1 Exp number, first 60 AAs:  0
```

```
# Cicerarietinum_XP_004496367.1 Total prob of N-in:        0.00657
Cicerarietinum_XP_004496367.1   TMHMM2.0        outside      1   316
```

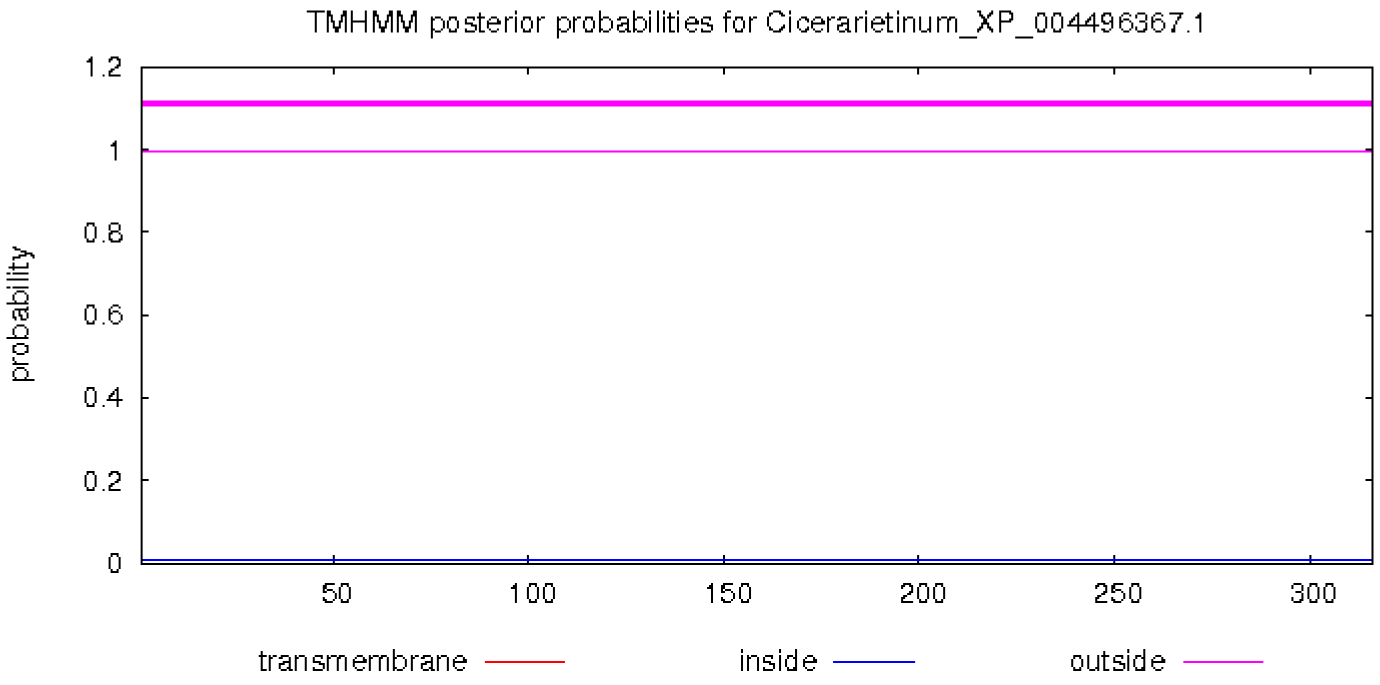

plot in postscript, script for making the plot in gnuplot, data for plot

---

```
# Cicerarietinum_XP_004496404.1 Length: 558
# Cicerarietinum_XP_004496404.1 Number of predicted TMHs:  1
# Cicerarietinum_XP_004496404.1 Exp number of AAs in TMHs: 21.93664
# Cicerarietinum_XP_004496404.1 Exp number, first 60 AAs:  0.00065
# Cicerarietinum_XP_004496404.1 Total prob of N-in:        0.03822
Cicerarietinum_XP_004496404.1   TMHMM2.0        outside      1   529
Cicerarietinum_XP_004496404.1   TMHMM2.0        TMhelix    530   552
Cicerarietinum_XP_004496404.1   TMHMM2.0        inside     553   558
```

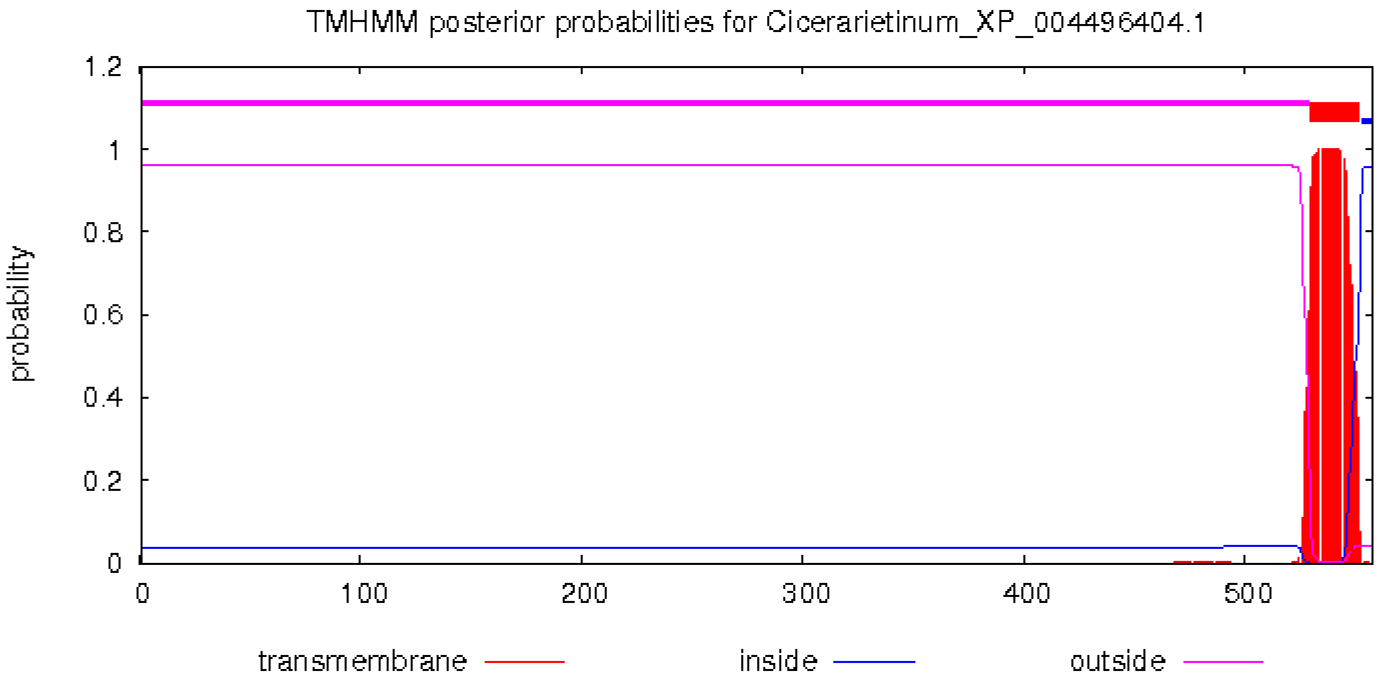

plot in postscript, script for making the plot in gnuplot, data for plot

---

```
# Cicerarietinum_XP_004498547.1 Length: 346
# Cicerarietinum_XP_004498547.1 Number of predicted TMHs:  0
```

```
# Cicerarietinum_XP_004498547.1 Exp number of AAs in TMHs: 0.0113
# Cicerarietinum_XP_004498547.1 Exp number, first 60 AAs:  0
# Cicerarietinum_XP_004498547.1 Total prob of N-in:        0.10681
Cicerarietinum_XP_004498547.1   TMHMM2.0        outside      1   346
```

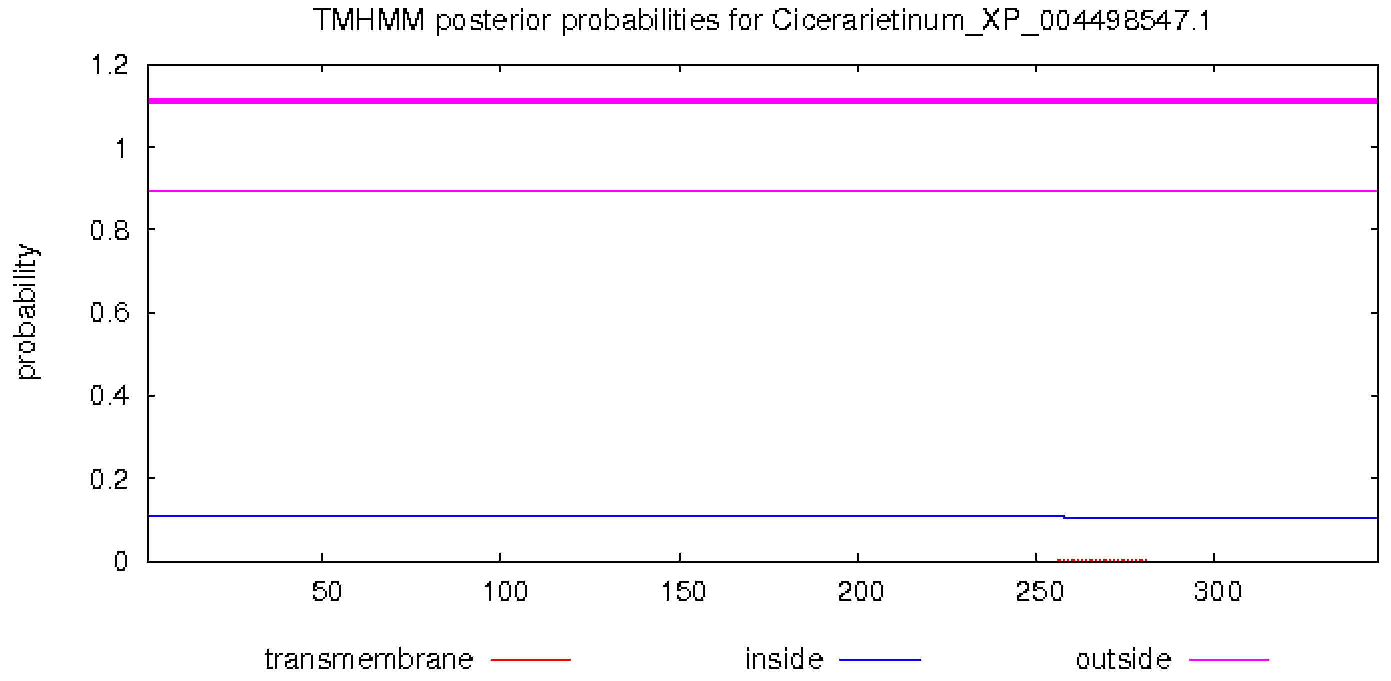

plot in postscript, script for making the plot in gnuplot, data for plot

---

```
# Cicerarietinum_XP_004499353.2 Length: 426
# Cicerarietinum_XP_004499353.2 Number of predicted TMHs:  0
# Cicerarietinum_XP_004499353.2 Exp number of AAs in TMHs: 0.03512
# Cicerarietinum_XP_004499353.2 Exp number, first 60 AAs:  0.00056
# Cicerarietinum_XP_004499353.2 Total prob of N-in:        0.01543
Cicerarietinum_XP_004499353.2   TMHMM2.0        outside      1   426
```

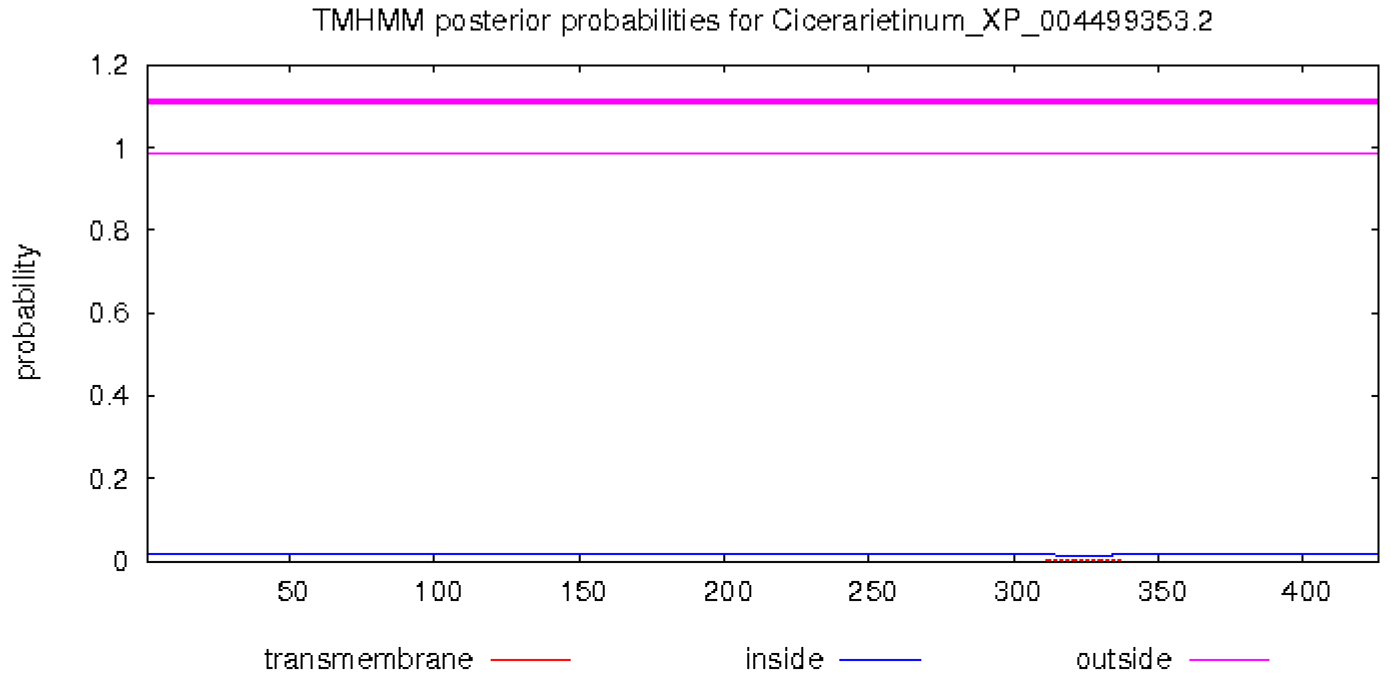

plot in postscript, script for making the plot in gnuplot, data for plot

---

```
# Cicerarietinum_XP_004499395.1 Length: 394
# Cicerarietinum_XP_004499395.1 Number of predicted TMHs:  0
```

```
# Cicerarietinum_XP_004499395.1 Exp number of AAs in TMHs: 0
# Cicerarietinum_XP_004499395.1 Exp number, first 60 AAs:  0
# Cicerarietinum_XP_004499395.1 Total prob of N-in:        0.00627
Cicerarietinum_XP_004499395.1	TMHMM2.0	outside	     1   394
```

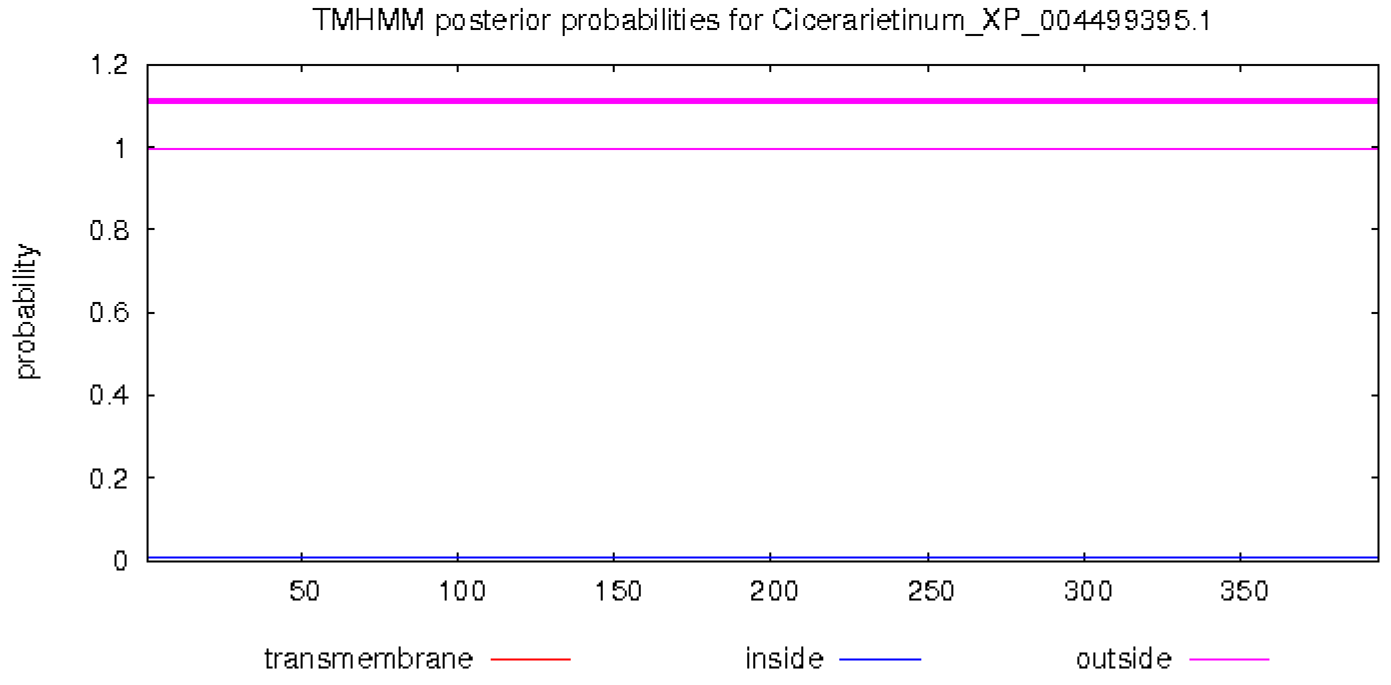

plot in postscript, script for making the plot in gnuplot, data for plot

---

```
# Cicerarietinum_XP_004500832.1 Length: 343
# Cicerarietinum_XP_004500832.1 Number of predicted TMHs:  0
# Cicerarietinum_XP_004500832.1 Exp number of AAs in TMHs: 0.01732
# Cicerarietinum_XP_004500832.1 Exp number, first 60 AAs:  0.00121
# Cicerarietinum_XP_004500832.1 Total prob of N-in:        0.00576
Cicerarietinum_XP_004500832.1	TMHMM2.0	outside	     1   343
```

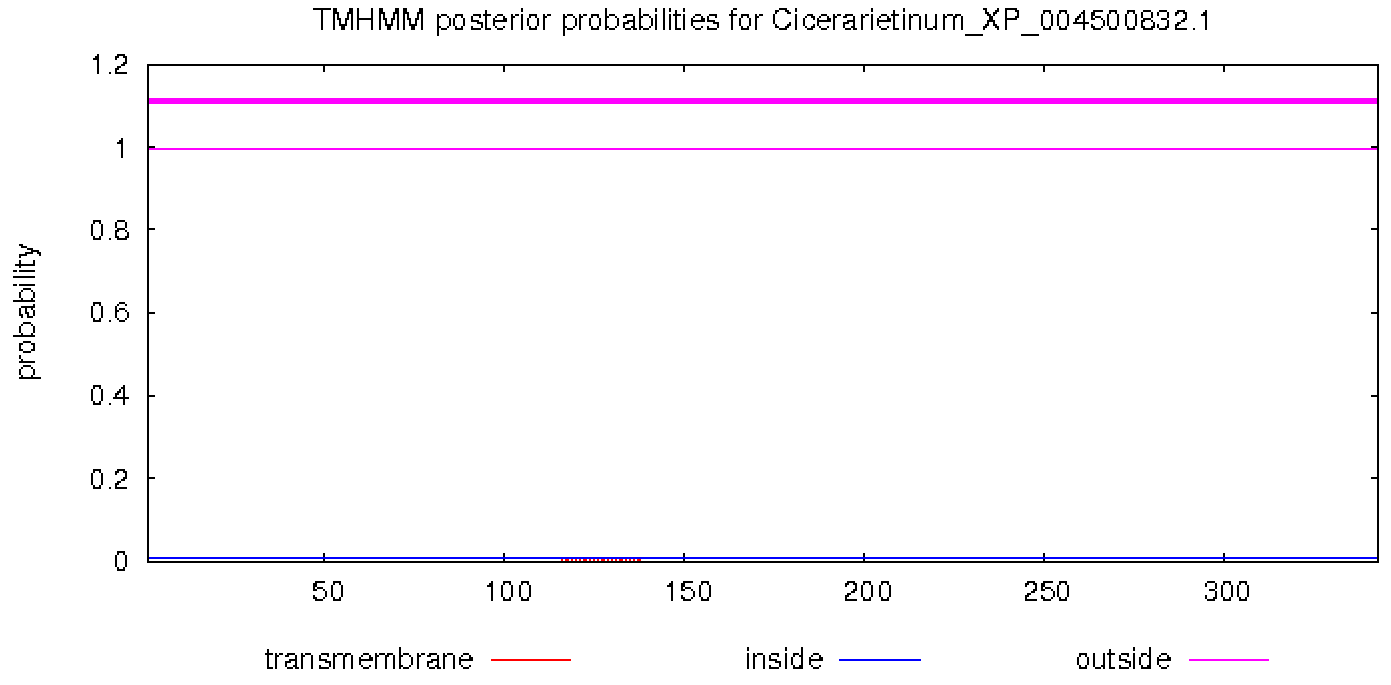

plot in postscript, script for making the plot in gnuplot, data for plot

---

```
# Cicerarietinum_XP_004501729.1 Length: 227
# Cicerarietinum_XP_004501729.1 Number of predicted TMHs:  0
```

```
# Cicerarietinum_XP_004501729.1 Exp number of AAs in TMHs: 0.00262
# Cicerarietinum_XP_004501729.1 Exp number, first 60 AAs:  0.00027
# Cicerarietinum_XP_004501729.1 Total prob of N-in:        0.09594
Cicerarietinum_XP_004501729.1   TMHMM2.0        outside      1   227
```

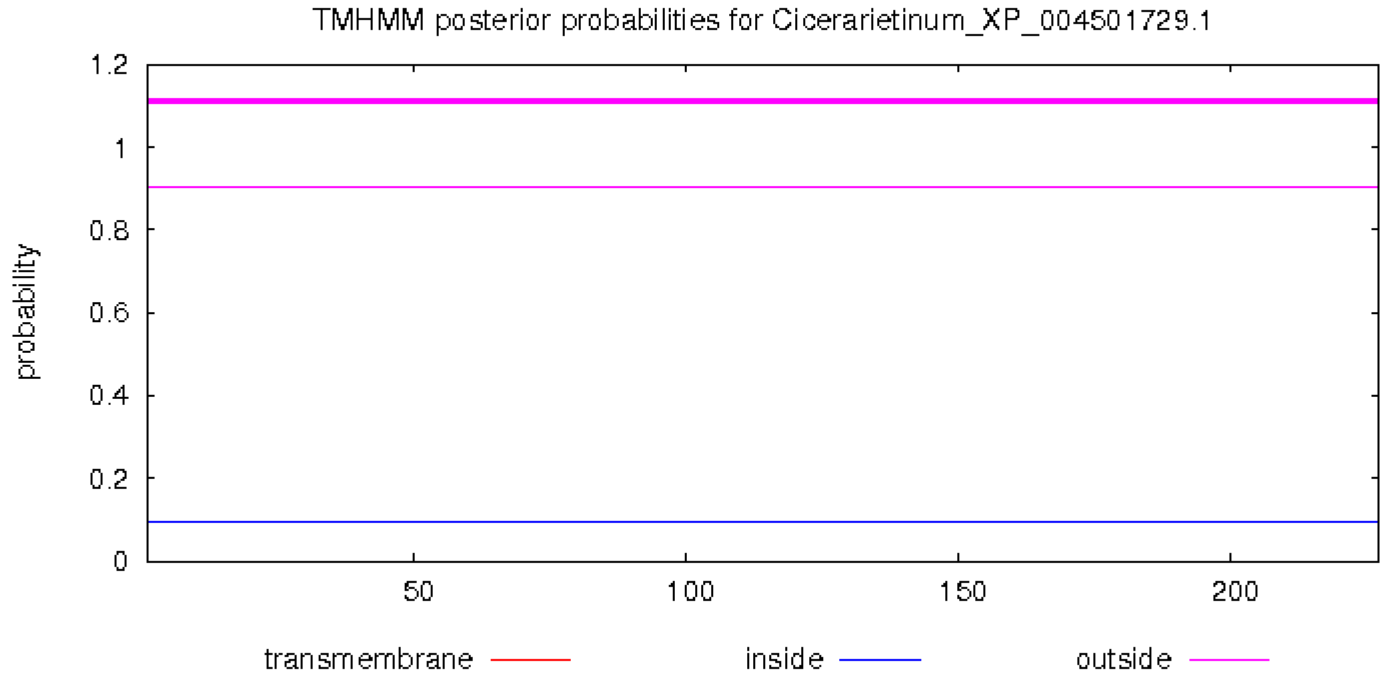

plot in postscript, script for making the plot in gnuplot, data for plot

---

```
# Cicerarietinum_XP_004502479.1 Length: 291
# Cicerarietinum_XP_004502479.1 Number of predicted TMHs:  0
# Cicerarietinum_XP_004502479.1 Exp number of AAs in TMHs: 0
# Cicerarietinum_XP_004502479.1 Exp number, first 60 AAs:  0
# Cicerarietinum_XP_004502479.1 Total prob of N-in:        0.00370
Cicerarietinum_XP_004502479.1   TMHMM2.0        outside      1   291
```

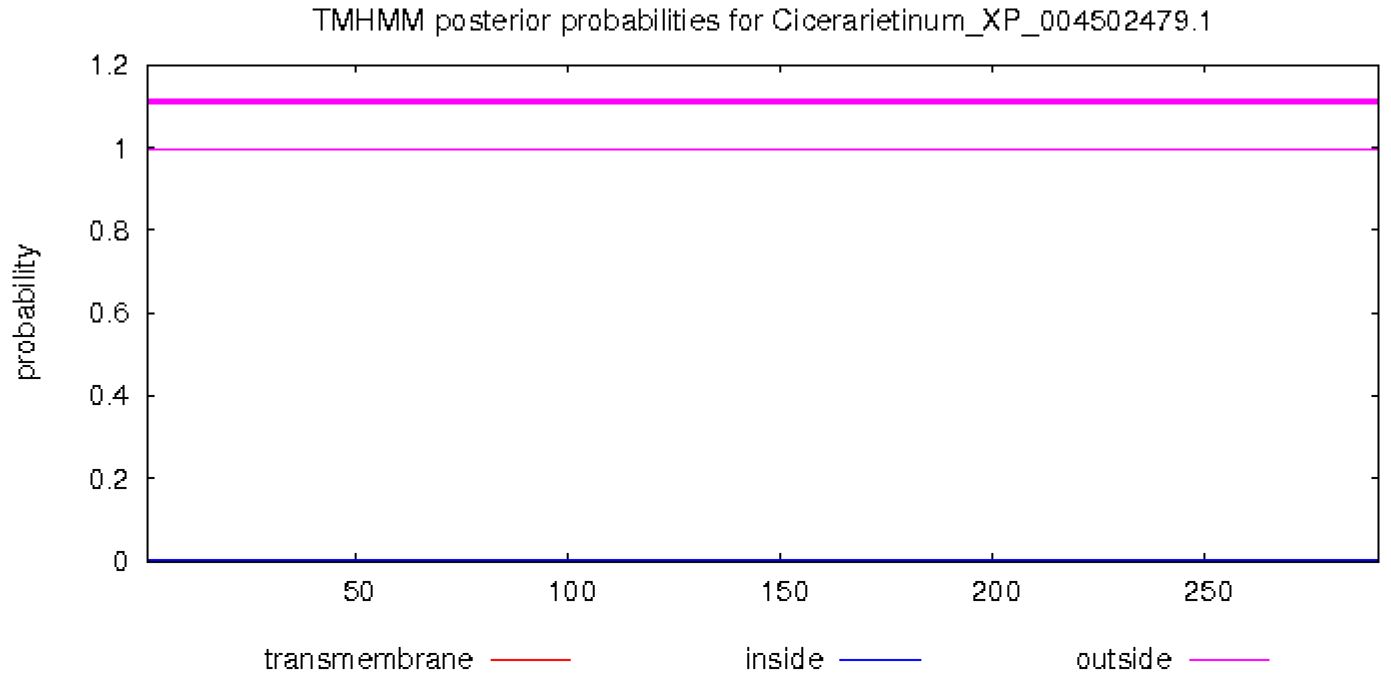

plot in postscript, script for making the plot in gnuplot, data for plot

---

```
# Cicerarietinum_XP_004502779.1 Length: 612
# Cicerarietinum_XP_004502779.1 Number of predicted TMHs:  1
```

```
# Cicerarietinum_XP_004502779.1 Exp number of AAs in TMHs: 22.28558
# Cicerarietinum_XP_004502779.1 Exp number, first 60 AAs:  0.00171
# Cicerarietinum_XP_004502779.1 Total prob of N-in:        0.00260
Cicerarietinum_XP_004502779.1   TMHMM2.0        outside      1   584
Cicerarietinum_XP_004502779.1   TMHMM2.0        TMhelix    585   607
Cicerarietinum_XP_004502779.1   TMHMM2.0        inside     608   612
```

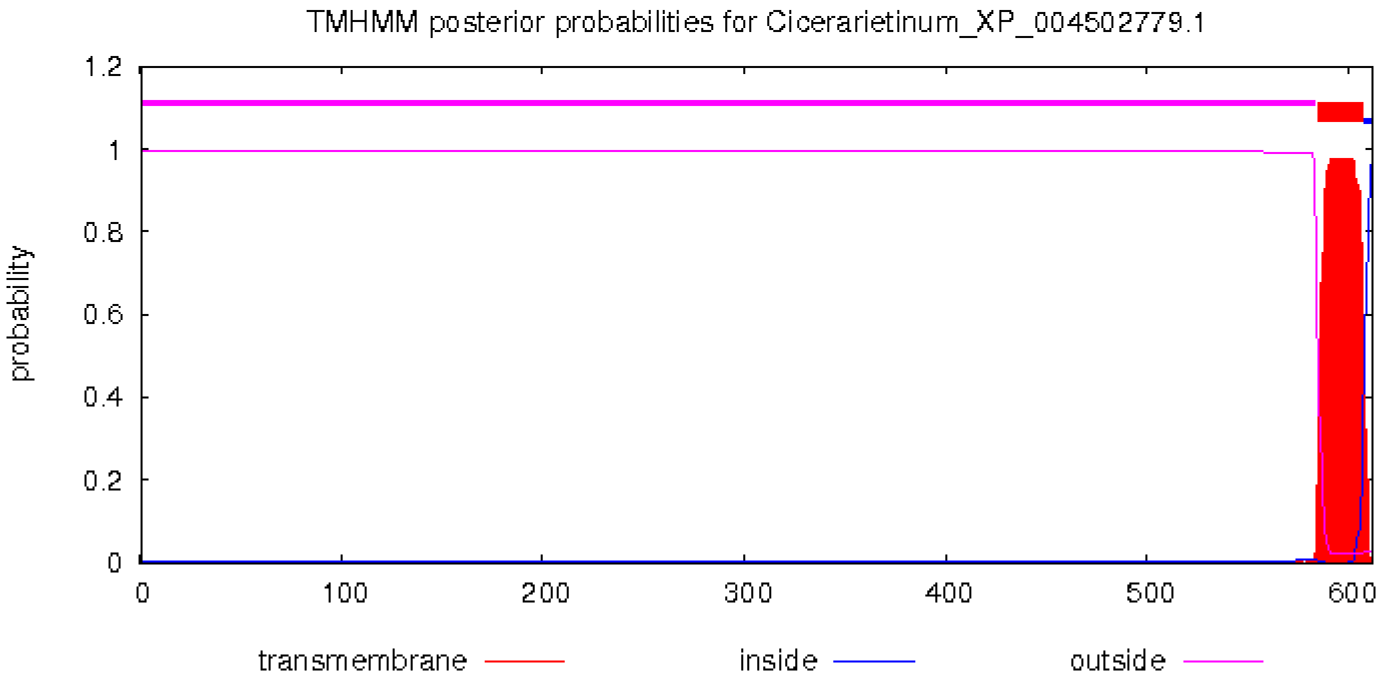

plot in postscript, script for making the plot in gnuplot, data for plot

---

```
# Cicerarietinum_XP_004502931.1 Length: 354
# Cicerarietinum_XP_004502931.1 Number of predicted TMHs:  0
# Cicerarietinum_XP_004502931.1 Exp number of AAs in TMHs: 0.00769
# Cicerarietinum_XP_004502931.1 Exp number, first 60 AAs:  0.00755
# Cicerarietinum_XP_004502931.1 Total prob of N-in:        0.00404
Cicerarietinum_XP_004502931.1   TMHMM2.0        outside      1   354
```

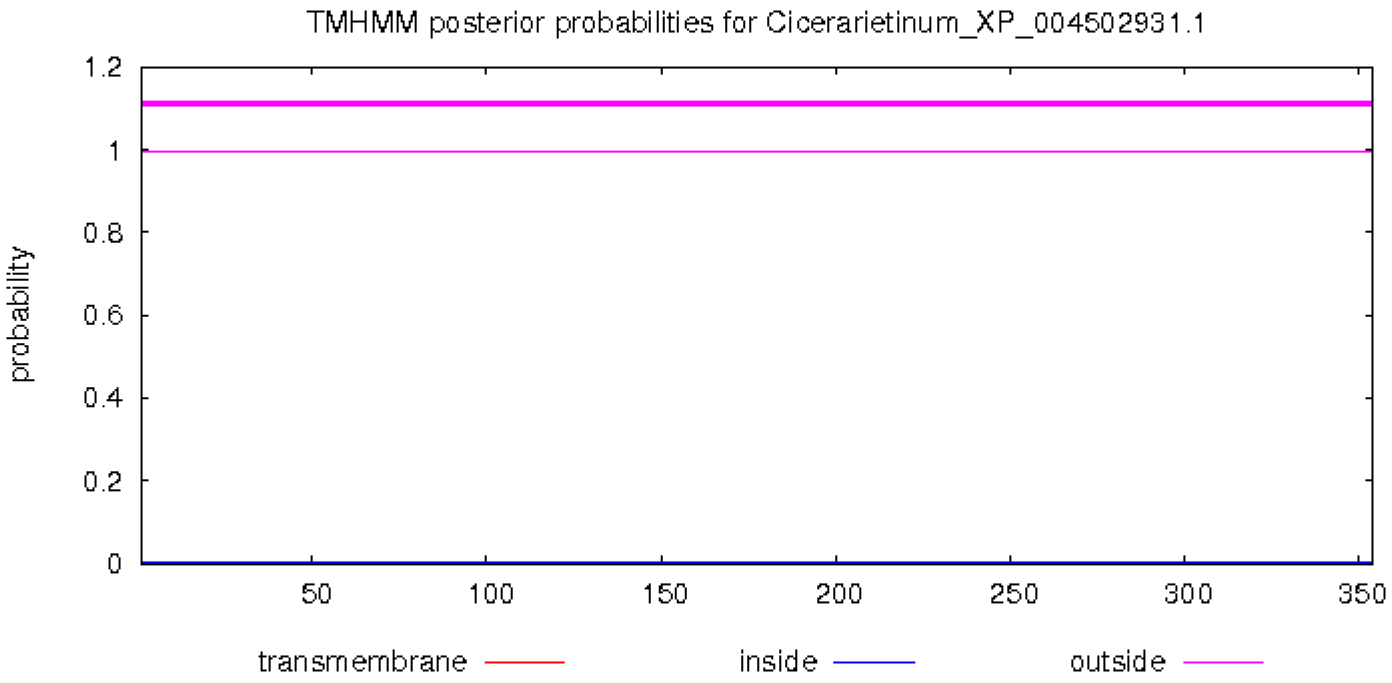

plot in postscript, script for making the plot in gnuplot, data for plot

---

```
# Ciceraríetinum_XP_004502932.1 Length: 334
# Cicerarietinum_XP_004502932.1 Number of predicted TMHs:  0
# Cicerarietinum_XP_004502932.1 Exp number of AAs in TMHs: 0.00055
# Cicerarietinum_XP_004502932.1 Exp number, first 60 AAs:  0.00019
# Cicerarietinum_XP_004502932.1 Total prob of N-in:        0.02197
Cicerarietinum_XP_004502932.1	TMHMM2.0	outside	     1   334
```

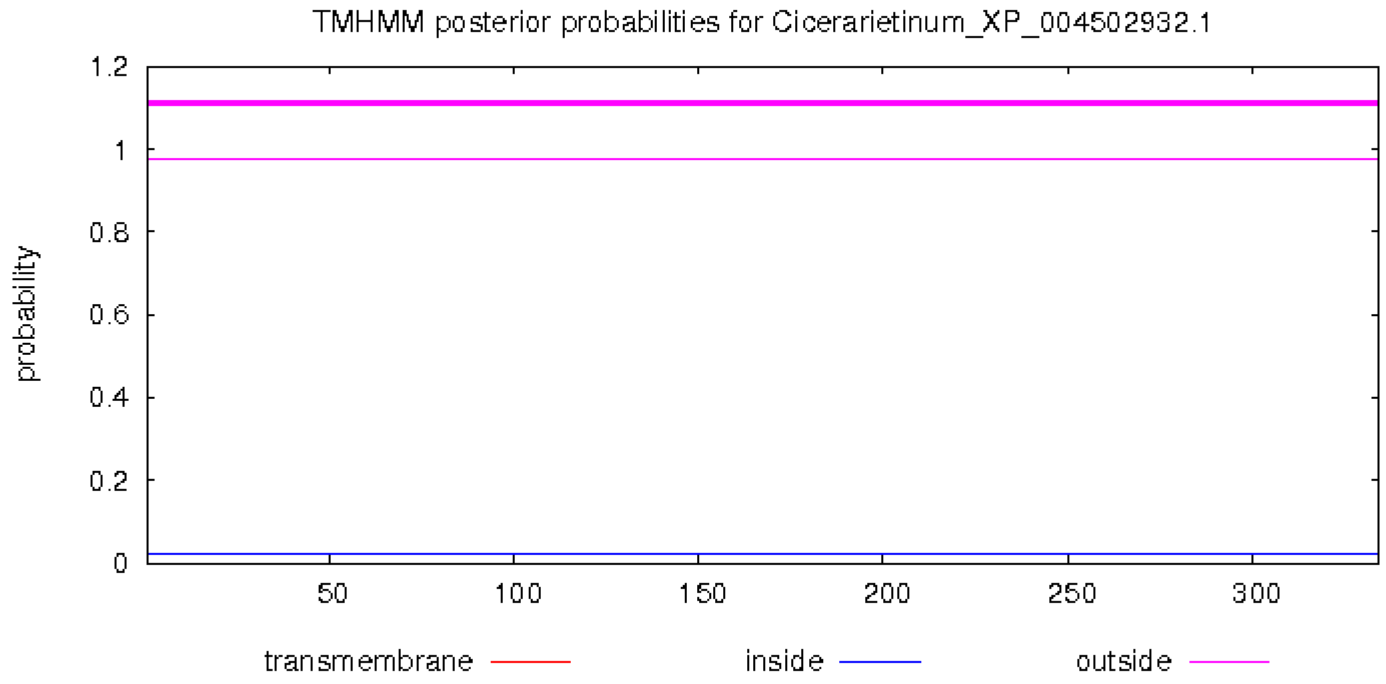

plot in postscript, script for making the plot in gnuplot, data for plot

```
# Cicerarietinum_XP_004502933.1 Length: 334
# Cicerarietinum_XP_004502933.1 Number of predicted TMHs:  0
# Cicerarietinum_XP_004502933.1 Exp number of AAs in TMHs: 0.00055
# Cicerarietinum_XP_004502933.1 Exp number, first 60 AAs:  0.00019
# Cicerarietinum_XP_004502933.1 Total prob of N-in:        0.02197
Cicerarietinum_XP_004502933.1	TMHMM2.0	outside	     1   334
```

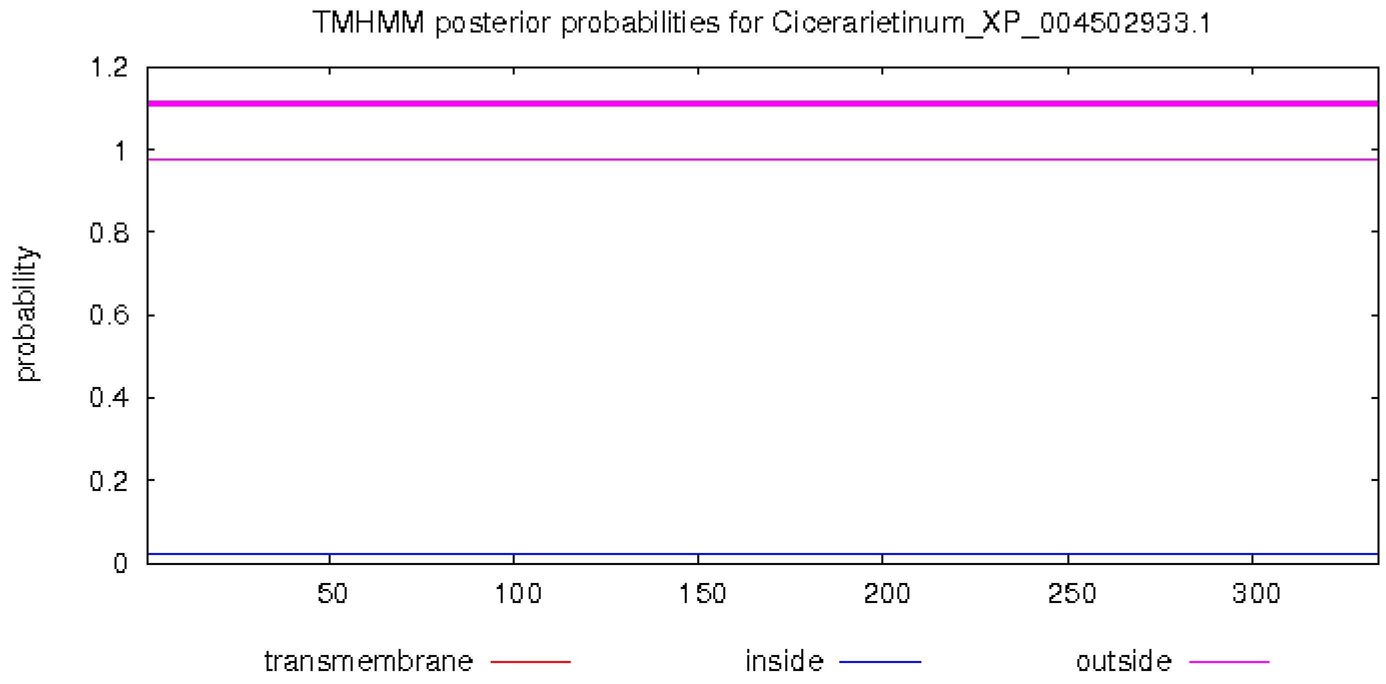

plot in postscript, script for making the plot in gnuplot, data for plot

```
# Cicerarietinum_XP_004502989.1 Length: 289
# Cicerarietinum_XP_004502989.1 Number of predicted TMHs:  0
# Cicerarietinum_XP_004502989.1 Exp number of AAs in TMHs: 0.00017
# Cicerarietinum_XP_004502989.1 Exp number, first 60 AAs:  0
# Cicerarietinum_XP_004502989.1 Total prob of N-in:        0.00297
Cicerarietinum_XP_004502989.1   TMHMM2.0        outside      1   289
```

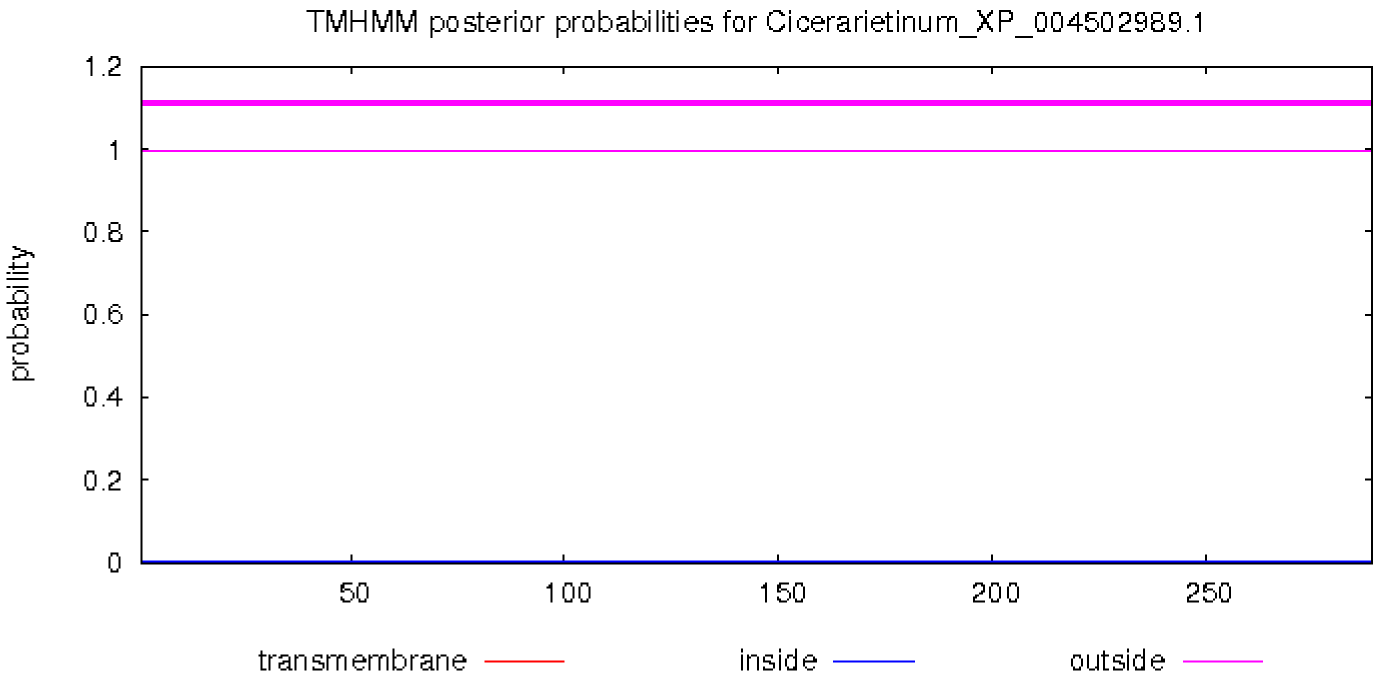

plot in postscript, script for making the plot in gnuplot, data for plot

---

```
# Cicerarietinum_XP_004503093.1 Length: 636
# Cicerarietinum_XP_004503093.1 Number of predicted TMHs:  2
# Cicerarietinum_XP_004503093.1 Exp number of AAs in TMHs: 40.15937
# Cicerarietinum_XP_004503093.1 Exp number, first 60 AAs:  0
# Cicerarietinum_XP_004503093.1 Total prob of N-in:        0.00021
Cicerarietinum_XP_004503093.1   TMHMM2.0        outside      1   538
Cicerarietinum_XP_004503093.1   TMHMM2.0        TMhelix    539   558
Cicerarietinum_XP_004503093.1   TMHMM2.0        inside     559   613
Cicerarietinum_XP_004503093.1   TMHMM2.0        TMhelix    614   633
Cicerarietinum_XP_004503093.1   TMHMM2.0        outside    634   636
```

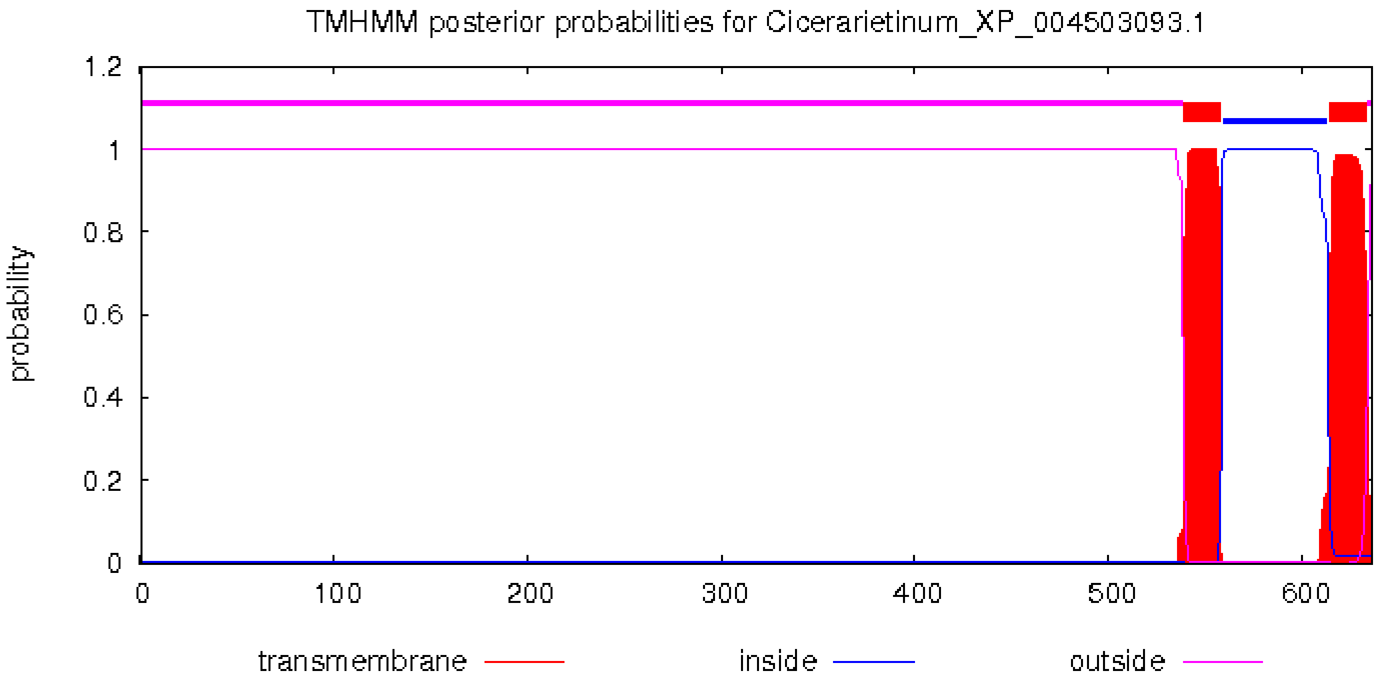

plot in postscript, script for making the plot in gnuplot, data for plot

---

```
# Cicerarietinum_XP_004503507.1 Length: 440
# Cicerarietinum_XP_004503507.1 Number of predicted TMHs:  0
# Cicerarietinum_XP_004503507.1 Exp number of AAs in TMHs: 0
# Cicerarietinum_XP_004503507.1 Exp number, first 60 AAs:  0
# Cicerarietinum_XP_004503507.1 Total prob of N-in:        0.02116
Cicerarietinum_XP_004503507.1   TMHMM2.0        outside      1   440
```

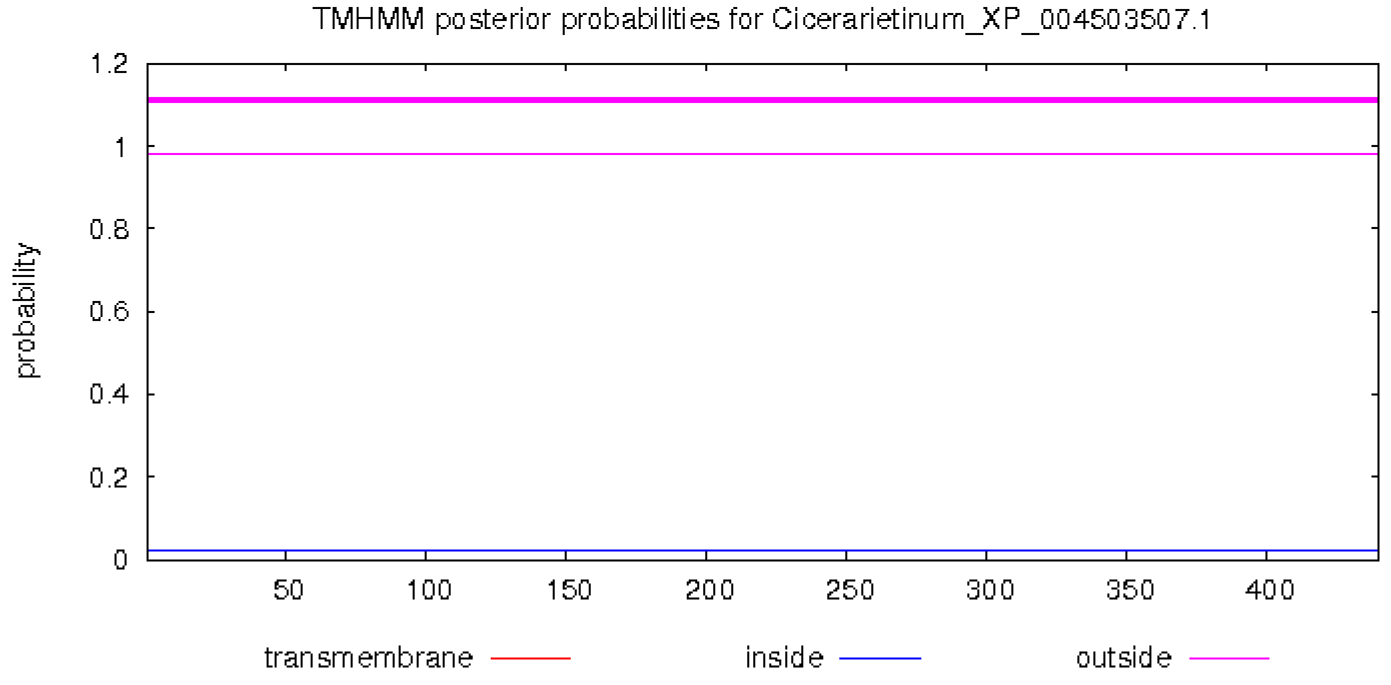

plot in postscript, script for making the plot in gnuplot, data for plot

---

```
# Cicerarietinum_XP_004503781.1 Length: 233
# Cicerarietinum_XP_004503781.1 Number of predicted TMHs:  0
# Cicerarietinum_XP_004503781.1 Exp number of AAs in TMHs: 0.00063
# Cicerarietinum_XP_004503781.1 Exp number, first 60 AAs:  0.00063
# Cicerarietinum_XP_004503781.1 Total prob of N-in:        0.05779
Cicerarietinum_XP_004503781.1   TMHMM2.0        outside      1   233
```

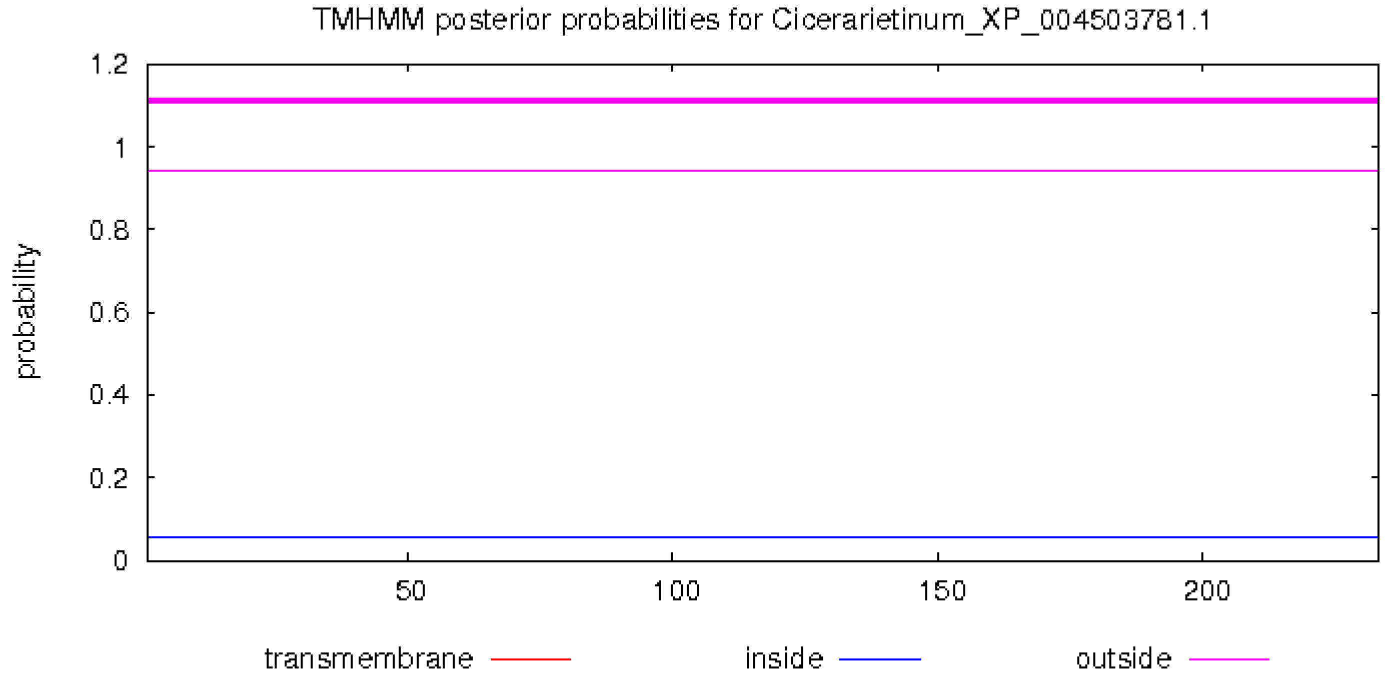

plot in postscript, script for making the plot in gnuplot, data for plot

---

```
# Cicerarietinum_XP_004503901.1 Length: 289
# Cicerarietinum_XP_004503901.1 Number of predicted TMHs:  0
# Cicerarietinum_XP_004503901.1 Exp number of AAs in TMHs: 0
# Cicerarietinum_XP_004503901.1 Exp number, first 60 AAs:  0
# Cicerarietinum_XP_004503901.1 Total prob of N-in:        0.00327
Cicerarietinum_XP_004503901.1	TMHMM2.0	outside	     1   289
```

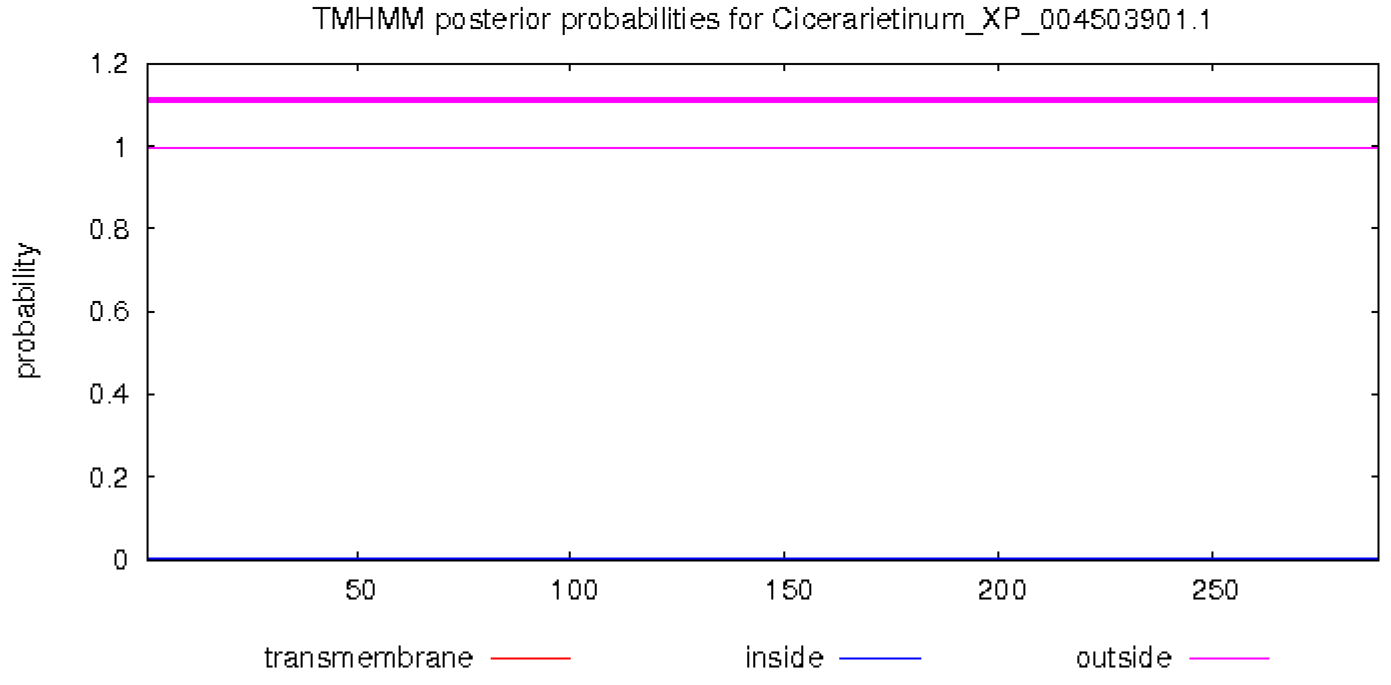

plot in postscript, script for making the plot in gnuplot, data for plot

---

```
# Cicerarietinum_XP_004504766.1 Length: 193
# Cicerarietinum_XP_004504766.1 Number of predicted TMHs:  0
# Cicerarietinum_XP_004504766.1 Exp number of AAs in TMHs: 0.00096
# Cicerarietinum_XP_004504766.1 Exp number, first 60 AAs:  0
# Cicerarietinum_XP_004504766.1 Total prob of N-in:        0.15867
Cicerarietinum_XP_004504766.1	TMHMM2.0	outside	     1   193
```

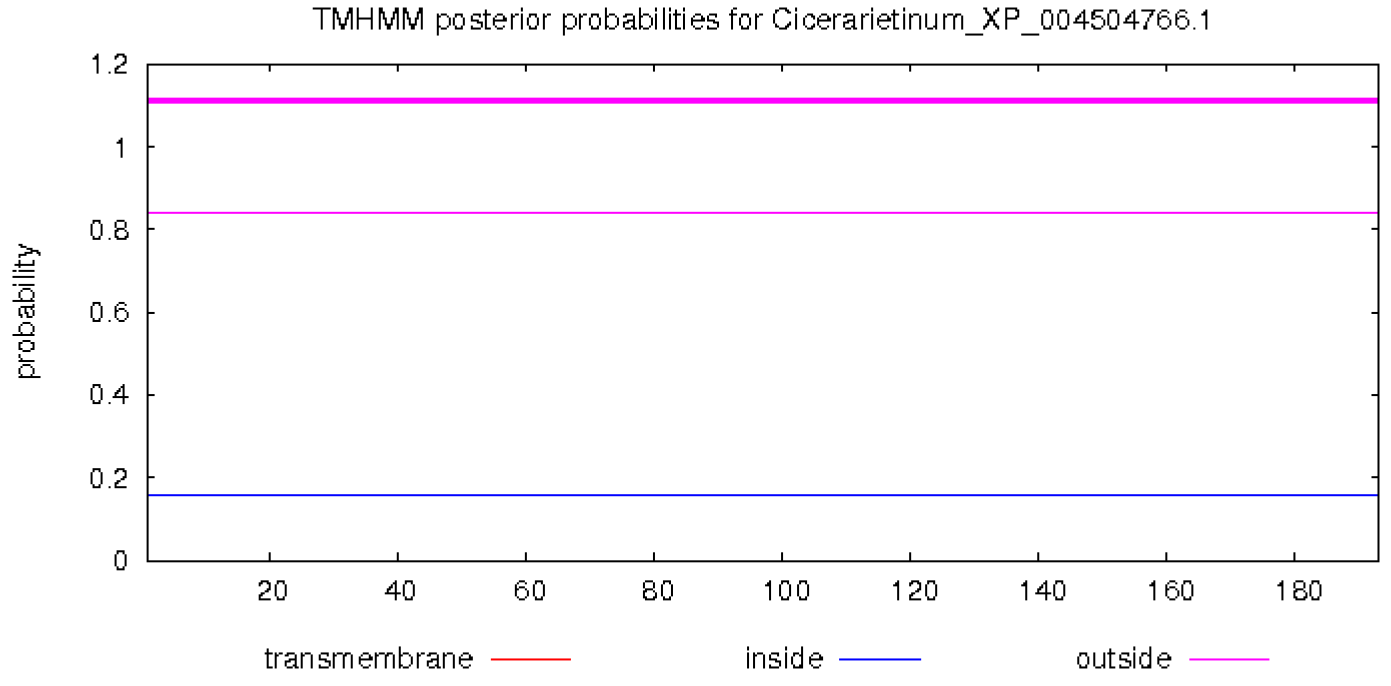

plot in postscript, script for making the plot in gnuplot, data for plot

---

```
# Cicerarietinum_XP_004505006.1 Length: 364
# Cicerarietinum_XP_004505006.1 Number of predicted TMHs:  0
# Cicerarietinum_XP_004505006.1 Exp number of AAs in TMHs: 0.00162
# Cicerarietinum_XP_004505006.1 Exp number, first 60 AAs:  0.00105
# Cicerarietinum_XP_004505006.1 Total prob of N-in:        0.01711
Cicerarietinum_XP_004505006.1   TMHMM2.0        outside      1   364
```

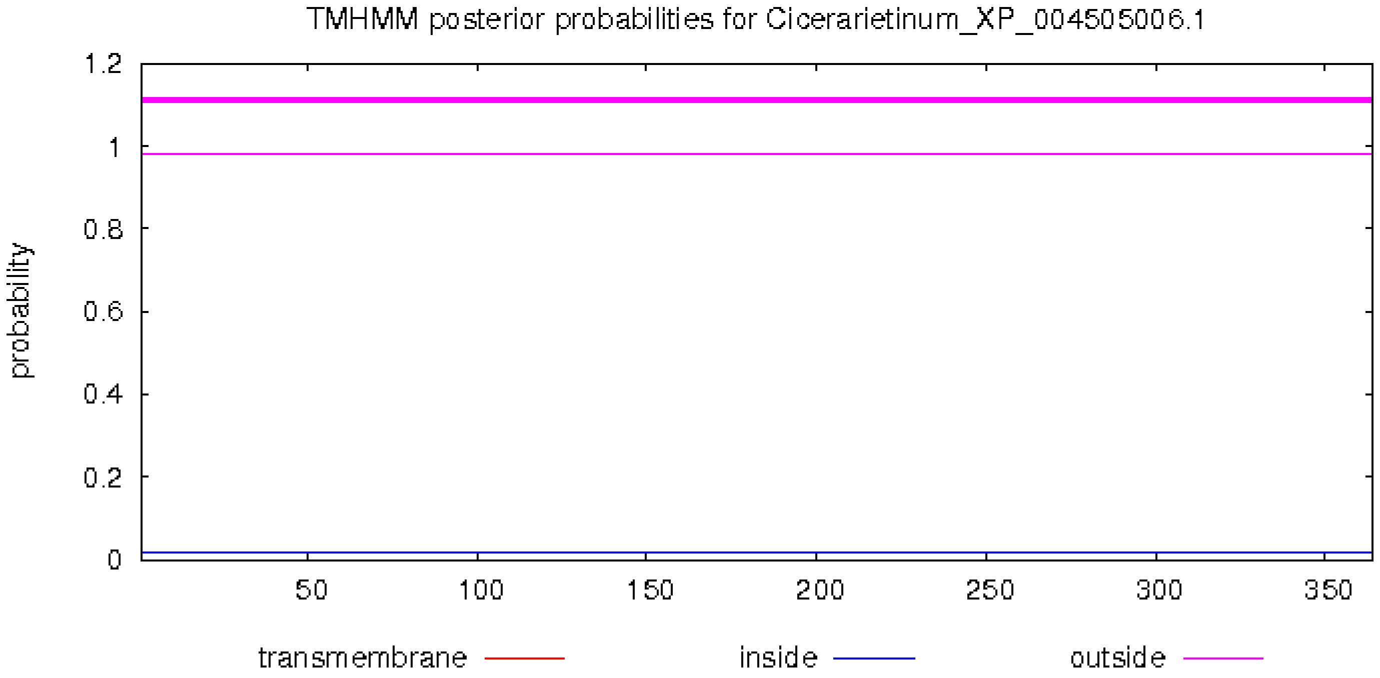

plot in postscript, script for making the plot in gnuplot, data for plot

---

```
# Cicerarietinum_XP_004505102.1 Length: 322
# Cicerarietinum_XP_004505102.1 Number of predicted TMHs:  0
# Cicerarietinum_XP_004505102.1 Exp number of AAs in TMHs: 0.08405
# Cicerarietinum_XP_004505102.1 Exp number, first 60 AAs:  0.0006
# Cicerarietinum_XP_004505102.1 Total prob of N-in:        0.10343
Cicerarietinum_XP_004505102.1   TMHMM2.0        outside      1   322
```

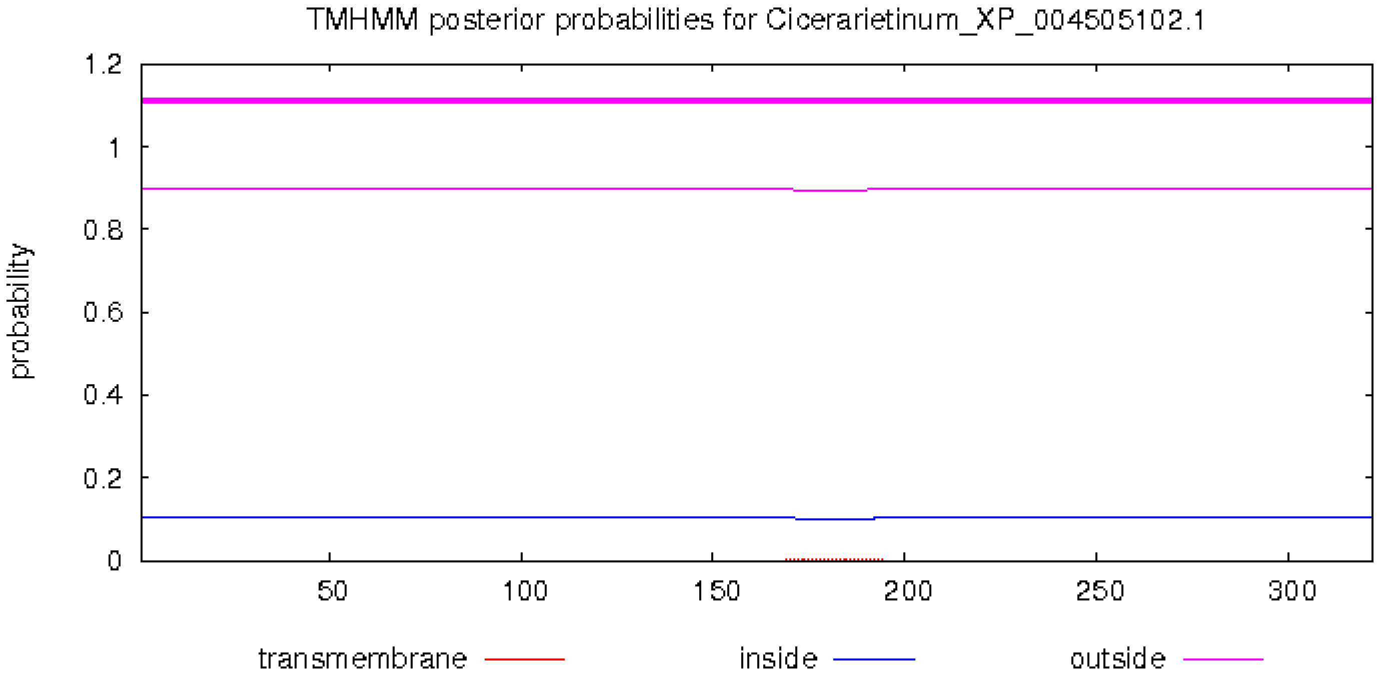

plot in postscript, script for making the plot in gnuplot, data for plot

---

```
# Cicerarietinum_XP_004505536.1 Length: 292
# Cicerarietinum_XP_004505536.1 Number of predicted TMHs:  0
# Cicerarietinum_XP_004505536.1 Exp number of AAs in TMHs: 0
# Cicerarietinum_XP_004505536.1 Exp number, first 60 AAs:  0
# Cicerarietinum_XP_004505536.1 Total prob of N-in:        0.02134
Cicerarietinum_XP_004505536.1   TMHMM2.0        outside      1   292
```

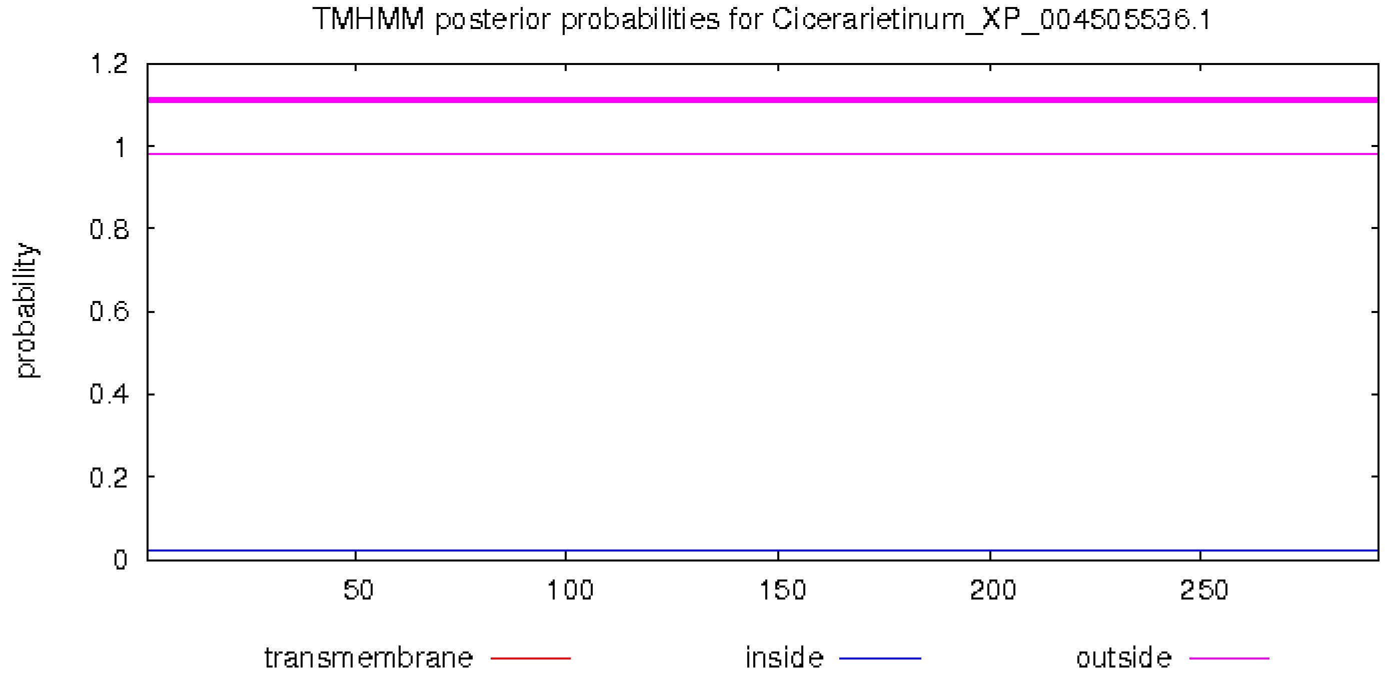

plot in postscript, script for making the plot in gnuplot, data for plot

---

```
# Cicerarietinum_XP_004505672.1 Length: 307
# Cicerarietinum_XP_004505672.1 Number of predicted TMHs:  0
# Cicerarietinum_XP_004505672.1 Exp number of AAs in TMHs: 0
# Cicerarietinum_XP_004505672.1 Exp number, first 60 AAs:  0
# Cicerarietinum_XP_004505672.1 Total prob of N-in:        0.02044
Cicerarietinum_XP_004505672.1   TMHMM2.0        outside      1   307
```

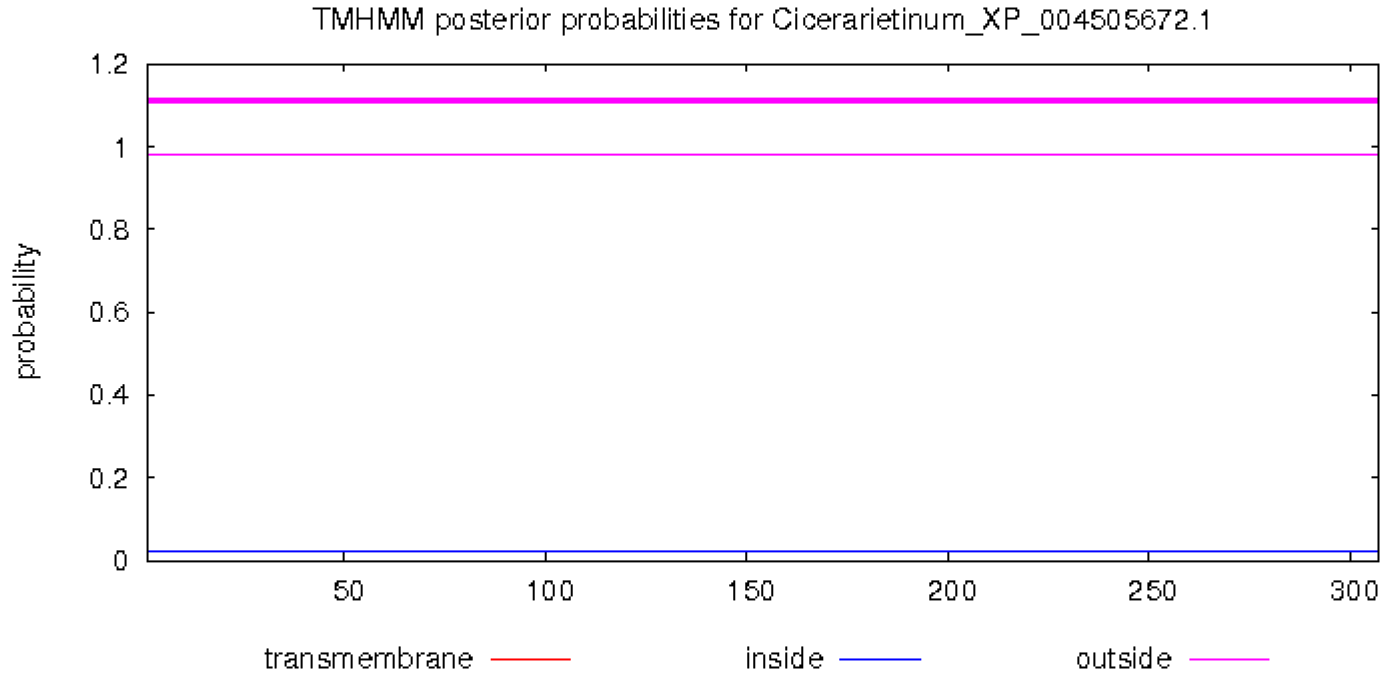

plot in postscript, script for making the plot in gnuplot, data for plot

```
# Cicerarietinum_XP_004506197.1 Length: 272
# Cicerarietinum_XP_004506197.1 Number of predicted TMHs:  0
# Cicerarietinum_XP_004506197.1 Exp number of AAs in TMHs: 0.00071
# Cicerarietinum_XP_004506197.1 Exp number, first 60 AAs:  0
# Cicerarietinum_XP_004506197.1 Total prob of N-in:        0.04064
Cicerarietinum_XP_004506197.1	TMHMM2.0	outside	     1   272
```

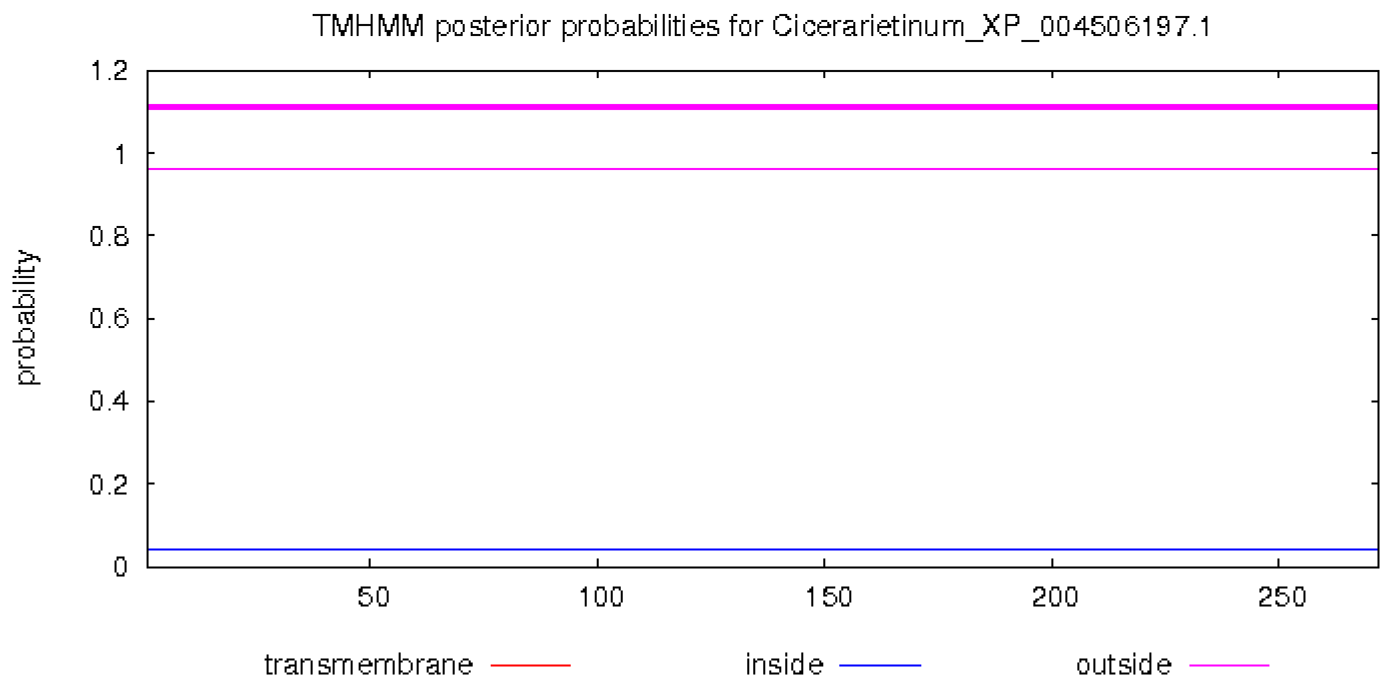

plot in postscript, script for making the plot in gnuplot, data for plot

```
# Cicerarietinum_XP_004506361.1 Length: 358
# Cicerarietinum_XP_004506361.1 Number of predicted TMHs:  0
# Cicerarietinum_XP_004506361.1 Exp number of AAs in TMHs: 0
# Cicerarietinum_XP_004506361.1 Exp number, first 60 AAs:  0
# Cicerarietinum_XP_004506361.1 Total prob of N-in:        0.00514
Cicerarietinum_XP_004506361.1	TMHMM2.0	outside	     1   358
```

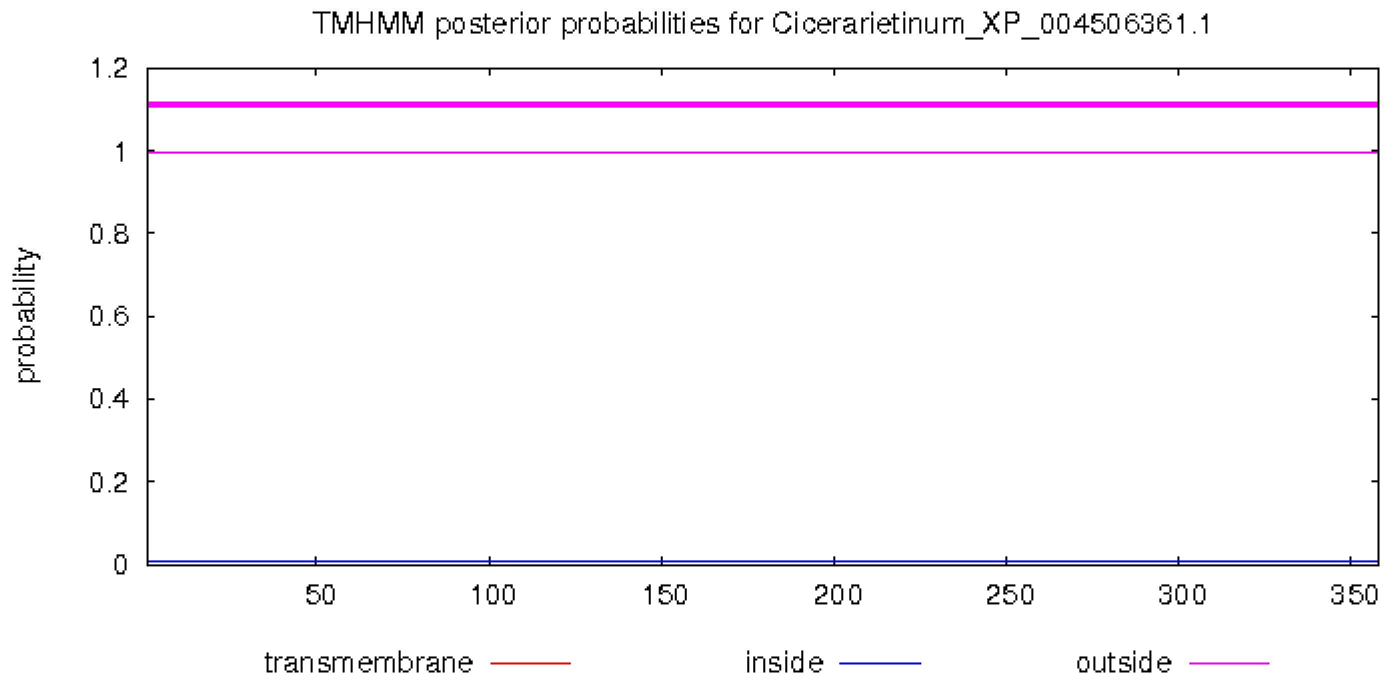

plot in postscript, script for making the plot in gnuplot, data for plot

```
# Cicerarietinum_XP_004508725.1 Length: 430
# Cicerarietinum_XP_004508725.1 Number of predicted TMHs:  2
# Cicerarietinum_XP_004508725.1 Exp number of AAs in TMHs: 39.78718
# Cicerarietinum_XP_004508725.1 Exp number, first 60 AAs:  35.9005
# Cicerarietinum_XP_004508725.1 Total prob of N-in:        0.11957
# Cicerarietinum_XP_004508725.1 POSSIBLE N-term signal sequence
Cicerarietinum_XP_004508725.1   TMHMM2.0        outside      1    14
Cicerarietinum_XP_004508725.1   TMHMM2.0        TMhelix     15    32
Cicerarietinum_XP_004508725.1   TMHMM2.0        inside      33    36
Cicerarietinum_XP_004508725.1   TMHMM2.0        TMhelix     37    59
Cicerarietinum_XP_004508725.1   TMHMM2.0        outside     60   430
```

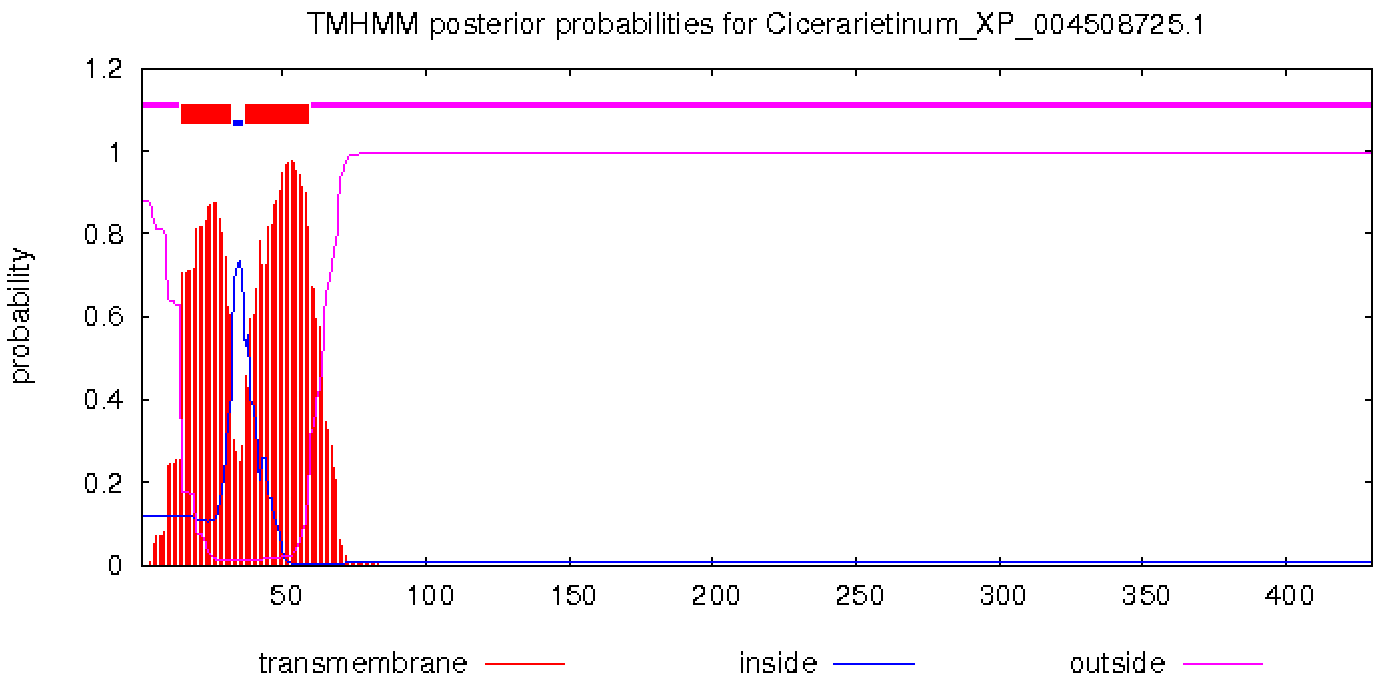

plot in postscript, script for making the plot in gnuplot, data for plot

```
# Cicerarietinum_XP_004509014.1 Length: 331
# Cicerarietinum_XP_004509014.1 Number of predicted TMHs:  0
# Cicerarietinum_XP_004509014.1 Exp number of AAs in TMHs: 0.0012
# Cicerarietinum_XP_004509014.1 Exp number, first 60 AAs:  0
# Cicerarietinum_XP_004509014.1 Total prob of N-in:        0.00064
Cicerarietinum_XP_004509014.1   TMHMM2.0        outside      1   331
```

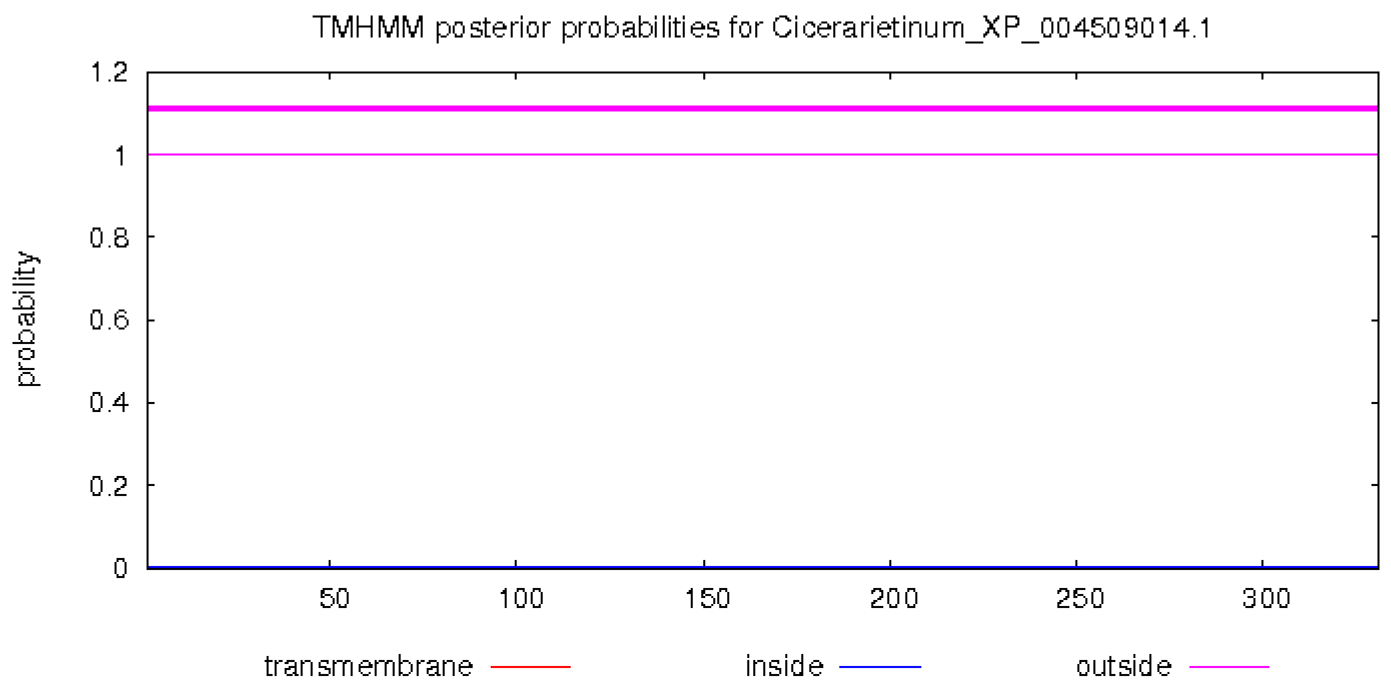

plot in postscript, script for making the plot in gnuplot, data for plot

---

```
# Cicerarietinum_XP_004509344.1 Length: 335
# Cicerarietinum_XP_004509344.1 Number of predicted TMHs:  0
# Cicerarietinum_XP_004509344.1 Exp number of AAs in TMHs: 0
# Cicerarietinum_XP_004509344.1 Exp number, first 60 AAs:  0
# Cicerarietinum_XP_004509344.1 Total prob of N-in:        0.00436
Cicerarietinum_XP_004509344.1   TMHMM2.0        outside      1   335
```

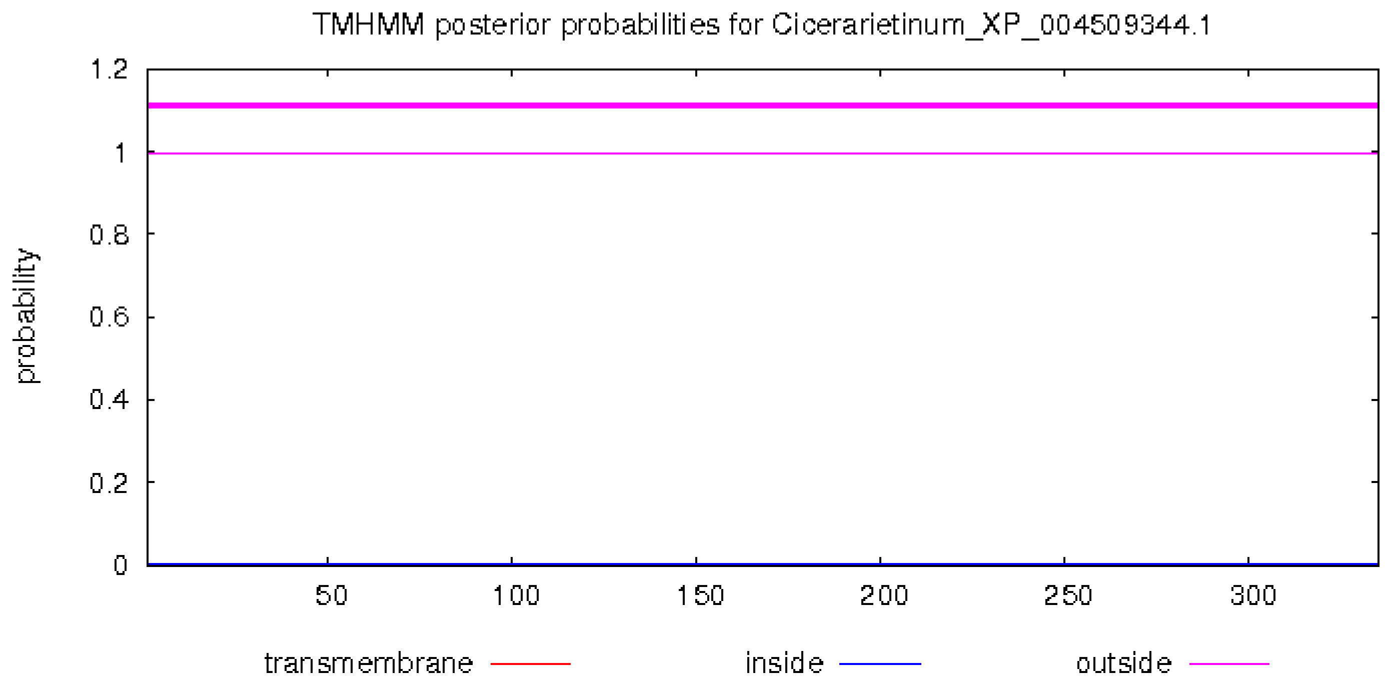

plot in postscript, script for making the plot in gnuplot, data for plot

---

```
# Cicerarietinum_XP_004510270.1 Length: 298
# Cicerarietinum_XP_004510270.1 Number of predicted TMHs:  0
# Cicerarietinum_XP_004510270.1 Exp number of AAs in TMHs: 0
# Cicerarietinum_XP_004510270.1 Exp number, first 60 AAs:  0
# Cicerarietinum_XP_004510270.1 Total prob of N-in:        0.10755
Cicerarietinum_XP_004510270.1   TMHMM2.0        outside      1   298
```

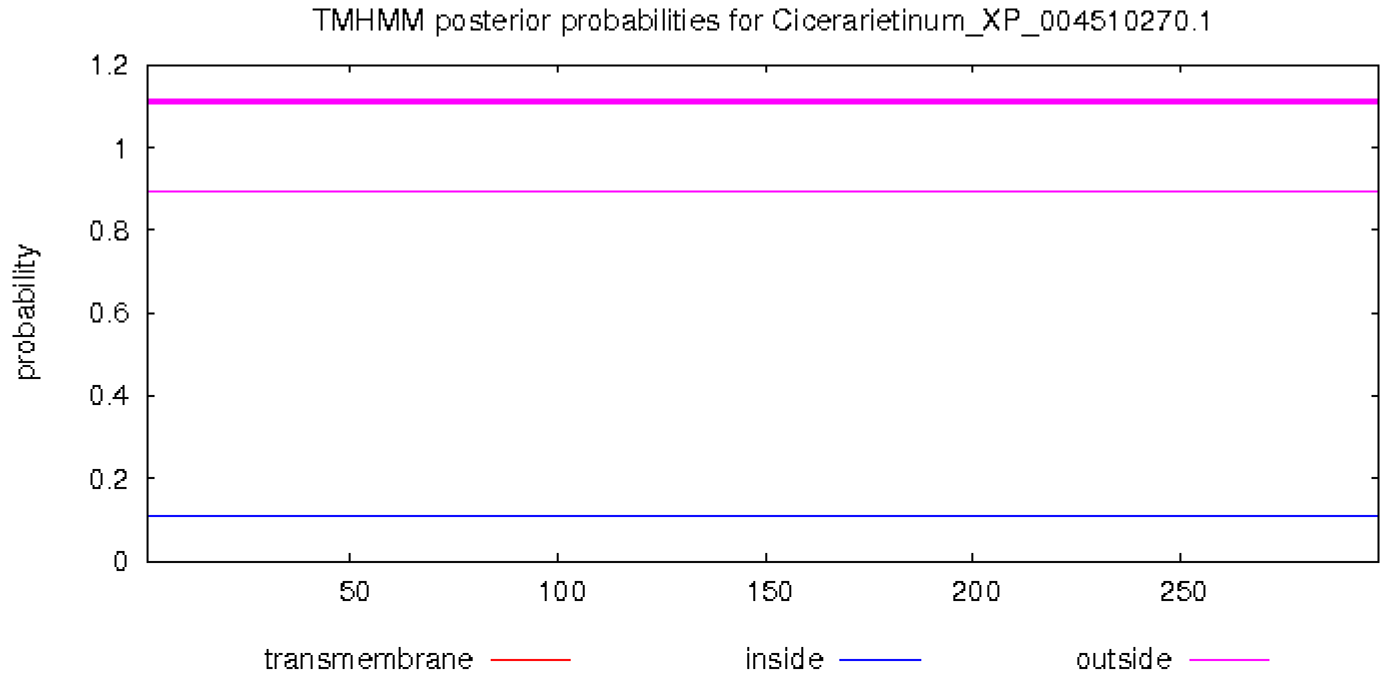

plot in postscript, script for making the plot in gnuplot, data for plot

```
# Cicerarietinum_XP_004511662.1 Length: 337
# Cicerarietinum_XP_004511662.1 Number of predicted TMHs:  0
# Cicerarietinum_XP_004511662.1 Exp number of AAs in TMHs: 0.00018
# Cicerarietinum_XP_004511662.1 Exp number, first 60 AAs:  0
# Cicerarietinum_XP_004511662.1 Total prob of N-in:        0.00583
Cicerarietinum_XP_004511662.1   TMHMM2.0        outside      1   337
```

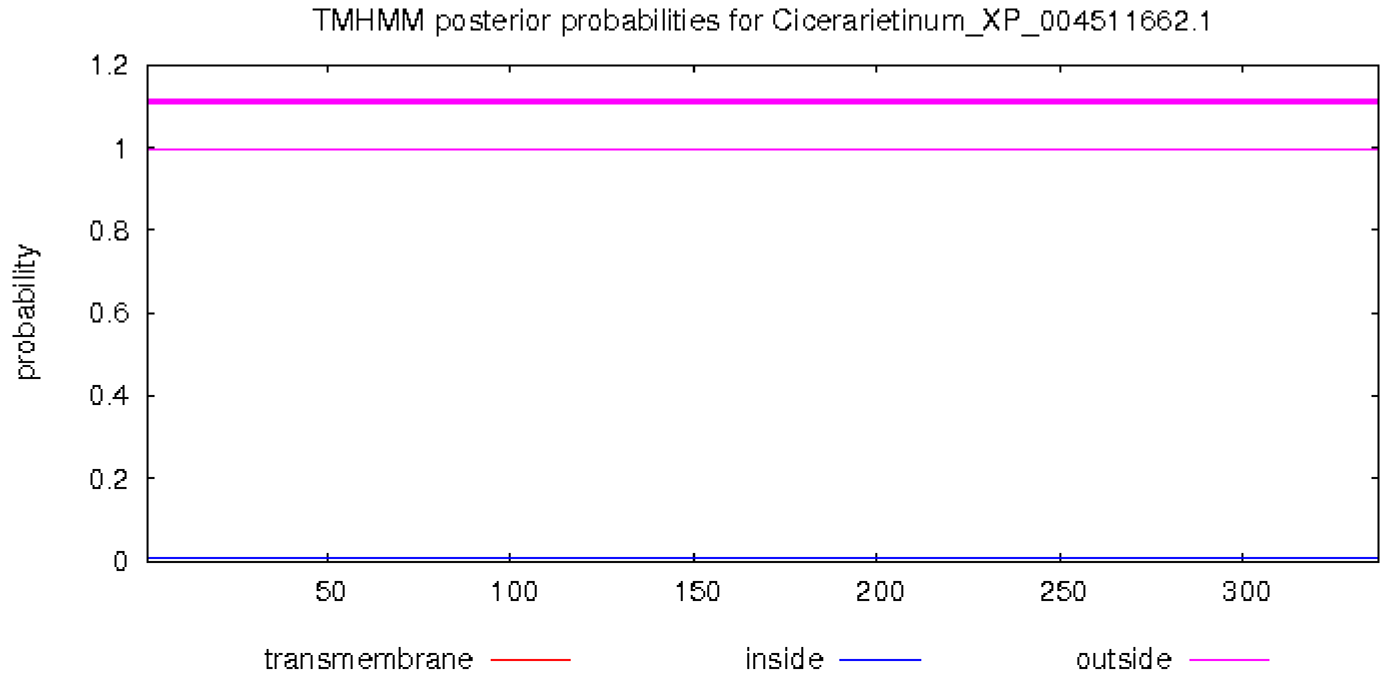

plot in postscript, script for making the plot in gnuplot, data for plot

```
# Cicerarietinum_XP_004511779.1 Length: 336
# Cicerarietinum_XP_004511779.1 Number of predicted TMHs:  0
# Cicerarietinum_XP_004511779.1 Exp number of AAs in TMHs: 0
# Cicerarietinum_XP_004511779.1 Exp number, first 60 AAs:  0
# Cicerarietinum_XP_004511779.1 Total prob of N-in:        0.00090
Cicerarietinum_XP_004511779.1   TMHMM2.0        outside      1   336
```

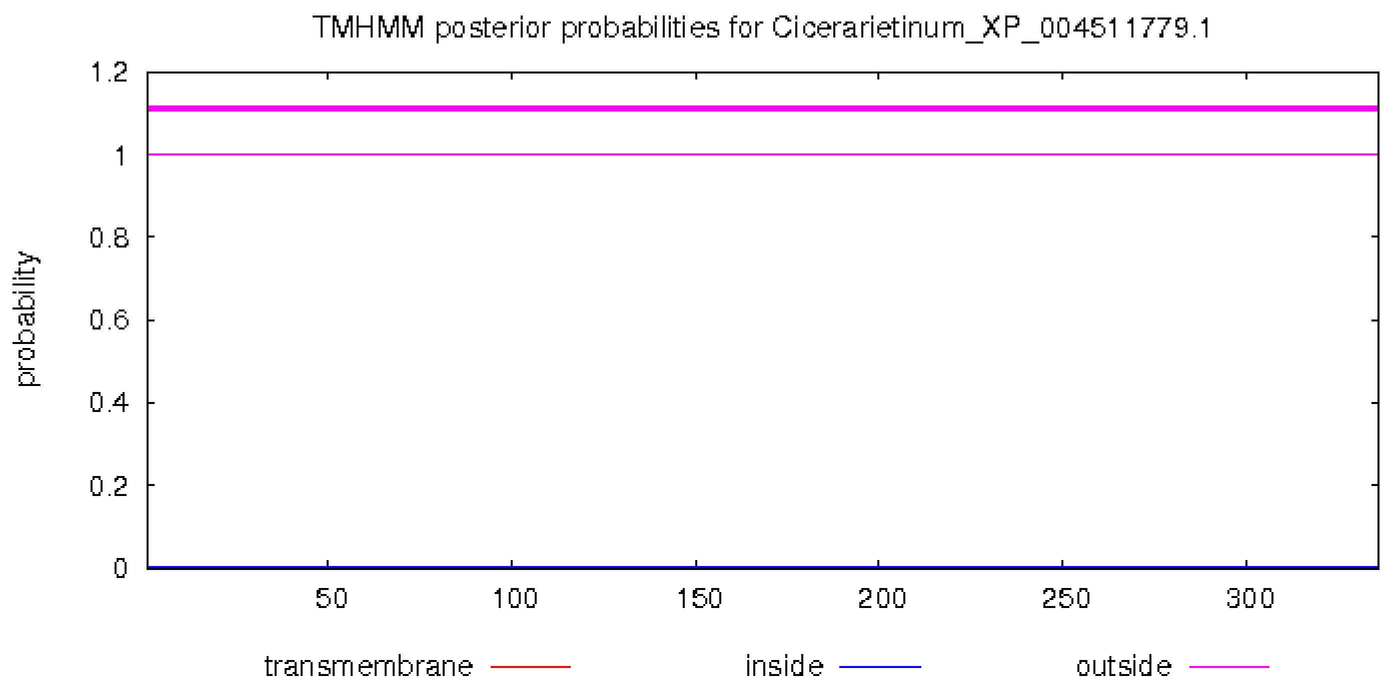

plot in postscript, script for making the plot in gnuplot, data for plot

---

```
# Cicerarietinum_XP_004512121.1 Length: 366
# Cicerarietinum_XP_004512121.1 Number of predicted TMHs:  0
# Cicerarietinum_XP_004512121.1 Exp number of AAs in TMHs: 0.00088
# Cicerarietinum_XP_004512121.1 Exp number, first 60 AAs:  0
# Cicerarietinum_XP_004512121.1 Total prob of N-in:        0.00653
Cicerarietinum_XP_004512121.1   TMHMM2.0        outside      1   366
```

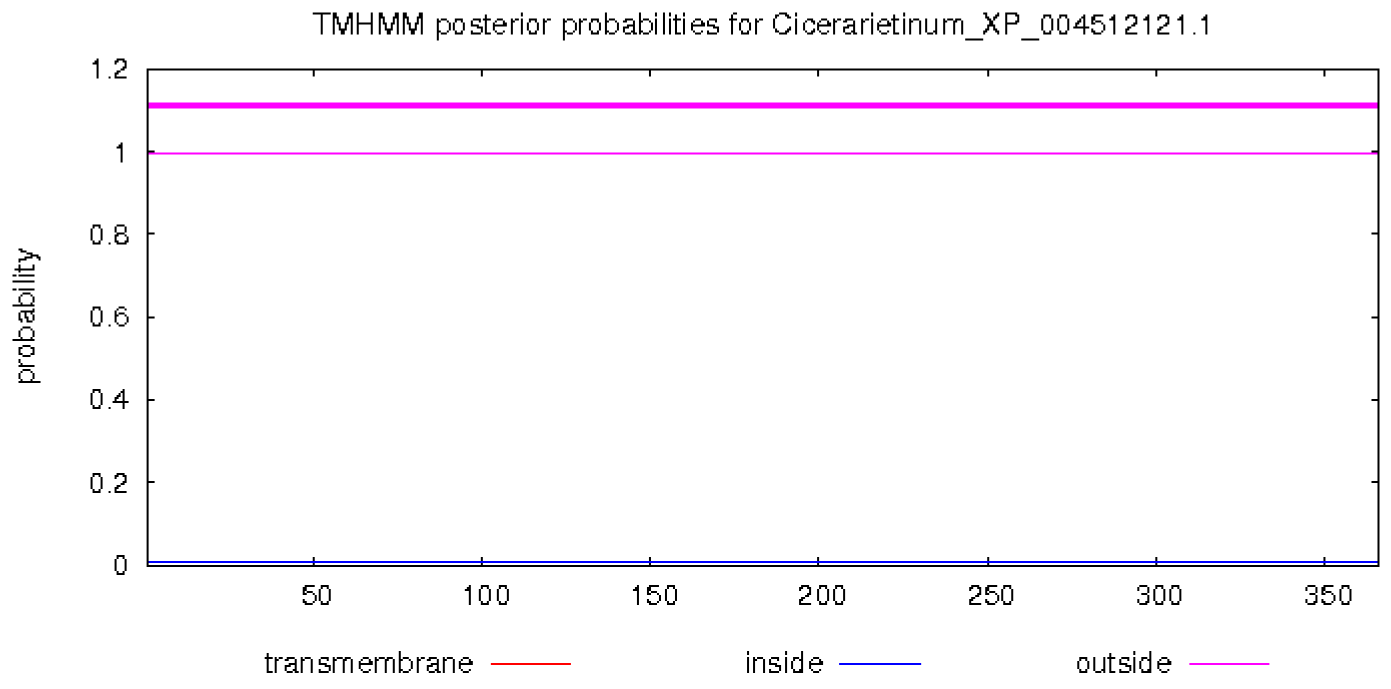

plot in postscript, script for making the plot in gnuplot, data for plot

---

```
# Cicerarietinum_XP_004512922.1 Length: 324
# Cicerarietinum_XP_004512922.1 Number of predicted TMHs:  0
# Cicerarietinum_XP_004512922.1 Exp number of AAs in TMHs: 0.000880000000000000001
# Cicerarietinum_XP_004512922.1 Exp number, first 60 AAs:  0
# Cicerarietinum_XP_004512922.1 Total prob of N-in:        0.00338
Cicerarietinum_XP_004512922.1   TMHMM2.0        outside      1   324
```

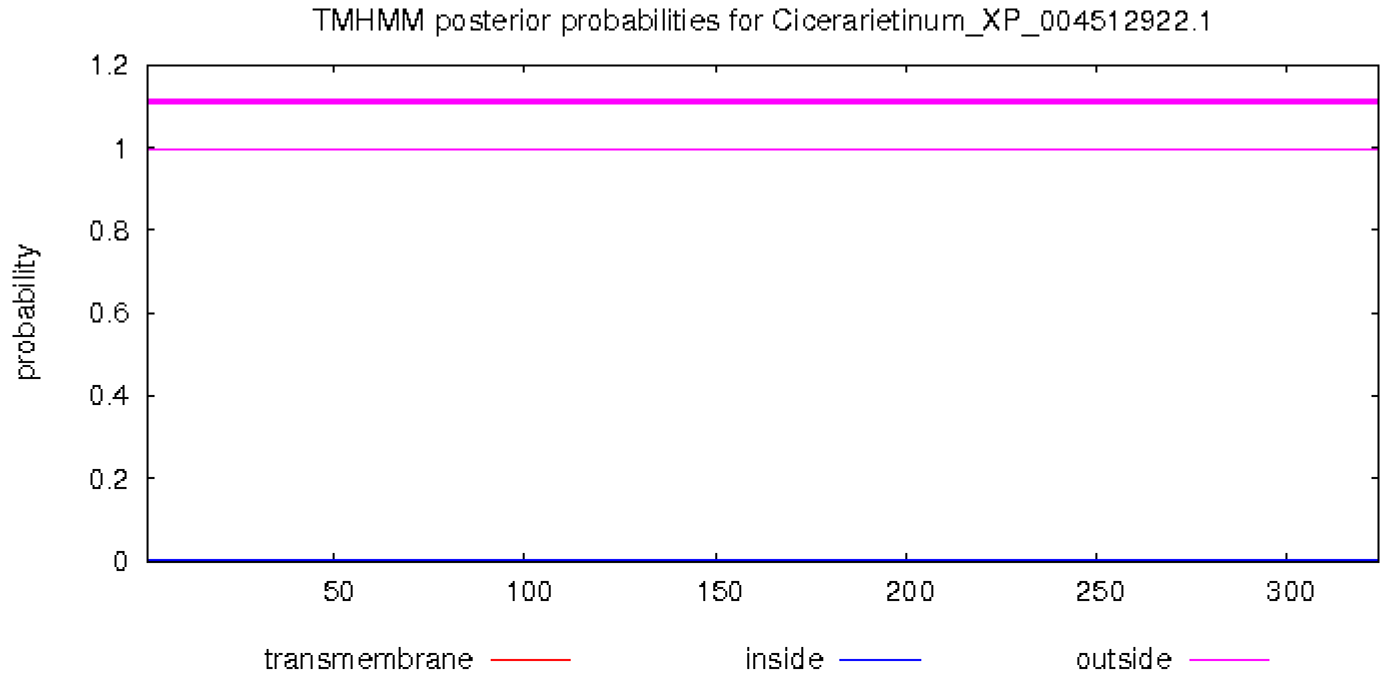

plot in postscript, script for making the plot in gnuplot, data for plot

---

```
# Cicerarietinum_XP_004513071.1 Length: 418
# Cicerarietinum_XP_004513071.1 Number of predicted TMHs:  0
# Cicerarietinum_XP_004513071.1 Exp number of AAs in TMHs: 0
# Cicerarietinum_XP_004513071.1 Exp number, first 60 AAs:  0
# Cicerarietinum_XP_004513071.1 Total prob of N-in:        0.01174
Cicerarietinum_XP_004513071.1   TMHMM2.0        outside      1   418
```

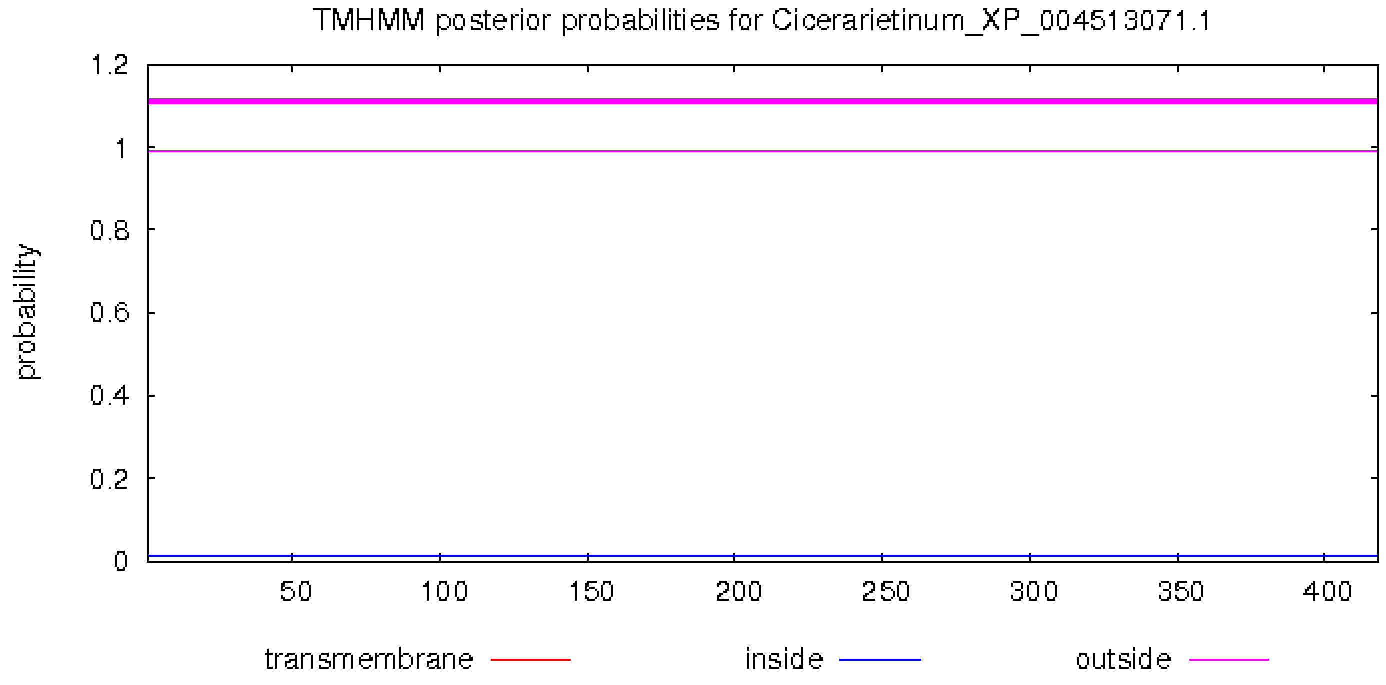

plot in postscript, script for making the plot in gnuplot, data for plot

---

```
# Cicerarietinum_XP_004513072.1 Length: 418
# Cicerarietinum_XP_004513072.1 Number of predicted TMHs:  0
# Cicerarietinum_XP_004513072.1 Exp number of AAs in TMHs: 0
# Cicerarietinum_XP_004513072.1 Exp number, first 60 AAs:  0
# Cicerarietinum_XP_004513072.1 Total prob of N-in:        0.01174
Cicerarietinum_XP_004513072.1   TMHMM2.0        outside      1   418
```

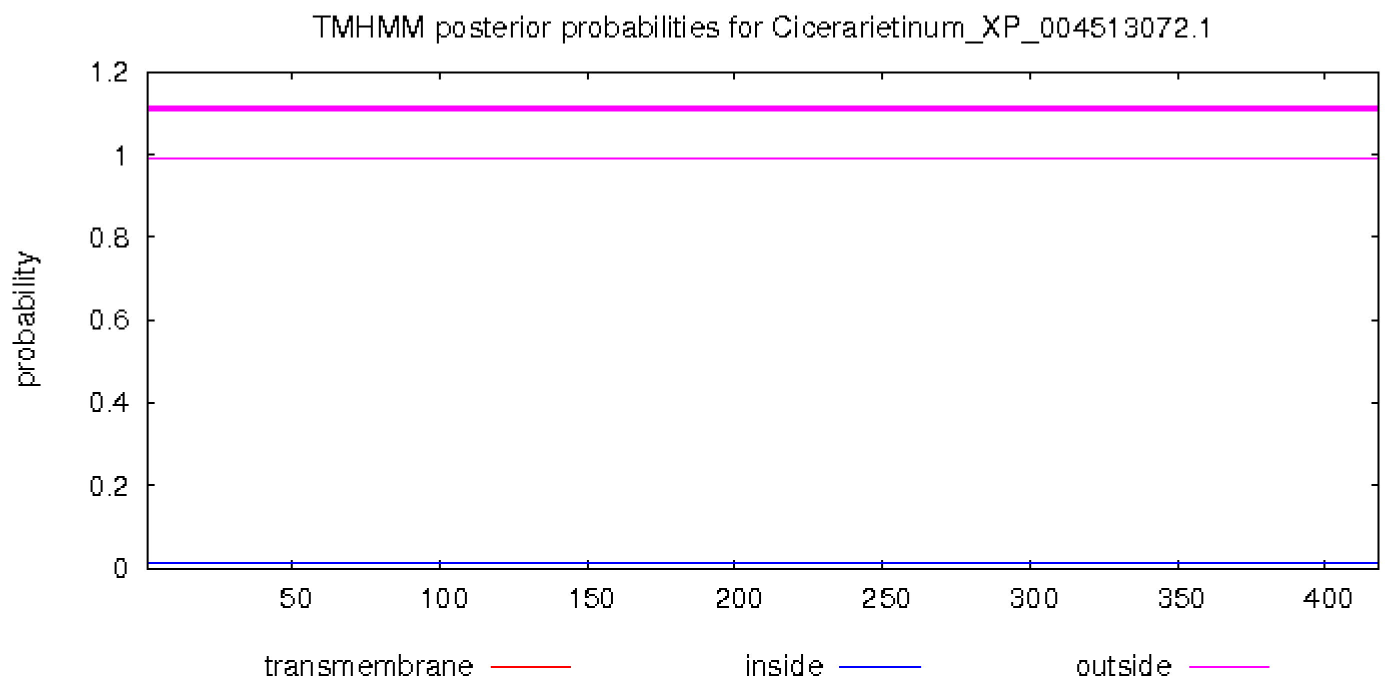

plot in postscript, script for making the plot in gnuplot, data for plot

---

```
# Cicerarietinum_XP_004513361.2 Length: 272
# Cicerarietinum_XP_004513361.2 Number of predicted TMHs:  0
# Cicerarietinum_XP_004513361.2 Exp number of AAs in TMHs: 0.00042
# Cicerarietinum_XP_004513361.2 Exp number, first 60 AAs:  0.00042
# Cicerarietinum_XP_004513361.2 Total prob of N-in:        0.00663
Cicerarietinum_XP_004513361.2   TMHMM2.0        outside      1   272
```

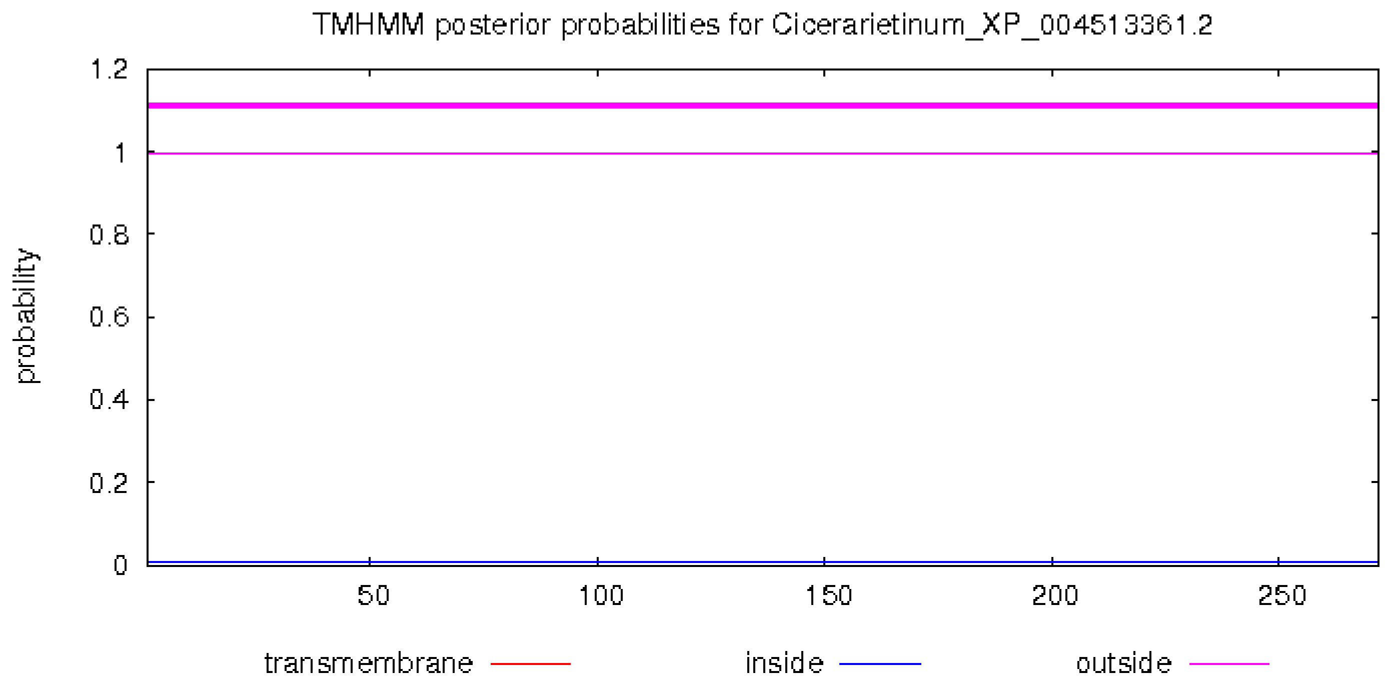

plot in postscript, script for making the plot in gnuplot, data for plot

---

```
# Cicerarietinum_XP_004514021.1 Length: 610
# Cicerarietinum_XP_004514021.1 Number of predicted TMHs:  1
# Cicerarietinum_XP_004514021.1 Exp number of AAs in TMHs: 22.60723
# Cicerarietinum_XP_004514021.1 Exp number, first 60 AAs:  0
# Cicerarietinum_XP_004514021.1 Total prob of N-in:        0.00220
Cicerarietinum_XP_004514021.1   TMHMM2.0        outside      1   581
```

```
Cicerarietinum_XP_004514021.1   TMHMM2.0        TMhelix    582   604
Cicerarietinum_XP_004514021.1   TMHMM2.0        inside     605   610
```

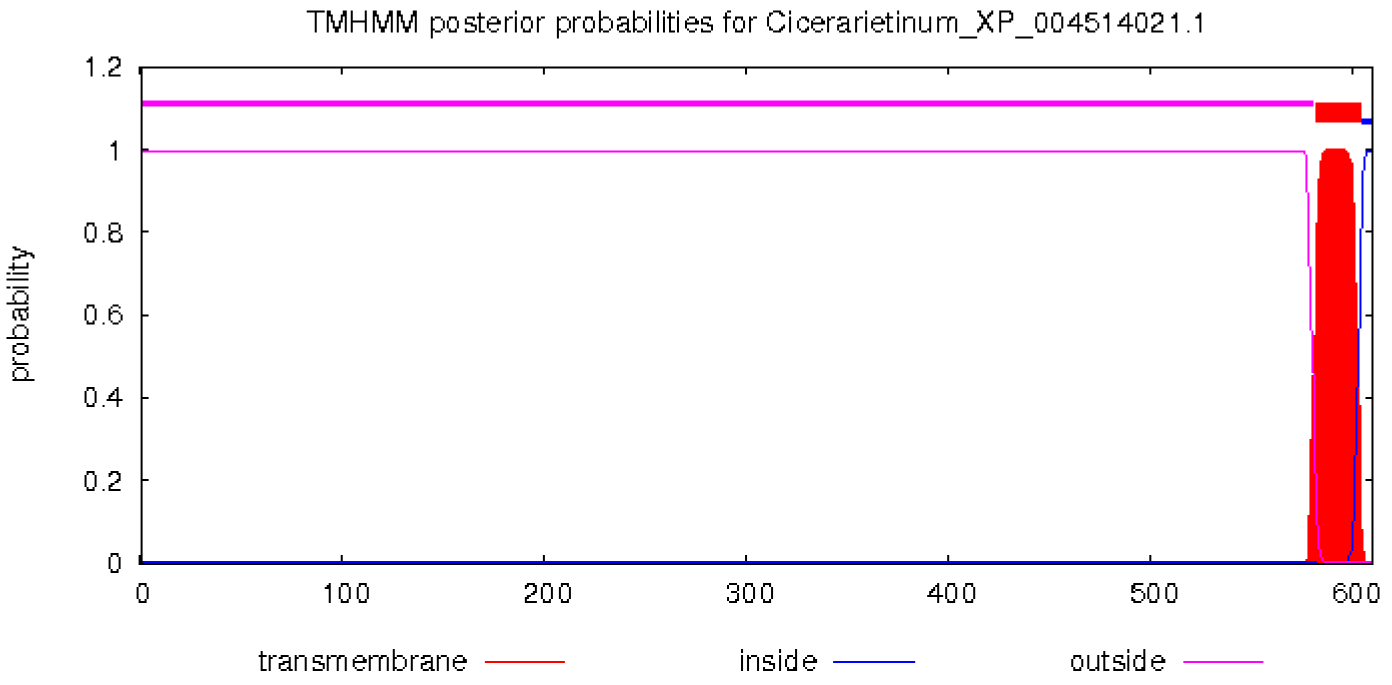

plot in postscript, script for making the plot in gnuplot, data for plot

---

```
# Cicerarietinum_XP_004514350.1 Length: 329
# Cicerarietinum_XP_004514350.1 Number of predicted TMHs:  0
# Cicerarietinum_XP_004514350.1 Exp number of AAs in TMHs: 0.00216
# Cicerarietinum_XP_004514350.1 Exp number, first 60 AAs:  0.00028
# Cicerarietinum_XP_004514350.1 Total prob of N-in:        0.00414
Cicerarietinum_XP_004514350.1   TMHMM2.0        outside      1   329
```

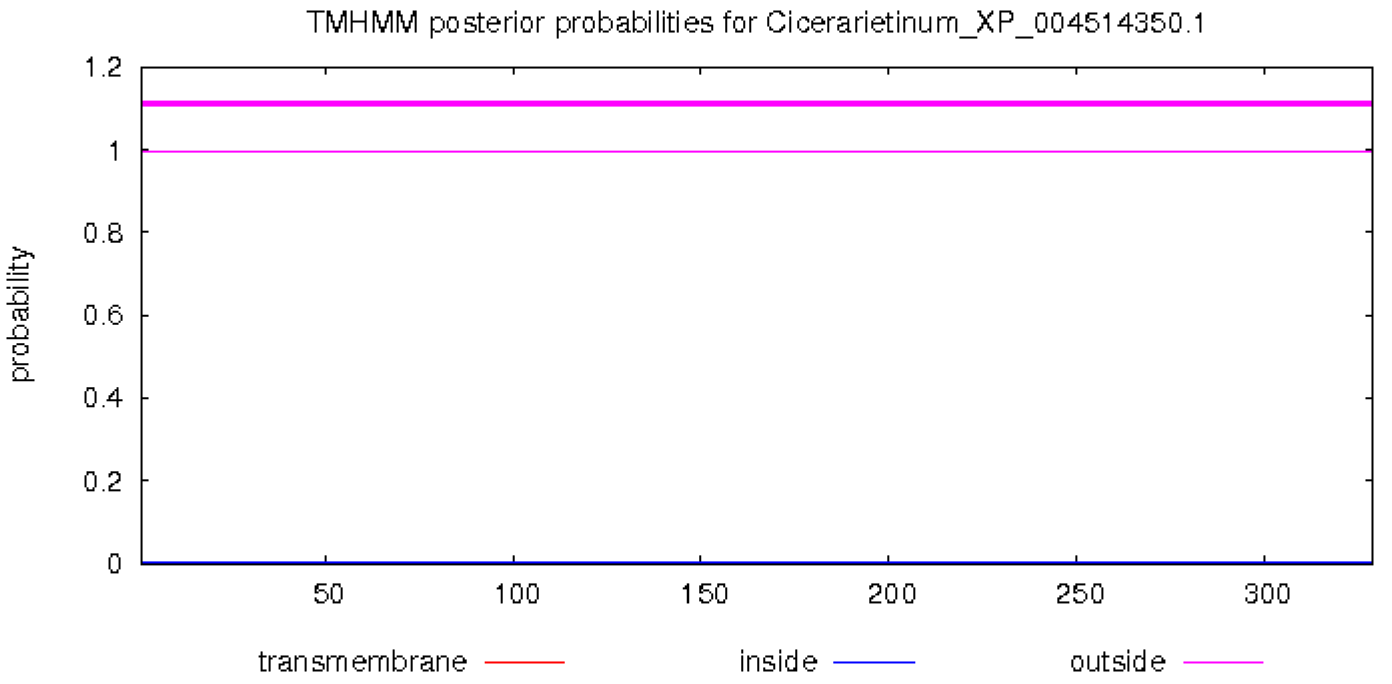

plot in postscript, script for making the plot in gnuplot, data for plot

---

```
# Cicerarietinum_XP_004514529.1 Length: 362
# Cicerarietinum_XP_004514529.1 Number of predicted TMHs:  0
# Cicerarietinum_XP_004514529.1 Exp number of AAs in TMHs: 0
# Cicerarietinum_XP_004514529.1 Exp number, first 60 AAs:  0
```

```
# Cicerarietinum_XP_004514529.1 Total prob of N-in:        0.00356
Cicerarietinum_XP_004514529.1   TMHMM2.0        outside      1   362
```

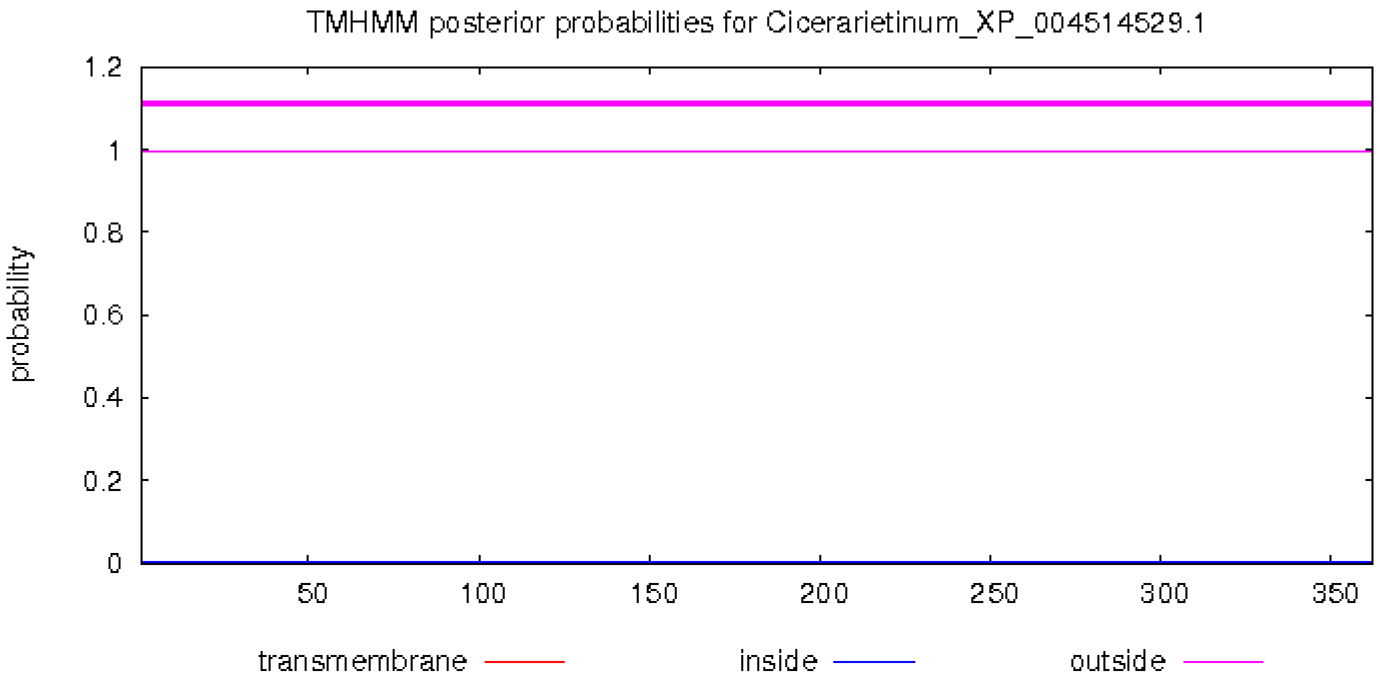

plot in postscript, script for making the plot in gnuplot, data for plot

---

```
# Cicerarietinum_XP_004514929.1 Length: 428
# Cicerarietinum_XP_004514929.1 Number of predicted TMHs:  0
# Cicerarietinum_XP_004514929.1 Exp number of AAs in TMHs: 0
# Cicerarietinum_XP_004514929.1 Exp number, first 60 AAs:  0
# Cicerarietinum_XP_004514929.1 Total prob of N-in:        0.00406
Cicerarietinum_XP_004514929.1   TMHMM2.0        outside      1   428
```

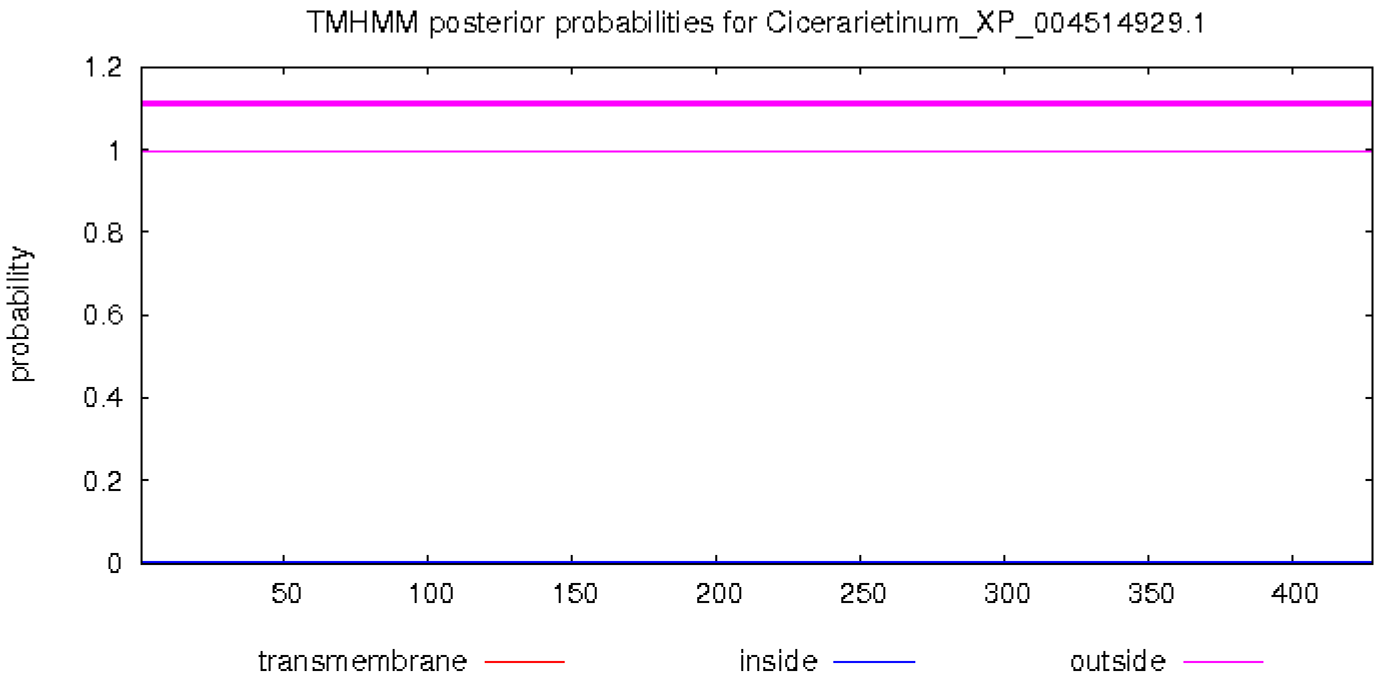

plot in postscript, script for making the plot in gnuplot, data for plot

---

```
# Cicerarietinum_XP_004514930.1 Length: 427
# Cicerarietinum_XP_004514930.1 Number of predicted TMHs:  0
# Cicerarietinum_XP_004514930.1 Exp number of AAs in TMHs: 0
# Cicerarietinum_XP_004514930.1 Exp number, first 60 AAs:  0
```

```
# Cicerarietinum_XP_004514930.1 Total prob of N-in:        0.00411
Cicerarietinum_XP_004514930.1   TMHMM2.0        outside      1   427
```

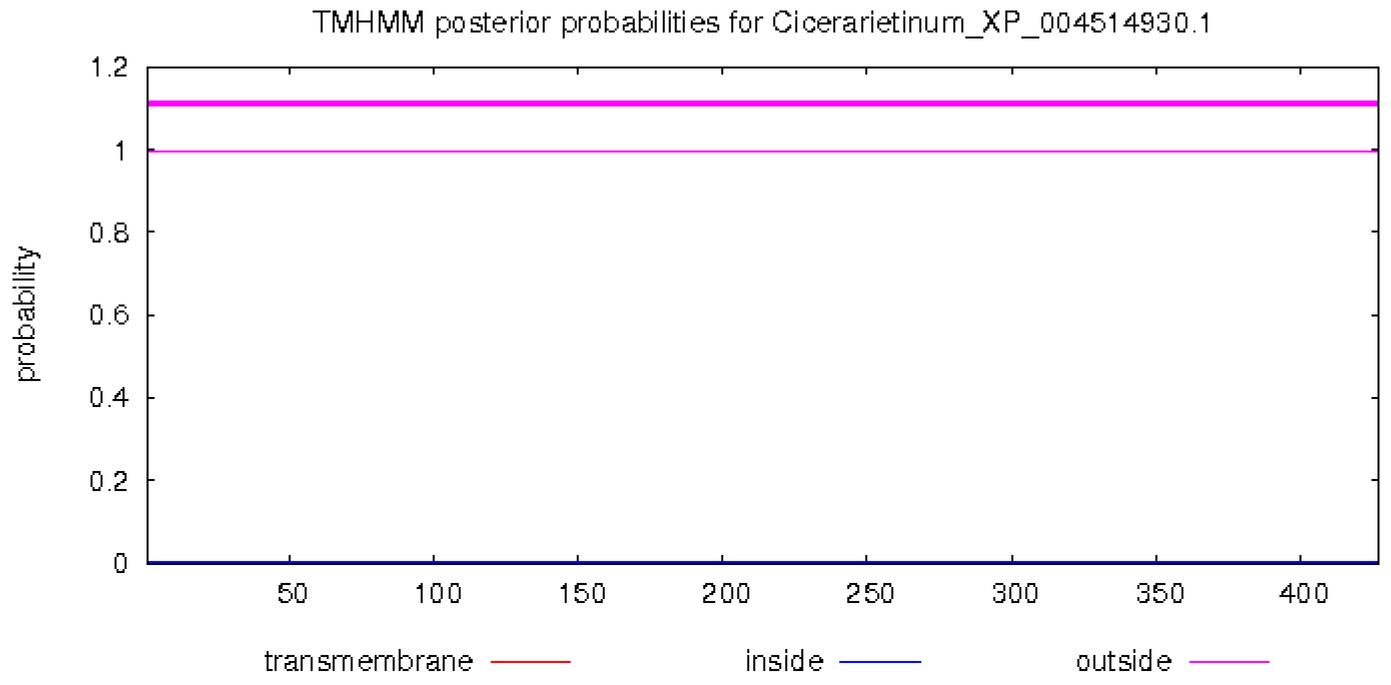

plot in postscript, script for making the plot in gnuplot, data for plot

---

```
# Cicerarietinum_XP_004515256.1 Length: 238
# Cicerarietinum_XP_004515256.1 Number of predicted TMHs:  0
# Cicerarietinum_XP_004515256.1 Exp number of AAs in TMHs: 0.0002
# Cicerarietinum_XP_004515256.1 Exp number, first 60 AAs:  0
# Cicerarietinum_XP_004515256.1 Total prob of N-in:        0.00221
Cicerarietinum_XP_004515256.1   TMHMM2.0        outside      1   238
```

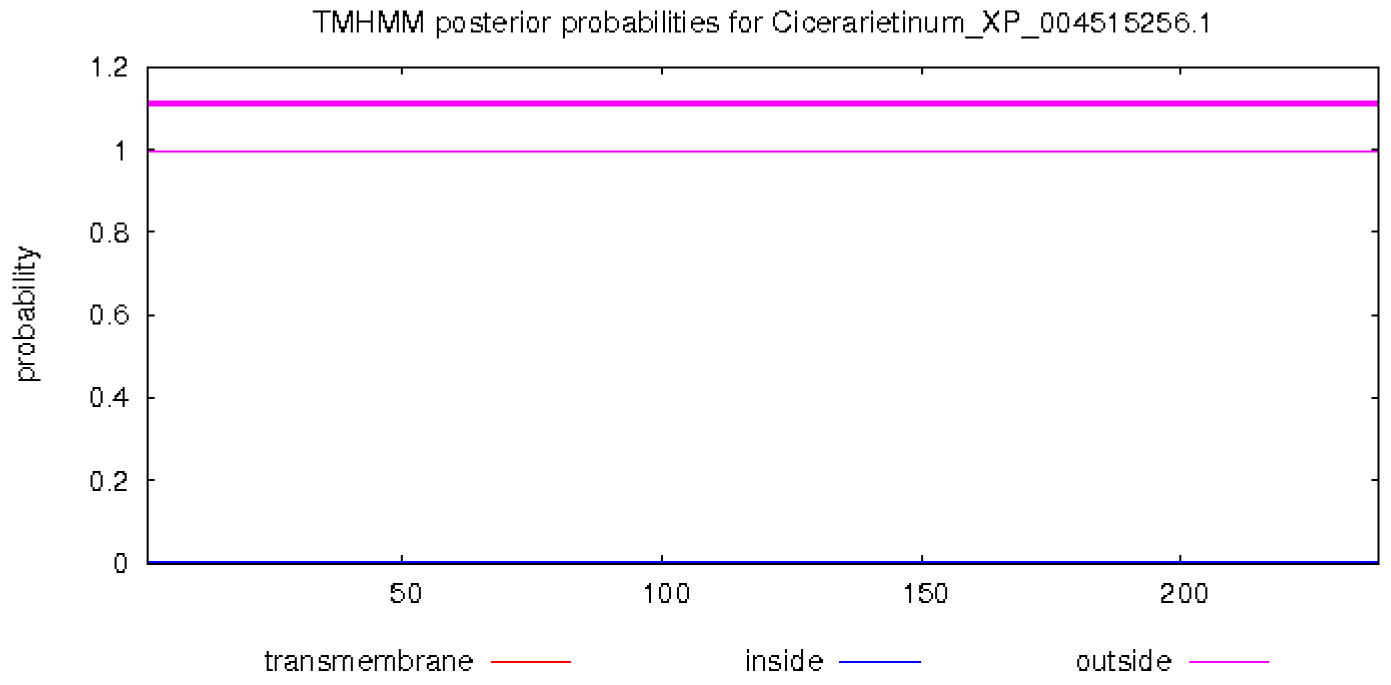

plot in postscript, script for making the plot in gnuplot, data for plot

---

```
# Cicerarietinum_XP_004515741.1 Length: 345
# Cicerarietinum_XP_004515741.1 Number of predicted TMHs:  0
# Cicerarietinum_XP_004515741.1 Exp number of AAs in TMHs: 0
# Cicerarietinum_XP_004515741.1 Exp number, first 60 AAs:  0
```

```
# Cicerarietinum_XP_004515741.1 Total prob of N-in:        0.00472
Cicerarietinum_XP_004515741.1   TMHMM2.0        outside      1   345
```

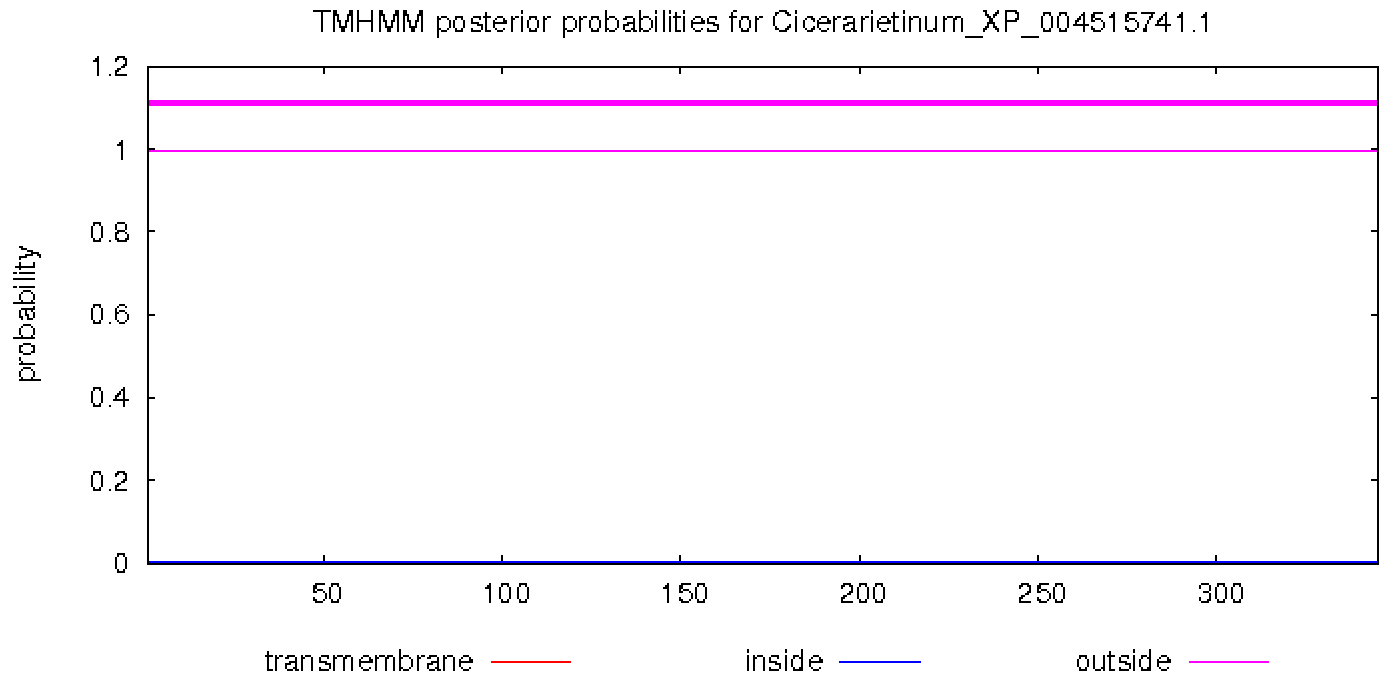

plot in postscript, script for making the plot in gnuplot, data for plot

---

```
# Cicerarietinum_XP_004516088.1 Length: 407
# Cicerarietinum_XP_004516088.1 Number of predicted TMHs:  0
# Cicerarietinum_XP_004516088.1 Exp number of AAs in TMHs: 0.00646
# Cicerarietinum_XP_004516088.1 Exp number, first 60 AAs:  0
# Cicerarietinum_XP_004516088.1 Total prob of N-in:        0.00879
Cicerarietinum_XP_004516088.1   TMHMM2.0        outside      1   407
```

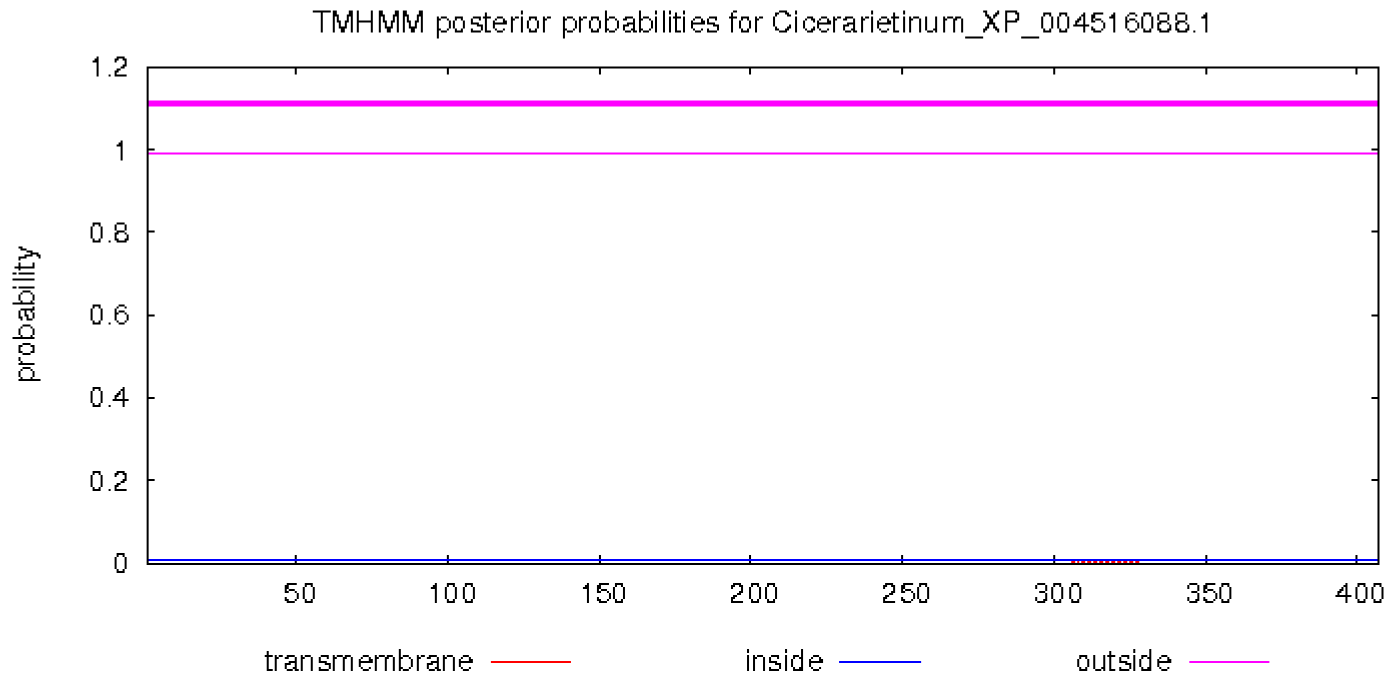

plot in postscript, script for making the plot in gnuplot, data for plot

---

```
# Cicerarietinum_XP_012567502.1 Length: 339
# Cicerarietinum_XP_012567502.1 Number of predicted TMHs:  0
# Cicerarietinum_XP_012567502.1 Exp number of AAs in TMHs: 0
# Cicerarietinum_XP_012567502.1 Exp number, first 60 AAs:  0
```

```
# Cicerarietinum_XP_012567502.1 Total prob of N-in:        0.01424
Cicerarietinum_XP_012567502.1   TMHMM2.0        outside      1   339
```

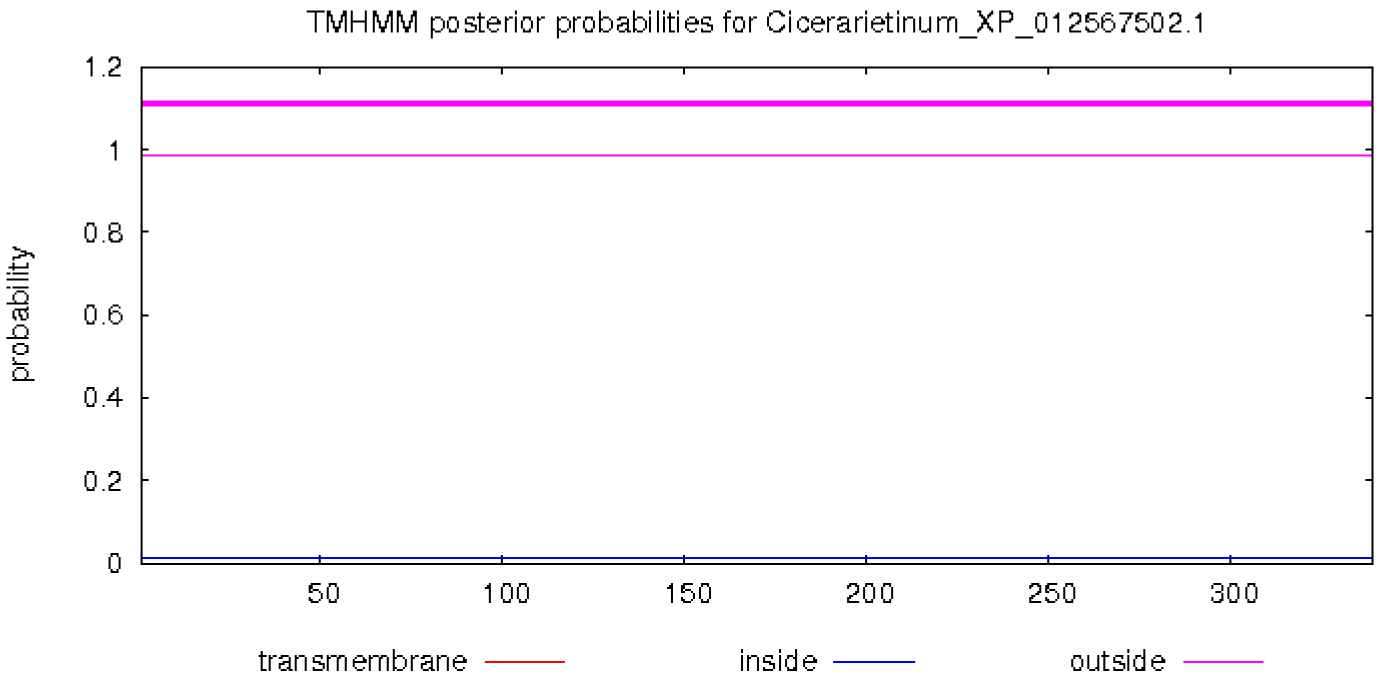

plot in postscript, script for making the plot in gnuplot, data for plot

---

```
# Cicerarietinum_XP_012568084.1 Length: 347
# Cicerarietinum_XP_012568084.1 Number of predicted TMHs:  0
# Cicerarietinum_XP_012568084.1 Exp number of AAs in TMHs: 0.00211
# Cicerarietinum_XP_012568084.1 Exp number, first 60 AAs:  0.00211
# Cicerarietinum_XP_012568084.1 Total prob of N-in:        0.06214
Cicerarietinum_XP_012568084.1   TMHMM2.0        outside      1   347
```

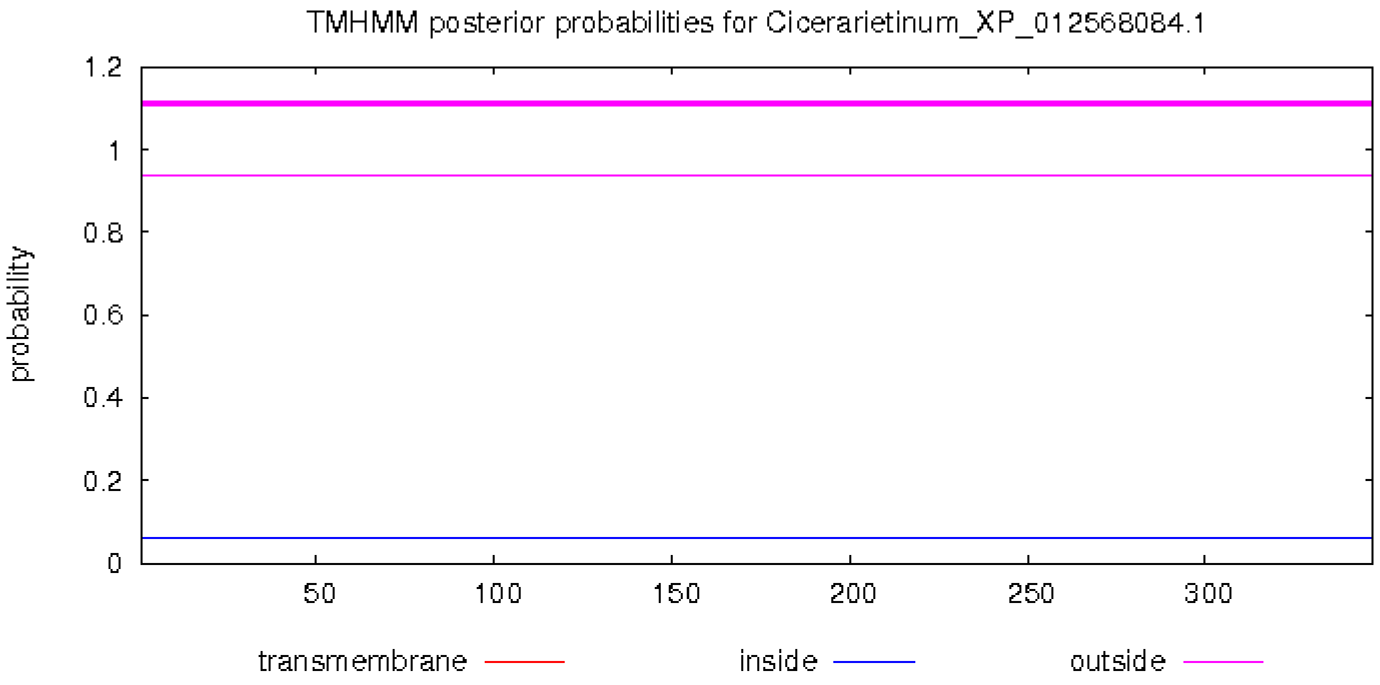

plot in postscript, script for making the plot in gnuplot, data for plot

---

```
# Cicerarietinum_XP_012568792.1 Length: 310
# Cicerarietinum_XP_012568792.1 Number of predicted TMHs:  0
# Cicerarietinum_XP_012568792.1 Exp number of AAs in TMHs: 0
# Cicerarietinum_XP_012568792.1 Exp number, first 60 AAs:  0
```

```
# Cicerarietinum_XP_012568792.1 Total prob of N-in:        0.01016
Cicerarietinum_XP_012568792.1   TMHMM2.0        outside      1   310
```

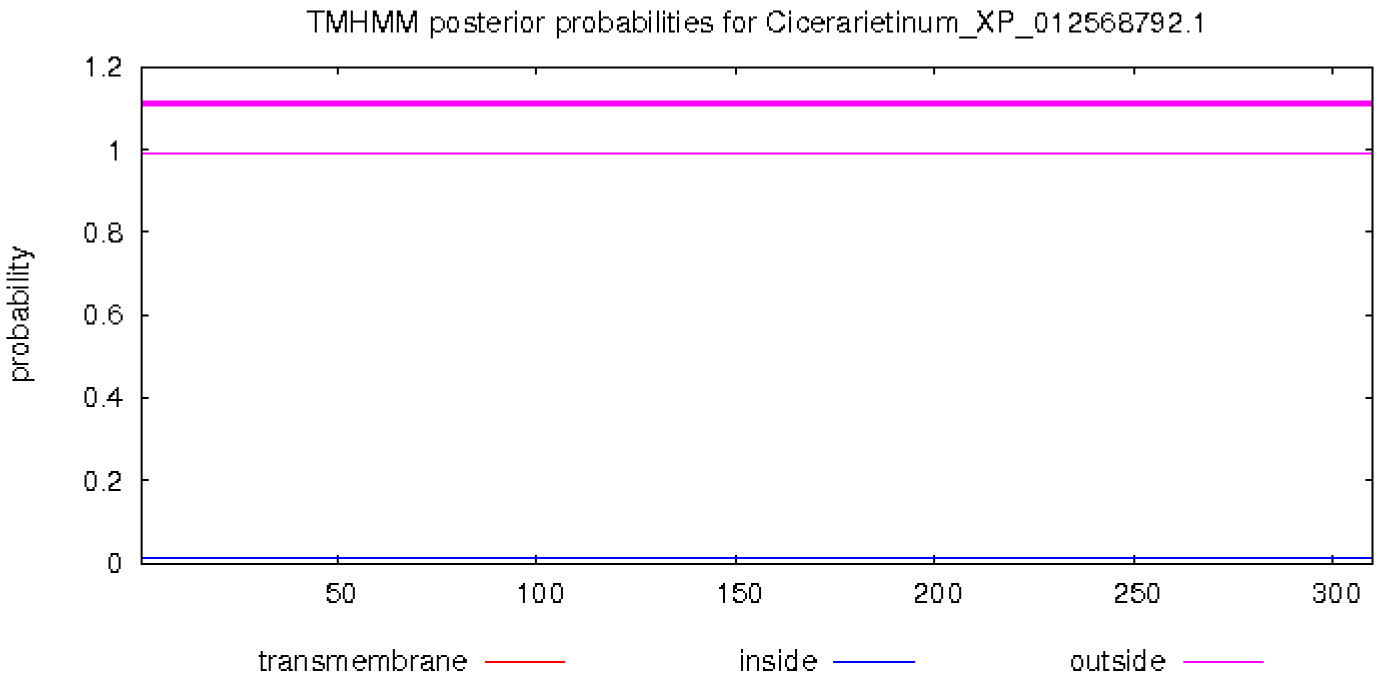

plot in postscript, script for making the plot in gnuplot, data for plot

---

```
# Cicerarietinum_XP_012570459.1 Length: 359
# Cicerarietinum_XP_012570459.1 Number of predicted TMHs:  0
# Cicerarietinum_XP_012570459.1 Exp number of AAs in TMHs: 0.0002
# Cicerarietinum_XP_012570459.1 Exp number, first 60 AAs:  0
# Cicerarietinum_XP_012570459.1 Total prob of N-in:        0.00672
Cicerarietinum_XP_012570459.1   TMHMM2.0        outside      1   359
```

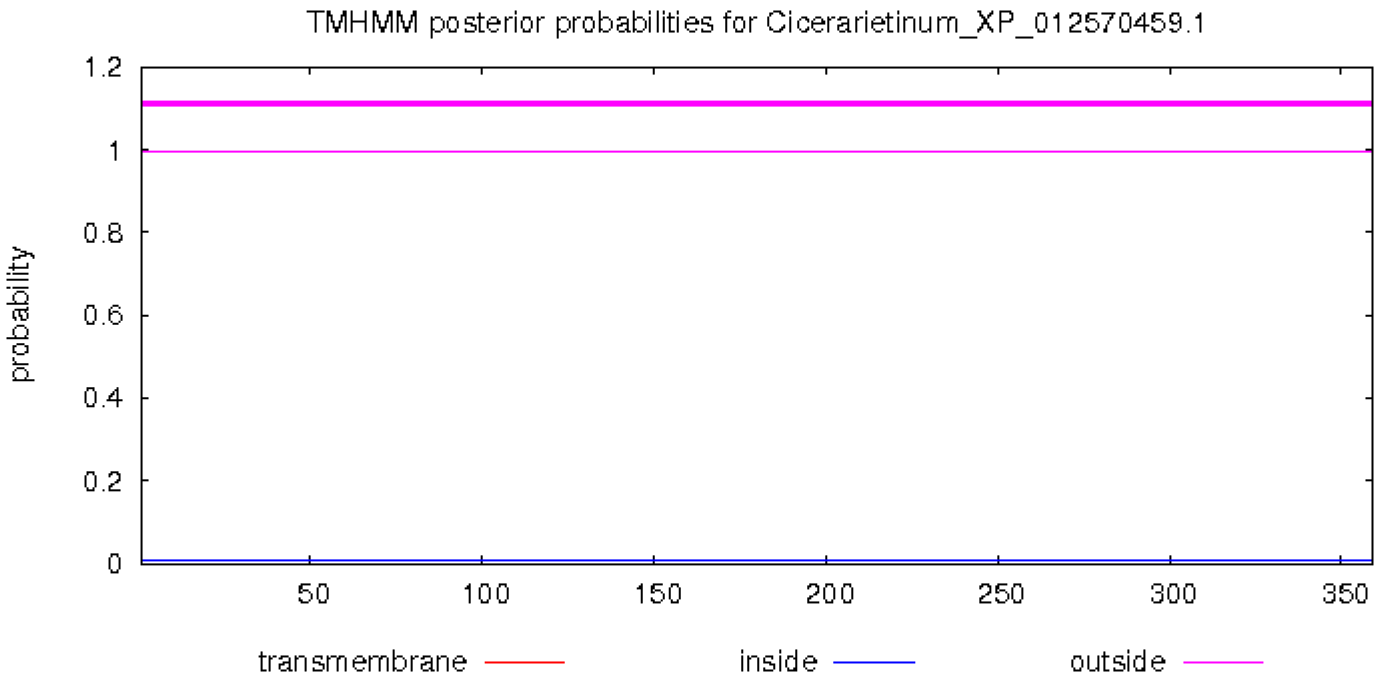

plot in postscript, script for making the plot in gnuplot, data for plot

---

```
# Cicerarietinum_XP_012571554.1 Length: 299
# Cicerarietinum_XP_012571554.1 Number of predicted TMHs:  0
# Cicerarietinum_XP_012571554.1 Exp number of AAs in TMHs: 0.00797
# Cicerarietinum_XP_012571554.1 Exp number, first 60 AAs:  0
```

```
# Cicerarietinum_XP_012571554.1 Total prob of N-in:        0.12712
Cicerarietinum_XP_012571554.1   TMHMM2.0        outside      1   299
```

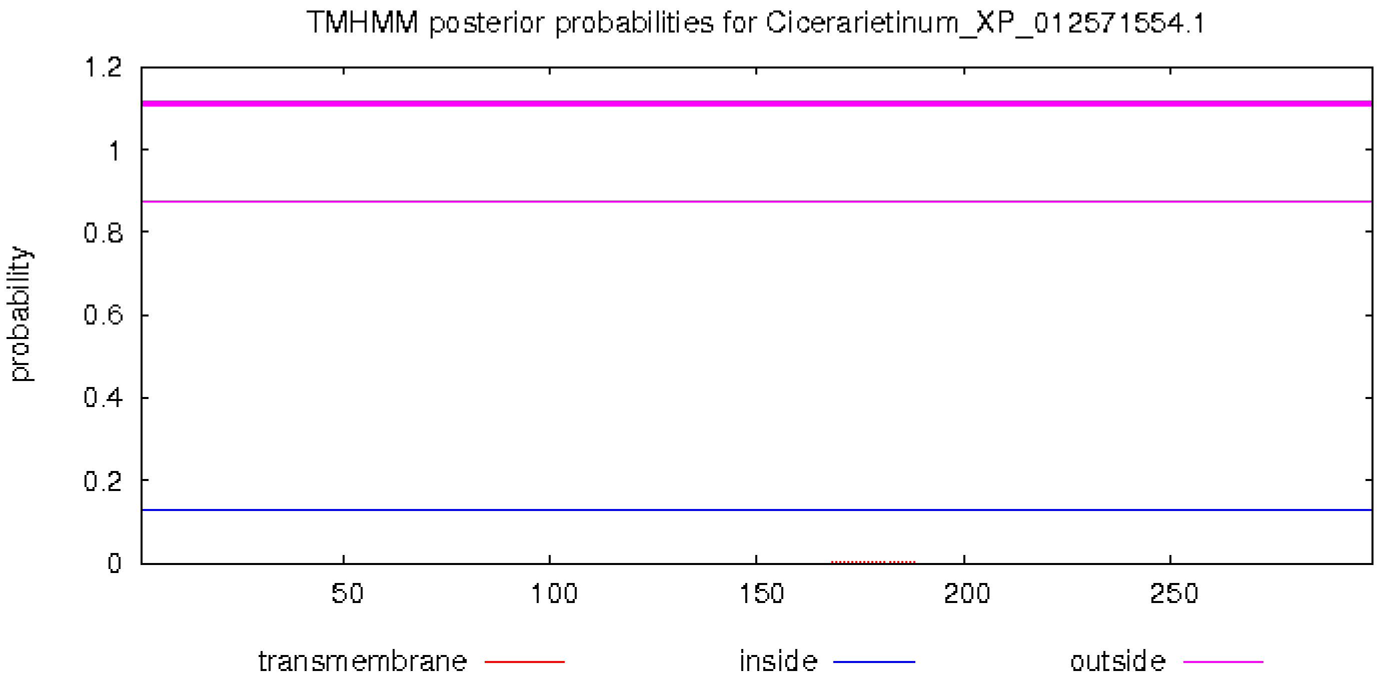

plot in postscript, script for making the plot in gnuplot, data for plot

---

```
# Cicerarietinum_XP_012571948.1 Length: 601
# Cicerarietinum_XP_012571948.1 Number of predicted TMHs:  1
# Cicerarietinum_XP_012571948.1 Exp number of AAs in TMHs: 22.28271
# Cicerarietinum_XP_012571948.1 Exp number, first 60 AAs:  0.00187
# Cicerarietinum_XP_012571948.1 Total prob of N-in:        0.00297
Cicerarietinum_XP_012571948.1   TMHMM2.0        outside      1   573
Cicerarietinum_XP_012571948.1   TMHMM2.0        TMhelix    574   596
Cicerarietinum_XP_012571948.1   TMHMM2.0        inside     597   601
```

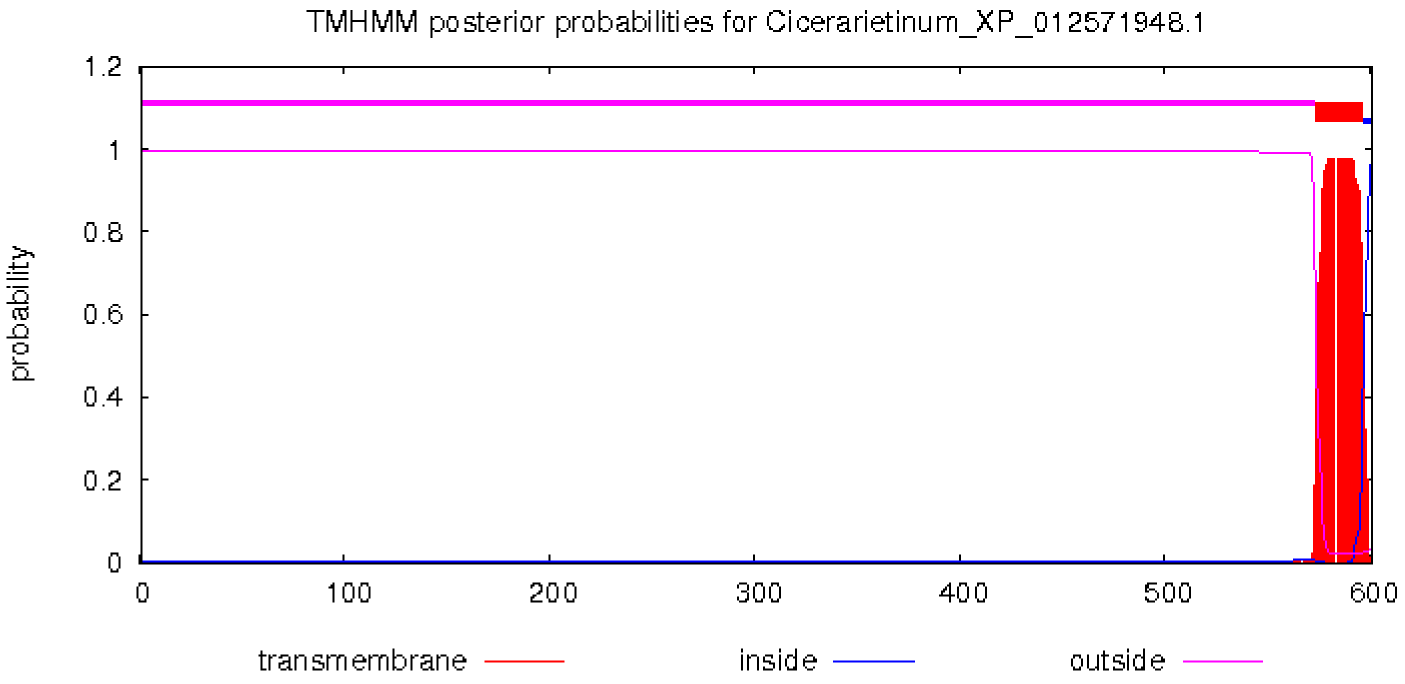

plot in postscript, script for making the plot in gnuplot, data for plot

---

```
# Cicerarietinum_XP_012572103.1 Length: 440
# Cicerarietinum_XP_012572103.1 Number of predicted TMHs:  0
```

```
# Cicerarietinum_XP_012572103.1 Exp number of AAs in TMHs: 0
# Cicerarietinum_XP_012572103.1 Exp number, first 60 AAs:  0
# Cicerarietinum_XP_012572103.1 Total prob of N-in:        0.02116
Cicerarietinum_XP_012572103.1	TMHMM2.0	outside	     1   440
```

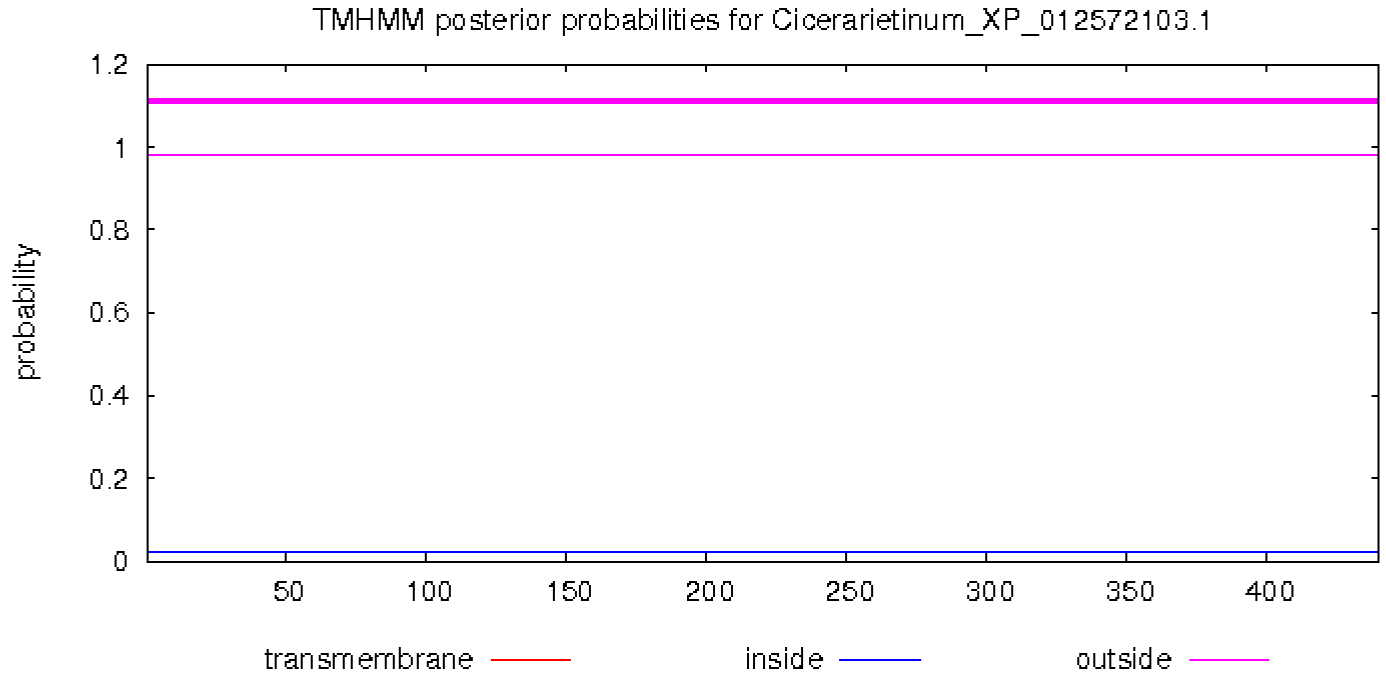

plot in postscript, script for making the plot in gnuplot, data for plot

---

```
# Cicerarietinum_XP_012572104.1 Length: 440
# Cicerarietinum_XP_012572104.1 Number of predicted TMHs:  0
# Cicerarietinum_XP_012572104.1 Exp number of AAs in TMHs: 0
# Cicerarietinum_XP_012572104.1 Exp number, first 60 AAs:  0
# Cicerarietinum_XP_012572104.1 Total prob of N-in:        0.02116
Cicerarietinum_XP_012572104.1	TMHMM2.0	outside	     1   440
```

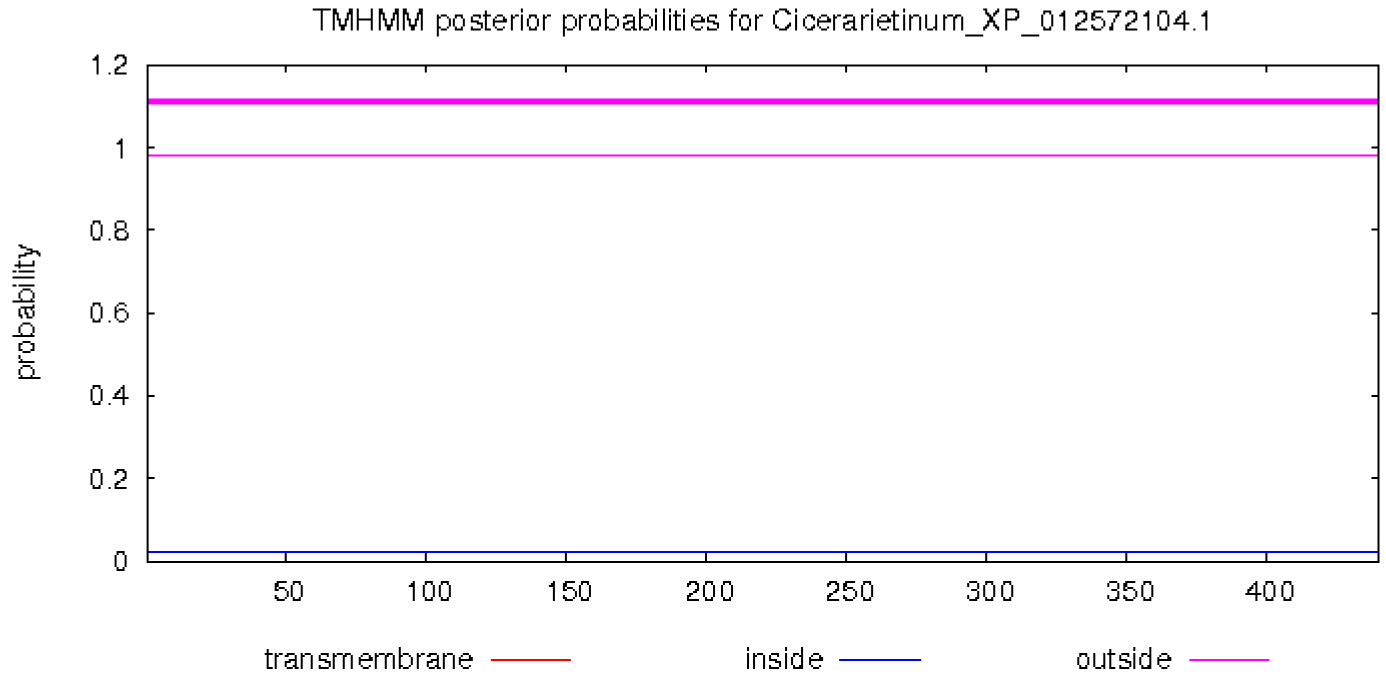

plot in postscript, script for making the plot in gnuplot, data for plot

---

```
# Cicerarietinum_XP_012572105.1 Length: 439
# Cicerarietinum_XP_012572105.1 Number of predicted TMHs:  0
```

```
# Cicerarietinum_XP_012572105.1 Exp number of AAs in TMHs: 0
# Cicerarietinum_XP_012572105.1 Exp number, first 60 AAs:  0
# Cicerarietinum_XP_012572105.1 Total prob of N-in:        0.02142
Cicerarietinum_XP_012572105.1   TMHMM2.0        outside      1   439
```

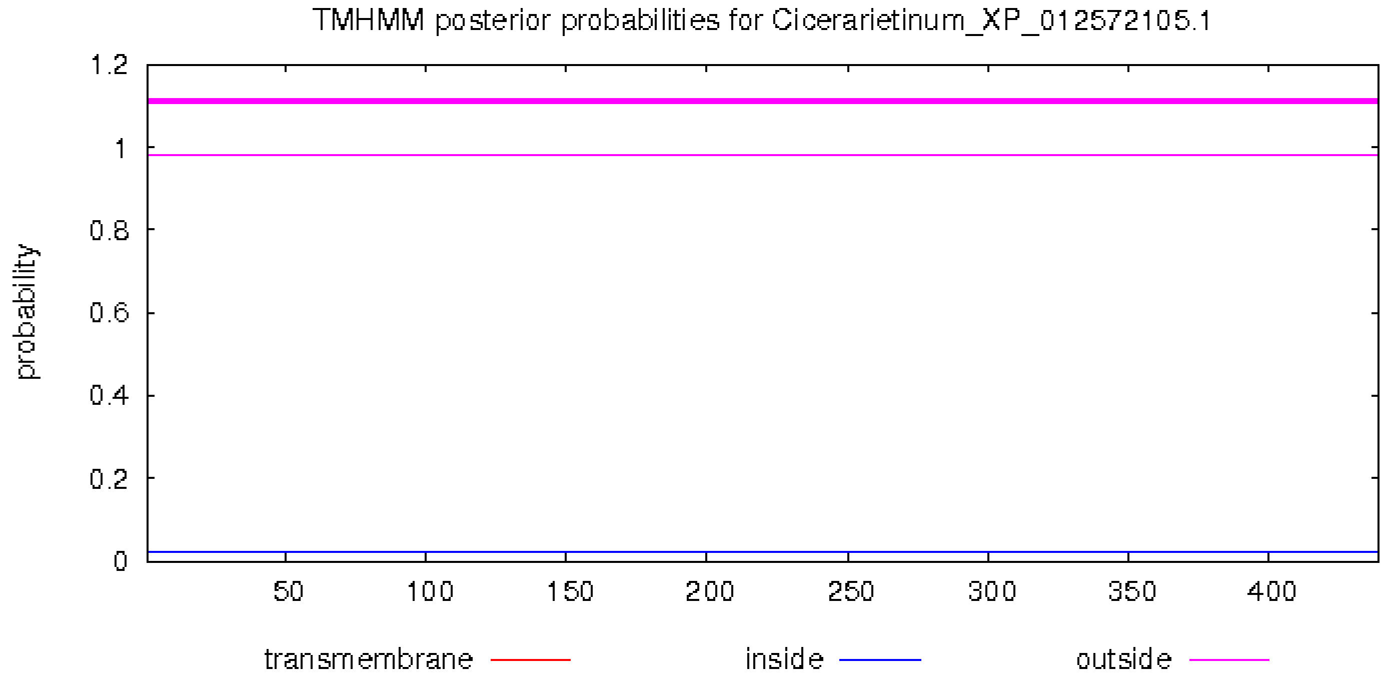

plot in postscript, script for making the plot in gnuplot, data for plot

---

```
# Cicerarietinum_XP_012572255.1 Length: 305
# Cicerarietinum_XP_012572255.1 Number of predicted TMHs:  0
# Cicerarietinum_XP_012572255.1 Exp number of AAs in TMHs: 0.1135
# Cicerarietinum_XP_012572255.1 Exp number, first 60 AAs:  0.00104
# Cicerarietinum_XP_012572255.1 Total prob of N-in:        0.00726
Cicerarietinum_XP_012572255.1   TMHMM2.0        outside      1   305
```

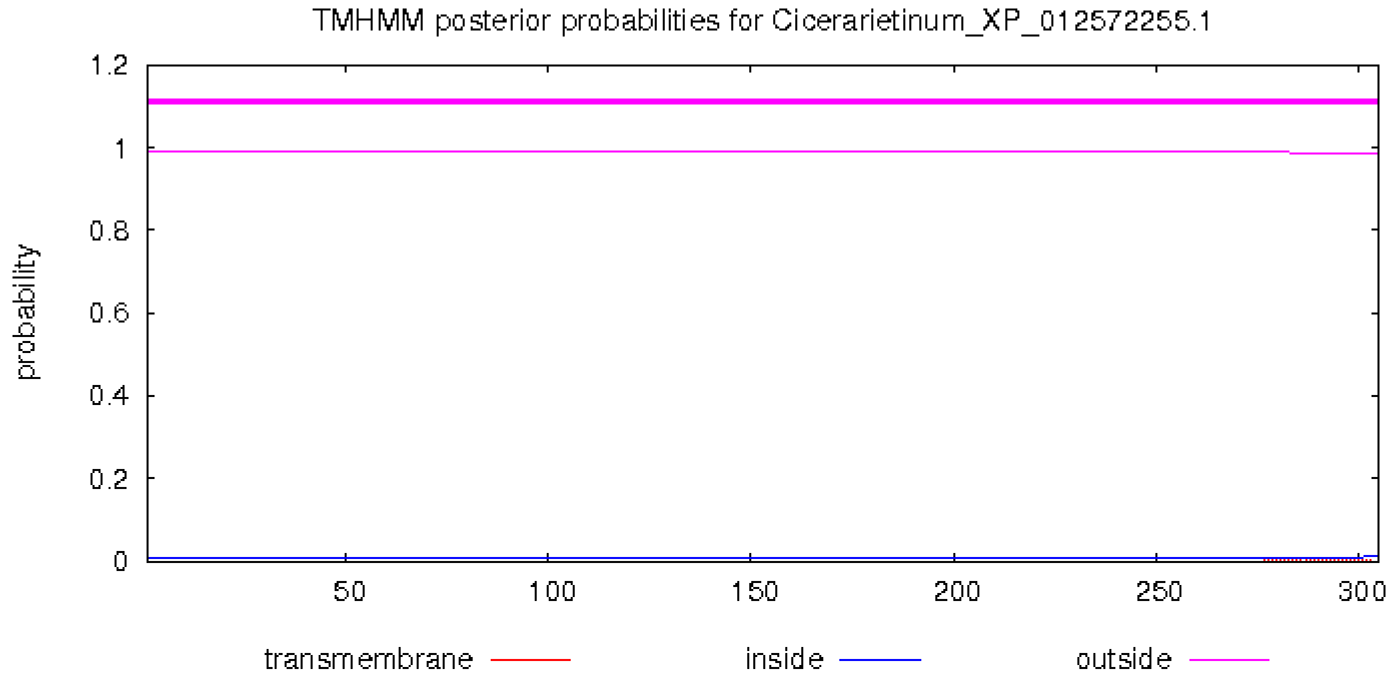

plot in postscript, script for making the plot in gnuplot, data for plot

---

```
# Cicerarietinum_XP_012572610.1 Length: 256
# Cicerarietinum_XP_012572610.1 Number of predicted TMHs:  0
```

```
# Cicerarietinum_XP_012572610.1 Exp number of AAs in TMHs: 0.00126
# Cicerarietinum_XP_012572610.1 Exp number, first 60 AAs:  0.0002
# Cicerarietinum_XP_012572610.1 Total prob of N-in:        0.02482
Cicerarietinum_XP_012572610.1   TMHMM2.0        outside      1   256
```

TMHMM posterior probabilities for Cicerarietinum_XP_012572610.1

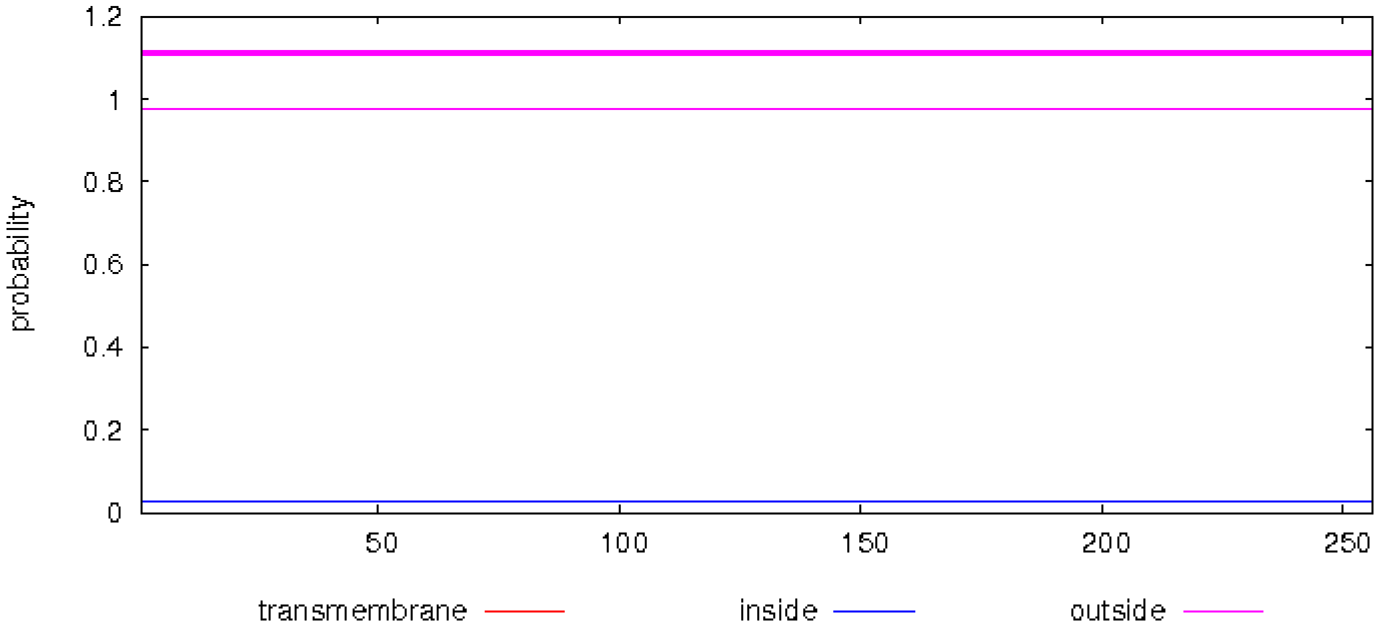

plot in postscript, script for making the plot in gnuplot, data for plot

---

```
# Cicerarietinum_XP_012572681.1 Length: 561
# Cicerarietinum_XP_012572681.1 Number of predicted TMHs:  2
# Cicerarietinum_XP_012572681.1 Exp number of AAs in TMHs: 43.57356
# Cicerarietinum_XP_012572681.1 Exp number, first 60 AAs:  0.00072
# Cicerarietinum_XP_012572681.1 Total prob of N-in:        0.06359
Cicerarietinum_XP_012572681.1   TMHMM2.0        outside      1   329
Cicerarietinum_XP_012572681.1   TMHMM2.0        TMhelix    330   352
Cicerarietinum_XP_012572681.1   TMHMM2.0        inside     353   448
Cicerarietinum_XP_012572681.1   TMHMM2.0        TMhelix    449   471
Cicerarietinum_XP_012572681.1   TMHMM2.0        outside    472   561
```

TMHMM posterior probabilities for Cicerarietinum_XP_012572681.1

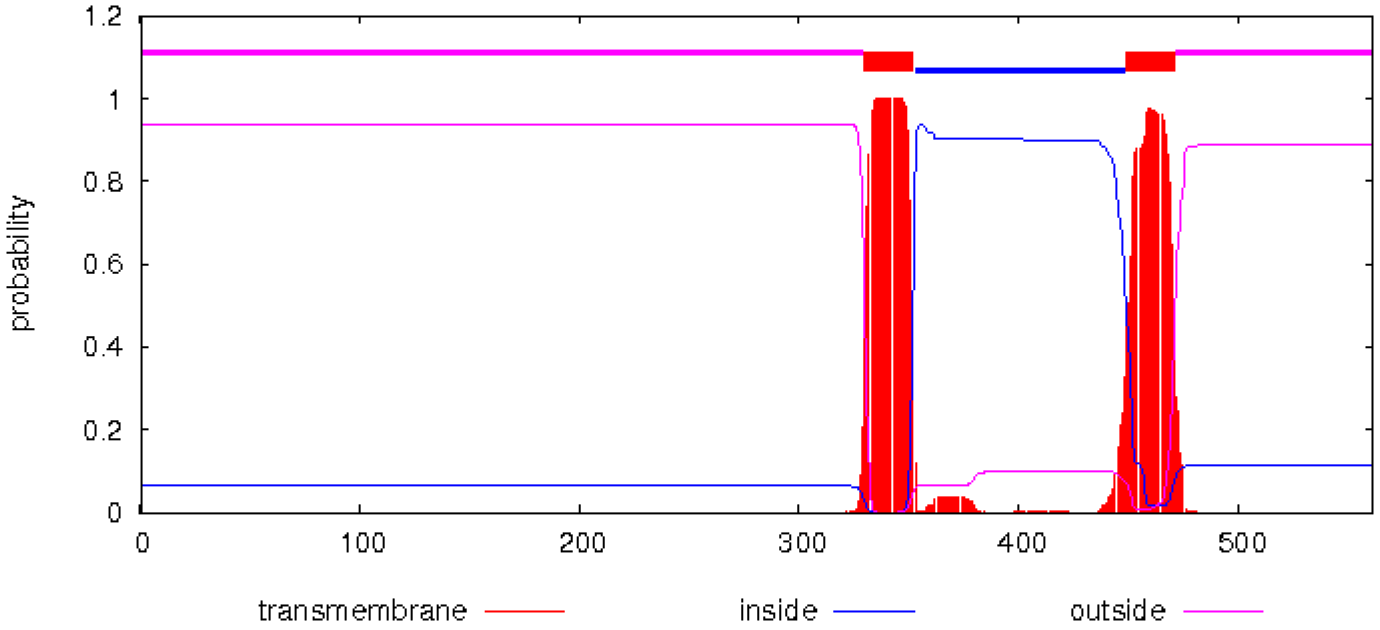

plot in postscript, script for making the plot in gnuplot, data for plot

```
# Cicerarietinum_XP_012574126.1 Length: 404
# Cicerarietinum_XP_012574126.1 Number of predicted TMHs:  0
# Cicerarietinum_XP_012574126.1 Exp number of AAs in TMHs: 0.00122
# Cicerarietinum_XP_012574126.1 Exp number, first 60 AAs:  0
# Cicerarietinum_XP_012574126.1 Total prob of N-in:        0.00218
Cicerarietinum_XP_012574126.1	TMHMM2.0	outside	     1   404
```

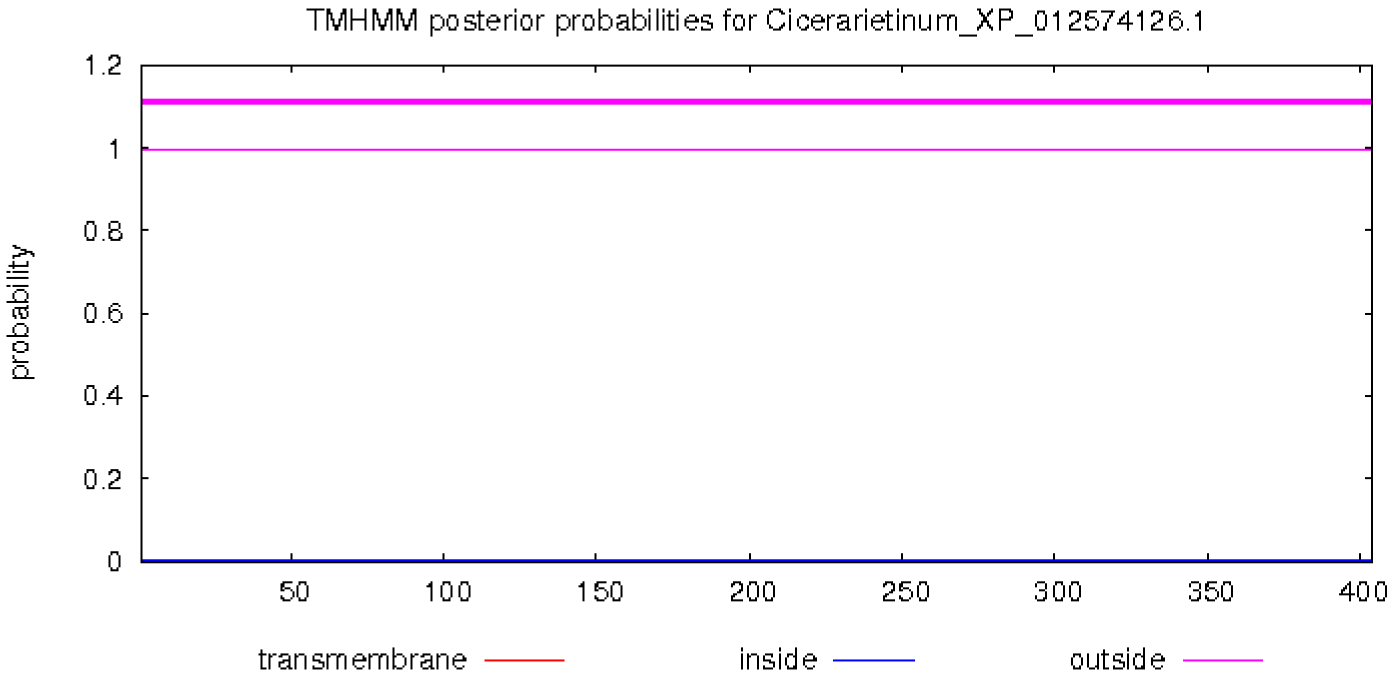

plot in postscript, script for making the plot in gnuplot, data for plot

```
# Cicerarietinum_XP_012574127.1 Length: 323
# Cicerarietinum_XP_012574127.1 Number of predicted TMHs:  0
# Cicerarietinum_XP_012574127.1 Exp number of AAs in TMHs: 0.00123
# Cicerarietinum_XP_012574127.1 Exp number, first 60 AAs:  0
# Cicerarietinum_XP_012574127.1 Total prob of N-in:        0.00589
Cicerarietinum_XP_012574127.1	TMHMM2.0	outside	     1   323
```

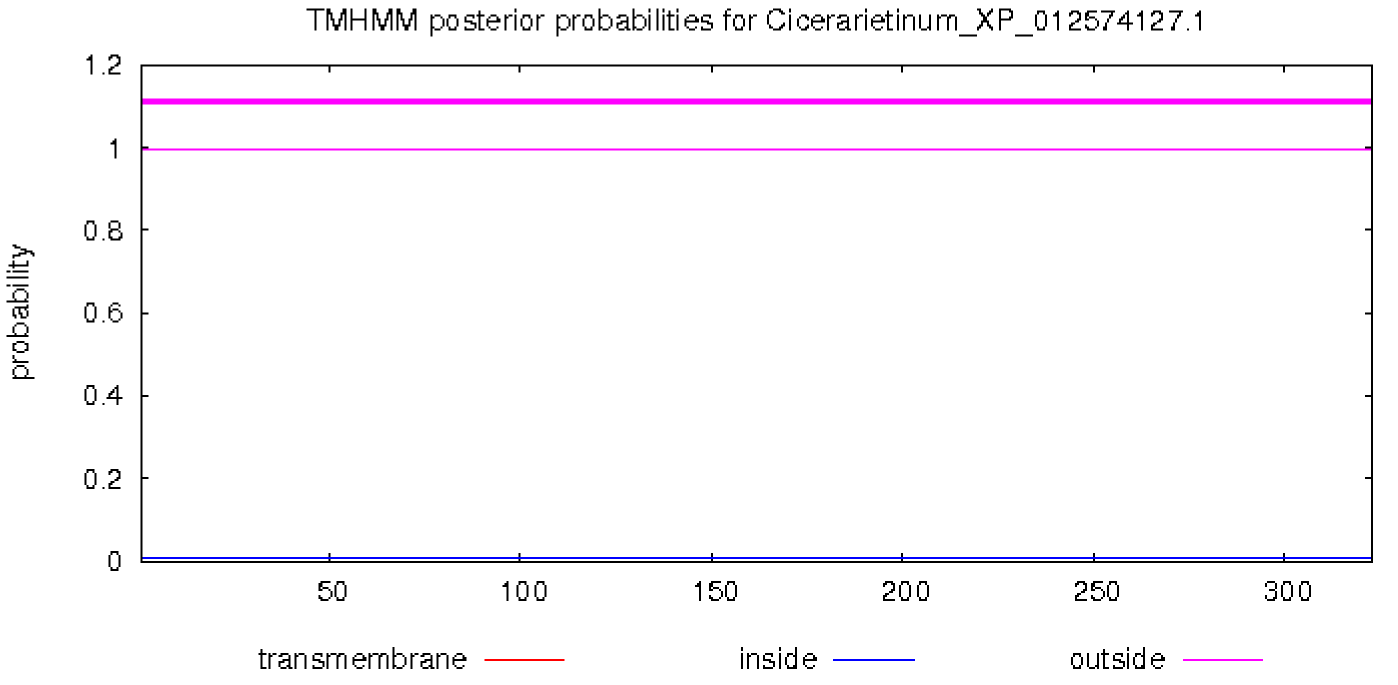

plot in postscript, script for making the plot in gnuplot, data for plot

```
# Cicerarietinum_XP_012574420.1 Length: 335
# Cicerarietinum_XP_012574420.1 Number of predicted TMHs:  0
# Cicerarietinum_XP_012574420.1 Exp number of AAs in TMHs: 0
# Cicerarietinum_XP_012574420.1 Exp number, first 60 AAs:  0
# Cicerarietinum_XP_012574420.1 Total prob of N-in:        0.00091
Cicerarietinum_XP_012574420.1   TMHMM2.0        outside      1   335
```

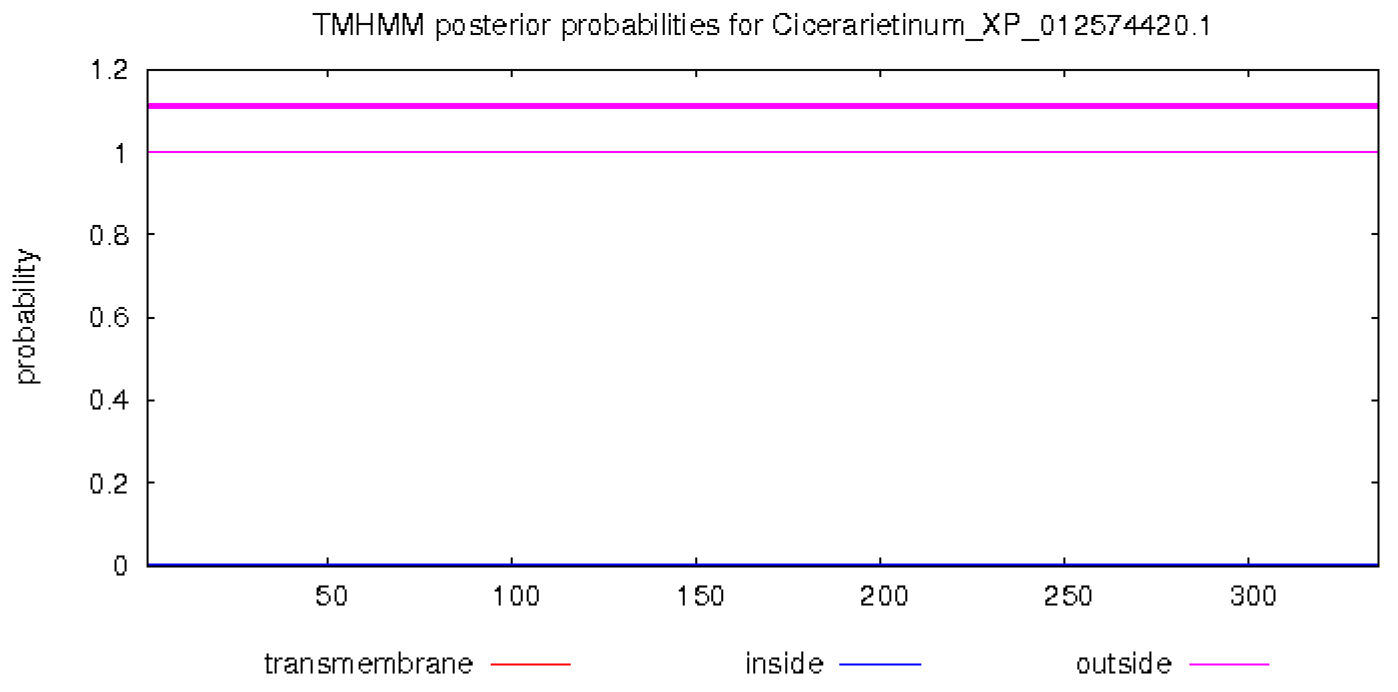

plot in postscript, script for making the plot in gnuplot, data for plot

---

```
# Cicerarietinum_XP_012574421.1 Length: 275
# Cicerarietinum_XP_012574421.1 Number of predicted TMHs:  0
# Cicerarietinum_XP_012574421.1 Exp number of AAs in TMHs: 0.00037
# Cicerarietinum_XP_012574421.1 Exp number, first 60 AAs:  0.00033
# Cicerarietinum_XP_012574421.1 Total prob of N-in:        0.01098
Cicerarietinum_XP_012574421.1   TMHMM2.0        outside      1   275
```

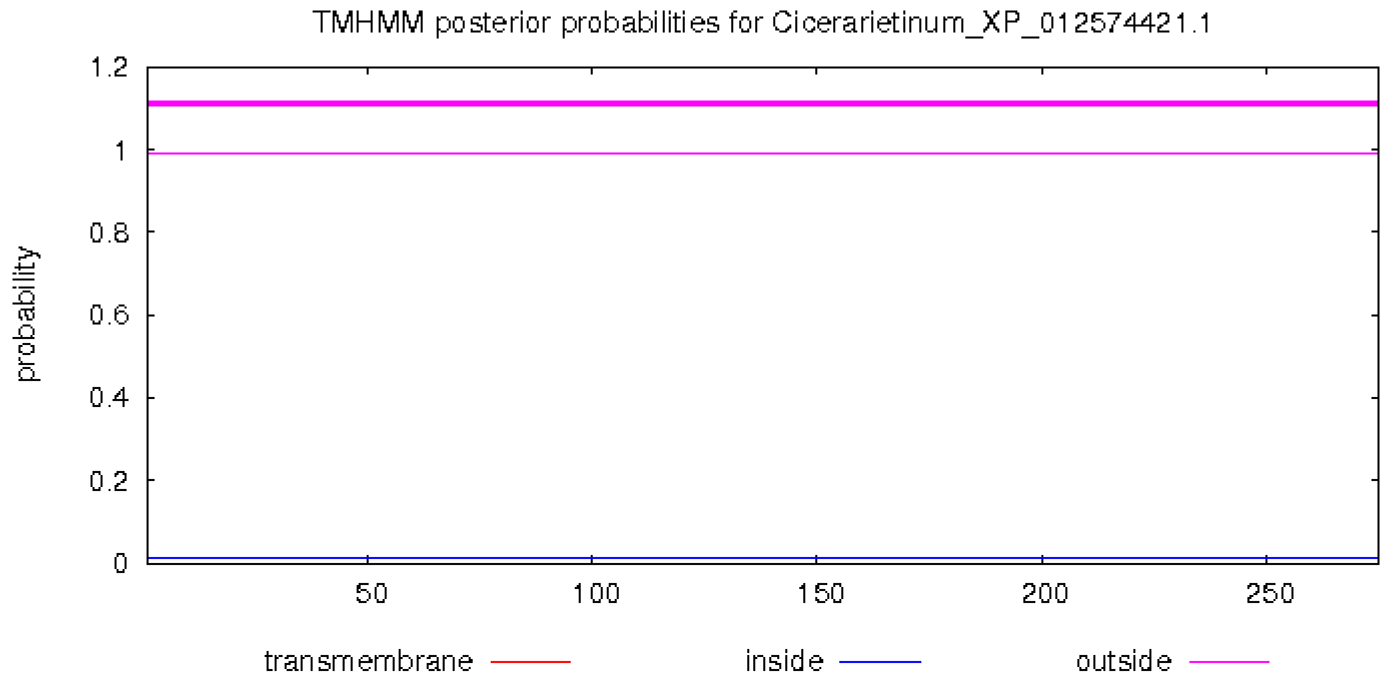

plot in postscript, script for making the plot in gnuplot, data for plot

---

```
# Cicerarietinum_XP_012574450.1 Length: 366
# Cicerarietinum_XP_012574450.1 Number of predicted TMHs:  0
# Cicerarietinum_XP_012574450.1 Exp number of AAs in TMHs: 0.00088
# Cicerarietinum_XP_012574450.1 Exp number, first 60 AAs:  0
# Cicerarietinum_XP_012574450.1 Total prob of N-in:        0.00653
Cicerarietinum_XP_012574450.1   TMHMM2.0        outside      1   366
```

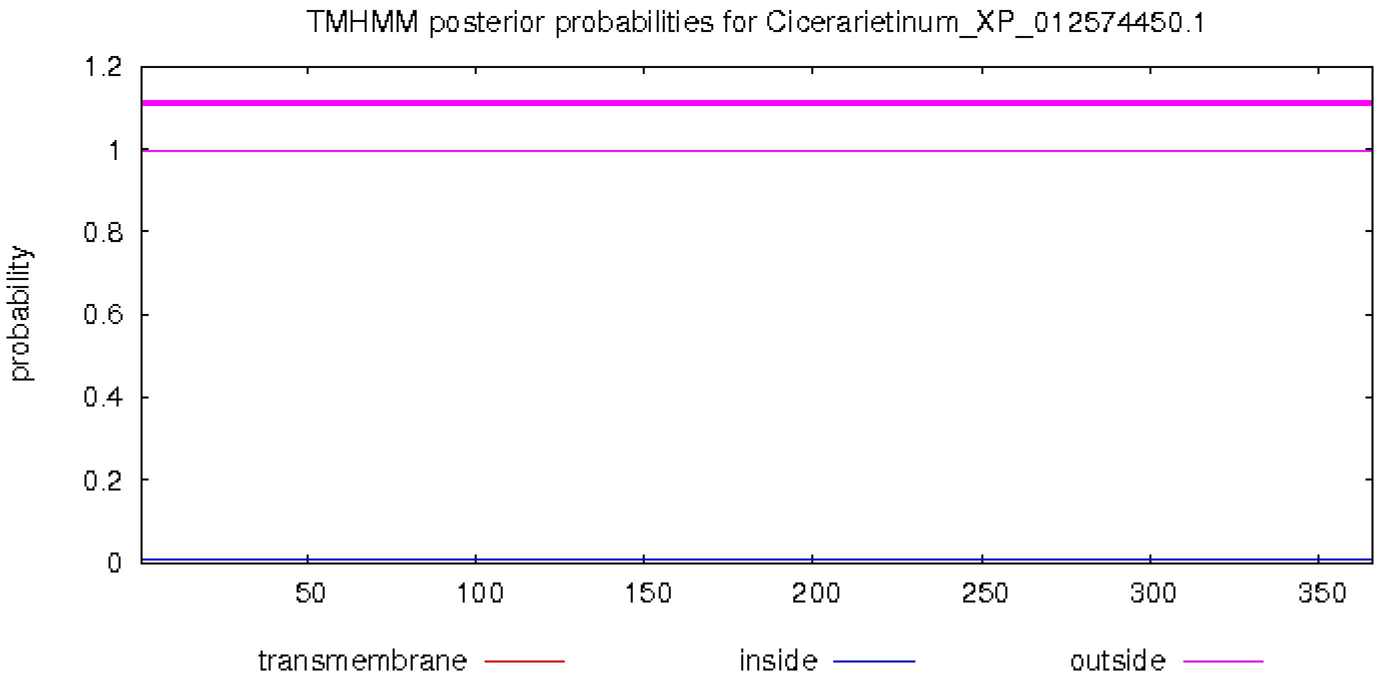

plot in postscript, script for making the plot in gnuplot, data for plot

---

```
```
[truncated: 608,686 more chars]
