# Supplementary material for: Genomics, molecular and evolutionary perspective of NAC transcription factors
Source: PLoS One. 2020 Apr 10;15(4):e0231425. doi: 10.1371/journal.pone.0231425 (PMC7147800; doi:10.1371/journal.pone.0231425)
Supplement: S2 Fig — (XZ) [file S2 Fig.pdf › S2 Fig.pdf]

Please cite this paper if NLStradamus was useful for your studies:
Nguyen Ba AN, Pogoutse A, Provart N, Moses AM. **NLStradamus: a simple Hidden Markov Model for nuclear localization signal prediction.** *BMC Bioinformatics.* 2009 Jun 29;**10(1)**:202.

The pubmed entry for this paper is found **here**.

- Program may resolve in an error if a large .fasta file is sent for prediction when images or graphics are desired. **Please only use the table display when using large predictions. (>100 sequences)**
- A standalone of the program is available **here**.
- Notice: NLStradamus is not a predictor of nuclear proteins; although better than random, it is a very poor classifier of nuclear proteins. Comparative approaches should be used for this problem. NLStradamus predicts NLSs in nuclear proteins that are transported by the import machinery of the cell.
- Click **here** for general help.

**Prediction Request**		
Sequence Text		Accepts :<br>- 1 protein sequence<br>- Multiple sequences in FASTA format.
File Upload	Choose File Actinidia chinensis.fasta	Accepts .fasta, .txt, and soon .gz, .bz2 and .zip.
**HMM state emission and transition frequencies.**		
Pre-loaded models	2 state HMM static / 2 state HMM dynamic / 4 state HMM static	Those are the models based on our characterized sequences.
**Prediction Type**		
Viterbi	☑	
Posterior	☑	0.4 Prediction Cutoff (]0,1])
**Prediction Display**		
Table	☑	A table that you can export with the start and stop positions of predicted NLS.
Image	☑	An image with the NLS boxed over a line representing the protein sequence.
Graphic	☑	Only applicable to posterior probability. A graphical representation of its distribution.
Predict		
Scroll down for predictions.		

**Predictions for Actinidiachinensis_Achn000661**	
Viterbi Path	

Viterbi Path

Posterior @ 0.4

Posterior @ 0.4

Posterior Graphic. Threshold Marked @ 0.4

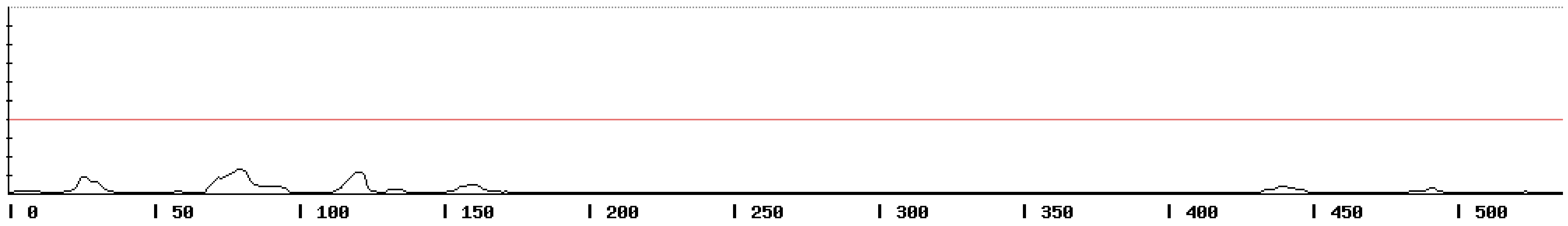

Predictions for Actinidiachinensis_Achn002691

Viterbi Path

Viterbi Path

Posterior @ 0.4

Posterior @ 0.4

Posterior Graphic. Threshold Marked @ 0.4

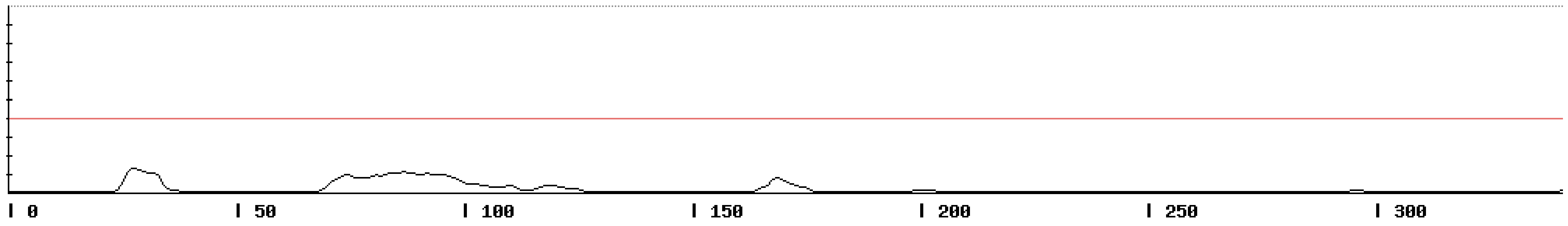

Predictions for Actinidiachinensis_Achn003771

Viterbi Path

Viterbi Path

Posterior @ 0.4

Posterior @ 0.4

Posterior Graphic. Threshold Marked @ 0.4

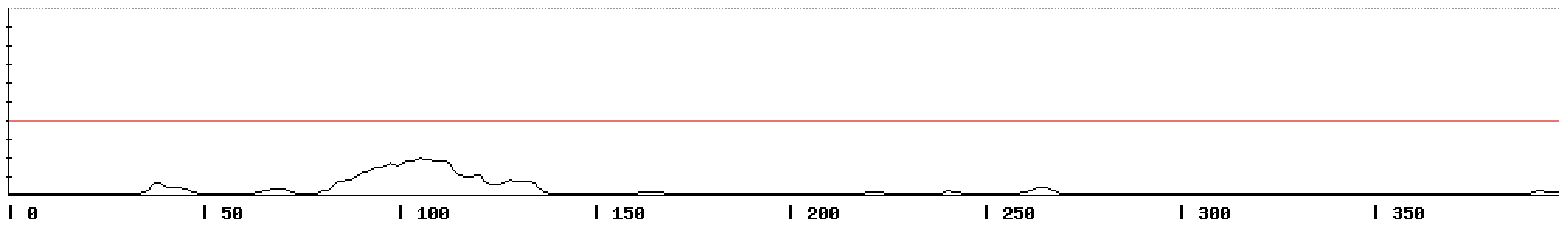

Predictions for Actinidiachinensis_Achn003781

Viterbi Path

Viterbi Path

Posterior @ 0.4

Posterior @ 0.4

Posterior Graphic. Threshold Marked @ 0.4

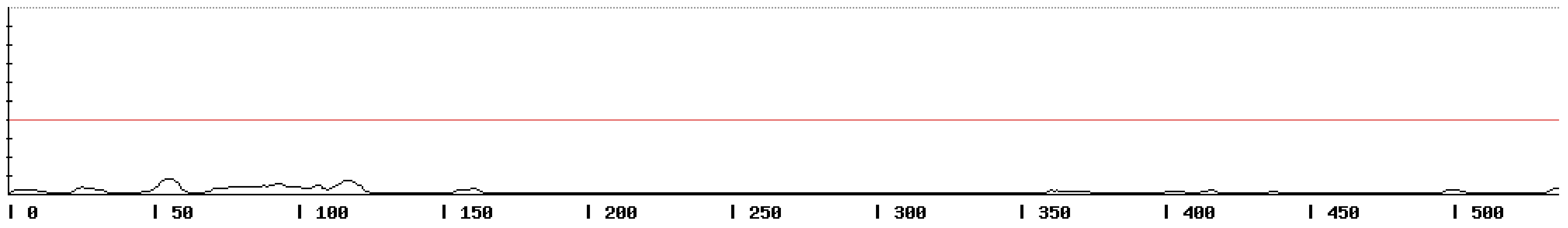

Predictions for Actinidiachinensis_Achn004191	
Viterbi Path	
Viterbi Path	
Posterior @ 0.4	159 - K - 159
Posterior @ 0.4	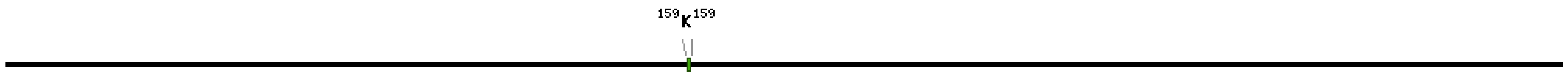
Posterior Graphic. Threshold Marked @ 0.4	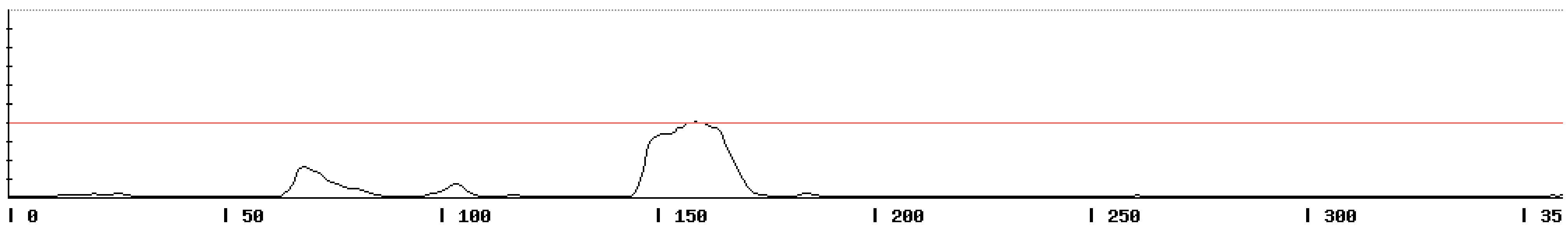

Predictions for Actinidiachinensis_Achn004841	
Viterbi Path	
Viterbi Path	
Posterior @ 0.4	
Posterior @ 0.4	
Posterior Graphic. Threshold Marked @ 0.4	

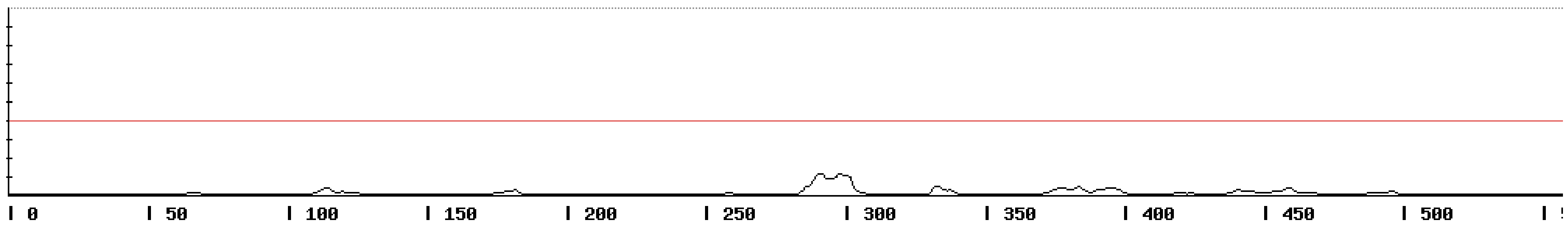

Predictions for Actinidiachinensis_Achn008531

Viterbi Path	
Viterbi Path	
Posterior @ 0.4	
Posterior @ 0.4	
Posterior Graphic. Threshold Marked @ 0.4	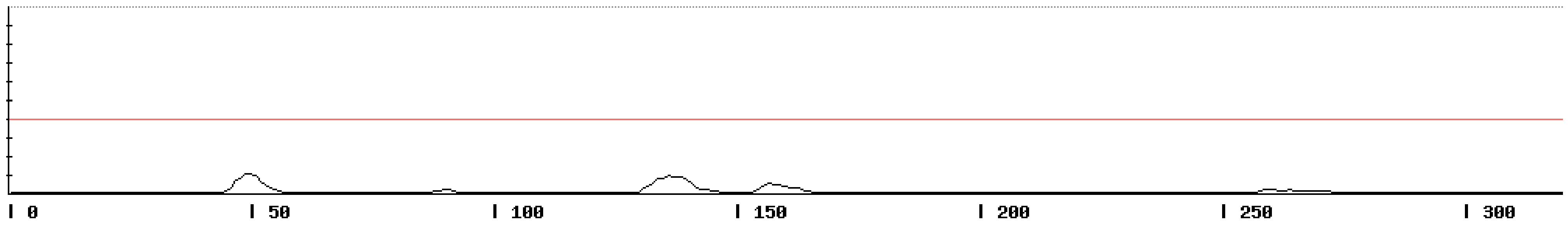

Predictions for Actinidiachinensis_Achn008541

Viterbi Path	
Viterbi Path	
Posterior @ 0.4	149 - KERAIKRDRQK - 159
Posterior @ 0.4	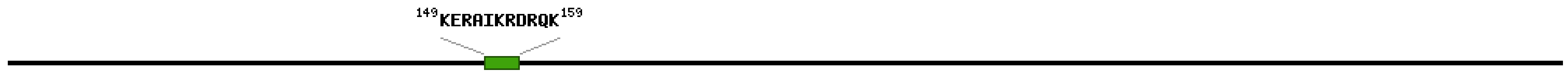

Posterior Graphic. Threshold Marked @ 0.4

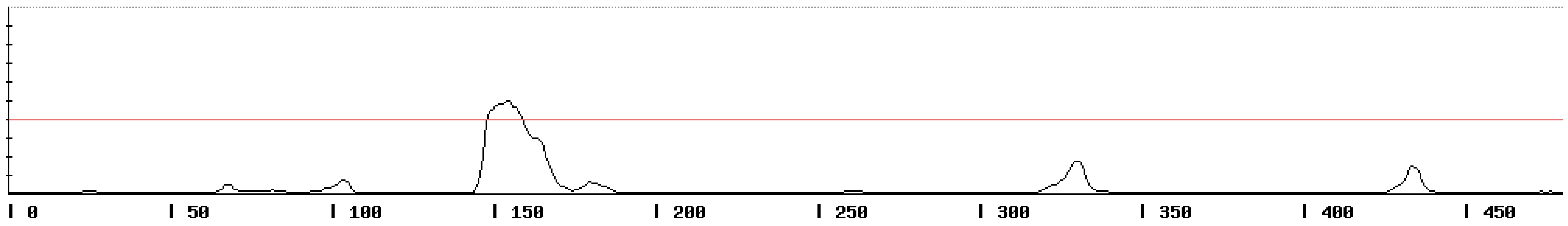

Predictions for Actinidiachinensis_Achn008561

Viterbi Path

Viterbi Path

Posterior @ 0.4

Posterior @ 0.4

Posterior Graphic. Threshold Marked @ 0.4

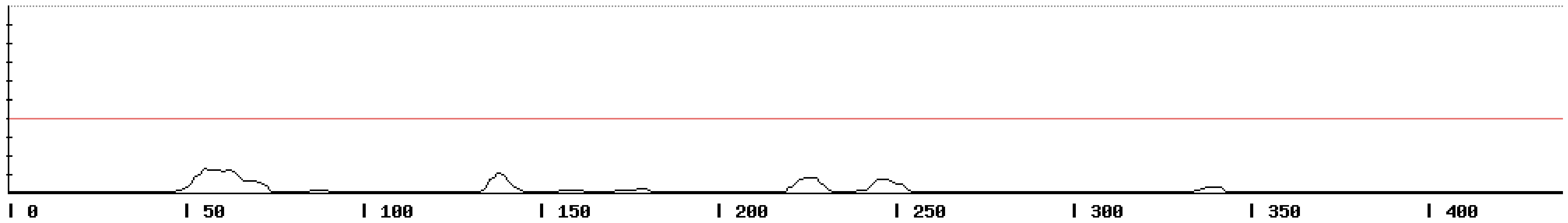

Predictions for Actinidiachinensis_Achn008571

Viterbi Path

Viterbi Path

Posterior @ 0.4

Posterior @ 0.4

Posterior Graphic. Threshold Marked @ 0.4

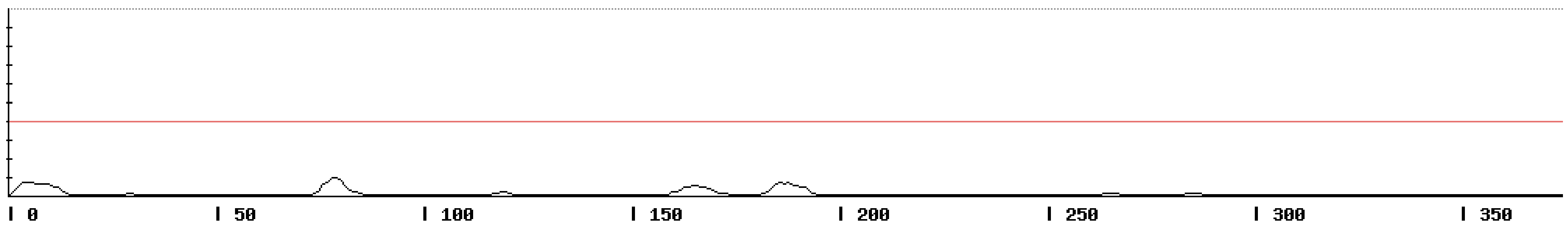

Predictions for Actinidiachinensis_Achn009451

Viterbi Path

Viterbi Path

Posterior @ 0.4 149 - KERAIKRDR - 157

Posterior @ 0.4

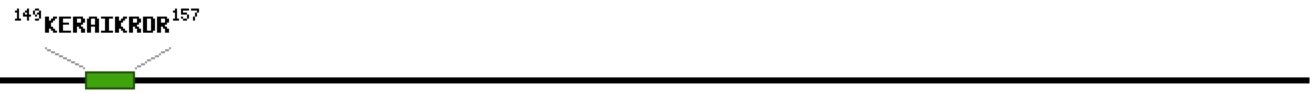

Posterior Graphic. Threshold Marked @ 0.4

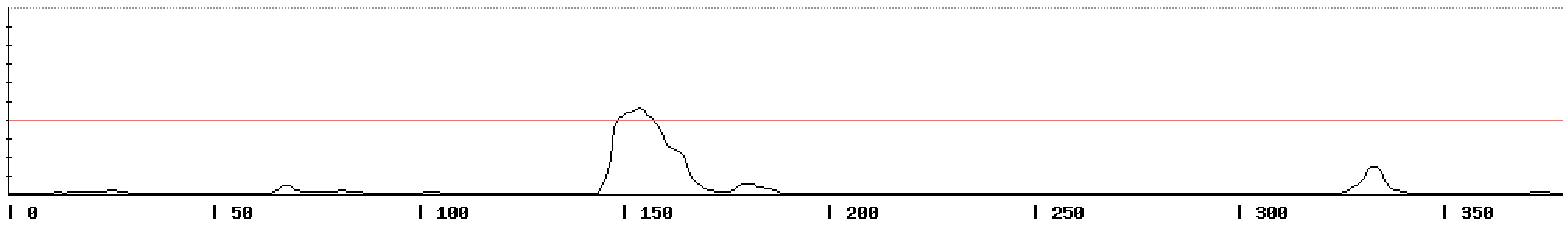

Predictions for Actinidiachinensis_Achn009461

Viterbi Path

Viterbi Path

Posterior @ 0.4

Posterior @ 0.4

Posterior Graphic. Threshold Marked @ 0.4

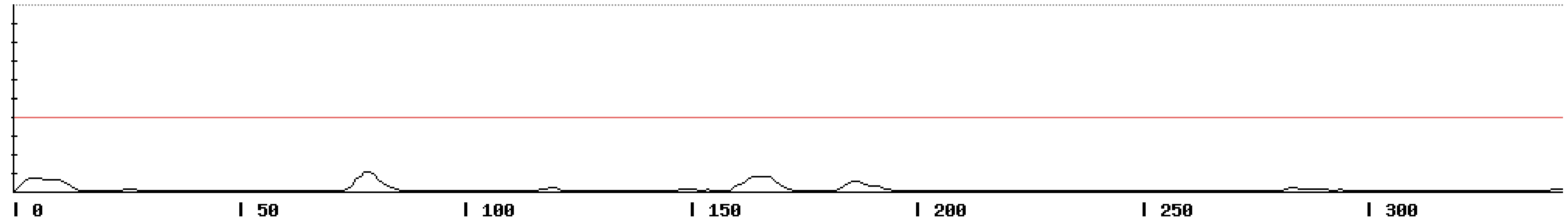

Predictions for Actinidiachinensis_Achn009471

Viterbi Path

Viterbi Path

Posterior @ 0.4 149 - KERAIKRDRQK - 159

Posterior @ 0.4

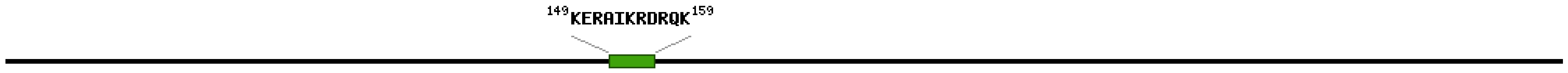

Posterior Graphic. Threshold Marked @ 0.4

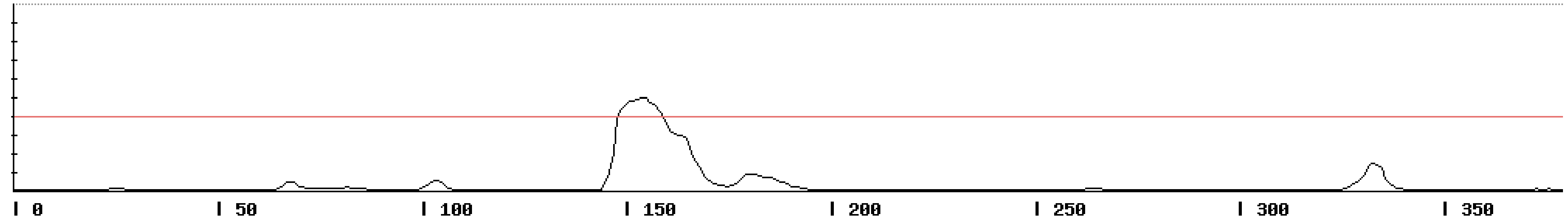

Predictions for Actinidiachinensis_Achn009481

Viterbi Path

Viterbi Path

Posterior @ 0.4

Posterior @ 0.4

Posterior Graphic. Threshold Marked @ 0.4

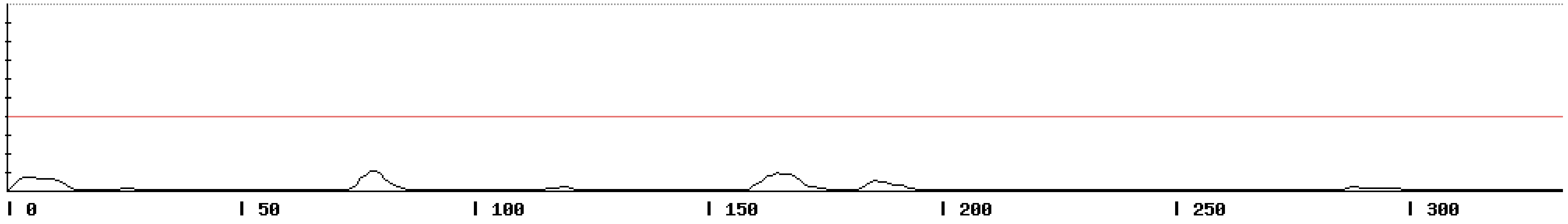

Predictions for Actinidiachinensis_Achn014031

Viterbi Path

Viterbi Path

Posterior @ 0.4

Posterior @ 0.4

Posterior Graphic. Threshold Marked @ 0.4

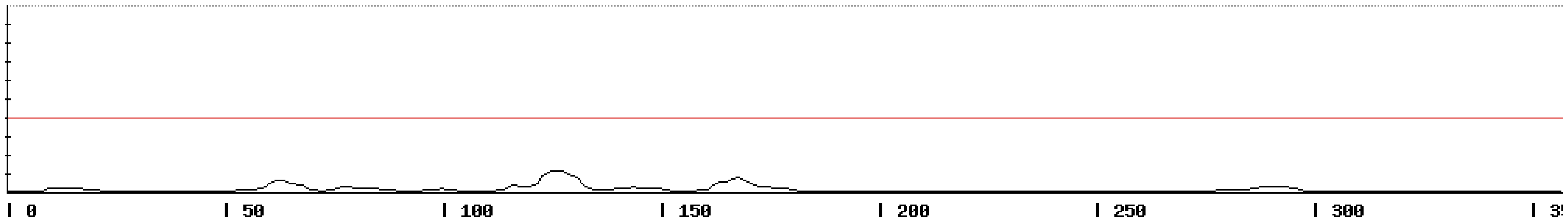

Predictions for Actinidiachinensis_Achn016981

Viterbi Path

Viterbi Path

Posterior @ 0.4

Posterior @ 0.4

Posterior Graphic. Threshold Marked @ 0.4

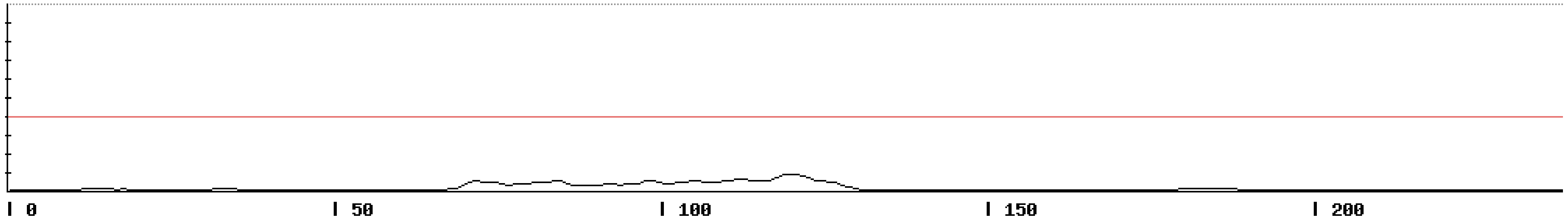

Predictions for Actinidiachinensis_Achn019581

Viterbi Path

Viterbi Path

Posterior @ 0.4

Posterior @ 0.4

Posterior Graphic. Threshold Marked @ 0.4

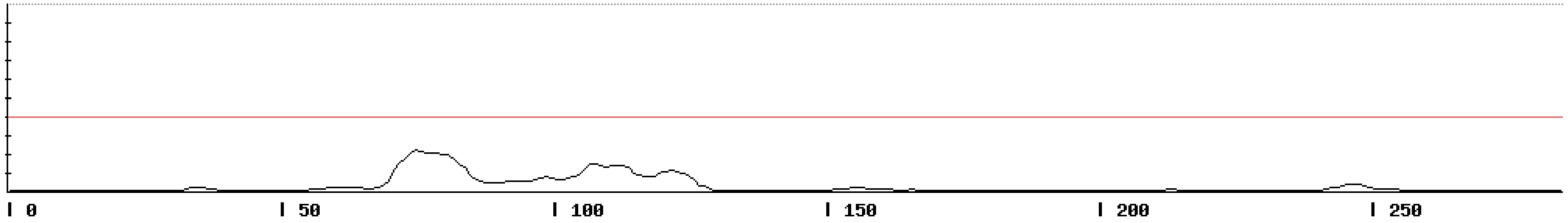

Predictions for Actinidiachinensis_Achn021021	
Viterbi Path	
Viterbi Path	
Posterior @ 0.4	85 - VLKQGGRRRAGGGYWRRRGKARDV - 108
Posterior @ 0.4	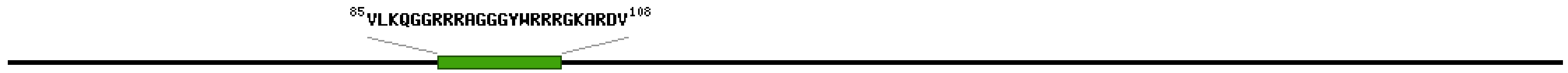
Posterior Graphic. Threshold Marked @ 0.4	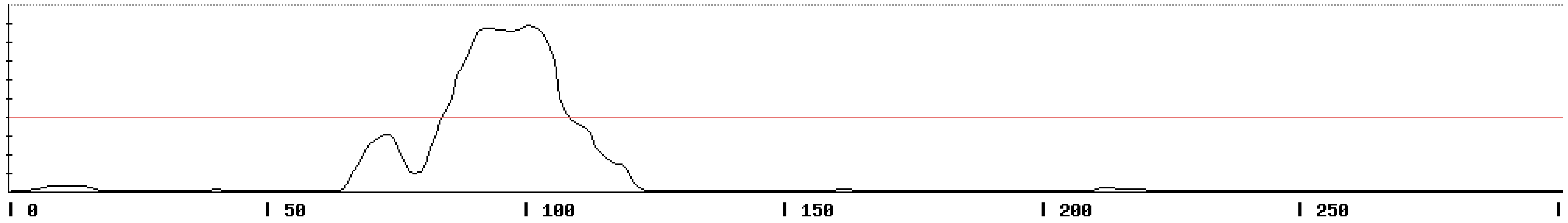

Predictions for Actinidiachinensis_Achn022601	
Viterbi Path	
Viterbi Path	
Posterior @ 0.4	
Posterior @ 0.4	
Posterior Graphic. Threshold Marked @ 0.4	

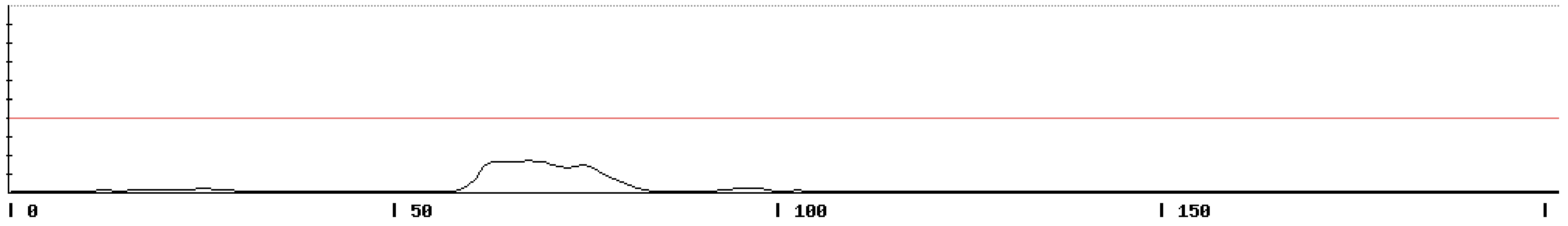

Predictions for Actinidiachinensis_Achn022611

Viterbi Path

Viterbi Path

Posterior @ 0.4

Posterior @ 0.4

Posterior Graphic. Threshold Marked @ 0.4

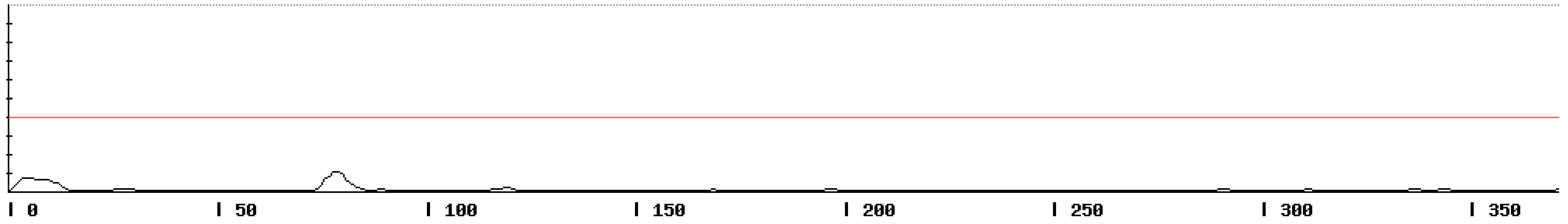

Predictions for Actinidiachinensis_Achn029231

Viterbi Path

Viterbi Path

Posterior @ 0.4 — 75 - PIKPTGHPKPVGIK - 88

Posterior @ 0.4

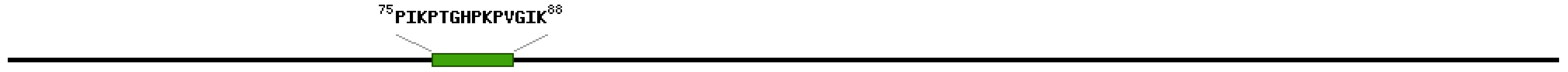

Posterior Graphic. Threshold Marked @ 0.4

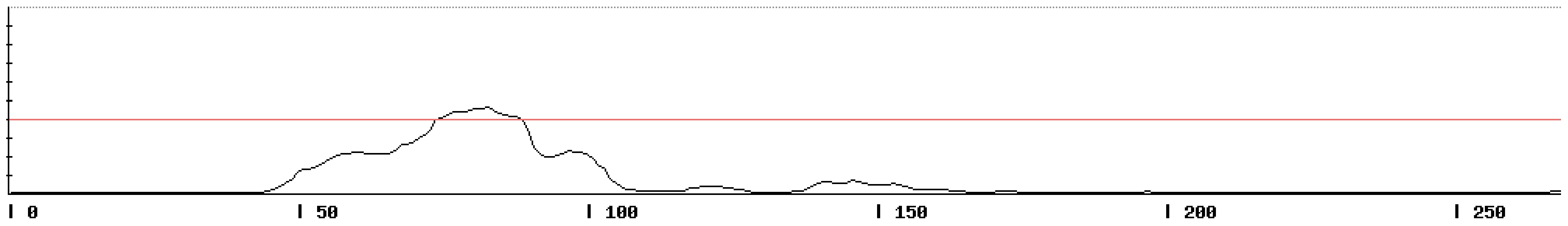

Predictions for Actinidiachinensis_Achn029521

Viterbi Path

Viterbi Path

Posterior @ 0.4

Posterior @ 0.4

Posterior Graphic. Threshold Marked @ 0.4

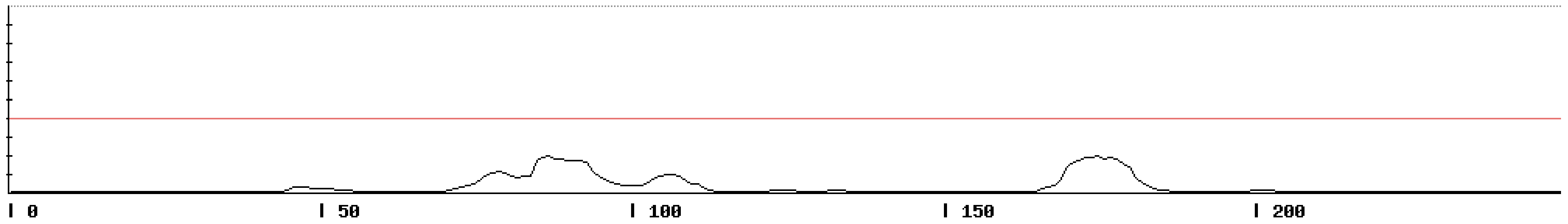

Predictions for Actinidiachinensis_Achn033651

Viterbi Path

Viterbi Path

Posterior @ 0.4

Posterior @ 0.4

Posterior Graphic. Threshold Marked @ 0.4

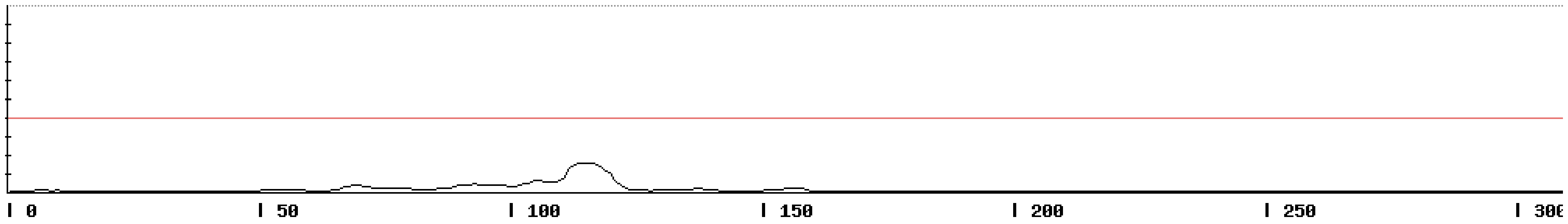

Predictions for Actinidiachinensis_Achn047821

Viterbi Path

Viterbi Path

Posterior @ 0.4

Posterior @ 0.4

Posterior Graphic. Threshold Marked @ 0.4

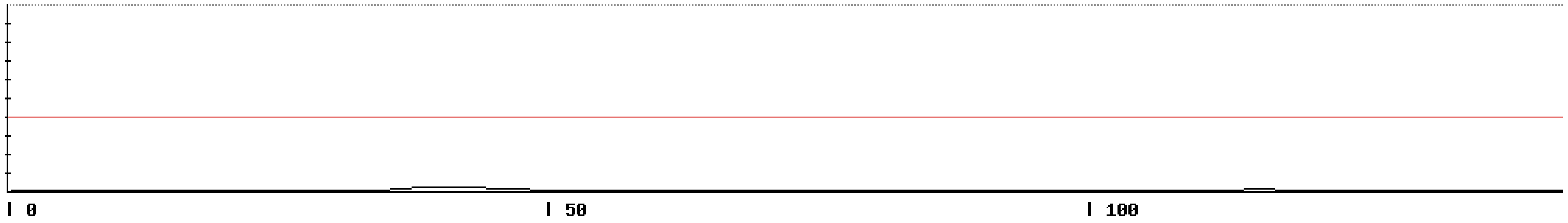

Predictions for Actinidiachinensis_Achn048661

Viterbi Path

Viterbi Path

Posterior @ 0.4

Posterior @ 0.4

Posterior Graphic. Threshold Marked @ 0.4

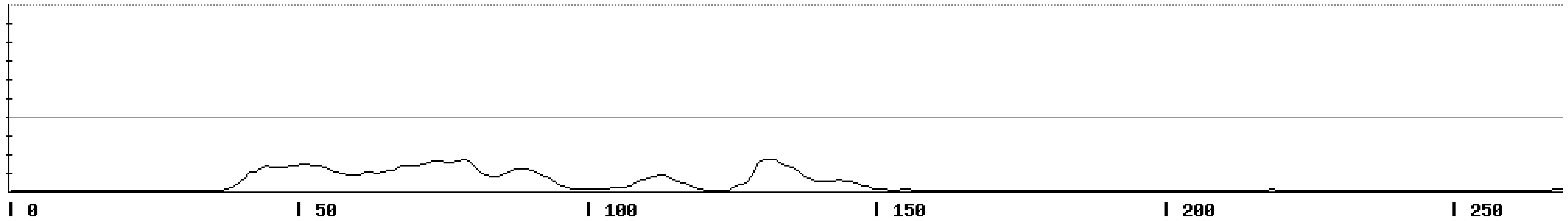

Predictions for Actinidiachinensis_Achn051151

Viterbi Path

Viterbi Path

Posterior @ 0.4

Posterior @ 0.4

Posterior Graphic. Threshold Marked @ 0.4

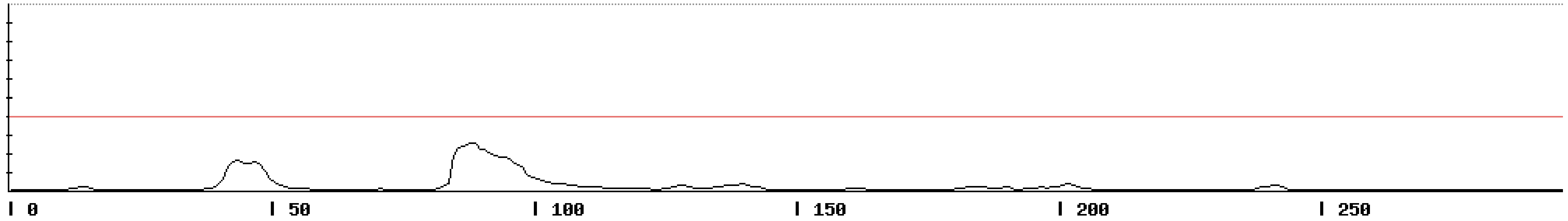

Predictions for Actinidiachinensis_Achn053241

Viterbi Path

Viterbi Path

Posterior @ 0.4	
Posterior @ 0.4	
Posterior Graphic. Threshold Marked @ 0.4	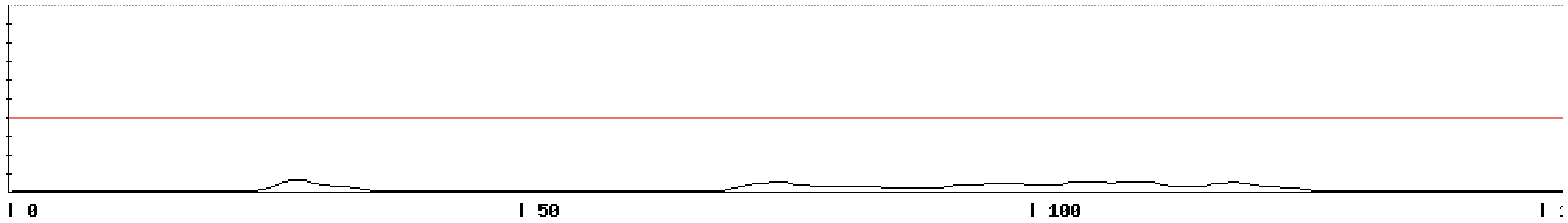

Predictions for Actinidiachinensis_Achn054031

Viterbi Path	
Viterbi Path	
Posterior @ 0.4	
Posterior @ 0.4	
Posterior Graphic. Threshold Marked @ 0.4	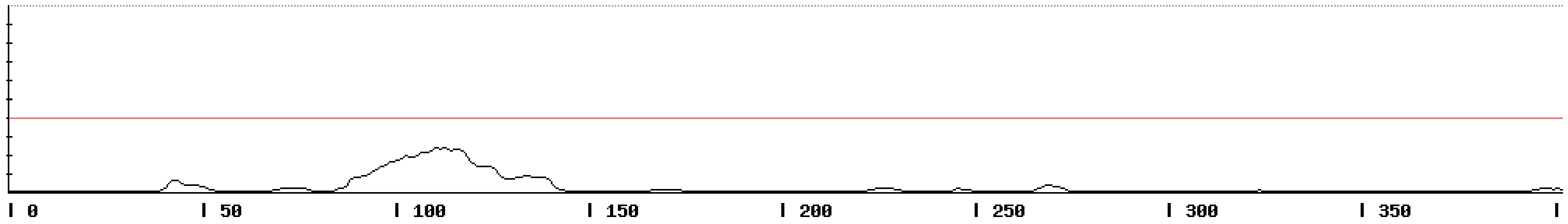

Predictions for Actinidiachinensis_Achn054041

Viterbi Path

Viterbi Path

Posterior @ 0.4

Posterior @ 0.4

Posterior Graphic. Threshold Marked @ 0.4

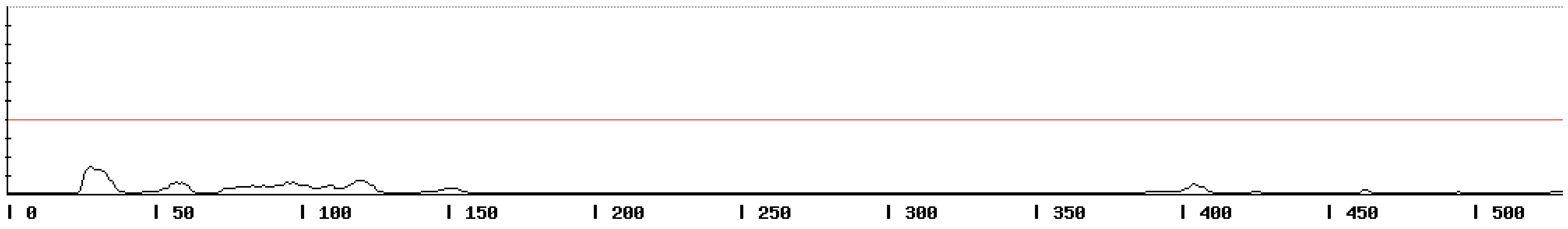

Predictions for Actinidiachinensis_Achn057581

Viterbi Path

Viterbi Path

Posterior @ 0.4

Posterior @ 0.4

Posterior Graphic. Threshold Marked @ 0.4

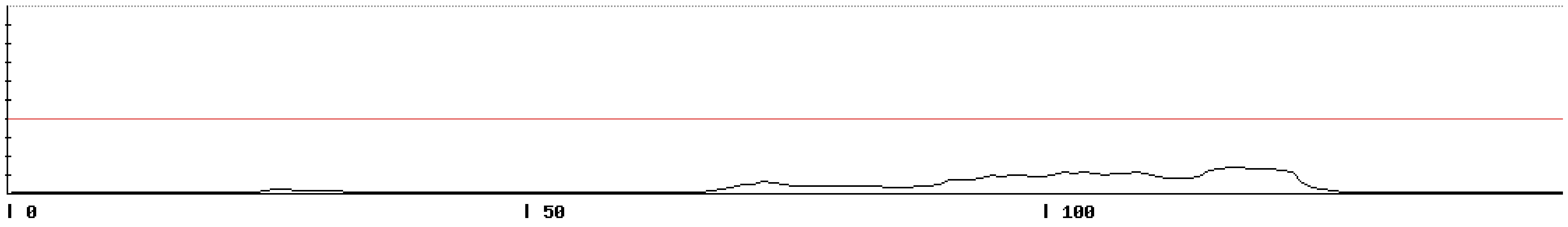

Predictions for Actinidiachinensis_Achn059641	
Viterbi Path	
Viterbi Path	
Posterior @ 0.4	81 - KKKG - 84
Posterior @ 0.4	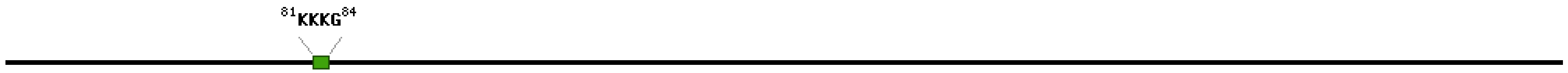
Posterior Graphic. Threshold Marked @ 0.4	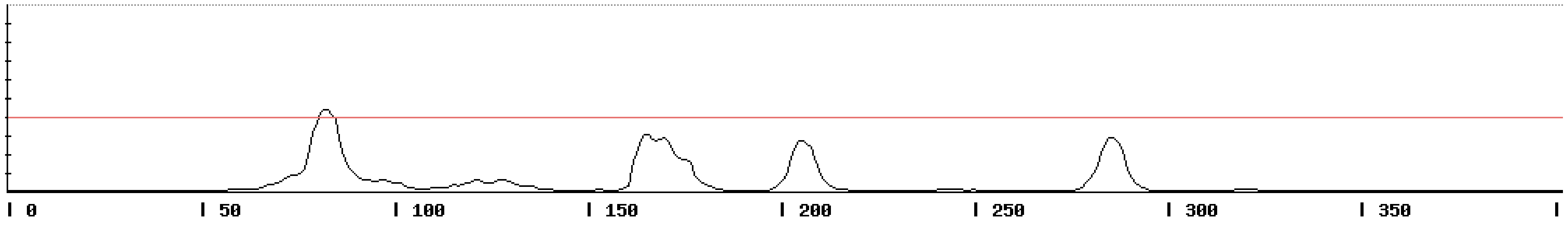

Predictions for Actinidiachinensis_Achn069281	
Viterbi Path	
Viterbi Path	
Posterior @ 0.4	
Posterior @ 0.4	
Posterior Graphic. Threshold Marked @ 0.4	

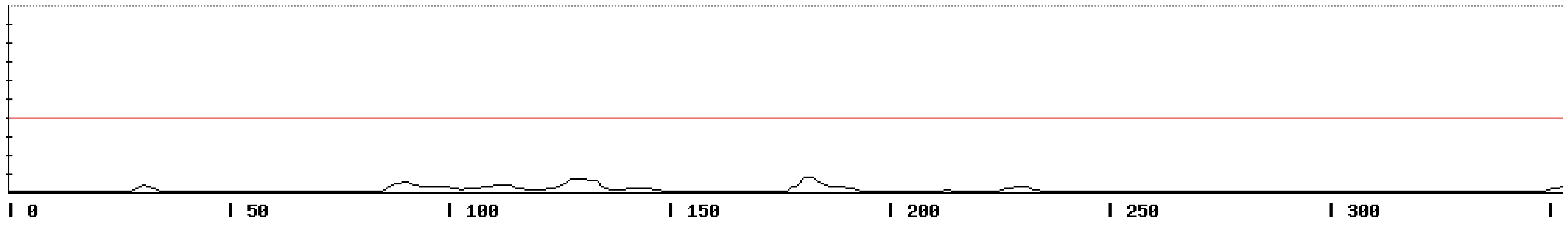

Predictions for Actinidiachinensis_Achn069791

Viterbi Path

Viterbi Path

Posterior @ 0.4

Posterior @ 0.4

Posterior Graphic. Threshold Marked @ 0.4

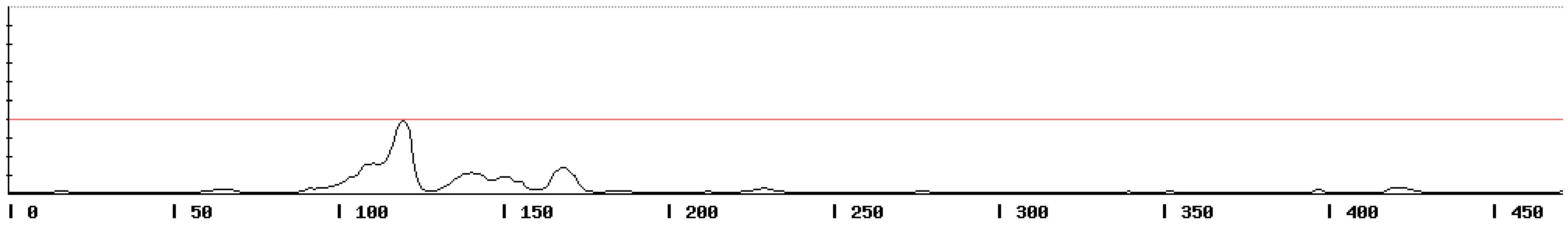

Predictions for Actinidiachinensis_Achn070531

Viterbi Path

Viterbi Path

Posterior @ 0.4

Posterior @ 0.4

Posterior Graphic. Threshold Marked @ 0.4

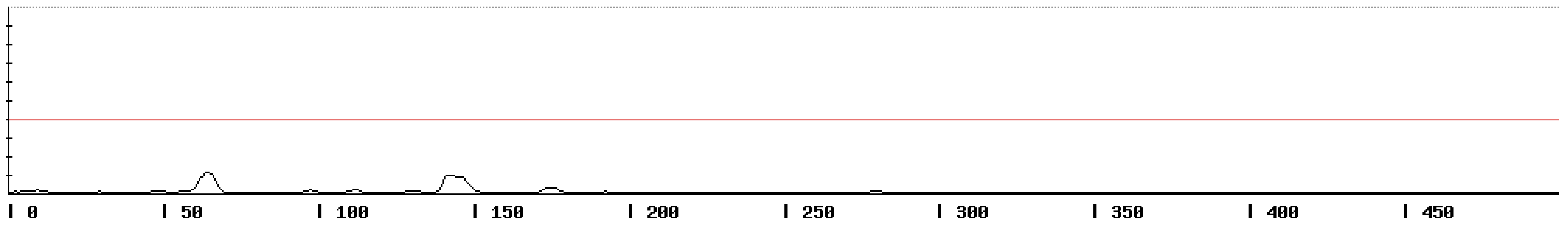

Predictions for Actinidiachinensis_Achn070691

Viterbi Path

Viterbi Path

Posterior @ 0.4

Posterior @ 0.4

Posterior Graphic. Threshold Marked @ 0.4

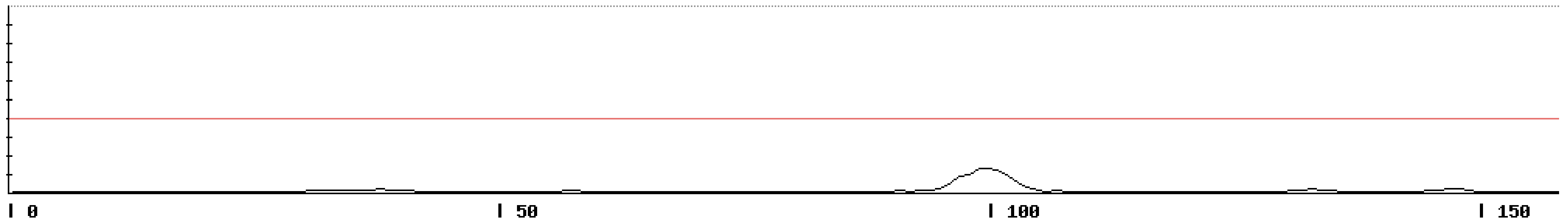

Predictions for Actinidiachinensis_Achn073421

Viterbi Path

Viterbi Path

Posterior @ 0.4

Posterior @ 0.4

Posterior Graphic. Threshold Marked @ 0.4

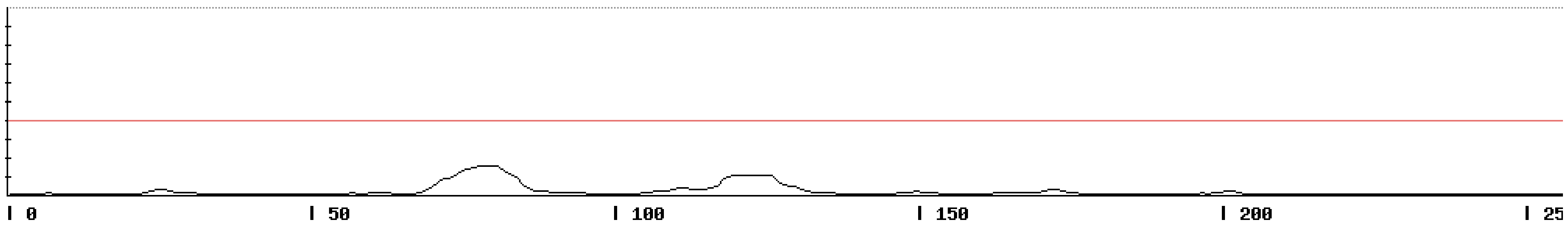

Predictions for Actinidiachinensis_Achn074521

Viterbi Path

Viterbi Path

Posterior @ 0.4

Posterior @ 0.4

Posterior Graphic. Threshold Marked @ 0.4

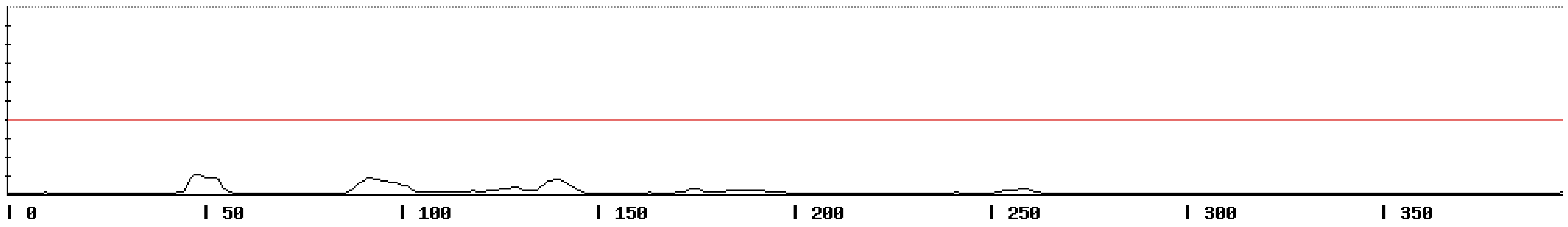

Predictions for Actinidiachinensis_Achn077531

Viterbi Path

Viterbi Path

Posterior @ 0.4	
Posterior @ 0.4	
Posterior Graphic. Threshold Marked @ 0.4	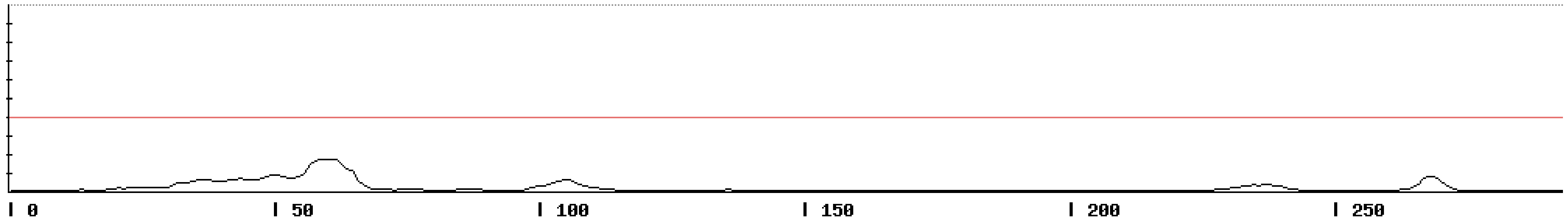

Predictions for Actinidiachinensis_Achn079241

Viterbi Path	
Viterbi Path	
Posterior @ 0.4	120 - RGRKYRNSIRPKR - 132
Posterior @ 0.4	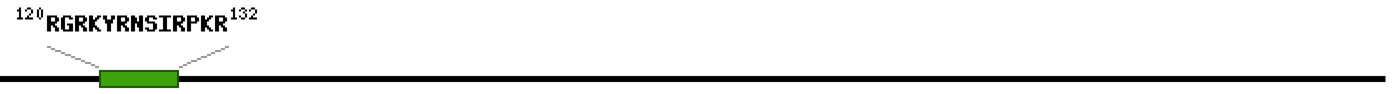
Posterior Graphic. Threshold Marked @ 0.4	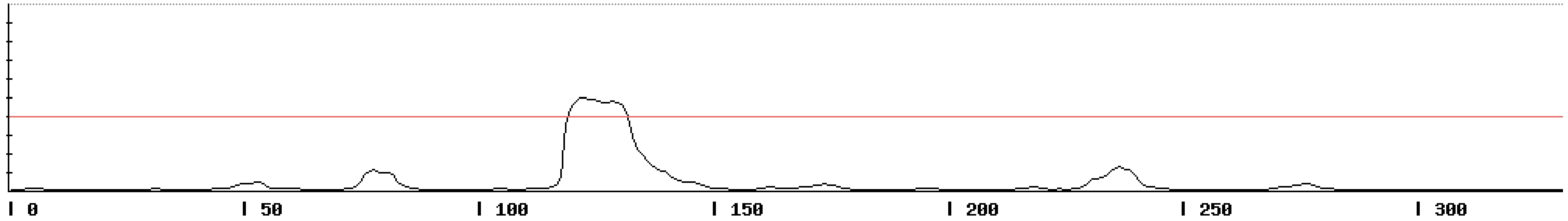

Predictions for Actinidiachinensis_Achn080521

Viterbi Path	
Viterbi Path	

Posterior @ 0.4

Posterior @ 0.4

Posterior Graphic. Threshold Marked @ 0.4

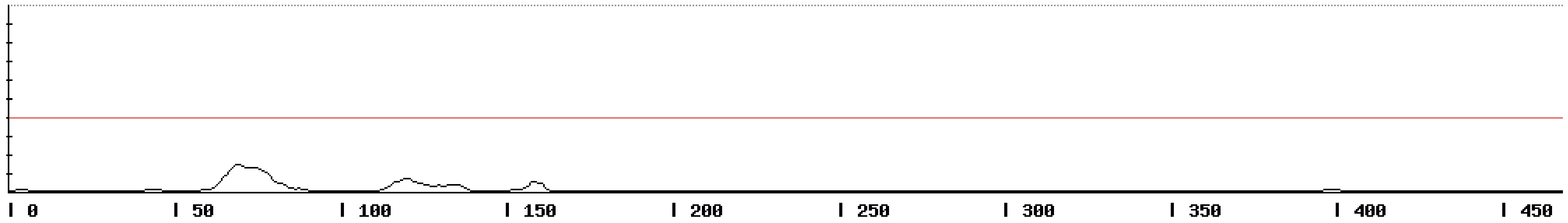

Predictions for Actinidiachinensis_Achn080531

Viterbi Path

Viterbi Path

Posterior @ 0.4

Posterior @ 0.4

Posterior Graphic. Threshold Marked @ 0.4

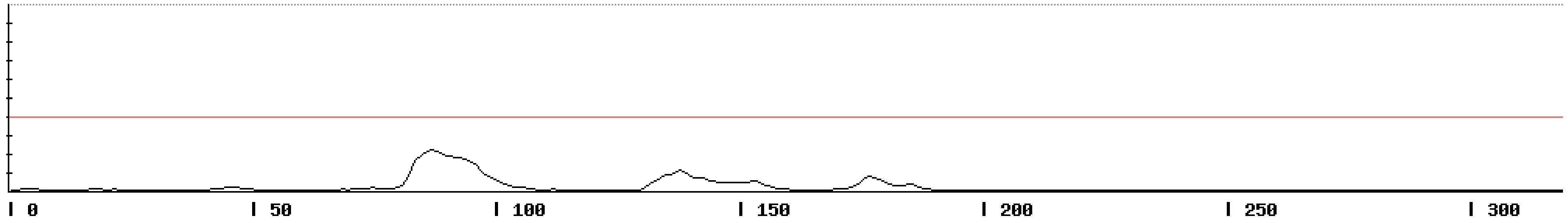

Predictions for Actinidiachinensis_Achn080541

Viterbi Path

Viterbi Path

Posterior @ 0.4

Posterior @ 0.4

Posterior Graphic. Threshold Marked @ 0.4

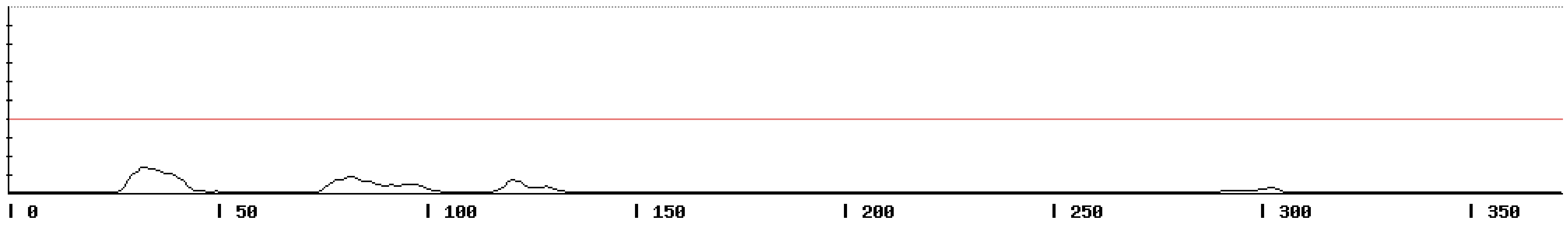

Predictions for Actinidiachinensis_Achn080851

Viterbi Path

Viterbi Path

Posterior @ 0.4

Posterior @ 0.4

Posterior Graphic. Threshold Marked @ 0.4

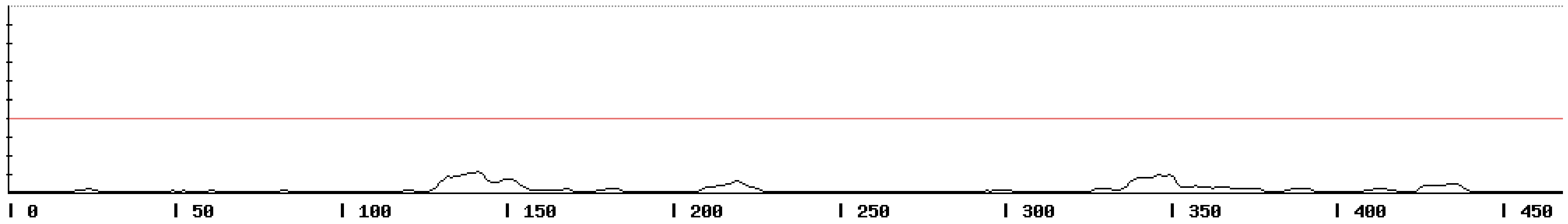

Predictions for Actinidiachinensis_Achn089081

Viterbi Path	646 - GRSMRSKVERRMRKESGKTLREIRRAKDLRKK - 677
Viterbi Path	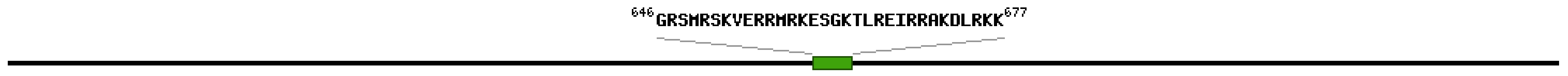
Posterior @ 0.4	347 - RKRRRIHNE - 355
	358 - MKKEG - 362
	647 - RSMRSKVERRMRKESGKTLREIRRAKDLRKKLM - 679
	689 - LRRAKKKV - 696
Posterior @ 0.4	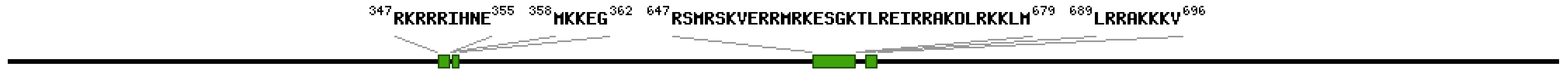
Posterior Graphic. Threshold Marked @ 0.4	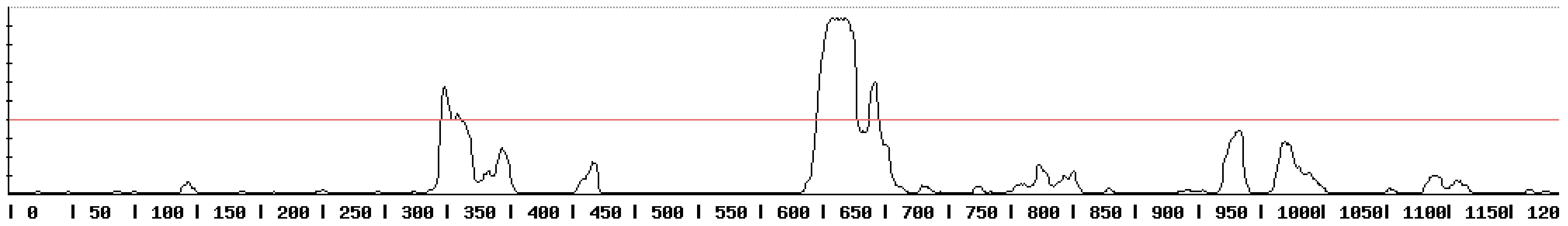

Predictions for Actinidiachinensis_Achn089521

Viterbi Path	
Viterbi Path	
Posterior @ 0.4	
Posterior @ 0.4	
Posterior Graphic. Threshold Marked @ 0.4	

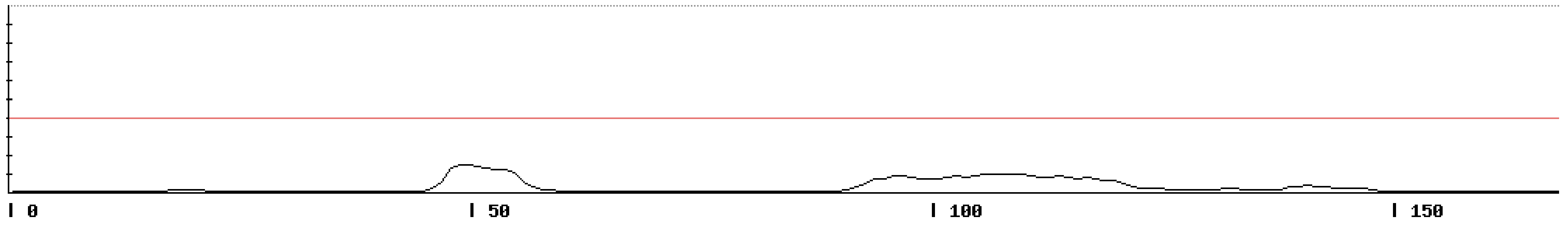

Predictions for Actinidiachinensis_Achn093311

Viterbi Path	
Viterbi Path	
Posterior @ 0.4	
Posterior @ 0.4	
Posterior Graphic. Threshold Marked @ 0.4	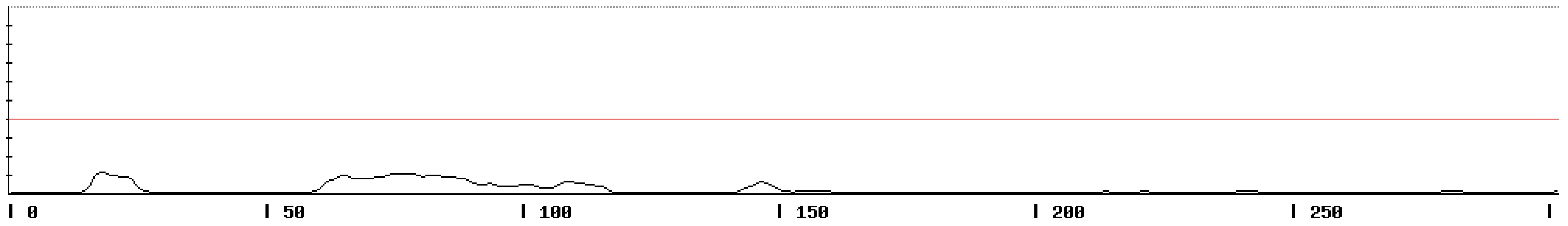

Predictions for Actinidiachinensis_Achn095321

Viterbi Path	
Viterbi Path	
Posterior @ 0.4	
Posterior @ 0.4	

Posterior Graphic. Threshold Marked @ 0.4	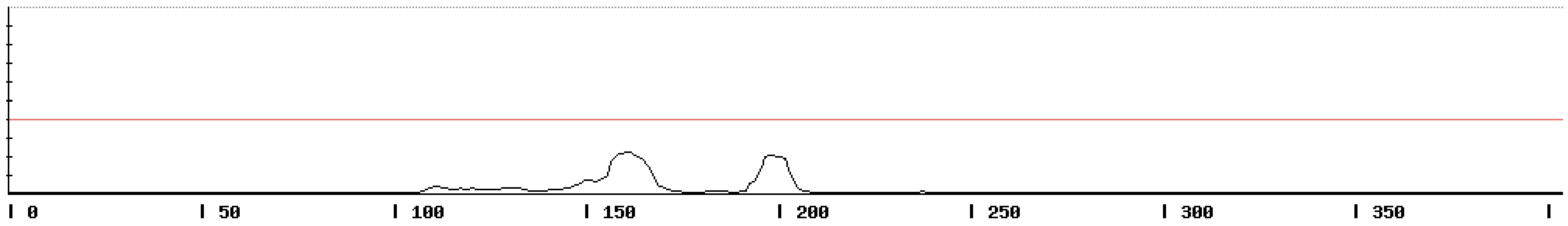

Predictions for Actinidiachinensis_Achn104221

Viterbi Path	
Viterbi Path	
Posterior @ 0.4	
Posterior @ 0.4	
Posterior Graphic. Threshold Marked @ 0.4	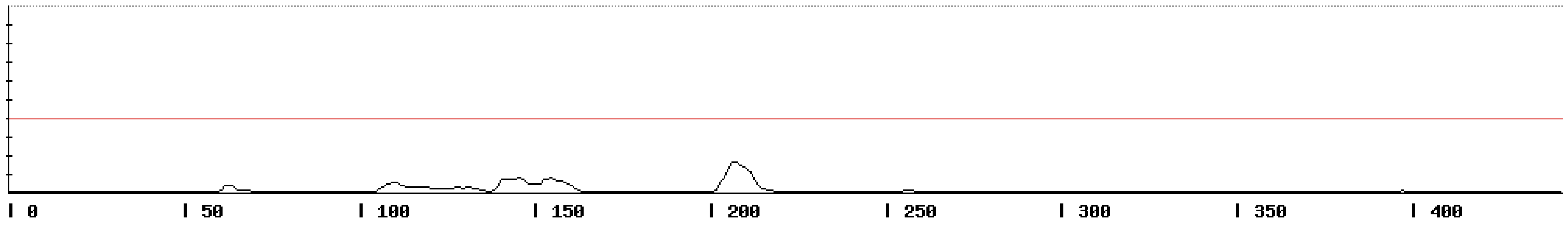

Predictions for Actinidiachinensis_Achn106991

Viterbi Path	
Viterbi Path	
Posterior @ 0.4	
Posterior @ 0.4	

Posterior Graphic. Threshold Marked @ 0.4	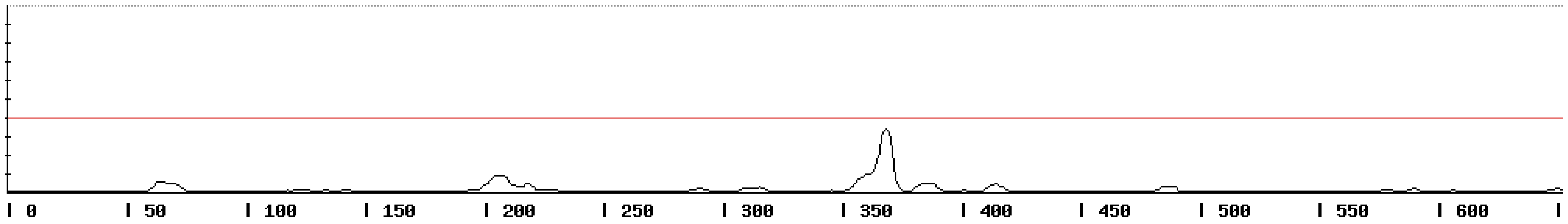

Predictions for Actinidiachinensis_Achn107641

Viterbi Path	
Viterbi Path	
Posterior @ 0.4	
Posterior @ 0.4	
Posterior Graphic. Threshold Marked @ 0.4	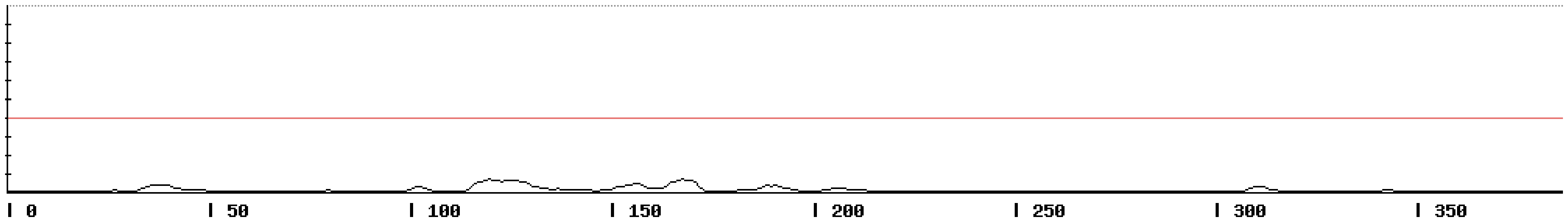

Predictions for Actinidiachinensis_Achn111051

Viterbi Path	
Viterbi Path	

Posterior @ 0.4

Posterior @ 0.4

Posterior Graphic. Threshold Marked @ 0.4

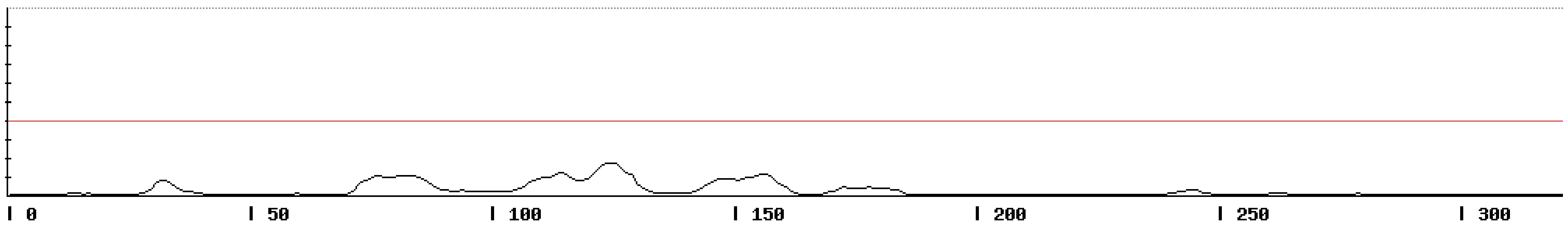

Predictions for Actinidiachinensis_Achn118631

Viterbi Path

Viterbi Path

Posterior @ 0.4

133 - RKRRK - 137

Posterior @ 0.4

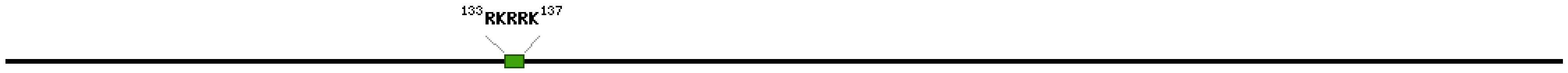

Posterior Graphic. Threshold Marked @ 0.4

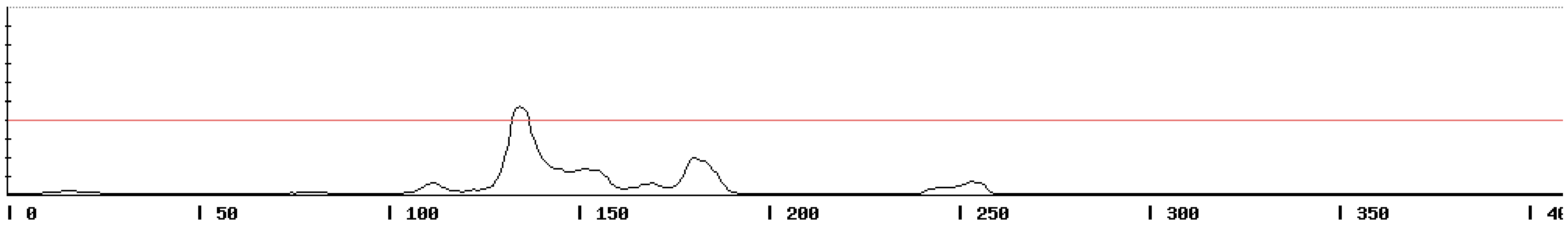

Predictions for Actinidiachinensis_Achn123251

Viterbi Path

Viterbi Path

Posterior @ 0.4 95 - KPIKPTGHPKPVGIKK - 110

Posterior @ 0.4

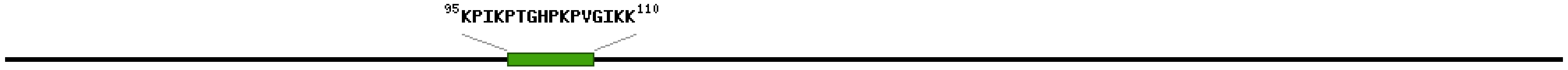

Posterior Graphic. Threshold Marked @ 0.4

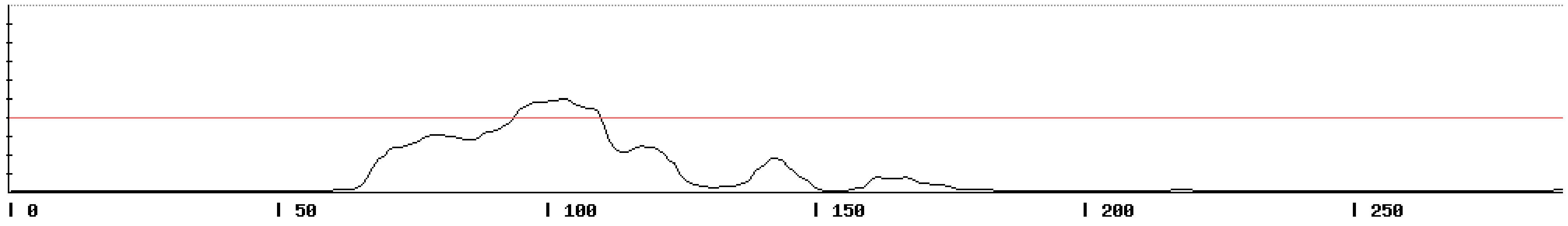

Predictions for Actinidiachinensis_Achn124361

Viterbi Path

Viterbi Path

Posterior @ 0.4

Posterior @ 0.4

Posterior Graphic. Threshold Marked @ 0.4

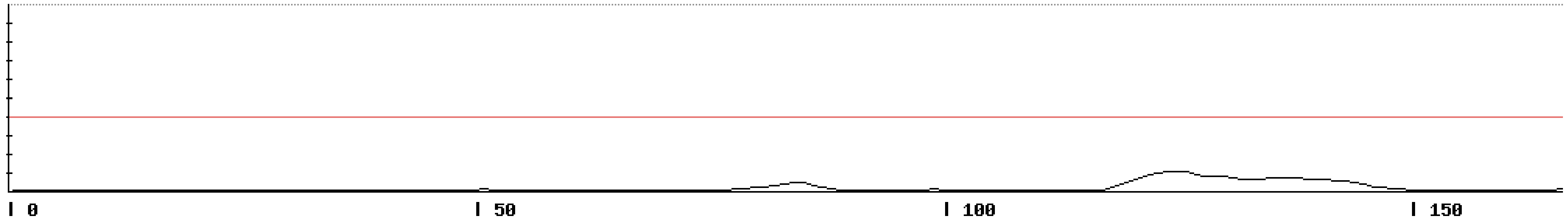

Predictions for Actinidiachinensis_Achn124381

Viterbi Path

Viterbi Path

Posterior @ 0.4

Posterior @ 0.4

Posterior Graphic. Threshold Marked @ 0.4

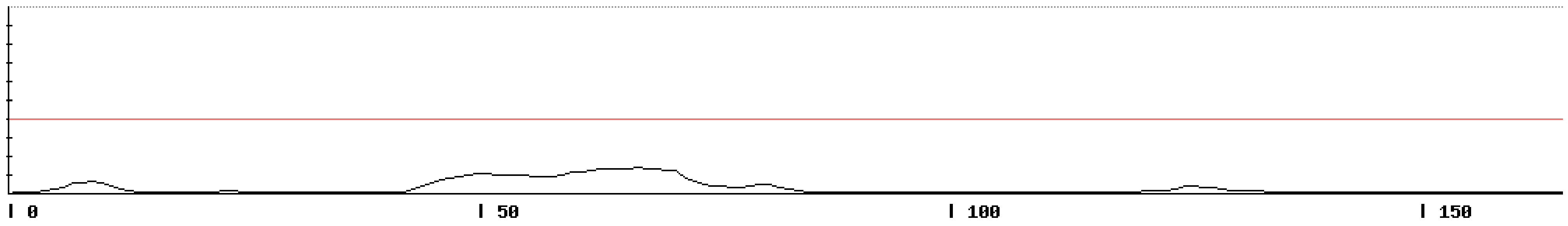

Predictions for Actinidiachinensis_Achn125861

Viterbi Path

Viterbi Path

Posterior @ 0.4

Posterior @ 0.4

Posterior Graphic. Threshold Marked @ 0.4

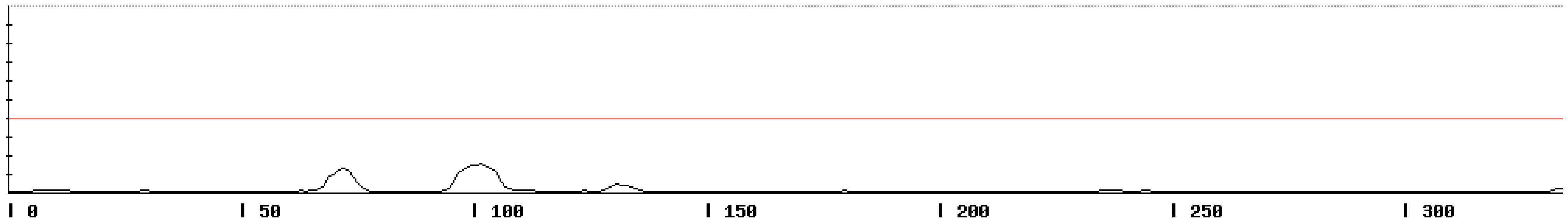

Predictions for Actinidiachinensis_Achn126291

Viterbi Path

Viterbi Path

Posterior @ 0.4

Posterior @ 0.4

Posterior Graphic. Threshold Marked @ 0.4

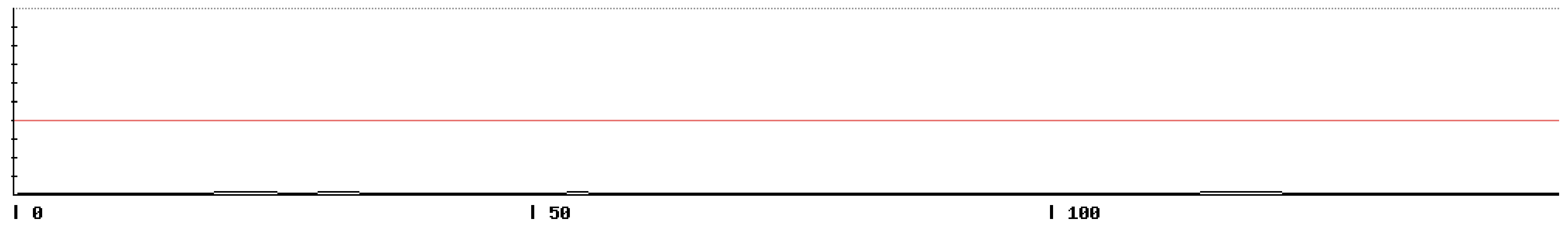

Predictions for Actinidiachinensis_Achn126301

Viterbi Path

Viterbi Path

Posterior @ 0.4

Posterior @ 0.4

Posterior Graphic. Threshold Marked @ 0.4

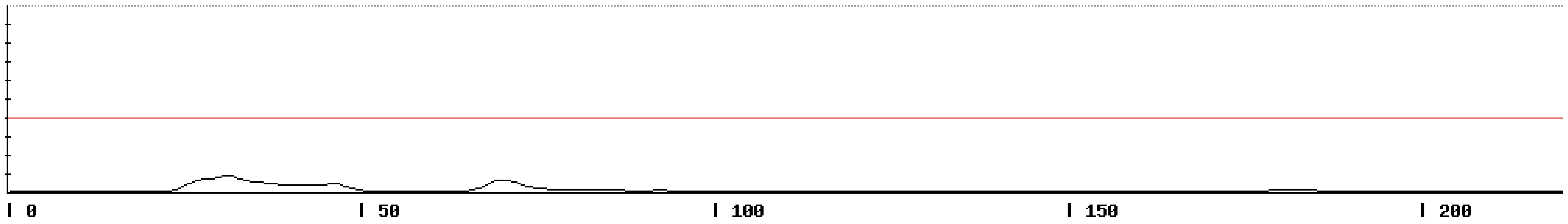

Predictions for Actinidiachinensis_Achn126311

Viterbi Path

Viterbi Path

Posterior @ 0.4

Posterior @ 0.4

Posterior Graphic. Threshold Marked @ 0.4

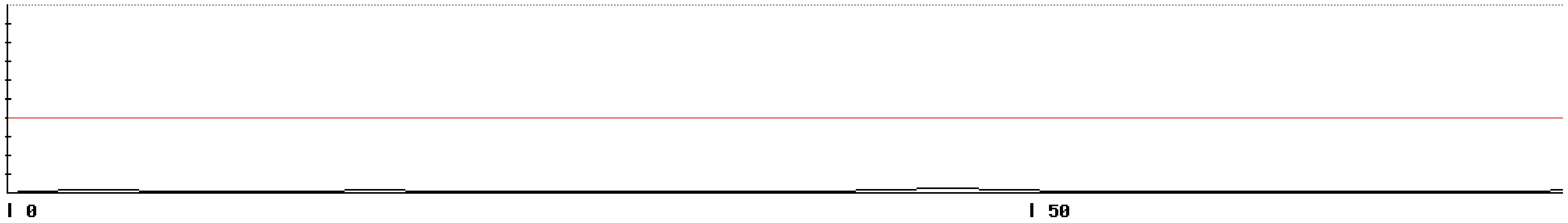

Predictions for Actinidiachinensis_Achn133971

Viterbi Path

Viterbi Path

Posterior @ 0.4

Posterior @ 0.4

Posterior Graphic. Threshold Marked @ 0.4

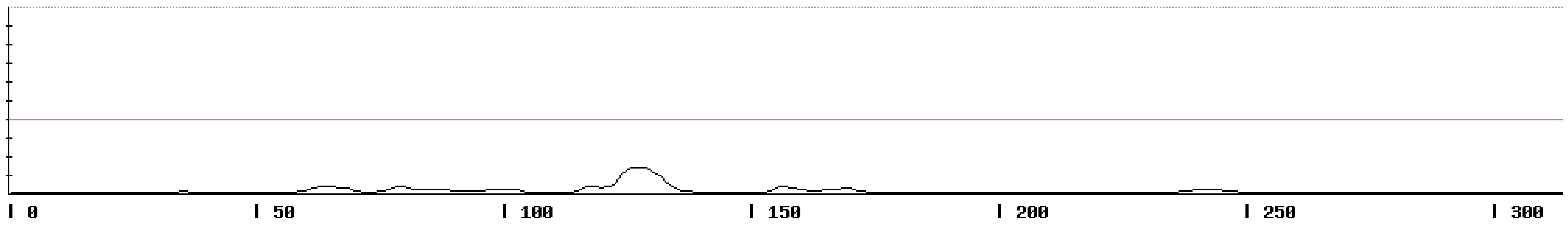

Predictions for Actinidiachinensis_Achn134171

Viterbi Path

Viterbi Path

Posterior @ 0.4

Posterior @ 0.4

Posterior Graphic. Threshold Marked @ 0.4

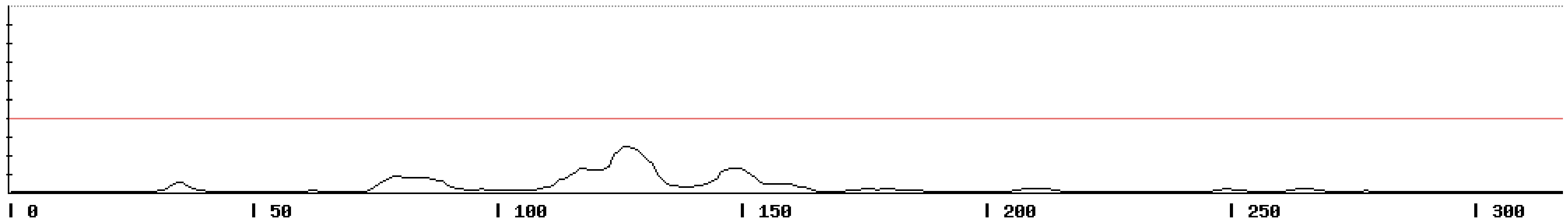

Predictions for Actinidiachinensis_Achn137931

Viterbi Path

Viterbi Path

Posterior @ 0.4

Posterior @ 0.4

Posterior Graphic. Threshold Marked @ 0.4	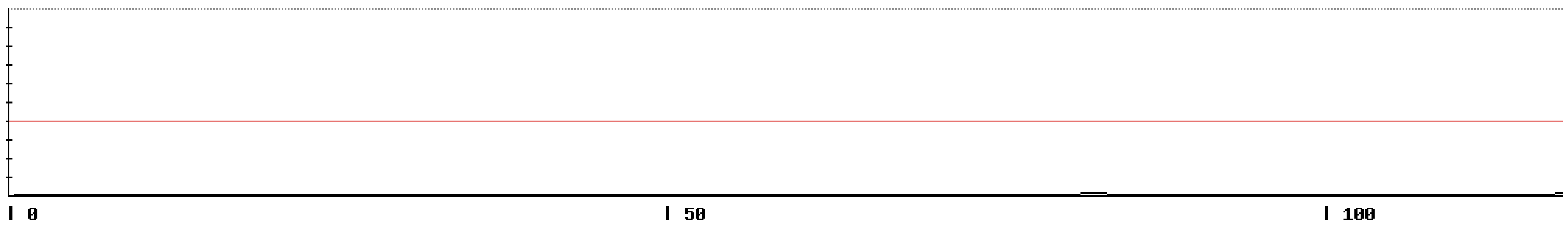

Predictions for Actinidiachinensis_Achn141961	
Viterbi Path	
Viterbi Path	
Posterior @ 0.4	
Posterior @ 0.4	
Posterior Graphic. Threshold Marked @ 0.4	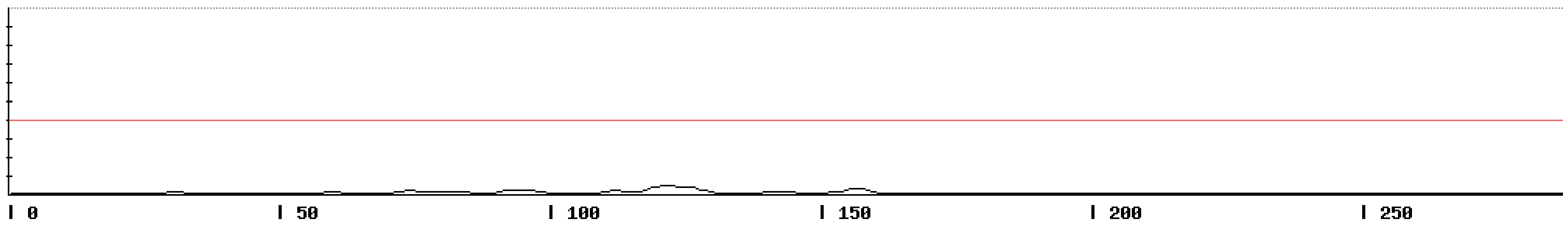

Predictions for Actinidiachinensis_Achn146511	
Viterbi Path	
Viterbi Path	

Posterior @ 0.4

Posterior @ 0.4

Posterior Graphic. Threshold Marked @ 0.4

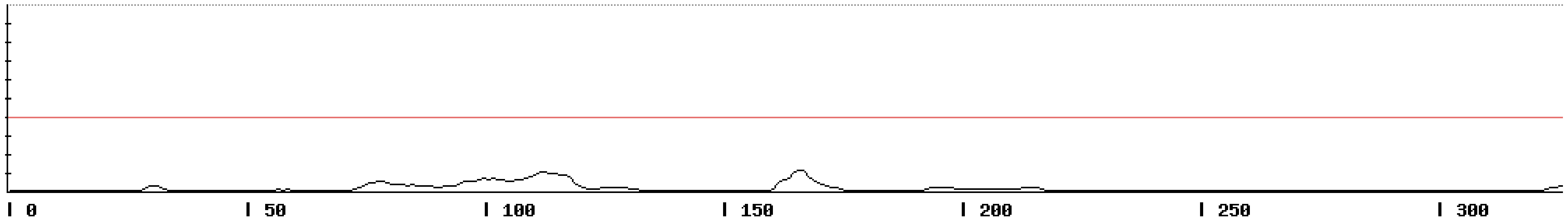

Predictions for Actinidiachinensis_Achn157011

Viterbi Path

Viterbi Path

Posterior @ 0.4

Posterior @ 0.4

Posterior Graphic. Threshold Marked @ 0.4

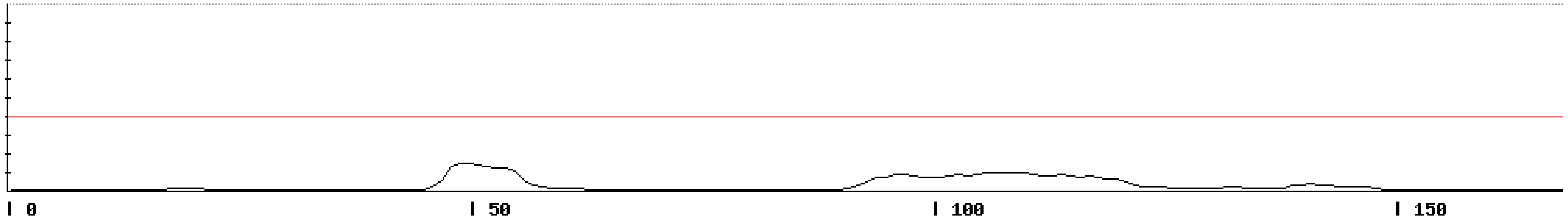

Predictions for Actinidiachinensis_Achn160251

Viterbi Path

Viterbi Path

Posterior @ 0.4

Posterior @ 0.4

Posterior Graphic. Threshold Marked @ 0.4

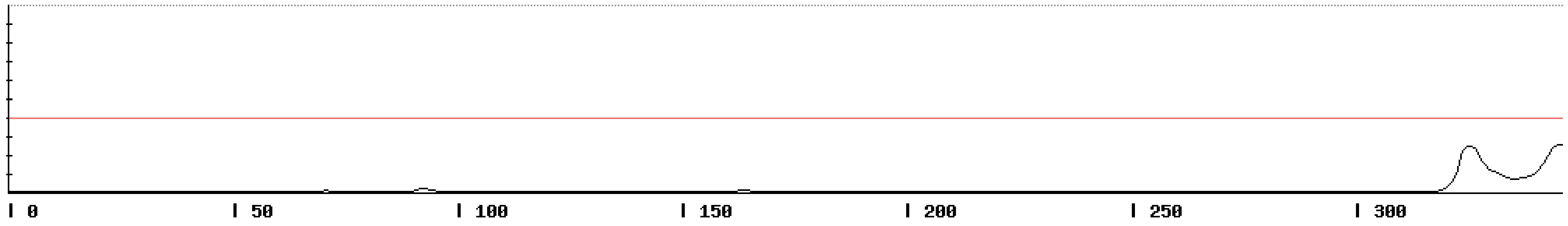

Predictions for Actinidiachinensis_Achn160261

Viterbi Path

Viterbi Path

Posterior @ 0.4

Posterior @ 0.4

Posterior Graphic. Threshold Marked @ 0.4

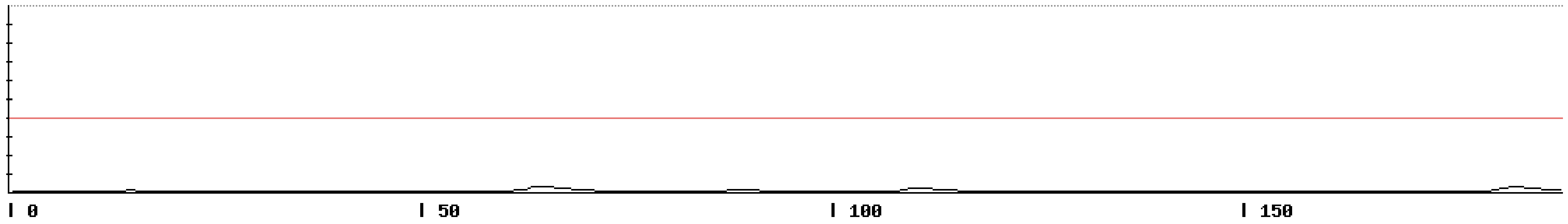

Predictions for Actinidiachinensis_Achn161331

Viterbi Path

Viterbi Path

Posterior @ 0.4

Posterior @ 0.4

Posterior Graphic. Threshold Marked @ 0.4

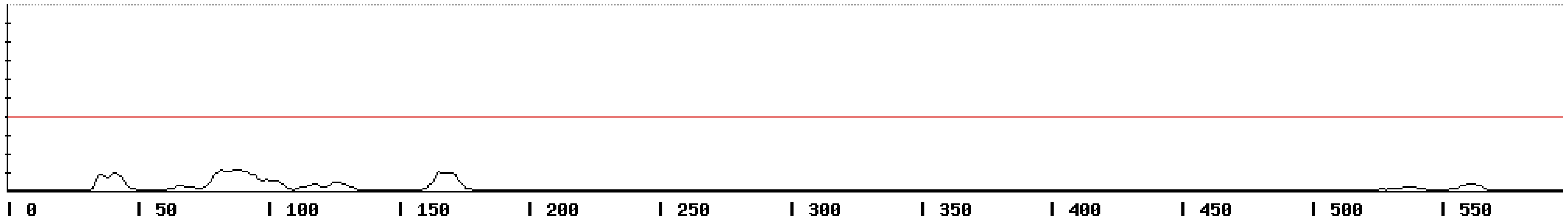

Predictions for Actinidiachinensis_Achn163571

Viterbi Path

Viterbi Path

Posterior @ 0.4

Posterior @ 0.4

Posterior Graphic. Threshold Marked @ 0.4

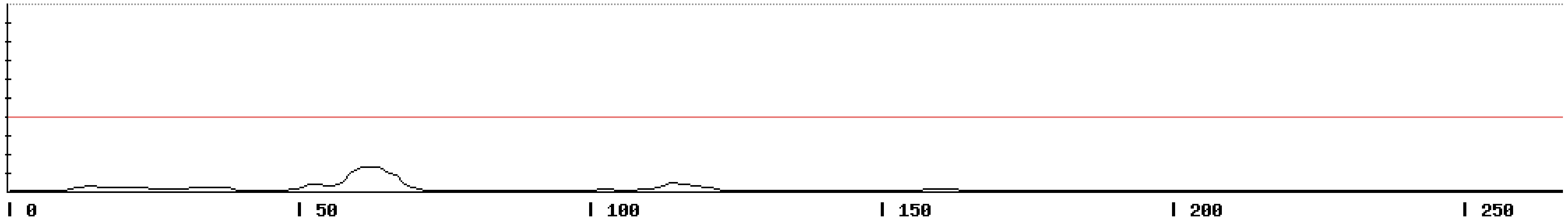

Predictions for Actinidiachinensis_Achn163771

Viterbi Path	
Viterbi Path	
Posterior @ 0.4	86 - RPK - 88
Posterior @ 0.4	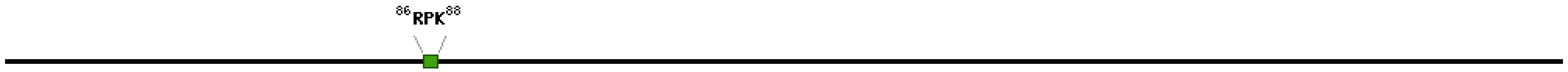
Posterior Graphic. Threshold Marked @ 0.4	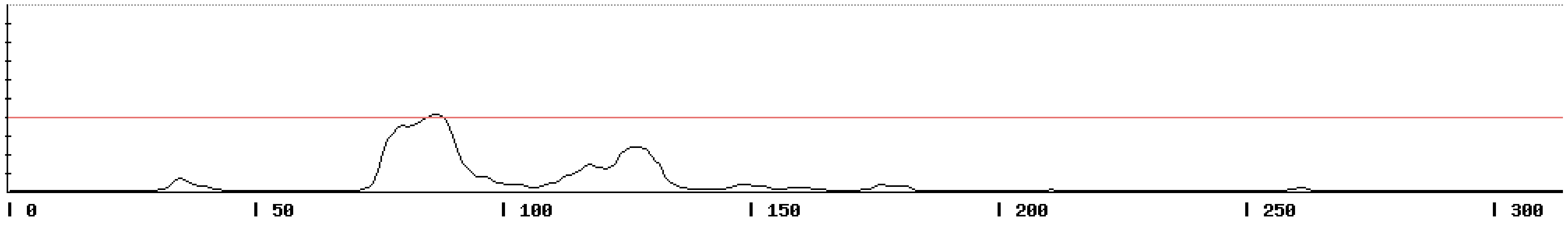

Predictions for Actinidiachinensis_Achn166541

Viterbi Path	
Viterbi Path	
Posterior @ 0.4	
Posterior @ 0.4	
Posterior Graphic. Threshold Marked @ 0.4	

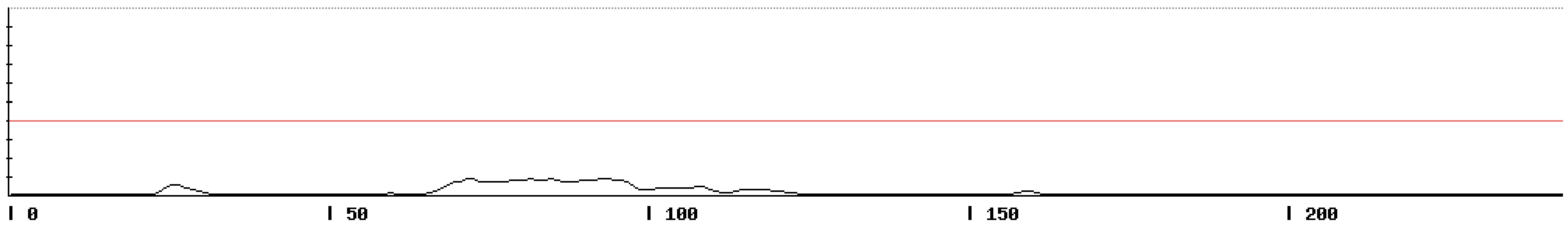

Predictions for Actinidiachinensis_Achn166731

Viterbi Path	
Viterbi Path	
Posterior @ 0.4	
Posterior @ 0.4	
Posterior Graphic. Threshold Marked @ 0.4	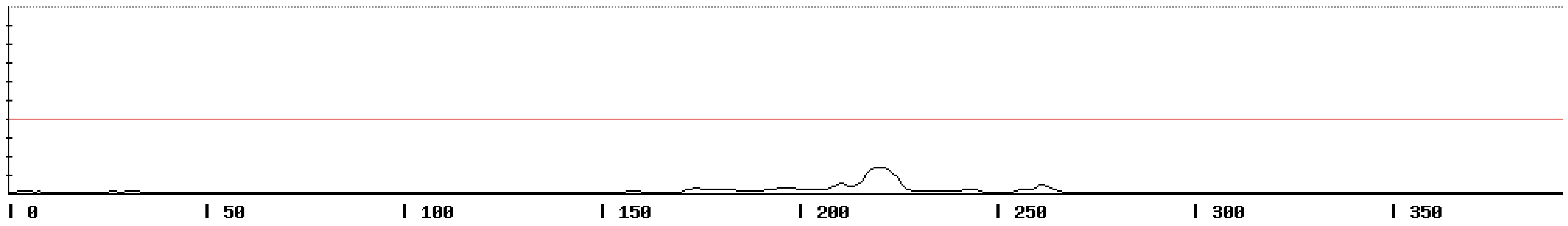

Predictions for Actinidiachinensis_Achn169421

Viterbi Path	
Viterbi Path	
Posterior @ 0.4	
Posterior @ 0.4	

Posterior Graphic. Threshold Marked @ 0.4

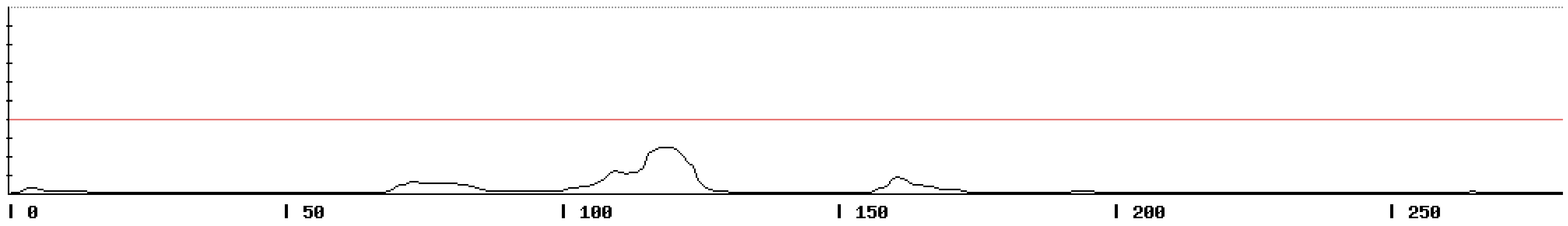

Predictions for Actinidiachinensis_Achn169681

Viterbi Path

Viterbi Path

Posterior @ 0.4

Posterior @ 0.4

Posterior Graphic. Threshold Marked @ 0.4

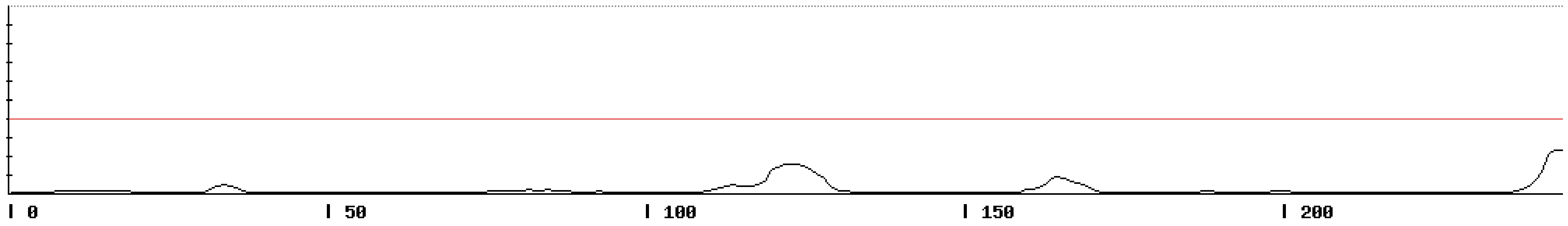

Predictions for Actinidiachinensis_Achn170411

Viterbi Path

Viterbi Path

Posterior @ 0.4

Posterior @ 0.4

Posterior Graphic. Threshold Marked @ 0.4

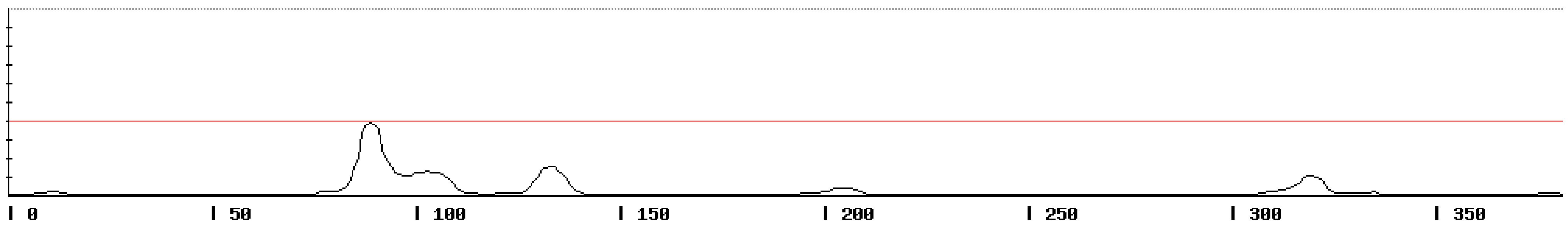

Predictions for Actinidiachinensis_Achn176031

Viterbi Path

Viterbi Path

Posterior @ 0.4

Posterior @ 0.4

Posterior Graphic. Threshold Marked @ 0.4

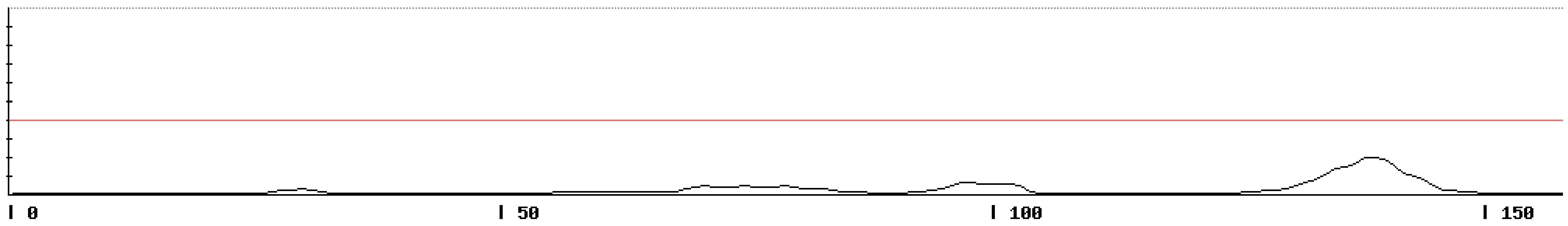

Predictions for Actinidiachinensis_Achn179541

Viterbi Path

Viterbi Path

Posterior @ 0.4

Posterior @ 0.4

Posterior Graphic. Threshold Marked @ 0.4

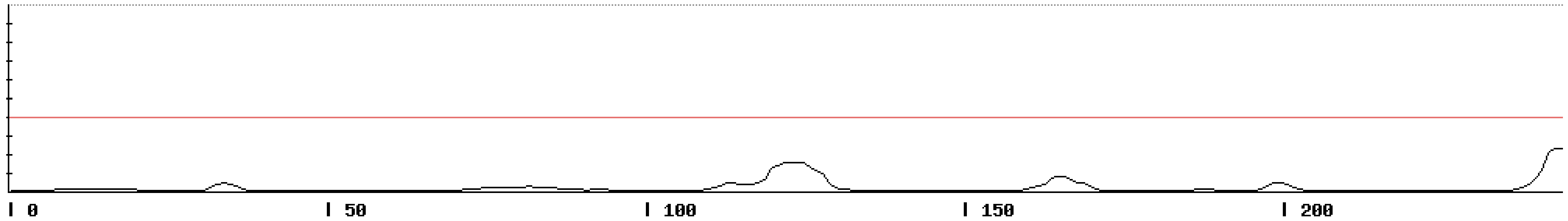

Predictions for Actinidiachinensis_Achn181121

Viterbi Path

Viterbi Path

Posterior @ 0.4

Posterior @ 0.4

Posterior Graphic. Threshold Marked @ 0.4

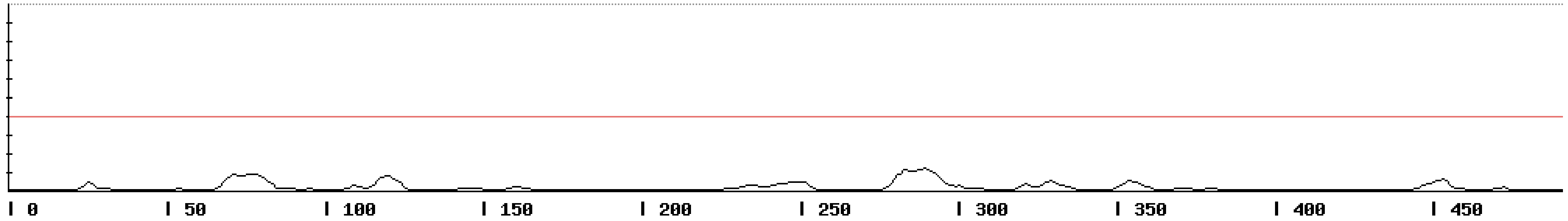

Predictions for Actinidiachinensis_Achn181381

Viterbi Path

Viterbi Path

Posterior @ 0.4	161 - RKRRK - 165
Posterior @ 0.4	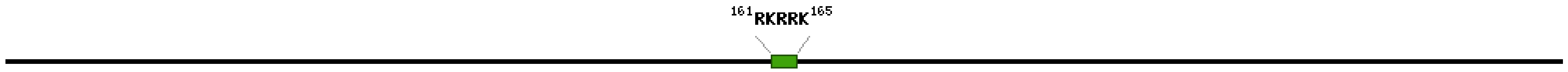
Posterior Graphic. Threshold Marked @ 0.4	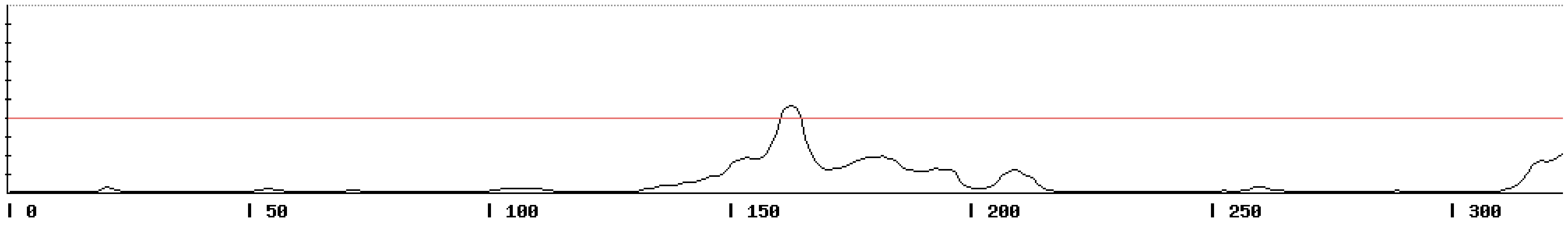

Predictions for Actinidiachinensis_Achn182861

Viterbi Path	
Viterbi Path	
Posterior @ 0.4	
Posterior @ 0.4	
Posterior Graphic. Threshold Marked @ 0.4	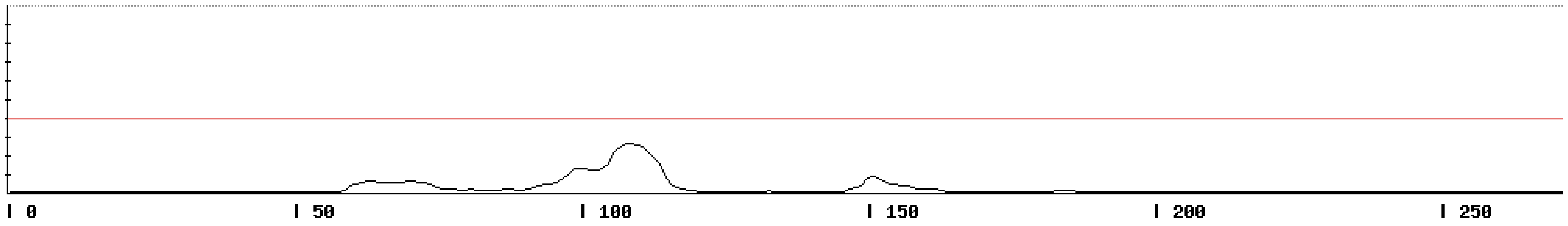

Predictions for Actinidiachinensis_Achn183781

Viterbi Path	
Viterbi Path	
Posterior @ 0.4	
Posterior @ 0.4	
Posterior Graphic. Threshold Marked @ 0.4	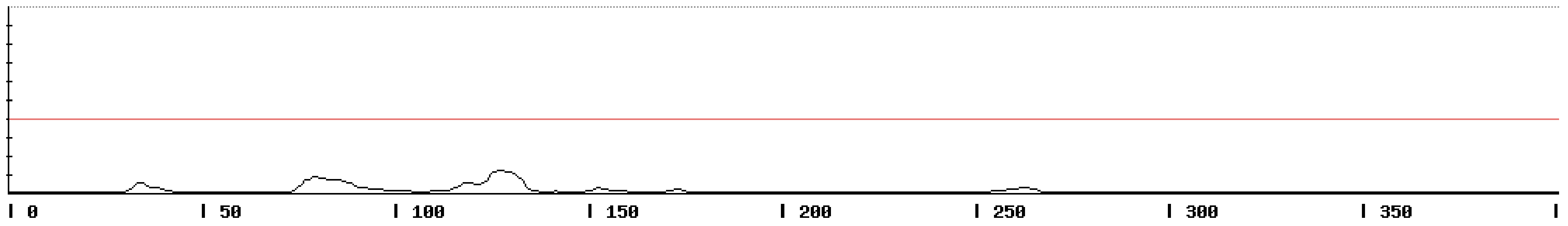

Predictions for Actinidiachinensis_Achn185001

Viterbi Path	
Viterbi Path	
Posterior @ 0.4	
Posterior @ 0.4	
Posterior Graphic. Threshold Marked @ 0.4	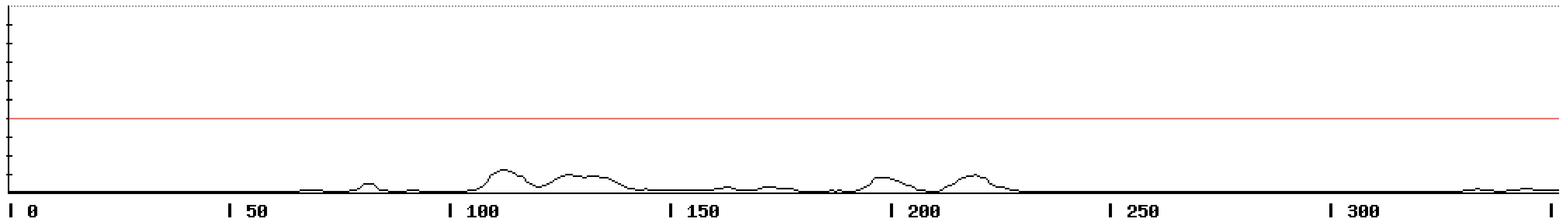

Predictions for Actinidiachinensis_Achn185081	
Viterbi Path	
Viterbi Path	
Posterior @ 0.4	
Posterior @ 0.4	
Posterior Graphic. Threshold Marked @ 0.4	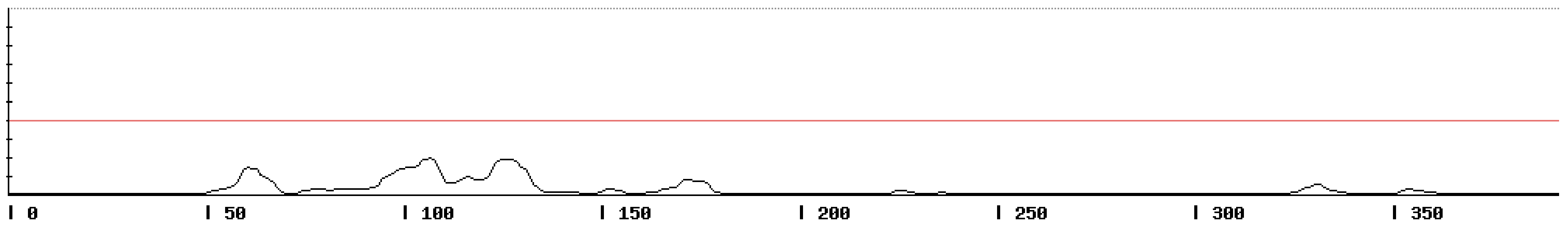

Predictions for Actinidiachinensis_Achn190001	
Viterbi Path	
Viterbi Path	
Posterior @ 0.4	
Posterior @ 0.4	
Posterior Graphic. Threshold Marked @ 0.4	

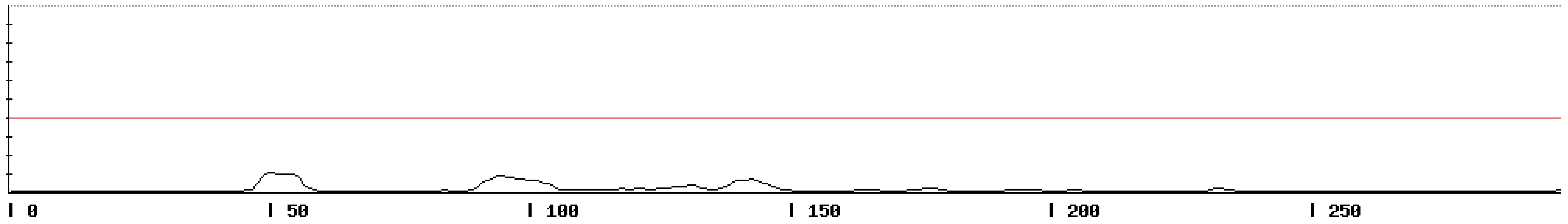

Predictions for Actinidiachinensis_Achn193461

Viterbi Path	
Viterbi Path	
Posterior @ 0.4	
Posterior @ 0.4	
Posterior Graphic. Threshold Marked @ 0.4	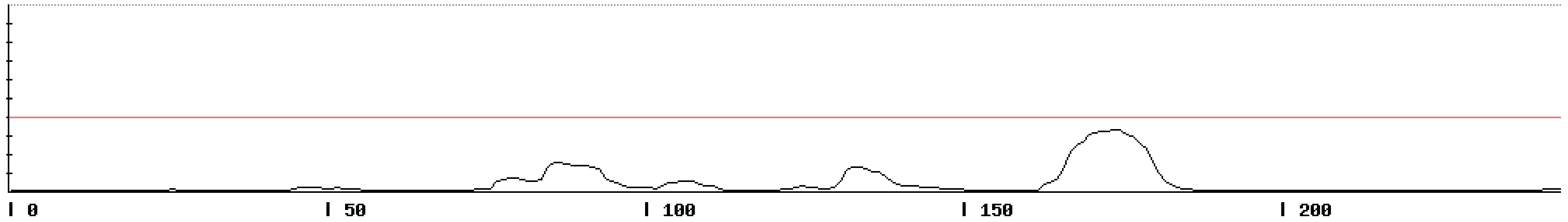

Predictions for Actinidiachinensis_Achn194231

Viterbi Path	
Viterbi Path	
Posterior @ 0.4	
Posterior @ 0.4	

Posterior Graphic. Threshold Marked @ 0.4

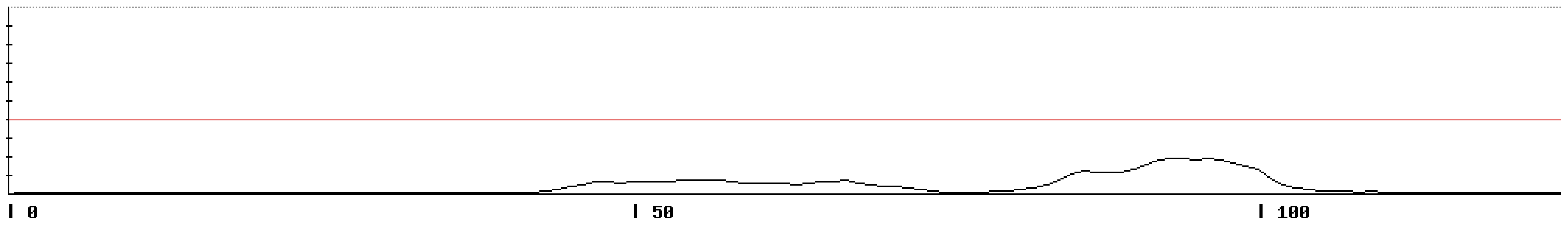

Predictions for Actinidiachinensis_Achn196961

Viterbi Path

Viterbi Path

Posterior @ 0.4

Posterior @ 0.4

Posterior Graphic. Threshold Marked @ 0.4

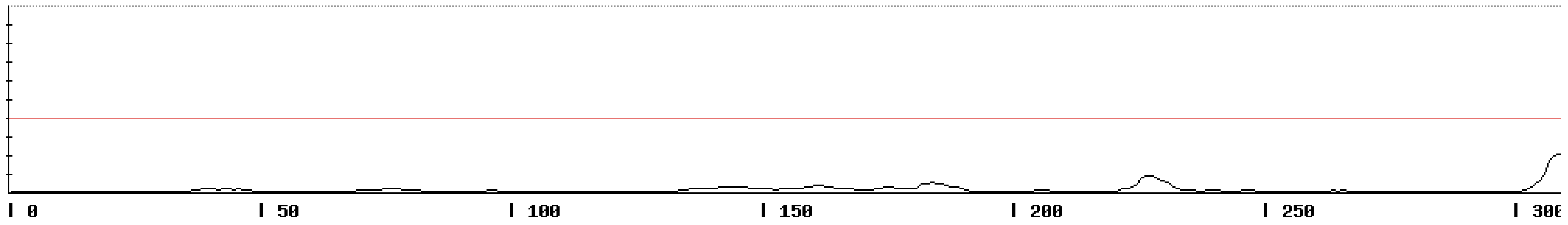

Predictions for Actinidiachinensis_Achn197411

Viterbi Path

Viterbi Path

Posterior @ 0.4

Posterior @ 0.4

Posterior Graphic. Threshold Marked @ 0.4

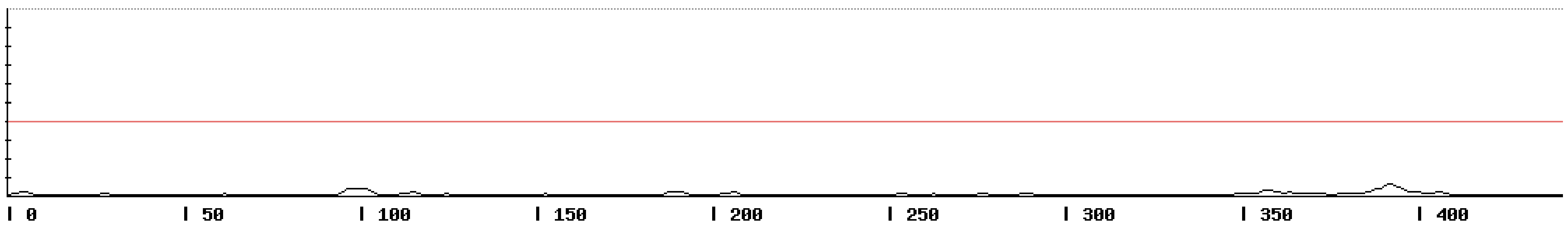

Predictions for Actinidiachinensis_Achn200291

Viterbi Path

Viterbi Path

Posterior @ 0.4 — 200 - KAPAGRKTK - 208

Posterior @ 0.4

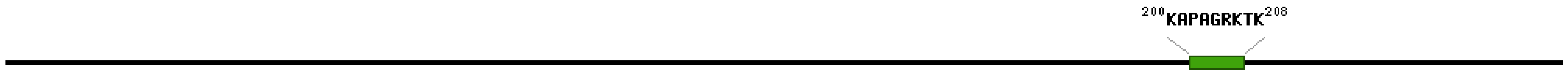

Posterior Graphic. Threshold Marked @ 0.4

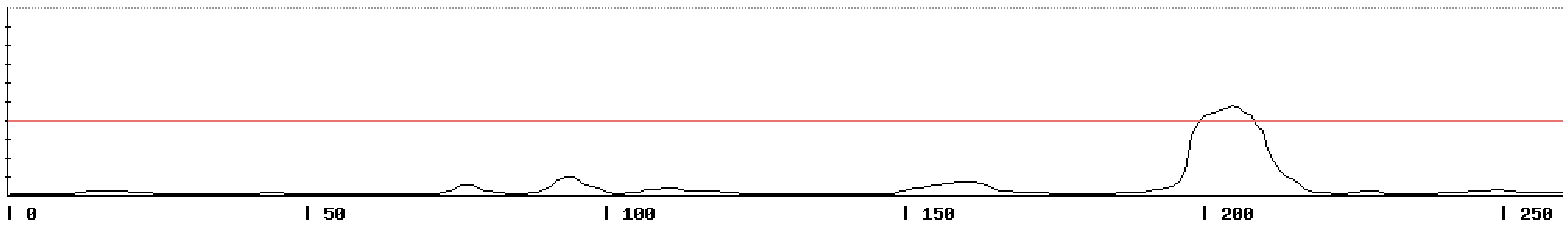

Predictions for Actinidiachinensis_Achn200301

Viterbi Path

Viterbi Path

Posterior @ 0.4

Posterior @ 0.4

Posterior Graphic. Threshold Marked @ 0.4

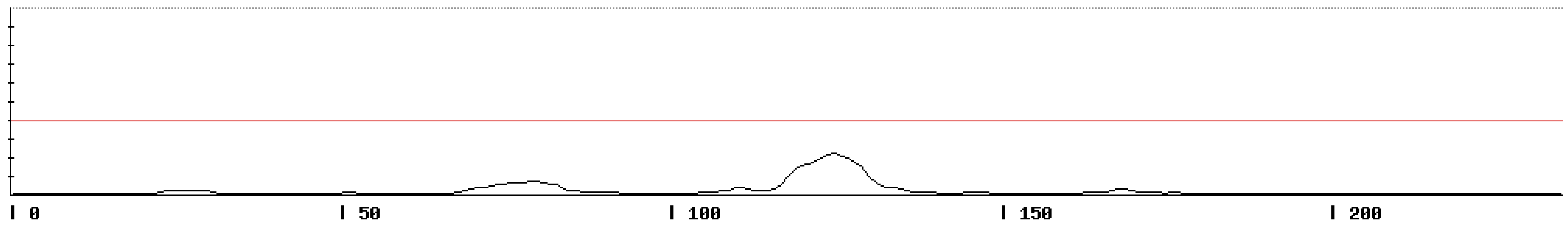

Predictions for Actinidiachinensis_Achn204171

Viterbi Path

Viterbi Path

Posterior @ 0.4

Posterior @ 0.4

Posterior Graphic. Threshold Marked @ 0.4

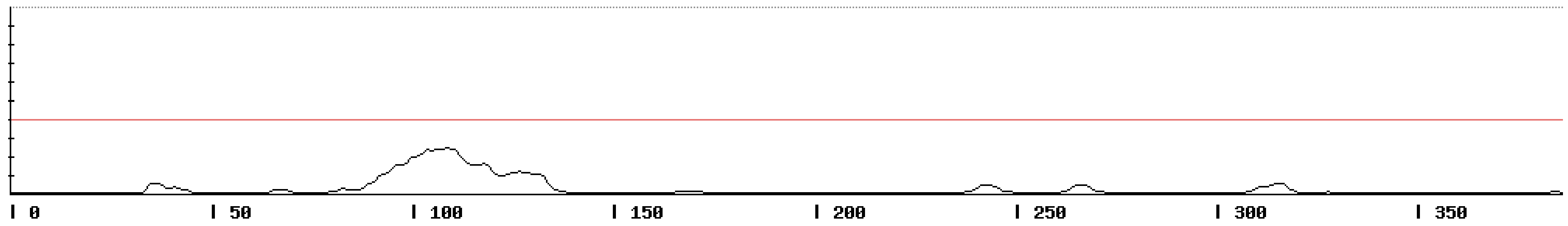

Predictions for Actinidiachinensis_Achn204181

Viterbi Path

Viterbi Path

Posterior @ 0.4

Posterior @ 0.4

Posterior Graphic. Threshold Marked @ 0.4

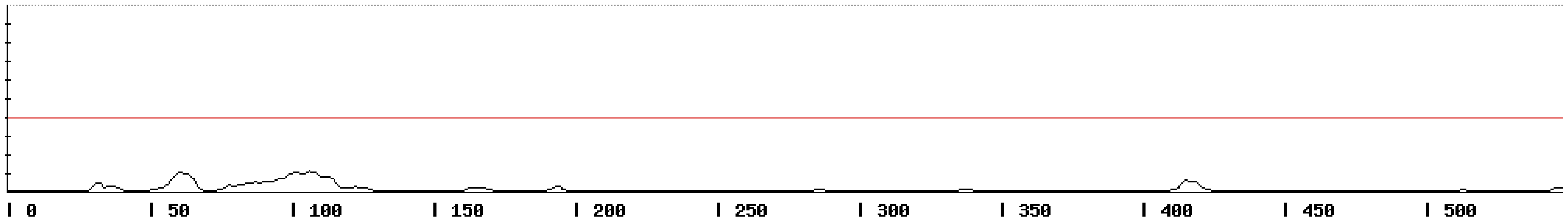

Predictions for Actinidiachinensis_Achn206771

Viterbi Path

Viterbi Path

Posterior @ 0.4

Posterior @ 0.4

Posterior Graphic. Threshold Marked @ 0.4

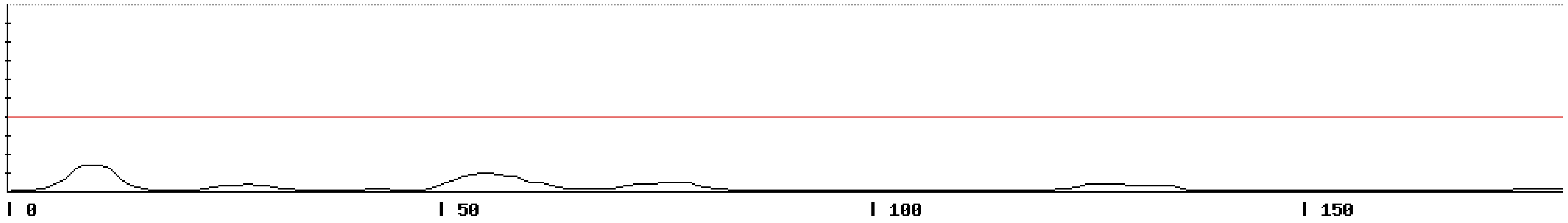

Predictions for Actinidiachinensis_Achn207401

Viterbi Path

Viterbi Path

Posterior @ 0.4

Posterior @ 0.4

Posterior Graphic. Threshold Marked @ 0.4

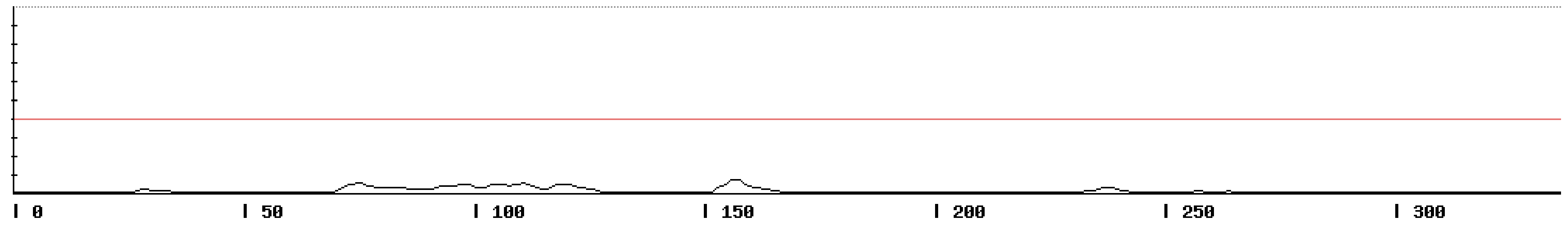

Predictions for Actinidiachinensis_Achn208181

Viterbi Path

Viterbi Path

Posterior @ 0.4

Posterior @ 0.4

Posterior Graphic. Threshold Marked @ 0.4

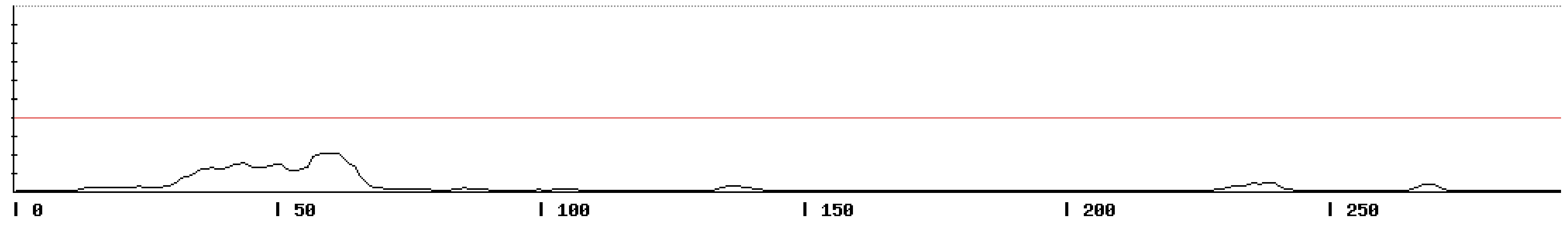

Predictions for Actinidiachinensis_Achn210451	
Viterbi Path	
Viterbi Path	
Posterior @ 0.4	
Posterior @ 0.4	
Posterior Graphic. Threshold Marked @ 0.4	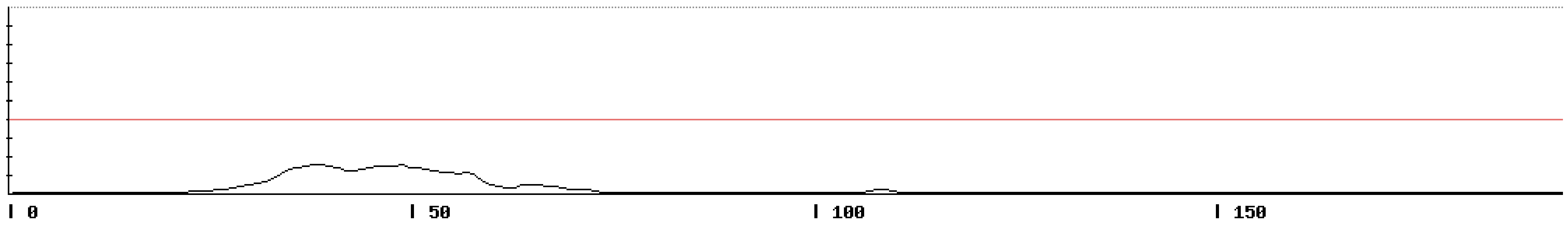

Predictions for Actinidiachinensis_Achn212001	
Viterbi Path	164 - KRREKRGGYKRGEGRV - 179
Viterbi Path	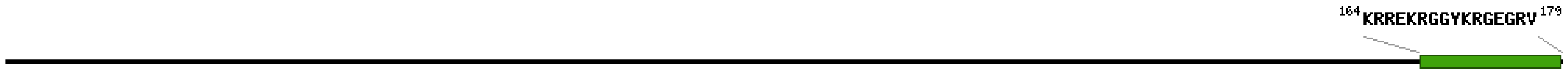
Posterior @ 0.4	164 - KRREKRGGYKRGEGRV - 179
Posterior @ 0.4	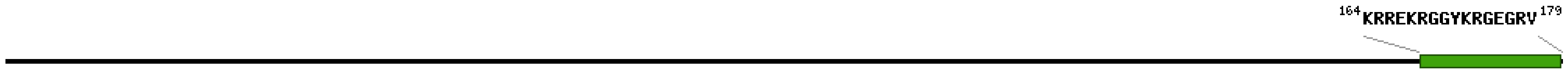
Posterior Graphic. Threshold Marked @ 0.4	

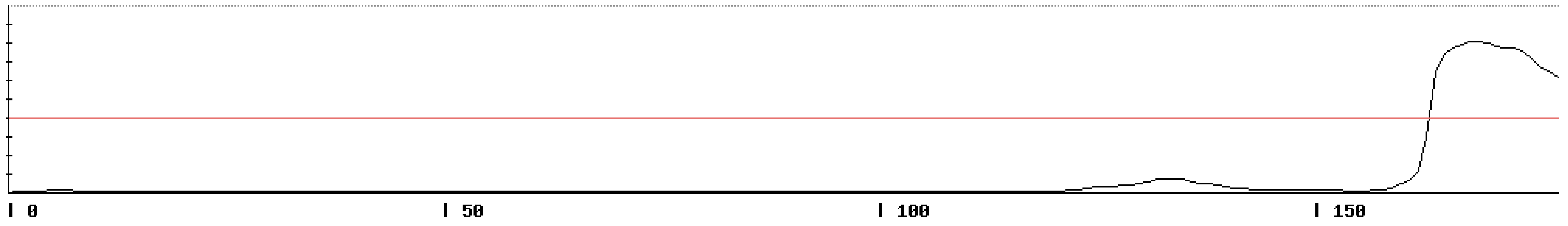

Predictions for Actinidiachinensis_Achn219111

Viterbi Path	
Viterbi Path	
Posterior @ 0.4	257 - RPPPRPKFGPRWRFQHHHNQRSHLPQRRDEEVEAKRRDAEKERARRDRLYH - 307
Posterior @ 0.4	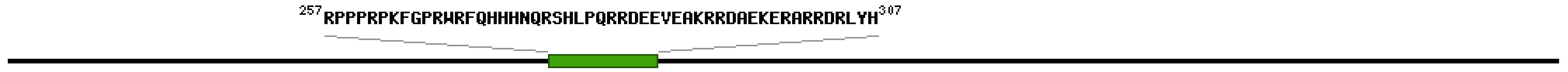
Posterior Graphic. Threshold Marked @ 0.4	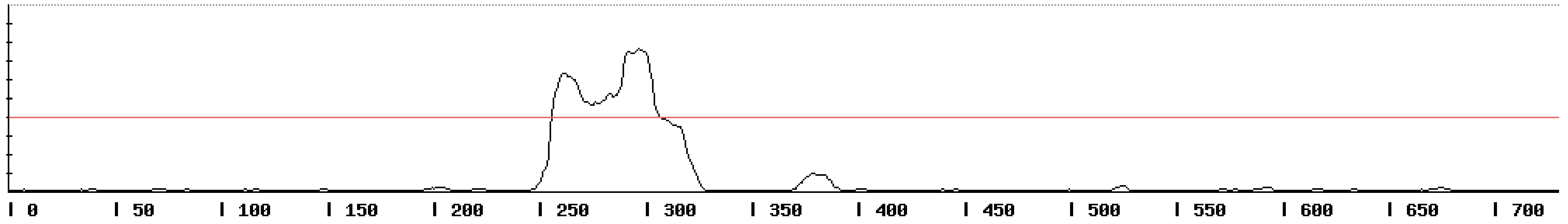

Predictions for Actinidiachinensis_Achn219211

Viterbi Path	
Viterbi Path	
Posterior @ 0.4	219 - RKRR - 222
Posterior @ 0.4	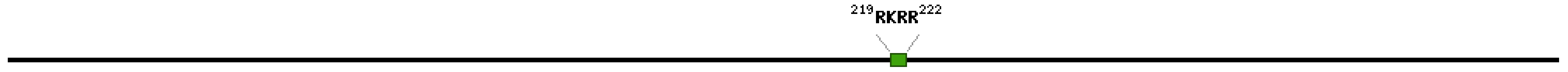

Posterior Graphic. Threshold Marked @ 0.4

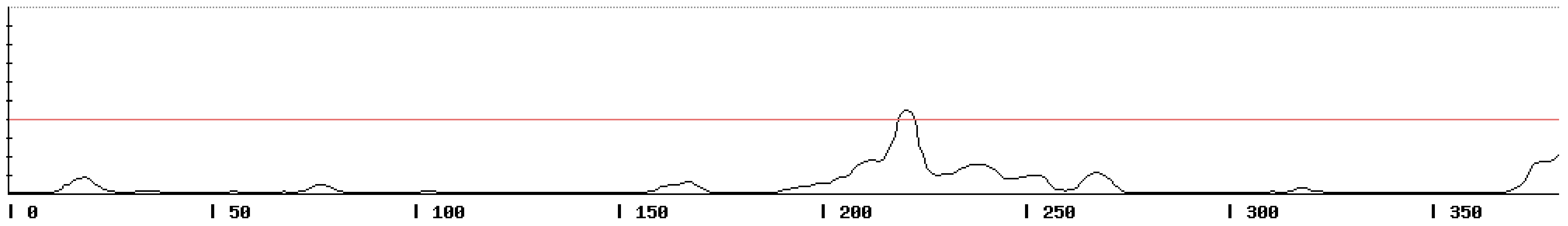

Predictions for Actinidiachinensis_Achn220201

Viterbi Path

Viterbi Path

Posterior @ 0.4

Posterior @ 0.4

Posterior Graphic. Threshold Marked @ 0.4

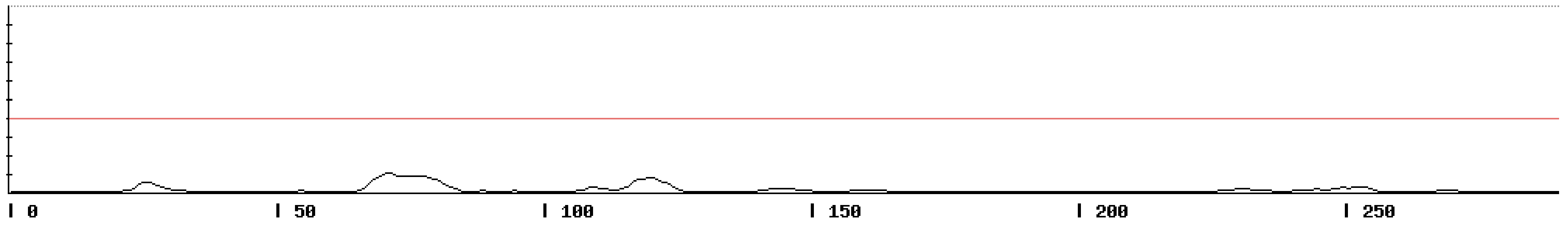

Predictions for Actinidiachinensis_Achn220211

Viterbi Path

Viterbi Path

Posterior @ 0.4

Posterior @ 0.4

Posterior Graphic. Threshold Marked @ 0.4

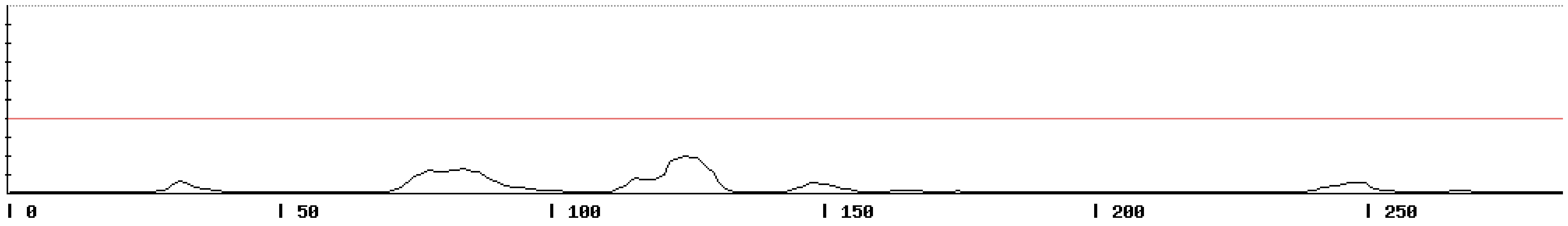

Predictions for Actinidiachinensis_Achn221871

Viterbi Path

Viterbi Path

Posterior @ 0.4 112 - RKRRK - 116

Posterior @ 0.4

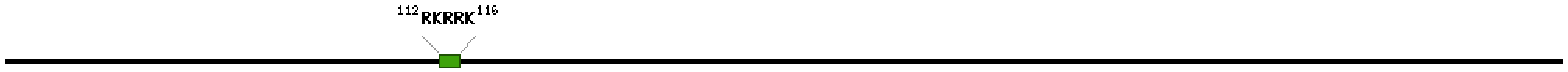

Posterior Graphic. Threshold Marked @ 0.4

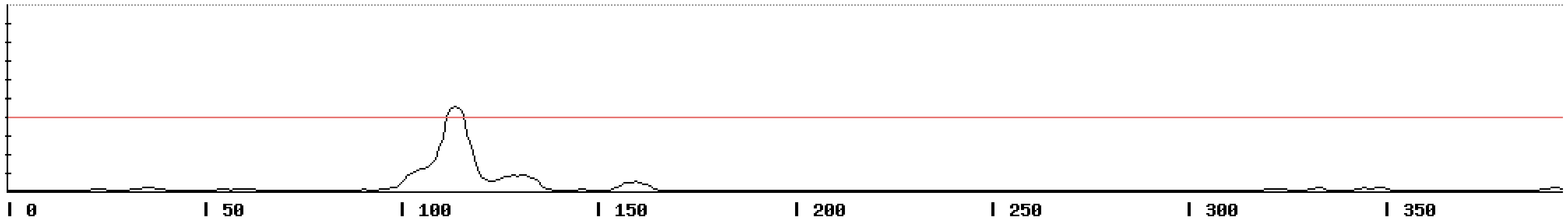

Predictions for Actinidiachinensis_Achn226931

Viterbi Path

Viterbi Path

Posterior @ 0.4

Posterior @ 0.4

Posterior Graphic. Threshold Marked @ 0.4

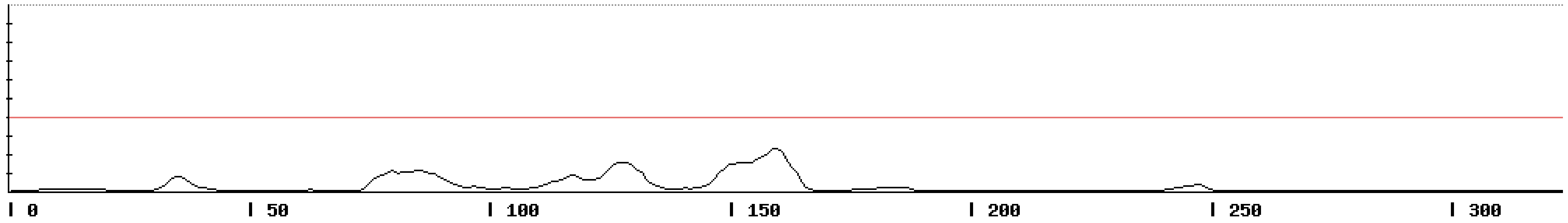

Predictions for Actinidiachinensis_Achn229031

Viterbi Path

Viterbi Path

Posterior @ 0.4

Posterior @ 0.4

Posterior Graphic. Threshold Marked @ 0.4

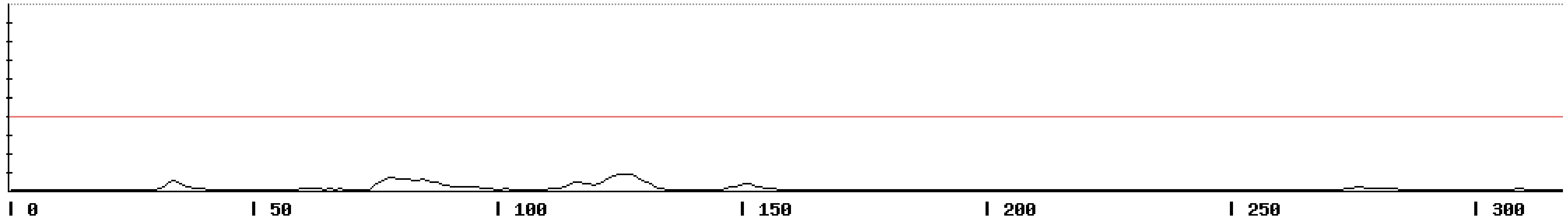

Predictions for Actinidiachinensis_Achn229061

Viterbi Path

Viterbi Path

Posterior @ 0.4

Posterior @ 0.4

Posterior Graphic. Threshold Marked @ 0.4

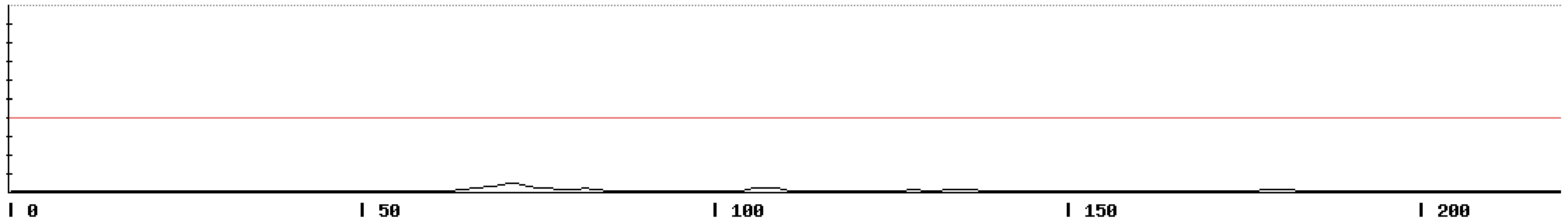

Predictions for Actinidiachinensis_Achn234861

Viterbi Path

Viterbi Path

Posterior @ 0.4

Posterior @ 0.4

Posterior Graphic. Threshold Marked @ 0.4

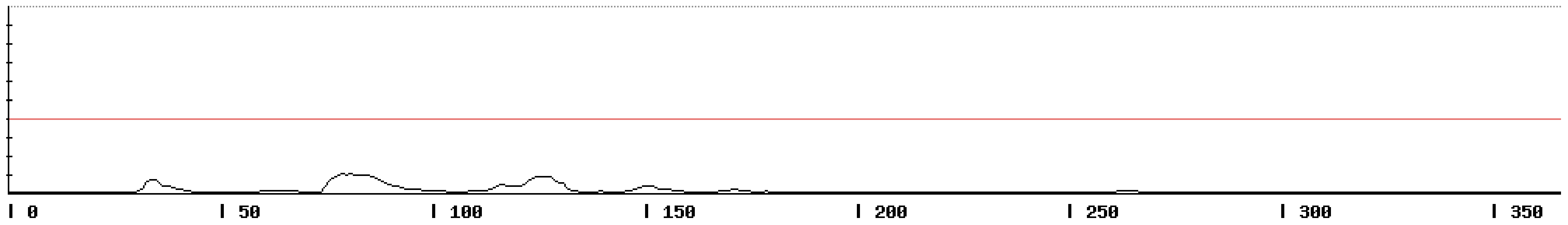

Predictions for Actinidiachinensis_Achn237291

Viterbi Path	
Viterbi Path	
Posterior @ 0.4	
Posterior @ 0.4	
Posterior Graphic. Threshold Marked @ 0.4	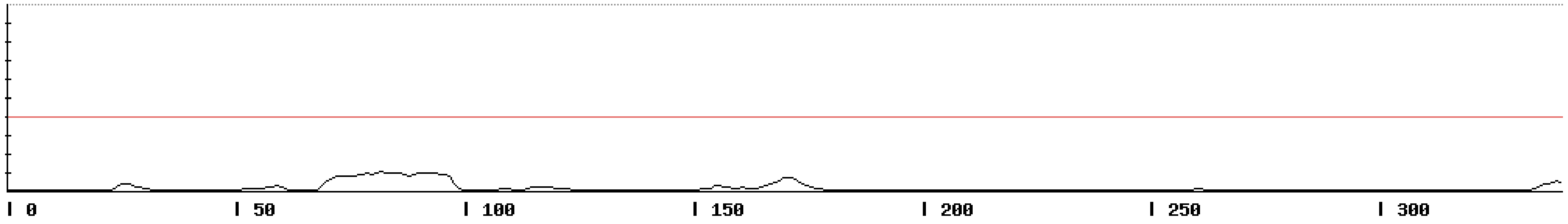

Predictions for Actinidiachinensis_Achn238591

Viterbi Path	
Viterbi Path	
Posterior @ 0.4	68 - KKKGK - 72
Posterior @ 0.4	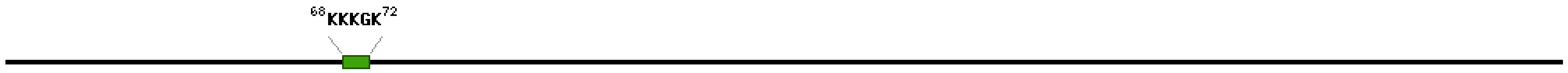
Posterior Graphic. Threshold Marked @ 0.4	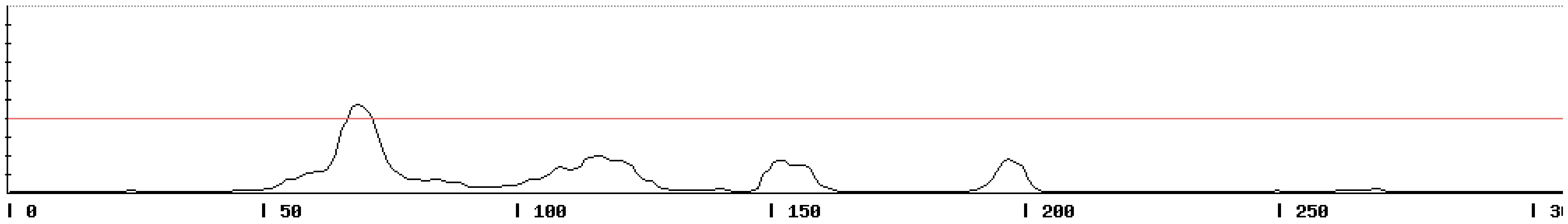

Predictions for Actinidiachinensis_Achn239781	
Viterbi Path	
Viterbi Path	
Posterior @ 0.4	67 - LKKKGK - 72
Posterior @ 0.4	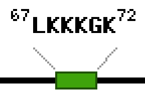
Posterior Graphic. Threshold Marked @ 0.4	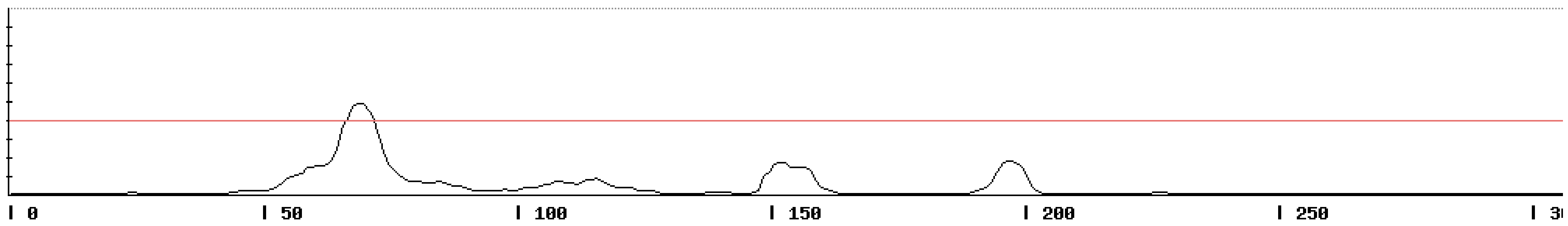

Predictions for Actinidiachinensis_Achn241171	
Viterbi Path	
Viterbi Path	
Posterior @ 0.4	
Posterior @ 0.4	
Posterior Graphic. Threshold Marked @ 0.4	

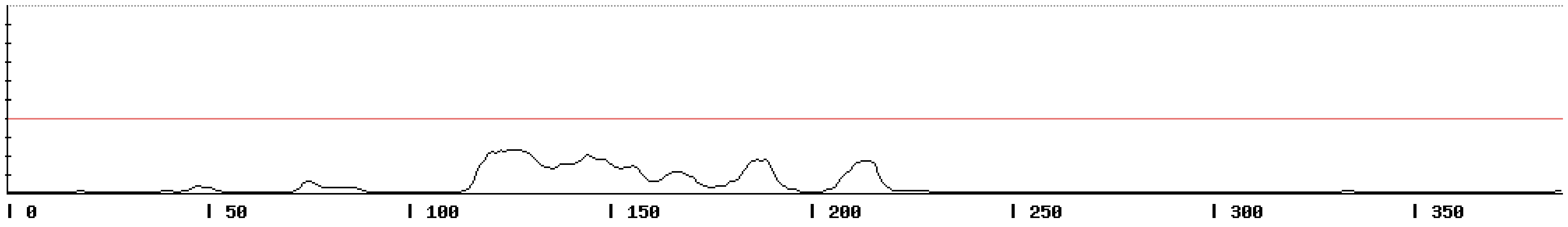

Predictions for Actinidiachinensis_Achn241181

Viterbi Path	
Viterbi Path	
Posterior @ 0.4	118 - RDRKYPKGKRPNRAA - 132
Posterior @ 0.4	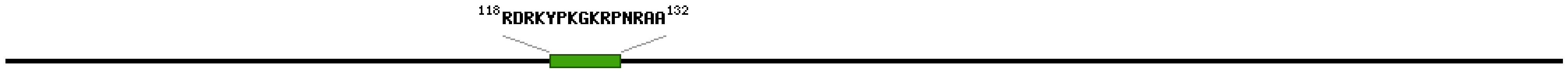
Posterior Graphic. Threshold Marked @ 0.4	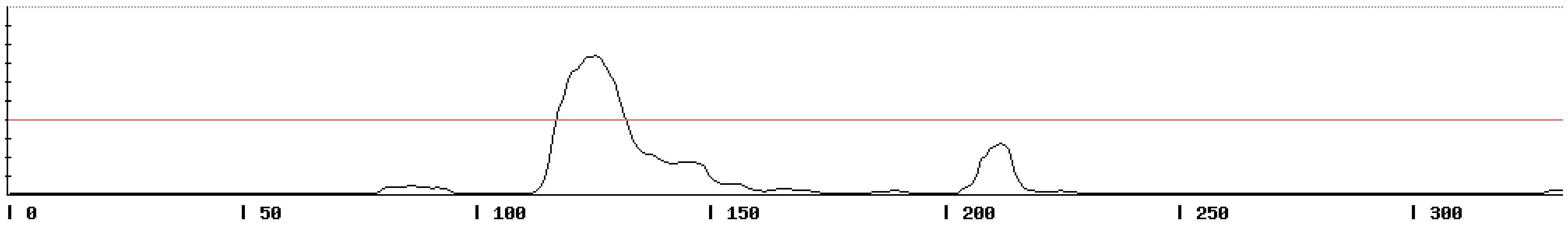

Predictions for Actinidiachinensis_Achn244831

Viterbi Path	
Viterbi Path	
Posterior @ 0.4	85 - KKKGK - 89
Posterior @ 0.4	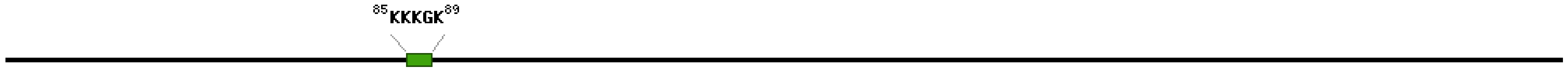

Posterior Graphic. Threshold Marked @ 0.4

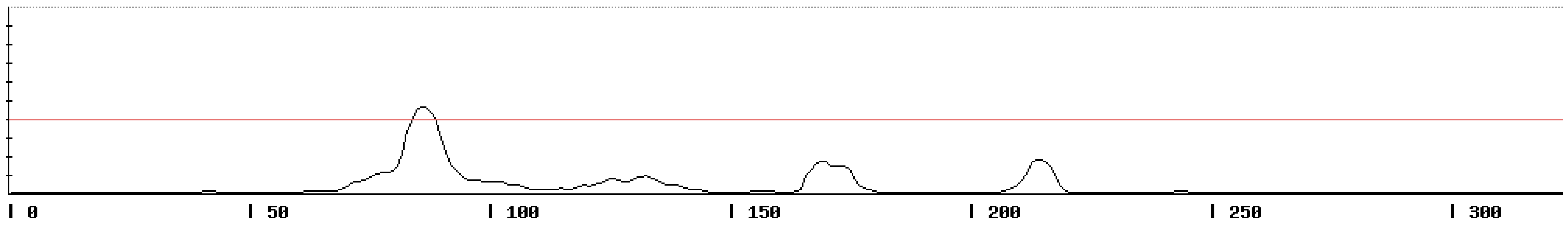

Predictions for Actinidiachinensis_Achn245061

Viterbi Path

Viterbi Path

Posterior @ 0.4

Posterior @ 0.4

Posterior Graphic. Threshold Marked @ 0.4

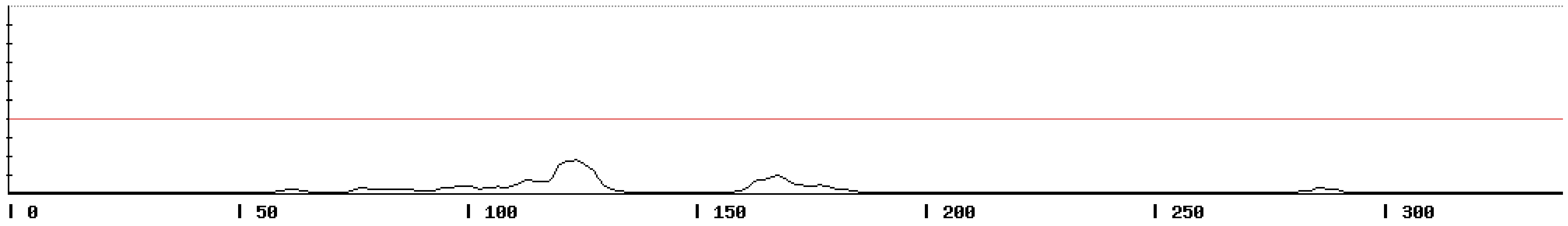

Predictions for Actinidiachinensis_Achn247931

Viterbi Path

Viterbi Path

Posterior @ 0.4

Posterior @ 0.4

Posterior Graphic. Threshold Marked @ 0.4

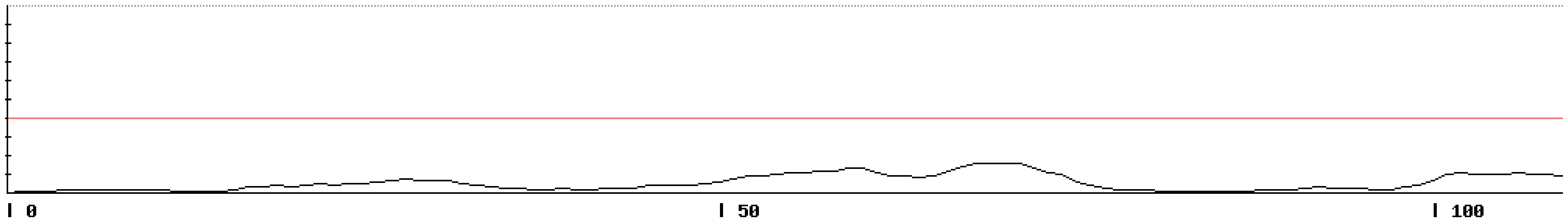

Predictions for Actinidiachinensis_Achn254041

Viterbi Path

Viterbi Path

Posterior @ 0.4 149 - KERAIKRDRQK - 159

Posterior @ 0.4

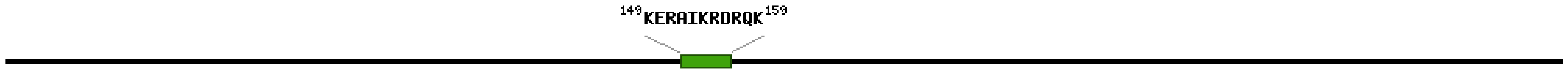

Posterior Graphic. Threshold Marked @ 0.4

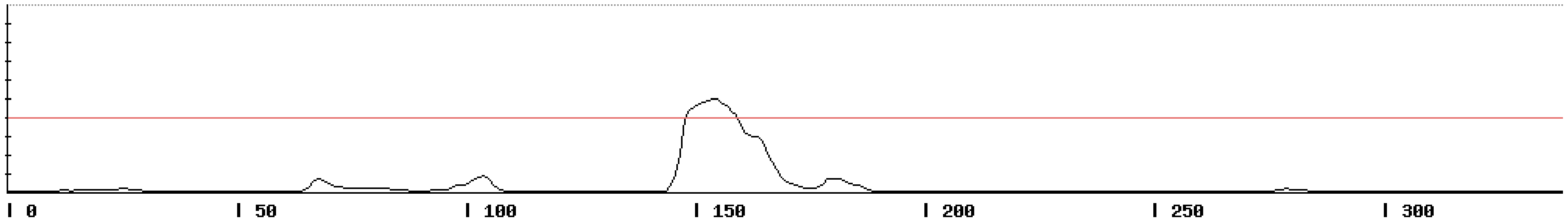

Predictions for Actinidiachinensis_Achn254051

Viterbi Path

Viterbi Path

Posterior @ 0.4

Posterior @ 0.4

Posterior Graphic. Threshold Marked @ 0.4

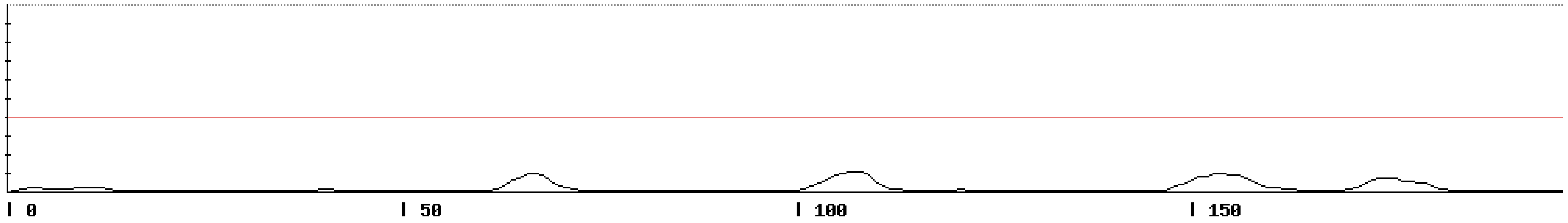

Predictions for Actinidiachinensis_Achn262621

Viterbi Path

Viterbi Path

Posterior @ 0.4

Posterior @ 0.4

Posterior Graphic. Threshold Marked @ 0.4

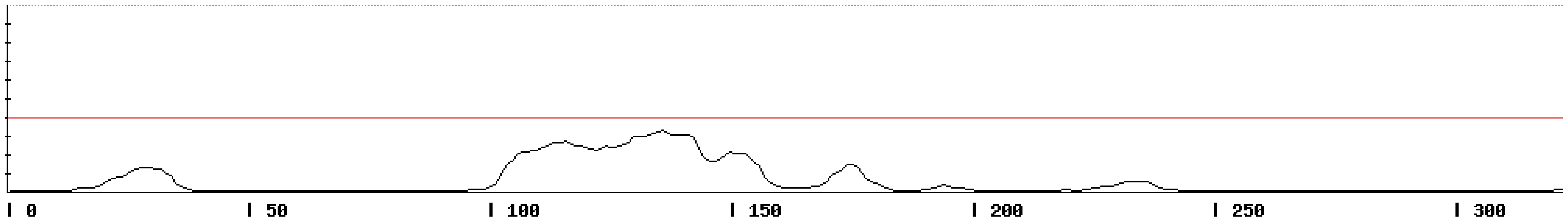

Predictions for Actinidiachinensis_Achn266441

Viterbi Path

Viterbi Path

Posterior @ 0.4

Posterior @ 0.4

Posterior Graphic. Threshold Marked @ 0.4

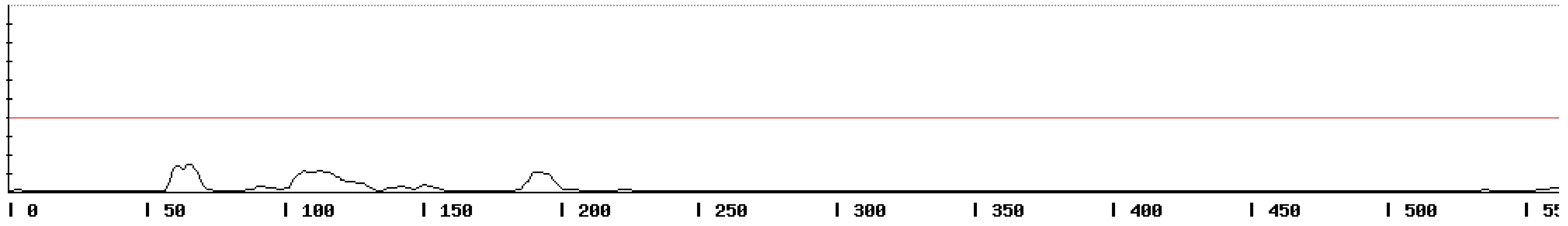

Predictions for Actinidiachinensis_Achn267671

Viterbi Path

Viterbi Path

Posterior @ 0.4 — 76 - KYKIGRRKNR - 85

Posterior @ 0.4

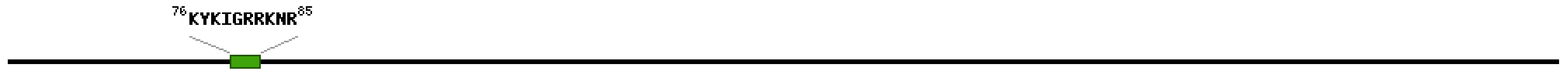

Posterior Graphic. Threshold Marked @ 0.4

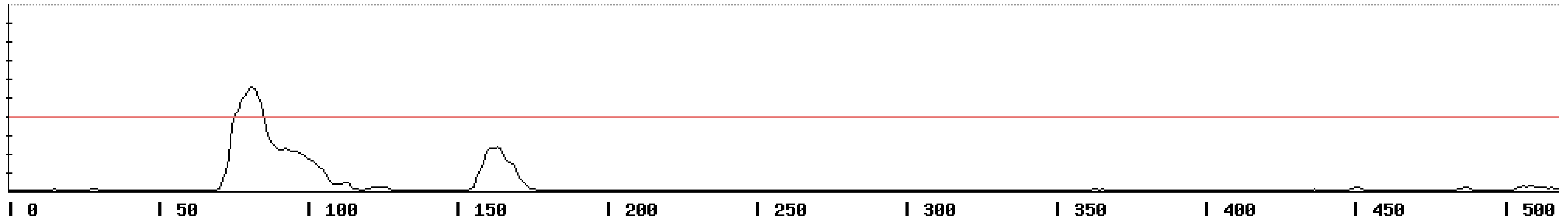

Predictions for Actinidiachinensis_Achn272561

Viterbi Path

Viterbi Path

Posterior @ 0.4

Posterior @ 0.4

Posterior Graphic. Threshold Marked @ 0.4

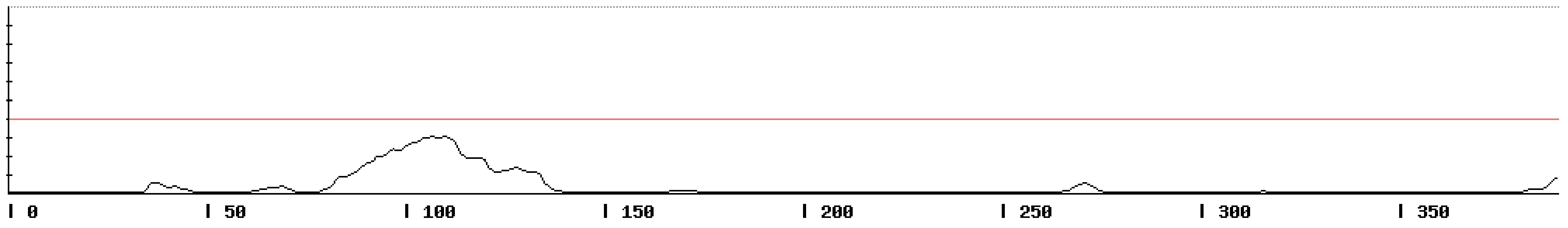

Predictions for Actinidiachinensis_Achn272571

Viterbi Path

Viterbi Path

Posterior @ 0.4

Posterior @ 0.4

Posterior Graphic. Threshold Marked @ 0.4

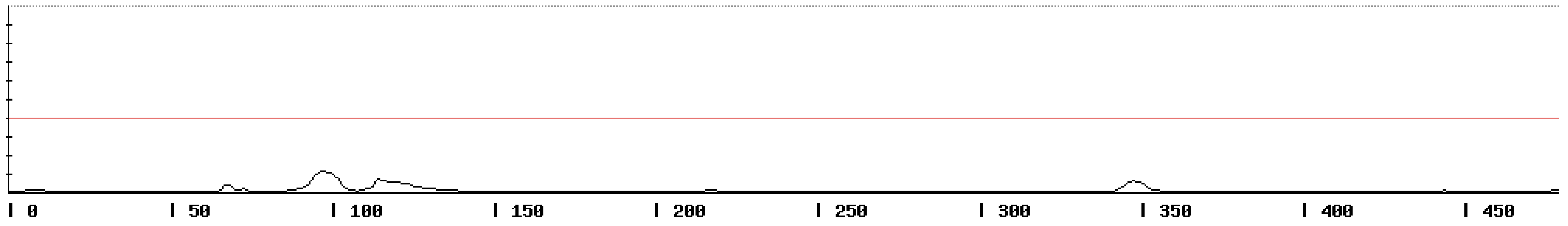

Predictions for Actinidiachinensis_Achn272901	
Viterbi Path	
Viterbi Path	
Posterior @ 0.4	160 - LKKKGK - 165
Posterior @ 0.4	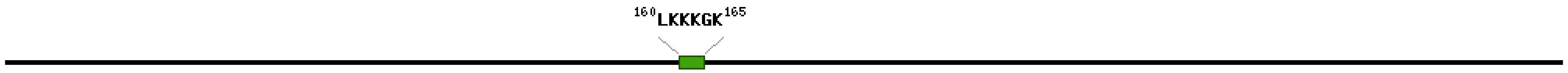
Posterior Graphic. Threshold Marked @ 0.4	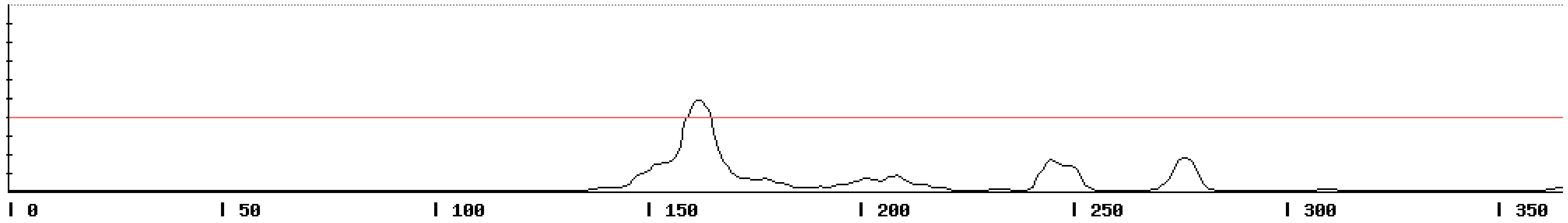

Predictions for Actinidiachinensis_Achn274891	
Viterbi Path	
Viterbi Path	
Posterior @ 0.4	
Posterior @ 0.4	
Posterior Graphic. Threshold Marked @ 0.4	

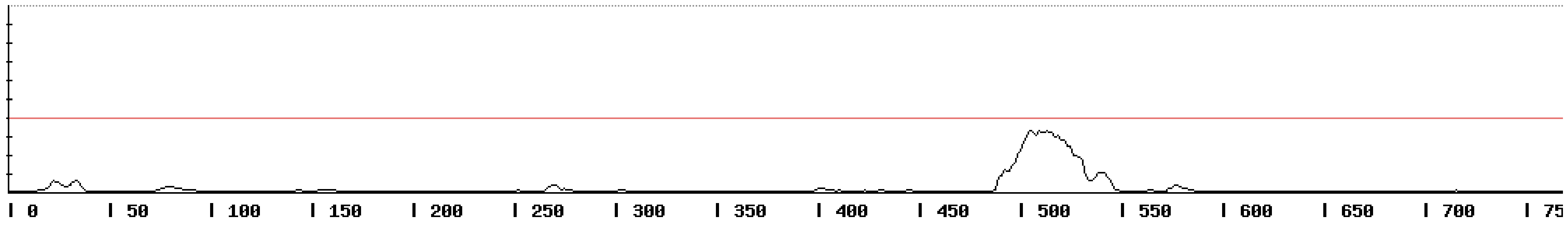

Predictions for Actinidiachinensis_Achn276021

Viterbi Path	
Viterbi Path	
Posterior @ 0.4	
Posterior @ 0.4	
Posterior Graphic. Threshold Marked @ 0.4	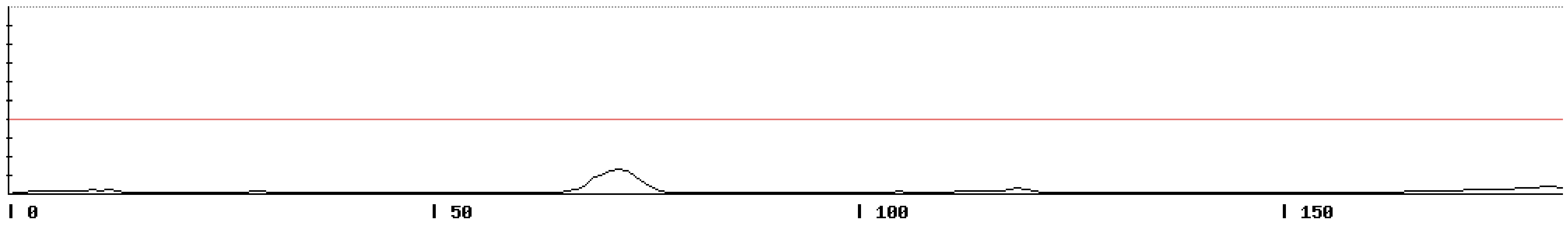

Predictions for Actinidiachinensis_Achn276961

Viterbi Path	
Viterbi Path	
Posterior @ 0.4	
Posterior @ 0.4	

Posterior Graphic. Threshold Marked @ 0.4

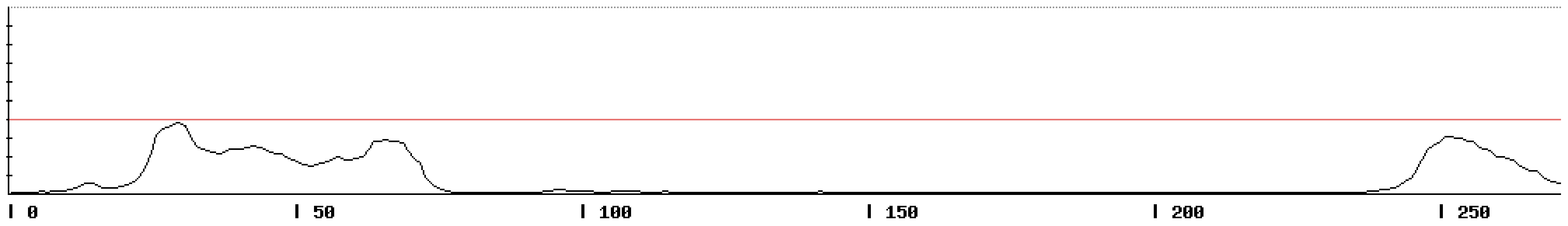

Predictions for Actinidiachinensis_Achn279951

Viterbi Path

Viterbi Path

Posterior @ 0.4

Posterior @ 0.4

Posterior Graphic. Threshold Marked @ 0.4

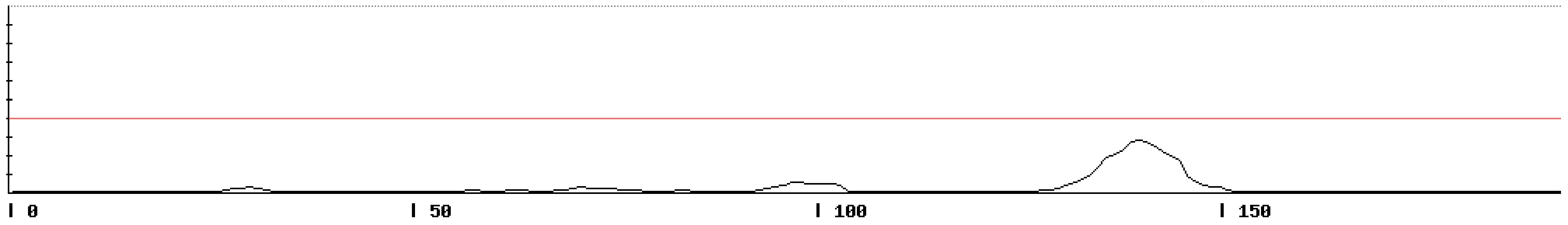

Predictions for Actinidiachinensis_Achn282481

Viterbi Path

Viterbi Path

Posterior @ 0.4

Posterior @ 0.4

Posterior Graphic. Threshold Marked @ 0.4

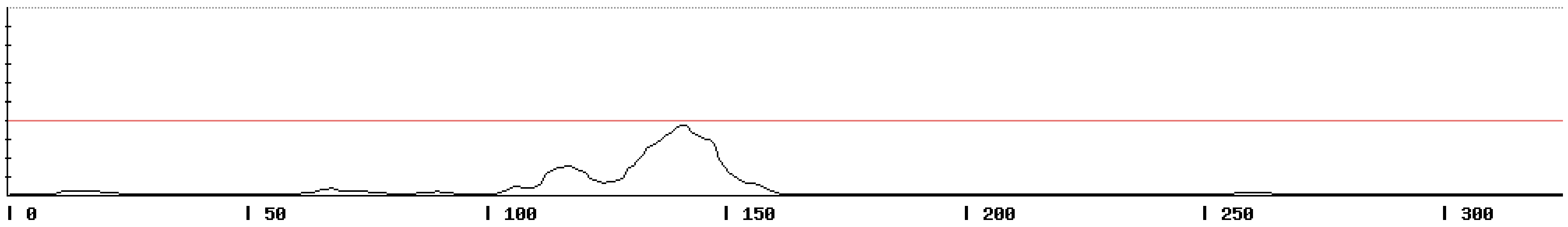

Predictions for Actinidiachinensis_Achn282651

Viterbi Path

Viterbi Path

Posterior @ 0.4

Posterior @ 0.4

Posterior Graphic. Threshold Marked @ 0.4

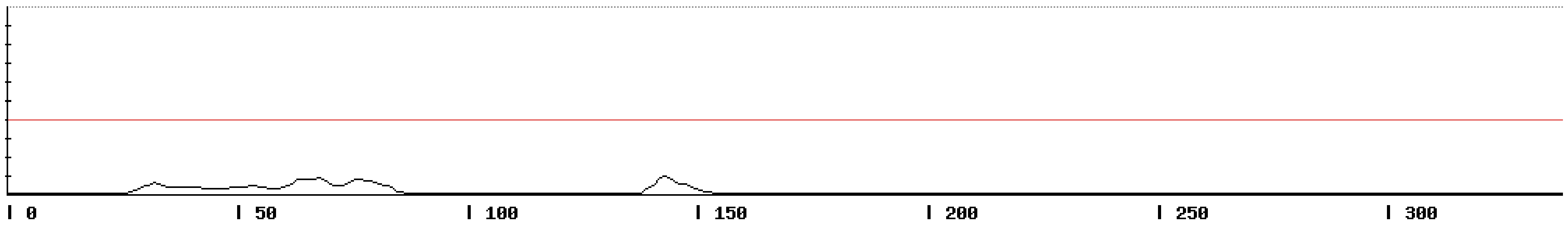

Predictions for Actinidiachinensis_Achn289231

Viterbi Path

Viterbi Path

Posterior @ 0.4	
Posterior @ 0.4	
Posterior Graphic. Threshold Marked @ 0.4	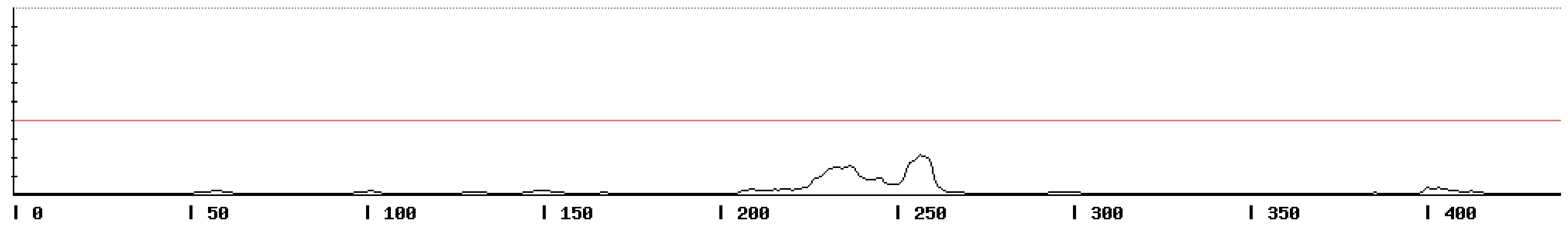

Predictions for Actinidiachinensis_Achn289291	
Viterbi Path	
Viterbi Path	
Posterior @ 0.4	
Posterior @ 0.4	
Posterior Graphic. Threshold Marked @ 0.4	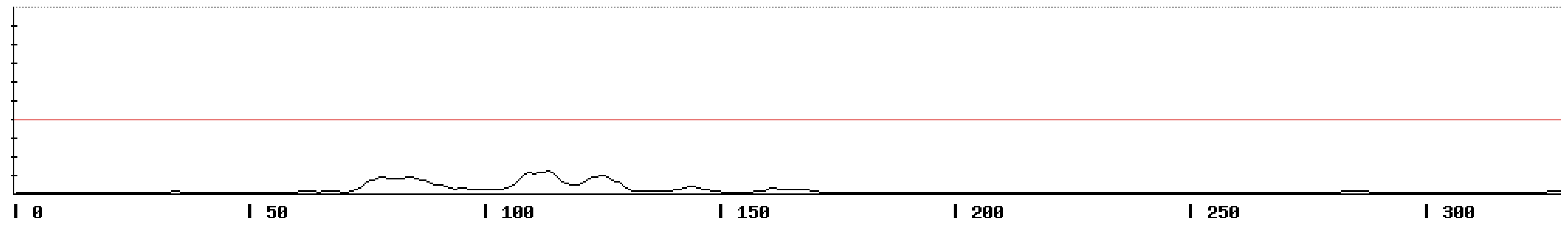

Predictions for Actinidiachinensis_Achn289301	
Viterbi Path	
Viterbi Path	

Posterior @ 0.4

Posterior @ 0.4

Posterior Graphic. Threshold Marked @ 0.4

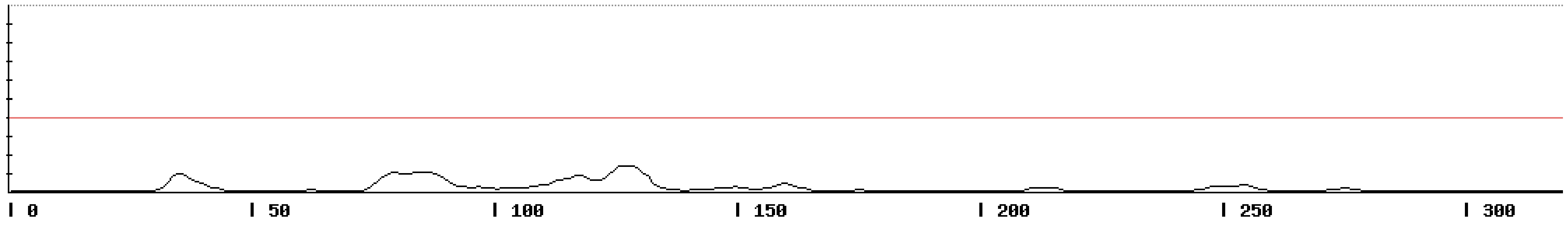

Predictions for Actinidiachinensis_Achn293411

Viterbi Path

Viterbi Path

Posterior @ 0.4

Posterior @ 0.4

Posterior Graphic. Threshold Marked @ 0.4

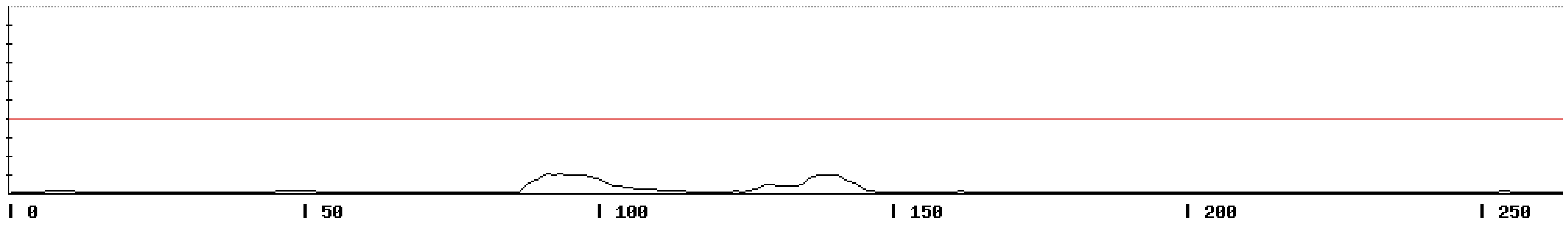

Predictions for Actinidiachinensis_Achn293491

Viterbi Path

Viterbi Path

Posterior @ 0.4

Posterior @ 0.4

Posterior Graphic. Threshold Marked @ 0.4

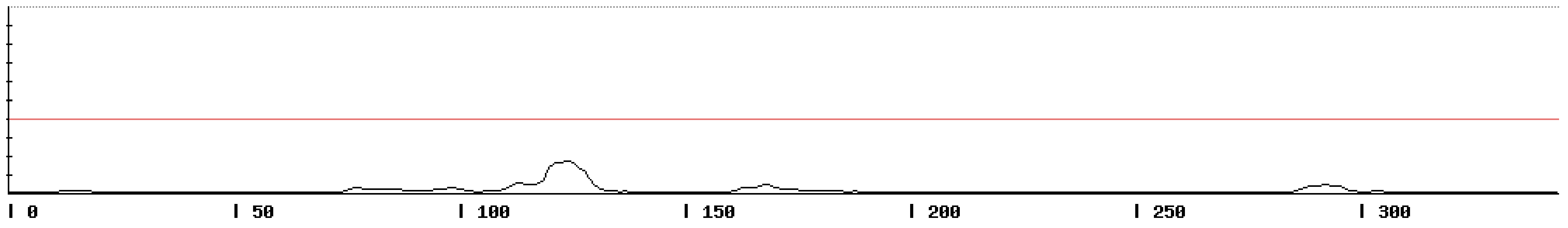

Predictions for Actinidiachinensis_Achn294661

Viterbi Path

Viterbi Path

Posterior @ 0.4

Posterior @ 0.4

Posterior Graphic. Threshold Marked @ 0.4

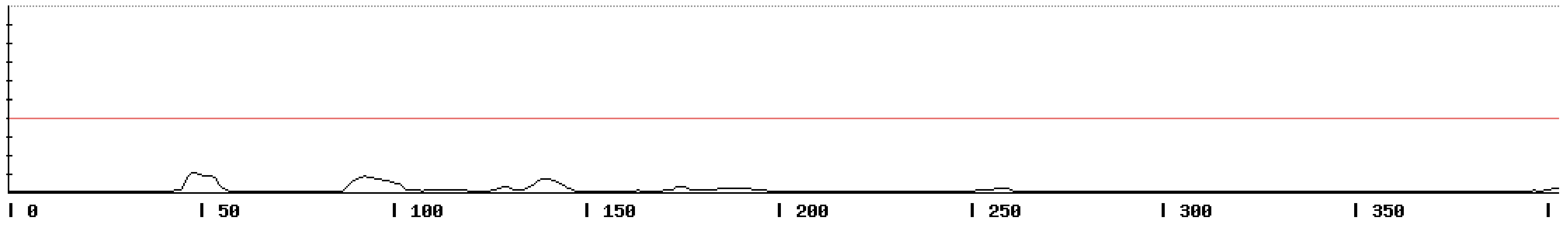

Predictions for Actinidiachinensis_Achn296141	
Viterbi Path	
Viterbi Path	
Posterior @ 0.4	
Posterior @ 0.4	
Posterior Graphic. Threshold Marked @ 0.4	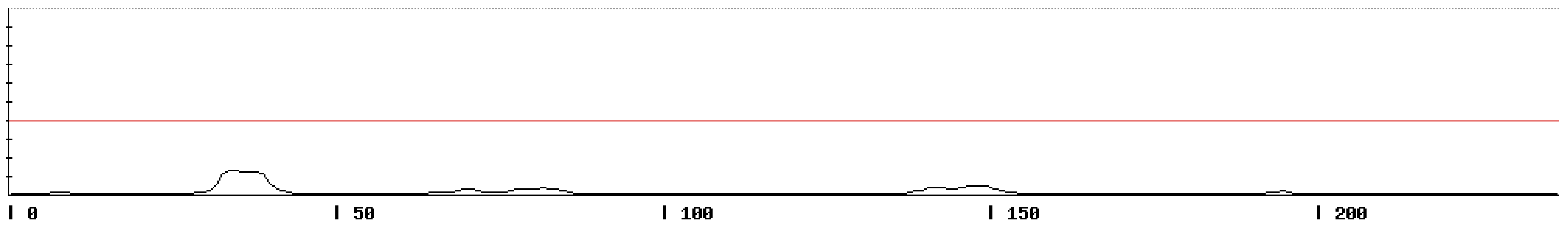

Predictions for Actinidiachinensis_Achn298161	
Viterbi Path	
Viterbi Path	
Posterior @ 0.4	
Posterior @ 0.4	
Posterior Graphic. Threshold Marked @ 0.4	

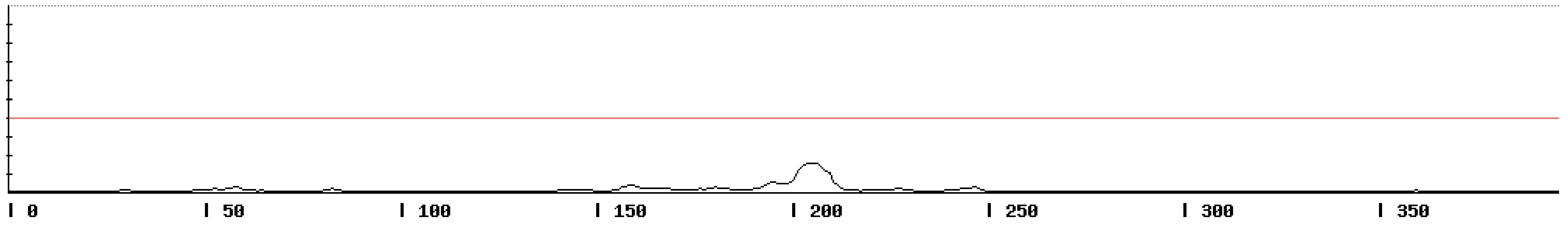

Predictions for Actinidiachinensis_Achn300931

Viterbi Path	
Viterbi Path	
Posterior @ 0.4	
Posterior @ 0.4	
Posterior Graphic. Threshold Marked @ 0.4	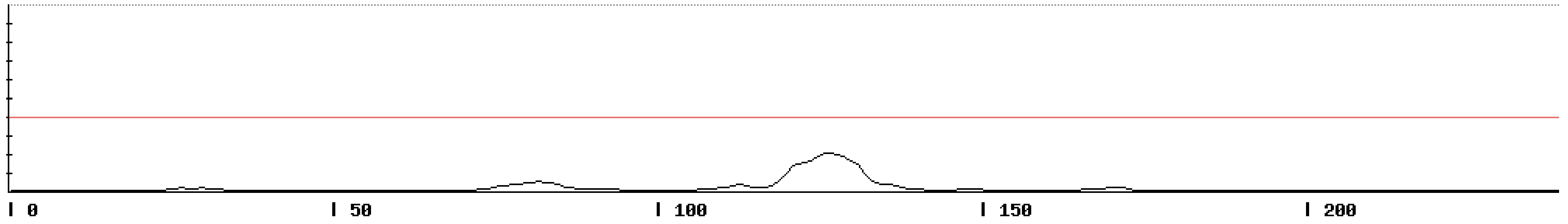

Predictions for Actinidiachinensis_Achn300981

Viterbi Path	
Viterbi Path	
Posterior @ 0.4	85 - GKAPAGRKTKW - 95
Posterior @ 0.4	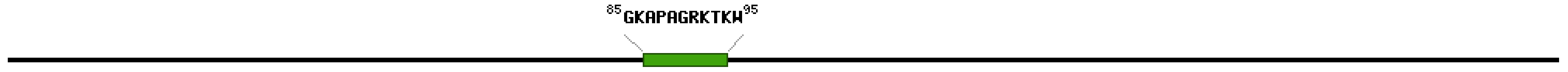

Posterior Graphic. Threshold Marked @ 0.4

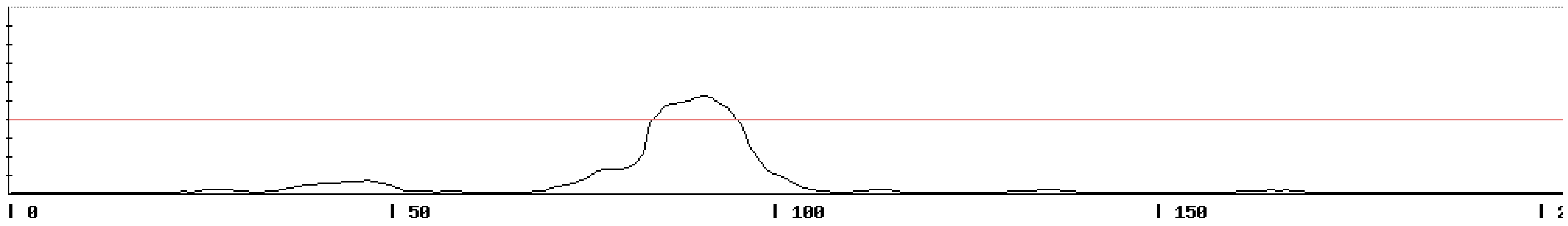

Predictions for Actinidiachinensis_Achn301081

Viterbi Path

Viterbi Path

Posterior @ 0.4

Posterior @ 0.4

Posterior Graphic. Threshold Marked @ 0.4

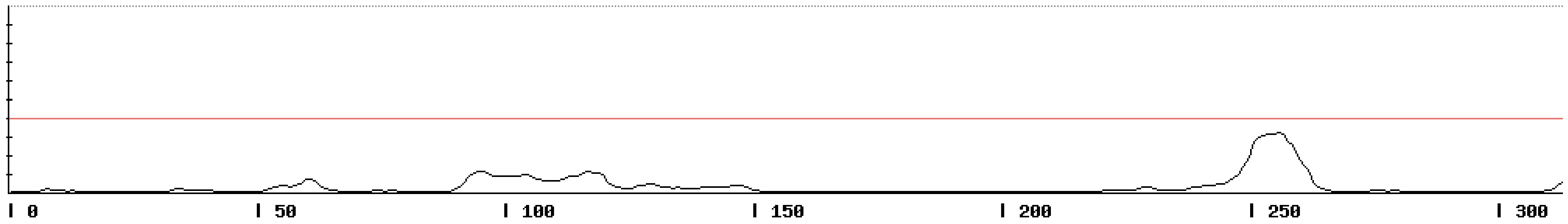

Predictions for Actinidiachinensis_Achn310421

Viterbi Path

Viterbi Path

Posterior @ 0.4

Posterior @ 0.4

Posterior Graphic. Threshold Marked @ 0.4

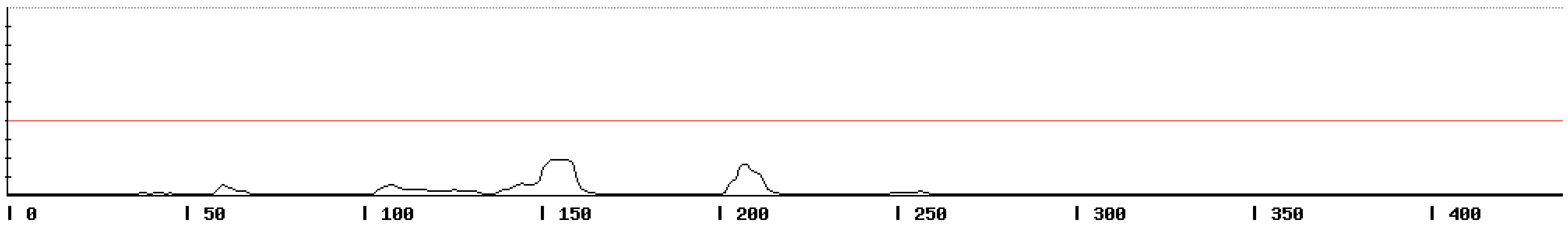

Predictions for Actinidiachinensis_Achn310791

Viterbi Path

Viterbi Path

Posterior @ 0.4

Posterior @ 0.4

Posterior Graphic. Threshold Marked @ 0.4

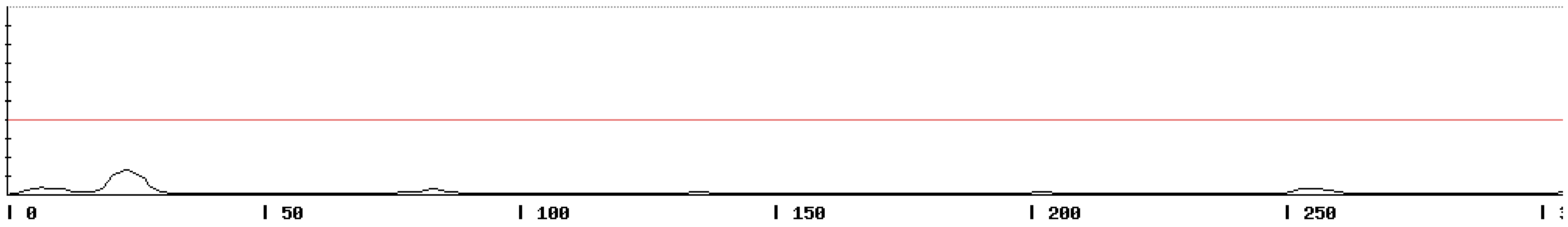

Predictions for Actinidiachinensis_Achn311471

Viterbi Path

Viterbi Path

Posterior @ 0.4	
Posterior @ 0.4	
Posterior Graphic. Threshold Marked @ 0.4	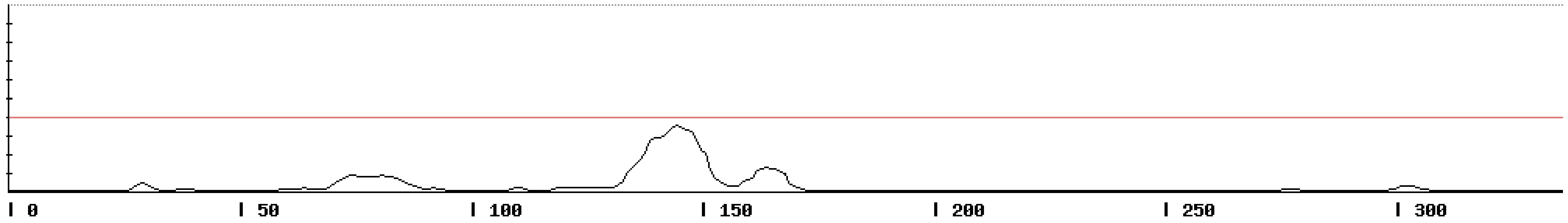

Predictions for Actinidiachinensis_Achn311531	
Viterbi Path	
Viterbi Path	
Posterior @ 0.4	
Posterior @ 0.4	
Posterior Graphic. Threshold Marked @ 0.4	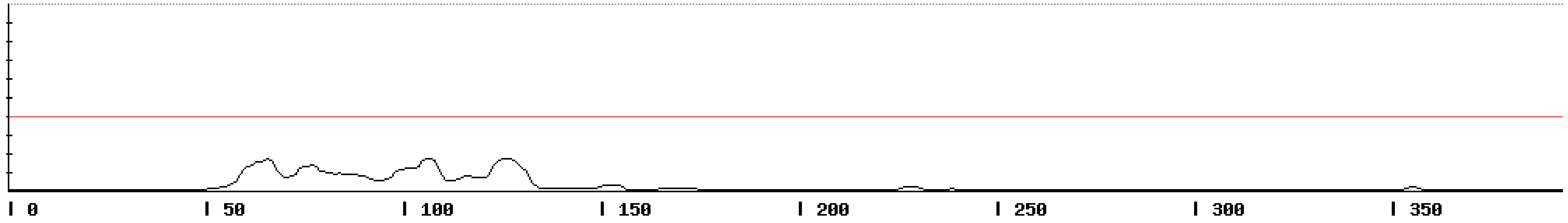

Predictions for Actinidiachinensis_Achn311801	
Viterbi Path	
Viterbi Path	

Posterior @ 0.4

Posterior @ 0.4

Posterior Graphic. Threshold Marked @ 0.4

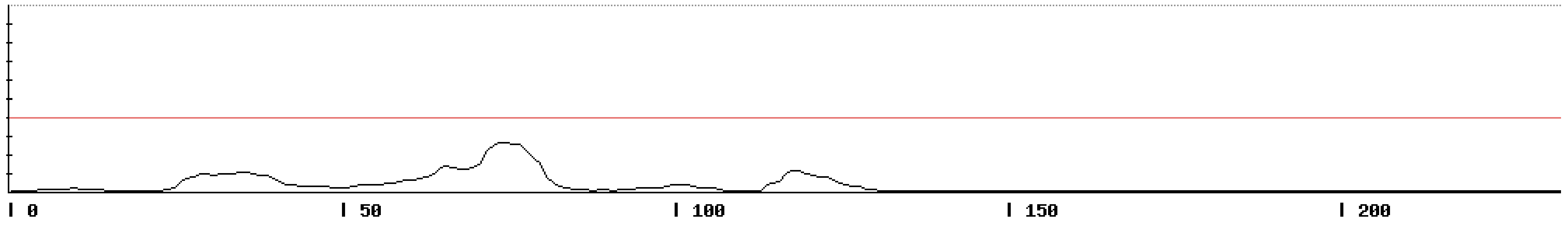

Predictions for Actinidiachinensis_Achn317511

Viterbi Path

Viterbi Path

Posterior @ 0.4

Posterior @ 0.4

Posterior Graphic. Threshold Marked @ 0.4

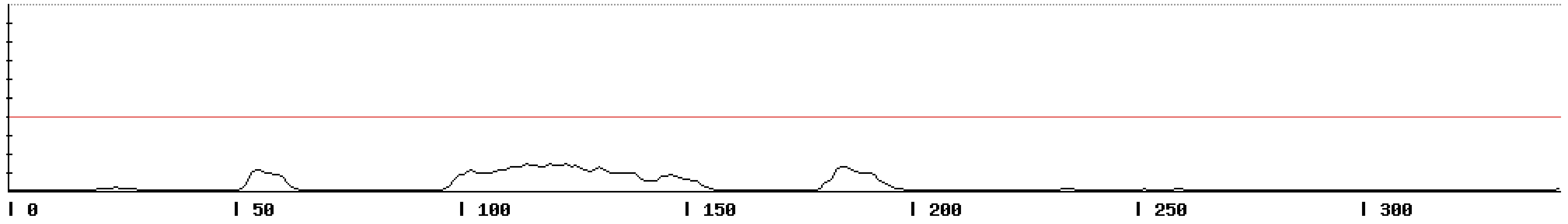

Predictions for Actinidiachinensis_Achn319671

Viterbi Path

Viterbi Path

Posterior @ 0.4

Posterior @ 0.4

Posterior Graphic. Threshold Marked @ 0.4

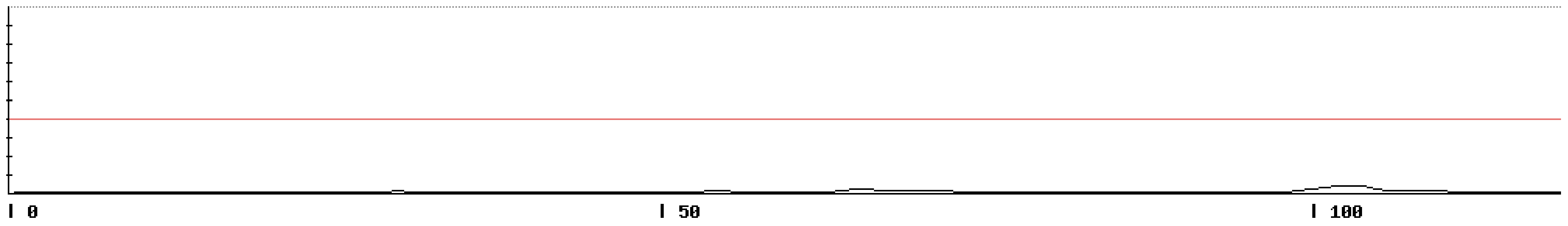

Predictions for Actinidiachinensis_Achn326001

Viterbi Path

Viterbi Path

Posterior @ 0.4

Posterior @ 0.4

Posterior Graphic. Threshold Marked @ 0.4

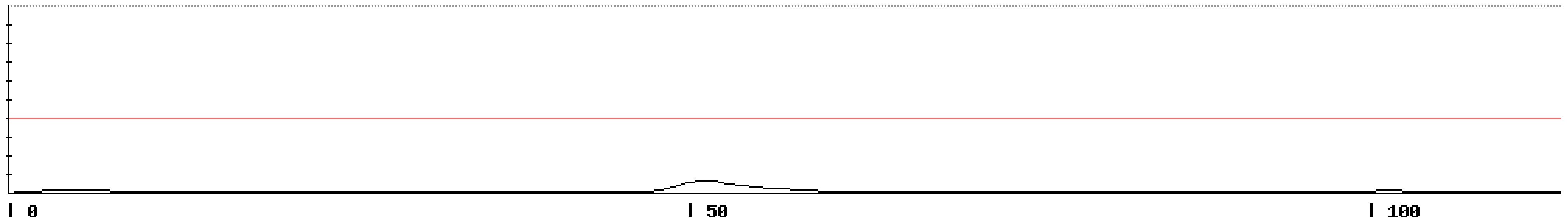

Predictions for Actinidiachinensis_Achn335281

Viterbi Path	
Viterbi Path	
Posterior @ 0.4	1181 - PSPKKSKGRTR - 1191
Posterior @ 0.4	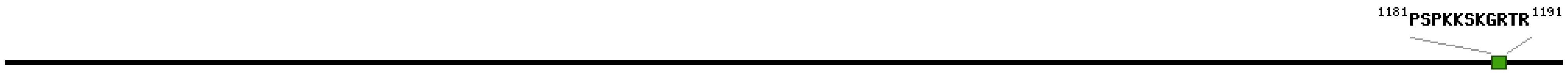
Posterior Graphic. Threshold Marked @ 0.4	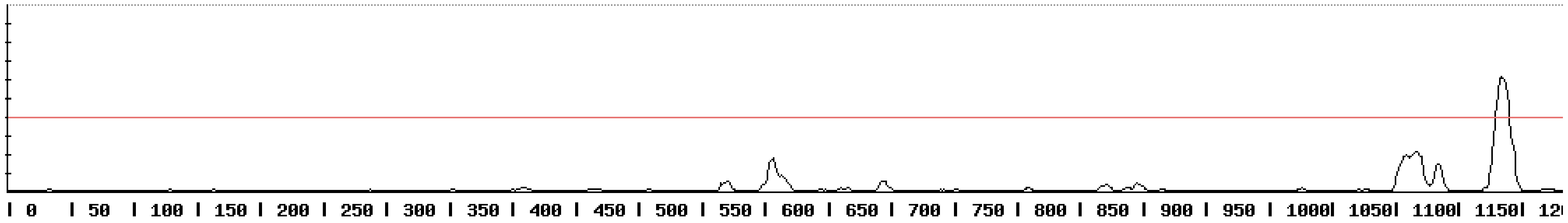

Predictions for Actinidiachinensis_Achn335731

Viterbi Path	
Viterbi Path	
Posterior @ 0.4	
Posterior @ 0.4	
Posterior Graphic. Threshold Marked @ 0.4	

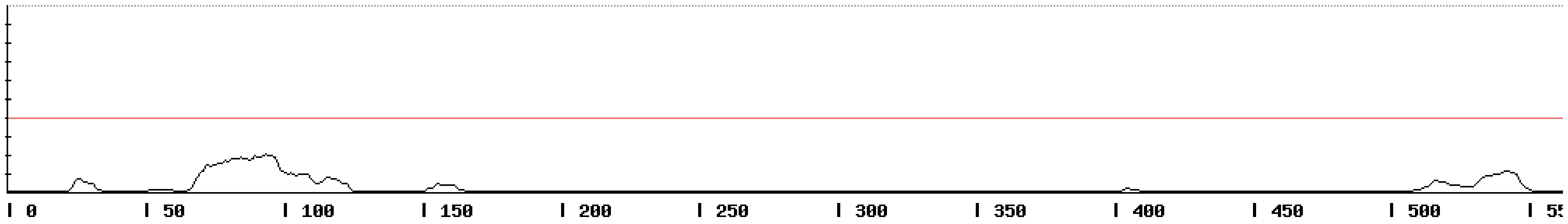

Predictions for Actinidiachinensis_Achn341031

Viterbi Path	
Viterbi Path	
Posterior @ 0.4	
Posterior @ 0.4	
Posterior Graphic. Threshold Marked @ 0.4	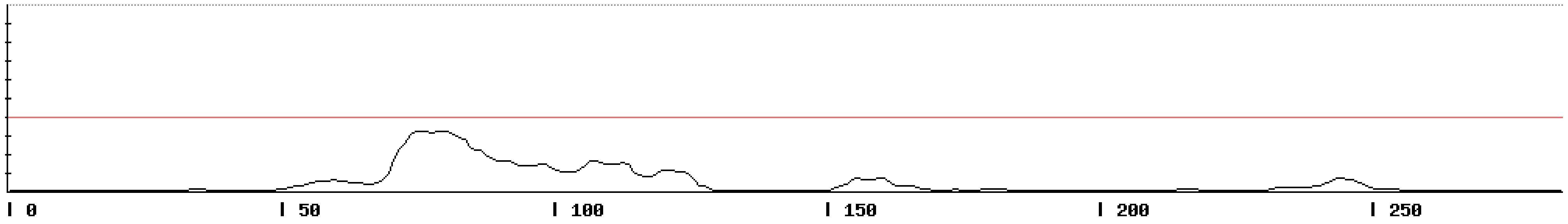

Predictions for Actinidiachinensis_Achn343671

Viterbi Path	
Viterbi Path	
Posterior @ 0.4	
Posterior @ 0.4	

Posterior Graphic. Threshold Marked @ 0.4	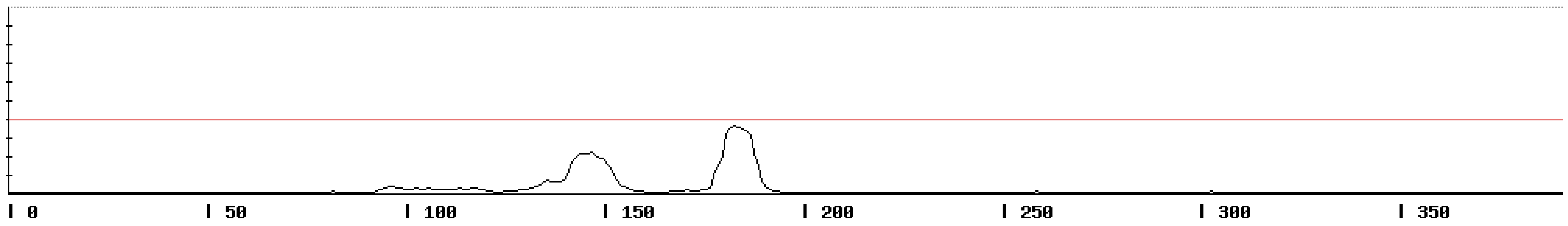

Predictions for Actinidiachinensis_Achn344701

Viterbi Path	
Viterbi Path	
Posterior @ 0.4	
Posterior @ 0.4	
Posterior Graphic. Threshold Marked @ 0.4	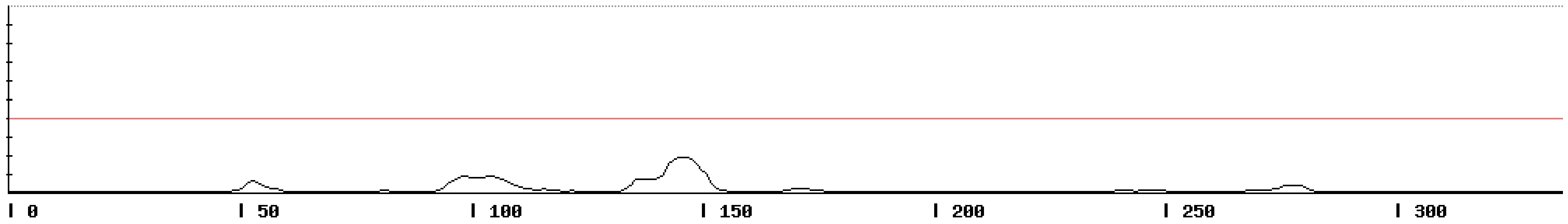

Predictions for Actinidiachinensis_Achn348191

Viterbi Path	
Viterbi Path	
Posterior @ 0.4	
Posterior @ 0.4	

Posterior Graphic. Threshold Marked @ 0.4

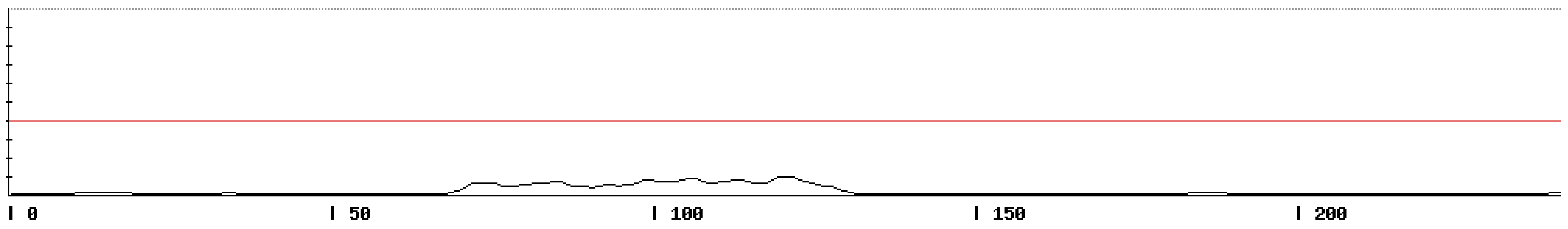

Predictions for Actinidiachinensis_Achn349691

Viterbi Path

Viterbi Path

Posterior @ 0.4

Posterior @ 0.4

Posterior Graphic. Threshold Marked @ 0.4

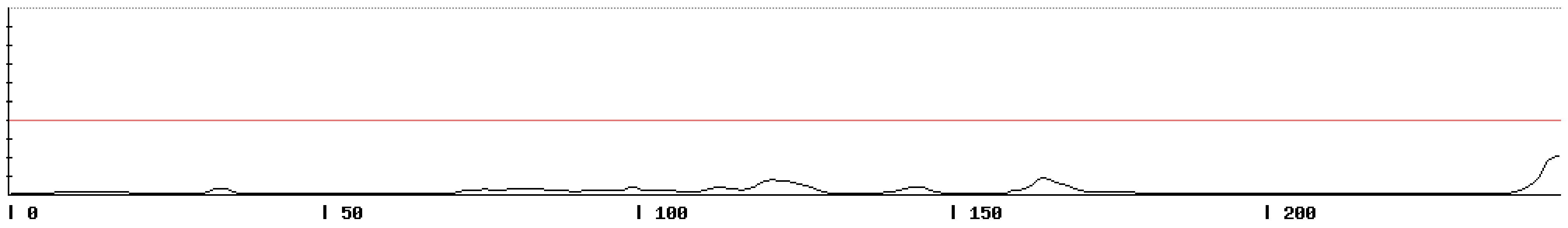

Predictions for Actinidiachinensis_Achn351611

Viterbi Path

Viterbi Path

Posterior @ 0.4

Posterior @ 0.4

Posterior Graphic. Threshold Marked @ 0.4

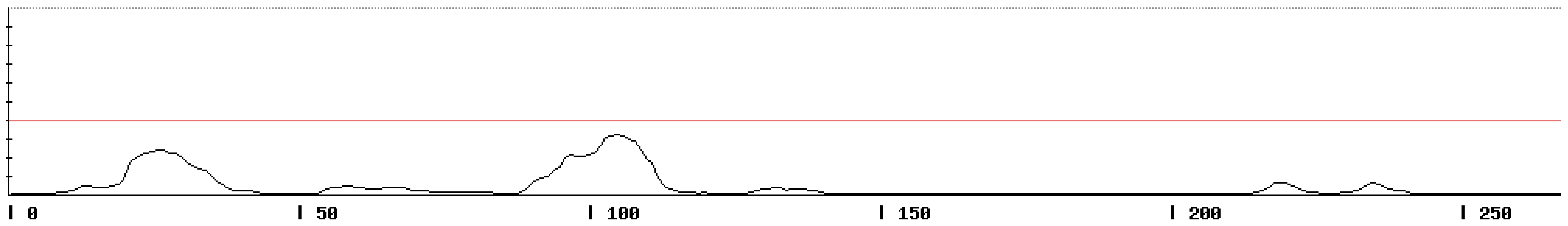

Predictions for Actinidiachinensis_Achn352721

Viterbi Path

Viterbi Path

Posterior @ 0.4

Posterior @ 0.4

Posterior Graphic. Threshold Marked @ 0.4

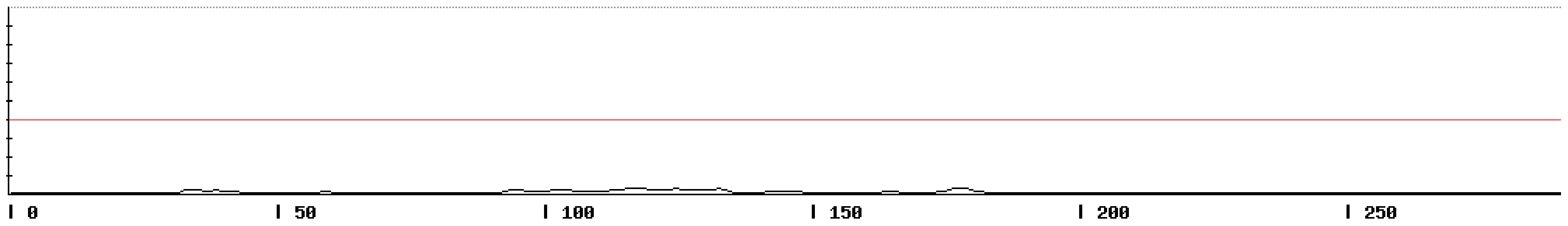

Predictions for Actinidiachinensis_Achn354611

Viterbi Path

Viterbi Path

Posterior @ 0.4

Posterior @ 0.4

Posterior Graphic. Threshold Marked @ 0.4

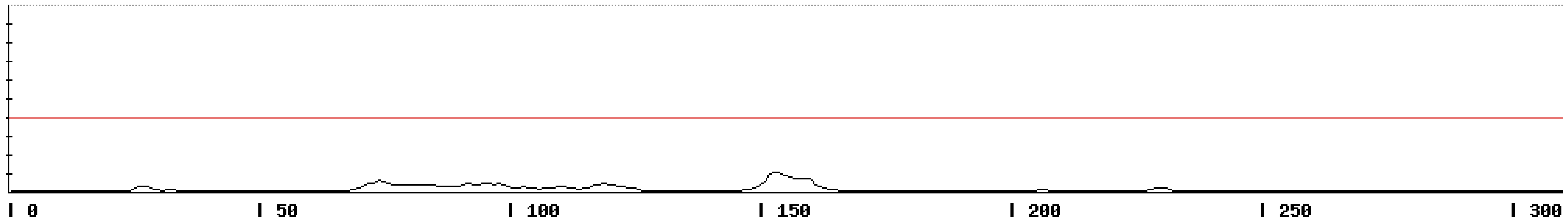

Predictions for Actinidiachinensis_Achn359751

Viterbi Path

Viterbi Path

Posterior @ 0.4

Posterior @ 0.4

Posterior Graphic. Threshold Marked @ 0.4

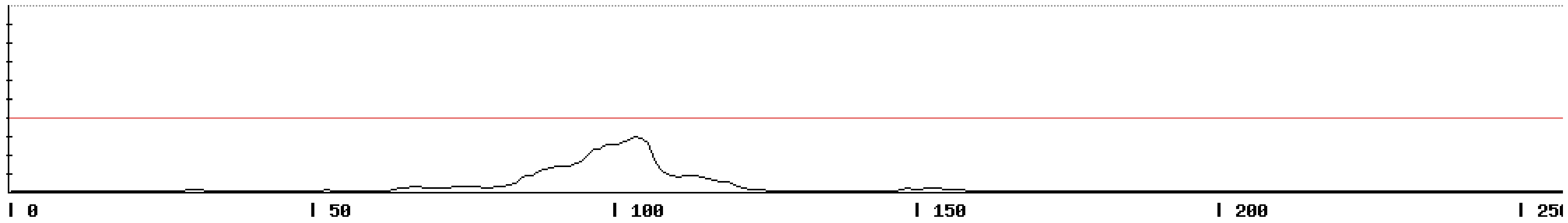

Predictions for Actinidiachinensis_Achn363171

Viterbi Path	
Viterbi Path	
Posterior @ 0.4	
Posterior @ 0.4	
Posterior Graphic. Threshold Marked @ 0.4	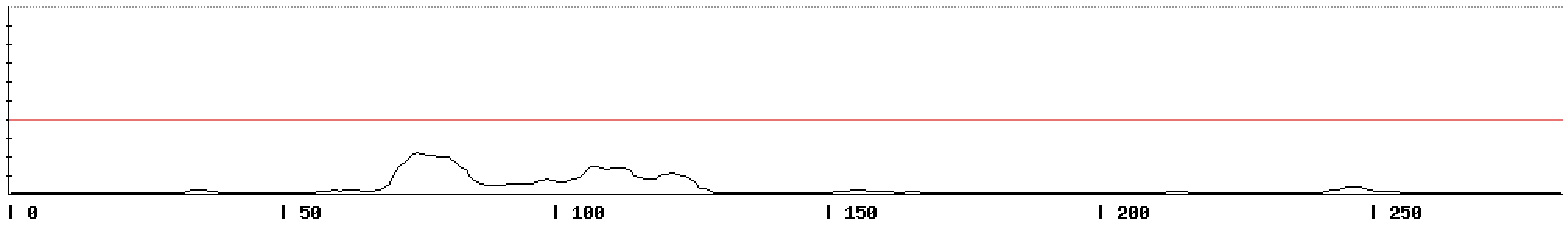

Predictions for Actinidiachinensis_Achn365331

Viterbi Path	
Viterbi Path	
Posterior @ 0.4	68 - KKKGK - 72
Posterior @ 0.4	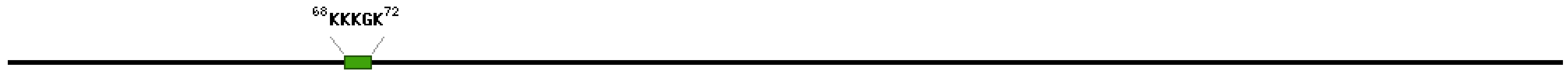
Posterior Graphic. Threshold Marked @ 0.4	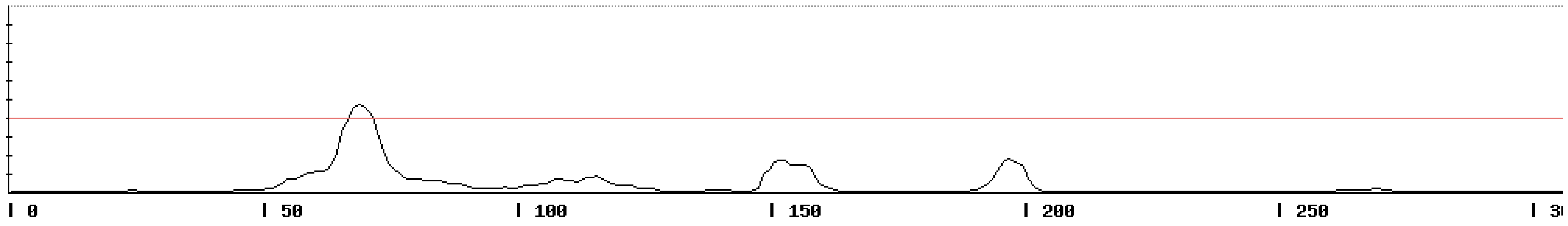

Predictions for Actinidiachinensis_Achn367821	
Viterbi Path	
Viterbi Path	
Posterior @ 0.4	
Posterior @ 0.4	
Posterior Graphic. Threshold Marked @ 0.4	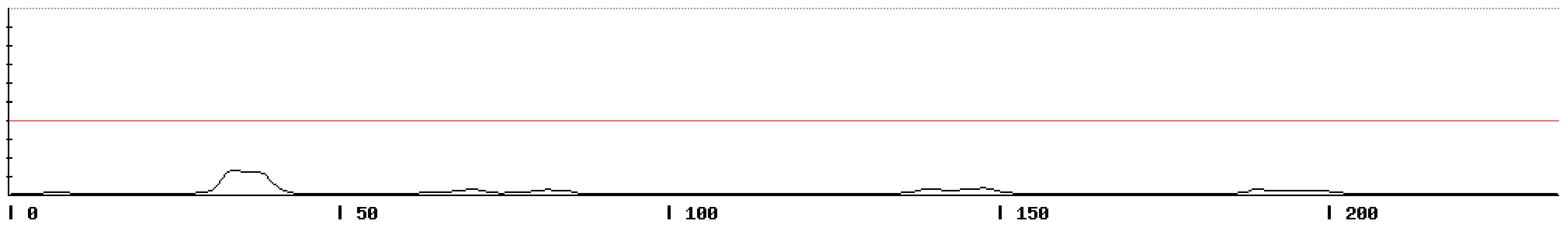

Predictions for Actinidiachinensis_Achn374931	
Viterbi Path	
Viterbi Path	
Posterior @ 0.4	
Posterior @ 0.4	
Posterior Graphic. Threshold Marked @ 0.4	

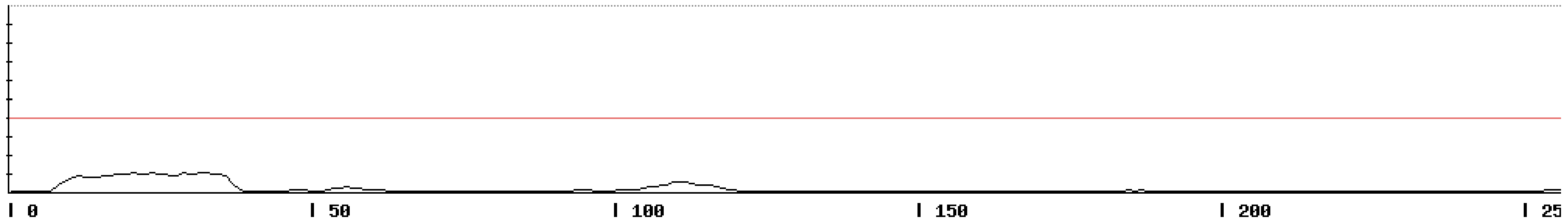

Predictions for Actinidiachinensis_Achn377511

Viterbi Path	
Viterbi Path	
Posterior @ 0.4	
Posterior @ 0.4	
Posterior Graphic. Threshold Marked @ 0.4	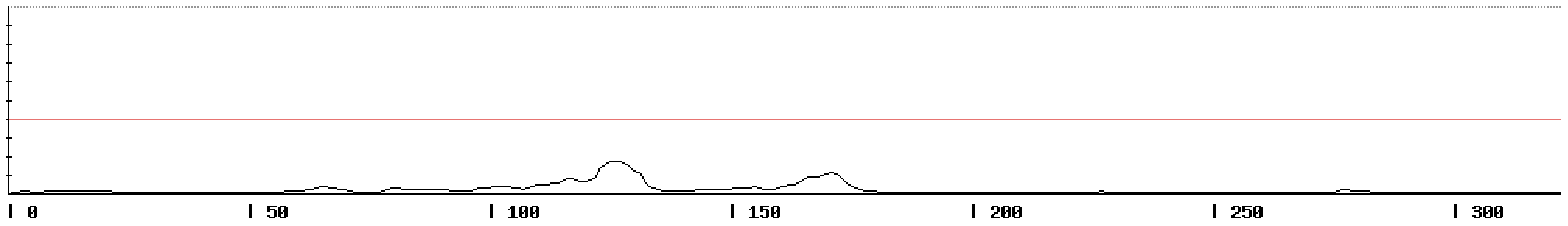

Predictions for Actinidiachinensis_Achn382311

Viterbi Path	
Viterbi Path	
Posterior @ 0.4	
Posterior @ 0.4	

Posterior Graphic. Threshold Marked @ 0.4

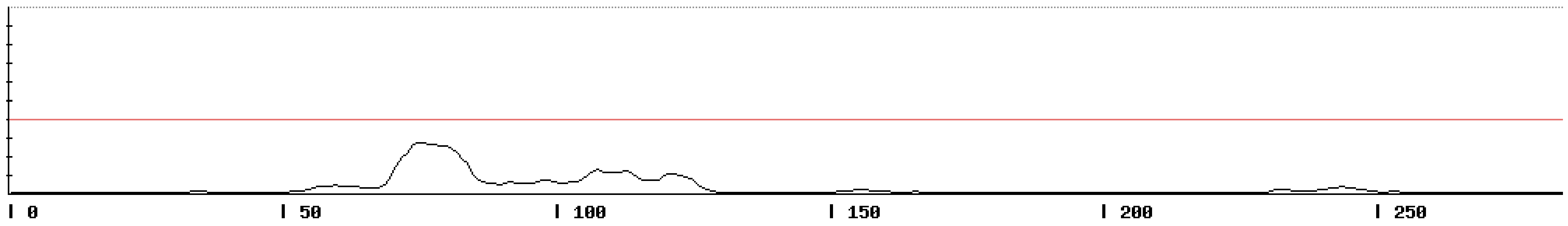

Predictions for Actinidiachinensis_Achn385691

Viterbi Path

Viterbi Path

Posterior @ 0.4

Posterior @ 0.4

Posterior Graphic. Threshold Marked @ 0.4

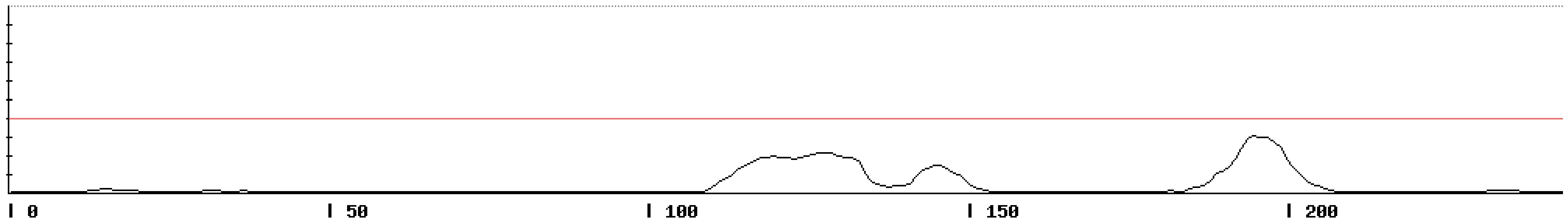

Predictions for Actinidiachinensis_Achn388561

Viterbi Path

Viterbi Path

Posterior @ 0.4

Posterior @ 0.4

Posterior Graphic. Threshold Marked @ 0.4

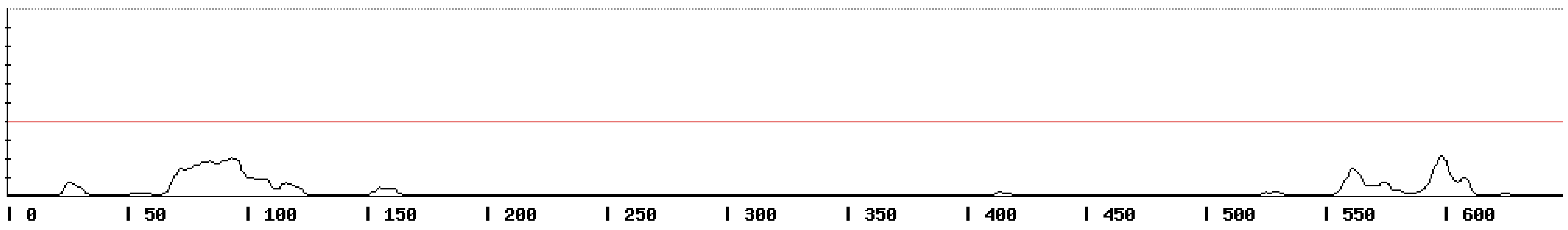

Predictions for Actinidiachinensis_Achn389881

Viterbi Path

Viterbi Path

Posterior @ 0.4 345 - RKRRKIH - 351

Posterior @ 0.4

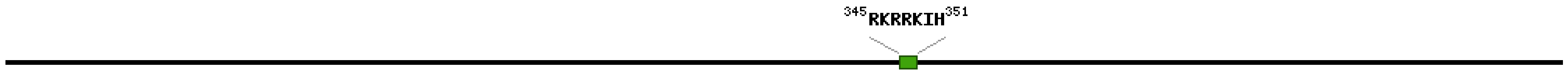

Posterior Graphic. Threshold Marked @ 0.4

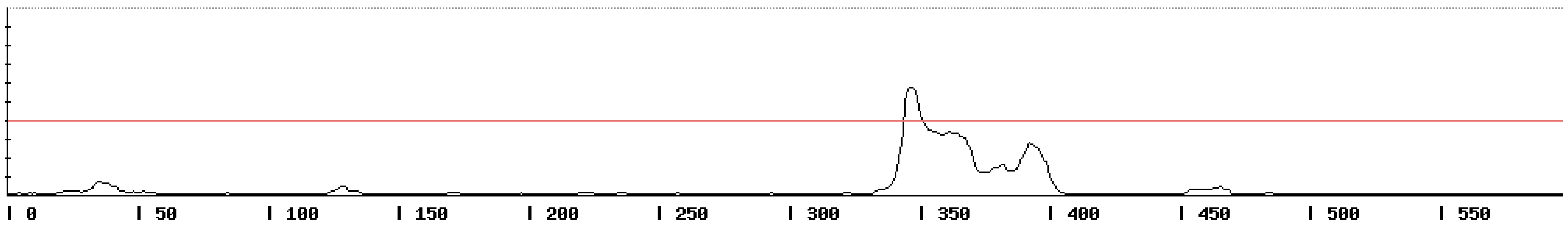

Revision r.9 - Last Updated on : February 24 2015 17:45:20. alex.nguyenba@utoronto.ca

Intended to be used with Firefox

Viterbi Path

Posterior @ 0.4

Posterior @ 0.4

Posterior Graphic. Threshold Marked @ 0.4

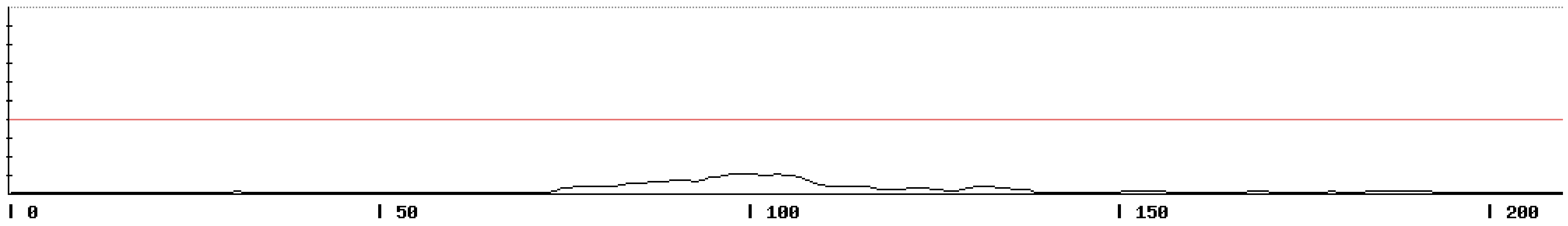

Predictions for Aegilopstauschii_EMT01861

Viterbi Path

Viterbi Path

Posterior @ 0.4

Posterior @ 0.4

Posterior Graphic. Threshold Marked @ 0.4

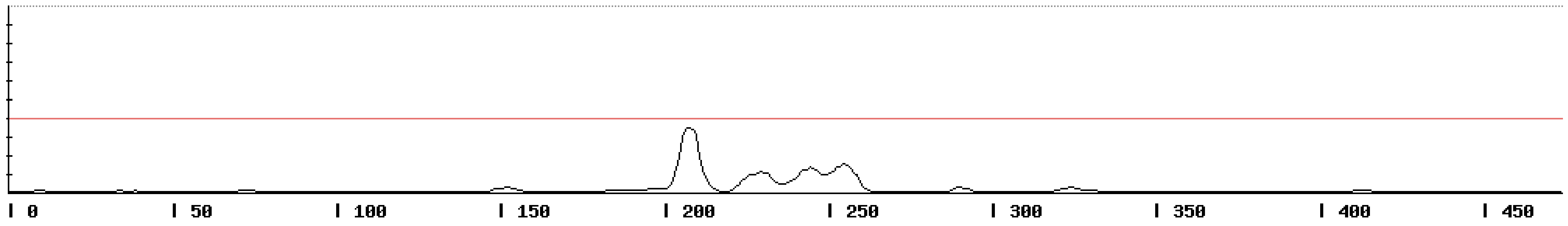

Predictions for Aegilopstauschii_EMT01908

Viterbi Path

Viterbi Path

Posterior @ 0.4 — 180 - SKNSASARKRKTPDDGPRLSPPSLHASAPKKSK - 212

Posterior @ 0.4

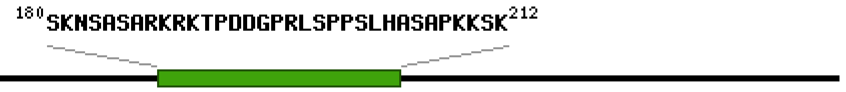

Posterior Graphic. Threshold Marked @ 0.4

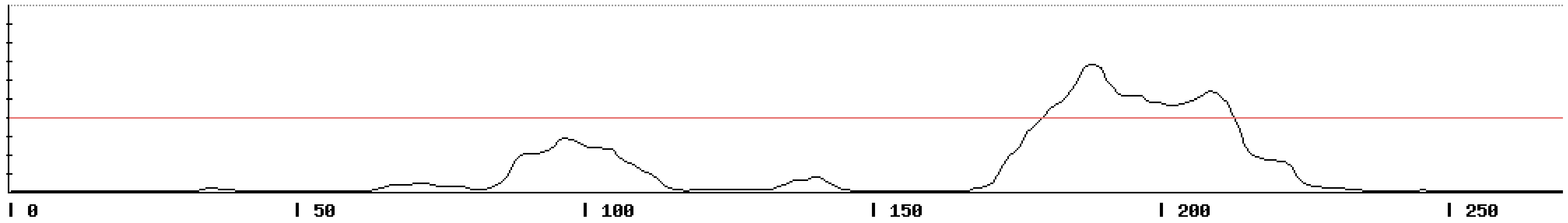

Predictions for Aegilopstauschii_EMT01994

Viterbi Path

Viterbi Path

Posterior @ 0.4

Posterior @ 0.4

Posterior Graphic. Threshold Marked @ 0.4

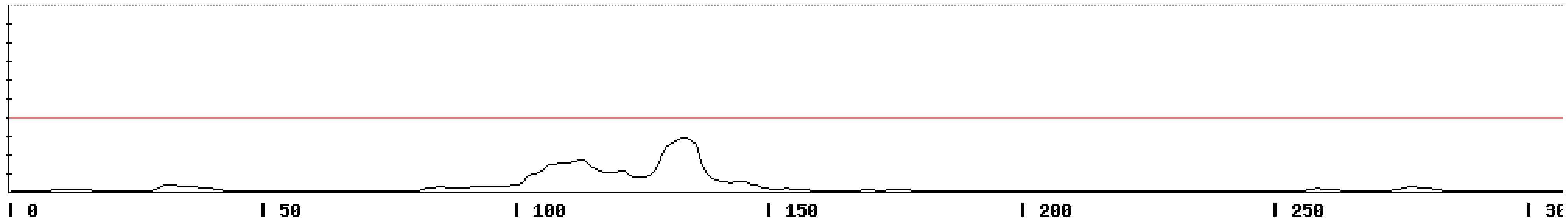

Predictions for Aegilopstauschii_EMT02241

Viterbi Path	
Viterbi Path	
Posterior @ 0.4	171 - KRK - 173
Posterior @ 0.4	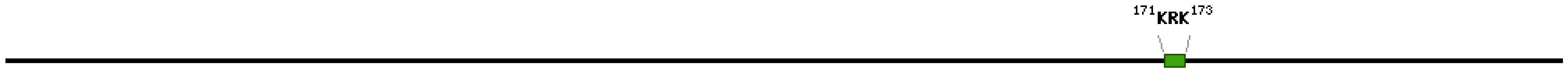
Posterior Graphic. Threshold Marked @ 0.4	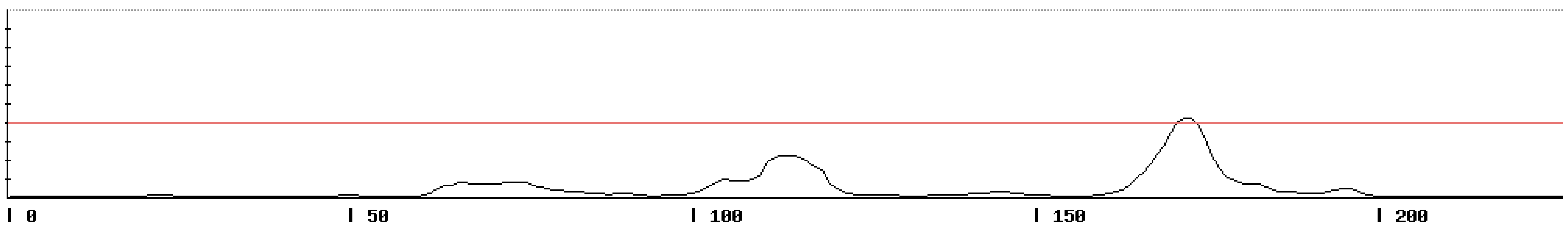

Predictions for Aegilopstauschii_EMT02809

Viterbi Path	
Viterbi Path	
Posterior @ 0.4	
Posterior @ 0.4	
Posterior Graphic. Threshold Marked @ 0.4	

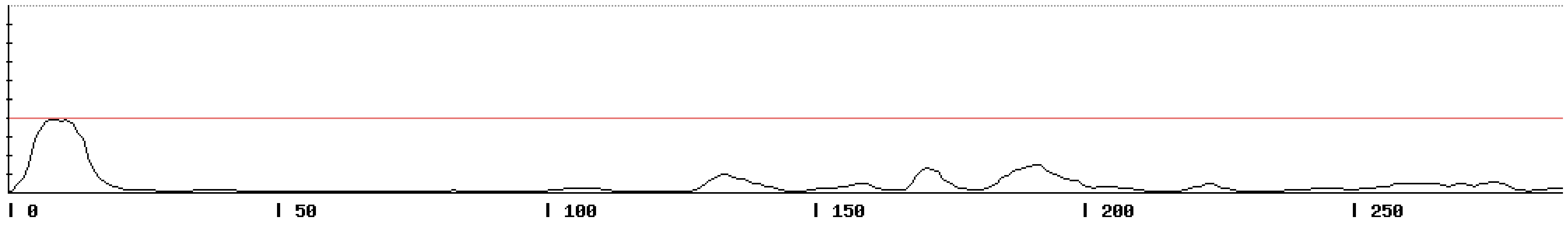

Predictions for Aegilopstauschii_EMT03210

Viterbi Path	
Viterbi Path	
Posterior @ 0.4	174 - RKPRSASVPTSPAYKRERKRKARDDSAPVKTVKRR - 208
Posterior @ 0.4	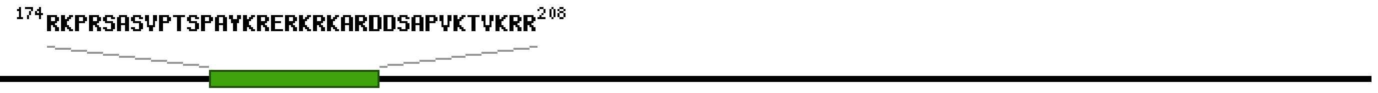
Posterior Graphic. Threshold Marked @ 0.4	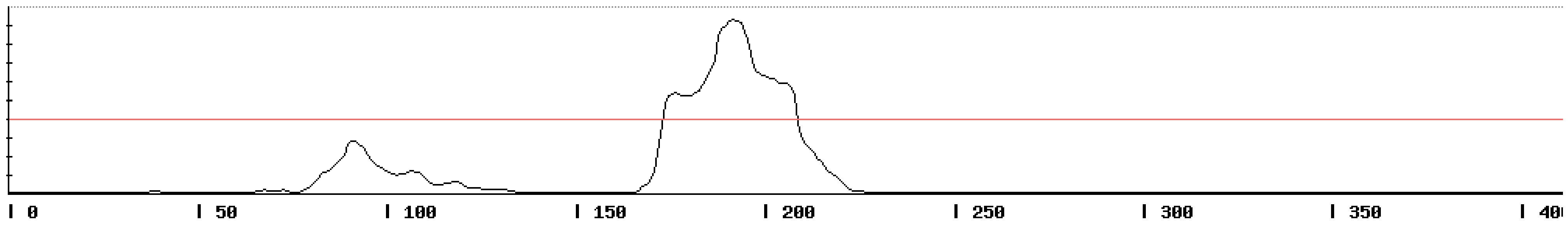

Predictions for Aegilopstauschii_EMT03350

Viterbi Path	
Viterbi Path	
Posterior @ 0.4	232 - KKKKQK - 237
Posterior @ 0.4	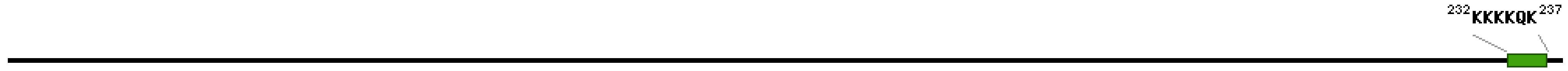

Posterior Graphic. Threshold Marked @ 0.4	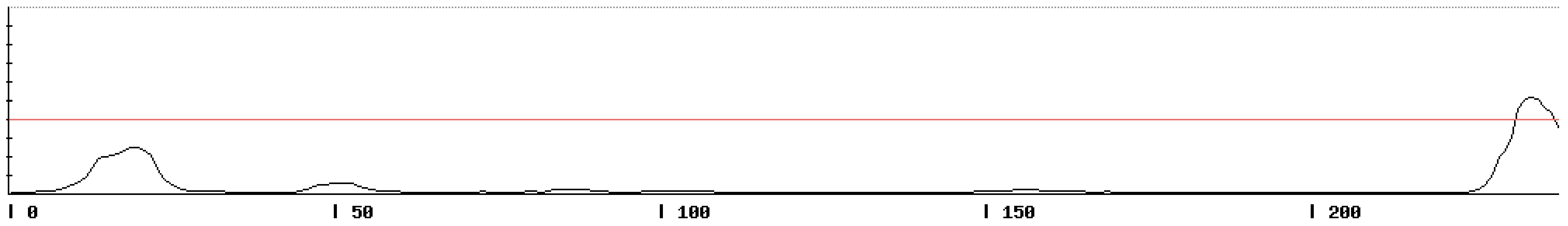

Predictions for Aegilopstauschii_EMT03425	
Viterbi Path	
Viterbi Path	
Posterior @ 0.4	
Posterior @ 0.4	
Posterior Graphic. Threshold Marked @ 0.4	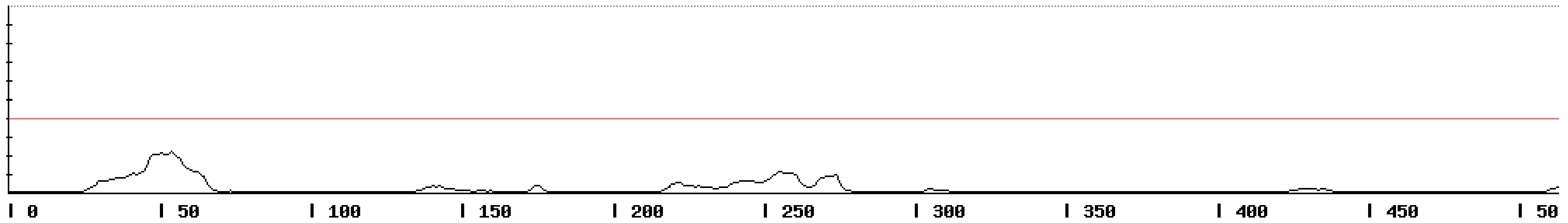

Predictions for Aegilopstauschii_EMT03563	
Viterbi Path	
Viterbi Path	
Posterior @ 0.4	
Posterior @ 0.4	

Posterior Graphic. Threshold Marked @ 0.4

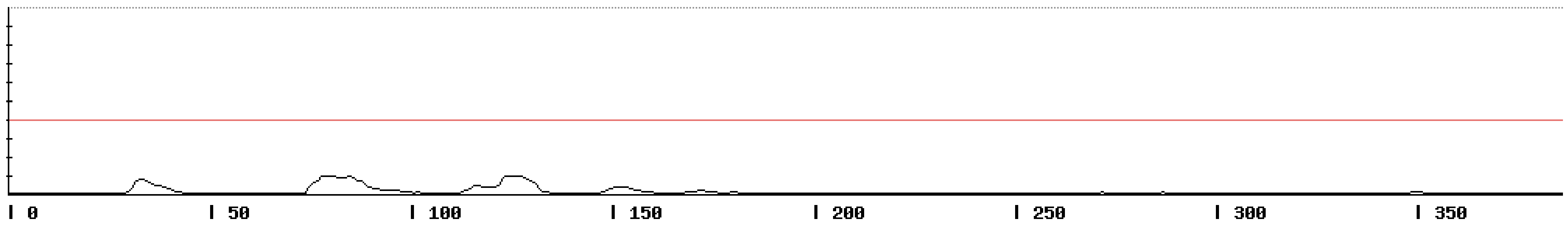

Predictions for Aegilopstauschii_EMT03564

Viterbi Path

Viterbi Path

Posterior @ 0.4

Posterior @ 0.4

Posterior Graphic. Threshold Marked @ 0.4

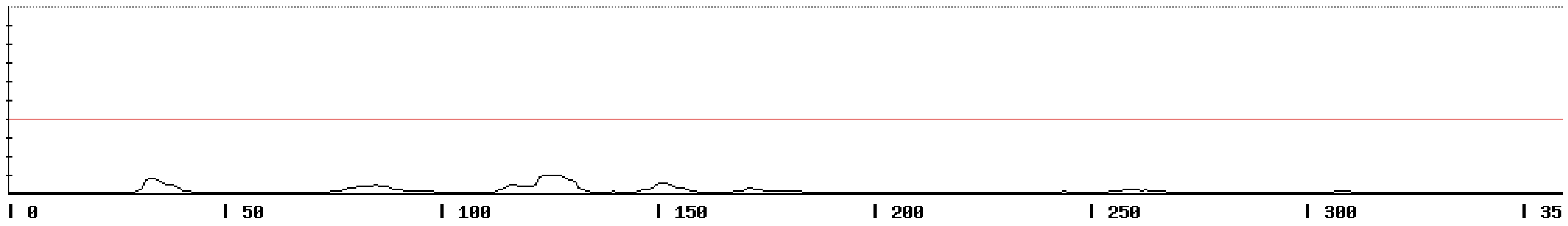

Predictions for Aegilopstauschii_EMT03885

Viterbi Path

Viterbi Path

Posterior @ 0.4

Posterior @ 0.4

Posterior Graphic. Threshold Marked @ 0.4

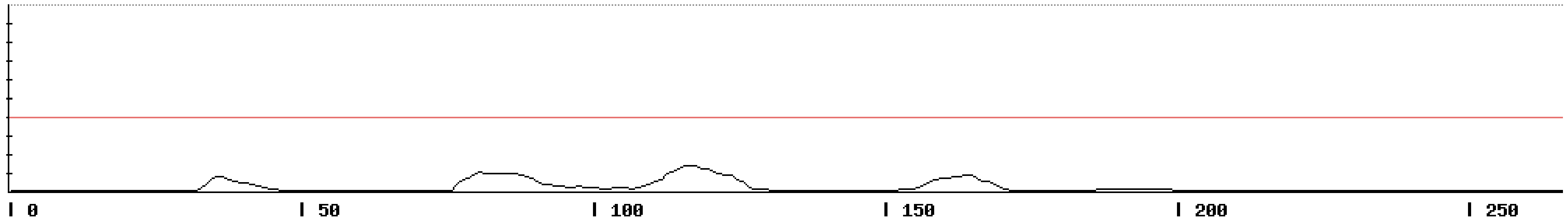

Predictions for Aegilopstauschii_EMT05112

Viterbi Path

Viterbi Path

Posterior @ 0.4 160 - KRKR - 163

Posterior @ 0.4

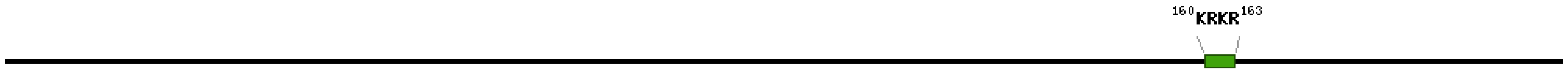

Posterior Graphic. Threshold Marked @ 0.4

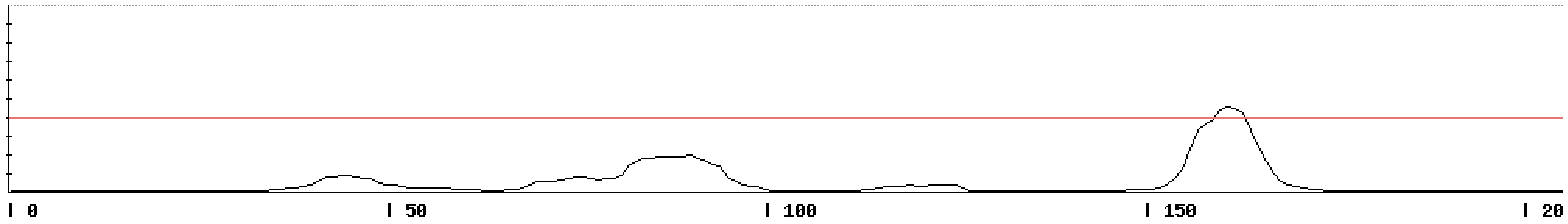

Predictions for Aegilopstauschii_EMT05113

Viterbi Path

Viterbi Path

Posterior @ 0.4

Posterior @ 0.4

Posterior Graphic. Threshold Marked @ 0.4

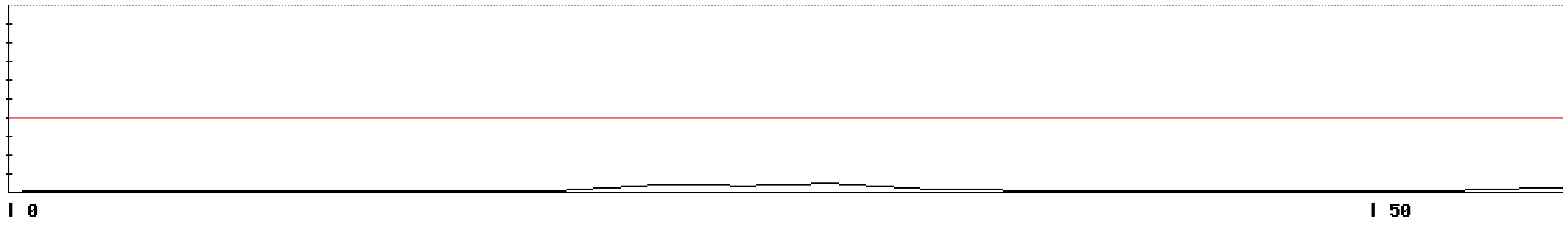

Predictions for Aegilopstauschii_EMT05147

Viterbi Path

Viterbi Path

Posterior @ 0.4 182 - PGSKKKRKAAADVQHPEAPRPKTPRR - 207

Posterior @ 0.4

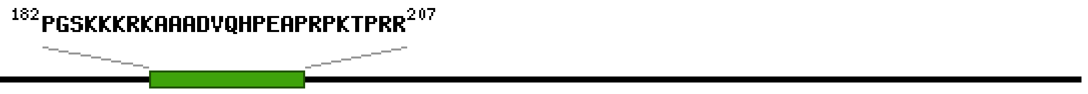

Posterior Graphic. Threshold Marked @ 0.4

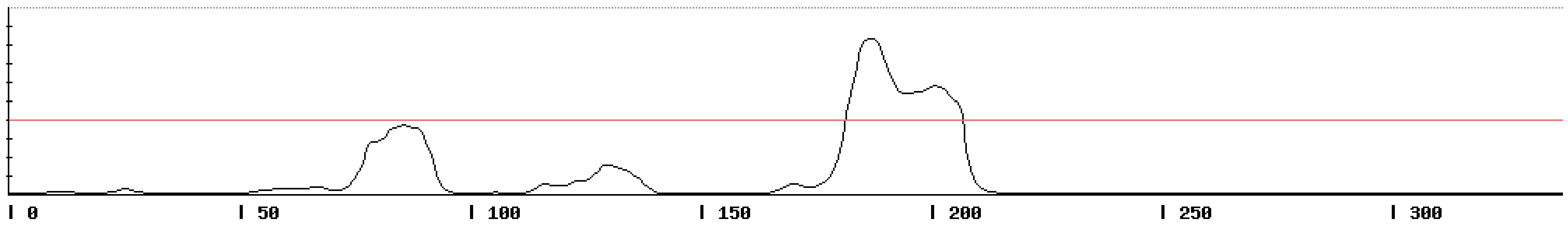

Predictions for Aegilopstauschii_EMT05508

Viterbi Path	369 - PPRPHEPRQPRVNKKPAFPRPQEARKPQVIQKPPPPRALDKRRVPTPTQQAPKRRAPPIAEPPRPKRIR - 437
Viterbi Path	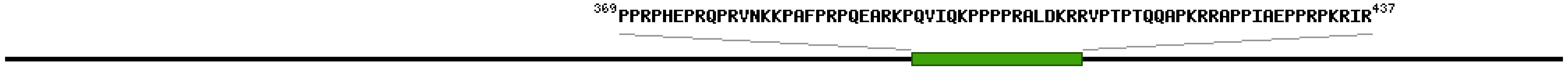
Posterior @ 0.4	15 - KKARDKGTDDGLPAPAKEKRRWSFRRP - 41
	369 - PPRPHEPRQPRVNKKPAFPRPQEARKPQVIQKPPPPRALDKRRVPTPTQQAPKRRAPPIAEPPRPKRIRAAA - 440
Posterior @ 0.4	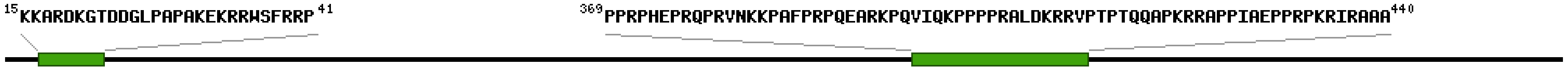
Posterior Graphic. Threshold Marked @ 0.4	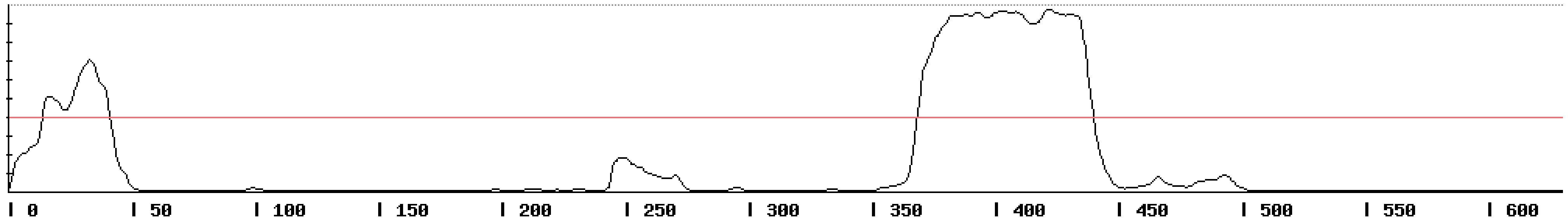

Predictions for Aegilopstauschii_EMT05947

Viterbi Path	
Viterbi Path	
Posterior @ 0.4	
Posterior @ 0.4	
Posterior Graphic. Threshold Marked @ 0.4	

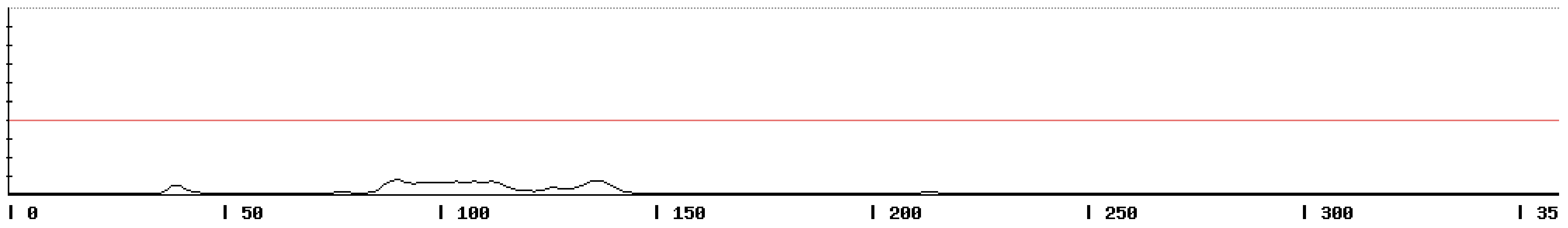

Predictions for Aegilopstauschii_EMT05948

Viterbi Path

Viterbi Path

Posterior @ 0.4

Posterior @ 0.4

Posterior Graphic. Threshold Marked @ 0.4

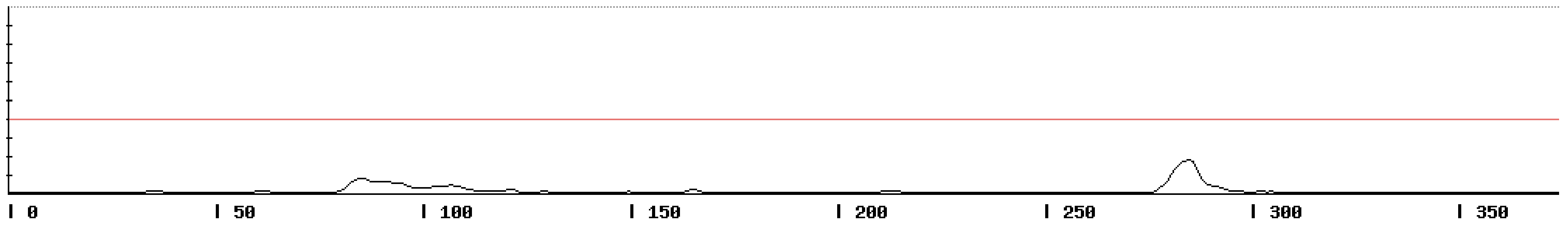

Predictions for Aegilopstauschii_EMT06396

Viterbi Path

Viterbi Path

Posterior @ 0.4

Posterior @ 0.4

Posterior Graphic. Threshold Marked @ 0.4

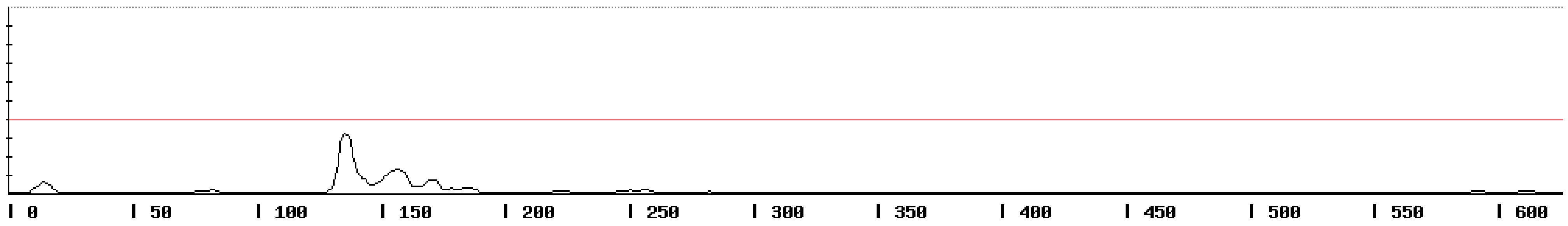

Predictions for Aegilopstauschii_EMT06613

Viterbi Path

Viterbi Path

Posterior @ 0.4

Posterior @ 0.4

Posterior Graphic. Threshold Marked @ 0.4

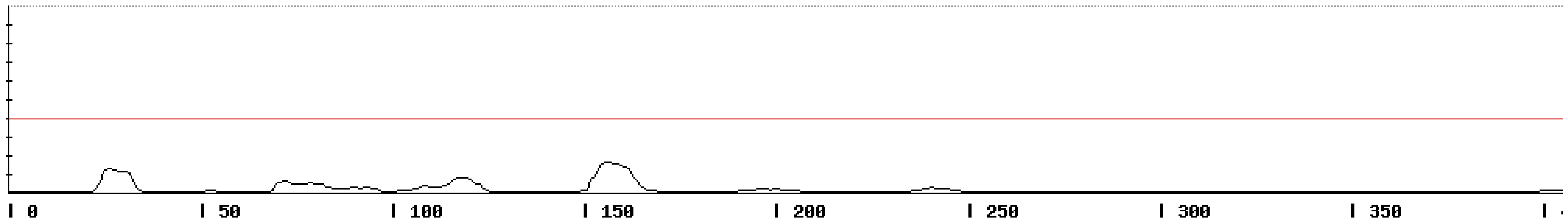

Predictions for Aegilopstauschii_EMT06950

Viterbi Path

Viterbi Path

Posterior @ 0.4

Posterior @ 0.4

Posterior Graphic. Threshold Marked @ 0.4

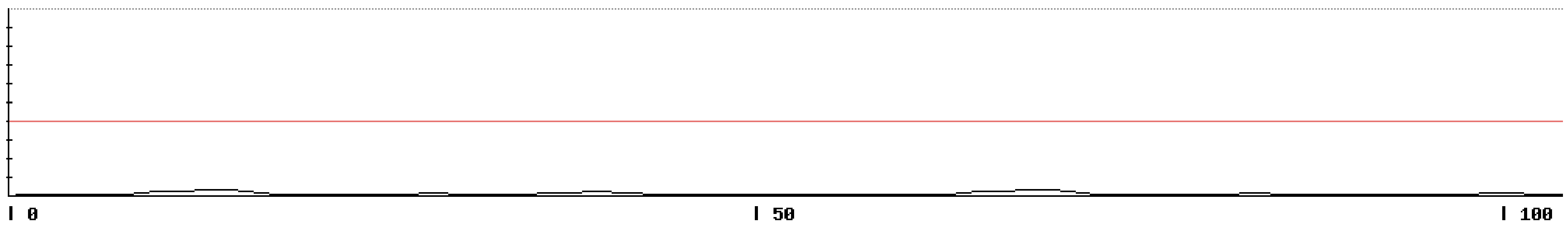

Predictions for Aegilopstauschii_EMT06951

Viterbi Path

Viterbi Path

Posterior @ 0.4

Posterior @ 0.4

Posterior Graphic. Threshold Marked @ 0.4

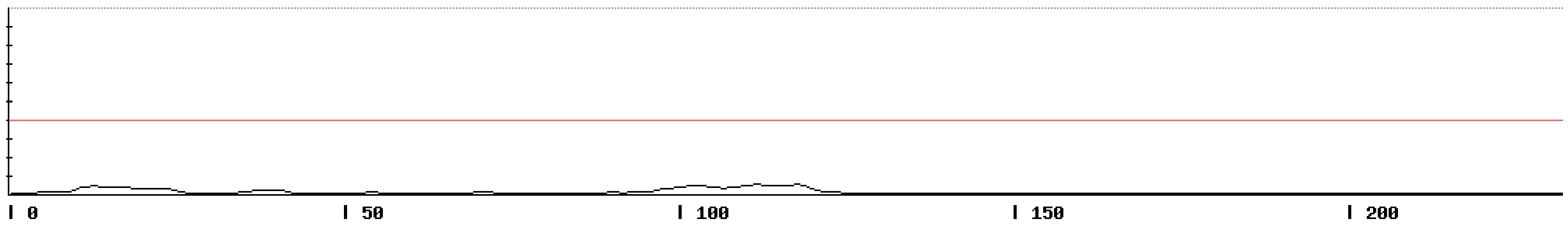

Predictions for Aegilopstauschii_EMT06992

Viterbi Path

Viterbi Path

Posterior @ 0.4

Posterior @ 0.4

Posterior Graphic. Threshold Marked @ 0.4

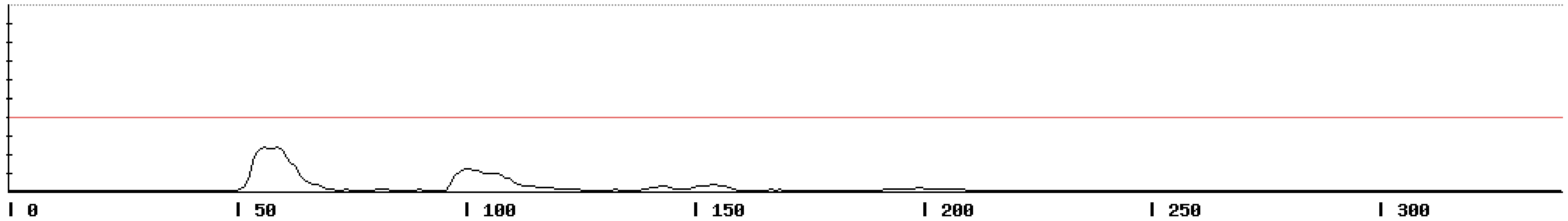

Predictions for Aegilopstauschii_EMT07032

Viterbi Path

Viterbi Path

Posterior @ 0.4

Posterior @ 0.4

Posterior Graphic. Threshold Marked @ 0.4

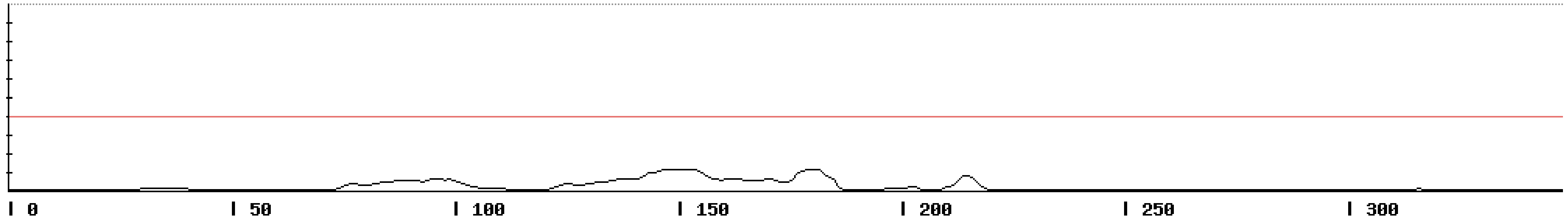

Predictions for Aegilopstauschii_EMT07070

Viterbi Path

Viterbi Path

Posterior @ 0.4	86 - PERARRRRRHL - 96
	162 - RPDRNRRRTSFKKRNQRP - 179

Posterior @ 0.4

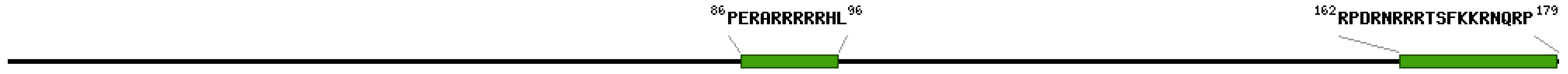

Posterior Graphic. Threshold Marked @ 0.4

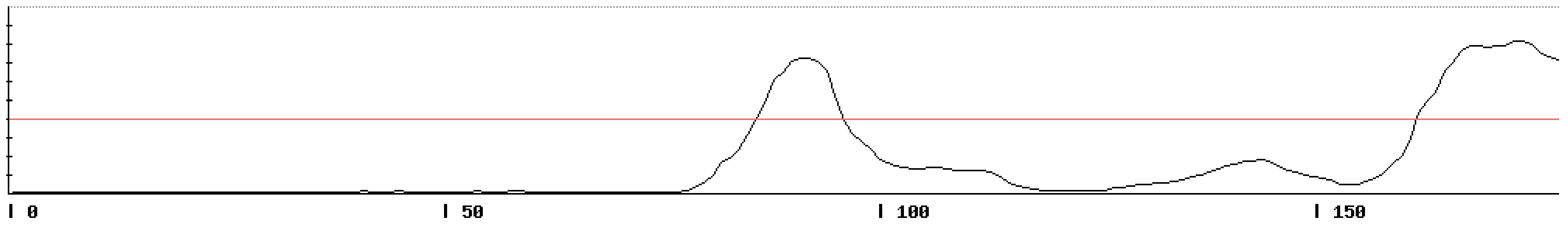

Predictions for Aegilopstauschii_EMT07151

Viterbi Path

Viterbi Path

Posterior @ 0.4

Posterior @ 0.4

Posterior Graphic. Threshold Marked @ 0.4

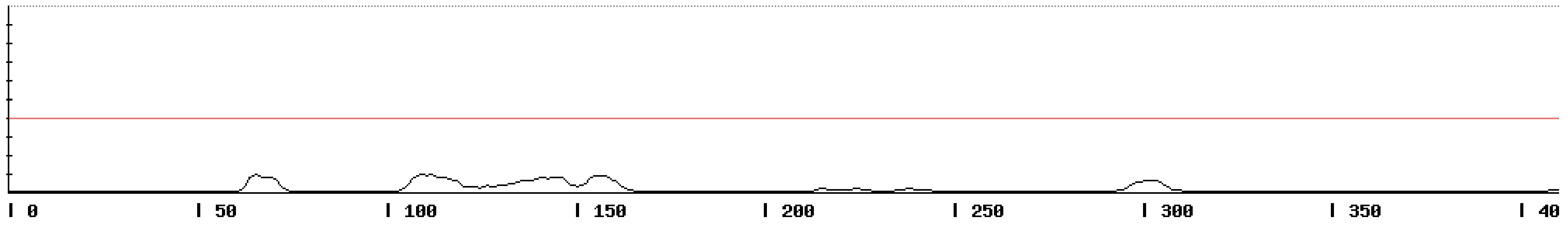

Predictions for Aegilopstauschii_EMT07371

Viterbi Path	
Viterbi Path	
Posterior @ 0.4	
Posterior @ 0.4	
Posterior Graphic. Threshold Marked @ 0.4	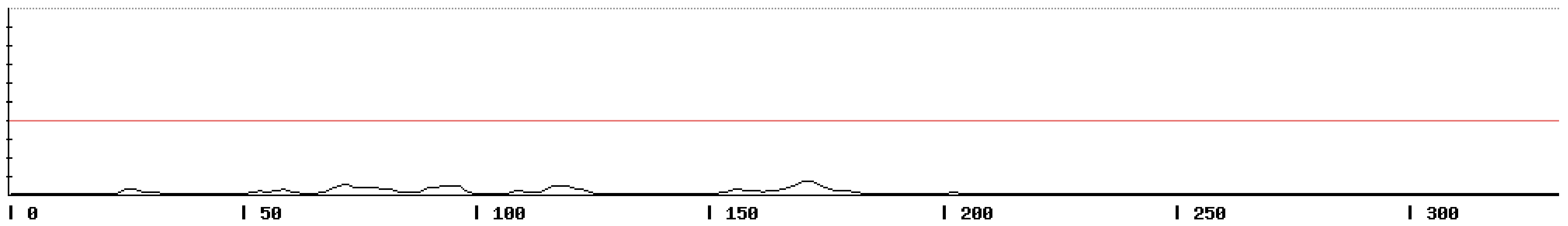

Predictions for Aegilopstauschii_EMT07892

Viterbi Path	
Viterbi Path	
Posterior @ 0.4	94 - KATGKDRVIKSKGEKKKQHMIGMKKTLVFHRGRAPKGE - 131
Posterior @ 0.4	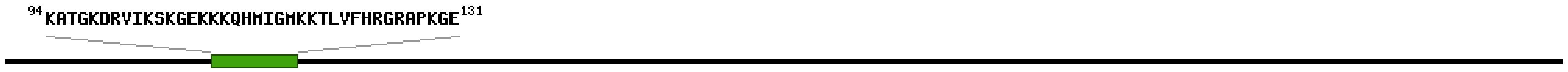
Posterior Graphic. Threshold Marked @ 0.4	

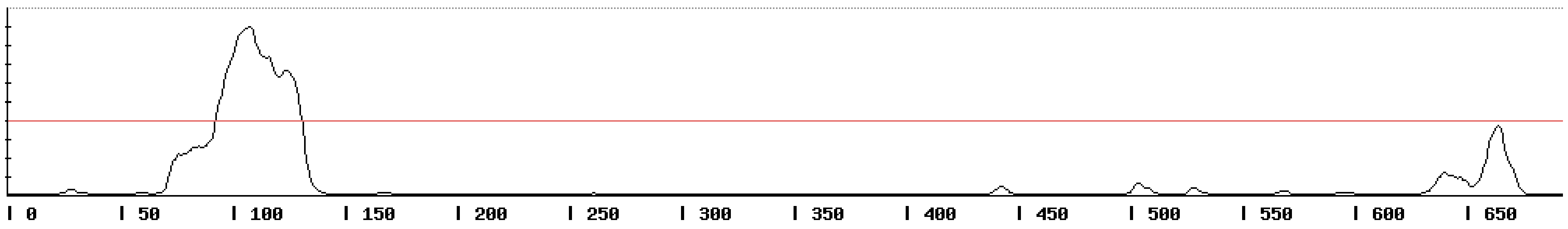

Predictions for Aegilopstauschii_EMT08095

Viterbi Path	
Viterbi Path	
Posterior @ 0.4	
Posterior @ 0.4	
Posterior Graphic. Threshold Marked @ 0.4	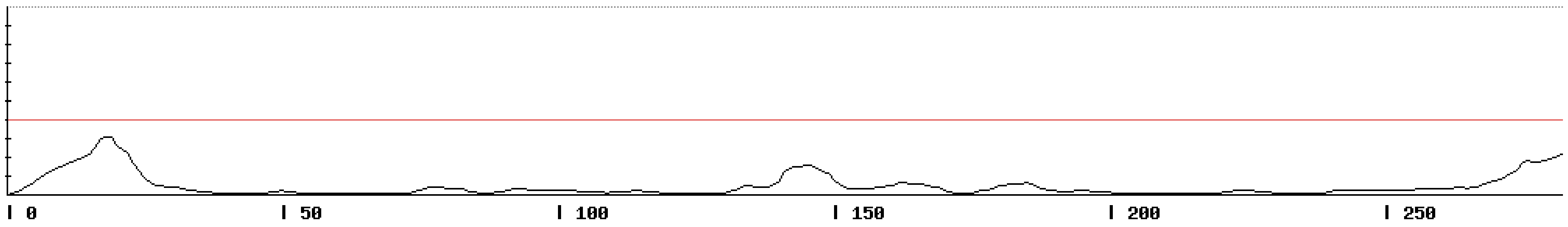

Predictions for Aegilopstauschii_EMT08988

Viterbi Path	
Viterbi Path	
Posterior @ 0.4	
Posterior @ 0.4	

Posterior Graphic. Threshold Marked @ 0.4

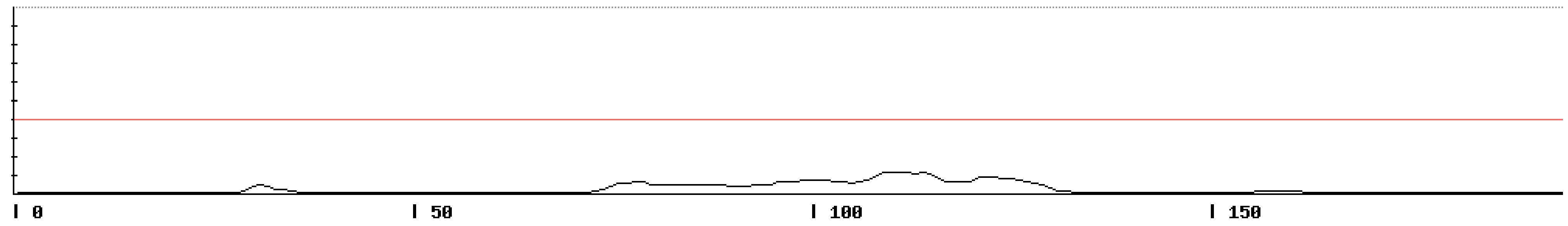

Predictions for Aegilopstauschii_EMT10359

Viterbi Path

Viterbi Path

Posterior @ 0.4

Posterior @ 0.4

Posterior Graphic. Threshold Marked @ 0.4

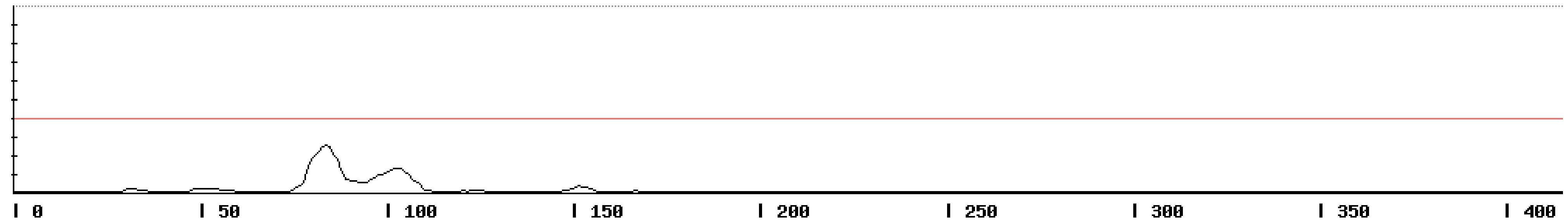

Predictions for Aegilopstauschii_EMT10902

Viterbi Path

Viterbi Path

Posterior @ 0.4

103 - KKR - 105

178 - PRGDKAKRKAKP - 189

Posterior @ 0.4

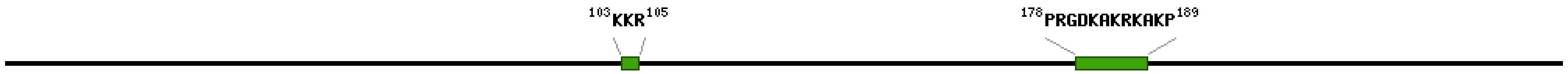

Posterior Graphic. Threshold Marked @ 0.4

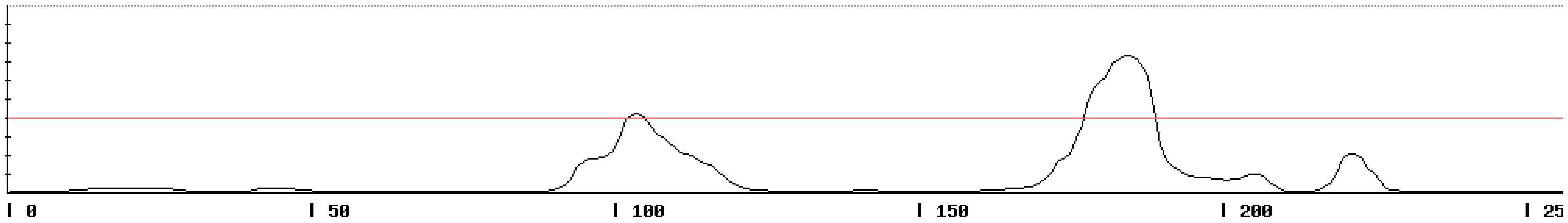

Predictions for Aegilopstauschii_EMT11023

Viterbi Path

Viterbi Path

Posterior @ 0.4

Posterior @ 0.4

Posterior Graphic. Threshold Marked @ 0.4

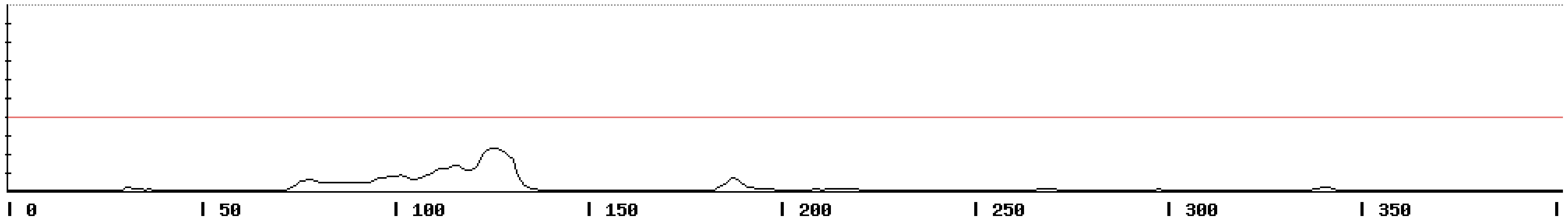

Predictions for Aegilopstauschii_EMT11045

Viterbi Path

Viterbi Path

Posterior @ 0.4

Posterior @ 0.4

Posterior Graphic. Threshold Marked @ 0.4

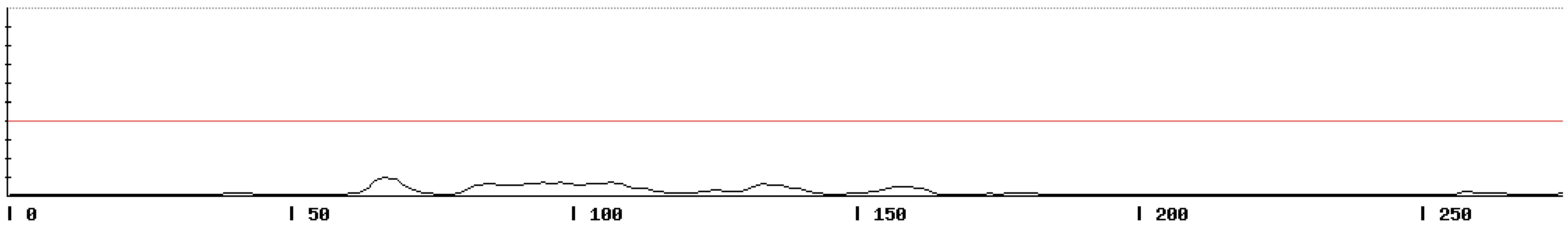

Predictions for Aegilopstauschii_EMT11046

Viterbi Path

Viterbi Path

Posterior @ 0.4

Posterior @ 0.4

Posterior Graphic. Threshold Marked @ 0.4

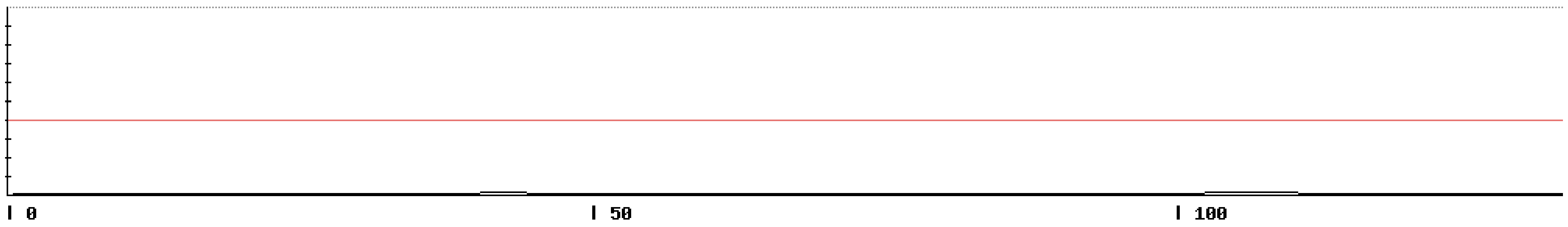

Predictions for Aegilopstauschii_EMT11192

Viterbi Path

Viterbi Path

Posterior @ 0.4	
Posterior @ 0.4	
Posterior Graphic. Threshold Marked @ 0.4	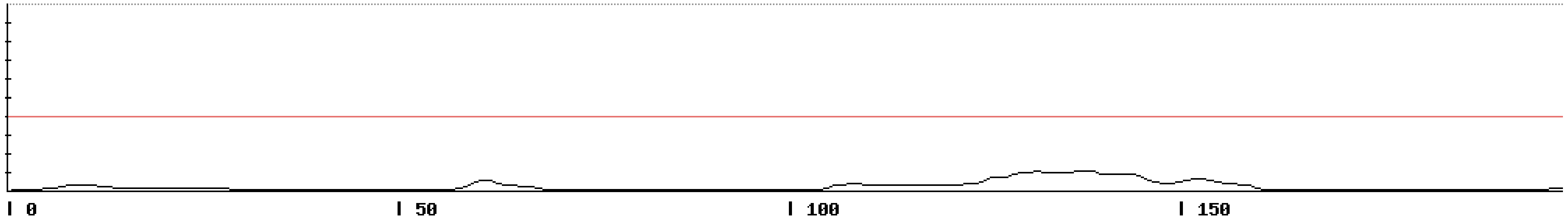

Predictions for Aegilopstauschii_EMT11879	
Viterbi Path	
Viterbi Path	
Posterior @ 0.4	
Posterior @ 0.4	
Posterior Graphic. Threshold Marked @ 0.4	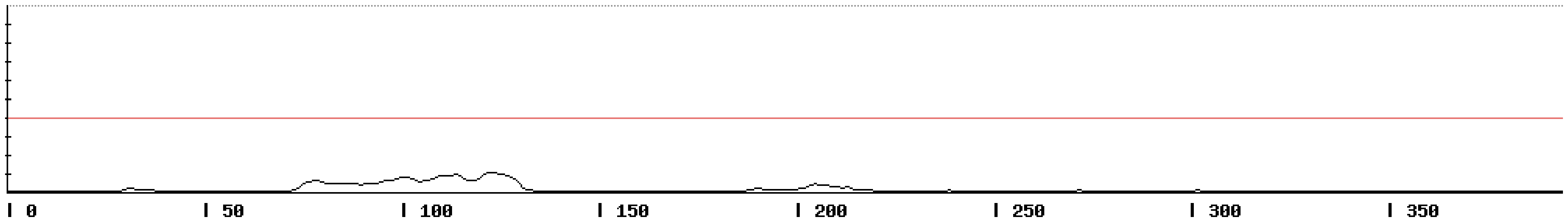

Predictions for Aegilopstauschii_EMT12034

Viterbi Path

Viterbi Path

Posterior @ 0.4

Posterior @ 0.4

Posterior Graphic. Threshold Marked @ 0.4

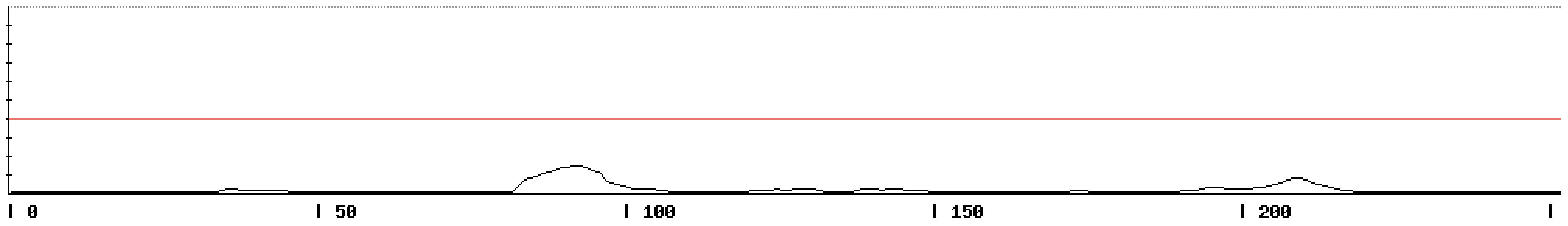

Predictions for Aegilopstauschii_EMT12401

Viterbi Path

Viterbi Path

Posterior @ 0.4

Posterior @ 0.4

Posterior Graphic. Threshold Marked @ 0.4

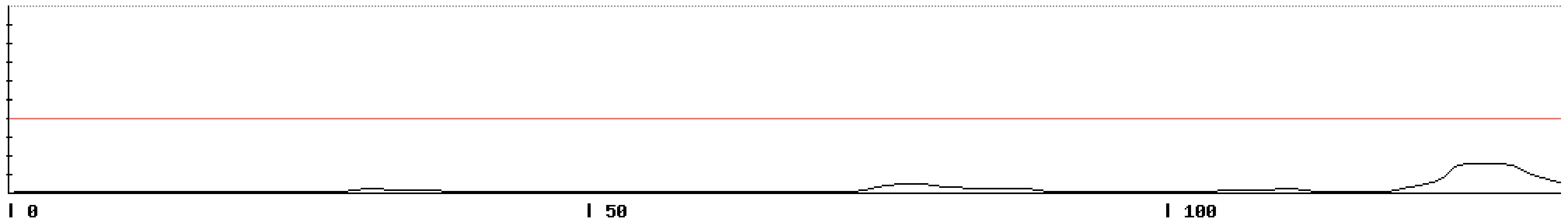

Predictions for Aegilopstauschii_EMT12583	
Viterbi Path	
Viterbi Path	
Posterior @ 0.4	
Posterior @ 0.4	
Posterior Graphic. Threshold Marked @ 0.4	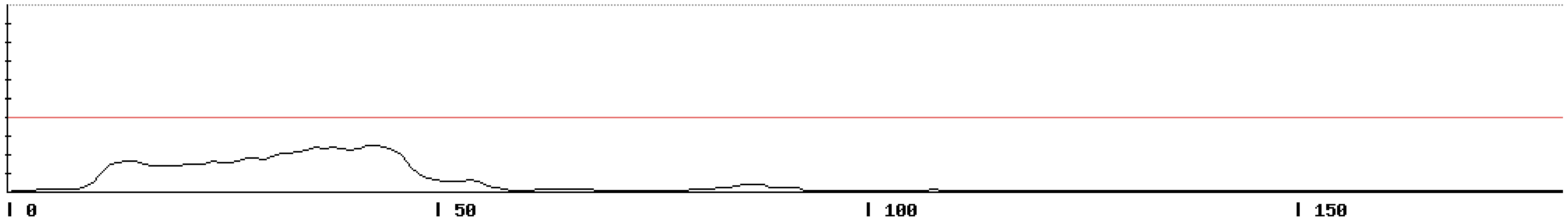

Predictions for Aegilopstauschii_EMT13314	
Viterbi Path	
Viterbi Path	
Posterior @ 0.4	155 - RKRRK - 159
Posterior @ 0.4	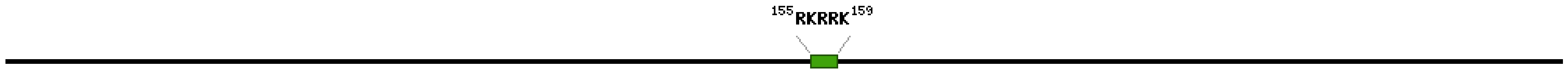
Posterior Graphic. Threshold Marked @ 0.4	

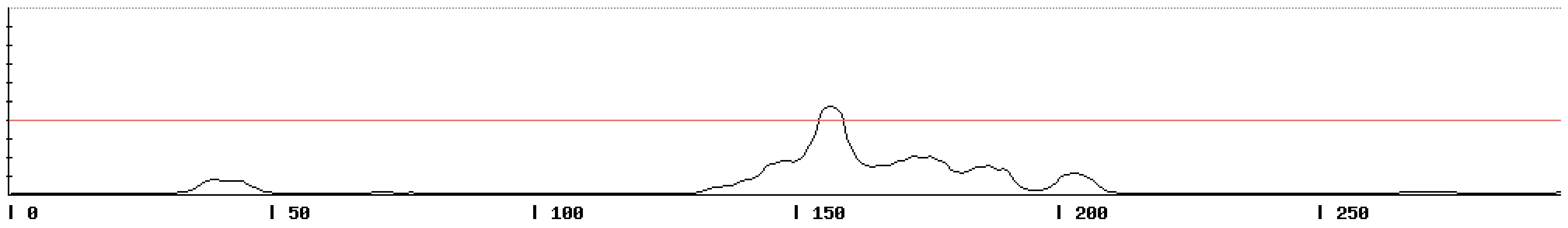

Predictions for Aegilopstauschii_EMT13683

Viterbi Path

Viterbi Path

Posterior @ 0.4

Posterior @ 0.4

Posterior Graphic. Threshold Marked @ 0.4

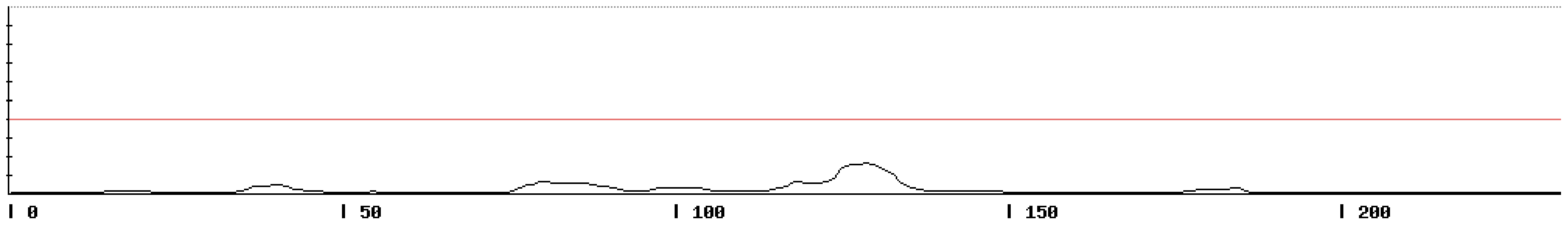

Predictions for Aegilopstauschii_EMT13710

Viterbi Path

Viterbi Path

Posterior @ 0.4

188 - KRPAPAAAADPHPPRPKKRMRGAGP - 212

Posterior @ 0.4

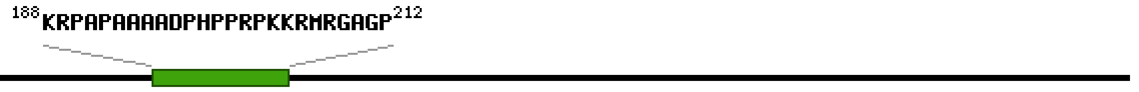

Posterior Graphic. Threshold Marked @ 0.4	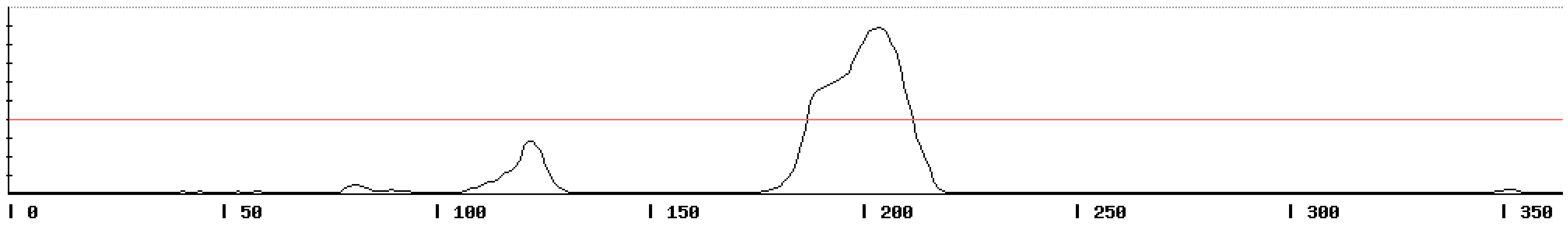

Predictions for Aegilopstauschii_EMT13722	
Viterbi Path	
Viterbi Path	
Posterior @ 0.4	187 - RDPGKKKRKQSG - 198
Posterior @ 0.4	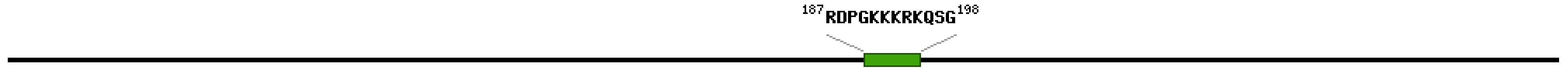
Posterior Graphic. Threshold Marked @ 0.4	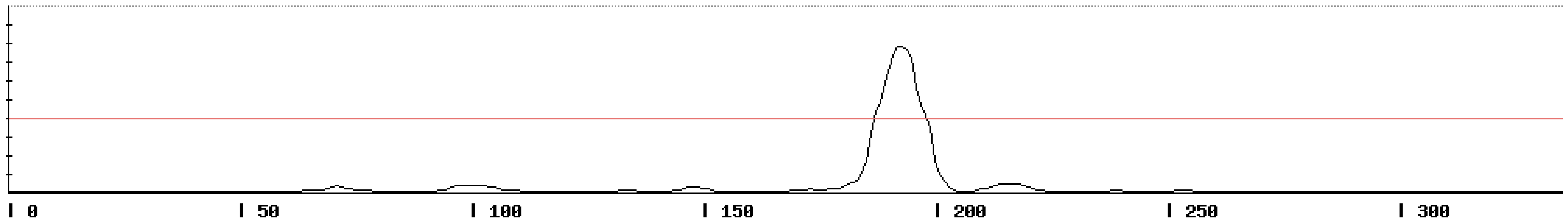

Predictions for Aegilopstauschii_EMT13761	
Viterbi Path	447 - KDKSRVKRKRRRYGA - 461
Viterbi Path	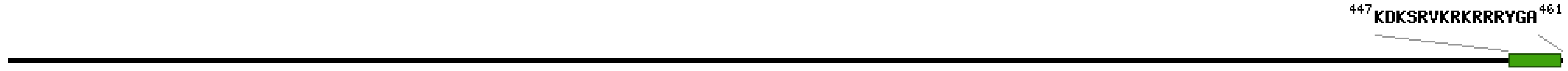
Posterior @ 0.4	447 - KDKSRVKRKRRRYGA - 461
Posterior @ 0.4	

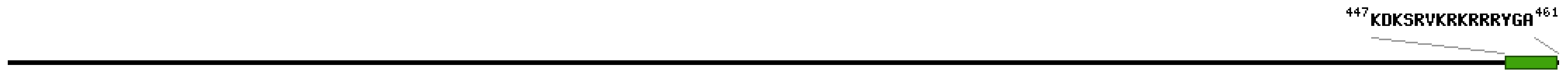

Posterior Graphic. Threshold Marked @ 0.4

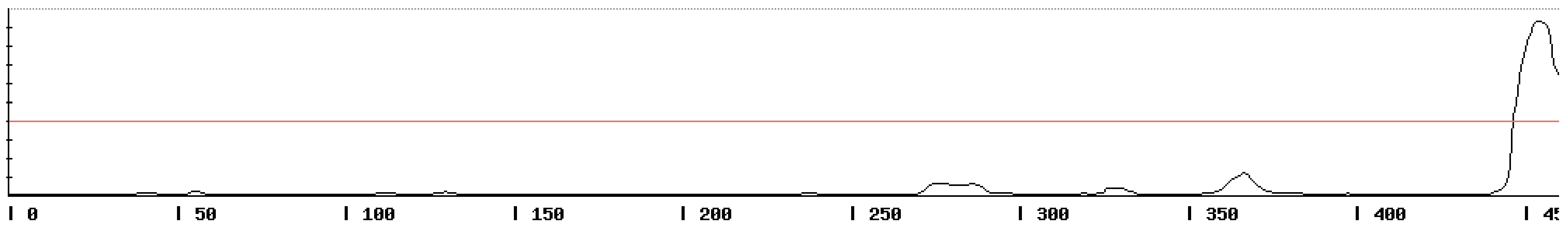

Predictions for Aegilopstauschii_EMT13975

Viterbi Path

Viterbi Path

Posterior @ 0.4

Posterior @ 0.4

Posterior Graphic. Threshold Marked @ 0.4

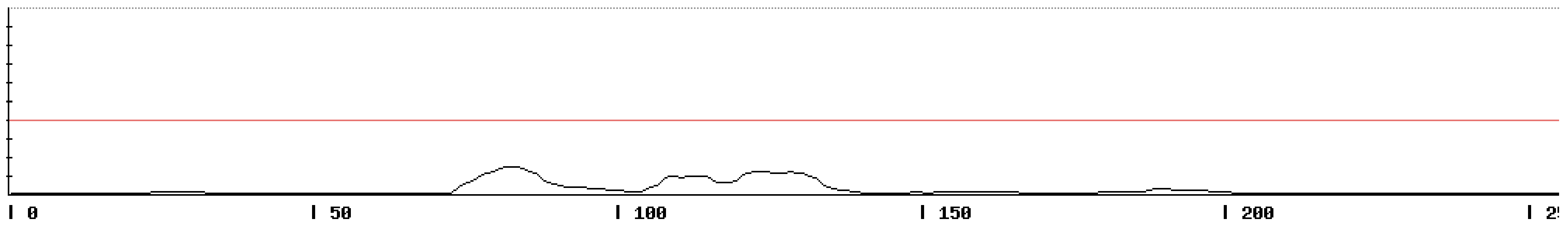

Predictions for Aegilopstauschii_EMT14094

Viterbi Path

Viterbi Path

Posterior @ 0.4

Posterior @ 0.4

Posterior Graphic. Threshold Marked @ 0.4

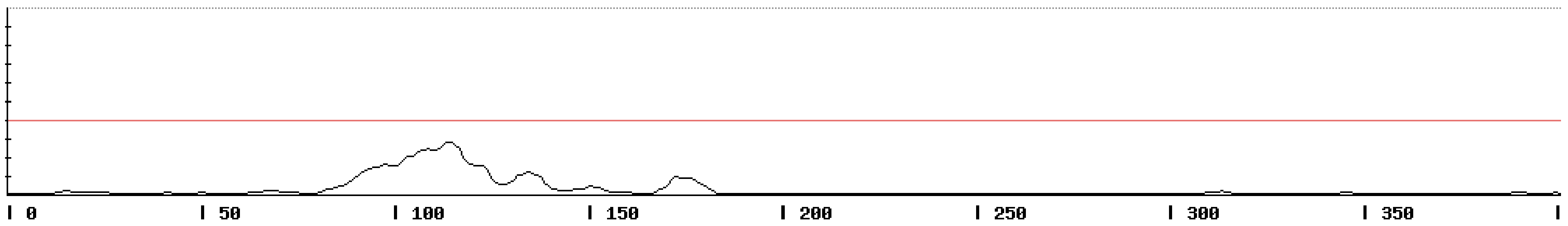

Predictions for Aegilopstauschii_EMT14095

Viterbi Path

Viterbi Path

Posterior @ 0.4

Posterior @ 0.4

Posterior Graphic. Threshold Marked @ 0.4

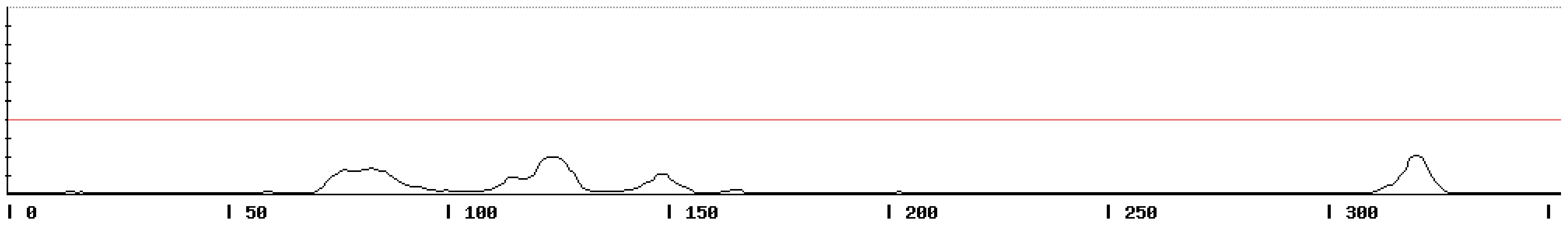

Predictions for Aegilopstauschii_EMT14186

Viterbi Path

Viterbi Path

Posterior @ 0.4

Posterior @ 0.4

Posterior Graphic. Threshold Marked @ 0.4

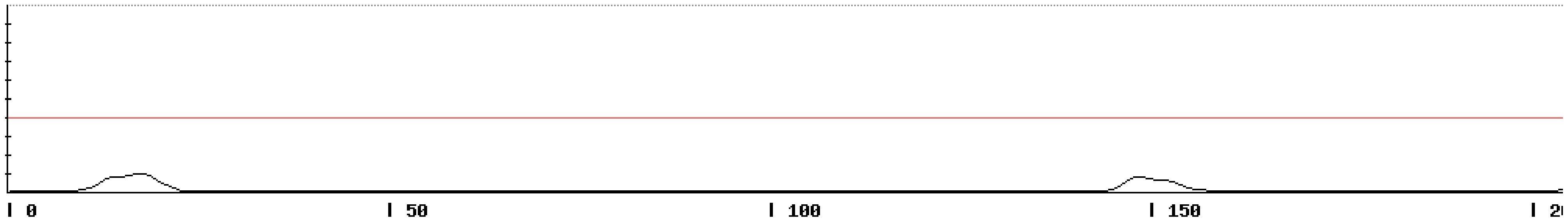

Predictions for Aegilopstauschii_EMT14221

Viterbi Path

Viterbi Path

Posterior @ 0.4

186 - GQRGARPGPARRP - 198

271 - AAGRKRK - 277

310 - GHKKRKTHSRNSGPVLRPARGVLKLTRPVTSGRKKNSS - 347

Posterior @ 0.4

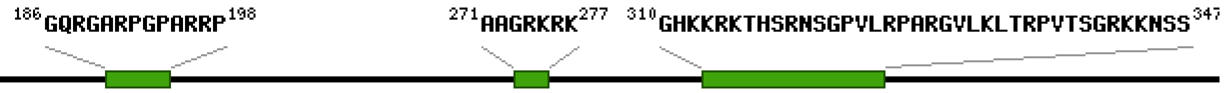

Posterior Graphic. Threshold Marked @ 0.4

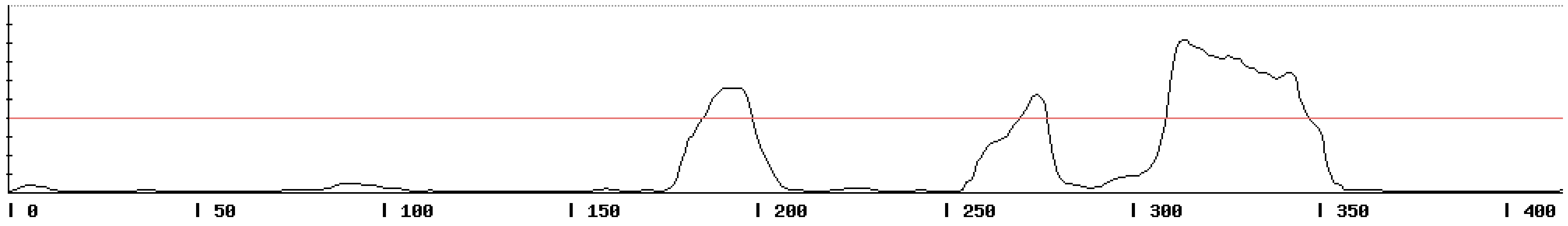

Predictions for Aegilopstauschii_EMT14569

Viterbi Path

Viterbi Path

Posterior @ 0.4

187 - KPEKKK - 192

362 - EHAKAIRKRKEHARASGDDAGKKGNAAVKKSSGRAK - 397

Posterior @ 0.4

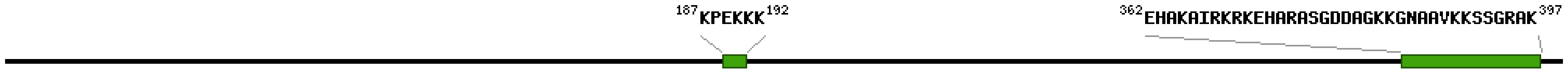

Posterior Graphic. Threshold Marked @ 0.4

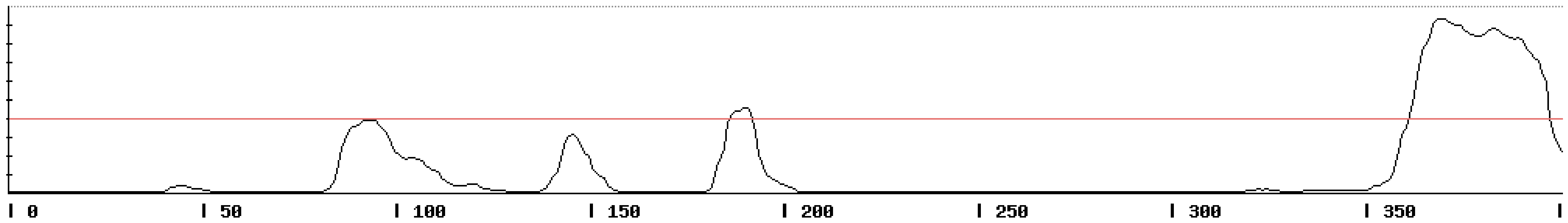

Predictions for Aegilopstauschii_EMT14807

Viterbi Path

Viterbi Path

Posterior @ 0.4

Posterior @ 0.4

Posterior Graphic. Threshold Marked @ 0.4

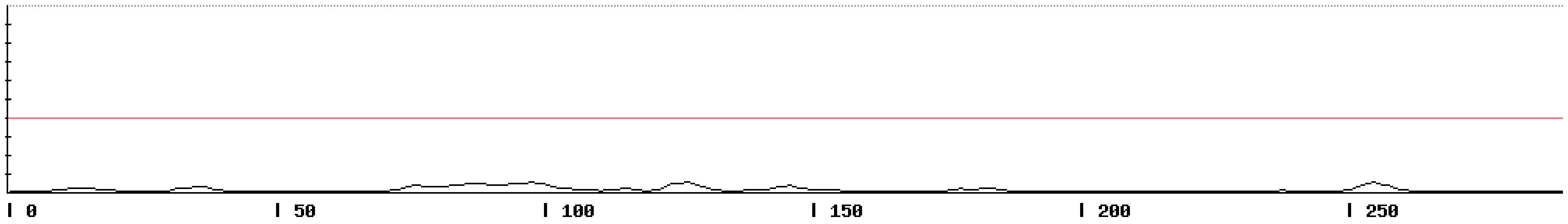

Predictions for Aegilopstauschii_EMT15405

Viterbi Path

Viterbi Path

Posterior @ 0.4 — 137 - SRKRSHKKKDH - 147

Posterior @ 0.4

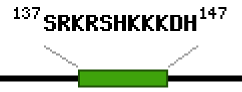

Posterior Graphic. Threshold Marked @ 0.4

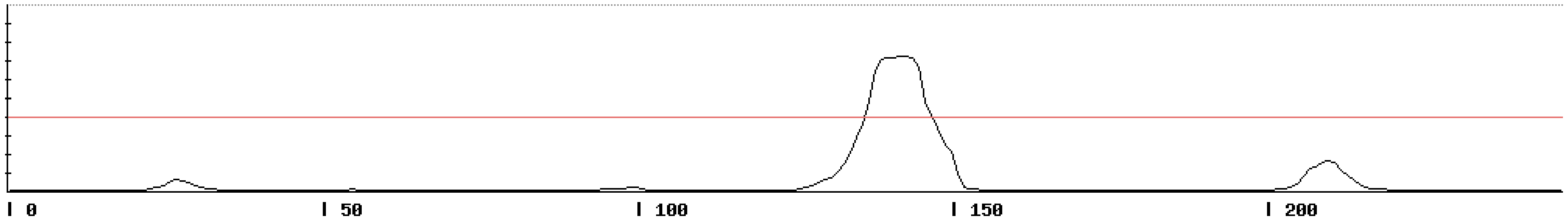

Predictions for Aegilopstauschii_EMT15453

Viterbi Path

Viterbi Path

Posterior @ 0.4

Posterior @ 0.4

Posterior Graphic. Threshold Marked @ 0.4

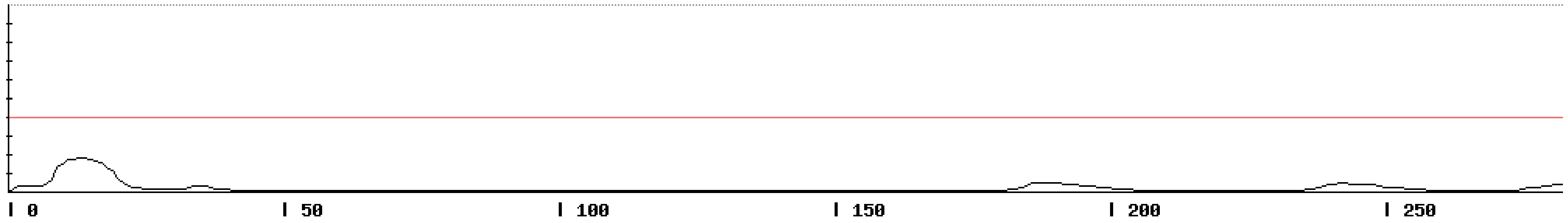

Predictions for Aegilopstauschii_EMT15468

Viterbi Path

Viterbi Path

Posterior @ 0.4 — 118 - PRERKYKNGVRPRRDTKDRLGRWKASTGKT - 147

Posterior @ 0.4

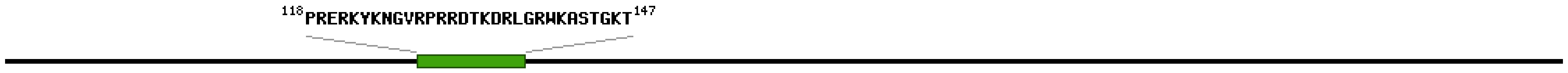

Posterior Graphic. Threshold Marked @ 0.4

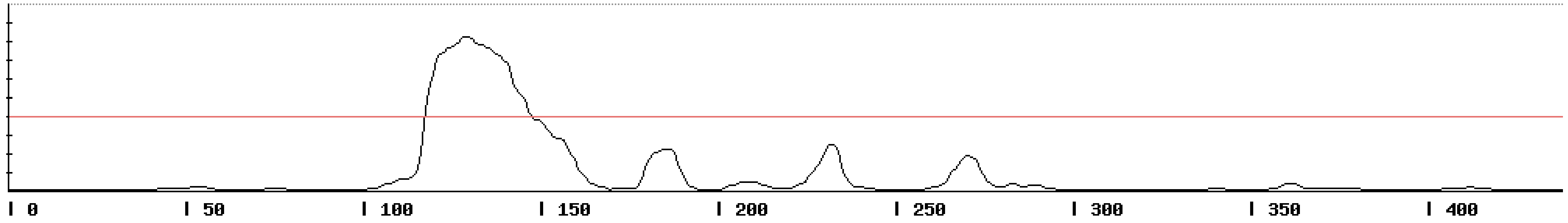

Predictions for Aegilopstauschii_EMT16679

Viterbi Path

Viterbi Path

Posterior @ 0.4

Posterior @ 0.4

Posterior Graphic. Threshold Marked @ 0.4

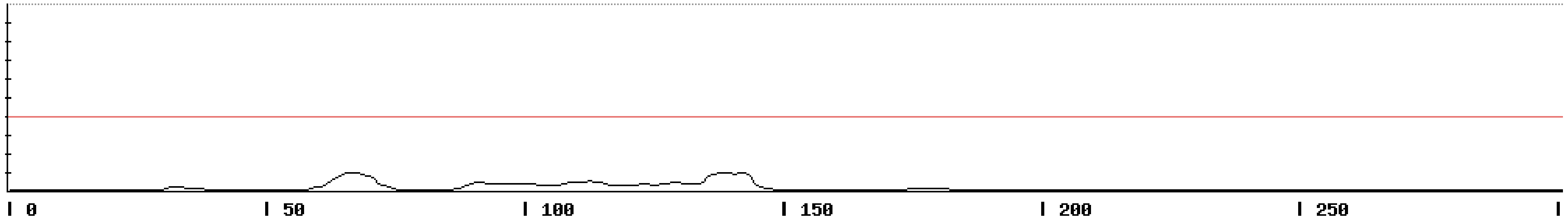

Predictions for Aegilopstauschii_EMT18249

Viterbi Path

Viterbi Path

Posterior @ 0.4

Posterior @ 0.4

Posterior Graphic. Threshold Marked @ 0.4

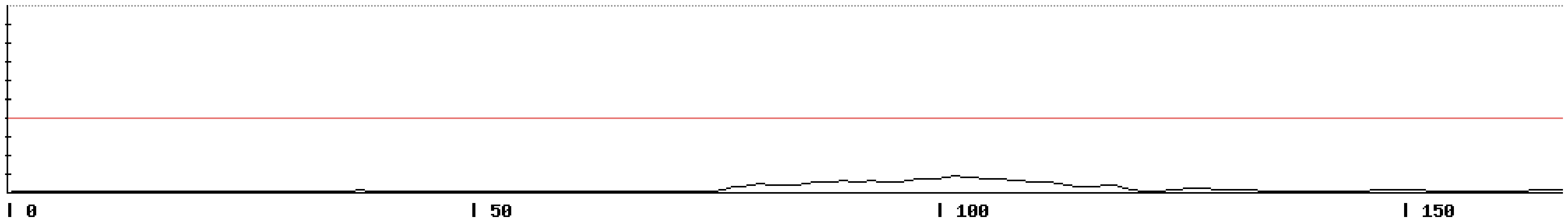

Predictions for Aegilopstauschii_EMT20495

Viterbi Path

Viterbi Path

Posterior @ 0.4

Posterior @ 0.4

Posterior Graphic. Threshold Marked @ 0.4

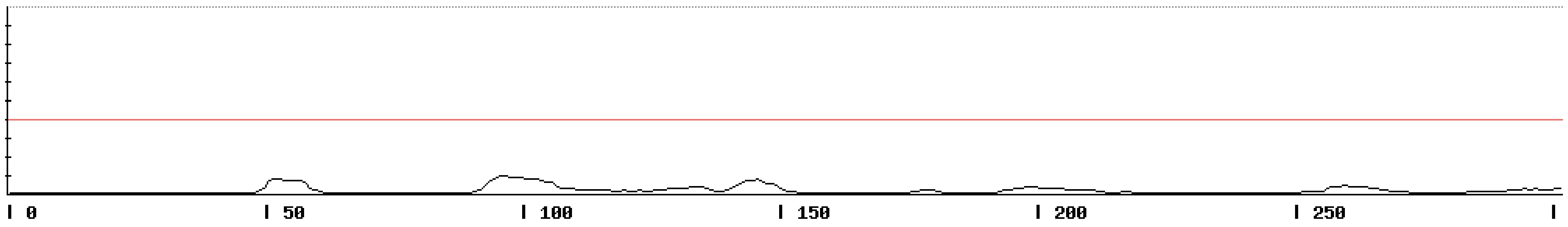

Predictions for Aegilopstauschii_EMT20593

Viterbi Path

Viterbi Path

Posterior @ 0.4

Posterior @ 0.4

Posterior Graphic. Threshold Marked @ 0.4

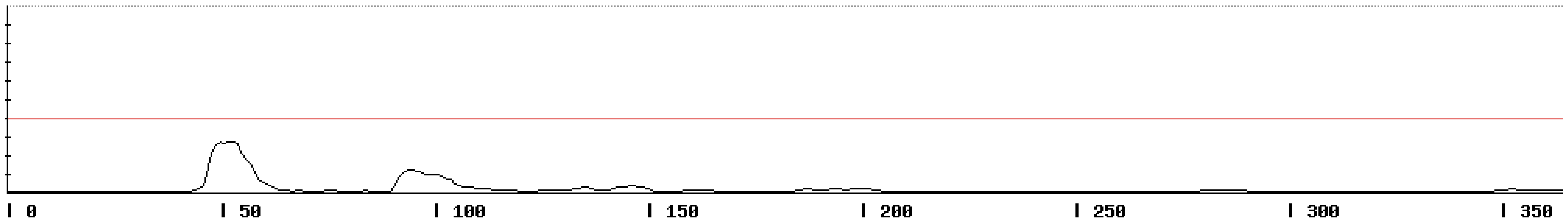

Predictions for Aegilopstauschii_EMT20647	
Viterbi Path	
Viterbi Path	
Posterior @ 0.4	
Posterior @ 0.4	
Posterior Graphic. Threshold Marked @ 0.4	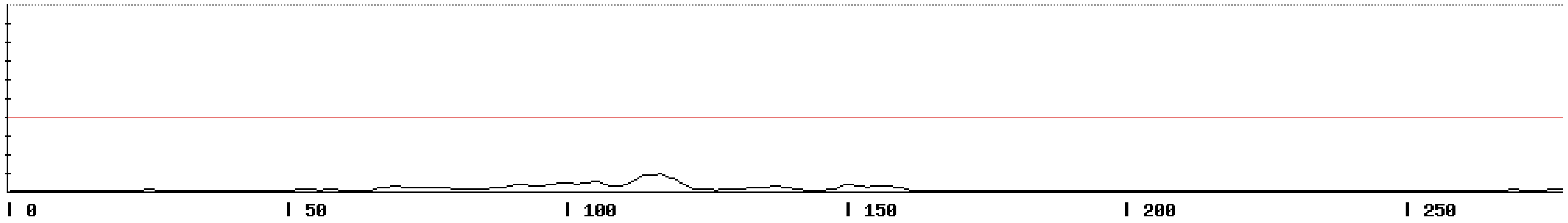

Predictions for Aegilopstauschii_EMT20851	
Viterbi Path	
Viterbi Path	
Posterior @ 0.4	
Posterior @ 0.4	
Posterior Graphic. Threshold Marked @ 0.4	

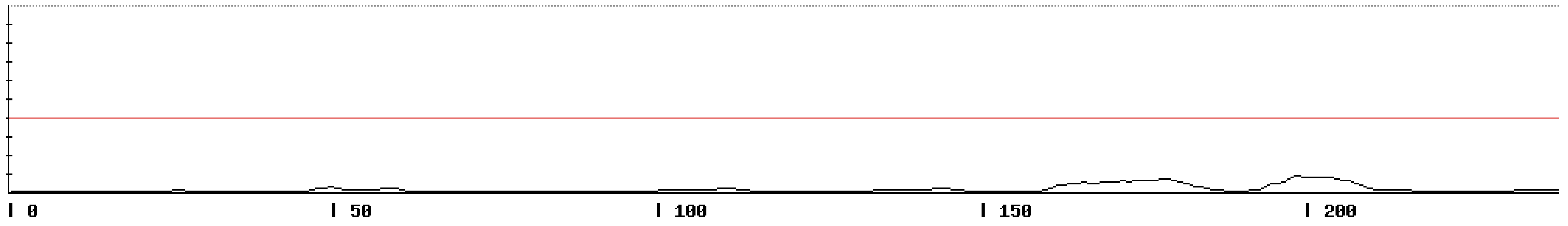

Predictions for Aegilopstauschii_EMT21053

Viterbi Path	
Viterbi Path	
Posterior @ 0.4	
Posterior @ 0.4	
Posterior Graphic. Threshold Marked @ 0.4	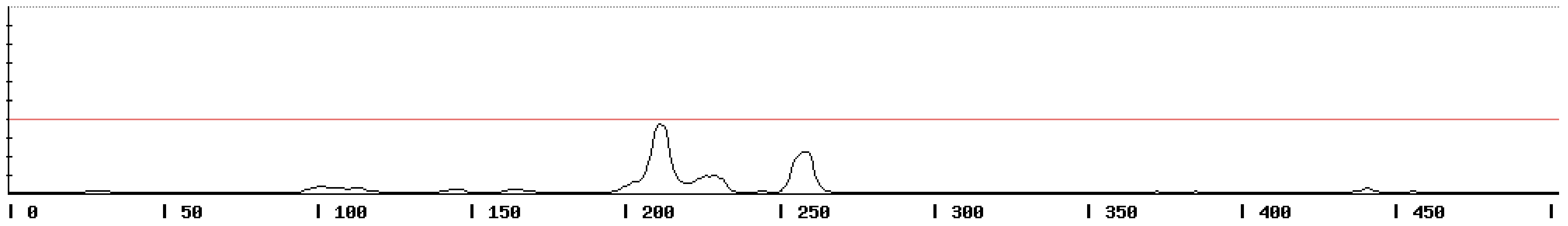

Predictions for Aegilopstauschii_EMT21398

Viterbi Path	
Viterbi Path	
Posterior @ 0.4	
Posterior @ 0.4	

Posterior Graphic. Threshold Marked @ 0.4	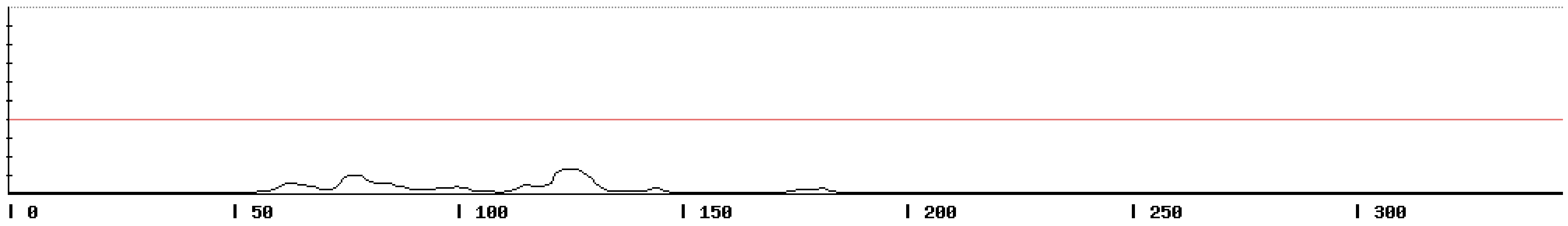

Predictions for Aegilopstauschii_EMT21400	
Viterbi Path	
Viterbi Path	
Posterior @ 0.4	
Posterior @ 0.4	
Posterior Graphic. Threshold Marked @ 0.4	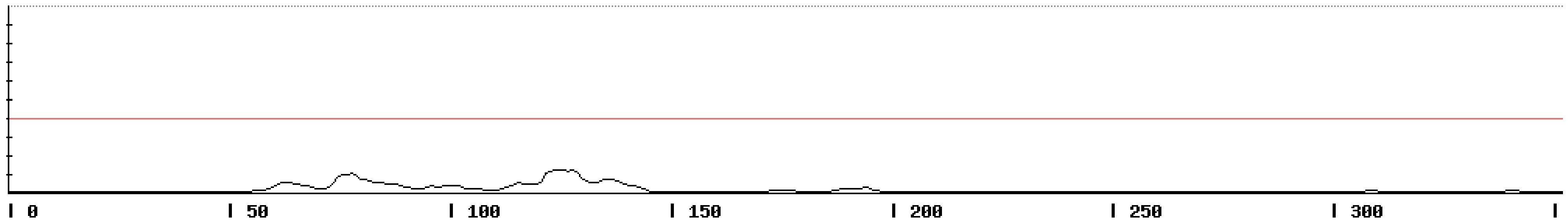

Predictions for Aegilopstauschii_EMT21589	
Viterbi Path	
Viterbi Path	
Posterior @ 0.4	
Posterior @ 0.4	

Posterior Graphic. Threshold Marked @ 0.4

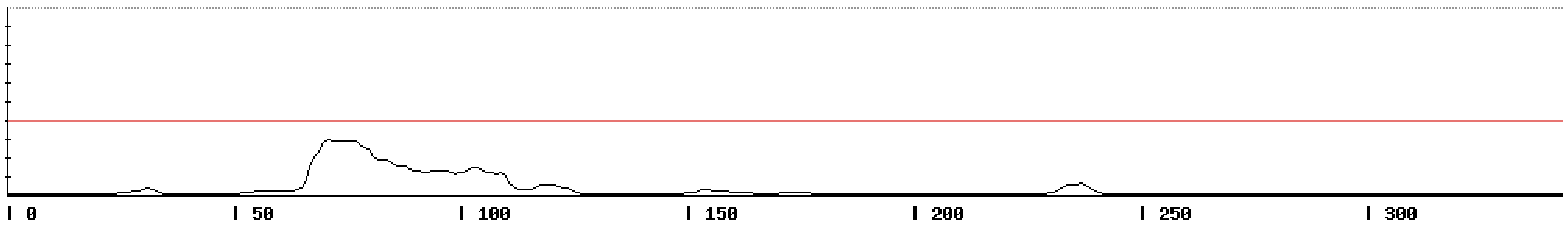

Predictions for Aegilopstauschii_EMT21626

Viterbi Path

Viterbi Path

Posterior @ 0.4

Posterior @ 0.4

Posterior Graphic. Threshold Marked @ 0.4

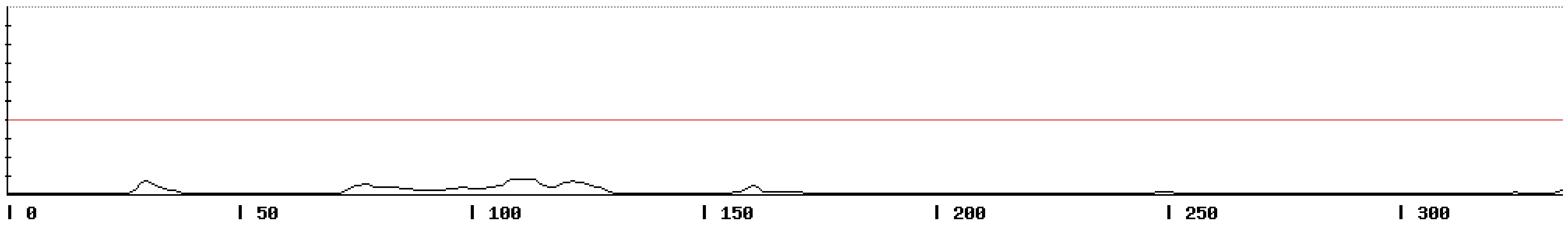

Predictions for Aegilopstauschii_EMT21706

Viterbi Path

Viterbi Path

Posterior @ 0.4

Posterior @ 0.4

Posterior Graphic. Threshold Marked @ 0.4

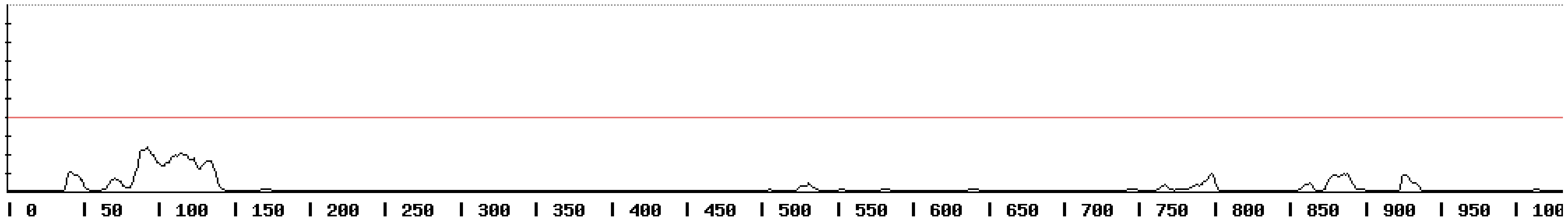

Predictions for Aegilopstauschii_EMT22193

Viterbi Path

Viterbi Path

Posterior @ 0.4

Posterior @ 0.4

Posterior Graphic. Threshold Marked @ 0.4

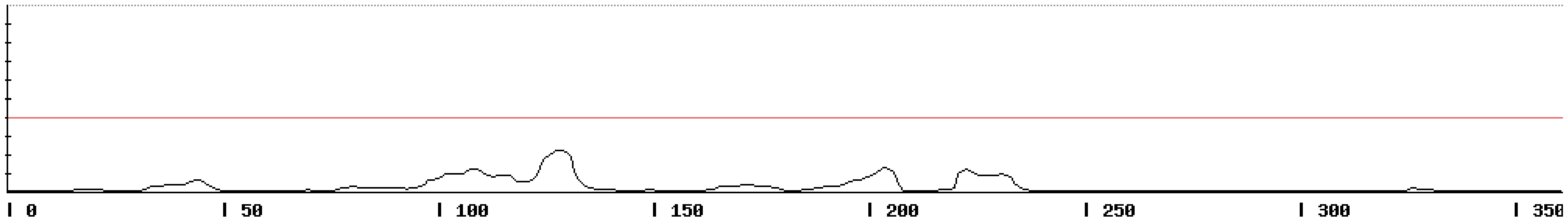

Predictions for Aegilopstauschii_EMT22201

Viterbi Path

Viterbi Path

Posterior @ 0.4

Posterior @ 0.4

Posterior Graphic. Threshold Marked @ 0.4

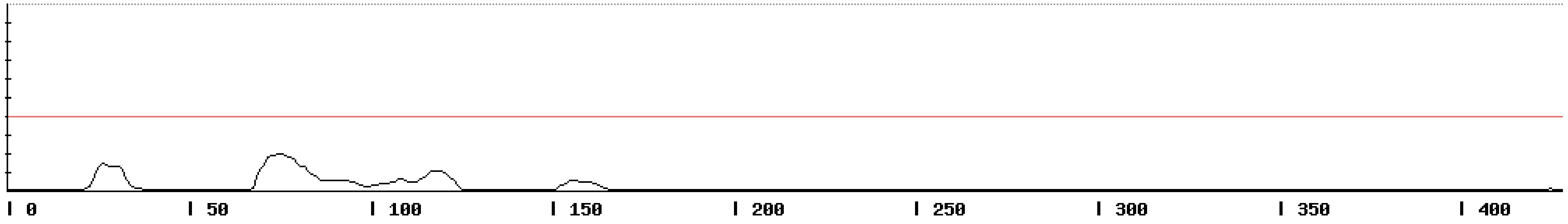

Predictions for Aegilopstauschii_EMT22922

Viterbi Path

Viterbi Path

Posterior @ 0.4

Posterior @ 0.4

Posterior Graphic. Threshold Marked @ 0.4

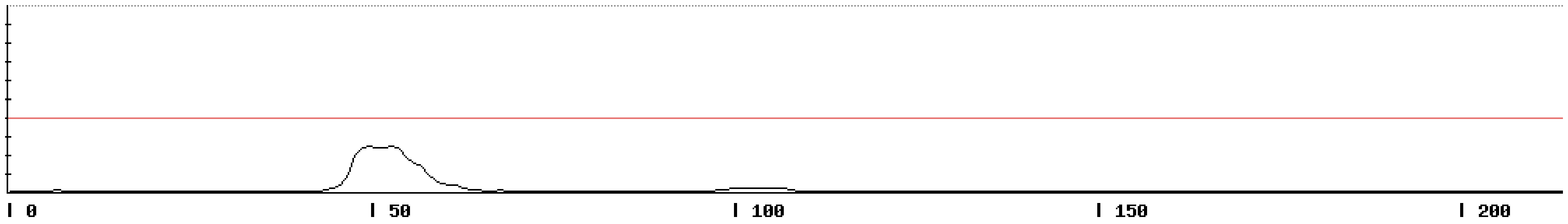

Predictions for Aegilopstauschii_EMT23410

Viterbi Path

Viterbi Path

Posterior @ 0.4

Posterior @ 0.4

Posterior Graphic. Threshold Marked @ 0.4

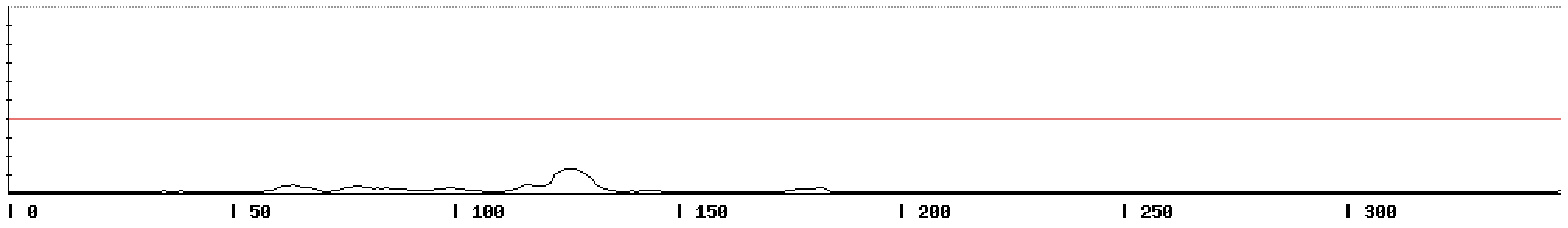

Predictions for Aegilopstauschii_EMT23614

Viterbi Path

Viterbi Path

Posterior @ 0.4

93 - GKDRKVN - 99

102 - K - 102

536 - SRKKAKKVKPGR - 547

Posterior @ 0.4

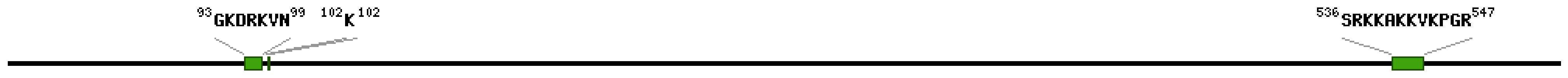

Posterior Graphic. Threshold Marked @ 0.4

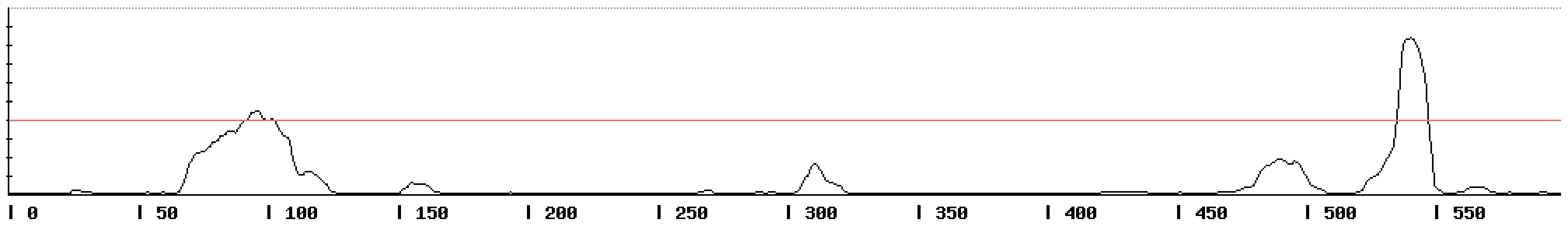

Predictions for Aegilopstauschii_EMT24079

Viterbi Path

Viterbi Path

Posterior @ 0.4

Posterior @ 0.4

Posterior Graphic. Threshold Marked @ 0.4

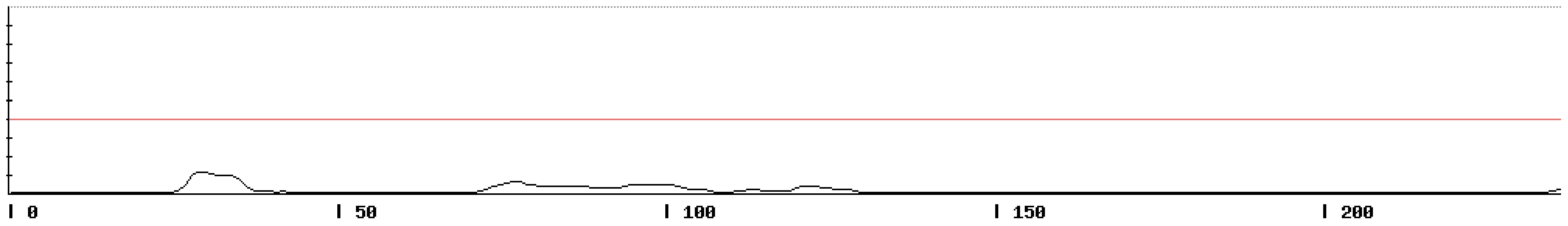

Predictions for Aegilopstauschii_EMT24093

Viterbi Path

Viterbi Path

Posterior @ 0.4

Posterior @ 0.4

Posterior Graphic. Threshold Marked @ 0.4

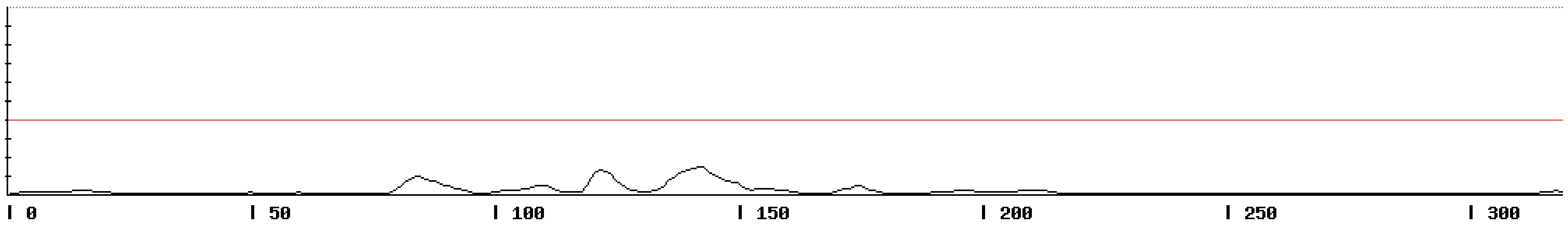

Predictions for Aegilopstauschii_EMT24300

Viterbi Path

Viterbi Path

Posterior @ 0.4

Posterior @ 0.4

Posterior Graphic. Threshold Marked @ 0.4

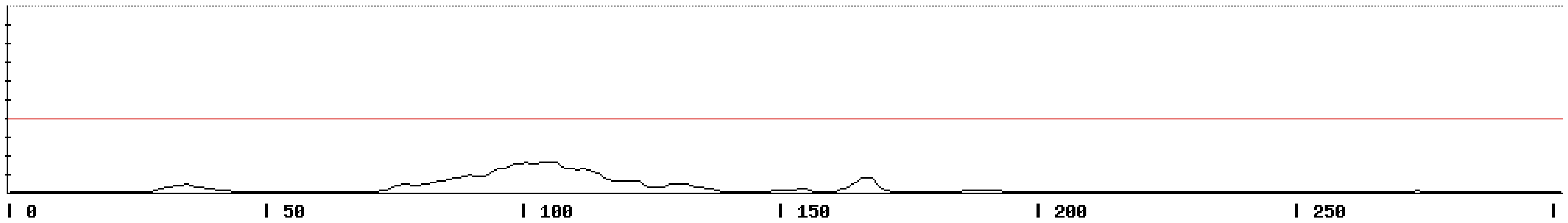

Predictions for Aegilopstauschii_EMT24618

Viterbi Path

Viterbi Path

Posterior @ 0.4

Posterior @ 0.4

Posterior Graphic. Threshold Marked @ 0.4

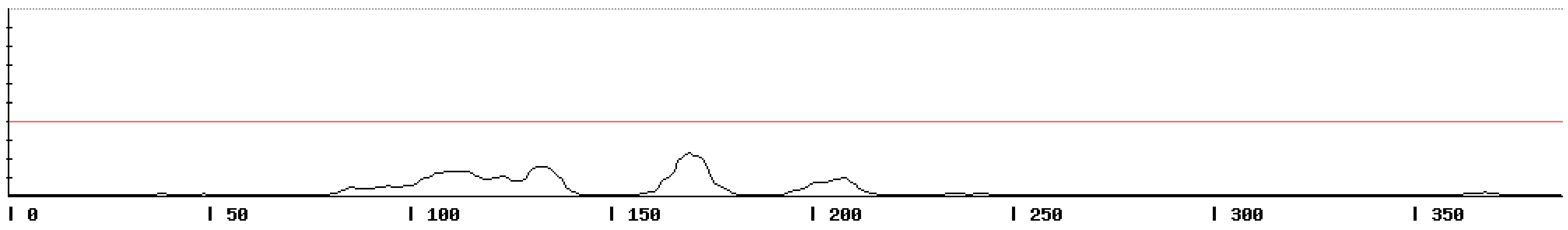

Predictions for Aegilopstauschii_EMT24735

Viterbi Path

Viterbi Path

Posterior @ 0.4

24 - RKILGKKLRR - 33

115 - KGRKE - 119

Posterior @ 0.4

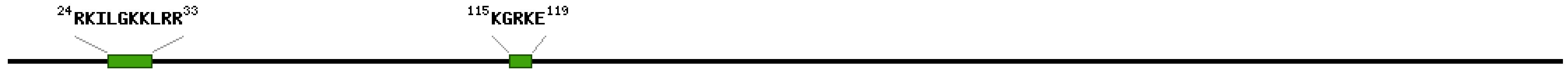

Posterior Graphic. Threshold Marked @ 0.4

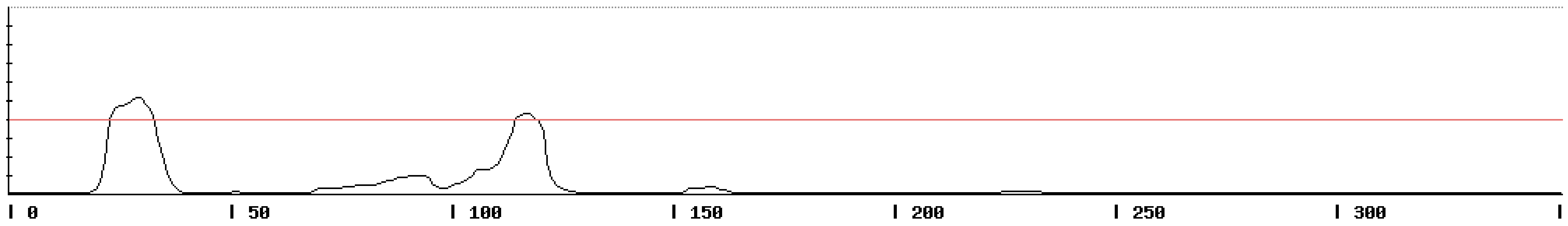

Predictions for Aegilopstauschii_EMT25084

Viterbi Path

Viterbi Path

Posterior @ 0.4

Posterior @ 0.4

Posterior Graphic. Threshold Marked @ 0.4

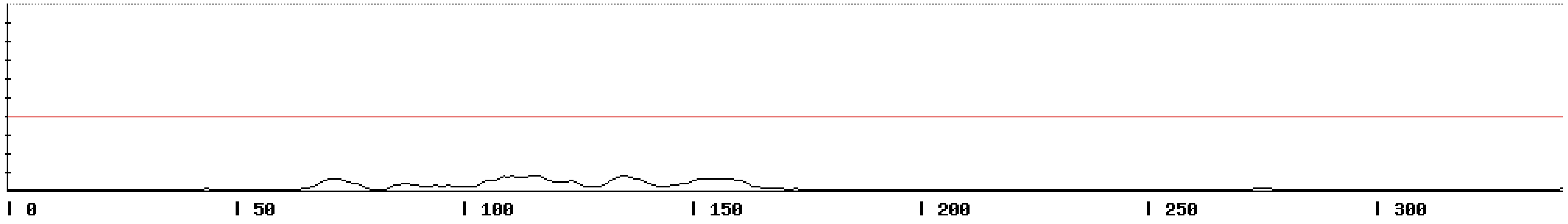

Predictions for Aegilopstauschii_EMT25749

Viterbi Path

Viterbi Path

Posterior @ 0.4

Posterior @ 0.4

Posterior Graphic. Threshold Marked @ 0.4

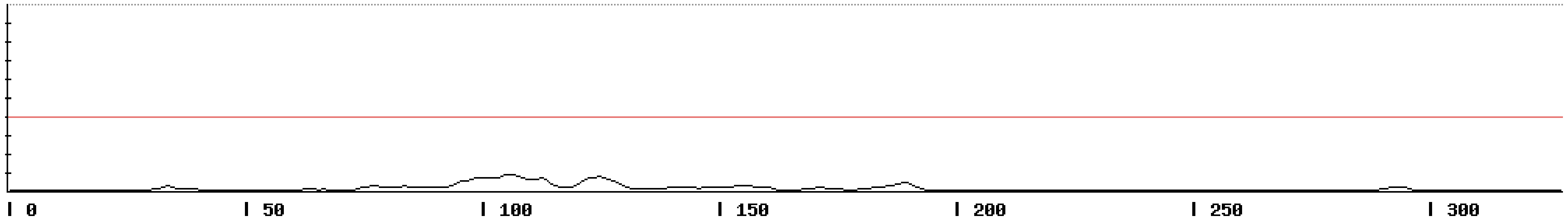

Predictions for Aegilopstauschii_EMT26194

Viterbi Path

Viterbi Path

Posterior @ 0.4

Posterior @ 0.4

Posterior Graphic. Threshold Marked @ 0.4

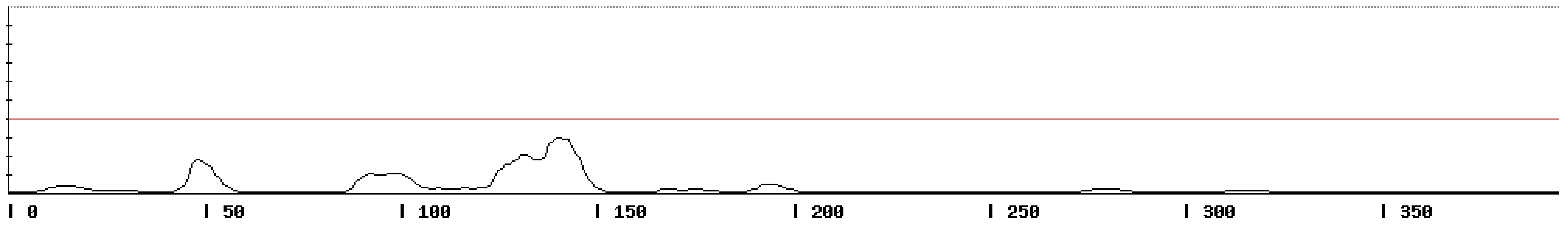

Predictions for Aegilopstauschii_EMT26627

Viterbi Path

Viterbi Path

Posterior @ 0.4

Posterior @ 0.4

Posterior Graphic. Threshold Marked @ 0.4

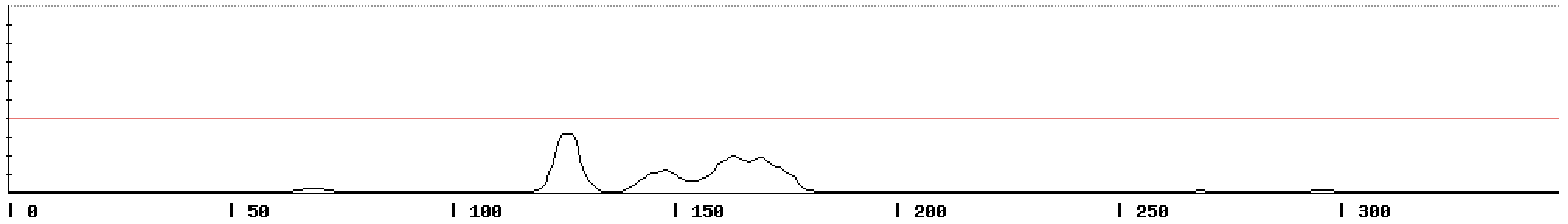

Predictions for Aegilopstauschii_EMT26799

Viterbi Path

Viterbi Path

Posterior @ 0.4

Posterior @ 0.4

Posterior Graphic. Threshold Marked @ 0.4

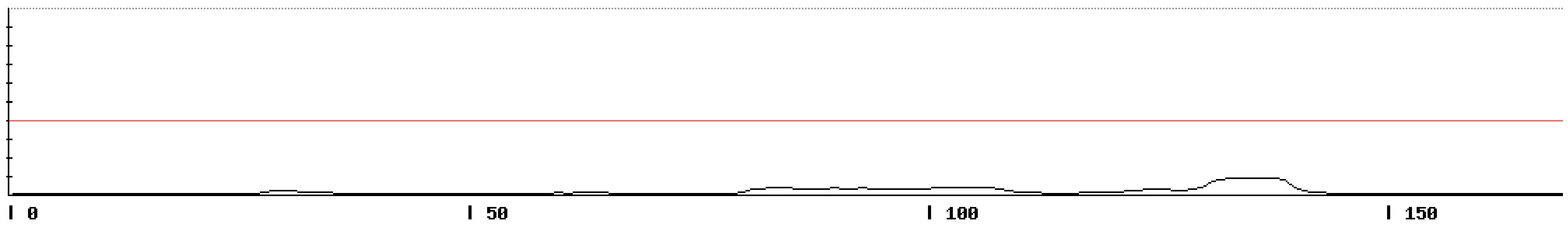

Predictions for Aegilopstauschii_EMT27068

Viterbi Path

Viterbi Path

Posterior @ 0.4

Posterior @ 0.4

Posterior Graphic. Threshold Marked @ 0.4

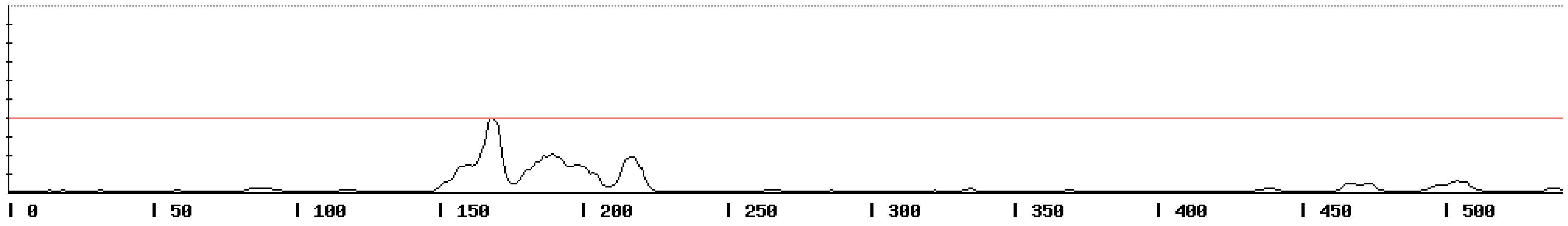

Predictions for Aegilopstauschii_EMT27197

Viterbi Path

Viterbi Path

Posterior @ 0.4

Posterior @ 0.4

Posterior Graphic. Threshold Marked @ 0.4

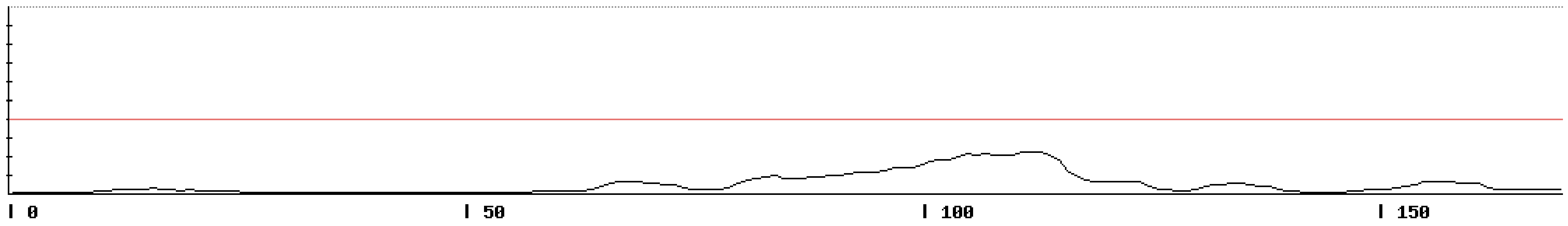

Predictions for Aegilopstauschii_EMT27450

Viterbi Path

Viterbi Path

Posterior @ 0.4

Posterior @ 0.4

Posterior Graphic. Threshold Marked @ 0.4

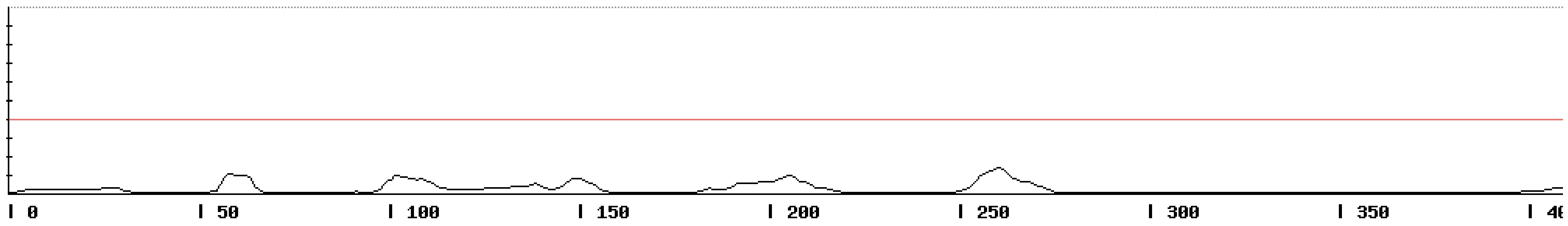

Predictions for Aegilopstauschii_EMT27713

Viterbi Path

Viterbi Path

Posterior @ 0.4

27 - PAPRRGRPLRPPPAPRVEPSPPAP - 50

597 - RQKRKKK - 603

Posterior @ 0.4

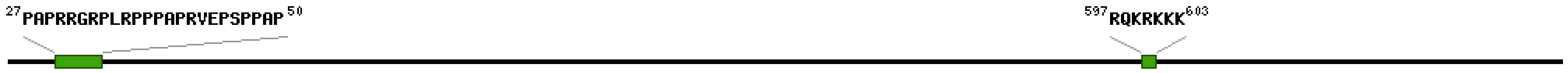

Posterior Graphic. Threshold Marked @ 0.4

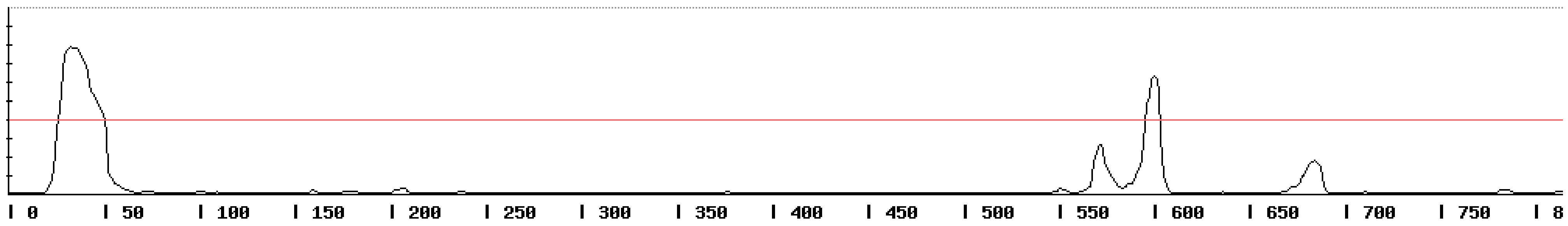

Predictions for Aegilopstauschii_EMT27961

Viterbi Path

Viterbi Path

Posterior @ 0.4

Posterior @ 0.4

Posterior Graphic. Threshold Marked @ 0.4	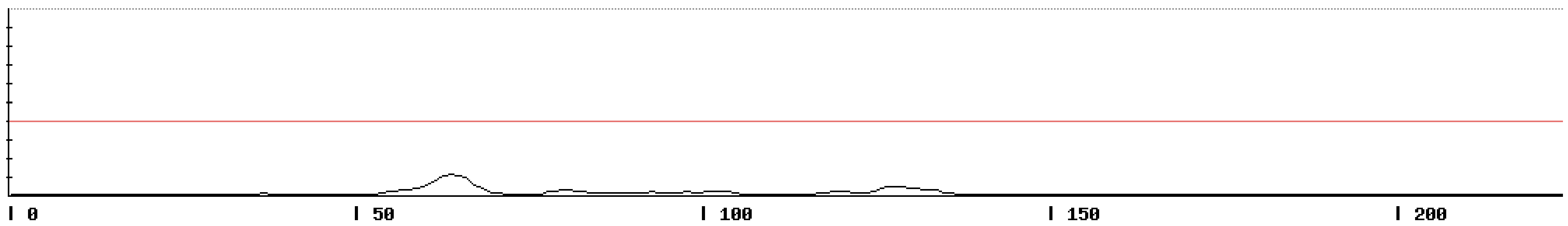

Predictions for Aegilopstauschii_EMT28110

Viterbi Path	
Viterbi Path	
Posterior @ 0.4	
Posterior @ 0.4	
Posterior Graphic. Threshold Marked @ 0.4	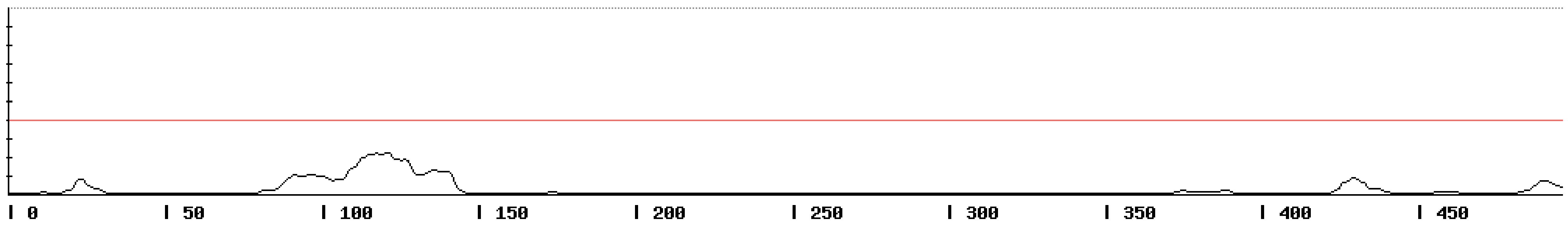

Predictions for Aegilopstauschii_EMT28157

Viterbi Path	
Viterbi Path	

Posterior @ 0.4

Posterior @ 0.4

Posterior Graphic. Threshold Marked @ 0.4

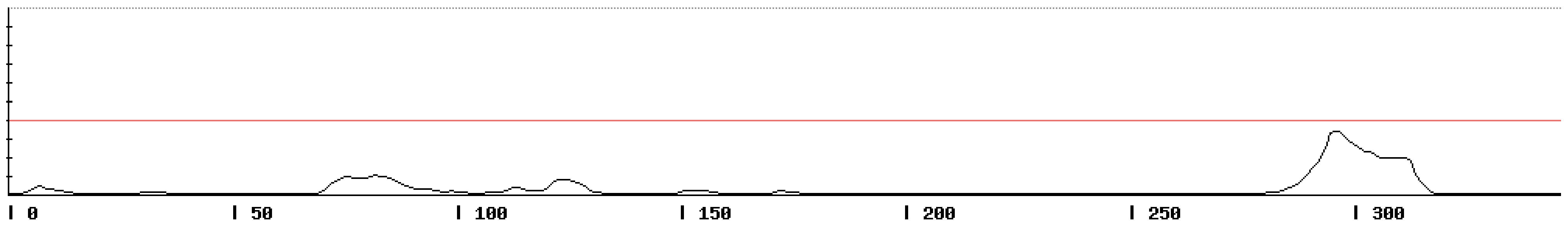

Predictions for Aegilopstauschii_EMT28257

Viterbi Path

Viterbi Path

Posterior @ 0.4

Posterior @ 0.4

Posterior Graphic. Threshold Marked @ 0.4

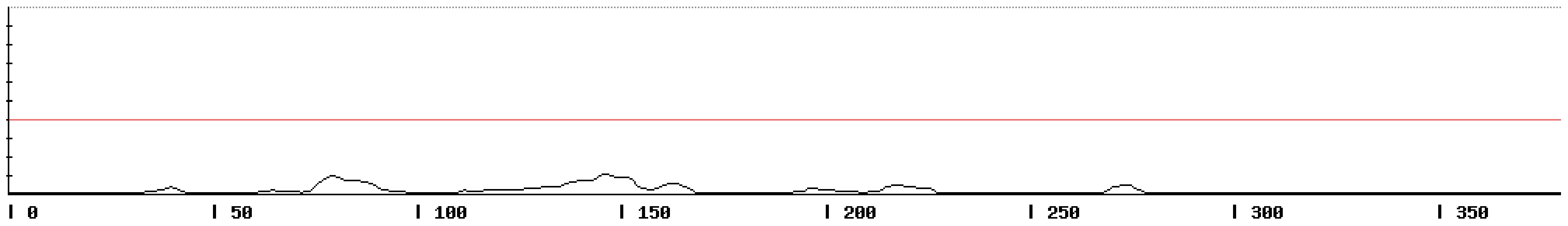

Predictions for Aegilopstauschii_EMT28295

Viterbi Path

Viterbi Path

Posterior @ 0.4

Posterior @ 0.4

Posterior Graphic. Threshold Marked @ 0.4

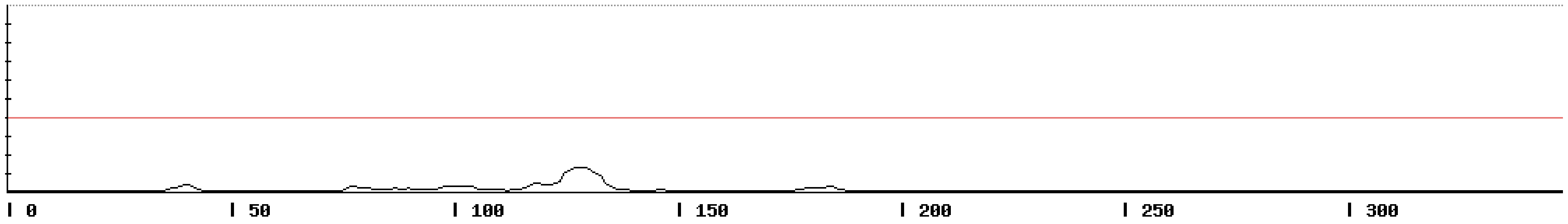

Predictions for Aegilopstauschii_EMT28787

Viterbi Path

Viterbi Path

Posterior @ 0.4 388 - KKRKK - 392

Posterior @ 0.4

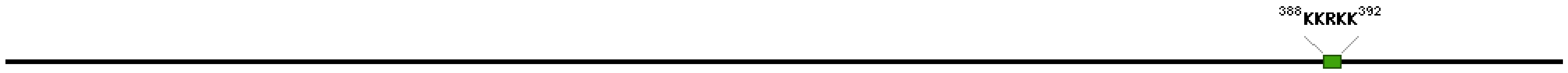

Posterior Graphic. Threshold Marked @ 0.4

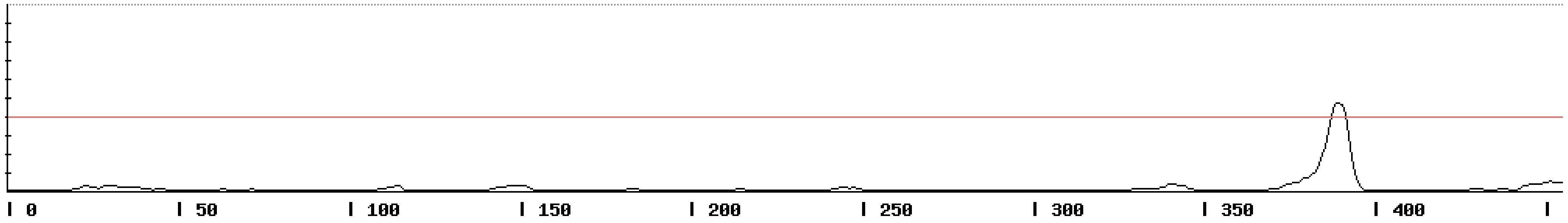

Predictions for Aegilopstauschii_EMT28823

Viterbi Path

Viterbi Path

Posterior @ 0.4

Posterior @ 0.4

Posterior Graphic. Threshold Marked @ 0.4

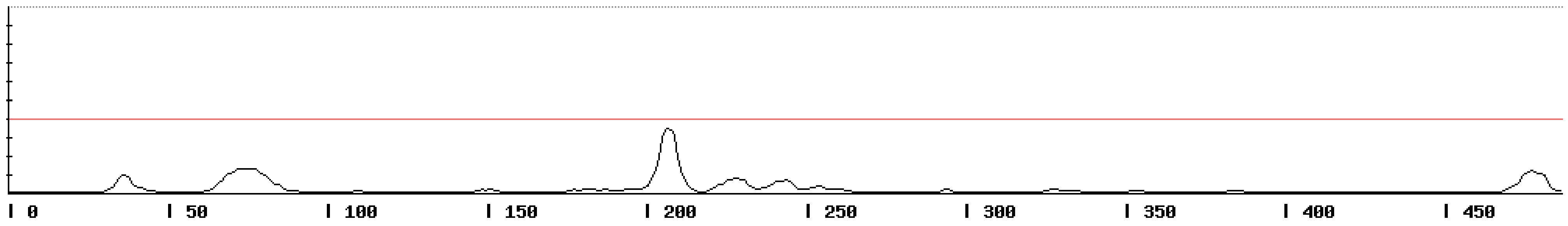

Predictions for Aegilopstauschii_EMT28859

Viterbi Path

Viterbi Path

Posterior @ 0.4

Posterior @ 0.4

Posterior Graphic. Threshold Marked @ 0.4

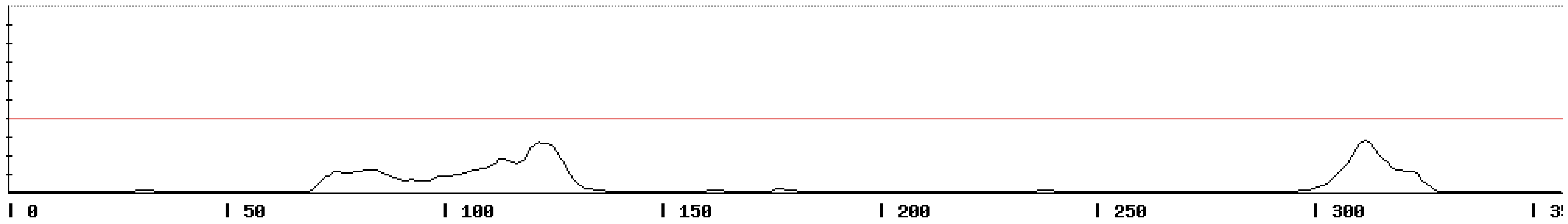

Predictions for Aegilopstauschii_EMT29234	
Viterbi Path	
Viterbi Path	
Posterior @ 0.4	
Posterior @ 0.4	
Posterior Graphic. Threshold Marked @ 0.4	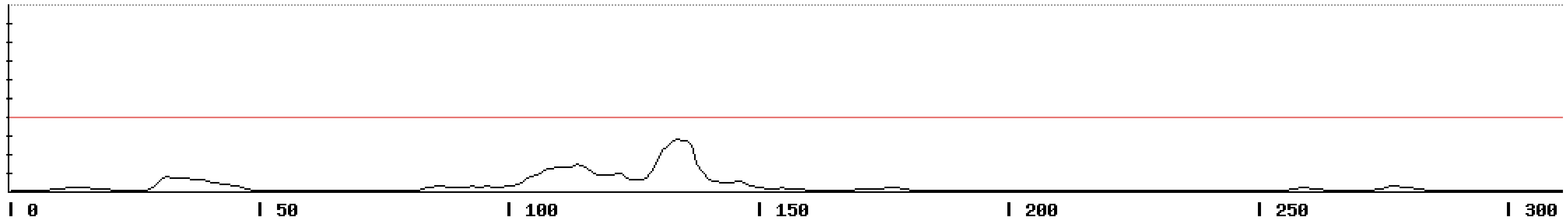

Predictions for Aegilopstauschii_EMT29472	
Viterbi Path	
Viterbi Path	
Posterior @ 0.4	
Posterior @ 0.4	
Posterior Graphic. Threshold Marked @ 0.4	

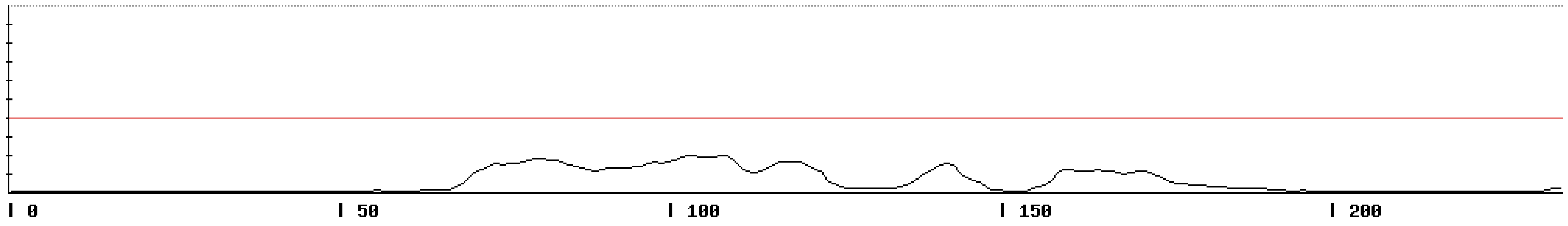

Predictions for Aegilopstauschii_EMT29816

Viterbi Path	
Viterbi Path	
Posterior @ 0.4	
Posterior @ 0.4	
Posterior Graphic. Threshold Marked @ 0.4	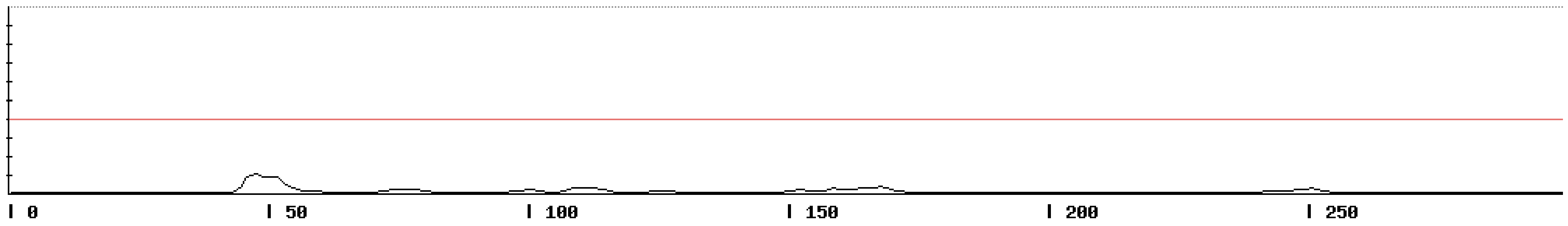

Predictions for Aegilopstauschii_EMT29965

Viterbi Path	
Viterbi Path	
Posterior @ 0.4	
Posterior @ 0.4	

Posterior Graphic. Threshold Marked @ 0.4

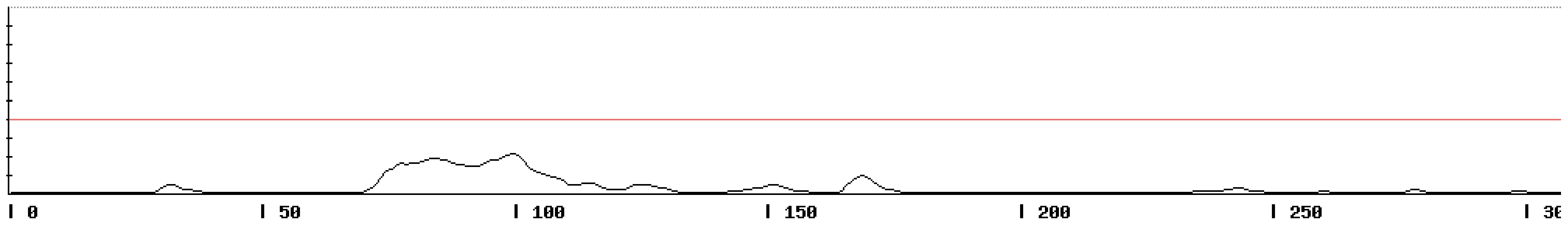

Predictions for Aegilopstauschii_EMT30422

Viterbi Path

Viterbi Path

Posterior @ 0.4

Posterior @ 0.4

Posterior Graphic. Threshold Marked @ 0.4

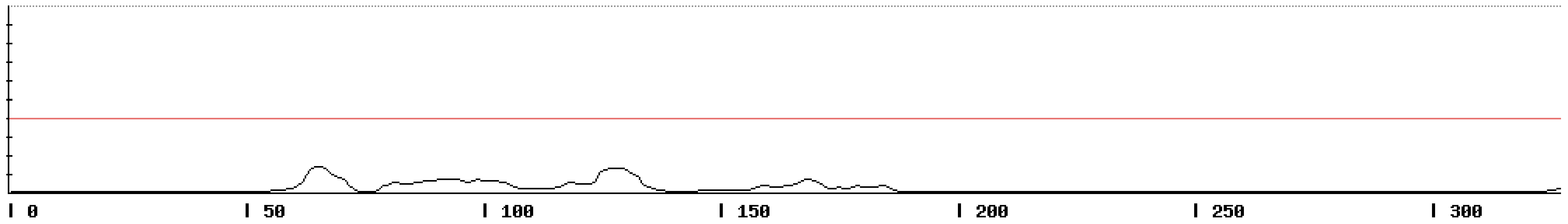

Predictions for Aegilopstauschii_EMT30514

Viterbi Path

Viterbi Path

Posterior @ 0.4

Posterior @ 0.4

Posterior Graphic. Threshold Marked @ 0.4

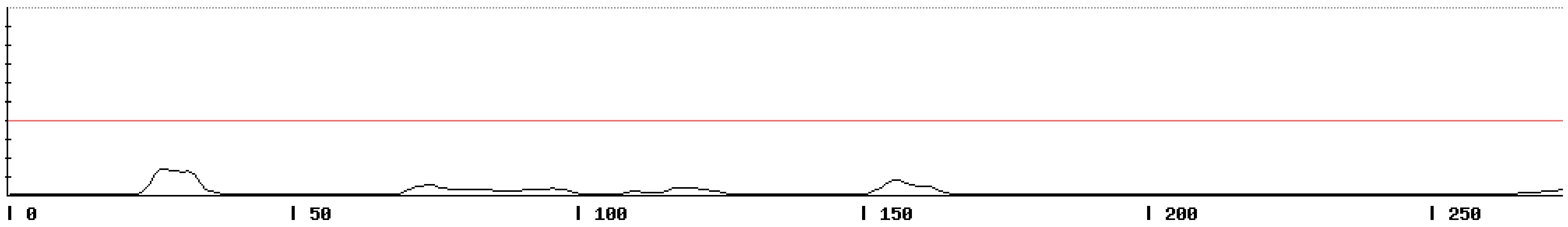

Predictions for Aegilopstauschii_EMT30661

Viterbi Path

Viterbi Path

Posterior @ 0.4

Posterior @ 0.4

Posterior Graphic. Threshold Marked @ 0.4

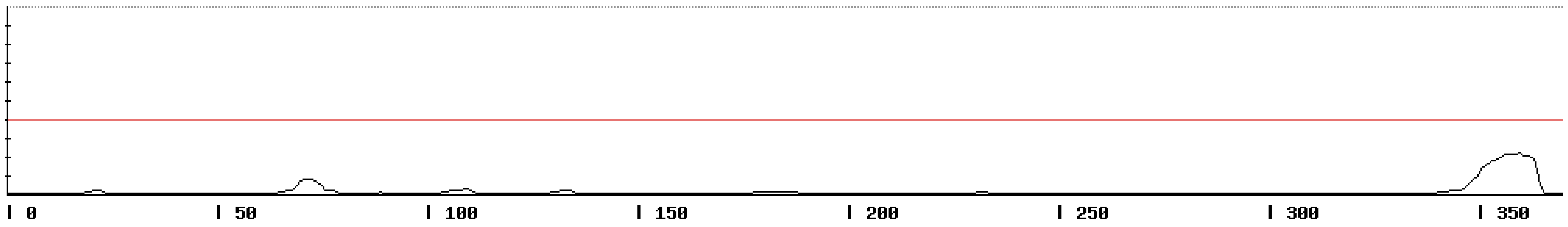

Predictions for Aegilopstauschii_EMT30898

Viterbi Path

Viterbi Path

Posterior @ 0.4

Posterior @ 0.4

Posterior Graphic. Threshold Marked @ 0.4

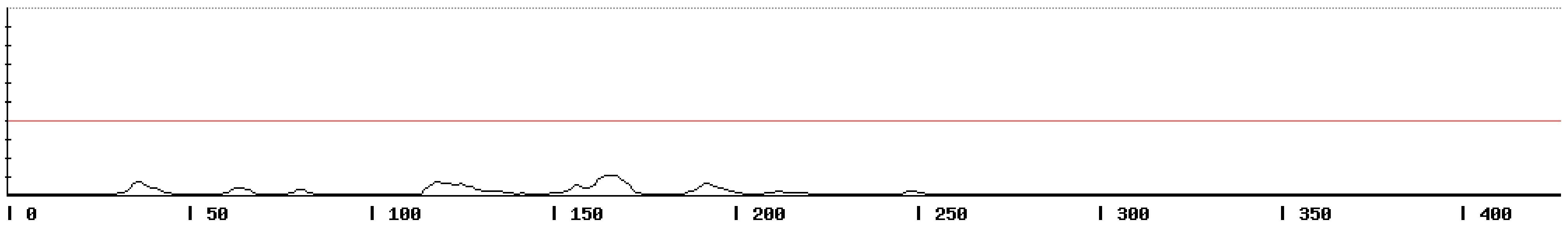

Predictions for Aegilopstauschii_EMT31805

Viterbi Path

Viterbi Path

Posterior @ 0.4 — 120 - RTRRAAAGRAPR - 131

Posterior @ 0.4

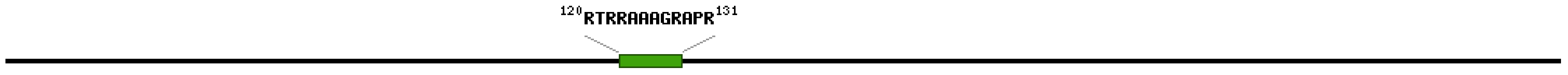

Posterior Graphic. Threshold Marked @ 0.4

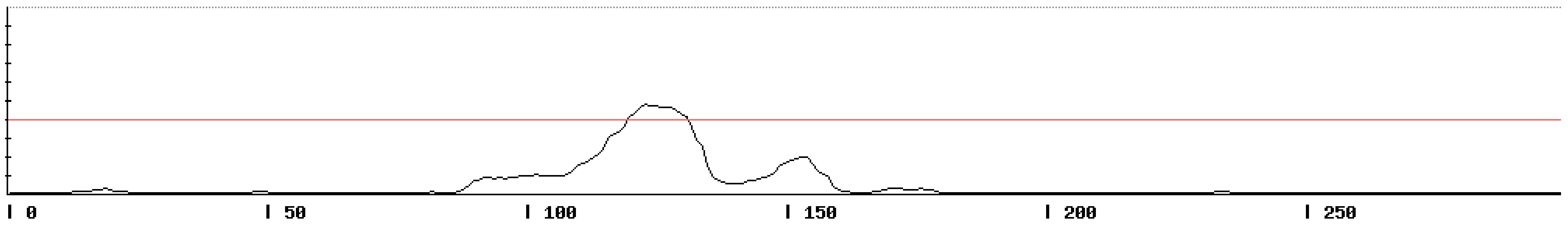

Predictions for Aegilopstauschii_EMT32184

Viterbi Path

Viterbi Path

Posterior @ 0.4

Posterior @ 0.4

Posterior Graphic. Threshold Marked @ 0.4

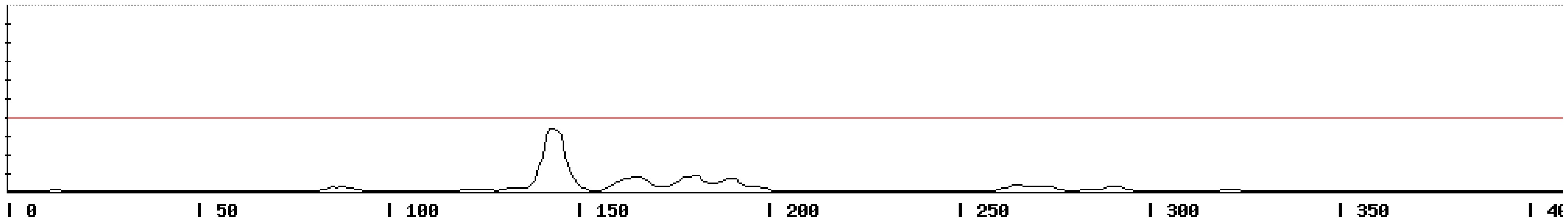

Predictions for Aegilopstauschii_EMT32450

Viterbi Path

Viterbi Path

Posterior @ 0.4

Posterior @ 0.4

Posterior Graphic. Threshold Marked @ 0.4

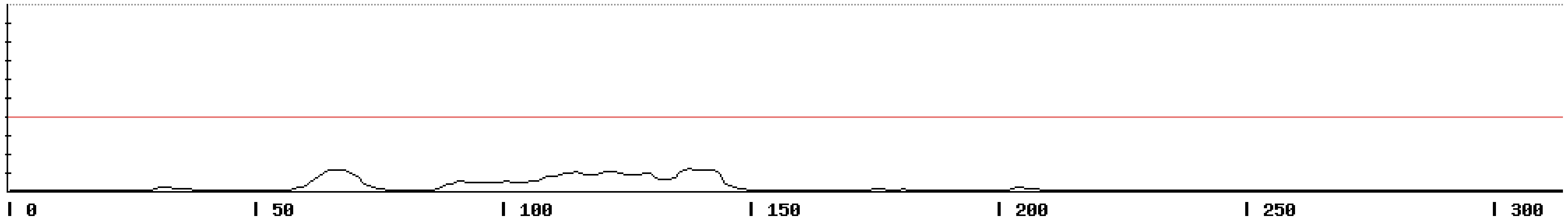

Predictions for Aegilopstauschii_EMT32451

Viterbi Path

Viterbi Path

Posterior @ 0.4

Posterior @ 0.4

Posterior Graphic. Threshold Marked @ 0.4

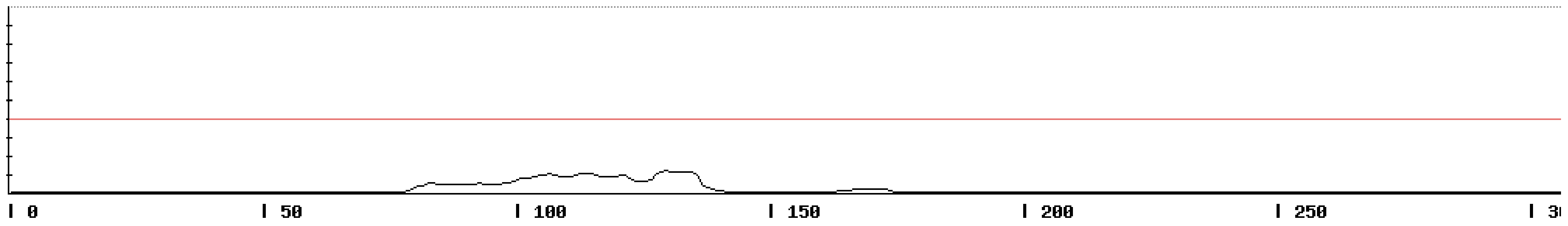

Predictions for Aegilopstauschii_EMT32771

Viterbi Path

Viterbi Path

Posterior @ 0.4

Posterior @ 0.4

Posterior Graphic. Threshold Marked @ 0.4

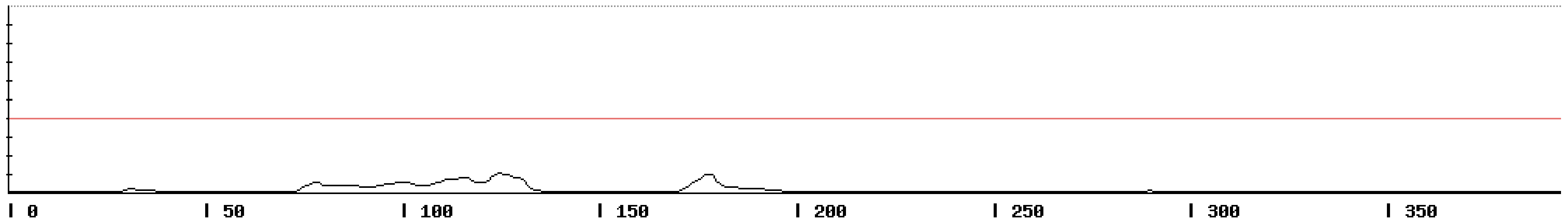

Predictions for Aegilopstauschii_EMT32772	
Viterbi Path	
Viterbi Path	
Posterior @ 0.4	
Posterior @ 0.4	
Posterior Graphic. Threshold Marked @ 0.4	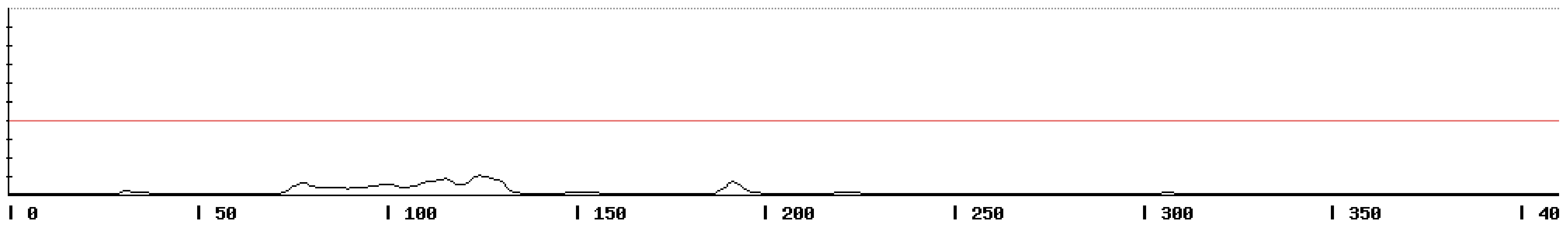

Predictions for Aegilopstauschii_EMT32940	
Viterbi Path	
Viterbi Path	
Posterior @ 0.4	
Posterior @ 0.4	
Posterior Graphic. Threshold Marked @ 0.4	

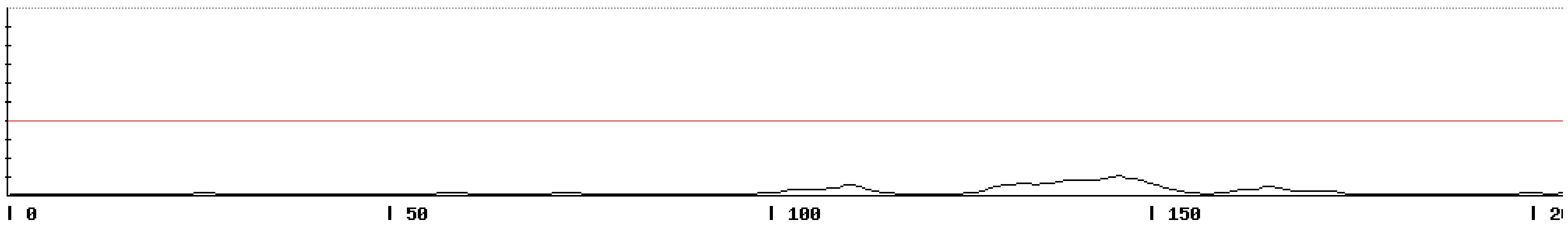

Predictions for Aegilopstauschii_EMT32999	
Viterbi Path	
Viterbi Path	
Posterior @ 0.4	123 - SLAFVPRRGKGKGSKAPP - 140
Posterior @ 0.4	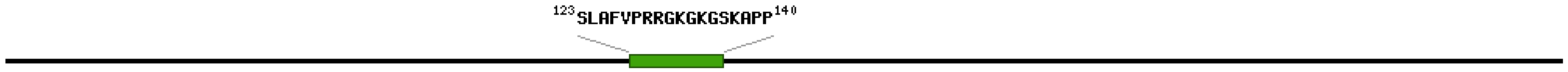
Posterior Graphic. Threshold Marked @ 0.4	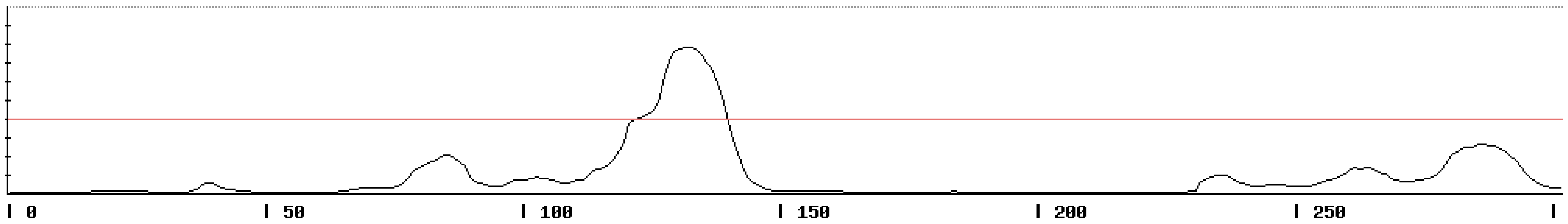

Predictions for Aegilopstauschii_EMT33003	
Viterbi Path	
Viterbi Path	
Posterior @ 0.4	
Posterior @ 0.4	

Posterior Graphic. Threshold Marked @ 0.4	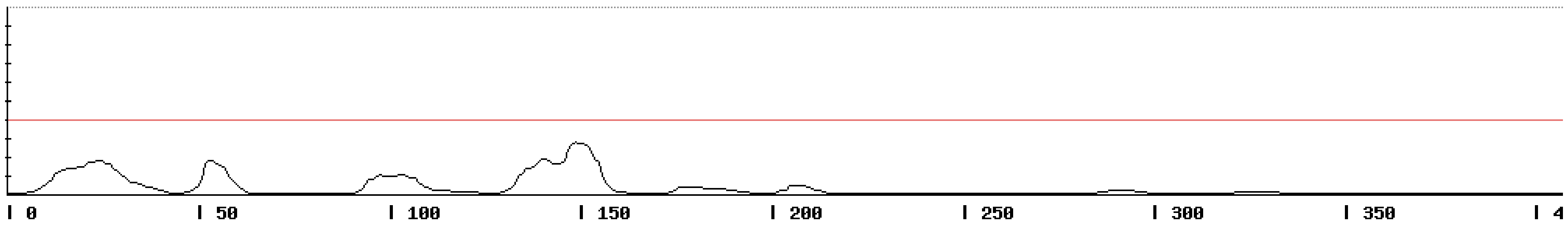

Predictions for Aegilopstauschii_EMT33279	
Viterbi Path	
Viterbi Path	
Posterior @ 0.4	
Posterior @ 0.4	
Posterior Graphic. Threshold Marked @ 0.4	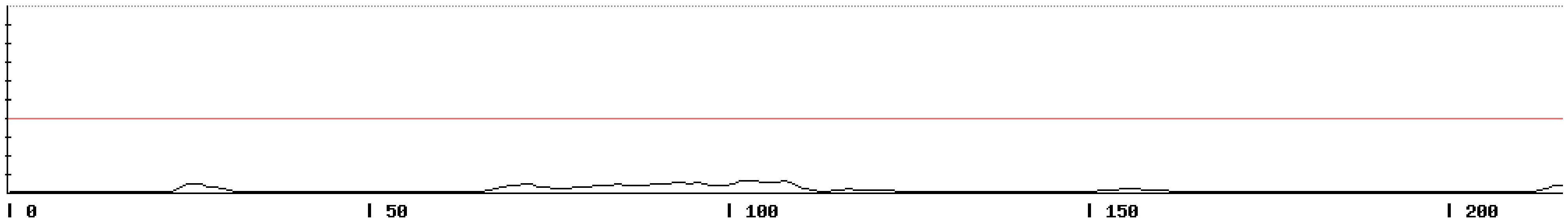

Predictions for Aegilopstauschii_EMT33280	
Viterbi Path	
Viterbi Path	
Posterior @ 0.4	
Posterior @ 0.4	

Posterior Graphic. Threshold Marked @ 0.4

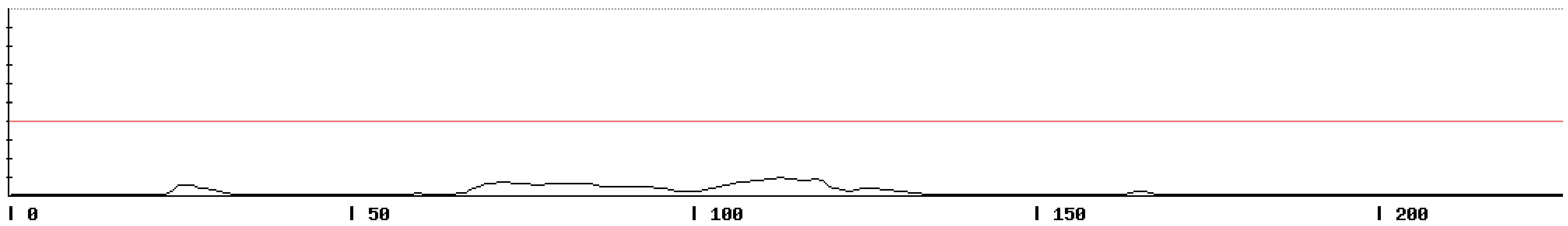

Predictions for Aegilopstauschii_EMT33580

Viterbi Path

Viterbi Path

Posterior @ 0.4

Posterior @ 0.4

Posterior Graphic. Threshold Marked @ 0.4

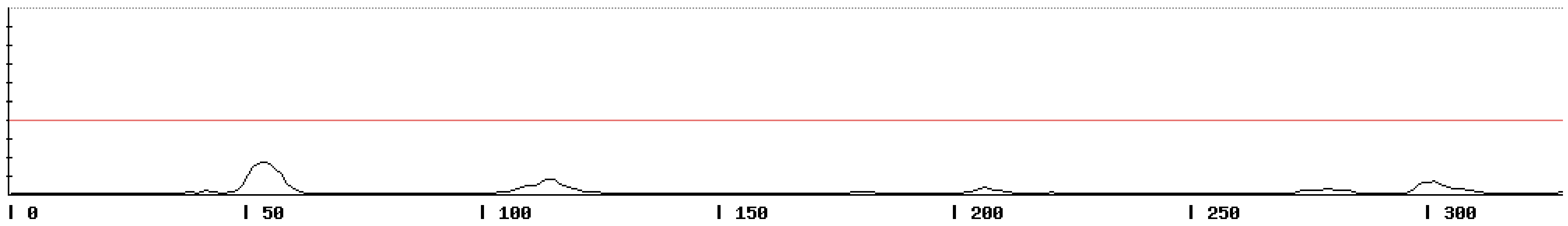

Predictions for Aegilopstauschii_EMT33674

Viterbi Path

Viterbi Path

Posterior @ 0.4	4 - KKPKKEARKLTPR - 16

Revision r.9 - Last Updated on : February 24 2015 17:45:20.

alex.nguyenba@utoronto.ca

Intended to be used with Firefox

Viterbi Path	
Posterior @ 0.4	93 - RKRRK - 97
Posterior @ 0.4	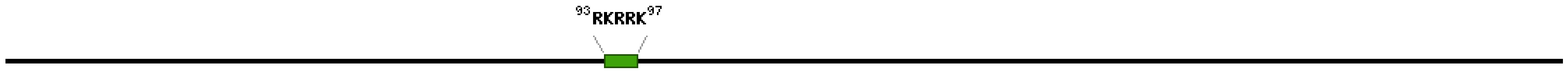
Posterior Graphic. Threshold Marked @ 0.4	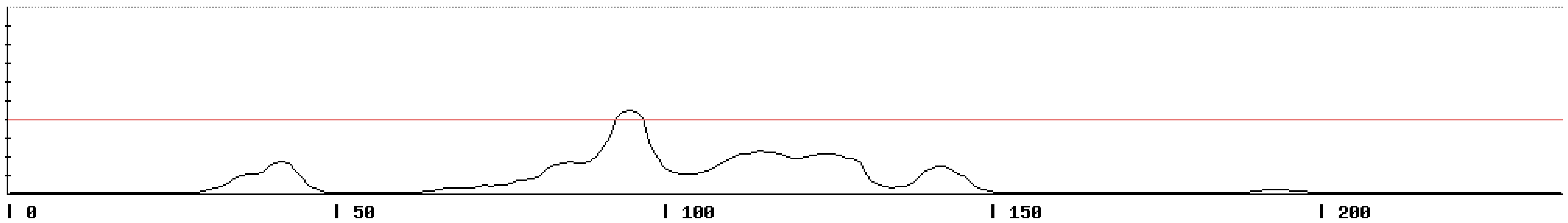

Predictions for Aethionemaarabicum_AA102G00010

Viterbi Path	
Viterbi Path	
Posterior @ 0.4	
Posterior @ 0.4	
Posterior Graphic. Threshold Marked @ 0.4	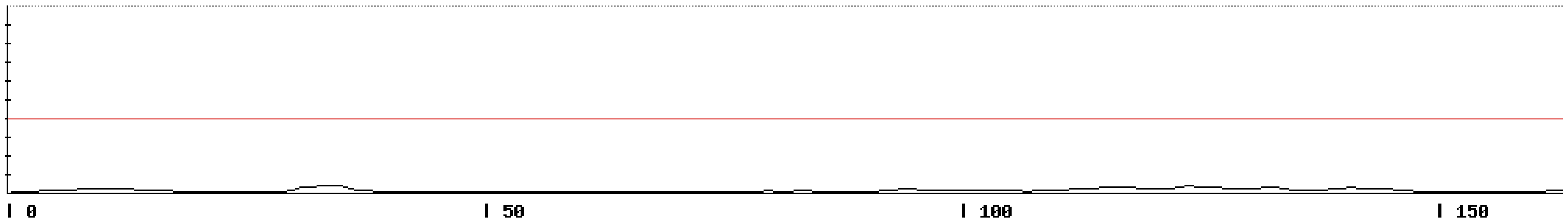

Predictions for Aethionemaarabicum_AA102G00073

Viterbi Path	
Viterbi Path	
Posterior @ 0.4	
Posterior @ 0.4	
Posterior Graphic. Threshold Marked @ 0.4	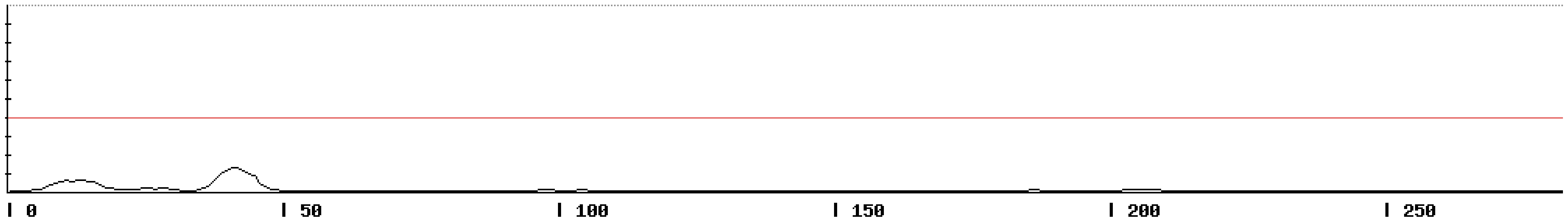

Predictions for Aethionemaarabicum_AA10G00196

Viterbi Path	
Viterbi Path	
Posterior @ 0.4	
Posterior @ 0.4	
Posterior Graphic. Threshold Marked @ 0.4	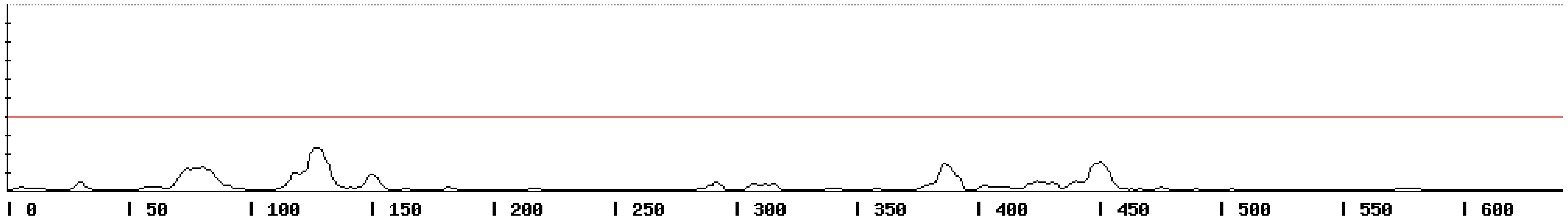

Predictions for Aethionemaarabicum_AA10G00295

Viterbi Path

Viterbi Path

Posterior @ 0.4

Posterior @ 0.4

Posterior Graphic. Threshold Marked @ 0.4

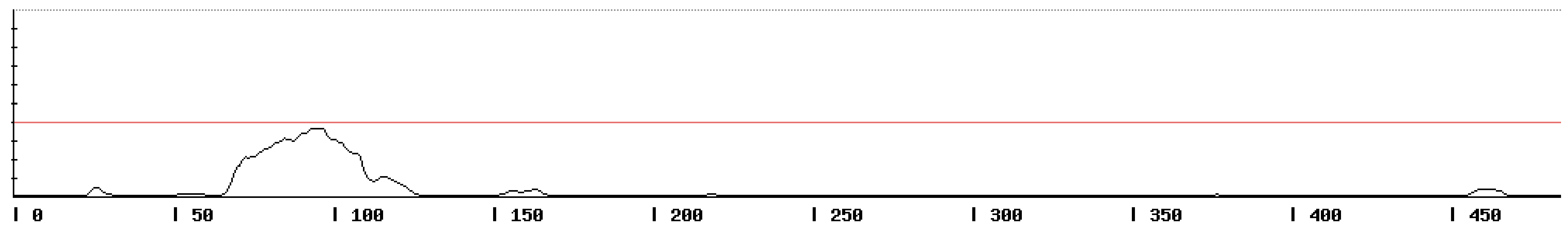

Predictions for Aethionemaarabicum_AA11G00017

Viterbi Path

Viterbi Path

Posterior @ 0.4

Posterior @ 0.4

Posterior Graphic. Threshold Marked @ 0.4

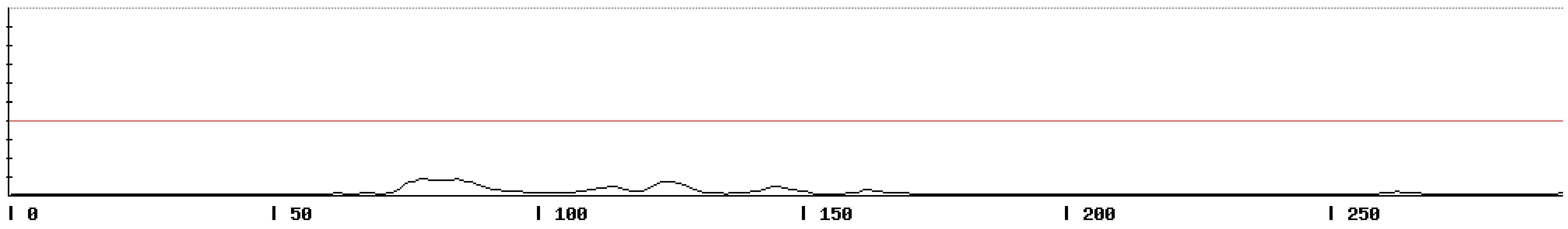

Predictions for Aethionemaarabicum_AA11G00058

Viterbi Path

Viterbi Path

Posterior @ 0.4

Posterior @ 0.4

Posterior Graphic. Threshold Marked @ 0.4

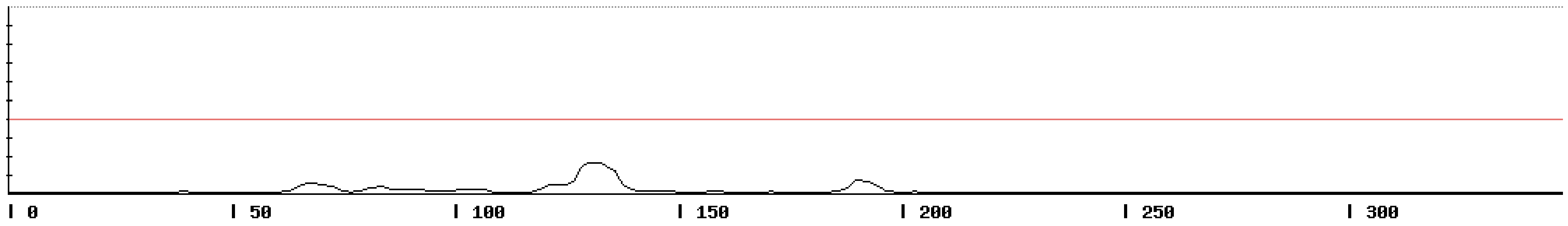

Predictions for Aethionemaarabicum_AA128G00002

Viterbi Path

Viterbi Path

Posterior @ 0.4

Posterior @ 0.4

Posterior Graphic. Threshold Marked @ 0.4

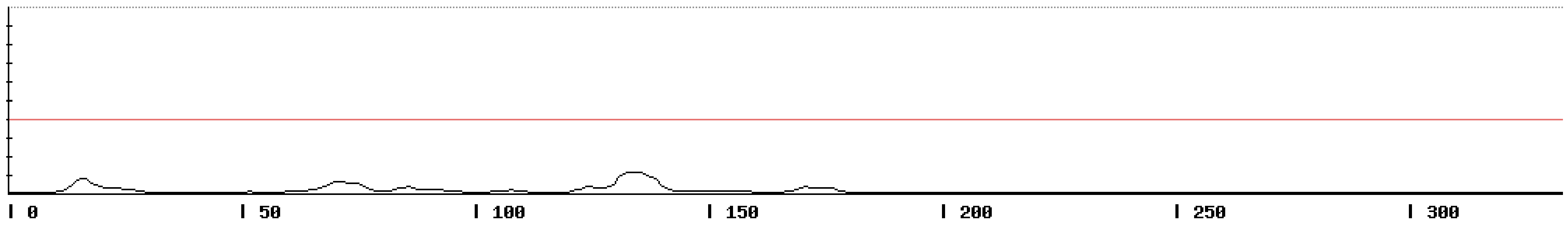

Predictions for Aethionemaarabicum_AA12G00161

Viterbi Path

Viterbi Path

Posterior @ 0.4

Posterior @ 0.4

Posterior Graphic. Threshold Marked @ 0.4

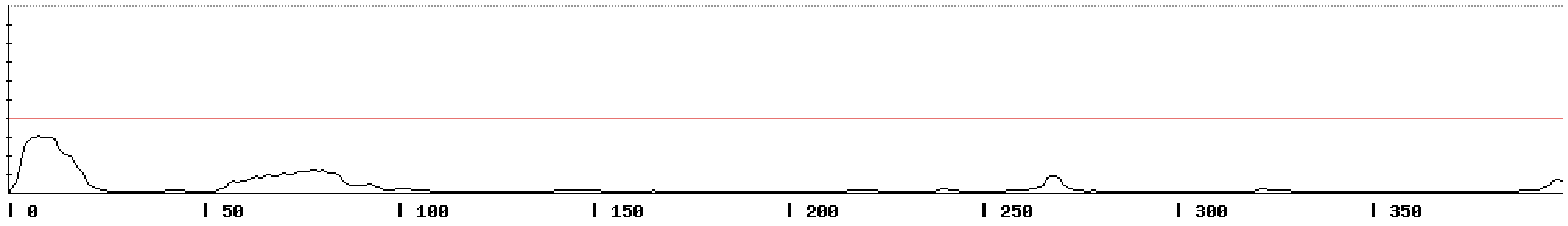

Predictions for Aethionemaarabicum_AA13G00126

Viterbi Path

Viterbi Path

Posterior @ 0.4 98 - ATGKDRKIKSGKTKIIGVKR - 117

Posterior @ 0.4

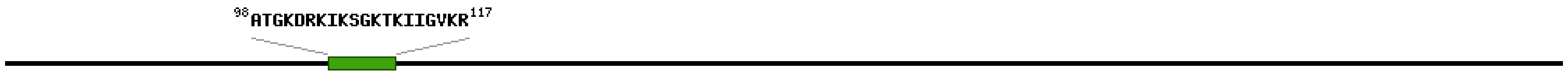

Posterior Graphic. Threshold Marked @ 0.4

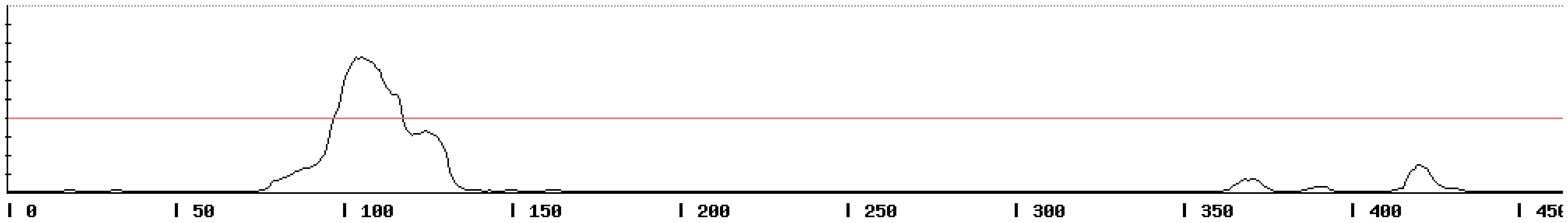

Predictions for Aethionemaarabicum_AA15G00071

Viterbi Path

Viterbi Path

Posterior @ 0.4

Posterior @ 0.4

Posterior Graphic. Threshold Marked @ 0.4

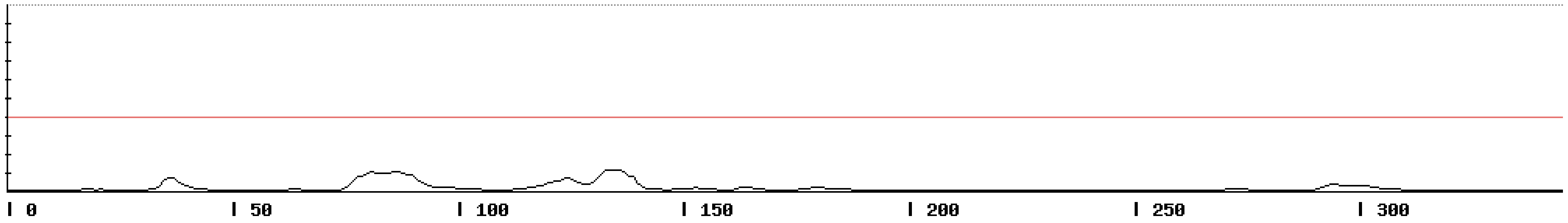

Predictions for Aethionemaarabicum_AA15G00326

Viterbi Path 190 - KEEKKKKKRKWRSRSRSR - 207

Viterbi Path

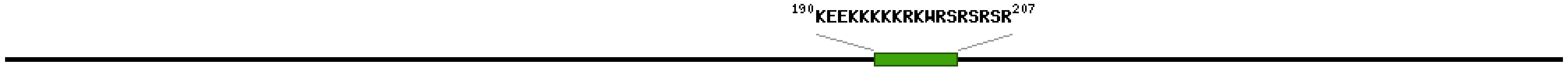

Posterior @ 0.4	103 - SKKVK - 107
	189 - EKEEKKKKKRKWRSRSRSR - 207
Posterior @ 0.4	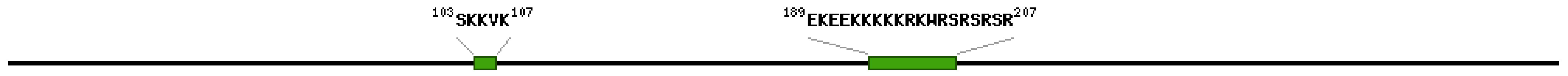
Posterior Graphic. Threshold Marked @ 0.4	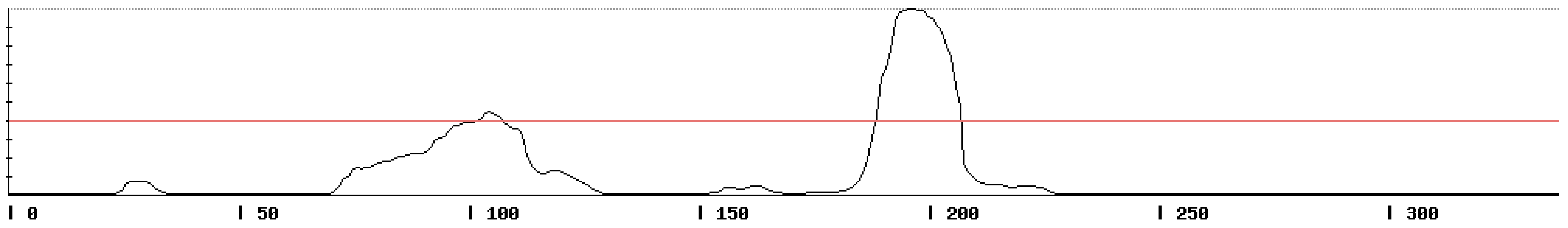

Predictions for Aethionemaarabicum_AA1G00022

Viterbi Path	
Viterbi Path	
Posterior @ 0.4	
Posterior @ 0.4	
Posterior Graphic. Threshold Marked @ 0.4	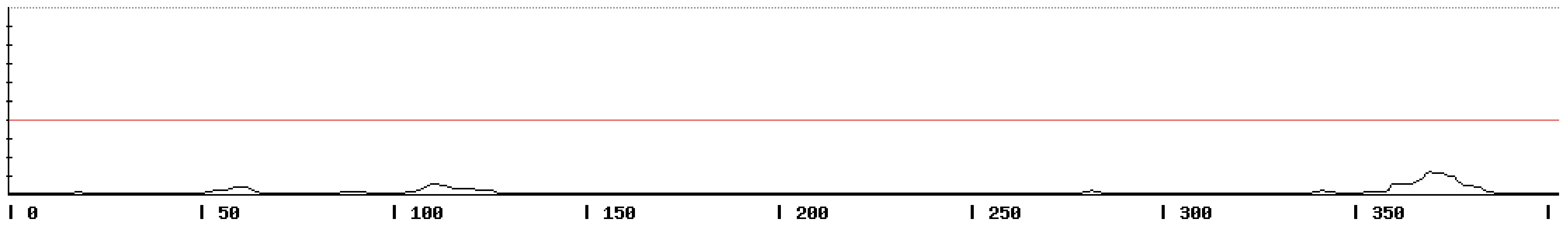

Predictions for Aethionemaarabicum_AA1G00023

Viterbi Path	
Viterbi Path	

Posterior @ 0.4	401 - KIEKKSHKS - 409
Posterior @ 0.4	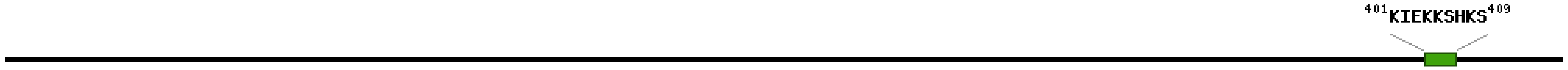
Posterior Graphic. Threshold Marked @ 0.4	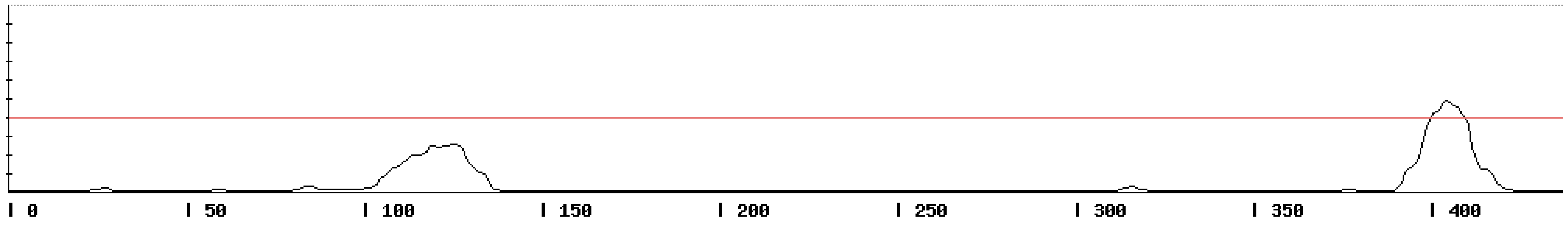

Predictions for Aethionemaarabicum_AA1G00024	
Viterbi Path	
Viterbi Path	
Posterior @ 0.4	
Posterior @ 0.4	
Posterior Graphic. Threshold Marked @ 0.4	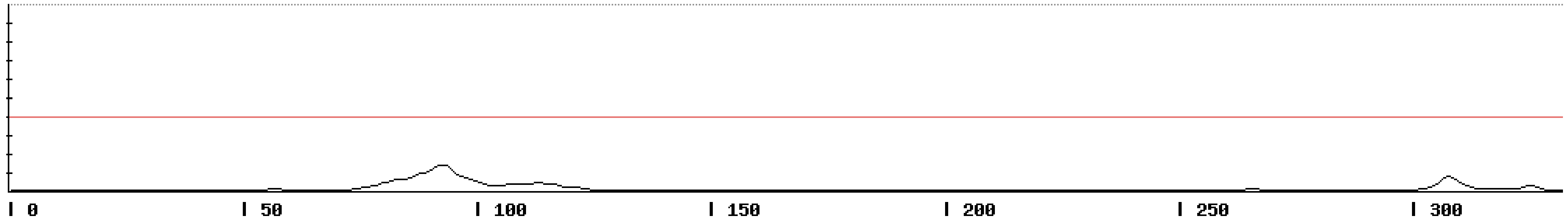

Predictions for Aethionemaarabicum_AA1G00025

Viterbi Path

Viterbi Path

Posterior @ 0.4

Posterior @ 0.4

Posterior Graphic. Threshold Marked @ 0.4

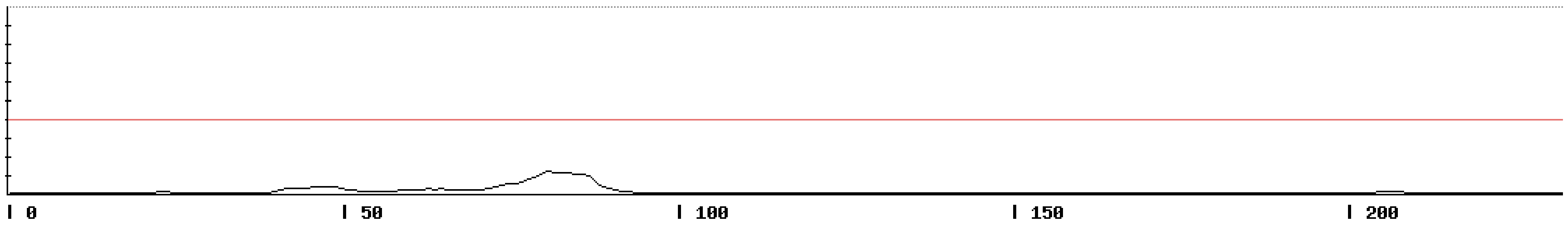

Predictions for Aethionemaarabicum_AA1G00027

Viterbi Path

Viterbi Path

Posterior @ 0.4

Posterior @ 0.4

Posterior Graphic. Threshold Marked @ 0.4

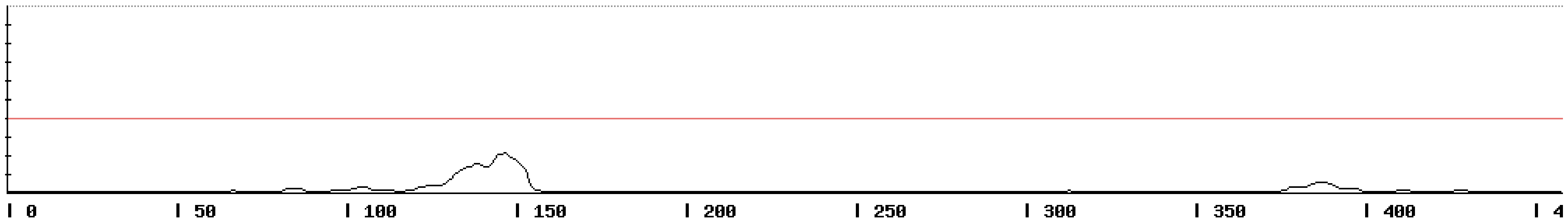

Predictions for Aethionemaarabicum_AA1G00029	
Viterbi Path	
Viterbi Path	
Posterior @ 0.4	
Posterior @ 0.4	
Posterior Graphic. Threshold Marked @ 0.4	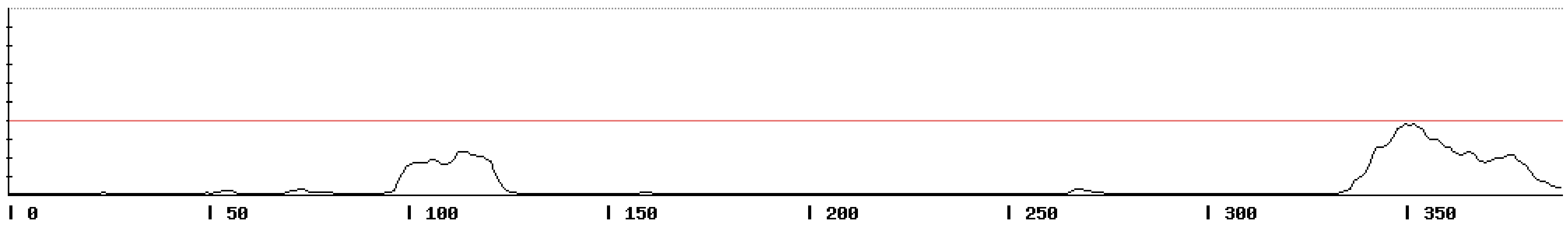

Predictions for Aethionemaarabicum_AA1G00030	
Viterbi Path	217 - KGKEKMKRRWR - 227
Viterbi Path	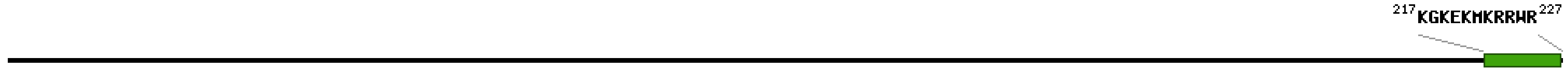
Posterior @ 0.4	215 - ELKGKEKMKRRWR - 227
Posterior @ 0.4	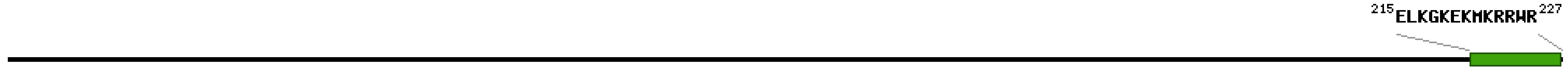
Posterior Graphic. Threshold Marked @ 0.4	

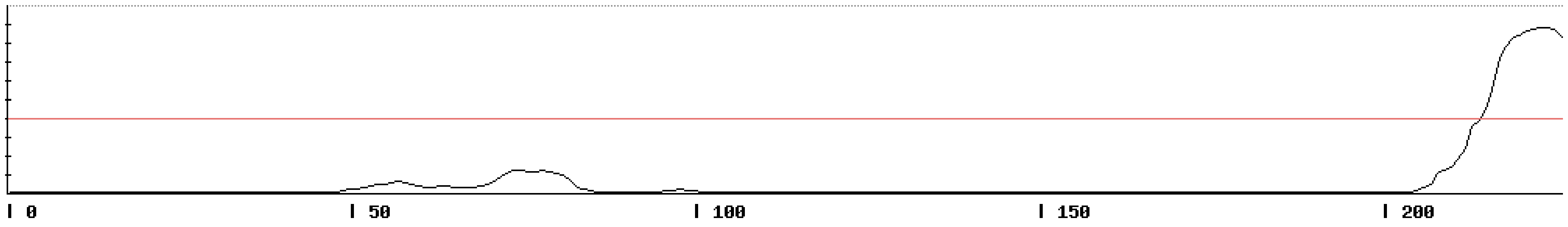

Predictions for Aethionemaarabicum_AA1G00032	
Viterbi Path	
Viterbi Path	
Posterior @ 0.4	71 - KNNRRKRTSRTGHWKITGRDRTIKDFNRKPIGLK - 104
Posterior @ 0.4	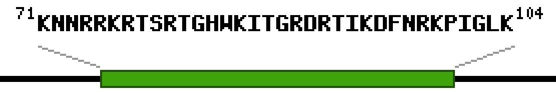
Posterior Graphic. Threshold Marked @ 0.4	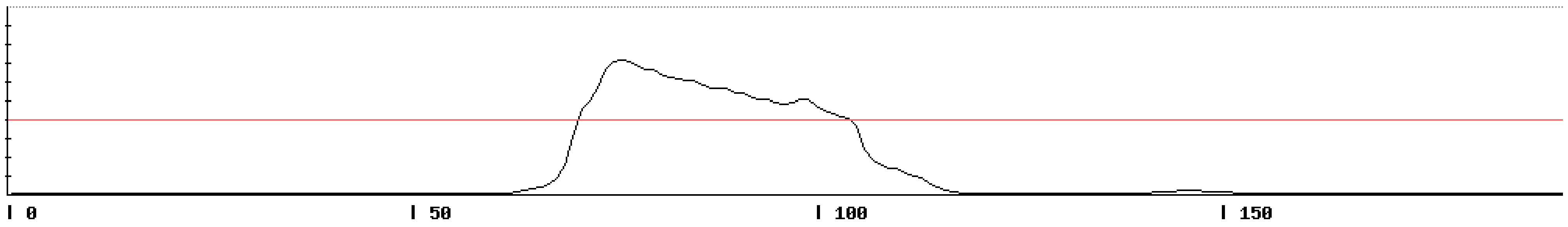

Predictions for Aethionemaarabicum_AA1G00033	
Viterbi Path	
Viterbi Path	
Posterior @ 0.4	105 - KKILRFHKGRIARRGK - 120
Posterior @ 0.4	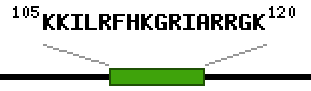

Posterior Graphic. Threshold Marked @ 0.4

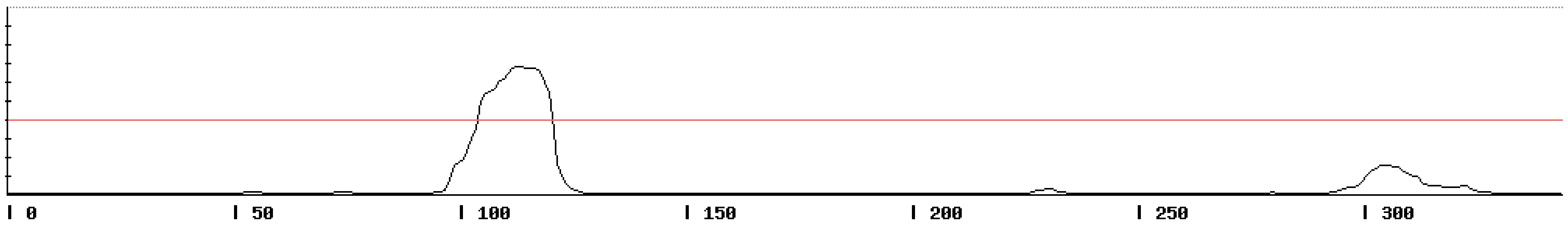

Predictions for Aethionemaarabicum_AA1G00095

Viterbi Path	
Viterbi Path	
Posterior @ 0.4	
Posterior @ 0.4	
Posterior Graphic. Threshold Marked @ 0.4	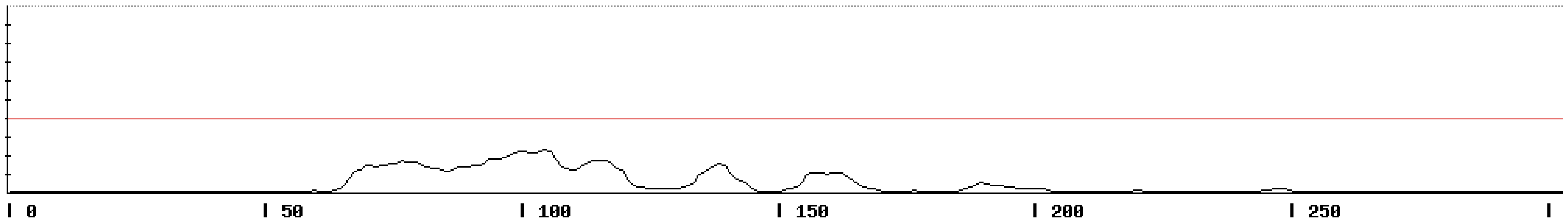

Predictions for Aethionemaarabicum_AA204G00012

Viterbi Path	
Viterbi Path	
Posterior @ 0.4	195 - KGRR - 198
Posterior @ 0.4	

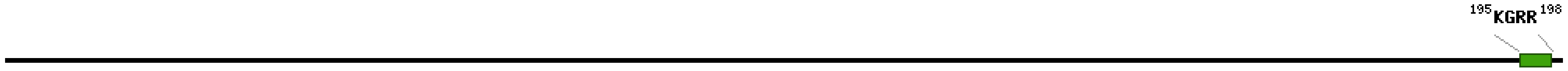

Posterior Graphic. Threshold Marked @ 0.4

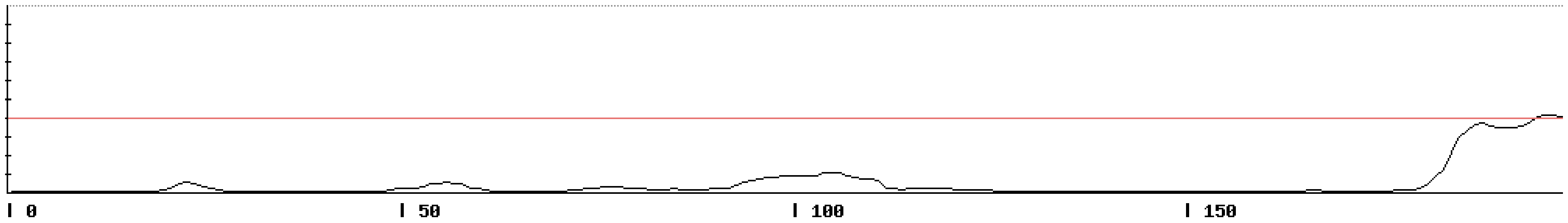

Predictions for Aethionemaarabicum_AA2093G00001

Viterbi Path

Viterbi Path

Posterior @ 0.4

Posterior @ 0.4

Posterior Graphic. Threshold Marked @ 0.4

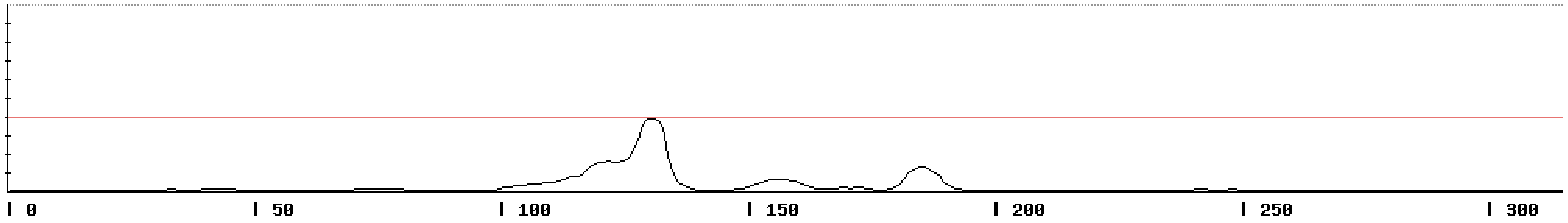

Predictions for Aethionemaarabicum_AA21G00088

Viterbi Path

Viterbi Path

Posterior @ 0.4

Posterior @ 0.4

Posterior Graphic. Threshold Marked @ 0.4

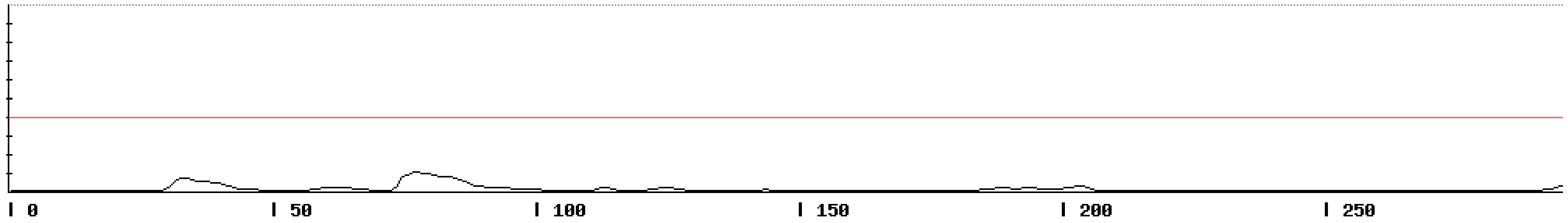

Predictions for Aethionemaarabicum_AA21G00405

Viterbi Path

Viterbi Path

Posterior @ 0.4

Posterior @ 0.4

Posterior Graphic. Threshold Marked @ 0.4

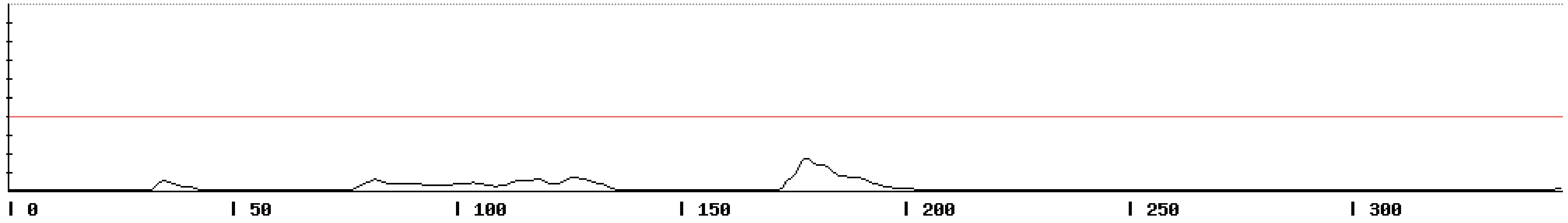

Predictions for Aethionemaarabicum_AA24G00045

Viterbi Path

Viterbi Path

Posterior @ 0.4

Posterior @ 0.4

Posterior Graphic. Threshold Marked @ 0.4

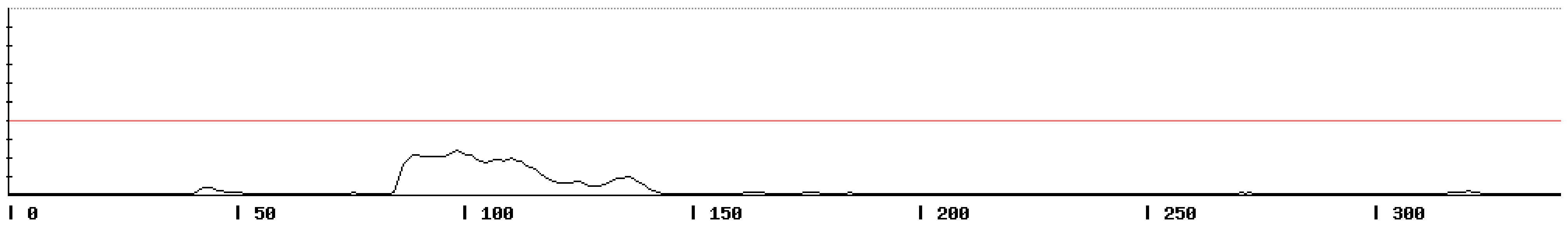

Predictions for Aethionemaarabicum_AA26G00012

Viterbi Path

Viterbi Path

Posterior @ 0.4

Posterior @ 0.4

Posterior Graphic. Threshold Marked @ 0.4

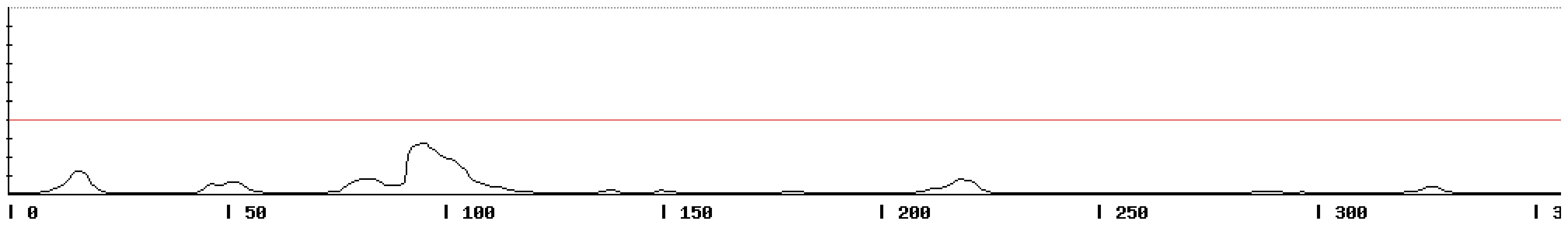

Predictions for Aethionemaarabicum_AA26G00019

Viterbi Path

Viterbi Path

Posterior @ 0.4

Posterior @ 0.4

Posterior Graphic. Threshold Marked @ 0.4

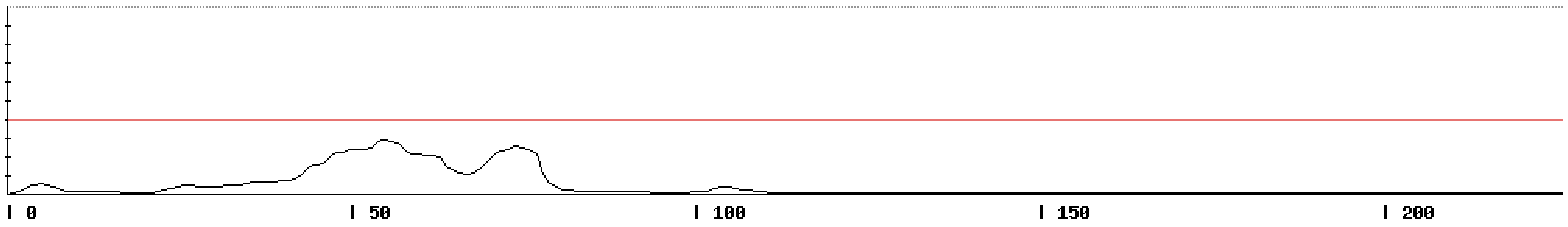

Predictions for Aethionemaarabicum_AA26G00226

Viterbi Path

Viterbi Path

Posterior @ 0.4

Posterior @ 0.4

Posterior Graphic. Threshold Marked @ 0.4

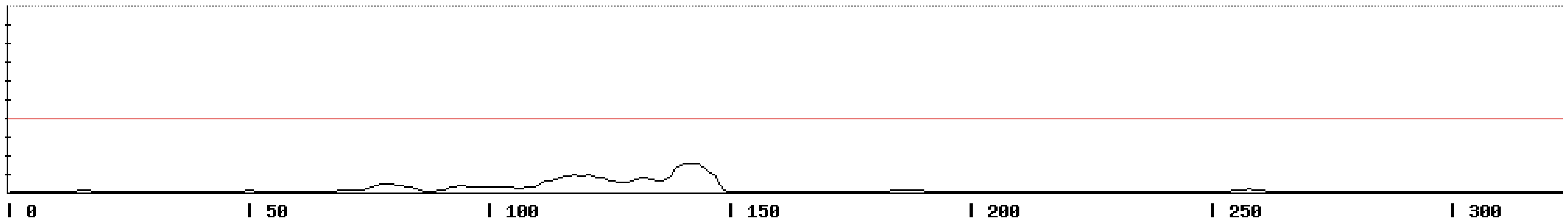

Predictions for Aethionemaarabicum_AA26G00262	
Viterbi Path	
Viterbi Path	
Posterior @ 0.4	
Posterior @ 0.4	
Posterior Graphic. Threshold Marked @ 0.4	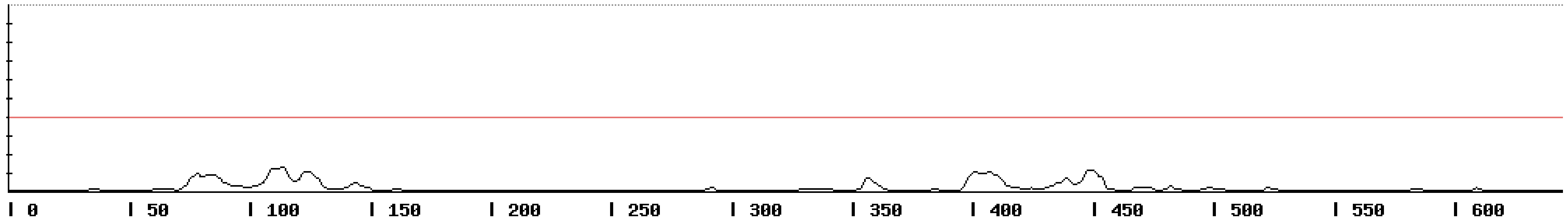

Predictions for Aethionemaarabicum_AA26G00448	
Viterbi Path	
Viterbi Path	
Posterior @ 0.4	
Posterior @ 0.4	
Posterior Graphic. Threshold Marked @ 0.4	

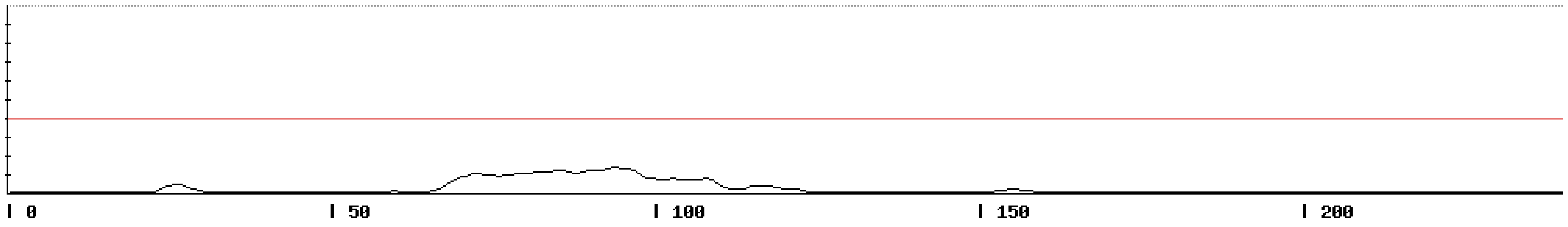

Predictions for Aethionemaarabicum_AA26G00510

Viterbi Path

Viterbi Path

Posterior @ 0.4

Posterior @ 0.4

Posterior Graphic. Threshold Marked @ 0.4

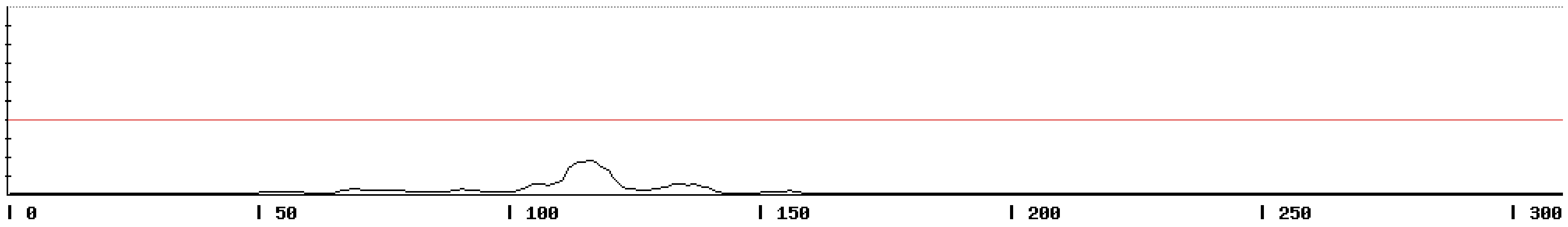

Predictions for Aethionemaarabicum_AA30G00135

Viterbi Path

Viterbi Path

Posterior @ 0.4

Posterior @ 0.4

Posterior Graphic. Threshold Marked @ 0.4	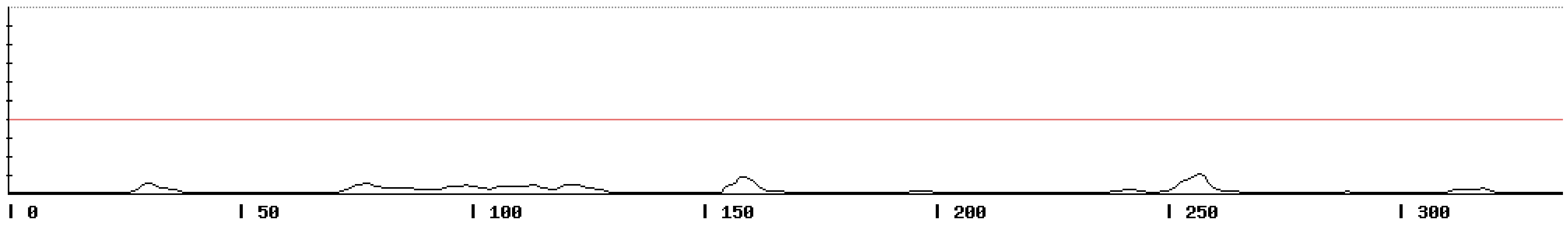

Predictions for Aethionemaarabicum_AA31G00119

Viterbi Path	
Viterbi Path	
Posterior @ 0.4	
Posterior @ 0.4	
Posterior Graphic. Threshold Marked @ 0.4	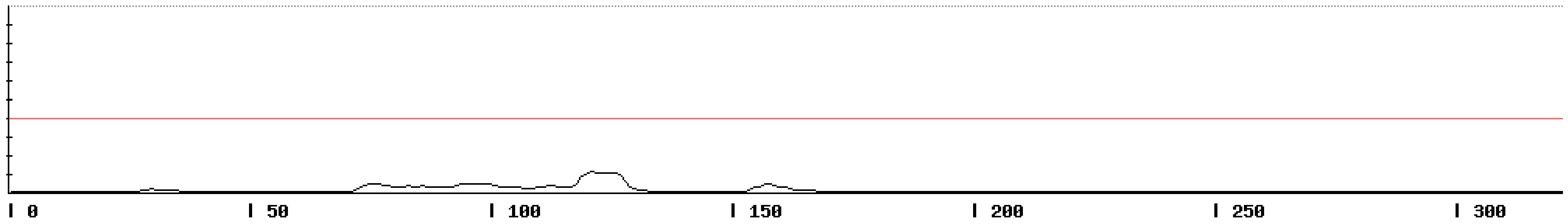

Predictions for Aethionemaarabicum_AA31G00236

Viterbi Path	
Viterbi Path	
Posterior @ 0.4	111 - GADKPIGRPKPVGIKKA - 127
Posterior @ 0.4	

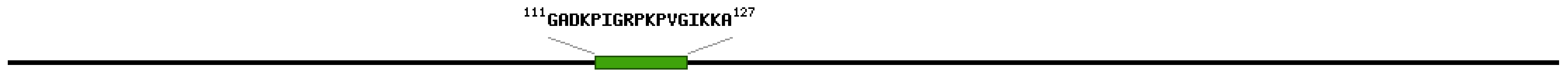

Posterior Graphic. Threshold Marked @ 0.4

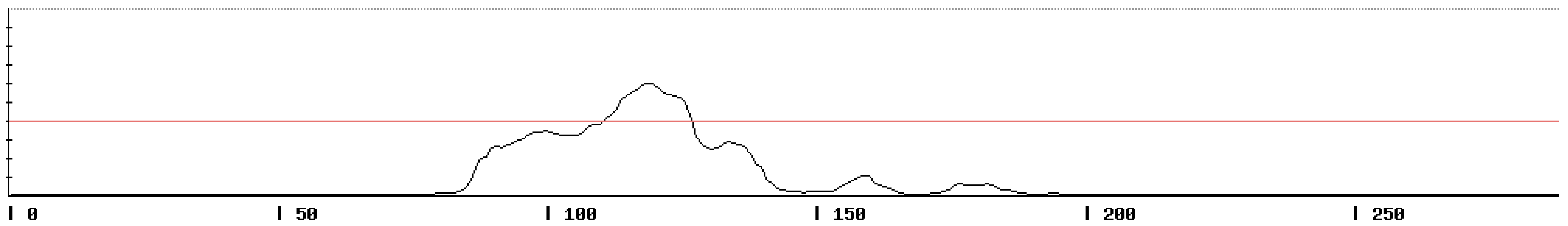

Predictions for Aethionemaarabicum_AA31G00322	
Viterbi Path	
Viterbi Path	
Posterior @ 0.4	
Posterior @ 0.4	
Posterior Graphic. Threshold Marked @ 0.4	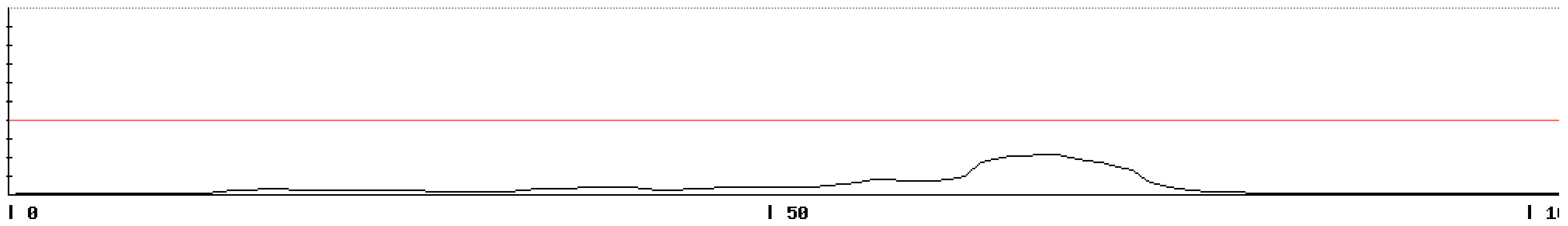

Predictions for Aethionemaarabicum_AA31G00323	
Viterbi Path	
Viterbi Path	

Posterior @ 0.4

Posterior @ 0.4

Posterior Graphic. Threshold Marked @ 0.4

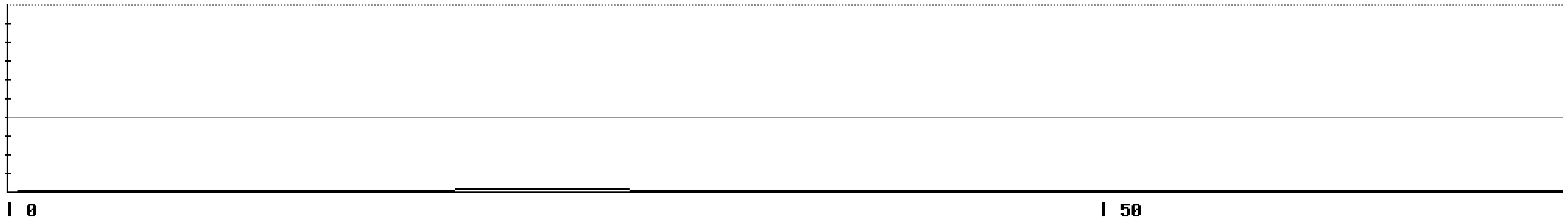

Predictions for Aethionemaarabicum_AA31G00823

Viterbi Path

Viterbi Path

Posterior @ 0.4

Posterior @ 0.4

Posterior Graphic. Threshold Marked @ 0.4

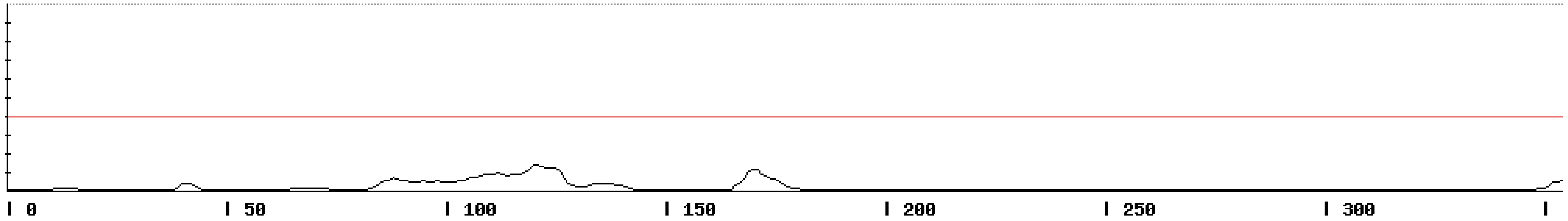

Predictions for Aethionemaarabicum_AA32G00197

Viterbi Path

Viterbi Path

Posterior @ 0.4	82 - GKKKGTKRTTRSGYWKATGVDRKIKDKRGNRSEIG - 116
Posterior @ 0.4	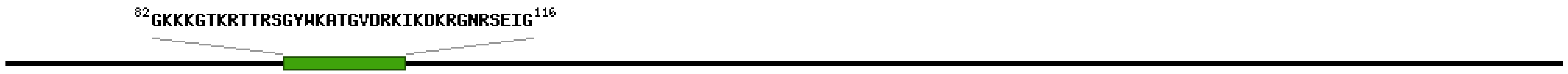
Posterior Graphic. Threshold Marked @ 0.4	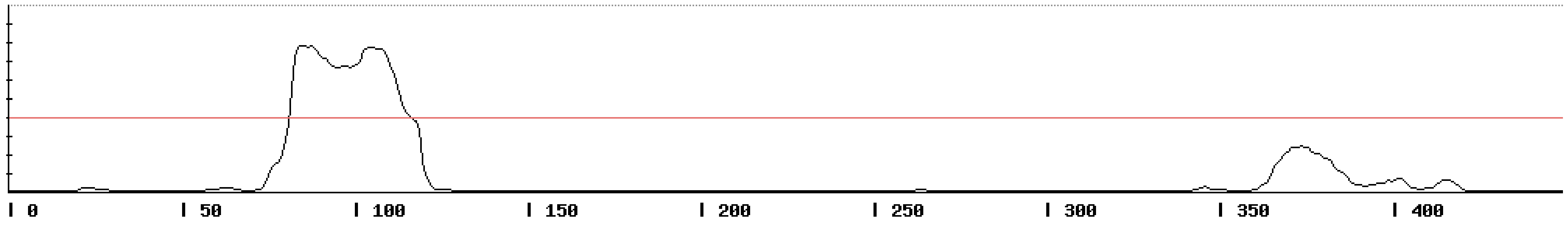

Predictions for Aethionemaarabicum_AA32G00958

Viterbi Path	
Viterbi Path	
Posterior @ 0.4	
Posterior @ 0.4	
Posterior Graphic. Threshold Marked @ 0.4	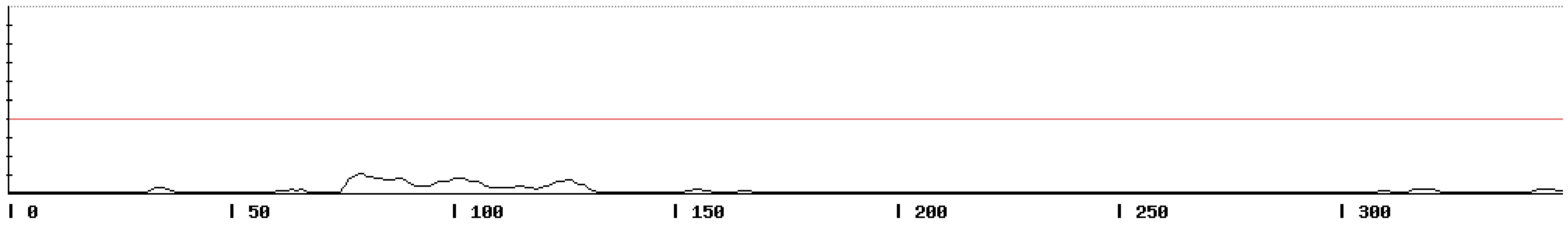

Predictions for Aethionemaarabicum_AA32G01100

Viterbi Path

Viterbi Path

Posterior @ 0.4

Posterior @ 0.4

Posterior Graphic. Threshold Marked @ 0.4

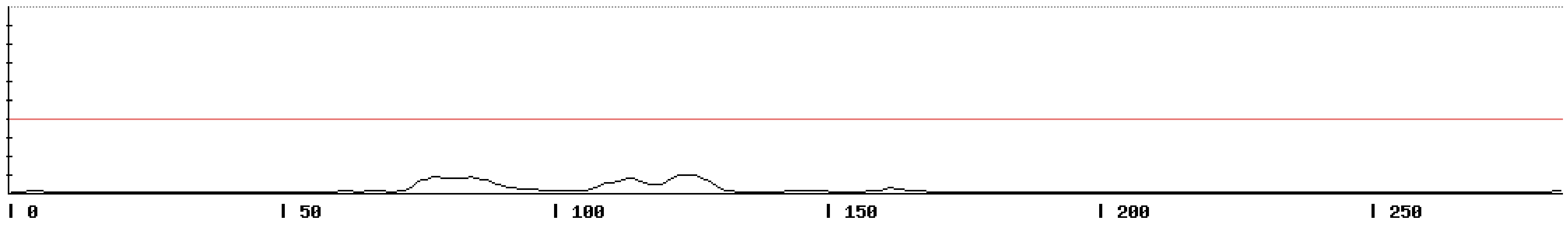

Predictions for Aethionemaarabicum_AA32G01165

Viterbi Path

Viterbi Path

Posterior @ 0.4

Posterior @ 0.4

Posterior Graphic. Threshold Marked @ 0.4

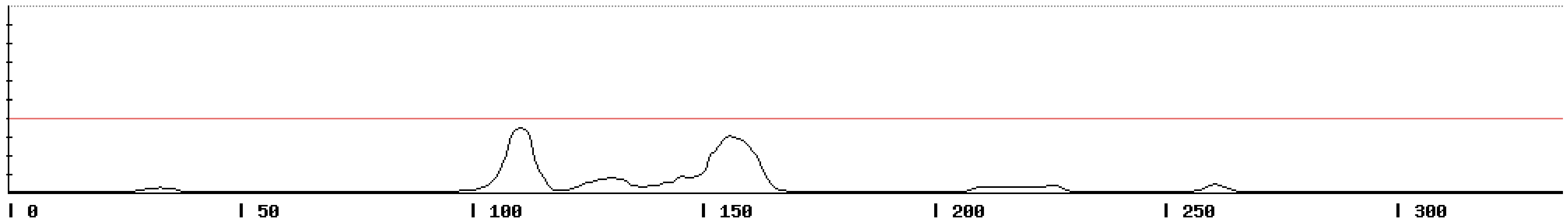

Predictions for Aethionemaarabicum_AA33G00018	
Viterbi Path	
Viterbi Path	
Posterior @ 0.4	
Posterior @ 0.4	
Posterior Graphic. Threshold Marked @ 0.4	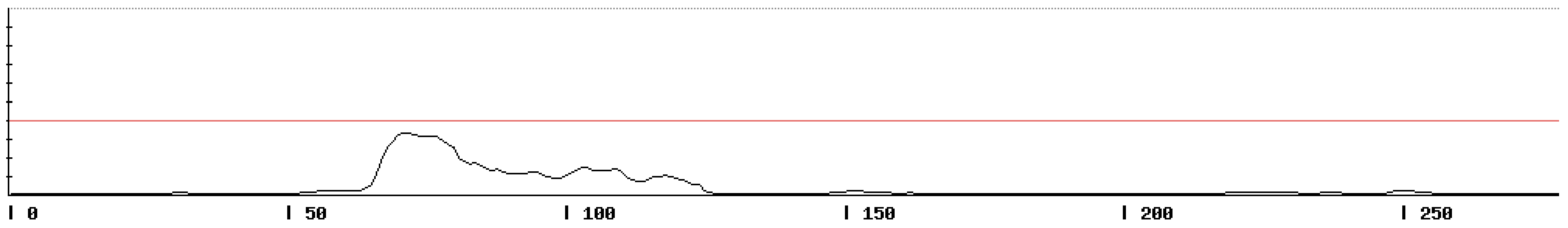

Predictions for Aethionemaarabicum_AA33G00102	
Viterbi Path	
Viterbi Path	
Posterior @ 0.4	244 - IKKKKM - 249
Posterior @ 0.4	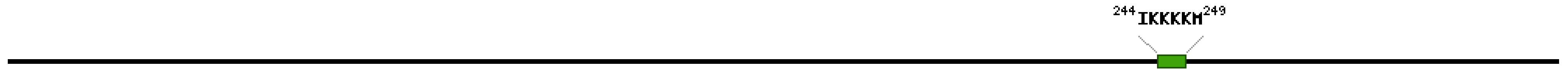
Posterior Graphic. Threshold Marked @ 0.4	

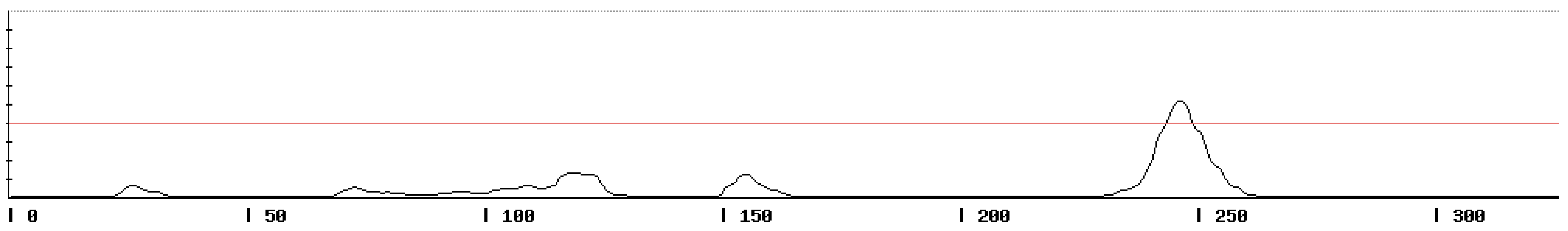

Predictions for Aethionemaarabicum_AA361G00005	
Viterbi Path	
Viterbi Path	
Posterior @ 0.4	154 - R - 154
Posterior @ 0.4	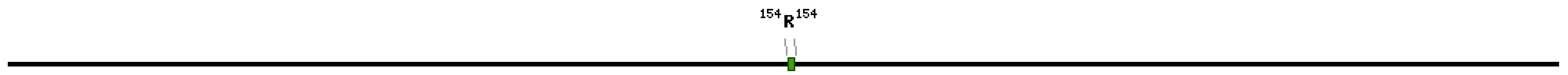
Posterior Graphic. Threshold Marked @ 0.4	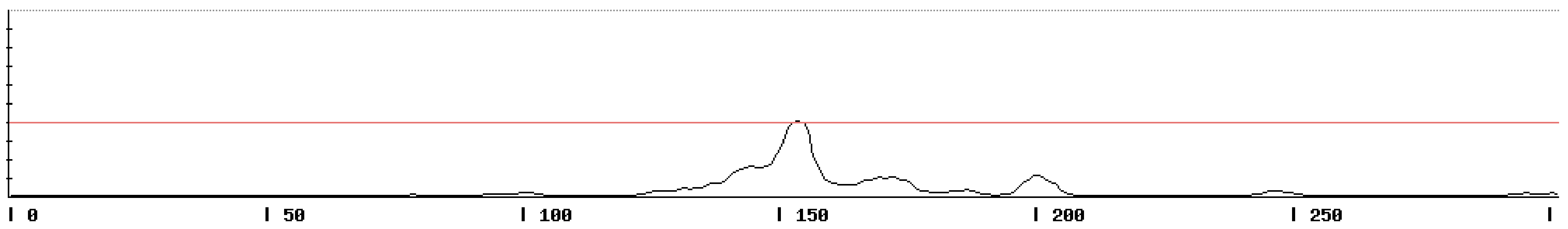

Predictions for Aethionemaarabicum_AA39G00465	
Viterbi Path	
Viterbi Path	
Posterior @ 0.4	470 - SMKRRENNKIKKNK - 483
Posterior @ 0.4	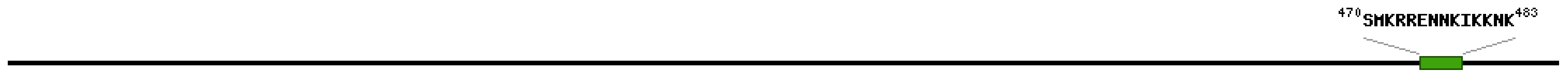

Posterior Graphic. Threshold Marked @ 0.4

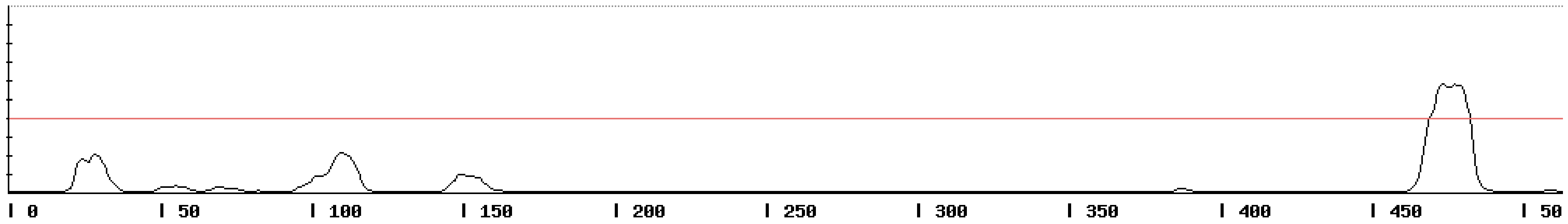

Predictions for Aethionemaarabicum_AA39G00617

Viterbi Path

Viterbi Path

Posterior @ 0.4

Posterior @ 0.4

Posterior Graphic. Threshold Marked @ 0.4

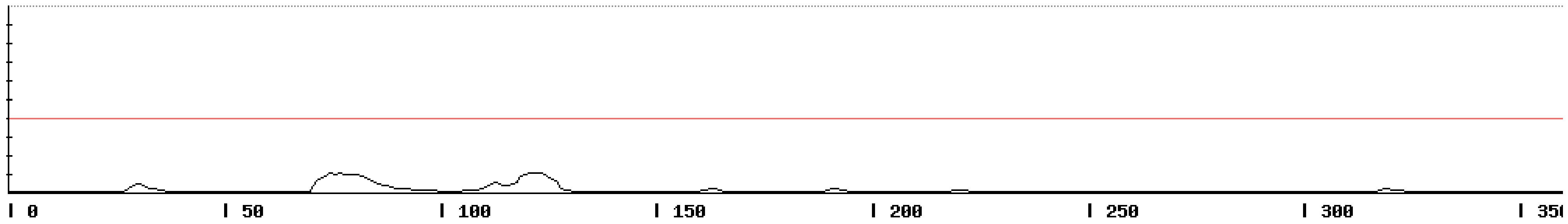

Predictions for Aethionemaarabicum_AA39G00676

Viterbi Path

Viterbi Path

Posterior @ 0.4

Posterior @ 0.4

Posterior Graphic. Threshold Marked @ 0.4

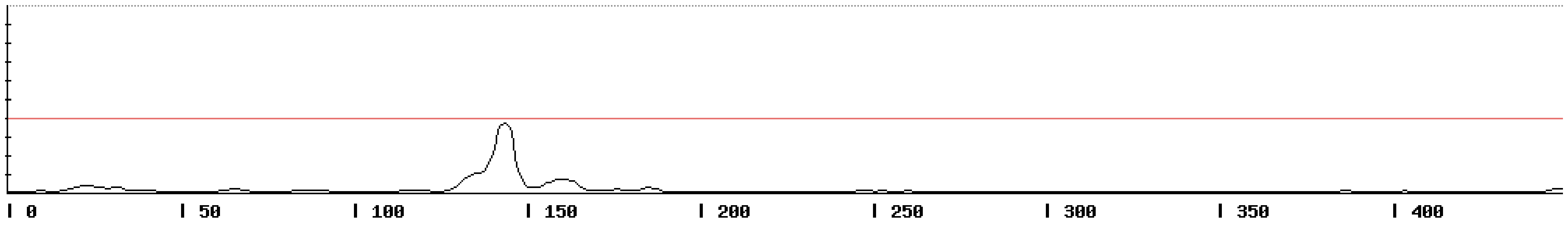

Predictions for Aethionemaarabicum_AA3G00107

Viterbi Path

Viterbi Path

Posterior @ 0.4

Posterior @ 0.4

Posterior Graphic. Threshold Marked @ 0.4

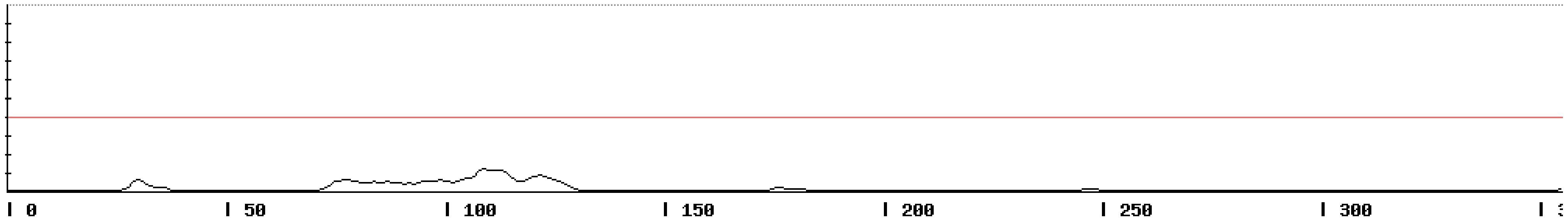

Predictions for Aethionemaarabicum_AA3G00274

Viterbi Path

Viterbi Path

Posterior @ 0.4

Posterior @ 0.4

Posterior Graphic. Threshold Marked @ 0.4

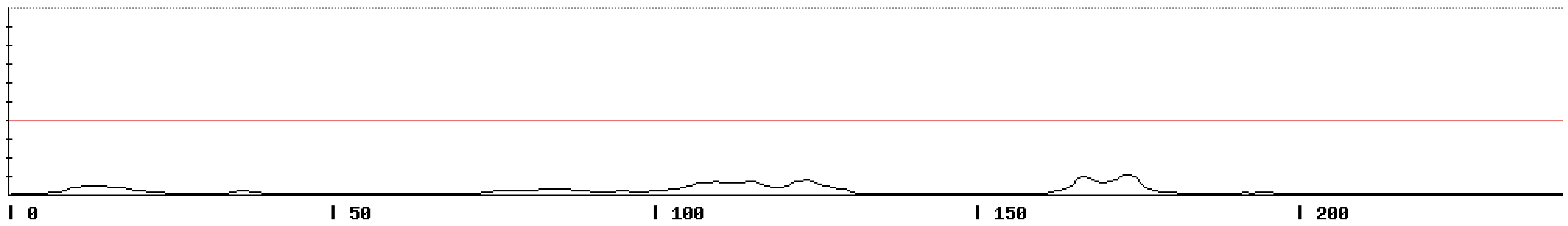

Predictions for Aethionemaarabicum_AA40G00281

Viterbi Path

Viterbi Path

Posterior @ 0.4

Posterior @ 0.4

Posterior Graphic. Threshold Marked @ 0.4

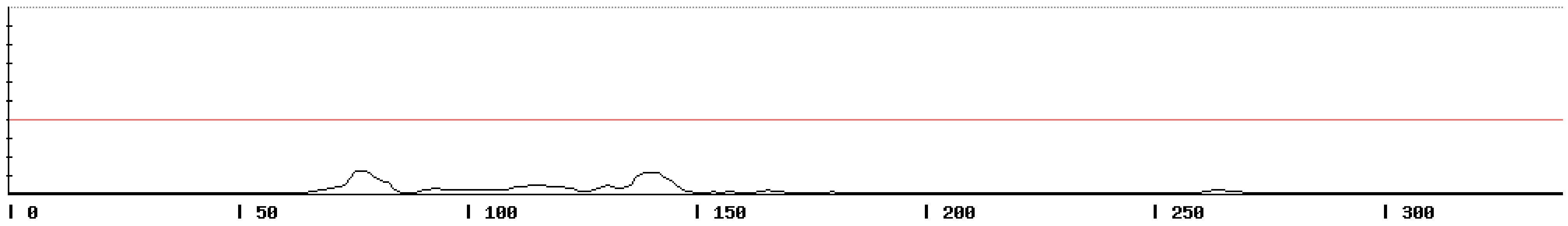

Predictions for Aethionemaarabicum_AA40G00382

Viterbi Path

Viterbi Path

Posterior @ 0.4

Posterior @ 0.4

Posterior Graphic. Threshold Marked @ 0.4

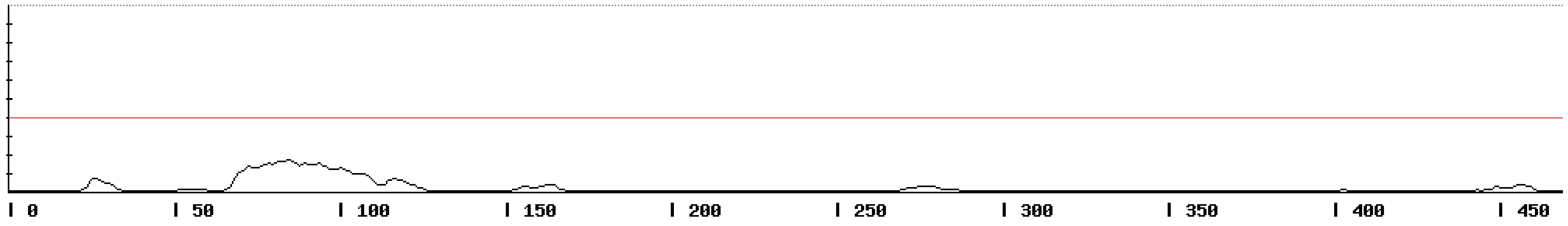

Predictions for Aethionemaarabicum_AA44G00040

Viterbi Path

Viterbi Path

Posterior @ 0.4

Posterior @ 0.4

Posterior Graphic. Threshold Marked @ 0.4

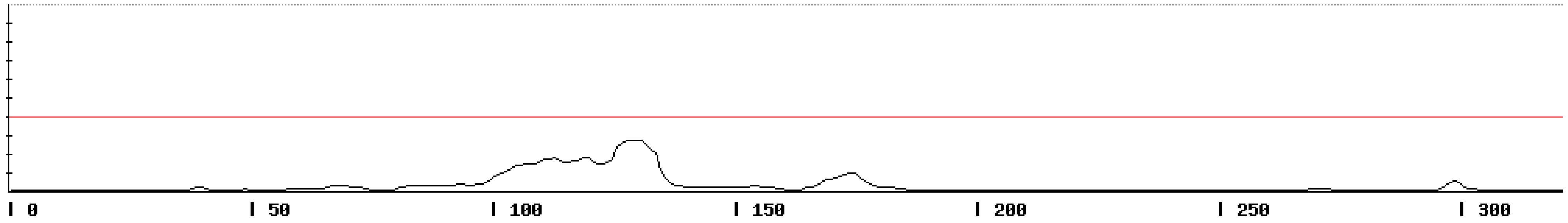

Predictions for Aethionemaarabicum_AA44G00202

Viterbi Path	
Viterbi Path	
Posterior @ 0.4	98 - GKPK - 101
Posterior @ 0.4	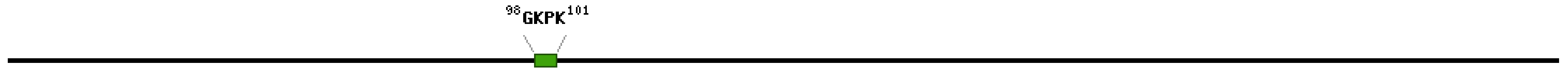
Posterior Graphic. Threshold Marked @ 0.4	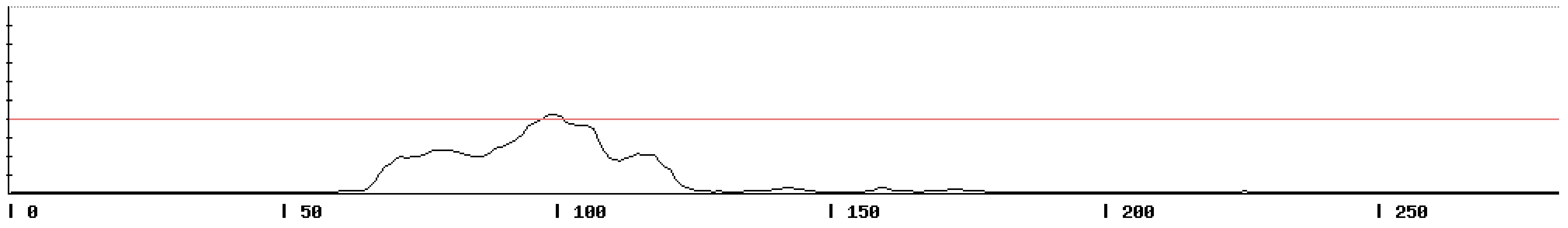

Predictions for Aethionemaarabicum_AA44G00210

Viterbi Path	
Viterbi Path	
Posterior @ 0.4	
Posterior @ 0.4	
Posterior Graphic. Threshold Marked @ 0.4	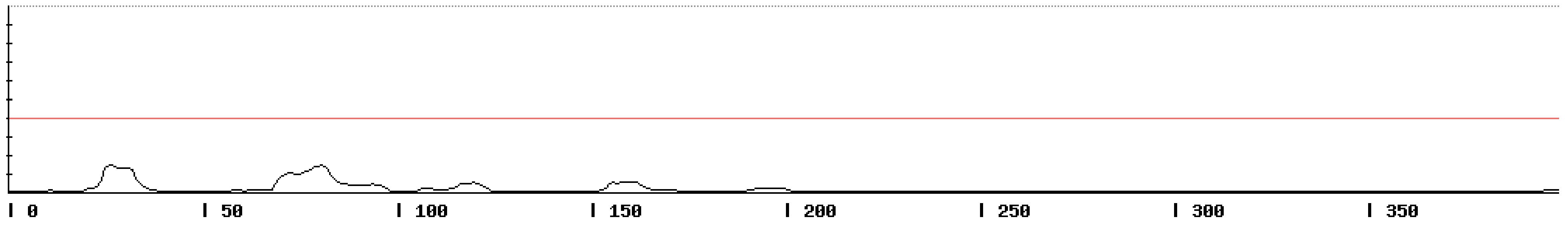

Predictions for Aethionemaarabicum_AA44G00526	
Viterbi Path	
Viterbi Path	
Posterior @ 0.4	
Posterior @ 0.4	
Posterior Graphic. Threshold Marked @ 0.4	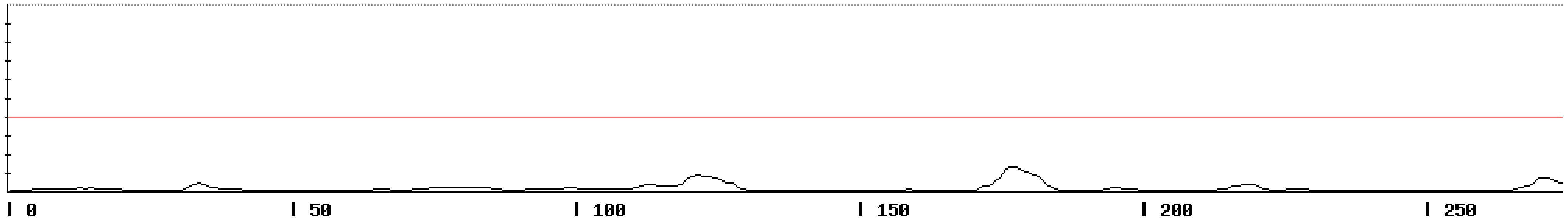

Predictions for Aethionemaarabicum_AA44G00721	
Viterbi Path	
Viterbi Path	
Posterior @ 0.4	146 - RKRRK - 150
Posterior @ 0.4	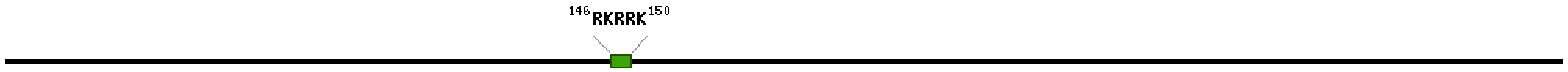
Posterior Graphic. Threshold Marked @ 0.4	

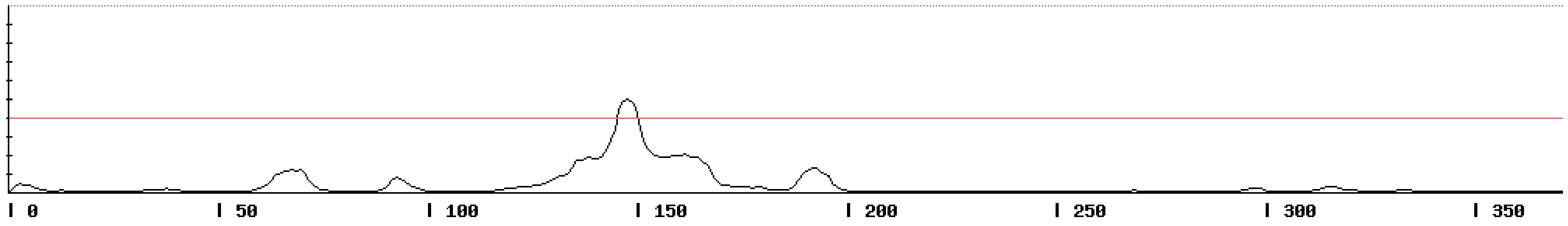

Predictions for Aethionemaarabicum_AA47G00012

Viterbi Path

Viterbi Path

Posterior @ 0.4

Posterior @ 0.4

Posterior Graphic. Threshold Marked @ 0.4

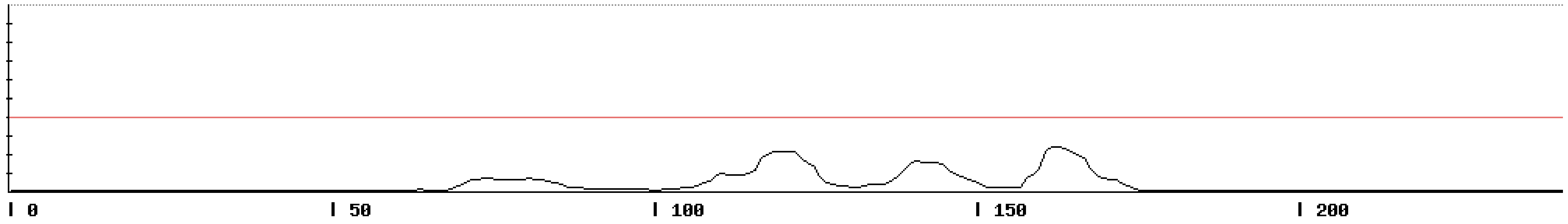

Predictions for Aethionemaarabicum_AA4G00113

Viterbi Path

Viterbi Path

Posterior @ 0.4

Posterior @ 0.4

Posterior Graphic. Threshold Marked @ 0.4

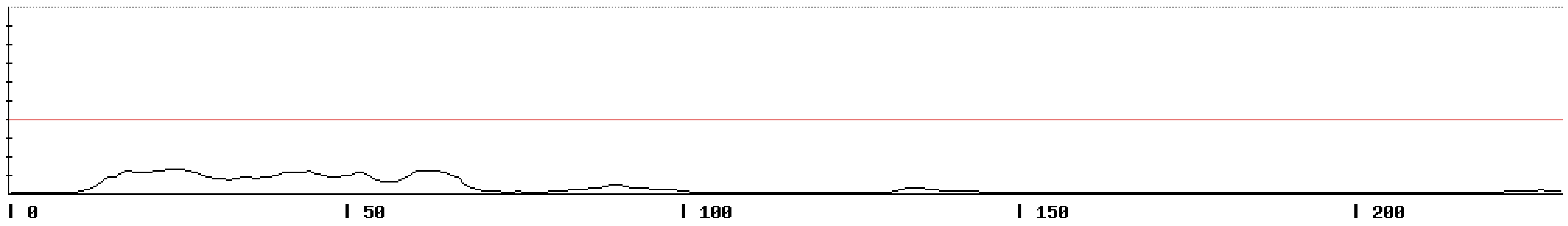

Predictions for Aethionemaarabicum_AA4G00115

Viterbi Path

Viterbi Path

Posterior @ 0.4

Posterior @ 0.4

Posterior Graphic. Threshold Marked @ 0.4

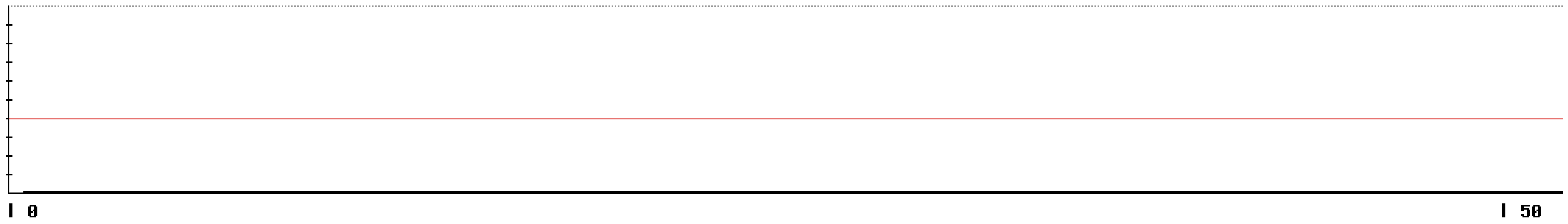

Predictions for Aethionemaarabicum_AA4G00191

Viterbi Path

Viterbi Path

Posterior @ 0.4

Posterior @ 0.4

Posterior Graphic. Threshold Marked @ 0.4

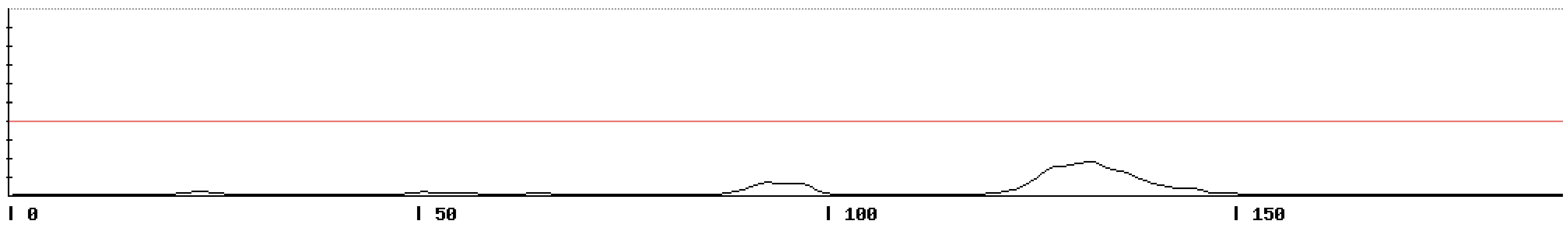

Predictions for Aethionemaarabicum_AA53G00237

Viterbi Path

Viterbi Path

Posterior @ 0.4

Posterior @ 0.4

Posterior Graphic. Threshold Marked @ 0.4

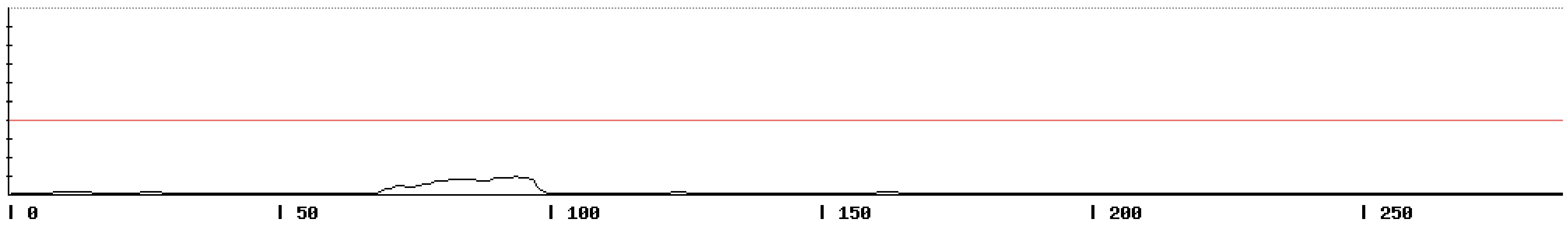

Predictions for Aethionemaarabicum_AA53G00532

Viterbi Path

Viterbi Path

Posterior @ 0.4

Posterior @ 0.4

Posterior Graphic. Threshold Marked @ 0.4

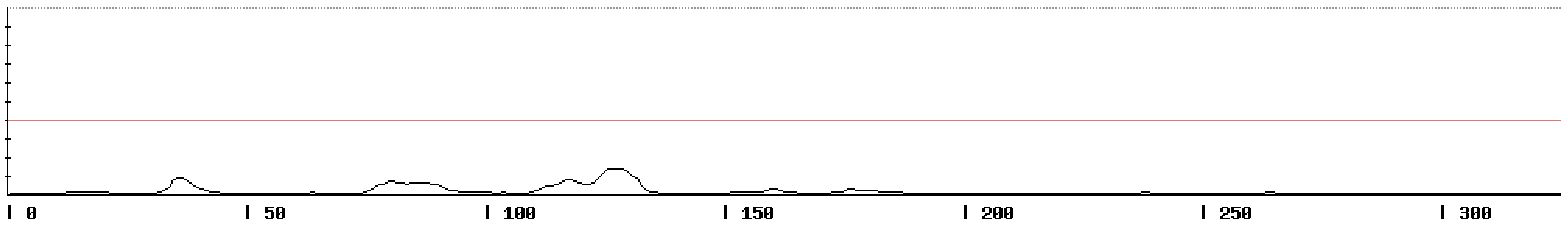

Predictions for Aethionemaarabicum_AA53G00706

Viterbi Path

Viterbi Path

Posterior @ 0.4

Posterior @ 0.4

Posterior Graphic. Threshold Marked @ 0.4

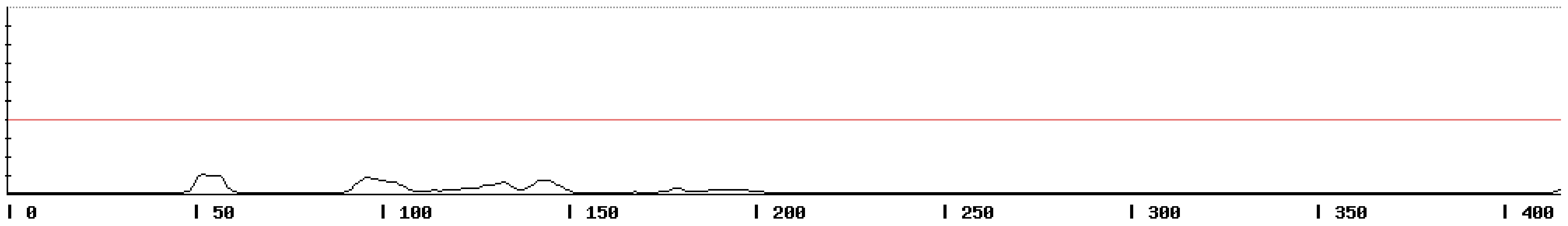

Predictions for Aethionemaarabicum_AA53G01090

Viterbi Path

Viterbi Path

Posterior @ 0.4

Posterior @ 0.4

Posterior Graphic. Threshold Marked @ 0.4

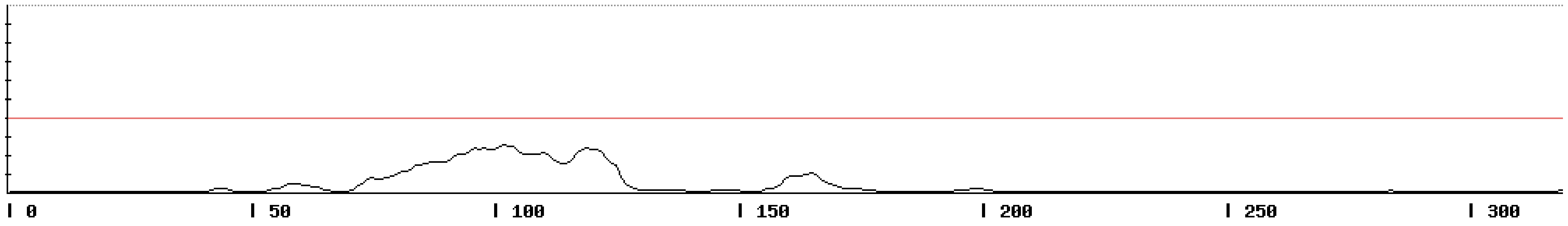

Predictions for Aethionemaarabicum_AA53G01143

Viterbi Path

Viterbi Path

Posterior @ 0.4

Posterior @ 0.4

Posterior Graphic. Threshold Marked @ 0.4

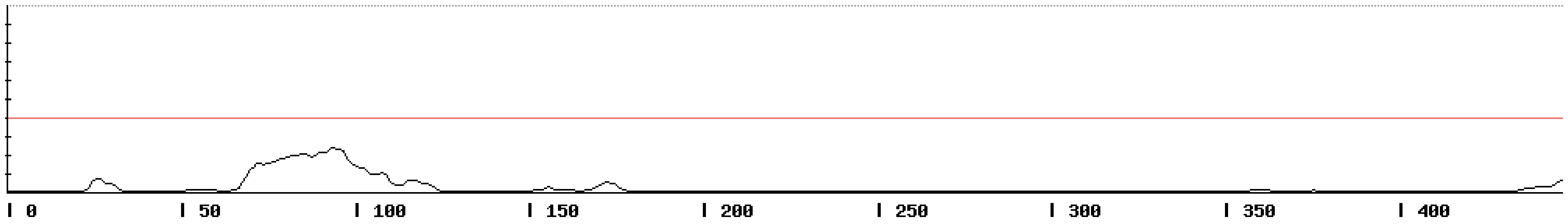

Predictions for Aethionemaarabicum_AA54G00138

Viterbi Path

Viterbi Path

Posterior @ 0.4

Posterior @ 0.4

Posterior Graphic. Threshold Marked @ 0.4

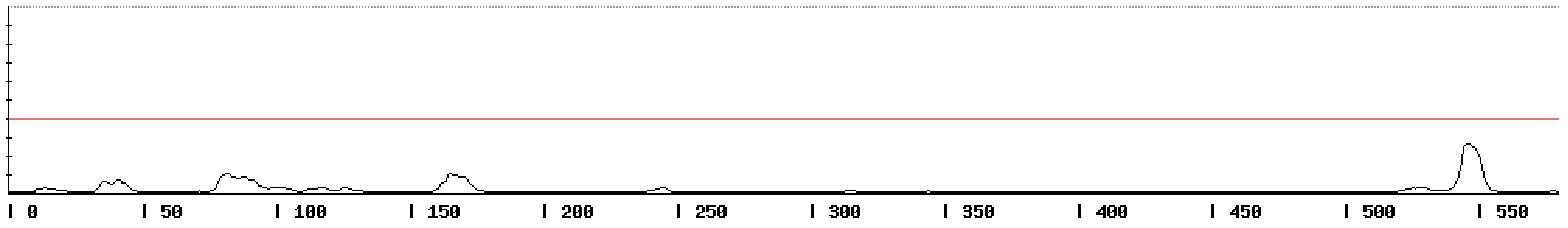

Predictions for Aethionemaarabicum_AA54G00190

Viterbi Path

Viterbi Path

Posterior @ 0.4

Posterior @ 0.4

Posterior Graphic. Threshold Marked @ 0.4

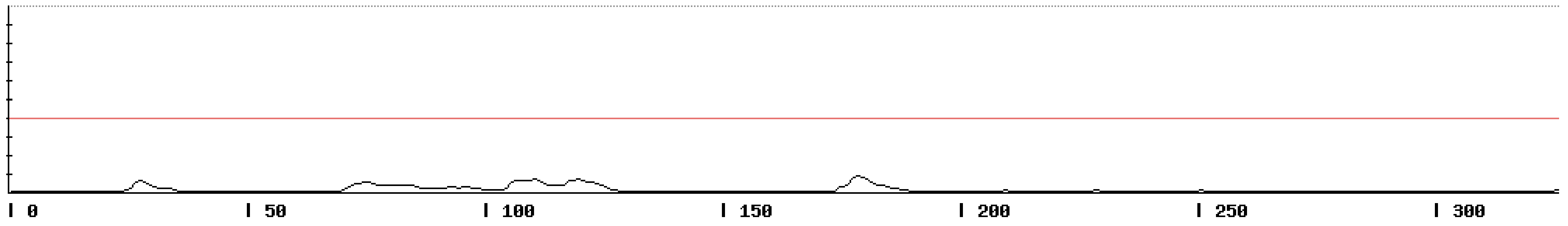

Predictions for Aethionemaarabicum_AA54G00203	
Viterbi Path	
Viterbi Path	
Posterior @ 0.4	
Posterior @ 0.4	
Posterior Graphic. Threshold Marked @ 0.4	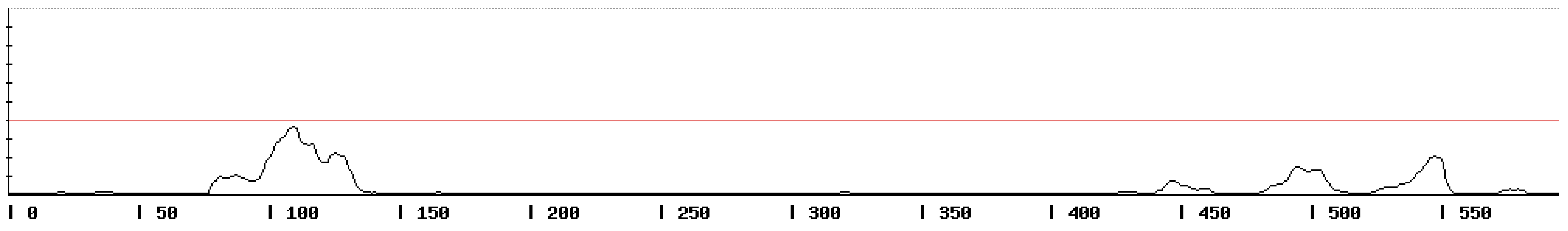

Predictions for Aethionemaarabicum_AA54G00226	
Viterbi Path	
Viterbi Path	
Posterior @ 0.4	
Posterior @ 0.4	
Posterior Graphic. Threshold Marked @ 0.4	

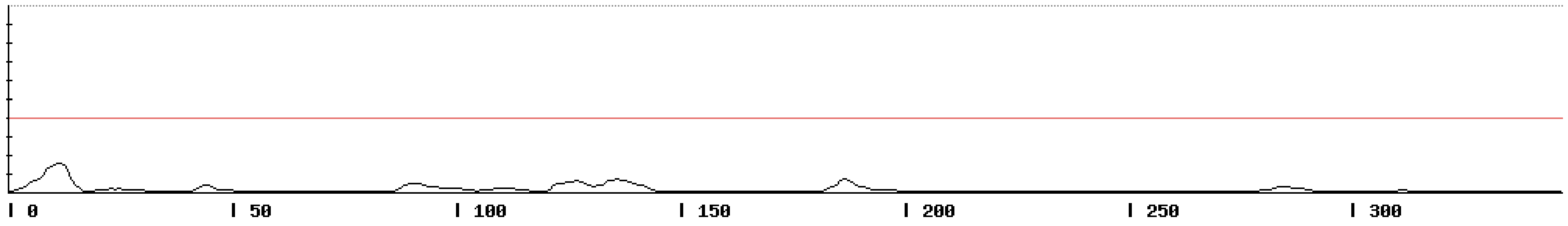

Predictions for Aethionemaarabicum_AA56G00025

Viterbi Path

Viterbi Path

Posterior @ 0.4

Posterior @ 0.4

Posterior Graphic. Threshold Marked @ 0.4

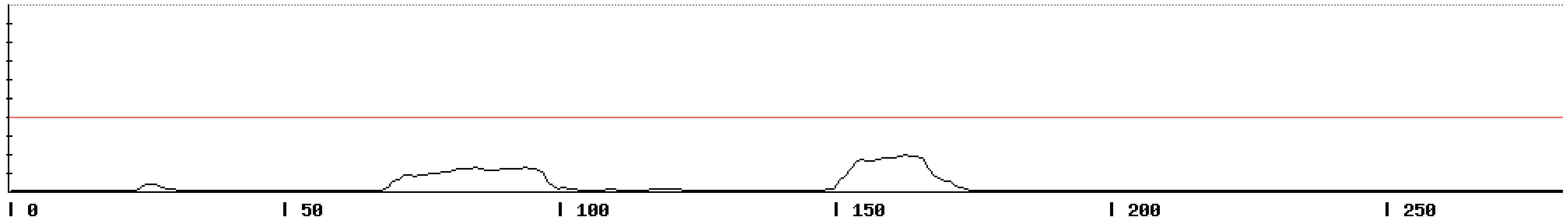

Predictions for Aethionemaarabicum_AA57G00073

Viterbi Path

Viterbi Path

Posterior @ 0.4

Posterior @ 0.4

Posterior Graphic. Threshold Marked @ 0.4

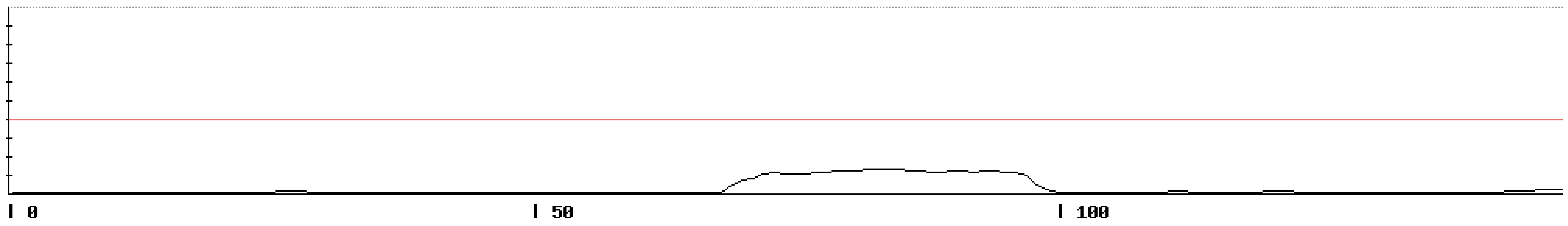

Predictions for Aethionemaarabicum_AA5G00075

Viterbi Path

Viterbi Path

Posterior @ 0.4

Posterior @ 0.4

Posterior Graphic. Threshold Marked @ 0.4

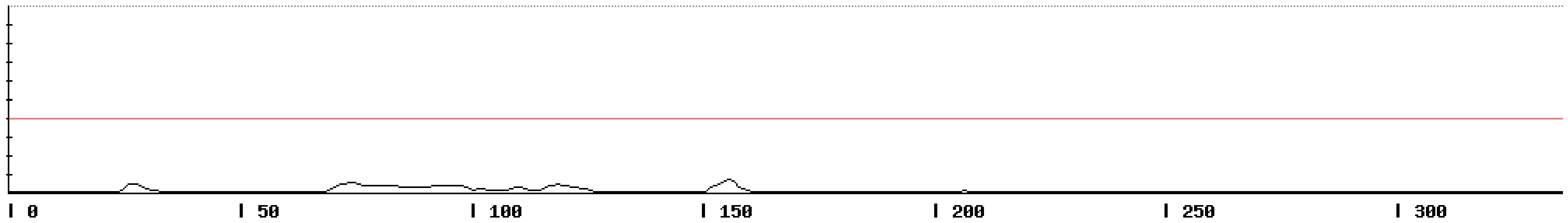

Predictions for Aethionemaarabicum_AA5G00148

Viterbi Path

Viterbi Path

Posterior @ 0.4

Posterior @ 0.4

Posterior Graphic. Threshold Marked @ 0.4

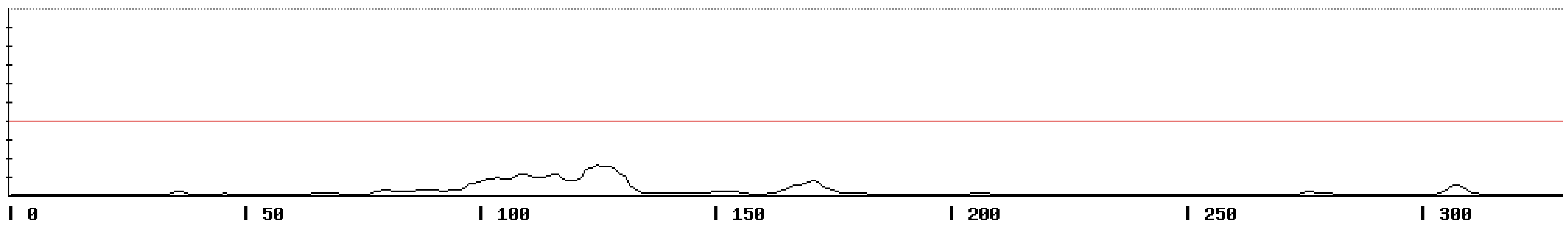

Predictions for Aethionemaarabicum_AA61G00547

Viterbi Path

Viterbi Path

Posterior @ 0.4

Posterior @ 0.4

Posterior Graphic. Threshold Marked @ 0.4

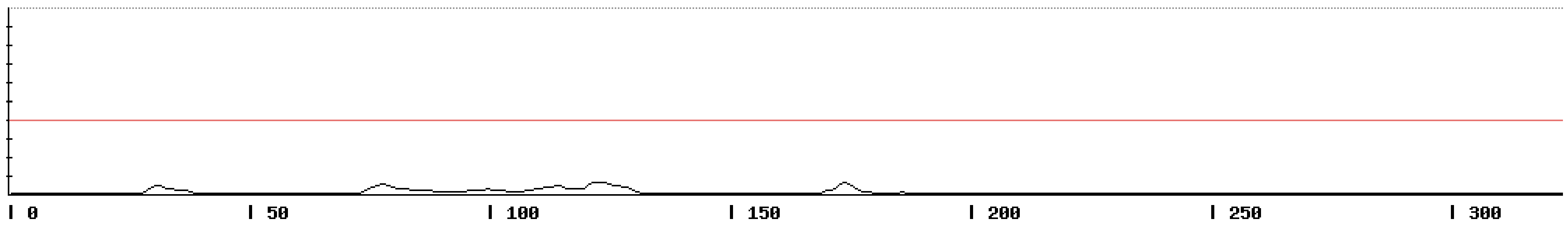

Predictions for Aethionemaarabicum_AA65G00271

Viterbi Path

Viterbi Path

Posterior @ 0.4

Posterior @ 0.4

Posterior Graphic. Threshold Marked @ 0.4

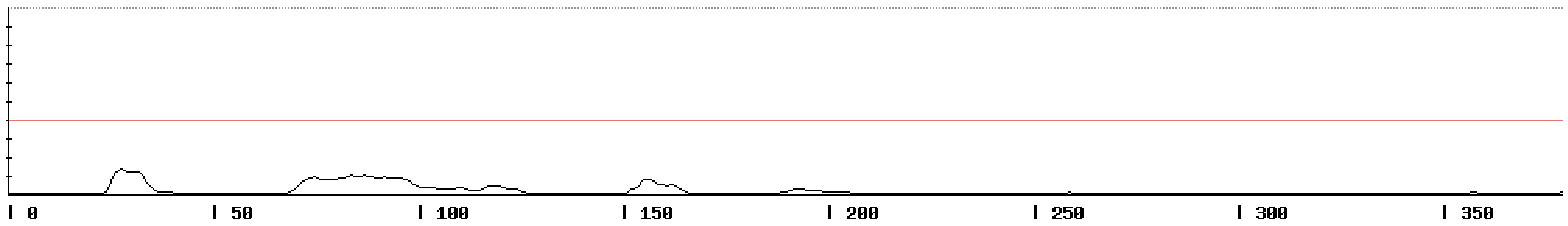

Predictions for Aethionemaarabicum_AA75G00035

Viterbi Path

Viterbi Path

Posterior @ 0.4

Posterior @ 0.4

Posterior Graphic. Threshold Marked @ 0.4

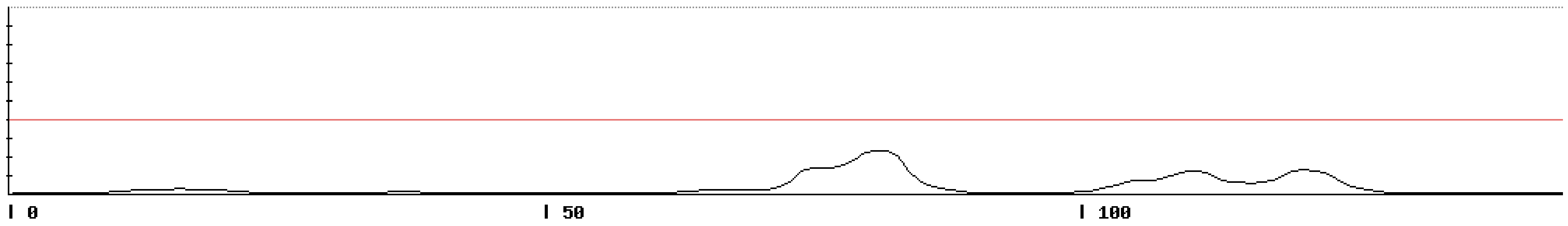

Predictions for Aethionemaarabicum_AA87G00007

Viterbi Path

Viterbi Path

Posterior @ 0.4

Posterior @ 0.4

Posterior Graphic. Threshold Marked @ 0.4

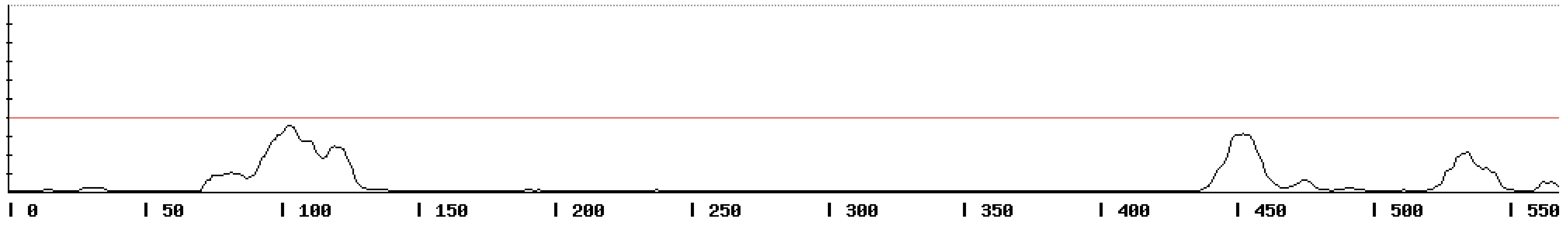

Predictions for Aethionemaarabicum_AA87G00083

Viterbi Path

Viterbi Path

Posterior @ 0.4

Posterior @ 0.4

Posterior Graphic. Threshold Marked @ 0.4

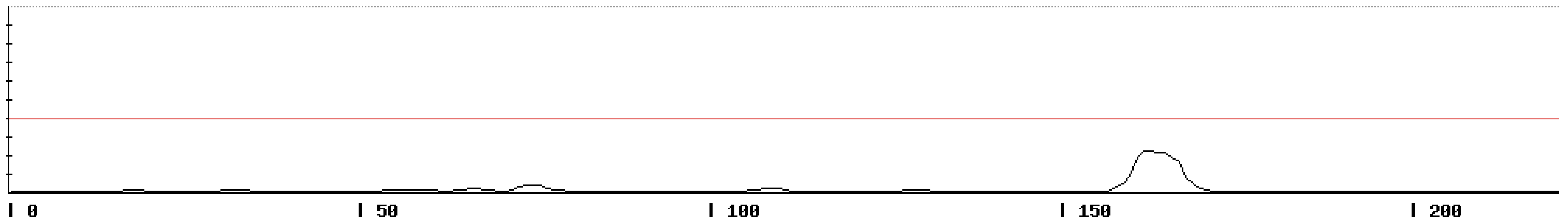

Predictions for Aethionemaarabicum_AA8G00098

Viterbi Path

Viterbi Path

Posterior @ 0.4

158 - KKRSNVGRKRR - 168

Posterior @ 0.4

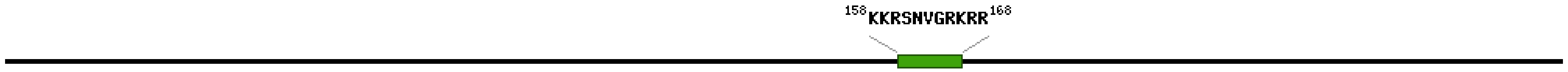

Posterior Graphic. Threshold Marked @ 0.4

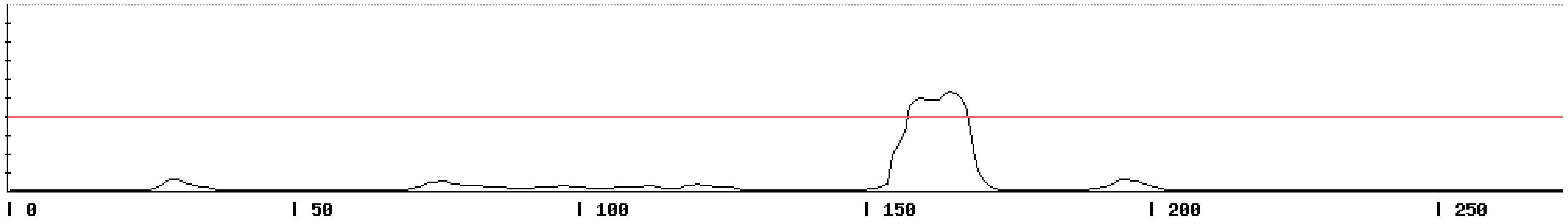

Predictions for Aethionemaarabicum_AA8G00264

Viterbi Path

Viterbi Path

Posterior @ 0.4

Posterior @ 0.4

Posterior Graphic. Threshold Marked @ 0.4

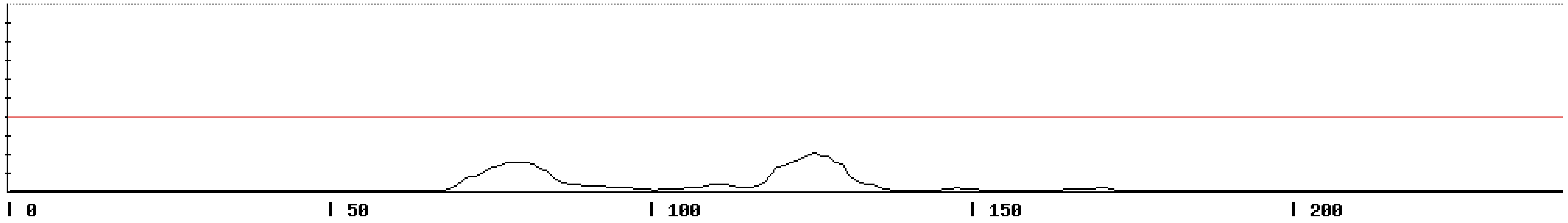

Predictions for Aethionemaarabicum_AA93G00231

Revision r.9 - Last Updated on : February 24 2015 17:45:20.

alex.nguyenba@utoronto.ca

Intended to be used with Firefox

Viterbi Path

Posterior @ 0.4

Posterior @ 0.4

Posterior Graphic. Threshold Marked @ 0.4

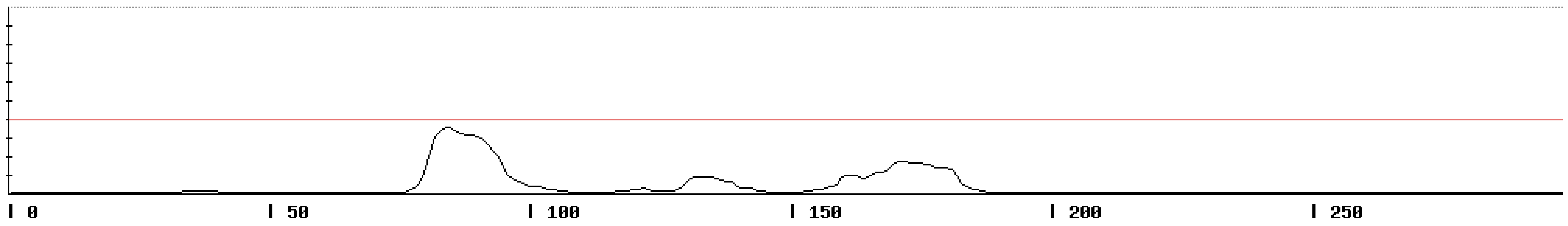

Predictions for Amaranthushypochondriacus_AHYPO_001663

Viterbi Path

Viterbi Path

Posterior @ 0.4

Posterior @ 0.4

Posterior Graphic. Threshold Marked @ 0.4

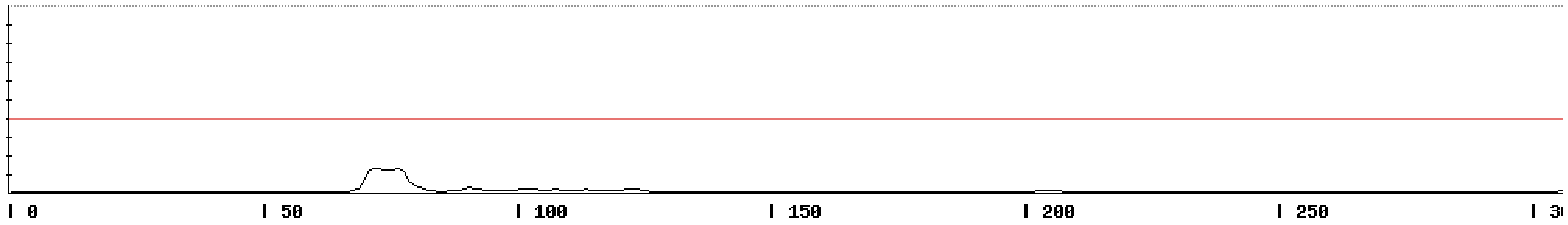

Predictions for Amaranthushypochondriacus_AHYPO_001938

Viterbi Path

Viterbi Path

Posterior @ 0.4

Posterior @ 0.4

Posterior Graphic. Threshold Marked @ 0.4

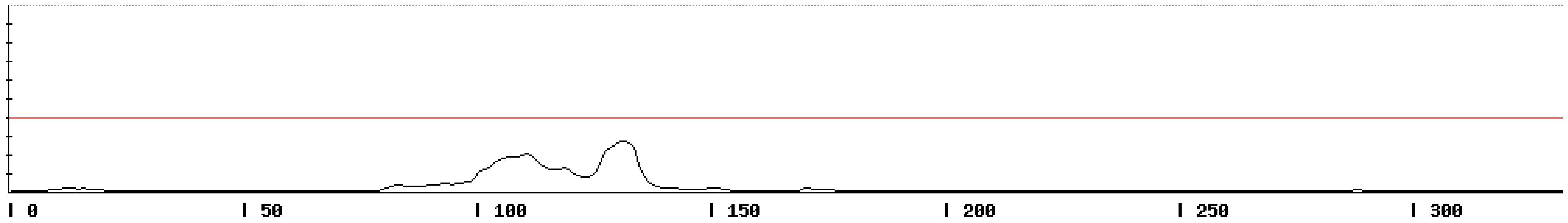

Predictions for Amaranthushypochondriacus_AHYPO_002189

Viterbi Path

Viterbi Path

Posterior @ 0.4

Posterior @ 0.4

Posterior Graphic. Threshold Marked @ 0.4

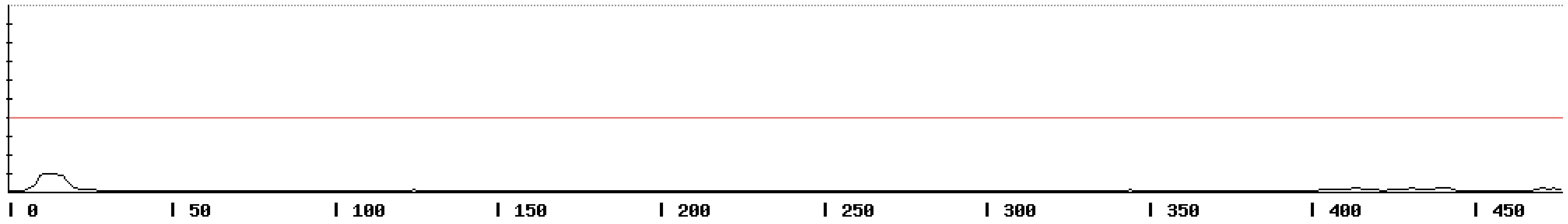

Predictions for Amaranthushypochondriacus_AHYPO_002480

Viterbi Path

Viterbi Path

Posterior @ 0.4

Posterior @ 0.4

Posterior Graphic. Threshold Marked @ 0.4

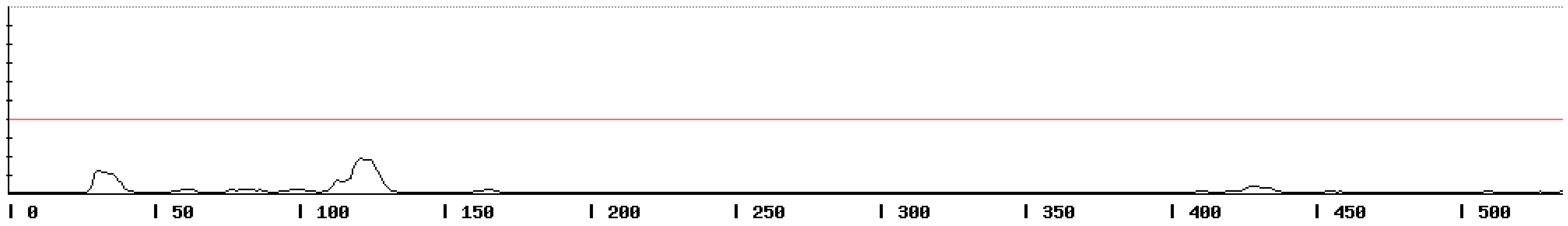

Predictions for Amaranthushypochondriacus_AHYPO_003129

Viterbi Path

Viterbi Path

Posterior @ 0.4

Posterior @ 0.4

Posterior Graphic. Threshold Marked @ 0.4

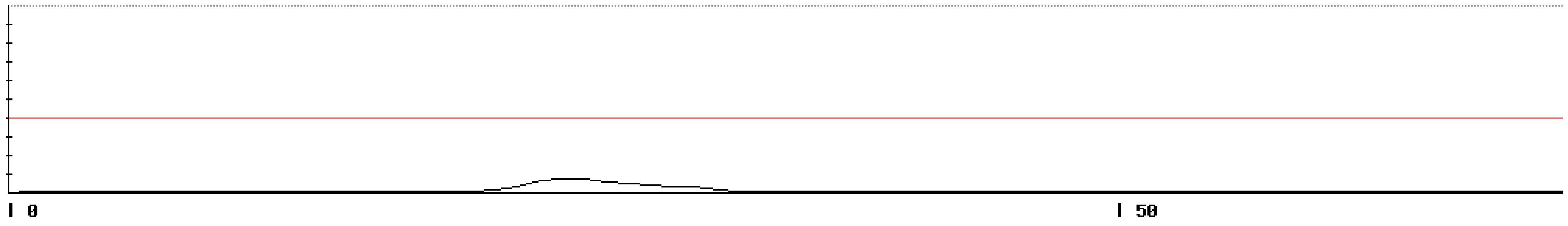

Predictions for Amaranthushypochondriacus_AHYPO_003454

Viterbi Path	
Viterbi Path	
Posterior @ 0.4	
Posterior @ 0.4	
Posterior Graphic. Threshold Marked @ 0.4	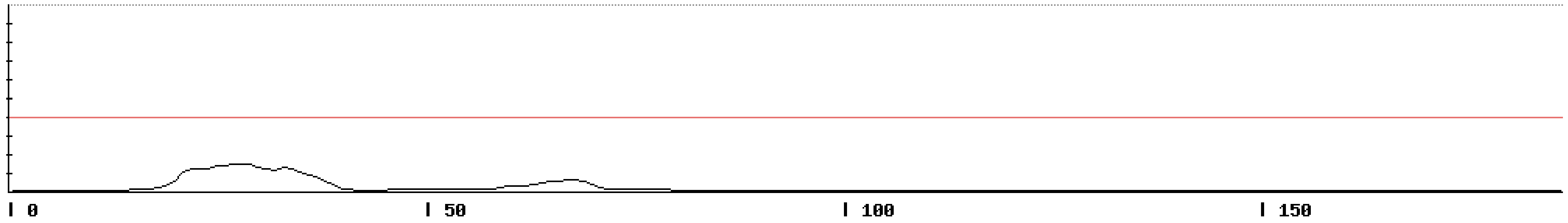

Predictions for Amaranthushypochondriacus_AHYPO_004169

Viterbi Path	
Viterbi Path	
Posterior @ 0.4	
Posterior @ 0.4	

Posterior Graphic. Threshold Marked @ 0.4

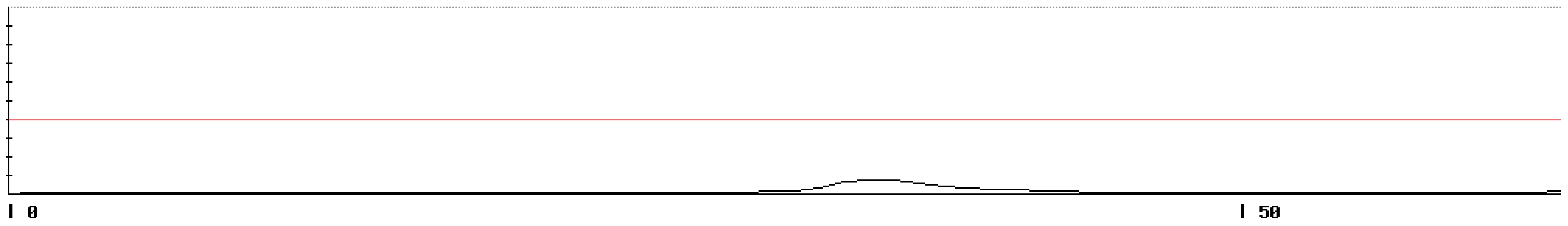

Predictions for Amaranthushypochondriacus_AHYPO_004861

Viterbi Path

Viterbi Path

Posterior @ 0.4

Posterior @ 0.4

Posterior Graphic. Threshold Marked @ 0.4

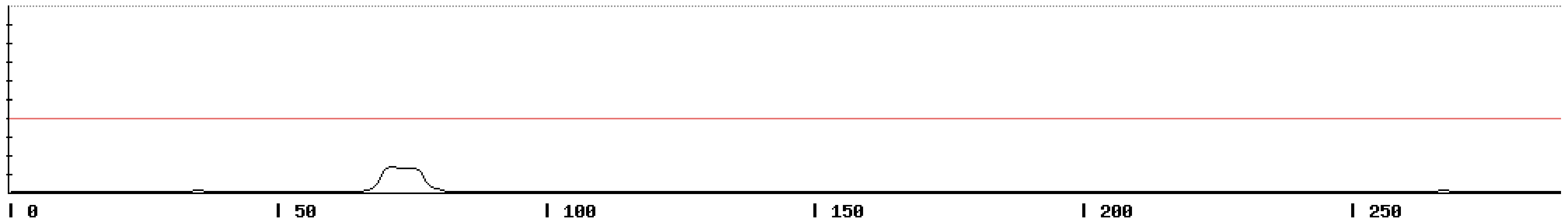

Predictions for Amaranthushypochondriacus_AHYPO_005582

Viterbi Path

Viterbi Path

Posterior @ 0.4 136 - RKRRK - 140

Posterior @ 0.4

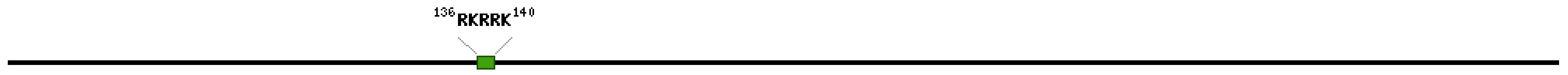

Posterior Graphic. Threshold Marked @ 0.4

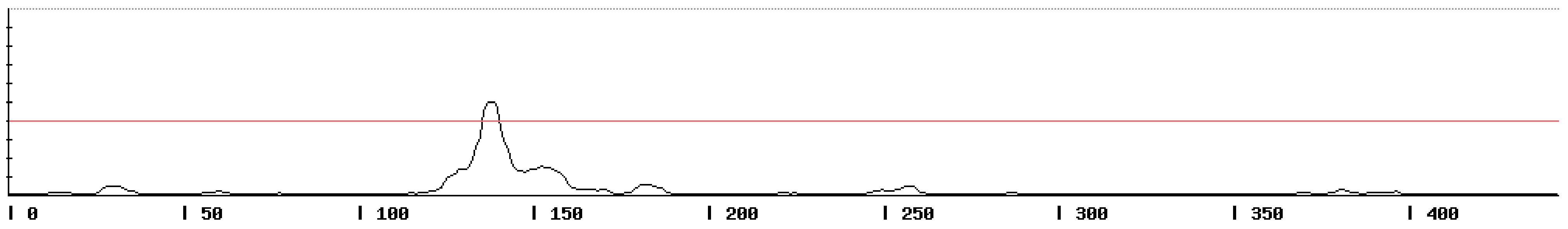

Predictions for Amaranthushypochondriacus_AHYPO_005721

Viterbi Path	
Viterbi Path	
Posterior @ 0.4	
Posterior @ 0.4	
Posterior Graphic. Threshold Marked @ 0.4	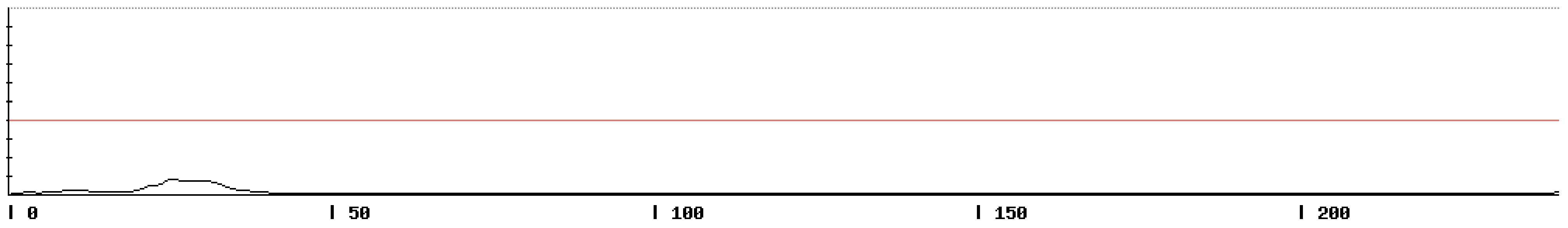

Predictions for Amaranthushypochondriacus_AHYPO_005917

Viterbi Path	
Viterbi Path	

Posterior @ 0.4

Posterior @ 0.4

Posterior Graphic. Threshold Marked @ 0.4

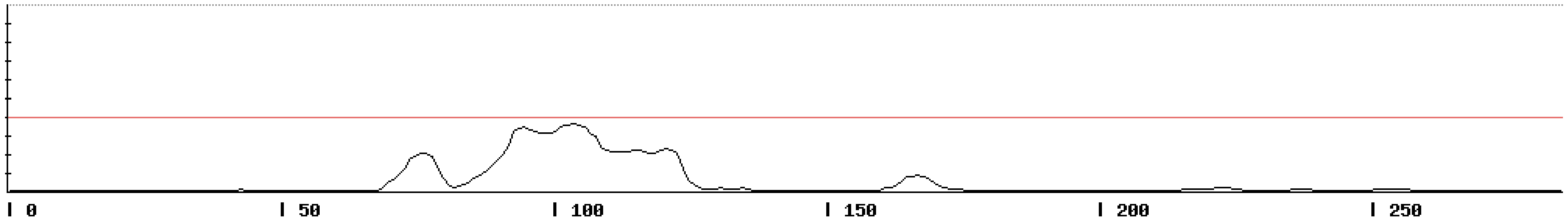

Predictions for Amaranthushypochondriacus_AHYPO_006792

Viterbi Path

Viterbi Path

Posterior @ 0.4

Posterior @ 0.4

Posterior Graphic. Threshold Marked @ 0.4

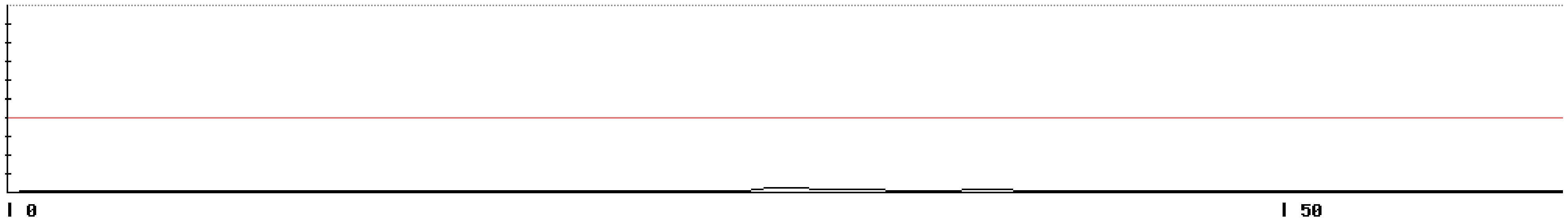

Predictions for Amaranthushypochondriacus_AHYPO_007255

Viterbi Path

Viterbi Path

Posterior @ 0.4

Posterior @ 0.4

Posterior Graphic. Threshold Marked @ 0.4

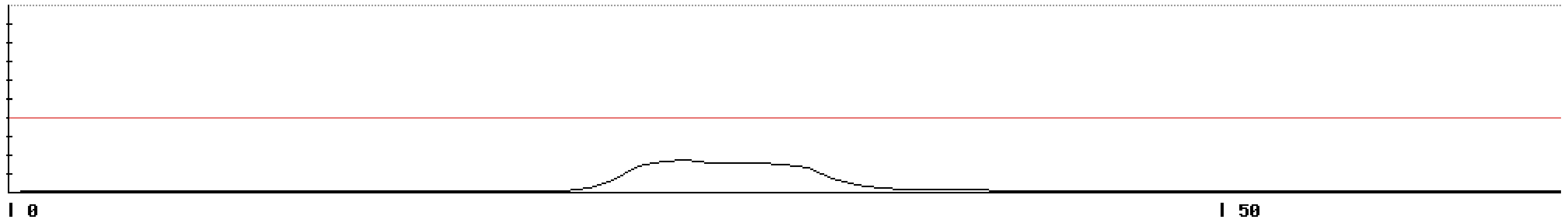

Predictions for Amaranthushypochondriacus_AHYPO_007548

Viterbi Path

Viterbi Path

Posterior @ 0.4

Posterior @ 0.4

Posterior Graphic. Threshold Marked @ 0.4

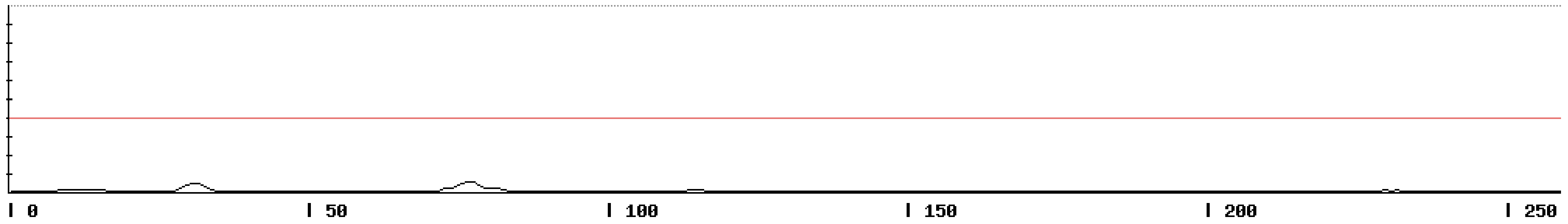

Predictions for Amaranthushypochondriacus_AHYPO_007963

Viterbi Path	
Viterbi Path	
Posterior @ 0.4	
Posterior @ 0.4	
Posterior Graphic. Threshold Marked @ 0.4	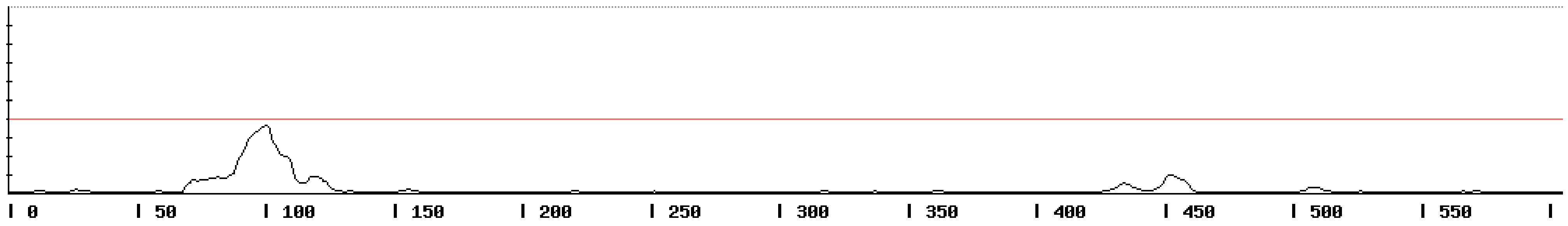

Predictions for Amaranthushypochondriacus_AHYPO_008111

Viterbi Path	
Viterbi Path	
Posterior @ 0.4	
Posterior @ 0.4	
Posterior Graphic. Threshold Marked @ 0.4	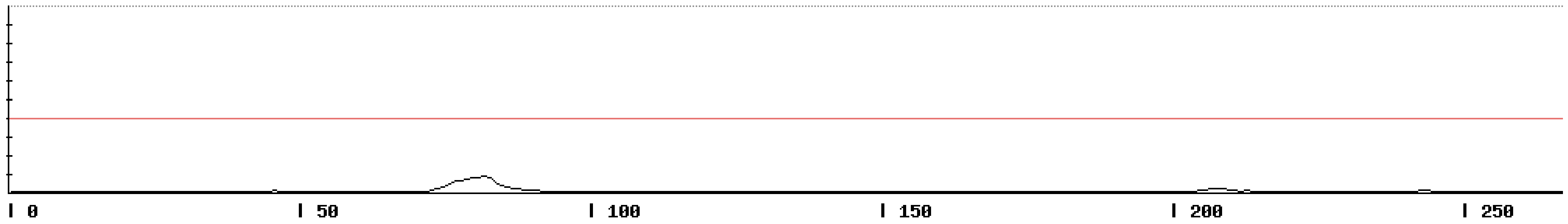

Predictions for Amaranthushypochondriacus_AHYPO_008532

Viterbi Path	
Viterbi Path	
Posterior @ 0.4	
Posterior @ 0.4	
Posterior Graphic. Threshold Marked @ 0.4	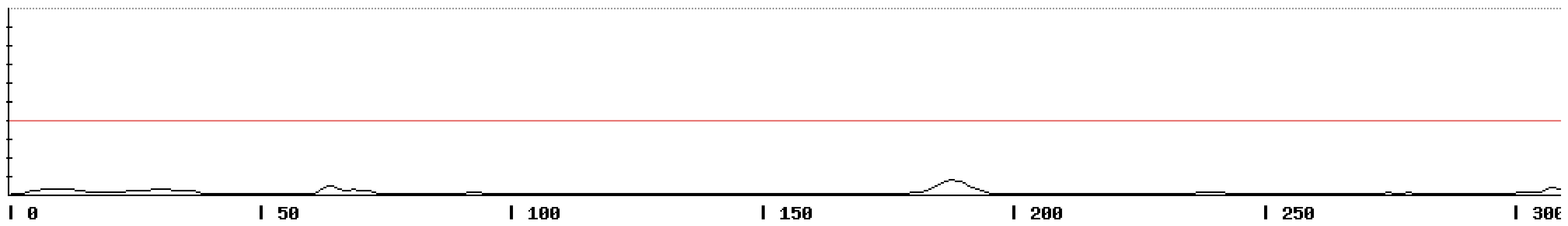

Predictions for Amaranthushypochondriacus_AHYPO_008586

Viterbi Path	
Viterbi Path	
Posterior @ 0.4	
Posterior @ 0.4	
Posterior Graphic. Threshold Marked @ 0.4	

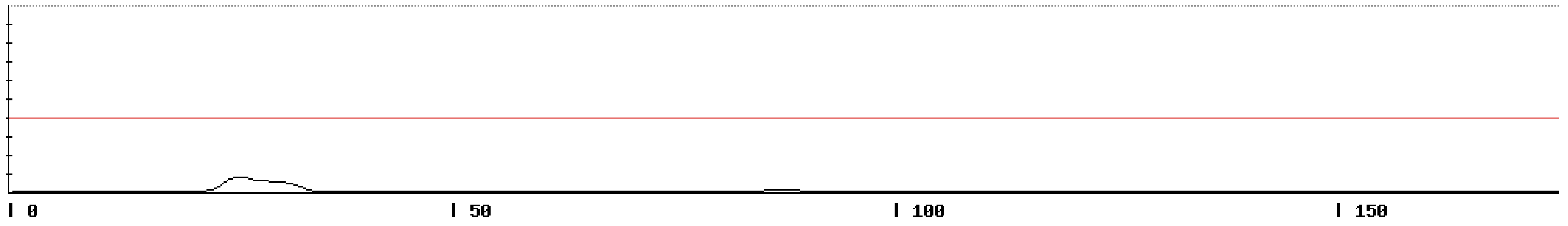

Predictions for Amaranthushypochondriacus_AHYPO_009365

Viterbi Path

Viterbi Path

Posterior @ 0.4

Posterior @ 0.4

Posterior Graphic. Threshold Marked @ 0.4

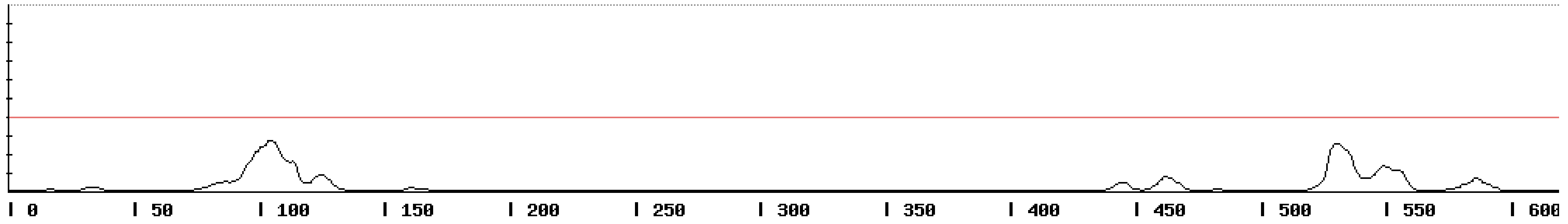

Predictions for Amaranthushypochondriacus_AHYPO_009543

Viterbi Path

Viterbi Path

Posterior @ 0.4

Posterior @ 0.4

Posterior Graphic. Threshold Marked @ 0.4

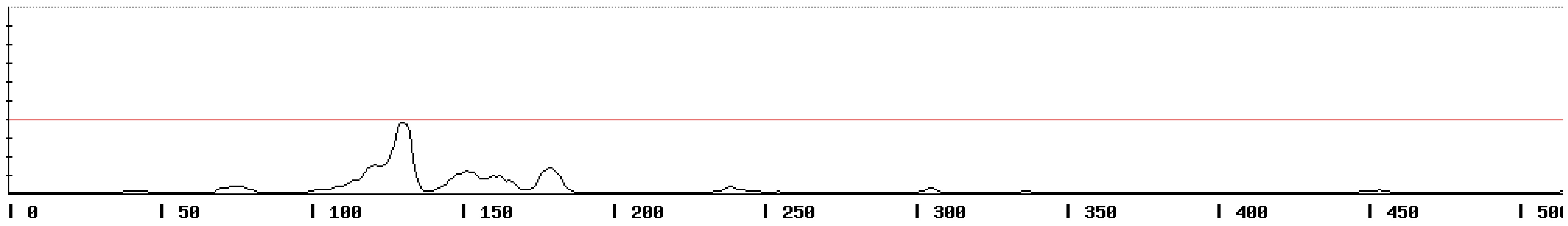

Predictions for Amaranthushypochondriacus_AHYPO_009690

Viterbi Path

Viterbi Path

Posterior @ 0.4

Posterior @ 0.4

Posterior Graphic. Threshold Marked @ 0.4

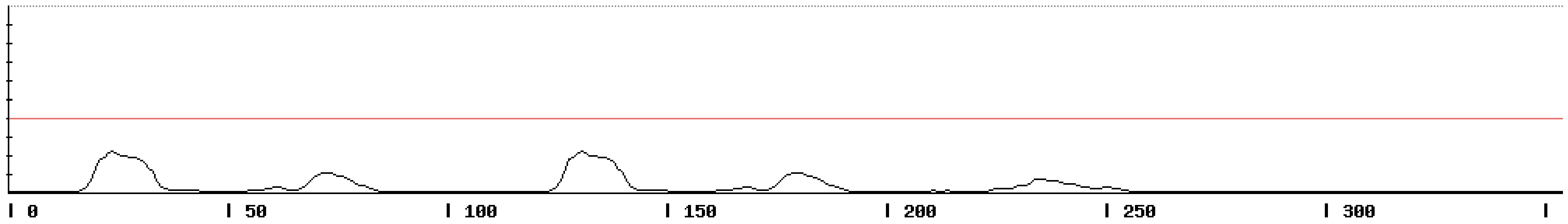

Predictions for Amaranthushypochondriacus_AHYPO_010449

Viterbi Path

Viterbi Path

Posterior @ 0.4

Posterior @ 0.4

Posterior Graphic. Threshold Marked @ 0.4

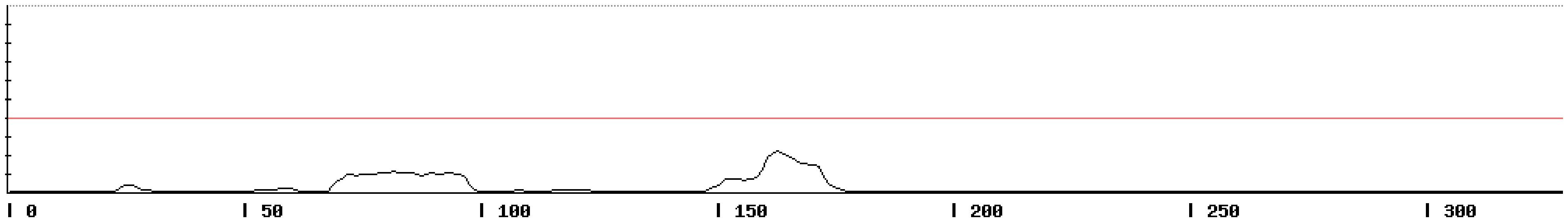

Predictions for Amaranthushypochondriacus_AHYPO_010500

Viterbi Path

Viterbi Path

Posterior @ 0.4

Posterior @ 0.4

Posterior Graphic. Threshold Marked @ 0.4

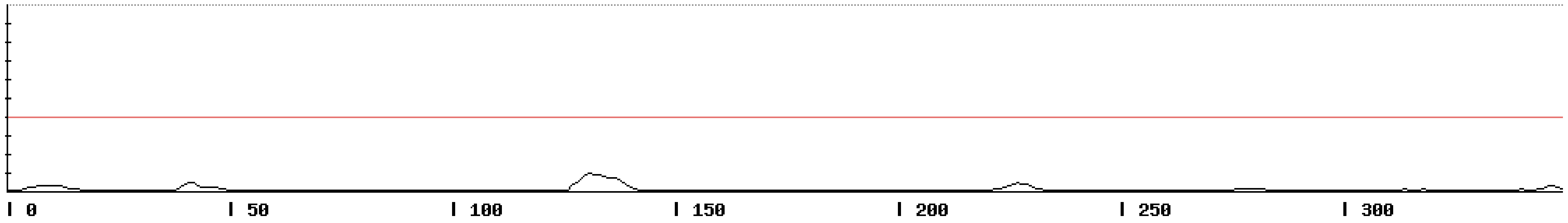

Predictions for Amaranthushypochondriacus_AHYPO_010646

Viterbi Path

Viterbi Path

Posterior @ 0.4	152 - HKRRSKRPDAKK - 163
Posterior @ 0.4	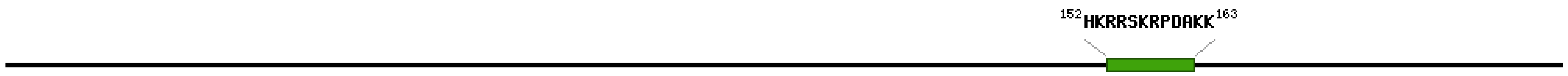
Posterior Graphic. Threshold Marked @ 0.4	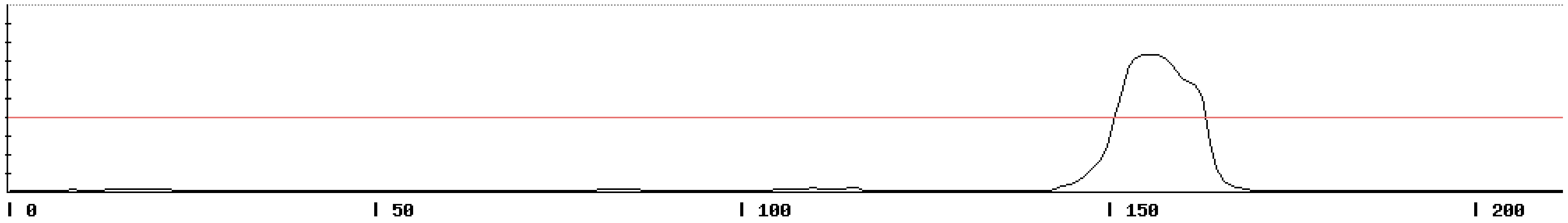

Predictions for Amaranthushypochondriacus_AHYPO_011135

Viterbi Path	
Viterbi Path	
Posterior @ 0.4	
Posterior @ 0.4	
Posterior Graphic. Threshold Marked @ 0.4	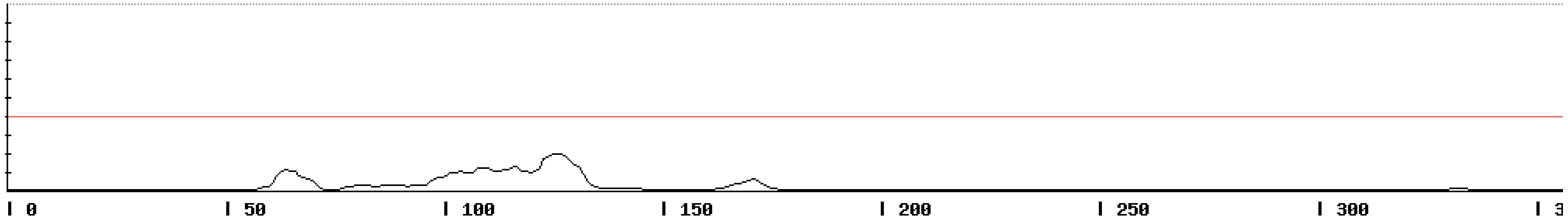

Predictions for Amaranthushypochondriacus_AHYPO_011586

Viterbi Path	
Viterbi Path	

Posterior @ 0.4	
Posterior @ 0.4	
Posterior Graphic. Threshold Marked @ 0.4	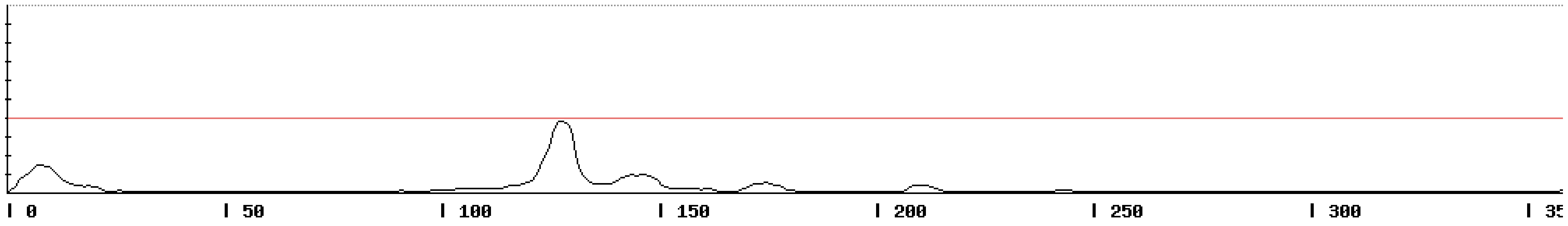

Predictions for Amaranthushypochondriacus_AHYPO_012201

Viterbi Path	
Viterbi Path	
Posterior @ 0.4	
Posterior @ 0.4	
Posterior Graphic. Threshold Marked @ 0.4	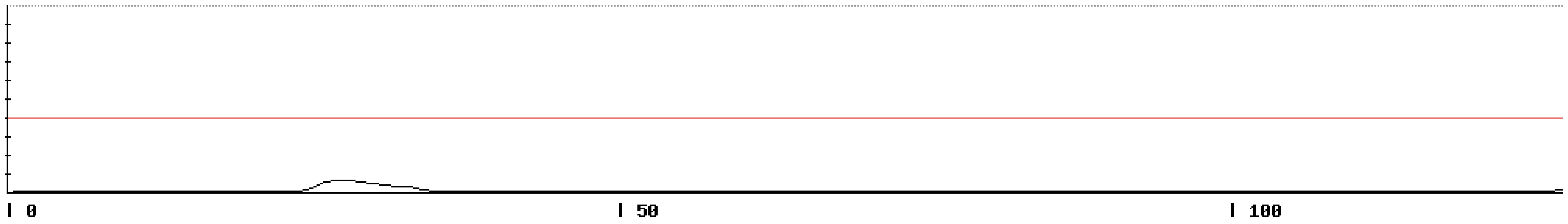

Predictions for Amaranthushypochondriacus_AHYPO_013086

Viterbi Path

Viterbi Path

Posterior @ 0.4

96 - ERKSRRKAGFGFWKMKGKGKDVIGNGGKVVLGRR - 129

Posterior @ 0.4

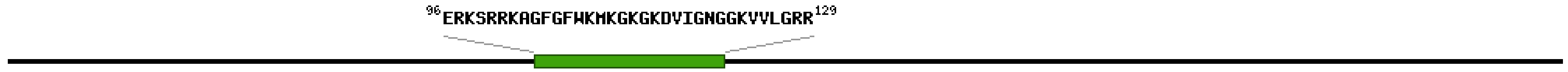

Posterior Graphic. Threshold Marked @ 0.4

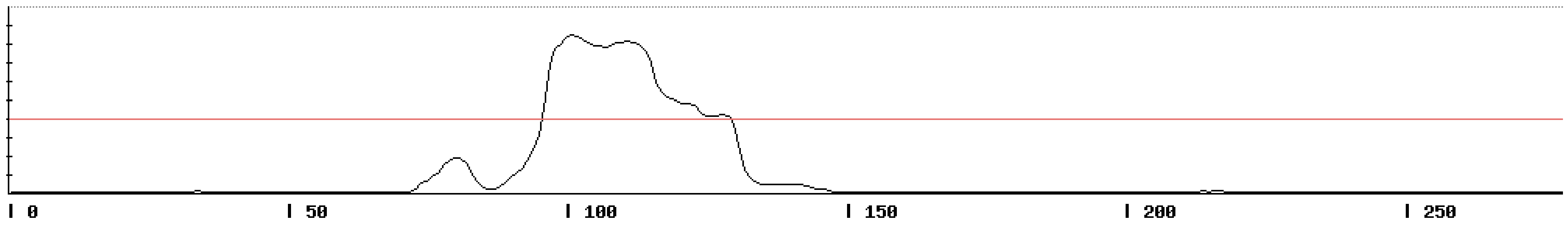

Predictions for Amaranthushypochondriacus_AHYPO_013259

Viterbi Path

Viterbi Path

Posterior @ 0.4

Posterior @ 0.4

Posterior Graphic. Threshold Marked @ 0.4

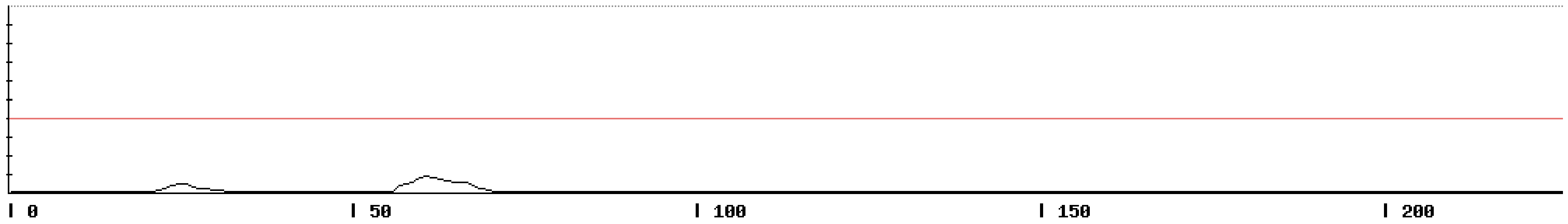

Predictions for Amaranthushypochondriacus_AHYPO_013998	
Viterbi Path	
Viterbi Path	
Posterior @ 0.4	
Posterior @ 0.4	
Posterior Graphic. Threshold Marked @ 0.4	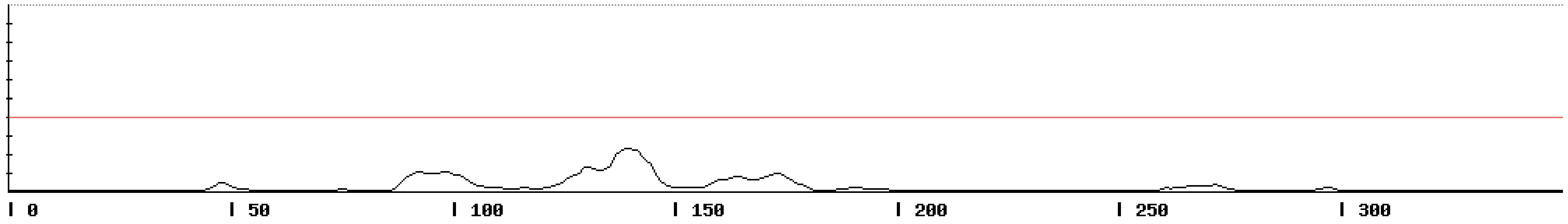

Predictions for Amaranthushypochondriacus_AHYPO_014151	
Viterbi Path	
Viterbi Path	
Posterior @ 0.4	
Posterior @ 0.4	
Posterior Graphic. Threshold Marked @ 0.4	

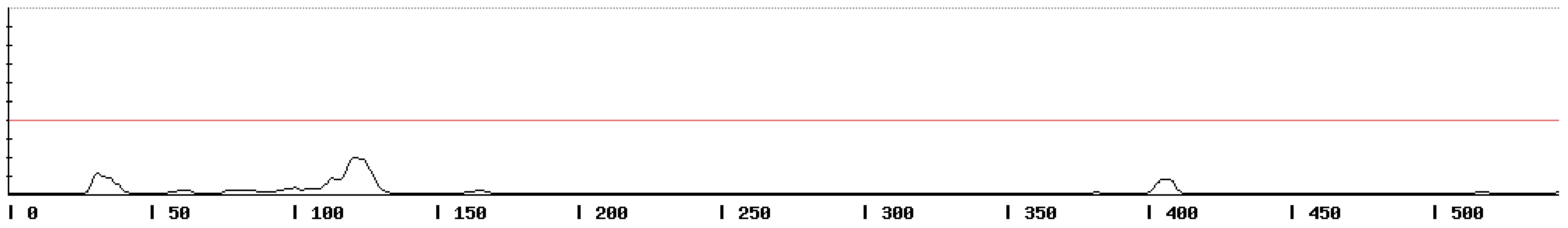

Predictions for Amaranthushypochondriacus_AHYPO_014758

Viterbi Path

Viterbi Path

Posterior @ 0.4

Posterior @ 0.4

Posterior Graphic. Threshold Marked @ 0.4

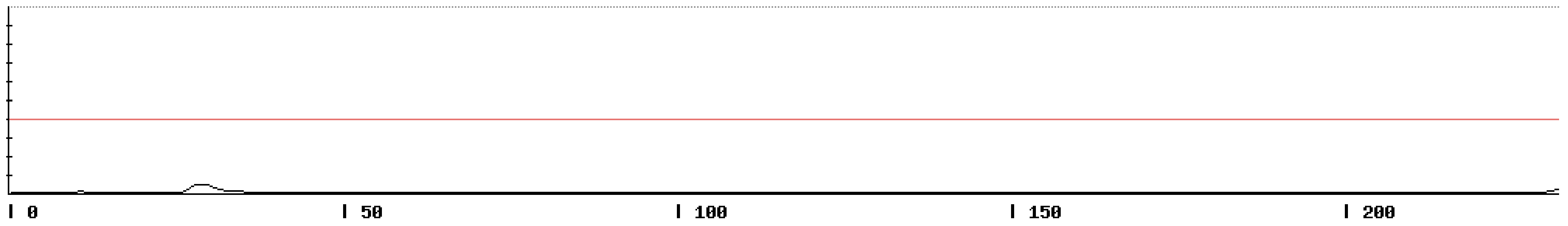

Predictions for Amaranthushypochondriacus_AHYPO_015602

Viterbi Path

Viterbi Path

Posterior @ 0.4

Posterior @ 0.4

Posterior Graphic. Threshold Marked @ 0.4

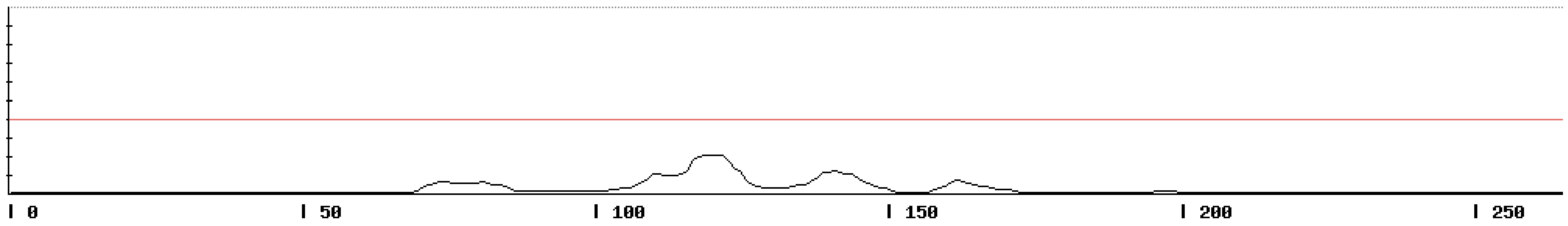

Predictions for Amaranthushypochondriacus_AHYPO_015615

Viterbi Path

Viterbi Path

Posterior @ 0.4

Posterior @ 0.4

Posterior Graphic. Threshold Marked @ 0.4

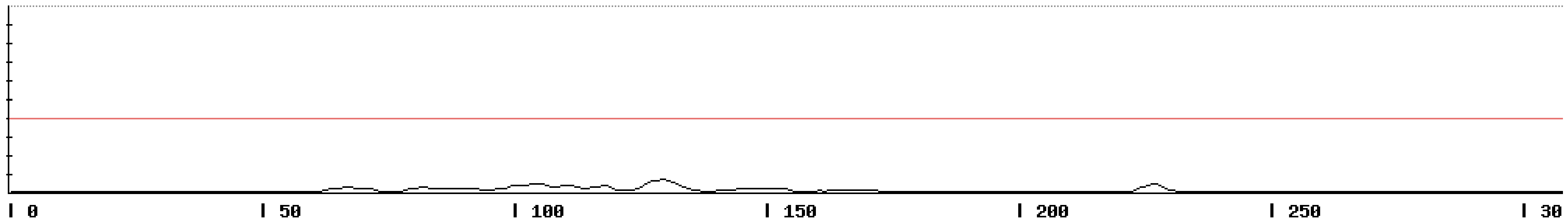

Predictions for Amaranthushypochondriacus_AHYPO_015940

Viterbi Path

Viterbi Path

Posterior @ 0.4

Posterior @ 0.4

Posterior Graphic. Threshold Marked @ 0.4

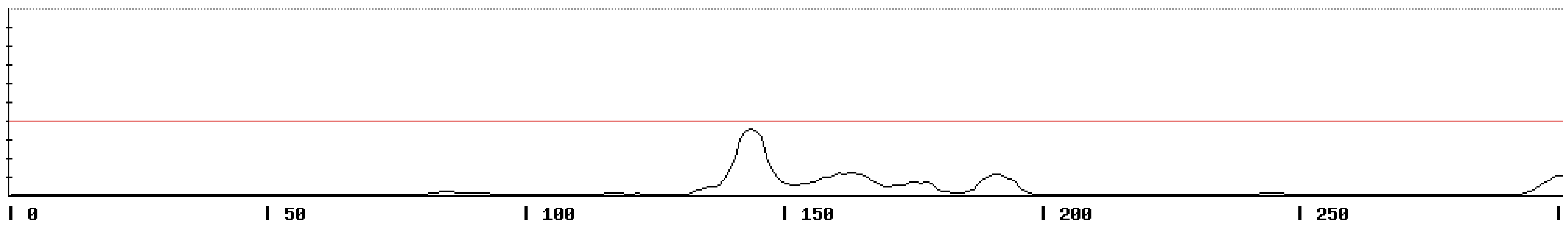

Predictions for Amaranthushypochondriacus_AHYPO_017377

Viterbi Path

Viterbi Path

Posterior @ 0.4

Posterior @ 0.4

Posterior Graphic. Threshold Marked @ 0.4

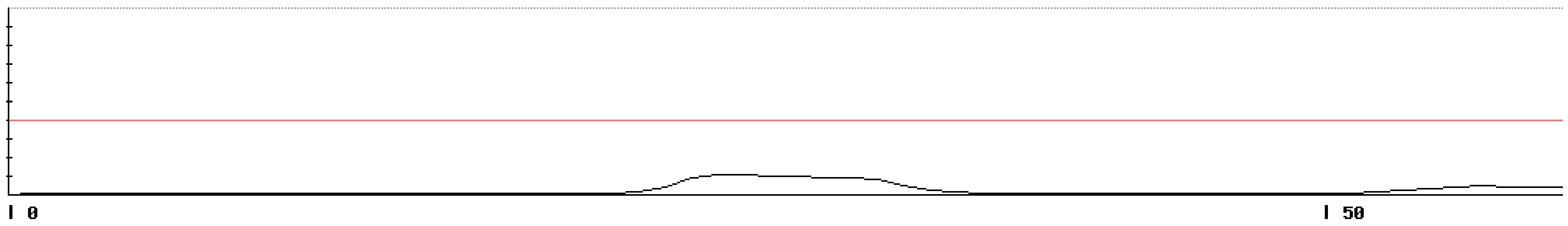

Predictions for Amaranthushypochondriacus_AHYPO_017491

Viterbi Path

Viterbi Path

Posterior @ 0.4

Posterior @ 0.4

Posterior Graphic. Threshold Marked @ 0.4

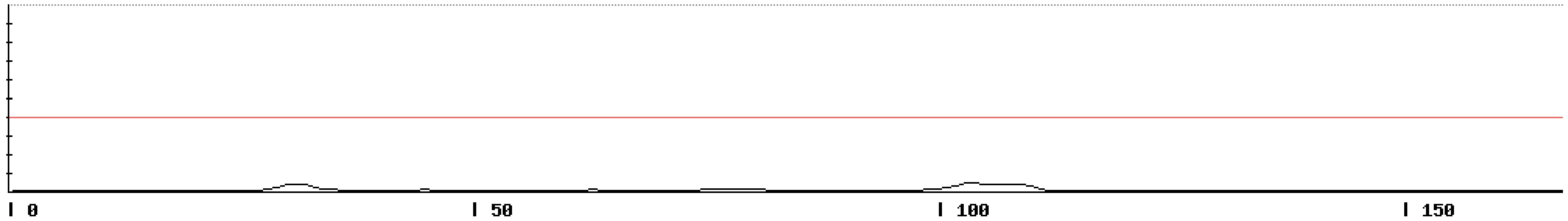

Predictions for Amaranthushypochondriacus_AHYPO_019273

Viterbi Path

Viterbi Path

Posterior @ 0.4

Posterior @ 0.4

Posterior Graphic. Threshold Marked @ 0.4

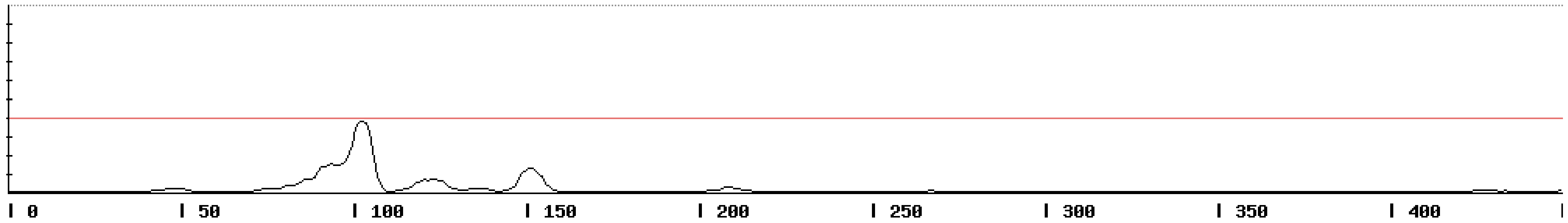

Predictions for Amaranthushypochondriacus_AHYPO_019388

Viterbi Path

Viterbi Path

Posterior @ 0.4	136 - RKRRK - 140
Posterior @ 0.4	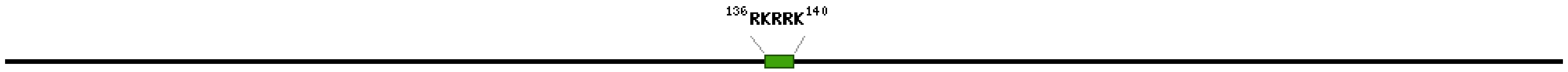
Posterior Graphic. Threshold Marked @ 0.4	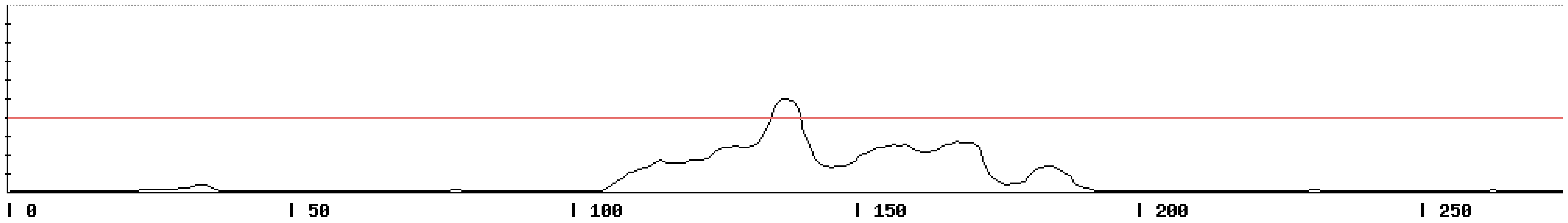

Predictions for Amaranthushypochondriacus_AHYPO_019896

Viterbi Path	
Viterbi Path	
Posterior @ 0.4	
Posterior @ 0.4	
Posterior Graphic. Threshold Marked @ 0.4	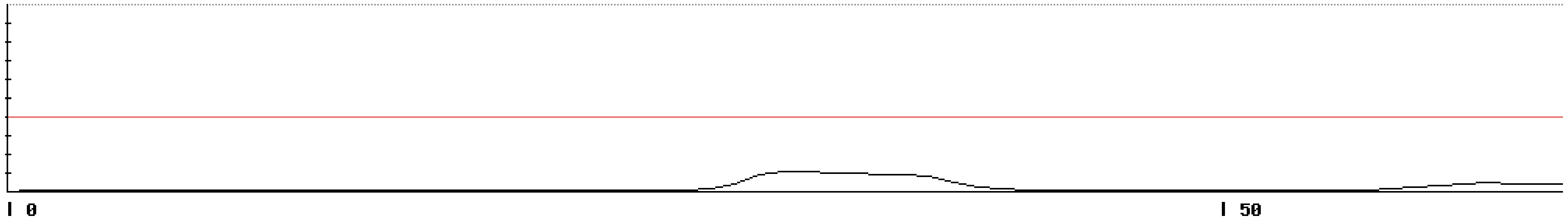

Predictions for Amaranthushypochondriacus_AHYPO_020247

Viterbi Path

Viterbi Path

Posterior @ 0.4

Posterior @ 0.4

Posterior Graphic. Threshold Marked @ 0.4

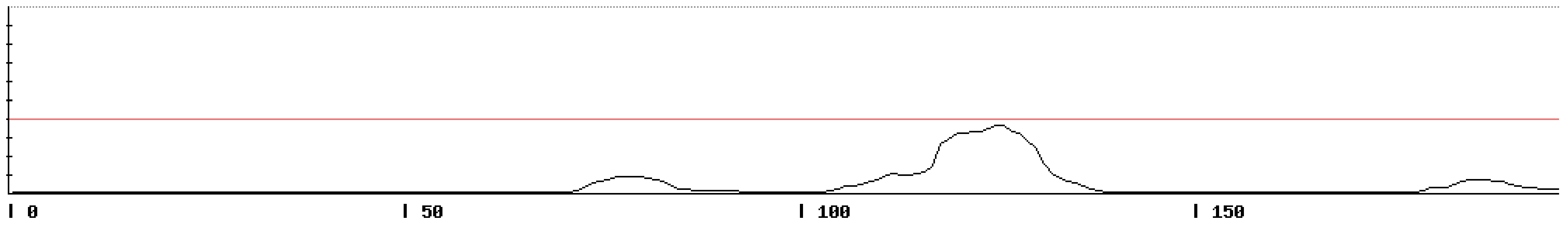

Predictions for Amaranthushypochondriacus_AHYPO_020702

Viterbi Path

Viterbi Path

Posterior @ 0.4

332 - NRRIRLKKLKI - 342

Posterior @ 0.4

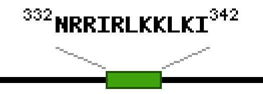

Posterior Graphic. Threshold Marked @ 0.4

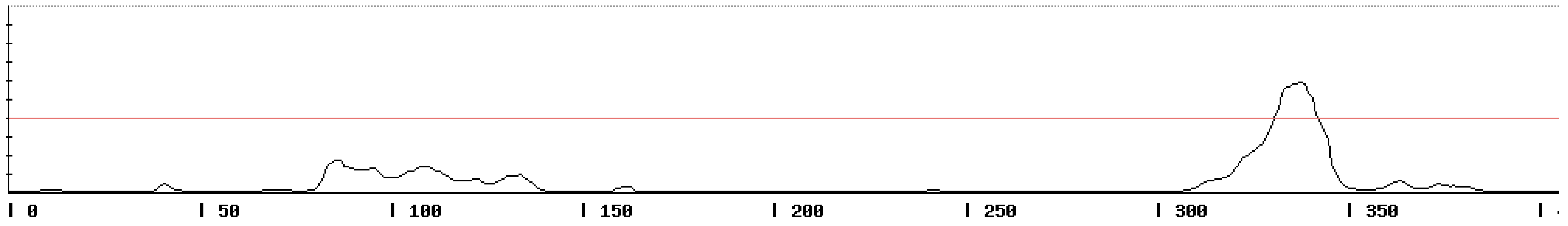

Predictions for Amaranthushypochondriacus_AHYPO_021403	
Viterbi Path	
Viterbi Path	
Posterior @ 0.4	
Posterior @ 0.4	

Revision r.9 - Last Updated on : February 24 2015 17:45:20.

alex.nguyenba@utoronto.ca

Intended to be used with Firefox

Viterbi Path	
Posterior @ 0.4	
Posterior @ 0.4	
Posterior Graphic. Threshold Marked @ 0.4	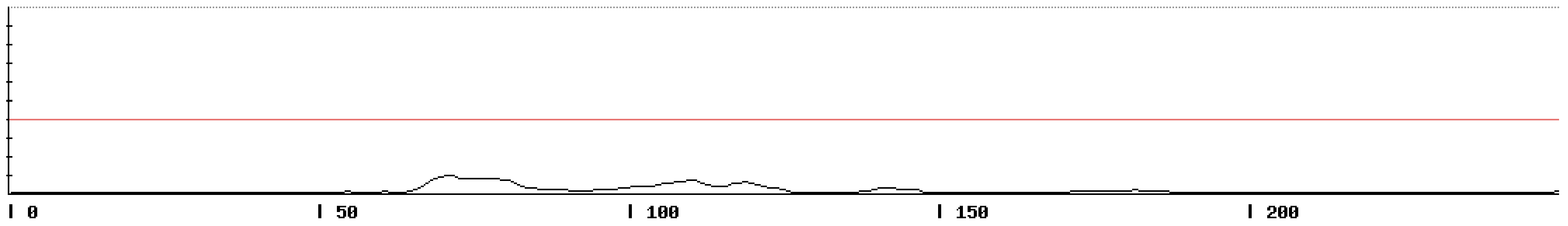

Predictions for Amborellatrichopoda_evm_27.model.AmTr_v1.0_scaffold00001.280	
Viterbi Path	
Viterbi Path	
Posterior @ 0.4	70 - KHPNGGRP - 77
Posterior @ 0.4	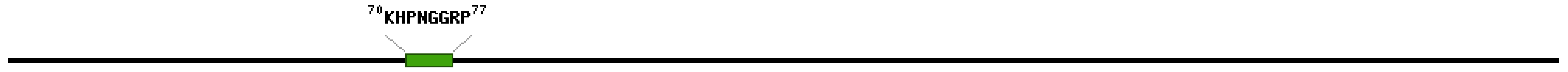
Posterior Graphic. Threshold Marked @ 0.4	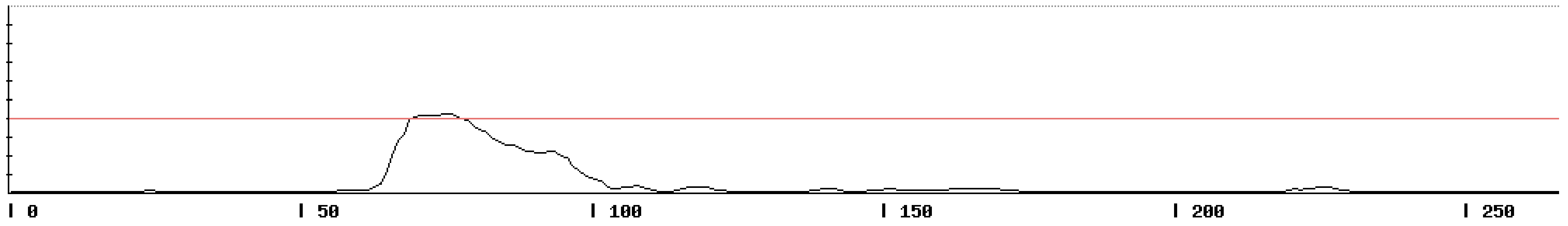

Predictions for Amborellatrichopoda_evm_27.model.AmTr_v1.0_scaffold00003.302

Viterbi Path

Viterbi Path

Posterior @ 0.4

Posterior @ 0.4

Posterior Graphic. Threshold Marked @ 0.4

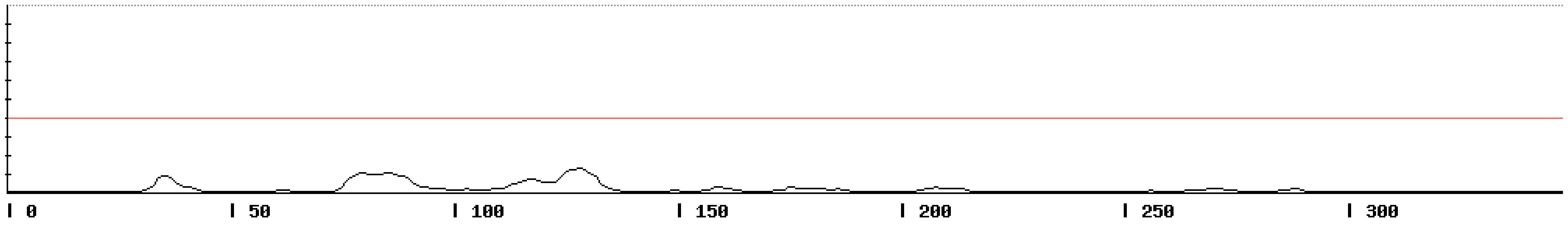

Predictions for Amborellatrichopoda_evm_27.model.AmTr_v1.0_scaffold00007.33

Viterbi Path

Viterbi Path

Posterior @ 0.4

Posterior @ 0.4

Posterior Graphic. Threshold Marked @ 0.4

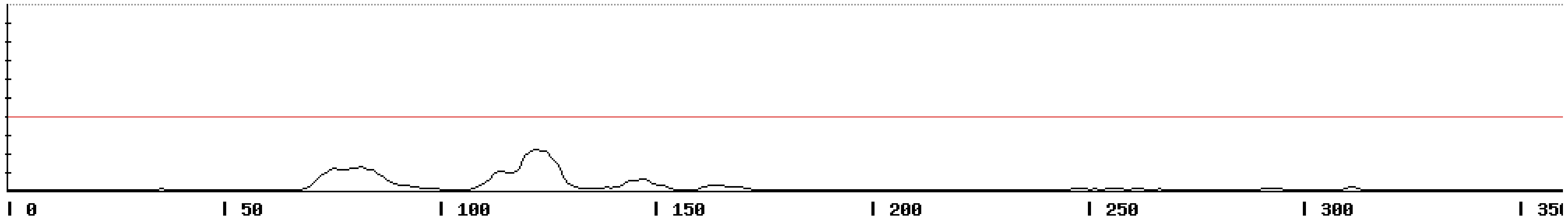

Predictions for Amborellatrichopoda_evm_27.model.AmTr_v1.0_scaffold00009.309	
Viterbi Path	
Viterbi Path	
Posterior @ 0.4	106 - NKKAV - 110
Posterior @ 0.4	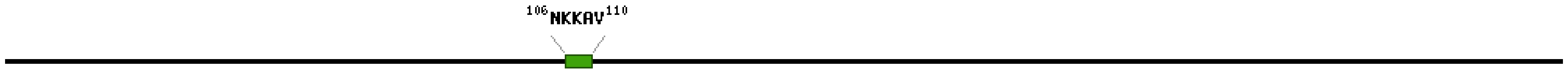
Posterior Graphic. Threshold Marked @ 0.4	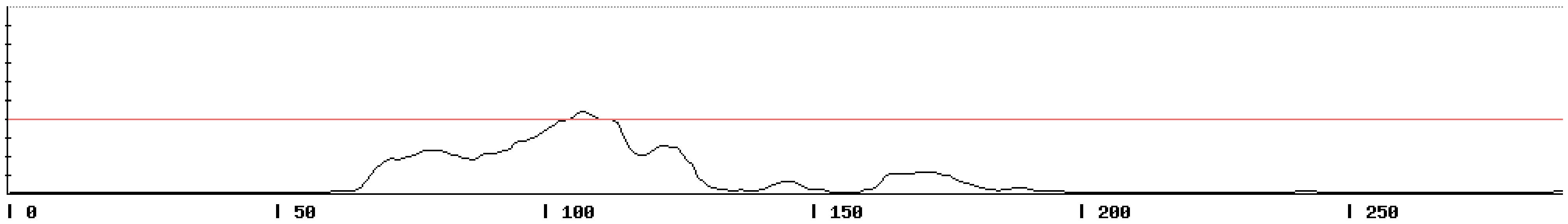

Predictions for Amborellatrichopoda_evm_27.model.AmTr_v1.0_scaffold00010.432	
Viterbi Path	
Viterbi Path	
Posterior @ 0.4	
Posterior @ 0.4	
Posterior Graphic. Threshold Marked @ 0.4	

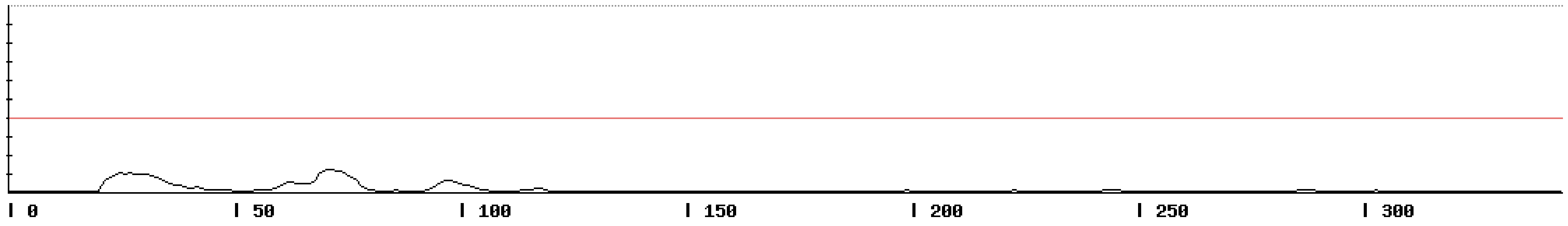

Predictions for Amborellatrichopoda_evm_27.model.AmTr_v1.0_scaffold00010.523	
Viterbi Path	
Viterbi Path	
Posterior @ 0.4	
Posterior @ 0.4	
Posterior Graphic. Threshold Marked @ 0.4	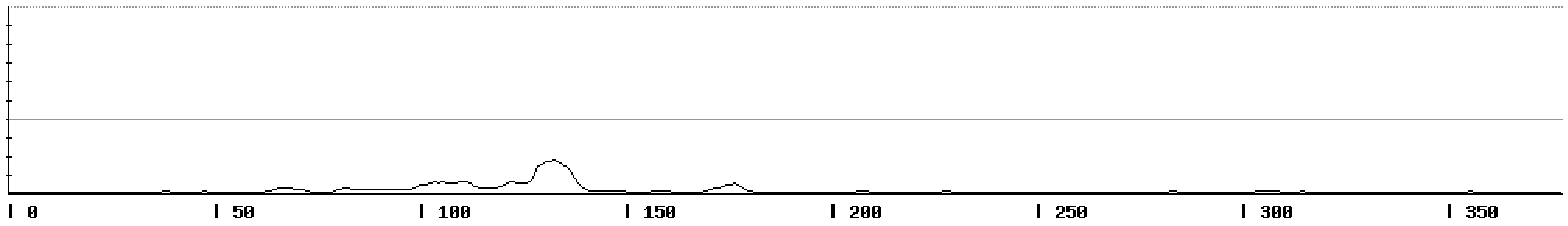

Predictions for Amborellatrichopoda_evm_27.model.AmTr_v1.0_scaffold00011.156	
Viterbi Path	
Viterbi Path	
Posterior @ 0.4	
Posterior @ 0.4	

Posterior Graphic. Threshold Marked @ 0.4

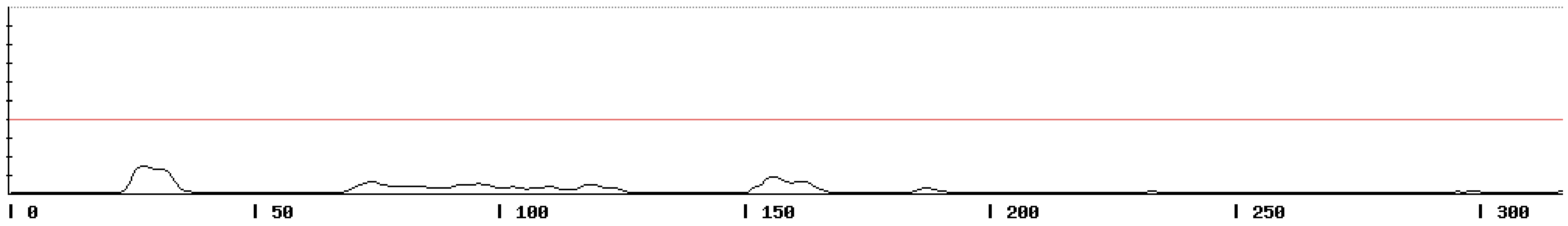

Predictions for Amborellatrichopoda_evm_27.model.AmTr_v1.0_scaffold00016.162

Viterbi Path	
Viterbi Path	
Posterior @ 0.4	173 - KKRGPKREA - 181
Posterior @ 0.4	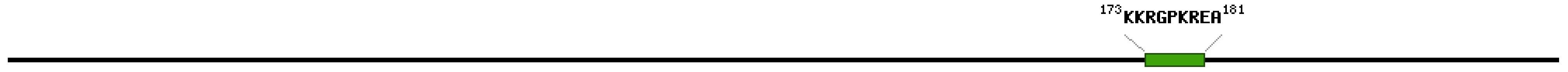
Posterior Graphic. Threshold Marked @ 0.4	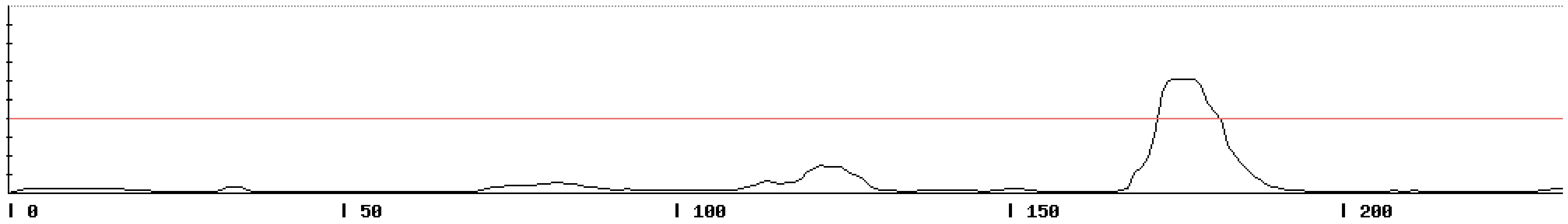

Predictions for Amborellatrichopoda_evm_27.model.AmTr_v1.0_scaffold00017.277

Viterbi Path	
Viterbi Path	
Posterior @ 0.4	70 - KRRQVKG - 76
Posterior @ 0.4	

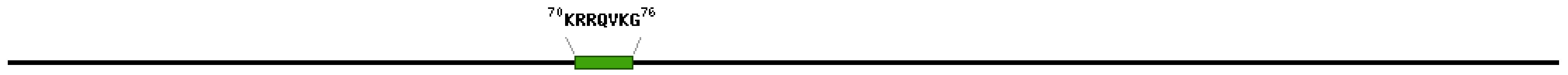

Posterior Graphic. Threshold Marked @ 0.4

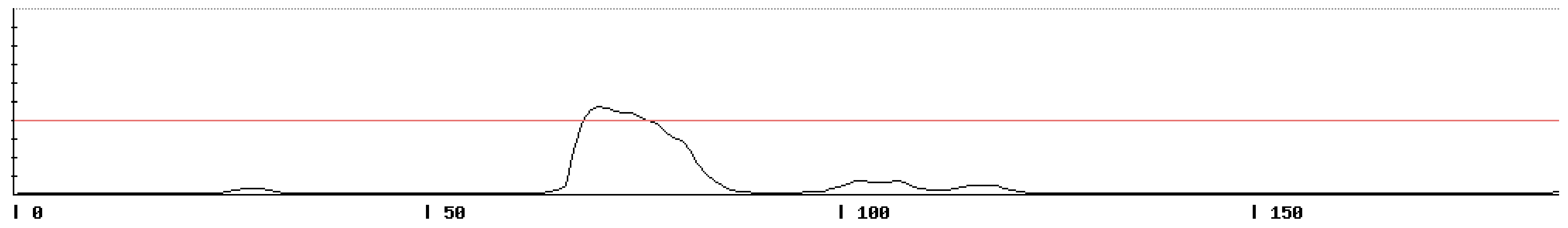

Predictions for Amborellatrichopoda_evm_27.model.AmTr_v1.0_scaffold00017.45

Viterbi Path

Viterbi Path

Posterior @ 0.4

Posterior @ 0.4

Posterior Graphic. Threshold Marked @ 0.4

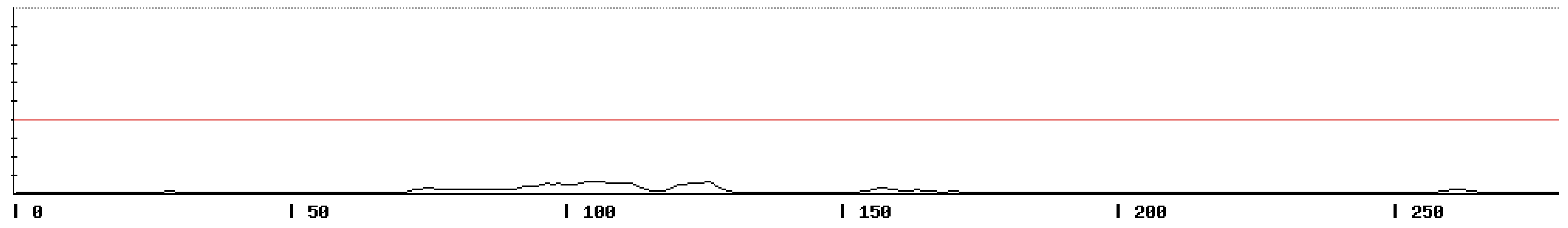

Predictions for Amborellatrichopoda_evm_27.model.AmTr_v1.0_scaffold00018.24

Viterbi Path

Viterbi Path

Posterior @ 0.4	80 - RRSNRA - 85
Posterior @ 0.4	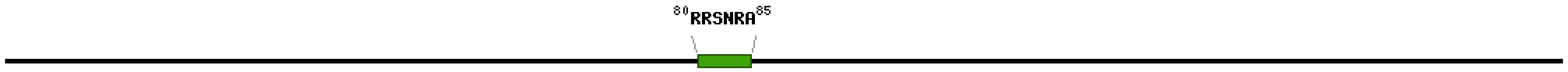
Posterior Graphic. Threshold Marked @ 0.4	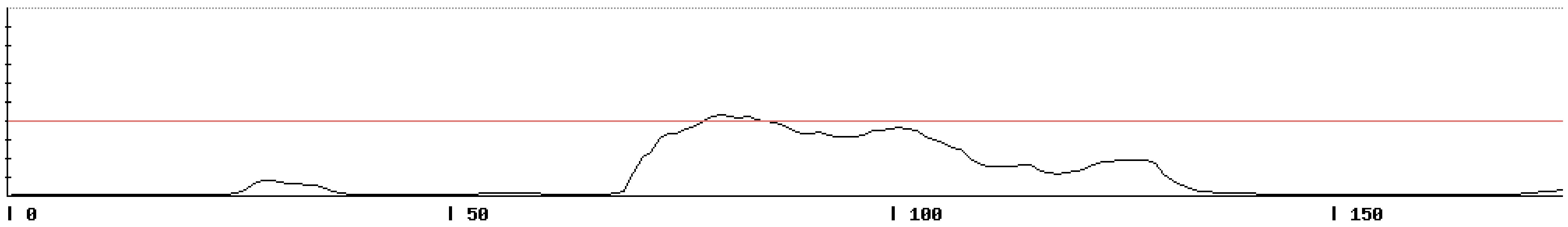

Predictions for Amborellatrichopoda_evm_27.model.AmTr_v1.0_scaffold00018.27	
Viterbi Path	
Viterbi Path	
Posterior @ 0.4	94 - TGKDRTIKSKSRRS - 107
	450 - LARRRPKKKVLRRSPTRAM - 468
Posterior @ 0.4	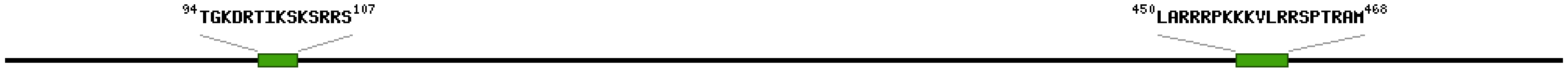
Posterior Graphic. Threshold Marked @ 0.4	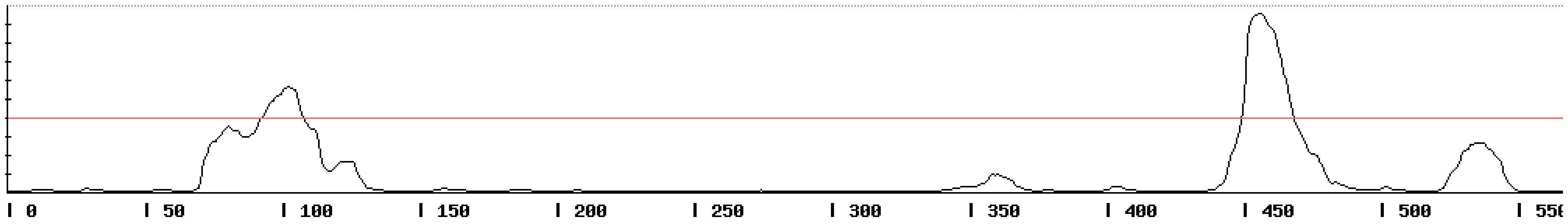

Predictions for Amborellatrichopoda_evm_27.model.AmTr_v1.0_scaffold00018.28	
Viterbi Path	
Viterbi Path	

Posterior @ 0.4	87 - WKATGKARKIKSKFH - 101
Posterior @ 0.4	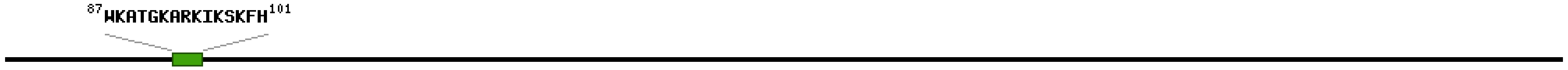
Posterior Graphic. Threshold Marked @ 0.4	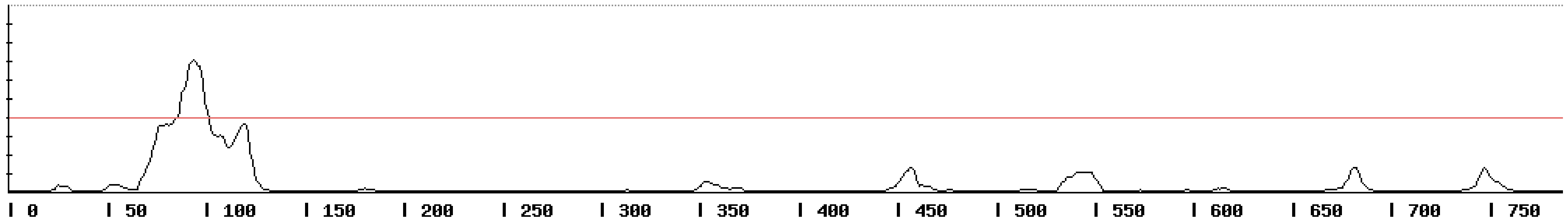

Predictions for Amborellatrichopoda_evm_27.model.AmTr_v1.0_scaffold00021.181

Viterbi Path	
Viterbi Path	
Posterior @ 0.4	144 - GTRKRRKVHTGPQ - 156
Posterior @ 0.4	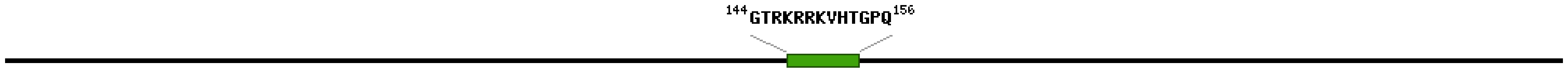
Posterior Graphic. Threshold Marked @ 0.4	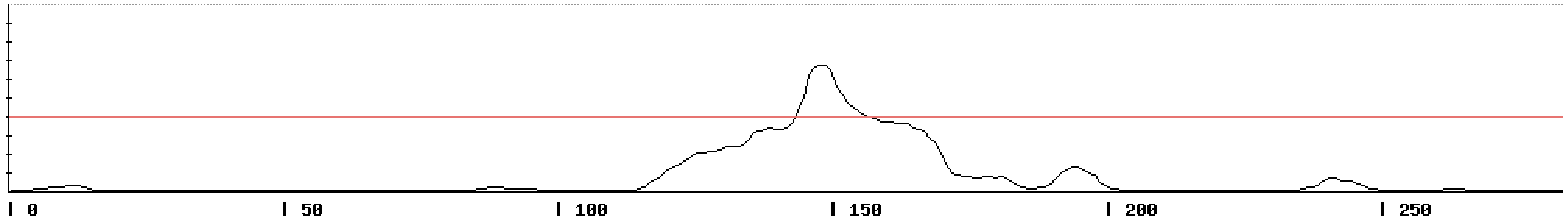

Predictions for Amborellatrichopoda_evm_27.model.AmTr_v1.0_scaffold00022.163

Viterbi Path

Viterbi Path

Posterior @ 0.4

Posterior @ 0.4

Posterior Graphic. Threshold Marked @ 0.4

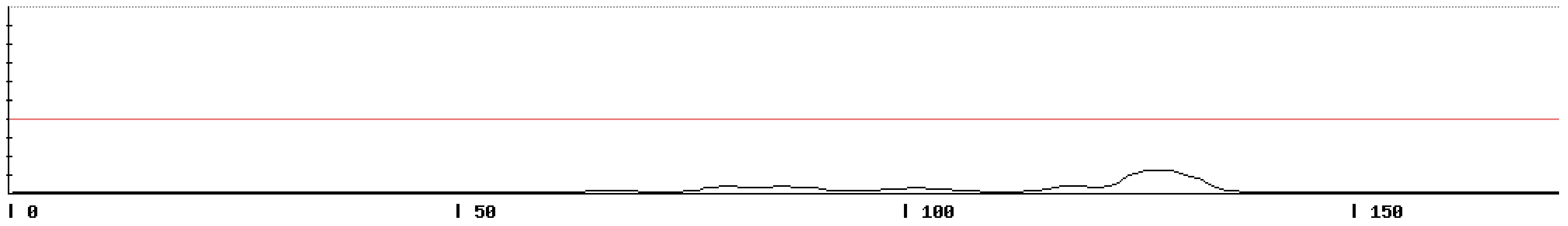

Predictions for Amborellatrichopoda_evm_27.model.AmTr_v1.0_scaffold00022.74

Viterbi Path

Viterbi Path

Posterior @ 0.4

Posterior @ 0.4

Posterior Graphic. Threshold Marked @ 0.4

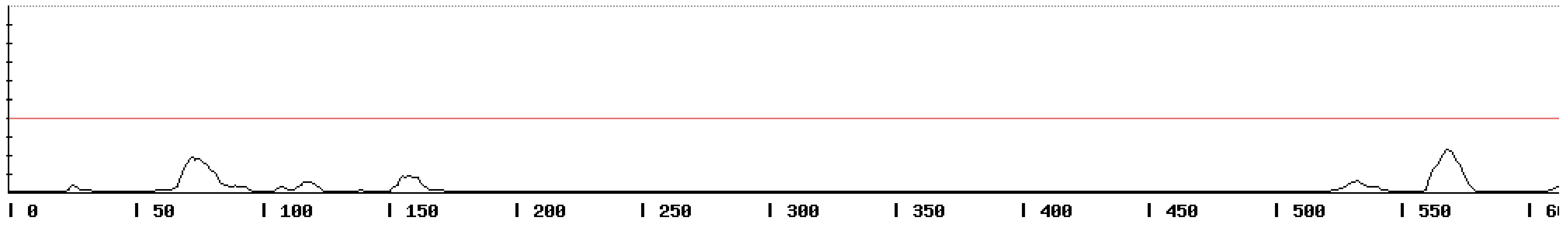

Predictions for Amborellatrichopoda_evm_27.model.AmTr_v1.0_scaffold00025.209	
Viterbi Path	
Viterbi Path	
Posterior @ 0.4	
Posterior @ 0.4	
Posterior Graphic. Threshold Marked @ 0.4	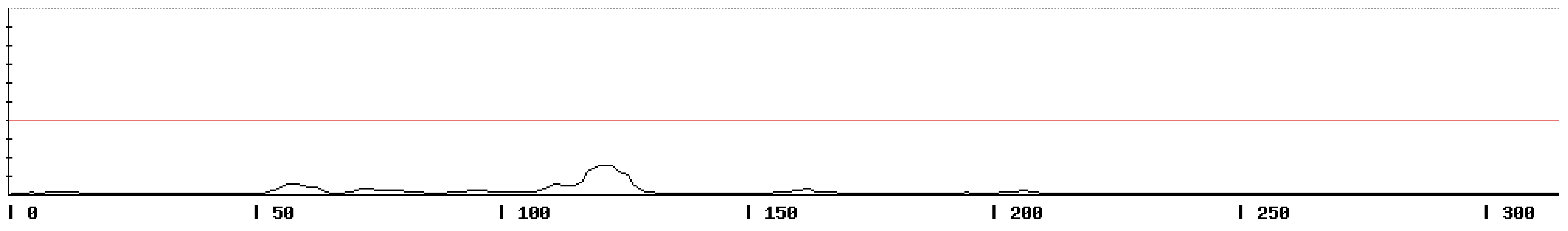

Predictions for Amborellatrichopoda_evm_27.model.AmTr_v1.0_scaffold00032.87	
Viterbi Path	
Viterbi Path	
Posterior @ 0.4	
Posterior @ 0.4	
Posterior Graphic. Threshold Marked @ 0.4	

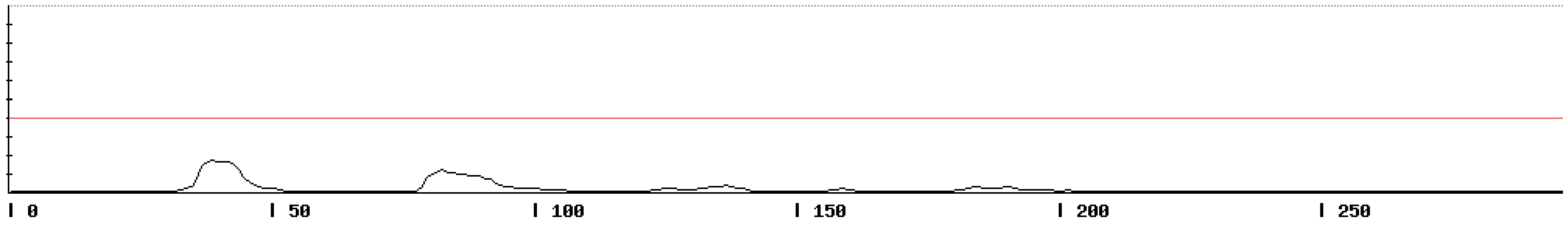

Predictions for Amborellatrichopoda_evm_27.model.AmTr_v1.0_scaffold00037.62

Viterbi Path

Viterbi Path

Posterior @ 0.4

Posterior @ 0.4

Posterior Graphic. Threshold Marked @ 0.4

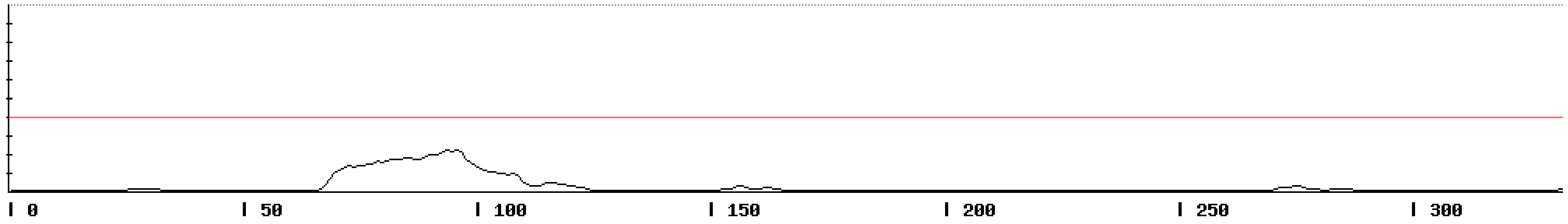

Predictions for Amborellatrichopoda_evm_27.model.AmTr_v1.0_scaffold00040.86

Viterbi Path

Viterbi Path

Posterior @ 0.4

Posterior @ 0.4

Posterior Graphic. Threshold Marked @ 0.4	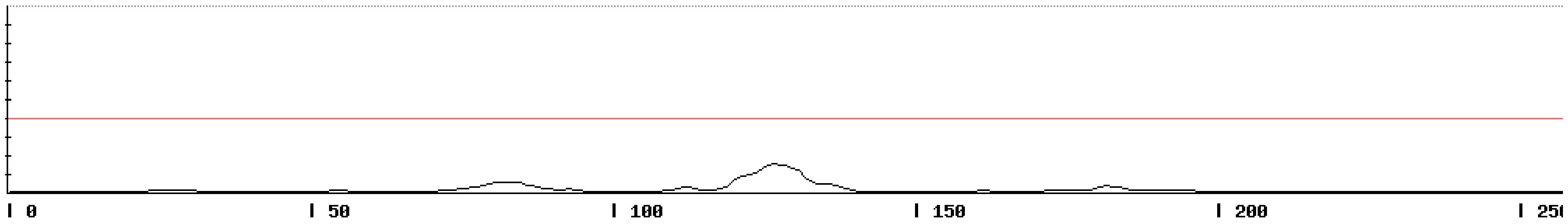

Predictions for Amborellatrichopoda_evm_27.model.AmTr_v1.0_scaffold00040.87

Viterbi Path	
Viterbi Path	
Posterior @ 0.4	
Posterior @ 0.4	
Posterior Graphic. Threshold Marked @ 0.4	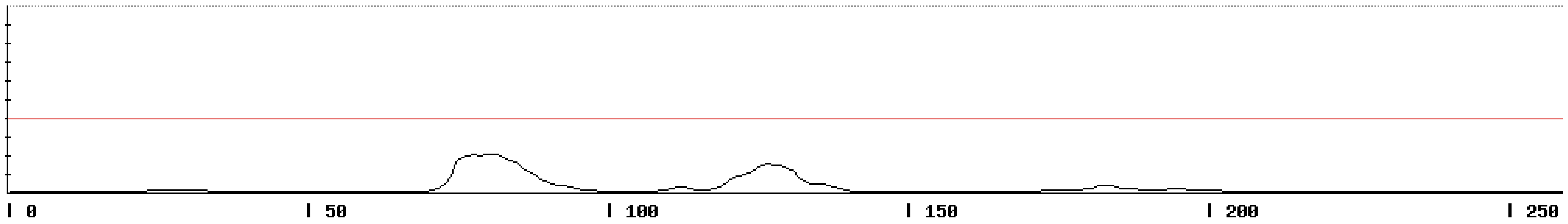

Predictions for Amborellatrichopoda_evm_27.model.AmTr_v1.0_scaffold00044.29

Viterbi Path	
Viterbi Path	
Posterior @ 0.4	
Posterior @ 0.4	

Posterior Graphic. Threshold Marked @ 0.4

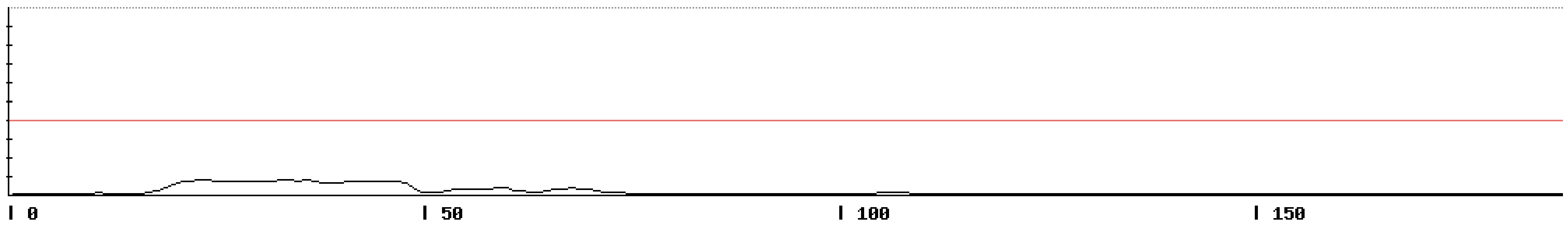

Predictions for Amborellatrichopoda_evm_27.model.AmTr_v1.0_scaffold00044.30

Viterbi Path

Viterbi Path

Posterior @ 0.4 | 743 - KR - 744

Posterior @ 0.4

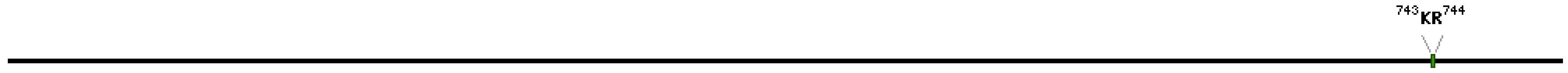

Posterior Graphic. Threshold Marked @ 0.4

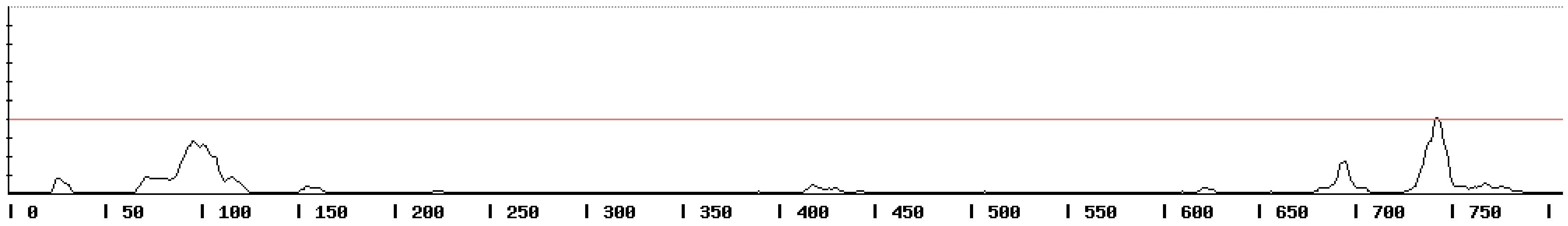

Predictions for Amborellatrichopoda_evm_27.model.AmTr_v1.0_scaffold00046.19

Viterbi Path

Viterbi Path

Posterior @ 0.4	
Posterior @ 0.4	
Posterior Graphic. Threshold Marked @ 0.4	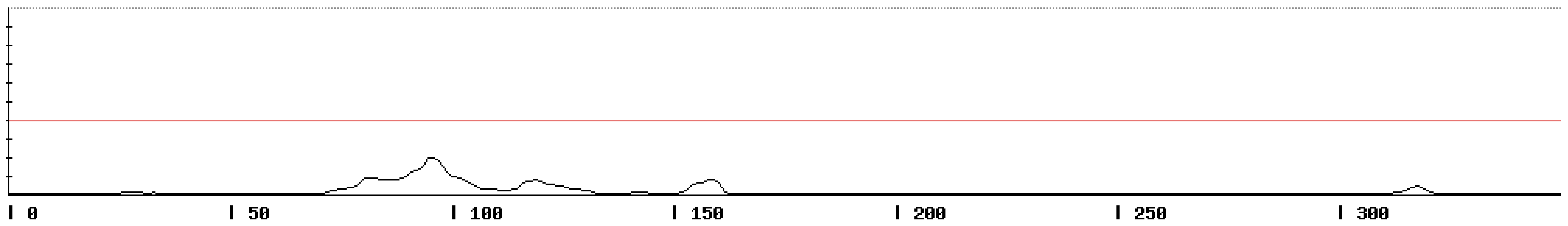

Predictions for Amborellatrichopoda_evm_27.model.AmTr_v1.0_scaffold00049.251

Viterbi Path	
Viterbi Path	
Posterior @ 0.4	135 - RKRRKI - 140
Posterior @ 0.4	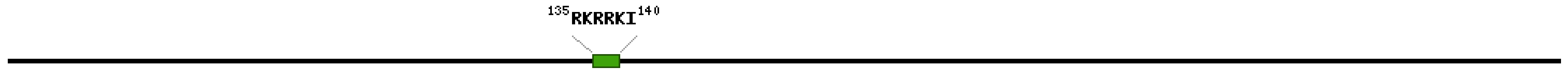
Posterior Graphic. Threshold Marked @ 0.4	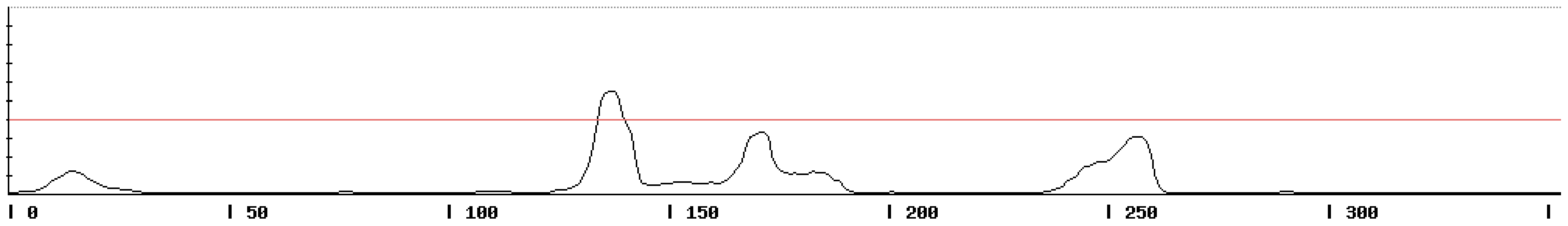

Predictions for Amborellatrichopoda_evm_27.model.AmTr_v1.0_scaffold00058.98

Viterbi Path	
Viterbi Path	

Posterior @ 0.4	
Posterior @ 0.4	
Posterior Graphic. Threshold Marked @ 0.4	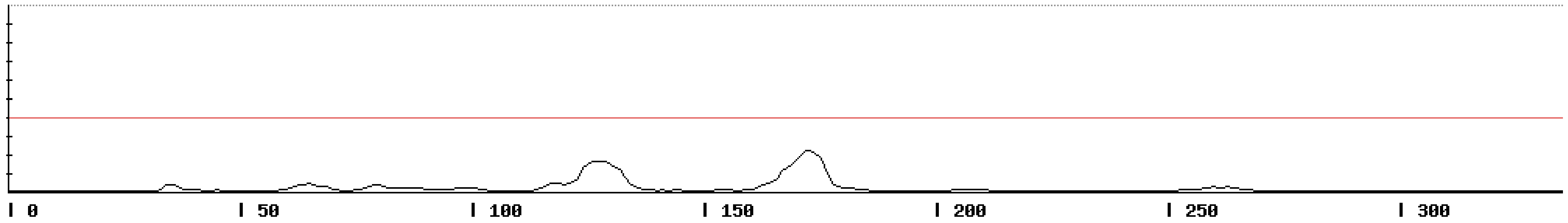

Predictions for Amborellatrichopoda_evm_27.model.AmTr_v1.0_scaffold00059.206	
Viterbi Path	
Viterbi Path	
Posterior @ 0.4	
Posterior @ 0.4	
Posterior Graphic. Threshold Marked @ 0.4	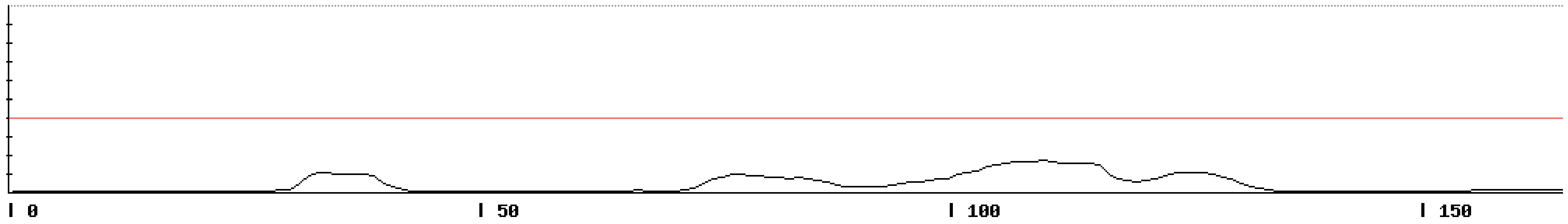

Predictions for Amborellatrichopoda_evm_27.model.AmTr_v1.0_scaffold00060.14

Viterbi Path

Viterbi Path

Posterior @ 0.4

Posterior @ 0.4

Posterior Graphic. Threshold Marked @ 0.4

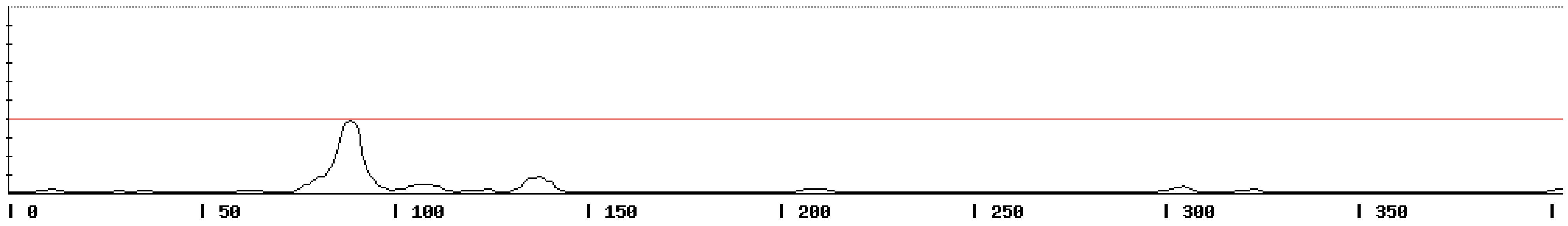

Predictions for Amborellatrichopoda_evm_27.model.AmTr_v1.0_scaffold00067.40

Viterbi Path

Viterbi Path

Posterior @ 0.4

Posterior @ 0.4

Posterior Graphic. Threshold Marked @ 0.4

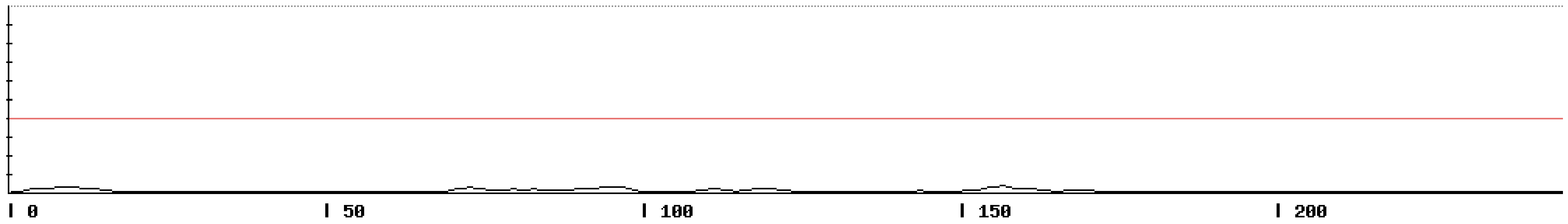

Predictions for Amborellatrichopoda_evm_27.model.AmTr_v1.0_scaffold00068.186	
Viterbi Path	
Viterbi Path	
Posterior @ 0.4	
Posterior @ 0.4	
Posterior Graphic. Threshold Marked @ 0.4	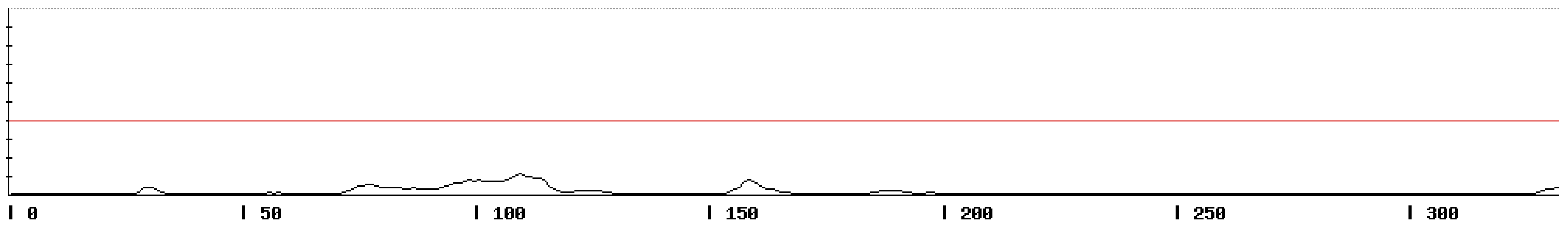

Predictions for Amborellatrichopoda_evm_27.model.AmTr_v1.0_scaffold00071.30	
Viterbi Path	
Viterbi Path	
Posterior @ 0.4	
Posterior @ 0.4	
Posterior Graphic. Threshold Marked @ 0.4	

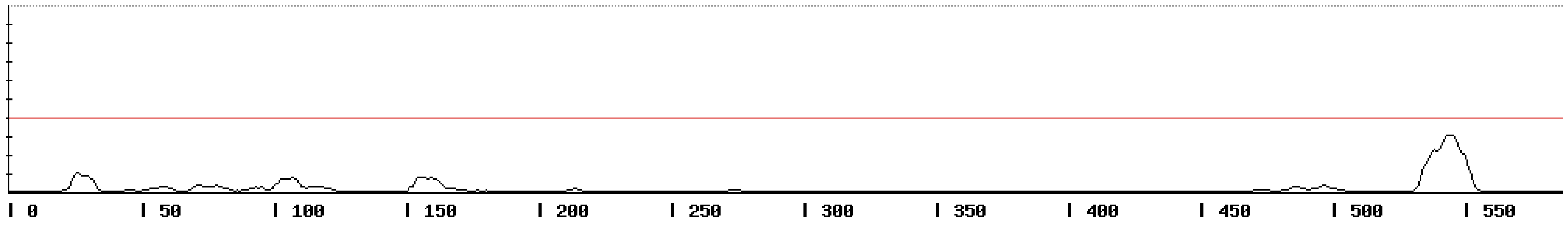

Predictions for Amborellatrichopoda_evm_27.model.AmTr_v1.0_scaffold00079.33

Viterbi Path	
Viterbi Path	
Posterior @ 0.4	
Posterior @ 0.4	
Posterior Graphic. Threshold Marked @ 0.4	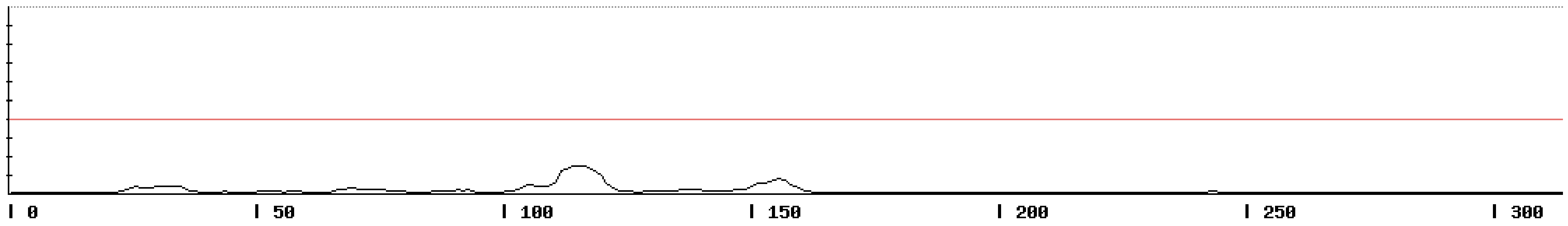

Predictions for Amborellatrichopoda_evm_27.model.AmTr_v1.0_scaffold00082.23

Viterbi Path	482 - KVKSRQKGRKARRPPKENVSLTKKQVKK - 509
Viterbi Path	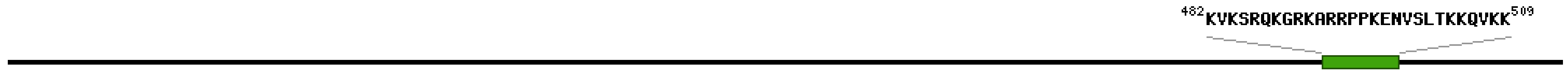
Posterior @ 0.4	80 - KKYPNGSRTNRATKAGFWKATGKDRNVKFRRRTIGTKK - 117
	480 - VAKVKSRQKGRKARRPPKENVSLTKKQVKK - 509
Posterior @ 0.4	

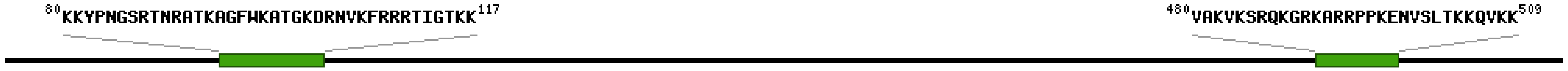

Posterior Graphic. Threshold Marked @ 0.4

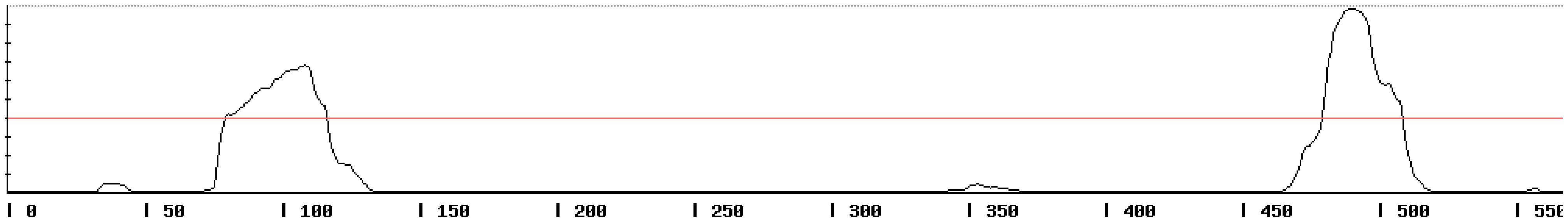

Predictions for Amborellatrichopoda_evm_27.model.AmTr_v1.0_scaffold00092.111

Viterbi Path

Viterbi Path

Posterior @ 0.4

Posterior @ 0.4

Posterior Graphic. Threshold Marked @ 0.4

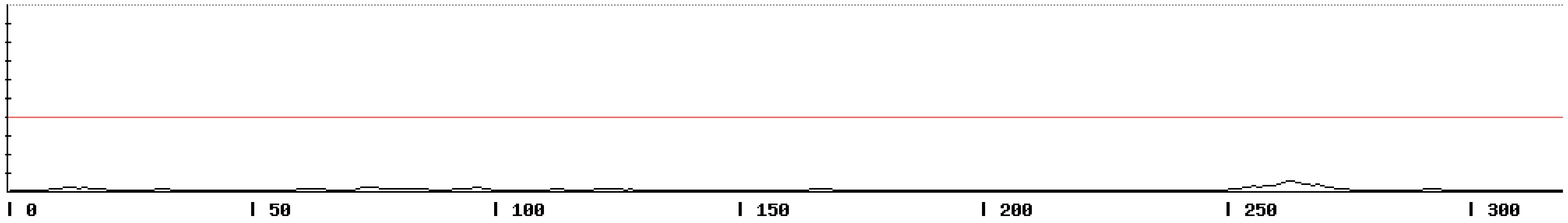

Predictions for Amborellatrichopoda_evm_27.model.AmTr_v1.0_scaffold00099.117

Viterbi Path

Viterbi Path

Posterior @ 0.4

Posterior @ 0.4

Posterior Graphic. Threshold Marked @ 0.4

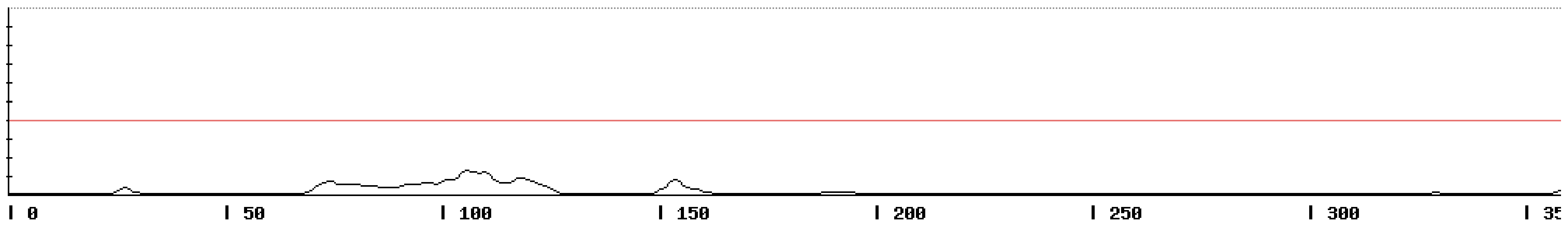

Predictions for Amborellatrichopoda_evm_27.model.AmTr_v1.0_scaffold00099.164

Viterbi Path

Viterbi Path

Posterior @ 0.4

Posterior @ 0.4

Posterior Graphic. Threshold Marked @ 0.4

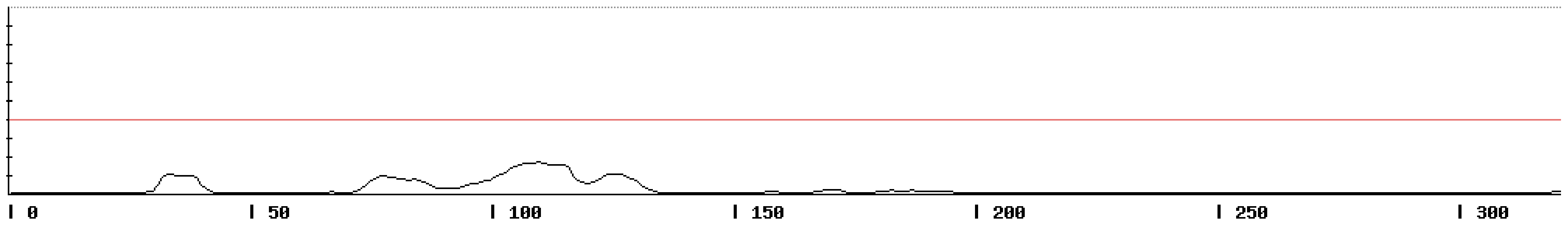

Predictions for Amborellatrichopoda_evm_27.model.AmTr_v1.0_scaffold00113.16

Viterbi Path

Viterbi Path

Posterior @ 0.4

Posterior @ 0.4

Posterior Graphic. Threshold Marked @ 0.4

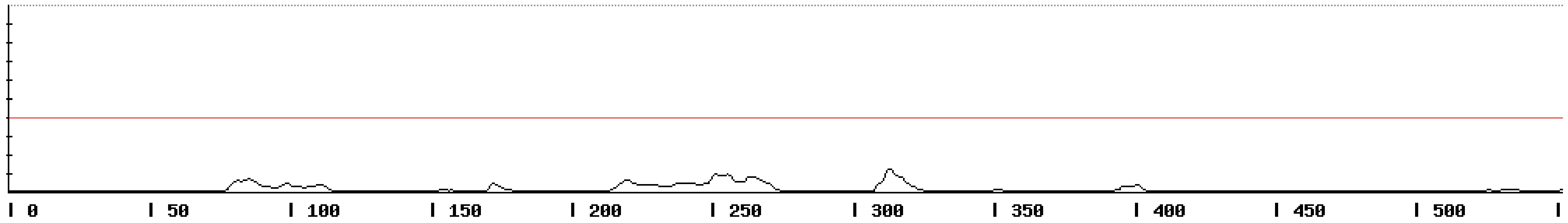

Predictions for Amborellatrichopoda_evm_27.model.AmTr_v1.0_scaffold00119.12

Viterbi Path

Viterbi Path

Posterior @ 0.4

Posterior @ 0.4

Posterior Graphic. Threshold Marked @ 0.4

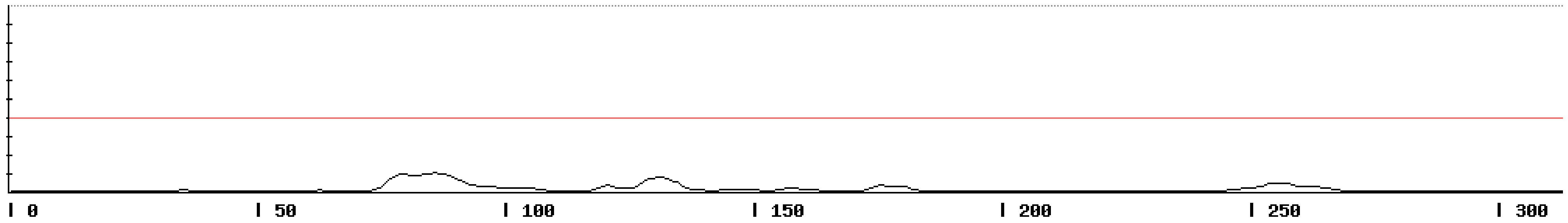

Predictions for Amborellatrichopoda_evm_27.model.AmTr_v1.0_scaffold00119.21

Viterbi Path

Viterbi Path

Posterior @ 0.4

Posterior @ 0.4

Posterior Graphic. Threshold Marked @ 0.4

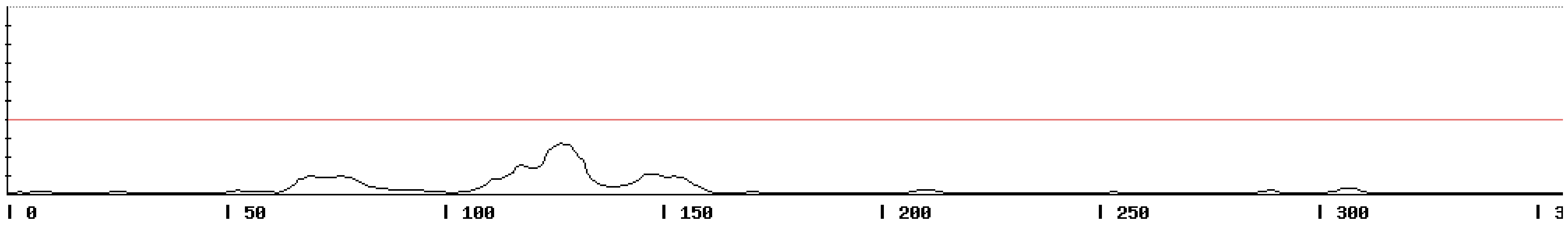

Predictions for Amborellatrichopoda_evm_27.model.AmTr_v1.0_scaffold00119.25

Viterbi Path

Viterbi Path

Posterior @ 0.4

Posterior @ 0.4

Posterior Graphic. Threshold Marked @ 0.4

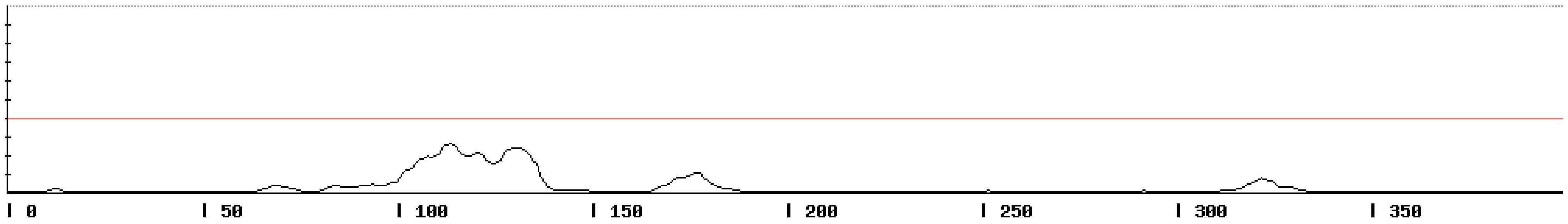

Predictions for Amborellatrichopoda_evm_27.model.AmTr_v1.0_scaffold00140.39	
Viterbi Path	
Viterbi Path	
Posterior @ 0.4	96 - RPIRR - 100
Posterior @ 0.4	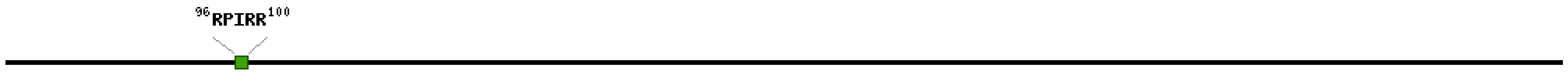
Posterior Graphic. Threshold Marked @ 0.4	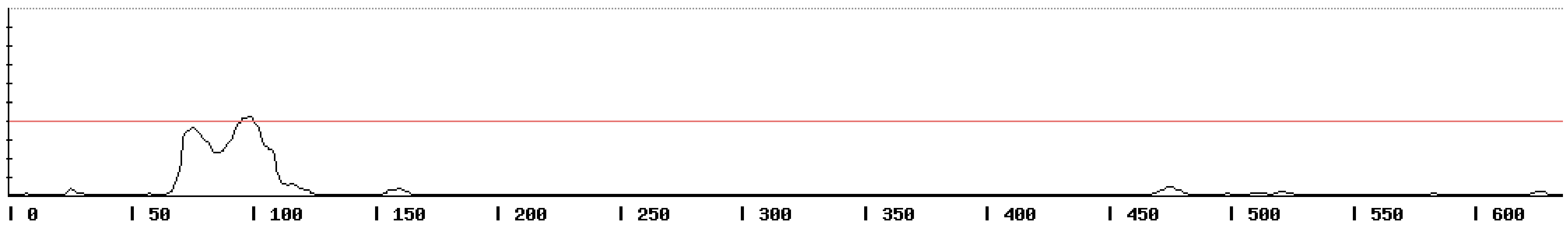

Predictions for Amborellatrichopoda_evm_27.model.AmTr_v1.0_scaffold00149.46	
Viterbi Path	
Viterbi Path	
Posterior @ 0.4	
Posterior @ 0.4	
Posterior Graphic. Threshold Marked @ 0.4	

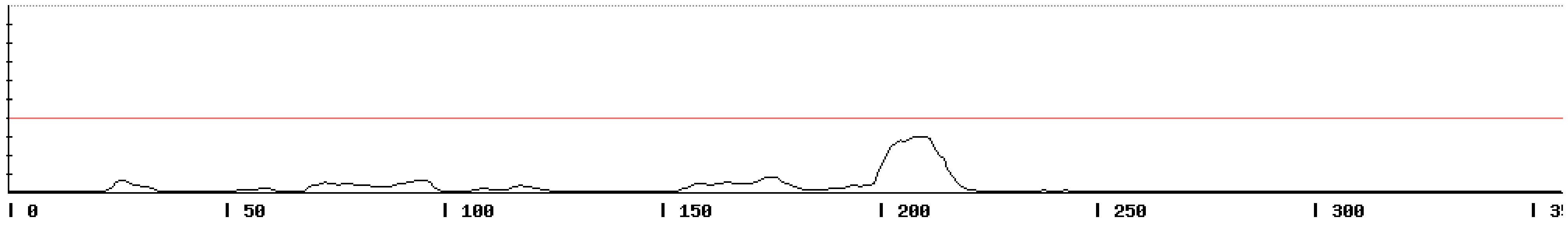

Predictions for Amborellatrichopoda_evm_27.model.AmTr_v1.0_scaffold00210.5

Revision r.9 - Last Updated on : February 24 2015 17:45:20. alex.nguyenba@utoronto.ca

Intended to be used with Firefox

Viterbi Path

Posterior @ 0.4

Posterior @ 0.4

Posterior Graphic. Threshold Marked @ 0.4

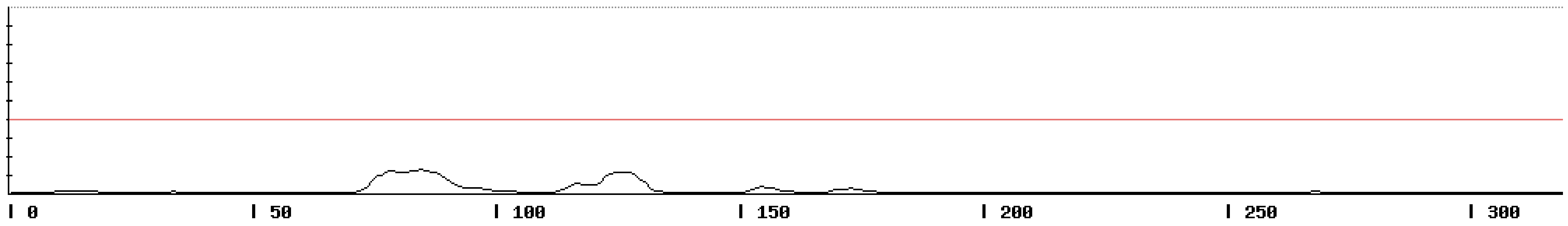

Predictions for Ananascomosus_Aco000364.1

Viterbi Path

Viterbi Path

Posterior @ 0.4

Posterior @ 0.4

Posterior Graphic. Threshold Marked @ 0.4

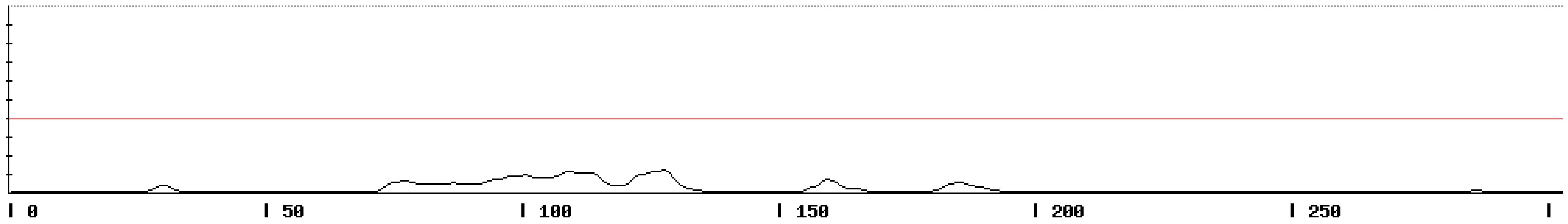

Predictions for Ananascomosus_Aco000744.1

Viterbi Path	
Viterbi Path	
Posterior @ 0.4	
Posterior @ 0.4	
Posterior Graphic. Threshold Marked @ 0.4	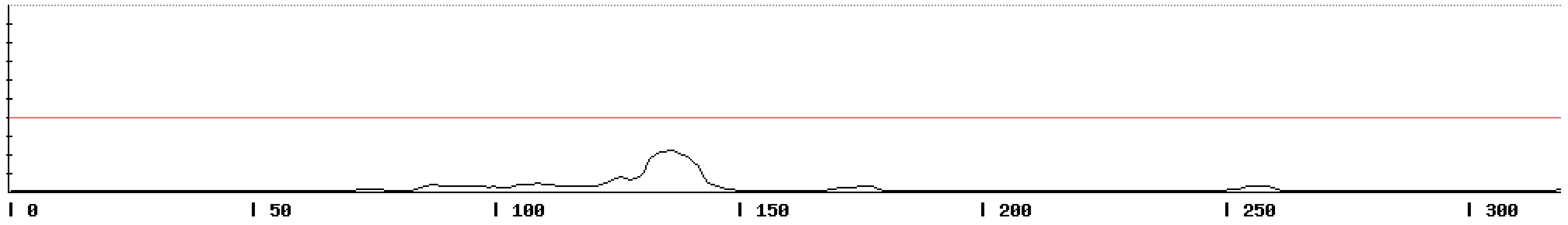

Predictions for Ananascomosus_Aco001109.1

Viterbi Path	
Viterbi Path	
Posterior @ 0.4	
Posterior @ 0.4	
Posterior Graphic. Threshold Marked @ 0.4	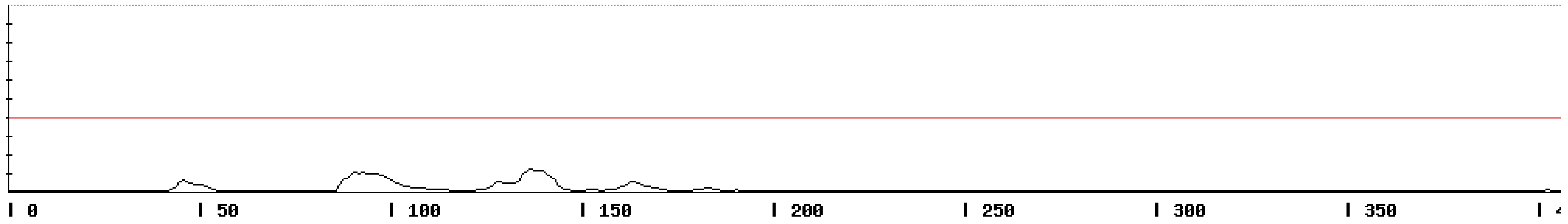

Predictions for Ananascomosus_Aco001674.1

Viterbi Path

Viterbi Path

Posterior @ 0.4

Posterior @ 0.4

Posterior Graphic. Threshold Marked @ 0.4

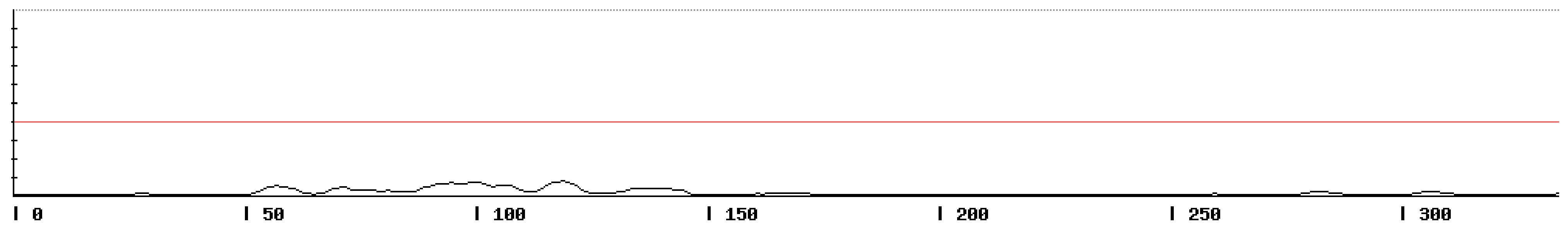

Predictions for Ananascomosus_Aco002066.1

Viterbi Path

Viterbi Path

Posterior @ 0.4

175 - KKSAREIARSHKRKREEAA - 193

Posterior @ 0.4

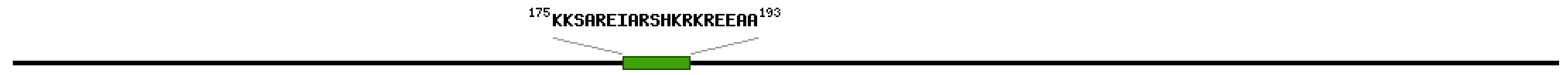

Posterior Graphic. Threshold Marked @ 0.4

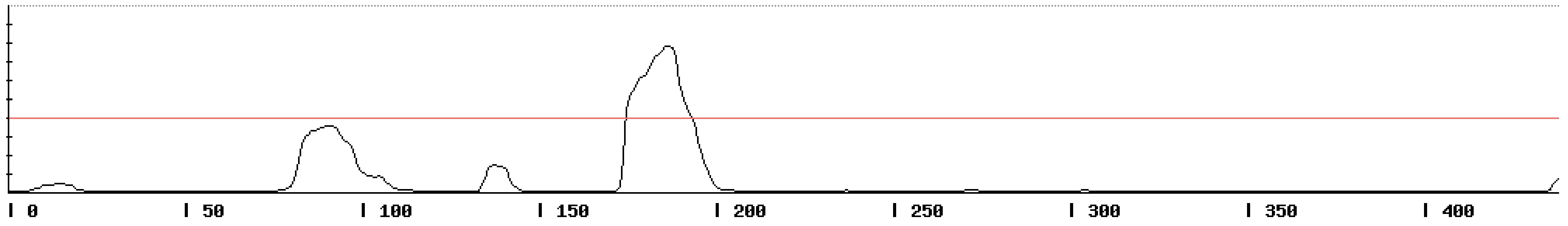

Predictions for Ananascomosus_Aco002068.1

Viterbi Path	
Viterbi Path	
Posterior @ 0.4	102 - KKSAREIARSHKRKREEAA - 120
Posterior @ 0.4	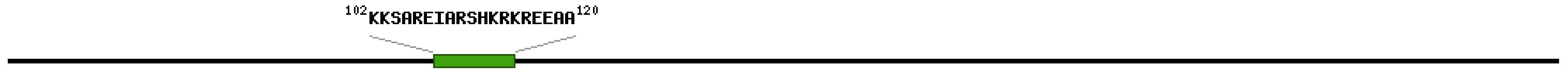
Posterior Graphic. Threshold Marked @ 0.4	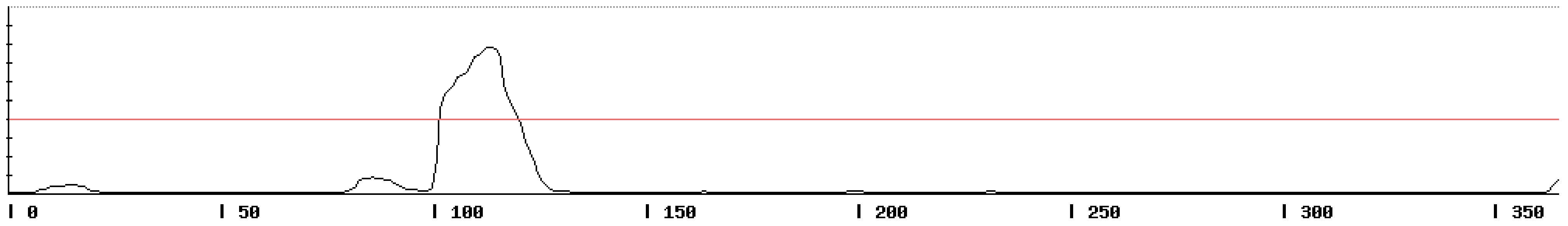

Predictions for Ananascomosus_Aco002878.1

Viterbi Path	
Viterbi Path	
Posterior @ 0.4	
Posterior @ 0.4	

Posterior Graphic. Threshold Marked @ 0.4

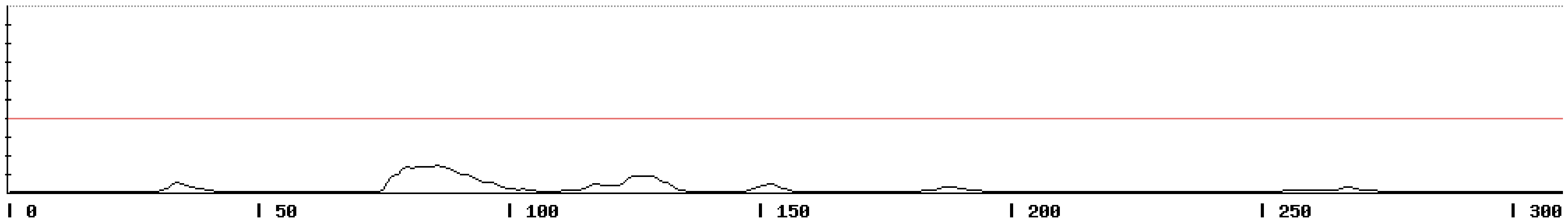

Predictions for Ananascomosus_Aco003015.1

Viterbi Path

Viterbi Path

Posterior @ 0.4

Posterior @ 0.4

Posterior Graphic. Threshold Marked @ 0.4

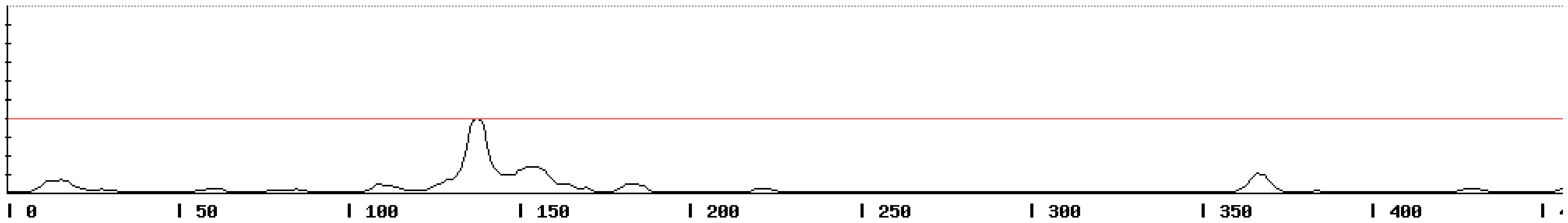

Predictions for Ananascomosus_Aco003302.1

Viterbi Path

Viterbi Path

Posterior @ 0.4

Posterior @ 0.4

Posterior Graphic. Threshold Marked @ 0.4

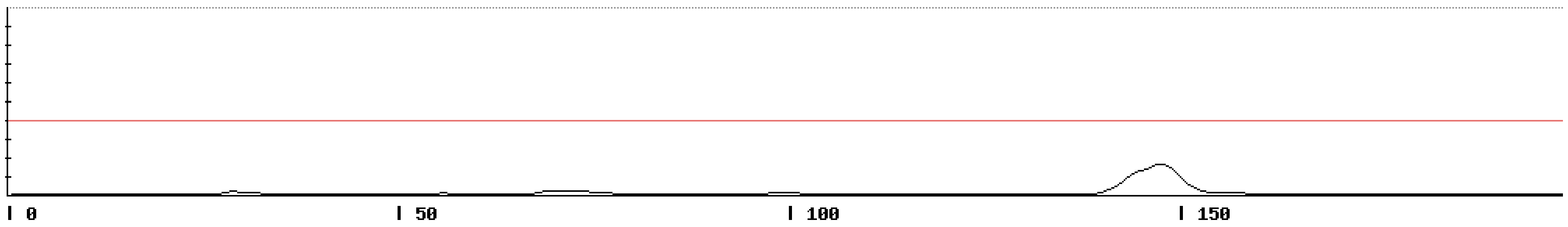

Predictions for Ananascomosus_Aco003626.1

Viterbi Path

Viterbi Path

Posterior @ 0.4

Posterior @ 0.4

Posterior Graphic. Threshold Marked @ 0.4

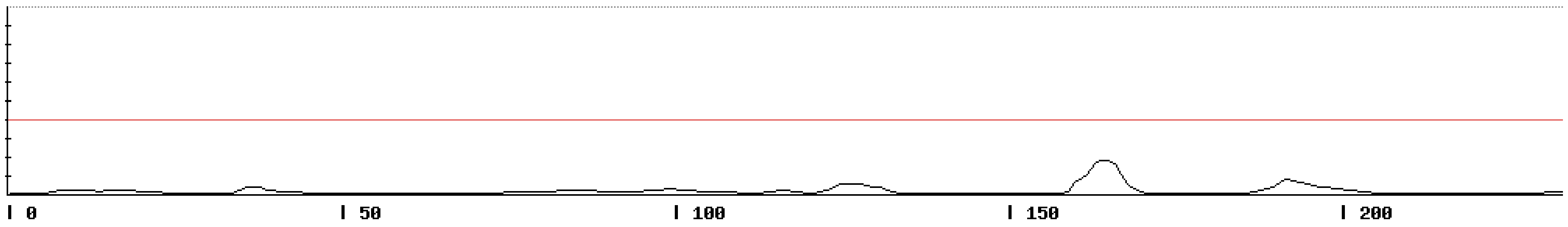

Predictions for Ananascomosus_Aco003781.1

Viterbi Path

Viterbi Path

Posterior @ 0.4

Posterior @ 0.4

Posterior Graphic. Threshold Marked @ 0.4

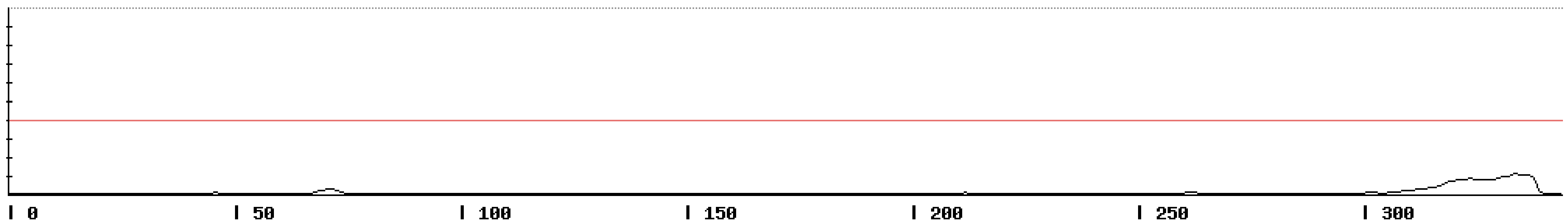

Predictions for Ananascomosus_Aco004364.1

Viterbi Path

Viterbi Path

Posterior @ 0.4

Posterior @ 0.4

Posterior Graphic. Threshold Marked @ 0.4

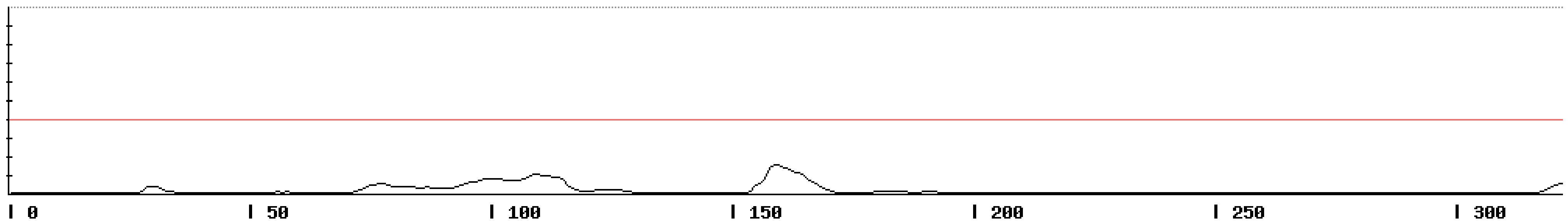

Predictions for Ananascomosus_Aco004632.1

Viterbi Path

Viterbi Path

Posterior @ 0.4

Posterior @ 0.4

Posterior Graphic. Threshold Marked @ 0.4

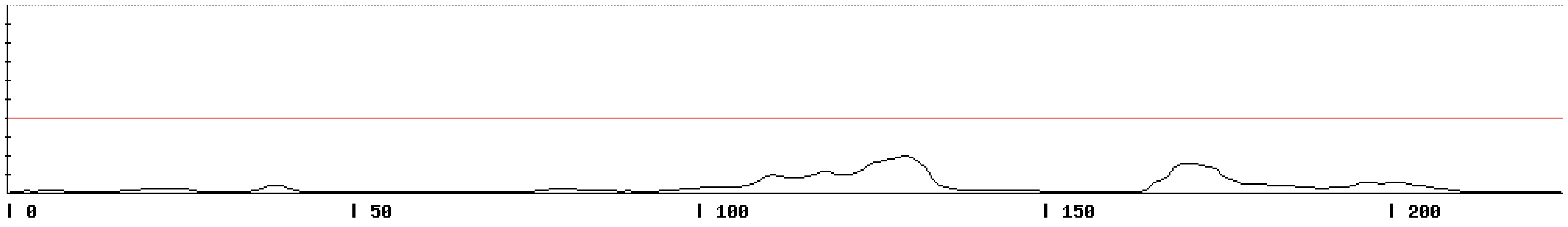

Predictions for Ananascomosus_Aco004776.1

Viterbi Path

Viterbi Path

Posterior @ 0.4

Posterior @ 0.4

Posterior Graphic. Threshold Marked @ 0.4

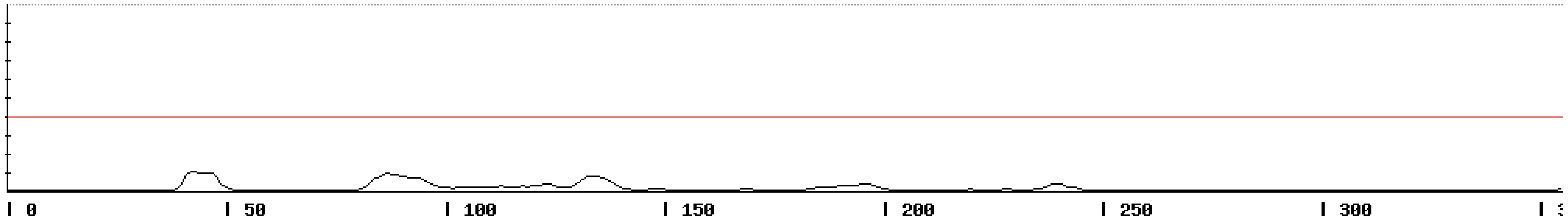

Predictions for Ananascomosus_Aco005019.1

Viterbi Path

Viterbi Path

Posterior @ 0.4

Posterior @ 0.4

Posterior Graphic. Threshold Marked @ 0.4

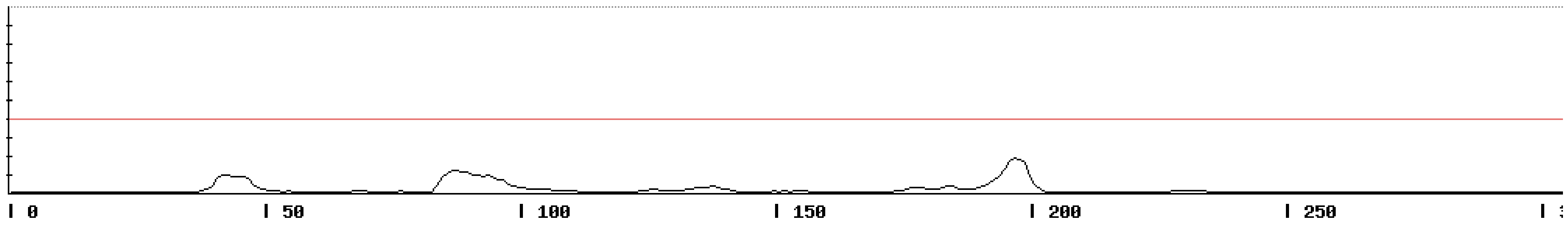

Predictions for Ananascomosus_Aco005059.1

Viterbi Path

Viterbi Path

Posterior @ 0.4

Posterior @ 0.4

Posterior Graphic. Threshold Marked @ 0.4

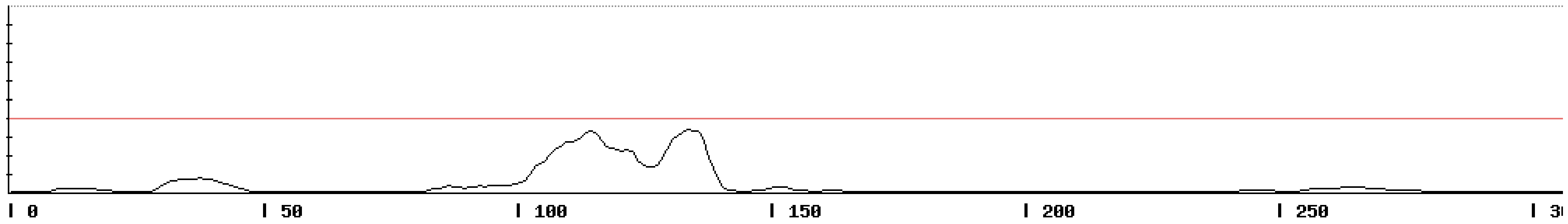

Predictions for Ananascomosus_Aco006854.1	
Viterbi Path	
Viterbi Path	
Posterior @ 0.4	
Posterior @ 0.4	
Posterior Graphic. Threshold Marked @ 0.4	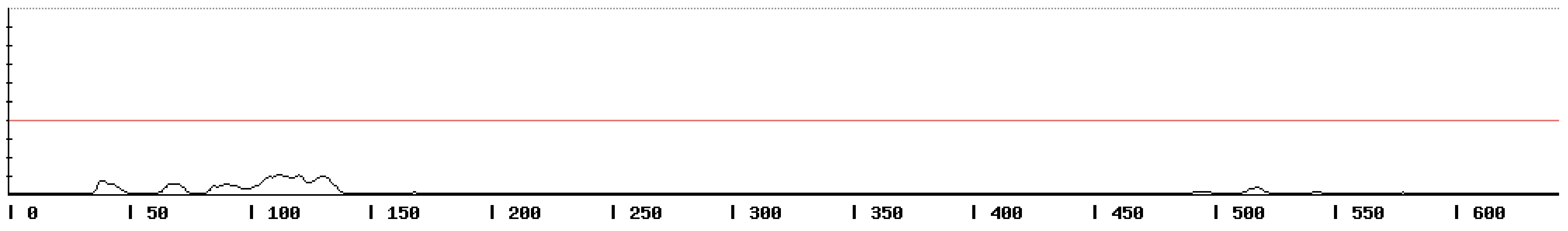

Predictions for Ananascomosus_Aco006979.1	
Viterbi Path	
Viterbi Path	
Posterior @ 0.4	
Posterior @ 0.4	
Posterior Graphic. Threshold Marked @ 0.4	

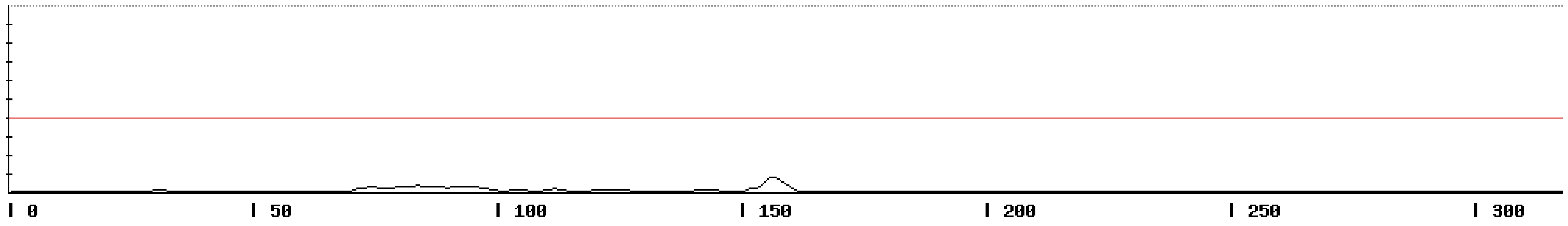

Predictions for Ananascomosus_Aco007448.1

Viterbi Path

Viterbi Path

Posterior @ 0.4

Posterior @ 0.4

Posterior Graphic. Threshold Marked @ 0.4

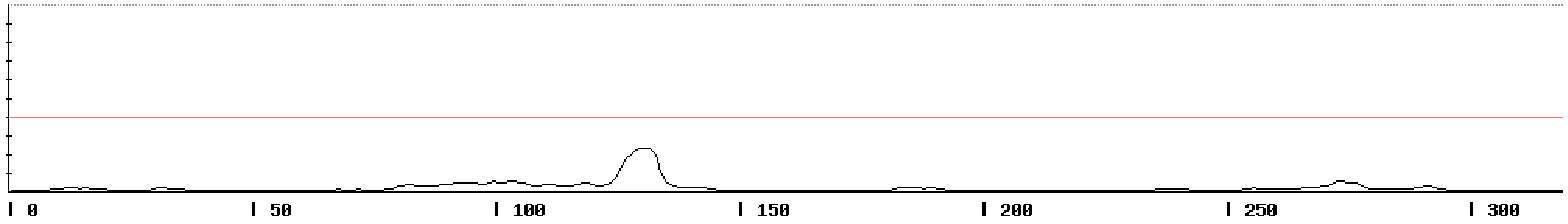

Predictions for Ananascomosus_Aco007609.1

Viterbi Path

Viterbi Path

Posterior @ 0.4

Posterior @ 0.4

Posterior Graphic. Threshold Marked @ 0.4

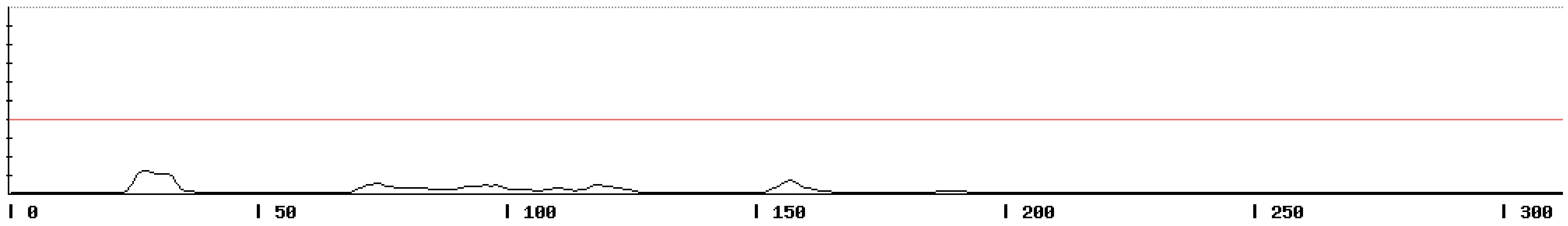

Predictions for Ananascomosus_Aco007742.1	
Viterbi Path	
Viterbi Path	
Posterior @ 0.4	
Posterior @ 0.4	
Posterior Graphic. Threshold Marked @ 0.4	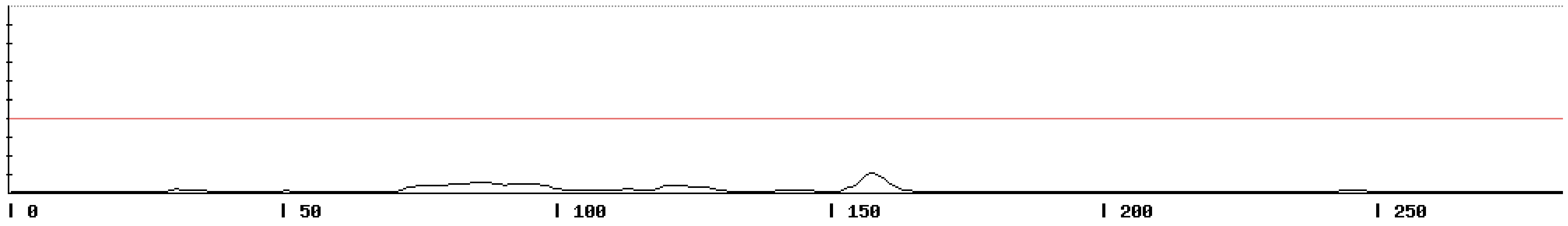

Predictions for Ananascomosus_Aco007925.1	
Viterbi Path	
Viterbi Path	
Posterior @ 0.4	146 - KKR - 148
Posterior @ 0.4	

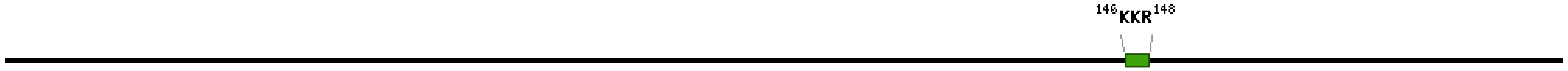

Posterior Graphic. Threshold Marked @ 0.4

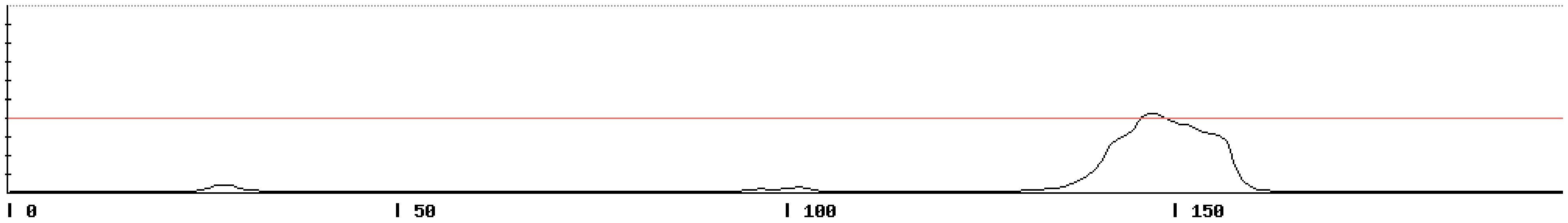

Predictions for Ananascomosus_Aco008000.1

Viterbi Path

Viterbi Path

Posterior @ 0.4

Posterior @ 0.4

Posterior Graphic. Threshold Marked @ 0.4

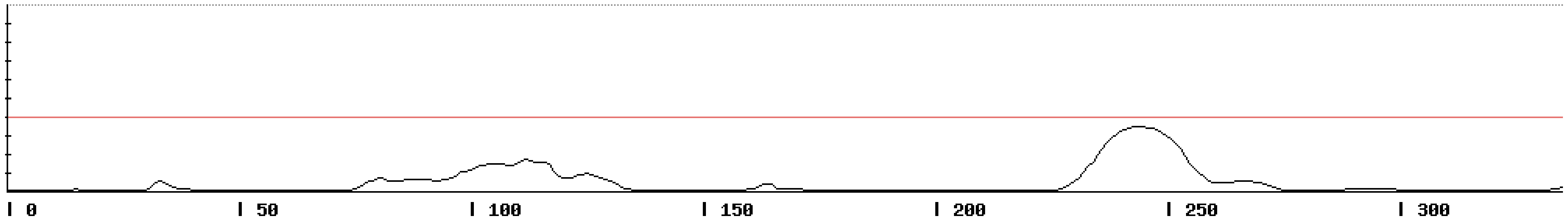

Predictions for Ananascomosus_Aco008654.1

Viterbi Path

Viterbi Path

Posterior @ 0.4

Posterior @ 0.4

Posterior Graphic. Threshold Marked @ 0.4

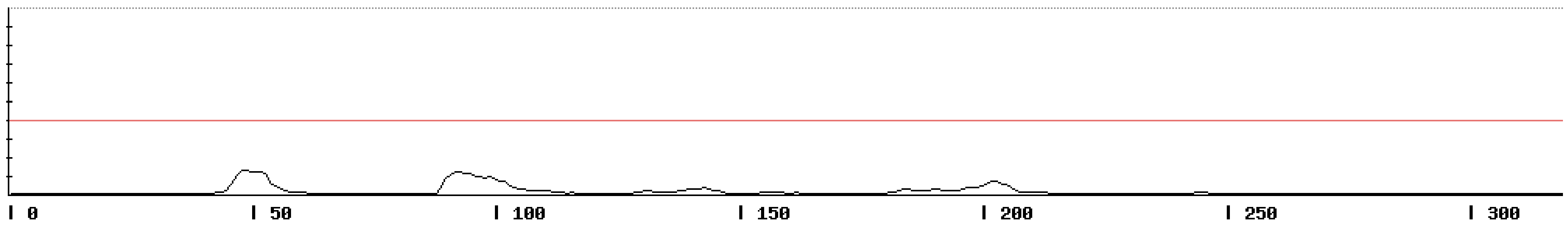

Predictions for Ananascomosus_Aco009152.1

Viterbi Path

Viterbi Path

Posterior @ 0.4

Posterior @ 0.4

Posterior Graphic. Threshold Marked @ 0.4

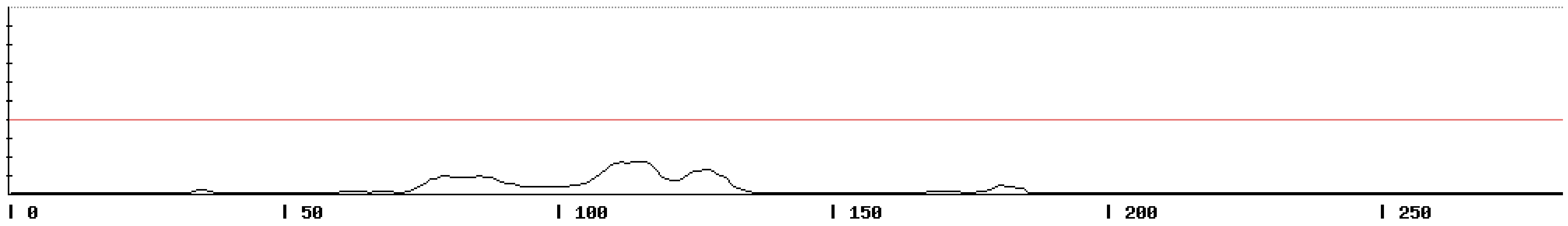

Predictions for Ananascomosus_Aco010231.1

Viterbi Path

Viterbi Path

Posterior @ 0.4

Posterior @ 0.4

Posterior Graphic. Threshold Marked @ 0.4

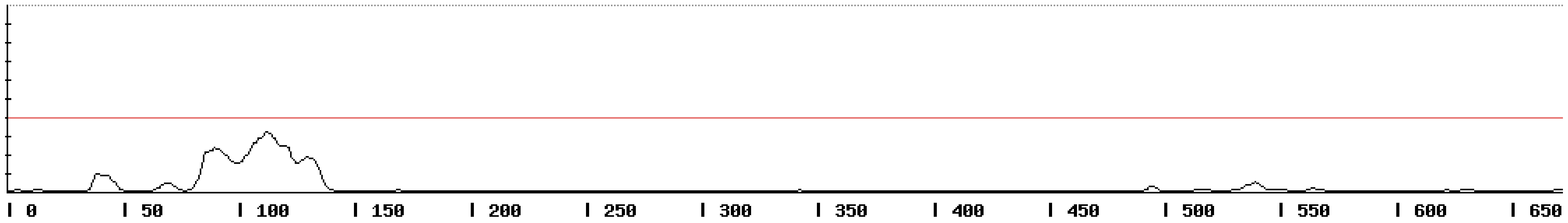

Predictions for Ananascomosus_Aco010558.1

Viterbi Path

Viterbi Path

Posterior @ 0.4 — 98 - KDRTIRSKKNPATIGMKKTLVFHKGRAPK - 126

Posterior @ 0.4

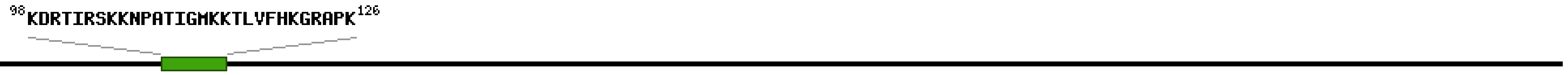

Posterior Graphic. Threshold Marked @ 0.4

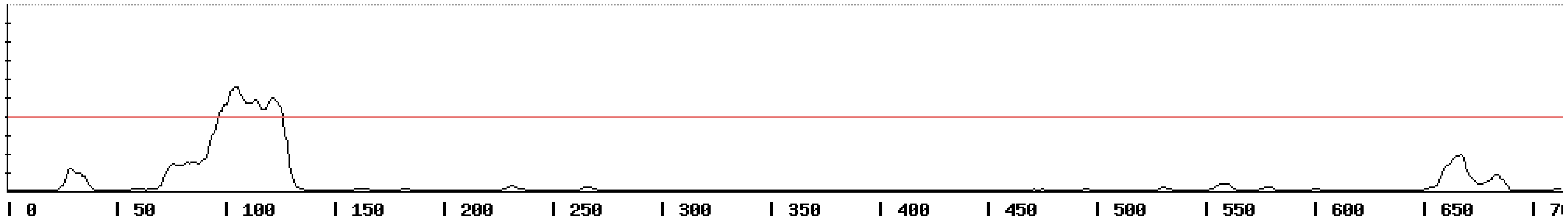

Predictions for Ananascomosus_Aco011352.1

Viterbi Path	
Viterbi Path	
Posterior @ 0.4	
Posterior @ 0.4	
Posterior Graphic. Threshold Marked @ 0.4	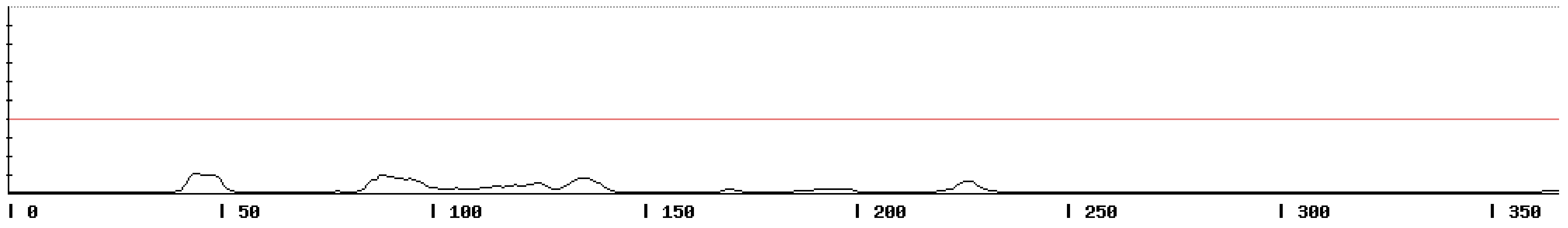

Predictions for Ananascomosus_Aco011825.1

Viterbi Path	
Viterbi Path	
Posterior @ 0.4	
Posterior @ 0.4	
Posterior Graphic. Threshold Marked @ 0.4	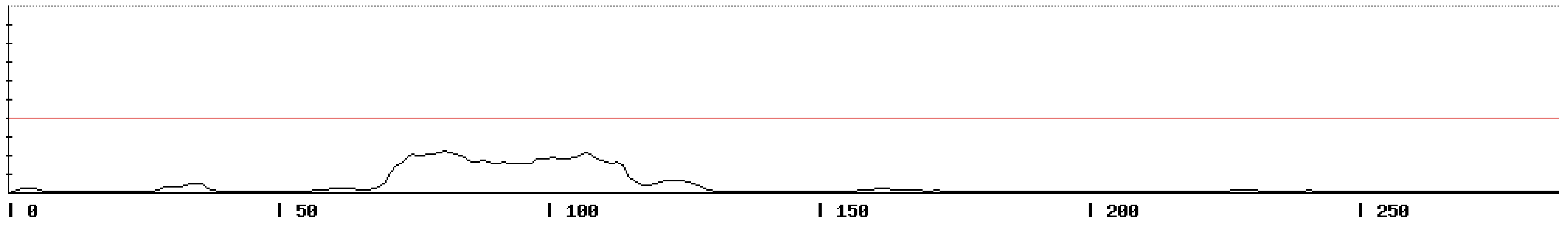

Predictions for Ananascomosus_Aco011880.1

Viterbi Path

Viterbi Path

Posterior @ 0.4

Posterior @ 0.4

Posterior Graphic. Threshold Marked @ 0.4

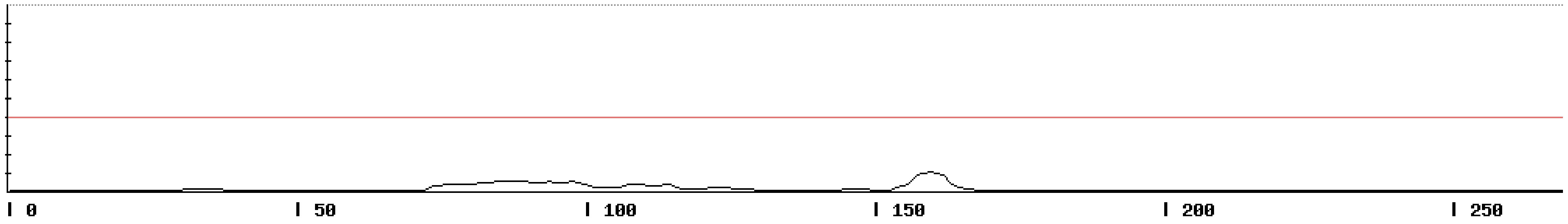

Predictions for Ananascomosus_Aco011895.1

Viterbi Path

Viterbi Path

Posterior @ 0.4

94 - KDRK - 97

Posterior @ 0.4

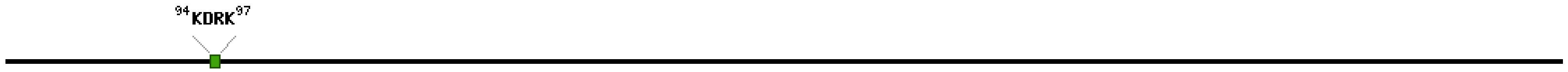

Posterior Graphic. Threshold Marked @ 0.4

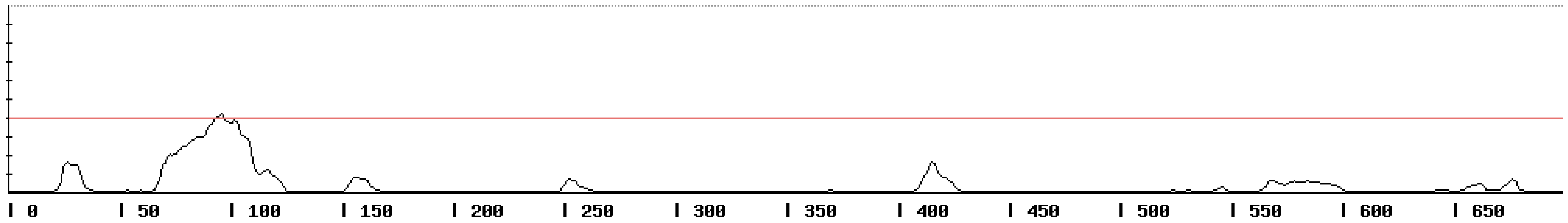

Predictions for Ananascomosus_Aco012211.1	
Viterbi Path	
Viterbi Path	
Posterior @ 0.4	
Posterior @ 0.4	
Posterior Graphic. Threshold Marked @ 0.4	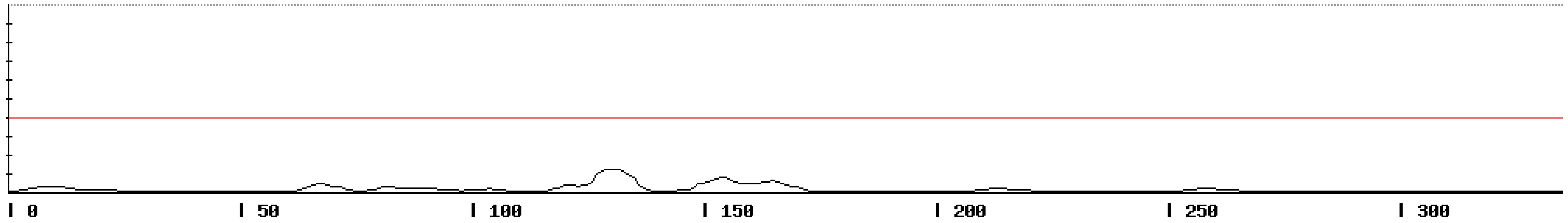

Predictions for Ananascomosus_Aco012598.1	
Viterbi Path	
Viterbi Path	
Posterior @ 0.4	88 - PNRAAGR - 94
	109 - K - 109
Posterior @ 0.4	

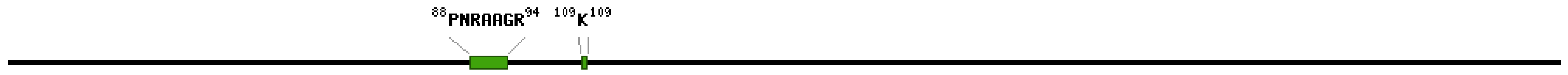

Posterior Graphic. Threshold Marked @ 0.4

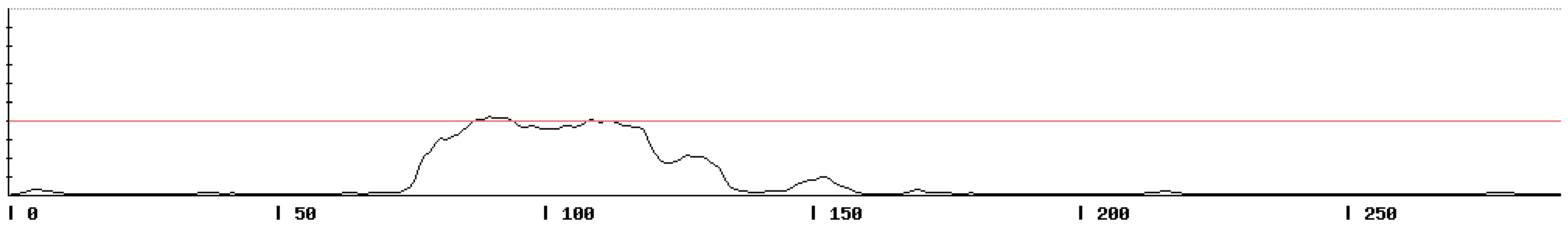

Predictions for Ananascomosus_Aco012599.1

Viterbi Path

Viterbi Path

Posterior @ 0.4

Posterior @ 0.4

Posterior Graphic. Threshold Marked @ 0.4

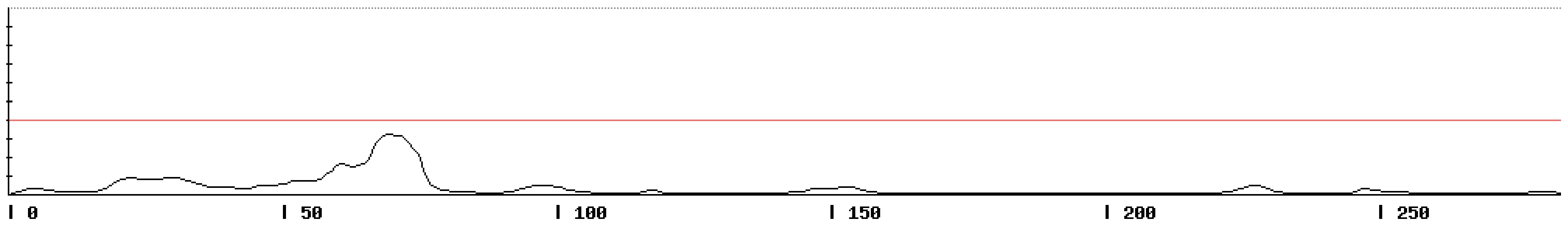

Predictions for Ananascomosus_Aco012600.1

Viterbi Path

Viterbi Path

Posterior @ 0.4

Posterior @ 0.4

Posterior Graphic. Threshold Marked @ 0.4

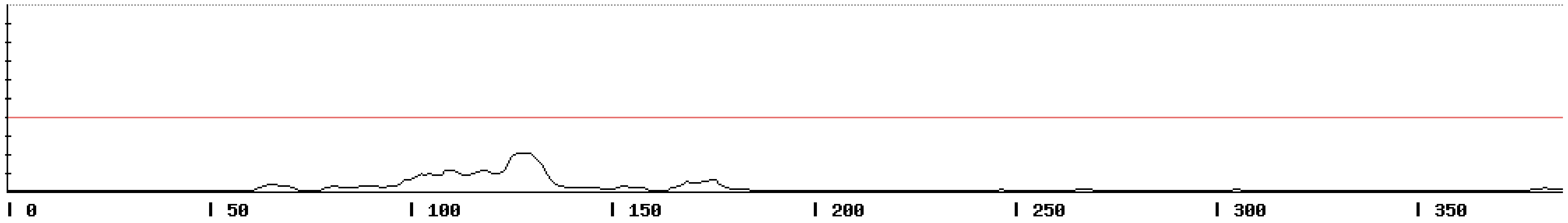

Predictions for Ananascomosus_Aco012922.1

Viterbi Path

Viterbi Path

Posterior @ 0.4

Posterior @ 0.4

Posterior Graphic. Threshold Marked @ 0.4

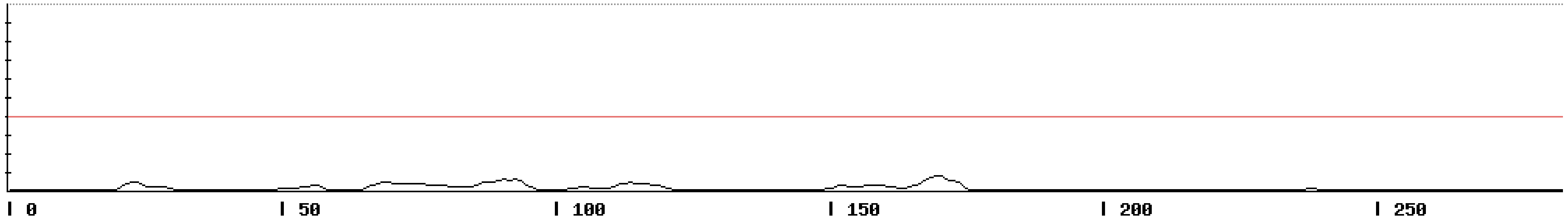

Predictions for Ananascomosus_Aco013021.1

Viterbi Path

Viterbi Path

Posterior @ 0.4

Posterior @ 0.4

Posterior Graphic. Threshold Marked @ 0.4

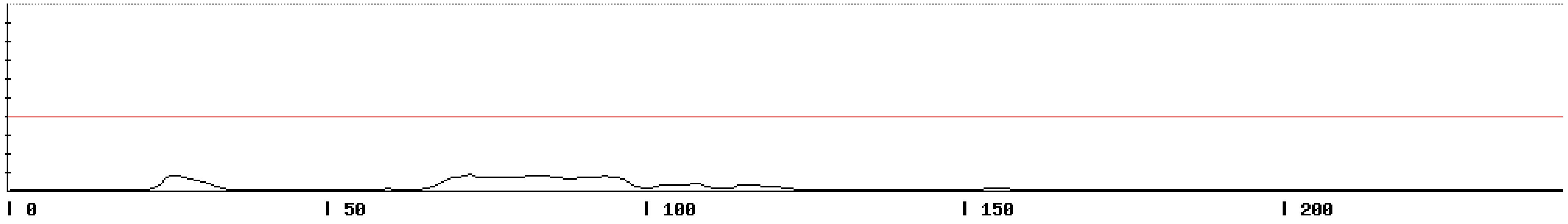

Predictions for Ananascomosus_Aco013059.1

Viterbi Path

Viterbi Path

Posterior @ 0.4

Posterior @ 0.4

Posterior Graphic. Threshold Marked @ 0.4

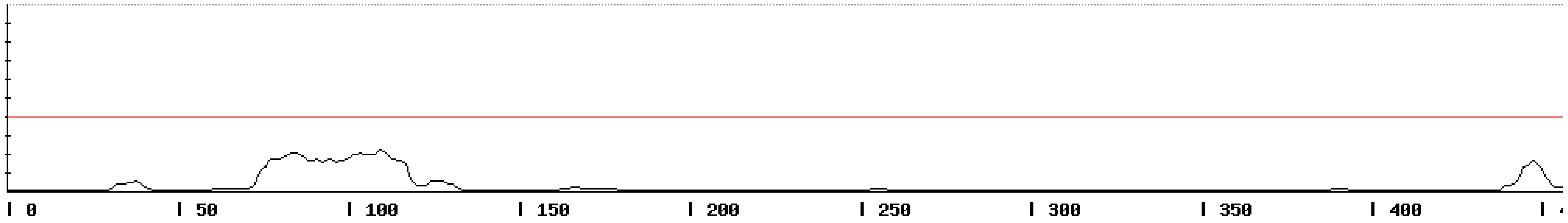

Predictions for Ananascomosus_Aco013223.1

Viterbi Path

Viterbi Path

Posterior @ 0.4 — 80 - GHRSNRATEKGYWKATGKDRIIRAKNAATGSKKHKPAVIGMKKTLVFHRGRAPRG - 134

Posterior @ 0.4

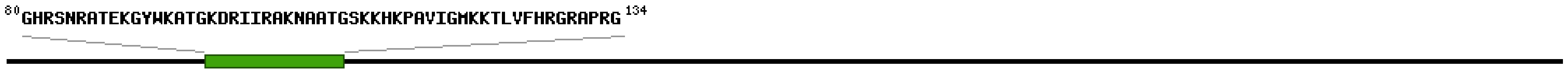

Posterior Graphic. Threshold Marked @ 0.4

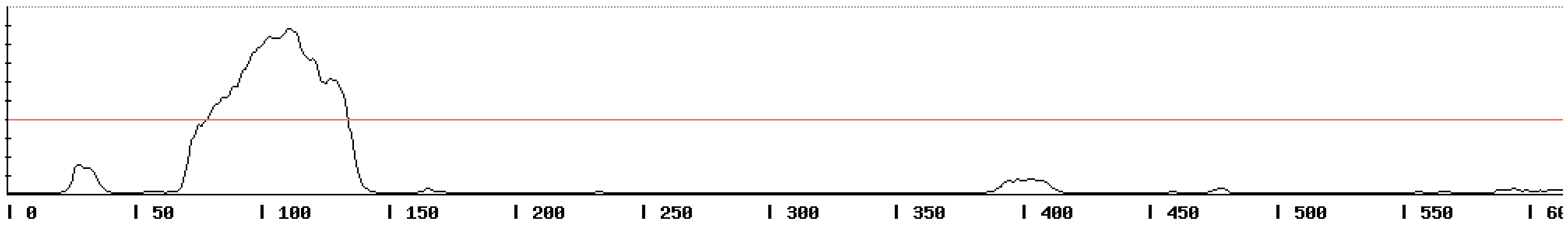

Predictions for Ananascomosus_Aco013436.1

Viterbi Path

Viterbi Path

Posterior @ 0.4 — 174 - RSTKGGKRKDRLRQ - 187

771 - RRKRKR - 776

Posterior @ 0.4

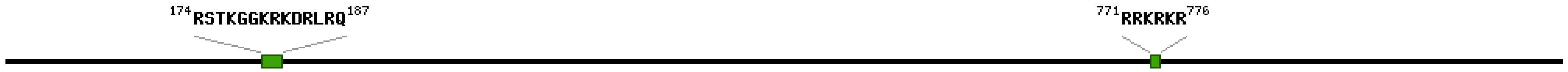

Posterior Graphic. Threshold Marked @ 0.4

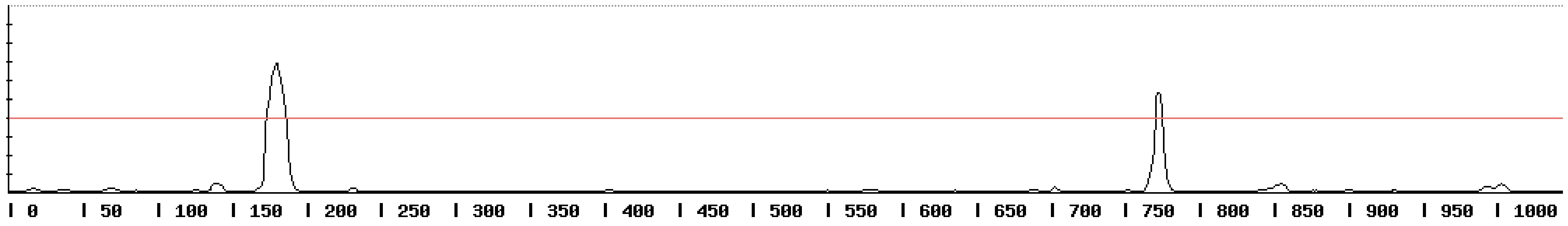

Predictions for Ananascomosus_Aco013595.1

Viterbi Path

Viterbi Path

Posterior @ 0.4

Posterior @ 0.4

Posterior Graphic. Threshold Marked @ 0.4

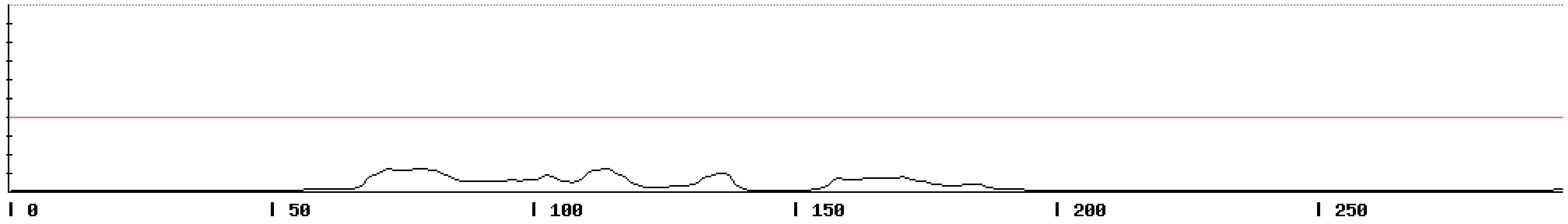

Predictions for Ananascomosus_Aco014680.1

Viterbi Path

Viterbi Path

Posterior @ 0.4

Posterior @ 0.4

Posterior Graphic. Threshold Marked @ 0.4	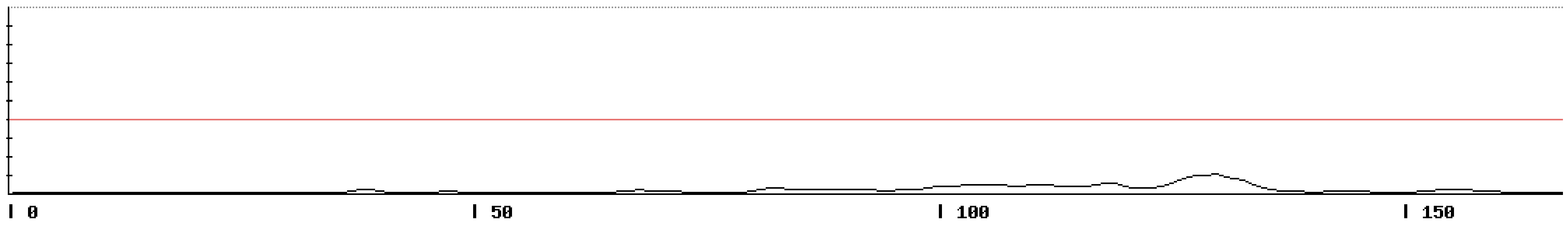

Predictions for Ananascomosus_Aco014816.1

Viterbi Path	
Viterbi Path	
Posterior @ 0.4	
Posterior @ 0.4	
Posterior Graphic. Threshold Marked @ 0.4	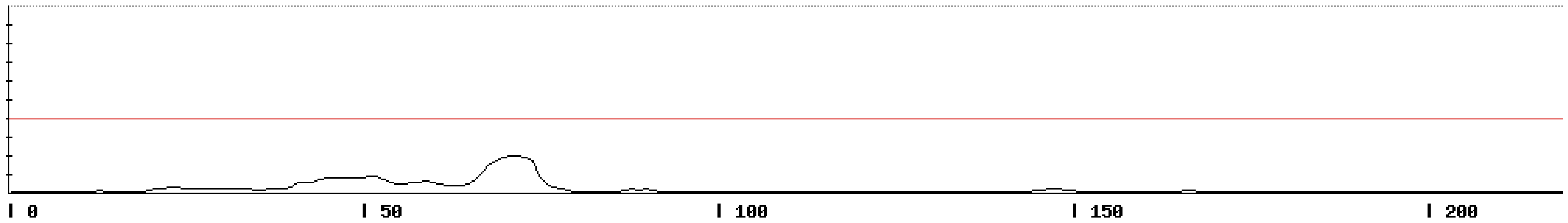

Predictions for Ananascomosus_Aco015086.1

Viterbi Path	
Viterbi Path	
Posterior @ 0.4	
Posterior @ 0.4	

Posterior Graphic. Threshold Marked @ 0.4

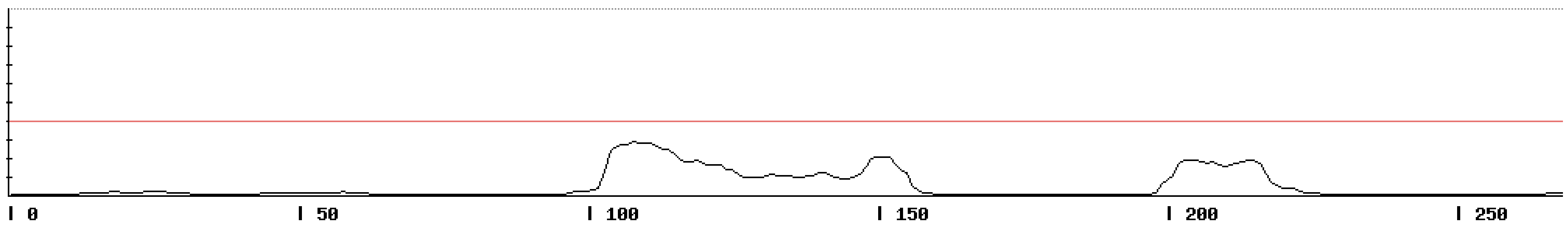

Predictions for Ananascomosus_Aco015181.1

Viterbi Path

Viterbi Path

Posterior @ 0.4 124 - RKRR - 127

Posterior @ 0.4

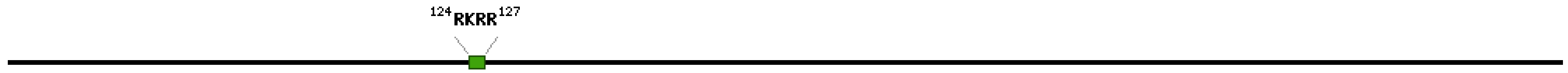

Posterior Graphic. Threshold Marked @ 0.4

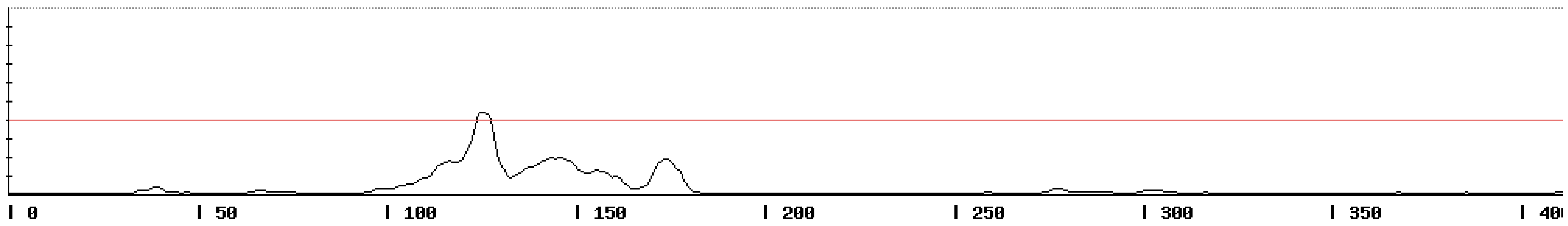

Predictions for Ananascomosus_Aco015539.1

Viterbi Path

Viterbi Path

Posterior @ 0.4

Posterior @ 0.4

Posterior Graphic. Threshold Marked @ 0.4

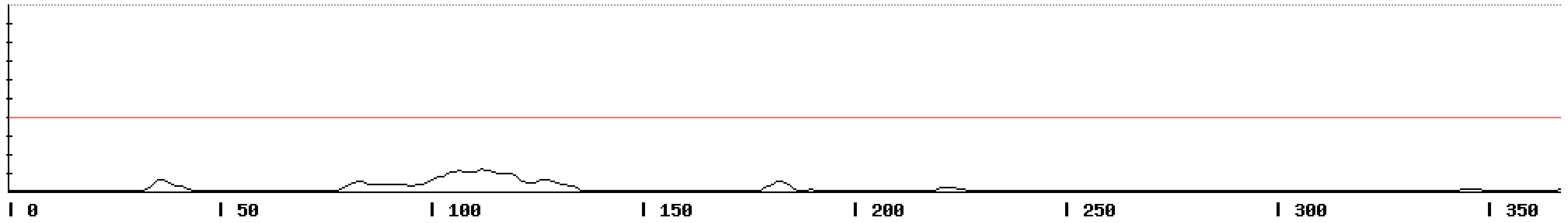

Predictions for Ananascomosus_Aco015958.1

Viterbi Path

Viterbi Path

Posterior @ 0.4

Posterior @ 0.4

Posterior Graphic. Threshold Marked @ 0.4

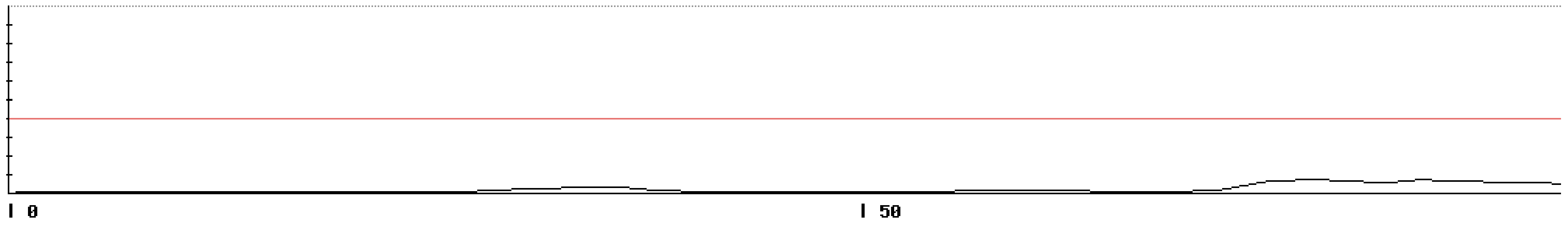

Predictions for Ananascomosus_Aco016738.1

Viterbi Path

Viterbi Path

Posterior @ 0.4

Posterior @ 0.4

Posterior Graphic. Threshold Marked @ 0.4

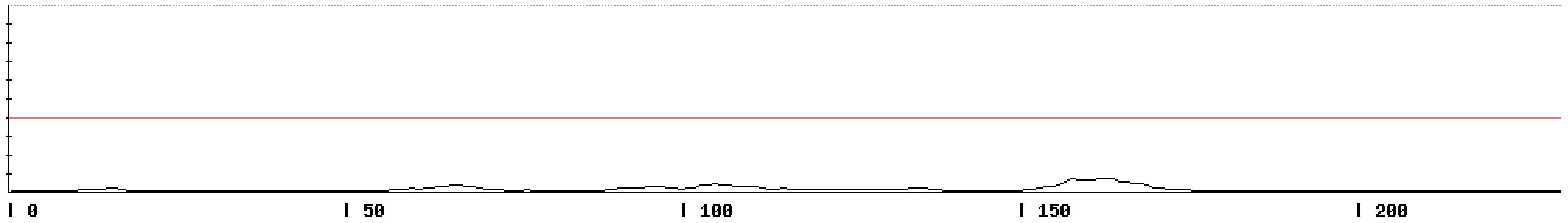

Predictions for Ananascomosus_Aco017708.1

Viterbi Path

Viterbi Path

Posterior @ 0.4

Posterior @ 0.4

Posterior Graphic. Threshold Marked @ 0.4

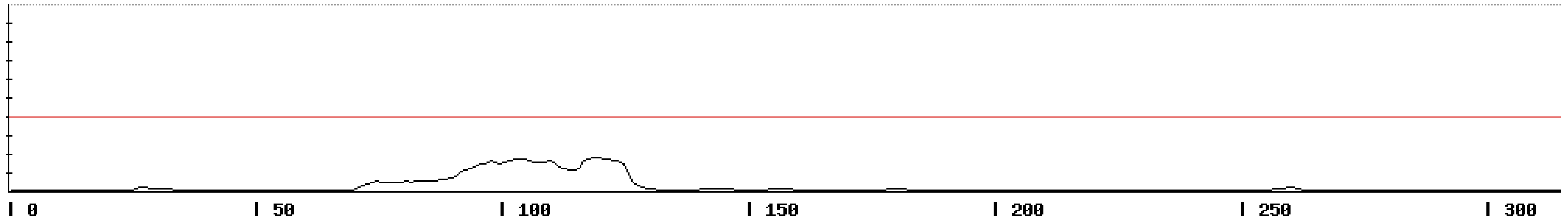

Predictions for Ananascomosus_Aco018062.1

Viterbi Path	
Viterbi Path	
Posterior @ 0.4	106 - KRR - 108
Posterior @ 0.4	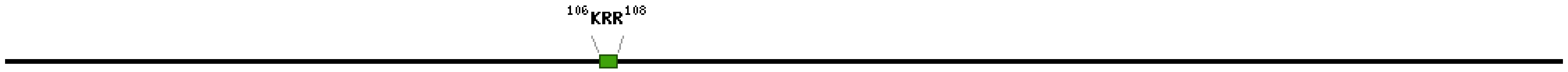
Posterior Graphic. Threshold Marked @ 0.4	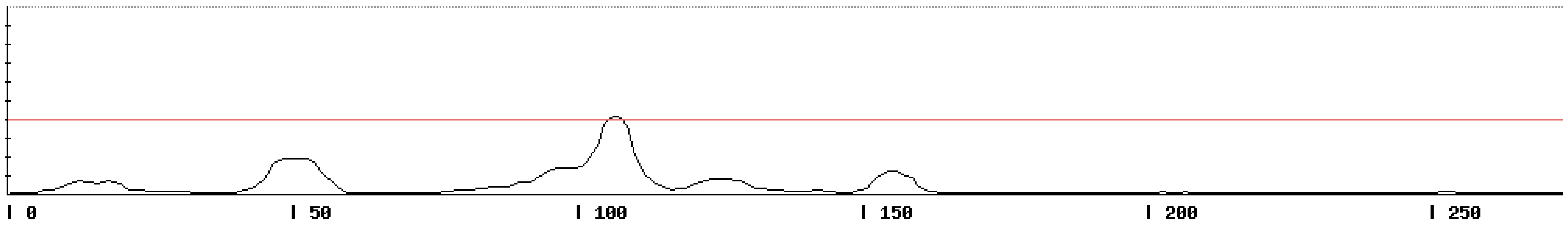

Predictions for Ananascomosus_Aco018555.1

Viterbi Path	
Viterbi Path	
Posterior @ 0.4	
Posterior @ 0.4	
Posterior Graphic. Threshold Marked @ 0.4	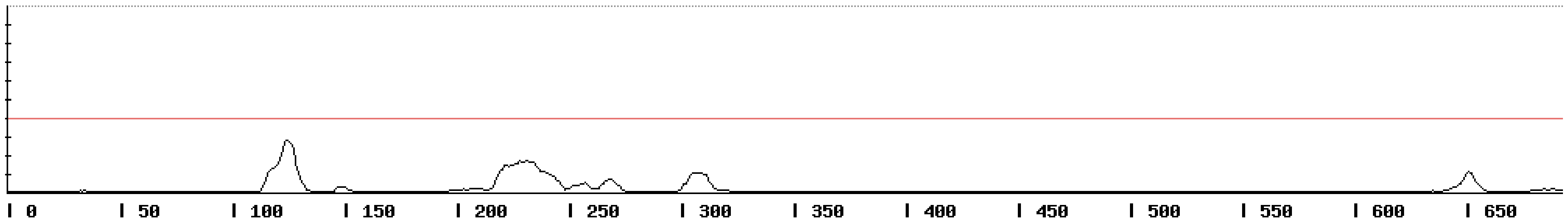

Predictions for Ananascomosus_Aco018659.1	
Viterbi Path	
Viterbi Path	
Posterior @ 0.4	
Posterior @ 0.4	
Posterior Graphic. Threshold Marked @ 0.4	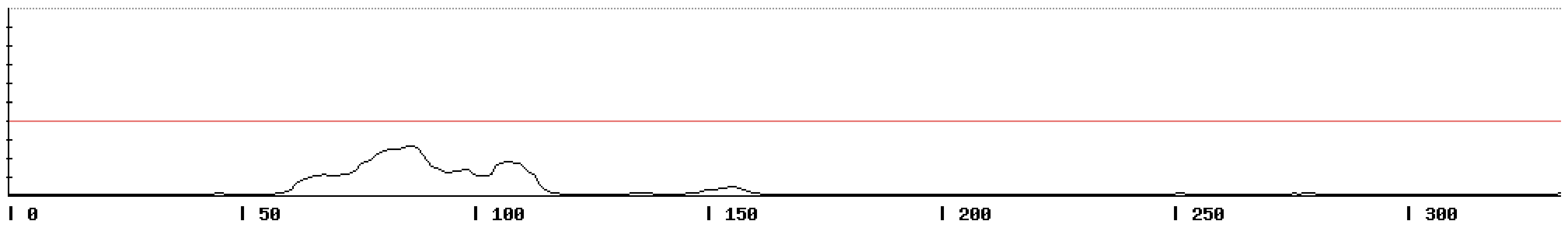

Predictions for Ananascomosus_Aco018846.1	
Viterbi Path	
Viterbi Path	
Posterior @ 0.4	
Posterior @ 0.4	
Posterior Graphic. Threshold Marked @ 0.4	

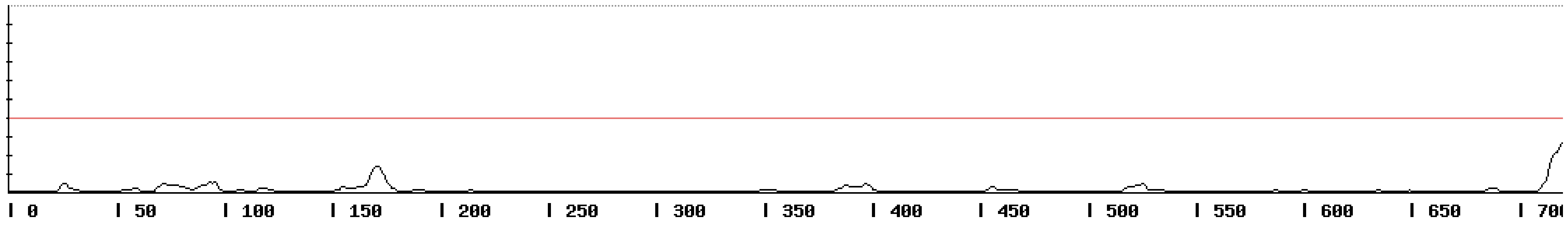

Predictions for Ananascomosus_Aco019352.1

Viterbi Path	
Viterbi Path	
Posterior @ 0.4	
Posterior @ 0.4	
Posterior Graphic. Threshold Marked @ 0.4	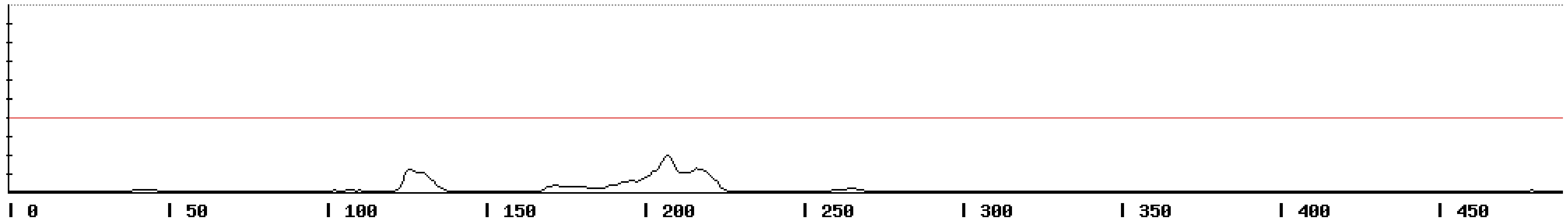

Predictions for Ananascomosus_Aco019379.1

Viterbi Path	
Viterbi Path	
Posterior @ 0.4	
Posterior @ 0.4	

Posterior Graphic. Threshold Marked @ 0.4

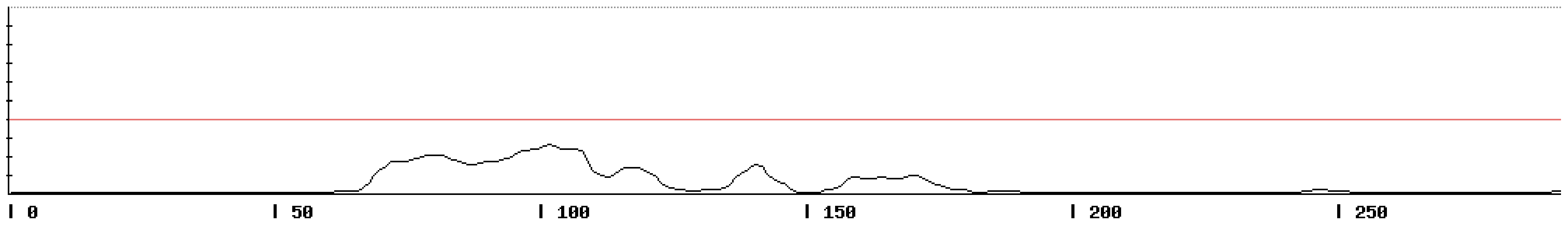

Predictions for Ananascomosus_Aco020033.1

Viterbi Path

Viterbi Path

Posterior @ 0.4

Posterior @ 0.4

Posterior Graphic. Threshold Marked @ 0.4

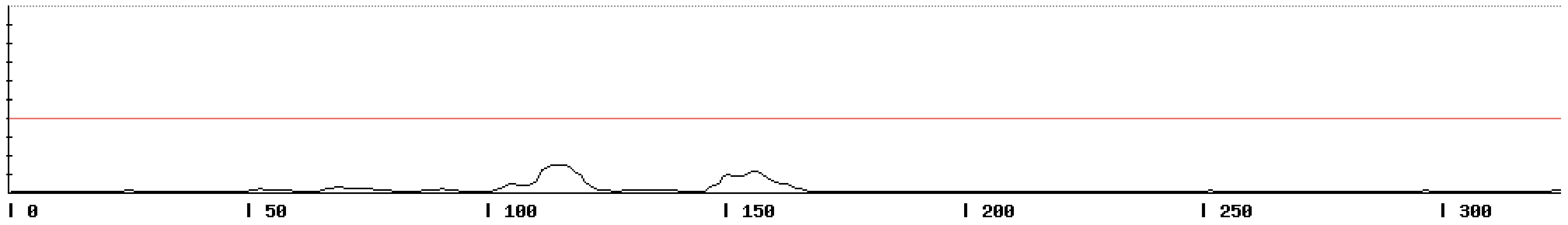

Predictions for Ananascomosus_Aco020094.1

Viterbi Path

Viterbi Path

Posterior @ 0.4

Posterior @ 0.4

Posterior Graphic. Threshold Marked @ 0.4	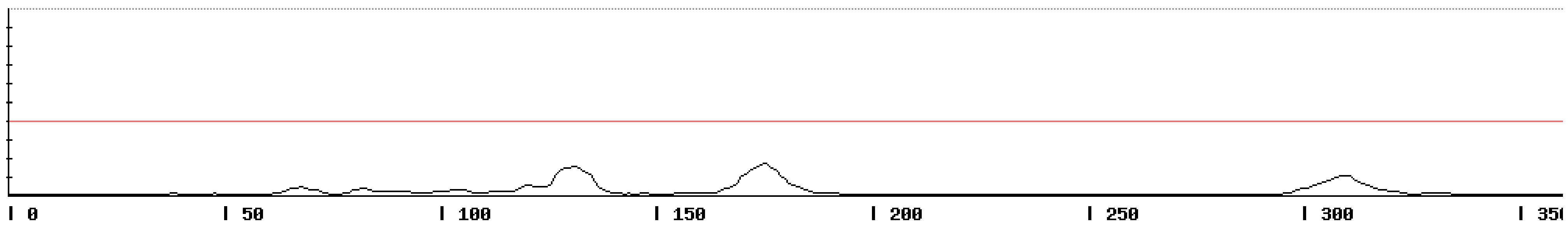

Predictions for Ananascomosus_Aco020562.1

Viterbi Path	
Viterbi Path	
Posterior @ 0.4	334 - RKRHKK - 339
Posterior @ 0.4	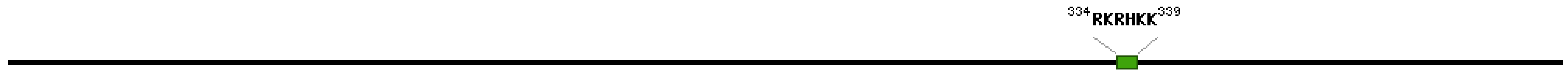
Posterior Graphic. Threshold Marked @ 0.4	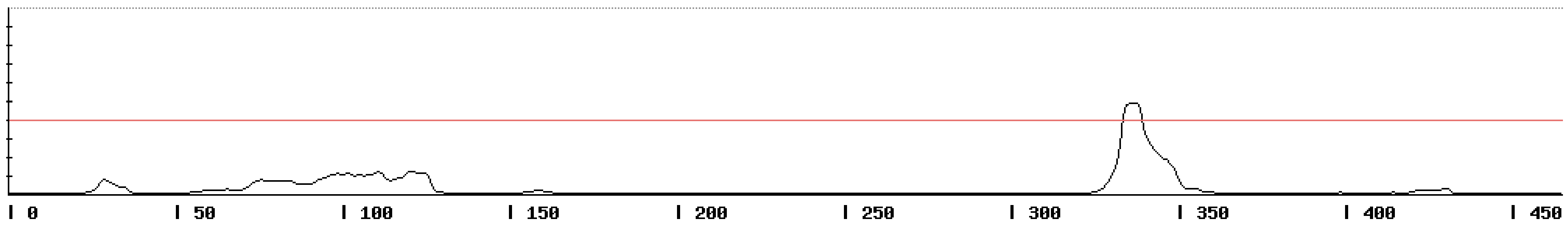

Predictions for Ananascomosus_Aco021379.1

Viterbi Path	
Viterbi Path	

Posterior @ 0.4	139 - GTRKRRKVHADPHGGETRWHKTGKTRP - 165
	187 - KKRK - 190

Posterior @ 0.4

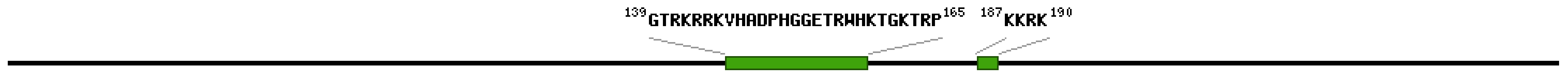

Posterior Graphic. Threshold Marked @ 0.4

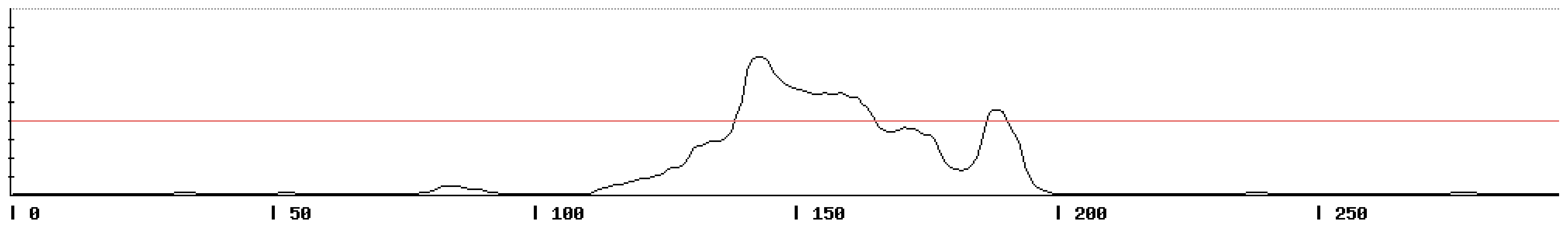

Predictions for Ananascomosus_Aco022043.1

Viterbi Path

Viterbi Path

Posterior @ 0.4	87 - KRRKI - 91
	120 - RKGWKK - 125
	211 - KK - 212

Posterior @ 0.4

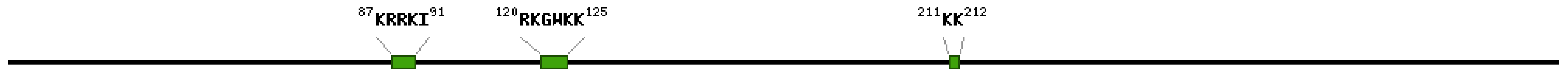

Posterior Graphic. Threshold Marked @ 0.4

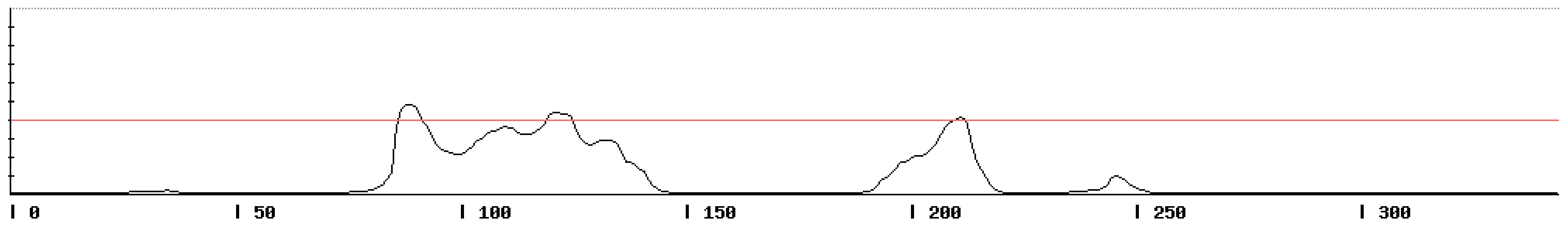

Predictions for Ananascomosus_Aco022129.1

Viterbi Path	
Viterbi Path	
Posterior @ 0.4	
Posterior @ 0.4	
Posterior Graphic. Threshold Marked @ 0.4	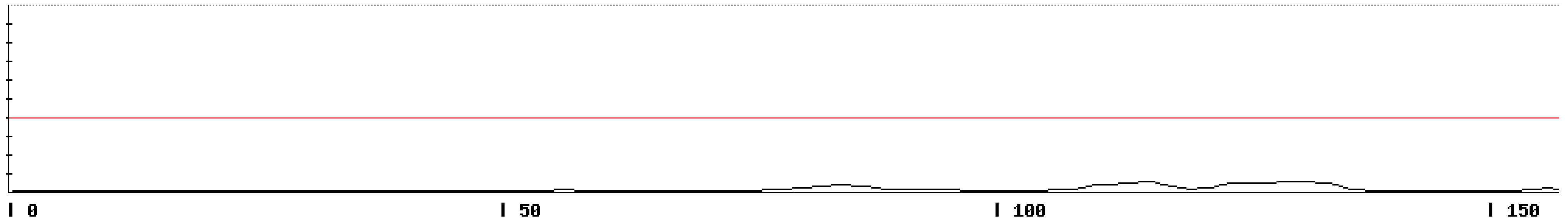

Predictions for Ananascomosus_Aco023285.1

Viterbi Path	
Viterbi Path	
Posterior @ 0.4	86 - SRPNRAAGRGYWKATGADKPIRPSGSNRPVGIK - 118
Posterior @ 0.4	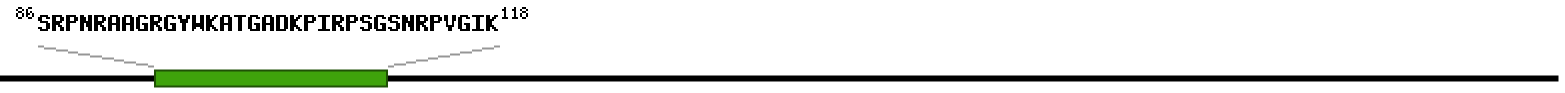
Posterior Graphic. Threshold Marked @ 0.4	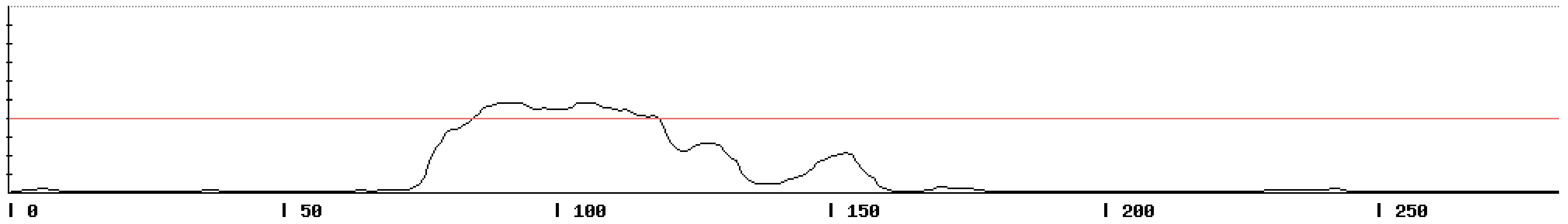

Predictions for Ananascomosus_Aco023288.1

Viterbi Path	
Viterbi Path	
Posterior @ 0.4	
Posterior @ 0.4	
Posterior Graphic. Threshold Marked @ 0.4	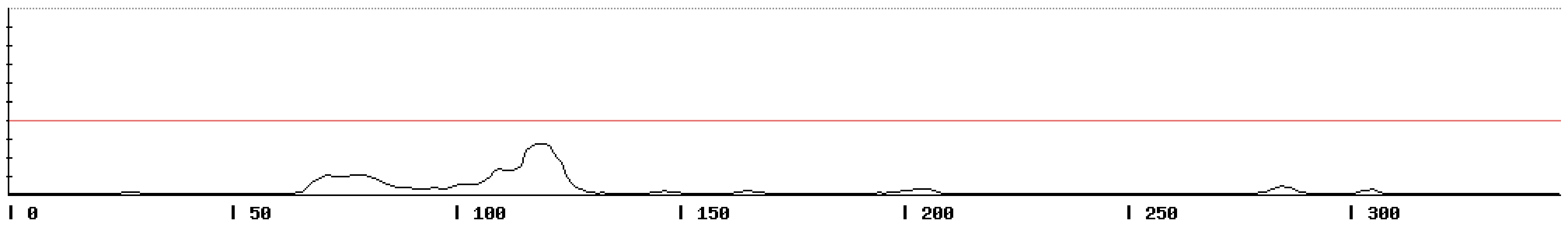

Predictions for Ananascomosus_Aco023290.1

Viterbi Path	
Viterbi Path	
Posterior @ 0.4	
Posterior @ 0.4	
Posterior Graphic. Threshold Marked @ 0.4	

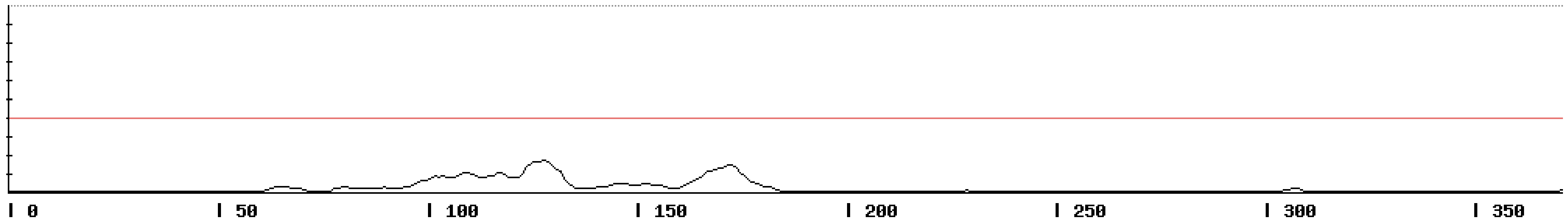

Predictions for Ananascomosus_Aco023481.1

Viterbi Path

Viterbi Path

Posterior @ 0.4

Posterior @ 0.4

Posterior Graphic. Threshold Marked @ 0.4

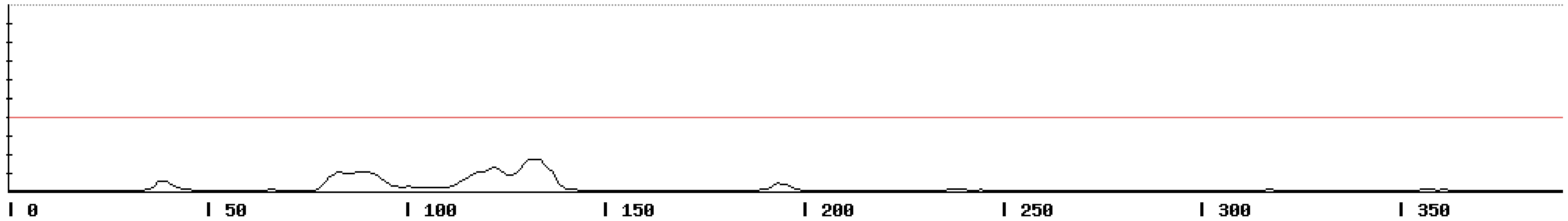

Predictions for Ananascomosus_Aco024095.1

Viterbi Path

Viterbi Path

Posterior @ 0.4

74 - KGKRARCGGRDDHQEARRGDGFKRGRKERSVR - 105

Posterior @ 0.4

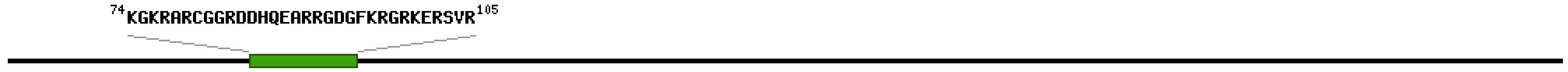

Posterior Graphic. Threshold Marked @ 0.4

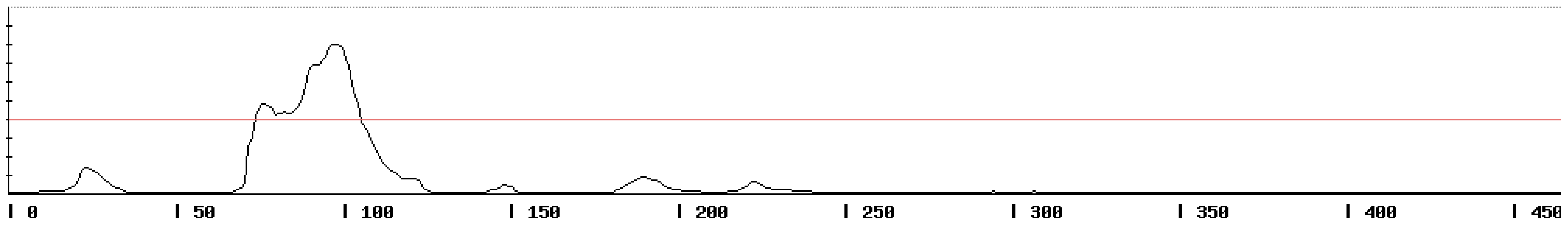

Predictions for Ananascomosus_Aco027001.1

Viterbi Path

Viterbi Path

Posterior @ 0.4

111 - KDRK - 114

Posterior @ 0.4

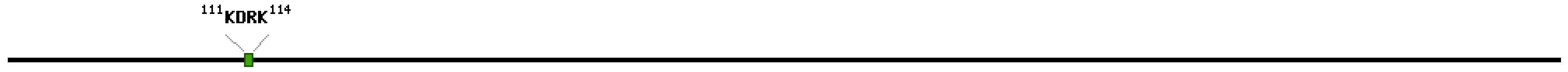

Posterior Graphic. Threshold Marked @ 0.4

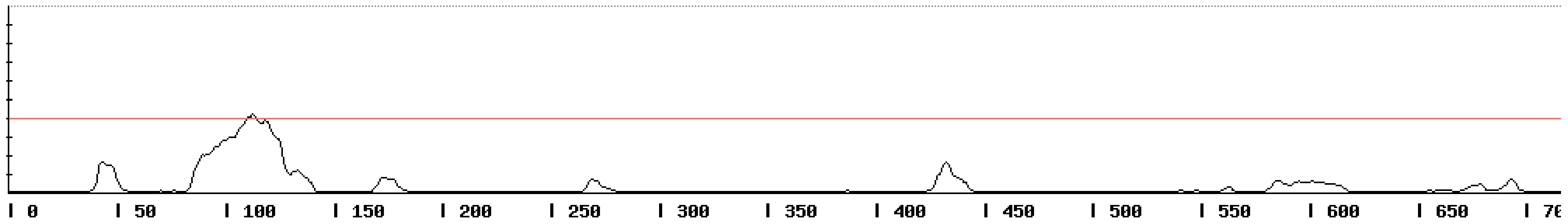

Predictions for Ananascomosus_Aco028749.1

Viterbi Path

Viterbi Path

Posterior @ 0.4

Posterior @ 0.4

Posterior Graphic. Threshold Marked @ 0.4

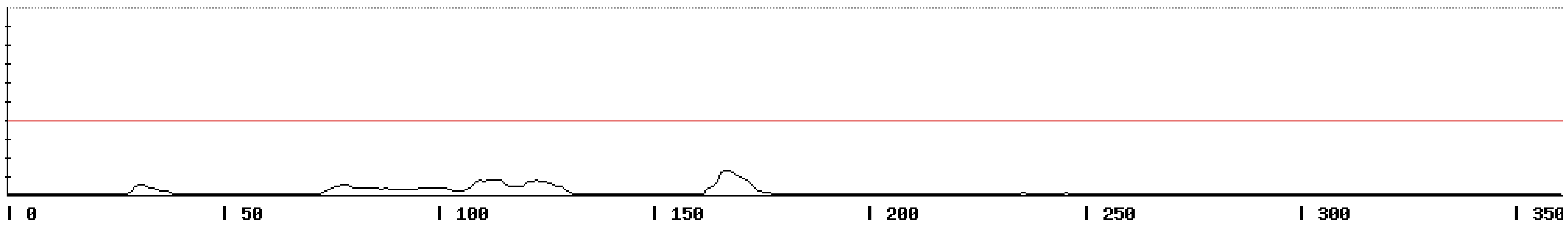

Predictions for Ananascomosus_Aco029272.1

Viterbi Path

Viterbi Path

Posterior @ 0.4 | 205 - KKSAREIARSHKRKREEAA - 223

Posterior @ 0.4

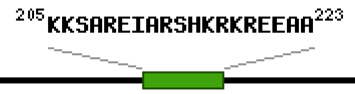

Posterior Graphic. Threshold Marked @ 0.4

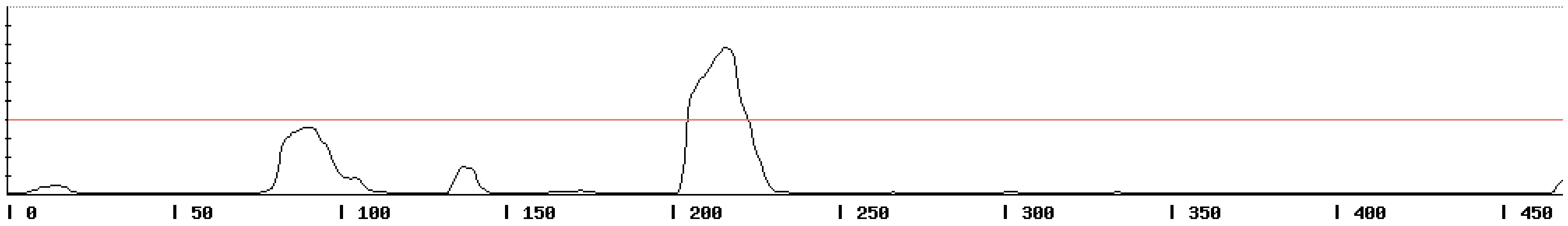

Predictions for Ananascomosus_Aco030916.1

Viterbi Path

Viterbi Path

Posterior @ 0.4	
Posterior @ 0.4	
Posterior Graphic. Threshold Marked @ 0.4	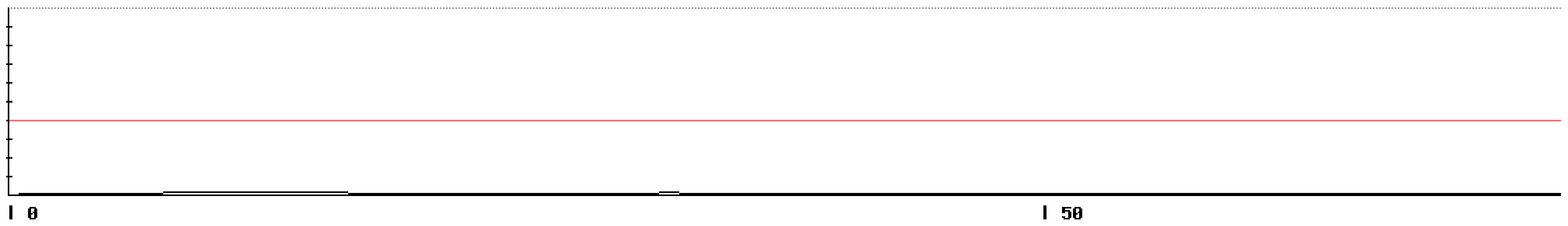

Predictions for Ananascomosus_Aco031295.1

Viterbi Path	
Viterbi Path	
Posterior @ 0.4	115 - RPNRAAGR - 122
	137 - KG - 138
	140 - HR - 141
Posterior @ 0.4	

Revision r.9 - Last Updated on : February 24 2015 17:45:20. alex.nguyenba@utoronto.ca

Intended to be used with Firefox

Viterbi Path

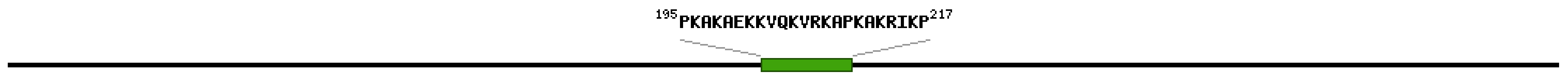

Posterior @ 0.4: 195 - PKAKAEKKVQKVRKAPKAKRIKP - 217

Posterior @ 0.4

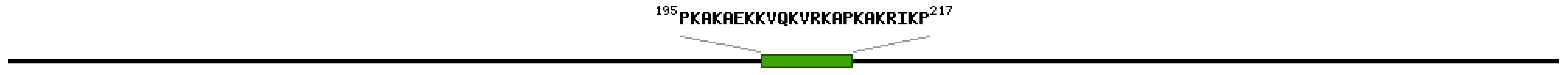

Posterior Graphic. Threshold Marked @ 0.4

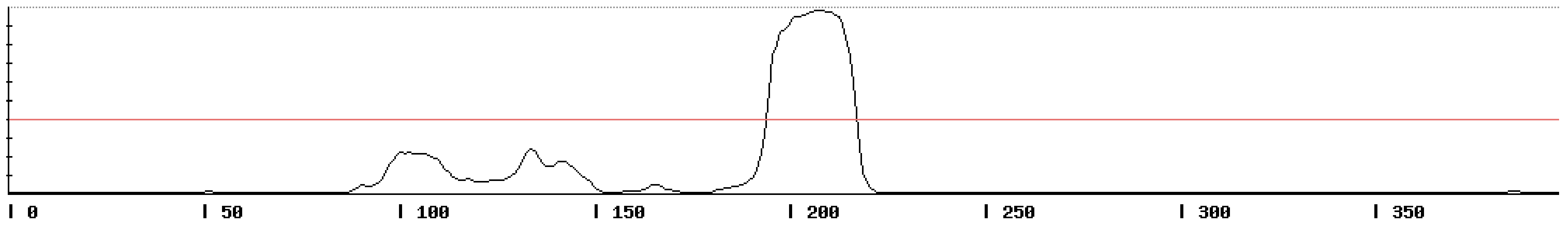

Predictions for Aquilegiacoerulea_Aqcoe1G058100.1.p

Viterbi Path: 461 - KKRKRTKKQ - 469

Viterbi Path

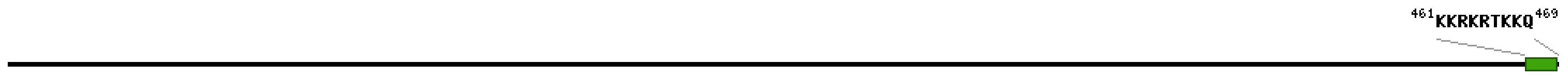

Posterior @ 0.4: 196 - QKRKAKGKKRV - 206

458 - KLTKKRKRTKKQ - 469

Posterior @ 0.4

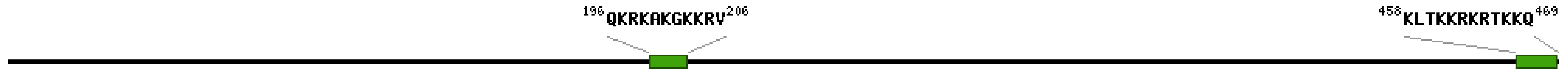

Posterior Graphic. Threshold Marked @ 0.4

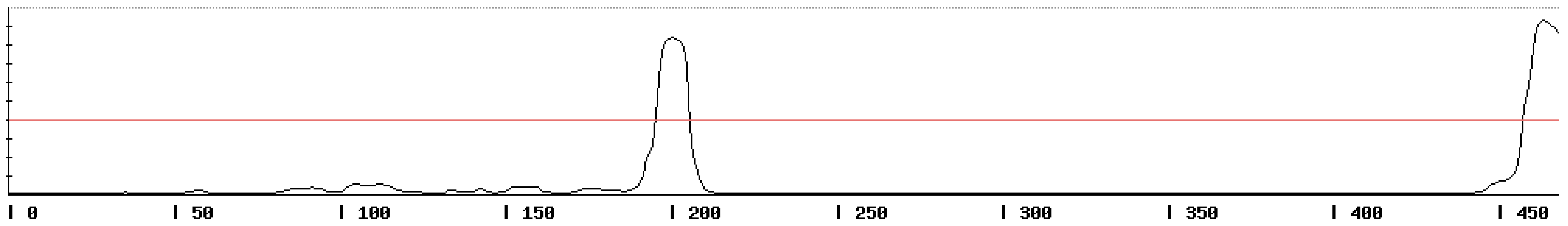

Predictions for Aquilegiacoerulea_Aqcoe1G110100.1.p

Viterbi Path

Viterbi Path

Posterior @ 0.4

103 - RERKYPKGSRA - 113

503 - RSKNKRRR - 510

Posterior @ 0.4

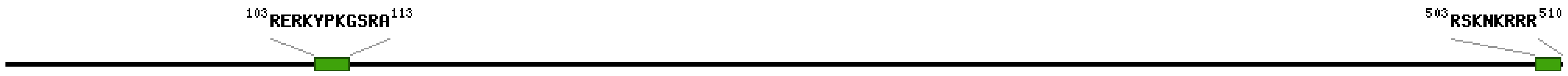

Posterior Graphic. Threshold Marked @ 0.4

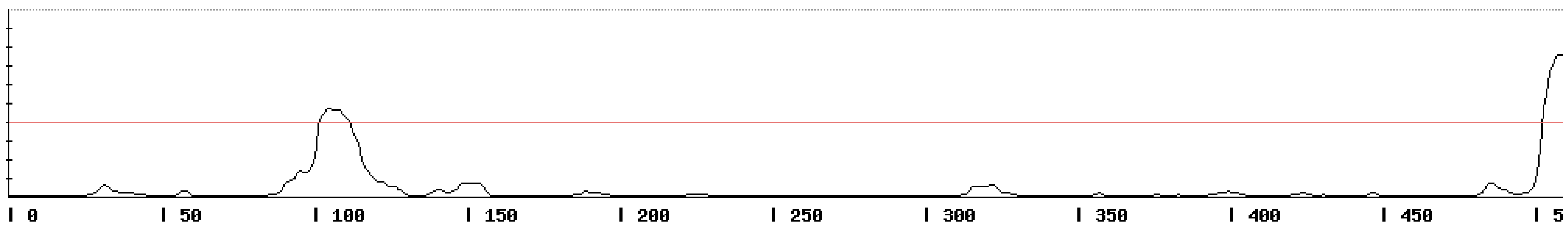

Predictions for Aquilegiacoerulea_Aqcoe1G126700.1.p

Viterbi Path

Viterbi Path

Posterior @ 0.4

Posterior @ 0.4

Posterior Graphic. Threshold Marked @ 0.4

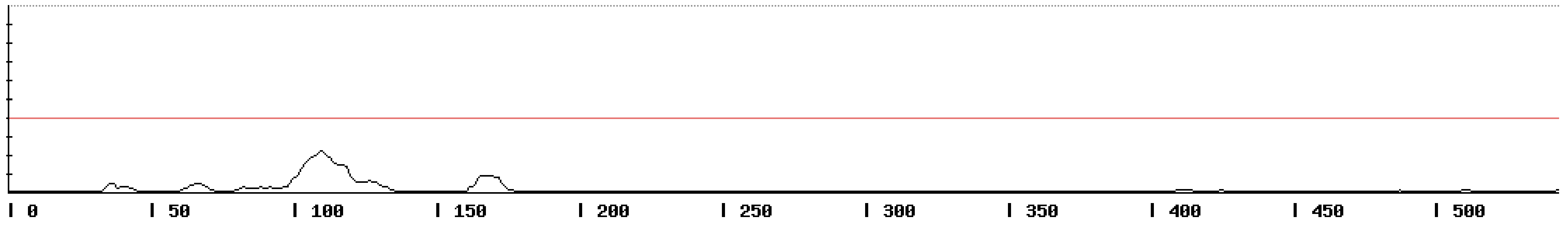

Predictions for Aquilegiacoerulea_Aqcoe1G154800.1.p

Viterbi Path	
Viterbi Path	
Posterior @ 0.4	
Posterior @ 0.4	
Posterior Graphic. Threshold Marked @ 0.4	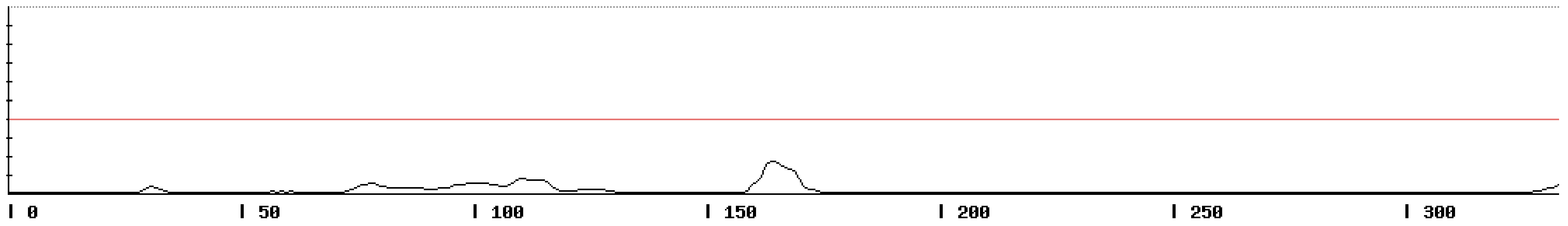

Predictions for Aquilegiacoerulea_Aqcoe1G177900.1.p

Viterbi Path	
Viterbi Path	
Posterior @ 0.4	
Posterior @ 0.4	

Posterior Graphic. Threshold Marked @ 0.4

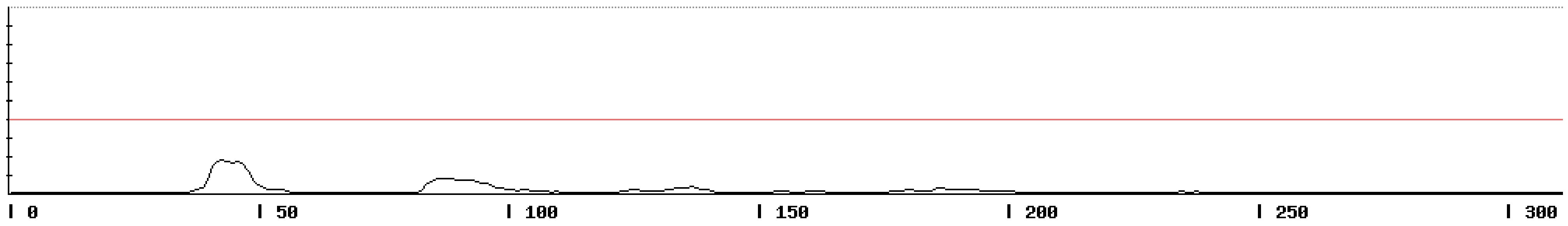

Predictions for Aquilegiacoerulea_Aqcoe1G182600.1.p

Viterbi Path

Viterbi Path

Posterior @ 0.4

Posterior @ 0.4

Posterior Graphic. Threshold Marked @ 0.4

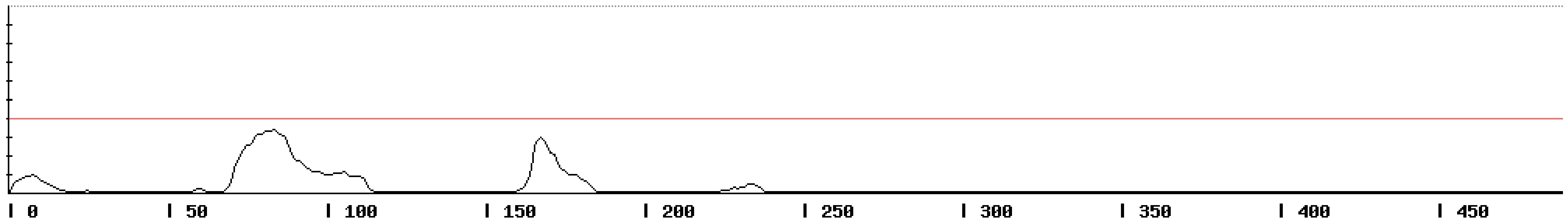

Predictions for Aquilegiacoerulea_Aqcoe1G251800.1.p

Viterbi Path

Viterbi Path

Posterior @ 0.4

133 - TRKRRKVH - 140

149 - RWHKTGKTRPVFIGGKIKG - 167

180 - KKRKP - 184

Posterior @ 0.4	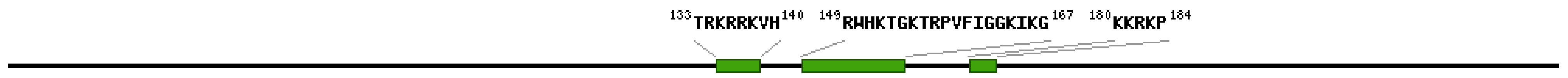
Posterior Graphic. Threshold Marked @ 0.4	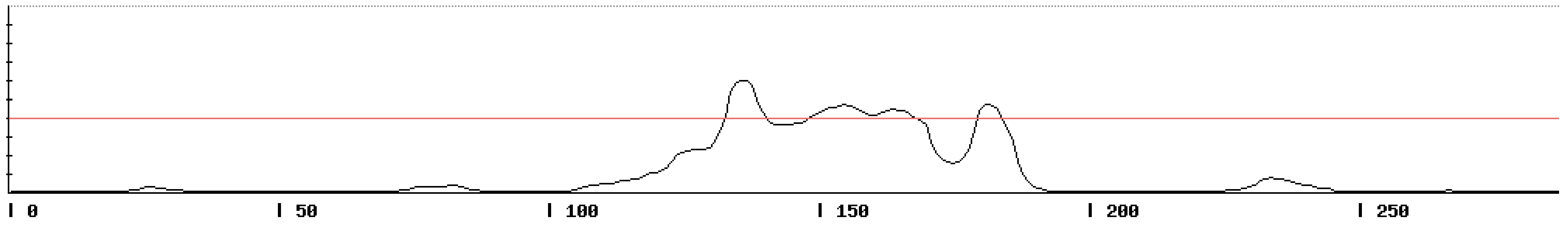

Predictions for Aquilegiacoerulea_Aqcoe1G321200.1.p

Viterbi Path	
Viterbi Path	
Posterior @ 0.4	
Posterior @ 0.4	
Posterior Graphic. Threshold Marked @ 0.4	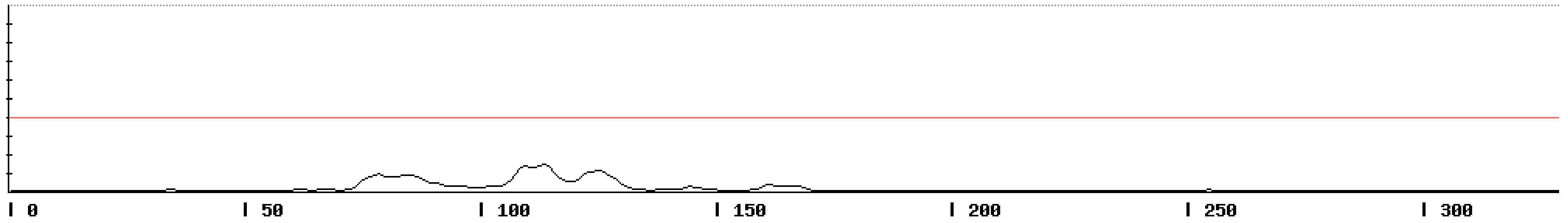

Predictions for Aquilegiacoerulea_Aqcoe1G321300.1.p

Viterbi Path	
Viterbi Path	

Posterior @ 0.4	
Posterior @ 0.4	
Posterior Graphic. Threshold Marked @ 0.4	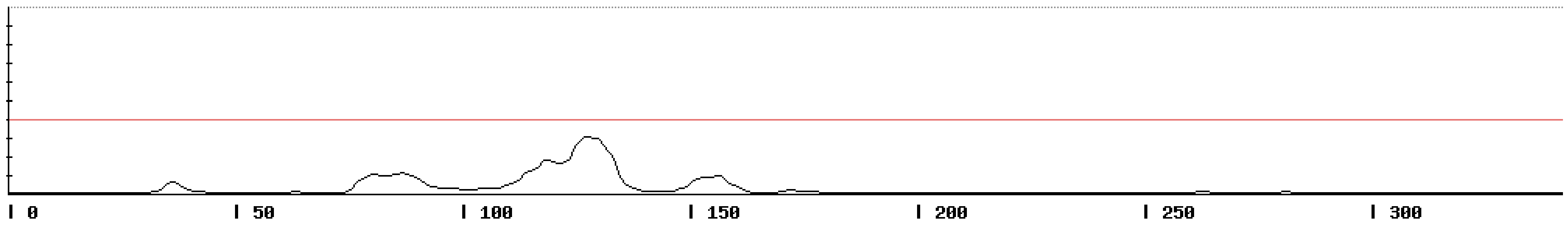

Predictions for Aquilegiacoerulea_Aqcoe1G351500.1.p

Viterbi Path	
Viterbi Path	
Posterior @ 0.4	201 - KKKKKRR - 207
Posterior @ 0.4	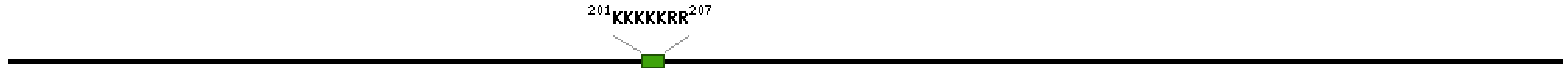
Posterior Graphic. Threshold Marked @ 0.4	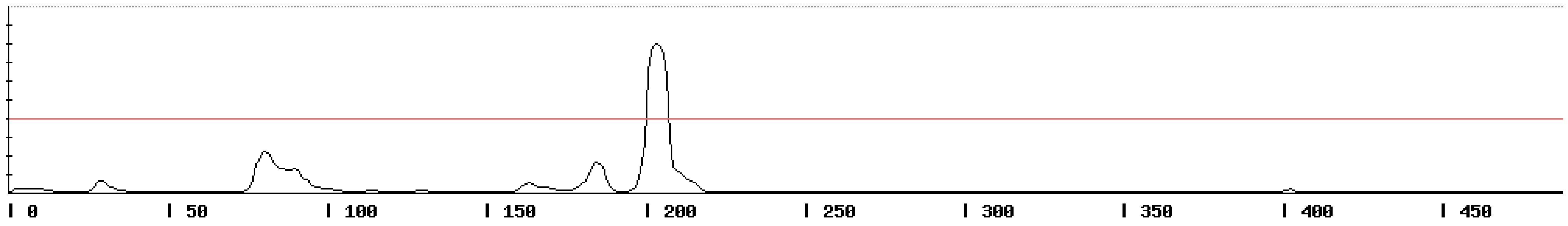

Predictions for Aquilegiacoerulea_Aqcoe1G372100.1.p

Viterbi Path	
Viterbi Path	

Posterior @ 0.4	201 - KK - 202
Posterior @ 0.4	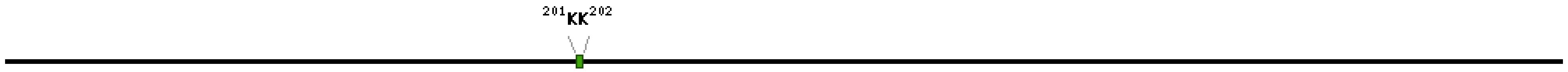
Posterior Graphic. Threshold Marked @ 0.4	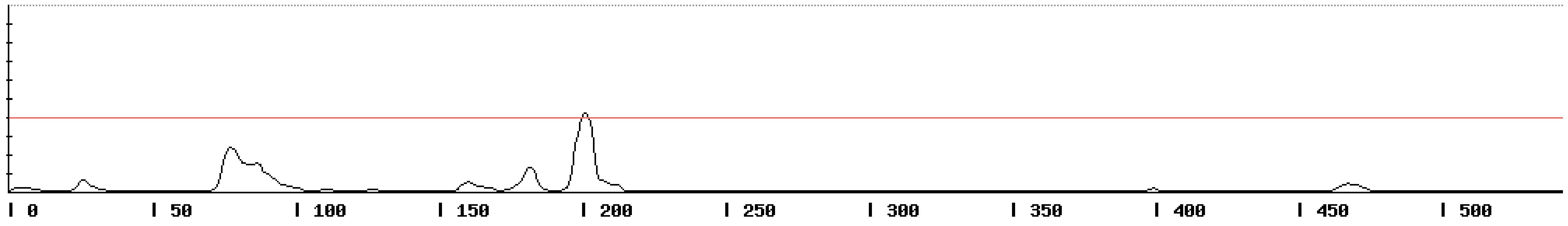

Predictions for Aquilegiacoerulea_Aqcoe1G373200.1.p

Viterbi Path	
Viterbi Path	
Posterior @ 0.4	167 - RKGRLLKDSHHNPPRSLKRKR - 187
Posterior @ 0.4	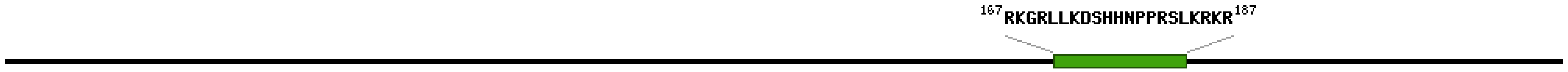
Posterior Graphic. Threshold Marked @ 0.4	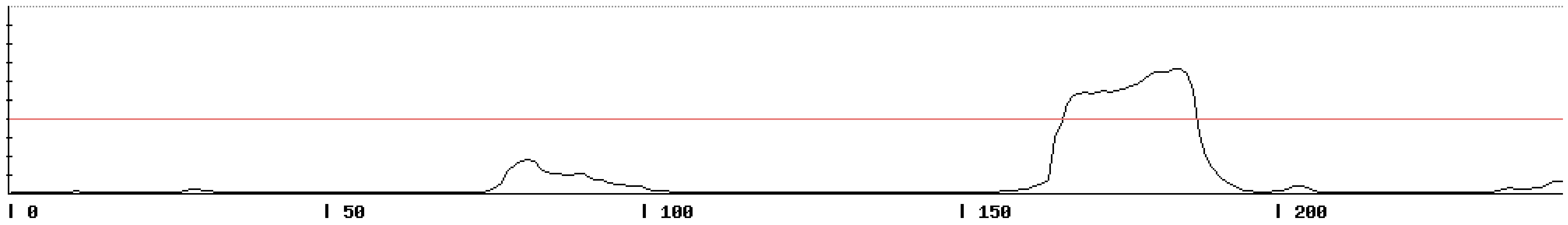

Predictions for Aquilegiacoerulea_Aqcoe1G373300.1.p

Viterbi Path

Viterbi Path

Posterior @ 0.4

215 - RKRKLLK - 221

228 - PRRSLKR - 234

Posterior @ 0.4

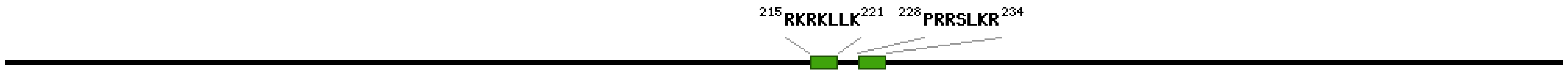

Posterior Graphic. Threshold Marked @ 0.4

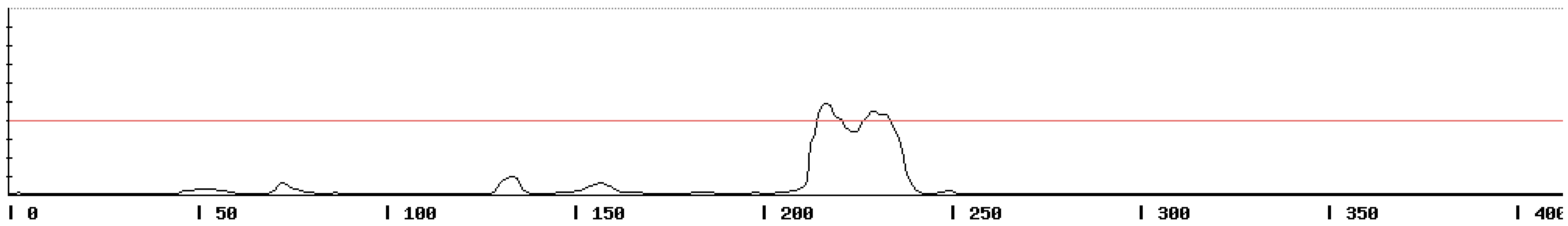

Predictions for Aquilegiacoerulea_Aqcoe1G373400.1.p

Viterbi Path

Viterbi Path

Posterior @ 0.4

197 - KKKKR - 201

224 - KK - 225

Posterior @ 0.4

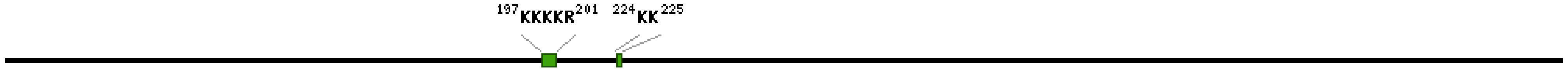

Posterior Graphic. Threshold Marked @ 0.4

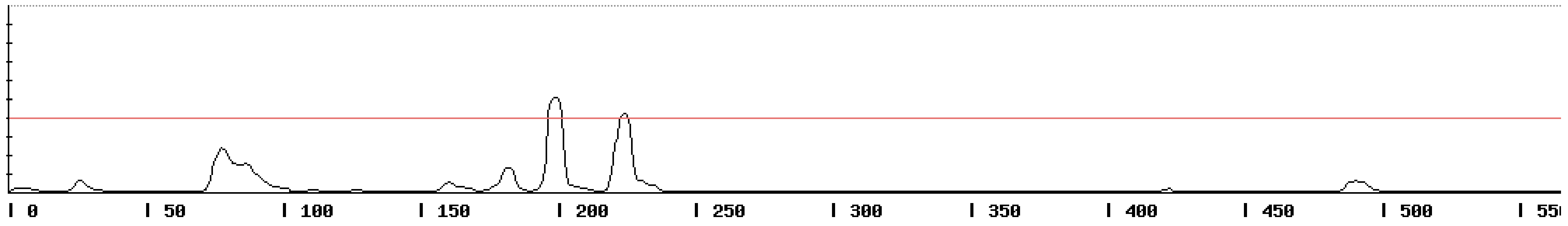

Predictions for Aquilegiacoerulea_Aqcoe1G398000.1.p

Viterbi Path

Viterbi Path

Posterior @ 0.4

Posterior @ 0.4

Posterior Graphic. Threshold Marked @ 0.4

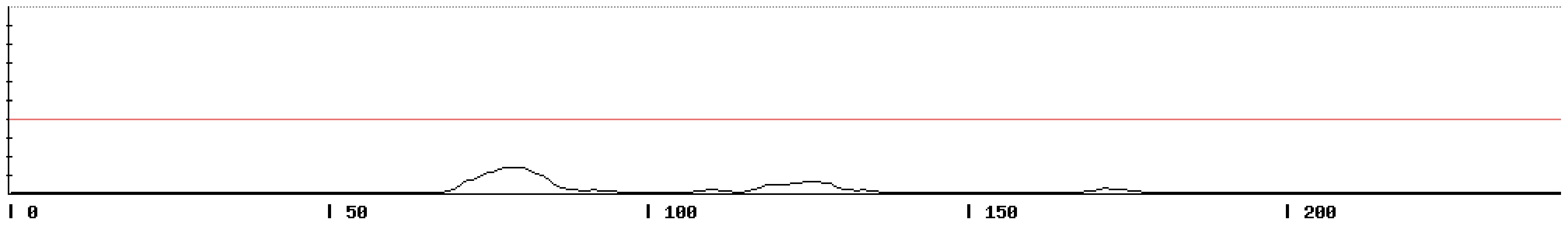

Predictions for Aquilegiacoerulea_Aqcoe1G439000.1.p

Viterbi Path

Viterbi Path

Posterior @ 0.4

Posterior @ 0.4

Posterior Graphic. Threshold Marked @ 0.4	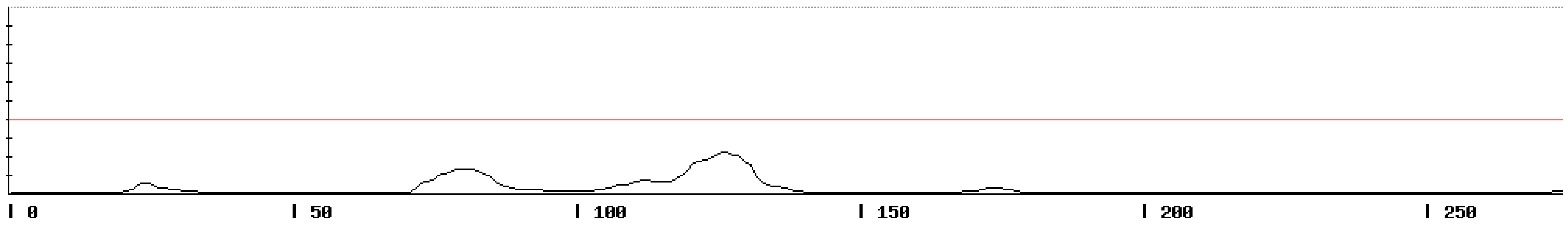

Predictions for Aquilegiacoerulea_Aqcoe1G457300.1.p	
Viterbi Path	
Viterbi Path	
Posterior @ 0.4	
Posterior @ 0.4	
Posterior Graphic. Threshold Marked @ 0.4	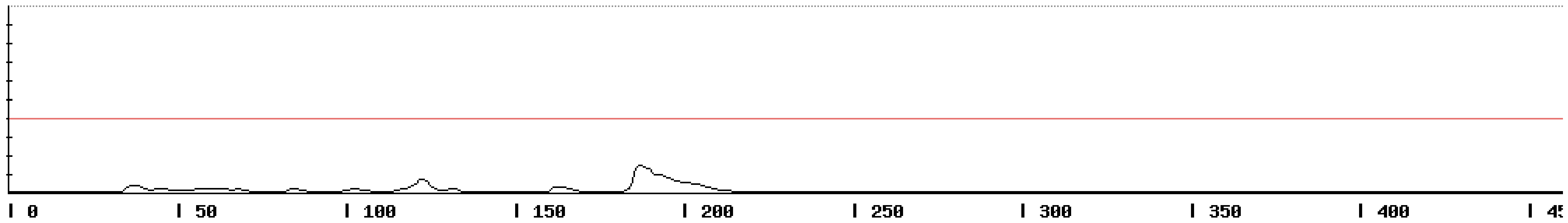

Predictions for Aquilegiacoerulea_Aqcoe1G463400.1.p	
Viterbi Path	
Viterbi Path	
Posterior @ 0.4	
Posterior @ 0.4	

Posterior Graphic. Threshold Marked @ 0.4

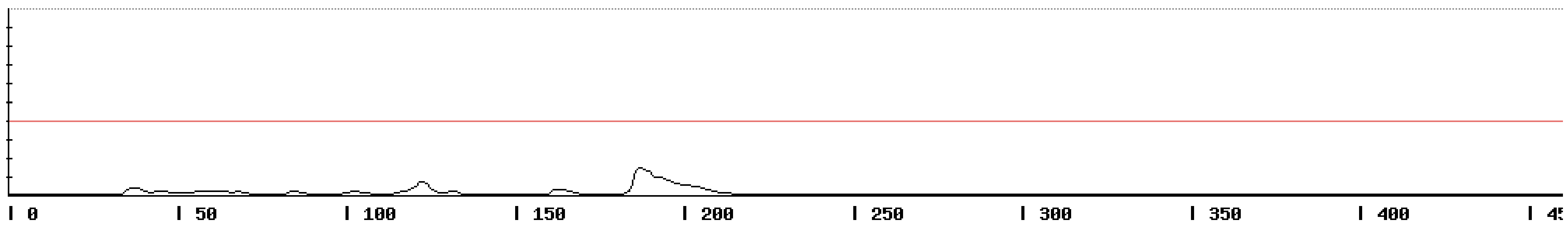

Predictions for Aquilegiacoerulea_Aqcoe1G496900.1.p

Viterbi Path

Viterbi Path

Posterior @ 0.4

Posterior @ 0.4

Posterior Graphic. Threshold Marked @ 0.4

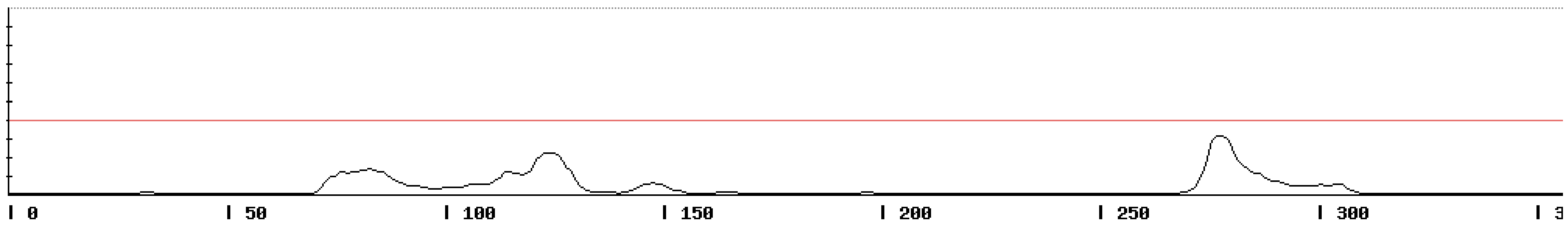

Predictions for Aquilegiacoerulea_Aqcoe2G001300.1.p

Viterbi Path

Viterbi Path

Posterior @ 0.4

Posterior @ 0.4

Posterior Graphic. Threshold Marked @ 0.4

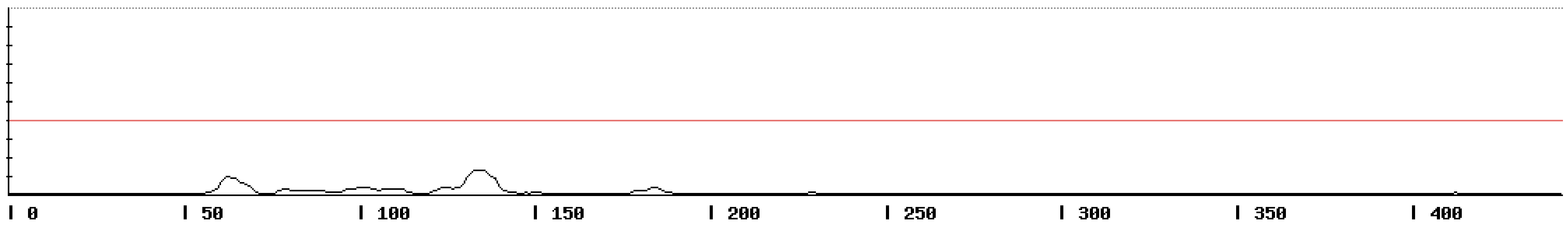

Predictions for Aquilegiacoerulea_Aqcoe2G001600.1.p

Viterbi Path

Viterbi Path

Posterior @ 0.4

Posterior @ 0.4

Posterior Graphic. Threshold Marked @ 0.4

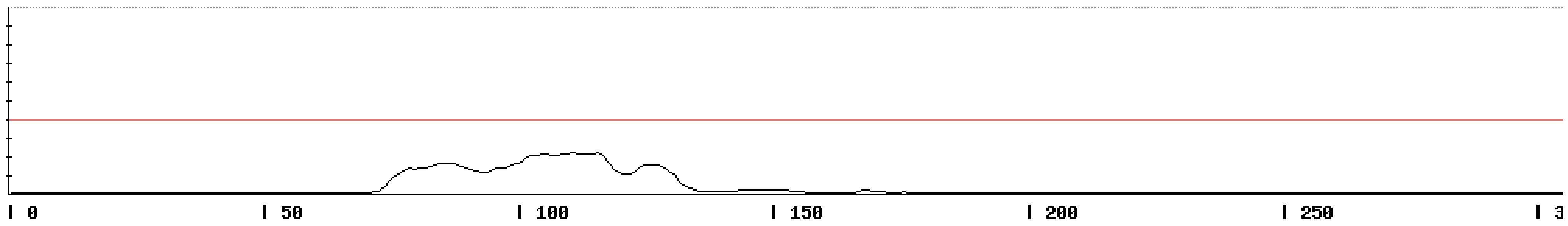

Predictions for Aquilegiacoerulea_Aqcoe2G158200.1.p

Viterbi Path

Viterbi Path

Posterior @ 0.4	194 - K - 194
Posterior @ 0.4	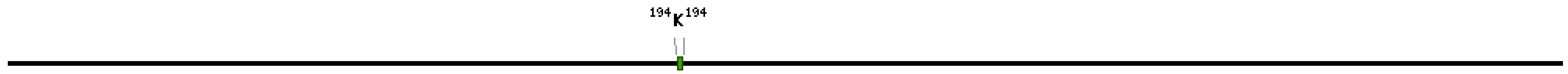
Posterior Graphic. Threshold Marked @ 0.4	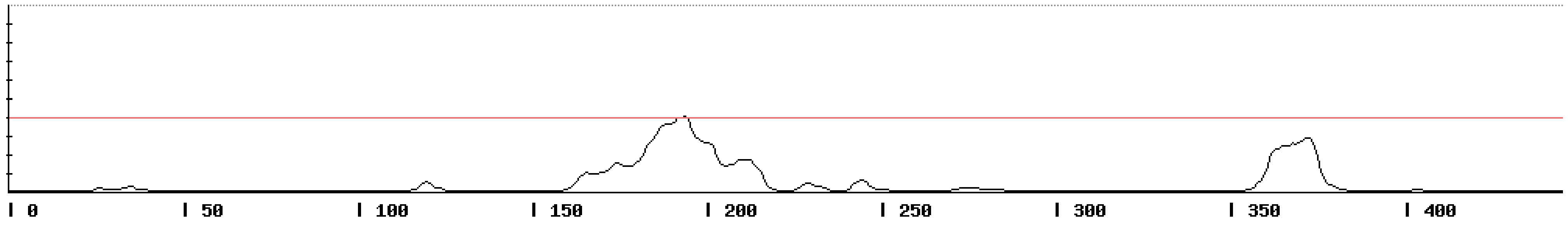

Predictions for Aquilegiacoerulea_Aqcoe2G306500.1.p	
Viterbi Path	
Viterbi Path	
Posterior @ 0.4	
Posterior @ 0.4	
Posterior Graphic. Threshold Marked @ 0.4	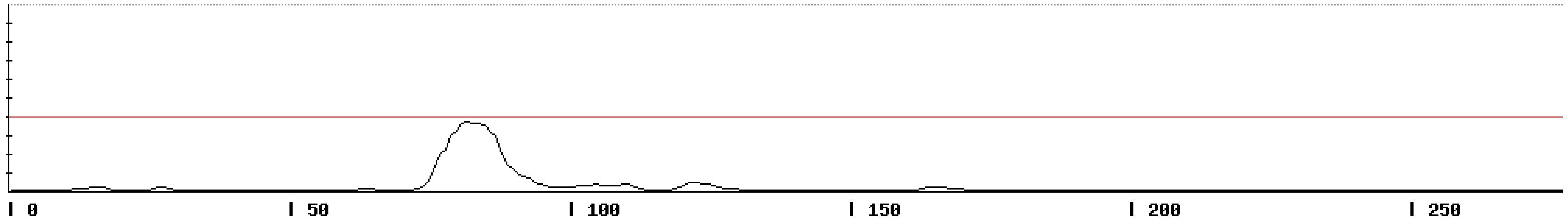

Predictions for Aquilegiacoerulea_Aqcoe2G308700.1.p

Viterbi Path

Viterbi Path

Posterior @ 0.4

Posterior @ 0.4

Posterior Graphic. Threshold Marked @ 0.4

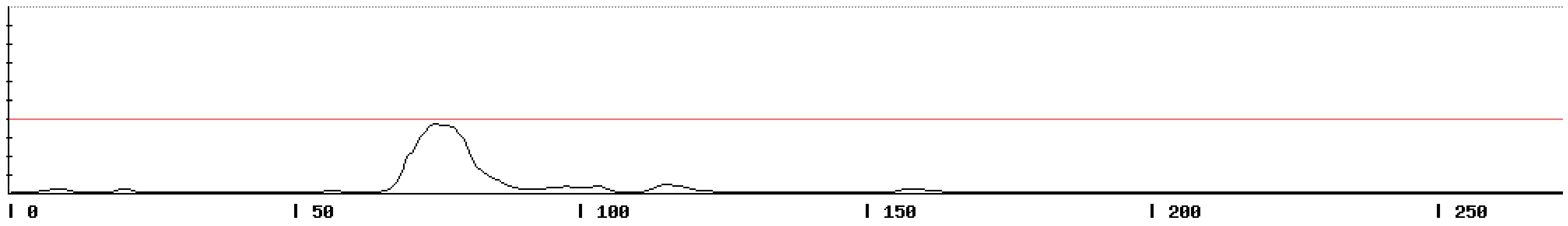

Predictions for Aquilegiacoerulea_Aqcoe2G312600.1.p

Viterbi Path

Viterbi Path

Posterior @ 0.4

Posterior @ 0.4

Posterior Graphic. Threshold Marked @ 0.4

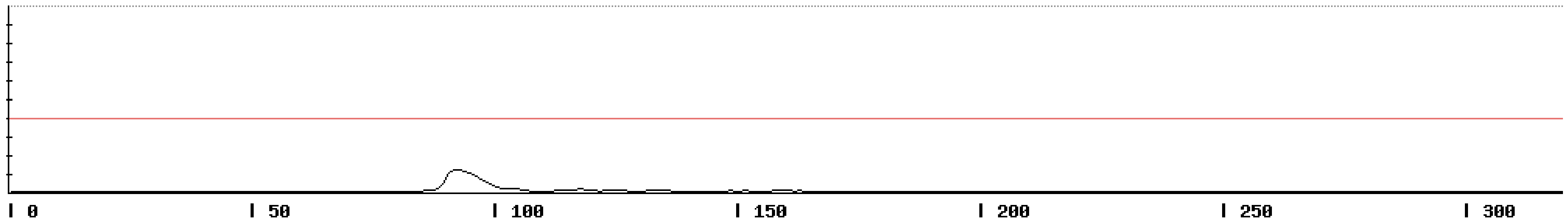

Predictions for Aquilegiacoerulea_Aqcoe2G366500.1.p	
Viterbi Path	
Viterbi Path	
Posterior @ 0.4	
Posterior @ 0.4	
Posterior Graphic. Threshold Marked @ 0.4	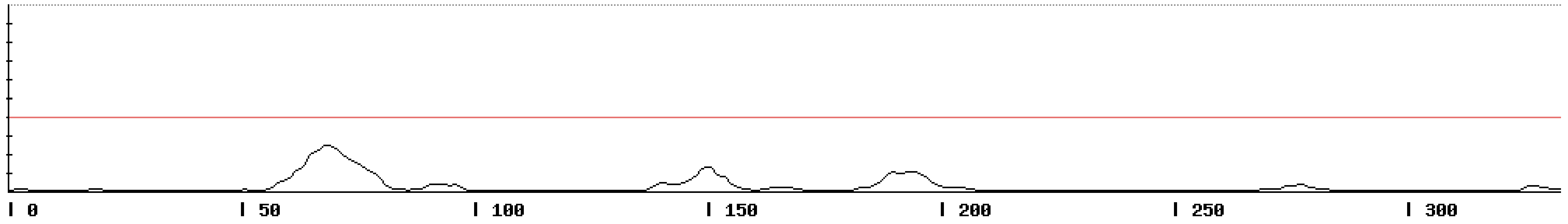

Predictions for Aquilegiacoerulea_Aqcoe2G375500.1.p	
Viterbi Path	
Viterbi Path	
Posterior @ 0.4	
Posterior @ 0.4	
Posterior Graphic. Threshold Marked @ 0.4	

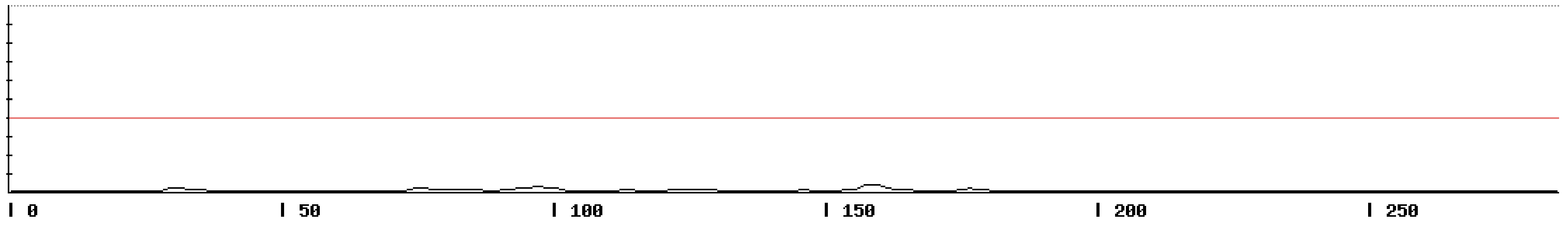

Predictions for Aquilegiacoerulea_Aqcoe3G037800.1.p

Viterbi Path

Viterbi Path

Posterior @ 0.4

Posterior @ 0.4

Posterior Graphic. Threshold Marked @ 0.4

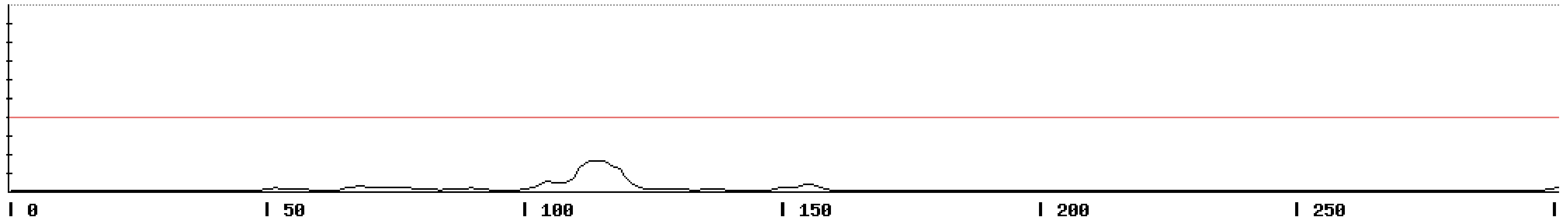

Predictions for Aquilegiacoerulea_Aqcoe3G079400.1.p

Viterbi Path

Viterbi Path

Posterior @ 0.4

Posterior @ 0.4

Posterior Graphic. Threshold Marked @ 0.4

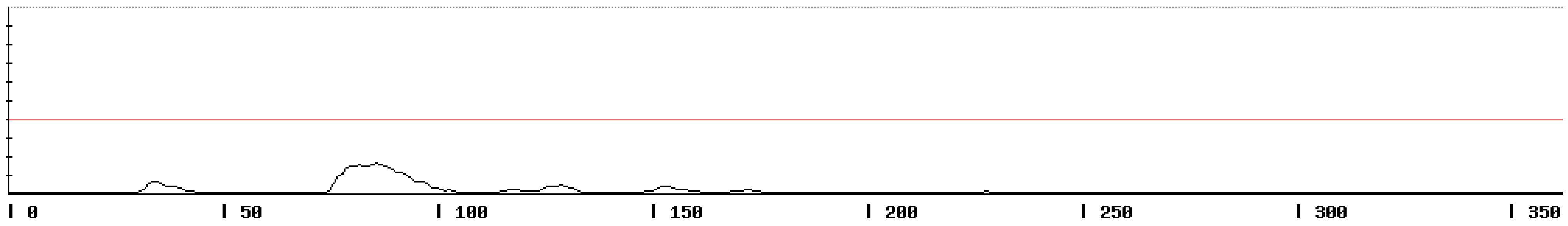

Predictions for Aquilegiacoerulea_Aqcoe3G130600.1.p

Viterbi Path

Viterbi Path

Posterior @ 0.4

Posterior @ 0.4

Posterior Graphic. Threshold Marked @ 0.4

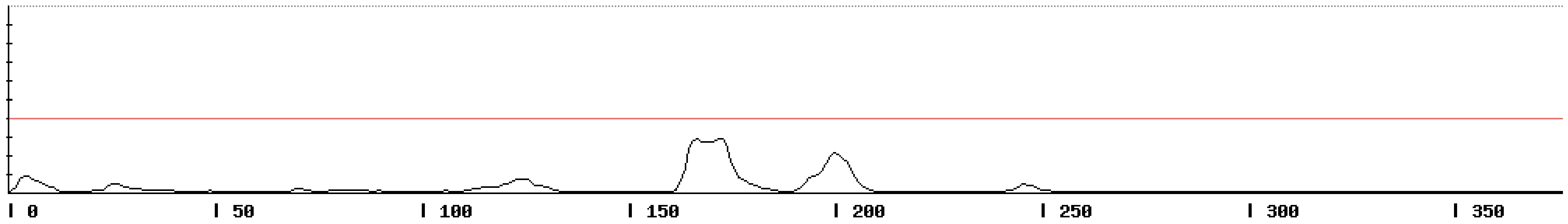

Predictions for Aquilegiacoerulea_Aqcoe3G136600.1.p

Viterbi Path

Viterbi Path

Posterior @ 0.4 216 - KRK - 218

Posterior @ 0.4

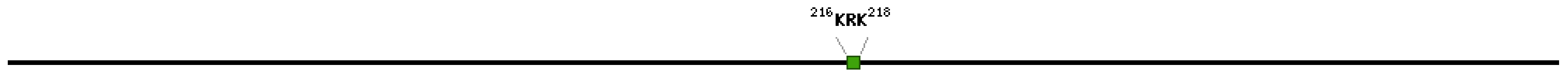

Posterior Graphic. Threshold Marked @ 0.4

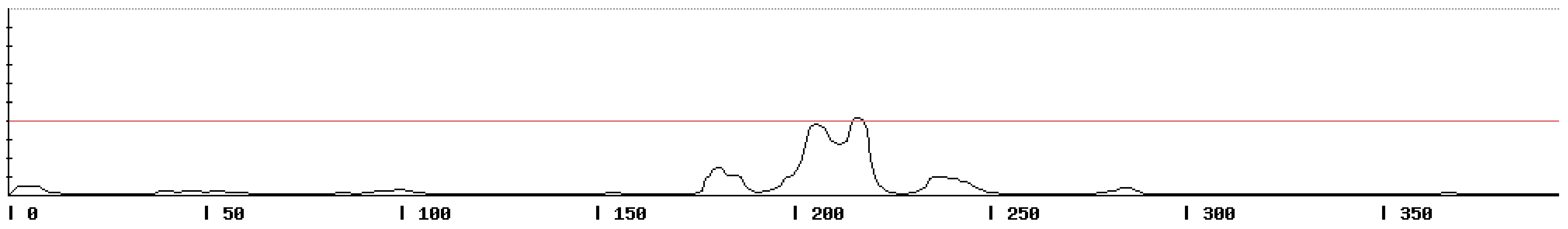

Predictions for Aquilegiacoerulea_Aqcoe3G142100.1.p

Viterbi Path

Viterbi Path

Posterior @ 0.4 — 132 - HRKRRK - 137

Posterior @ 0.4

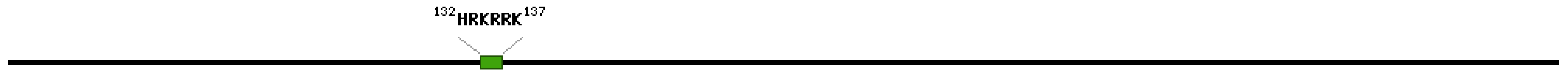

Posterior Graphic. Threshold Marked @ 0.4

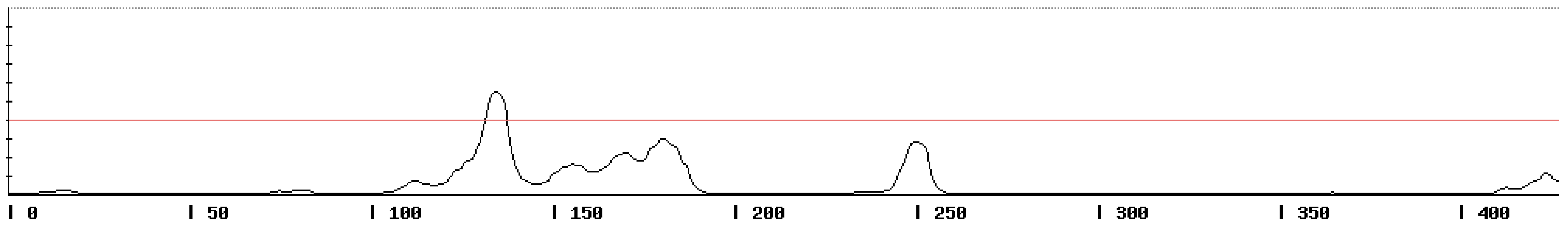

Predictions for Aquilegiacoerulea_Aqcoe3G179900.1.p

Viterbi Path

Viterbi Path

Posterior @ 0.4	
Posterior @ 0.4	
Posterior Graphic. Threshold Marked @ 0.4	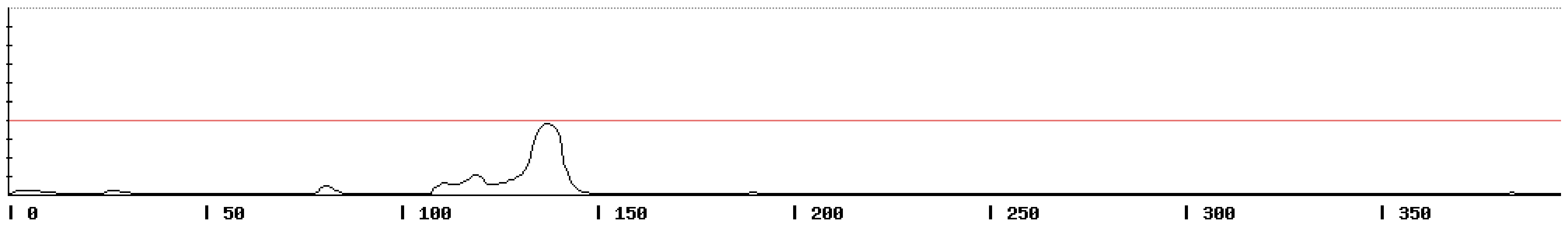

Predictions for Aquilegiacoerulea_Aqcoe3G199000.1.p

Viterbi Path	
Viterbi Path	
Posterior @ 0.4	87 - KKKYGKGSRPSRSTAGDGFWKATGRDKQVLSKGKKLVIGLKKTLVFYRGDPKRKKDAKK - 145
Posterior @ 0.4	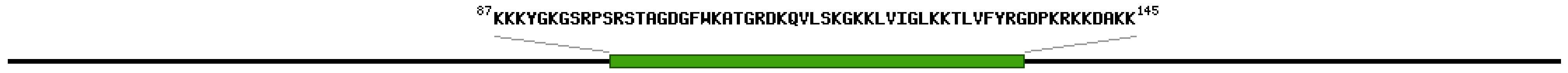
Posterior Graphic. Threshold Marked @ 0.4	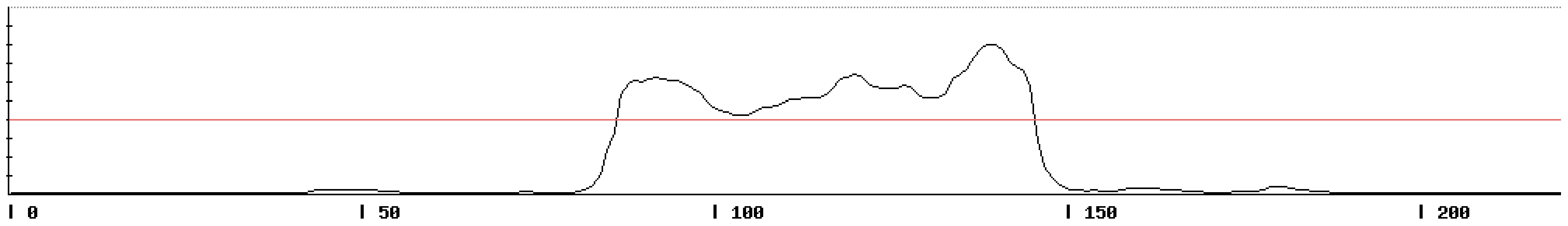

Predictions for Aquilegiacoerulea_Aqcoe3G236400.1.p

Viterbi Path	
Viterbi Path	

Posterior @ 0.4

Posterior @ 0.4

Posterior Graphic. Threshold Marked @ 0.4

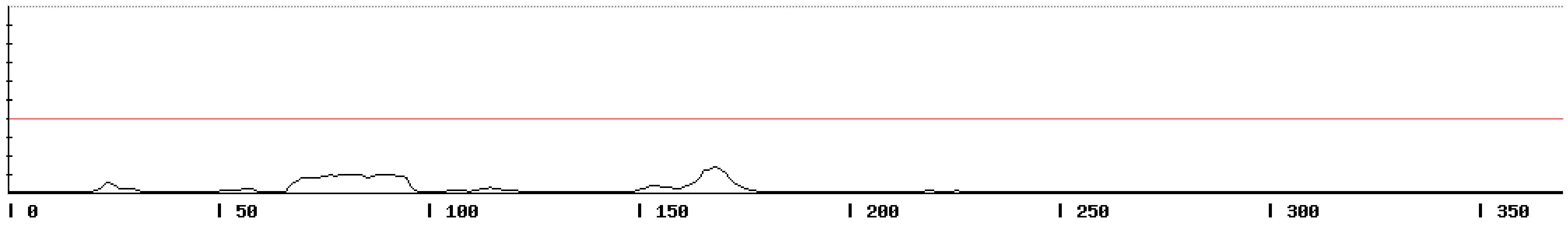

Predictions for Aquilegiacoerulea_Aqcoe3G337400.1.p

Viterbi Path

Viterbi Path

Posterior @ 0.4

Posterior @ 0.4

Posterior Graphic. Threshold Marked @ 0.4

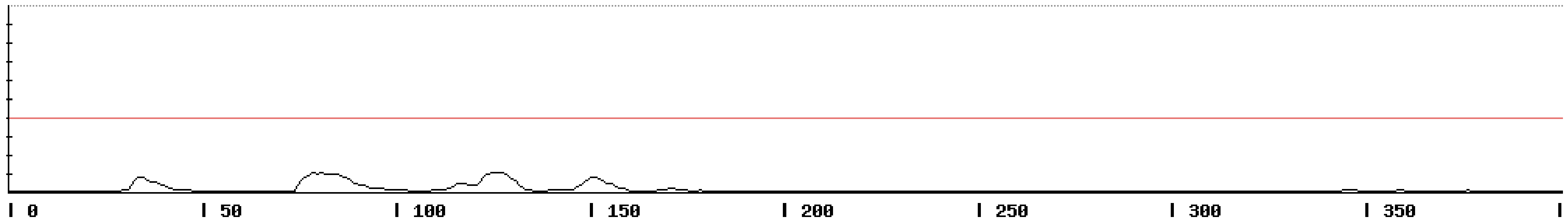

Predictions for Aquilegiacoerulea_Aqcoe3G345800.1.p

Viterbi Path	
Viterbi Path	
Posterior @ 0.4	
Posterior @ 0.4	
Posterior Graphic. Threshold Marked @ 0.4	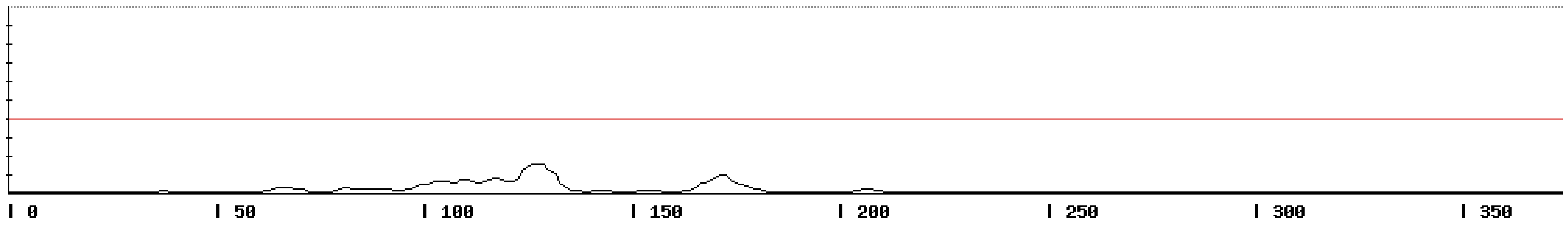

Predictions for Aquilegiacoerulea_Aqcoe3G345900.1.p	
Viterbi Path	
Viterbi Path	
Posterior @ 0.4	
Posterior @ 0.4	
Posterior Graphic. Threshold Marked @ 0.4	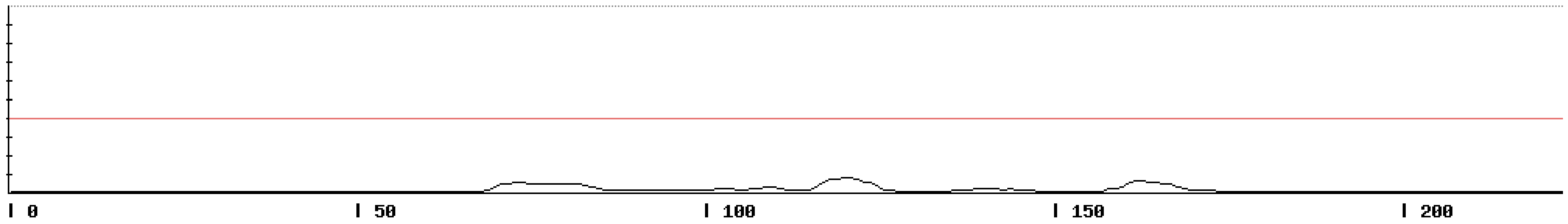

Predictions for Aquilegiacoerulea_Aqcoe3G361700.1.p	
Viterbi Path	
Viterbi Path	
Posterior @ 0.4	
Posterior @ 0.4	
Posterior Graphic. Threshold Marked @ 0.4	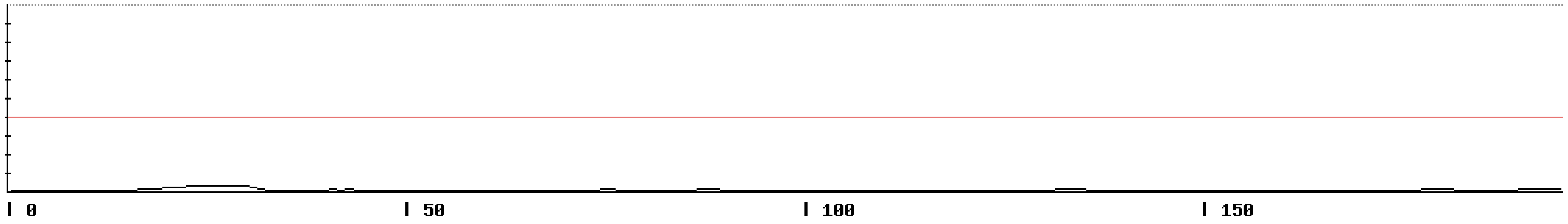

Predictions for Aquilegiacoerulea_Aqcoe3G368500.1.p	
Viterbi Path	209 - KTKATKAVTNKKKKNVRKKKNDKP - 232
Viterbi Path	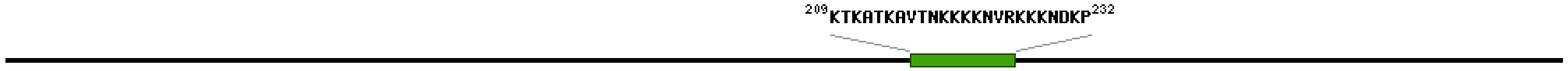
Posterior @ 0.4	159 - KPPKGRKT - 166<br>210 - TKATKAVTNKKKKNVRKKKNDKP - 232
Posterior @ 0.4	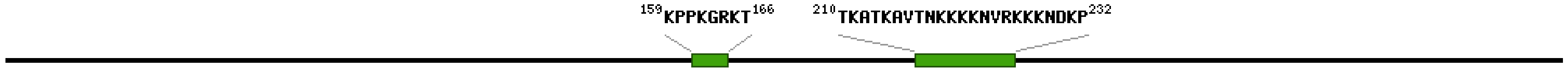
Posterior Graphic. Threshold Marked @ 0.4	

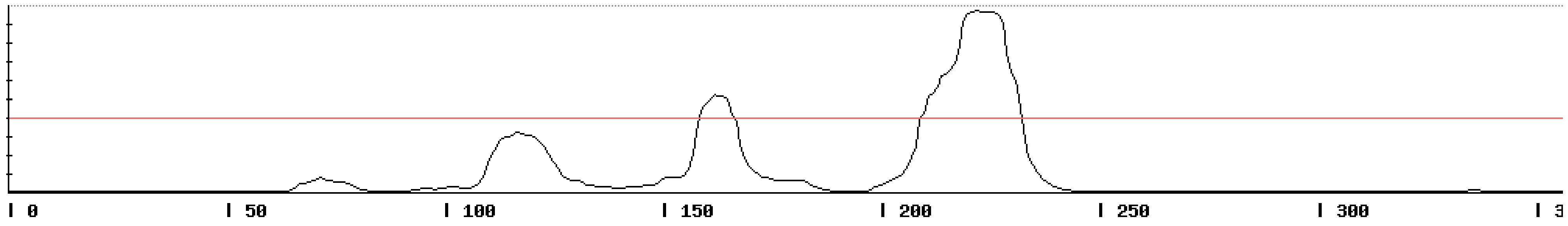

Predictions for Aquilegiacoerulea_Aqcoe3G396000.1.p

Viterbi Path	
Viterbi Path	
Posterior @ 0.4	136 - RKRRK - 140
Posterior @ 0.4	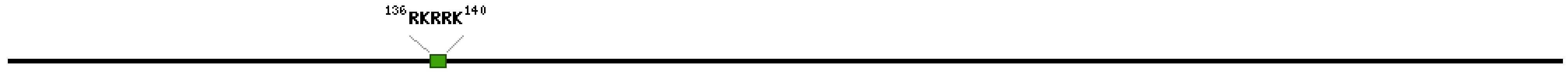
Posterior Graphic. Threshold Marked @ 0.4	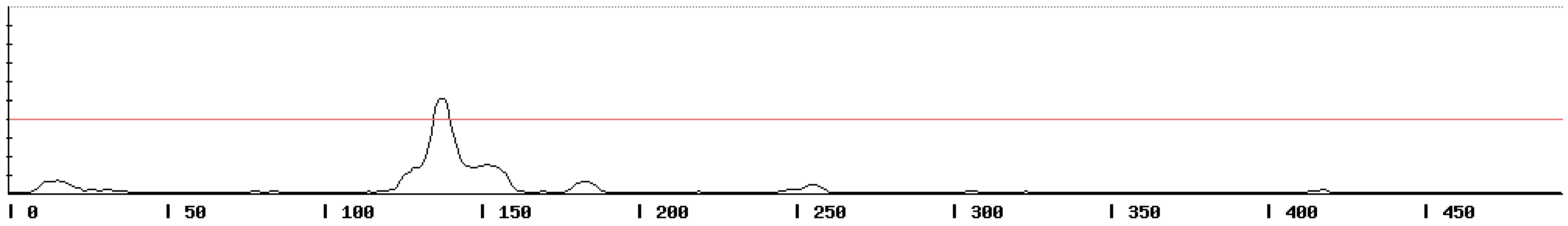

Predictions for Aquilegiacoerulea_Aqcoe3G424400.1.p

Viterbi Path	
Viterbi Path	
Posterior @ 0.4	
Posterior @ 0.4	

Posterior Graphic. Threshold Marked @ 0.4

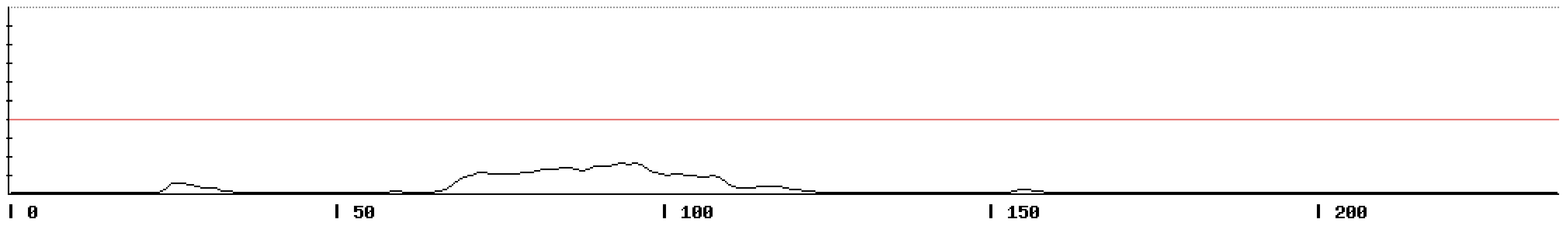

Predictions for Aquilegiacoerulea_Aqcoe3G424500.1.p

Viterbi Path

Viterbi Path

Posterior @ 0.4

Posterior @ 0.4

Posterior Graphic. Threshold Marked @ 0.4

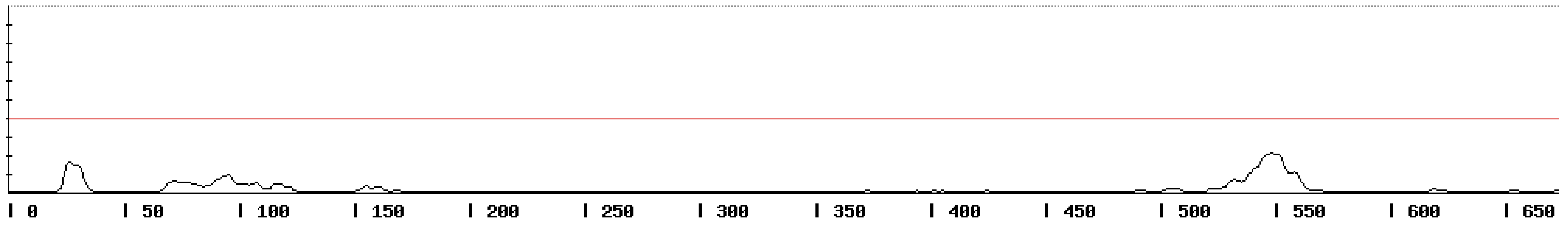

Predictions for Aquilegiacoerulea_Aqcoe4G076900.1.p

Viterbi Path

Viterbi Path

Posterior @ 0.4	138 - KR - 139
	167 - KLKGHK - 172

Posterior @ 0.4

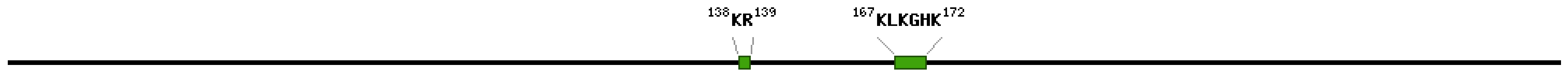

Posterior Graphic. Threshold Marked @ 0.4

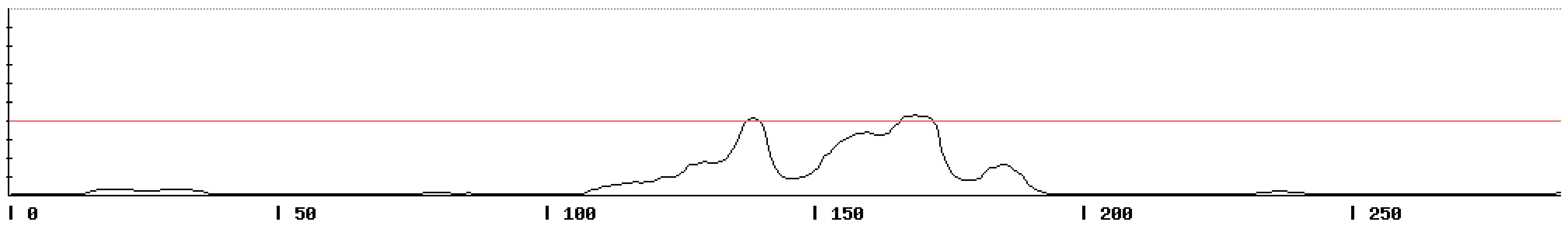

Predictions for Aquilegiacoerulea_Aqcoe4G090300.1.p

Viterbi Path

Viterbi Path

Posterior @ 0.4

Posterior @ 0.4

Posterior Graphic. Threshold Marked @ 0.4

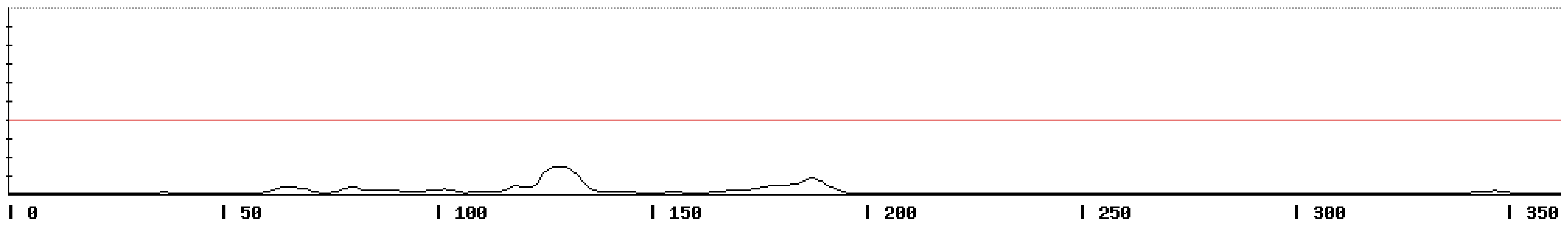

Predictions for Aquilegiacoerulea_Aqcoe4G132100.1.p

Viterbi Path

Viterbi Path

Posterior @ 0.4	77 - KKHGRK - 82
Posterior @ 0.4	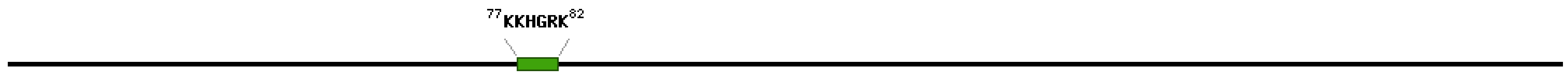
Posterior Graphic. Threshold Marked @ 0.4	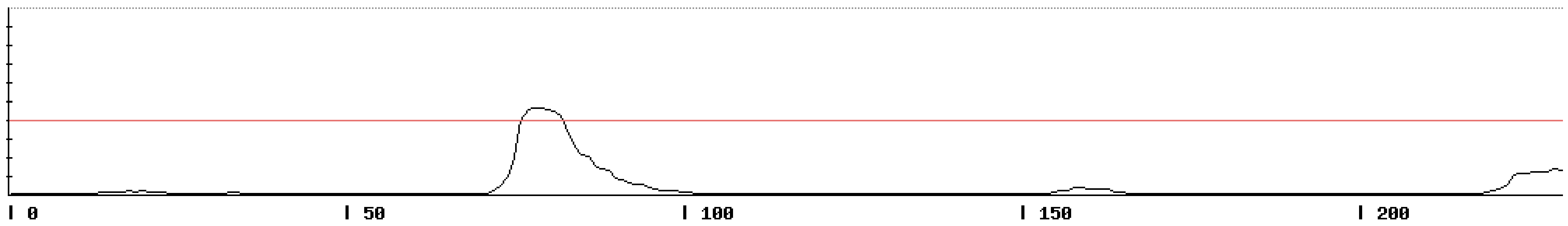

Predictions for Aquilegiacoerulea_Aqcoe4G152500.1.p

Viterbi Path	
Viterbi Path	
Posterior @ 0.4	
Posterior @ 0.4	
Posterior Graphic. Threshold Marked @ 0.4	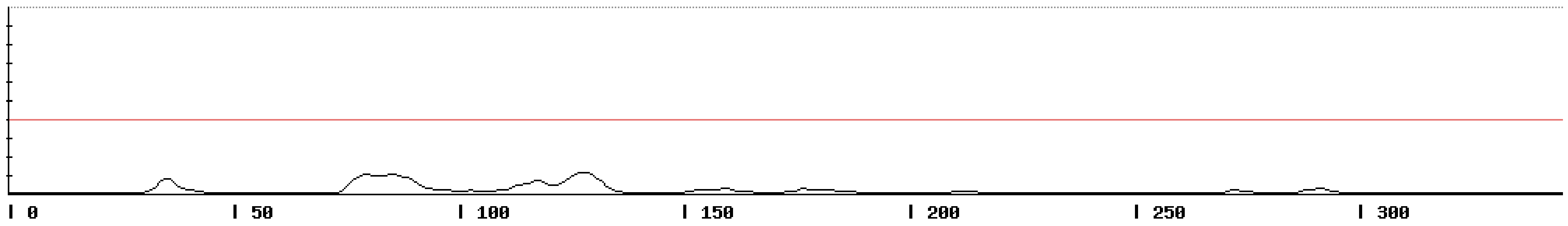

Predictions for Aquilegiacoerulea_Aqcoe5G024200.1.p

Viterbi Path	
Viterbi Path	

Posterior @ 0.4	
Posterior @ 0.4	
Posterior Graphic. Threshold Marked @ 0.4	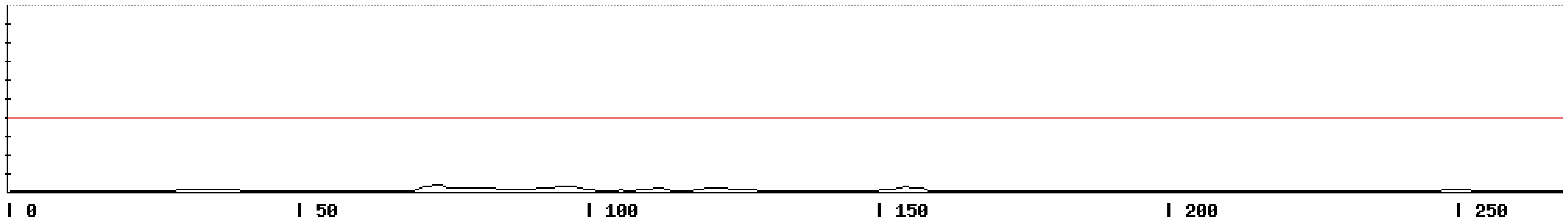

Predictions for Aquilegiacoerulea_Aqcoe5G084800.1.p

Viterbi Path	
Viterbi Path	
Posterior @ 0.4	138 - DKKSNKMNSKVRHGKRESKKIKE - 160
Posterior @ 0.4	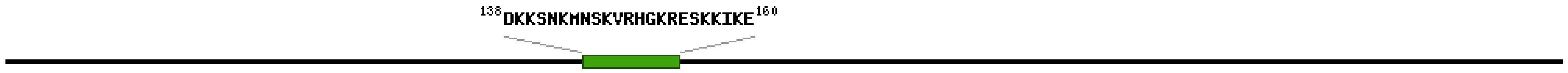
Posterior Graphic. Threshold Marked @ 0.4	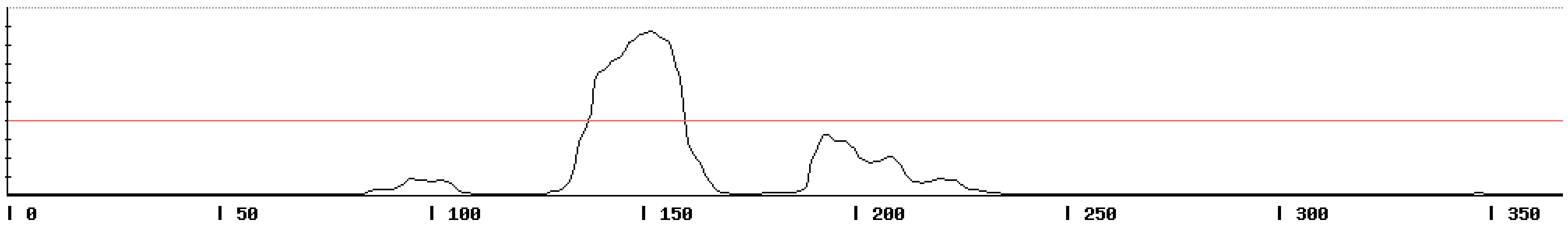

Predictions for Aquilegiacoerulea_Aqcoe5G095700.1.p

Viterbi Path	
Viterbi Path	
Posterior @ 0.4	53 - KVKKKHNKSIGKKLERRAG - 71
Posterior @ 0.4	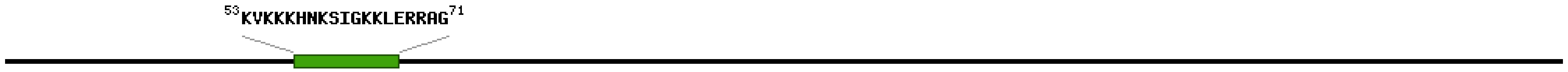
Posterior Graphic. Threshold Marked @ 0.4	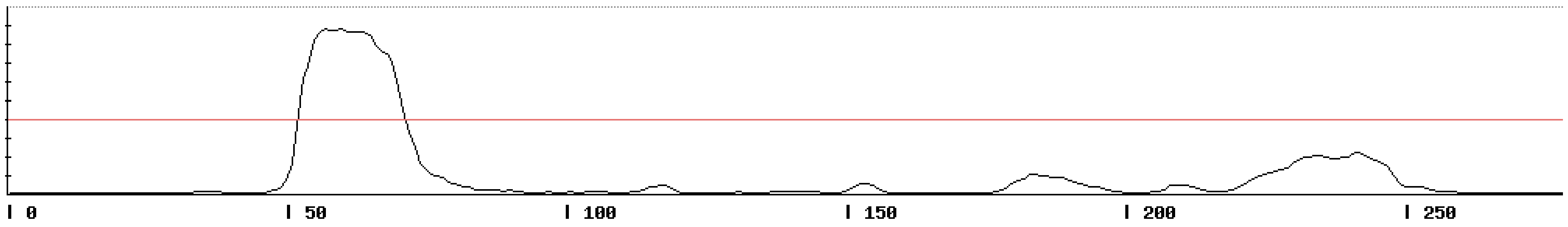

Predictions for Aquilegiacoerulea_Aqcoe5G095800.1.p	
Viterbi Path	
Viterbi Path	
Posterior @ 0.4	
Posterior @ 0.4	
Posterior Graphic. Threshold Marked @ 0.4	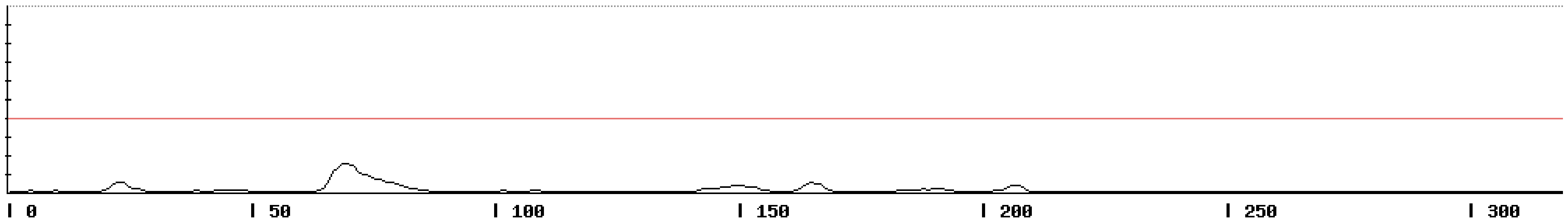

Predictions for Aquilegiacoerulea_Aqcoe5G106600.1.p	
Viterbi Path	
Viterbi Path	
Posterior @ 0.4	
Posterior @ 0.4	
Posterior Graphic. Threshold Marked @ 0.4	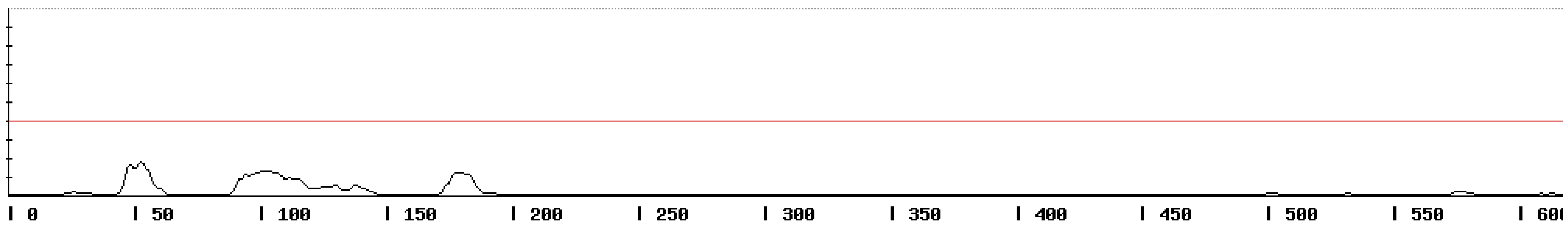

Predictions for Aquilegiacoerulea_Aqcoe5G113900.1.p	
Viterbi Path	
Viterbi Path	
Posterior @ 0.4	
Posterior @ 0.4	
Posterior Graphic. Threshold Marked @ 0.4	

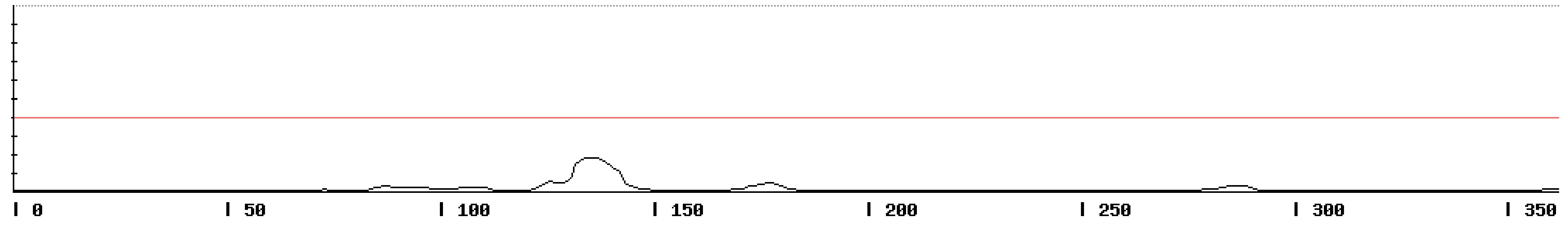

Predictions for Aquilegiacoerulea_Aqcoe5G177100.1.p

Viterbi Path	
Viterbi Path	
Posterior @ 0.4	
Posterior @ 0.4	
Posterior Graphic. Threshold Marked @ 0.4	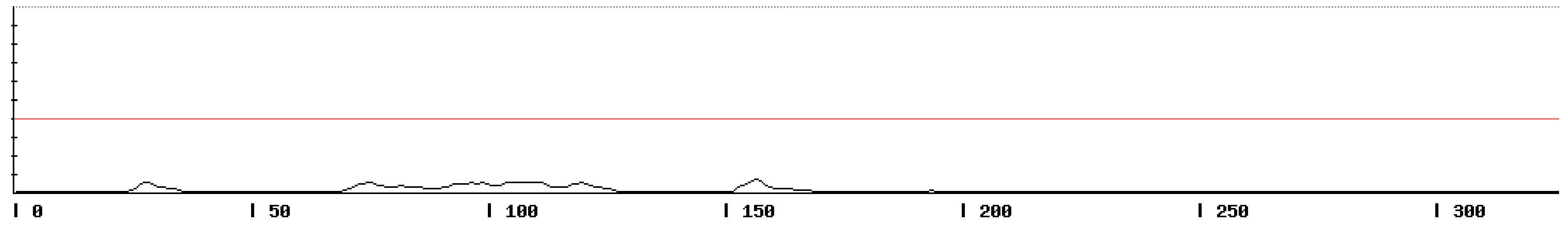

Predictions for Aquilegiacoerulea_Aqcoe5G198100.1.p

Viterbi Path	
Viterbi Path	
Posterior @ 0.4	
Posterior @ 0.4	

Posterior Graphic. Threshold Marked @ 0.4	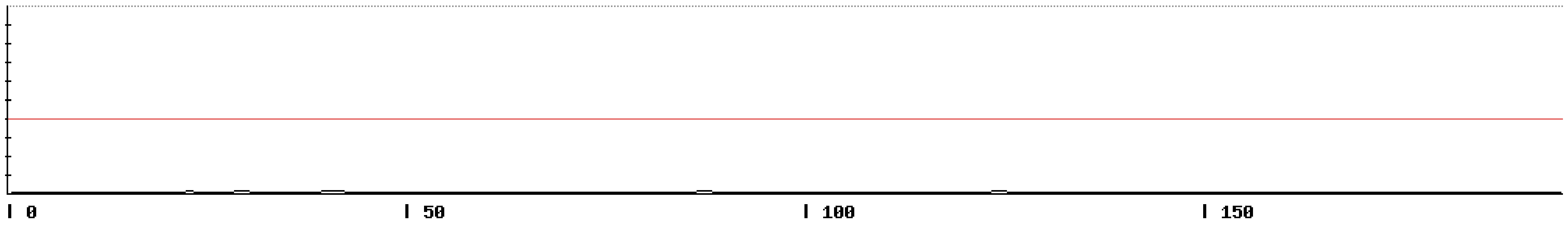

Predictions for Aquilegiacoerulea_Aqcoe5G198200.1.p	
Viterbi Path	
Viterbi Path	
Posterior @ 0.4	
Posterior @ 0.4	
Posterior Graphic. Threshold Marked @ 0.4	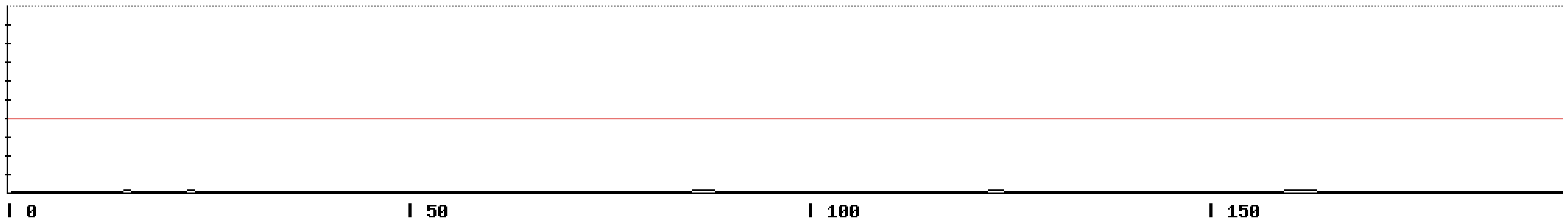

Predictions for Aquilegiacoerulea_Aqcoe5G225900.1.p	
Viterbi Path	
Viterbi Path	
Posterior @ 0.4	
Posterior @ 0.4	

Posterior Graphic. Threshold Marked @ 0.4

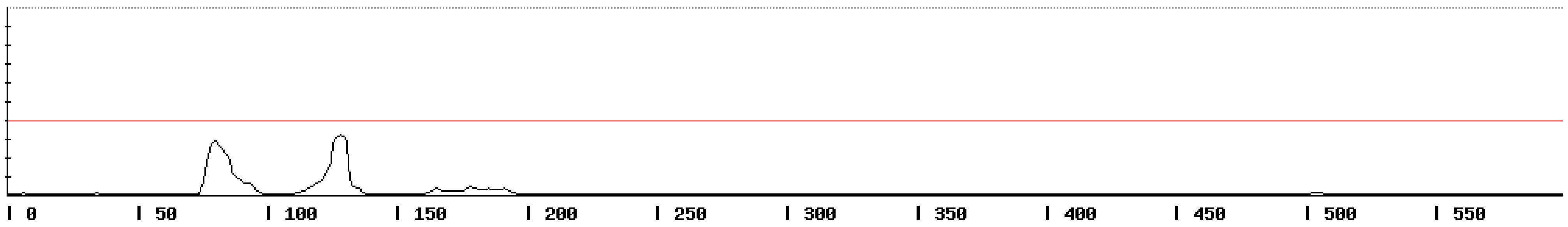

Predictions for Aquilegiacoerulea_Aqcoe5G233600.1.p

Viterbi Path

Viterbi Path

Posterior @ 0.4

Posterior @ 0.4

Posterior Graphic. Threshold Marked @ 0.4

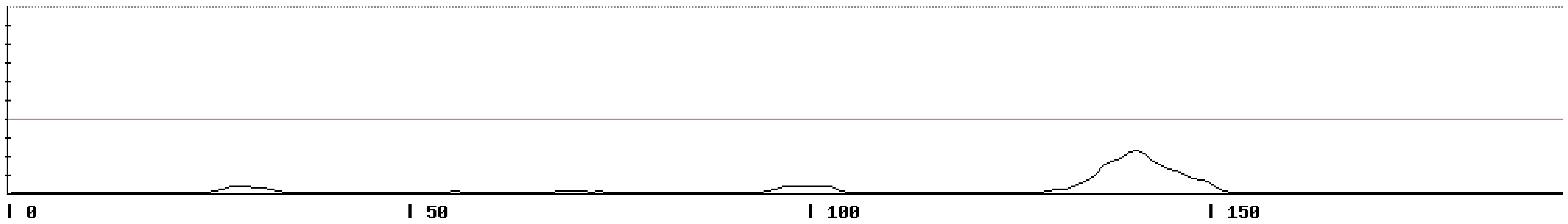

Predictions for Aquilegiacoerulea_Aqcoe5G234900.1.p

Viterbi Path

Viterbi Path

Posterior @ 0.4

Posterior @ 0.4

Posterior Graphic. Threshold Marked @ 0.4

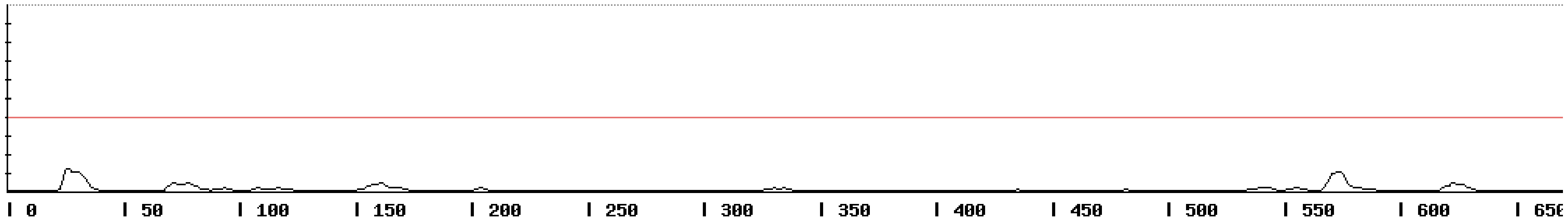

Predictions for Aquilegiacoerulea_Aqcoe5G240700.1.p

Viterbi Path

Viterbi Path

Posterior @ 0.4

Posterior @ 0.4

Posterior Graphic. Threshold Marked @ 0.4

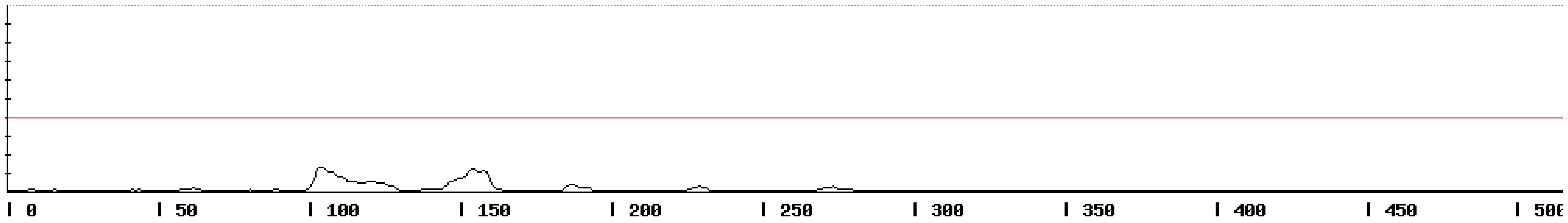

Predictions for Aquilegiacoerulea_Aqcoe5G304500.1.p

Viterbi Path

Viterbi Path

Posterior @ 0.4

Posterior @ 0.4

Posterior Graphic. Threshold Marked @ 0.4

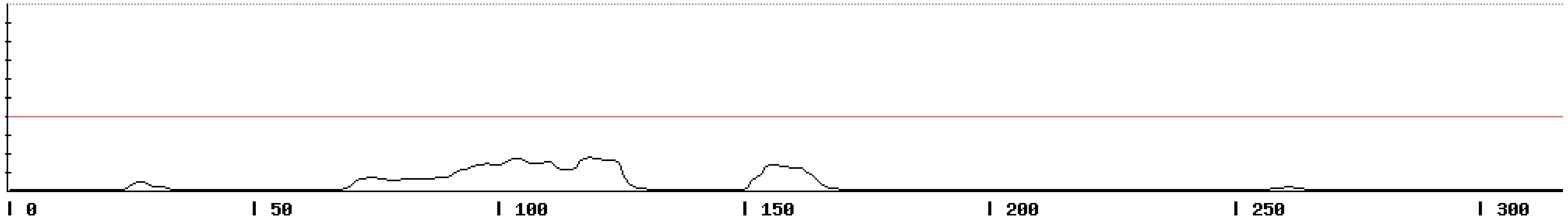

Predictions for Aquilegiacoerulea_Aqcoe5G344500.1.p

Viterbi Path

Viterbi Path

Posterior @ 0.4

Posterior @ 0.4

Posterior Graphic. Threshold Marked @ 0.4

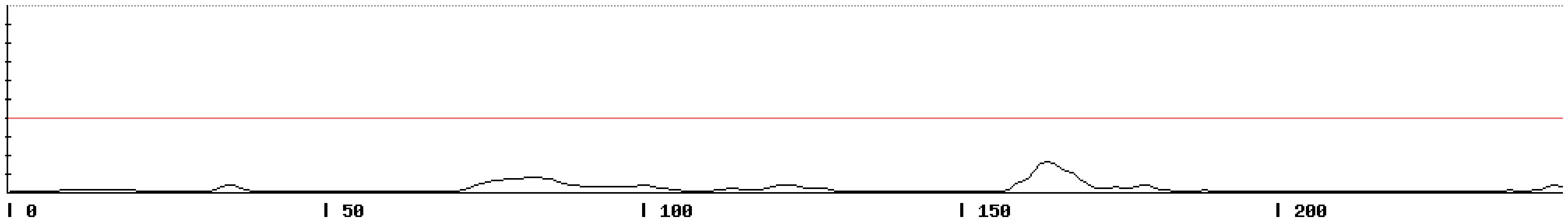

Predictions for Aquilegiacoerulea_Aqcoe5G470900.1.p

Viterbi Path

Viterbi Path

Posterior @ 0.4

Posterior @ 0.4

Posterior Graphic. Threshold Marked @ 0.4

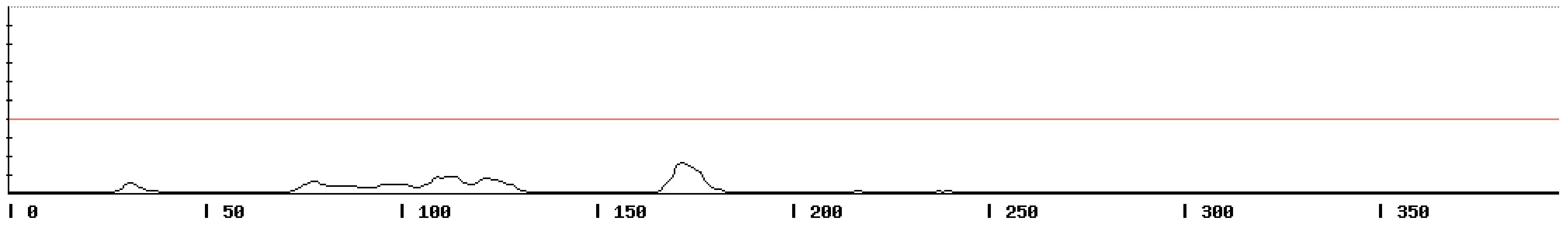

Predictions for Aquilegiacoerulea_Aqcoe6G001700.1.p

Viterbi Path

Viterbi Path

Posterior @ 0.4

Posterior @ 0.4

Posterior Graphic. Threshold Marked @ 0.4

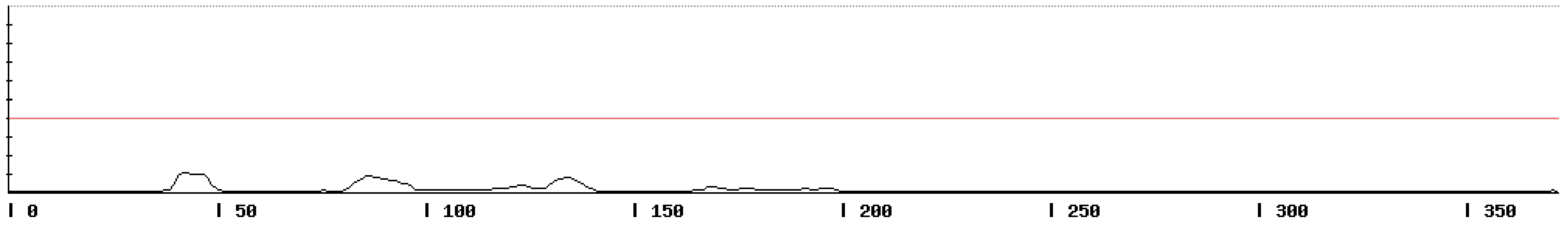

Predictions for Aquilegiacoerulea_Aqcoe6G003200.1.p	
Viterbi Path	
Viterbi Path	
Posterior @ 0.4	
Posterior @ 0.4	
Posterior Graphic. Threshold Marked @ 0.4	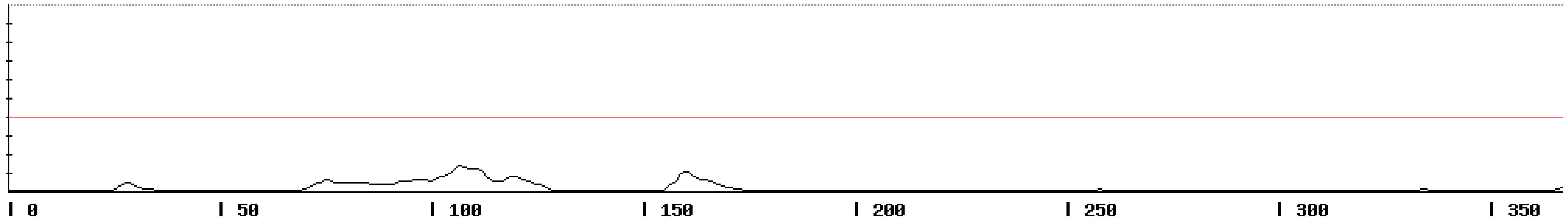

Predictions for Aquilegiacoerulea_Aqcoe6G026500.1.p	
Viterbi Path	
Viterbi Path	
Posterior @ 0.4	
Posterior @ 0.4	
Posterior Graphic. Threshold Marked @ 0.4	

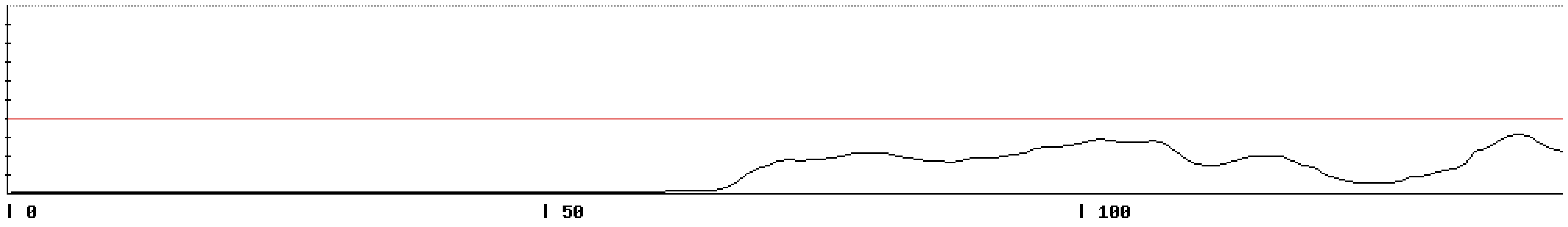

Predictions for Aquilegiacoerulea_Aqcoe6G109900.1.p	
Viterbi Path	
Viterbi Path	
Posterior @ 0.4	
Posterior @ 0.4	
Posterior Graphic. Threshold Marked @ 0.4	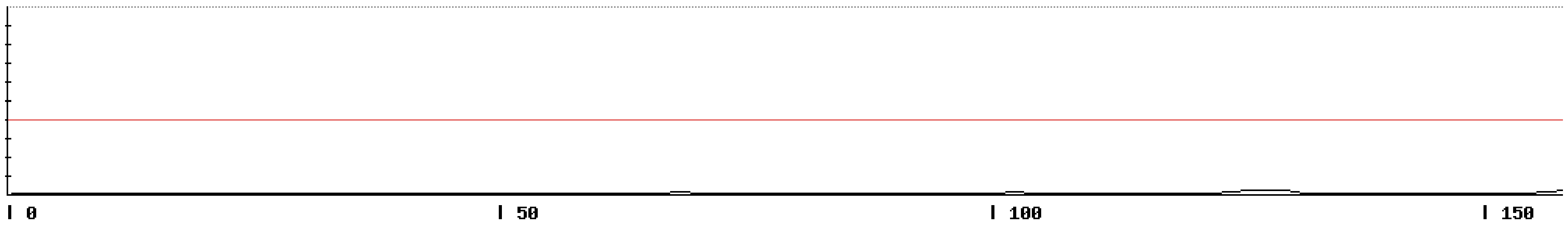

Predictions for Aquilegiacoerulea_Aqcoe6G137000.1.p	
Viterbi Path	
Viterbi Path	
Posterior @ 0.4	96 - RKGSLVKRKSKAGFWKRKCKPR - 117
Posterior @ 0.4	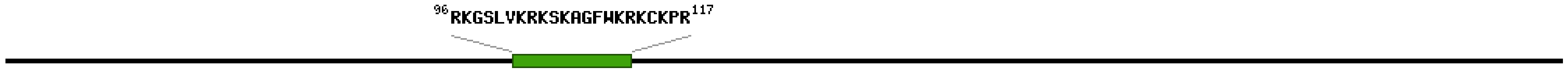

Posterior Graphic. Threshold Marked @ 0.4

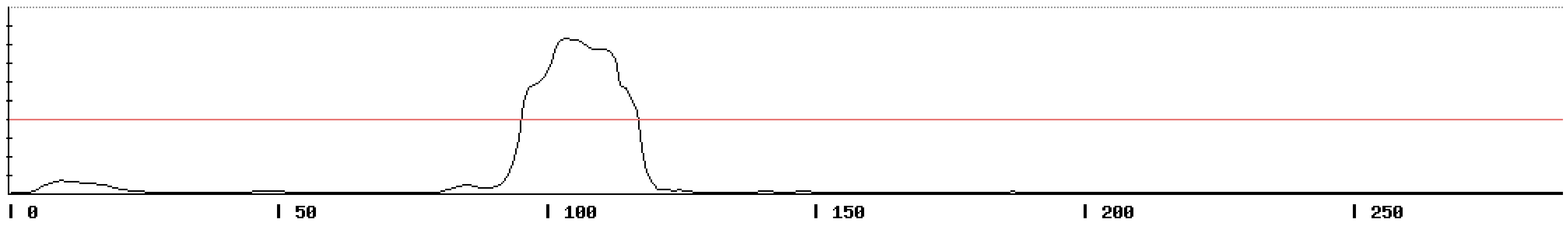

Predictions for Aquilegiacoerulea_Aqcoe6G165500.1.p

Viterbi Path

Viterbi Path

Posterior @ 0.4

Posterior @ 0.4

Posterior Graphic. Threshold Marked @ 0.4

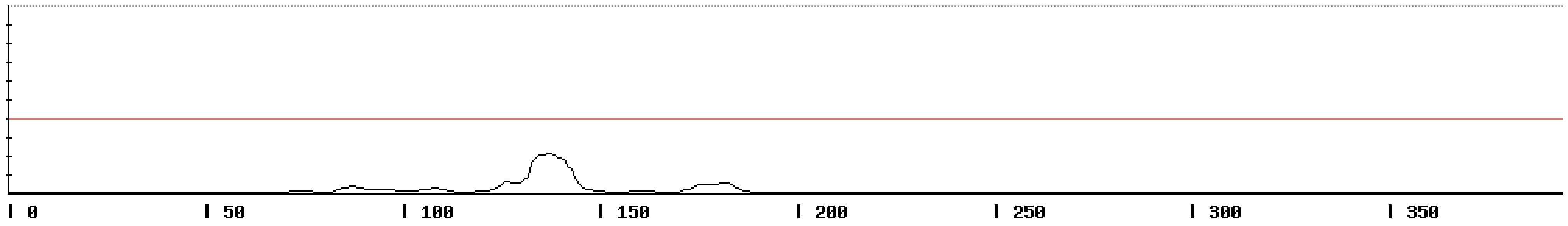

Predictions for Aquilegiacoerulea_Aqcoe6G193500.1.p

Viterbi Path

Viterbi Path

Posterior @ 0.4

Posterior @ 0.4

Posterior Graphic. Threshold Marked @ 0.4

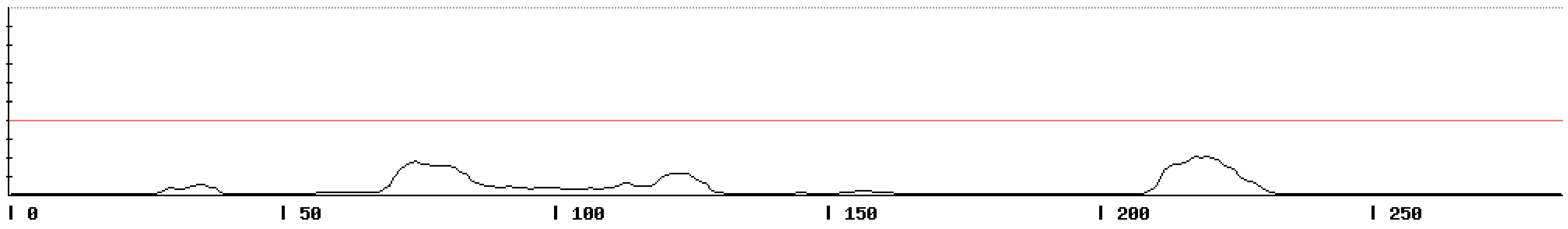

Predictions for Aquilegiacoerulea_Aqcoe6G244500.1.p

Viterbi Path

Viterbi Path

Posterior @ 0.4

Posterior @ 0.4

Posterior Graphic. Threshold Marked @ 0.4

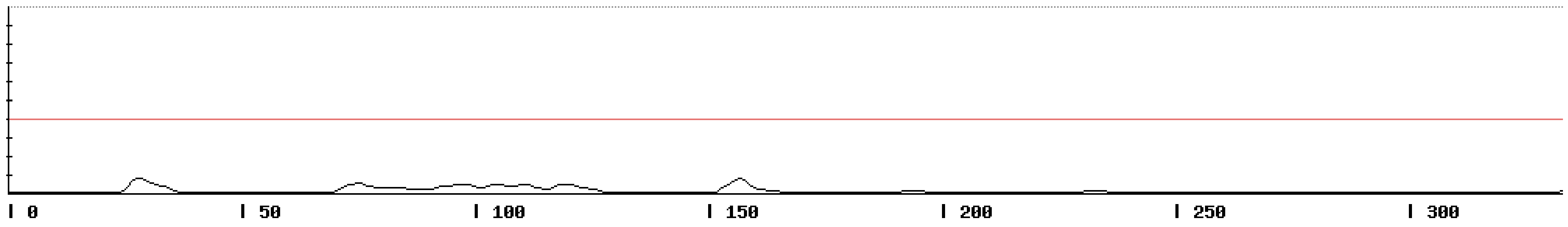

Predictions for Aquilegiacoerulea_Aqcoe7G033600.1.p

Viterbi Path

Viterbi Path

Posterior @ 0.4

Posterior @ 0.4

Posterior Graphic. Threshold Marked @ 0.4

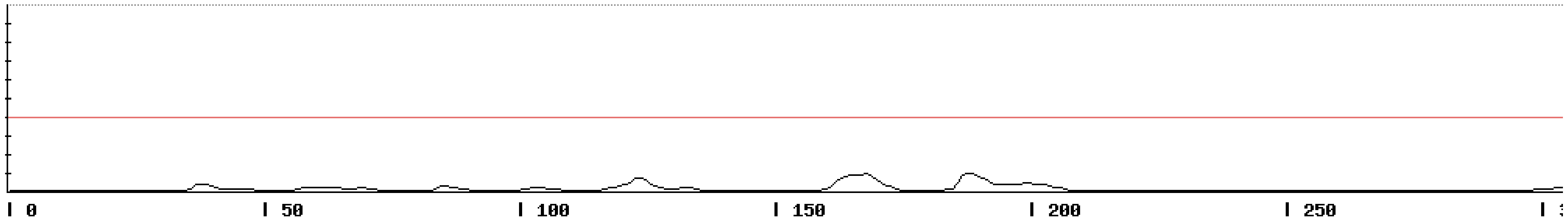

Predictions for Aquilegiacoerulea_Aqcoe7G078200.1.p

Viterbi Path

Viterbi Path

Posterior @ 0.4

Posterior @ 0.4

Posterior Graphic. Threshold Marked @ 0.4

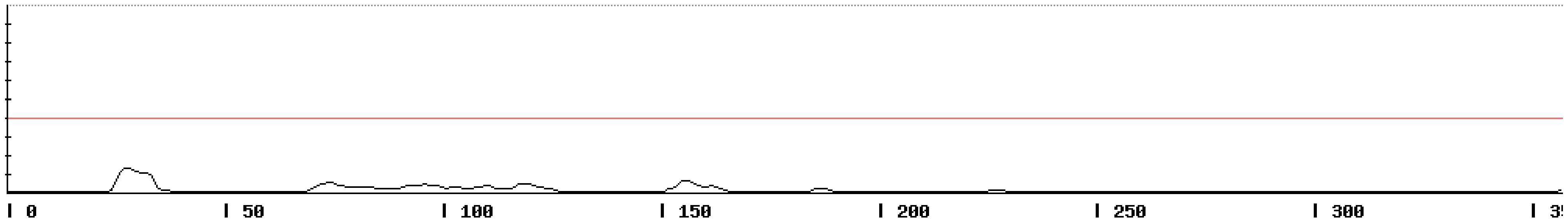

Predictions for Aquilegiacoerulea_Aqcoe7G145000.1.p

Viterbi Path

Viterbi Path

Posterior @ 0.4

Posterior @ 0.4

Posterior Graphic. Threshold Marked @ 0.4

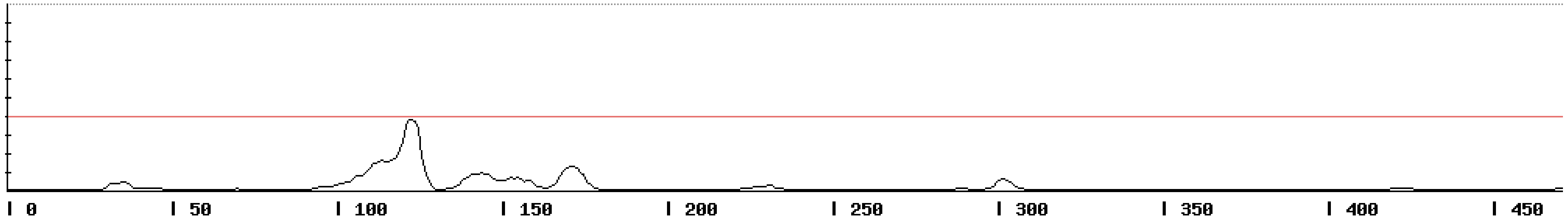

Predictions for Aquilegiacoerulea_Aqcoe7G292300.1.p

Viterbi Path

Viterbi Path

Posterior @ 0.4

Posterior @ 0.4

Posterior Graphic. Threshold Marked @ 0.4

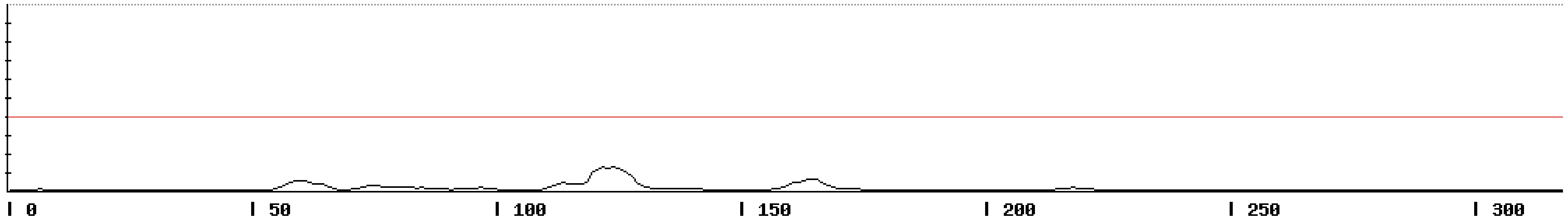

Predictions for Aquilegiacoerulea_Aqcoe7G342900.1.p

Viterbi Path	
Viterbi Path	
Posterior @ 0.4	75 - RKRRYPKGGRPQ - 86
Posterior @ 0.4	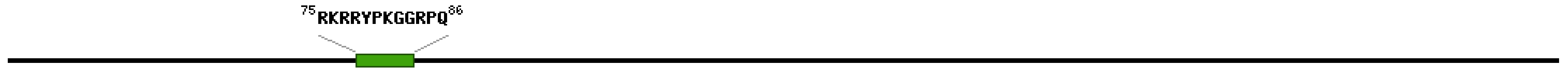
Posterior Graphic. Threshold Marked @ 0.4	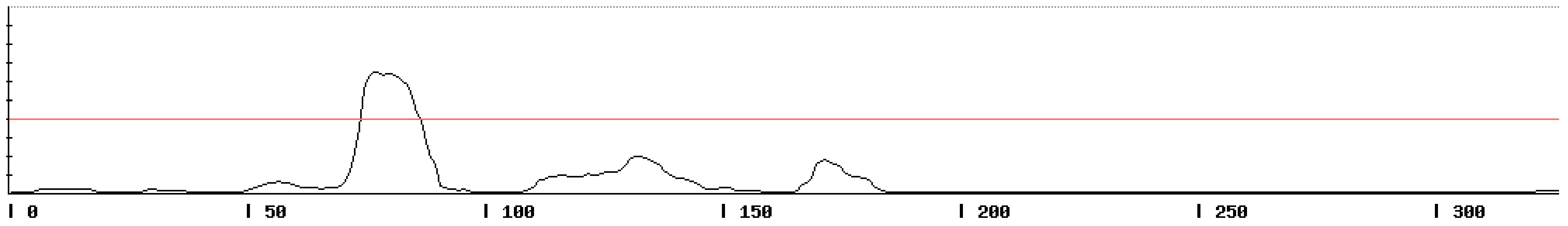

Predictions for Aquilegiacoerulea_Aqcoe7G346600.1.p

Viterbi Path	
Viterbi Path	
Posterior @ 0.4	
Posterior @ 0.4	
Posterior Graphic. Threshold Marked @ 0.4	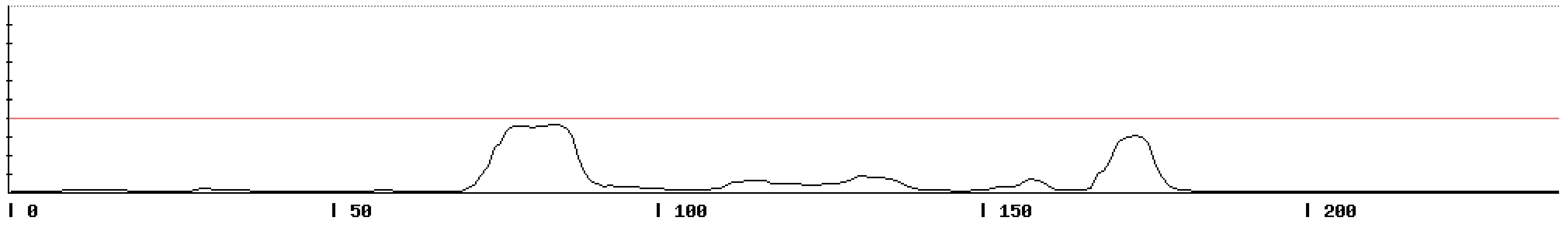

Predictions for Aquilegiacoerulea_Aqcoe7G377900.1.p

Viterbi Path	
Viterbi Path	
Posterior @ 0.4	76 - KYPKGRRSNRRTDKGFWKATGKDRTIKARTSGMKKIGMKK - 115<br>447 - QKKLIR - 452
Posterior @ 0.4	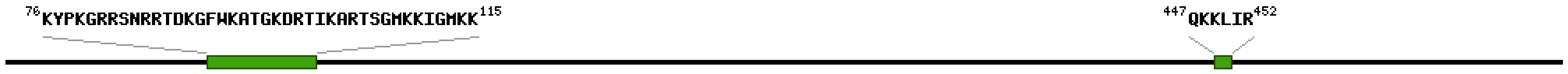
Posterior Graphic. Threshold Marked @ 0.4	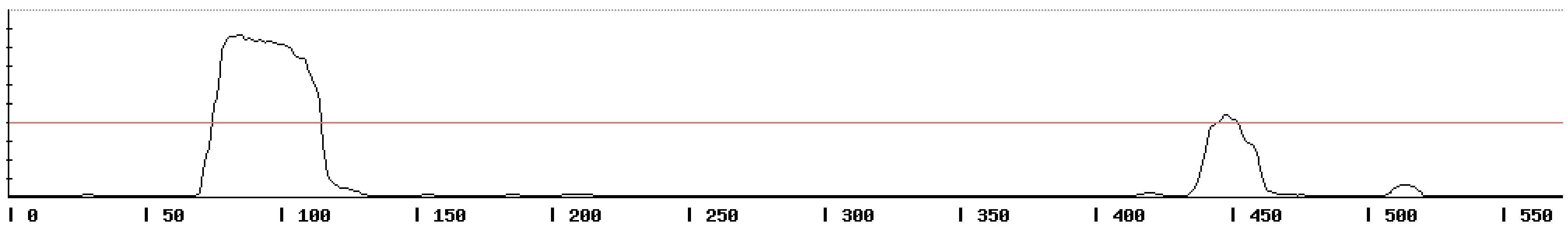

Predictions for Aquilegiacoerulea_Aqcoe7G378100.1.p

Revision r.9 - Last Updated on : February 24 2015 17:45:20. alex.nguyenba@utoronto.ca

Intended to be used with Firefox

Viterbi Path

Posterior @ 0.4

Posterior @ 0.4

Posterior Graphic. Threshold Marked @ 0.4

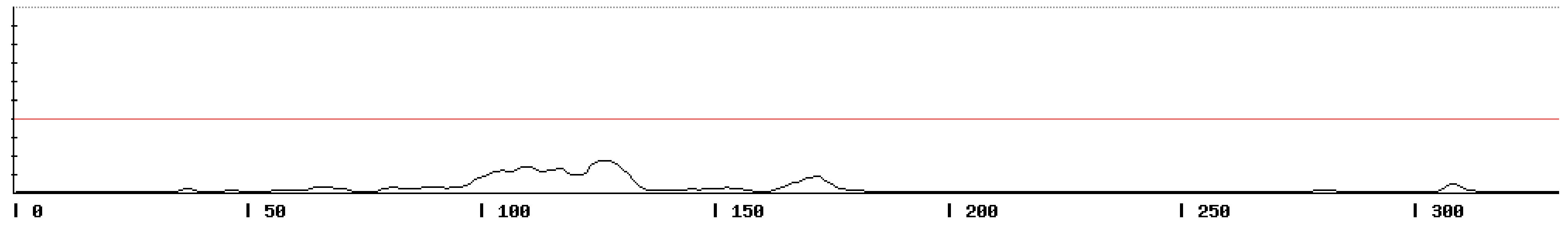

Predictions for Arabidopsishalleri_Araha.0129s0005.1.p

Viterbi Path

Viterbi Path

Posterior @ 0.4

Posterior @ 0.4

Posterior Graphic. Threshold Marked @ 0.4

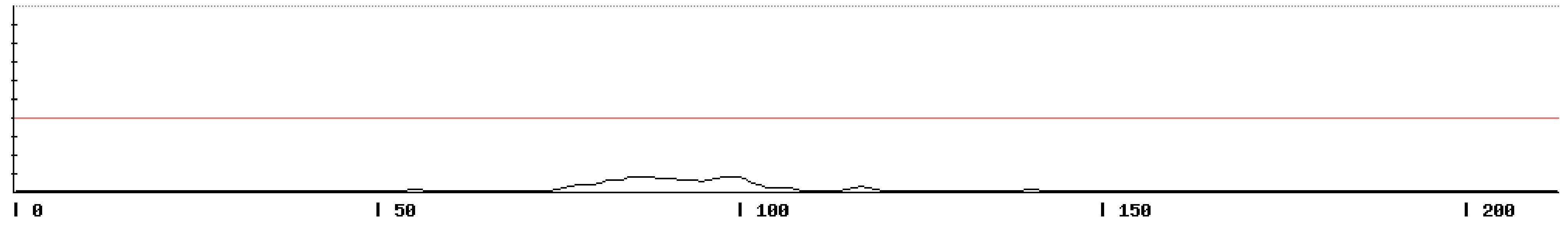

Predictions for Arabidopsishalleri_Araha.0173s0016.1.p

Viterbi Path

Viterbi Path

Posterior @ 0.4

Posterior @ 0.4

Posterior Graphic. Threshold Marked @ 0.4

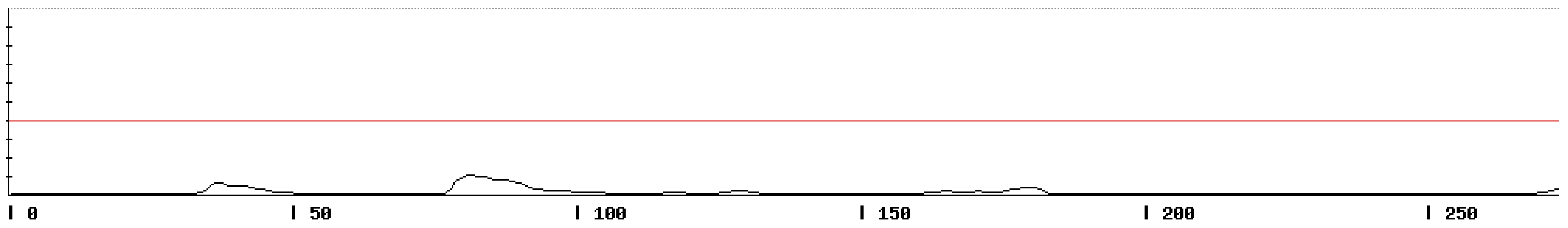

Predictions for Arabidopsishalleri_Araha.0299s0007.1.p

Viterbi Path

Viterbi Path

Posterior @ 0.4

Posterior @ 0.4

Posterior Graphic. Threshold Marked @ 0.4

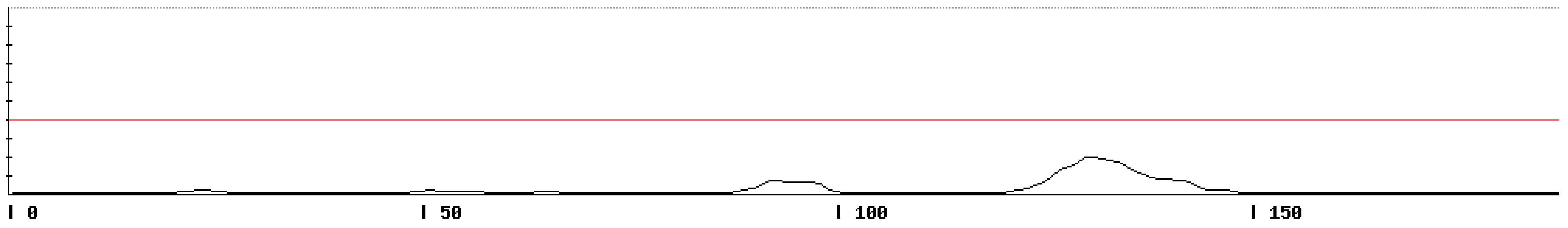

Predictions for Arabidopsishalleri_Araha.0650s0016.1.p

Viterbi Path	
Viterbi Path	
Posterior @ 0.4	
Posterior @ 0.4	
Posterior Graphic. Threshold Marked @ 0.4	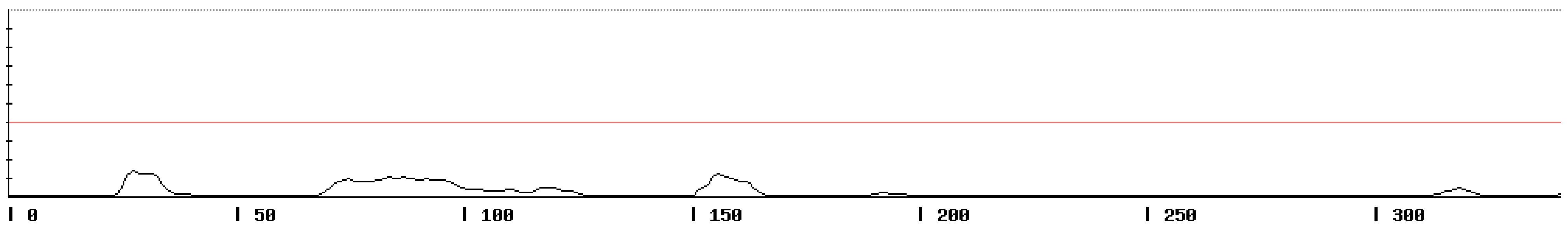

Predictions for Arabidopsishalleri_Araha.0709s0001.1.p

Viterbi Path	
Viterbi Path	
Posterior @ 0.4	
Posterior @ 0.4	
Posterior Graphic. Threshold Marked @ 0.4	

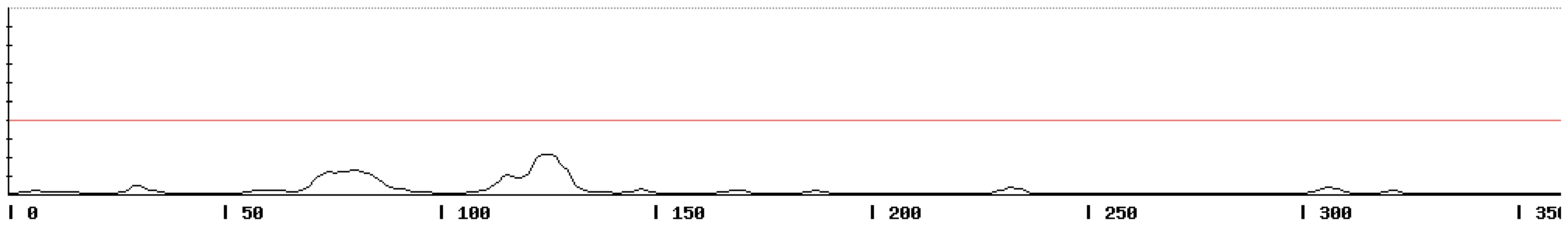

Predictions for Arabidopsishalleri_Araha.10341s0001.1.p

Viterbi Path	
Viterbi Path	
Posterior @ 0.4	
Posterior @ 0.4	
Posterior Graphic. Threshold Marked @ 0.4	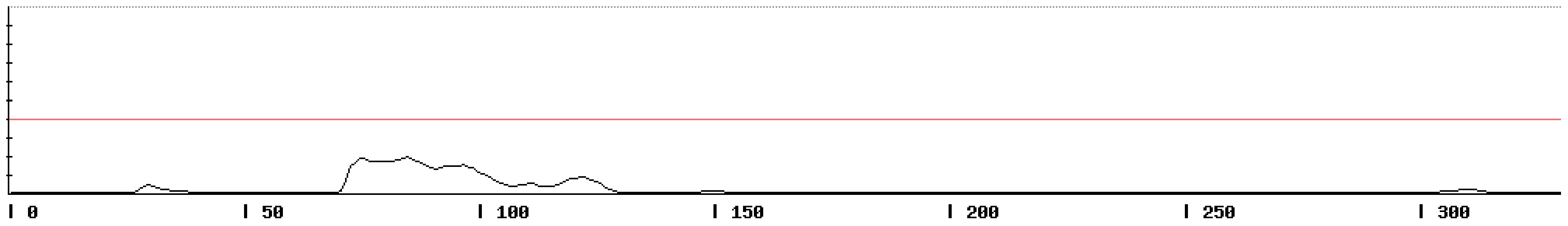

Predictions for Arabidopsishalleri_Araha.10656s0016.1.p

Viterbi Path	
Viterbi Path	
Posterior @ 0.4	122 - PIGKPKT - 128
Posterior @ 0.4	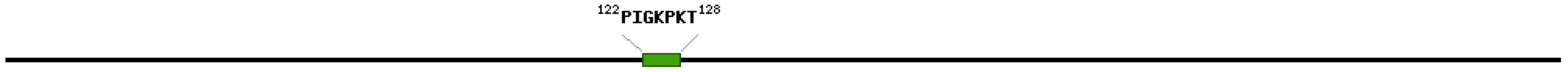

Posterior Graphic. Threshold Marked @ 0.4

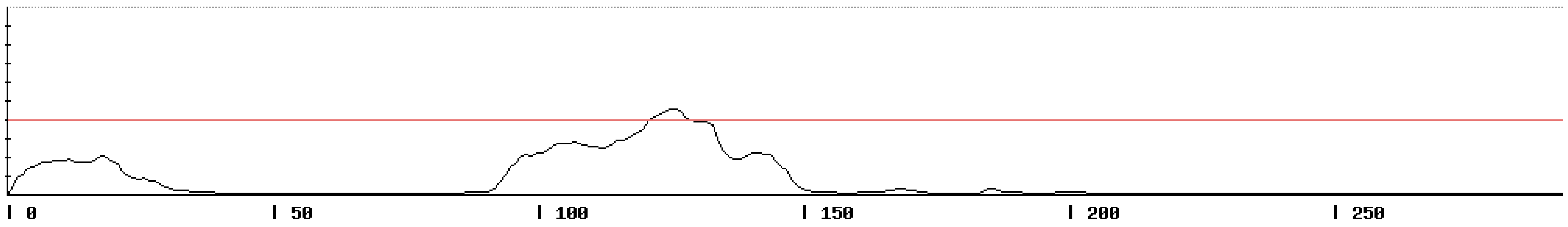

Predictions for Arabidopsishalleri_Araha.1084s0026.1.p

Viterbi Path

Viterbi Path

Posterior @ 0.4

Posterior @ 0.4

Posterior Graphic. Threshold Marked @ 0.4

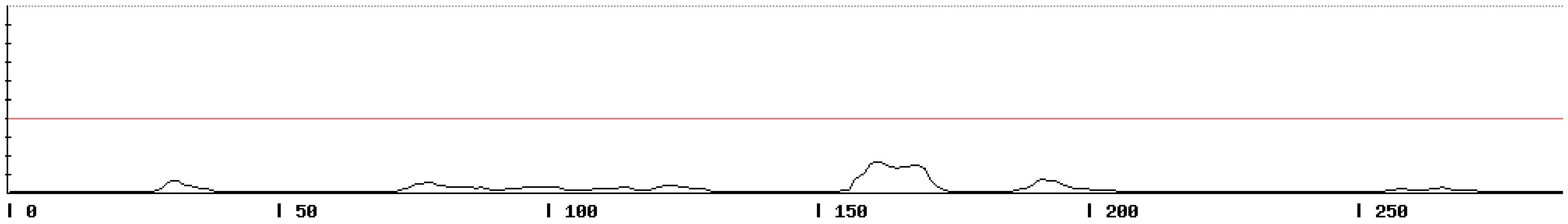

Predictions for Arabidopsishalleri_Araha.1146s0008.1.p

Viterbi Path

Viterbi Path

Posterior @ 0.4 62 - DRKIKDKRGNRGEI - 75

Posterior @ 0.4

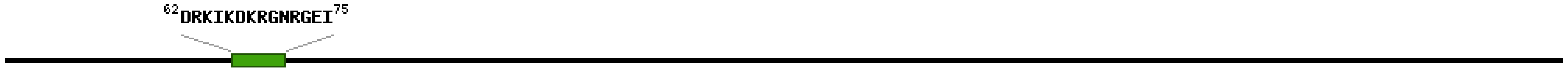

Posterior Graphic. Threshold Marked @ 0.4

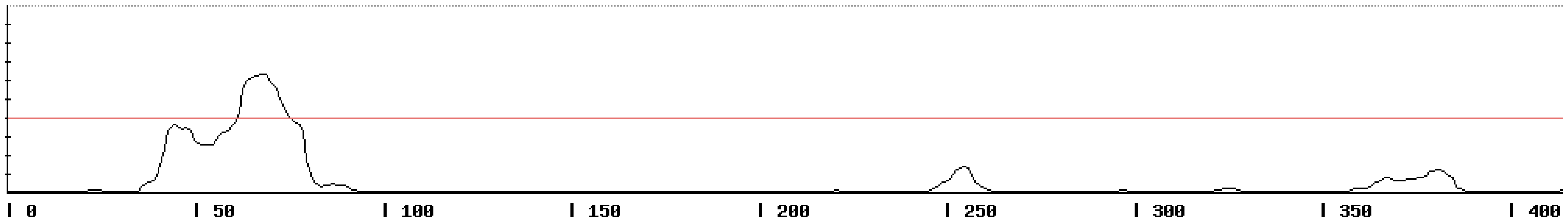

Predictions for Arabidopsishalleri_Araha.1146s0009.1.p

Viterbi Path

Viterbi Path

Posterior @ 0.4

Posterior @ 0.4

Posterior Graphic. Threshold Marked @ 0.4

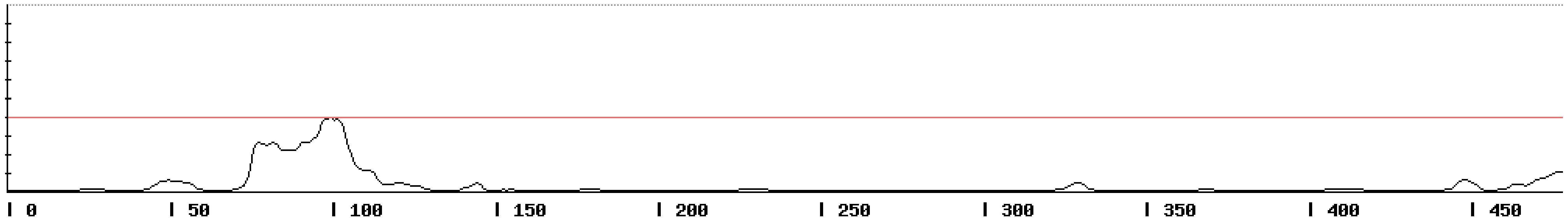

Predictions for Arabidopsishalleri_Araha.1212s0002.1.p

Viterbi Path

Viterbi Path

Posterior @ 0.4 | 99 - TGKDRKIKSGKTKII - 113

Posterior @ 0.4

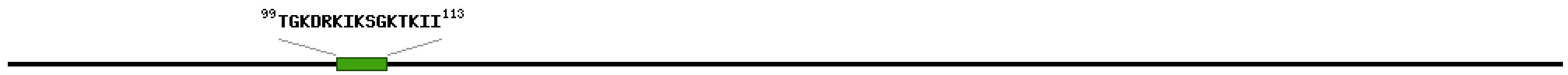

Posterior Graphic. Threshold Marked @ 0.4

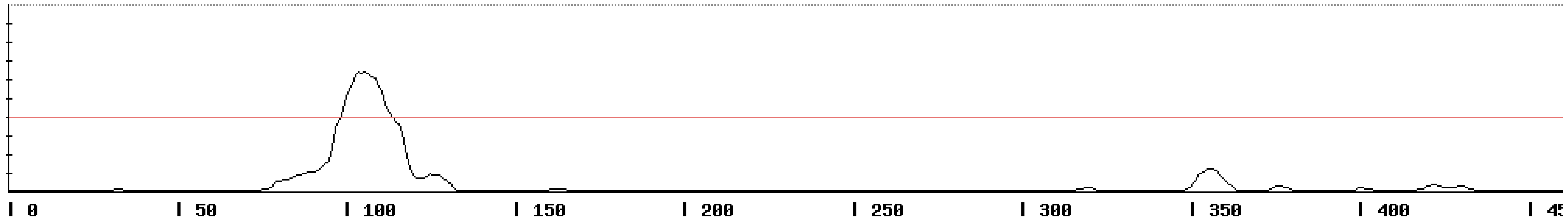

Predictions for Arabidopsishalleri_Araha.12281s0001.1.p

Viterbi Path

Viterbi Path

Posterior @ 0.4

Posterior @ 0.4

Posterior Graphic. Threshold Marked @ 0.4

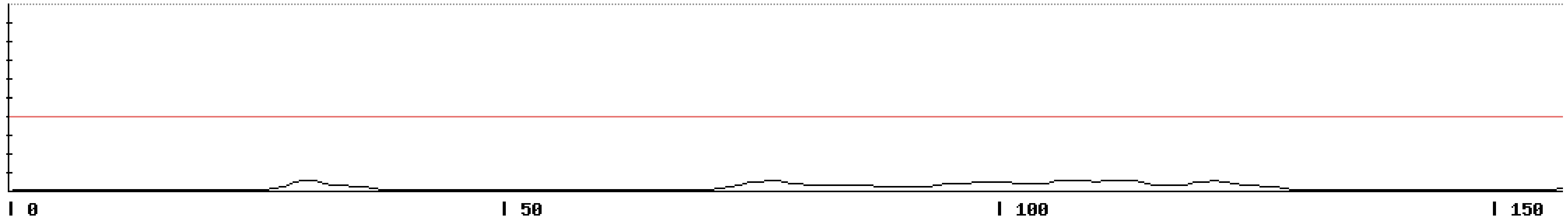

Predictions for Arabidopsishalleri_Araha.13692s0015.1.p

Viterbi Path

Viterbi Path

Posterior @ 0.4	
Posterior @ 0.4	
Posterior Graphic. Threshold Marked @ 0.4	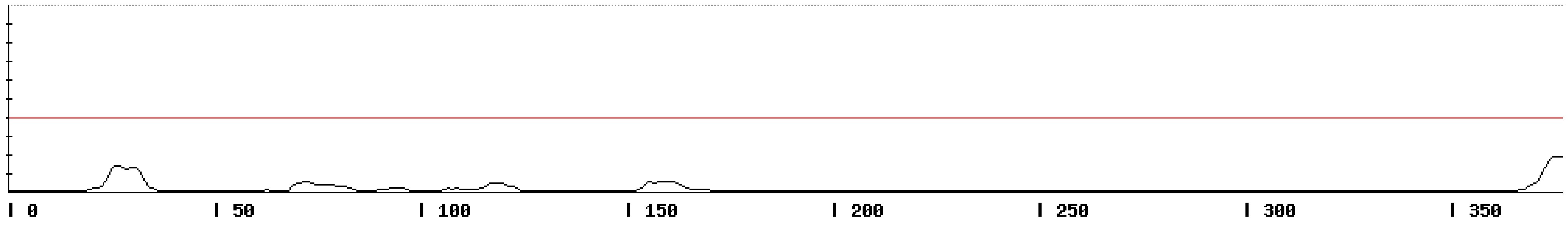

Predictions for Arabidopsishalleri_Araha.13695s0002.1.p	
Viterbi Path	
Viterbi Path	
Posterior @ 0.4	
Posterior @ 0.4	
Posterior Graphic. Threshold Marked @ 0.4	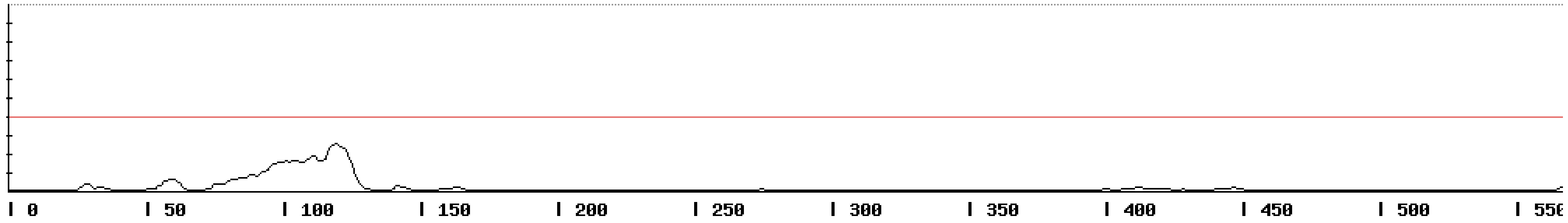

Predictions for Arabidopsishalleri_Araha.1448s0011.1.p

Viterbi Path	
Viterbi Path	
Posterior @ 0.4	
Posterior @ 0.4	
Posterior Graphic. Threshold Marked @ 0.4	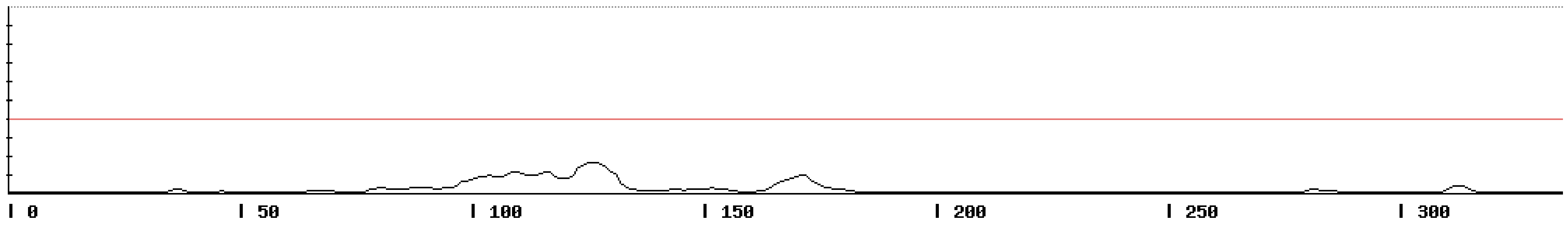

Predictions for Arabidopsishalleri_Araha.16028s0003.1.p

Viterbi Path	
Viterbi Path	
Posterior @ 0.4	82 - LIKSKATGKILGFKKILKFCKKTKPRE - 108
Posterior @ 0.4	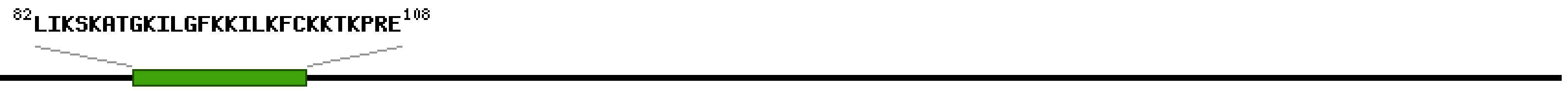
Posterior Graphic. Threshold Marked @ 0.4	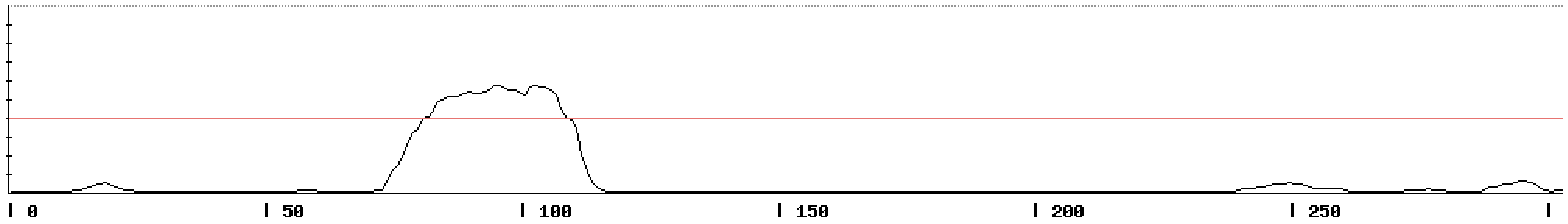

Predictions for Arabidopsishalleri_Araha.16094s0001.1.p	
Viterbi Path	
Viterbi Path	
Posterior @ 0.4	103 - RKIK - 106
Posterior @ 0.4	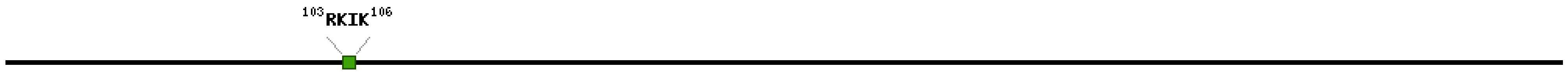
Posterior Graphic. Threshold Marked @ 0.4	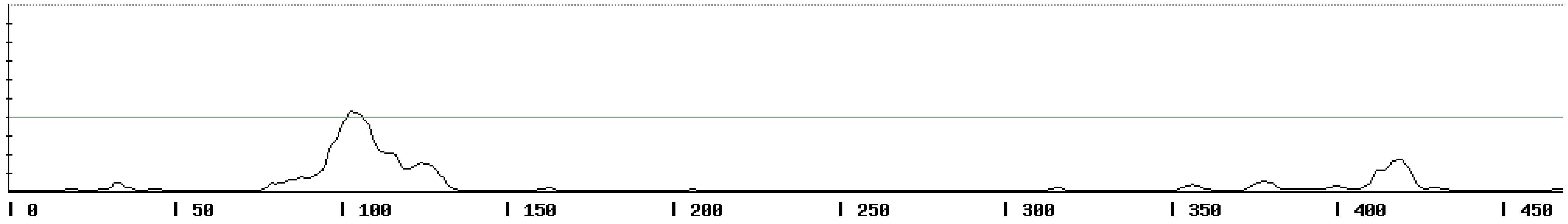

Predictions for Arabidopsishalleri_Araha.16225s0003.1.p	
Viterbi Path	
Viterbi Path	
Posterior @ 0.4	275 - RENKRKKKK - 283
Posterior @ 0.4	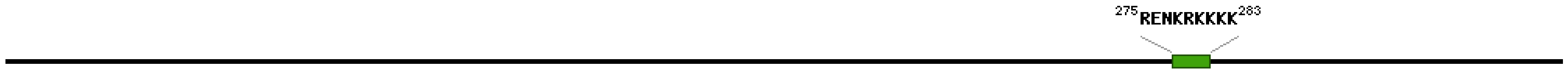
Posterior Graphic. Threshold Marked @ 0.4	

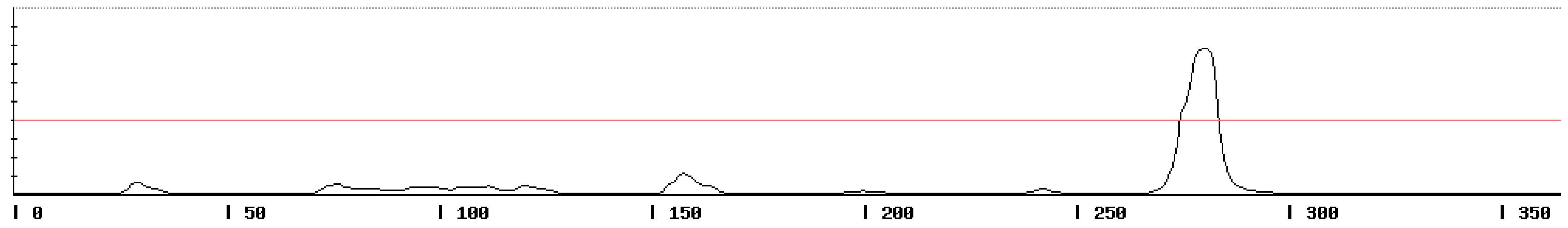

Predictions for Arabidopsishalleri_Araha.16261s0010.1.p

Viterbi Path	
Viterbi Path	
Posterior @ 0.4	
Posterior @ 0.4	
Posterior Graphic. Threshold Marked @ 0.4	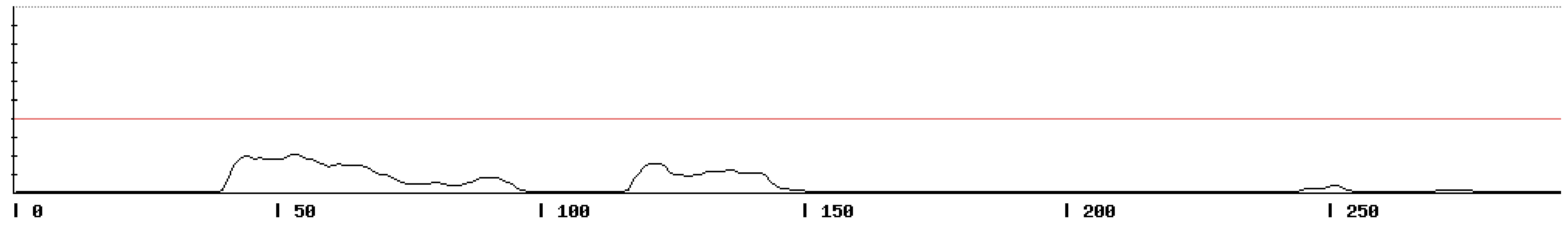

Predictions for Arabidopsishalleri_Araha.16477s0001.1.p

Viterbi Path	
Viterbi Path	
Posterior @ 0.4	
Posterior @ 0.4	

Posterior Graphic. Threshold Marked @ 0.4

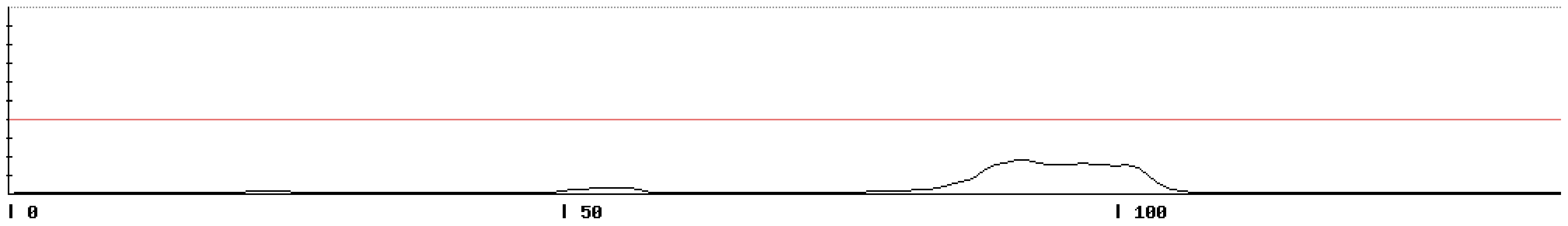

Predictions for Arabidopsishalleri_Araha.17797s0002.1.p

Viterbi Path

Viterbi Path

Posterior @ 0.4

Posterior @ 0.4

Posterior Graphic. Threshold Marked @ 0.4

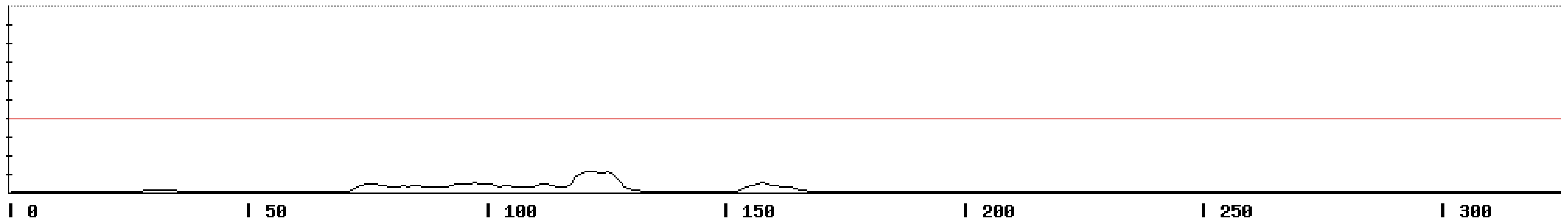

Predictions for Arabidopsishalleri_Araha.18637s0005.1.p

Viterbi Path

Viterbi Path

Posterior @ 0.4

Posterior @ 0.4

Posterior Graphic. Threshold Marked @ 0.4

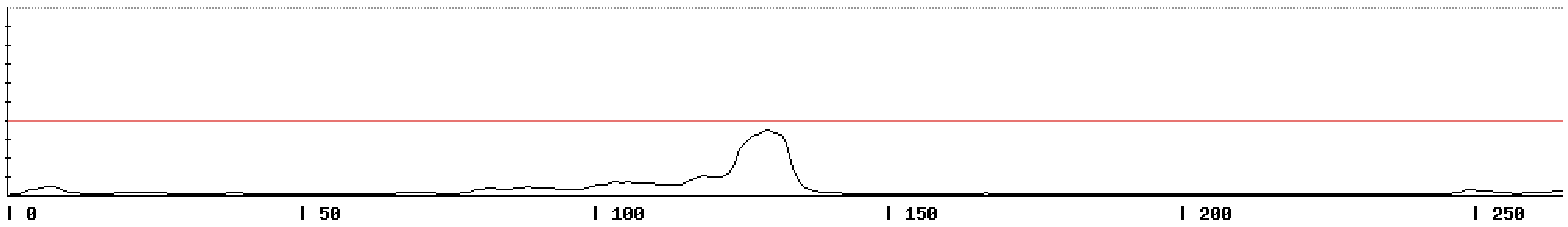

Predictions for Arabidopsishalleri_Araha.19157s0013.1.p

Viterbi Path

Viterbi Path

Posterior @ 0.4

101 - IKSKK - 105

Posterior @ 0.4

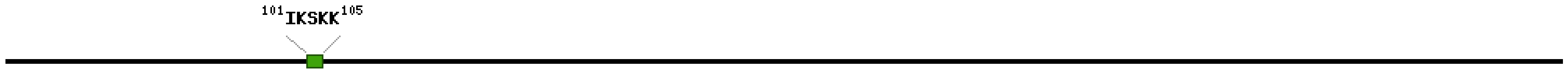

Posterior Graphic. Threshold Marked @ 0.4

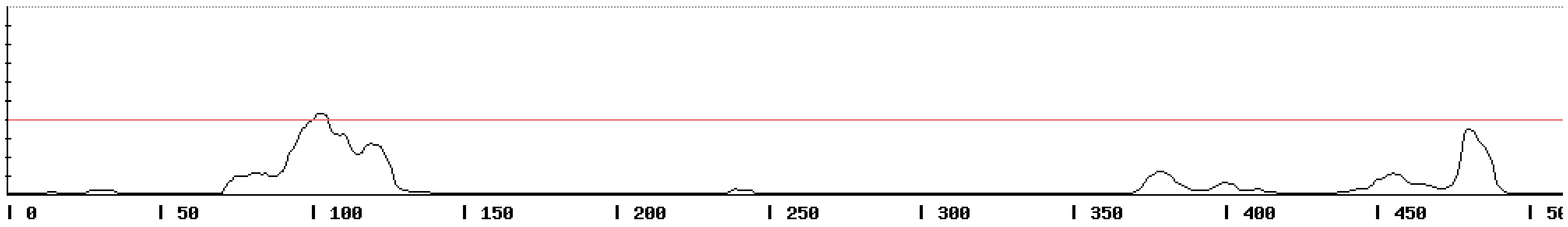

Predictions for Arabidopsishalleri_Araha.19457s0003.1.p

Viterbi Path

Viterbi Path

Posterior @ 0.4

Posterior @ 0.4

Posterior Graphic. Threshold Marked @ 0.4

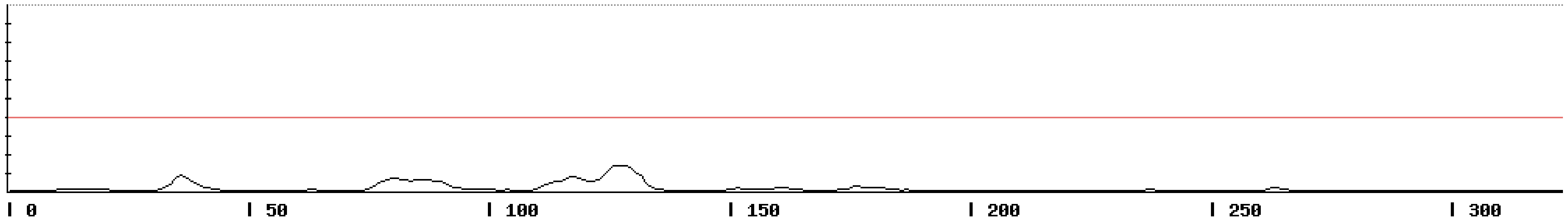

Predictions for Arabidopsishalleri_Araha.20405s0001.1.p

Viterbi Path

Viterbi Path

Posterior @ 0.4

Posterior @ 0.4

Posterior Graphic. Threshold Marked @ 0.4

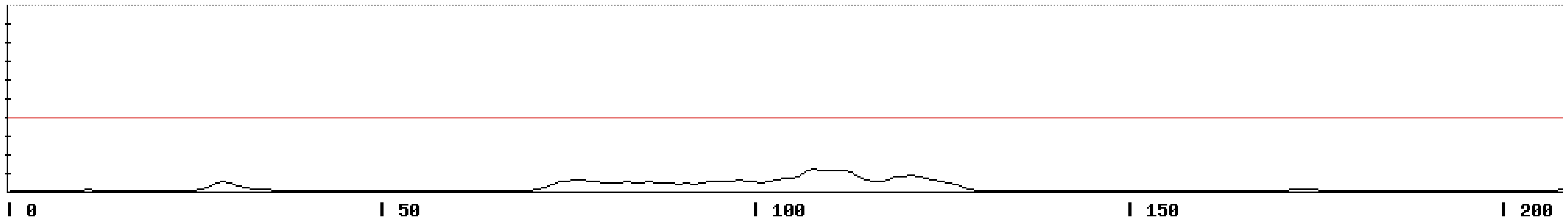

Predictions for Arabidopsishalleri_Araha.21058s0001.1.p

Viterbi Path

Viterbi Path

Posterior @ 0.4

Posterior @ 0.4

Posterior Graphic. Threshold Marked @ 0.4

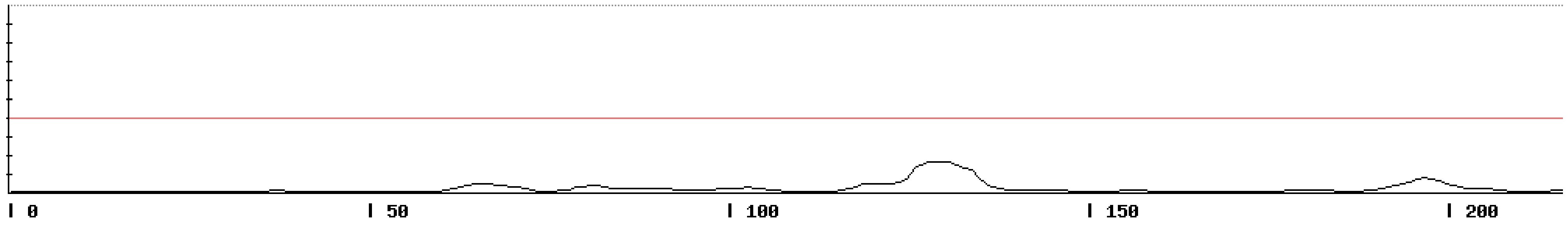

Predictions for Arabidopsishalleri_Araha.21283s0004.1.p

Viterbi Path

Viterbi Path

Posterior @ 0.4

Posterior @ 0.4

Posterior Graphic. Threshold Marked @ 0.4

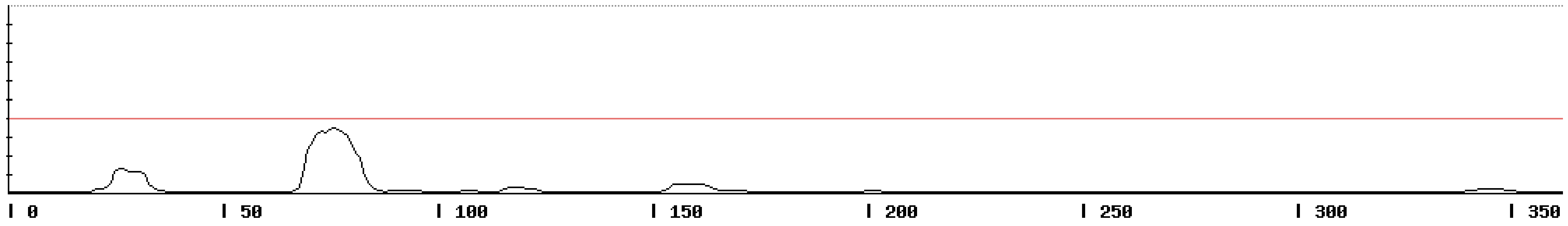

Predictions for Arabidopsishalleri_Araha.21478s0011.1.p

Viterbi Path	
Viterbi Path	
Posterior @ 0.4	
Posterior @ 0.4	
Posterior Graphic. Threshold Marked @ 0.4	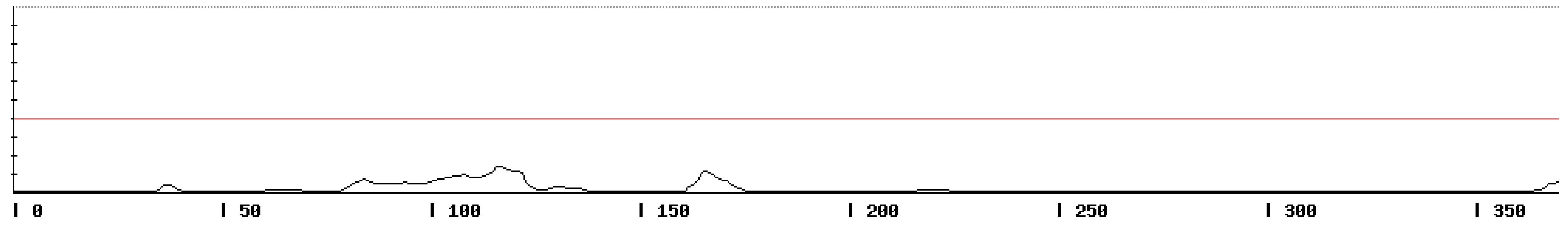

Predictions for Arabidopsishalleri_Araha.21527s0003.1.p

Viterbi Path	148 - PKAAKKKKAEEKKNEKLKK - 166
Viterbi Path	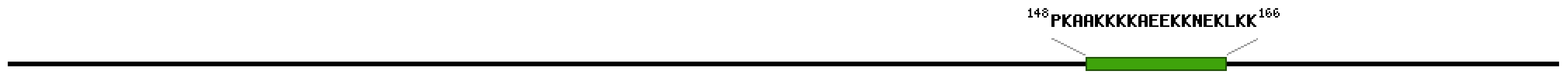
Posterior @ 0.4	148 - PKAAKKKKAEEKKNEKLKKEV - 168
Posterior @ 0.4	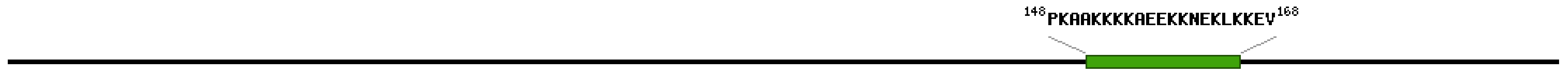
Posterior Graphic. Threshold Marked @ 0.4	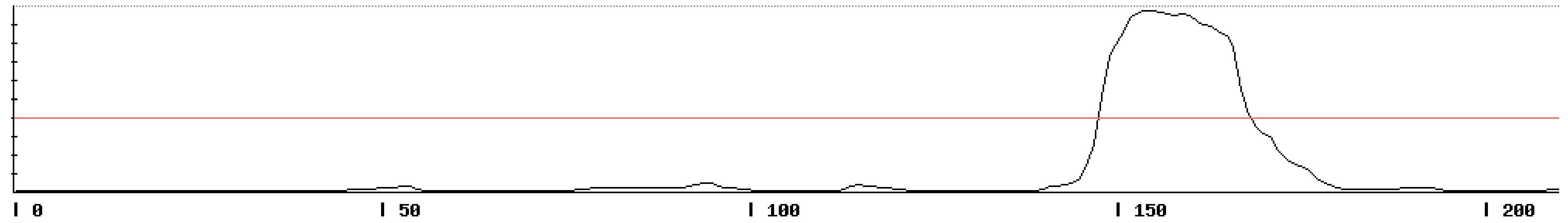

Predictions for Arabidopsishalleri_Araha.23250s0004.1.p	
Viterbi Path	
Viterbi Path	
Posterior @ 0.4	185 - KKK - 187
Posterior @ 0.4	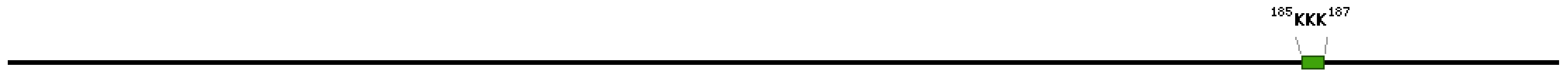
Posterior Graphic. Threshold Marked @ 0.4	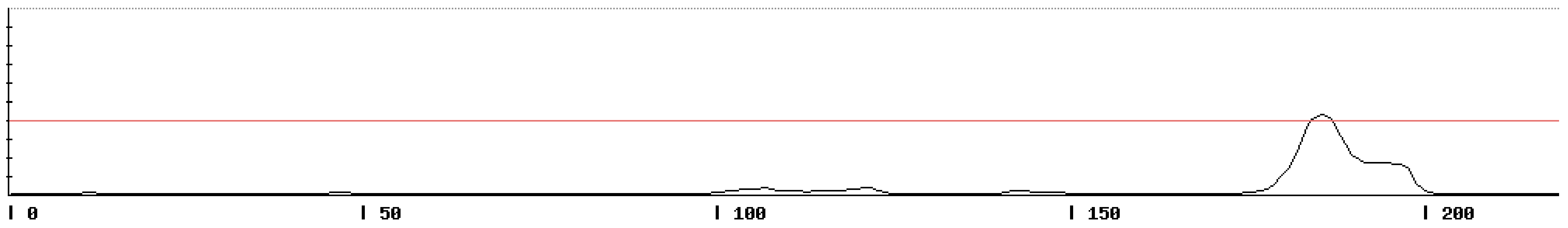

Predictions for Arabidopsishalleri_Araha.23250s0005.1.p	
Viterbi Path	
Viterbi Path	
Posterior @ 0.4	
Posterior @ 0.4	
Posterior Graphic. Threshold Marked @ 0.4	

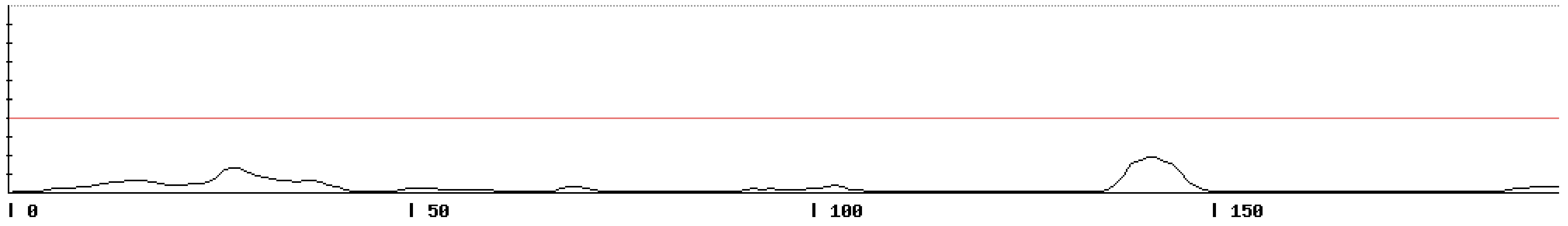

Predictions for Arabidopsishalleri_Araha.24359s0001.1.p

Viterbi Path	
Viterbi Path	
Posterior @ 0.4	
Posterior @ 0.4	
Posterior Graphic. Threshold Marked @ 0.4	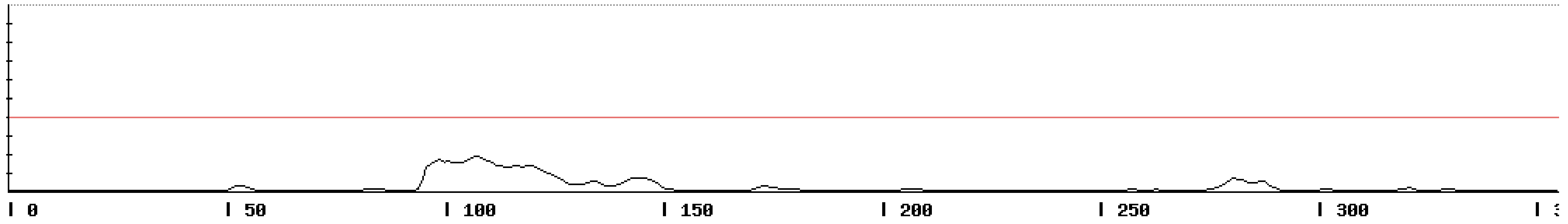

Predictions for Arabidopsishalleri_Araha.24857s0004.1.p

Viterbi Path	
Viterbi Path	
Posterior @ 0.4	
Posterior @ 0.4	

Posterior Graphic. Threshold Marked @ 0.4

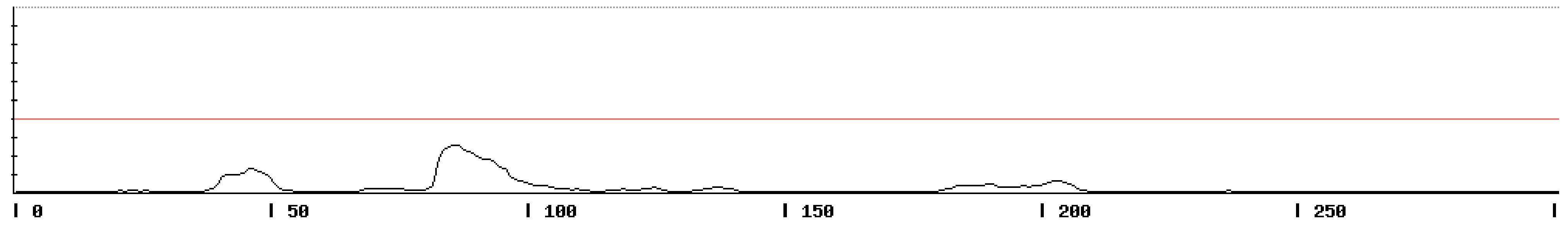

Predictions for Arabidopsishalleri_Araha.25334s0001.1.p

Viterbi Path

Viterbi Path

Posterior @ 0.4

Posterior @ 0.4

Posterior Graphic. Threshold Marked @ 0.4

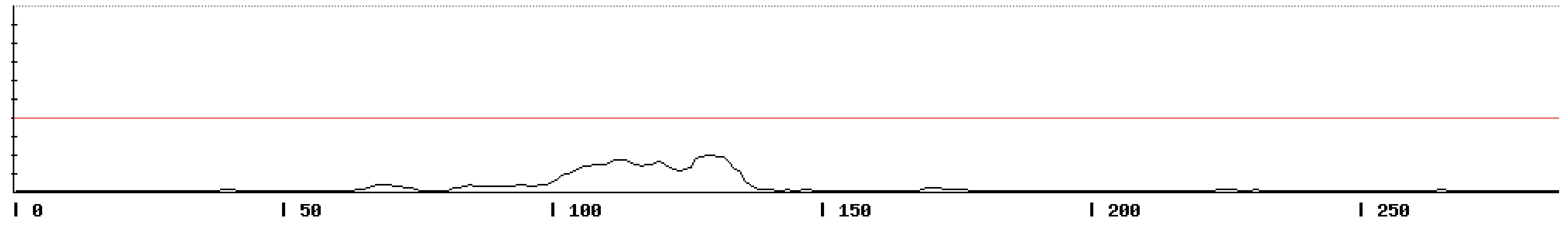

Predictions for Arabidopsishalleri_Araha.27238s0003.1.p

Viterbi Path

Viterbi Path

Posterior @ 0.4

Posterior @ 0.4

Posterior Graphic. Threshold Marked @ 0.4	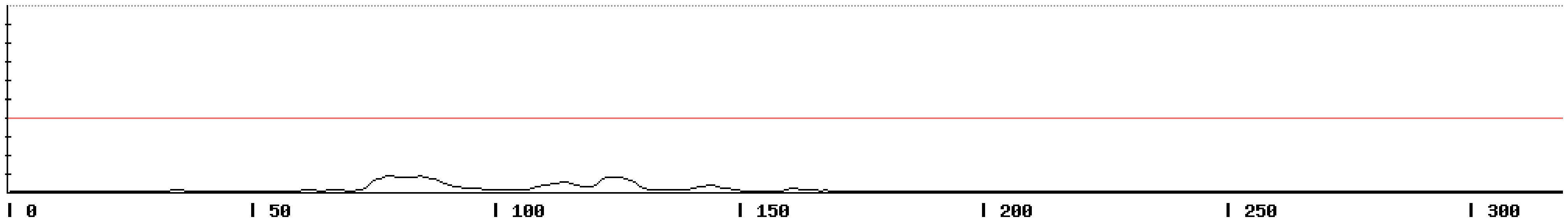

Predictions for Arabidopsishalleri_Araha.3034s0046.1.p	
Viterbi Path	
Viterbi Path	
Posterior @ 0.4	
Posterior @ 0.4	
Posterior Graphic. Threshold Marked @ 0.4	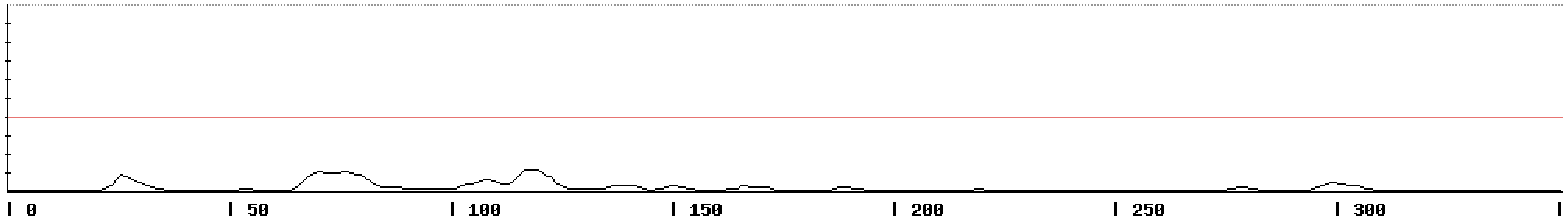

Predictions for Arabidopsishalleri_Araha.3105s0045.1.p	
Viterbi Path	
Viterbi Path	

Posterior @ 0.4	160 - RKGSKGRKQSKS - 171
Posterior @ 0.4	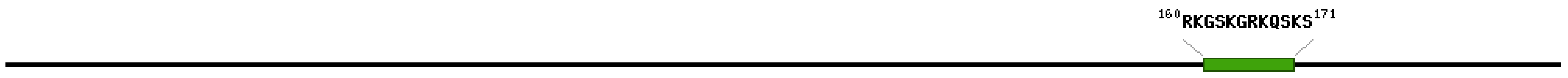
Posterior Graphic. Threshold Marked @ 0.4	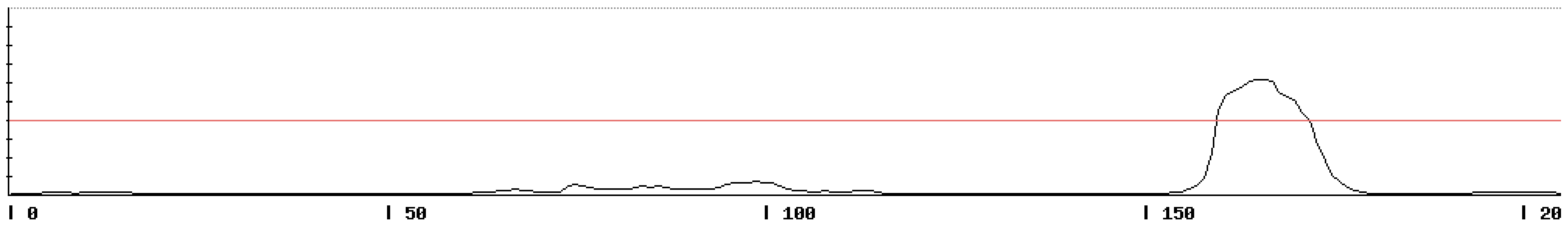

Predictions for Arabidopsishalleri_Araha.3134s0014.1.p

Viterbi Path	
Viterbi Path	
Posterior @ 0.4	
Posterior @ 0.4	
Posterior Graphic. Threshold Marked @ 0.4	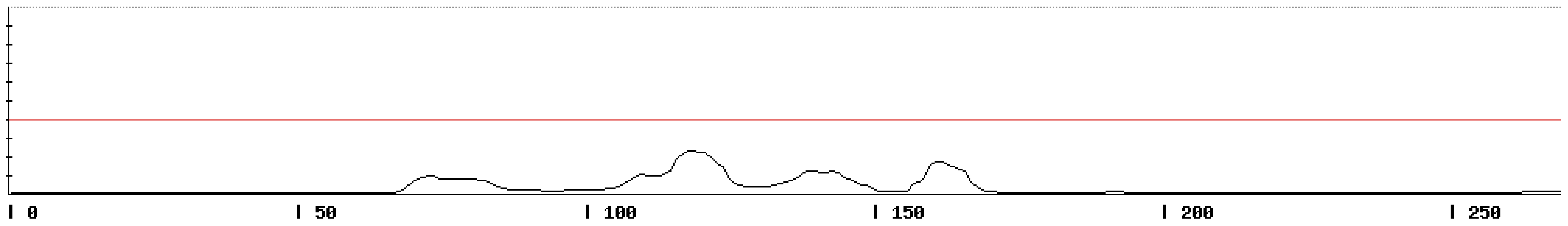

Predictions for Arabidopsishalleri_Araha.32489s0002.1.p

Viterbi Path	
Viterbi Path	

Posterior @ 0.4	
Posterior @ 0.4	
Posterior Graphic. Threshold Marked @ 0.4	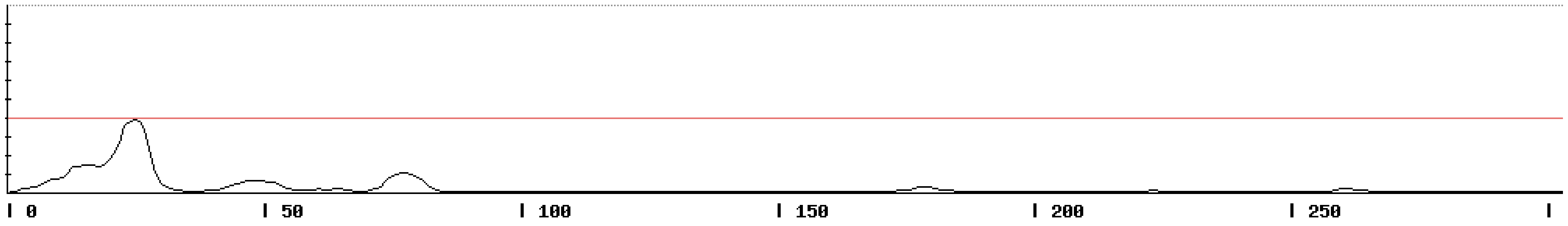

Predictions for Arabidopsishalleri_Araha.3353s0007.1.p

Viterbi Path	
Viterbi Path	
Posterior @ 0.4	
Posterior @ 0.4	
Posterior Graphic. Threshold Marked @ 0.4	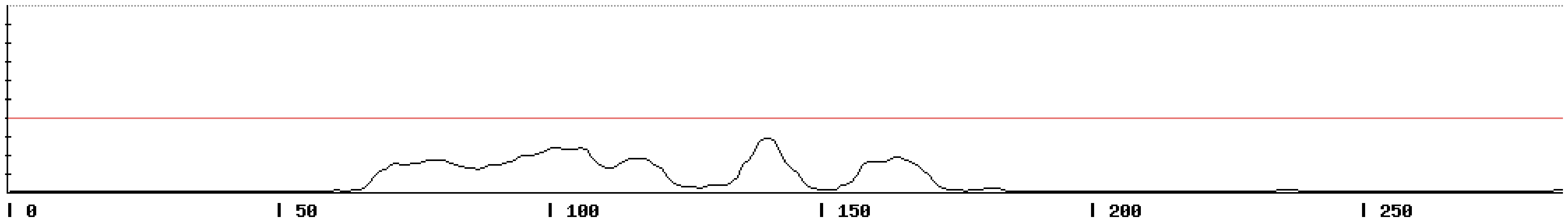

Predictions for Arabidopsishalleri_Araha.3365s0004.1.p

Viterbi Path	
Viterbi Path	
Posterior @ 0.4	137 - RKRRK - 141
Posterior @ 0.4	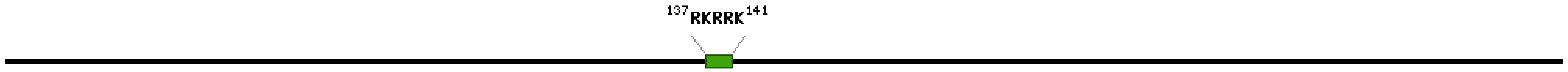
Posterior Graphic. Threshold Marked @ 0.4	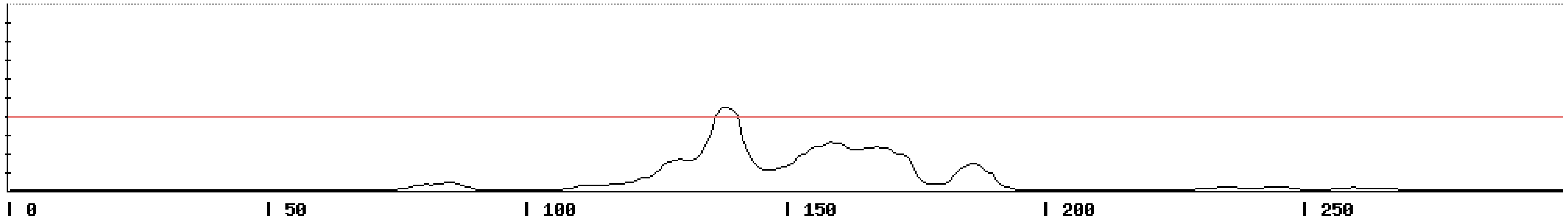

Predictions for Arabidopsishalleri_Araha.3365s0007.1.p

Viterbi Path	
Viterbi Path	
Posterior @ 0.4	
Posterior @ 0.4	
Posterior Graphic. Threshold Marked @ 0.4	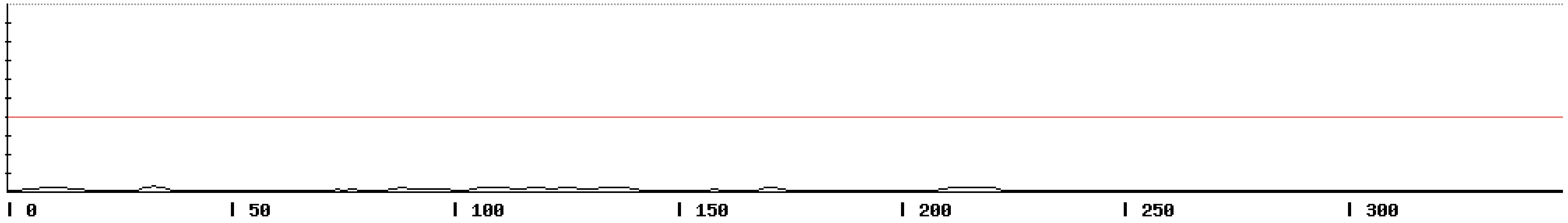

Predictions for Arabidopsishalleri_Araha.34065s0002.1.p	
Viterbi Path	
Viterbi Path	
Posterior @ 0.4	
Posterior @ 0.4	
Posterior Graphic. Threshold Marked @ 0.4	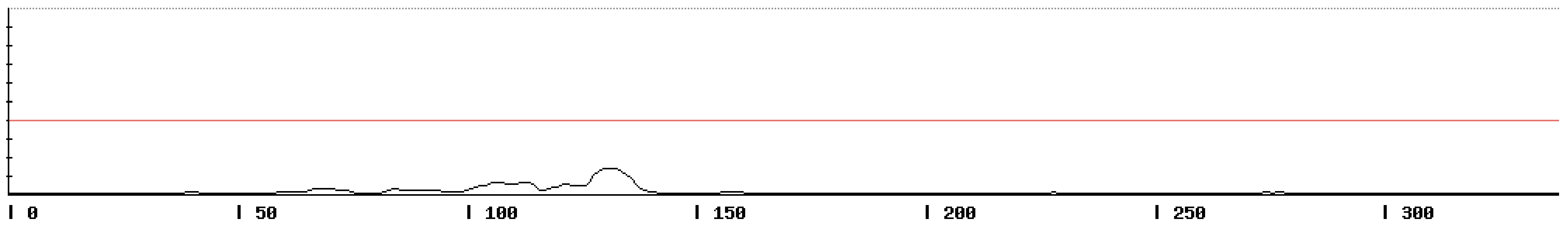

Predictions for Arabidopsishalleri_Araha.34090s0001.1.p	
Viterbi Path	
Viterbi Path	
Posterior @ 0.4	
Posterior @ 0.4	
Posterior Graphic. Threshold Marked @ 0.4	

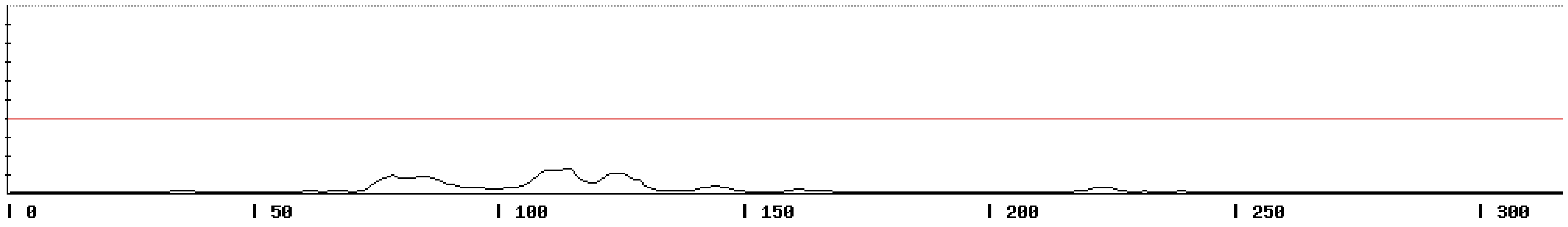

Predictions for Arabidopsishalleri_Araha.3440s0003.1.p

Viterbi Path

Viterbi Path

Posterior @ 0.4

20 - NRGDRQMRKTKSGFWKKTGVTINIMRKRGNREKIGEKK - 57

Posterior @ 0.4

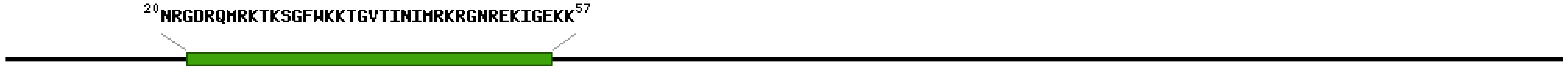

Posterior Graphic. Threshold Marked @ 0.4

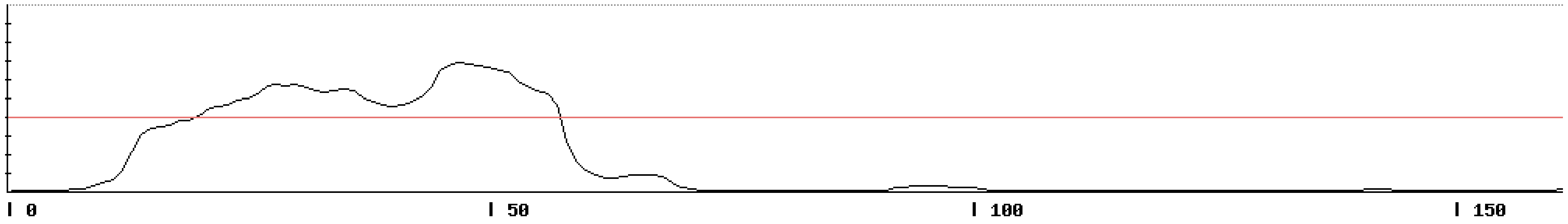

Predictions for Arabidopsishalleri_Araha.3440s0004.1.p

Viterbi Path

Viterbi Path

Posterior @ 0.4

88 - WKKTGKTMTIMRKRGNREKIGEKR - 111

Posterior @ 0.4

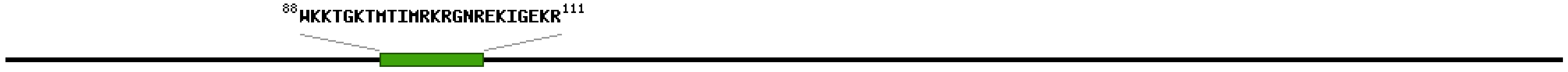

Posterior Graphic. Threshold Marked @ 0.4	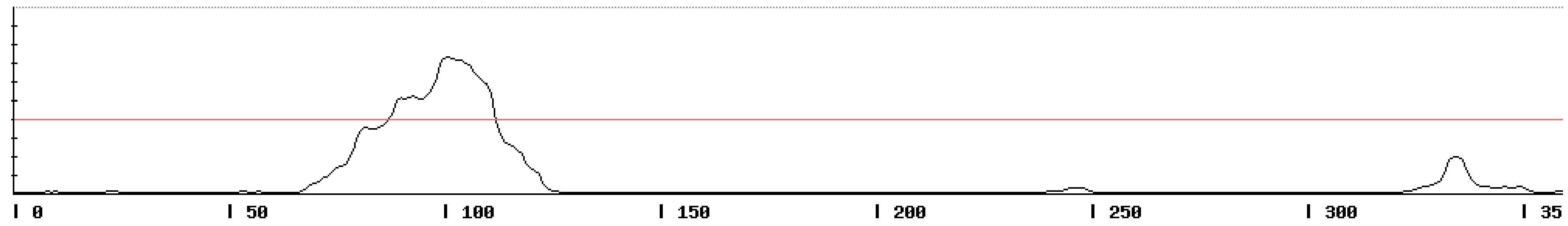

Predictions for Arabidopsishalleri_Araha.3440s0005.1.p	
Viterbi Path	
Viterbi Path	
Posterior @ 0.4	
Posterior @ 0.4	
Posterior Graphic. Threshold Marked @ 0.4	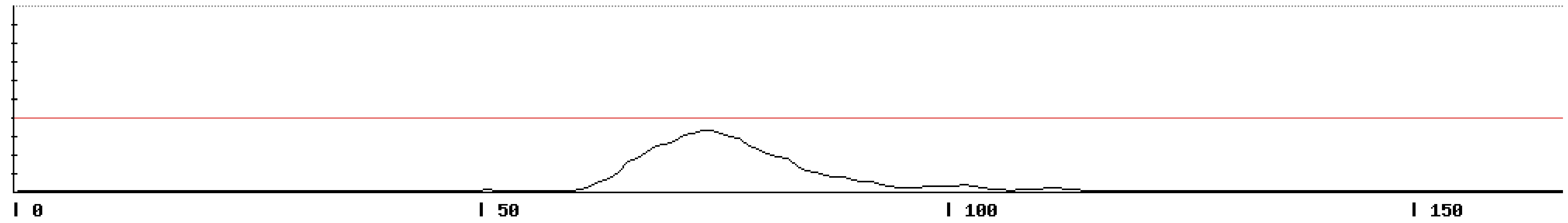

Predictions for Arabidopsishalleri_Araha.3538s0001.1.p	
Viterbi Path	
Viterbi Path	
Posterior @ 0.4	
Posterior @ 0.4	

Posterior Graphic. Threshold Marked @ 0.4

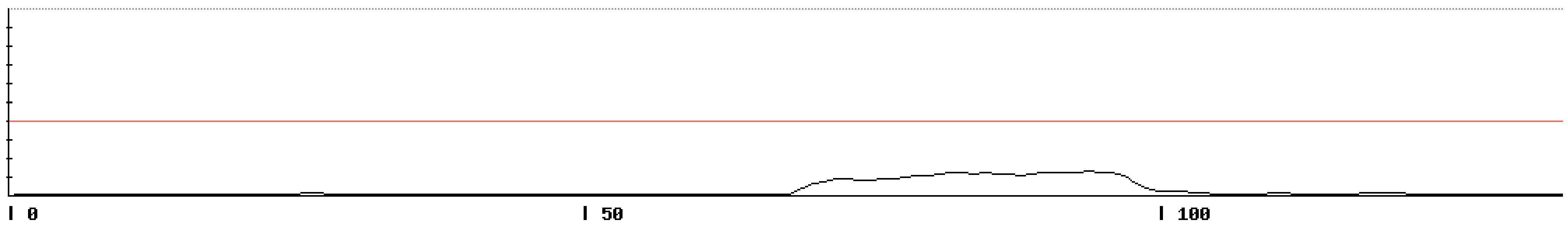

Predictions for Arabidopsishalleri_Araha.35607s0009.1.p

Viterbi Path

Viterbi Path

Posterior @ 0.4

Posterior @ 0.4

Posterior Graphic. Threshold Marked @ 0.4

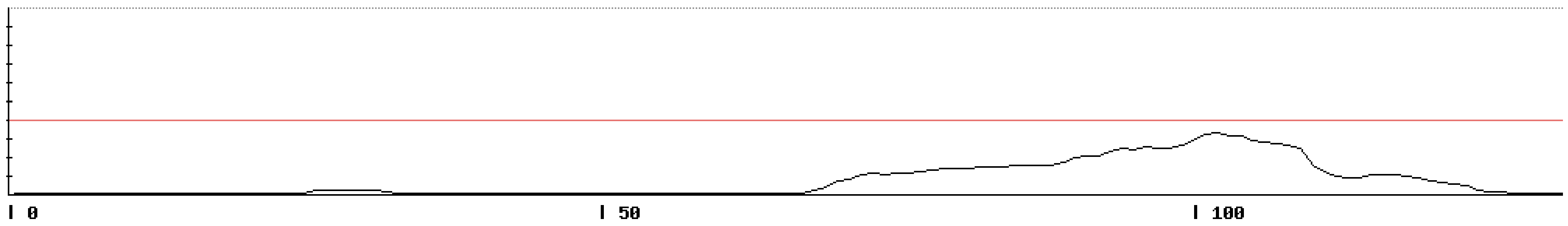

Predictions for Arabidopsishalleri_Araha.35673s0001.1.p

Viterbi Path

Viterbi Path

Posterior @ 0.4

Posterior @ 0.4

Posterior Graphic. Threshold Marked @ 0.4

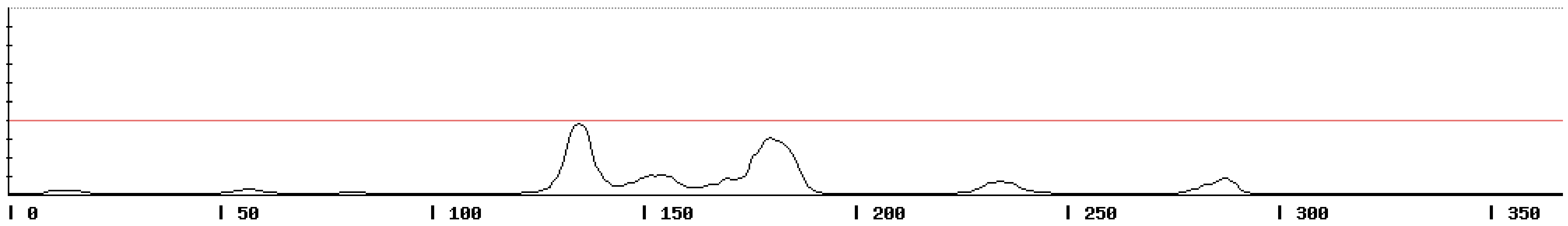

Predictions for Arabidopsishalleri_Araha.3578s0006.1.p

Viterbi Path

Viterbi Path

Posterior @ 0.4

Posterior @ 0.4

Posterior Graphic. Threshold Marked @ 0.4

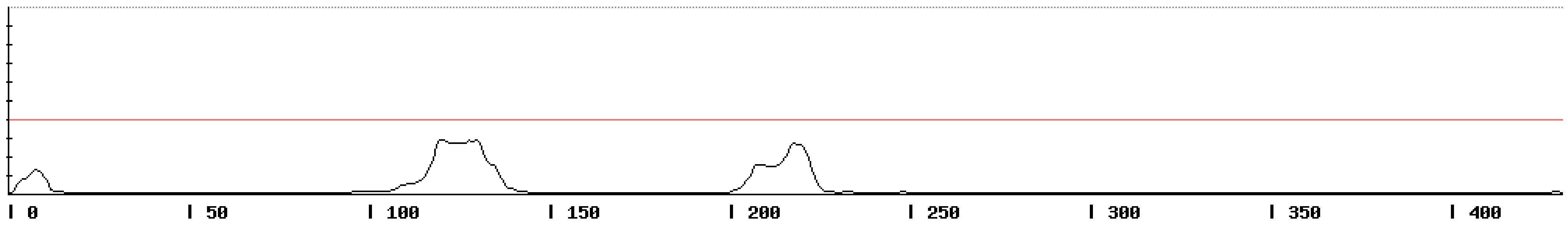

Predictions for Arabidopsishalleri_Araha.37102s0001.1.p

Viterbi Path

Viterbi Path

Posterior @ 0.4	137 - KRR - 139
Posterior @ 0.4	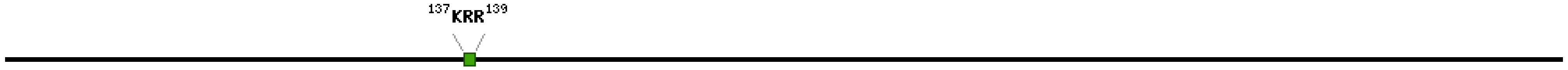
Posterior Graphic. Threshold Marked @ 0.4	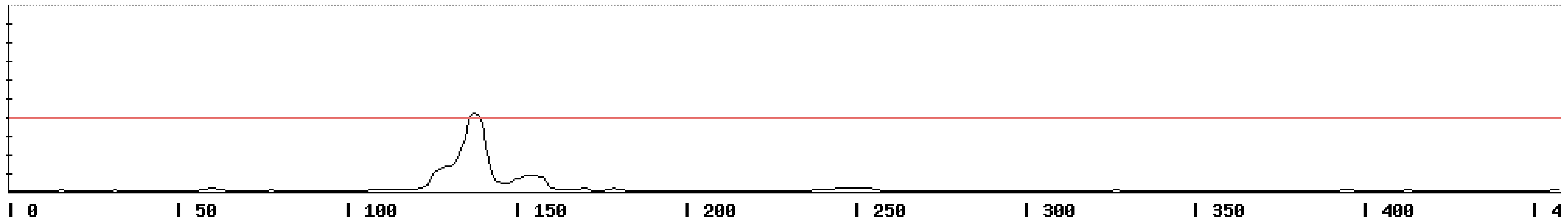

Predictions for Arabidopsishalleri_Araha.3726s0007.1.p

Viterbi Path	
Viterbi Path	
Posterior @ 0.4	
Posterior @ 0.4	
Posterior Graphic. Threshold Marked @ 0.4	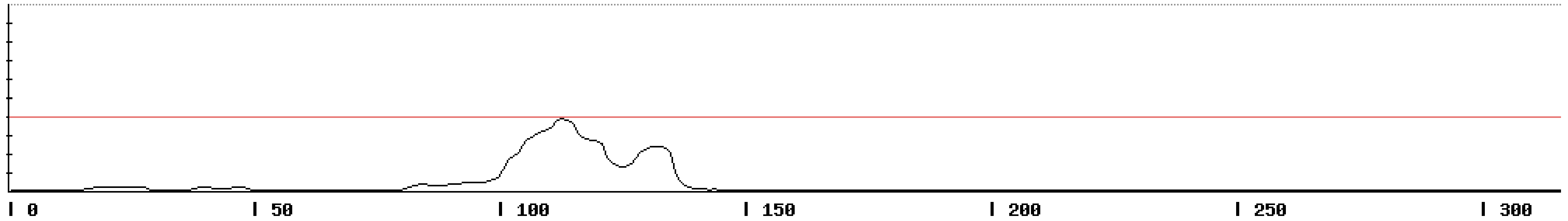

Predictions for Arabidopsishalleri_Araha.37771s0001.1.p

Viterbi Path	
Viterbi Path	
Posterior @ 0.4	
Posterior @ 0.4	
Posterior Graphic. Threshold Marked @ 0.4	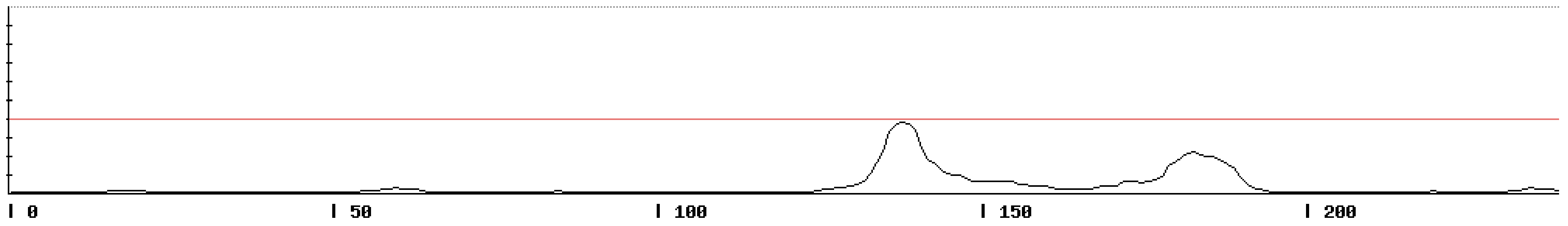

Predictions for Arabidopsishalleri_Araha.40634s0001.1.p	
Viterbi Path	
Viterbi Path	
Posterior @ 0.4	
Posterior @ 0.4	
Posterior Graphic. Threshold Marked @ 0.4	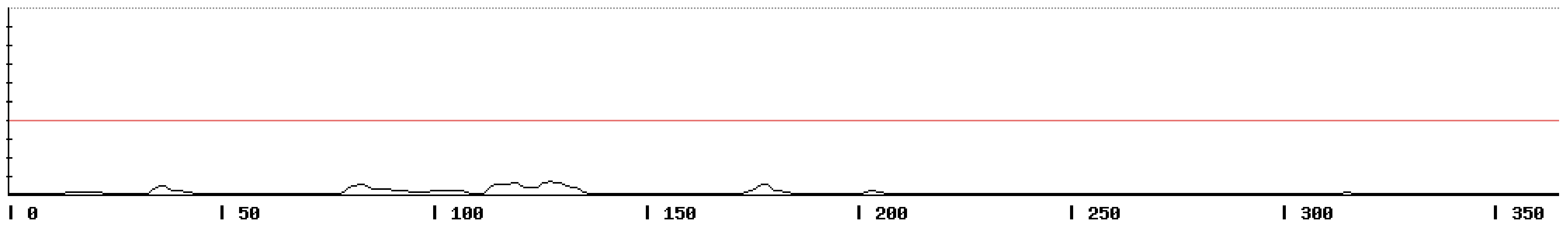

Predictions for Arabidopsishalleri_Araha.41818s0002.1.p	
Viterbi Path	
Viterbi Path	
Posterior @ 0.4	7 - NKRKARSSPGRK - 18
Posterior @ 0.4	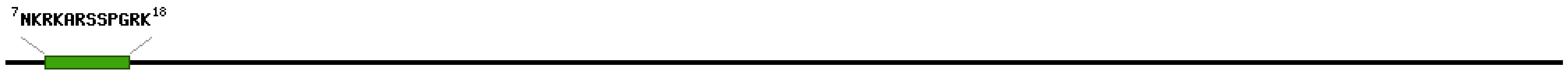
Posterior Graphic. Threshold Marked @ 0.4	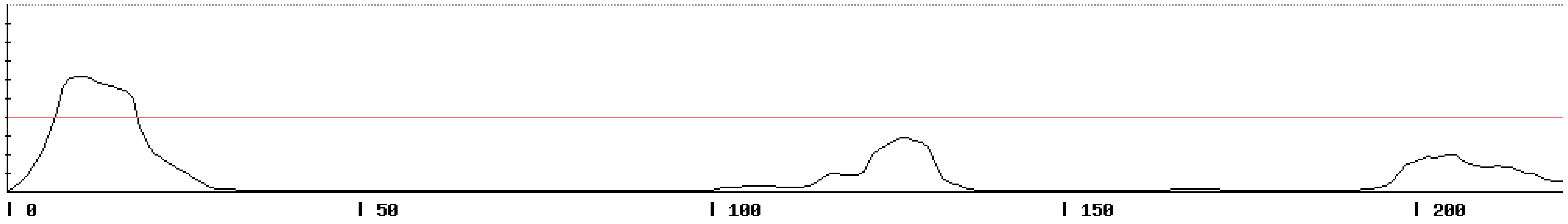

Predictions for Arabidopsishalleri_Araha.43003s0010.1.p	
Viterbi Path	
Viterbi Path	
Posterior @ 0.4	
Posterior @ 0.4	
Posterior Graphic. Threshold Marked @ 0.4	

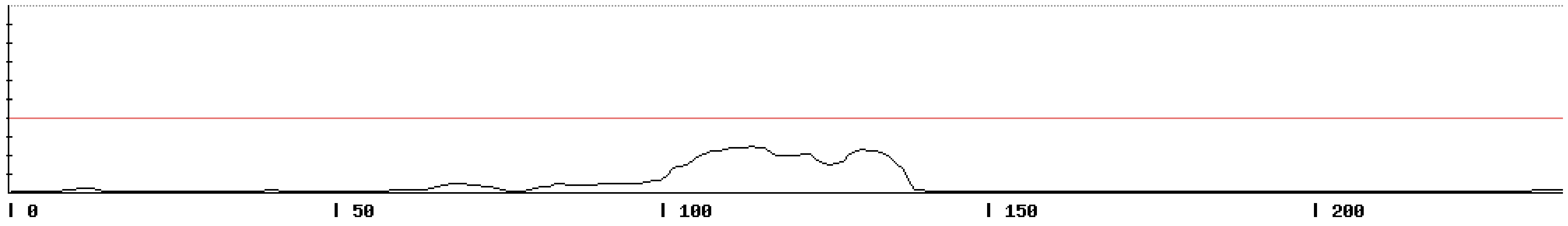

Predictions for Arabidopsishalleri_Araha.4353s0004.1.p

Viterbi Path	
Viterbi Path	
Posterior @ 0.4	
Posterior @ 0.4	
Posterior Graphic. Threshold Marked @ 0.4	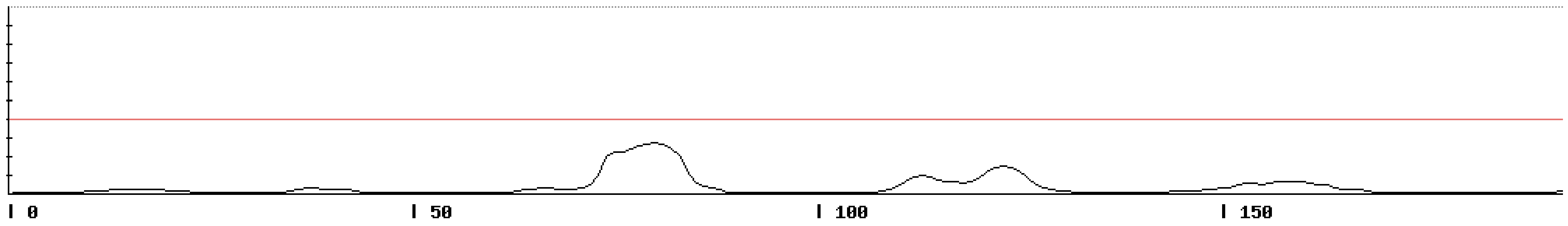

Predictions for Arabidopsishalleri_Araha.4375s0001.1.p

Viterbi Path	
Viterbi Path	
Posterior @ 0.4	
Posterior @ 0.4	

Posterior Graphic. Threshold Marked @ 0.4

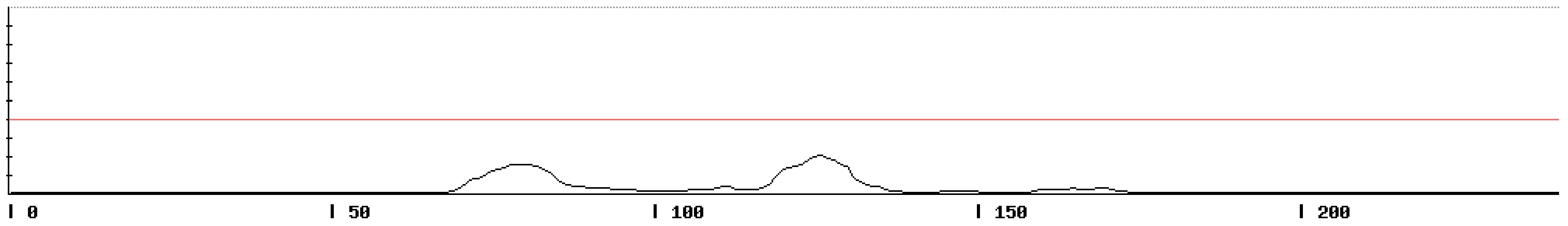

Predictions for Arabidopsishalleri_Araha.4414s0025.1.p

Viterbi Path

Viterbi Path

Posterior @ 0.4

Posterior @ 0.4

Posterior Graphic. Threshold Marked @ 0.4

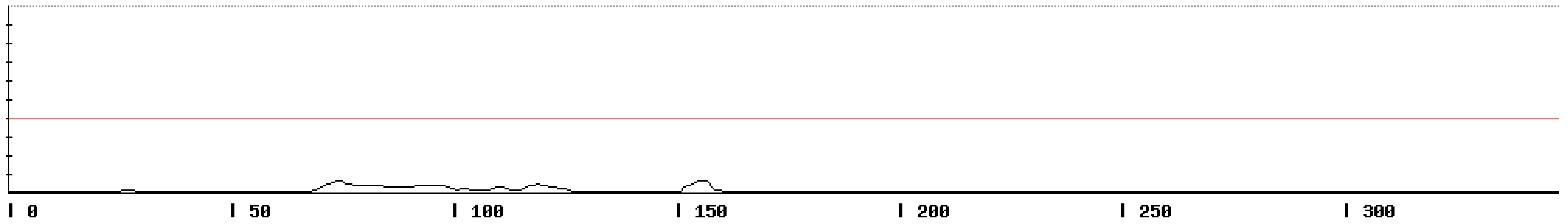

Predictions for Arabidopsishalleri_Araha.46415s0001.1.p

Viterbi Path

Viterbi Path

Posterior @ 0.4

Posterior @ 0.4

Posterior Graphic. Threshold Marked @ 0.4	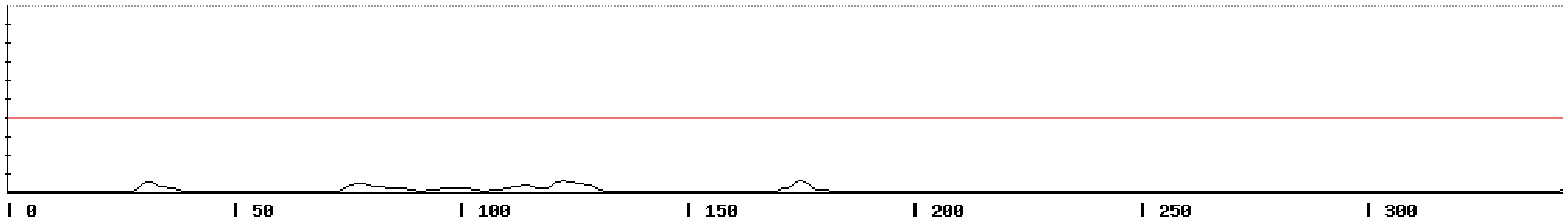

Predictions for Arabidopsishalleri_Araha.4814s0003.1.p	
Viterbi Path	205 - RKDMKKKRRRRRR - 217
Viterbi Path	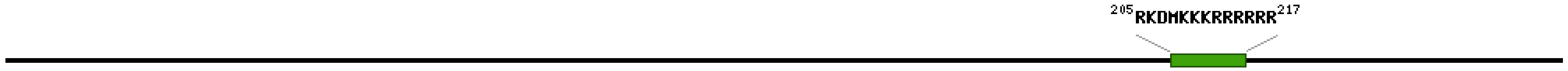
Posterior @ 0.4	205 - RKDMKKKRRRRRR - 217
Posterior @ 0.4	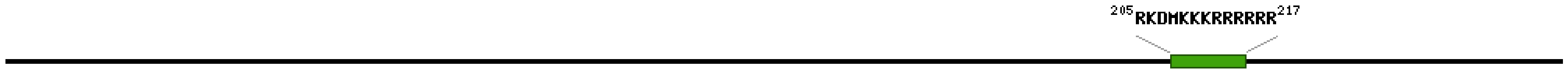
Posterior Graphic. Threshold Marked @ 0.4	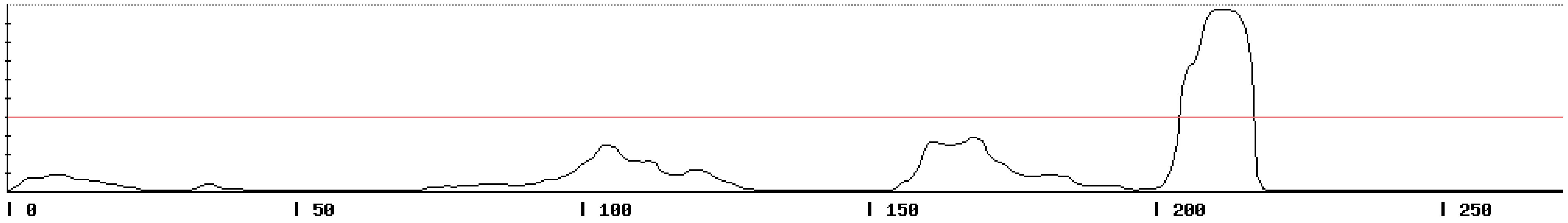

Predictions for Arabidopsishalleri_Araha.4954s0010.1.p	
Viterbi Path	
Viterbi Path	

Posterior @ 0.4

Posterior @ 0.4

Posterior Graphic. Threshold Marked @ 0.4

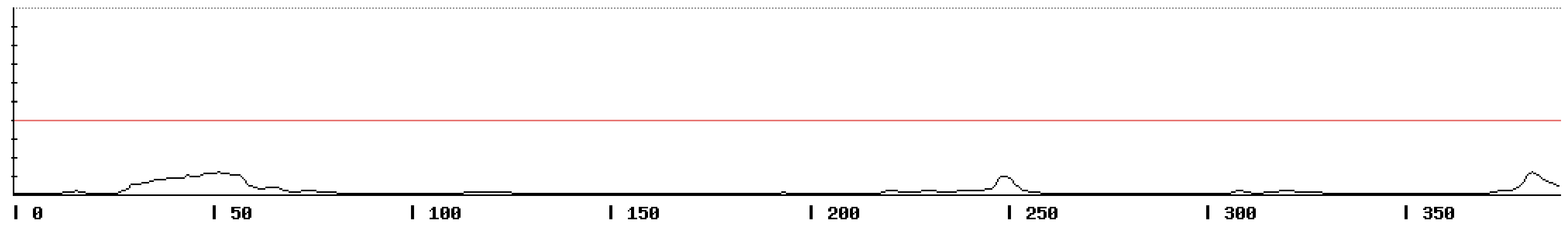

Predictions for Arabidopsishalleri_Araha.4954s0011.1.p

Viterbi Path

Viterbi Path

Posterior @ 0.4

Posterior @ 0.4

Posterior Graphic. Threshold Marked @ 0.4

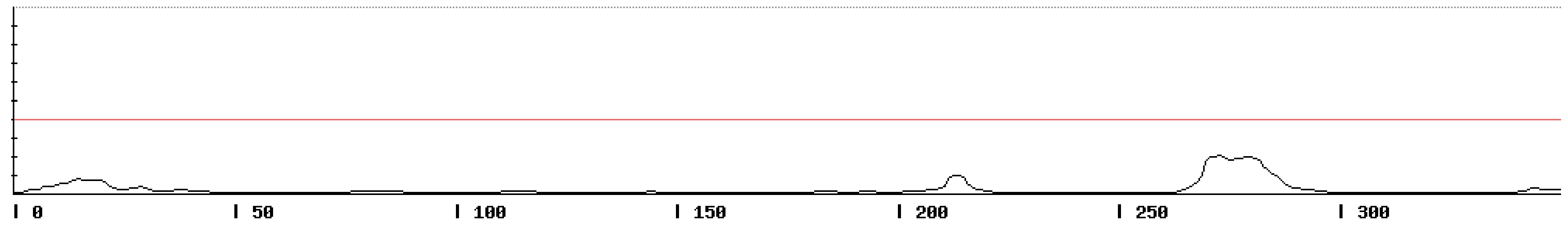

Predictions for Arabidopsishalleri_Araha.4954s0012.1.p

Viterbi Path

Viterbi Path

Posterior @ 0.4

Posterior @ 0.4

Posterior Graphic. Threshold Marked @ 0.4

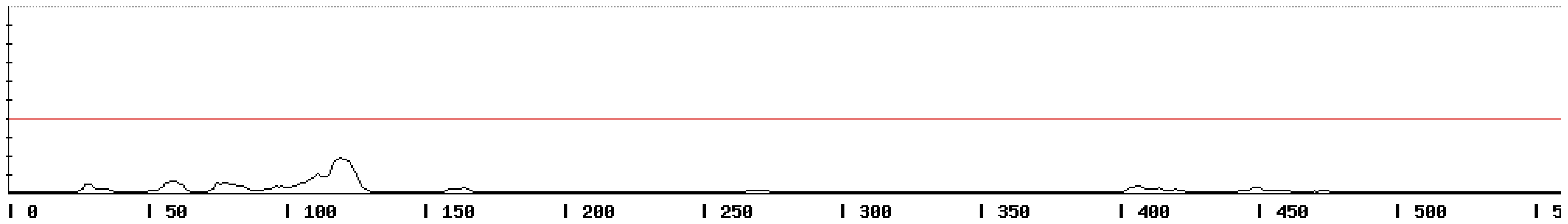

Predictions for Arabidopsishalleri_Araha.4956s0002.1.p

Viterbi Path

Viterbi Path

Posterior @ 0.4

Posterior @ 0.4

Posterior Graphic. Threshold Marked @ 0.4

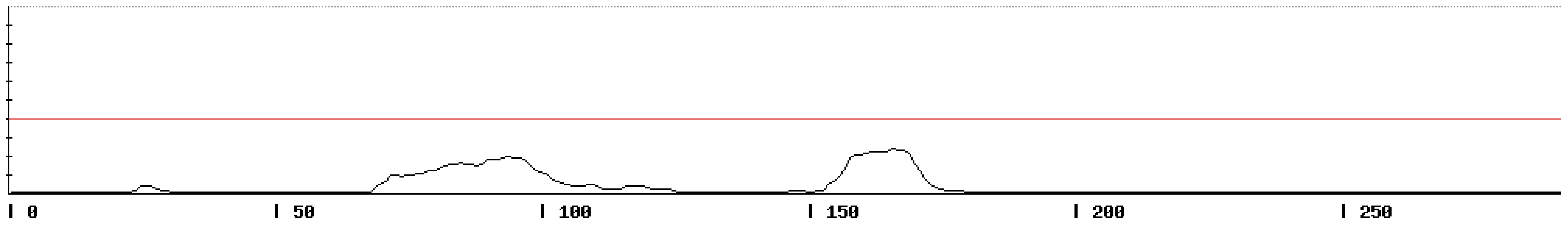

Predictions for Arabidopsishalleri_Araha.5052s0042.1.p

Viterbi Path	
Viterbi Path	
Posterior @ 0.4	121 - IRYKKKKKVNKIRR - 134
Posterior @ 0.4	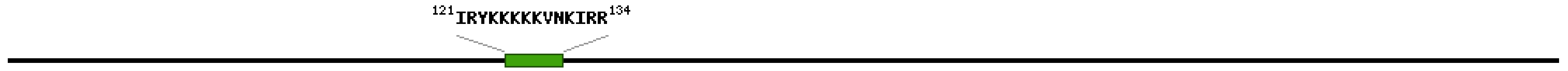
Posterior Graphic. Threshold Marked @ 0.4	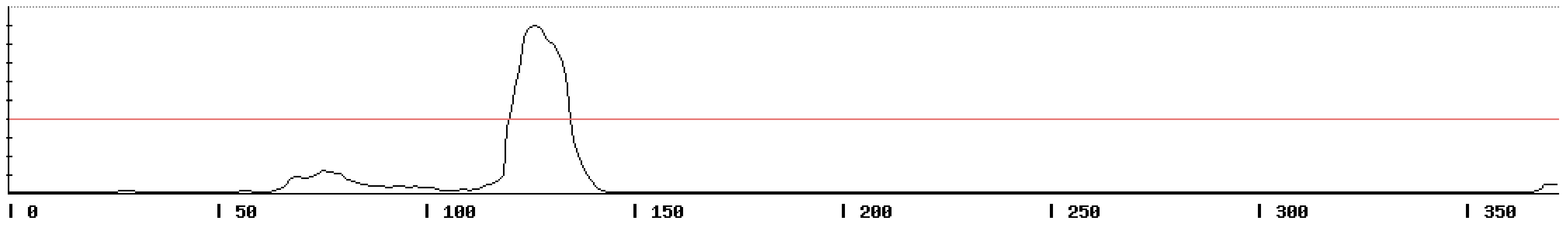

Predictions for Arabidopsishalleri_Araha.5099s0006.1.p

Viterbi Path	
Viterbi Path	
Posterior @ 0.4	
Posterior @ 0.4	
Posterior Graphic. Threshold Marked @ 0.4	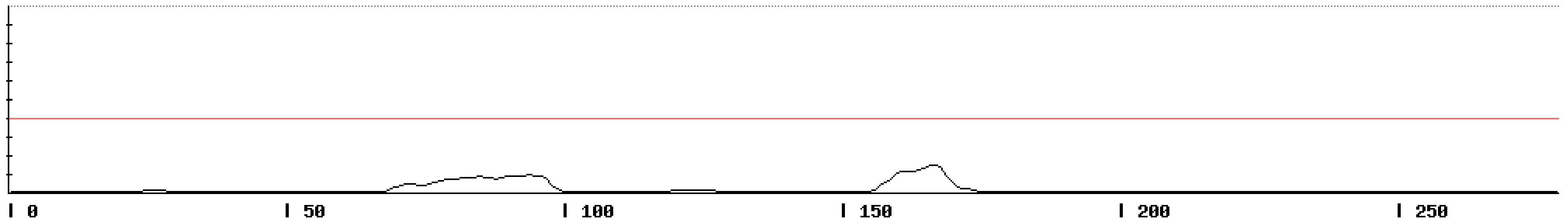

Predictions for Arabidopsishalleri_Araha.5101s0001.1.p

Viterbi Path	
Viterbi Path	
Posterior @ 0.4	
Posterior @ 0.4	
Posterior Graphic. Threshold Marked @ 0.4	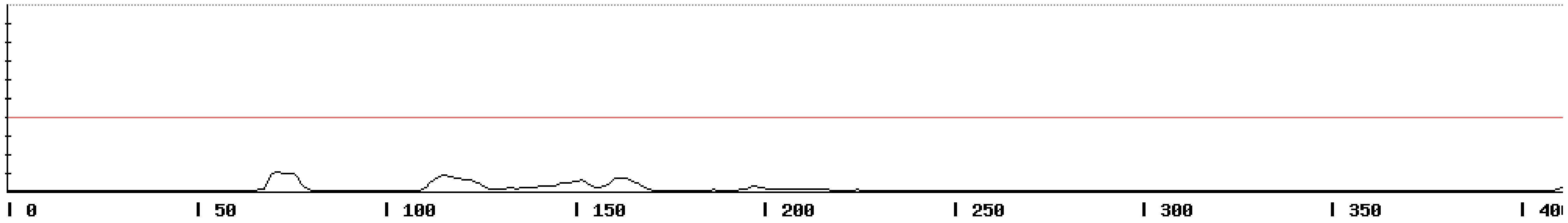

Predictions for Arabidopsishalleri_Araha.55234s0001.1.p

Viterbi Path	
Viterbi Path	
Posterior @ 0.4	25 - HK - 26
	106 - KK - 107
Posterior @ 0.4	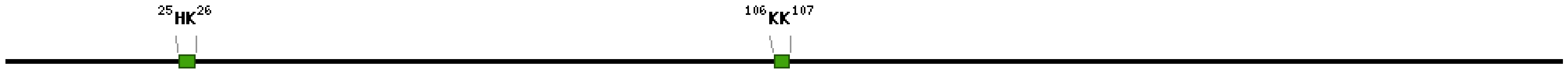
Posterior Graphic. Threshold Marked @ 0.4	

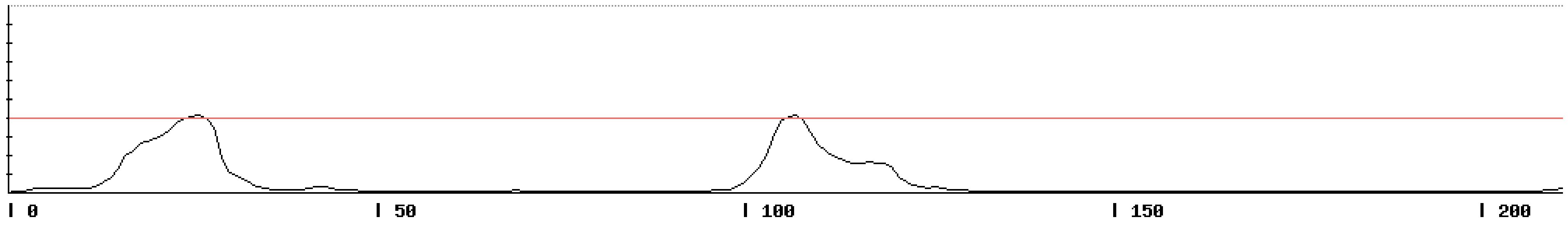

Predictions for Arabidopsishalleri_Araha.5579s0003.1.p

Viterbi Path	
Viterbi Path	
Posterior @ 0.4	
Posterior @ 0.4	
Posterior Graphic. Threshold Marked @ 0.4	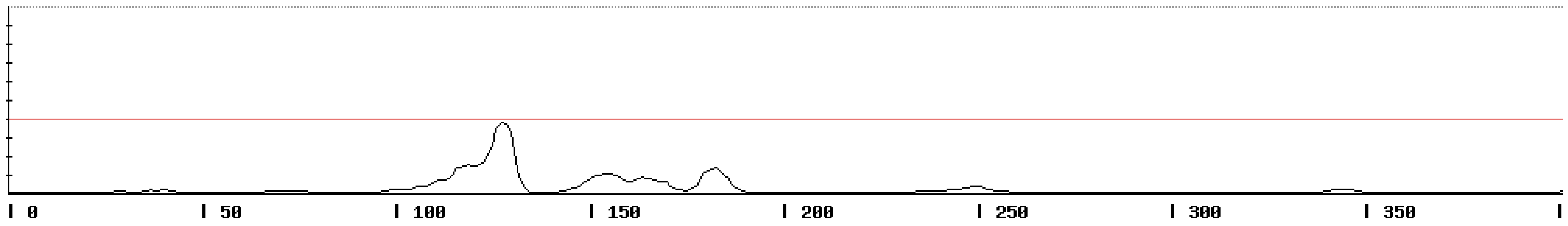

Predictions for Arabidopsishalleri_Araha.5599s0009.1.p

Viterbi Path	
Viterbi Path	
Posterior @ 0.4	166 - KRR - 168
Posterior @ 0.4	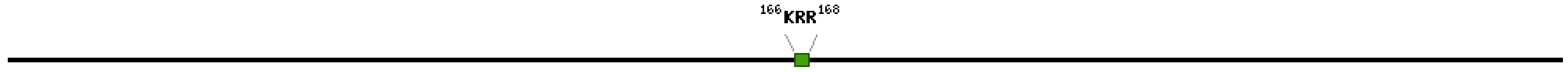

Posterior Graphic. Threshold Marked @ 0.4

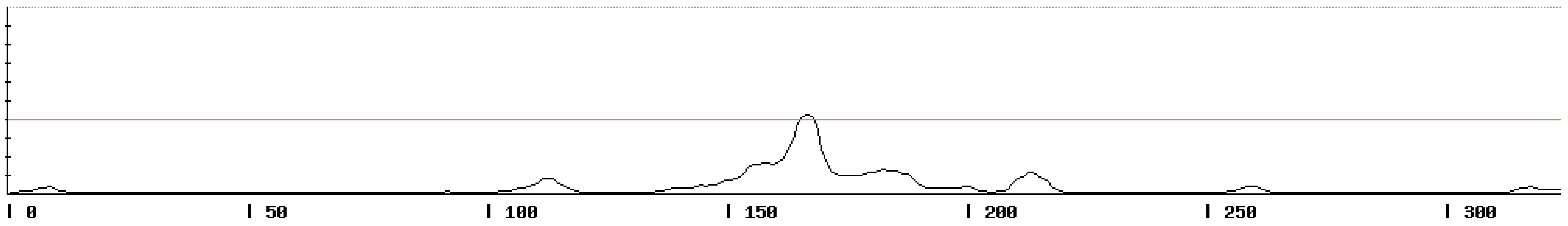

Predictions for Arabidopsishalleri_Araha.57496s0006.1.p

Viterbi Path

Viterbi Path

Posterior @ 0.4

78 - KKTNRKVKGWKATGGLKDVLSKKNGKVIGKKRNR - 111

Posterior @ 0.4

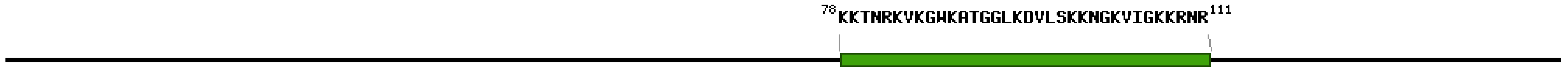

Posterior Graphic. Threshold Marked @ 0.4

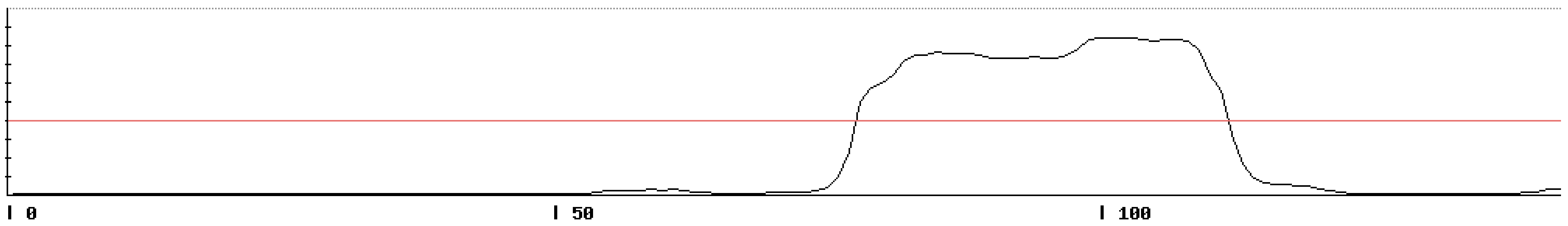

Predictions for Arabidopsishalleri_Araha.5892s0003.1.p

Viterbi Path

Viterbi Path

Posterior @ 0.4

Posterior @ 0.4

Posterior Graphic. Threshold Marked @ 0.4

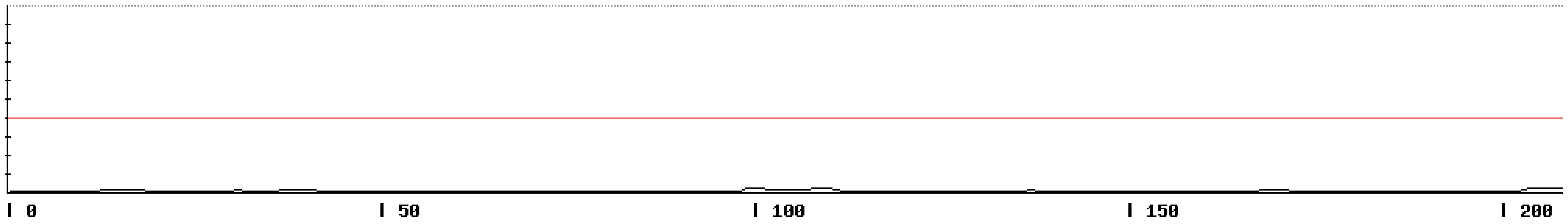

Predictions for Arabidopsishalleri_Araha.59992s0001.1.p

Viterbi Path

Viterbi Path

Posterior @ 0.4

Posterior @ 0.4

Posterior Graphic. Threshold Marked @ 0.4

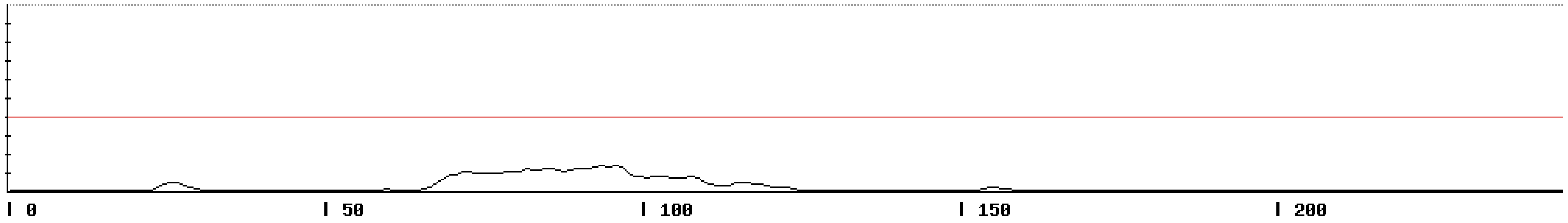

Predictions for Arabidopsishalleri_Araha.6161s0007.1.p

Viterbi Path

Viterbi Path

Posterior @ 0.4

Posterior @ 0.4

Posterior Graphic. Threshold Marked @ 0.4

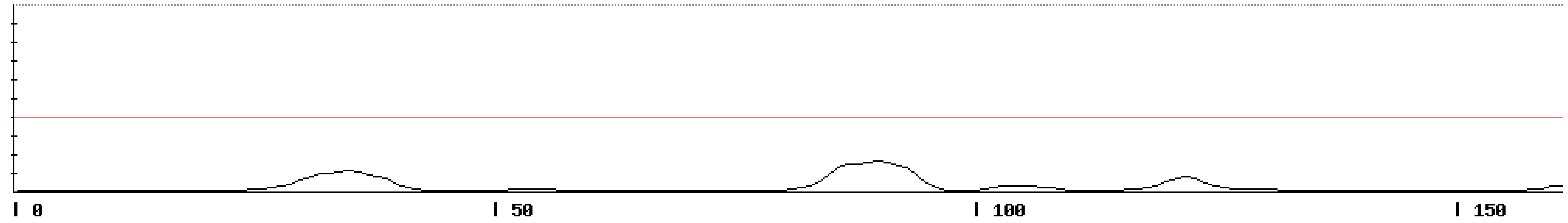

Predictions for Arabidopsishalleri_Araha.61839s0001.1.p

Viterbi Path

Viterbi Path

Posterior @ 0.4 — 96 - KSKETGKILGFKKILKFCKKTKPRE - 120

Posterior @ 0.4

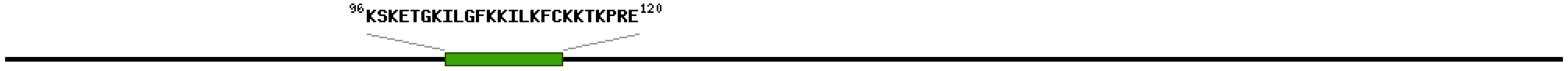

Posterior Graphic. Threshold Marked @ 0.4

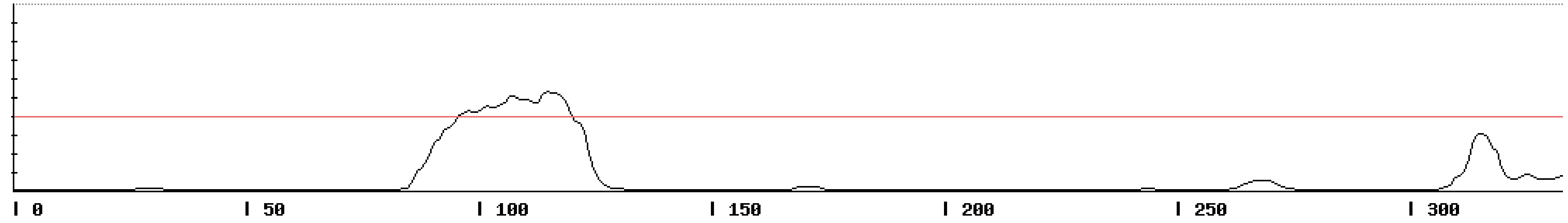

Predictions for Arabidopsishalleri_Araha.6475s0002.1.p

Viterbi Path

Viterbi Path

Posterior @ 0.4	
Posterior @ 0.4	
Posterior Graphic. Threshold Marked @ 0.4	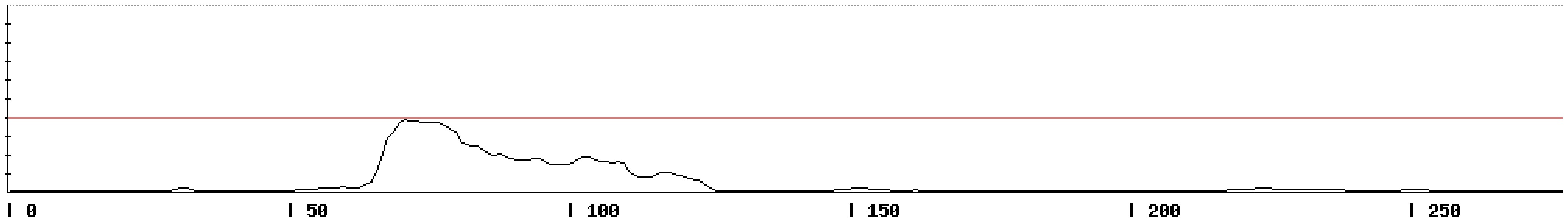

Predictions for Arabidopsishalleri_Araha.65799s0001.1.p

Viterbi Path	
Viterbi Path	
Posterior @ 0.4	
Posterior @ 0.4	
Posterior Graphic. Threshold Marked @ 0.4	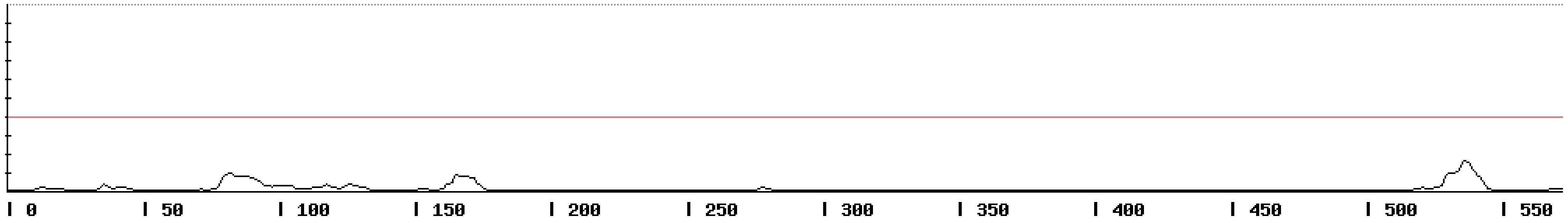

Predictions for Arabidopsishalleri_Araha.6755s0003.1.p

Viterbi Path

Viterbi Path

Posterior @ 0.4

Posterior @ 0.4

Posterior Graphic. Threshold Marked @ 0.4

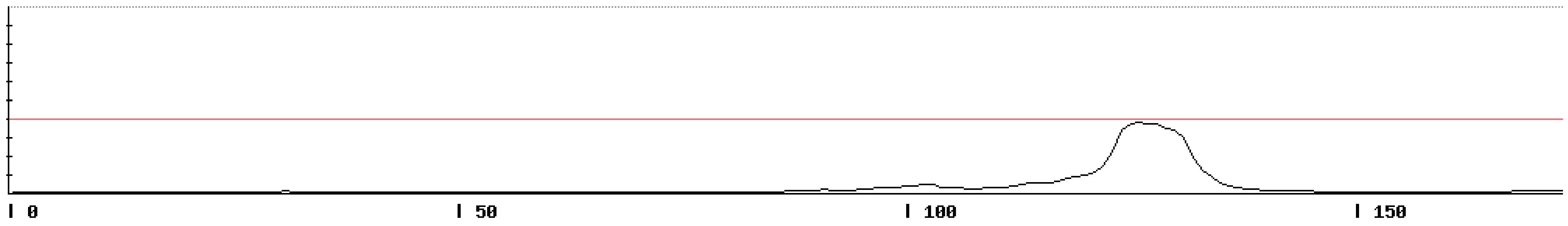

Predictions for Arabidopsishalleri_Araha.68360s0001.1.p

Viterbi Path

Viterbi Path

Posterior @ 0.4

Posterior @ 0.4

Posterior Graphic. Threshold Marked @ 0.4

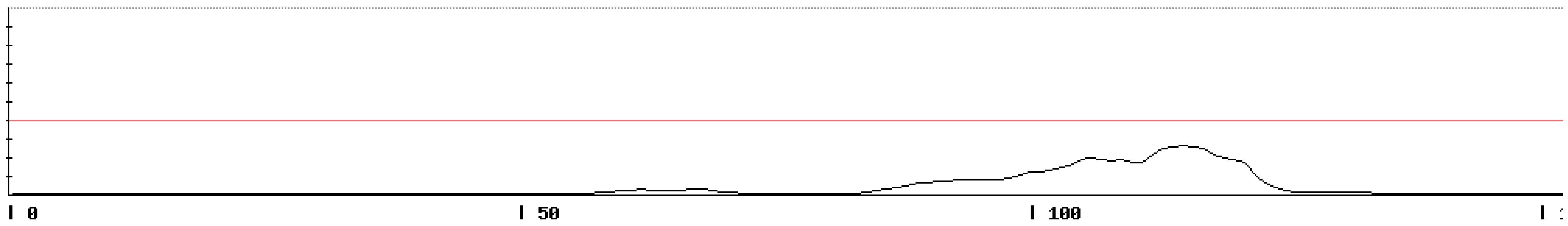

Predictions for Arabidopsishalleri_Araha.7513s0013.1.p	
Viterbi Path	
Viterbi Path	
Posterior @ 0.4	
Posterior @ 0.4	
Posterior Graphic. Threshold Marked @ 0.4	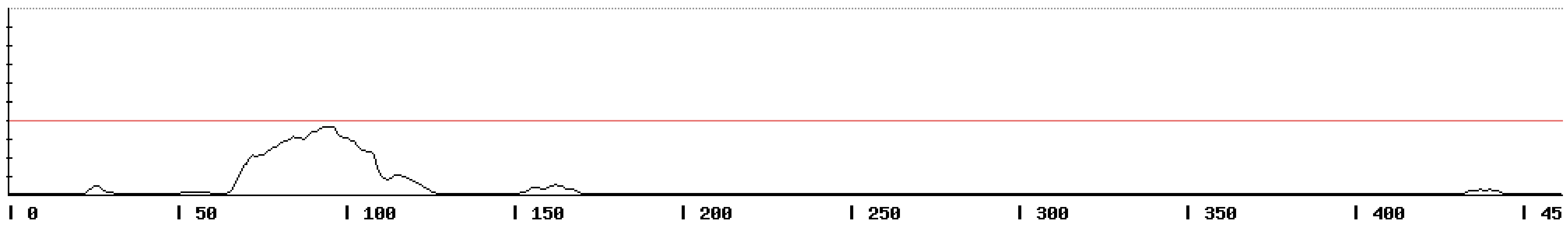

Predictions for Arabidopsishalleri_Araha.7640s0010.1.p	
Viterbi Path	
Viterbi Path	
Posterior @ 0.4	98 - GKPK - 101
Posterior @ 0.4	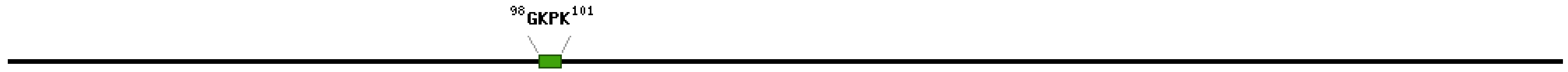
Posterior Graphic. Threshold Marked @ 0.4	

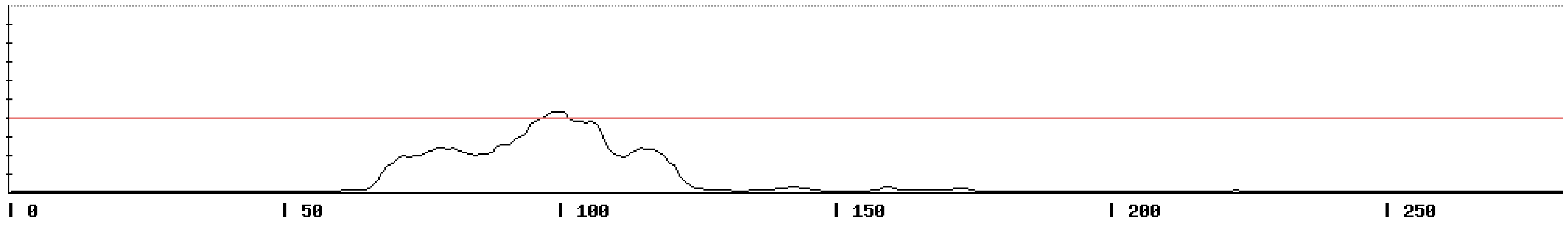

Predictions for Arabidopsishalleri_Araha.8686s0001.1.p

Viterbi Path	
Viterbi Path	
Posterior @ 0.4	
Posterior @ 0.4	
Posterior Graphic. Threshold Marked @ 0.4	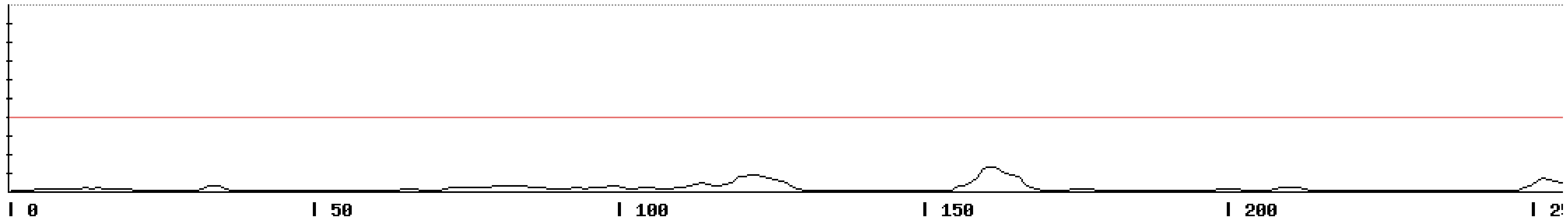

Predictions for Arabidopsishalleri_Araha.8689s0023.1.p

Viterbi Path	
Viterbi Path	
Posterior @ 0.4	
Posterior @ 0.4	

Posterior Graphic. Threshold Marked @ 0.4

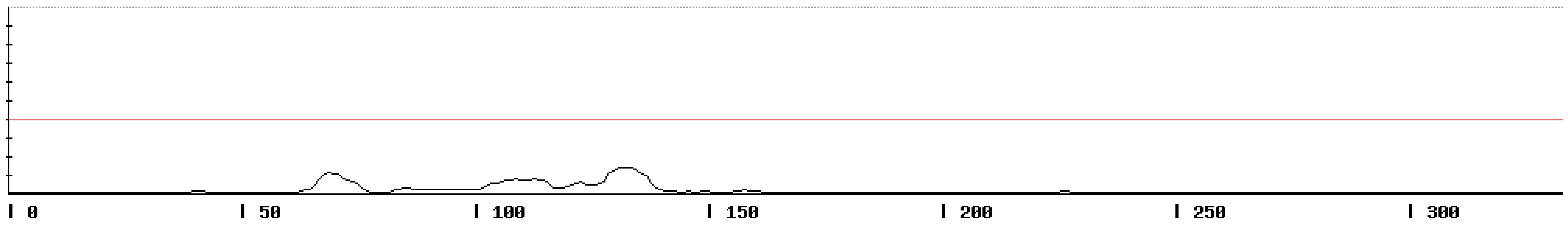

Predictions for Arabidopsishalleri_Araha.8824s0002.1.p

Viterbi Path

Viterbi Path

Posterior @ 0.4

Posterior @ 0.4

Posterior Graphic. Threshold Marked @ 0.4

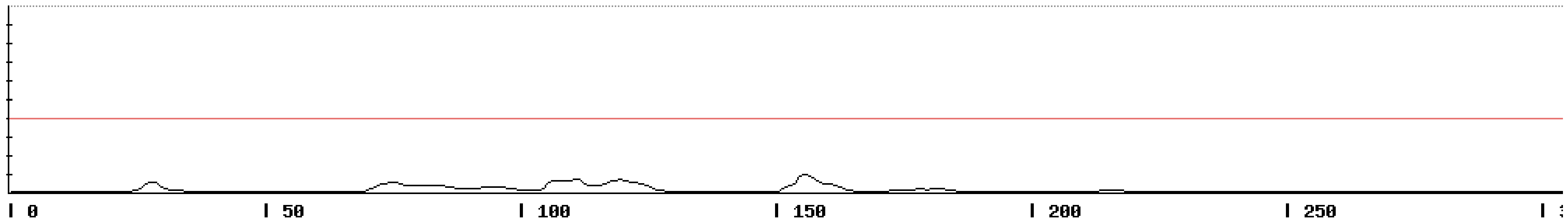

Predictions for Arabidopsishalleri_Araha.8989s0001.1.p

Viterbi Path

Viterbi Path

Posterior @ 0.4

Posterior @ 0.4

Posterior Graphic. Threshold Marked @ 0.4

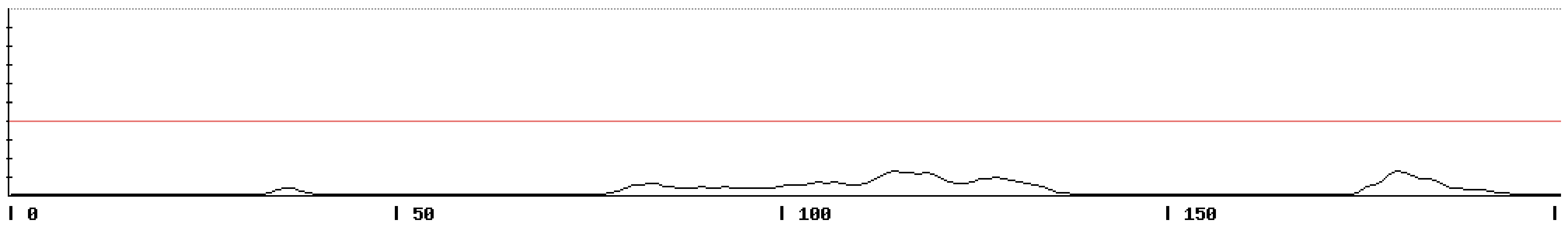

Predictions for Arabidopsishalleri_Araha.9027s0002.1.p

Viterbi Path

Viterbi Path

Posterior @ 0.4

Posterior @ 0.4

Posterior Graphic. Threshold Marked @ 0.4

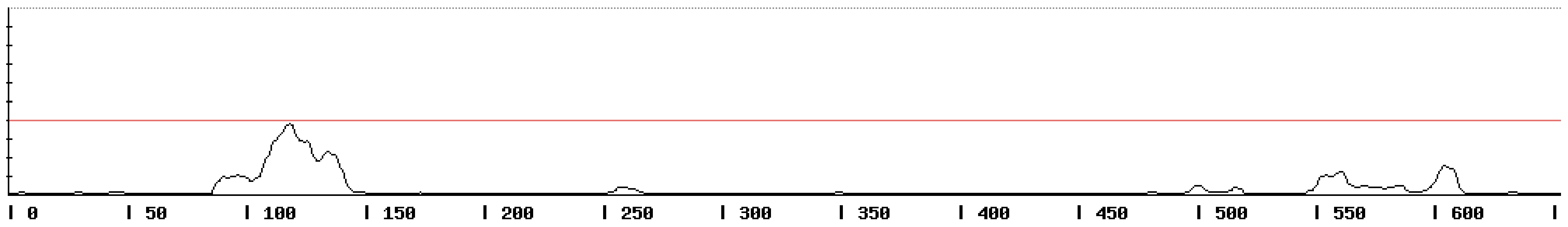

Predictions for Arabidopsishalleri_Araha.9424s0001.1.p

Viterbi Path

Viterbi Path

Posterior @ 0.4	
Posterior @ 0.4	
Posterior Graphic. Threshold Marked @ 0.4	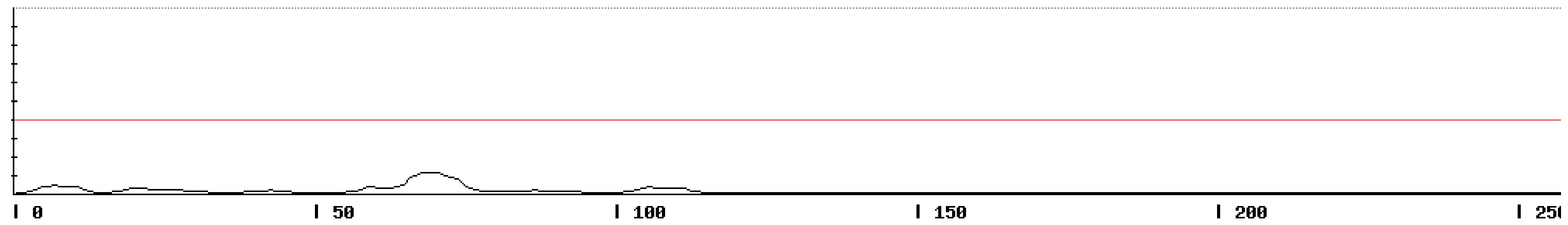

Predictions for Arabidopsishalleri_Araha.9431s0002.1.p

Viterbi Path	
Viterbi Path	
Posterior @ 0.4	
Posterior @ 0.4	
Posterior Graphic. Threshold Marked @ 0.4	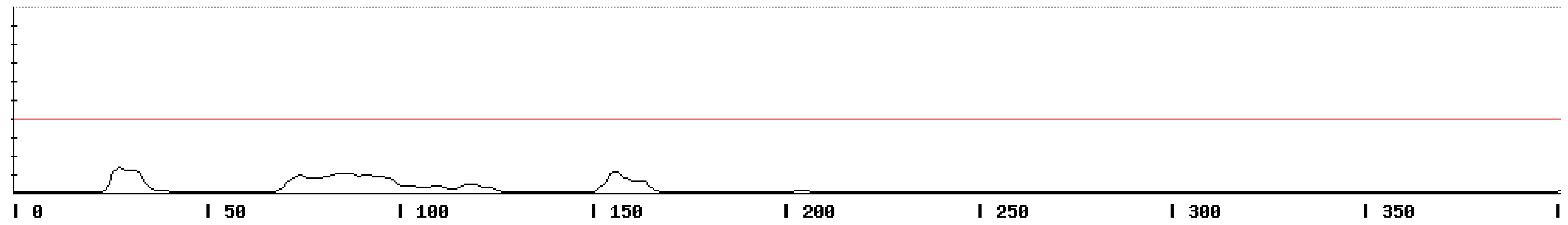

Predictions for Arabidopsishalleri_Araha.9570s0009.1.p

Viterbi Path	
Viterbi Path	

Posterior @ 0.4	
Posterior @ 0.4	
Posterior Graphic. Threshold Marked @ 0.4	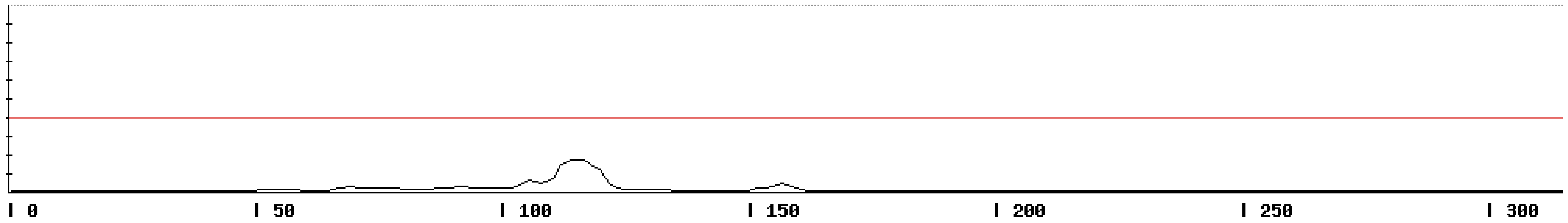

Predictions for Arabidopsishalleri_Araha.9776s0006.1.p	
Viterbi Path	
Viterbi Path	
Posterior @ 0.4	
Posterior @ 0.4	
Posterior Graphic. Threshold Marked @ 0.4	

Revision r.9 - Last Updated on : February 24 2015 17:45:20. alex.nguyenba@utoronto.ca

Intended to be used with Firefox

Viterbi Path

Posterior @ 0.4

Posterior @ 0.4

Posterior Graphic. Threshold Marked @ 0.4

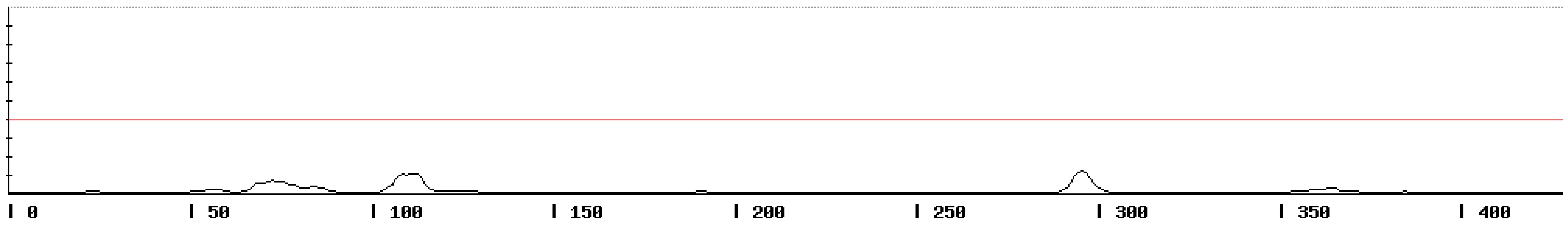

Predictions for Arabidopsisthaliana_AT1G01720.1

Viterbi Path

Viterbi Path

Posterior @ 0.4

Posterior @ 0.4

Posterior Graphic. Threshold Marked @ 0.4

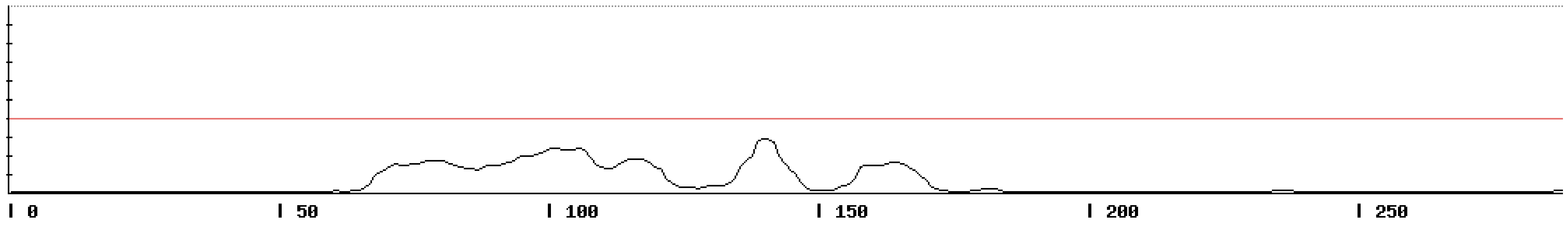

Predictions for Arabidopsisthaliana_AT1G02210.1

Viterbi Path	
Viterbi Path	
Posterior @ 0.4	
Posterior @ 0.4	
Posterior Graphic. Threshold Marked @ 0.4	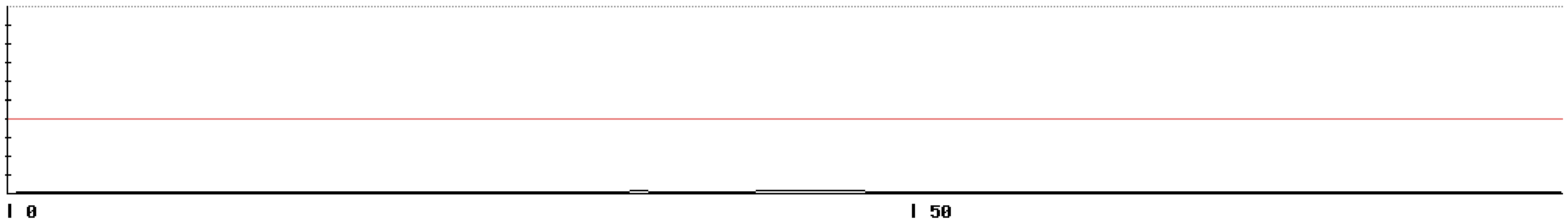

Predictions for Arabidopsisthaliana_AT1G02220.1	
Viterbi Path	
Viterbi Path	
Posterior @ 0.4	89 - KSTGRPKP - 96
Posterior @ 0.4	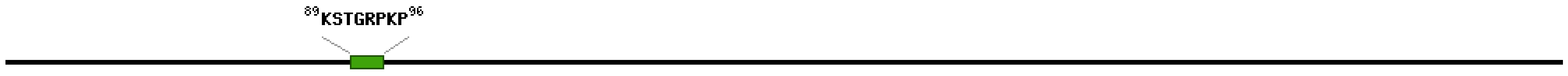
Posterior Graphic. Threshold Marked @ 0.4	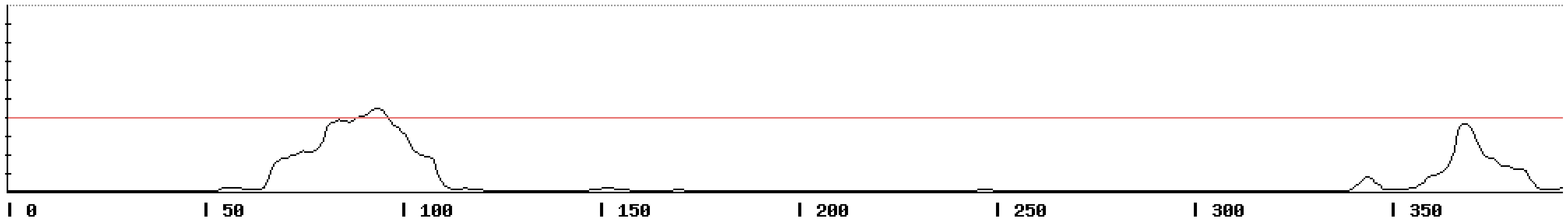

Predictions for Arabidopsisthaliana_AT1G02230.1	
Viterbi Path	
Viterbi Path	
Posterior @ 0.4	89 - KKTGKTMTVMRKRGNREKIGEKR - 111
Posterior @ 0.4	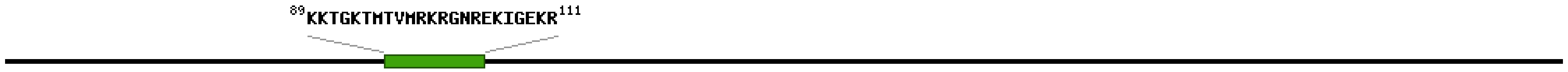
Posterior Graphic. Threshold Marked @ 0.4	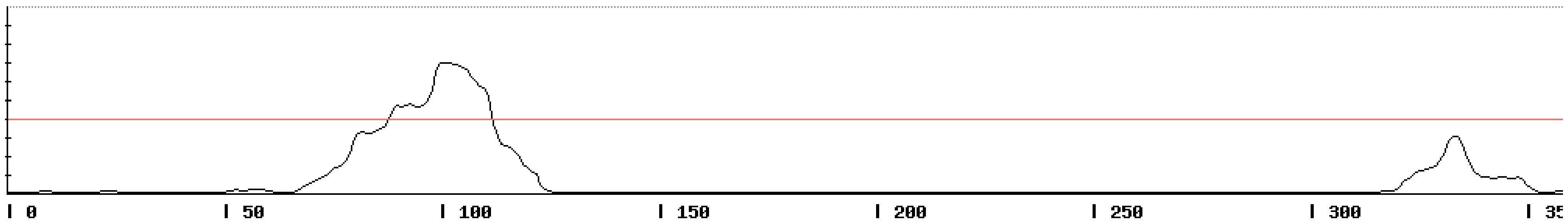

Predictions for Arabidopsisthaliana_AT1G02250.1	
Viterbi Path	
Viterbi Path	
Posterior @ 0.4	72 - KYGKGDRQIRKTKSG - 86
Posterior @ 0.4	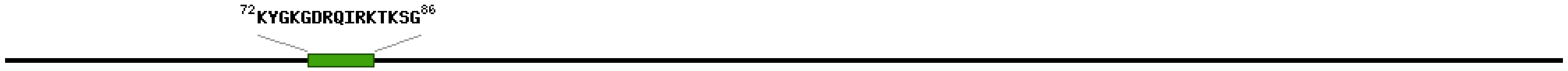
Posterior Graphic. Threshold Marked @ 0.4	

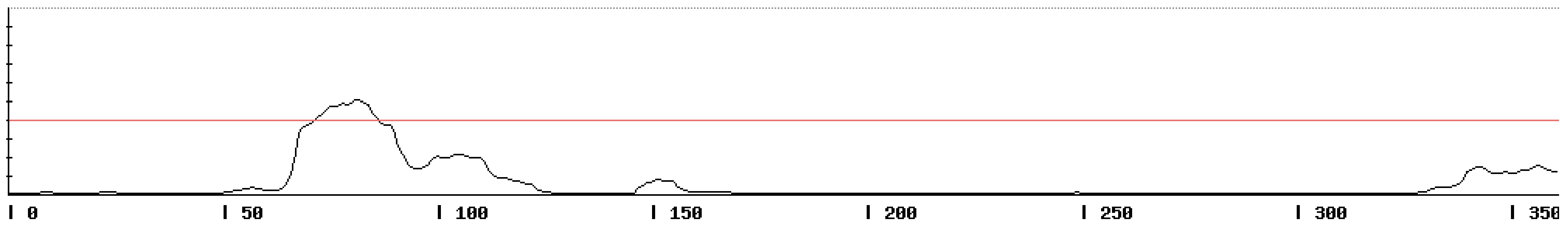

Predictions for Arabidopsisthaliana_AT1G03490.1

Viterbi Path

Viterbi Path

Posterior @ 0.4

Posterior @ 0.4

Posterior Graphic. Threshold Marked @ 0.4

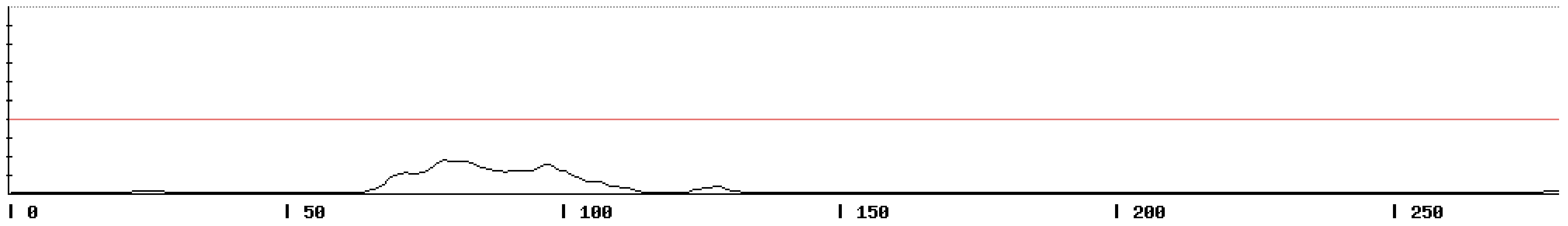

Predictions for Arabidopsisthaliana_AT1G12260.1

Viterbi Path

Viterbi Path

Posterior @ 0.4

Posterior @ 0.4

Posterior Graphic. Threshold Marked @ 0.4

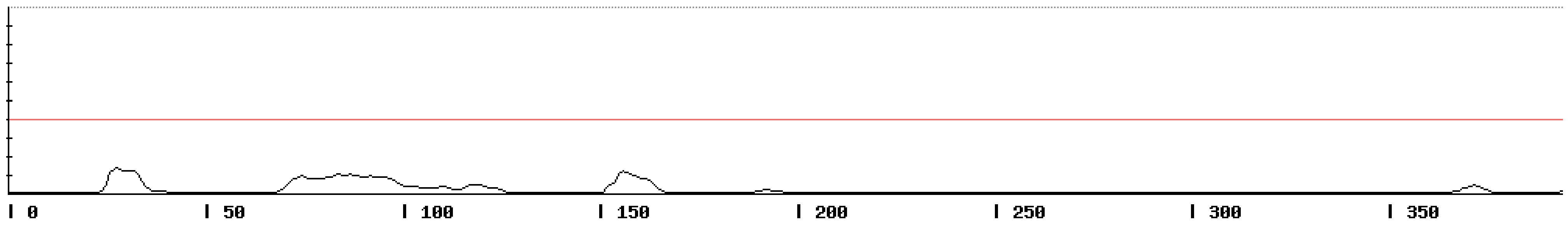

Predictions for Arabidopsisthaliana_AT1G19040.1

Viterbi Path	
Viterbi Path	
Posterior @ 0.4	
Posterior @ 0.4	
Posterior Graphic. Threshold Marked @ 0.4	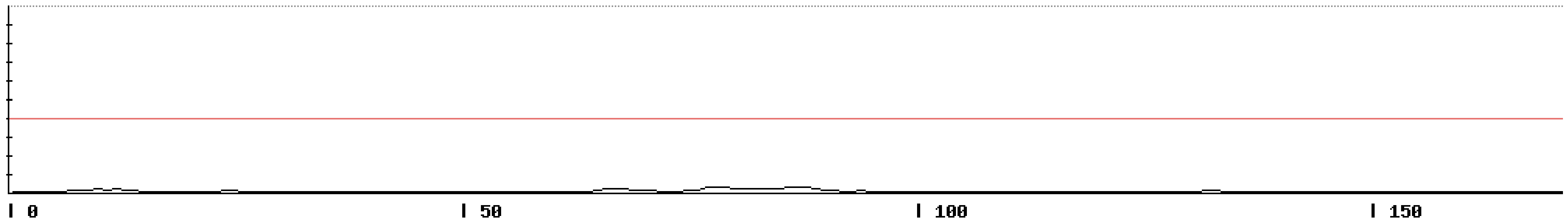

Predictions for Arabidopsisthaliana_AT1G25580.1

Viterbi Path	
Viterbi Path	
Posterior @ 0.4	137 - KRR - 139
Posterior @ 0.4	

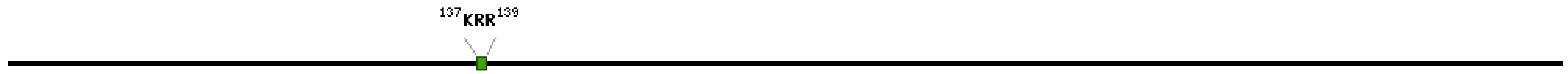

Posterior Graphic. Threshold Marked @ 0.4

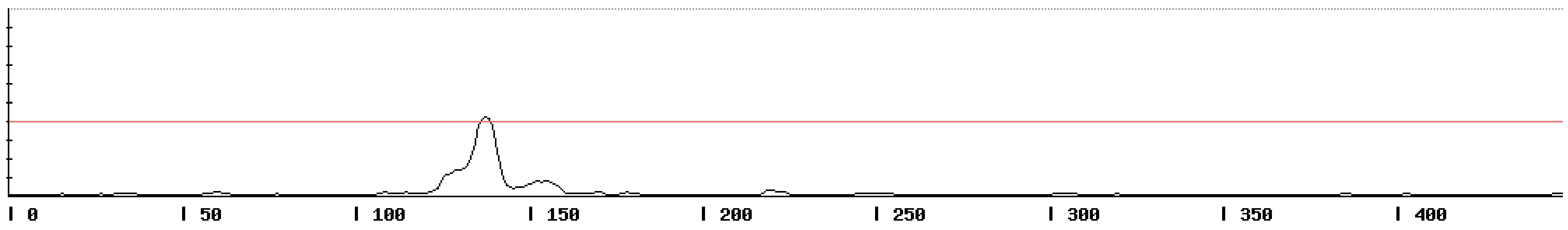

Predictions for Arabidopsisthaliana_AT1G26870.1

Viterbi Path

Viterbi Path

Posterior @ 0.4

Posterior @ 0.4

Posterior Graphic. Threshold Marked @ 0.4

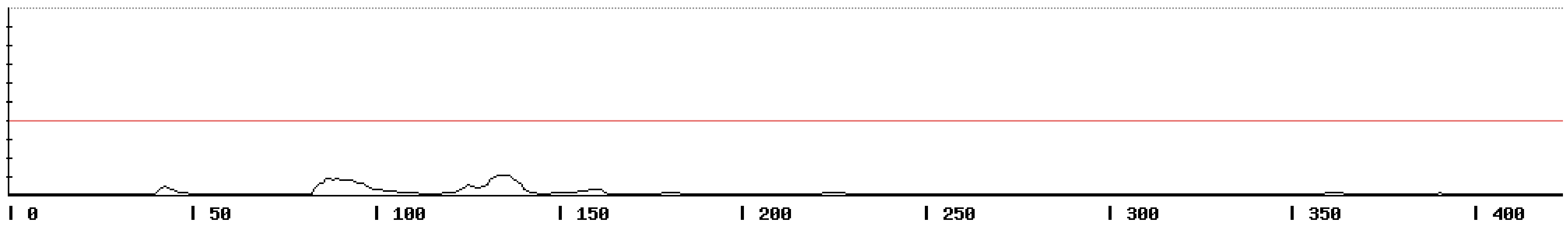

Predictions for Arabidopsisthaliana_AT1G28470.1

Viterbi Path

Viterbi Path

Posterior @ 0.4 | 156 - KRR - 158

Posterior @ 0.4

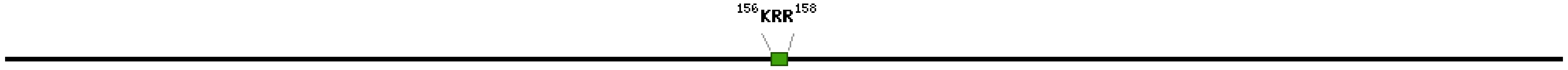

Posterior Graphic. Threshold Marked @ 0.4

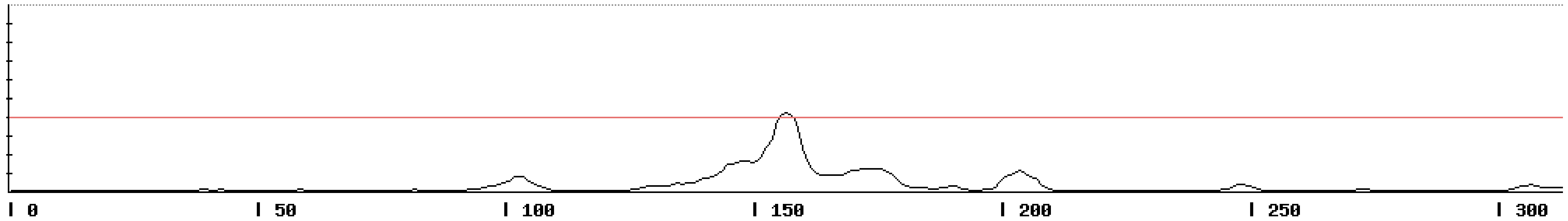

Predictions for Arabidopsisthaliana_AT1G32510.1

Viterbi Path

Viterbi Path

Posterior @ 0.4

Posterior @ 0.4

Posterior Graphic. Threshold Marked @ 0.4

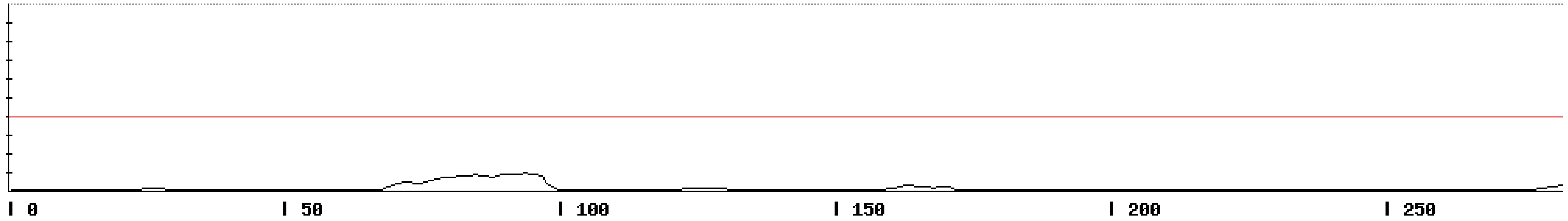

Predictions for Arabidopsisthaliana_AT1G32770.1

Viterbi Path

Viterbi Path

Posterior @ 0.4

Posterior @ 0.4

Posterior Graphic. Threshold Marked @ 0.4

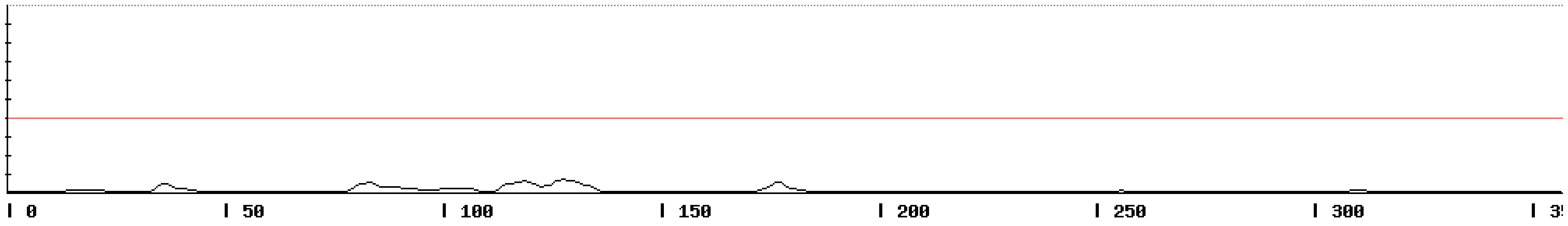

Predictions for Arabidopsisthaliana_AT1G32870.1

Viterbi Path

Viterbi Path

Posterior @ 0.4

29 - KRKIRRKKLR - 38

481 - KRNKSGGNNDKKKKKNKG - 498

Posterior @ 0.4

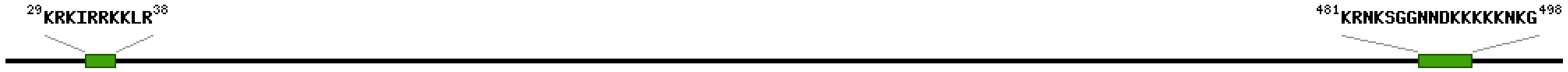

Posterior Graphic. Threshold Marked @ 0.4

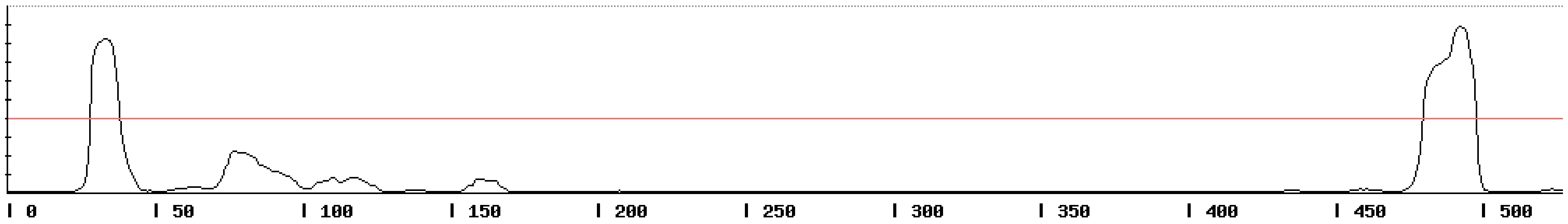

Predictions for Arabidopsisthaliana_AT1G33060.1	
Viterbi Path	
Viterbi Path	
Posterior @ 0.4	
Posterior @ 0.4	
Posterior Graphic. Threshold Marked @ 0.4	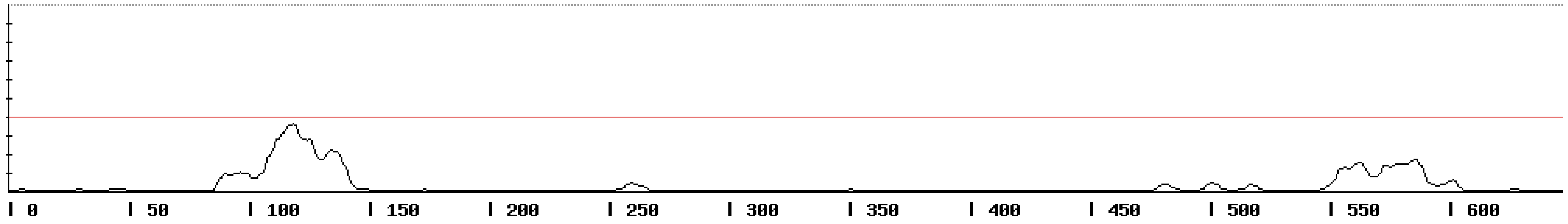

Predictions for Arabidopsisthaliana_AT1G33280.1	
Viterbi Path	
Viterbi Path	
Posterior @ 0.4	
Posterior @ 0.4	
Posterior Graphic. Threshold Marked @ 0.4	

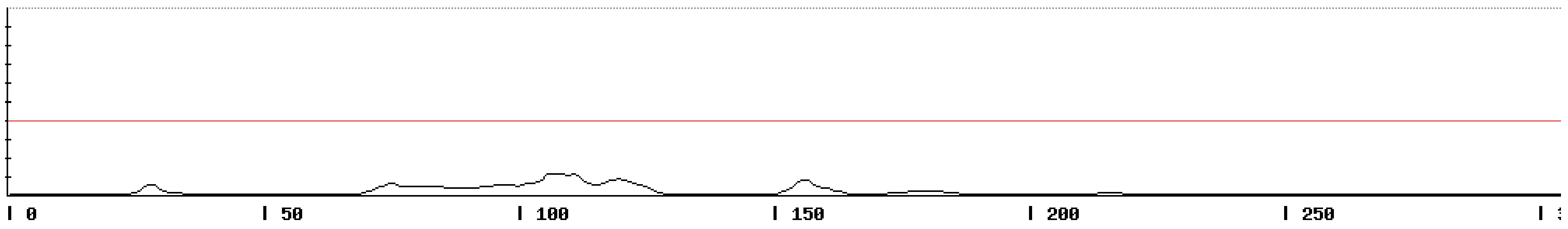

Predictions for Arabidopsisthaliana_AT1G34180.1

Viterbi Path	
Viterbi Path	
Posterior @ 0.4	
Posterior @ 0.4	
Posterior Graphic. Threshold Marked @ 0.4	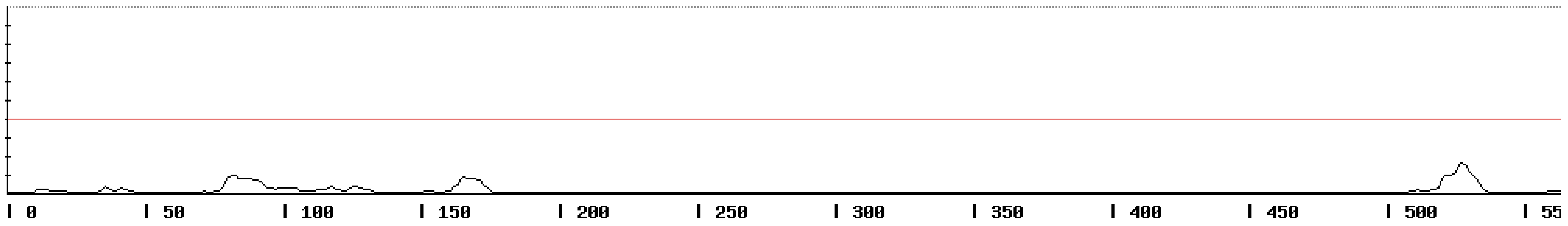

Predictions for Arabidopsisthaliana_AT1G34190.1

Viterbi Path	
Viterbi Path	
Posterior @ 0.4	
Posterior @ 0.4	

Posterior Graphic. Threshold Marked @ 0.4	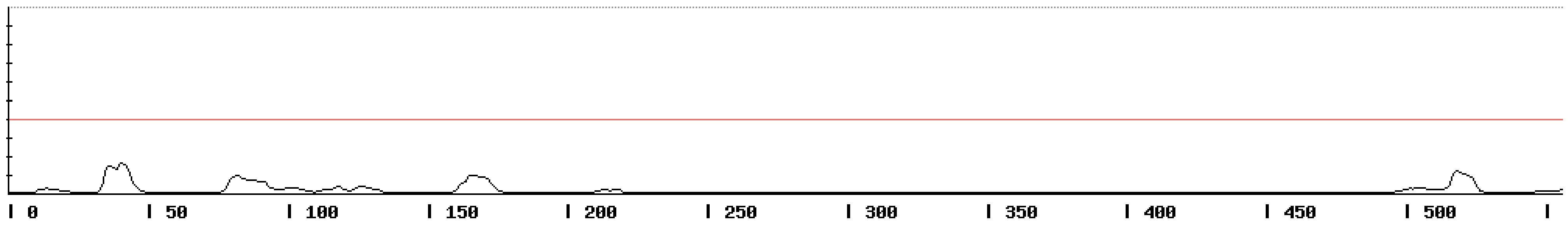

Predictions for Arabidopsisthaliana_AT1G52880.1

Viterbi Path	
Viterbi Path	
Posterior @ 0.4	
Posterior @ 0.4	
Posterior Graphic. Threshold Marked @ 0.4	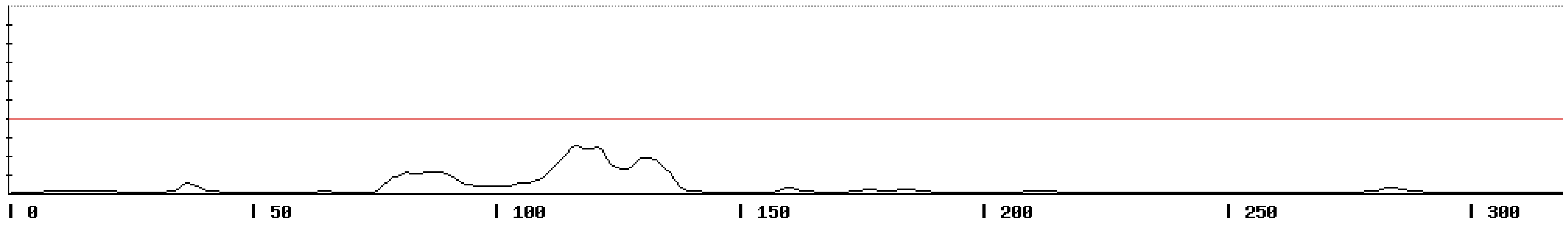

Predictions for Arabidopsisthaliana_AT1G52890.1

Viterbi Path	
Viterbi Path	
Posterior @ 0.4	
Posterior @ 0.4	

Posterior Graphic. Threshold Marked @ 0.4

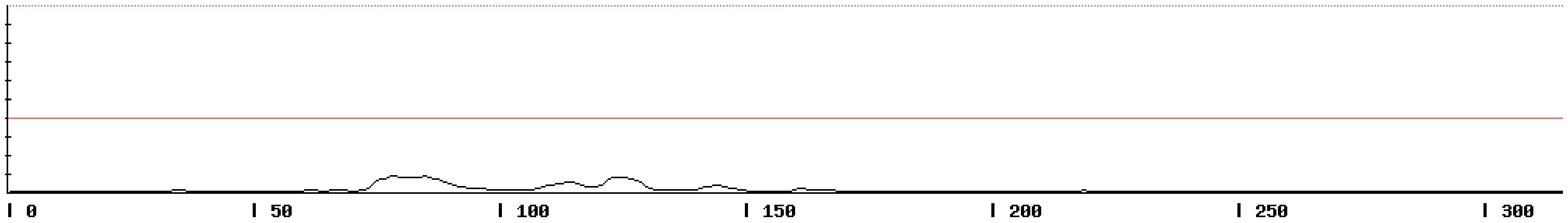

Predictions for Arabidopsisthaliana_AT1G54330.1

Viterbi Path

Viterbi Path

Posterior @ 0.4

Posterior @ 0.4

Posterior Graphic. Threshold Marked @ 0.4

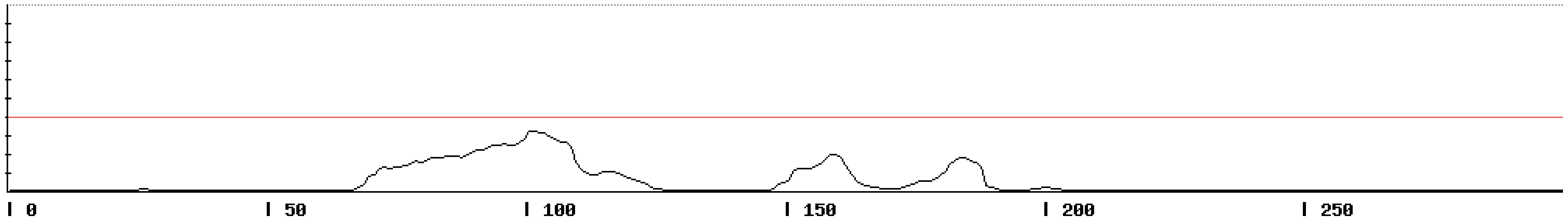

Predictions for Arabidopsisthaliana_AT1G56010.1

Viterbi Path

Viterbi Path

Posterior @ 0.4

Posterior @ 0.4

Posterior Graphic. Threshold Marked @ 0.4

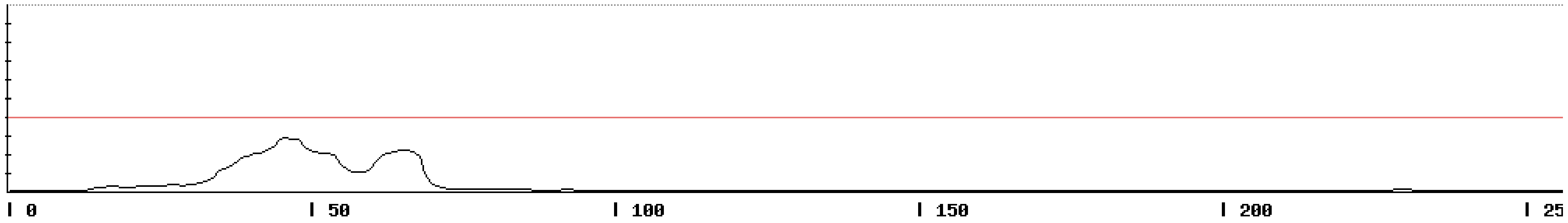

Predictions for Arabidopsisthaliana_AT1G60240.1

Viterbi Path

Viterbi Path

Posterior @ 0.4 — 113 - KPKSVFKFLEKEKRRVRDRR - 132

Posterior @ 0.4

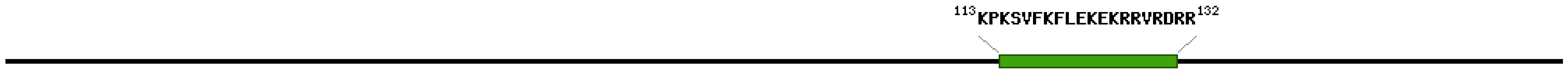

Posterior Graphic. Threshold Marked @ 0.4

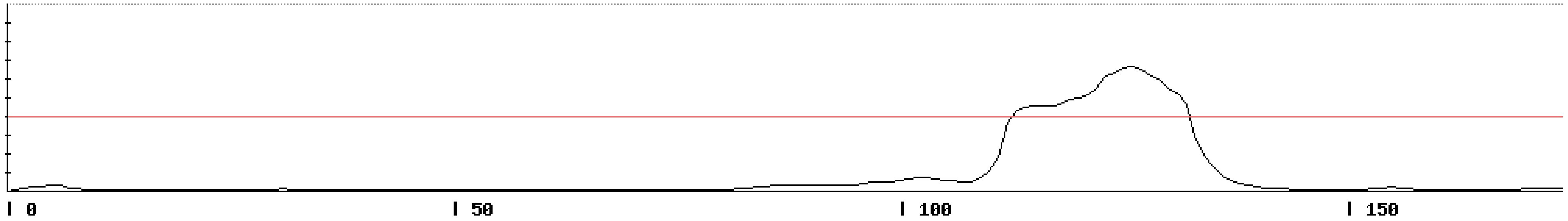

Predictions for Arabidopsisthaliana_AT1G60280.1

Viterbi Path

Viterbi Path

Posterior @ 0.4	114 - KETGKILGFKKILKFCLKKKPREYK - 138
Posterior @ 0.4	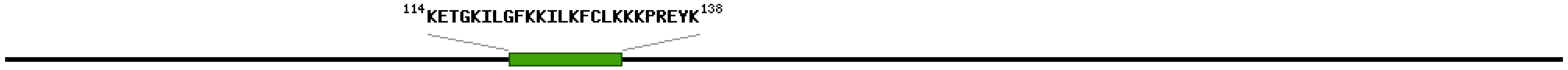
Posterior Graphic. Threshold Marked @ 0.4	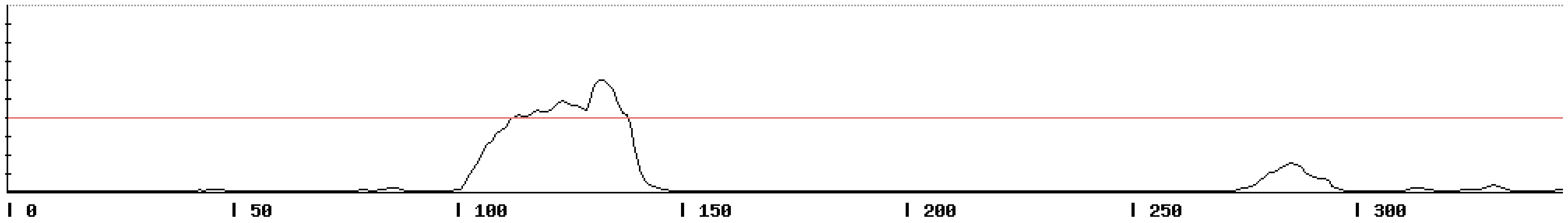

Predictions for Arabidopsisthaliana_AT1G60300.1

Viterbi Path	
Viterbi Path	
Posterior @ 0.4	
Posterior @ 0.4	
Posterior Graphic. Threshold Marked @ 0.4	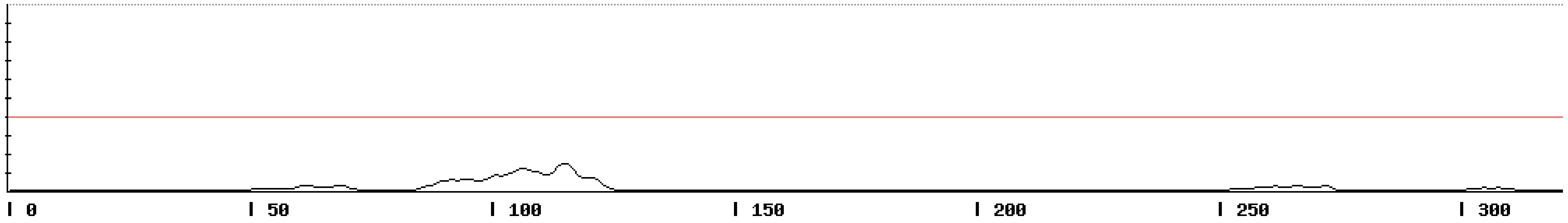

Predictions for Arabidopsisthaliana_AT1G60340.1

Viterbi Path	
Viterbi Path	
Posterior @ 0.4	
Posterior @ 0.4	
Posterior Graphic. Threshold Marked @ 0.4	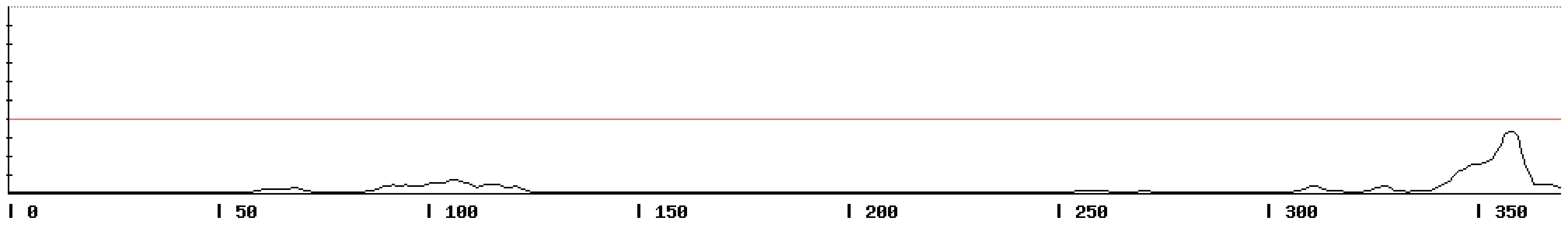

Predictions for Arabidopsisthaliana_AT1G60350.1

Viterbi Path	
Viterbi Path	
Posterior @ 0.4	113 - KRKPRGY - 119
Posterior @ 0.4	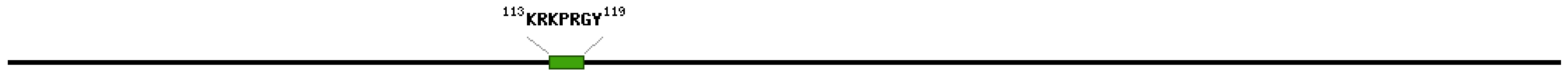
Posterior Graphic. Threshold Marked @ 0.4	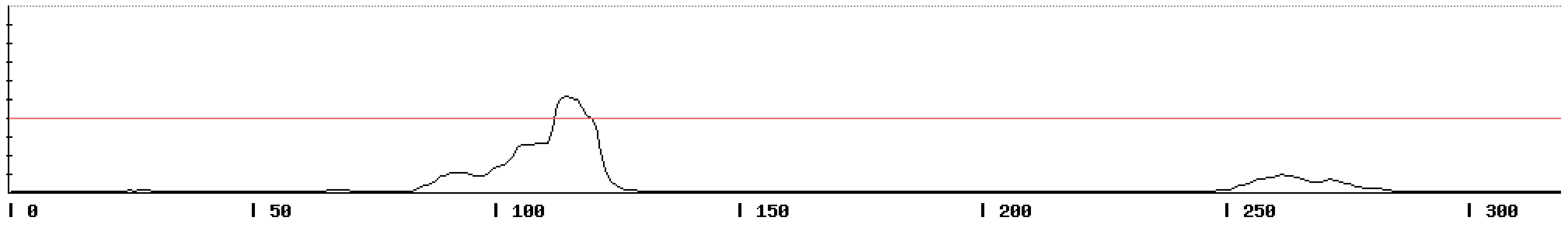

Predictions for Arabidopsisthaliana_AT1G60380.1

Viterbi Path	
Viterbi Path	
Posterior @ 0.4	
Posterior @ 0.4	
Posterior Graphic. Threshold Marked @ 0.4	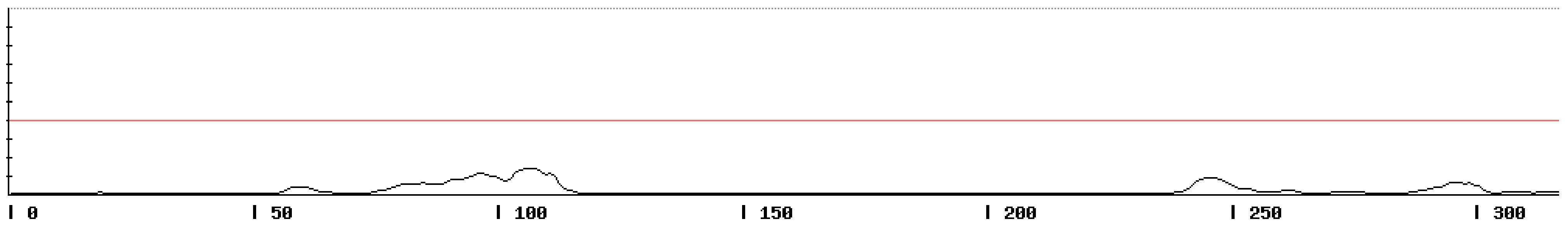

Predictions for Arabidopsisthaliana_AT1G61110.1

Viterbi Path	
Viterbi Path	
Posterior @ 0.4	
Posterior @ 0.4	
Posterior Graphic. Threshold Marked @ 0.4	

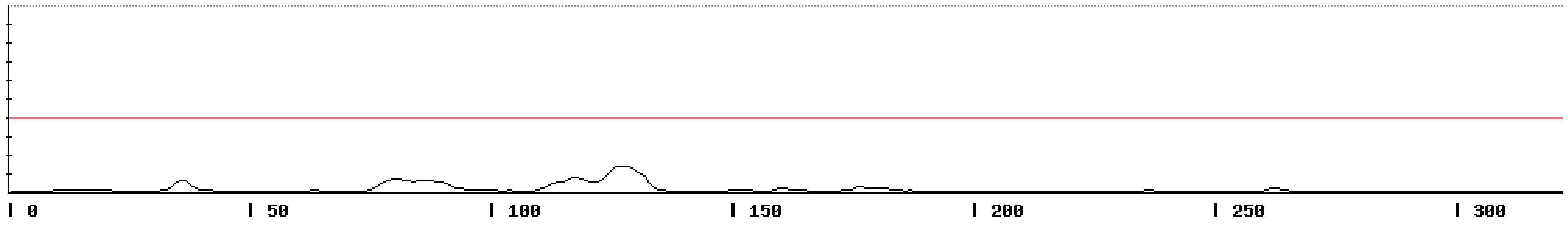

Predictions for Arabidopsisthaliana_AT1G62700.1	
Viterbi Path	
Viterbi Path	
Posterior @ 0.4	
Posterior @ 0.4	
Posterior Graphic. Threshold Marked @ 0.4	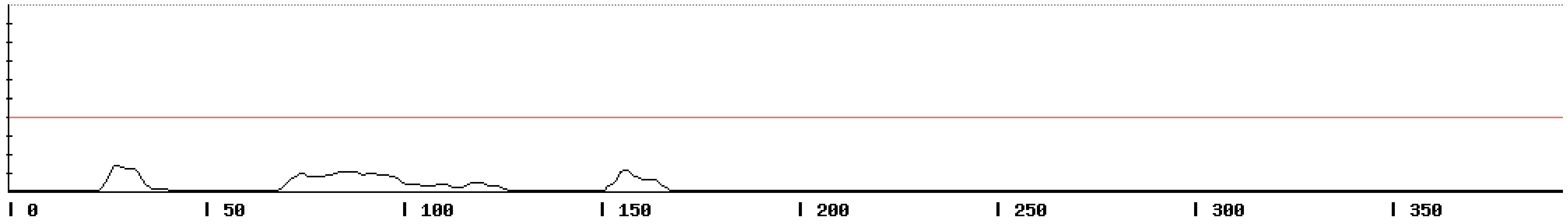

Predictions for Arabidopsisthaliana_AT1G64100.2	
Viterbi Path	
Viterbi Path	
Posterior @ 0.4	
Posterior @ 0.4	

Posterior Graphic. Threshold Marked @ 0.4

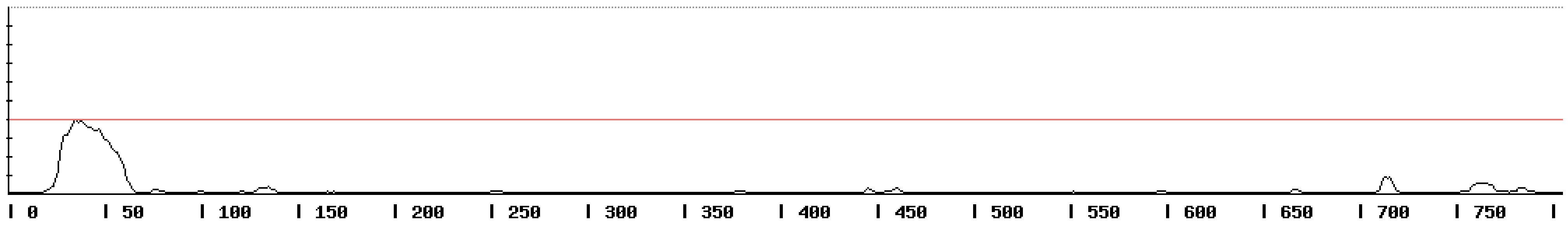

Predictions for Arabidopsisthaliana_AT1G64105.1

Viterbi Path

Viterbi Path

Posterior @ 0.4

Posterior @ 0.4

Posterior Graphic. Threshold Marked @ 0.4

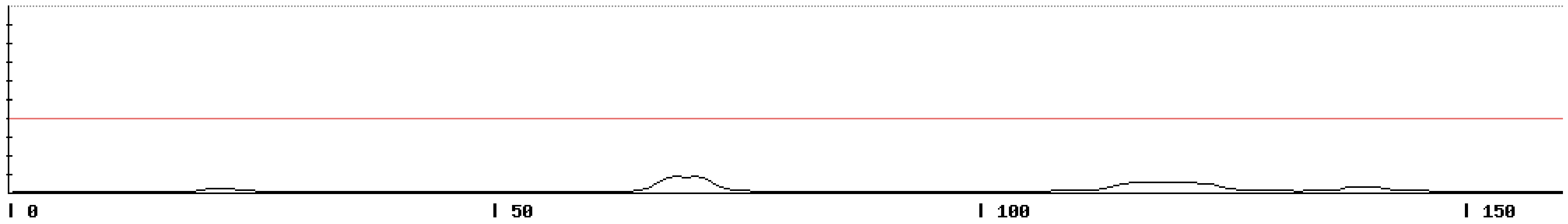

Predictions for Arabidopsisthaliana_AT1G65910.1

Viterbi Path

Viterbi Path

Posterior @ 0.4 586 - RRKIWKKKKEK - 596

Posterior @ 0.4

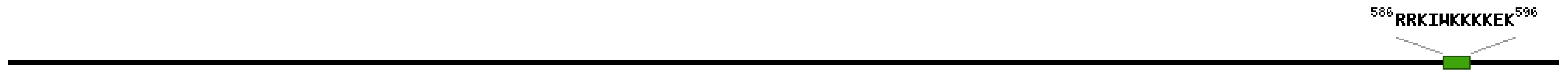

Posterior Graphic. Threshold Marked @ 0.4

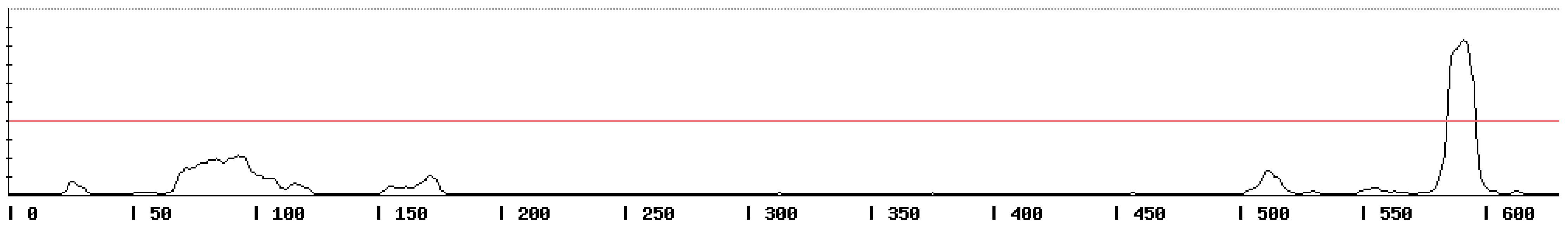

Predictions for Arabidopsisthaliana_AT1G69490.1

Viterbi Path

Viterbi Path

Posterior @ 0.4

Posterior @ 0.4

Posterior Graphic. Threshold Marked @ 0.4

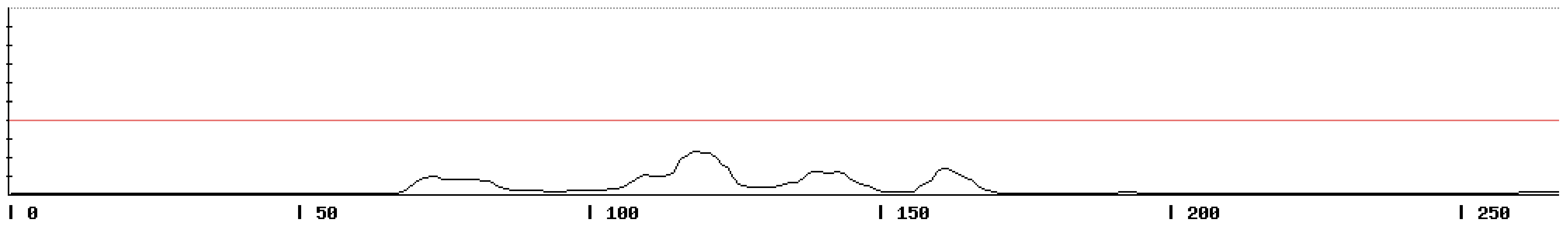

Predictions for Arabidopsisthaliana_AT1G71930.1

Viterbi Path

Viterbi Path

Posterior @ 0.4

Posterior @ 0.4

Posterior Graphic. Threshold Marked @ 0.4

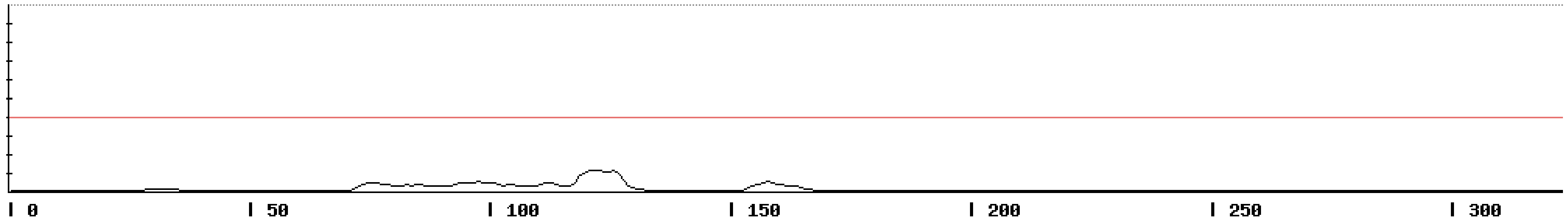

Predictions for Arabidopsisthaliana_AT1G76420.1

Viterbi Path

Viterbi Path

Posterior @ 0.4

Posterior @ 0.4

Posterior Graphic. Threshold Marked @ 0.4

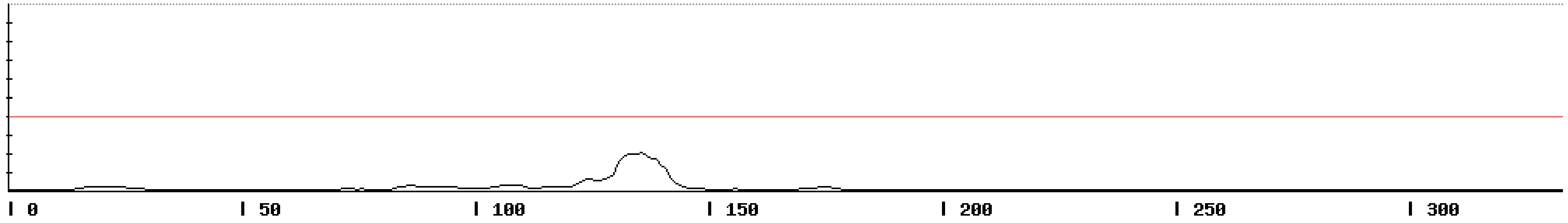

Predictions for Arabidopsisthaliana_AT1G77450.1

Viterbi Path

Viterbi Path

Posterior @ 0.4 | 98 - KPIGRPKPVGIK - 109

Posterior @ 0.4

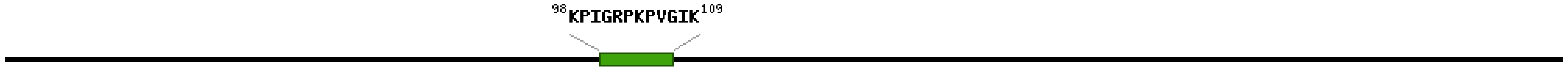

Posterior Graphic. Threshold Marked @ 0.4

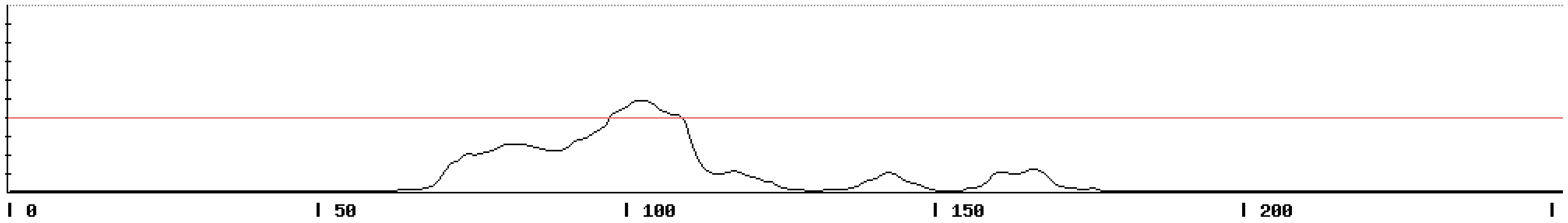

Predictions for Arabidopsisthaliana_AT1G79580.1

Viterbi Path

Viterbi Path

Posterior @ 0.4

Posterior @ 0.4

Posterior Graphic. Threshold Marked @ 0.4

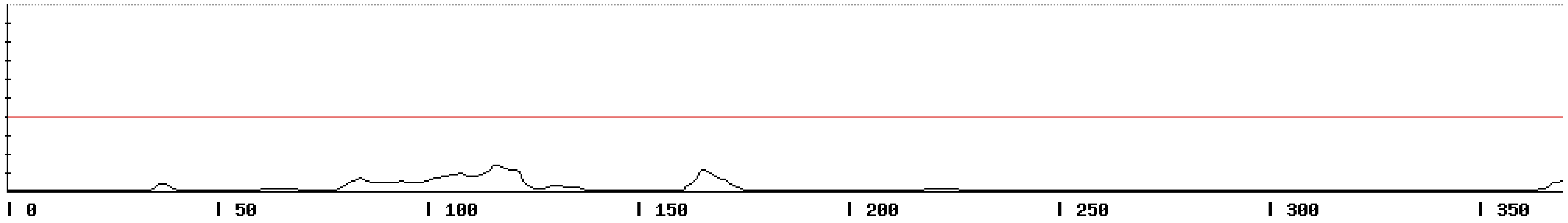

Predictions for Arabidopsisthaliana_AT2G02450.1

Viterbi Path	
Viterbi Path	
Posterior @ 0.4	
Posterior @ 0.4	
Posterior Graphic. Threshold Marked @ 0.4	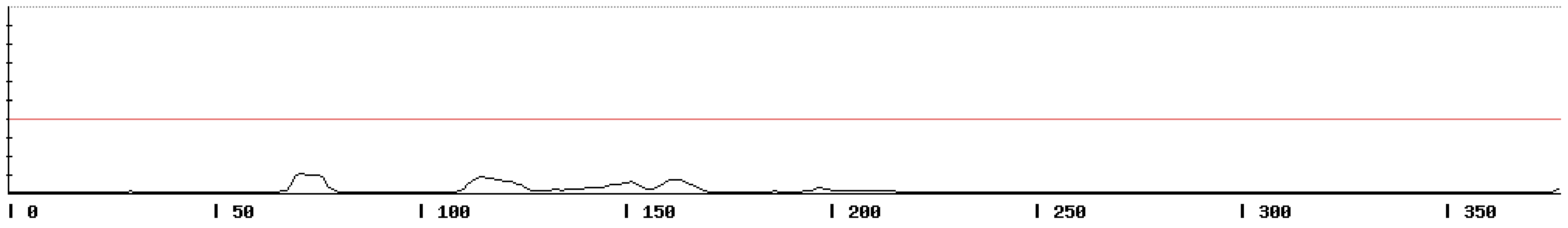

Predictions for Arabidopsisthaliana_AT2G17040.1

Viterbi Path	
Viterbi Path	
Posterior @ 0.4	
Posterior @ 0.4	
Posterior Graphic. Threshold Marked @ 0.4	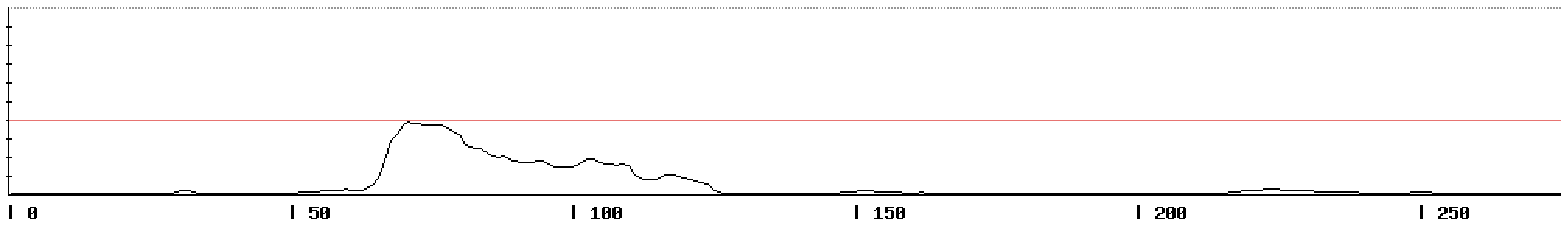

Predictions for Arabidopsisthaliana_AT2G18060.1

Viterbi Path

Viterbi Path

Posterior @ 0.4 — 277 - DRRKKKK - 283

Posterior @ 0.4

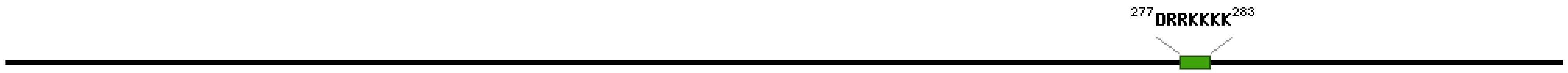

Posterior Graphic. Threshold Marked @ 0.4

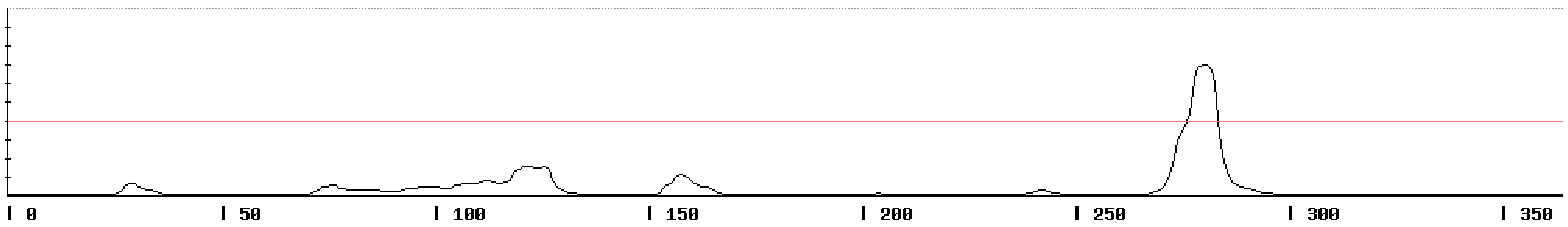

Predictions for Arabidopsisthaliana_AT2G24430.1

Viterbi Path

Viterbi Path

Posterior @ 0.4

Posterior @ 0.4

Posterior Graphic. Threshold Marked @ 0.4

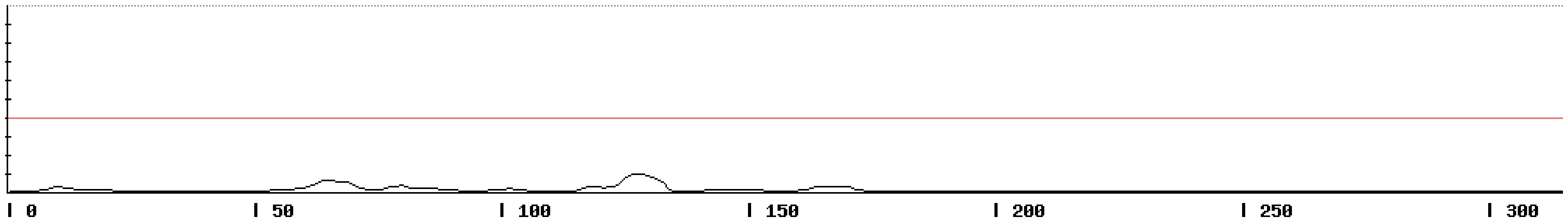

Predictions for Arabidopsisthaliana_AT2G27300.1

Viterbi Path	
Viterbi Path	
Posterior @ 0.4	
Posterior @ 0.4	
Posterior Graphic. Threshold Marked @ 0.4	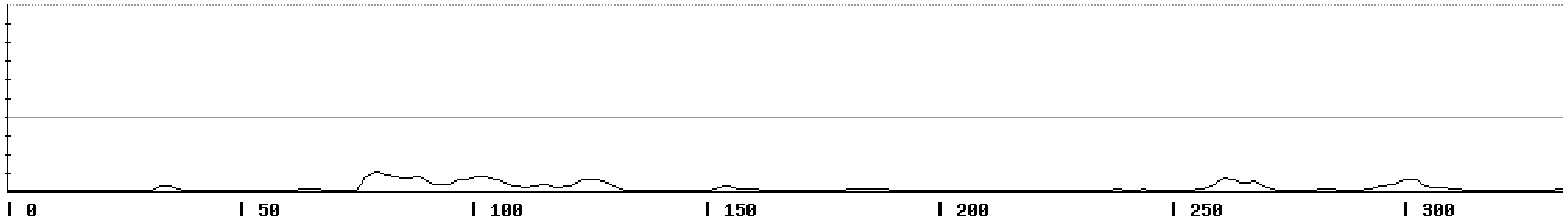

Predictions for Arabidopsisthaliana_AT2G33480.1

Viterbi Path	206 - RKDTKKKRRRRR - 217
Viterbi Path	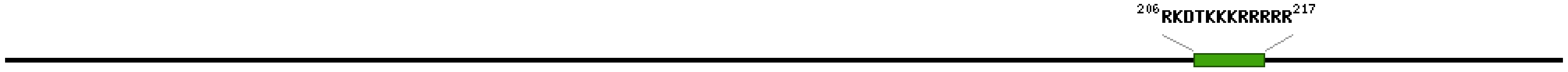
Posterior @ 0.4	206 - RKDTKKKRRRRR - 217
Posterior @ 0.4	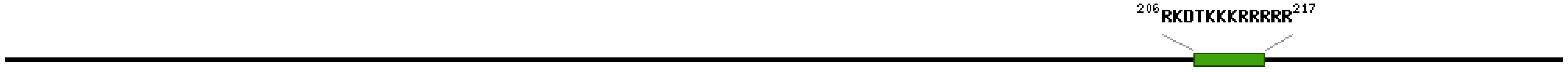

Posterior Graphic. Threshold Marked @ 0.4

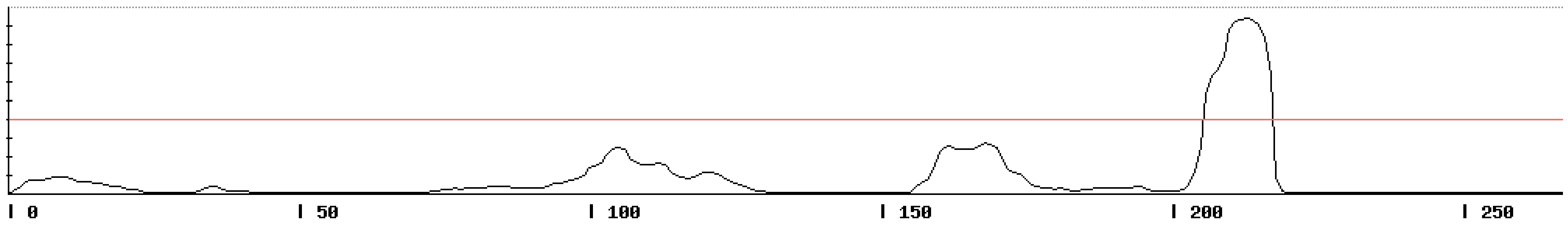

Predictions for Arabidopsisthaliana_AT2G43000.1

Viterbi Path

Viterbi Path

Posterior @ 0.4

Posterior @ 0.4

Posterior Graphic. Threshold Marked @ 0.4

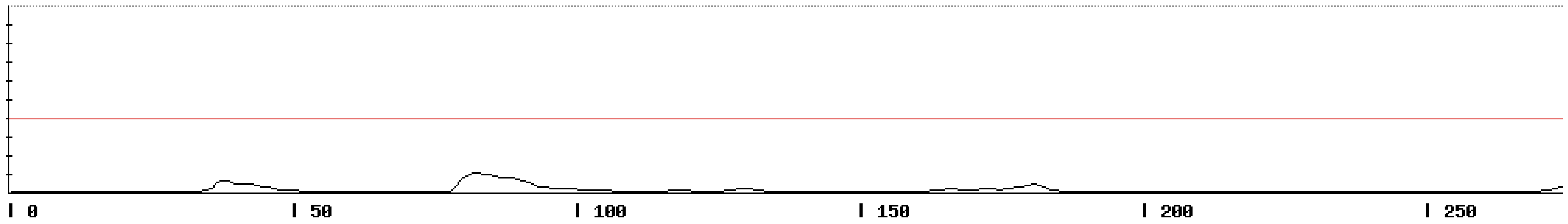

Predictions for Arabidopsisthaliana_AT2G46770.1

Viterbi Path

Viterbi Path

Posterior @ 0.4

Posterior @ 0.4

Posterior Graphic. Threshold Marked @ 0.4

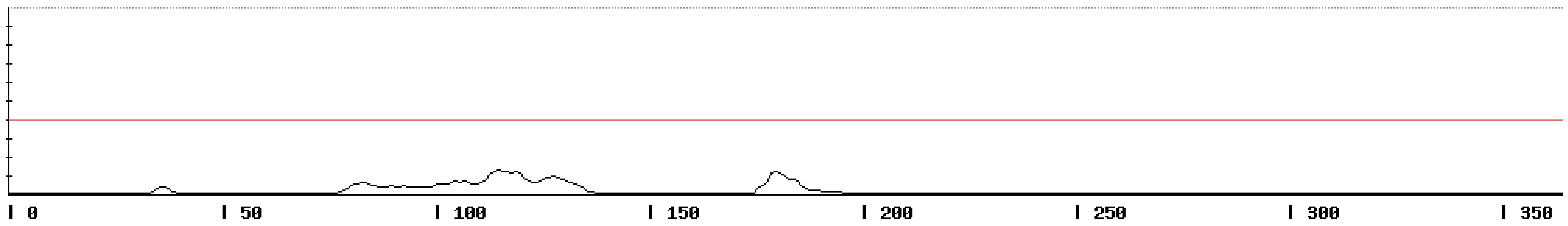

Predictions for Arabidopsisthaliana_AT3G01600.1

Viterbi Path

Viterbi Path

Posterior @ 0.4

Posterior @ 0.4

Posterior Graphic. Threshold Marked @ 0.4

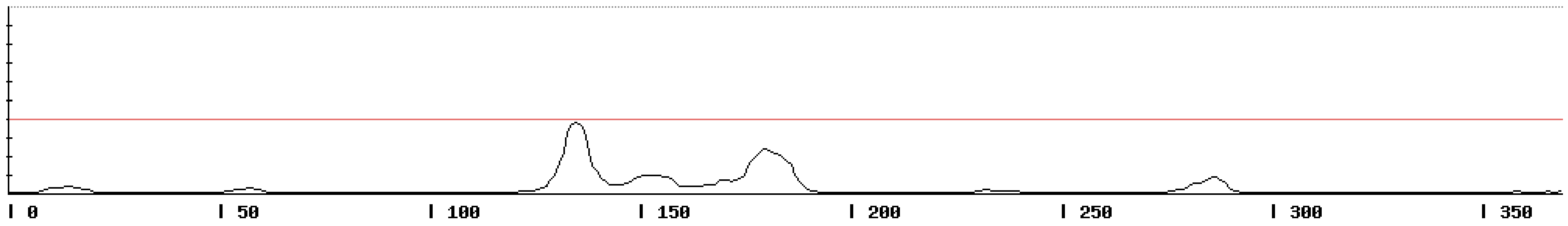

Predictions for Arabidopsisthaliana_AT3G03200.1

Viterbi Path

Viterbi Path

Posterior @ 0.4

Posterior @ 0.4

Posterior Graphic. Threshold Marked @ 0.4

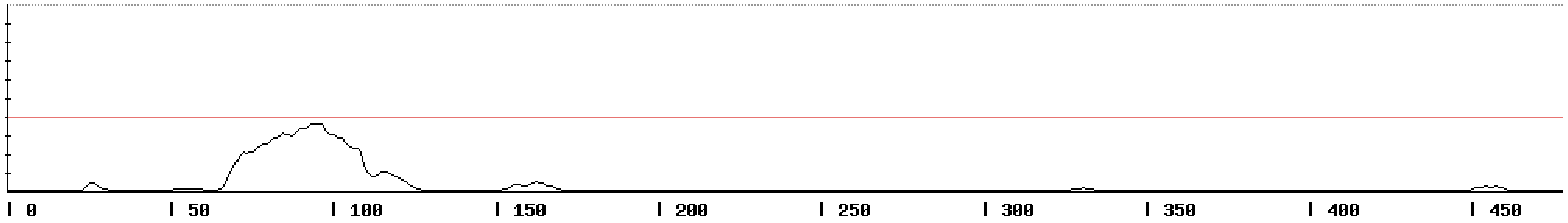

Predictions for Arabidopsisthaliana_AT3G04060.1

Viterbi Path

Viterbi Path

Posterior @ 0.4

Posterior @ 0.4

Posterior Graphic. Threshold Marked @ 0.4

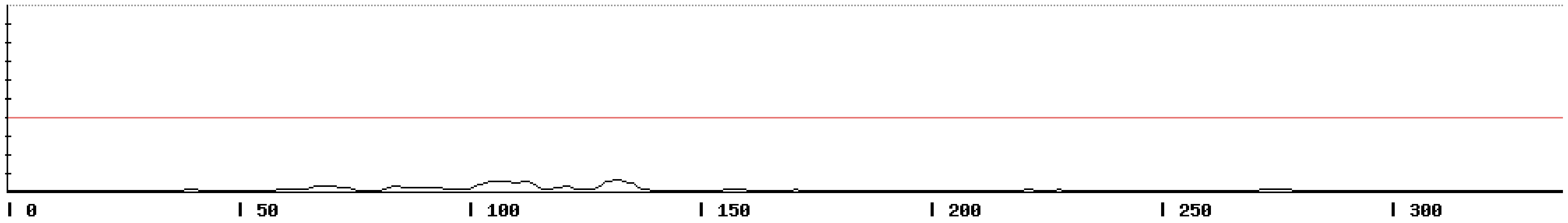

Predictions for Arabidopsisthaliana_AT3G04070.1

Viterbi Path

Viterbi Path

Posterior @ 0.4

Posterior @ 0.4

Posterior Graphic. Threshold Marked @ 0.4

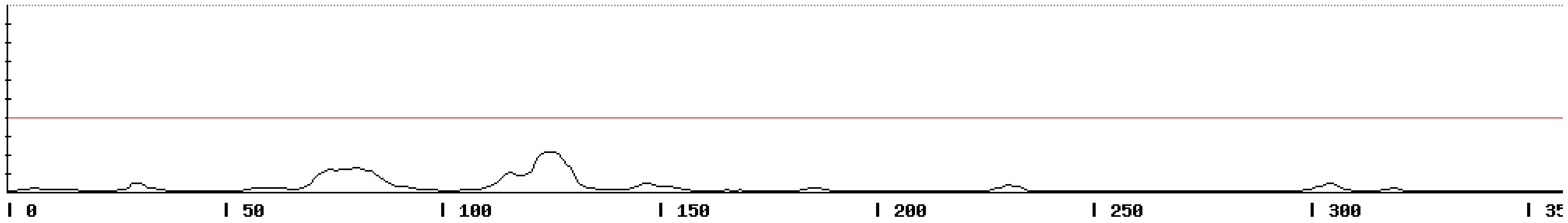

Predictions for Arabidopsisthaliana_AT3G04410.1

Viterbi Path

Viterbi Path

Posterior @ 0.4

Posterior @ 0.4

Posterior Graphic. Threshold Marked @ 0.4

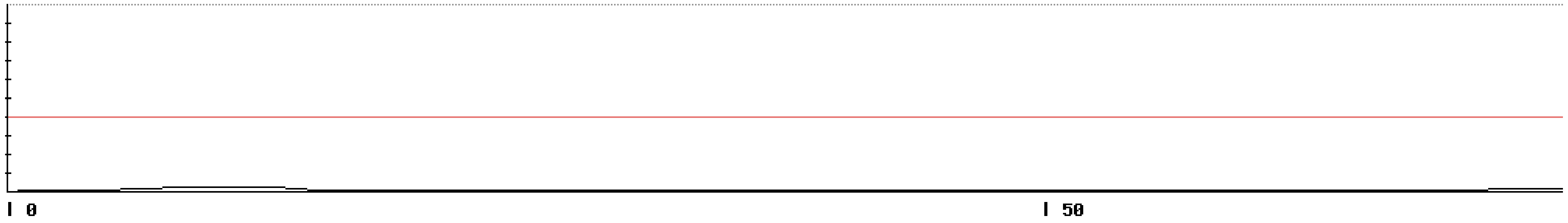

Predictions for Arabidopsisthaliana_AT3G04420.1

Viterbi Path

Viterbi Path

Posterior @ 0.4

Posterior @ 0.4

Posterior Graphic. Threshold Marked @ 0.4

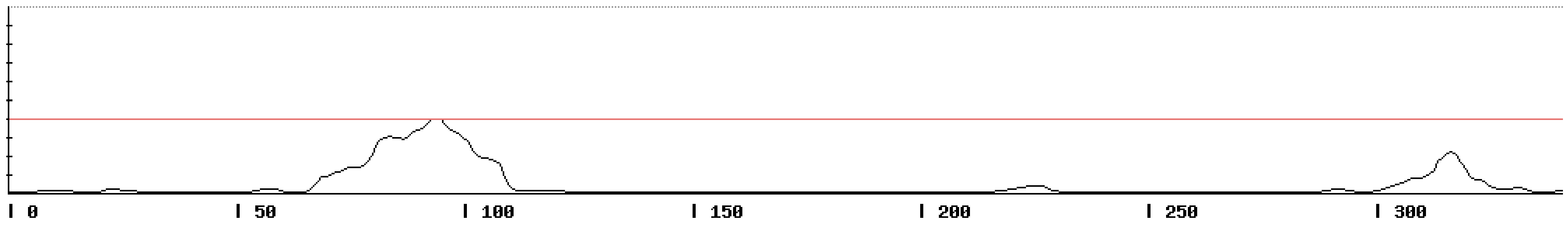

Predictions for Arabidopsisthaliana_AT3G04430.1

Viterbi Path

Viterbi Path

Posterior @ 0.4

Posterior @ 0.4

Posterior Graphic. Threshold Marked @ 0.4

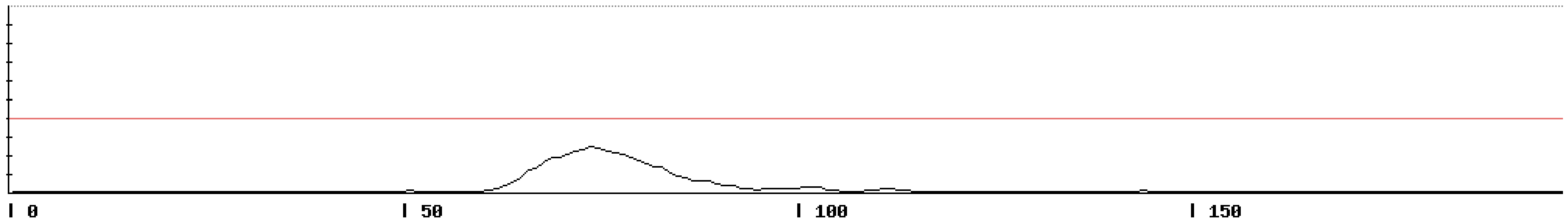

Predictions for Arabidopsisthaliana_AT3G10480.1	
Viterbi Path	
Viterbi Path	
Posterior @ 0.4	
Posterior @ 0.4	
Posterior Graphic. Threshold Marked @ 0.4	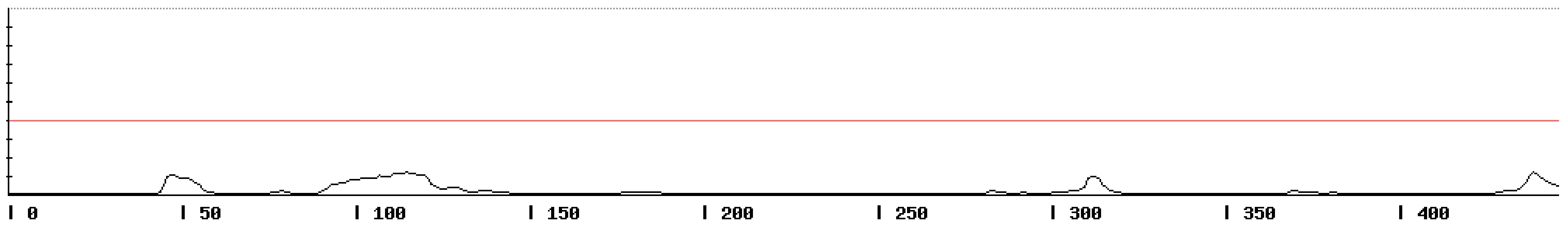

Predictions for Arabidopsisthaliana_AT3G10490.1	
Viterbi Path	
Viterbi Path	
Posterior @ 0.4	
Posterior @ 0.4	
Posterior Graphic. Threshold Marked @ 0.4	

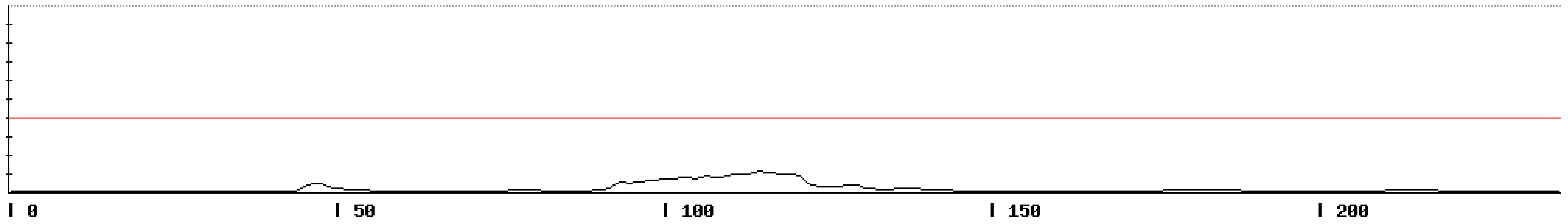

Predictions for Arabidopsisthaliana_AT3G10500.1

Viterbi Path

Viterbi Path

Posterior @ 0.4

Posterior @ 0.4

Posterior Graphic. Threshold Marked @ 0.4

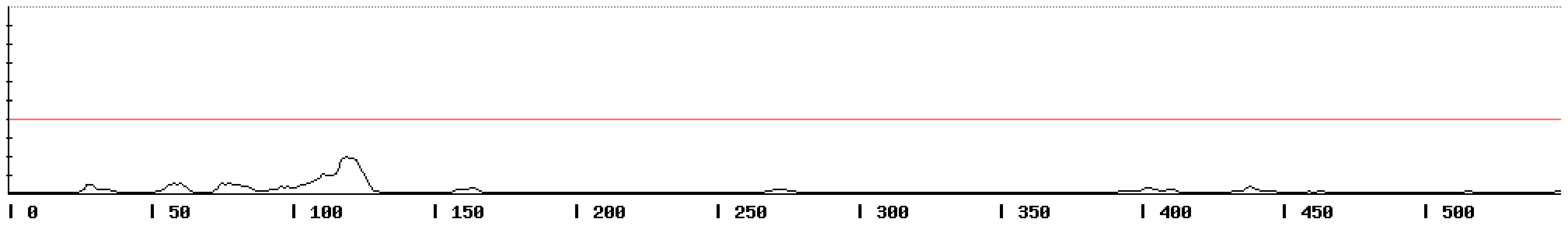

Predictions for Arabidopsisthaliana_AT3G12910.1

Viterbi Path

Viterbi Path

Posterior @ 0.4

Posterior @ 0.4

Posterior Graphic. Threshold Marked @ 0.4

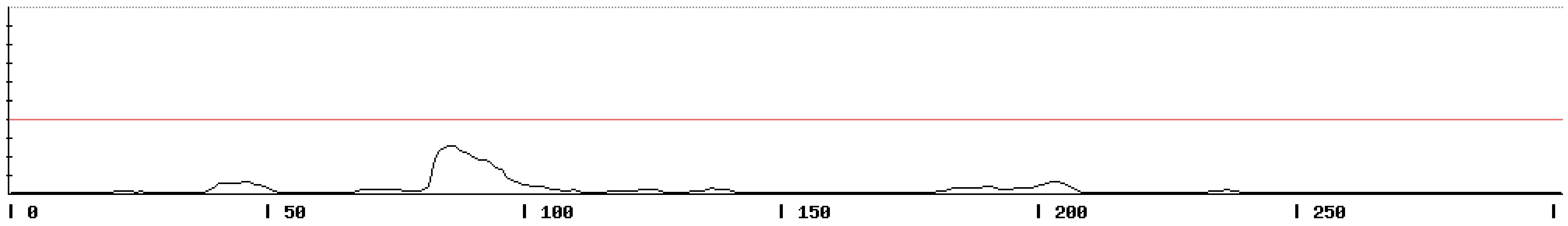

Predictions for Arabidopsisthaliana_AT3G12977.1

Viterbi Path

Viterbi Path

Posterior @ 0.4

Posterior @ 0.4

Posterior Graphic. Threshold Marked @ 0.4

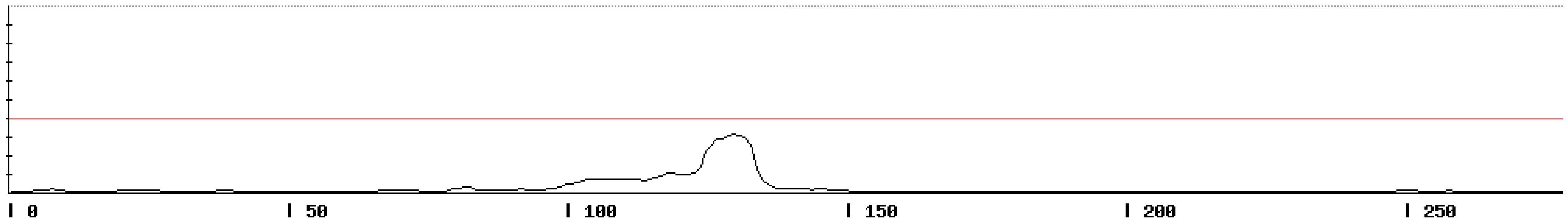

Predictions for Arabidopsisthaliana_AT3G15170.1

Viterbi Path

Viterbi Path

Posterior @ 0.4

Posterior @ 0.4

Posterior Graphic. Threshold Marked @ 0.4

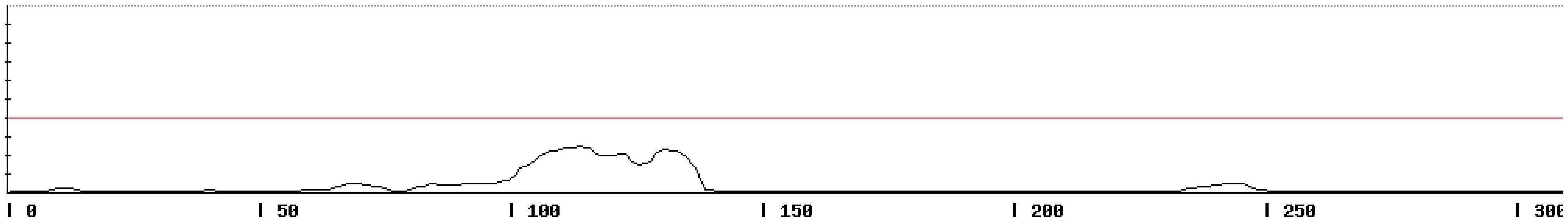

Predictions for Arabidopsisthaliana_AT3G15500.1

Viterbi Path

Viterbi Path

Posterior @ 0.4

Posterior @ 0.4

Posterior Graphic. Threshold Marked @ 0.4

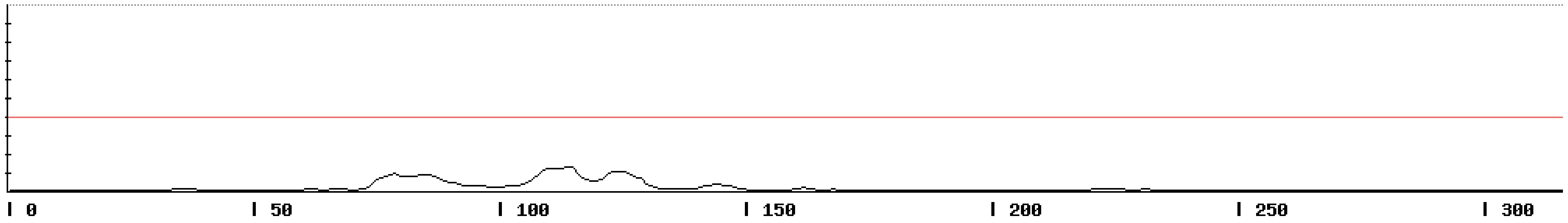

Predictions for Arabidopsisthaliana_AT3G15510.1

Viterbi Path

Viterbi Path

Posterior @ 0.4

Posterior @ 0.4

Posterior Graphic. Threshold Marked @ 0.4

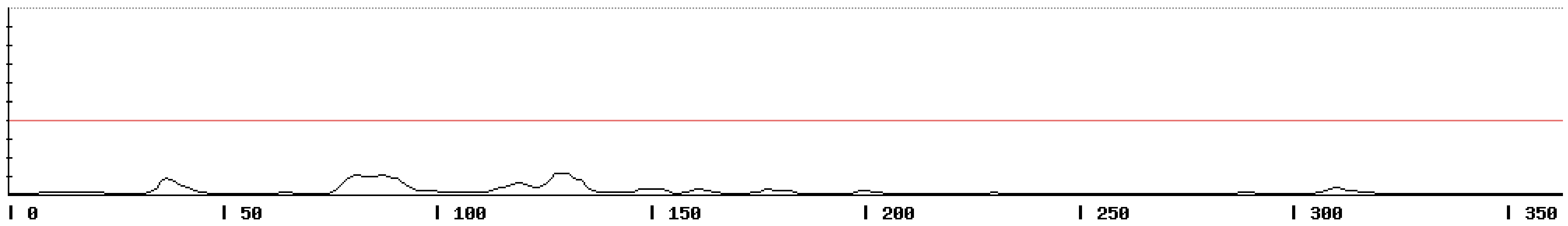

Predictions for Arabidopsisthaliana_AT3G17730.1

Viterbi Path

Viterbi Path

Posterior @ 0.4

Posterior @ 0.4

Posterior Graphic. Threshold Marked @ 0.4

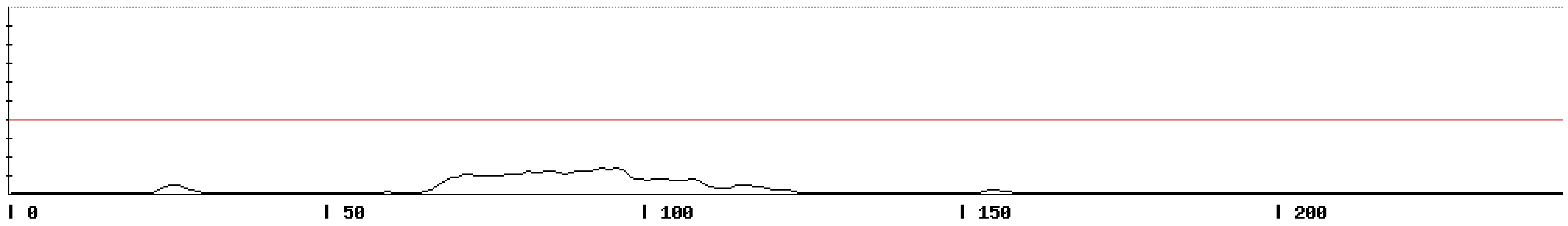

Predictions for Arabidopsisthaliana_AT3G18400.1

Viterbi Path

Viterbi Path

Posterior @ 0.4

Posterior @ 0.4

Posterior Graphic. Threshold Marked @ 0.4

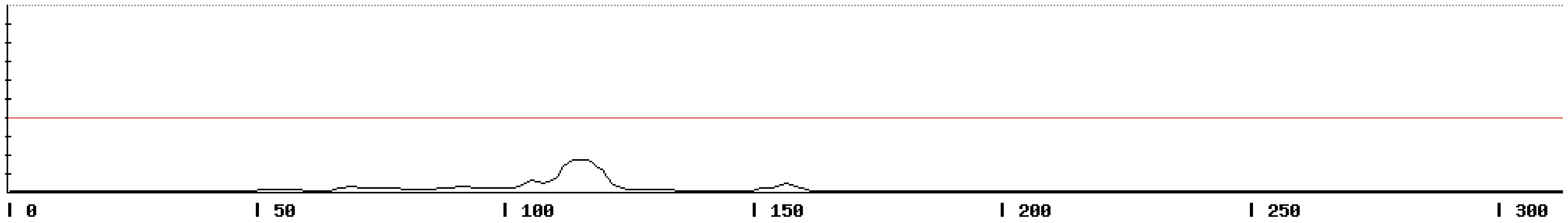

Predictions for Arabidopsisthaliana_AT3G29035.1

Viterbi Path

Viterbi Path

Posterior @ 0.4

Posterior @ 0.4

Posterior Graphic. Threshold Marked @ 0.4

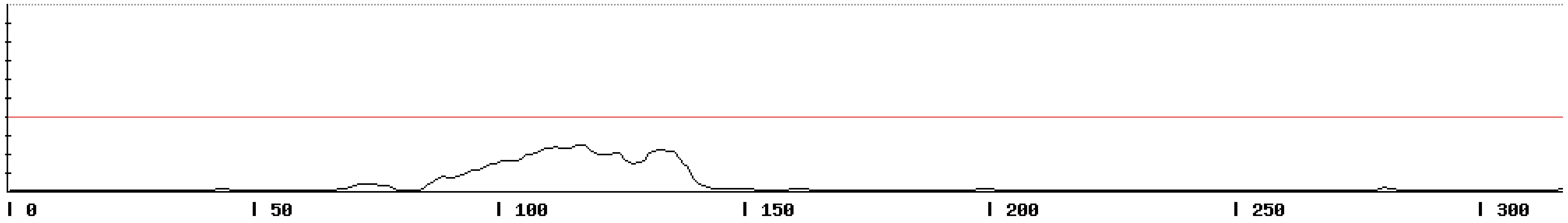

Predictions for Arabidopsisthaliana_AT3G44290.1

Viterbi Path	
Viterbi Path	
Posterior @ 0.4	
Posterior @ 0.4	
Posterior Graphic. Threshold Marked @ 0.4	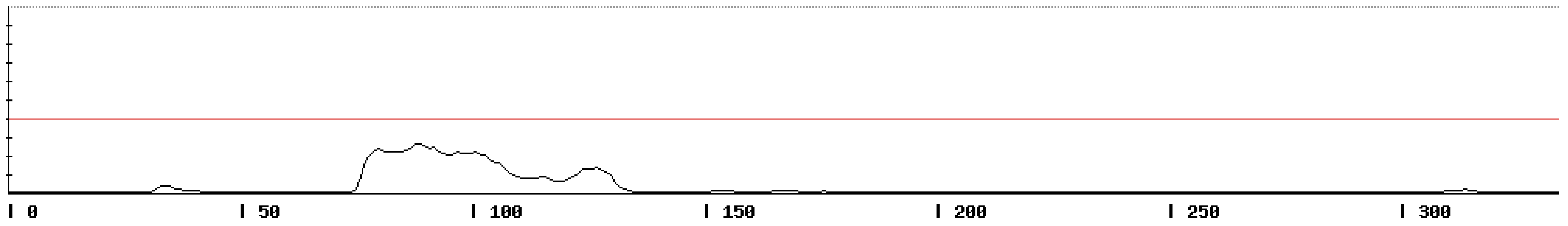

Predictions for Arabidopsisthaliana_AT3G44350.1

Viterbi Path	
Viterbi Path	
Posterior @ 0.4	
Posterior @ 0.4	
Posterior Graphic. Threshold Marked @ 0.4	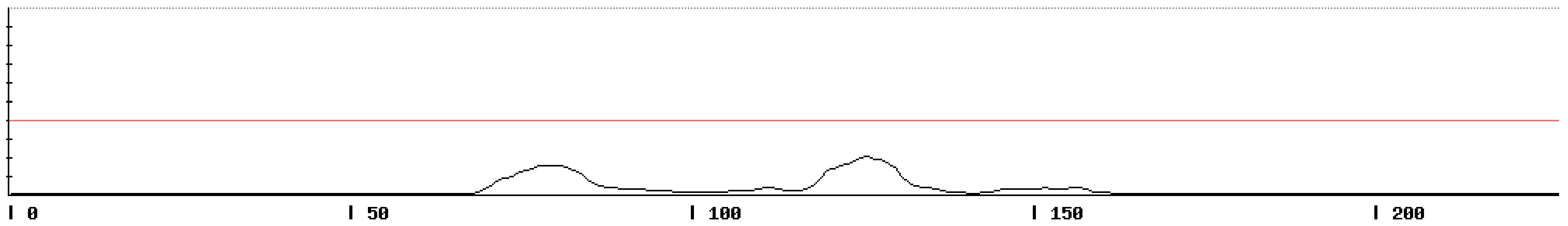

Predictions for Arabidopsisthaliana_AT3G49530.1	
Viterbi Path	
Viterbi Path	
Posterior @ 0.4	103 - RKIK - 106
Posterior @ 0.4	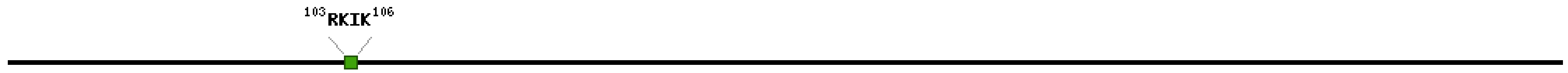
Posterior Graphic. Threshold Marked @ 0.4	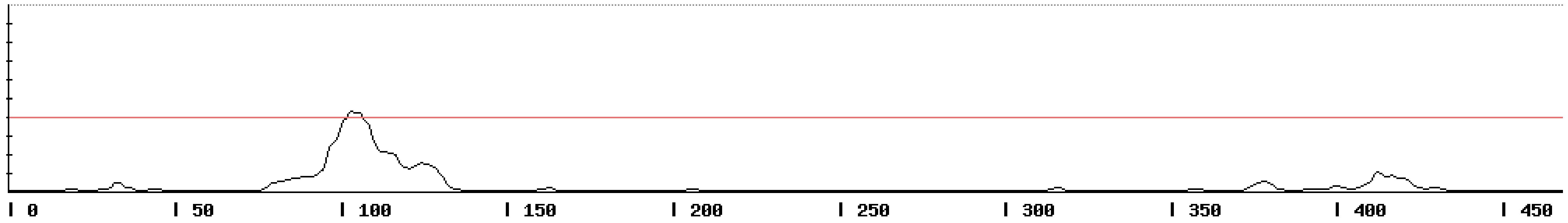

Predictions for Arabidopsisthaliana_AT3G55210.1	
Viterbi Path	
Viterbi Path	
Posterior @ 0.4	183 - KKKQKK - 188
Posterior @ 0.4	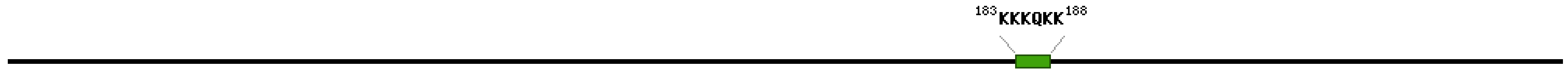
Posterior Graphic. Threshold Marked @ 0.4	

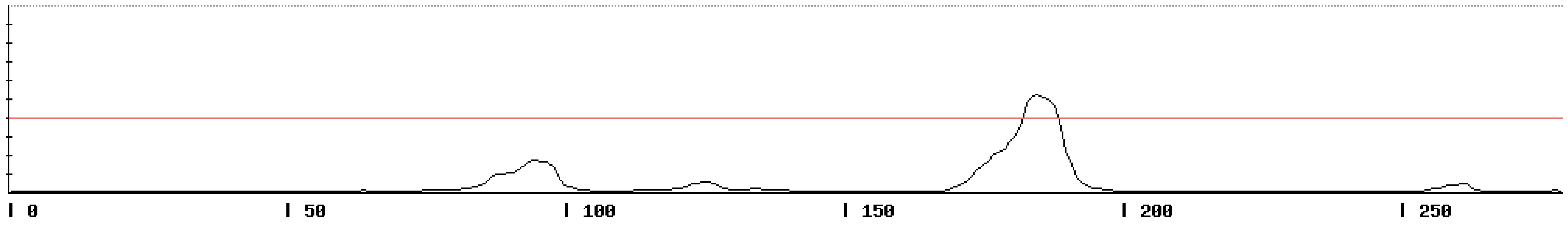

Predictions for Arabidopsisthaliana_AT3G56520.1

Viterbi Path	
Viterbi Path	
Posterior @ 0.4	
Posterior @ 0.4	
Posterior Graphic. Threshold Marked @ 0.4	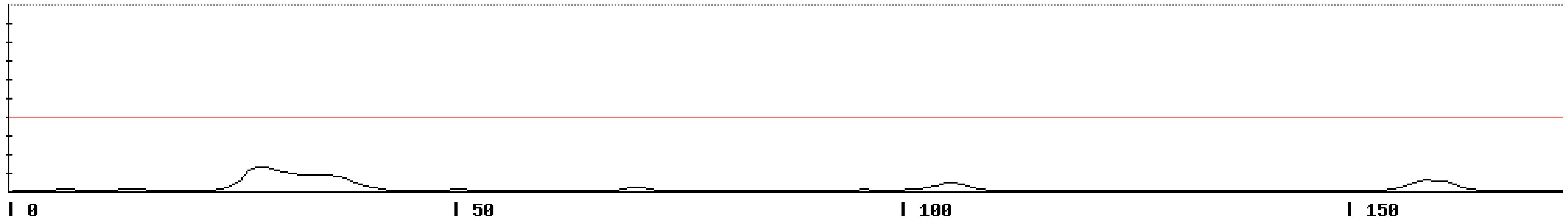

Predictions for Arabidopsisthaliana_AT3G56530.1

Viterbi Path	
Viterbi Path	
Posterior @ 0.4	8 - KRKA - 11
	206 - KKKKA - 210
Posterior @ 0.4	

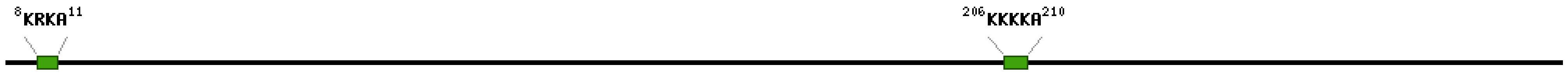

Posterior Graphic. Threshold Marked @ 0.4

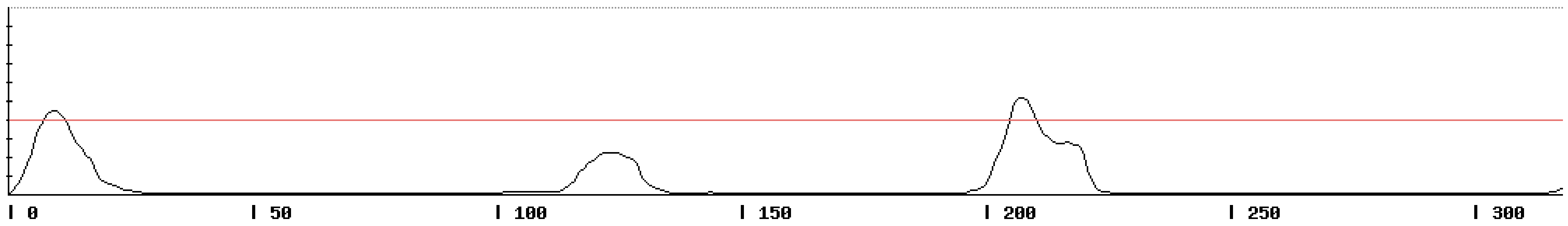

Predictions for Arabidopsisthaliana_AT3G56560.1

Viterbi Path

Viterbi Path

Posterior @ 0.4

Posterior @ 0.4

Posterior Graphic. Threshold Marked @ 0.4

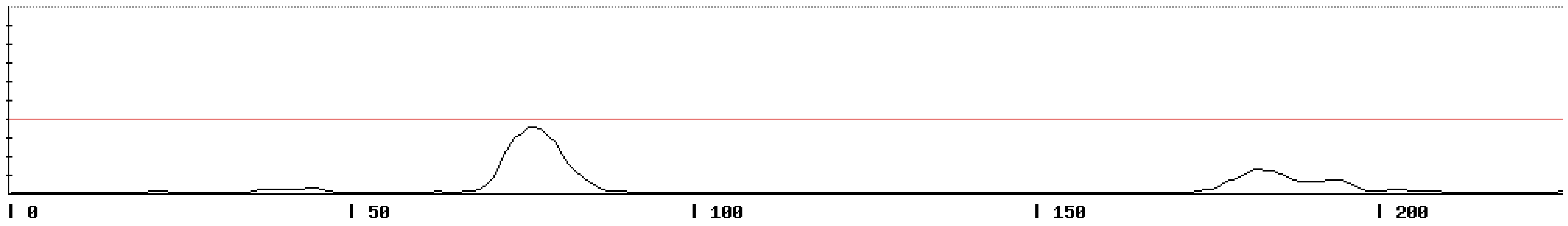

Predictions for Arabidopsisthaliana_AT3G61910.1

Viterbi Path

Viterbi Path

Posterior @ 0.4

Posterior @ 0.4

Posterior Graphic. Threshold Marked @ 0.4

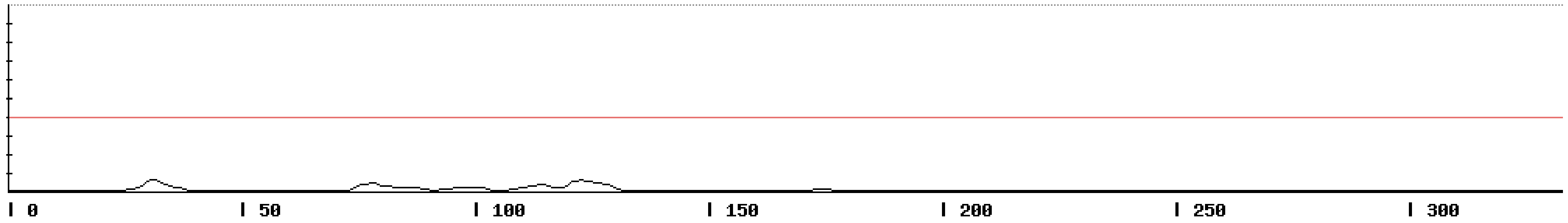

Predictions for Arabidopsisthaliana_AT4G01520.1

Viterbi Path

Viterbi Path

Posterior @ 0.4

Posterior @ 0.4

Posterior Graphic. Threshold Marked @ 0.4

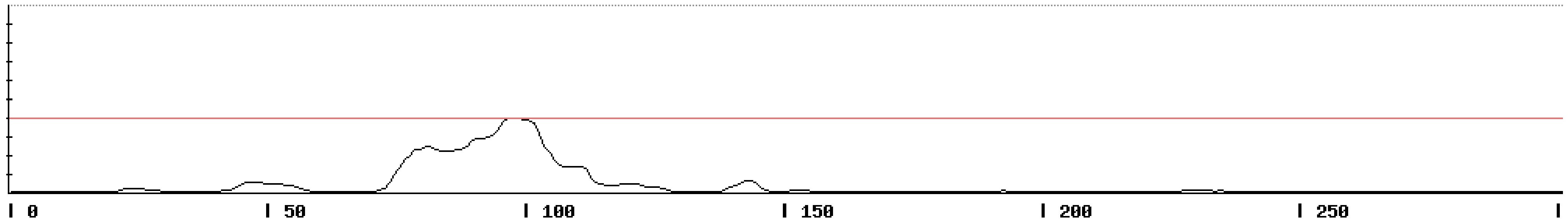

Predictions for Arabidopsisthaliana_AT4G01540.1

Viterbi Path

Viterbi Path

Posterior @ 0.4

Posterior @ 0.4

Posterior Graphic. Threshold Marked @ 0.4

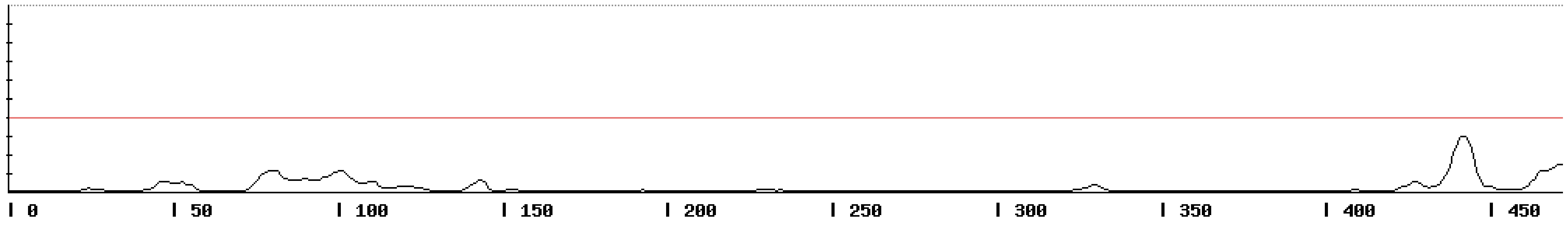

Predictions for Arabidopsisthaliana_AT4G01550.1

Viterbi Path

Viterbi Path

Posterior @ 0.4 — 94 - DRKIKDKRGNRGEI - 107

Posterior @ 0.4

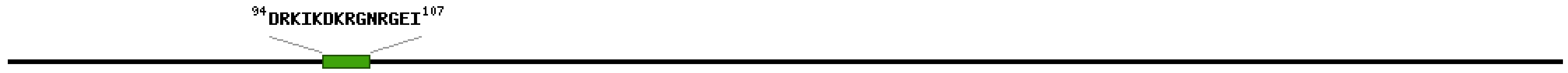

Posterior Graphic. Threshold Marked @ 0.4

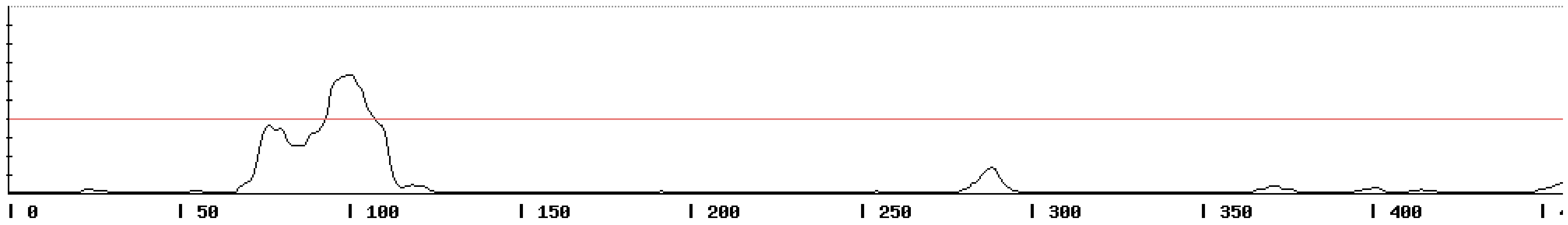

Predictions for Arabidopsisthaliana_AT4G10350.1

Viterbi Path

Viterbi Path

Posterior @ 0.4

Posterior @ 0.4

Posterior Graphic. Threshold Marked @ 0.4

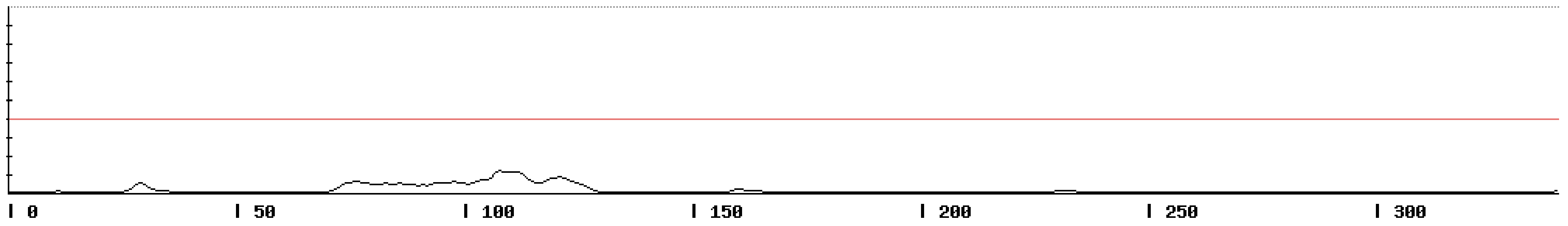

Predictions for Arabidopsisthaliana_AT4G17980.1

Viterbi Path

Viterbi Path

Posterior @ 0.4

Posterior @ 0.4

Posterior Graphic. Threshold Marked @ 0.4

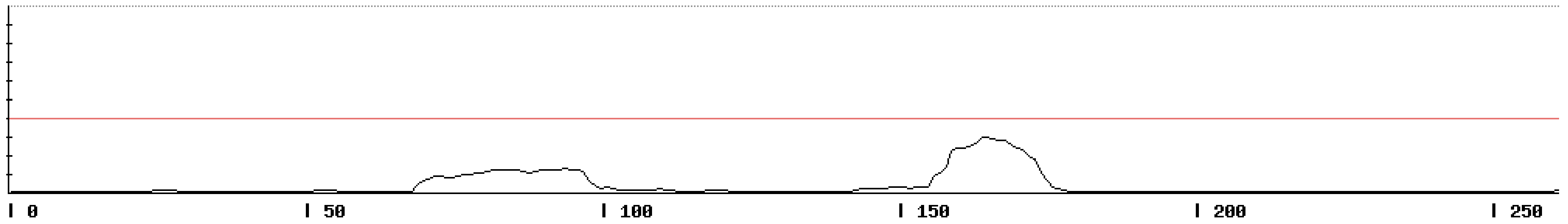

Predictions for Arabidopsisthaliana_AT4G27410.2	
Viterbi Path	
Viterbi Path	
Posterior @ 0.4	
Posterior @ 0.4	
Posterior Graphic. Threshold Marked @ 0.4	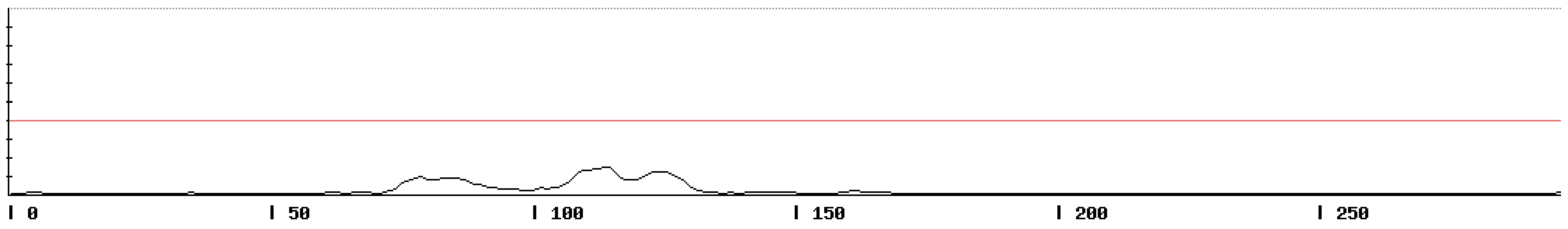

Predictions for Arabidopsisthaliana_AT4G28500.1	
Viterbi Path	
Viterbi Path	
Posterior @ 0.4	137 - RKRRK - 141
Posterior @ 0.4	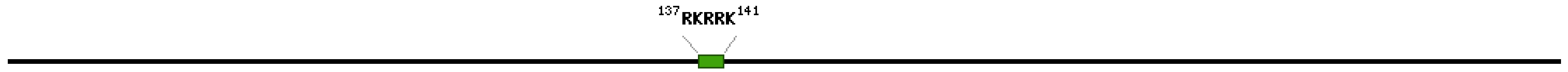
Posterior Graphic. Threshold Marked @ 0.4	

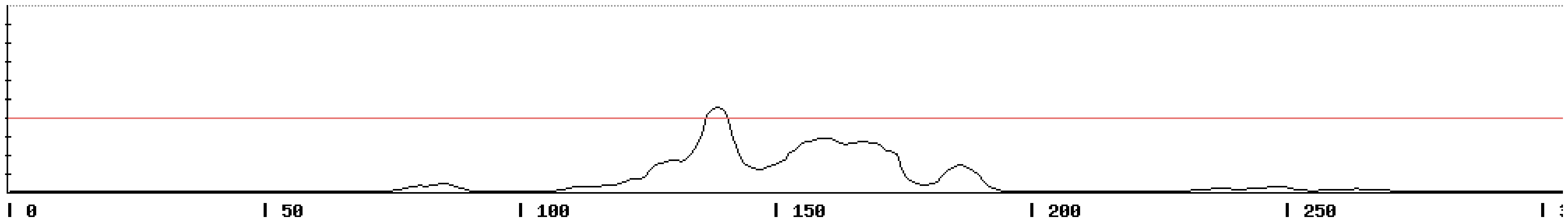

Predictions for Arabidopsisthaliana_AT4G28530.1

Viterbi Path

Viterbi Path

Posterior @ 0.4

Posterior @ 0.4

Posterior Graphic. Threshold Marked @ 0.4

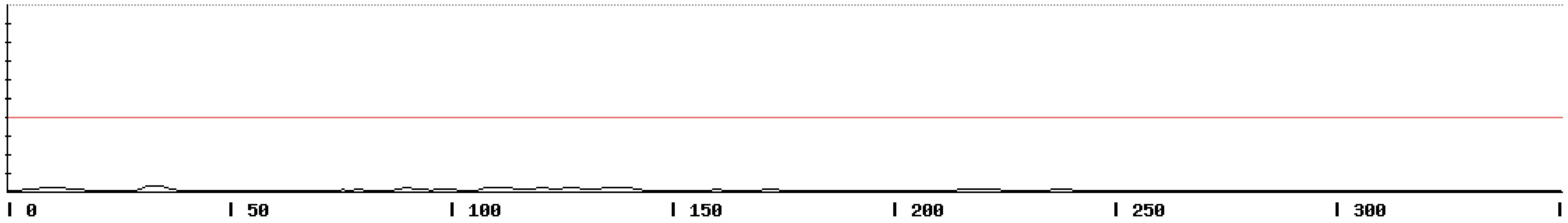

Predictions for Arabidopsisthaliana_AT4G29230.1

Viterbi Path

Viterbi Path

Posterior @ 0.4

Posterior @ 0.4

Posterior Graphic. Threshold Marked @ 0.4

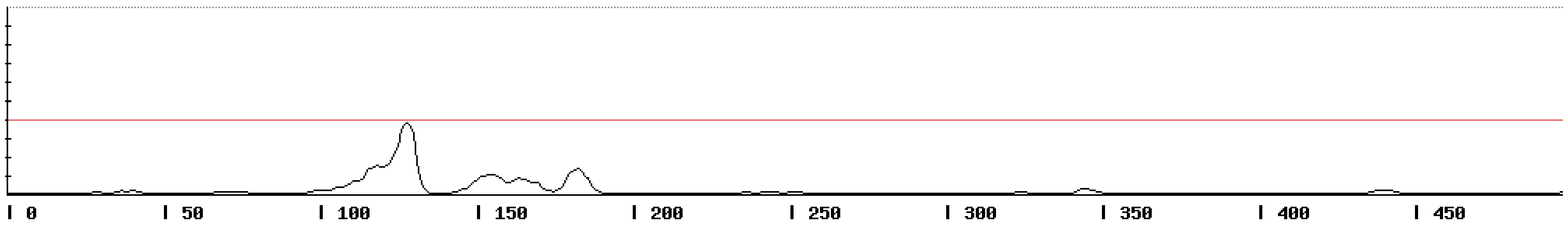

Predictions for Arabidopsisthaliana_AT4G35580.1

Viterbi Path

Viterbi Path

Posterior @ 0.4

101 - IKSKK - 105

479 - RKRRGGKRWK - 488

Posterior @ 0.4

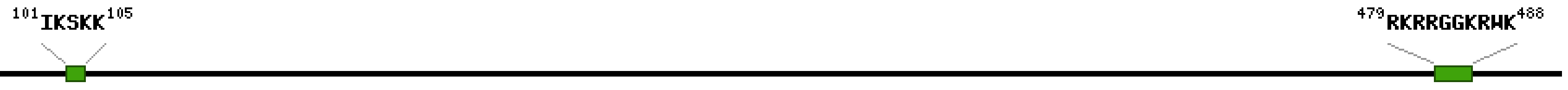

Posterior Graphic. Threshold Marked @ 0.4

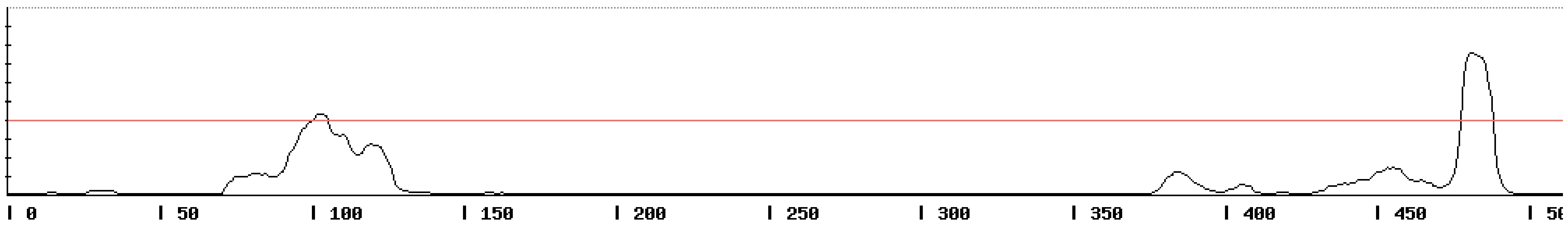

Predictions for Arabidopsisthaliana_AT4G36160.1

Viterbi Path

Viterbi Path

Posterior @ 0.4

281 - KDKKKKK - 287

Posterior @ 0.4

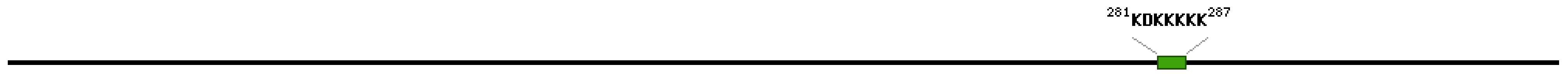

Posterior Graphic. Threshold Marked @ 0.4

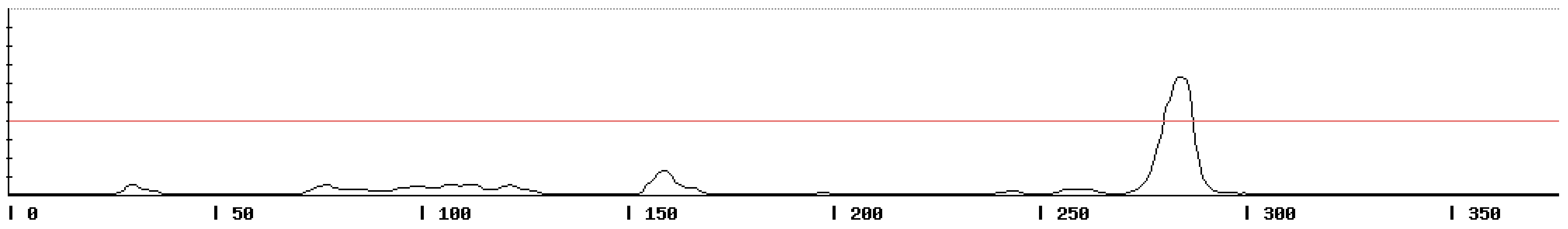

Predictions for Arabidopsisthaliana_AT5G04400.1

Viterbi Path

Viterbi Path

Posterior @ 0.4

Posterior @ 0.4

Posterior Graphic. Threshold Marked @ 0.4

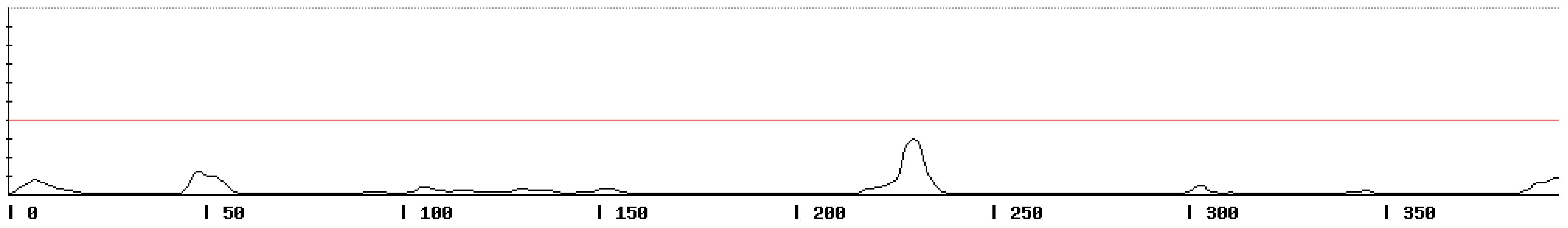

Predictions for Arabidopsisthaliana_AT5G04410.1

Viterbi Path

Viterbi Path

Posterior @ 0.4

Posterior @ 0.4

Posterior Graphic. Threshold Marked @ 0.4

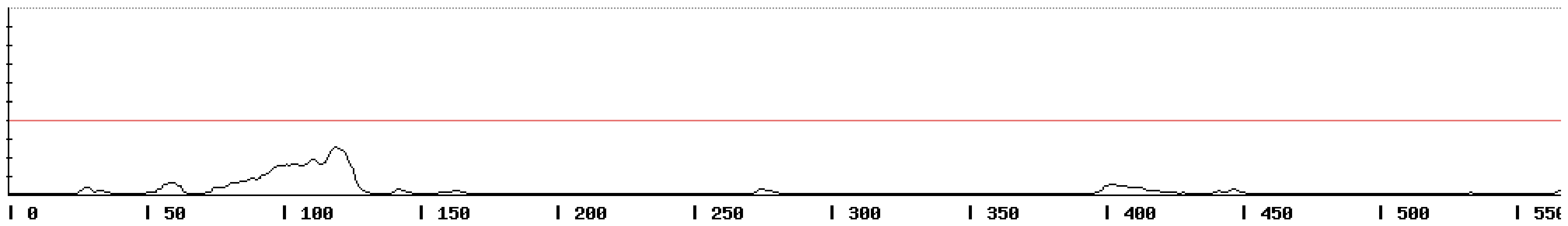

Predictions for Arabidopsisthaliana_AT5G07680.1

Viterbi Path

Viterbi Path

Posterior @ 0.4

Posterior @ 0.4

Posterior Graphic. Threshold Marked @ 0.4

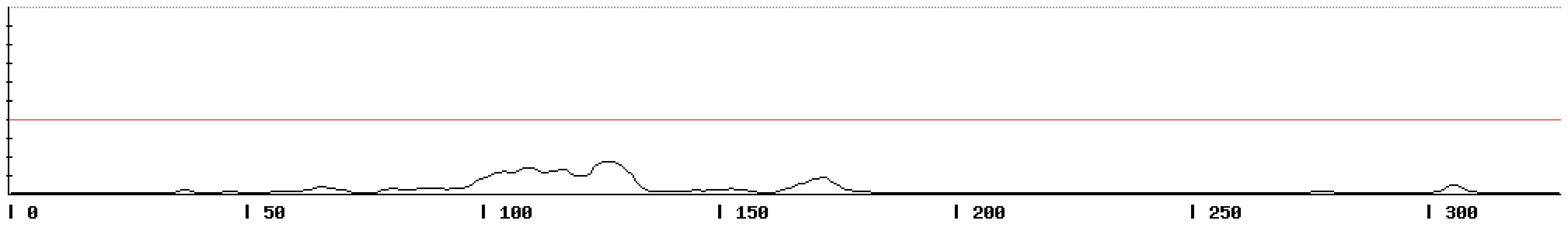

Predictions for Arabidopsisthaliana_AT5G08790.1

Viterbi Path

Viterbi Path

Posterior @ 0.4	98 - GKPK - 101
Posterior @ 0.4	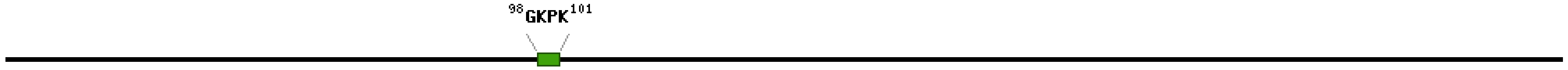
Posterior Graphic. Threshold Marked @ 0.4	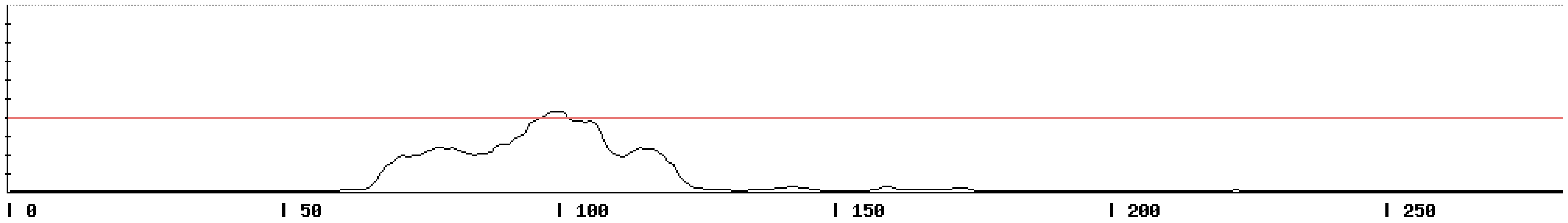

Predictions for Arabidopsisthaliana_AT5G09330.1

Viterbi Path	
Viterbi Path	
Posterior @ 0.4	
Posterior @ 0.4	
Posterior Graphic. Threshold Marked @ 0.4	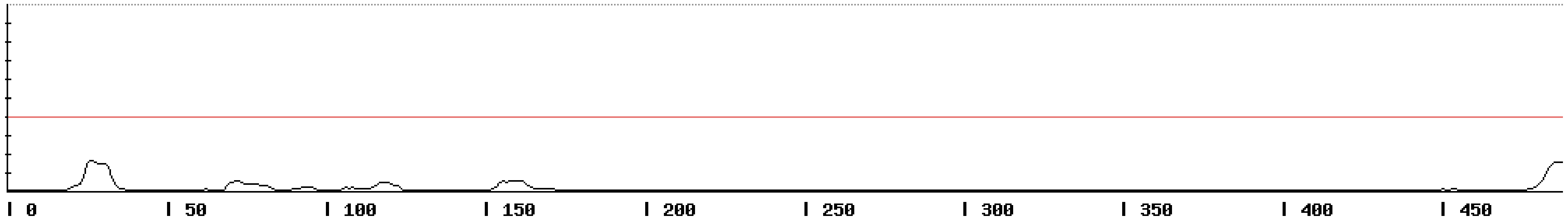

Predictions for Arabidopsisthaliana_AT5G13180.1

Viterbi Path	
Viterbi Path	
Posterior @ 0.4	
Posterior @ 0.4	
Posterior Graphic. Threshold Marked @ 0.4	

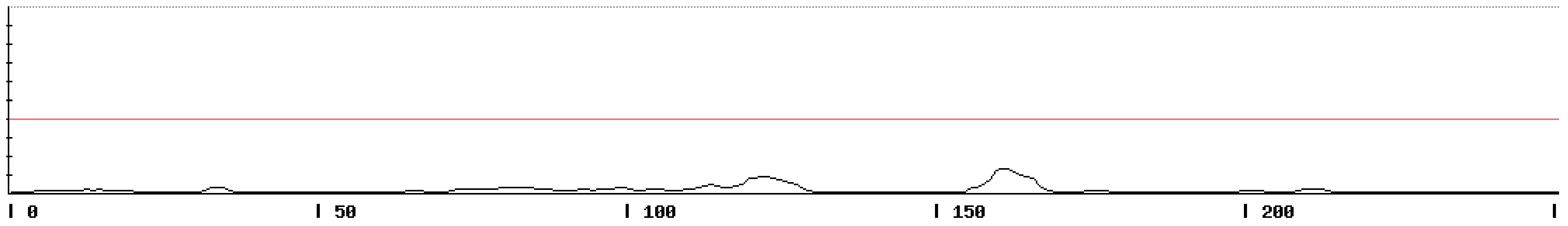

Predictions for Arabidopsisthaliana_AT5G14000.1

Viterbi Path	
Viterbi Path	
Posterior @ 0.4	160 - RKGSKGRKQRKSR - 172
Posterior @ 0.4	
Posterior Graphic. Threshold Marked @ 0.4	

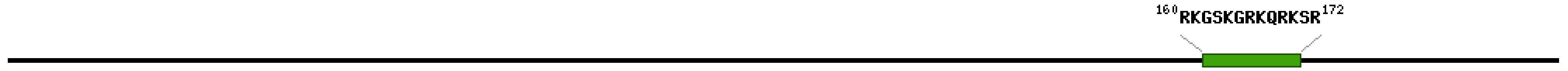

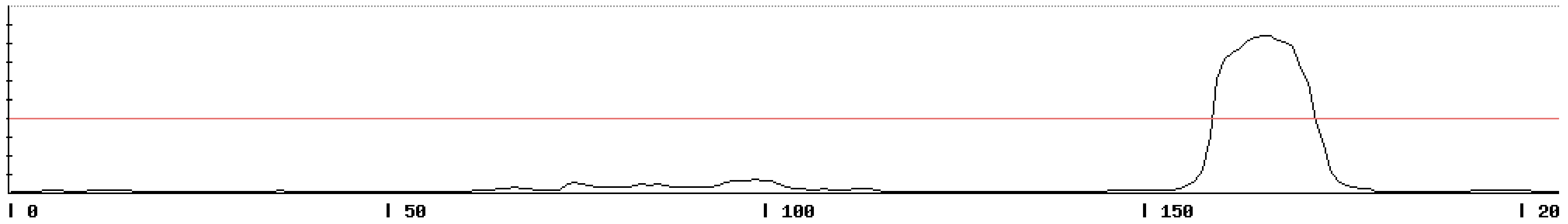

Predictions for Arabidopsisthaliana_AT5G14490.1	
Viterbi Path	
Viterbi Path	
Posterior @ 0.4	
Posterior @ 0.4	
Posterior Graphic. Threshold Marked @ 0.4	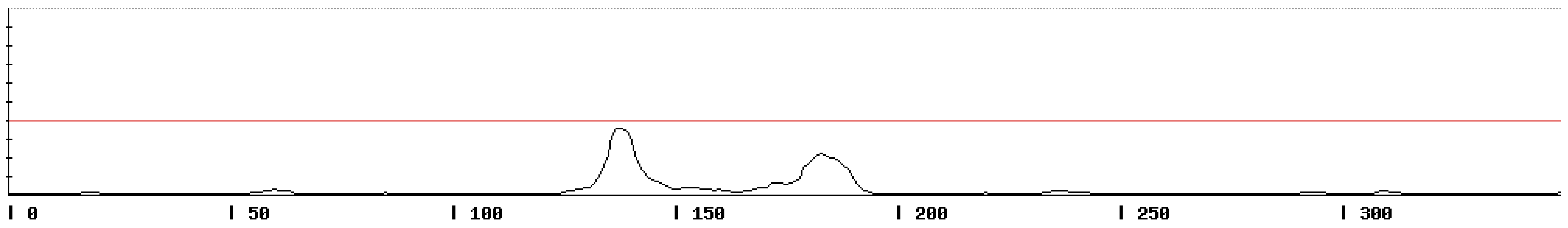

Predictions for Arabidopsisthaliana_AT5G17260.1	
Viterbi Path	
Viterbi Path	
Posterior @ 0.4	
Posterior @ 0.4	
Posterior Graphic. Threshold Marked @ 0.4	

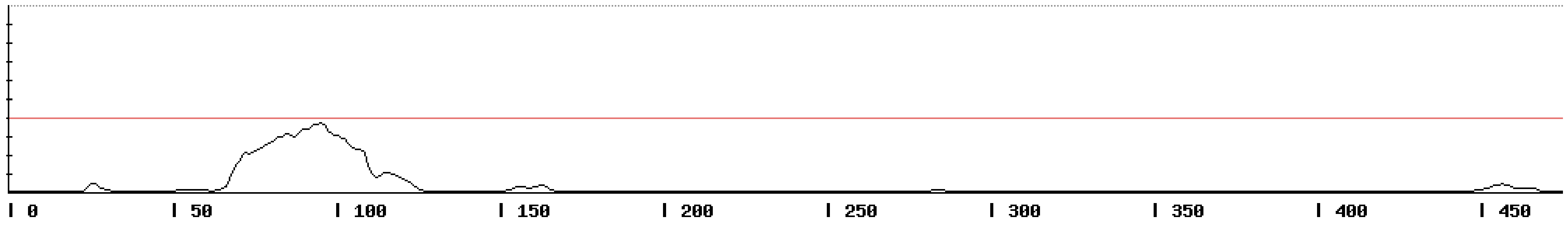

Predictions for Arabidopsisthaliana_AT5G18037.1

Viterbi Path

Viterbi Path

Posterior @ 0.4

Posterior @ 0.4

Posterior Graphic. Threshold Marked @ 0.4

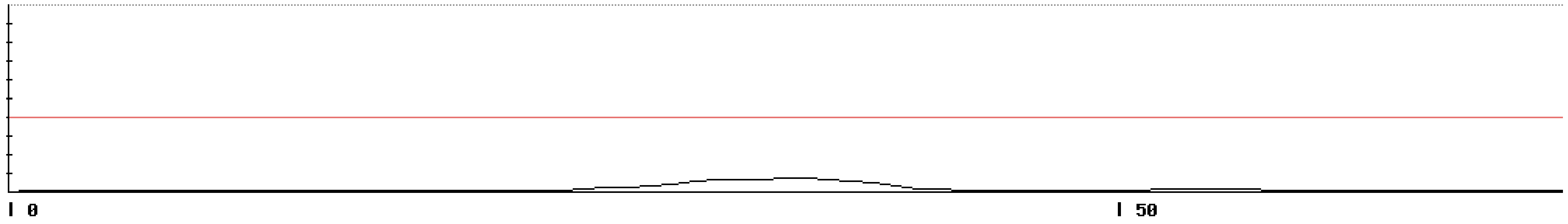

Predictions for Arabidopsisthaliana_AT5G18270.1

Viterbi Path

Viterbi Path

Posterior @ 0.4

Posterior @ 0.4

Posterior Graphic. Threshold Marked @ 0.4

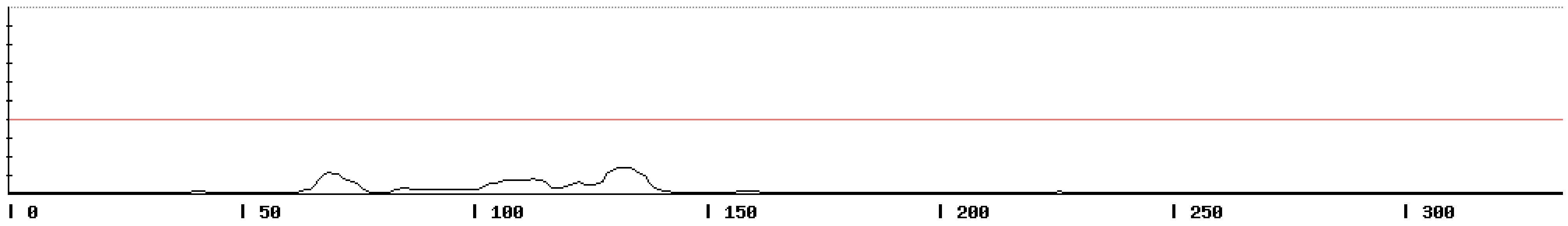

Predictions for Arabidopsisthaliana_AT5G18300.1

Viterbi Path

Viterbi Path

Posterior @ 0.4

Posterior @ 0.4

Posterior Graphic. Threshold Marked @ 0.4

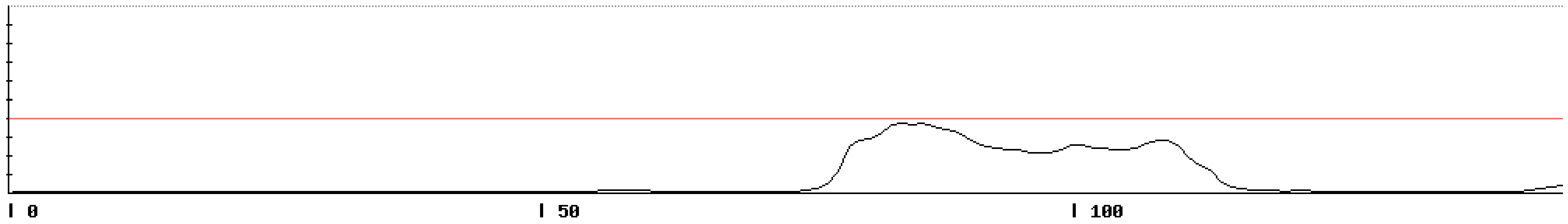

Predictions for Arabidopsisthaliana_AT5G22290.1

Viterbi Path

Viterbi Path

Posterior @ 0.4

Posterior @ 0.4

Posterior Graphic. Threshold Marked @ 0.4

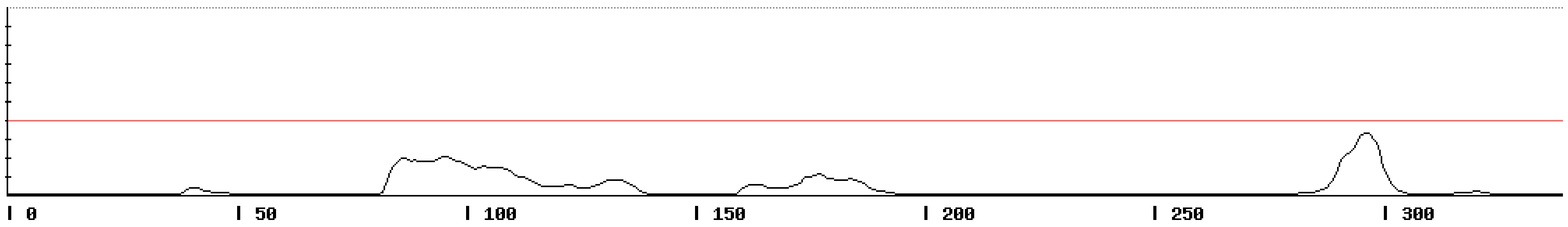

Predictions for Arabidopsisthaliana_AT5G22380.1

Viterbi Path

Viterbi Path

Posterior @ 0.4

Posterior @ 0.4

Posterior Graphic. Threshold Marked @ 0.4

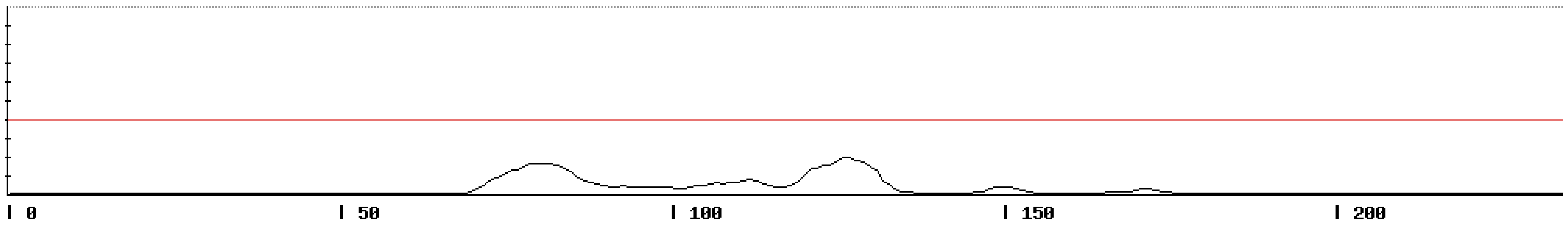

Predictions for Arabidopsisthaliana_AT5G24590.2

Viterbi Path

Viterbi Path

Posterior @ 0.4	99 - TGKDRKIKSGKTKII - 113
	341 - GIKIRAR - 347

Posterior @ 0.4

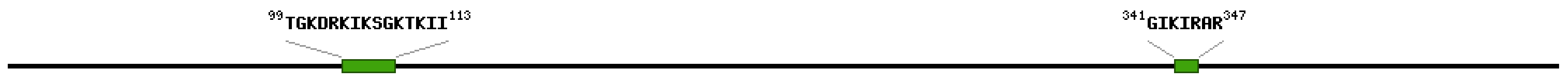

Posterior Graphic. Threshold Marked @ 0.4

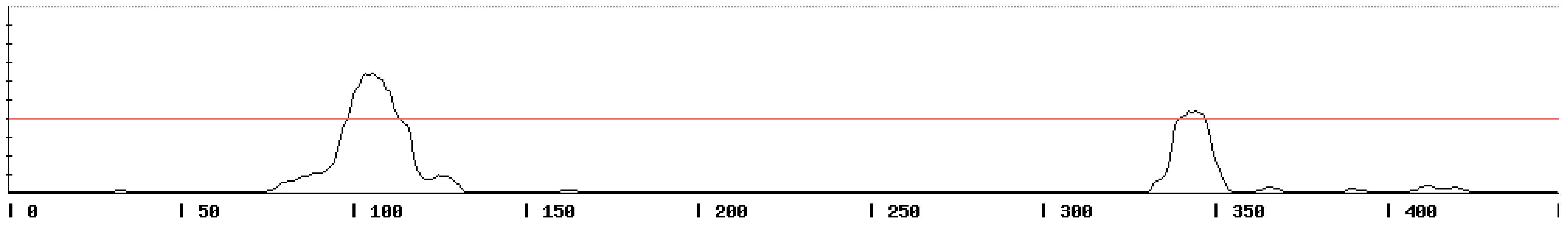

Predictions for Arabidopsisthaliana_AT5G39610.1

Viterbi Path

Viterbi Path

Posterior @ 0.4

Posterior @ 0.4

Posterior Graphic. Threshold Marked @ 0.4

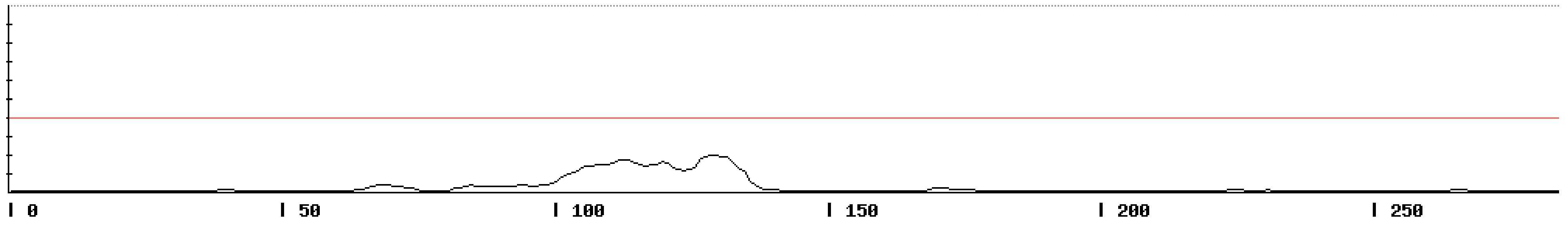

Predictions for Arabidopsisthaliana_AT5G39690.1

Viterbi Path

Viterbi Path

Posterior @ 0.4 — 156 - KKKQKK - 161

Posterior @ 0.4

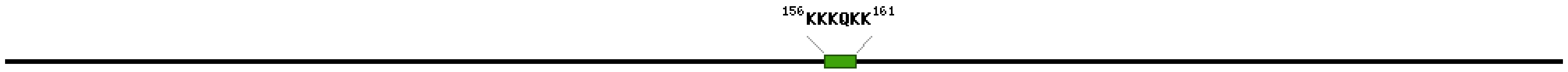

Posterior Graphic. Threshold Marked @ 0.4

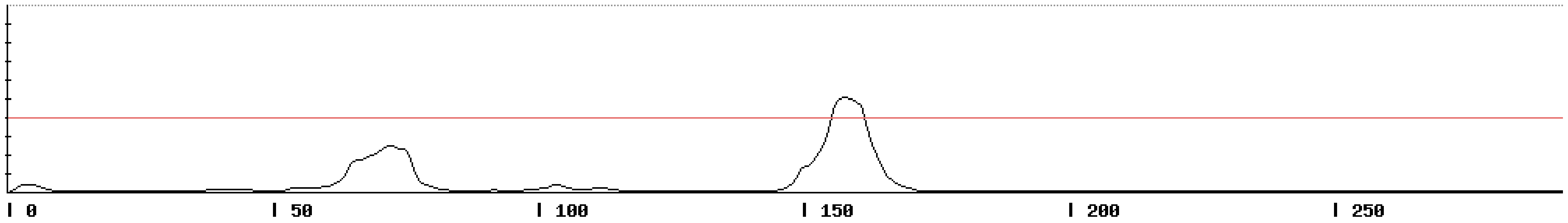

Predictions for Arabidopsisthaliana_AT5G39820.1

Viterbi Path

Viterbi Path

Posterior @ 0.4

Posterior @ 0.4

Posterior Graphic. Threshold Marked @ 0.4

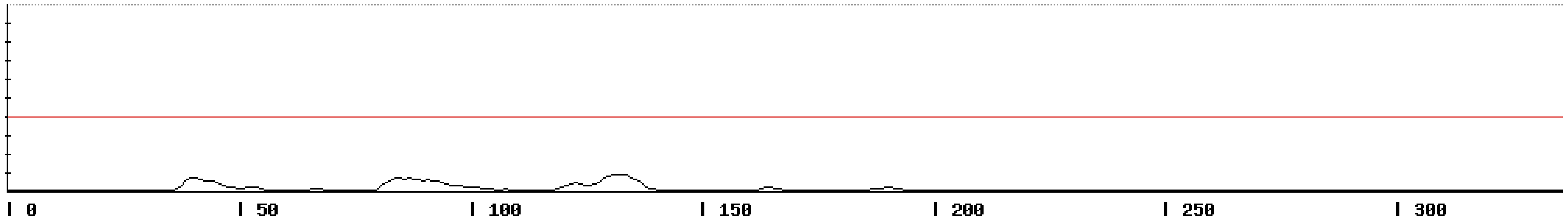

Predictions for Arabidopsisthaliana_AT5G41090.1

Viterbi Path

Viterbi Path

Posterior @ 0.4

Posterior @ 0.4

Posterior Graphic. Threshold Marked @ 0.4

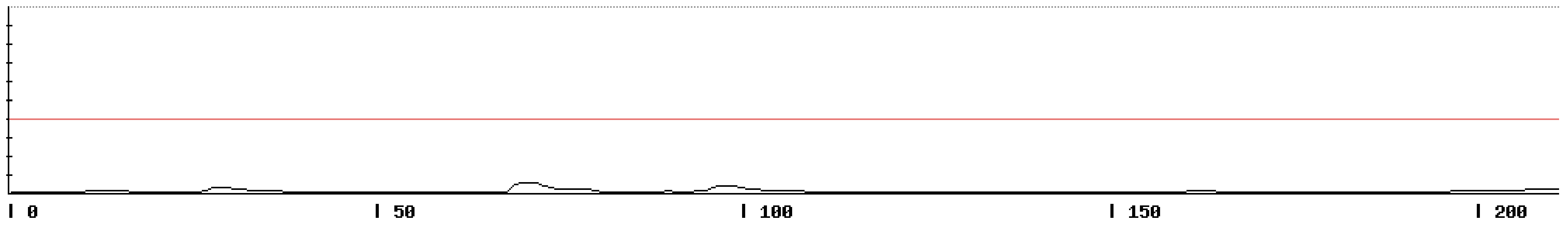

Predictions for Arabidopsisthaliana_AT5G46590.1

Viterbi Path

Viterbi Path

Posterior @ 0.4

Posterior @ 0.4

Posterior Graphic. Threshold Marked @ 0.4

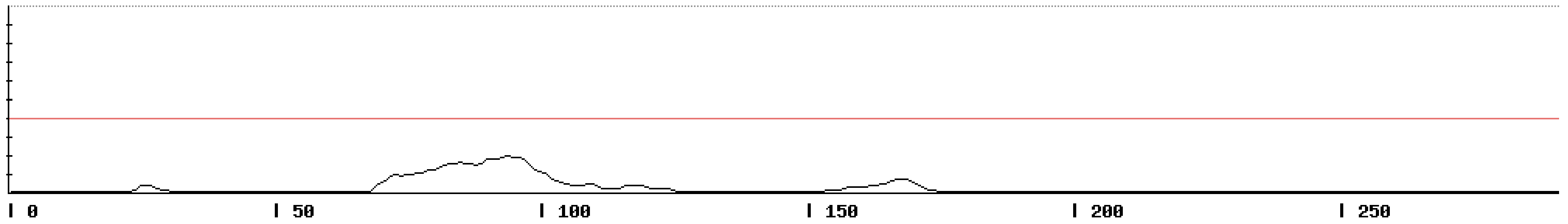

Predictions for Arabidopsisthaliana_AT5G50820.1

Viterbi Path	
Viterbi Path	
Posterior @ 0.4	
Posterior @ 0.4	
Posterior Graphic. Threshold Marked @ 0.4	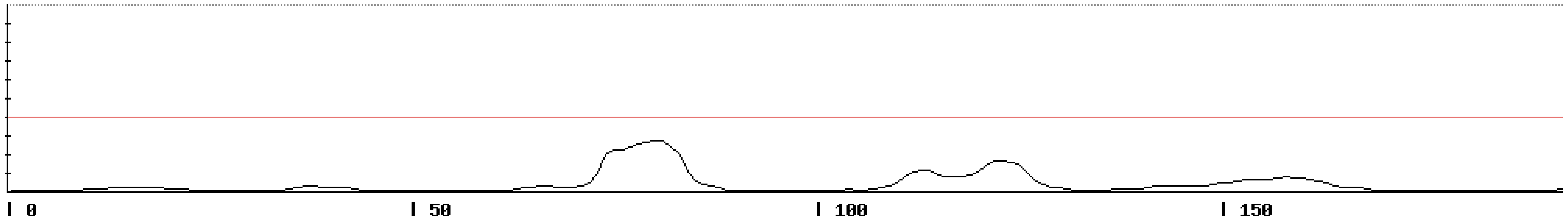

Predictions for Arabidopsisthaliana_AT5G53950.1

Viterbi Path	
Viterbi Path	
Posterior @ 0.4	
Posterior @ 0.4	
Posterior Graphic. Threshold Marked @ 0.4	

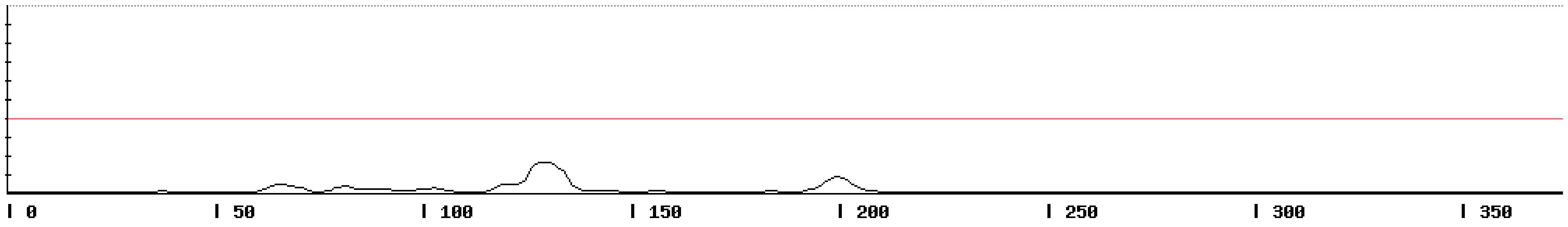

Predictions for Arabidopsisthaliana_AT5G56620.1

Viterbi Path

Viterbi Path

Posterior @ 0.4

Posterior @ 0.4

Posterior Graphic. Threshold Marked @ 0.4

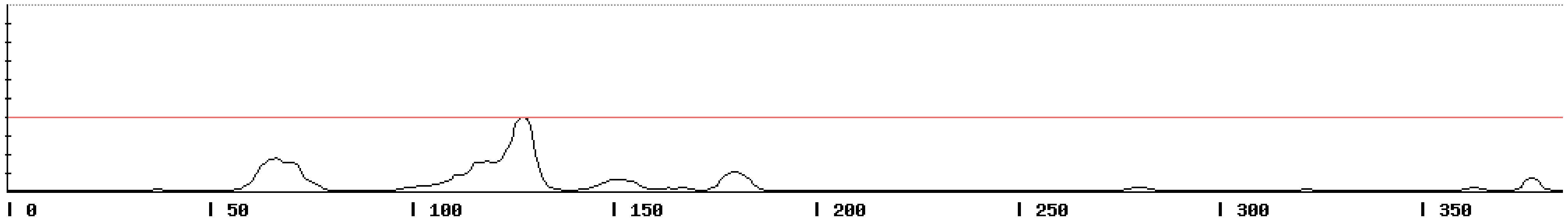

Predictions for Arabidopsisthaliana_AT5G61430.1

Viterbi Path

Viterbi Path

Posterior @ 0.4

Posterior @ 0.4

Posterior Graphic. Threshold Marked @ 0.4

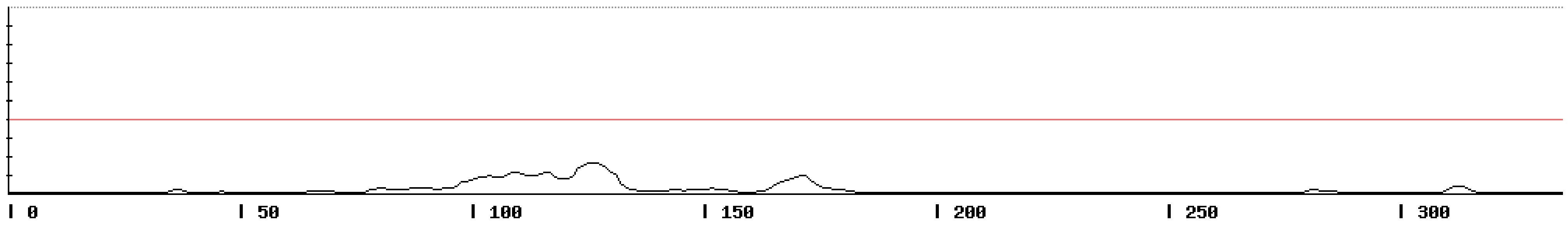

Predictions for Arabidopsisthaliana_AT5G62380.1

Viterbi Path	
Viterbi Path	
Posterior @ 0.4	
Posterior @ 0.4	
Posterior Graphic. Threshold Marked @ 0.4	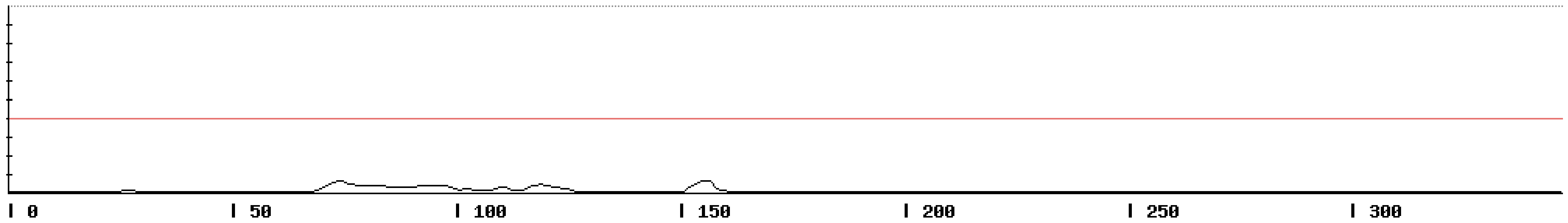

Predictions for Arabidopsisthaliana_AT5G63790.1

Viterbi Path	
Viterbi Path	
Posterior @ 0.4	141 - GKPK - 144
Posterior @ 0.4	

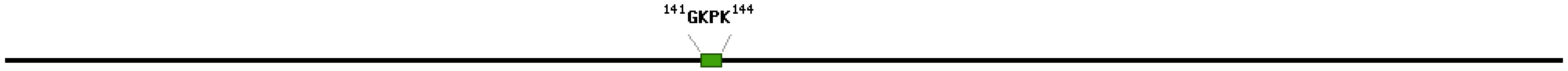

Posterior Graphic. Threshold Marked @ 0.4

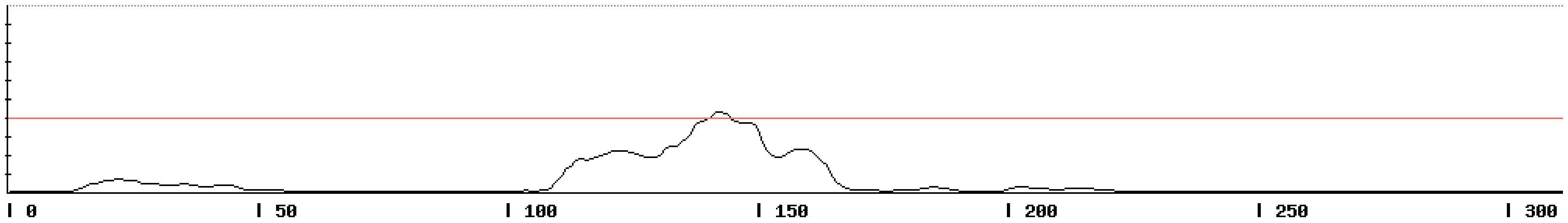

Predictions for Arabidopsisthaliana_AT5G64060.1	
Viterbi Path	
Viterbi Path	
Posterior @ 0.4	
Posterior @ 0.4	
Posterior Graphic. Threshold Marked @ 0.4	

Revision r.9 - Last Updated on : February 24 2015 17:45:20. alex.nguyenba@utoronto.ca

Intended to be used with Firefox

Viterbi Path	
Posterior @ 0.4	89 - RGALKR - 94
Posterior @ 0.4	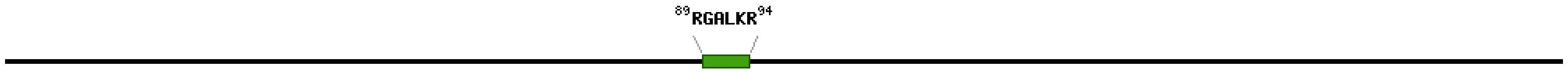
Posterior Graphic. Threshold Marked @ 0.4	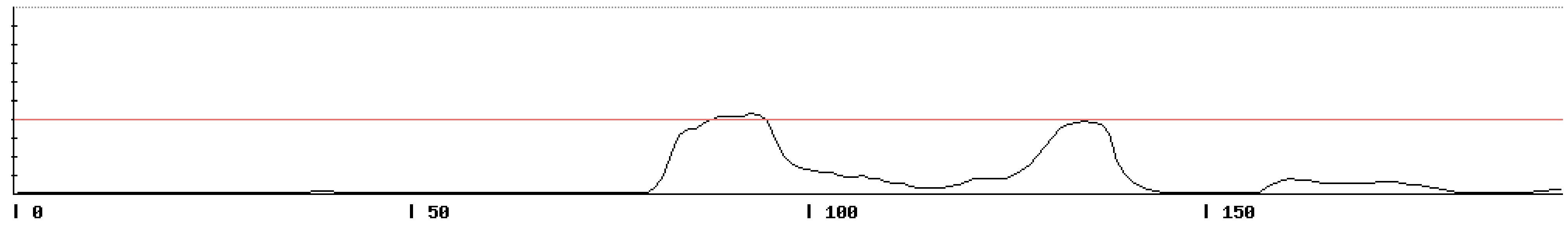

Predictions for Arabisalpina_KFK22783.1

Viterbi Path	
Viterbi Path	
Posterior @ 0.4	24 - RKTGKIIGKKQ - 34
	38 - YTRRNKKEKR - 47
Posterior @ 0.4	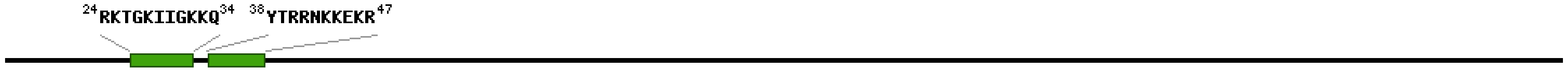
Posterior Graphic. Threshold Marked @ 0.4	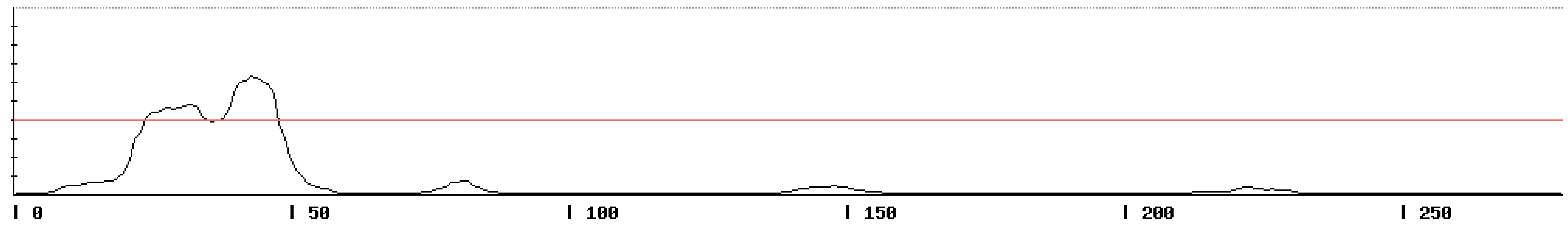

Predictions for Arabisalpina_KFK23146.1	
Viterbi Path	
Viterbi Path	
Posterior @ 0.4	
Posterior @ 0.4	
Posterior Graphic. Threshold Marked @ 0.4	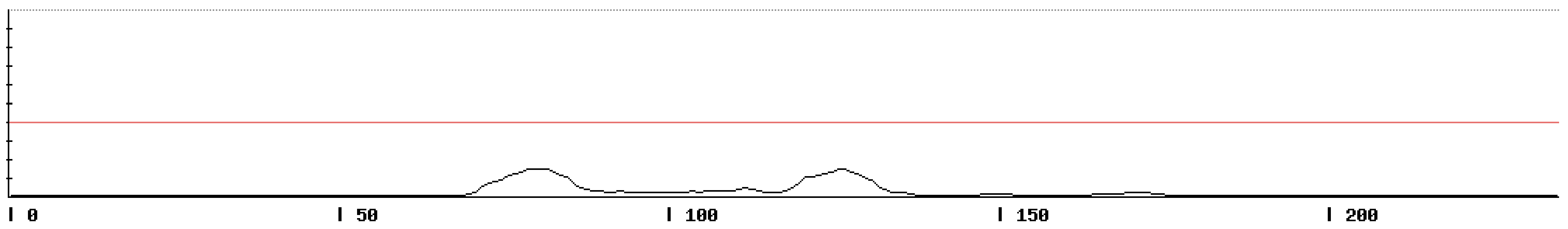

Predictions for Arabisalpina_KFK24130.1	
Viterbi Path	
Viterbi Path	
Posterior @ 0.4	
Posterior @ 0.4	
Posterior Graphic. Threshold Marked @ 0.4	

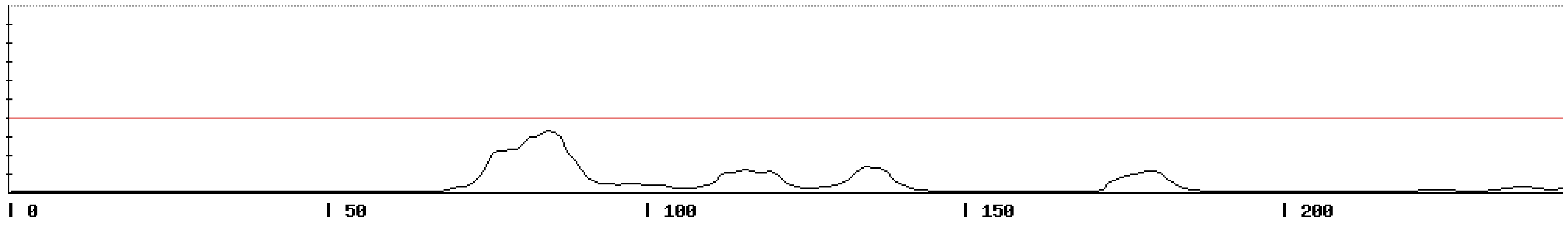

Predictions for Arabisalpina_KFK24890.1

Viterbi Path	
Viterbi Path	
Posterior @ 0.4	268 - PKQYKRRRSS - 277
Posterior @ 0.4	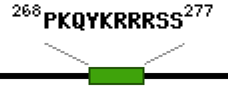
Posterior Graphic. Threshold Marked @ 0.4	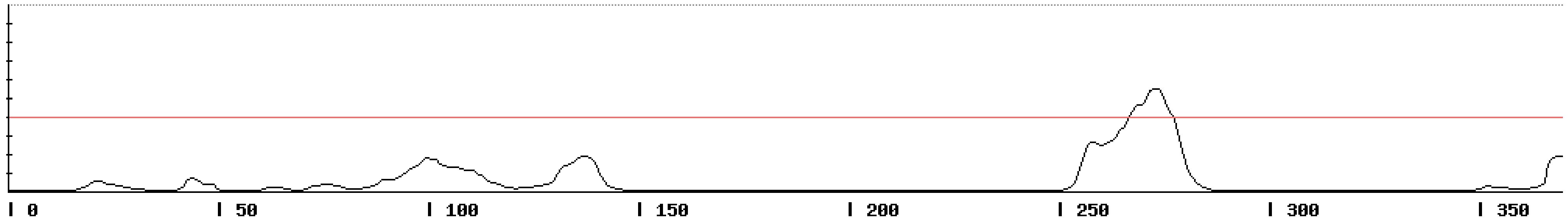

Predictions for Arabisalpina_KFK24891.1

Viterbi Path	
Viterbi Path	
Posterior @ 0.4	
Posterior @ 0.4	

Posterior Graphic. Threshold Marked @ 0.4

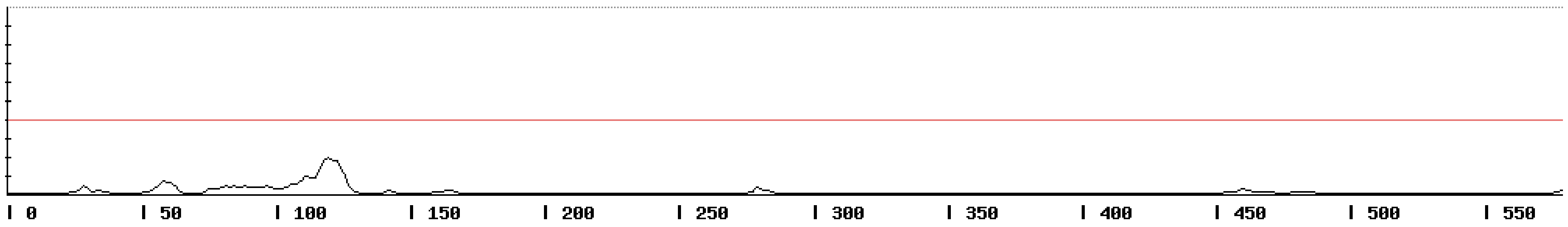

Predictions for Arabisalpina_KFK25161.1

Viterbi Path

Viterbi Path

Posterior @ 0.4

Posterior @ 0.4

Posterior Graphic. Threshold Marked @ 0.4

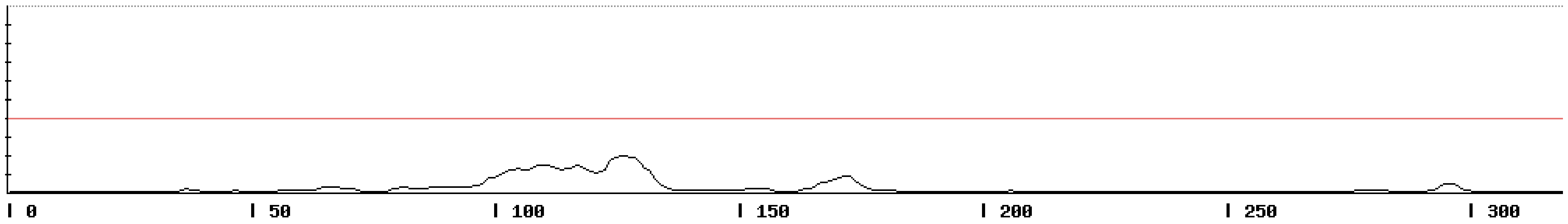

Predictions for Arabisalpina_KFK25187.1

Viterbi Path

Viterbi Path

Posterior @ 0.4

Posterior @ 0.4

Posterior Graphic. Threshold Marked @ 0.4

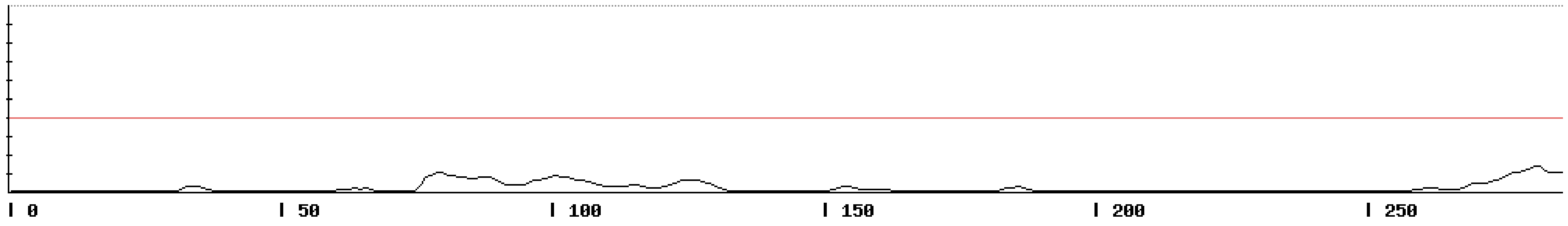

Predictions for Arabisalpina_KFK25233.1

Viterbi Path

Viterbi Path

Posterior @ 0.4 | 98 - GKPK - 101

Posterior @ 0.4

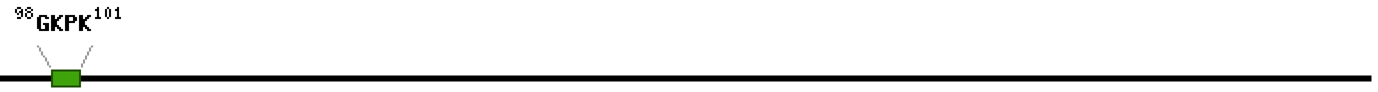

Posterior Graphic. Threshold Marked @ 0.4

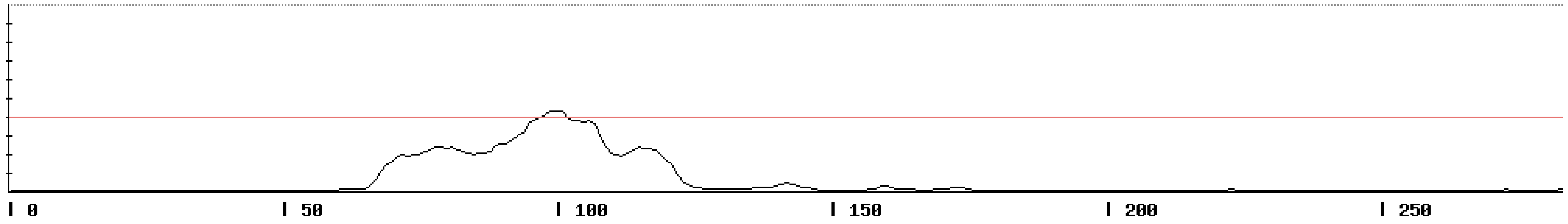

Predictions for Arabisalpina_KFK25634.1

Viterbi Path

Viterbi Path

Posterior @ 0.4	152 - RKRSKVSKQRKSRK - 165
Posterior @ 0.4	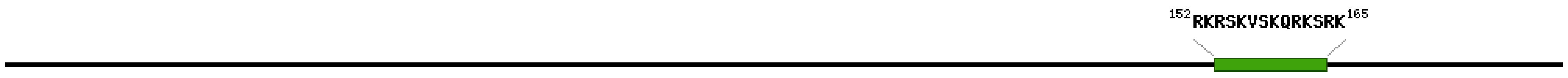
Posterior Graphic. Threshold Marked @ 0.4	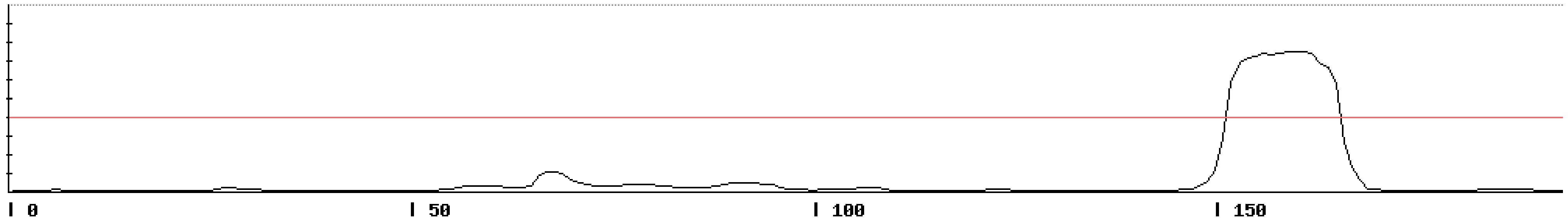

Predictions for Arabisalpina_KFK25688.1

Viterbi Path	52 - KKSKKKKKKKKKDRSEKK - 69
Viterbi Path	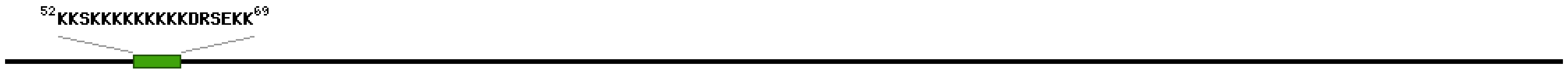
Posterior @ 0.4	52 - KKSKKKKKKKKKDRSEKKEDTKLSPASEK - 80
	96 - KKRP - 99
	107 - VKEKRVKTEEKKR - 119
	396 - KGERKRRKI - 404
Posterior @ 0.4	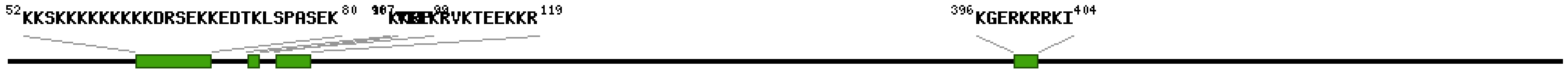
Posterior Graphic. Threshold Marked @ 0.4	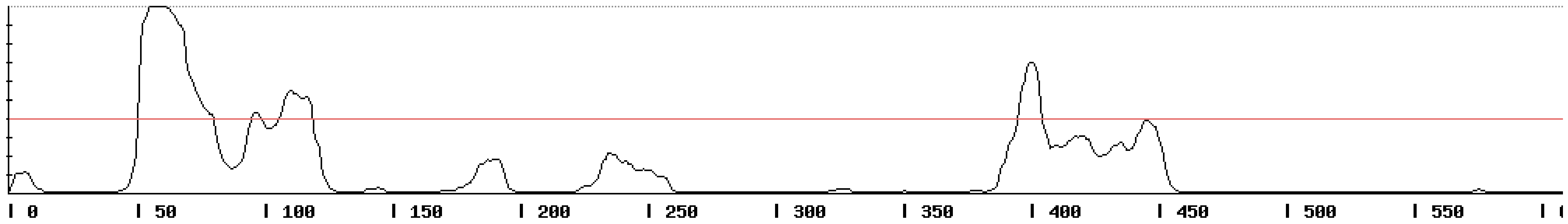

Predictions for Arabisalpina_KFK26065.1

Viterbi Path

Viterbi Path

Posterior @ 0.4

Posterior @ 0.4

Posterior Graphic. Threshold Marked @ 0.4

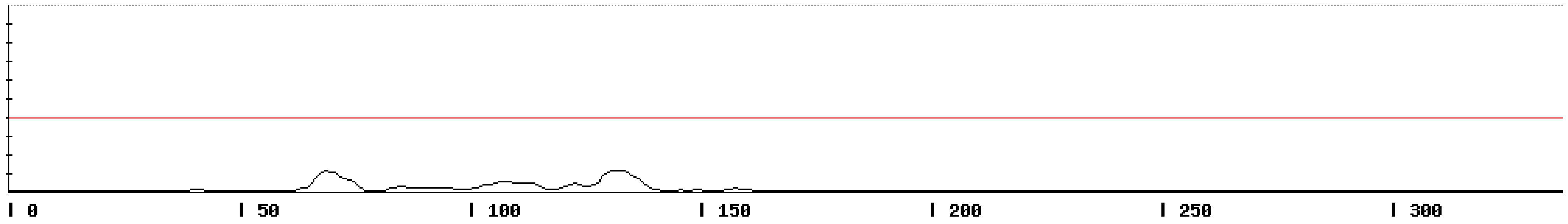

Predictions for Arabisalpina_KFK26331.1

Viterbi Path

Viterbi Path

Posterior @ 0.4

Posterior @ 0.4

Posterior Graphic. Threshold Marked @ 0.4

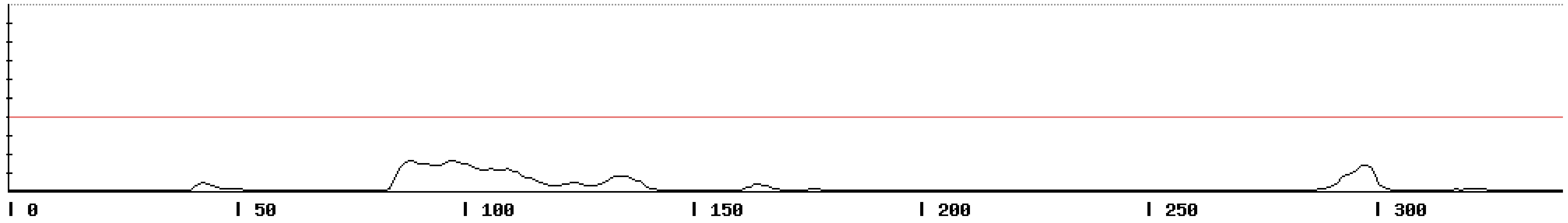

Predictions for Arabisalpina_KFK26711.1

Viterbi Path	
Viterbi Path	
Posterior @ 0.4	
Posterior @ 0.4	
Posterior Graphic. Threshold Marked @ 0.4	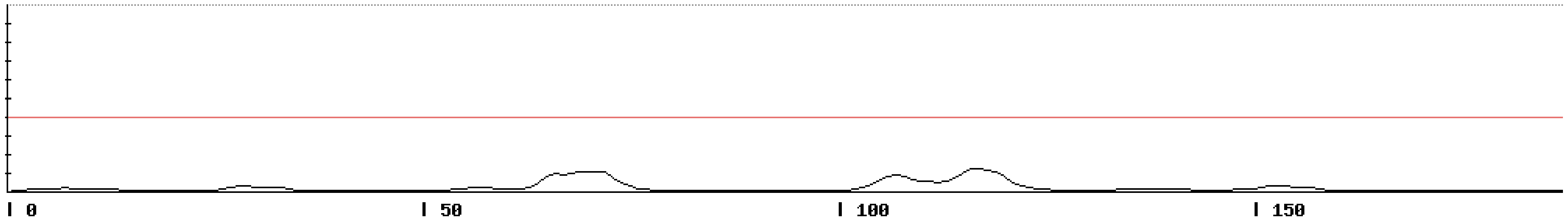

Predictions for Arabisalpina_KFK26712.1

Viterbi Path	
Viterbi Path	
Posterior @ 0.4	
Posterior @ 0.4	
Posterior Graphic. Threshold Marked @ 0.4	

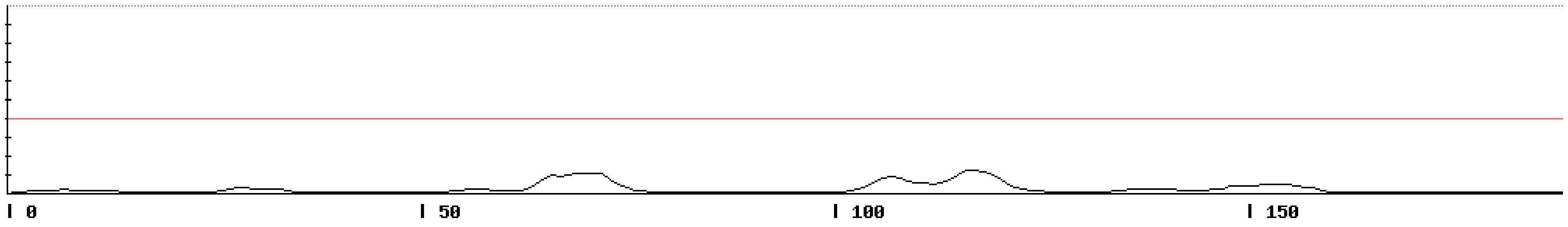

Predictions for Arabisalpina_KFK27011.1

Viterbi Path	
Viterbi Path	
Posterior @ 0.4	
Posterior @ 0.4	
Posterior Graphic. Threshold Marked @ 0.4	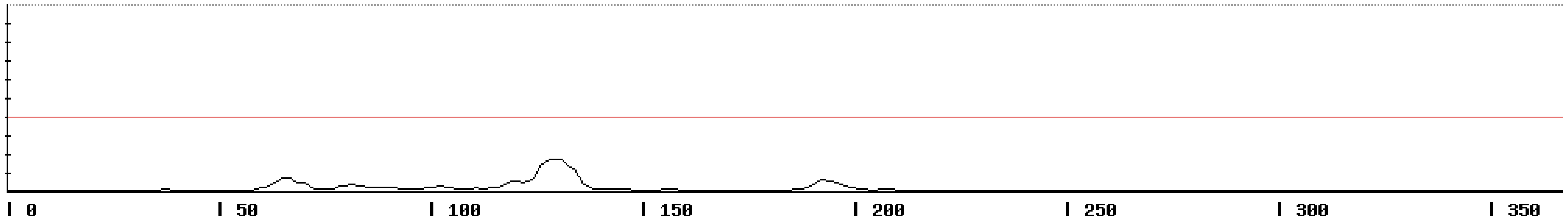

Predictions for Arabisalpina_KFK27422.1

Viterbi Path	
Viterbi Path	
Posterior @ 0.4	
Posterior @ 0.4	

Posterior Graphic. Threshold Marked @ 0.4

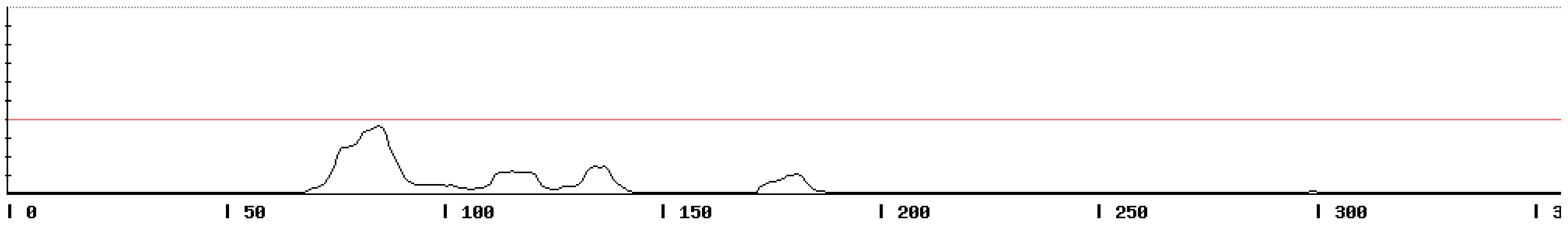

Predictions for Arabisalpina_KFK27438.1

Viterbi Path	
Viterbi Path	
Posterior @ 0.4	82 - KK - 83
	113 - KTGKIIGRK - 121
	127 - TRRNKKEKR - 135
Posterior @ 0.4	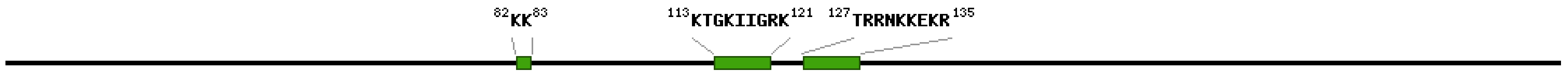
Posterior Graphic. Threshold Marked @ 0.4	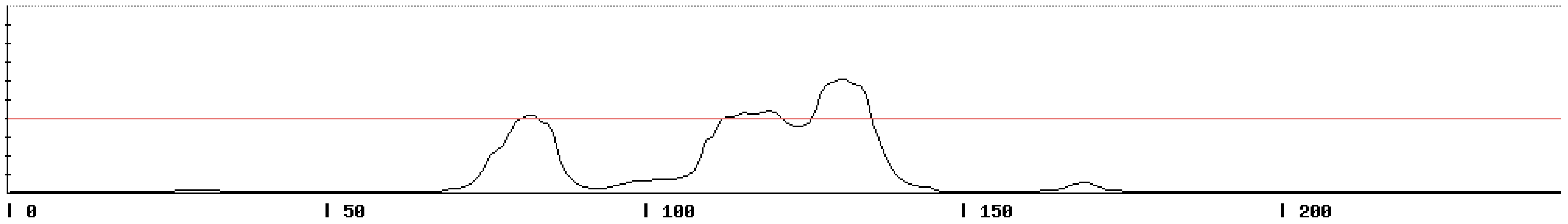

Predictions for Arabisalpina_KFK27441.1

Viterbi Path	
Viterbi Path	
Posterior @ 0.4	

Posterior @ 0.4

Posterior Graphic. Threshold Marked @ 0.4

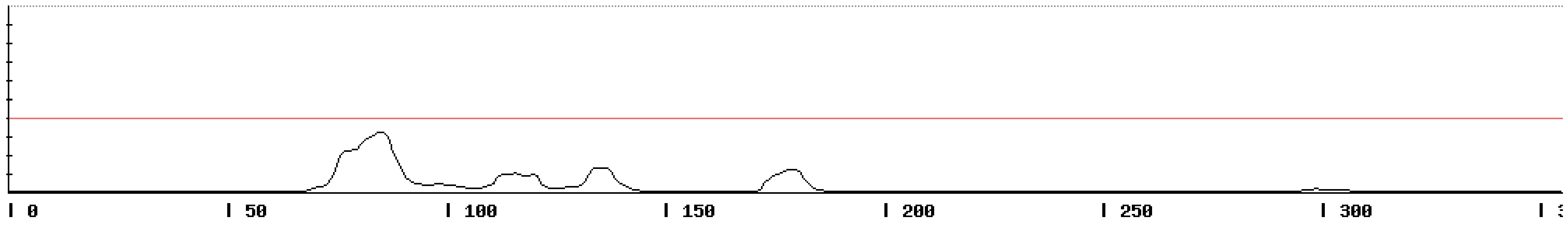

Predictions for Arabisalpina_KFK27735.1

Viterbi Path

Viterbi Path

Posterior @ 0.4

Posterior @ 0.4

Posterior Graphic. Threshold Marked @ 0.4

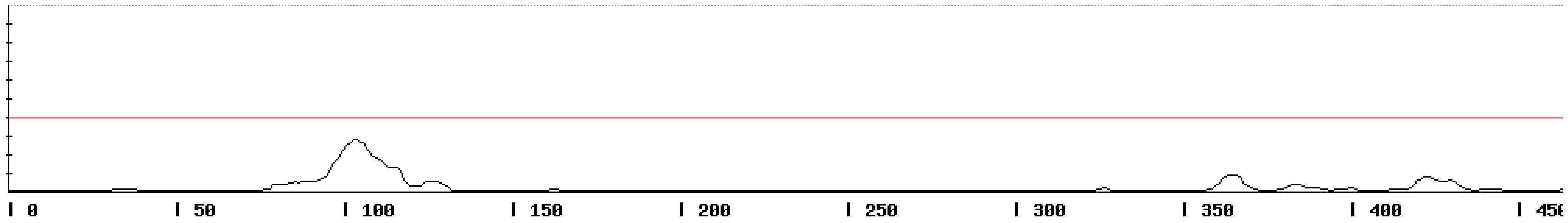

Predictions for Arabisalpina_KFK27900.1

Viterbi Path

Viterbi Path

Posterior @ 0.4	
Posterior @ 0.4	
Posterior Graphic. Threshold Marked @ 0.4	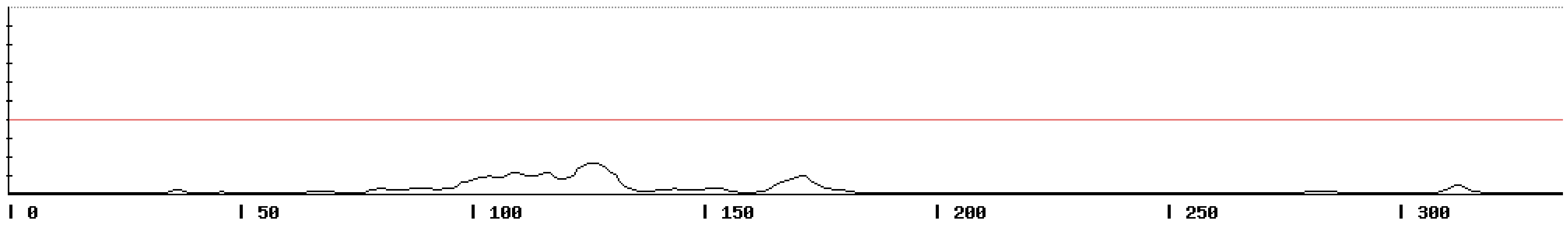

Predictions for Arabisalpina_KFK28073.1	
Viterbi Path	
Viterbi Path	
Posterior @ 0.4	99 - KPK - 101
Posterior @ 0.4	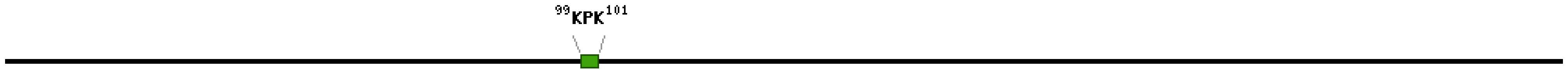
Posterior Graphic. Threshold Marked @ 0.4	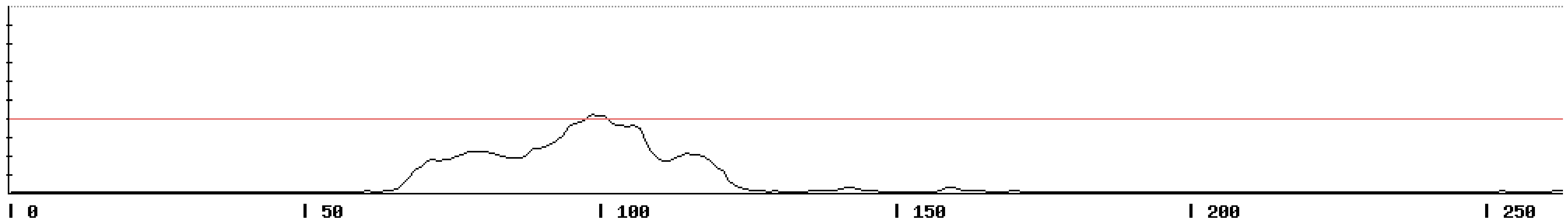

Predictions for Arabisalpina_KFK28146.1	
Viterbi Path	
Viterbi Path	

Posterior @ 0.4

Posterior @ 0.4

Posterior Graphic. Threshold Marked @ 0.4

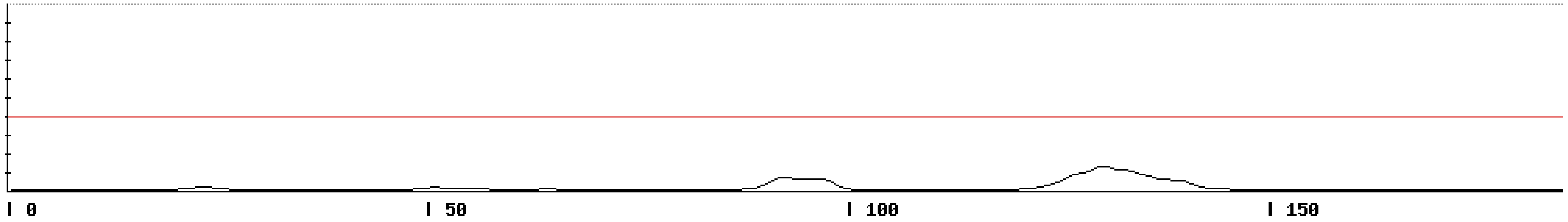

Predictions for Arabisalpina_KFK28483.1

Viterbi Path

Viterbi Path

Posterior @ 0.4

Posterior @ 0.4

Posterior Graphic. Threshold Marked @ 0.4

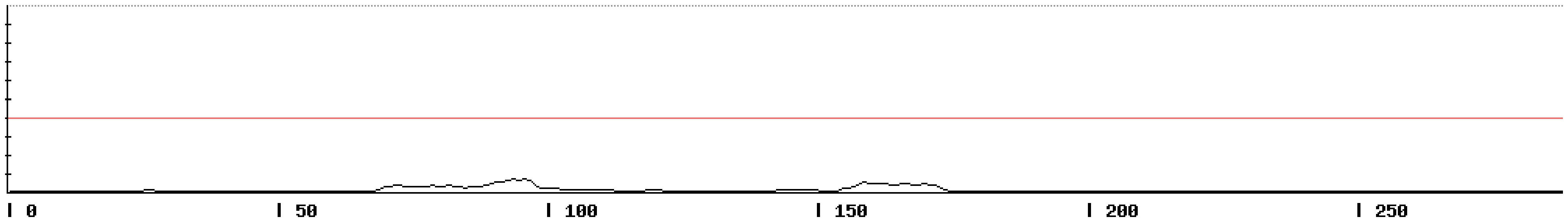

Predictions for Arabisalpina_KFK29255.1

Viterbi Path	
Viterbi Path	
Posterior @ 0.4	
Posterior @ 0.4	
Posterior Graphic. Threshold Marked @ 0.4	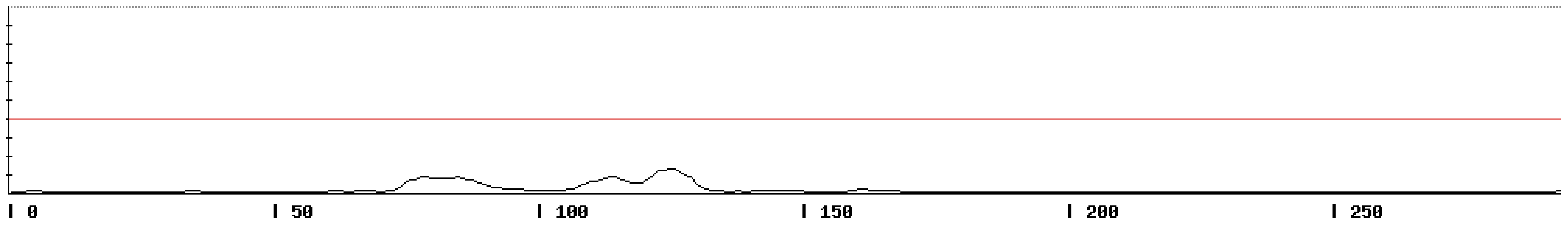

Predictions for Arabisalpina_KFK29384.1

Viterbi Path	
Viterbi Path	
Posterior @ 0.4	137 - RKRRK - 141
Posterior @ 0.4	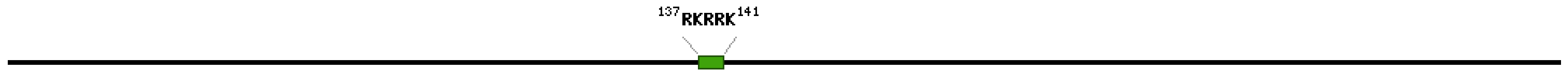
Posterior Graphic. Threshold Marked @ 0.4	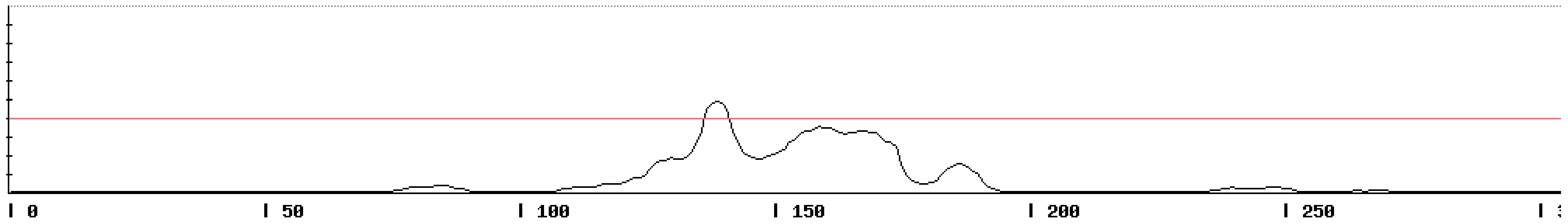

Predictions for Arabisalpina_KFK29397.1

Viterbi Path

Viterbi Path

Posterior @ 0.4

Posterior @ 0.4

Posterior Graphic. Threshold Marked @ 0.4

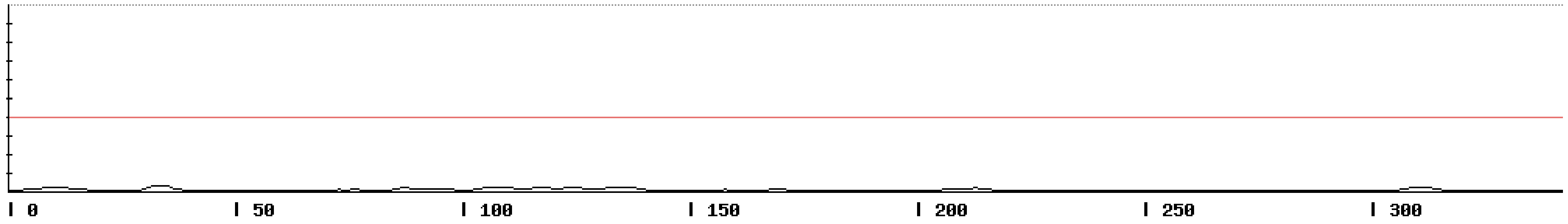

Predictions for Arabisalpina_KFK30695.1

Viterbi Path

Viterbi Path

Posterior @ 0.4

Posterior @ 0.4

Posterior Graphic. Threshold Marked @ 0.4

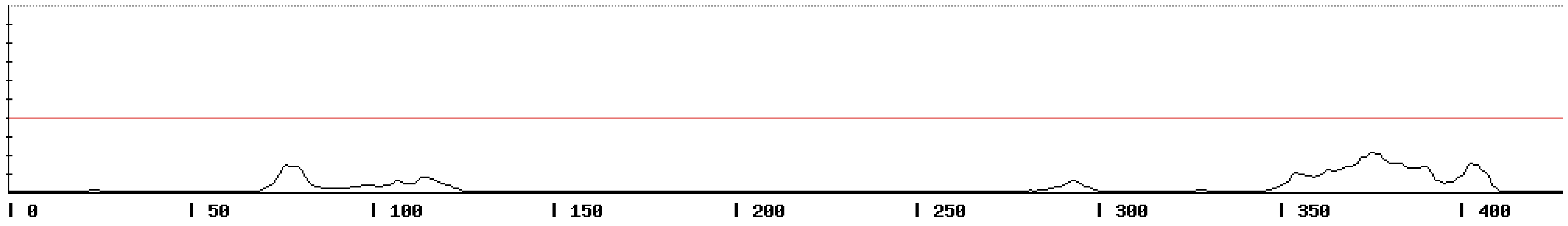

Predictions for Arabisalpina_KFK30696.1

Viterbi Path	
Viterbi Path	
Posterior @ 0.4	
Posterior @ 0.4	
Posterior Graphic. Threshold Marked @ 0.4	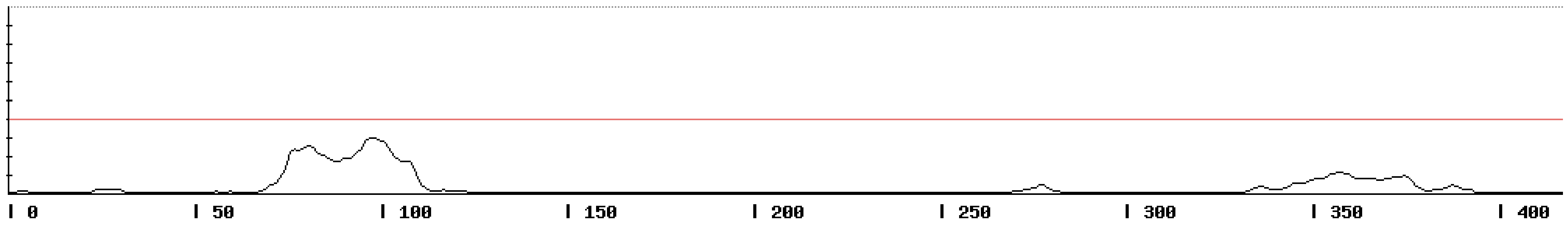

Predictions for Arabisalpina_KFK30743.1

Viterbi Path	
Viterbi Path	
Posterior @ 0.4	72 - RKRPAKIKNPNSKR - 85
Posterior @ 0.4	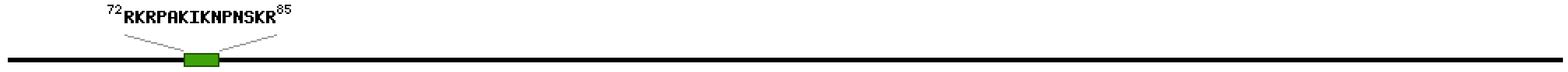

Posterior Graphic. Threshold Marked @ 0.4

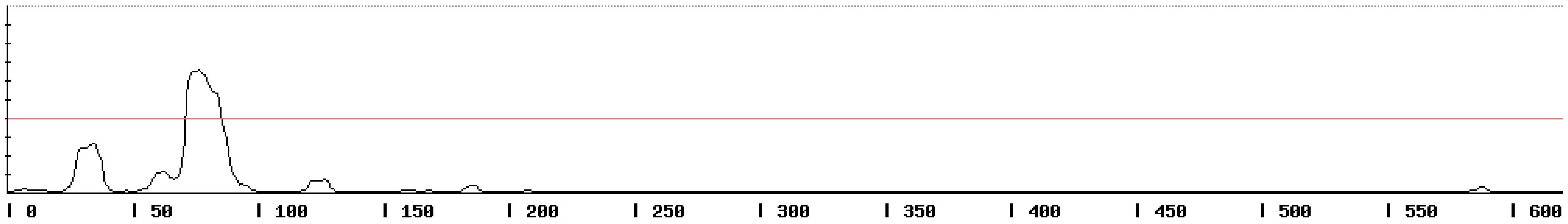

Predictions for Arabisalpina_KFK31456.1

Viterbi Path

Viterbi Path

Posterior @ 0.4

Posterior @ 0.4

Posterior Graphic. Threshold Marked @ 0.4

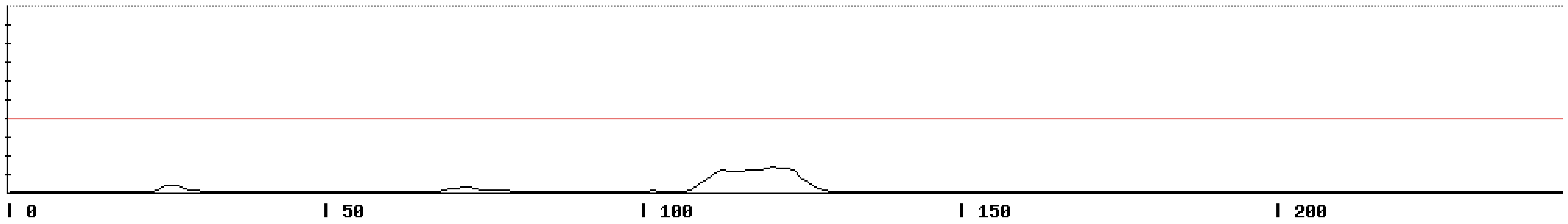

Predictions for Arabisalpina_KFK31989.1

Viterbi Path

Viterbi Path

Posterior @ 0.4

Posterior @ 0.4

Posterior Graphic. Threshold Marked @ 0.4

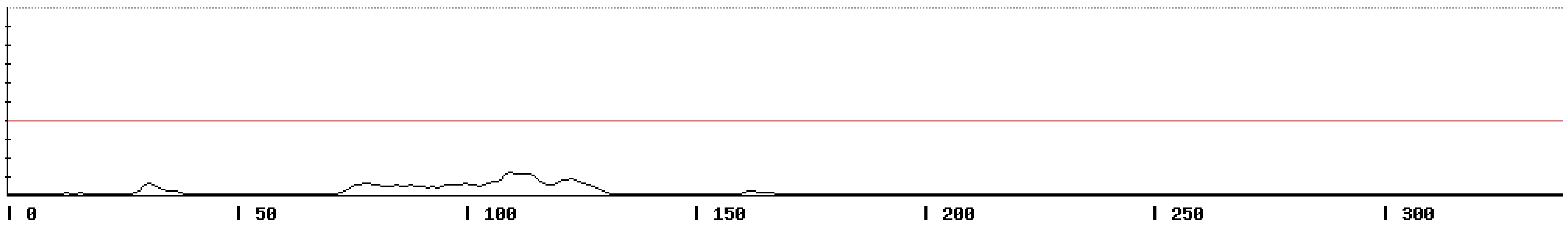

Predictions for Arabisalpina_KFK32634.1

Viterbi Path

Viterbi Path

Posterior @ 0.4

Posterior @ 0.4

Posterior Graphic. Threshold Marked @ 0.4

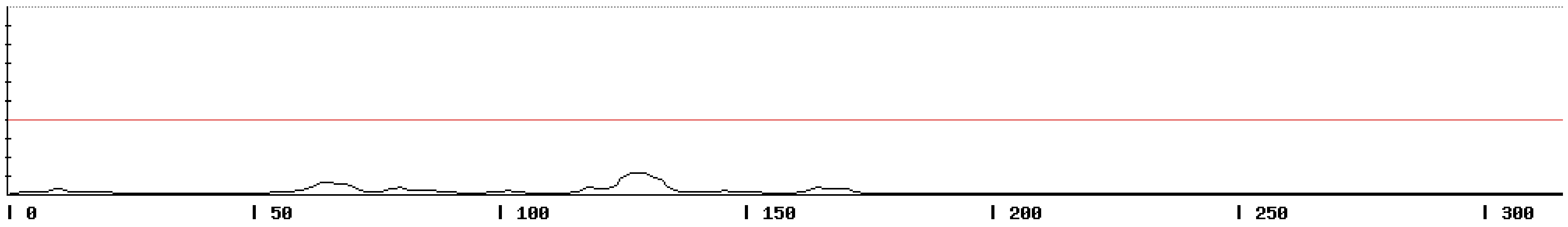

Predictions for Arabisalpina_KFK33032.1

Viterbi Path

Viterbi Path

Posterior @ 0.4

Posterior @ 0.4

Posterior Graphic. Threshold Marked @ 0.4

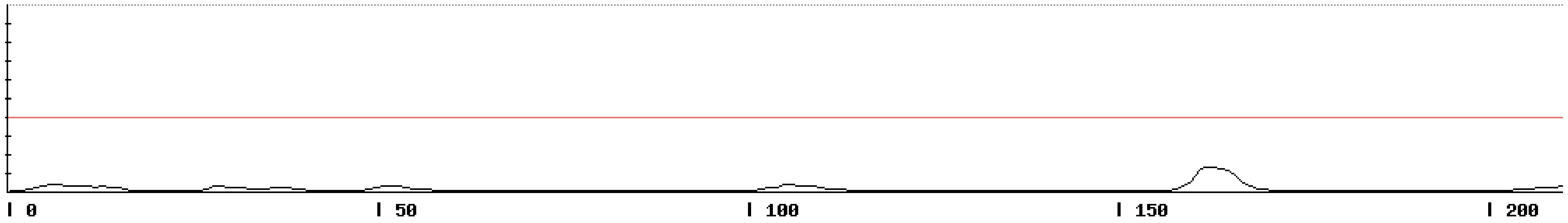

Predictions for Arabisalpina_KFK33194.1

Viterbi Path

Viterbi Path

Posterior @ 0.4

Posterior @ 0.4

Posterior Graphic. Threshold Marked @ 0.4

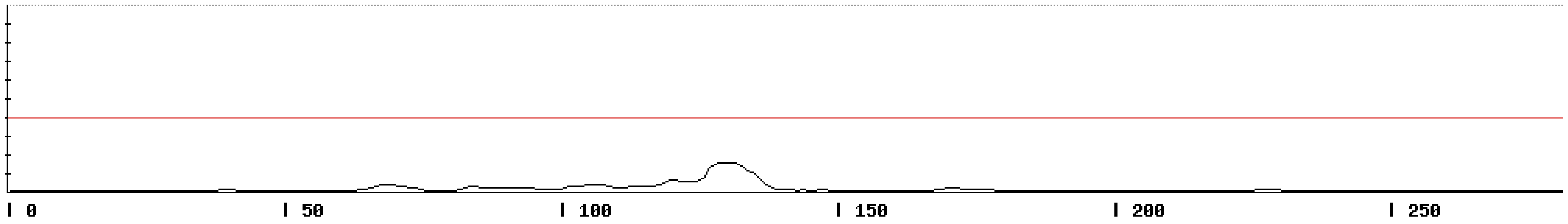

Predictions for Arabisalpina_KFK33442.1

Viterbi Path

Viterbi Path

Posterior @ 0.4

Posterior @ 0.4

Posterior Graphic. Threshold Marked @ 0.4

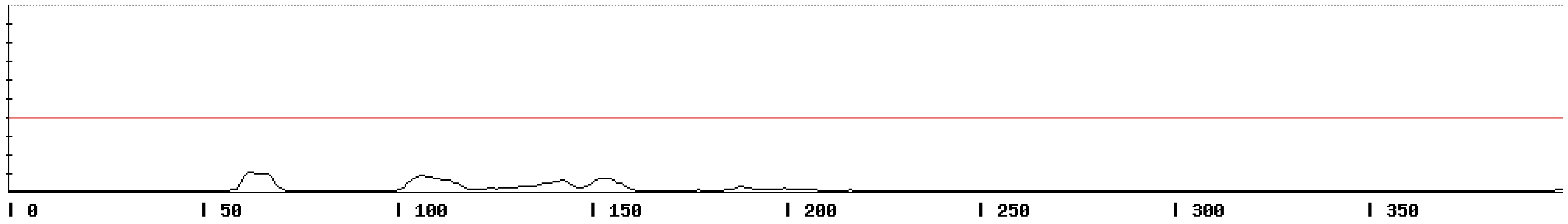

Predictions for Arabisalpina_KFK33777.1

Viterbi Path

Viterbi Path

Posterior @ 0.4

Posterior @ 0.4

Posterior Graphic. Threshold Marked @ 0.4

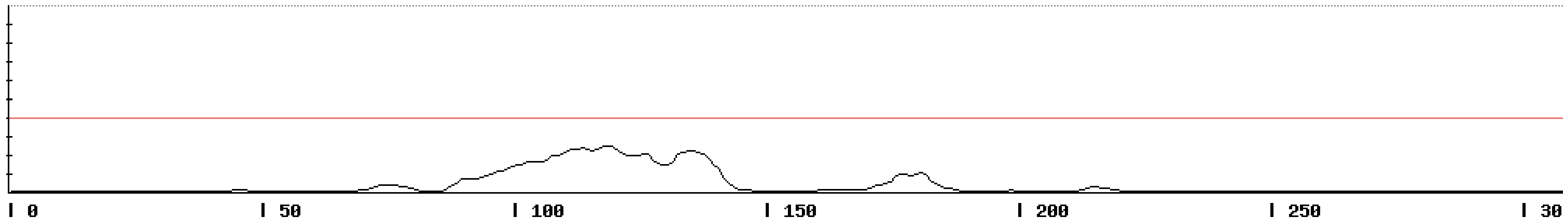

Predictions for Arabisalpina_KFK33874.1

Viterbi Path	
Viterbi Path	
Posterior @ 0.4	
Posterior @ 0.4	
Posterior Graphic. Threshold Marked @ 0.4	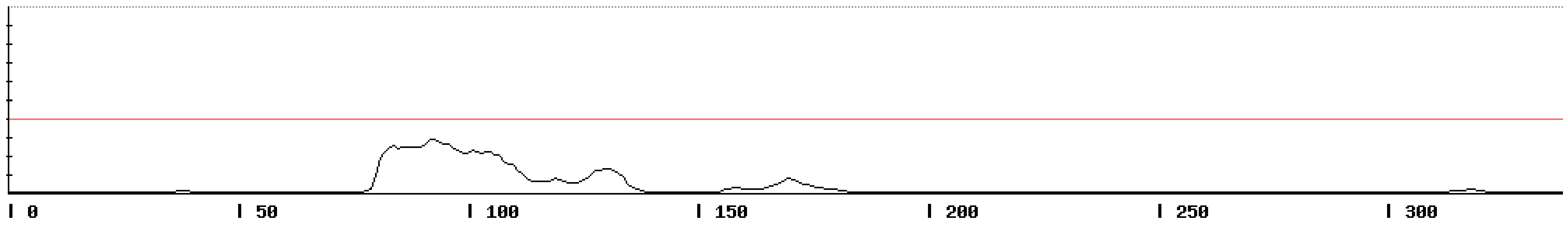

Predictions for Arabisalpina_KFK33878.1

Viterbi Path	
Viterbi Path	
Posterior @ 0.4	
Posterior @ 0.4	
Posterior Graphic. Threshold Marked @ 0.4	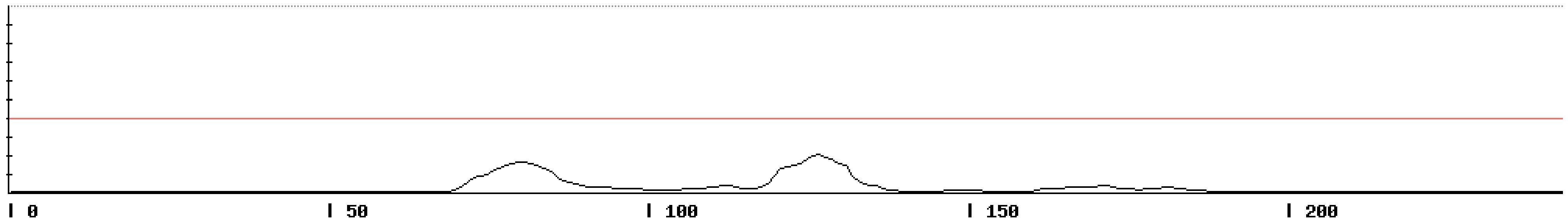

Predictions for Arabisalpina_KFK34231.1	
Viterbi Path	
Viterbi Path	
Posterior @ 0.4	98 - ATGKDRKIKSAKKNIIGVKR - 117
Posterior @ 0.4	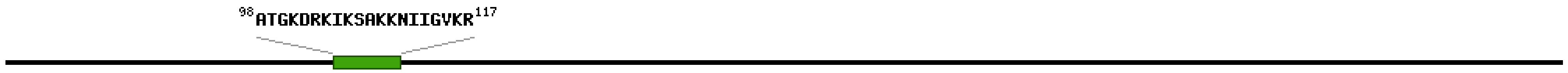
Posterior Graphic. Threshold Marked @ 0.4	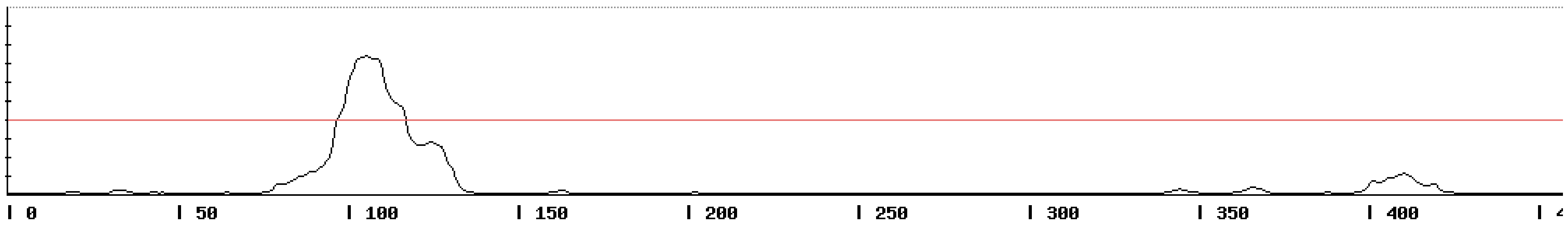

Predictions for Arabisalpina_KFK35377.1	
Viterbi Path	
Viterbi Path	
Posterior @ 0.4	
Posterior @ 0.4	
Posterior Graphic. Threshold Marked @ 0.4	

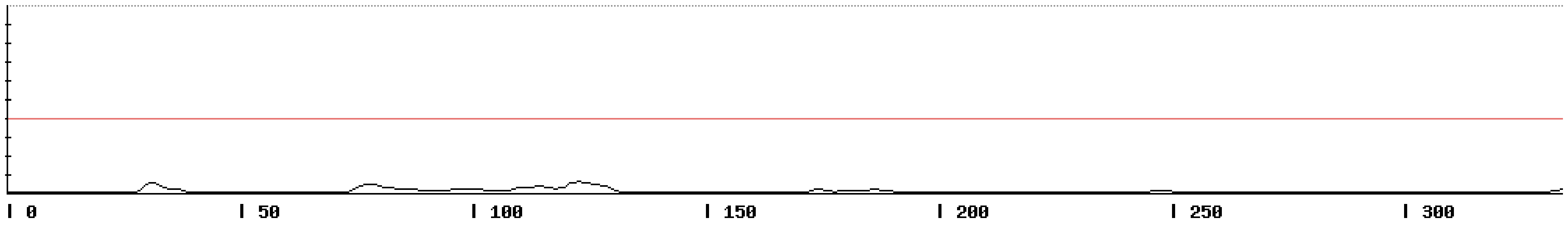

Predictions for Arabisalpina_KFK35527.1

Viterbi Path	
Viterbi Path	
Posterior @ 0.4	186 - KKKKWR - 191
Posterior @ 0.4	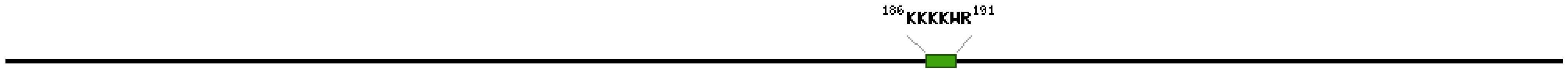
Posterior Graphic. Threshold Marked @ 0.4	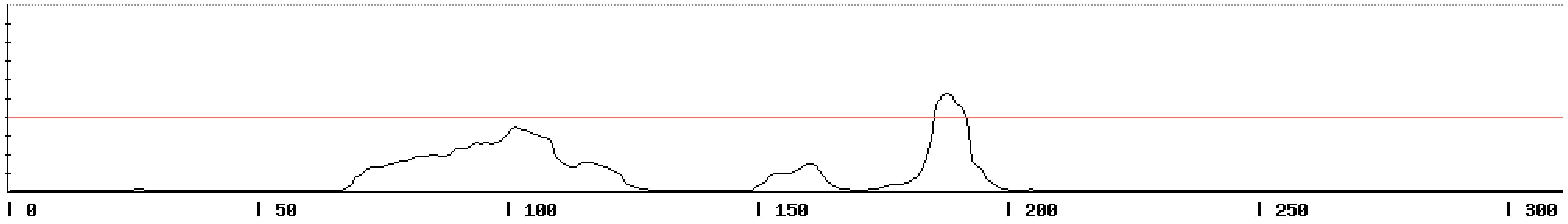

Predictions for Arabisalpina_KFK35543.1

Viterbi Path	
Viterbi Path	
Posterior @ 0.4	
Posterior @ 0.4	

Posterior Graphic. Threshold Marked @ 0.4

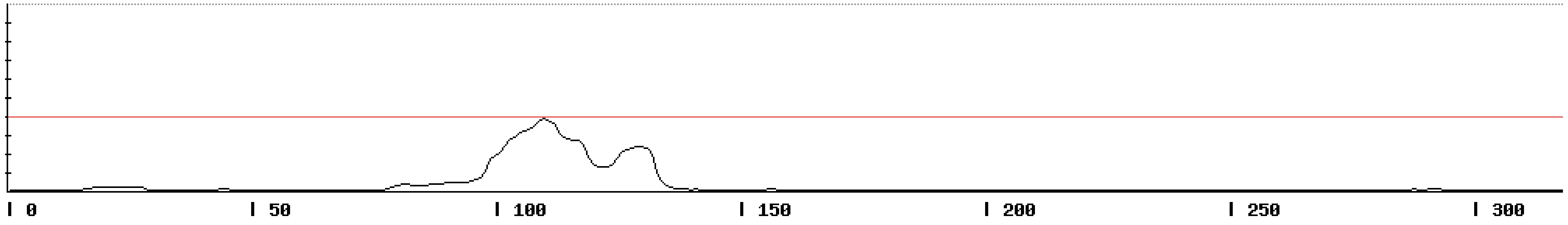

Predictions for Arabisalpina_KFK35760.1

Viterbi Path	
Viterbi Path	
Posterior @ 0.4	
Posterior @ 0.4	
Posterior Graphic. Threshold Marked @ 0.4	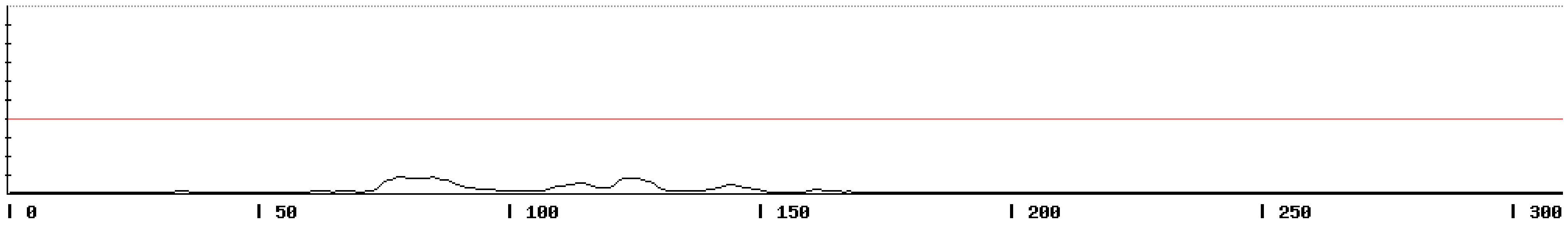

Predictions for Arabisalpina_KFK35761.1

Viterbi Path	
Viterbi Path	
Posterior @ 0.4	
Posterior @ 0.4	

Posterior Graphic. Threshold Marked @ 0.4	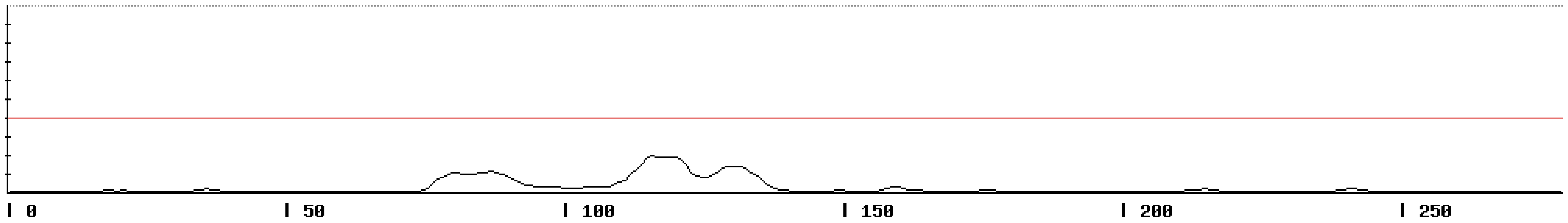

Predictions for Arabisalpina_KFK37142.1

Viterbi Path	
Viterbi Path	
Posterior @ 0.4	
Posterior @ 0.4	
Posterior Graphic. Threshold Marked @ 0.4	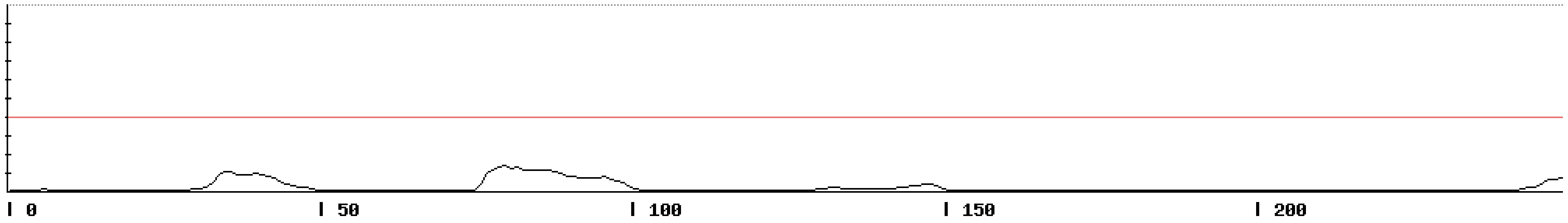

Predictions for Arabisalpina_KFK37442.1

Viterbi Path	
Viterbi Path	

Posterior @ 0.4

Posterior @ 0.4

Posterior Graphic. Threshold Marked @ 0.4

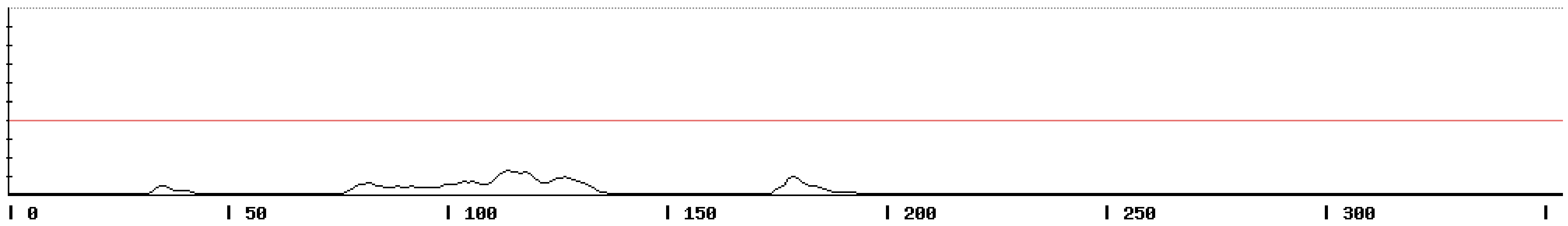

Predictions for Arabisalpina_KFK37625.1

Viterbi Path

Viterbi Path

Posterior @ 0.4

Posterior @ 0.4

Posterior Graphic. Threshold Marked @ 0.4

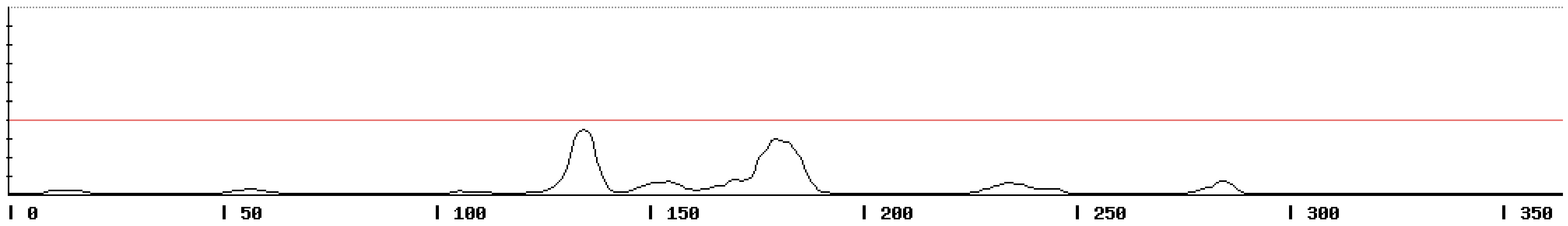

Predictions for Arabisalpina_KFK37763.1

Viterbi Path

Viterbi Path

Posterior @ 0.4

Posterior @ 0.4

Posterior Graphic. Threshold Marked @ 0.4

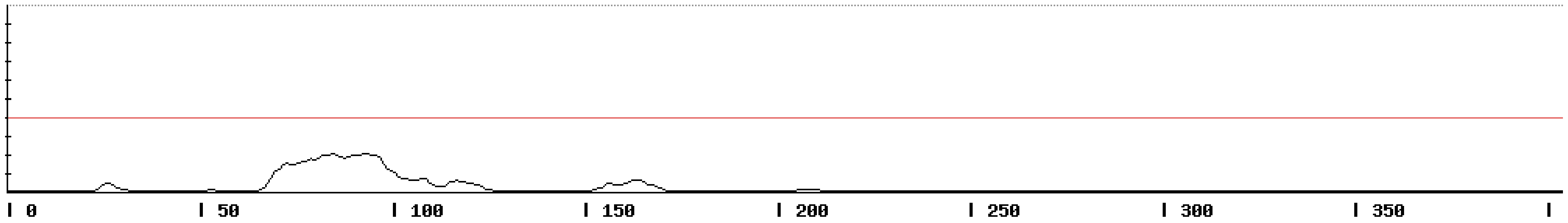

Predictions for Arabisalpina_KFK37841.1

Viterbi Path

Viterbi Path

Posterior @ 0.4

Posterior @ 0.4

Posterior Graphic. Threshold Marked @ 0.4

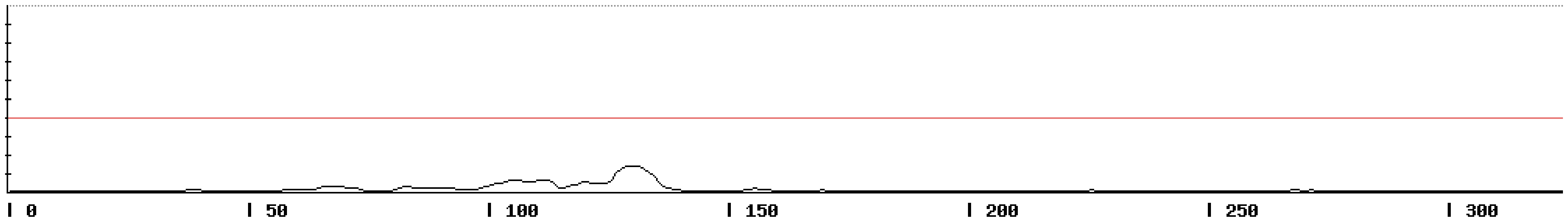

Predictions for Arabisalpina_KFK37842.1

Viterbi Path

Viterbi Path

Posterior @ 0.4

Posterior @ 0.4

Posterior Graphic. Threshold Marked @ 0.4

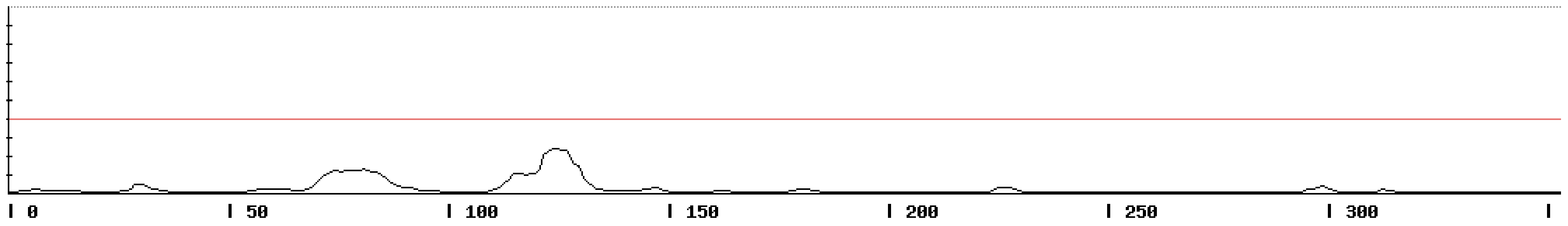

Predictions for Arabisalpina_KFK38436.1

Viterbi Path

Viterbi Path

Posterior @ 0.4

Posterior @ 0.4

Posterior Graphic. Threshold Marked @ 0.4

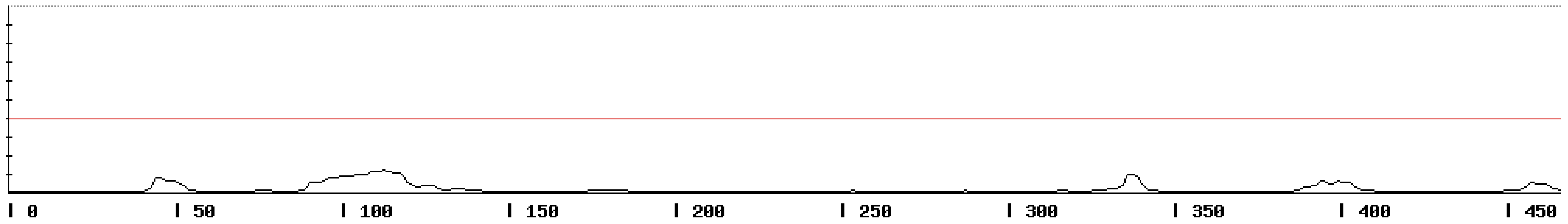

Predictions for Arabisalpina_KFK38437.1	
Viterbi Path	
Viterbi Path	
Posterior @ 0.4	
Posterior @ 0.4	
Posterior Graphic. Threshold Marked @ 0.4	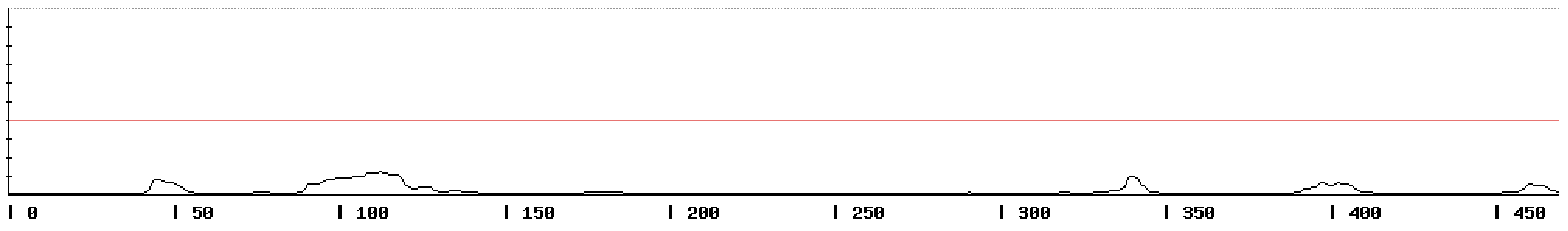

Predictions for Arabisalpina_KFK38438.1	
Viterbi Path	
Viterbi Path	
Posterior @ 0.4	
Posterior @ 0.4	
Posterior Graphic. Threshold Marked @ 0.4	

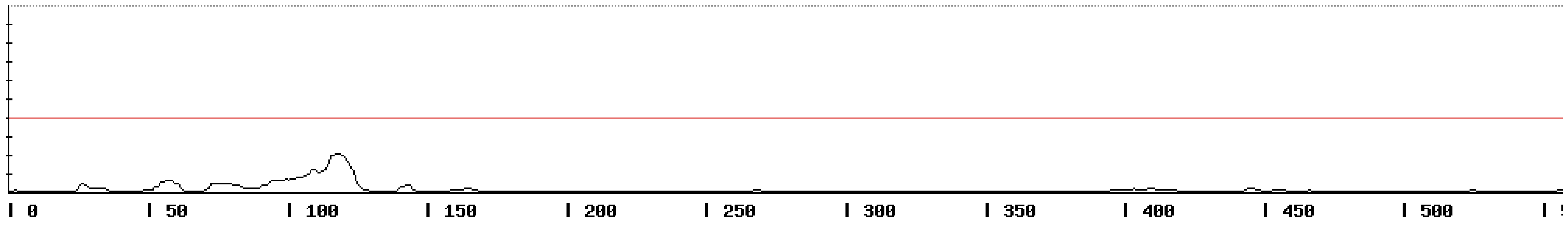

Predictions for Arabisalpina_KFK38633.1

Viterbi Path

Viterbi Path

Posterior @ 0.4

Posterior @ 0.4

Posterior Graphic. Threshold Marked @ 0.4

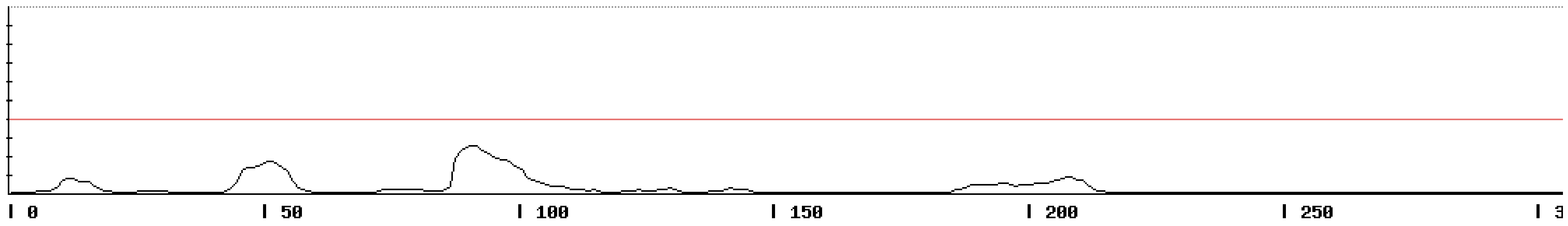

Predictions for Arabisalpina_KFK38638.1

Viterbi Path

Viterbi Path

Posterior @ 0.4

Posterior @ 0.4

Posterior Graphic. Threshold Marked @ 0.4

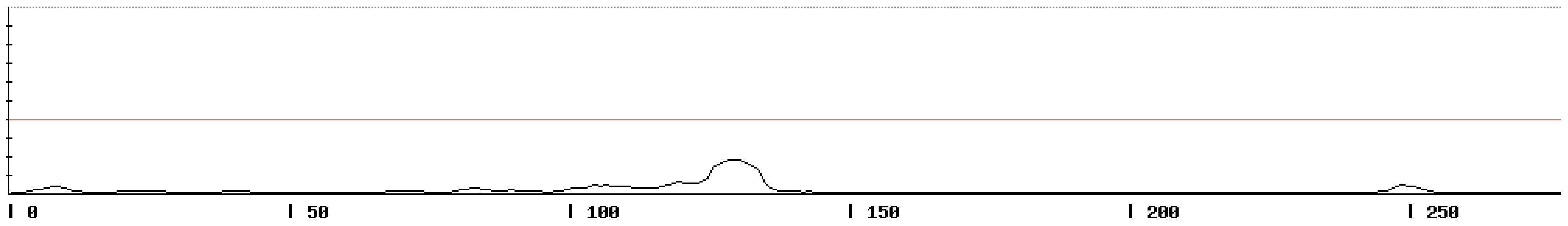

Predictions for Arabisalpina_KFK38892.1

Viterbi Path

Viterbi Path

Posterior @ 0.4

Posterior @ 0.4

Posterior Graphic. Threshold Marked @ 0.4

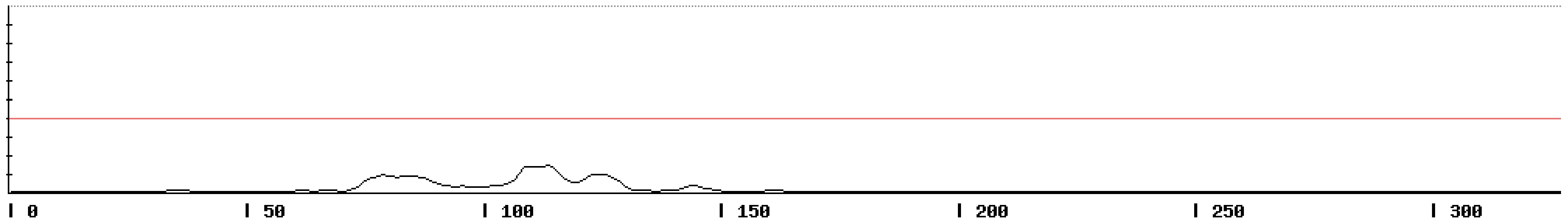

Predictions for Arabisalpina_KFK38893.1

Viterbi Path

Viterbi Path

Posterior @ 0.4

Posterior @ 0.4

Posterior Graphic. Threshold Marked @ 0.4

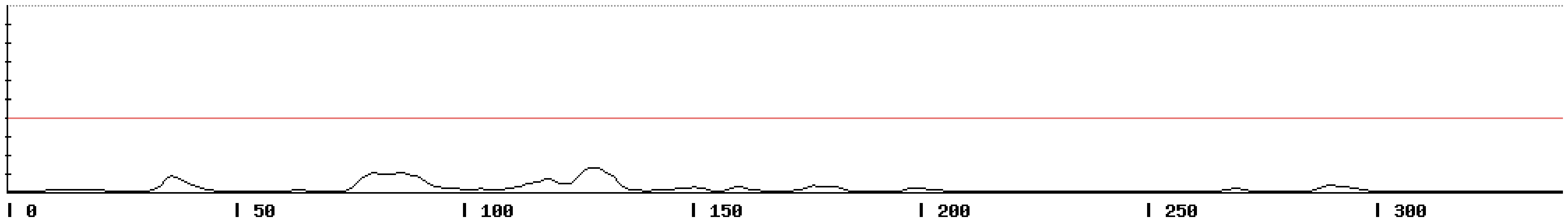

Predictions for Arabisalpina_KFK39102.1

Viterbi Path

Viterbi Path

Posterior @ 0.4

Posterior @ 0.4

Posterior Graphic. Threshold Marked @ 0.4

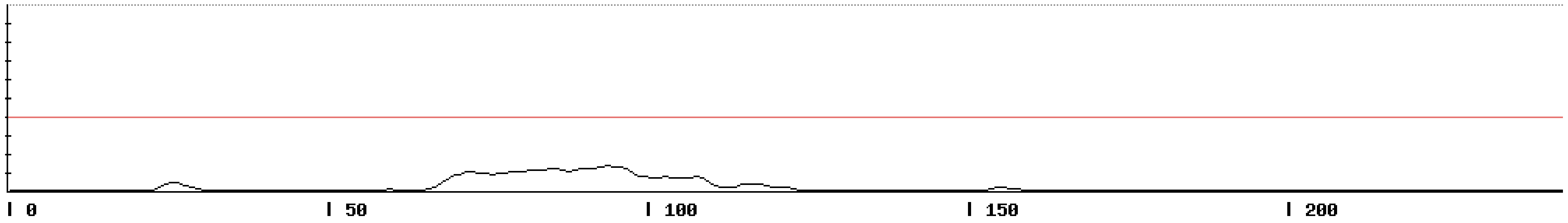

Predictions for Arabisalpina_KFK40091.1

Viterbi Path

Viterbi Path

Posterior @ 0.4

Posterior @ 0.4

Posterior Graphic. Threshold Marked @ 0.4

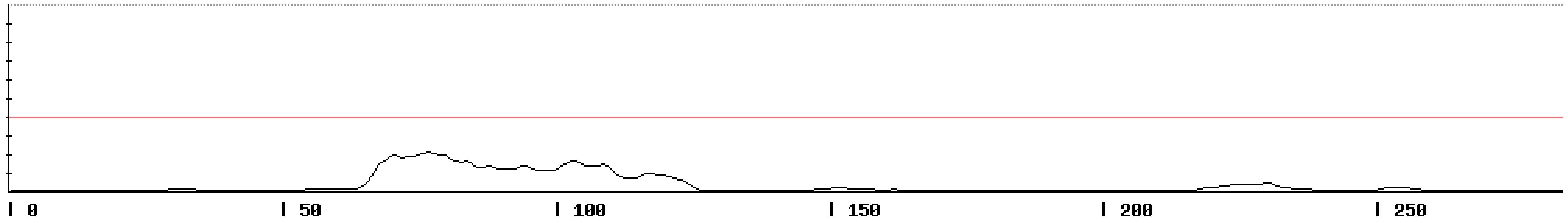

Predictions for Arabisalpina_KFK41373.1

Viterbi Path

Viterbi Path

Posterior @ 0.4

Posterior @ 0.4

Posterior Graphic. Threshold Marked @ 0.4

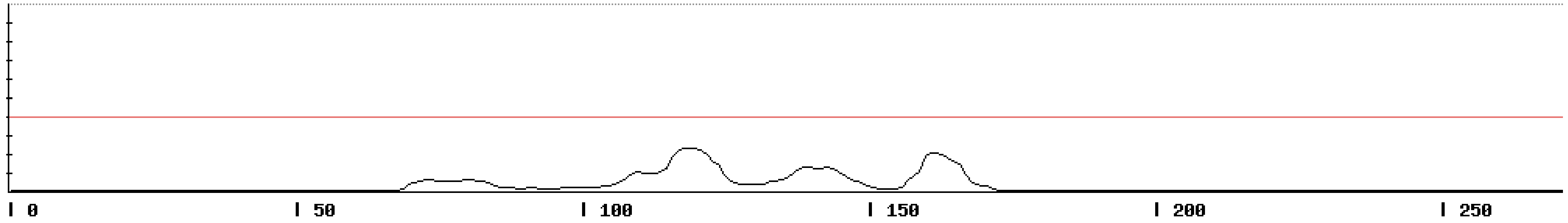

Predictions for Arabisalpina_KFK41460.1

Viterbi Path

Viterbi Path

Posterior @ 0.4	
Posterior @ 0.4	
Posterior Graphic. Threshold Marked @ 0.4	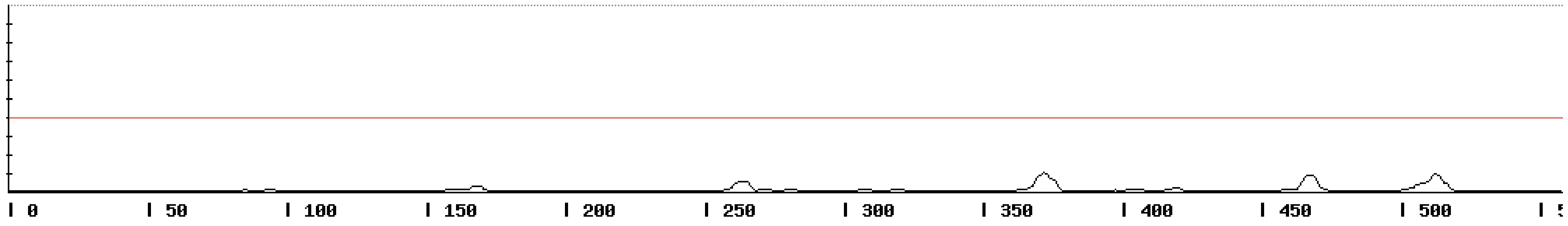

Predictions for Arabisalpina_KFK42062.1

Viterbi Path	
Viterbi Path	
Posterior @ 0.4	
Posterior @ 0.4	
Posterior Graphic. Threshold Marked @ 0.4	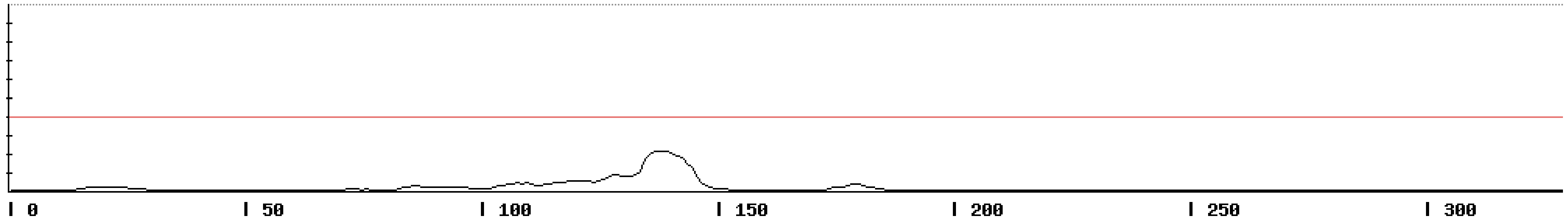

Predictions for Arabisalpina_KFK42139.1

Viterbi Path

Viterbi Path

Posterior @ 0.4 — 98 - KPIGRPKPVGIKK - 110

Posterior @ 0.4

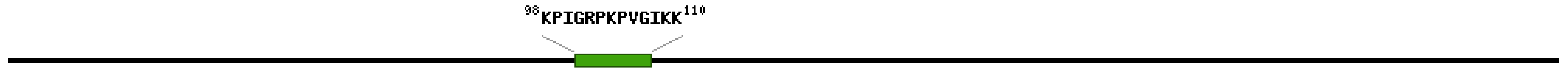

Posterior Graphic. Threshold Marked @ 0.4

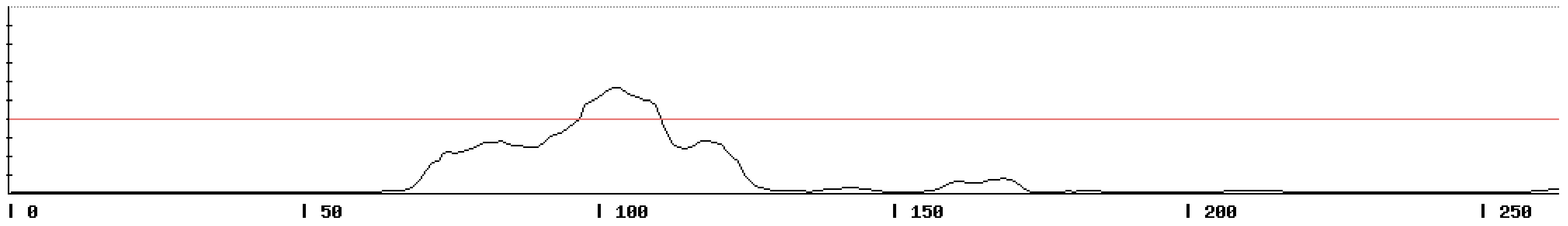

Predictions for Arabisalpina_KFK42489.1

Viterbi Path

Viterbi Path

Posterior @ 0.4 — 91 - KKTGKPTEIKRKSGNREK - 108

286 - RKENKYNRGDRQIRKTKSGYWKKTGKPTEIKRKNGEKIGEK - 326

Posterior @ 0.4

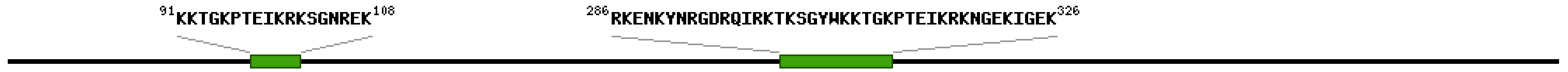

Posterior Graphic. Threshold Marked @ 0.4

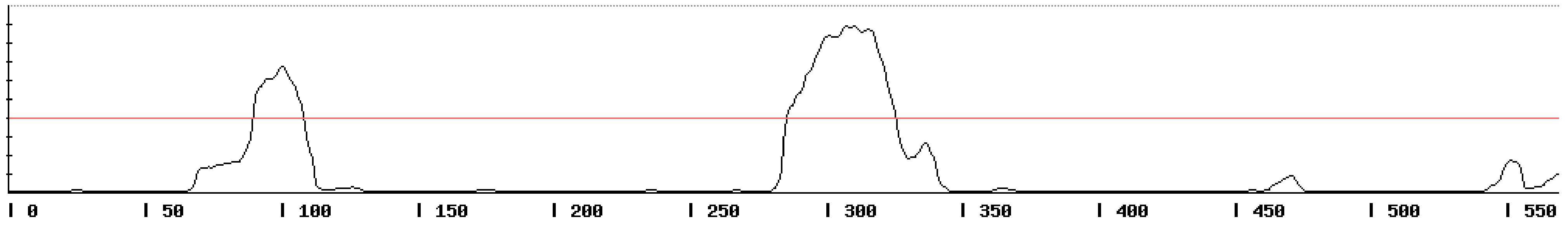

Predictions for Arabisalpina_KFK42490.1

Viterbi Path	
Viterbi Path	
Posterior @ 0.4	68 - GKLKRTKRASR - 78
Posterior @ 0.4	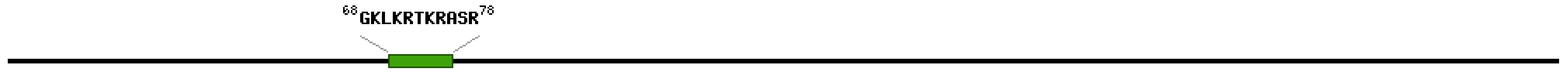
Posterior Graphic. Threshold Marked @ 0.4	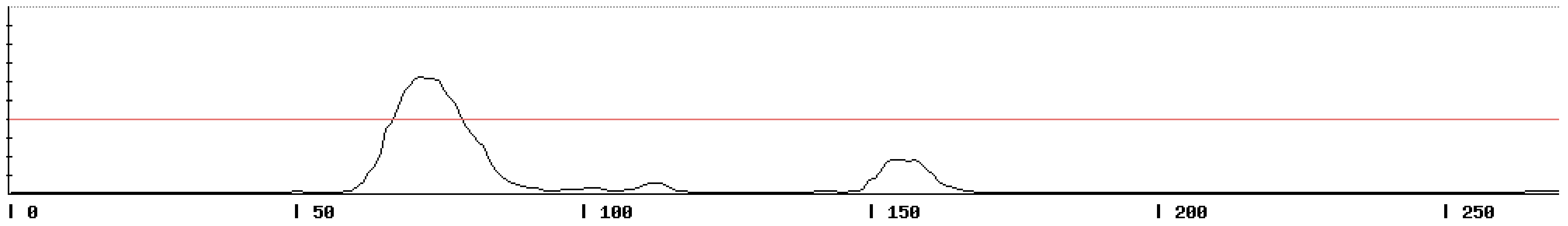

Predictions for Arabisalpina_KFK42491.1

Viterbi Path	
Viterbi Path	
Posterior @ 0.4	
Posterior @ 0.4	

Posterior Graphic. Threshold Marked @ 0.4

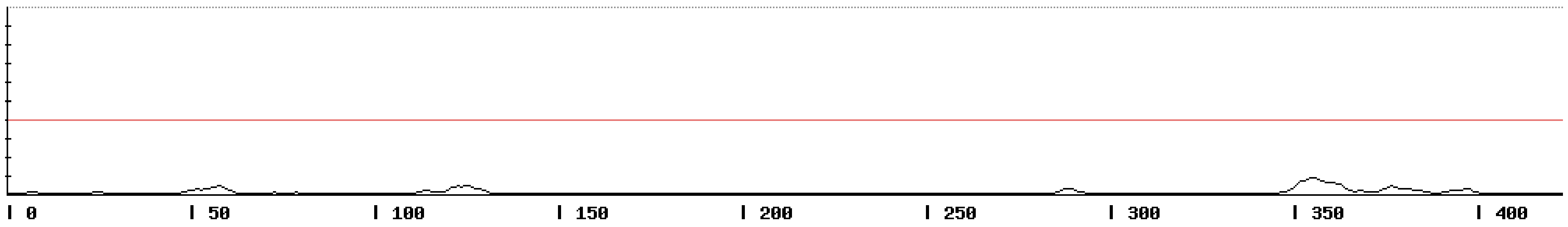

Predictions for Arabisalpina_KFK42525.1

Viterbi Path

Viterbi Path

Posterior @ 0.4 76 - KENKYNRGDRQMRKTKSGFWKKTGKPTEIKRKNGEKI - 112

Posterior @ 0.4

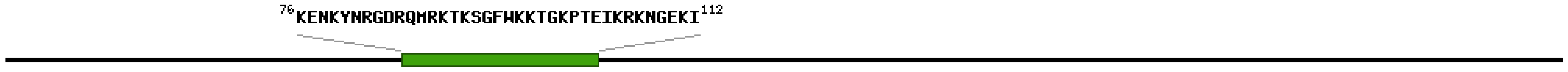

Posterior Graphic. Threshold Marked @ 0.4

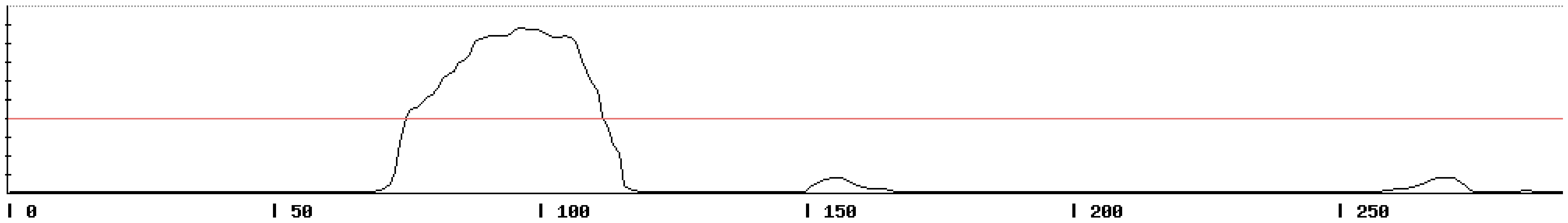

Predictions for Arabisalpina_KFK42526.1

Viterbi Path

Viterbi Path

Posterior @ 0.4 70 - KENKYNRGDRQIRKTKSGYWKKTGKPTEIKRKNGEKIGEK - 109

Posterior @ 0.4

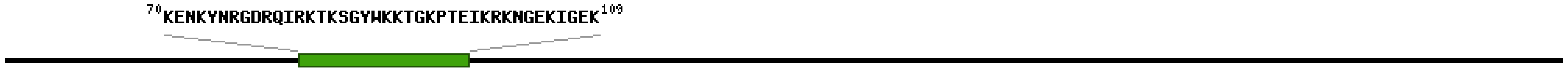

Posterior Graphic. Threshold Marked @ 0.4

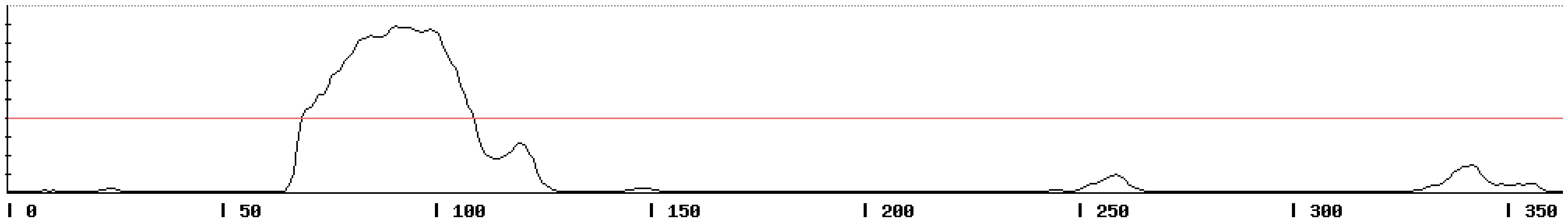

Predictions for Arabisalpina_KFK42547.1

Viterbi Path

Viterbi Path

Posterior @ 0.4

Posterior @ 0.4

Posterior Graphic. Threshold Marked @ 0.4

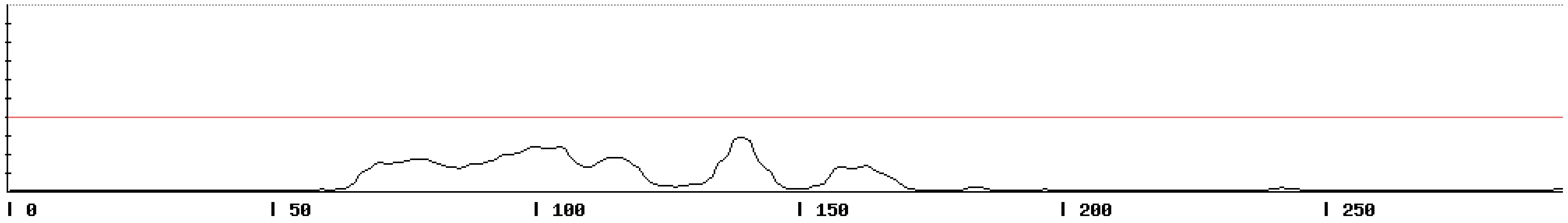

Predictions for Arabisalpina_KFK42739.1

Viterbi Path

Viterbi Path

Posterior @ 0.4

Posterior @ 0.4

Posterior Graphic. Threshold Marked @ 0.4

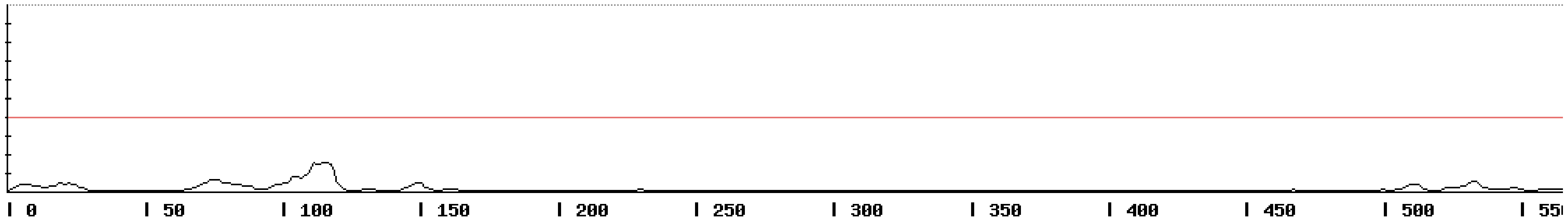

Predictions for Arabisalpina_KFK42926.1

Viterbi Path

Viterbi Path

Posterior @ 0.4

Posterior @ 0.4

Posterior Graphic. Threshold Marked @ 0.4

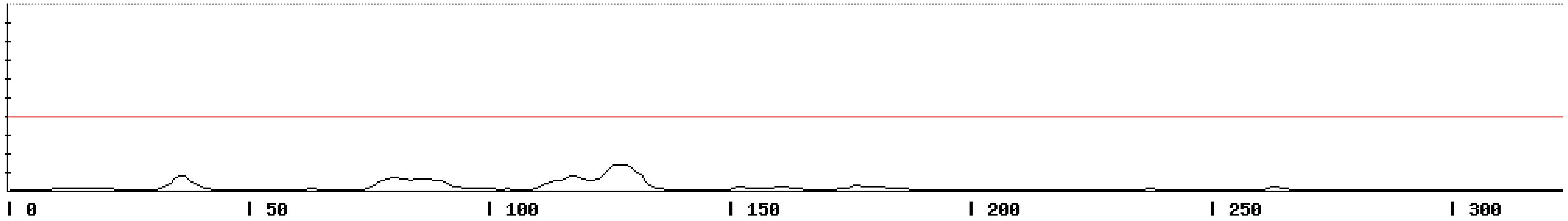

Predictions for Arabisalpina_KFK44551.1

Viterbi Path

Viterbi Path

Posterior @ 0.4	138 - KRR - 140
Posterior @ 0.4	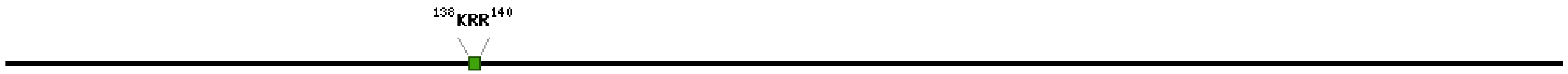
Posterior Graphic. Threshold Marked @ 0.4	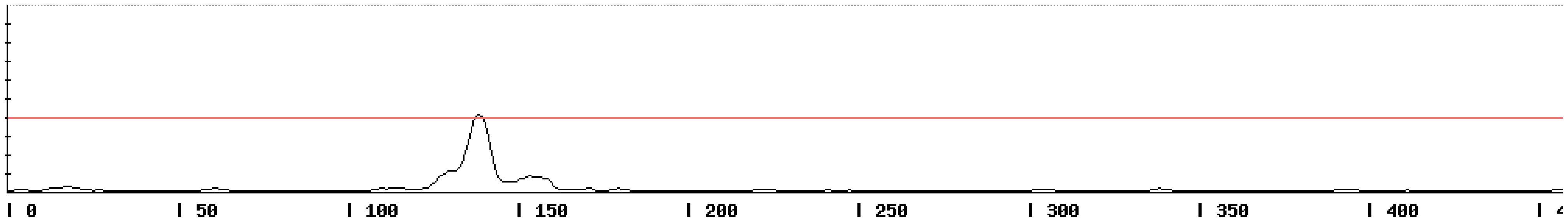

Predictions for Arabisalpina_KFK44718.1

Viterbi Path	
Viterbi Path	
Posterior @ 0.4	155 - KRR - 157
Posterior @ 0.4	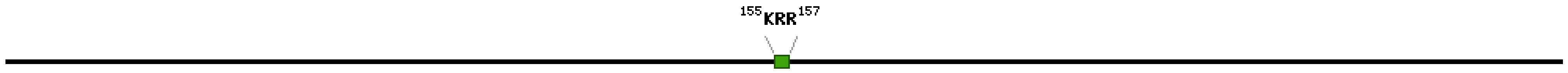
Posterior Graphic. Threshold Marked @ 0.4	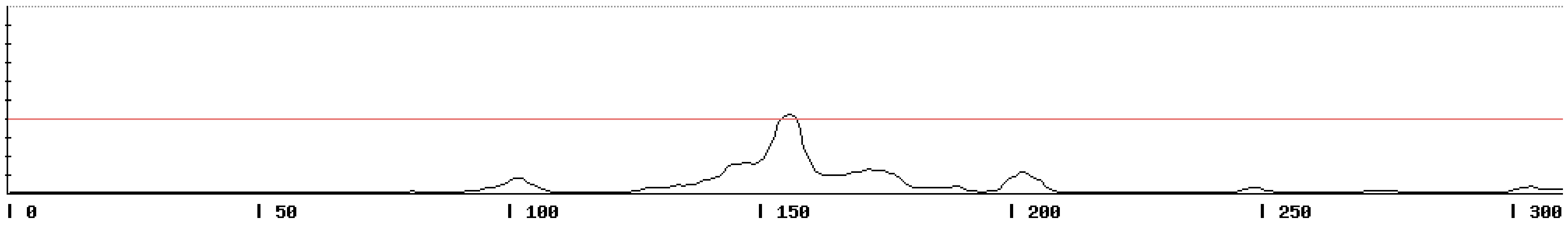

Predictions for Arabisalpina_KFK44992.1

Viterbi Path

Viterbi Path

Posterior @ 0.4

Posterior @ 0.4

Posterior Graphic. Threshold Marked @ 0.4

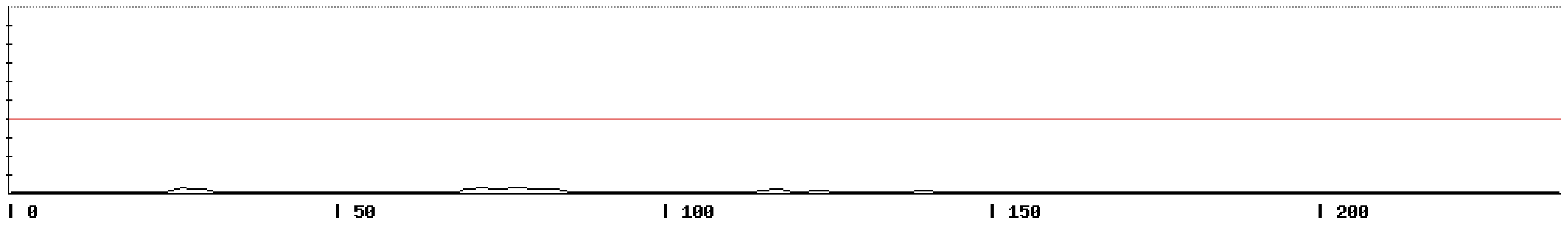

Predictions for Arabisalpina_KFK45025.1

Viterbi Path

Viterbi Path

Posterior @ 0.4

Posterior @ 0.4

Posterior Graphic. Threshold Marked @ 0.4

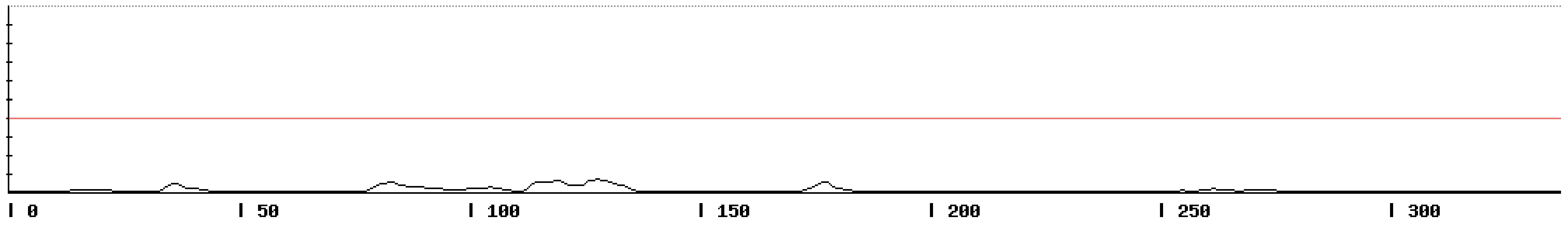

Predictions for Arabisalpina_KFK45026.1

Viterbi Path	
Viterbi Path	
Posterior @ 0.4	
Posterior @ 0.4	
Posterior Graphic. Threshold Marked @ 0.4	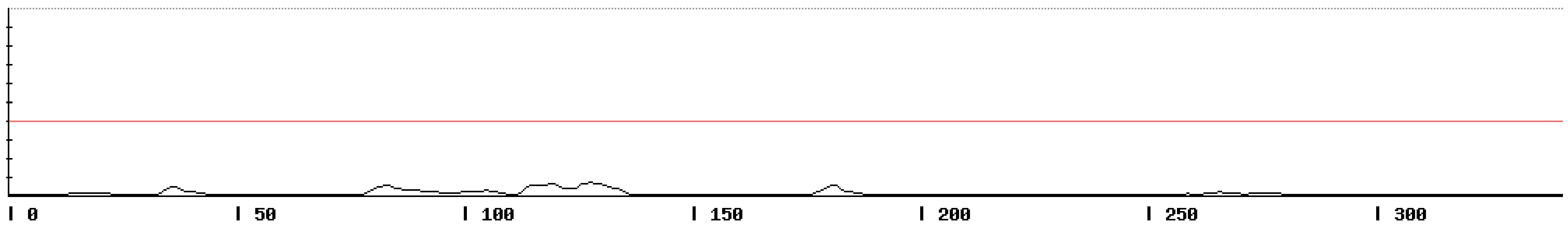

Predictions for Arabisalpina_KFK45049.1

Viterbi Path	
Viterbi Path	
Posterior @ 0.4	490 - NKKKERPADIKKKER - 504
Posterior @ 0.4	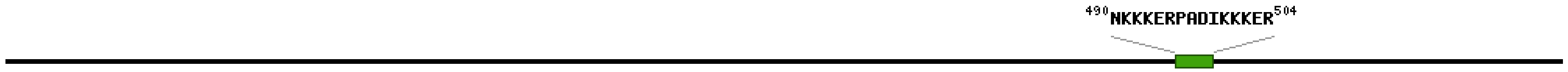
Posterior Graphic. Threshold Marked @ 0.4	

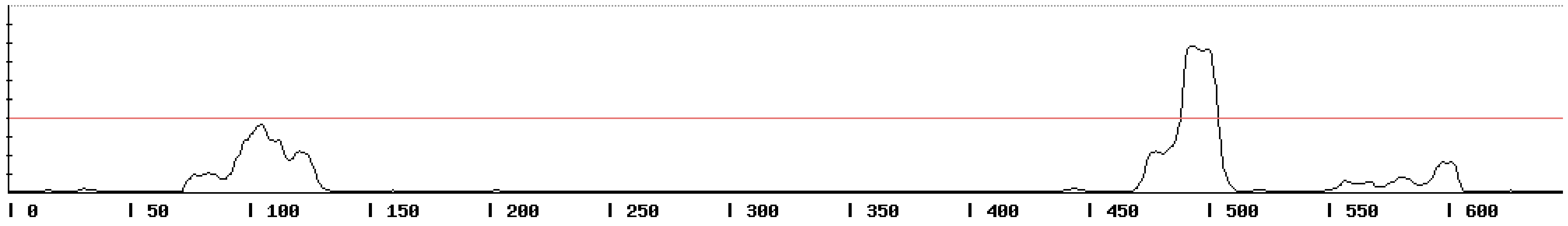

Predictions for Arabisalpina_KFK45050.1

Viterbi Path

Viterbi Path

Posterior @ 0.4

491 - NKKKERPADIKKKER - 505

Posterior @ 0.4

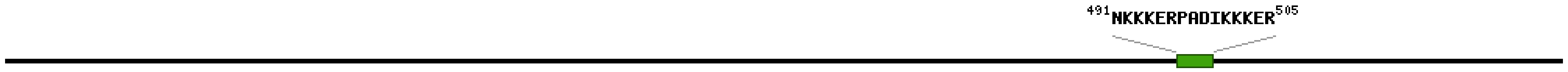

Posterior Graphic. Threshold Marked @ 0.4

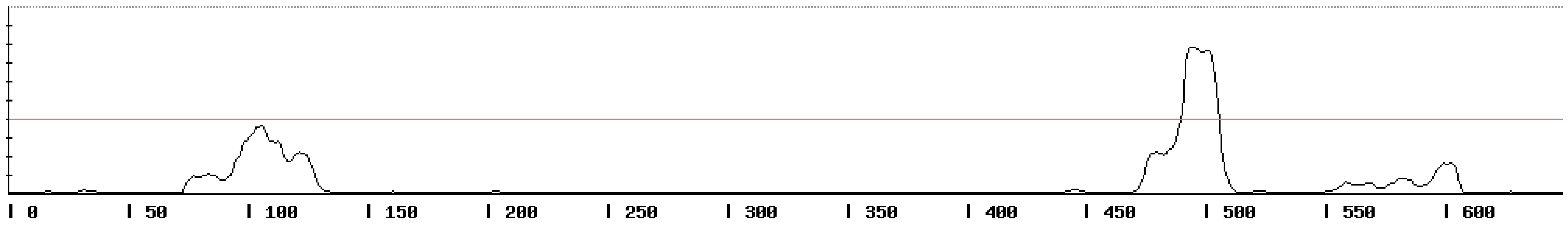

Predictions for Arabisalpina_KFK45065.1

Viterbi Path

Viterbi Path

Posterior @ 0.4

Posterior @ 0.4

Posterior Graphic. Threshold Marked @ 0.4

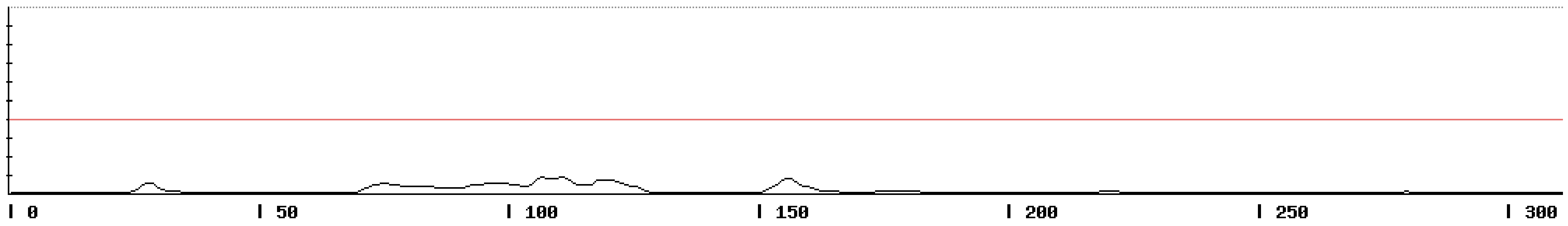

Predictions for Arabisalpina_KFK45122.1

Viterbi Path

Viterbi Path

Posterior @ 0.4

Posterior @ 0.4

Posterior Graphic. Threshold Marked @ 0.4

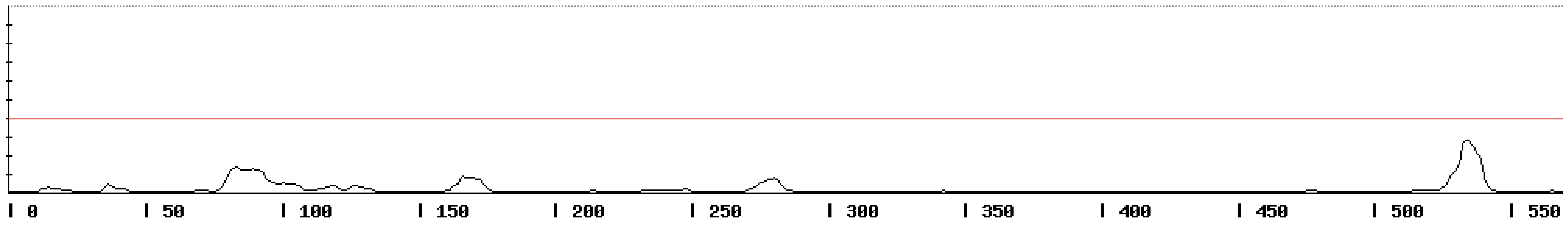

Predictions for Arabisalpina_KFK45123.1

Viterbi Path

Viterbi Path

Revision r.9 - Last Updated on : February 24 2015 17:45:20.

alex.nguyenba@utoronto.ca

Intended to be used with Firefox

Viterbi Path	
Posterior @ 0.4	
Posterior @ 0.4	
Posterior Graphic. Threshold Marked @ 0.4	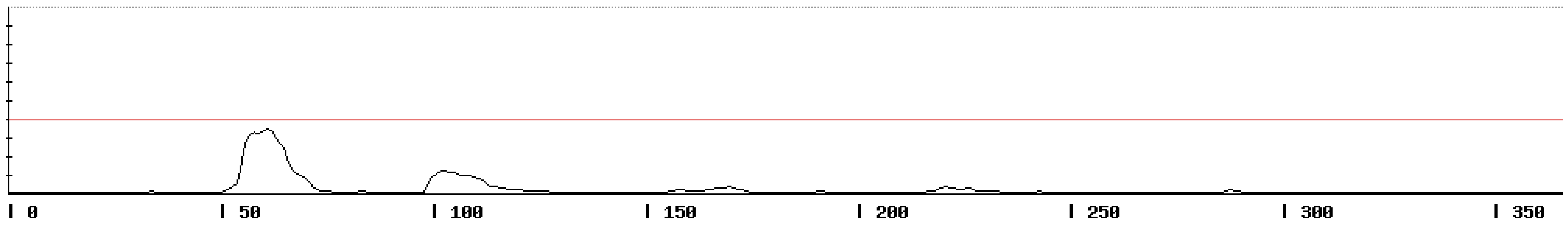

Predictions for Arachisduranensis_Aradu.08TAH

Viterbi Path	
Viterbi Path	
Posterior @ 0.4	
Posterior @ 0.4	
Posterior Graphic. Threshold Marked @ 0.4	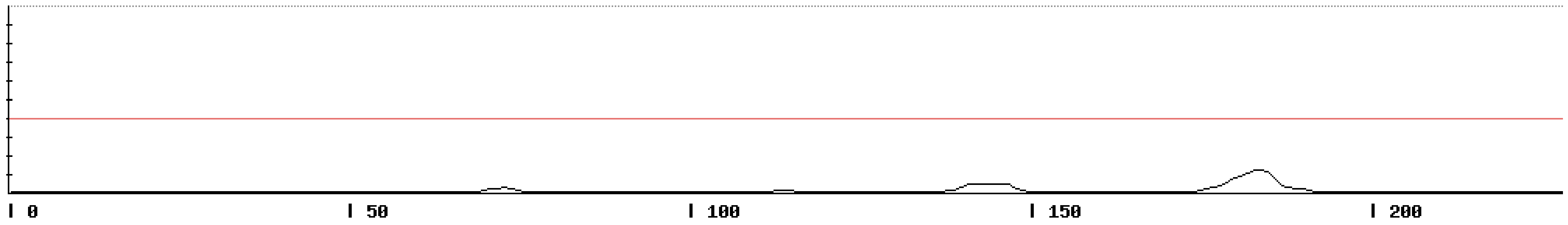

Predictions for Arachisduranensis_Aradu.0MJ0X

Viterbi Path

Viterbi Path

Posterior @ 0.4

Posterior @ 0.4

Posterior Graphic. Threshold Marked @ 0.4

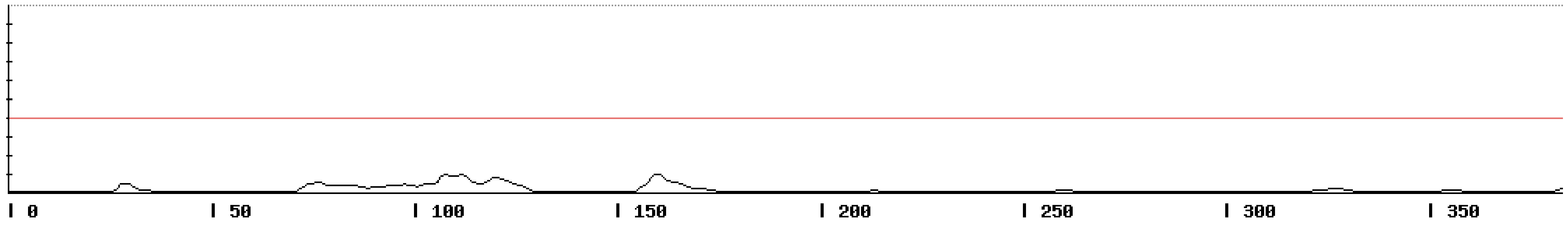

Predictions for Arachisduranensis_Aradu.13D06

Viterbi Path

Viterbi Path

Posterior @ 0.4

Posterior @ 0.4

Posterior Graphic. Threshold Marked @ 0.4

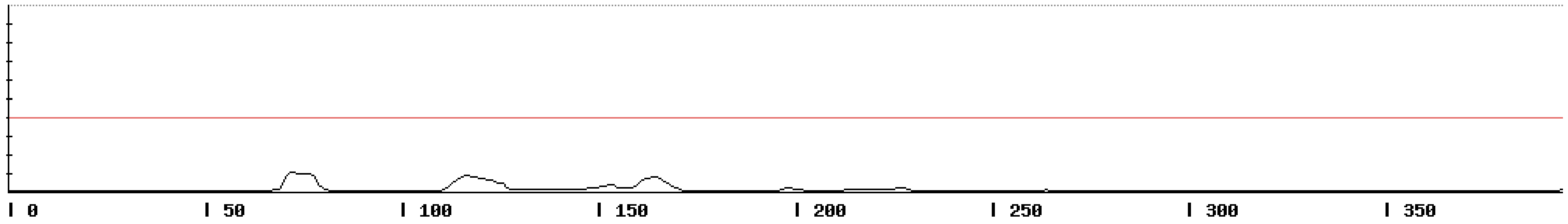

Predictions for Arachisduranensis_Aradu.15JI0	
Viterbi Path	
Viterbi Path	
Posterior @ 0.4	
Posterior @ 0.4	
Posterior Graphic. Threshold Marked @ 0.4	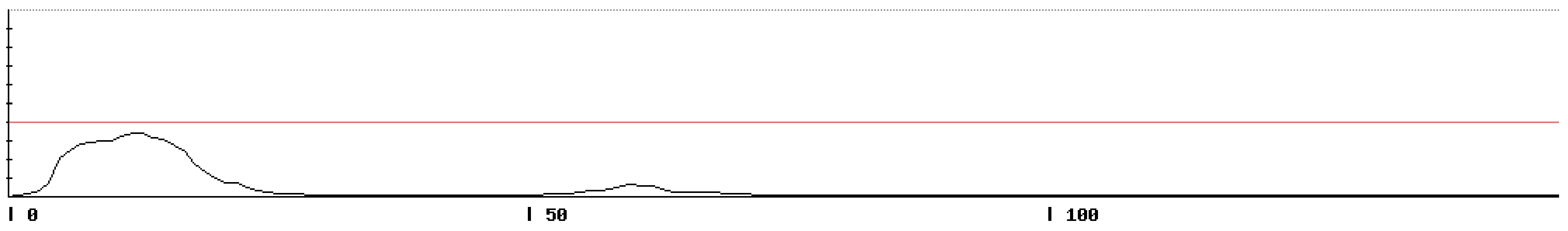

Predictions for Arachisduranensis_Aradu.15QQT	
Viterbi Path	
Viterbi Path	
Posterior @ 0.4	
Posterior @ 0.4	
Posterior Graphic. Threshold Marked @ 0.4	

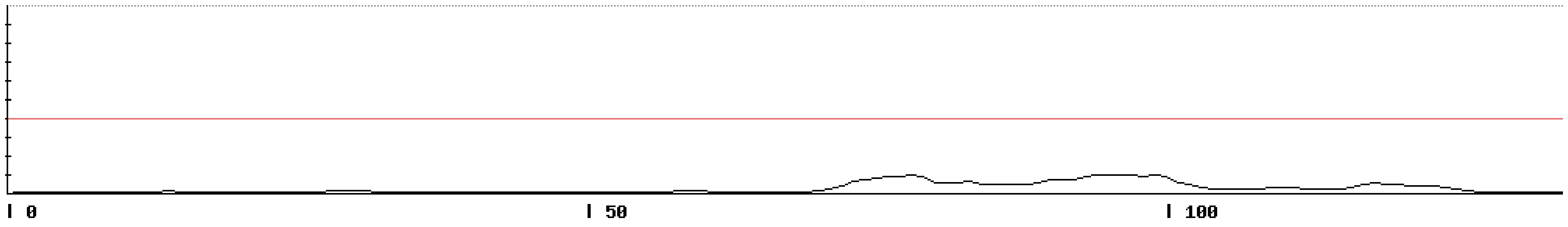

Predictions for Arachisduranensis_Aradu.1AJ4F

Viterbi Path	
Viterbi Path	
Posterior @ 0.4	
Posterior @ 0.4	
Posterior Graphic. Threshold Marked @ 0.4	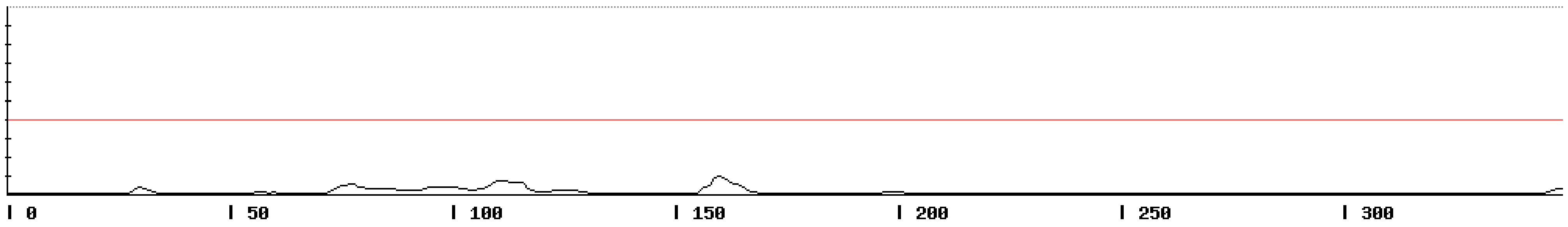

Predictions for Arachisduranensis_Aradu.215DG

Viterbi Path	
Viterbi Path	
Posterior @ 0.4	135 - RKRRK - 139
Posterior @ 0.4	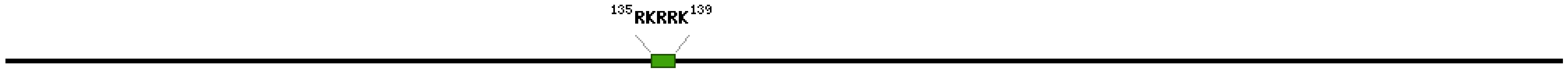

Posterior Graphic. Threshold Marked @ 0.4

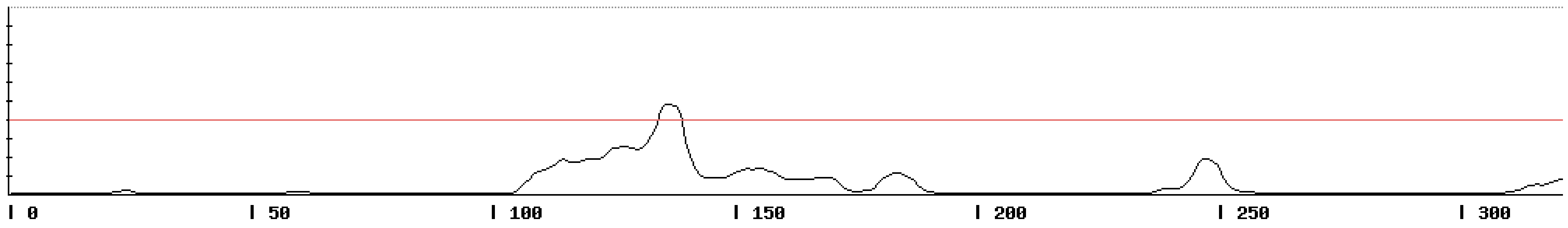

Predictions for Arachisduranensis_Aradu.22647

Viterbi Path	
Viterbi Path	
Posterior @ 0.4	
Posterior @ 0.4	
Posterior Graphic. Threshold Marked @ 0.4	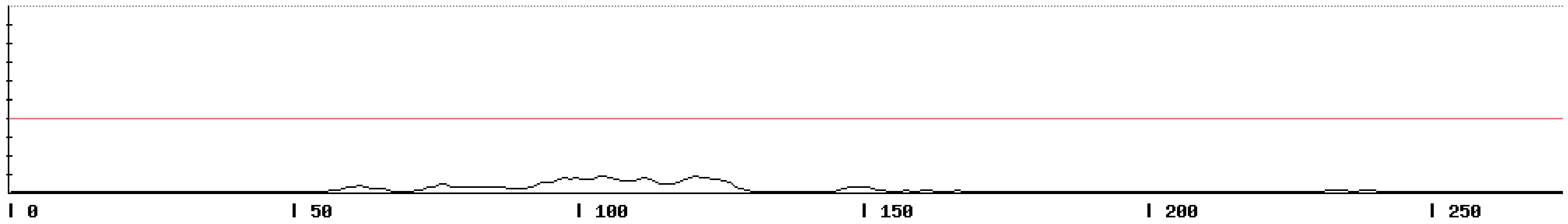

Predictions for Arachisduranensis_Aradu.30S8W

Viterbi Path	
Viterbi Path	
Posterior @ 0.4	125 - HKVFKKK - 131
Posterior @ 0.4	

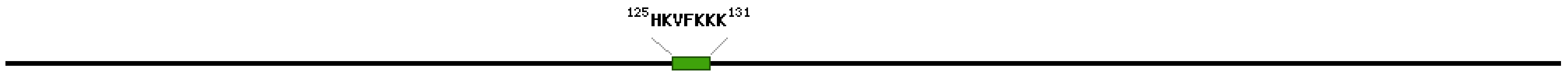

Posterior Graphic. Threshold Marked @ 0.4

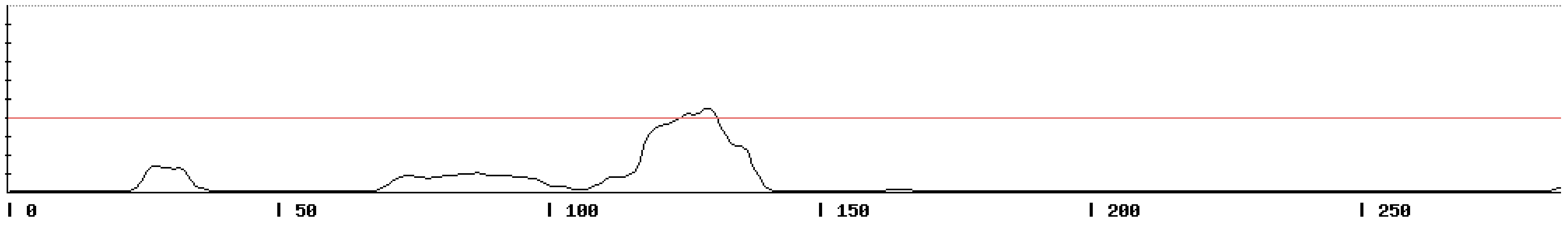

Predictions for Arachisduranensis_Aradu.3R7A3

Viterbi Path

Viterbi Path

Posterior @ 0.4 262 - KK - 263

Posterior @ 0.4

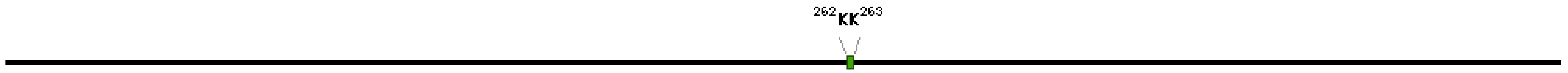

Posterior Graphic. Threshold Marked @ 0.4

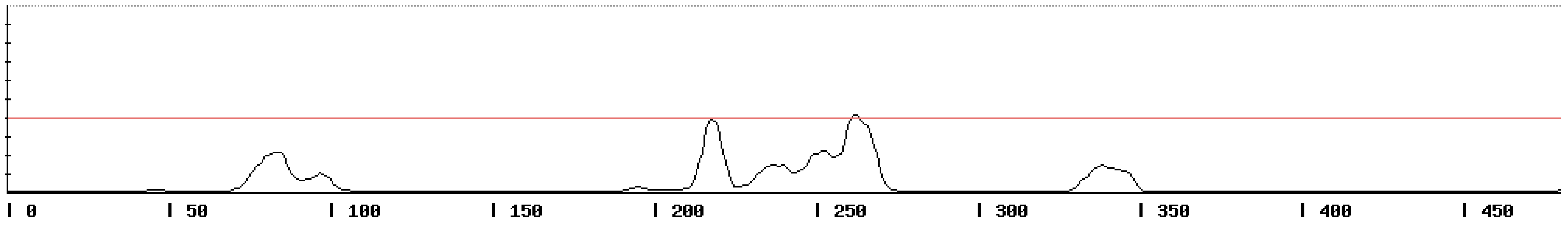

Predictions for Arachisduranensis_Aradu.46U1T

Viterbi Path

Viterbi Path

Posterior @ 0.4

Posterior @ 0.4

Posterior Graphic. Threshold Marked @ 0.4

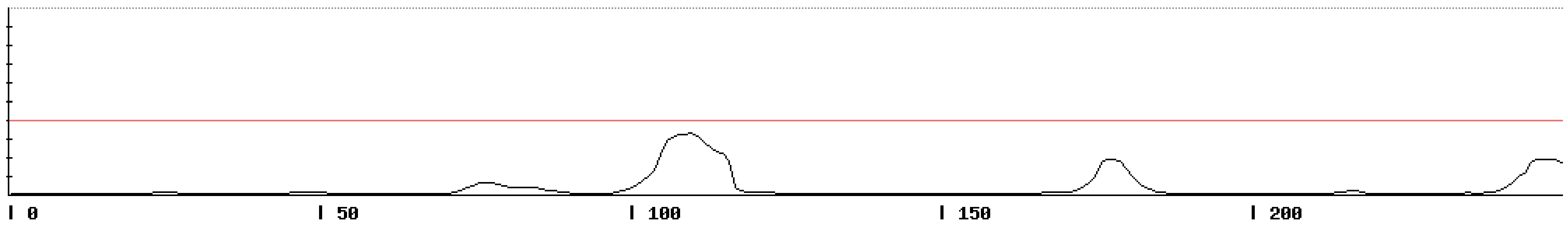

Predictions for Arachisduranensis_Aradu.47JQU

Viterbi Path

Viterbi Path

Posterior @ 0.4

Posterior @ 0.4

Posterior Graphic. Threshold Marked @ 0.4

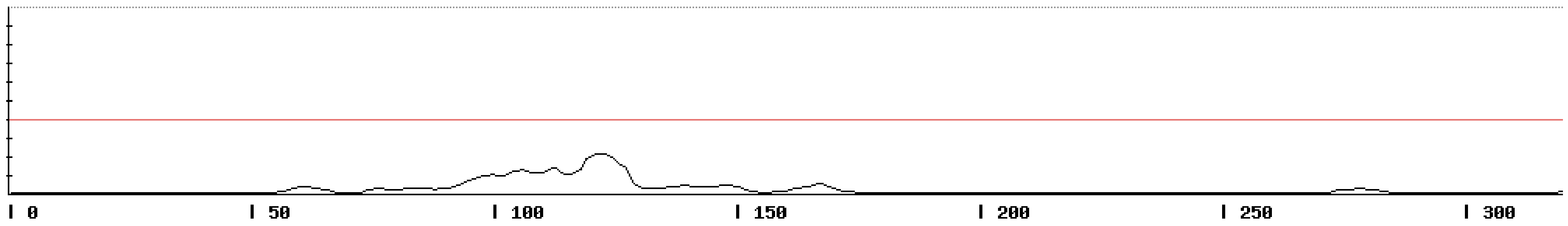

Predictions for Arachisduranensis_Aradu.4RJ0E

Viterbi Path

Viterbi Path

Posterior @ 0.4

Posterior @ 0.4

Posterior Graphic. Threshold Marked @ 0.4

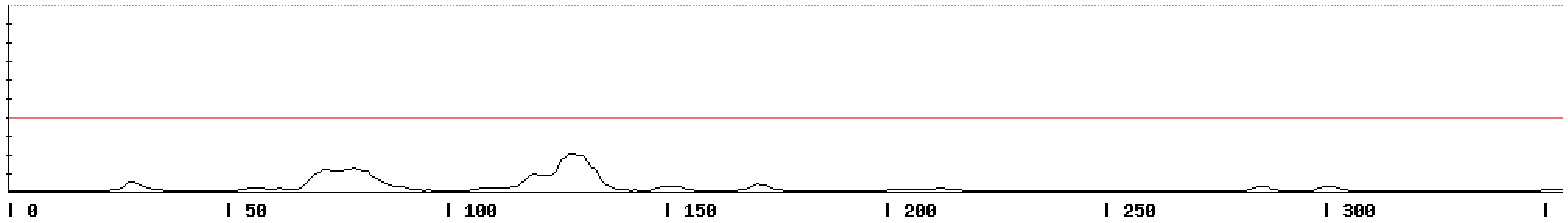

Predictions for Arachisduranensis_Aradu.58D1A

Viterbi Path

Viterbi Path

Posterior @ 0.4

Posterior @ 0.4

Posterior Graphic. Threshold Marked @ 0.4

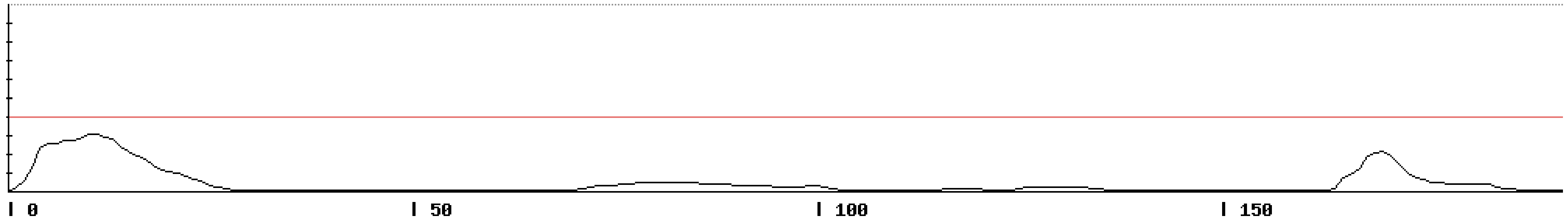

Predictions for Arachisduranensis_Aradu.5D5JN

Viterbi Path

Viterbi Path

Posterior @ 0.4

Posterior @ 0.4

Posterior Graphic. Threshold Marked @ 0.4

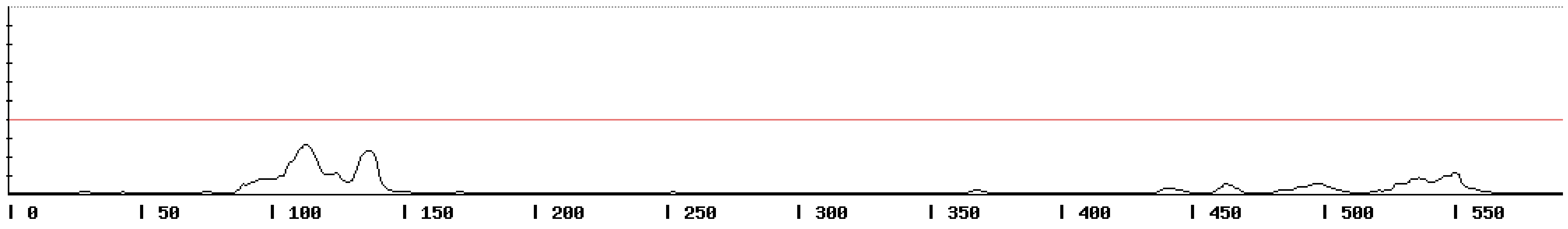

Predictions for Arachisduranensis_Aradu.60U13

Viterbi Path

Viterbi Path

Posterior @ 0.4

Posterior @ 0.4

Posterior Graphic. Threshold Marked @ 0.4

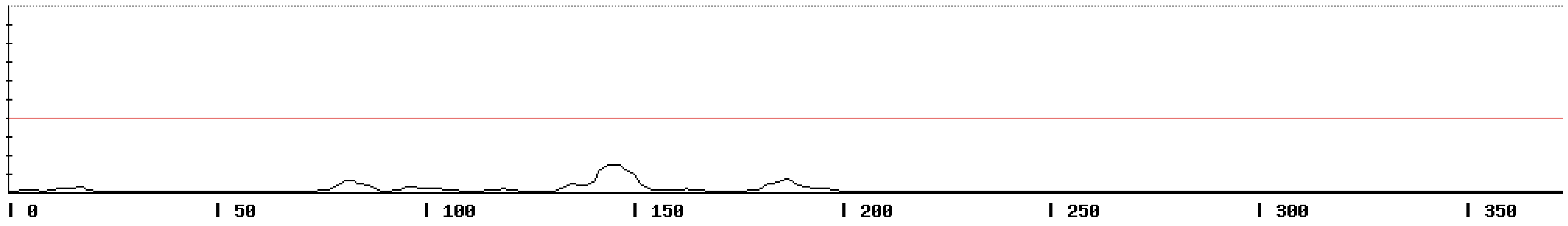

Predictions for Arachisduranensis_Aradu.66XRP

Viterbi Path	
Viterbi Path	
Posterior @ 0.4	
Posterior @ 0.4	
Posterior Graphic. Threshold Marked @ 0.4	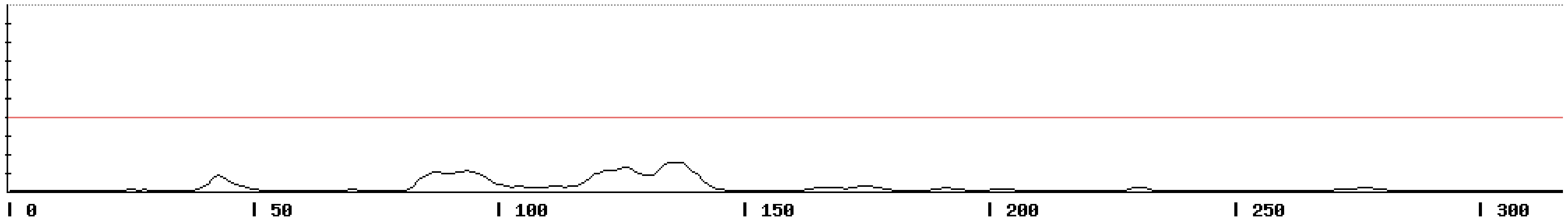

Predictions for Arachisduranensis_Aradu.6H4PP

Viterbi Path	
Viterbi Path	
Posterior @ 0.4	
Posterior @ 0.4	
Posterior Graphic. Threshold Marked @ 0.4	

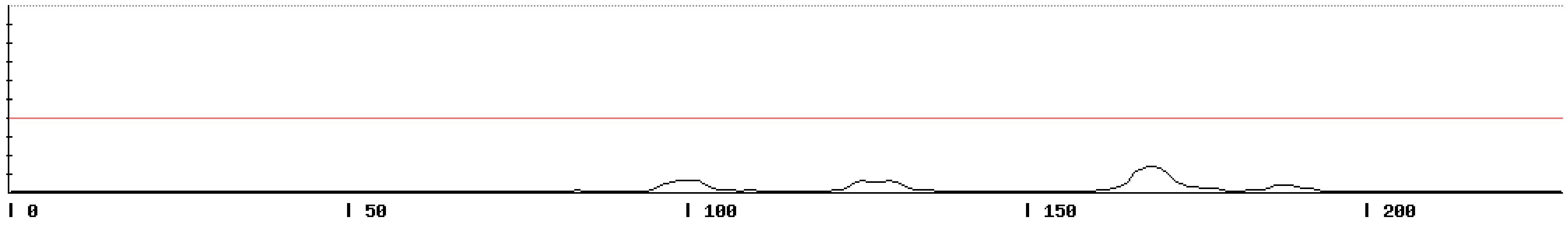

Predictions for Arachisduranensis_Aradu.79PL2	
Viterbi Path	
Viterbi Path	
Posterior @ 0.4	
Posterior @ 0.4	
Posterior Graphic. Threshold Marked @ 0.4	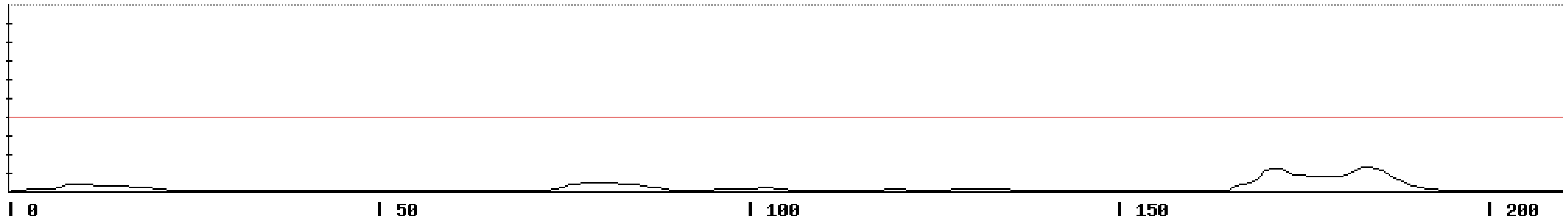

Predictions for Arachisduranensis_Aradu.7NI41	
Viterbi Path	
Viterbi Path	
Posterior @ 0.4	127 - RKRRK - 131
	155 - RGKLK - 159
Posterior @ 0.4	

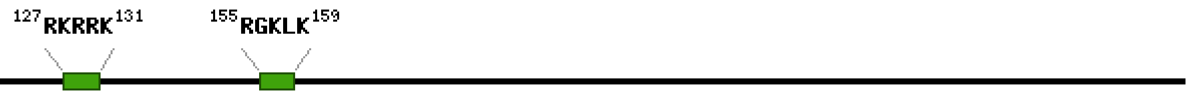

Posterior Graphic. Threshold Marked @ 0.4

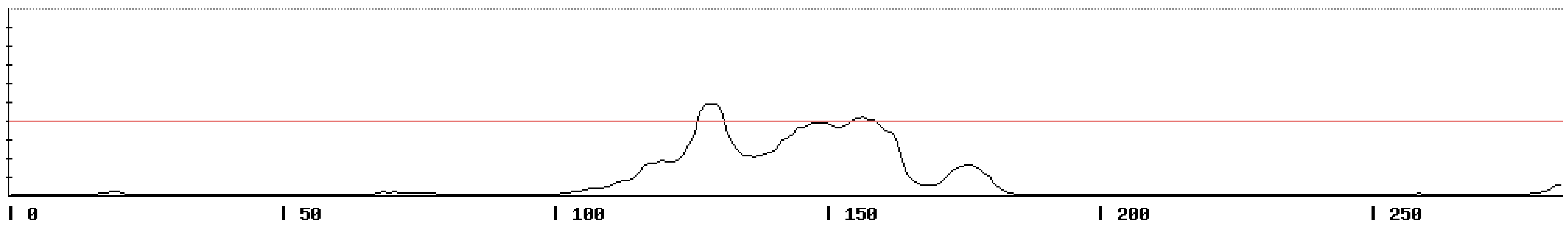

Predictions for Arachisduranensis_Aradu.7X5EV

Viterbi Path

Viterbi Path

Posterior @ 0.4

Posterior @ 0.4

Posterior Graphic. Threshold Marked @ 0.4

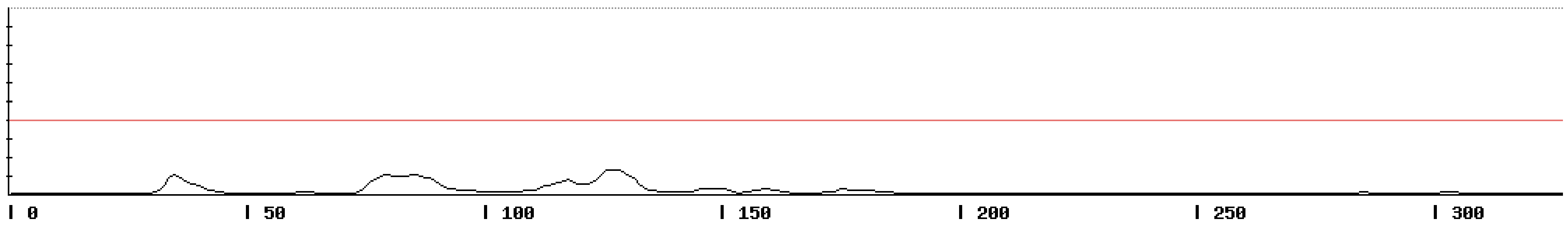

Predictions for Arachisduranensis_Aradu.895HT

Viterbi Path

Viterbi Path

Posterior @ 0.4

Posterior @ 0.4

Posterior Graphic. Threshold Marked @ 0.4

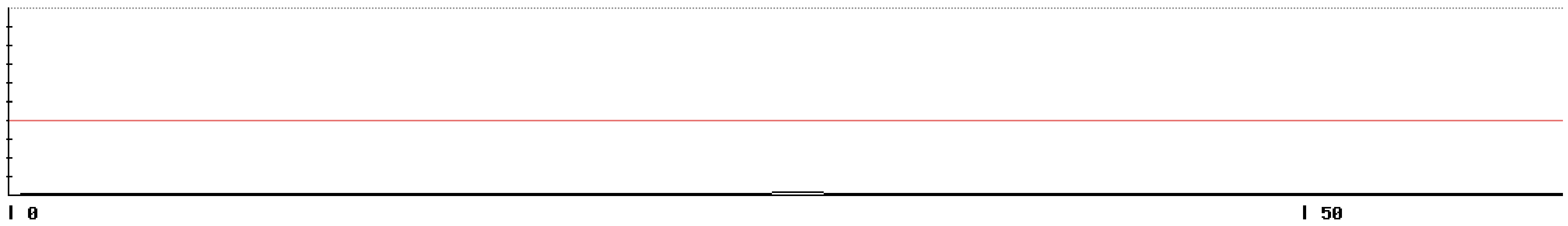

Predictions for Arachisduranensis_Aradu.8Q7DY

Viterbi Path

Viterbi Path

Posterior @ 0.4

Posterior @ 0.4

Posterior Graphic. Threshold Marked @ 0.4

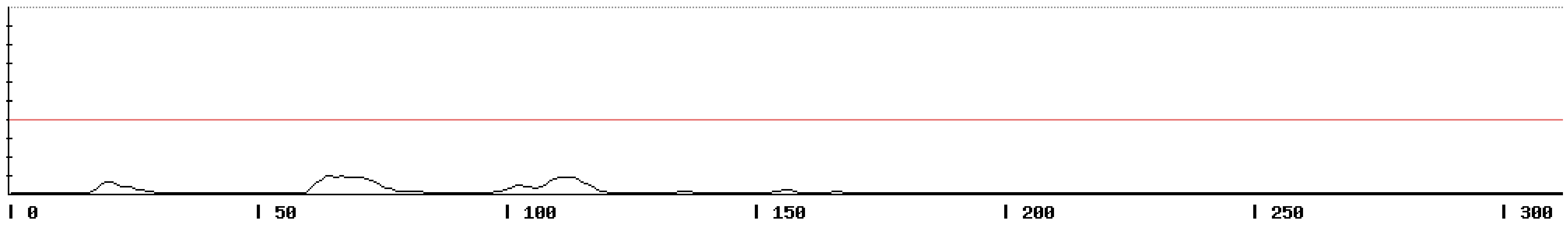

Predictions for Arachisduranensis_Aradu.9FF24

Viterbi Path

Viterbi Path

Posterior @ 0.4

Posterior @ 0.4

Posterior Graphic. Threshold Marked @ 0.4

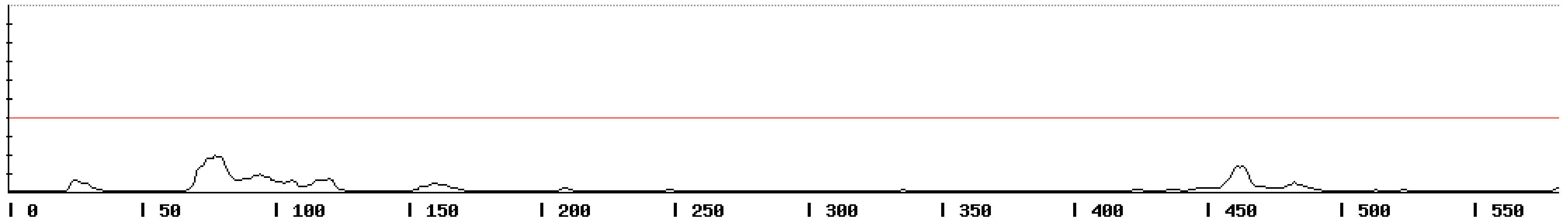

Predictions for Arachisduranensis_Aradu.9T4H8

Viterbi Path

Viterbi Path

Posterior @ 0.4

Posterior @ 0.4

Posterior Graphic. Threshold Marked @ 0.4

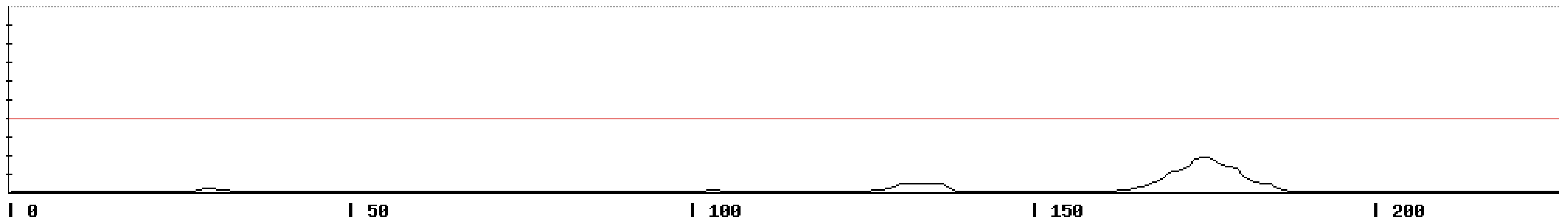

Predictions for Arachisduranensis_Aradu.9Y6NH

Viterbi Path

Viterbi Path

Posterior @ 0.4

Posterior @ 0.4

Posterior Graphic. Threshold Marked @ 0.4

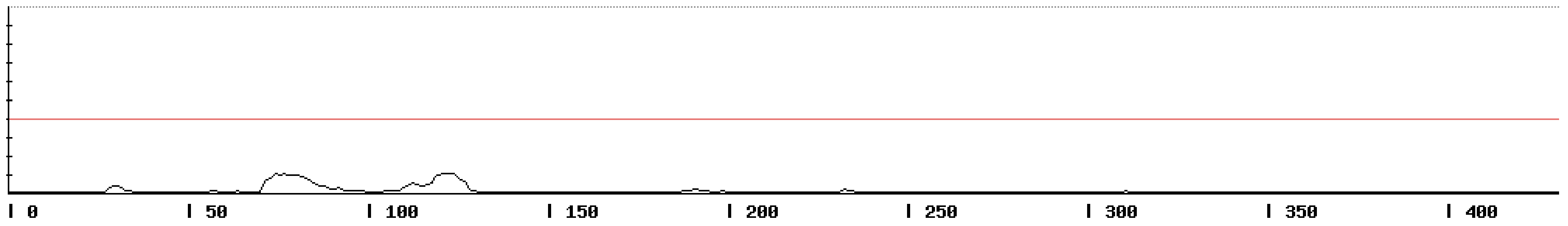

Predictions for Arachisduranensis_Aradu.AF9FZ

Viterbi Path

Viterbi Path

Posterior @ 0.4

Posterior @ 0.4

Posterior Graphic. Threshold Marked @ 0.4

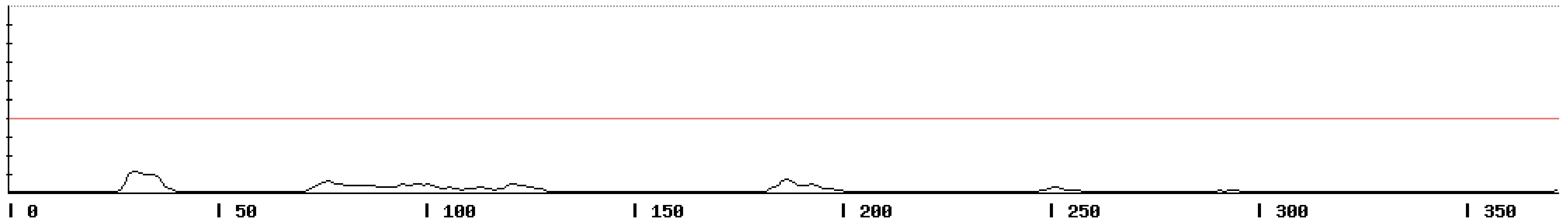

Predictions for Arachisduranensis_Aradu.B5XXI	
Viterbi Path	
Viterbi Path	
Posterior @ 0.4	
Posterior @ 0.4	
Posterior Graphic. Threshold Marked @ 0.4	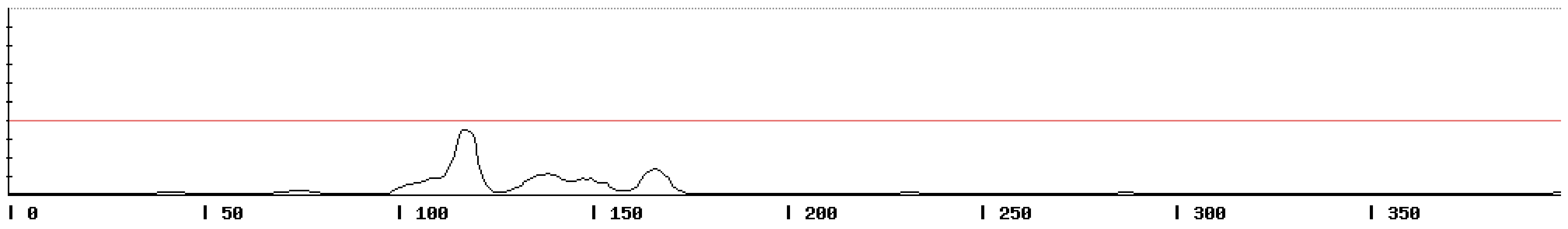

Predictions for Arachisduranensis_Aradu.BPK98	
Viterbi Path	
Viterbi Path	
Posterior @ 0.4	
Posterior @ 0.4	
Posterior Graphic. Threshold Marked @ 0.4	

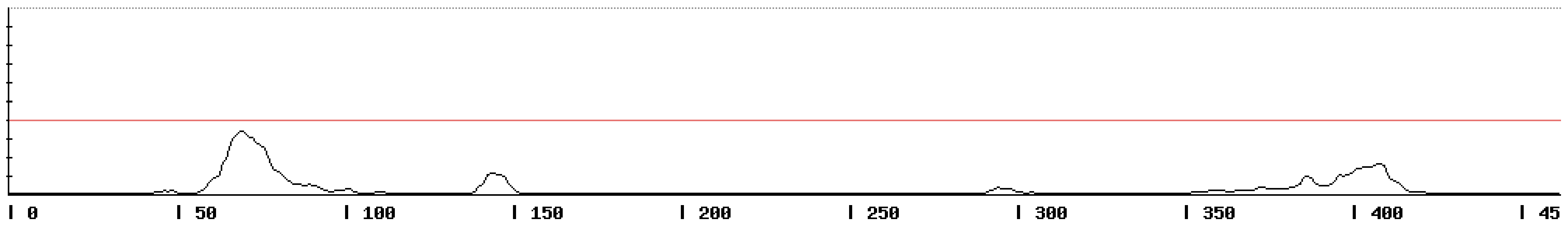

Predictions for Arachisduranensis_Aradu.BS3JU

Viterbi Path

Viterbi Path

Posterior @ 0.4 138 - RKRRK - 142

Posterior @ 0.4

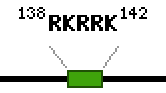

Posterior Graphic. Threshold Marked @ 0.4

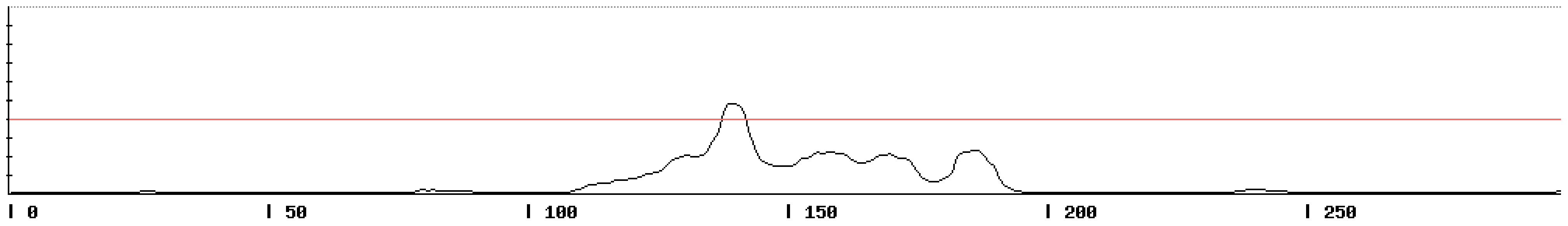

Predictions for Arachisduranensis_Aradu.C1Q0A

Viterbi Path

Viterbi Path

Posterior @ 0.4

Posterior @ 0.4

Posterior Graphic. Threshold Marked @ 0.4

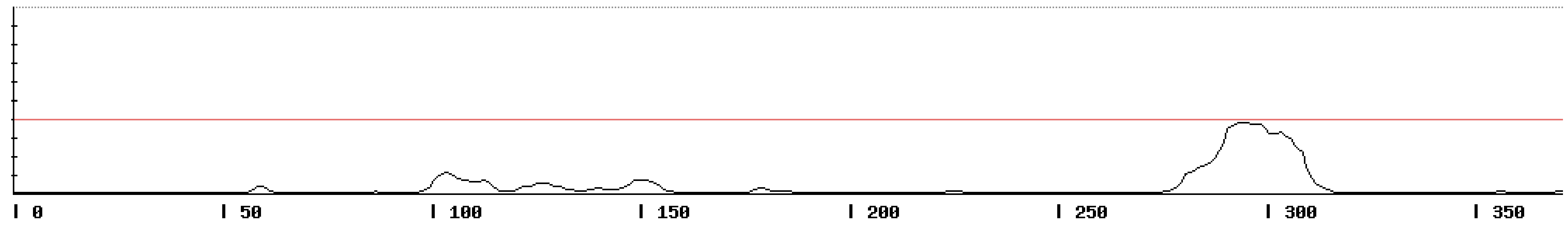

Predictions for Arachisduranensis_Aradu.DII8L

Viterbi Path

Viterbi Path

Posterior @ 0.4

Posterior @ 0.4

Posterior Graphic. Threshold Marked @ 0.4

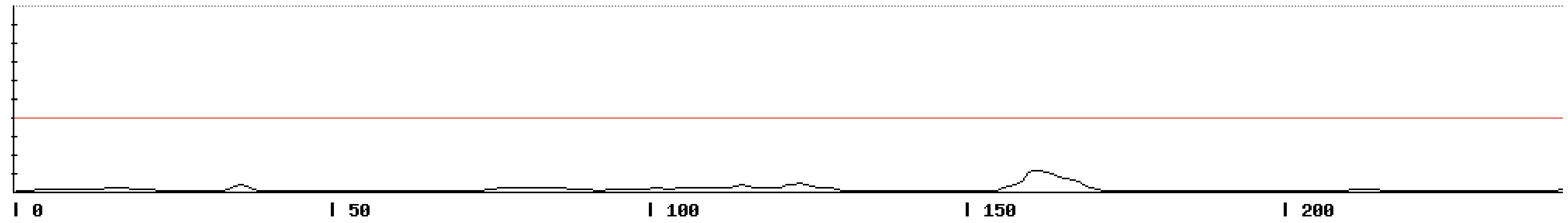

Predictions for Arachisduranensis_Aradu.DQR3M

Viterbi Path

Viterbi Path

Posterior @ 0.4

Posterior @ 0.4

Posterior Graphic. Threshold Marked @ 0.4

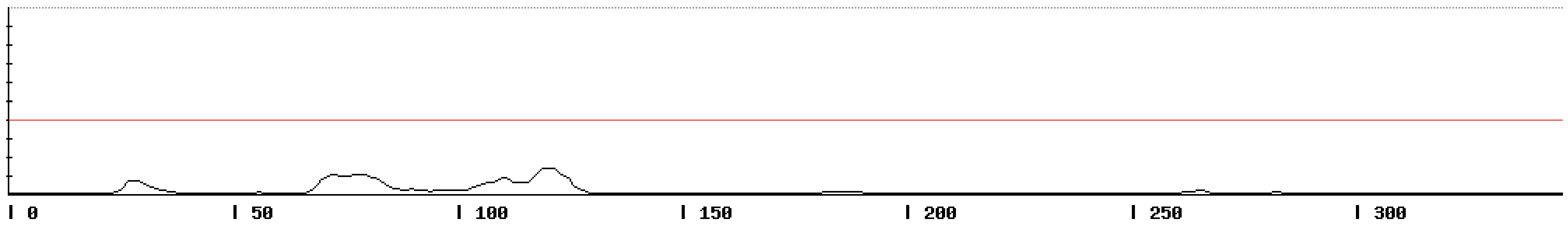

Predictions for Arachisduranensis_Aradu.EP425

Viterbi Path

Viterbi Path

Posterior @ 0.4

Posterior @ 0.4

Posterior Graphic. Threshold Marked @ 0.4

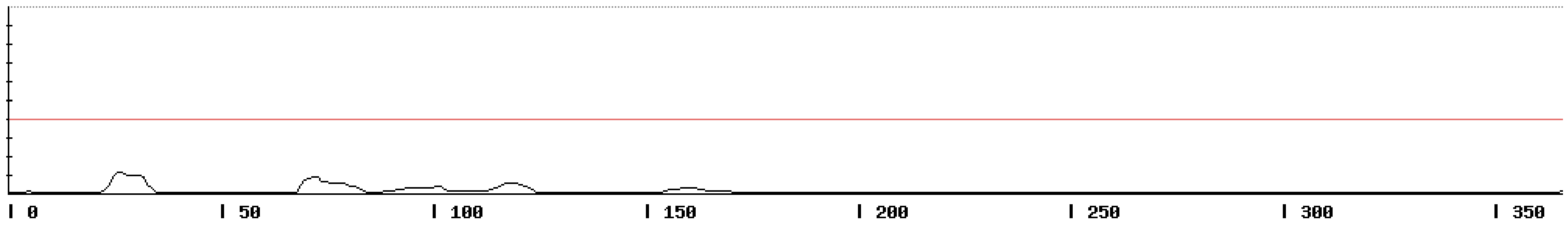

Predictions for Arachisduranensis_Aradu.ETZ8K

Viterbi Path

Viterbi Path

Posterior @ 0.4

Posterior @ 0.4

Posterior Graphic. Threshold Marked @ 0.4

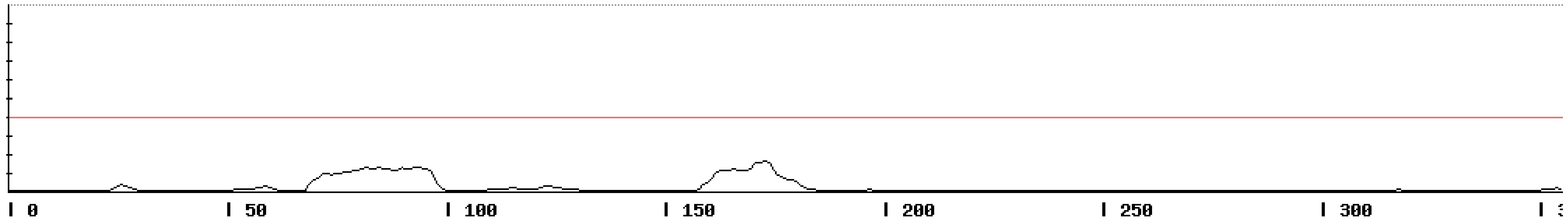

Predictions for Arachisduranensis_Aradu.F2DT2

Viterbi Path

Viterbi Path

Posterior @ 0.4

Posterior @ 0.4

Posterior Graphic. Threshold Marked @ 0.4

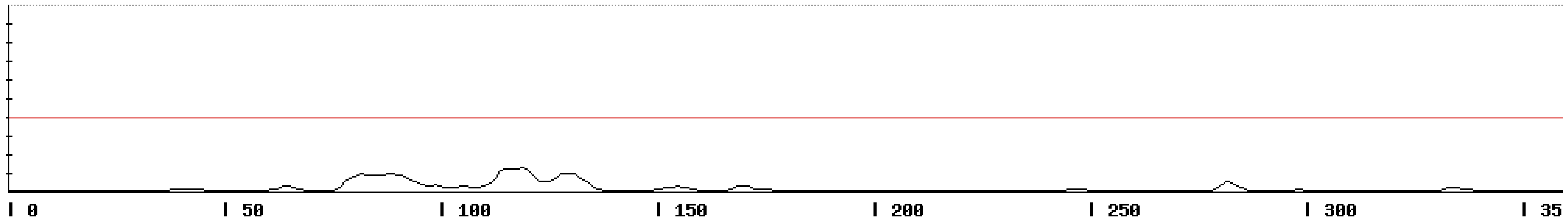

Predictions for Arachisduranensis_Aradu.F48KW

Viterbi Path

Viterbi Path

Posterior @ 0.4

Posterior @ 0.4

Posterior Graphic. Threshold Marked @ 0.4

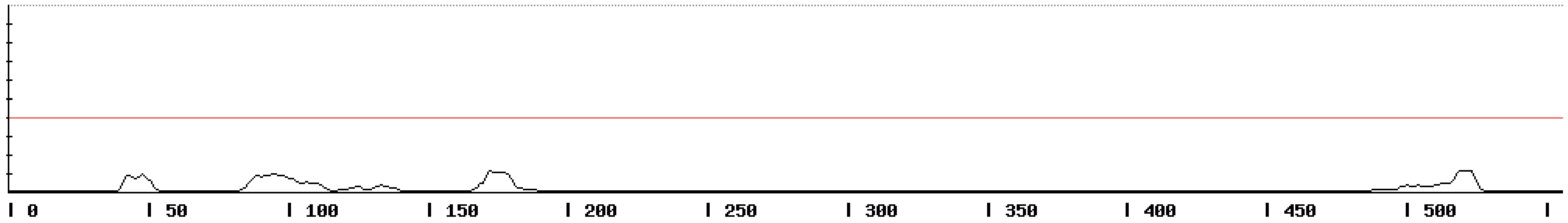

Predictions for Arachisduranensis_Aradu.F6Z4G

Viterbi Path

Viterbi Path

Posterior @ 0.4

Posterior @ 0.4

Posterior Graphic. Threshold Marked @ 0.4

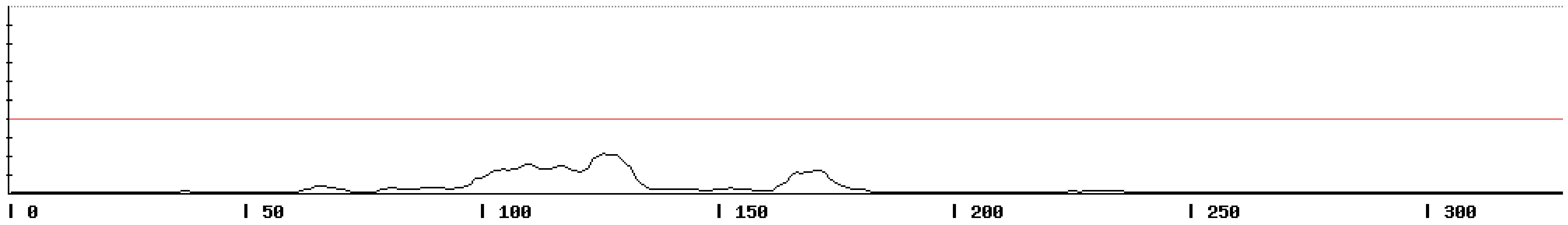

Predictions for Arachisduranensis_Aradu.F8VRL

Viterbi Path	
Viterbi Path	
Posterior @ 0.4	123 - RKRRK - 127
Posterior @ 0.4	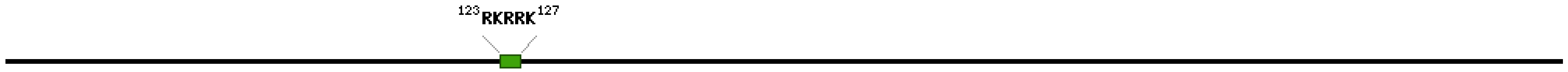
Posterior Graphic. Threshold Marked @ 0.4	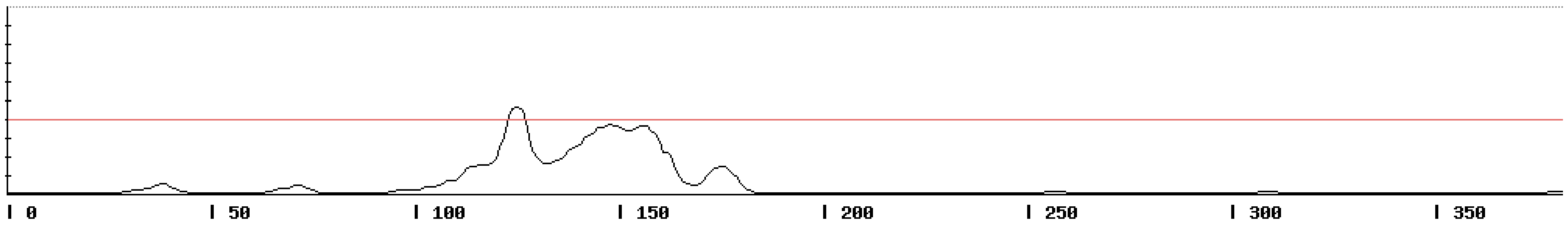

Predictions for Arachisduranensis_Aradu.H2YS3

Viterbi Path	
Viterbi Path	
Posterior @ 0.4	
Posterior @ 0.4	
Posterior Graphic. Threshold Marked @ 0.4	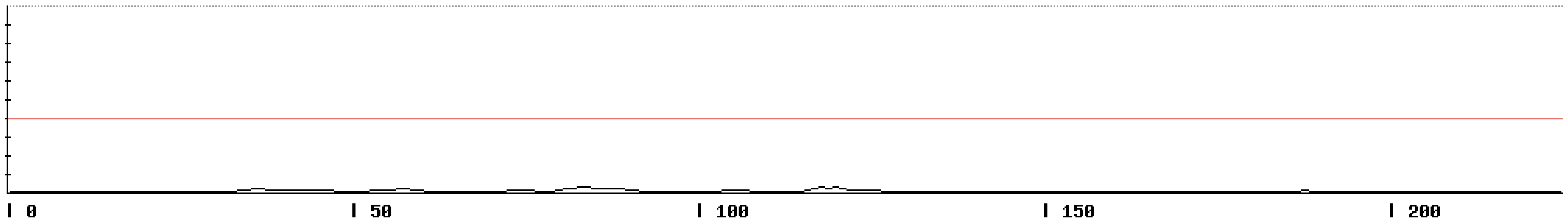

Predictions for Arachisduranensis_Aradu.H5KV7	
Viterbi Path	
Viterbi Path	
Posterior @ 0.4	
Posterior @ 0.4	
Posterior Graphic. Threshold Marked @ 0.4	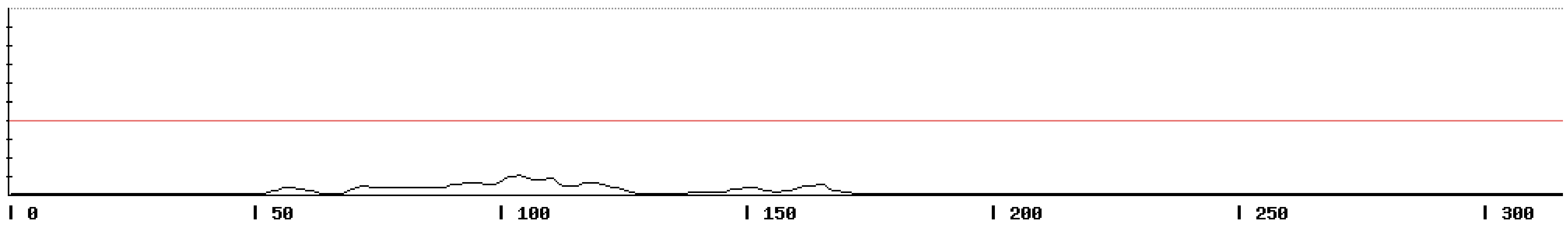

Predictions for Arachisduranensis_Aradu.JV7AK	
Viterbi Path	
Viterbi Path	
Posterior @ 0.4	156 - RKRRK - 160
Posterior @ 0.4	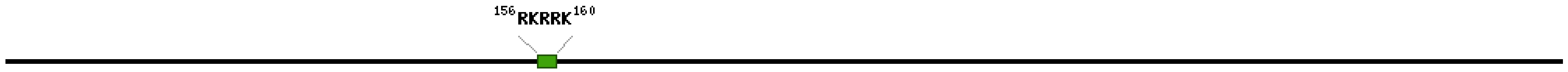
Posterior Graphic. Threshold Marked @ 0.4	

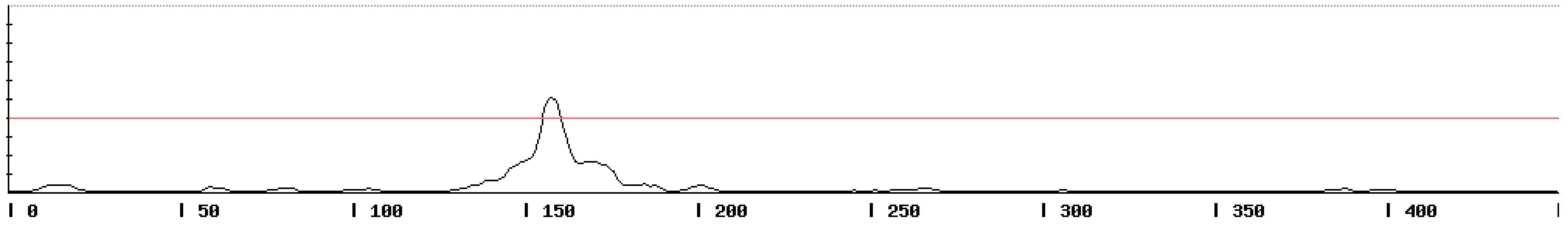

Predictions for Arachisduranensis_Aradu.JZK1S

Viterbi Path

Viterbi Path

Posterior @ 0.4

Posterior @ 0.4

Posterior Graphic. Threshold Marked @ 0.4

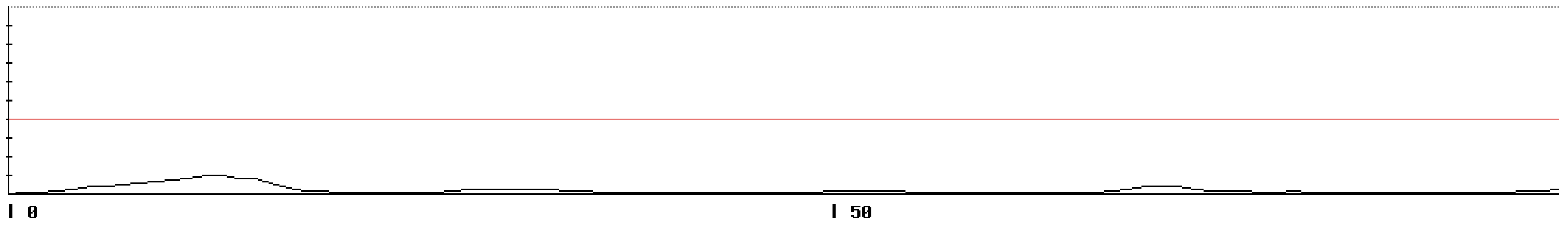

Predictions for Arachisduranensis_Aradu.K2UJH

Viterbi Path

Viterbi Path

Posterior @ 0.4

Posterior @ 0.4

Posterior Graphic. Threshold Marked @ 0.4

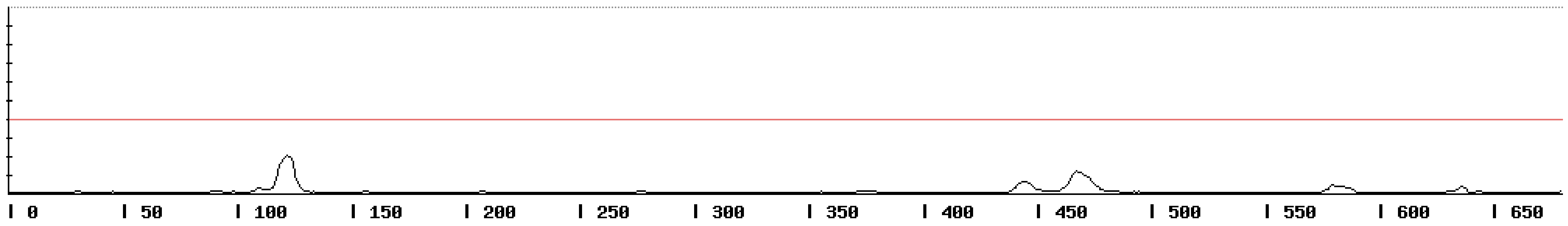

Predictions for Arachisduranensis_Aradu.KF8UQ

Viterbi Path

Viterbi Path

Posterior @ 0.4

Posterior @ 0.4

Posterior Graphic. Threshold Marked @ 0.4

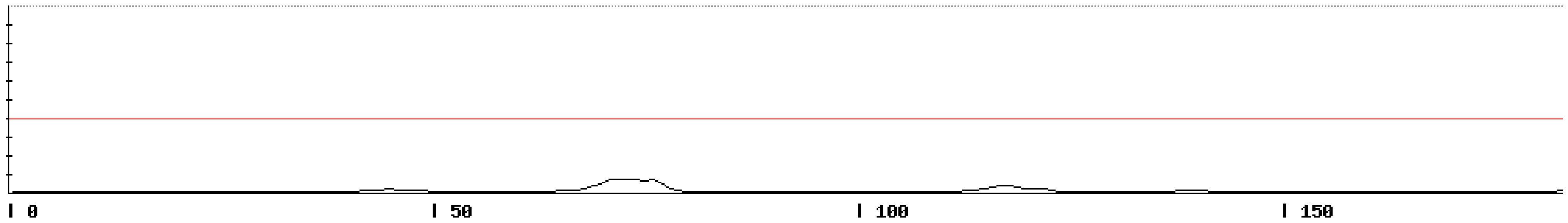

Predictions for Arachisduranensis_Aradu.L3QY1

Viterbi Path

Viterbi Path

Posterior @ 0.4

Posterior @ 0.4

Posterior Graphic. Threshold Marked @ 0.4

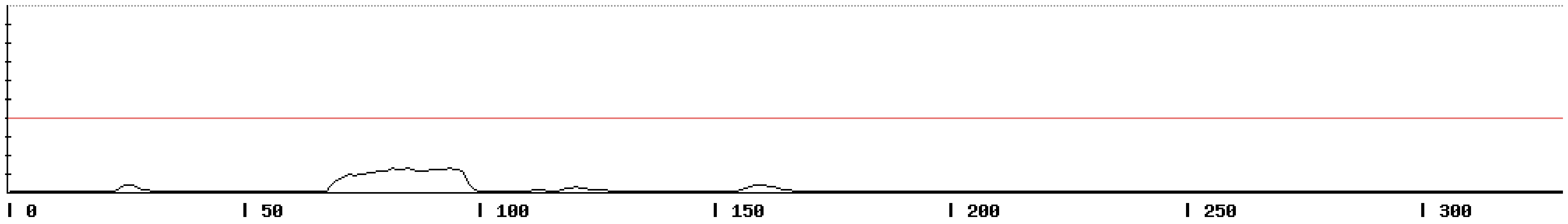

Predictions for Arachisduranensis_Aradu.L6S7Y

Viterbi Path

Viterbi Path

Posterior @ 0.4

Posterior @ 0.4

Posterior Graphic. Threshold Marked @ 0.4

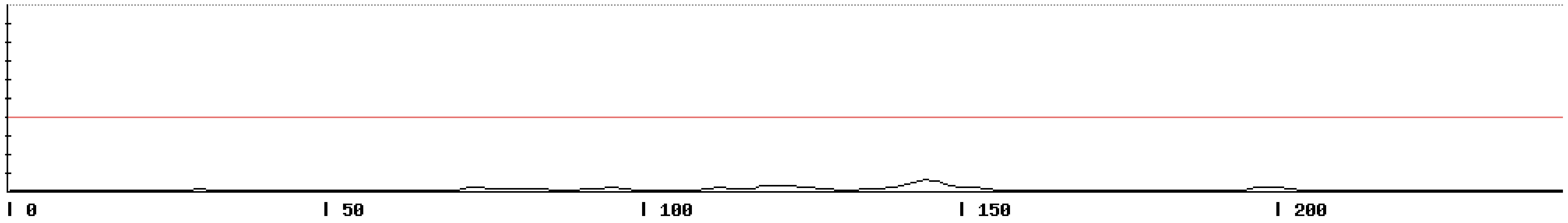

Predictions for Arachisduranensis_Aradu.L8SVN

Viterbi Path

Viterbi Path

Posterior @ 0.4	
Posterior @ 0.4	
Posterior Graphic. Threshold Marked @ 0.4	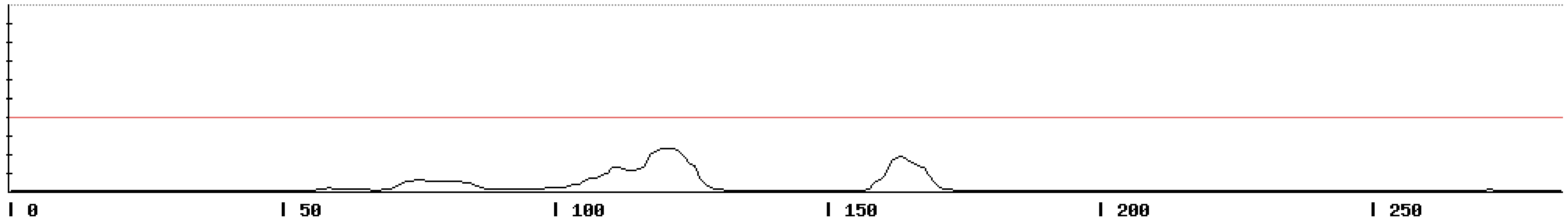

Predictions for Arachisduranensis_Aradu.LG4RX

Viterbi Path	
Viterbi Path	
Posterior @ 0.4	161 - LKKDKRRNKKAK - 172
Posterior @ 0.4	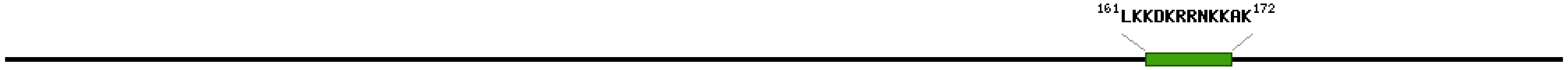
Posterior Graphic. Threshold Marked @ 0.4	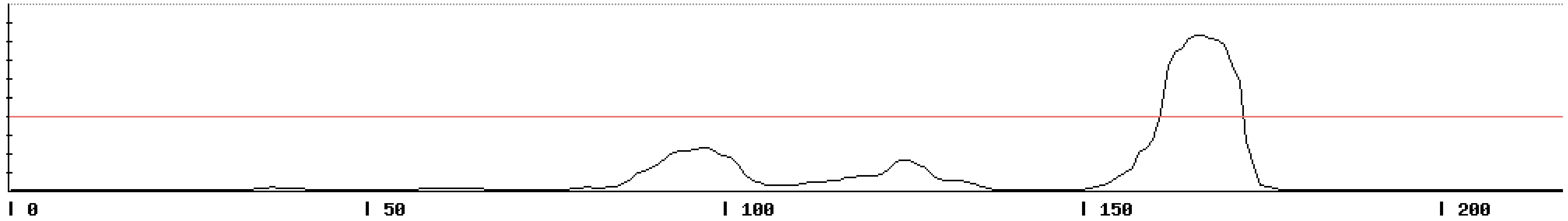

Predictions for Arachisduranensis_Aradu.LZ0D8

Viterbi Path	
Viterbi Path	

Posterior @ 0.4

Posterior @ 0.4

Posterior Graphic. Threshold Marked @ 0.4

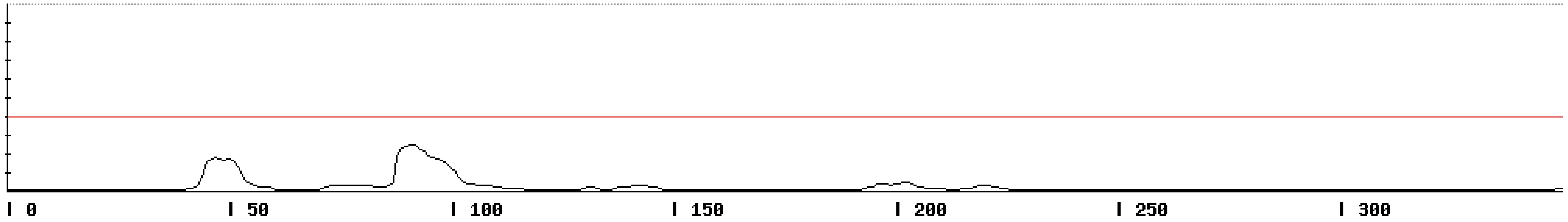

Predictions for Arachisduranensis_Aradu.M7213

Viterbi Path

Viterbi Path

Posterior @ 0.4

Posterior @ 0.4

Posterior Graphic. Threshold Marked @ 0.4

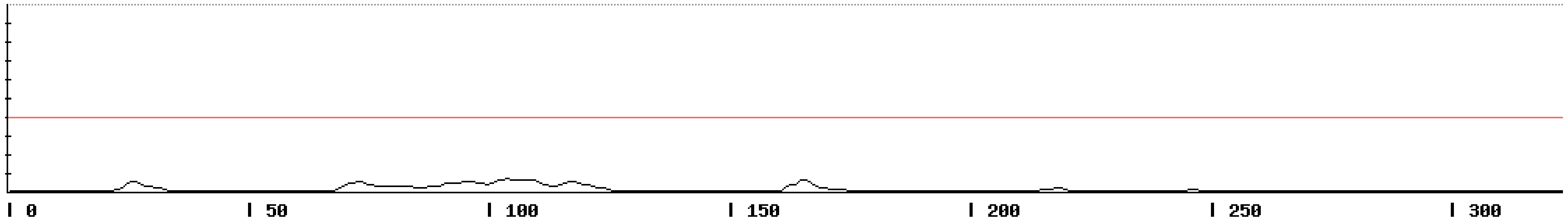

Predictions for Arachisduranensis_Aradu.M8PFR

Viterbi Path

Viterbi Path

Posterior @ 0.4

Posterior @ 0.4

Posterior Graphic. Threshold Marked @ 0.4

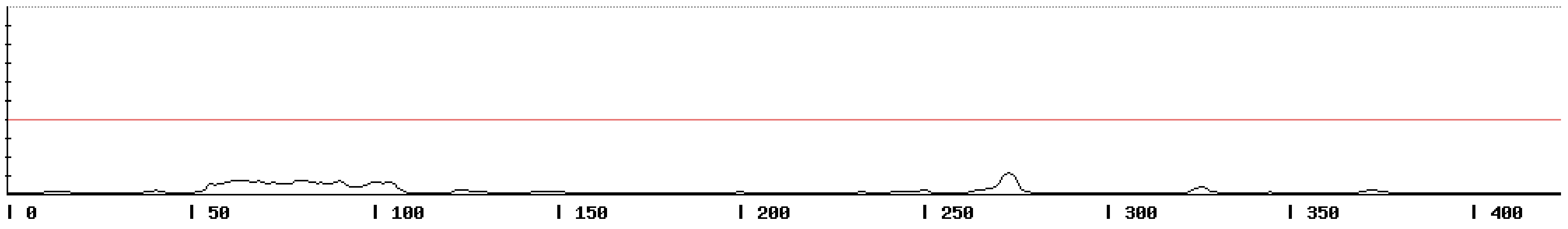

Predictions for Arachisduranensis_Aradu.M9GL4

Viterbi Path

Viterbi Path

Posterior @ 0.4

Posterior @ 0.4

Posterior Graphic. Threshold Marked @ 0.4

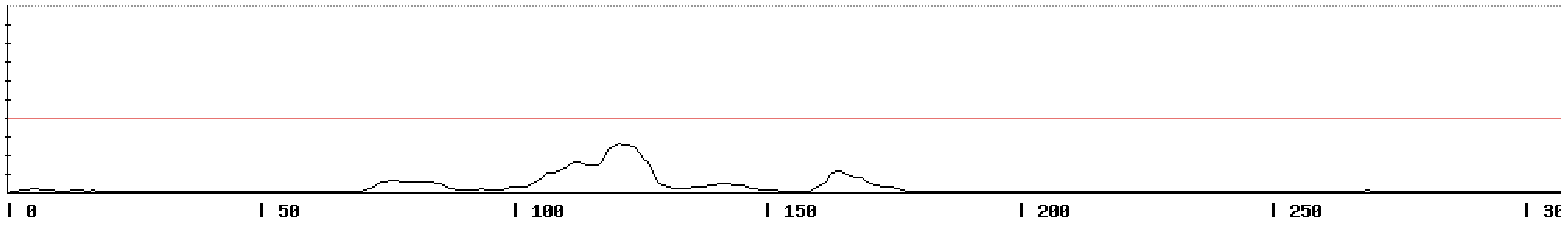

Predictions for Arachisduranensis_Aradu.N8F6V

Viterbi Path

Viterbi Path

Posterior @ 0.4

Posterior @ 0.4

Posterior Graphic. Threshold Marked @ 0.4

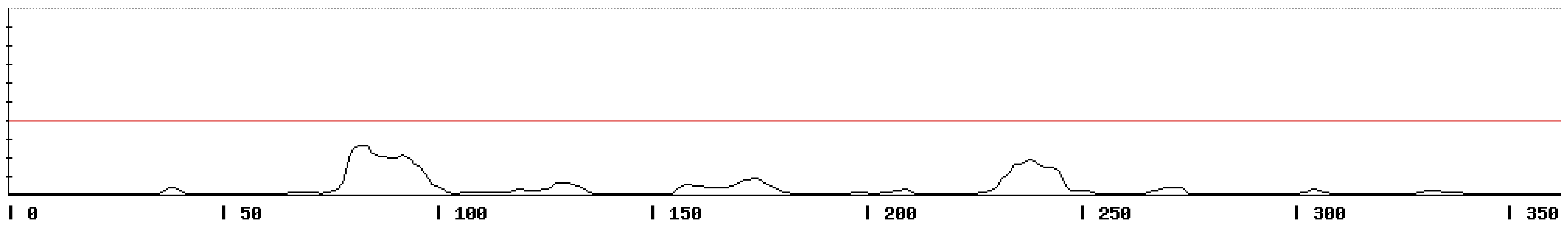

Predictions for Arachisduranensis_Aradu.N8MU8

Viterbi Path

Viterbi Path

Posterior @ 0.4

Posterior @ 0.4

Posterior Graphic. Threshold Marked @ 0.4

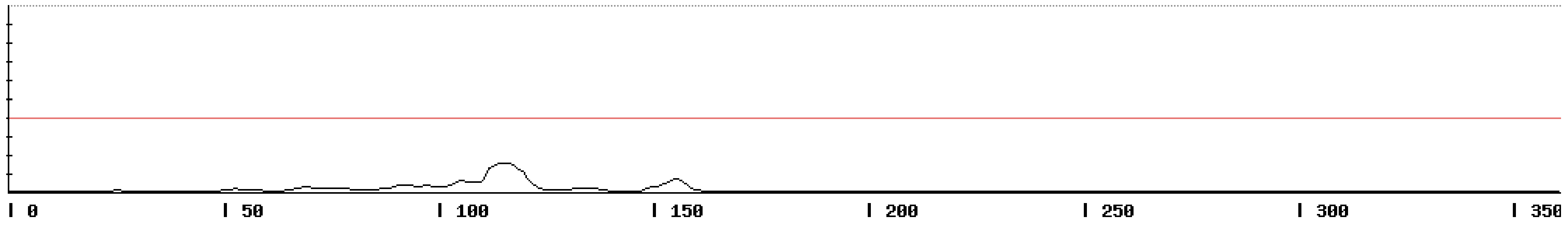

Predictions for Arachisduranensis_Aradu.NEU1C

Viterbi Path	
Viterbi Path	
Posterior @ 0.4	
Posterior @ 0.4	
Posterior Graphic. Threshold Marked @ 0.4	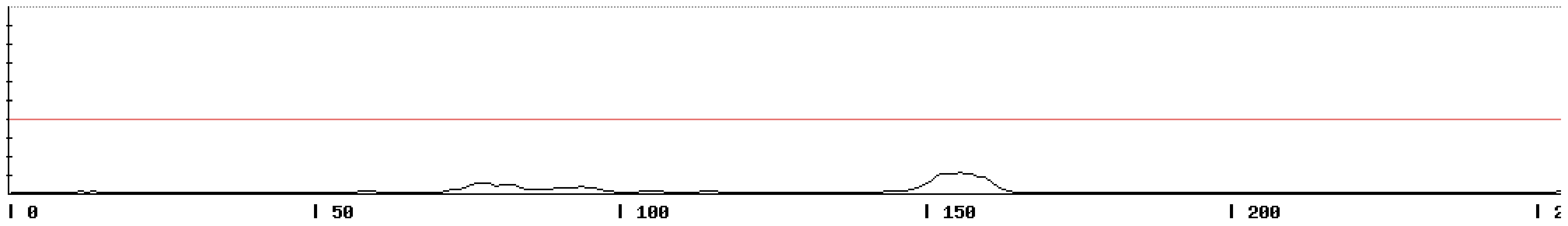

Predictions for Arachisduranensis_Aradu.R9F07

Viterbi Path	
Viterbi Path	
Posterior @ 0.4	
Posterior @ 0.4	

Posterior Graphic. Threshold Marked @ 0.4

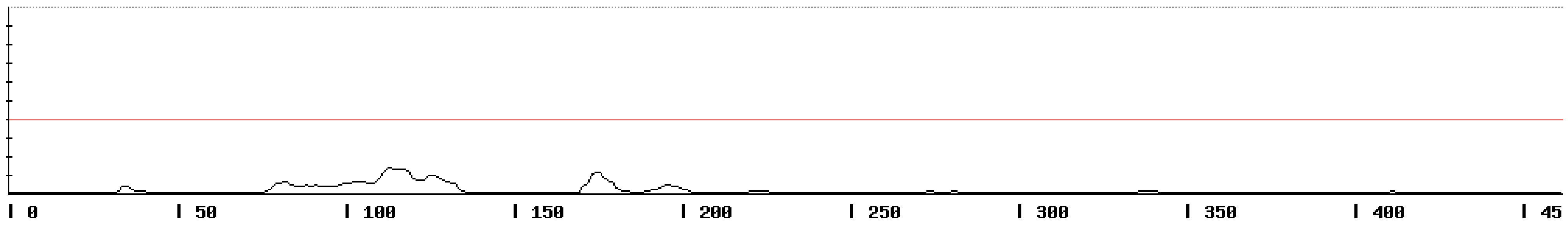

Predictions for Arachisduranensis_Aradu.RP61F

Viterbi Path	
Viterbi Path	
Posterior @ 0.4	104 - SRGERWKRKRKVKSGFWLKRGR - 125
Posterior @ 0.4	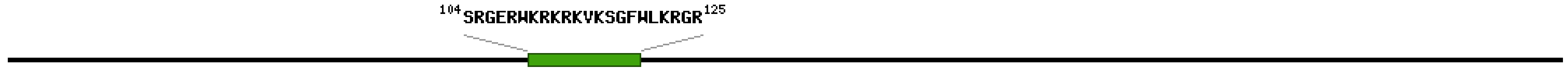
Posterior Graphic. Threshold Marked @ 0.4	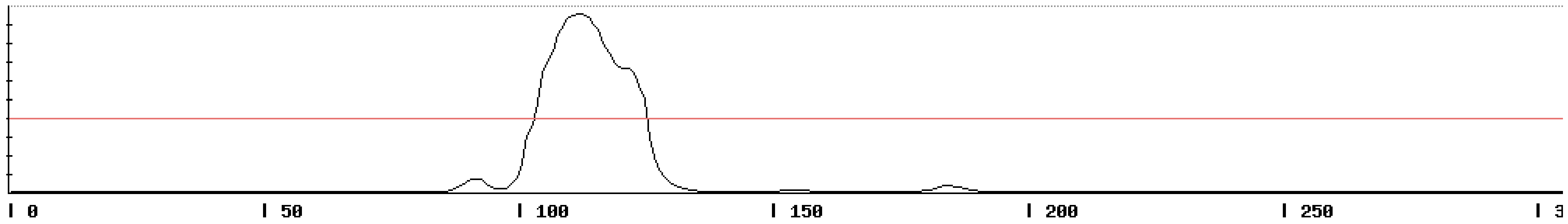

Predictions for Arachisduranensis_Aradu.RRT20

Viterbi Path	
Viterbi Path	
Posterior @ 0.4	
Posterior @ 0.4	

Posterior Graphic. Threshold Marked @ 0.4

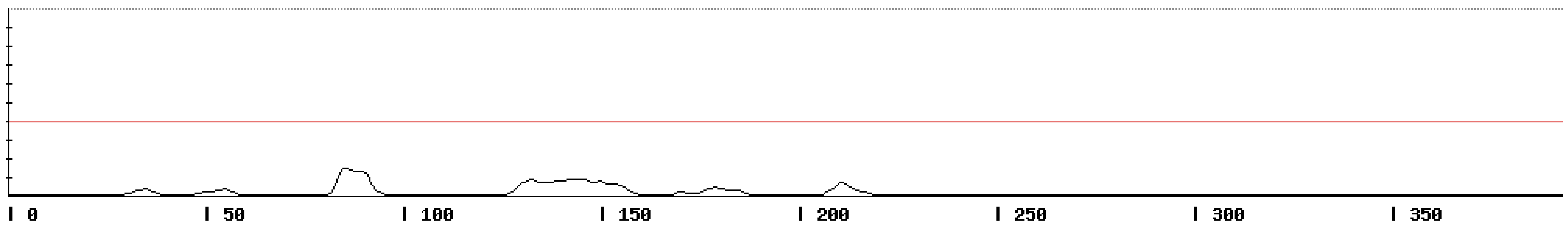

Predictions for Arachisduranensis_Aradu.S13QQ

Viterbi Path

Viterbi Path

Posterior @ 0.4

Posterior @ 0.4

Posterior Graphic. Threshold Marked @ 0.4

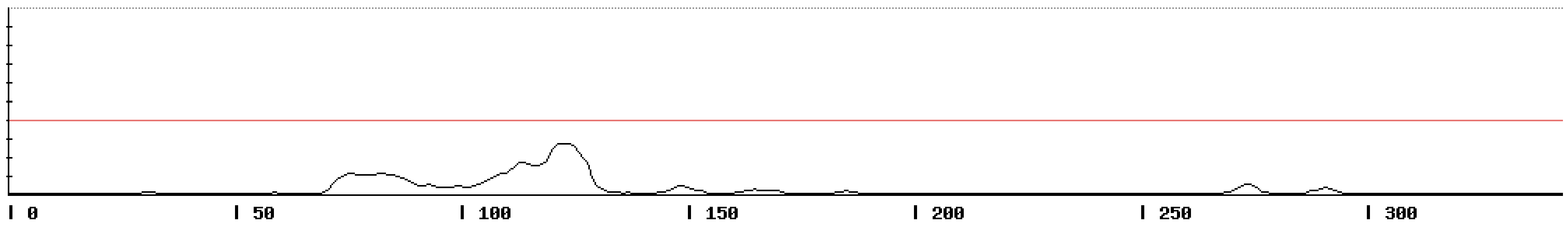

Predictions for Arachisduranensis_Aradu.TGA11

Viterbi Path

Viterbi Path

Posterior @ 0.4

Posterior @ 0.4

Posterior Graphic. Threshold Marked @ 0.4

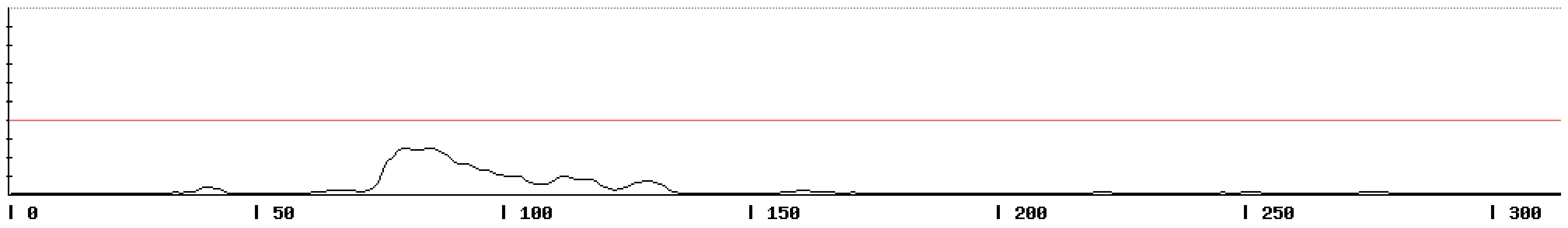

Predictions for Arachisduranensis_Aradu.TI0Z7

Viterbi Path

Viterbi Path

Posterior @ 0.4

Posterior @ 0.4

Posterior Graphic. Threshold Marked @ 0.4

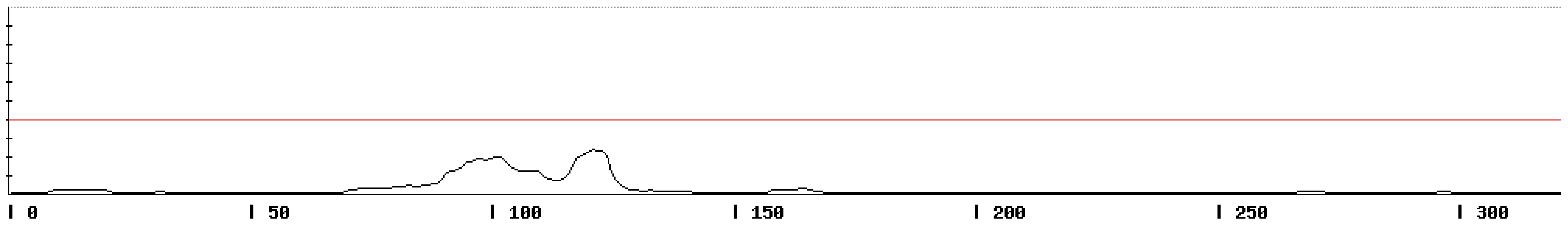

Predictions for Arachisduranensis_Aradu.U974Q

Viterbi Path

Viterbi Path

Posterior @ 0.4

Posterior @ 0.4

Posterior Graphic. Threshold Marked @ 0.4

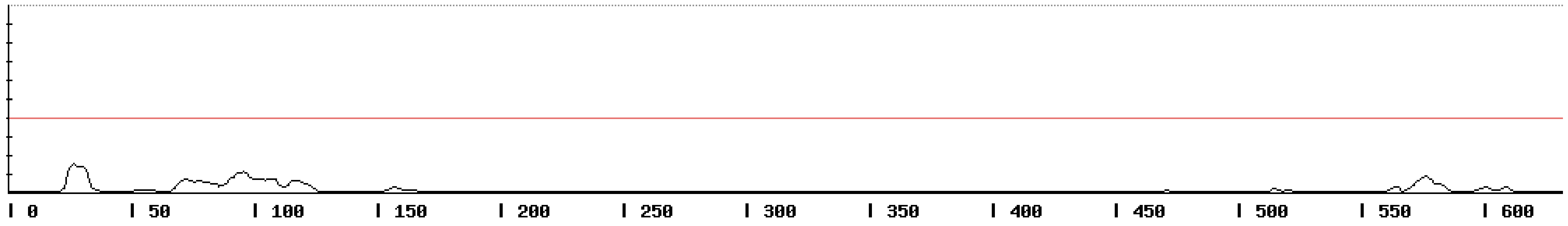

Predictions for Arachisduranensis_Aradu.USH95

Viterbi Path

Viterbi Path

Posterior @ 0.4

Posterior @ 0.4

Posterior Graphic. Threshold Marked @ 0.4

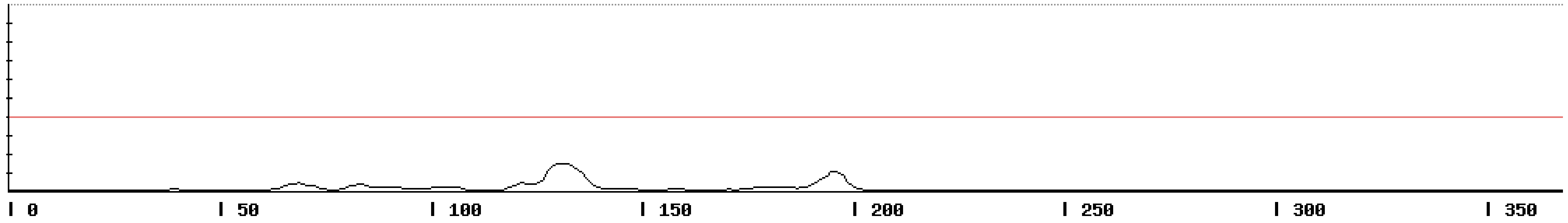

Predictions for Arachisduranensis_Aradu.UXN6T

Viterbi Path

Viterbi Path

Posterior @ 0.4: 92 - GADKPIGKPKALGIKK - 107

Posterior @ 0.4

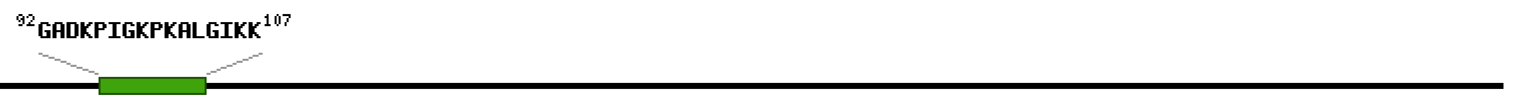

Posterior Graphic. Threshold Marked @ 0.4

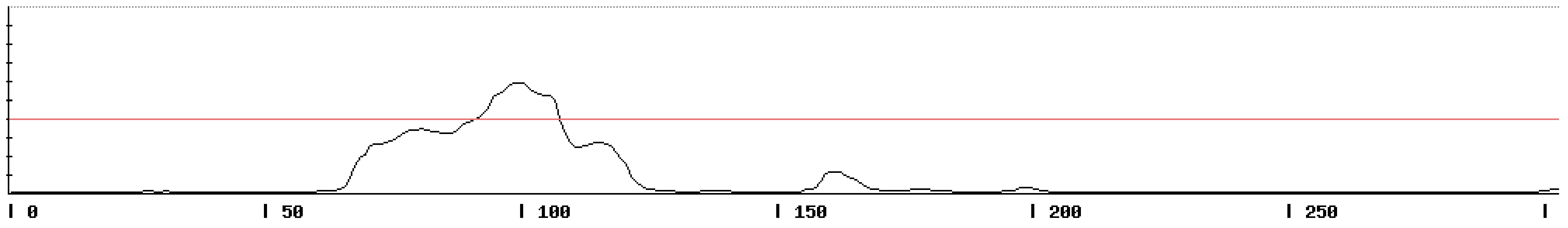

Predictions for Arachisduranensis_Aradu.VUC67

Viterbi Path

Viterbi Path

Posterior @ 0.4

Posterior @ 0.4

Posterior Graphic. Threshold Marked @ 0.4

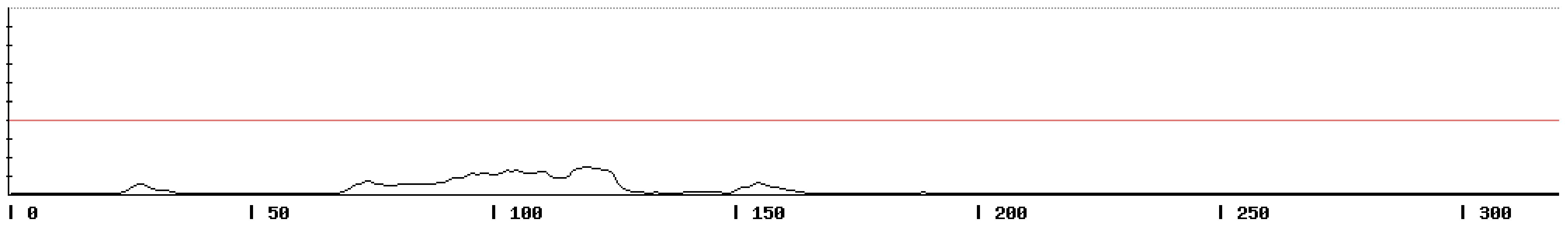

Predictions for Arachisduranensis_Aradu.W3GLH	
Viterbi Path	
Viterbi Path	
Posterior @ 0.4	
Posterior @ 0.4	
Posterior Graphic. Threshold Marked @ 0.4	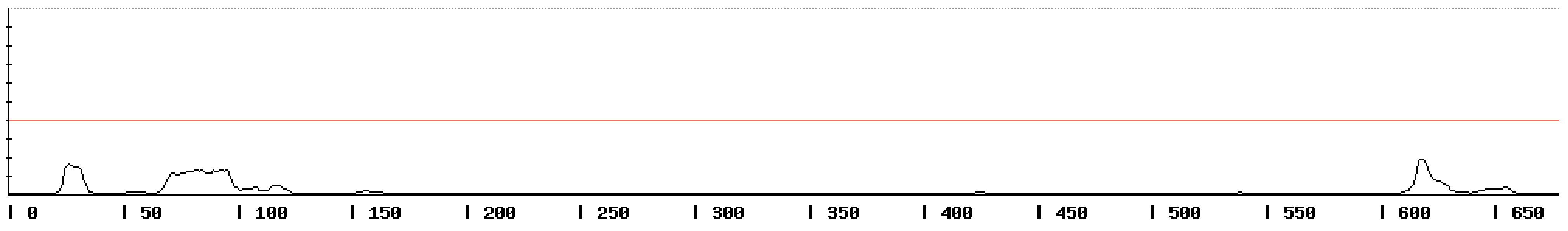

Predictions for Arachisduranensis_Aradu.WIT0W	
Viterbi Path	
Viterbi Path	
Posterior @ 0.4	
Posterior @ 0.4	
Posterior Graphic. Threshold Marked @ 0.4	

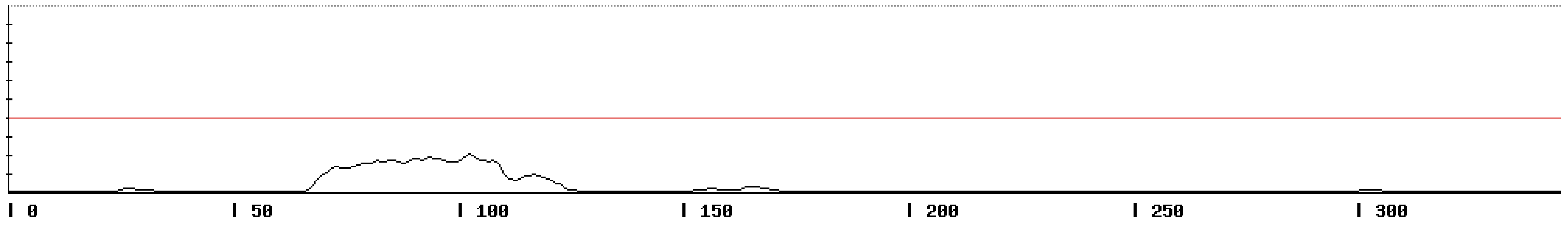

Predictions for Arachisduranensis_Aradu.WS3DN

Viterbi Path	
Viterbi Path	
Posterior @ 0.4	
Posterior @ 0.4	
Posterior Graphic. Threshold Marked @ 0.4	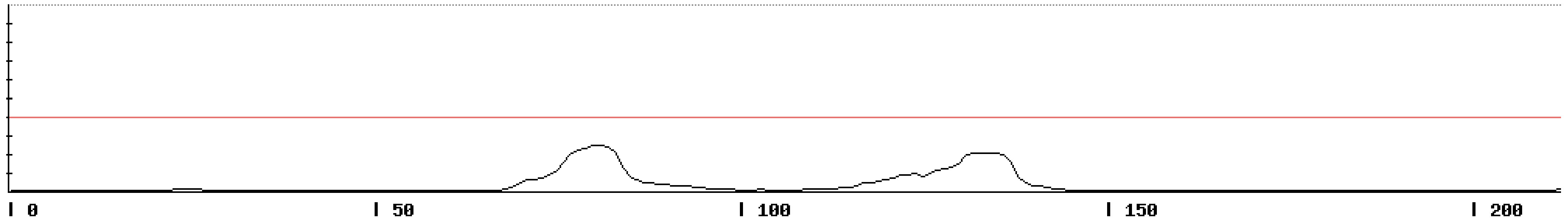

Predictions for Arachisduranensis_Aradu.XE8WZ

Viterbi Path	
Viterbi Path	
Posterior @ 0.4	
Posterior @ 0.4	

Posterior Graphic. Threshold Marked @ 0.4

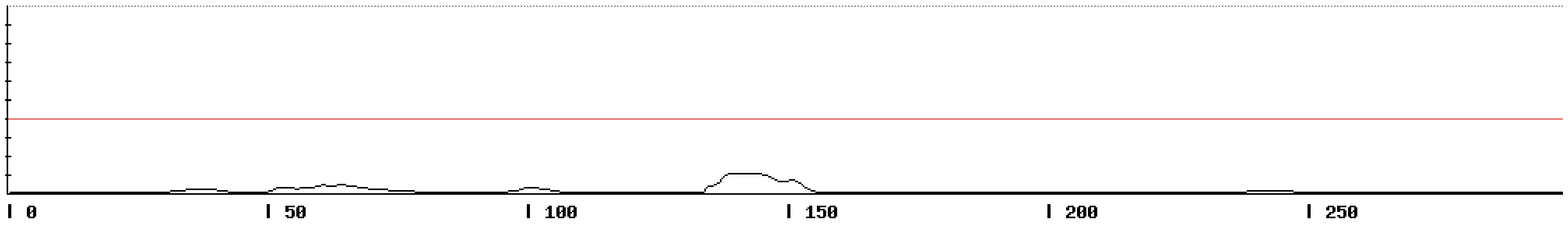

Predictions for Arachisduranensis_Aradu.XJF09

Viterbi Path

Viterbi Path

Posterior @ 0.4

134 - KRRK - 137

177 - VRPKRGAKPNKSK - 189

Posterior @ 0.4

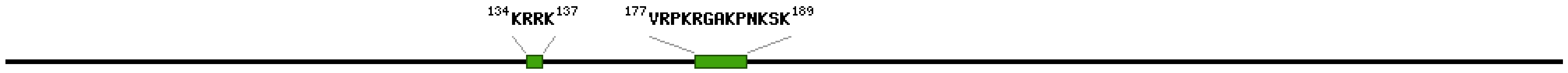

Posterior Graphic. Threshold Marked @ 0.4

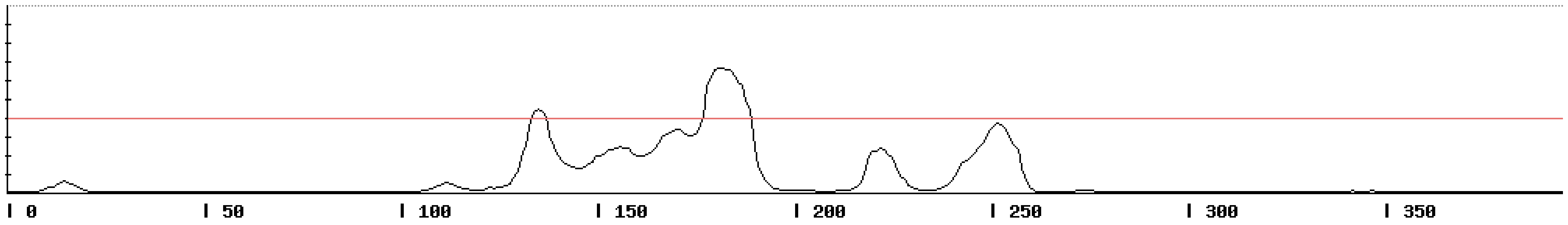

Predictions for Arachisduranensis_Aradu.XQ4VP

Viterbi Path

Viterbi Path

Posterior @ 0.4

Posterior @ 0.4

Posterior Graphic. Threshold Marked @ 0.4

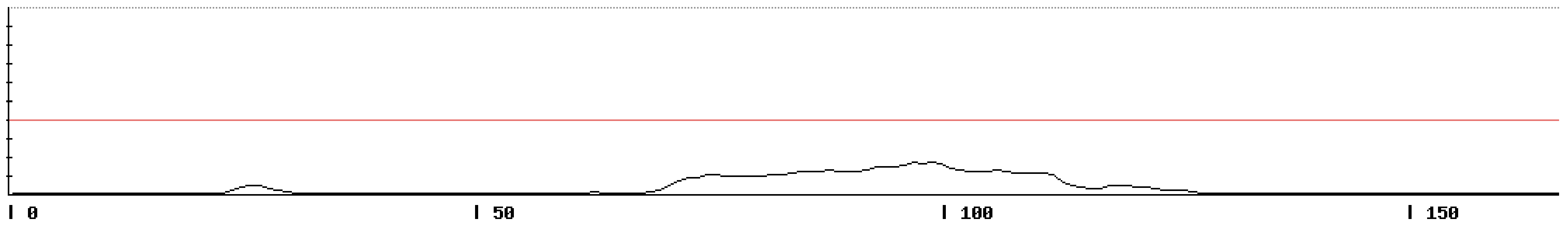

Predictions for Arachisduranensis_Aradu.Y1DM8

Viterbi Path

Viterbi Path

Posterior @ 0.4

Posterior @ 0.4

Posterior Graphic. Threshold Marked @ 0.4

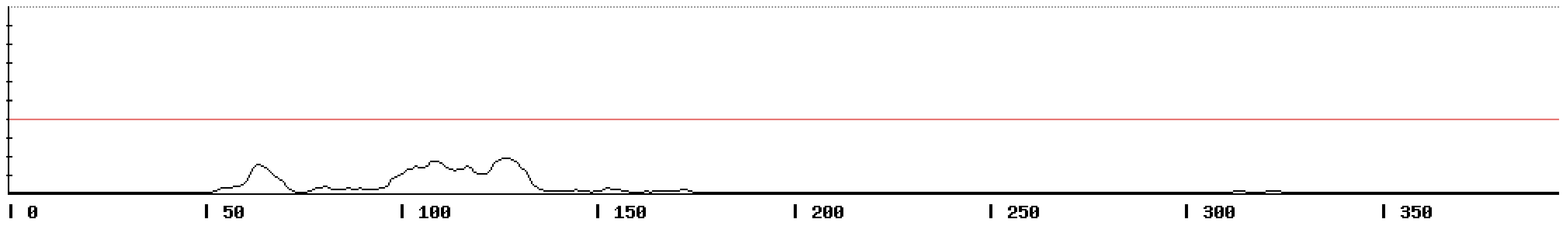

Predictions for Arachisduranensis_Aradu.Y9JNS

Viterbi Path

Viterbi Path

Posterior @ 0.4

Posterior @ 0.4

Posterior Graphic. Threshold Marked @ 0.4

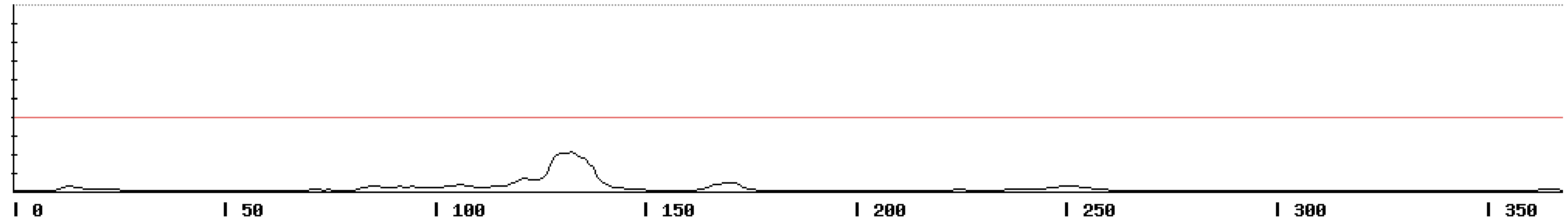

Predictions for Arachisduranensis_Aradu.YFQ3P

Viterbi Path

Viterbi Path

Posterior @ 0.4 — 95 - KPIGKPKALAIKK - 107

Posterior @ 0.4

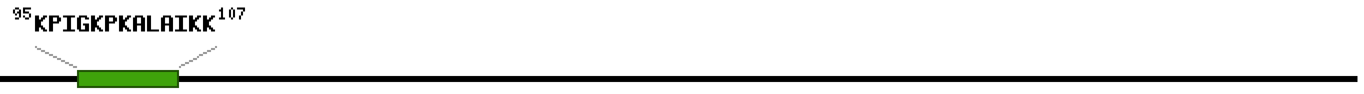

Posterior Graphic. Threshold Marked @ 0.4

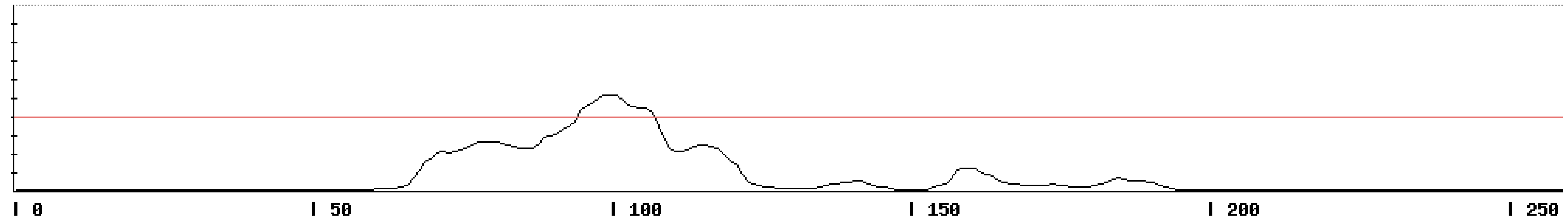

Predictions for Arachisduranensis_Aradu.YIQ80

Viterbi Path

Viterbi Path

Posterior @ 0.4

Posterior @ 0.4

Posterior Graphic. Threshold Marked @ 0.4

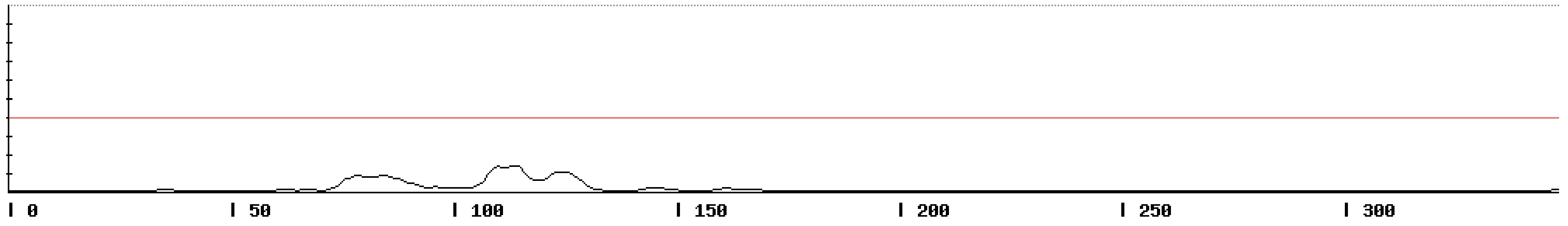

Predictions for Arachisduranensis_Aradu.YXW0Z

Viterbi Path

Viterbi Path

Posterior @ 0.4 | 169 - RKRRK - 173

Posterior @ 0.4

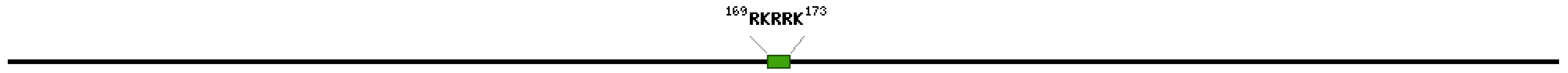

Posterior Graphic. Threshold Marked @ 0.4

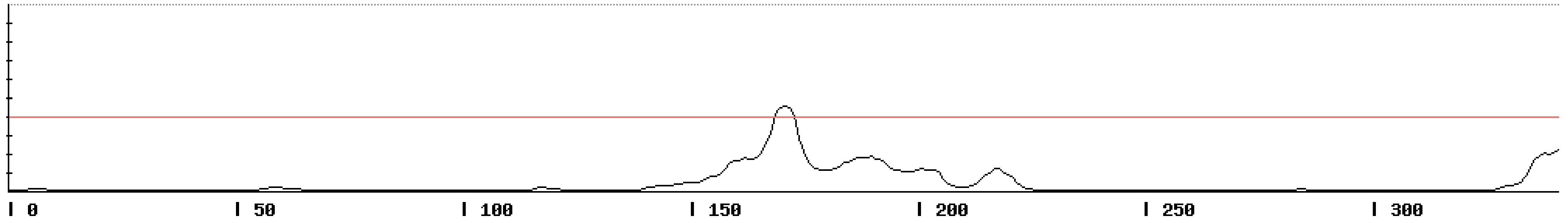

Predictions for Arachisduranensis_Aradu.Z4K97

Viterbi Path

Posterior @ 0.4

Posterior @ 0.4

Posterior Graphic. Threshold Marked @ 0.4

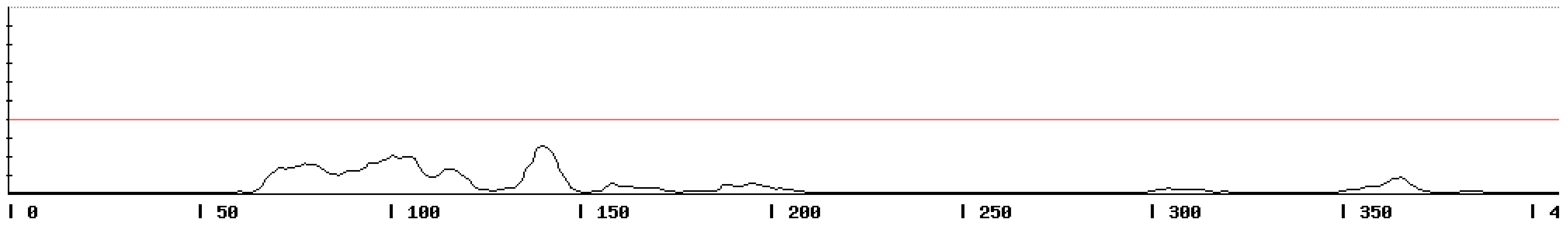

Predictions for Arachishypogaea_Ahy004174

Viterbi Path

Viterbi Path

Posterior @ 0.4 — 93 - ADKPIGKPKALAIKK - 107

Posterior @ 0.4

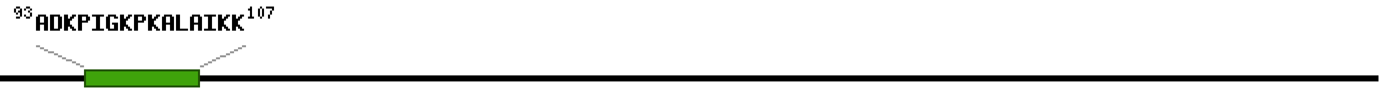

Posterior Graphic. Threshold Marked @ 0.4

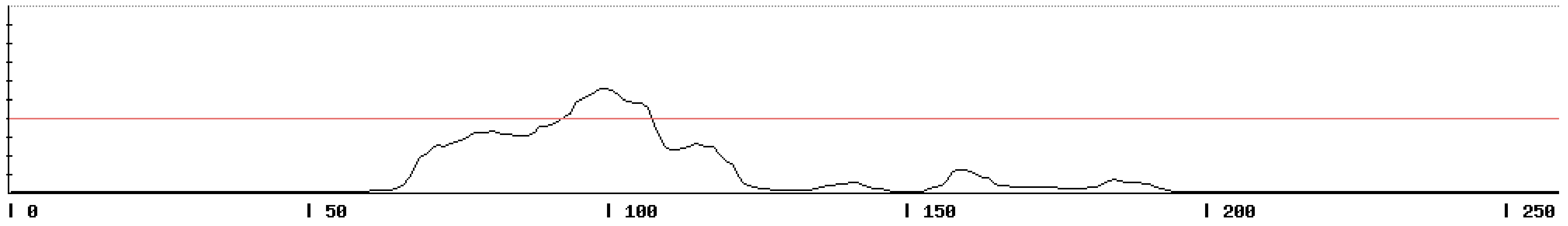

Predictions for Arachishypogaea_Ahy007630

Viterbi Path	
Viterbi Path	
Posterior @ 0.4	178 - KK - 179
Posterior @ 0.4	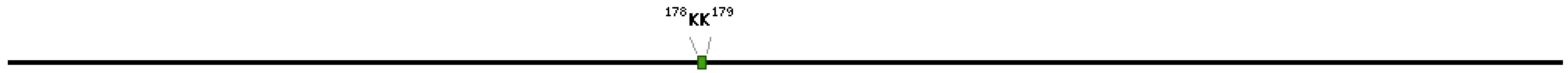
Posterior Graphic. Threshold Marked @ 0.4	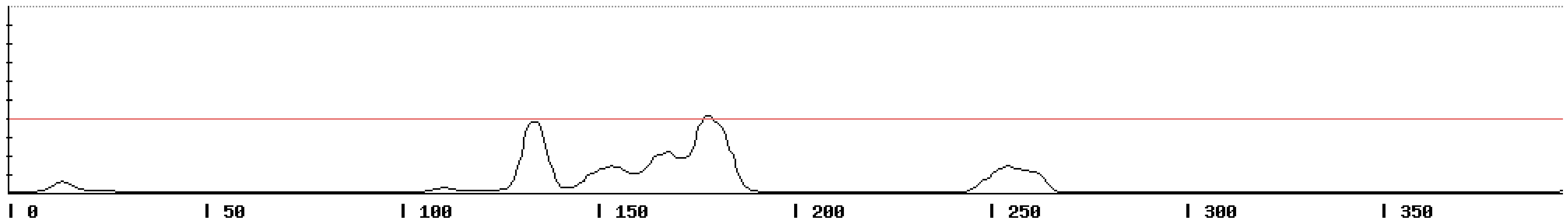

Predictions for Arachishypogaea_Ahy008073

Viterbi Path	
Viterbi Path	
Posterior @ 0.4	
Posterior @ 0.4	
Posterior Graphic. Threshold Marked @ 0.4	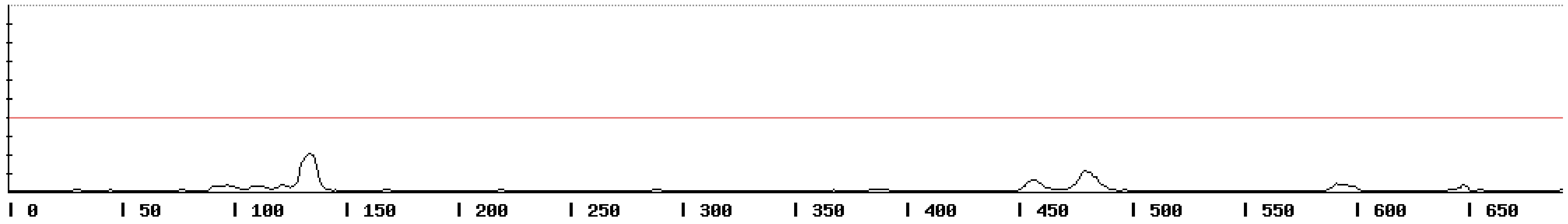

Predictions for Arachishypogaea_Ahy008395

Viterbi Path

Viterbi Path

Posterior @ 0.4

Posterior @ 0.4

Posterior Graphic. Threshold Marked @ 0.4

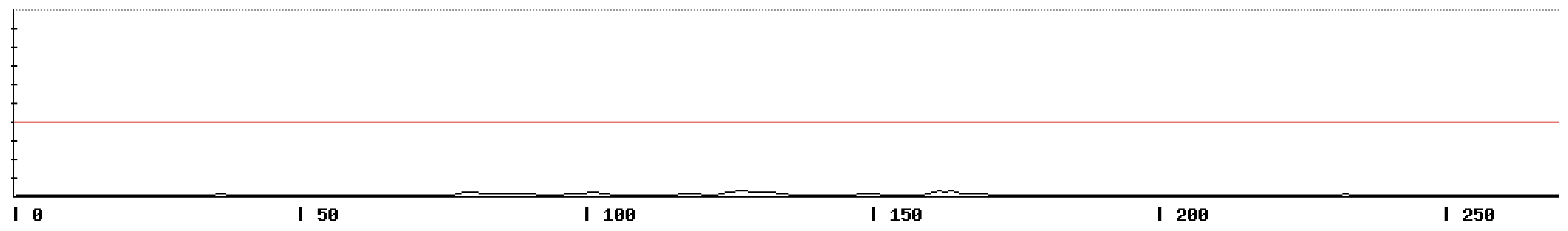

Predictions for Arachishypogaea_Ahy008545

Viterbi Path

Viterbi Path

Posterior @ 0.4

Posterior @ 0.4

Posterior Graphic. Threshold Marked @ 0.4

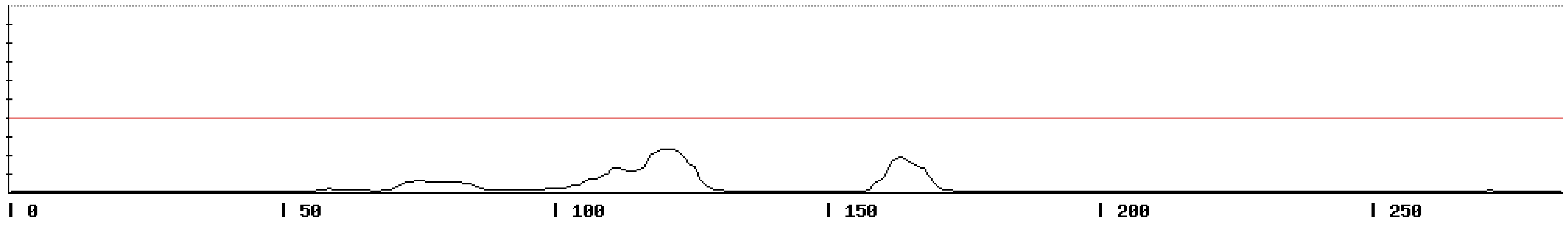

Predictions for Arachishypogaea_Ahy008969	
Viterbi Path	
Viterbi Path	
Posterior @ 0.4	
Posterior @ 0.4	
Posterior Graphic. Threshold Marked @ 0.4	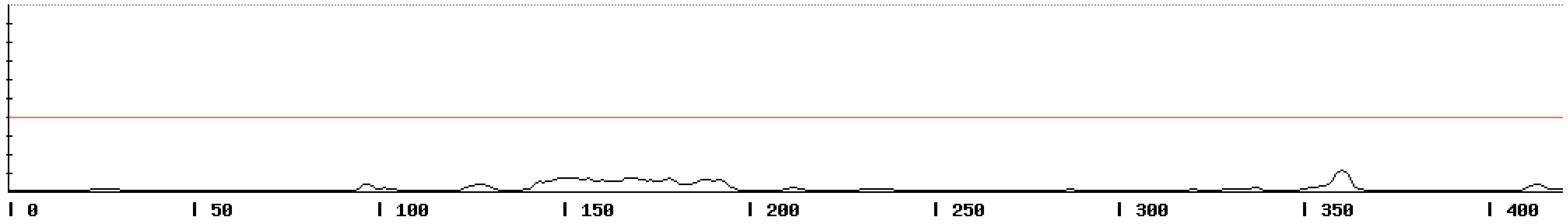

Predictions for Arachishypogaea_Ahy009563	
Viterbi Path	
Viterbi Path	
Posterior @ 0.4	
Posterior @ 0.4	

Posterior Graphic. Threshold Marked @ 0.4

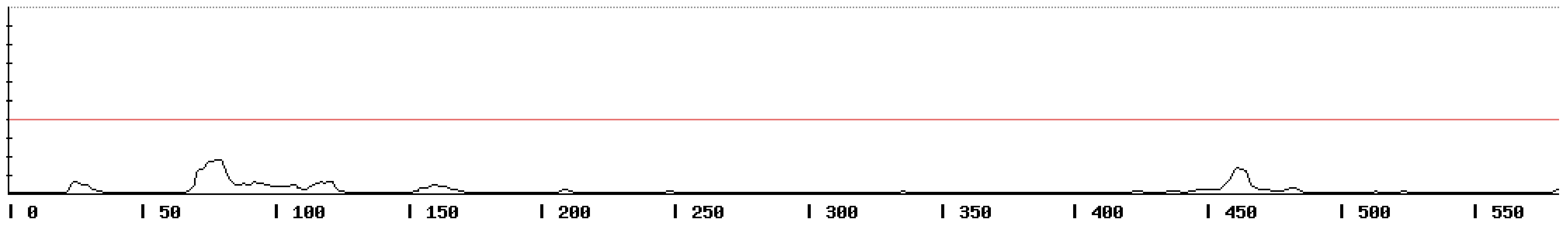

Predictions for Arachishypogaea_Ahy009594

Viterbi Path	
Viterbi Path	
Posterior @ 0.4	104 - KRR - 106
Posterior @ 0.4	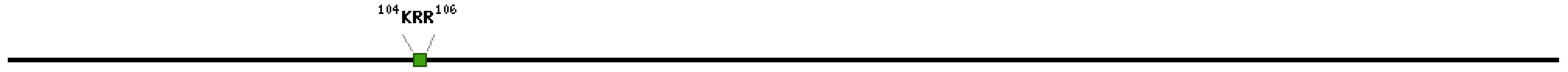
Posterior Graphic. Threshold Marked @ 0.4	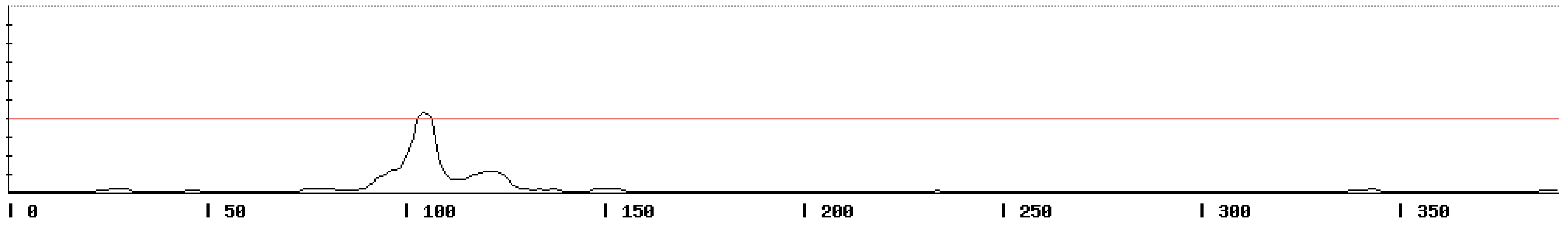

Predictions for Arachishypogaea_Ahy009762

Viterbi Path	
Viterbi Path	
Posterior @ 0.4	
Posterior @ 0.4	

Posterior Graphic. Threshold Marked @ 0.4

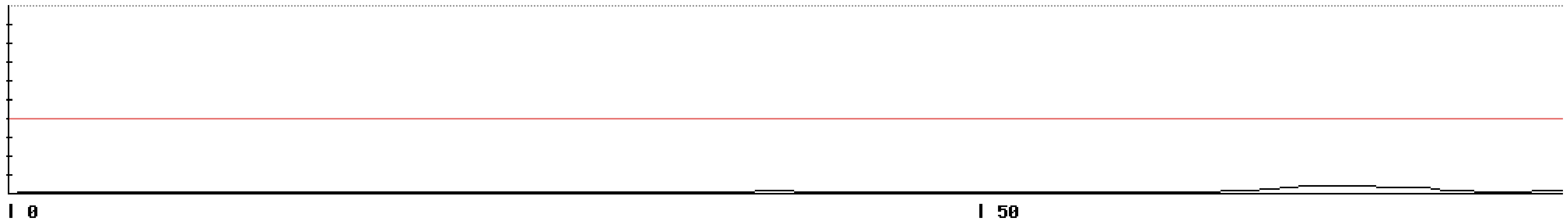

Predictions for Arachishypogaea_Ahy010013

Viterbi Path

Viterbi Path

Posterior @ 0.4

Posterior @ 0.4

Posterior Graphic. Threshold Marked @ 0.4

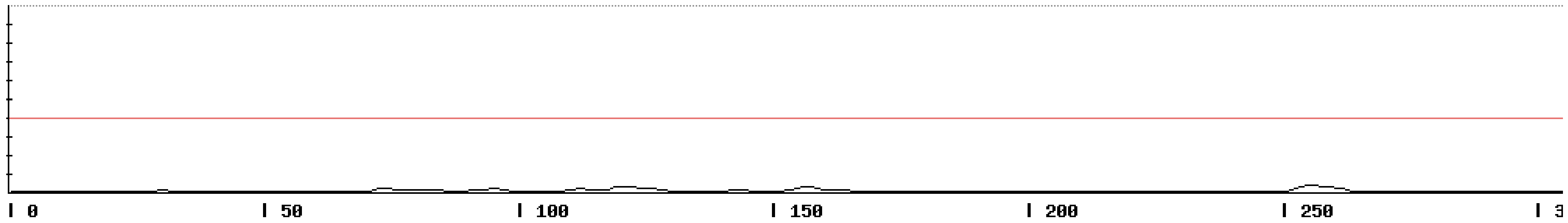

Predictions for Arachishypogaea_Ahy010059

Viterbi Path

Viterbi Path

Posterior @ 0.4

Posterior @ 0.4

Posterior Graphic. Threshold Marked @ 0.4

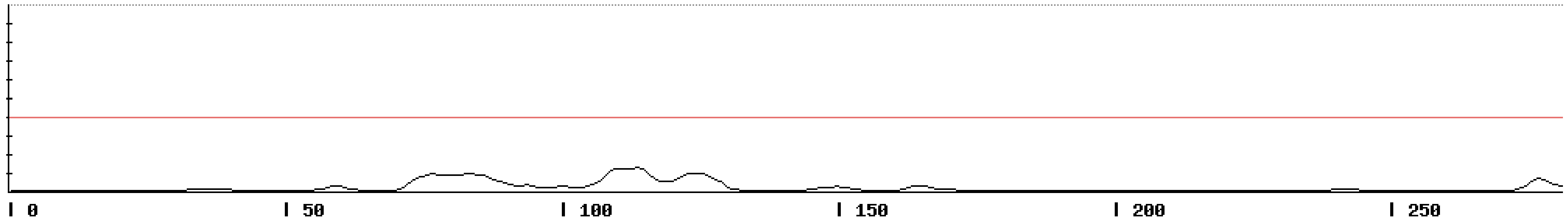

Predictions for Arachishypogaea_Ahy010486

Viterbi Path

Viterbi Path

Posterior @ 0.4

104 - SRGERWKRKRKVKSGFWLRRGR - 125

Posterior @ 0.4

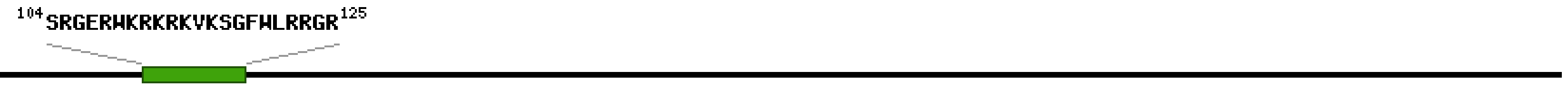

Posterior Graphic. Threshold Marked @ 0.4

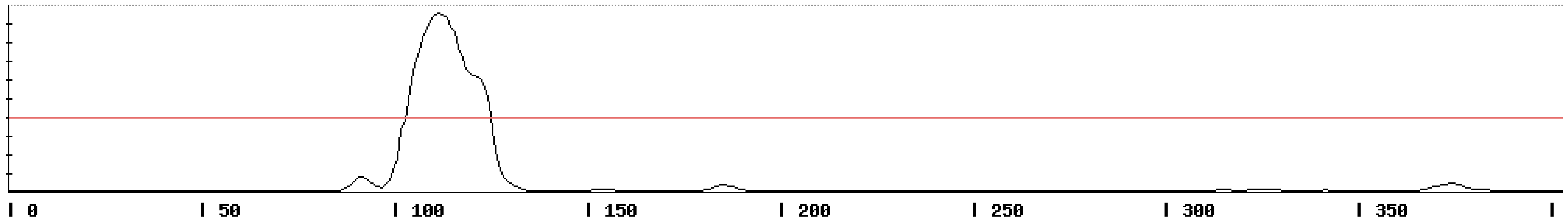

Predictions for Arachishypogaea_Ahy011698

Viterbi Path

Viterbi Path

Posterior @ 0.4

Posterior @ 0.4

Posterior Graphic. Threshold Marked @ 0.4

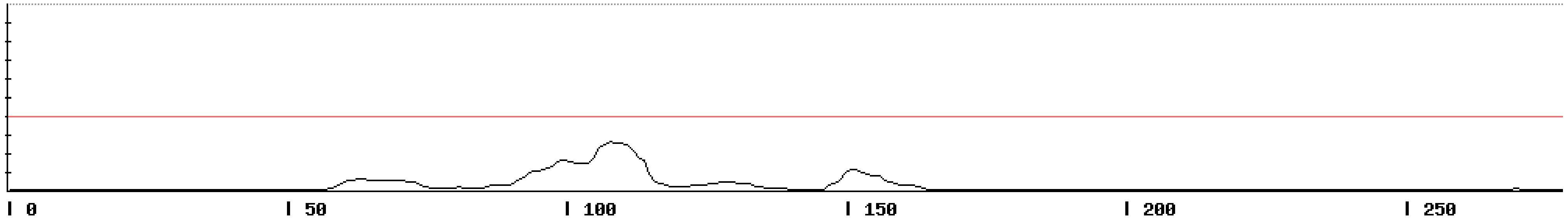

Predictions for Arachishypogaea_Ahy012574

Viterbi Path

Viterbi Path

Posterior @ 0.4

Posterior @ 0.4

Posterior Graphic. Threshold Marked @ 0.4

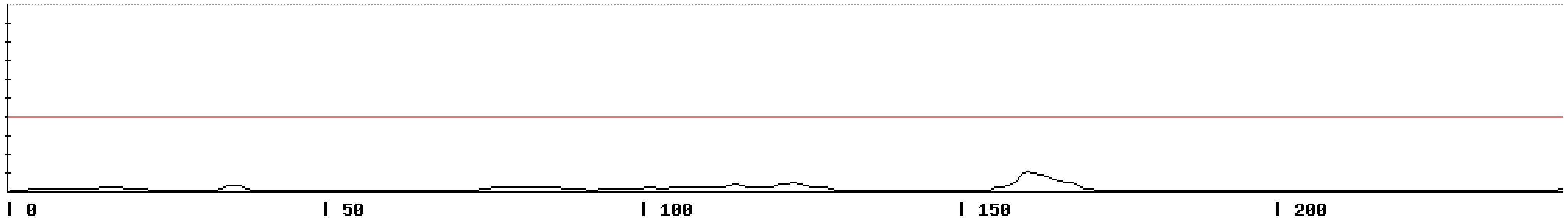

Predictions for Arachishypogaea_Ahy012663

Viterbi Path	
Viterbi Path	
Posterior @ 0.4	136 - RKRRK - 140
Posterior @ 0.4	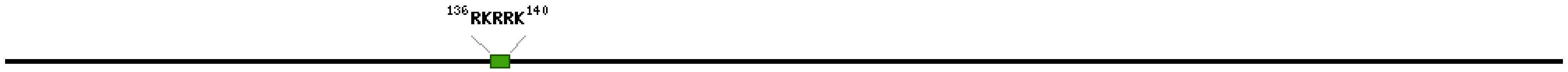
Posterior Graphic. Threshold Marked @ 0.4	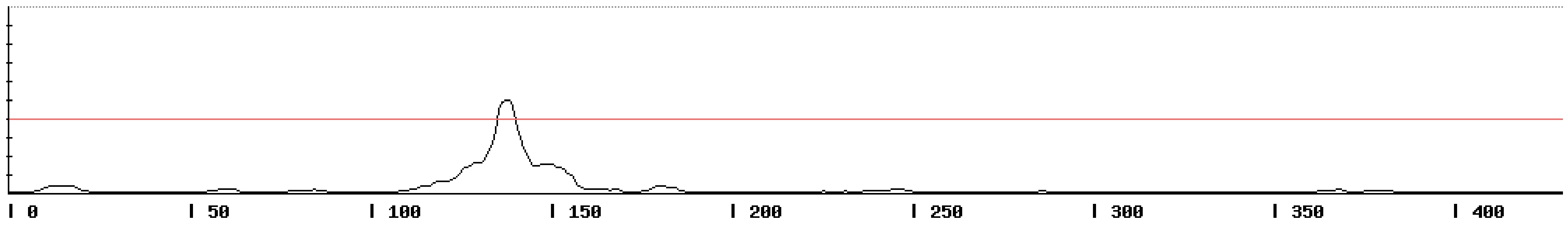

Predictions for Arachishypogaea_Ahy012805

Viterbi Path	
Viterbi Path	
Posterior @ 0.4	
Posterior @ 0.4	
Posterior Graphic. Threshold Marked @ 0.4	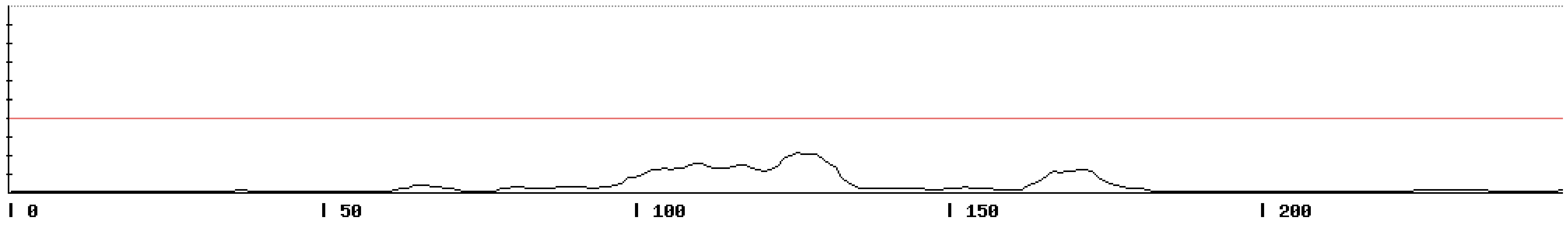

Predictions for Arachishypogaea_Ahy014891	
Viterbi Path	
Viterbi Path	
Posterior @ 0.4	
Posterior @ 0.4	
Posterior Graphic. Threshold Marked @ 0.4	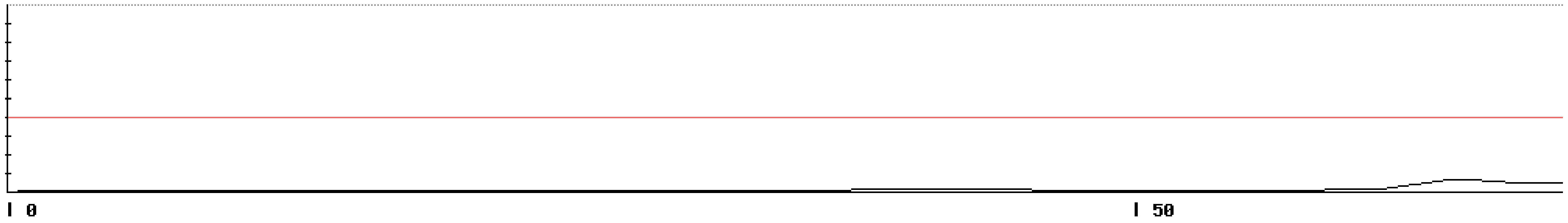

Predictions for Arachishypogaea_Ahy016041	
Viterbi Path	
Viterbi Path	
Posterior @ 0.4	105 - KRRK - 108
	148 - IRPKRGAKPNKSK - 160
	189 - KKKTKKN - 195
	220 - PPKRPRTGK - 228
Posterior @ 0.4	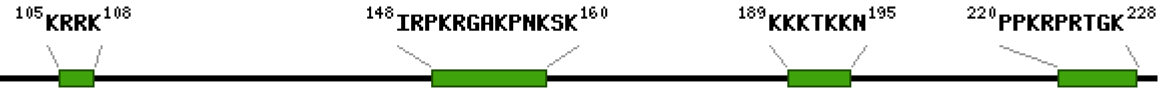
Posterior Graphic. Threshold Marked @ 0.4	

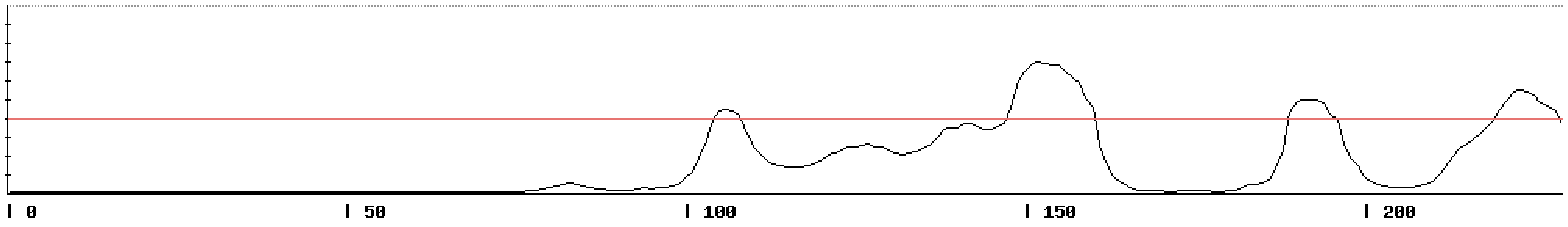

Predictions for Arachishypogaea_Ahy016633

Viterbi Path	
Viterbi Path	
Posterior @ 0.4	37 - GADKPIGKPKALGIKK - 52
Posterior @ 0.4	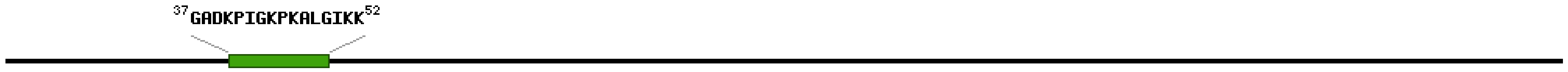
Posterior Graphic. Threshold Marked @ 0.4	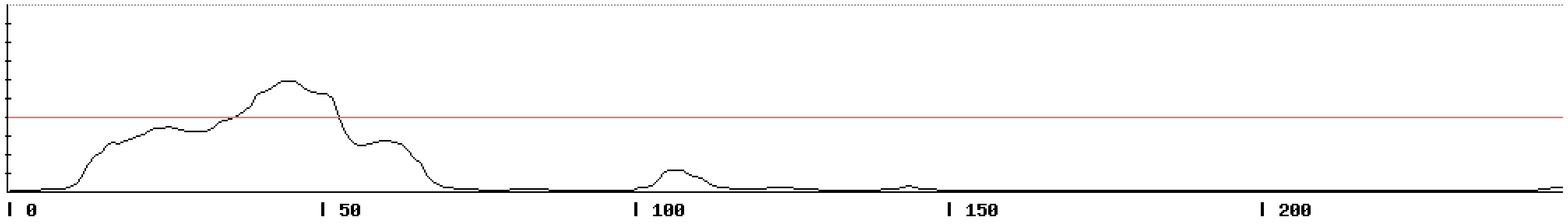

Predictions for Arachishypogaea_Ahy016999

Viterbi Path	
Viterbi Path	
Posterior @ 0.4	
Posterior @ 0.4	

Posterior Graphic. Threshold Marked @ 0.4

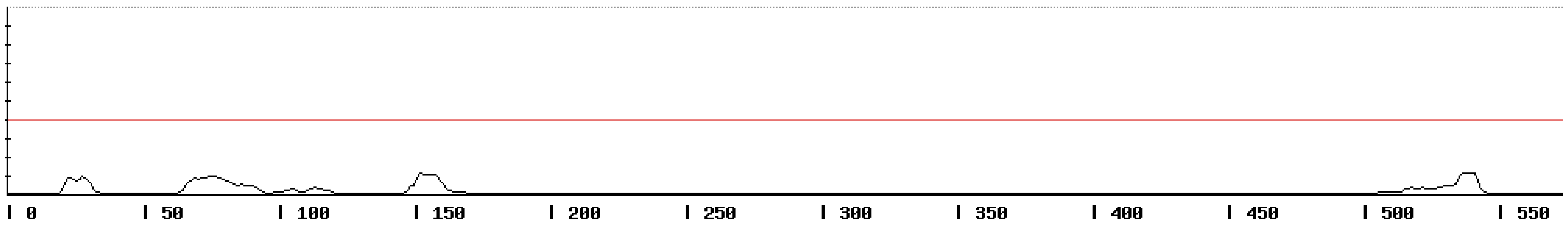

Predictions for Arachishypogaea_Ahy018843

Viterbi Path

Viterbi Path

Posterior @ 0.4

Posterior @ 0.4

Posterior Graphic. Threshold Marked @ 0.4

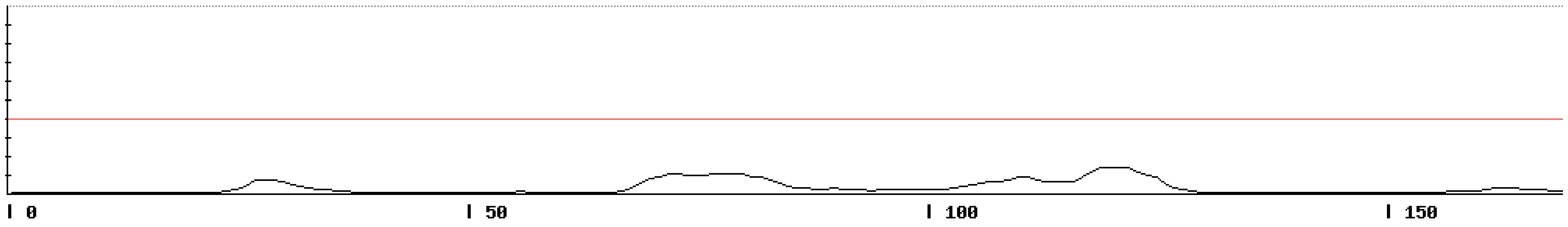

Predictions for Arachishypogaea_Ahy020393

Viterbi Path

Viterbi Path

Posterior @ 0.4

Posterior @ 0.4

Posterior Graphic. Threshold Marked @ 0.4

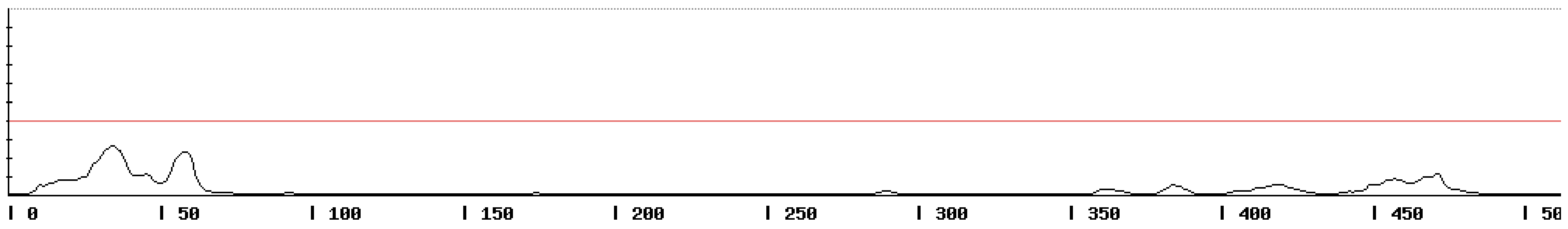

Predictions for Arachishypogaea_Ahy020431

Viterbi Path

Viterbi Path

Posterior @ 0.4

Posterior @ 0.4

Posterior Graphic. Threshold Marked @ 0.4

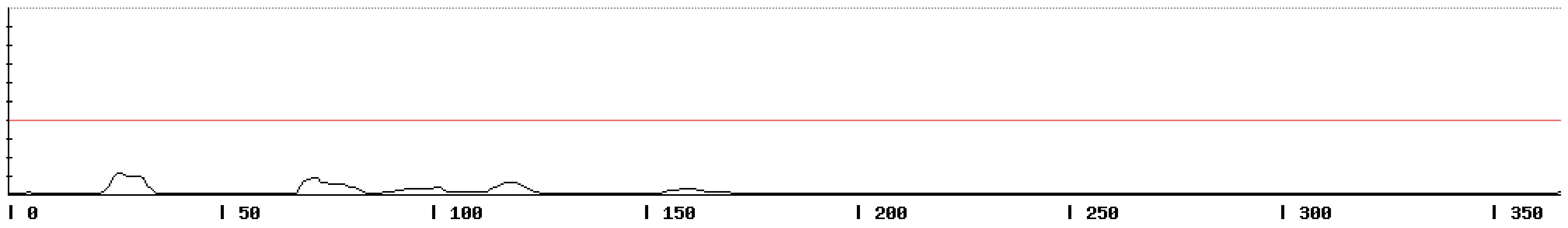

Predictions for Arachishypogaea_Ahy020455

Viterbi Path

Viterbi Path

Posterior @ 0.4

Posterior @ 0.4

Posterior Graphic. Threshold Marked @ 0.4

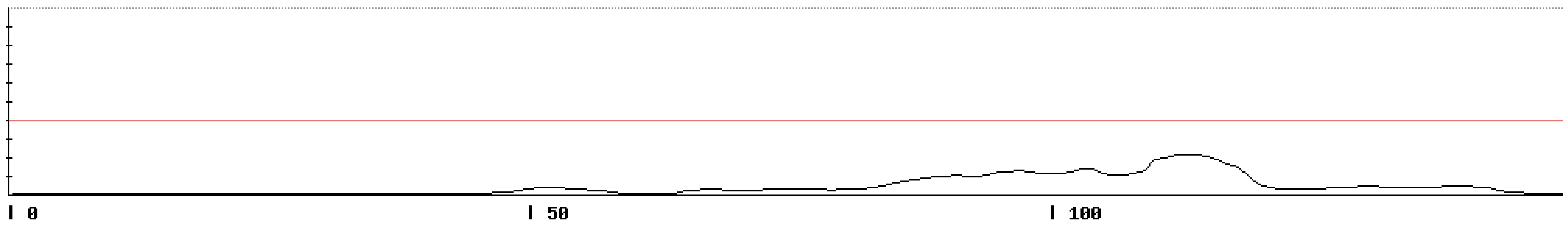

Predictions for Arachishypogaea_Ahy020949

Viterbi Path

Viterbi Path

Posterior @ 0.4

Posterior @ 0.4

Posterior Graphic. Threshold Marked @ 0.4

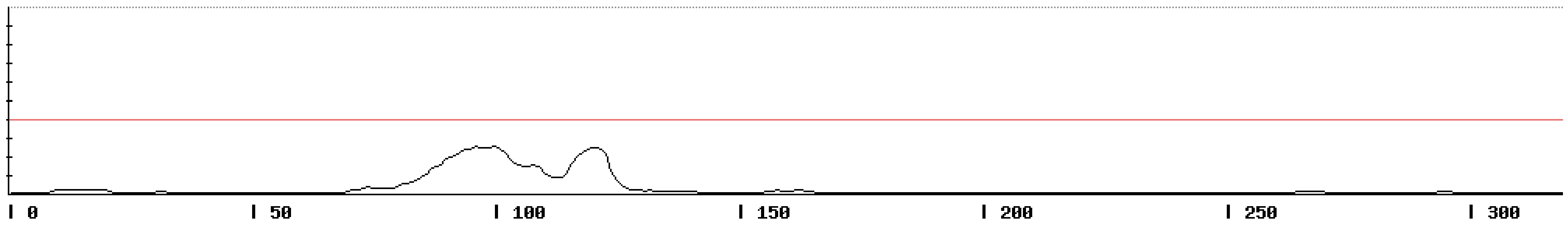

Predictions for Arachishypogaea_Ahy021814

Viterbi Path

Viterbi Path

Posterior @ 0.4

Posterior @ 0.4

Posterior Graphic. Threshold Marked @ 0.4

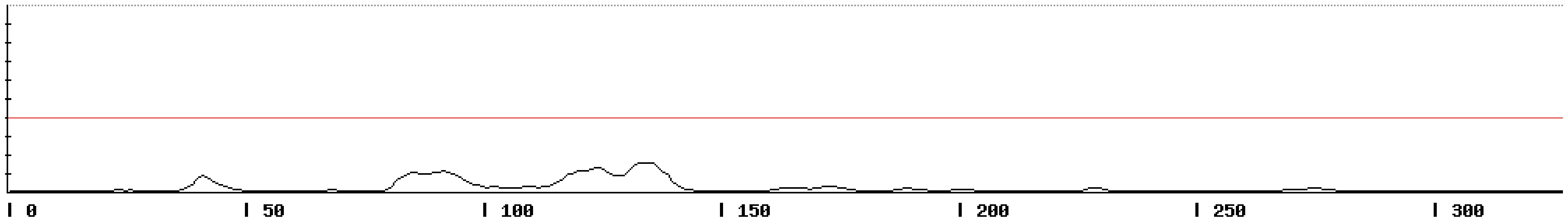

Predictions for Arachishypogaea_Ahy021850

Viterbi Path

Viterbi Path

Posterior @ 0.4

Posterior @ 0.4

Posterior Graphic. Threshold Marked @ 0.4

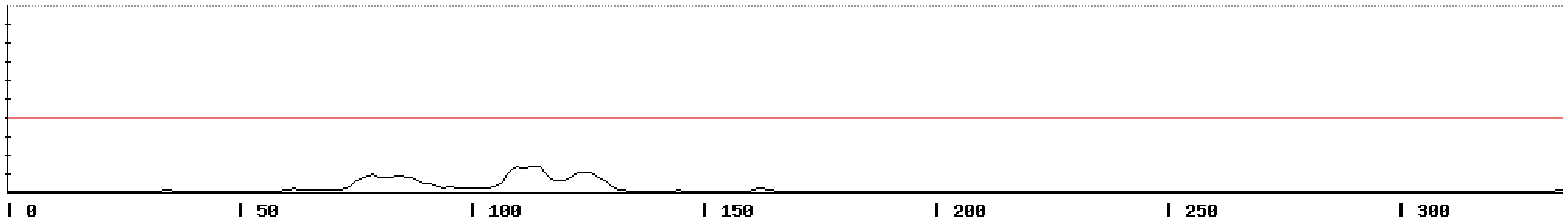

Predictions for Arachishypogaea_Ahy022124

Viterbi Path	
Viterbi Path	
Posterior @ 0.4	
Posterior @ 0.4	
Posterior Graphic. Threshold Marked @ 0.4	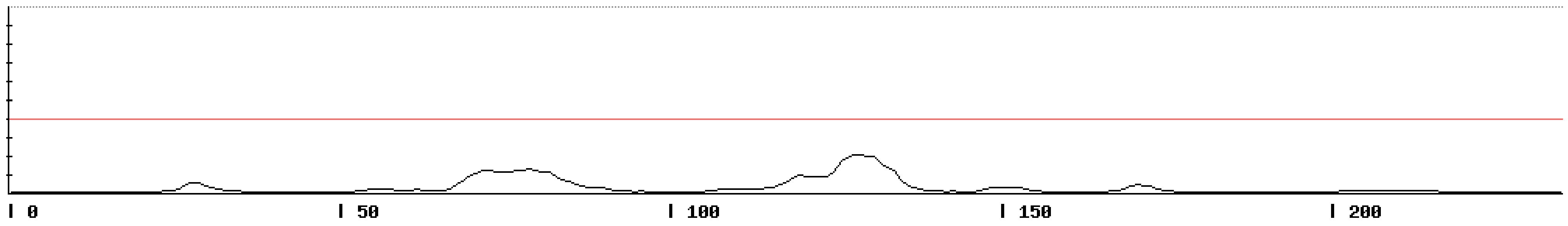

Predictions for Arachishypogaea_Ahy022375	
Viterbi Path	236 - KKKKKKKSER - 245
Viterbi Path	

Revision r.9 - Last Updated on : February 24 2015 17:45:20.

alex.nguyenba@utoronto.ca

Intended to be used with Firefox

Viterbi Path	
Posterior @ 0.4	
Posterior @ 0.4	
Posterior Graphic. Threshold Marked @ 0.4	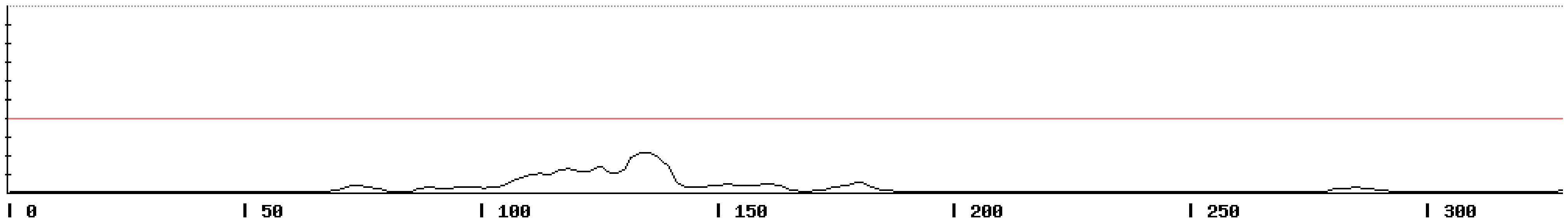

Predictions for Arachisipaensis_Araip.0S3JI

Viterbi Path	
Viterbi Path	
Posterior @ 0.4	
Posterior @ 0.4	
Posterior Graphic. Threshold Marked @ 0.4	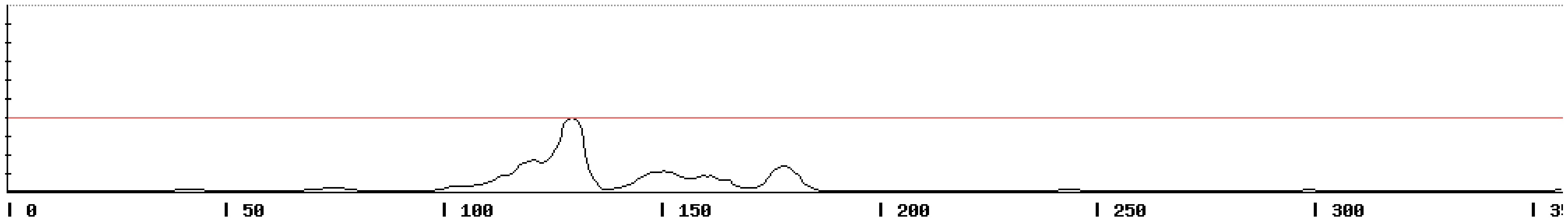

Predictions for Arachisipaensis_Araip.1N7IP

Viterbi Path	
Viterbi Path	
Posterior @ 0.4	132 - RKRRK - 136
Posterior @ 0.4	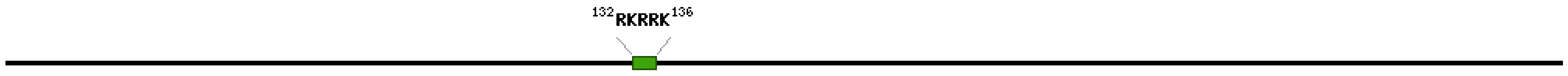
Posterior Graphic. Threshold Marked @ 0.4	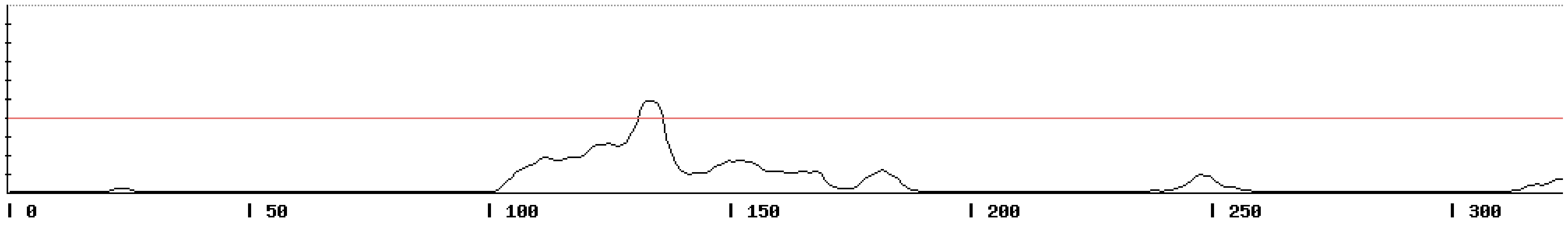

Predictions for Arachisipaensis_Araip.1Z0SD

Viterbi Path	
Viterbi Path	
Posterior @ 0.4	123 - RKRRK - 127
Posterior @ 0.4	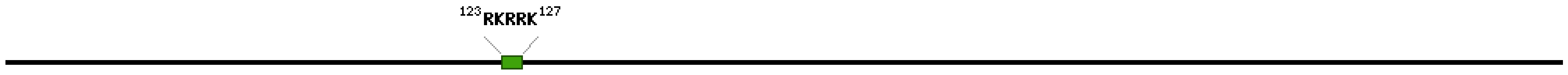
Posterior Graphic. Threshold Marked @ 0.4	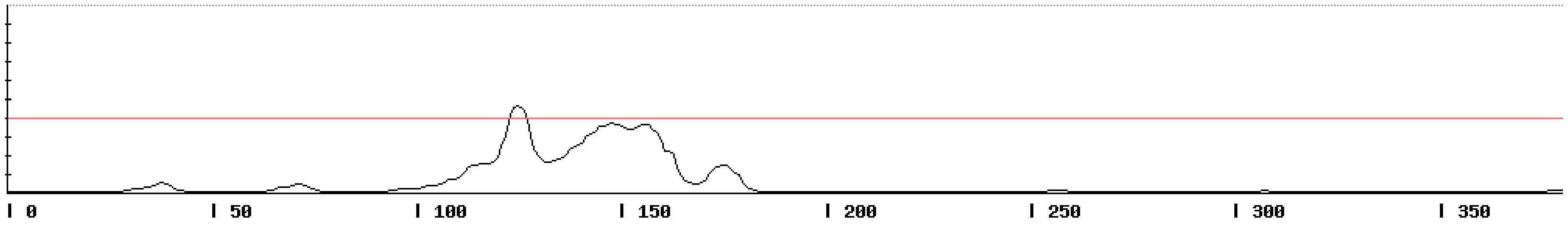

Predictions for Arachisipaensis_Araip.2BL8E

Viterbi Path	
Viterbi Path	
Posterior @ 0.4	241 - RRIRLRVPKSTVPKSRVPK - 259
Posterior @ 0.4	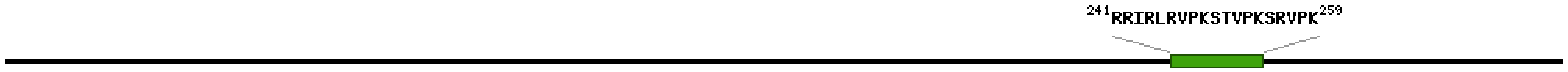
Posterior Graphic. Threshold Marked @ 0.4	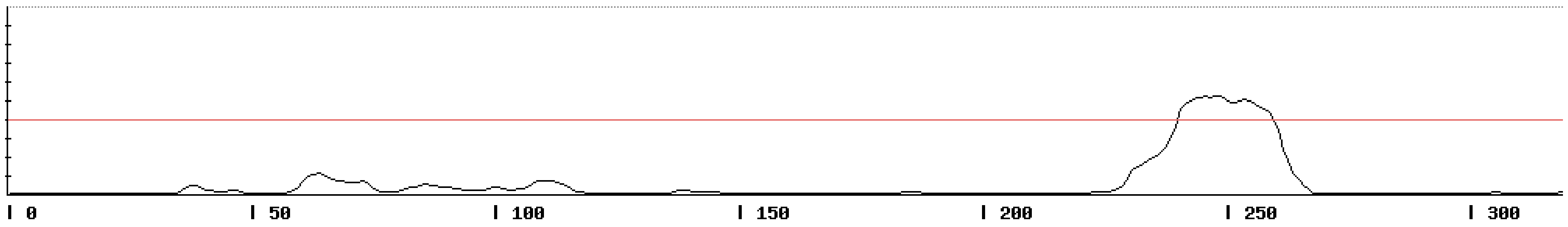

Predictions for Arachisipaensis_Araip.2W5R5

Viterbi Path	
Viterbi Path	
Posterior @ 0.4	
Posterior @ 0.4	
Posterior Graphic. Threshold Marked @ 0.4	

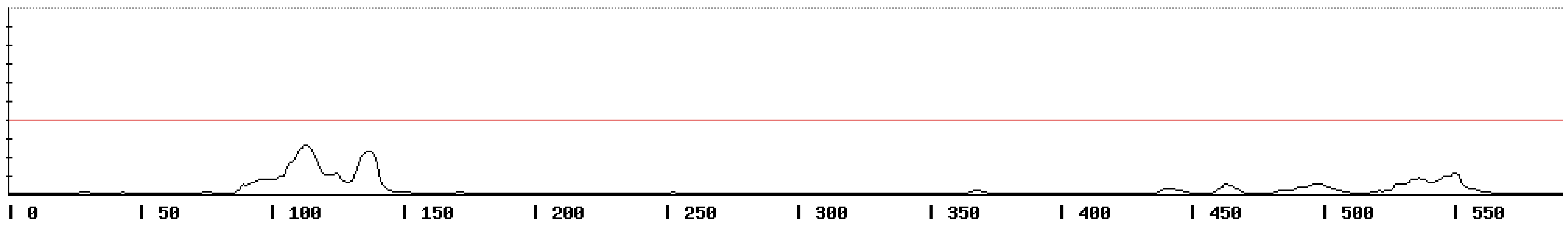

Predictions for Arachisipaensis_Araip.310T2

Viterbi Path	
Viterbi Path	
Posterior @ 0.4	
Posterior @ 0.4	
Posterior Graphic. Threshold Marked @ 0.4	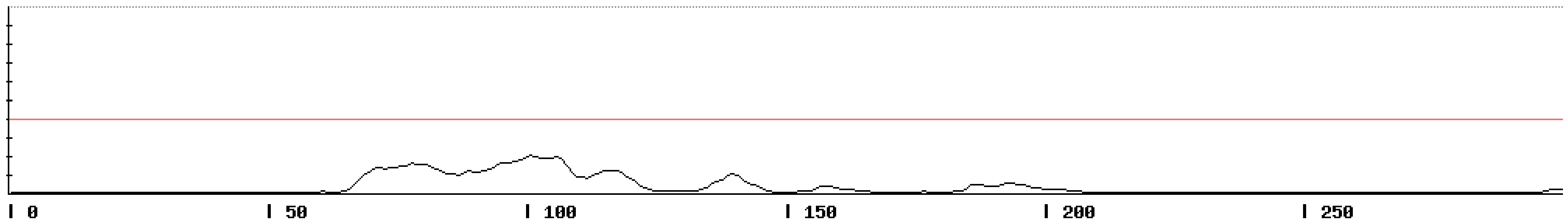

Predictions for Arachisipaensis_Araip.31EFM

Viterbi Path	
Viterbi Path	
Posterior @ 0.4	133 - KRRK - 136
	176 - IRPKRGAKPNKSK - 188
	217 - KKKTKKN - 223
Posterior @ 0.4	

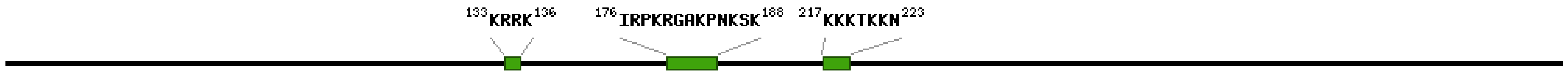

Posterior Graphic. Threshold Marked @ 0.4

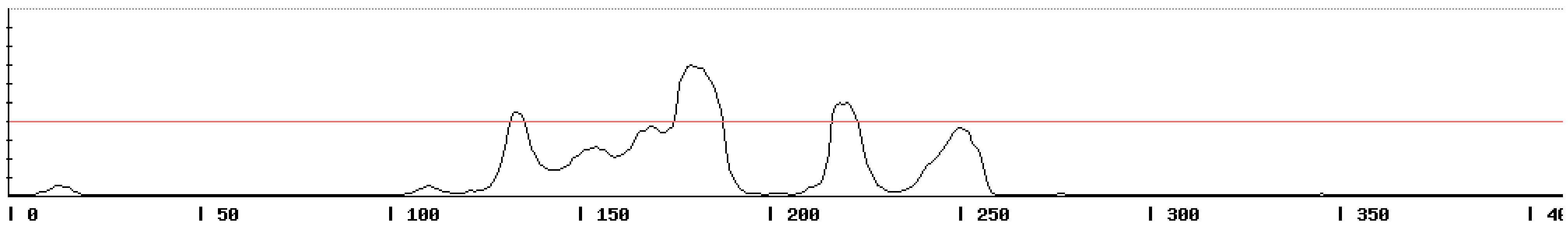

Predictions for Arachisipaensis_Araip.333QY

Viterbi Path

Viterbi Path

Posterior @ 0.4

Posterior @ 0.4

Posterior Graphic. Threshold Marked @ 0.4

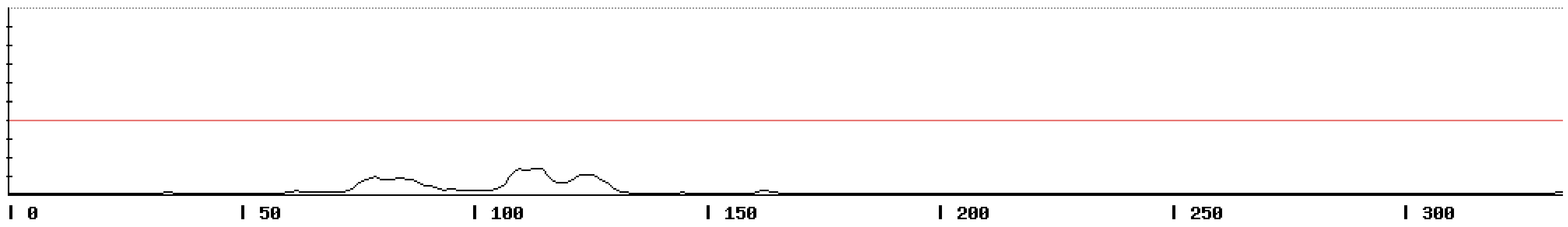

Predictions for Arachisipaensis_Araip.4A49L

Viterbi Path

Viterbi Path

Posterior @ 0.4

Posterior @ 0.4

Posterior Graphic. Threshold Marked @ 0.4

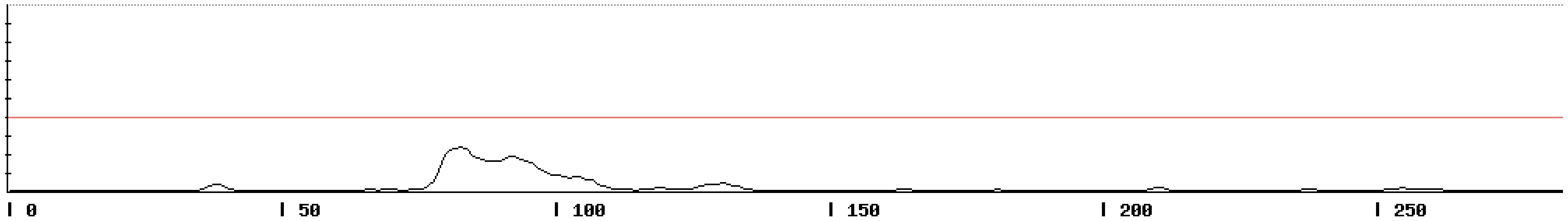

Predictions for Arachisipaensis_Araip.4R4C1

Viterbi Path

Viterbi Path

Posterior @ 0.4

Posterior @ 0.4

Posterior Graphic. Threshold Marked @ 0.4

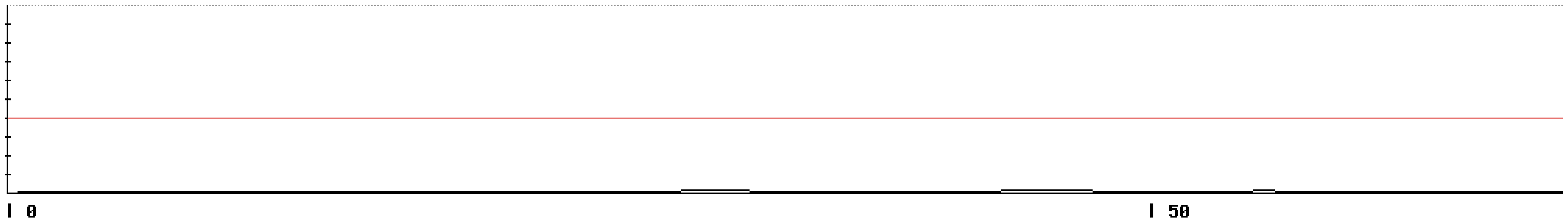

Predictions for Arachisipaensis_Araip.609WS

Viterbi Path

Viterbi Path

Posterior @ 0.4

Posterior @ 0.4

Posterior Graphic. Threshold Marked @ 0.4

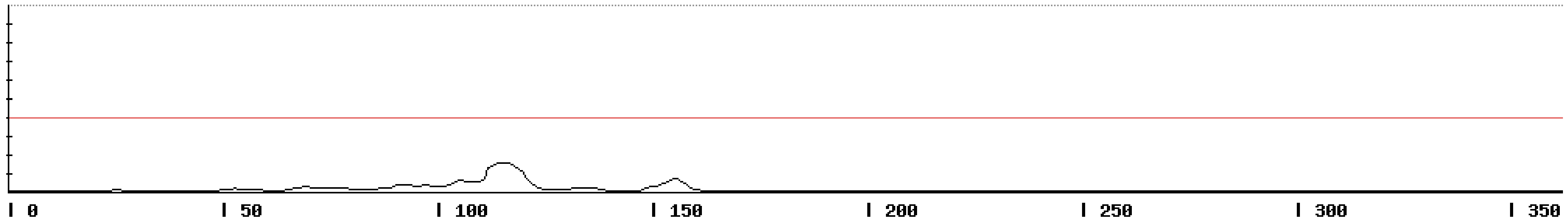

Predictions for Arachisipaensis_Araip.64GCN

Viterbi Path

Viterbi Path

Posterior @ 0.4

Posterior @ 0.4

Posterior Graphic. Threshold Marked @ 0.4

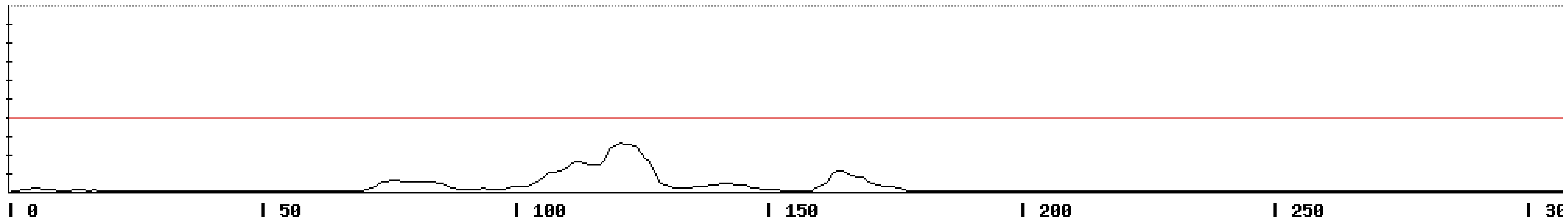

Predictions for Arachisipaensis_Araip.67R8V

Viterbi Path	
Viterbi Path	
Posterior @ 0.4	252 - KK - 253
Posterior @ 0.4	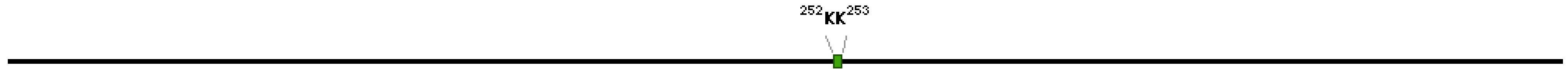
Posterior Graphic. Threshold Marked @ 0.4	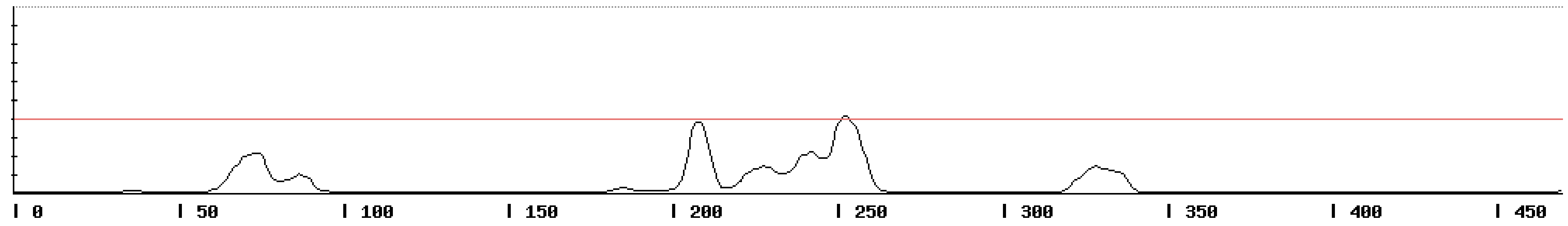

Predictions for Arachisipaensis_Araip.6CI1F

Viterbi Path	
Viterbi Path	
Posterior @ 0.4	
Posterior @ 0.4	
Posterior Graphic. Threshold Marked @ 0.4	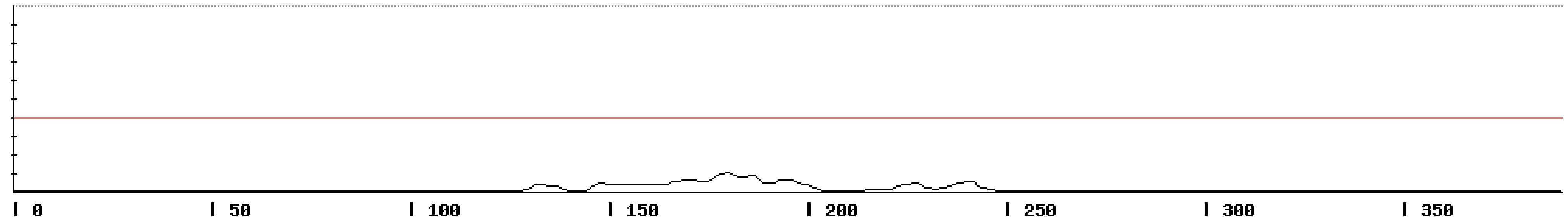

Predictions for Arachisipaensis_Araip.6Y0GY

Viterbi Path	
Viterbi Path	
Posterior @ 0.4	
Posterior @ 0.4	
Posterior Graphic. Threshold Marked @ 0.4	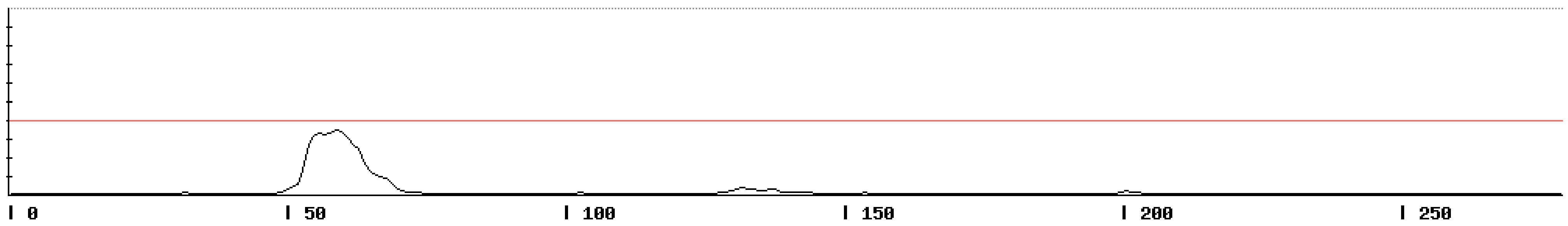

Predictions for Arachisipaensis_Araip.714GL

Viterbi Path	
Viterbi Path	
Posterior @ 0.4	
Posterior @ 0.4	
Posterior Graphic. Threshold Marked @ 0.4	

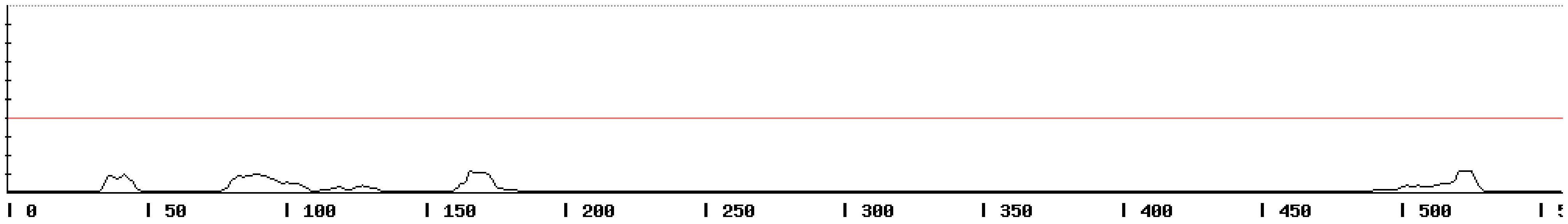

Predictions for Arachisipaensis_Araip.71CS3

Viterbi Path	
Viterbi Path	
Posterior @ 0.4	
Posterior @ 0.4	
Posterior Graphic. Threshold Marked @ 0.4	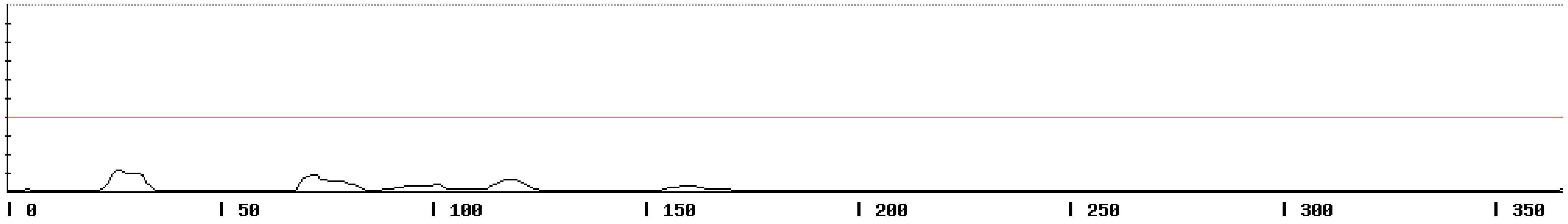

Predictions for Arachisipaensis_Araip.77ISR

Viterbi Path	
Viterbi Path	
Posterior @ 0.4	
Posterior @ 0.4	

Posterior Graphic. Threshold Marked @ 0.4

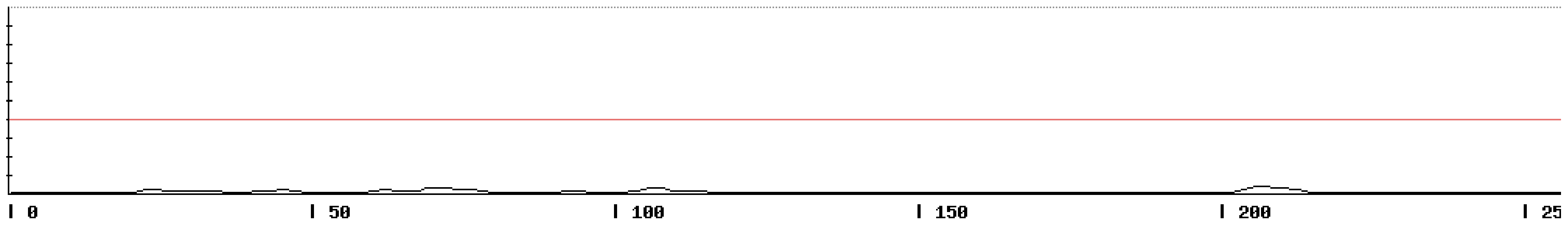

Predictions for Arachisipaensis_Araip.78PTT

Viterbi Path

Viterbi Path

Posterior @ 0.4

Posterior @ 0.4

Posterior Graphic. Threshold Marked @ 0.4

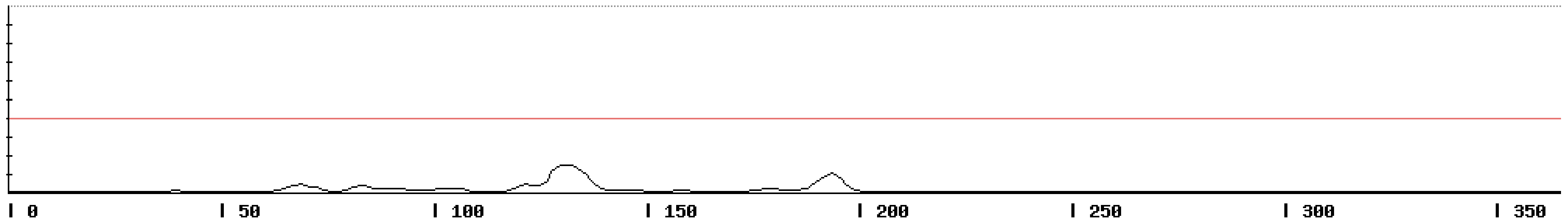

Predictions for Arachisipaensis_Araip.79TDF

Viterbi Path

Viterbi Path

Posterior @ 0.4

Posterior @ 0.4

Posterior Graphic. Threshold Marked @ 0.4

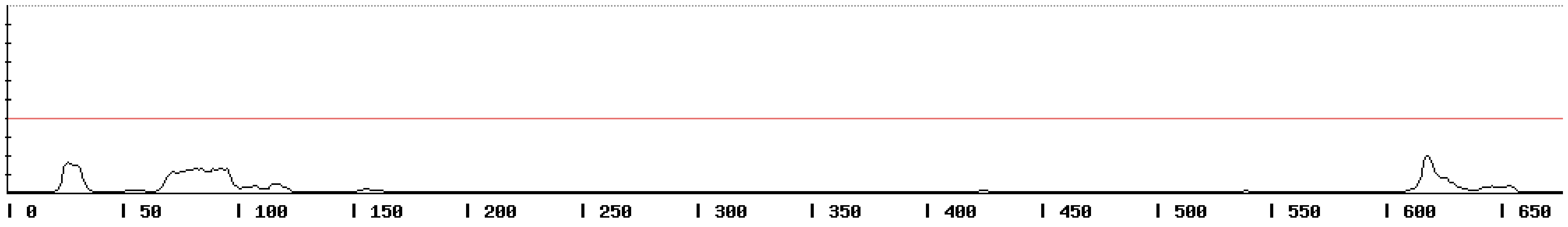

Predictions for Arachisipaensis_Araip.7L9YW

Viterbi Path	
Viterbi Path	
Posterior @ 0.4	146 - RKRRK - 150
Posterior @ 0.4	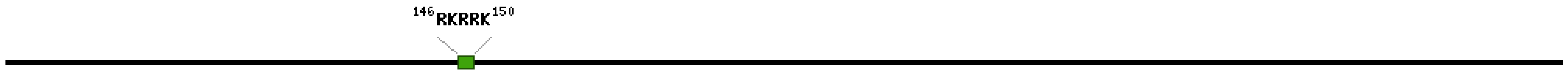
Posterior Graphic. Threshold Marked @ 0.4	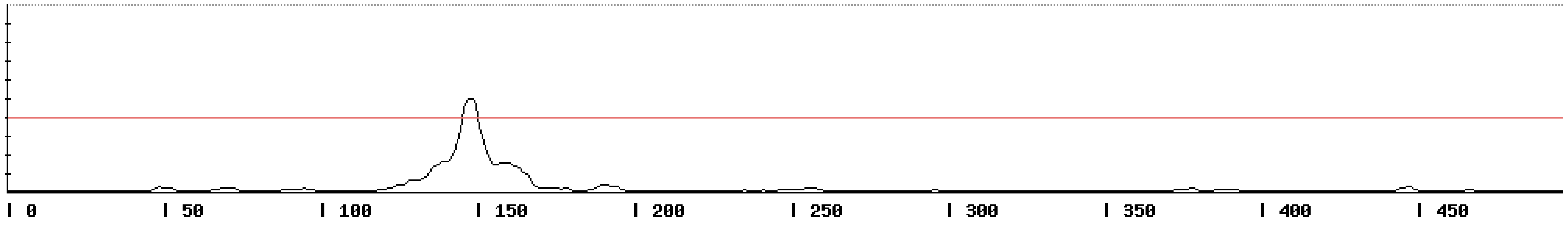

Predictions for Arachisipaensis_Araip.8NR3H

Viterbi Path	
Viterbi Path	

Posterior @ 0.4 93 - ADKPIGKPKALAIKK - 107

Posterior @ 0.4

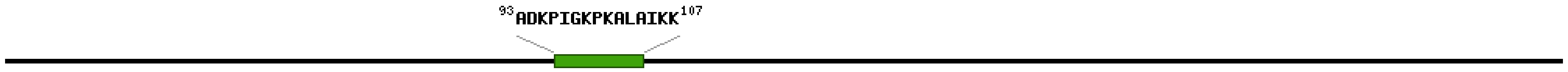

Posterior Graphic. Threshold Marked @ 0.4

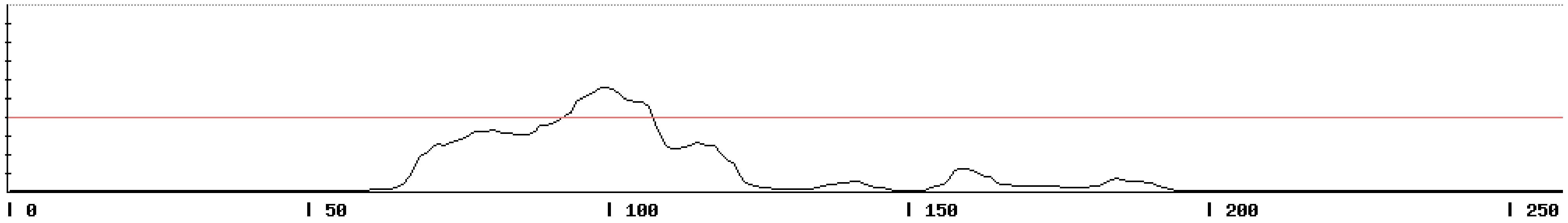

Predictions for Arachisipaensis_Araip.92BTQ

Viterbi Path

Viterbi Path

Posterior @ 0.4

Posterior @ 0.4

Posterior Graphic. Threshold Marked @ 0.4

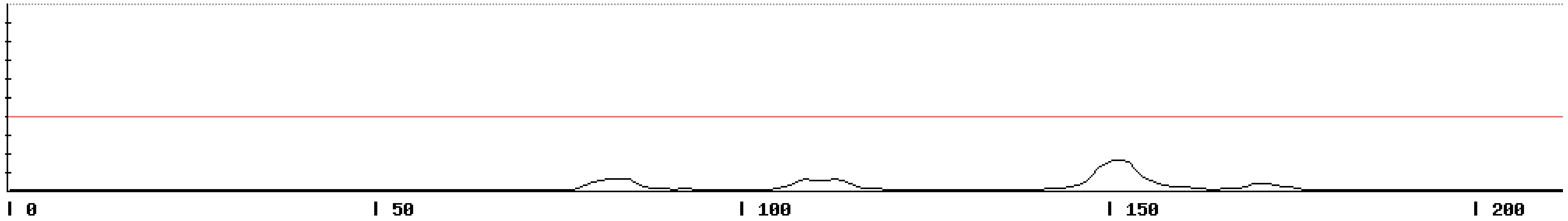

Predictions for Arachisipaensis_Araip.9BR1Z

Viterbi Path

Viterbi Path

Posterior @ 0.4

Posterior @ 0.4

Posterior Graphic. Threshold Marked @ 0.4

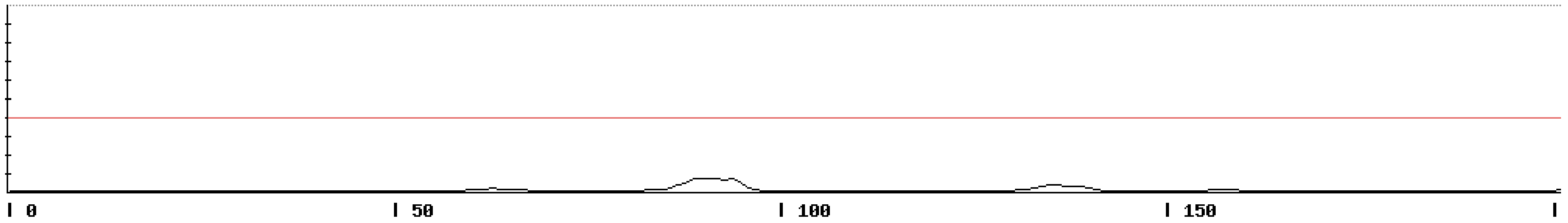

Predictions for Arachisipaensis_Araip.9MG9F

Viterbi Path

Viterbi Path

Posterior @ 0.4

163 - RKRRK - 167

191 - RGKLK - 195

Posterior @ 0.4

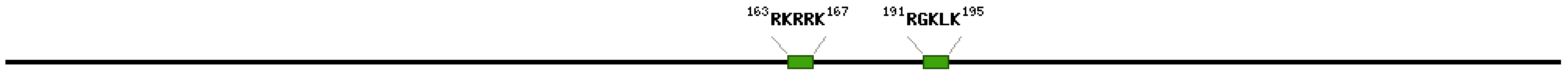

Posterior Graphic. Threshold Marked @ 0.4

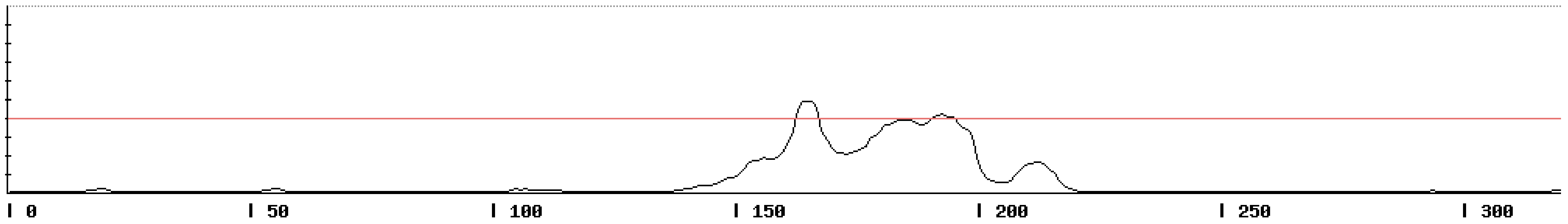

Predictions for Arachisipaensis_Araip.9N5S4	
Viterbi Path	
Viterbi Path	
Posterior @ 0.4	
Posterior @ 0.4	
Posterior Graphic. Threshold Marked @ 0.4	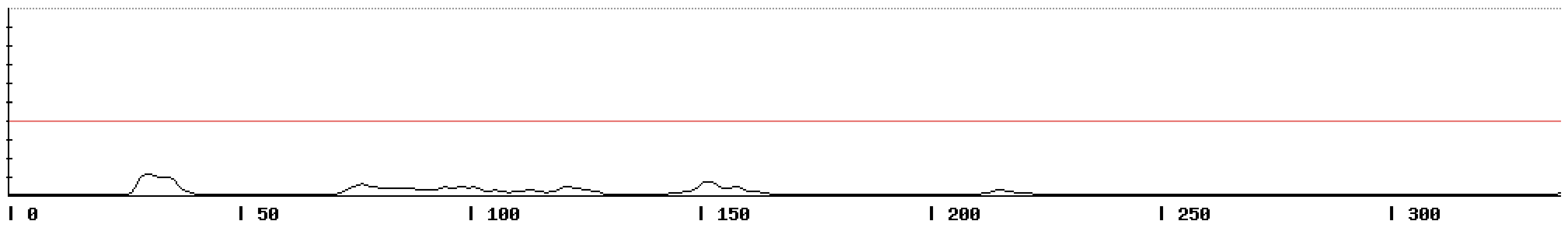

Predictions for Arachisipaensis_Araip.9W6SR	
Viterbi Path	
Viterbi Path	
Posterior @ 0.4	
Posterior @ 0.4	
Posterior Graphic. Threshold Marked @ 0.4	

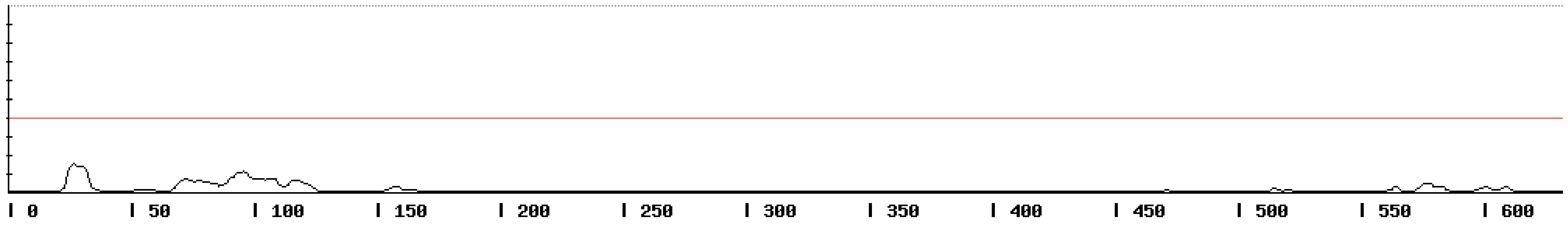

Predictions for Arachisipaensis_Araip.A6QWC

Viterbi Path

Viterbi Path

Posterior @ 0.4: 419 - KARKTSPGK - 427

Posterior @ 0.4

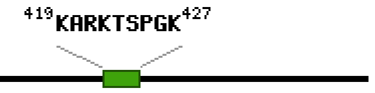

Posterior Graphic. Threshold Marked @ 0.4

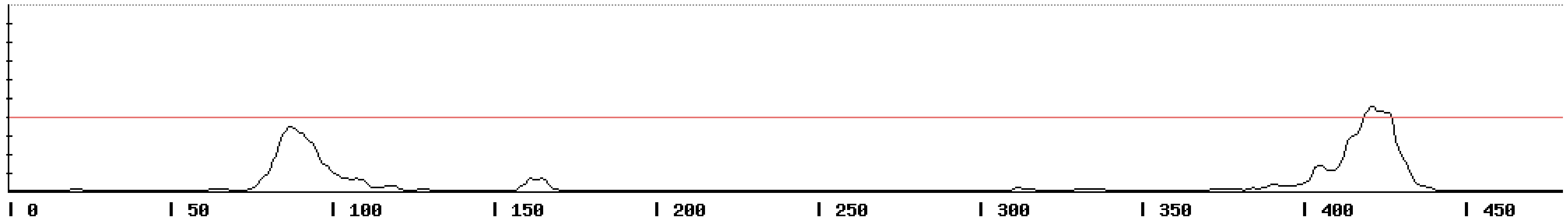

Predictions for Arachisipaensis_Araip.AVV74

Viterbi Path

Viterbi Path

Posterior @ 0.4

Posterior @ 0.4

Posterior Graphic. Threshold Marked @ 0.4	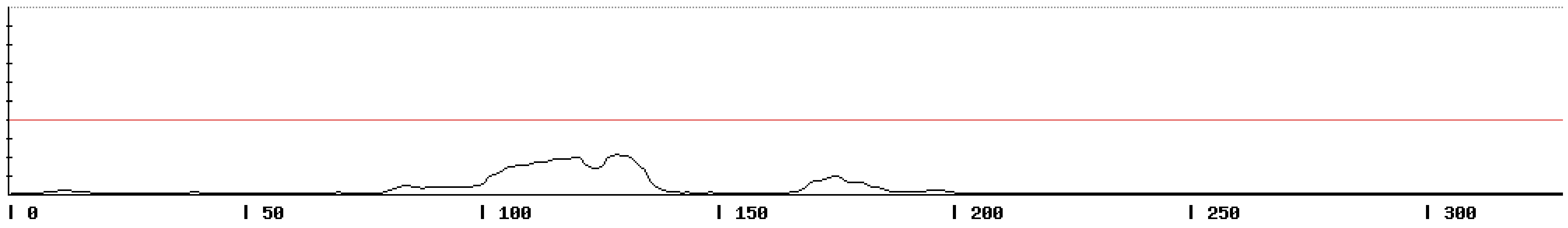

Predictions for Arachisipaensis_Araip.AWF0A	
Viterbi Path	
Viterbi Path	
Posterior @ 0.4	
Posterior @ 0.4	
Posterior Graphic. Threshold Marked @ 0.4	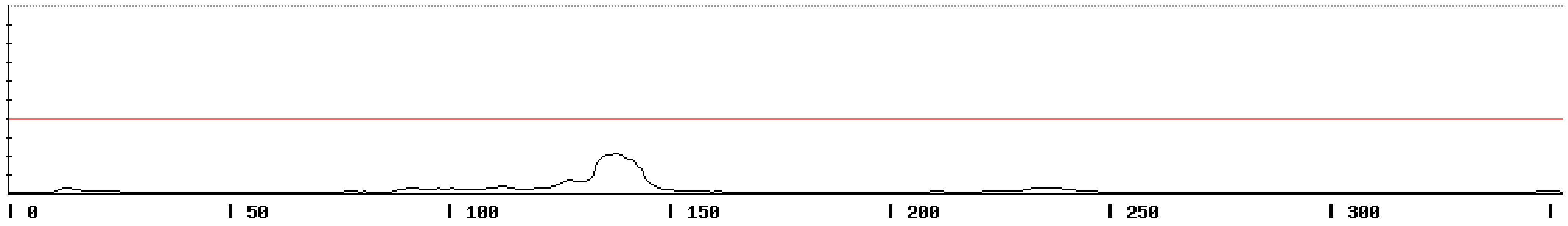

Predictions for Arachisipaensis_Araip.CC7W1	
Viterbi Path	
Viterbi Path	
Posterior @ 0.4	
Posterior @ 0.4	

Posterior Graphic. Threshold Marked @ 0.4

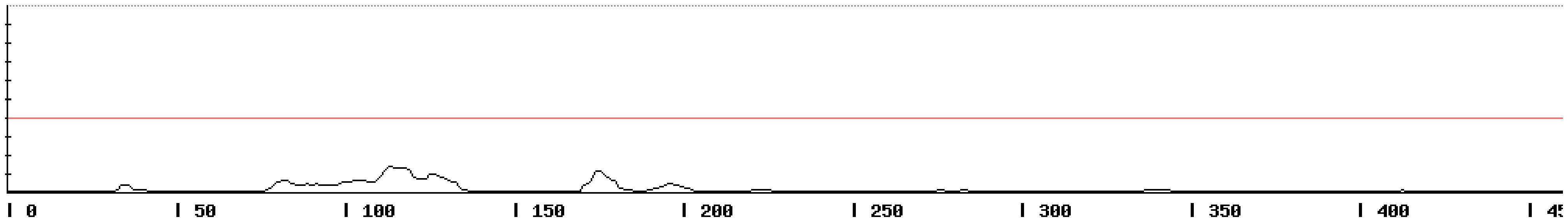

Predictions for Arachisipaensis_Araip.D25HB

Viterbi Path

Viterbi Path

Posterior @ 0.4

Posterior @ 0.4

Posterior Graphic. Threshold Marked @ 0.4

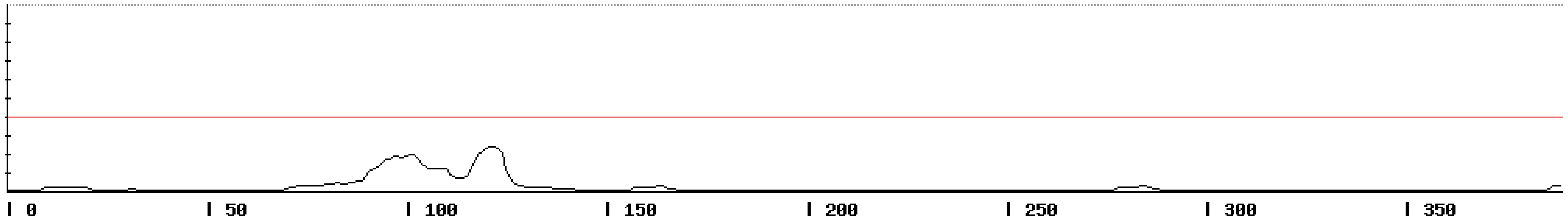

Predictions for Arachisipaensis_Araip.D7N1Q

Viterbi Path

Viterbi Path

Posterior @ 0.4

Posterior @ 0.4

Posterior Graphic. Threshold Marked @ 0.4

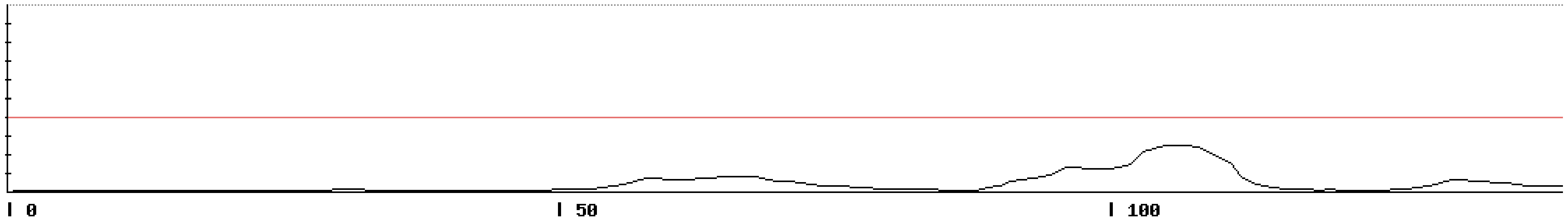

Predictions for Arachisipaensis_Araip.DEH65

Viterbi Path

Viterbi Path

Posterior @ 0.4

Posterior @ 0.4

Posterior Graphic. Threshold Marked @ 0.4

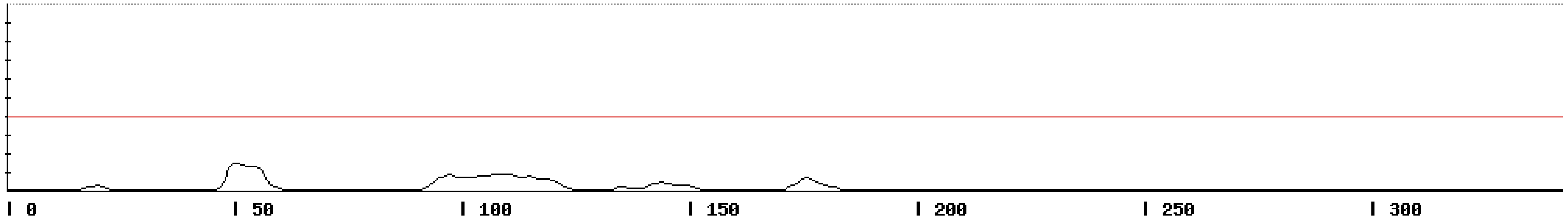

Predictions for Arachisipaensis_Araip.DL86S

Viterbi Path

Viterbi Path

Posterior @ 0.4

Posterior @ 0.4

Posterior Graphic. Threshold Marked @ 0.4

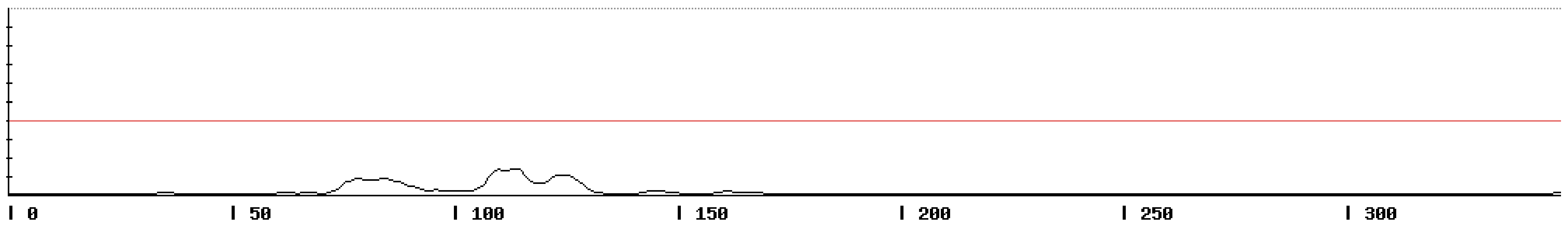

Predictions for Arachisipaensis_Araip.DR280

Viterbi Path

Viterbi Path

Posterior @ 0.4

Posterior @ 0.4

Posterior Graphic. Threshold Marked @ 0.4

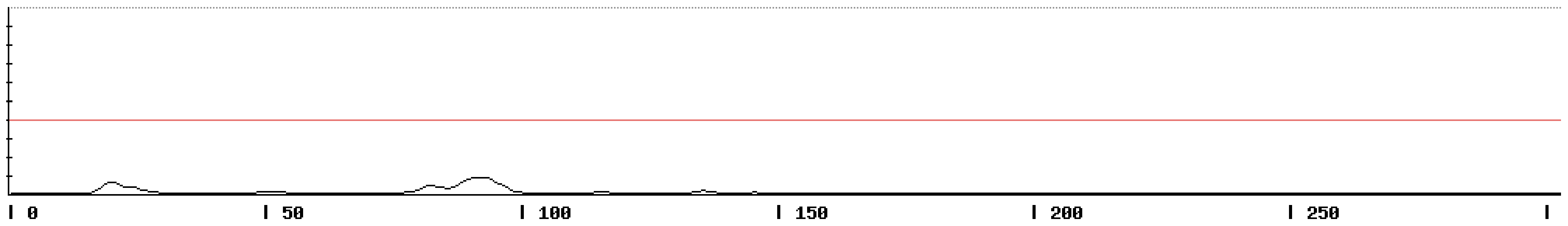

Predictions for Arachisipaensis_Araip.E0NQ0

Viterbi Path	
Viterbi Path	
Posterior @ 0.4	207 - KRR - 209
Posterior @ 0.4	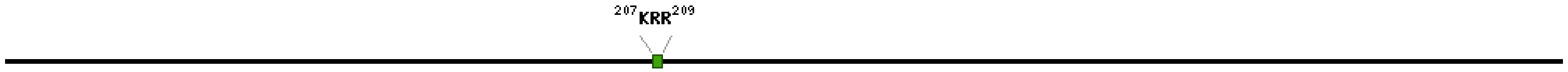
Posterior Graphic. Threshold Marked @ 0.4	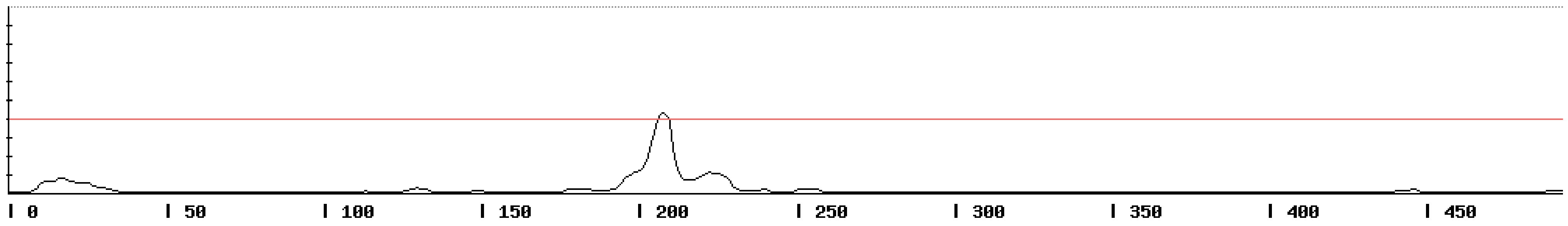

Predictions for Arachisipaensis_Araip.F5AGL

Viterbi Path	
Viterbi Path	
Posterior @ 0.4	
Posterior @ 0.4	
Posterior Graphic. Threshold Marked @ 0.4	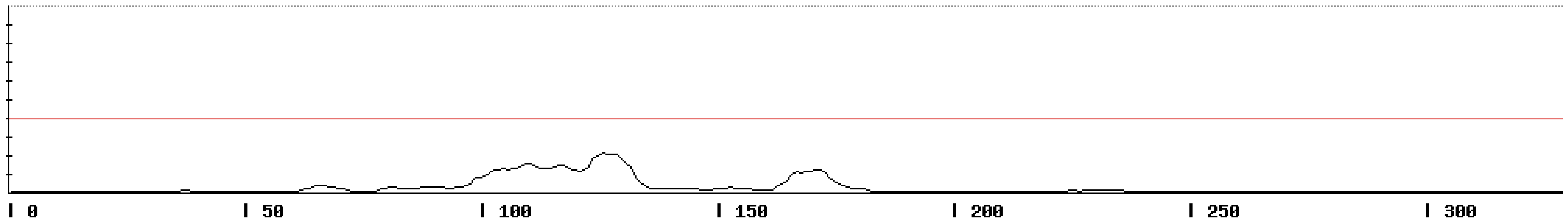

Predictions for Arachisipaensis_Araip.F8I62	
Viterbi Path	
Viterbi Path	
Posterior @ 0.4	
Posterior @ 0.4	
Posterior Graphic. Threshold Marked @ 0.4	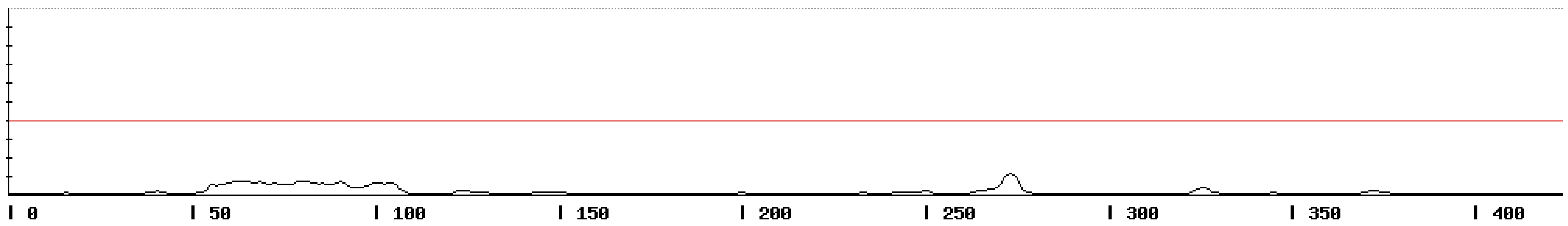

Predictions for Arachisipaensis_Araip.FR0NA	
Viterbi Path	
Viterbi Path	
Posterior @ 0.4	
Posterior @ 0.4	
Posterior Graphic. Threshold Marked @ 0.4	

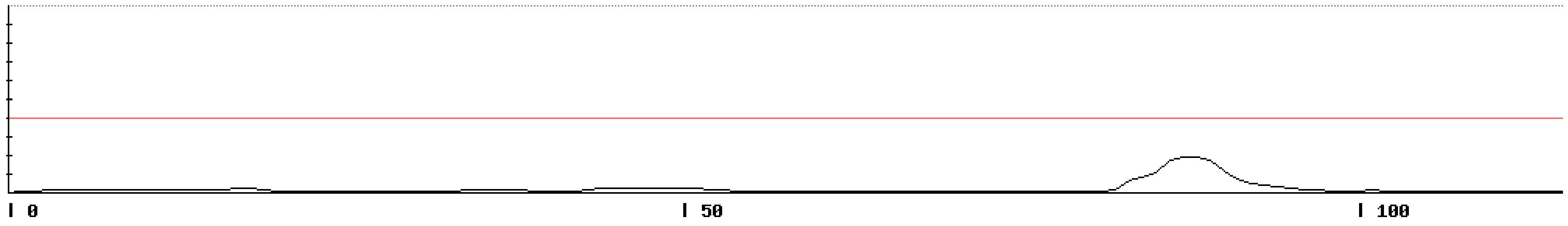

Predictions for Arachisipaensis_Araip.FRS32

Viterbi Path	
Viterbi Path	
Posterior @ 0.4	
Posterior @ 0.4	
Posterior Graphic. Threshold Marked @ 0.4	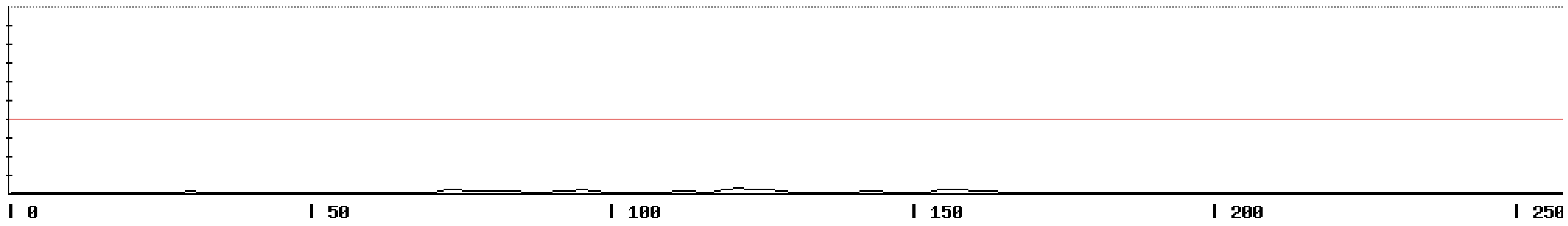

Predictions for Arachisipaensis_Araip.G3ZLR

Viterbi Path	
Viterbi Path	
Posterior @ 0.4	
Posterior @ 0.4	

Posterior Graphic. Threshold Marked @ 0.4

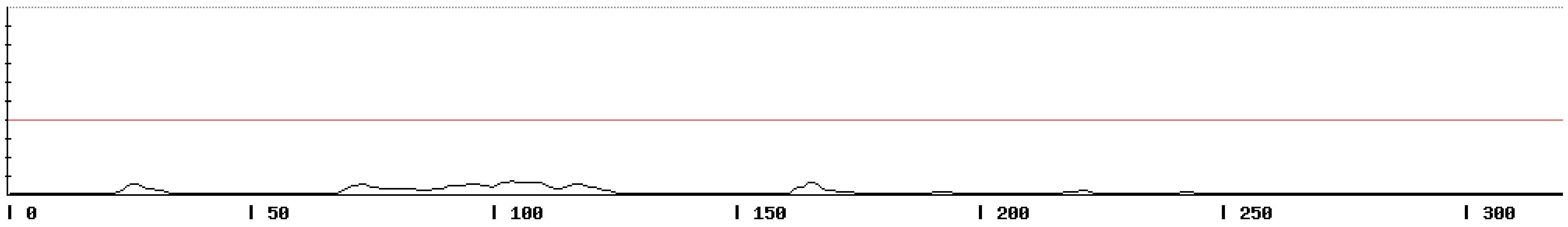

Predictions for Arachisipaensis_Araip.G88UP

Viterbi Path

Viterbi Path

Posterior @ 0.4: 159 - RKRRK - 163

Posterior @ 0.4

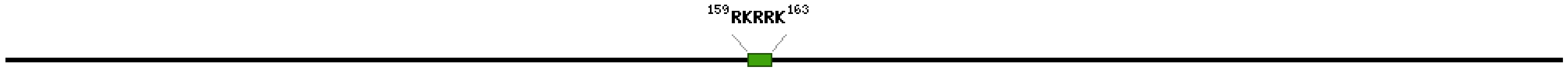

Posterior Graphic. Threshold Marked @ 0.4

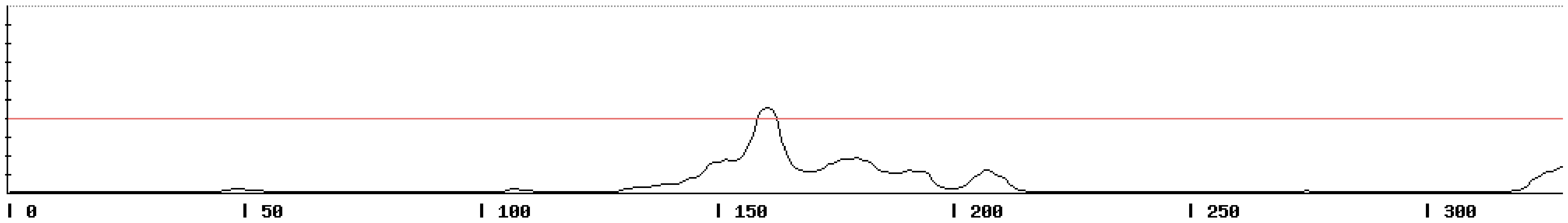

Predictions for Arachisipaensis_Araip.HIJ9F

Viterbi Path

Viterbi Path

Posterior @ 0.4

Posterior @ 0.4

Posterior Graphic. Threshold Marked @ 0.4

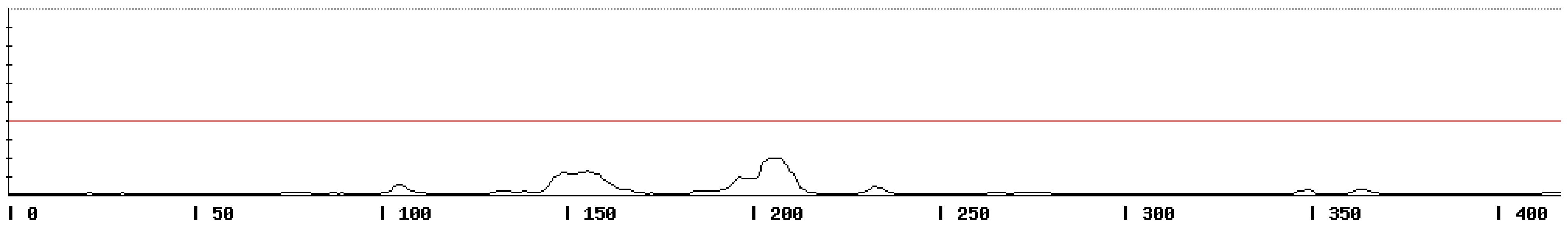

Predictions for Arachisipaensis_Araip.HYM8C

Viterbi Path

Viterbi Path

Posterior @ 0.4

Posterior @ 0.4

Posterior Graphic. Threshold Marked @ 0.4

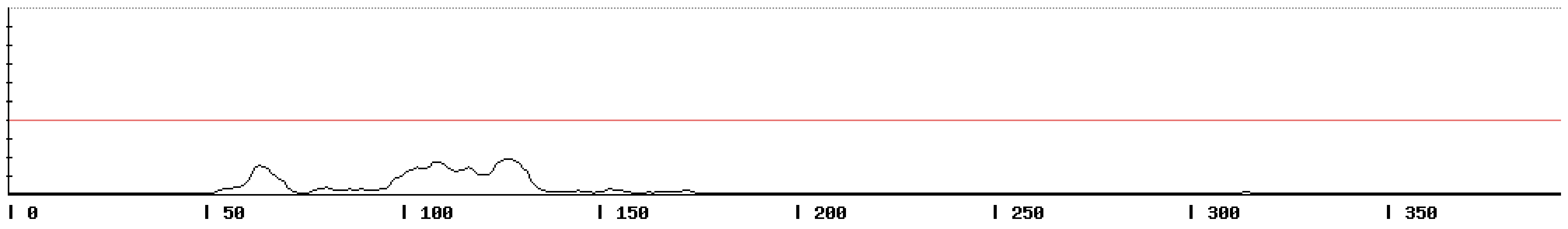

Predictions for Arachisipaensis_Araip.I60BC

Viterbi Path

Viterbi Path

Posterior @ 0.4	89 - SGKAPTGRKTK - 99
Posterior @ 0.4	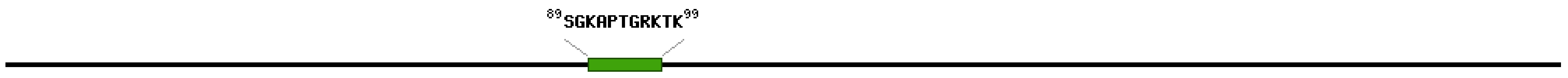
Posterior Graphic. Threshold Marked @ 0.4	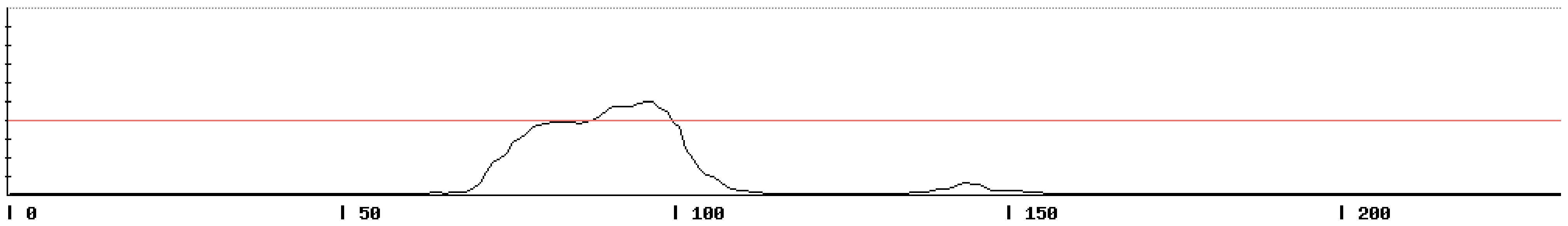

Predictions for Arachisipaensis_Araip.I6LH9

Viterbi Path	
Viterbi Path	
Posterior @ 0.4	106 - GADKPIGKPKALGIKK - 121
Posterior @ 0.4	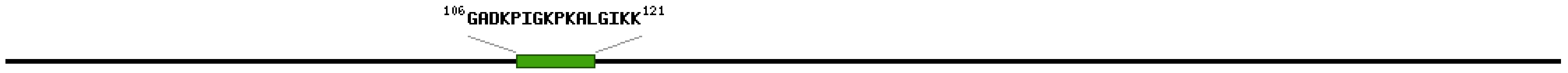
Posterior Graphic. Threshold Marked @ 0.4	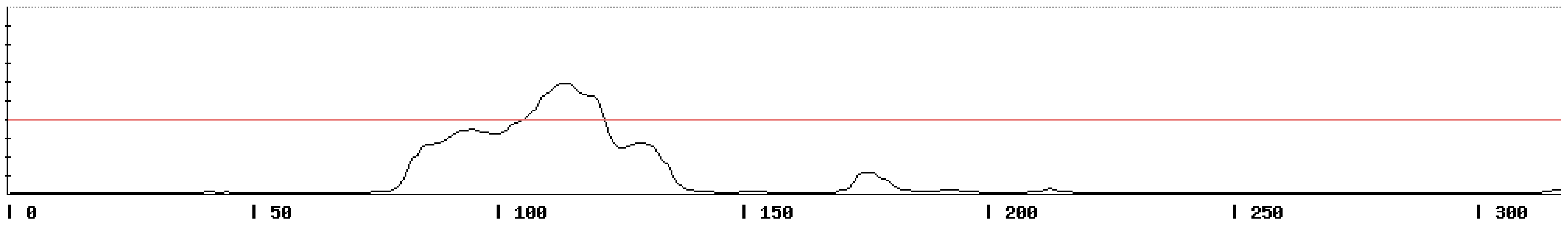

Predictions for Arachisipaensis_Araip.J93FI

Viterbi Path	
Viterbi Path	

Posterior @ 0.4	132 - RKRRK - 136
Posterior @ 0.4	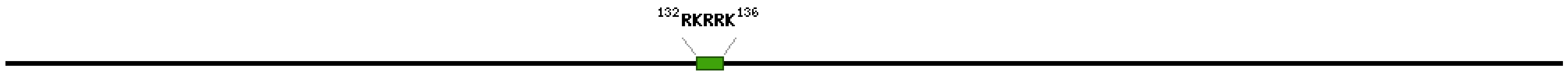
Posterior Graphic. Threshold Marked @ 0.4	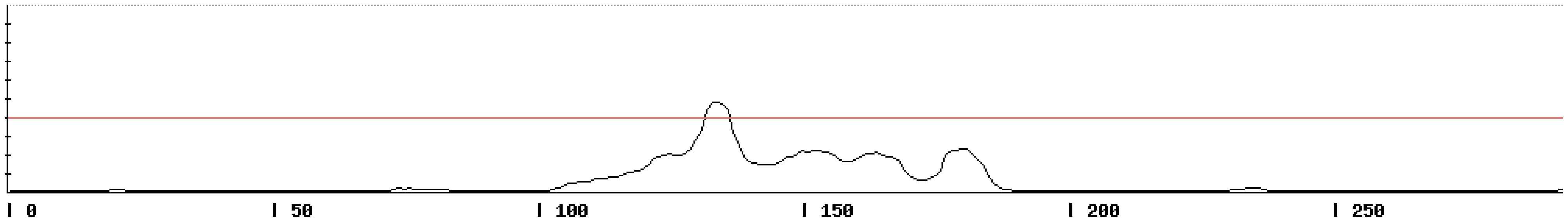

Predictions for Arachisipaensis_Araip.J9WH5

Viterbi Path	
Viterbi Path	
Posterior @ 0.4	
Posterior @ 0.4	
Posterior Graphic. Threshold Marked @ 0.4	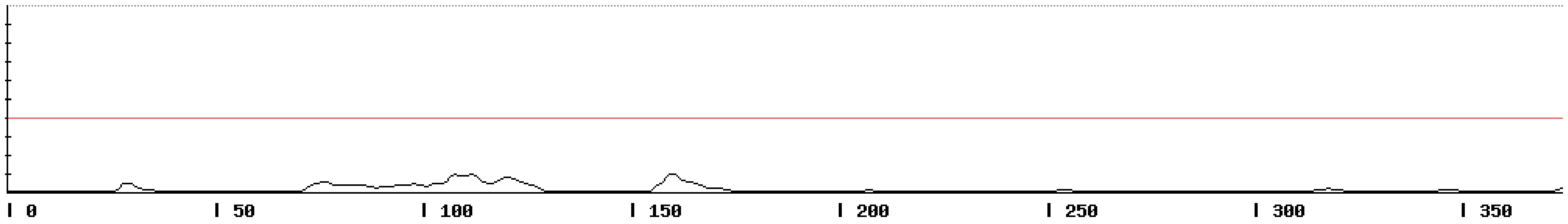

Predictions for Arachisipaensis_Araip.KI83M

Viterbi Path

Viterbi Path

Posterior @ 0.4

Posterior @ 0.4

Posterior Graphic. Threshold Marked @ 0.4

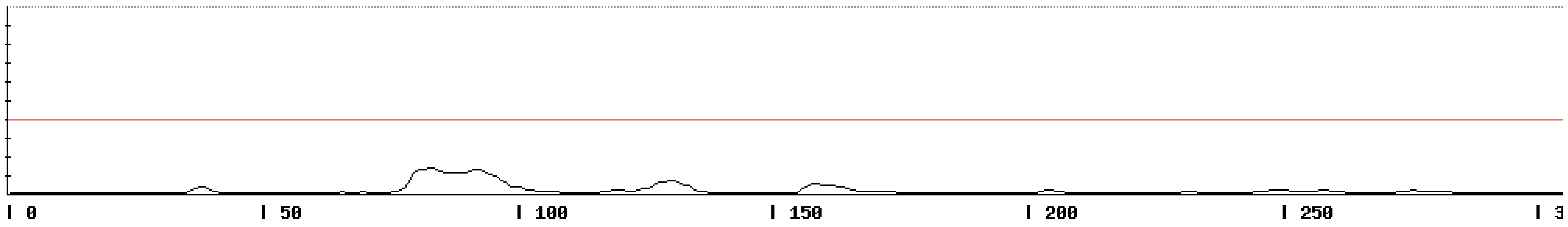

Predictions for Arachisipaensis_Araip.KM0ZG

Viterbi Path

Viterbi Path

Posterior @ 0.4

Posterior @ 0.4

Posterior Graphic. Threshold Marked @ 0.4

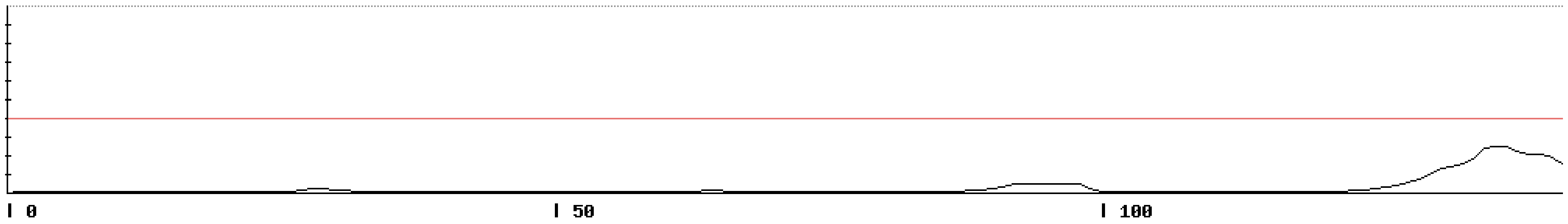

Predictions for Arachisipaensis_Araip.KP5QZ	
Viterbi Path	
Viterbi Path	
Posterior @ 0.4	
Posterior @ 0.4	
Posterior Graphic. Threshold Marked @ 0.4	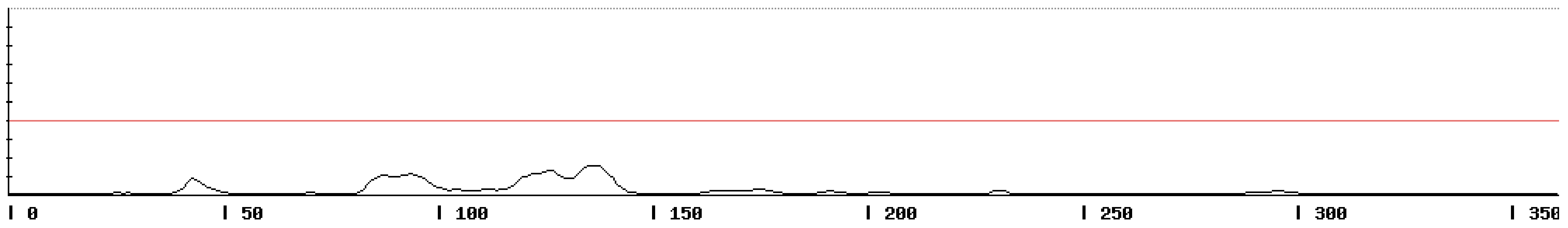

Predictions for Arachisipaensis_Araip.KTL6D	
Viterbi Path	
Viterbi Path	
Posterior @ 0.4	
Posterior @ 0.4	
Posterior Graphic. Threshold Marked @ 0.4	

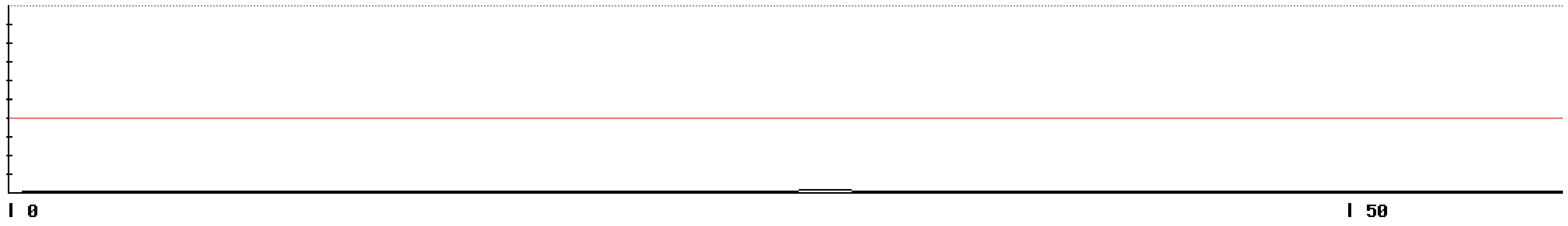

Predictions for Arachisipaensis_Araip.KXD50|Arachis_ipaensis|NAC|Araip.KXD50

Viterbi Path

Viterbi Path

Posterior @ 0.4

Posterior @ 0.4

Posterior Graphic. Threshold Marked @ 0.4

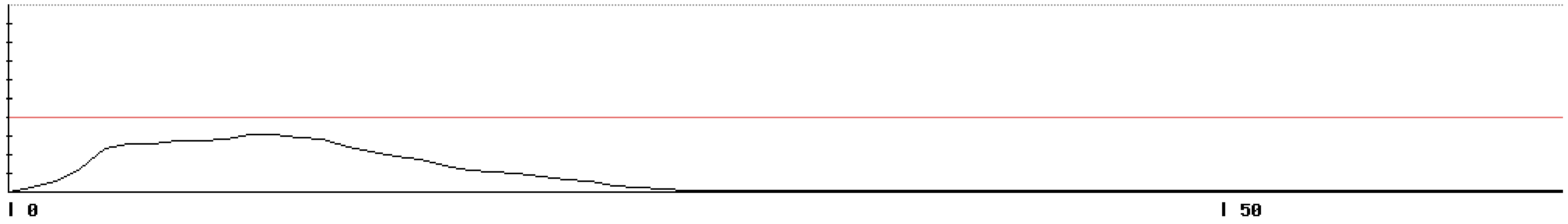

Predictions for Arachisipaensis_Araip.L222I

Viterbi Path

Viterbi Path

Posterior @ 0.4

Posterior @ 0.4

Posterior Graphic. Threshold Marked @ 0.4	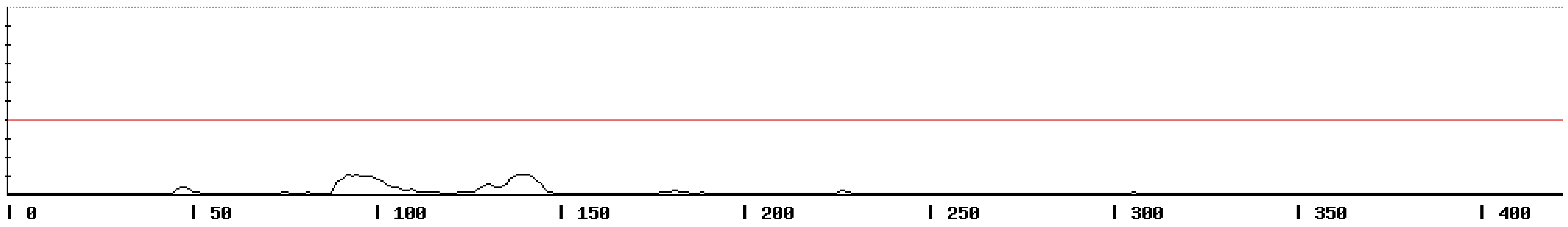

Predictions for Arachisipaensis_Araip.MQD5S

Viterbi Path	
Viterbi Path	
Posterior @ 0.4	158 - EKK - 160
Posterior @ 0.4	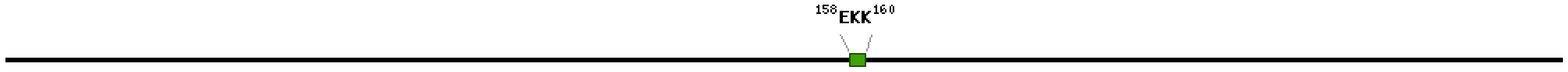
Posterior Graphic. Threshold Marked @ 0.4	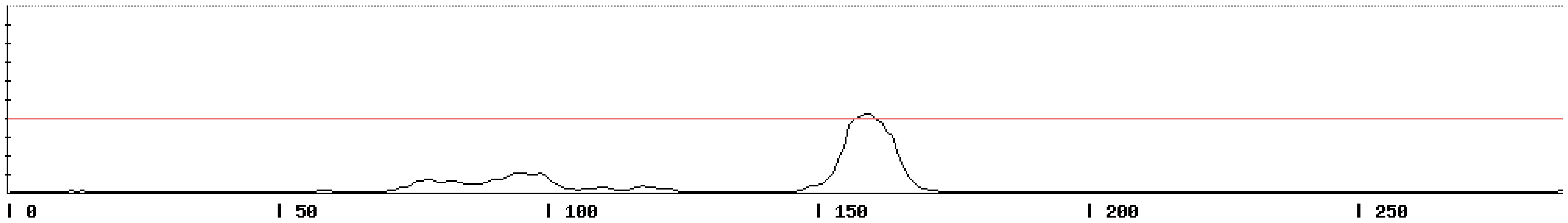

Predictions for Arachisipaensis_Araip.NB7HU

Viterbi Path	
Viterbi Path	
Posterior @ 0.4	
Posterior @ 0.4	

Posterior Graphic. Threshold Marked @ 0.4

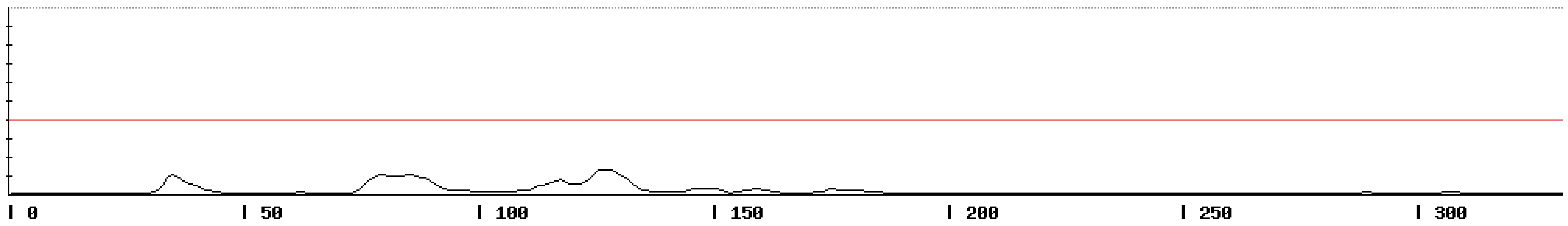

Predictions for Arachisipaensis_Araip.NL359

Viterbi Path

Viterbi Path

Posterior @ 0.4

Posterior @ 0.4

Posterior Graphic. Threshold Marked @ 0.4

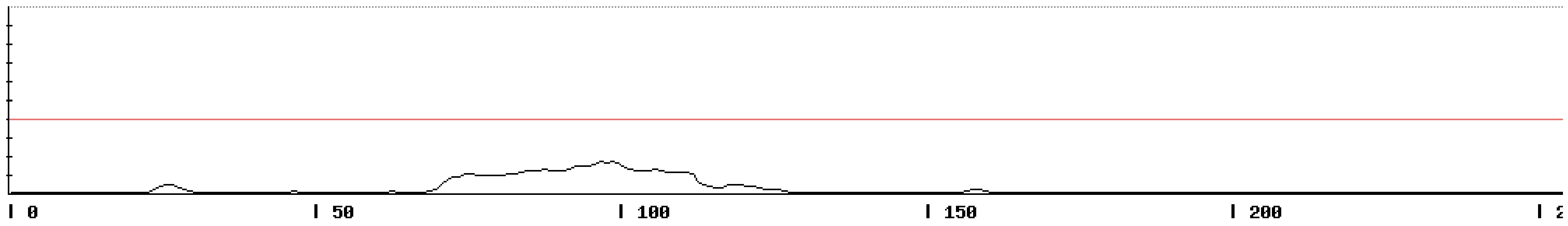

Predictions for Arachisipaensis_Araip.PNX61

Viterbi Path

Viterbi Path

Posterior @ 0.4	104 - SRGERWKRKRKVKSGFWLRRGR - 125
Posterior @ 0.4	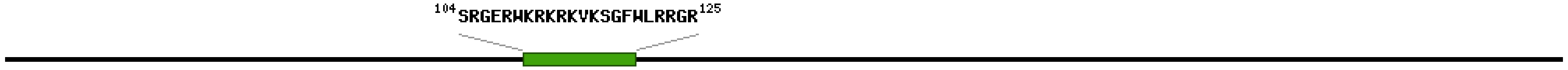
Posterior Graphic. Threshold Marked @ 0.4	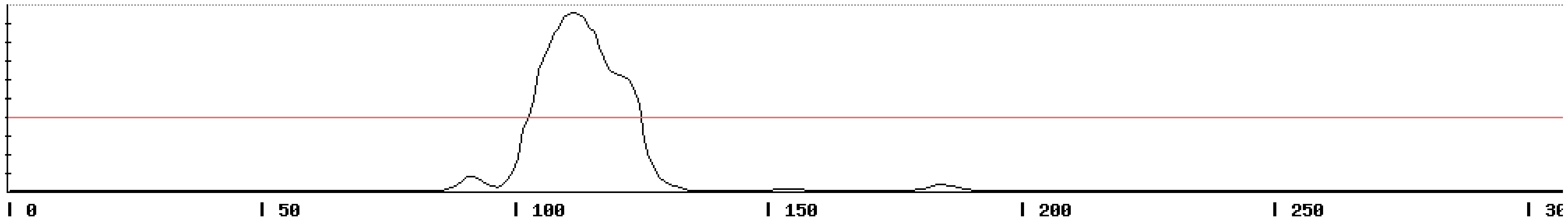

Predictions for Arachisipaensis_Araip.PT231

Viterbi Path	
Viterbi Path	
Posterior @ 0.4	
Posterior @ 0.4	
Posterior Graphic. Threshold Marked @ 0.4	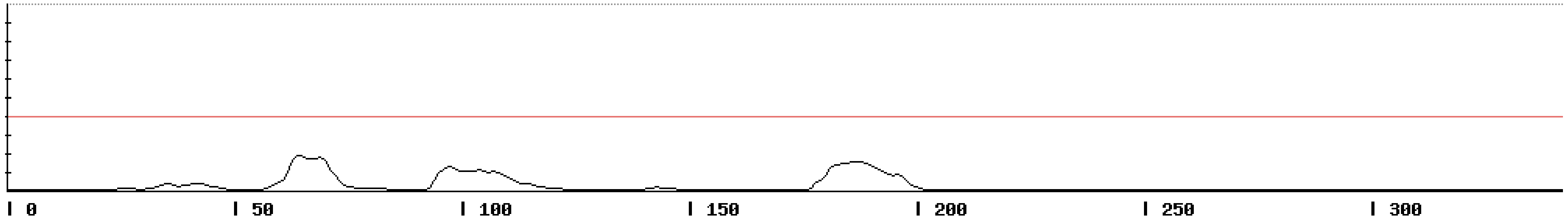

Predictions for Arachisipaensis_Araip.PW8UQ

Viterbi Path	
Viterbi Path	

Posterior @ 0.4

Posterior @ 0.4

Posterior Graphic. Threshold Marked @ 0.4

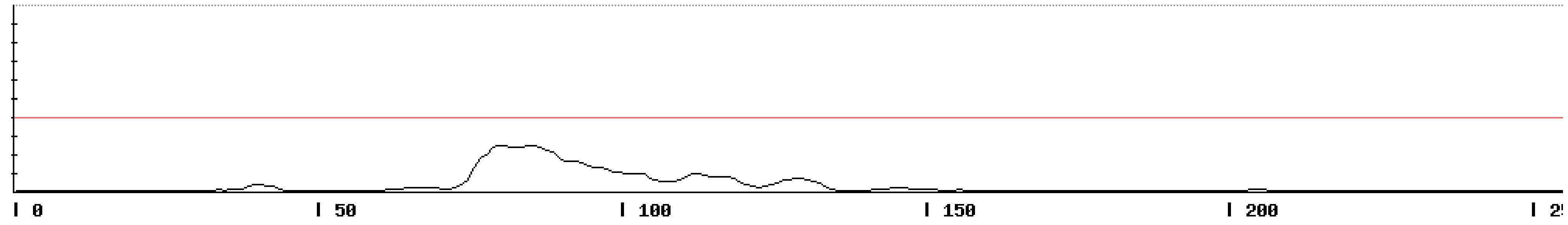

Predictions for Arachisipaensis_Araip.PX0QP

Viterbi Path

Viterbi Path

Posterior @ 0.4

Posterior @ 0.4

Posterior Graphic. Threshold Marked @ 0.4

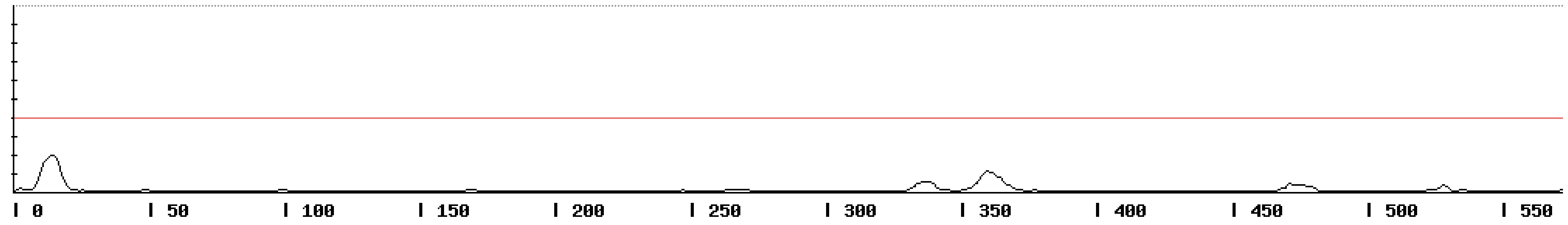

Predictions for Arachisipaensis_Araip.Q1JTJ

Viterbi Path

Viterbi Path

Posterior @ 0.4

Posterior @ 0.4

Posterior Graphic. Threshold Marked @ 0.4

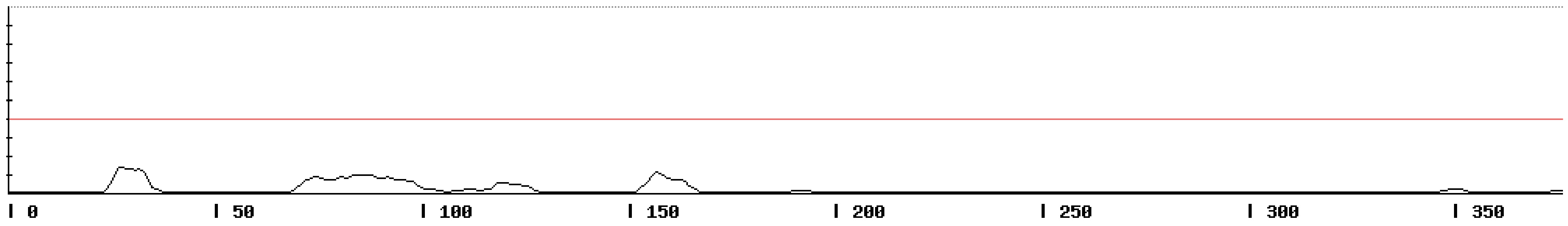

Predictions for Arachisipaensis_Araip.Q3R6H

Viterbi Path

Viterbi Path

Posterior @ 0.4

Posterior @ 0.4

Posterior Graphic. Threshold Marked @ 0.4

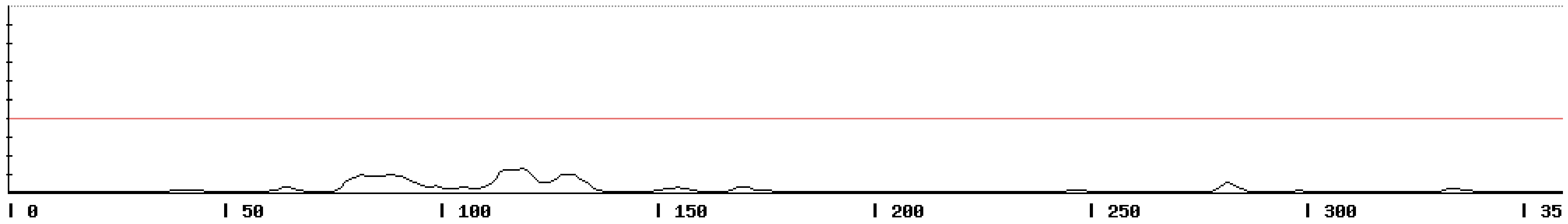

Predictions for Arachisipaensis_Araip.QS7JY	
Viterbi Path	
Viterbi Path	
Posterior @ 0.4	
Posterior @ 0.4	
Posterior Graphic. Threshold Marked @ 0.4	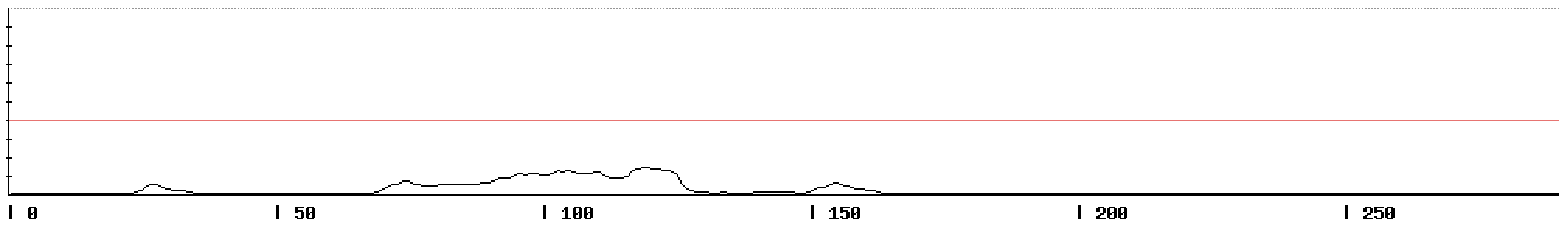

Predictions for Arachisipaensis_Araip.R0657	
Viterbi Path	
Viterbi Path	
Posterior @ 0.4	
Posterior @ 0.4	
Posterior Graphic. Threshold Marked @ 0.4	

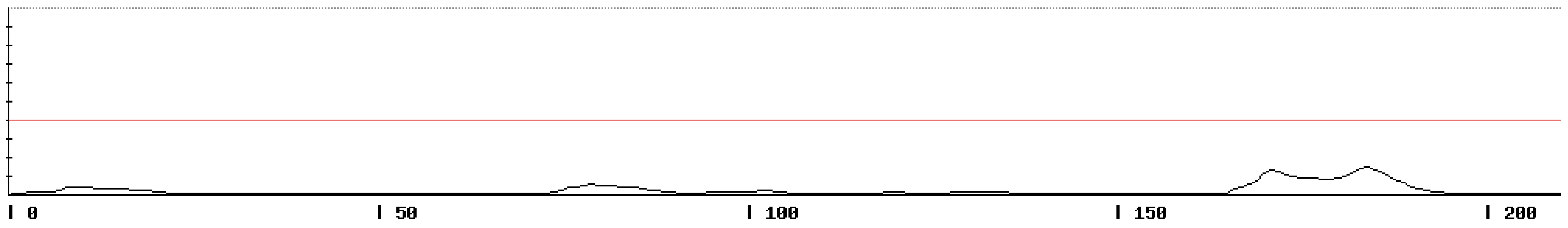

Predictions for Arachisipaensis_Araip.T6ICI

Viterbi Path

Viterbi Path

Posterior @ 0.4

Posterior @ 0.4

Posterior Graphic. Threshold Marked @ 0.4

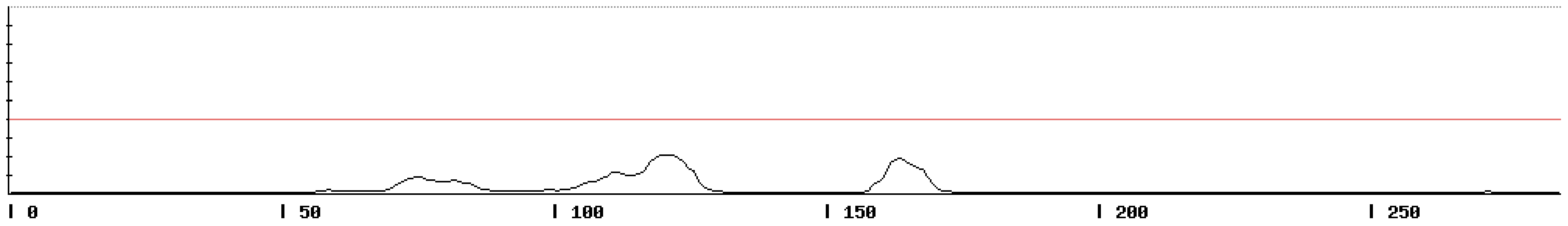

Predictions for Arachisipaensis_Araip.TL0B5

Viterbi Path

Viterbi Path

Posterior @ 0.4

117 - KNVEKREKKVKRKA - 130

Posterior @ 0.4

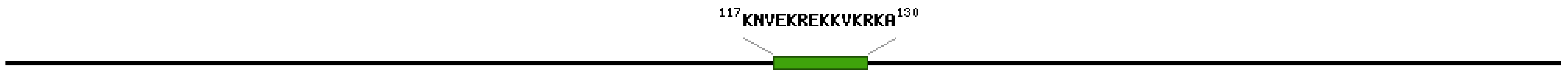

Posterior Graphic. Threshold Marked @ 0.4

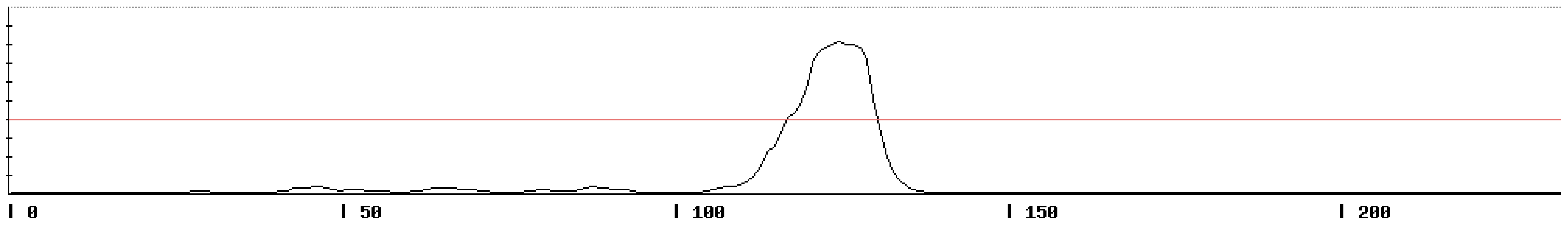

Predictions for Arachisipaensis_Araip.U9RGH

Viterbi Path

Viterbi Path

Posterior @ 0.4

Posterior @ 0.4

Posterior Graphic. Threshold Marked @ 0.4

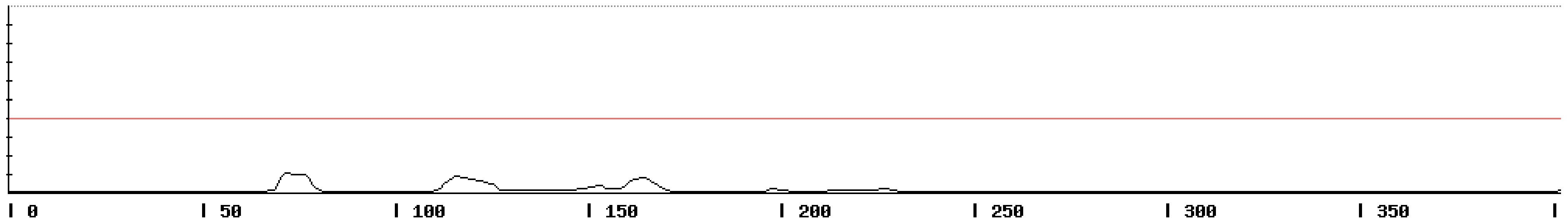

Predictions for Arachisipaensis_Araip.UA0W9

Viterbi Path

Viterbi Path

Posterior @ 0.4

Posterior @ 0.4

Posterior Graphic. Threshold Marked @ 0.4

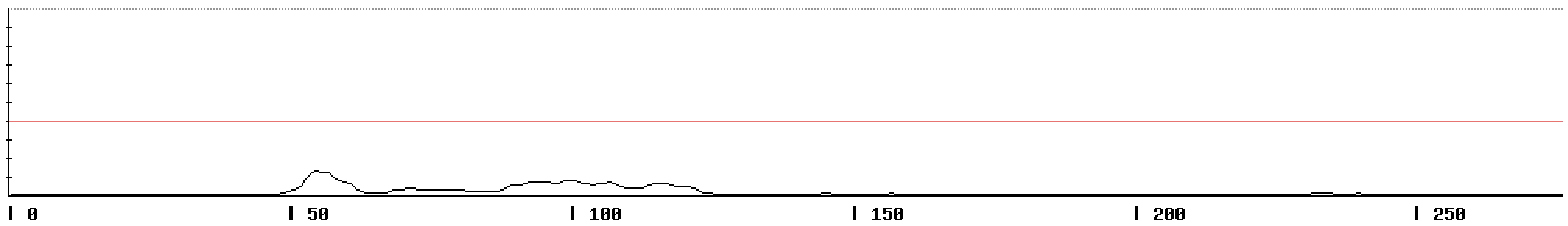

Predictions for Arachisipaensis_Araip.WV14F

Viterbi Path

Viterbi Path

Posterior @ 0.4

Posterior @ 0.4

Posterior Graphic. Threshold Marked @ 0.4

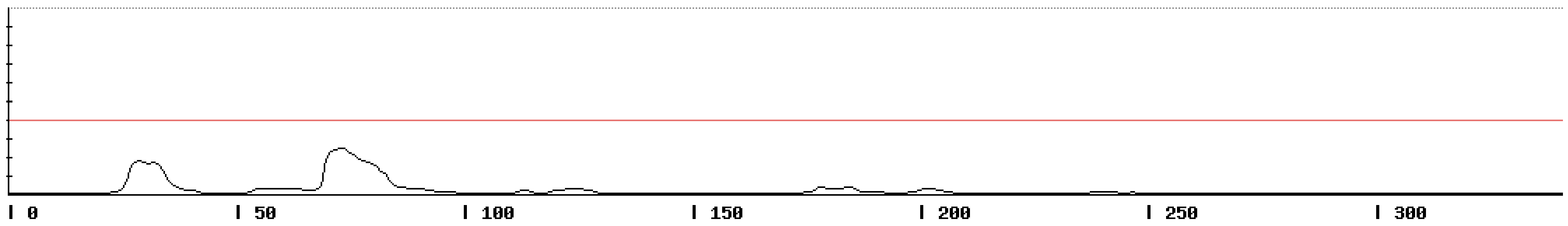

Predictions for Arachisipaensis_Araip.X2KK1

Viterbi Path

Viterbi Path

Posterior @ 0.4

Posterior @ 0.4

Posterior Graphic. Threshold Marked @ 0.4

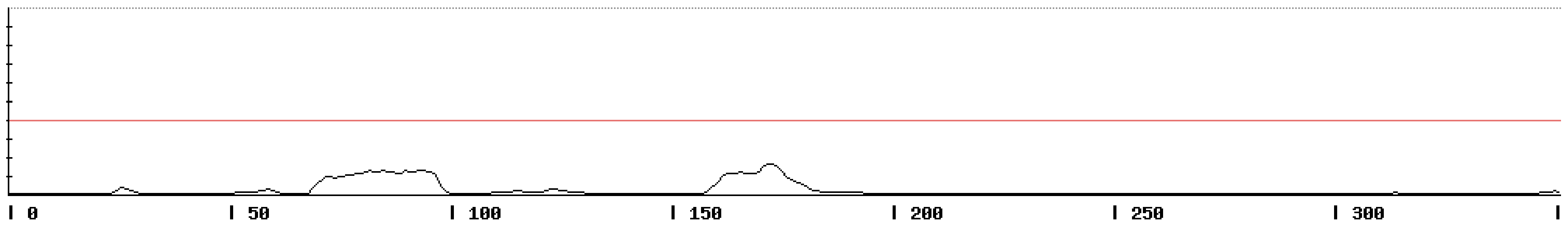

Predictions for Arachisipaensis_Araip.XJ3T4

Viterbi Path

Viterbi Path

Posterior @ 0.4

Posterior @ 0.4

Posterior Graphic. Threshold Marked @ 0.4

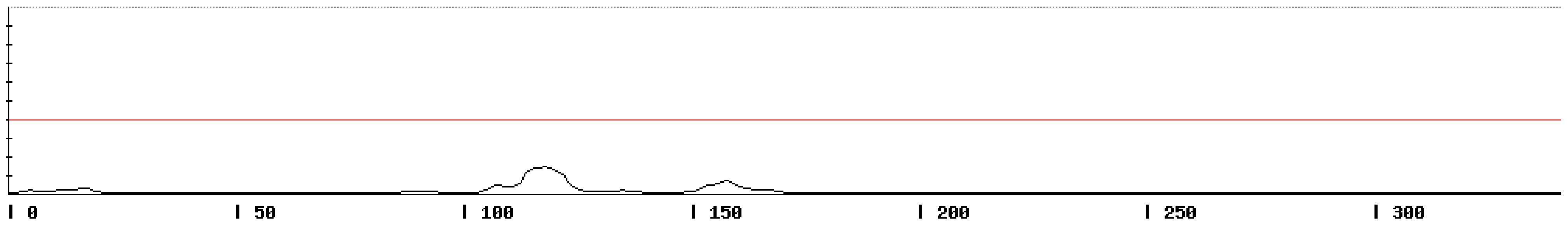

Predictions for Arachisipaensis_Araip.XJX1I

Viterbi Path

Viterbi Path

Posterior @ 0.4

Posterior @ 0.4

Posterior Graphic. Threshold Marked @ 0.4

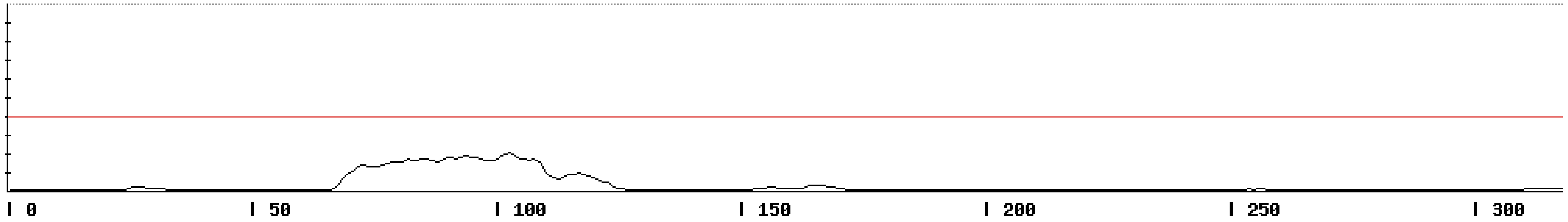

Predictions for Arachisipaensis_Araip.XK9AB

Viterbi Path

Viterbi Path

Posterior @ 0.4

Posterior @ 0.4

Posterior Graphic. Threshold Marked @ 0.4

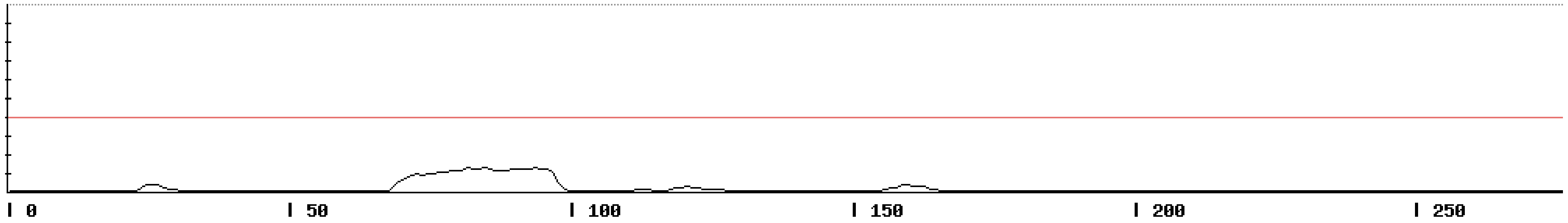

Predictions for Arachisipaensis_Araip.XQA0A

Viterbi Path

Viterbi Path

Posterior @ 0.4

Posterior @ 0.4

Posterior Graphic. Threshold Marked @ 0.4

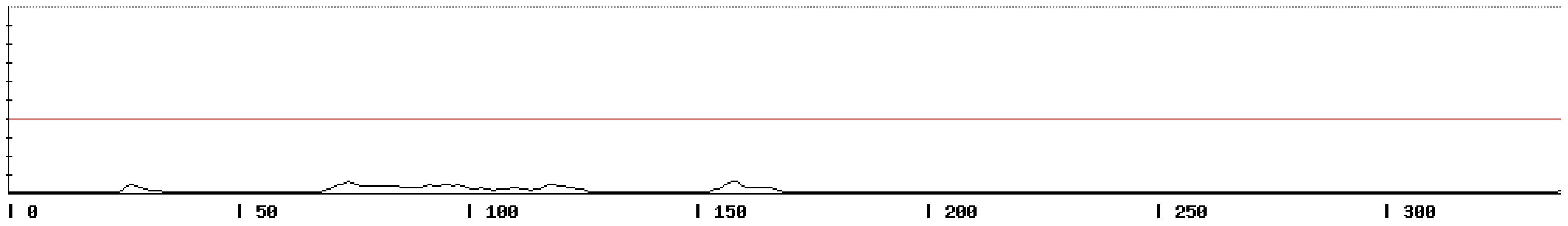

Predictions for Arachisipaensis_Araip.XT8UZ

Viterbi Path

Viterbi Path

Posterior @ 0.4

Posterior @ 0.4

Posterior Graphic. Threshold Marked @ 0.4

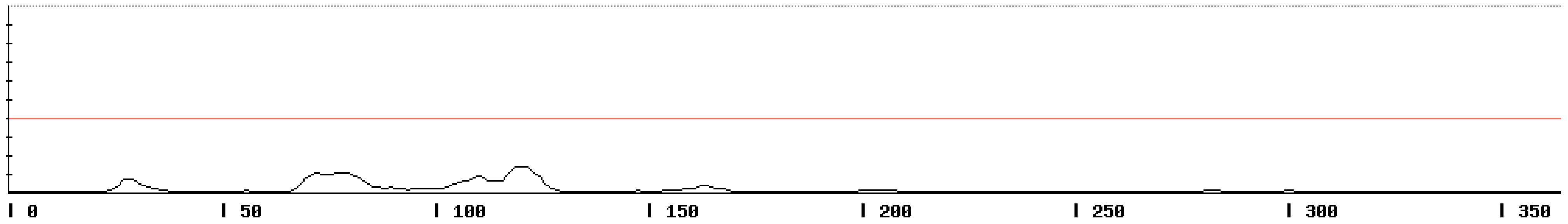

Predictions for Arachisipaensis_Araip.YL288	
Viterbi Path	
Viterbi Path	
Posterior @ 0.4	
Posterior @ 0.4	
Posterior Graphic. Threshold Marked @ 0.4	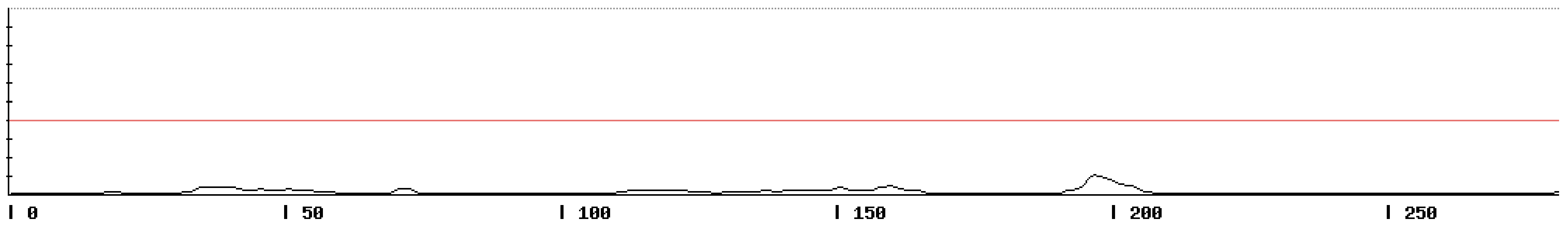

Predictions for Arachisipaensis_Araip.YS3WM	
Viterbi Path	
Viterbi Path	
Posterior @ 0.4	
Posterior @ 0.4	
Posterior Graphic. Threshold Marked @ 0.4	

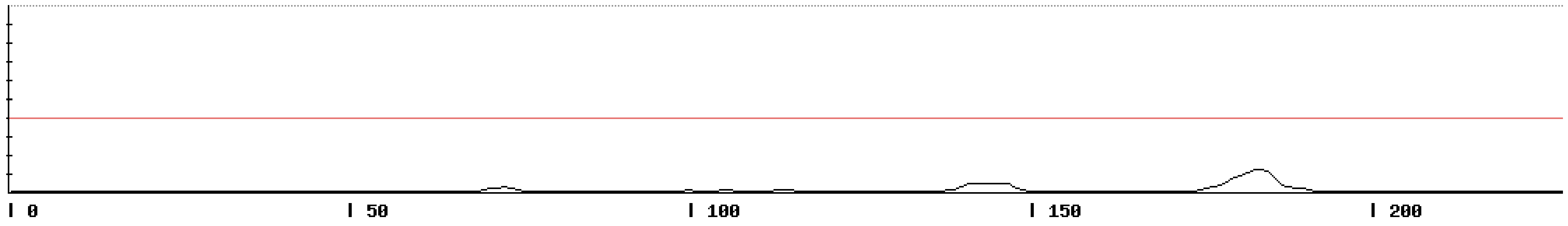

Predictions for Arachisipaensis_Araip.Z57SD

Viterbi Path	
Viterbi Path	
Posterior @ 0.4	
Posterior @ 0.4	
Posterior Graphic. Threshold Marked @ 0.4	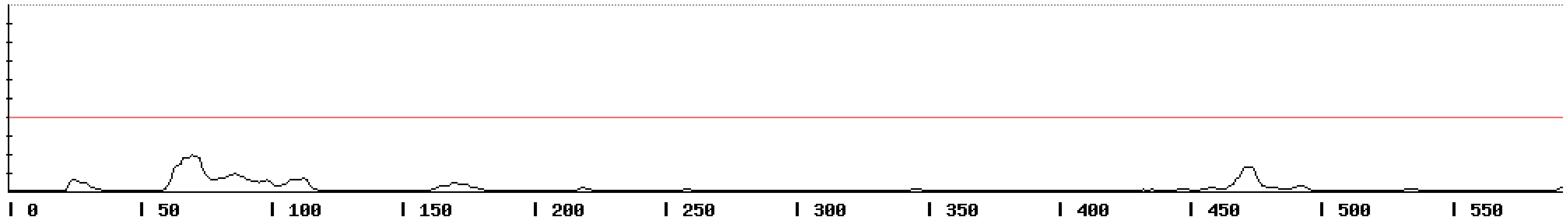

Predictions for Arachisipaensis_Araip.ZUP60

Viterbi Path	
Viterbi Path	
Posterior @ 0.4	
Posterior @ 0.4	

Viterbi Path

Posterior @ 0.4

Posterior @ 0.4

Posterior Graphic. Threshold Marked @ 0.4

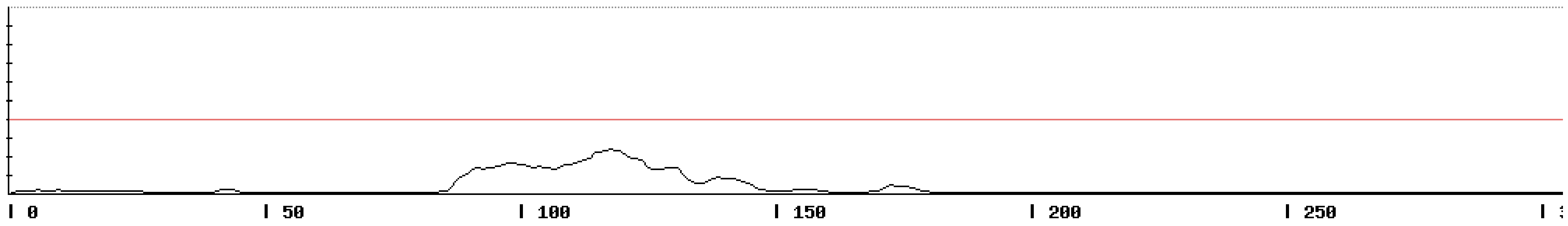

Predictions for Artemisiaannua_Aan001843

Viterbi Path

Viterbi Path

Posterior @ 0.4

Posterior @ 0.4

Posterior Graphic. Threshold Marked @ 0.4

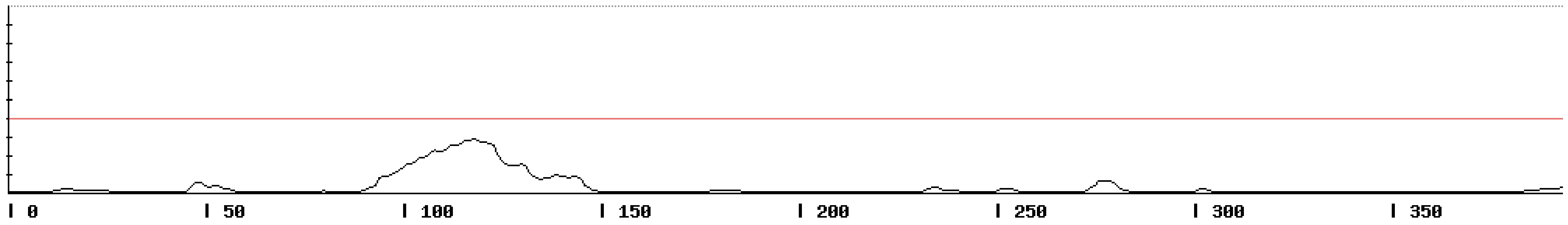

Predictions for Artemisiaannua_Aan002468

Viterbi Path

Viterbi Path

Posterior @ 0.4

Posterior @ 0.4

Posterior Graphic. Threshold Marked @ 0.4

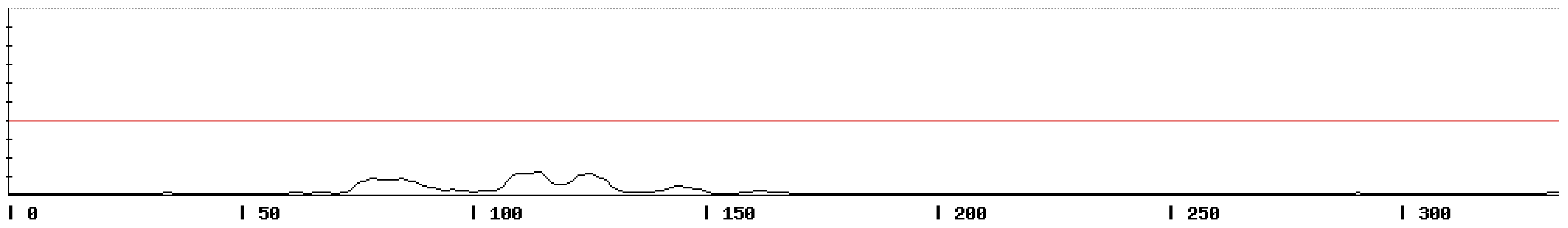

Predictions for Artemisiaannua_Aan006201

Viterbi Path

Viterbi Path

Posterior @ 0.4

Posterior @ 0.4

Posterior Graphic. Threshold Marked @ 0.4

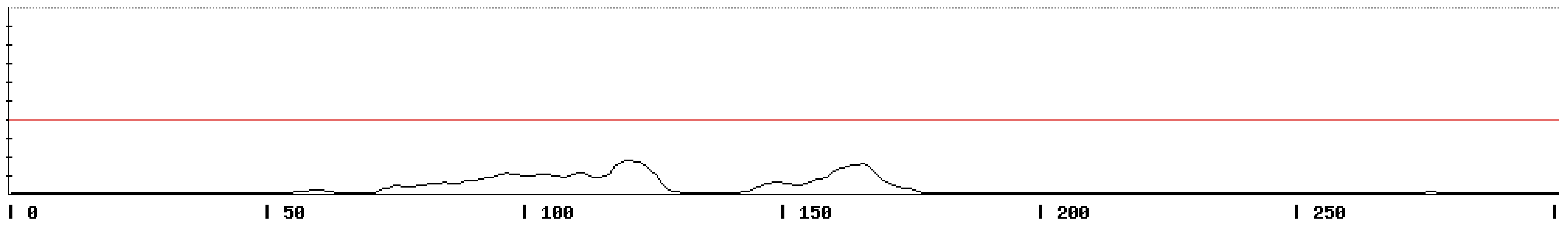

Predictions for Artemisiaannua_Aan006361

Viterbi Path	
Viterbi Path	
Posterior @ 0.4	
Posterior @ 0.4	
Posterior Graphic. Threshold Marked @ 0.4	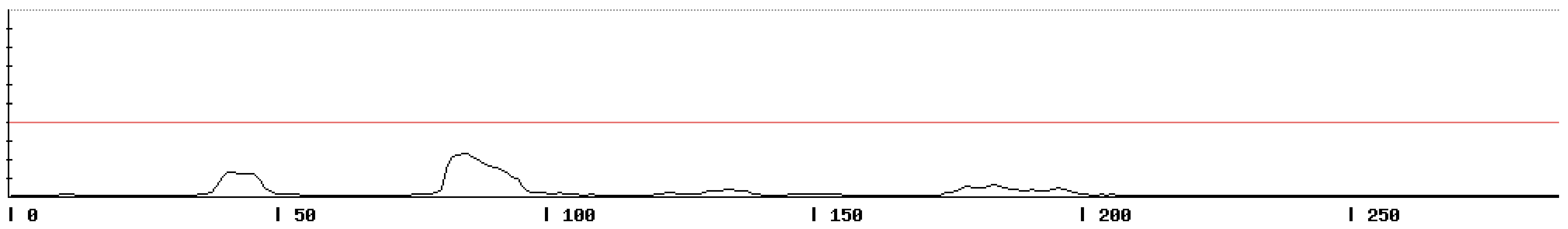

Predictions for Artemisiaannua_Aan008548

Viterbi Path	
Viterbi Path	
Posterior @ 0.4	
Posterior @ 0.4	
Posterior Graphic. Threshold Marked @ 0.4	

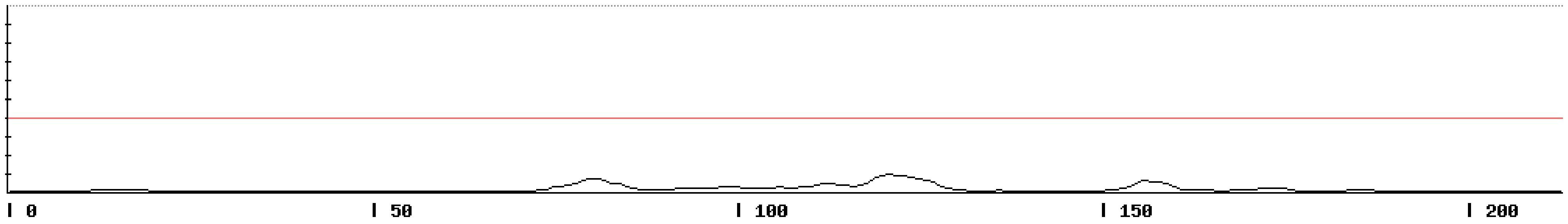

Predictions for Artemisiaannua_Aan009721

Viterbi Path

Viterbi Path

Posterior @ 0.4 18 - RRKRRK - 23

Posterior @ 0.4

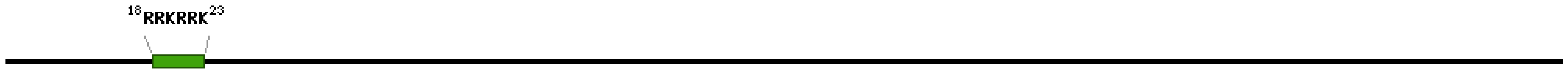

Posterior Graphic. Threshold Marked @ 0.4

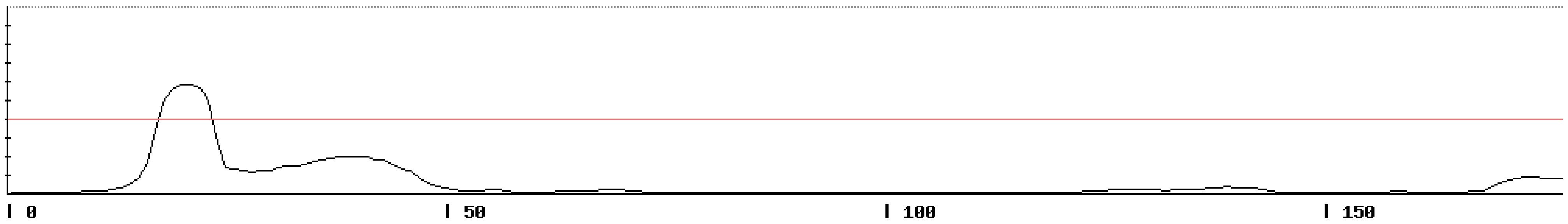

Predictions for Artemisiaannua_Aan010235

Viterbi Path

Viterbi Path

Posterior @ 0.4

Posterior @ 0.4

Posterior Graphic. Threshold Marked @ 0.4	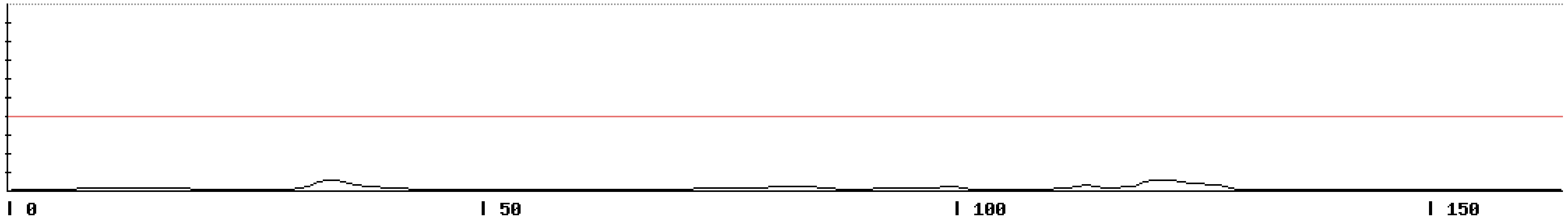

Predictions for Artemisiaannua_Aan010386	
Viterbi Path	
Viterbi Path	
Posterior @ 0.4	
Posterior @ 0.4	
Posterior Graphic. Threshold Marked @ 0.4	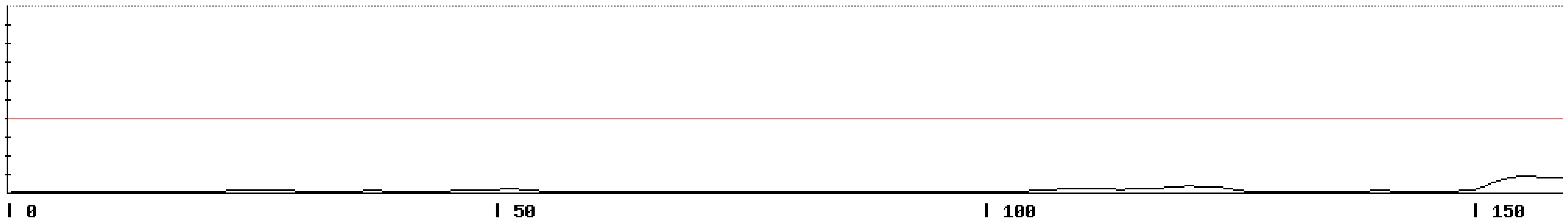

Predictions for Artemisiaannua_Aan010878	
Viterbi Path	
Viterbi Path	
Posterior @ 0.4	104 - RRIRRKKP - 111
Posterior @ 0.4	

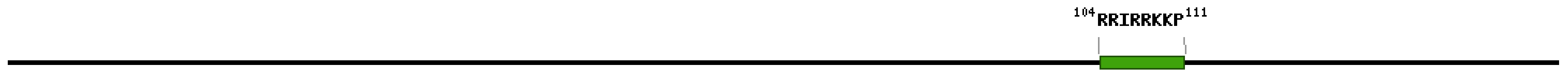

Posterior Graphic. Threshold Marked @ 0.4

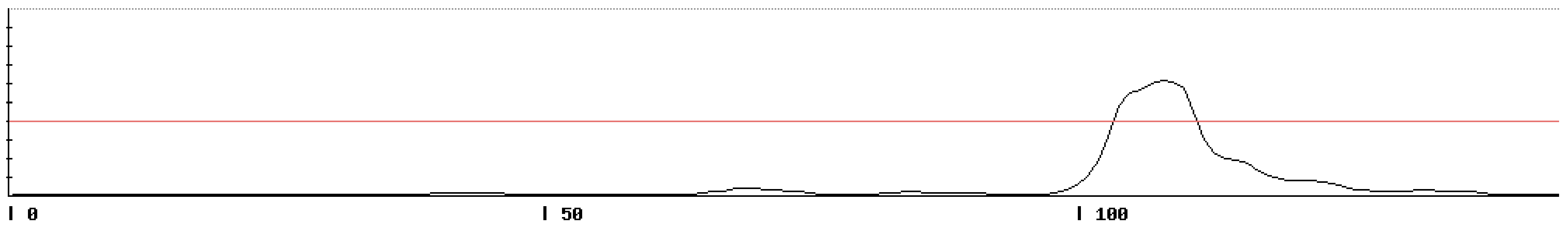

Predictions for Artemisiaannua_Aan012884

Viterbi Path

Viterbi Path

Posterior @ 0.4

Posterior @ 0.4

Posterior Graphic. Threshold Marked @ 0.4

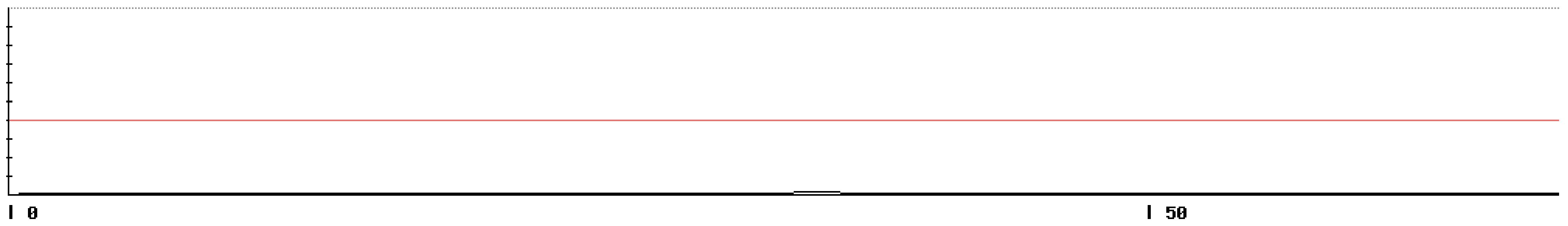

Predictions for Artemisiaannua_Aan012975

Viterbi Path

Viterbi Path

Posterior @ 0.4	54 - K - 54
Posterior @ 0.4	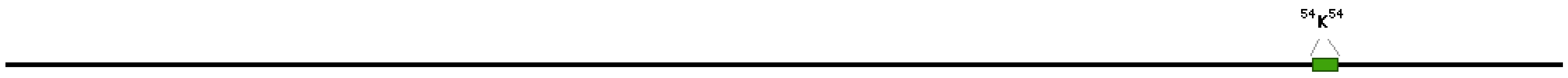
Posterior Graphic. Threshold Marked @ 0.4	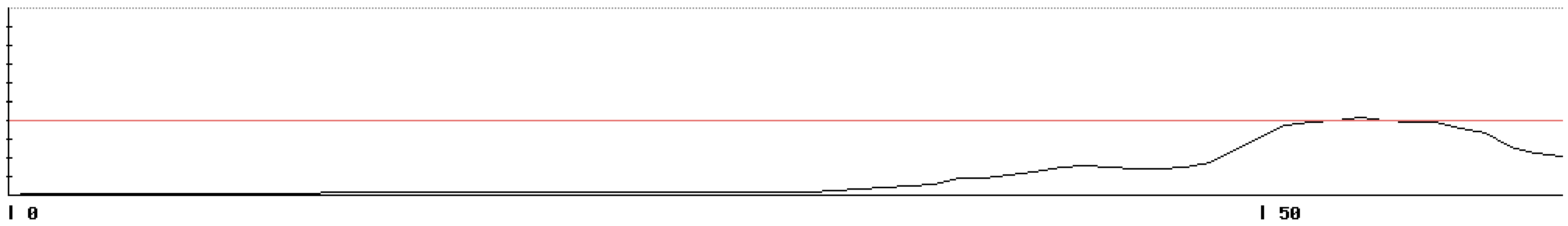

Predictions for Artemisiaannua_Aan013239

Viterbi Path	
Viterbi Path	
Posterior @ 0.4	
Posterior @ 0.4	
Posterior Graphic. Threshold Marked @ 0.4	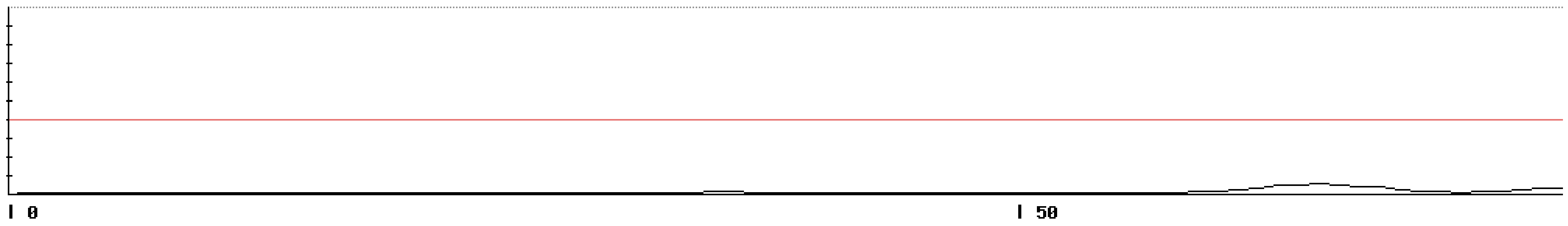

Predictions for Artemisiaannua_Aan013749

Viterbi Path	
Viterbi Path	

Posterior @ 0.4	
Posterior @ 0.4	
Posterior Graphic. Threshold Marked @ 0.4	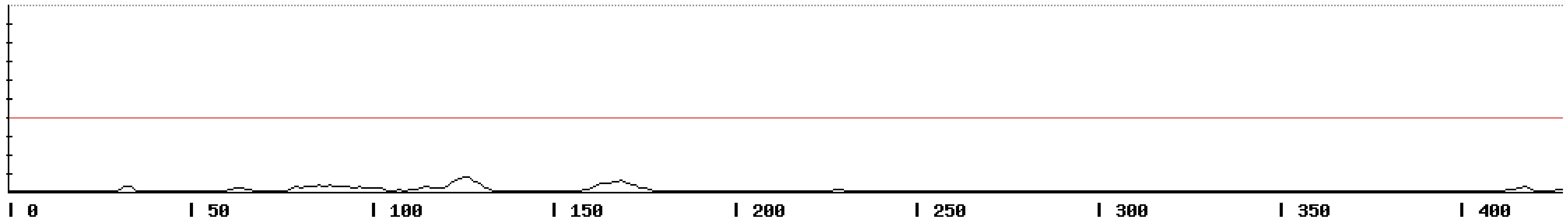

Predictions for Artemisiaannua_Aan014755

Viterbi Path	
Viterbi Path	
Posterior @ 0.4	171 - KRGNK - 175
Posterior @ 0.4	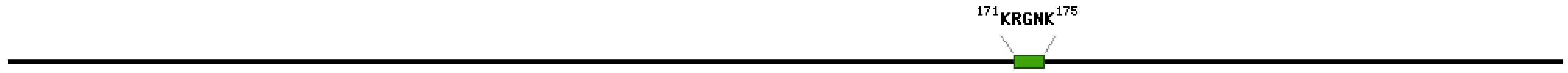
Posterior Graphic. Threshold Marked @ 0.4	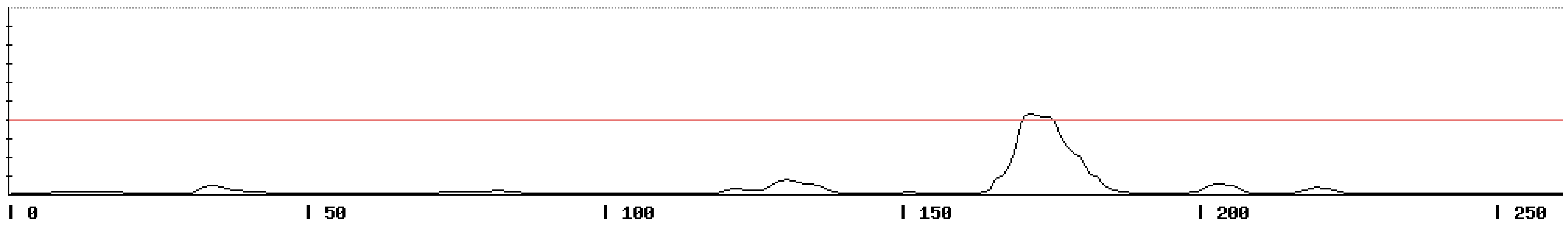

Predictions for Artemisiaannua_Aan015209

Viterbi Path

Viterbi Path

Posterior @ 0.4

Posterior @ 0.4

Posterior Graphic. Threshold Marked @ 0.4

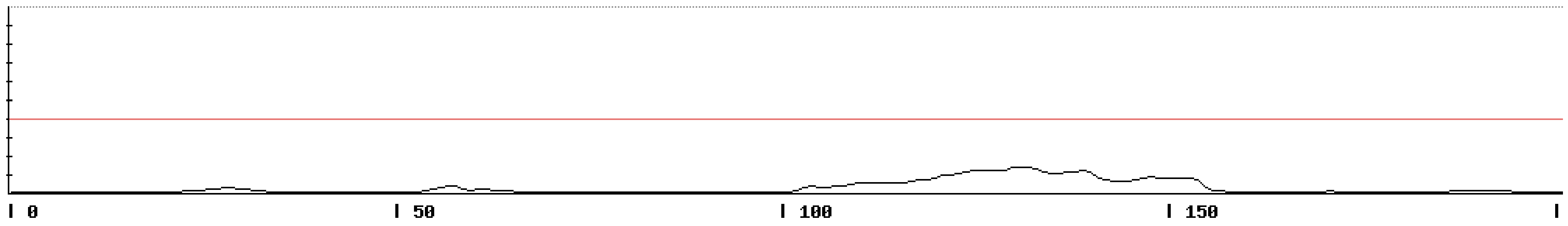

Predictions for Artemisiaannua_Aan015985

Viterbi Path

Revision r.9 - Last Updated on : February 24 2015 17:45:20.

alex.nguyenba@utoronto.ca

Intended to be used with Firefox

Viterbi Path

Posterior @ 0.4

Posterior @ 0.4

Posterior Graphic. Threshold Marked @ 0.4

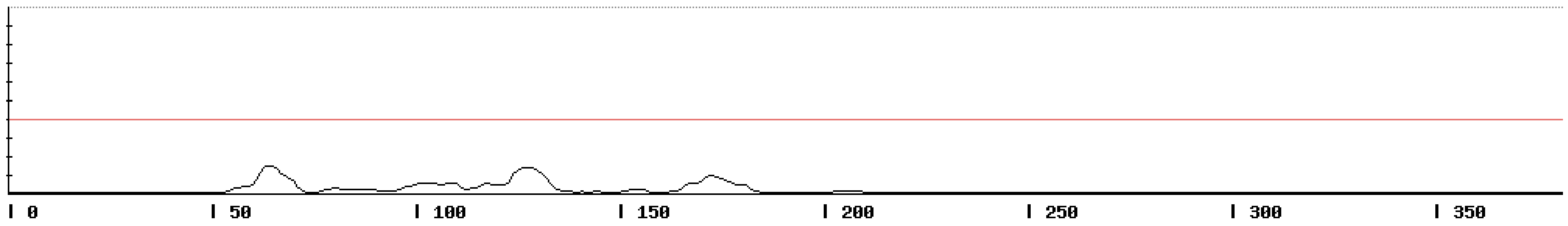

Predictions for Azadirachtaindica_Neem_10068_f_1

Viterbi Path

Viterbi Path

Posterior @ 0.4

Posterior @ 0.4

Posterior Graphic. Threshold Marked @ 0.4

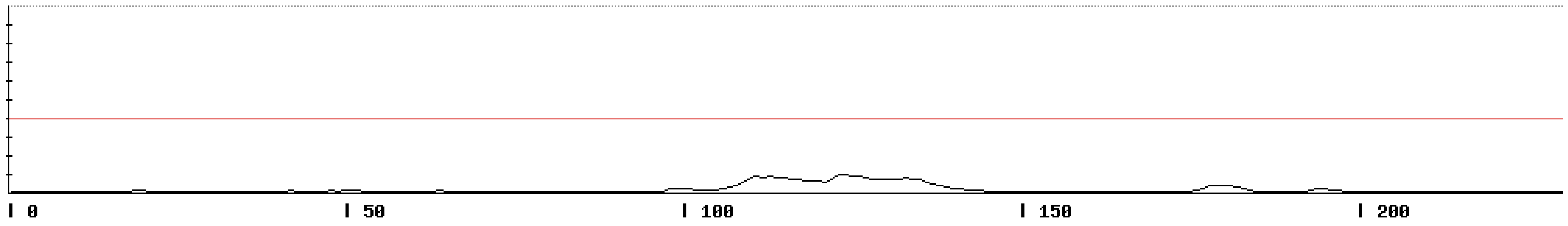

Predictions for Azadirachtaindica_Neem_10068_f_2

Viterbi Path

Viterbi Path

Posterior @ 0.4

Posterior @ 0.4

Posterior Graphic. Threshold Marked @ 0.4

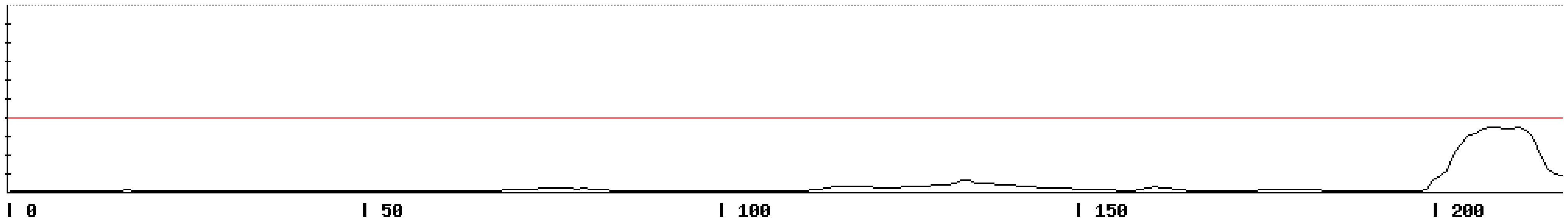

Predictions for Azadirachtaindica_Neem_10194_f_1

Viterbi Path

Viterbi Path

Posterior @ 0.4

Posterior @ 0.4

Posterior Graphic. Threshold Marked @ 0.4

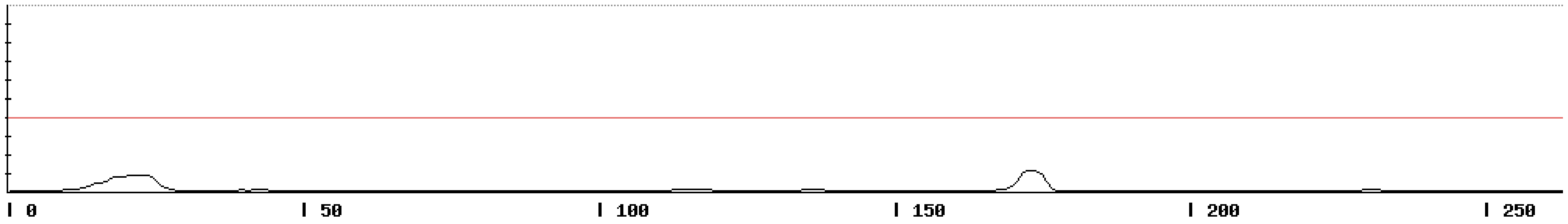

Predictions for Azadirachtaindica_Neem_10248_f_2	
Viterbi Path	
Viterbi Path	
Posterior @ 0.4	38 - GADKPIGKPKALGIKKA - 54
Posterior @ 0.4	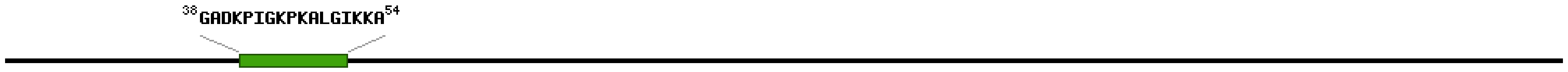
Posterior Graphic. Threshold Marked @ 0.4	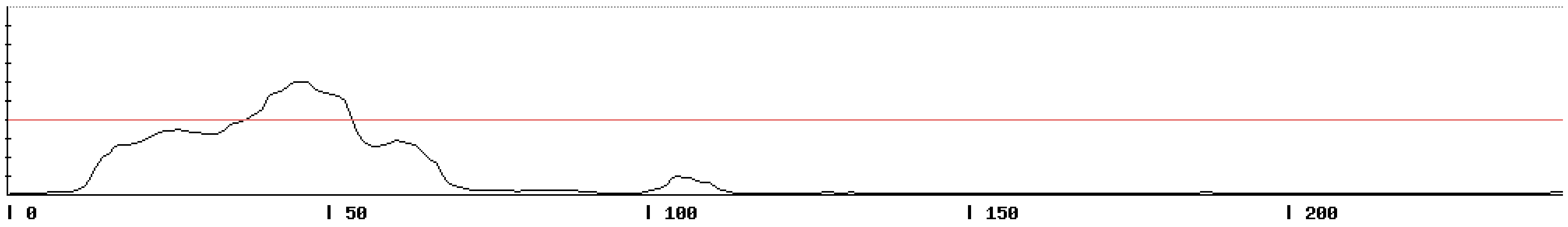

Predictions for Azadirachtaindica_Neem_10408_f_4	
Viterbi Path	
Viterbi Path	
Posterior @ 0.4	
Posterior @ 0.4	
Posterior Graphic. Threshold Marked @ 0.4	

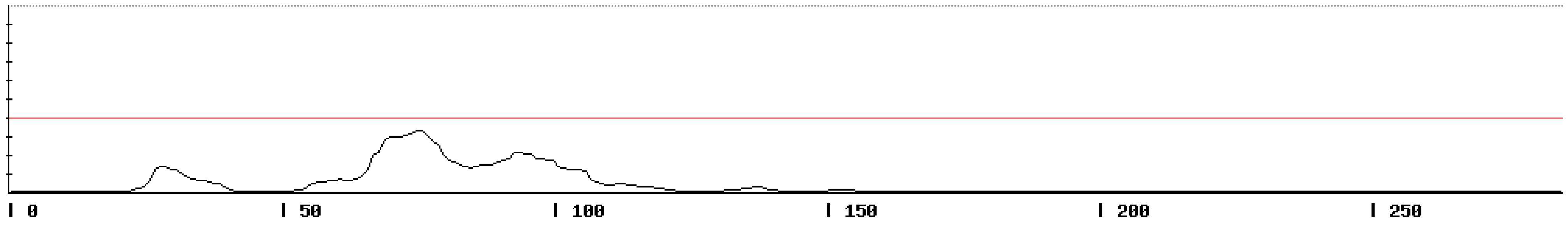

Predictions for Azadirachtaindica_Neem_10484_f_1

Viterbi Path

Viterbi Path

Posterior @ 0.4

Posterior @ 0.4

Posterior Graphic. Threshold Marked @ 0.4

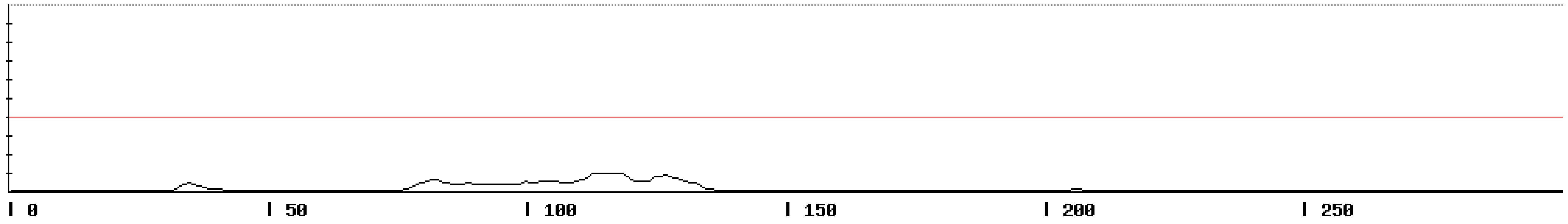

Predictions for Azadirachtaindica_Neem_10791_f_1

Viterbi Path

Viterbi Path

Posterior @ 0.4

Posterior @ 0.4

Posterior Graphic. Threshold Marked @ 0.4	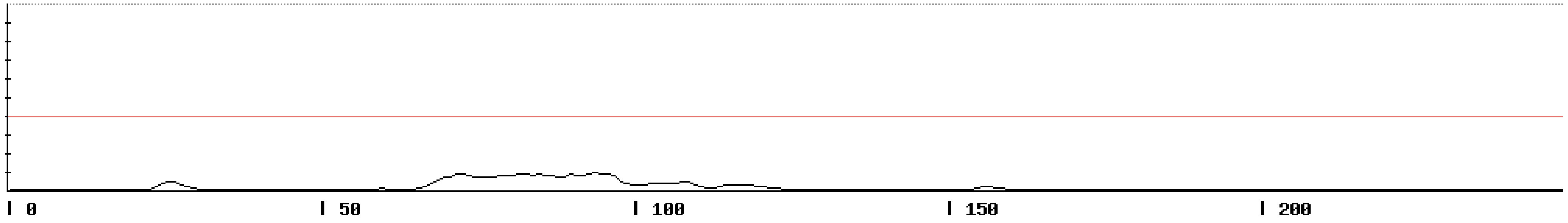

Predictions for Azadirachtaindica_Neem_10795_f_2	
Viterbi Path	
Viterbi Path	
Posterior @ 0.4	
Posterior @ 0.4	
Posterior Graphic. Threshold Marked @ 0.4	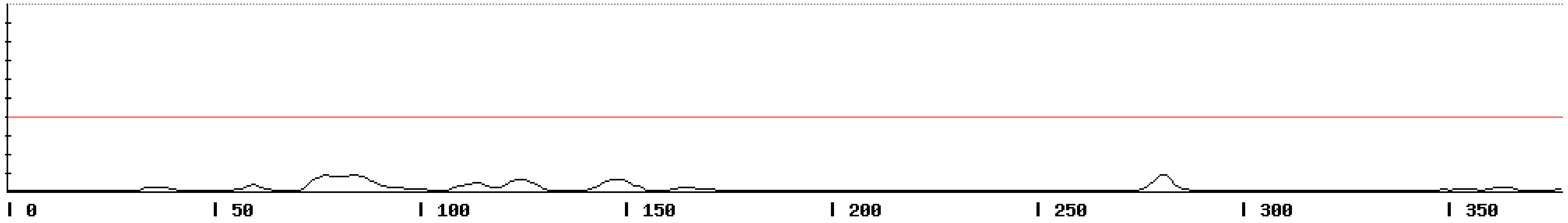

Predictions for Azadirachtaindica_Neem_10879_f_1	
Viterbi Path	
Viterbi Path	
Posterior @ 0.4	85 - GYWKVTGKPRRIRDKRGKKEIGYKR - 109
Posterior @ 0.4	

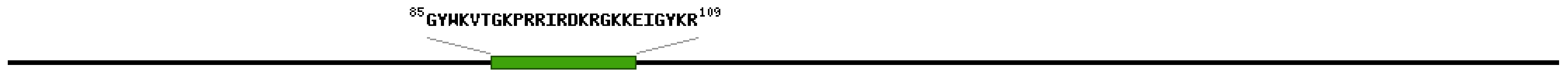

Posterior Graphic. Threshold Marked @ 0.4

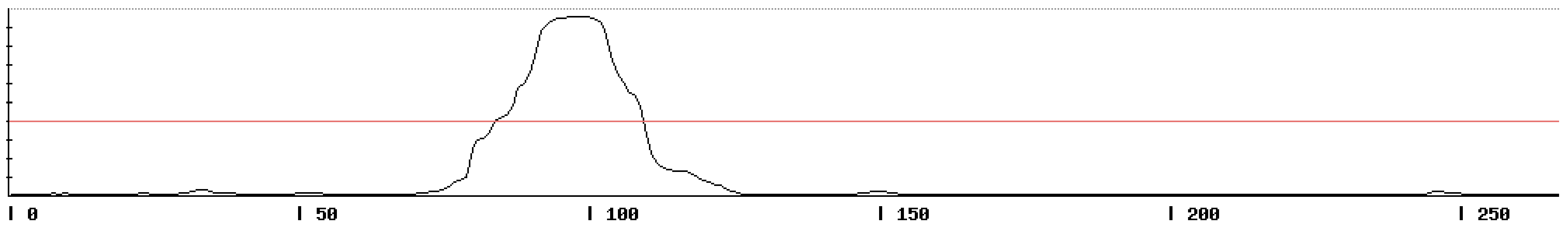

Predictions for Azadirachtaindica_Neem_10938_f_1

Viterbi Path

Viterbi Path

Posterior @ 0.4

Posterior @ 0.4

Posterior Graphic. Threshold Marked @ 0.4

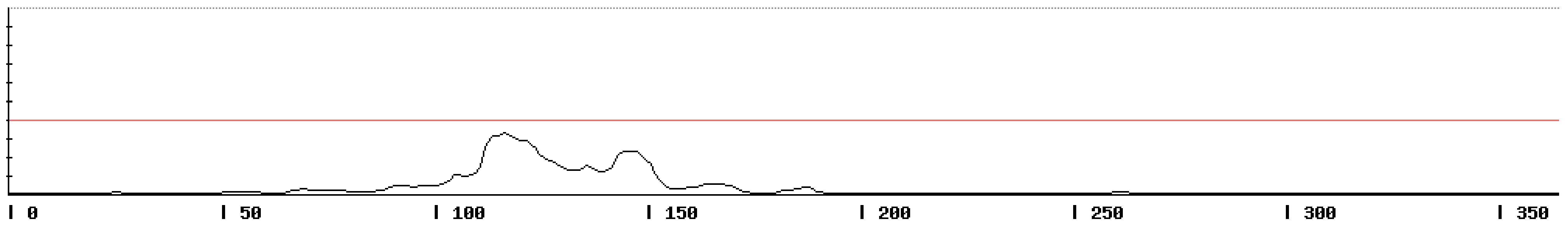

Predictions for Azadirachtaindica_Neem_11412_f_1

Viterbi Path

Viterbi Path

Posterior @ 0.4

Posterior @ 0.4

Posterior Graphic. Threshold Marked @ 0.4

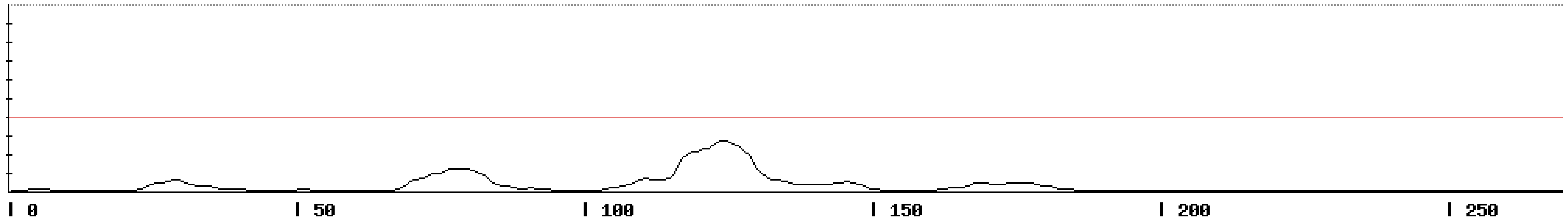

Predictions for Azadirachtaindica_Neem_11486_f_1

Viterbi Path

Viterbi Path

Posterior @ 0.4

Posterior @ 0.4

Posterior Graphic. Threshold Marked @ 0.4

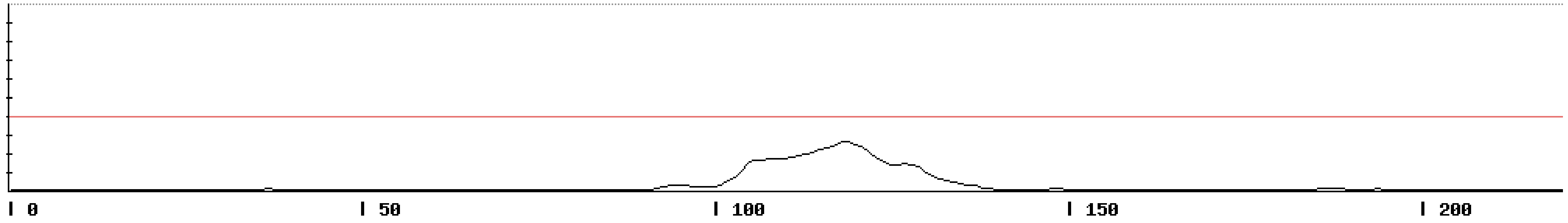

Predictions for Azadirachtaindica_Neem_11567_f_1

Viterbi Path

Viterbi Path

Posterior @ 0.4

Posterior @ 0.4

Posterior Graphic. Threshold Marked @ 0.4

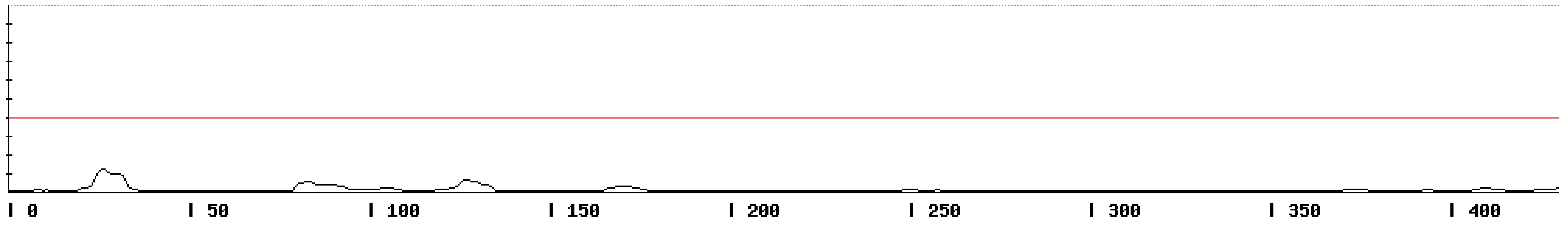

Predictions for Azadirachtaindica_Neem_11643_f_3

Viterbi Path

Viterbi Path

Posterior @ 0.4

Posterior @ 0.4

Posterior Graphic. Threshold Marked @ 0.4

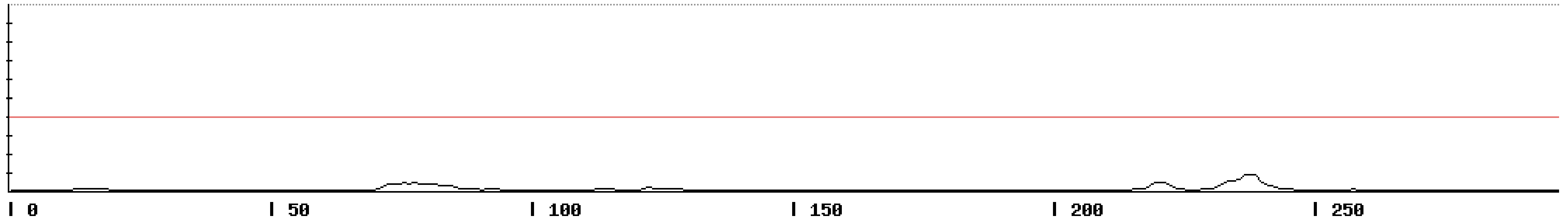

Predictions for Azadirachtaindica_Neem_11660_f_5

Viterbi Path	
Viterbi Path	
Posterior @ 0.4	
Posterior @ 0.4	
Posterior Graphic. Threshold Marked @ 0.4	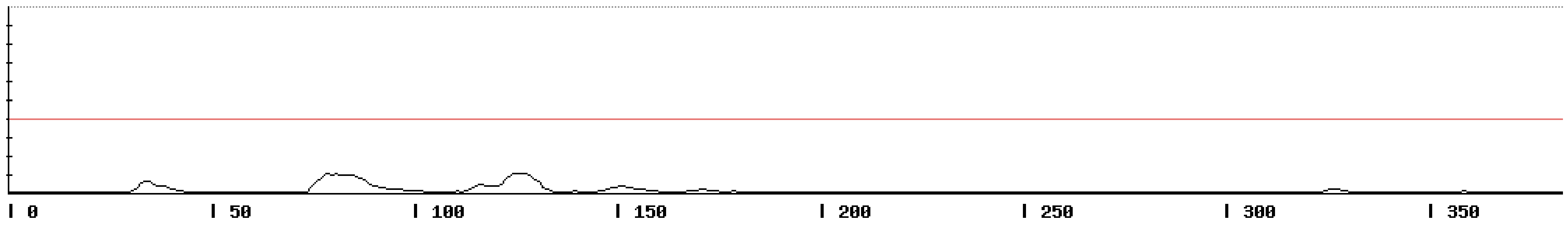

Predictions for Azadirachtaindica_Neem_11713_f_2

Viterbi Path	
Viterbi Path	
Posterior @ 0.4	
Posterior @ 0.4	
Posterior Graphic. Threshold Marked @ 0.4	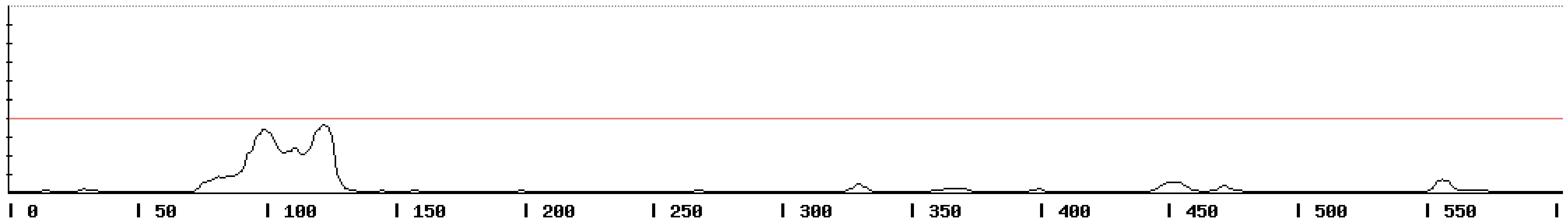

Predictions for Azadirachtaindica_Neem_11719_f_1

Viterbi Path	
Viterbi Path	
Posterior @ 0.4	
Posterior @ 0.4	
Posterior Graphic. Threshold Marked @ 0.4	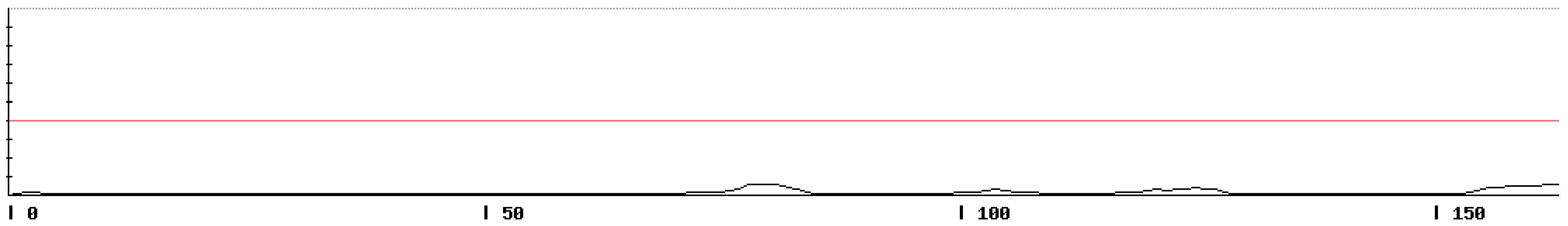

Predictions for Azadirachtaindica_Neem_12089_f_2

Viterbi Path	
Viterbi Path	
Posterior @ 0.4	138 - RKRRK - 142
Posterior @ 0.4	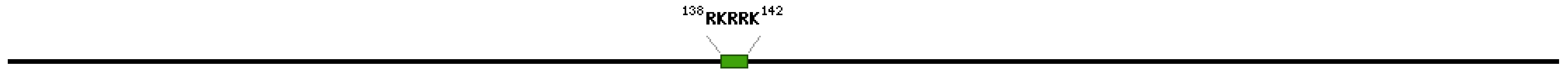
Posterior Graphic. Threshold Marked @ 0.4	

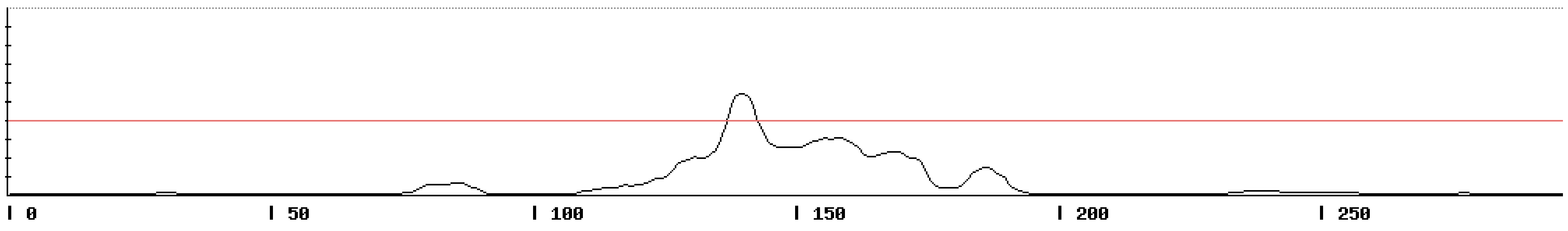

Predictions for Azadirachtaindica_Neem_12411_f_2

Viterbi Path

Viterbi Path

Posterior @ 0.4

76 - RKYKNGKRPNR - 86

162 - KKKEKDRSRR - 171

Posterior @ 0.4

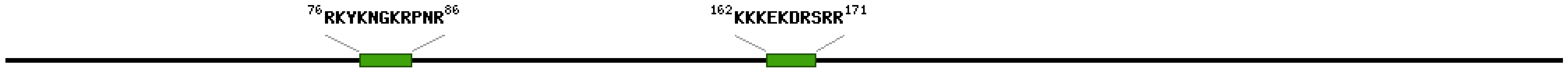

Posterior Graphic. Threshold Marked @ 0.4

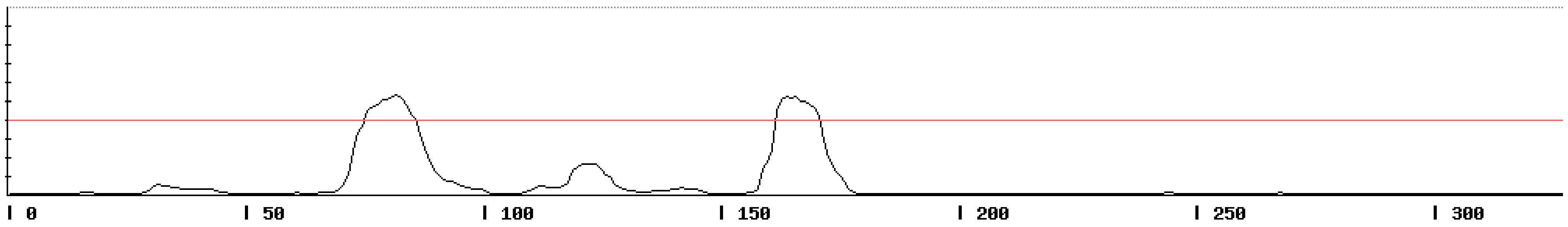

Predictions for Azadirachtaindica_Neem_12411_f_3

Viterbi Path

Viterbi Path

Posterior @ 0.4

Posterior @ 0.4

Posterior Graphic. Threshold Marked @ 0.4

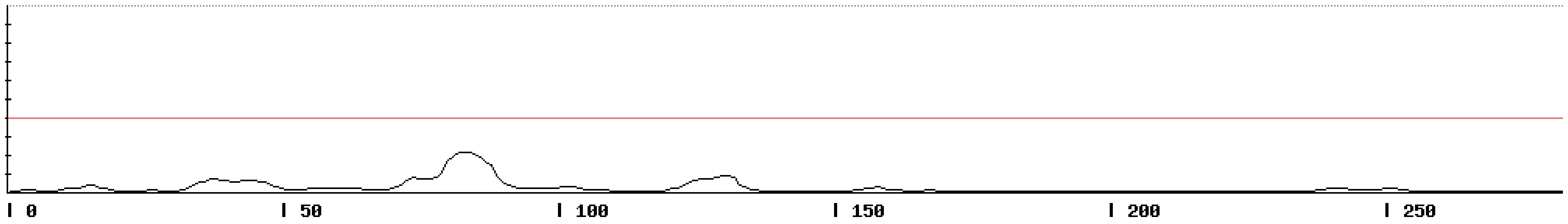

Predictions for Azadirachtaindica_Neem_12571_f_1

Viterbi Path

Viterbi Path

Posterior @ 0.4

Posterior @ 0.4

Posterior Graphic. Threshold Marked @ 0.4

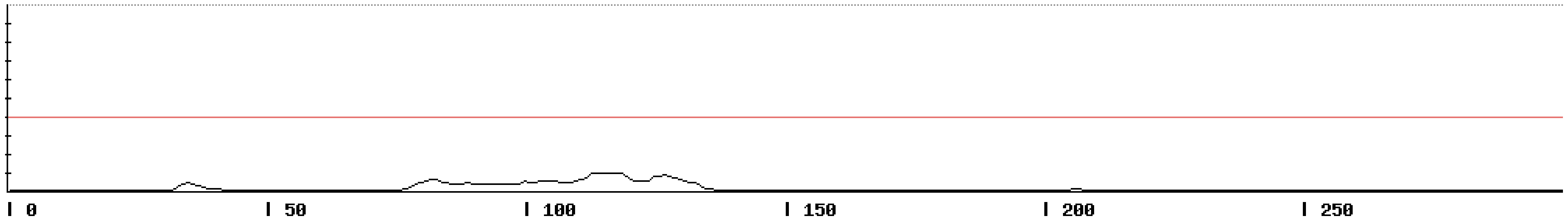

Predictions for Azadirachtaindica_Neem_12632_f_1

Viterbi Path

Viterbi Path

Posterior @ 0.4

Posterior @ 0.4

Posterior Graphic. Threshold Marked @ 0.4

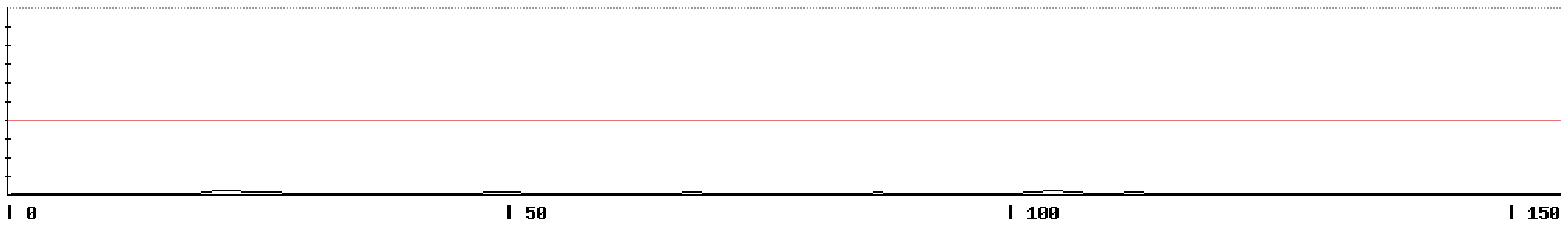

Predictions for Azadirachtaindica_Neem_12730_f_1

Viterbi Path

Viterbi Path

Posterior @ 0.4

Posterior @ 0.4

Posterior Graphic. Threshold Marked @ 0.4

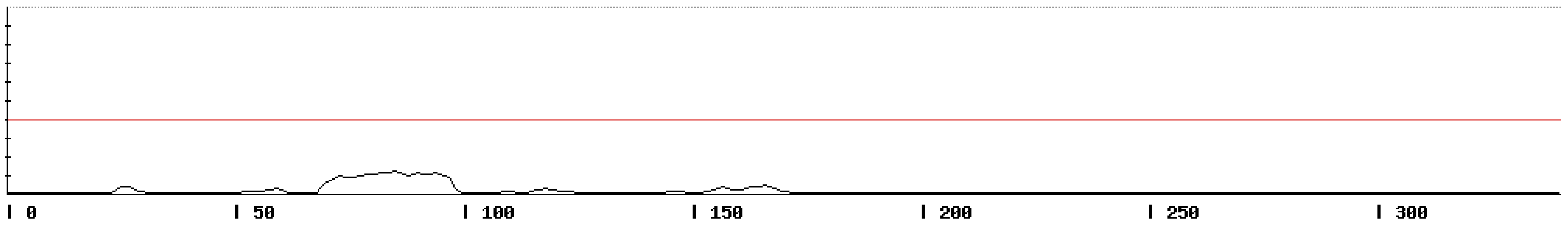

Predictions for Azadirachtaindica_Neem_12856_f_1

Viterbi Path

Viterbi Path

Posterior @ 0.4

Posterior @ 0.4

Posterior Graphic. Threshold Marked @ 0.4

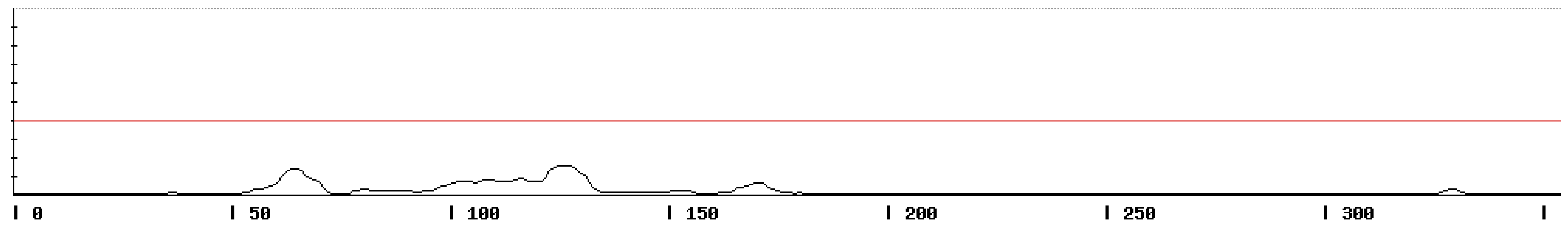

Predictions for Azadirachtaindica_Neem_1311_f_4

Viterbi Path

Viterbi Path

Posterior @ 0.4

Posterior @ 0.4

Posterior Graphic. Threshold Marked @ 0.4

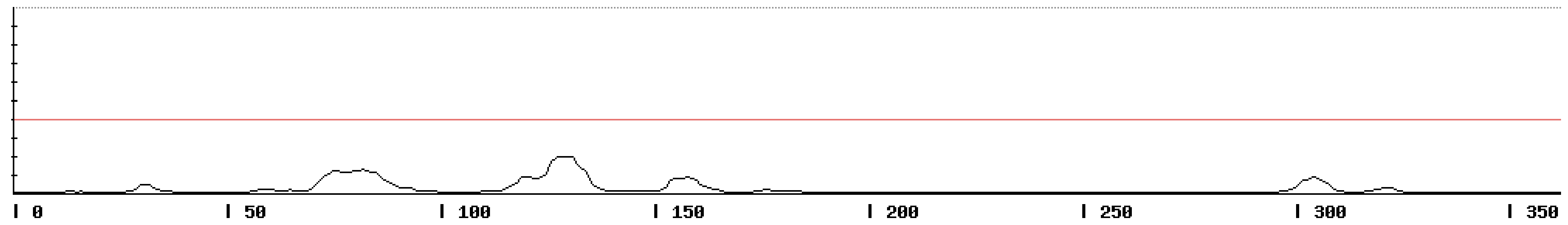

Predictions for Azadirachtaindica_Neem_1368_f_4

Viterbi Path

Viterbi Path

Posterior @ 0.4

Posterior @ 0.4

Posterior Graphic. Threshold Marked @ 0.4

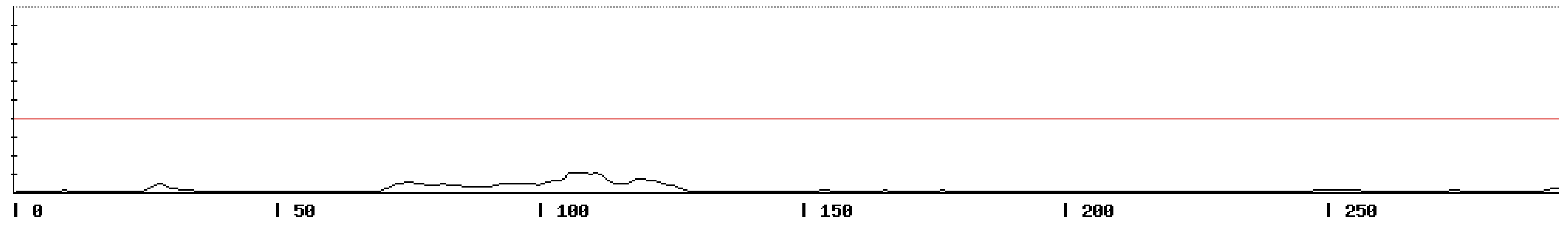

Predictions for Azadirachtaindica_Neem_1375_f_2

Viterbi Path

Viterbi Path

Posterior @ 0.4

Posterior @ 0.4

Posterior Graphic. Threshold Marked @ 0.4

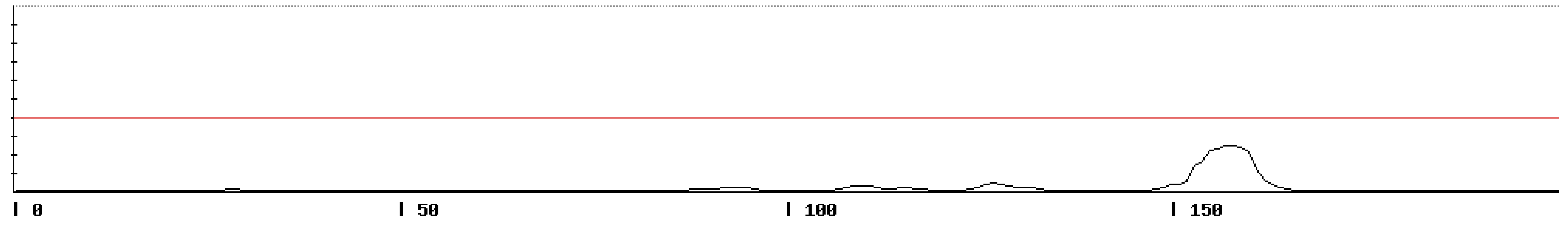

Predictions for Azadirachtaindica_Neem_13837_f_1	
Viterbi Path	
Viterbi Path	
Posterior @ 0.4	
Posterior @ 0.4	
Posterior Graphic. Threshold Marked @ 0.4	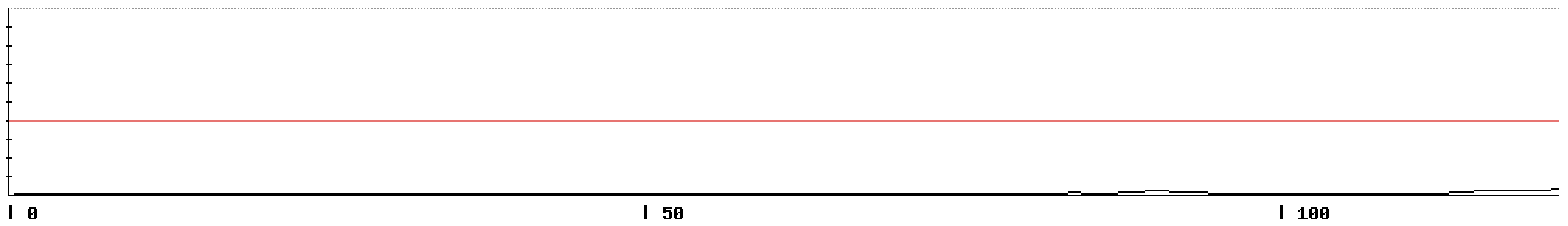

Predictions for Azadirachtaindica_Neem_13860_f_1	
Viterbi Path	
Viterbi Path	
Posterior @ 0.4	
Posterior @ 0.4	
Posterior Graphic. Threshold Marked @ 0.4	

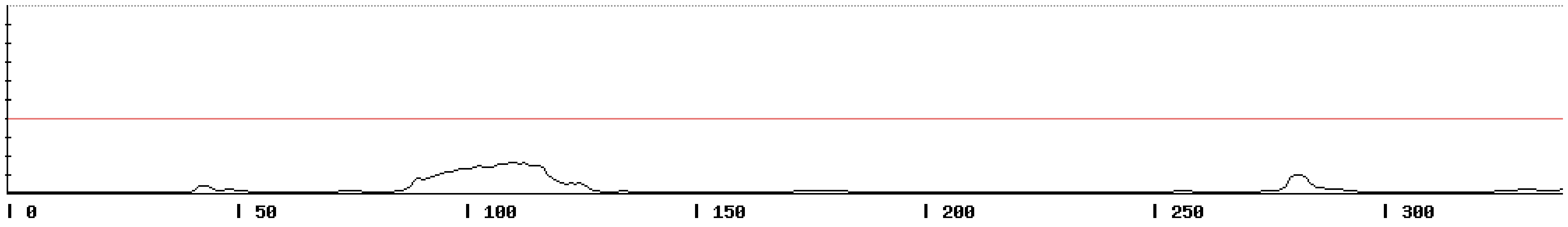

Predictions for Azadirachtaindica_Neem_13873_f_2	
Viterbi Path	187 - GKKKSKSKQIGKKNGSGEGATGRKRKLK - 214
Viterbi Path	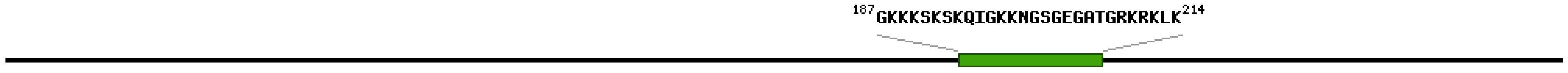
Posterior @ 0.4	185 - DLGKKKSKSKQIGKKNGSGEGATGRKRKLK - 214
Posterior @ 0.4	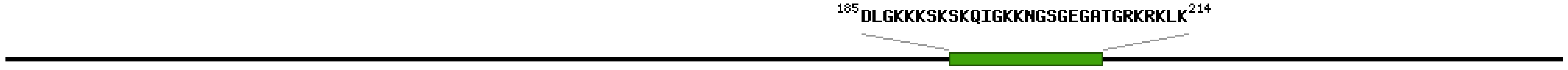
Posterior Graphic. Threshold Marked @ 0.4	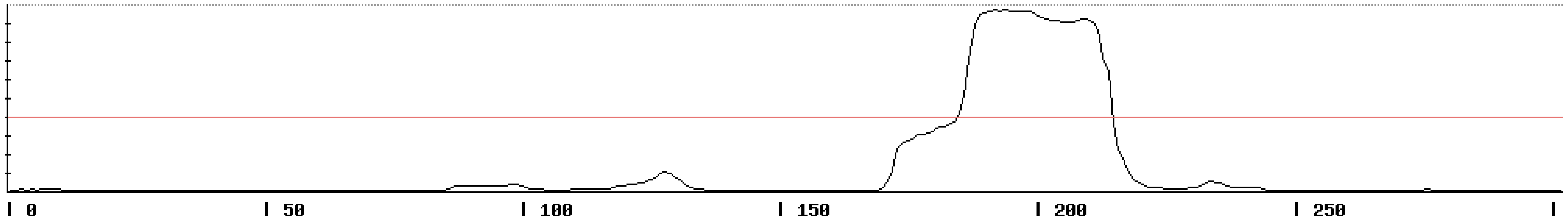

Predictions for Azadirachtaindica_Neem_14406_f_1	
Viterbi Path	
Viterbi Path	
Posterior @ 0.4	
Posterior @ 0.4	

Posterior Graphic. Threshold Marked @ 0.4

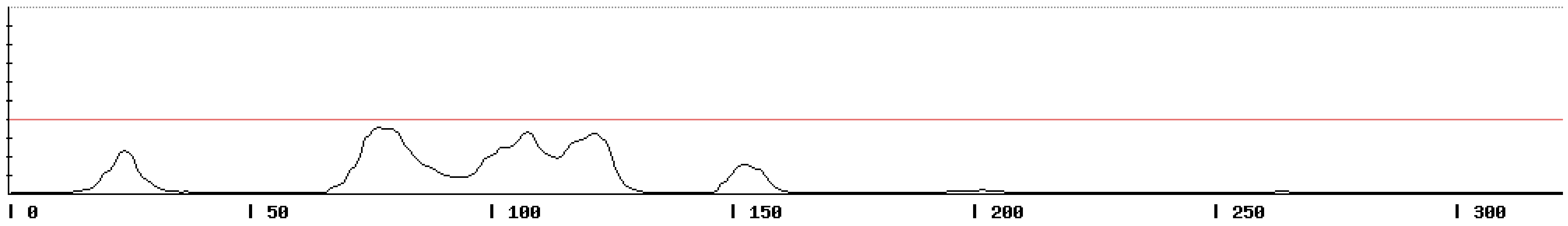

Predictions for Azadirachtaindica_Neem_14495_f_1

Viterbi Path	
Viterbi Path	
Posterior @ 0.4	
Posterior @ 0.4	
Posterior Graphic. Threshold Marked @ 0.4	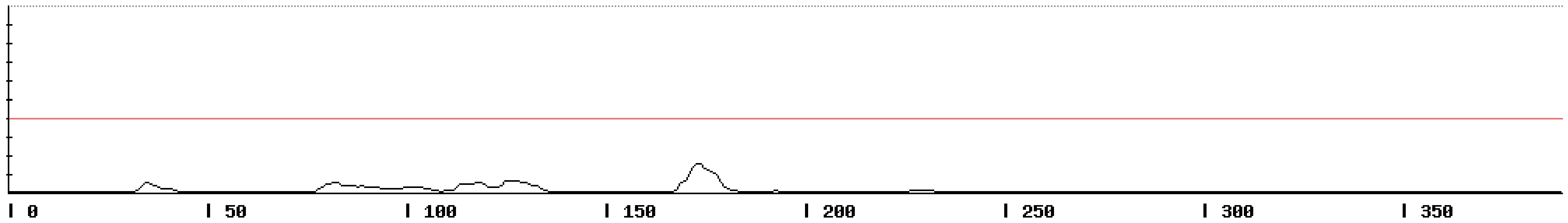

Predictions for Azadirachtaindica_Neem_14534_f_2

Viterbi Path	
Viterbi Path	
Posterior @ 0.4	138 - RKRRK - 142
Posterior @ 0.4	

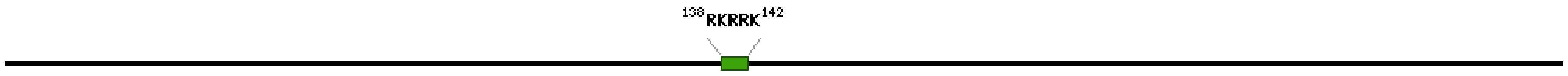

Posterior Graphic. Threshold Marked @ 0.4

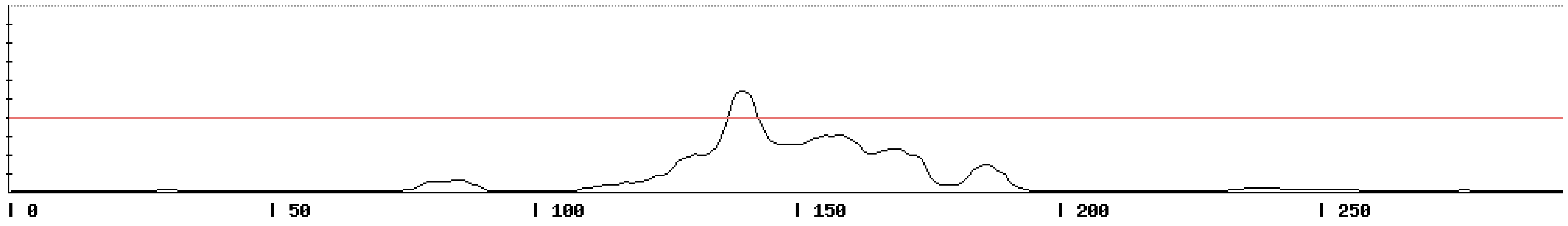

Predictions for Azadirachtaindica_Neem_14749_f_1

Viterbi Path

Viterbi Path

Posterior @ 0.4

Posterior @ 0.4

Posterior Graphic. Threshold Marked @ 0.4

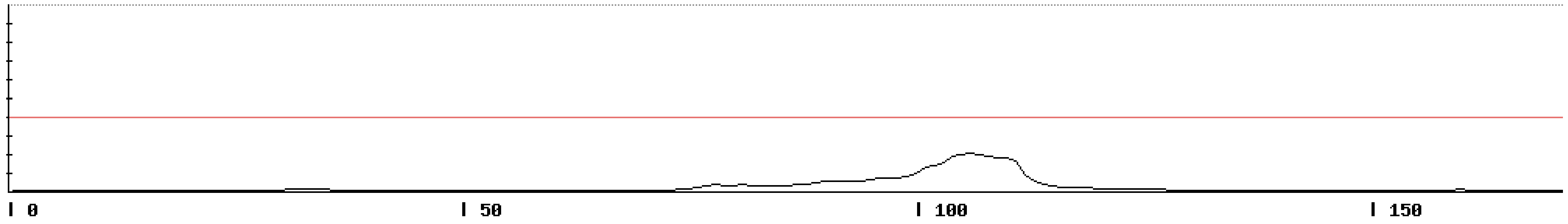

Predictions for Azadirachtaindica_Neem_15575_f_1

Viterbi Path

Viterbi Path

Posterior @ 0.4

Posterior @ 0.4

Posterior Graphic. Threshold Marked @ 0.4

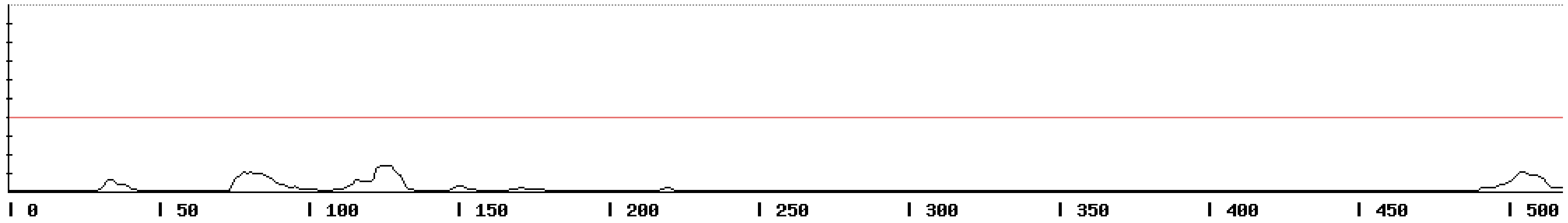

Predictions for Azadirachtaindica_Neem_15803_f_1

Viterbi Path

Viterbi Path

Posterior @ 0.4

76 - RKYKNGKRPNR - 86

162 - KKKEKDRSRR - 171

Posterior @ 0.4

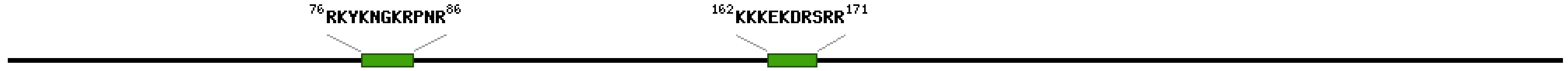

Posterior Graphic. Threshold Marked @ 0.4

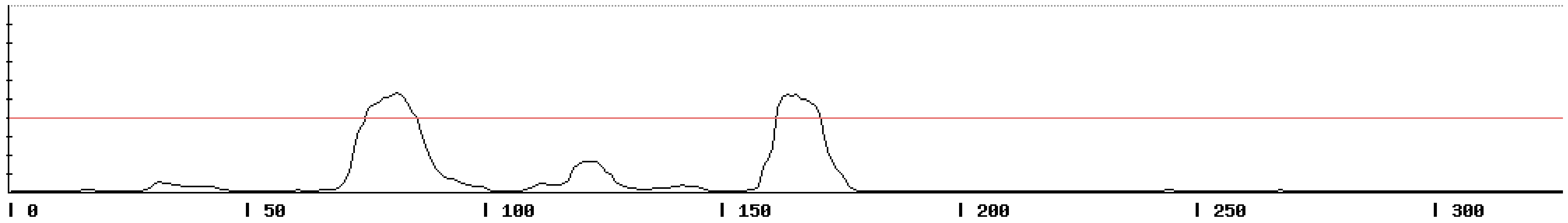

Predictions for Azadirachtaindica_Neem_16015_f_1

Viterbi Path

Viterbi Path

Posterior @ 0.4

Posterior @ 0.4

Posterior Graphic. Threshold Marked @ 0.4

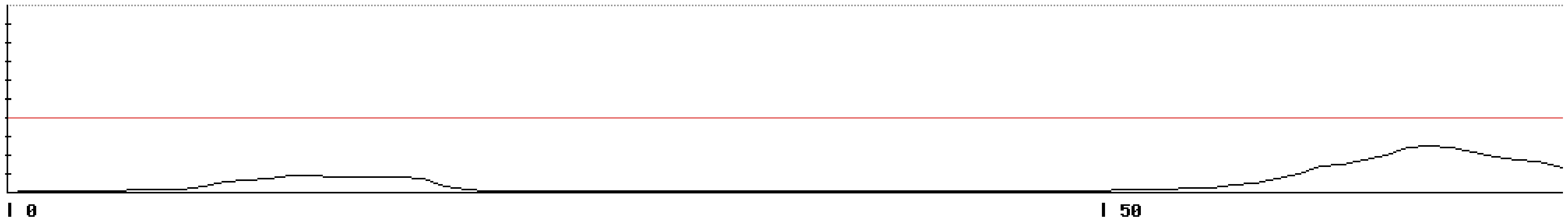

Predictions for Azadirachtaindica_Neem_16434_f_1

Viterbi Path

Viterbi Path

Posterior @ 0.4

Posterior @ 0.4

Posterior Graphic. Threshold Marked @ 0.4

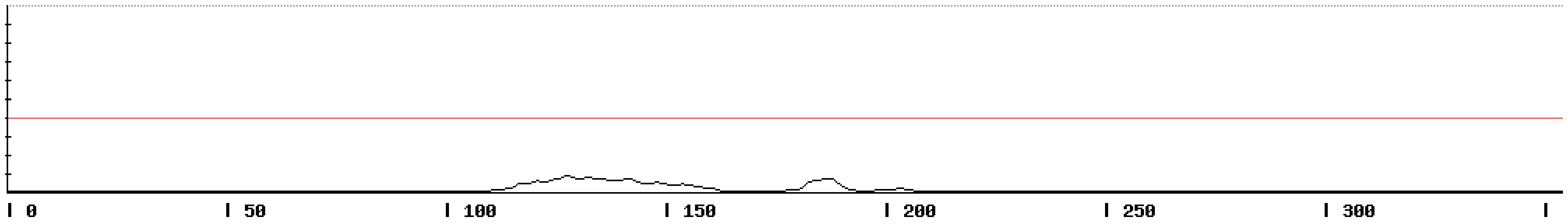

Predictions for Azadirachtaindica_Neem_16489_f_1

Viterbi Path

Viterbi Path

Posterior @ 0.4

Posterior @ 0.4

Posterior Graphic. Threshold Marked @ 0.4

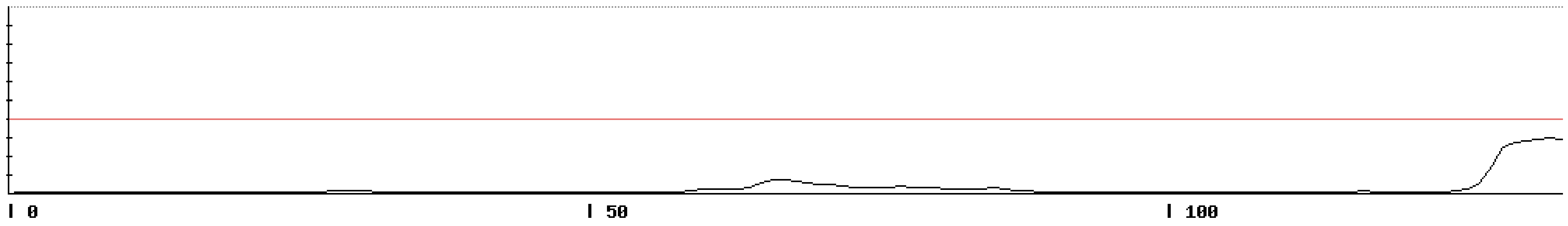

Predictions for Azadirachtaindica_Neem_1648_f_2

Viterbi Path

Viterbi Path

Posterior @ 0.4

Posterior @ 0.4

Posterior Graphic. Threshold Marked @ 0.4

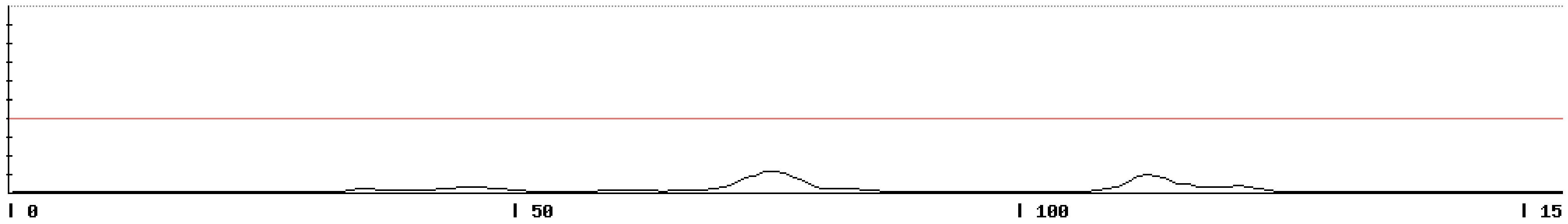

Predictions for Azadirachtaindica_Neem_16822_f_1

Viterbi Path	76 - PRSKGENGKPRPKGKNGKLFHRKTTKGCWKINGKGSKVNRIGRKR - 120
Viterbi Path	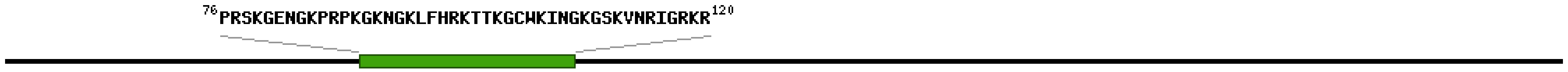
Posterior @ 0.4	76 - PRSKGENGKPRPKGKNGKLFHRKTTKGCWKINGKGSKVNRIGRKRTLSFR - 125<br>157 - RKKRKWDKKRG - 167
Posterior @ 0.4	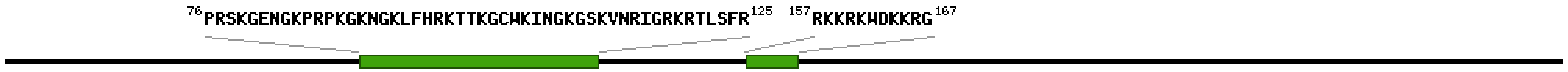
Posterior Graphic. Threshold Marked @ 0.4	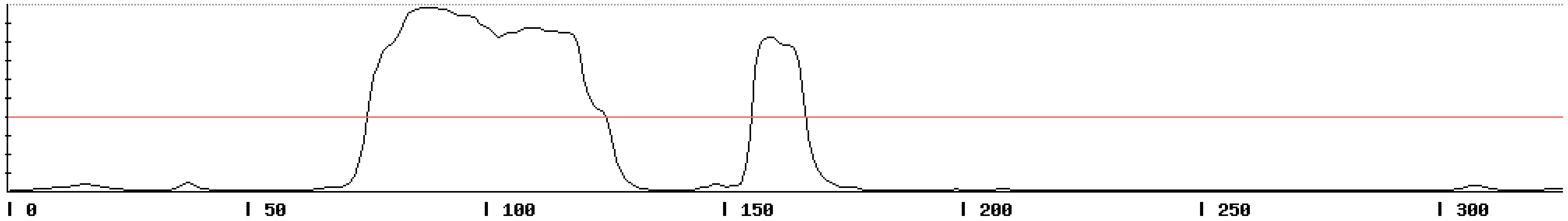

Predictions for Azadirachtaindica_Neem_17060_f_1

Viterbi Path	
Viterbi Path	
Posterior @ 0.4	
Posterior @ 0.4	
Posterior Graphic. Threshold Marked @ 0.4	

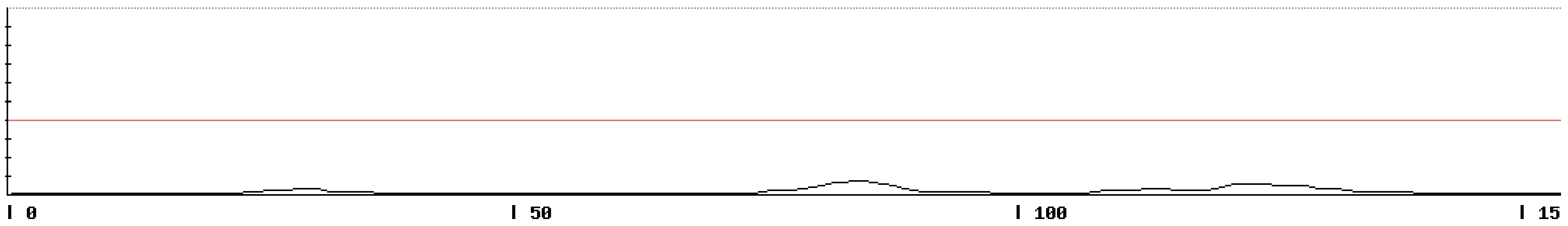

Predictions for Azadirachtaindica_Neem_17097_f_1

Viterbi Path	
Viterbi Path	
Posterior @ 0.4	
Posterior @ 0.4	
Posterior Graphic. Threshold Marked @ 0.4	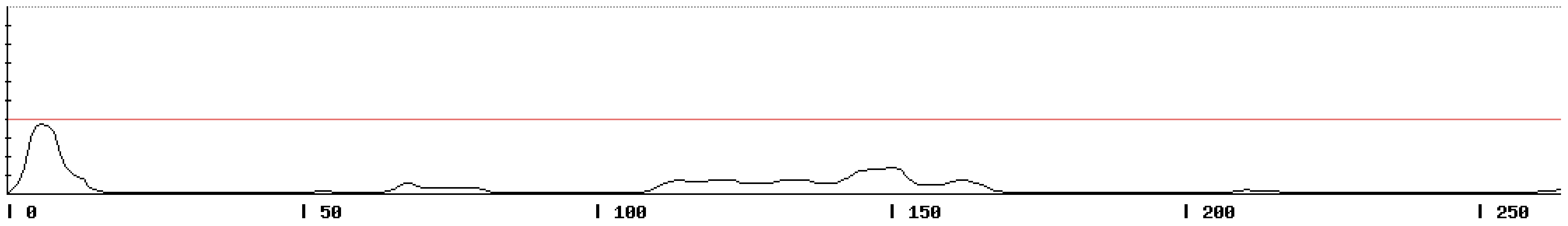

Predictions for Azadirachtaindica_Neem_17126_f_1

Viterbi Path	
Viterbi Path	
Posterior @ 0.4	
Posterior @ 0.4	

Posterior Graphic. Threshold Marked @ 0.4	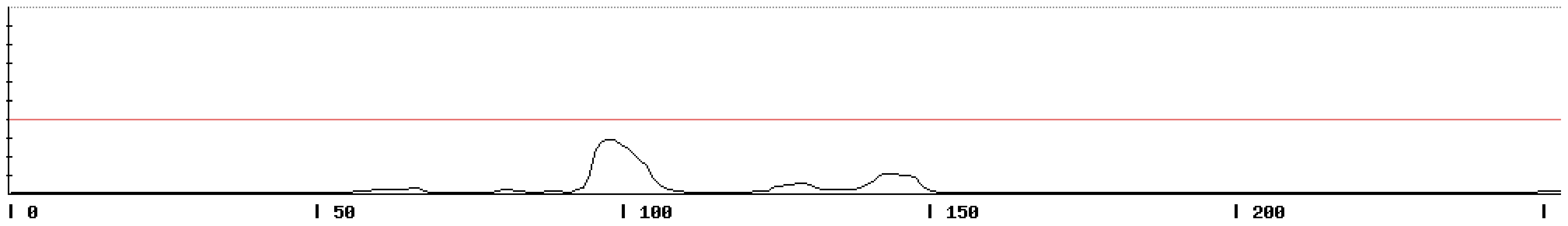

Predictions for Azadirachtaindica_Neem_17557_f_3	
Viterbi Path	
Viterbi Path	
Posterior @ 0.4	
Posterior @ 0.4	
Posterior Graphic. Threshold Marked @ 0.4	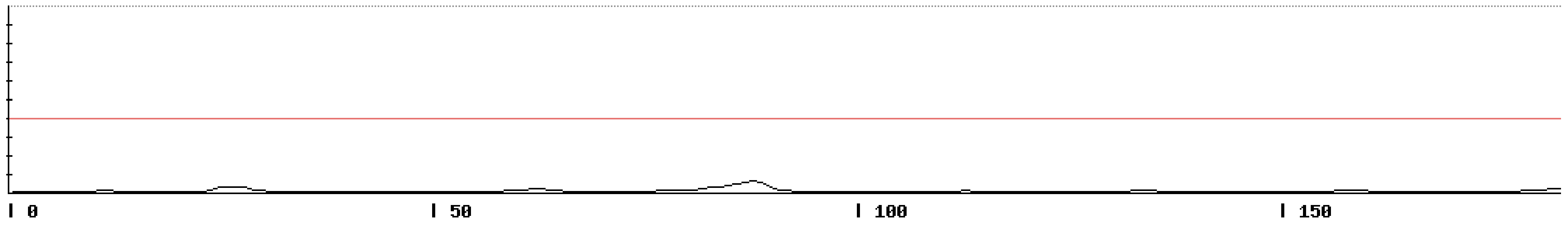

Predictions for Azadirachtaindica_Neem_17601_f_1	
Viterbi Path	
Viterbi Path	
Posterior @ 0.4	182 - KVNKSDKKKREG - 193
Posterior @ 0.4	

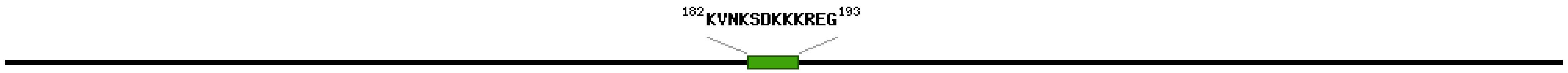

Posterior Graphic. Threshold Marked @ 0.4

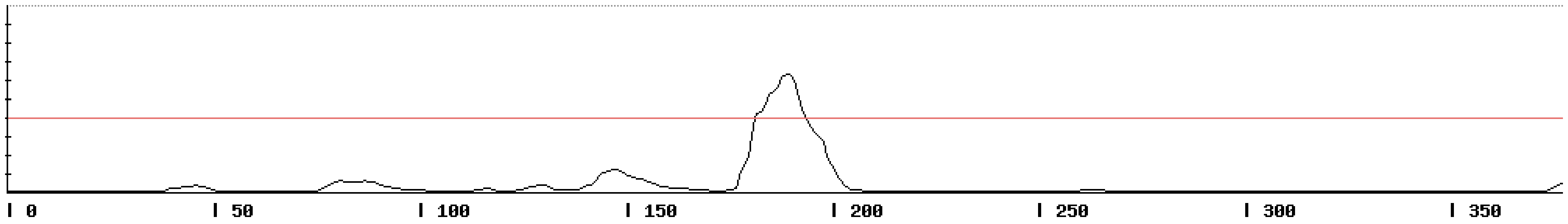

Predictions for Azadirachtaindica_Neem_17750_f_1

Viterbi Path

Viterbi Path

Posterior @ 0.4 — 143 - KRR - 145

Posterior @ 0.4

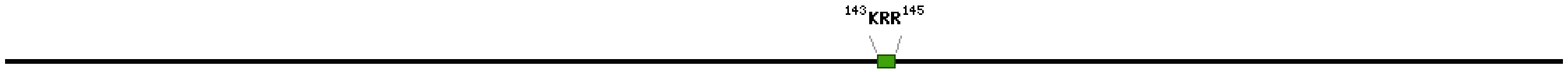

Posterior Graphic. Threshold Marked @ 0.4

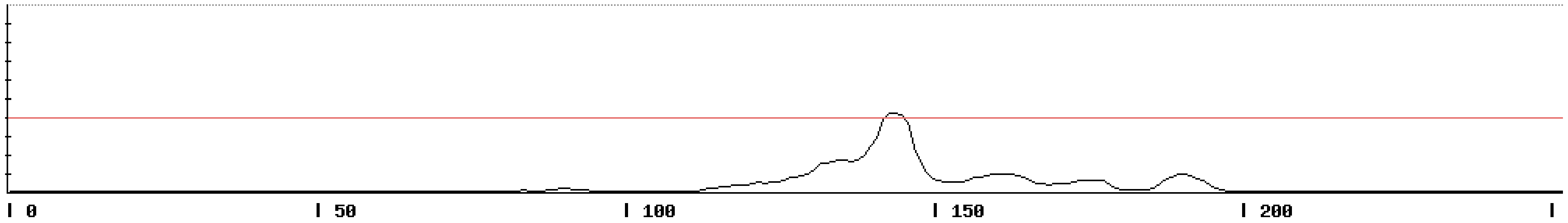

Predictions for Azadirachtaindica_Neem_17887_f_1

Viterbi Path

Viterbi Path

Posterior @ 0.4

Posterior @ 0.4

Posterior Graphic. Threshold Marked @ 0.4

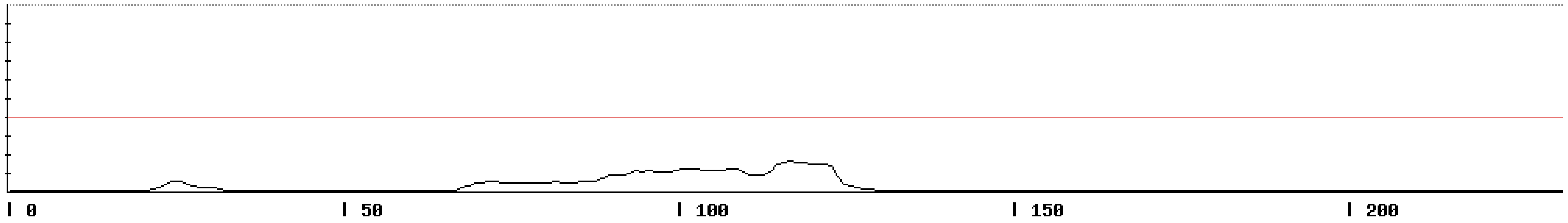

Predictions for Azadirachtaindica_Neem_17921_f_1

Viterbi Path

Viterbi Path

Posterior @ 0.4

Posterior @ 0.4

Posterior Graphic. Threshold Marked @ 0.4

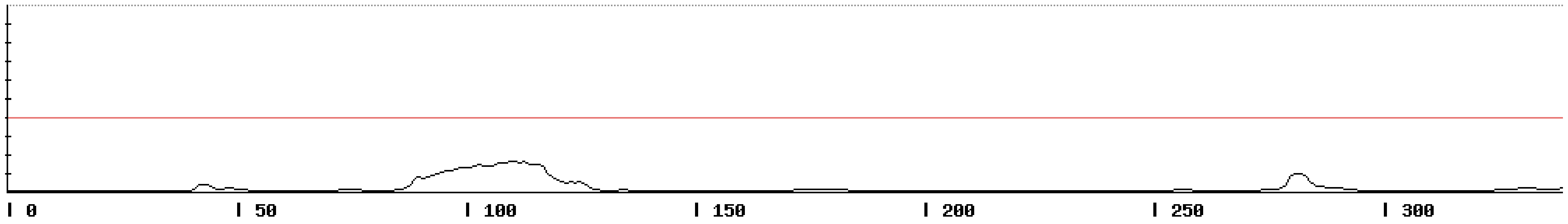

Predictions for Azadirachtaindica_Neem_17921_f_2

Viterbi Path

Viterbi Path

Posterior @ 0.4	
Posterior @ 0.4	
Posterior Graphic. Threshold Marked @ 0.4	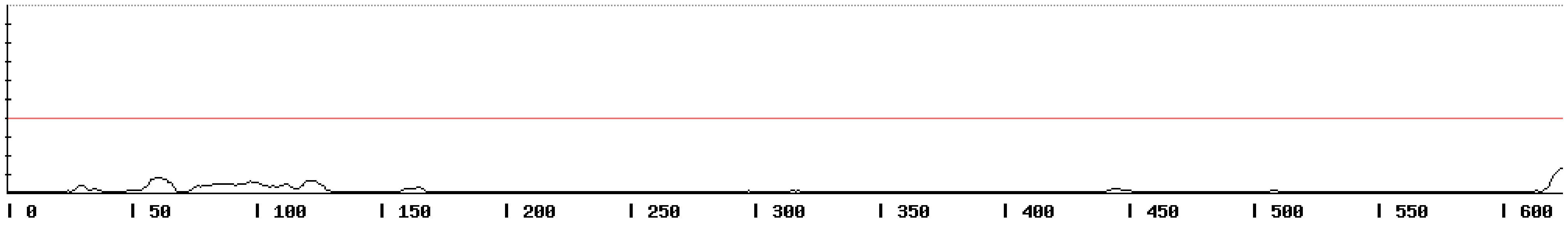

Predictions for Azadirachtaindica_Neem_18453_f_1

Viterbi Path	
Viterbi Path	
Posterior @ 0.4	
Posterior @ 0.4	
Posterior Graphic. Threshold Marked @ 0.4	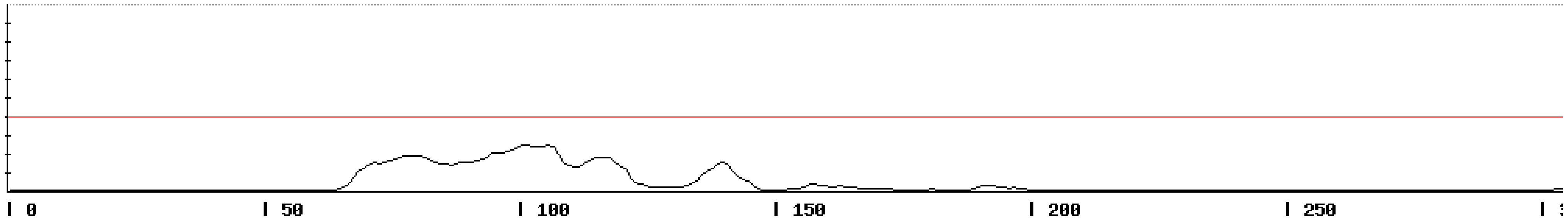

Predictions for Azadirachtaindica_Neem_18705_f_2

Viterbi Path

Viterbi Path

Posterior @ 0.4

Posterior @ 0.4

Posterior Graphic. Threshold Marked @ 0.4

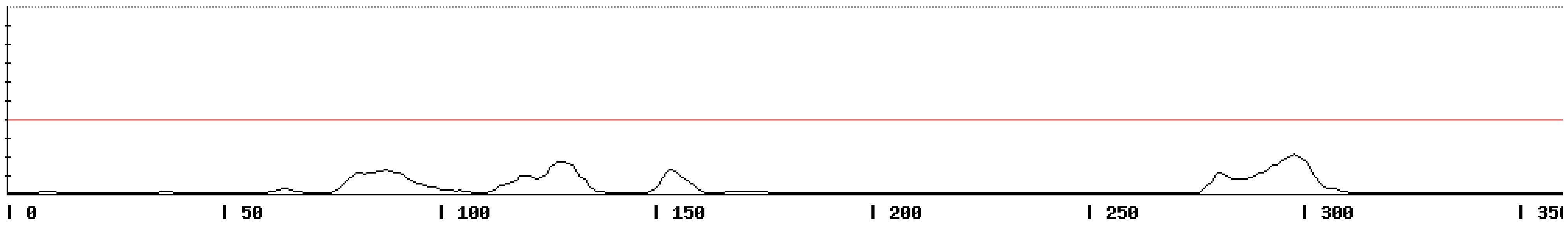

Predictions for Azadirachtaindica_Neem_19267_f_1

Viterbi Path

Viterbi Path

Posterior @ 0.4

Posterior @ 0.4

Posterior Graphic. Threshold Marked @ 0.4

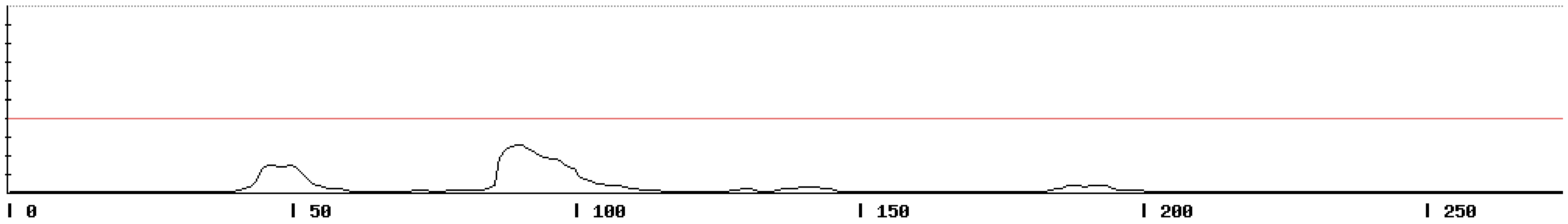

Predictions for Azadirachtaindica_Neem_20502_f_1	
Viterbi Path	
Viterbi Path	
Posterior @ 0.4	
Posterior @ 0.4	
Posterior Graphic. Threshold Marked @ 0.4	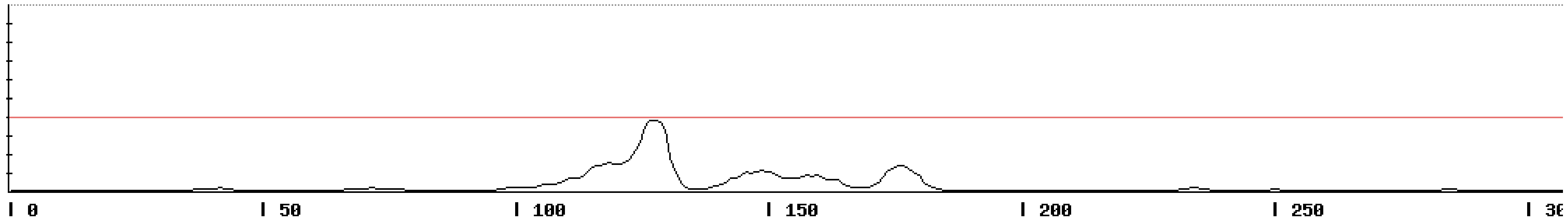

Predictions for Azadirachtaindica_Neem_20955_f_1	
Viterbi Path	
Viterbi Path	
Posterior @ 0.4	
Posterior @ 0.4	
Posterior Graphic. Threshold Marked @ 0.4	

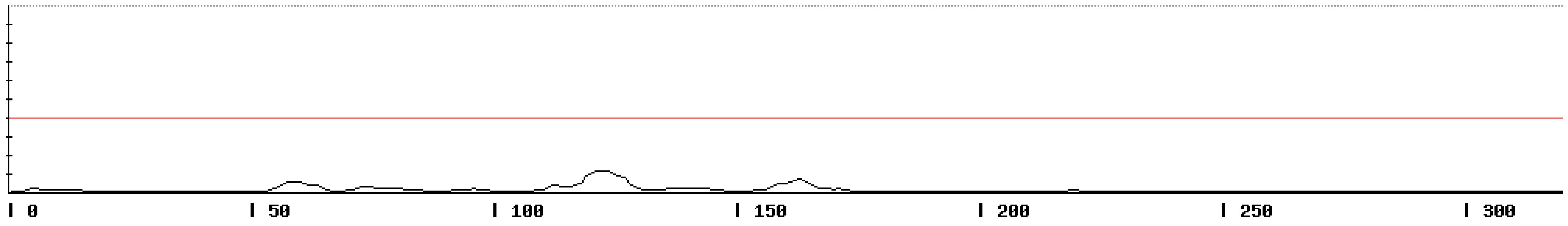

Predictions for Azadirachtaindica_Neem_21394_f_3

Viterbi Path

Viterbi Path

Posterior @ 0.4

Posterior @ 0.4

Posterior Graphic. Threshold Marked @ 0.4

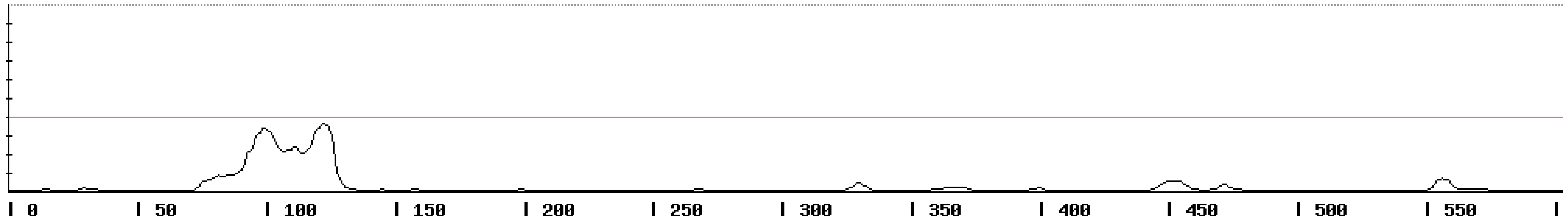

Predictions for Azadirachtaindica_Neem_2174_f_6

Viterbi Path

Viterbi Path

Posterior @ 0.4

Posterior @ 0.4

Posterior Graphic. Threshold Marked @ 0.4

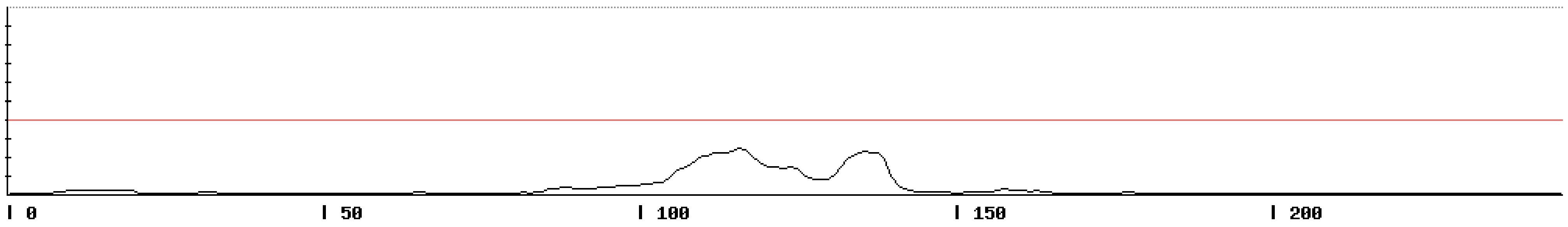

Predictions for Azadirachtaindica_Neem_21789_f_1

Viterbi Path

Viterbi Path

Posterior @ 0.4

Posterior @ 0.4

Posterior Graphic. Threshold Marked @ 0.4

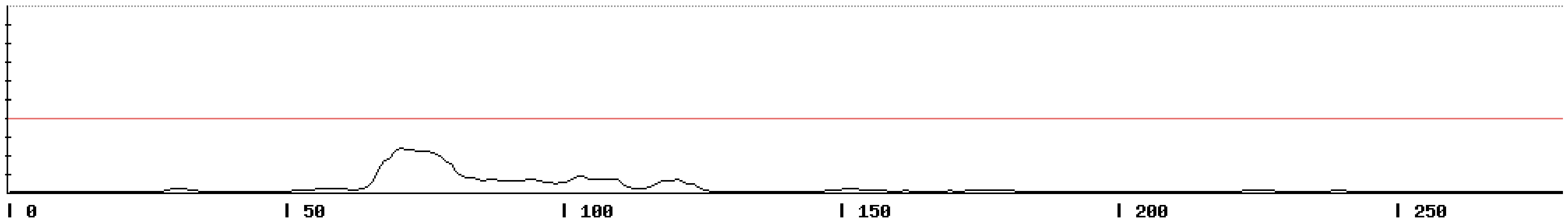

Predictions for Azadirachtaindica_Neem_22363_f_2

Viterbi Path

Viterbi Path

Posterior @ 0.4 — 169 - QRKRKPKKHRA - 179

Posterior @ 0.4

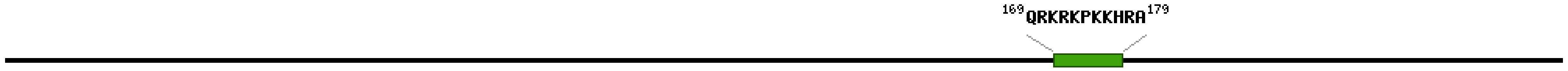

Posterior Graphic. Threshold Marked @ 0.4

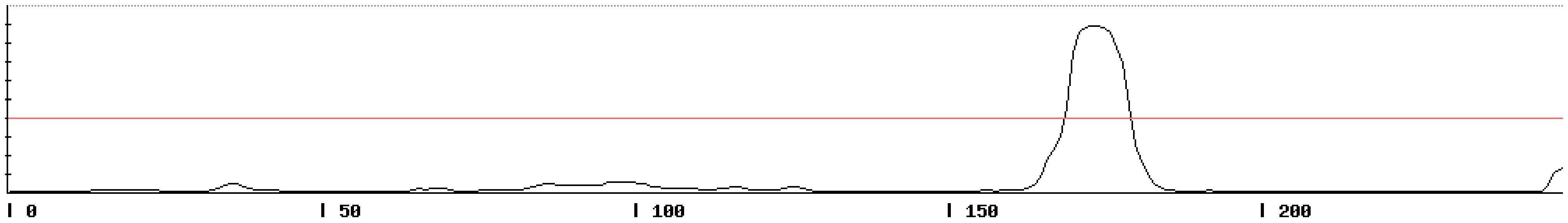

Predictions for Azadirachtaindica_Neem_22839_f_1

Viterbi Path

Viterbi Path

Posterior @ 0.4

Posterior @ 0.4

Posterior Graphic. Threshold Marked @ 0.4

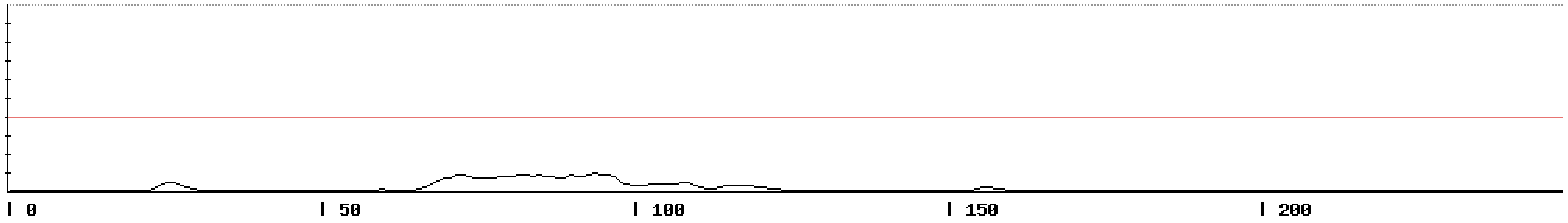

Predictions for Azadirachtaindica_Neem_23091_f_1

Viterbi Path

Viterbi Path

Posterior @ 0.4

Posterior @ 0.4

Posterior Graphic. Threshold Marked @ 0.4

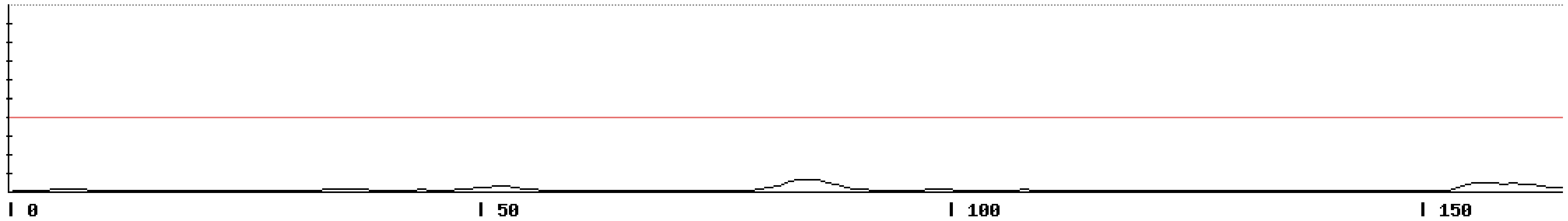

Predictions for Azadirachtaindica_Neem_2314_f_2

Viterbi Path

Viterbi Path

Posterior @ 0.4 184 - KRR - 186

Posterior @ 0.4

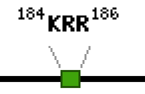

Posterior Graphic. Threshold Marked @ 0.4

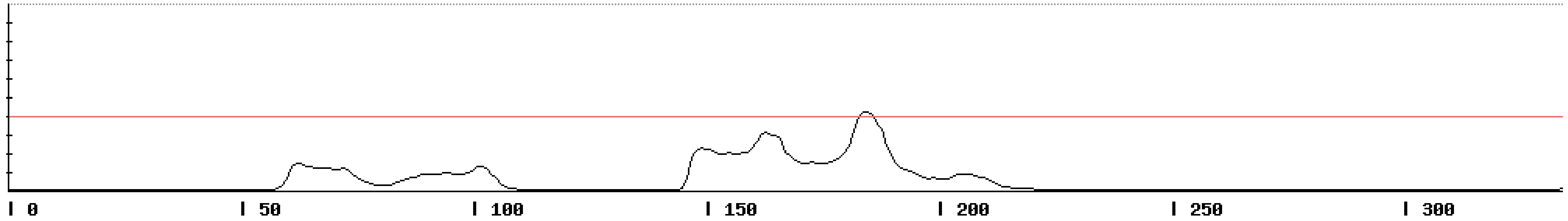

Predictions for Azadirachtaindica_Neem_23158_f_1

Viterbi Path

Viterbi Path

Posterior @ 0.4

Posterior @ 0.4

Posterior Graphic. Threshold Marked @ 0.4

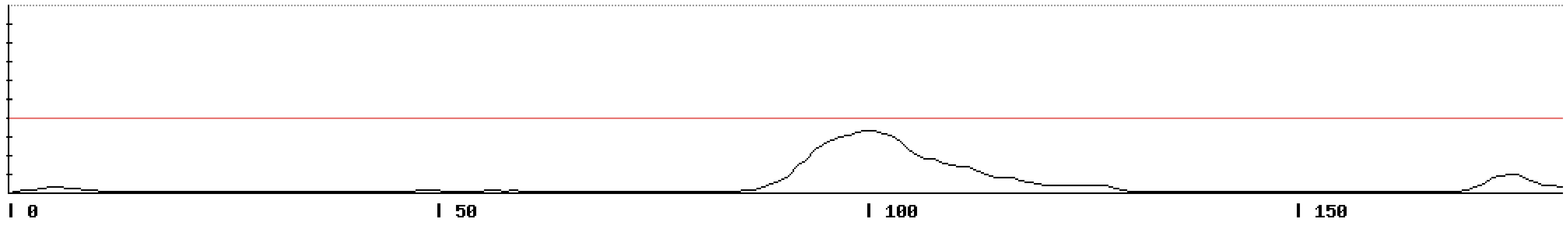

Predictions for Azadirachtaindica_Neem_2328_f_1

Viterbi Path

Viterbi Path

Posterior @ 0.4

Posterior @ 0.4

Posterior Graphic. Threshold Marked @ 0.4

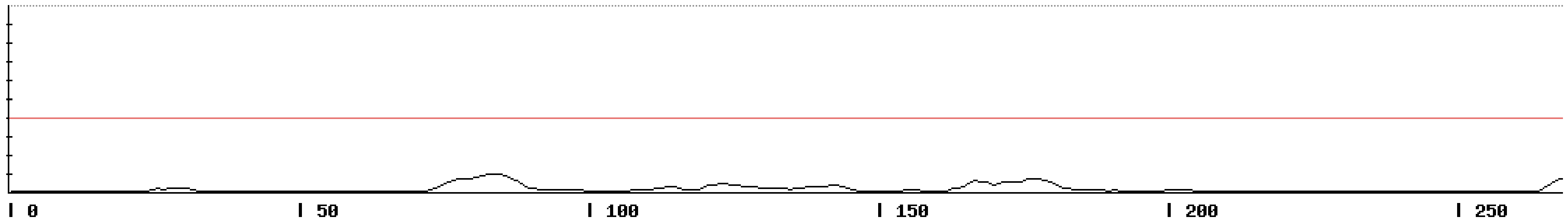

Predictions for Azadirachtaindica_Neem_23413_f_1

Viterbi Path	
Viterbi Path	
Posterior @ 0.4	38 - GADKPIGKPKALGIKKA - 54
Posterior @ 0.4	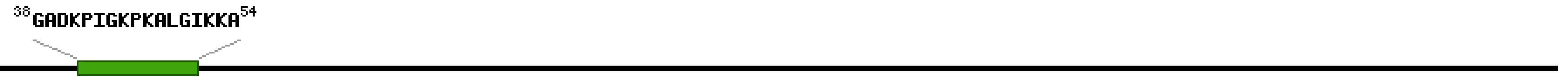
Posterior Graphic. Threshold Marked @ 0.4	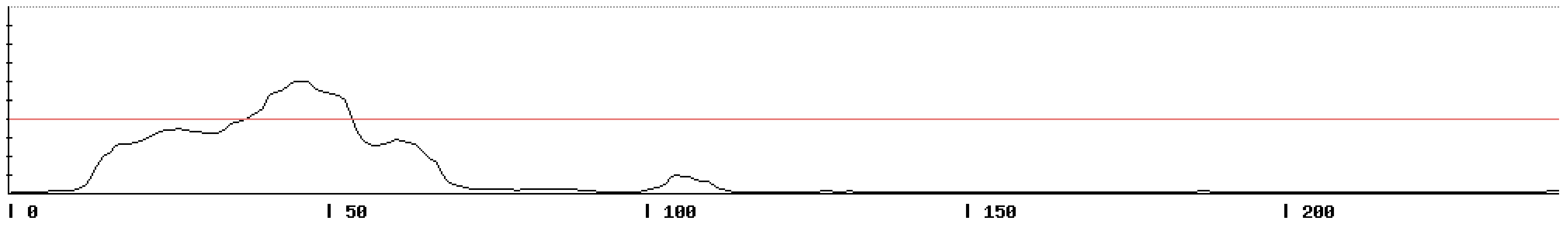

Predictions for Azadirachtaindica_Neem_23449_f_2

Viterbi Path	
Viterbi Path	
Posterior @ 0.4	
Posterior @ 0.4	
Posterior Graphic. Threshold Marked @ 0.4	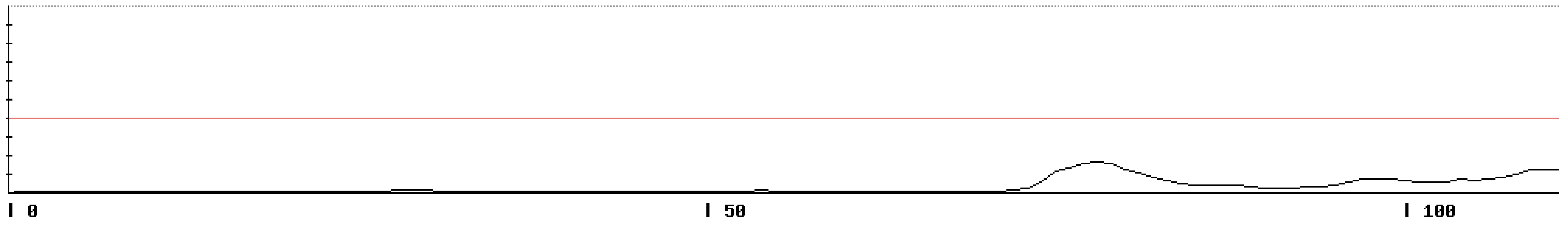

Predictions for Azadirachtaindica_Neem_2345_f_3	
Viterbi Path	
Viterbi Path	
Posterior @ 0.4	599 - KMKKSTTRVKGNNKK - 613
Posterior @ 0.4	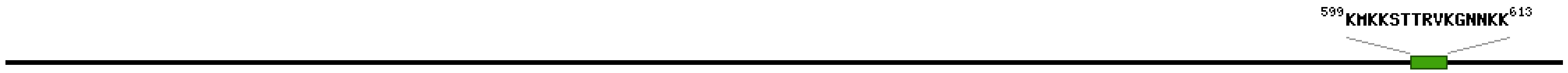
Posterior Graphic. Threshold Marked @ 0.4	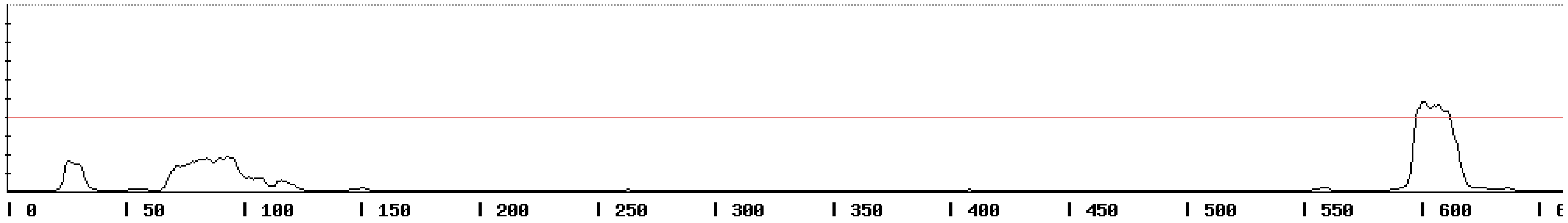

Predictions for Azadirachtaindica_Neem_23729_f_1	
Viterbi Path	
Viterbi Path	
Posterior @ 0.4	
Posterior @ 0.4	
Posterior Graphic. Threshold Marked @ 0.4	

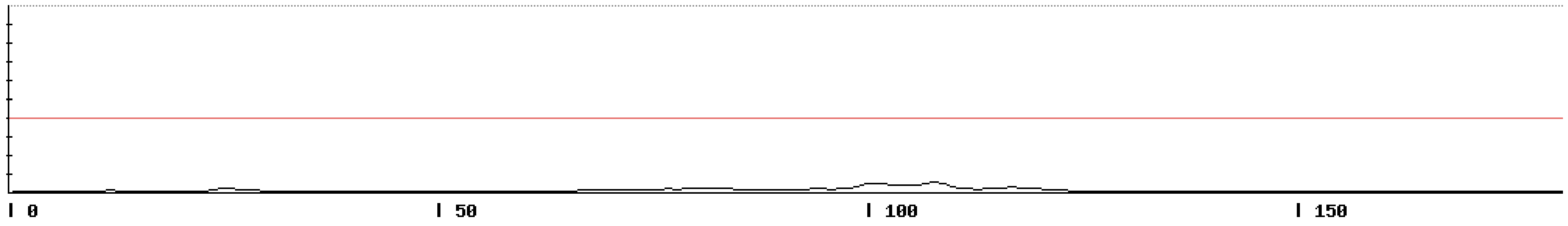

Predictions for Azadirachtaindica_Neem_2377_f_1

Viterbi Path	
Viterbi Path	
Posterior @ 0.4	
Posterior @ 0.4	
Posterior Graphic. Threshold Marked @ 0.4	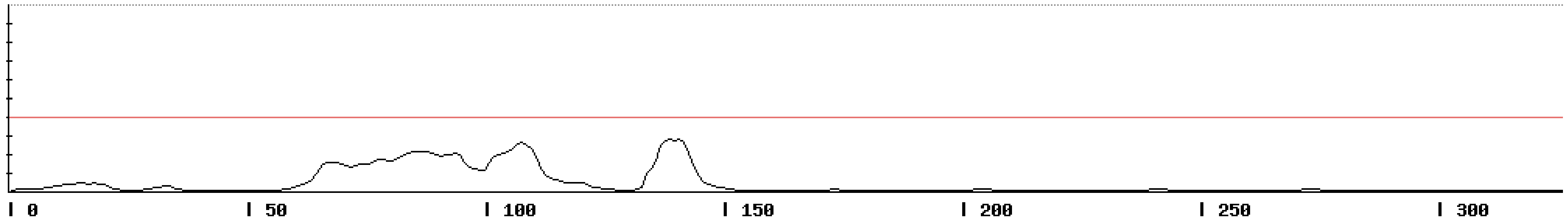

Predictions for Azadirachtaindica_Neem_24521_f_3

Viterbi Path	
Viterbi Path	
Posterior @ 0.4	81 - KK - 82
Posterior @ 0.4	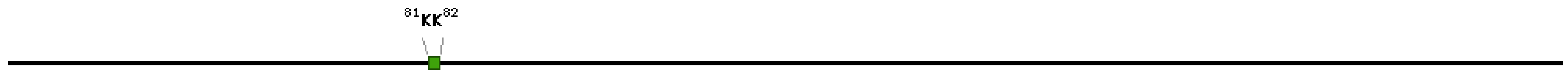

Posterior Graphic. Threshold Marked @ 0.4

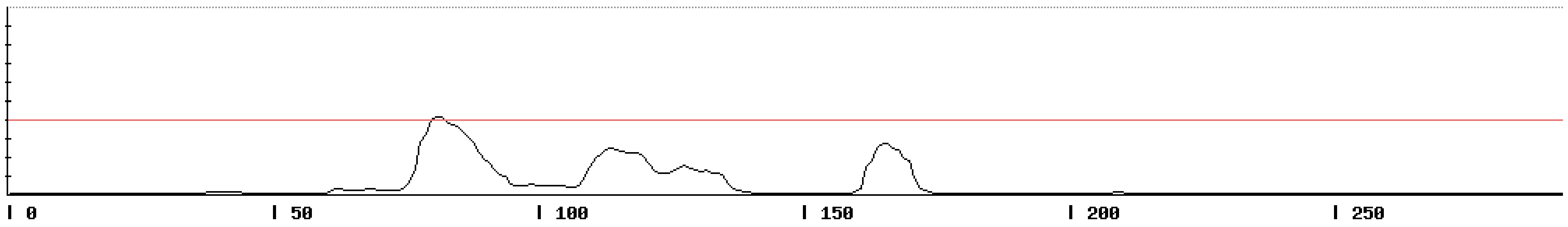

Predictions for Azadirachtaindica_Neem_2455_f_1

Viterbi Path

Viterbi Path

Posterior @ 0.4 124 - RRRKKA - 129

Posterior @ 0.4

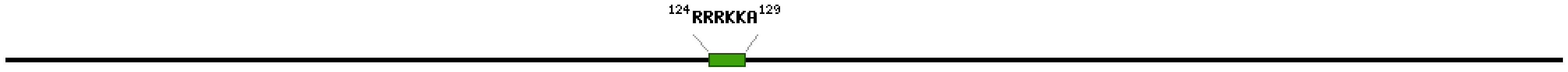

Posterior Graphic. Threshold Marked @ 0.4

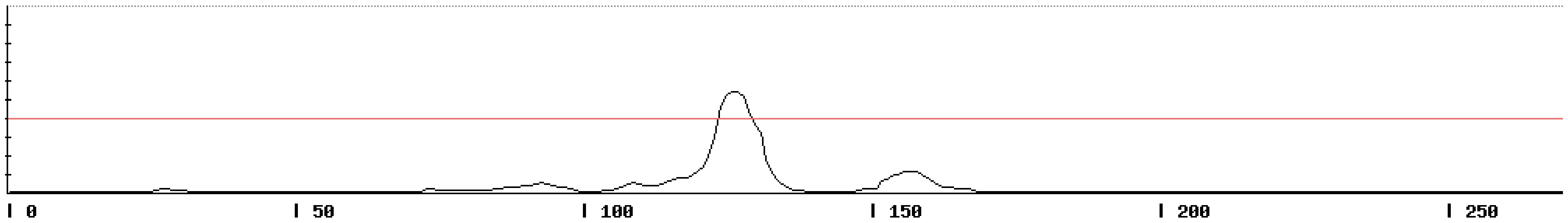

Predictions for Azadirachtaindica_Neem_2493_f_1

Viterbi Path

Viterbi Path

Posterior @ 0.4

Posterior @ 0.4

Posterior Graphic. Threshold Marked @ 0.4

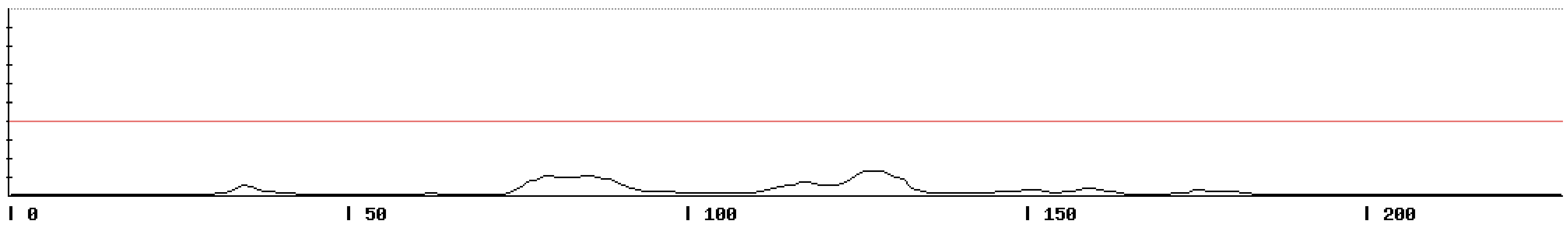

Predictions for Azadirachtaindica_Neem_24960_f_1

Viterbi Path

Viterbi Path

Posterior @ 0.4

Posterior @ 0.4

Posterior Graphic. Threshold Marked @ 0.4

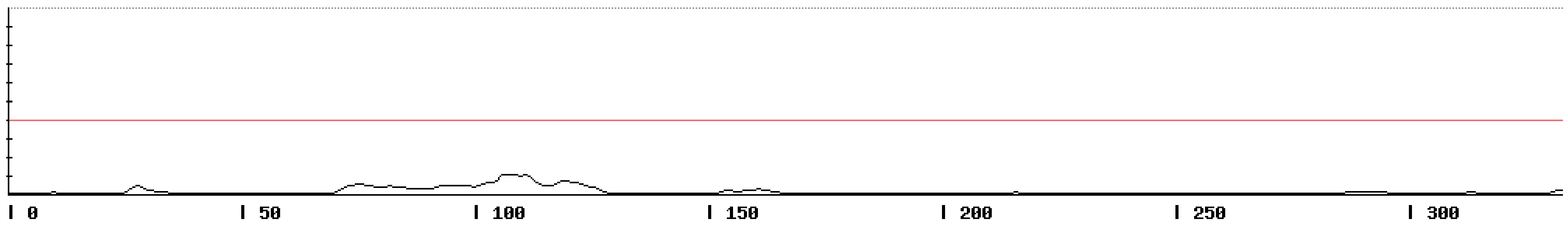

Predictions for Azadirachtaindica_Neem_2507_f_1

Viterbi Path

Viterbi Path

Posterior @ 0.4

Posterior @ 0.4

Posterior Graphic. Threshold Marked @ 0.4

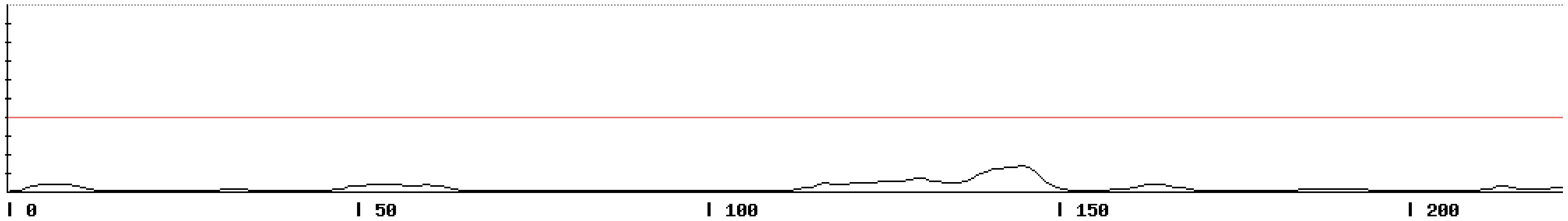

Predictions for Azadirachtaindica_Neem_25879_f_1

Viterbi Path

Viterbi Path

Posterior @ 0.4

Posterior @ 0.4

Posterior Graphic. Threshold Marked @ 0.4

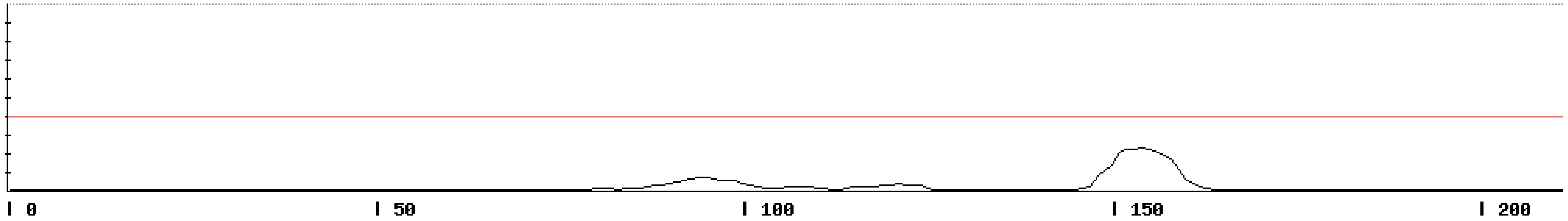

Predictions for Azadirachtaindica_Neem_2659_f_2

Viterbi Path

Viterbi Path

Posterior @ 0.4

123 - KKNEKGQRNEKADLGKKQRKSKQIGENNEPGKGATGWKRKLK - 164

Posterior @ 0.4

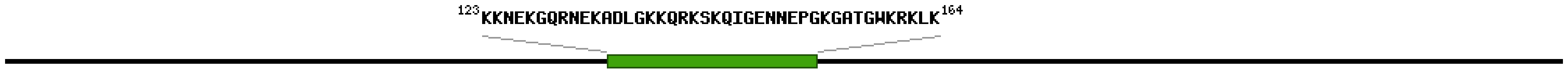

Posterior Graphic. Threshold Marked @ 0.4

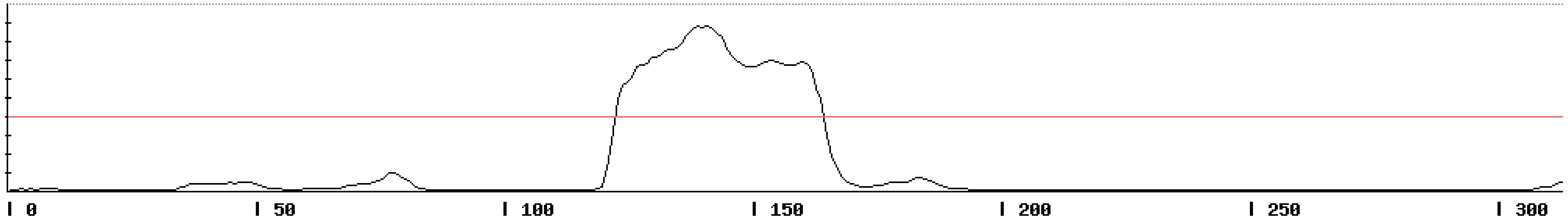

Predictions for Azadirachtaindica_Neem_26821_f_2

Viterbi Path

Viterbi Path

Posterior @ 0.4

Posterior @ 0.4

Posterior Graphic. Threshold Marked @ 0.4

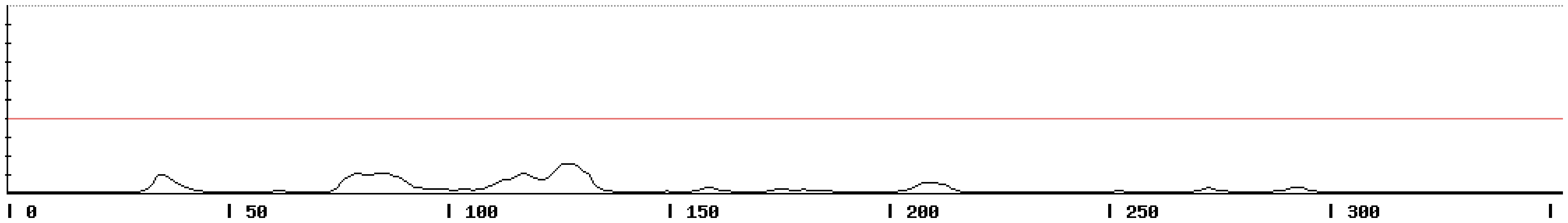

Predictions for Azadirachtaindica_Neem_27088_f_1

Viterbi Path

Viterbi Path

Posterior @ 0.4

Posterior @ 0.4

Posterior Graphic. Threshold Marked @ 0.4

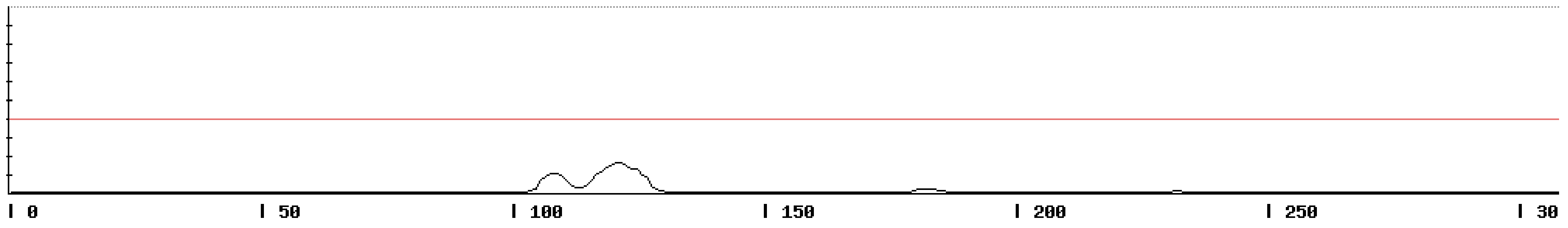

Predictions for Azadirachtaindica_Neem_27137_f_3

Viterbi Path

Viterbi Path

Posterior @ 0.4

Posterior @ 0.4

Posterior Graphic. Threshold Marked @ 0.4

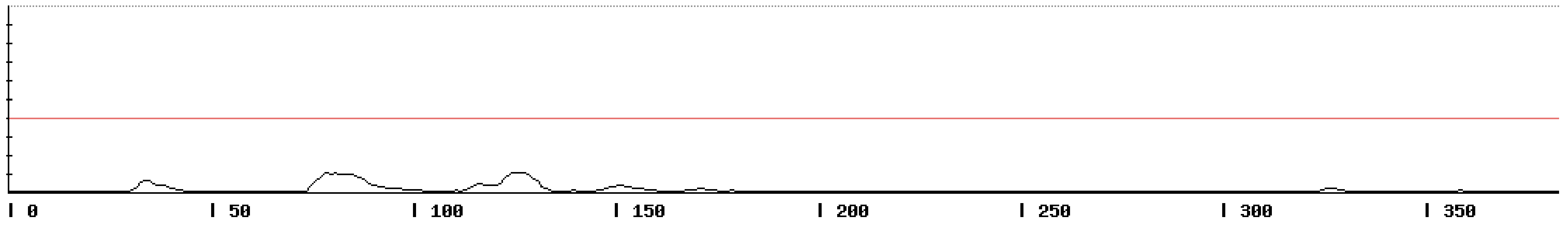

Predictions for Azadirachtaindica_Neem_2718_f_2	
Viterbi Path	
Viterbi Path	
Posterior @ 0.4	
Posterior @ 0.4	
Posterior Graphic. Threshold Marked @ 0.4	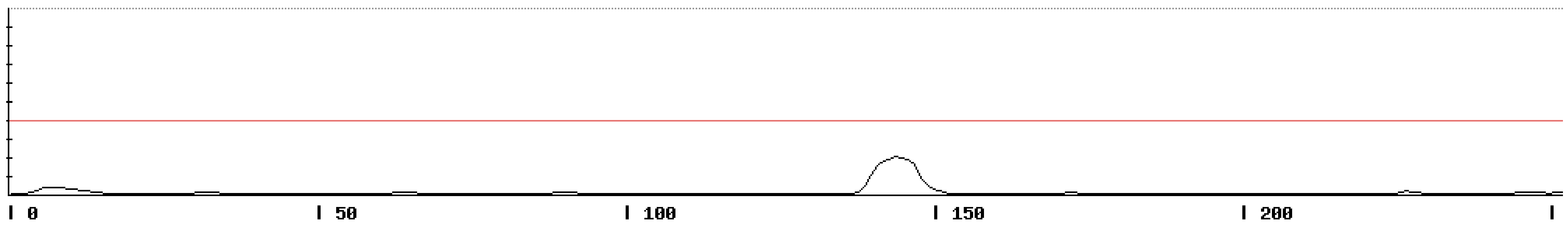

Predictions for Azadirachtaindica_Neem_27369_f_1	
Viterbi Path	
Viterbi Path	
Posterior @ 0.4	
Posterior @ 0.4	
Posterior Graphic. Threshold Marked @ 0.4	

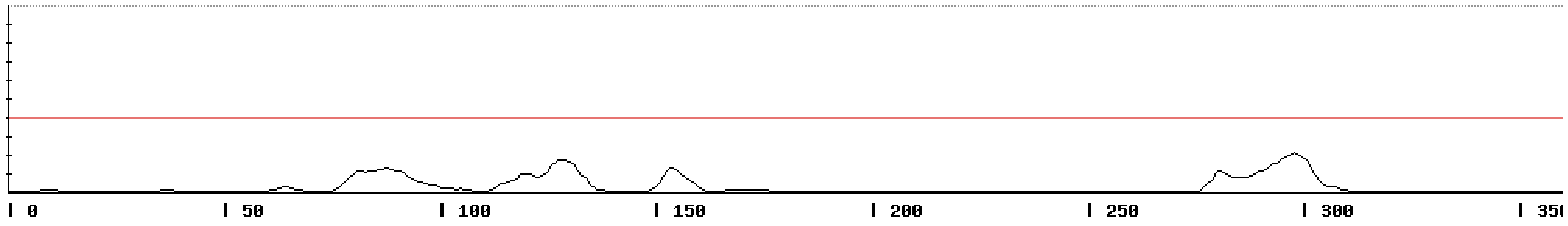

Predictions for Azadirachtaindica_Neem_27894_f_1	
Viterbi Path	
Viterbi Path	
Posterior @ 0.4	
Posterior @ 0.4	
Posterior Graphic. Threshold Marked @ 0.4	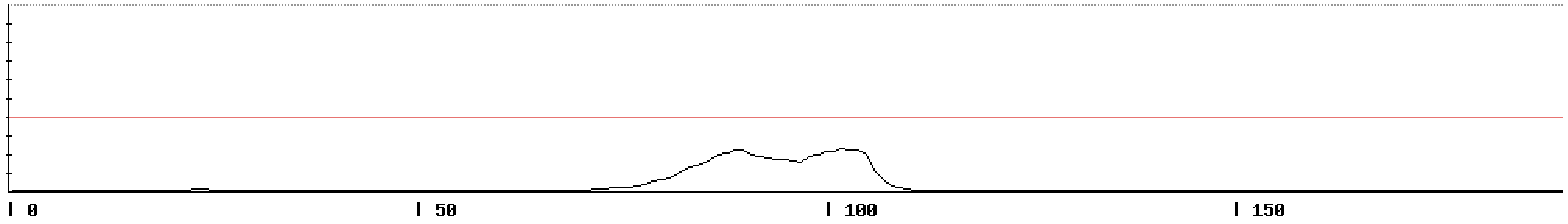

Predictions for Azadirachtaindica_Neem_28050_f_1	
Viterbi Path	
Viterbi Path	
Posterior @ 0.4	
Posterior @ 0.4	

Posterior Graphic. Threshold Marked @ 0.4	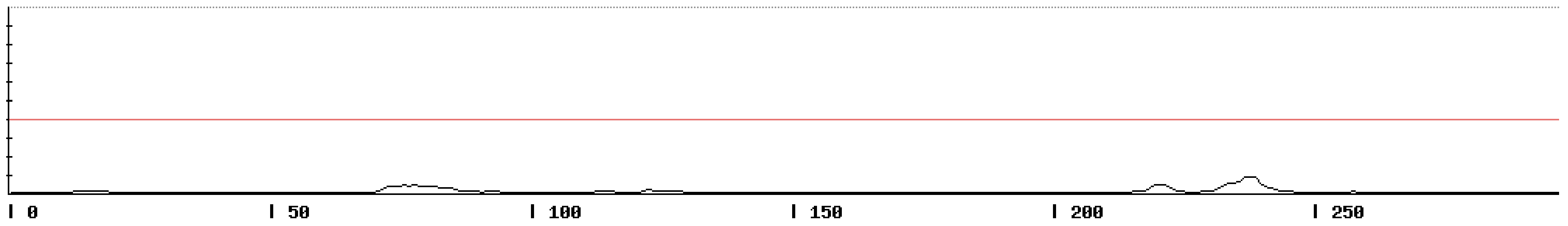

Predictions for Azadirachtaindica_Neem_28263_f_1

Viterbi Path	
Viterbi Path	
Posterior @ 0.4	
Posterior @ 0.4	
Posterior Graphic. Threshold Marked @ 0.4	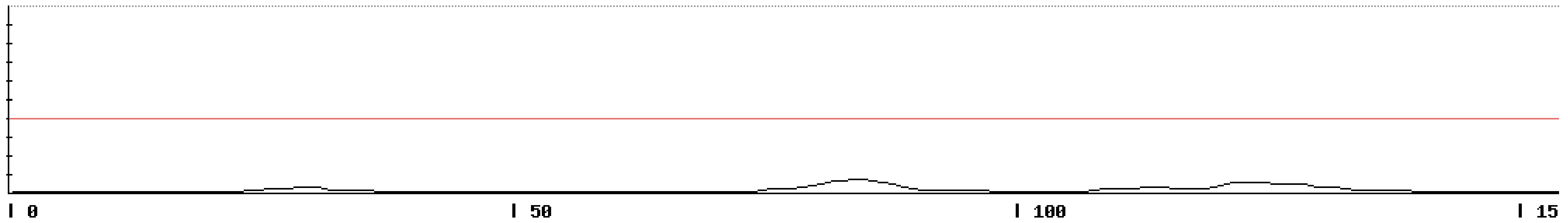

Predictions for Azadirachtaindica_Neem_28678_f_1

Viterbi Path	
Viterbi Path	
Posterior @ 0.4	397 - KKAAVGRMKKLKRK - 410
Posterior @ 0.4	

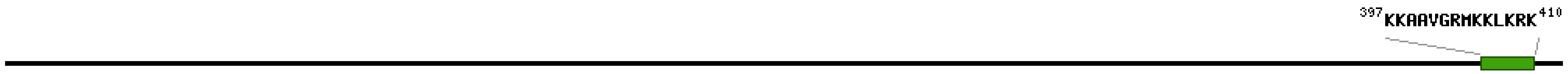

Posterior Graphic. Threshold Marked @ 0.4

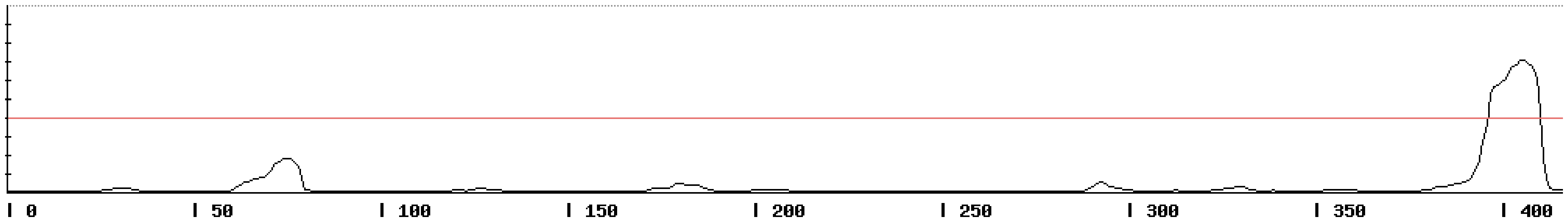

Predictions for Azadirachtaindica_Neem_2867_f_1

Viterbi Path

Viterbi Path

Posterior @ 0.4

Posterior @ 0.4

Posterior Graphic. Threshold Marked @ 0.4

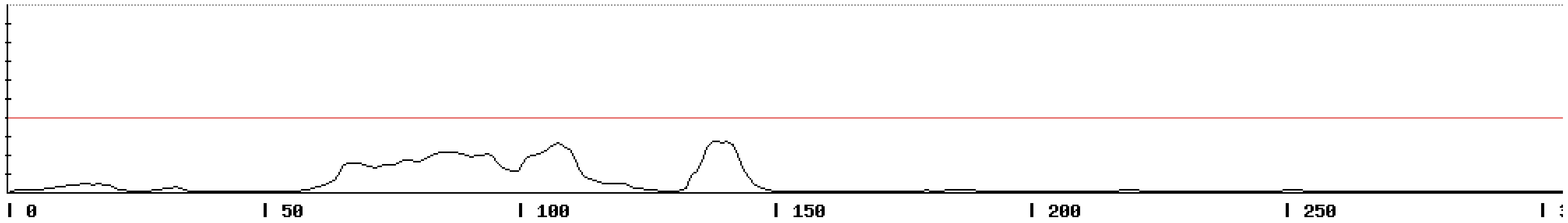

Predictions for Azadirachtaindica_Neem_28837_f_1

Viterbi Path

Viterbi Path

Posterior @ 0.4

Posterior @ 0.4

Posterior Graphic. Threshold Marked @ 0.4

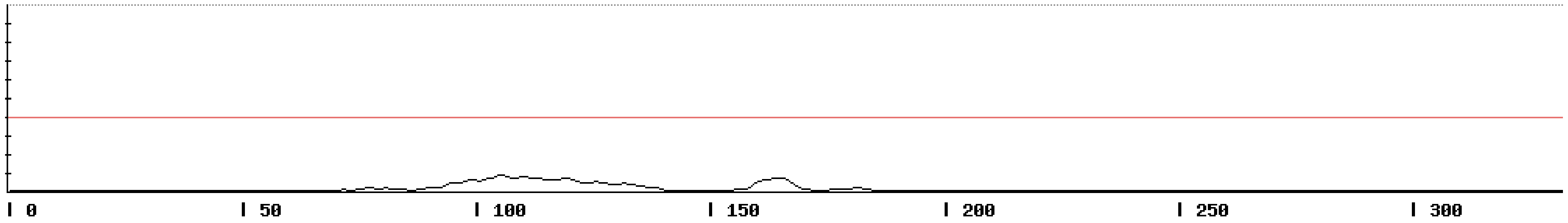

Predictions for Azadirachtaindica_Neem_29032_f_1

Viterbi Path

Viterbi Path

Posterior @ 0.4

Posterior @ 0.4

Posterior Graphic. Threshold Marked @ 0.4

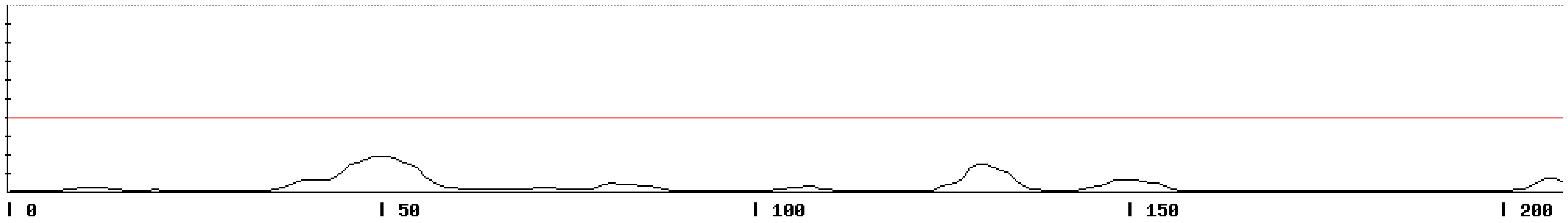

Predictions for Azadirachtaindica_Neem_29819_f_1

Viterbi Path

Viterbi Path

Posterior @ 0.4

Posterior @ 0.4

Posterior Graphic. Threshold Marked @ 0.4

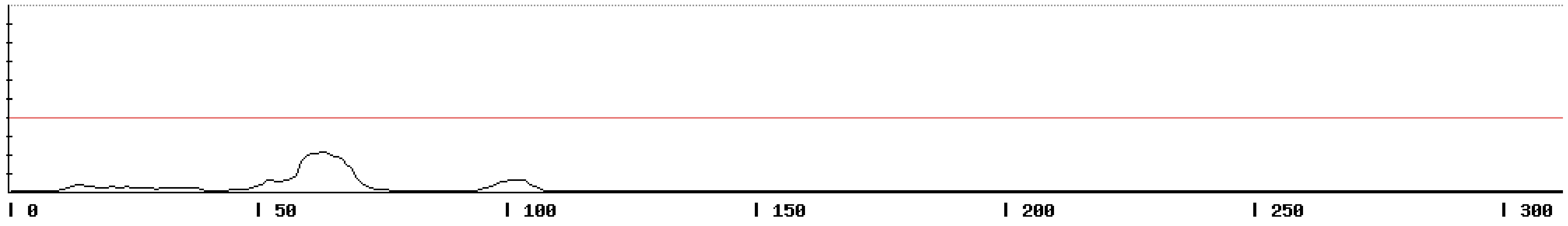

Predictions for Azadirachtaindica_Neem_2986_a_1

Viterbi Path

Viterbi Path

Posterior @ 0.4

68 - KLKKKTKGSRINRK - 81

105 - KLKKKTPKGSRINRK - 119

189 - KNQPTWKKNQTGKNNKSGKSKKRKL - 213

Posterior @ 0.4

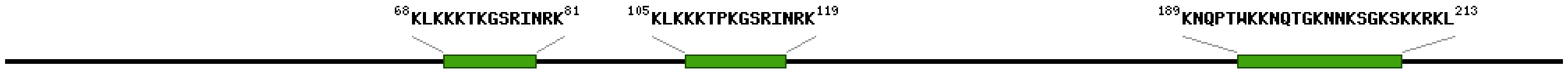

Posterior Graphic. Threshold Marked @ 0.4

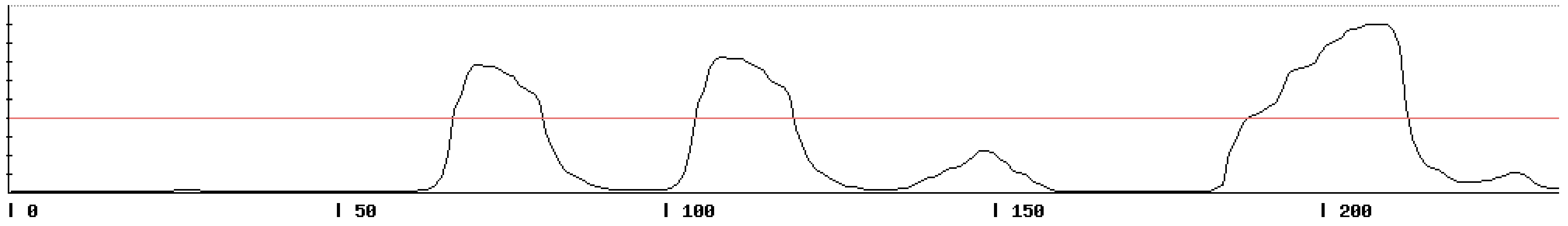

Predictions for Azadirachtaindica_Neem_29920_f_1

Viterbi Path	
Viterbi Path	
Posterior @ 0.4	
Posterior @ 0.4	
Posterior Graphic. Threshold Marked @ 0.4	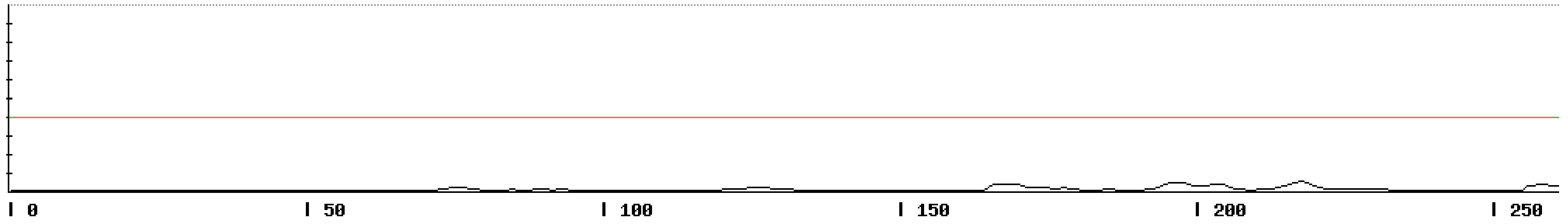

Predictions for Azadirachtaindica_Neem_30138_f_1

Viterbi Path	
Viterbi Path	
Posterior @ 0.4	
Posterior @ 0.4	

Posterior Graphic. Threshold Marked @ 0.4

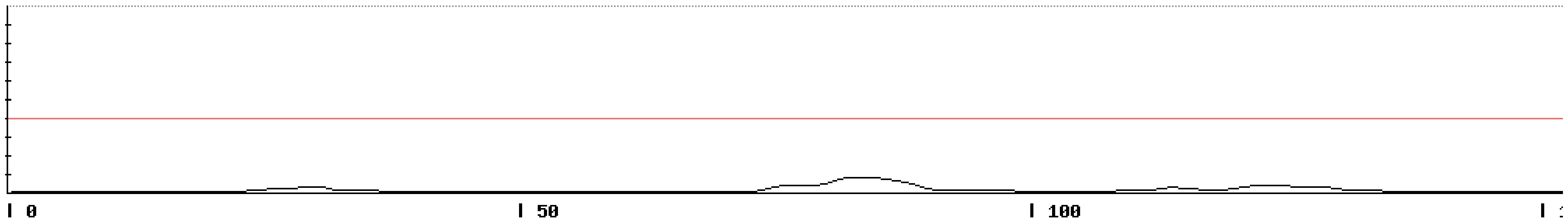

Predictions for Azadirachtaindica_Neem_30361_f_1

Viterbi Path

Viterbi Path

Posterior @ 0.4

Posterior @ 0.4

Posterior Graphic. Threshold Marked @ 0.4

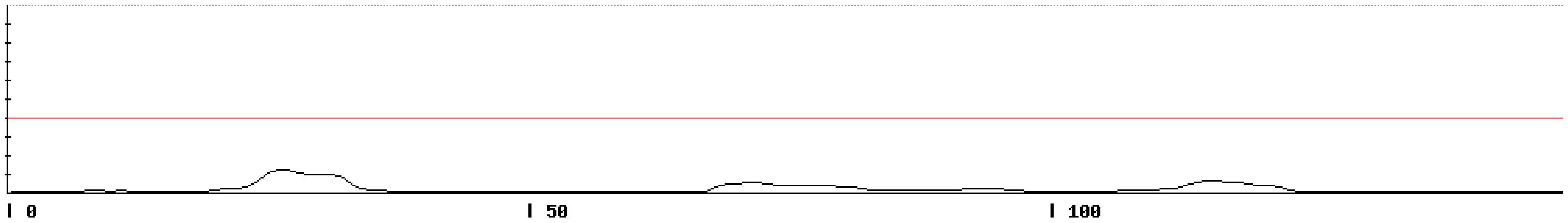

Predictions for Azadirachtaindica_Neem_3043_f_3

Viterbi Path

Viterbi Path

Posterior @ 0.4

Posterior @ 0.4

Posterior Graphic. Threshold Marked @ 0.4

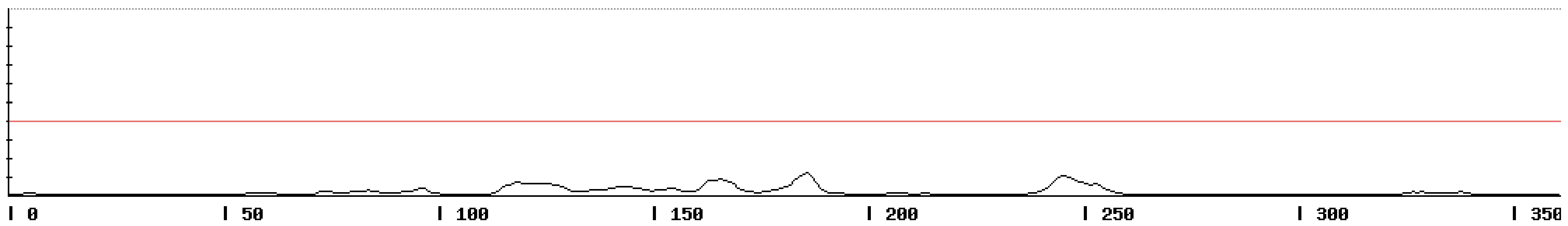

Predictions for Azadirachtaindica_Neem_30599_f_1

Viterbi Path

Viterbi Path

Posterior @ 0.4 — 45 - KLKKKTPKGSRINRKV - 60

Posterior @ 0.4

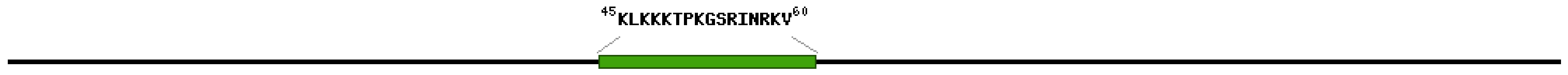

Posterior Graphic. Threshold Marked @ 0.4

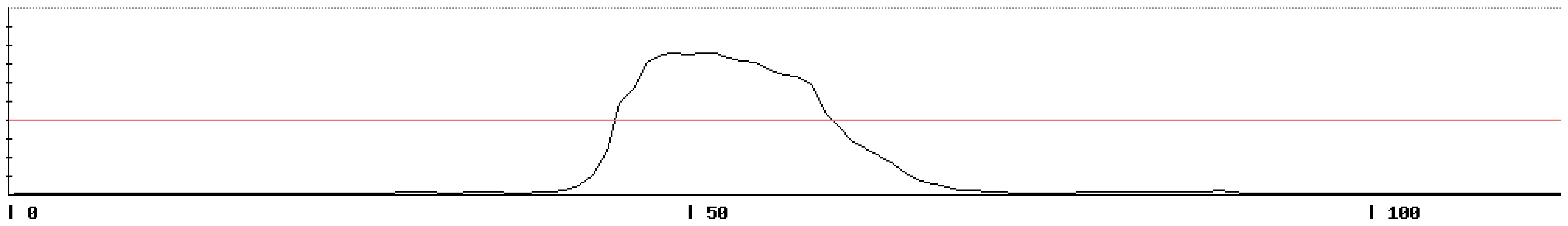

Predictions for Azadirachtaindica_Neem_30788_f_1

Viterbi Path

Viterbi Path

Posterior @ 0.4	
Posterior @ 0.4	
Posterior Graphic. Threshold Marked @ 0.4	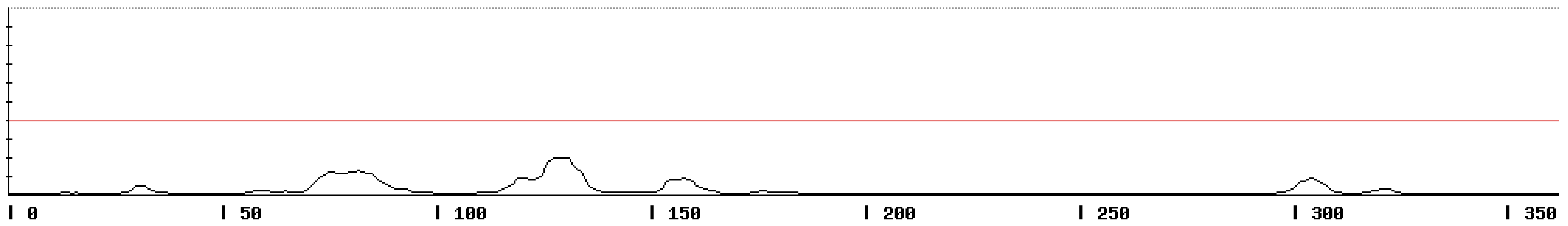

Predictions for Azadirachtaindica_Neem_30861_f_1

Viterbi Path	
Viterbi Path	
Posterior @ 0.4	
Posterior @ 0.4	
Posterior Graphic. Threshold Marked @ 0.4	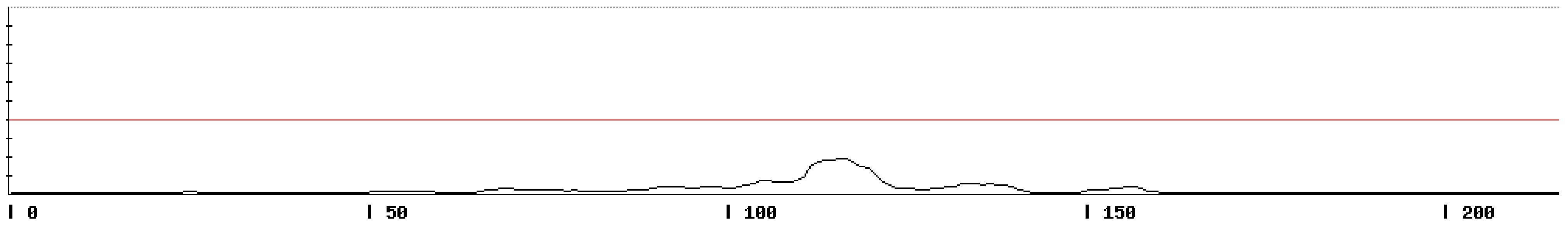

Predictions for Azadirachtaindica_Neem_30988_f_2

Viterbi Path	
Viterbi Path	

Posterior @ 0.4	
Posterior @ 0.4	
Posterior Graphic. Threshold Marked @ 0.4	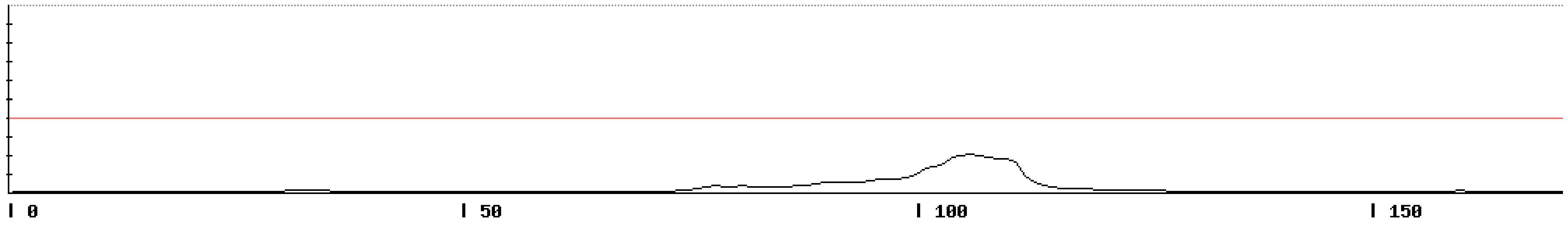

Predictions for Azadirachtaindica_Neem_3103_f_14

Viterbi Path	
Viterbi Path	
Posterior @ 0.4	
Posterior @ 0.4	
Posterior Graphic. Threshold Marked @ 0.4	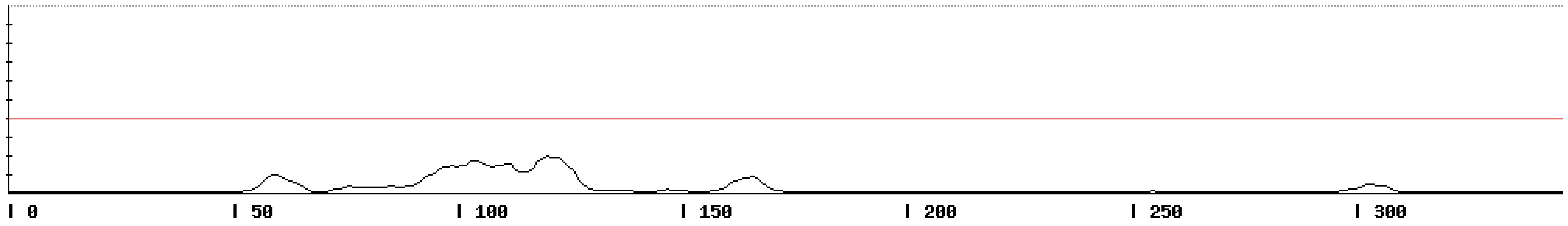

Predictions for Azadirachtaindica_Neem_31069_f_2

Viterbi Path

Viterbi Path

Posterior @ 0.4

Posterior @ 0.4

Posterior Graphic. Threshold Marked @ 0.4

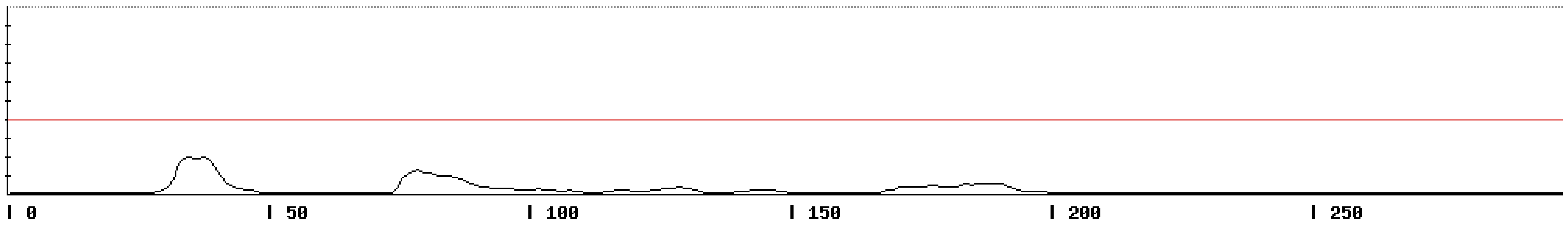

Predictions for Azadirachtaindica_Neem_31746_f_1

Viterbi Path

Viterbi Path

Posterior @ 0.4

Posterior @ 0.4

Posterior Graphic. Threshold Marked @ 0.4

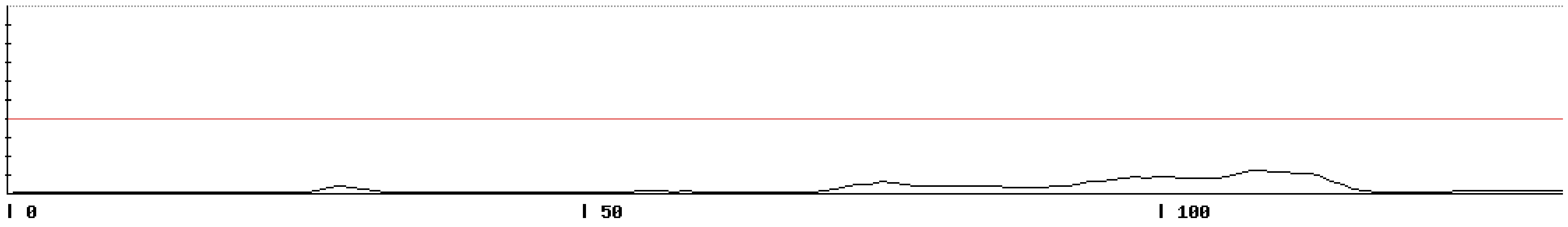

Predictions for Azadirachtaindica_Neem_31990_f_1	
Viterbi Path	
Viterbi Path	
Posterior @ 0.4	
Posterior @ 0.4	
Posterior Graphic. Threshold Marked @ 0.4	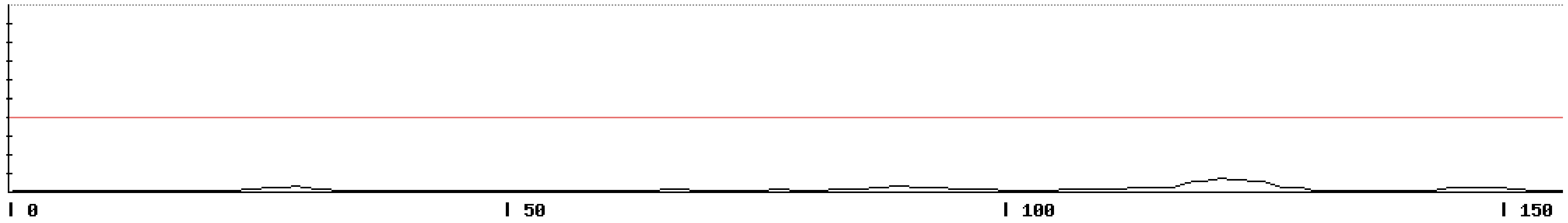

Predictions for Azadirachtaindica_Neem_32411_f_1	
Viterbi Path	
Viterbi Path	
Posterior @ 0.4	
Posterior @ 0.4	
Posterior Graphic. Threshold Marked @ 0.4	

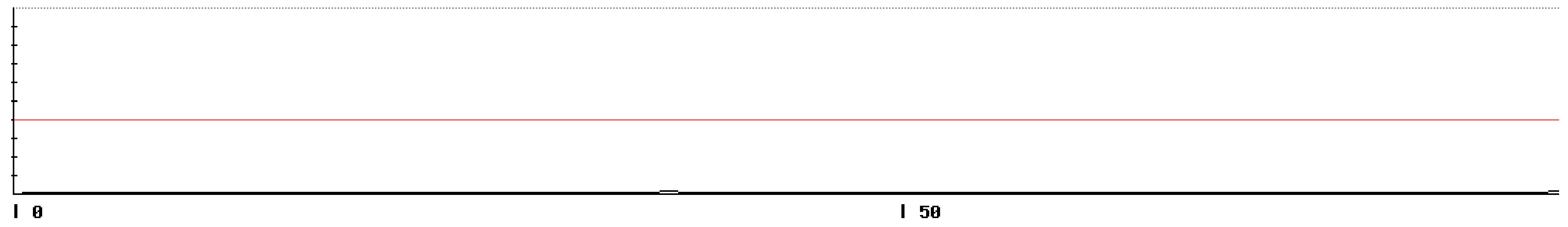

Predictions for Azadirachtaindica_Neem_32518_f_2

Viterbi Path

Viterbi Path

Posterior @ 0.4

Posterior @ 0.4

Posterior Graphic. Threshold Marked @ 0.4

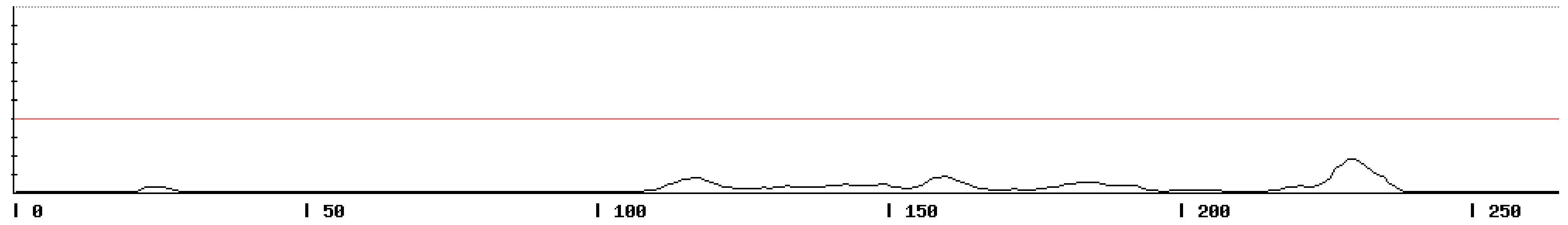

Predictions for Azadirachtaindica_Neem_32626_f_1

Viterbi Path

Viterbi Path

Posterior @ 0.4

Posterior @ 0.4

Posterior Graphic. Threshold Marked @ 0.4

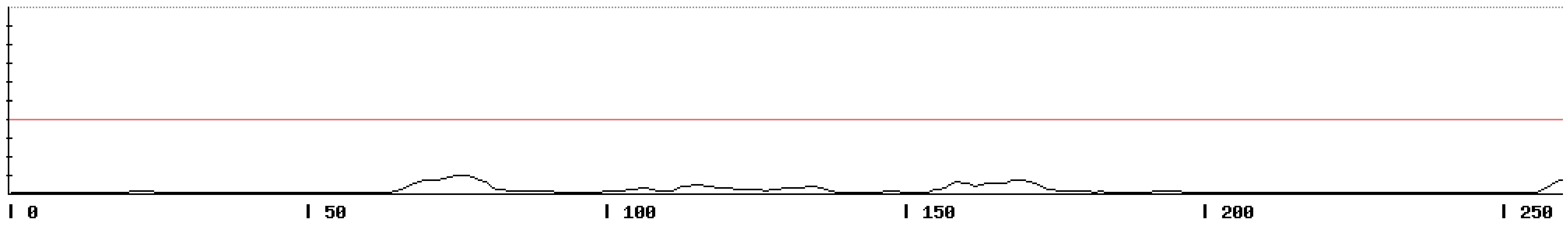

Predictions for Azadirachtaindica_Neem_32792_f_2

Viterbi Path

Viterbi Path

Posterior @ 0.4

Posterior @ 0.4

Posterior Graphic. Threshold Marked @ 0.4

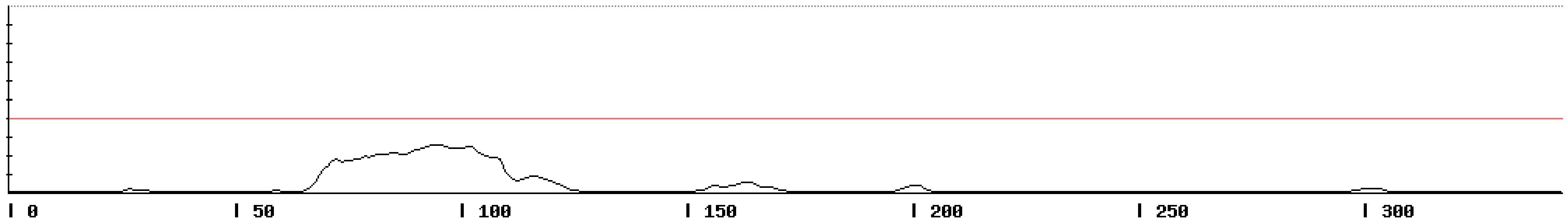

Predictions for Azadirachtaindica_Neem_327_f_1

Viterbi Path

Viterbi Path

Posterior @ 0.4

Posterior @ 0.4

Posterior Graphic. Threshold Marked @ 0.4

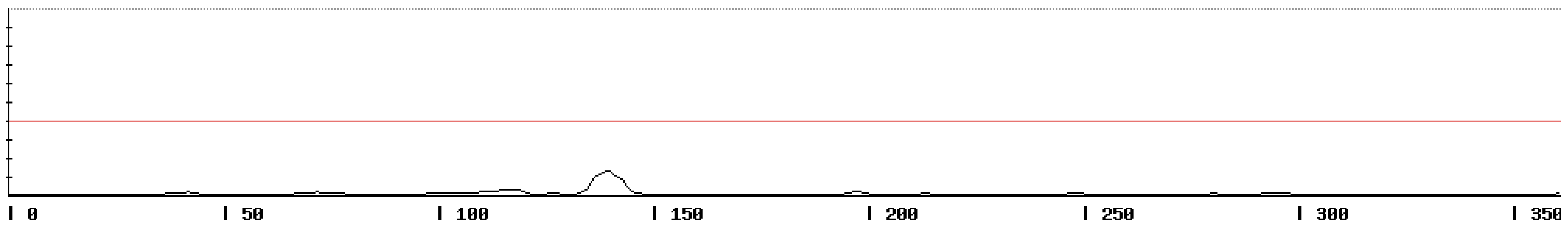

Predictions for Azadirachtaindica_Neem_3287_a_1

Viterbi Path

Viterbi Path

Posterior @ 0.4

154 - KERERTAKREGR - 165

Posterior @ 0.4

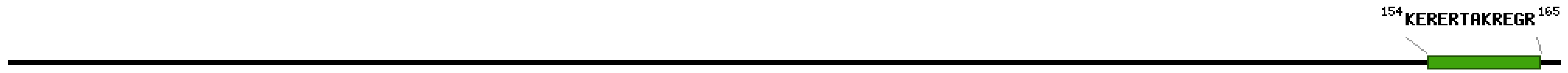

Posterior Graphic. Threshold Marked @ 0.4

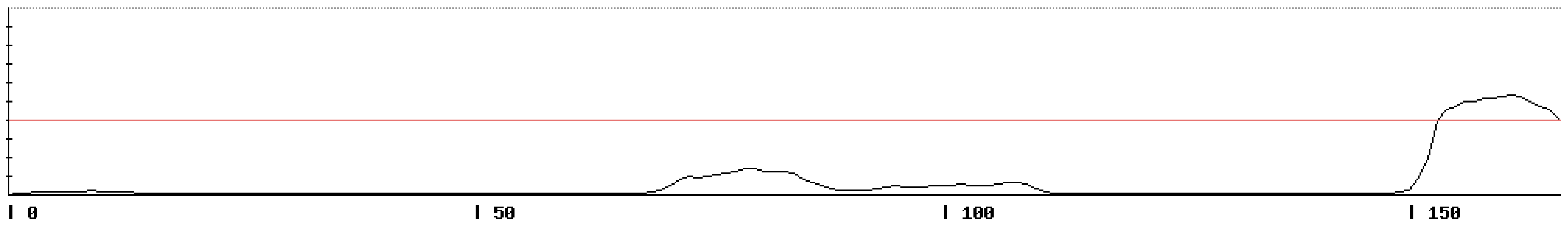

Predictions for Azadirachtaindica_Neem_32940_f_1

Viterbi Path

Viterbi Path

Posterior @ 0.4	
Posterior @ 0.4	
Posterior Graphic. Threshold Marked @ 0.4	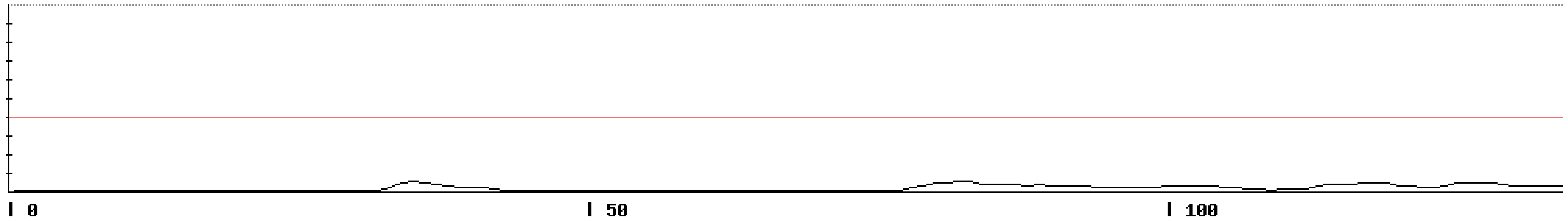

Predictions for Azadirachtaindica_Neem_33457_f_1	
Viterbi Path	
Viterbi Path	
Posterior @ 0.4	
Posterior @ 0.4	
Posterior Graphic. Threshold Marked @ 0.4	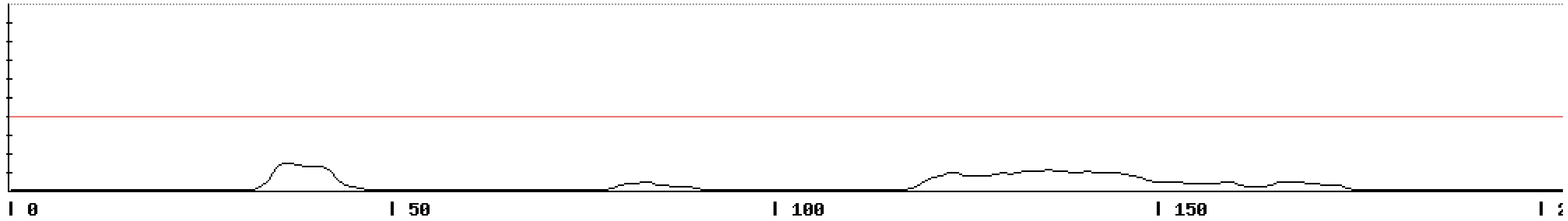

Predictions for Azadirachtaindica_Neem_33765_f_1	
Viterbi Path	
Viterbi Path	

Posterior @ 0.4

Posterior @ 0.4

Posterior Graphic. Threshold Marked @ 0.4

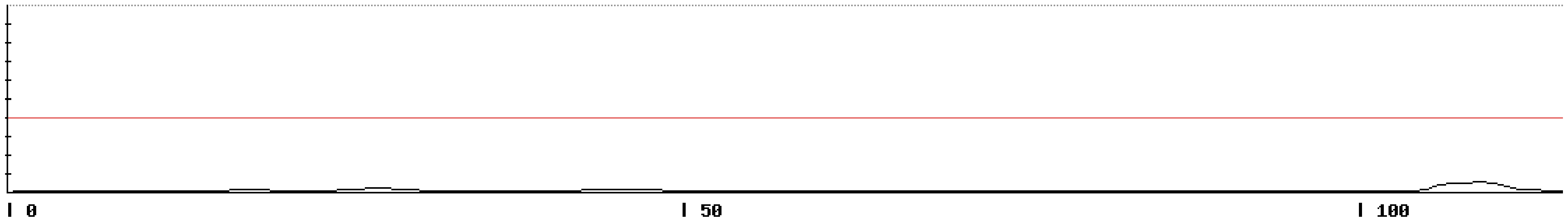

Predictions for Azadirachtaindica_Neem_34010_f_1

Viterbi Path

Viterbi Path

Posterior @ 0.4

Posterior @ 0.4

Posterior Graphic. Threshold Marked @ 0.4

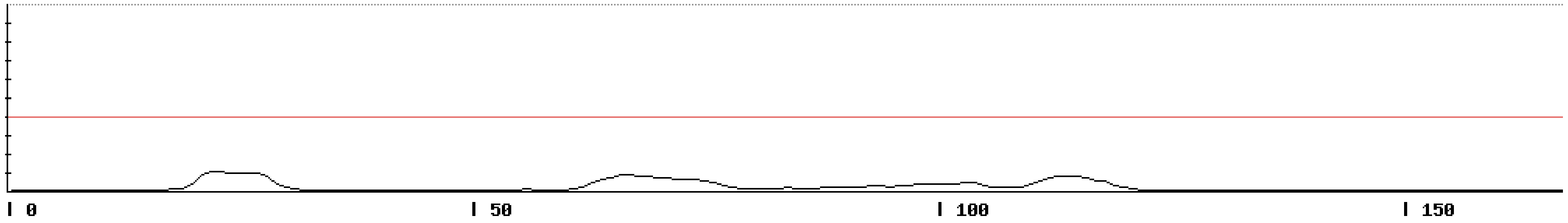

Predictions for Azadirachtaindica_Neem_34120_f_1

Viterbi Path

Viterbi Path

Posterior @ 0.4

Posterior @ 0.4

Posterior Graphic. Threshold Marked @ 0.4

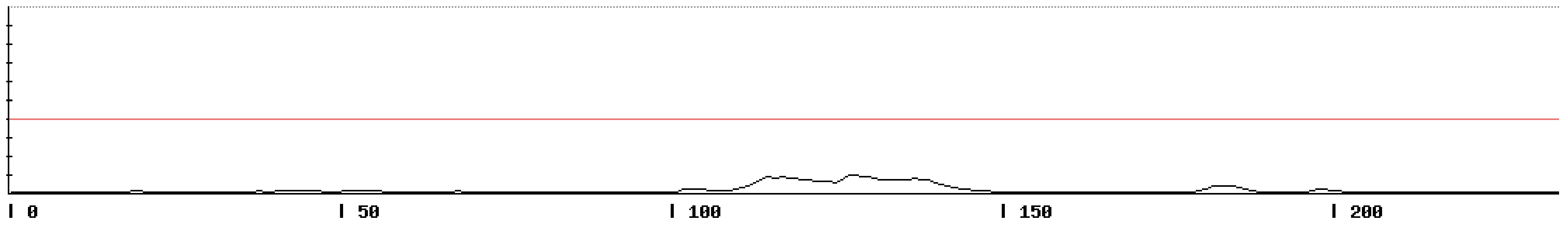

Predictions for Azadirachtaindica_Neem_34330_f_2

Viterbi Path 76 - PRSKGENGKPRPKGKNGKLFHRKTTKGCWKINGKGSKVNRIGRKR - 120

Viterbi Path

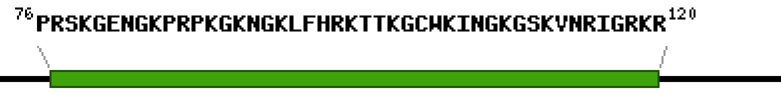

Posterior @ 0.4 76 - PRSKGENGKPRPKGKNGKLFHRKTTKGCWKINGKGSKVNRIGRKRTLSFR - 125

Posterior @ 0.4

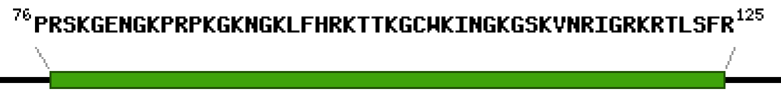

Posterior Graphic. Threshold Marked @ 0.4

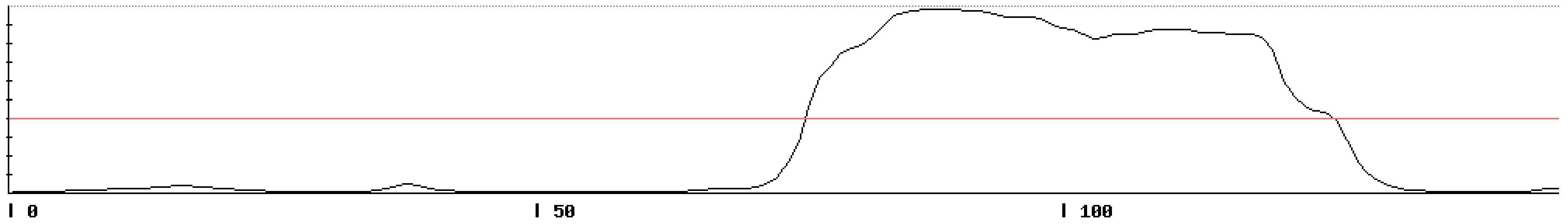

Predictions for Azadirachtaindica_Neem_34778_f_1

Viterbi Path	
Viterbi Path	
Posterior @ 0.4	
Posterior @ 0.4	
Posterior Graphic. Threshold Marked @ 0.4	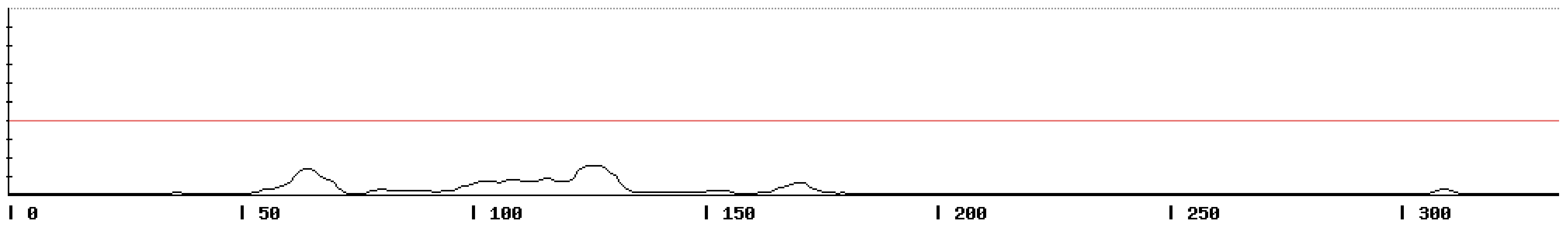

Predictions for Azadirachtaindica_Neem_34841_f_1

Viterbi Path	
Viterbi Path	
Posterior @ 0.4	137 - KSHKRKGNRK - 146
Posterior @ 0.4	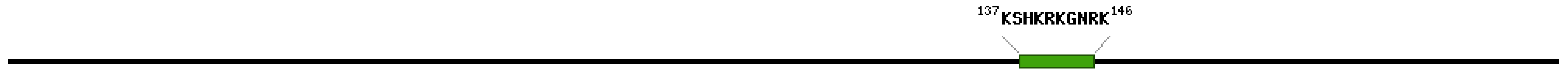
Posterior Graphic. Threshold Marked @ 0.4	

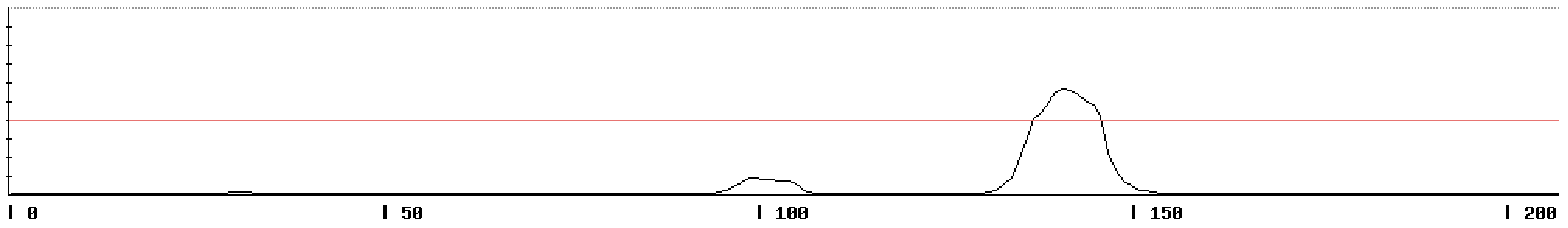

Predictions for Azadirachtaindica_Neem_34950_f_1

Viterbi Path

Viterbi Path

Posterior @ 0.4

Posterior @ 0.4

Posterior Graphic. Threshold Marked @ 0.4

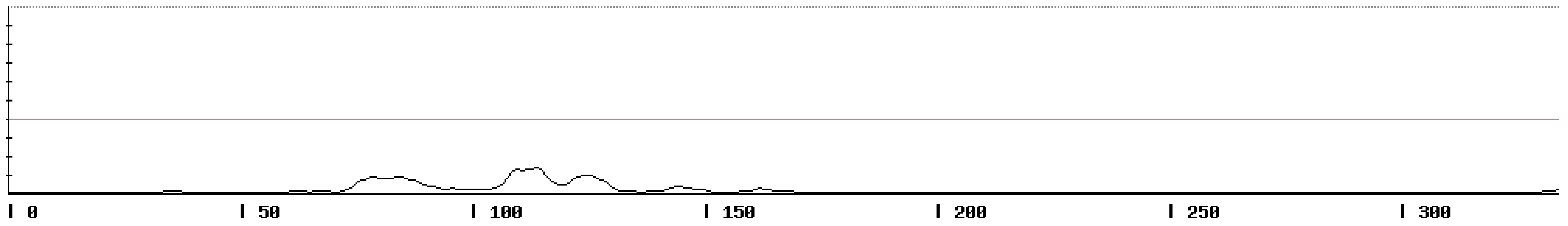

Predictions for Azadirachtaindica_Neem_35390_f_1

Viterbi Path

Viterbi Path

Posterior @ 0.4

Posterior @ 0.4

Posterior Graphic. Threshold Marked @ 0.4

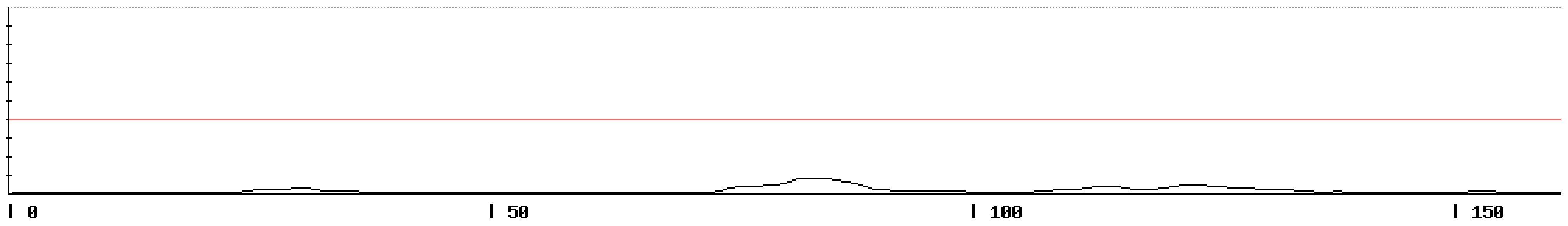

Predictions for Azadirachtaindica_Neem_35472_f_1

Viterbi Path

Viterbi Path

Posterior @ 0.4

Posterior @ 0.4

Posterior Graphic. Threshold Marked @ 0.4

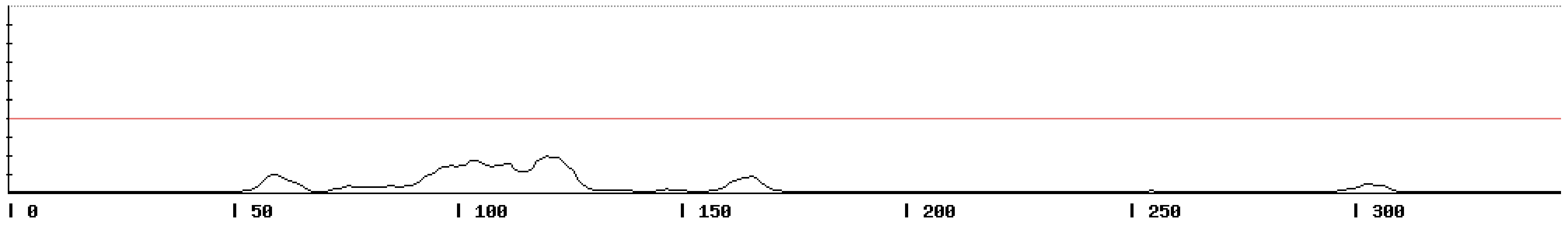

Predictions for Azadirachtaindica_Neem_35720_f_1

Viterbi Path

Viterbi Path

Posterior @ 0.4

Posterior @ 0.4

Posterior Graphic. Threshold Marked @ 0.4

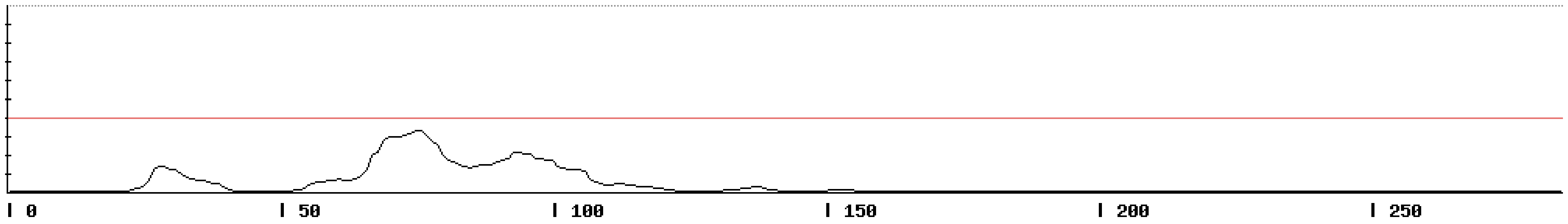

Predictions for Azadirachtaindica_Neem_3589_f_8

Viterbi Path

Viterbi Path

Posterior @ 0.4

Posterior @ 0.4

Posterior Graphic. Threshold Marked @ 0.4

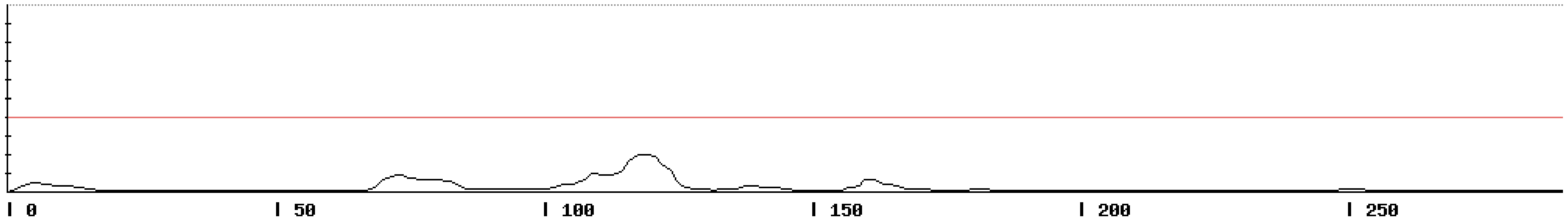

Predictions for Azadirachtaindica_Neem_36116_a_1

Viterbi Path

Viterbi Path

Posterior @ 0.4

Posterior @ 0.4

Posterior Graphic. Threshold Marked @ 0.4

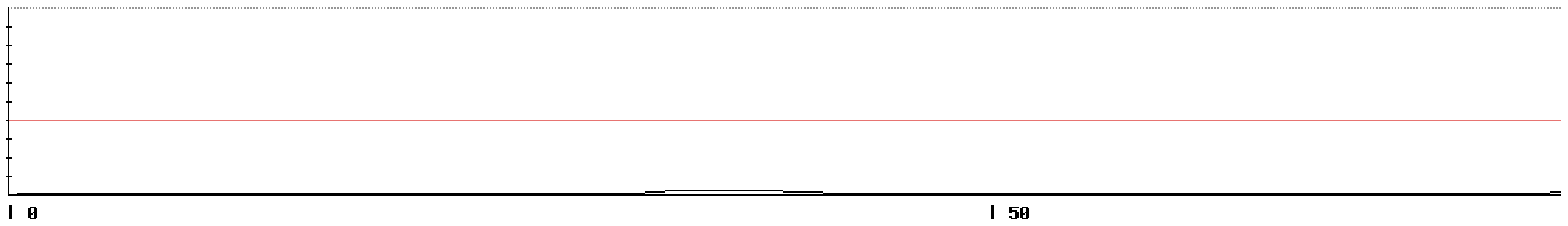

Predictions for Azadirachtaindica_Neem_3629_f_1

Viterbi Path

Viterbi Path

Posterior @ 0.4

Posterior @ 0.4

Posterior Graphic. Threshold Marked @ 0.4

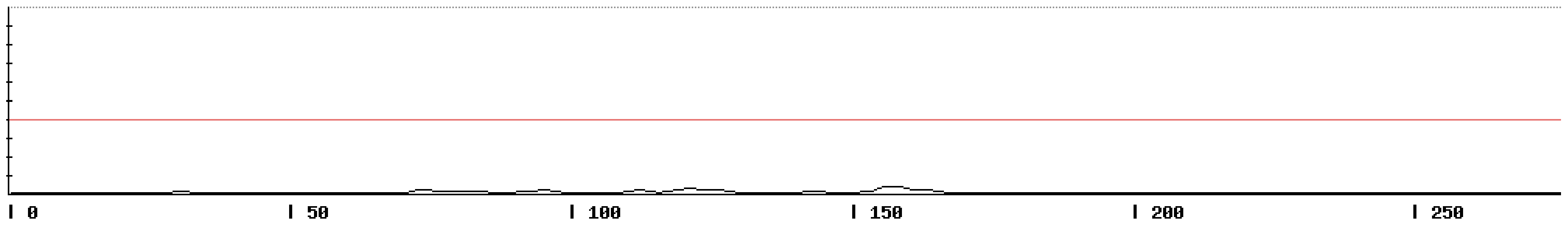

Predictions for Azadirachtaindica_Neem_36363_f_1

Viterbi Path

Viterbi Path

Posterior @ 0.4

Posterior @ 0.4

Posterior Graphic. Threshold Marked @ 0.4

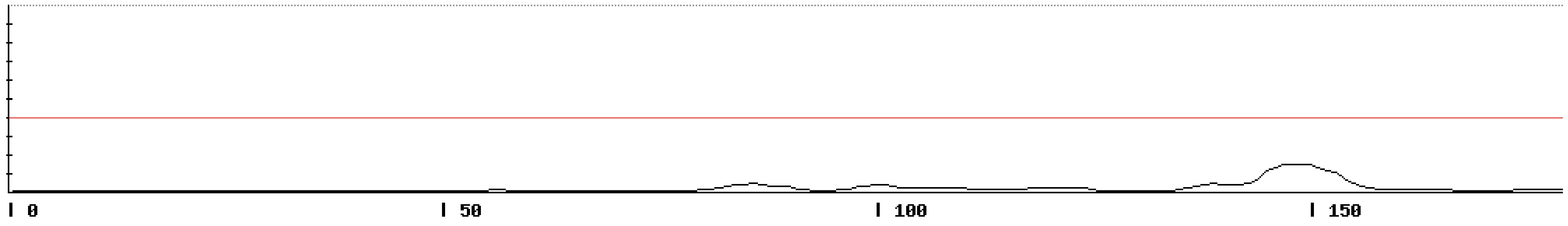

Predictions for Azadirachtaindica_Neem_37635_f_3

Viterbi Path

Viterbi Path

Posterior @ 0.4

Posterior @ 0.4

Posterior Graphic. Threshold Marked @ 0.4

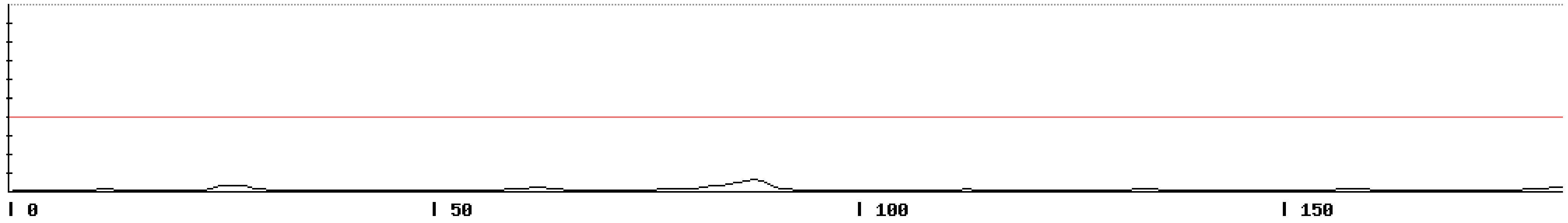

Predictions for Azadirachtaindica_Neem_38603_f_1

Viterbi Path

Viterbi Path

Posterior @ 0.4

Posterior @ 0.4

Posterior Graphic. Threshold Marked @ 0.4

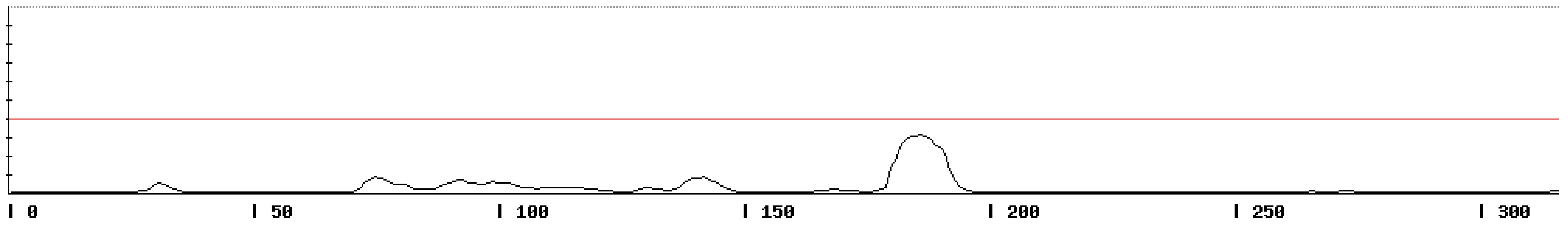

Predictions for Azadirachtaindica_Neem_39437_f_1

Viterbi Path

Viterbi Path

Posterior @ 0.4

Posterior @ 0.4

Posterior Graphic. Threshold Marked @ 0.4

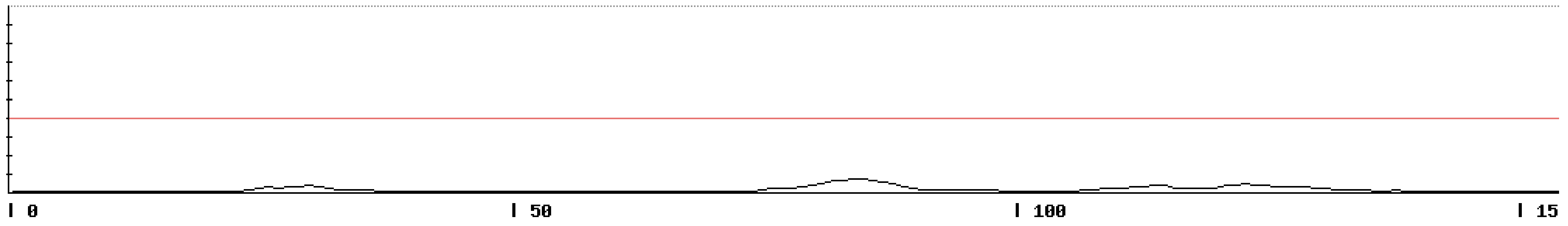

Predictions for Azadirachtaindica_Neem_39493_f_2	
Viterbi Path	
Viterbi Path	
Posterior @ 0.4	85 - GYWKVTGKPRRIRDKRGKKEIGYKR - 109
Posterior @ 0.4	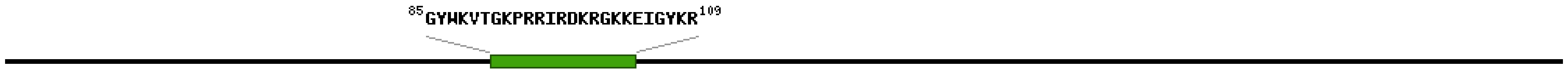
Posterior Graphic. Threshold Marked @ 0.4	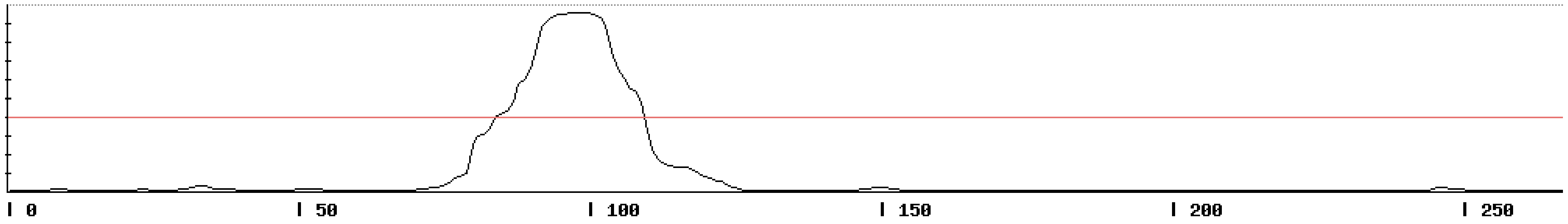

Predictions for Azadirachtaindica_Neem_3950_f_1	
Viterbi Path	
Viterbi Path	
Posterior @ 0.4	
Posterior @ 0.4	
Posterior Graphic. Threshold Marked @ 0.4	

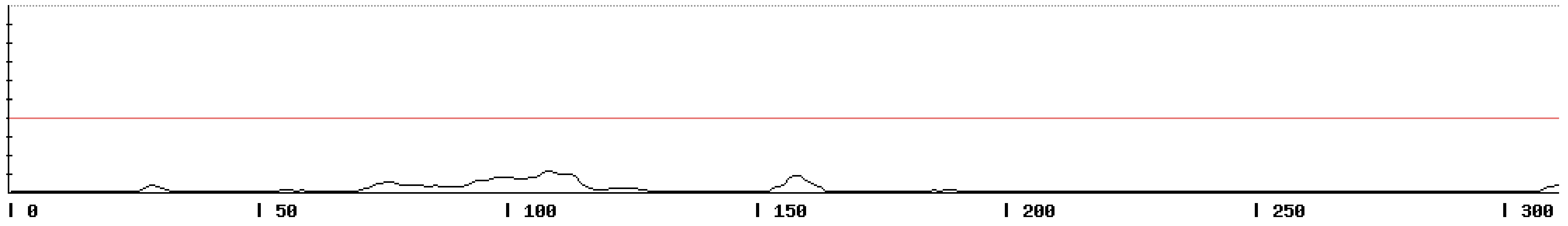

Predictions for Azadirachtaindica_Neem_39610_f_1

Viterbi Path

Viterbi Path

Posterior @ 0.4

Posterior @ 0.4

Posterior Graphic. Threshold Marked @ 0.4

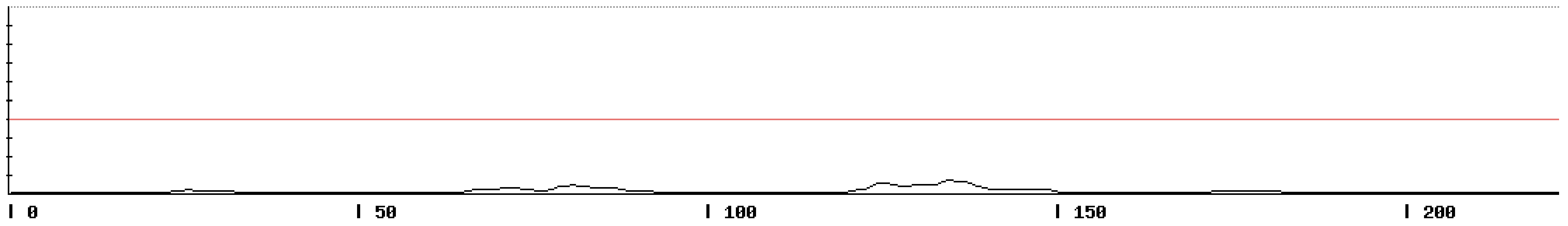

Predictions for Azadirachtaindica_Neem_39628_f_1

Viterbi Path

Viterbi Path

Posterior @ 0.4

Posterior @ 0.4

Posterior Graphic. Threshold Marked @ 0.4

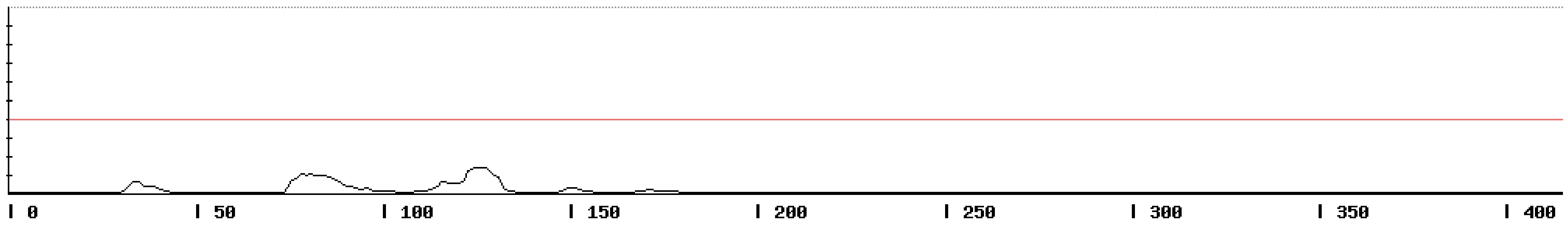

Predictions for Azadirachtaindica_Neem_39666_f_1

Viterbi Path

Viterbi Path

Posterior @ 0.4

4 - RRSKKRL - 10

17 - SLAKVTKKRRKSNRKDRNRIG - 37

352 - RRIKLRKQRPGL - 363

Posterior @ 0.4

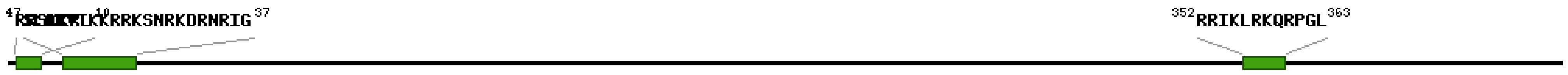

Posterior Graphic. Threshold Marked @ 0.4

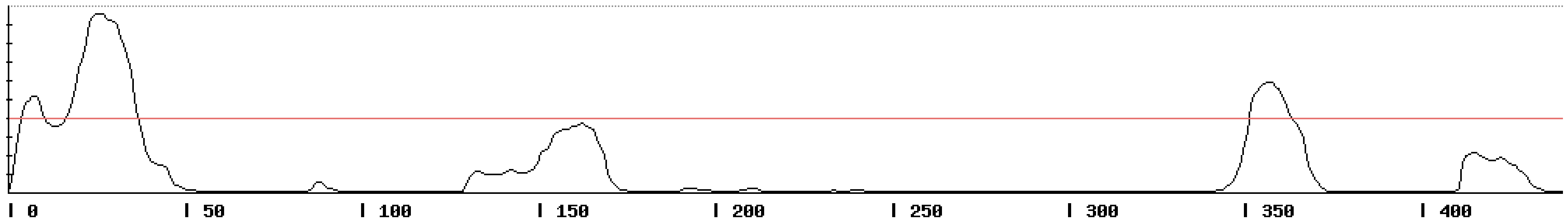

Predictions for Azadirachtaindica_Neem_409_f_2

Viterbi Path

Viterbi Path

Posterior @ 0.4

Posterior @ 0.4

Posterior Graphic. Threshold Marked @ 0.4

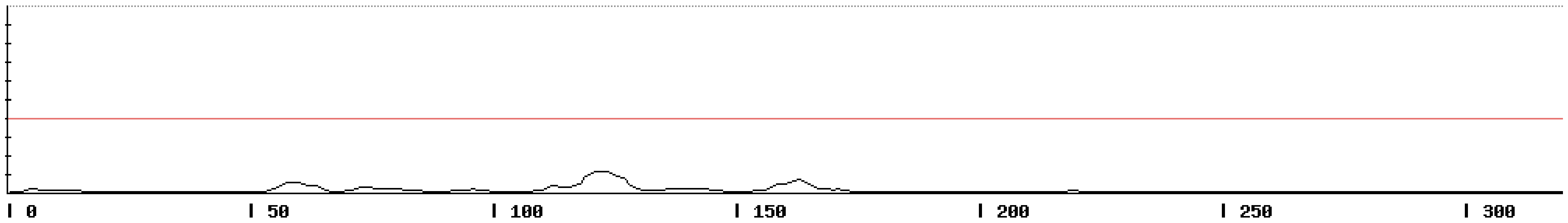

Predictions for Azadirachtaindica_Neem_4377_a_2

Viterbi Path

Viterbi Path

Posterior @ 0.4

Posterior @ 0.4

Posterior Graphic. Threshold Marked @ 0.4

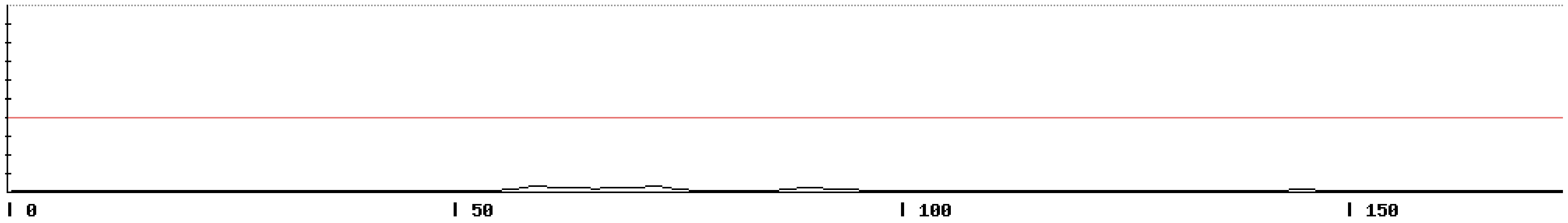

Predictions for Azadirachtaindica_Neem_4530_f_1

Viterbi Path

Viterbi Path

Posterior @ 0.4

Posterior @ 0.4

Posterior Graphic. Threshold Marked @ 0.4

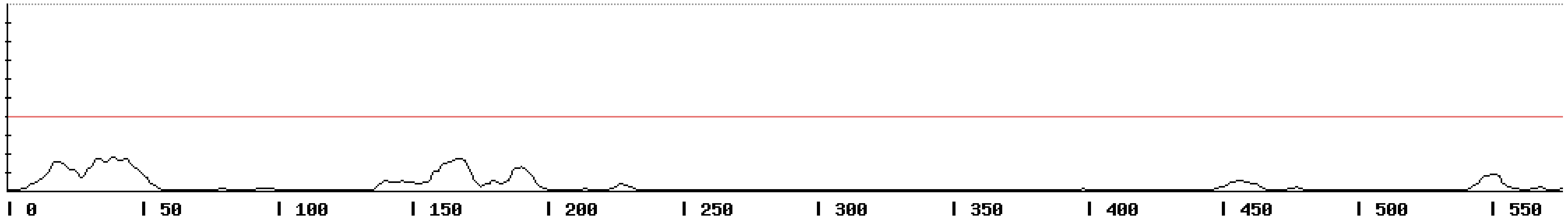

Predictions for Azadirachtaindica_Neem_4815_f_1

Viterbi Path

Viterbi Path

Posterior @ 0.4

Posterior @ 0.4

Posterior Graphic. Threshold Marked @ 0.4

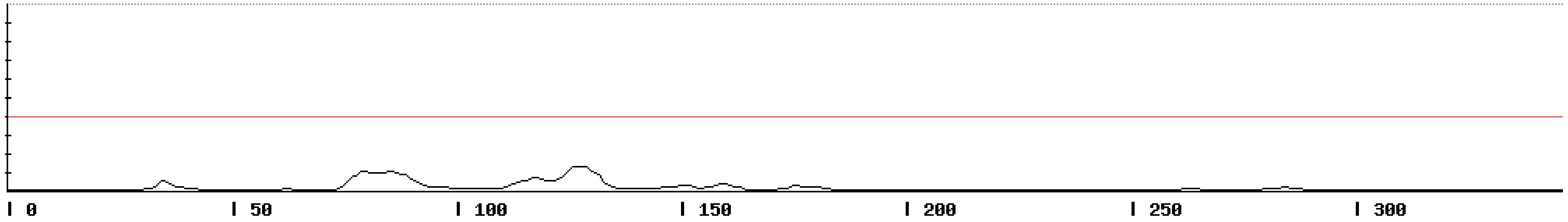

Predictions for Azadirachtaindica_Neem_4983_f_1

Viterbi Path

Viterbi Path

Posterior @ 0.4	
Posterior @ 0.4	
Posterior Graphic. Threshold Marked @ 0.4	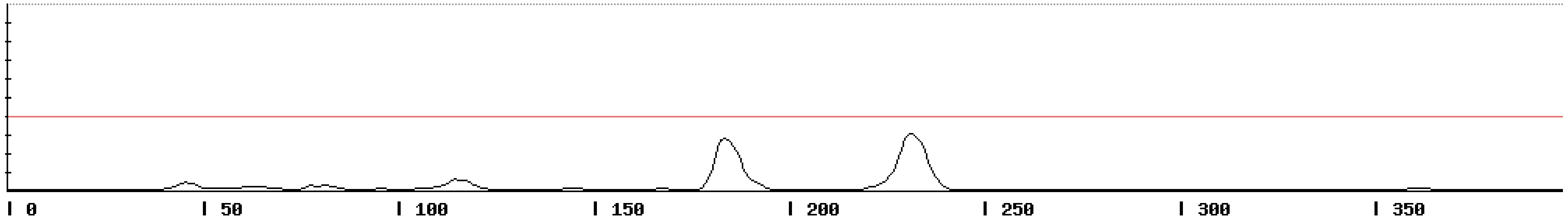

Predictions for Azadirachtaindica_Neem_517_f_8

Viterbi Path	
Viterbi Path	
Posterior @ 0.4	169 - QRKRKPKKHRA - 179
Posterior @ 0.4	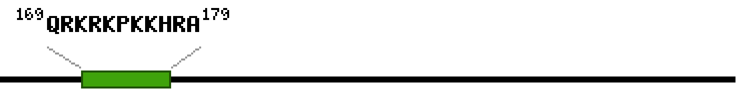

Posterior Graphic. Threshold Marked @ 0.4	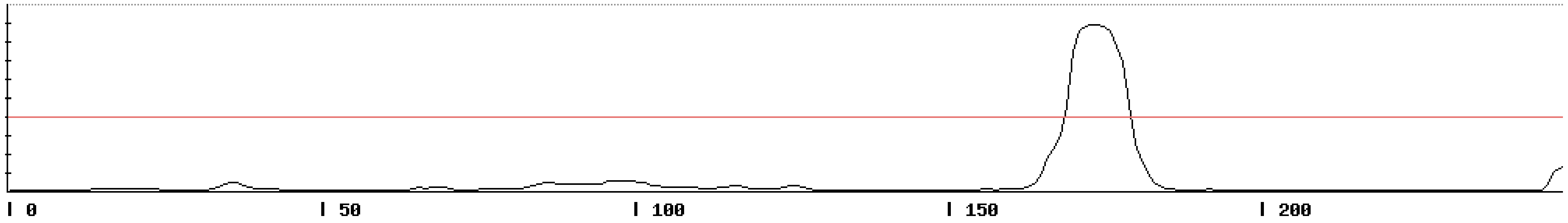

Predictions for Azadirachtaindica_Neem_5254_f_1

Viterbi Path

Viterbi Path

Posterior @ 0.4

Posterior @ 0.4

Posterior Graphic. Threshold Marked @ 0.4

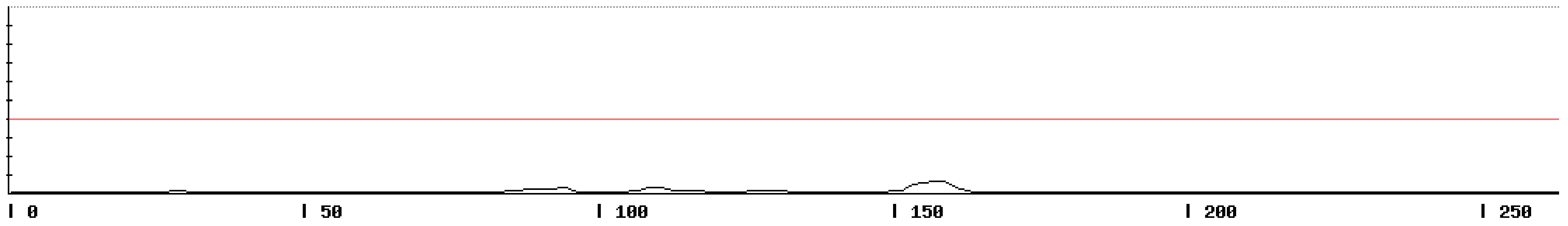

Predictions for Azadirachtaindica_Neem_530_f_5

Viterbi Path

Viterbi Path

Posterior @ 0.4

90 - RKRRK - 94

Posterior @ 0.4

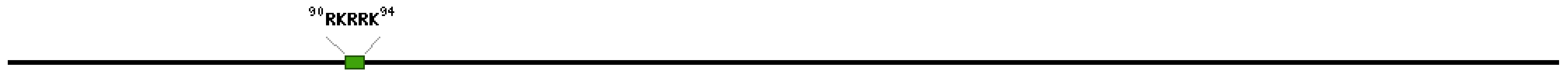

Posterior Graphic. Threshold Marked @ 0.4

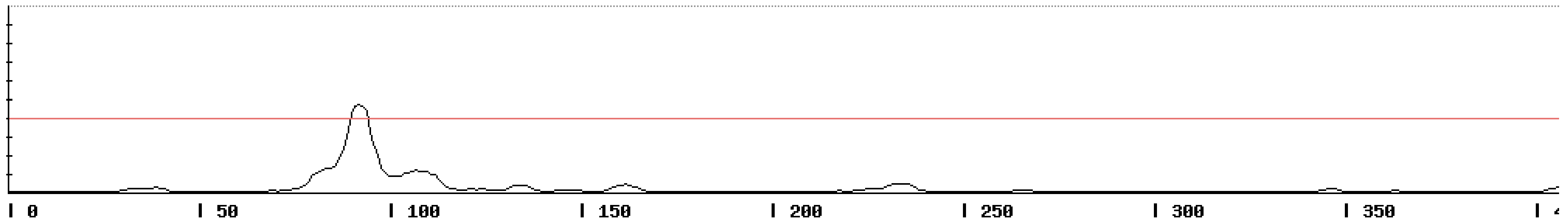

Predictions for Azadirachtaindica_Neem_5527_a_2

Viterbi Path

Viterbi Path

Posterior @ 0.4

Posterior @ 0.4

Posterior Graphic. Threshold Marked @ 0.4

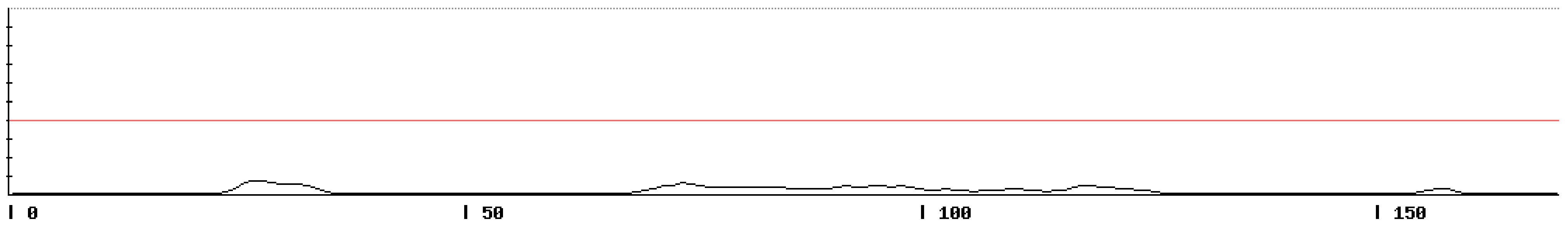

Predictions for Azadirachtaindica_Neem_5527_f_1

Viterbi Path

Viterbi Path

Posterior @ 0.4

Posterior @ 0.4

Posterior Graphic. Threshold Marked @ 0.4

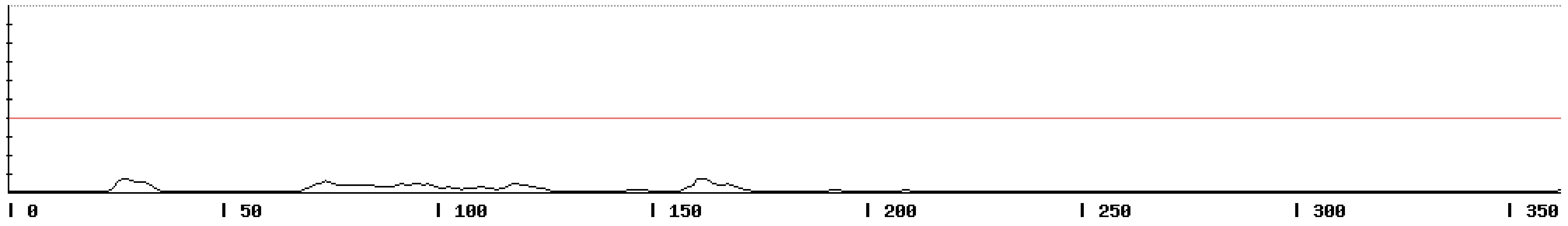

Predictions for Azadirachtaindica_Neem_5630_f_1

Viterbi Path

Viterbi Path

Posterior @ 0.4

Posterior @ 0.4

Posterior Graphic. Threshold Marked @ 0.4

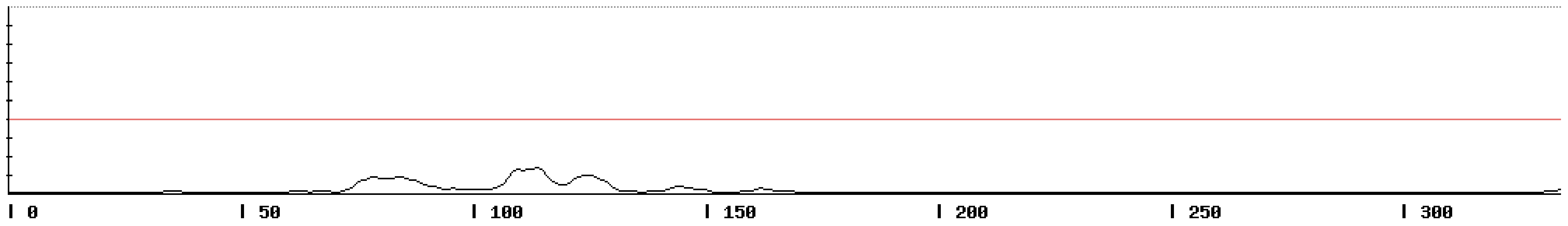

Predictions for Azadirachtaindica_Neem_5679_f_1

Viterbi Path

Viterbi Path

Posterior @ 0.4

Posterior @ 0.4

Posterior Graphic. Threshold Marked @ 0.4

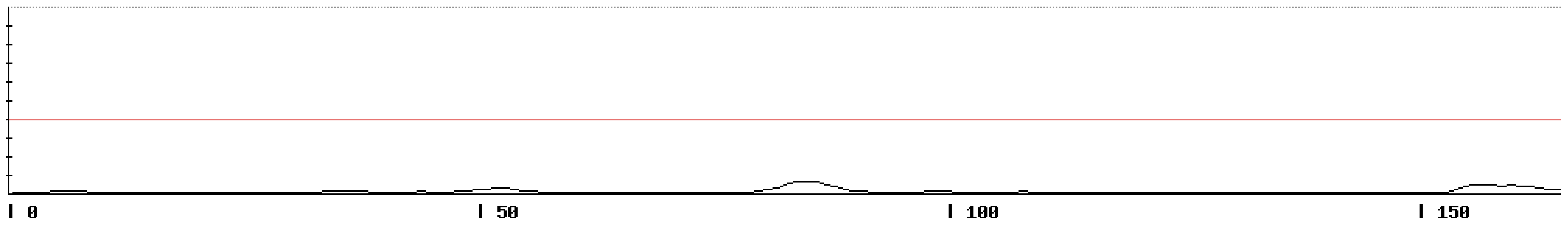

Predictions for Azadirachtaindica_Neem_5801_f_1

Viterbi Path

Viterbi Path

Posterior @ 0.4

Posterior @ 0.4

Posterior Graphic. Threshold Marked @ 0.4

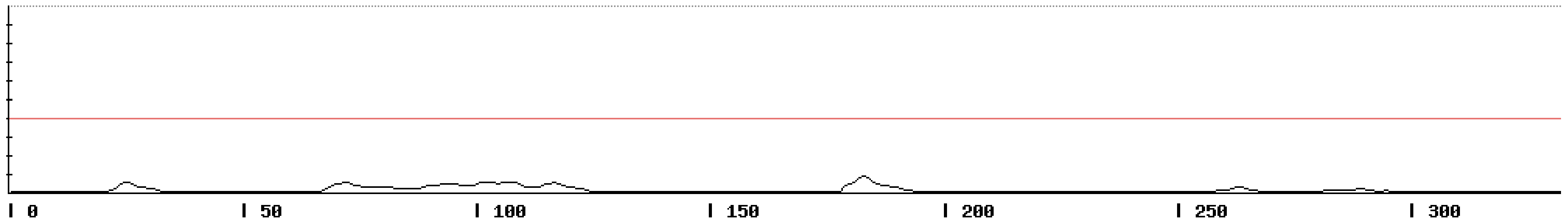

Predictions for Azadirachtaindica_Neem_585_f_6

Viterbi Path

Viterbi Path

Posterior @ 0.4

Posterior @ 0.4

Posterior Graphic. Threshold Marked @ 0.4

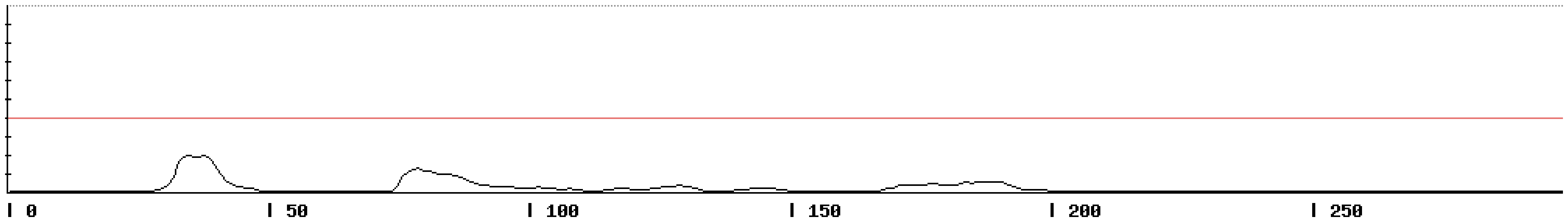

Predictions for Azadirachtaindica_Neem_5959_f_1

Viterbi Path

Viterbi Path

Posterior @ 0.4

Posterior @ 0.4

Posterior Graphic. Threshold Marked @ 0.4

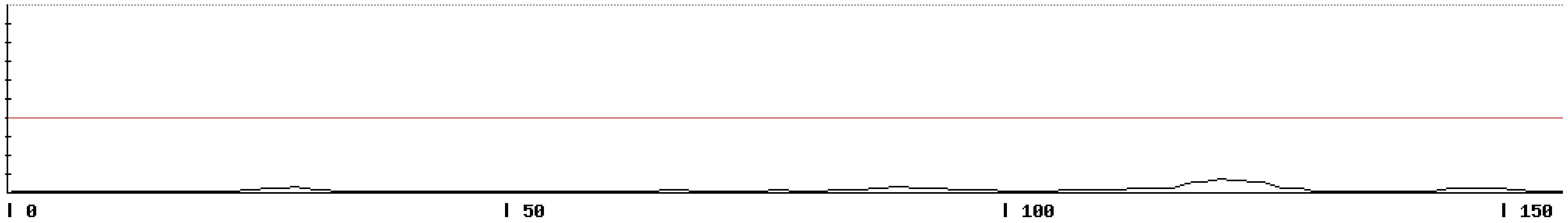

Predictions for Azadirachtaindica_Neem_6031_f_1

Viterbi Path

Viterbi Path

Posterior @ 0.4

Posterior @ 0.4

Posterior Graphic. Threshold Marked @ 0.4

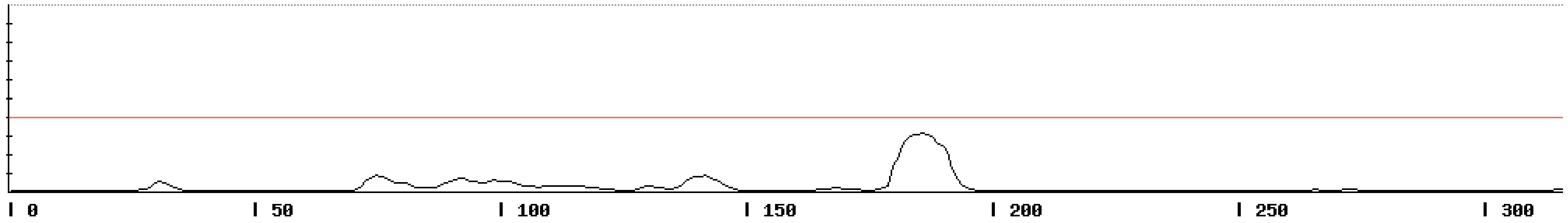

Predictions for Azadirachtaindica_Neem_6040_a_4

Viterbi Path

Viterbi Path

Posterior @ 0.4

4 - RRSKKRL - 10

17 - SLAKVTKKRRKSNRKDRNRIG - 37

Posterior @ 0.4

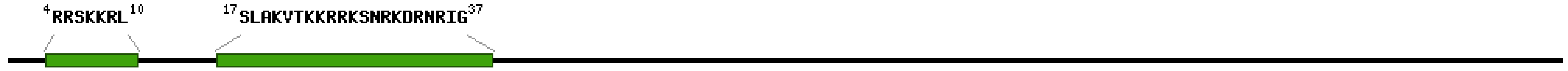

Posterior Graphic. Threshold Marked @ 0.4

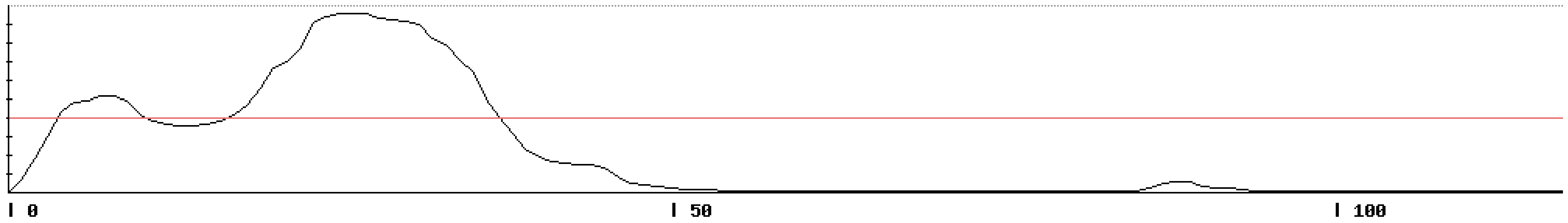

Predictions for Azadirachtaindica_Neem_6403_f_1

Viterbi Path

Viterbi Path

Posterior @ 0.4	81 - KK - 82
Posterior @ 0.4	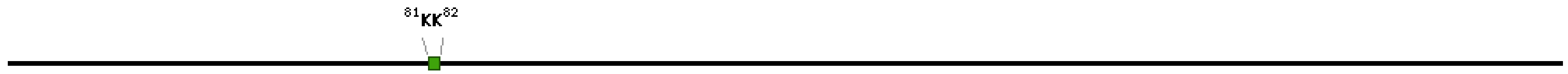
Posterior Graphic. Threshold Marked @ 0.4	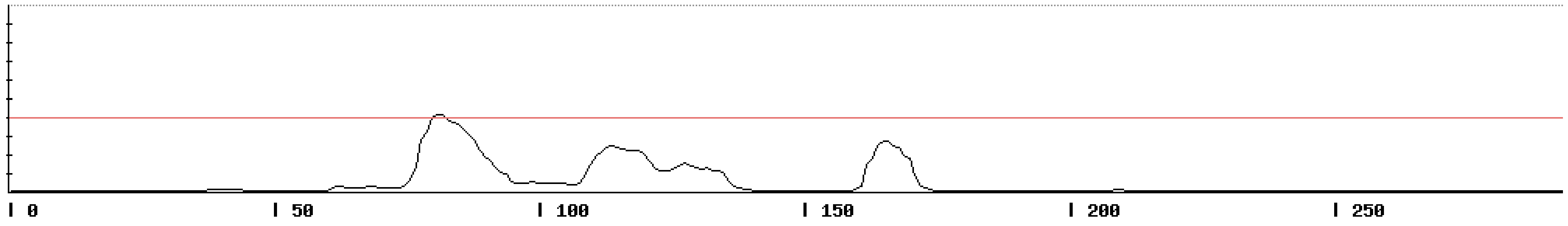

Predictions for Azadirachtaindica_Neem_6492_f_1

Viterbi Path	
Viterbi Path	
Posterior @ 0.4	
Posterior @ 0.4	
Posterior Graphic. Threshold Marked @ 0.4	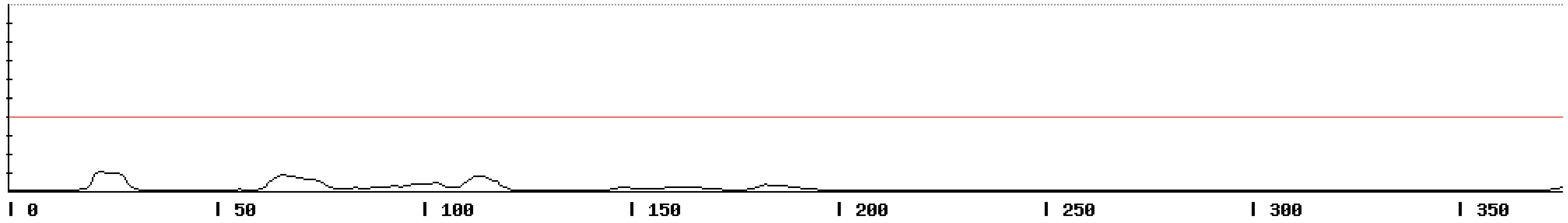

Predictions for Azadirachtaindica_Neem_6716_f_1

Viterbi Path

Viterbi Path

Posterior @ 0.4

Posterior @ 0.4

Posterior Graphic. Threshold Marked @ 0.4

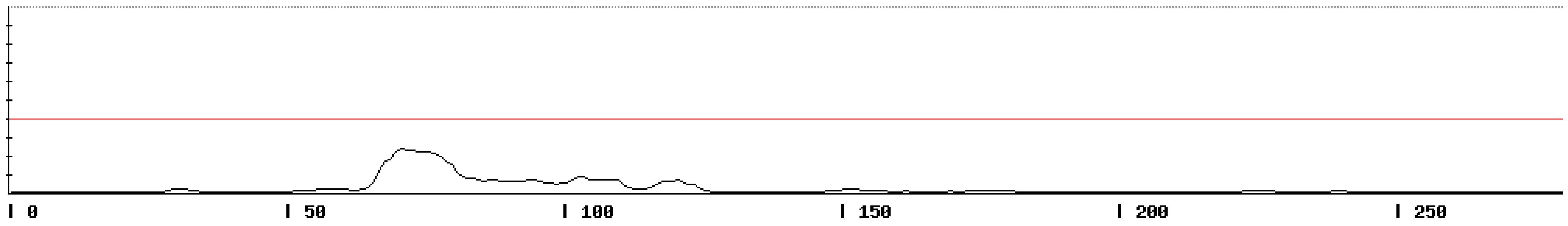

Predictions for Azadirachtaindica_Neem_6790_f_1

Viterbi Path

Viterbi Path

Posterior @ 0.4

Posterior @ 0.4

Posterior Graphic. Threshold Marked @ 0.4

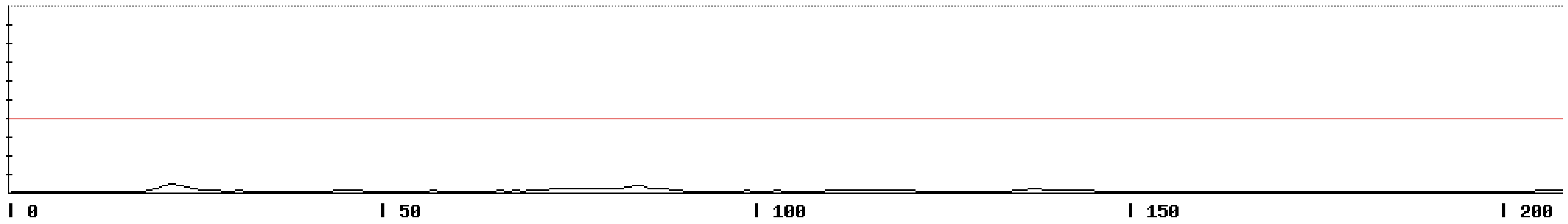

Predictions for Azadirachtaindica_Neem_7002_f_4	
Viterbi Path	
Viterbi Path	
Posterior @ 0.4	
Posterior @ 0.4	
Posterior Graphic. Threshold Marked @ 0.4	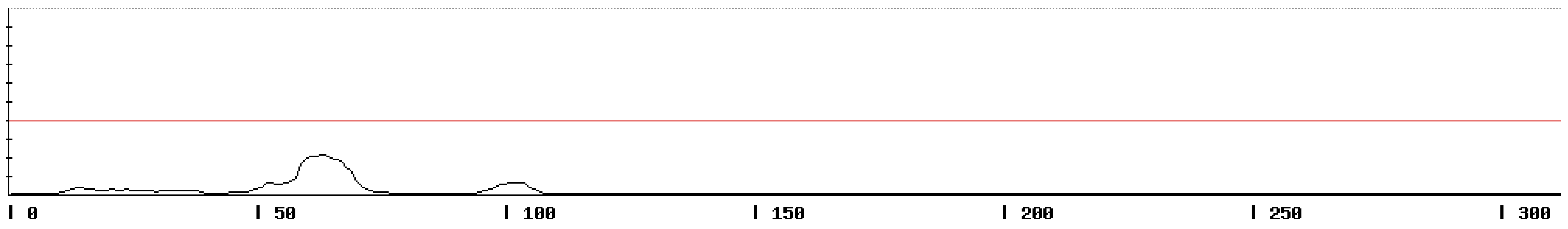

Predictions for Azadirachtaindica_Neem_7195_f_2	
Viterbi Path	
Viterbi Path	
Posterior @ 0.4	397 - KKAAVGRMKKLKRK - 410
Posterior @ 0.4	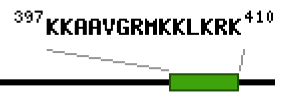
Posterior Graphic. Threshold Marked @ 0.4	

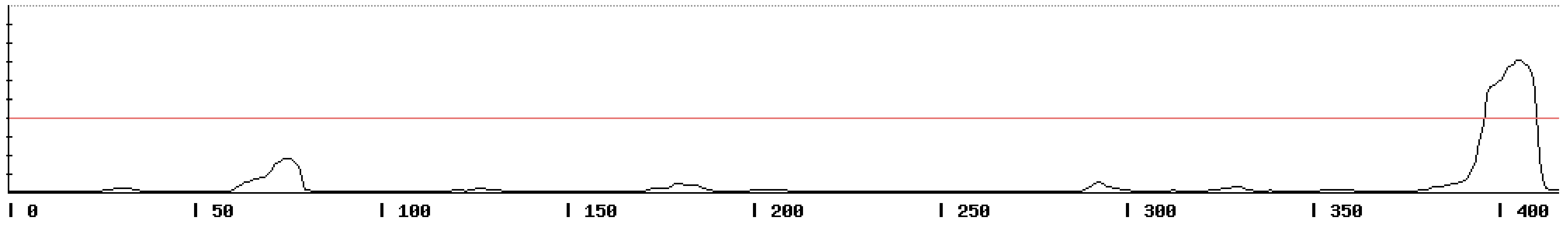

Predictions for Azadirachtaindica_Neem_7280_f_1

Viterbi Path

Viterbi Path

Posterior @ 0.4

Posterior @ 0.4

Posterior Graphic. Threshold Marked @ 0.4

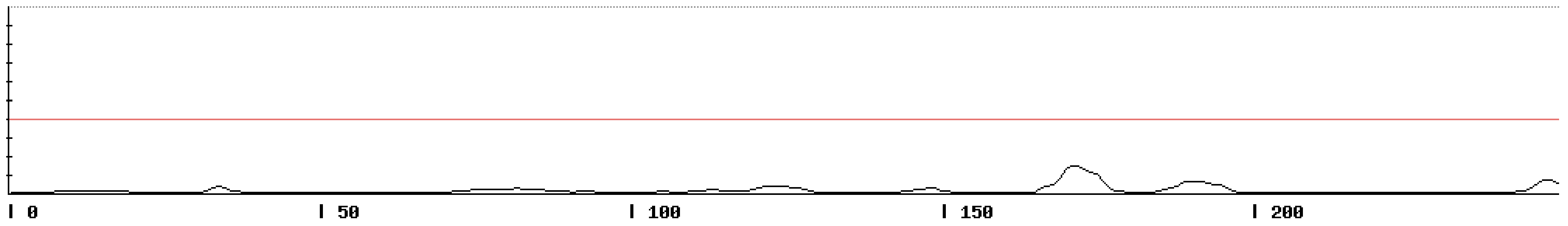

Predictions for Azadirachtaindica_Neem_7328_f_1

Viterbi Path

Viterbi Path

Posterior @ 0.4

Posterior @ 0.4

Posterior Graphic. Threshold Marked @ 0.4

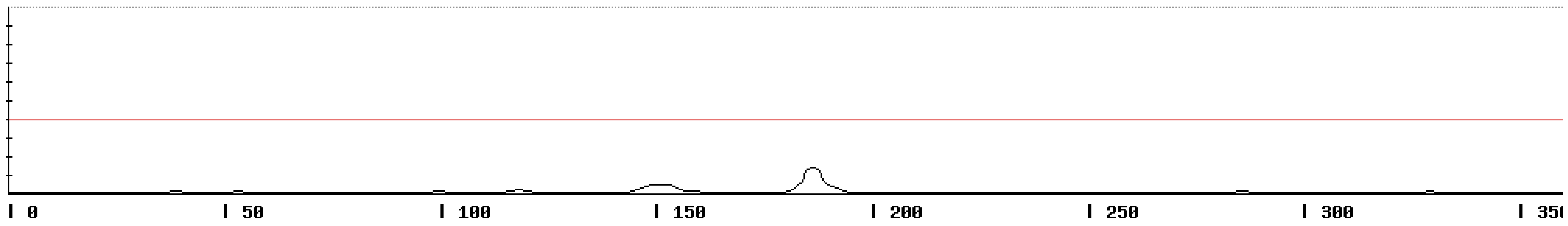

Predictions for Azadirachtaindica_Neem_7400_f_1

Viterbi Path	
Viterbi Path	
Posterior @ 0.4	92 - RRKK - 95
Posterior @ 0.4	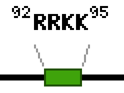
Posterior Graphic. Threshold Marked @ 0.4	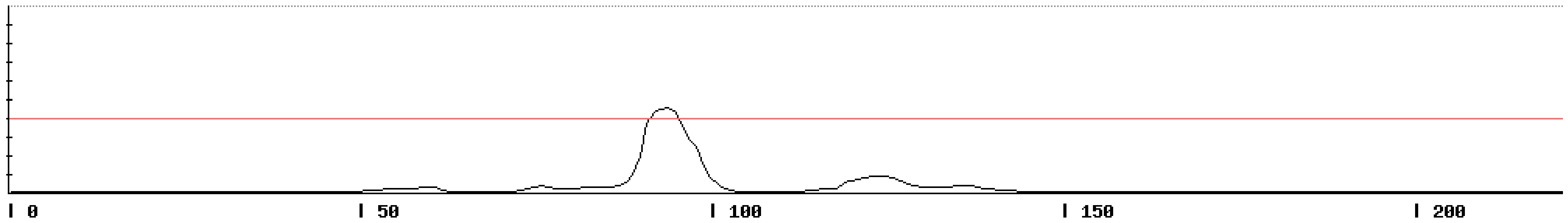

Predictions for Azadirachtaindica_Neem_7585_f_1

Viterbi Path	
Viterbi Path	
Posterior @ 0.4	
Posterior @ 0.4	

Posterior Graphic. Threshold Marked @ 0.4

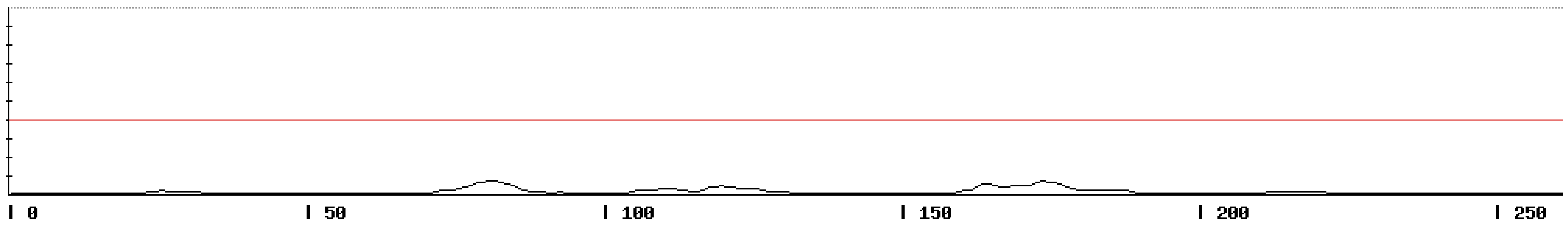

Predictions for Azadirachtaindica_Neem_7585_f_2

Viterbi Path

Viterbi Path

Posterior @ 0.4

Posterior @ 0.4

Posterior Graphic. Threshold Marked @ 0.4

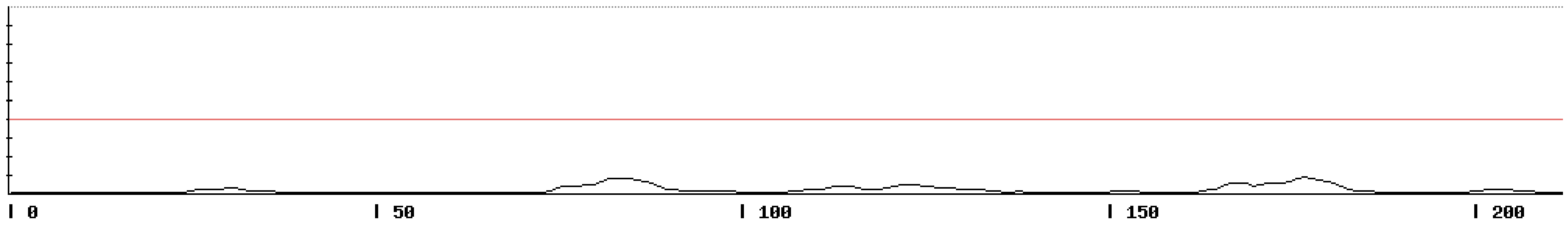

Predictions for Azadirachtaindica_Neem_7585_f_4

Viterbi Path

Viterbi Path

Posterior @ 0.4

Posterior @ 0.4

Posterior Graphic. Threshold Marked @ 0.4

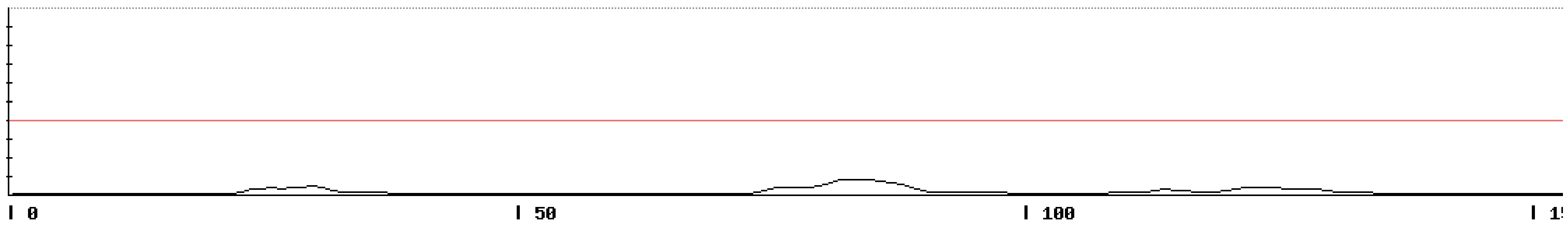

Predictions for Azadirachtaindica_Neem_7585_f_7

Viterbi Path

Viterbi Path

Posterior @ 0.4

Posterior @ 0.4

Posterior Graphic. Threshold Marked @ 0.4

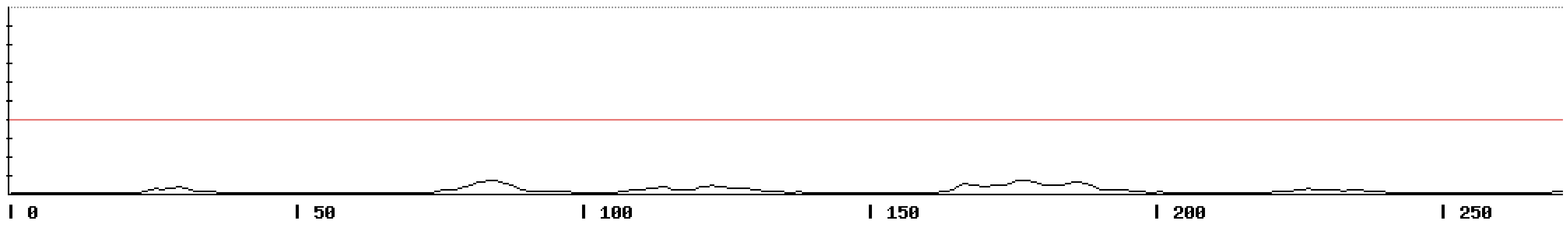

Predictions for Azadirachtaindica_Neem_7680_f_1

Viterbi Path

Viterbi Path

Posterior @ 0.4

Posterior @ 0.4

Posterior Graphic. Threshold Marked @ 0.4

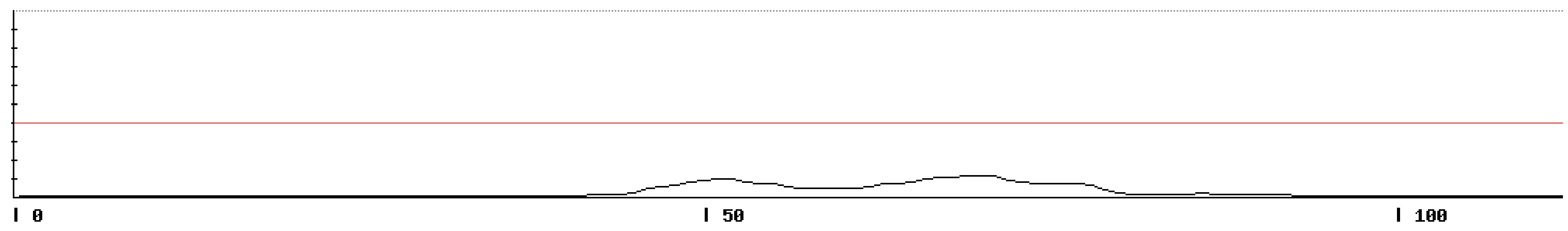

Predictions for Azadirachtaindica_Neem_7766_f_1

Viterbi Path

Viterbi Path

Posterior @ 0.4

Posterior @ 0.4

Posterior Graphic. Threshold Marked @ 0.4

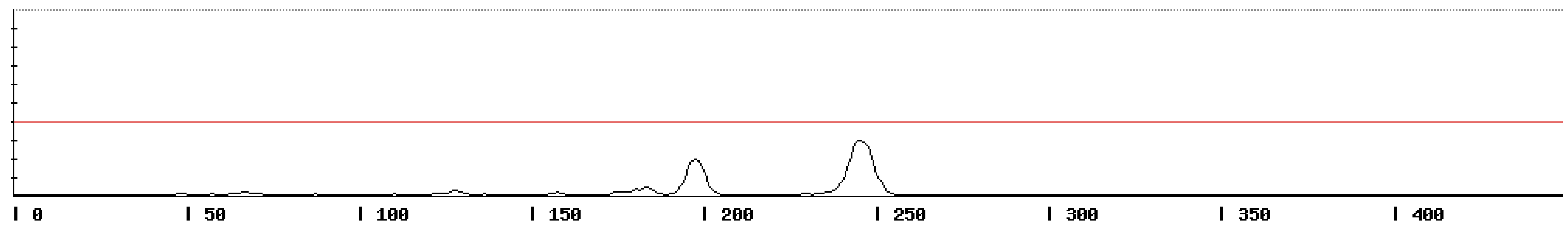

Predictions for Azadirachtaindica_Neem_8133_f_4

Viterbi Path	
Viterbi Path	
Posterior @ 0.4	
Posterior @ 0.4	
Posterior Graphic. Threshold Marked @ 0.4	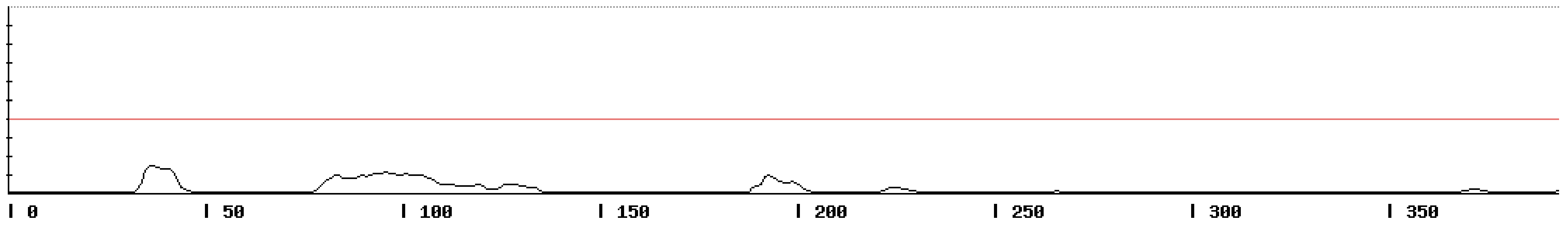

Predictions for Azadirachtaindica_Neem_8272_f_2

Viterbi Path	
Viterbi Path	
Posterior @ 0.4	
Posterior @ 0.4	
Posterior Graphic. Threshold Marked @ 0.4	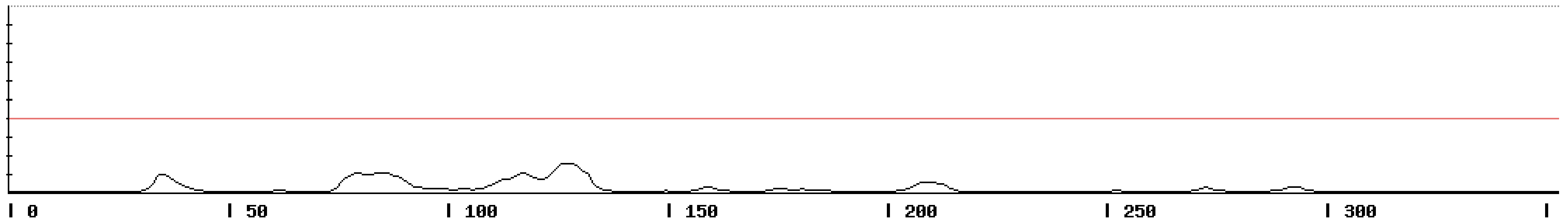

Predictions for Azadirachtaindica_Neem_8368_f_2	
Viterbi Path	
Viterbi Path	
Posterior @ 0.4	
Posterior @ 0.4	
Posterior Graphic. Threshold Marked @ 0.4	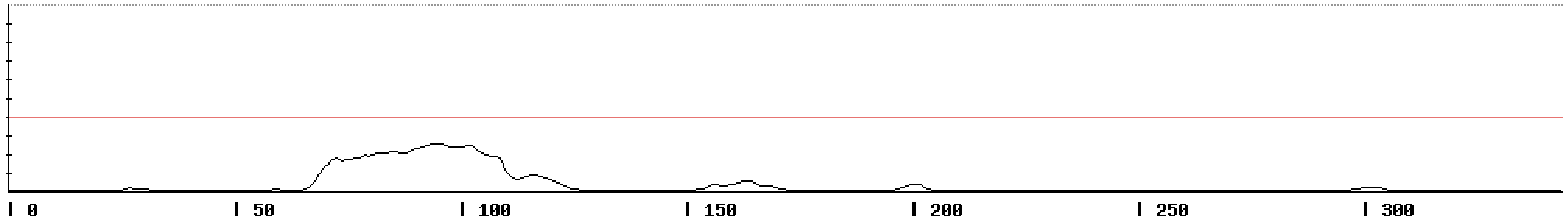

Predictions for Azadirachtaindica_Neem_8411_f_2	
Viterbi Path	
Viterbi Path	
Posterior @ 0.4	
Posterior @ 0.4	
Posterior Graphic. Threshold Marked @ 0.4	

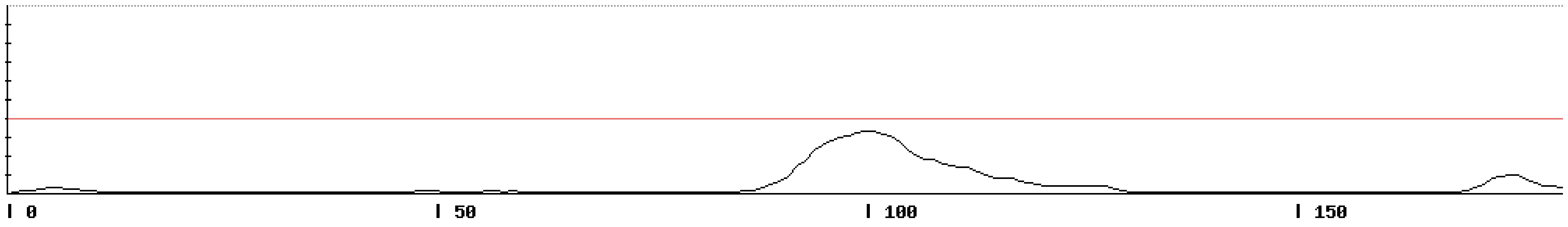

Predictions for Azadirachtaindica_Neem_8434_f_2

Viterbi Path	
Viterbi Path	
Posterior @ 0.4	174 - RKG - 176
Posterior @ 0.4	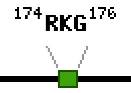
Posterior Graphic. Threshold Marked @ 0.4	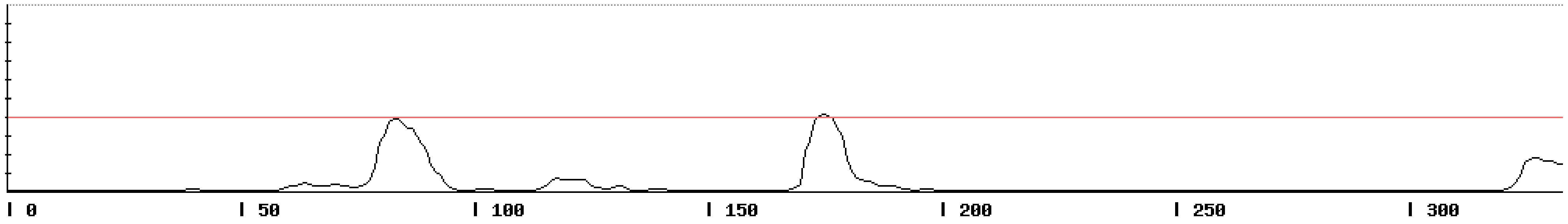

Predictions for Azadirachtaindica_Neem_8476_f_1

Viterbi Path	
Viterbi Path	
Posterior @ 0.4	
Posterior @ 0.4	

Posterior Graphic. Threshold Marked @ 0.4	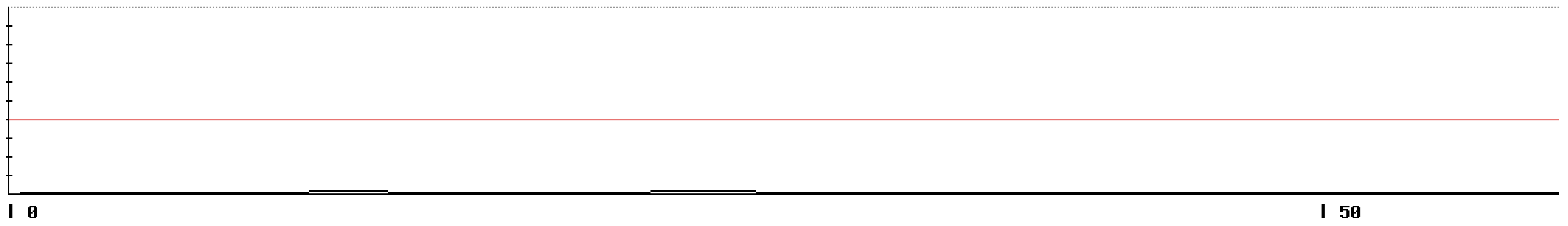

Predictions for Azadirachtaindica_Neem_8476_f_2	
Viterbi Path	
Viterbi Path	
Posterior @ 0.4	
Posterior @ 0.4	
Posterior Graphic. Threshold Marked @ 0.4	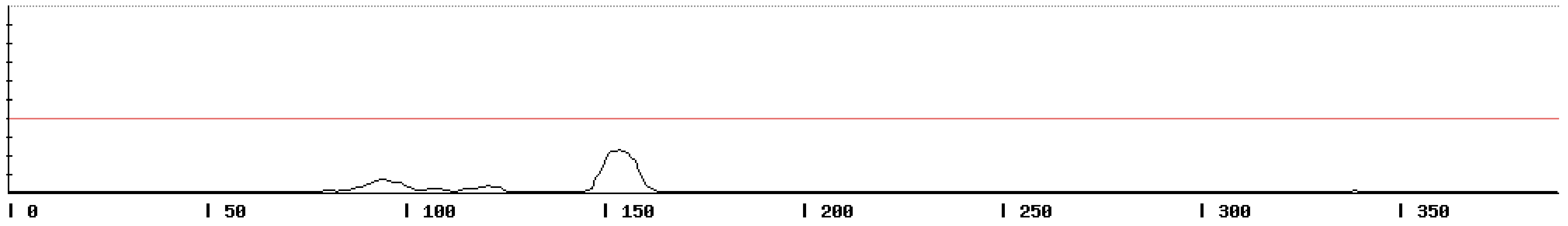

Predictions for Azadirachtaindica_Neem_9030_f_1	
Viterbi Path	
Viterbi Path	
Posterior @ 0.4	137 - KSHKRKGNRK - 146
Posterior @ 0.4	

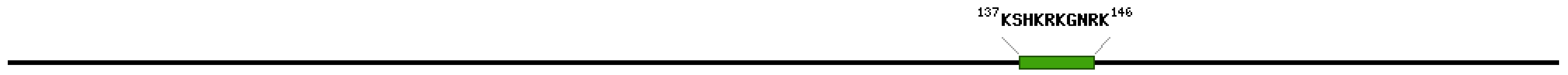

Posterior Graphic. Threshold Marked @ 0.4

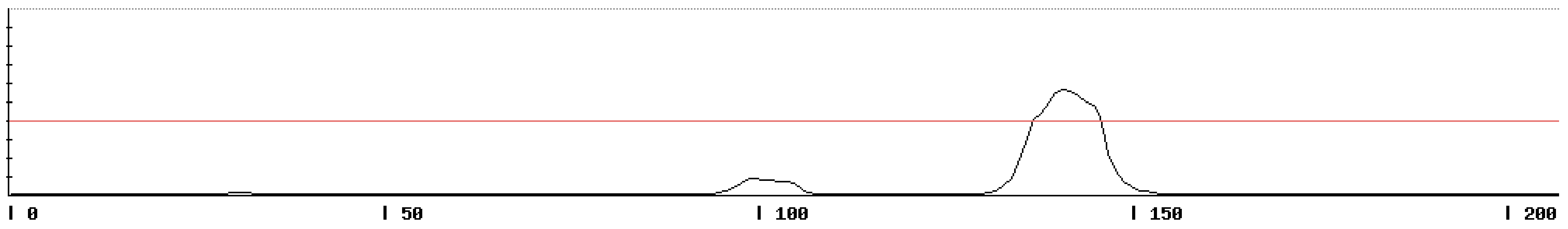

Predictions for Azadirachtaindica_Neem_9151_f_6

Viterbi Path

Viterbi Path

Posterior @ 0.4

Posterior @ 0.4

Posterior Graphic. Threshold Marked @ 0.4

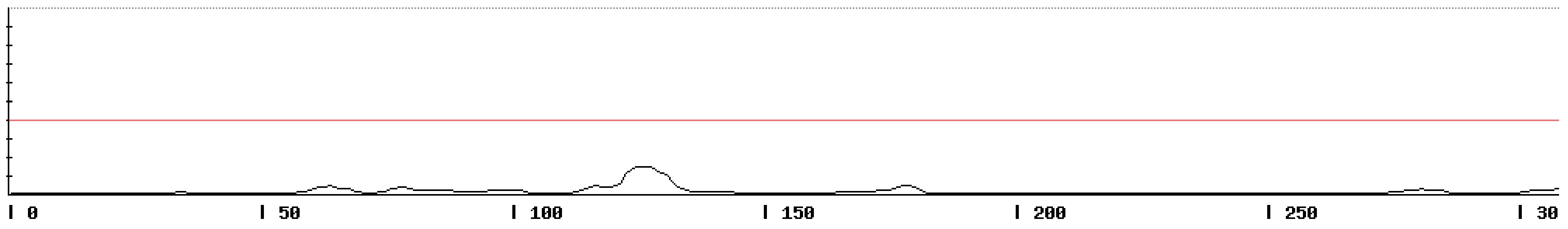

Predictions for Azadirachtaindica_Neem_923_f_1

Viterbi Path

Viterbi Path

Posterior @ 0.4

Posterior @ 0.4

Posterior Graphic. Threshold Marked @ 0.4

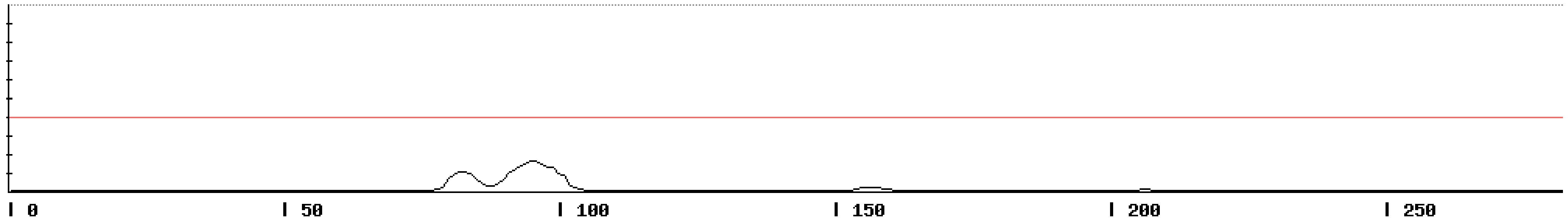

Predictions for Azadirachtaindica_Neem_9263_f_10

Viterbi Path

Viterbi Path

Posterior @ 0.4

Posterior @ 0.4

Posterior Graphic. Threshold Marked @ 0.4

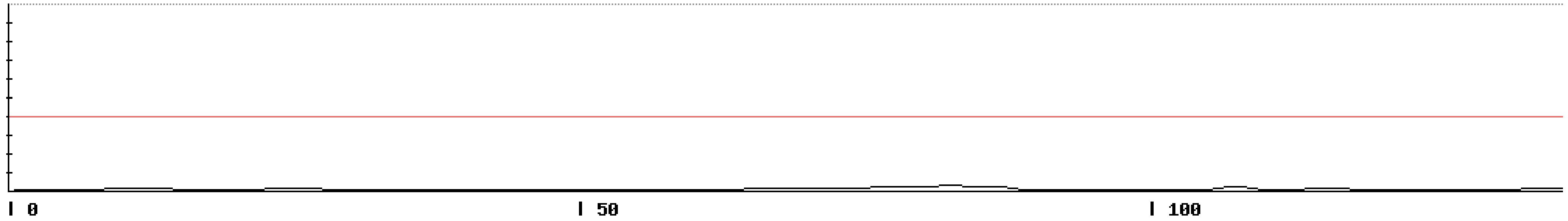

Predictions for Azadirachtaindica_Neem_9263_f_12

Viterbi Path

Viterbi Path

Posterior @ 0.4

Posterior @ 0.4

Posterior Graphic. Threshold Marked @ 0.4

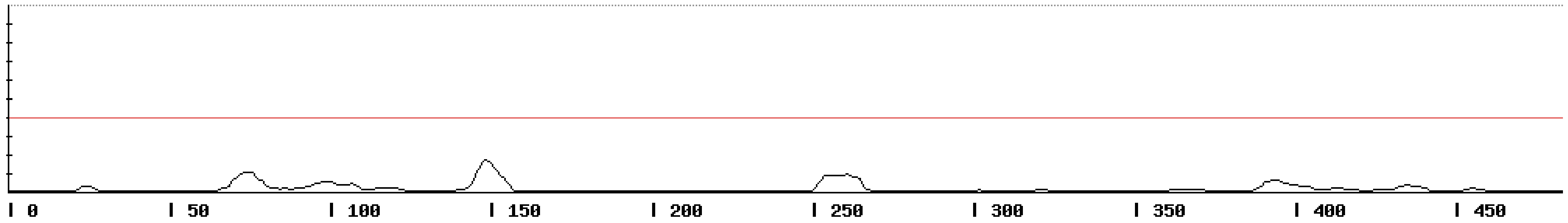

Predictions for Azadirachtaindica_Neem_9263_f_8

Viterbi Path

Viterbi Path

Posterior @ 0.4

Posterior @ 0.4

Posterior Graphic. Threshold Marked @ 0.4

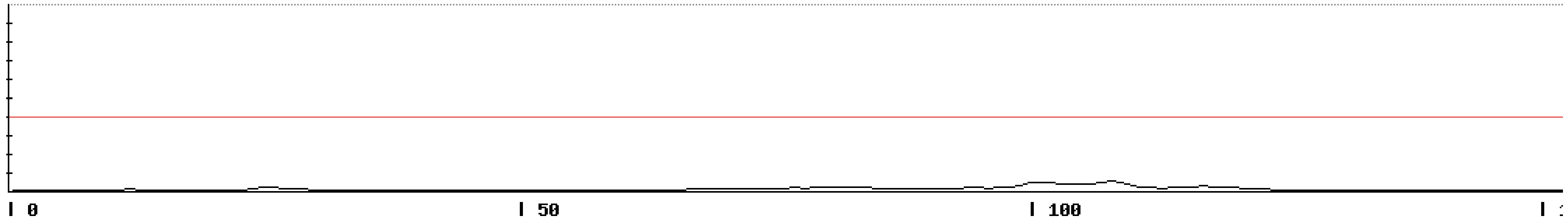

Predictions for Azadirachtaindica_Neem_9263_f_9

Viterbi Path

Viterbi Path

Posterior @ 0.4

Posterior @ 0.4

Posterior Graphic. Threshold Marked @ 0.4

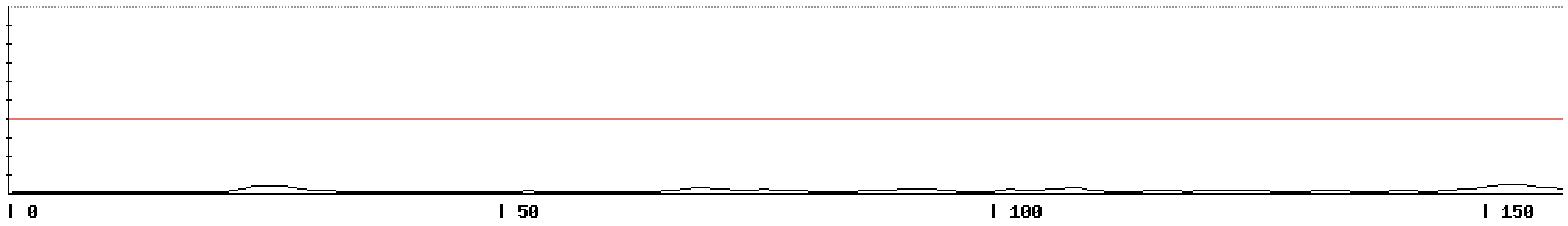

Predictions for Azadirachtaindica_Neem_9411_f_1

Viterbi Path

Viterbi Path

Posterior @ 0.4

Posterior @ 0.4

Posterior Graphic. Threshold Marked @ 0.4

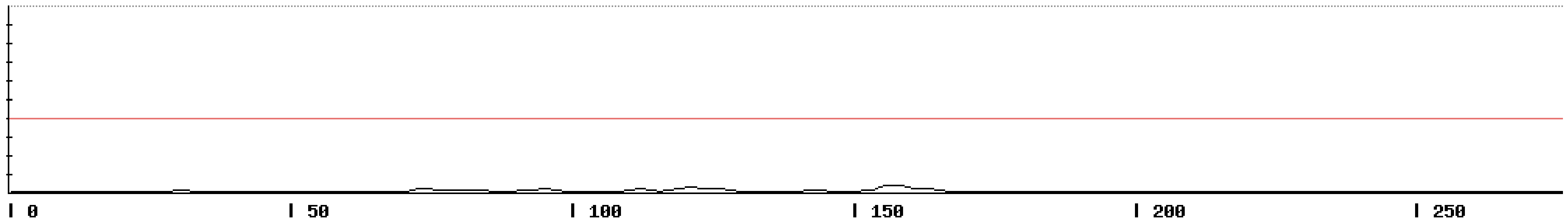

Predictions for Azadirachtaindica_Neem_9517_f_1	
Viterbi Path	
Viterbi Path	
Posterior @ 0.4	
Posterior @ 0.4	
Posterior Graphic. Threshold Marked @ 0.4	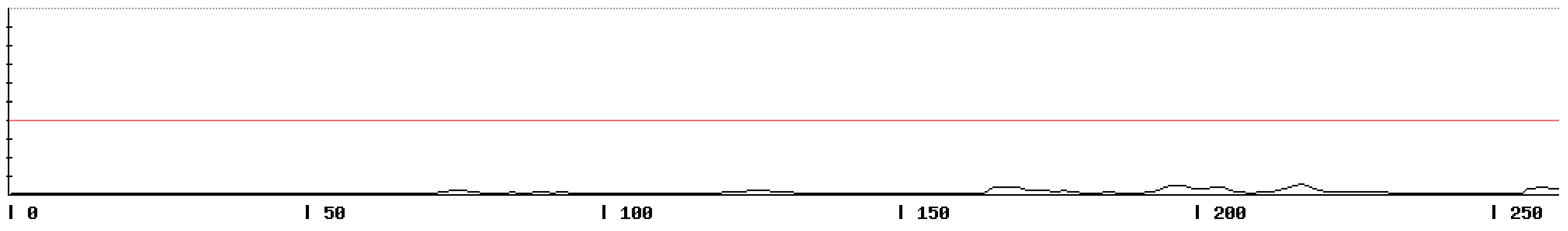

Predictions for Azadirachtaindica_Neem_9779_f_3	
Viterbi Path	
Viterbi Path	
Posterior @ 0.4	
Posterior @ 0.4	
Posterior Graphic. Threshold Marked @ 0.4	

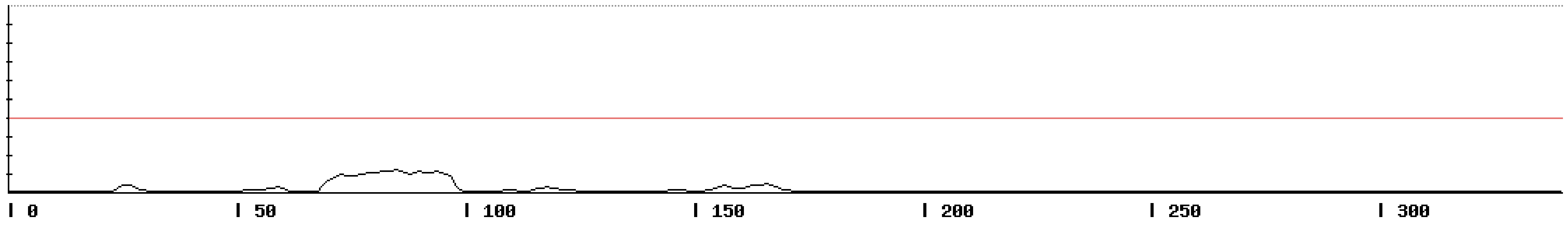

Revision r.9 - Last Updated on : February 24 2015 17:45:20.

alex.nguyenba@utoronto.ca

Intended to be used with Firefox

Viterbi Path	
Posterior @ 0.4	
Posterior @ 0.4	
Posterior Graphic. Threshold Marked @ 0.4	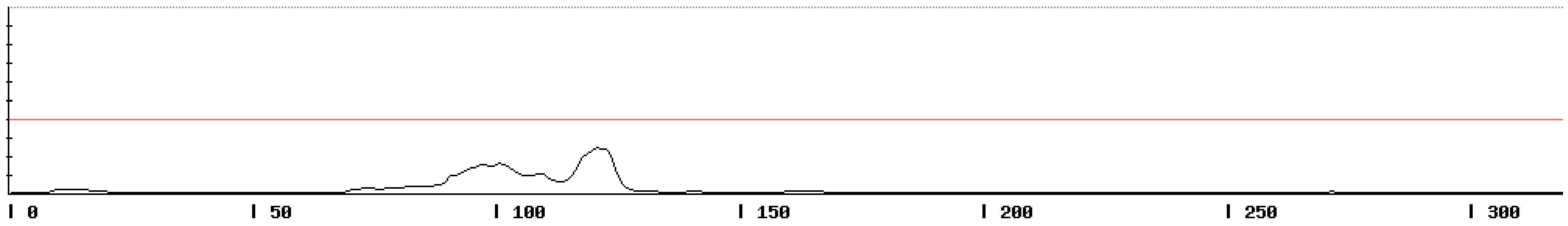

Predictions for Betavulgaris_Bv1_001030_strk.t1

Viterbi Path	
Viterbi Path	
Posterior @ 0.4	
Posterior @ 0.4	
Posterior Graphic. Threshold Marked @ 0.4	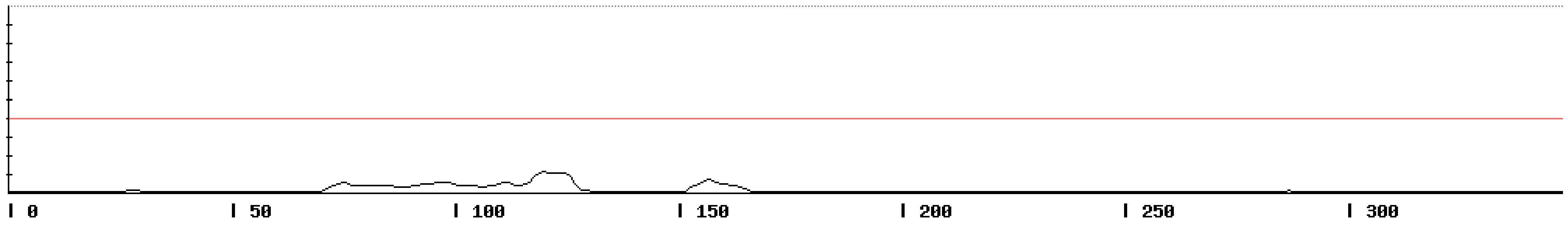

Predictions for Betavulgaris_Bv1_001950_ekgh.t1

Viterbi Path	
Viterbi Path	
Posterior @ 0.4	
Posterior @ 0.4	
Posterior Graphic. Threshold Marked @ 0.4	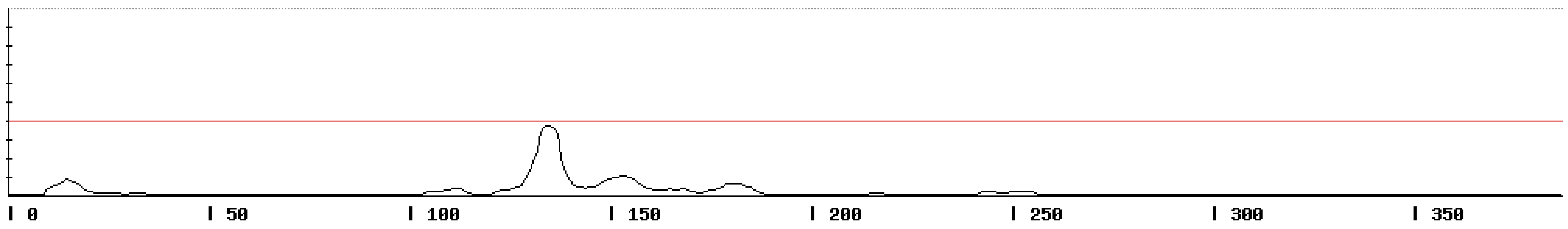

Predictions for Betavulgaris_Bv1_002240_rapp.t1

Viterbi Path	
Viterbi Path	
Posterior @ 0.4	
Posterior @ 0.4	
Posterior Graphic. Threshold Marked @ 0.4	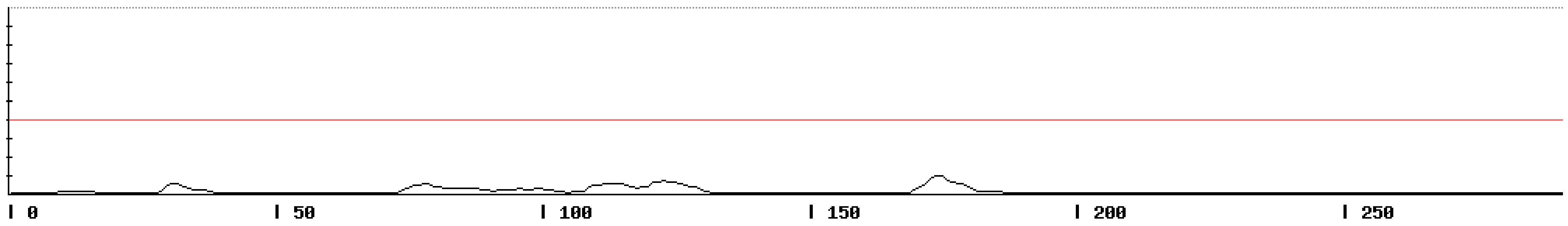

Predictions for Betavulgaris_Bv1_011210_zihs.t1	
Viterbi Path	
Viterbi Path	
Posterior @ 0.4	
Posterior @ 0.4	
Posterior Graphic. Threshold Marked @ 0.4	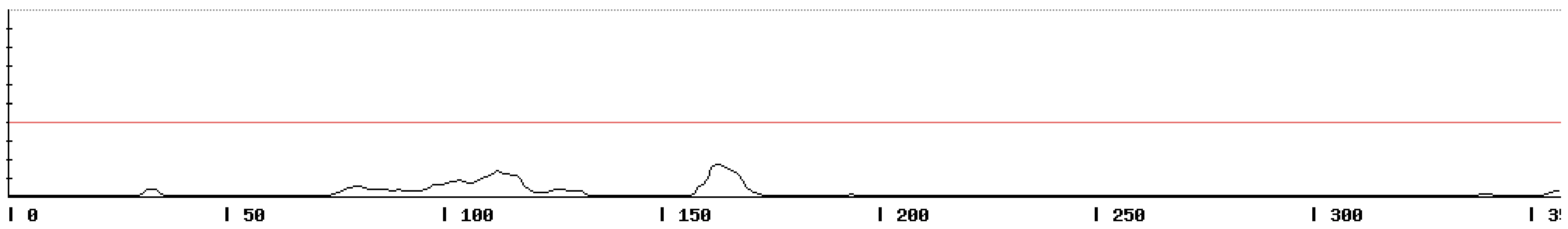

Predictions for Betavulgaris_Bv1_011220_zfhk.t1	
Viterbi Path	
Viterbi Path	
Posterior @ 0.4	
Posterior @ 0.4	
Posterior Graphic. Threshold Marked @ 0.4	

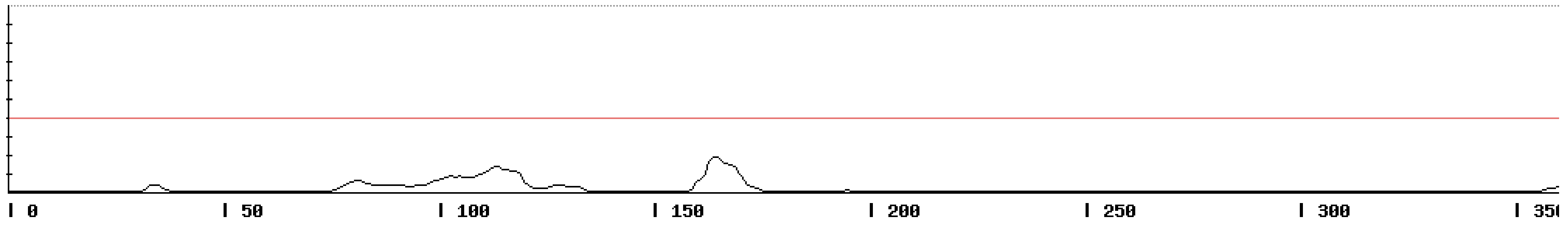

Predictions for Betavulgaris_Bv1_014170_yzgr.t1

Viterbi Path	
Viterbi Path	
Posterior @ 0.4	
Posterior @ 0.4	
Posterior Graphic. Threshold Marked @ 0.4	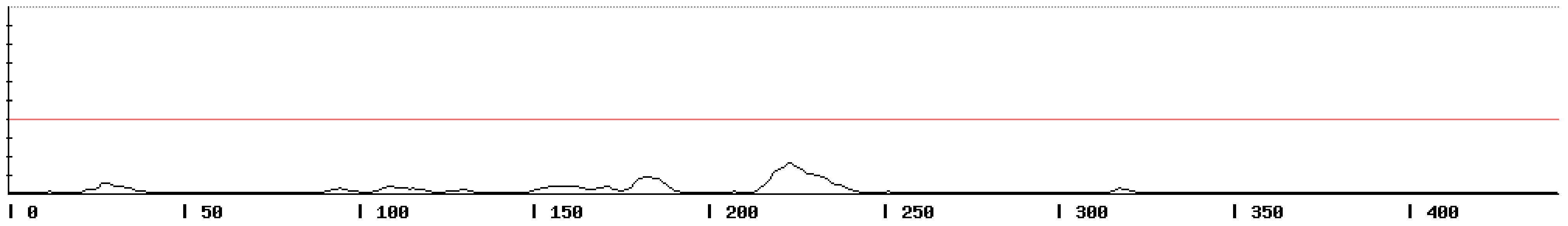

Predictions for Betavulgaris_Bv1_022030_ueac.t1

Viterbi Path	
Viterbi Path	
Posterior @ 0.4	316 - RIRLKRLR - 323
Posterior @ 0.4	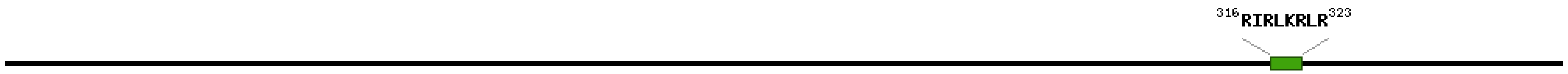

Posterior Graphic. Threshold Marked @ 0.4

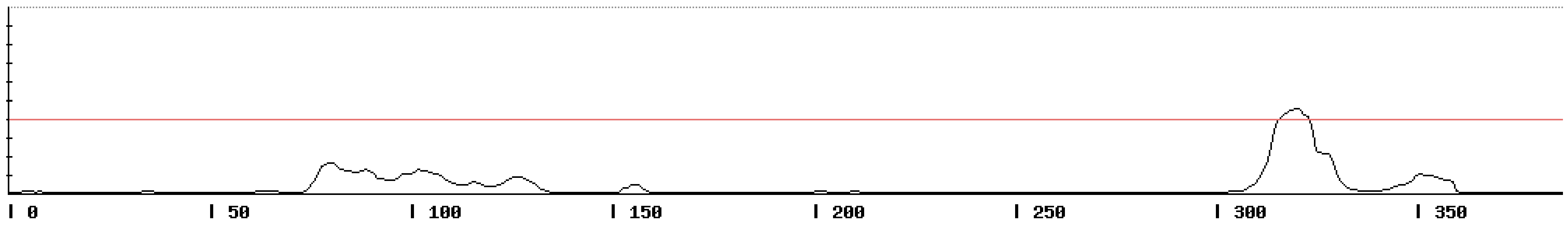

Predictions for Betavulgaris_Bv2_032870_nige.t1

Viterbi Path

Viterbi Path

Posterior @ 0.4 317 - NRRIRLRKLRRFSPRKE - 333

Posterior @ 0.4

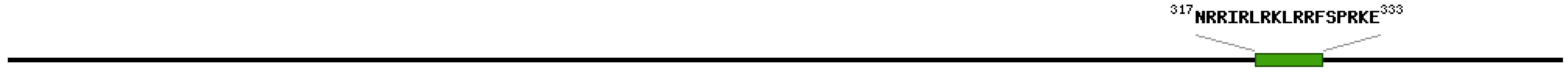

Posterior Graphic. Threshold Marked @ 0.4

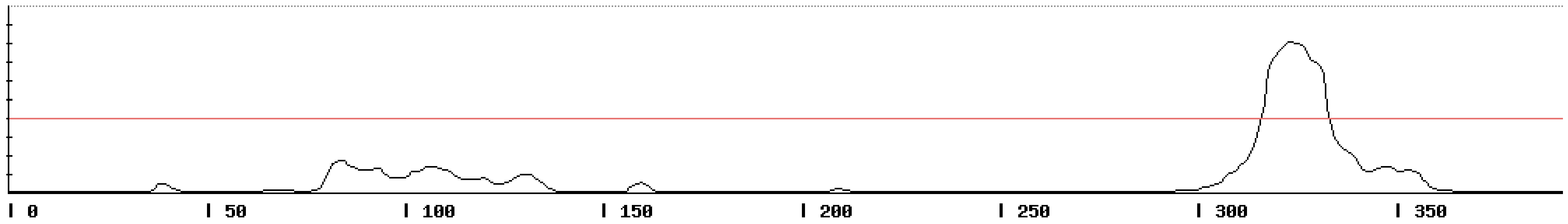

Predictions for Betavulgaris_Bv2_035010_gcaa.t1

Viterbi Path

Viterbi Path

Posterior @ 0.4

Posterior @ 0.4

Posterior Graphic. Threshold Marked @ 0.4	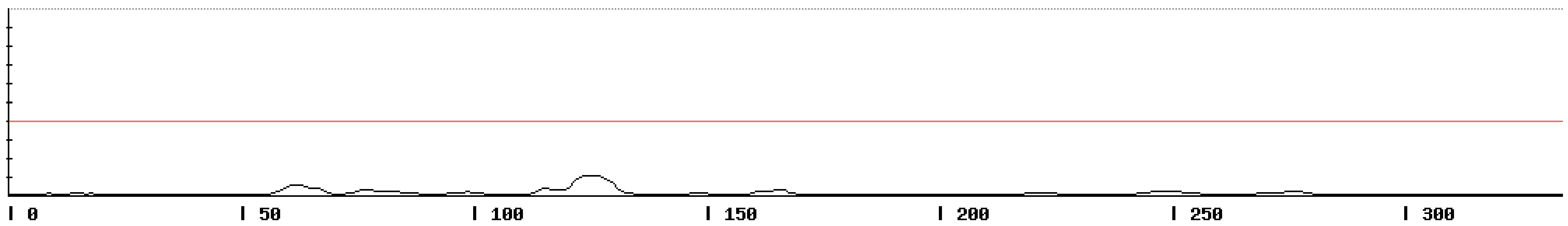

Predictions for Betavulgaris_Bv2_040580_mnyx.t1

Viterbi Path	
Viterbi Path	
Posterior @ 0.4	
Posterior @ 0.4	
Posterior Graphic. Threshold Marked @ 0.4	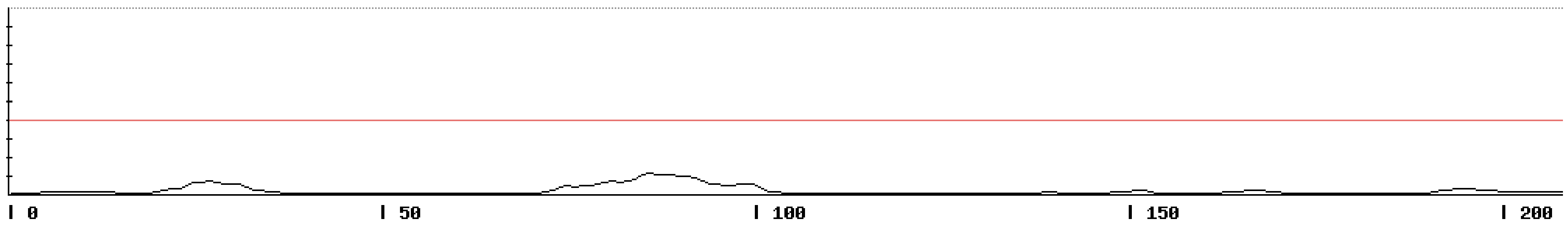

Predictions for Betavulgaris_Bv2_046230_sgnn.t1

Viterbi Path	
Viterbi Path	

Posterior @ 0.4

Posterior @ 0.4

Posterior Graphic. Threshold Marked @ 0.4

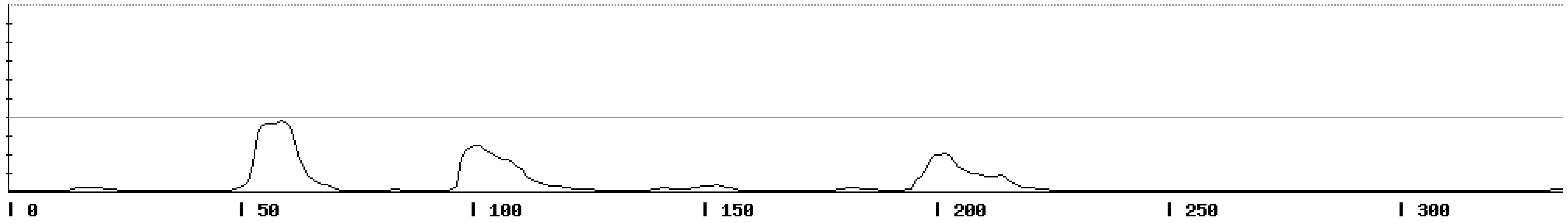

Predictions for Betavulgaris_Bv2_046650_xzdt.t1

Viterbi Path

Viterbi Path

Posterior @ 0.4

Posterior @ 0.4

Posterior Graphic. Threshold Marked @ 0.4

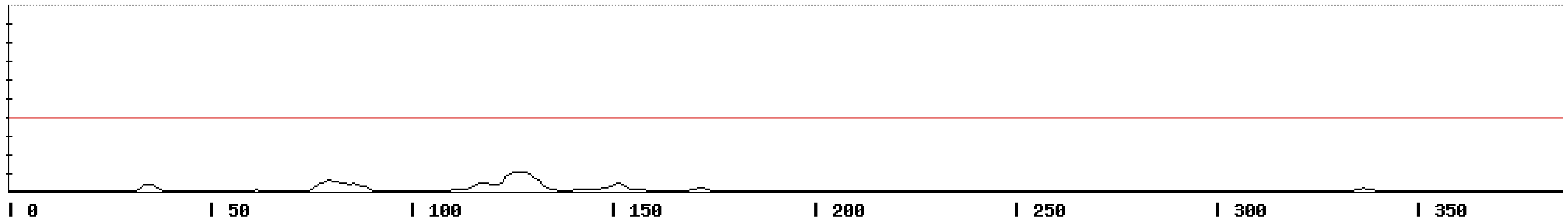

Predictions for Betavulgaris_Bv2_046950_hasi.t1

Viterbi Path

Viterbi Path

Posterior @ 0.4	
Posterior @ 0.4	
Posterior Graphic. Threshold Marked @ 0.4	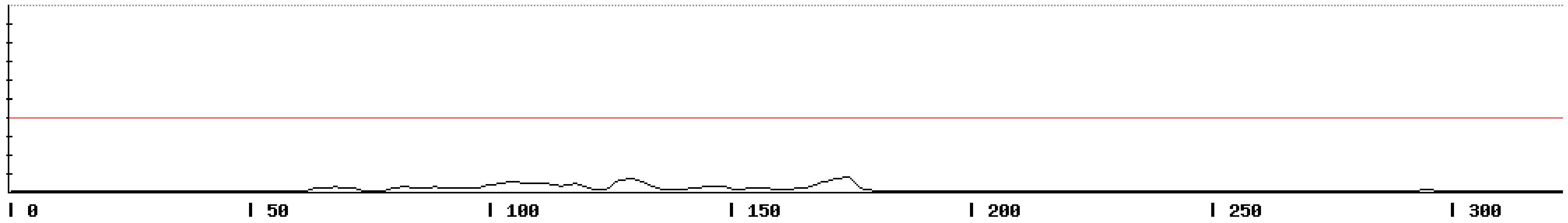

Predictions for Betavulgaris_Bv2_047470_qmgy.t1

Viterbi Path	
Viterbi Path	
Posterior @ 0.4	
Posterior @ 0.4	
Posterior Graphic. Threshold Marked @ 0.4	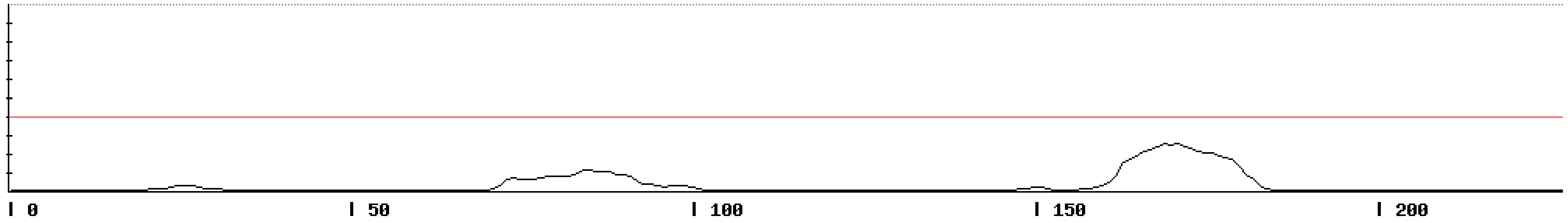

Predictions for Betavulgaris_Bv3_050780_usim.t1

Viterbi Path	
Viterbi Path	
Posterior @ 0.4	113 - SIKSRK - 118
Posterior @ 0.4	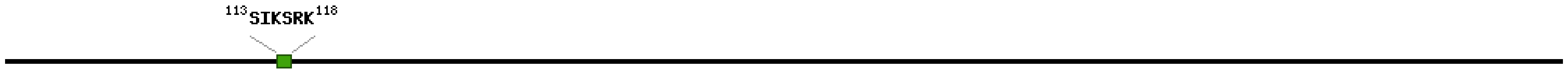
Posterior Graphic. Threshold Marked @ 0.4	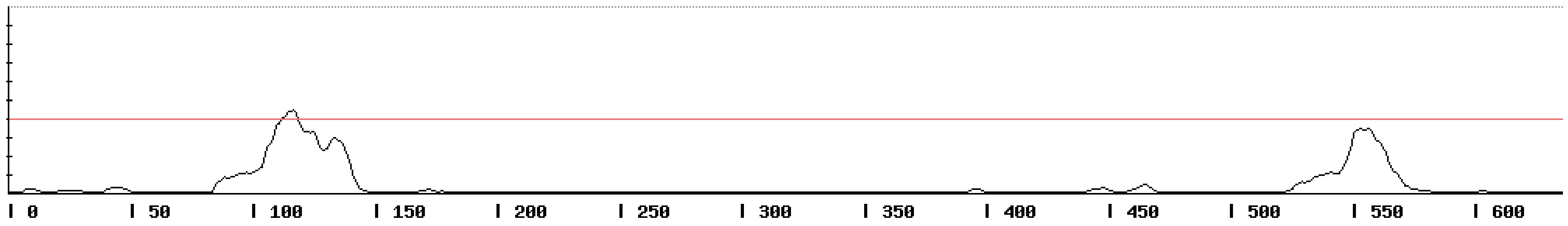

Predictions for Betavulgaris_Bv3_056020_uozo.t1

Viterbi Path	
Viterbi Path	
Posterior @ 0.4	
Posterior @ 0.4	
Posterior Graphic. Threshold Marked @ 0.4	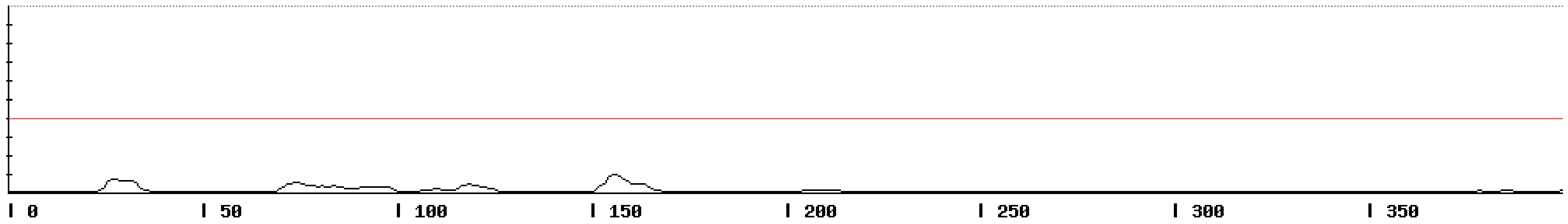

Predictions for Betavulgaris_Bv4_078190_hzcx.t1	
Viterbi Path	
Viterbi Path	
Posterior @ 0.4	99 - GKPK - 102
Posterior @ 0.4	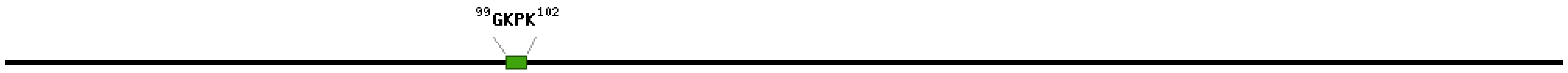
Posterior Graphic. Threshold Marked @ 0.4	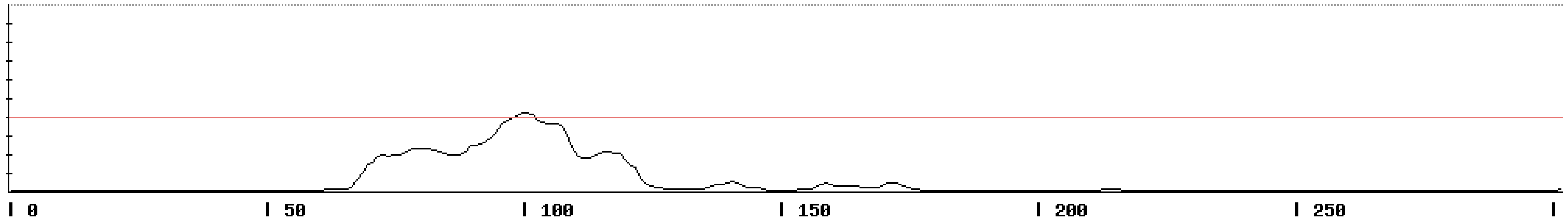

Predictions for Betavulgaris_Bv4_079110_tgus.t1	
Viterbi Path	
Viterbi Path	
Posterior @ 0.4	
Posterior @ 0.4	
Posterior Graphic. Threshold Marked @ 0.4	

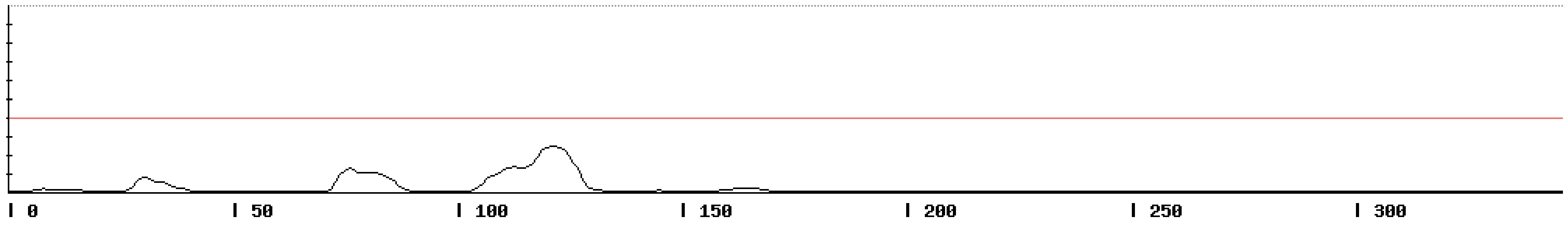

Predictions for Betavulgaris_Bv4_086610_cugw.t1

Viterbi Path	
Viterbi Path	
Posterior @ 0.4	
Posterior @ 0.4	
Posterior Graphic. Threshold Marked @ 0.4	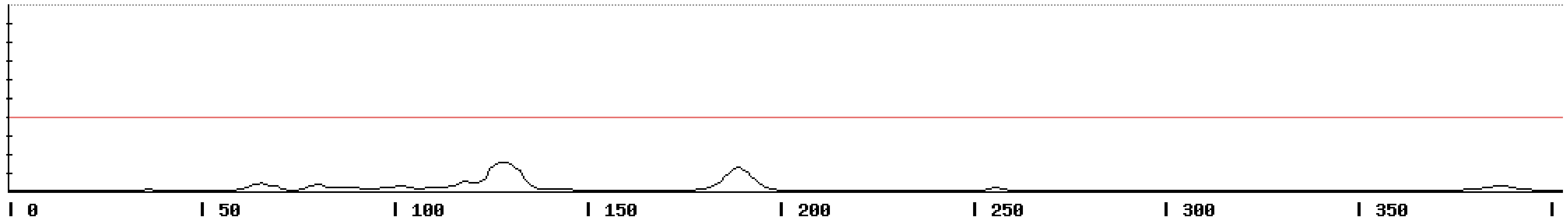

Predictions for Betavulgaris_Bv4_087410_qejj.t1

Viterbi Path	
Viterbi Path	
Posterior @ 0.4	
Posterior @ 0.4	

Posterior Graphic. Threshold Marked @ 0.4	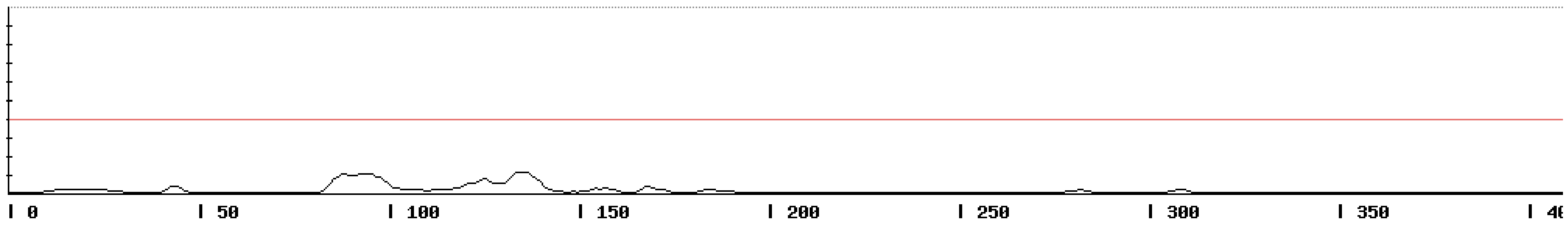

Predictions for Betavulgaris_Bv4_087440_twas.t1	
Viterbi Path	
Viterbi Path	
Posterior @ 0.4	
Posterior @ 0.4	
Posterior Graphic. Threshold Marked @ 0.4	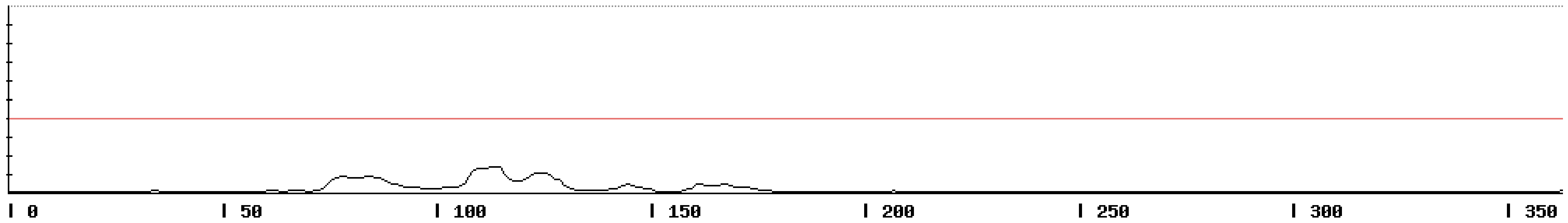

Predictions for Betavulgaris_Bv4_093610_xiqu.t1	
Viterbi Path	
Viterbi Path	
Posterior @ 0.4	
Posterior @ 0.4	

Posterior Graphic. Threshold Marked @ 0.4

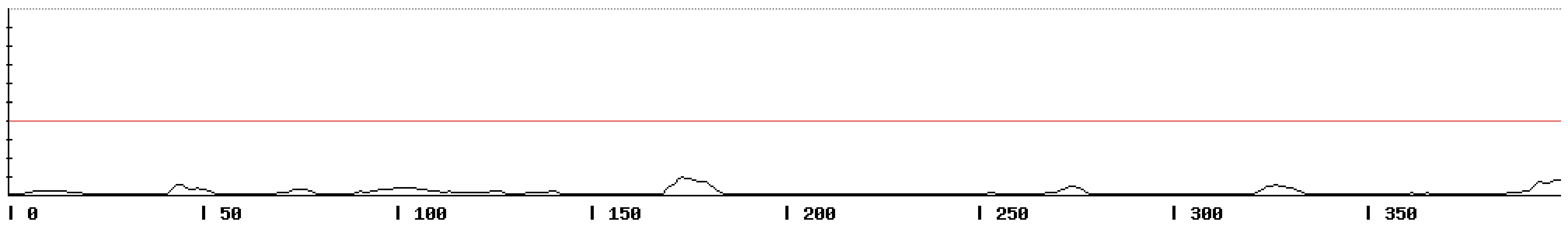

Predictions for Betavulgaris_Bv4_093720_guiw.t1

Viterbi Path

Viterbi Path

Posterior @ 0.4

Posterior @ 0.4

Posterior Graphic. Threshold Marked @ 0.4

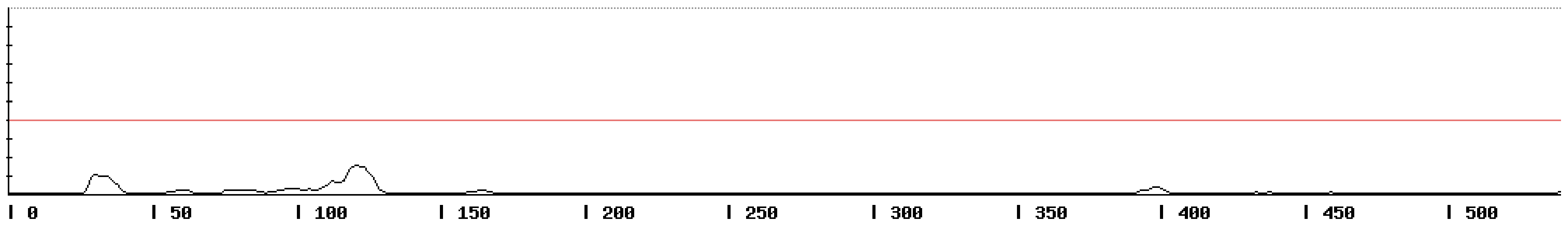

Predictions for Betavulgaris_Bv4_095900_twpc.t1

Viterbi Path

Viterbi Path

Posterior @ 0.4	114 - RKSPR - 118
Posterior @ 0.4	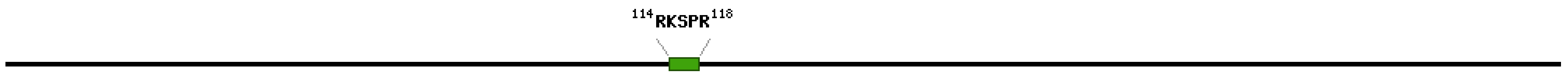
Posterior Graphic. Threshold Marked @ 0.4	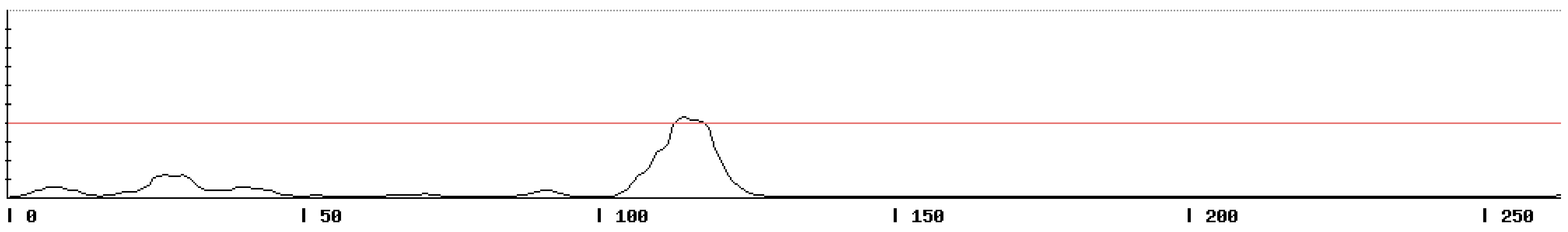

Predictions for Betavulgaris_Bv4_096620_mhnx.t1	
Viterbi Path	
Viterbi Path	
Posterior @ 0.4	
Posterior @ 0.4	
Posterior Graphic. Threshold Marked @ 0.4	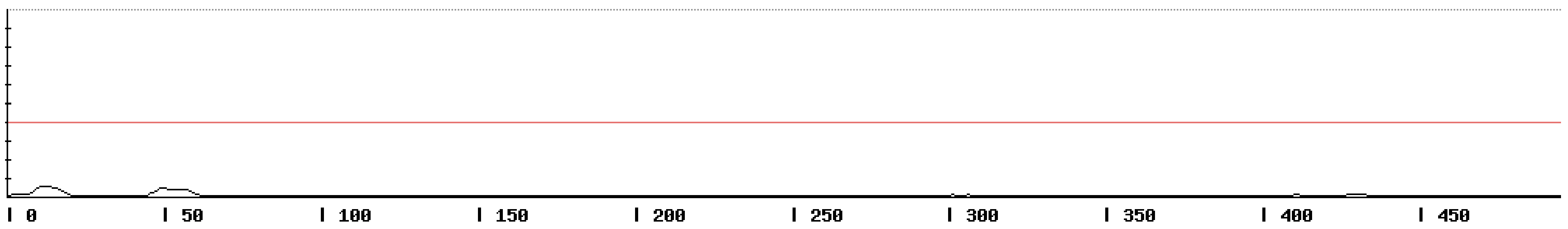

Predictions for Betavulgaris_Bv4_096650_oxms.t1	
Viterbi Path	
Viterbi Path	

Posterior @ 0.4

Posterior @ 0.4

Posterior Graphic. Threshold Marked @ 0.4

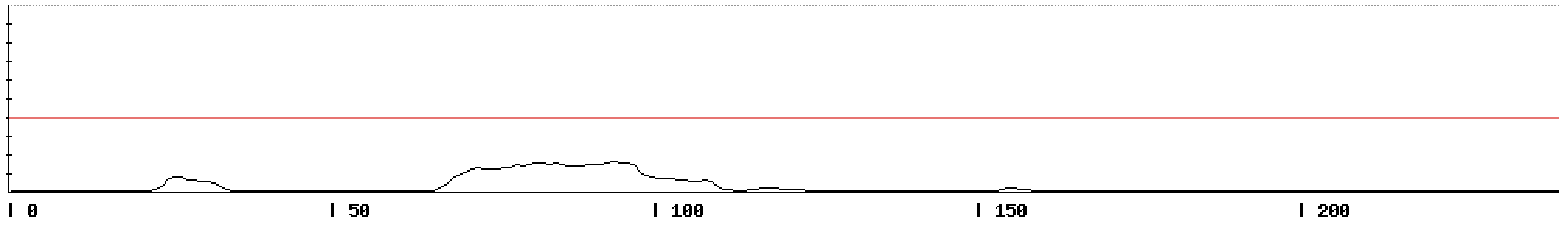

Predictions for Betavulgaris_Bv5_097990_fpfn.t1

Viterbi Path

Viterbi Path

Posterior @ 0.4

Posterior @ 0.4

Posterior Graphic. Threshold Marked @ 0.4

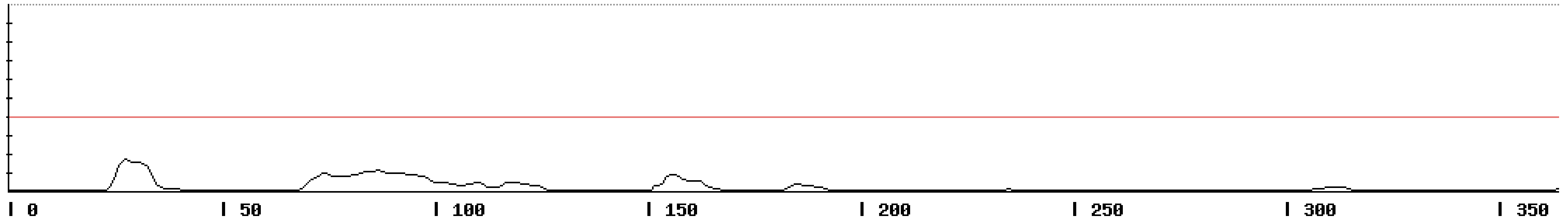

Predictions for Betavulgaris_Bv5_104170_xrgj.t1

Viterbi Path

Viterbi Path

Posterior @ 0.4

Posterior @ 0.4

Posterior Graphic. Threshold Marked @ 0.4

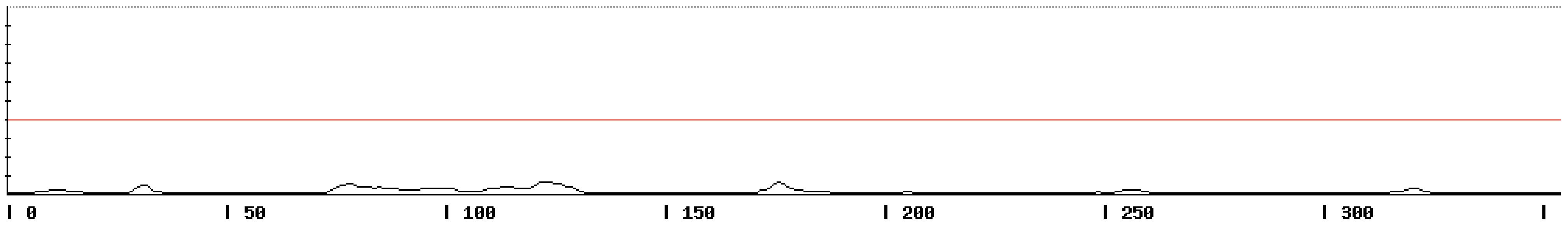

Predictions for Betavulgaris_Bv5_106480_gzwx.t1

Viterbi Path

Viterbi Path

Posterior @ 0.4

Posterior @ 0.4

Posterior Graphic. Threshold Marked @ 0.4

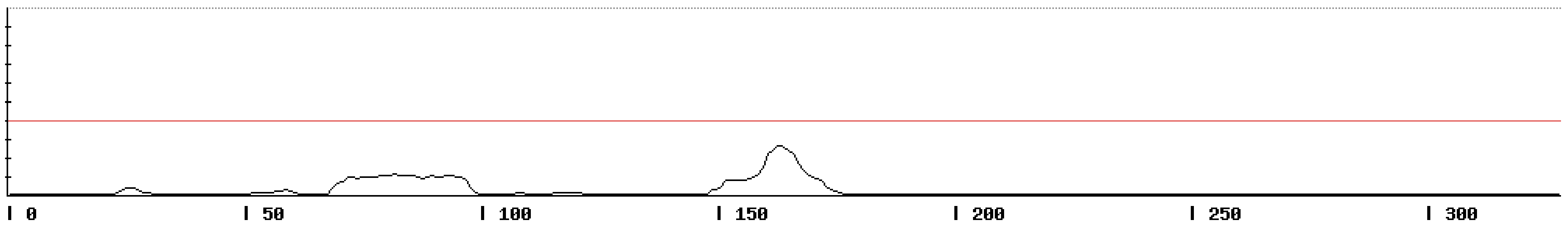

Predictions for Betavulgaris_Bv5_114130_gzxk.t1	
Viterbi Path	
Viterbi Path	
Posterior @ 0.4	
Posterior @ 0.4	
Posterior Graphic. Threshold Marked @ 0.4	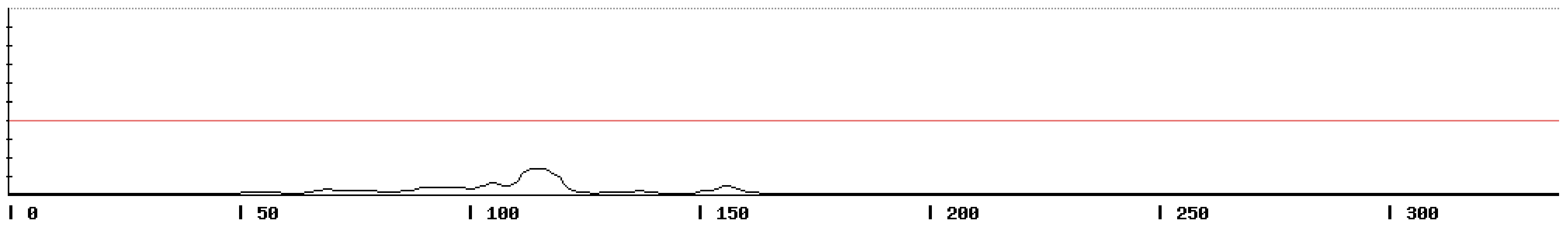

Predictions for Betavulgaris_Bv5_114390_pjnp.t1	
Viterbi Path	
Viterbi Path	
Posterior @ 0.4	
Posterior @ 0.4	
Posterior Graphic. Threshold Marked @ 0.4	

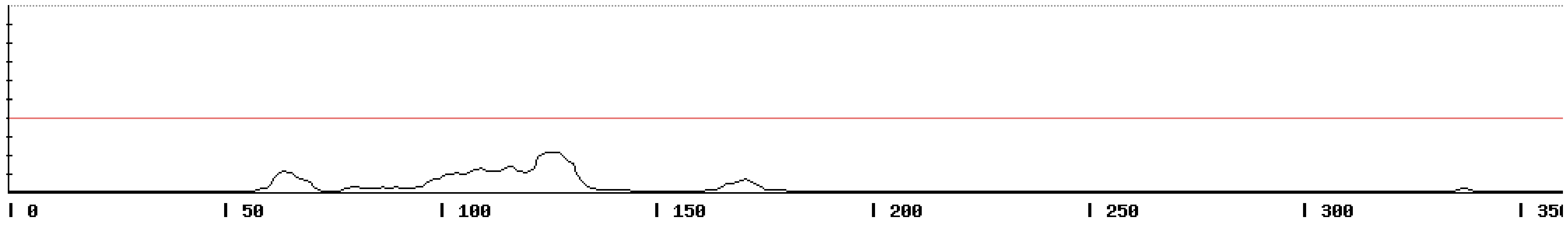

Predictions for Betavulgaris_Bv5_121330_zrdc.t1

Viterbi Path	
Viterbi Path	
Posterior @ 0.4	
Posterior @ 0.4	
Posterior Graphic. Threshold Marked @ 0.4	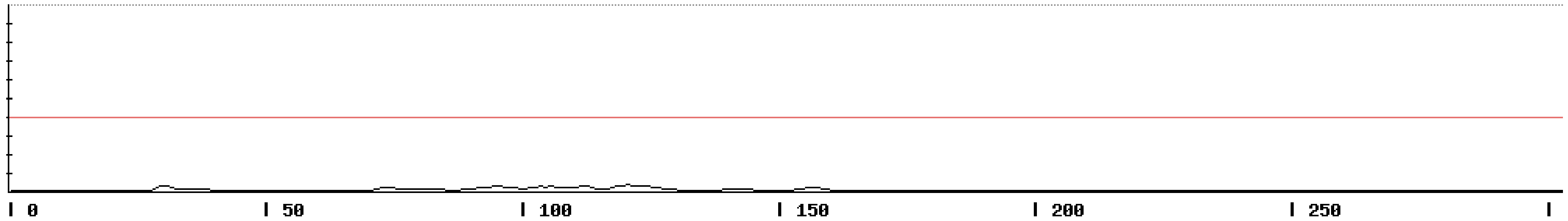

Predictions for Betavulgaris_Bv6_129910_cmip.t1

Viterbi Path	
Viterbi Path	
Posterior @ 0.4	
Posterior @ 0.4	

Posterior Graphic. Threshold Marked @ 0.4

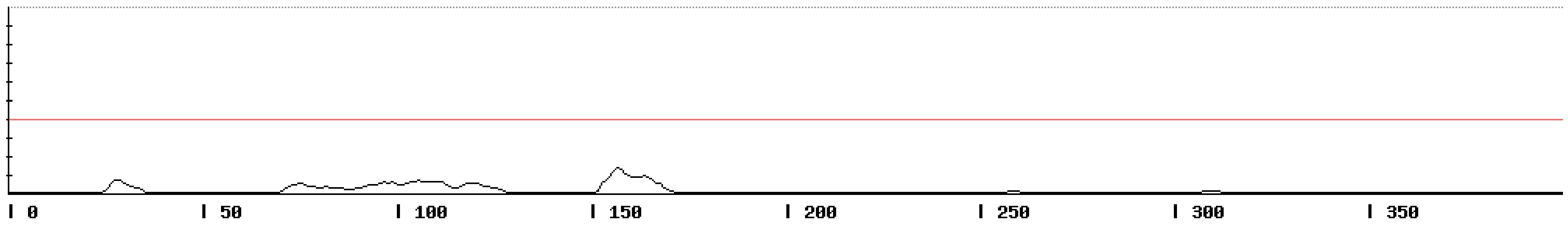

Predictions for Betavulgaris_Bv6_132760_wkcm.t2

Viterbi Path

Viterbi Path

Posterior @ 0.4

Posterior @ 0.4

Posterior Graphic. Threshold Marked @ 0.4

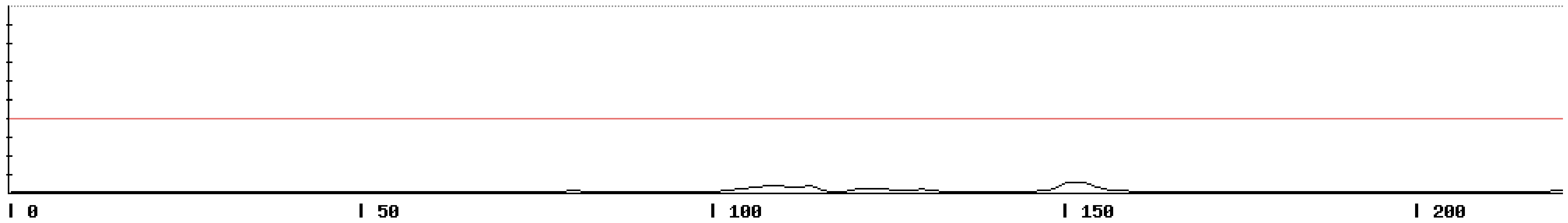

Predictions for Betavulgaris_Bv6_132770_wshi.t1

Viterbi Path

Viterbi Path

Posterior @ 0.4

Posterior @ 0.4

Posterior Graphic. Threshold Marked @ 0.4

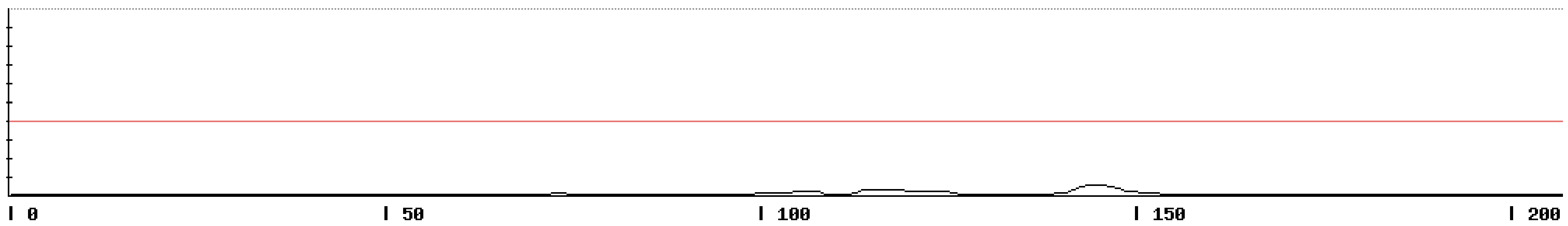

Predictions for Betavulgaris_Bv6_136600_detz.t1

Viterbi Path

Viterbi Path

Posterior @ 0.4 — 99 - RDKRHKNGSRPSRRAKDGYWKA - 120

Posterior @ 0.4

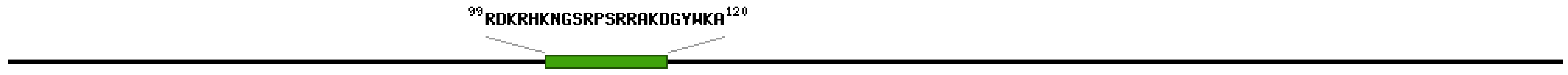

Posterior Graphic. Threshold Marked @ 0.4

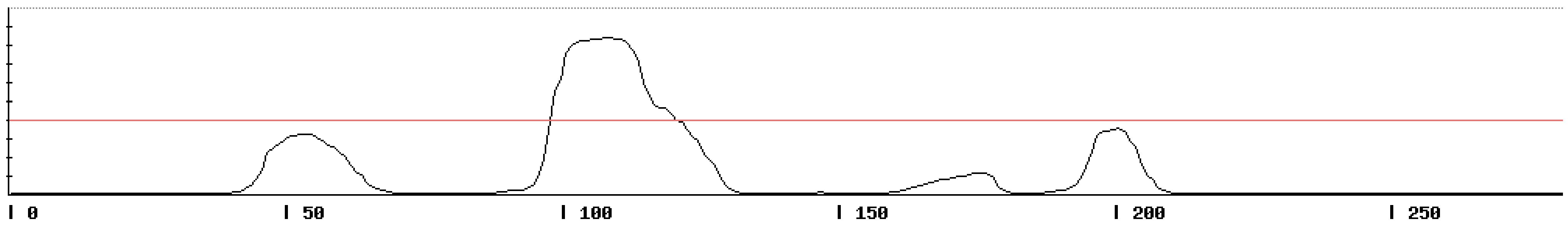

Predictions for Betavulgaris_Bv6_136620_pzjj.t1

Viterbi Path

Viterbi Path

Posterior @ 0.4	95 - VKRYKKGSRPN - 105
Posterior @ 0.4	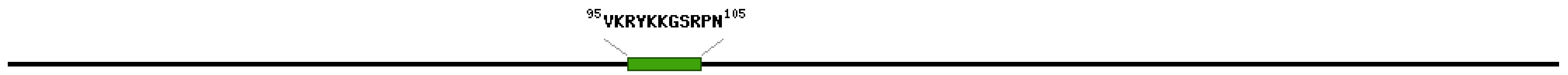
Posterior Graphic. Threshold Marked @ 0.4	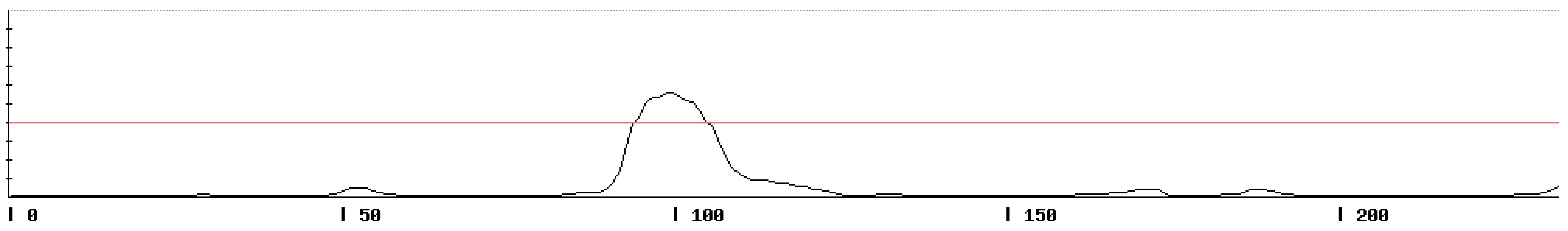

Predictions for Betavulgaris_Bv6_145550_jkxz.t1

Viterbi Path	
Viterbi Path	
Posterior @ 0.4	
Posterior @ 0.4	
Posterior Graphic. Threshold Marked @ 0.4	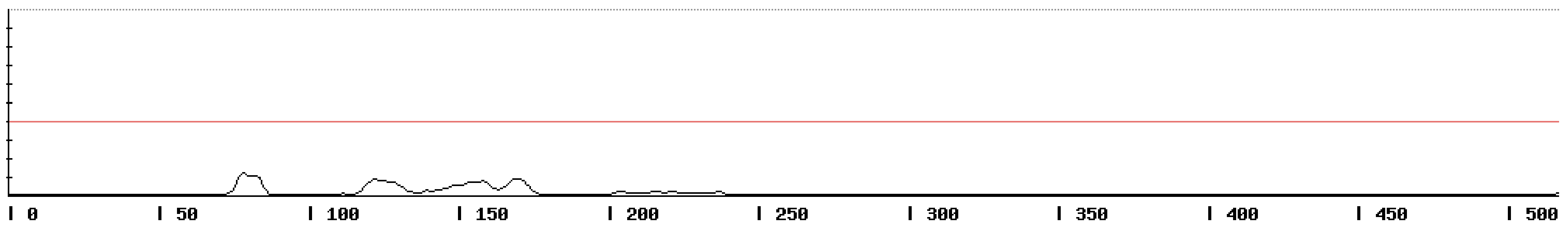

Predictions for Betavulgaris_Bv6_150040_hdfh.t1

Viterbi Path	
Viterbi Path	

Posterior @ 0.4

Posterior @ 0.4

Posterior Graphic. Threshold Marked @ 0.4

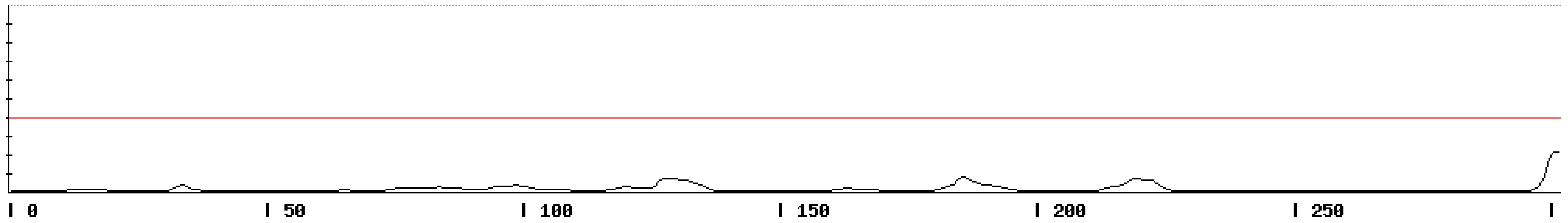

Predictions for Betavulgaris_Bv6_153540_qoyx.t1

Viterbi Path

Viterbi Path

Posterior @ 0.4

Posterior @ 0.4

Posterior Graphic. Threshold Marked @ 0.4

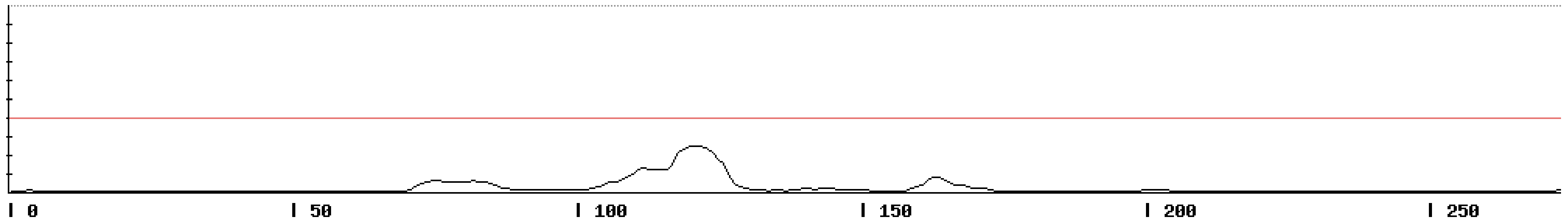

Predictions for Betavulgaris_Bv6_155570_rpmu.t1

Viterbi Path

Viterbi Path

Posterior @ 0.4 — 135 - TRKRRKIHGDKLGDVRWHK - 153

Posterior @ 0.4

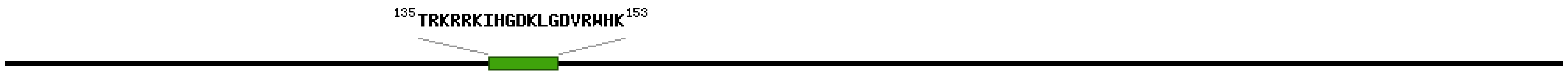

Posterior Graphic. Threshold Marked @ 0.4

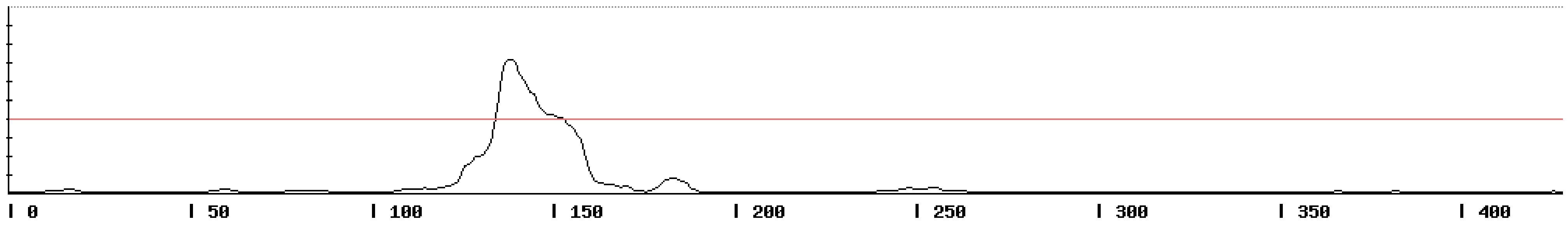

Predictions for Betavulgaris_Bv7_159010_tzwj.t1

Viterbi Path

Viterbi Path

Posterior @ 0.4

Posterior @ 0.4

Posterior Graphic. Threshold Marked @ 0.4

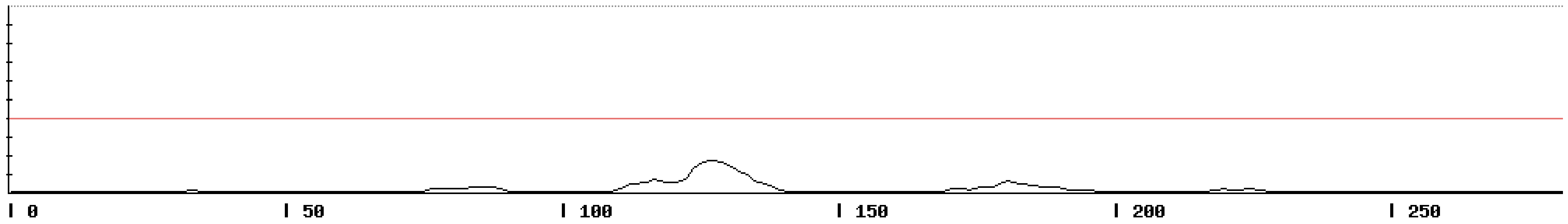

Predictions for Betavulgaris_Bv7_179460_yqtj.t1

Viterbi Path	
Viterbi Path	
Posterior @ 0.4	
Posterior @ 0.4	
Posterior Graphic. Threshold Marked @ 0.4	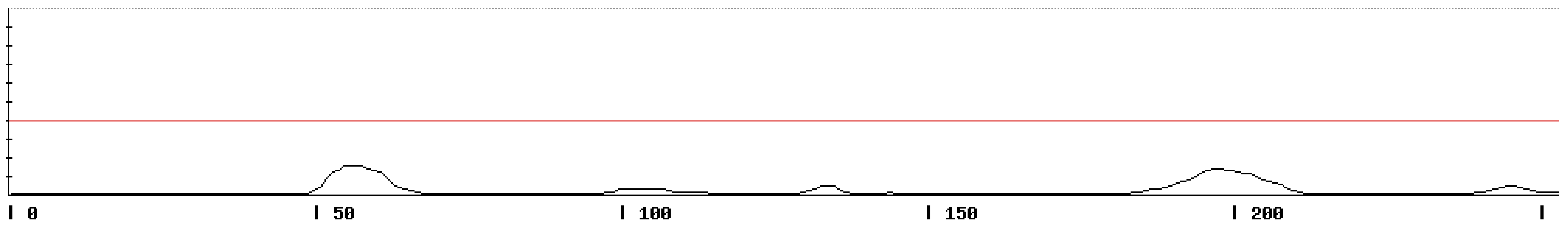

Predictions for Betavulgaris_Bv8_182450_hisu.t1

Viterbi Path	
Viterbi Path	
Posterior @ 0.4	
Posterior @ 0.4	
Posterior Graphic. Threshold Marked @ 0.4	

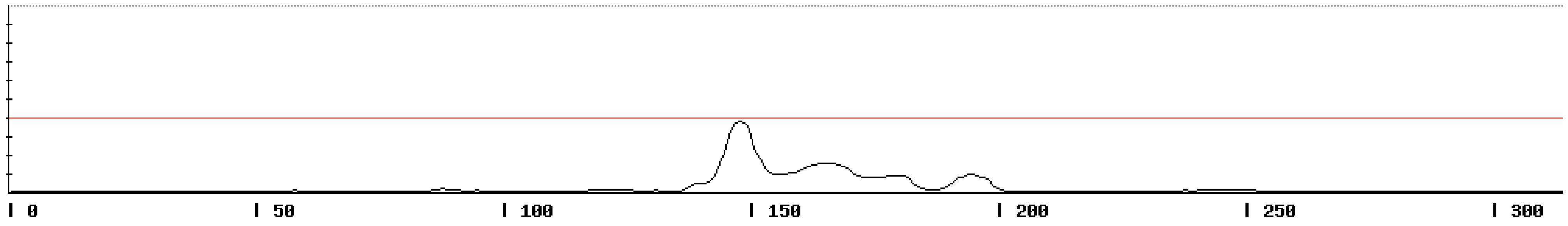

Predictions for Betavulgaris_Bv8_184020_pfso.t1

Viterbi Path	
Viterbi Path	
Posterior @ 0.4	
Posterior @ 0.4	
Posterior Graphic. Threshold Marked @ 0.4	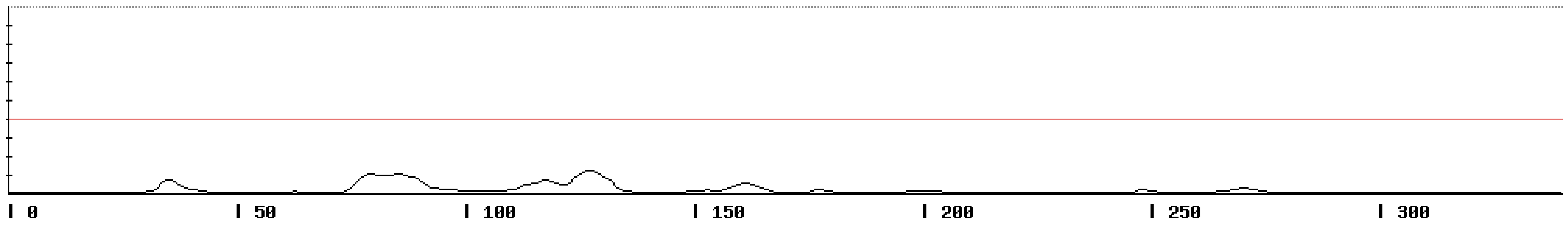

Predictions for Betavulgaris_Bv8_190180_ktgn.t1

Viterbi Path	
Viterbi Path	
Posterior @ 0.4	
Posterior @ 0.4	

Posterior Graphic. Threshold Marked @ 0.4	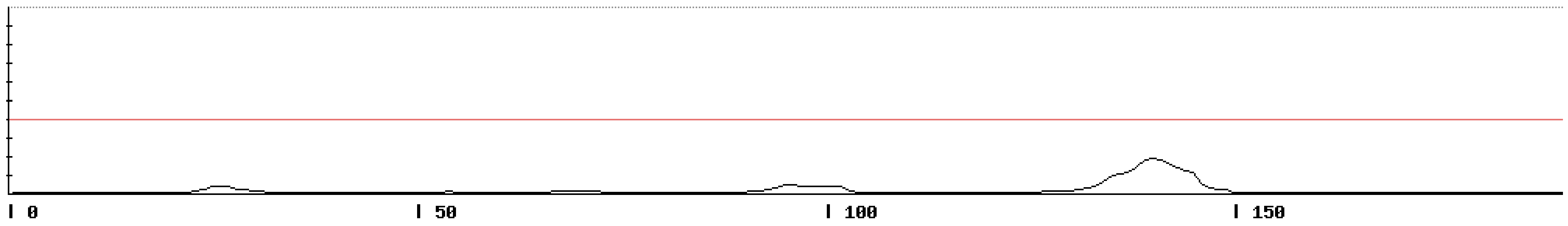

Predictions for Betavulgaris_Bv8_197850_tdfd.t1

Viterbi Path	
Viterbi Path	
Posterior @ 0.4	
Posterior @ 0.4	
Posterior Graphic. Threshold Marked @ 0.4	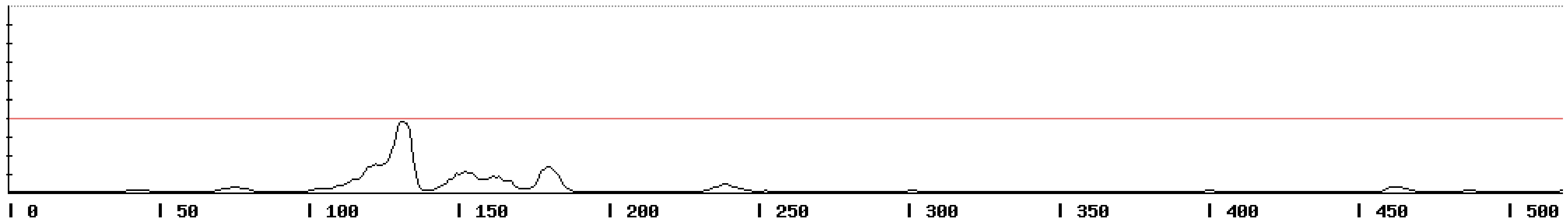

Predictions for Betavulgaris_Bv8_199060_znkf.t1

Viterbi Path	
Viterbi Path	
Posterior @ 0.4	
Posterior @ 0.4	

Posterior Graphic. Threshold Marked @ 0.4

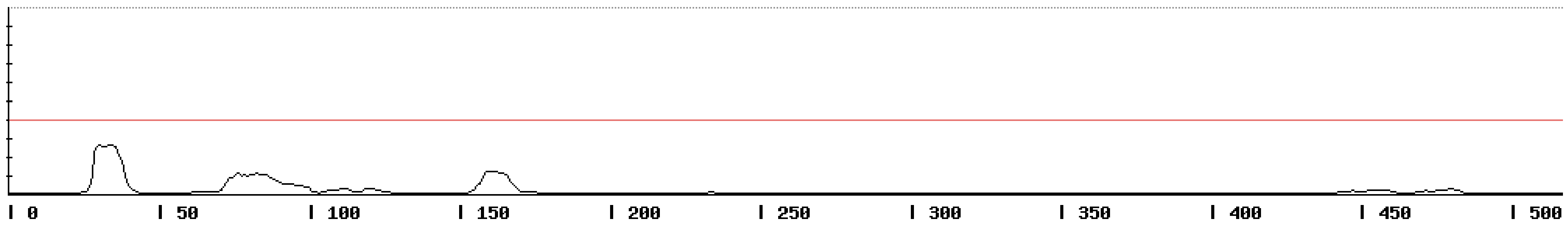

Predictions for Betavulgaris_Bv9_203240_qoku.t1

Viterbi Path

Viterbi Path

Posterior @ 0.4

Posterior @ 0.4

Posterior Graphic. Threshold Marked @ 0.4

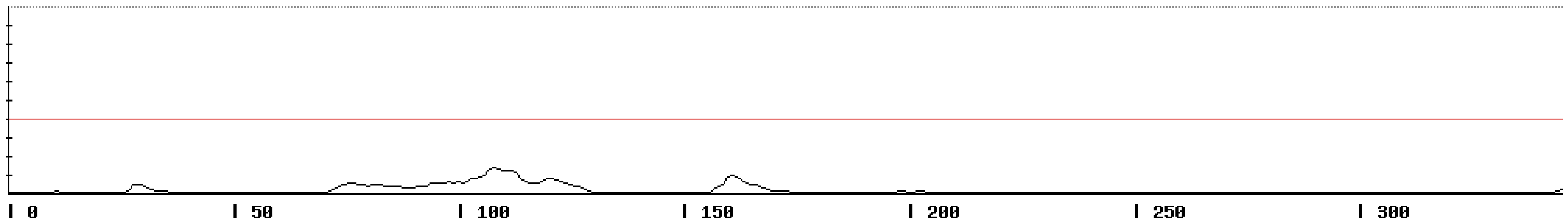

Predictions for Betavulgaris_Bv9_209530_aktr.t1

Viterbi Path

Viterbi Path

Posterior @ 0.4	131 - RKRR - 134
Posterior @ 0.4	
Posterior Graphic. Threshold Marked @ 0.4	

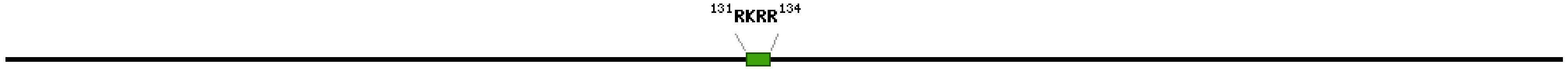

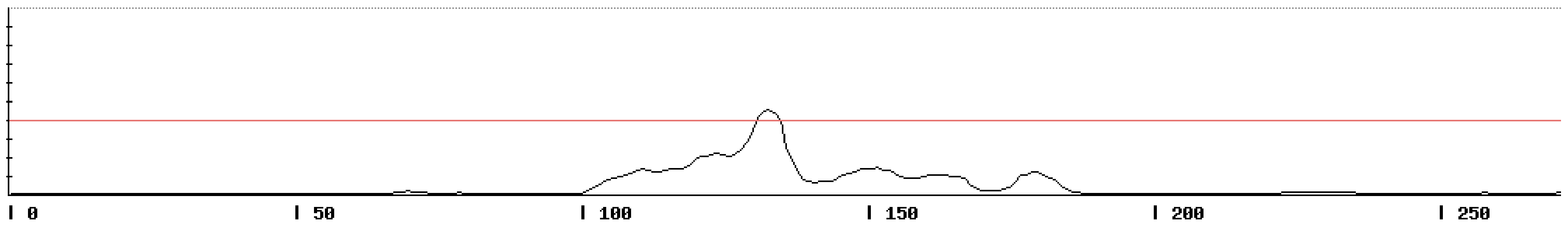

Predictions for Betavulgaris_Bv9_212910_kjzy.t1

Viterbi Path

Viterbi Path

Posterior @ 0.4

Posterior @ 0.4

Posterior Graphic. Threshold Marked @ 0.4

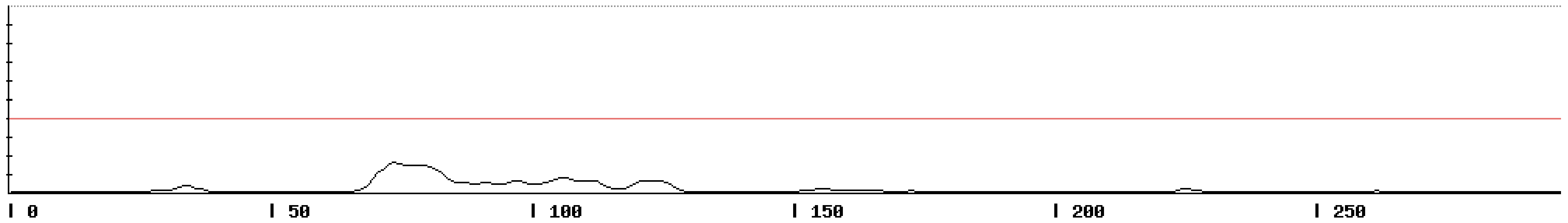

Predictions for Betavulgaris_Bv9_214490_ofji.t1

Viterbi Path

Viterbi Path

Viterbi Path

Posterior @ 0.4

Posterior @ 0.4

Posterior Graphic. Threshold Marked @ 0.4

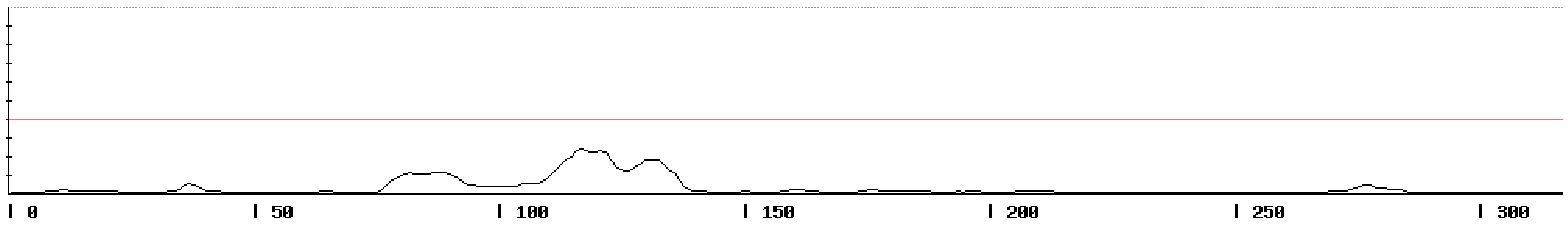

Predictions for Boecherastricta_Bostr.0124s0082.1.p

Viterbi Path

Viterbi Path

Posterior @ 0.4

Posterior @ 0.4

Posterior Graphic. Threshold Marked @ 0.4

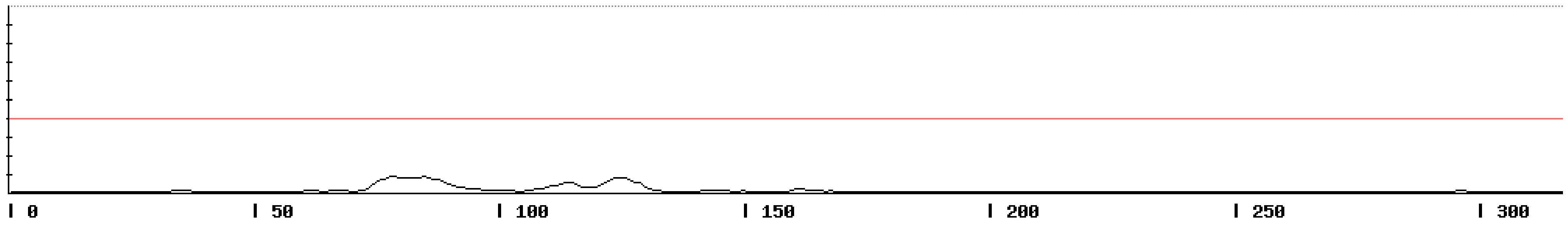

Predictions for Boecherastricta_Bostr.0556s0069.1.p

Viterbi Path

Viterbi Path

Posterior @ 0.4

Posterior @ 0.4

Posterior Graphic. Threshold Marked @ 0.4

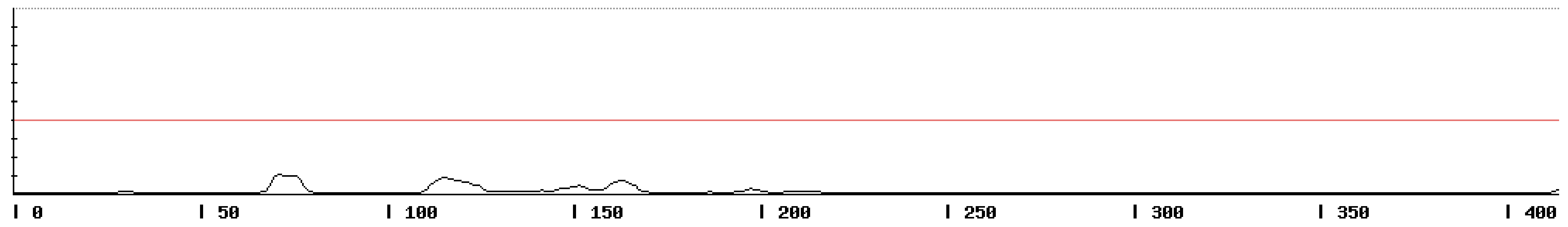

Predictions for Boecherastricta_Bostr.0556s0555.1.p

Viterbi Path

Viterbi Path

Posterior @ 0.4

Posterior @ 0.4

Posterior Graphic. Threshold Marked @ 0.4

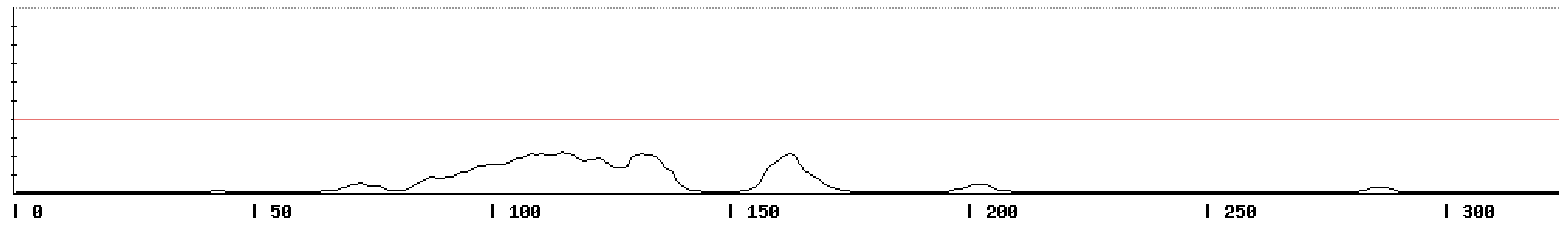

Predictions for Boecherastricta_Bostr.0556s0726.1.p

Viterbi Path

Viterbi Path

Posterior @ 0.4

Posterior @ 0.4

Posterior Graphic. Threshold Marked @ 0.4

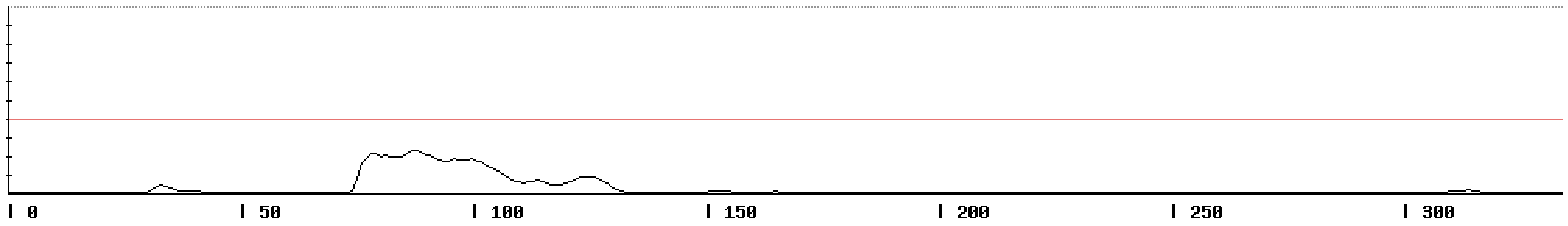

Predictions for Boecherastricta_Bostr.0556s0732.1.p

Viterbi Path

Viterbi Path

Posterior @ 0.4

Posterior @ 0.4

Posterior Graphic. Threshold Marked @ 0.4

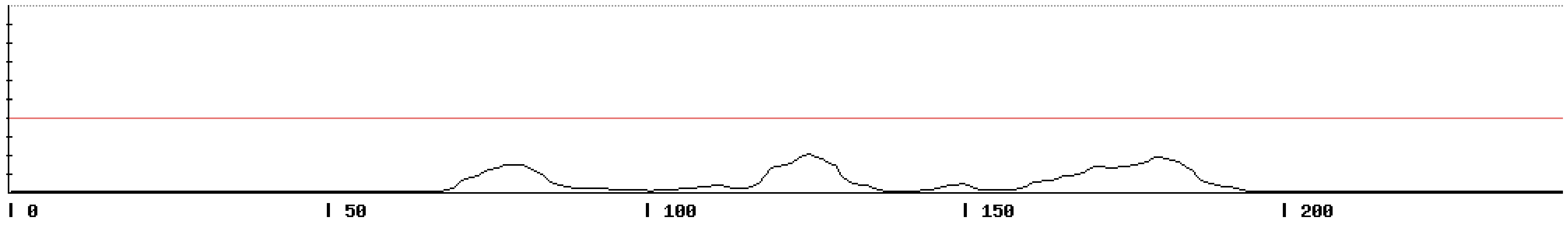

Predictions for Boecherastricta_Bostr.0556s0784.1.p

Viterbi Path	
Viterbi Path	
Posterior @ 0.4	
Posterior @ 0.4	
Posterior Graphic. Threshold Marked @ 0.4	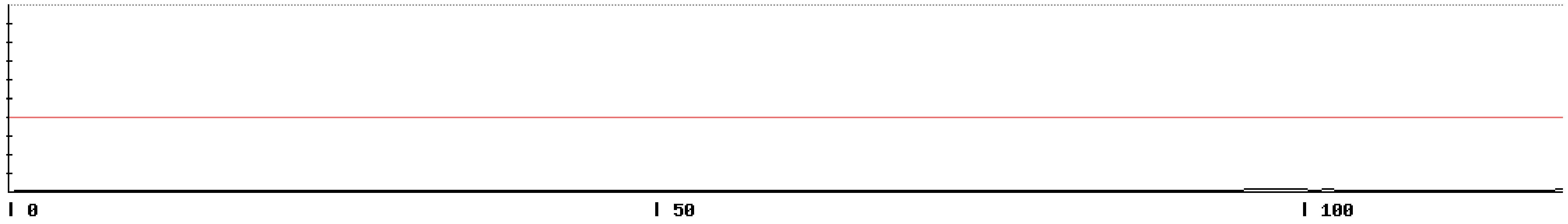

Predictions for Boecherastricta_Bostr.0568s0042.1.p

Viterbi Path	
Viterbi Path	
Posterior @ 0.4	
Posterior @ 0.4	

Posterior Graphic. Threshold Marked @ 0.4

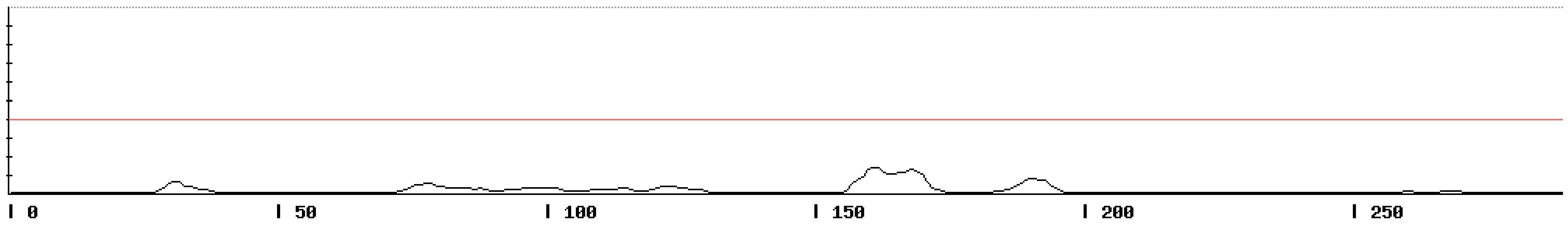

Predictions for Boecherastricta_Bostr.0568s0345.1.p

Viterbi Path

Viterbi Path

Posterior @ 0.4

Posterior @ 0.4

Posterior Graphic. Threshold Marked @ 0.4

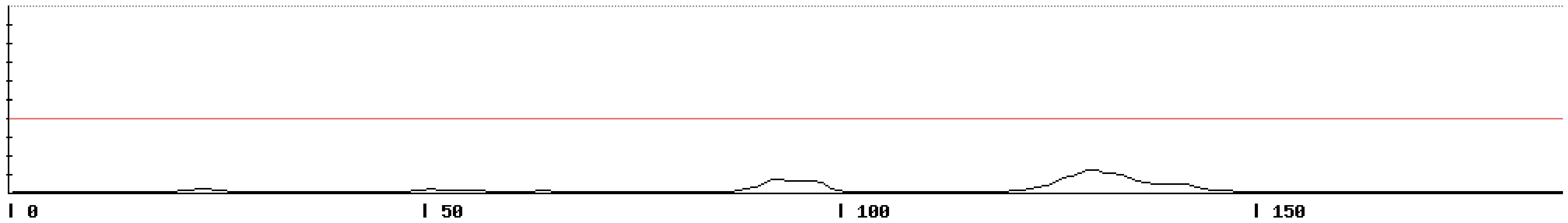

Predictions for Boecherastricta_Bostr.0568s0398.1.p

Viterbi Path

Viterbi Path

Posterior @ 0.4

Posterior @ 0.4

Posterior Graphic. Threshold Marked @ 0.4

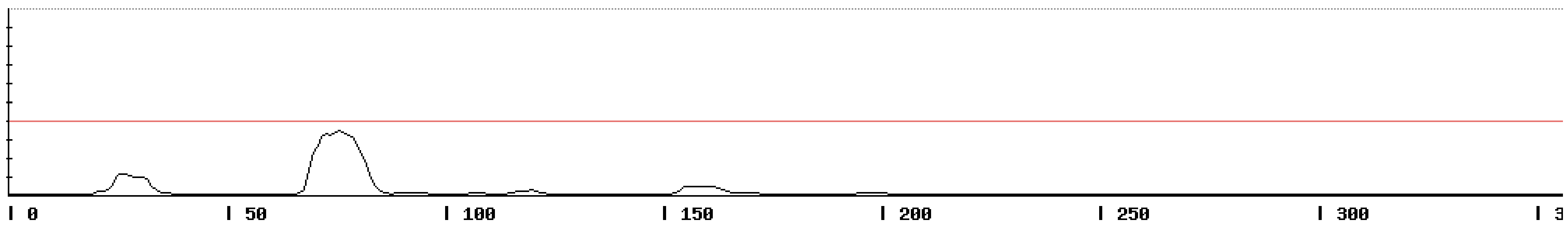

Predictions for Boecherastricta_Bostr.0568s0429.1.p

Viterbi Path

Viterbi Path

Posterior @ 0.4 98 - GKPK - 101

Posterior @ 0.4

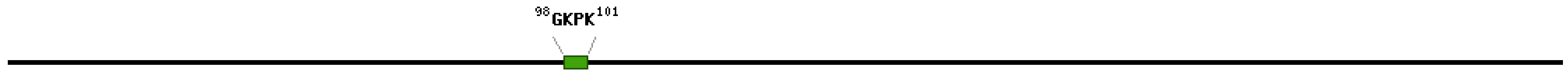

Posterior Graphic. Threshold Marked @ 0.4

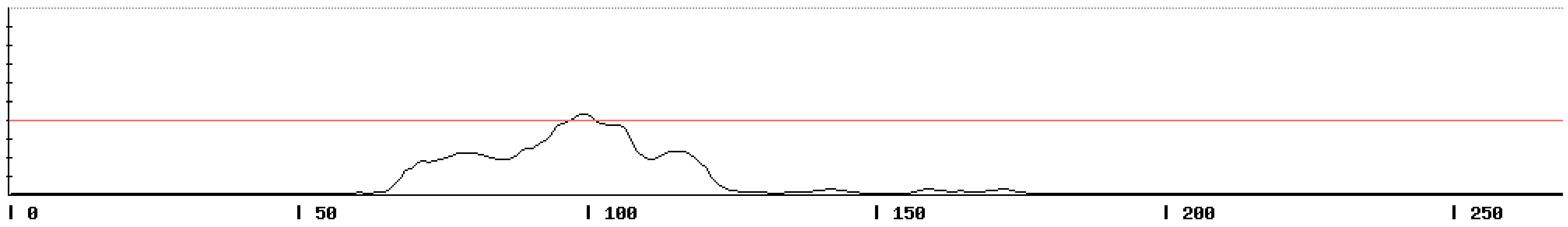

Predictions for Boecherastricta_Bostr.0568s0569.1.p

Viterbi Path

Viterbi Path

Posterior @ 0.4	
Posterior @ 0.4	
Posterior Graphic. Threshold Marked @ 0.4	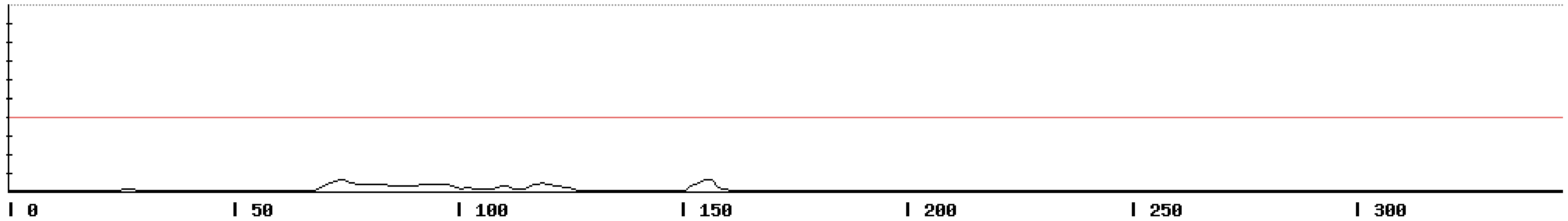

Predictions for Boecherastricta_Bostr.10040s0089.1.p

Viterbi Path	
Viterbi Path	
Posterior @ 0.4	
Posterior @ 0.4	
Posterior Graphic. Threshold Marked @ 0.4	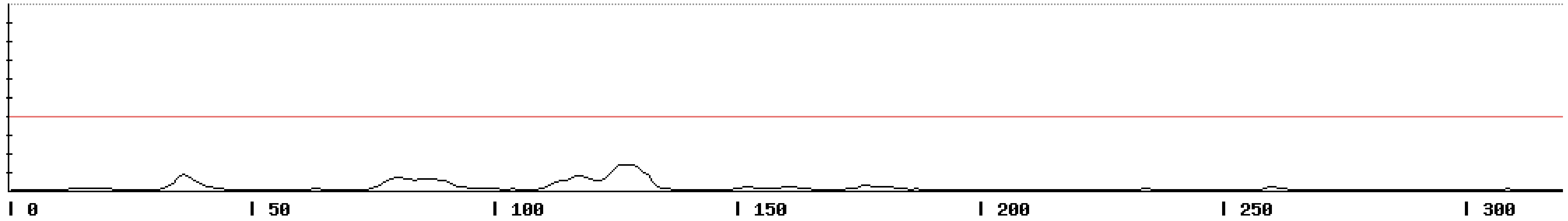

Predictions for Boecherastricta_Bostr.10040s0187.1.p

Viterbi Path	
Viterbi Path	

Posterior @ 0.4

94 - K - 94

97 - GKVLGFKKILKFCEKRKPRE - 116

Posterior @ 0.4

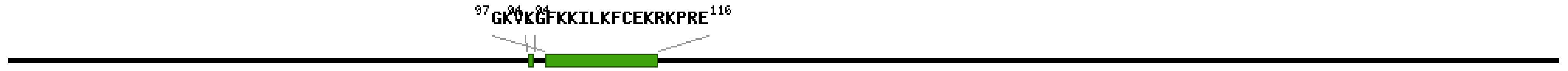

Posterior Graphic. Threshold Marked @ 0.4

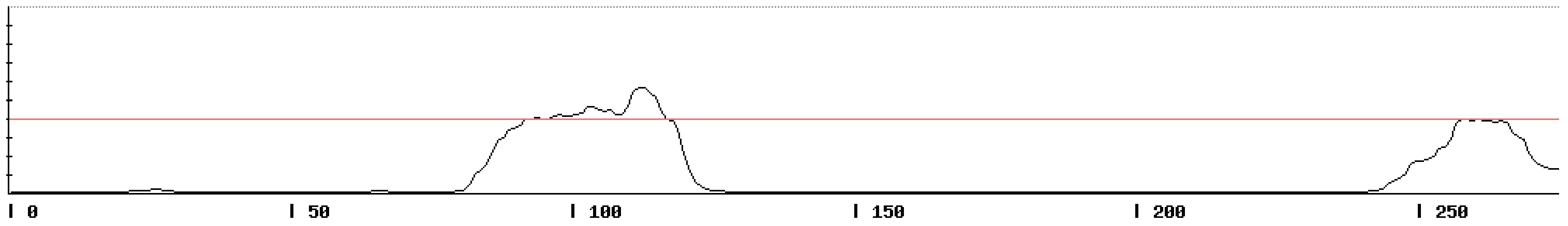

Predictions for Boecherastricta_Bostr.10040s0190.1.p

Viterbi Path

Viterbi Path

Posterior @ 0.4

115 - KKKKPREYK - 123

Posterior @ 0.4

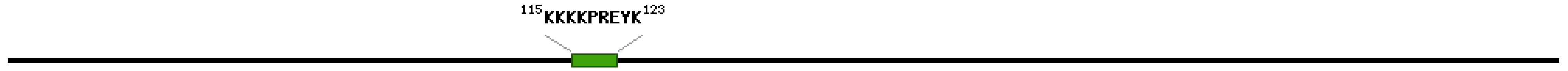

Posterior Graphic. Threshold Marked @ 0.4

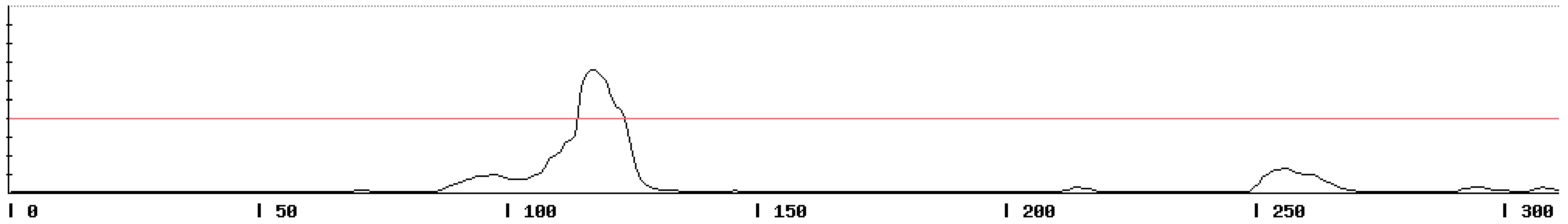

Predictions for Boecherastricta_Bostr.10040s0194.1.p	
Viterbi Path	
Viterbi Path	
Posterior @ 0.4	122 - EKRRVR - 127
Posterior @ 0.4	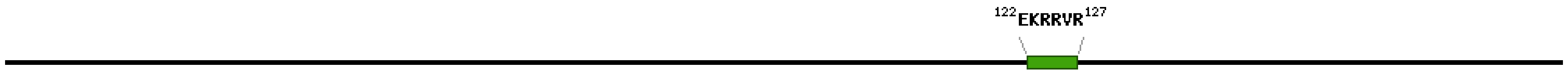
Posterior Graphic. Threshold Marked @ 0.4	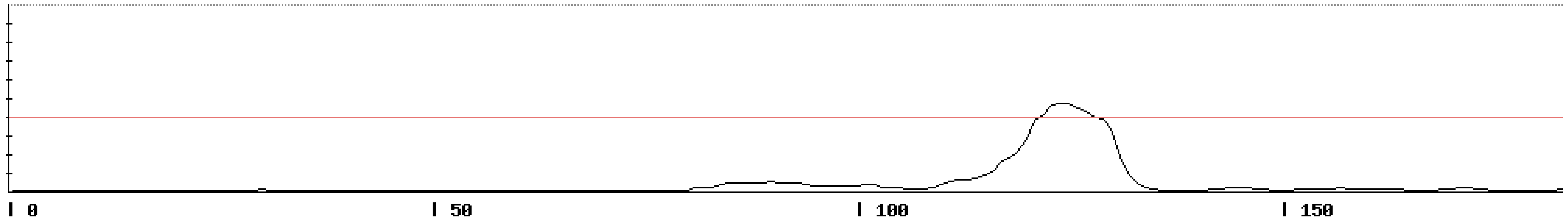

Predictions for Boecherastricta_Bostr.10040s0202.1.p	
Viterbi Path	
Viterbi Path	
Posterior @ 0.4	75 - KKILK - 79<br>82 - EKRKPRE - 88
Posterior @ 0.4	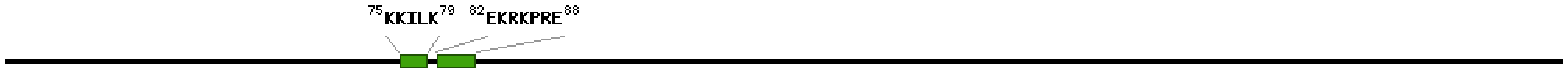
Posterior Graphic. Threshold Marked @ 0.4	

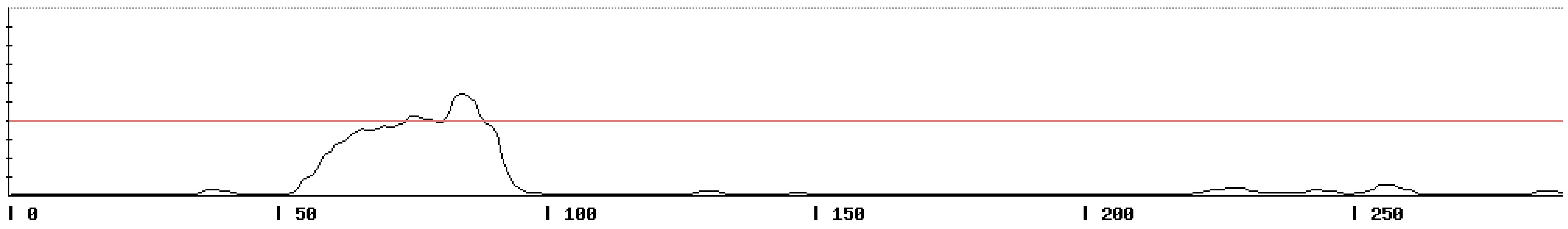

Predictions for Boecherastricta_Bostr.10058s0013.1.p

Viterbi Path

Viterbi Path

Posterior @ 0.4

Posterior @ 0.4

Posterior Graphic. Threshold Marked @ 0.4

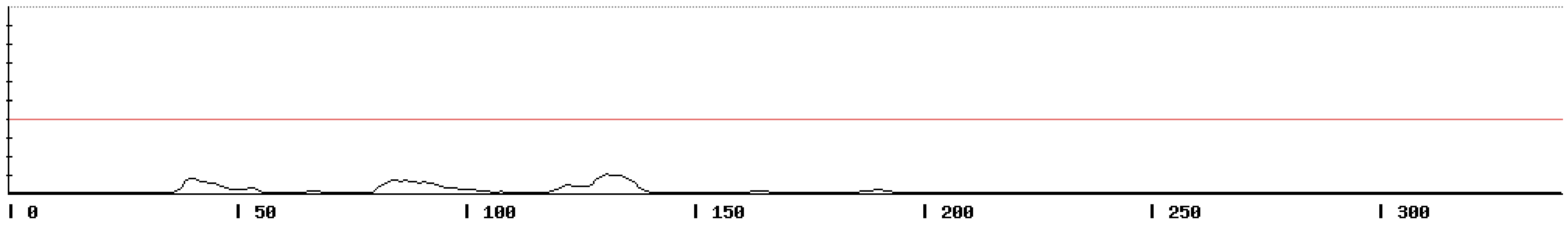

Predictions for Boecherastricta_Bostr.10058s0039.1.p

Viterbi Path

Viterbi Path

Posterior @ 0.4

Posterior @ 0.4

Posterior Graphic. Threshold Marked @ 0.4

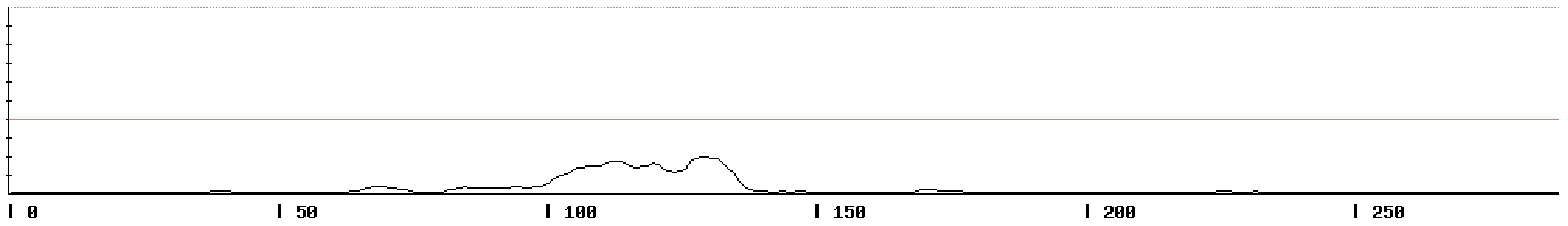

Predictions for Boecherastricta_Bostr.10064s0010.1.p

Viterbi Path

Viterbi Path

Posterior @ 0.4

Posterior @ 0.4

Posterior Graphic. Threshold Marked @ 0.4

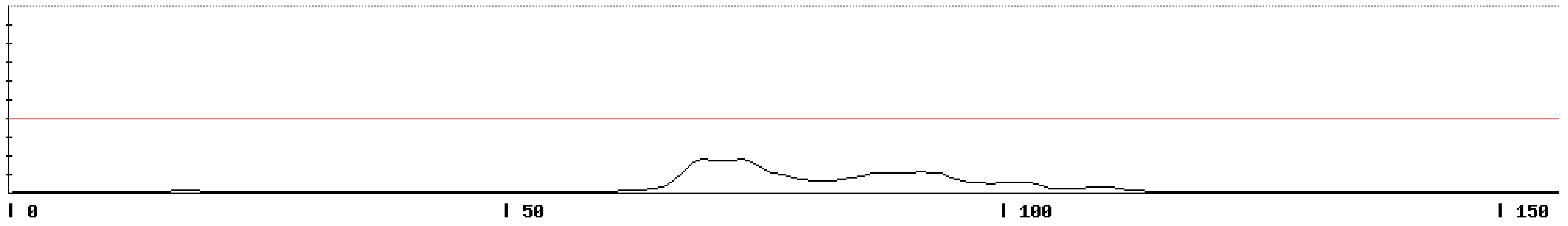

Predictions for Boecherastricta_Bostr.10273s0160.1.p

Viterbi Path

Viterbi Path

Posterior @ 0.4

Posterior @ 0.4

Posterior Graphic. Threshold Marked @ 0.4

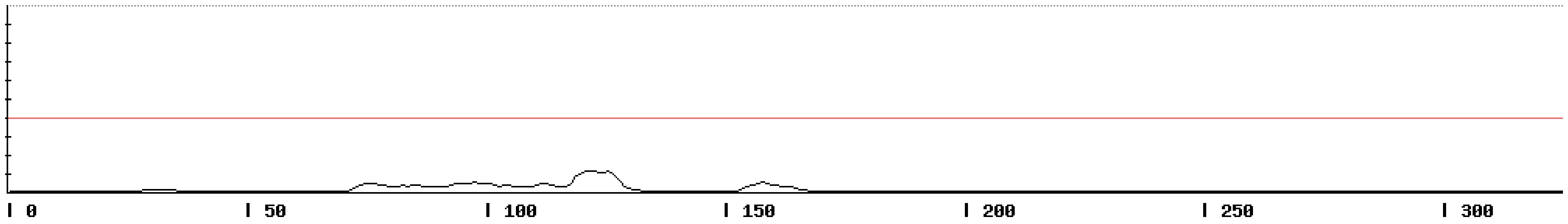

Predictions for Boecherastricta_Bostr.10273s0400.1.p

Viterbi Path

Viterbi Path

Posterior @ 0.4

Posterior @ 0.4

Posterior Graphic. Threshold Marked @ 0.4

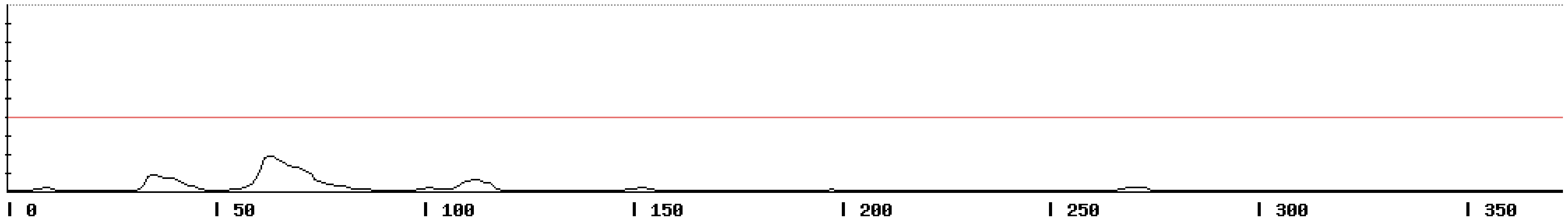

Predictions for Boecherastricta_Bostr.10273s0418.1.p

Viterbi Path

Viterbi Path

Posterior @ 0.4

Posterior @ 0.4

Posterior Graphic. Threshold Marked @ 0.4

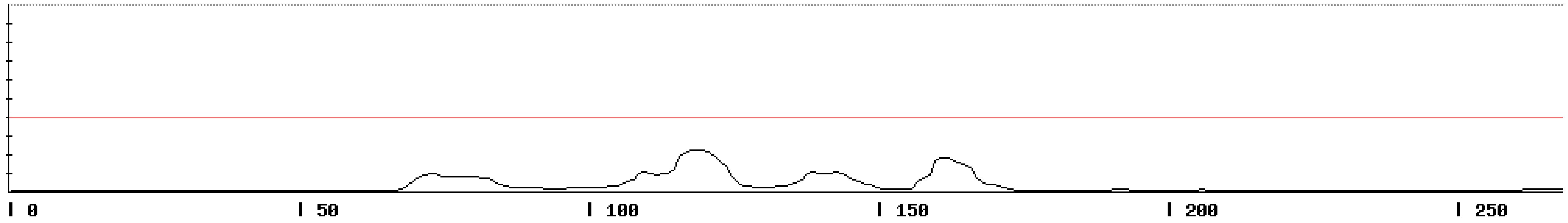

Predictions for Boecherastricta_Bostr.1040s0050.1.p

Viterbi Path

Viterbi Path

Posterior @ 0.4

Posterior @ 0.4

Posterior Graphic. Threshold Marked @ 0.4

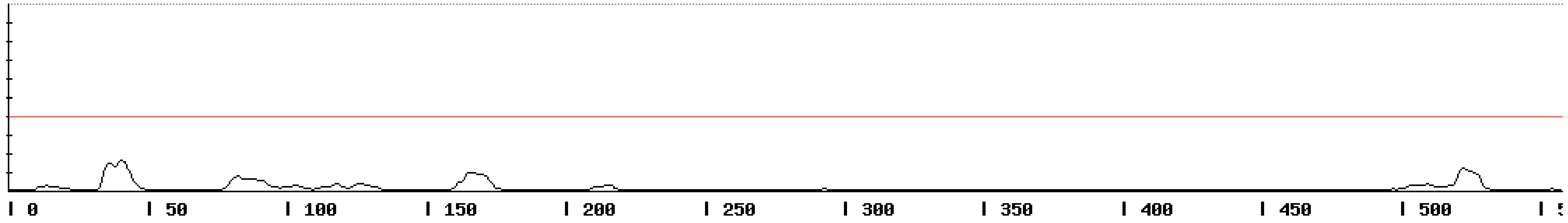

Predictions for Boecherastricta_Bostr.1040s0051.1.p

Viterbi Path

Viterbi Path

Posterior @ 0.4

Posterior @ 0.4

Posterior Graphic. Threshold Marked @ 0.4

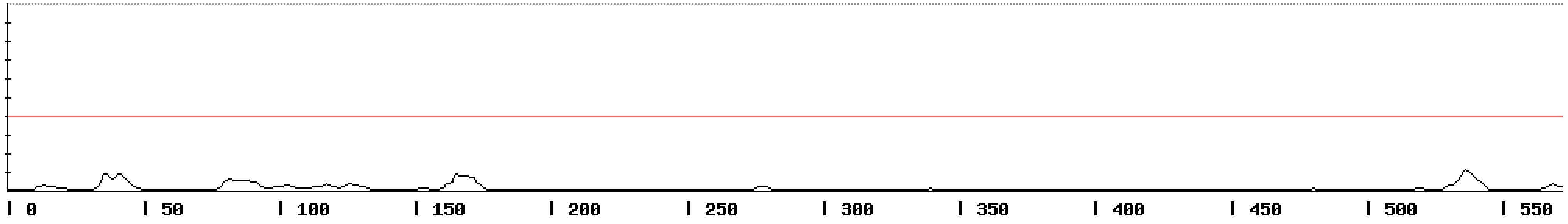

Predictions for Boecherastricta_Bostr.12659s0312.1.p

Viterbi Path

Viterbi Path

Posterior @ 0.4

Posterior @ 0.4

Posterior Graphic. Threshold Marked @ 0.4

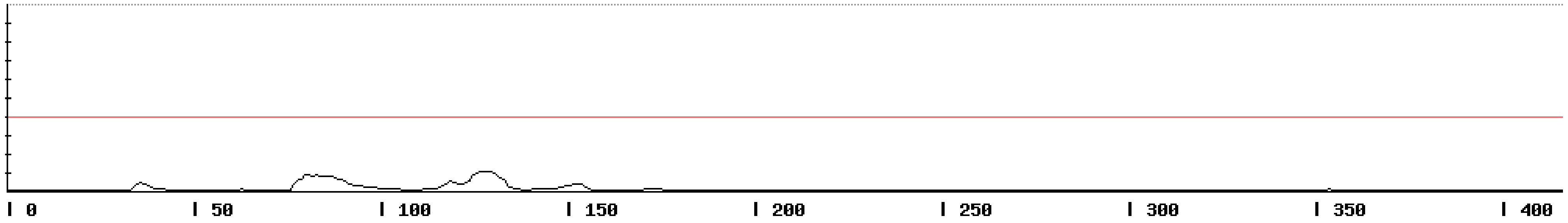

Predictions for Boecherastricta_Bostr.12659s0396.1.p

Viterbi Path	
Viterbi Path	
Posterior @ 0.4	137 - KRR - 139
Posterior @ 0.4	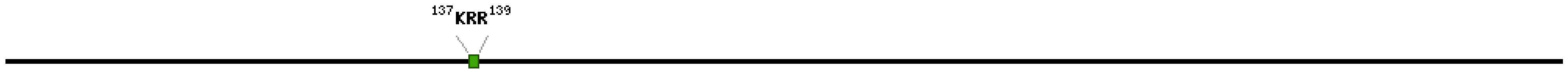
Posterior Graphic. Threshold Marked @ 0.4	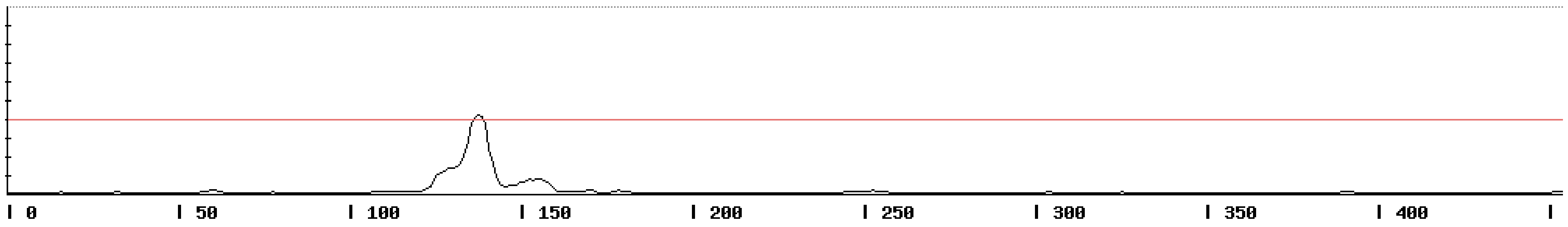

Predictions for Boecherastricta_Bostr.13129s0062.1.p

Viterbi Path	
Viterbi Path	
Posterior @ 0.4	
Posterior @ 0.4	
Posterior Graphic. Threshold Marked @ 0.4	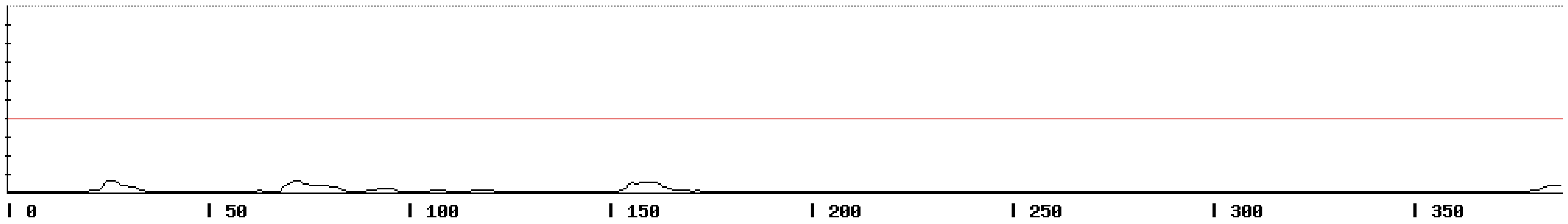

Predictions for Boecherastricta_Bostr.13129s0073.1.p

Viterbi Path	
Viterbi Path	
Posterior @ 0.4	98 - GKPK - 101
Posterior @ 0.4	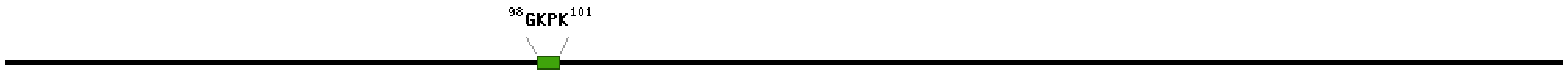
Posterior Graphic. Threshold Marked @ 0.4	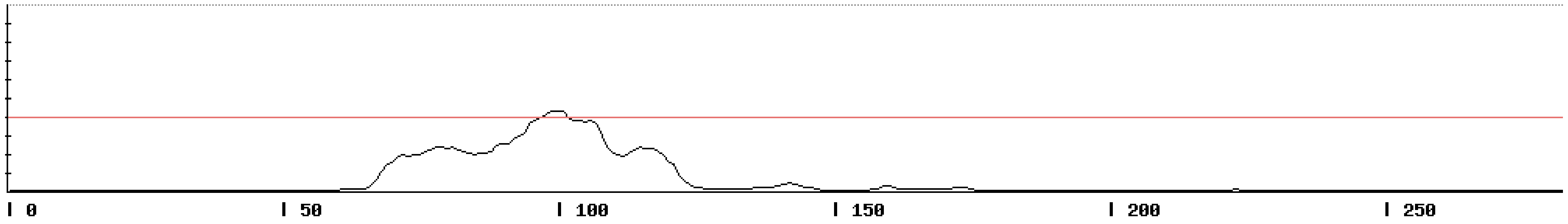

Predictions for Boecherastricta_Bostr.13129s0198.1.p

Viterbi Path	
Viterbi Path	
Posterior @ 0.4	
Posterior @ 0.4	
Posterior Graphic. Threshold Marked @ 0.4	

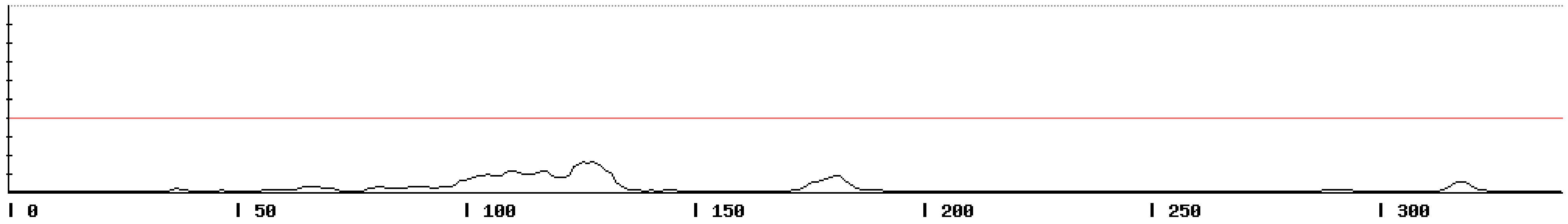

Predictions for Boecherastricta_Bostr.13158s0196.1.p	
Viterbi Path	
Viterbi Path	
Posterior @ 0.4	
Posterior @ 0.4	
Posterior Graphic. Threshold Marked @ 0.4	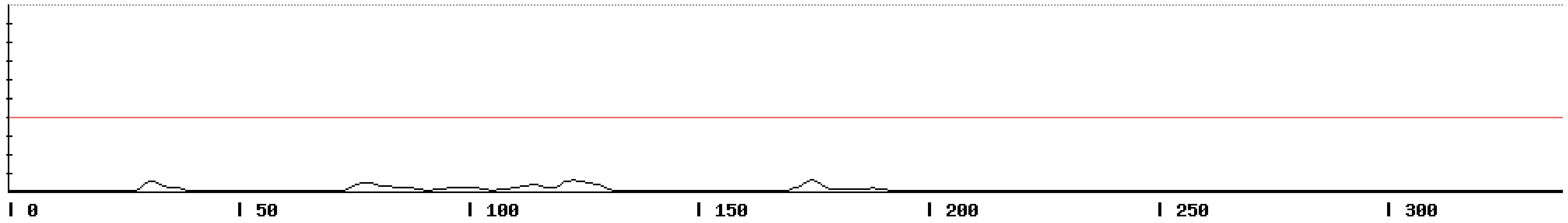

Predictions for Boecherastricta_Bostr.13671s0233.1.p	
Viterbi Path	
Viterbi Path	
Posterior @ 0.4	
Posterior @ 0.4	

Posterior Graphic. Threshold Marked @ 0.4

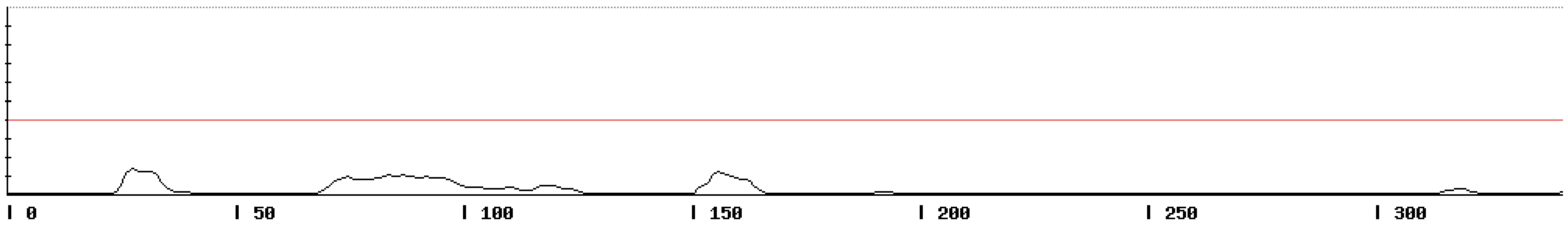

Predictions for Boecherastricta_Bostr.1460s0050.1.p

Viterbi Path	
Viterbi Path	
Posterior @ 0.4	
Posterior @ 0.4	
Posterior Graphic. Threshold Marked @ 0.4	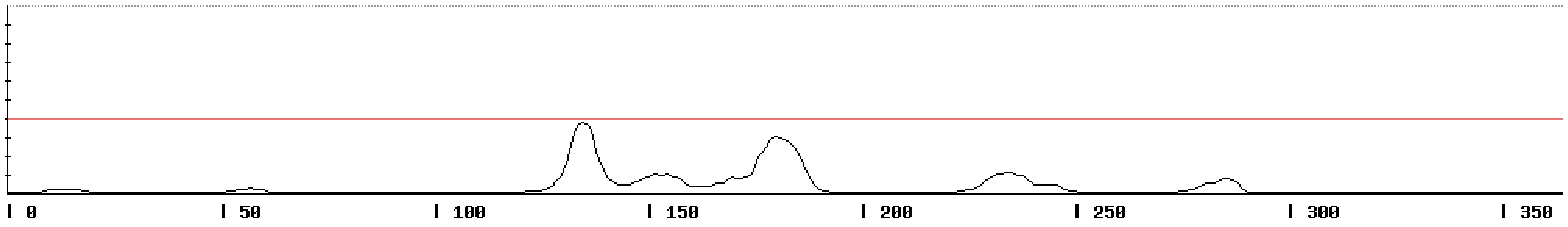

Predictions for Boecherastricta_Bostr.15697s0168.1.p

Viterbi Path	
Viterbi Path	
Posterior @ 0.4	156 - RKRR - 159
Posterior @ 0.4	

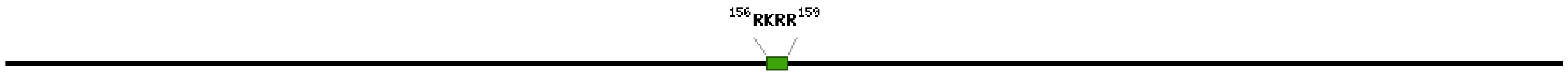

Posterior Graphic. Threshold Marked @ 0.4

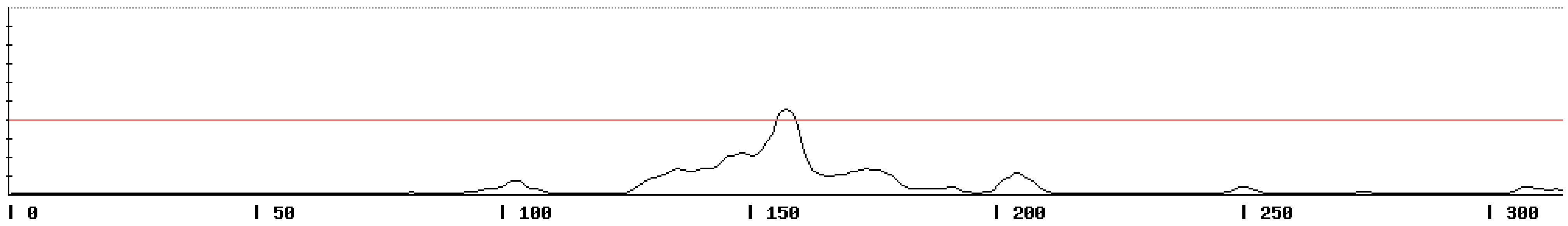

Predictions for Boecherastricta_Bostr.15697s0466.1.p

Viterbi Path

Viterbi Path

Posterior @ 0.4

Posterior @ 0.4

Posterior Graphic. Threshold Marked @ 0.4

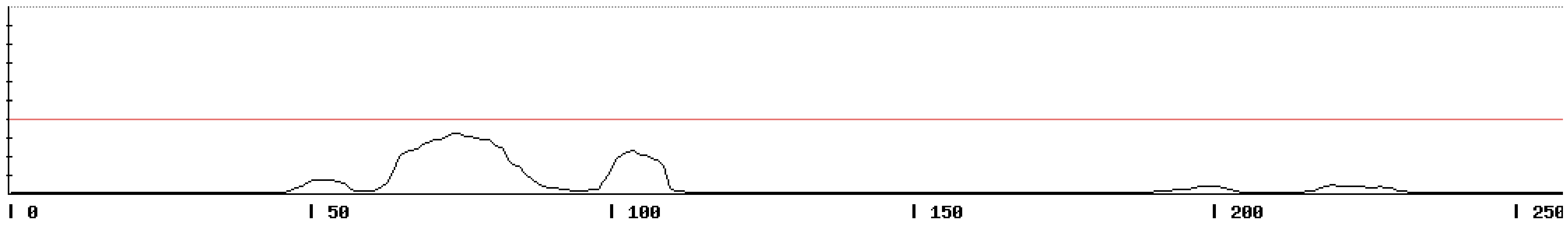

Predictions for Boecherastricta_Bostr.15774s0177.1.p

Viterbi Path

Viterbi Path

Posterior @ 0.4	
Posterior @ 0.4	
Posterior Graphic. Threshold Marked @ 0.4	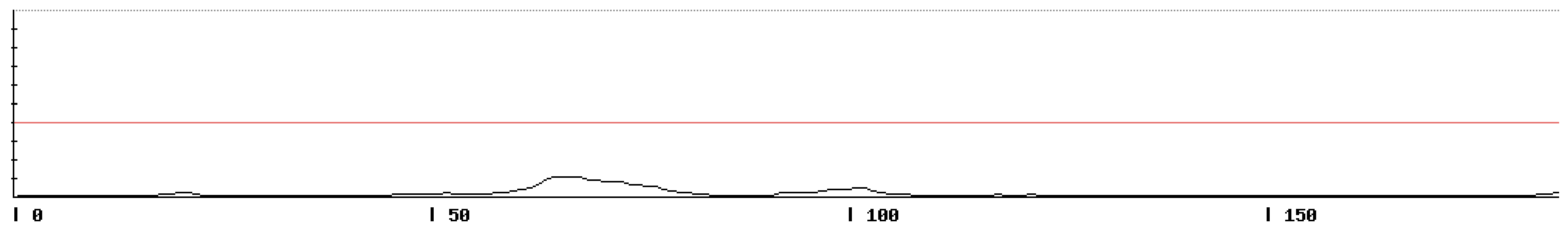

Predictions for Boecherastricta_Bostr.15774s0178.1.p

Viterbi Path	
Viterbi Path	
Posterior @ 0.4	71 - GRHQRRRRASRTGIWKVTGKK - 91
Posterior @ 0.4	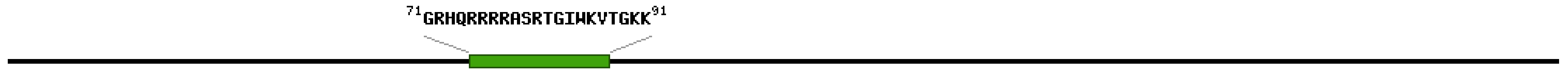
Posterior Graphic. Threshold Marked @ 0.4	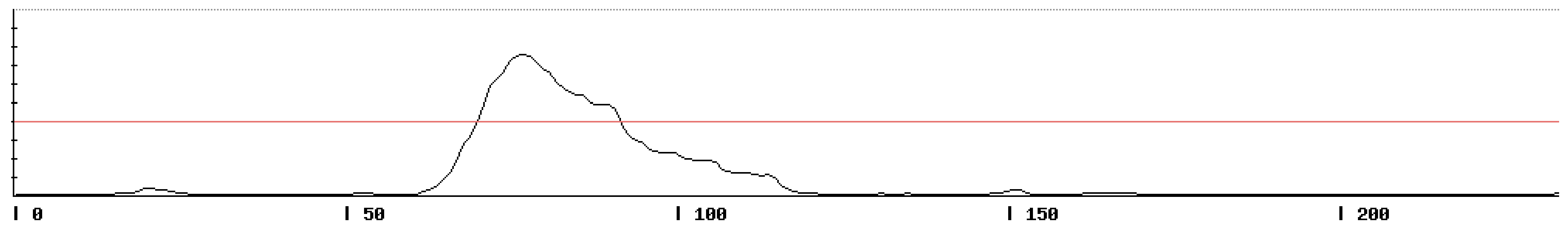

Predictions for Boecherastricta_Bostr.15774s0222.1.p

Viterbi Path	
Viterbi Path	

Posterior @ 0.4

Posterior @ 0.4

Posterior Graphic. Threshold Marked @ 0.4

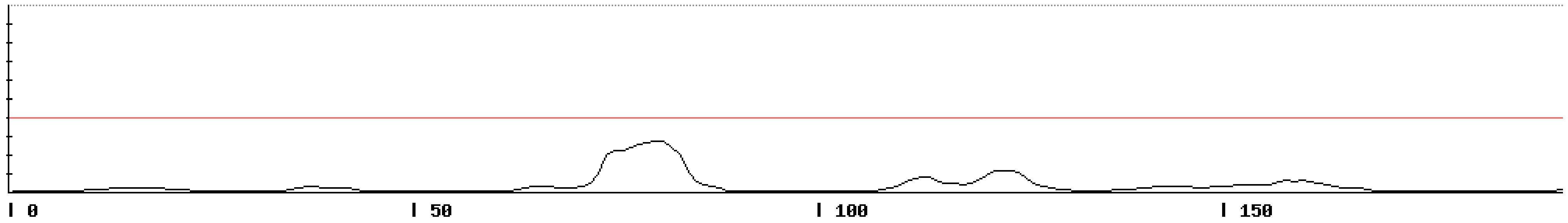

Predictions for Boecherastricta_Bostr.18351s0021.1.p

Viterbi Path

Viterbi Path

Posterior @ 0.4

196 - PKAVKRKRAEMKEDEKLKKEKG - 217

273 - PKAAKRKKAEMKEDEKLKK - 291

350 - PKAAKKKKADIKEDEKLKK - 368

Posterior @ 0.4

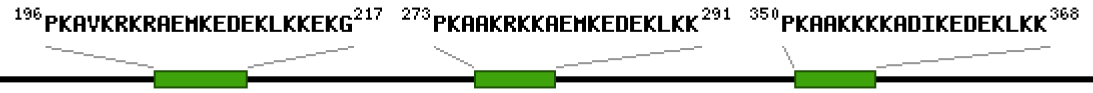

Posterior Graphic. Threshold Marked @ 0.4

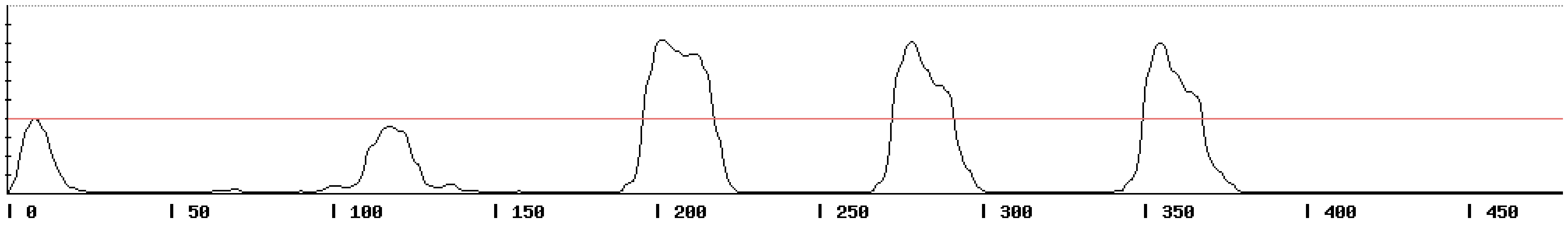

Predictions for Boecherastricta_Bostr.18351s0165.1.p	
Viterbi Path	
Viterbi Path	
Posterior @ 0.4	
Posterior @ 0.4	
Posterior Graphic. Threshold Marked @ 0.4	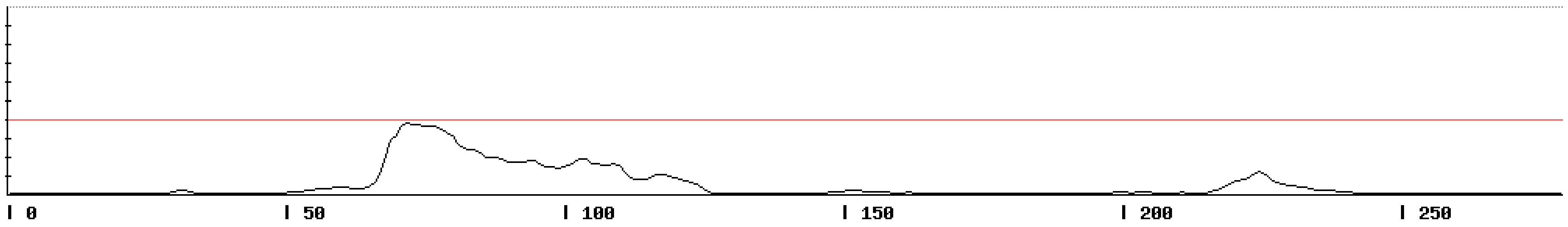

Predictions for Boecherastricta_Bostr.18351s0273.1.p	
Viterbi Path	
Viterbi Path	
Posterior @ 0.4	275 - RENKRKKKK - 283
Posterior @ 0.4	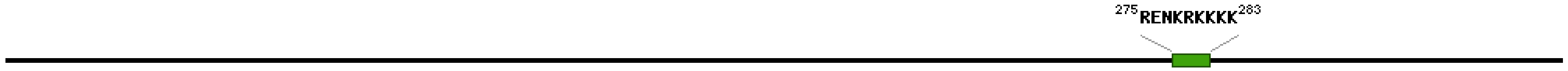

Posterior Graphic. Threshold Marked @ 0.4

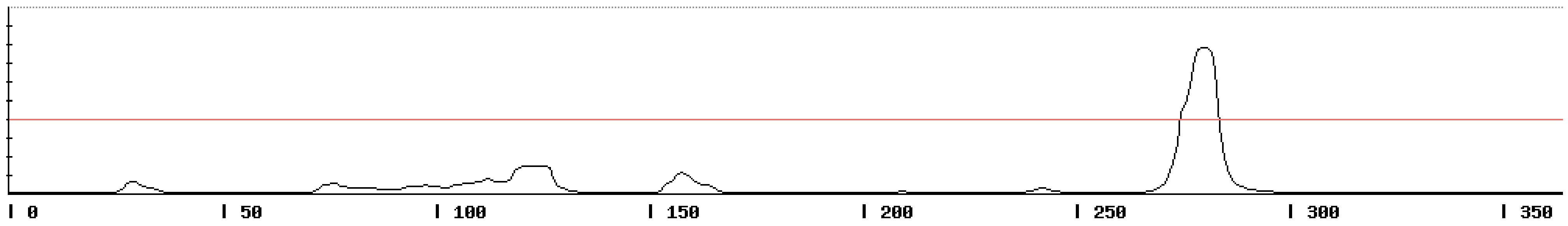

Predictions for Boecherastricta_Bostr.19046s0031.1.p

Viterbi Path

Viterbi Path

Posterior @ 0.4

Posterior @ 0.4

Posterior Graphic. Threshold Marked @ 0.4

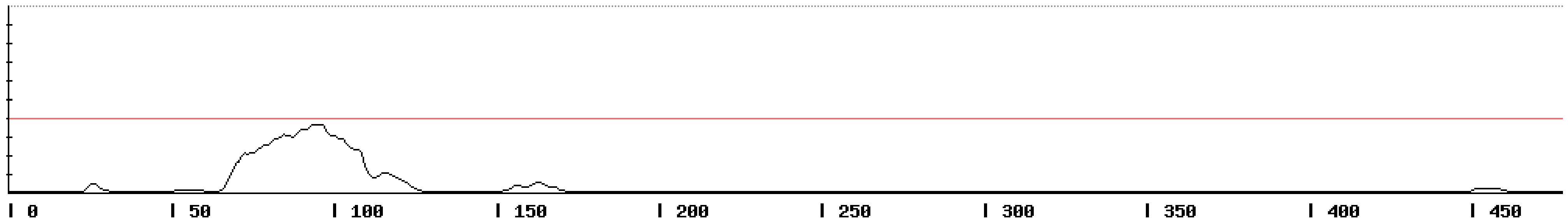

Predictions for Boecherastricta_Bostr.19424s0822.1.p

Viterbi Path

Viterbi Path

Posterior @ 0.4

Posterior @ 0.4

Posterior Graphic. Threshold Marked @ 0.4

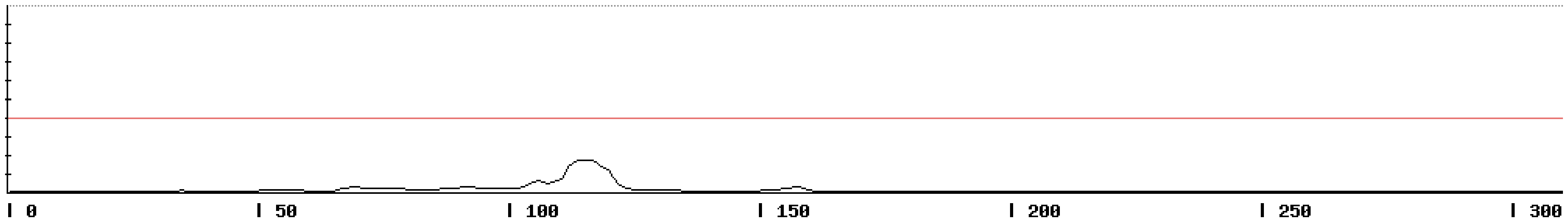

Predictions for Boecherastricta_Bostr.19424s0897.1.p

Viterbi Path

Viterbi Path

Posterior @ 0.4

Posterior @ 0.4

Posterior Graphic. Threshold Marked @ 0.4

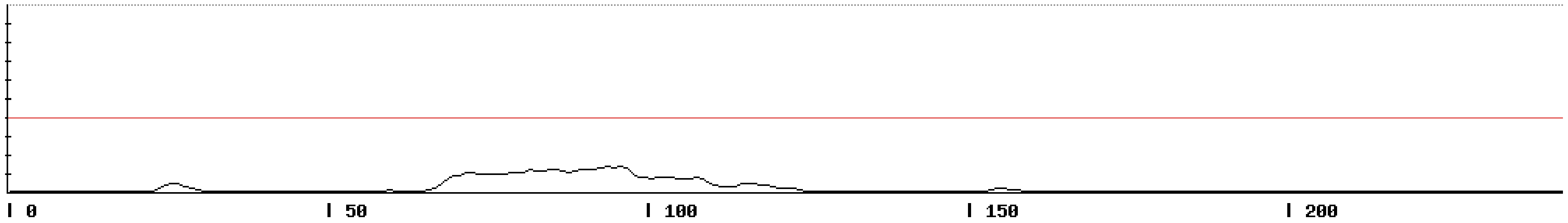

Predictions for Boecherastricta_Bostr.20129s0079.1.p

Viterbi Path

Viterbi Path

Posterior @ 0.4	
Posterior @ 0.4	
Posterior Graphic. Threshold Marked @ 0.4	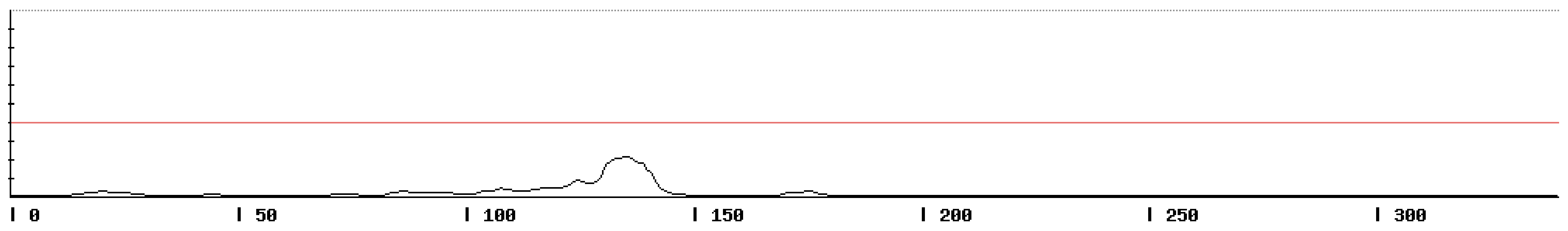

Predictions for Boecherastricta_Bostr.20129s0180.1.p

Viterbi Path	
Viterbi Path	
Posterior @ 0.4	100 - IGRPKPVG - 107
Posterior @ 0.4	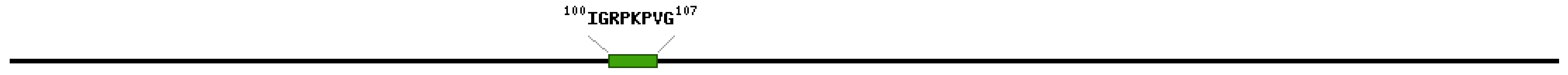
Posterior Graphic. Threshold Marked @ 0.4	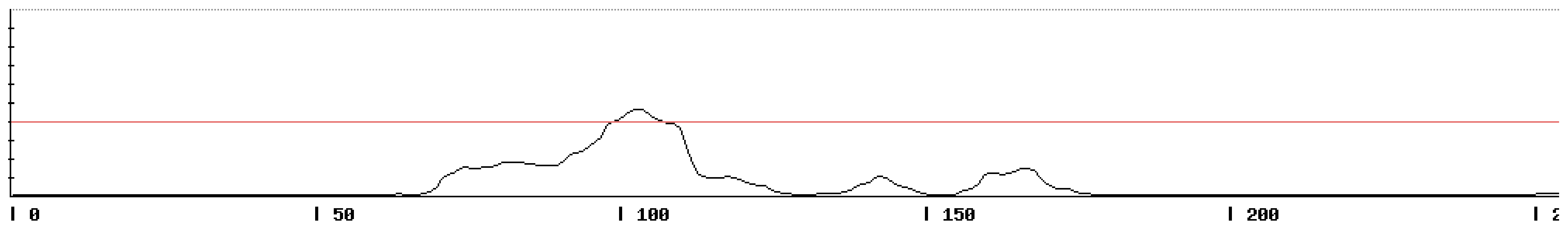

Predictions for Boecherastricta_Bostr.20129s0396.1.p

Viterbi Path	
Viterbi Path	

Posterior @ 0.4

Posterior @ 0.4

Posterior Graphic. Threshold Marked @ 0.4

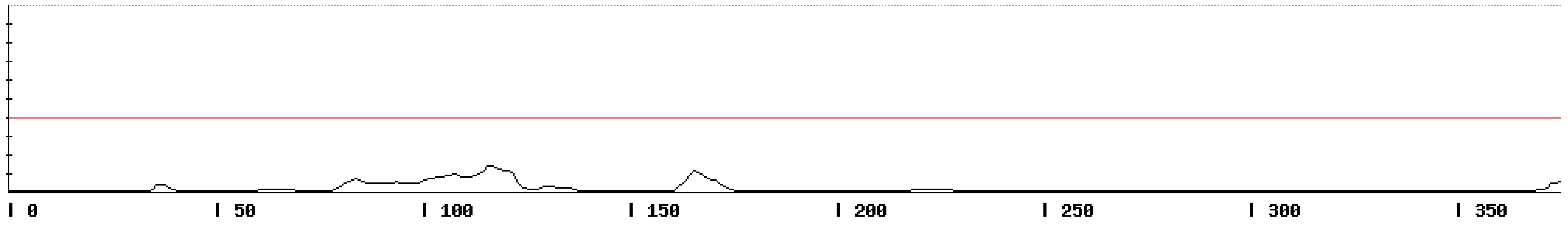

Predictions for Boecherastricta_Bostr.20505s0101.1.p

Viterbi Path

Viterbi Path

Posterior @ 0.4

8 - KRK - 10

120 - IGKGGRMKR - 128

202 - KAAKKKKA - 209

Posterior @ 0.4

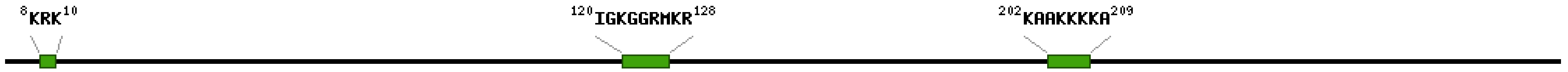

Posterior Graphic. Threshold Marked @ 0.4

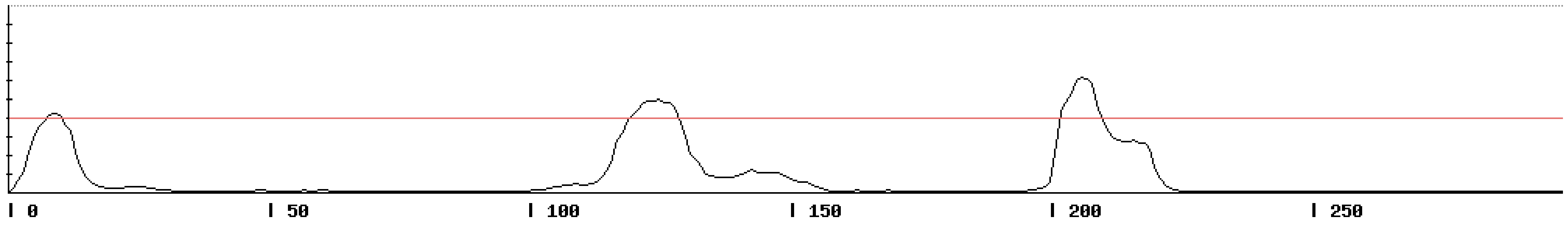

Predictions for Boecherastricta_Bostr.20505s0102.1.p

Viterbi Path

Viterbi Path

Posterior @ 0.4

Posterior @ 0.4

Posterior Graphic. Threshold Marked @ 0.4

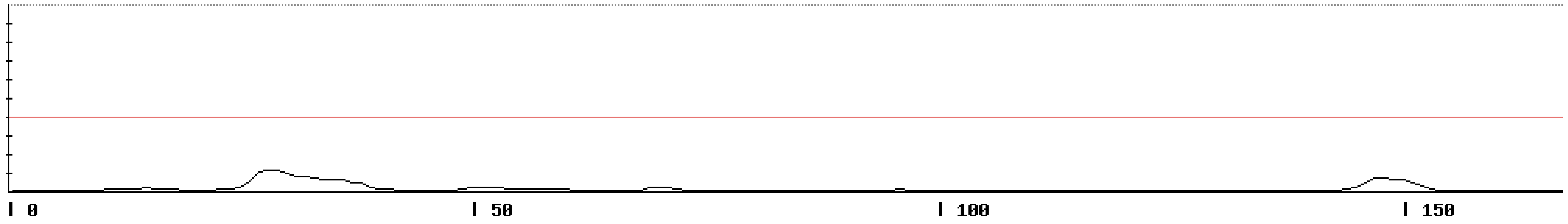

Predictions for Boecherastricta_Bostr.21132s0013.1.p

Viterbi Path

Viterbi Path

Posterior @ 0.4

136 - KIHKKKKSKKDKDH - 149

161 - SKSHKKKNSKKEKAH - 175

Posterior @ 0.4

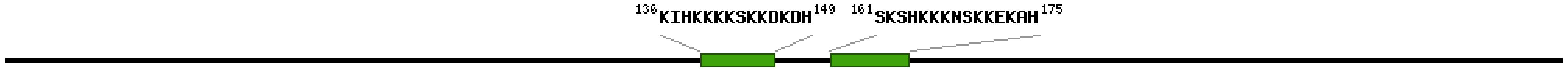

Posterior Graphic. Threshold Marked @ 0.4

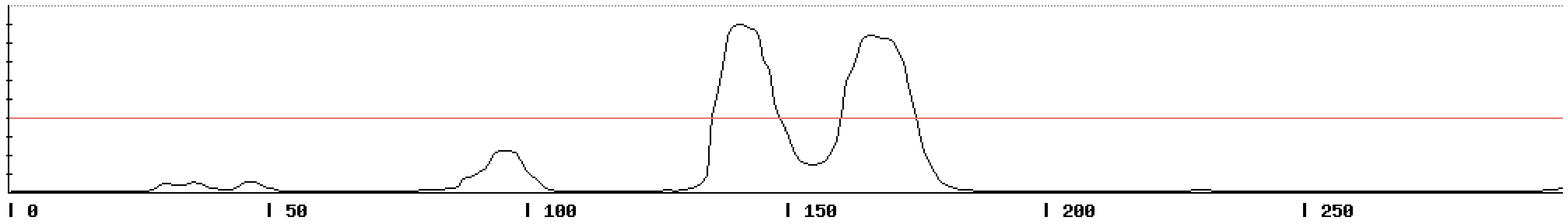

Predictions for Boecherastricta_Bostr.2128s0007.1.p

Viterbi Path

Viterbi Path

Posterior @ 0.4

Posterior @ 0.4

Posterior Graphic. Threshold Marked @ 0.4

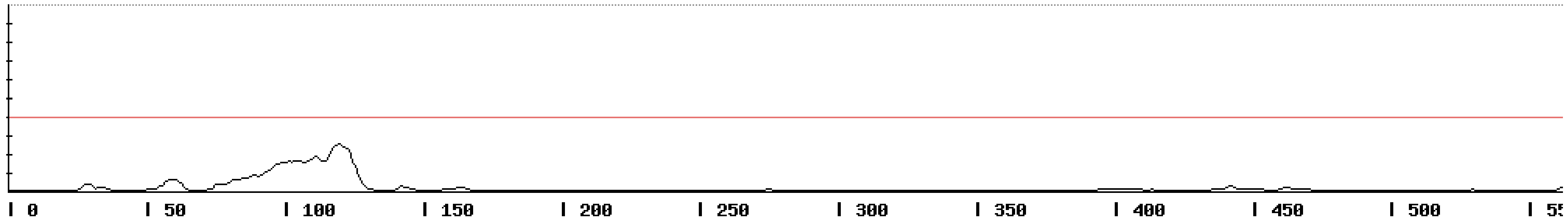

Predictions for Boecherastricta_Bostr.2128s0008.1.p

Viterbi Path

Viterbi Path

Posterior @ 0.4

Posterior @ 0.4

Posterior Graphic. Threshold Marked @ 0.4

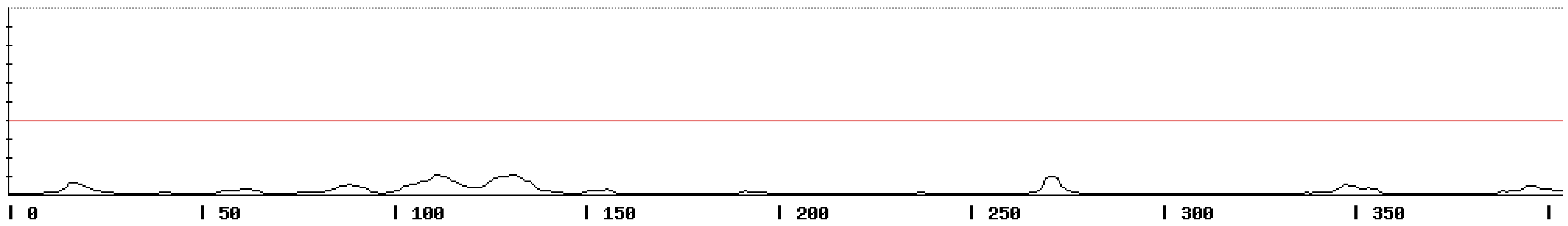

Predictions for Boecherastricta_Bostr.22157s0066.1.p

Viterbi Path

Viterbi Path

Posterior @ 0.4

Posterior @ 0.4

Posterior Graphic. Threshold Marked @ 0.4

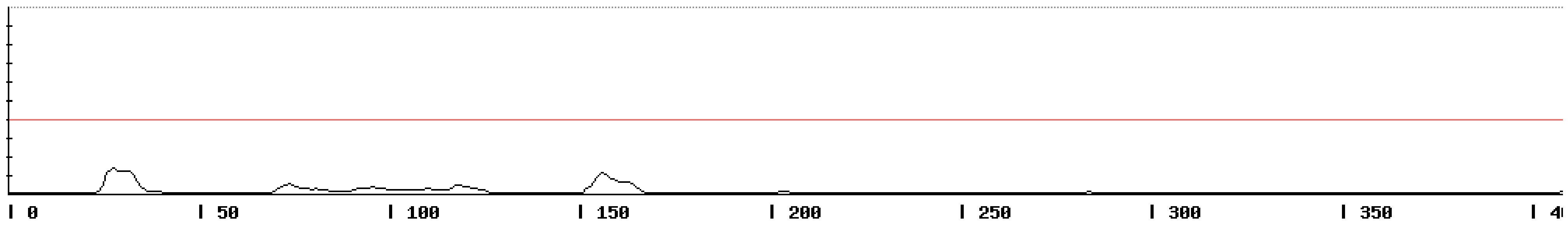

Predictions for Boecherastricta_Bostr.22252s0010.1.p

Viterbi Path

Viterbi Path

Posterior @ 0.4

Posterior @ 0.4

Posterior Graphic. Threshold Marked @ 0.4

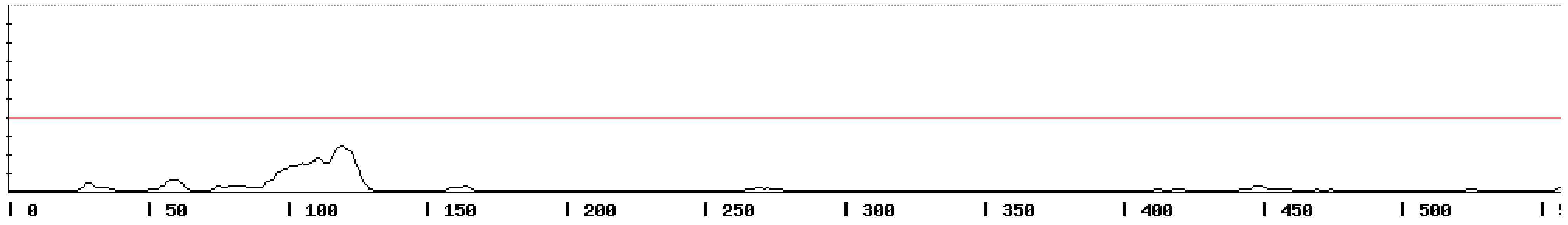

Predictions for Boecherastricta_Bostr.22252s0011.1.p

Viterbi Path

Viterbi Path

Posterior @ 0.4

Posterior @ 0.4

Posterior Graphic. Threshold Marked @ 0.4

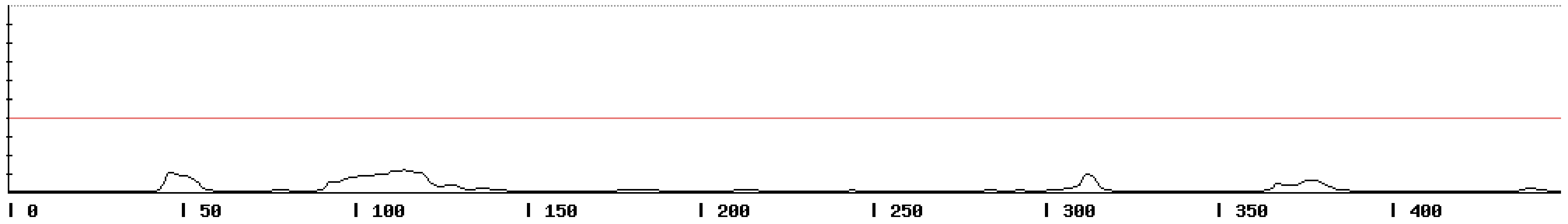

Predictions for Boecherastricta_Bostr.22252s0011.2.p

Viterbi Path

Viterbi Path

Posterior @ 0.4

Posterior @ 0.4

Posterior Graphic. Threshold Marked @ 0.4

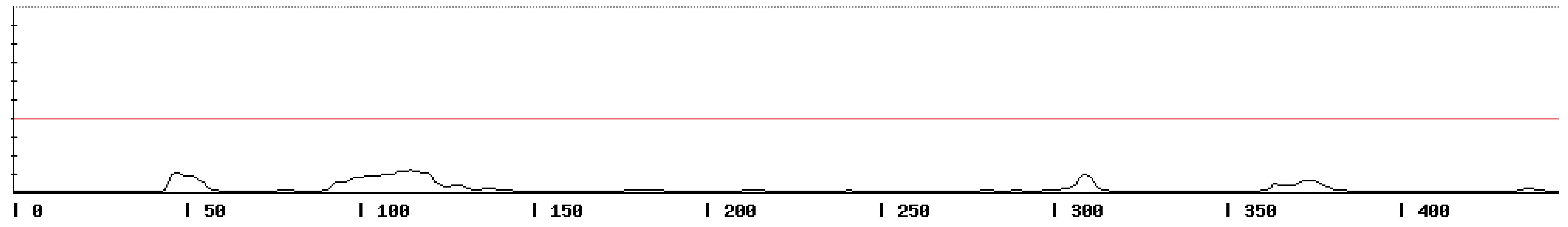

Predictions for Boecherastricta_Bostr.22252s0012.1.p

Viterbi Path

Viterbi Path

Posterior @ 0.4

Posterior @ 0.4

Posterior Graphic. Threshold Marked @ 0.4

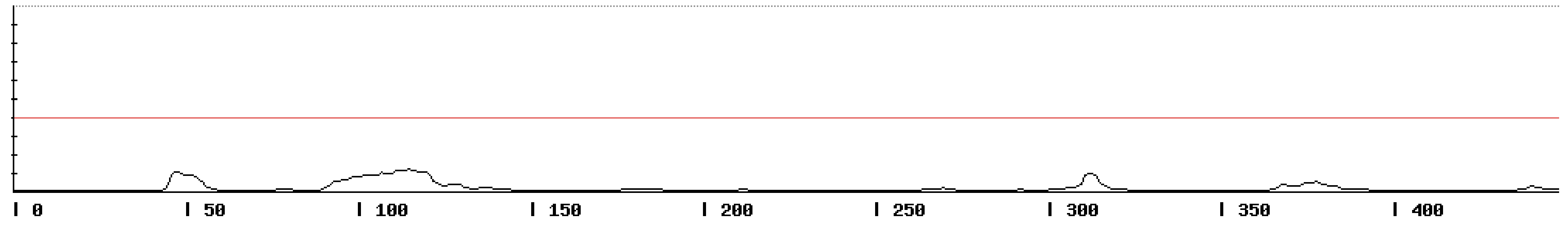

Predictions for Boecherastricta_Bostr.23794s0753.1.p

Viterbi Path	
Viterbi Path	
Posterior @ 0.4	161 - KRSNSNNKRKK - 171<br>204 - RKDKKTRRRR - 213
Posterior @ 0.4	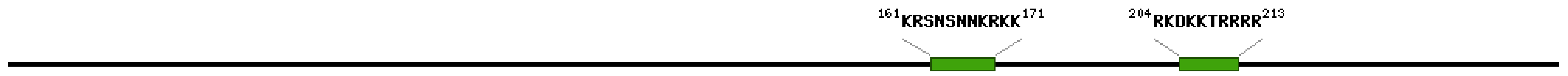
Posterior Graphic. Threshold Marked @ 0.4	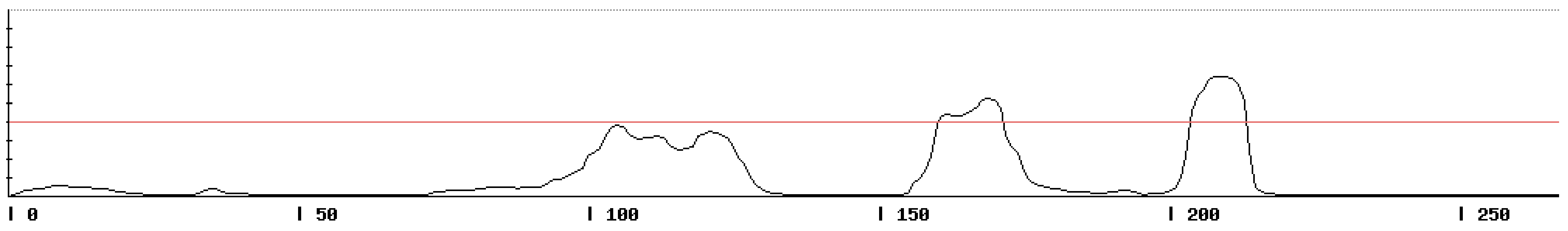

Predictions for Boecherastricta_Bostr.25375s0002.1.p

Viterbi Path	
Viterbi Path	
Posterior @ 0.4	
Posterior @ 0.4	
Posterior Graphic. Threshold Marked @ 0.4	

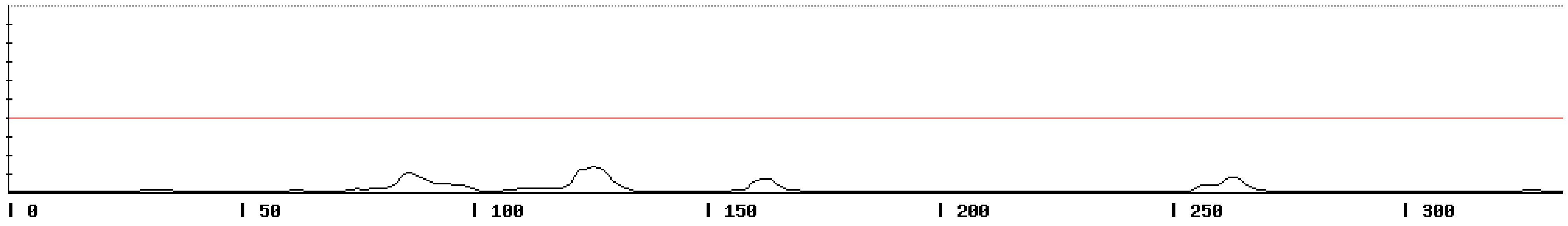

Predictions for Boecherastricta_Bostr.25463s0283.1.p

Viterbi Path	
Viterbi Path	
Posterior @ 0.4	
Posterior @ 0.4	
Posterior Graphic. Threshold Marked @ 0.4	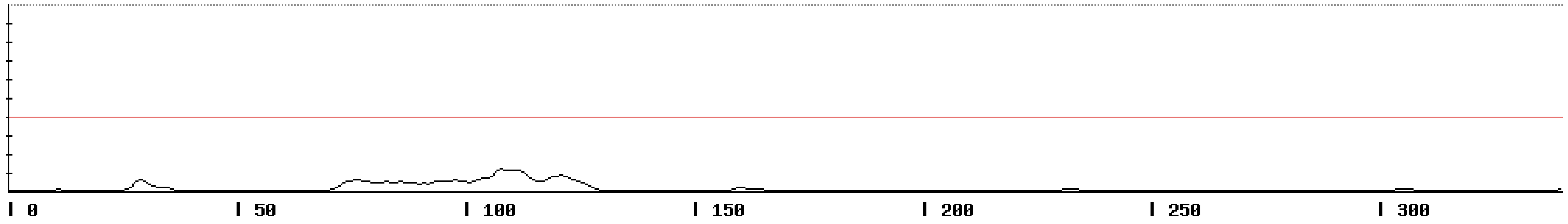

Predictions for Boecherastricta_Bostr.25463s0411.1.p

Viterbi Path	
Viterbi Path	
Posterior @ 0.4	65 - RNKLRGKIPRRTVPGR - 80
Posterior @ 0.4	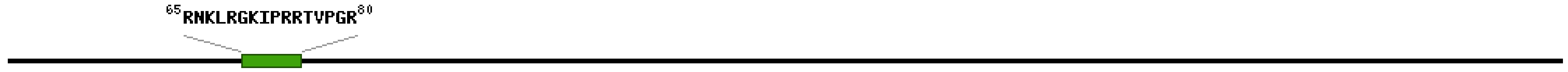

Posterior Graphic. Threshold Marked @ 0.4

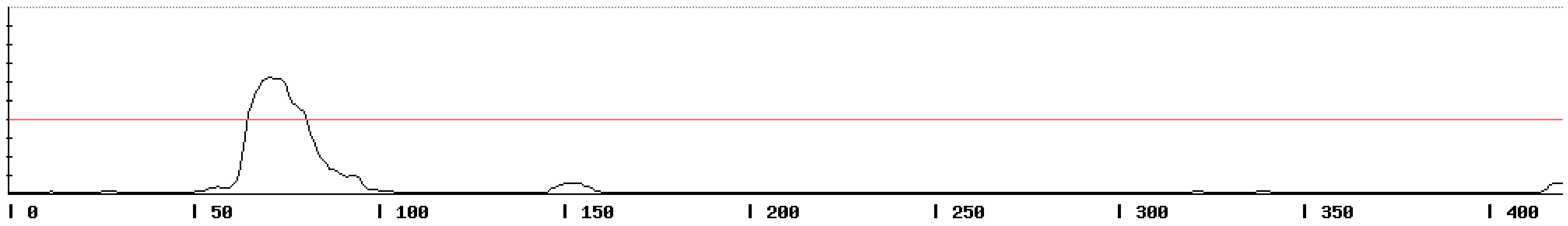

Predictions for Boecherastricta_Bostr.2570s0301.1.p

Viterbi Path

Viterbi Path

Posterior @ 0.4

Posterior @ 0.4

Posterior Graphic. Threshold Marked @ 0.4

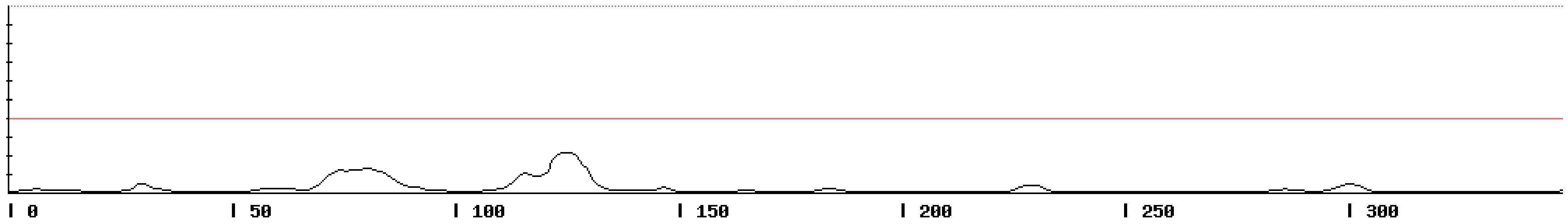

Predictions for Boecherastricta_Bostr.2570s0302.1.p

Viterbi Path

Viterbi Path

Posterior @ 0.4

Posterior @ 0.4

Posterior Graphic. Threshold Marked @ 0.4

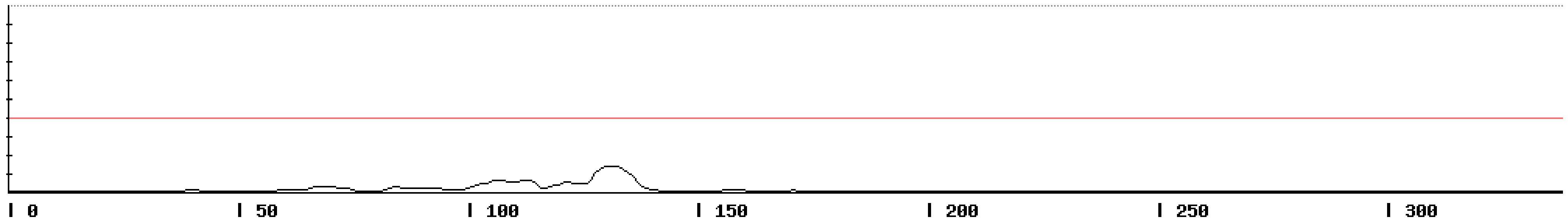

Predictions for Boecherastricta_Bostr.25993s0090.1.p

Viterbi Path

Viterbi Path

Posterior @ 0.4

Posterior @ 0.4

Posterior Graphic. Threshold Marked @ 0.4

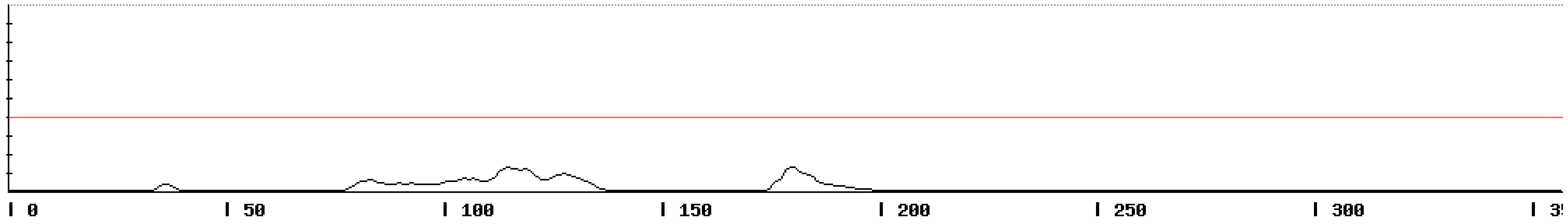

Predictions for Boecherastricta_Bostr.25993s0479.1.p

Viterbi Path

Viterbi Path

Posterior @ 0.4

Posterior @ 0.4

Posterior Graphic. Threshold Marked @ 0.4

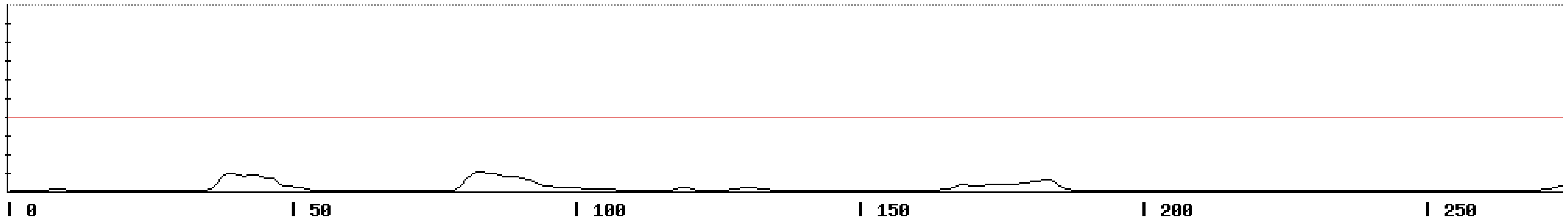

Predictions for Boecherastricta_Bostr.2618s0061.1.p

Viterbi Path

Viterbi Path

Posterior @ 0.4

Posterior @ 0.4

Posterior Graphic. Threshold Marked @ 0.4

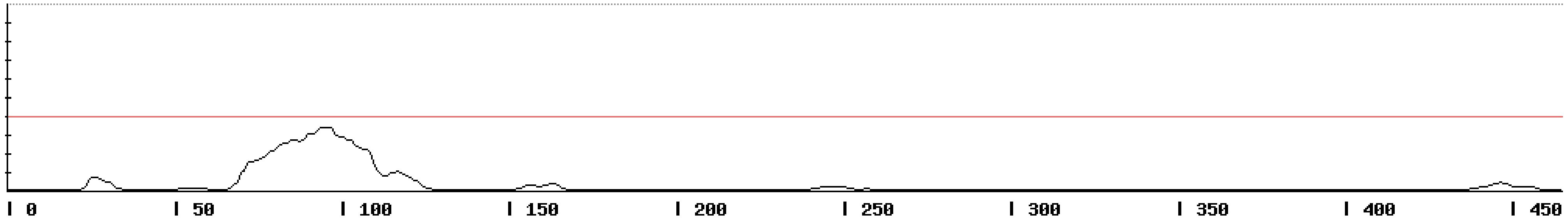

Predictions for Boecherastricta_Bostr.26527s0039.1.p

Viterbi Path

Viterbi Path

Posterior @ 0.4

Posterior @ 0.4

Posterior Graphic. Threshold Marked @ 0.4

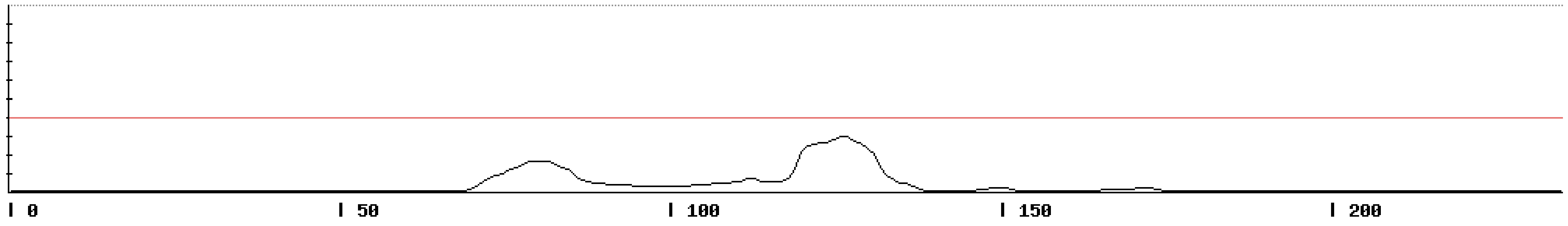

Predictions for Boecherastricta_Bostr.26527s0049.1.p

Viterbi Path

Viterbi Path

Posterior @ 0.4

Posterior @ 0.4

Posterior Graphic. Threshold Marked @ 0.4

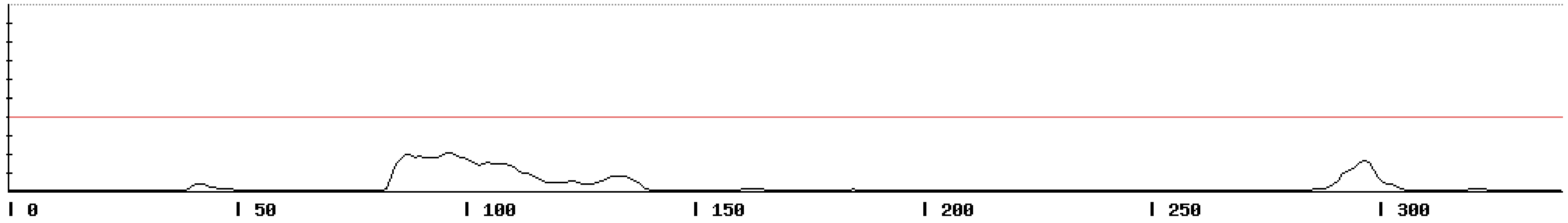

Predictions for Boecherastricta_Bostr.26527s0378.1.p

Viterbi Path

Viterbi Path

Posterior @ 0.4

Posterior @ 0.4

Posterior Graphic. Threshold Marked @ 0.4

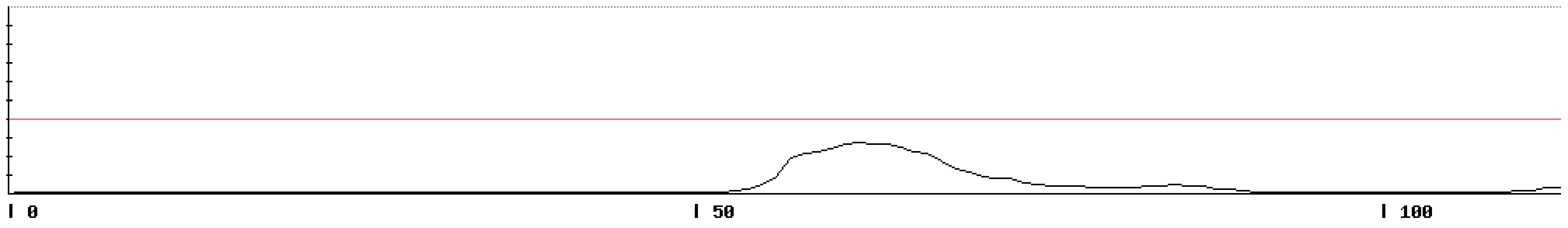

Predictions for Boecherastricta_Bostr.26527s0381.1.p

Viterbi Path

Viterbi Path

Posterior @ 0.4

Posterior @ 0.4

Posterior Graphic. Threshold Marked @ 0.4

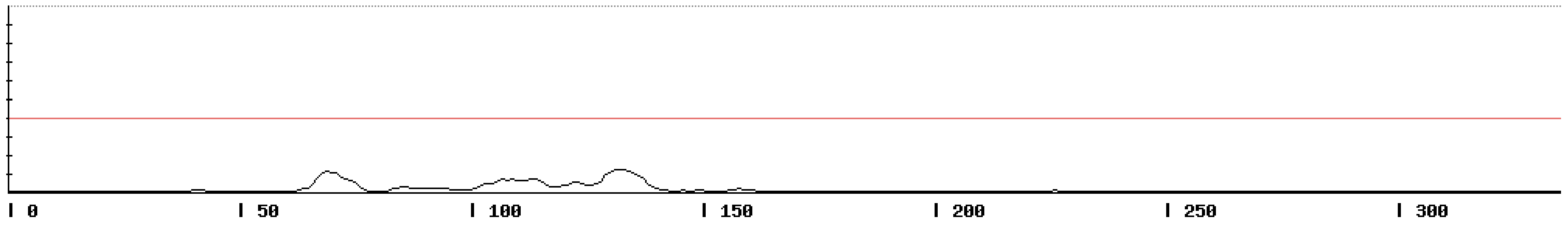

Predictions for Boecherastricta_Bostr.26527s0404.1.p

Viterbi Path

Viterbi Path

Posterior @ 0.4

Posterior @ 0.4

Posterior Graphic. Threshold Marked @ 0.4

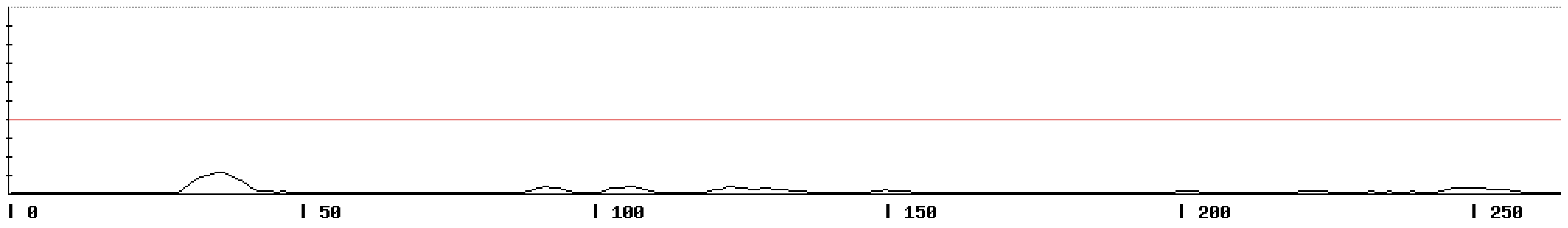

Predictions for Boecherastricta_Bostr.26675s0262.1.p

Viterbi Path

Viterbi Path

Posterior @ 0.4

Posterior @ 0.4

Posterior Graphic. Threshold Marked @ 0.4

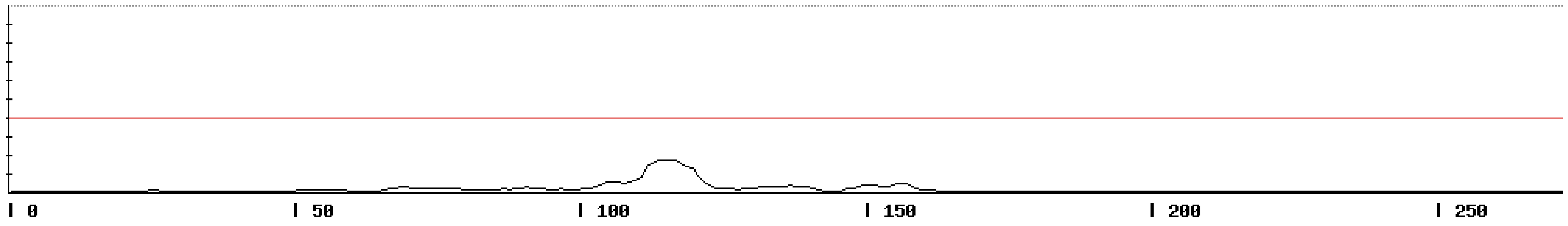

Predictions for Boecherastricta_Bostr.26833s0095.1.p

Viterbi Path	
Viterbi Path	
Posterior @ 0.4	
Posterior @ 0.4	
Posterior Graphic. Threshold Marked @ 0.4	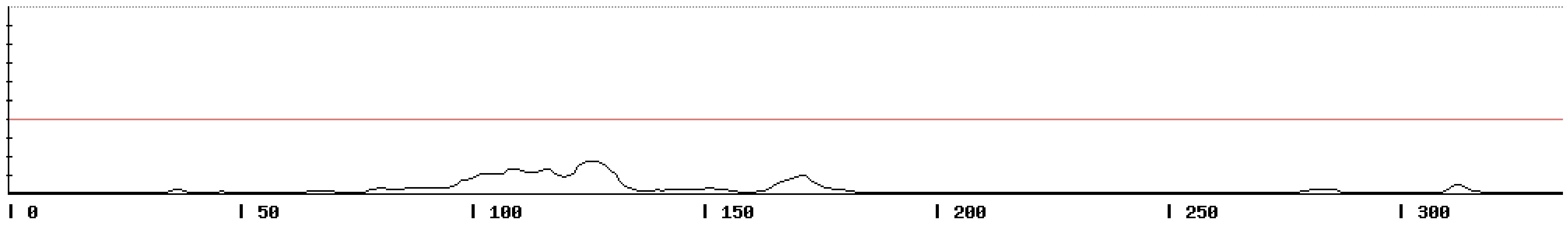

Predictions for Boecherastricta_Bostr.26833s0275.1.p

Viterbi Path	
Viterbi Path	
Posterior @ 0.4	137 - KKKHKR - 142
	181 - TKKKNPKKK - 189
Posterior @ 0.4	

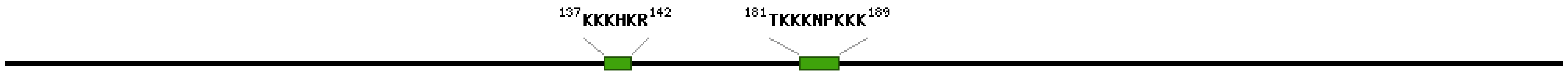

Posterior Graphic. Threshold Marked @ 0.4

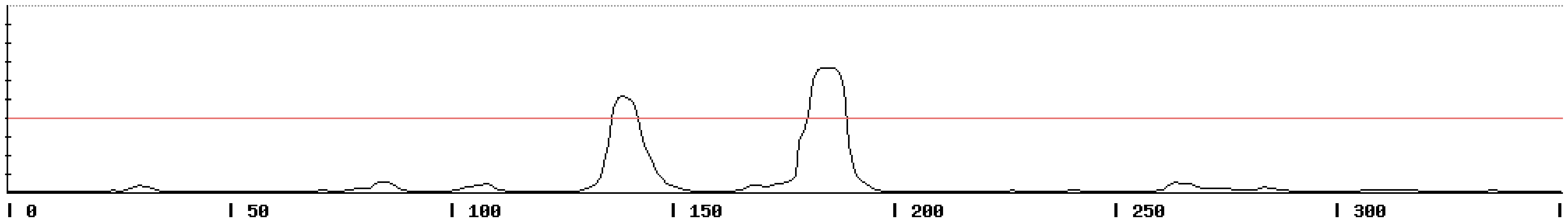

Predictions for Boecherastricta_Bostr.26833s0491.1.p

Viterbi Path

Viterbi Path

Posterior @ 0.4

Posterior @ 0.4

Posterior Graphic. Threshold Marked @ 0.4

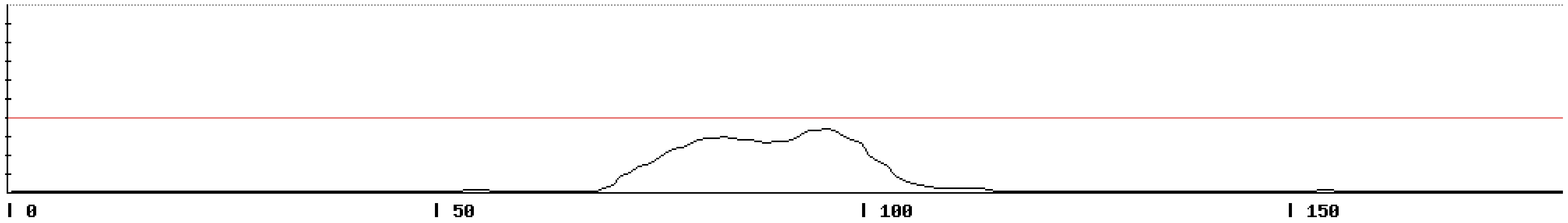

Predictions for Boecherastricta_Bostr.26833s0596.1.p

Viterbi Path

Viterbi Path

Posterior @ 0.4

Posterior @ 0.4

Posterior Graphic. Threshold Marked @ 0.4

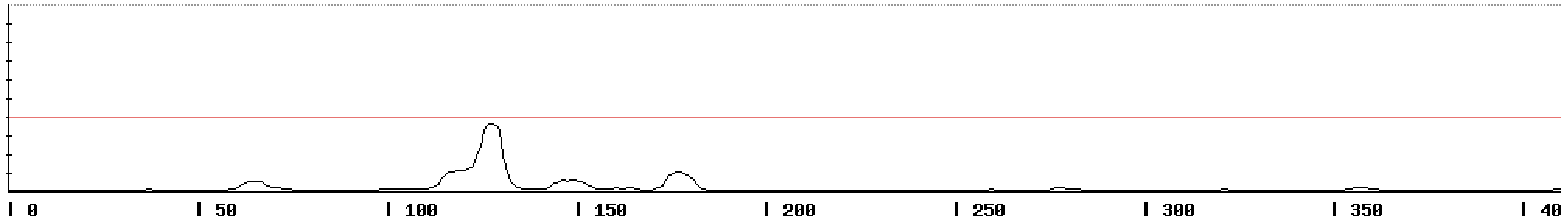

Predictions for Boecherastricta_Bostr.26833s0880.1.p

Viterbi Path

Viterbi Path

Posterior @ 0.4

Posterior @ 0.4

Posterior Graphic. Threshold Marked @ 0.4

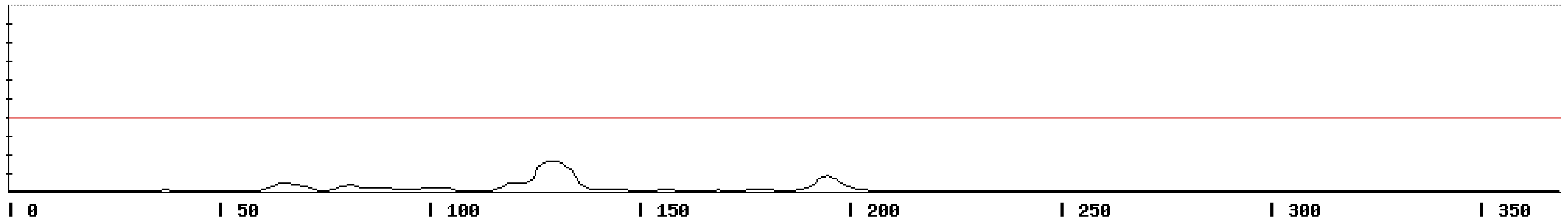

Predictions for Boecherastricta_Bostr.26959s0323.1.p

Viterbi Path

Viterbi Path

Posterior @ 0.4	586 - RKICKKKKE - 594
Posterior @ 0.4	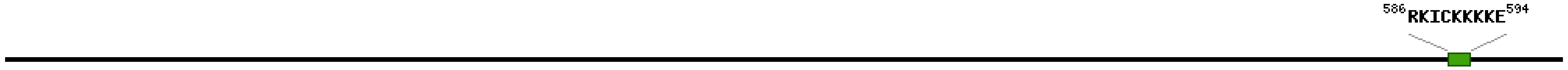
Posterior Graphic. Threshold Marked @ 0.4	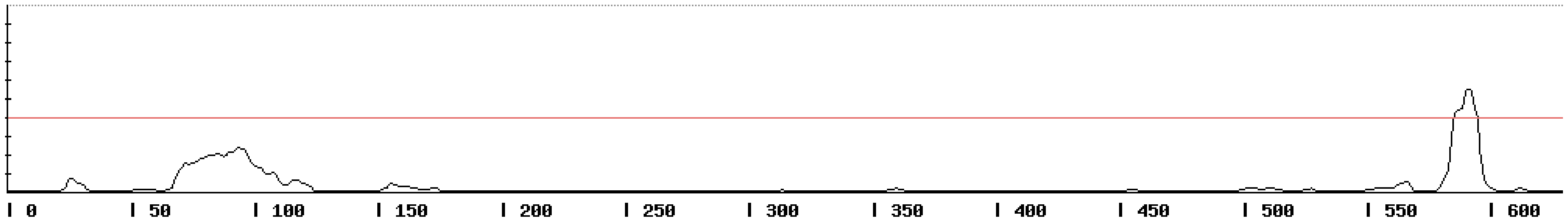

Predictions for Boecherastricta_Bostr.27895s0039.1.p

Viterbi Path	
Viterbi Path	
Posterior @ 0.4	
Posterior @ 0.4	
Posterior Graphic. Threshold Marked @ 0.4	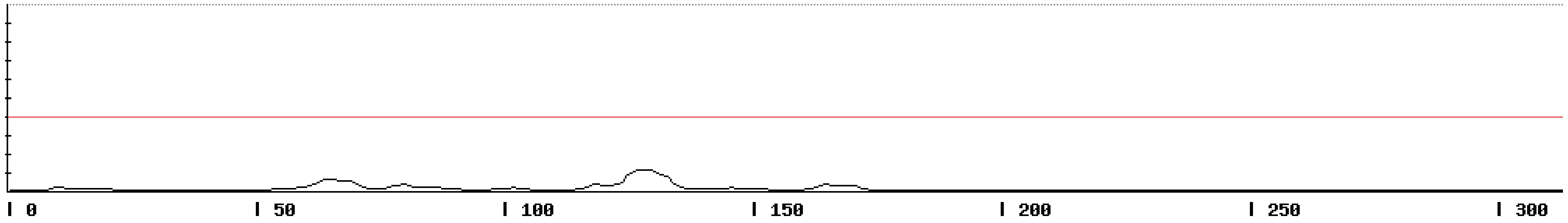

Predictions for Boecherastricta_Bostr.28625s0148.1.p

Viterbi Path

Viterbi Path

Posterior @ 0.4

Posterior @ 0.4

Posterior Graphic. Threshold Marked @ 0.4

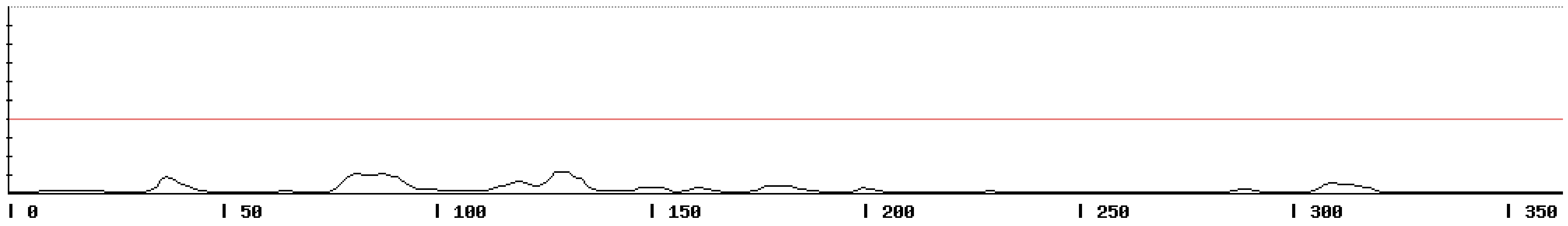

Predictions for Boecherastricta_Bostr.28625s0149.1.p

Viterbi Path

Viterbi Path

Posterior @ 0.4

Posterior @ 0.4

Posterior Graphic. Threshold Marked @ 0.4

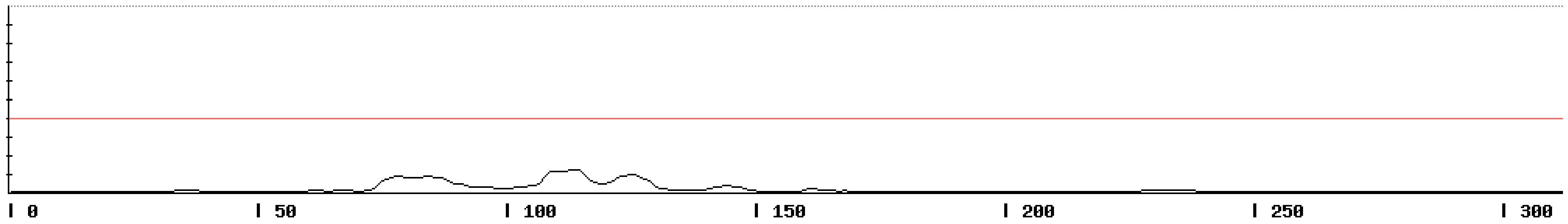

Predictions for Boecherastricta_Bostr.28625s0180.1.p	
Viterbi Path	
Viterbi Path	
Posterior @ 0.4	
Posterior @ 0.4	
Posterior Graphic. Threshold Marked @ 0.4	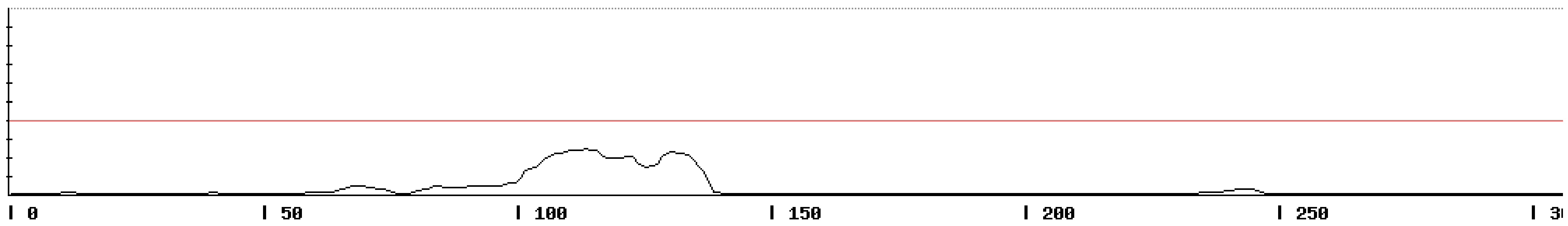

Predictions for Boecherastricta_Bostr.28794s0029.1.p	
Viterbi Path	
Viterbi Path	
Posterior @ 0.4	
Posterior @ 0.4	
Posterior Graphic. Threshold Marked @ 0.4	

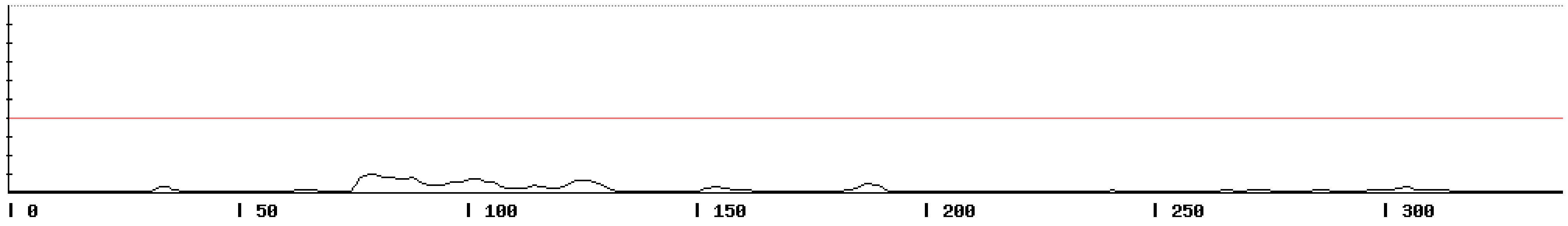

Predictions for Boecherastricta_Bostr.2902s0053.1.p

Viterbi Path

Viterbi Path

Posterior @ 0.4

Posterior @ 0.4

Posterior Graphic. Threshold Marked @ 0.4

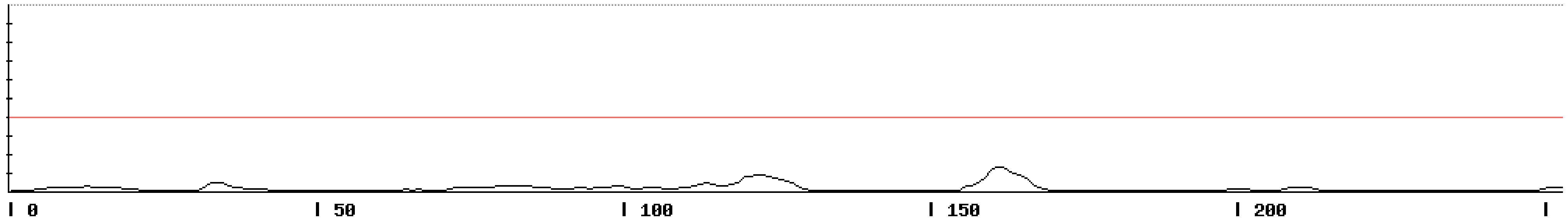

Predictions for Boecherastricta_Bostr.2902s0136.1.p

Viterbi Path

Viterbi Path

Posterior @ 0.4

161 - RKGSKARKQRKSR - 173

Posterior @ 0.4

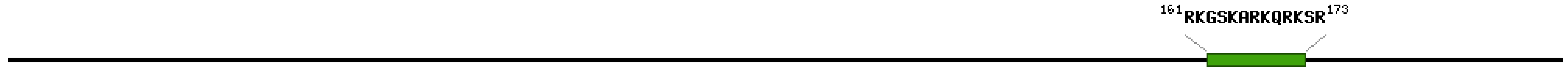

Posterior Graphic. Threshold Marked @ 0.4

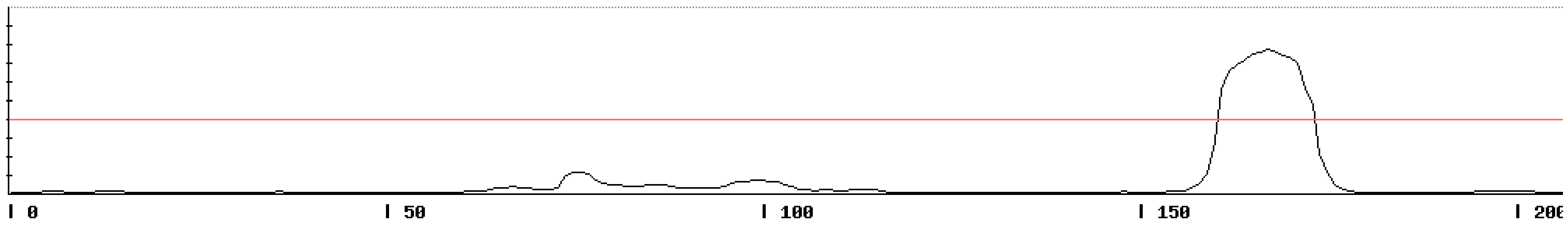

Predictions for Boecherastricta_Bostr.2902s0186.1.p

Viterbi Path

Viterbi Path

Posterior @ 0.4

Posterior @ 0.4

Posterior Graphic. Threshold Marked @ 0.4

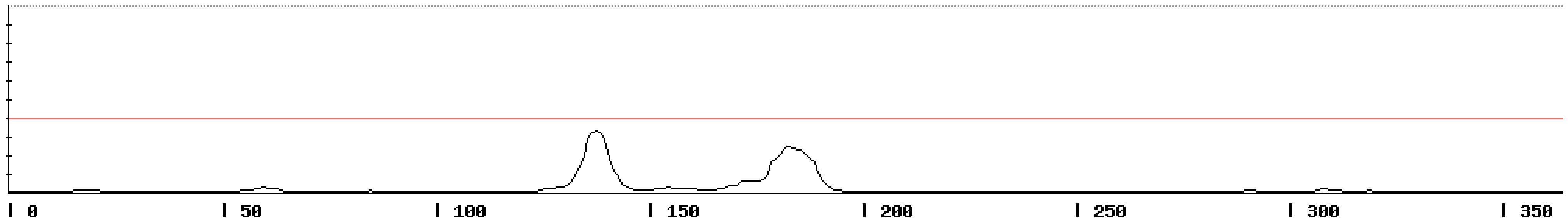

Predictions for Boecherastricta_Bostr.30275s0267.1.p

Viterbi Path

Viterbi Path

Posterior @ 0.4

Posterior @ 0.4

Posterior Graphic. Threshold Marked @ 0.4

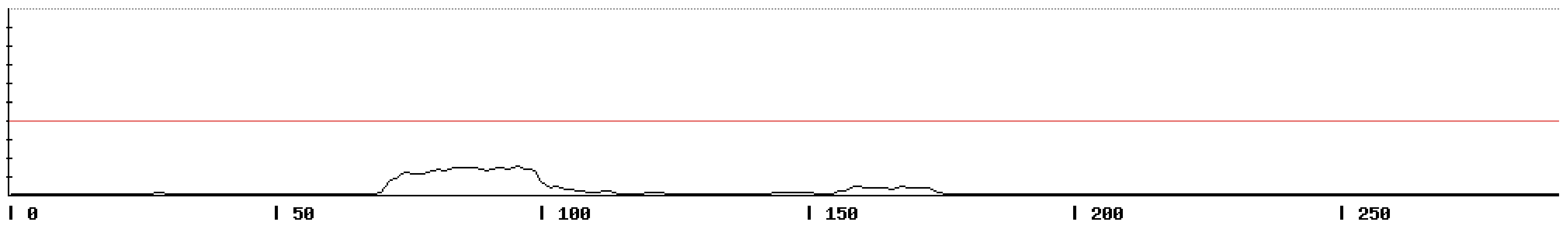

Predictions for Boecherastricta_Bostr.3359s0042.1.p

Viterbi Path	
Viterbi Path	
Posterior @ 0.4	
Posterior @ 0.4	
Posterior Graphic. Threshold Marked @ 0.4	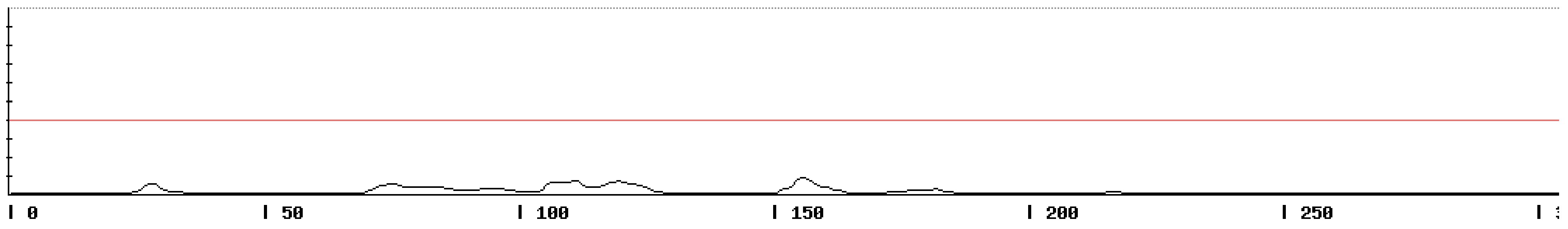

Predictions for Boecherastricta_Bostr.3359s0059.1.p

Viterbi Path	
Viterbi Path	

Posterior @ 0.4	
Posterior @ 0.4	
Posterior Graphic. Threshold Marked @ 0.4	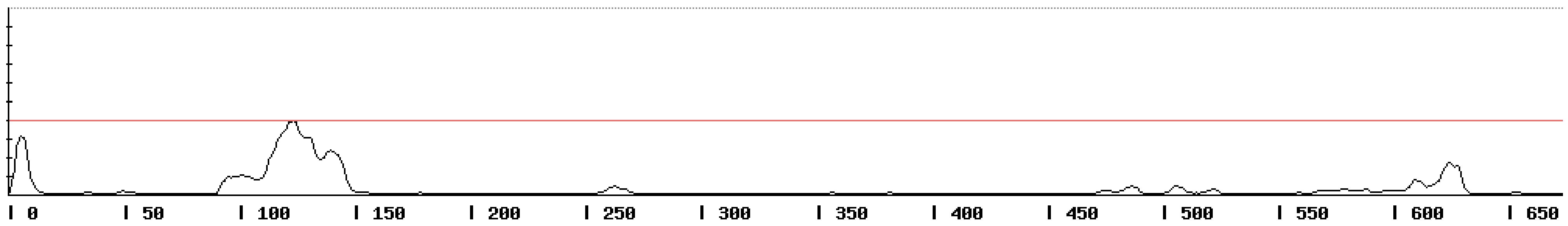

Predictions for Boecherastricta_Bostr.3359s0074.1.p

Viterbi Path	
Viterbi Path	
Posterior @ 0.4	29 - KRKIRRKKLR - 38
	486 - AKRKKSGGNNDKKNKNK - 502
Posterior @ 0.4	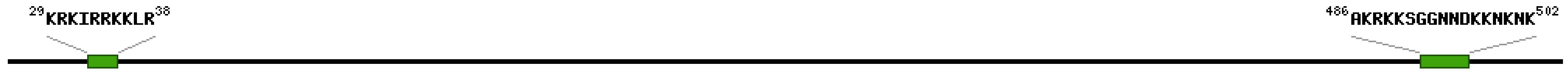
Posterior Graphic. Threshold Marked @ 0.4	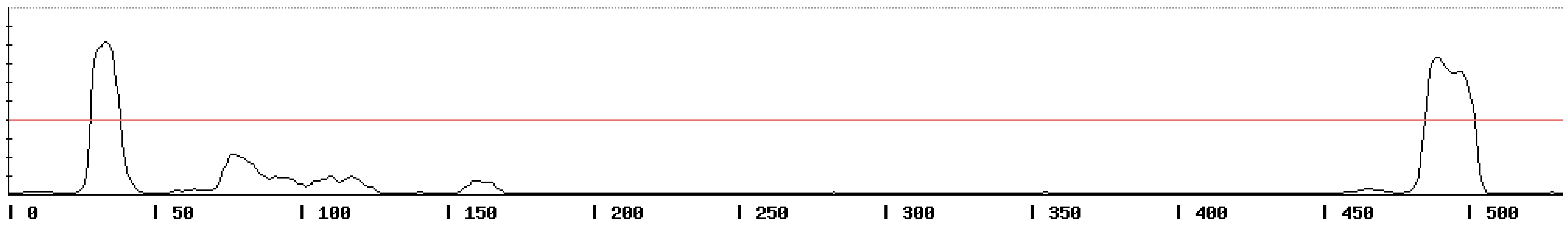

Predictions for Boecherastricta_Bostr.3359s0081.1.p

Viterbi Path	
Viterbi Path	

Posterior @ 0.4	
Posterior @ 0.4	
Posterior Graphic. Threshold Marked @ 0.4	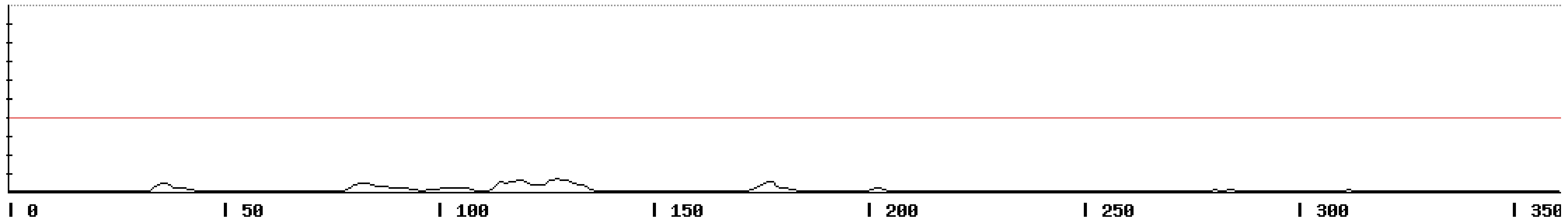

Predictions for Boecherastricta_Bostr.3359s0109.1.p

Viterbi Path	
Viterbi Path	
Posterior @ 0.4	
Posterior @ 0.4	
Posterior Graphic. Threshold Marked @ 0.4	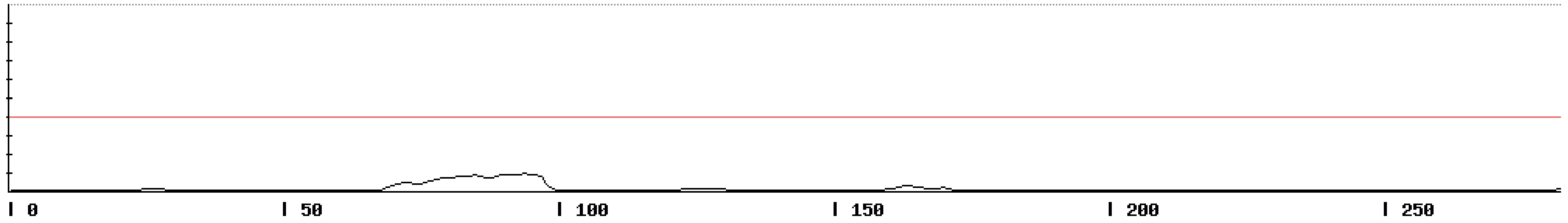

Predictions for Boecherastricta_Bostr.3640s0064.1.p

Viterbi Path

Viterbi Path

Posterior @ 0.4

Posterior @ 0.4

Posterior Graphic. Threshold Marked @ 0.4

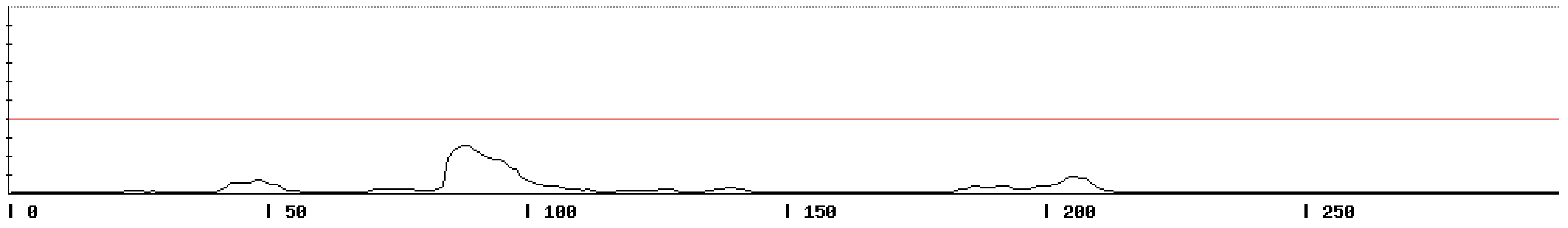

Predictions for Boecherastricta_Bostr.3640s0073.1.p

Viterbi Path

Viterbi Path

Posterior @ 0.4

Posterior @ 0.4

Posterior Graphic. Threshold Marked @ 0.4

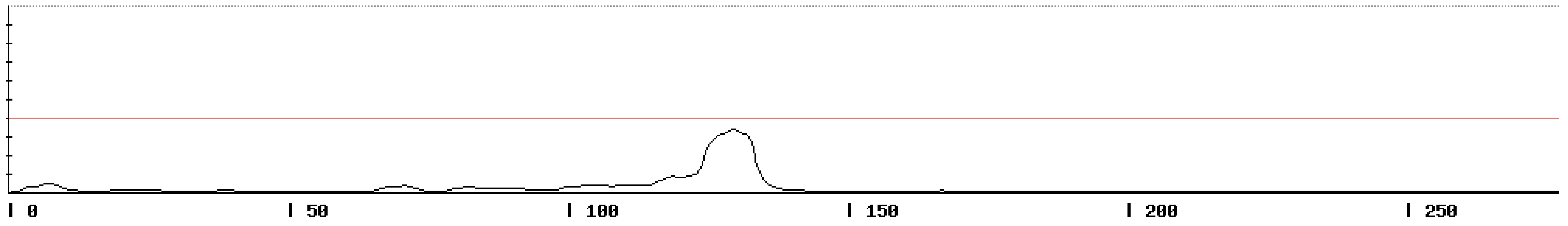

Predictions for Boecherastricta_Bostr.4485s0006.1.p

Viterbi Path	
Viterbi Path	
Posterior @ 0.4	
Posterior @ 0.4	
Posterior Graphic. Threshold Marked @ 0.4	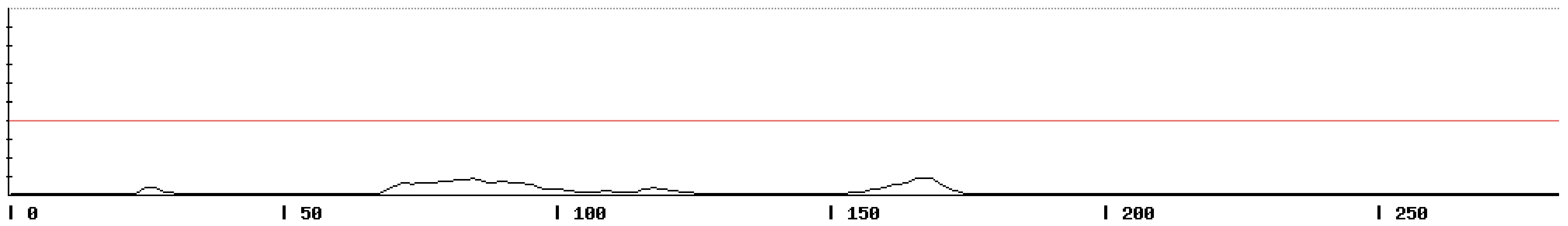

Predictions for Boecherastricta_Bostr.4825s0021.1.p

Viterbi Path	
Viterbi Path	
Posterior @ 0.4	74 - AKKKAMKRSTGSGFWKPTGKDRIIRDKRRNG - 104
Posterior @ 0.4	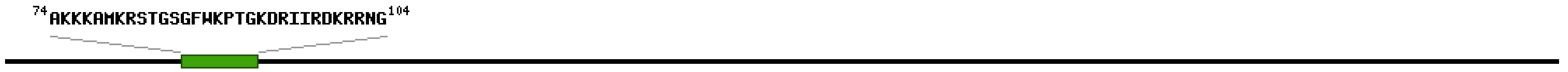
Posterior Graphic. Threshold Marked @ 0.4	

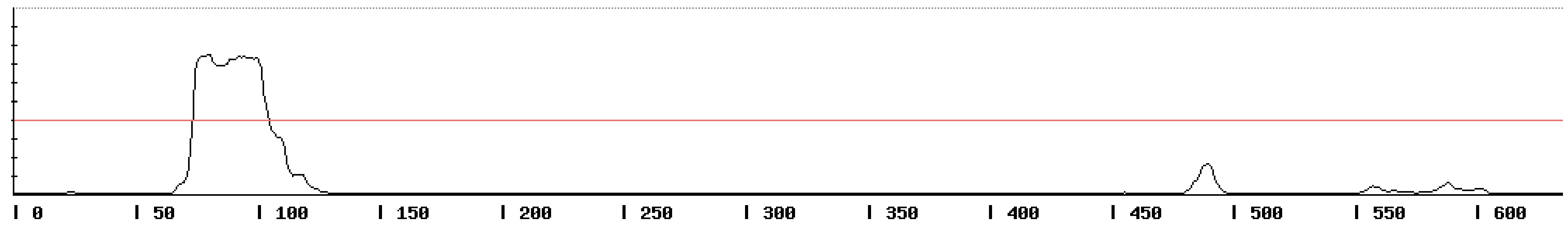

Predictions for Boecherastricta_Bostr.4825s0023.1.p

Viterbi Path	
Viterbi Path	
Posterior @ 0.4	95 - RKIKDKRGNRGE - 106
Posterior @ 0.4	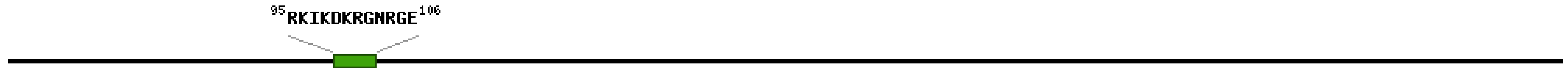
Posterior Graphic. Threshold Marked @ 0.4	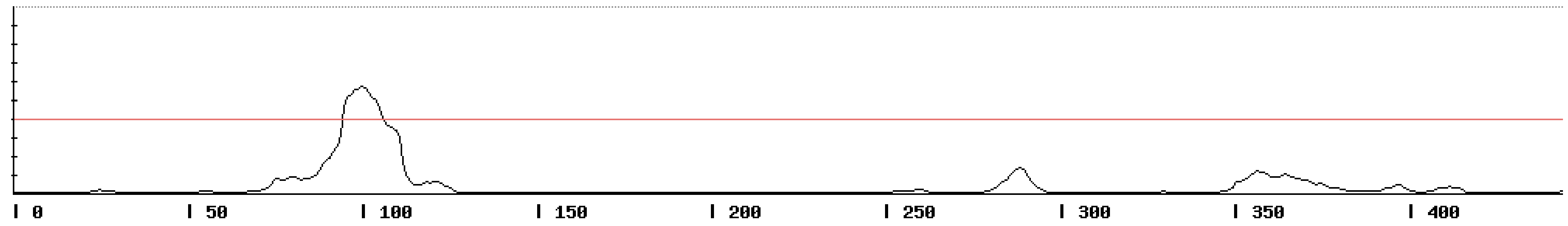

Predictions for Boecherastricta_Bostr.4825s0024.1.p

Viterbi Path	12 - KKKKKKKK - 19
Viterbi Path	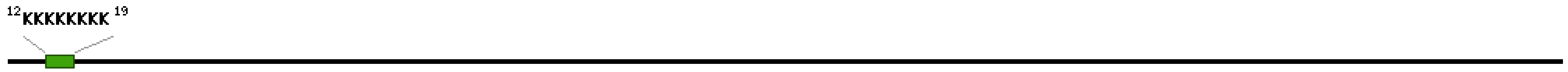
Posterior @ 0.4	11 - QKKKKKKKKDPI - 22
Posterior @ 0.4	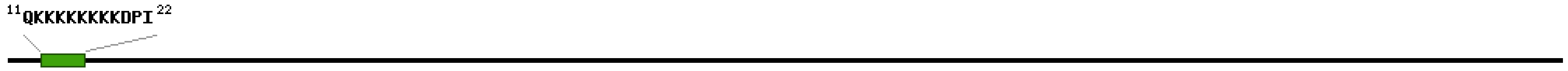

Posterior Graphic. Threshold Marked @ 0.4

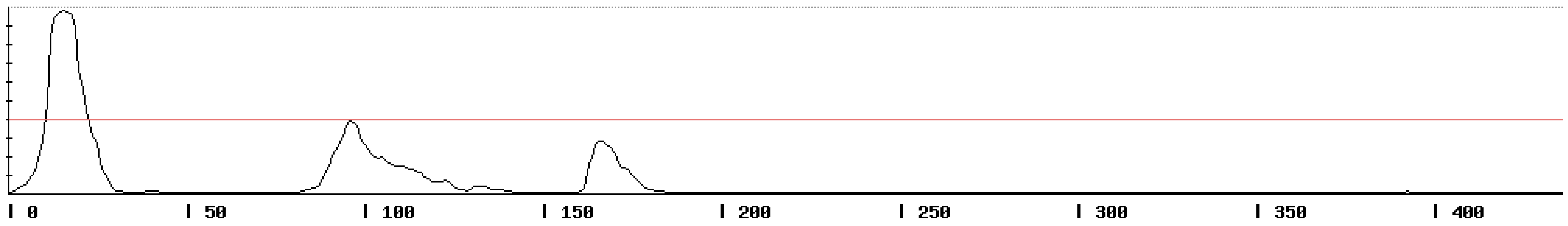

Predictions for Boecherastricta_Bostr.5325s0006.1.p

Viterbi Path

Viterbi Path

Posterior @ 0.4

Posterior @ 0.4

Posterior Graphic. Threshold Marked @ 0.4

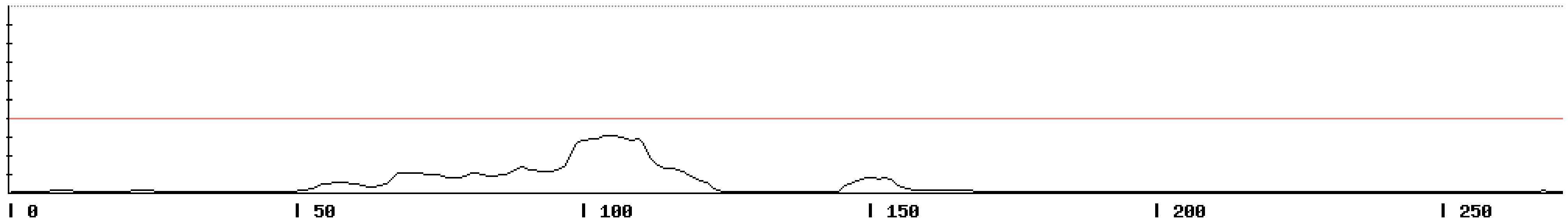

Predictions for Boecherastricta_Bostr.5325s0007.1.p

Viterbi Path

Viterbi Path

Posterior @ 0.4

Posterior @ 0.4

Posterior Graphic. Threshold Marked @ 0.4	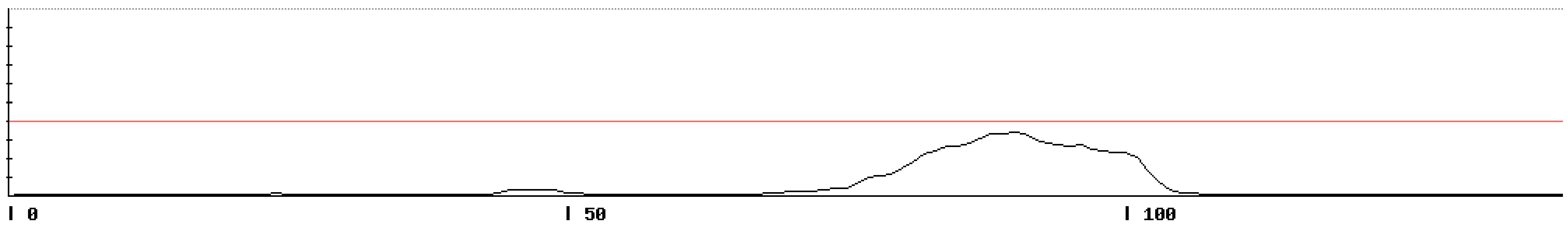

Predictions for Boecherastricta_Bostr.5325s0008.1.p	
Viterbi Path	
Viterbi Path	
Posterior @ 0.4	
Posterior @ 0.4	
Posterior Graphic. Threshold Marked @ 0.4	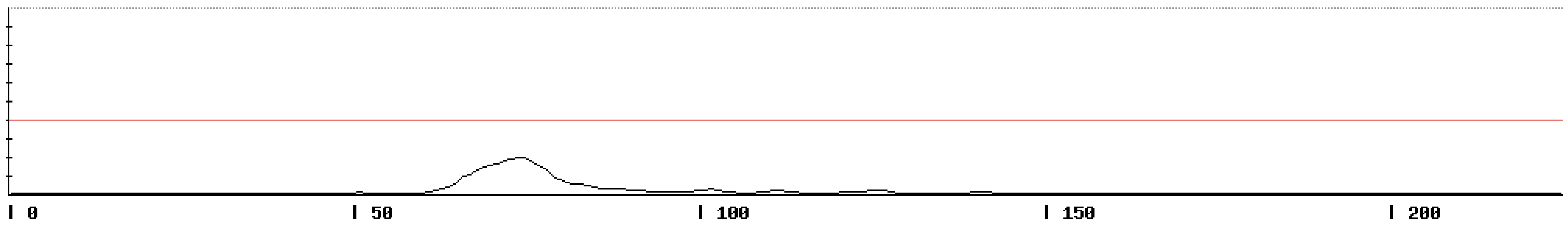

Predictions for Boecherastricta_Bostr.5325s0009.1.p	
Viterbi Path	
Viterbi Path	

Posterior @ 0.4

Posterior @ 0.4

Posterior Graphic. Threshold Marked @ 0.4

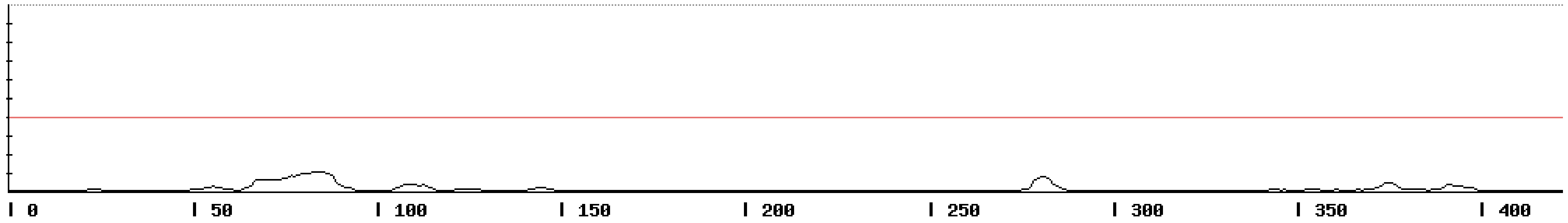

Predictions for Boecherastricta_Bostr.5325s0010.1.p

Viterbi Path

Viterbi Path

Posterior @ 0.4 — 59 - RRKNKYGTGDRRNRKTPSGK - 78

Posterior @ 0.4

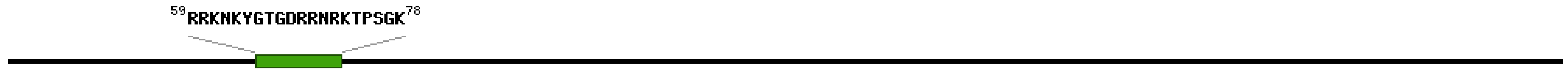

Posterior Graphic. Threshold Marked @ 0.4

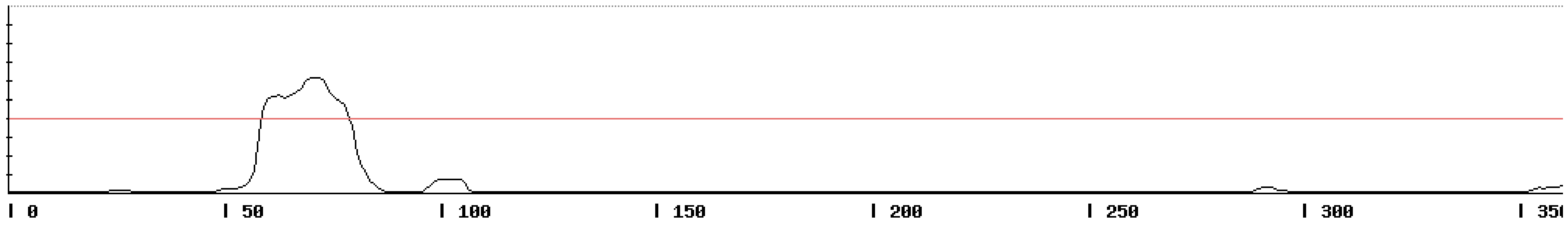

Predictions for Boecherastricta_Bostr.5325s0011.1.p

Viterbi Path

Viterbi Path

Posterior @ 0.4	
Posterior @ 0.4	
Posterior Graphic. Threshold Marked @ 0.4	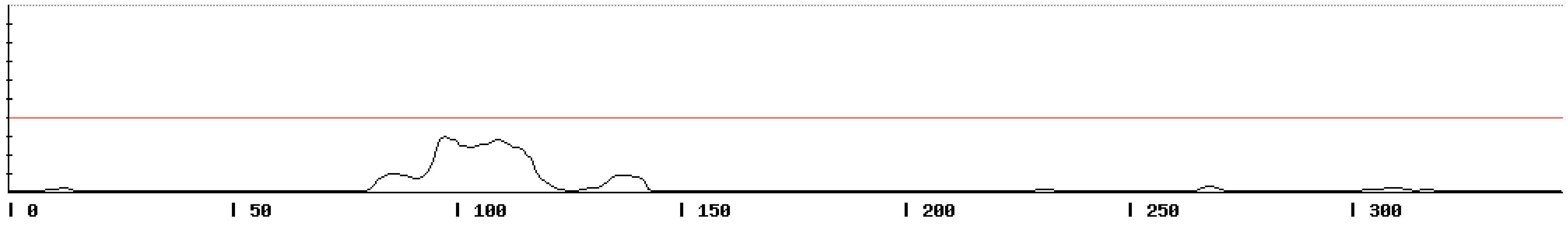

Predictions for Boecherastricta_Bostr.5325s0013.1.p

Viterbi Path	
Viterbi Path	
Posterior @ 0.4	
Posterior @ 0.4	
Posterior Graphic. Threshold Marked @ 0.4	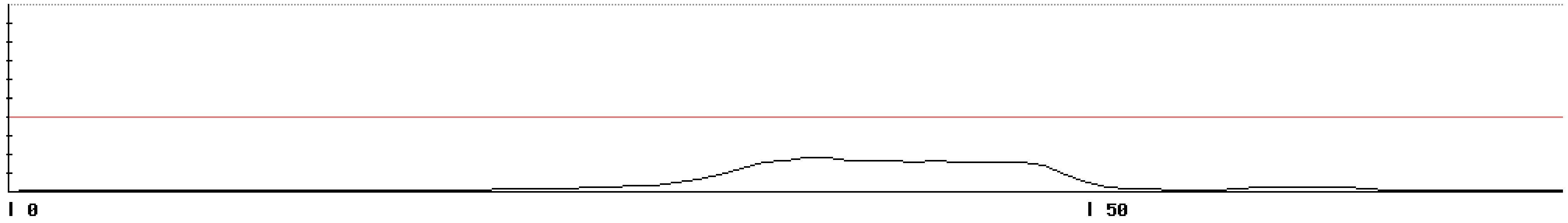

Predictions for Boecherastricta_Bostr.5325s0014.1.p

Viterbi Path	
Viterbi Path	
Posterior @ 0.4	
Posterior @ 0.4	
Posterior Graphic. Threshold Marked @ 0.4	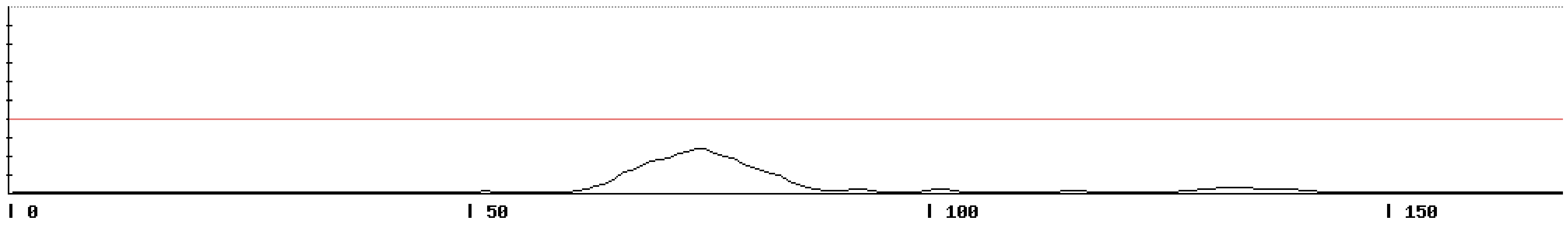

Predictions for Boecherastricta_Bostr.5325s0016.1.p

Viterbi Path	
Viterbi Path	
Posterior @ 0.4	
Posterior @ 0.4	
Posterior Graphic. Threshold Marked @ 0.4	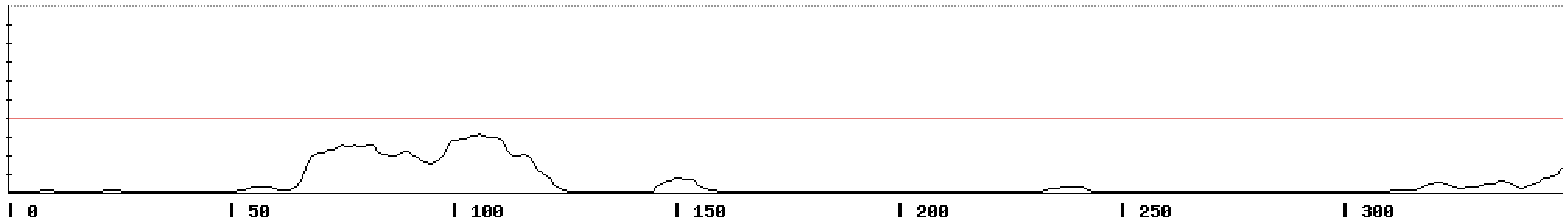

Predictions for Boecherastricta_Bostr.5325s0017.1.p	
Viterbi Path	208 - KKHQRKRRR - 216
Viterbi Path	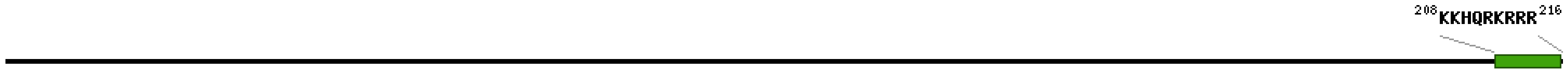
Posterior @ 0.4	83 - RKT - 85
	207 - IKKHQRKRRR - 216
Posterior @ 0.4	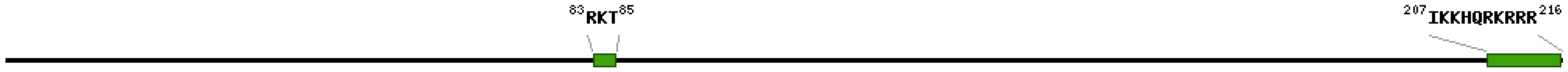
Posterior Graphic. Threshold Marked @ 0.4	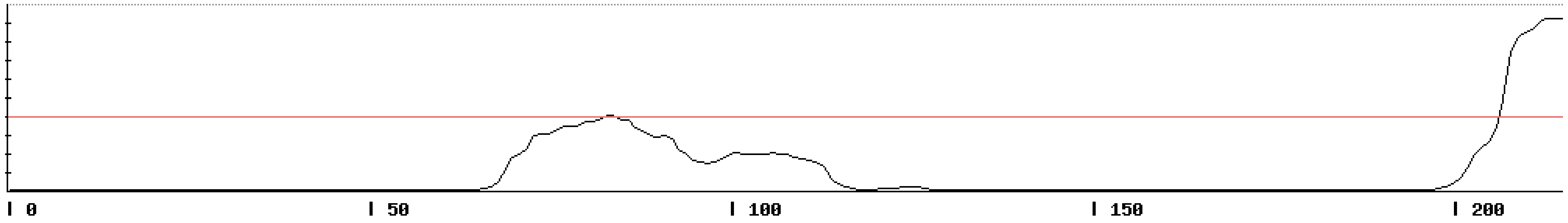

Predictions for Boecherastricta_Bostr.5325s0018.1.p	
Viterbi Path	
Viterbi Path	
Posterior @ 0.4	
Posterior @ 0.4	
Posterior Graphic. Threshold Marked @ 0.4	

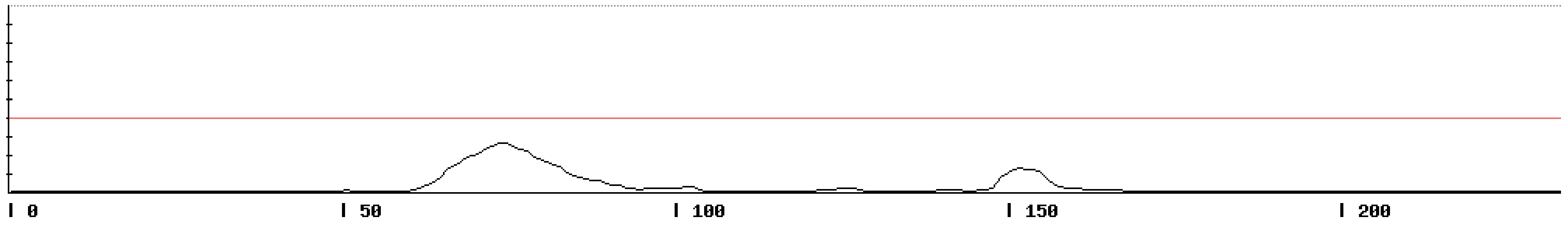

Predictions for Boecherastricta_Bostr.5325s0019.1.p

Viterbi Path

Viterbi Path

Posterior @ 0.4

Posterior @ 0.4

Posterior Graphic. Threshold Marked @ 0.4

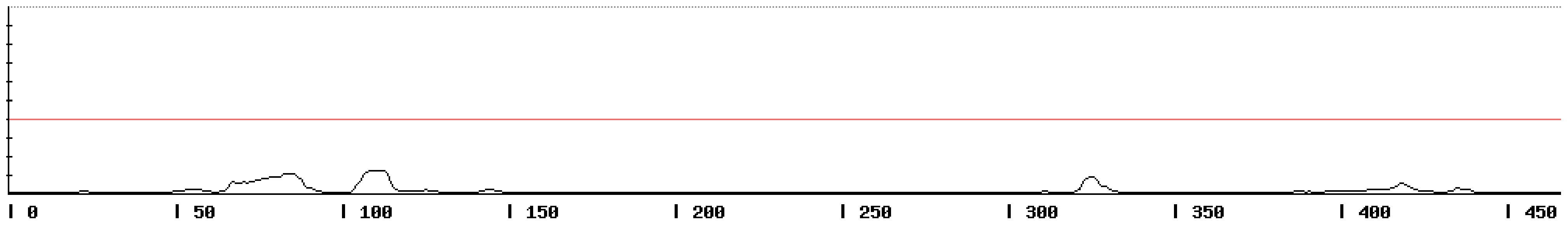

Predictions for Boecherastricta_Bostr.5325s0094.1.p

Viterbi Path

Viterbi Path

Posterior @ 0.4

Posterior @ 0.4

Posterior Graphic. Threshold Marked @ 0.4

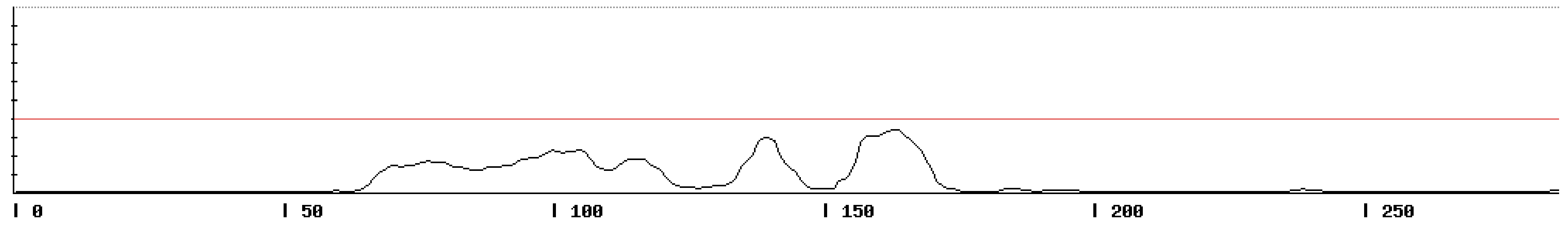

Predictions for Boecherastricta_Bostr.5763s0059.1.p

Viterbi Path

Viterbi Path

Posterior @ 0.4

Posterior @ 0.4

Posterior Graphic. Threshold Marked @ 0.4

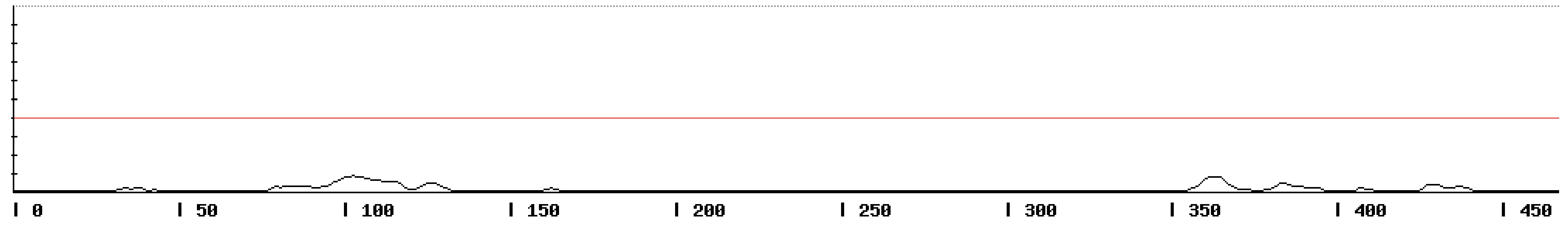

Predictions for Boecherastricta_Bostr.6758s0055.1.p

Viterbi Path

Viterbi Path

Posterior @ 0.4

Posterior @ 0.4

Posterior Graphic. Threshold Marked @ 0.4	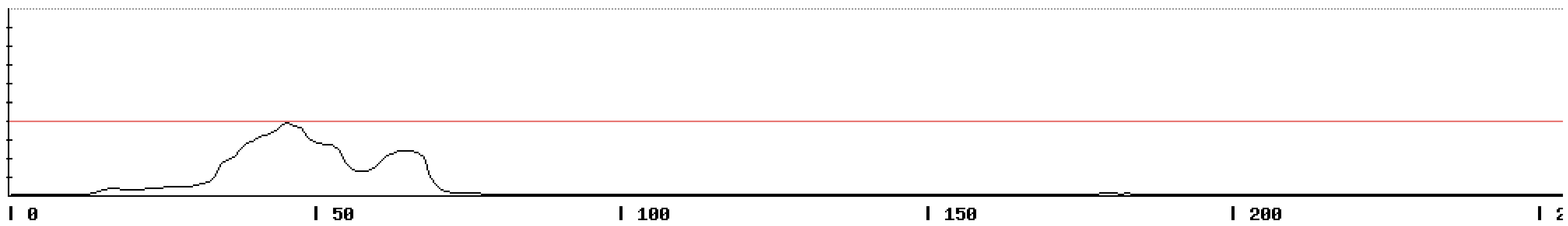

Predictions for Boecherastricta_Bostr.6758s0078.1.p

Viterbi Path	
Viterbi Path	
Posterior @ 0.4	
Posterior @ 0.4	
Posterior Graphic. Threshold Marked @ 0.4	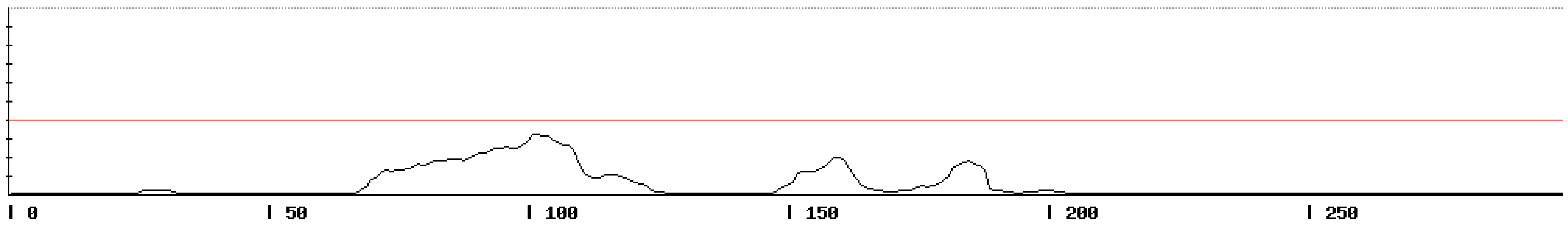

Predictions for Boecherastricta_Bostr.6864s0295.1.p

Viterbi Path	
Viterbi Path	

Posterior @ 0.4

Posterior @ 0.4

Posterior Graphic. Threshold Marked @ 0.4

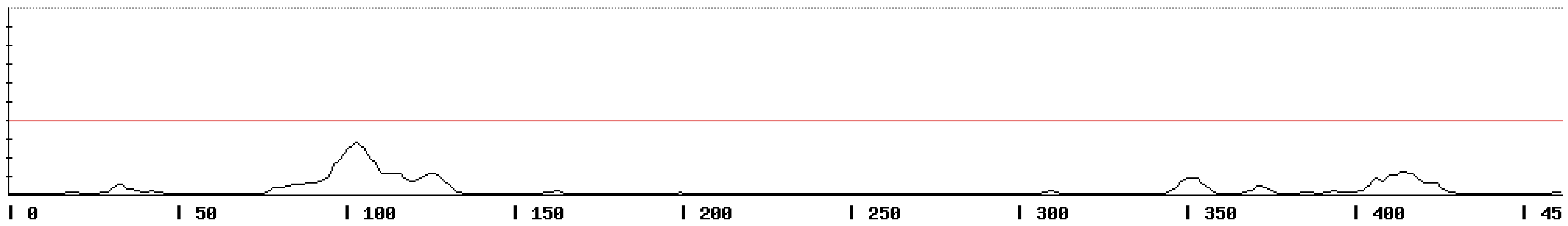

Predictions for Boecherastricta_Bostr.7200s0128.1.p

Viterbi Path

Viterbi Path

Posterior @ 0.4

Posterior @ 0.4

Posterior Graphic. Threshold Marked @ 0.4

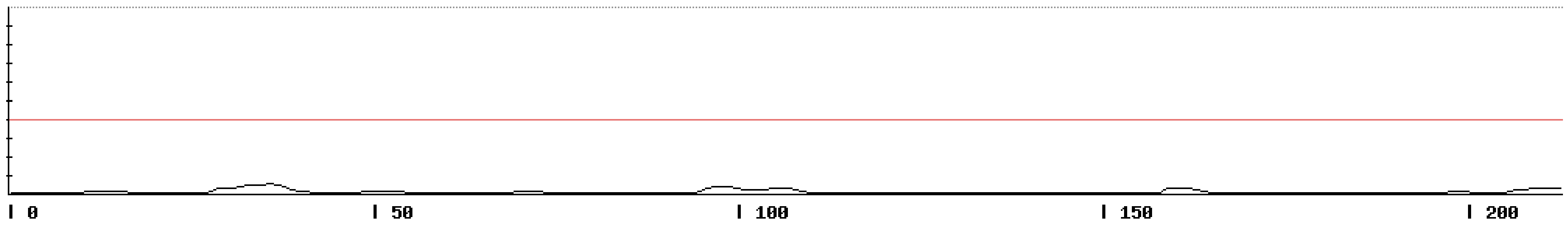

Predictions for Boecherastricta_Bostr.7867s0562.1.p

Viterbi Path

Viterbi Path

Posterior @ 0.4

Posterior @ 0.4

Posterior Graphic. Threshold Marked @ 0.4

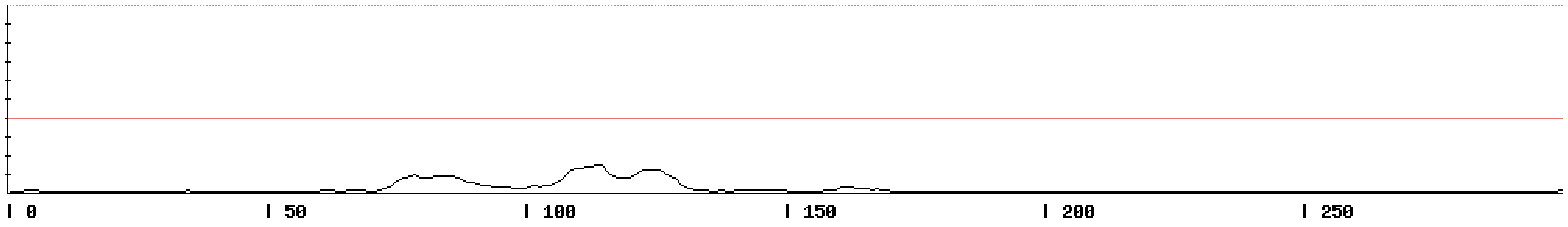

Predictions for Boecherastricta_Bostr.7867s0691.1.p

Viterbi Path

Viterbi Path

Posterior @ 0.4 | 137 - RKRRK - 141

Posterior @ 0.4

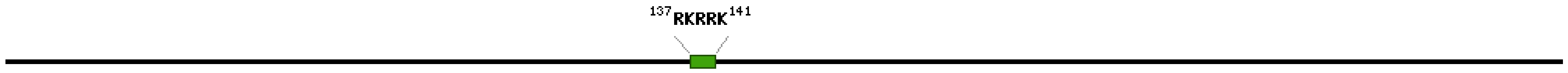

Posterior Graphic. Threshold Marked @ 0.4

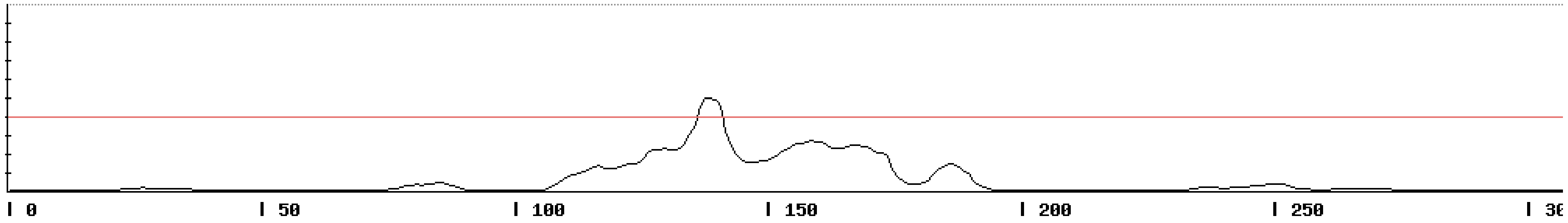

Predictions for Boecherastricta_Bostr.7867s0694.1.p

Viterbi Path

Viterbi Path

Posterior @ 0.4

Posterior @ 0.4

Posterior Graphic. Threshold Marked @ 0.4

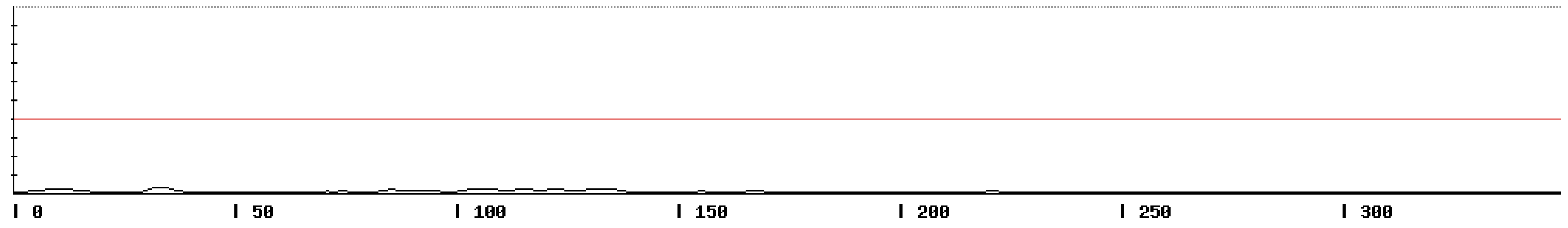

Predictions for Boecherastricta_Bostr.7867s0767.1.p

Viterbi Path

Viterbi Path

Posterior @ 0.4

Posterior @ 0.4

Posterior Graphic. Threshold Marked @ 0.4

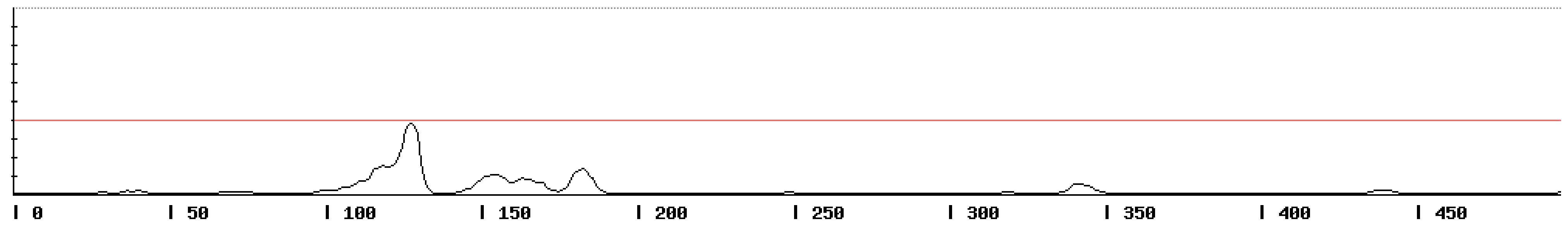

Predictions for Boecherastricta_Bostr.7867s1416.1.p

Viterbi Path	
Viterbi Path	
Posterior @ 0.4	101 - IKSKK - 105
	490 - RKRREGKRWK - 499
Posterior @ 0.4	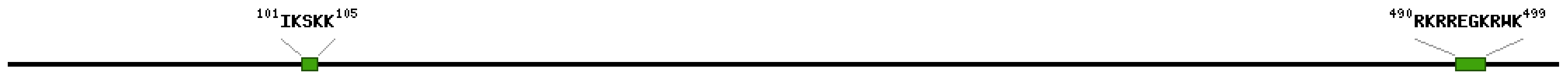
Posterior Graphic. Threshold Marked @ 0.4	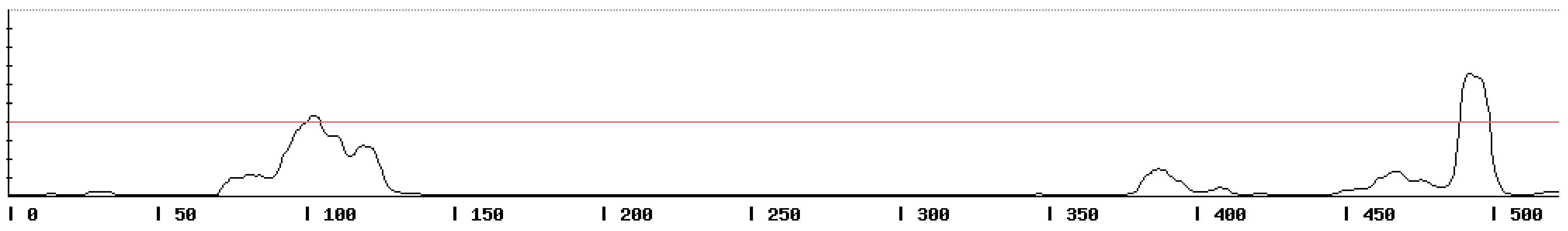

Predictions for Boecherastricta_Bostr.7867s1483.1.p

Viterbi Path	
Viterbi Path	

Revision r.9 - Last Updated on : February 24 2015 17:45:20. alex.nguyenba@utoronto.ca

Intended to be used with Firefox

Viterbi Path

Posterior @ 0.4 235 - PARPRAAAAQKRK - 247

Posterior @ 0.4

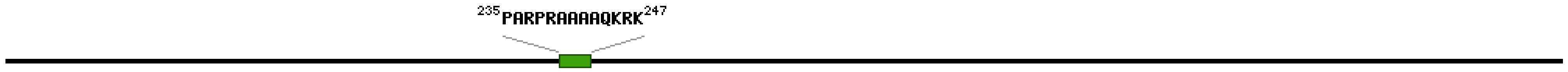

Posterior Graphic. Threshold Marked @ 0.4

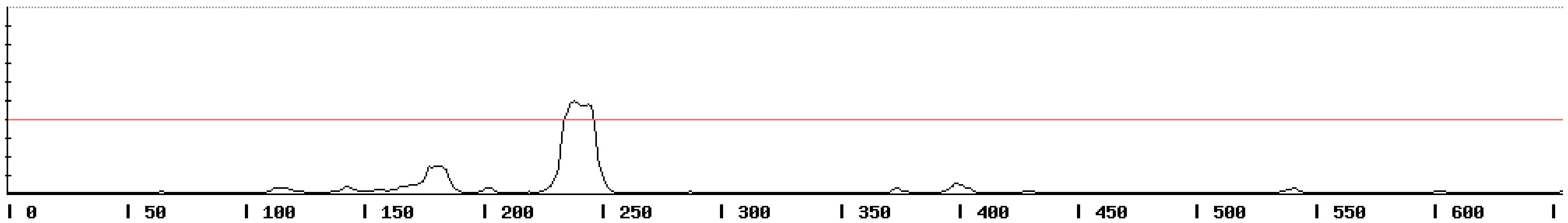

Predictions for Brachypodiumdistachyon_Bradi1g01650.1.p

Viterbi Path

Viterbi Path

Posterior @ 0.4 137 - KKRFSYKKSRPEKGGK - 152

Posterior @ 0.4

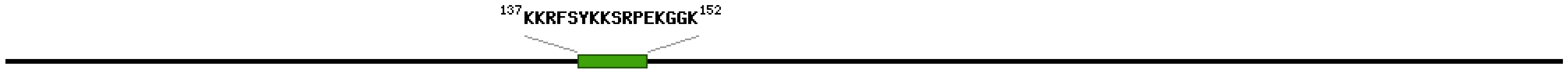

Posterior Graphic. Threshold Marked @ 0.4

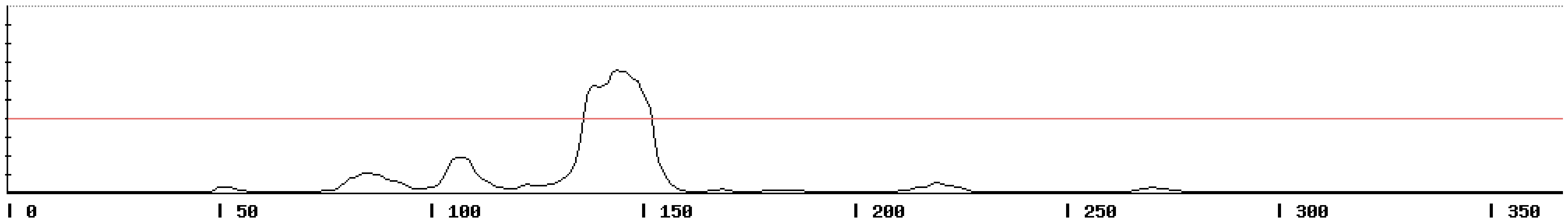

Predictions for Brachypodiumdistachyon_Bradi1g01683.1.p

Viterbi Path

Viterbi Path

Posterior @ 0.4 — 77 - KKKNSGRRARII - 88

Posterior @ 0.4

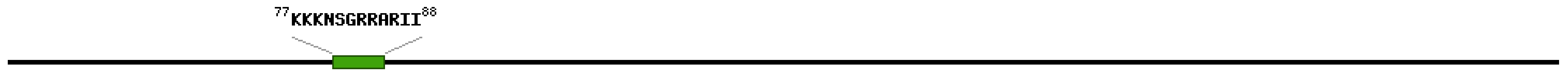

Posterior Graphic. Threshold Marked @ 0.4

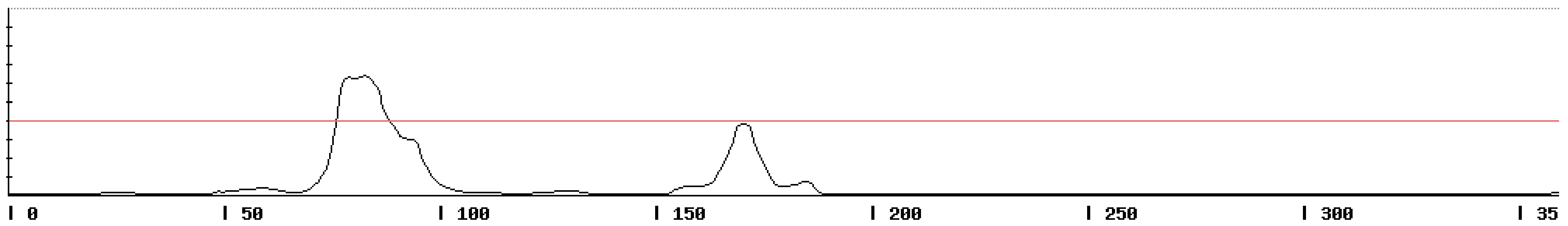

Predictions for Brachypodiumdistachyon_Bradi1g01695.1.p

Viterbi Path

Viterbi Path

Posterior @ 0.4 — 77 - KKKNSGRRAR - 86

Posterior @ 0.4

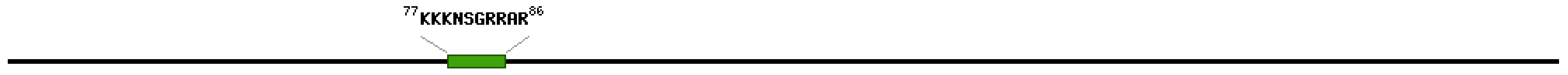

Posterior Graphic. Threshold Marked @ 0.4

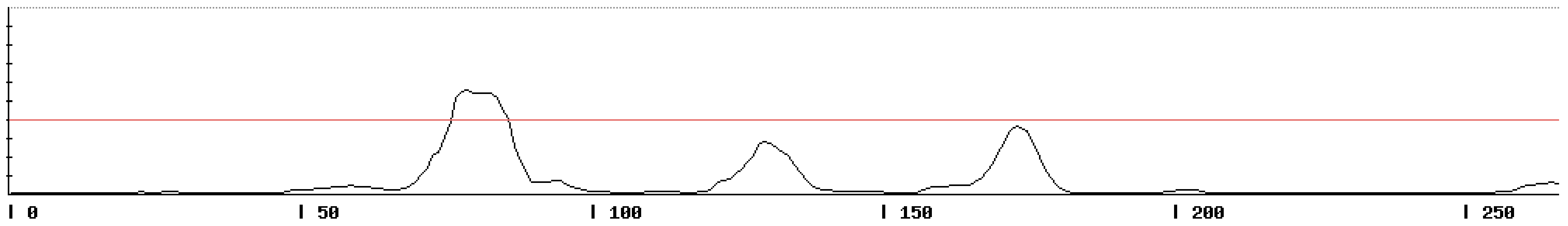

Predictions for Brachypodiumdistachyon_Bradi1g01801.1.p

Viterbi Path	
Viterbi Path	
Posterior @ 0.4	
Posterior @ 0.4	
Posterior Graphic. Threshold Marked @ 0.4	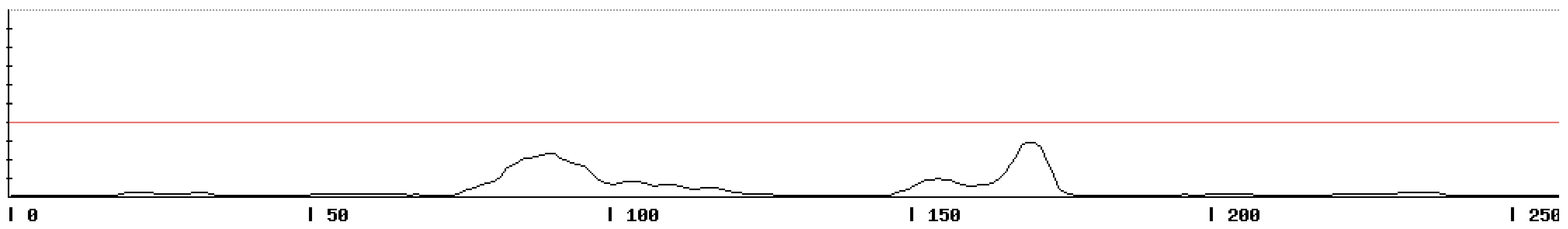

Predictions for Brachypodiumdistachyon_Bradi1g01807.1.p

Viterbi Path	
Viterbi Path	
Posterior @ 0.4	77 - KKKNSGRRARII - 88
Posterior @ 0.4	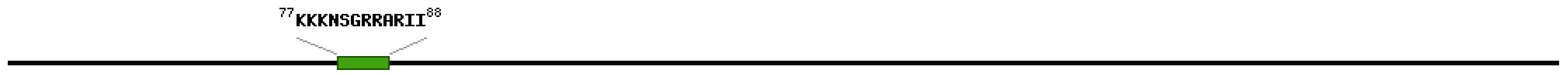
Posterior Graphic. Threshold Marked @ 0.4	

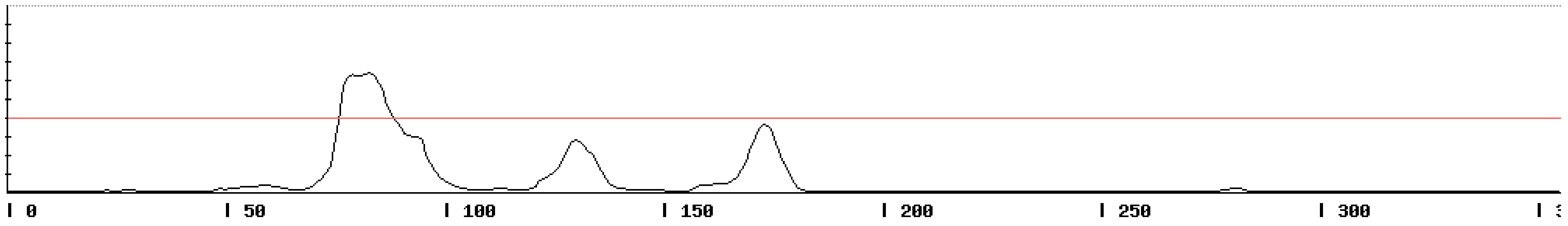

Predictions for Brachypodiumdistachyon_Bradi1g03146.1.p

Viterbi Path

Viterbi Path

Posterior @ 0.4

106 - AGKKKSSGRRSR - 117

Posterior @ 0.4

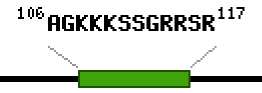

Posterior Graphic. Threshold Marked @ 0.4

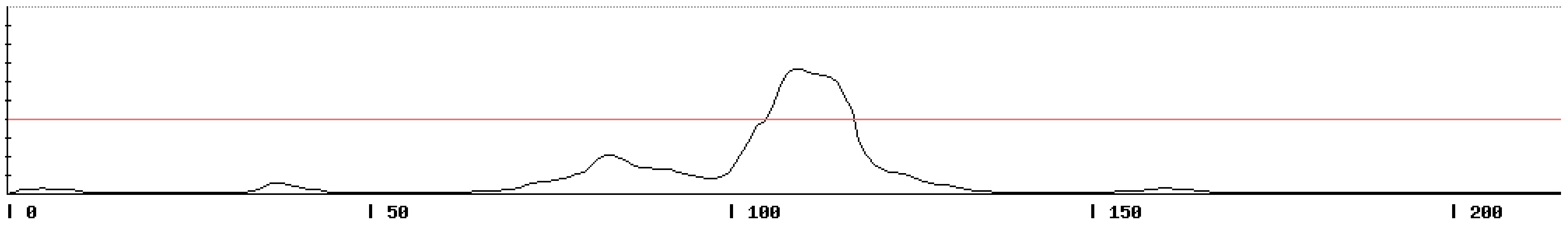

Predictions for Brachypodiumdistachyon_Bradi1g03245.1.p

Viterbi Path

Viterbi Path

Posterior @ 0.4

Posterior @ 0.4

Posterior Graphic. Threshold Marked @ 0.4

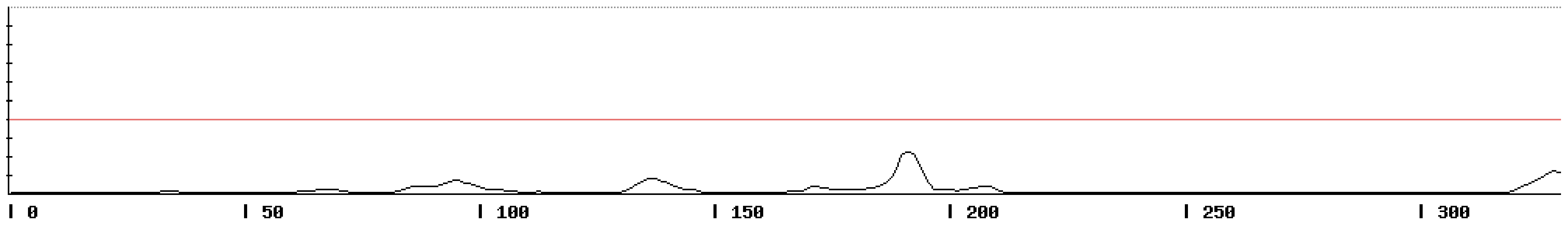

Predictions for Brachypodiumdistachyon_Bradi1g03313.1.p

Viterbi Path

Viterbi Path

Posterior @ 0.4

55 - RYAGKKKSSGRRSR - 68

157 - KFKKRKA - 163

Posterior @ 0.4

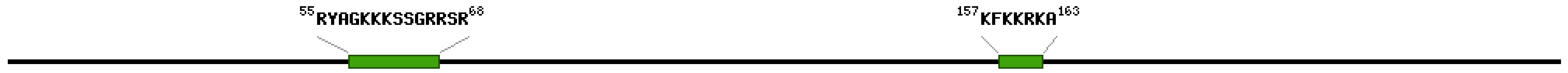

Posterior Graphic. Threshold Marked @ 0.4

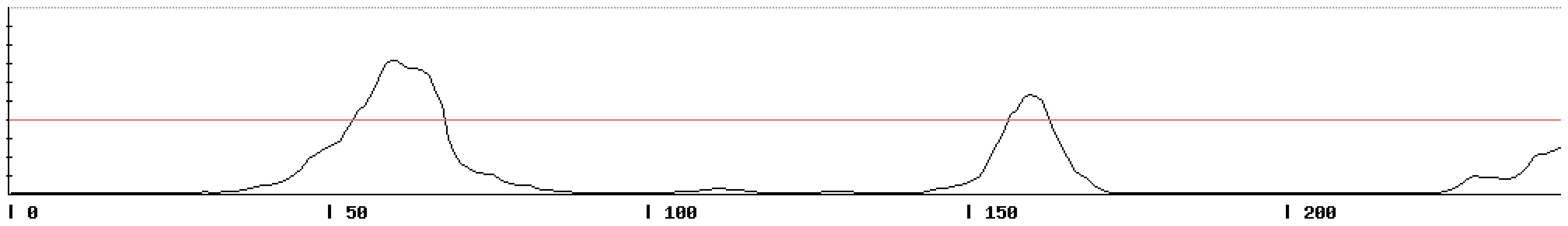

Predictions for Brachypodiumdistachyon_Bradi1g04150.1.p

Viterbi Path

Viterbi Path

Posterior @ 0.4

Posterior @ 0.4

Posterior Graphic. Threshold Marked @ 0.4

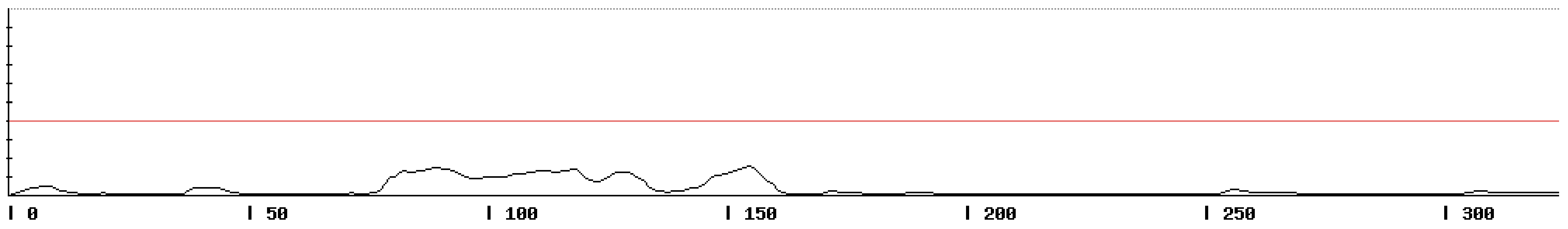

Predictions for Brachypodiumdistachyon_Bradi1g04229.1.p

Viterbi Path

131 - PPAGRRPRGRPRGSTAAKSRSRAKPK - 156

Viterbi Path

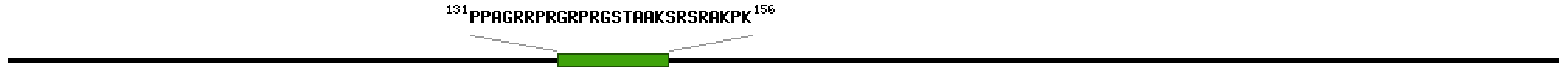

Posterior @ 0.4

128 - PPNPPAGRRPRGRPRGSTAAKSRSRAKPKL - 157

290 - RRYSRGKRPAR - 300

Posterior @ 0.4

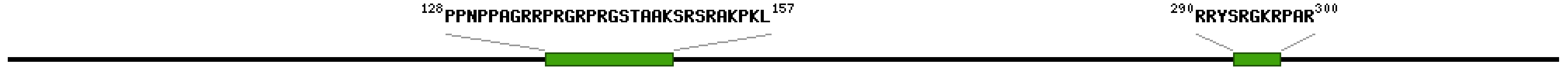

Posterior Graphic. Threshold Marked @ 0.4

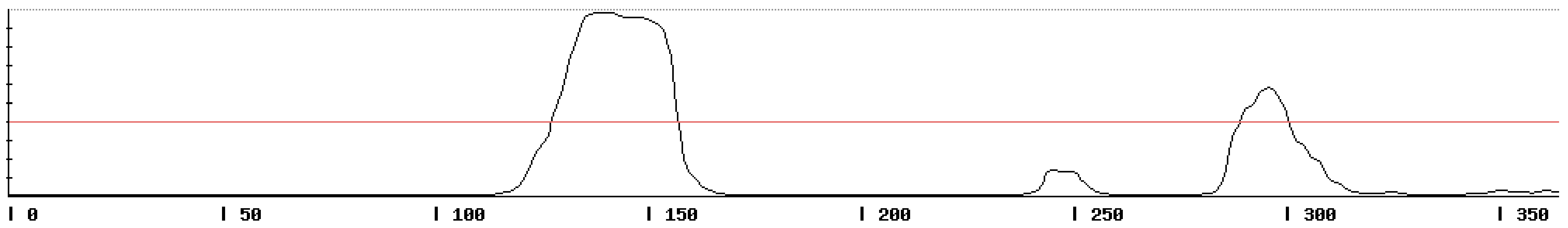

Predictions for Brachypodiumdistachyon_Bradi1g06970.1.p

Viterbi Path

Viterbi Path

Posterior @ 0.4

Posterior @ 0.4

Posterior Graphic. Threshold Marked @ 0.4

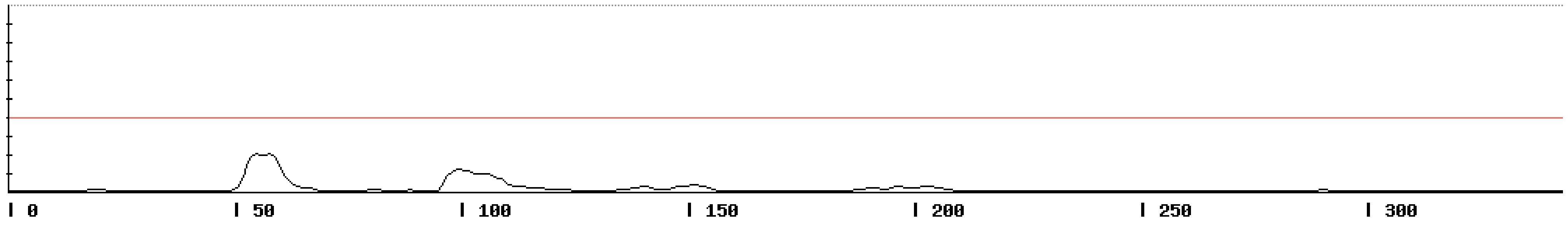

Predictions for Brachypodiumdistachyon_Bradi1g14467.1.p

Viterbi Path

Viterbi Path

Posterior @ 0.4

Posterior @ 0.4

Posterior Graphic. Threshold Marked @ 0.4

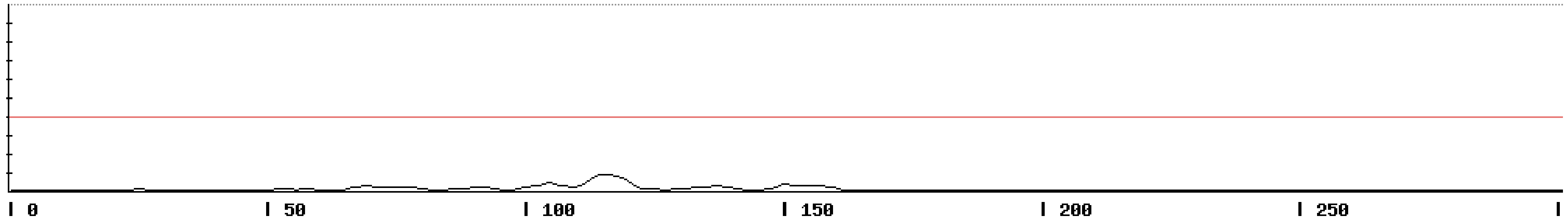

Predictions for Brachypodiumdistachyon_Bradi1g17440.1.p

Viterbi Path	
Viterbi Path	
Posterior @ 0.4	
Posterior @ 0.4	
Posterior Graphic. Threshold Marked @ 0.4	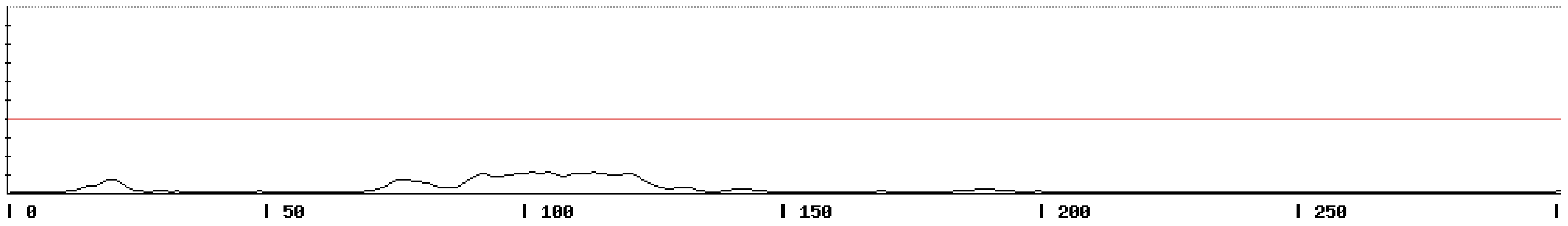

Predictions for Brachypodiumdistachyon_Bradi1g17480.1.p

Viterbi Path	
Viterbi Path	
Posterior @ 0.4	
Posterior @ 0.4	
Posterior Graphic. Threshold Marked @ 0.4	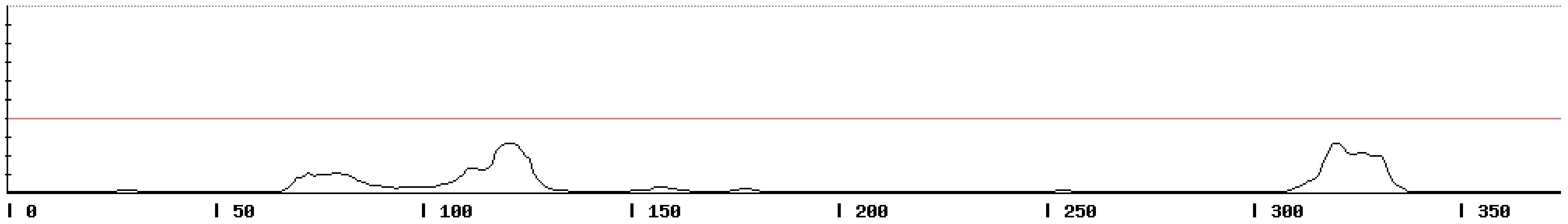

Predictions for Brachypodiumdistachyon_Bradi1g20063.2.p	
Viterbi Path	2 - GRRRGRPRGSTKPMLANKAAAKPKPKHAKSAR - 33
Viterbi Path	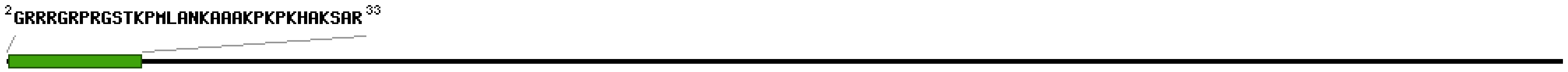
Posterior @ 0.4	2 - GRRRGRPRGSTKPMLANKAAAKPKPKHAKSARQ - 34<br>222 - WRMKEYRIPDNRRKRDPKRRKPSQR - 246
Posterior @ 0.4	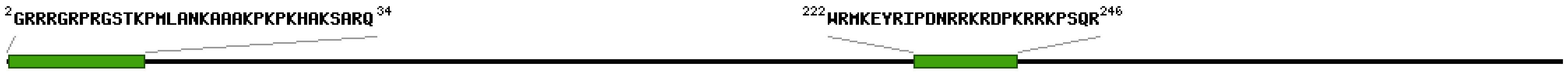
Posterior Graphic. Threshold Marked @ 0.4	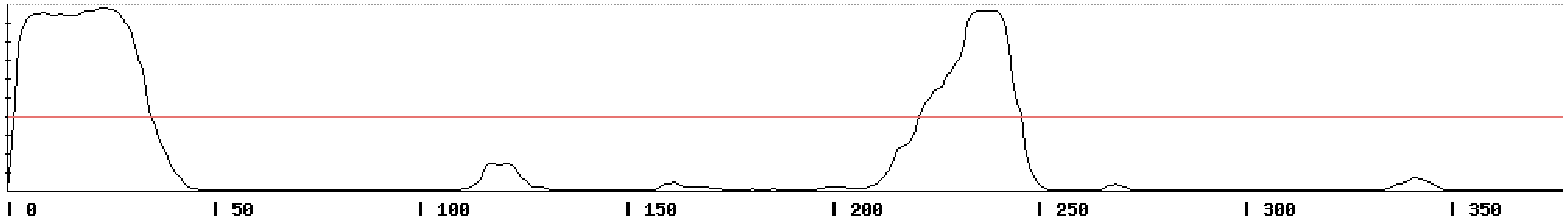

Predictions for Brachypodiumdistachyon_Bradi1g20801.1.p	
Viterbi Path	
Viterbi Path	
Posterior @ 0.4	
Posterior @ 0.4	
Posterior Graphic. Threshold Marked @ 0.4	

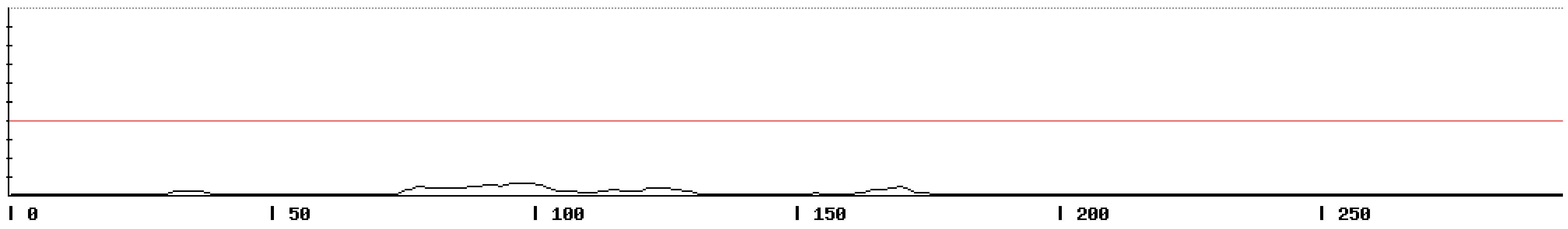

Predictions for Brachypodiumdistachyon_Bradi1g20805.1.p

Viterbi Path

Viterbi Path

Posterior @ 0.4

Posterior @ 0.4

Posterior Graphic. Threshold Marked @ 0.4

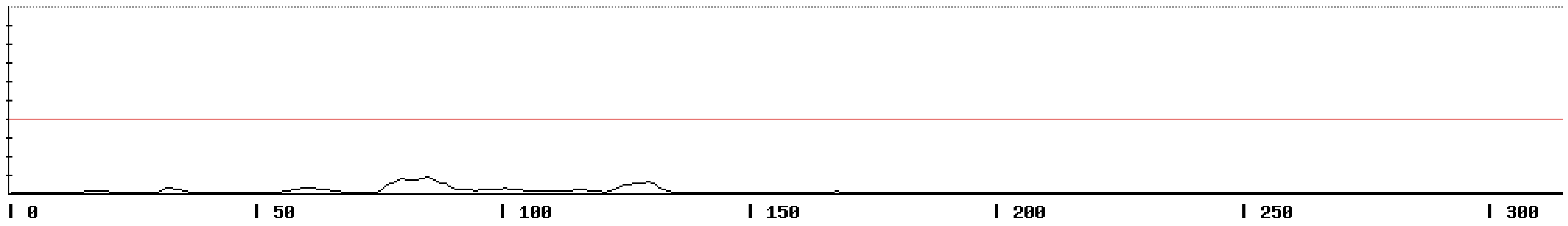

Predictions for Brachypodiumdistachyon_Bradi1g24390.1.p

Viterbi Path

Viterbi Path

Posterior @ 0.4

Posterior @ 0.4

Posterior Graphic. Threshold Marked @ 0.4

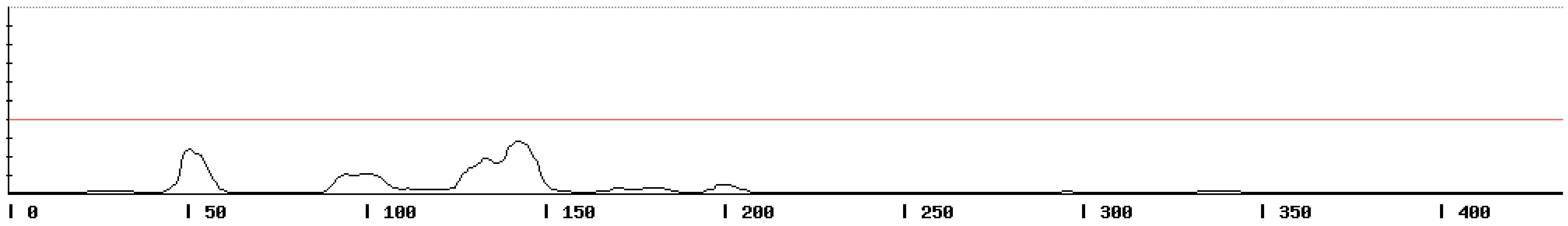

Predictions for Brachypodiumdistachyon_Bradi1g29857.1.p

Viterbi Path

Viterbi Path

Posterior @ 0.4

Posterior @ 0.4

Posterior Graphic. Threshold Marked @ 0.4

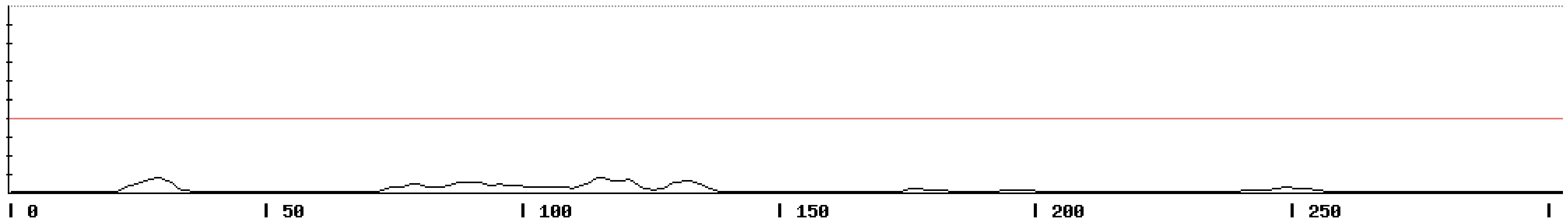

Predictions for Brachypodiumdistachyon_Bradi1g32660.1.p

Viterbi Path

Viterbi Path

Posterior @ 0.4

Posterior @ 0.4

Posterior Graphic. Threshold Marked @ 0.4	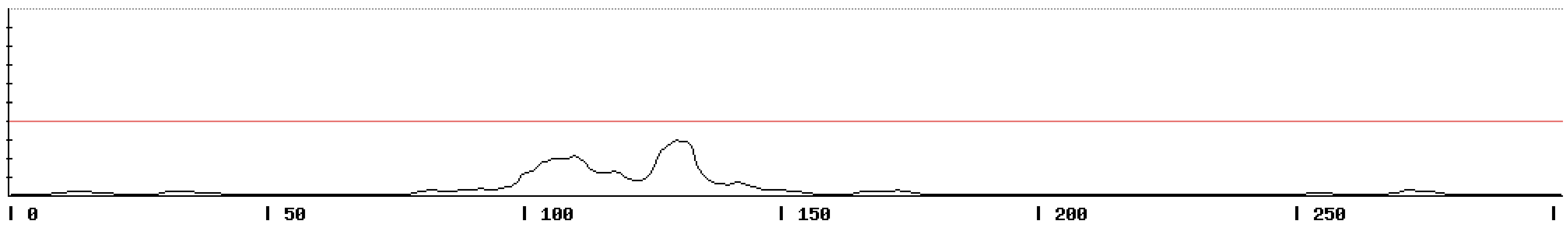

Predictions for Brachypodiumdistachyon_Bradi1g36200.1.p

Viterbi Path	
Viterbi Path	
Posterior @ 0.4	165 - DKAKERRAAKLAKESVRAG - 183
Posterior @ 0.4	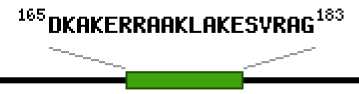
Posterior Graphic. Threshold Marked @ 0.4	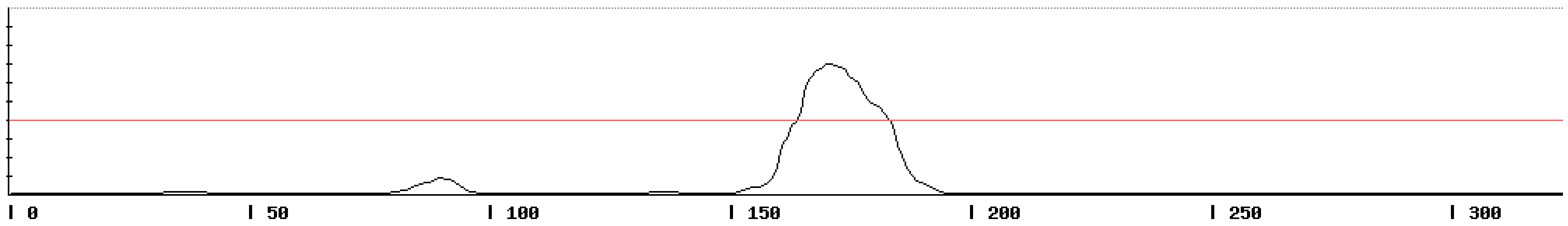

Predictions for Brachypodiumdistachyon_Bradi1g37898.1.p

Viterbi Path	
Viterbi Path	

Posterior @ 0.4	142 - RKRRK - 146
Posterior @ 0.4	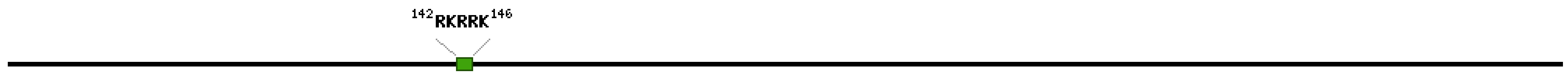
Posterior Graphic. Threshold Marked @ 0.4	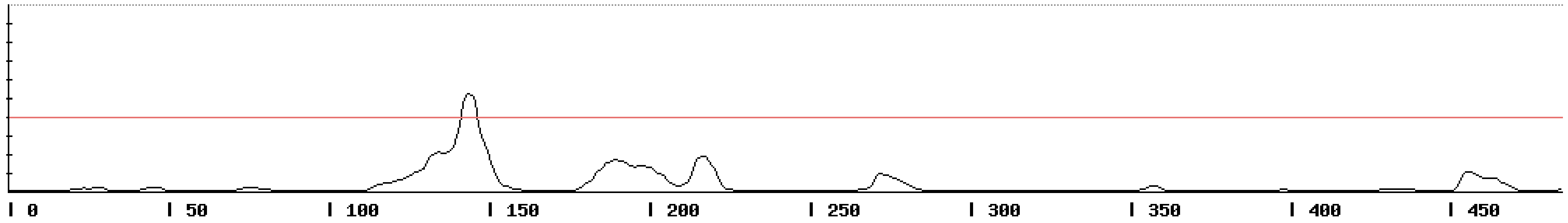

Predictions for Brachypodiumdistachyon_Bradi1g38730.1.p

Viterbi Path	
Viterbi Path	
Posterior @ 0.4	
Posterior @ 0.4	
Posterior Graphic. Threshold Marked @ 0.4	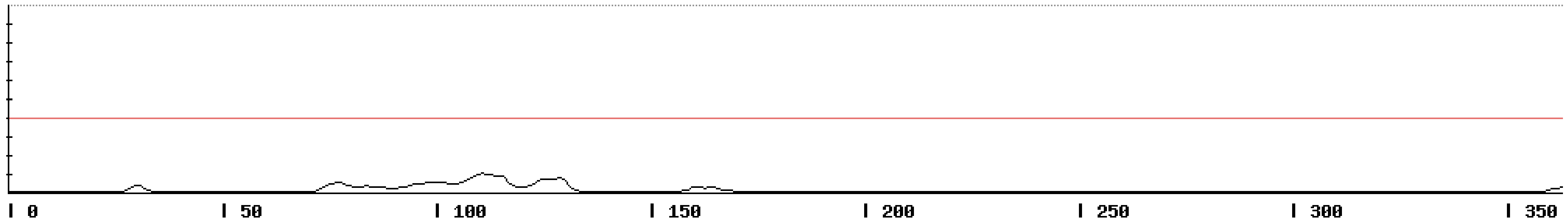

Predictions for Brachypodiumdistachyon_Bradi1g41712.2.p

Viterbi Path	
Viterbi Path	

Posterior @ 0.4

Posterior @ 0.4

Posterior Graphic. Threshold Marked @ 0.4

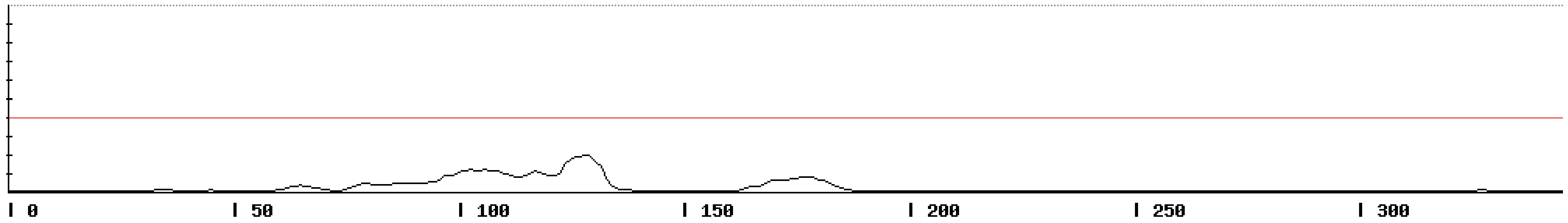

Predictions for Brachypodiumdistachyon_Bradi1g43840.1.p

Viterbi Path

Viterbi Path

Posterior @ 0.4

Posterior @ 0.4

Posterior Graphic. Threshold Marked @ 0.4

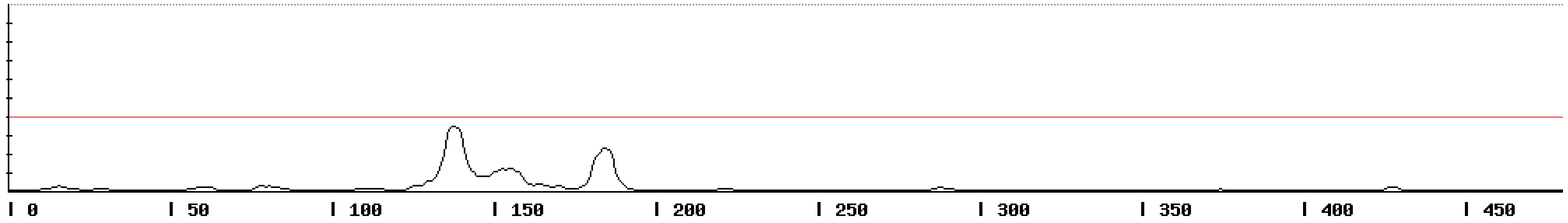

Predictions for Brachypodiumdistachyon_Bradi1g50057.1.p

Viterbi Path	
Viterbi Path	
Posterior @ 0.4	
Posterior @ 0.4	
Posterior Graphic. Threshold Marked @ 0.4	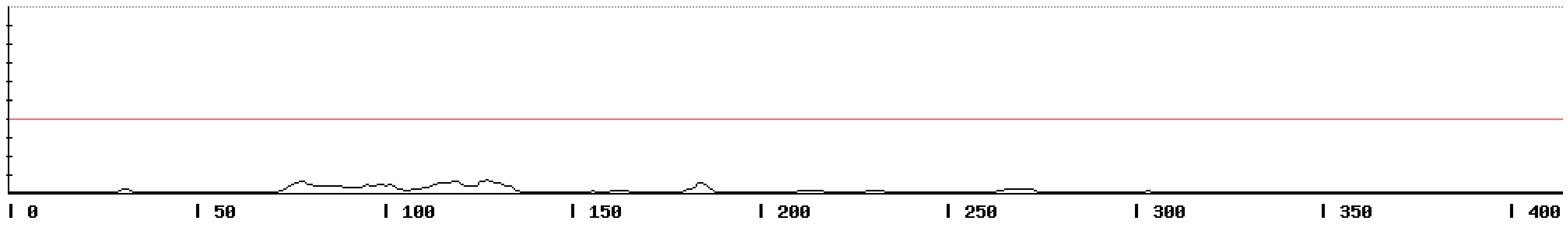

Predictions for Brachypodiumdistachyon_Bradi1g52187.2.p

Viterbi Path	
Viterbi Path	
Posterior @ 0.4	
Posterior @ 0.4	
Posterior Graphic. Threshold Marked @ 0.4	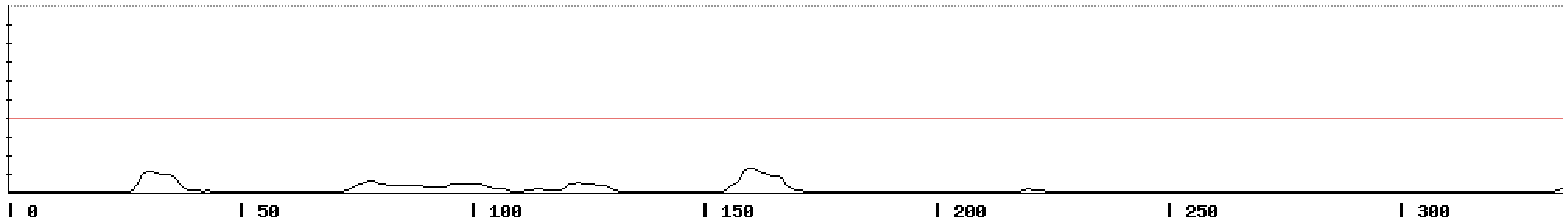

Predictions for Brachypodiumdistachyon_Bradi1g52480.2.p	
Viterbi Path	
Viterbi Path	
Posterior @ 0.4	80 - DRPKYPGKSPR - 90
Posterior @ 0.4	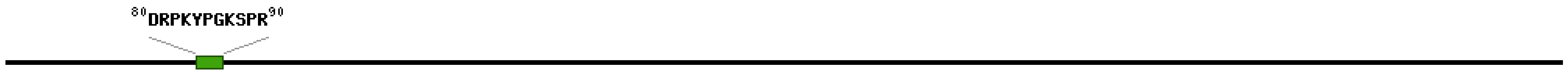
Posterior Graphic. Threshold Marked @ 0.4	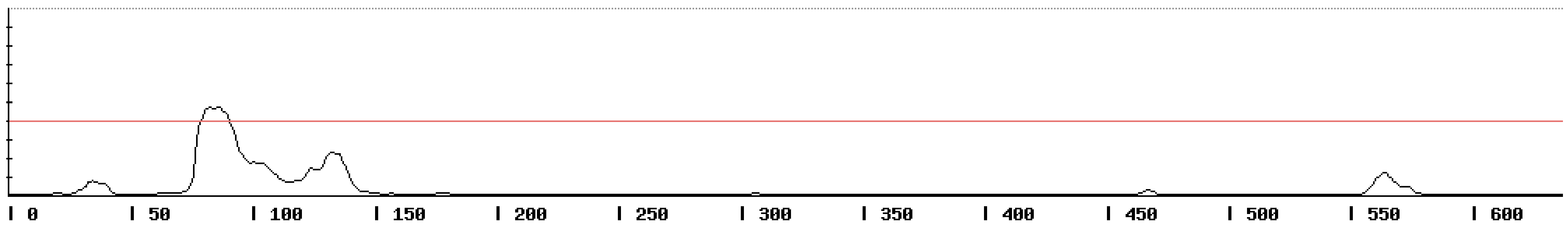

Predictions for Brachypodiumdistachyon_Bradi1g53656.1.p	
Viterbi Path	
Viterbi Path	
Posterior @ 0.4	
Posterior @ 0.4	
Posterior Graphic. Threshold Marked @ 0.4	

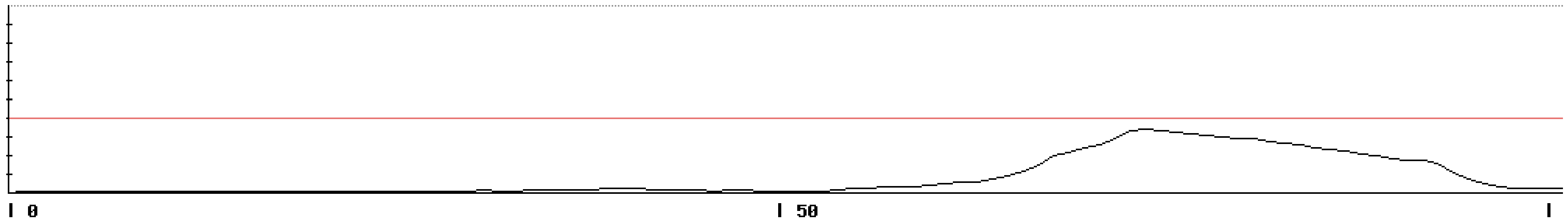

Predictions for Brachypodiumdistachyon_Bradi1g53770.1.p

Viterbi Path

Viterbi Path

Posterior @ 0.4 109 - KGR - 111

Posterior @ 0.4

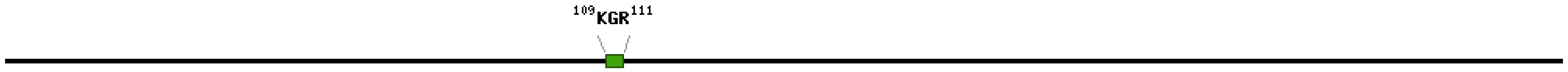

Posterior Graphic. Threshold Marked @ 0.4

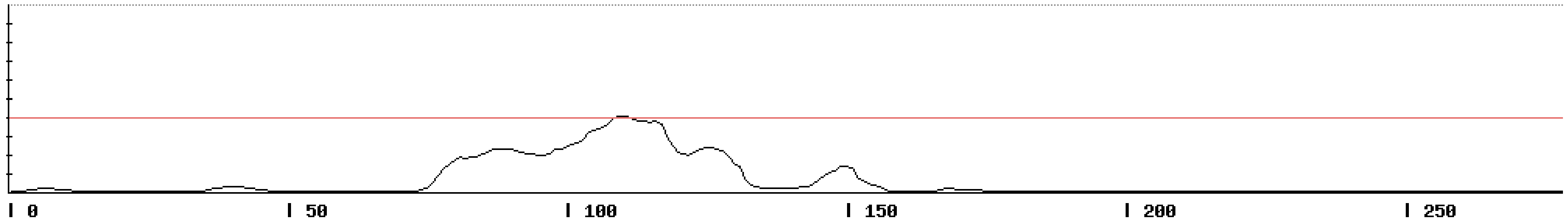

Predictions for Brachypodiumdistachyon_Bradi1g58057.1.p

Viterbi Path

Viterbi Path

Posterior @ 0.4

Posterior @ 0.4

Posterior Graphic. Threshold Marked @ 0.4

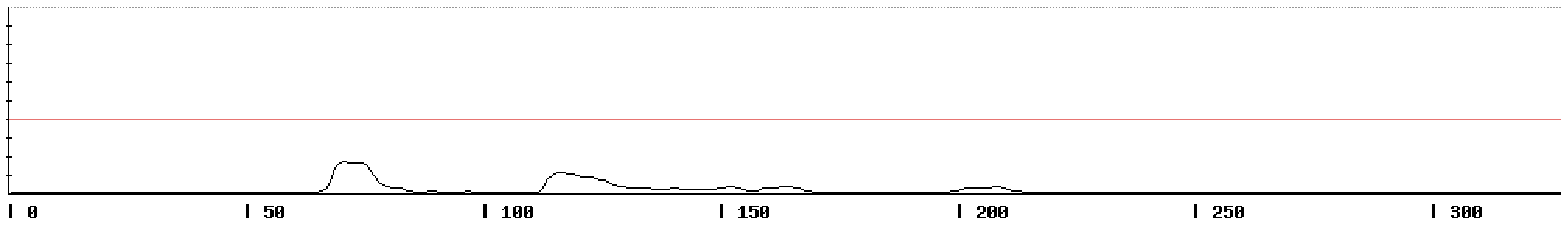

Predictions for Brachypodiumdistachyon_Bradi1g61525.1.p

Viterbi Path

Viterbi Path

Posterior @ 0.4

194 - RRRCNGKEKGRG - 205

Posterior @ 0.4

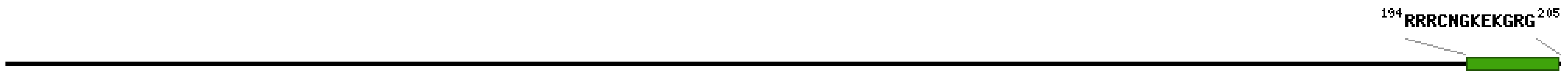

Posterior Graphic. Threshold Marked @ 0.4

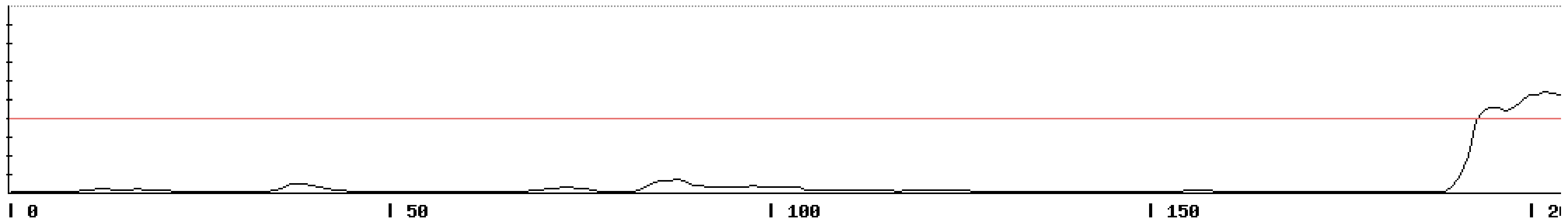

Predictions for Brachypodiumdistachyon_Bradi1g63600.1.p

Viterbi Path

Viterbi Path

Posterior @ 0.4

295 - RK - 296

Posterior @ 0.4

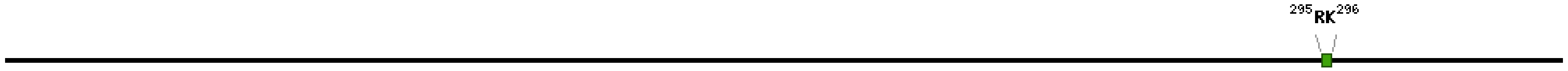

Posterior Graphic. Threshold Marked @ 0.4

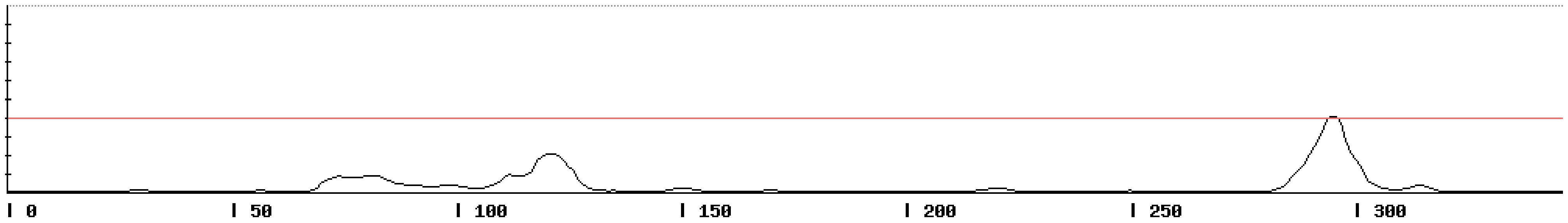

Predictions for Brachypodiumdistachyon_Bradi1g63630.1.p

Viterbi Path

Viterbi Path

Posterior @ 0.4

Posterior @ 0.4

Posterior Graphic. Threshold Marked @ 0.4

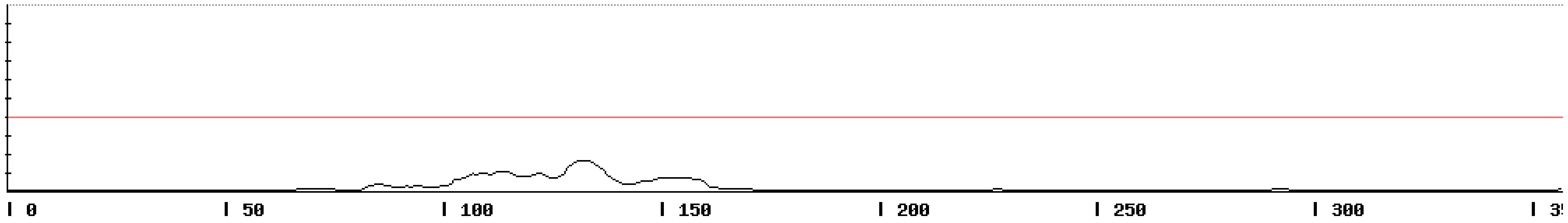

Predictions for Brachypodiumdistachyon_Bradi1g69617.2.p

Viterbi Path

Viterbi Path

Posterior @ 0.4	545 - RKKRK - 549
	574 - SRRRRKKKT - 582
Posterior @ 0.4	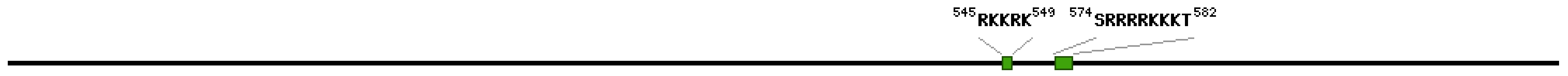
Posterior Graphic. Threshold Marked @ 0.4	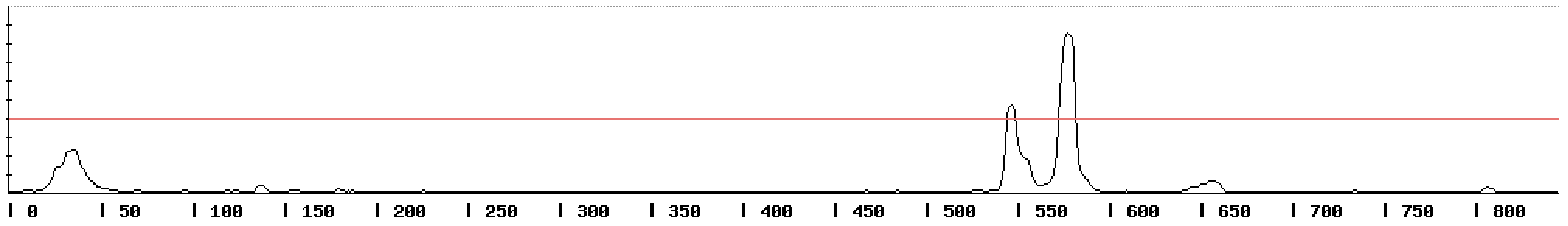

Predictions for Brachypodiumdistachyon_Bradi1g76207.2.p

Viterbi Path	
Viterbi Path	
Posterior @ 0.4	
Posterior @ 0.4	
Posterior Graphic. Threshold Marked @ 0.4	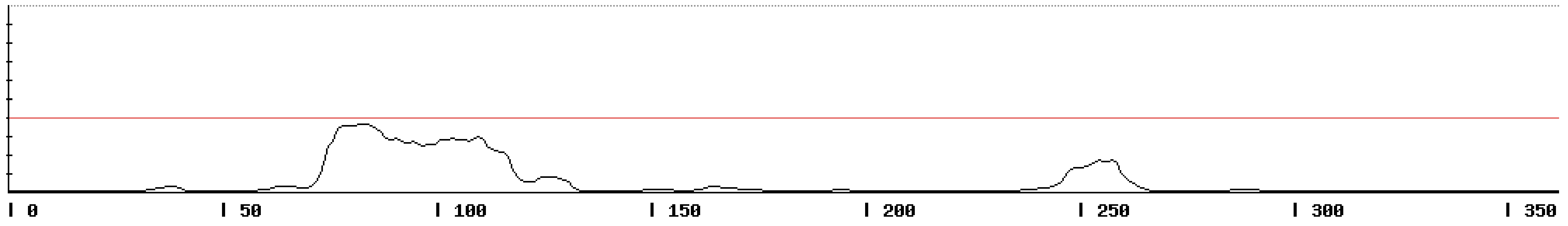

Predictions for Brachypodiumdistachyon_Bradi1g76732.1.p

Viterbi Path	
Viterbi Path	

Posterior @ 0.4 — 244 - ATKKKTKNGRK - 254

Posterior @ 0.4

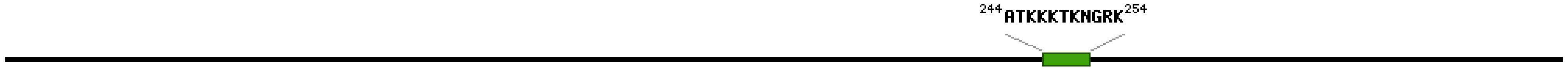

Posterior Graphic. Threshold Marked @ 0.4

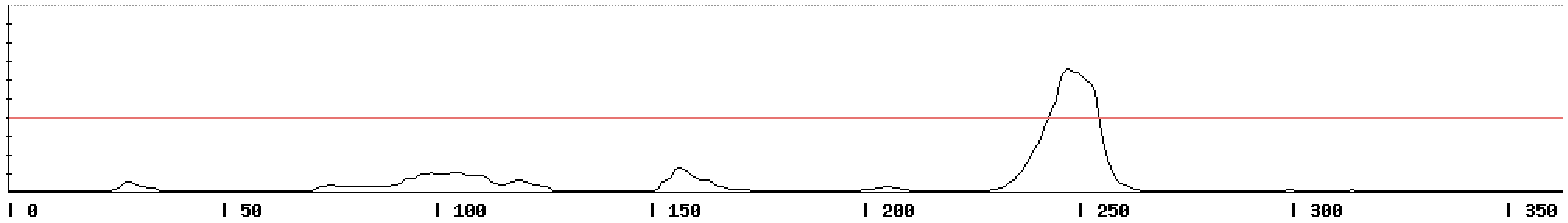

Predictions for Brachypodiumdistachyon_Bradi1g77217.1.p

Viterbi Path

Viterbi Path

Posterior @ 0.4 — 94 - K - 94

96 - RK - 97

Posterior @ 0.4

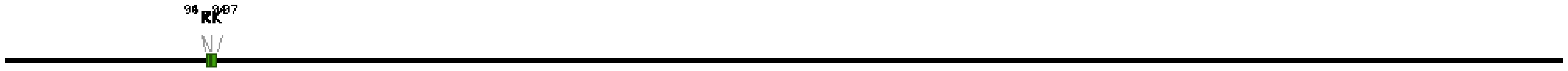

Posterior Graphic. Threshold Marked @ 0.4

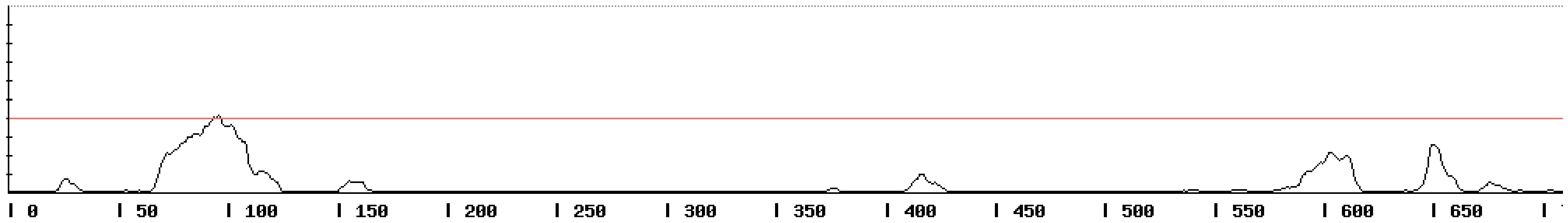

Predictions for Brachypodiumdistachyon_Bradi2g03448.1.p

Viterbi Path

Viterbi Path

Posterior @ 0.4

Posterior @ 0.4

Posterior Graphic. Threshold Marked @ 0.4

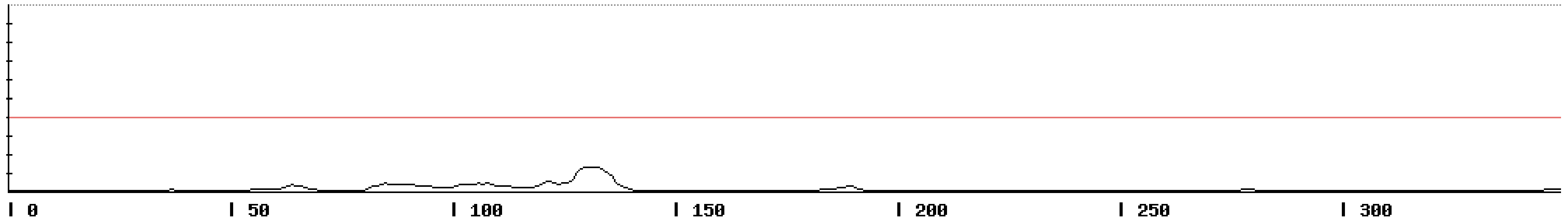

Predictions for Brachypodiumdistachyon_Bradi2g03467.1.p

Viterbi Path

Viterbi Path

Posterior @ 0.4

Posterior @ 0.4

Posterior Graphic. Threshold Marked @ 0.4

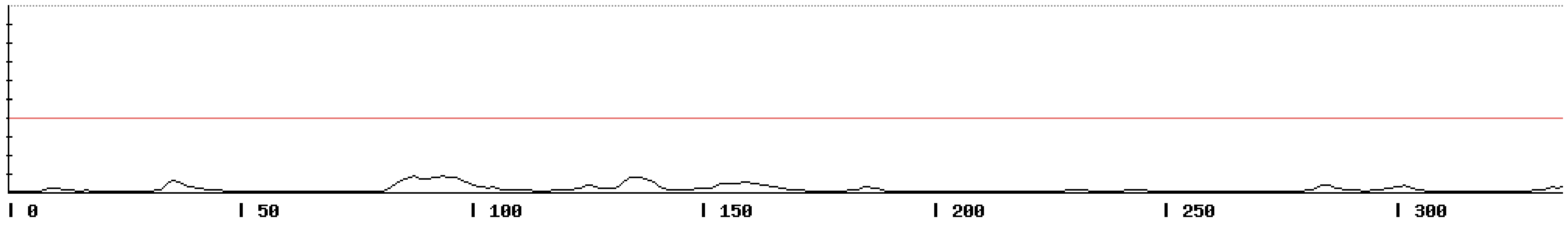

Predictions for Brachypodiumdistachyon_Bradi2g05700.2.p

Viterbi Path

Viterbi Path

Posterior @ 0.4

Posterior @ 0.4

Posterior Graphic. Threshold Marked @ 0.4

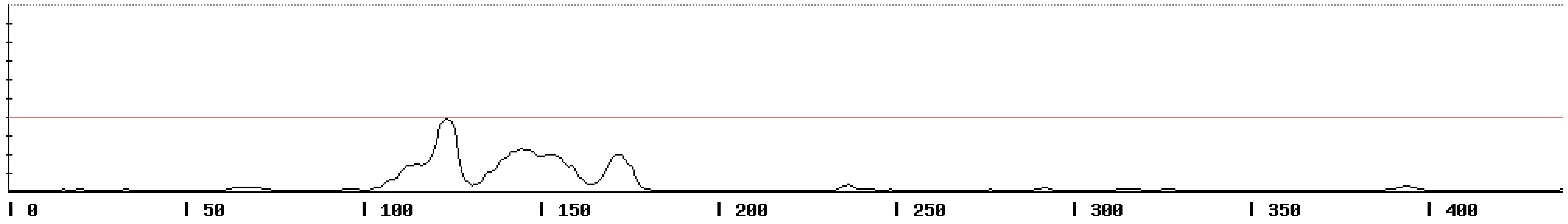

Predictions for Brachypodiumdistachyon_Bradi2g08366.1.p

Viterbi Path

Viterbi Path

Posterior @ 0.4

Posterior @ 0.4

Posterior Graphic. Threshold Marked @ 0.4

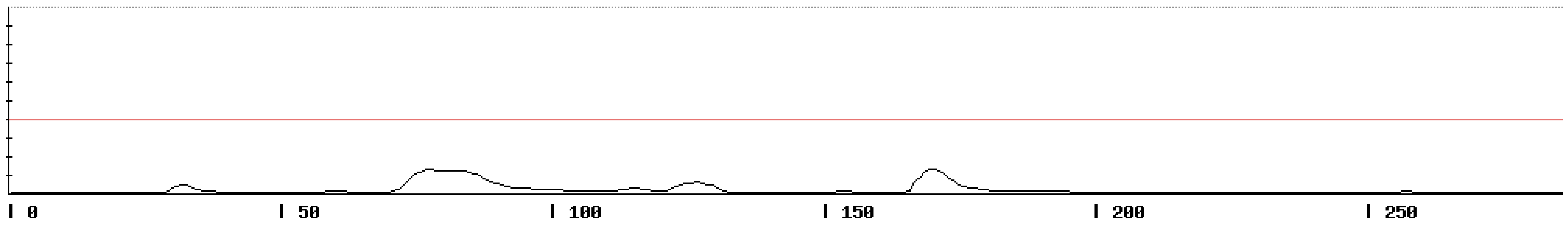

Predictions for Brachypodiumdistachyon_Bradi2g09530.1.p

Viterbi Path

Viterbi Path

Posterior @ 0.4

Posterior @ 0.4

Posterior Graphic. Threshold Marked @ 0.4

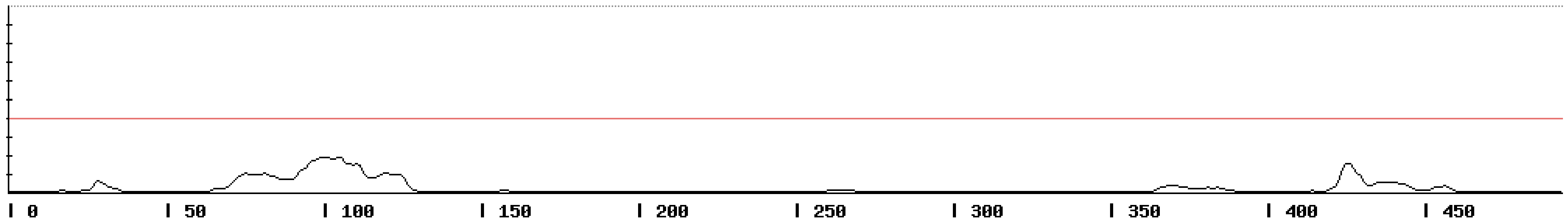

Predictions for Brachypodiumdistachyon_Bradi2g20590.1.p

Viterbi Path

Viterbi Path

Posterior @ 0.4

169 - RKRRSA - 174

182 - RSALKRK - 188

Posterior @ 0.4

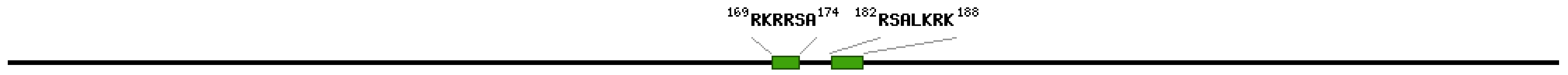

Posterior Graphic. Threshold Marked @ 0.4

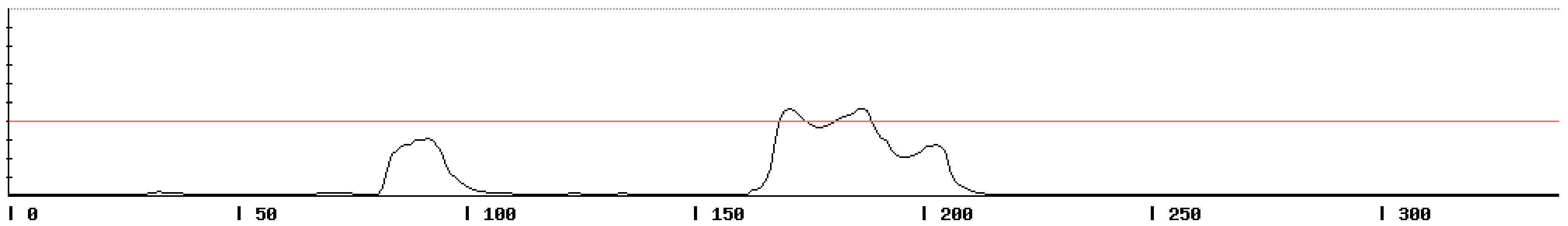

Predictions for Brachypodiumdistachyon_Bradi2g23967.1.p

Viterbi Path

Viterbi Path

Posterior @ 0.4

Posterior @ 0.4

Posterior Graphic. Threshold Marked @ 0.4

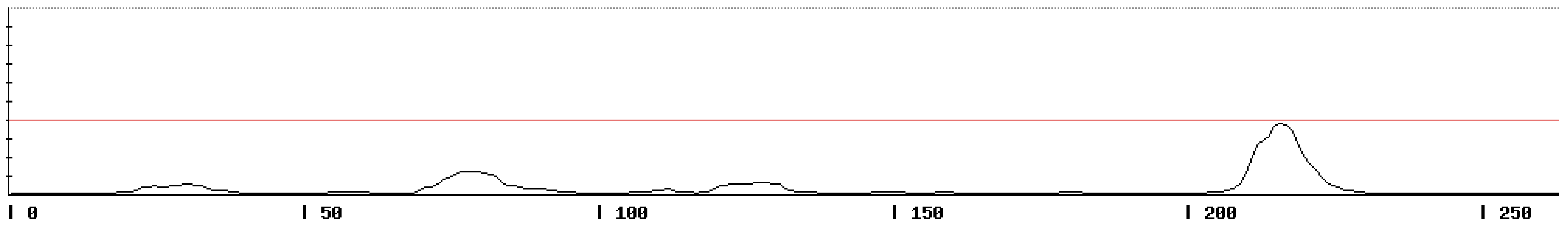

Predictions for Brachypodiumdistachyon_Bradi2g24790.1.p

Viterbi Path

Viterbi Path

Posterior @ 0.4	
Posterior @ 0.4	
Posterior Graphic. Threshold Marked @ 0.4	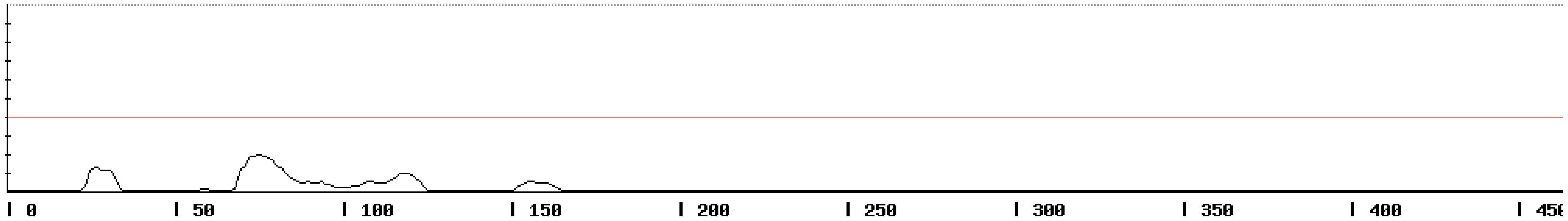

Predictions for Brachypodiumdistachyon_Bradi2g24987.1.p

Viterbi Path	
Viterbi Path	
Posterior @ 0.4	
Posterior @ 0.4	
Posterior Graphic. Threshold Marked @ 0.4	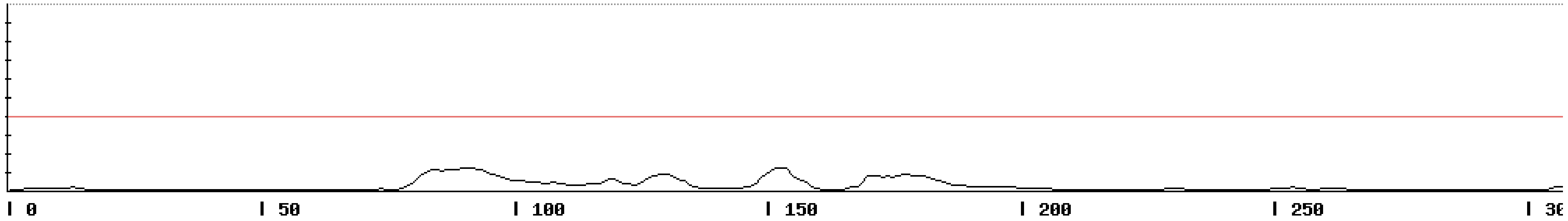

Predictions for Brachypodiumdistachyon_Bradi2g25150.1.p

Viterbi Path	
Viterbi Path	

Posterior @ 0.4

Posterior @ 0.4

Posterior Graphic. Threshold Marked @ 0.4

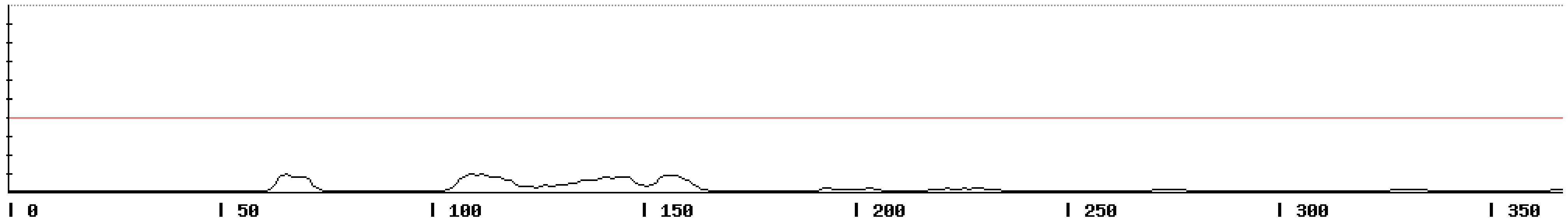

Predictions for Brachypodiumdistachyon_Bradi2g28020.1.p

Viterbi Path

Viterbi Path

Posterior @ 0.4 — 112 - KGEMKHLSFKKGIKKEK - 128

Posterior @ 0.4

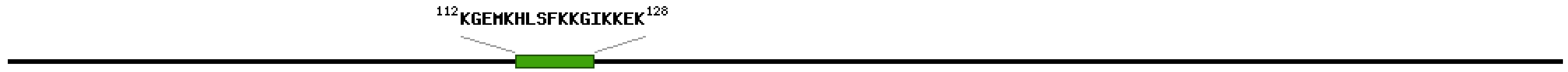

Posterior Graphic. Threshold Marked @ 0.4

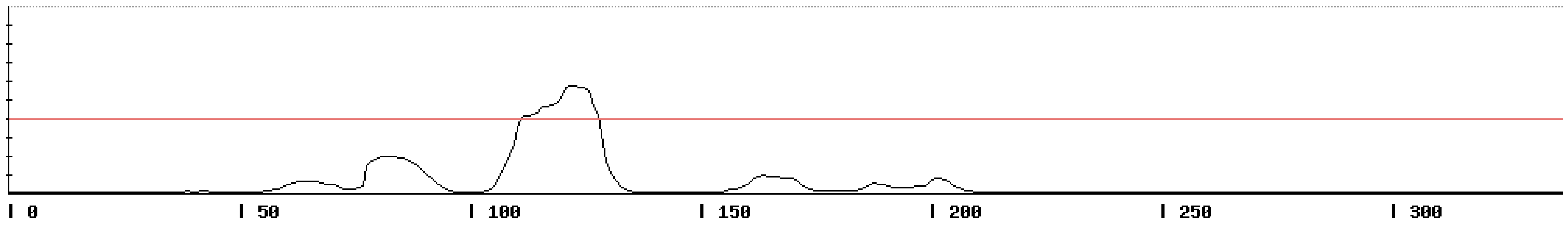

Predictions for Brachypodiumdistachyon_Bradi2g33307.2.p

Viterbi Path	
Viterbi Path	
Posterior @ 0.4	123 - R - 123
Posterior @ 0.4	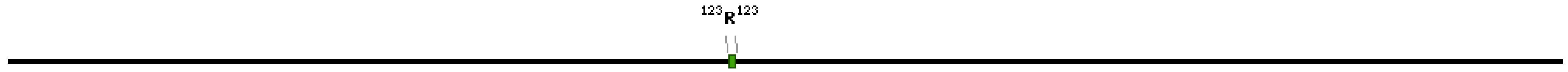
Posterior Graphic. Threshold Marked @ 0.4	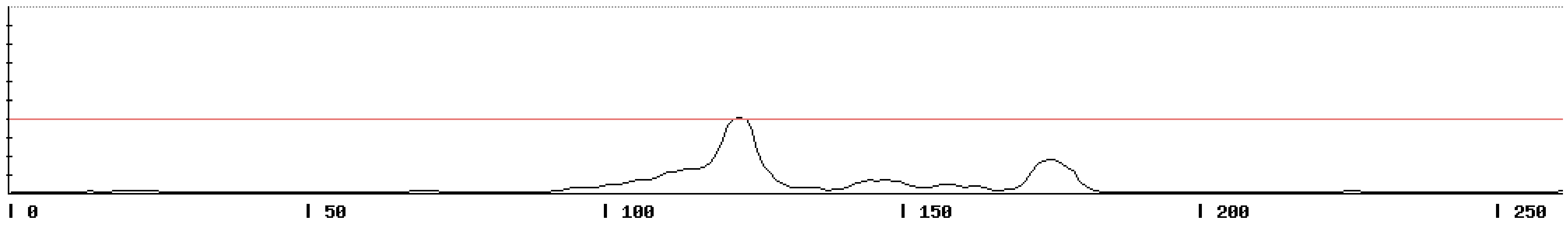

**Predictions for Brachypodiumdistachyon_Bradi2g40026.1.p**	
Viterbi Path	309 - RKKKKRHQPVEEMKLGAAARDGGIKSQRGERSQEPEMRKKRHTNKV - 354
Viterbi Path	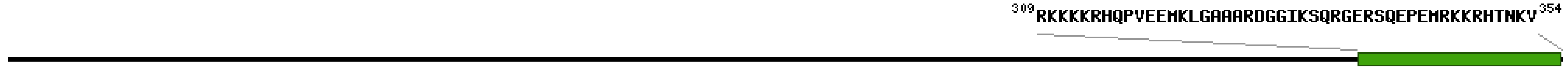
Posterior @ 0.4	78 - KRSR - 81<br>308 - IRKKKKRHQPVEEMKLGAAARDGGIKSQRGERSQEPEMRKKRHTNKV - 354
Posterior @ 0.4	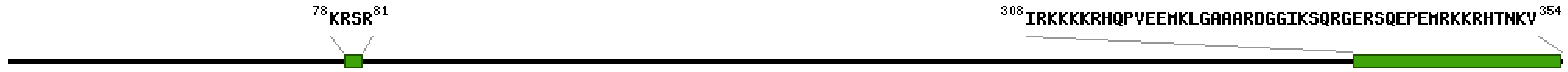
Posterior Graphic. Threshold Marked @ 0.4	

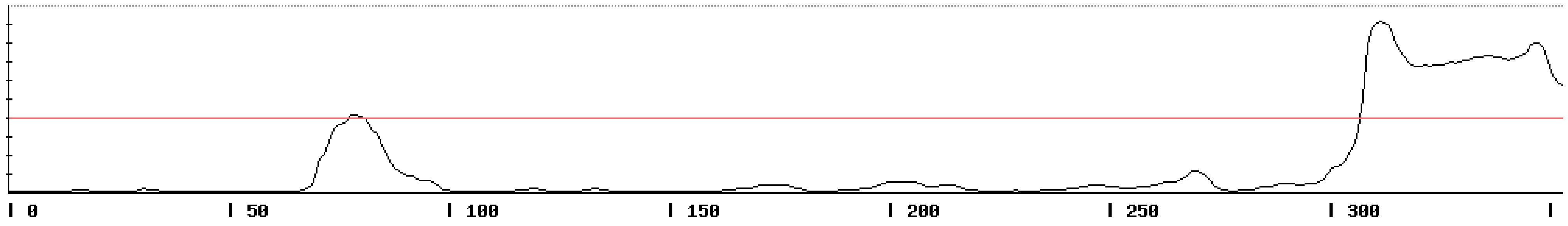

Predictions for Brachypodiumdistachyon_Bradi2g46020.1.p

Viterbi Path	
Viterbi Path	
Posterior @ 0.4	182 - RKKP - 185
	197 - RKR - 199
Posterior @ 0.4	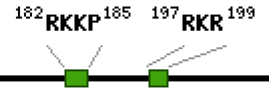
Posterior Graphic. Threshold Marked @ 0.4	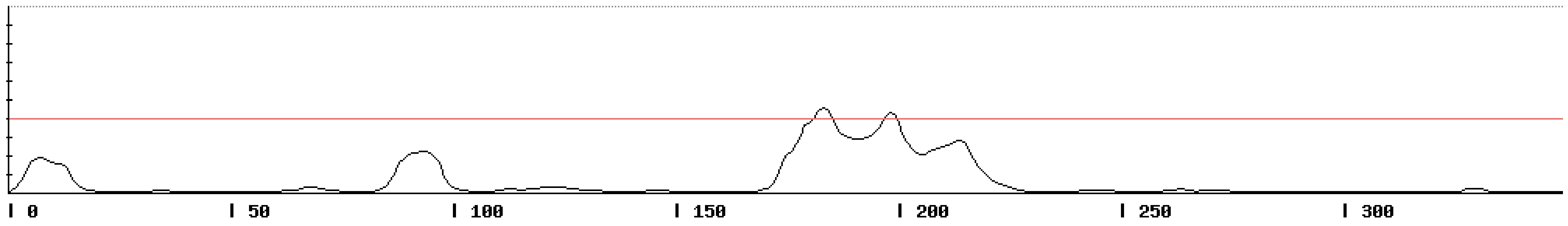

Predictions for Brachypodiumdistachyon_Bradi2g46197.2.p

Viterbi Path	
Viterbi Path	
Posterior @ 0.4	151 - RKRRK - 155
Posterior @ 0.4	

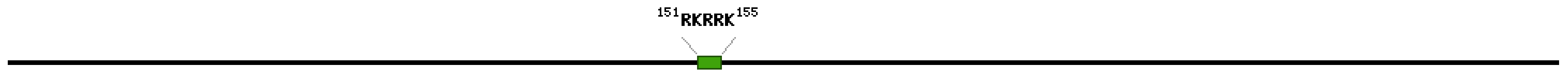

Posterior Graphic. Threshold Marked @ 0.4

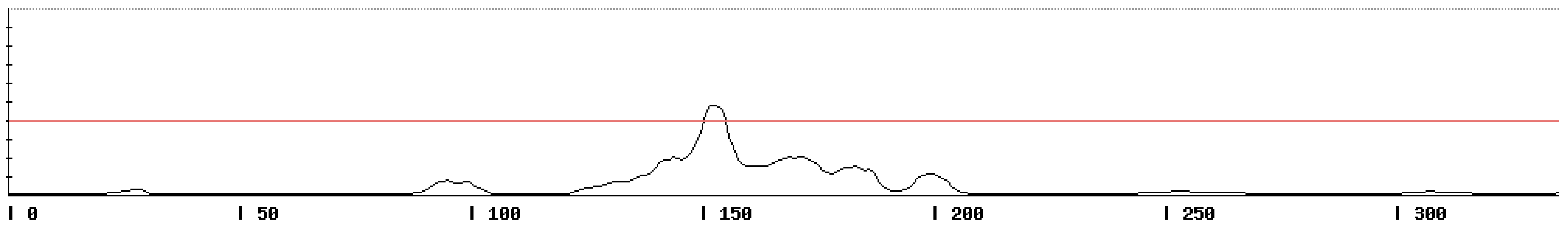

Predictions for Brachypodiumdistachyon_Bradi2g46427.1.p

Viterbi Path

Viterbi Path

Posterior @ 0.4

77 - RAKSRRGQRKS - 87

345 - RIRGQRR - 351

448 - RKRKASEENSGAESPARGTKKSTAPAASGRKK - 479

Posterior @ 0.4

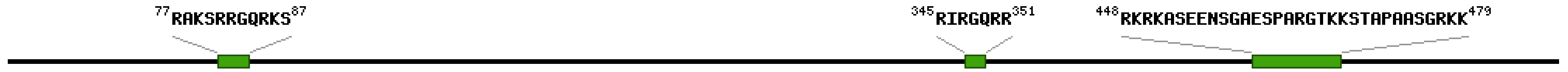

Posterior Graphic. Threshold Marked @ 0.4

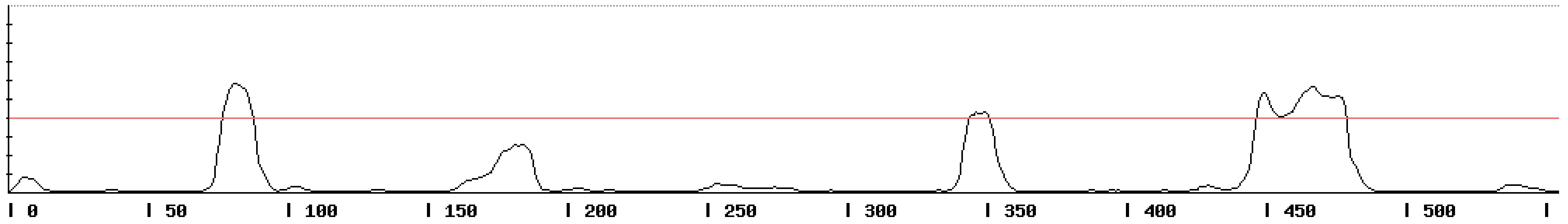

Predictions for Brachypodiumdistachyon_Bradi2g46483.1.p

Viterbi Path

Viterbi Path

Posterior @ 0.4

Posterior @ 0.4

Posterior Graphic. Threshold Marked @ 0.4

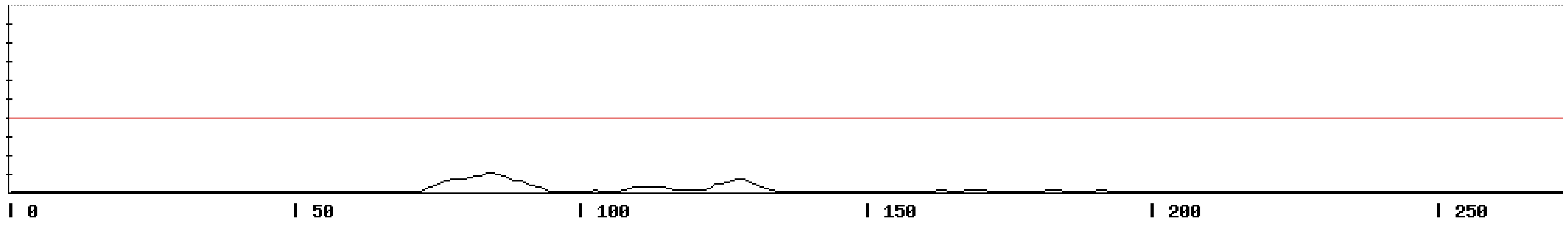

Predictions for Brachypodiumdistachyon_Bradi2g46980.1.p

Viterbi Path

Viterbi Path

Posterior @ 0.4

Posterior @ 0.4

Posterior Graphic. Threshold Marked @ 0.4

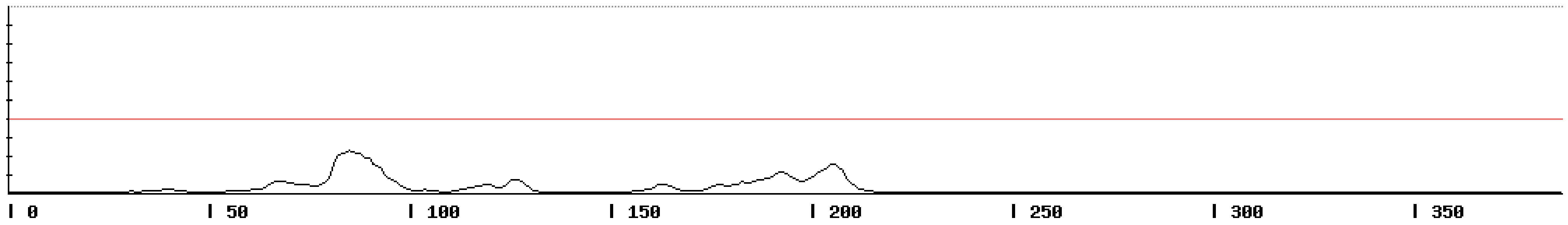

Predictions for Brachypodiumdistachyon_Bradi2g53260.1.p

Viterbi Path

Viterbi Path

Posterior @ 0.4

Posterior @ 0.4

Posterior Graphic. Threshold Marked @ 0.4

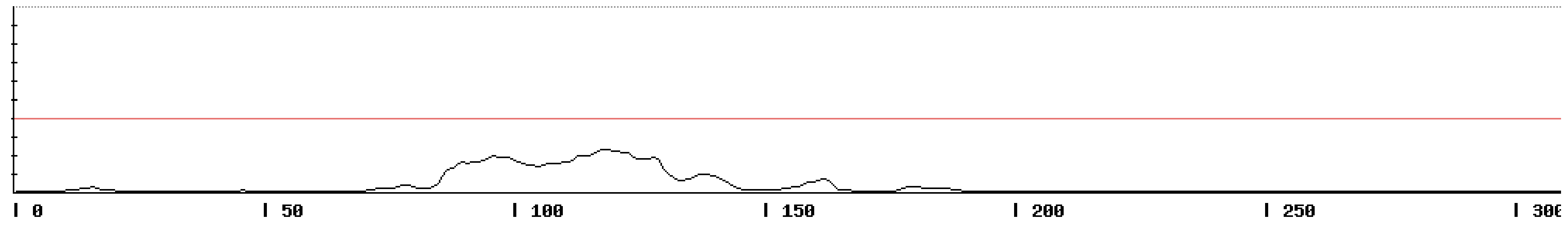

Predictions for Brachypodiumdistachyon_Bradi2g55780.1.p

Viterbi Path

Viterbi Path

Posterior @ 0.4

Posterior @ 0.4

Posterior Graphic. Threshold Marked @ 0.4

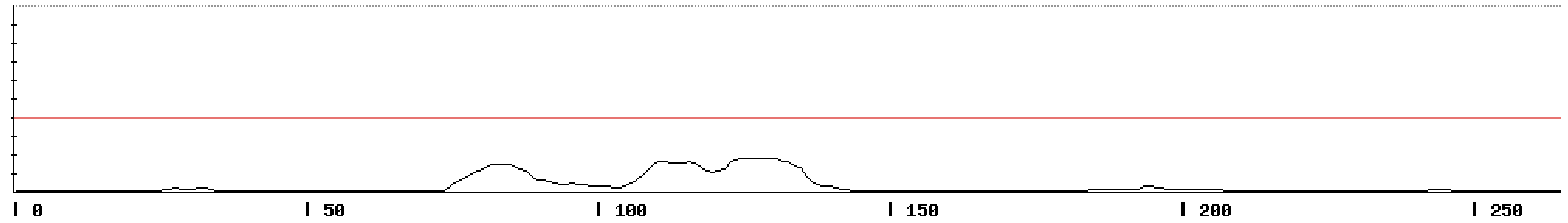

Predictions for Brachypodiumdistachyon_Bradi2g57100.1.p	
Viterbi Path	
Viterbi Path	
Posterior @ 0.4	
Posterior @ 0.4	
Posterior Graphic. Threshold Marked @ 0.4	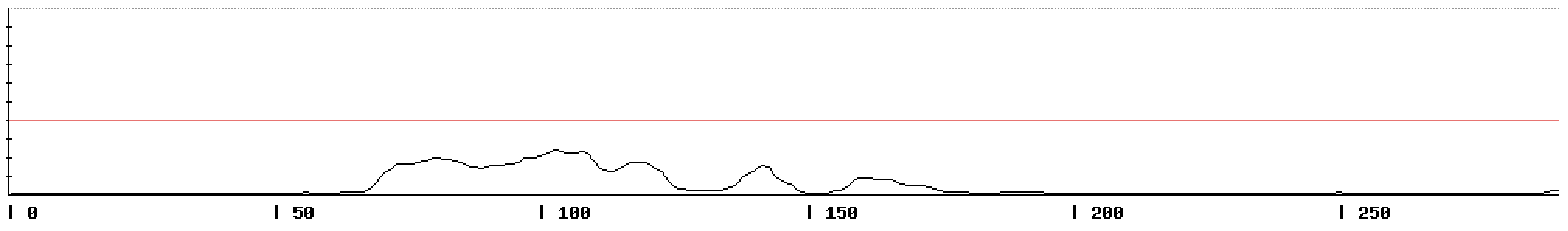

Predictions for Brachypodiumdistachyon_Bradi2g57297.1.p	
Viterbi Path	
Viterbi Path	
Posterior @ 0.4	
Posterior @ 0.4	
Posterior Graphic. Threshold Marked @ 0.4	

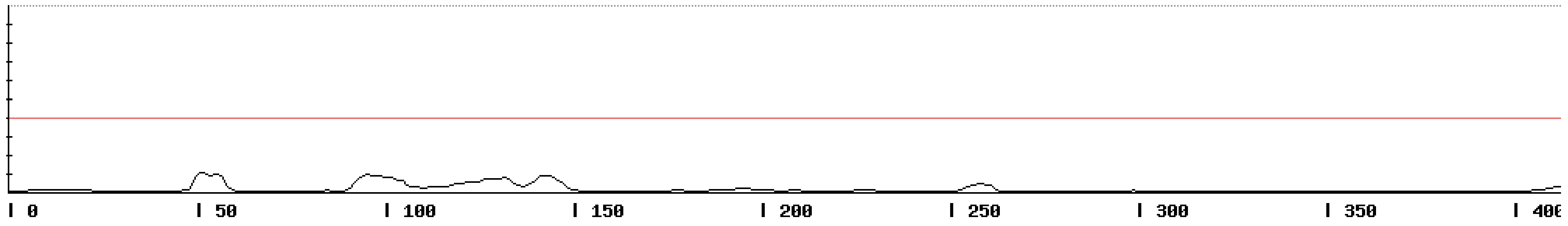

Predictions for Brachypodiumdistachyon_Bradi2g60620.1.p

Viterbi Path	
Viterbi Path	
Posterior @ 0.4	81 - RHGLRRRGRGYFFAPRRRRGGTVRRSPAGGKGGSWMHR - 118
	190 - SRAAAAAAAKPRARR - 203
Posterior @ 0.4	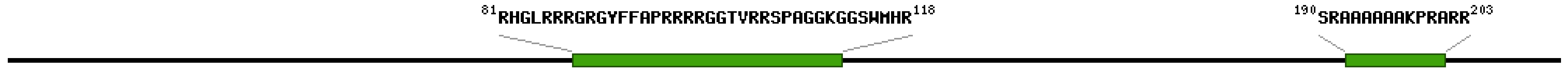
Posterior Graphic. Threshold Marked @ 0.4	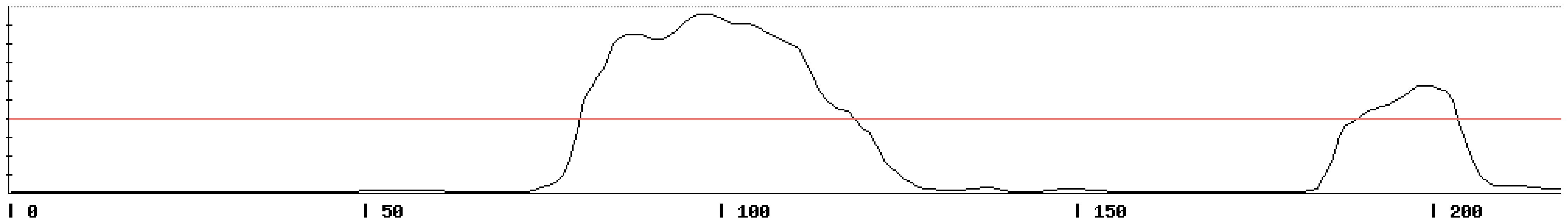

Predictions for Brachypodiumdistachyon_Bradi3g09520.2.p

Viterbi Path	
Viterbi Path	
Posterior @ 0.4	
Posterior @ 0.4	

Posterior Graphic. Threshold Marked @ 0.4

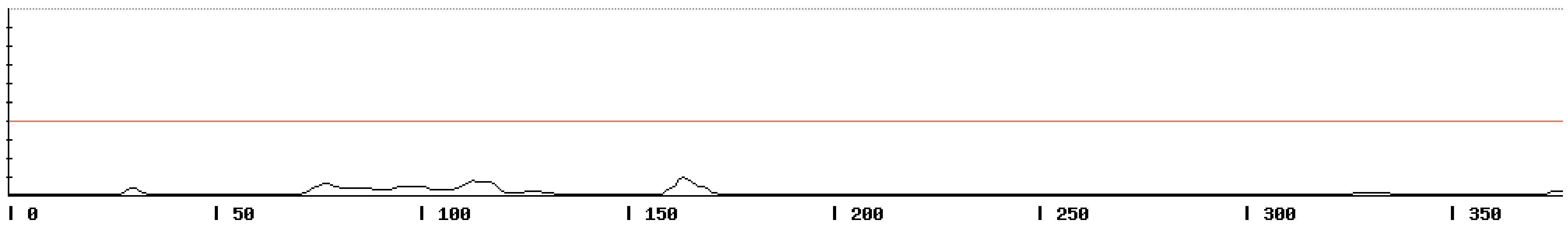

Predictions for Brachypodiumdistachyon_Bradi3g10414.1.p

Viterbi Path

Viterbi Path

Posterior @ 0.4

Posterior @ 0.4

Posterior Graphic. Threshold Marked @ 0.4

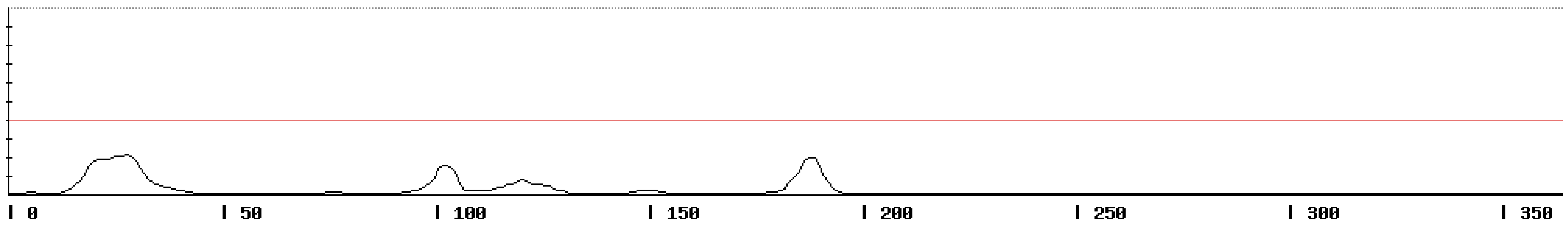

Predictions for Brachypodiumdistachyon_Bradi3g10540.2.p

Viterbi Path

Viterbi Path

Posterior @ 0.4	
Posterior @ 0.4	
Posterior Graphic. Threshold Marked @ 0.4	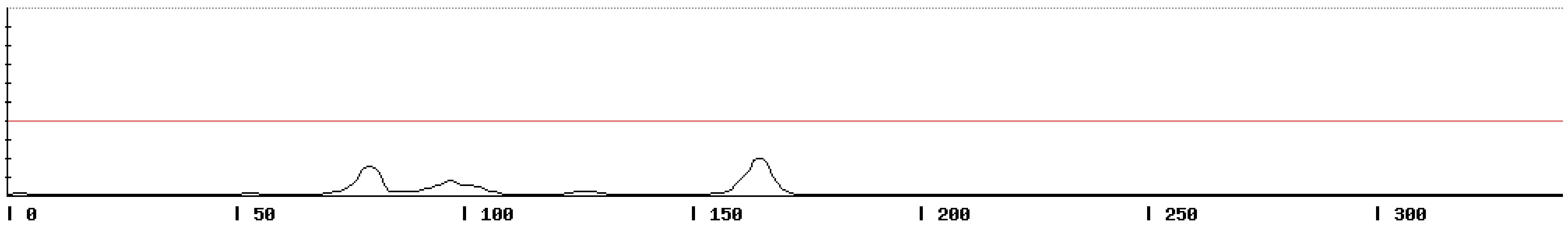

Predictions for Brachypodiumdistachyon_Bradi3g12470.1.p

Viterbi Path	
Viterbi Path	
Posterior @ 0.4	
Posterior @ 0.4	
Posterior Graphic. Threshold Marked @ 0.4	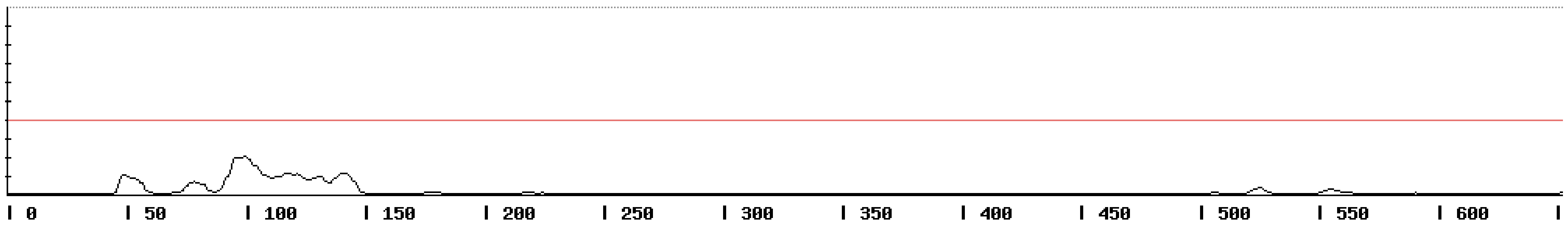

Predictions for Brachypodiumdistachyon_Bradi3g13117.1.p

Viterbi Path	
Viterbi Path	

Posterior @ 0.4

Posterior @ 0.4

Posterior Graphic. Threshold Marked @ 0.4

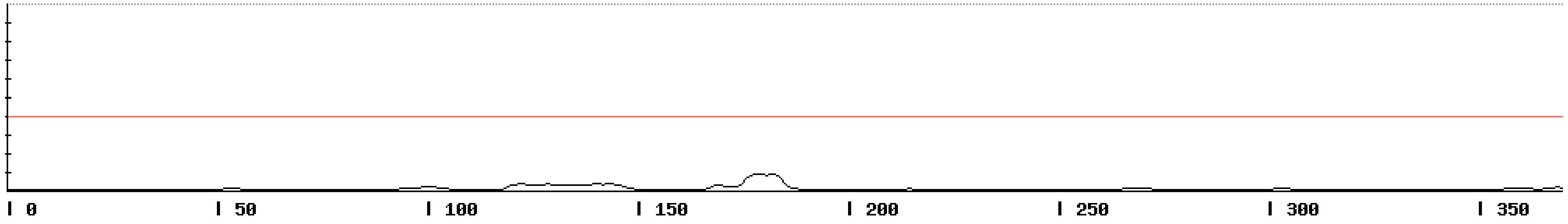

Predictions for Brachypodiumdistachyon_Bradi3g13630.1.p

Viterbi Path

Viterbi Path

Posterior @ 0.4

Posterior @ 0.4

Posterior Graphic. Threshold Marked @ 0.4

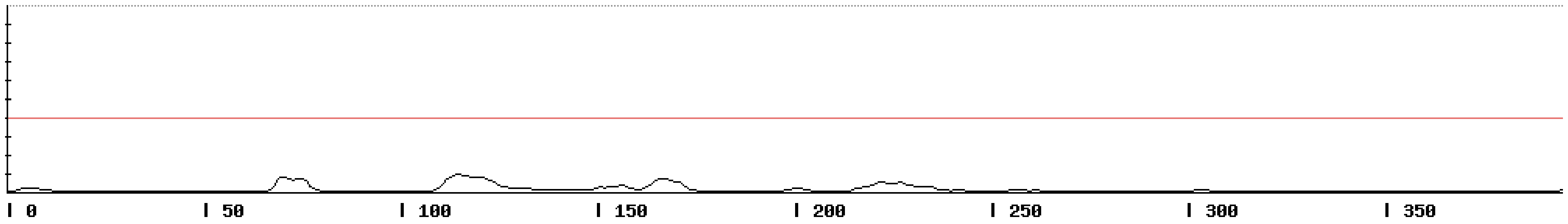

Predictions for Brachypodiumdistachyon_Bradi3g13727.1.p

Viterbi Path

Viterbi Path

Posterior @ 0.4

Posterior @ 0.4

Posterior Graphic. Threshold Marked @ 0.4

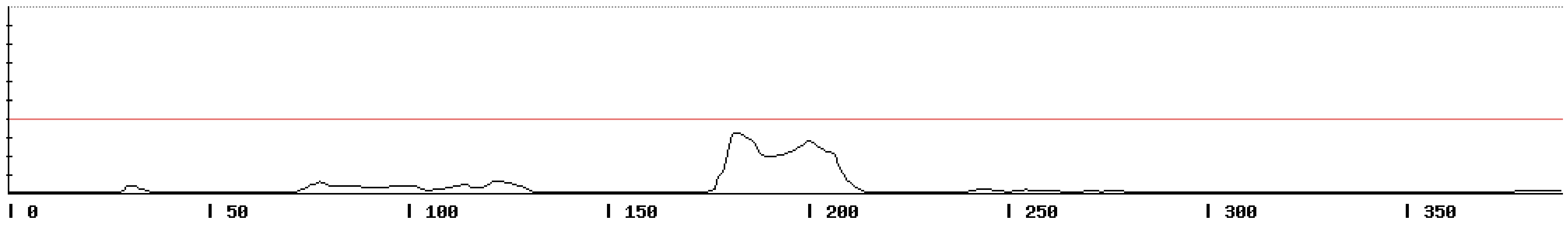

Predictions for Brachypodiumdistachyon_Bradi3g16480.1.p

Viterbi Path

Viterbi Path

Posterior @ 0.4

94 - KATGKDRIIKSKGEKKKQHMIGMKKTLVFHRGRAPKGE - 131

Posterior @ 0.4

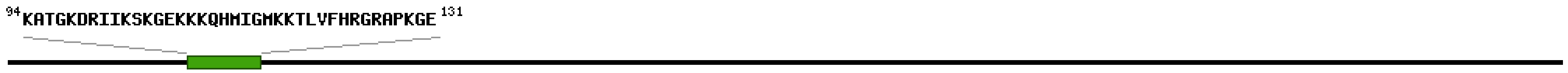

Posterior Graphic. Threshold Marked @ 0.4

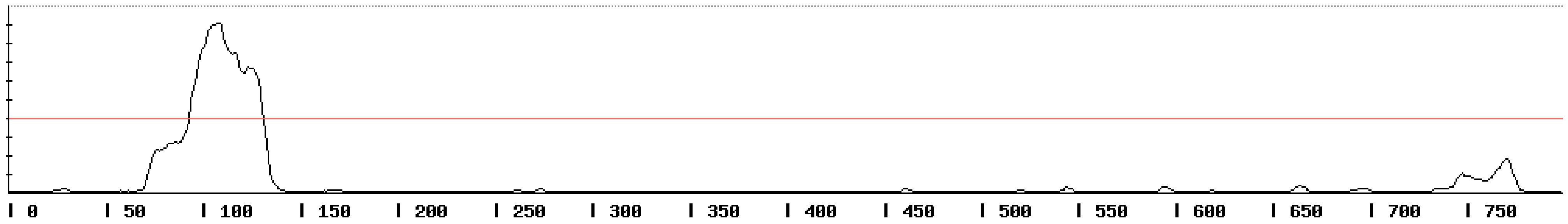

Predictions for Brachypodiumdistachyon_Bradi3g17287.1.p

Viterbi Path

Viterbi Path

Posterior @ 0.4

Posterior @ 0.4

Posterior Graphic. Threshold Marked @ 0.4

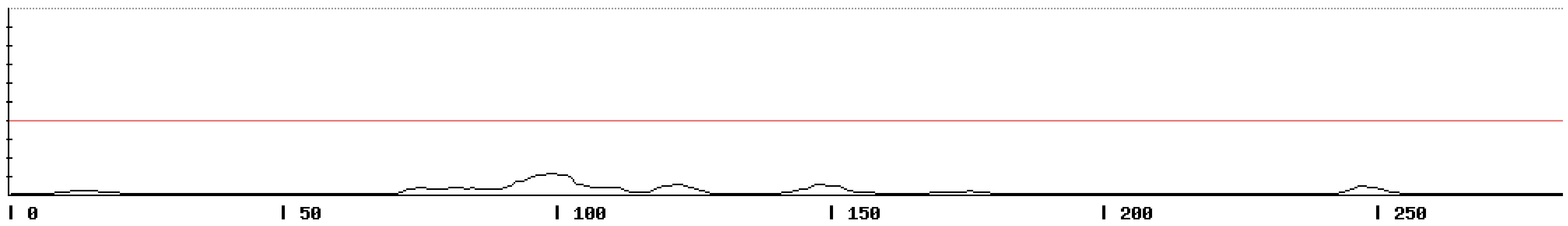

Predictions for Brachypodiumdistachyon_Bradi3g17573.1.p

Viterbi Path

Viterbi Path

Posterior @ 0.4

Posterior @ 0.4

Posterior Graphic. Threshold Marked @ 0.4

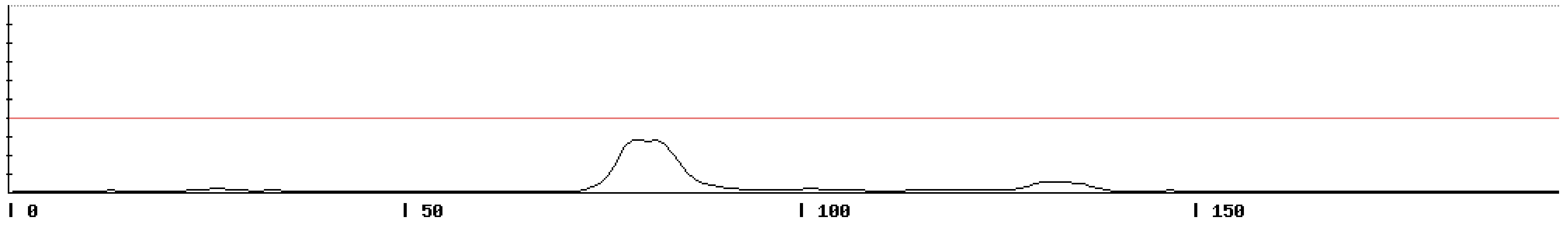

Predictions for Brachypodiumdistachyon_Bradi3g26665.1.p

Viterbi Path	
Viterbi Path	
Posterior @ 0.4	83 - RGNKQRPARGAKHGAWKASGGG - 104
	174 - SRGKKNKMKAELR - 186
Posterior @ 0.4	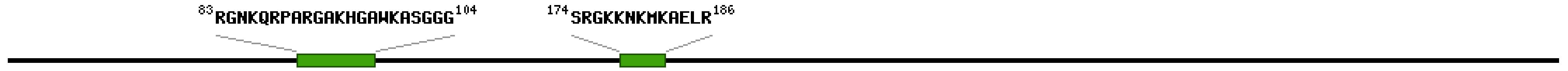
Posterior Graphic. Threshold Marked @ 0.4	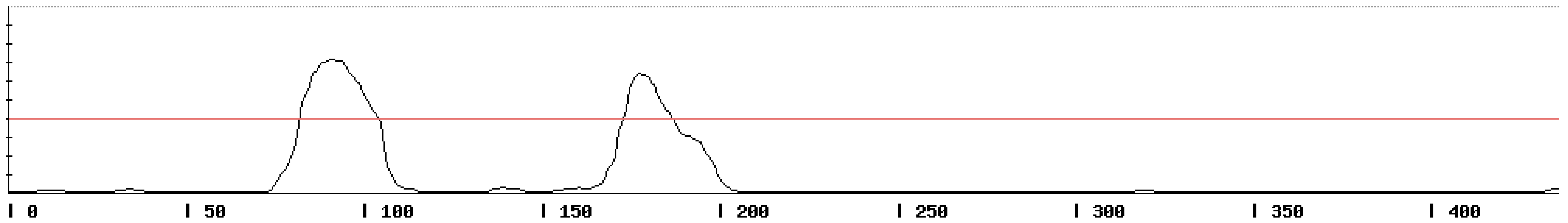

Predictions for Brachypodiumdistachyon_Bradi3g33320.3.p

Viterbi Path	
Viterbi Path	
Posterior @ 0.4	
Posterior @ 0.4	

Posterior Graphic. Threshold Marked @ 0.4

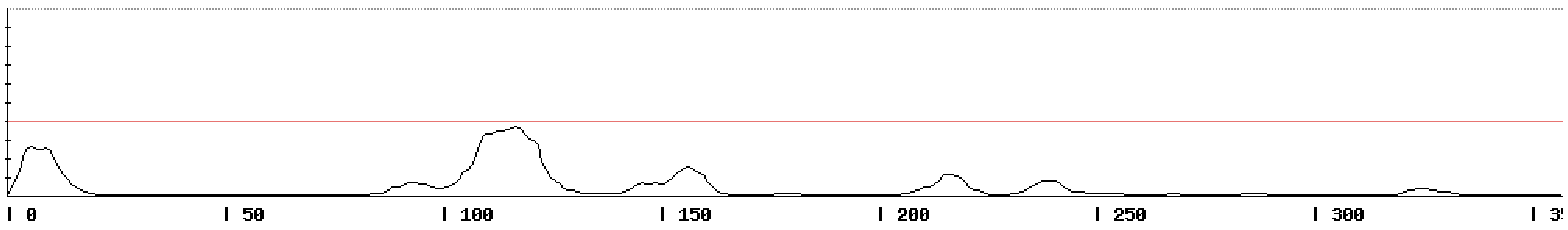

Predictions for Brachypodiumdistachyon_Bradi3g33680.2.p

Viterbi Path

Viterbi Path

Posterior @ 0.4 171 - RLSGHGKKRKR - 181

Posterior @ 0.4

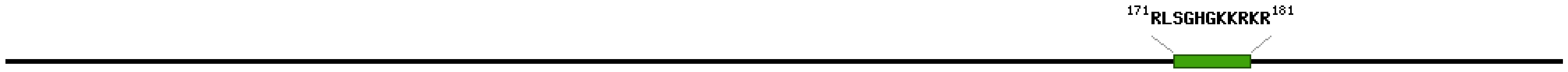

Posterior Graphic. Threshold Marked @ 0.4

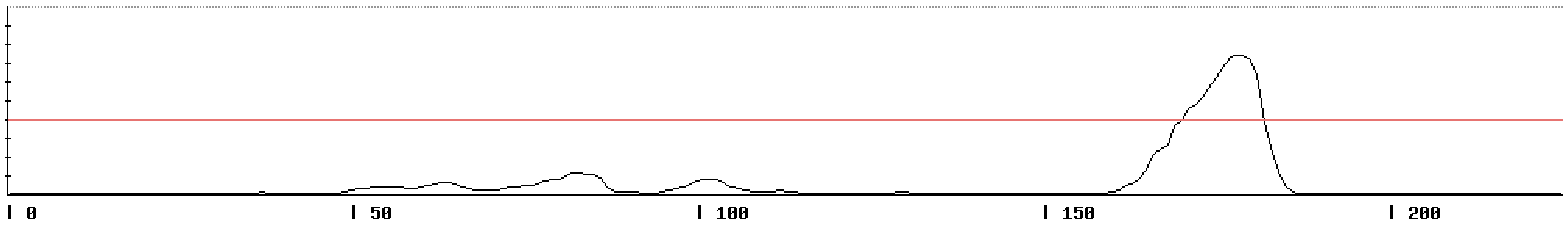

Predictions for Brachypodiumdistachyon_Bradi3g33957.2.p

Viterbi Path

Viterbi Path

Posterior @ 0.4

Posterior @ 0.4

Posterior Graphic. Threshold Marked @ 0.4

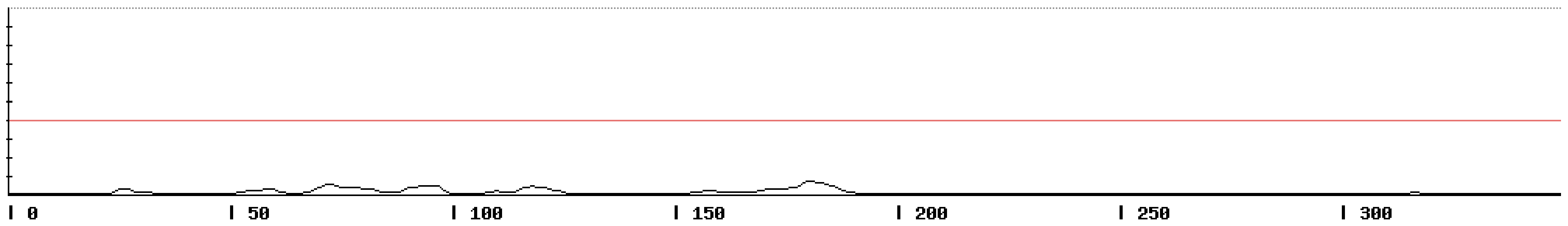

Predictions for Brachypodiumdistachyon_Bradi3g36670.1.p

Viterbi Path

Viterbi Path

Posterior @ 0.4

Posterior @ 0.4

Posterior Graphic. Threshold Marked @ 0.4

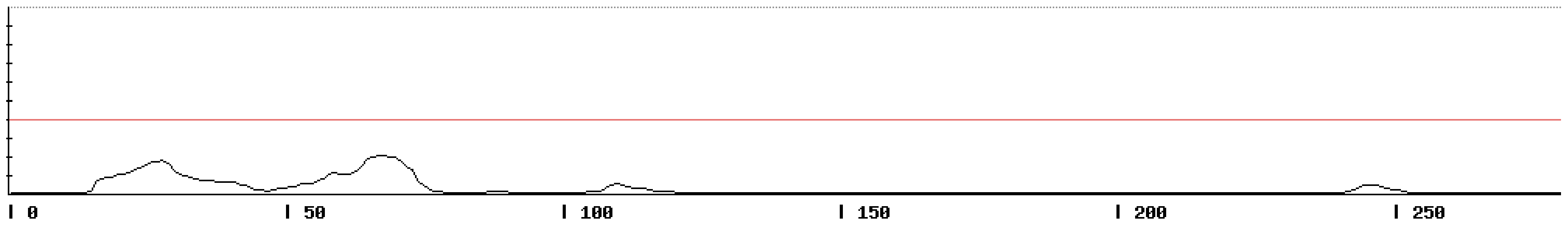

Predictions for Brachypodiumdistachyon_Bradi3g37067.1.p

Viterbi Path

Viterbi Path

Posterior @ 0.4

Posterior @ 0.4

Posterior Graphic. Threshold Marked @ 0.4

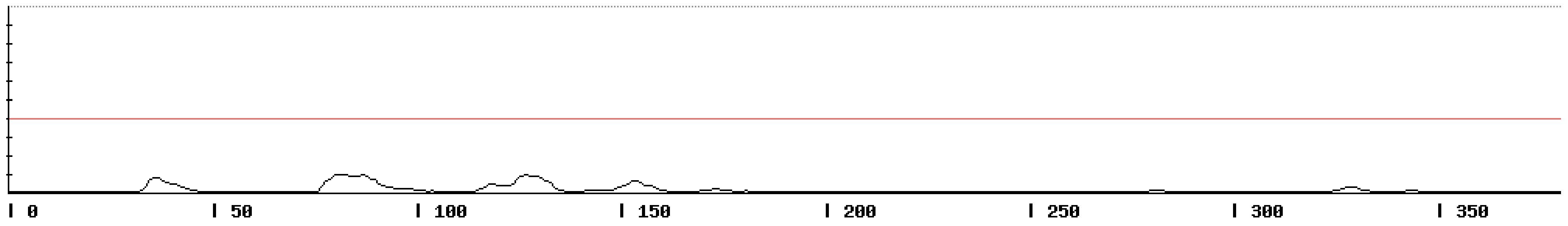

Predictions for Brachypodiumdistachyon_Bradi3g40085.1.p

Viterbi Path

Viterbi Path

Posterior @ 0.4

Posterior @ 0.4

Posterior Graphic. Threshold Marked @ 0.4

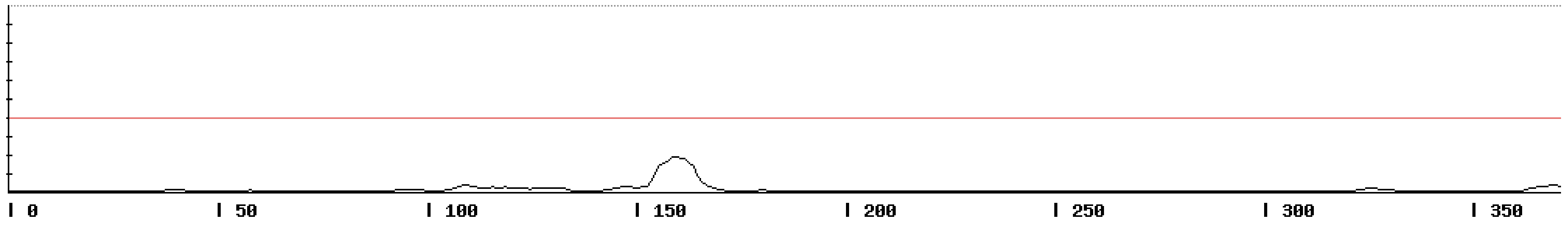

Predictions for Brachypodiumdistachyon_Bradi3g41470.1.p

Viterbi Path	
Viterbi Path	
Posterior @ 0.4	133 - AHRGKK - 138
Posterior @ 0.4	
Posterior Graphic. Threshold Marked @ 0.4	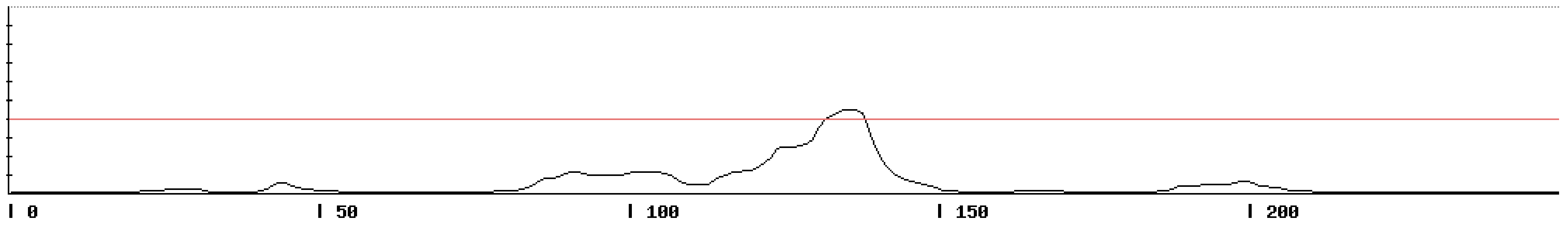

Predictions for Brachypodiumdistachyon_Bradi3g46900.1.p

Viterbi Path	
Viterbi Path	
Posterior @ 0.4	
Posterior @ 0.4	
Posterior Graphic. Threshold Marked @ 0.4	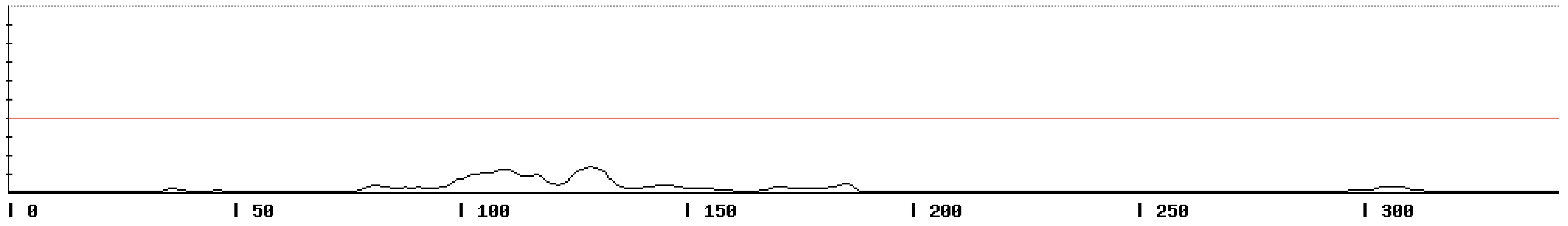

Predictions for Brachypodiumdistachyon_Bradi3g47627.1.p	
Viterbi Path	
Viterbi Path	
Posterior @ 0.4	133 - KRKRRR - 138
Posterior @ 0.4	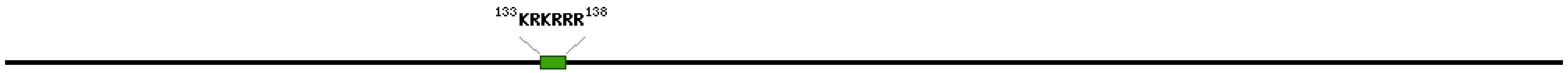
Posterior Graphic. Threshold Marked @ 0.4	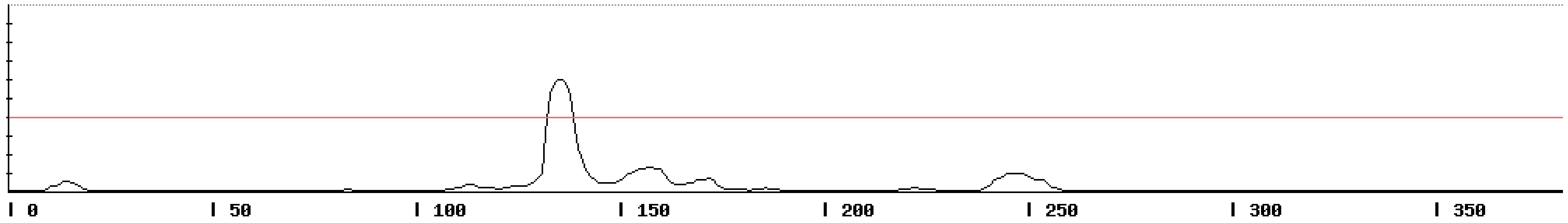

Predictions for Brachypodiumdistachyon_Bradi3g50067.1.p	
Viterbi Path	
Viterbi Path	
Posterior @ 0.4	
Posterior @ 0.4	
Posterior Graphic. Threshold Marked @ 0.4	

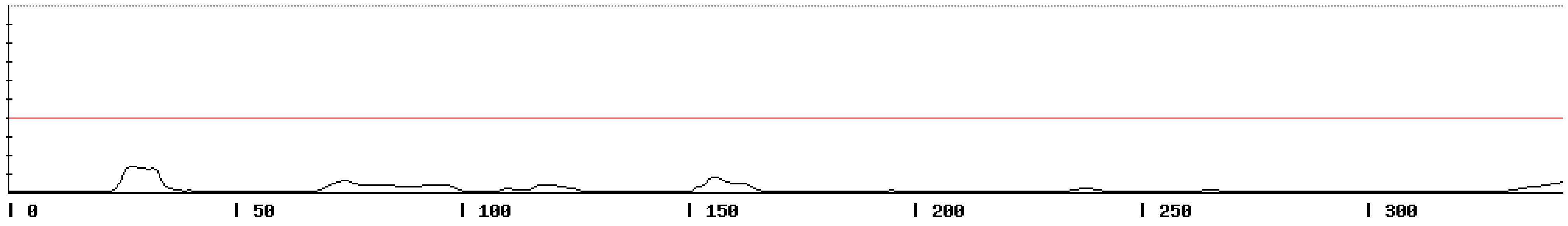

Predictions for Brachypodiumdistachyon_Bradi3g54817.1.p

Viterbi Path	
Viterbi Path	
Posterior @ 0.4	
Posterior @ 0.4	
Posterior Graphic. Threshold Marked @ 0.4	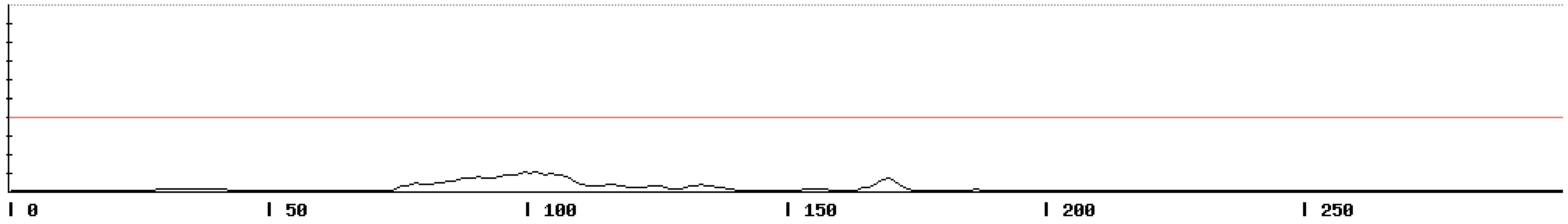

Predictions for Brachypodiumdistachyon_Bradi3g56080.1.p

Viterbi Path	
Viterbi Path	
Posterior @ 0.4	89 - GRGGPGNR - 96
Posterior @ 0.4	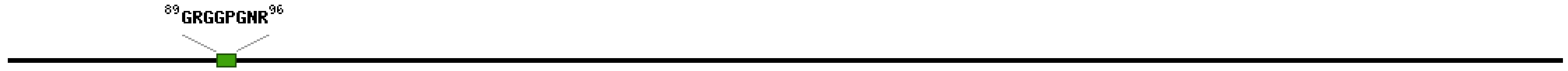

Posterior Graphic. Threshold Marked @ 0.4

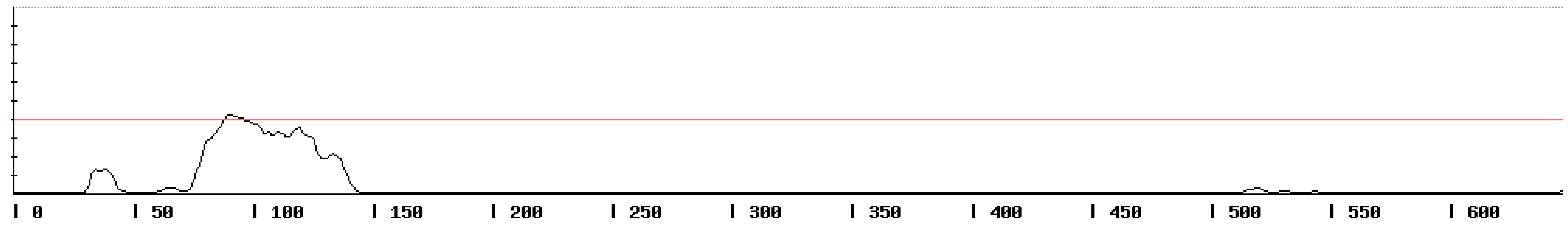

Predictions for Brachypodiumdistachyon_Bradi3g59380.1.p

Viterbi Path

Viterbi Path

Posterior @ 0.4

Posterior @ 0.4

Posterior Graphic. Threshold Marked @ 0.4

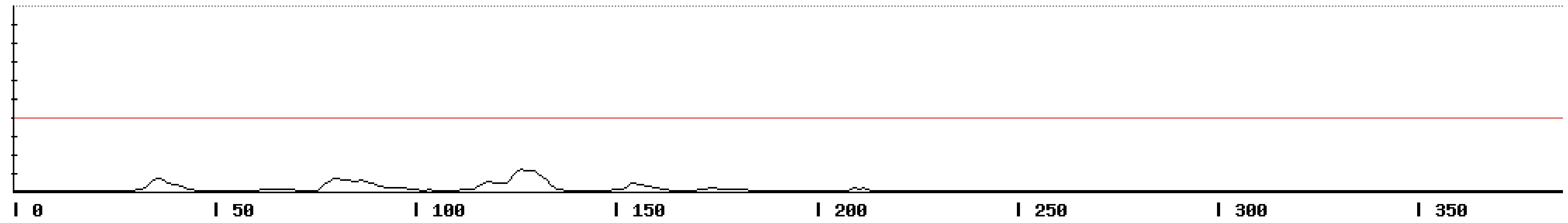

Predictions for Brachypodiumdistachyon_Bradi3g59866.1.p

Viterbi Path

Viterbi Path

Posterior @ 0.4

Posterior @ 0.4

Posterior Graphic. Threshold Marked @ 0.4

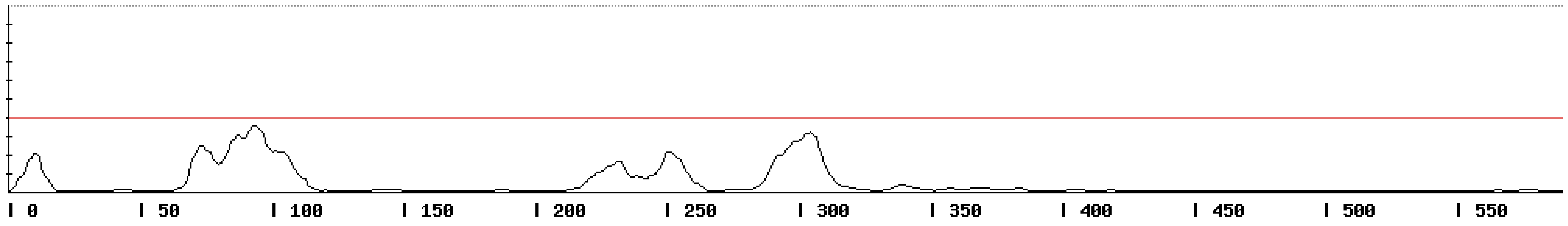

Predictions for Brachypodiumdistachyon_Bradi4g02060.2.p

Viterbi Path

Viterbi Path

Posterior @ 0.4

Posterior @ 0.4

Posterior Graphic. Threshold Marked @ 0.4

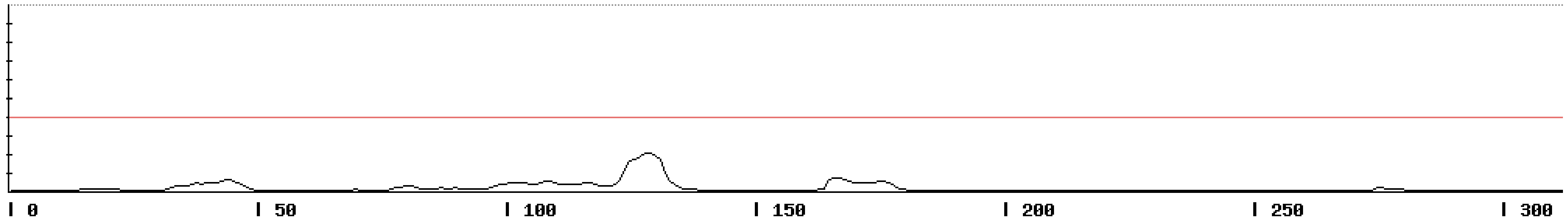

Predictions for Brachypodiumdistachyon_Bradi4g03620.1.p

Viterbi Path

Viterbi Path

Posterior @ 0.4

Posterior @ 0.4

Posterior Graphic. Threshold Marked @ 0.4

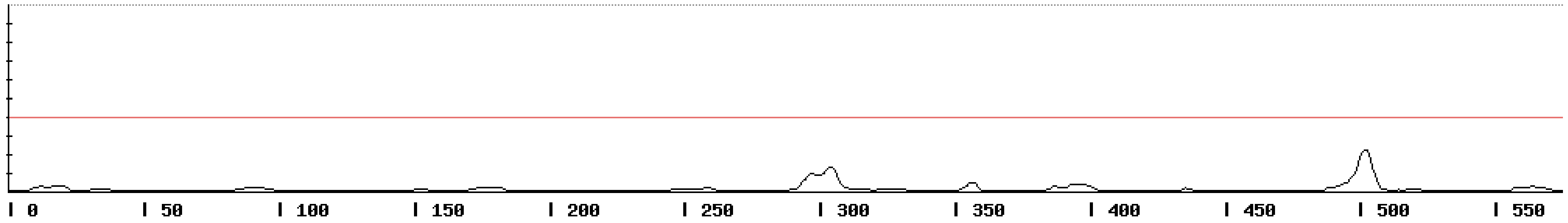

Predictions for Brachypodiumdistachyon_Bradi4g07527.1.p

Viterbi Path

Viterbi Path

Posterior @ 0.4

Posterior @ 0.4

Posterior Graphic. Threshold Marked @ 0.4

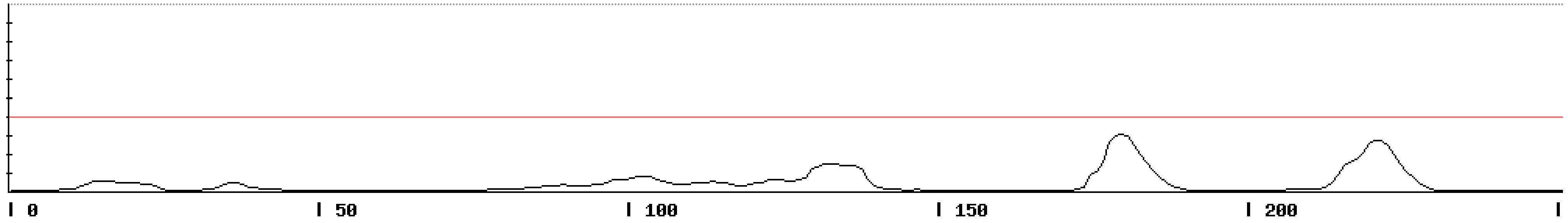

Predictions for Brachypodiumdistachyon_Bradi4g12065.1.p

Viterbi Path

Viterbi Path

Posterior @ 0.4	
Posterior @ 0.4	
Posterior Graphic. Threshold Marked @ 0.4	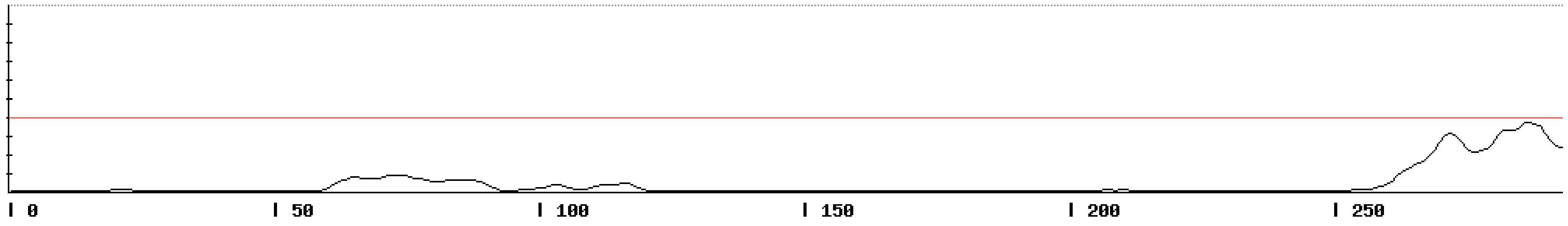

Predictions for Brachypodiumdistachyon_Bradi4g13562.1.p

Viterbi Path	
Viterbi Path	
Posterior @ 0.4	
Posterior @ 0.4	
Posterior Graphic. Threshold Marked @ 0.4	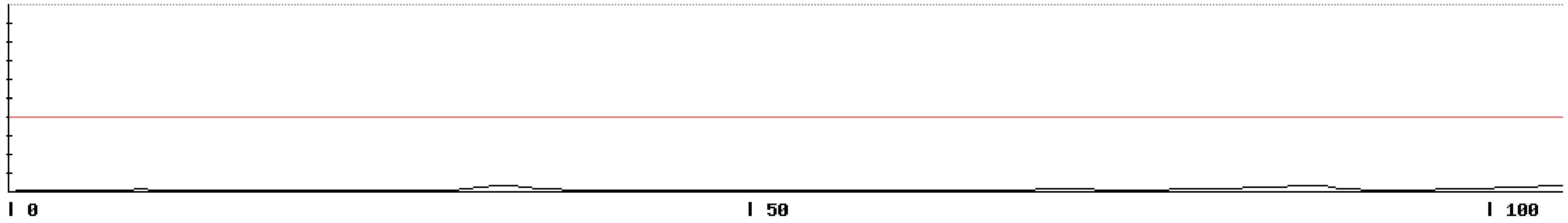

Predictions for Brachypodiumdistachyon_Bradi4g13570.1.p

Viterbi Path

Viterbi Path

Posterior @ 0.4

Posterior @ 0.4

Posterior Graphic. Threshold Marked @ 0.4

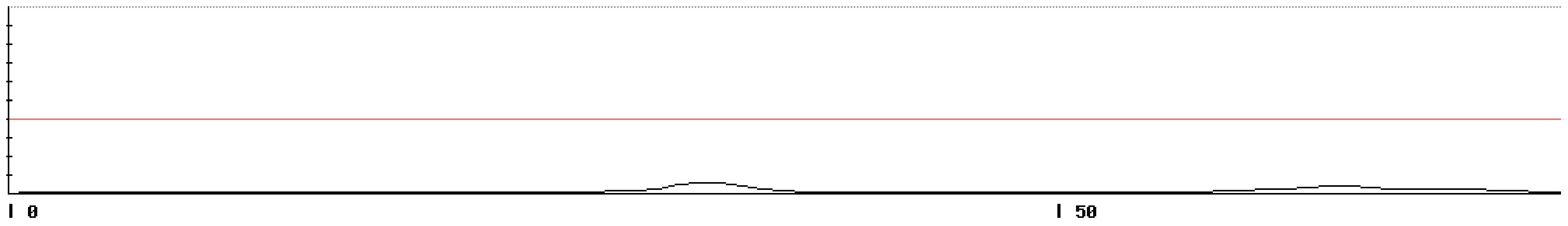

Predictions for Brachypodiumdistachyon_Bradi4g13575.1.p

Viterbi Path

Viterbi Path

Posterior @ 0.4

Posterior @ 0.4

Posterior Graphic. Threshold Marked @ 0.4

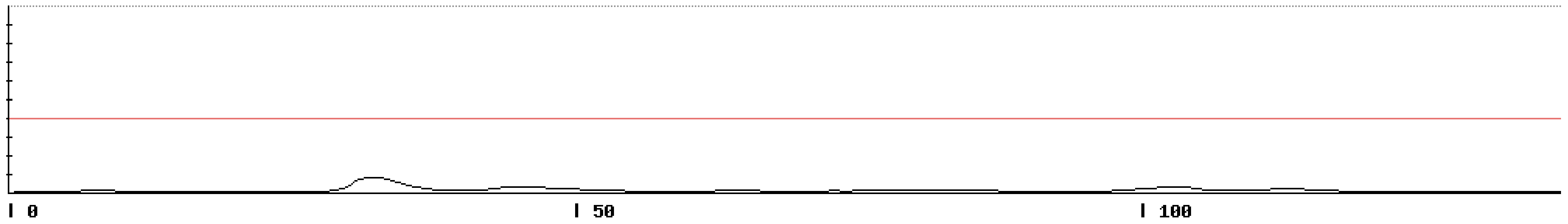

Predictions for Brachypodiumdistachyon_Bradi4g13586.1.p	
Viterbi Path	
Viterbi Path	
Posterior @ 0.4	
Posterior @ 0.4	
Posterior Graphic. Threshold Marked @ 0.4	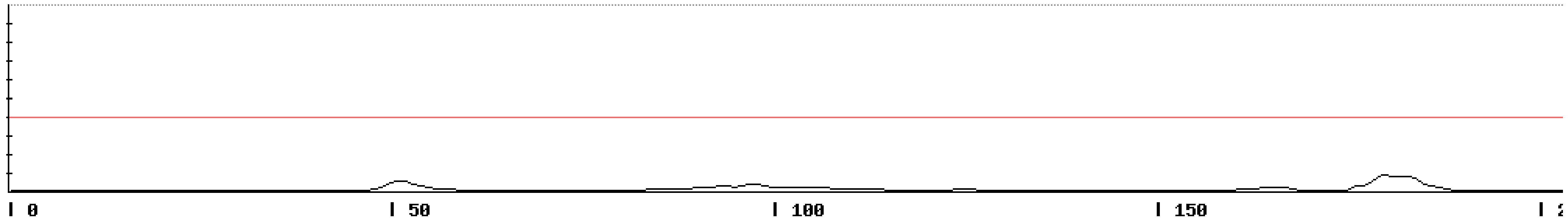

Predictions for Brachypodiumdistachyon_Bradi4g22195.1.p	
Viterbi Path	
Viterbi Path	
Posterior @ 0.4	
Posterior @ 0.4	
Posterior Graphic. Threshold Marked @ 0.4	

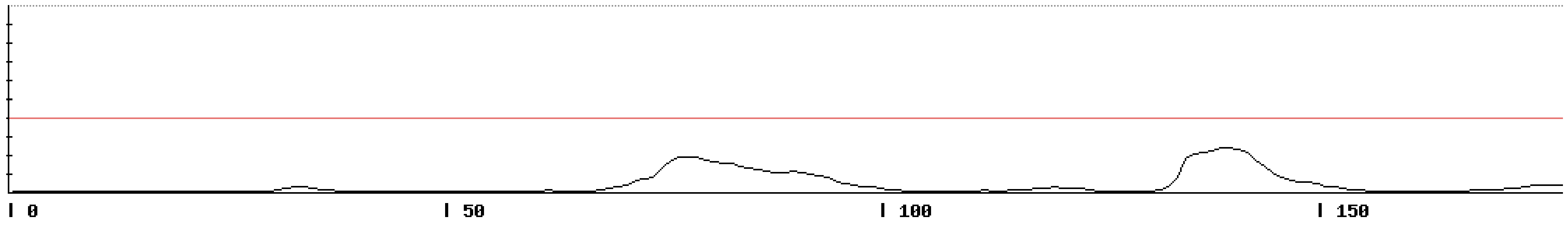

Predictions for Brachypodiumdistachyon_Bradi4g22216.2.p

Viterbi Path

Viterbi Path

Posterior @ 0.4

Posterior @ 0.4

Posterior Graphic. Threshold Marked @ 0.4

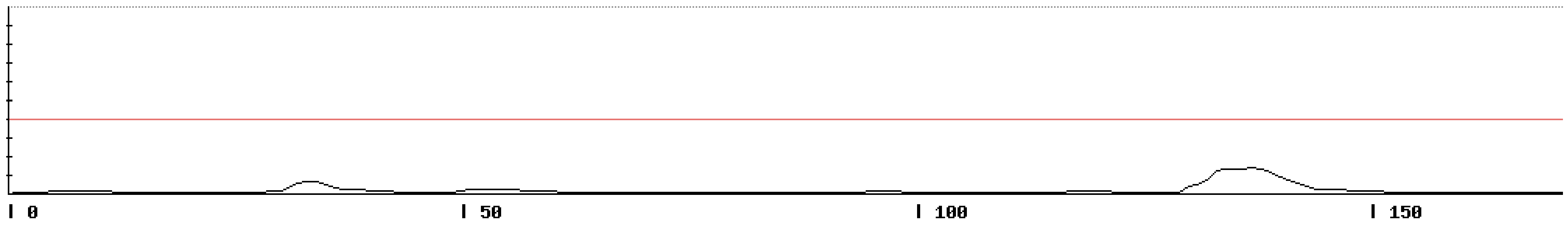

Predictions for Brachypodiumdistachyon_Bradi4g22681.1.p

Viterbi Path

Viterbi Path

Posterior @ 0.4

Posterior @ 0.4

Posterior Graphic. Threshold Marked @ 0.4

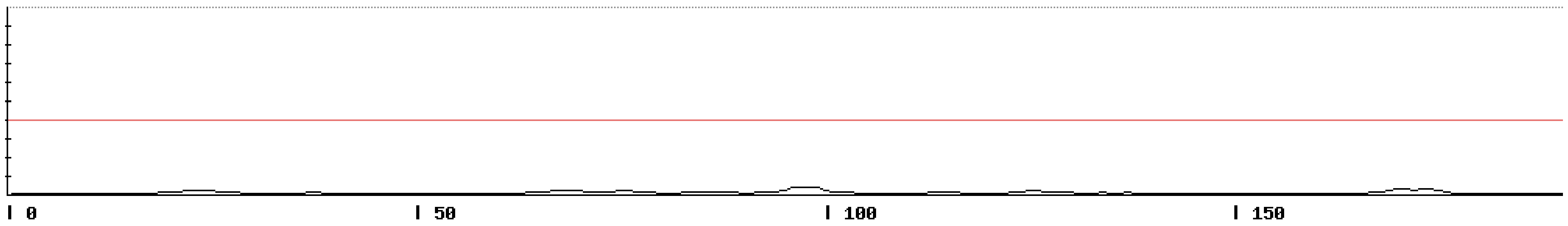

Predictions for Brachypodiumdistachyon_Bradi4g23640.1.p

Viterbi Path

Viterbi Path

Posterior @ 0.4 214 - KK - 215

Posterior @ 0.4

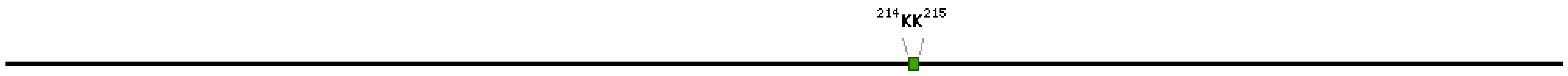

Posterior Graphic. Threshold Marked @ 0.4

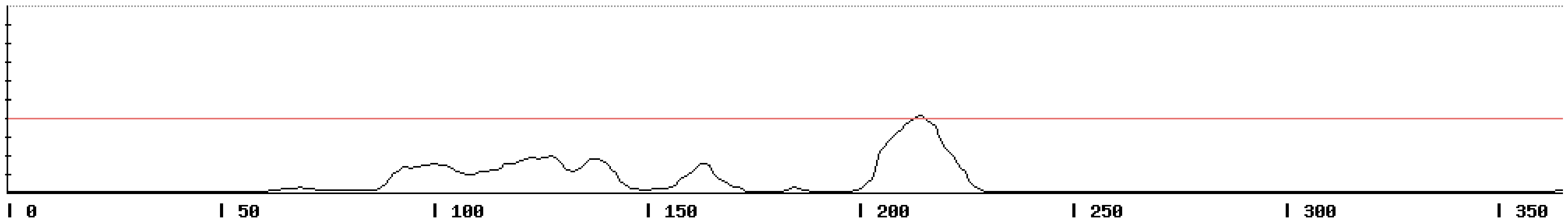

Predictions for Brachypodiumdistachyon_Bradi4g24060.1.p

Viterbi Path

Viterbi Path

Posterior @ 0.4

Posterior @ 0.4

Posterior Graphic. Threshold Marked @ 0.4

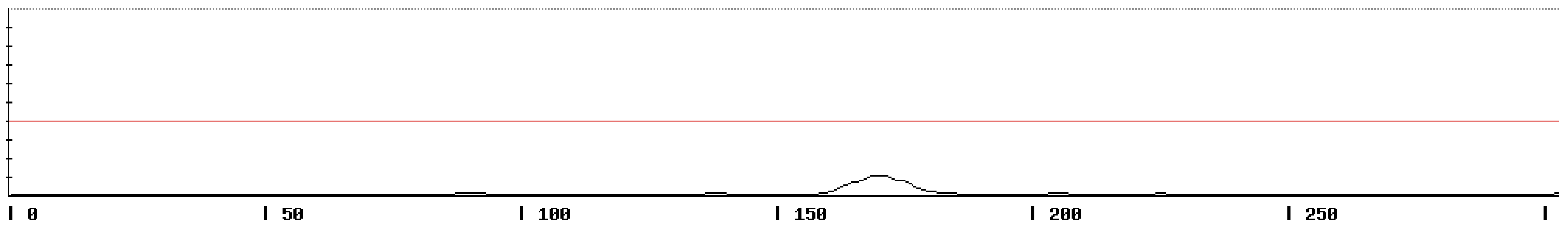

Predictions for Brachypodiumdistachyon_Bradi4g24100.1.p

Viterbi Path

Viterbi Path

Posterior @ 0.4 | 176 - KAREKRAAK - 184

Posterior @ 0.4

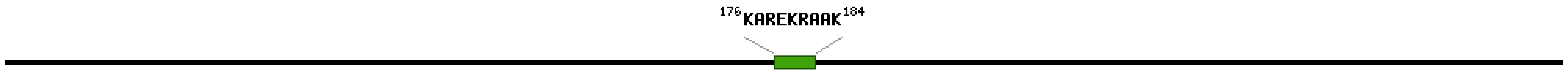

Posterior Graphic. Threshold Marked @ 0.4

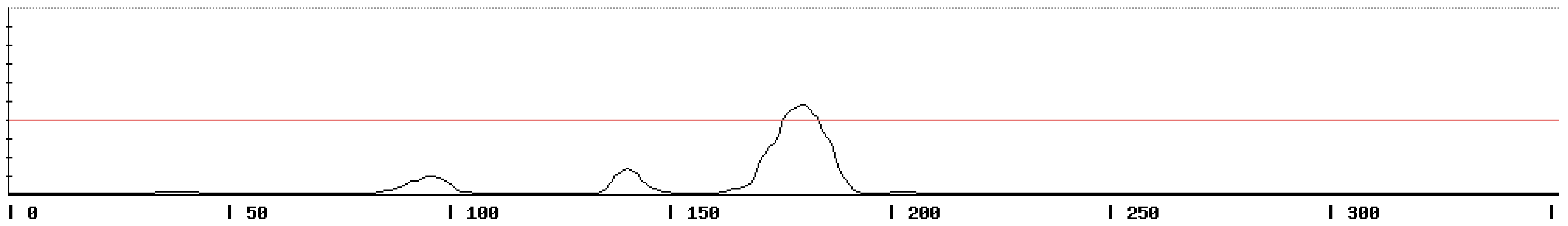

Predictions for Brachypodiumdistachyon_Bradi4g26275.1.p

Viterbi Path

Viterbi Path

Posterior @ 0.4 233 - DAPRPKKRARAAAAPAA - 249

Posterior @ 0.4

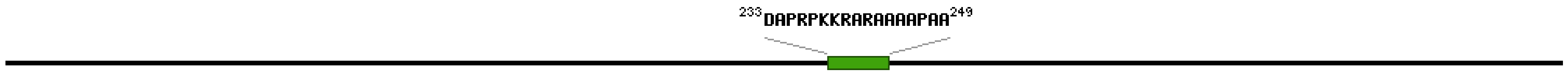

Posterior Graphic. Threshold Marked @ 0.4

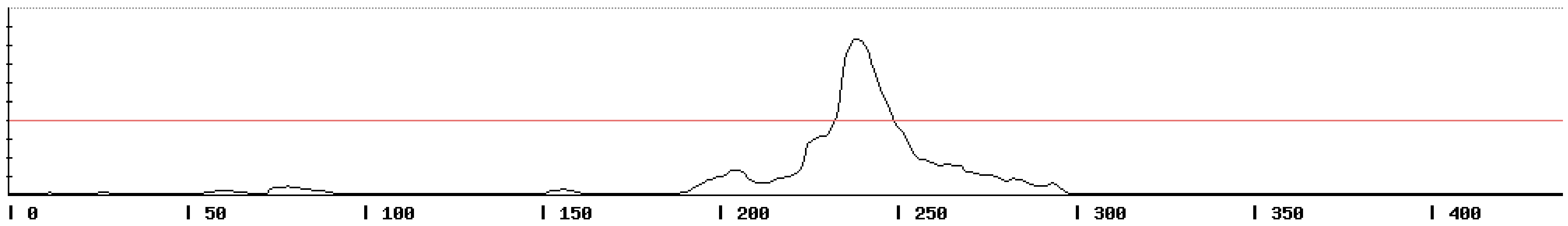

Predictions for Brachypodiumdistachyon_Bradi4g26470.2.p

Viterbi Path

Viterbi Path

Posterior @ 0.4 227 - APRPKKRTRAAAPA - 240

Posterior @ 0.4

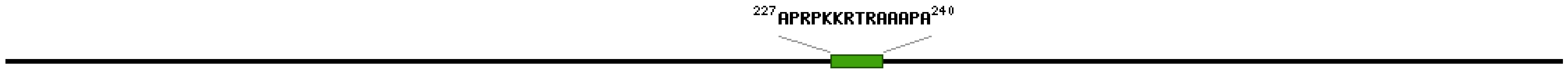

Posterior Graphic. Threshold Marked @ 0.4

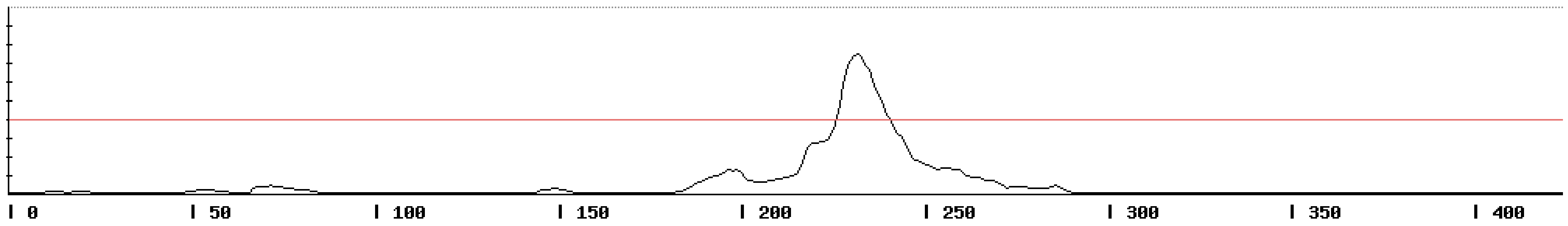

Predictions for Brachypodiumdistachyon_Bradi4g26600.1.p

Viterbi Path

Viterbi Path

Posterior @ 0.4	35 - GSGKKKKKMLMKKEKH - 50<br>215 - KRRKRISDGSKEPSTRKKKT - 234
Posterior @ 0.4	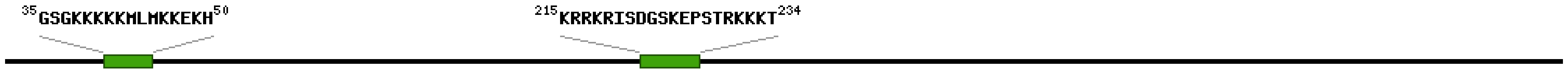
Posterior Graphic. Threshold Marked @ 0.4	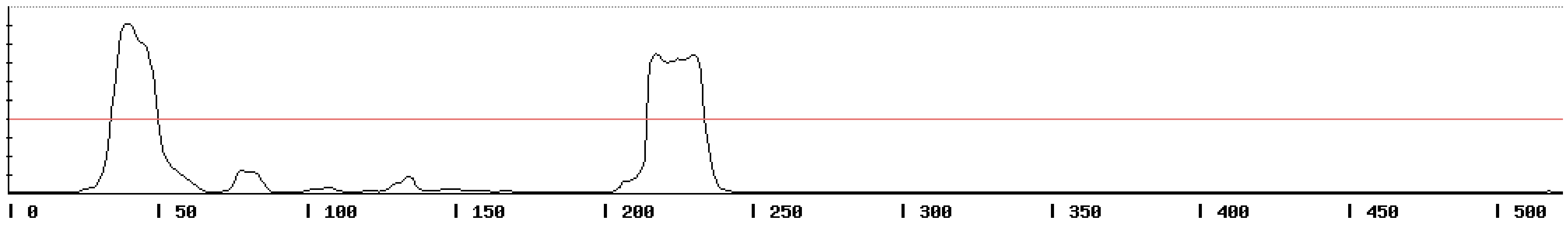

Predictions for Brachypodiumdistachyon_Bradi4g26605.1.p

Viterbi Path	208 - GKRRKRPAKELLPKRKK - 224
Viterbi Path	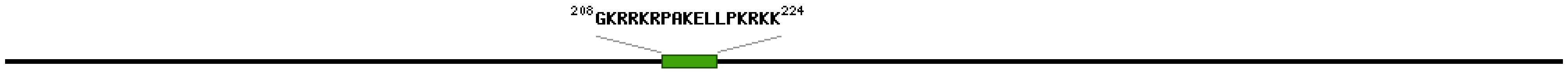
Posterior @ 0.4	39 - EKKKKEKEK - 47<br>208 - GKRRKRPAKELLPKRKKEE - 226
Posterior @ 0.4	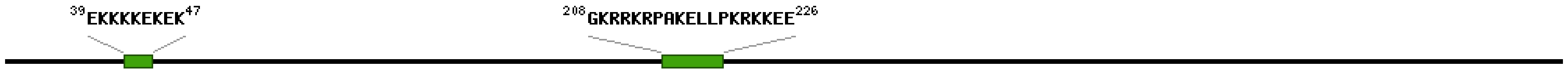
Posterior Graphic. Threshold Marked @ 0.4	

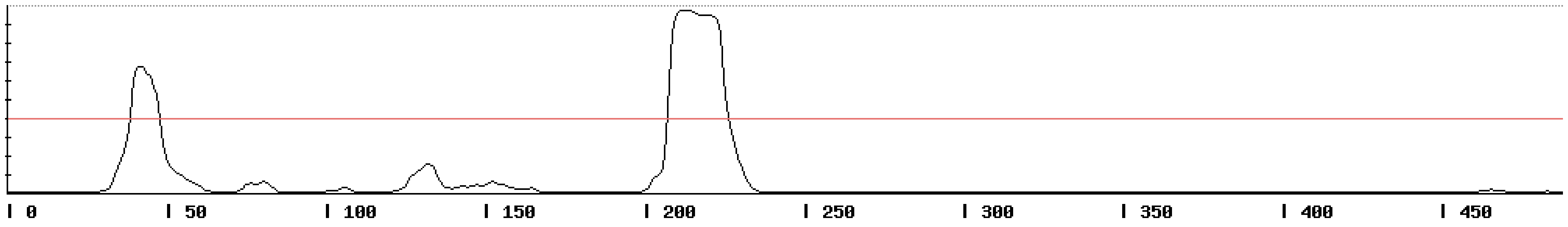

Predictions for Brachypodiumdistachyon_Bradi4g30177.1.p

Viterbi Path

Viterbi Path

Posterior @ 0.4

Posterior @ 0.4

Posterior Graphic. Threshold Marked @ 0.4

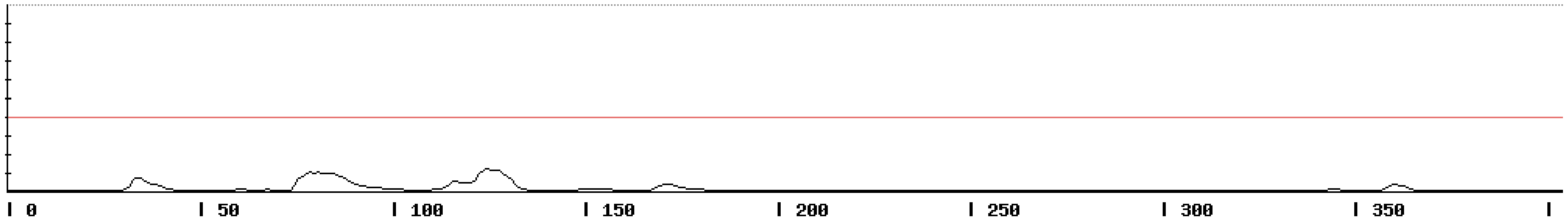

Predictions for Brachypodiumdistachyon_Bradi4g34022.1.p

Viterbi Path

Viterbi Path

Posterior @ 0.4

Posterior @ 0.4

Posterior Graphic. Threshold Marked @ 0.4

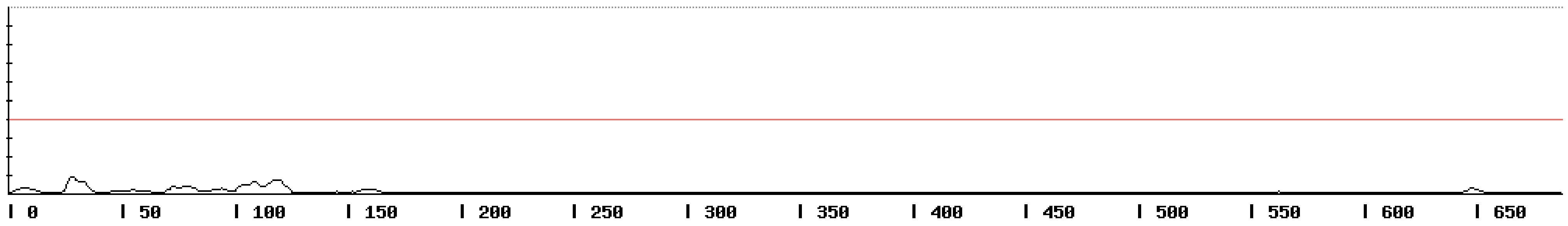

Predictions for Brachypodiumdistachyon_Bradi4g34157.1.p

Viterbi Path

Viterbi Path

Posterior @ 0.4

Posterior @ 0.4

Posterior Graphic. Threshold Marked @ 0.4

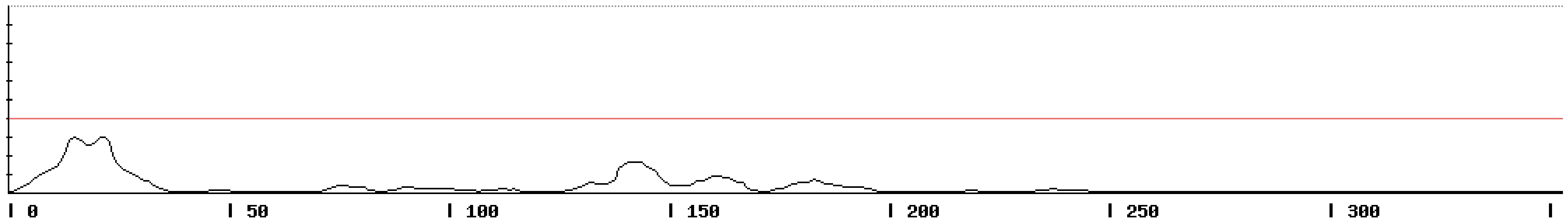

Predictions for Brachypodiumdistachyon_Bradi4g34157.2.p

Viterbi Path

Viterbi Path

Posterior @ 0.4

Posterior @ 0.4

Posterior Graphic. Threshold Marked @ 0.4

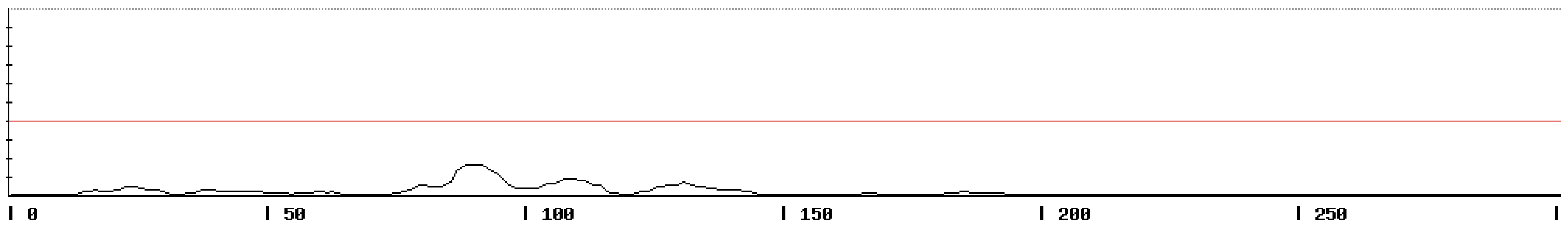

Predictions for Brachypodiumdistachyon_Bradi4g34800.1.p

Viterbi Path

Viterbi Path

Posterior @ 0.4

Posterior @ 0.4

Posterior Graphic. Threshold Marked @ 0.4

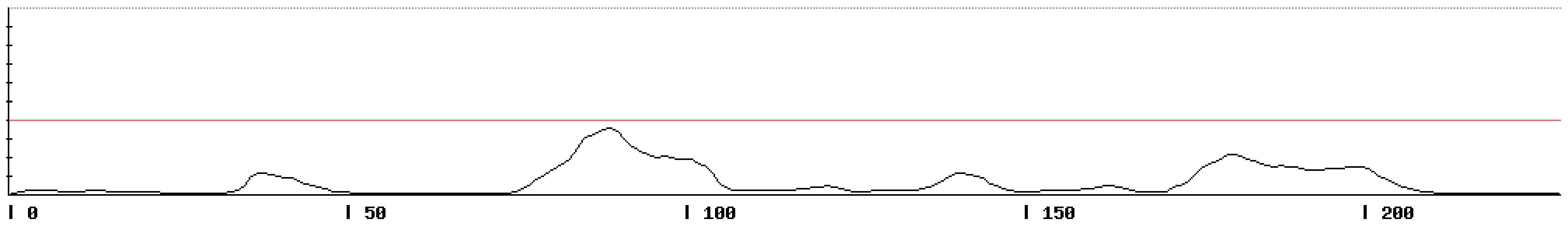

Predictions for Brachypodiumdistachyon_Bradi4g37318.1.p

Viterbi Path

Viterbi Path

Posterior @ 0.4

Posterior @ 0.4

Posterior Graphic. Threshold Marked @ 0.4

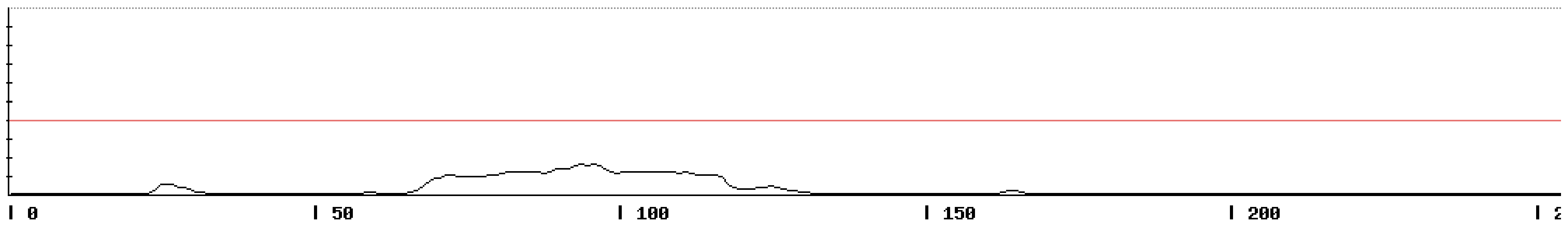

Predictions for Brachypodiumdistachyon_Bradi4g37327.1.p

Viterbi Path

Viterbi Path

Posterior @ 0.4

Posterior @ 0.4

Posterior Graphic. Threshold Marked @ 0.4

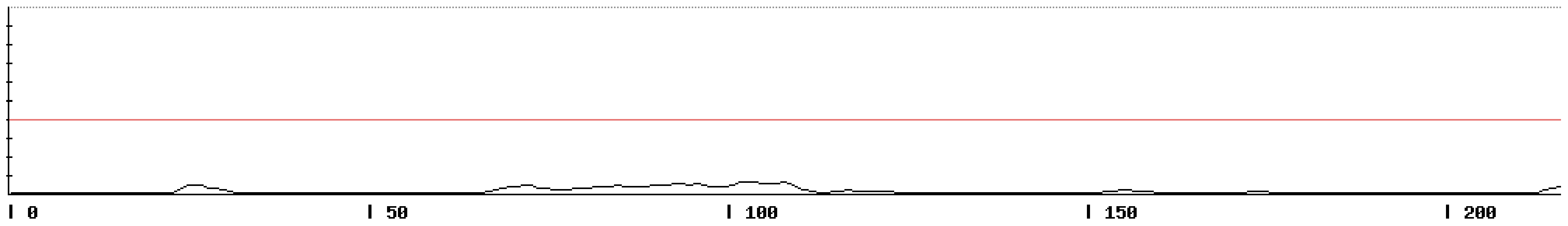

Predictions for Brachypodiumdistachyon_Bradi4g40880.1.p

Viterbi Path

Viterbi Path

Posterior @ 0.4	
Posterior @ 0.4	
Posterior Graphic. Threshold Marked @ 0.4	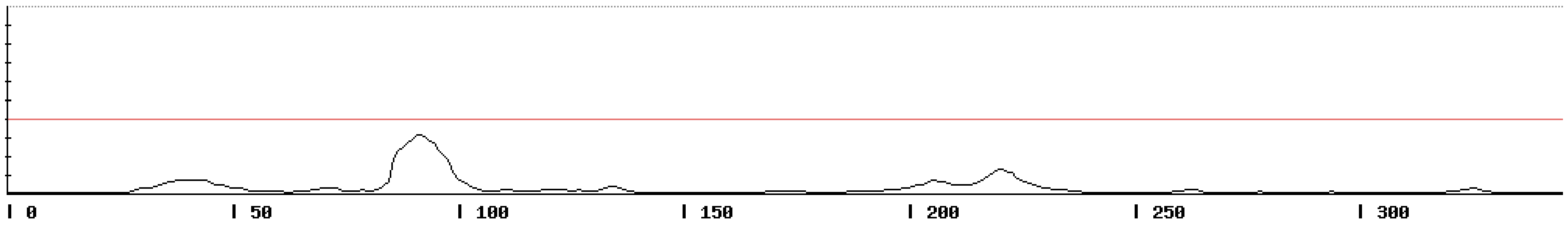

Predictions for Brachypodiumdistachyon_Bradi4g41200.1.p	
Viterbi Path	
Viterbi Path	
Posterior @ 0.4	4 - KNKNRKKRA - 12
Posterior @ 0.4	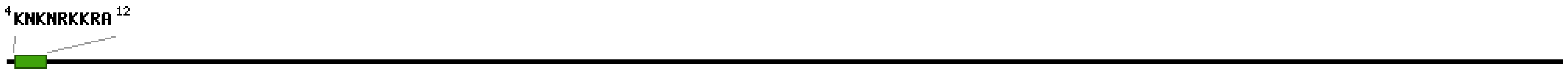
Posterior Graphic. Threshold Marked @ 0.4	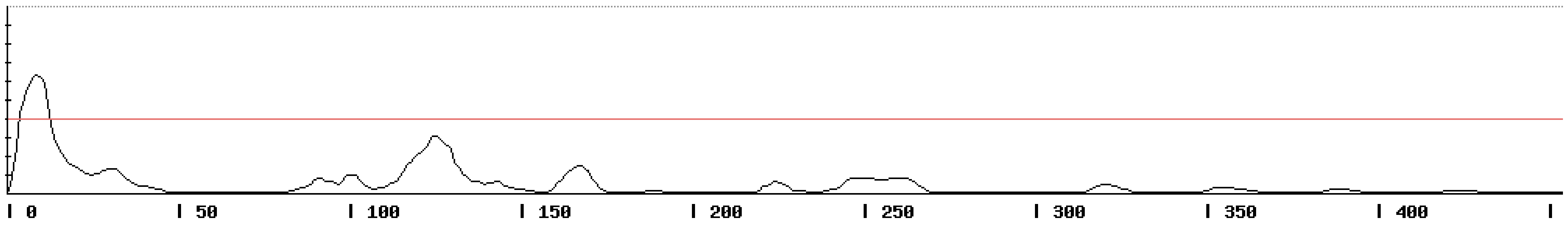

Predictions for Brachypodiumdistachyon_Bradi4g42180.1.p

Viterbi Path	
Viterbi Path	
Posterior @ 0.4	
Posterior @ 0.4	
Posterior Graphic. Threshold Marked @ 0.4	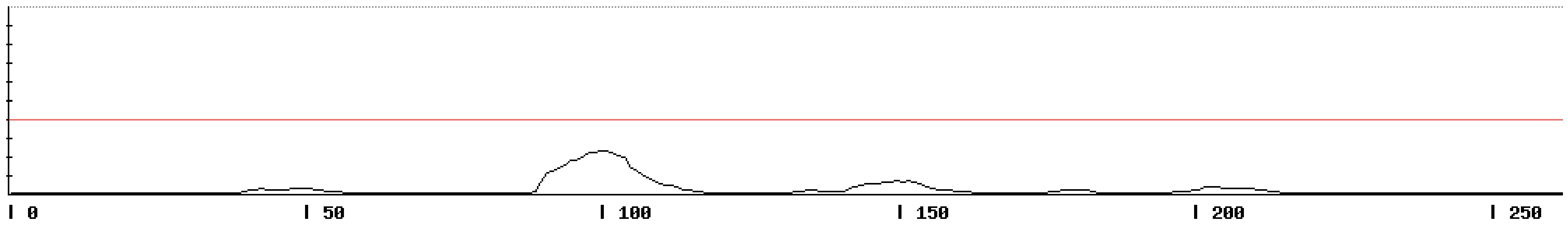

Predictions for Brachypodiumdistachyon_Bradi4g43010.1.p	
Viterbi Path	
Viterbi Path	
Posterior @ 0.4	110 - VKKGEVKKLSFKKGIKKEK - 128
Posterior @ 0.4	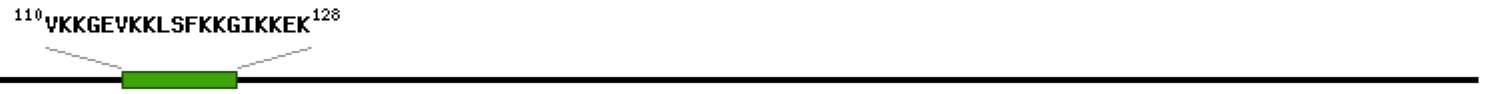
Posterior Graphic. Threshold Marked @ 0.4	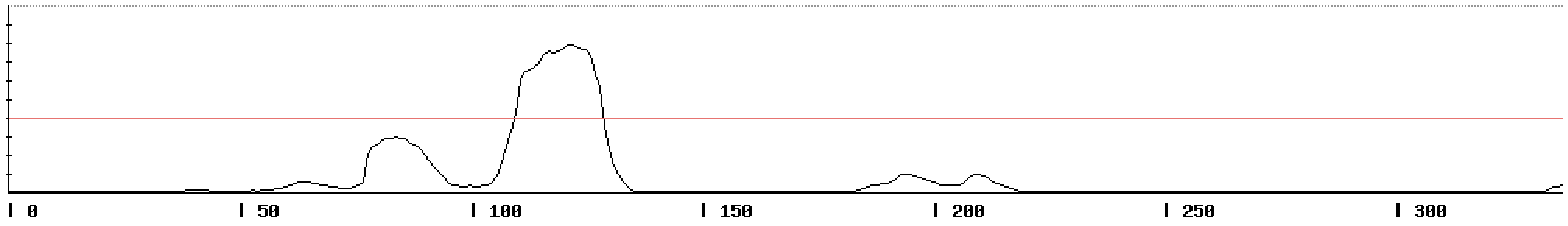

Predictions for Brachypodiumdistachyon_Bradi4g43020.1.p	
Viterbi Path	
Viterbi Path	
Posterior @ 0.4	
Posterior @ 0.4	
Posterior Graphic. Threshold Marked @ 0.4	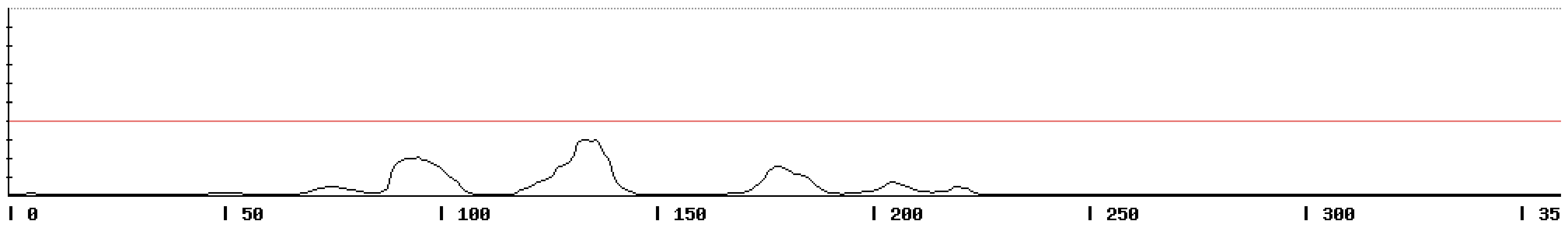

Predictions for Brachypodiumdistachyon_Bradi4g43330.1.p	
Viterbi Path	
Viterbi Path	
Posterior @ 0.4	
Posterior @ 0.4	
Posterior Graphic. Threshold Marked @ 0.4	

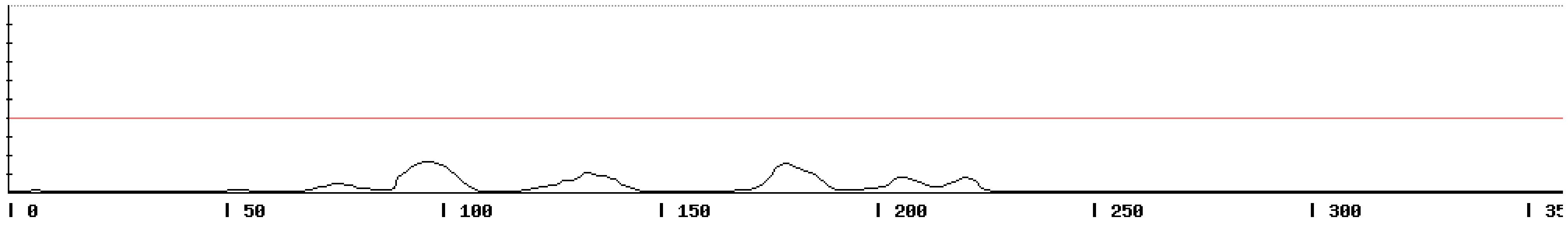

Predictions for Brachypodiumdistachyon_Bradi4g43346.1.p

Viterbi Path	
Viterbi Path	
Posterior @ 0.4	103 - VKKGEVKKLSFKKGIKKEK - 121
Posterior @ 0.4	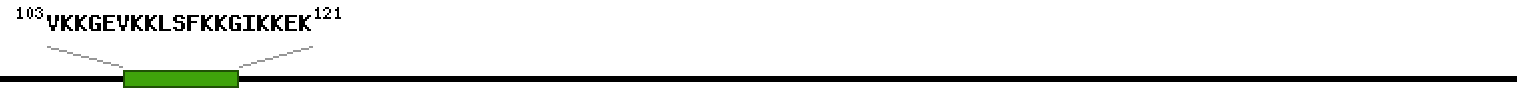
Posterior Graphic. Threshold Marked @ 0.4	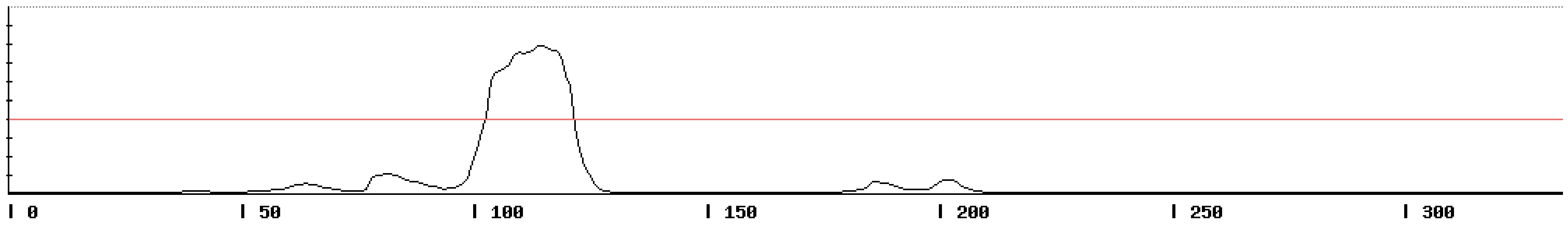

Predictions for Brachypodiumdistachyon_Bradi4g43995.1.p

Viterbi Path	
Viterbi Path	
Posterior @ 0.4	
Posterior @ 0.4	

Posterior Graphic. Threshold Marked @ 0.4

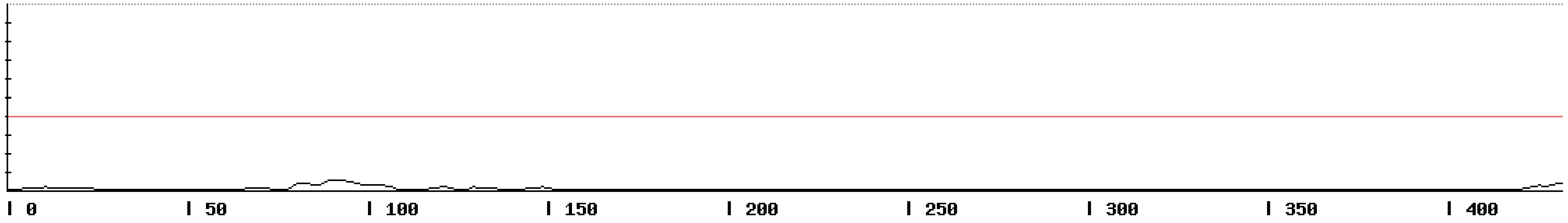

Predictions for Brachypodiumdistachyon_Bradi4g44000.1.p

Viterbi Path	
Viterbi Path	
Posterior @ 0.4	
Posterior @ 0.4	
Posterior Graphic. Threshold Marked @ 0.4	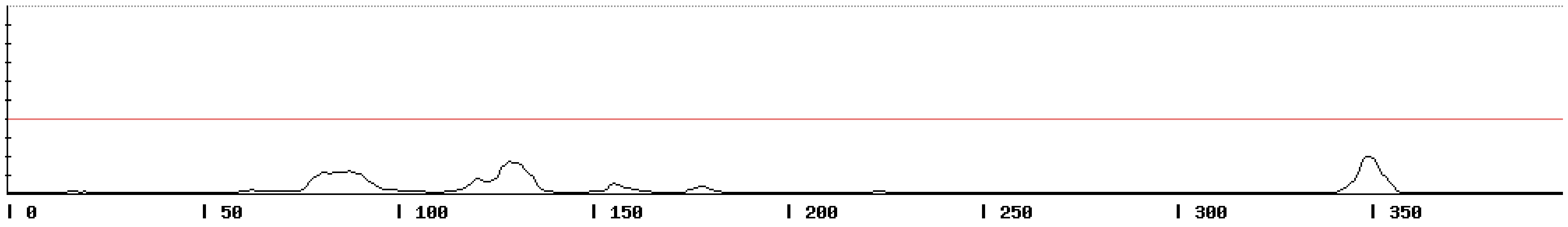

Predictions for Brachypodiumdistachyon_Bradi5g10640.1.p

Viterbi Path	
Viterbi Path	
Posterior @ 0.4	138 - GKRSHKKKGHS - 148
Posterior @ 0.4	

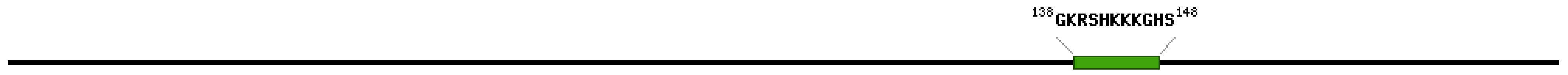

Posterior Graphic. Threshold Marked @ 0.4

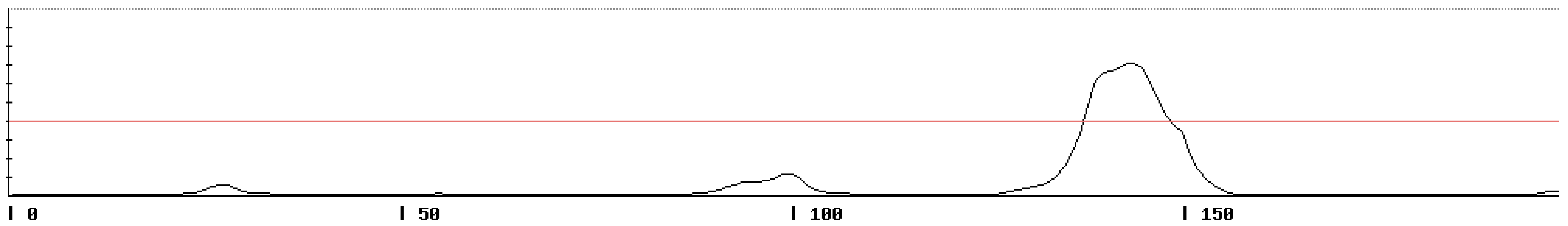

Predictions for Brachypodiumdistachyon_Bradi5g11247.1.p

Viterbi Path

Viterbi Path

Posterior @ 0.4

Posterior @ 0.4

Posterior Graphic. Threshold Marked @ 0.4

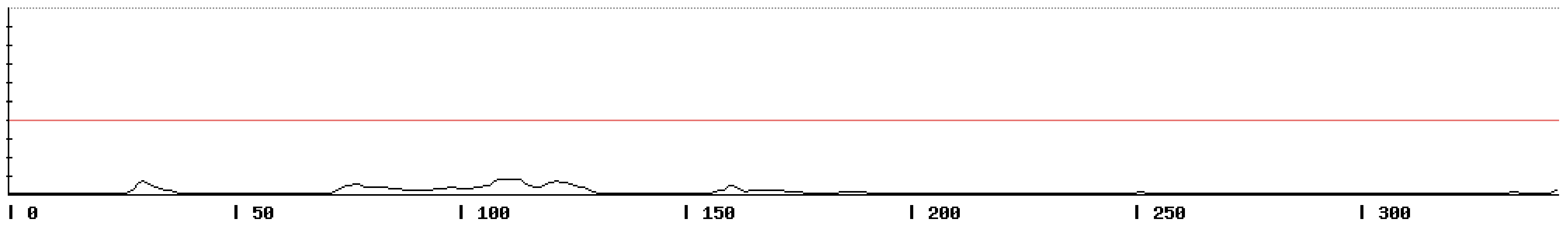

Predictions for Brachypodiumdistachyon_Bradi5g12407.4.p

Viterbi Path

Viterbi Path

Posterior @ 0.4

Posterior @ 0.4

Posterior Graphic. Threshold Marked @ 0.4

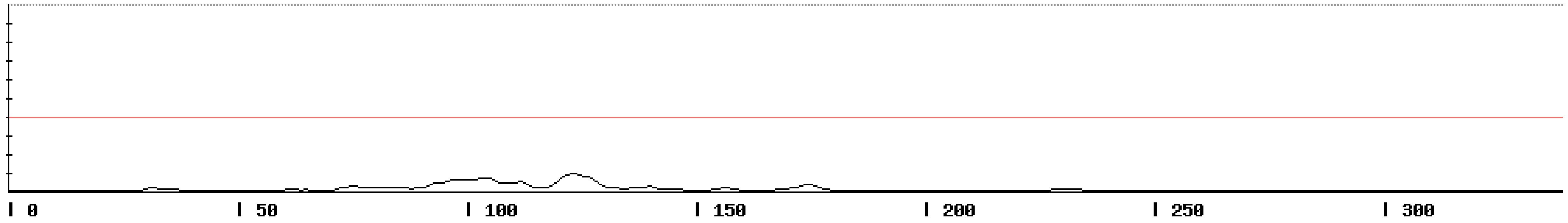

Predictions for Brachypodiumdistachyon_Bradi5g13300.1.p

Viterbi Path

Viterbi Path

Posterior @ 0.4

Posterior @ 0.4

Posterior Graphic. Threshold Marked @ 0.4

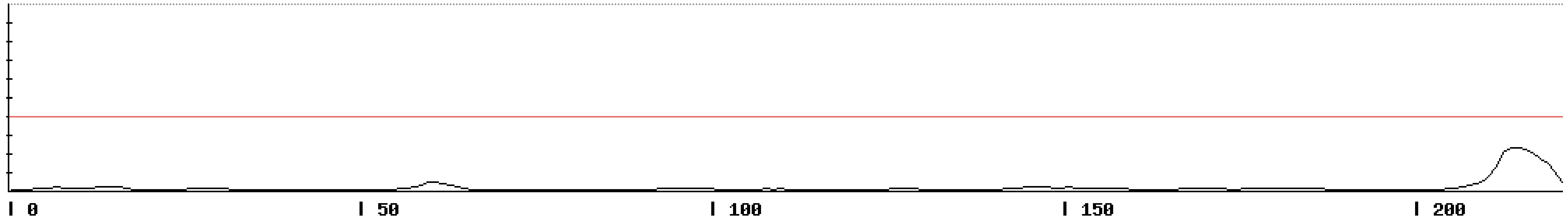

Predictions for Brachypodiumdistachyon_Bradi5g13450.1.p

Viterbi Path

Viterbi Path

Posterior @ 0.4

Posterior @ 0.4

Posterior Graphic. Threshold Marked @ 0.4

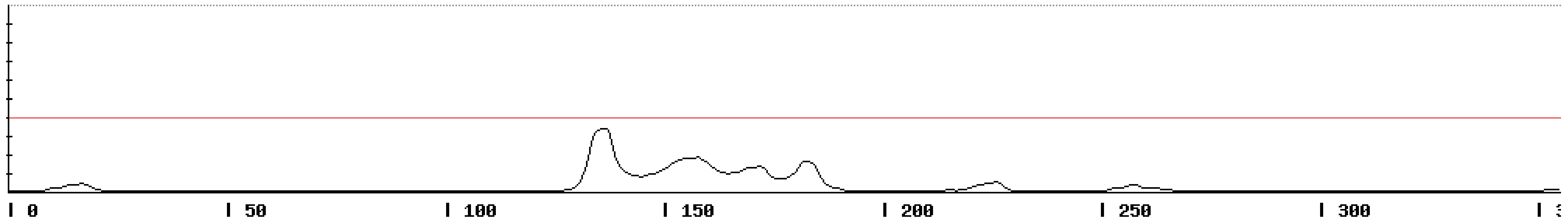

Predictions for Brachypodiumdistachyon_Bradi5g13816.1.p

Viterbi Path

Viterbi Path

Posterior @ 0.4

Posterior @ 0.4

Posterior Graphic. Threshold Marked @ 0.4

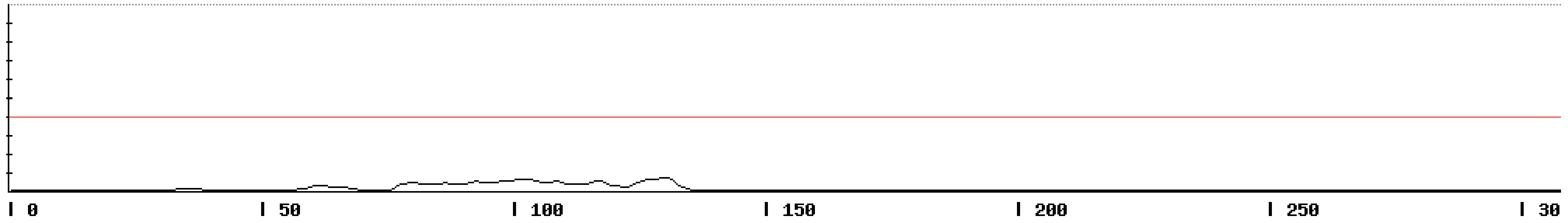

Predictions for Brachypodiumdistachyon_Bradi5g14736.1.p

Viterbi Path

Viterbi Path

Posterior @ 0.4

Posterior @ 0.4

Posterior Graphic. Threshold Marked @ 0.4

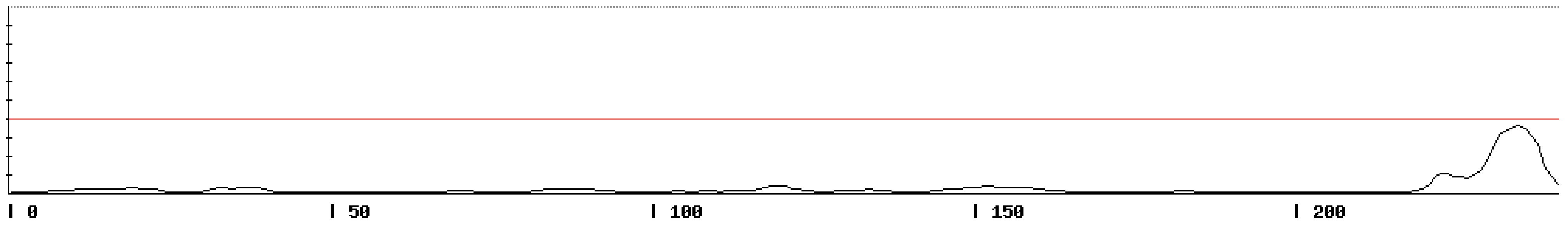

Predictions for Brachypodiumdistachyon_Bradi5g14952.1.p

Viterbi Path

Viterbi Path

Posterior @ 0.4

Posterior @ 0.4

Posterior Graphic. Threshold Marked @ 0.4

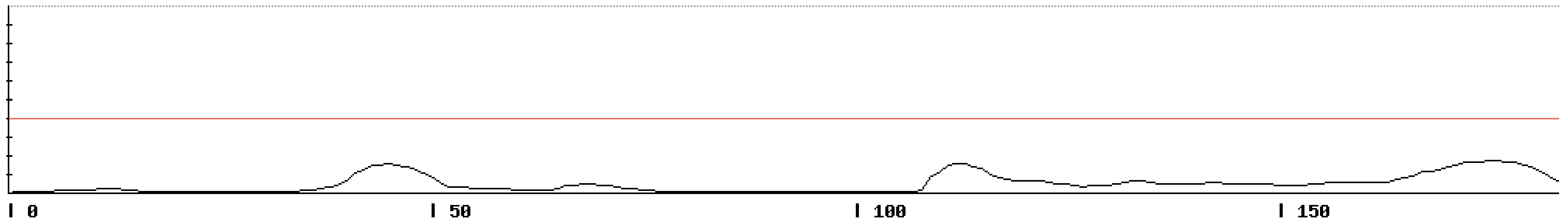

Predictions for Brachypodiumdistachyon_Bradi5g15230.1.p

Viterbi Path

Viterbi Path

Posterior @ 0.4

30 - KPRTRDKPPRTPPPR - 44

291 - RRKRAANG - 298

302 - ERPRR - 306

Posterior @ 0.4

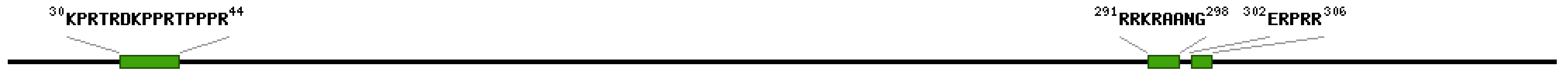

Posterior Graphic. Threshold Marked @ 0.4

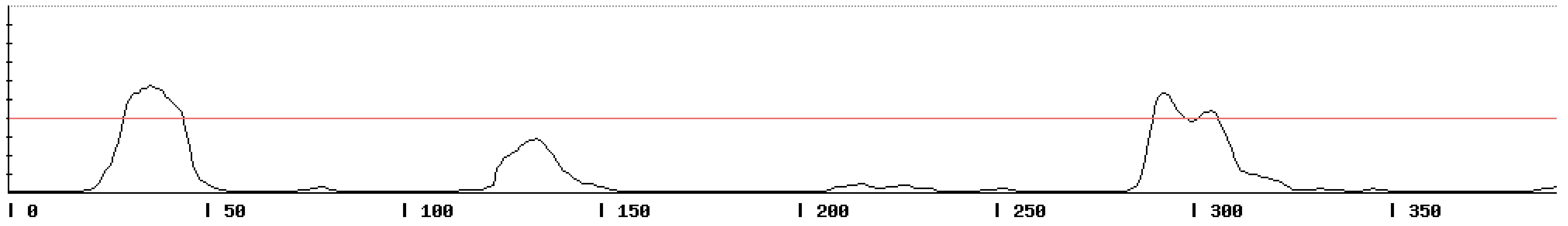

Predictions for Brachypodiumdistachyon_Bradi5g15587.1.p

Viterbi Path

Viterbi Path

Posterior @ 0.4

Posterior @ 0.4

Posterior Graphic. Threshold Marked @ 0.4

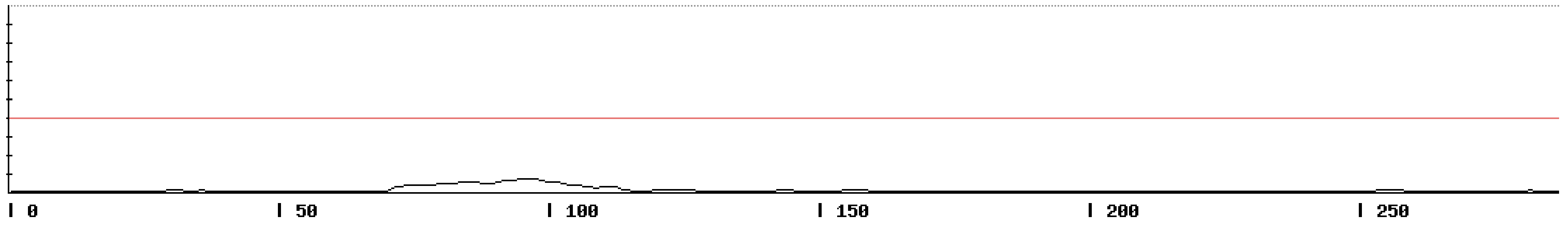

Predictions for Brachypodiumdistachyon_Bradi5g16917.1.p

Viterbi Path	
Viterbi Path	
Posterior @ 0.4	
Posterior @ 0.4	
Posterior Graphic. Threshold Marked @ 0.4	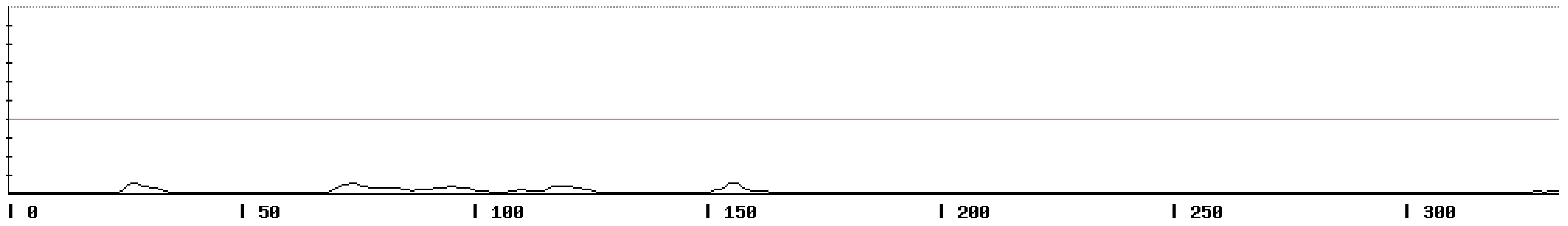

Predictions for Brachypodiumdistachyon_Bradi5g18044.2.p

Viterbi Path	
Viterbi Path	
Posterior @ 0.4	226 - NKLSARQKGKRPVEAMRRGPMATPKR - 251
Posterior @ 0.4	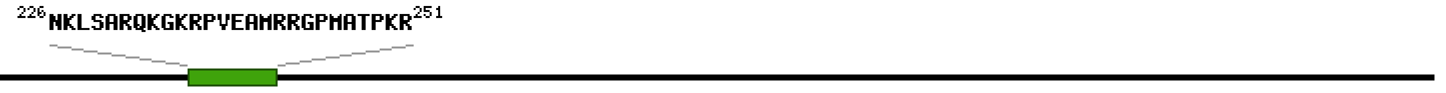

Posterior Graphic. Threshold Marked @ 0.4

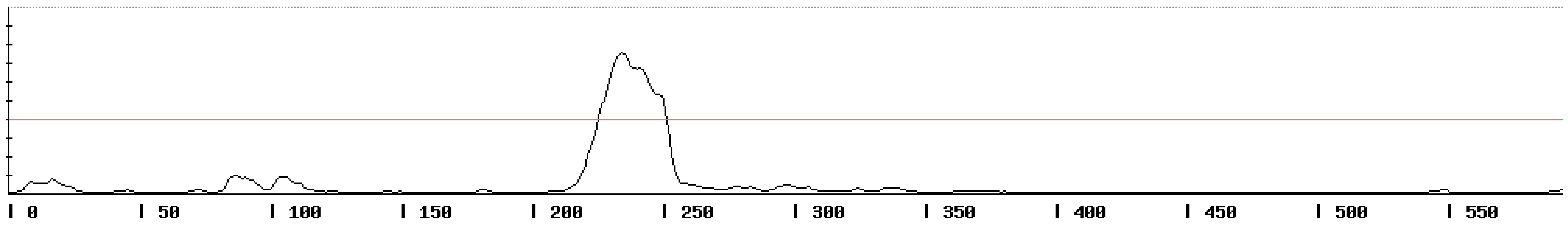

Predictions for Brachypodiumdistachyon_Bradi5g21010.1.p

Viterbi Path

Viterbi Path

Posterior @ 0.4

Posterior @ 0.4

Posterior Graphic. Threshold Marked @ 0.4

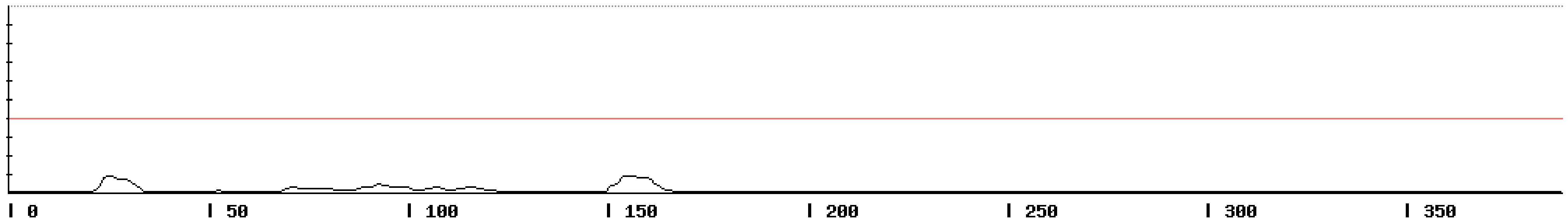

Predictions for Brachypodiumdistachyon_Bradi5g21983.1.p

<

alex.nguyenba@utoronto.ca

Intended to be used with Firefox

Viterbi Path

Posterior @ 0.4

99 - APRRRRGGAVRRS - 111

193 - RRSSRAPAARRPPARRAHL - 211

Posterior @ 0.4

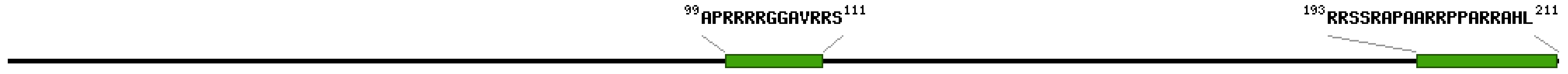

Posterior Graphic. Threshold Marked @ 0.4

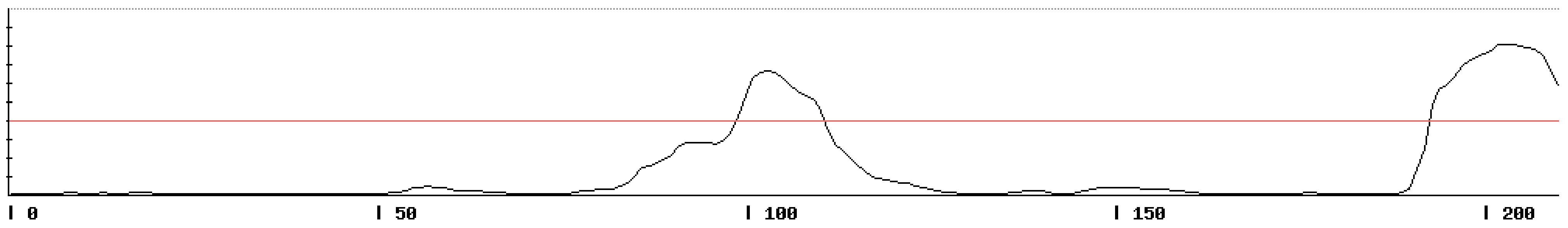

Predictions for Brachypodiumstacei_Brast01G038400.1.p

Viterbi Path

Viterbi Path

Posterior @ 0.4

80 - RARR - 83

Posterior @ 0.4

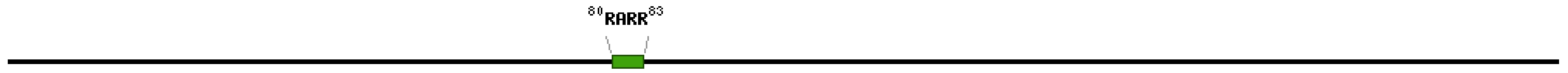

Posterior Graphic. Threshold Marked @ 0.4

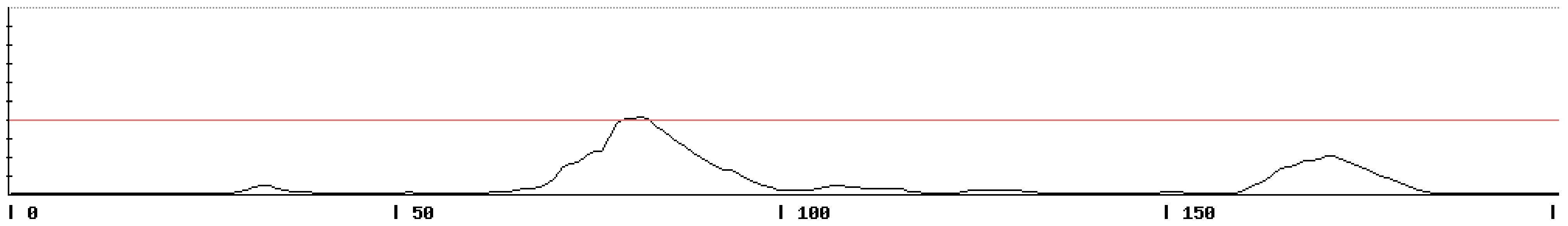

Predictions for Brachypodiumstacei_Brast01G038600.1.p

Viterbi Path

Viterbi Path

Posterior @ 0.4

Posterior @ 0.4

Posterior Graphic. Threshold Marked @ 0.4

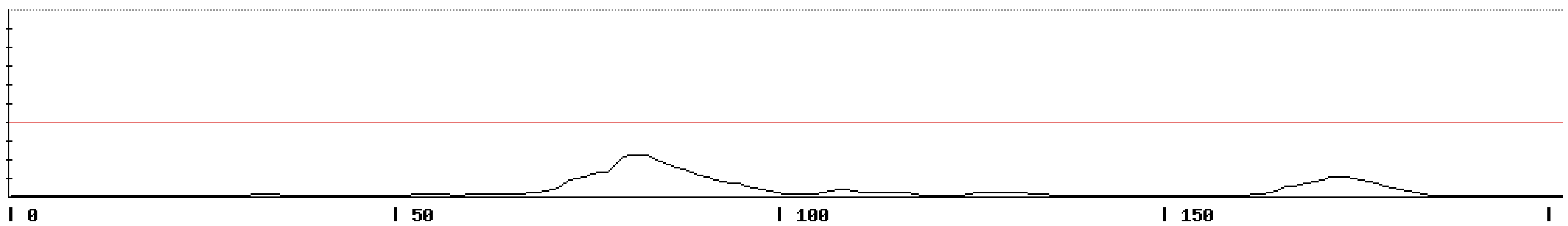

Predictions for Brachypodiumstacei_Brast01G063400.1.p

Viterbi Path

Viterbi Path

Posterior @ 0.4

Posterior @ 0.4

Posterior Graphic. Threshold Marked @ 0.4

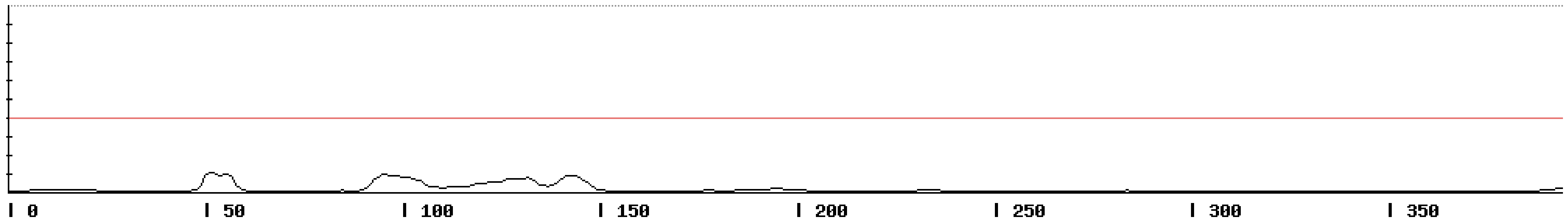

Predictions for Brachypodiumstacei_Brast01G065900.1.p	
Viterbi Path	
Viterbi Path	
Posterior @ 0.4	
Posterior @ 0.4	
Posterior Graphic. Threshold Marked @ 0.4	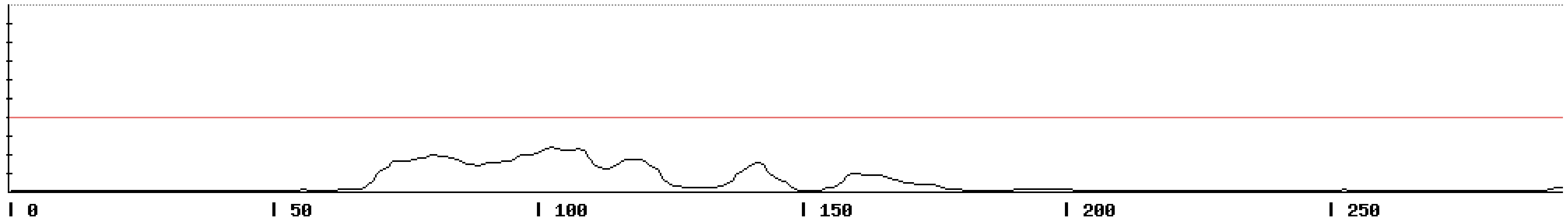

Predictions for Brachypodiumstacei_Brast01G080400.1.p	
Viterbi Path	
Viterbi Path	
Posterior @ 0.4	
Posterior @ 0.4	

Posterior Graphic. Threshold Marked @ 0.4

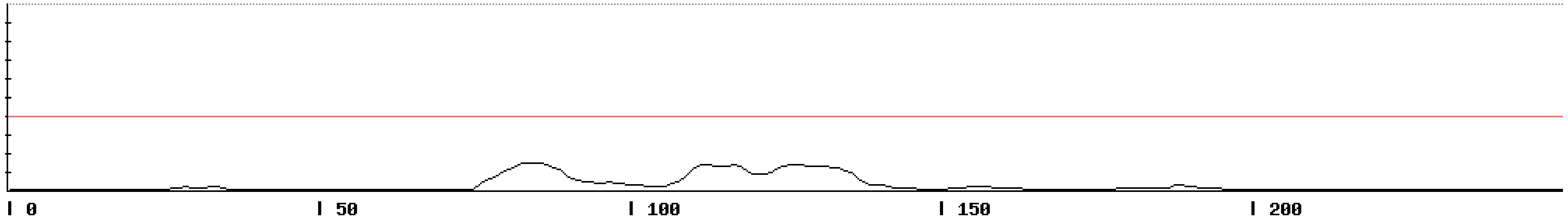

Predictions for Brachypodiumstacei_Brast01G108000.1.p

Viterbi Path	
Viterbi Path	
Posterior @ 0.4	
Posterior @ 0.4	
Posterior Graphic. Threshold Marked @ 0.4	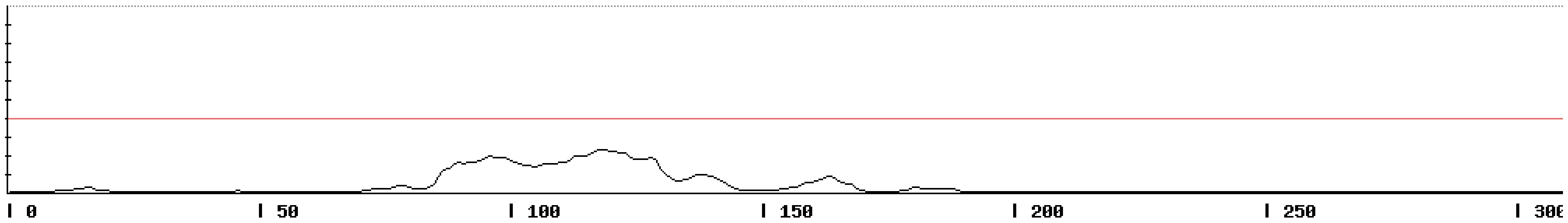

Predictions for Brachypodiumstacei_Brast01G111300.1.p

Viterbi Path	
Viterbi Path	
Posterior @ 0.4	93 - RNRKYPKGKRPSRNTGTAGRWK - 114
Posterior @ 0.4	

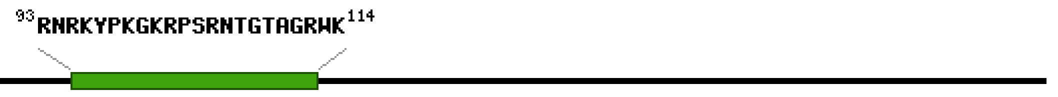

Posterior Graphic. Threshold Marked @ 0.4

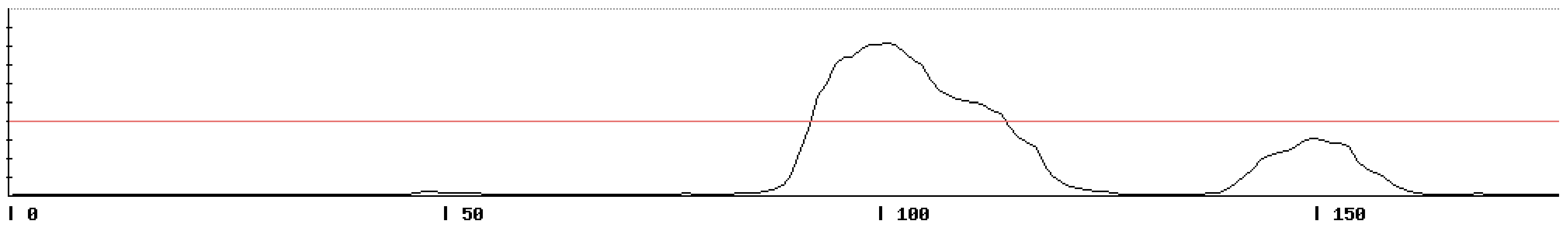

Predictions for Brachypodiumstacei_Brast01G184700.1.p

Viterbi Path

Viterbi Path

Posterior @ 0.4

Posterior @ 0.4

Posterior Graphic. Threshold Marked @ 0.4

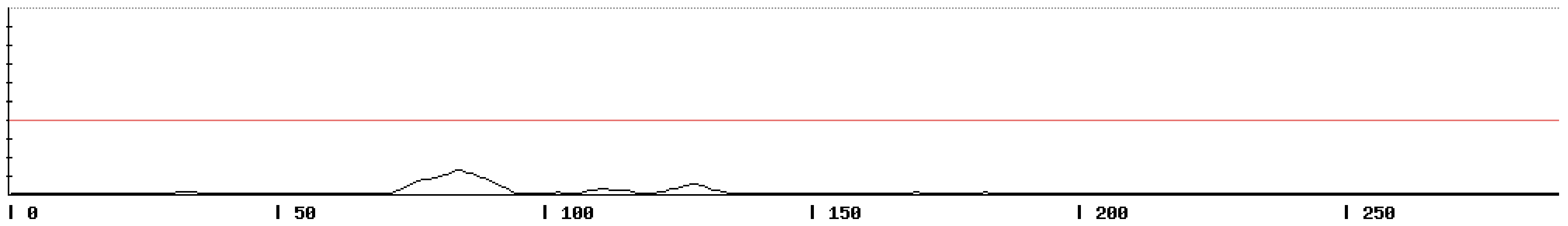

Predictions for Brachypodiumstacei_Brast01G185400.1.p

Viterbi Path

Viterbi Path

Posterior @ 0.4	77 - RAKSRRGQRKS - 87
	373 - AKRIRGQRRPR - 383
	495 - RGTKKSTALAASGRKKK - 511
Posterior @ 0.4	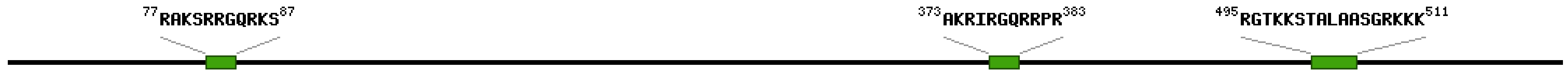
Posterior Graphic. Threshold Marked @ 0.4	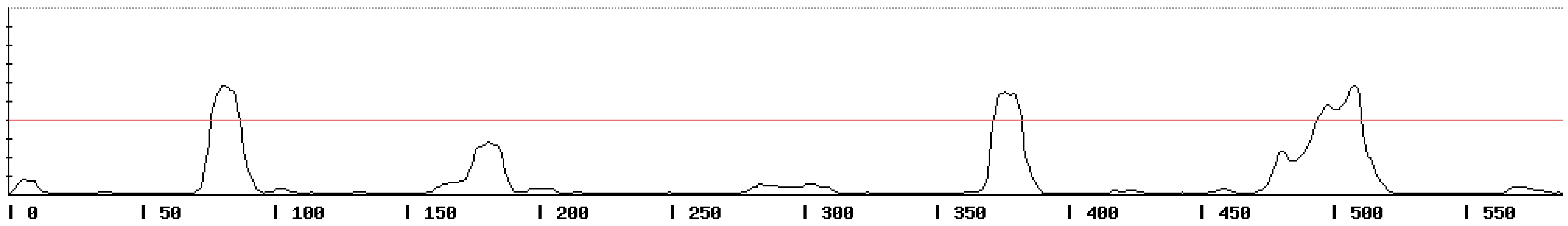

Predictions for Brachypodiumstacei_Brast01G188200.1.p

Viterbi Path	
Viterbi Path	
Posterior @ 0.4	151 - RKRRK - 155
Posterior @ 0.4	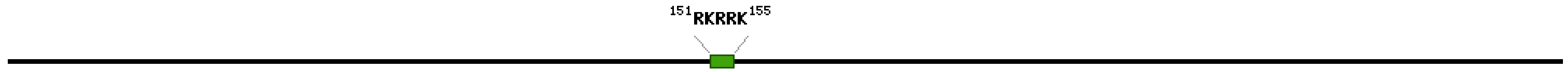
Posterior Graphic. Threshold Marked @ 0.4	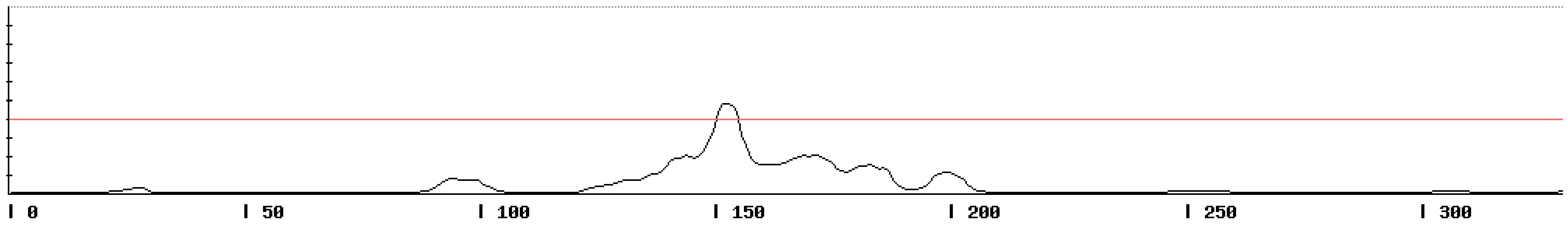

Predictions for Brachypodiumstacei_Brast01G190200.1.p

Viterbi Path	

Viterbi Path

Posterior @ 0.4 — 161 - AAAKNQHRKPASSSSPSATRKRKAAAPADARPPAGQRRRHA - 201

Posterior @ 0.4

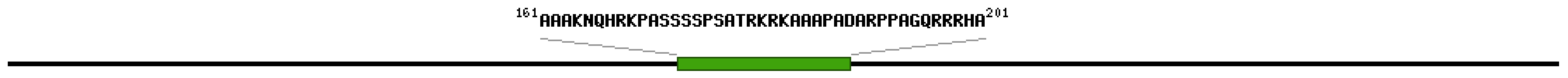

Posterior Graphic. Threshold Marked @ 0.4

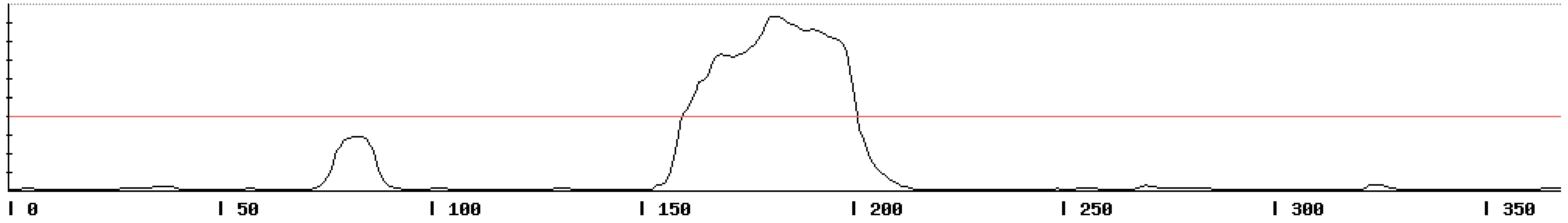

Predictions for Brachypodiumstacei_Brast01G304500.1.p

Viterbi Path

Viterbi Path

Posterior @ 0.4 — 76 - GRRARGAAAETRRPRSRPA - 94

Posterior @ 0.4

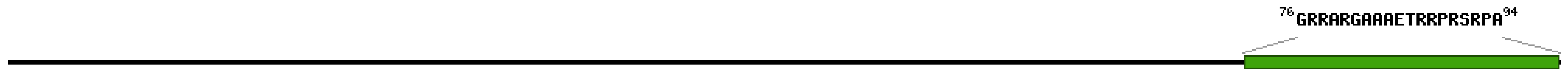

Posterior Graphic. Threshold Marked @ 0.4

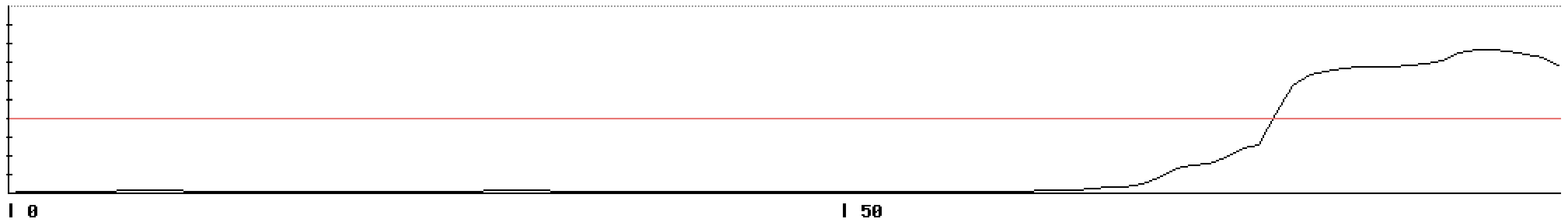

Predictions for Brachypodiumstacei_Brast01G307800.1.p

Viterbi Path

Viterbi Path

Posterior @ 0.4

Posterior @ 0.4

Posterior Graphic. Threshold Marked @ 0.4

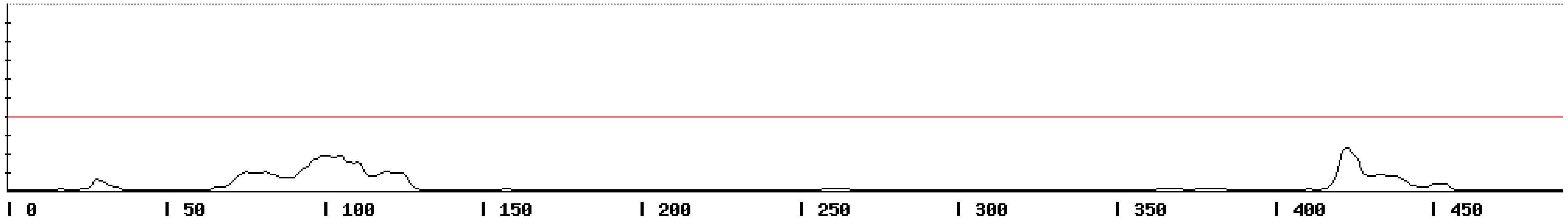

Predictions for Brachypodiumstacei_Brast01G322200.1.p

Viterbi Path

Viterbi Path

Posterior @ 0.4

Posterior @ 0.4

Posterior Graphic. Threshold Marked @ 0.4

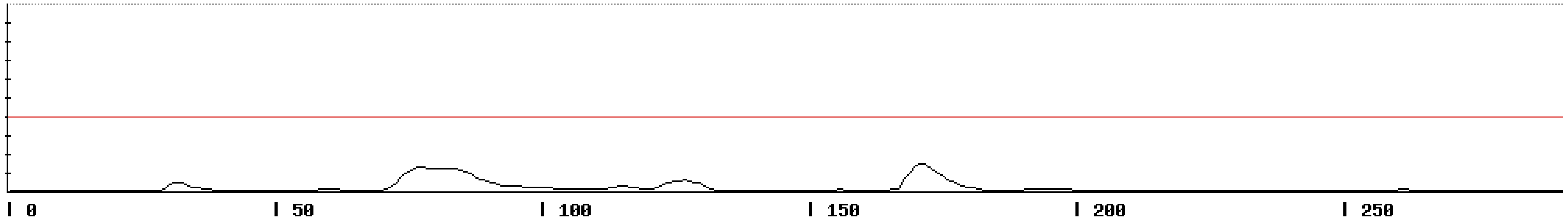

Predictions for Brachypodiumstacei_Brast01G345300.1.p	
Viterbi Path	
Viterbi Path	
Posterior @ 0.4	123 - KKRKK - 127
Posterior @ 0.4	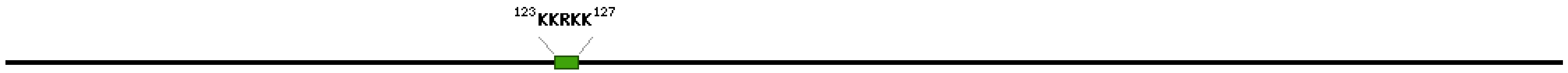
Posterior Graphic. Threshold Marked @ 0.4	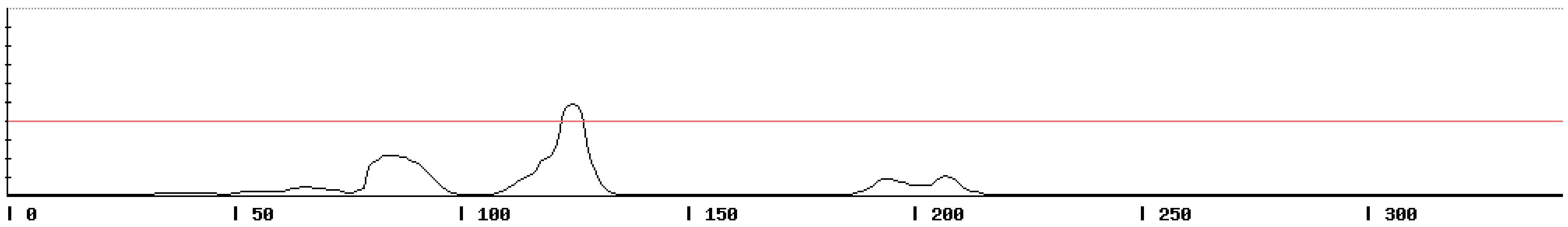

Predictions for Brachypodiumstacei_Brast01G355100.1.p	
Viterbi Path	
Viterbi Path	
Posterior @ 0.4	
Posterior @ 0.4	
Posterior Graphic. Threshold Marked @ 0.4	

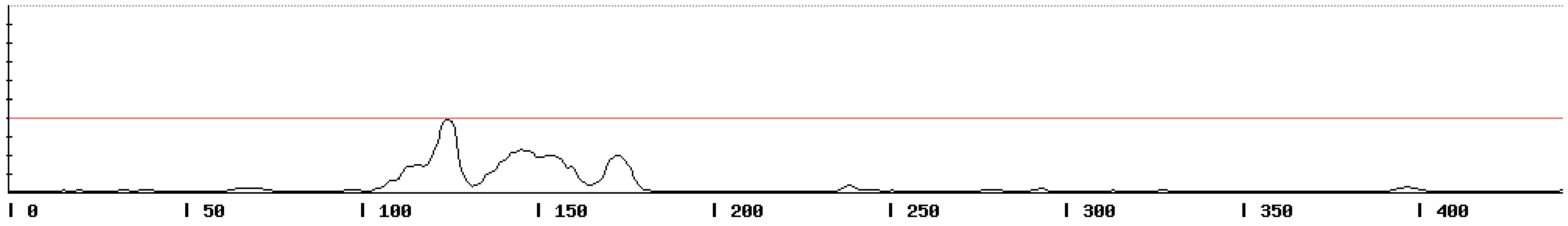

Predictions for Brachypodiumstacei_Brast01G380000.1.p	
Viterbi Path	
Viterbi Path	
Posterior @ 0.4	
Posterior @ 0.4	
Posterior Graphic. Threshold Marked @ 0.4	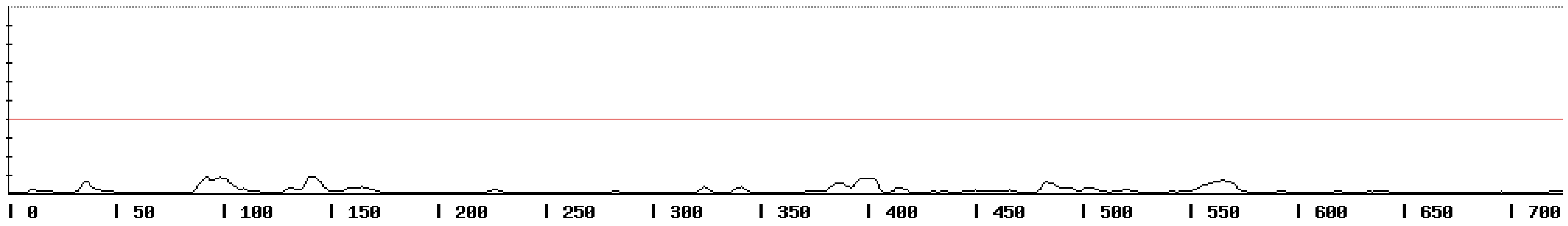

Predictions for Brachypodiumstacei_Brast01G380100.1.p	
Viterbi Path	
Viterbi Path	
Posterior @ 0.4	
Posterior @ 0.4	

Posterior Graphic. Threshold Marked @ 0.4

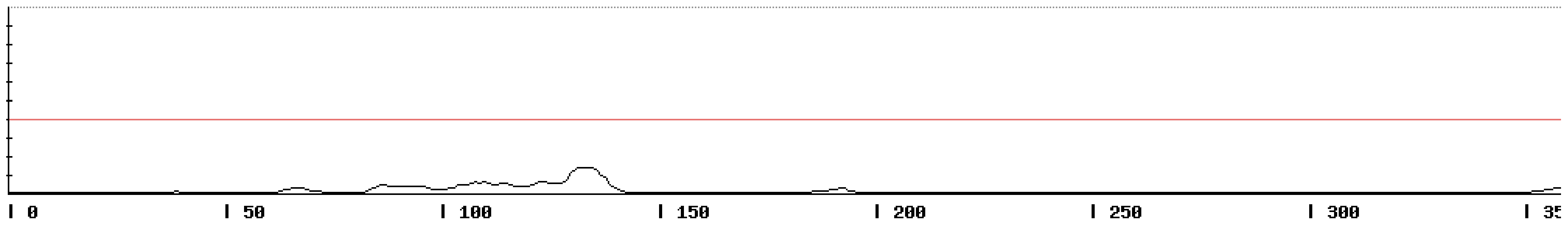

Predictions for Brachypodiumstacei_Brast02G009100.1.p

Viterbi Path

Viterbi Path

Posterior @ 0.4 — 140 - GGKKGKGKGKAPA - 152

Posterior @ 0.4

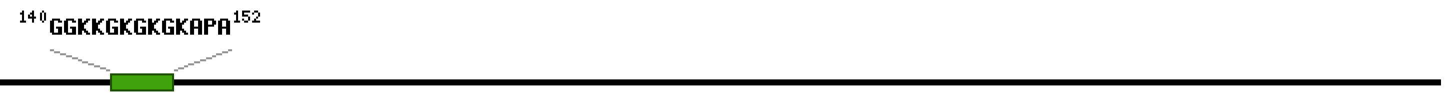

Posterior Graphic. Threshold Marked @ 0.4

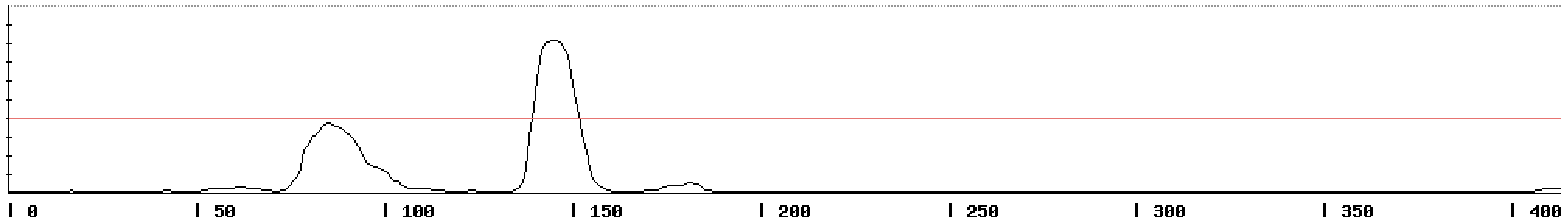

Predictions for Brachypodiumstacei_Brast02G018500.1.p

Viterbi Path

Viterbi Path

Posterior @ 0.4

94 - K - 94

96 - RK - 97

Posterior @ 0.4

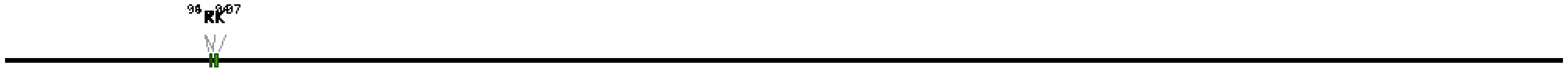

Posterior Graphic. Threshold Marked @ 0.4

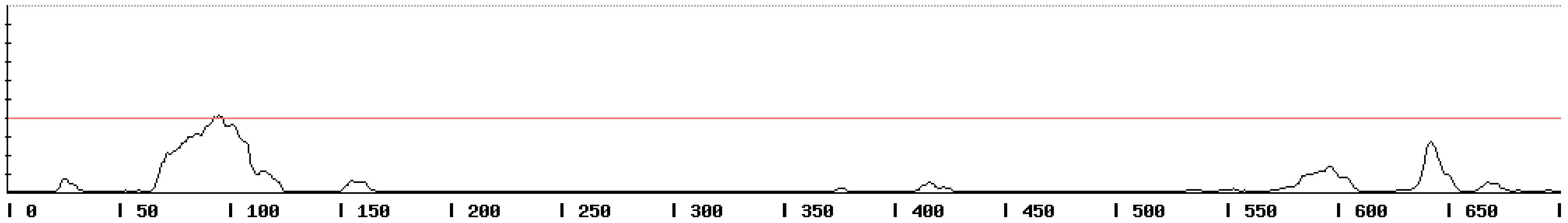

Predictions for Brachypodiumstacei_Brast02G024100.1.p

Viterbi Path	
Viterbi Path	
Posterior @ 0.4	245 - KKKAK - 249
Posterior @ 0.4	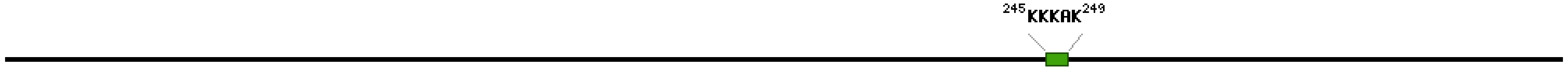
Posterior Graphic. Threshold Marked @ 0.4	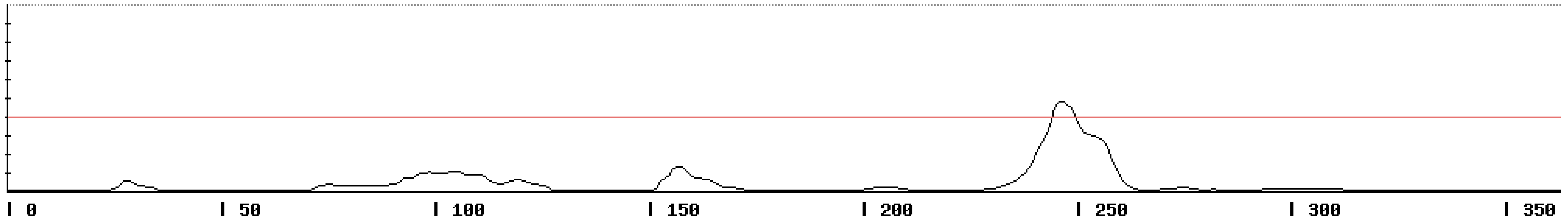

Predictions for Brachypodiumstacei_Brast02G030400.1.p

Viterbi Path	
Viterbi Path	

Posterior @ 0.4

Posterior @ 0.4

Posterior Graphic. Threshold Marked @ 0.4

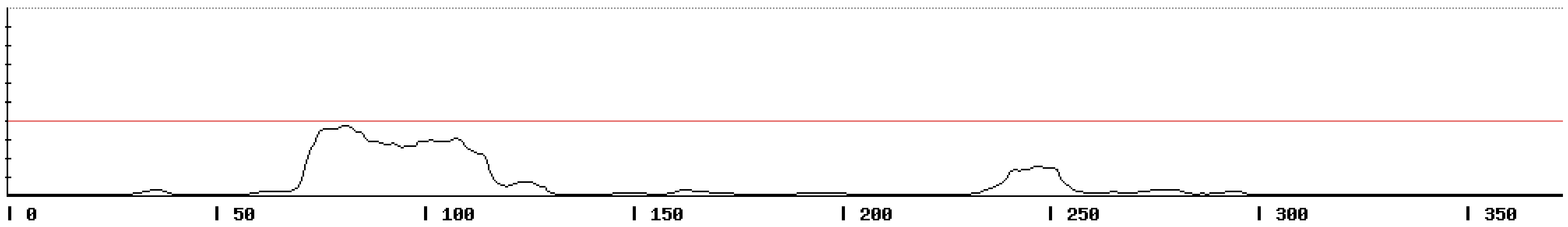

Predictions for Brachypodiumstacei_Brast02G031500.1.p

Viterbi Path

Viterbi Path

Posterior @ 0.4

110 - GKKKRKR - 116

188 - RRPRRKGDAPARE - 200

Posterior @ 0.4

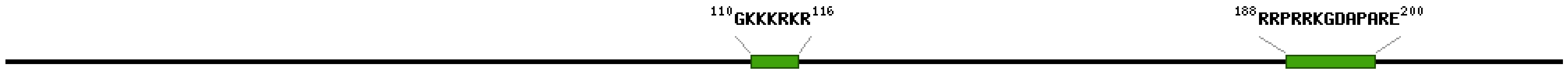

Posterior Graphic. Threshold Marked @ 0.4

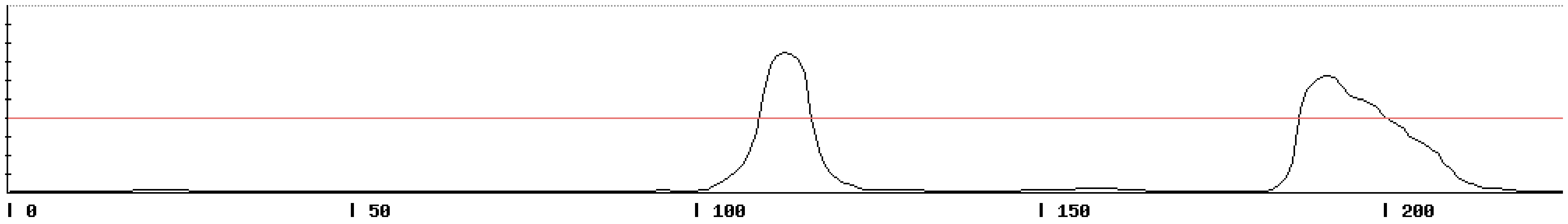

Predictions for Brachypodiumstacei_Brast02G098700.1.p

Viterbi Path

Viterbi Path

Posterior @ 0.4

574 - SRRRRKKKT - 582

Posterior @ 0.4

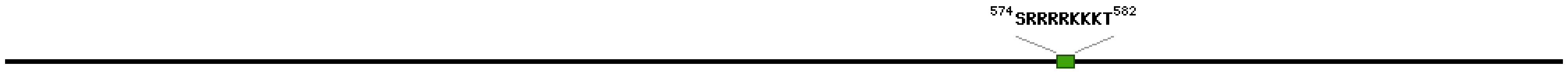

Posterior Graphic. Threshold Marked @ 0.4

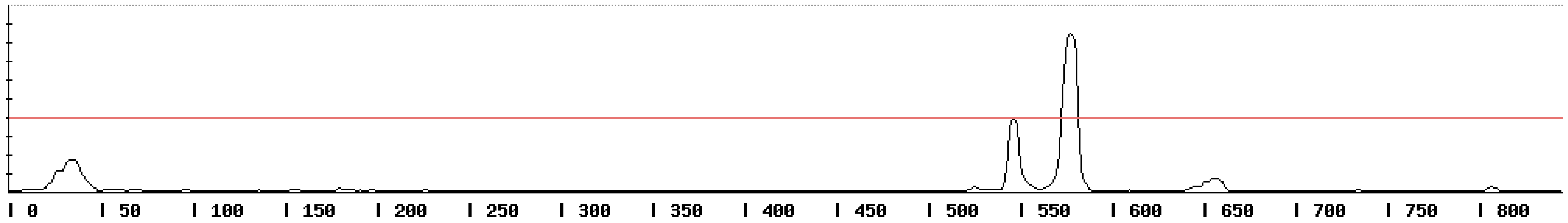

Predictions for Brachypodiumstacei_Brast02G110400.1.p

Viterbi Path

Viterbi Path

Posterior @ 0.4

202 - RQKRAAPMAEPPRPKKRTRASPA - 224

Posterior @ 0.4

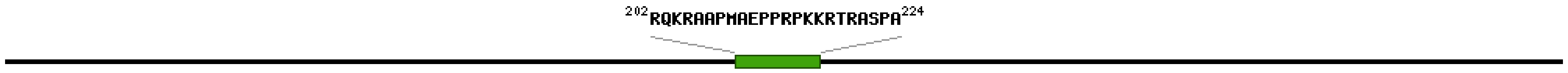

Posterior Graphic. Threshold Marked @ 0.4

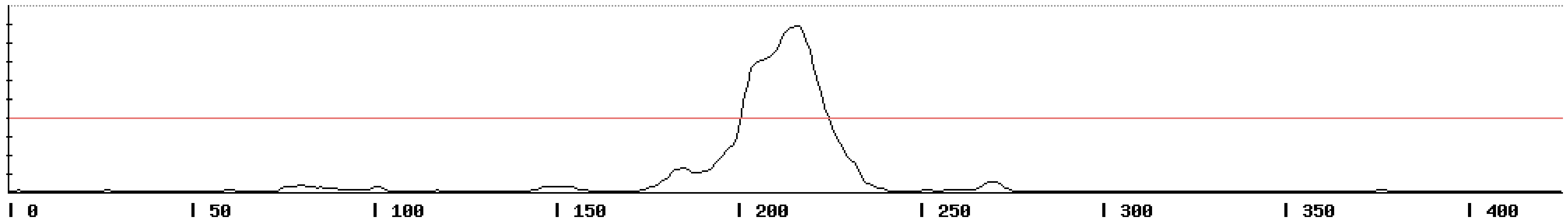

Predictions for Brachypodiumstacei_Brast02G166800.1.p

Viterbi Path

Viterbi Path

Posterior @ 0.4

Posterior @ 0.4

Posterior Graphic. Threshold Marked @ 0.4

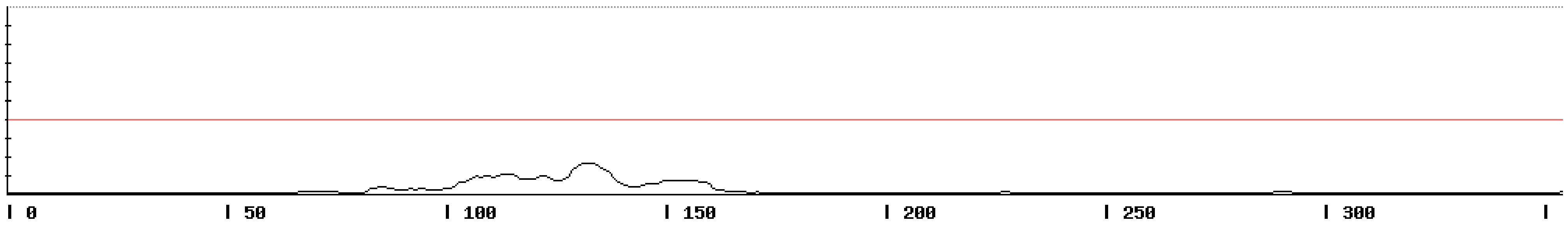

Predictions for Brachypodiumstacei_Brast02G167100.1.p

Viterbi Path

Viterbi Path

Posterior @ 0.4

Posterior @ 0.4

Posterior Graphic. Threshold Marked @ 0.4

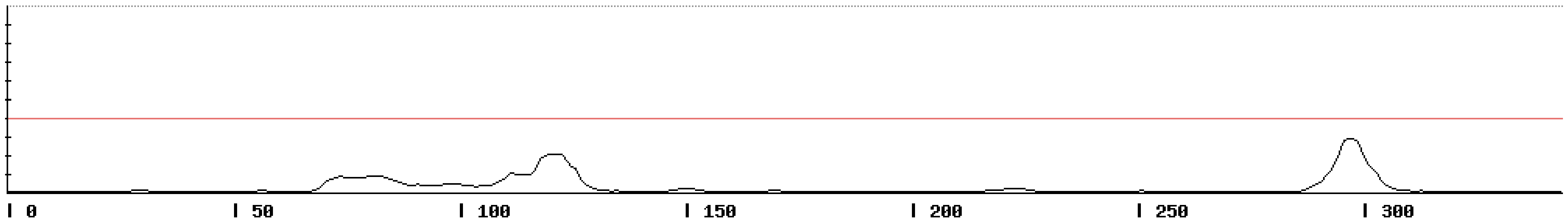

Predictions for Brachypodiumstacei_Brast02G192700.1.p	
Viterbi Path	206 - RAGGKKIKGKREINVGVKKRKREEK - 230
Viterbi Path	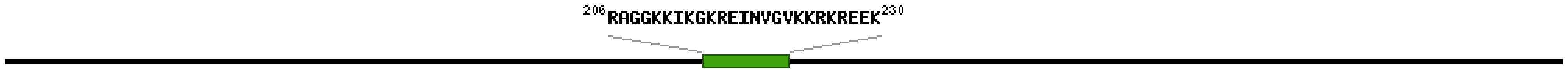
Posterior @ 0.4	205 - DRAGGKKIKGKREINVGVKKRKREEKIE - 232
Posterior @ 0.4	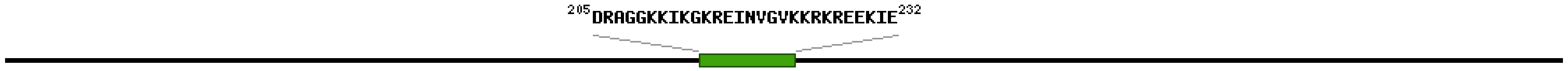
Posterior Graphic. Threshold Marked @ 0.4	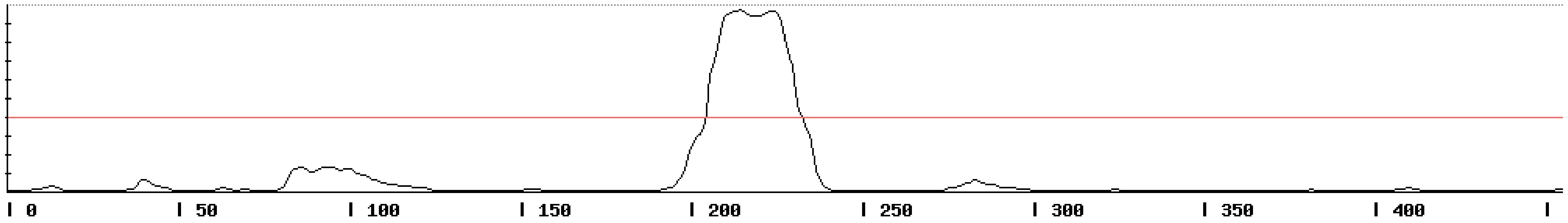

Predictions for Brachypodiumstacei_Brast02G236200.1.p	
Viterbi Path	
Viterbi Path	
Posterior @ 0.4	
Posterior @ 0.4	
Posterior Graphic. Threshold Marked @ 0.4	

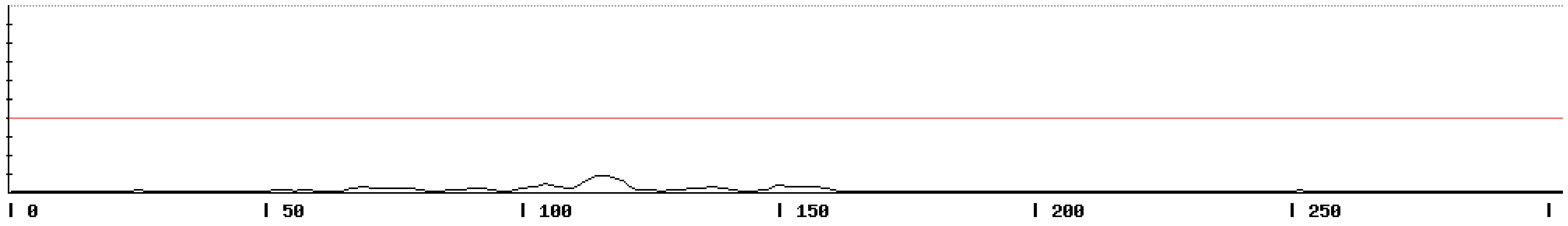

Predictions for Brachypodiumstacei_Brast02G325400.1.p	
Viterbi Path	
Viterbi Path	
Posterior @ 0.4	
Posterior @ 0.4	
Posterior Graphic. Threshold Marked @ 0.4	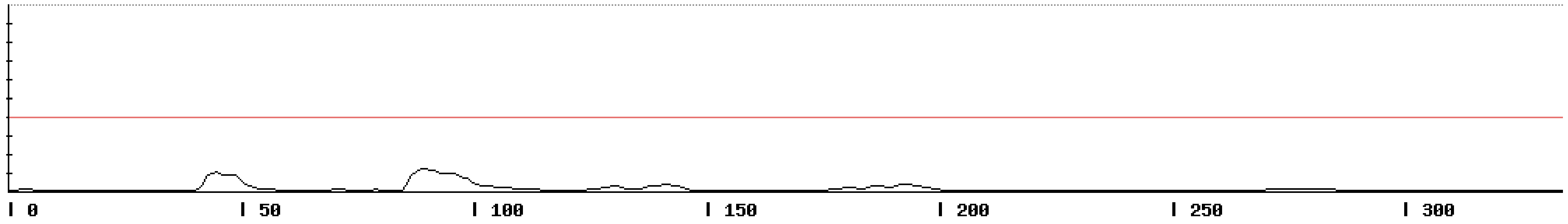

Predictions for Brachypodiumstacei_Brast02G355100.1.p	
Viterbi Path	227 - PPKPPTGRRPRGRPRGSTAAKSRSKAKAKAKPKPK - 261
Viterbi Path	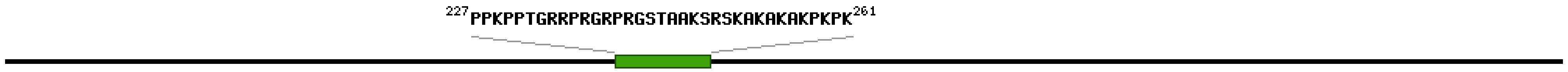
Posterior @ 0.4	224 - PADPPKPPTGRRPRGRPRGSTAAKSRSKAKAKAKPKPKL - 262
Posterior @ 0.4	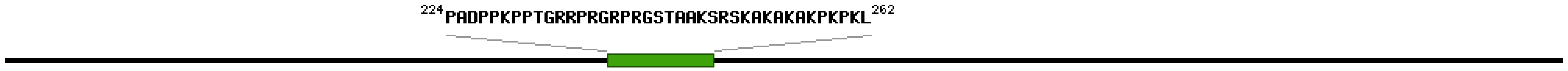

Posterior Graphic. Threshold Marked @ 0.4

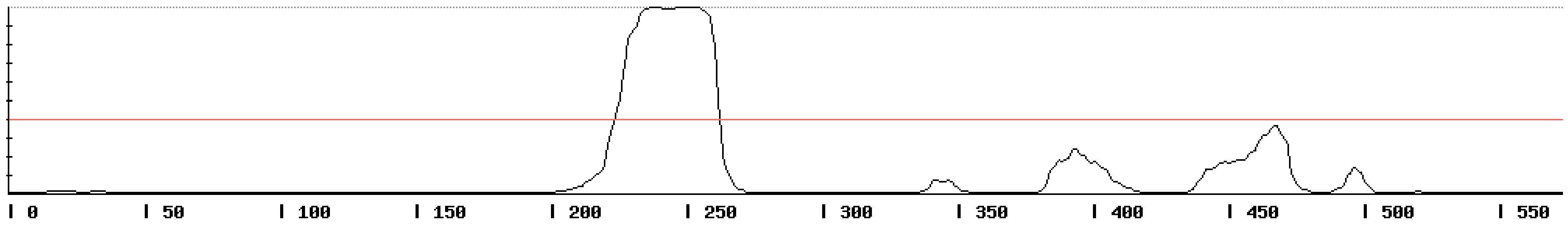

Predictions for Brachypodiumstacei_Brast02G356100.1.p

Viterbi Path

Viterbi Path

Posterior @ 0.4

Posterior @ 0.4

Posterior Graphic. Threshold Marked @ 0.4

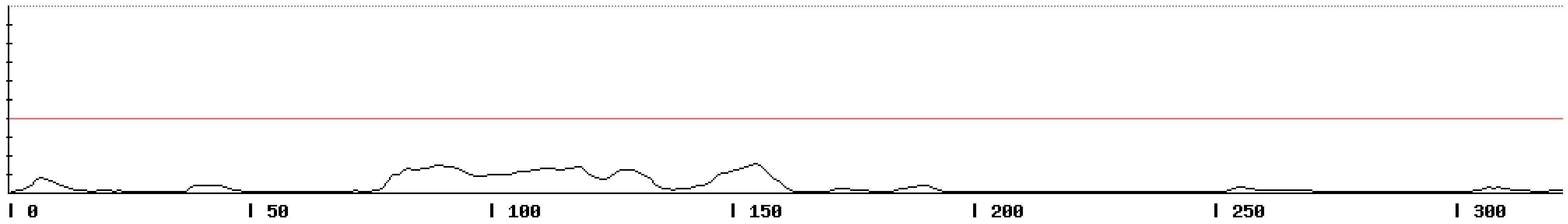

Predictions for Brachypodiumstacei_Brast02G382000.1.p

Viterbi Path

Viterbi Path

Posterior @ 0.4 — 94 - RRAKRRS - 100

Posterior @ 0.4

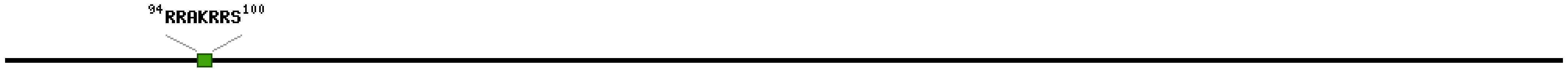

Posterior Graphic. Threshold Marked @ 0.4

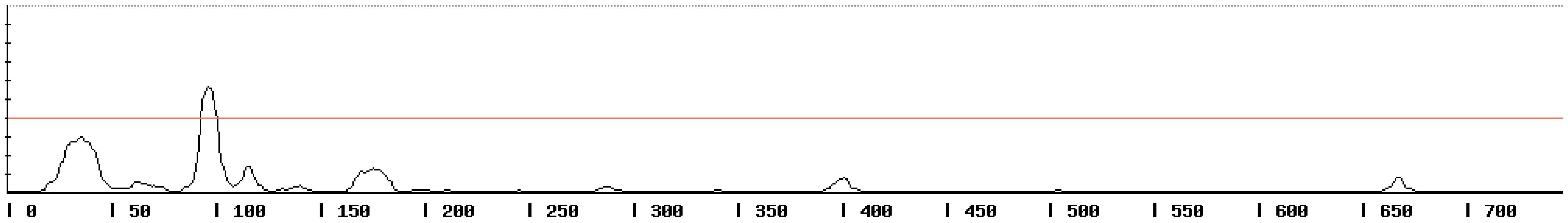

Predictions for Brachypodiumstacei_Brast02G383500.1.p

Viterbi Path

Viterbi Path

Posterior @ 0.4

78 - SKKKKSGRRARAVGGDSGKK - 97

167 - KEEAAAGSSKKRKA - 180

Posterior @ 0.4

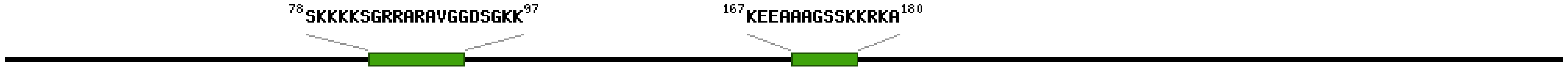

Posterior Graphic. Threshold Marked @ 0.4

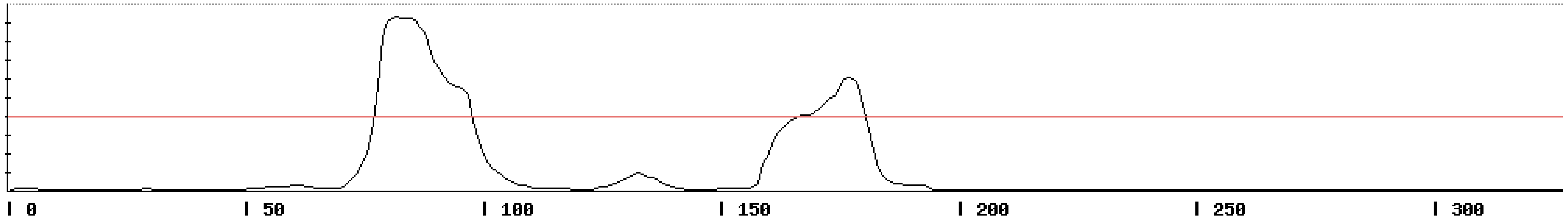

Predictions for Brachypodiumstacei_Brast02G383700.1.p

Viterbi Path

399 - RPAPPHRLLARSAPPNRRAAHVARHGRSSLRFGKRRKSRR - 438

Viterbi Path

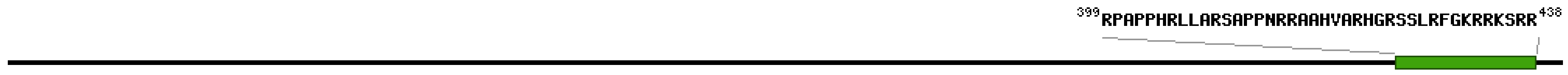

Posterior @ 0.4

62 - RAKYGKGGKRQRKAG - 76

398 - ARPAPPHRLLARSAPPNRRAAHVARHGRSSLRFGKRRKSRRL - 439

Posterior @ 0.4

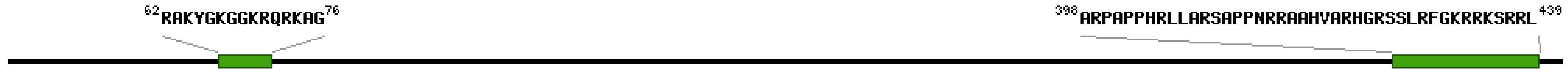

Posterior Graphic. Threshold Marked @ 0.4

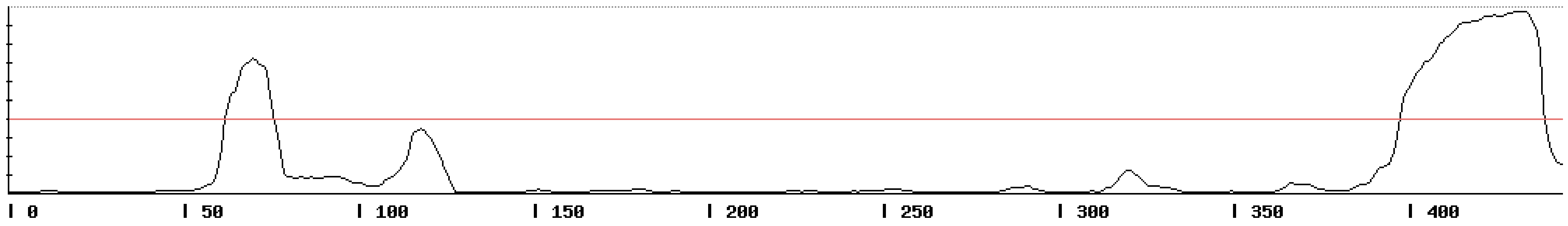

Predictions for Brachypodiumstacei_Brast03G008200.1.p

Viterbi Path

Viterbi Path

Posterior @ 0.4

Posterior @ 0.4

Posterior Graphic. Threshold Marked @ 0.4

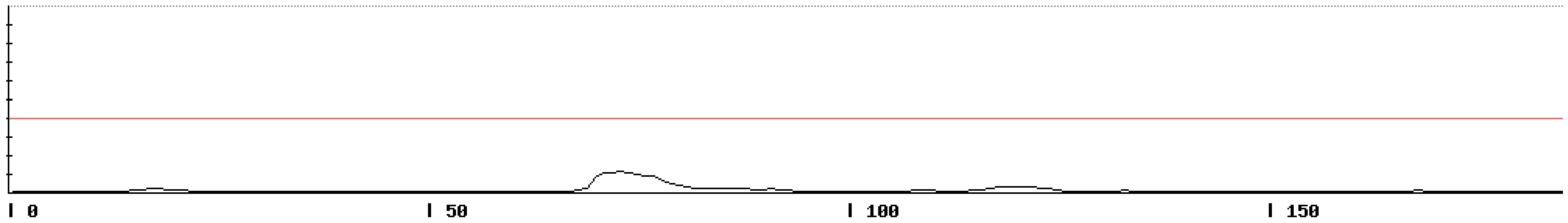

Predictions for Brachypodiumstacei_Brast03G010700.1.p

Viterbi Path	
Viterbi Path	
Posterior @ 0.4	
Posterior @ 0.4	
Posterior Graphic. Threshold Marked @ 0.4	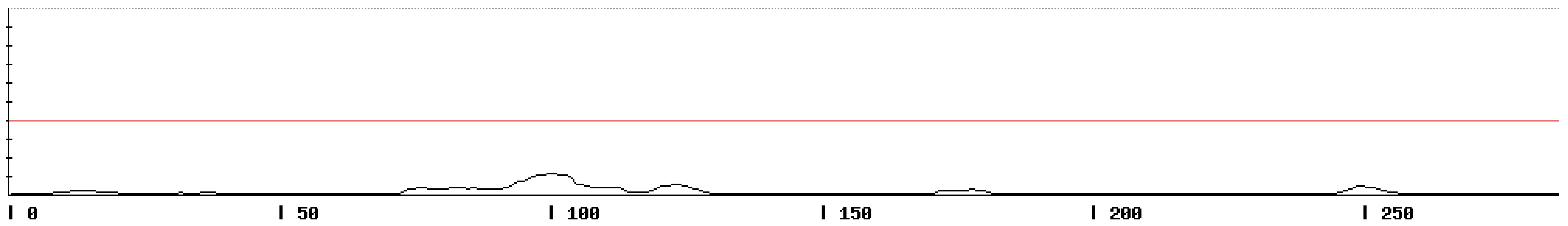

Predictions for Brachypodiumstacei_Brast03G018400.1.p

Viterbi Path	
Viterbi Path	
Posterior @ 0.4	94 - KATGKDRIIKSKGEKKKQHMIGMKKTLVFHRGRAPKGE - 131
	766 - RAPRKK - 771
Posterior @ 0.4	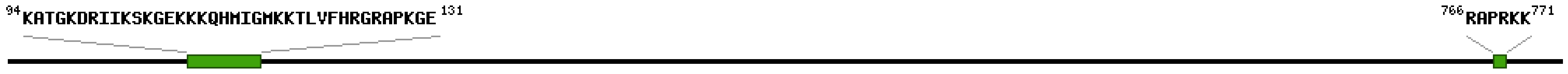
Posterior Graphic. Threshold Marked @ 0.4	

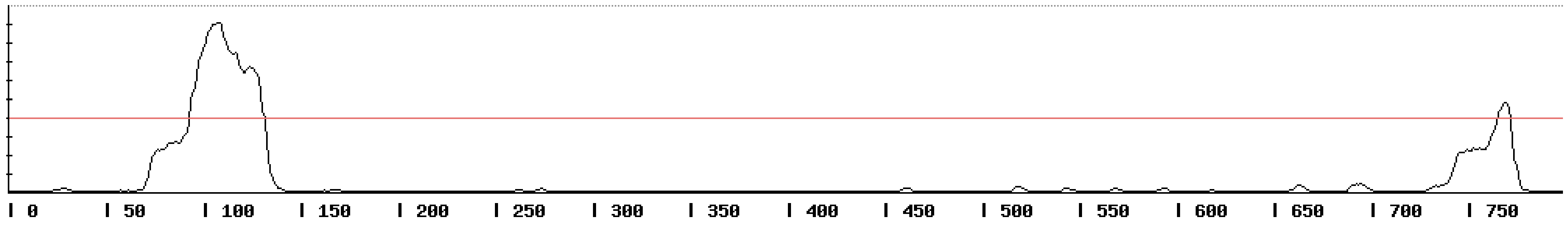

Predictions for Brachypodiumstacei_Brast03G048700.1.p

Viterbi Path	
Viterbi Path	
Posterior @ 0.4	178 - KKH - 180
Posterior @ 0.4	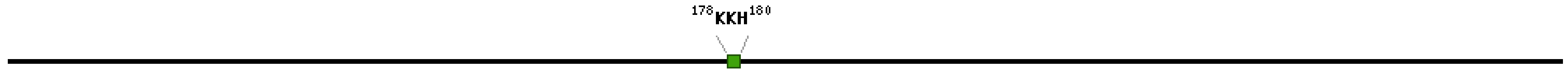
Posterior Graphic. Threshold Marked @ 0.4	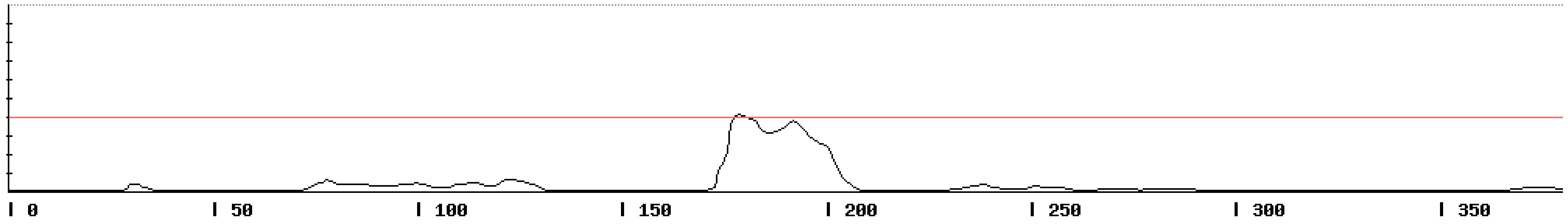

Predictions for Brachypodiumstacei_Brast03G050200.1.p

Viterbi Path	
Viterbi Path	
Posterior @ 0.4	
Posterior @ 0.4	

Posterior Graphic. Threshold Marked @ 0.4

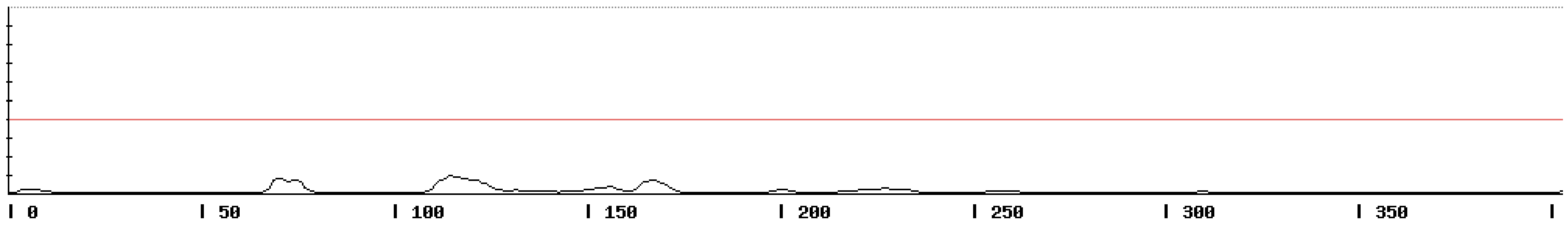

Predictions for Brachypodiumstacei_Brast03G055500.1.p

Viterbi Path

Viterbi Path

Posterior @ 0.4

Posterior @ 0.4

Posterior Graphic. Threshold Marked @ 0.4

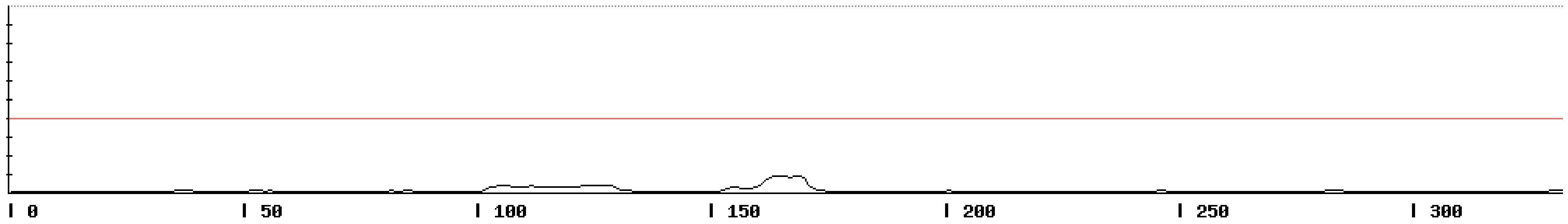

Predictions for Brachypodiumstacei_Brast03G114000.1.p

Viterbi Path

Viterbi Path

Posterior @ 0.4

83 - RGSKQRPARGAKNGAWKASG - 102

163 - KMKKSKKKKMK - 173

Posterior @ 0.4

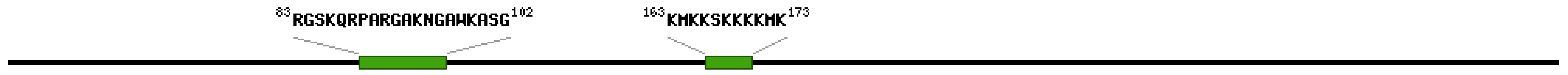

Posterior Graphic. Threshold Marked @ 0.4

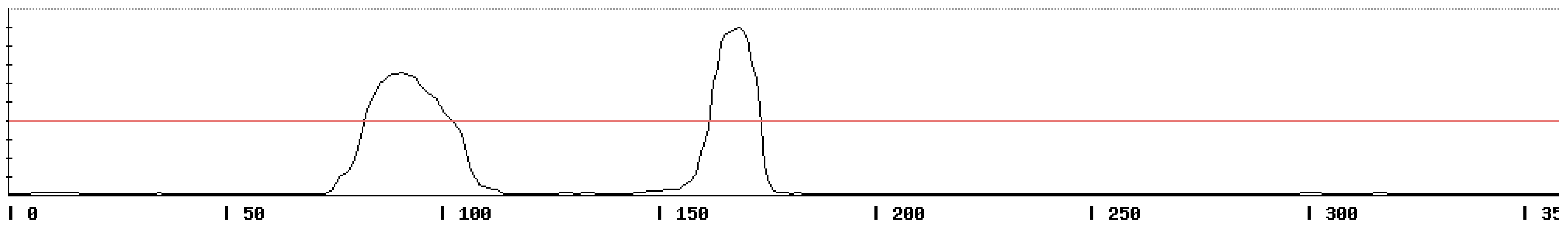

Predictions for Brachypodiumstacei_Brast03G115600.1.p

Viterbi Path

Viterbi Path

Posterior @ 0.4

Posterior @ 0.4

Posterior Graphic. Threshold Marked @ 0.4

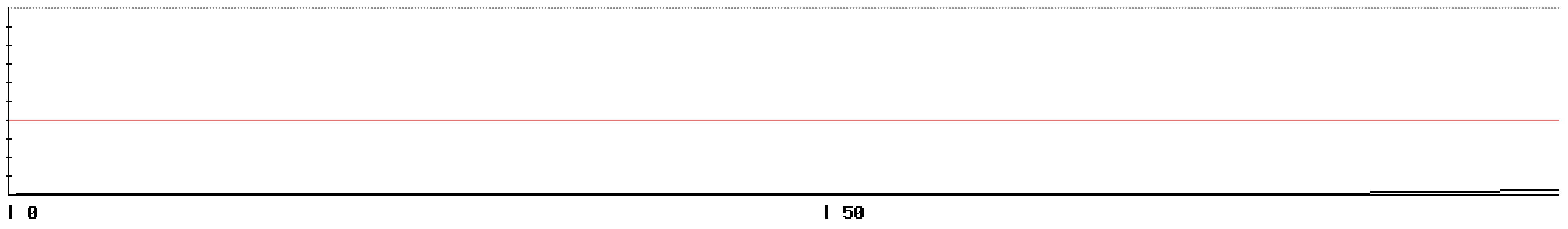

Predictions for Brachypodiumstacei_Brast03G197400.1.p

Viterbi Path

Viterbi Path

Posterior @ 0.4	113 - RKNPRASRRADR - 124
Posterior @ 0.4	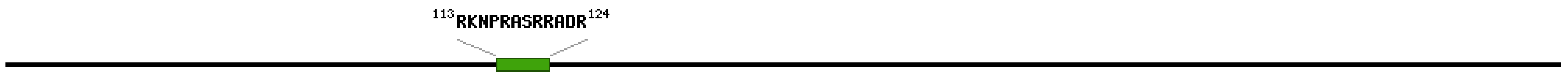
Posterior Graphic. Threshold Marked @ 0.4	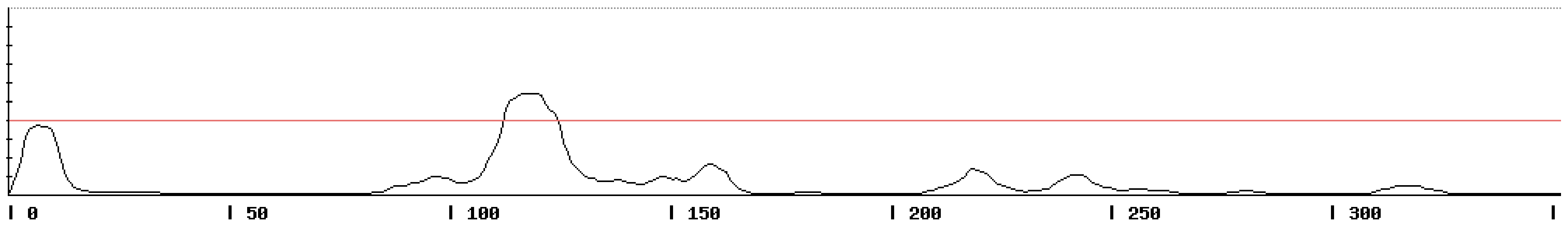

Predictions for Brachypodiumstacei_Brast03G197500.1.p

Viterbi Path	
Viterbi Path	
Posterior @ 0.4	169 - EHGKKRKR - 176
Posterior @ 0.4	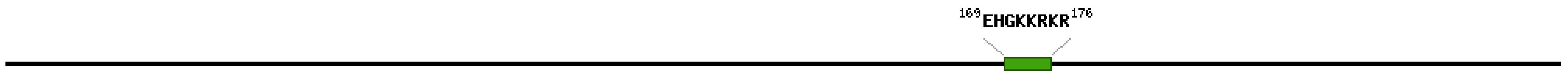
Posterior Graphic. Threshold Marked @ 0.4	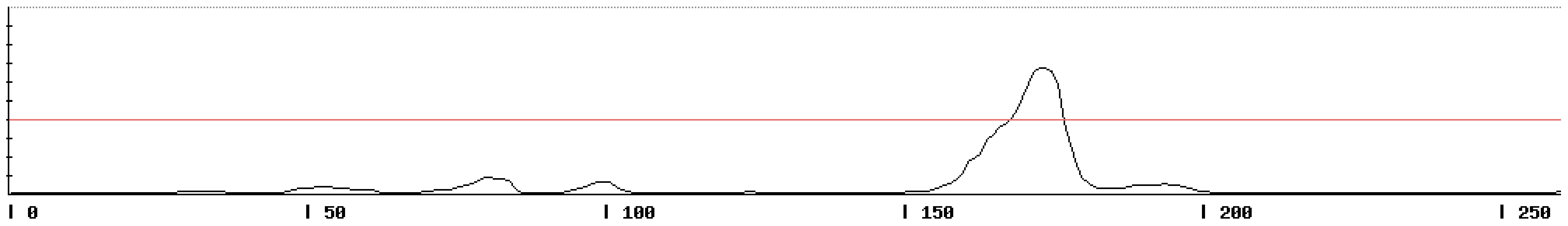

Predictions for Brachypodiumstacei_Brast03G202100.1.p

Viterbi Path	
Viterbi Path	

Posterior @ 0.4	166 - RLGEHGKKRKRE - 177
Posterior @ 0.4	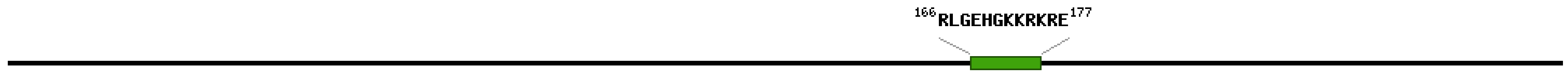
Posterior Graphic. Threshold Marked @ 0.4	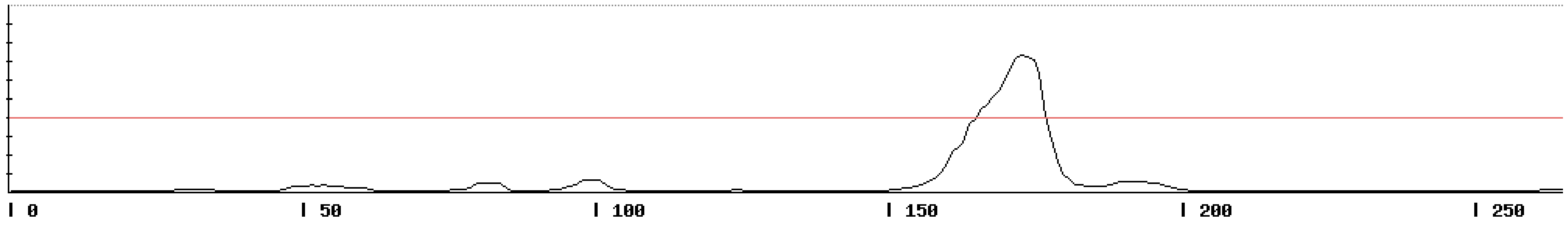

Predictions for Brachypodiumstacei_Brast03G205400.1.p

Viterbi Path	
Viterbi Path	
Posterior @ 0.4	
Posterior @ 0.4	
Posterior Graphic. Threshold Marked @ 0.4	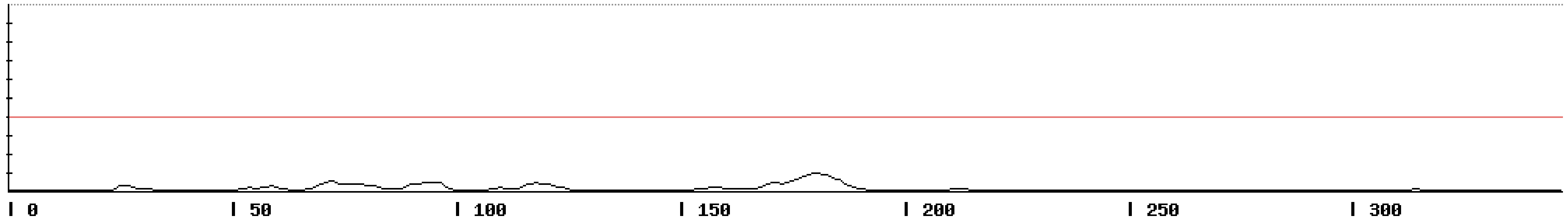

Predictions for Brachypodiumstacei_Brast03G237200.1.p

Viterbi Path

Viterbi Path

Posterior @ 0.4

Posterior @ 0.4

Posterior Graphic. Threshold Marked @ 0.4

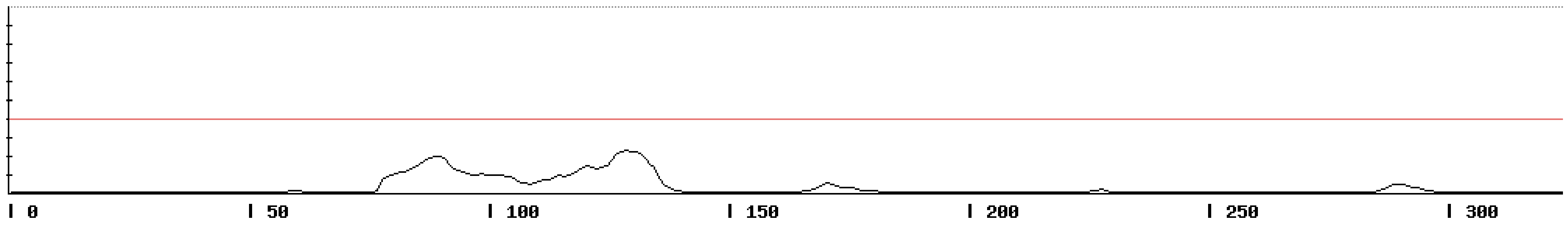

Predictions for Brachypodiumstacei_Brast03G241500.1.p

Viterbi Path

Viterbi Path

Posterior @ 0.4

Posterior @ 0.4

Posterior Graphic. Threshold Marked @ 0.4

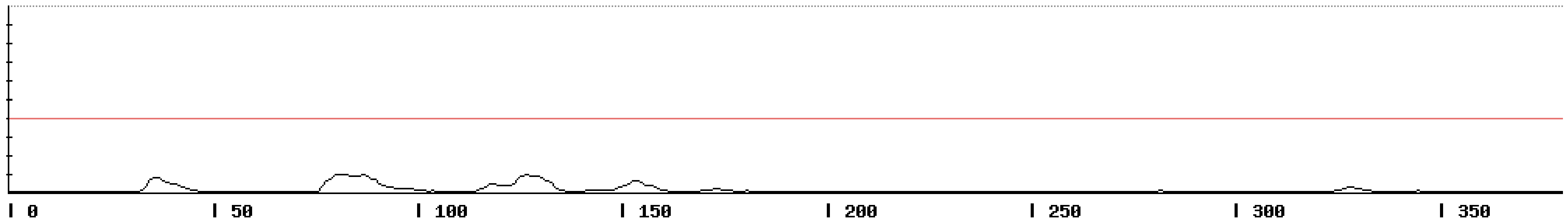

Predictions for Brachypodiumstacei_Brast03G275500.1.p	
Viterbi Path	
Viterbi Path	
Posterior @ 0.4	
Posterior @ 0.4	
Posterior Graphic. Threshold Marked @ 0.4	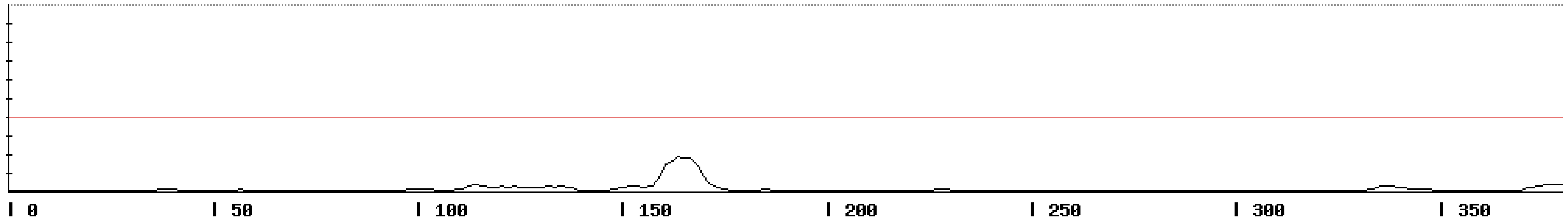

Predictions for Brachypodiumstacei_Brast03G291700.1.p	
Viterbi Path	
Viterbi Path	
Posterior @ 0.4	138 - PRARRGKRG - 146
Posterior @ 0.4	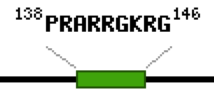
Posterior Graphic. Threshold Marked @ 0.4	

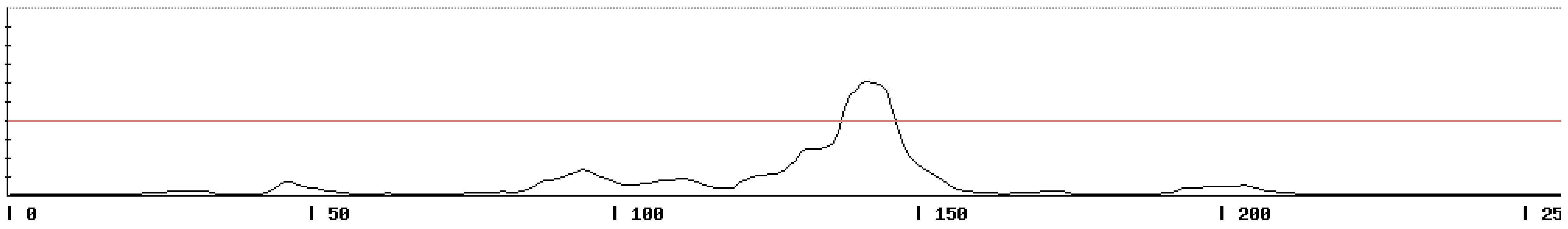

Predictions for Brachypodiumstacei_Brast03G309200.1.p

Viterbi Path	
Viterbi Path	
Posterior @ 0.4	
Posterior @ 0.4	
Posterior Graphic. Threshold Marked @ 0.4	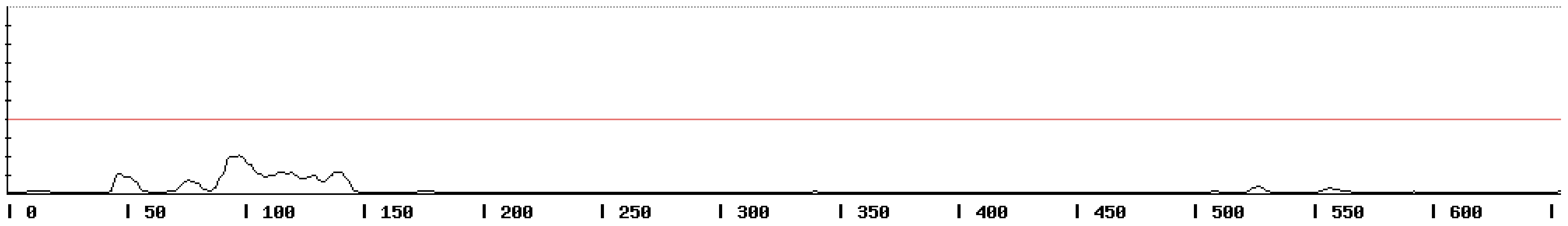

Predictions for Brachypodiumstacei_Brast04G010800.1.p

Viterbi Path	
Viterbi Path	
Posterior @ 0.4	57 - PRERKYEHGKRPSRKTGDRLGRWK - 80
Posterior @ 0.4	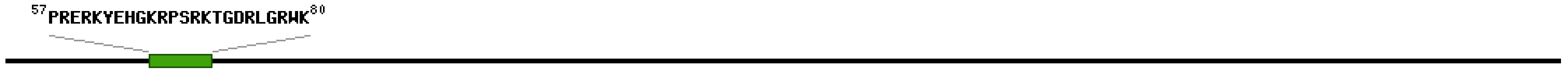

Posterior Graphic. Threshold Marked @ 0.4

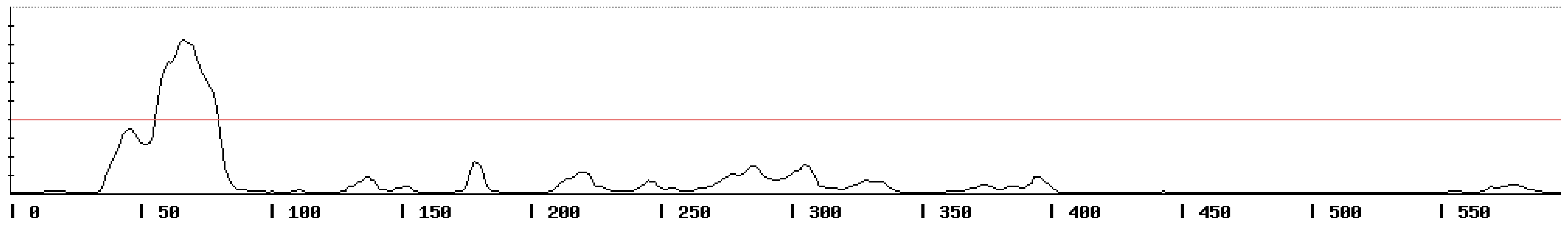

Predictions for Brachypodiumstacei_Brast04G016800.1.p

Viterbi Path	
Viterbi Path	
Posterior @ 0.4	
Posterior @ 0.4	
Posterior Graphic. Threshold Marked @ 0.4	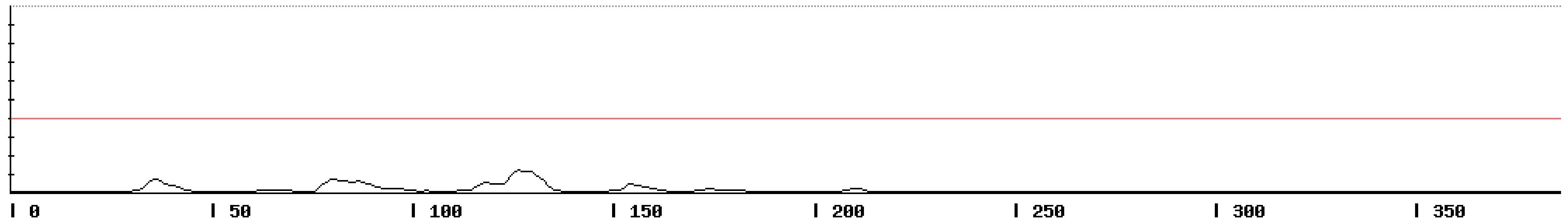

Predictions for Brachypodiumstacei_Brast04G052200.1.p

Viterbi Path	
Viterbi Path	
Posterior @ 0.4	89 - GRGGPGNR - 96
Posterior @ 0.4	

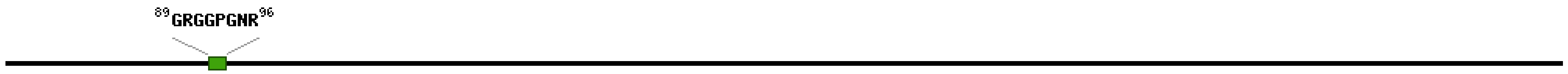

Posterior Graphic. Threshold Marked @ 0.4

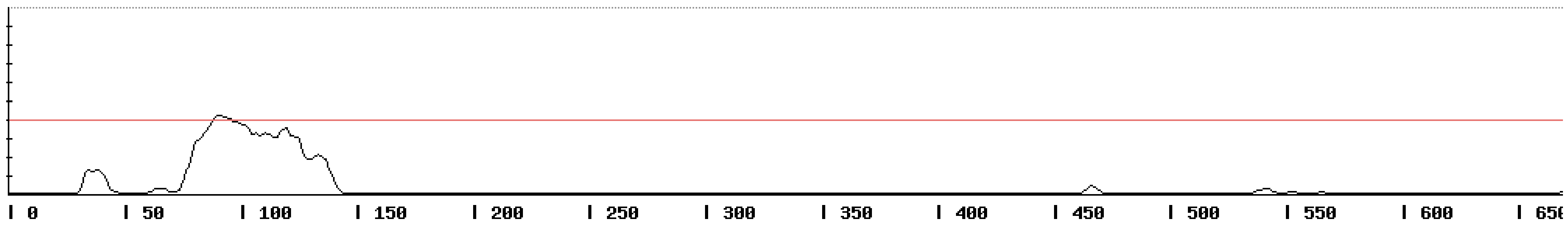

Predictions for Brachypodiumstacei_Brast04G066800.1.p

Viterbi Path	
Viterbi Path	
Posterior @ 0.4	
Posterior @ 0.4	

Posterior Graphic. Threshold Marked @ 0.4

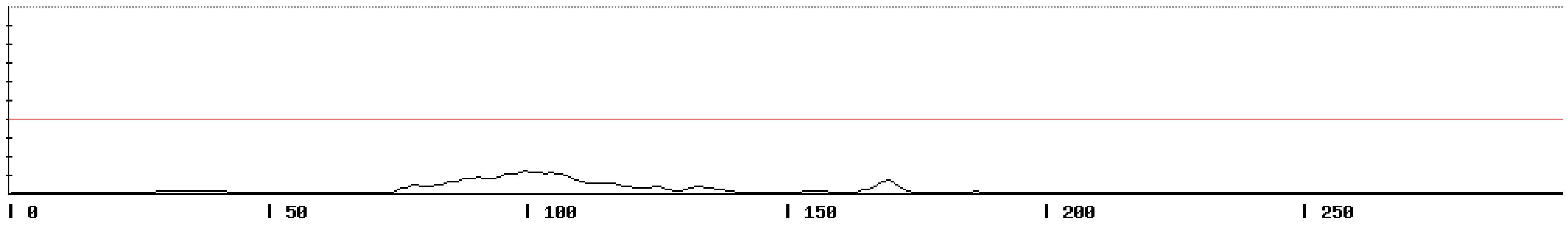

Predictions for Brachypodiumstacei_Brast04G123400.1.p

Viterbi Path	
Viterbi Path	

Posterior @ 0.4	
Posterior @ 0.4	
Posterior Graphic. Threshold Marked @ 0.4	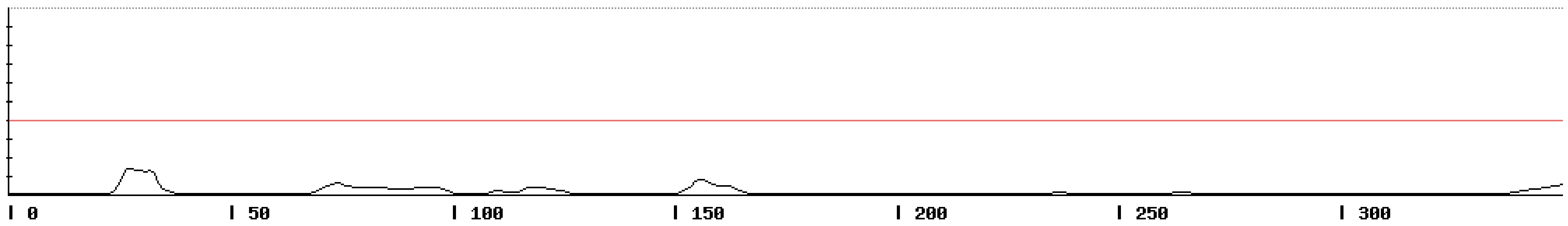

Predictions for Brachypodiumstacei_Brast04G151000.1.p

Viterbi Path	
Viterbi Path	
Posterior @ 0.4	133 - KRKRRR - 138
Posterior @ 0.4	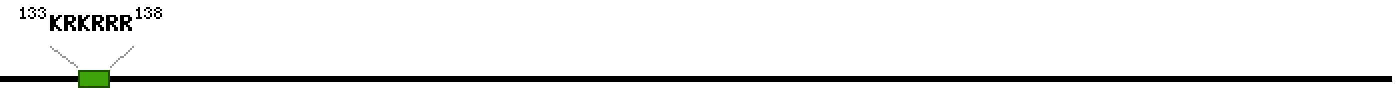
Posterior Graphic. Threshold Marked @ 0.4	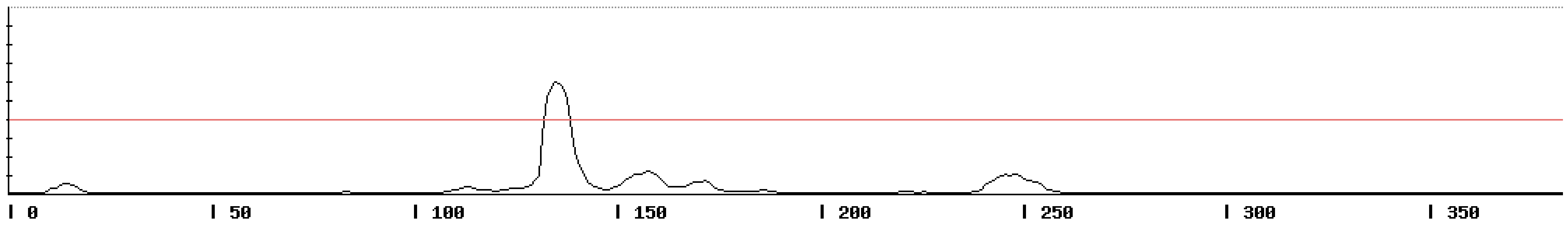

Predictions for Brachypodiumstacei_Brast04G158600.1.p

Viterbi Path	
Viterbi Path	

Posterior @ 0.4

Posterior @ 0.4

Posterior Graphic. Threshold Marked @ 0.4

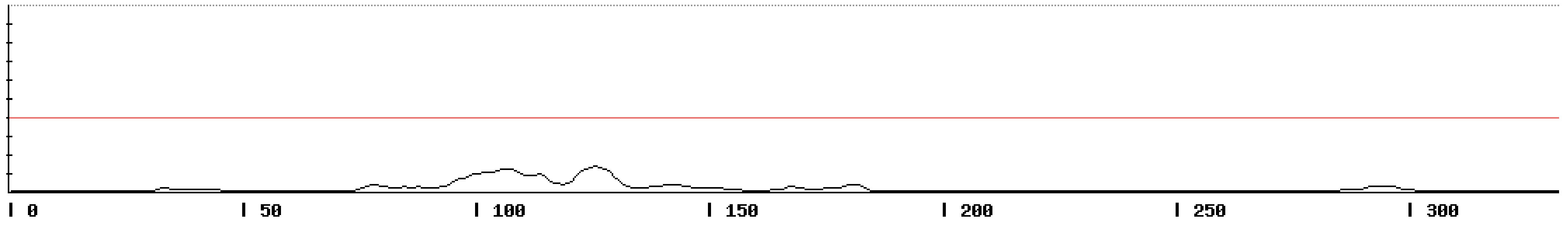

Predictions for Brachypodiumstacei_Brast04G217600.1.p

Viterbi Path

Viterbi Path

Posterior @ 0.4

Posterior @ 0.4

Posterior Graphic. Threshold Marked @ 0.4

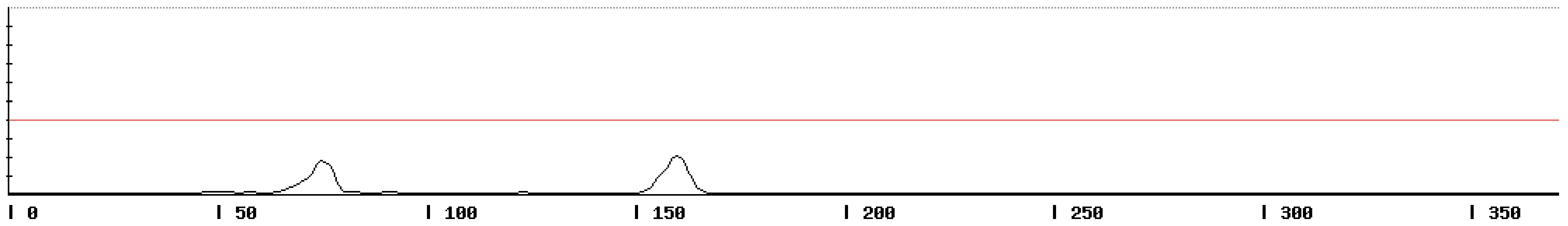

Predictions for Brachypodiumstacei_Brast04G233200.1.p

Viterbi Path

Viterbi Path

Posterior @ 0.4

Posterior @ 0.4

Posterior Graphic. Threshold Marked @ 0.4

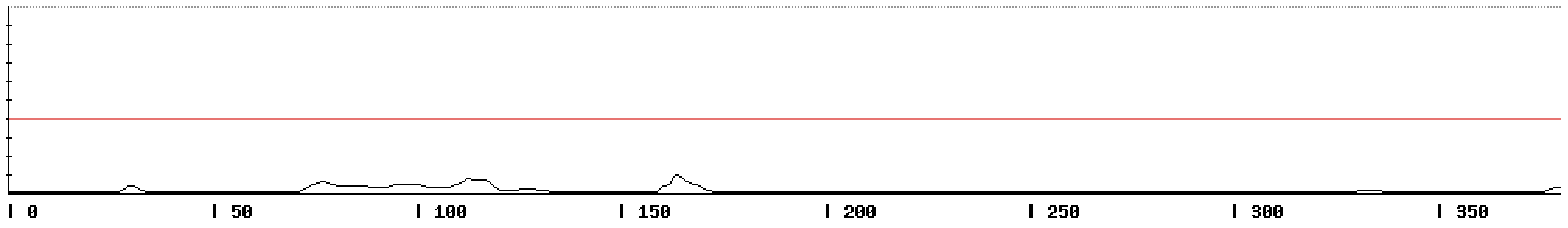

Predictions for Brachypodiumstacei_Brast05G004300.1.p

Viterbi Path

44 - KKKEKKKEEKEKHK - 57

210 - KMHRDGKRRKRPAKELLPKRNNKK - 233

Viterbi Path

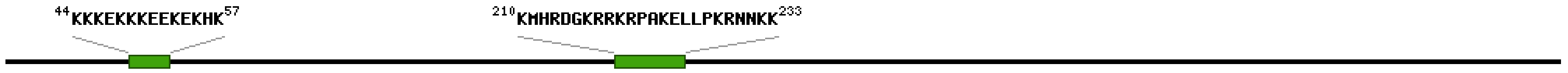

Posterior @ 0.4

43 - EKKKEKKKEEKEKHKH - 58

210 - KMHRDGKRRKRPAKELLPKRNNKKAA - 235

Posterior @ 0.4

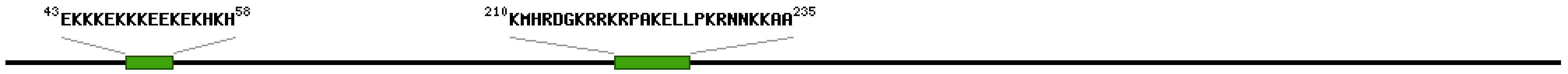

Posterior Graphic. Threshold Marked @ 0.4

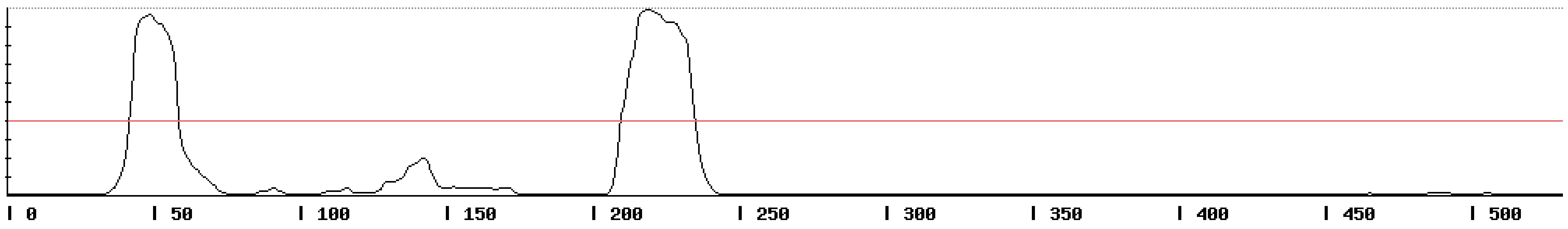

Predictions for Brachypodiumstacei_Brast05G004400.1.p	
Viterbi Path	166 - KKHTDAKGKRRERISDGSNKETPKRKKK - 193
Viterbi Path	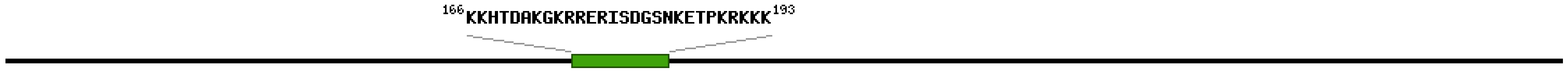
Posterior @ 0.4	166 - KKHTDAKGKRRERISDGSNKETPKRKKKTP - 195
Posterior @ 0.4	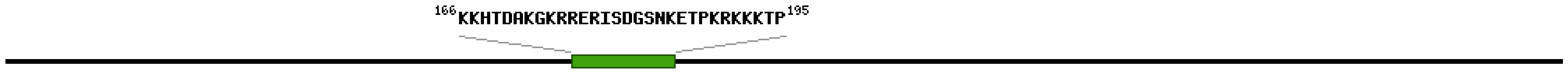
Posterior Graphic. Threshold Marked @ 0.4	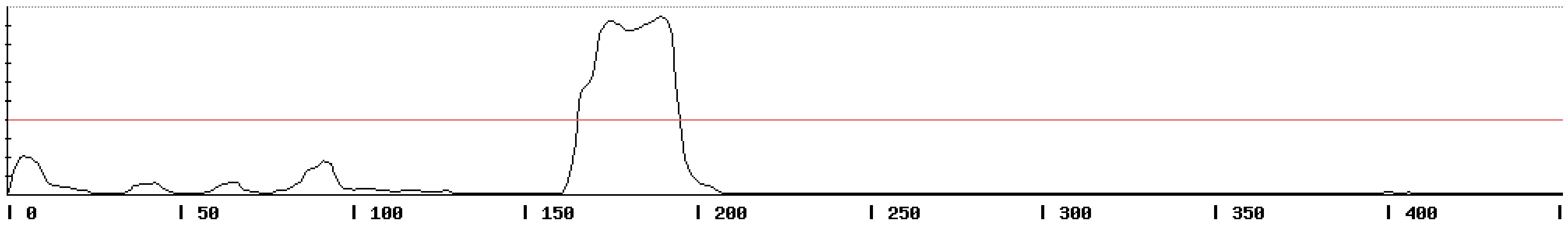

Predictions for Brachypodiumstacei_Brast05G005600.1.p	
Viterbi Path	
Viterbi Path	
Posterior @ 0.4	210 - EPAPVKKRAAPVAEPPRPKKRTRAAAPA - 237
Posterior @ 0.4	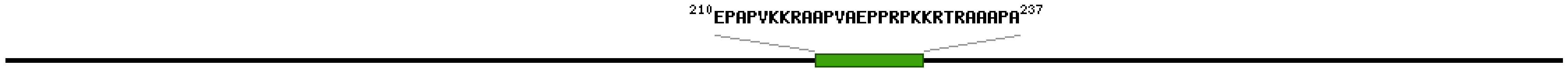

Posterior Graphic. Threshold Marked @ 0.4

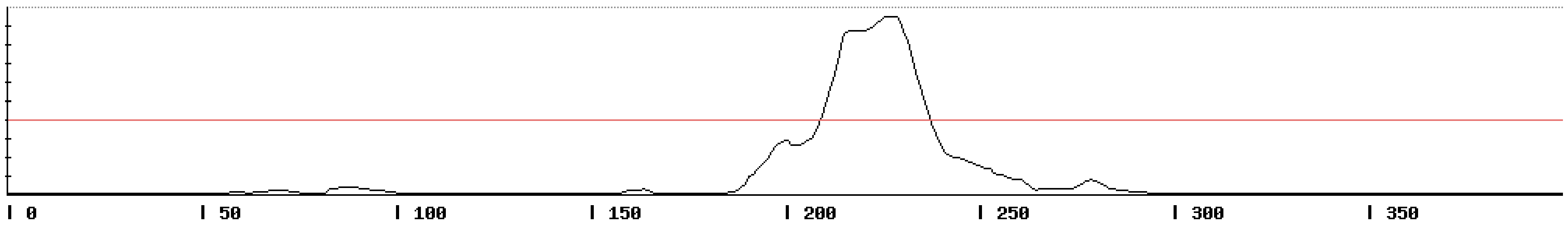

Predictions for Brachypodiumstacei_Brast05G007900.1.p

Viterbi Path	
Viterbi Path	
Posterior @ 0.4	181 - EPAPVKKRAAPVAEPPRPKKRTRAAAPA - 208
Posterior @ 0.4	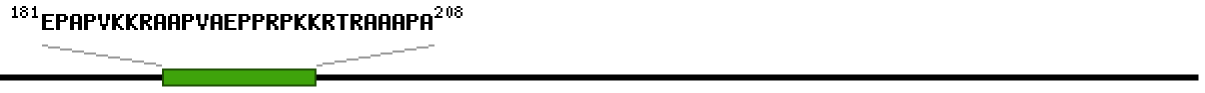
Posterior Graphic. Threshold Marked @ 0.4	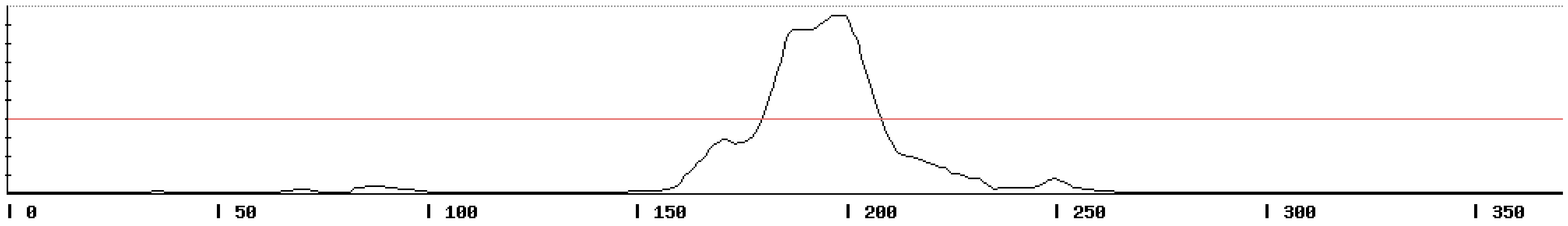

Predictions for Brachypodiumstacei_Brast05G029300.1.p

Viterbi Path	
Viterbi Path	
Posterior @ 0.4	164 - RFEKARGRRAGKL - 176
Posterior @ 0.4	

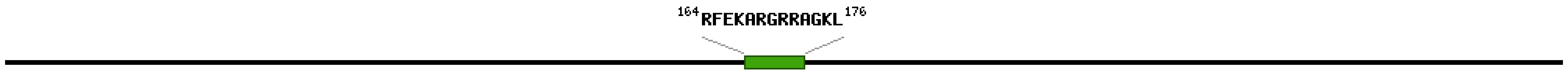

Posterior Graphic. Threshold Marked @ 0.4

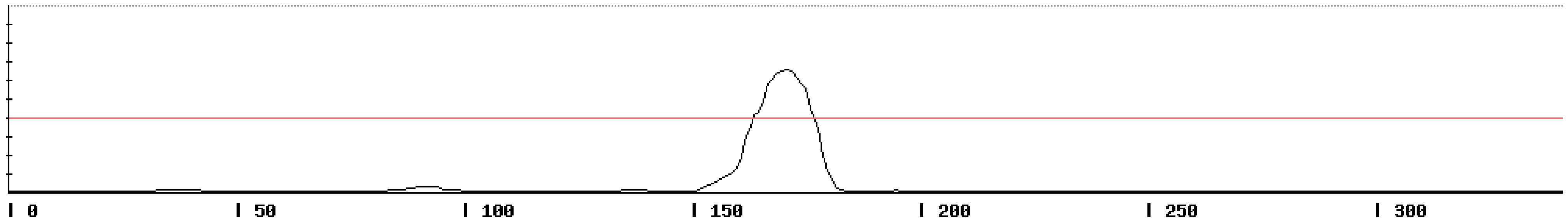

Predictions for Brachypodiumstacei_Brast05G029400.1.p

Viterbi Path

Viterbi Path

Posterior @ 0.4

Posterior @ 0.4

Posterior Graphic. Threshold Marked @ 0.4

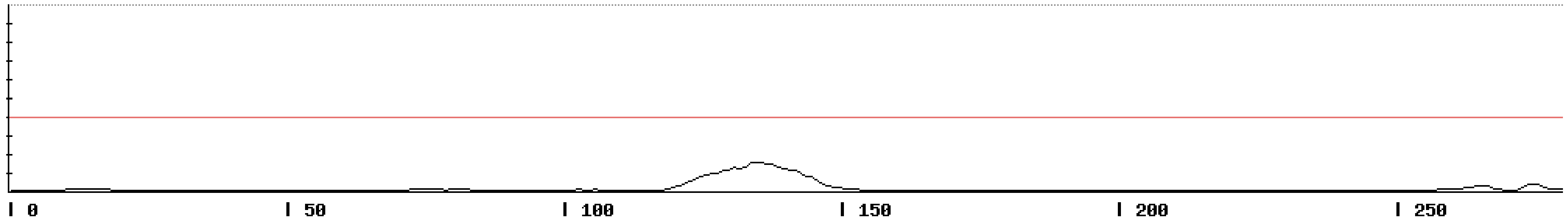

Predictions for Brachypodiumstacei_Brast05G030000.1.p

Viterbi Path

Viterbi Path

Posterior @ 0.4 164 - RFEKARGRRAGKL - 176

Posterior @ 0.4

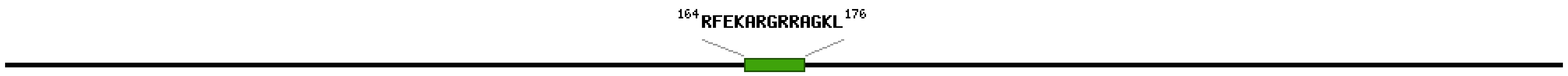

Posterior Graphic. Threshold Marked @ 0.4

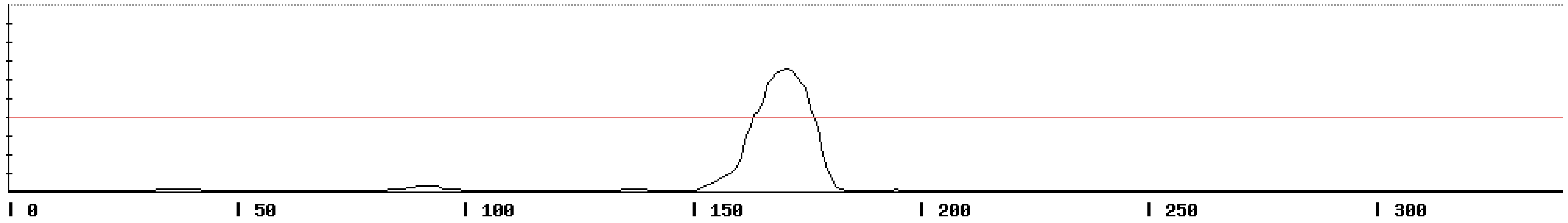

Predictions for Brachypodiumstacei_Brast05G035500.1.p

Viterbi Path

Viterbi Path

Posterior @ 0.4 209 - AKKNPR - 214

Posterior @ 0.4

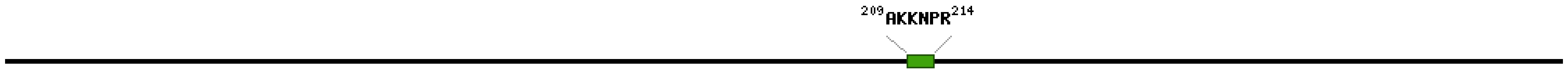

Posterior Graphic. Threshold Marked @ 0.4

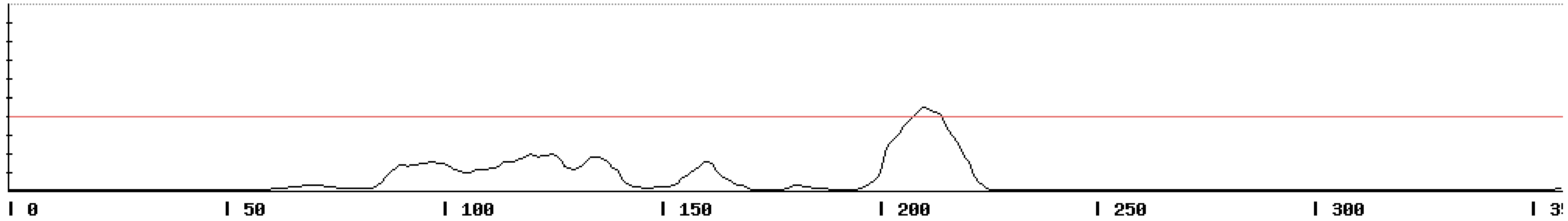

Predictions for Brachypodiumstacei_Brast05G067100.1.p

Viterbi Path

Viterbi Path

Posterior @ 0.4

Posterior @ 0.4

Posterior Graphic. Threshold Marked @ 0.4

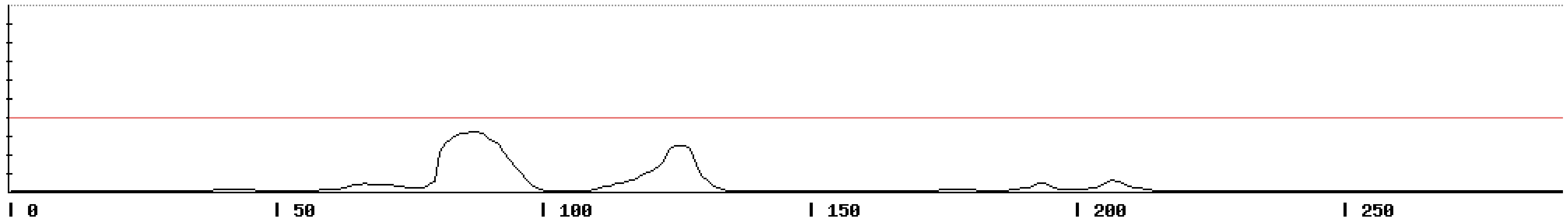

Predictions for Brachypodiumstacei_Brast05G124400.1.p

Viterbi Path

Viterbi Path

Posterior @ 0.4

Posterior @ 0.4

Posterior Graphic. Threshold Marked @ 0.4

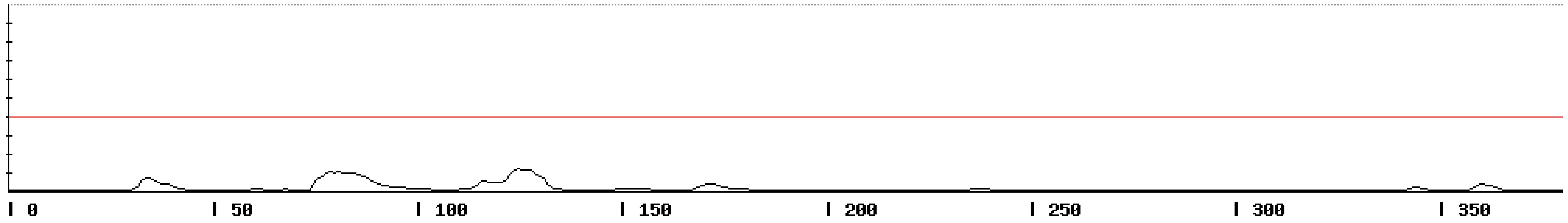

Predictions for Brachypodiumstacei_Brast05G168300.1.p

Viterbi Path	
Viterbi Path	
Posterior @ 0.4	
Posterior @ 0.4	
Posterior Graphic. Threshold Marked @ 0.4	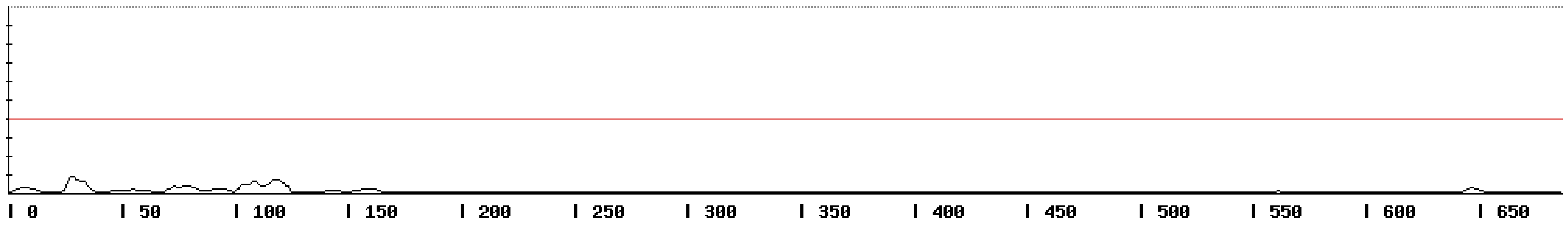

Predictions for Brachypodiumstacei_Brast05G169900.1.p

Viterbi Path	
Viterbi Path	
Posterior @ 0.4	
Posterior @ 0.4	
Posterior Graphic. Threshold Marked @ 0.4	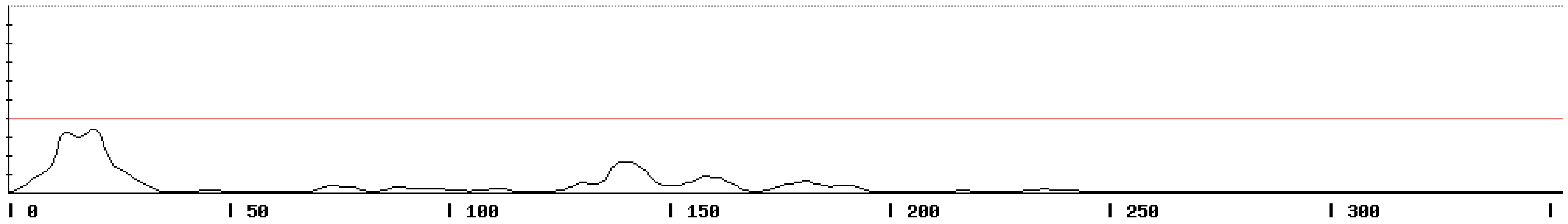

Predictions for Brachypodiumstacei_Brast05G177000.1.p

Viterbi Path	
Viterbi Path	
Posterior @ 0.4	89 - GSRRR - 93
Posterior @ 0.4	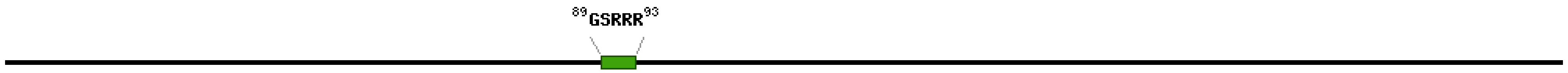
Posterior Graphic. Threshold Marked @ 0.4	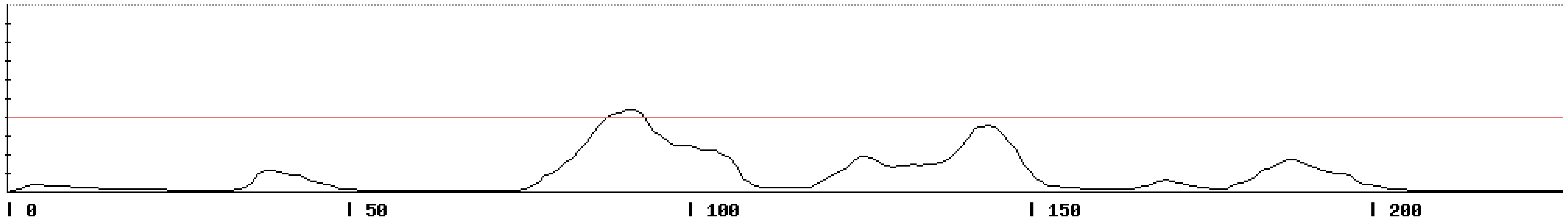

Predictions for Brachypodiumstacei_Brast05G207900.1.p

Viterbi Path	
Viterbi Path	
Posterior @ 0.4	
Posterior @ 0.4	
Posterior Graphic. Threshold Marked @ 0.4	

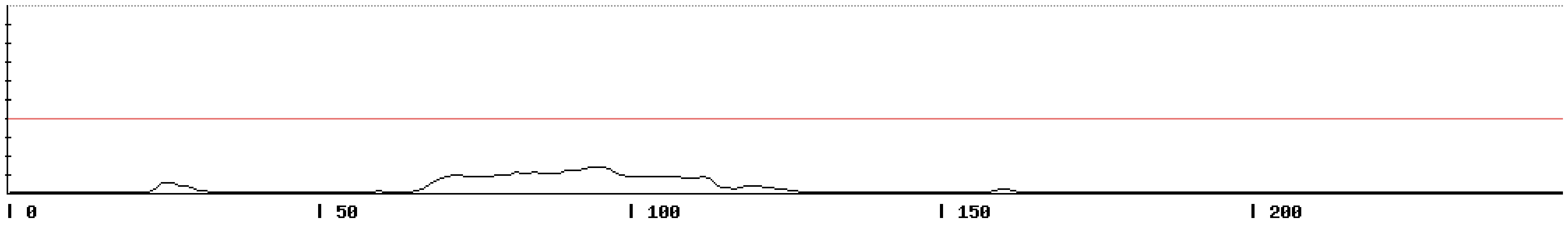

Predictions for Brachypodiumstacei_Brast05G208100.1.p

Viterbi Path

Viterbi Path

Posterior @ 0.4

Posterior @ 0.4

Posterior Graphic. Threshold Marked @ 0.4

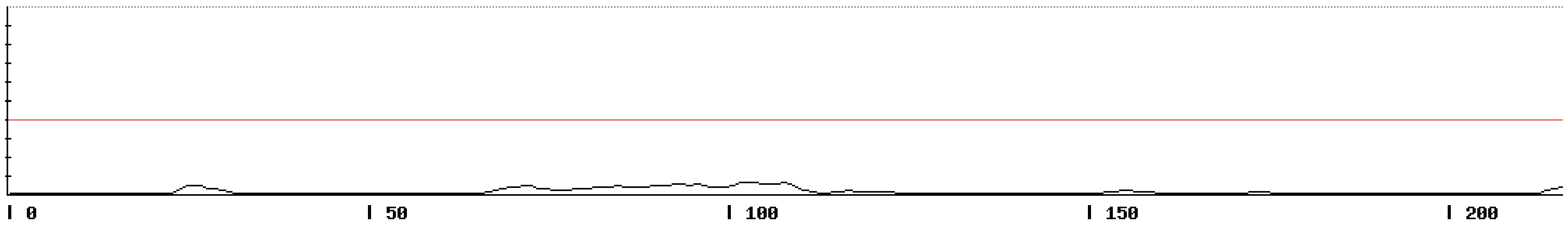

Predictions for Brachypodiumstacei_Brast05G253000.1.p

Viterbi Path

Viterbi Path

Posterior @ 0.4

4 - KNKNRKKRA - 12

114 - SKKPPAPGARRRADRR - 129

Posterior @ 0.4

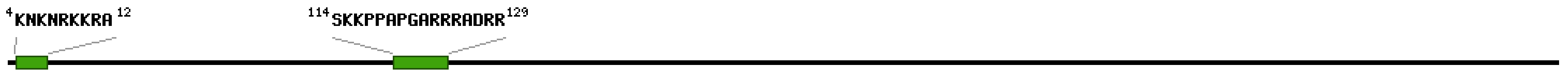

Posterior Graphic. Threshold Marked @ 0.4

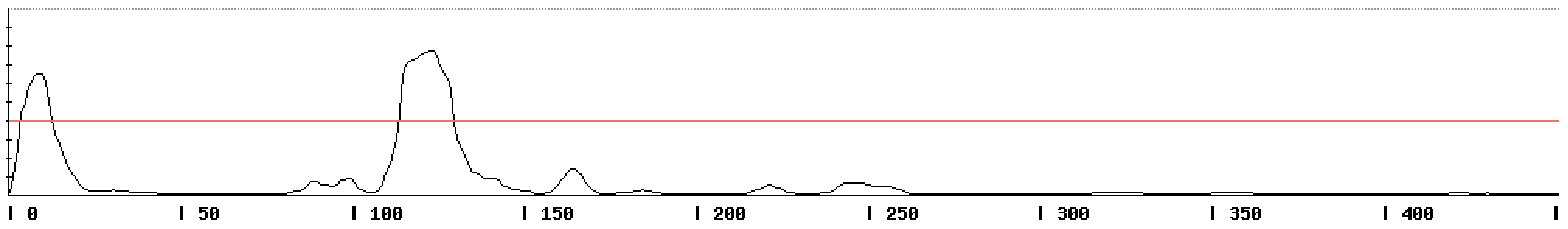

Predictions for Brachypodiumstacei_Brast05G265600.1.p

Viterbi Path

Viterbi Path

Posterior @ 0.4

Posterior @ 0.4

Posterior Graphic. Threshold Marked @ 0.4

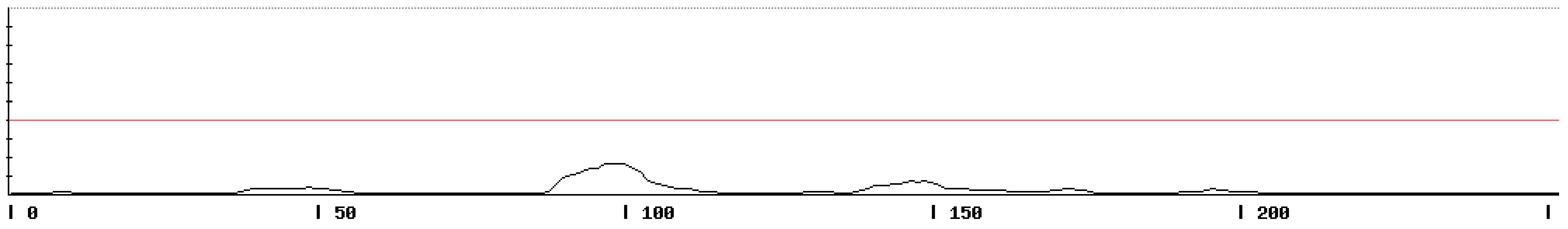

Predictions for Brachypodiumstacei_Brast05G275400.1.p

Viterbi Path

Viterbi Path

Posterior @ 0.4	111 - KKGEEKKLSFKSKKD - 125
Posterior @ 0.4	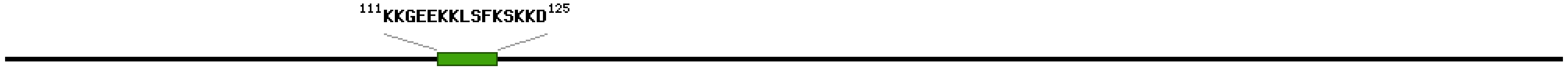
Posterior Graphic. Threshold Marked @ 0.4	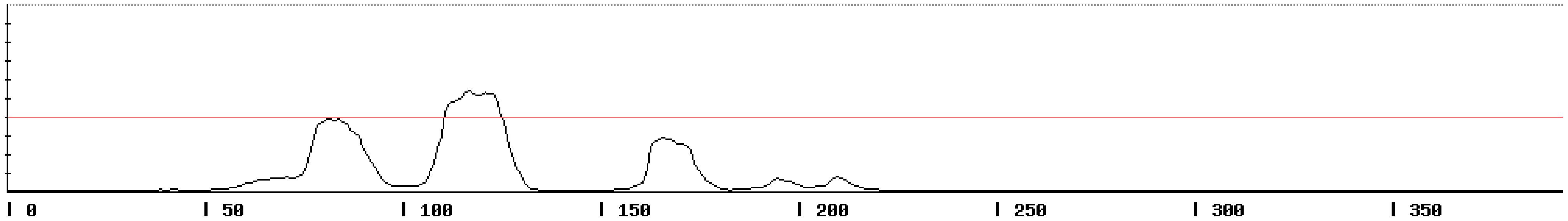

Predictions for Brachypodiumstacei_Brast05G276000.1.p	
Viterbi Path	
Viterbi Path	
Posterior @ 0.4	111 - KKGEEKKLSFKSKKD - 125
Posterior @ 0.4	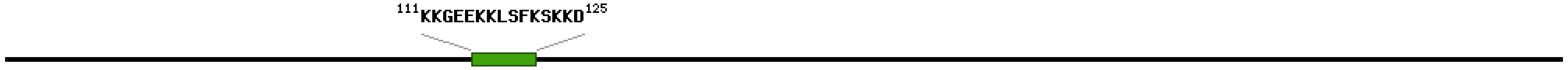
Posterior Graphic. Threshold Marked @ 0.4	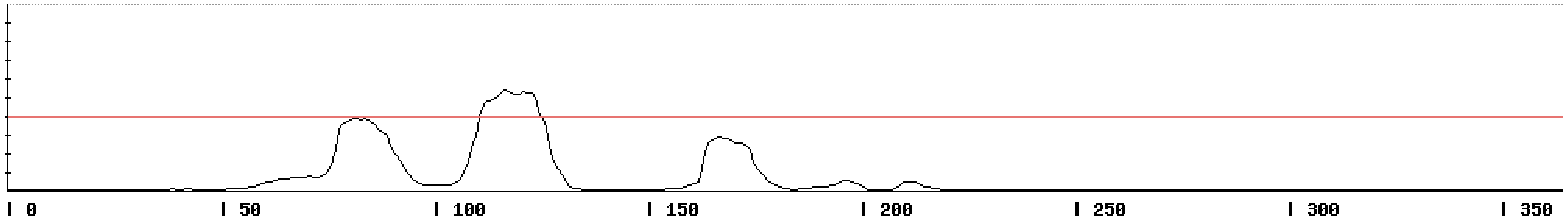

Predictions for Brachypodiumstacei_Brast05G276100.1.p	
Viterbi Path	
Viterbi Path	

Posterior @ 0.4

Posterior @ 0.4

Posterior Graphic. Threshold Marked @ 0.4

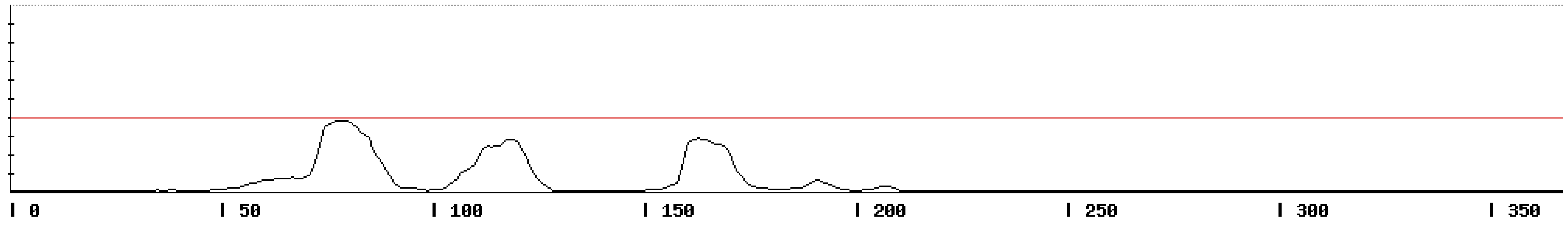

Predictions for Brachypodiumstacei_Brast05G280400.1.p

Viterbi Path

Viterbi Path

Posterior @ 0.4

Posterior @ 0.4

Posterior Graphic. Threshold Marked @ 0.4

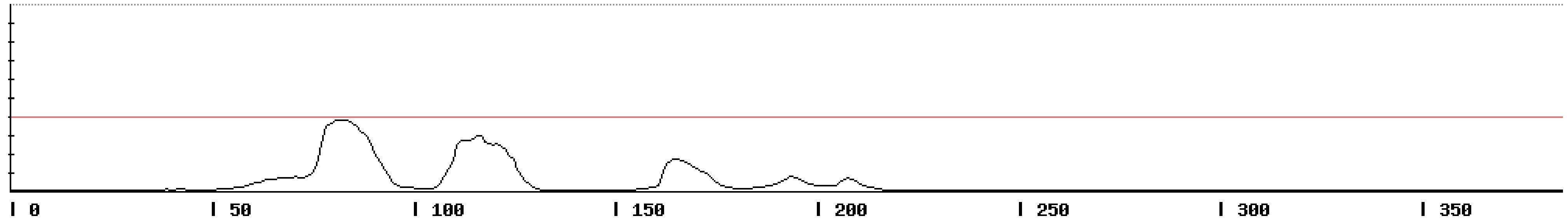

Predictions for Brachypodiumstacei_Brast05G287500.1.p

Viterbi Path

Viterbi Path

Posterior @ 0.4

Posterior @ 0.4

Posterior Graphic. Threshold Marked @ 0.4

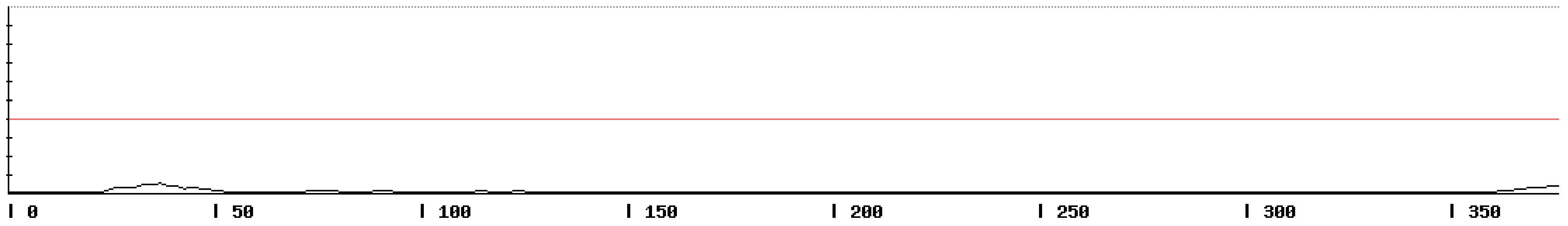

Predictions for Brachypodiumstacei_Brast05G287600.1.p

Viterbi Path

Viterbi Path

Posterior @ 0.4

Posterior @ 0.4

Posterior Graphic. Threshold Marked @ 0.4

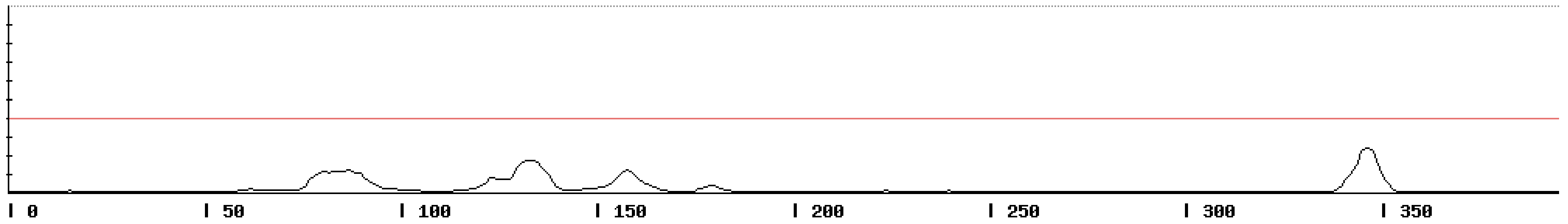

Predictions for Brachypodiumstacei_Brast06G009100.1.p

Viterbi Path	
Viterbi Path	
Posterior @ 0.4	
Posterior @ 0.4	
Posterior Graphic. Threshold Marked @ 0.4	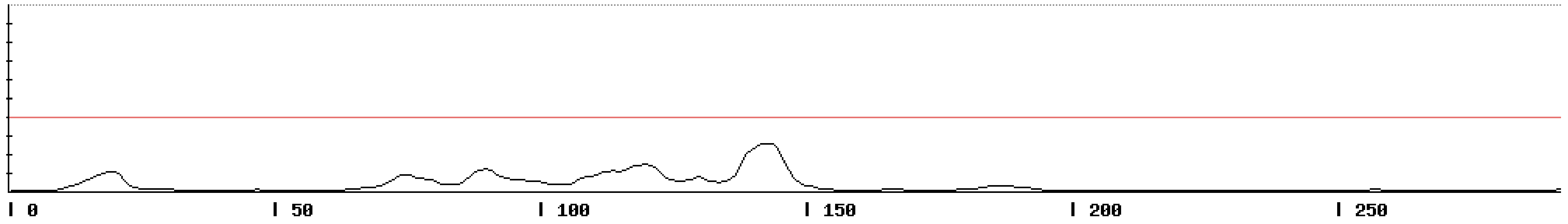

Predictions for Brachypodiumstacei_Brast06G009400.1.p

Viterbi Path	
Viterbi Path	
Posterior @ 0.4	
Posterior @ 0.4	
Posterior Graphic. Threshold Marked @ 0.4	

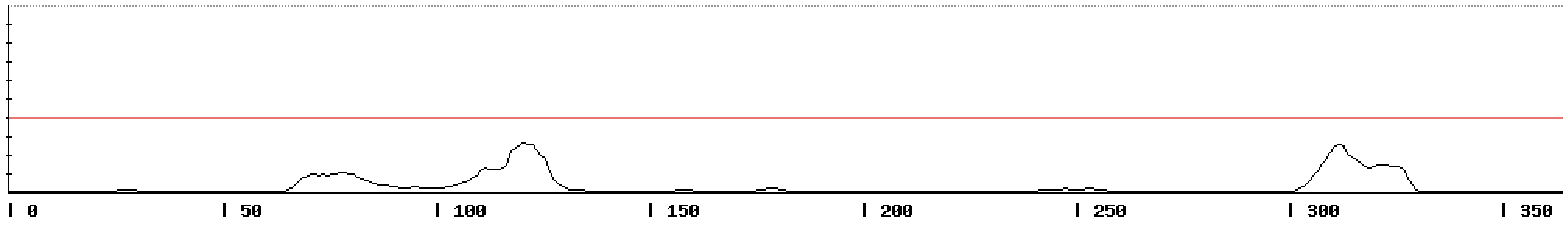

Predictions for Brachypodiumstacei_Brast06G038200.1.p

Viterbi Path

60 - PPAGRRRGRPRGSTNKPKVANKAAAKPKPK - 89

Viterbi Path

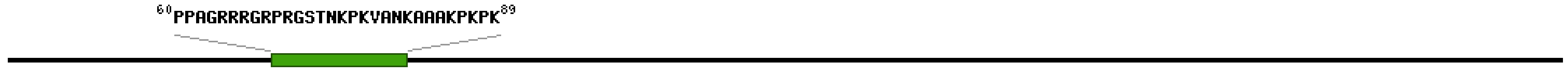

Posterior @ 0.4

57 - PGNPPAGRRRGRPRGSTNKPKVANKAAAKPKPKLD - 91

280 - ERDPKRRKASRRN - 292

Posterior @ 0.4

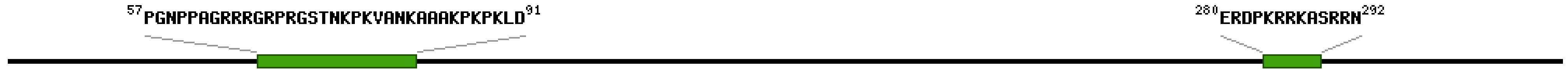

Posterior Graphic. Threshold Marked @ 0.4

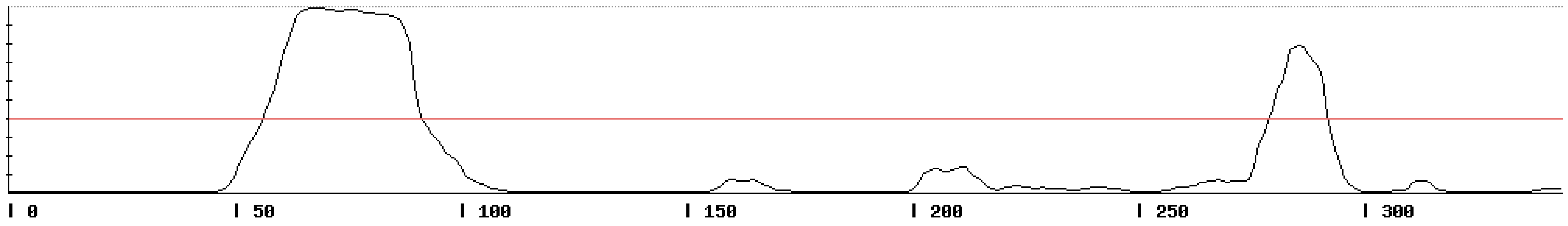

Predictions for Brachypodiumstacei_Brast06G046700.1.p

Viterbi Path

Viterbi Path

Posterior @ 0.4

Posterior @ 0.4

Posterior Graphic. Threshold Marked @ 0.4

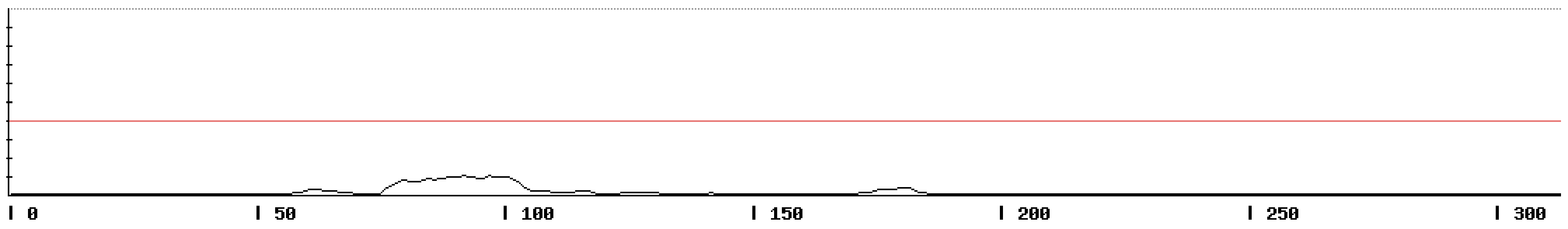

Predictions for Brachypodiumstacei_Brast06G046900.1.p

Viterbi Path

Viterbi Path

Posterior @ 0.4

Posterior @ 0.4

Posterior Graphic. Threshold Marked @ 0.4

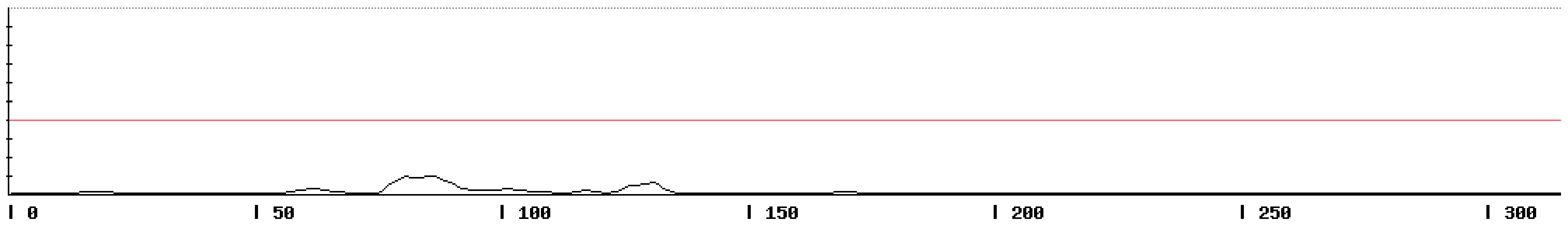

Predictions for Brachypodiumstacei_Brast06G088900.1.p

Viterbi Path

Viterbi Path

Posterior @ 0.4	
Posterior @ 0.4	
Posterior Graphic. Threshold Marked @ 0.4	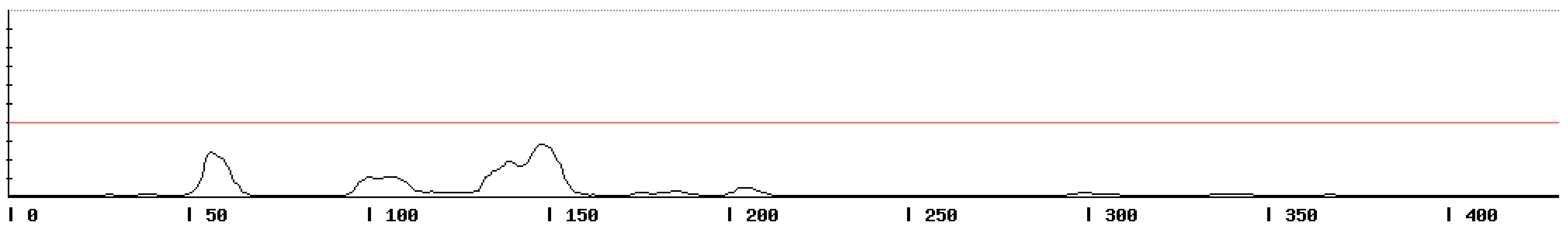

Predictions for Brachypodiumstacei_Brast06G167100.1.p

Viterbi Path	
Viterbi Path	
Posterior @ 0.4	
Posterior @ 0.4	
Posterior Graphic. Threshold Marked @ 0.4	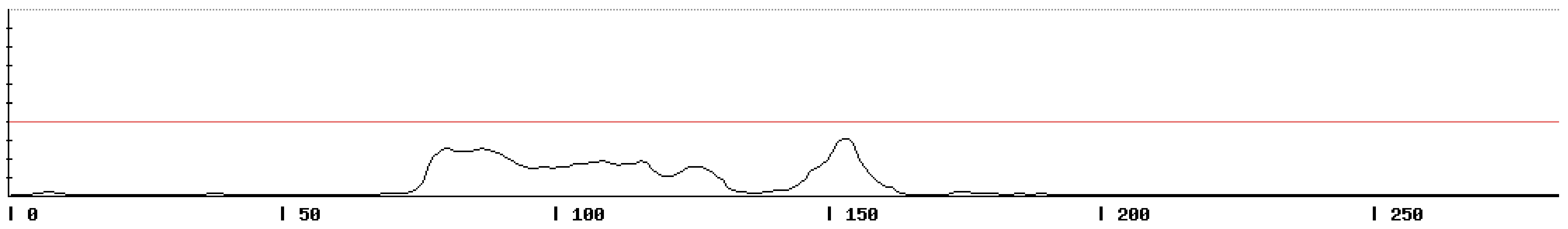

Predictions for Brachypodiumstacei_Brast06G224900.1.p

Viterbi Path	
Viterbi Path	

Posterior @ 0.4

Posterior @ 0.4

Posterior Graphic. Threshold Marked @ 0.4

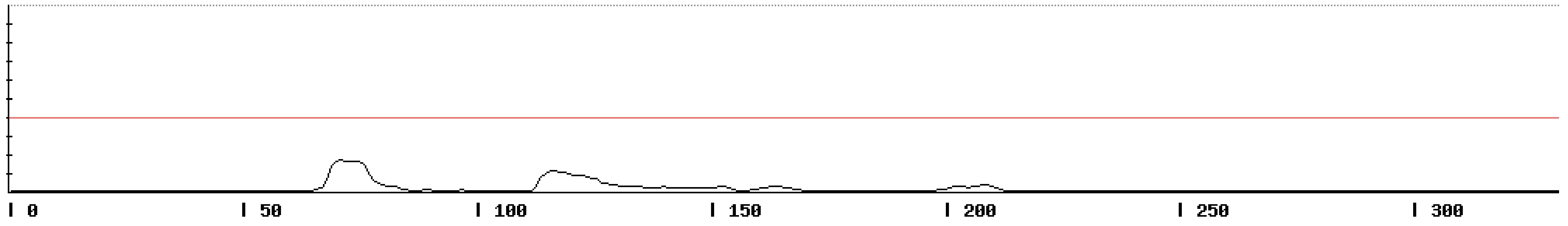

Predictions for Brachypodiumstacei_Brast07G003500.1.p

Viterbi Path

Viterbi Path

Posterior @ 0.4

Posterior @ 0.4

Posterior Graphic. Threshold Marked @ 0.4

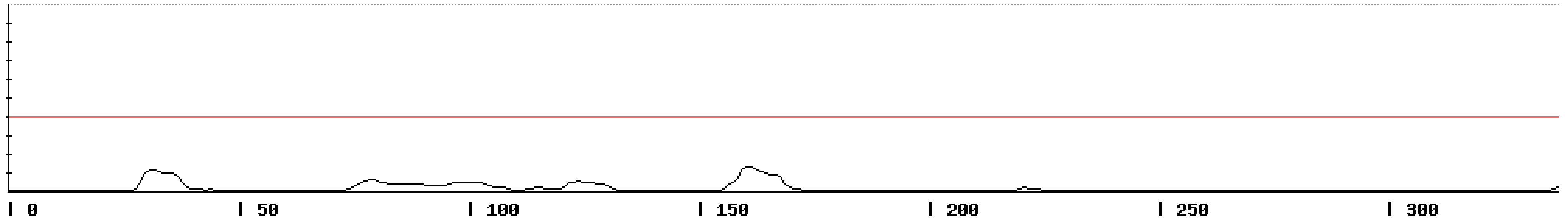

Predictions for Brachypodiumstacei_Brast07G026300.1.p

Viterbi Path	
Viterbi Path	
Posterior @ 0.4	
Posterior @ 0.4	
Posterior Graphic. Threshold Marked @ 0.4	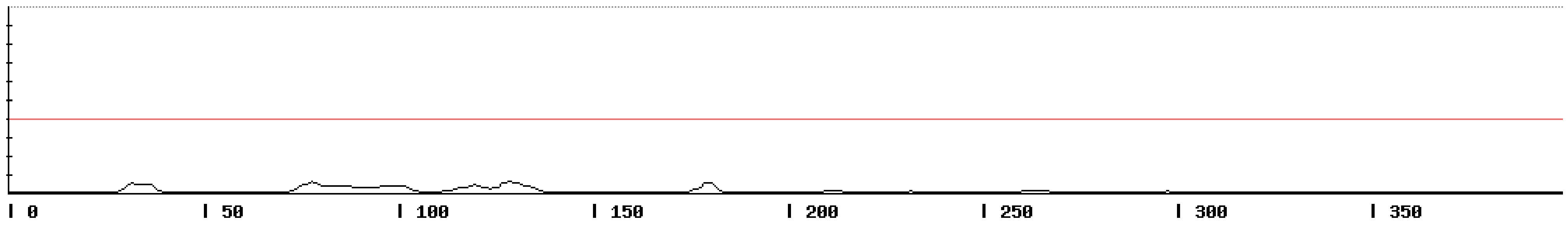

Predictions for Brachypodiumstacei_Brast07G098400.1.p

Viterbi Path	
Viterbi Path	
Posterior @ 0.4	
Posterior @ 0.4	
Posterior Graphic. Threshold Marked @ 0.4	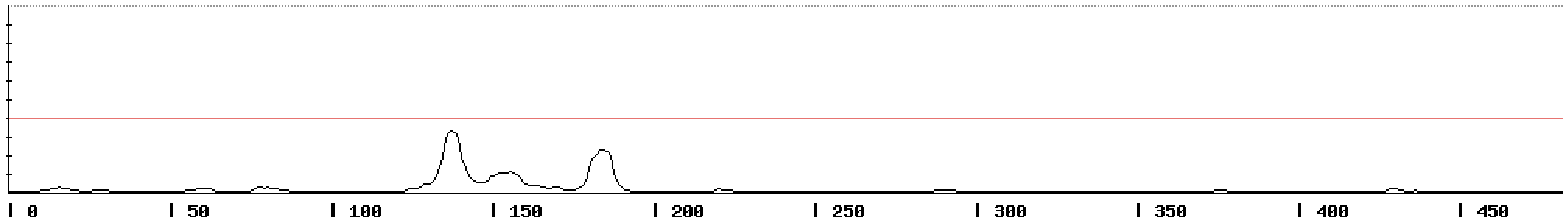

Predictions for Brachypodiumstacei_Brast07G127300.1.p

Viterbi Path	
Viterbi Path	
Posterior @ 0.4	
Posterior @ 0.4	
Posterior Graphic. Threshold Marked @ 0.4	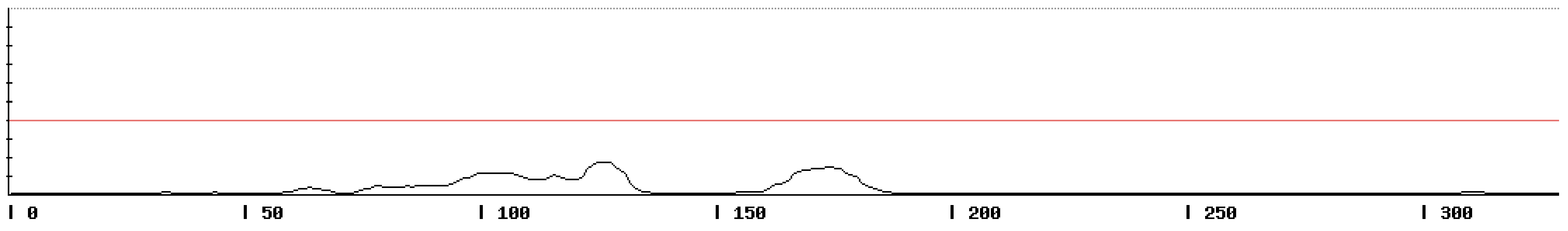

Predictions for Brachypodiumstacei_Brast07G134300.1.p

Viterbi Path	
Viterbi Path	
Posterior @ 0.4	
Posterior @ 0.4	
Posterior Graphic. Threshold Marked @ 0.4	

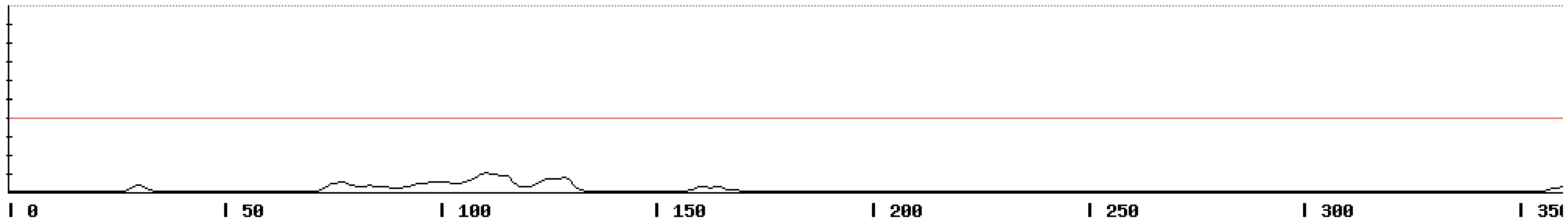

Predictions for Brachypodiumstacei_Brast07G145000.1.p

Viterbi Path	
Viterbi Path	
Posterior @ 0.4	140 - RKRRK - 144
Posterior @ 0.4	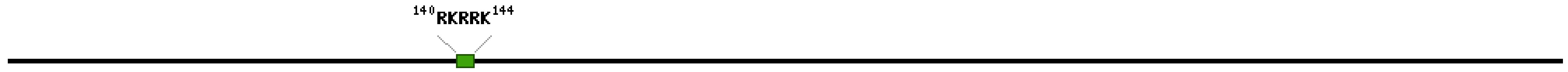
Posterior Graphic. Threshold Marked @ 0.4	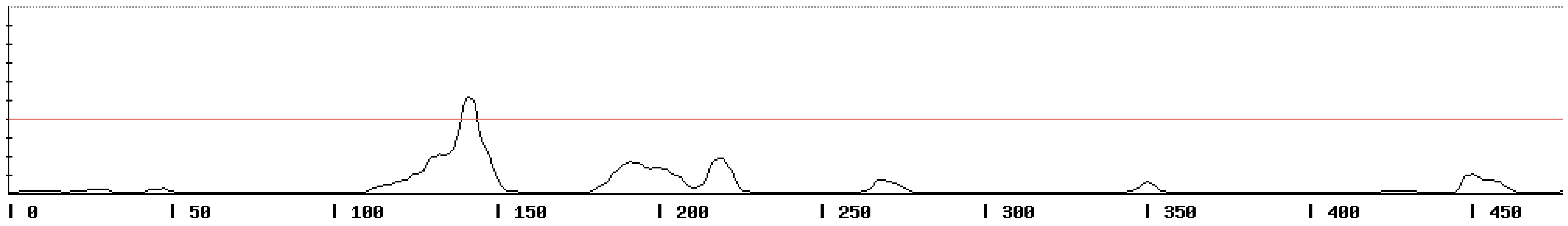

Predictions for Brachypodiumstacei_Brast07G209700.1.p

Viterbi Path	
Viterbi Path	
Posterior @ 0.4	
Posterior @ 0.4	

Posterior Graphic. Threshold Marked @ 0.4

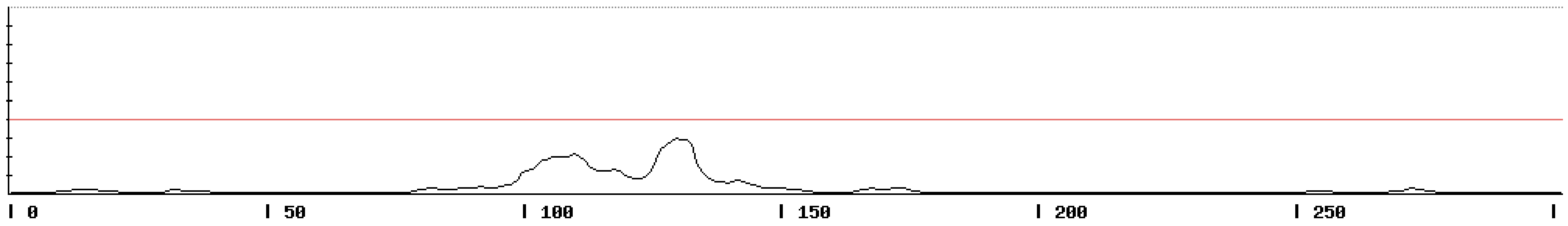

Predictions for Brachypodiumstacei_Brast07G238700.1.p

Viterbi Path

Viterbi Path

Posterior @ 0.4

Posterior @ 0.4

Posterior Graphic. Threshold Marked @ 0.4

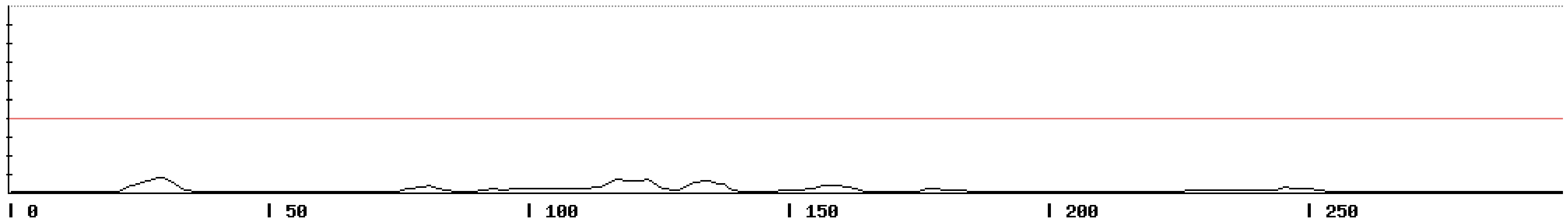

Predictions for Brachypodiumstacei_Brast08G069400.1.p

Viterbi Path

Viterbi Path

Posterior @ 0.4 180 - RSALKRK - 186

Posterior @ 0.4

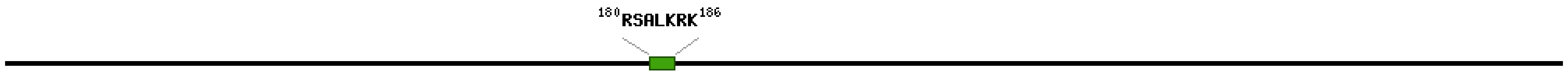

Posterior Graphic. Threshold Marked @ 0.4	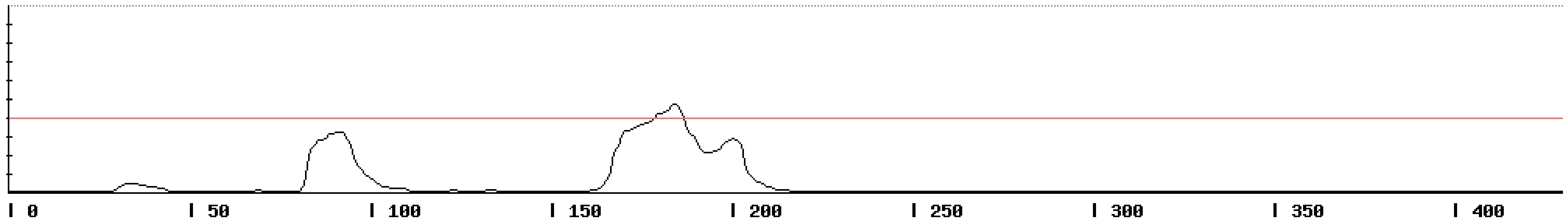

Predictions for Brachypodiumstacei_Brast08G105300.1.p	
Viterbi Path	
Viterbi Path	
Posterior @ 0.4	203 - RGQKRKRRA - 211
Posterior @ 0.4	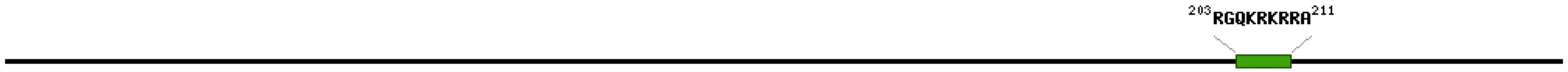
Posterior Graphic. Threshold Marked @ 0.4	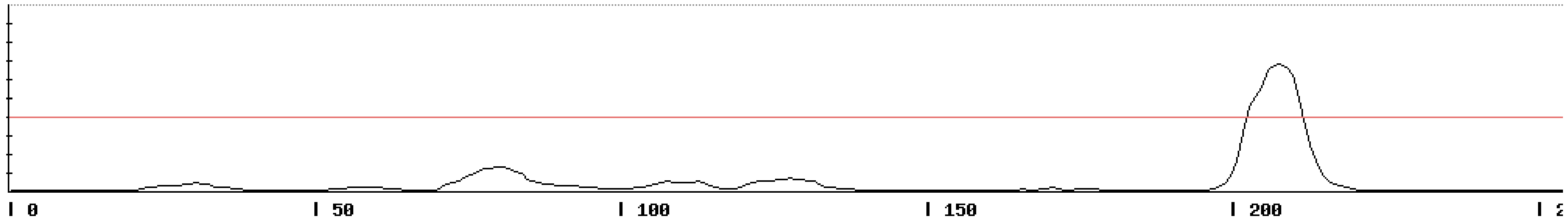

Predictions for Brachypodiumstacei_Brast08G113200.1.p	
Viterbi Path	
Viterbi Path	

Posterior @ 0.4

Posterior @ 0.4

Posterior Graphic. Threshold Marked @ 0.4

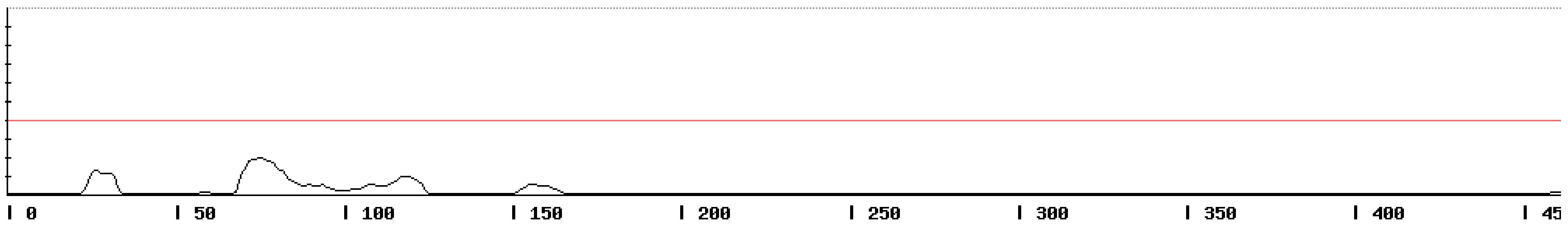

Predictions for Brachypodiumstacei_Brast08G114900.1.p

Viterbi Path

Viterbi Path

Posterior @ 0.4

Posterior @ 0.4

Posterior Graphic. Threshold Marked @ 0.4

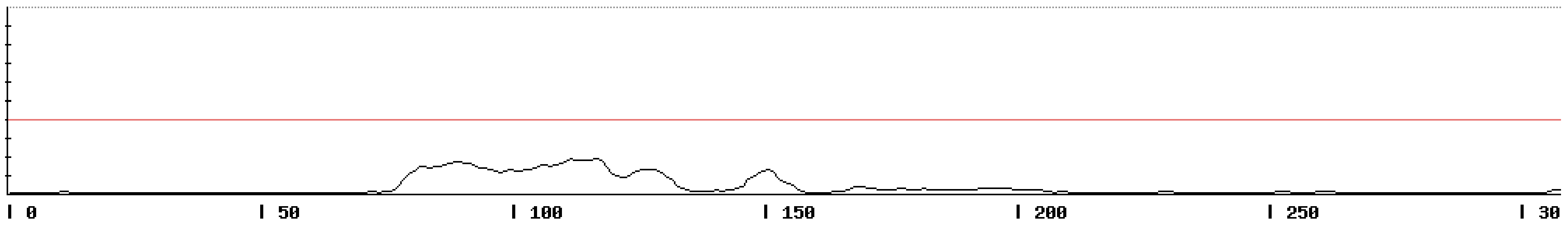

Predictions for Brachypodiumstacei_Brast08G116600.1.p

Viterbi Path

Viterbi Path

Posterior @ 0.4

Posterior @ 0.4

Posterior Graphic. Threshold Marked @ 0.4

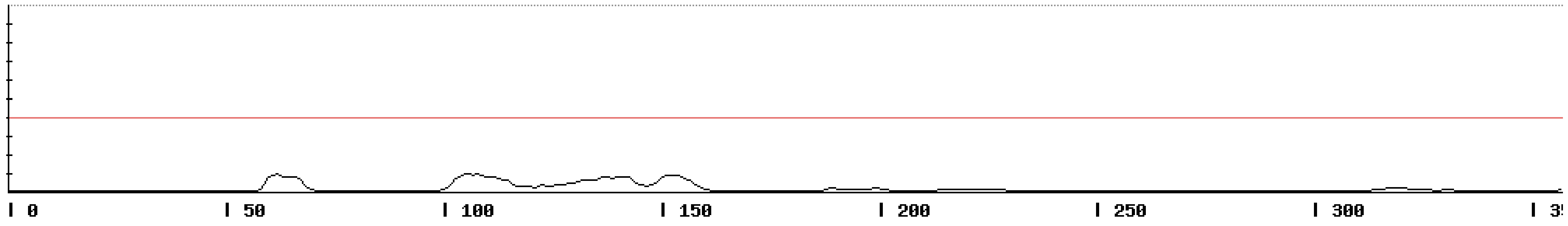

Predictions for Brachypodiumstacei_Brast08G178700.1.p

Viterbi Path

Viterbi Path

Posterior @ 0.4

Posterior @ 0.4

Posterior Graphic. Threshold Marked @ 0.4

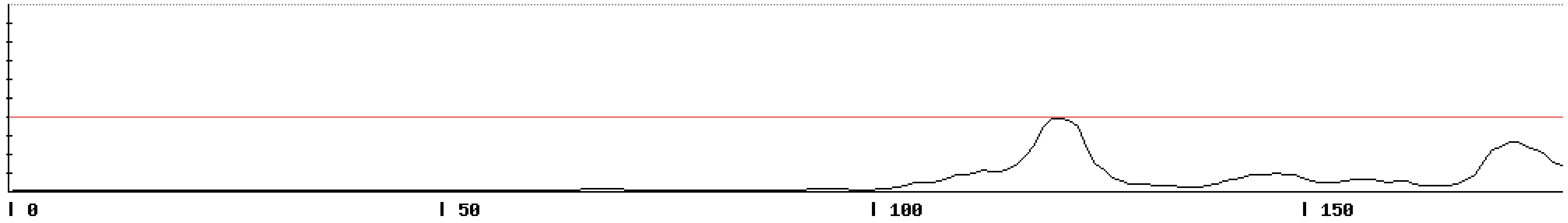

Predictions for Brachypodiumstacei_Brast08G249300.1.p

Viterbi Path

Viterbi Path

Posterior @ 0.4 — 75 - HRKYSKRPRPNR - 86

Posterior @ 0.4

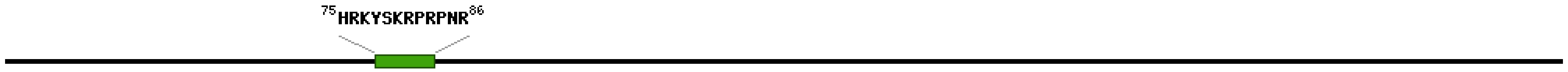

Posterior Graphic. Threshold Marked @ 0.4

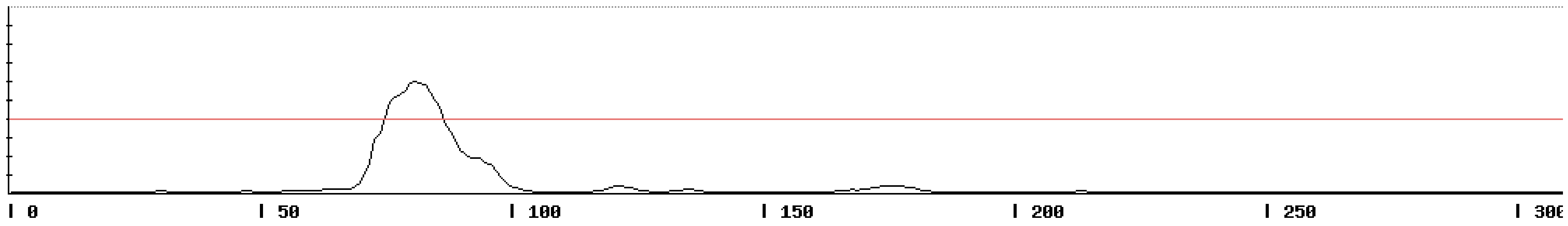

Predictions for Brachypodiumstacei_Brast09G087300.1.p

Viterbi Path

Viterbi Path

Posterior @ 0.4 — 138 - GKRSHKKKGHS - 148

Posterior @ 0.4

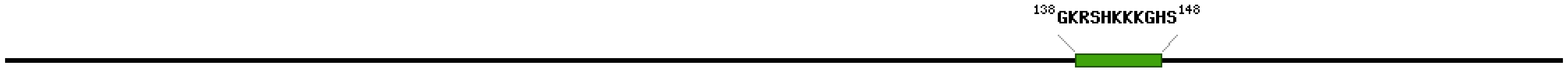

Posterior Graphic. Threshold Marked @ 0.4

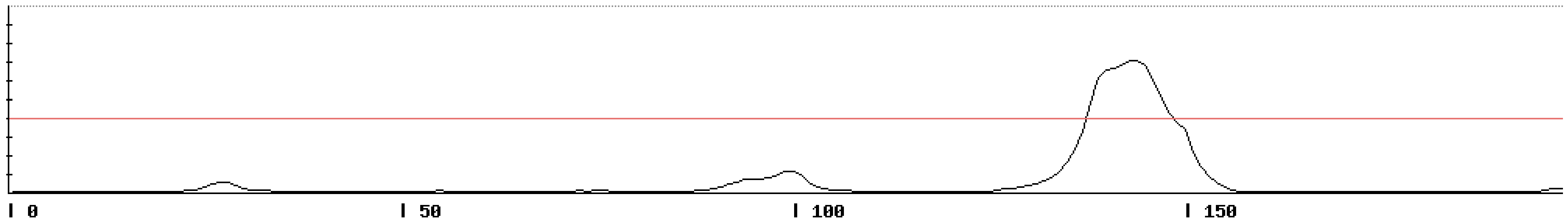

Predictions for Brachypodiumstacei_Brast09G093500.1.p	
Viterbi Path	
Viterbi Path	
Posterior @ 0.4	
Posterior @ 0.4	
Posterior Graphic. Threshold Marked @ 0.4	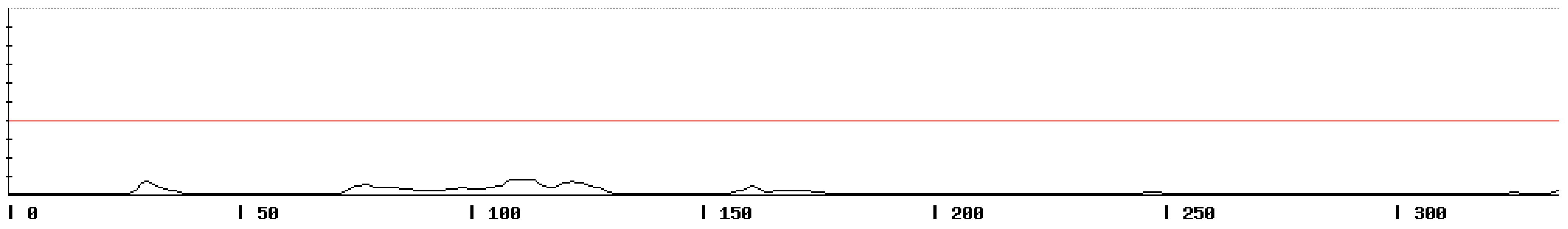

Predictions for Brachypodiumstacei_Brast09G106400.1.p	
Viterbi Path	
Viterbi Path	
Posterior @ 0.4	
Posterior @ 0.4	
Posterior Graphic. Threshold Marked @ 0.4	

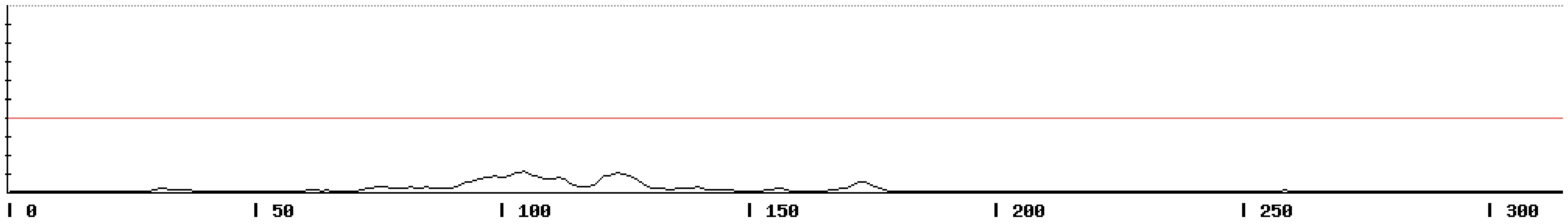

Predictions for Brachypodiumstacei_Brast09G117500.1.p

Viterbi Path

Viterbi Path

Posterior @ 0.4: 212 - RKRARFR - 218

Posterior @ 0.4

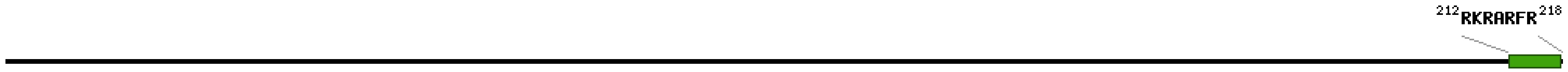

Posterior Graphic. Threshold Marked @ 0.4

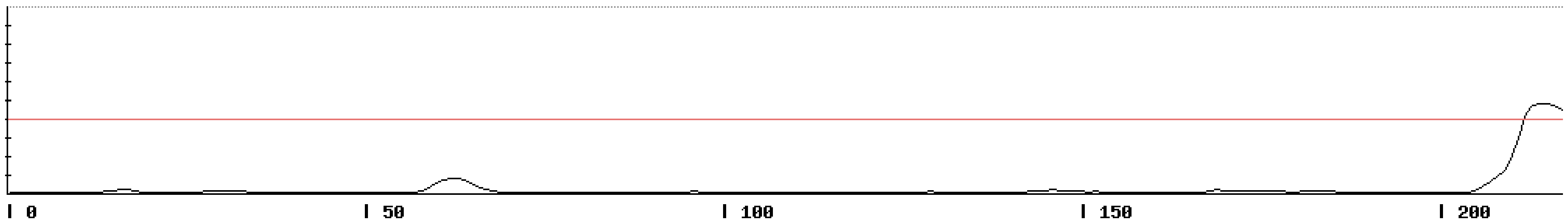

Predictions for Brachypodiumstacei_Brast09G117600.1.p

Viterbi Path: 221 - RRGGSRLPPRAARPPRPPKASKKPK - 245

Viterbi Path

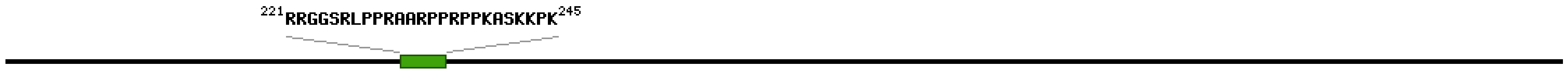

Posterior @ 0.4: 220 - LRRGGSRLPPRAARPPRPPKASKKPKVAPPPPAGPV - 255

Posterior @ 0.4

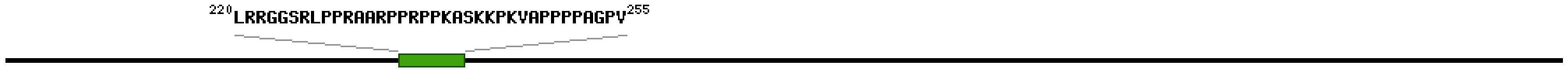

Posterior Graphic. Threshold Marked @ 0.4

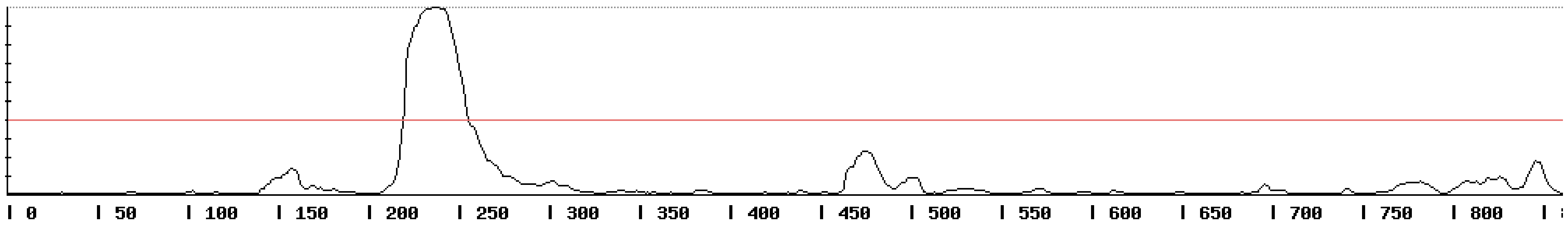

Predictions for Brachypodiumstacei_Brast09G119200.1.p

Viterbi Path

Viterbi Path

Posterior @ 0.4 — 156 - KRKRRK - 161

Posterior @ 0.4

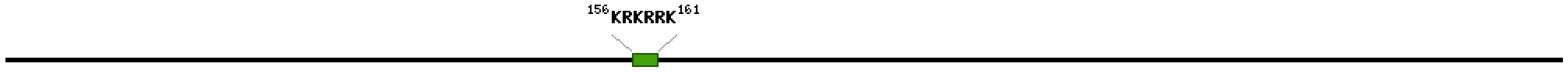

Posterior Graphic. Threshold Marked @ 0.4

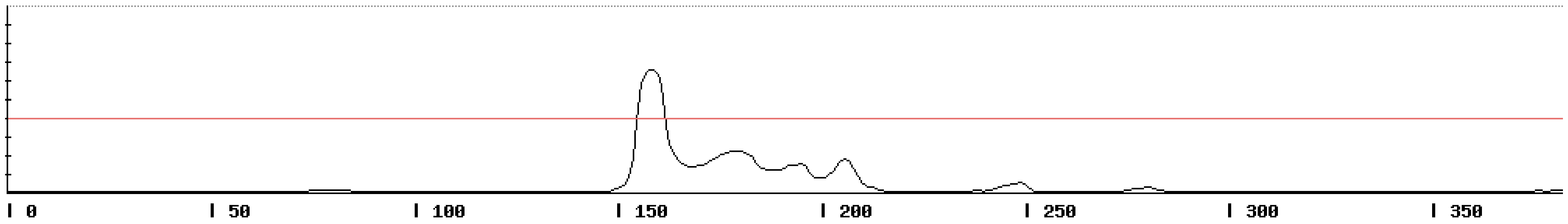

Predictions for Brachypodiumstacei_Brast09G119300.1.p

Viterbi Path

Viterbi Path

Posterior @ 0.4

Posterior @ 0.4

Posterior Graphic. Threshold Marked @ 0.4

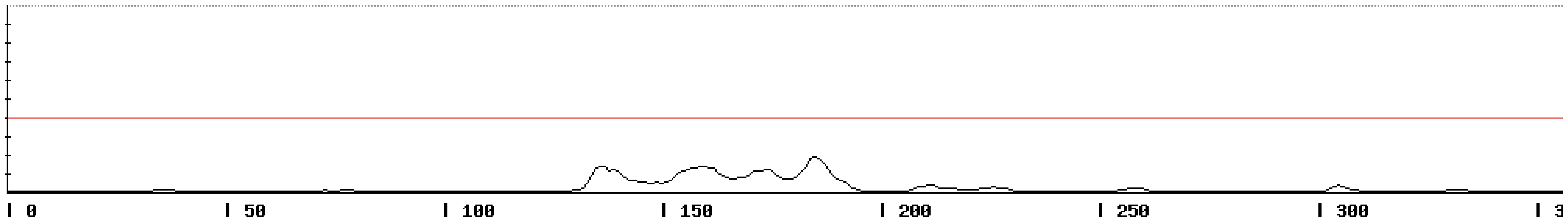

Predictions for Brachypodiumstacei_Brast09G123100.1.p

Viterbi Path

Viterbi Path

Posterior @ 0.4

Posterior @ 0.4

Posterior Graphic. Threshold Marked @ 0.4

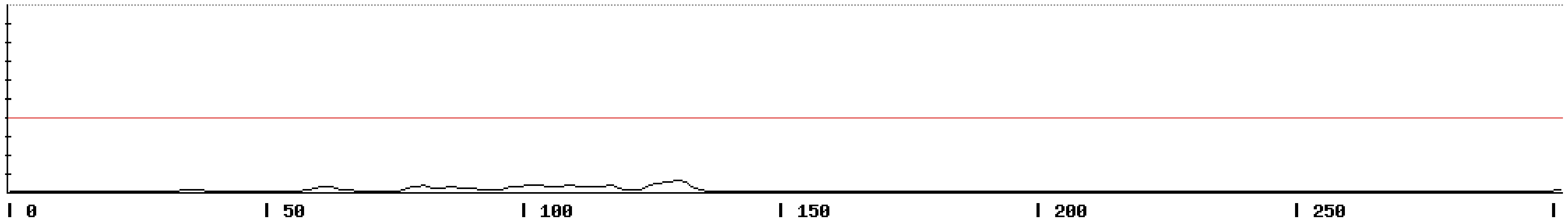

Predictions for Brachypodiumstacei_Brast09G133100.1.p

Viterbi Path

Viterbi Path

Posterior @ 0.4	232 - KRAR - 235
Posterior @ 0.4	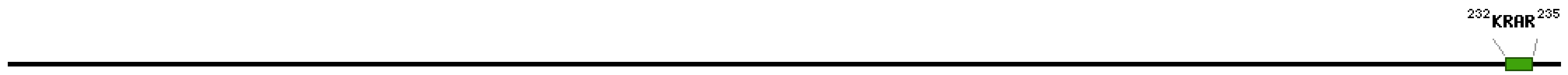
Posterior Graphic. Threshold Marked @ 0.4	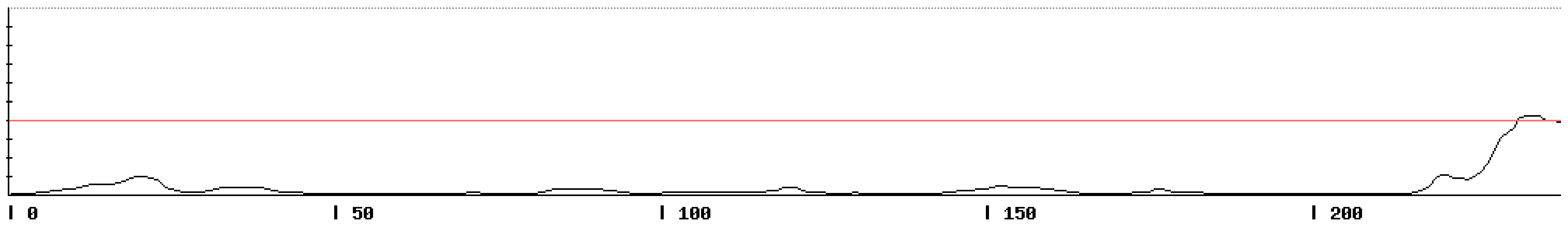

Predictions for Brachypodiumstacei_Brast09G135700.1.p

Viterbi Path	
Viterbi Path	
Posterior @ 0.4	
Posterior @ 0.4	
Posterior Graphic. Threshold Marked @ 0.4	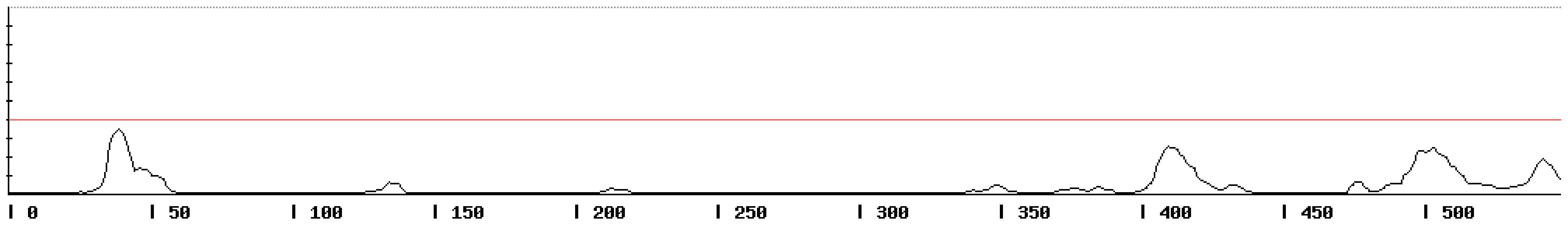

Predictions for Brachypodiumstacei_Brast09G138900.1.p

Viterbi Path	
Viterbi Path	

Posterior @ 0.4

32 - PRGKPLRPRPR - 42

273 - ARRPKHKRAANGDEEERPRRA - 293

Posterior @ 0.4

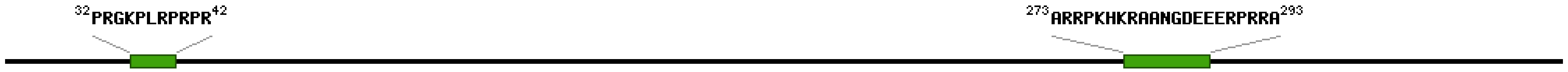

Posterior Graphic. Threshold Marked @ 0.4

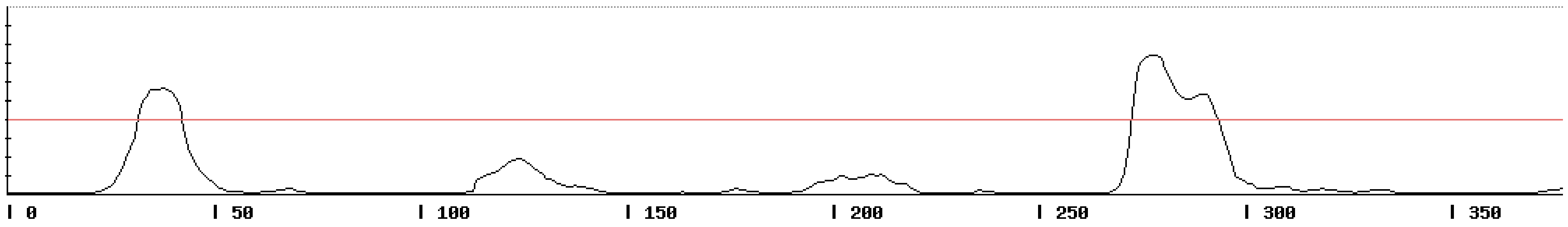

Predictions for Brachypodiumstacei_Brast09G142600.1.p

Viterbi Path

Viterbi Path

Posterior @ 0.4

Posterior @ 0.4

Posterior Graphic. Threshold Marked @ 0.4

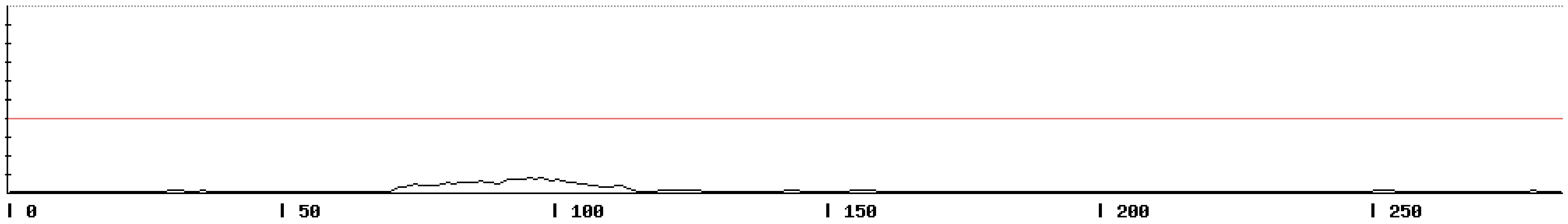

Predictions for Brachypodiumstacei_Brast09G156400.1.p	
Viterbi Path	
Viterbi Path	
Posterior @ 0.4	
Posterior @ 0.4	
Posterior Graphic. Threshold Marked @ 0.4	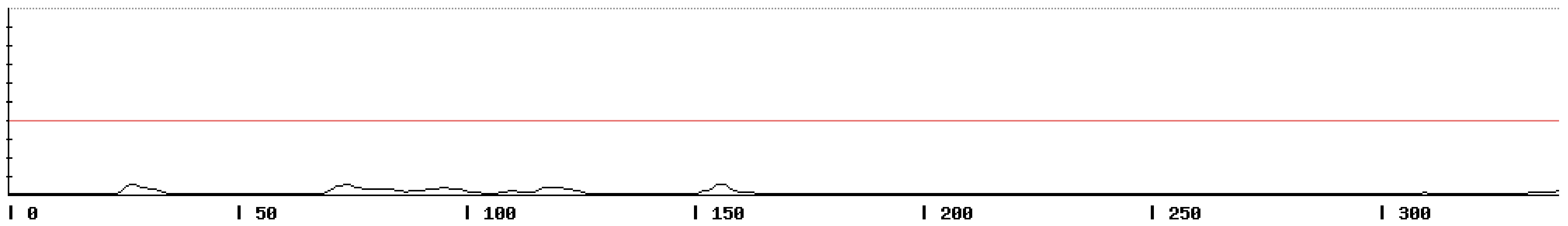

Predictions for Brachypodiumstacei_Brast09G168600.1.p	
Viterbi Path	
Viterbi Path	
Posterior @ 0.4	86 - RSRKYTKGKRPDRGTDDHRGR - 106
	233 - ARQKGKRPIEAMRRGPMATPKR - 254
Posterior @ 0.4	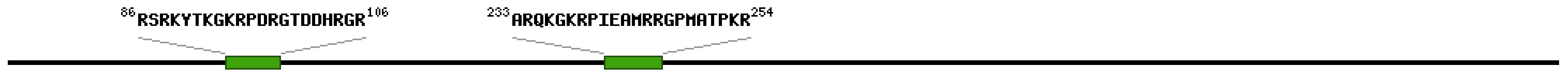
Posterior Graphic. Threshold Marked @ 0.4	

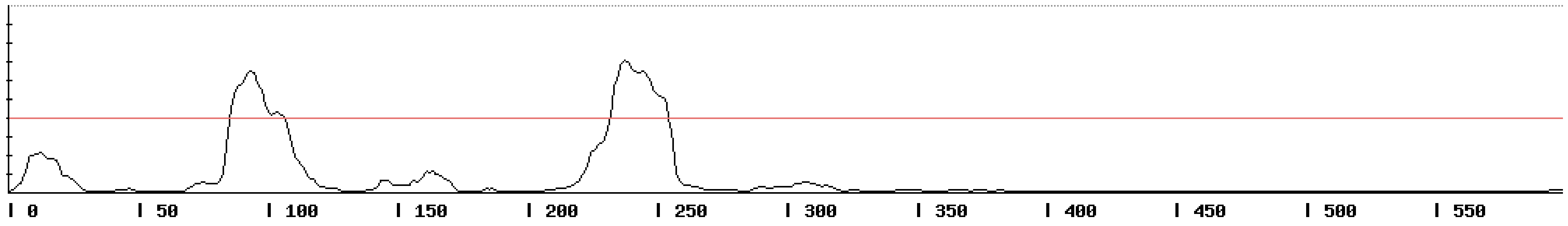

Predictions for Brachypodiumstacei_Brast09G200100.1.p

Viterbi Path	
Viterbi Path	
Posterior @ 0.4	
Posterior @ 0.4	
Posterior Graphic. Threshold Marked @ 0.4	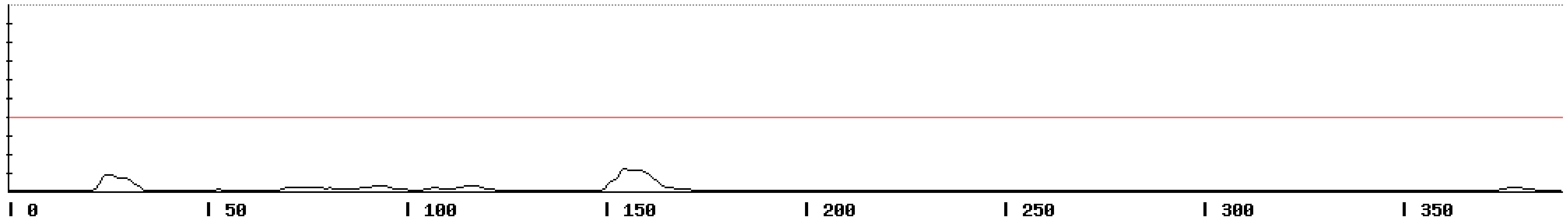

Predictions for Brachypodiumstacei_Brast09G210800.1.p

Viterbi Path	
Viterbi Path	
Posterior @ 0.4	
Posterior @ 0.4	

Posterior Graphic. Threshold Marked @ 0.4

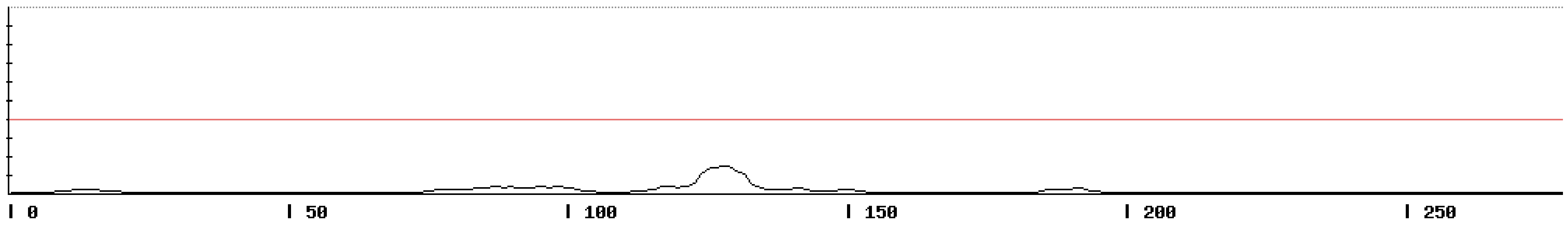

Predictions for Brachypodiumstacei_Brast09G268800.1.p

Viterbi Path

Viterbi Path

Posterior @ 0.4

Posterior @ 0.4

Posterior Graphic. Threshold Marked @ 0.4

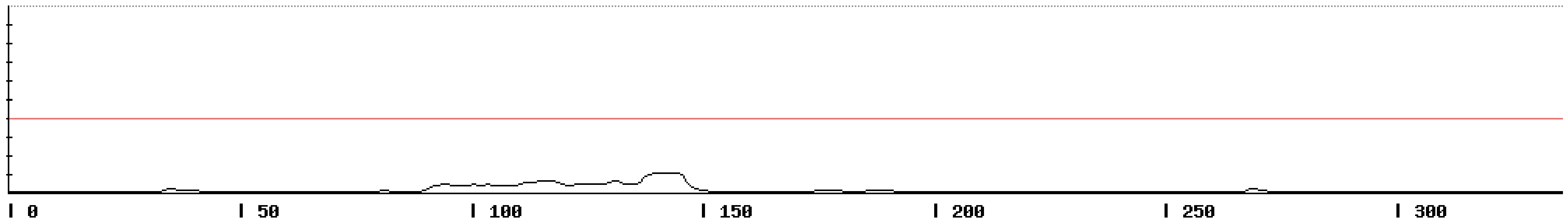

Predictions for Brachypodiumstacei_Brast10G019700.1.p

Viterbi Path

Viterbi Path

Posterior @ 0.4

Posterior @ 0.4

Posterior Graphic. Threshold Marked @ 0.4	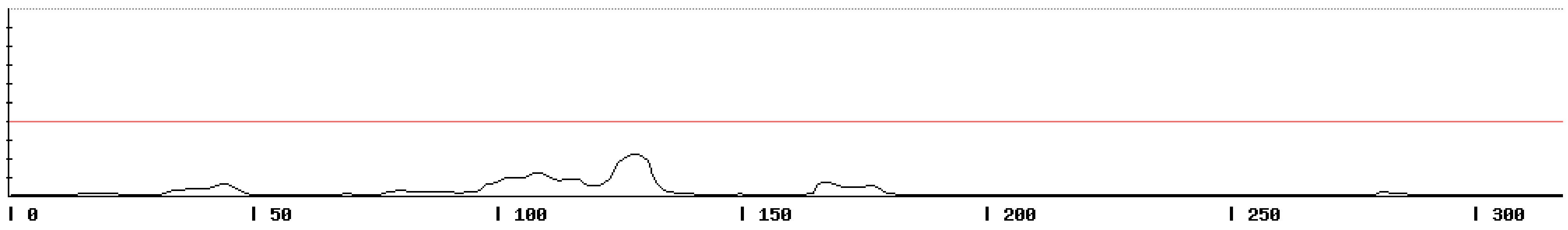

Predictions for Brachypodiumstacei_Brast10G036500.1.p

Viterbi Path	
Viterbi Path	
Posterior @ 0.4	
Posterior @ 0.4	
Posterior Graphic. Threshold Marked @ 0.4	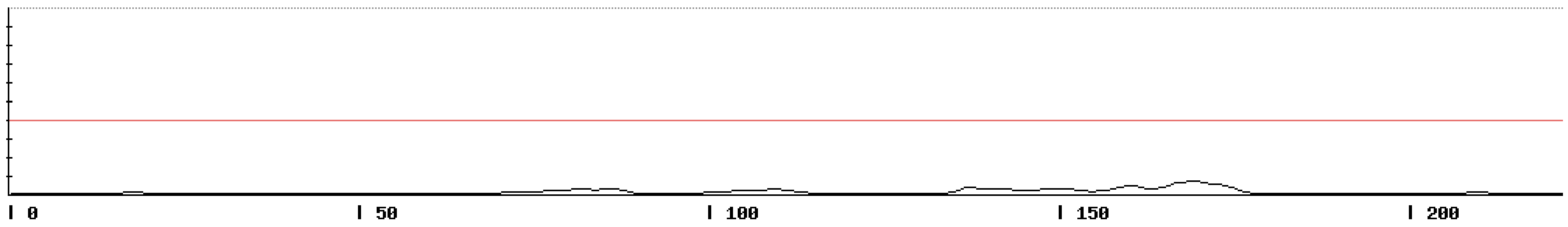

Predictions for Brachypodiumstacei_Brast10G085400.1.p

Viterbi Path	
Posterior @ 0.4	
Posterior @ 0.4	
Posterior Graphic. Threshold Marked @ 0.4	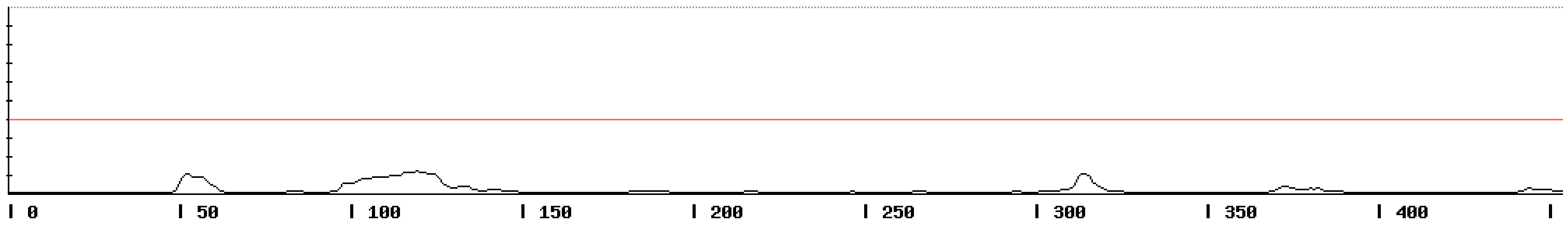

Predictions for Brassicanapus_GSBRNA2T00000580001

Viterbi Path	
Viterbi Path	
Posterior @ 0.4	
Posterior @ 0.4	
Posterior Graphic. Threshold Marked @ 0.4	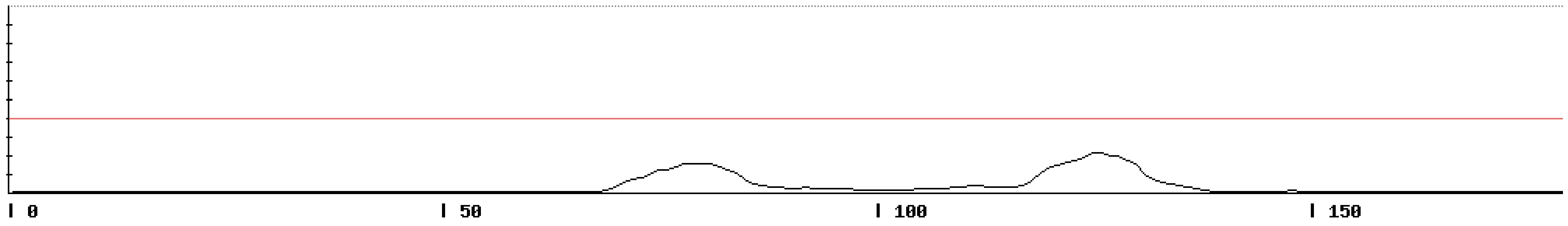

Predictions for Brassicanapus_GSBRNA2T00001356001

Viterbi Path

Viterbi Path

Posterior @ 0.4

Posterior @ 0.4

Posterior Graphic. Threshold Marked @ 0.4

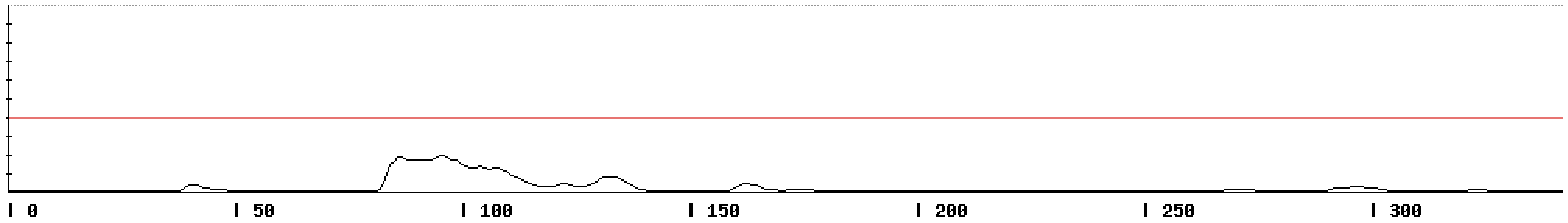

Predictions for Brassicanapus_GSBRNA2T00001362001

Viterbi Path

Viterbi Path

Posterior @ 0.4

Posterior @ 0.4

Posterior Graphic. Threshold Marked @ 0.4

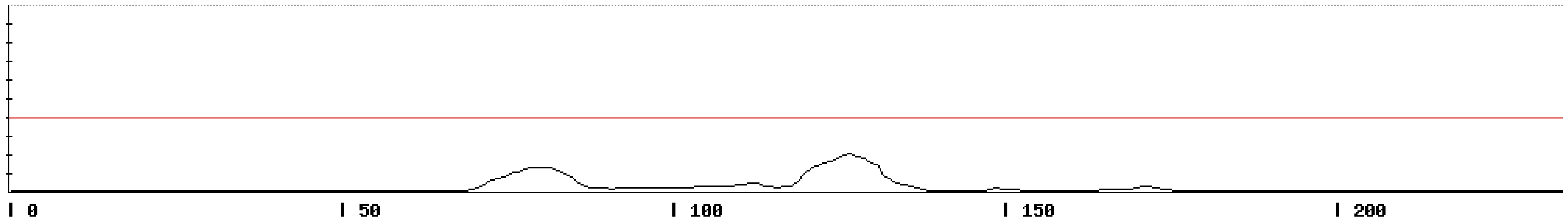

Predictions for Brassicanapus_GSBRNA2T00001799001	
Viterbi Path	
Viterbi Path	
Posterior @ 0.4	
Posterior @ 0.4	
Posterior Graphic. Threshold Marked @ 0.4	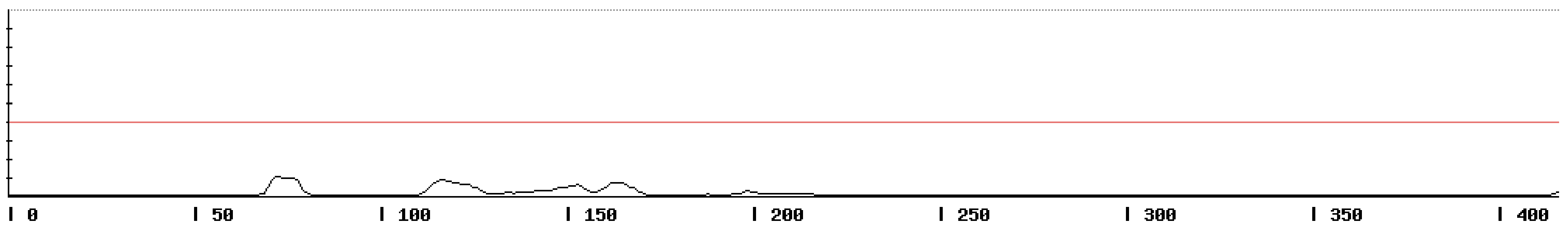

Predictions for Brassicanapus_GSBRNA2T00002998001	
Viterbi Path	
Viterbi Path	
Posterior @ 0.4	
Posterior @ 0.4	
Posterior Graphic. Threshold Marked @ 0.4	

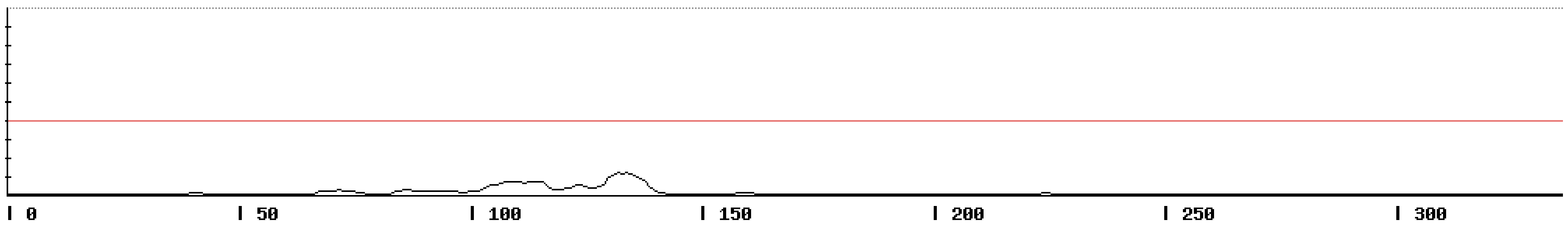

Predictions for Brassicanapus_GSBRNA2T00003061001

Viterbi Path	
Viterbi Path	
Posterior @ 0.4	
Posterior @ 0.4	
Posterior Graphic. Threshold Marked @ 0.4	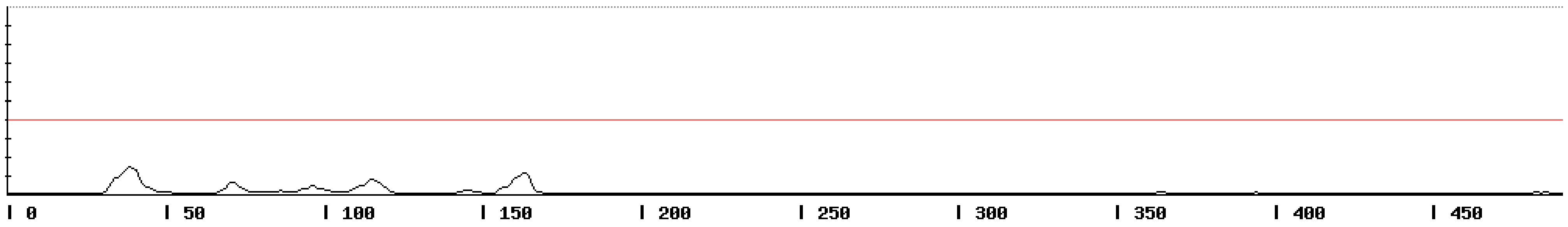

Predictions for Brassicanapus_GSBRNA2T00003098001

Viterbi Path	
Viterbi Path	
Posterior @ 0.4	
Posterior @ 0.4	

Posterior Graphic. Threshold Marked @ 0.4

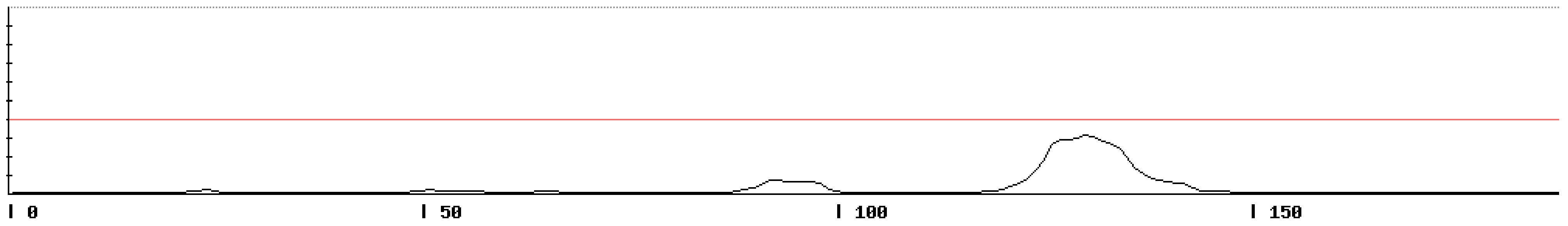

Predictions for Brassicanapus_GSBRNA2T00003406001

Viterbi Path

Viterbi Path

Posterior @ 0.4 — 94 - KRR - 96

Posterior @ 0.4

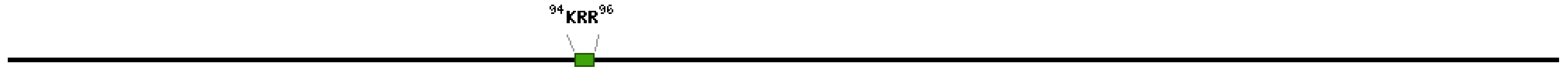

Posterior Graphic. Threshold Marked @ 0.4

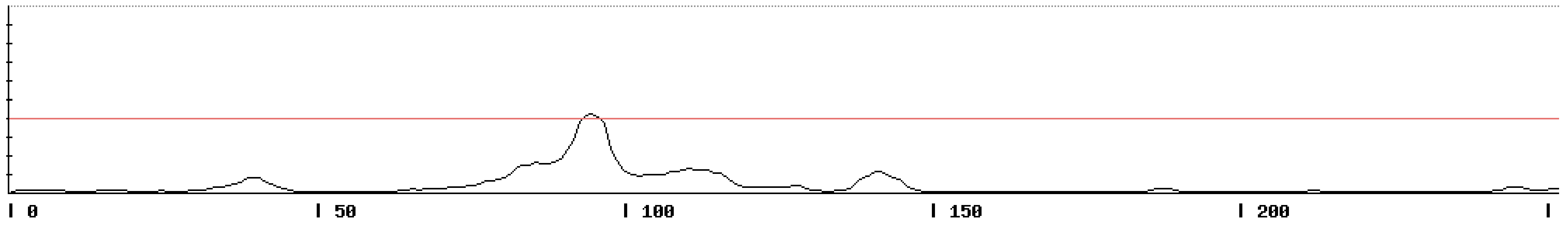

Predictions for Brassicanapus_GSBRNA2T00003593001

Viterbi Path

Viterbi Path

Posterior @ 0.4

Posterior @ 0.4

Posterior Graphic. Threshold Marked @ 0.4	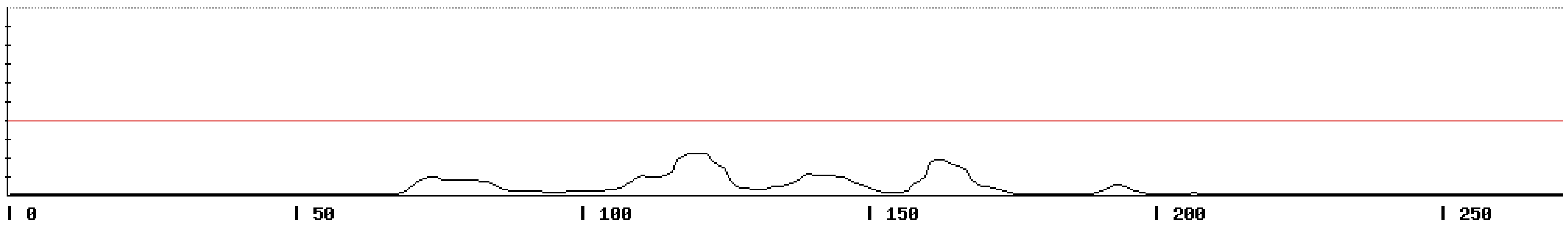

Predictions for Brassicanapus_GSBRNA2T00004056001	
Viterbi Path	
Viterbi Path	
Posterior @ 0.4	
Posterior @ 0.4	
Posterior Graphic. Threshold Marked @ 0.4	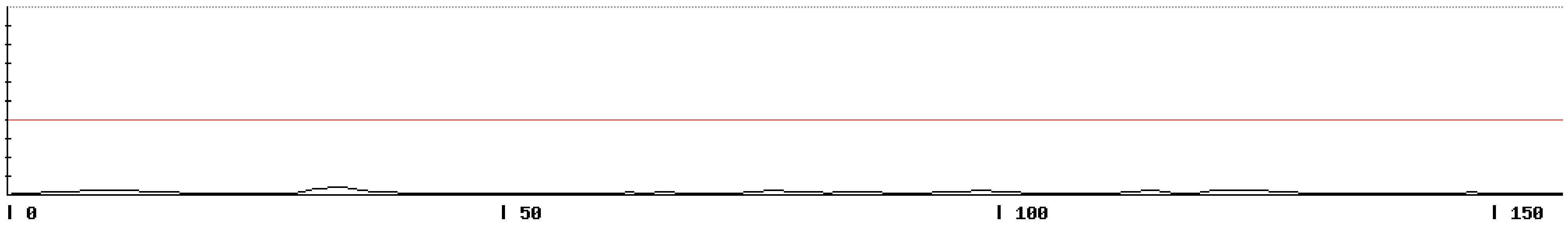

Predictions for Brassicanapus_GSBRNA2T00004489001	
Viterbi Path	
Viterbi Path	

Posterior @ 0.4

Posterior @ 0.4

Posterior Graphic. Threshold Marked @ 0.4

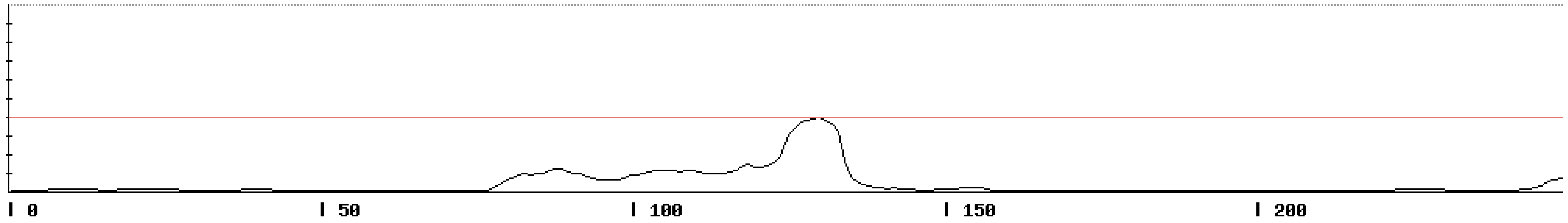

Predictions for Brassicanapus_GSBRNA2T00004530001

Viterbi Path

Viterbi Path

Posterior @ 0.4

Posterior @ 0.4

Posterior Graphic. Threshold Marked @ 0.4

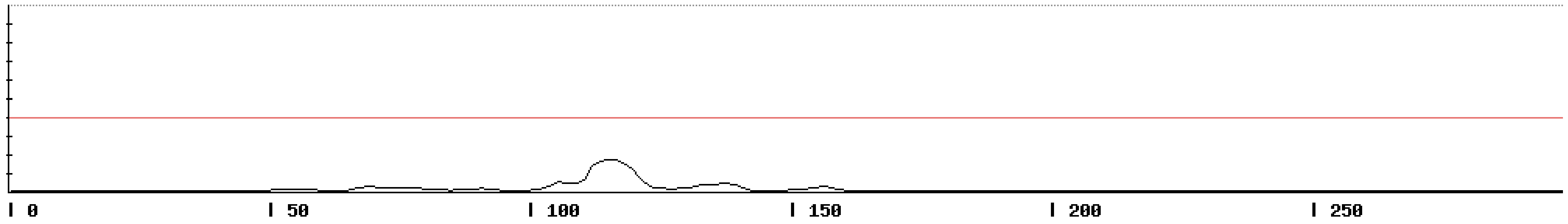

Predictions for Brassicanapus_GSBRNA2T00004862001

Viterbi Path

Viterbi Path

Posterior @ 0.4

Posterior @ 0.4

Posterior Graphic. Threshold Marked @ 0.4

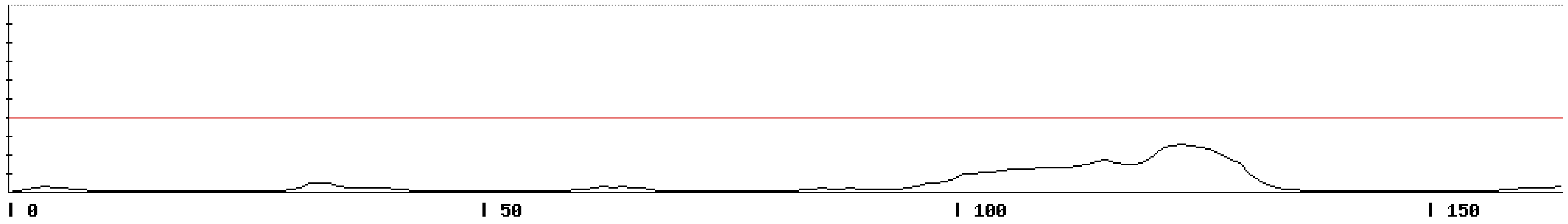

Predictions for Brassicanapus_GSBRNA2T00004863001

Viterbi Path

Viterbi Path

Posterior @ 0.4

Posterior @ 0.4

Posterior Graphic. Threshold Marked @ 0.4

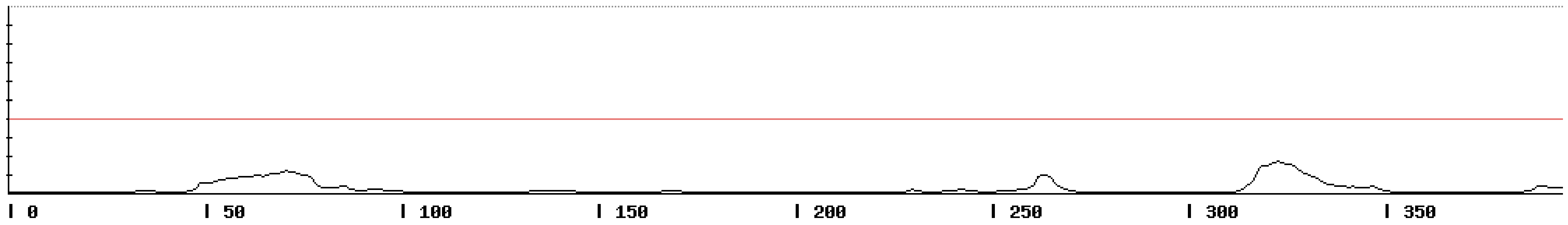

Predictions for Brassicanapus_GSBRNA2T00005539001

Viterbi Path

Viterbi Path

Posterior @ 0.4

Posterior @ 0.4

Posterior Graphic. Threshold Marked @ 0.4

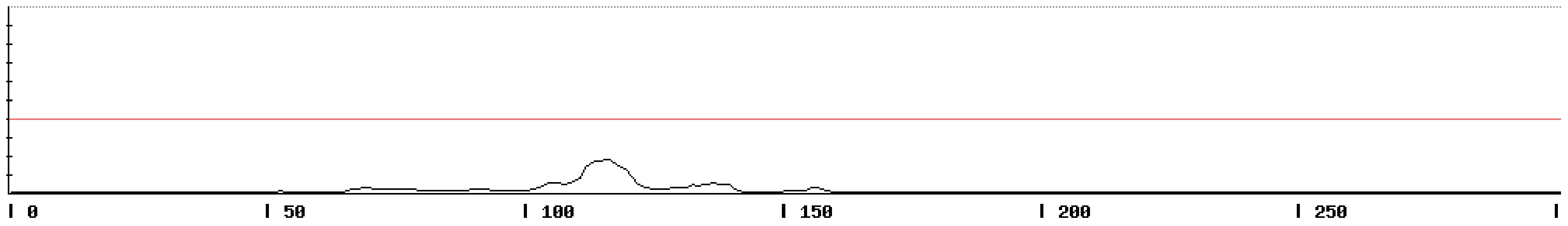

Predictions for Brassicanapus_GSBRNA2T00005792001

Viterbi Path

Viterbi Path

Posterior @ 0.4

Posterior @ 0.4

Posterior Graphic. Threshold Marked @ 0.4

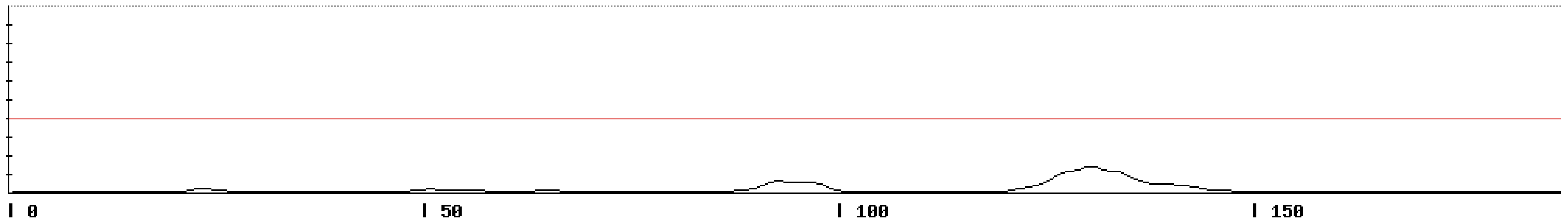

Predictions for Brassicanapus_GSBRNA2T00007082001	
Viterbi Path	
Viterbi Path	
Posterior @ 0.4	
Posterior @ 0.4	
Posterior Graphic. Threshold Marked @ 0.4	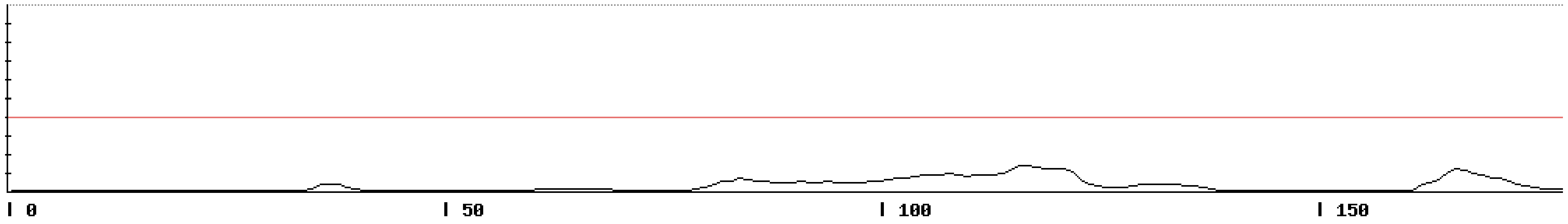

Predictions for Brassicanapus_GSBRNA2T00009098001	
Viterbi Path	156 - KKRSNNNKKKKKEDEREKK - 174
Viterbi Path	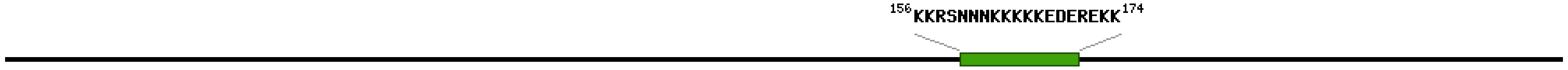
Posterior @ 0.4	156 - KKRSNNNKKKKKEDEREKK - 174
Posterior @ 0.4	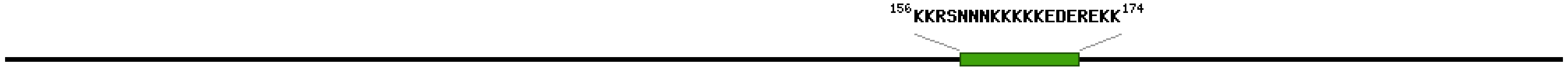
Posterior Graphic. Threshold Marked @ 0.4	

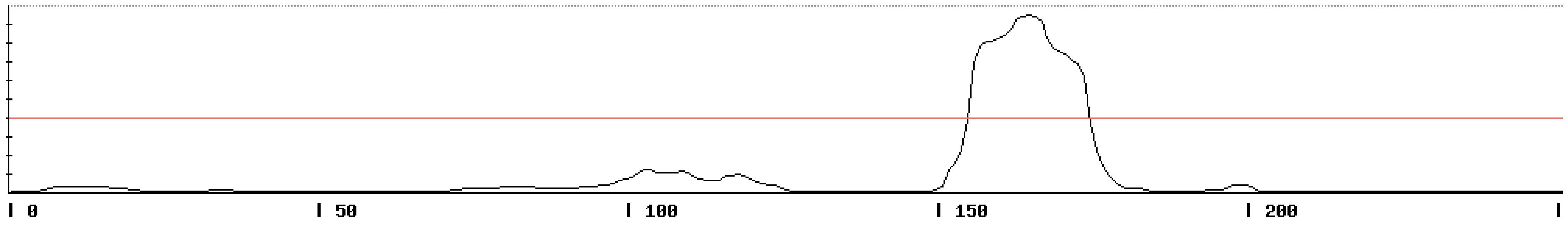

Predictions for Brassicanapus_GSBRNA2T00009100001	
Viterbi Path	156 - KKRSNNNKKKKKEDEREKK - 174
Viterbi Path	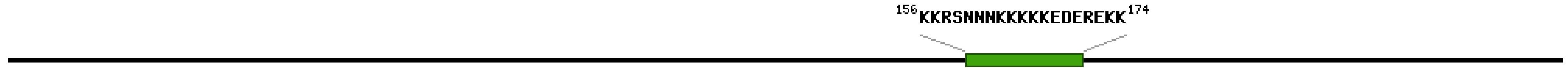
Posterior @ 0.4	156 - KKRSNNNKKKKKEDEREKK - 174
Posterior @ 0.4	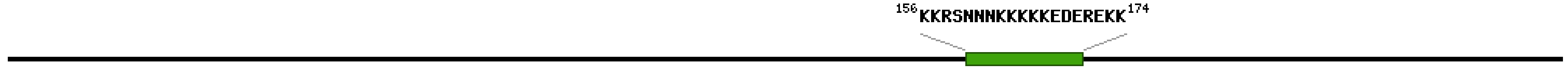
Posterior Graphic. Threshold Marked @ 0.4	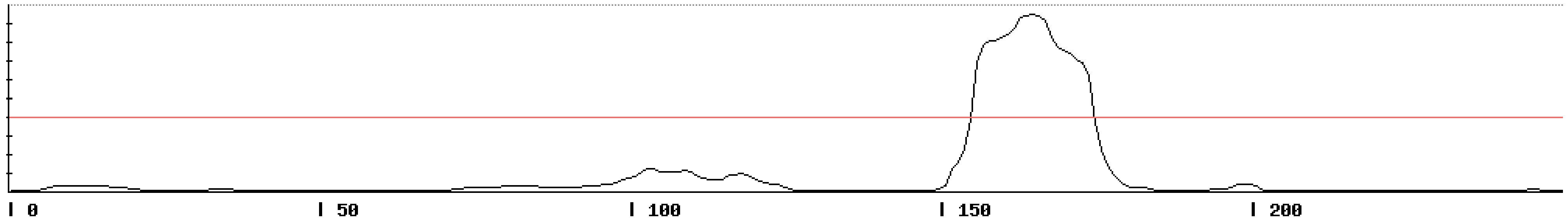

Predictions for Brassicanapus_GSBRNA2T00009589001	
Viterbi Path	
Viterbi Path	
Posterior @ 0.4	
Posterior @ 0.4	

Posterior Graphic. Threshold Marked @ 0.4	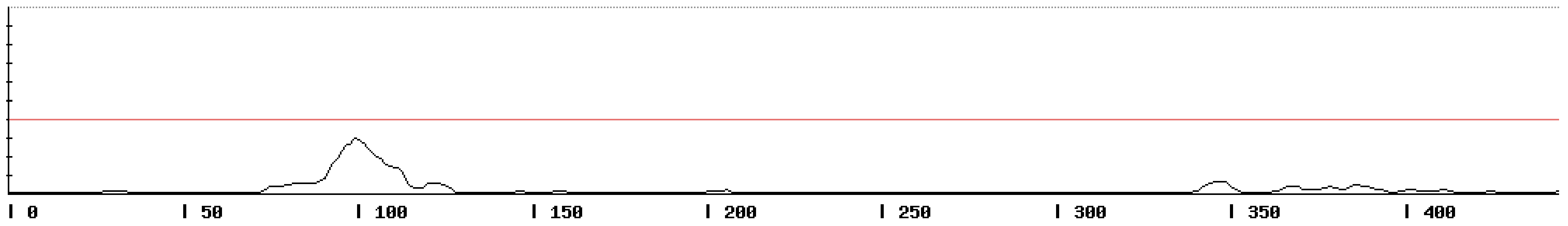

Predictions for Brassicanapus_GSBRNA2T00009829001

Viterbi Path	
Viterbi Path	
Posterior @ 0.4	
Posterior @ 0.4	
Posterior Graphic. Threshold Marked @ 0.4	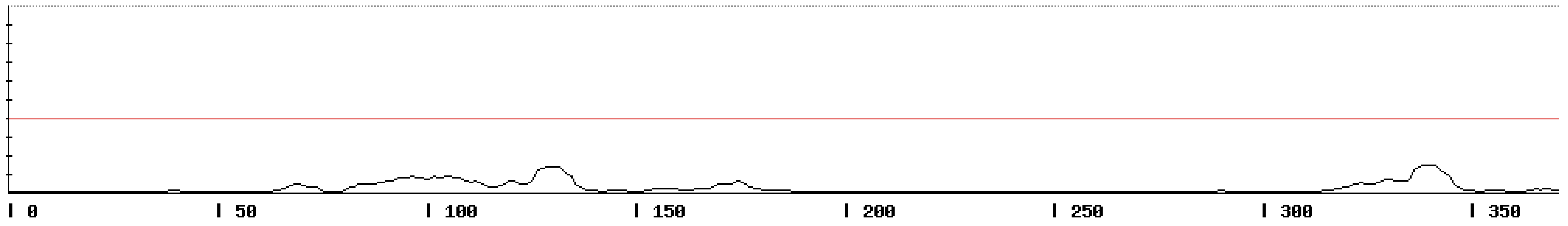

Predictions for Brassicanapus_GSBRNA2T00009956001

Viterbi Path	
Viterbi Path	
Posterior @ 0.4	
Posterior @ 0.4	

Posterior Graphic. Threshold Marked @ 0.4

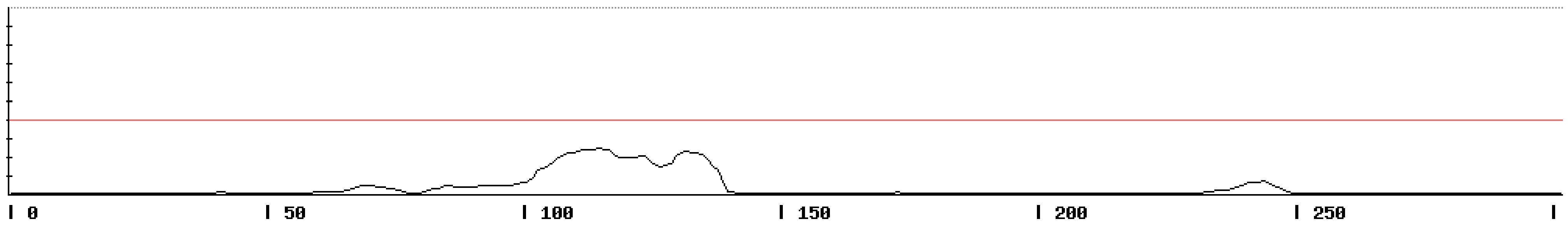

Predictions for Brassicanapus_GSBRNA2T00009965001

Viterbi Path

Viterbi Path

Posterior @ 0.4

Posterior @ 0.4

Posterior Graphic. Threshold Marked @ 0.4

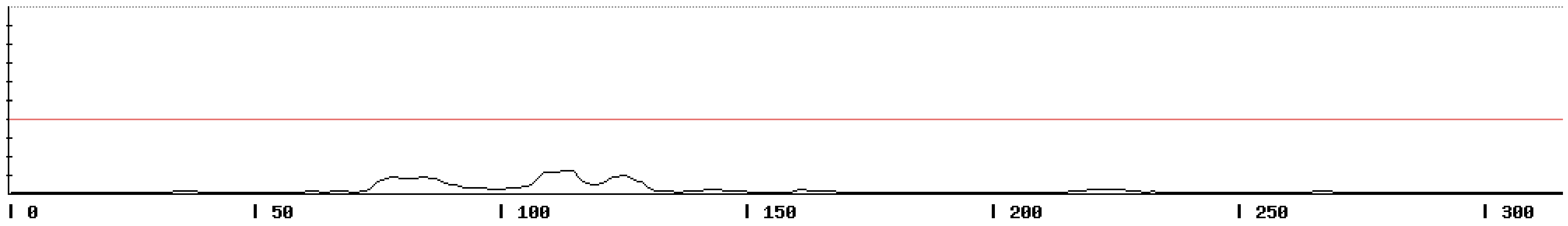

Predictions for Brassicanapus_GSBRNA2T00009990001

Viterbi Path

Viterbi Path

Posterior @ 0.4	
Posterior @ 0.4	
Posterior Graphic. Threshold Marked @ 0.4	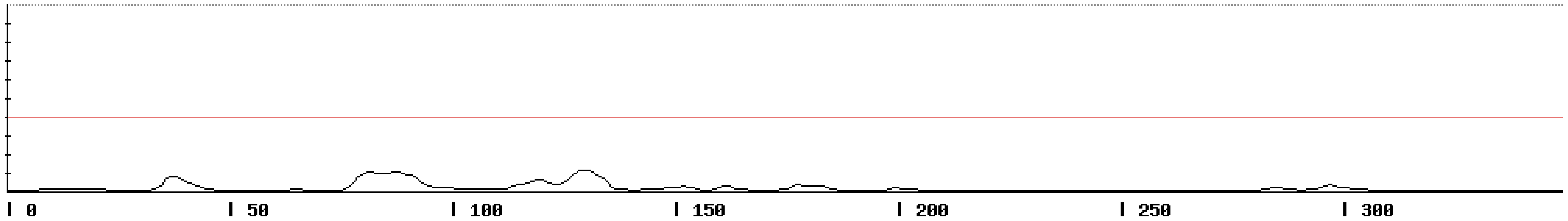

Predictions for Brassicanapus_GSBRNA2T00010274001

Viterbi Path	
Viterbi Path	
Posterior @ 0.4	
Posterior @ 0.4	
Posterior Graphic. Threshold Marked @ 0.4	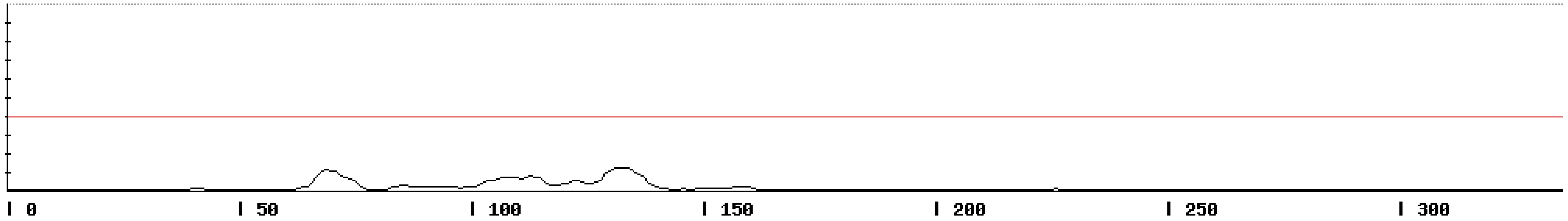

Predictions for Brassicanapus_GSBRNA2T00010843001

Viterbi Path	
Viterbi Path	

Posterior @ 0.4

Posterior @ 0.4

Posterior Graphic. Threshold Marked @ 0.4

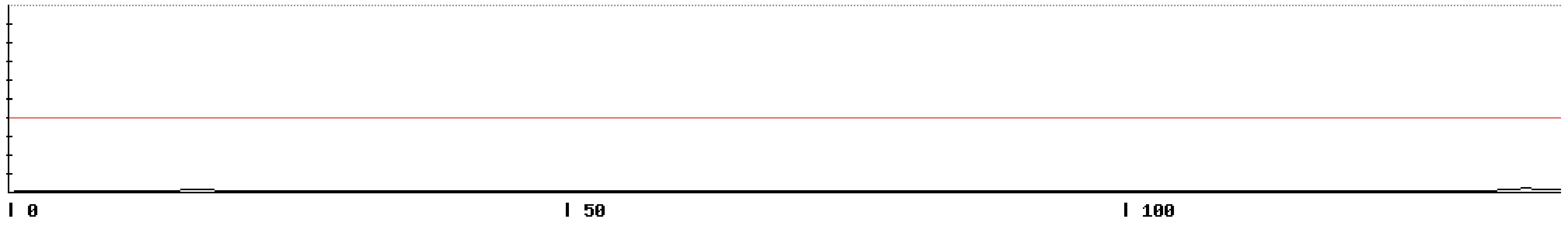

Predictions for Brassicanapus_GSBRNA2T00011094001

Viterbi Path

Viterbi Path

Posterior @ 0.4

Posterior @ 0.4

Posterior Graphic. Threshold Marked @ 0.4

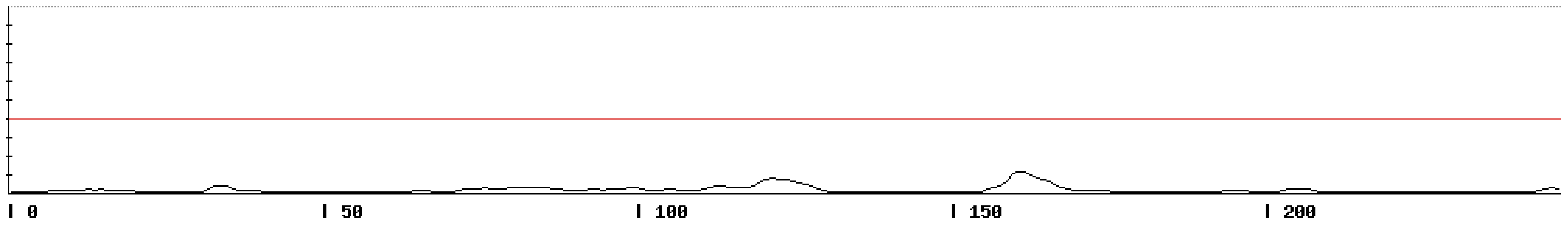

Predictions for Brassicanapus_GSBRNA2T00011138001

Viterbi Path

Viterbi Path

Posterior @ 0.4

Posterior @ 0.4

Posterior Graphic. Threshold Marked @ 0.4

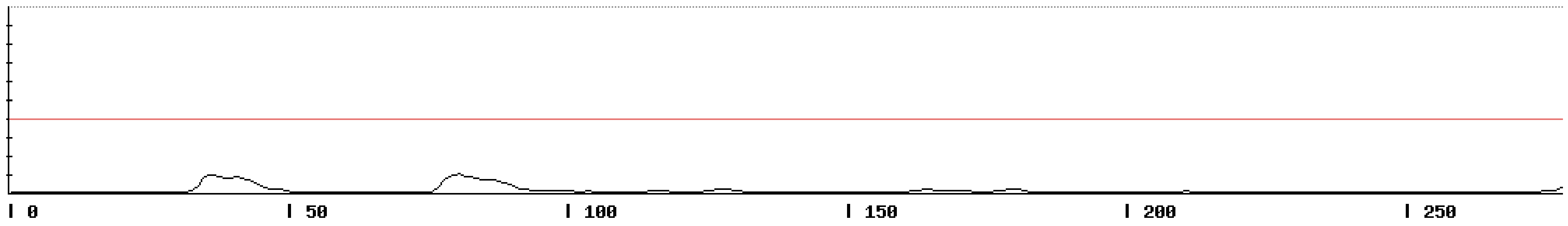

Predictions for Brassicanapus_GSBRNA2T00011481001

Viterbi Path

Viterbi Path

Posterior @ 0.4

Posterior @ 0.4

Posterior Graphic. Threshold Marked @ 0.4

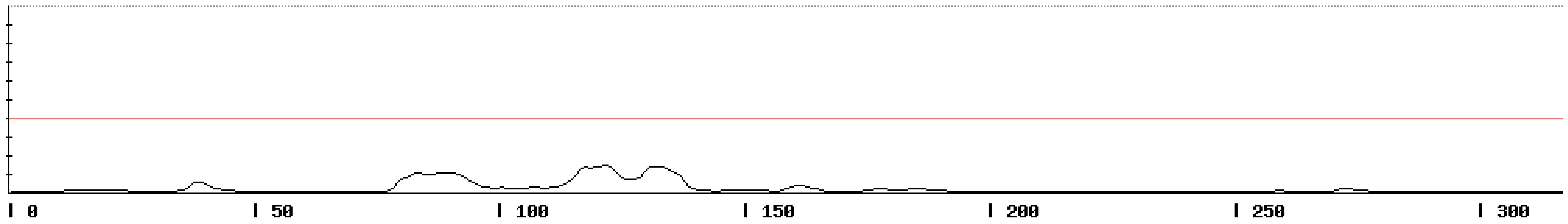

Predictions for Brassicanapus_GSBRNA2T00011918001	
Viterbi Path	
Viterbi Path	
Posterior @ 0.4	
Posterior @ 0.4	
Posterior Graphic. Threshold Marked @ 0.4	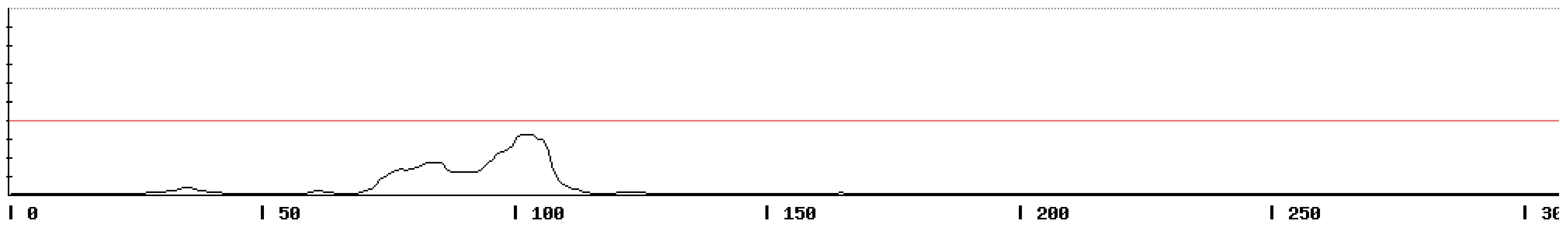

Predictions for Brassicanapus_GSBRNA2T00012247001	
Viterbi Path	
Viterbi Path	
Posterior @ 0.4	
Posterior @ 0.4	
Posterior Graphic. Threshold Marked @ 0.4	

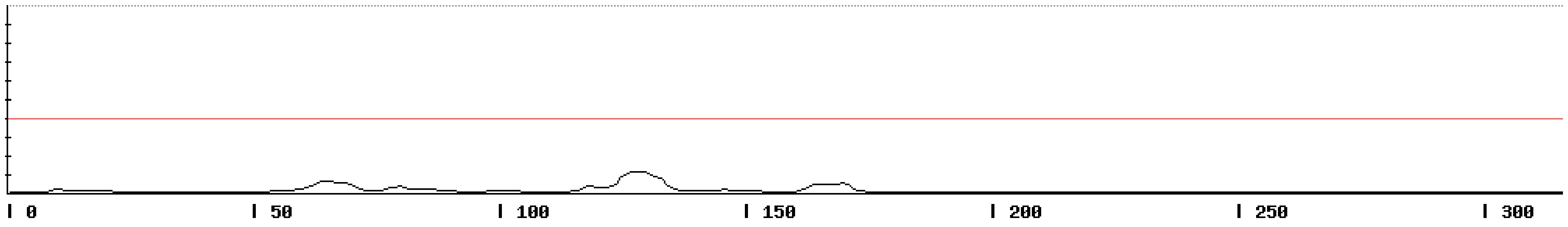

Predictions for Brassicanapus_GSBRNA2T00012336001

Viterbi Path	
Viterbi Path	
Posterior @ 0.4	
Posterior @ 0.4	
Posterior Graphic. Threshold Marked @ 0.4	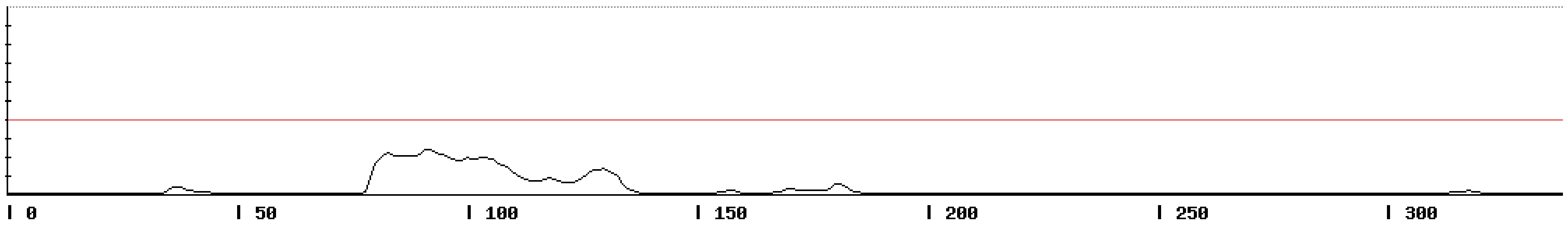

Predictions for Brassicanapus_GSBRNA2T00012468001

Viterbi Path	
Viterbi Path	
Posterior @ 0.4	
Posterior @ 0.4	

Posterior Graphic. Threshold Marked @ 0.4

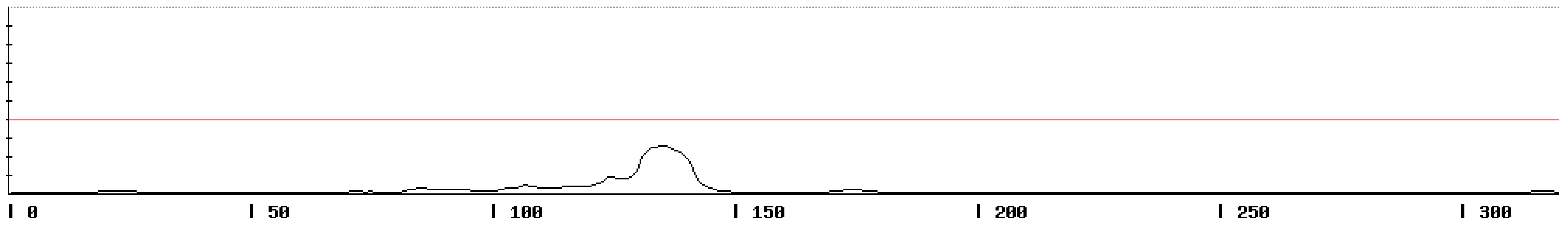

Predictions for Brassicanapus_GSBRNA2T00012643001

Viterbi Path

Viterbi Path

Posterior @ 0.4 134 - KR - 135

Posterior @ 0.4

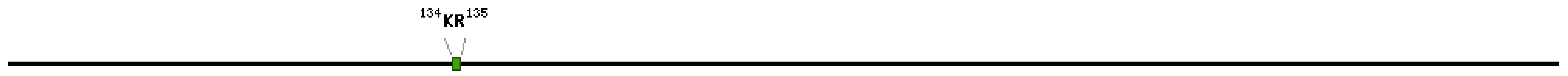

Posterior Graphic. Threshold Marked @ 0.4

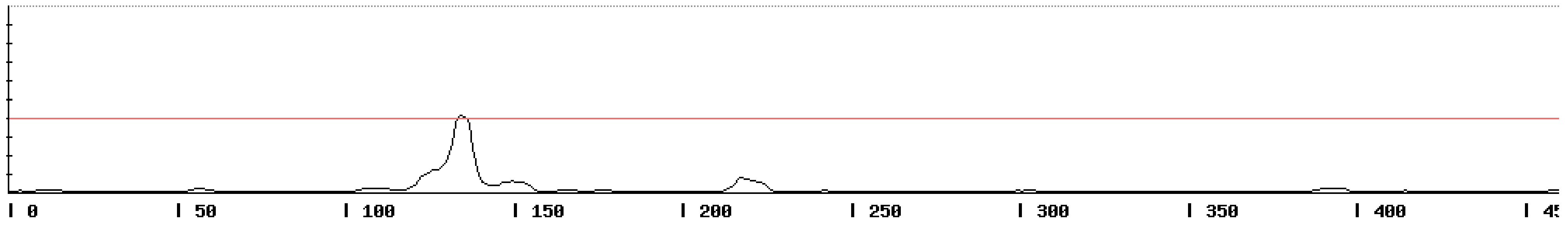

Predictions for Brassicanapus_GSBRNA2T00013424001

Viterbi Path

Viterbi Path

Posterior @ 0.4

Posterior @ 0.4

Posterior Graphic. Threshold Marked @ 0.4

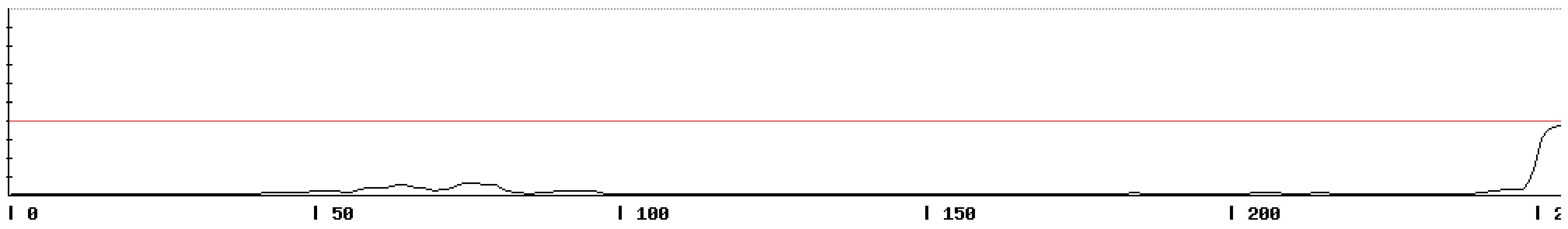

Predictions for Brassicanapus_GSBRNA2T00013675001

Viterbi Path	
Viterbi Path	
Posterior @ 0.4	82 - RATRAGYWKATGKDRAVKSKKMKLGMKK - 109
Posterior @ 0.4	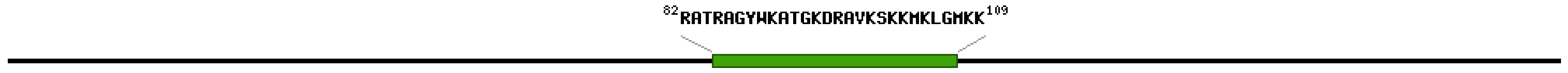
Posterior Graphic. Threshold Marked @ 0.4	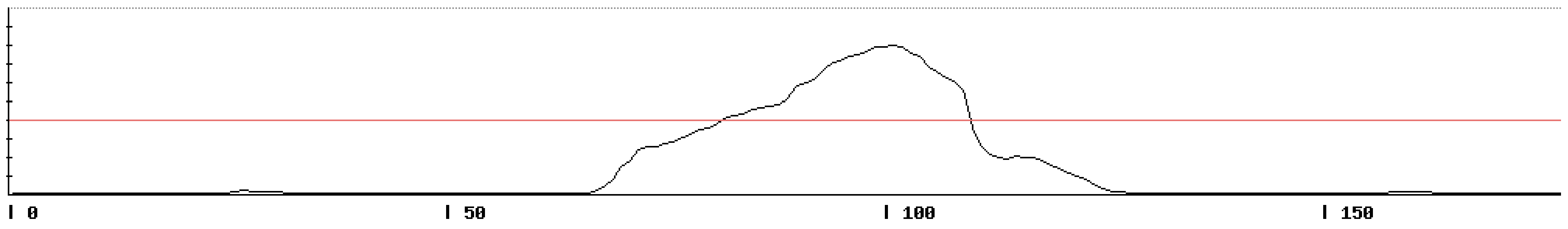

Predictions for Brassicanapus_GSBRNA2T00014031001

Viterbi Path	
Viterbi Path	

Posterior @ 0.4

Posterior @ 0.4

Posterior Graphic. Threshold Marked @ 0.4

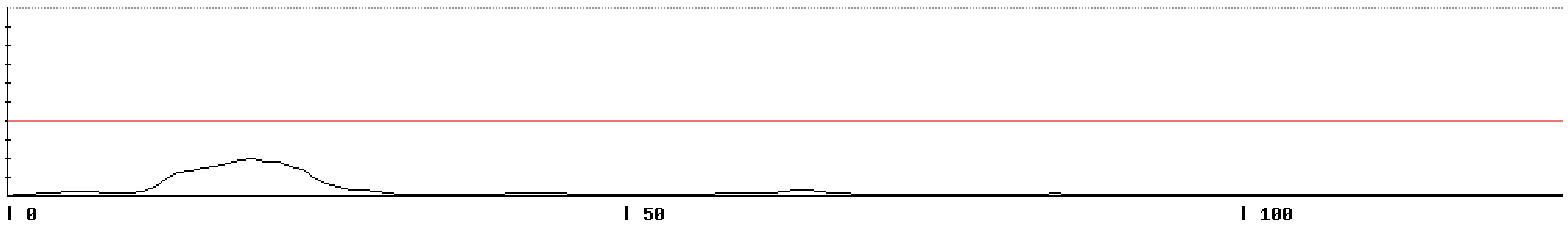

Predictions for Brassicanapus_GSBRNA2T00015204001

Viterbi Path

Viterbi Path

Posterior @ 0.4

Posterior @ 0.4

Posterior Graphic. Threshold Marked @ 0.4

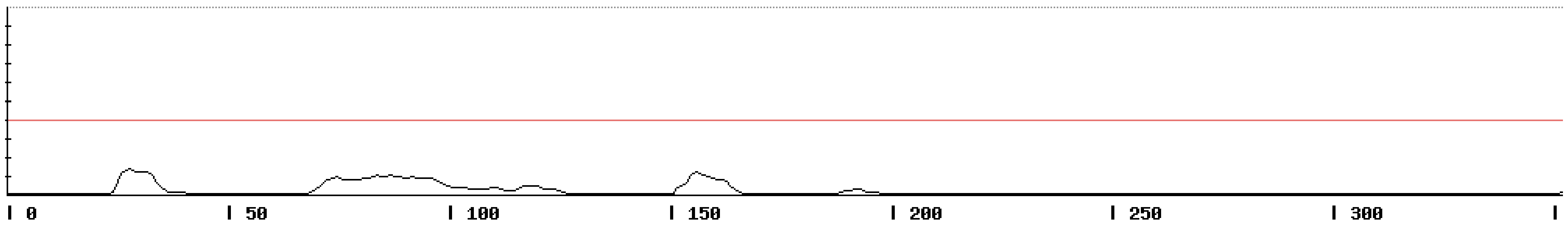

Predictions for Brassicanapus_GSBRNA2T00015428001

Viterbi Path

Viterbi Path

Posterior @ 0.4	
Posterior @ 0.4	
Posterior Graphic. Threshold Marked @ 0.4	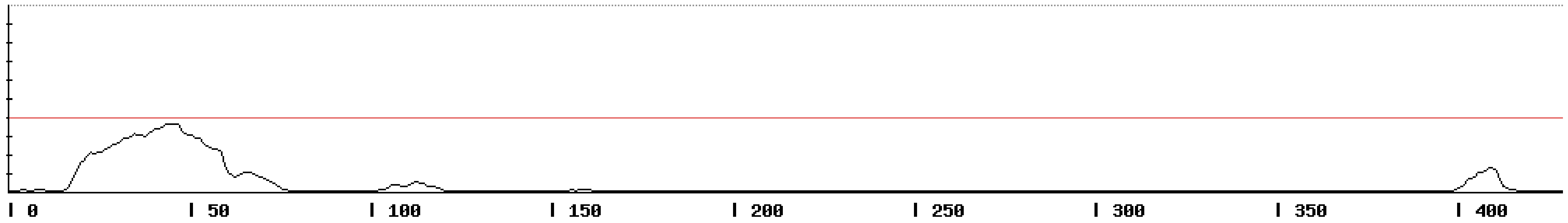

Predictions for Brassicanapus_GSBRNA2T00015476001

Viterbi Path	
Viterbi Path	
Posterior @ 0.4	121 - KAPKGRKTKWK - 131
Posterior @ 0.4	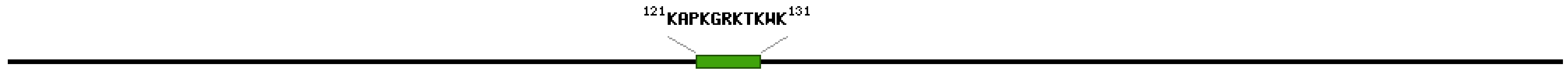
Posterior Graphic. Threshold Marked @ 0.4	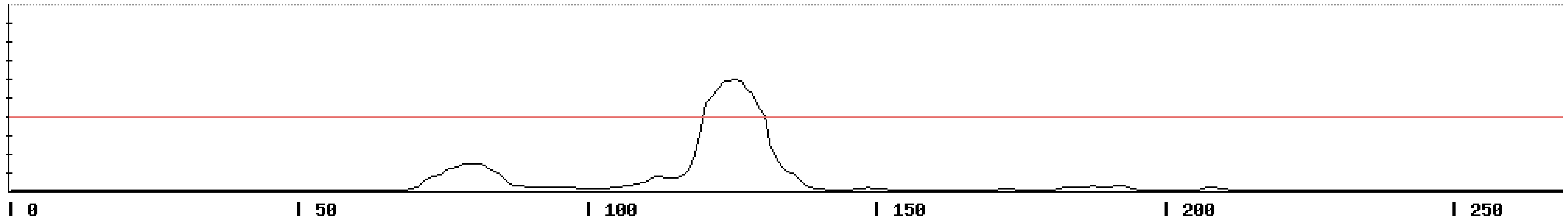

Predictions for Brassicanapus_GSBRNA2T00016028001

Viterbi Path	
Viterbi Path	
Posterior @ 0.4	98 - GKPK - 101
Posterior @ 0.4	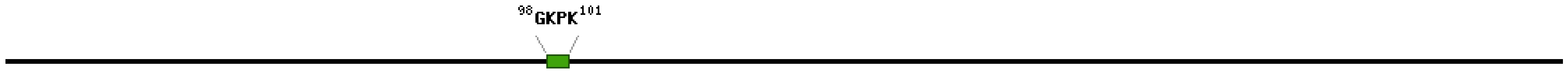
Posterior Graphic. Threshold Marked @ 0.4	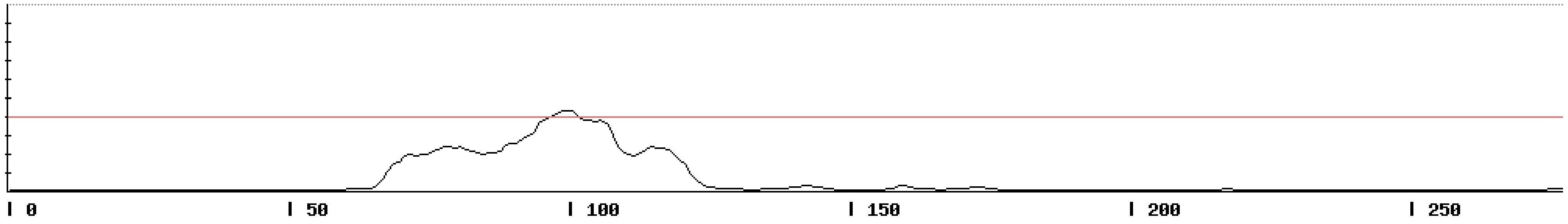

Predictions for Brassicanapus_GSBRNA2T00016038001

Viterbi Path	
Viterbi Path	
Posterior @ 0.4	
Posterior @ 0.4	
Posterior Graphic. Threshold Marked @ 0.4	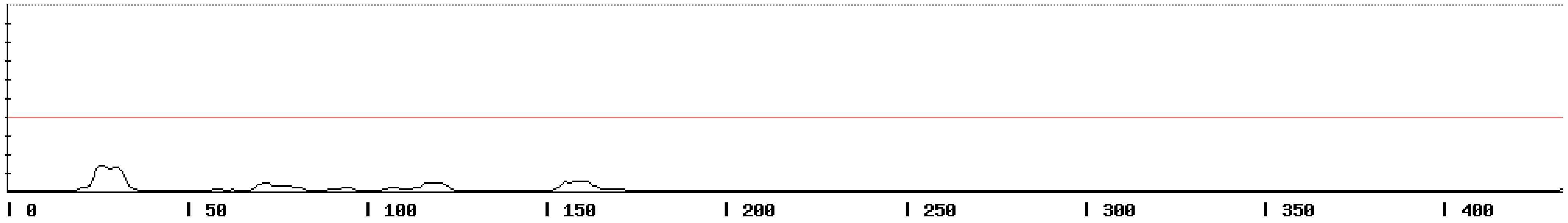

Predictions for Brassicanapus_GSBRNA2T00016676001	
Viterbi Path	
Viterbi Path	
Posterior @ 0.4	72 - RKESRYNRGERQKRKTKSGYWKKTGNT - 98<br>103 - RK - 104
Posterior @ 0.4	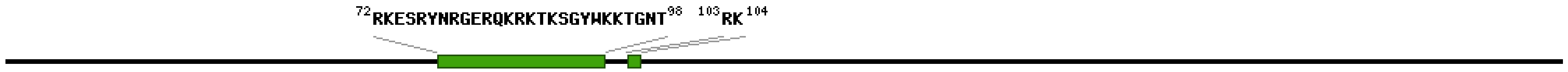
Posterior Graphic. Threshold Marked @ 0.4	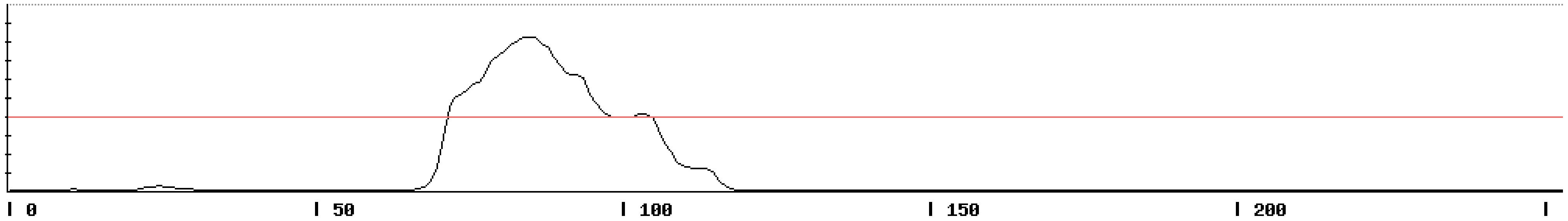

Predictions for Brassicanapus_GSBRNA2T00016677001	
Viterbi Path	68 - GRKESKYNKGERQKRKTKSGFWKKTGNTLPIKRKRGNRQKIGEK - 111
Viterbi Path	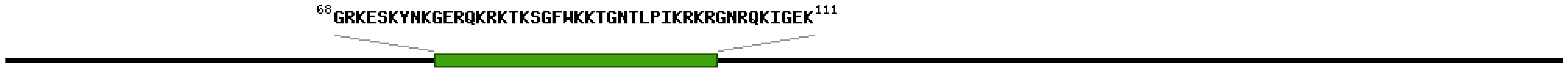
Posterior @ 0.4	69 - RKESKYNKGERQKRKTKSGFWKKTGNTLPIKRKRGNRQKIGE - 110
Posterior @ 0.4	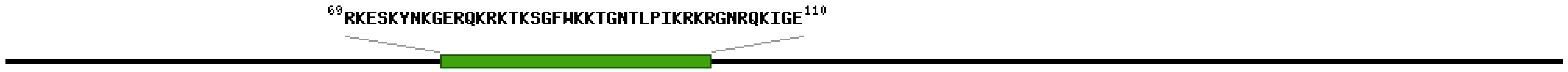
Posterior Graphic. Threshold Marked @ 0.4	

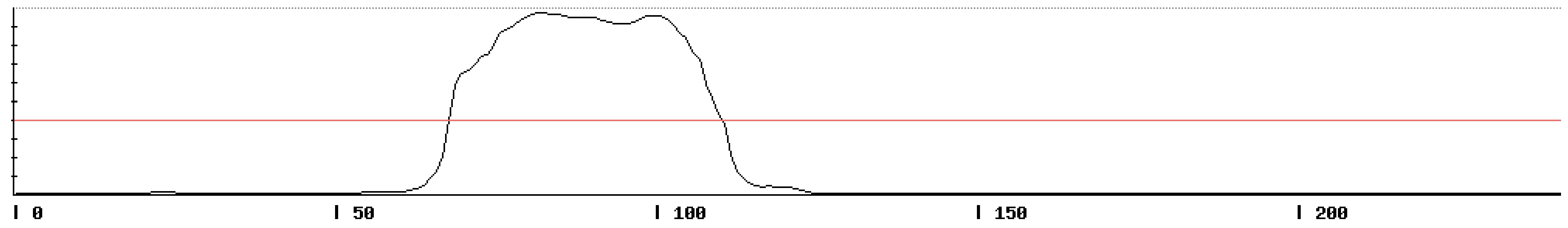

Predictions for Brassicanapus_GSBRNA2T00016973001

Viterbi Path	
Viterbi Path	
Posterior @ 0.4	
Posterior @ 0.4	
Posterior Graphic. Threshold Marked @ 0.4	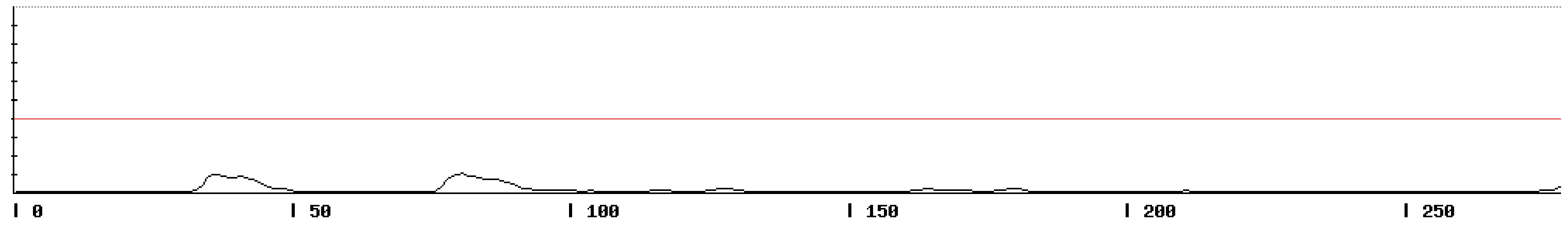

Predictions for Brassicanapus_GSBRNA2T00018140001

Viterbi Path	
Viterbi Path	
Posterior @ 0.4	
Posterior @ 0.4	

Posterior Graphic. Threshold Marked @ 0.4

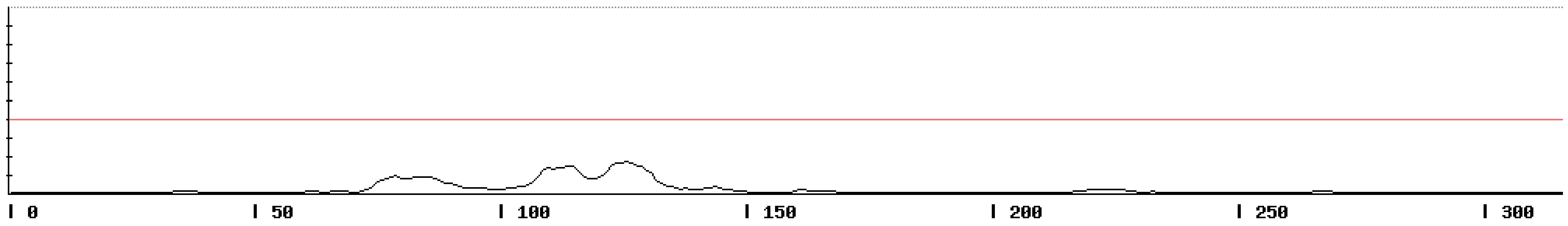

Predictions for Brassicanapus_GSBRNA2T00018364001

Viterbi Path	
Viterbi Path	
Posterior @ 0.4	69 - PAIKKIDSNRPSRKVAKSGRW - 89
Posterior @ 0.4	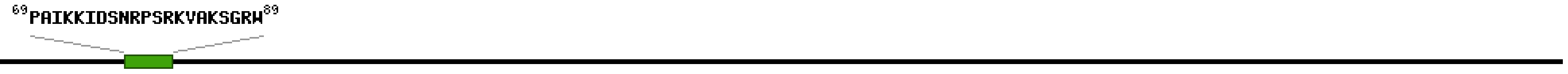
Posterior Graphic. Threshold Marked @ 0.4	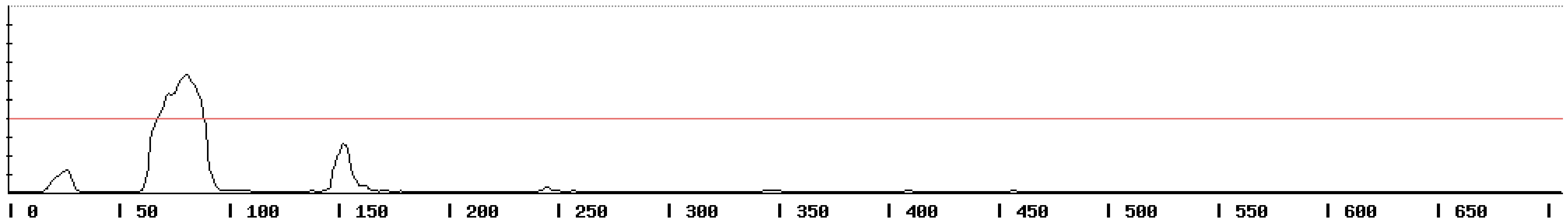

Predictions for Brassicanapus_GSBRNA2T00018373001

Viterbi Path	
Viterbi Path	
Posterior @ 0.4	63 - KKAADSKRPSRKHF - 76
Posterior @ 0.4	

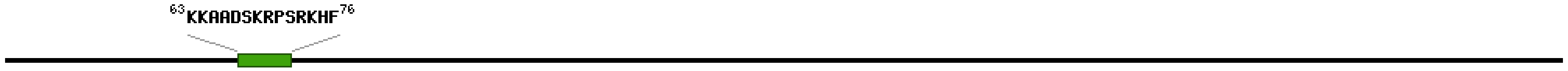

Posterior Graphic. Threshold Marked @ 0.4

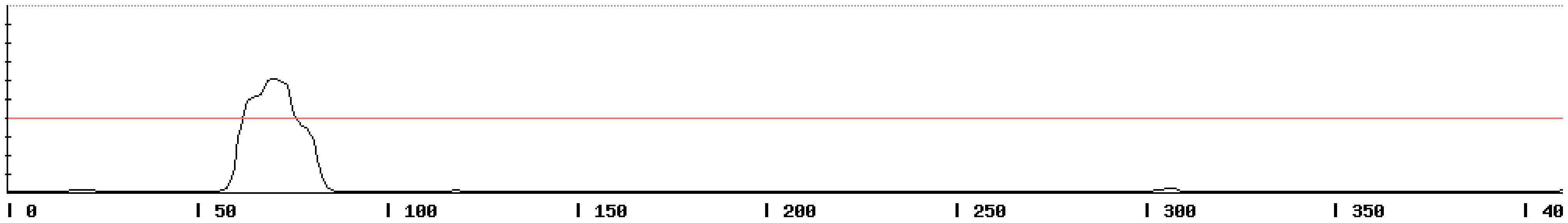

Predictions for Brassicanapus_GSBRNA2T00018374001

Viterbi Path

Viterbi Path

Posterior @ 0.4

Posterior @ 0.4

Posterior Graphic. Threshold Marked @ 0.4

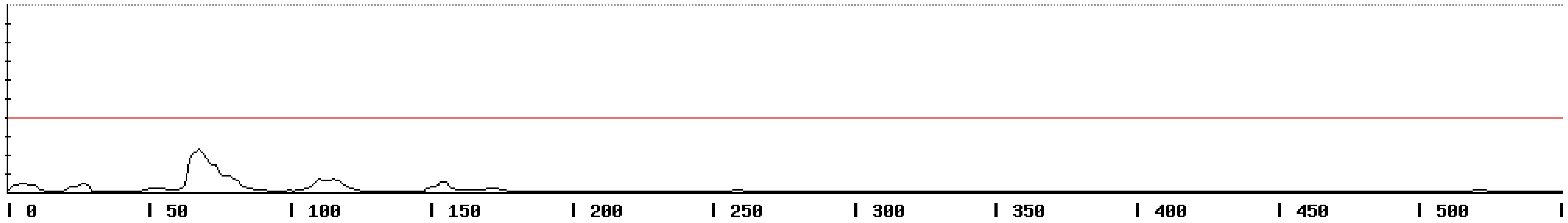

Predictions for Brassicanapus_GSBRNA2T00018746001

Viterbi Path

Viterbi Path

Posterior @ 0.4	
Posterior @ 0.4	
Posterior Graphic. Threshold Marked @ 0.4	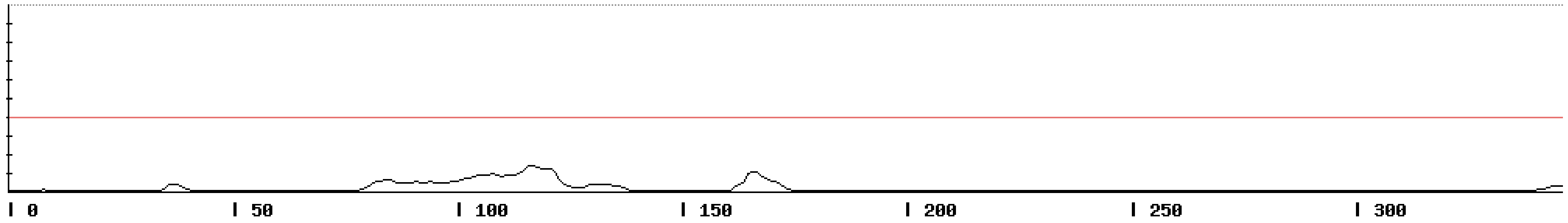

Predictions for Brassicanapus_GSBRNA2T00019258001

Viterbi Path	
Viterbi Path	
Posterior @ 0.4	
Posterior @ 0.4	
Posterior Graphic. Threshold Marked @ 0.4	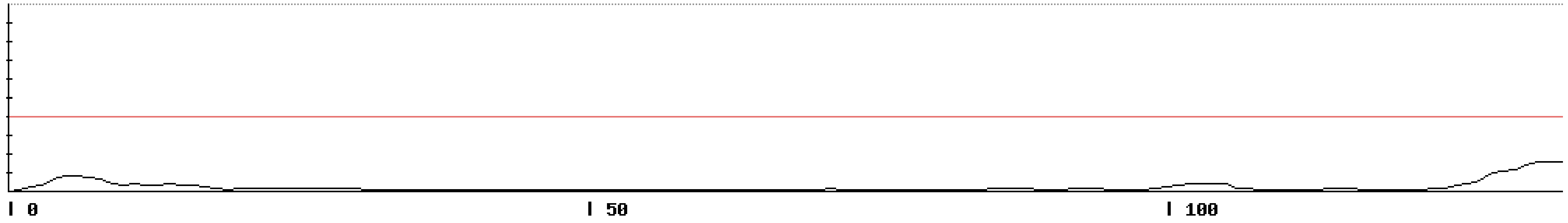

Predictions for Brassicanapus_GSBRNA2T00019342001

Viterbi Path	
Viterbi Path	

Posterior @ 0.4

Posterior @ 0.4

Posterior Graphic. Threshold Marked @ 0.4

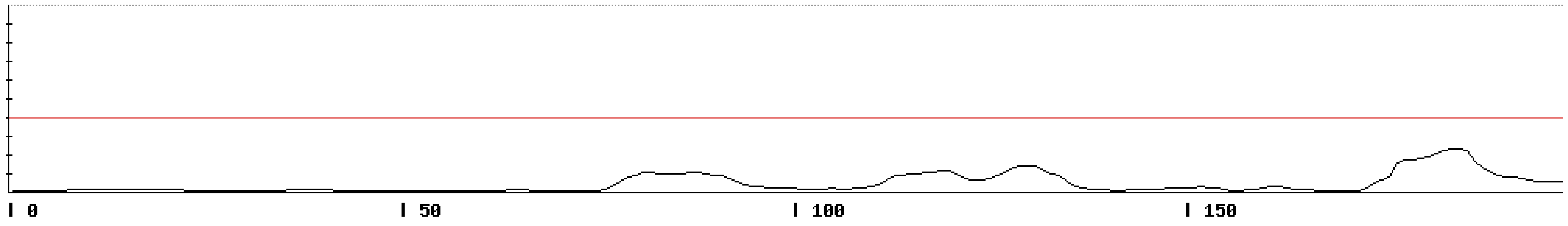

Predictions for Brassicanapus_GSBRNA2T00019578001

Viterbi Path

Viterbi Path

Posterior @ 0.4

Posterior @ 0.4

Posterior Graphic. Threshold Marked @ 0.4

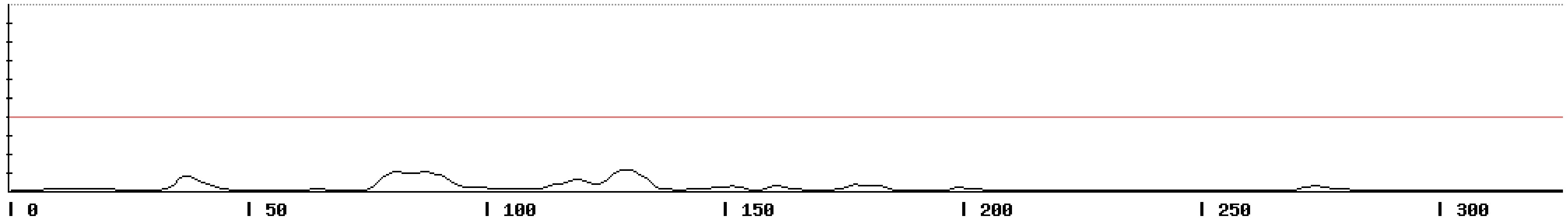

Predictions for Brassicanapus_GSBRNA2T00019640001

Viterbi Path

Viterbi Path

Posterior @ 0.4

Posterior @ 0.4

Posterior Graphic. Threshold Marked @ 0.4

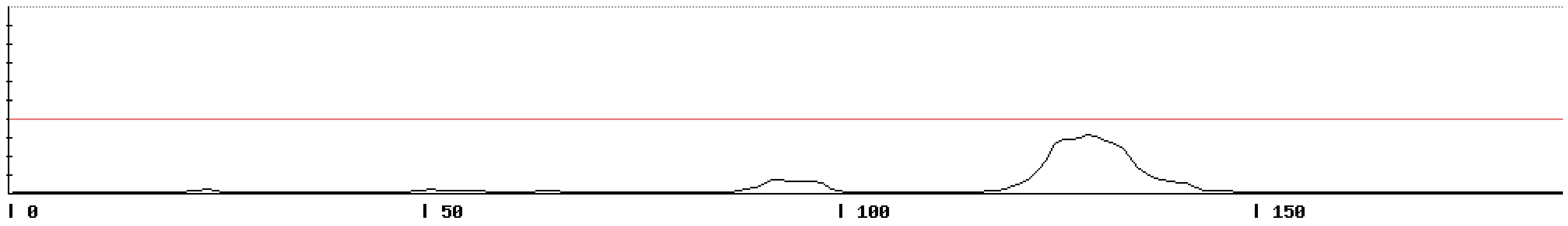

Predictions for Brassicanapus_GSBRNA2T00020292001

Viterbi Path

Viterbi Path

Posterior @ 0.4

Posterior @ 0.4

Posterior Graphic. Threshold Marked @ 0.4

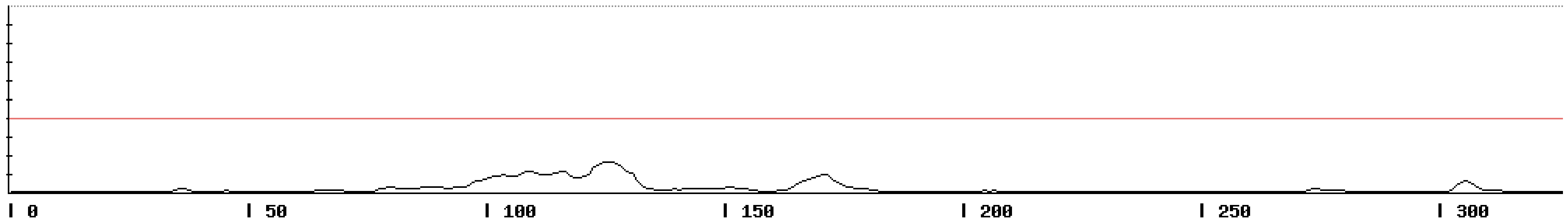

Predictions for Brassicanapus_GSBRNA2T00020965001	
Viterbi Path	
Viterbi Path	
Posterior @ 0.4	
Posterior @ 0.4	
Posterior Graphic. Threshold Marked @ 0.4	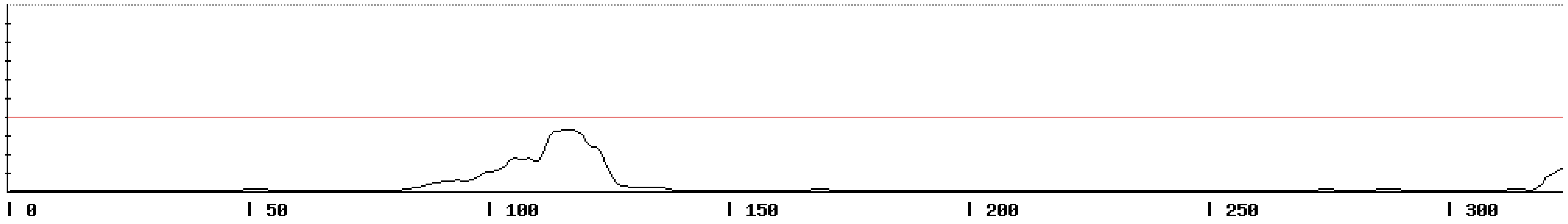

Predictions for Brassicanapus_GSBRNA2T00021663001	
Viterbi Path	
Viterbi Path	
Posterior @ 0.4	233 - KKRLRRD - 239
Posterior @ 0.4	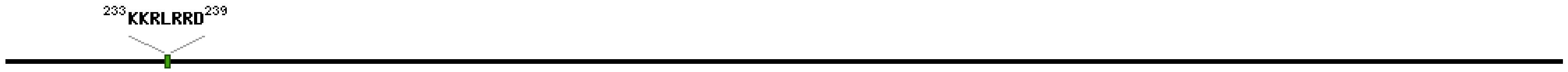
Posterior Graphic. Threshold Marked @ 0.4	

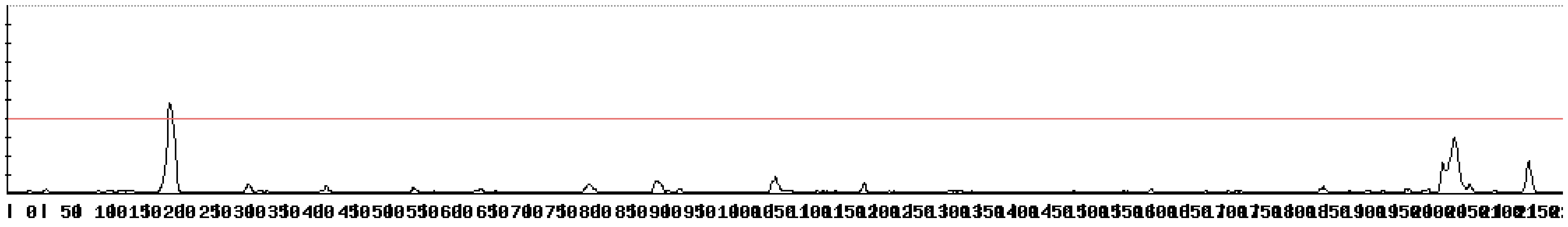

Predictions for Brassicanapus_GSBRNA2T00022937001

Viterbi Path	
Viterbi Path	
Posterior @ 0.4	
Posterior @ 0.4	
Posterior Graphic. Threshold Marked @ 0.4	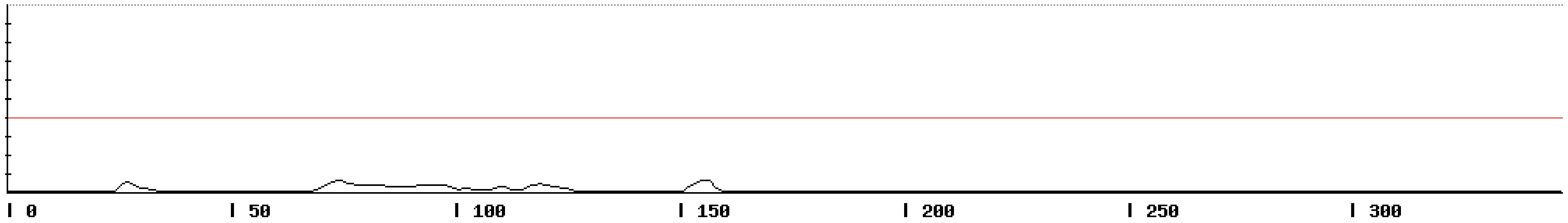

Predictions for Brassicanapus_GSBRNA2T00022943001

Viterbi Path	
Viterbi Path	
Posterior @ 0.4	
Posterior @ 0.4	

Posterior Graphic. Threshold Marked @ 0.4

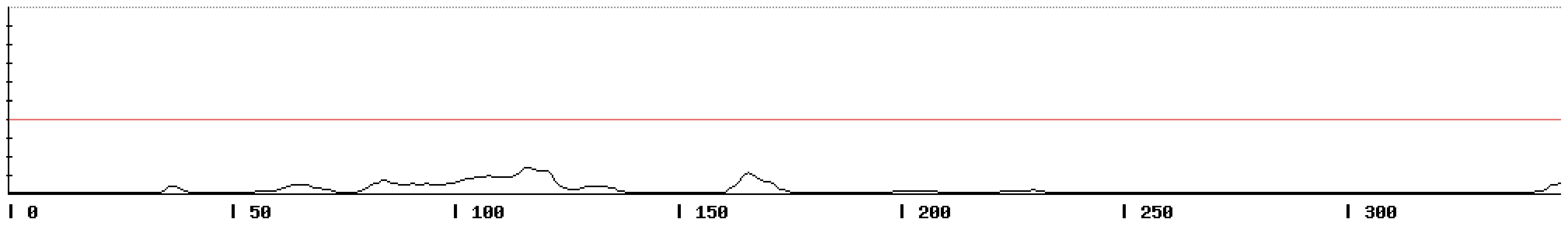

Predictions for Brassicanapus_GSBRNA2T00023614001

Viterbi Path

Viterbi Path

Posterior @ 0.4

Posterior @ 0.4

Posterior Graphic. Threshold Marked @ 0.4

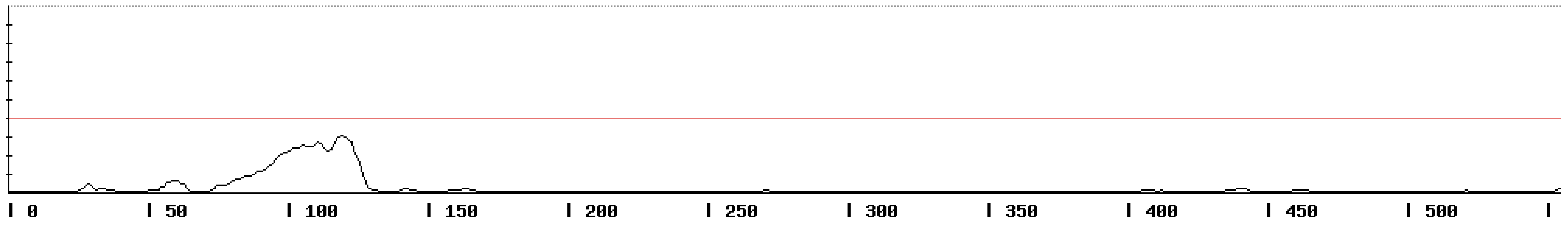

Predictions for Brassicanapus_GSBRNA2T00023738001

Viterbi Path

Viterbi Path

Posterior @ 0.4

Posterior @ 0.4

Posterior Graphic. Threshold Marked @ 0.4

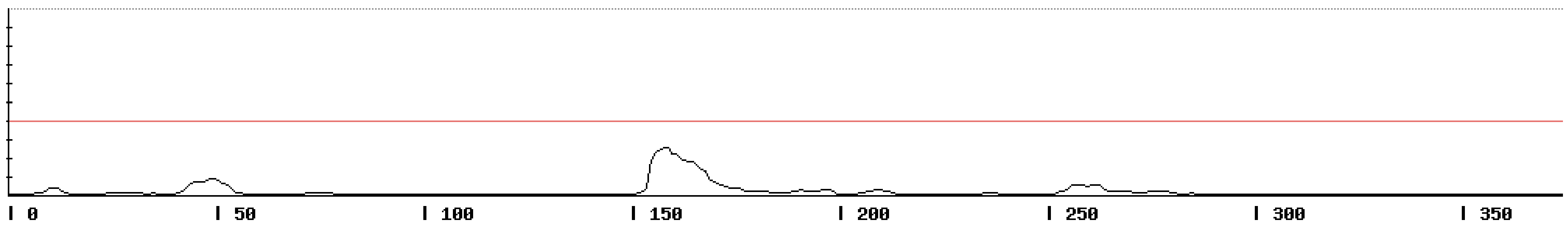

Predictions for Brassicanapus_GSBRNA2T00023746001

Viterbi Path

Viterbi Path

Posterior @ 0.4

Posterior @ 0.4

Posterior Graphic. Threshold Marked @ 0.4

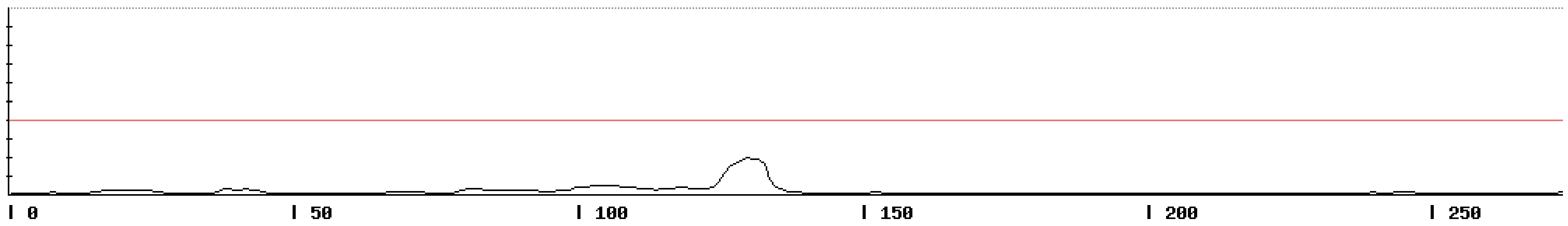

Predictions for Brassicanapus_GSBRNA2T00024057001

Viterbi Path

Viterbi Path

Posterior @ 0.4	197 - KDKKRK - 202
Posterior @ 0.4	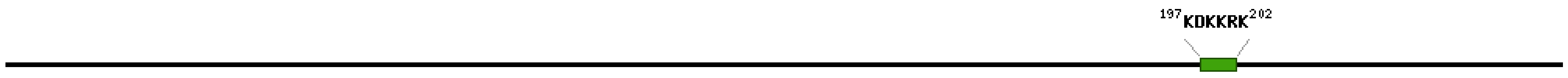
Posterior Graphic. Threshold Marked @ 0.4	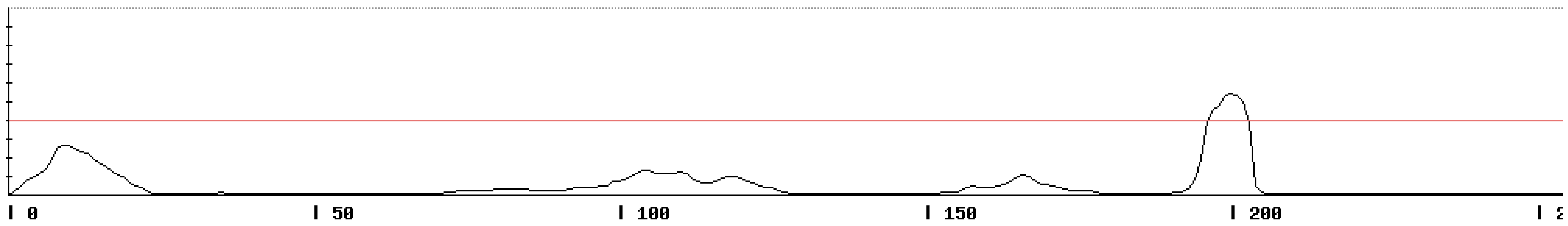

Predictions for Brassicanapus_GSBRNA2T00024330001

Viterbi Path	
Viterbi Path	
Posterior @ 0.4	
Posterior @ 0.4	
Posterior Graphic. Threshold Marked @ 0.4	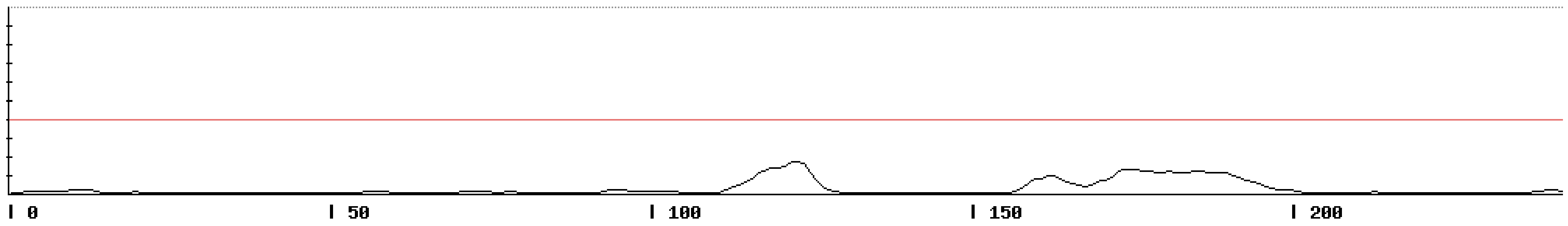

Predictions for Brassicanapus_GSBRNA2T00024336001

Viterbi Path	
Viterbi Path	

Posterior @ 0.4

Posterior @ 0.4

Posterior Graphic. Threshold Marked @ 0.4

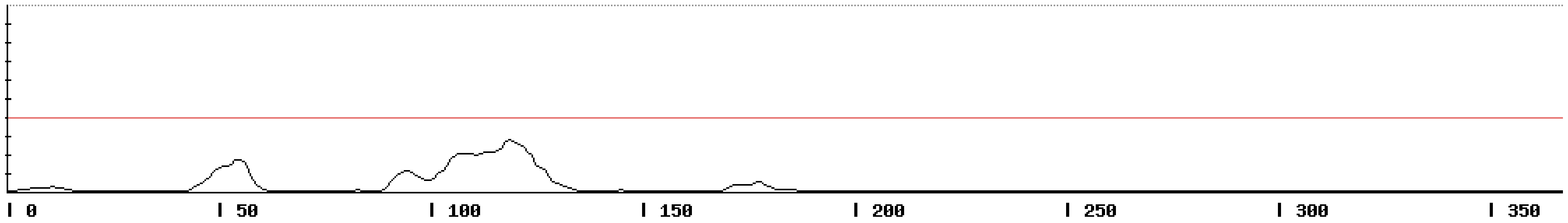

Predictions for Brassicanapus_GSBRNA2T00026353001

Viterbi Path

Viterbi Path

Posterior @ 0.4

Posterior @ 0.4

Posterior Graphic. Threshold Marked @ 0.4

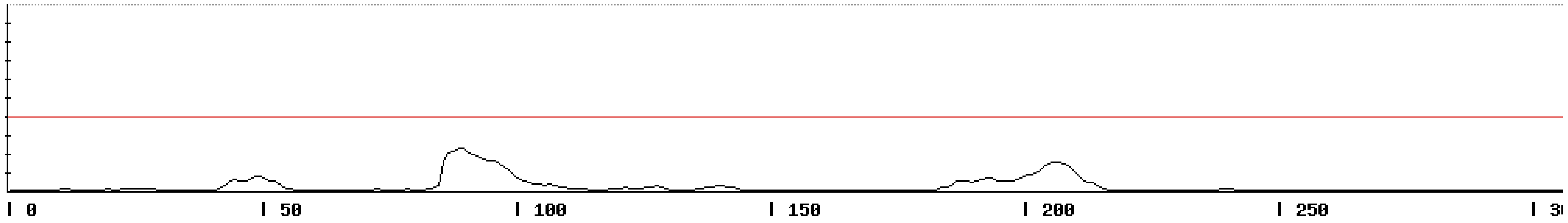

Predictions for Brassicanapus_GSBRNA2T00026805001

Viterbi Path

Viterbi Path

Posterior @ 0.4

Posterior @ 0.4

Posterior Graphic. Threshold Marked @ 0.4

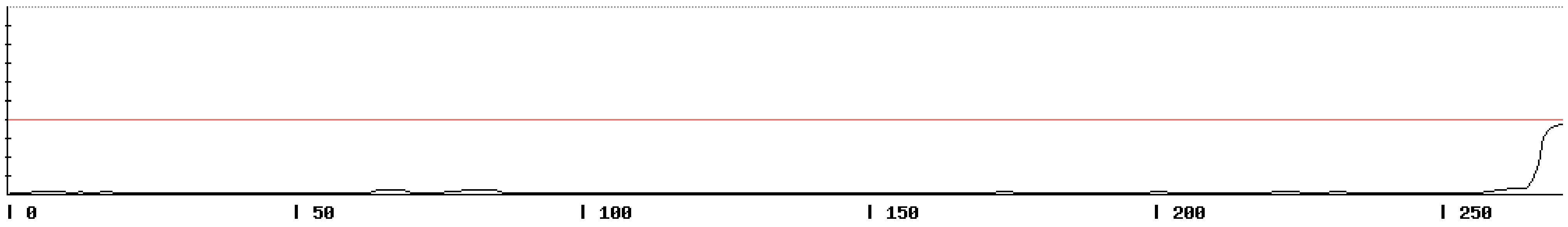

Predictions for Brassicanapus_GSBRNA2T00026833001

Viterbi Path

Viterbi Path

Posterior @ 0.4

Posterior @ 0.4

Posterior Graphic. Threshold Marked @ 0.4

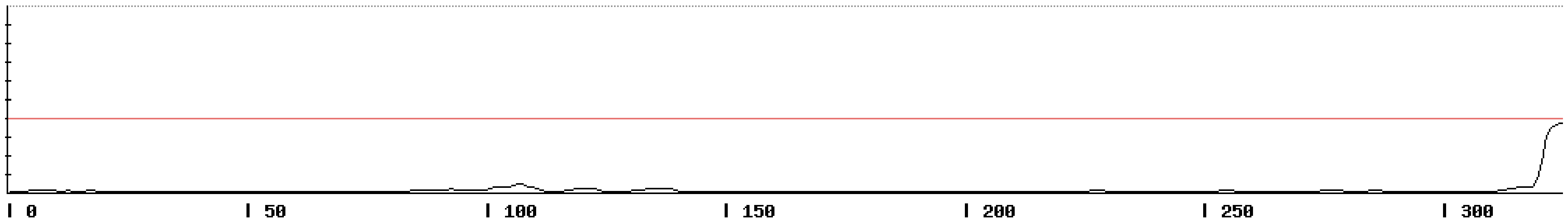

Predictions for Brassicanapus_GSBRNA2T00027379001

Viterbi Path	
Viterbi Path	
Posterior @ 0.4	118 - KVKGKP - 123
Posterior @ 0.4	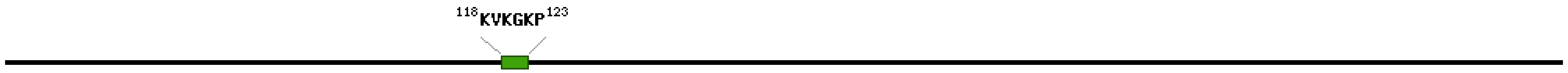
Posterior Graphic. Threshold Marked @ 0.4	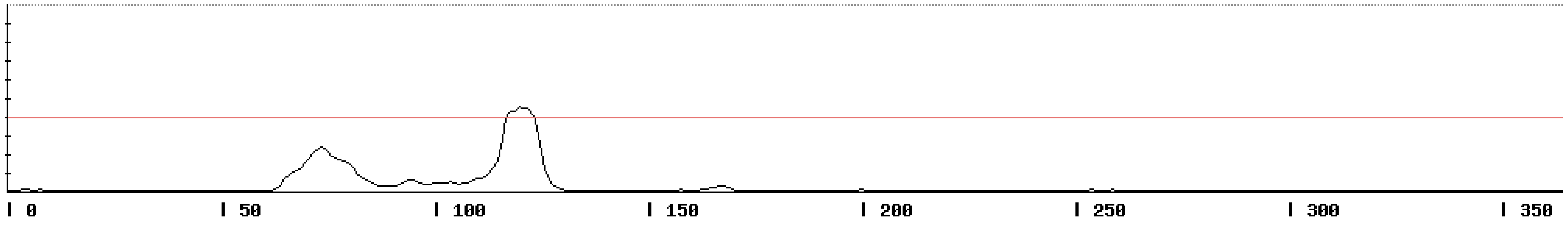

Predictions for Brassicanapus_GSBRNA2T00027399001

Viterbi Path	
Viterbi Path	
Posterior @ 0.4	
Posterior @ 0.4	
Posterior Graphic. Threshold Marked @ 0.4	

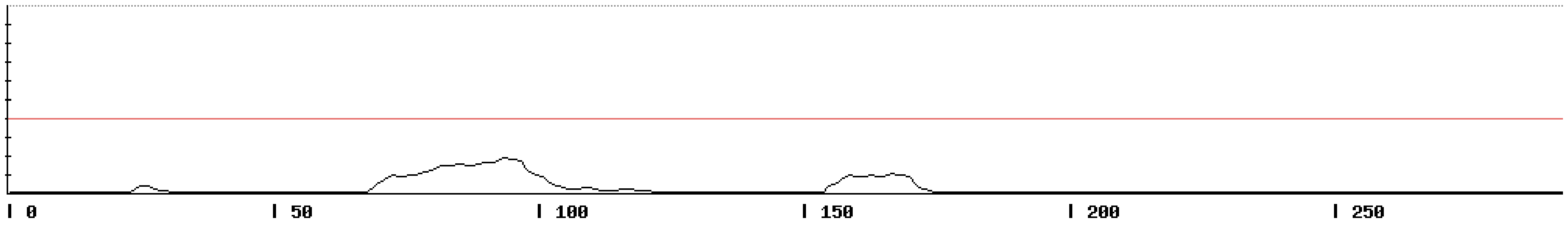

Predictions for Brassicanapus_GSBRNA2T00027750001

Viterbi Path

Viterbi Path

Posterior @ 0.4

77 - KKGTK - 81

366 - KVKKKTRKPIKK - 377

Posterior @ 0.4

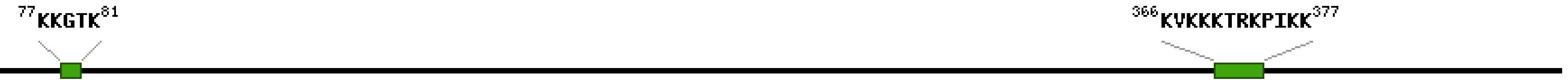

Posterior Graphic. Threshold Marked @ 0.4

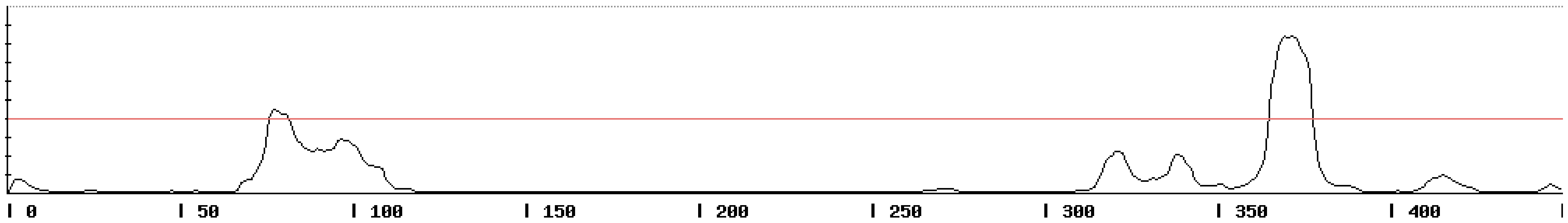

Predictions for Brassicanapus_GSBRNA2T00027751001

Viterbi Path

Viterbi Path

Posterior @ 0.4

75 - EKKKGTKRTTPSGFWKATGKDRIIRDKRGH - 104

389 - RNRKKP - 394

Posterior @ 0.4

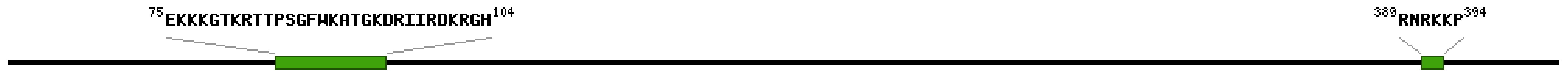

Posterior Graphic. Threshold Marked @ 0.4

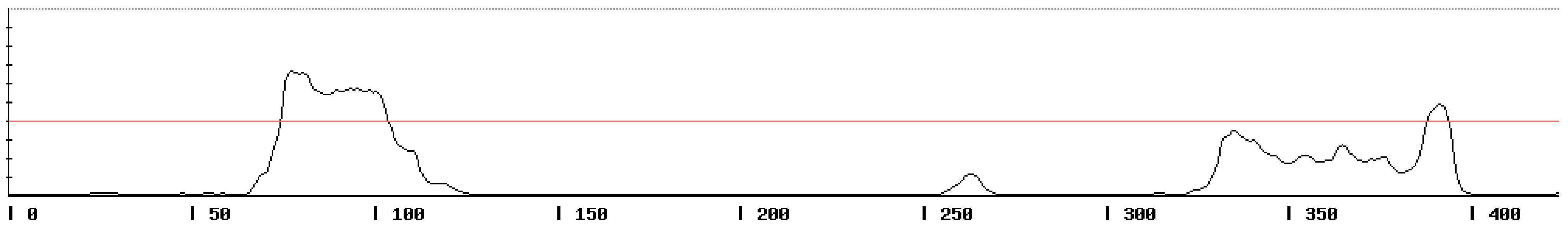

Predictions for Brassicanapus_GSBRNA2T00027754001

Viterbi Path	
Viterbi Path	
Posterior @ 0.4	72 - KSPKKSLTKRTTPSGFWKSTGKDRKIKEDKRRD - 104
Posterior @ 0.4	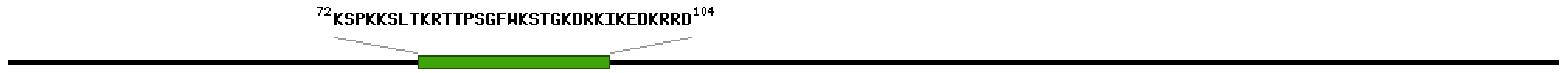
Posterior Graphic. Threshold Marked @ 0.4	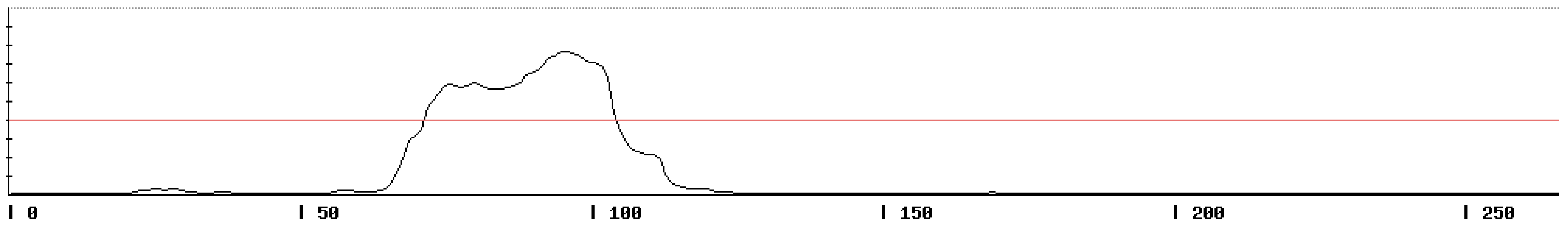

Predictions for Brassicanapus_GSBRNA2T00028210001

Viterbi Path	
Viterbi Path	

Posterior @ 0.4

Posterior @ 0.4

Posterior Graphic. Threshold Marked @ 0.4

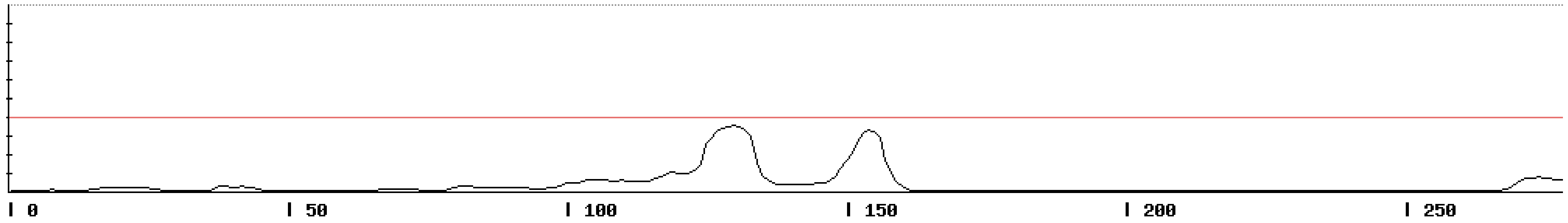

Predictions for Brassicanapus_GSBRNA2T00028217001

Viterbi Path

Viterbi Path

Posterior @ 0.4

Posterior @ 0.4

Posterior Graphic. Threshold Marked @ 0.4

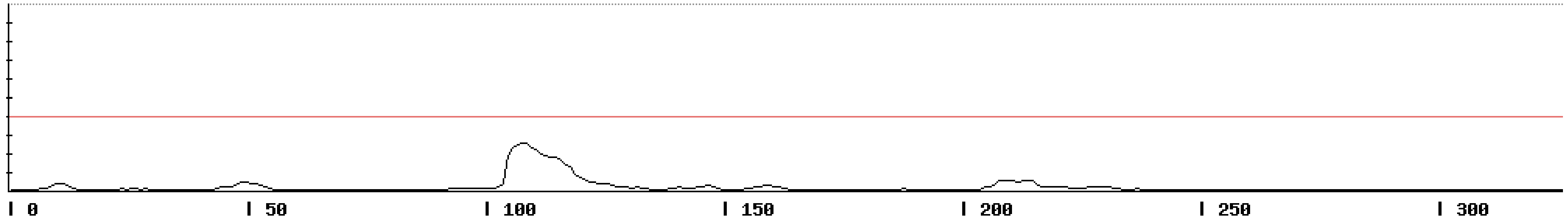

Predictions for Brassicanapus_GSBRNA2T00028681001

Viterbi Path

Viterbi Path

Posterior @ 0.4	55 - RRK - 57
Posterior @ 0.4	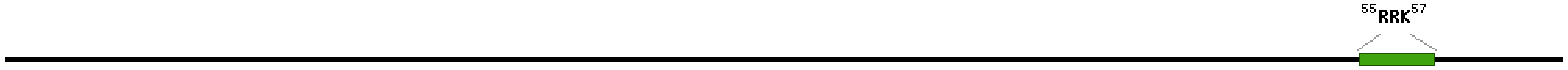
Posterior Graphic. Threshold Marked @ 0.4	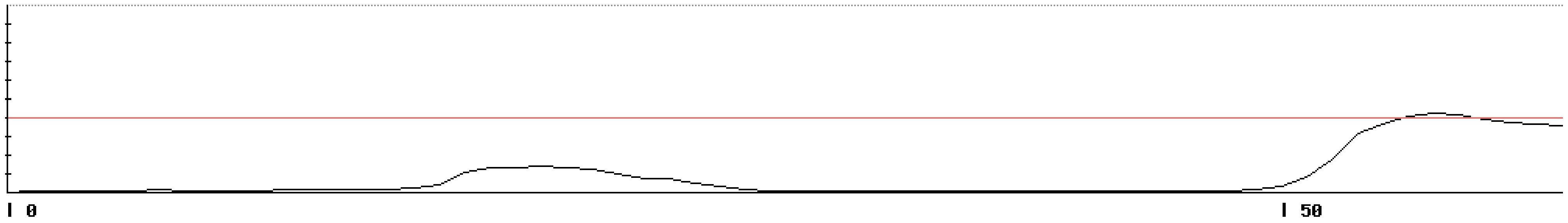

Predictions for Brassicanapus_GSBRNA2T00028935001

Viterbi Path	
Viterbi Path	
Posterior @ 0.4	
Posterior @ 0.4	
Posterior Graphic. Threshold Marked @ 0.4	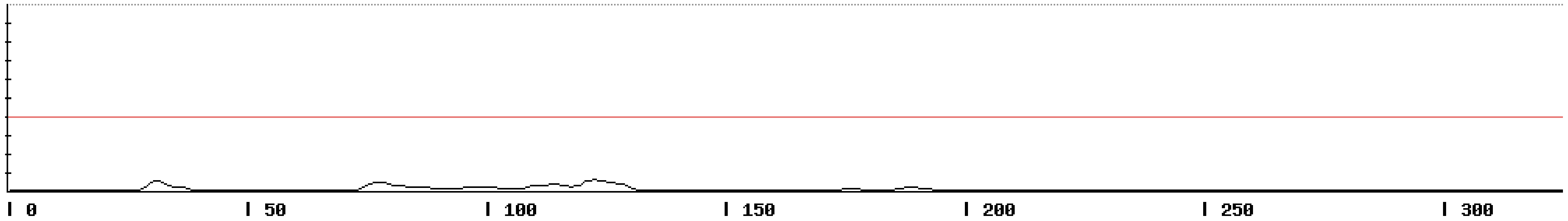

Predictions for Brassicanapus_GSBRNA2T00030157001

Viterbi Path

Viterbi Path

Posterior @ 0.4

Posterior @ 0.4

Posterior Graphic. Threshold Marked @ 0.4

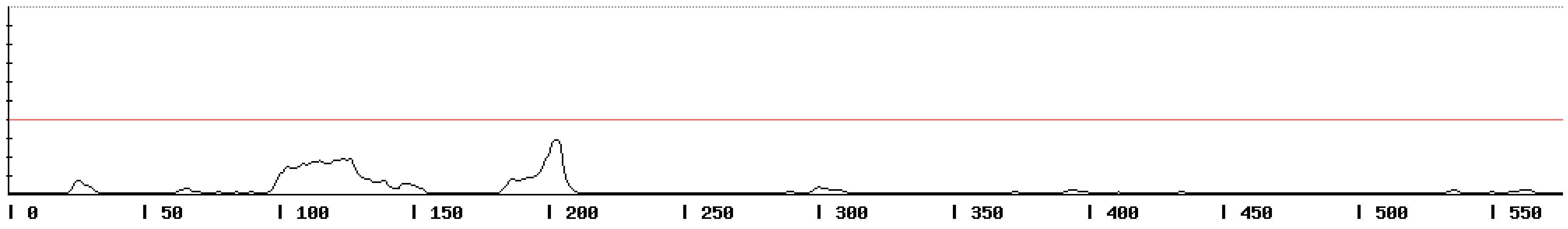

Predictions for Brassicanapus_GSBRNA2T00030633001

Viterbi Path

Viterbi Path

Posterior @ 0.4

Posterior @ 0.4

Posterior Graphic. Threshold Marked @ 0.4

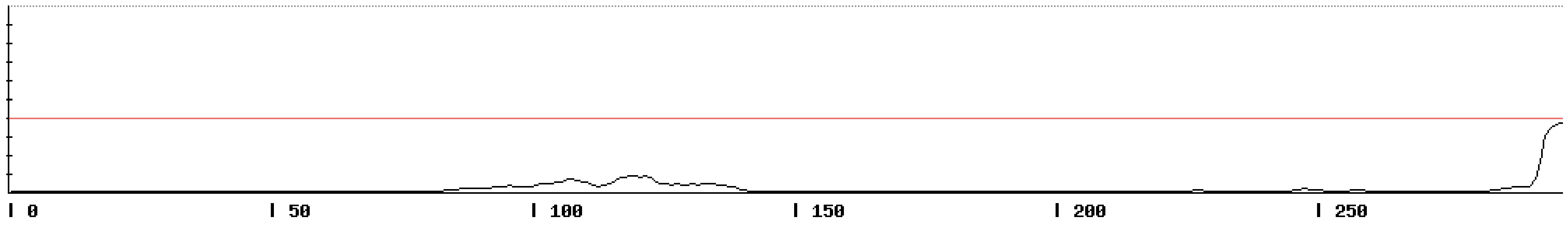

Predictions for Brassicanapus_GSBRNA2T00030840001	
Viterbi Path	
Viterbi Path	
Posterior @ 0.4	
Posterior @ 0.4	
Posterior Graphic. Threshold Marked @ 0.4	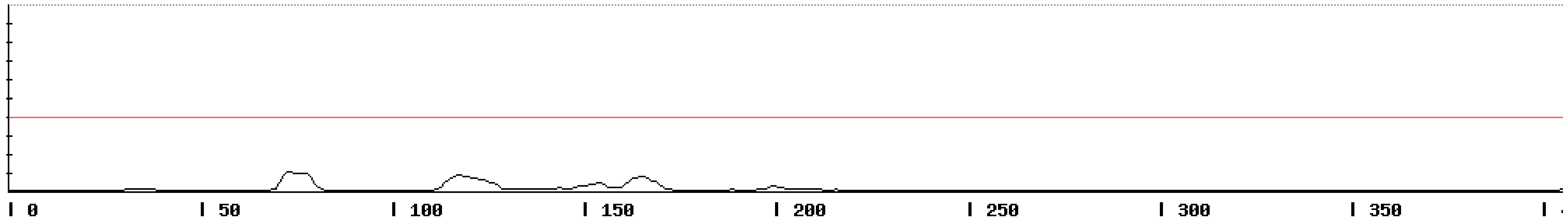

Predictions for Brassicanapus_GSBRNA2T00031473001	
Viterbi Path	
Viterbi Path	
Posterior @ 0.4	
Posterior @ 0.4	
Posterior Graphic. Threshold Marked @ 0.4	

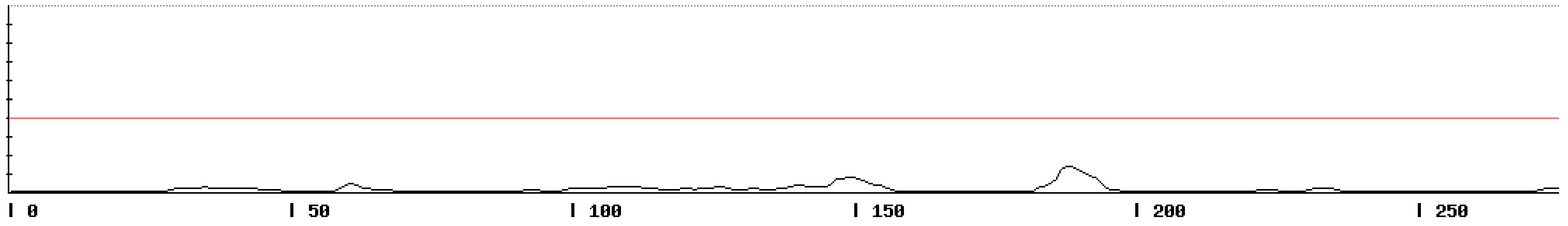

Predictions for Brassicanapus_GSBRNA2T00031577001

Viterbi Path	146 - RKASKGKRQRKSRGR - 160
Viterbi Path	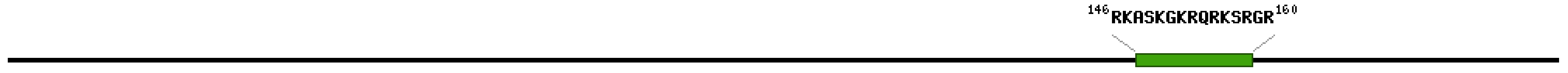
Posterior @ 0.4	63 - KR - 64<br>145 - ERKASKGKRQRKSRGR - 160
Posterior @ 0.4	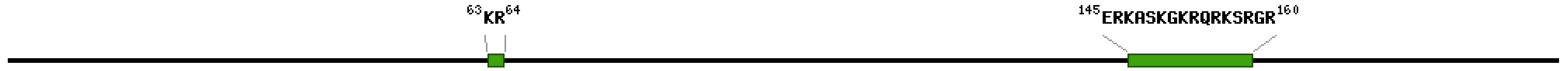
Posterior Graphic. Threshold Marked @ 0.4	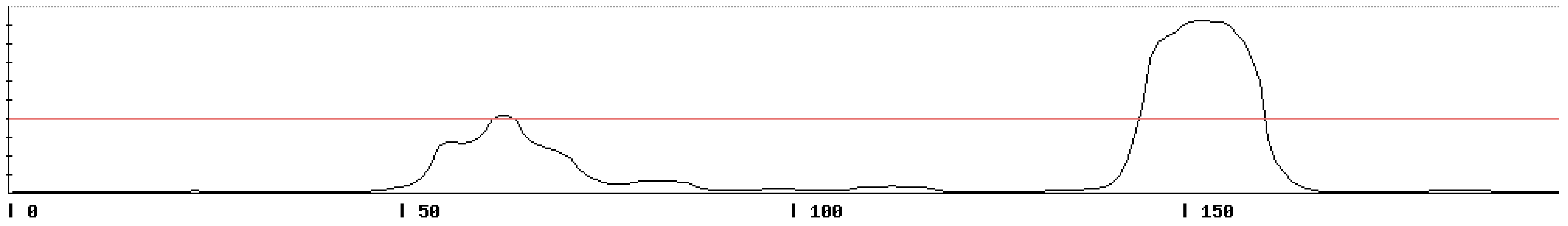

Predictions for Brassicanapus_GSBRNA2T00032449001

Viterbi Path	
Viterbi Path	
Posterior @ 0.4	178 - KKKWK - 182
Posterior @ 0.4	

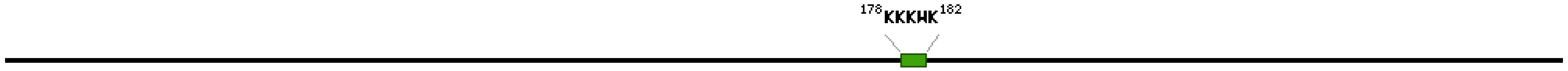

Posterior Graphic. Threshold Marked @ 0.4

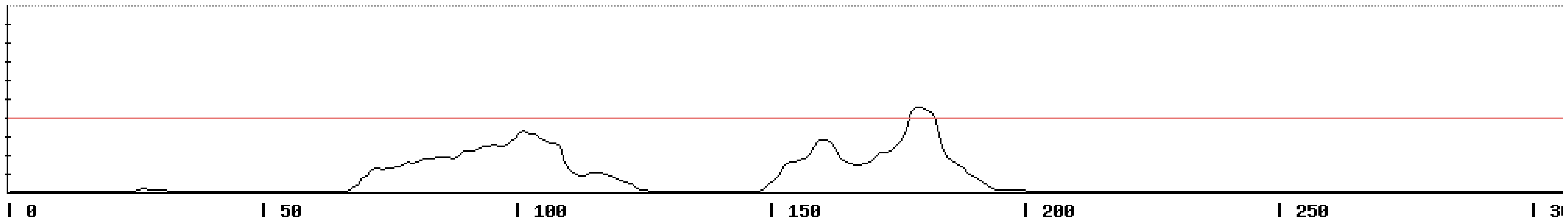

Predictions for Brassicanapus_GSBRNA2T00032467001

Viterbi Path	
Viterbi Path	
Posterior @ 0.4	
Posterior @ 0.4	
Posterior Graphic. Threshold Marked @ 0.4	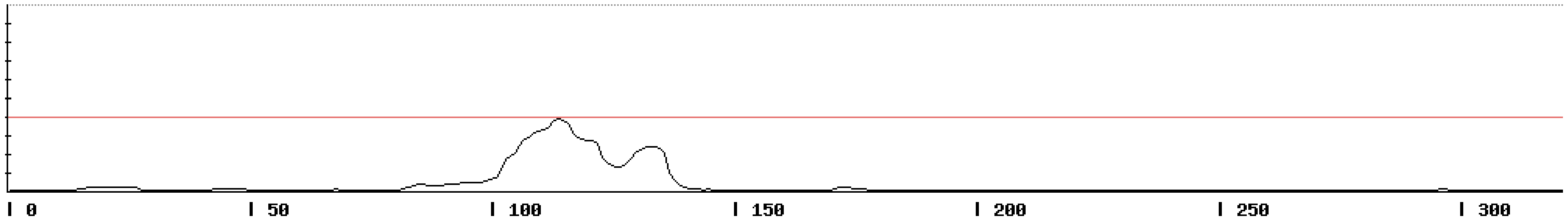

Predictions for Brassicanapus_GSBRNA2T00033138001

Viterbi Path	
Viterbi Path	

Posterior @ 0.4	35 - KRKIRRKKLR - 44
Posterior @ 0.4	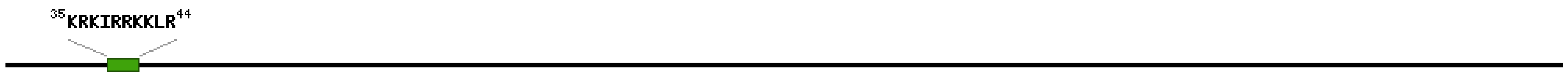
Posterior Graphic. Threshold Marked @ 0.4	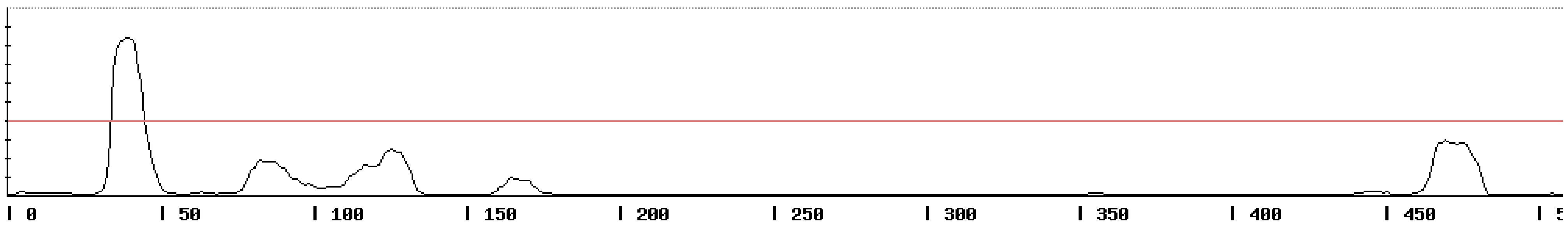

Predictions for Brassicanapus_GSBRNA2T00033572001	
Viterbi Path	
Viterbi Path	
Posterior @ 0.4	
Posterior @ 0.4	
Posterior Graphic. Threshold Marked @ 0.4	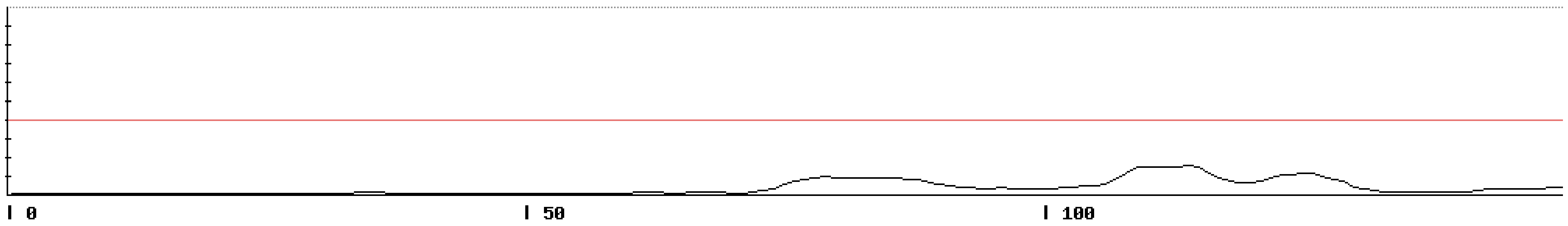

Predictions for Brassicanapus_GSBRNA2T00033573001	
Viterbi Path	
Viterbi Path	

Posterior @ 0.4

Posterior @ 0.4

Posterior Graphic. Threshold Marked @ 0.4

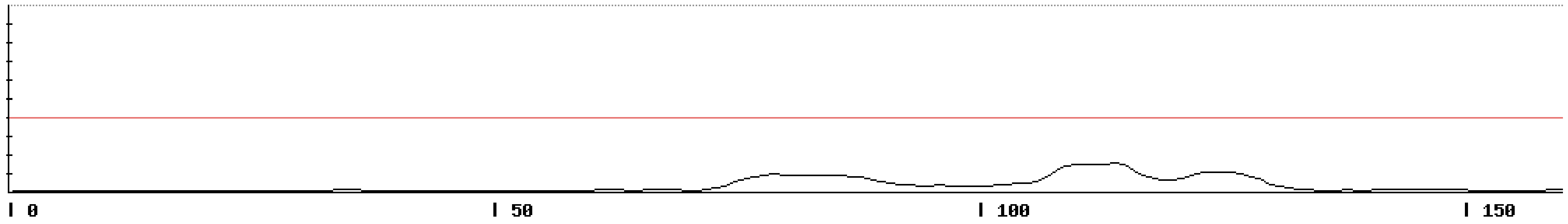

Predictions for Brassicanapus_GSBRNA2T00034156001

Viterbi Path

Viterbi Path

Posterior @ 0.4

Posterior @ 0.4

Posterior Graphic. Threshold Marked @ 0.4

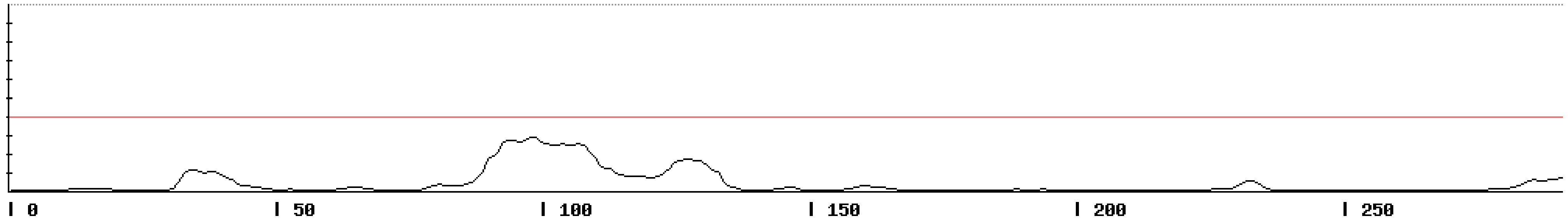

Predictions for Brassicanapus_GSBRNA2T00034316001

Viterbi Path

Viterbi Path

Posterior @ 0.4

Posterior @ 0.4

Posterior Graphic. Threshold Marked @ 0.4

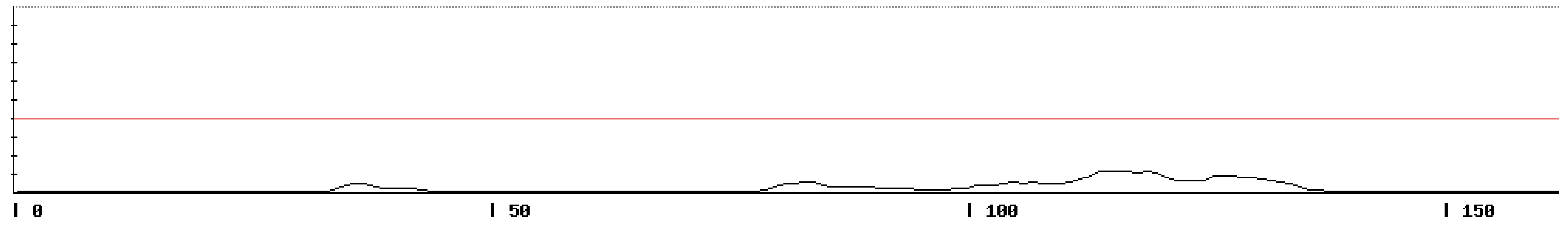

Predictions for Brassicanapus_GSBRNA2T00034345001

Viterbi Path

Viterbi Path

Posterior @ 0.4

Posterior @ 0.4

Posterior Graphic. Threshold Marked @ 0.4

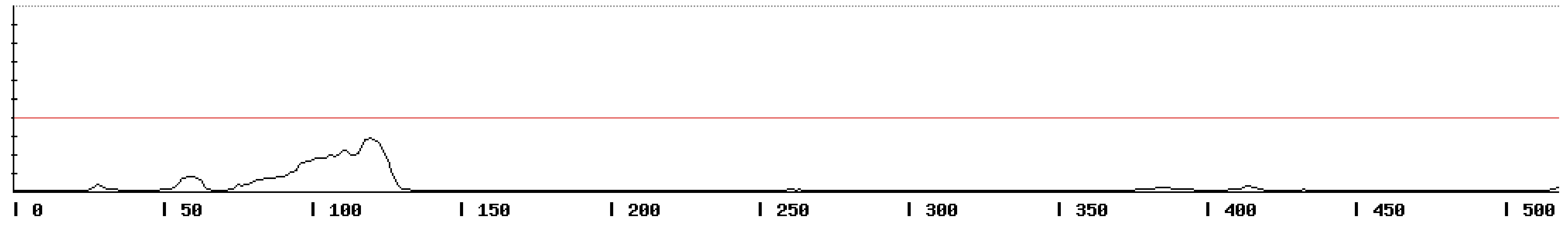

Predictions for Brassicanapus_GSBRNA2T00035413001	
Viterbi Path	
Viterbi Path	
Posterior @ 0.4	
Posterior @ 0.4	
Posterior Graphic. Threshold Marked @ 0.4	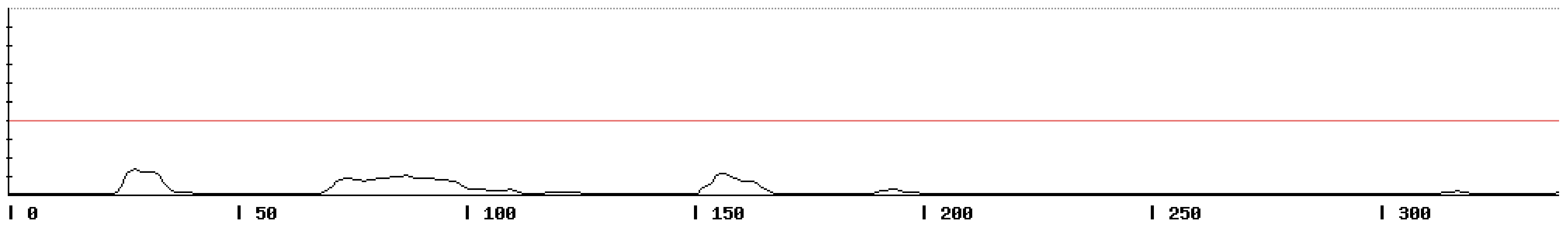

Predictions for Brassicanapus_GSBRNA2T00035731001	
Viterbi Path	
Viterbi Path	
Posterior @ 0.4	
Posterior @ 0.4	
Posterior Graphic. Threshold Marked @ 0.4	

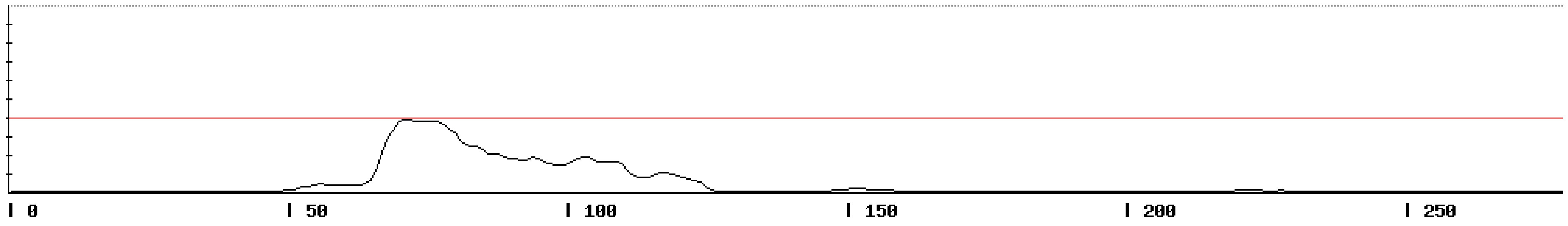

Predictions for Brassicanapus_GSBRNA2T00036630001

Viterbi Path	
Viterbi Path	
Posterior @ 0.4	
Posterior @ 0.4	
Posterior Graphic. Threshold Marked @ 0.4	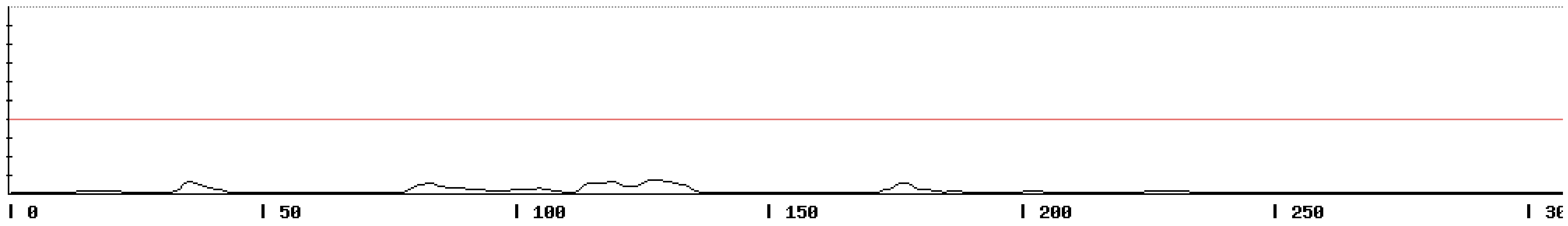

Predictions for Brassicanapus_GSBRNA2T00036642001

Viterbi Path	
Viterbi Path	
Posterior @ 0.4	
Posterior @ 0.4	

Posterior Graphic. Threshold Marked @ 0.4	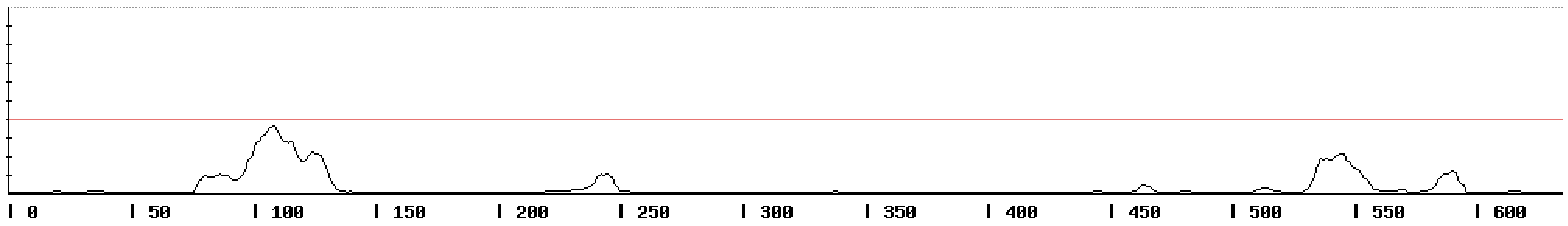

Predictions for Brassicanapus_GSBRNA2T00037075001

Viterbi Path	
Viterbi Path	
Posterior @ 0.4	
Posterior @ 0.4	
Posterior Graphic. Threshold Marked @ 0.4	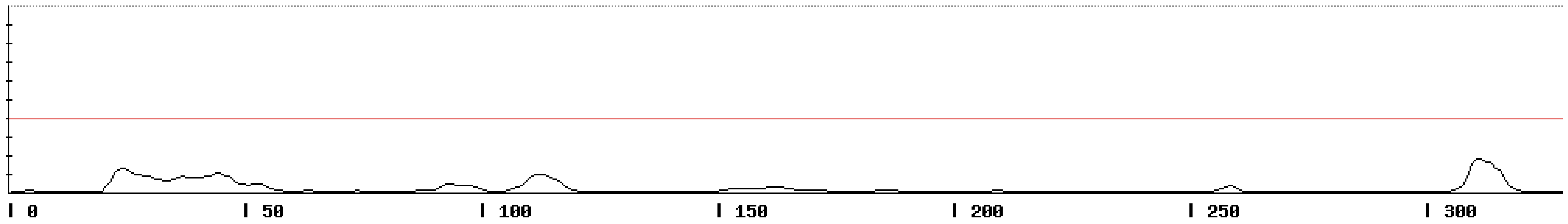

Predictions for Brassicanapus_GSBRNA2T00037593001

Viterbi Path	
Viterbi Path	
Posterior @ 0.4	
Posterior @ 0.4	

Posterior Graphic. Threshold Marked @ 0.4

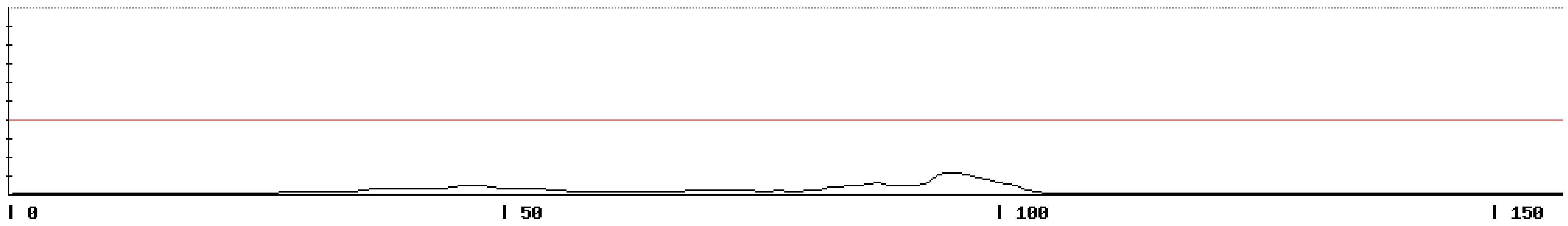

Predictions for Brassicanapus_GSBRNA2T00037877001

Viterbi Path

Viterbi Path

Posterior @ 0.4

Posterior @ 0.4

Posterior Graphic. Threshold Marked @ 0.4

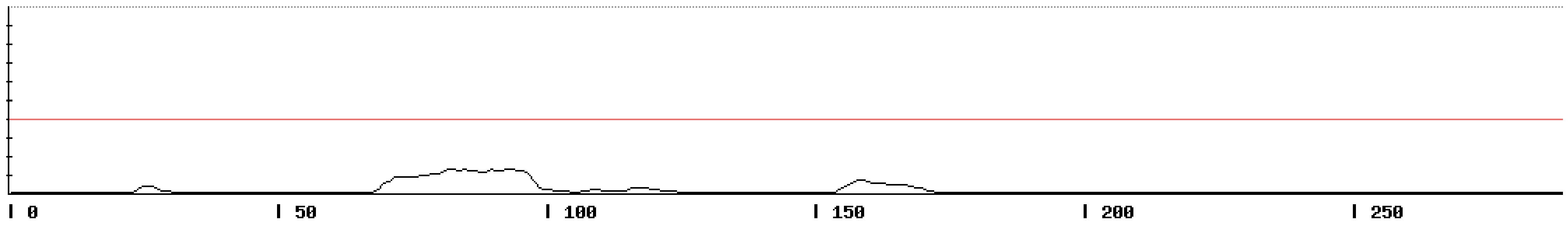

Predictions for Brassicanapus_GSBRNA2T00038201001

Viterbi Path

Viterbi Path

Posterior @ 0.4

Posterior @ 0.4

Posterior Graphic. Threshold Marked @ 0.4

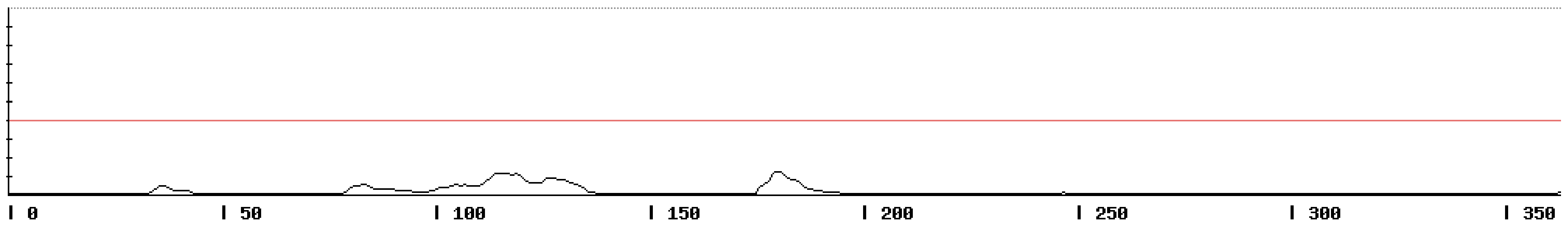

Predictions for Brassicanapus_GSBRNA2T00038789001

Viterbi Path

Viterbi Path

Posterior @ 0.4

Posterior @ 0.4

Posterior Graphic. Threshold Marked @ 0.4

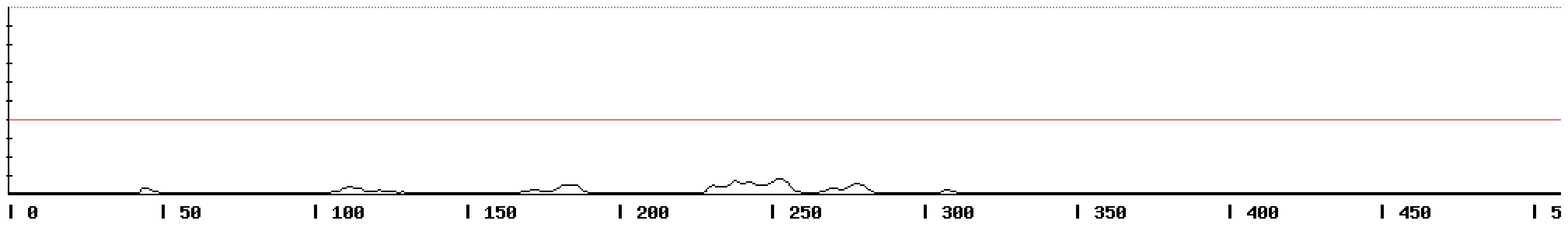

Predictions for Brassicanapus_GSBRNA2T00040227001

Viterbi Path

Viterbi Path

Posterior @ 0.4

Posterior @ 0.4

Posterior Graphic. Threshold Marked @ 0.4

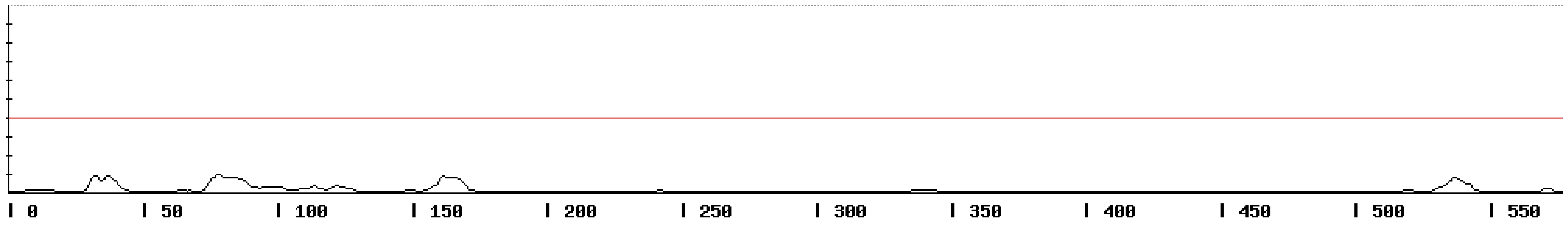

Predictions for Brassicanapus_GSBRNA2T00040233001

Viterbi Path

Viterbi Path

Posterior @ 0.4

Posterior @ 0.4

Posterior Graphic. Threshold Marked @ 0.4

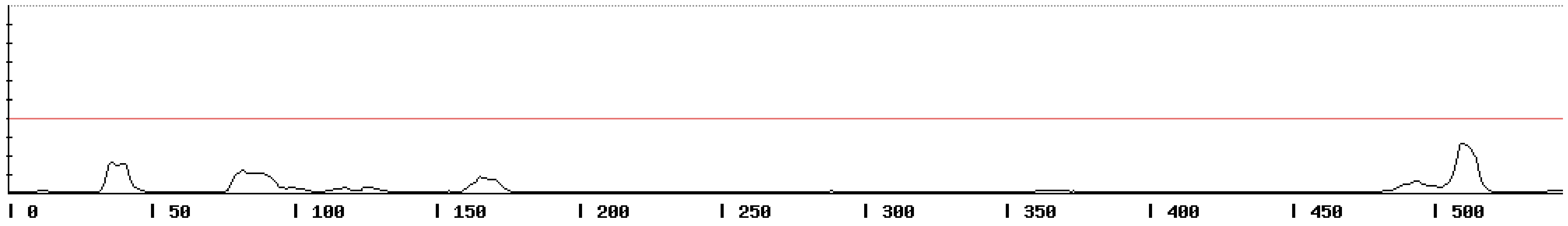

Predictions for Brassicanapus_GSBRNA2T00040675001

Viterbi Path

Viterbi Path

Posterior @ 0.4

Posterior @ 0.4

Posterior Graphic. Threshold Marked @ 0.4

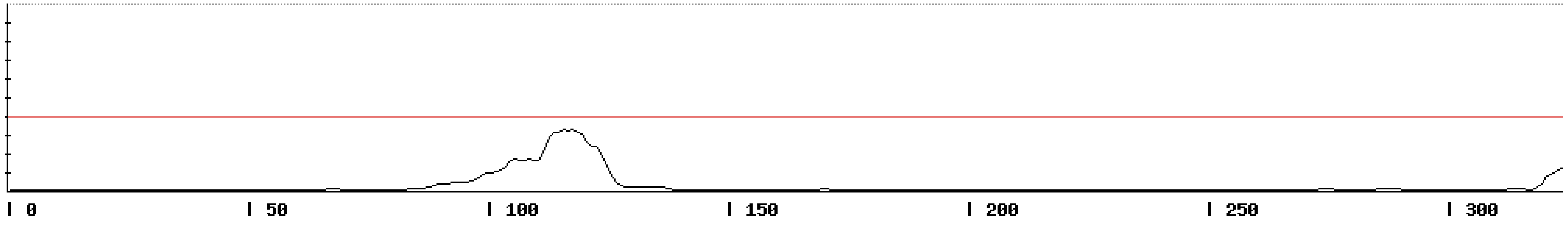

Predictions for Brassicanapus_GSBRNA2T00040908001

Viterbi Path

Viterbi Path

Posterior @ 0.4

90 - KSIGKRKEIKKKDK - 103

Posterior @ 0.4

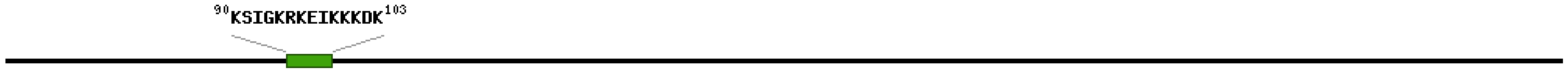

Posterior Graphic. Threshold Marked @ 0.4

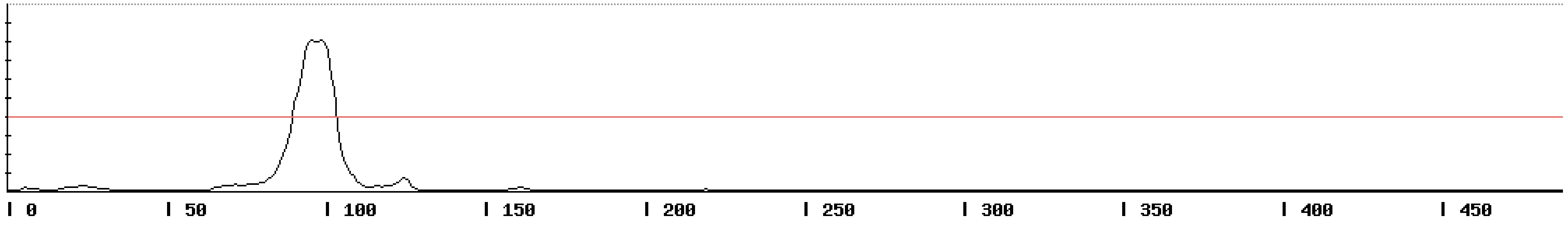

Predictions for Brassicanapus_GSBRNA2T00041385001	
Viterbi Path	
Viterbi Path	
Posterior @ 0.4	
Posterior @ 0.4	
Posterior Graphic. Threshold Marked @ 0.4	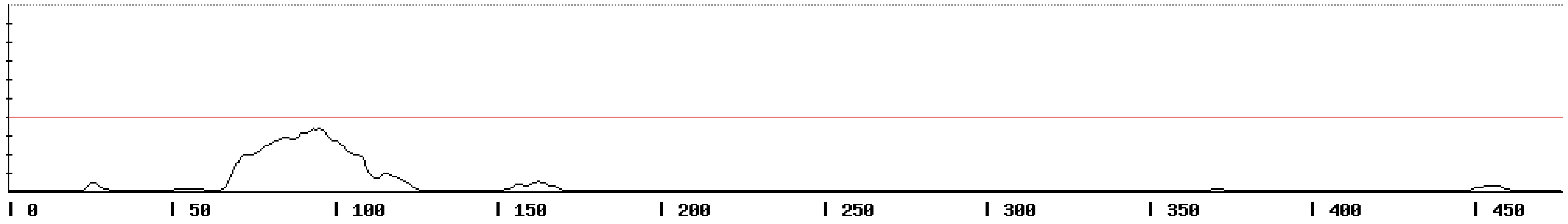

Predictions for Brassicanapus_GSBRNA2T00041459001	
Viterbi Path	
Viterbi Path	
Posterior @ 0.4	
Posterior @ 0.4	
Posterior Graphic. Threshold Marked @ 0.4	

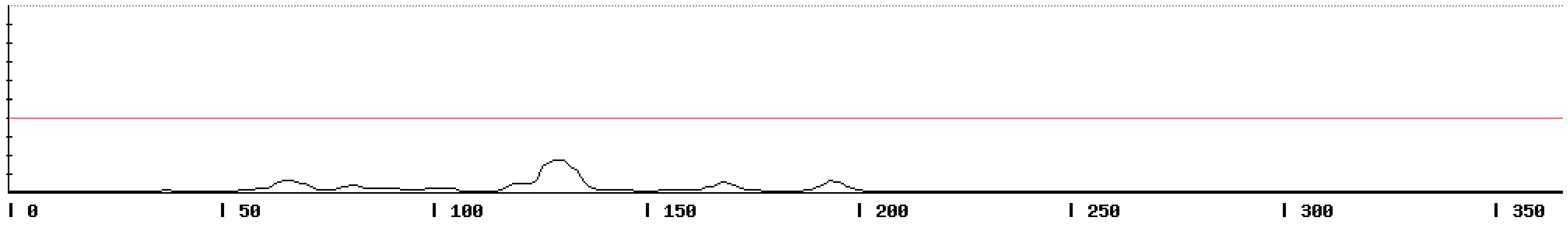

Predictions for Brassicanapus_GSBRNA2T00042092001	
Viterbi Path	
Viterbi Path	
Posterior @ 0.4	
Posterior @ 0.4	
Posterior Graphic. Threshold Marked @ 0.4	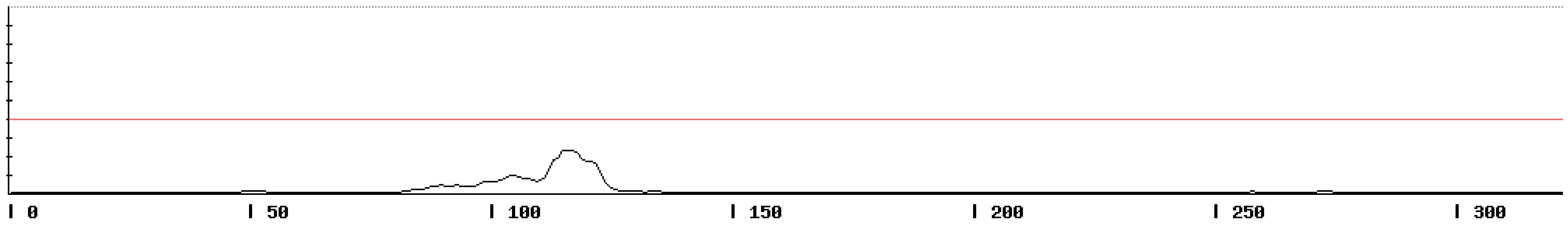

Predictions for Brassicanapus_GSBRNA2T00042260001	
Viterbi Path	
Viterbi Path	
Posterior @ 0.4	
Posterior @ 0.4	

Posterior Graphic. Threshold Marked @ 0.4

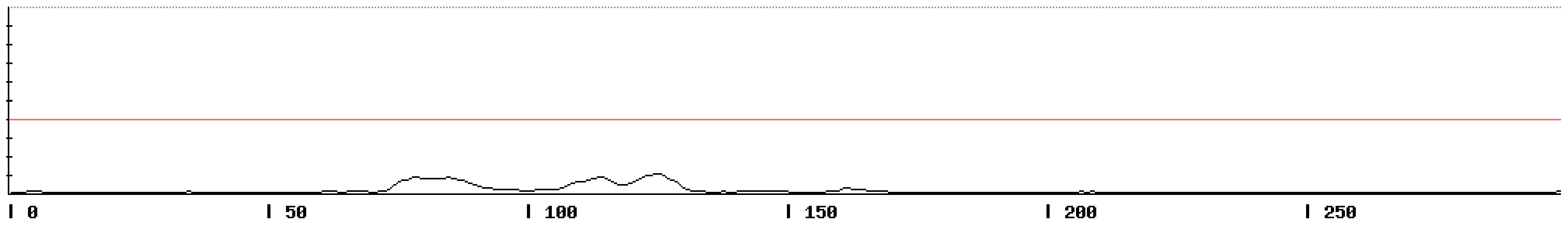

Predictions for Brassicanapus_GSBRNA2T00042350001

Viterbi Path

Viterbi Path

Posterior @ 0.4

Posterior @ 0.4

Posterior Graphic. Threshold Marked @ 0.4

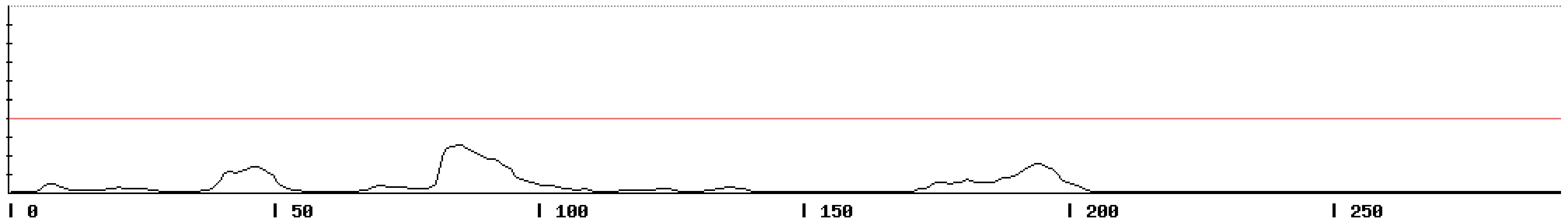

Predictions for Brassicanapus_GSBRNA2T00042526001

Viterbi Path

Viterbi Path

Posterior @ 0.4

Posterior @ 0.4

Posterior Graphic. Threshold Marked @ 0.4

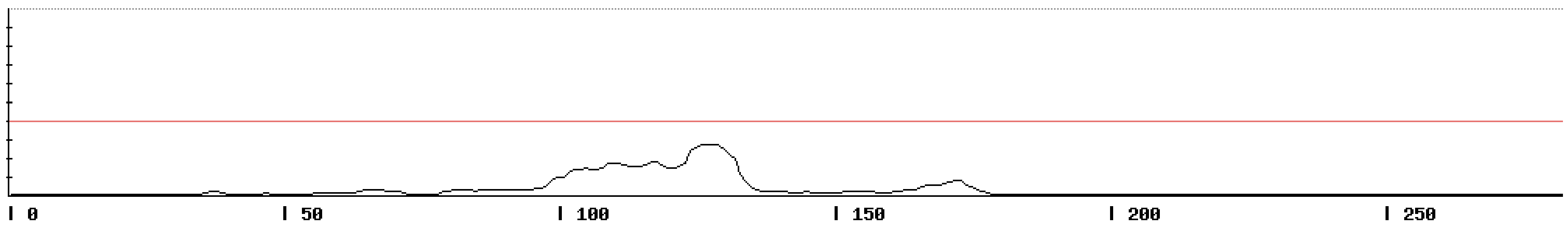

Predictions for Brassicanapus_GSBRNA2T00043949001

Viterbi Path	
Viterbi Path	
Posterior @ 0.4	127 - R - 127
Posterior @ 0.4	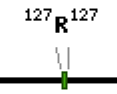
Posterior Graphic. Threshold Marked @ 0.4	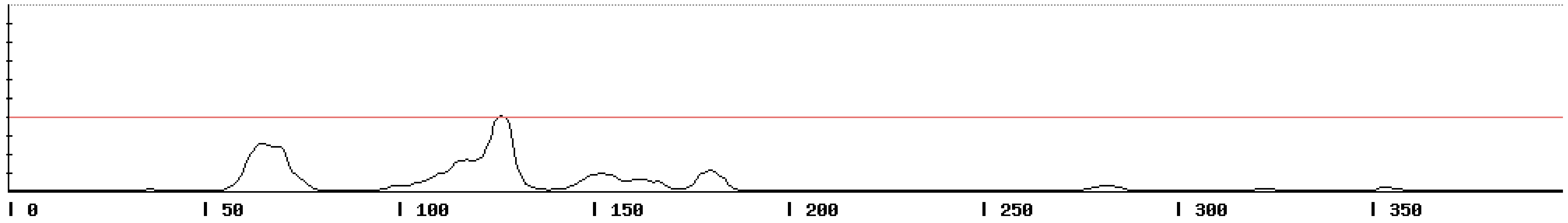

Predictions for Brassicanapus_GSBRNA2T00044271001

Viterbi Path	
Viterbi Path	

Posterior @ 0.4

Posterior @ 0.4

Posterior Graphic. Threshold Marked @ 0.4

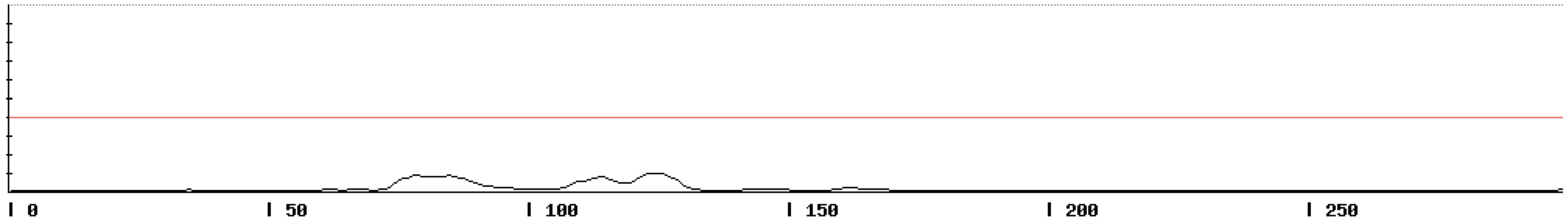

Predictions for Brassicanapus_GSBRNA2T00044371001

Viterbi Path

Viterbi Path

Posterior @ 0.4 — 138 - KRR - 140

Posterior @ 0.4

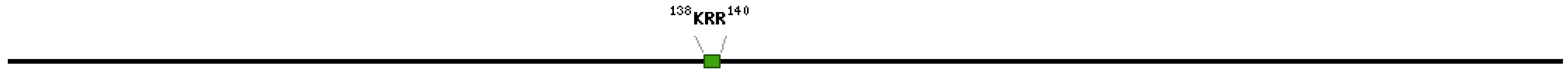

Posterior Graphic. Threshold Marked @ 0.4

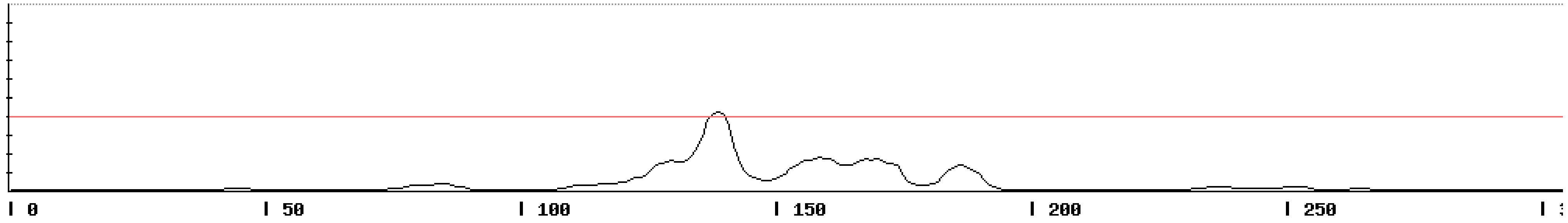

Predictions for Brassicanapus_GSBRNA2T00044503001

Viterbi Path

Viterbi Path

Posterior @ 0.4

Posterior @ 0.4

Posterior Graphic. Threshold Marked @ 0.4

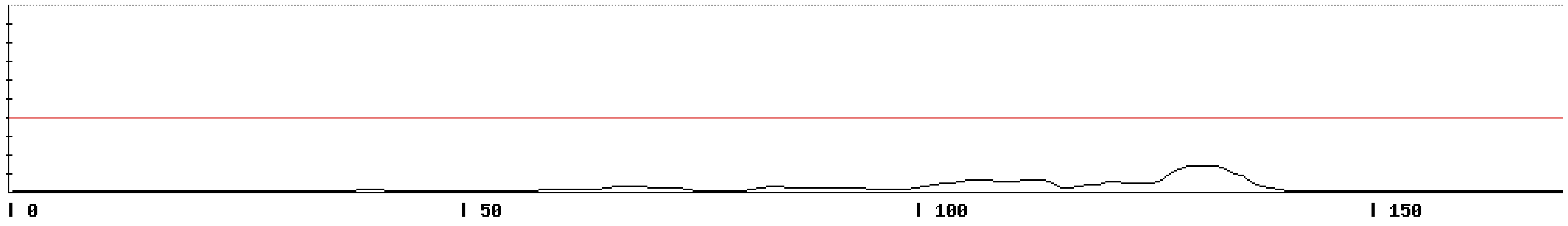

Predictions for Brassicanapus_GSBRNA2T00044505001

Viterbi Path

Viterbi Path

Posterior @ 0.4

Posterior @ 0.4

Posterior Graphic. Threshold Marked @ 0.4

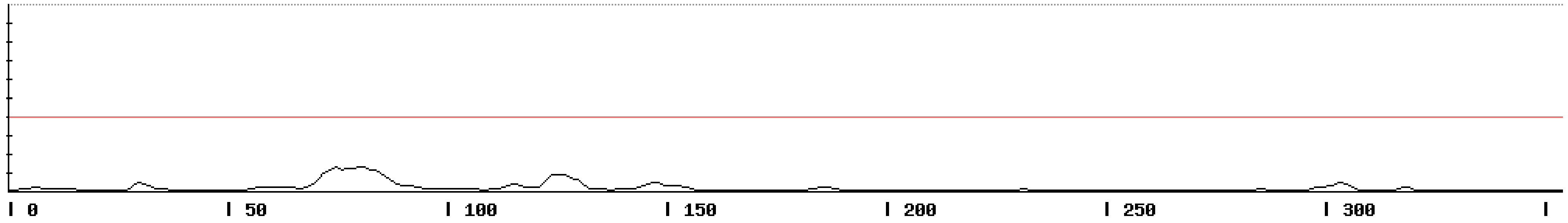

Predictions for Brassicanapus_GSBRNA2T00044605001

Viterbi Path	
Viterbi Path	
Posterior @ 0.4	
Posterior @ 0.4	
Posterior Graphic. Threshold Marked @ 0.4	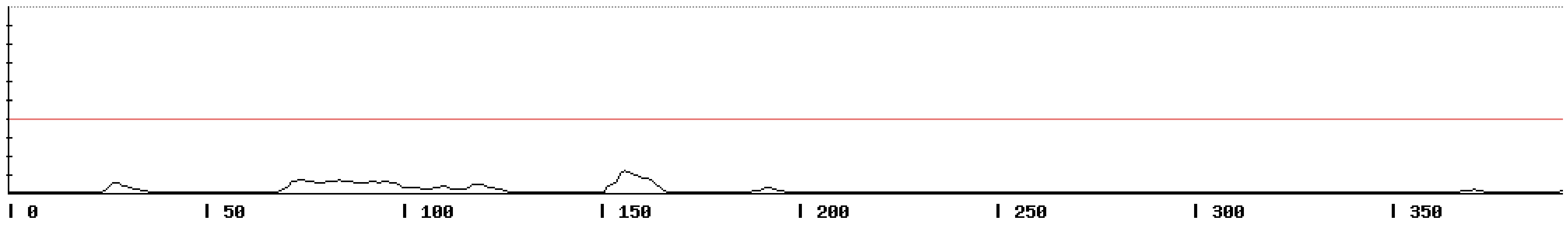

Predictions for Brassicanapus_GSBRNA2T00044843001

Viterbi Path	
Viterbi Path	
Posterior @ 0.4	
Posterior @ 0.4	
Posterior Graphic. Threshold Marked @ 0.4	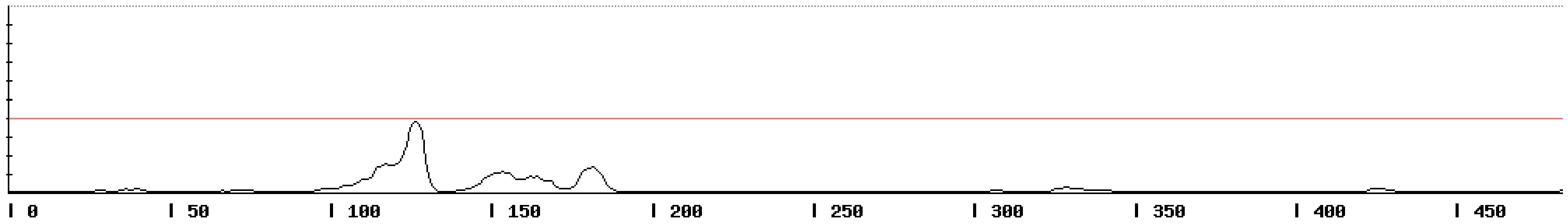

Predictions for Brassicanapus_GSBRNA2T00044895001	
Viterbi Path	
Viterbi Path	
Posterior @ 0.4	
Posterior @ 0.4	
Posterior Graphic. Threshold Marked @ 0.4	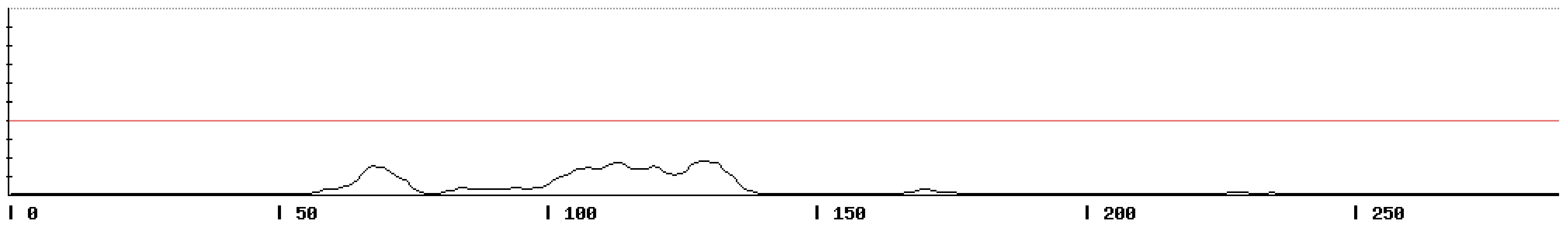

Predictions for Brassicanapus_GSBRNA2T00045062001	
Viterbi Path	
Viterbi Path	
Posterior @ 0.4	
Posterior @ 0.4	
Posterior Graphic. Threshold Marked @ 0.4	

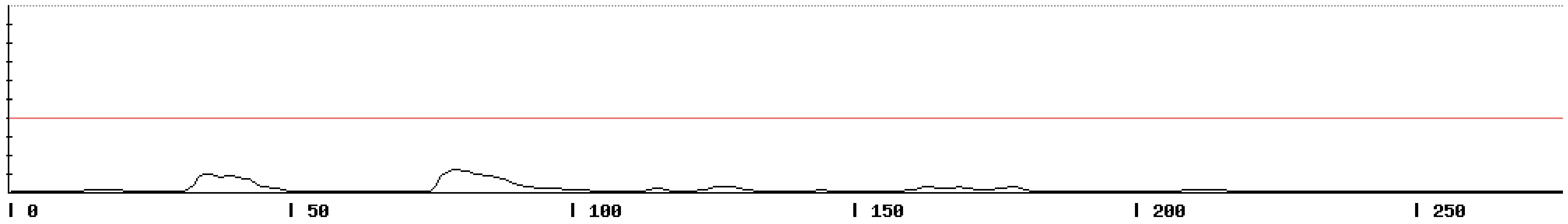

Predictions for Brassicanapus_GSBRNA2T00045496001	
Viterbi Path	
Viterbi Path	
Posterior @ 0.4	
Posterior @ 0.4	
Posterior Graphic. Threshold Marked @ 0.4	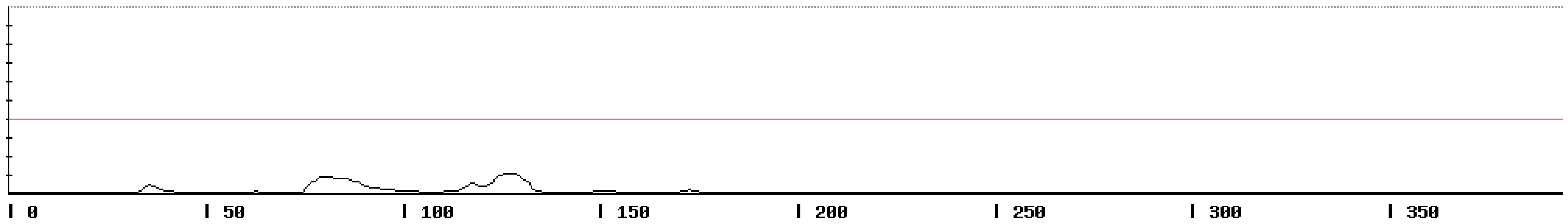

Predictions for Brassicanapus_GSBRNA2T00046056001	
Viterbi Path	
Viterbi Path	
Posterior @ 0.4	
Posterior @ 0.4	

Posterior Graphic. Threshold Marked @ 0.4	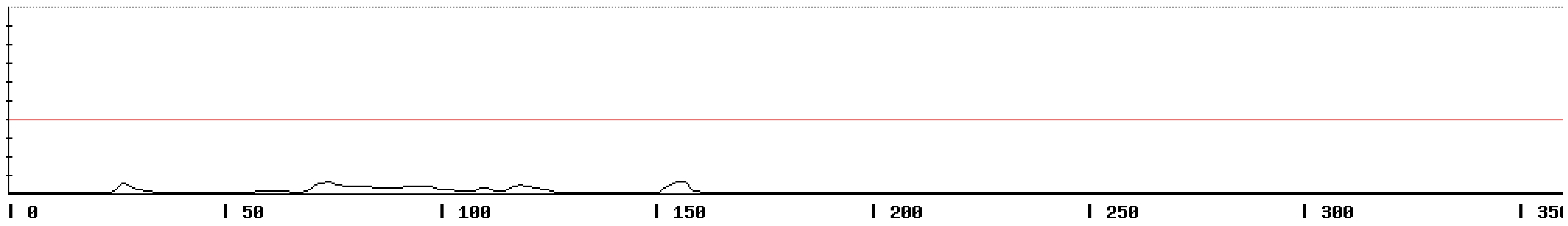

Predictions for Brassicanapus_GSBRNA2T00046205001	
Viterbi Path	
Viterbi Path	
Posterior @ 0.4	
Posterior @ 0.4	
Posterior Graphic. Threshold Marked @ 0.4	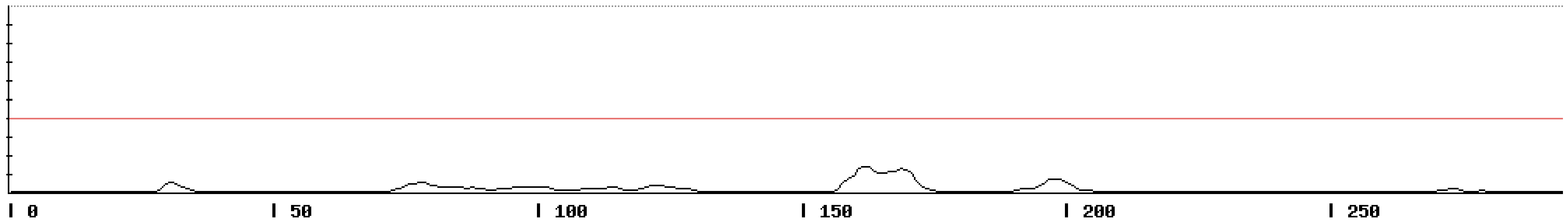

Predictions for Brassicanapus_GSBRNA2T00046259001	
Viterbi Path	
Viterbi Path	
Posterior @ 0.4	153 - KRR - 155
Posterior @ 0.4	

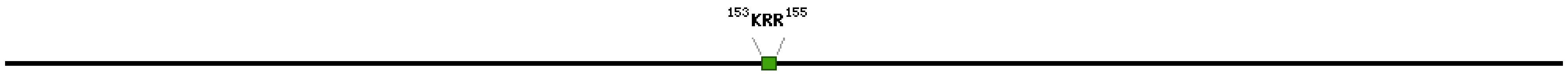

Posterior Graphic. Threshold Marked @ 0.4

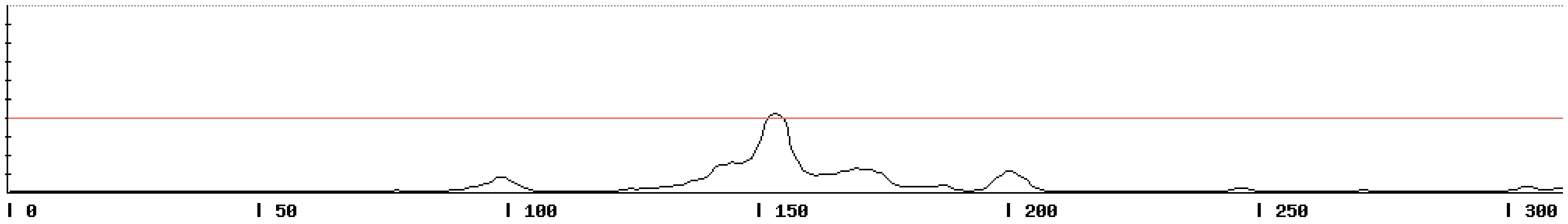

Predictions for Brassicanapus_GSBRNA2T00046501001

Viterbi Path

Viterbi Path

Posterior @ 0.4: 121 - KAPKGRKTKWK - 131

Posterior @ 0.4

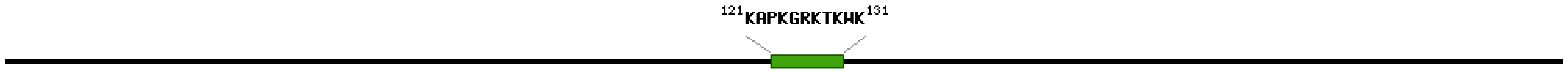

Posterior Graphic. Threshold Marked @ 0.4

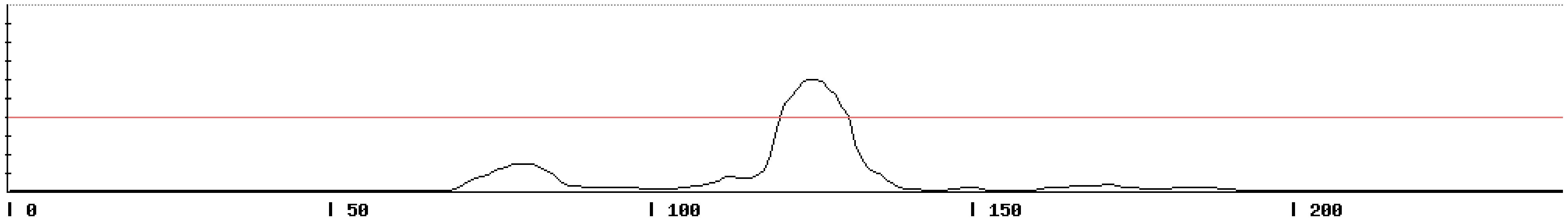

Predictions for Brassicanapus_GSBRNA2T00047395001

Viterbi Path

Viterbi Path

Posterior @ 0.4

Posterior @ 0.4

Posterior Graphic. Threshold Marked @ 0.4

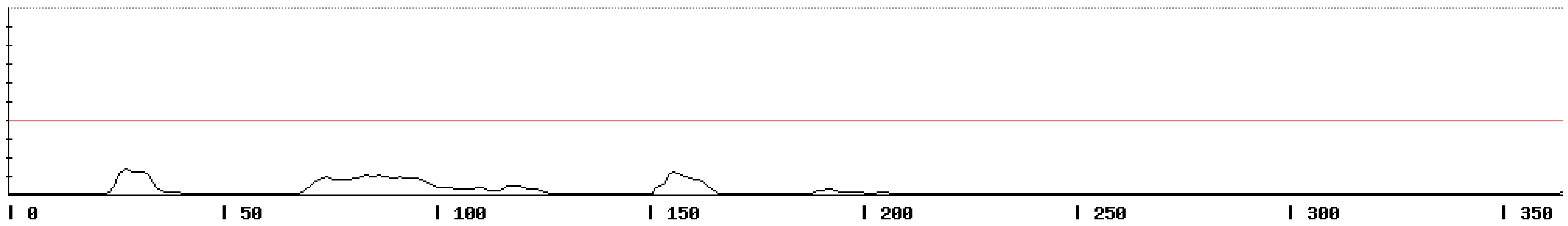

Predictions for Brassicanapus_GSBRNA2T00047488001

Viterbi Path

Viterbi Path

Posterior @ 0.4

Posterior @ 0.4

Posterior Graphic. Threshold Marked @ 0.4

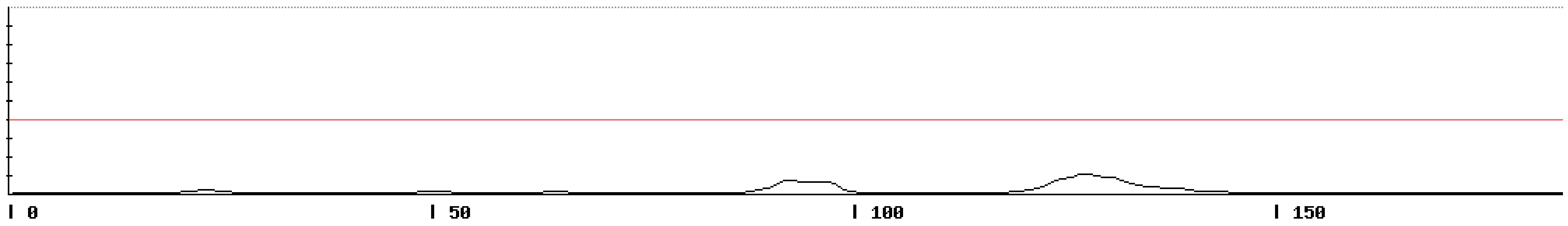

Predictions for Brassicanapus_GSBRNA2T00047541001

Viterbi Path

Viterbi Path

Posterior @ 0.4	99 - KP - 100
Posterior @ 0.4	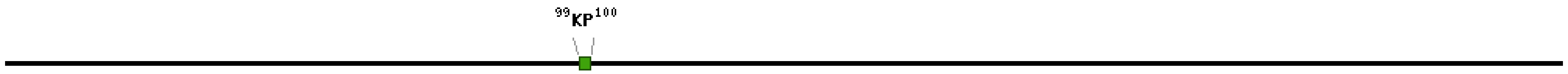
Posterior Graphic. Threshold Marked @ 0.4	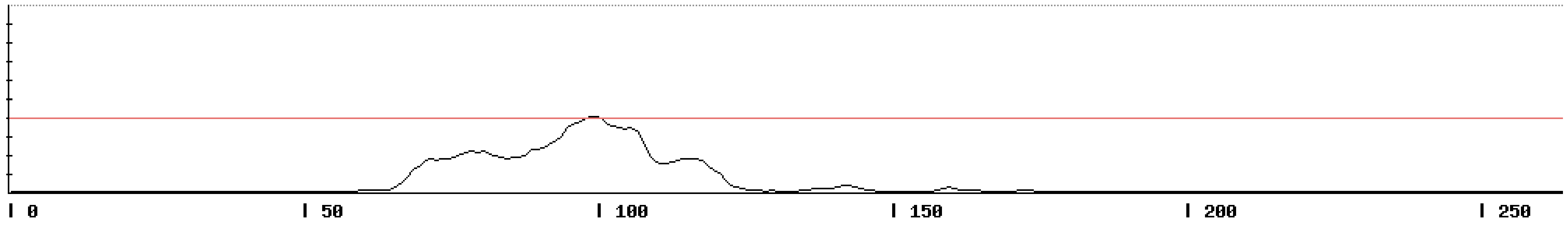

Predictions for Brassicanapus_GSBRNA2T00047591001

Viterbi Path	
Viterbi Path	
Posterior @ 0.4	
Posterior @ 0.4	
Posterior Graphic. Threshold Marked @ 0.4	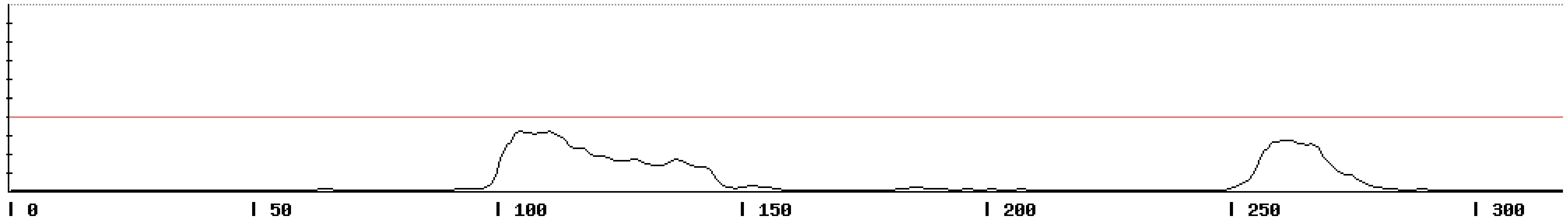

Predictions for Brassicanapus_GSBRNA2T00049322001

Viterbi Path	
Viterbi Path	
Posterior @ 0.4	
Posterior @ 0.4	
Posterior Graphic. Threshold Marked @ 0.4	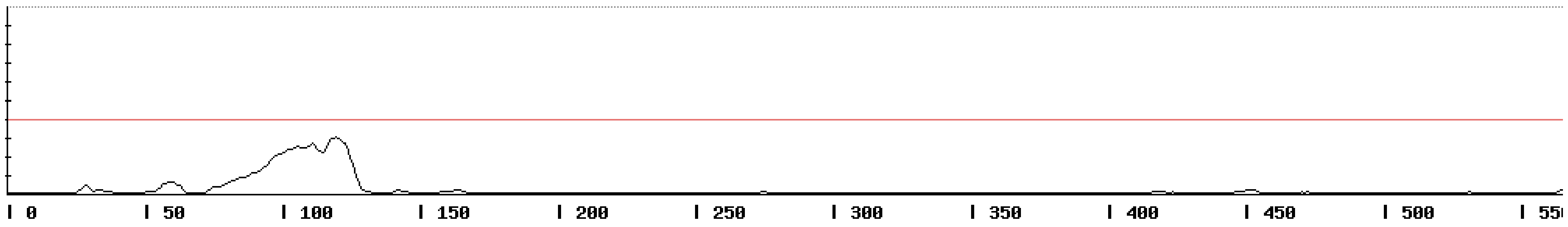

Predictions for Brassicanapus_GSBRNA2T00049441001

Viterbi Path	
Viterbi Path	
Posterior @ 0.4	
Posterior @ 0.4	
Posterior Graphic. Threshold Marked @ 0.4	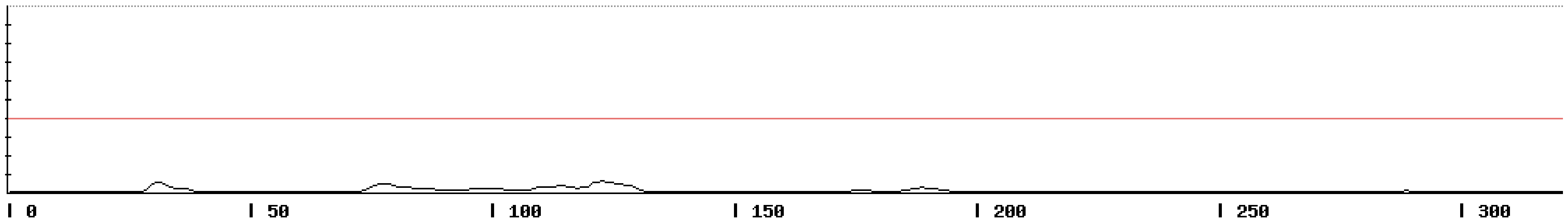

Predictions for Brassicanapus_GSBRNA2T00049808001	
Viterbi Path	
Viterbi Path	
Posterior @ 0.4	
Posterior @ 0.4	
Posterior Graphic. Threshold Marked @ 0.4	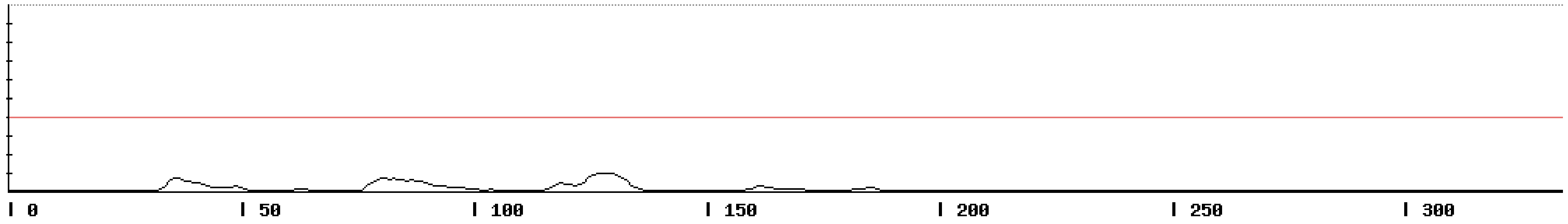

Predictions for Brassicanapus_GSBRNA2T00049948001	
Viterbi Path	
Viterbi Path	
Posterior @ 0.4	
Posterior @ 0.4	
Posterior Graphic. Threshold Marked @ 0.4	

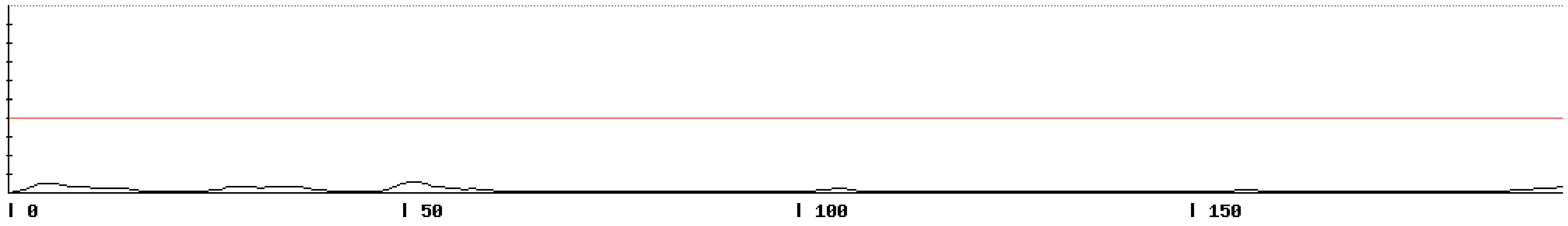

Predictions for Brassicanapus_GSBRNA2T00049955001

Viterbi Path: 135 - RKKKK - 139

Viterbi Path:

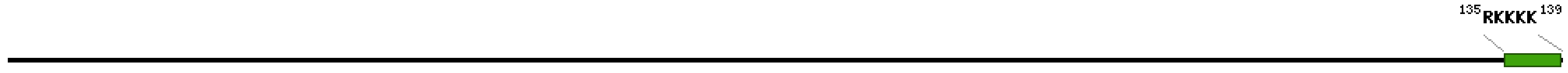

Posterior @ 0.4:

94 - SKKKDKYNRGGRQSRKKSSGFWKKT - 118

135 - RKKKK - 139

Posterior @ 0.4:

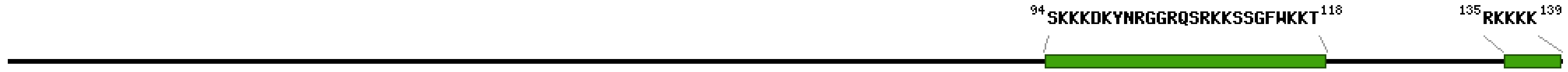

Posterior Graphic. Threshold Marked @ 0.4

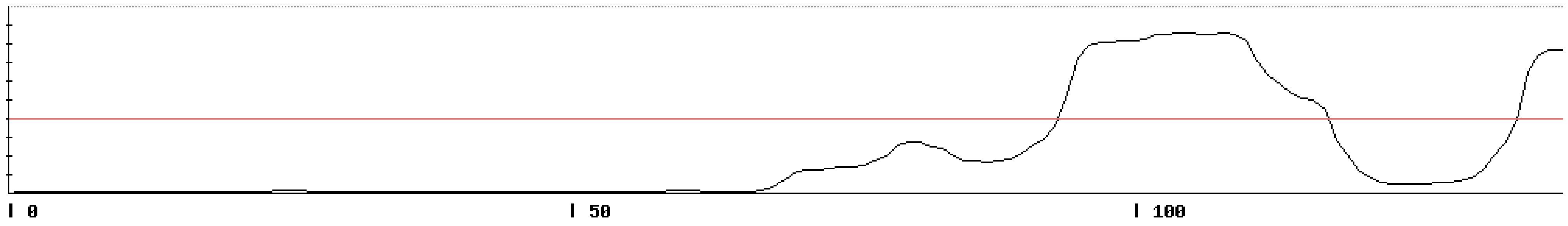

Predictions for Brassicanapus_GSBRNA2T00050536001

Viterbi Path:

Viterbi Path:

Posterior @ 0.4:

Posterior @ 0.4:

Posterior Graphic. Threshold Marked @ 0.4

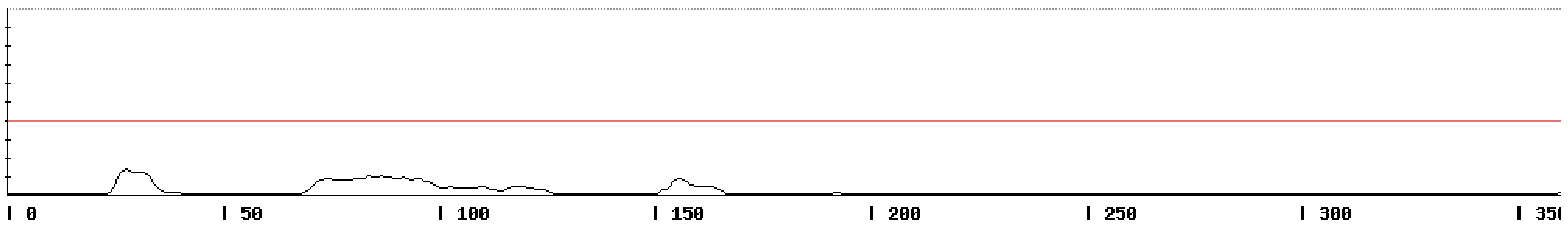

Predictions for Brassicanapus_GSBRNA2T00050859001

Viterbi Path

Viterbi Path

Posterior @ 0.4

Posterior @ 0.4

Posterior Graphic. Threshold Marked @ 0.4

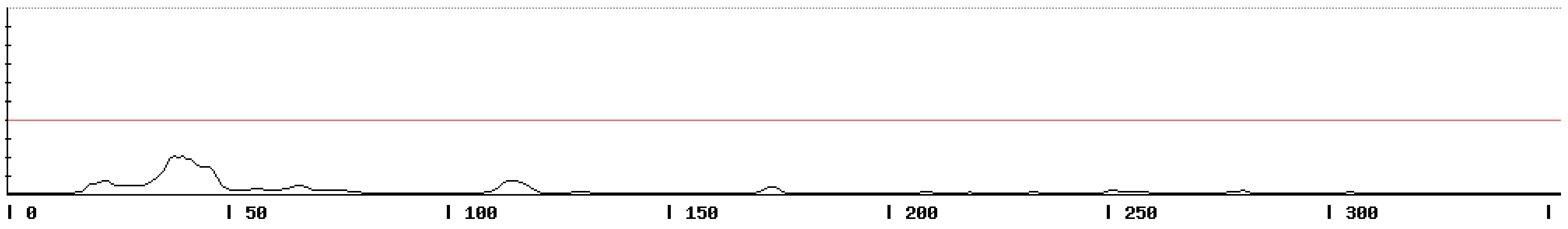

Predictions for Brassicanapus_GSBRNA2T00050907001

Viterbi Path

Viterbi Path

Posterior @ 0.4

Posterior @ 0.4

Posterior Graphic. Threshold Marked @ 0.4

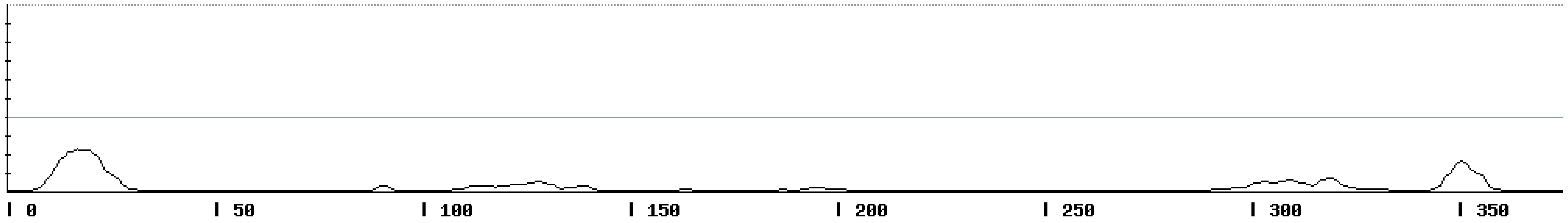

Predictions for Brassicanapus_GSBRNA2T00051194001

Viterbi Path

Viterbi Path

Posterior @ 0.4

Posterior @ 0.4

Posterior Graphic. Threshold Marked @ 0.4

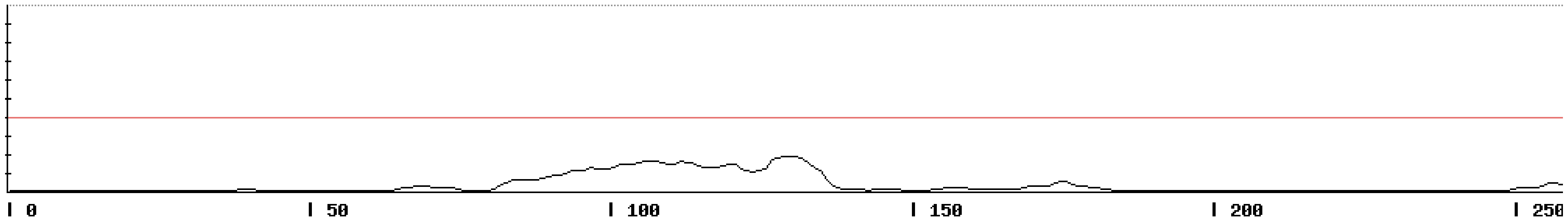

Predictions for Brassicanapus_GSBRNA2T00051253001

Viterbi Path

Viterbi Path

Posterior @ 0.4

Posterior @ 0.4

Posterior Graphic. Threshold Marked @ 0.4

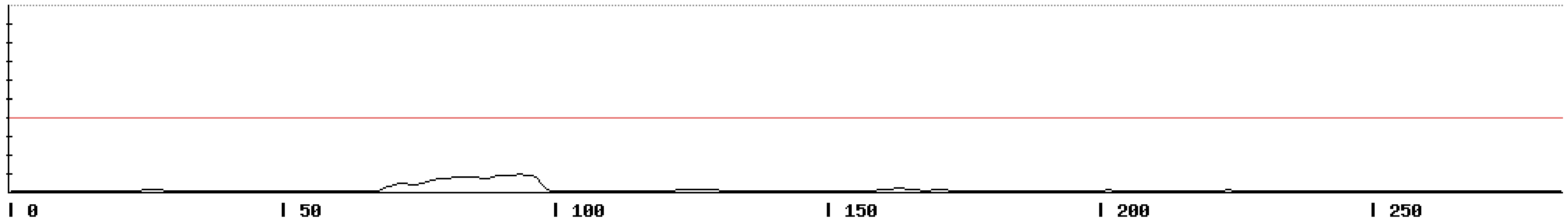

Predictions for Brassicanapus_GSBRNA2T00051813001

Viterbi Path

Viterbi Path

Posterior @ 0.4

Posterior @ 0.4

Posterior Graphic. Threshold Marked @ 0.4

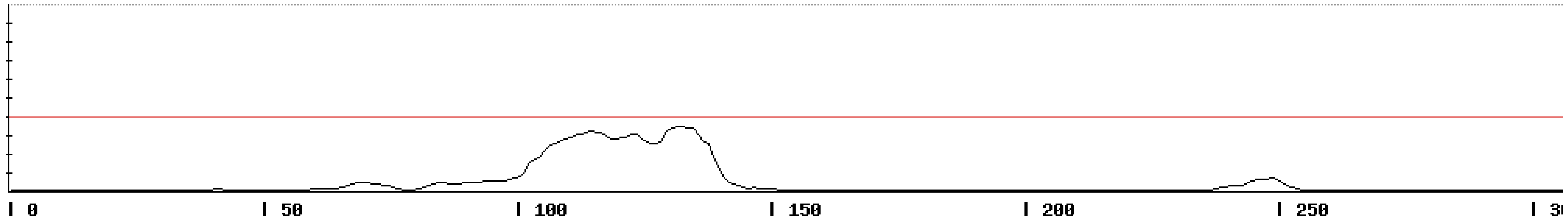

Predictions for Brassicanapus_GSBRNA2T00052195001

Viterbi Path	
Viterbi Path	
Posterior @ 0.4	
Posterior @ 0.4	
Posterior Graphic. Threshold Marked @ 0.4	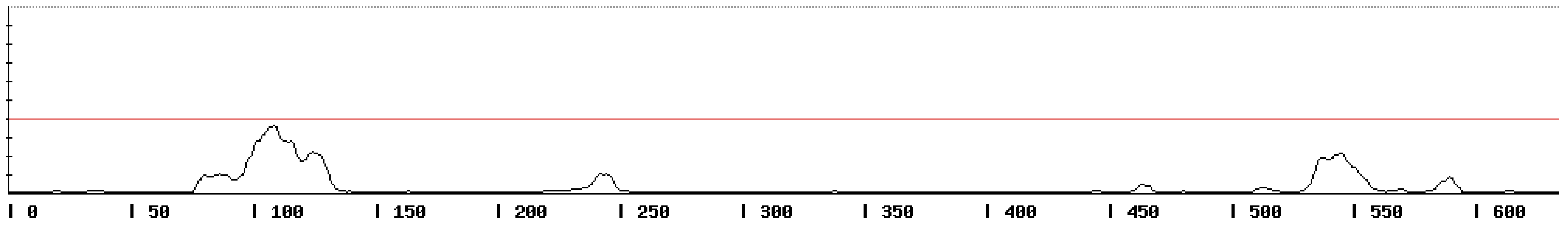

Predictions for Brassicanapus_GSBRNA2T00052418001

Viterbi Path	
Viterbi Path	
Posterior @ 0.4	69 - RKESRYNRGERQKRKTKSGYWKKTGKTLPITRK - 101
Posterior @ 0.4	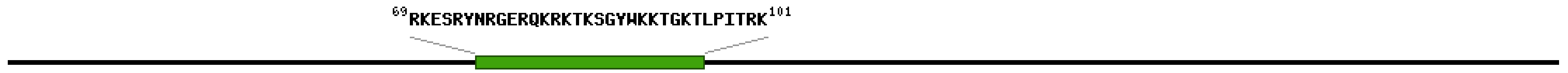
Posterior Graphic. Threshold Marked @ 0.4	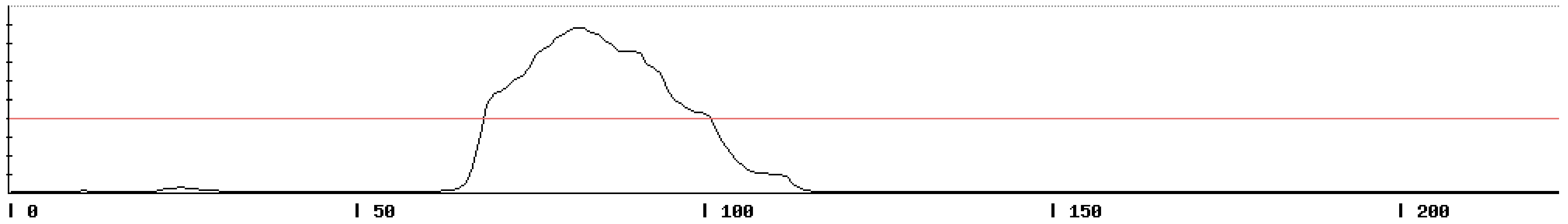

Predictions for Brassicanapus_GSBRNA2T00052419001	
Viterbi Path	
Viterbi Path	
Posterior @ 0.4	69 - RKESRYNRGERQKRKTKSGYWKKTGKTLPITRK - 101
Posterior @ 0.4	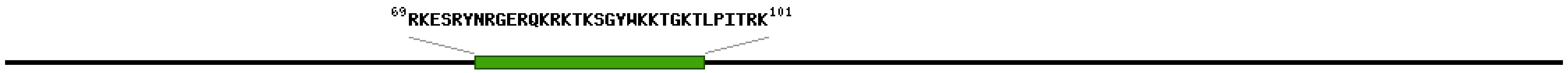
Posterior Graphic. Threshold Marked @ 0.4	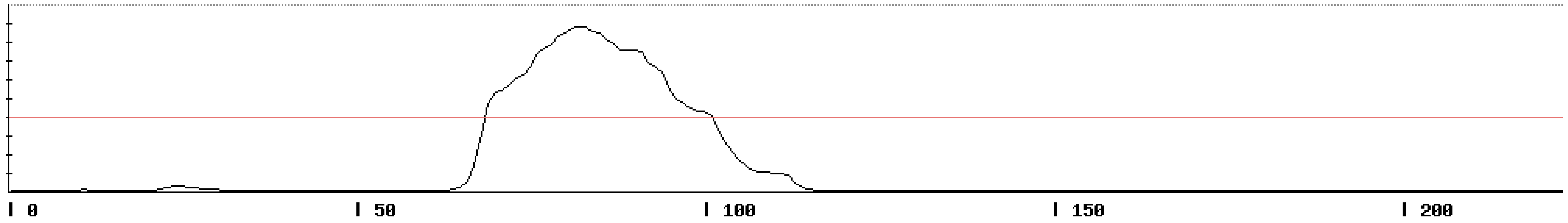

Predictions for Brassicanapus_GSBRNA2T00053001001	
Viterbi Path	
Viterbi Path	
Posterior @ 0.4	126 - KR - 127
Posterior @ 0.4	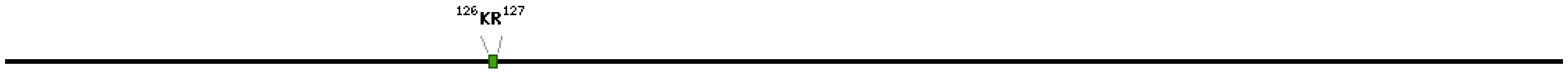
Posterior Graphic. Threshold Marked @ 0.4	

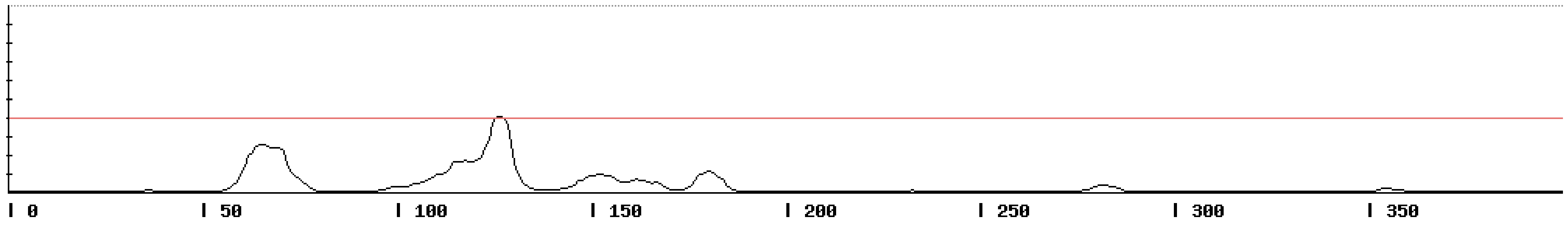

Predictions for Brassicanapus_GSBRNA2T00056083001

Viterbi Path

Viterbi Path

Posterior @ 0.4 121 - EKAEEKEKRRARDKR - 135

Posterior @ 0.4

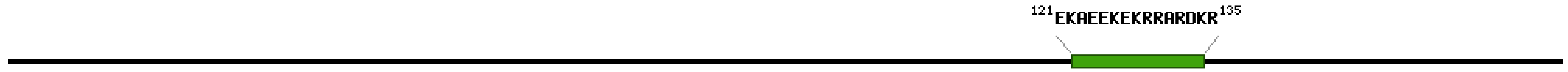

Posterior Graphic. Threshold Marked @ 0.4

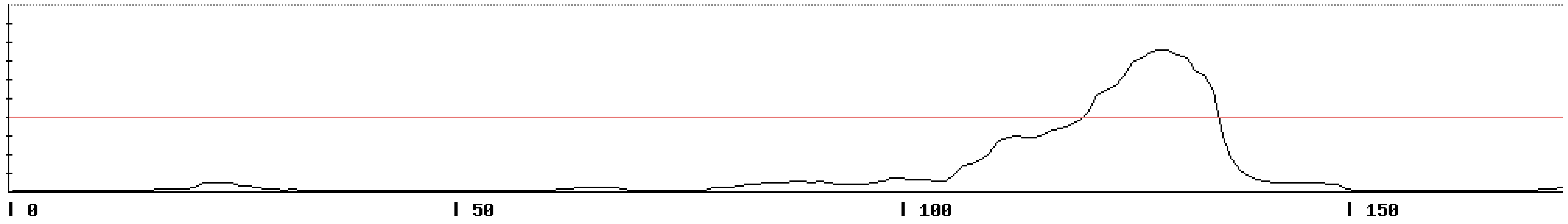

Predictions for Brassicanapus_GSBRNA2T00057235001

Viterbi Path

Viterbi Path

Posterior @ 0.4

Posterior @ 0.4

Posterior Graphic. Threshold Marked @ 0.4

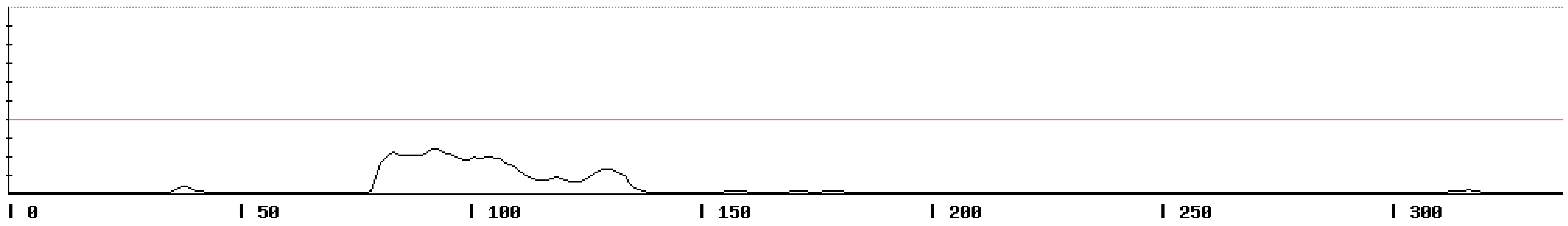

Predictions for Brassicanapus_GSBRNA2T00057237001

Viterbi Path

Viterbi Path

Posterior @ 0.4

Posterior @ 0.4

Posterior Graphic. Threshold Marked @ 0.4

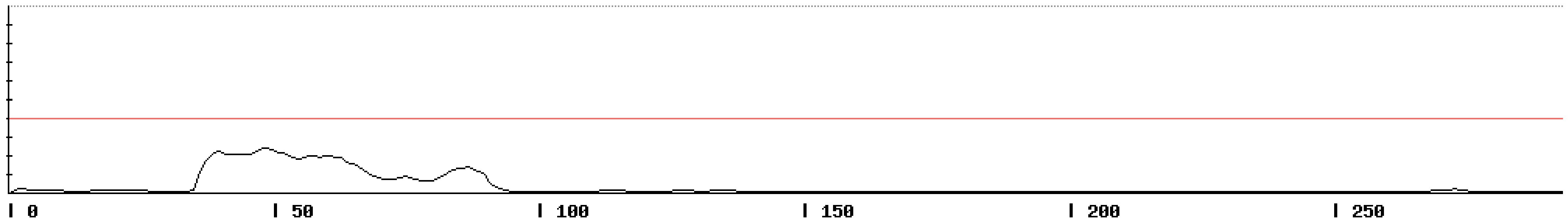

Predictions for Brassicanapus_GSBRNA2T00057317001

Viterbi Path

Viterbi Path

Posterior @ 0.4

Posterior @ 0.4

Posterior Graphic. Threshold Marked @ 0.4

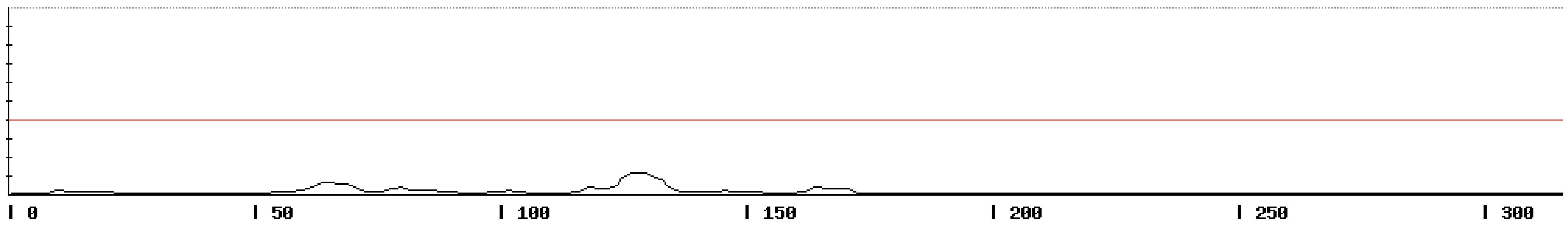

Predictions for Brassicanapus_GSBRNA2T00057875001

Viterbi Path

Viterbi Path

Posterior @ 0.4

Posterior @ 0.4

Posterior Graphic. Threshold Marked @ 0.4

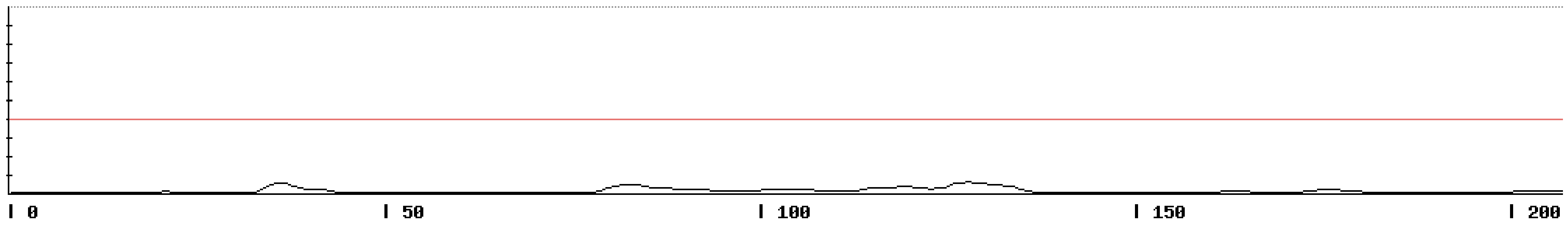

Predictions for Brassicanapus_GSBRNA2T00058097001

Viterbi Path

Viterbi Path

Posterior @ 0.4	137 - RKRRK - 141
Posterior @ 0.4	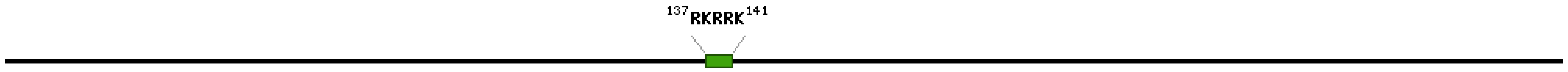
Posterior Graphic. Threshold Marked @ 0.4	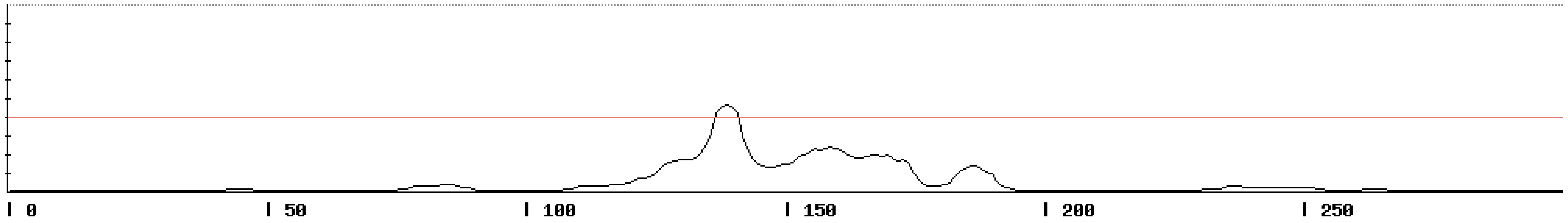

Predictions for Brassicanapus_GSBRNA2T00058100001

Viterbi Path	
Viterbi Path	
Posterior @ 0.4	
Posterior @ 0.4	
Posterior Graphic. Threshold Marked @ 0.4	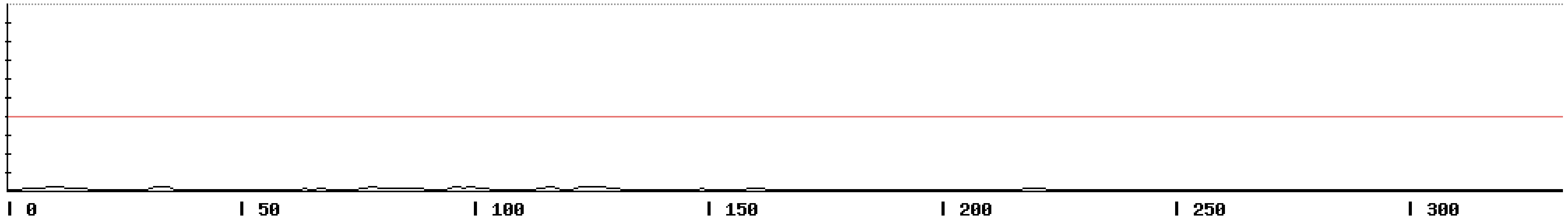

Predictions for Brassicanapus_GSBRNA2T00059078001

Viterbi Path	
Viterbi Path	

Posterior @ 0.4

Posterior @ 0.4

Posterior Graphic. Threshold Marked @ 0.4

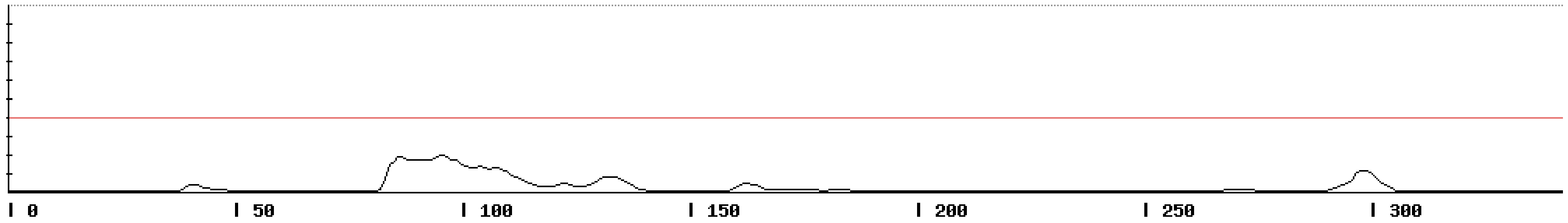

Predictions for Brassicanapus_GSBRNA2T00059754001

Viterbi Path

Viterbi Path

Posterior @ 0.4

Posterior @ 0.4

Posterior Graphic. Threshold Marked @ 0.4

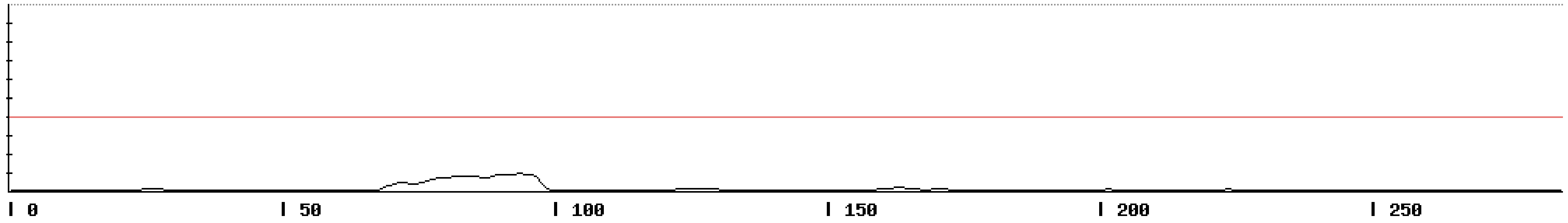

Predictions for Brassicanapus_GSBRNA2T00061848001

Viterbi Path	
Viterbi Path	
Posterior @ 0.4	64 - KRKIRRKKLR - 73
Posterior @ 0.4	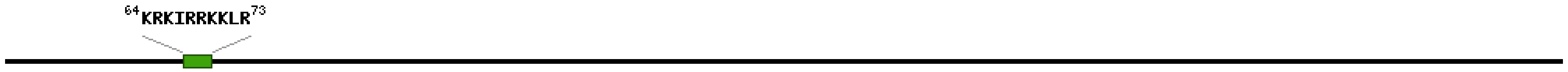
Posterior Graphic. Threshold Marked @ 0.4	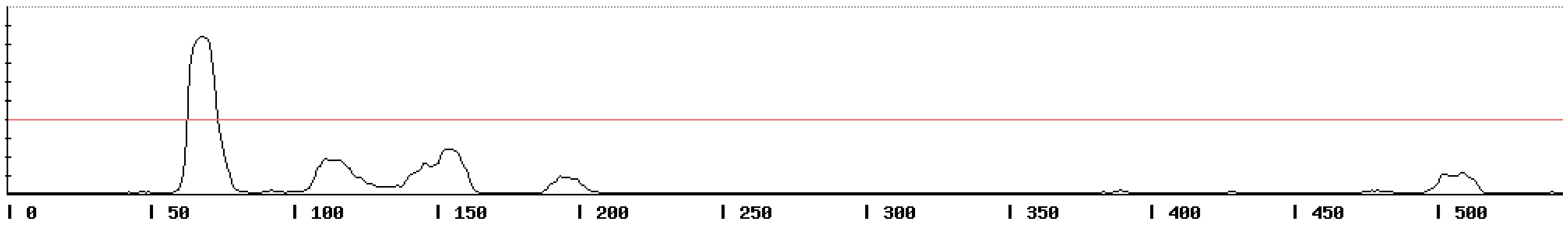

Predictions for Brassicanapus_GSBRNA2T00063201001

Viterbi Path	
Viterbi Path	
Posterior @ 0.4	
Posterior @ 0.4	
Posterior Graphic. Threshold Marked @ 0.4	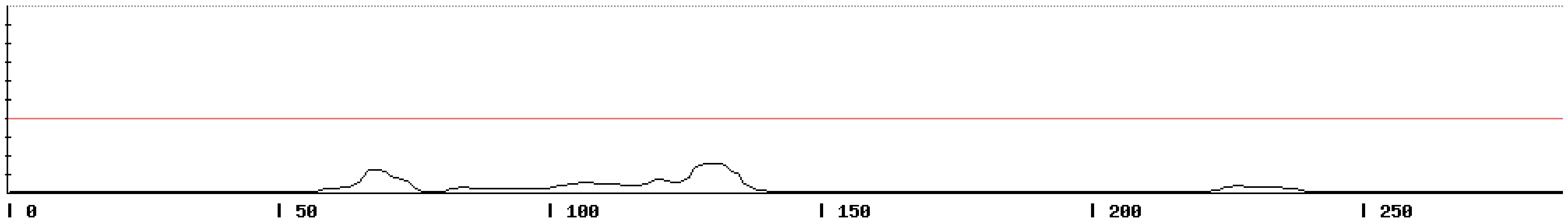

**Predictions for Brassicanapus_GSBRNA2T00064615001**	
Viterbi Path	
Viterbi Path	
Posterior @ 0.4	197 - KDKKRK - 202
Posterior @ 0.4	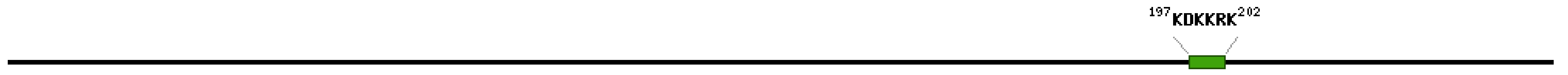
Posterior Graphic. Threshold Marked @ 0.4	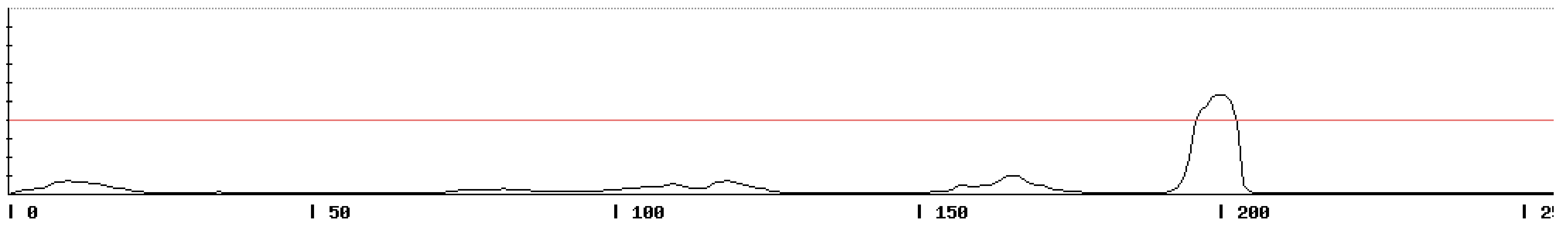

**Predictions for Brassicanapus_GSBRNA2T00065160001**	
Viterbi Path	
Viterbi Path	
Posterior @ 0.4	
Posterior @ 0.4	
Posterior Graphic. Threshold Marked @ 0.4	

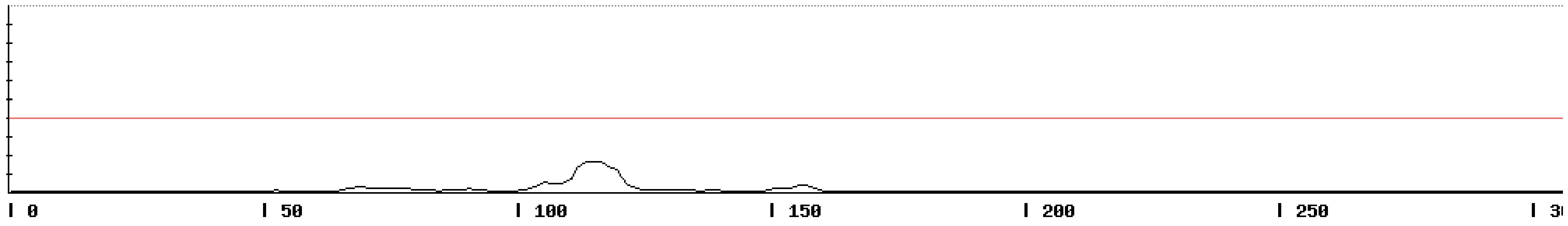

Predictions for Brassicanapus_GSBRNA2T00065262001

Viterbi Path	
Viterbi Path	
Posterior @ 0.4	118 - KVKGKP - 123
Posterior @ 0.4	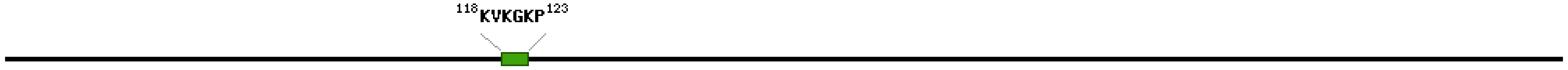
Posterior Graphic. Threshold Marked @ 0.4	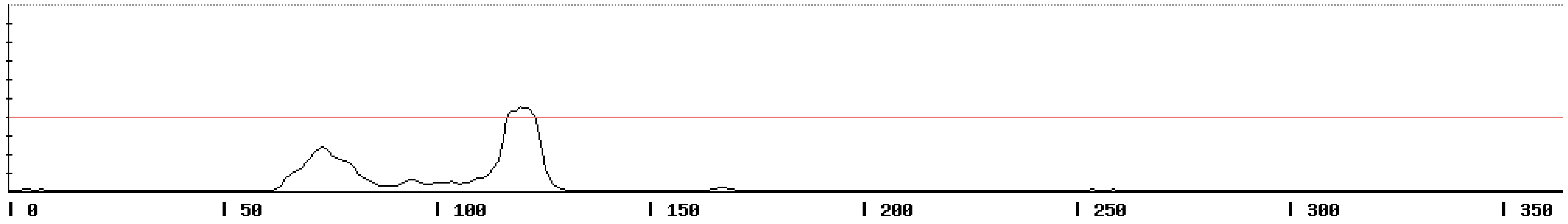

Predictions for Brassicanapus_GSBRNA2T00065483001

Viterbi Path	
Viterbi Path	
Posterior @ 0.4	275 - NKRKNKKKQVK - 285
Posterior @ 0.4	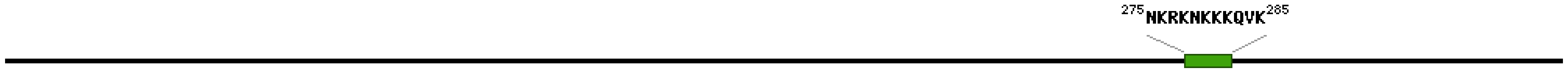

Posterior Graphic. Threshold Marked @ 0.4

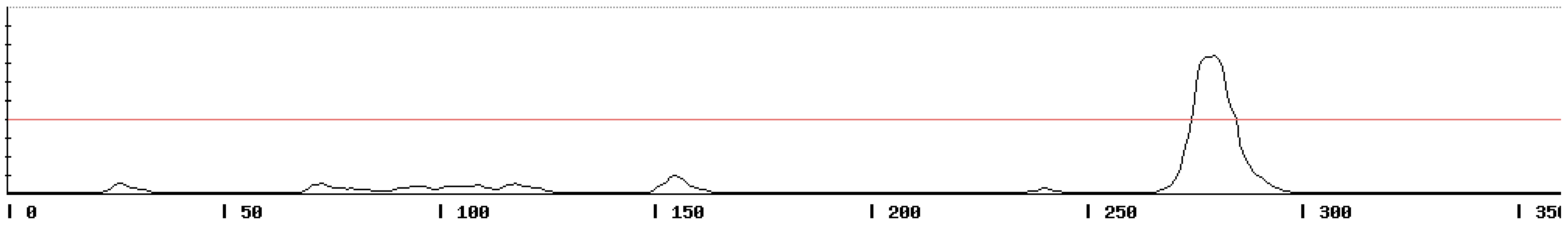

Predictions for Brassicanapus_GSBRNA2T00065750001

Viterbi Path

Viterbi Path

Posterior @ 0.4

98 - KPIGRPKPVGIKK - 110

Posterior @ 0.4

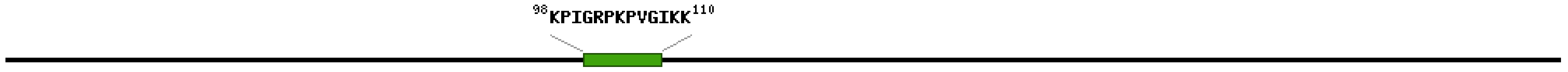

Posterior Graphic. Threshold Marked @ 0.4

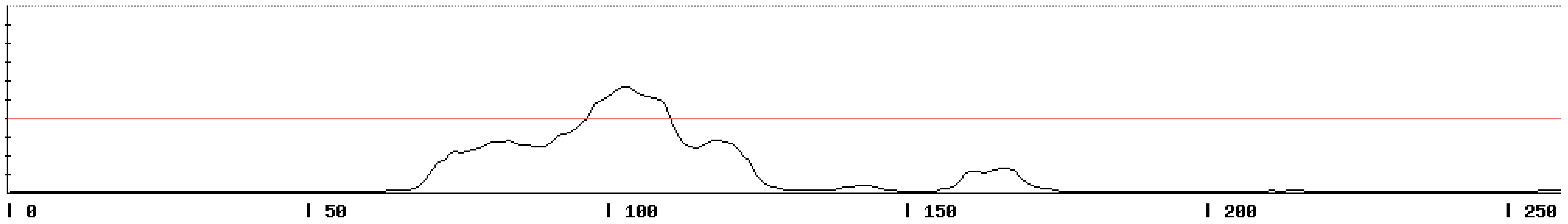

Predictions for Brassicanapus_GSBRNA2T00065801001

Viterbi Path

Viterbi Path

Posterior @ 0.4

Posterior @ 0.4

Posterior Graphic. Threshold Marked @ 0.4

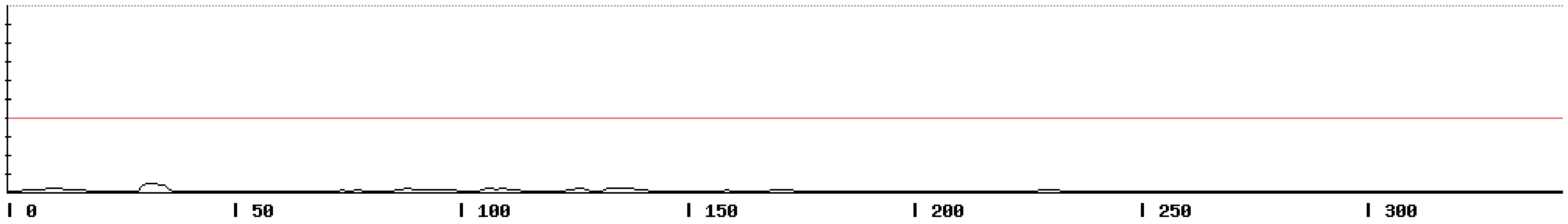

Predictions for Brassicanapus_GSBRNA2T00065806001

Viterbi Path

Viterbi Path

Posterior @ 0.4 — 138 - KRR - 140

Posterior @ 0.4

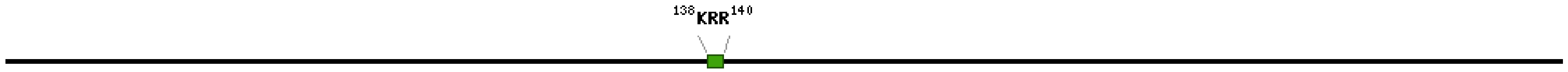

Posterior Graphic. Threshold Marked @ 0.4

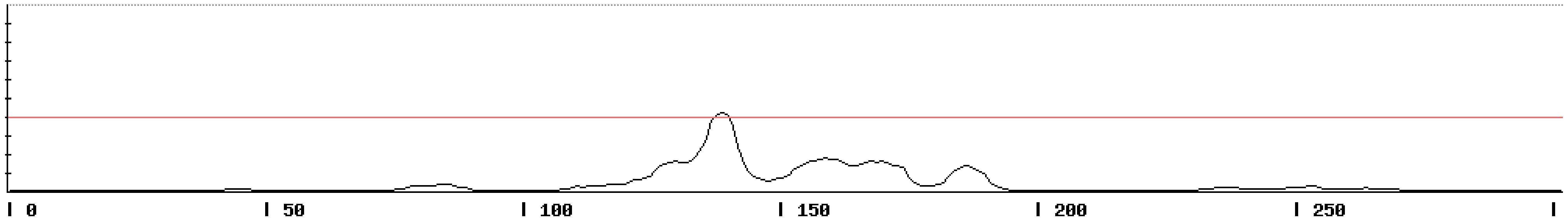

Predictions for Brassicanapus_GSBRNA2T00067987001

Viterbi Path

Viterbi Path

Posterior @ 0.4	
Posterior @ 0.4	
Posterior Graphic. Threshold Marked @ 0.4	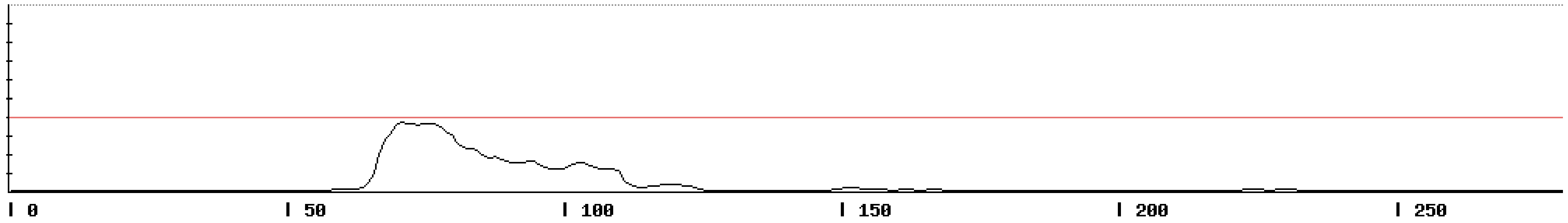

Predictions for Brassicanapus_GSBRNA2T00068670001	
Viterbi Path	
Viterbi Path	
Posterior @ 0.4	129 - KP - 130
Posterior @ 0.4	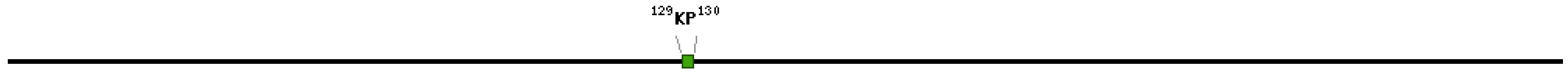
Posterior Graphic. Threshold Marked @ 0.4	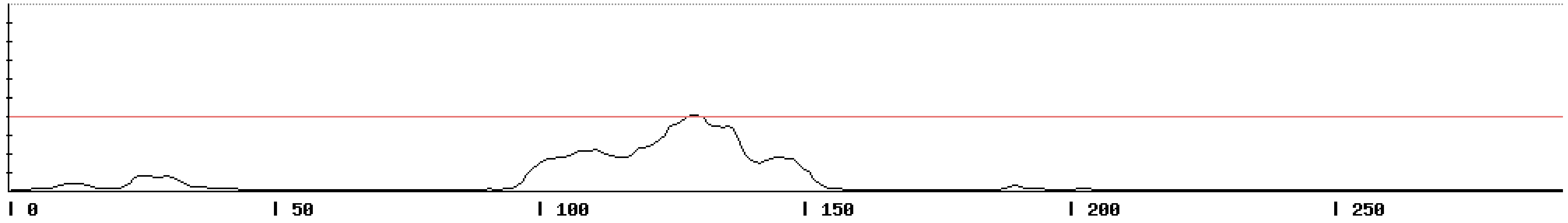

Predictions for Brassicanapus_GSBRNA2T00070546001	
Viterbi Path	
Viterbi Path	

Posterior @ 0.4	
Posterior @ 0.4	
Posterior Graphic. Threshold Marked @ 0.4	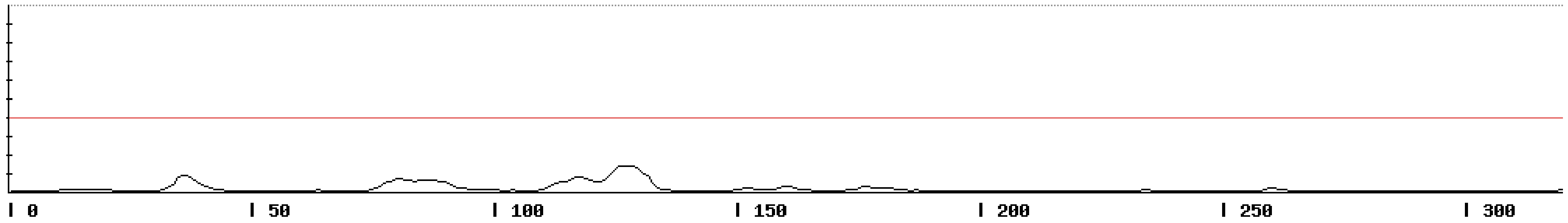

Predictions for Brassicanapus_GSBRNA2T00071627001

Viterbi Path	
Viterbi Path	
Posterior @ 0.4	98 - GKPK - 101
Posterior @ 0.4	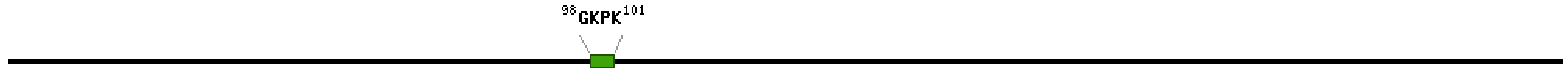
Posterior Graphic. Threshold Marked @ 0.4	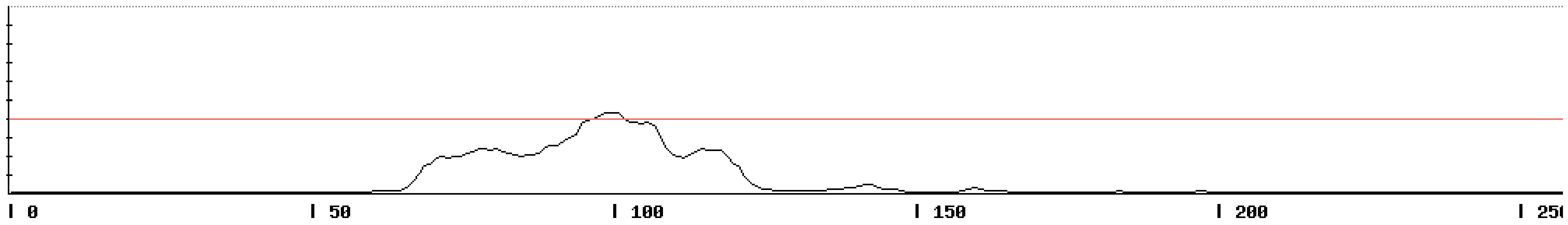

Predictions for Brassicanapus_GSBRNA2T00071676001

Viterbi Path

Viterbi Path

Posterior @ 0.4

Posterior @ 0.4

Posterior Graphic. Threshold Marked @ 0.4

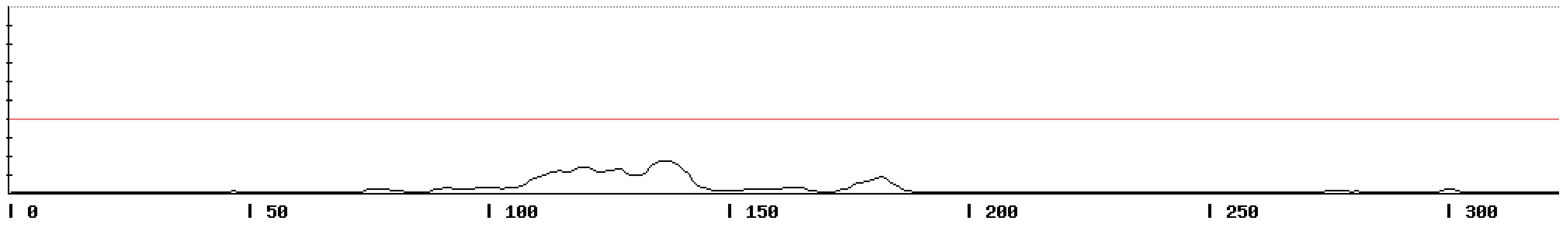

Predictions for Brassicanapus_GSBRNA2T00071930001

Viterbi Path

Viterbi Path

Posterior @ 0.4

98 - KPIGRPKPVGIKK - 110

Posterior @ 0.4

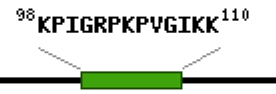

Posterior Graphic. Threshold Marked @ 0.4

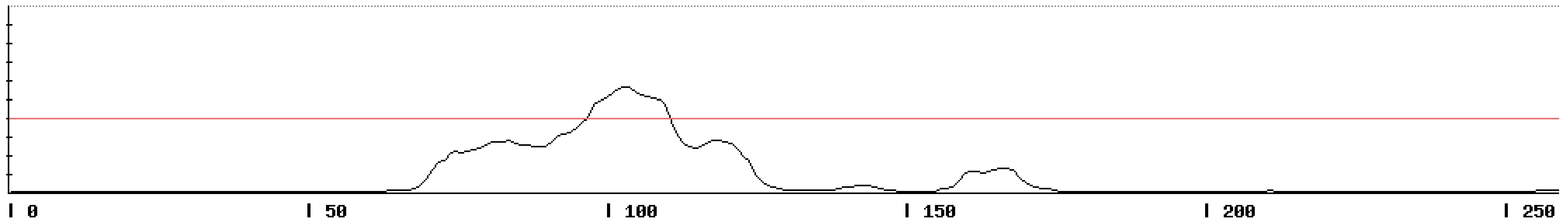

Predictions for Brassicanapus_GSBRNA2T00071983001	
Viterbi Path	
Viterbi Path	
Posterior @ 0.4	
Posterior @ 0.4	
Posterior Graphic. Threshold Marked @ 0.4	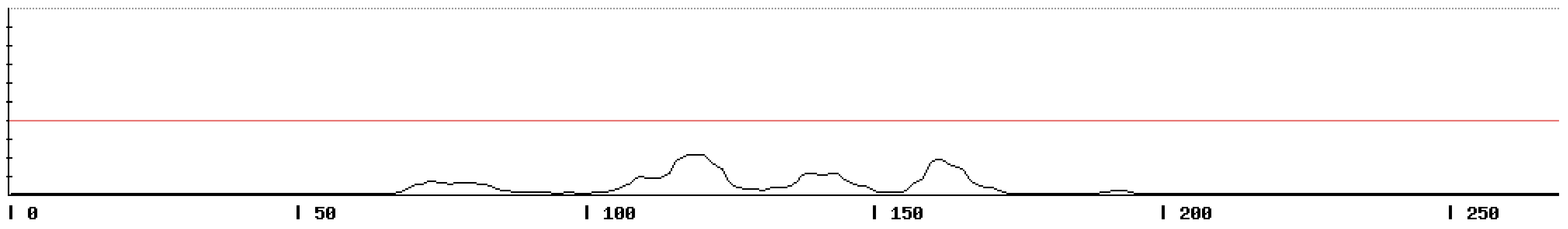

Predictions for Brassicanapus_GSBRNA2T00072232001	
Viterbi Path	
Viterbi Path	
Posterior @ 0.4	
Posterior @ 0.4	
Posterior Graphic. Threshold Marked @ 0.4	

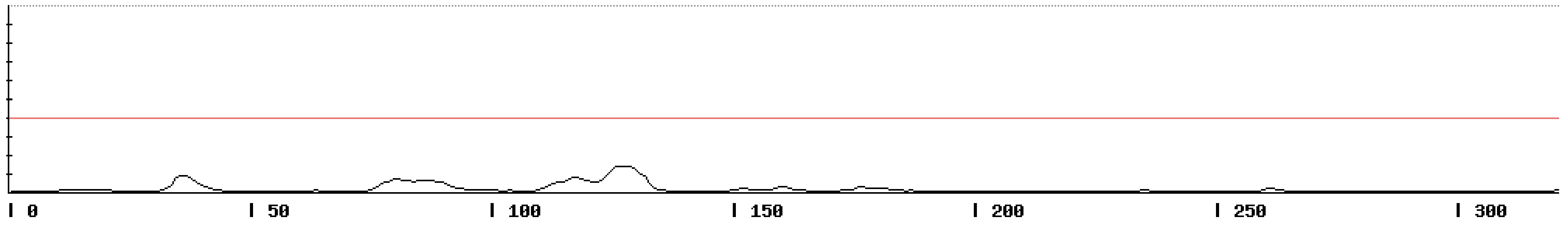

Predictions for Brassicanapus_GSBRNA2T00072911001

Viterbi Path	
Viterbi Path	
Posterior @ 0.4	
Posterior @ 0.4	
Posterior Graphic. Threshold Marked @ 0.4	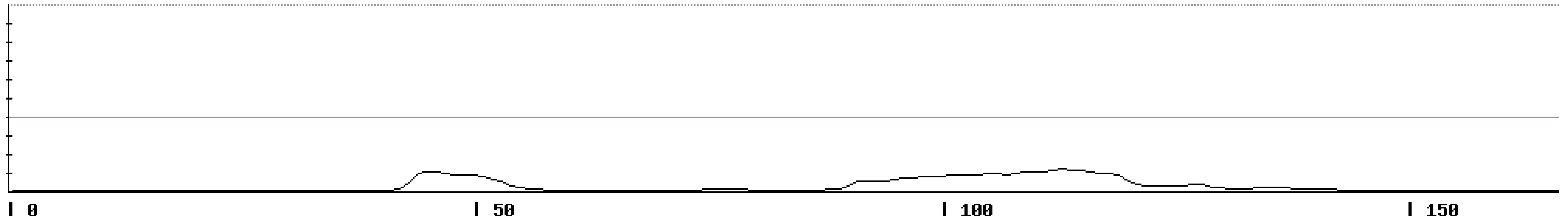

Predictions for Brassicanapus_GSBRNA2T00072912001

Viterbi Path	
Viterbi Path	
Posterior @ 0.4	
Posterior @ 0.4	

Posterior Graphic. Threshold Marked @ 0.4	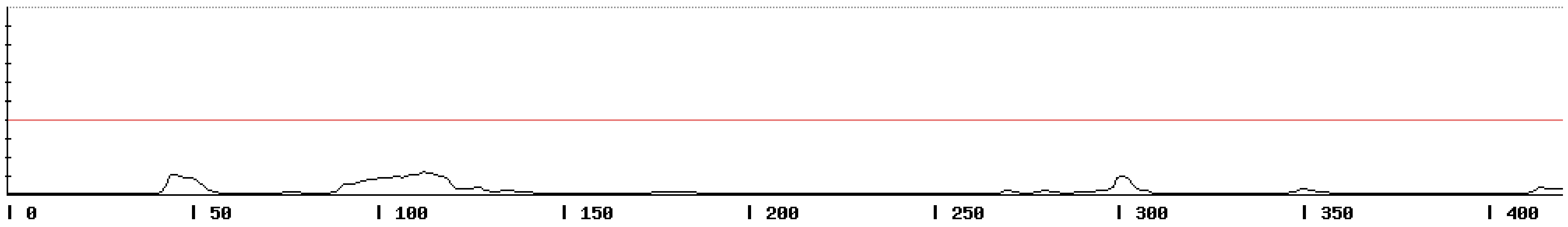

Predictions for Brassicanapus_GSBRNA2T00072913001

Viterbi Path	
Viterbi Path	
Posterior @ 0.4	
Posterior @ 0.4	
Posterior Graphic. Threshold Marked @ 0.4	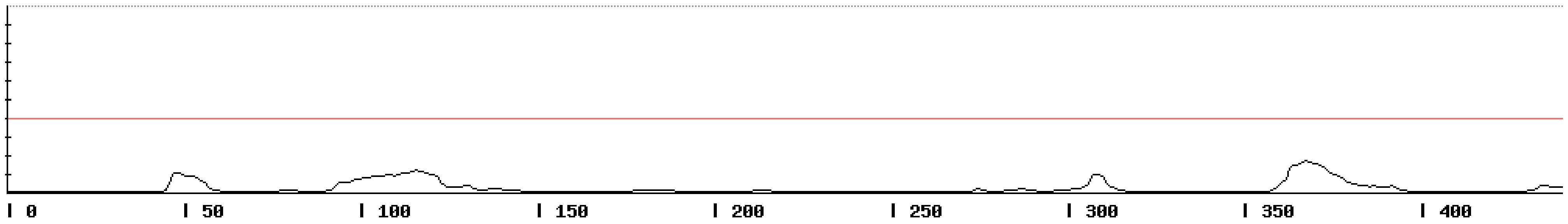

Predictions for Brassicanapus_GSBRNA2T00072914001

Viterbi Path	
Viterbi Path	
Posterior @ 0.4	
Posterior @ 0.4	

Posterior Graphic. Threshold Marked @ 0.4

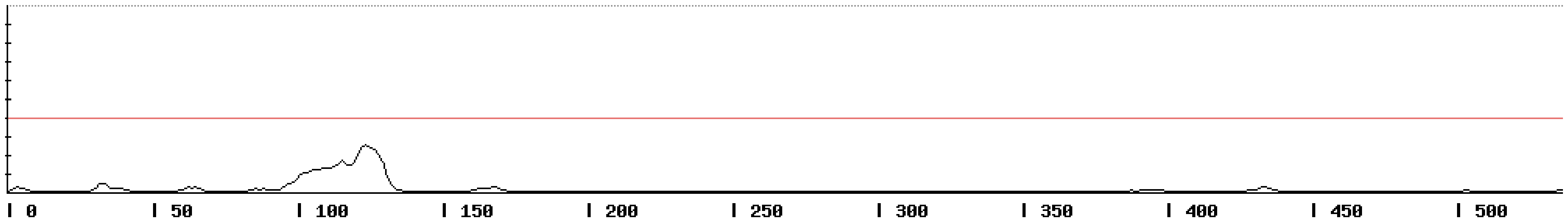

Predictions for Brassicanapus_GSBRNA2T00073164001

Viterbi Path

Viterbi Path

Posterior @ 0.4

Posterior @ 0.4

Posterior Graphic. Threshold Marked @ 0.4

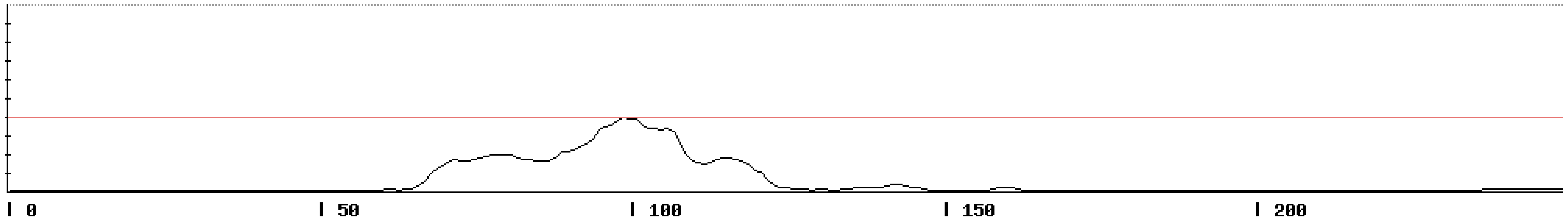

Predictions for Brassicanapus_GSBRNA2T00073188001

Viterbi Path

Viterbi Path

Posterior @ 0.4

Posterior @ 0.4

Posterior Graphic. Threshold Marked @ 0.4

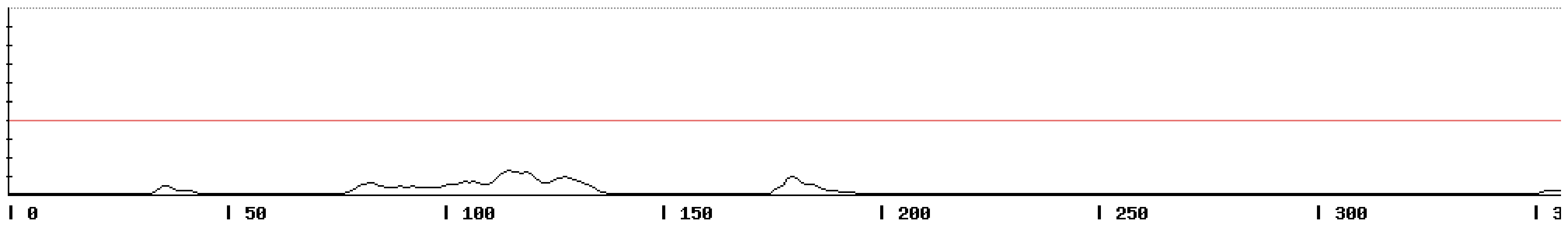

Predictions for Brassicanapus_GSBRNA2T00074690001

Viterbi Path

Viterbi Path

Posterior @ 0.4

Posterior @ 0.4

Posterior Graphic. Threshold Marked @ 0.4

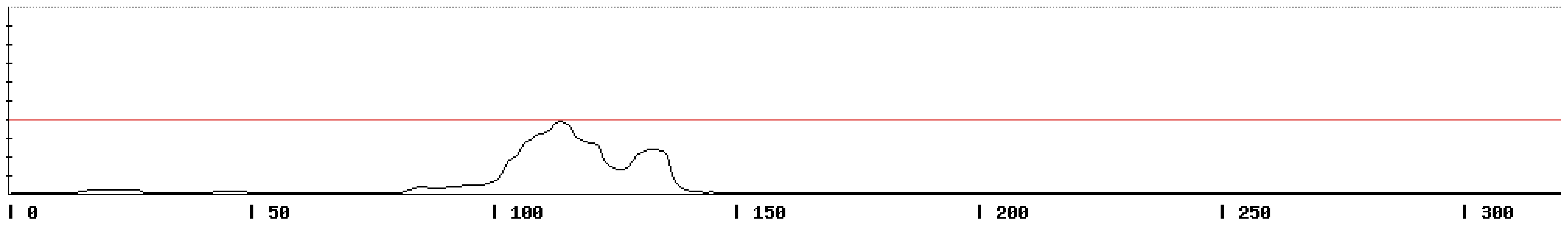

Predictions for Brassicanapus_GSBRNA2T00074713001

Viterbi Path

Viterbi Path

Posterior @ 0.4

Posterior @ 0.4

Posterior Graphic. Threshold Marked @ 0.4

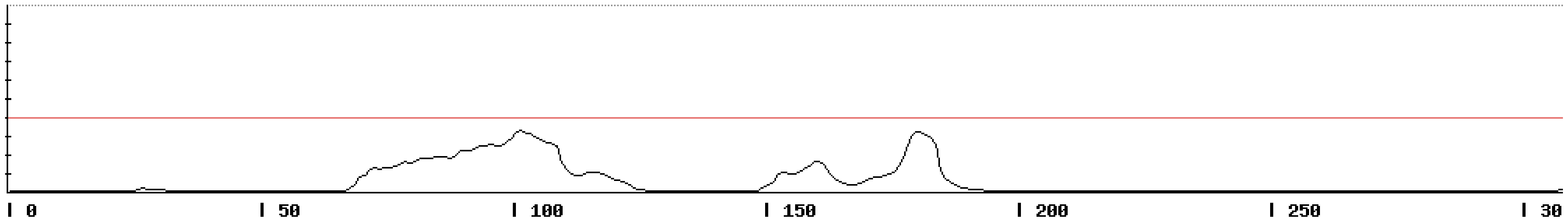

Predictions for Brassicanapus_GSBRNA2T00075489001

Viterbi Path

Viterbi Path

Posterior @ 0.4

305 - VKRPRGRPAGSKNKPKPP - 322

Posterior @ 0.4

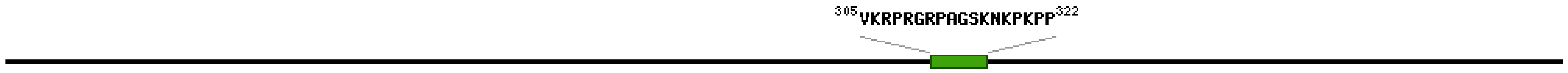

Posterior Graphic. Threshold Marked @ 0.4

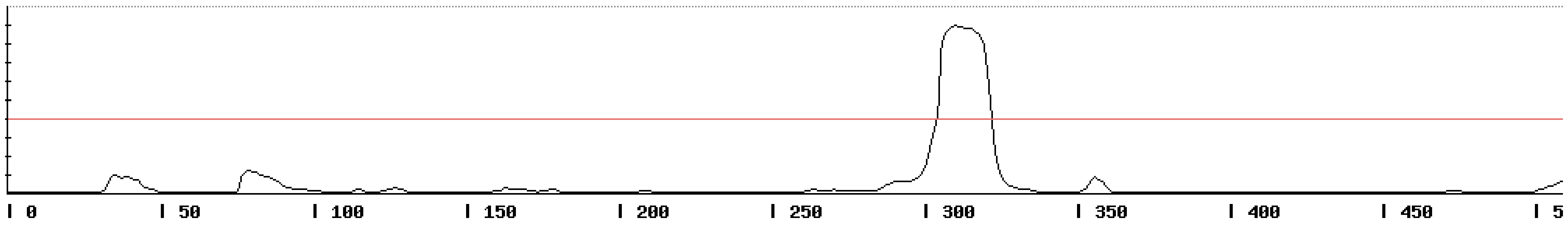

Predictions for Brassicanapus_GSBRNA2T00075573001

Viterbi Path	
Viterbi Path	
Posterior @ 0.4	
Posterior @ 0.4	
Posterior Graphic. Threshold Marked @ 0.4	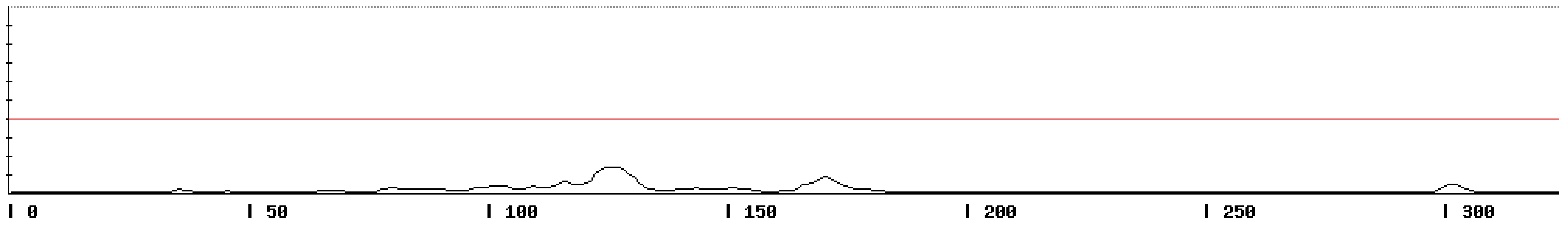

Predictions for Brassicanapus_GSBRNA2T00075596001

Viterbi Path	
Viterbi Path	
Posterior @ 0.4	188 - RKRRK - 192
Posterior @ 0.4	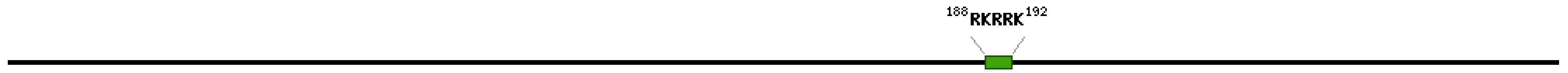
Posterior Graphic. Threshold Marked @ 0.4	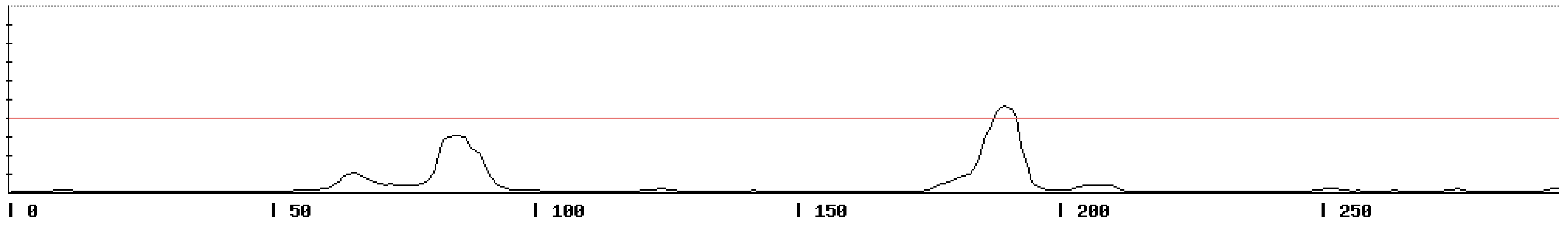

Predictions for Brassicanapus_GSBRNA2T00075630001	
Viterbi Path	
Viterbi Path	
Posterior @ 0.4	
Posterior @ 0.4	
Posterior Graphic. Threshold Marked @ 0.4	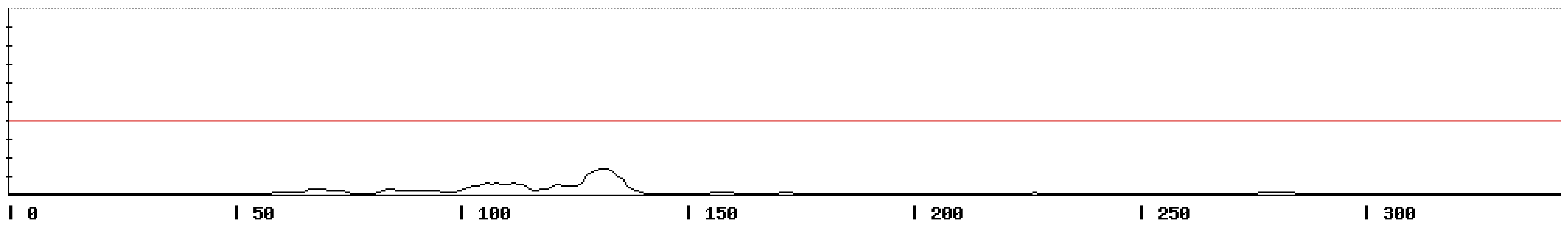

Predictions for Brassicanapus_GSBRNA2T00075632001	
Viterbi Path	
Viterbi Path	
Posterior @ 0.4	
Posterior @ 0.4	
Posterior Graphic. Threshold Marked @ 0.4	

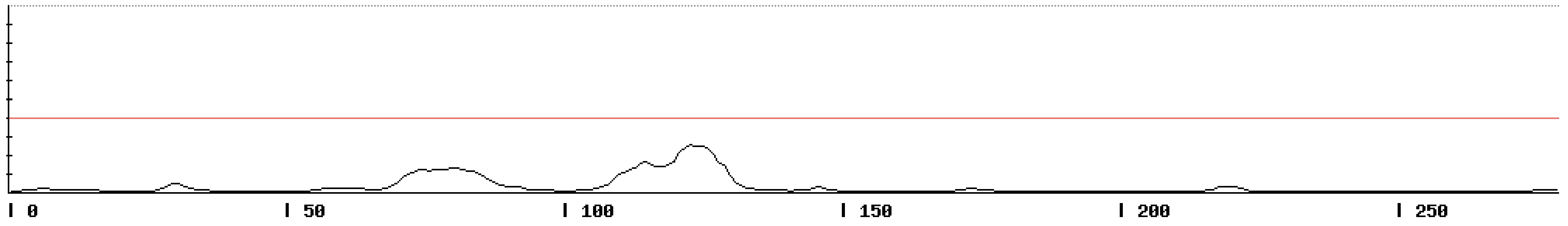

Predictions for Brassicanapus_GSBRNA2T00076116001

Viterbi Path

Viterbi Path

Posterior @ 0.4

Posterior @ 0.4

Posterior Graphic. Threshold Marked @ 0.4

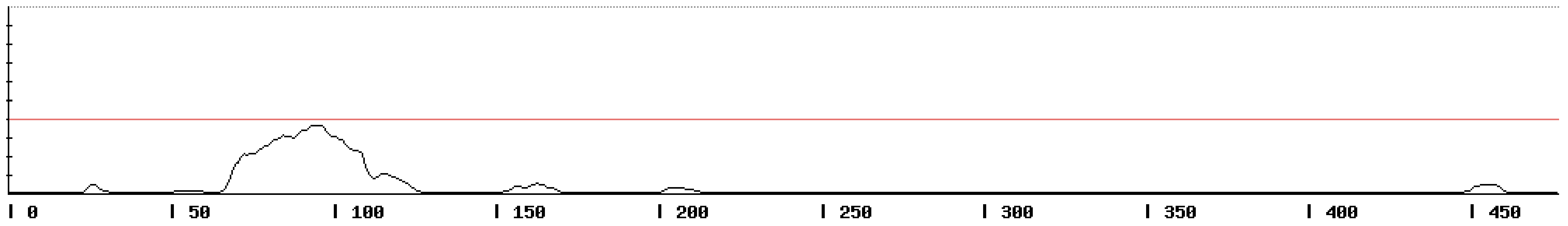

Predictions for Brassicanapus_GSBRNA2T00076462001

Viterbi Path

Viterbi Path

Posterior @ 0.4

Posterior @ 0.4

Posterior Graphic. Threshold Marked @ 0.4	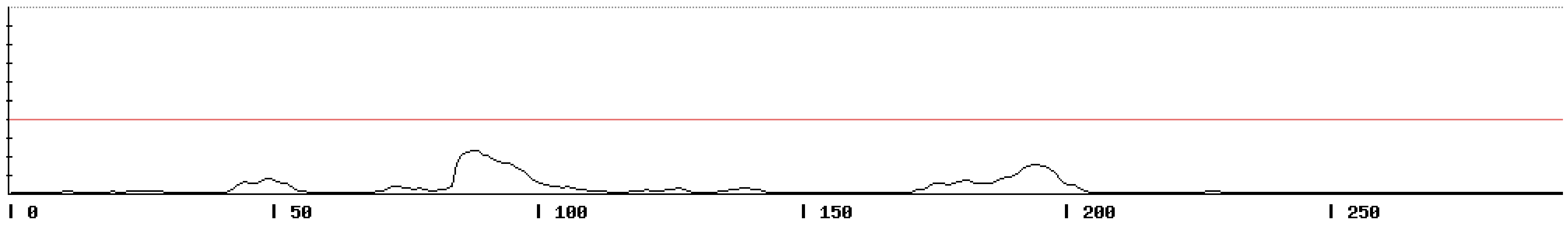

Predictions for Brassicanapus_GSBRNA2T00077057001	
Viterbi Path	
Viterbi Path	
Posterior @ 0.4	
Posterior @ 0.4	
Posterior Graphic. Threshold Marked @ 0.4	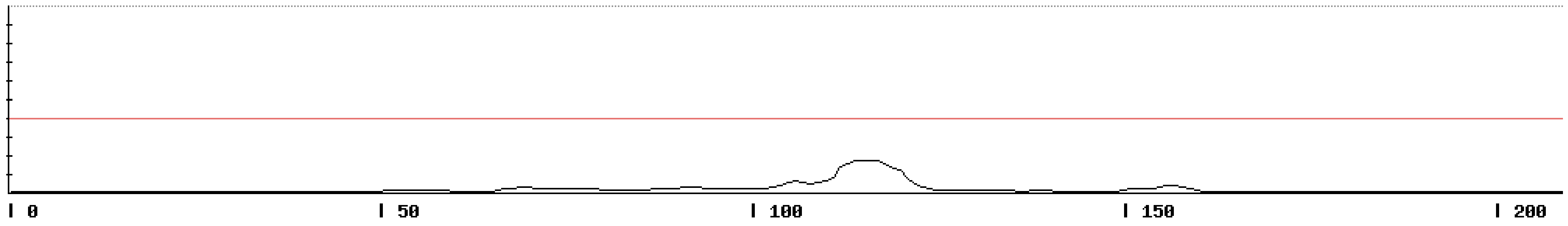

Predictions for Brassicanapus_GSBRNA2T00077286001	
Viterbi Path	
Viterbi Path	
Posterior @ 0.4	
Posterior @ 0.4	

Posterior Graphic. Threshold Marked @ 0.4	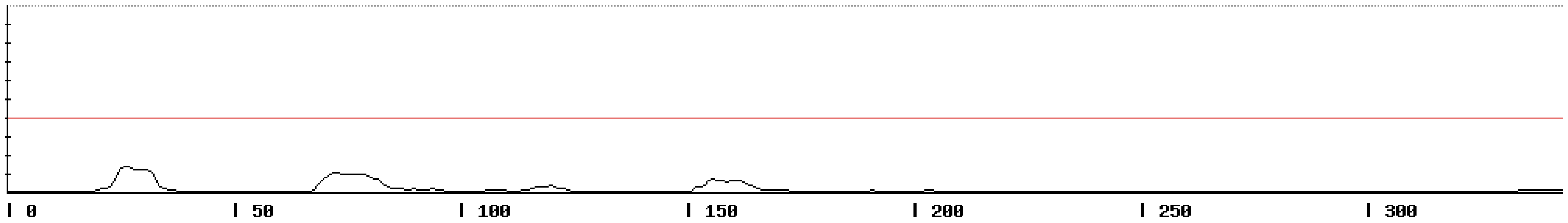

Predictions for Brassicanapus_GSBRNA2T00078555001	
Viterbi Path	
Viterbi Path	
Posterior @ 0.4	
Posterior @ 0.4	
Posterior Graphic. Threshold Marked @ 0.4	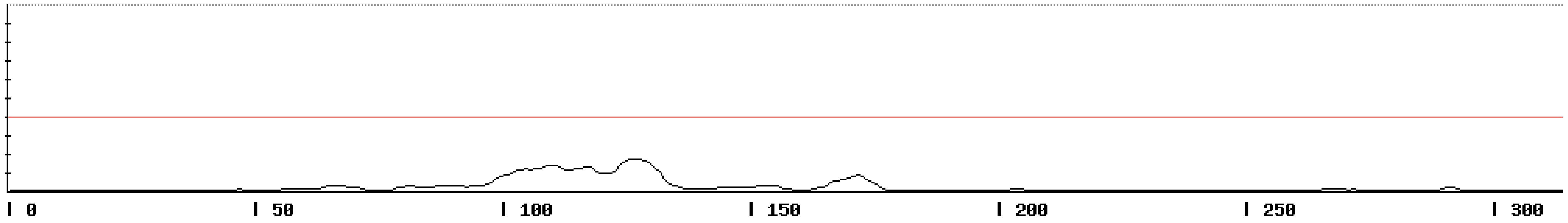

Predictions for Brassicanapus_GSBRNA2T00078706001	
Viterbi Path	
Viterbi Path	

Posterior @ 0.4	
Posterior @ 0.4	
Posterior Graphic. Threshold Marked @ 0.4	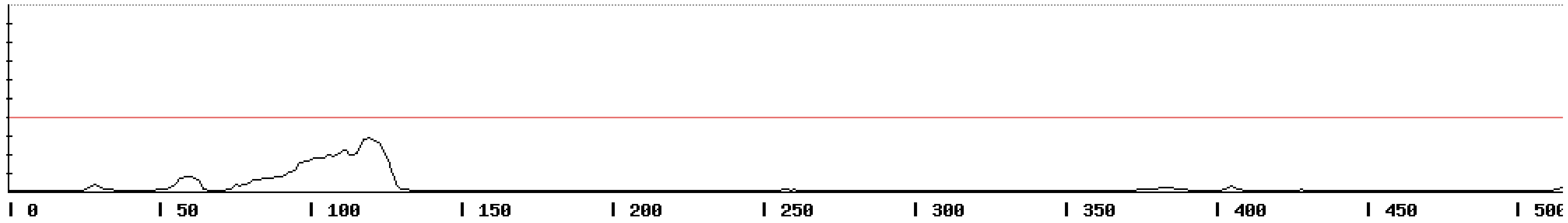

Predictions for Brassicanapus_GSBRNA2T00078811001	
Viterbi Path	
Viterbi Path	
Posterior @ 0.4	
Posterior @ 0.4	
Posterior Graphic. Threshold Marked @ 0.4	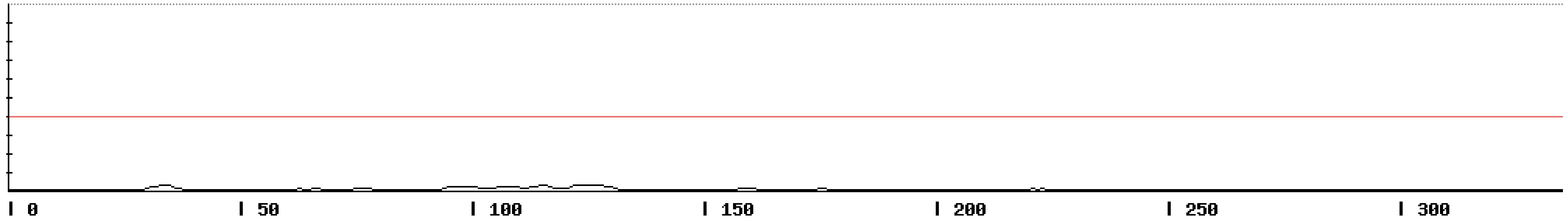

Predictions for Brassicanapus_GSBRNA2T00080256001	
Viterbi Path	
Viterbi Path	

Posterior @ 0.4	
Posterior @ 0.4	
Posterior Graphic. Threshold Marked @ 0.4	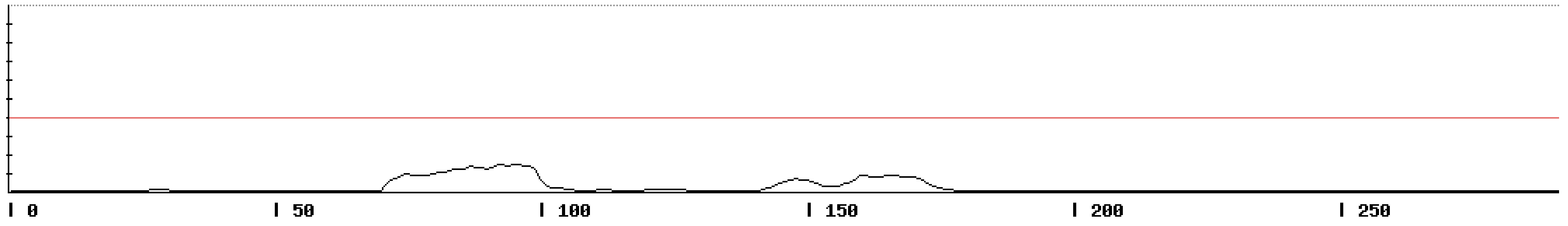

Predictions for Brassicanapus_GSBRNA2T00080395001

Viterbi Path	
Viterbi Path	
Posterior @ 0.4	
Posterior @ 0.4	
Posterior Graphic. Threshold Marked @ 0.4	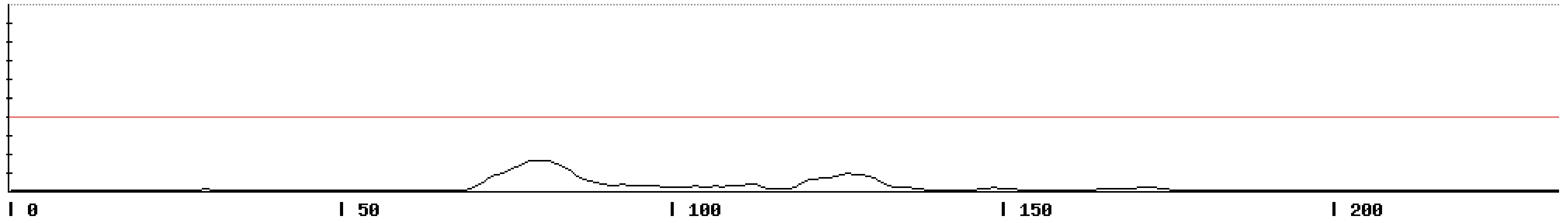

Predictions for Brassicanapus_GSBRNA2T00080792001

Viterbi Path	
Viterbi Path	
Posterior @ 0.4	98 - GKPK - 101
Posterior @ 0.4	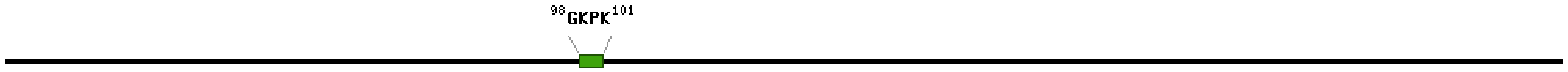
Posterior Graphic. Threshold Marked @ 0.4	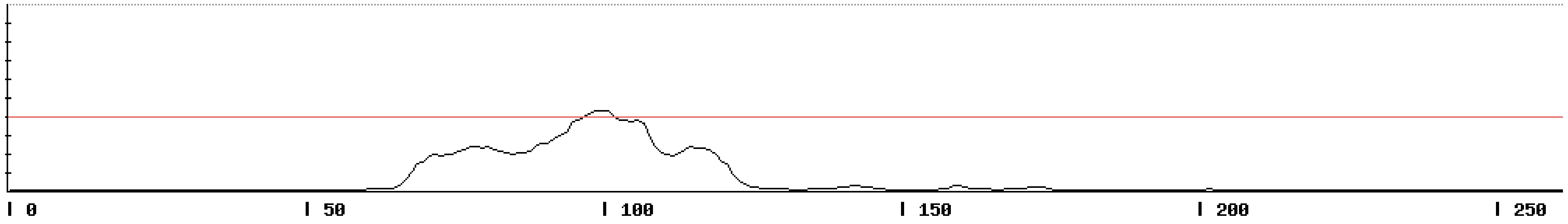

Predictions for Brassicanapus_GSBRNA2T00080799001

Viterbi Path	
Viterbi Path	
Posterior @ 0.4	
Posterior @ 0.4	
Posterior Graphic. Threshold Marked @ 0.4	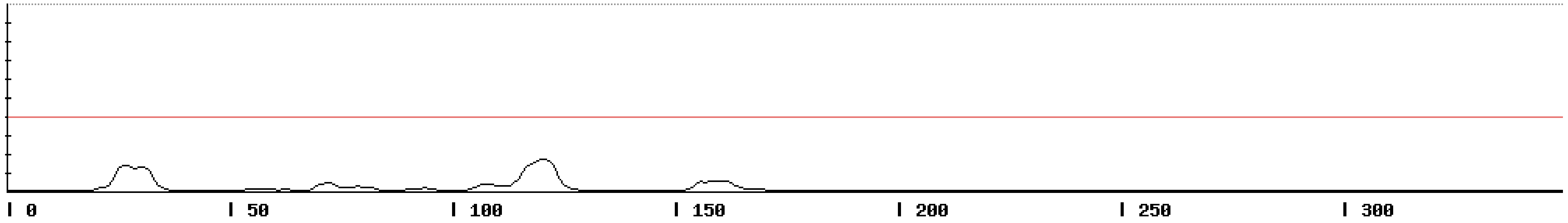

Predictions for Brassicanapus_GSBRNA2T00080904001	
Viterbi Path	
Viterbi Path	
Posterior @ 0.4	
Posterior @ 0.4	
Posterior Graphic. Threshold Marked @ 0.4	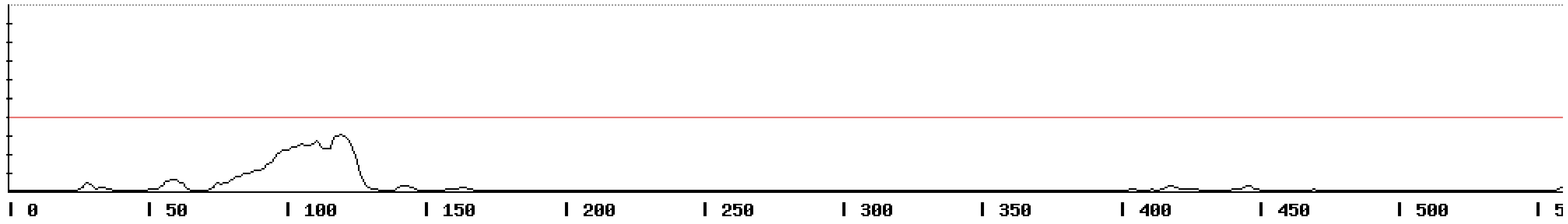

Predictions for Brassicanapus_GSBRNA2T00081655001	
Viterbi Path	
Viterbi Path	
Posterior @ 0.4	
Posterior @ 0.4	
Posterior Graphic. Threshold Marked @ 0.4	

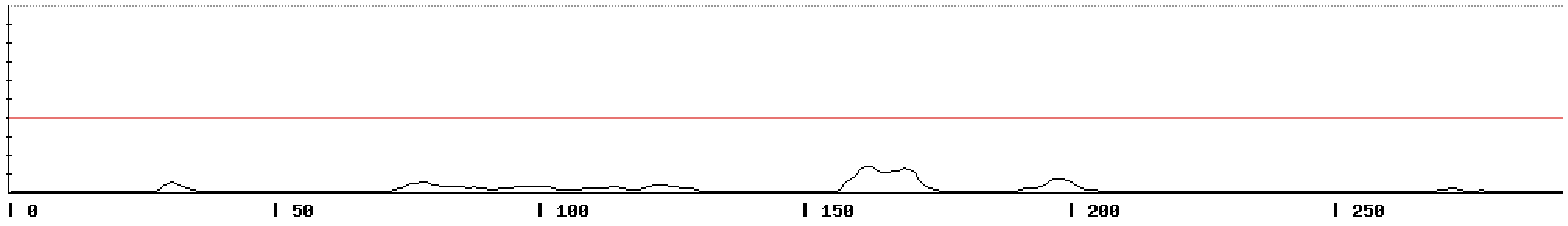

Predictions for Brassicanapus_GSBRNA2T00083095001

Viterbi Path	
Viterbi Path	
Posterior @ 0.4	
Posterior @ 0.4	
Posterior Graphic. Threshold Marked @ 0.4	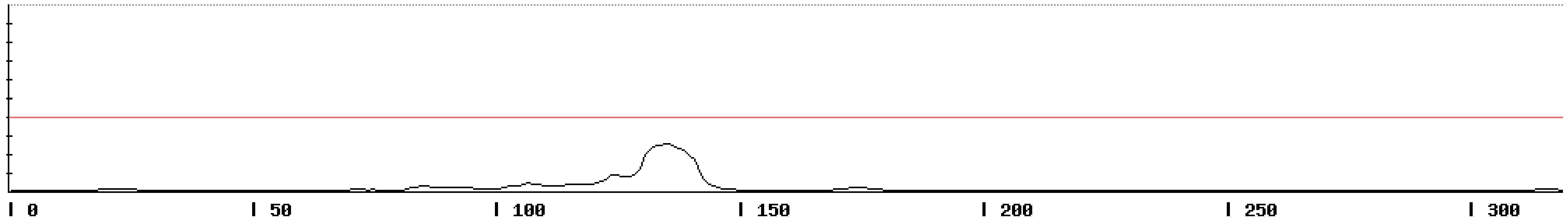

Predictions for Brassicanapus_GSBRNA2T00083327001

Viterbi Path	
Viterbi Path	
Posterior @ 0.4	
Posterior @ 0.4	

Posterior Graphic. Threshold Marked @ 0.4

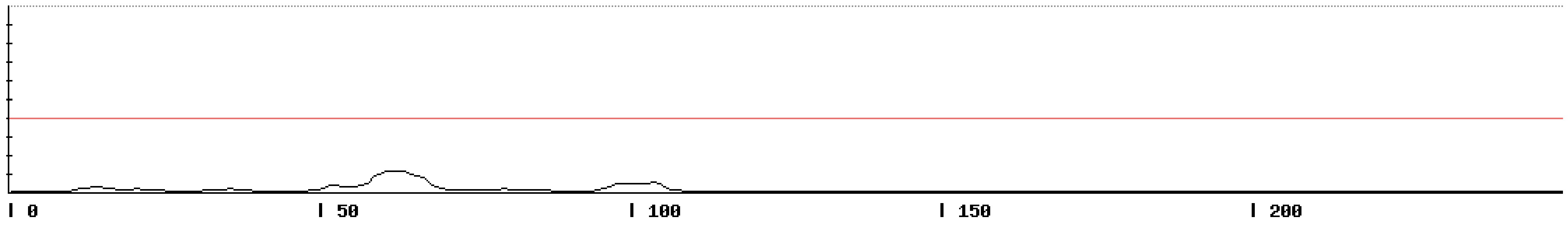

Predictions for Brassicanapus_GSBRNA2T00083814001

Viterbi Path

Viterbi Path

Posterior @ 0.4

Posterior @ 0.4

Posterior Graphic. Threshold Marked @ 0.4

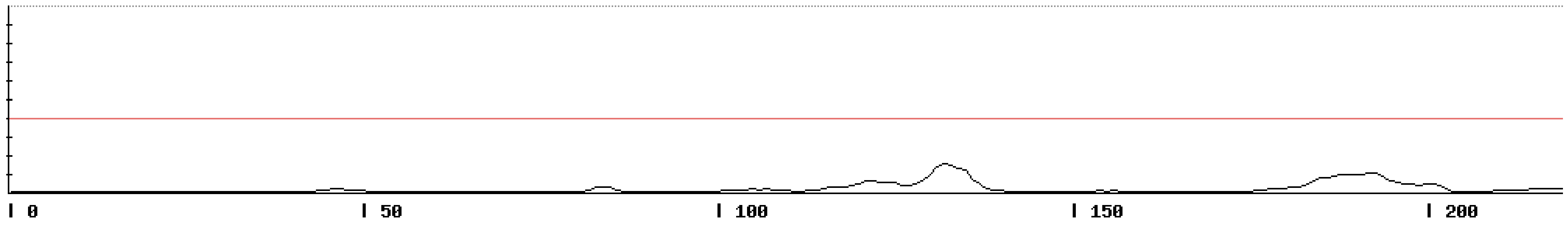

Predictions for Brassicanapus_GSBRNA2T00083815001

Viterbi Path

Viterbi Path

Posterior @ 0.4

Posterior @ 0.4

Posterior Graphic. Threshold Marked @ 0.4

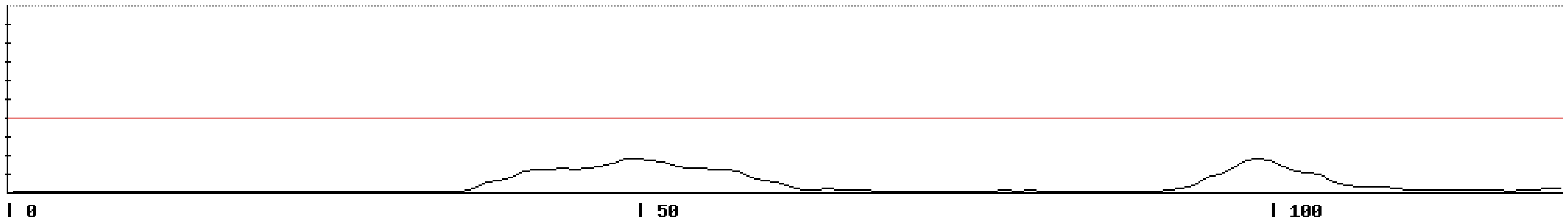

Predictions for Brassicanapus_GSBRNA2T00083944001

Viterbi Path

Viterbi Path

Posterior @ 0.4

Posterior @ 0.4

Posterior Graphic. Threshold Marked @ 0.4

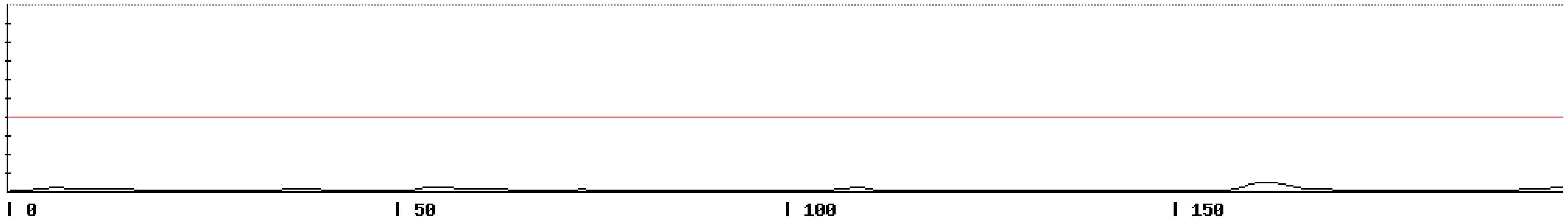

Predictions for Brassicanapus_GSBRNA2T00083984001

Viterbi Path

Viterbi Path

Posterior @ 0.4

Posterior @ 0.4

Posterior Graphic. Threshold Marked @ 0.4

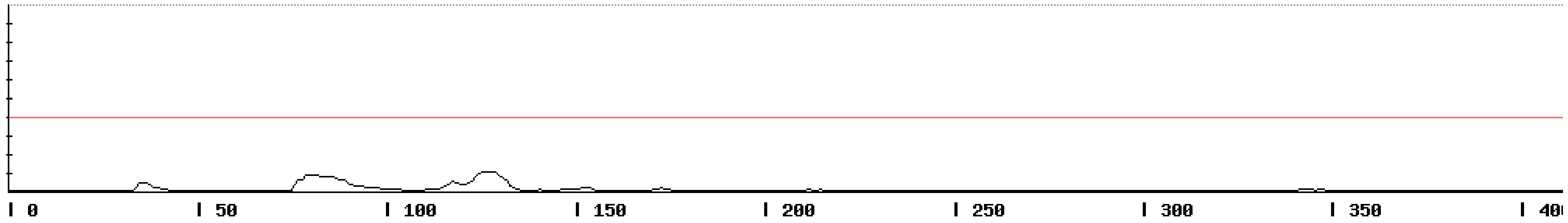

Predictions for Brassicanapus_GSBRNA2T00084044001

Viterbi Path

Viterbi Path

Posterior @ 0.4

Posterior @ 0.4

Posterior Graphic. Threshold Marked @ 0.4

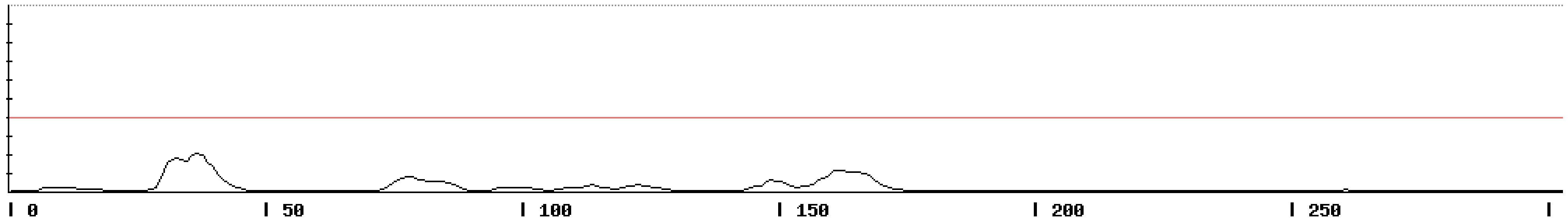

Predictions for Brassicanapus_GSBRNA2T00084755001

Viterbi Path

Viterbi Path

Posterior @ 0.4

Posterior @ 0.4

Posterior Graphic. Threshold Marked @ 0.4

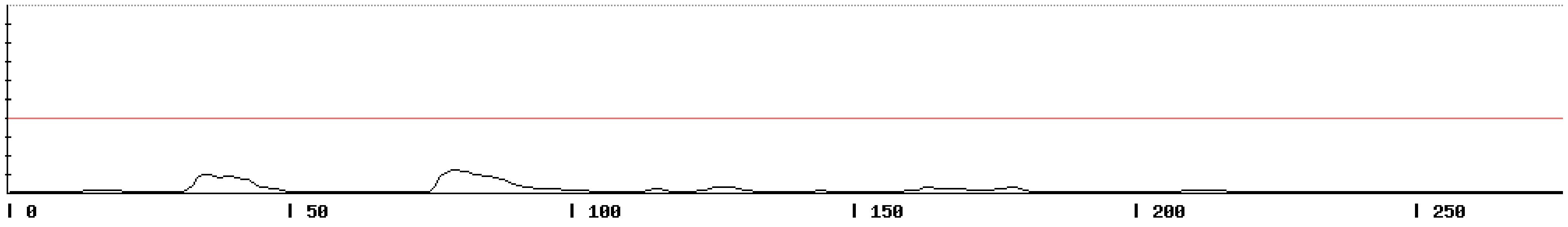

Predictions for Brassicanapus_GSBRNA2T00085330001

Viterbi Path

Viterbi Path

Posterior @ 0.4

Posterior @ 0.4

Posterior Graphic. Threshold Marked @ 0.4

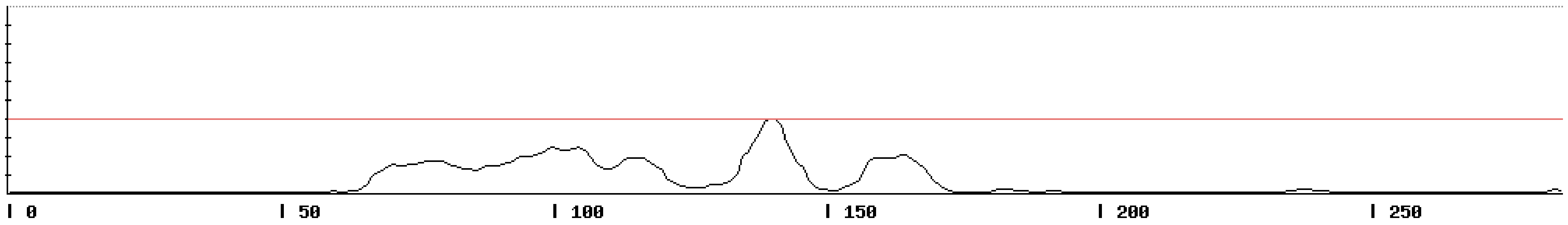

Predictions for Brassicanapus_GSBRNA2T00085398001

Viterbi Path

Viterbi Path

Posterior @ 0.4

Posterior @ 0.4

Posterior Graphic. Threshold Marked @ 0.4

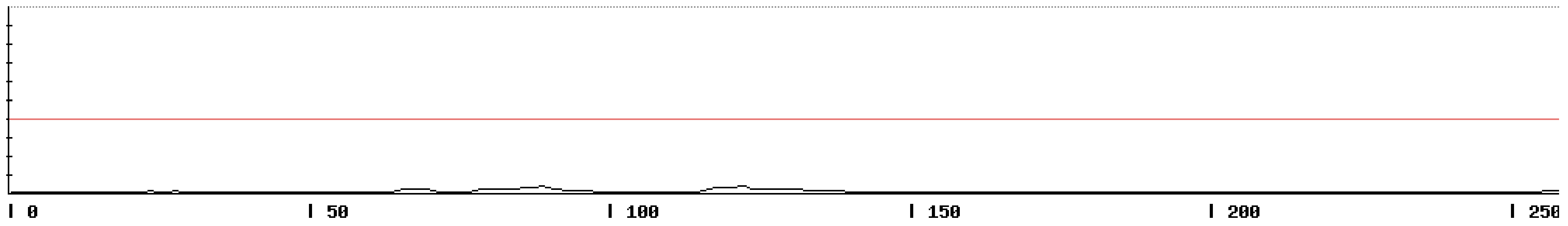

Predictions for Brassicanapus_GSBRNA2T00085404001

Viterbi Path

Viterbi Path

Posterior @ 0.4

Posterior @ 0.4

Posterior Graphic. Threshold Marked @ 0.4

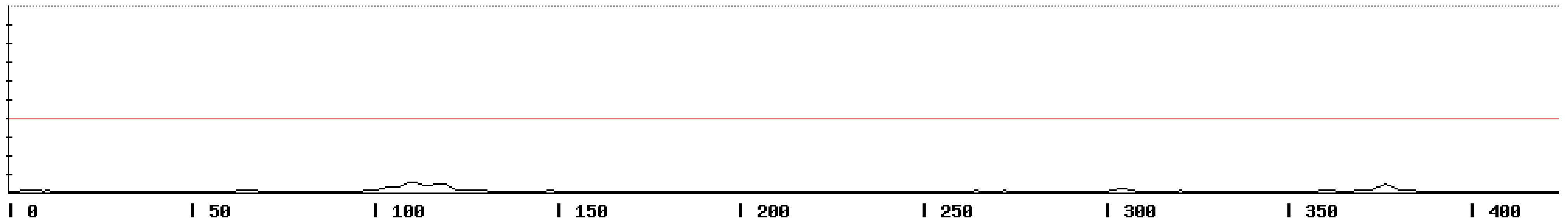

Predictions for Brassicanapus_GSBRNA2T00085406001

Viterbi Path	
Viterbi Path	
Posterior @ 0.4	
Posterior @ 0.4	
Posterior Graphic. Threshold Marked @ 0.4	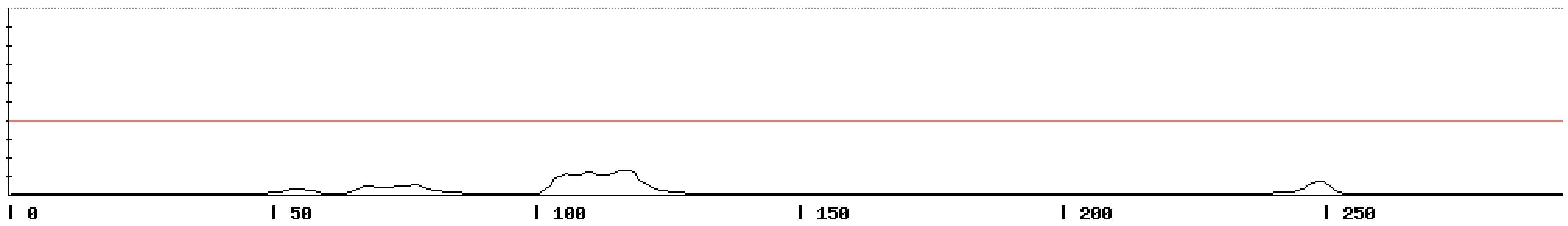

Predictions for Brassicanapus_GSBRNA2T00085407001

Viterbi Path	
Viterbi Path	
Posterior @ 0.4	
Posterior @ 0.4	
Posterior Graphic. Threshold Marked @ 0.4	

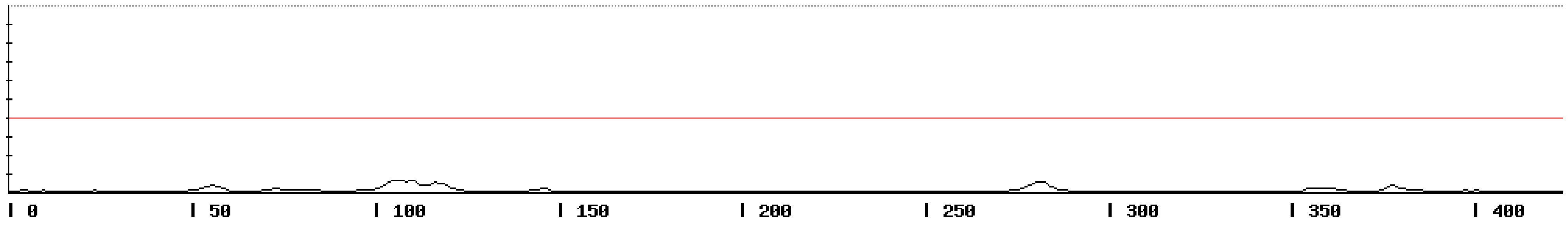

Predictions for Brassicanapus_GSBRNA2T00085409001

Viterbi Path	
Viterbi Path	
Posterior @ 0.4	64 - VKKFGKLNRRKRASKTGH - 81
Posterior @ 0.4	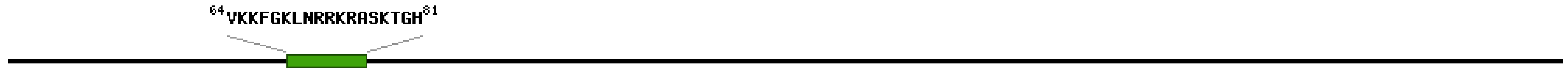
Posterior Graphic. Threshold Marked @ 0.4	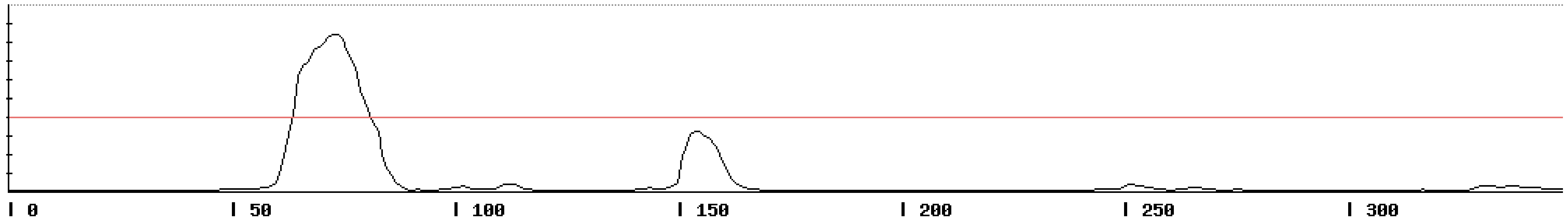

Predictions for Brassicanapus_GSBRNA2T00085411001

Viterbi Path	
Viterbi Path	
Posterior @ 0.4	93 - RKRGNREKIGE - 103
Posterior @ 0.4	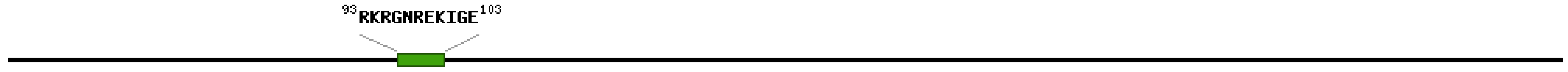

Posterior Graphic. Threshold Marked @ 0.4

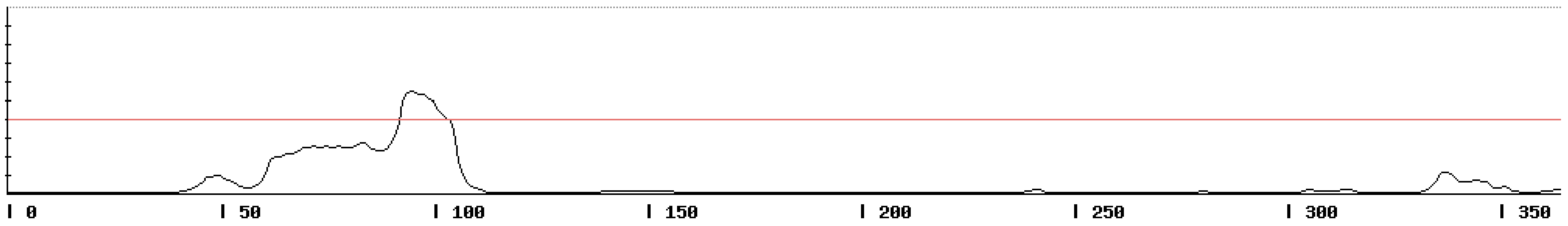

Predictions for Brassicanapus_GSBRNA2T00085413001

Viterbi Path	68 - GRKEKRYNRGERQIRKTKSGFWKKTGVTMDIKRKRSGHREKIGEKR - 113
Viterbi Path	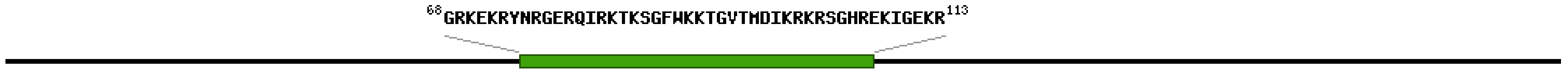
Posterior @ 0.4	68 - GRKEKRYNRGERQIRKTKSGFWKKTGVTMDIKRKRSGHREKIGEKR - 113
Posterior @ 0.4	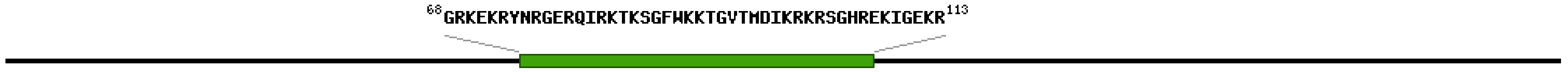
Posterior Graphic. Threshold Marked @ 0.4	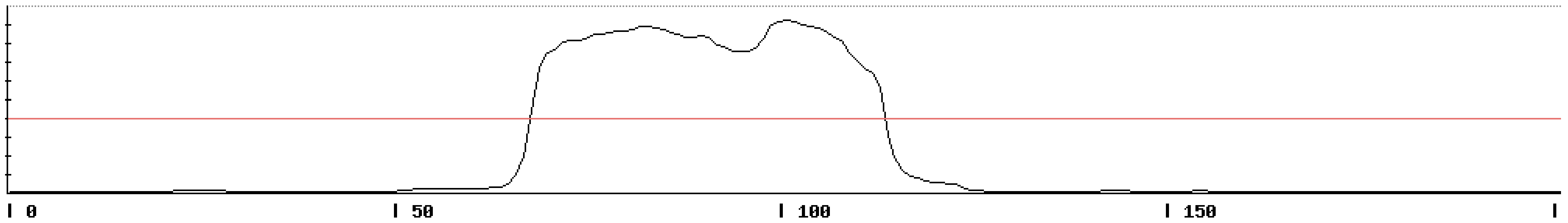

Predictions for Brassicanapus_GSBRNA2T00085414001

Viterbi Path	
Viterbi Path	
Posterior @ 0.4	94 - KPIIRKKGGGGDHEKIGEKRVFSKSKSKSK - 123
Posterior @ 0.4	

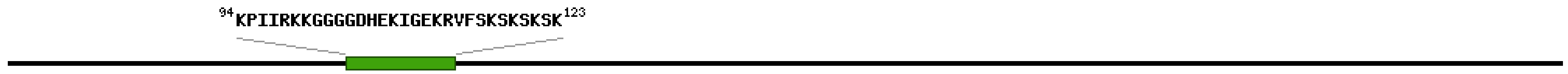

Posterior Graphic. Threshold Marked @ 0.4

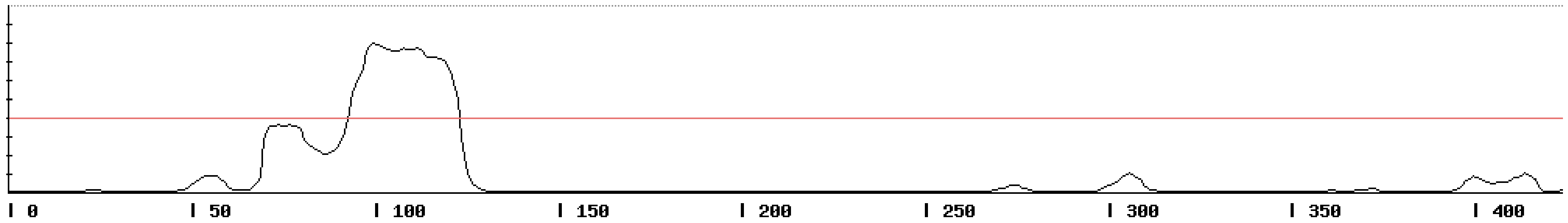

Predictions for Brassicanapus_GSBRNA2T00085416001

Viterbi Path

Viterbi Path

Posterior @ 0.4 — 78 - SRKTKS - 83

Posterior @ 0.4

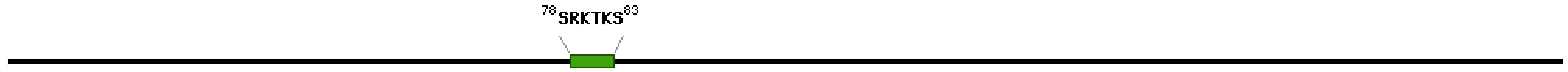

Posterior Graphic. Threshold Marked @ 0.4

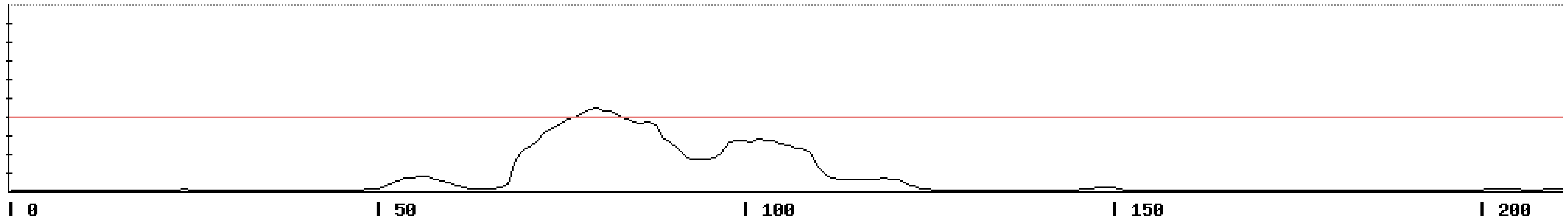

Predictions for Brassicanapus_GSBRNA2T00085419001

Viterbi Path

Viterbi Path

Posterior @ 0.4 — 85 - RKTK - 88

Posterior @ 0.4

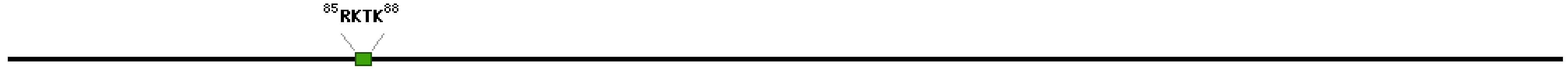

Posterior Graphic. Threshold Marked @ 0.4

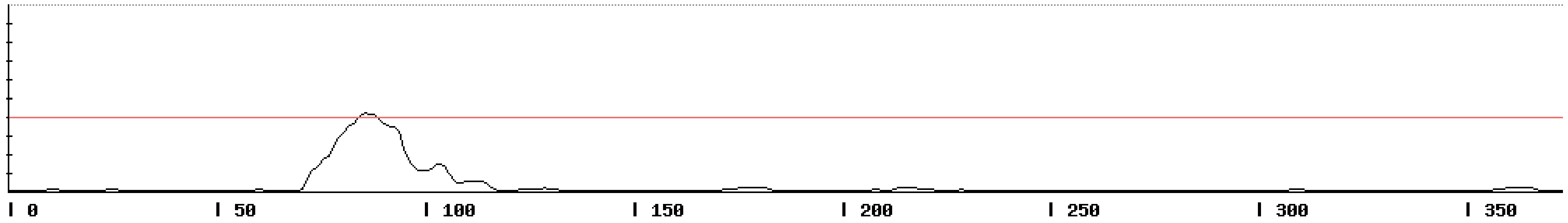

Predictions for Brassicanapus_GSBRNA2T00085420001

Viterbi Path

Viterbi Path

Posterior @ 0.4 — 76 - GERQSRRTKSGFWKKTGPTMDIMRKRGNREKIGEKR - 111

Posterior @ 0.4

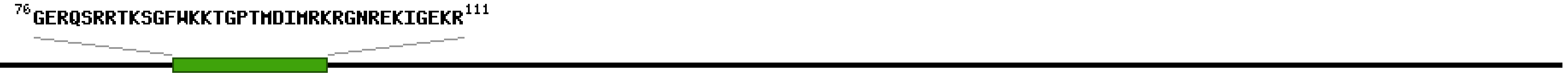

Posterior Graphic. Threshold Marked @ 0.4

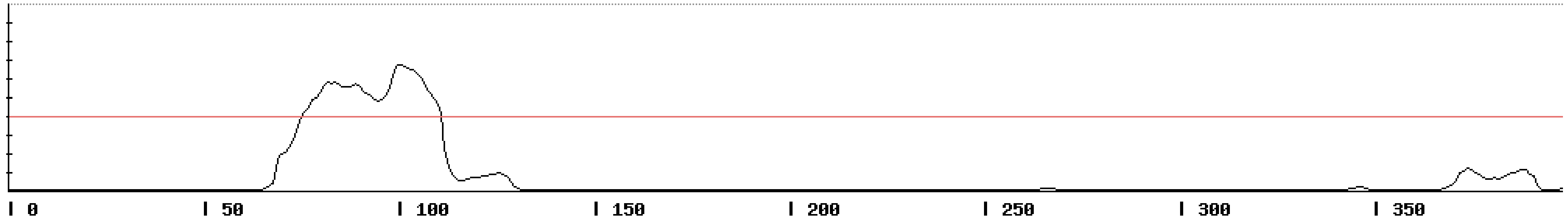

Predictions for Brassicanapus_GSBRNA2T00086242001

Viterbi Path

Viterbi Path

Posterior @ 0.4

Posterior @ 0.4

Posterior Graphic. Threshold Marked @ 0.4

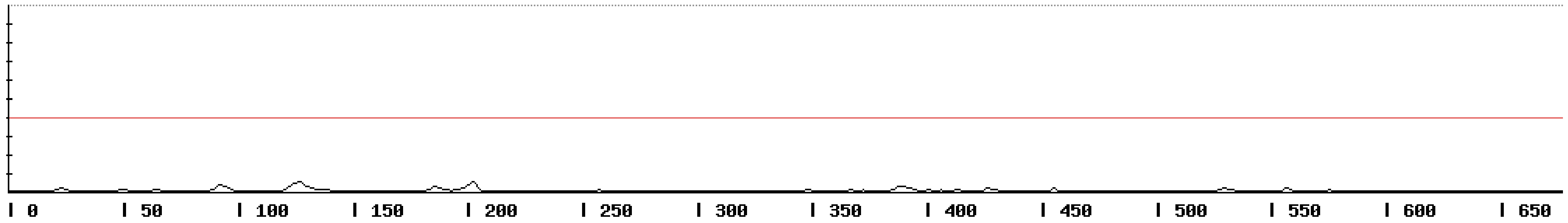

Predictions for Brassicanapus_GSBRNA2T00086386001

Viterbi Path

Viterbi Path

Posterior @ 0.4

Posterior @ 0.4

Posterior Graphic. Threshold Marked @ 0.4

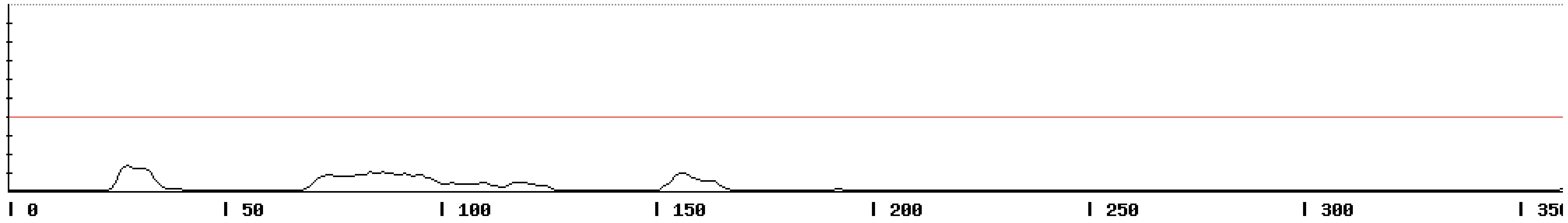

Predictions for Brassicanapus_GSBRNA2T00086664001

Viterbi Path	
Viterbi Path	
Posterior @ 0.4	238 - KDKKKK - 243
Posterior @ 0.4	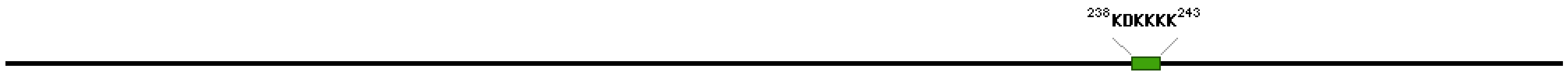
Posterior Graphic. Threshold Marked @ 0.4	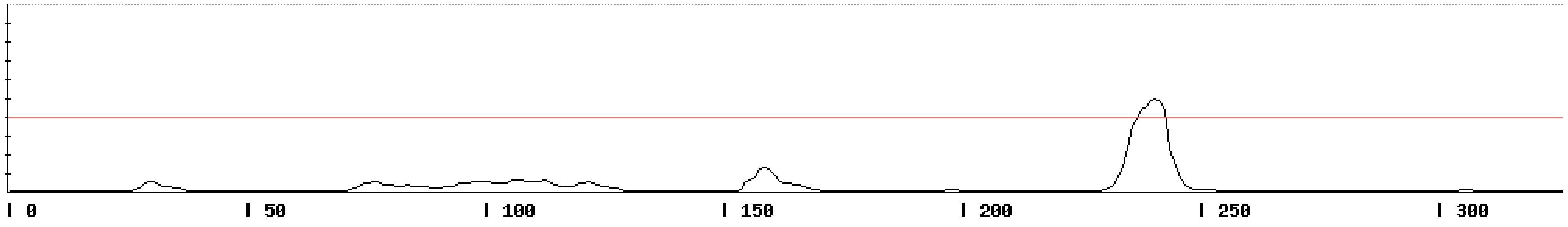

Predictions for Brassicanapus_GSBRNA2T00087046001

Viterbi Path	
Viterbi Path	
Posterior @ 0.4	
Posterior @ 0.4	
Posterior Graphic. Threshold Marked @ 0.4	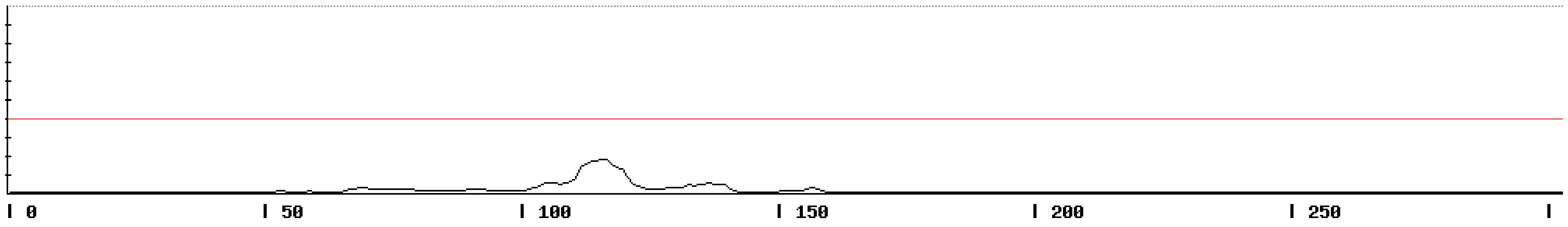

Predictions for Brassicanapus_GSBRNA2T00087136001

Viterbi Path

Viterbi Path

Posterior @ 0.4

Posterior @ 0.4

Posterior Graphic. Threshold Marked @ 0.4

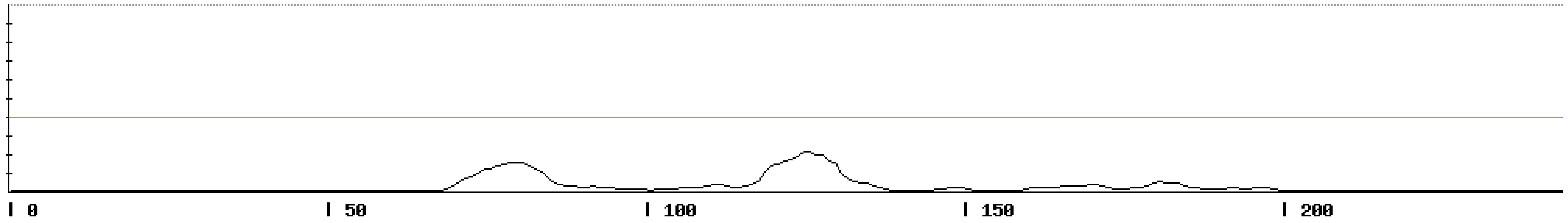

Predictions for Brassicanapus_GSBRNA2T00087538001

Viterbi Path

Viterbi Path

Posterior @ 0.4

Posterior @ 0.4

Posterior Graphic. Threshold Marked @ 0.4

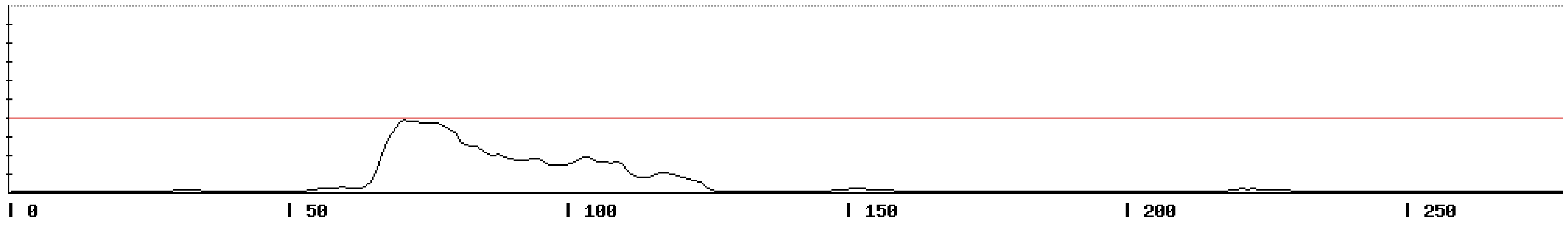

Predictions for Brassicanapus_GSBRNA2T00087539001

Viterbi Path	
Viterbi Path	
Posterior @ 0.4	
Posterior @ 0.4	
Posterior Graphic. Threshold Marked @ 0.4	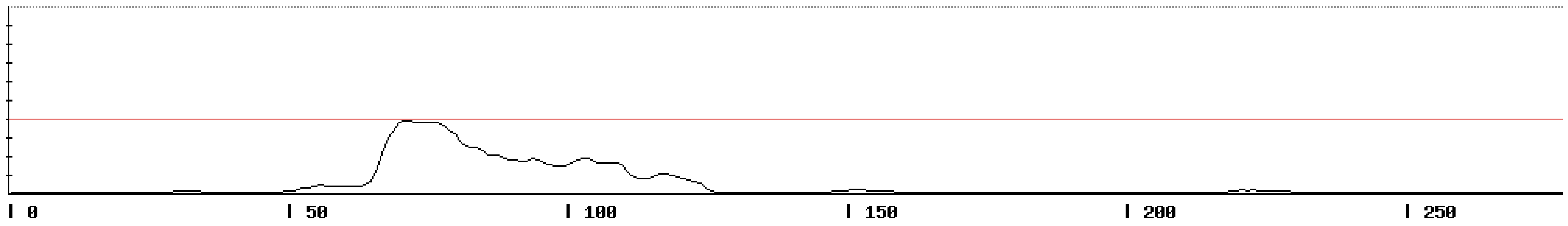

Predictions for Brassicanapus_GSBRNA2T00088073001

Viterbi Path	
Viterbi Path	
Posterior @ 0.4	
Posterior @ 0.4	

Posterior Graphic. Threshold Marked @ 0.4	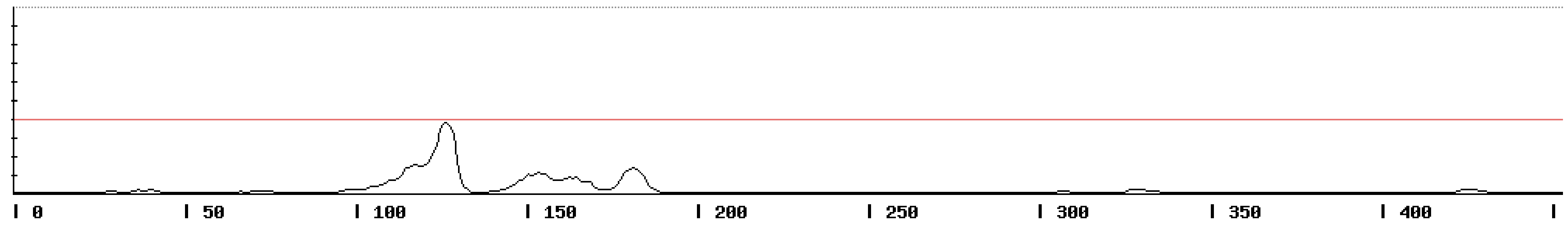

Predictions for Brassicanapus_GSBRNA2T00088528001	
Viterbi Path	
Viterbi Path	
Posterior @ 0.4	
Posterior @ 0.4	
Posterior Graphic. Threshold Marked @ 0.4	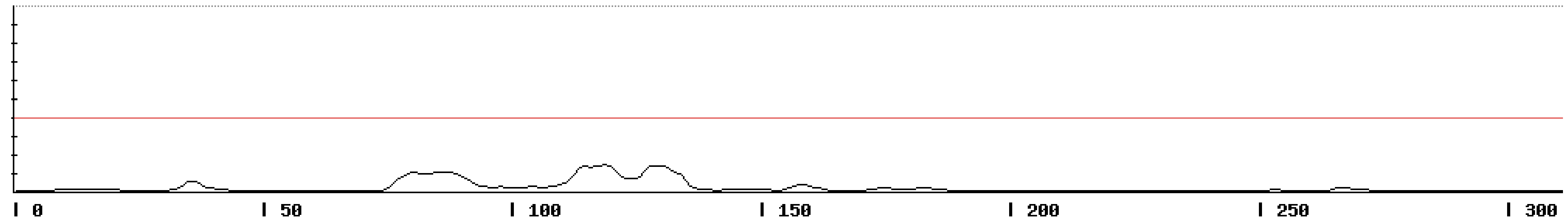

Predictions for Brassicanapus_GSBRNA2T00089877001	
Viterbi Path	
Viterbi Path	
Posterior @ 0.4	156 - KRR - 158
Posterior @ 0.4	

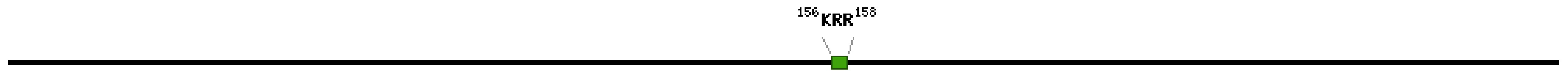

Posterior Graphic. Threshold Marked @ 0.4

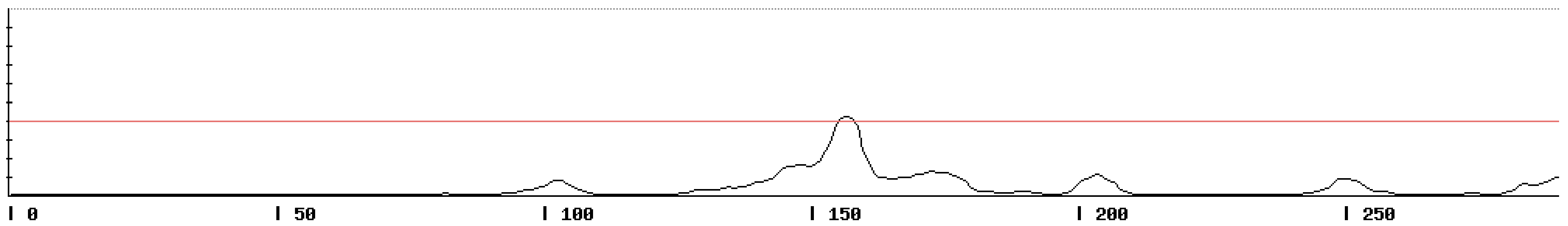

Predictions for Brassicanapus_GSBRNA2T00090171001

Viterbi Path

Viterbi Path

Posterior @ 0.4

Posterior @ 0.4

Posterior Graphic. Threshold Marked @ 0.4

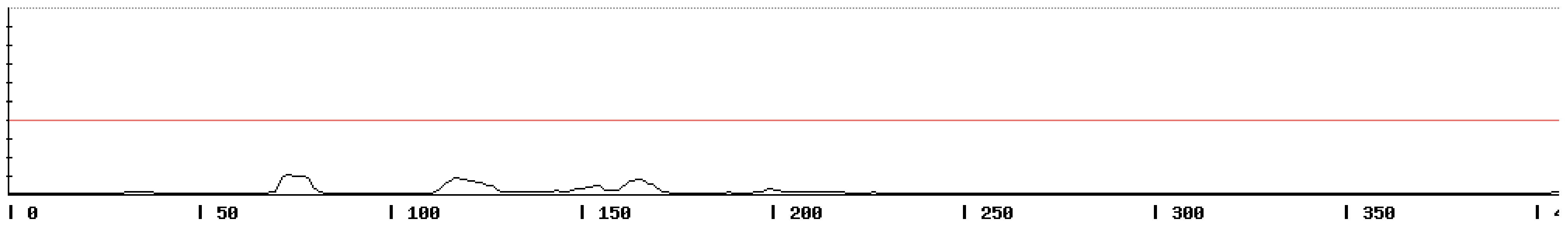

Predictions for Brassicanapus_GSBRNA2T00090371001

Viterbi Path

Viterbi Path

Posterior @ 0.4

Posterior @ 0.4

Posterior Graphic. Threshold Marked @ 0.4

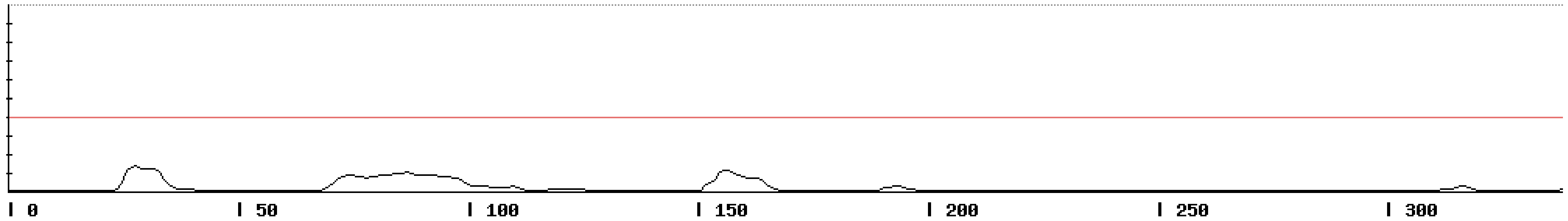

Predictions for Brassicanapus_GSBRNA2T00090572001

Viterbi Path

Viterbi Path

Posterior @ 0.4 | 137 - KRR - 139

Posterior @ 0.4

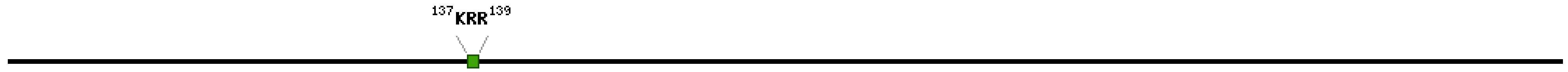

Posterior Graphic. Threshold Marked @ 0.4

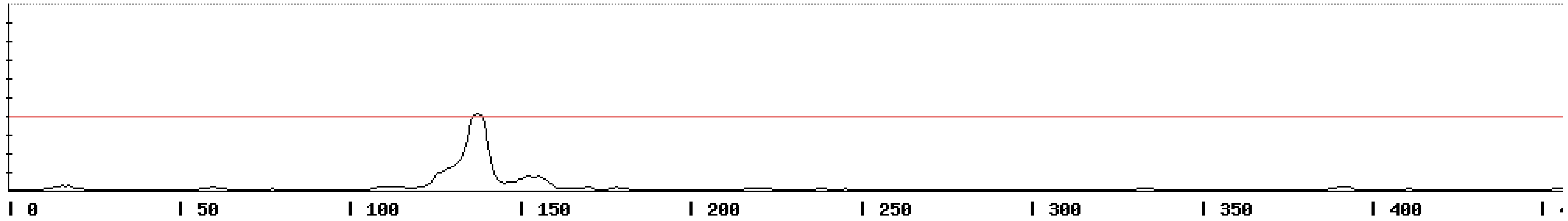

Predictions for Brassicanapus_GSBRNA2T00090982001

Viterbi Path

Viterbi Path

Posterior @ 0.4

Posterior @ 0.4

Posterior Graphic. Threshold Marked @ 0.4

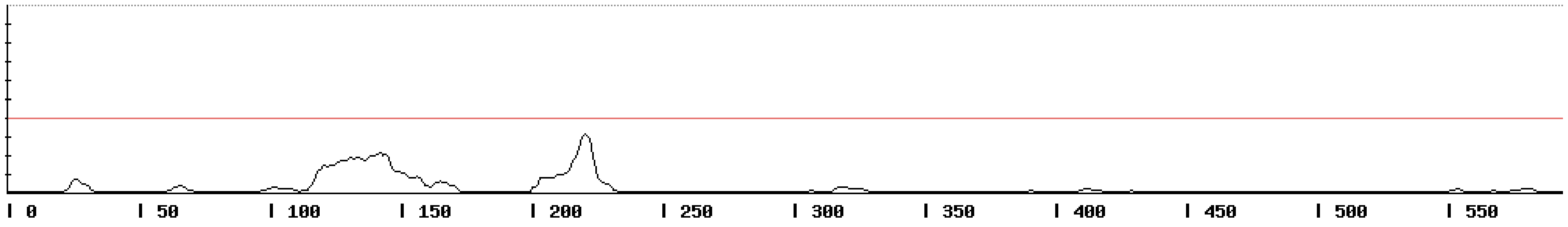

Predictions for Brassicanapus_GSBRNA2T00091365001

Viterbi Path

Viterbi Path

Posterior @ 0.4

Posterior @ 0.4

Posterior Graphic. Threshold Marked @ 0.4

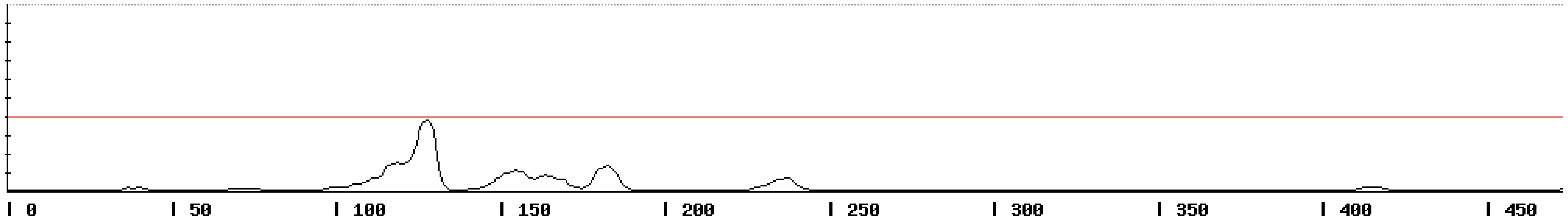

Predictions for Brassicanapus_GSBRNA2T00091404001

Viterbi Path

Viterbi Path

Posterior @ 0.4

Posterior @ 0.4

Posterior Graphic. Threshold Marked @ 0.4

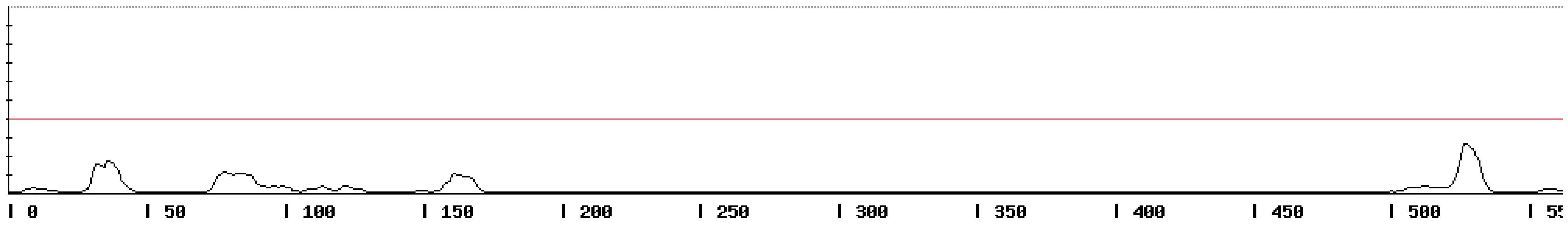

Predictions for Brassicanapus_GSBRNA2T00092085001

Viterbi Path

Viterbi Path

Posterior @ 0.4

Posterior @ 0.4

Posterior Graphic. Threshold Marked @ 0.4

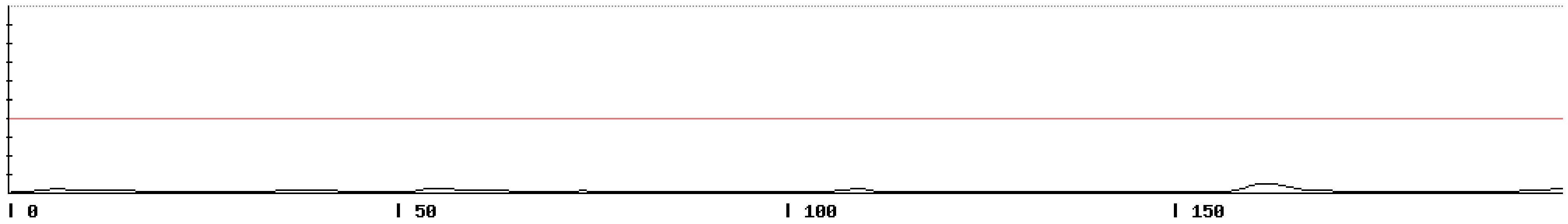

Predictions for Brassicanapus_GSBRNA2T00093660001

Viterbi Path

Viterbi Path

Posterior @ 0.4

Posterior @ 0.4

Posterior Graphic. Threshold Marked @ 0.4

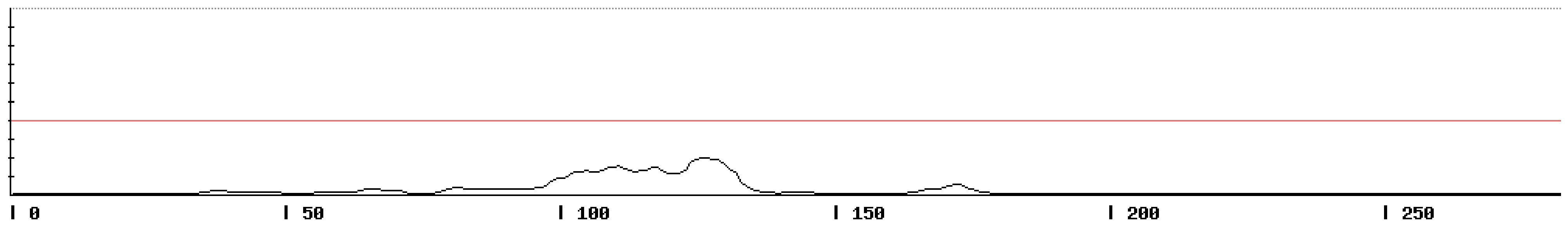

Predictions for Brassicanapus_GSBRNA2T00094099001

Viterbi Path

Viterbi Path

Posterior @ 0.4

Posterior @ 0.4

Posterior Graphic. Threshold Marked @ 0.4

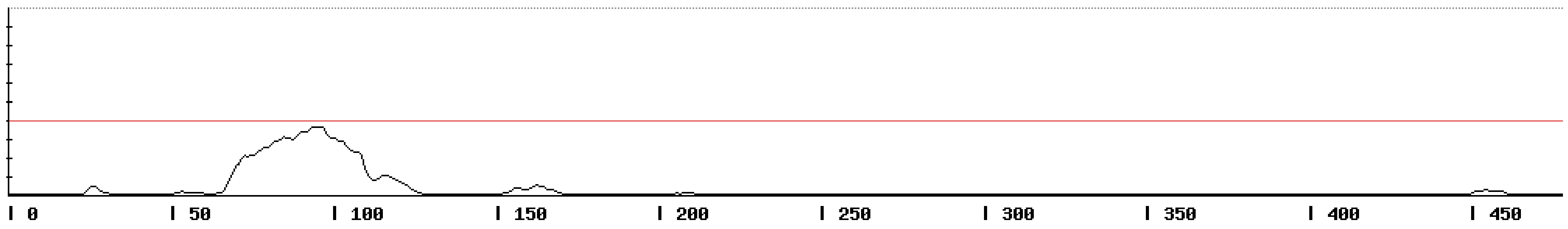

Predictions for Brassicanapus_GSBRNA2T00094309001

Viterbi Path

Viterbi Path

Posterior @ 0.4

Posterior @ 0.4

Posterior Graphic. Threshold Marked @ 0.4

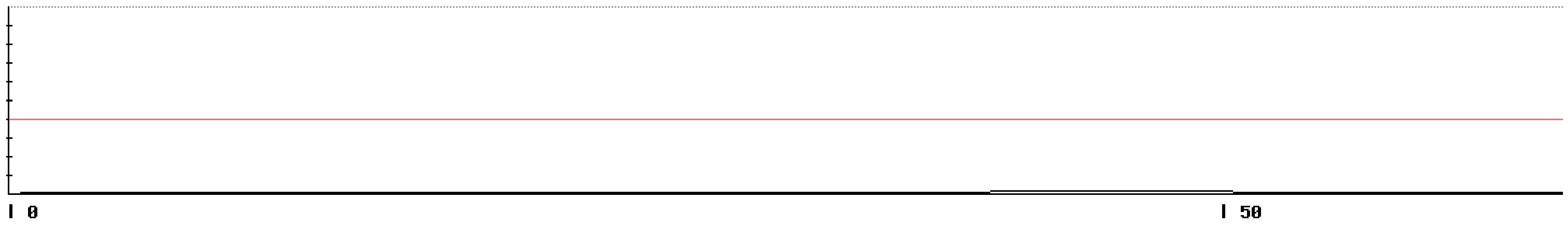

Predictions for Brassicanapus_GSBRNA2T00094654001

Viterbi Path

Viterbi Path

Posterior @ 0.4

Posterior @ 0.4

Posterior Graphic. Threshold Marked @ 0.4	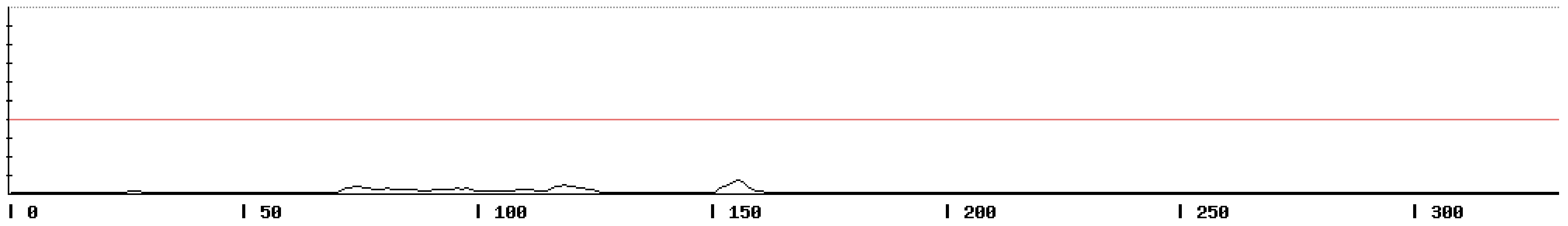

Predictions for Brassicanapus_GSBRNA2T00094866001

Viterbi Path	
Viterbi Path	
Posterior @ 0.4	
Posterior @ 0.4	
Posterior Graphic. Threshold Marked @ 0.4	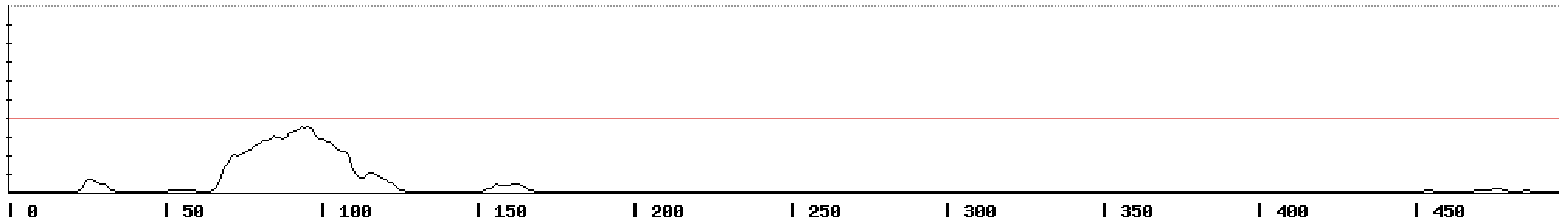

Predictions for Brassicanapus_GSBRNA2T00095240001

Viterbi Path	
Viterbi Path	
Posterior @ 0.4	156 - KKRSNNNNKKKKEDEREKK - 174
Posterior @ 0.4	

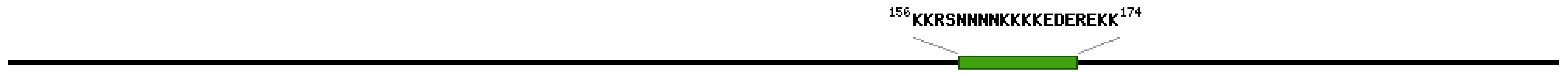

Posterior Graphic. Threshold Marked @ 0.4

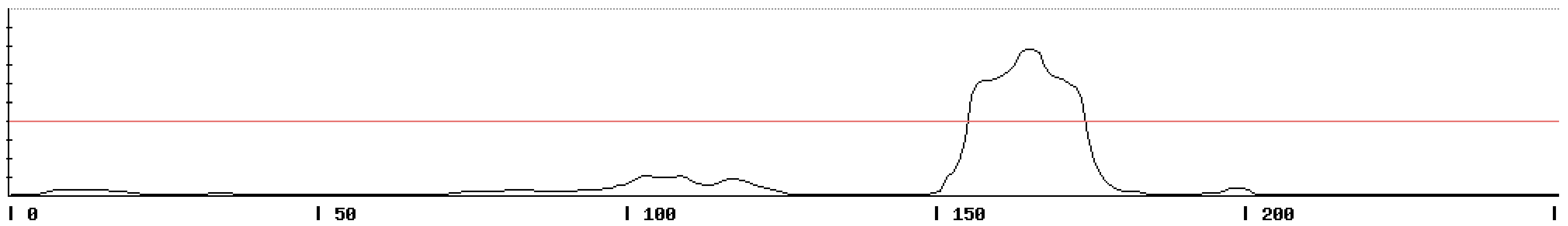

Predictions for Brassicanapus_GSBRNA2T00095484001

Viterbi Path

Viterbi Path

Posterior @ 0.4

Posterior @ 0.4

Posterior Graphic. Threshold Marked @ 0.4

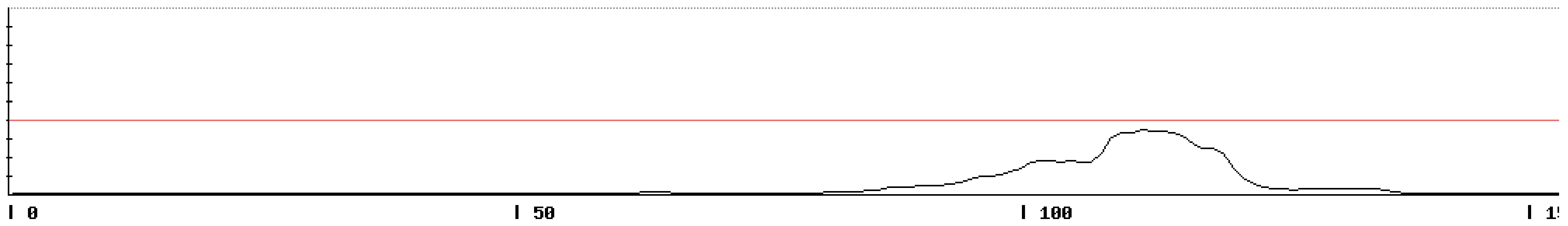

Predictions for Brassicanapus_GSBRNA2T00095486001

Viterbi Path

Viterbi Path

Posterior @ 0.4 | 113 - KKTKRGR - 119

Posterior @ 0.4

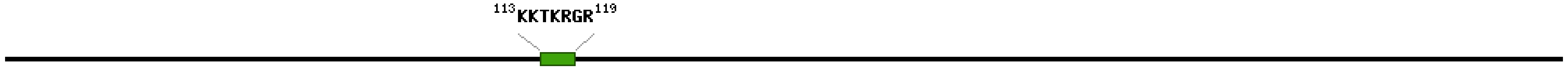

Posterior Graphic. Threshold Marked @ 0.4

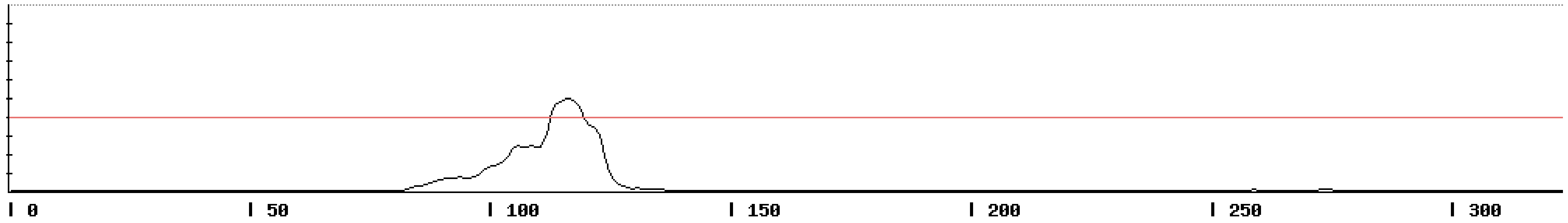

Predictions for Brassicanapus_GSBRNA2T00095503001

Viterbi Path

Viterbi Path

Posterior @ 0.4

Posterior @ 0.4

Posterior Graphic. Threshold Marked @ 0.4

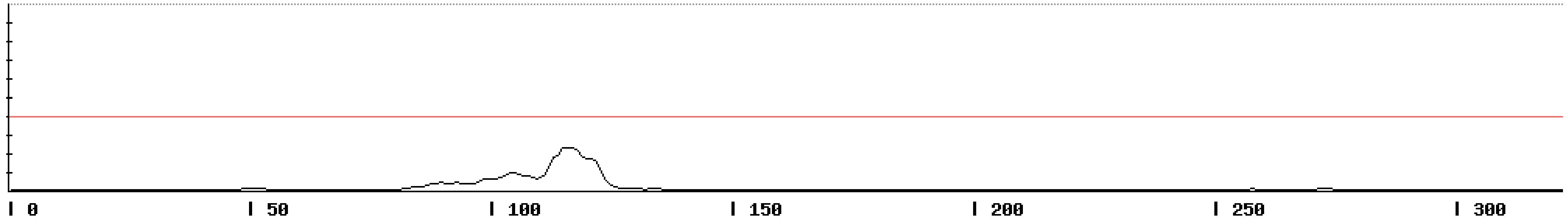

Predictions for Brassicanapus_GSBRNA2T00095580001

Viterbi Path

Viterbi Path

Posterior @ 0.4	
Posterior @ 0.4	
Posterior Graphic. Threshold Marked @ 0.4	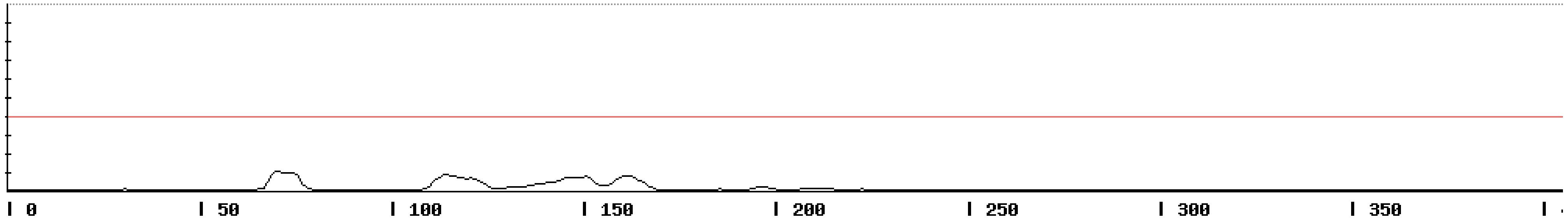

Predictions for Brassicanapus_GSBRNA2T00095993001

Viterbi Path	
Viterbi Path	
Posterior @ 0.4	
Posterior @ 0.4	
Posterior Graphic. Threshold Marked @ 0.4	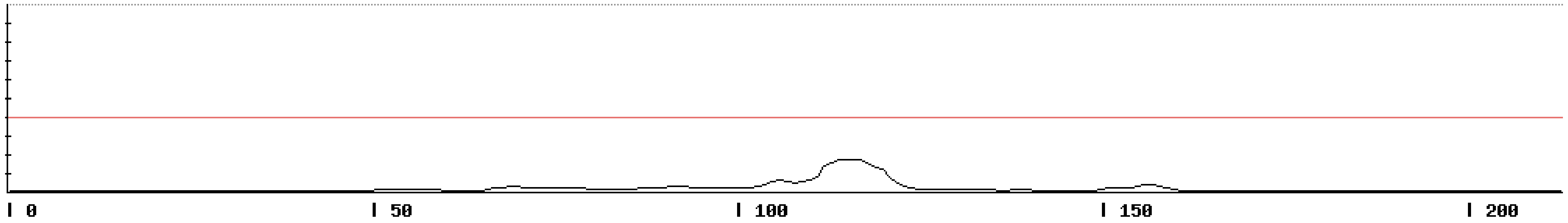

Predictions for Brassicanapus_GSBRNA2T00096027001

Viterbi Path	
Viterbi Path	
Posterior @ 0.4	
Posterior @ 0.4	
Posterior Graphic. Threshold Marked @ 0.4	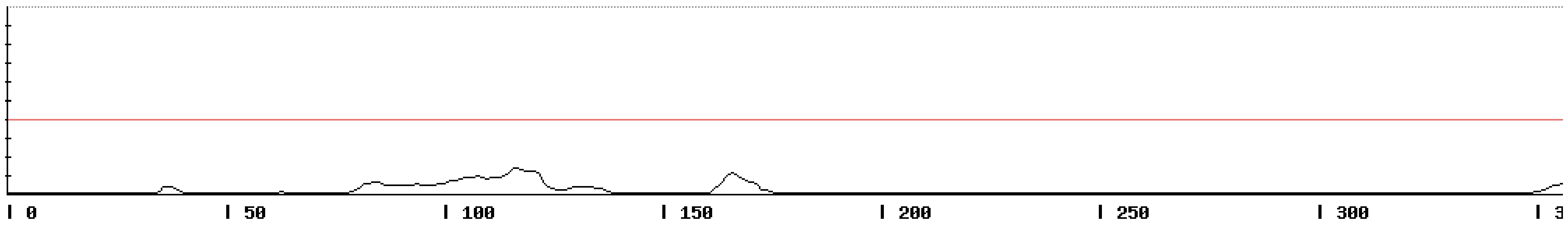

Predictions for Brassicanapus_GSBRNA2T00097708001

Viterbi Path	
Viterbi Path	
Posterior @ 0.4	
Posterior @ 0.4	
Posterior Graphic. Threshold Marked @ 0.4	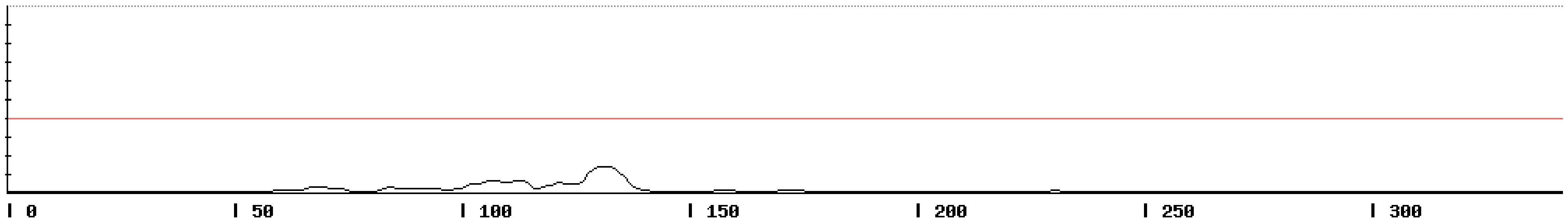

Predictions for Brassicanapus_GSBRNA2T00097709001	
Viterbi Path	
Viterbi Path	
Posterior @ 0.4	
Posterior @ 0.4	
Posterior Graphic. Threshold Marked @ 0.4	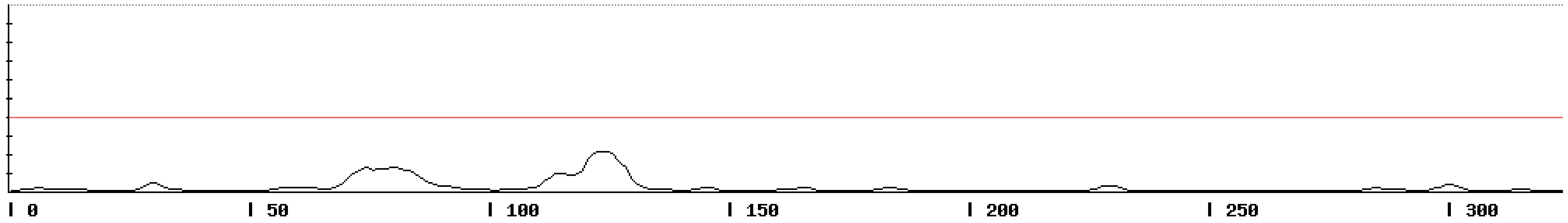

Predictions for Brassicanapus_GSBRNA2T00098287001	
Viterbi Path	
Viterbi Path	
Posterior @ 0.4	117 - GKKIKR - 122
Posterior @ 0.4	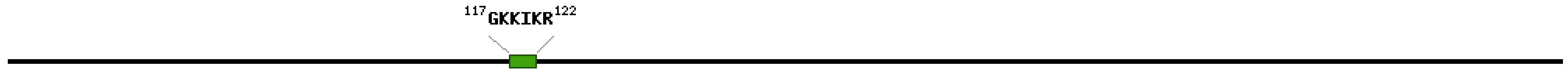
Posterior Graphic. Threshold Marked @ 0.4	

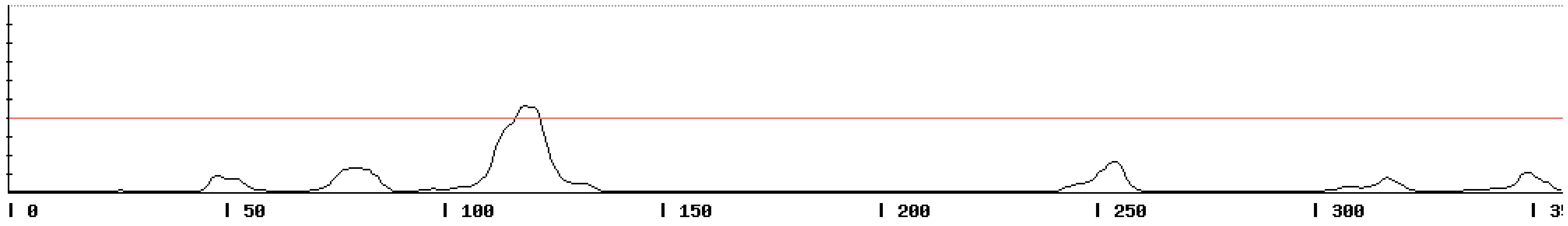

Predictions for Brassicanapus_GSBRNA2T00098300001

Viterbi Path

Viterbi Path

Posterior @ 0.4

Posterior @ 0.4

Posterior Graphic. Threshold Marked @ 0.4

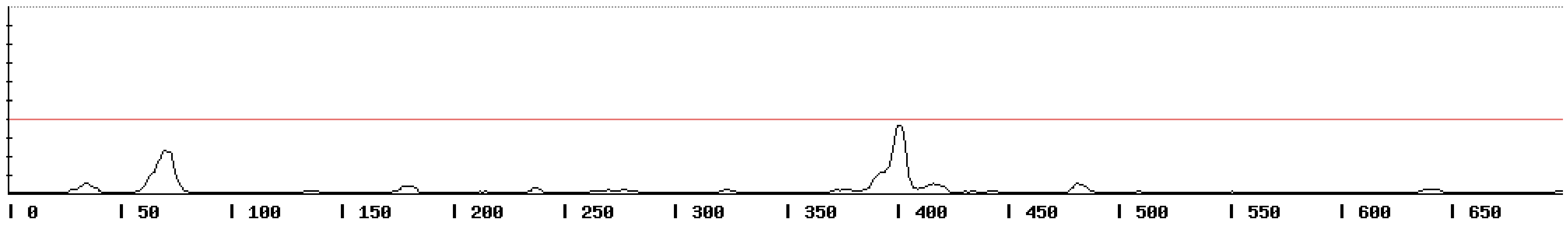

Predictions for Brassicanapus_GSBRNA2T00098528001

Viterbi Path

Viterbi Path

Posterior @ 0.4

Posterior @ 0.4

Posterior Graphic. Threshold Marked @ 0.4	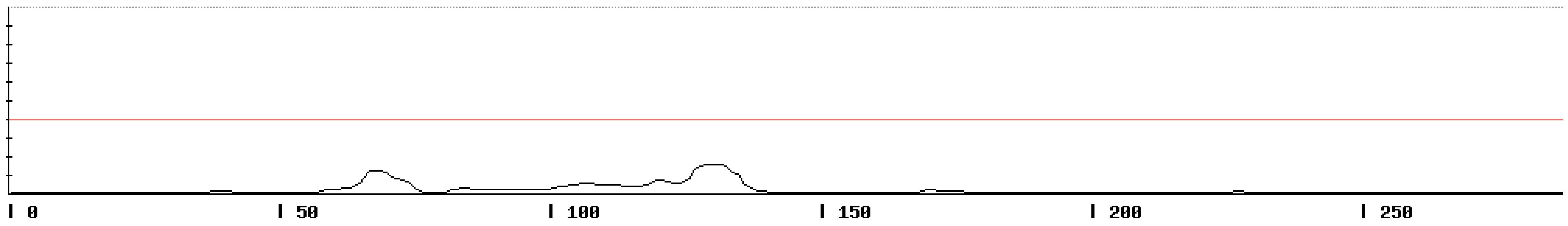

Predictions for Brassicanapus_GSBRNA2T00098539001

Viterbi Path	
Viterbi Path	
Posterior @ 0.4	
Posterior @ 0.4	
Posterior Graphic. Threshold Marked @ 0.4	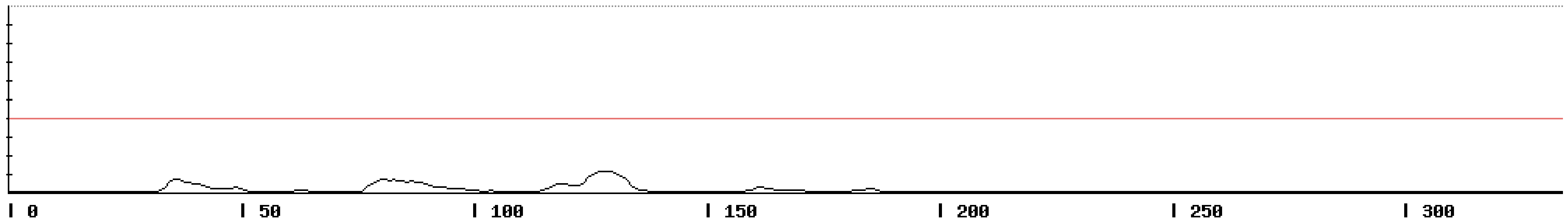

Predictions for Brassicanapus_GSBRNA2T00098598001

Viterbi Path	
Viterbi Path	
Posterior @ 0.4	
Posterior @ 0.4	

Posterior Graphic. Threshold Marked @ 0.4	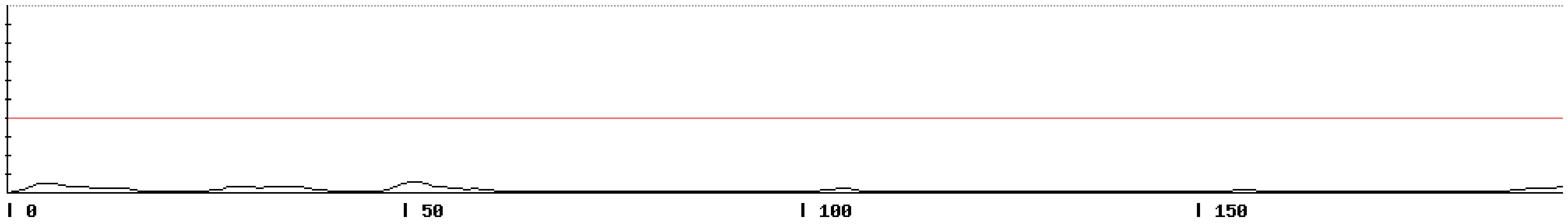

Predictions for Brassicanapus_GSBRNA2T00098805001	
Viterbi Path	
Viterbi Path	
Posterior @ 0.4	
Posterior @ 0.4	
Posterior Graphic. Threshold Marked @ 0.4	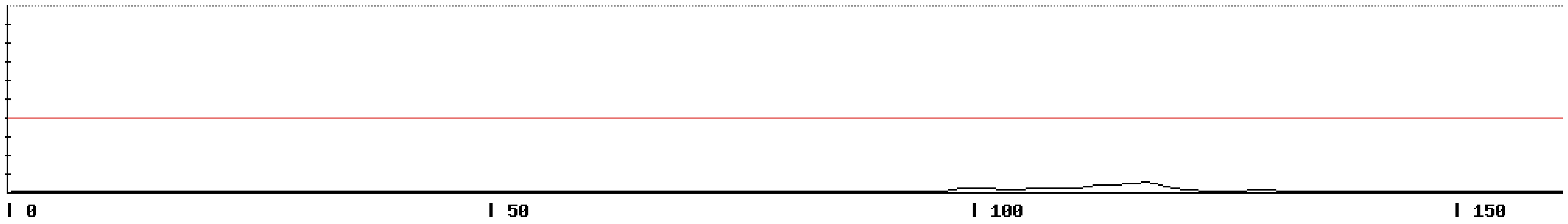

Predictions for Brassicanapus_GSBRNA2T00098817001	
Viterbi Path	
Viterbi Path	

Posterior @ 0.4

Posterior @ 0.4

Posterior Graphic. Threshold Marked @ 0.4

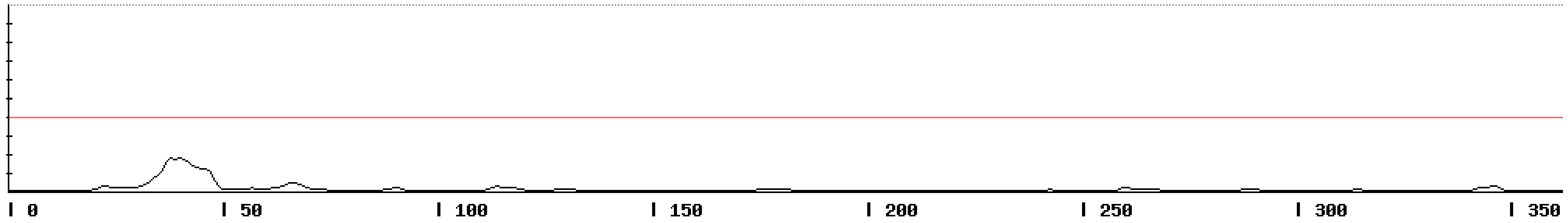

Predictions for Brassicanapus_GSBRNA2T00098926001

Viterbi Path

Viterbi Path

Posterior @ 0.4 — 100 - SKKMK - 104

Posterior @ 0.4

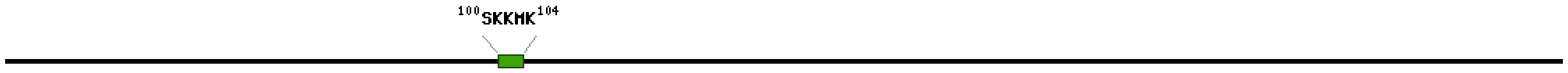

Posterior Graphic. Threshold Marked @ 0.4

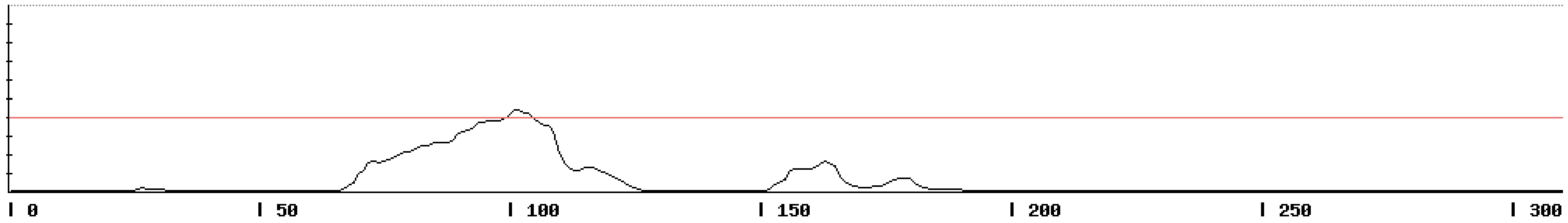

Predictions for Brassicanapus_GSBRNA2T00099070001

Viterbi Path

Viterbi Path

Posterior @ 0.4	
Posterior @ 0.4	
Posterior Graphic. Threshold Marked @ 0.4	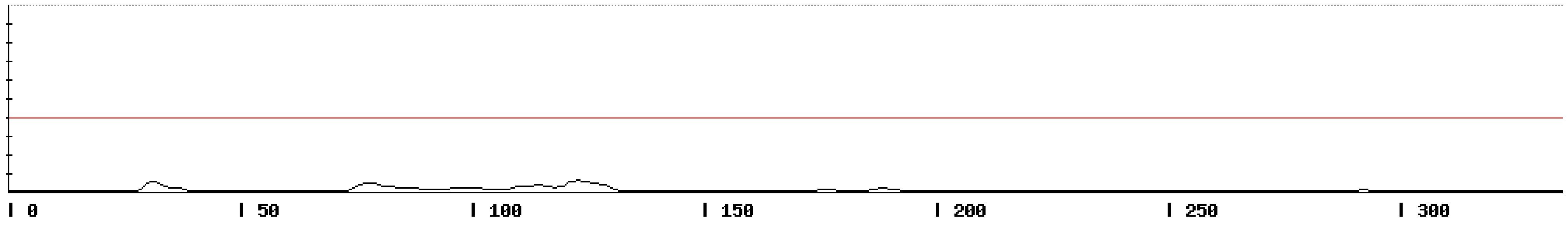

Predictions for Brassicanapus_GSBRNA2T00100118001

Viterbi Path	
Viterbi Path	
Posterior @ 0.4	249 - SKDKKKK - 255
Posterior @ 0.4	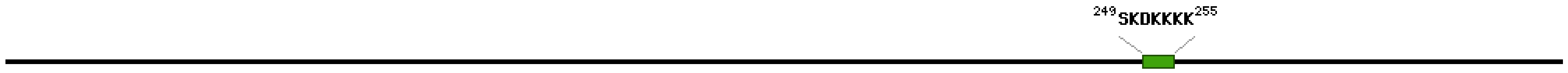
Posterior Graphic. Threshold Marked @ 0.4	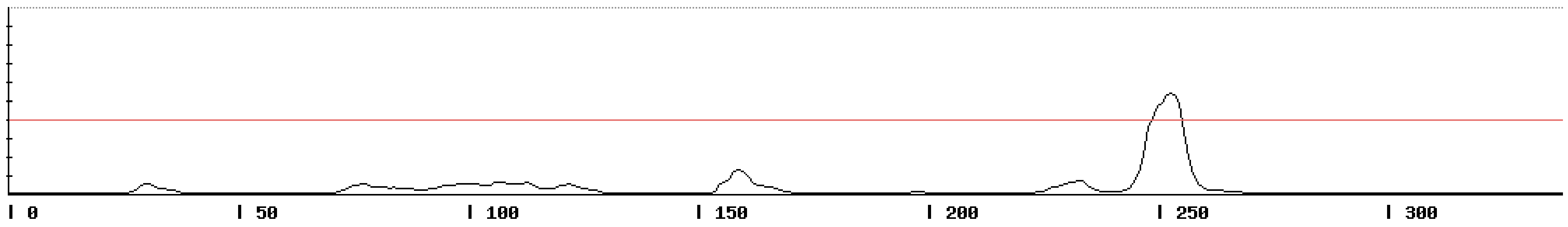

Predictions for Brassicanapus_GSBRNA2T00100375001

Viterbi Path	
Viterbi Path	
Posterior @ 0.4	223 - KIKRNGQKQKR - 233
	248 - KKIK - 251
Posterior @ 0.4	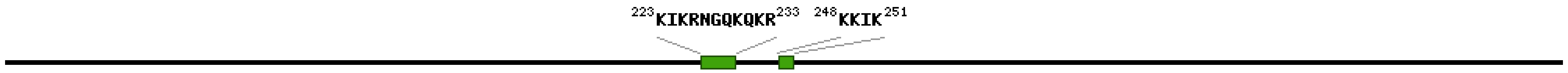
Posterior Graphic. Threshold Marked @ 0.4	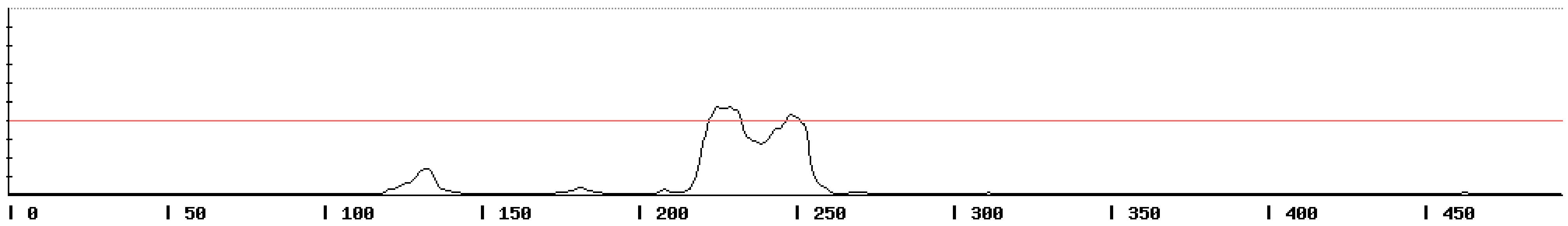

Predictions for Brassicanapus_GSBRNA2T00101780001

Viterbi Path	
Viterbi Path	
Posterior @ 0.4	
Posterior @ 0.4	
Posterior Graphic. Threshold Marked @ 0.4	

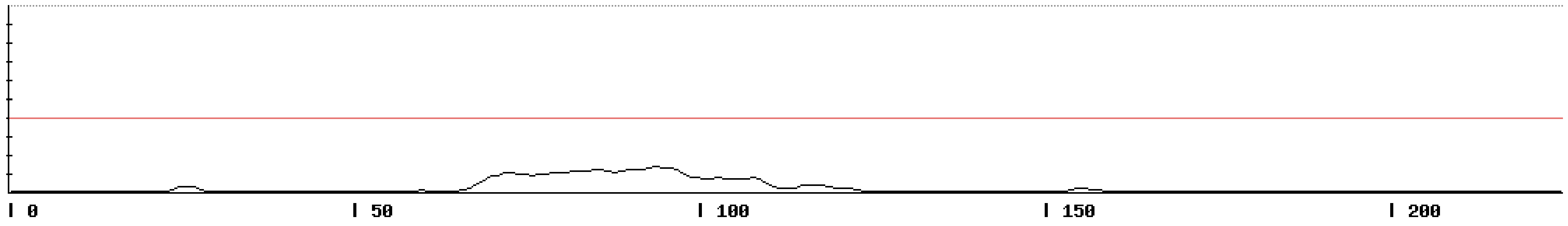

Predictions for Brassicanapus_GSBRNA2T00101917001

Viterbi Path

Viterbi Path

Posterior @ 0.4

Posterior @ 0.4

Posterior Graphic. Threshold Marked @ 0.4

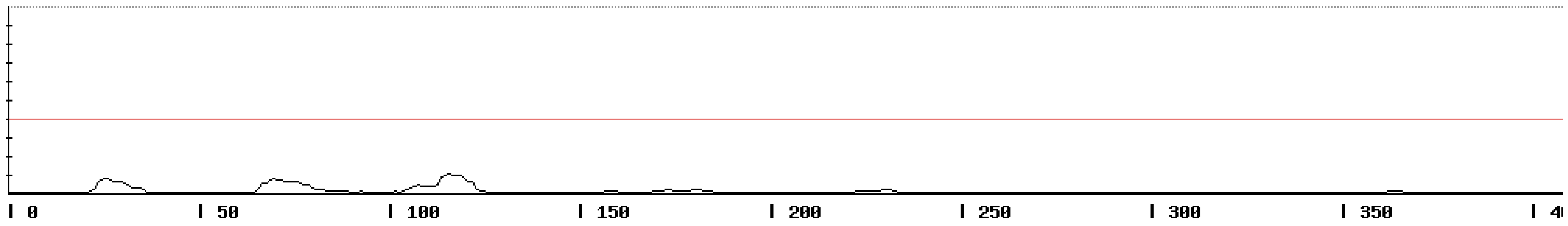

Predictions for Brassicanapus_GSBRNA2T00101944001

Viterbi Path

Viterbi Path

Posterior @ 0.4

Posterior @ 0.4

Posterior Graphic. Threshold Marked @ 0.4

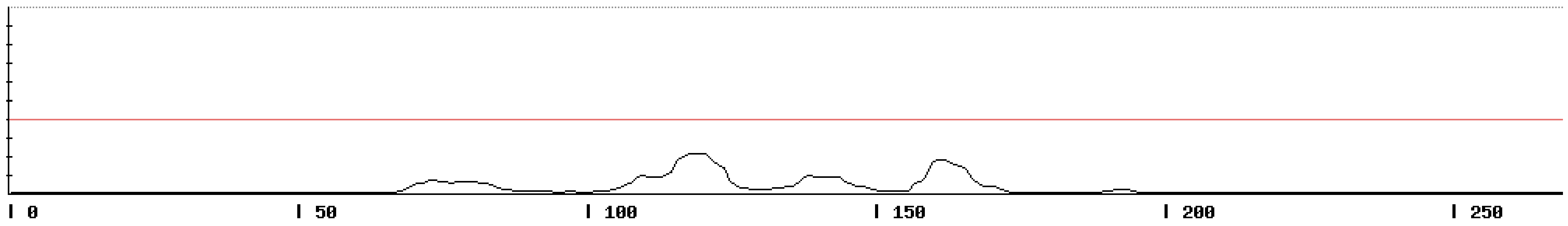

Predictions for Brassicanapus_GSBRNA2T00102391001

Viterbi Path

Viterbi Path

Posterior @ 0.4

Posterior @ 0.4

Posterior Graphic. Threshold Marked @ 0.4

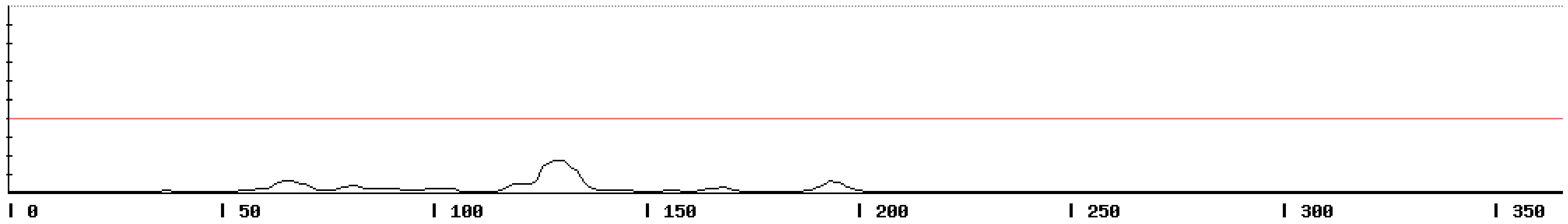

Predictions for Brassicanapus_GSBRNA2T00102514001

Viterbi Path

Viterbi Path

Posterior @ 0.4

Posterior @ 0.4

Posterior Graphic. Threshold Marked @ 0.4

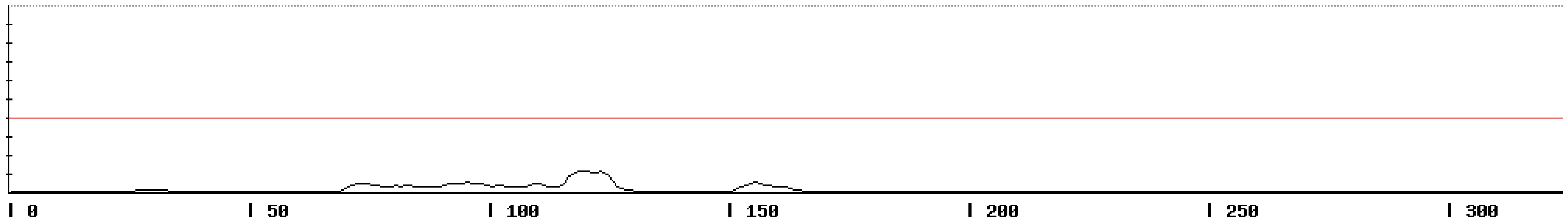

Predictions for Brassicanapus_GSBRNA2T00103244001

Viterbi Path

Viterbi Path

Posterior @ 0.4

Posterior @ 0.4

Posterior Graphic. Threshold Marked @ 0.4

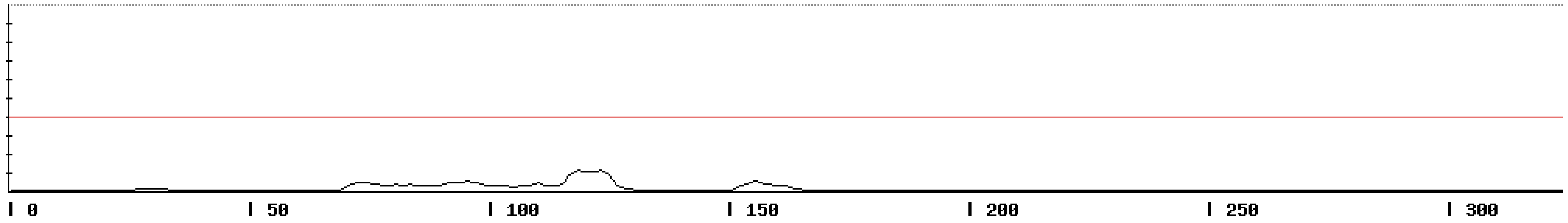

Predictions for Brassicanapus_GSBRNA2T00104250001

Viterbi Path

Viterbi Path

Posterior @ 0.4

Posterior @ 0.4

Posterior Graphic. Threshold Marked @ 0.4

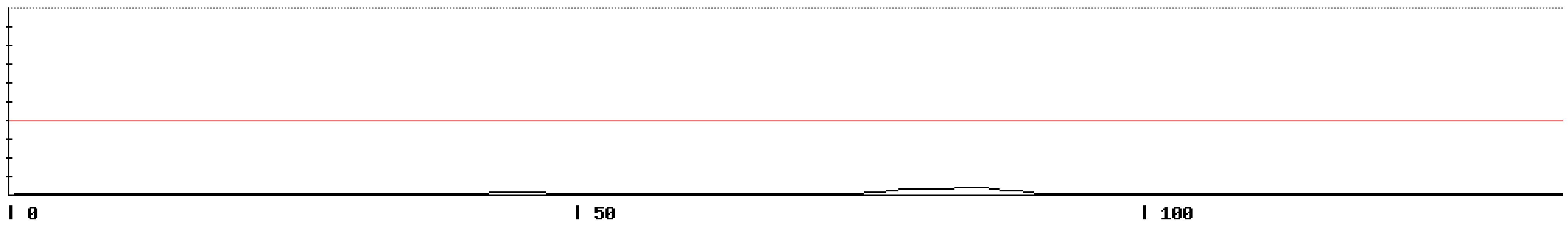

Predictions for Brassicanapus_GSBRNA2T00104269001

Viterbi Path

Viterbi Path

Posterior @ 0.4

Posterior @ 0.4

Posterior Graphic. Threshold Marked @ 0.4

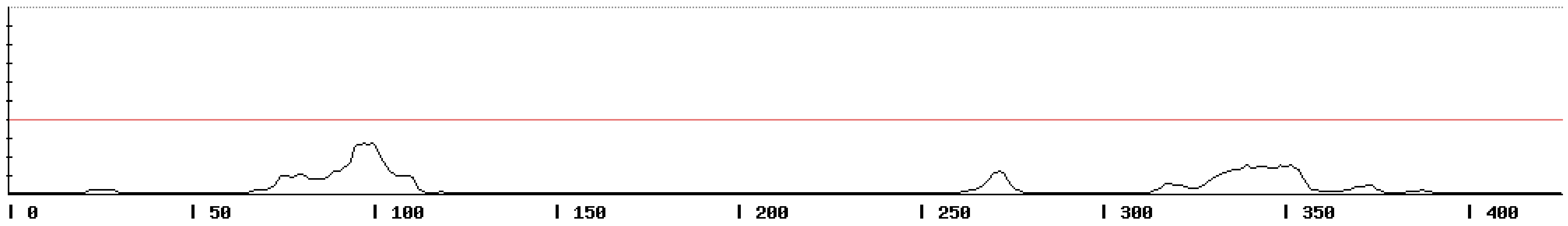

Predictions for Brassicanapus_GSBRNA2T00104607001

Viterbi Path

Viterbi Path

Posterior @ 0.4

Posterior @ 0.4

Posterior Graphic. Threshold Marked @ 0.4

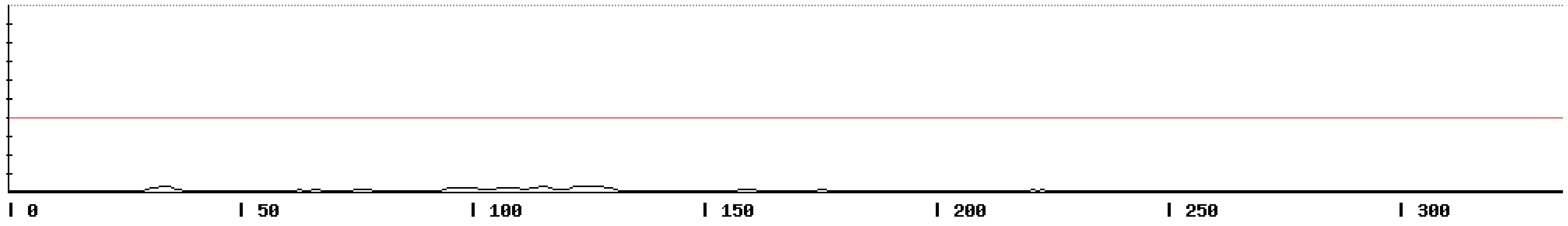

Predictions for Brassicanapus_GSBRNA2T00104909001

Viterbi Path

Viterbi Path

Posterior @ 0.4

Posterior @ 0.4

Posterior Graphic. Threshold Marked @ 0.4

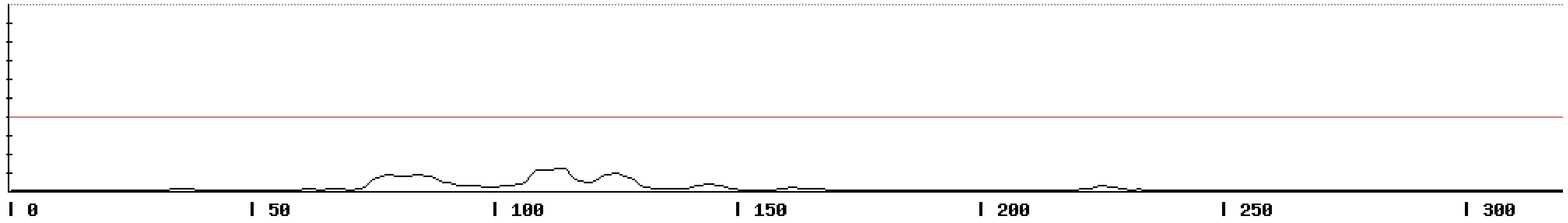

Predictions for Brassicanapus_GSBRNA2T00104912001

Viterbi Path

Viterbi Path

Posterior @ 0.4

Posterior @ 0.4

Posterior Graphic. Threshold Marked @ 0.4

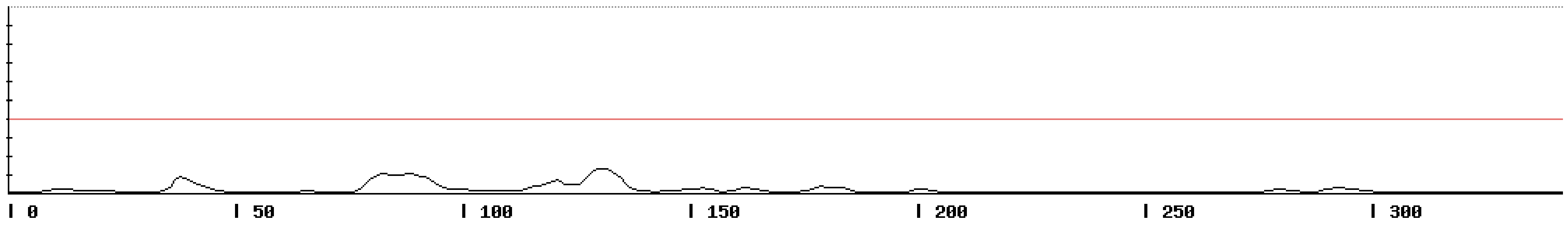

Predictions for Brassicanapus_GSBRNA2T00105458001

Viterbi Path

Viterbi Path

Posterior @ 0.4

Posterior @ 0.4

Posterior Graphic. Threshold Marked @ 0.4

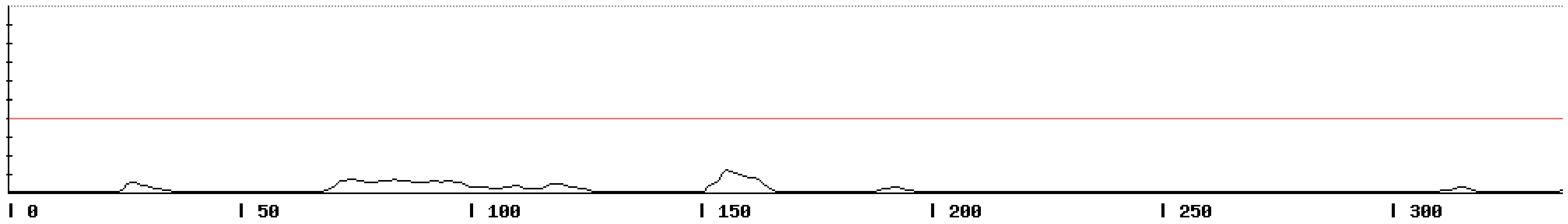

Predictions for Brassicanapus_GSBRNA2T00106173001

Viterbi Path	
Viterbi Path	
Posterior @ 0.4	
Posterior @ 0.4	
Posterior Graphic. Threshold Marked @ 0.4	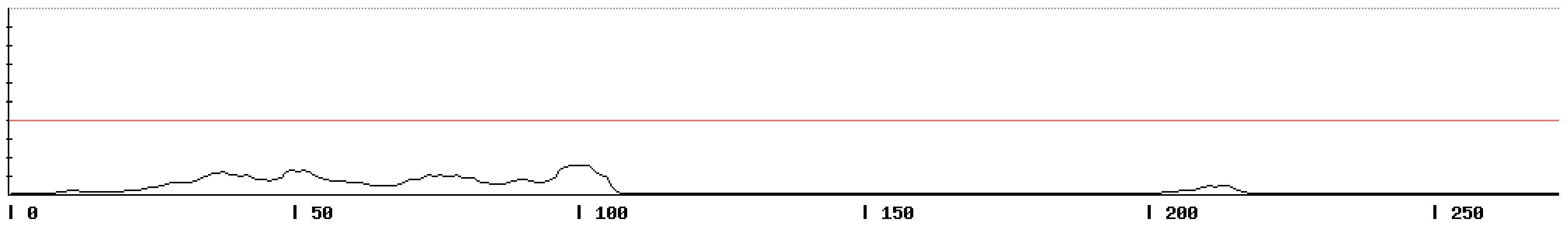

Predictions for Brassicanapus_GSBRNA2T00106217001

Viterbi Path	
Viterbi Path	
Posterior @ 0.4	
Posterior @ 0.4	
Posterior Graphic. Threshold Marked @ 0.4	

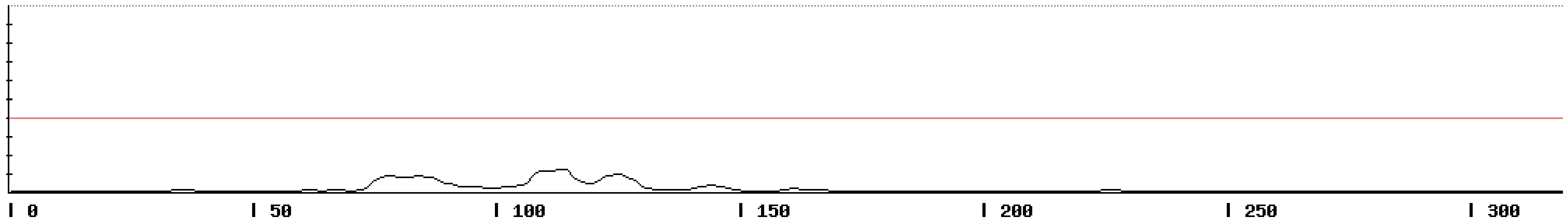

Predictions for Brassicanapus_GSBRNA2T00106219001

Viterbi Path	
Viterbi Path	
Posterior @ 0.4	
Posterior @ 0.4	
Posterior Graphic. Threshold Marked @ 0.4	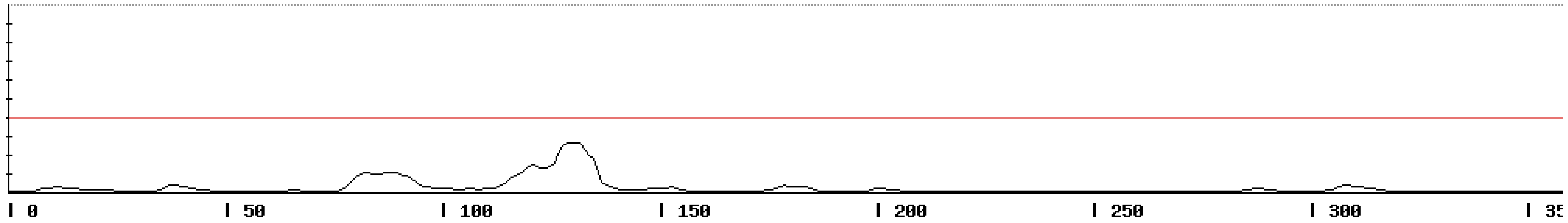

Predictions for Brassicanapus_GSBRNA2T00106315001

Viterbi Path	
Viterbi Path	
Posterior @ 0.4	
Posterior @ 0.4	

Posterior Graphic. Threshold Marked @ 0.4	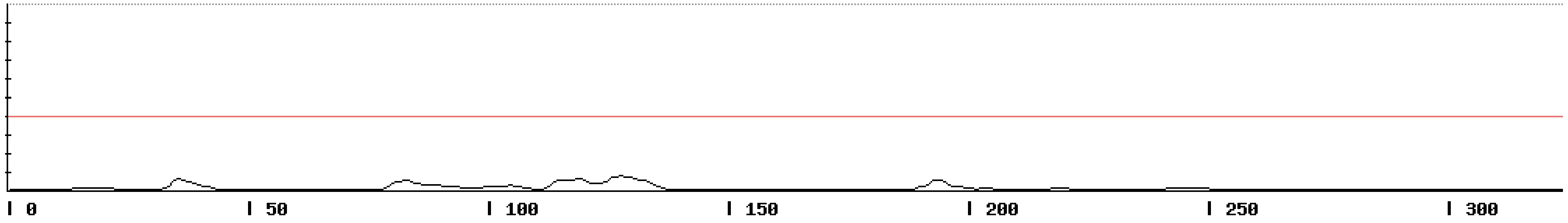

Predictions for Brassicanapus_GSBRNA2T00106395001

Viterbi Path	
Viterbi Path	
Posterior @ 0.4	
Posterior @ 0.4	
Posterior Graphic. Threshold Marked @ 0.4	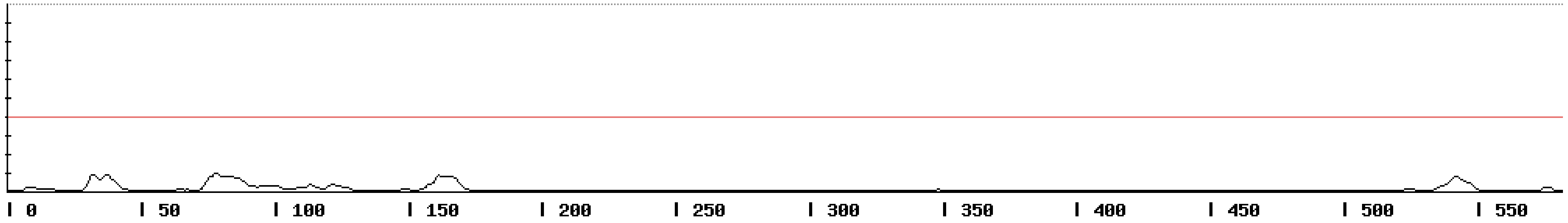

Predictions for Brassicanapus_GSBRNA2T00106396001

Viterbi Path	
Viterbi Path	
Posterior @ 0.4	
Posterior @ 0.4	

Posterior Graphic. Threshold Marked @ 0.4

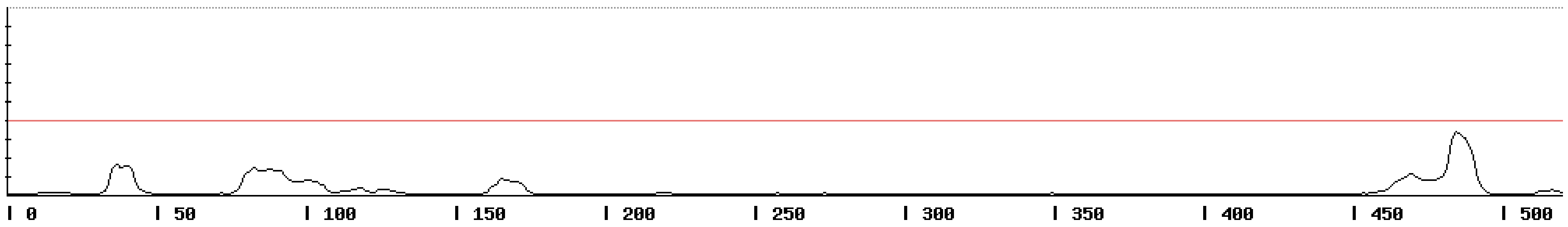

Predictions for Brassicanapus_GSBRNA2T00108261001

Viterbi Path

Viterbi Path

Posterior @ 0.4

Posterior @ 0.4

Posterior Graphic. Threshold Marked @ 0.4

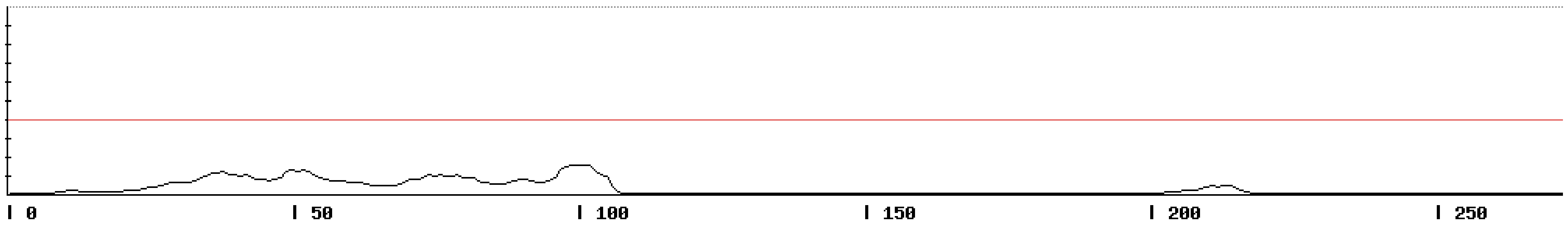

Predictions for Brassicanapus_GSBRNA2T00109745001

Viterbi Path

Viterbi Path

Posterior @ 0.4	98 - ATGKDRKIKSGK - 109
	374 - KAIKKGKLMRSKNR - 387
Posterior @ 0.4	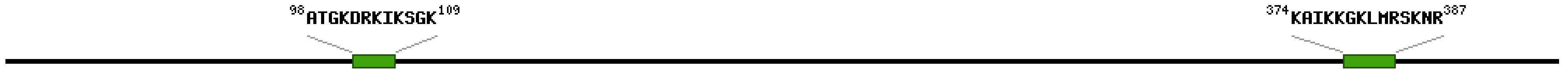
Posterior Graphic. Threshold Marked @ 0.4	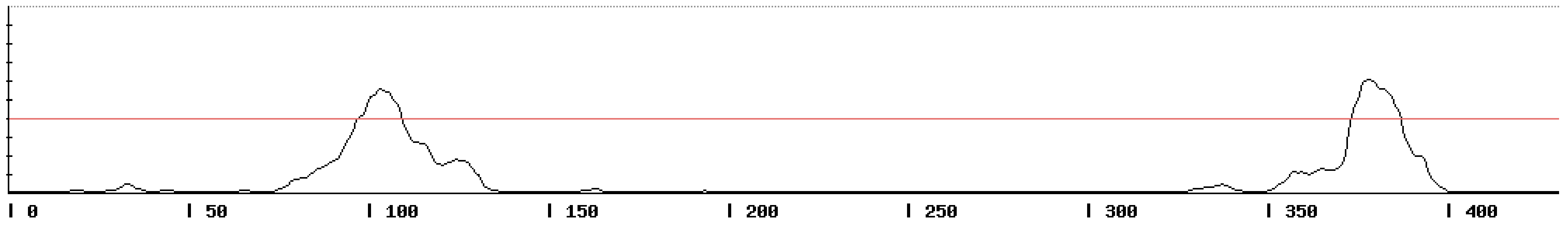

Predictions for Brassicanapus_GSBRNA2T00109854001

Viterbi Path	
Viterbi Path	
Posterior @ 0.4	
Posterior @ 0.4	
Posterior Graphic. Threshold Marked @ 0.4	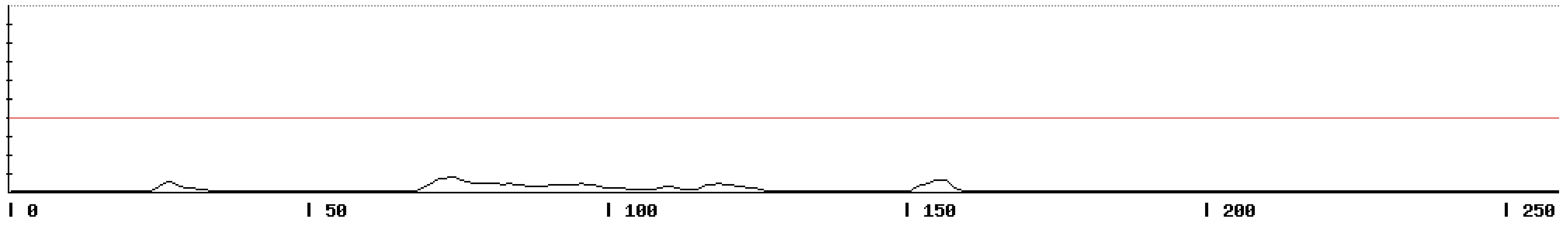

Predictions for Brassicanapus_GSBRNA2T00110506001

Viterbi Path	
Viterbi Path	

Posterior @ 0.4

Posterior @ 0.4

Posterior Graphic. Threshold Marked @ 0.4

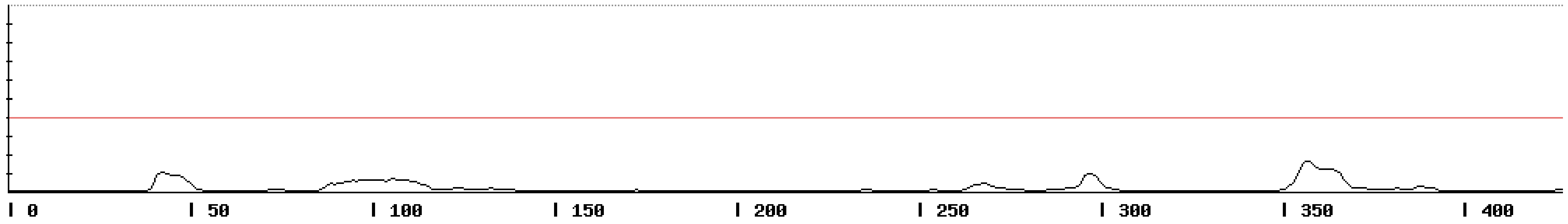

Predictions for Brassicanapus_GSBRNA2T00110507001

Viterbi Path

Viterbi Path

Posterior @ 0.4

Posterior @ 0.4

Posterior Graphic. Threshold Marked @ 0.4

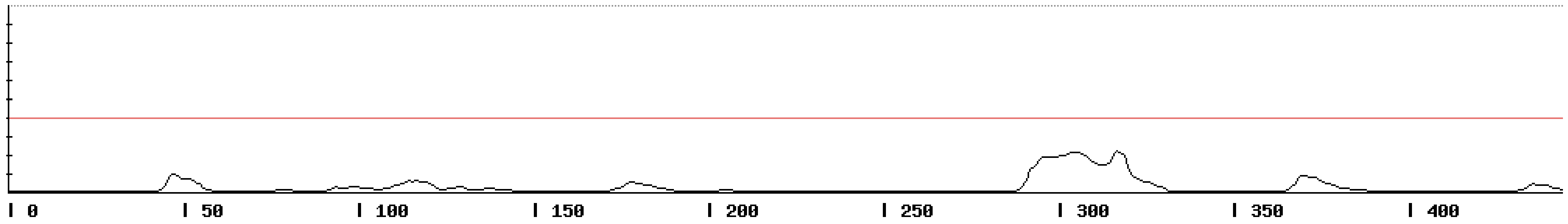

Predictions for Brassicanapus_GSBRNA2T00110954001

Viterbi Path

Viterbi Path

Posterior @ 0.4

Posterior @ 0.4

Posterior Graphic. Threshold Marked @ 0.4

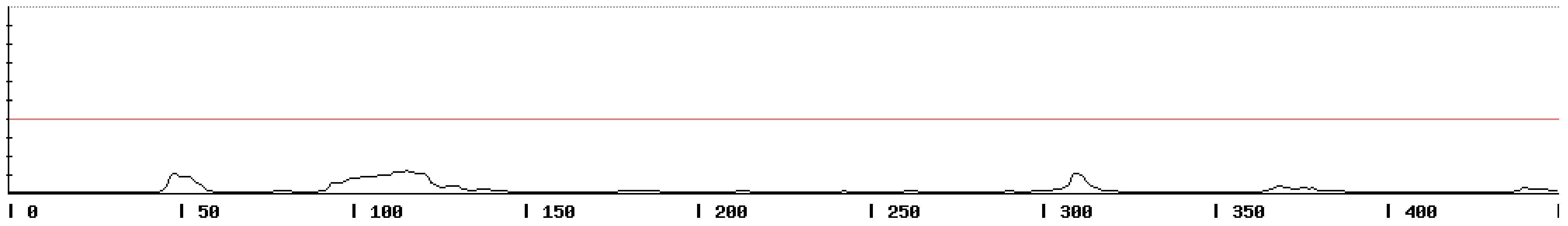

Predictions for Brassicanapus_GSBRNA2T00111390001

Viterbi Path

Viterbi Path

Posterior @ 0.4

Posterior @ 0.4

Posterior Graphic. Threshold Marked @ 0.4

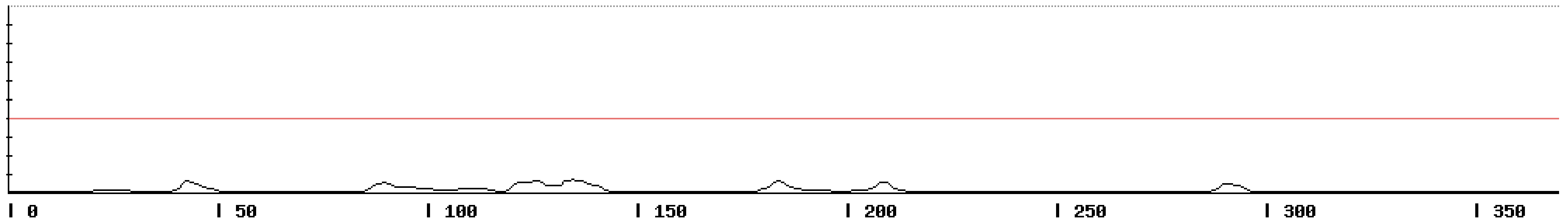

Predictions for Brassicanapus_GSBRNA2T00111648001	
Viterbi Path	
Viterbi Path	
Posterior @ 0.4	
Posterior @ 0.4	
Posterior Graphic. Threshold Marked @ 0.4	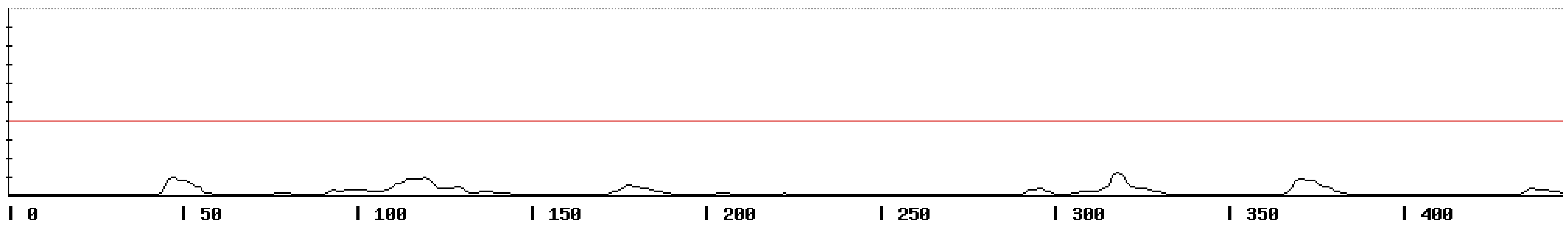

Predictions for Brassicanapus_GSBRNA2T00111649001	
Viterbi Path	
Viterbi Path	
Posterior @ 0.4	
Posterior @ 0.4	
Posterior Graphic. Threshold Marked @ 0.4	

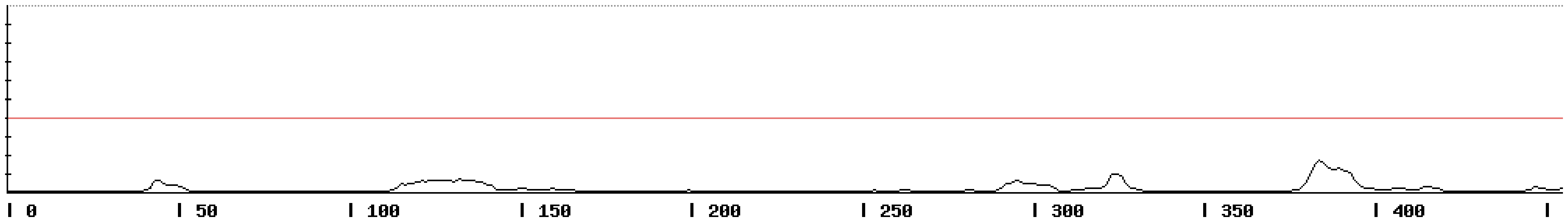

Predictions for Brassicanapus_GSBRNA2T00112100001

Viterbi Path

Viterbi Path

Posterior @ 0.4

487 - KRRK - 490

529 - KSARKGSKPEKS - 540

Posterior @ 0.4

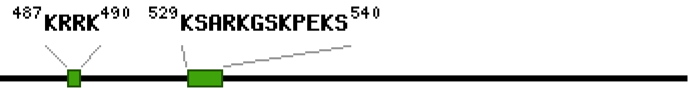

Posterior Graphic. Threshold Marked @ 0.4

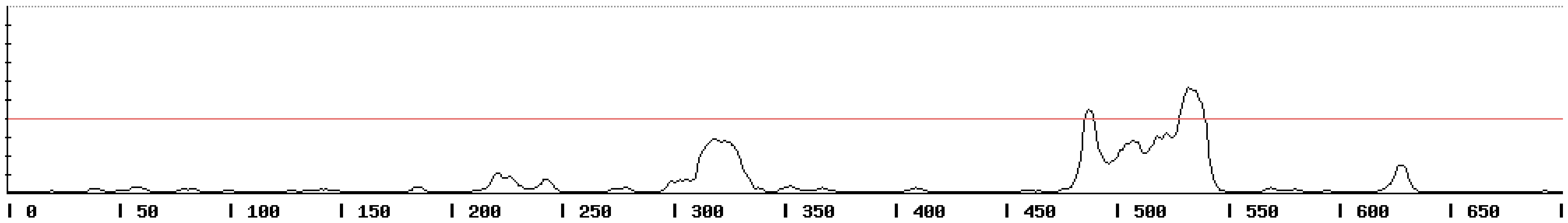

Predictions for Brassicanapus_GSBRNA2T00112525001

Viterbi Path

Viterbi Path

Posterior @ 0.4

Posterior @ 0.4

Posterior Graphic. Threshold Marked @ 0.4

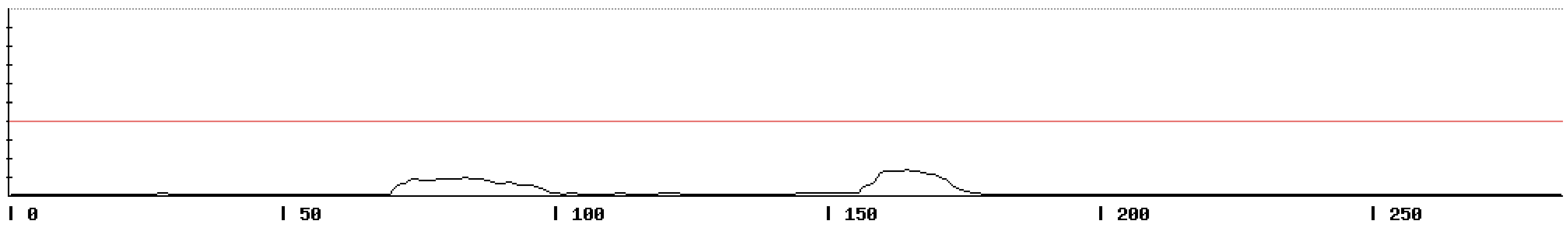

Predictions for Brassicanapus_GSBRNA2T00112879001

Viterbi Path

Viterbi Path

Posterior @ 0.4

Posterior @ 0.4

Posterior Graphic. Threshold Marked @ 0.4

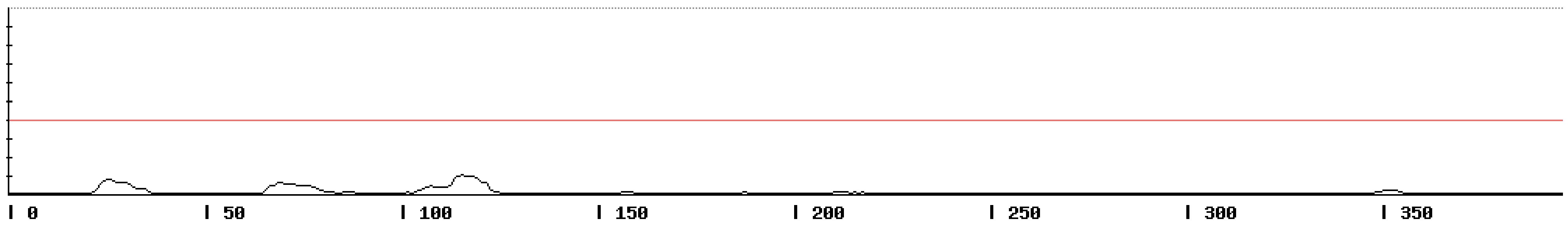

Predictions for Brassicanapus_GSBRNA2T00113154001

Viterbi Path

Viterbi Path

Posterior @ 0.4

Posterior @ 0.4

Posterior Graphic. Threshold Marked @ 0.4

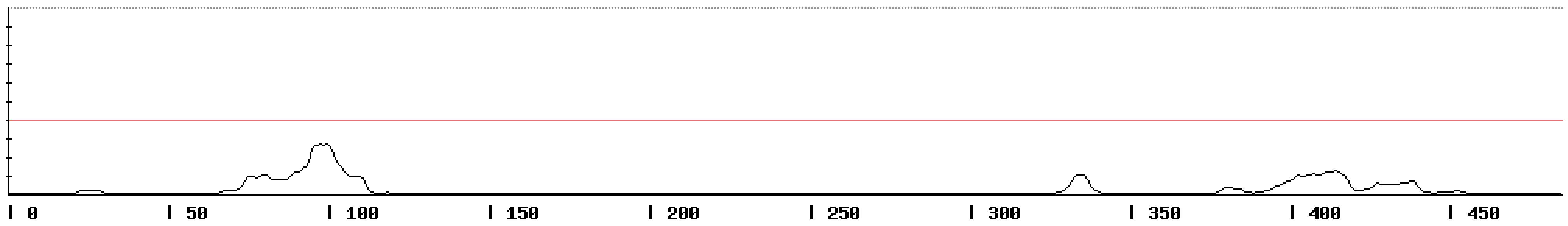

Predictions for Brassicanapus_GSBRNA2T00113639001

Viterbi Path

Viterbi Path

Posterior @ 0.4

Posterior @ 0.4

Posterior Graphic. Threshold Marked @ 0.4

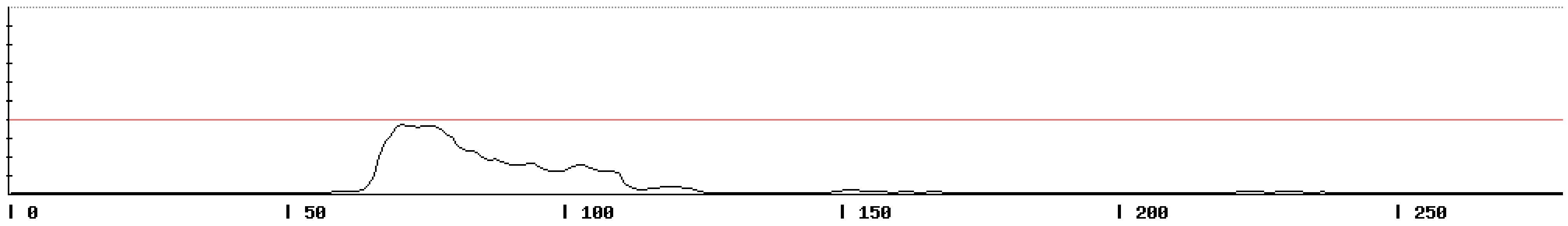

Predictions for Brassicanapus_GSBRNA2T00114310001

Viterbi Path

Viterbi Path

Posterior @ 0.4

Posterior @ 0.4

Posterior Graphic. Threshold Marked @ 0.4

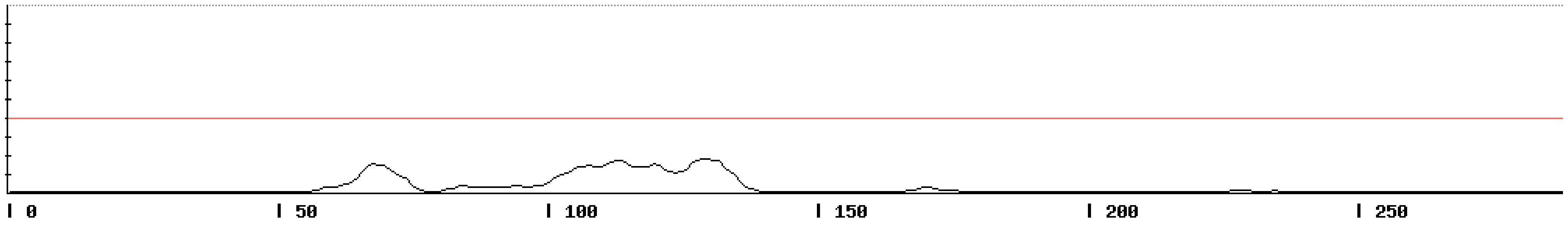

Predictions for Brassicanapus_GSBRNA2T00114768001

Viterbi Path

Viterbi Path

Posterior @ 0.4

Posterior @ 0.4

Posterior Graphic. Threshold Marked @ 0.4

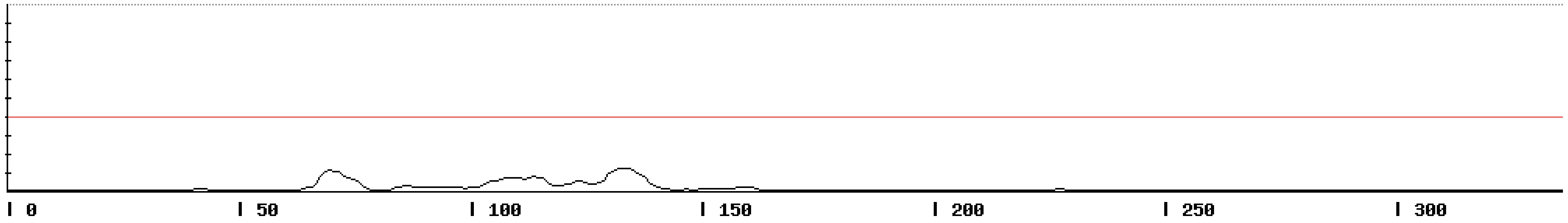

Predictions for Brassicanapus_GSBRNA2T00115065001

Viterbi Path

Viterbi Path

Posterior @ 0.4 — 104 - RGRNSKRR - 111

Posterior @ 0.4

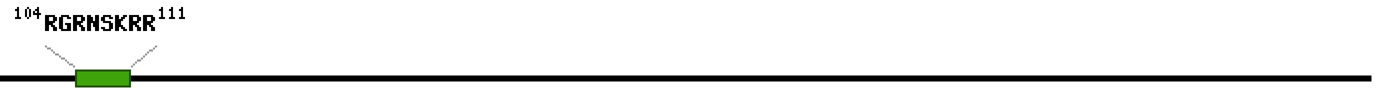

Posterior Graphic. Threshold Marked @ 0.4

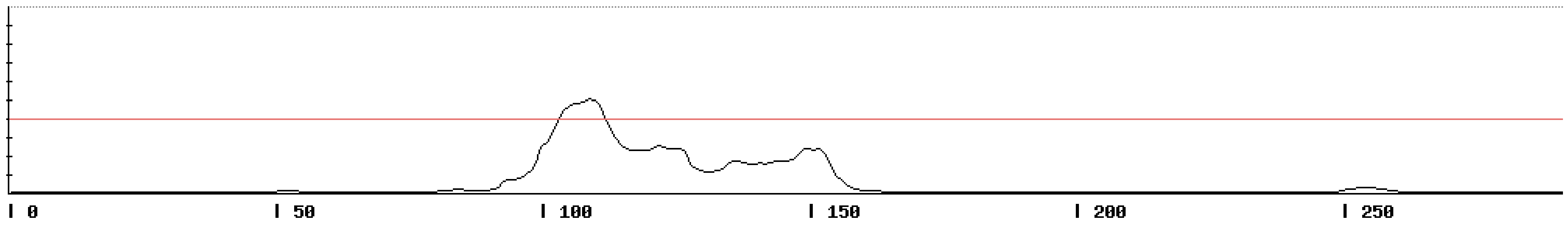

Predictions for Brassicanapus_GSBRNA2T00115069001

Viterbi Path

Viterbi Path

Posterior @ 0.4 — 41 - RGRNSKRR - 48

210 - RRGRLK - 215

Posterior @ 0.4

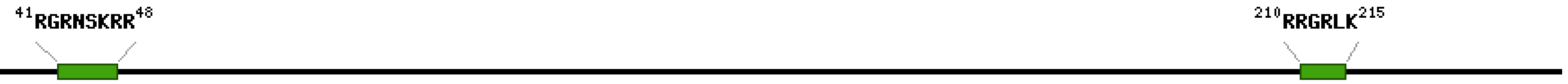

Posterior Graphic. Threshold Marked @ 0.4

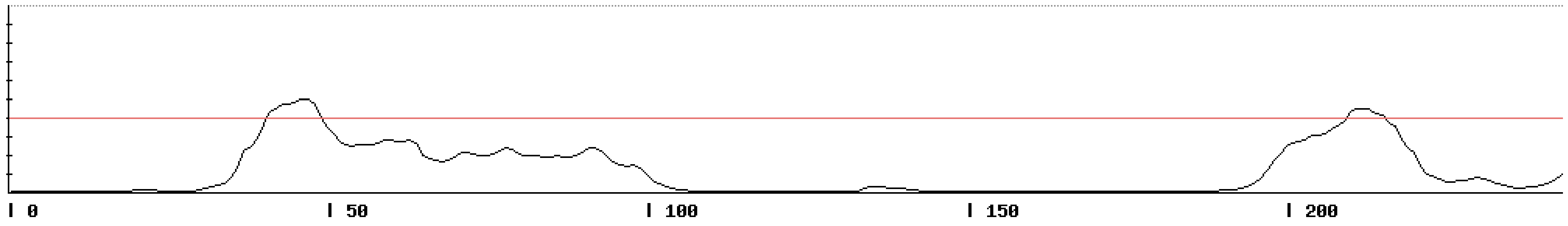

Predictions for Brassicanapus_GSBRNA2T00115095001

Viterbi Path

Viterbi Path

Posterior @ 0.4 41 - RGRNSKRR - 48

Posterior @ 0.4

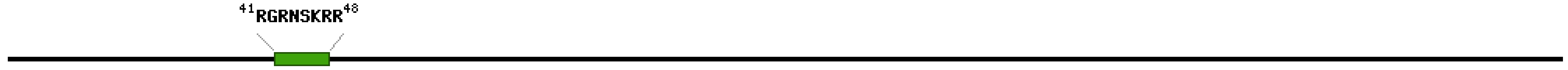

Posterior Graphic. Threshold Marked @ 0.4

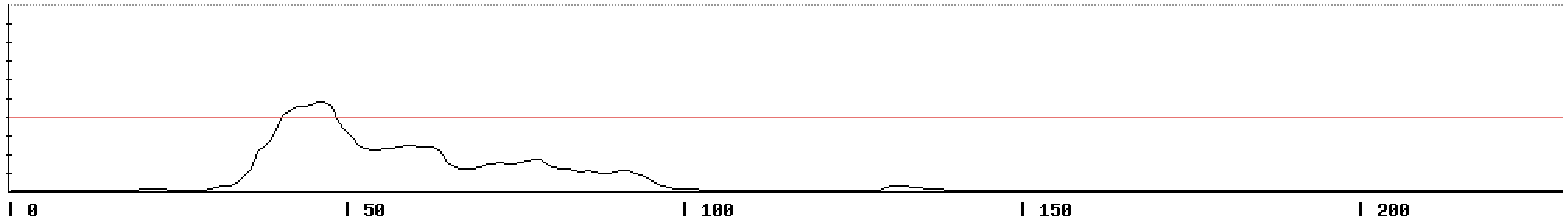

Predictions for Brassicanapus_GSBRNA2T00115382001

Viterbi Path

Viterbi Path

Posterior @ 0.4

Posterior @ 0.4

Posterior Graphic. Threshold Marked @ 0.4

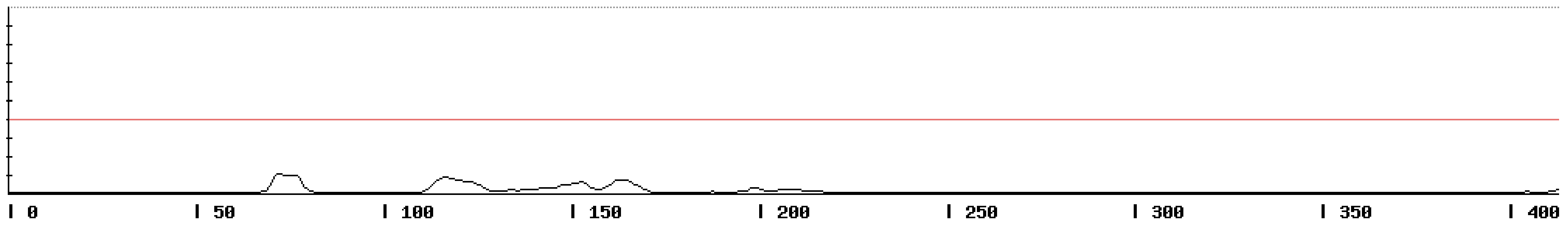

Predictions for Brassicanapus_GSBRNA2T00116457001

Viterbi Path

Viterbi Path

Posterior @ 0.4

Posterior @ 0.4

Posterior Graphic. Threshold Marked @ 0.4

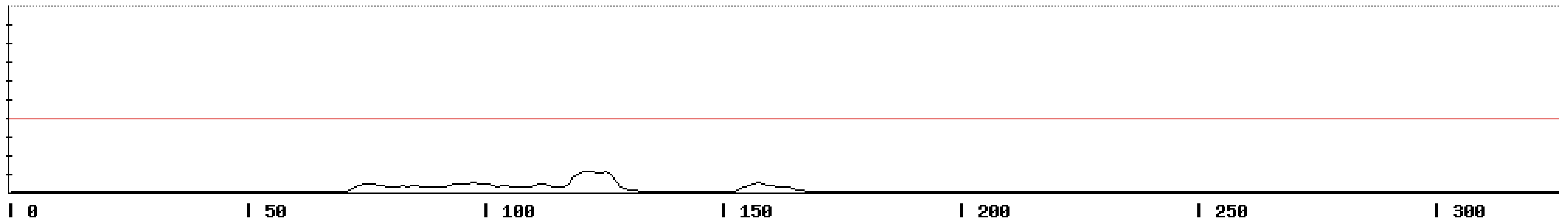

Predictions for Brassicanapus_GSBRNA2T00117792001

Viterbi Path

Viterbi Path

Posterior @ 0.4

Posterior @ 0.4

Posterior Graphic. Threshold Marked @ 0.4

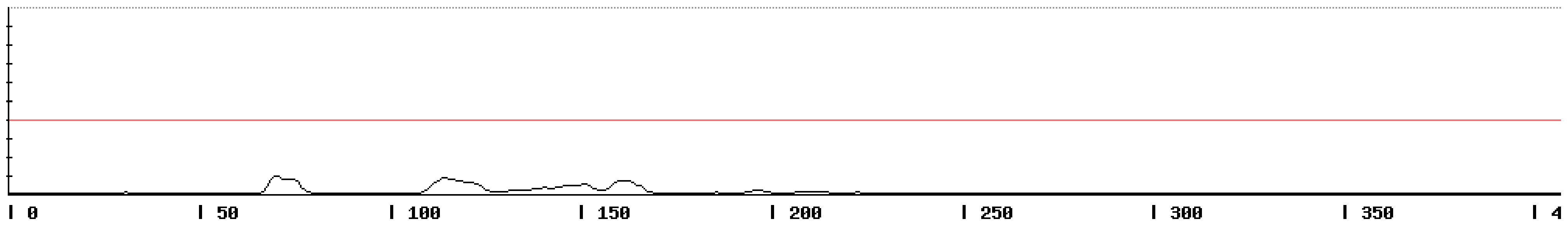

Predictions for Brassicanapus_GSBRNA2T00118651001

Viterbi Path

Viterbi Path

Posterior @ 0.4

Posterior @ 0.4

Posterior Graphic. Threshold Marked @ 0.4

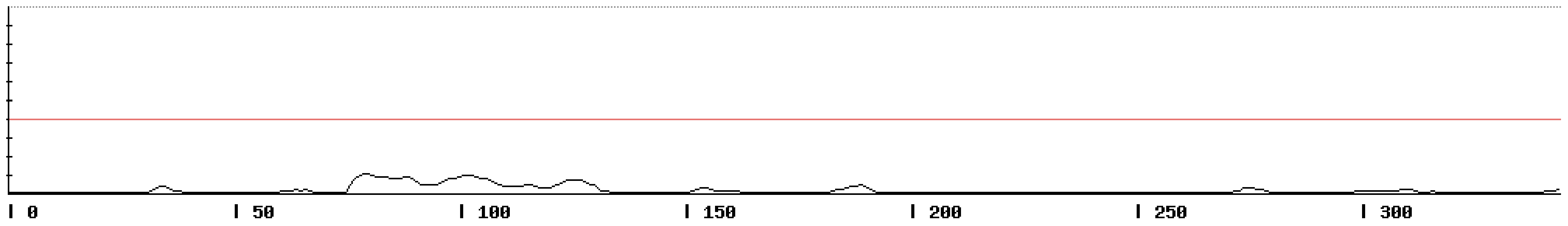

Predictions for Brassicanapus_GSBRNA2T00119863001

Viterbi Path

Viterbi Path

Posterior @ 0.4

Posterior @ 0.4

Posterior Graphic. Threshold Marked @ 0.4

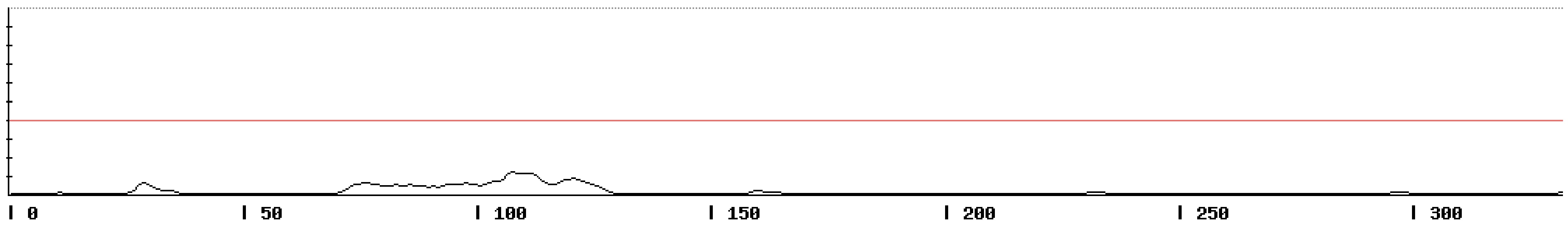

Predictions for Brassicanapus_GSBRNA2T00120947001

Viterbi Path

Viterbi Path

Posterior @ 0.4

Posterior @ 0.4

Posterior Graphic. Threshold Marked @ 0.4

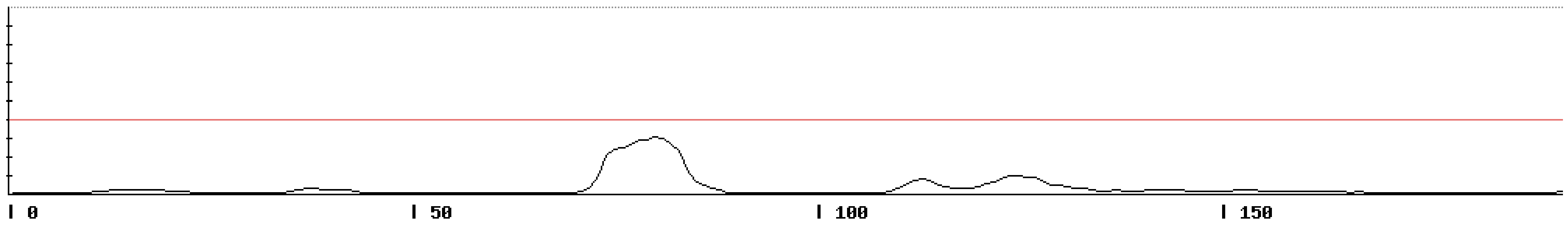

Predictions for Brassicanapus_GSBRNA2T00121461001

Viterbi Path

Viterbi Path

Posterior @ 0.4

Posterior @ 0.4

Posterior Graphic. Threshold Marked @ 0.4

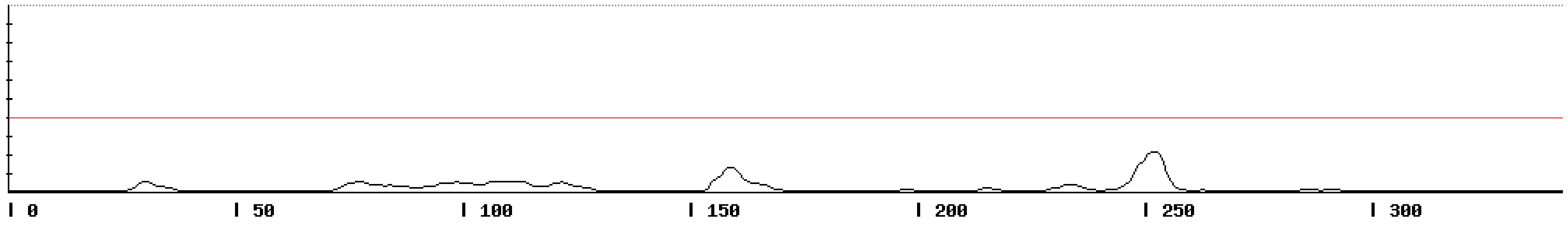

Predictions for Brassicanapus_GSBRNA2T00121760001

Viterbi Path

Viterbi Path

Posterior @ 0.4

104 - KDRKIKSGK - 112

Posterior @ 0.4

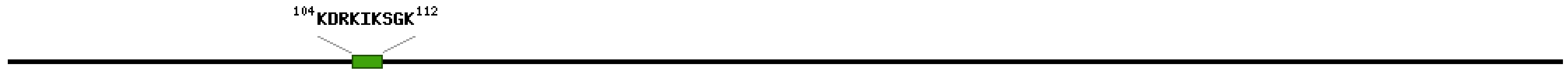

Posterior Graphic. Threshold Marked @ 0.4

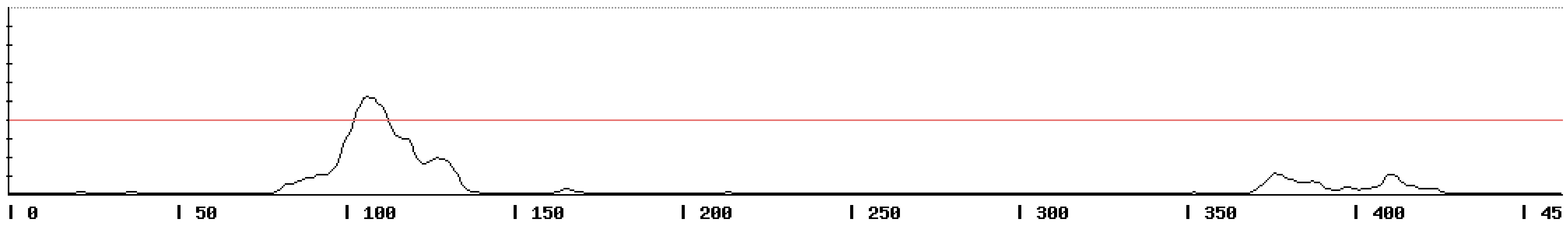

Predictions for Brassicanapus_GSBRNA2T00123077001

Viterbi Path

Viterbi Path

Posterior @ 0.4

Posterior @ 0.4

Posterior Graphic. Threshold Marked @ 0.4

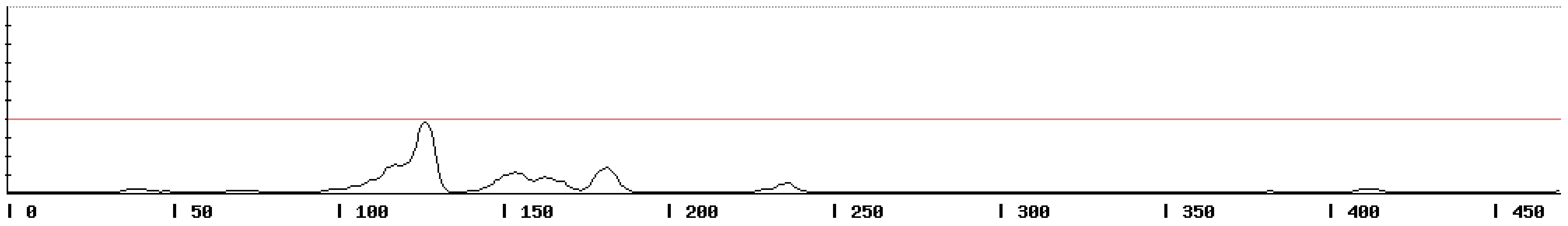

Predictions for Brassicanapus_GSBRNA2T00123451001

Viterbi Path

Viterbi Path

Posterior @ 0.4 65 - RRNKRPGKQKA - 75

Posterior @ 0.4

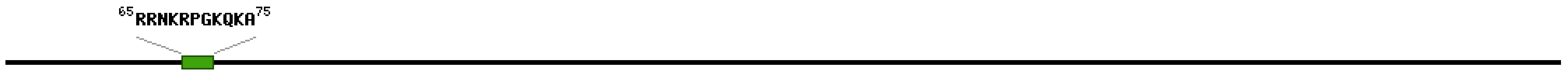

Posterior Graphic. Threshold Marked @ 0.4

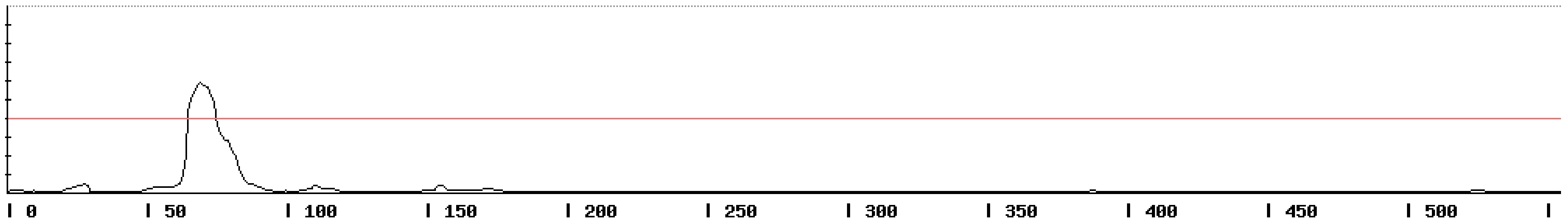

Predictions for Brassicanapus_GSBRNA2T00123464001	
Viterbi Path	65 - RKRSAIKKTDSKRPSRKVAKSGRWK - 89
Viterbi Path	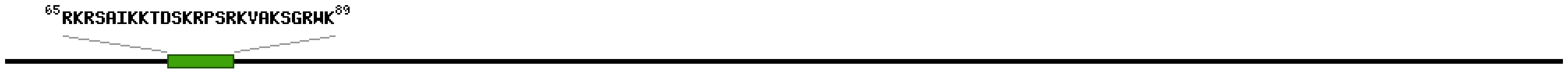
Posterior @ 0.4	65 - RKRSAIKKTDSKRPSRKVAKSGRWK - 89
Posterior @ 0.4	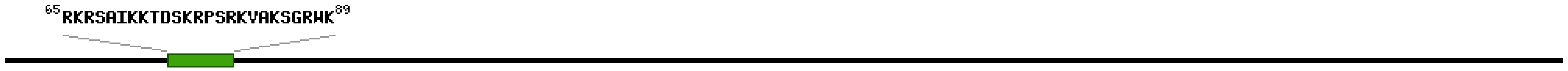
Posterior Graphic. Threshold Marked @ 0.4	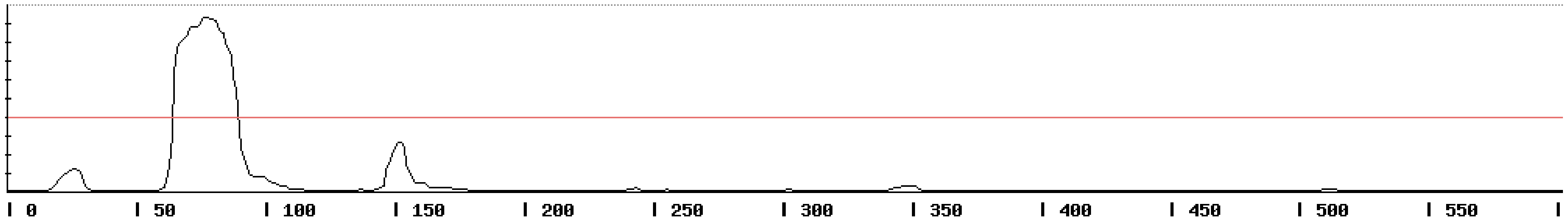

Predictions for Brassicanapus_GSBRNA2T00123484001	
Viterbi Path	
Viterbi Path	
Posterior @ 0.4	76 - KKKGTKR - 82
Posterior @ 0.4	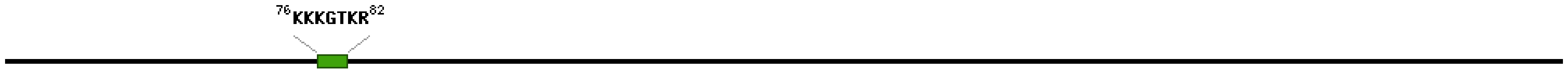
Posterior Graphic. Threshold Marked @ 0.4	

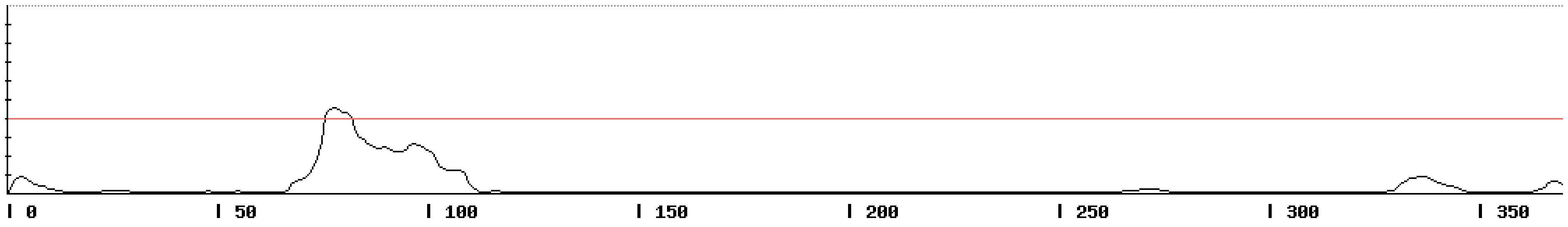

Predictions for Brassicanapus_GSBRNA2T00123485001

Viterbi Path

Viterbi Path

Posterior @ 0.4 75 - EKKKGTKRTTPSGFWKATGKDRIIRDKRGHGV - 106

Posterior @ 0.4

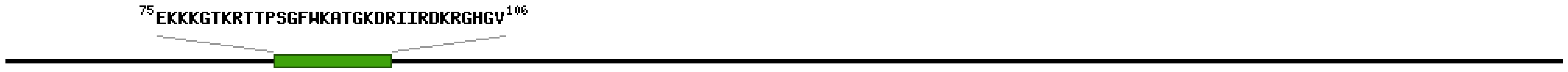

Posterior Graphic. Threshold Marked @ 0.4

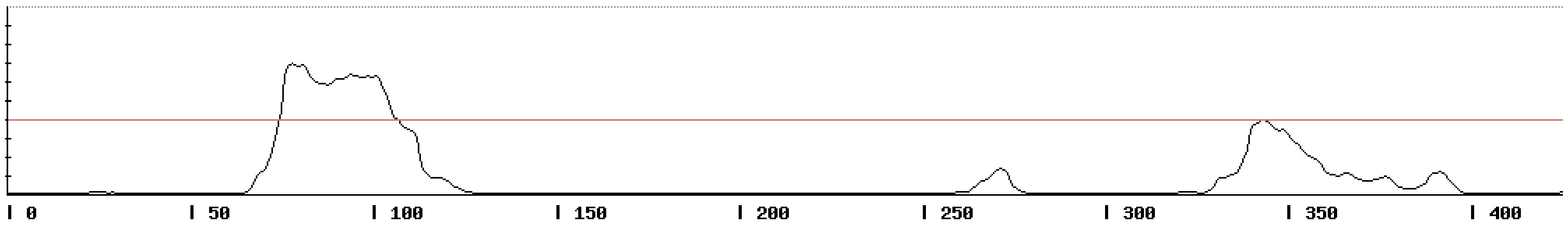

Predictions for Brassicanapus_GSBRNA2T00123489001

Viterbi Path

Viterbi Path

Posterior @ 0.4 72 - KSPKKSLTKRTTPSGFWKSTGKDRKVKEEKRRDG - 105

Posterior @ 0.4

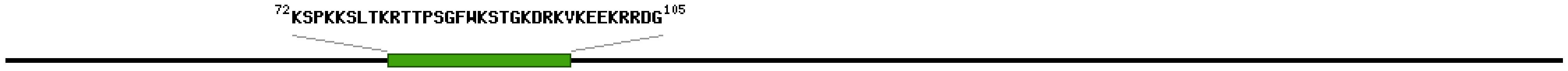

Posterior Graphic. Threshold Marked @ 0.4

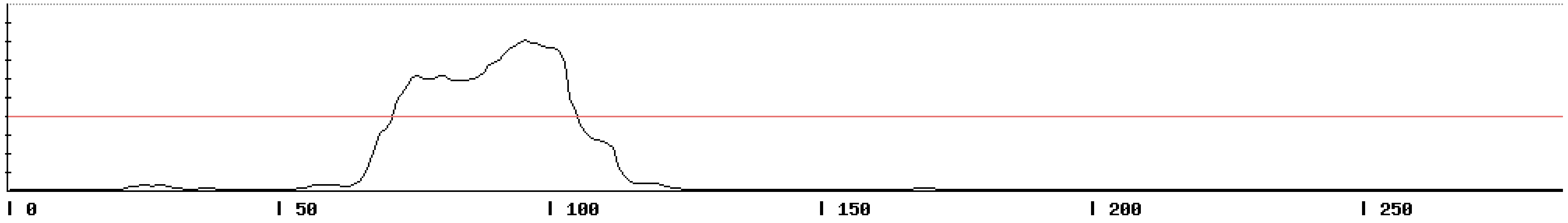

Predictions for Brassicanapus_GSBRNA2T00123768001

Viterbi Path

Viterbi Path

Posterior @ 0.4

Posterior @ 0.4

Posterior Graphic. Threshold Marked @ 0.4

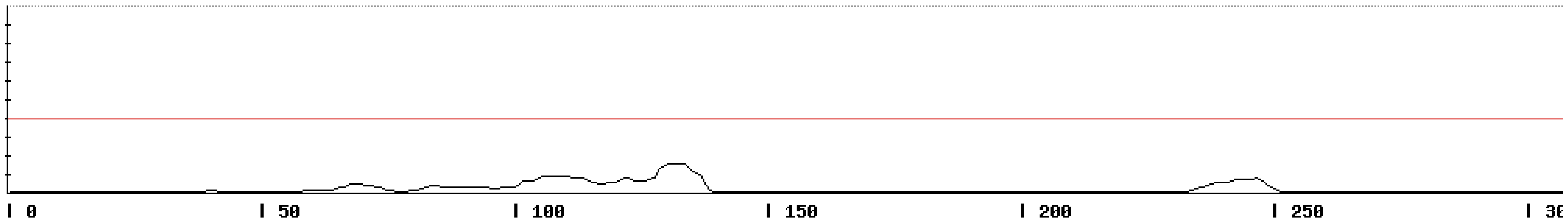

Predictions for Brassicanapus_GSBRNA2T00123793001

Viterbi Path

Viterbi Path

Posterior @ 0.4

Posterior @ 0.4

Posterior Graphic. Threshold Marked @ 0.4	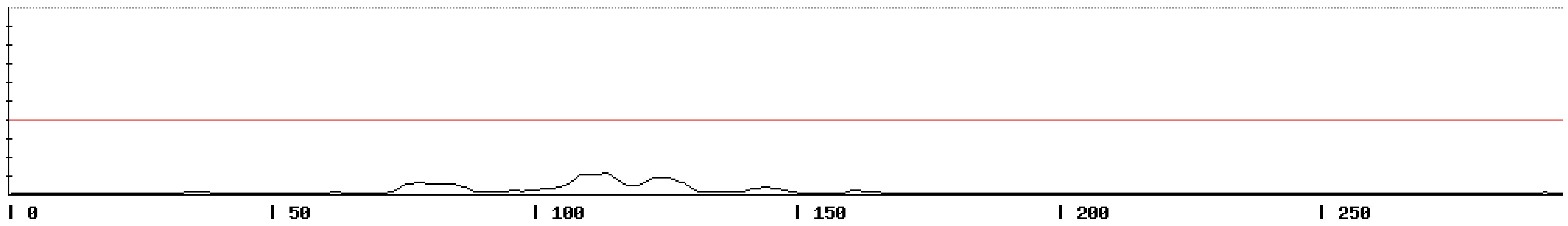

Predictions for Brassicanapus_GSBRNA2T00123958001	
Viterbi Path	
Viterbi Path	
Posterior @ 0.4	
Posterior @ 0.4	
Posterior Graphic. Threshold Marked @ 0.4	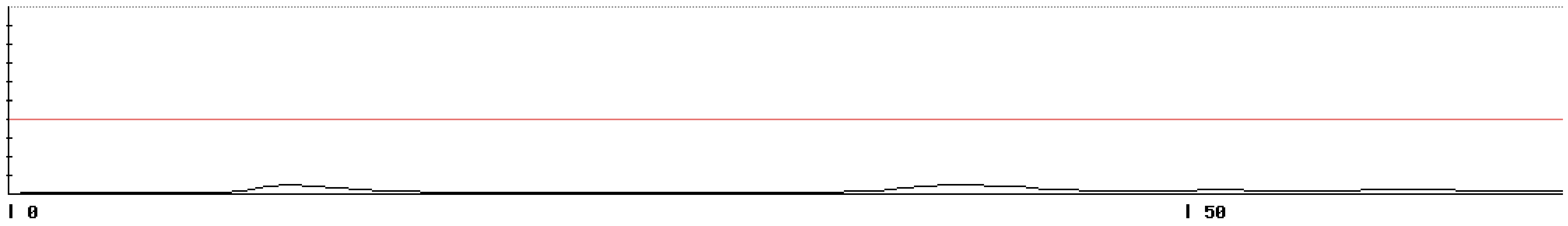

Predictions for Brassicanapus_GSBRNA2T00123999001	
Viterbi Path	
Viterbi Path	

Posterior @ 0.4

Posterior @ 0.4

Posterior Graphic. Threshold Marked @ 0.4

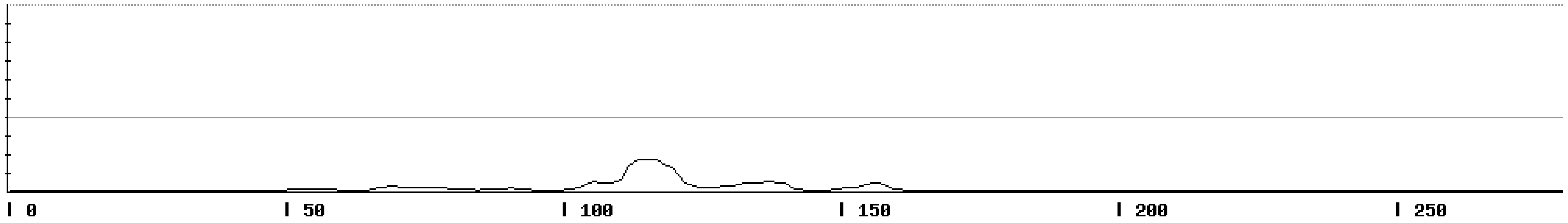

Predictions for Brassicanapus_GSBRNA2T00124306001

Viterbi Path

Viterbi Path

Posterior @ 0.4

Posterior @ 0.4

Posterior Graphic. Threshold Marked @ 0.4

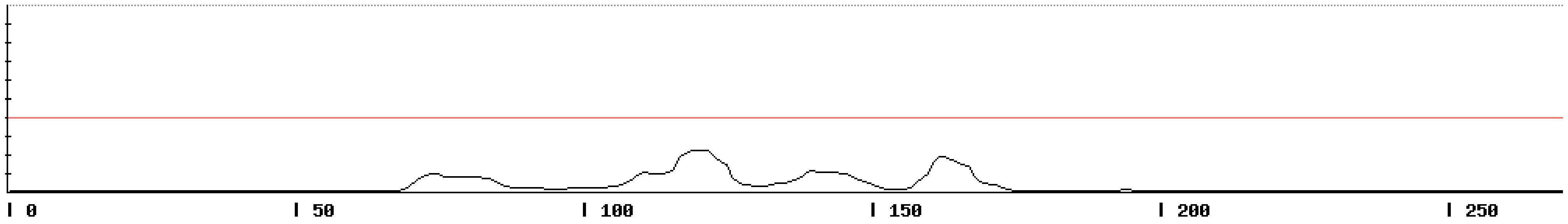

Predictions for Brassicanapus_GSBRNA2T00124696001

Viterbi Path

Viterbi Path

Posterior @ 0.4

Posterior @ 0.4

Posterior Graphic. Threshold Marked @ 0.4

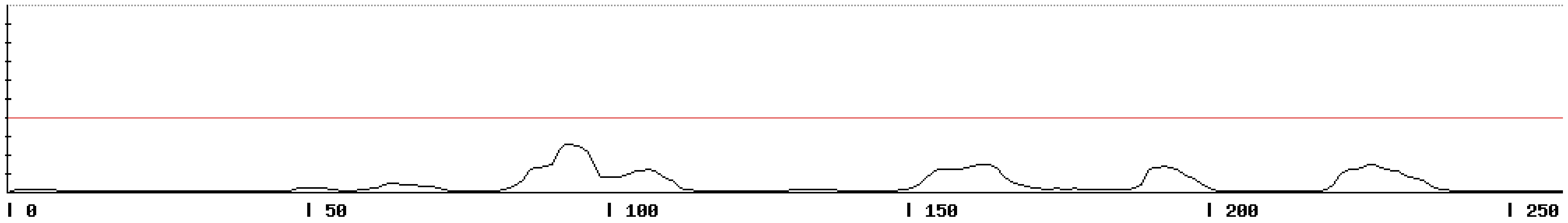

Predictions for Brassicanapus_GSBRNA2T00125430001

Viterbi Path

Viterbi Path

Posterior @ 0.4

104 - KDRKIKSGK - 112

410 - KKGKPSKNK - 418

Posterior @ 0.4

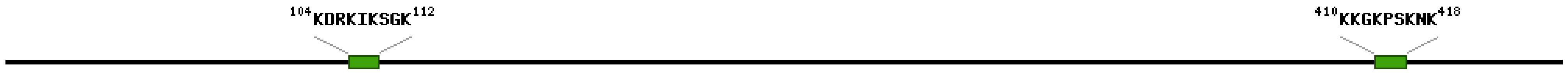

Posterior Graphic. Threshold Marked @ 0.4

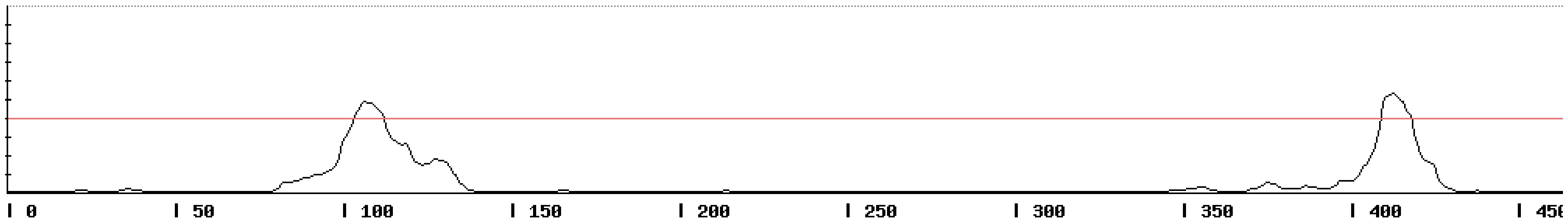

Predictions for Brassicanapus_GSBRNA2T00125625001	
Viterbi Path	
Viterbi Path	
Posterior @ 0.4	
Posterior @ 0.4	
Posterior Graphic. Threshold Marked @ 0.4	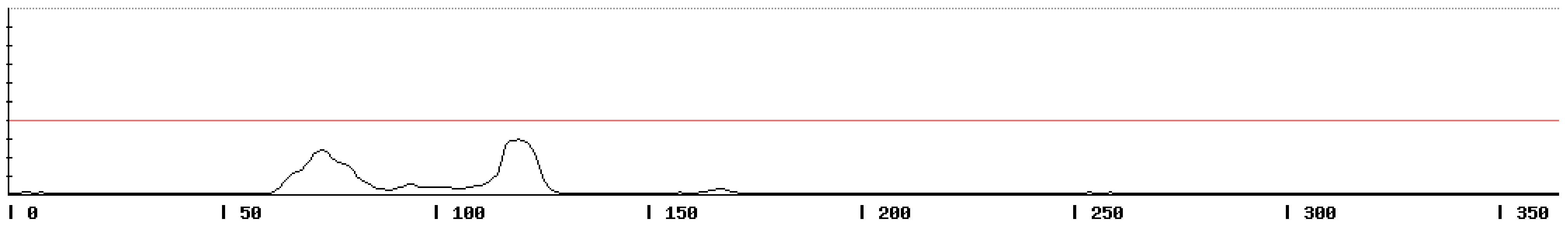

Predictions for Brassicanapus_GSBRNA2T00125630001	
Viterbi Path	
Viterbi Path	
Posterior @ 0.4	118 - KVKGKP - 123
Posterior @ 0.4	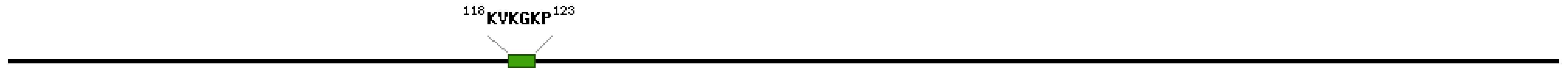
Posterior Graphic. Threshold Marked @ 0.4	

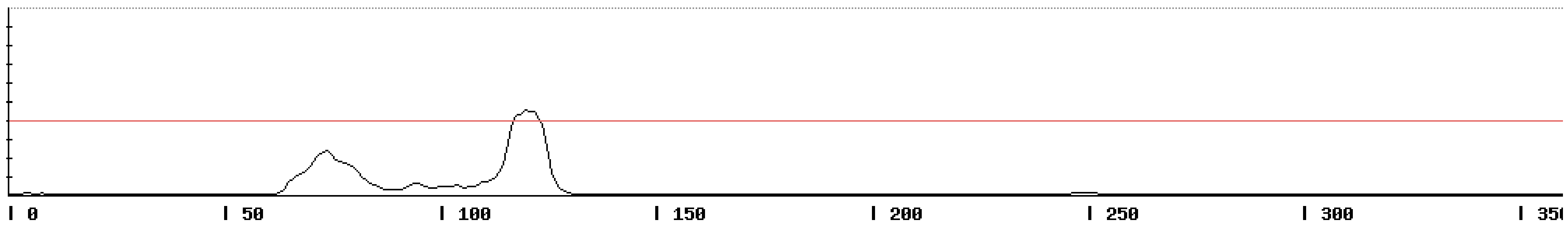

Predictions for Brassicanapus_GSBRNA2T00125806001

Viterbi Path

Viterbi Path

Posterior @ 0.4

Posterior @ 0.4

Posterior Graphic. Threshold Marked @ 0.4

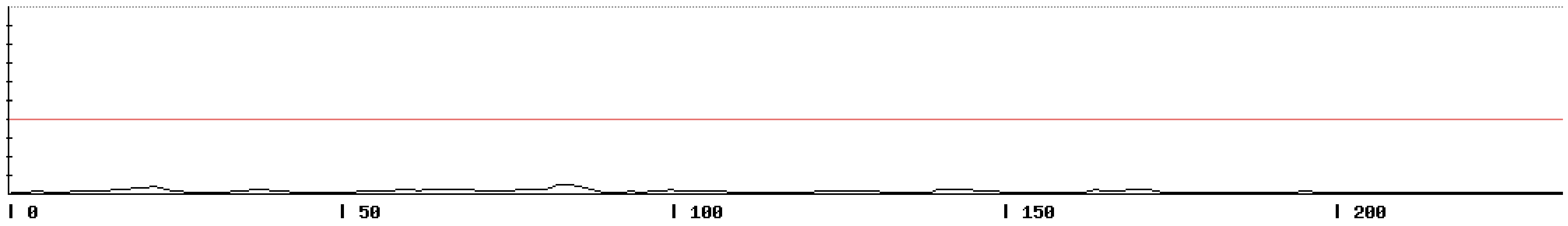

Predictions for Brassicanapus_GSBRNA2T00126210001

Viterbi Path

Viterbi Path

Posterior @ 0.4

Posterior @ 0.4

Posterior Graphic. Threshold Marked @ 0.4

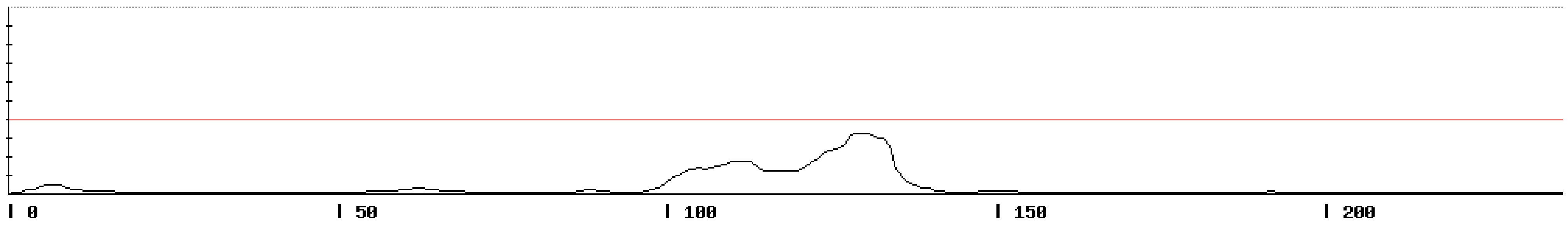

Predictions for Brassicanapus_GSBRNA2T00127050001	
Viterbi Path	
Viterbi Path	
Posterior @ 0.4	
Posterior @ 0.4	
Posterior Graphic. Threshold Marked @ 0.4	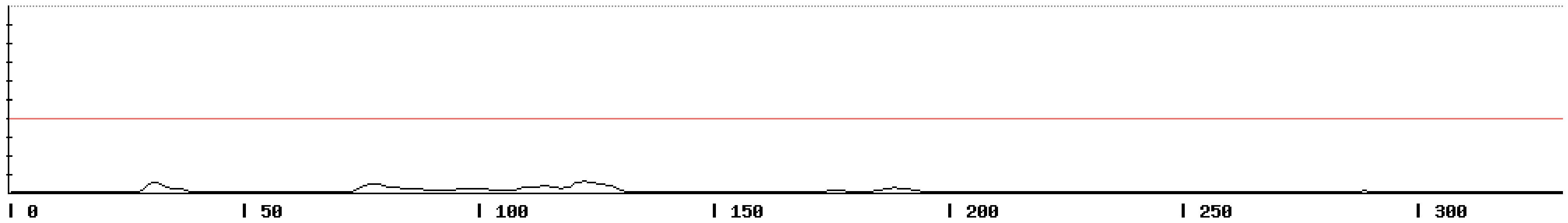

Predictions for Brassicanapus_GSBRNA2T00127318001	
Viterbi Path	
Viterbi Path	
Posterior @ 0.4	
Posterior @ 0.4	

Posterior Graphic. Threshold Marked @ 0.4

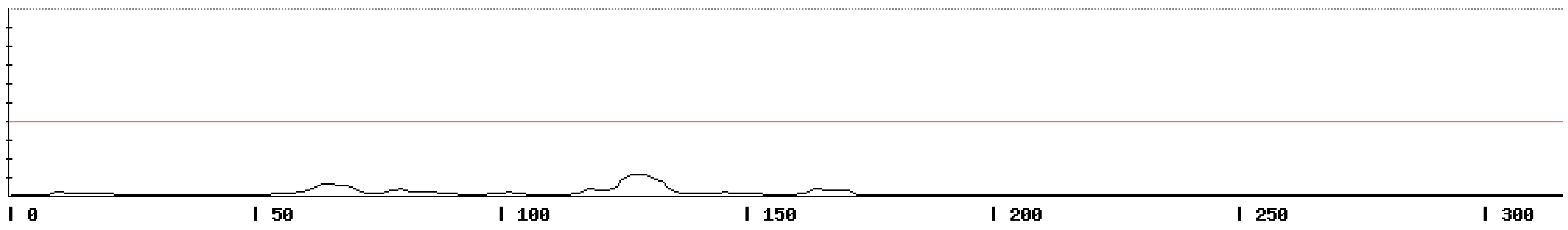

Predictions for Brassicanapus_GSBRNA2T00128635001

Viterbi Path

Viterbi Path

Posterior @ 0.4

Posterior @ 0.4

Posterior Graphic. Threshold Marked @ 0.4

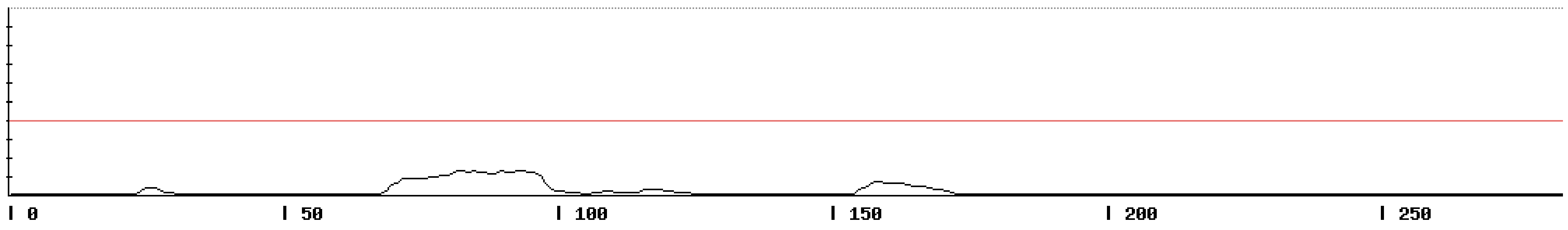

Predictions for Brassicanapus_GSBRNA2T00129716001

Viterbi Path

Viterbi Path

Posterior @ 0.4	98 - GKPK - 101
Posterior @ 0.4	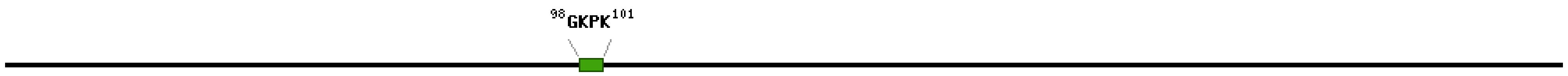
Posterior Graphic. Threshold Marked @ 0.4	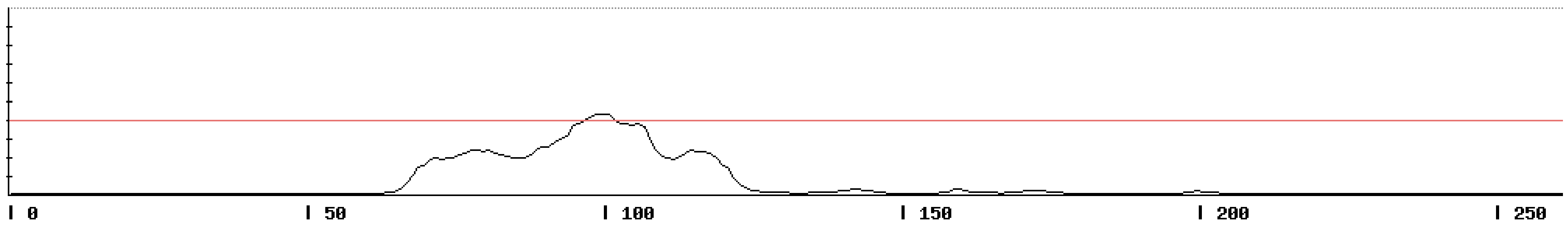

Predictions for Brassicanapus_GSBRNA2T00129721001

Viterbi Path	
Viterbi Path	
Posterior @ 0.4	
Posterior @ 0.4	
Posterior Graphic. Threshold Marked @ 0.4	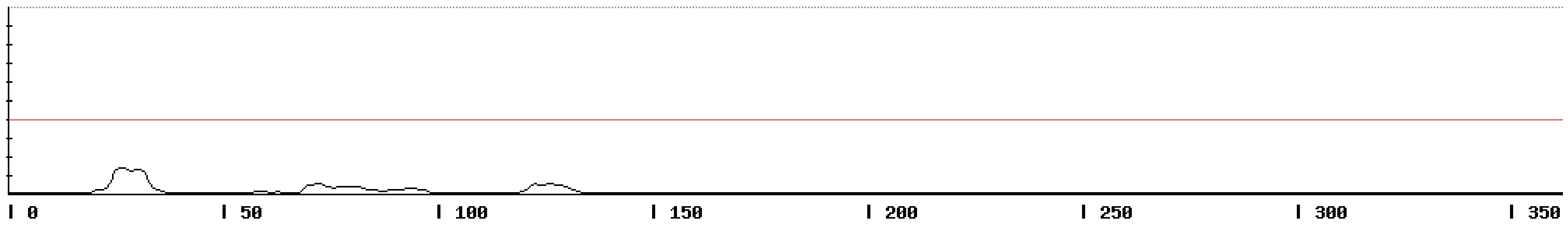

Predictions for Brassicanapus_GSBRNA2T00129913001

Viterbi Path	
Viterbi Path	

Posterior @ 0.4	
Posterior @ 0.4	
Posterior Graphic. Threshold Marked @ 0.4	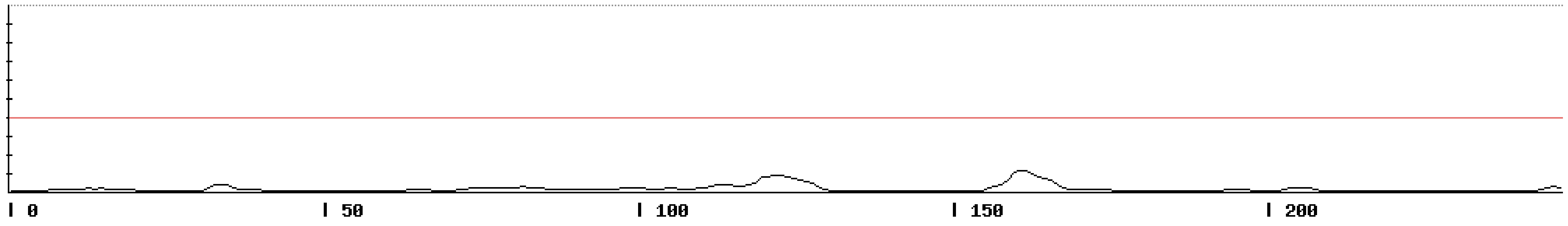

Predictions for Brassicanapus_GSBRNA2T00129957001	
Viterbi Path	160 - KRKEPKGRRQRKPRVR - 175
Viterbi Path	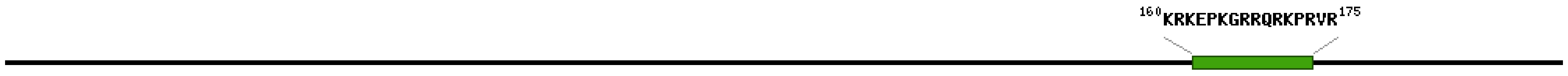
Posterior @ 0.4	159 - YKRKEPKGRRQRKPRVR - 175
Posterior @ 0.4	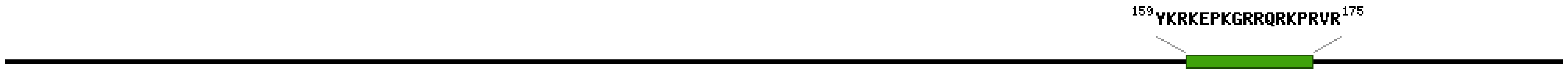
Posterior Graphic. Threshold Marked @ 0.4	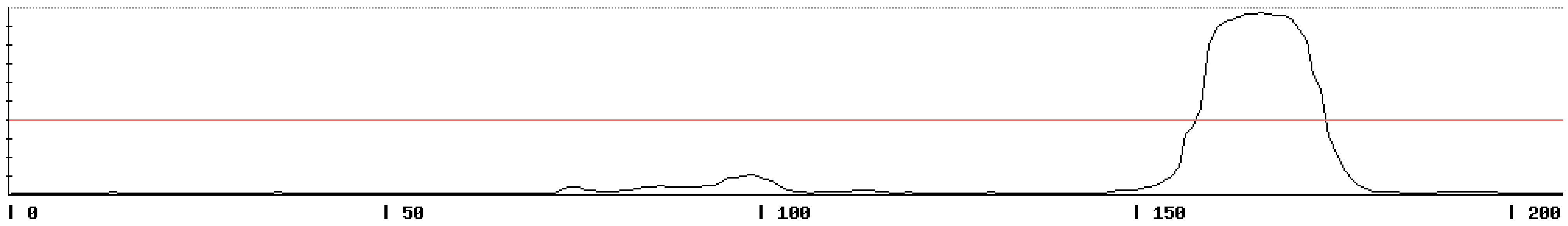

Predictions for Brassicanapus_GSBRNA2T00129990001

Viterbi Path

Viterbi Path

Posterior @ 0.4 | 184 - AKKGLKPEK - 192

Posterior @ 0.4

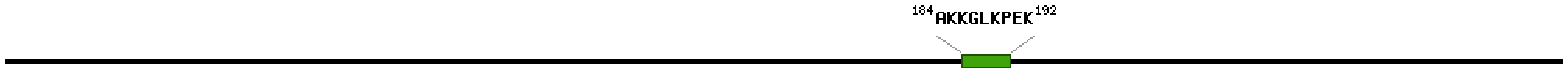

Posterior Graphic. Threshold Marked @ 0.4

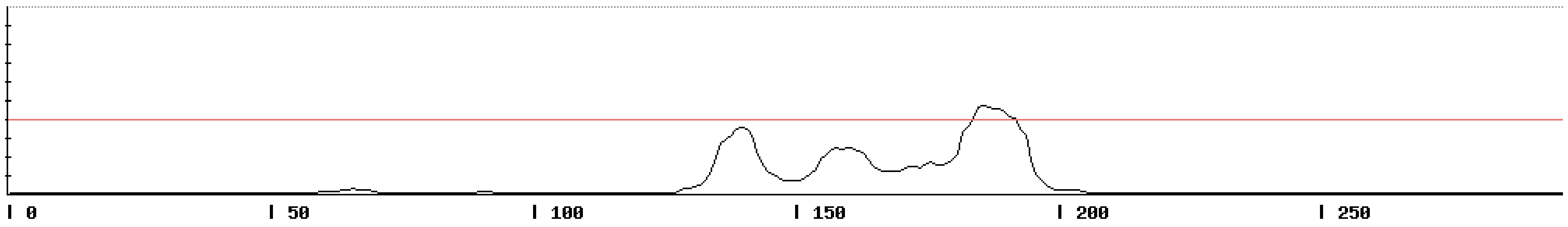

Predictions for Brassicanapus_GSBRNA2T00130163001

Viterbi Path

Viterbi Path

Posterior @ 0.4

Posterior @ 0.4

Posterior Graphic. Threshold Marked @ 0.4

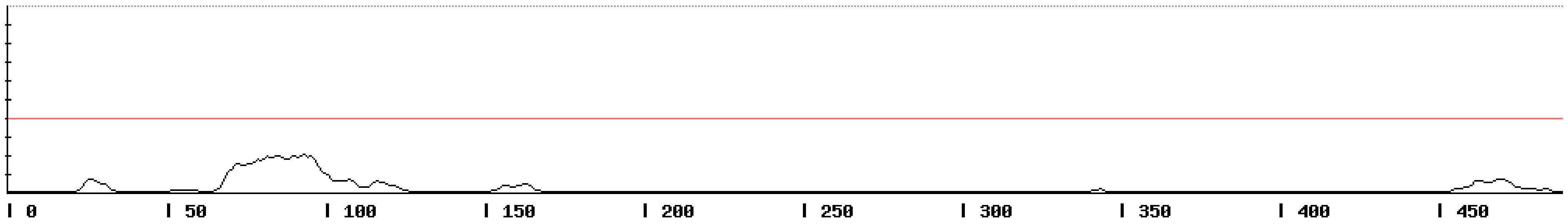

Predictions for Brassicanapus_GSBRNA2T00131011001	
Viterbi Path	
Viterbi Path	
Posterior @ 0.4	
Posterior @ 0.4	
Posterior Graphic. Threshold Marked @ 0.4	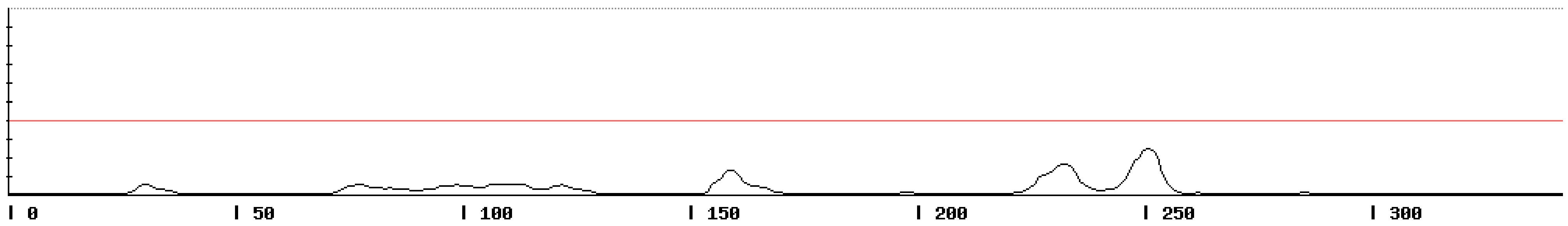

Predictions for Brassicanapus_GSBRNA2T00131012001	
Viterbi Path	
Viterbi Path	
Posterior @ 0.4	
Posterior @ 0.4	
Posterior Graphic. Threshold Marked @ 0.4	

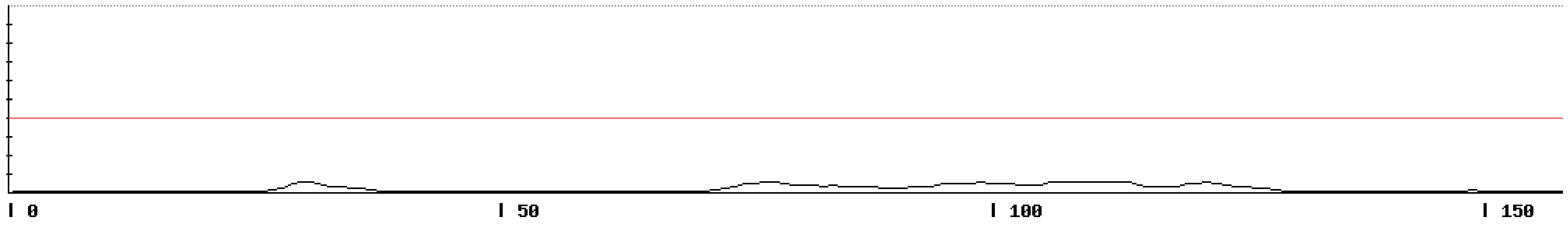

Predictions for Brassicanapus_GSBRNA2T00131286001

Viterbi Path

Viterbi Path

Posterior @ 0.4

Posterior @ 0.4

Posterior Graphic. Threshold Marked @ 0.4

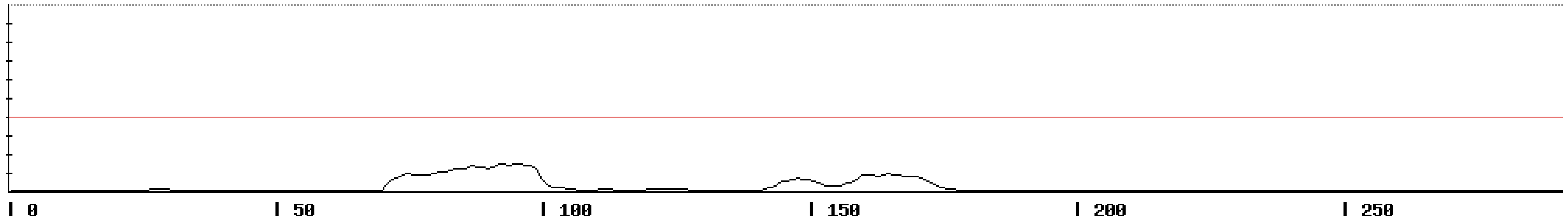

Predictions for Brassicanapus_GSBRNA2T00132845001

Viterbi Path

Viterbi Path

Posterior @ 0.4

Posterior @ 0.4

Posterior Graphic. Threshold Marked @ 0.4	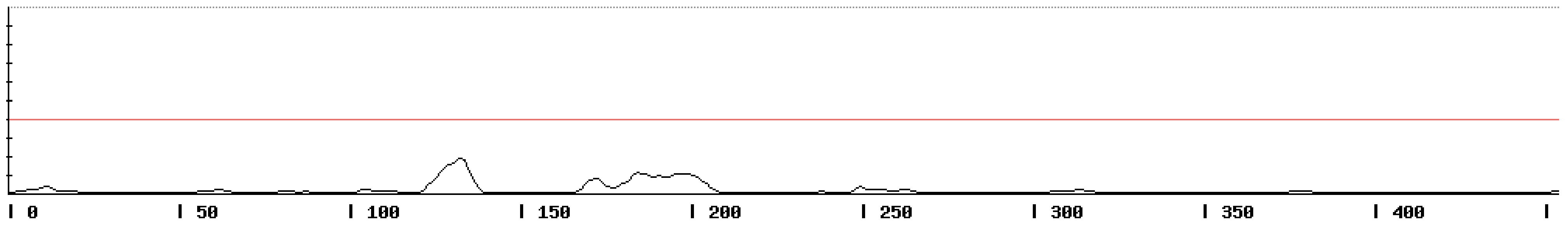

Predictions for Brassicanapus_GSBRNA2T00132849001

Viterbi Path	
Viterbi Path	
Posterior @ 0.4	
Posterior @ 0.4	
Posterior Graphic. Threshold Marked @ 0.4	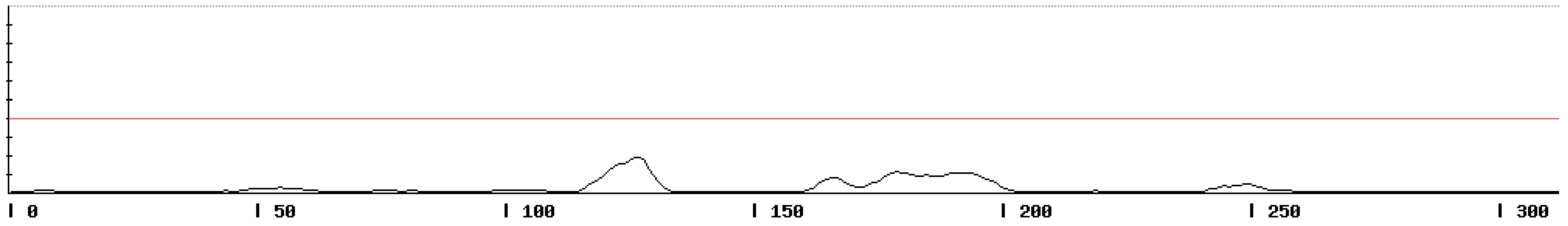

Predictions for Brassicanapus_GSBRNA2T00132929001

Viterbi Path	
Viterbi Path	
Posterior @ 0.4	
Posterior @ 0.4	

Posterior Graphic. Threshold Marked @ 0.4

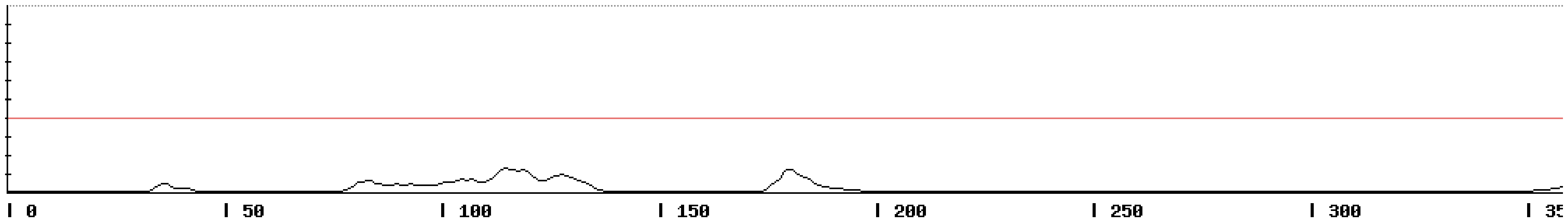

Predictions for Brassicanapus_GSBRNA2T00133340001

Viterbi Path

Viterbi Path

Posterior @ 0.4 74 - KKKIGCGKKVKRKI - 87

Posterior @ 0.4

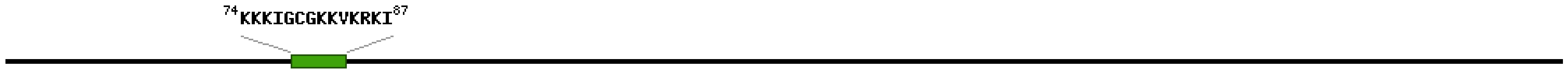

Posterior Graphic. Threshold Marked @ 0.4

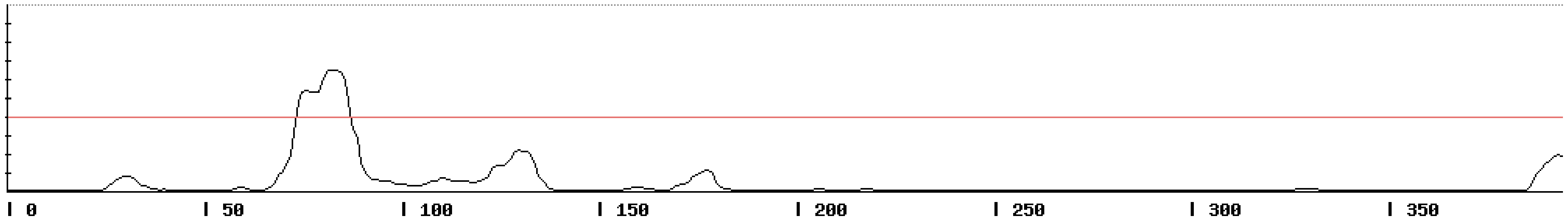

Predictions for Brassicanapus_GSBRNA2T00133341001

Viterbi Path

Viterbi Path

Posterior @ 0.4

Posterior @ 0.4

Posterior Graphic. Threshold Marked @ 0.4

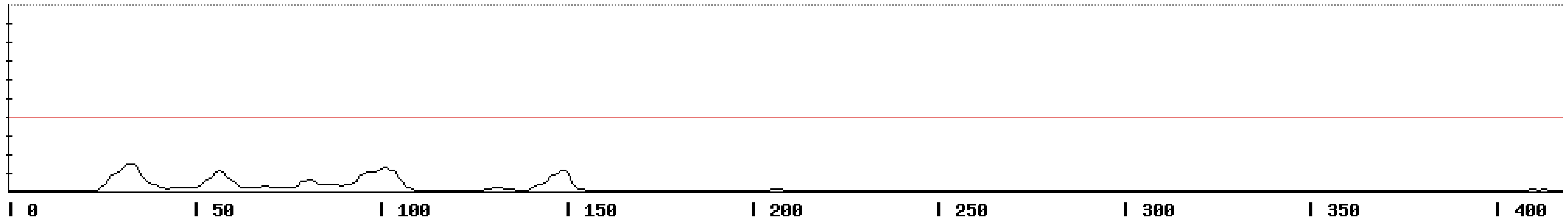

Predictions for Brassicanapus_GSBRNA2T00133539001

Viterbi Path

Viterbi Path

Posterior @ 0.4

Posterior @ 0.4

Posterior Graphic. Threshold Marked @ 0.4

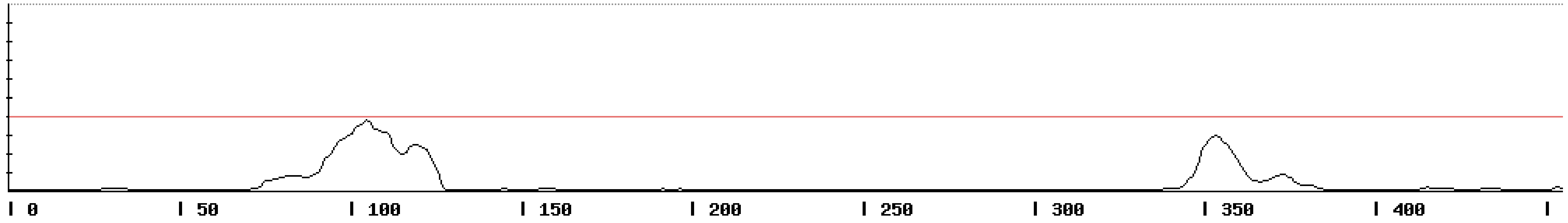

Predictions for Brassicanapus_GSBRNA2T00134569001

Viterbi Path

Viterbi Path

Posterior @ 0.4	78 - GKPK - 81
Posterior @ 0.4	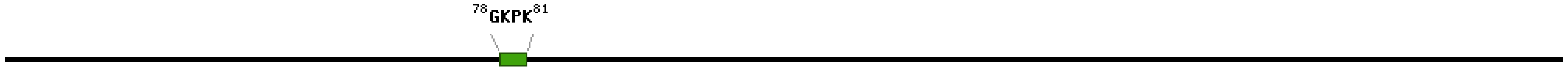
Posterior Graphic. Threshold Marked @ 0.4	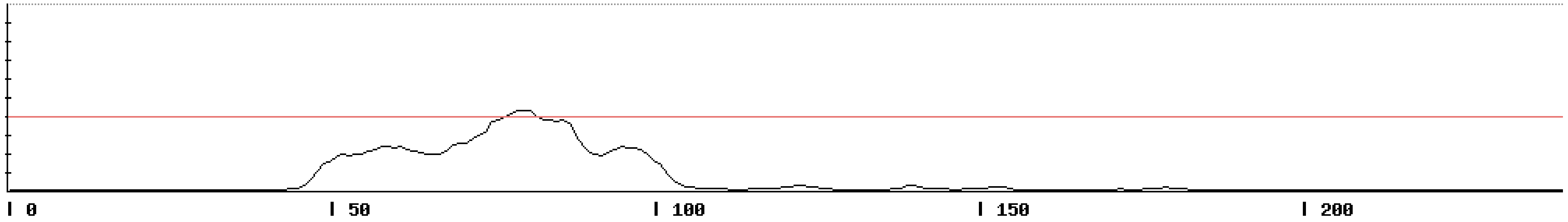

Predictions for Brassicanapus_GSBRNA2T00134573001

Viterbi Path	
Viterbi Path	
Posterior @ 0.4	
Posterior @ 0.4	
Posterior Graphic. Threshold Marked @ 0.4	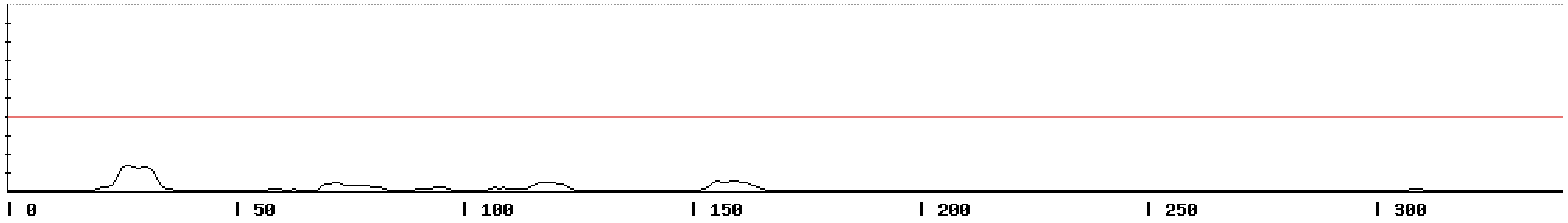

Predictions for Brassicanapus_GSBRNA2T00134697001

Viterbi Path

Viterbi Path

Posterior @ 0.4

Posterior @ 0.4

Posterior Graphic. Threshold Marked @ 0.4

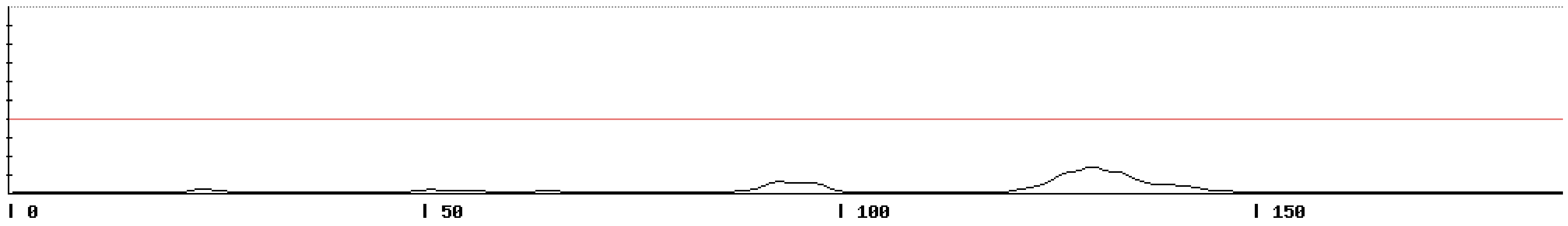

Predictions for Brassicanapus_GSBRNA2T00135630001

Viterbi Path 151 - RKASKGRRQRKSRGR - 165

Viterbi Path

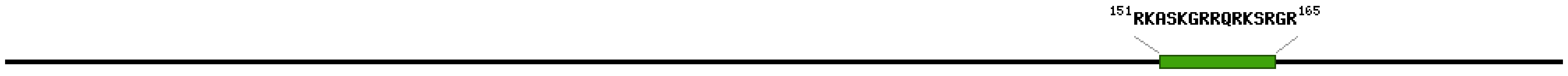

Posterior @ 0.4 151 - RKASKGRRQRKSRGR - 165

Posterior @ 0.4

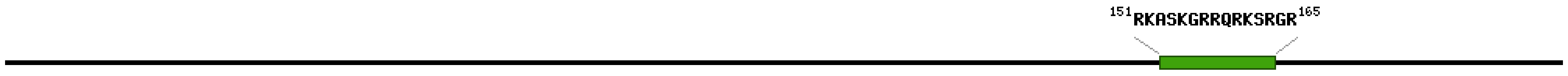

Posterior Graphic. Threshold Marked @ 0.4

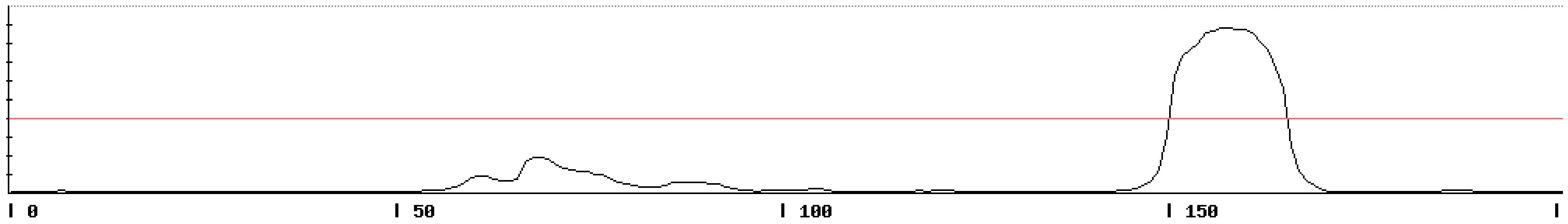

Predictions for Brassicanapus_GSBRNA2T00135689001

Viterbi Path	
Viterbi Path	
Posterior @ 0.4	
Posterior @ 0.4	
Posterior Graphic. Threshold Marked @ 0.4	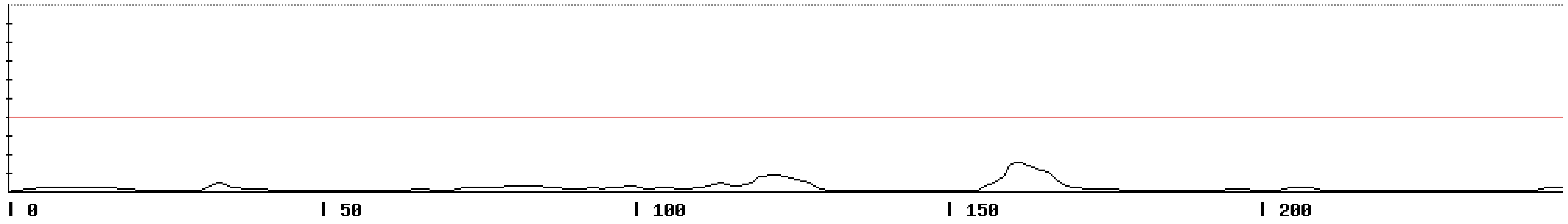

Predictions for Brassicanapus_GSBRNA2T00135820001

Viterbi Path	
Viterbi Path	
Posterior @ 0.4	
Posterior @ 0.4	
Posterior Graphic. Threshold Marked @ 0.4	

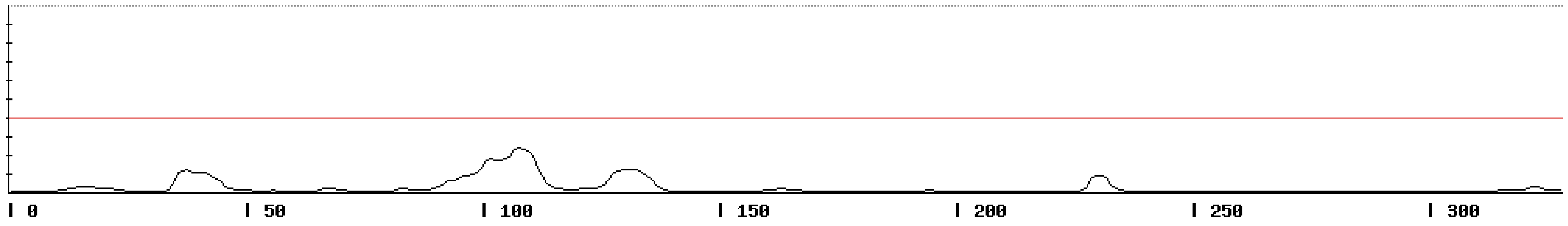

Predictions for Brassicanapus_GSBRNA2T00135984001

Viterbi Path	
Viterbi Path	
Posterior @ 0.4	
Posterior @ 0.4	
Posterior Graphic. Threshold Marked @ 0.4	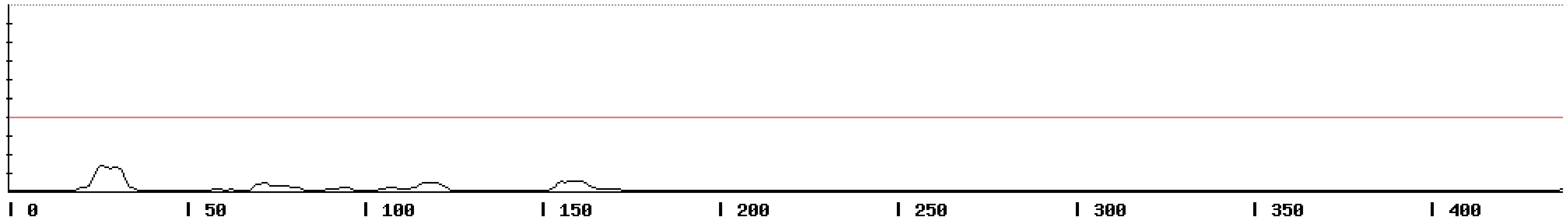

Predictions for Brassicanapus_GSBRNA2T00135993001

Viterbi Path	
Viterbi Path	
Posterior @ 0.4	98 - GKPK - 101
Posterior @ 0.4	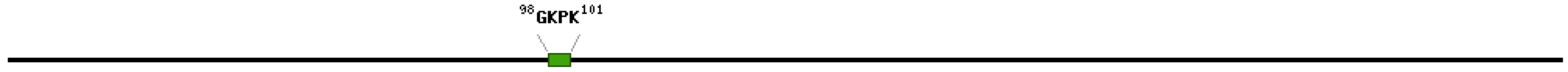

Posterior Graphic. Threshold Marked @ 0.4

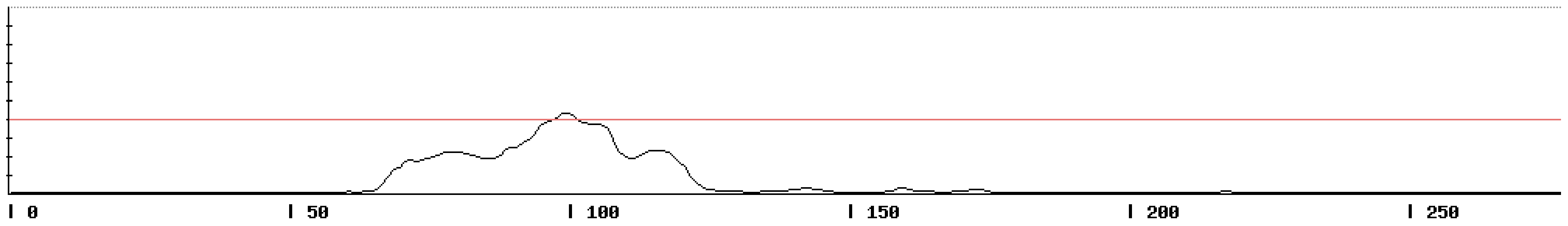

Predictions for Brassicanapus_GSBRNA2T00136356001

Viterbi Path

Viterbi Path

Posterior @ 0.4

Posterior @ 0.4

Posterior Graphic. Threshold Marked @ 0.4

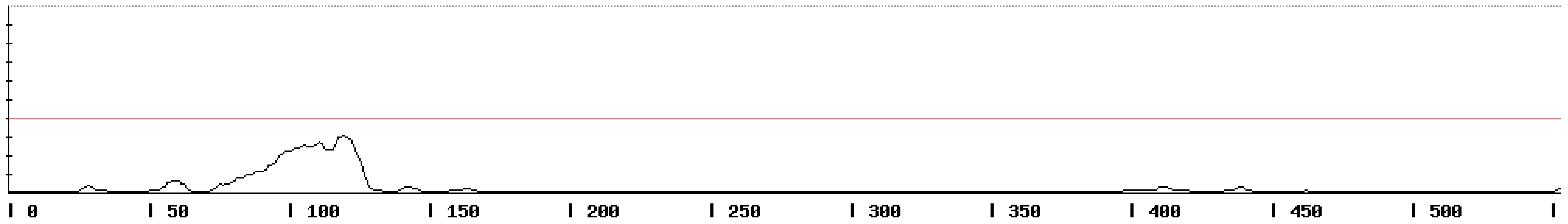

Predictions for Brassicanapus_GSBRNA2T00137289001

Viterbi Path

Viterbi Path

Posterior @ 0.4

Posterior @ 0.4

Posterior Graphic. Threshold Marked @ 0.4

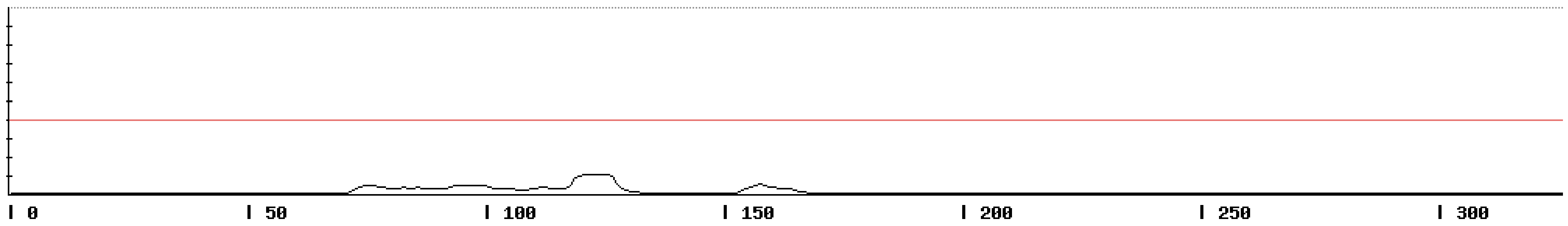

Predictions for Brassicanapus_GSBRNA2T00137562001

Viterbi Path

Viterbi Path

Posterior @ 0.4

Posterior @ 0.4

Posterior Graphic. Threshold Marked @ 0.4

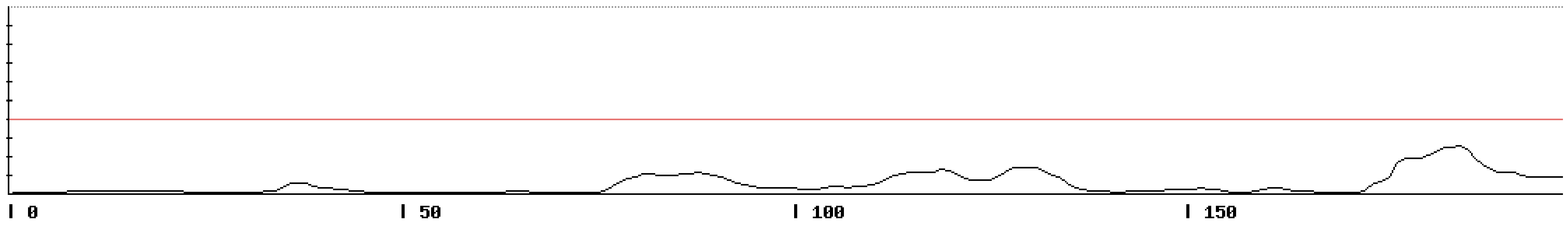

Predictions for Brassicanapus_GSBRNA2T00137565001

Viterbi Path

Viterbi Path

Posterior @ 0.4

Posterior @ 0.4

Posterior Graphic. Threshold Marked @ 0.4

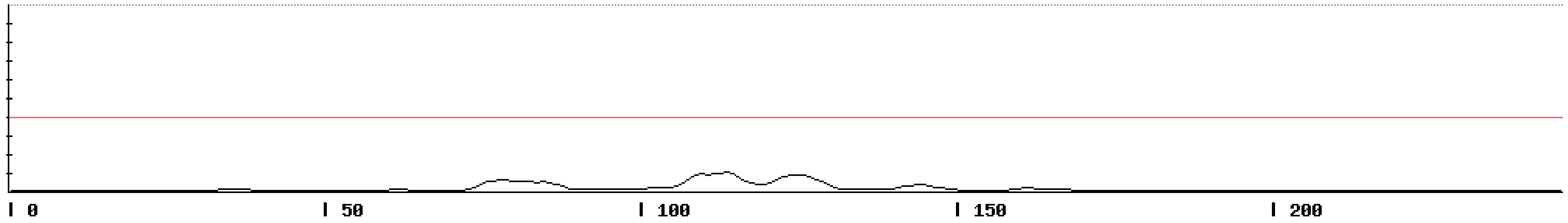

Predictions for Brassicanapus_GSBRNA2T00137582001

Viterbi Path

Viterbi Path

Posterior @ 0.4

Posterior @ 0.4

Posterior Graphic. Threshold Marked @ 0.4

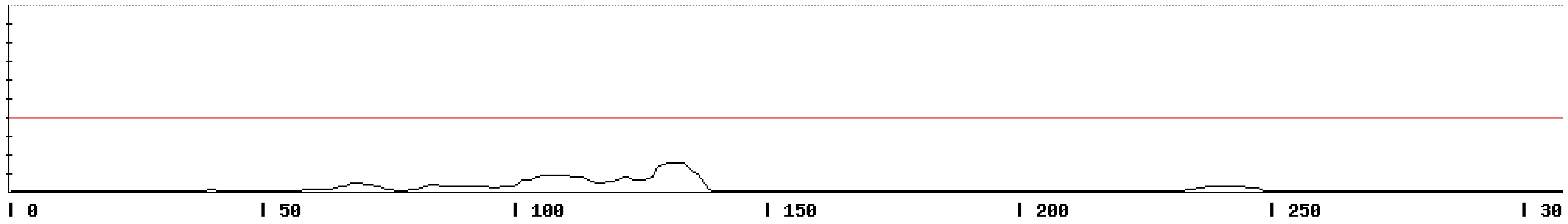

Predictions for Brassicanapus_GSBRNA2T00137735001

Viterbi Path

Viterbi Path

Posterior @ 0.4

Posterior @ 0.4

Posterior Graphic. Threshold Marked @ 0.4

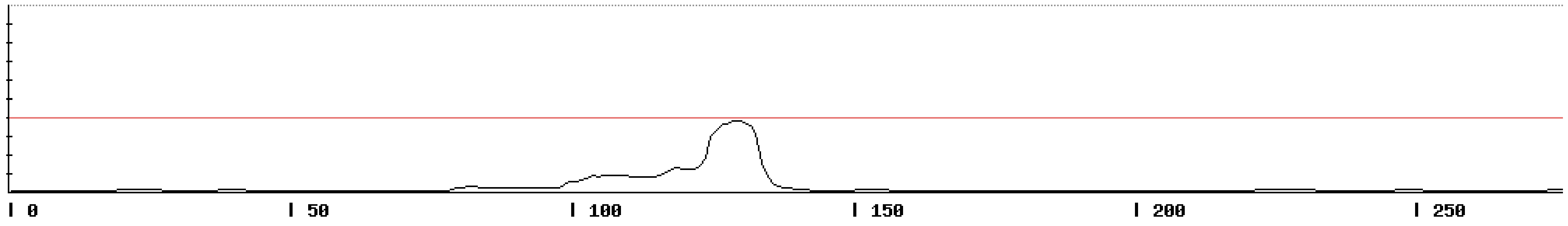

Predictions for Brassicanapus_GSBRNA2T00137740001

Viterbi Path

Viterbi Path

Posterior @ 0.4

Posterior @ 0.4

Posterior Graphic. Threshold Marked @ 0.4

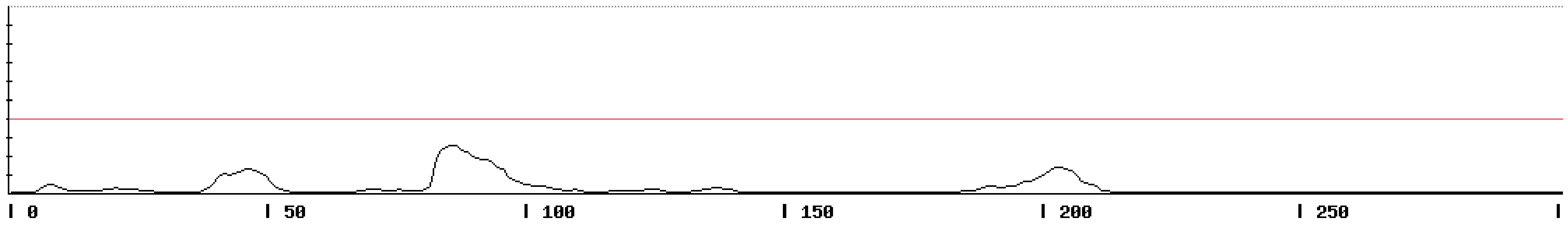

Predictions for Brassicanapus_GSBRNA2T00139105001

Viterbi Path	
Viterbi Path	
Posterior @ 0.4	305 - VKRPRGRPAGSKNKPKPP - 322
Posterior @ 0.4	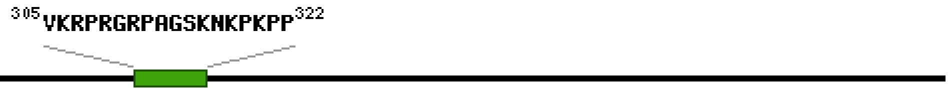
Posterior Graphic. Threshold Marked @ 0.4	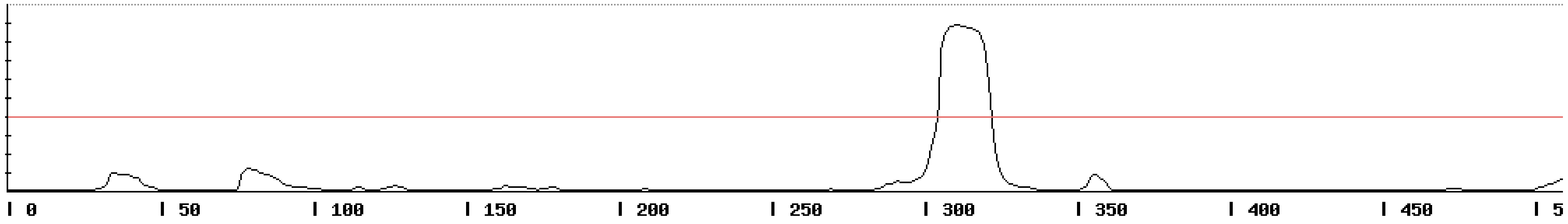

Predictions for Brassicanapus_GSBRNA2T00139538001

Viterbi Path	
Viterbi Path	
Posterior @ 0.4	
Posterior @ 0.4	
Posterior Graphic. Threshold Marked @ 0.4	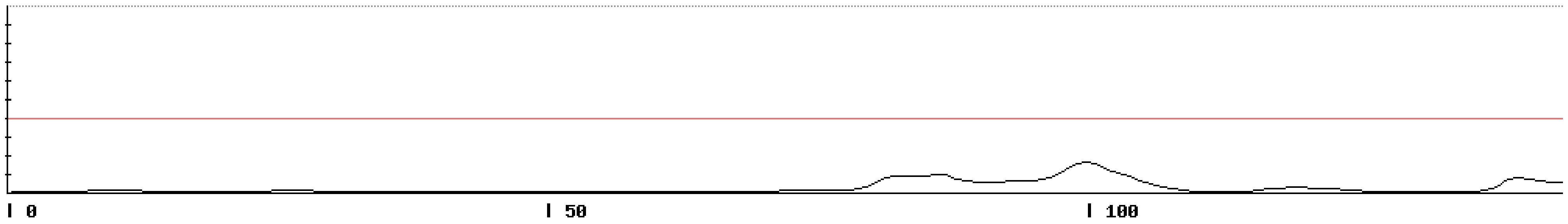

Predictions for Brassicanapus_GSBRNA2T00139904001	
Viterbi Path	
Viterbi Path	
Posterior @ 0.4	31 - KPIGRPKPVGIKK - 43
Posterior @ 0.4	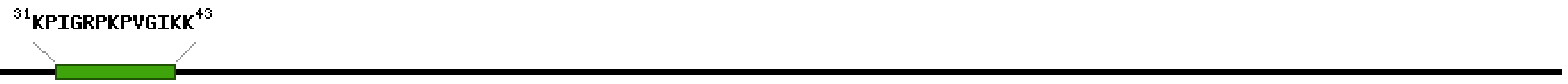
Posterior Graphic. Threshold Marked @ 0.4	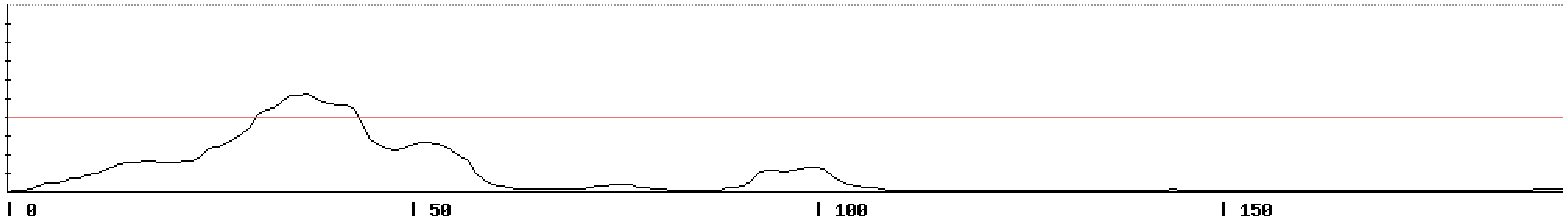

Predictions for Brassicanapus_GSBRNA2T00139965001	
Viterbi Path	
Viterbi Path	
Posterior @ 0.4	
Posterior @ 0.4	
Posterior Graphic. Threshold Marked @ 0.4	

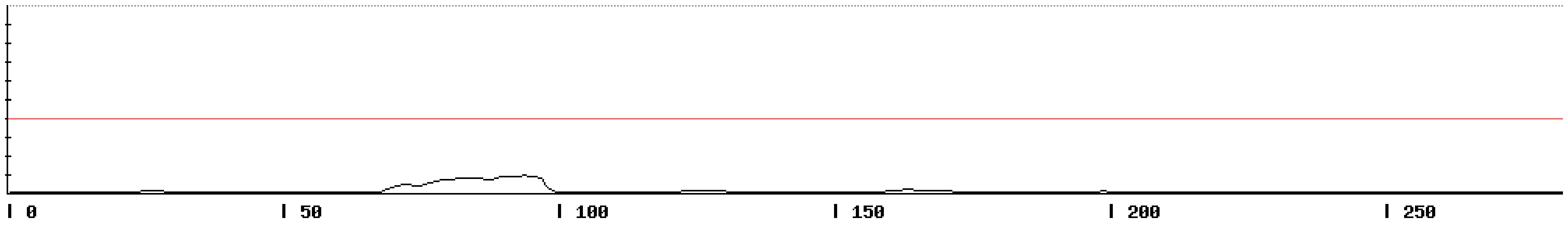

Predictions for Brassicanapus_GSBRNA2T00140364001

Viterbi Path: 160 - KRKEPKGRRQRKSRVR - 175

Viterbi Path

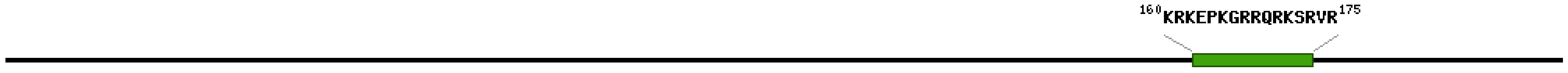

Posterior @ 0.4: 159 - YKRKEPKGRRQRKSRVRD - 176

Posterior @ 0.4

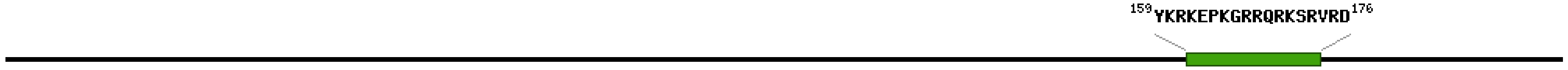

Posterior Graphic. Threshold Marked @ 0.4

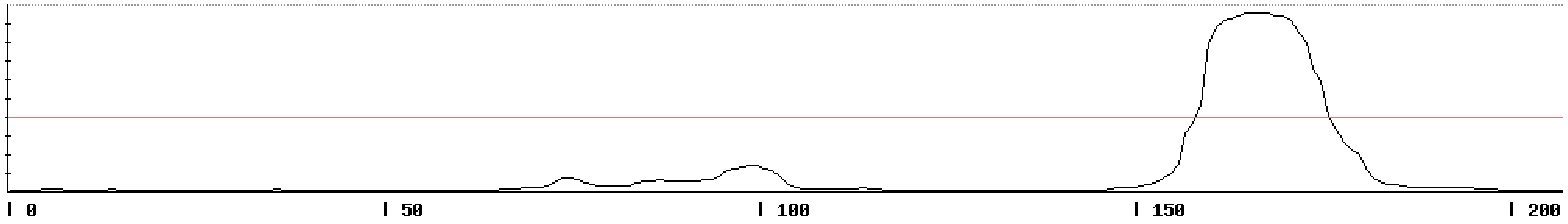

Predictions for Brassicanapus_GSBRNA2T00140409001

Viterbi Path

Viterbi Path

Posterior @ 0.4

Posterior @ 0.4

Posterior Graphic. Threshold Marked @ 0.4

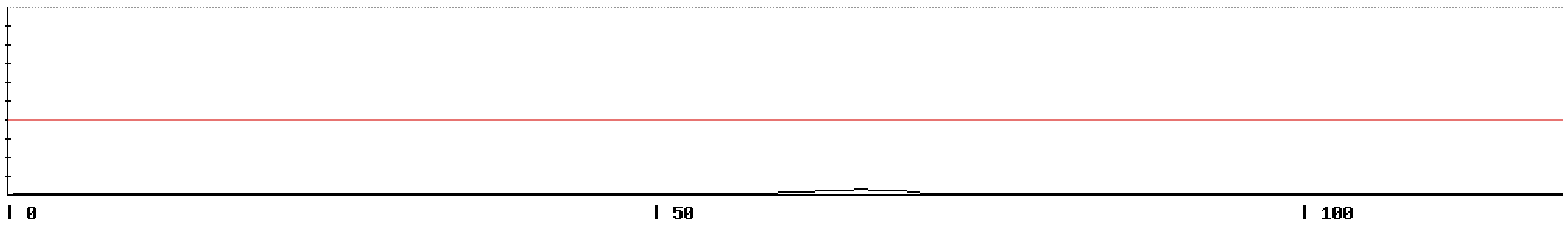

Predictions for Brassicanapus_GSBRNA2T00140620001

Viterbi Path

Viterbi Path

Posterior @ 0.4

Posterior @ 0.4

Posterior Graphic. Threshold Marked @ 0.4

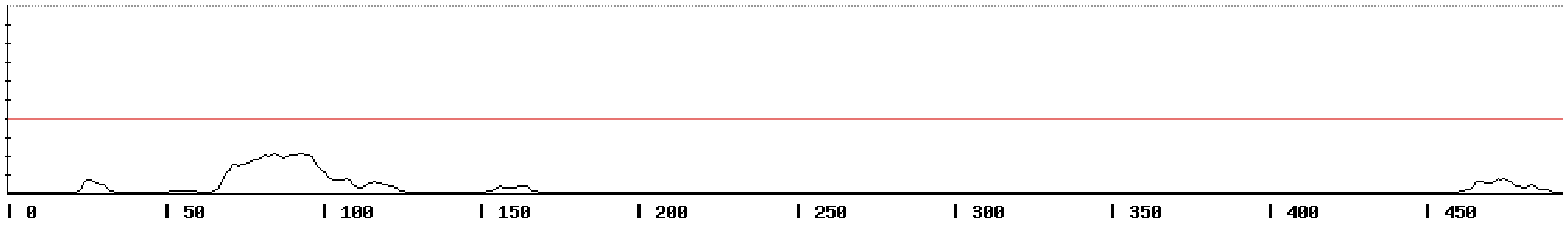

Predictions for Brassicanapus_GSBRNA2T00140686001

Viterbi Path

Viterbi Path

Posterior @ 0.4

Posterior @ 0.4

Posterior Graphic. Threshold Marked @ 0.4

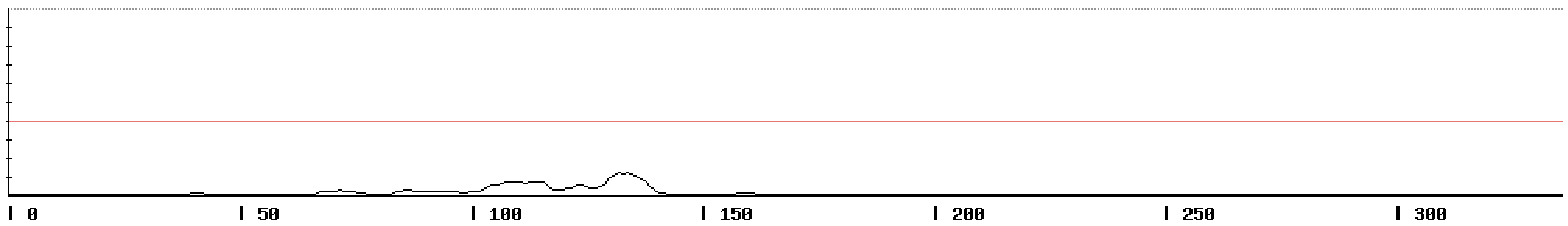

Predictions for Brassicanapus_GSBRNA2T00140926001

Viterbi Path

Viterbi Path

Posterior @ 0.4

Posterior @ 0.4

Posterior Graphic. Threshold Marked @ 0.4

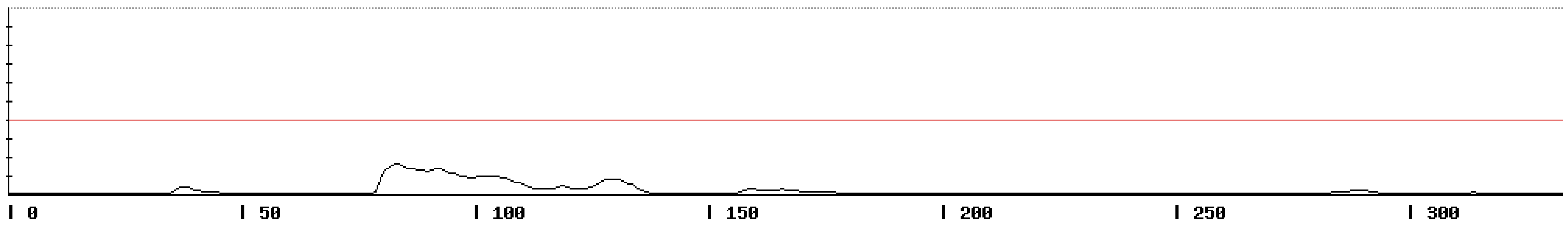

Predictions for Brassicanapus_GSBRNA2T00141573001

Viterbi Path

Viterbi Path

Posterior @ 0.4

Posterior @ 0.4

Posterior Graphic. Threshold Marked @ 0.4

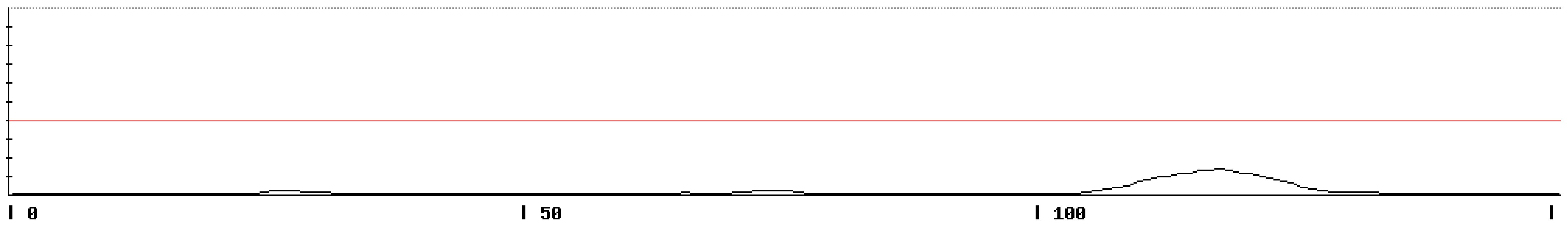

Predictions for Brassicanapus_GSBRNA2T00141649001

Viterbi Path

Viterbi Path

Posterior @ 0.4

Posterior @ 0.4

Posterior Graphic. Threshold Marked @ 0.4

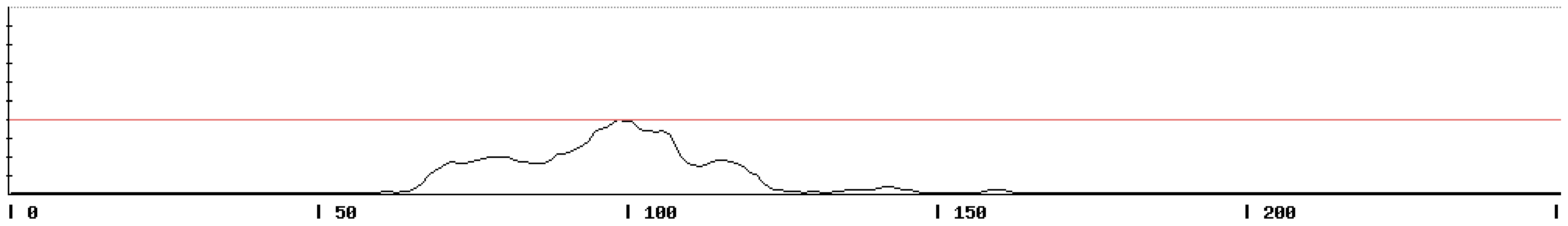

Predictions for Brassicanapus_GSBRNA2T00141706001

Viterbi Path

Viterbi Path

Posterior @ 0.4

Posterior @ 0.4

Posterior Graphic. Threshold Marked @ 0.4

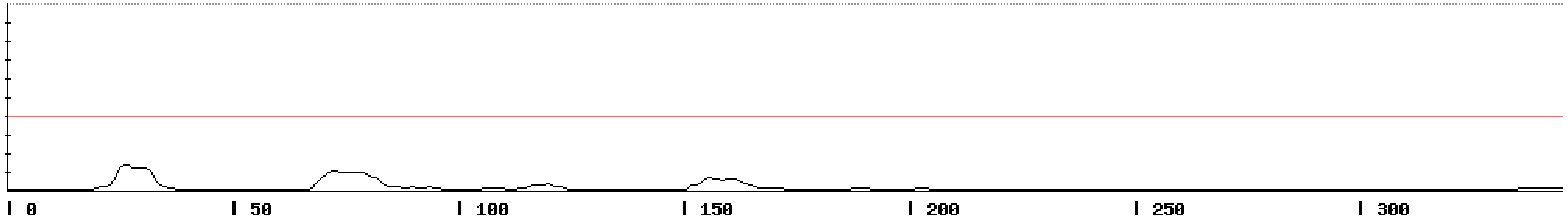

Predictions for Brassicanapus_GSBRNA2T00142424001

Viterbi Path

Viterbi Path

Posterior @ 0.4 — 138 - KRR - 140

Posterior @ 0.4

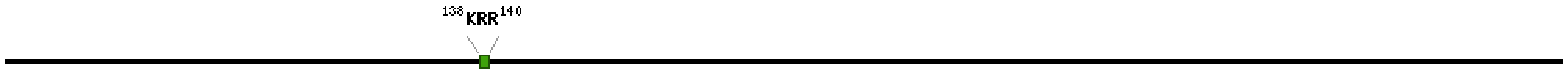

Posterior Graphic. Threshold Marked @ 0.4

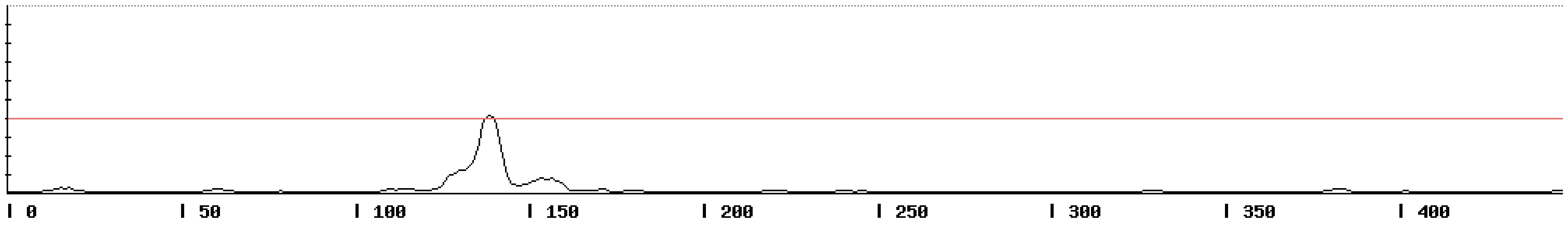

Predictions for Brassicanapus_GSBRNA2T00142881001

Viterbi Path

Viterbi Path

Posterior @ 0.4

Posterior @ 0.4

Posterior Graphic. Threshold Marked @ 0.4

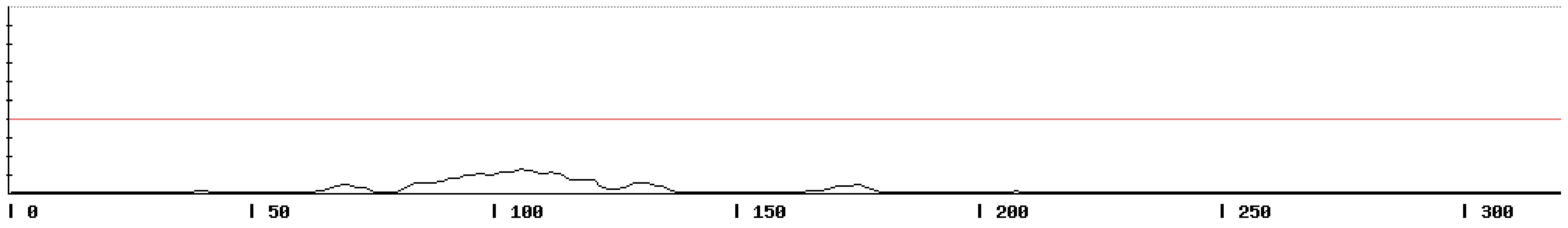

Predictions for Brassicanapus_GSBRNA2T00143403001

Viterbi Path

Viterbi Path

Posterior @ 0.4

Posterior @ 0.4

Posterior Graphic. Threshold Marked @ 0.4

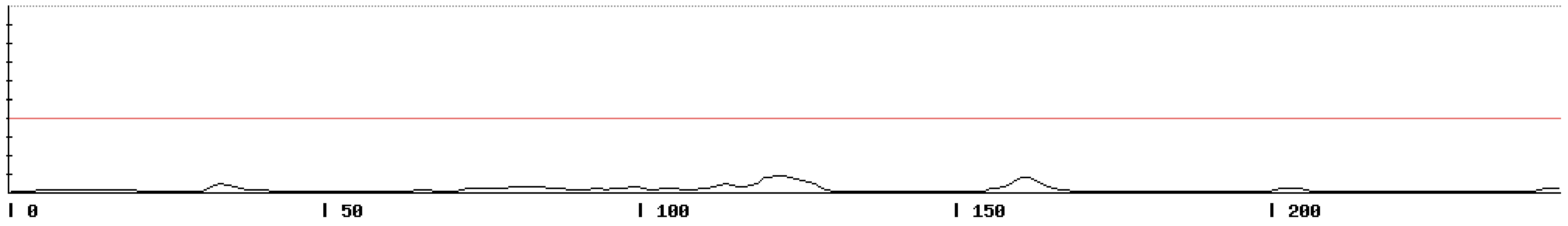

Predictions for Brassicanapus_GSBRNA2T00143470001	
Viterbi Path	
Viterbi Path	
Posterior @ 0.4	105 - AKGNWRERGREKKIKRGGDG - 124
Posterior @ 0.4	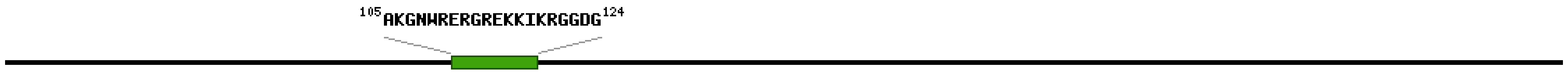
Posterior Graphic. Threshold Marked @ 0.4	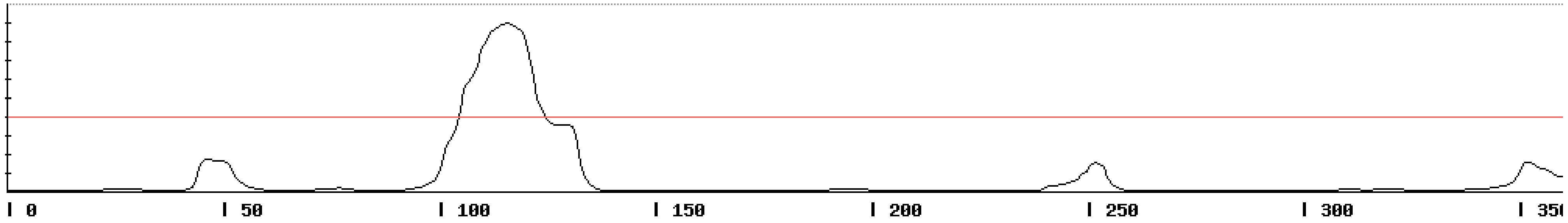

Predictions for Brassicanapus_GSBRNA2T00144521001	
Viterbi Path	
Viterbi Path	
Posterior @ 0.4	
Posterior @ 0.4	
Posterior Graphic. Threshold Marked @ 0.4	

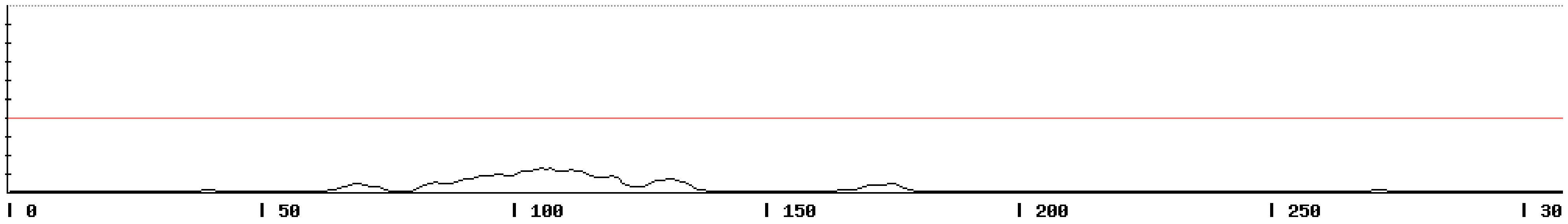

Predictions for Brassicanapus_GSBRNA2T00145006001	
Viterbi Path	
Viterbi Path	
Posterior @ 0.4	
Posterior @ 0.4	
Posterior Graphic. Threshold Marked @ 0.4	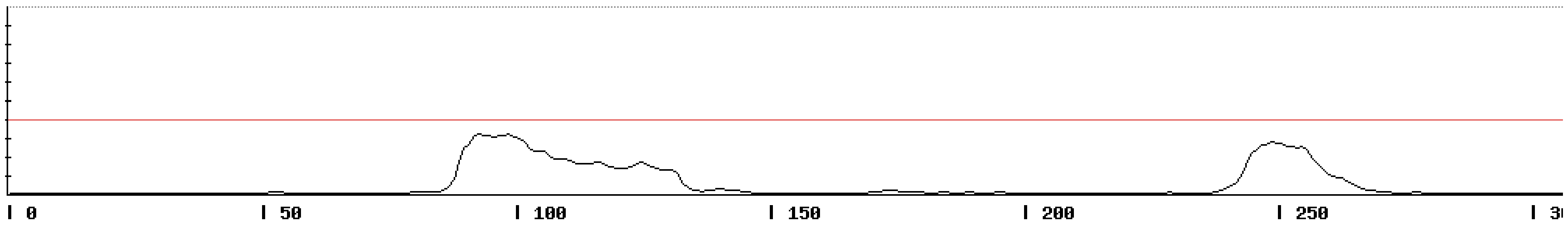

Predictions for Brassicanapus_GSBRNA2T00145796001	
Viterbi Path	
Viterbi Path	
Posterior @ 0.4	98 - ATGKDRKIKSGK - 109
Posterior @ 0.4	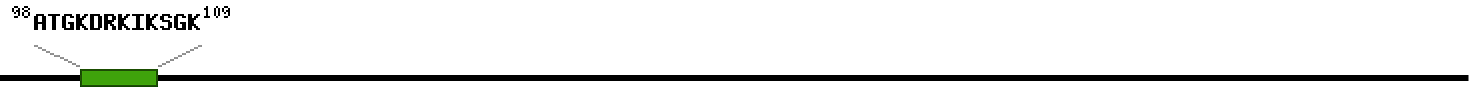

Posterior Graphic. Threshold Marked @ 0.4

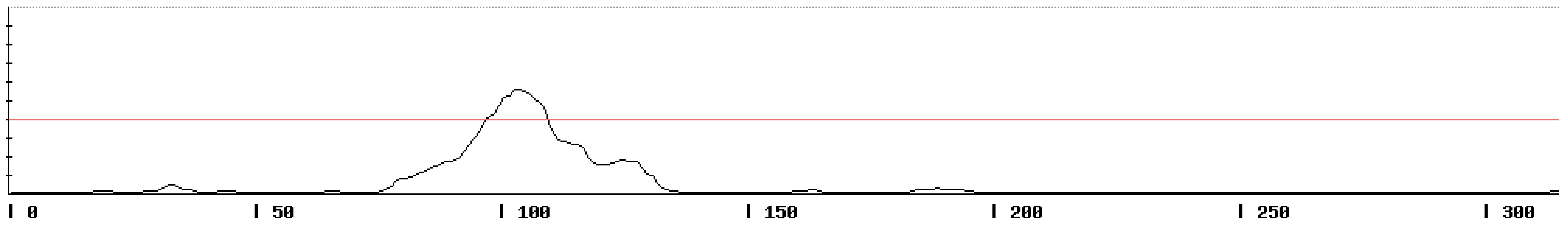

Predictions for Brassicanapus_GSBRNA2T00146022001

Viterbi Path

155 - KKAPKKKSPKK - 165

316 - KGSGKAKAKSENVVGKKRGRPSKEKQQQQQPAKKARKKKQK - 356

Viterbi Path

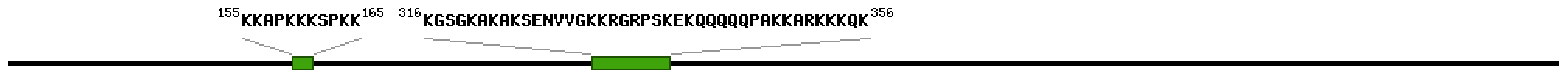

Posterior @ 0.4

153 - AVKKAPKKKSPKKN - 166

316 - KGSGKAKAKSENVVGKKRGRPSKEKQQQQQPAKKARKKKQKQVR - 359

Posterior @ 0.4

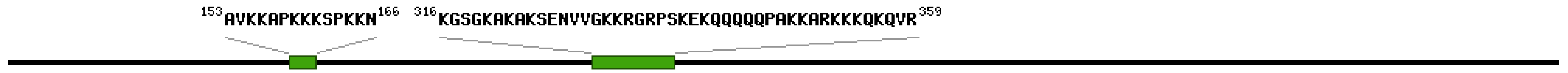

Posterior Graphic. Threshold Marked @ 0.4

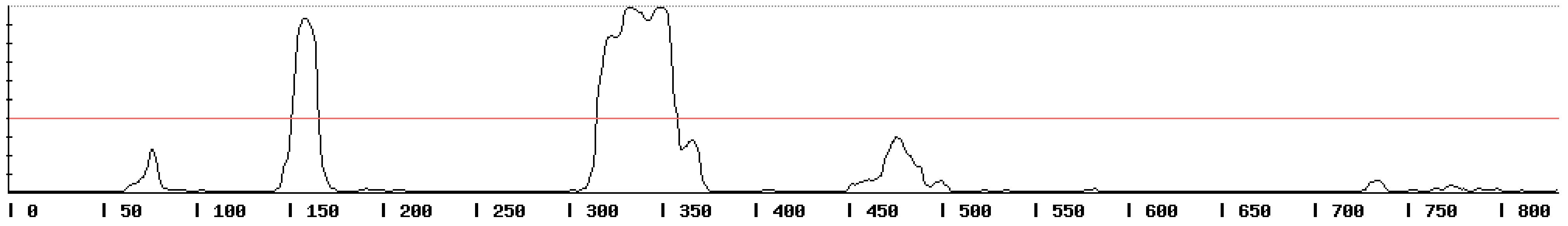

Predictions for Brassicanapus_GSBRNA2T00146186001

Viterbi Path

Viterbi Path

Posterior @ 0.4

Posterior @ 0.4

Posterior Graphic. Threshold Marked @ 0.4

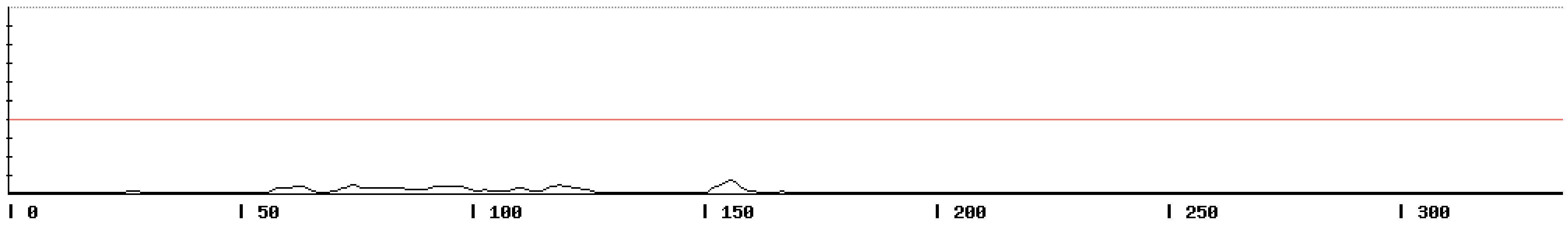

Predictions for Brassicanapus_GSBRNA2T00146341001

Viterbi Path

Viterbi Path

Posterior @ 0.4

Posterior @ 0.4

Posterior Graphic. Threshold Marked @ 0.4

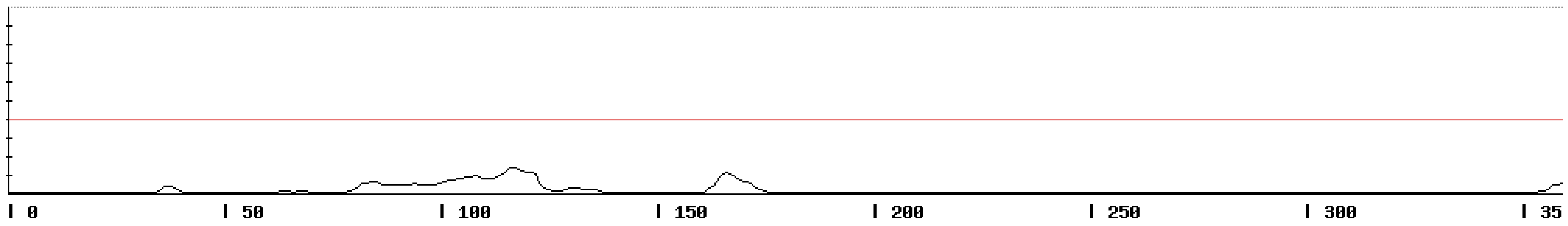

Predictions for Brassicanapus_GSBRNA2T00146607001

Viterbi Path

Viterbi Path

Posterior @ 0.4

Posterior @ 0.4

Posterior Graphic. Threshold Marked @ 0.4

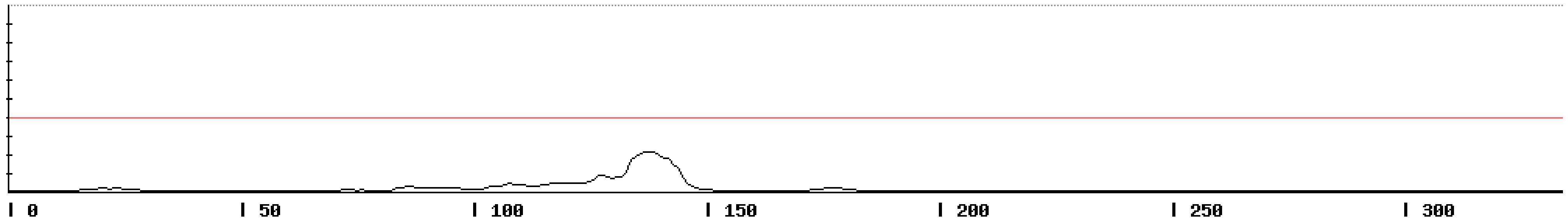

Predictions for Brassicanapus_GSBRNA2T00146726001

Viterbi Path

Viterbi Path

Posterior @ 0.4

Posterior @ 0.4

Posterior Graphic. Threshold Marked @ 0.4

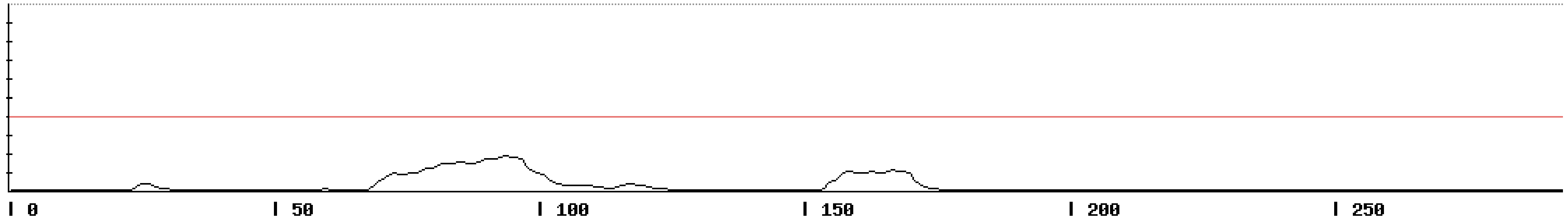

Predictions for Brassicanapus_GSBRNA2T00147549001

Viterbi Path

Viterbi Path

Posterior @ 0.4

Posterior @ 0.4

Posterior Graphic. Threshold Marked @ 0.4

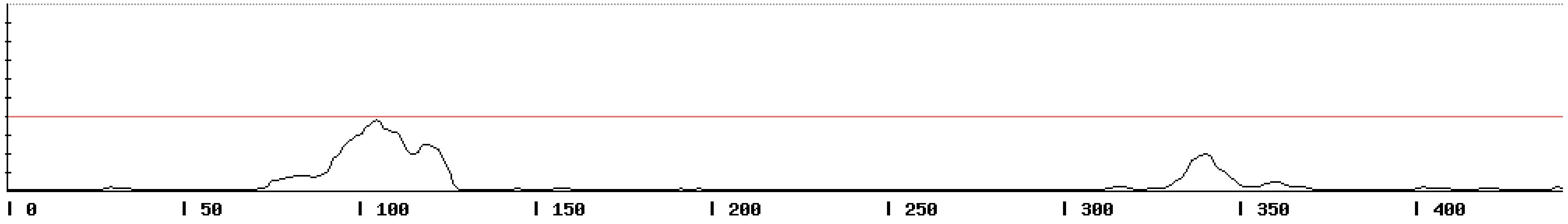

Predictions for Brassicanapus_GSBRNA2T00147768001

Viterbi Path

Viterbi Path

Posterior @ 0.4

Posterior @ 0.4

Posterior Graphic. Threshold Marked @ 0.4

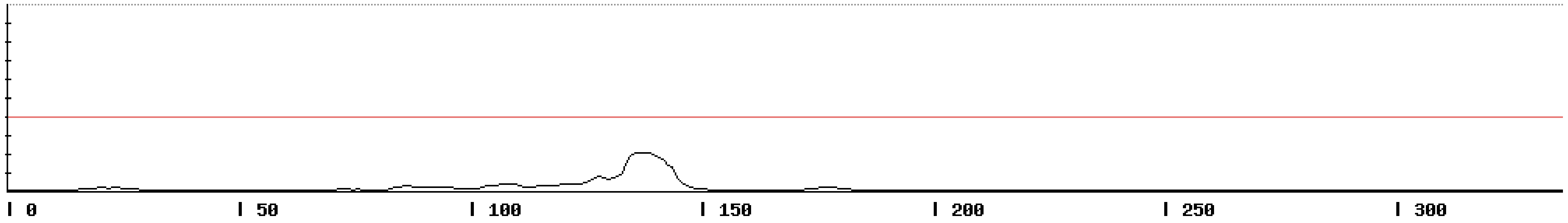

Predictions for Brassicanapus_GSBRNA2T00148111001

Viterbi Path

Viterbi Path

Posterior @ 0.4

Posterior @ 0.4

Posterior Graphic. Threshold Marked @ 0.4

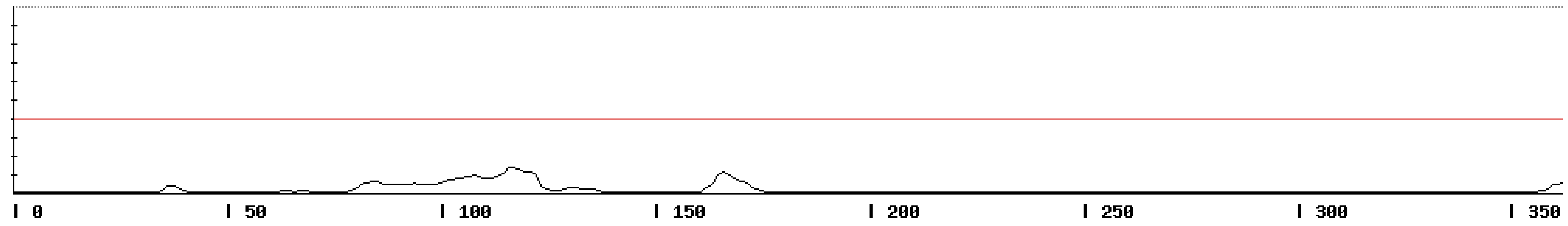

Predictions for Brassicanapus_GSBRNA2T00148321001

Viterbi Path

Viterbi Path

Posterior @ 0.4

Posterior @ 0.4

Posterior Graphic. Threshold Marked @ 0.4

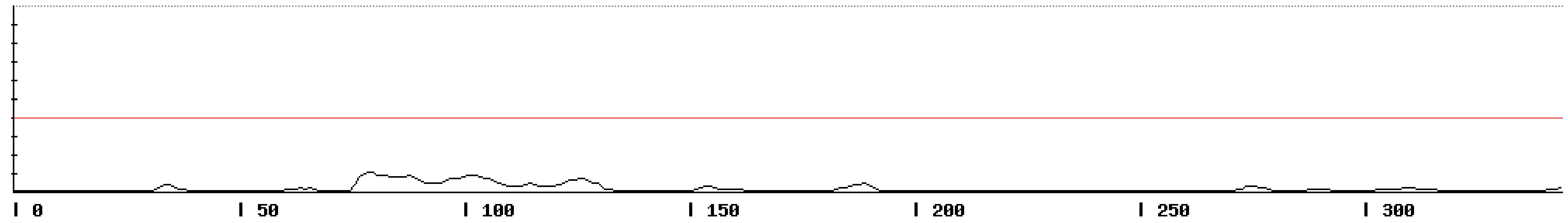

Predictions for Brassicanapus_GSBRNA2T00148862001

Viterbi Path	
Viterbi Path	
Posterior @ 0.4	
Posterior @ 0.4	
Posterior Graphic. Threshold Marked @ 0.4	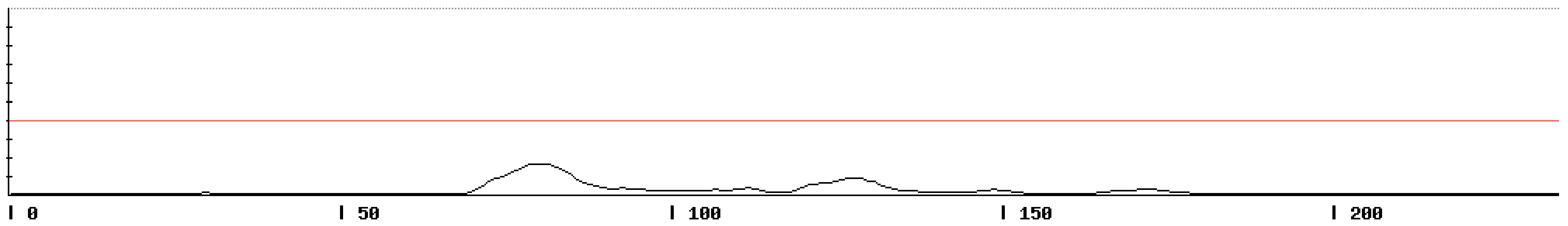

Predictions for Brassicanapus_GSBRNA2T00148867001

Viterbi Path	
Viterbi Path	
Posterior @ 0.4	
Posterior @ 0.4	
Posterior Graphic. Threshold Marked @ 0.4	

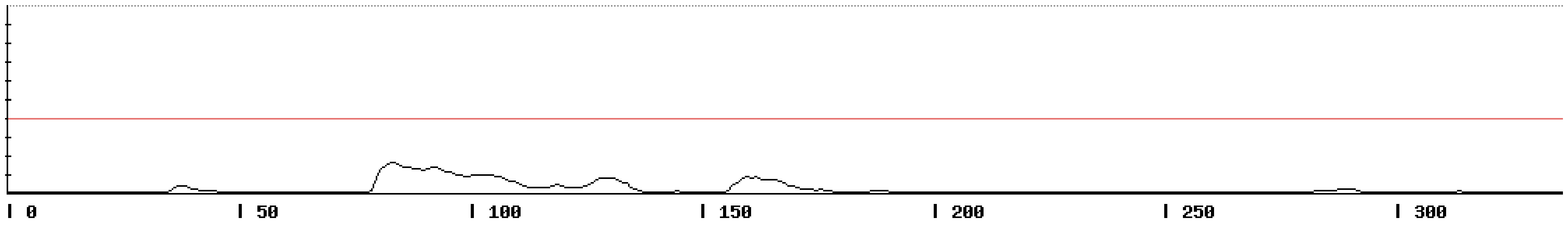

Predictions for Brassicanapus_GSBRNA2T00149455001

Viterbi Path	
Viterbi Path	
Posterior @ 0.4	
Posterior @ 0.4	
Posterior Graphic. Threshold Marked @ 0.4	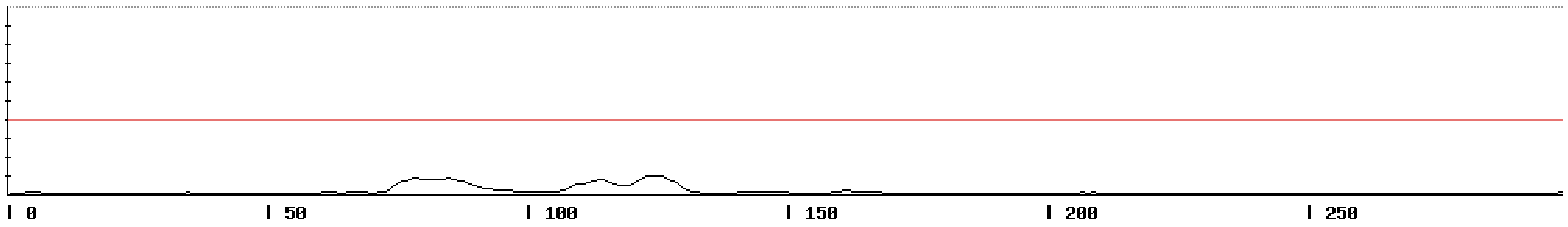

Predictions for Brassicanapus_GSBRNA2T00150485001

Viterbi Path	
Viterbi Path	
Posterior @ 0.4	100 - RKRGNREKIGE - 110
Posterior @ 0.4	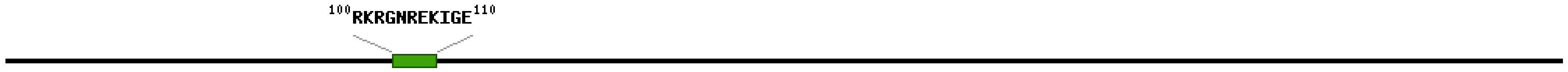

Posterior Graphic. Threshold Marked @ 0.4

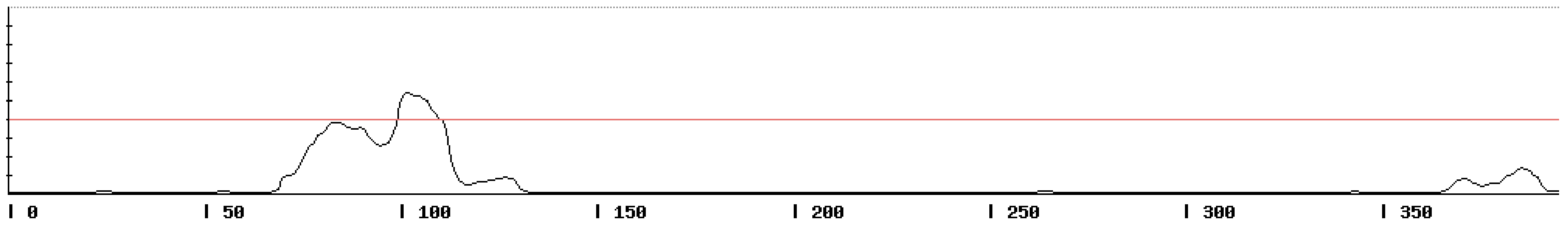

Predictions for Brassicanapus_GSBRNA2T00150489001

Viterbi Path

Viterbi Path

Posterior @ 0.4

78 - SRKTK - 82

Posterior @ 0.4

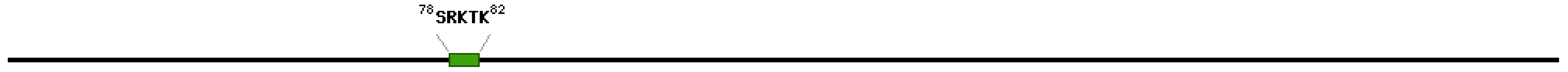

Posterior Graphic. Threshold Marked @ 0.4

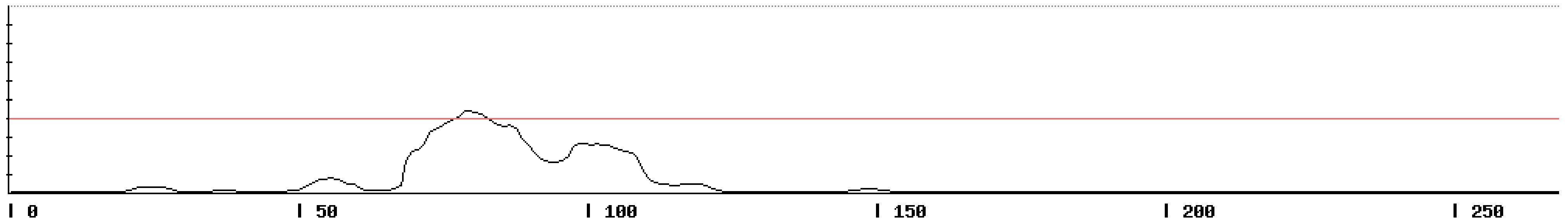

Predictions for Brassicanapus_GSBRNA2T00150491001

Viterbi Path

99 - RKKGDHEKIGEKRVFVFKYSSKIPGGSKSKSKSKSKSKSKSKSK - 142

Viterbi Path

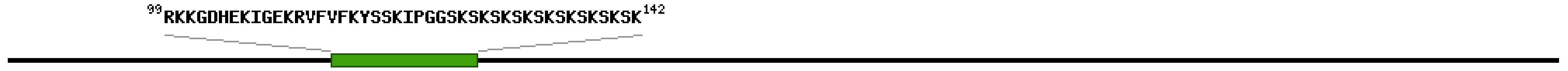

Posterior @ 0.4

99 - RKKGDHEKIGEKRVFVFKYSSKIPGGSKSKSKSKSKSKSKSKSK - 142

Posterior @ 0.4

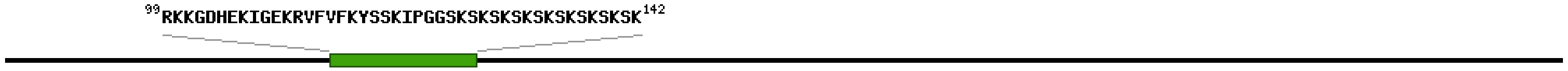

Posterior Graphic. Threshold Marked @ 0.4

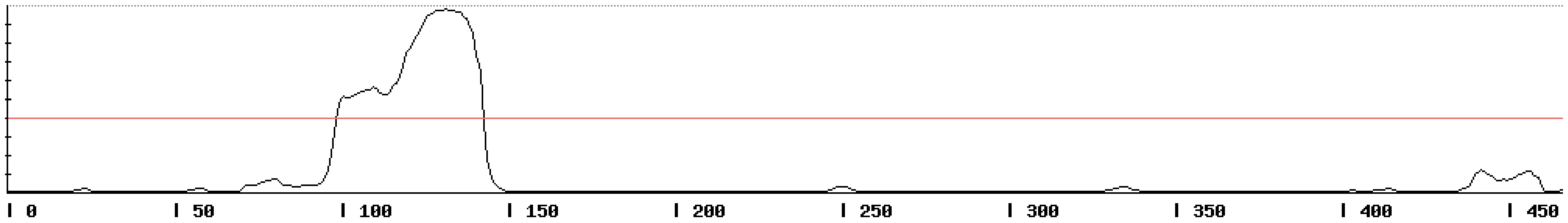

Predictions for Brassicanapus_GSBRNA2T00150492001

Viterbi Path	68 - GRKEKRYNRGERQIRKTKSGFWKKTGVTMDIKRKRSAHREKIGEKR - 113
Viterbi Path	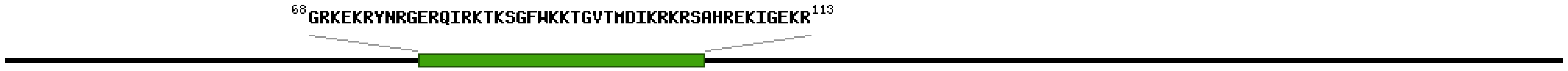
Posterior @ 0.4	68 - GRKEKRYNRGERQIRKTKSGFWKKTGVTMDIKRKRSAHREKIGEKR - 113
Posterior @ 0.4	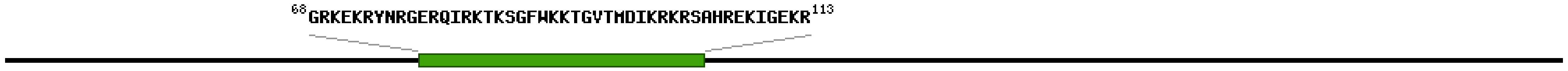
Posterior Graphic. Threshold Marked @ 0.4	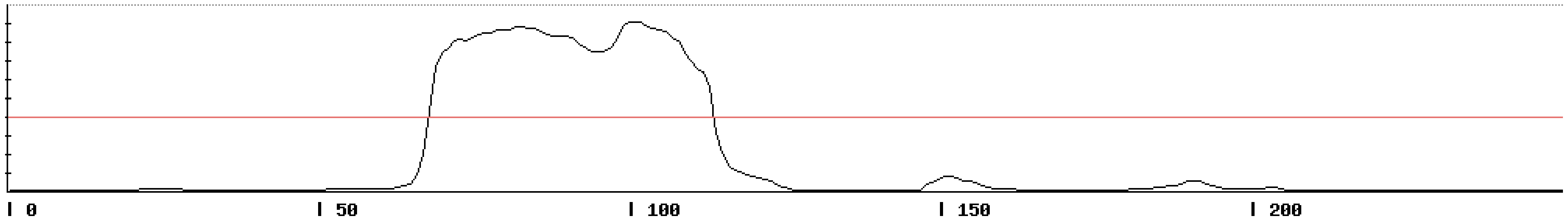

Predictions for Brassicanapus_GSBRNA2T00150493001

Viterbi Path	
Viterbi Path	

Posterior @ 0.4 | 70 - DNKYNRGERQSRKTKSGSWKKTGVTTEIIRKRGNREKIGEKR - 111

Posterior @ 0.4

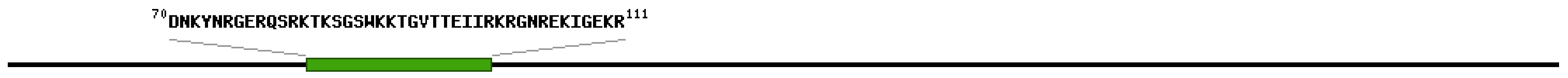

Posterior Graphic. Threshold Marked @ 0.4

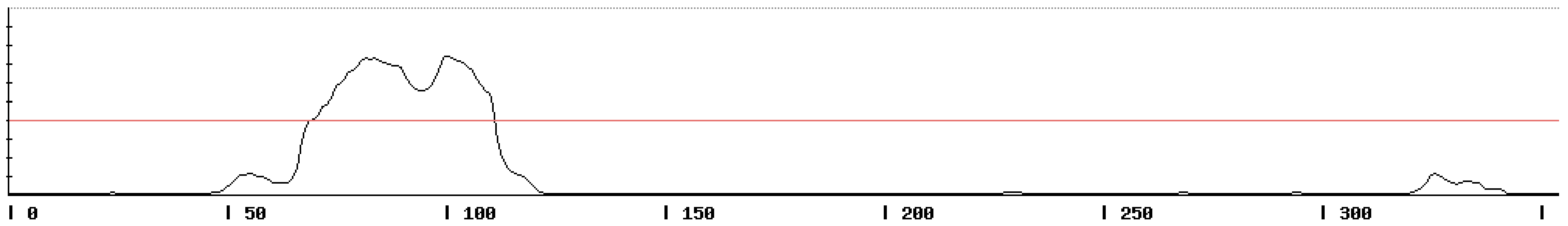

Predictions for Brassicanapus_GSBRNA2T00150494001

Viterbi Path

Viterbi Path

Posterior @ 0.4 | 68 - GKLNRRKRASKT - 79

Posterior @ 0.4

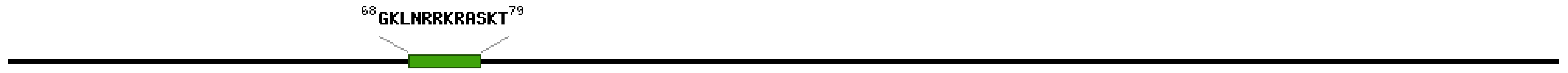

Posterior Graphic. Threshold Marked @ 0.4

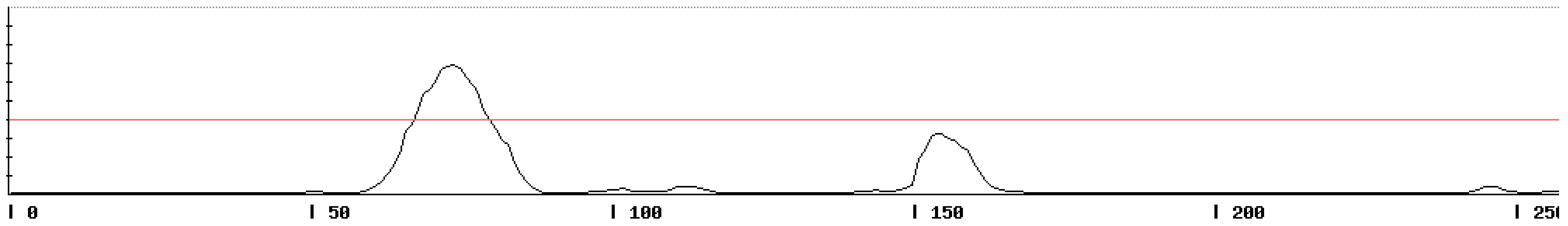

Predictions for Brassicanapus_GSBRNA2T00150497001

Viterbi Path

Viterbi Path

Posterior @ 0.4

Posterior @ 0.4

Posterior Graphic. Threshold Marked @ 0.4

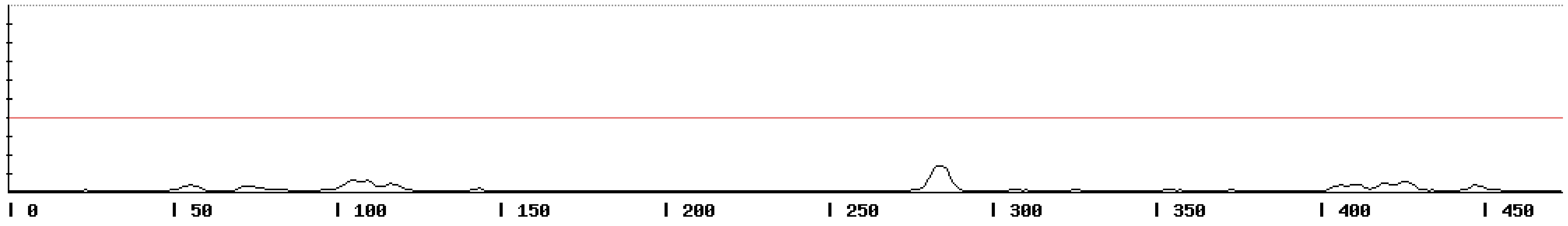

Predictions for Brassicanapus_GSBRNA2T00150558001

Viterbi Path

Viterbi Path

Posterior @ 0.4

Posterior @ 0.4

Posterior Graphic. Threshold Marked @ 0.4

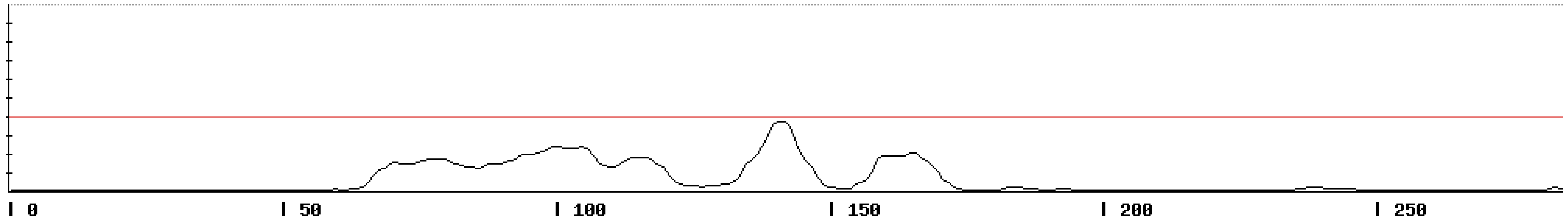

Predictions for Brassicanapus_GSBRNA2T00150656001

Viterbi Path	
Viterbi Path	
Posterior @ 0.4	80 - KEDKYNRGGRQSRKKSSGF - 98<br>121 - K - 121
Posterior @ 0.4	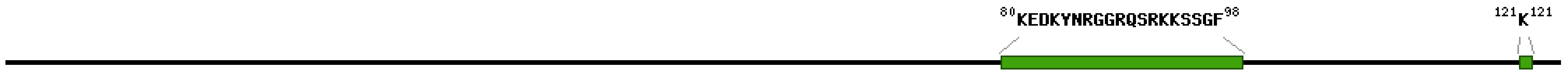
Posterior Graphic. Threshold Marked @ 0.4	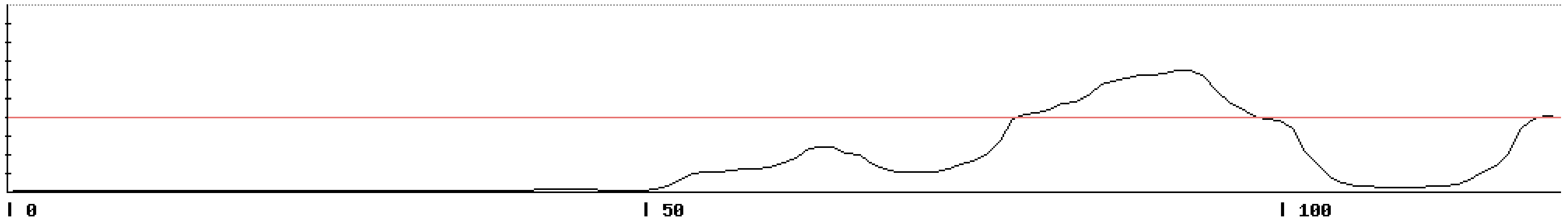

Predictions for Brassicanapus_GSBRNA2T00151041001

Viterbi Path	
Viterbi Path	
Posterior @ 0.4	
Posterior @ 0.4	
Posterior Graphic. Threshold Marked @ 0.4	

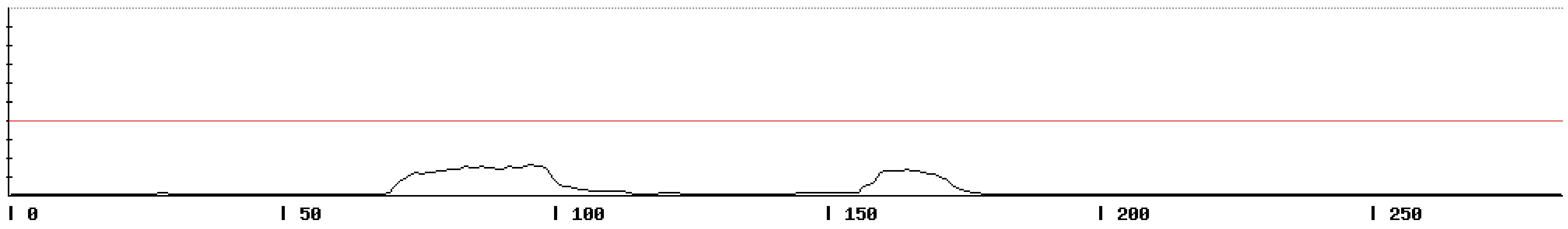

Predictions for Brassicanapus_GSBRNA2T00151449001

Viterbi Path

Viterbi Path

Posterior @ 0.4

Posterior @ 0.4

Posterior Graphic. Threshold Marked @ 0.4

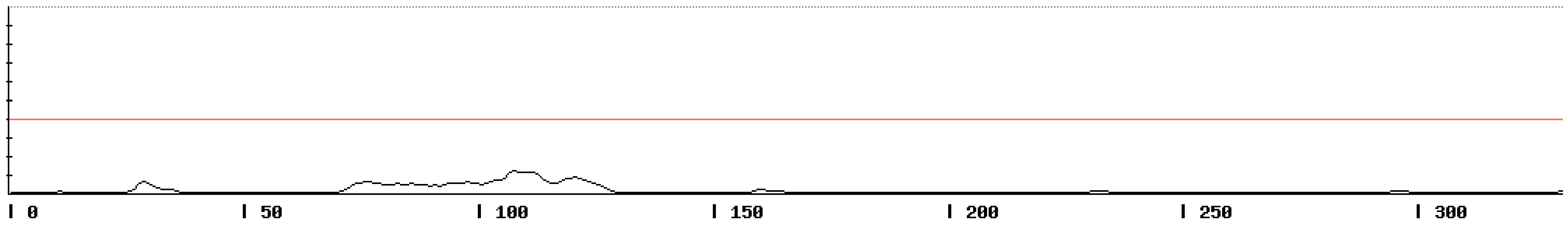

Predictions for Brassicanapus_GSBRNA2T00151934001

Viterbi Path

Viterbi Path

Posterior @ 0.4

Posterior @ 0.4

Posterior Graphic. Threshold Marked @ 0.4

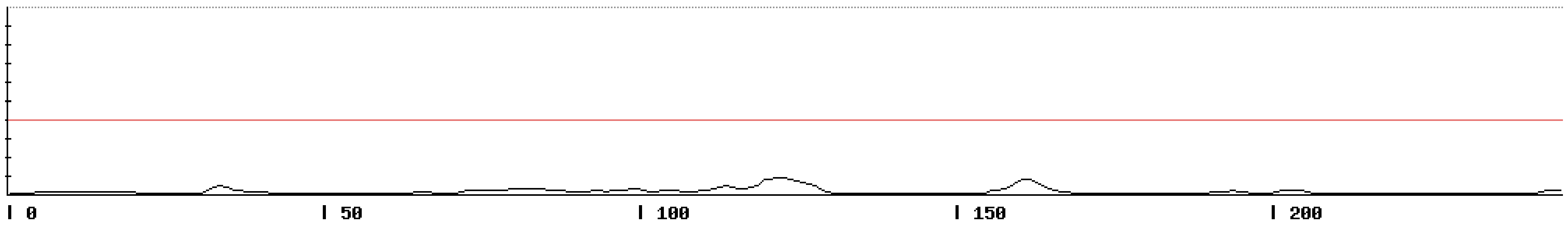

Predictions for Brassicanapus_GSBRNA2T00152277001

Viterbi Path

Viterbi Path

Posterior @ 0.4

Posterior @ 0.4

Posterior Graphic. Threshold Marked @ 0.4

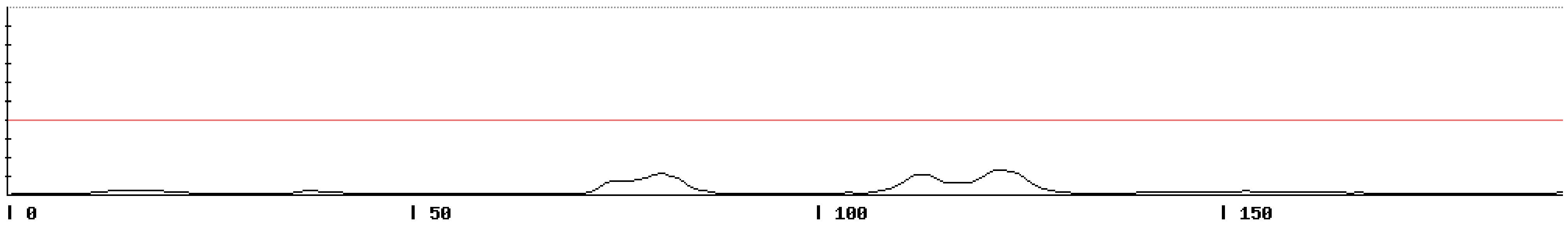

Predictions for Brassicanapus_GSBRNA2T00152426001

Viterbi Path

Viterbi Path

Posterior @ 0.4

Posterior @ 0.4

Posterior Graphic. Threshold Marked @ 0.4	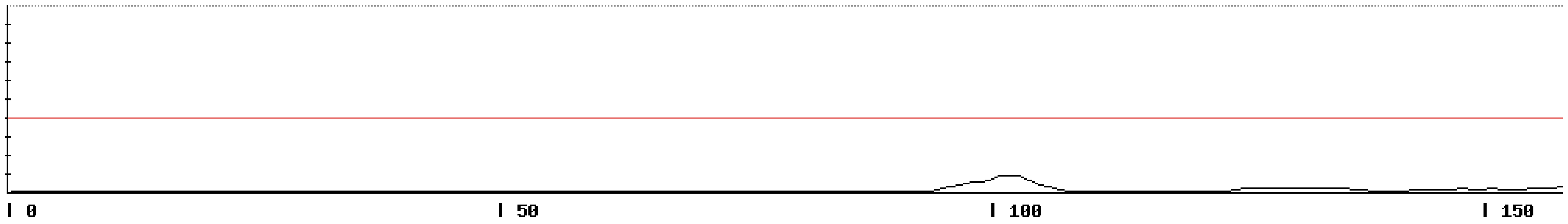

Predictions for Brassicanapus_GSBRNA2T00153359001

Viterbi Path	
Viterbi Path	
Posterior @ 0.4	
Posterior @ 0.4	
Posterior Graphic. Threshold Marked @ 0.4	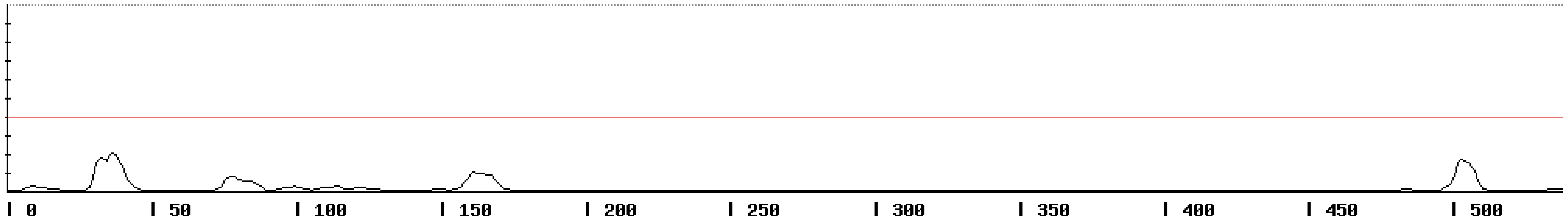

Predictions for Brassicanapus_GSBRNA2T00153449001

Viterbi Path	
Viterbi Path	

Posterior @ 0.4	
Posterior @ 0.4	
Posterior Graphic. Threshold Marked @ 0.4	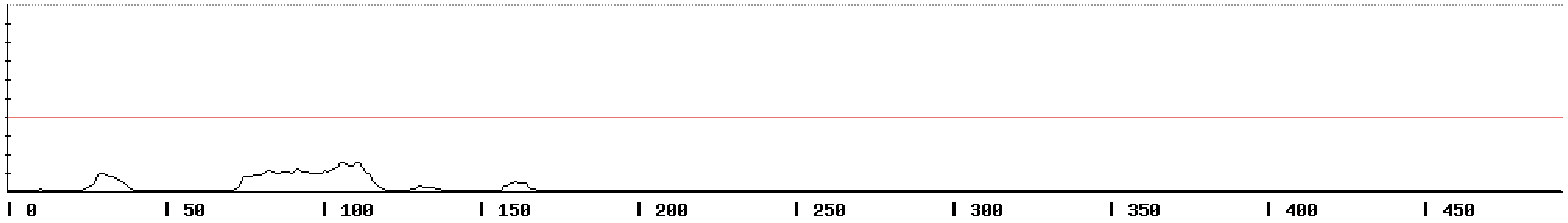

Predictions for Brassicanapus_GSBRNA2T00153576001

Viterbi Path	
Viterbi Path	
Posterior @ 0.4	510 - KSARKGSKPEK - 520
Posterior @ 0.4	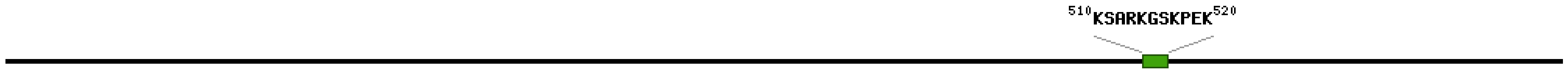
Posterior Graphic. Threshold Marked @ 0.4	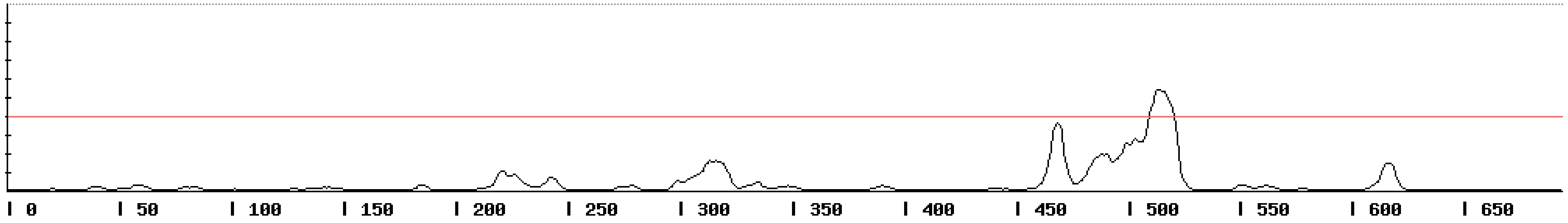

Predictions for Brassicanapus_GSBRNA2T00153852001

Viterbi Path	
Viterbi Path	

Posterior @ 0.4

Posterior @ 0.4

Posterior Graphic. Threshold Marked @ 0.4

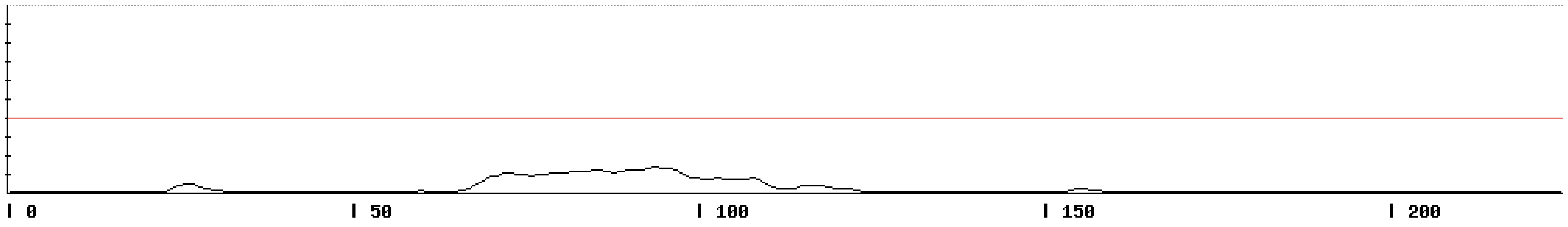

Predictions for Brassicanapus_GSBRNA2T00154092001

Viterbi Path

Viterbi Path

Posterior @ 0.4

Posterior @ 0.4

Posterior Graphic. Threshold Marked @ 0.4

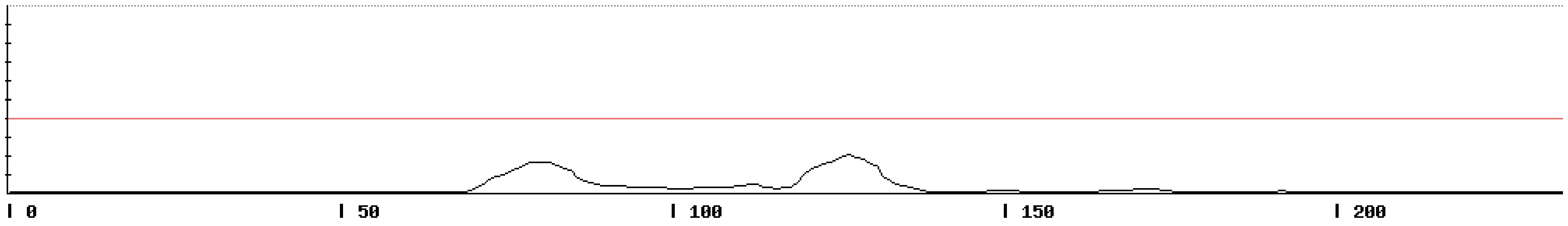

Predictions for Brassicanapus_GSBRNA2T00154116001

Viterbi Path

Viterbi Path

Posterior @ 0.4

Posterior @ 0.4

Posterior Graphic. Threshold Marked @ 0.4

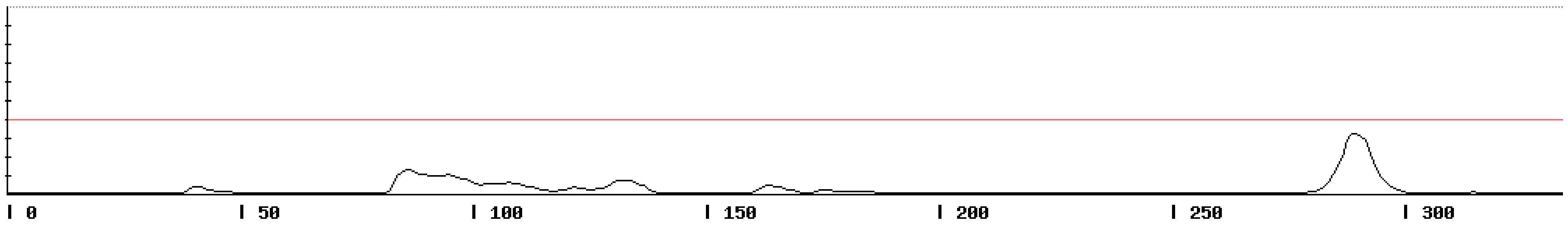

Predictions for Brassicanapus_GSBRNA2T00154660001

Viterbi Path

Viterbi Path

Posterior @ 0.4

Posterior @ 0.4

Posterior Graphic. Threshold Marked @ 0.4

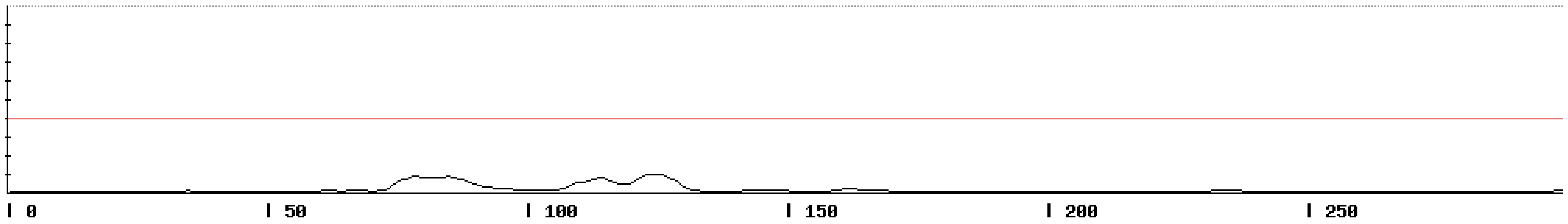

Predictions for Brassicanapus_GSBRNA2T00154717001	
Viterbi Path	
Viterbi Path	
Posterior @ 0.4	137 - RKRRK - 141
Posterior @ 0.4	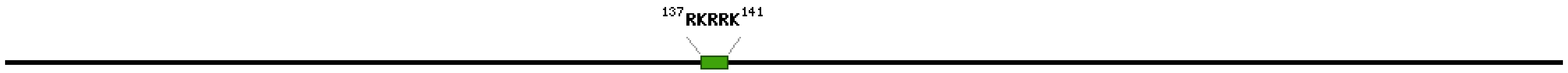
Posterior Graphic. Threshold Marked @ 0.4	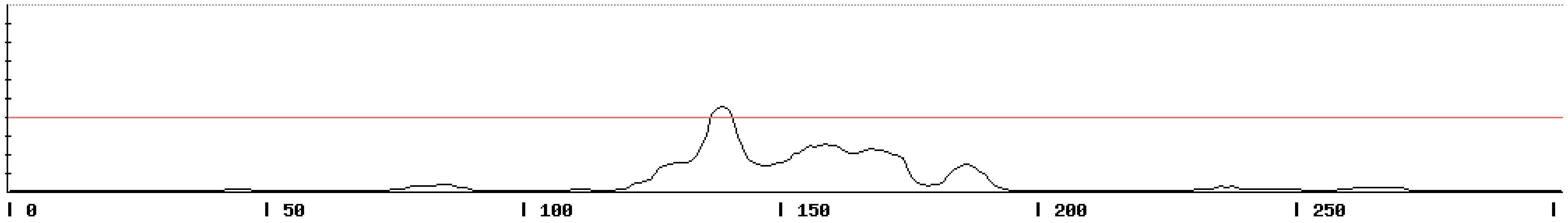

Predictions for Brassicanapus_GSBRNA2T00154720001	
Viterbi Path	
Viterbi Path	
Posterior @ 0.4	
Posterior @ 0.4	
Posterior Graphic. Threshold Marked @ 0.4	

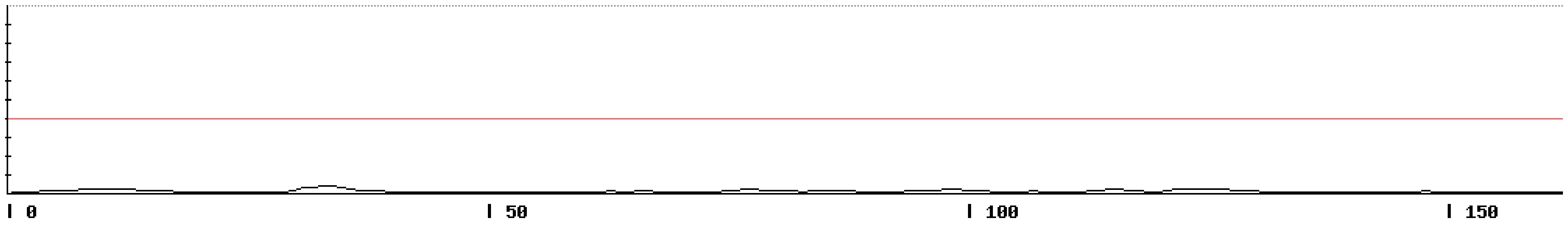

Predictions for Brassicanapus_GSBRNA2T00155943001

Viterbi Path	
Viterbi Path	
Posterior @ 0.4	335 - KKREKKK - 341
Posterior @ 0.4	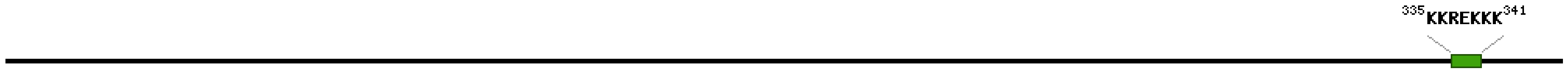
Posterior Graphic. Threshold Marked @ 0.4	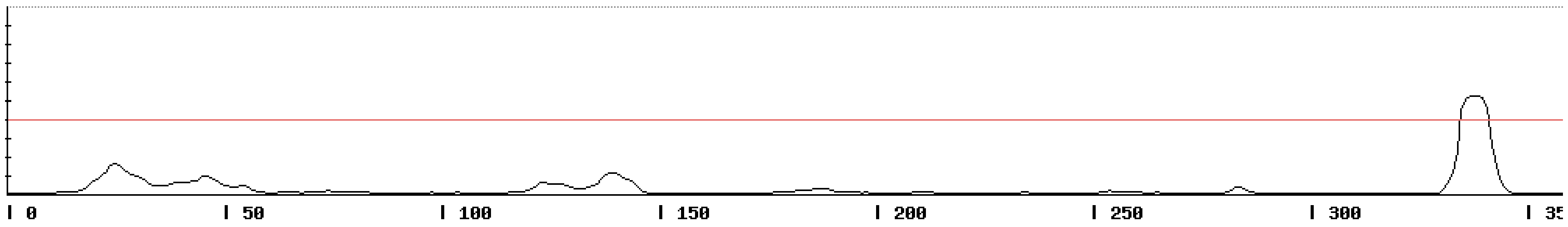

Predictions for Brassicanapus_GSBRNA2T00156429001

Viterbi Path	
Viterbi Path	
Posterior @ 0.4	
Posterior @ 0.4	

Posterior Graphic. Threshold Marked @ 0.4

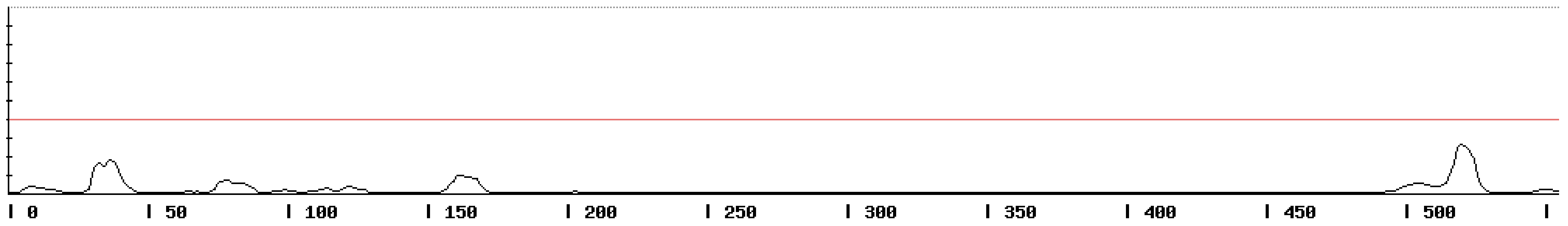

Predictions for Brassicanapus_GSBRNA2T00156597001

Viterbi Path	
Viterbi Path	
Posterior @ 0.4	
Posterior @ 0.4	

Posterior Graphic. Threshold Marked @ 0.4

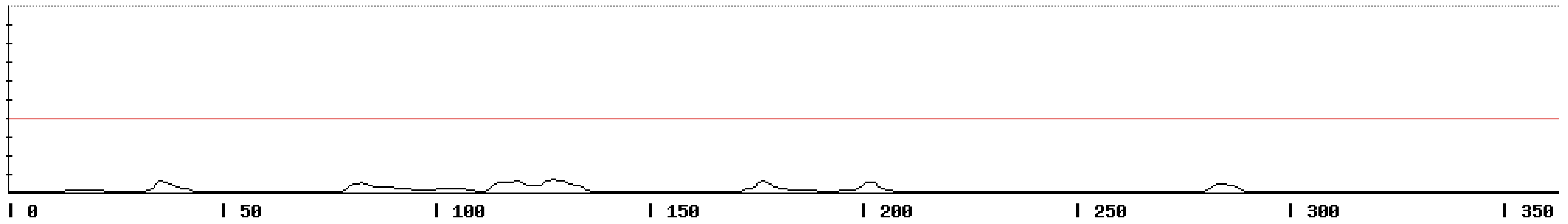

Predictions for Brassicanapus_GSBRNA2T00157940001

Viterbi Path	
Viterbi Path	
Posterior @ 0.4	154 - TRKRRKVR - 161
Posterior @ 0.4	

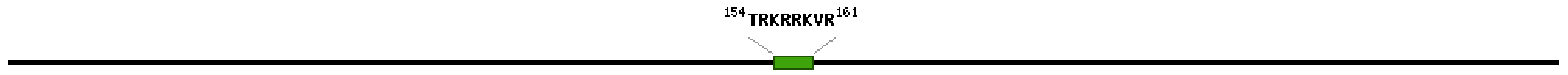

Posterior Graphic. Threshold Marked @ 0.4

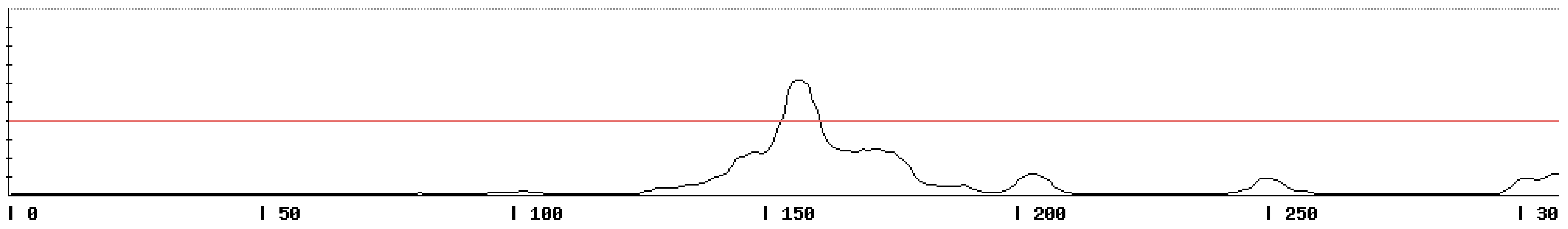

Viterbi Path

Posterior @ 0.4

Posterior @ 0.4

Posterior Graphic. Threshold Marked @ 0.4

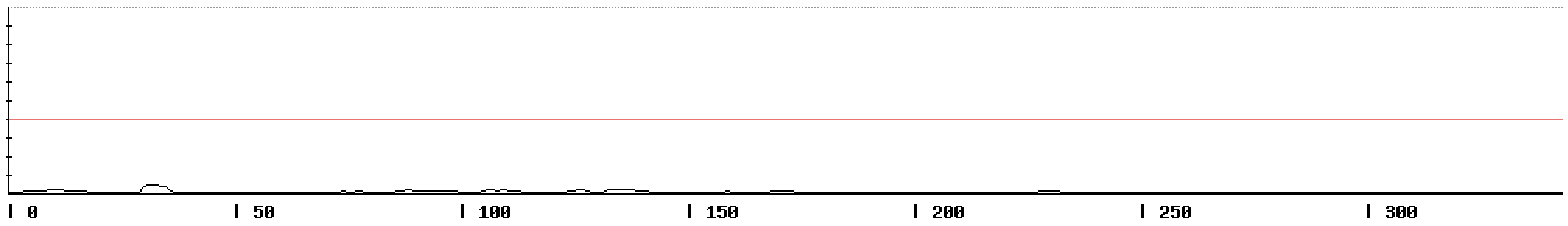

Predictions for Brassicaoleracea_XP_013583708.1

Viterbi Path

Viterbi Path

Posterior @ 0.4

Posterior @ 0.4

Posterior Graphic. Threshold Marked @ 0.4

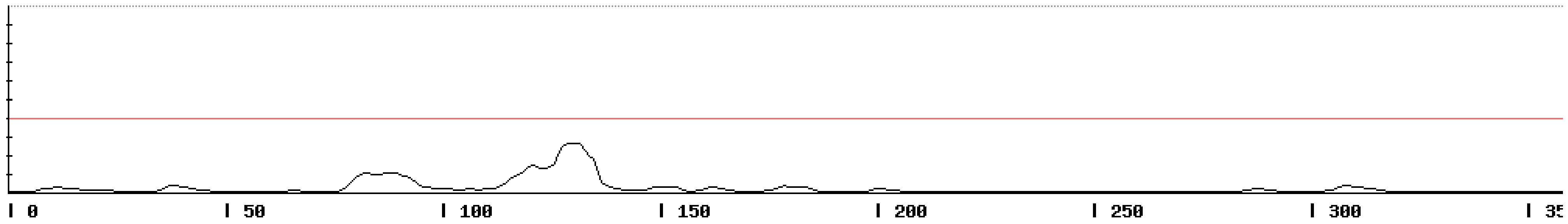

Predictions for Brassicaoleracea_XP_013584205.1

Viterbi Path	
Viterbi Path	
Posterior @ 0.4	
Posterior @ 0.4	
Posterior Graphic. Threshold Marked @ 0.4	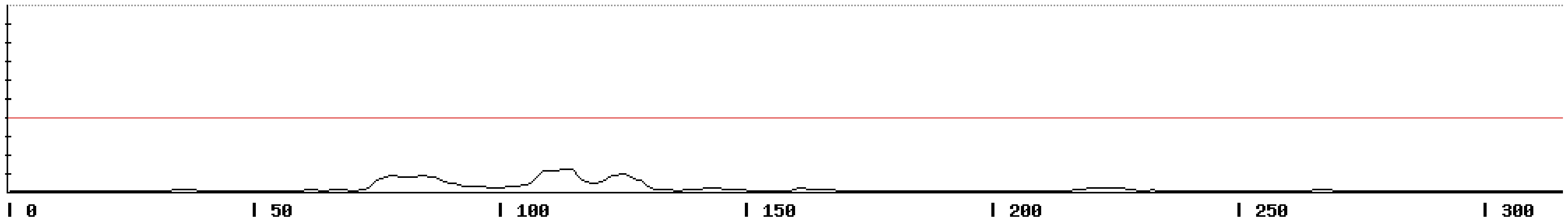

Predictions for Brassicaoleracea_XP_013584293.1

Viterbi Path	
Viterbi Path	
Posterior @ 0.4	
Posterior @ 0.4	
Posterior Graphic. Threshold Marked @ 0.4	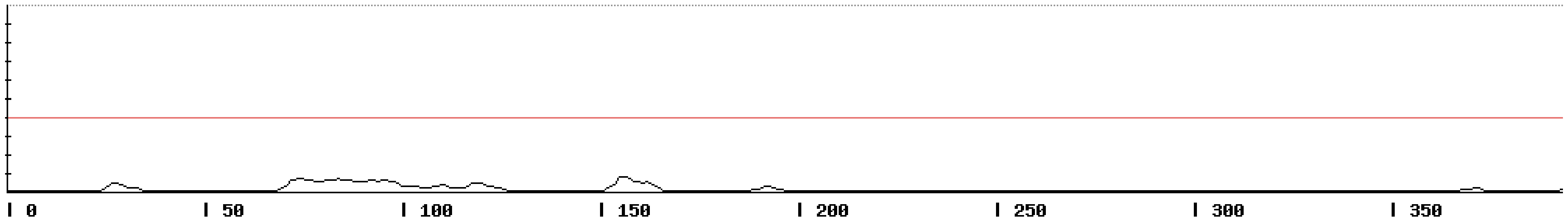

Predictions for Brassicaoleracea_XP_013584294.1	
Viterbi Path	
Viterbi Path	
Posterior @ 0.4	
Posterior @ 0.4	
Posterior Graphic. Threshold Marked @ 0.4	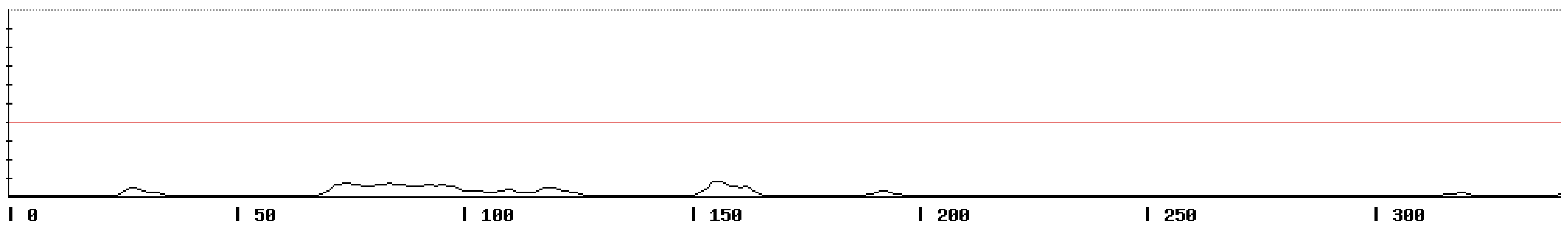

Predictions for Brassicaoleracea_XP_013584372.1	
Viterbi Path	
Viterbi Path	
Posterior @ 0.4	
Posterior @ 0.4	
Posterior Graphic. Threshold Marked @ 0.4	

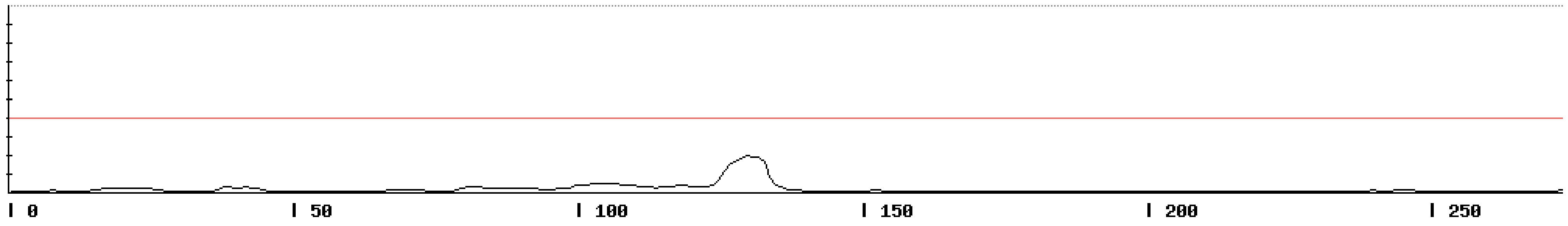

Predictions for Brassicaoleracea_XP_013584744.1

Viterbi Path

Viterbi Path

Posterior @ 0.4

Posterior @ 0.4

Posterior Graphic. Threshold Marked @ 0.4

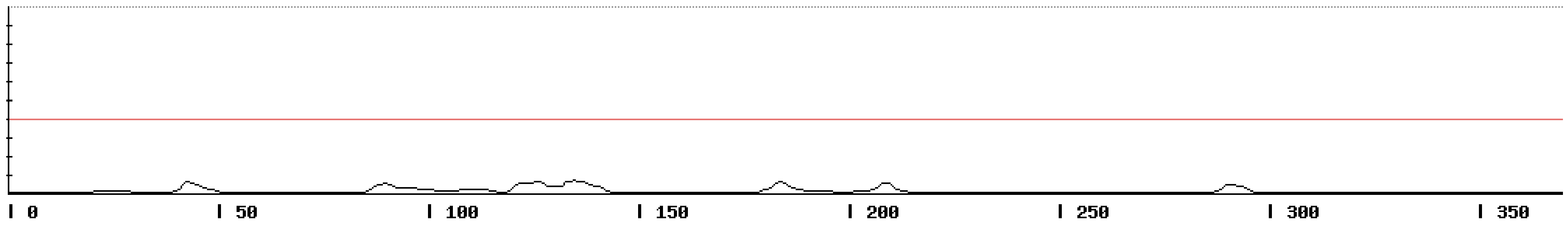

Predictions for Brassicaoleracea_XP_013584829.1

Viterbi Path

Viterbi Path

Posterior @ 0.4

Posterior @ 0.4

Posterior Graphic. Threshold Marked @ 0.4

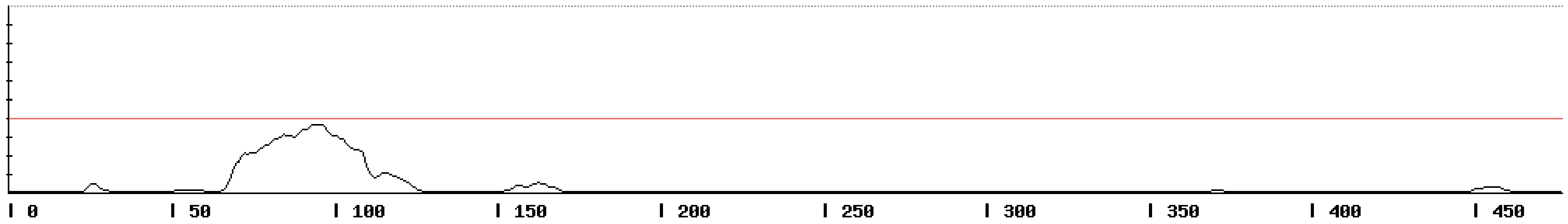

Predictions for Brassicaoleracea_XP_013585182.1

Viterbi Path

Viterbi Path

Posterior @ 0.4

Posterior @ 0.4

Posterior Graphic. Threshold Marked @ 0.4

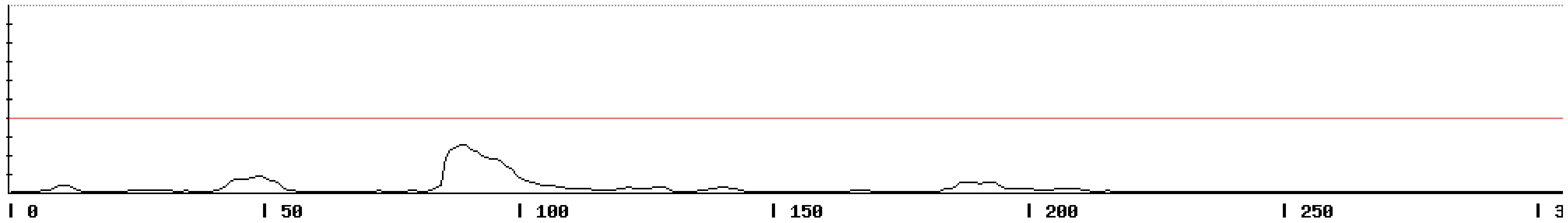

Predictions for Brassicaoleracea_XP_013585218.1

Viterbi Path

Viterbi Path

Posterior @ 0.4

Posterior @ 0.4

Posterior Graphic. Threshold Marked @ 0.4	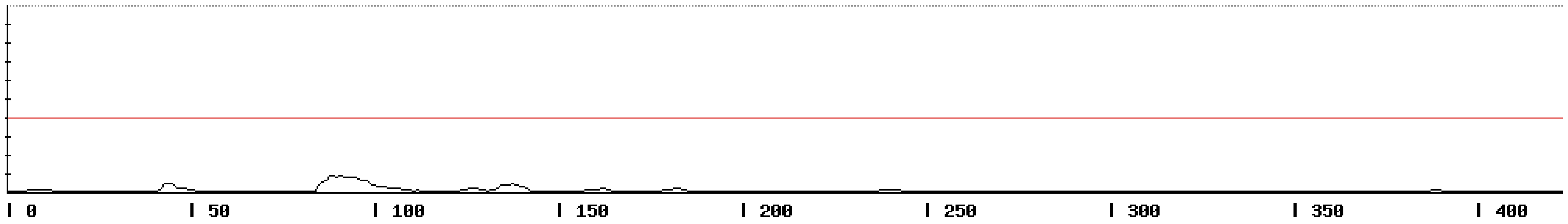

Predictions for Brassicaoleracea_XP_013585238.1

Viterbi Path	
Viterbi Path	
Posterior @ 0.4	
Posterior @ 0.4	
Posterior Graphic. Threshold Marked @ 0.4	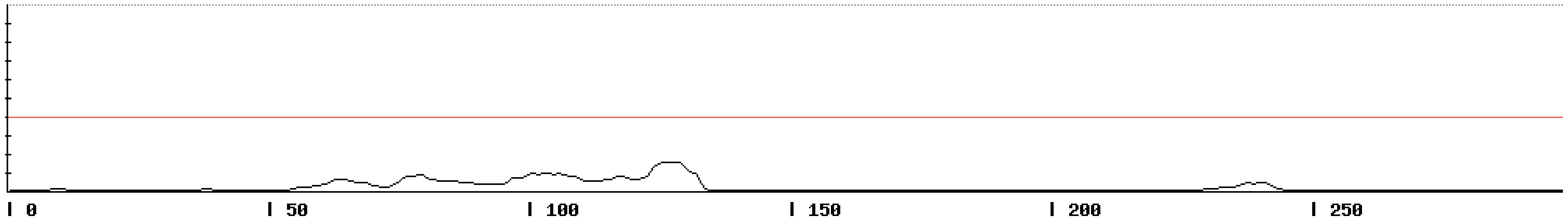

Predictions for Brassicaoleracea_XP_013585389.1

Viterbi Path	
Viterbi Path	

Posterior @ 0.4

Posterior @ 0.4

Posterior Graphic. Threshold Marked @ 0.4

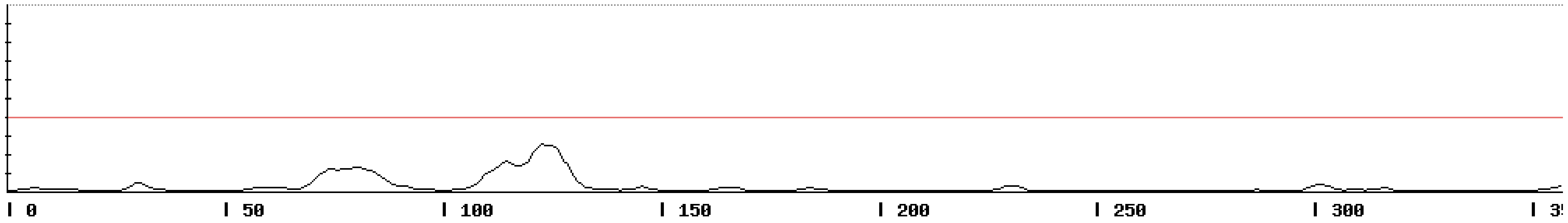

Predictions for Brassicaoleracea_XP_013585390.1

Viterbi Path

Viterbi Path

Posterior @ 0.4

Posterior @ 0.4

Posterior Graphic. Threshold Marked @ 0.4

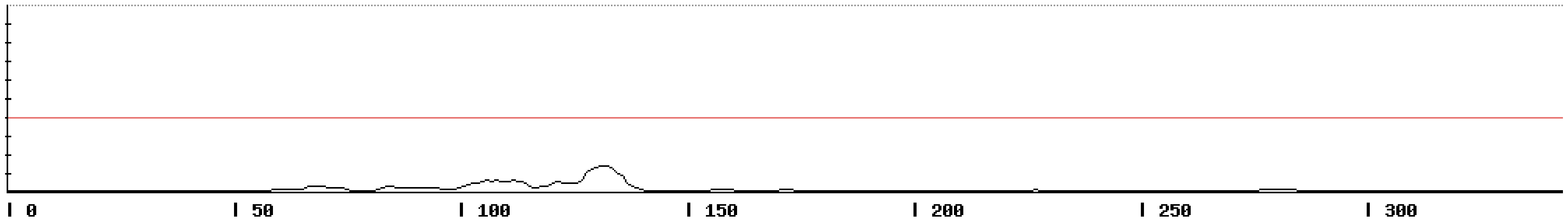

Predictions for Brassicaoleracea_XP_013585394.1

Viterbi Path 68 - GRKEKRYNRGERQIRKTKSGFWKKTGVTMDIKRKRSGHREKIGEKR - 113

Viterbi Path

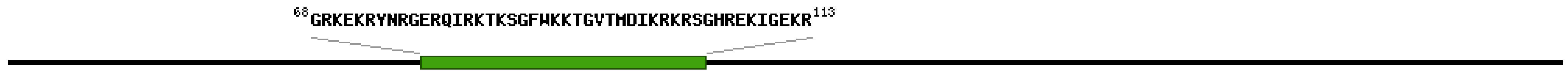

Posterior @ 0.4 | 68 - GRKEKRYNRGERQIRKTKSGFWKKTGVTMDIKRKRSGHREKIGEKR - 113

Posterior @ 0.4

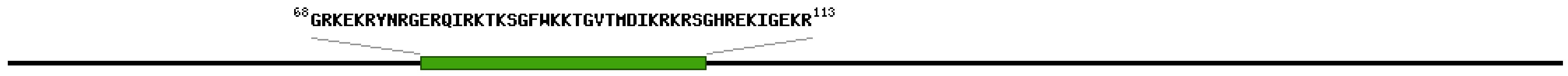

Posterior Graphic. Threshold Marked @ 0.4

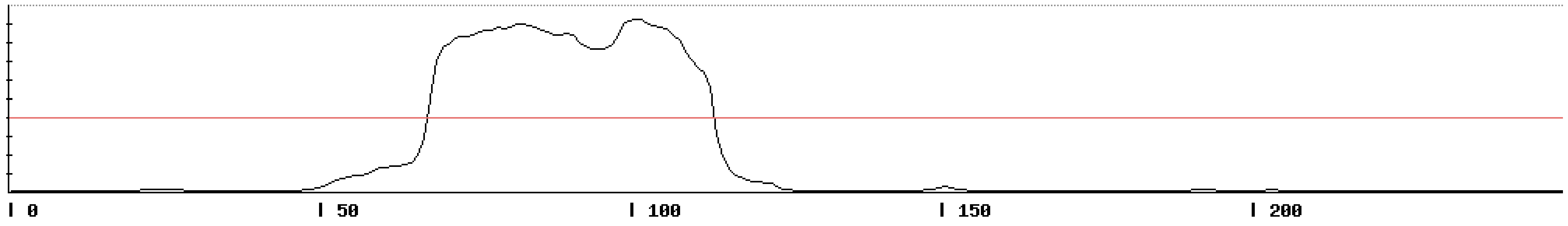

Predictions for Brassicaoleracea_XP_013585836.1

Viterbi Path

Viterbi Path

Posterior @ 0.4

Posterior @ 0.4

Posterior Graphic. Threshold Marked @ 0.4

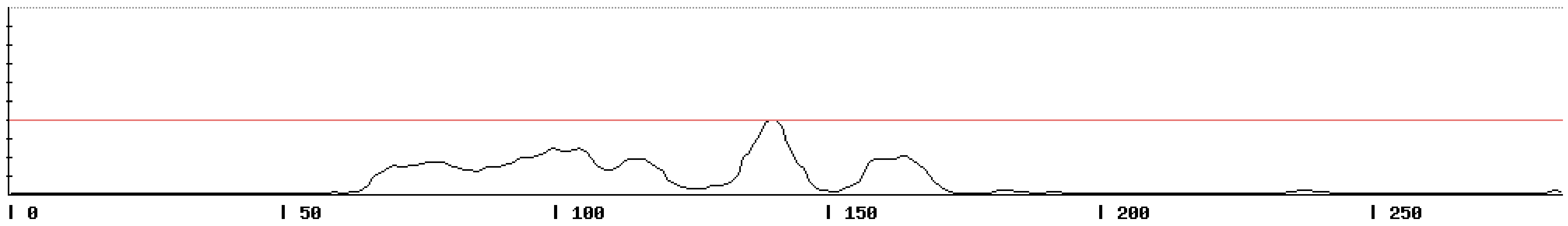

Predictions for Brassicaoleracea_XP_013586611.1

Viterbi Path

Viterbi Path

Posterior @ 0.4

Posterior @ 0.4

Posterior Graphic. Threshold Marked @ 0.4

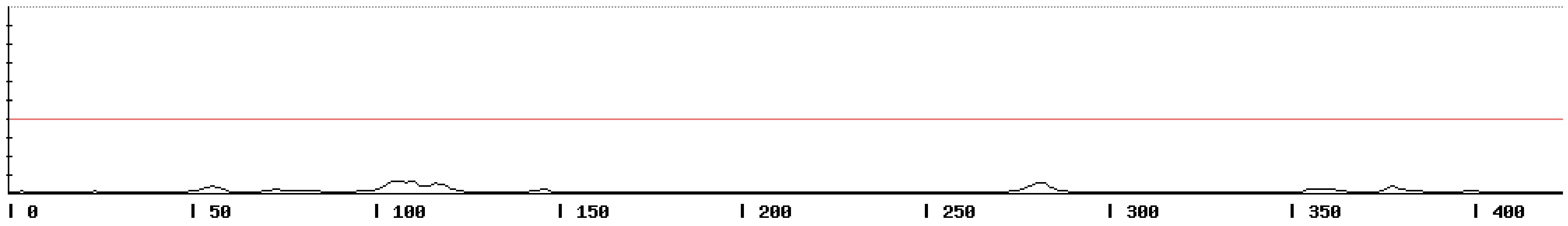

Predictions for Brassicaoleracea_XP_013586612.1

Viterbi Path

Viterbi Path

Posterior @ 0.4

Posterior @ 0.4

Posterior Graphic. Threshold Marked @ 0.4

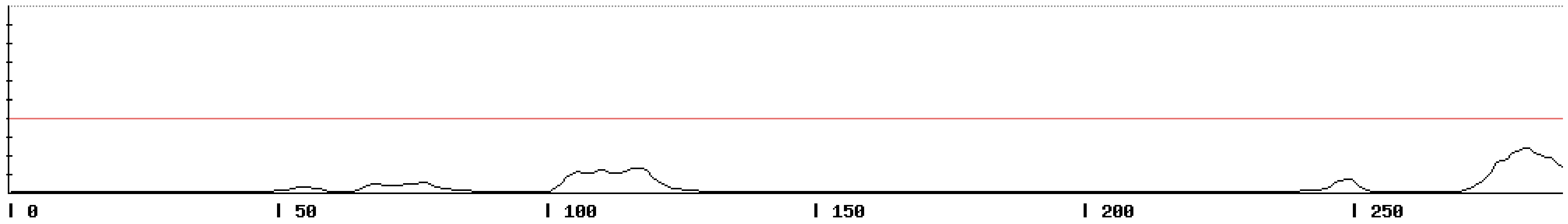

Predictions for Brassicaoleracea_XP_013586773.1	
Viterbi Path	
Viterbi Path	
Posterior @ 0.4	
Posterior @ 0.4	
Posterior Graphic. Threshold Marked @ 0.4	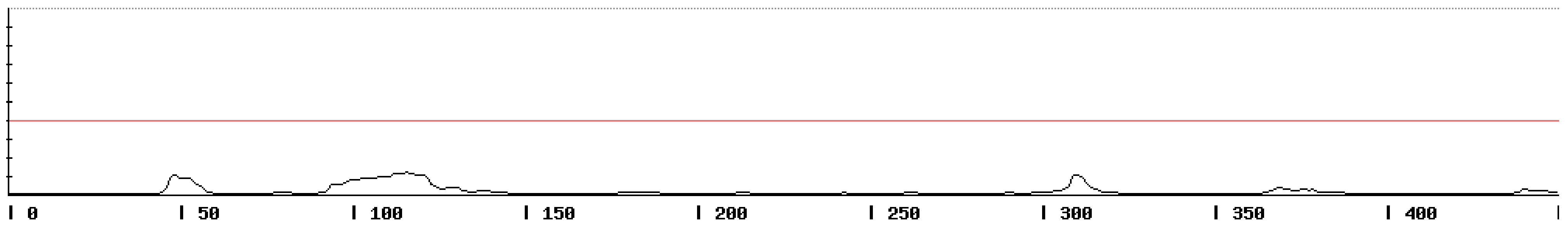

Predictions for Brassicaoleracea_XP_013588383.1	
Viterbi Path	
Viterbi Path	
Posterior @ 0.4	
Posterior @ 0.4	
Posterior Graphic. Threshold Marked @ 0.4	

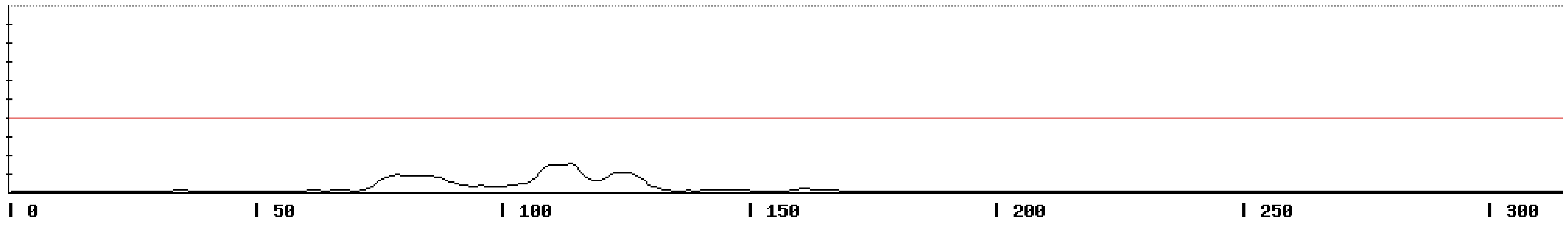

Predictions for Brassicaoleracea_XP_013588625.1	
Viterbi Path	
Viterbi Path	
Posterior @ 0.4	
Posterior @ 0.4	
Posterior Graphic. Threshold Marked @ 0.4	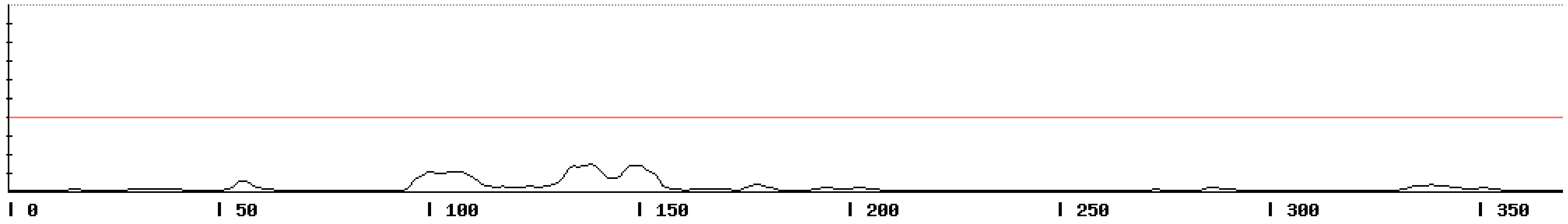

Predictions for Brassicaoleracea_XP_013588626.1	
Viterbi Path	
Viterbi Path	
Posterior @ 0.4	
Posterior @ 0.4	

Posterior Graphic. Threshold Marked @ 0.4

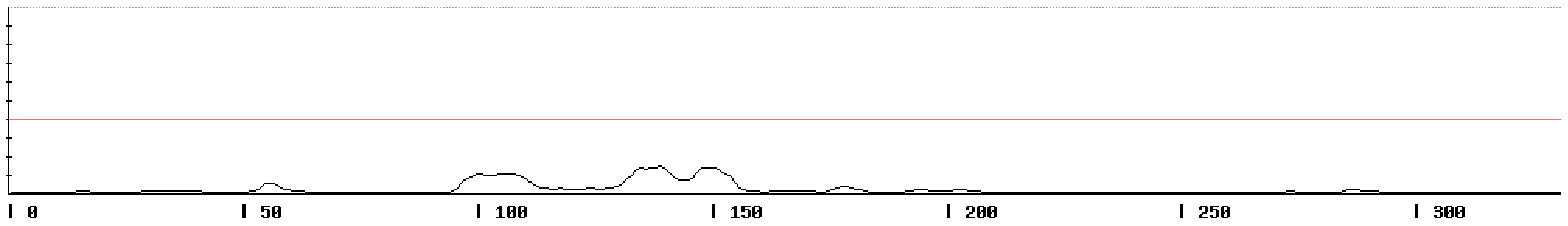

Predictions for Brassicaoleracea_XP_013588851.1

Viterbi Path

Viterbi Path

Posterior @ 0.4

Posterior @ 0.4

Posterior Graphic. Threshold Marked @ 0.4

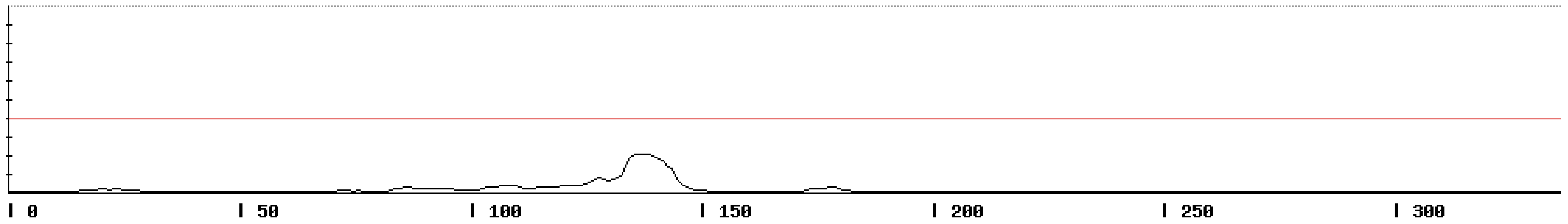

Predictions for Brassicaoleracea_XP_013589211.1

Viterbi Path

Viterbi Path

Posterior @ 0.4

Posterior @ 0.4

Posterior Graphic. Threshold Marked @ 0.4

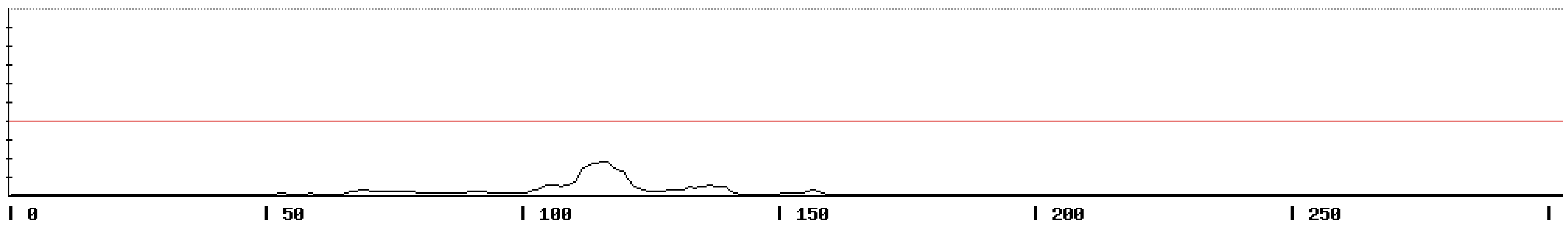

Predictions for Brassicaoleracea_XP_013589227.1

Viterbi Path	
Viterbi Path	
Posterior @ 0.4	118 - KVKGKP - 123
Posterior @ 0.4	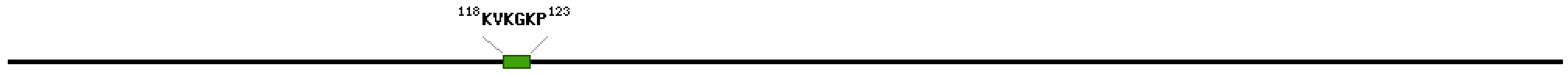
Posterior Graphic. Threshold Marked @ 0.4	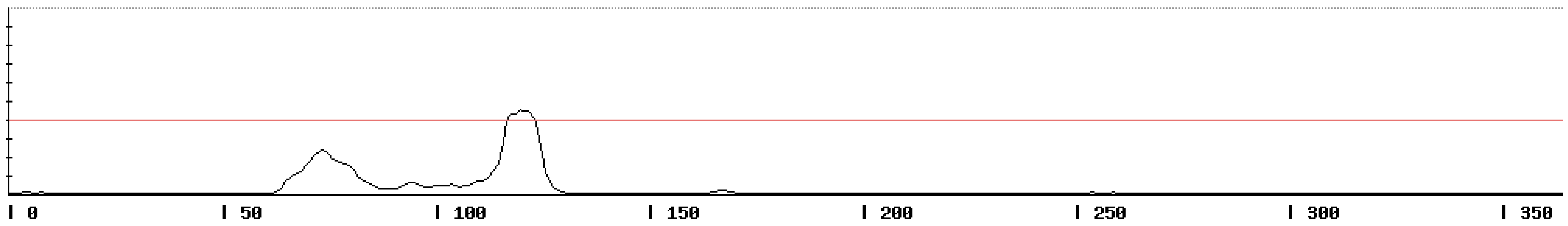

Predictions for Brassicaoleracea_XP_013589229.1

Viterbi Path	
Viterbi Path	

Posterior @ 0.4	118 - KVKGKP - 123
Posterior @ 0.4	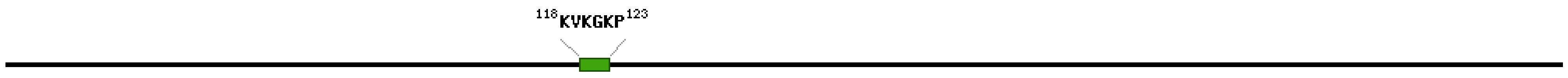
Posterior Graphic. Threshold Marked @ 0.4	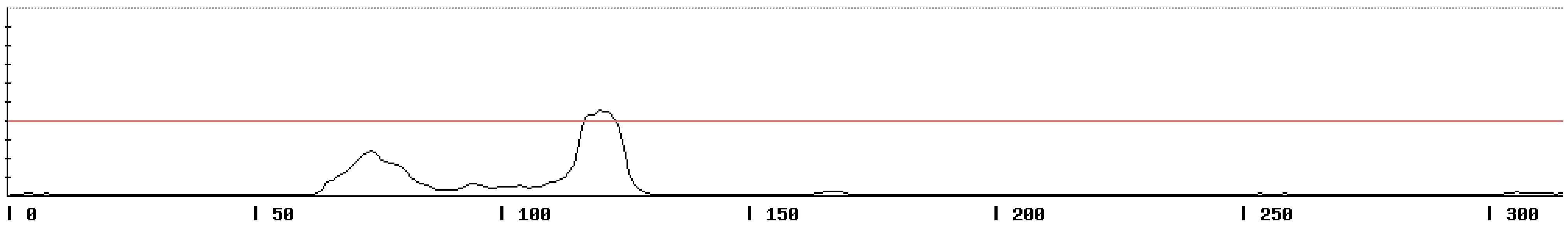

**Predictions for Brassicaoleracea_XP_013589248.1**	
Viterbi Path	
Viterbi Path	
Posterior @ 0.4	
Posterior @ 0.4	
Posterior Graphic. Threshold Marked @ 0.4	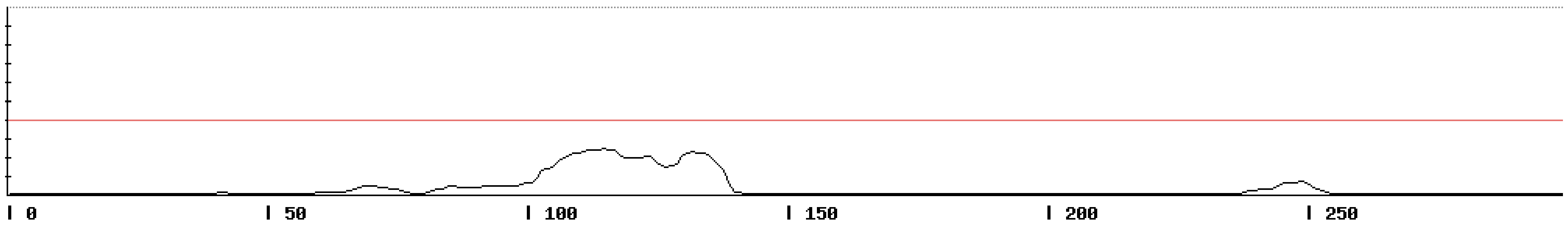

**Predictions for Brassicaoleracea_XP_013589257.1**	
Viterbi Path	
Viterbi Path	

Posterior @ 0.4

Posterior @ 0.4

Posterior Graphic. Threshold Marked @ 0.4

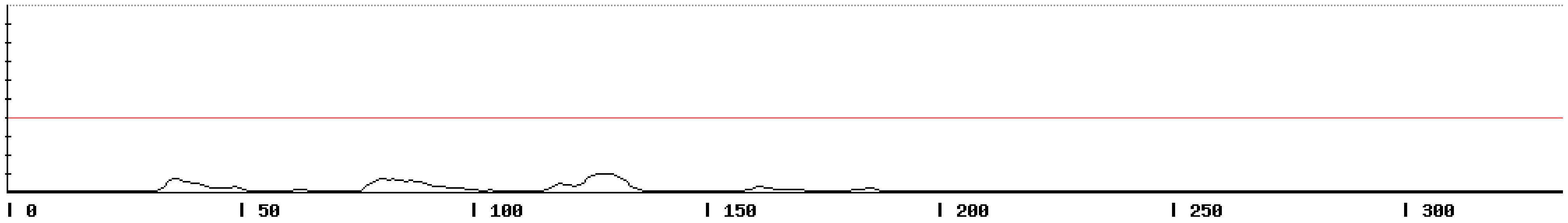

Predictions for Brassicaoleracea_XP_013589285.1

Viterbi Path

Viterbi Path

Posterior @ 0.4

Posterior @ 0.4

Posterior Graphic. Threshold Marked @ 0.4

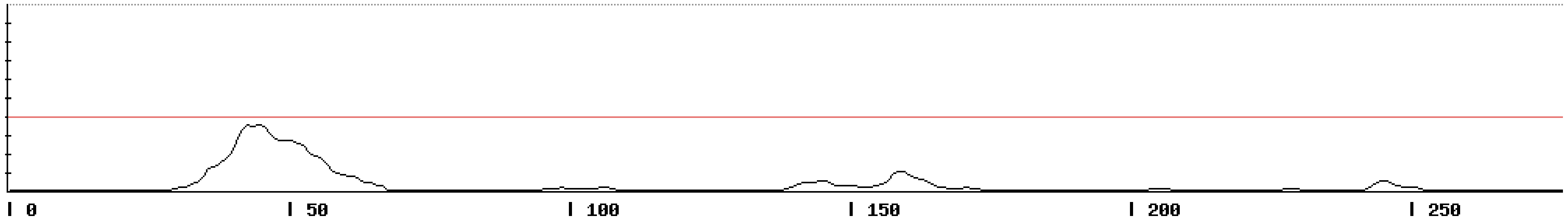

Predictions for Brassicaoleracea_XP_013589340.1

Viterbi Path

Viterbi Path

Posterior @ 0.4

Posterior @ 0.4

Posterior Graphic. Threshold Marked @ 0.4

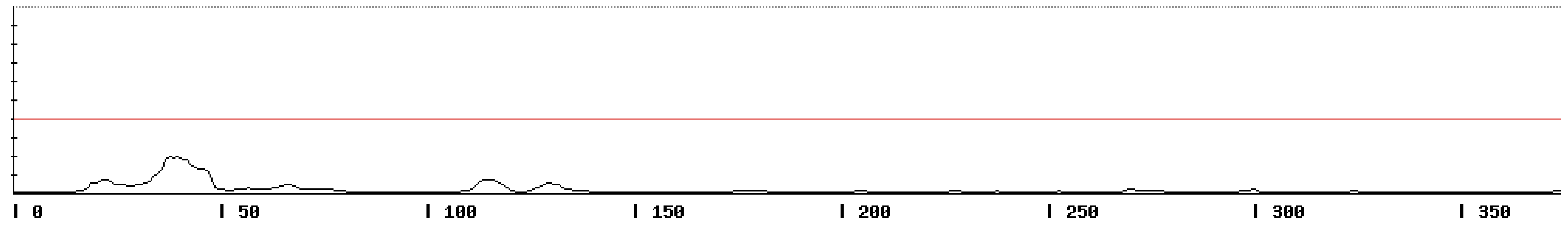

Predictions for Brassicaoleracea_XP_013589625.1

Viterbi Path

Viterbi Path

Posterior @ 0.4

Posterior @ 0.4

Posterior Graphic. Threshold Marked @ 0.4

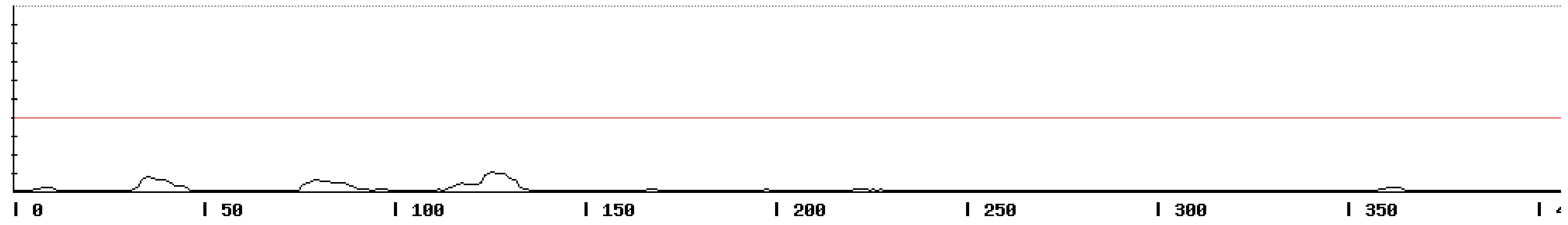

Predictions for Brassicaoleracea_XP_013589746.1	
Viterbi Path	
Viterbi Path	
Posterior @ 0.4	
Posterior @ 0.4	
Posterior Graphic. Threshold Marked @ 0.4	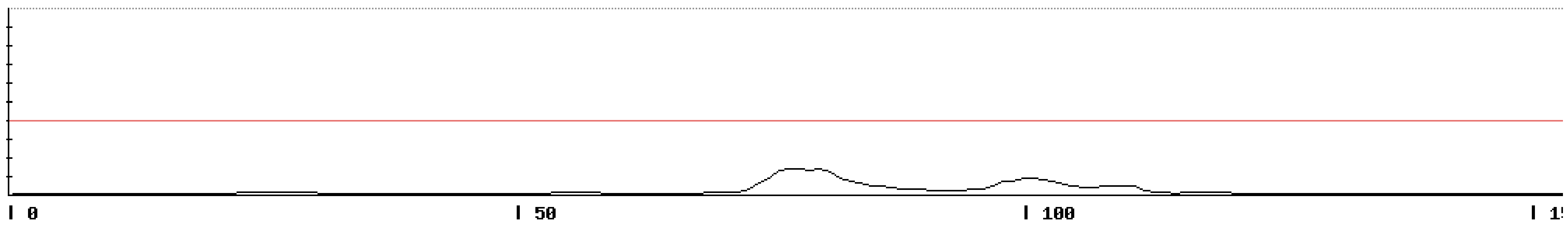

Predictions for Brassicaoleracea_XP_013589912.1	
Viterbi Path	
Viterbi Path	
Posterior @ 0.4	
Posterior @ 0.4	
Posterior Graphic. Threshold Marked @ 0.4	

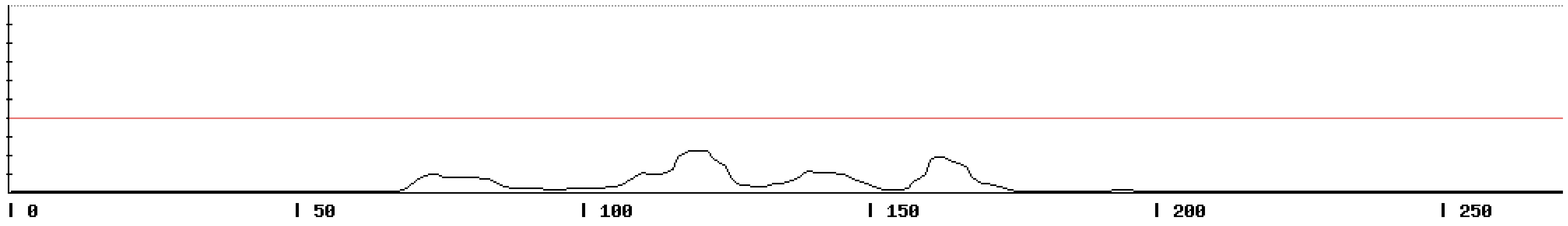

Predictions for Brassicaoleracea_XP_013590191.1

Viterbi Path	
Viterbi Path	
Posterior @ 0.4	
Posterior @ 0.4	
Posterior Graphic. Threshold Marked @ 0.4	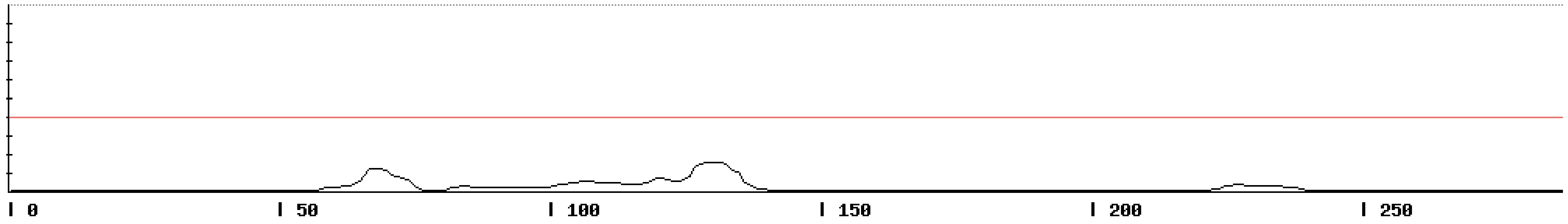

Predictions for Brassicaoleracea_XP_013590250.1

Viterbi Path	
Viterbi Path	
Posterior @ 0.4	82 - RATRAGYWKATGKDRAVKSKKMKLGMKK - 109
Posterior @ 0.4	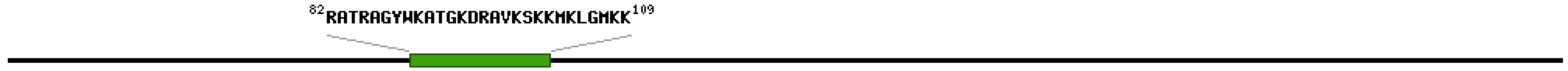

Posterior Graphic. Threshold Marked @ 0.4

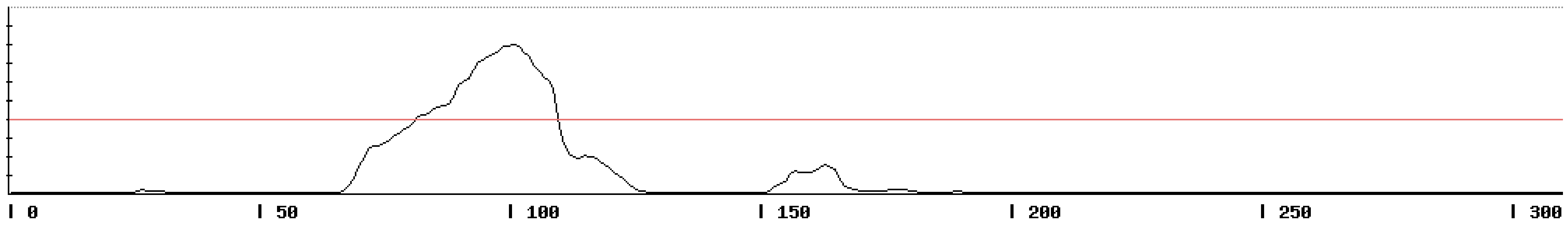

Predictions for Brassicaoleracea_XP_013590272.1

Viterbi Path

Viterbi Path

Posterior @ 0.4

Posterior @ 0.4

Posterior Graphic. Threshold Marked @ 0.4

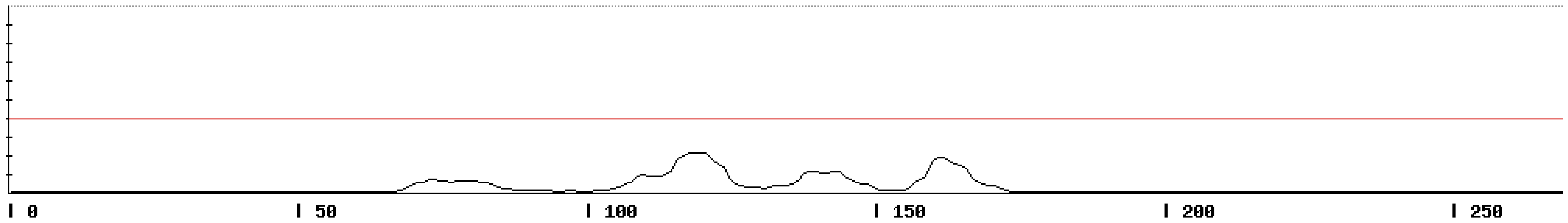

Predictions for Brassicaoleracea_XP_013590608.1

Viterbi Path

Viterbi Path

Posterior @ 0.4

Posterior @ 0.4

Posterior Graphic. Threshold Marked @ 0.4

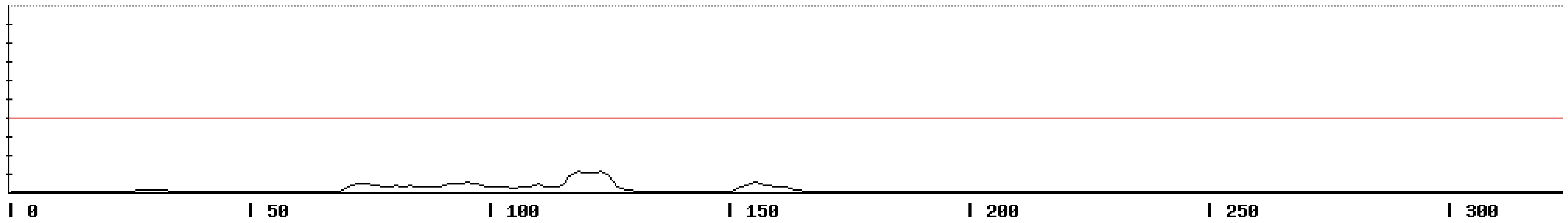

Predictions for Brassicaoleracea_XP_013590609.1

Viterbi Path

Viterbi Path

Posterior @ 0.4

Posterior @ 0.4

Posterior Graphic. Threshold Marked @ 0.4

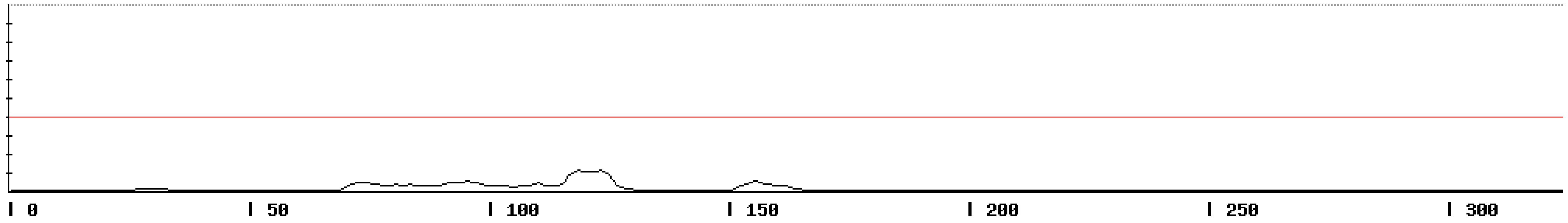

Predictions for Brassicaoleracea_XP_013590798.1

Viterbi Path

Viterbi Path

Posterior @ 0.4

Posterior @ 0.4

Posterior Graphic. Threshold Marked @ 0.4

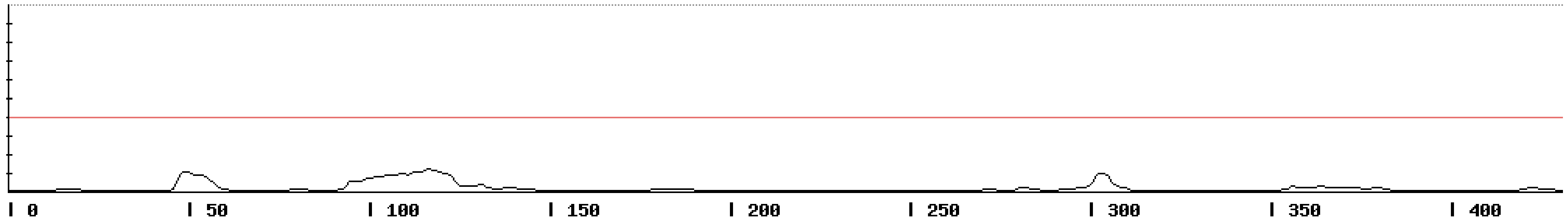

Predictions for Brassicaoleracea_XP_013590906.1

Viterbi Path

Viterbi Path

Posterior @ 0.4

Posterior @ 0.4

Posterior Graphic. Threshold Marked @ 0.4

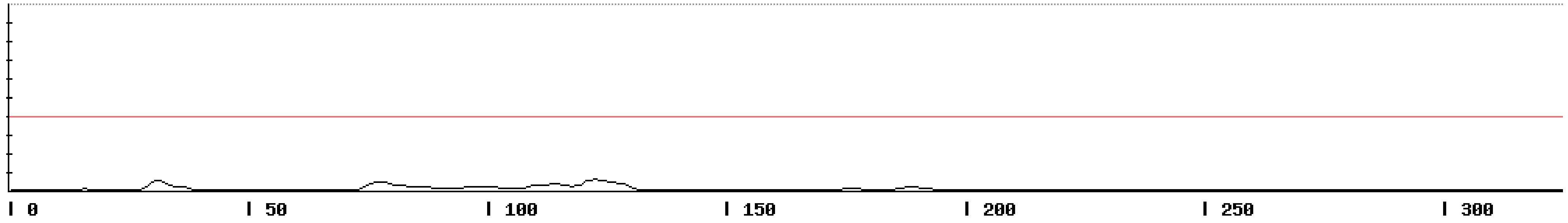

Predictions for Brassicaoleracea_XP_013590909.1

Viterbi Path

Viterbi Path

Posterior @ 0.4	
Posterior @ 0.4	
Posterior Graphic. Threshold Marked @ 0.4	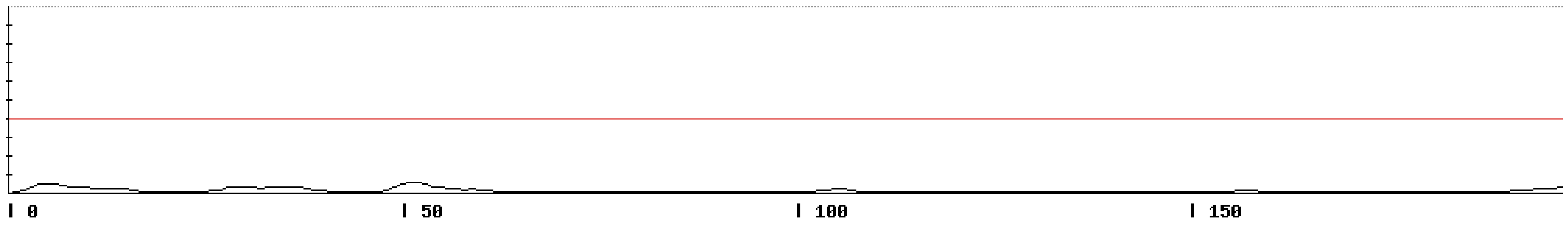

Predictions for Brassicaoleracea_XP_013590910.1

Viterbi Path	
Viterbi Path	
Posterior @ 0.4	
Posterior @ 0.4	
Posterior Graphic. Threshold Marked @ 0.4	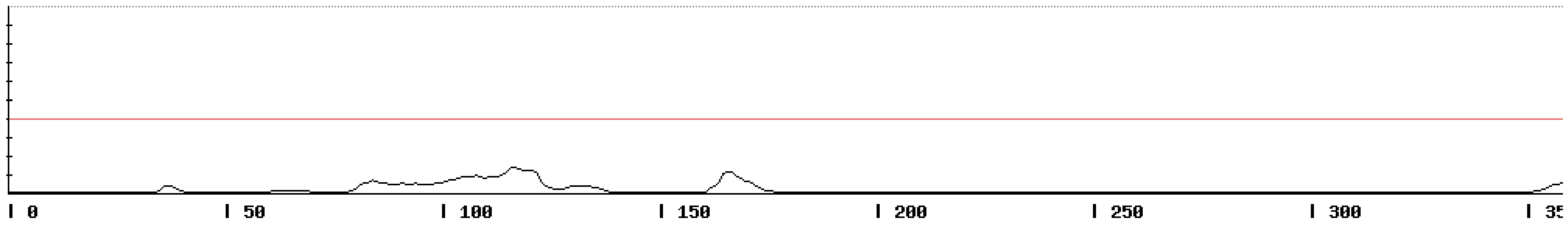

Predictions for Brassicaoleracea_XP_013591746.1

Viterbi Path

Viterbi Path

Posterior @ 0.4

Posterior @ 0.4

Posterior Graphic. Threshold Marked @ 0.4

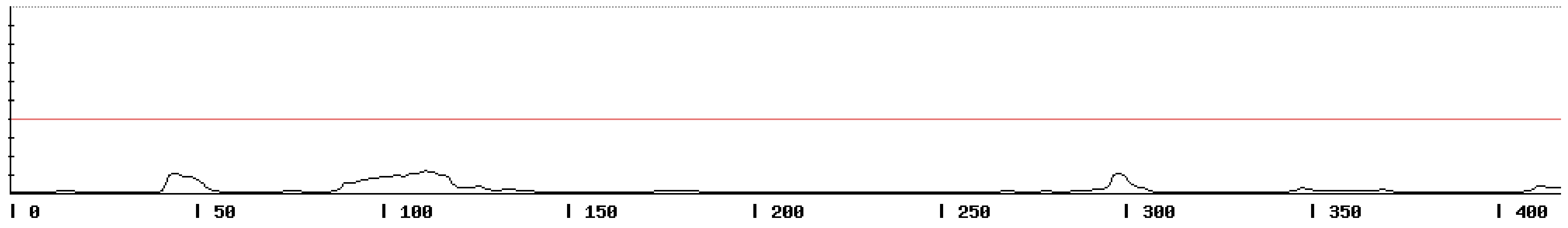

Predictions for Brassicaoleracea_XP_013592194.1

Viterbi Path

Viterbi Path

Posterior @ 0.4

Posterior @ 0.4

Posterior Graphic. Threshold Marked @ 0.4

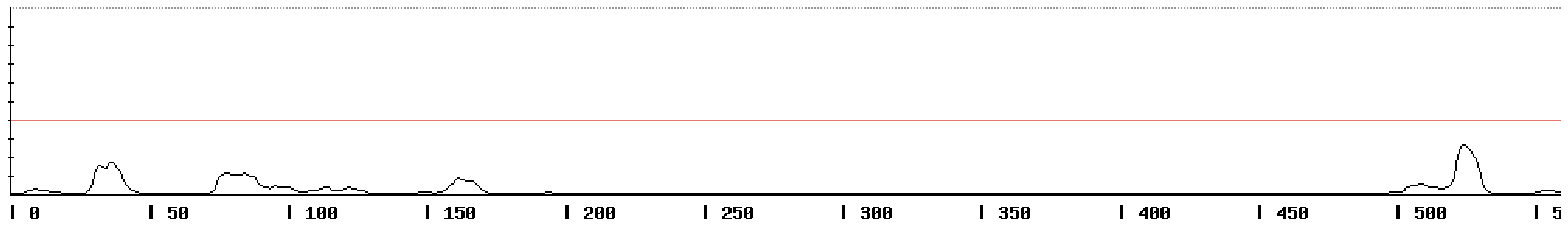

Predictions for Brassicaoleracea_XP_013592210.1	
Viterbi Path	
Viterbi Path	
Posterior @ 0.4	
Posterior @ 0.4	
Posterior Graphic. Threshold Marked @ 0.4	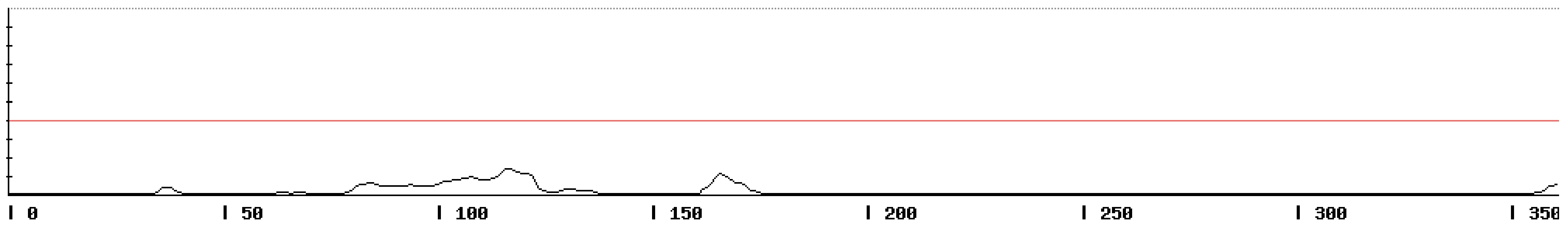

Predictions for Brassicaoleracea_XP_013592976.1	
Viterbi Path	
Viterbi Path	
Posterior @ 0.4	64 - KRKIRRKKLR - 73
Posterior @ 0.4	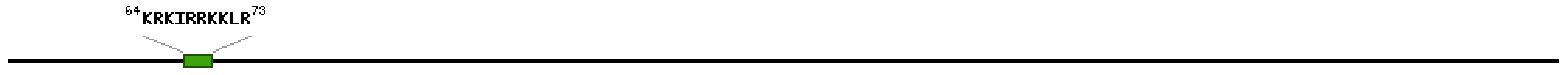
Posterior Graphic. Threshold Marked @ 0.4	

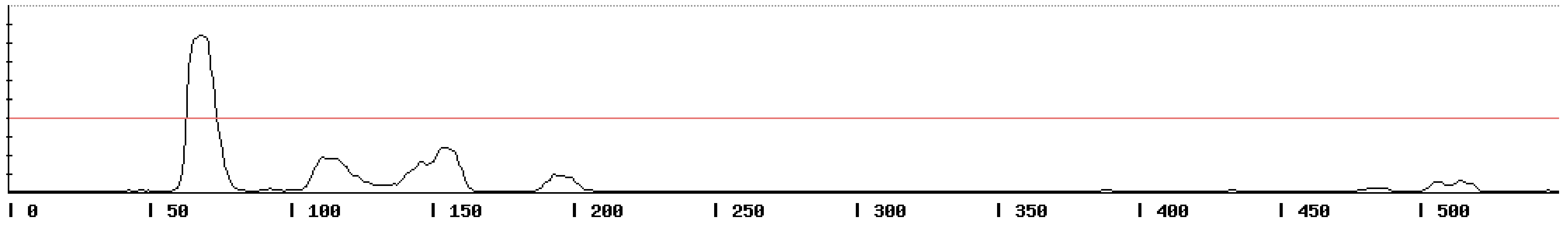

Predictions for Brassicaoleracea_XP_013593565.1

Viterbi Path	
Viterbi Path	
Posterior @ 0.4	
Posterior @ 0.4	
Posterior Graphic. Threshold Marked @ 0.4	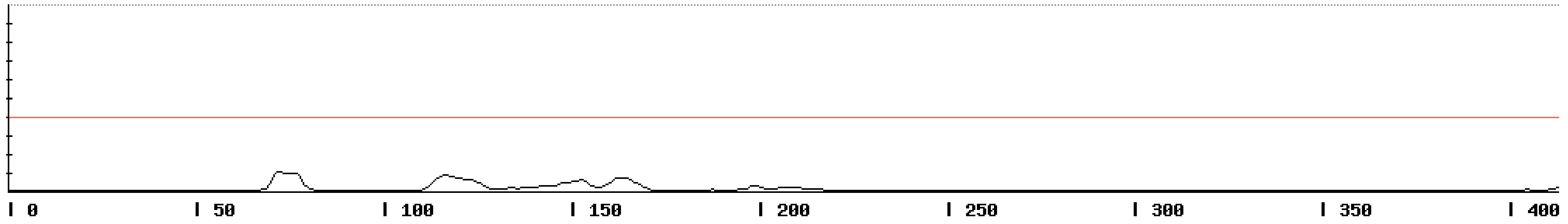

Predictions for Brassicaoleracea_XP_013593766.1

Viterbi Path	
Viterbi Path	
Posterior @ 0.4	134 - KR - 135
Posterior @ 0.4	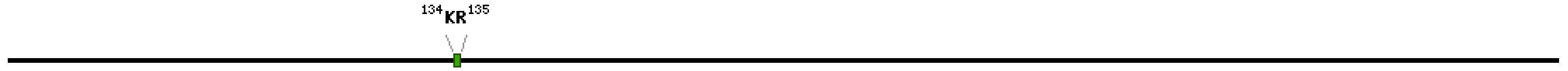

Posterior Graphic. Threshold Marked @ 0.4

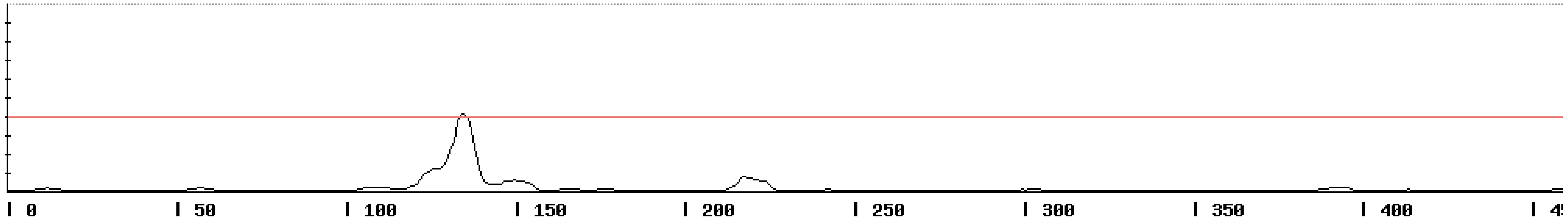

Predictions for Brassicaoleracea_XP_013594147.1

Viterbi Path

Viterbi Path

Posterior @ 0.4

Posterior @ 0.4

Posterior Graphic. Threshold Marked @ 0.4

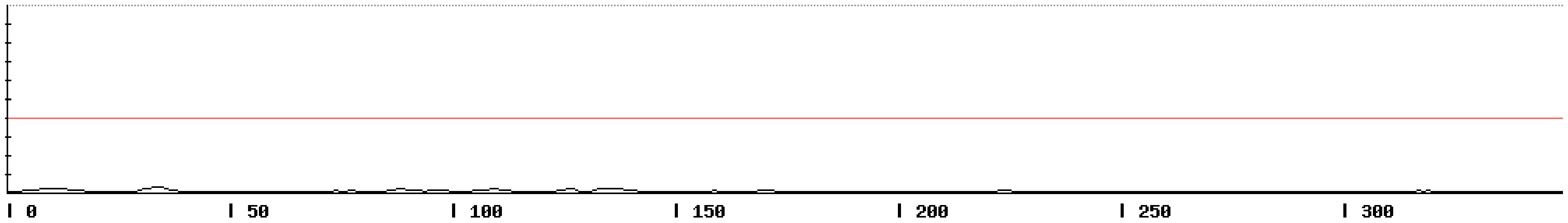

Predictions for Brassicaoleracea_XP_013594184.1

Viterbi Path

Viterbi Path

Posterior @ 0.4

Posterior @ 0.4

Posterior Graphic. Threshold Marked @ 0.4

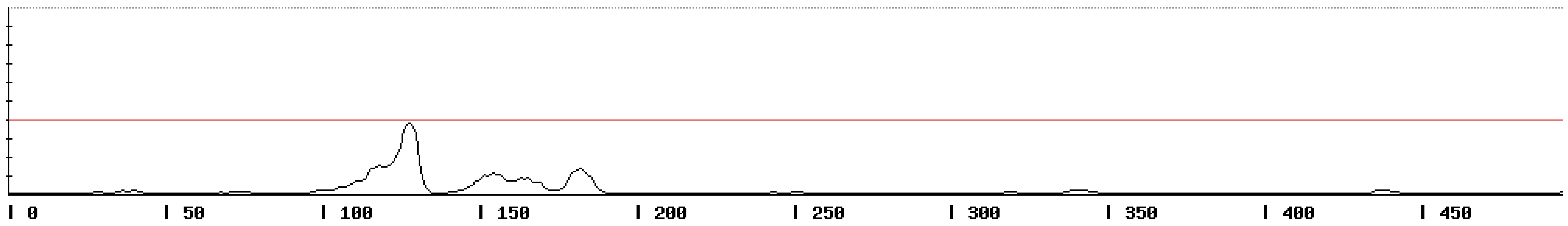

Predictions for Brassicaoleracea_XP_013594185.1

Viterbi Path

Viterbi Path

Posterior @ 0.4

Posterior @ 0.4

Posterior Graphic. Threshold Marked @ 0.4

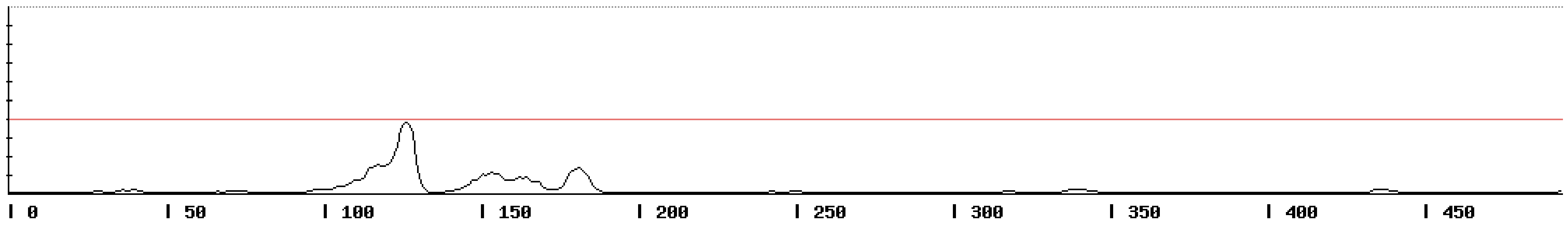

Predictions for Brassicaoleracea_XP_013594418.1

Viterbi Path

Viterbi Path

Posterior @ 0.4

Posterior @ 0.4

Posterior Graphic. Threshold Marked @ 0.4

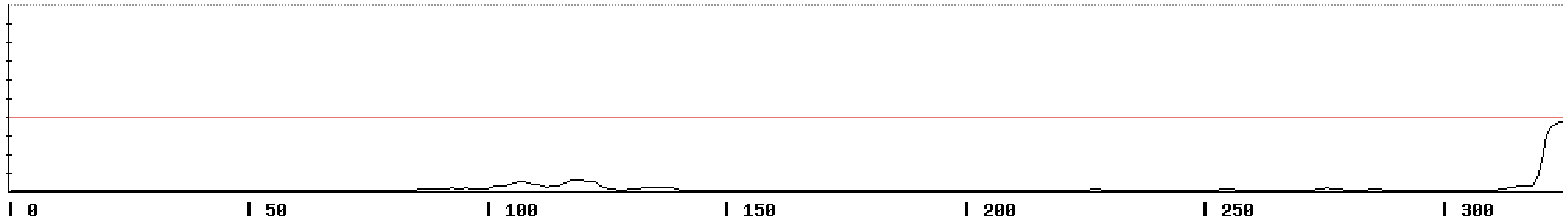

Predictions for Brassicaoleracea_XP_013595617.1

Viterbi Path

Viterbi Path

Posterior @ 0.4

Posterior @ 0.4

Posterior Graphic. Threshold Marked @ 0.4

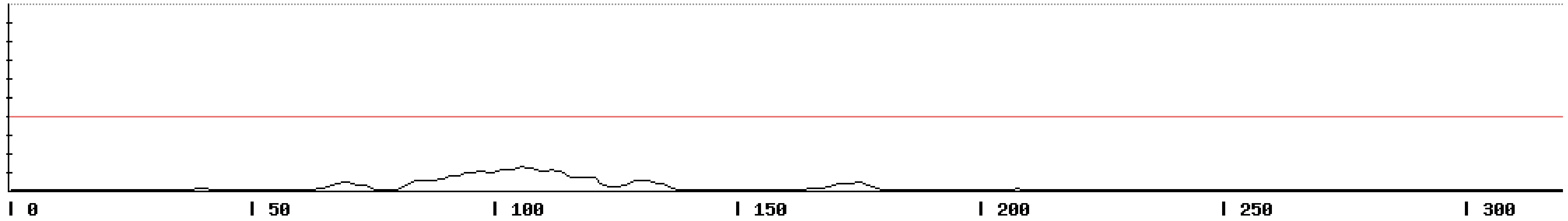

Predictions for Brassicaoleracea_XP_013595819.1

Viterbi Path

Viterbi Path

Posterior @ 0.4

Posterior @ 0.4

Posterior Graphic. Threshold Marked @ 0.4

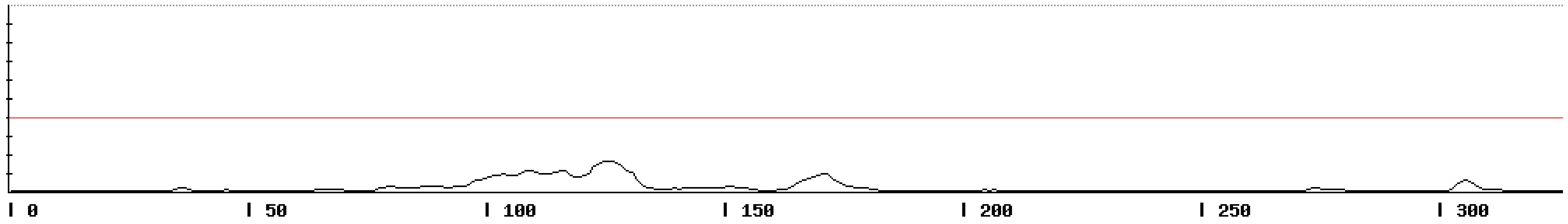

Predictions for Brassicaoleracea_XP_013595944.1

Viterbi Path

Viterbi Path

Posterior @ 0.4 — 278 - SKDKKKK - 284

Posterior @ 0.4

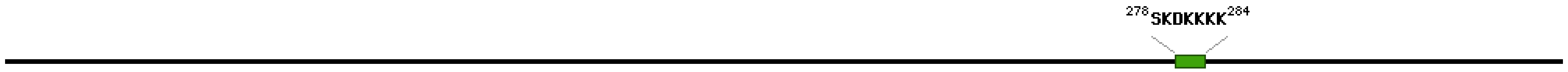

Posterior Graphic. Threshold Marked @ 0.4

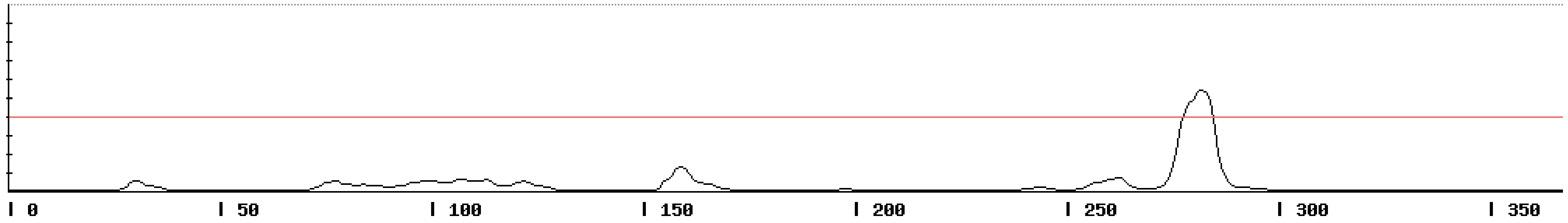

Predictions for Brassicaoleracea_XP_013595945.1

Viterbi Path	
Viterbi Path	
Posterior @ 0.4	278 - SKDKKKK - 284
Posterior @ 0.4	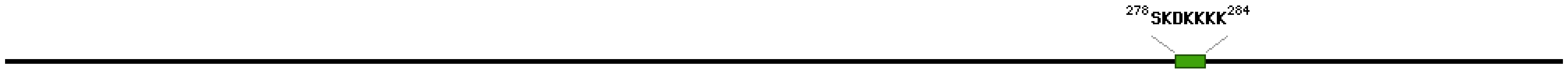
Posterior Graphic. Threshold Marked @ 0.4	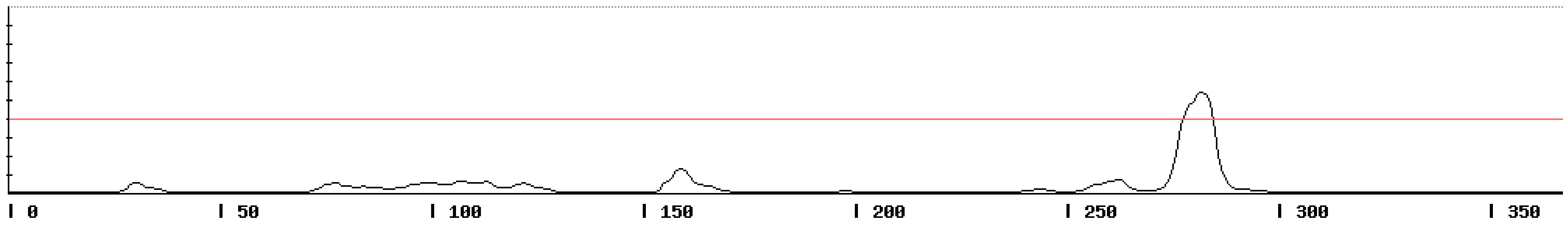

Predictions for Brassicaoleracea_XP_013596007.1

Viterbi Path	
Viterbi Path	
Posterior @ 0.4	
Posterior @ 0.4	
Posterior Graphic. Threshold Marked @ 0.4	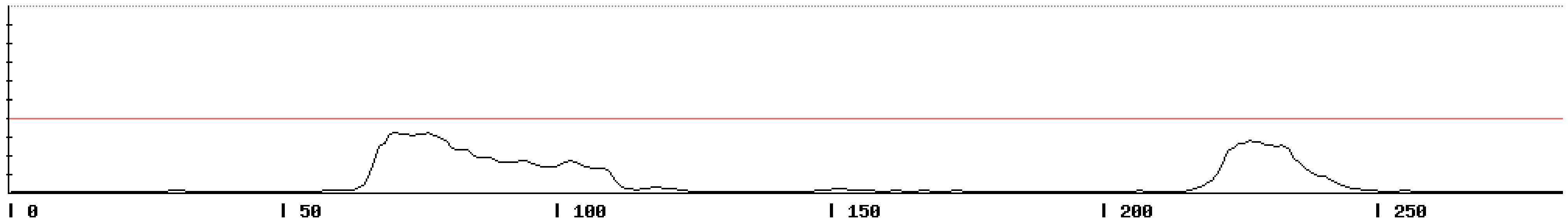

Predictions for Brassicaoleracea_XP_013596124.1

Viterbi Path	
Viterbi Path	
Posterior @ 0.4	158 - TRKRRKVR - 165
Posterior @ 0.4	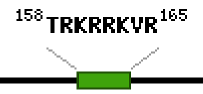
Posterior Graphic. Threshold Marked @ 0.4	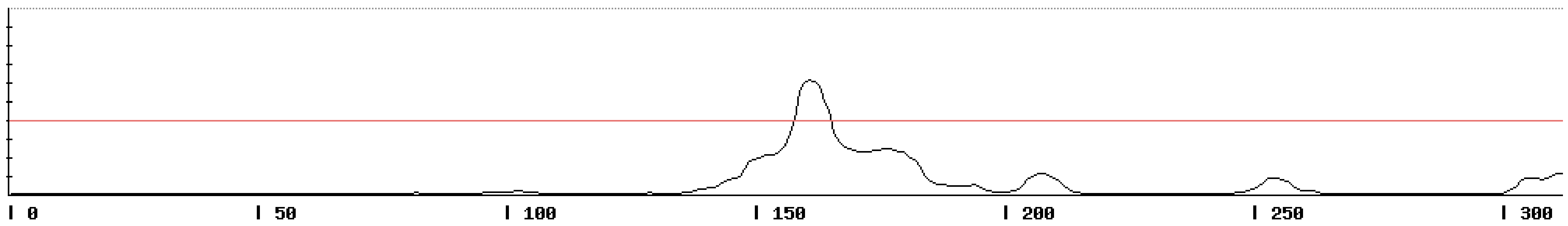

Predictions for Brassicaoleracea_XP_013596125.1

Viterbi Path	
Viterbi Path	
Posterior @ 0.4	154 - TRKRRKVR - 161
Posterior @ 0.4	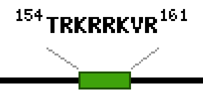
Posterior Graphic. Threshold Marked @ 0.4	

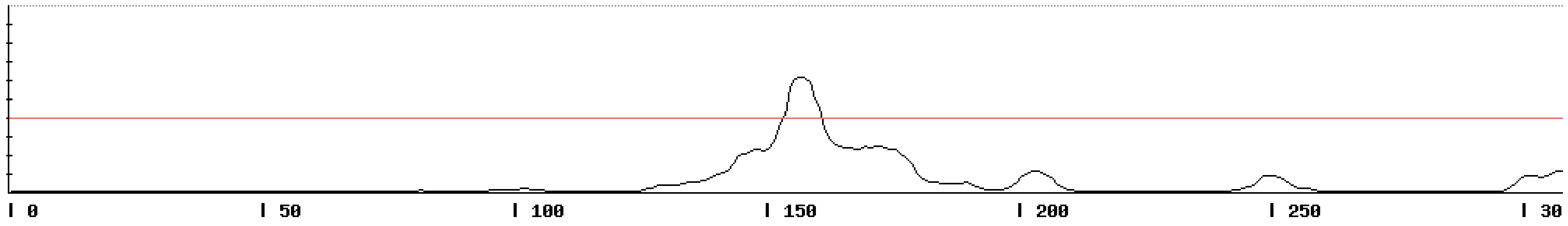

Predictions for Brassicaoleracea_XP_013596215.1	
Viterbi Path	
Viterbi Path	
Posterior @ 0.4	137 - RKRRK - 141
Posterior @ 0.4	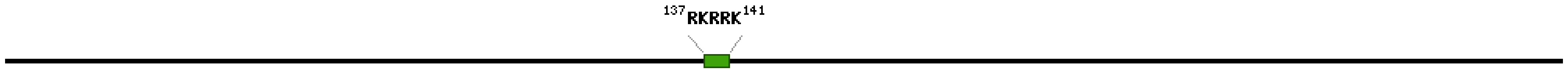
Posterior Graphic. Threshold Marked @ 0.4	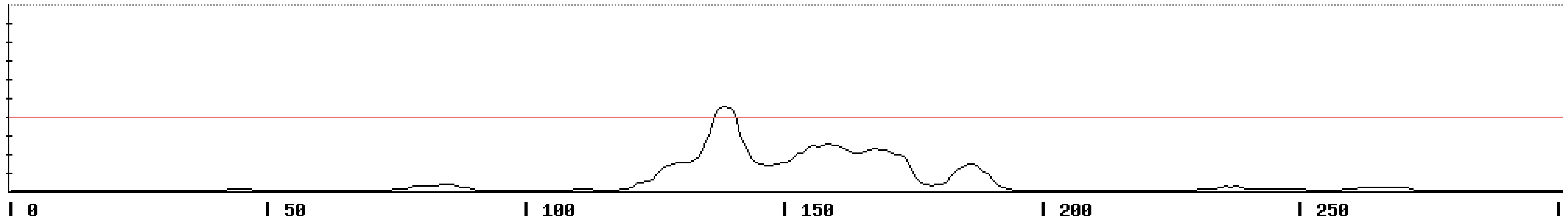

Predictions for Brassicaoleracea_XP_013596223.1	
Viterbi Path	
Viterbi Path	
Posterior @ 0.4	
Posterior @ 0.4	

Posterior Graphic. Threshold Marked @ 0.4

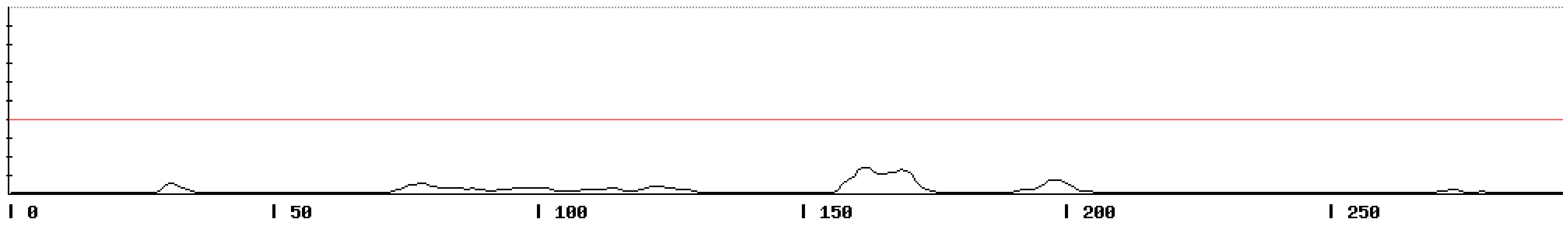

Predictions for Brassicaoleracea_XP_013596224.1

Viterbi Path

Viterbi Path

Posterior @ 0.4

Posterior @ 0.4

Posterior Graphic. Threshold Marked @ 0.4

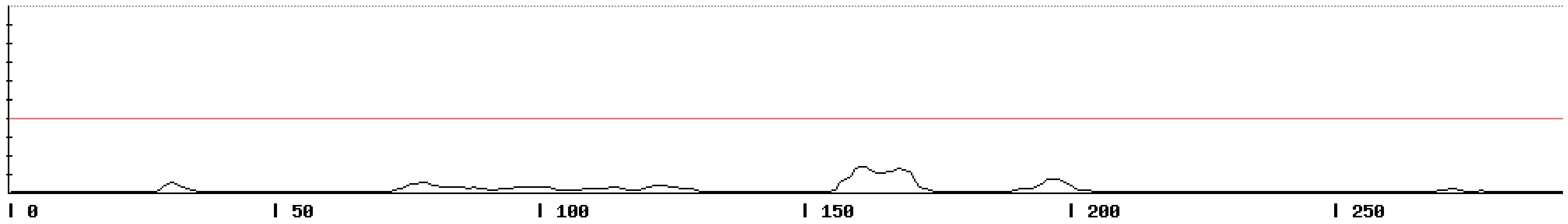

Predictions for Brassicaoleracea_XP_013596229.1

Viterbi Path

Viterbi Path

Posterior @ 0.4

Posterior @ 0.4

Posterior Graphic. Threshold Marked @ 0.4

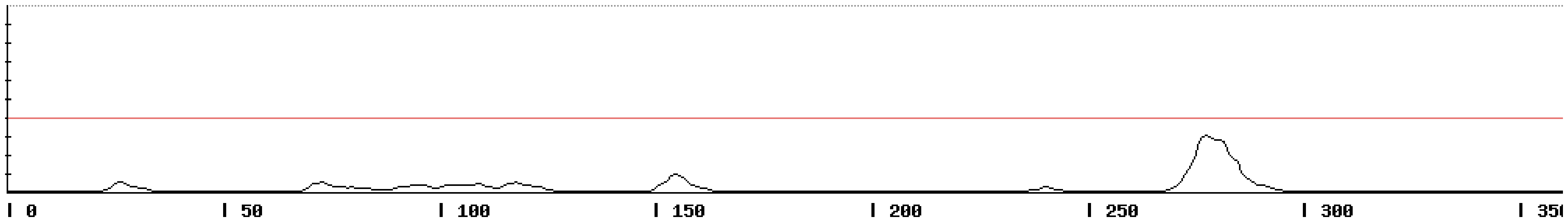

Predictions for Brassicaoleracea_XP_013596725.1

Viterbi Path

Viterbi Path

Posterior @ 0.4

Posterior @ 0.4

Posterior Graphic. Threshold Marked @ 0.4

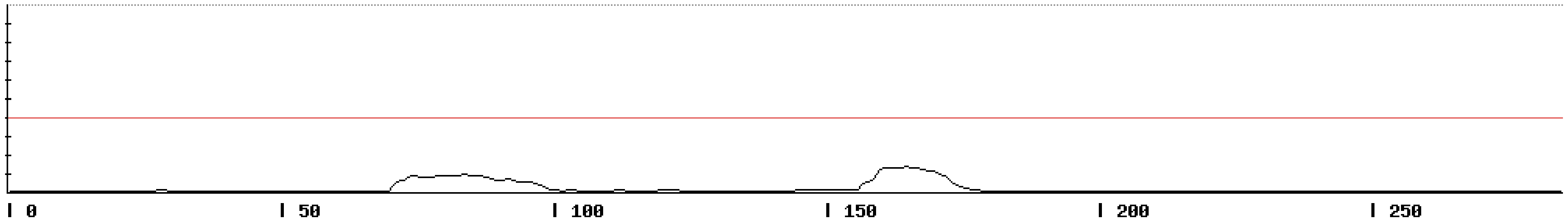

Predictions for Brassicaoleracea_XP_013596941.1

Viterbi Path

Viterbi Path

Posterior @ 0.4	
Posterior @ 0.4	
Posterior Graphic. Threshold Marked @ 0.4	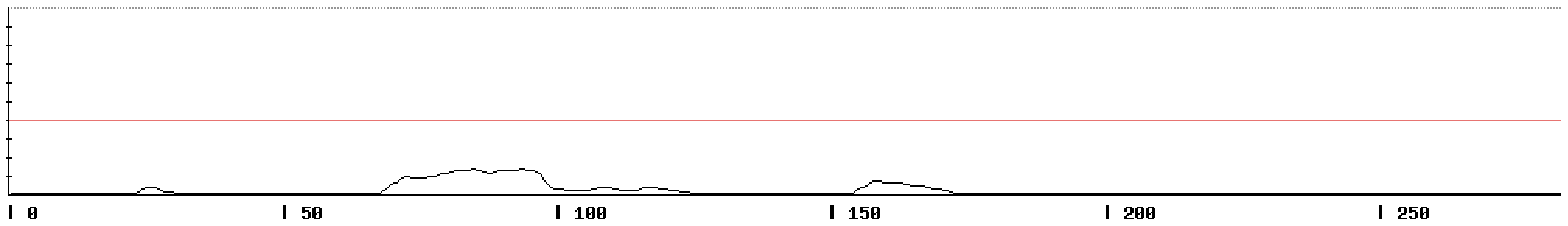

Predictions for Brassicaoleracea_XP_013597963.1

Viterbi Path	
Viterbi Path	
Posterior @ 0.4	
Posterior @ 0.4	
Posterior Graphic. Threshold Marked @ 0.4	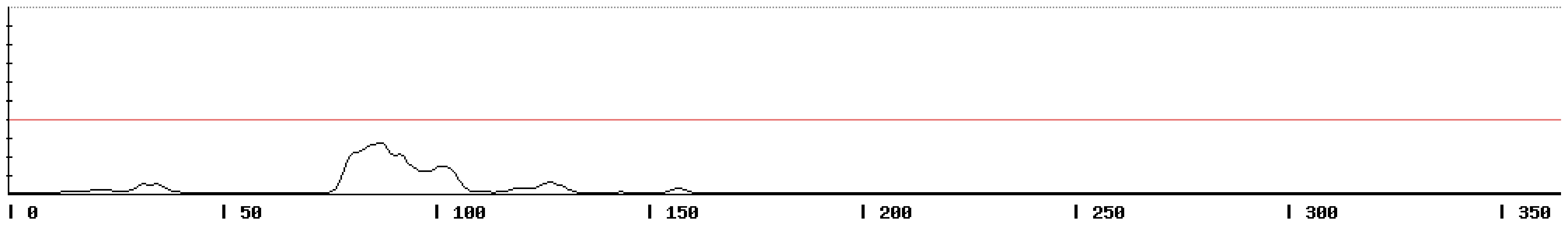

Predictions for Brassicaoleracea_XP_013597964.1

Viterbi Path	
Viterbi Path	

Posterior @ 0.4	
Posterior @ 0.4	
Posterior Graphic. Threshold Marked @ 0.4	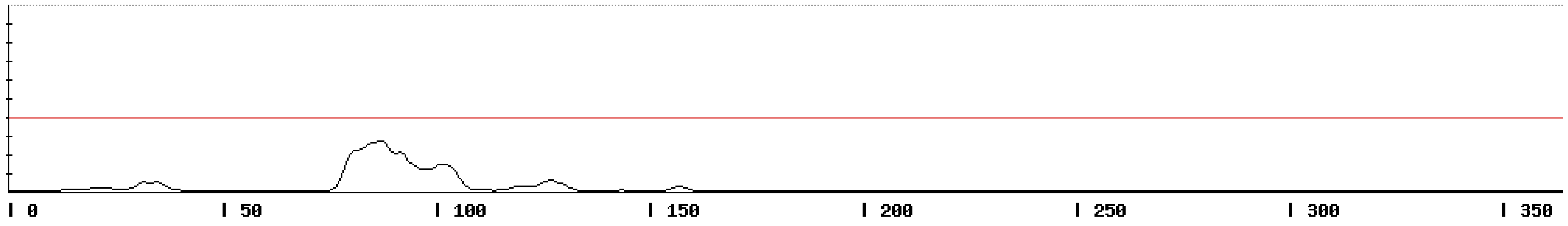

Predictions for Brassicaoleracea_XP_013597965.1	
Viterbi Path	
Viterbi Path	
Posterior @ 0.4	
Posterior @ 0.4	
Posterior Graphic. Threshold Marked @ 0.4	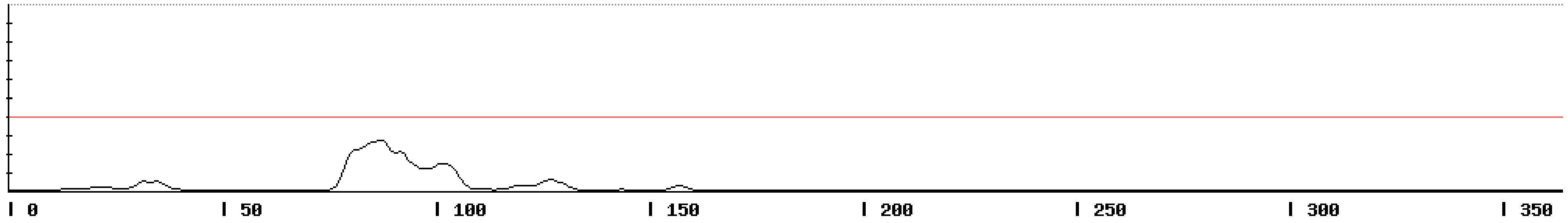

Predictions for Brassicaoleracea_XP_013597966.1

Viterbi Path	
Viterbi Path	
Posterior @ 0.4	
Posterior @ 0.4	
Posterior Graphic. Threshold Marked @ 0.4	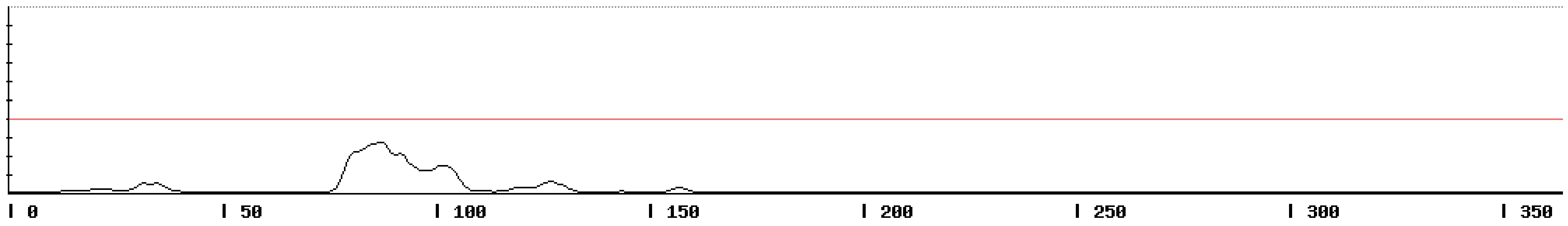

Predictions for Brassicaoleracea_XP_013597967.1

Viterbi Path	
Viterbi Path	
Posterior @ 0.4	
Posterior @ 0.4	
Posterior Graphic. Threshold Marked @ 0.4	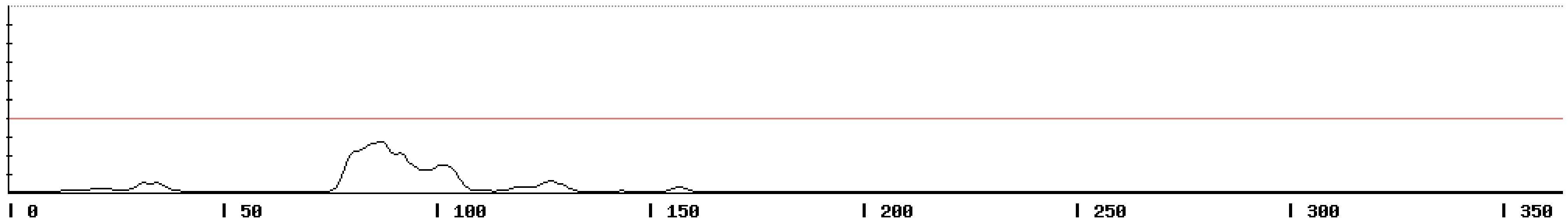

Predictions for Brassicaoleracea_XP_013597968.1

Viterbi Path

Viterbi Path

Posterior @ 0.4

Posterior @ 0.4

Posterior Graphic. Threshold Marked @ 0.4

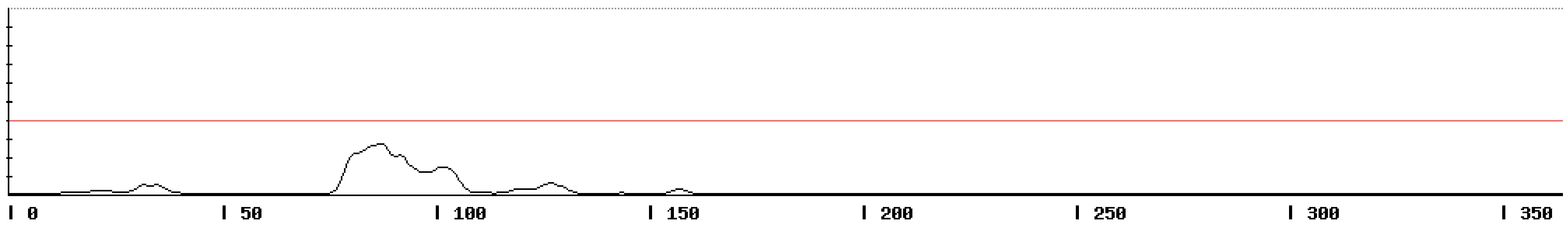

Predictions for Brassicaoleracea_XP_013597969.1

Viterbi Path

Viterbi Path

Posterior @ 0.4

Posterior @ 0.4

Posterior Graphic. Threshold Marked @ 0.4

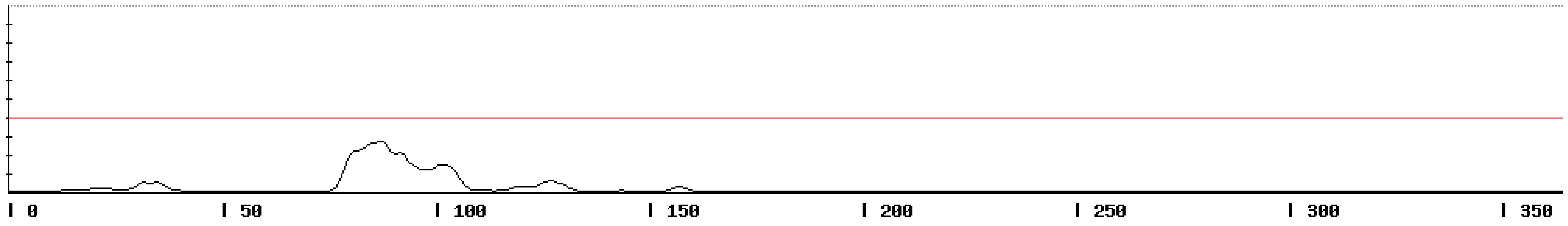

Predictions for Brassicaoleracea_XP_013597971.1	
Viterbi Path	
Viterbi Path	
Posterior @ 0.4	
Posterior @ 0.4	
Posterior Graphic. Threshold Marked @ 0.4	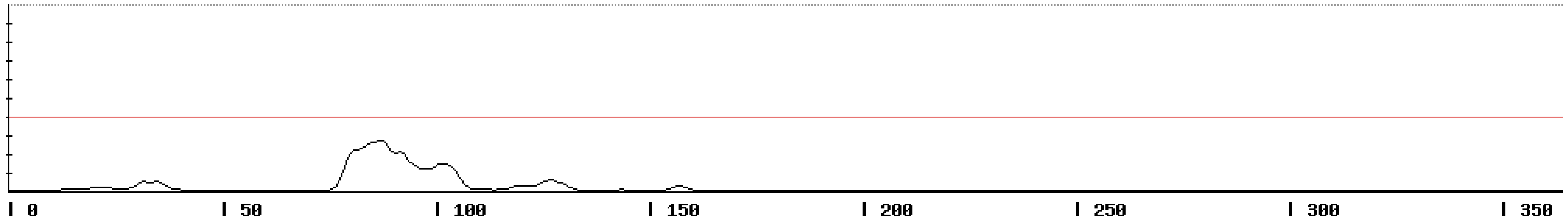

Predictions for Brassicaoleracea_XP_013598464.1	
Viterbi Path	
Viterbi Path	
Posterior @ 0.4	
Posterior @ 0.4	

Posterior Graphic. Threshold Marked @ 0.4	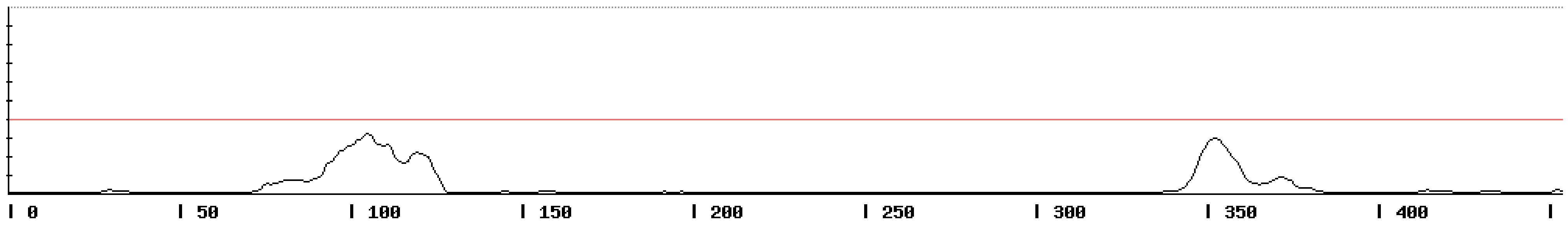

Predictions for Brassicaoleracea_XP_013598465.1

Viterbi Path	
Viterbi Path	
Posterior @ 0.4	
Posterior @ 0.4	
Posterior Graphic. Threshold Marked @ 0.4	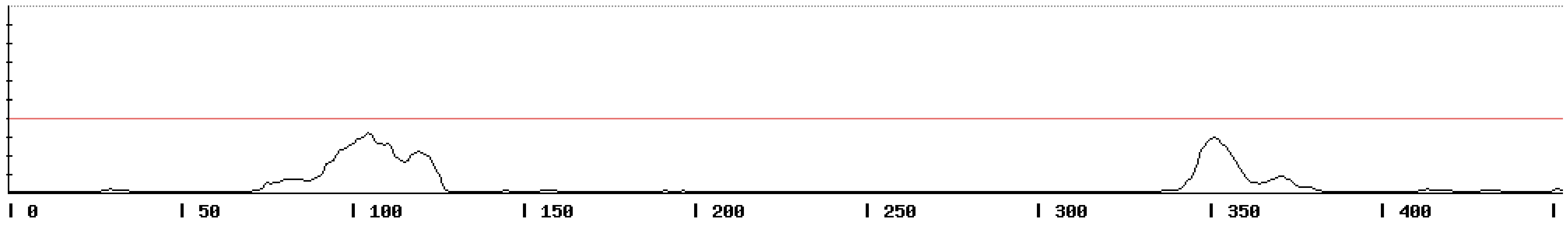

Predictions for Brassicaoleracea_XP_013598751.1

Viterbi Path	
Viterbi Path	
Posterior @ 0.4	
Posterior @ 0.4	

Posterior Graphic. Threshold Marked @ 0.4

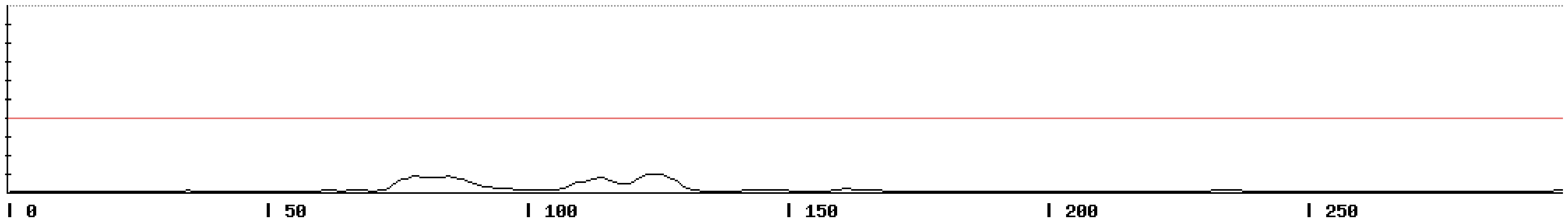

Predictions for Brassicaoleracea_XP_013599436.1

Viterbi Path

Viterbi Path

Posterior @ 0.4

Posterior @ 0.4

Posterior Graphic. Threshold Marked @ 0.4

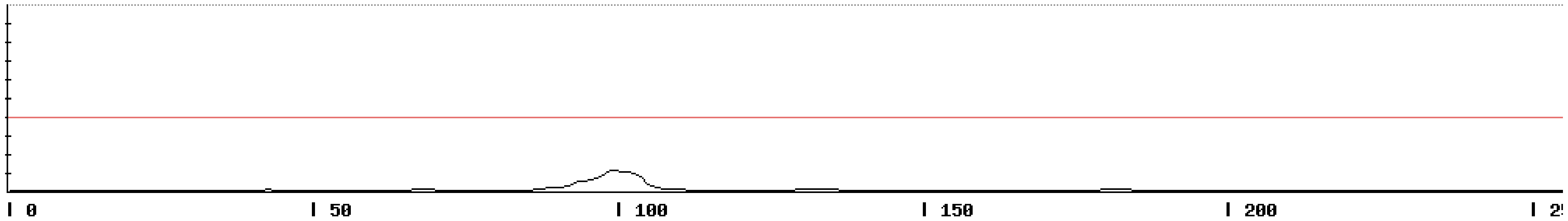

Predictions for Brassicaoleracea_XP_013601909.1

Viterbi Path: 68 - GRKESKYNKGERQKRKTKSGFWKKTGNTLPIKRKRGNRQKIGEK - 111

Viterbi Path

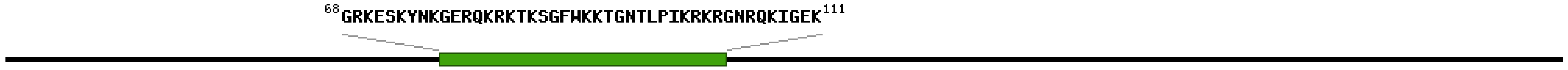

Posterior @ 0.4 — 69 - RKESKYNKGERQKRKTKSGFWKKTGNTLPIKRKRGNRQKIGE - 110

Posterior @ 0.4

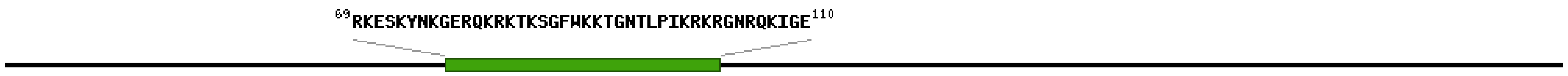

Posterior Graphic. Threshold Marked @ 0.4

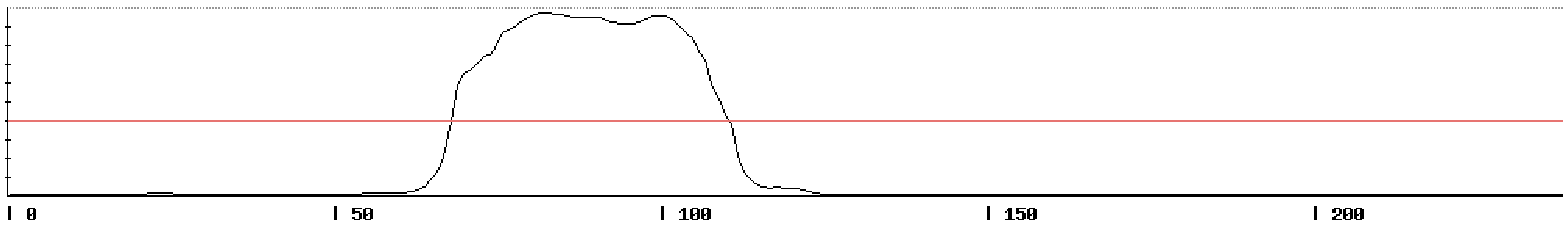

Predictions for Brassicaoleracea_XP_013601975.1

Viterbi Path

Viterbi Path

Posterior @ 0.4 — 337 - KKREKKK - 343

Posterior @ 0.4

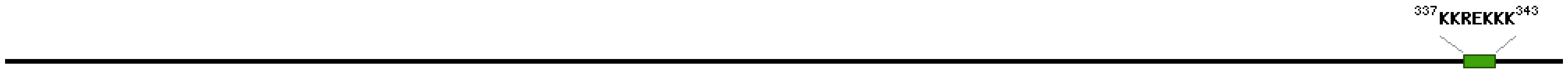

Posterior Graphic. Threshold Marked @ 0.4

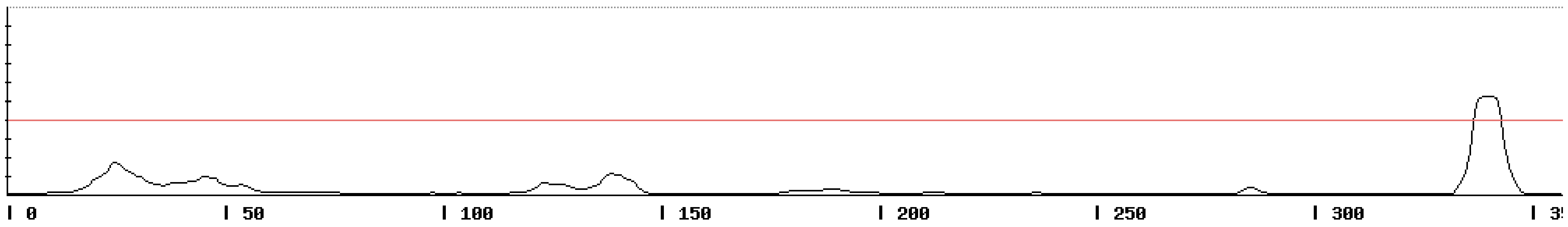

Predictions for Brassicaoleracea_XP_013602769.1

Viterbi Path

Viterbi Path

Posterior @ 0.4

Posterior @ 0.4

Posterior Graphic. Threshold Marked @ 0.4

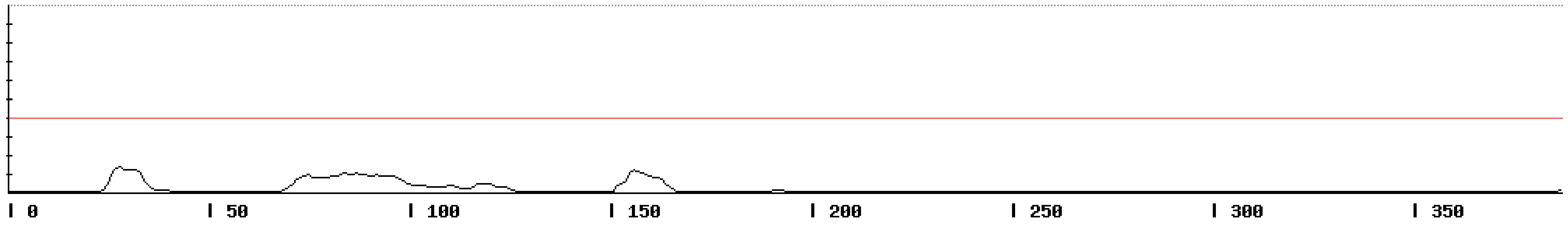

Predictions for Brassicaoleracea_XP_013602771.1

Viterbi Path

Viterbi Path

Posterior @ 0.4

Posterior @ 0.4

Posterior Graphic. Threshold Marked @ 0.4

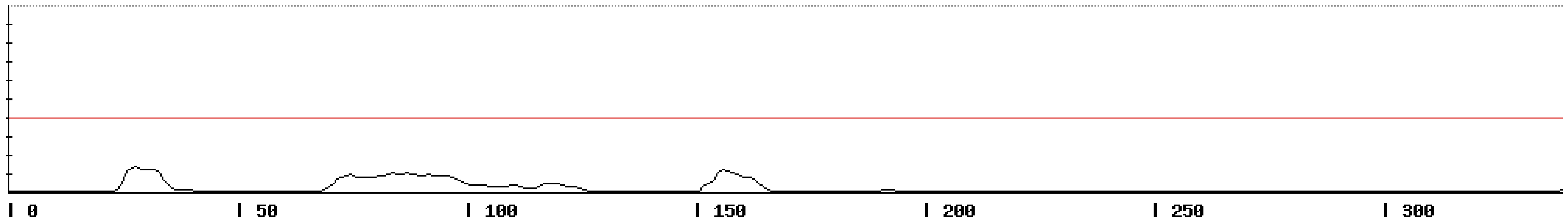

Predictions for Brassicaoleracea_XP_013602772.1

Viterbi Path	
Viterbi Path	
Posterior @ 0.4	
Posterior @ 0.4	
Posterior Graphic. Threshold Marked @ 0.4	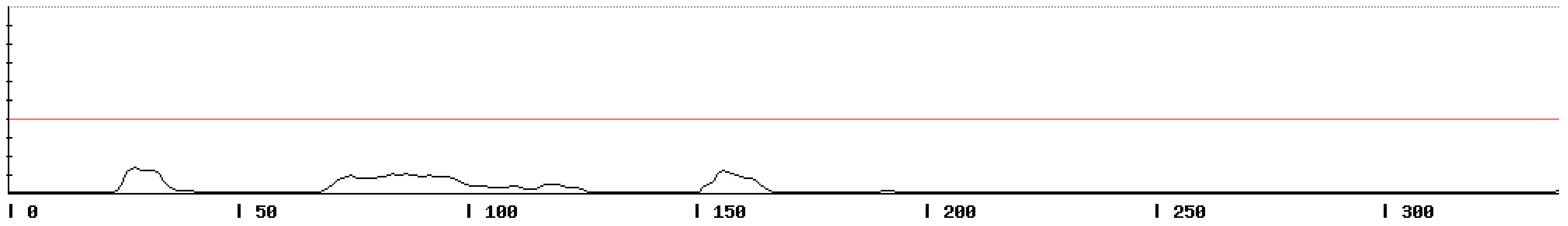

Predictions for Brassicaoleracea_XP_013602773.1

Viterbi Path	
Viterbi Path	
Posterior @ 0.4	
Posterior @ 0.4	
Posterior Graphic. Threshold Marked @ 0.4	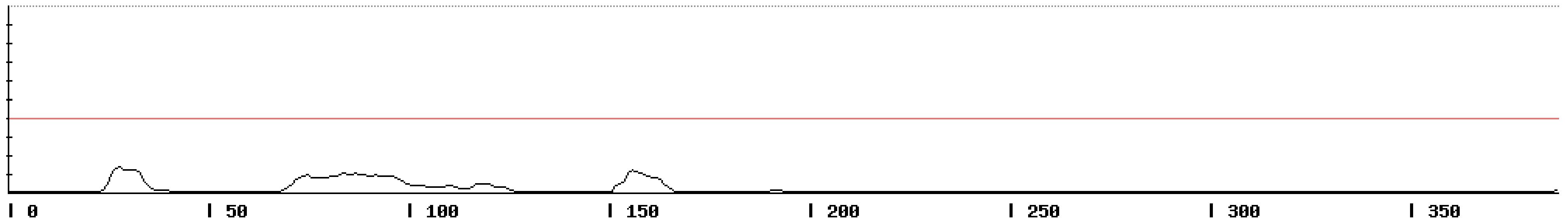

Predictions for Brassicaoleracea_XP_013603036.1

Viterbi Path

Viterbi Path

Posterior @ 0.4

Posterior @ 0.4

Posterior Graphic. Threshold Marked @ 0.4

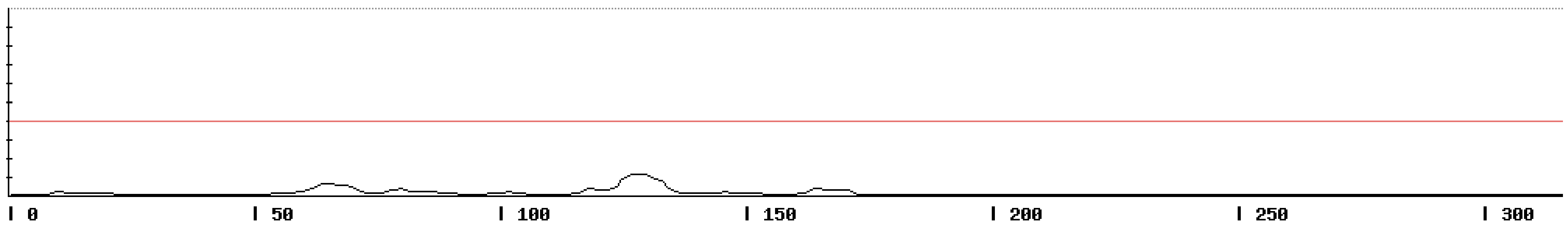

Predictions for Brassicaoleracea_XP_013603126.1

Viterbi Path

Viterbi Path

Posterior @ 0.4

Posterior @ 0.4

Posterior Graphic. Threshold Marked @ 0.4

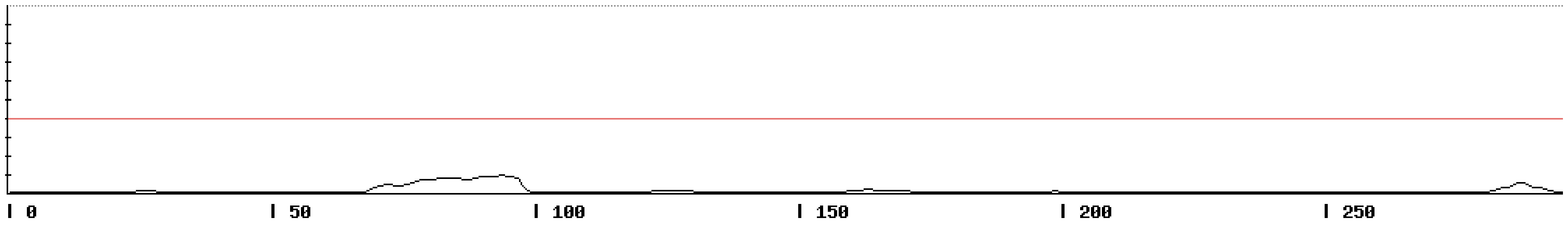

Predictions for Brassicaoleracea_XP_013603127.1

Viterbi Path	
Viterbi Path	
Posterior @ 0.4	
Posterior @ 0.4	
Posterior Graphic. Threshold Marked @ 0.4	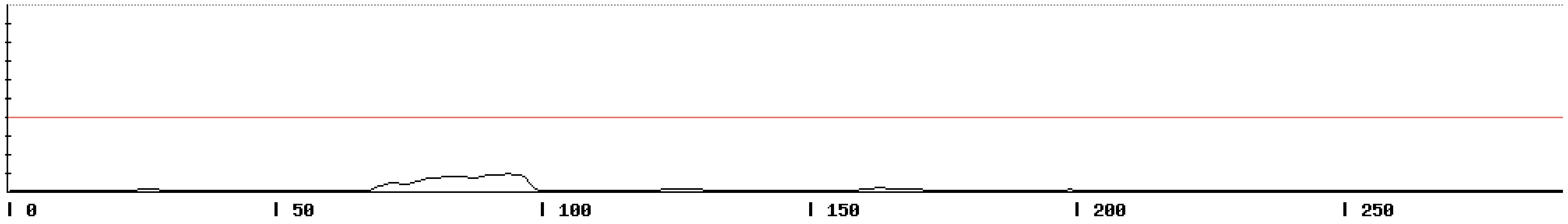

Predictions for Brassicaoleracea_XP_013603484.1

Viterbi Path	
Viterbi Path	
Posterior @ 0.4	
Posterior @ 0.4	

Posterior Graphic. Threshold Marked @ 0.4

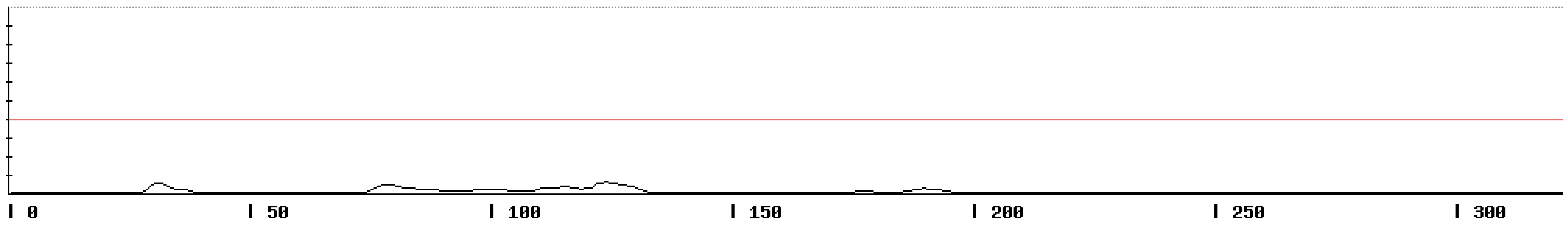

Predictions for Brassicaoleracea_XP_013603495.1

Viterbi Path	
Viterbi Path	
Posterior @ 0.4	
Posterior @ 0.4	
Posterior Graphic. Threshold Marked @ 0.4	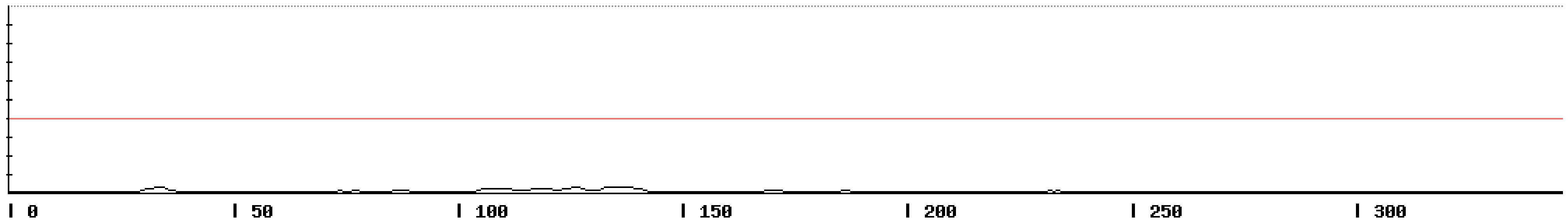

Predictions for Brassicaoleracea_XP_013603918.1

Viterbi Path	
Viterbi Path	
Posterior @ 0.4	72 - RKESRYNRGERQKRKTKSGYWKKTGNT - 98
	103 - RK - 104
Posterior @ 0.4	

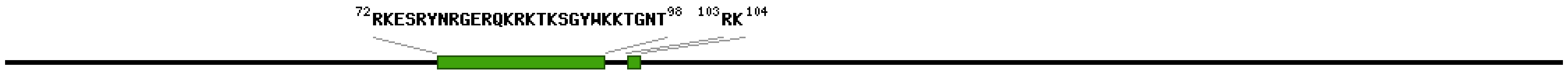

Posterior Graphic. Threshold Marked @ 0.4

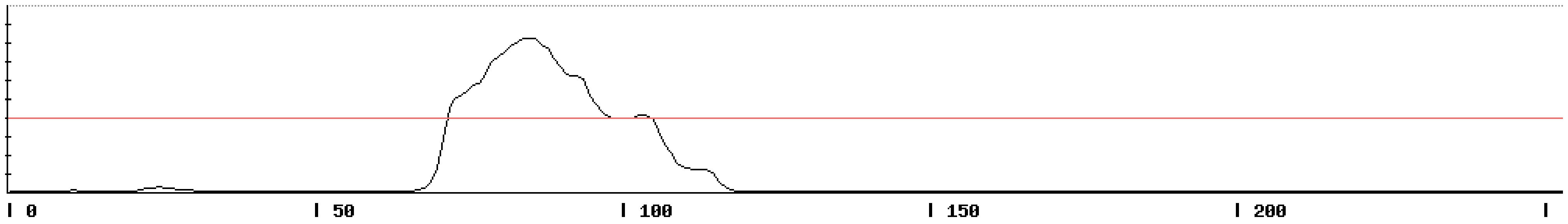

Predictions for Brassicaoleracea_XP_013603948.1

Viterbi Path

Viterbi Path

Posterior @ 0.4

Posterior @ 0.4

Posterior Graphic. Threshold Marked @ 0.4

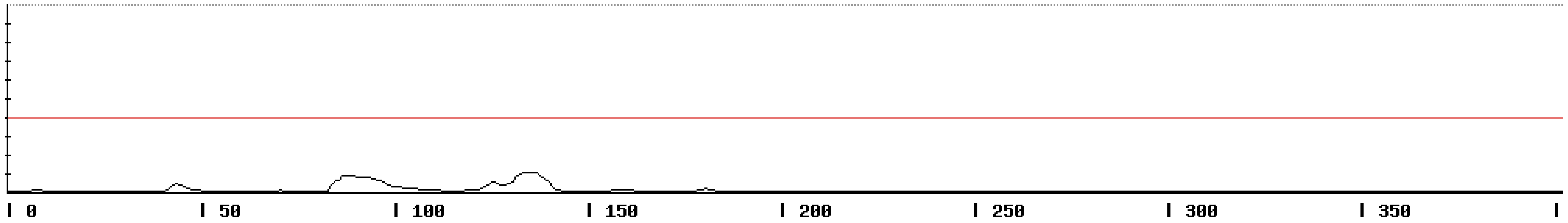

Predictions for Brassicaoleracea_XP_013604293.1

Viterbi Path

Viterbi Path

Posterior @ 0.4

Posterior @ 0.4

Posterior Graphic. Threshold Marked @ 0.4

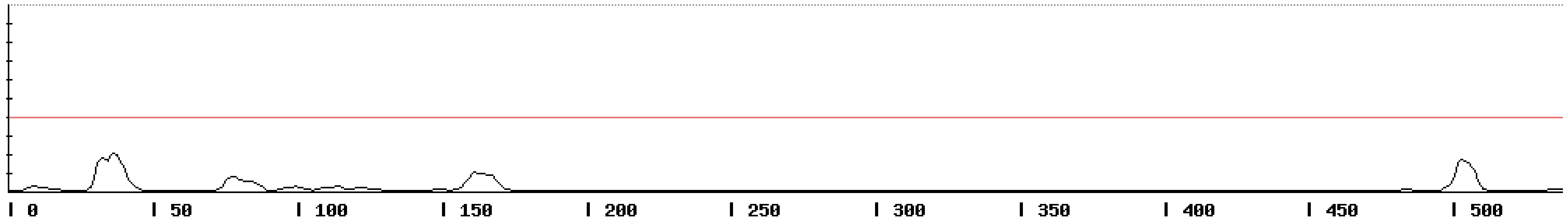

Predictions for Brassicaoleracea_XP_013605626.1

Viterbi Path

Viterbi Path

Posterior @ 0.4 — 105 - DRKIKS - 110

Posterior @ 0.4

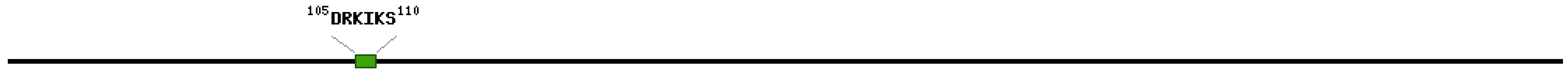

Posterior Graphic. Threshold Marked @ 0.4

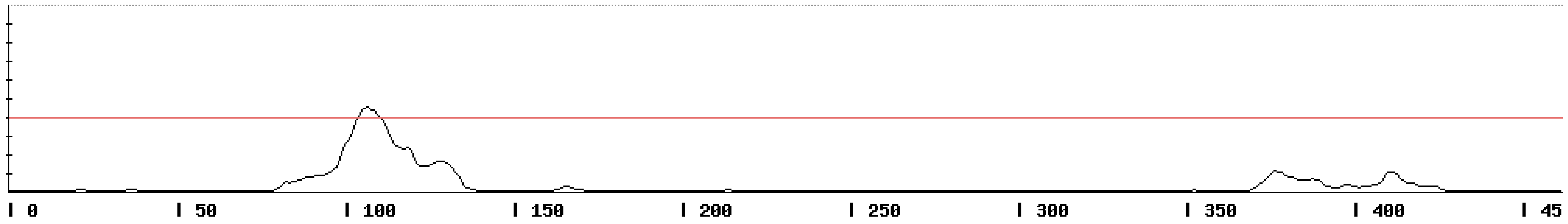

Predictions for Brassicaoleracea_XP_013605970.1

Viterbi Path

Viterbi Path

Posterior @ 0.4 | 75 - EKKKGTKRTTPSGFWKATGKDRIIRDKRGHGV - 106

Posterior @ 0.4

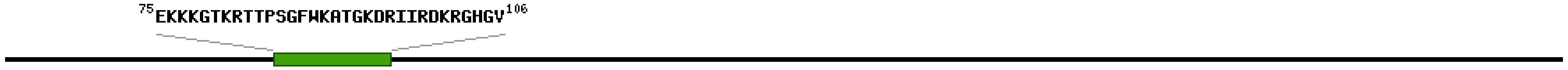

Posterior Graphic. Threshold Marked @ 0.4

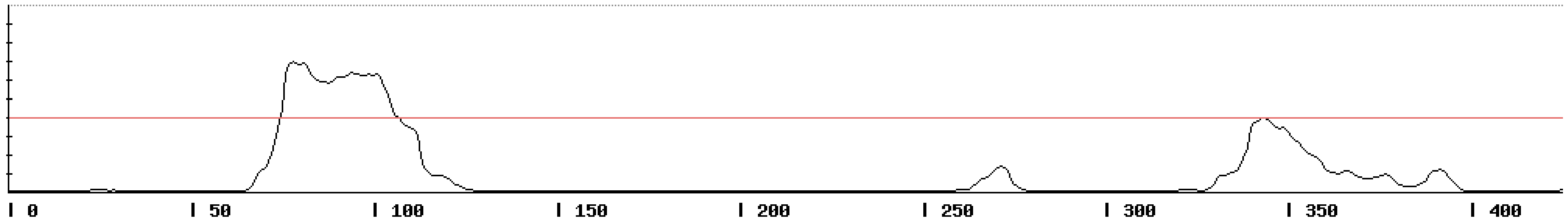

Predictions for Brassicaoleracea_XP_013605971.1

Viterbi Path

Viterbi Path

Posterior @ 0.4 | 75 - EKKKGTKRTTPSGFWKATGKDRIIRDKRGHGV - 106

Posterior @ 0.4

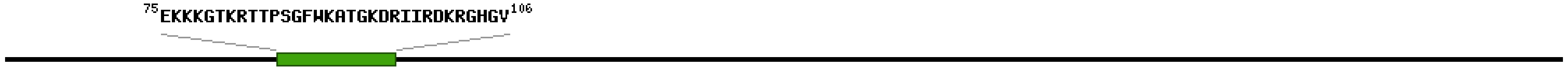

Posterior Graphic. Threshold Marked @ 0.4

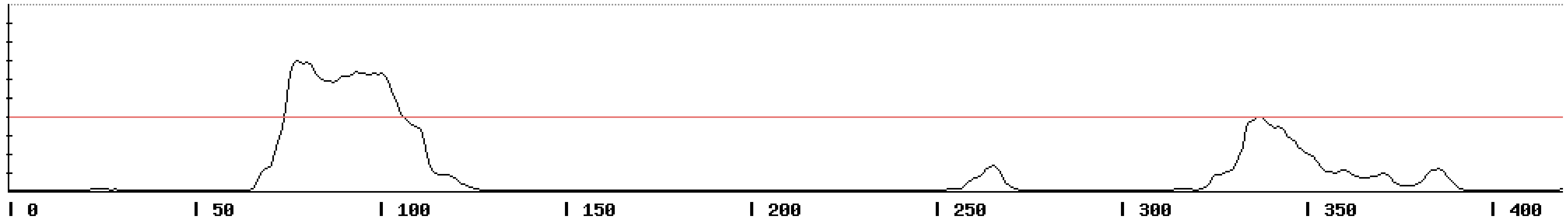

Predictions for Brassicaoleracea_XP_013606555.1

Viterbi Path

Viterbi Path

Posterior @ 0.4

Posterior @ 0.4

Posterior Graphic. Threshold Marked @ 0.4

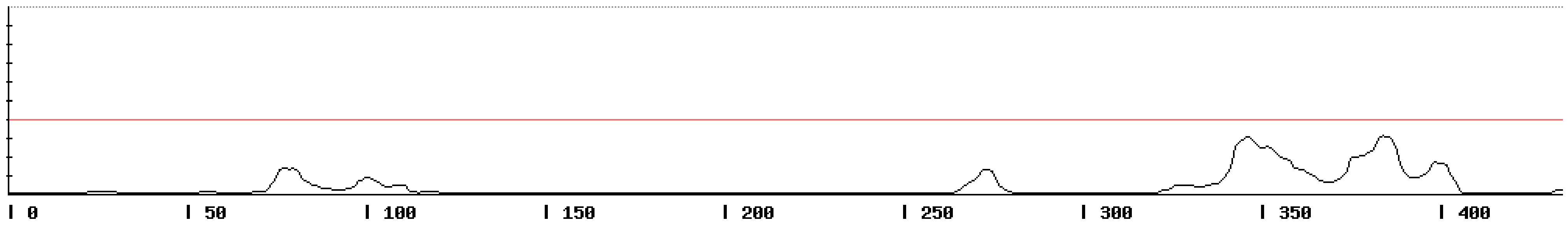

Predictions for Brassicaoleracea_XP_013606976.1

Viterbi Path

Viterbi Path

Posterior @ 0.4

Posterior @ 0.4

Posterior Graphic. Threshold Marked @ 0.4

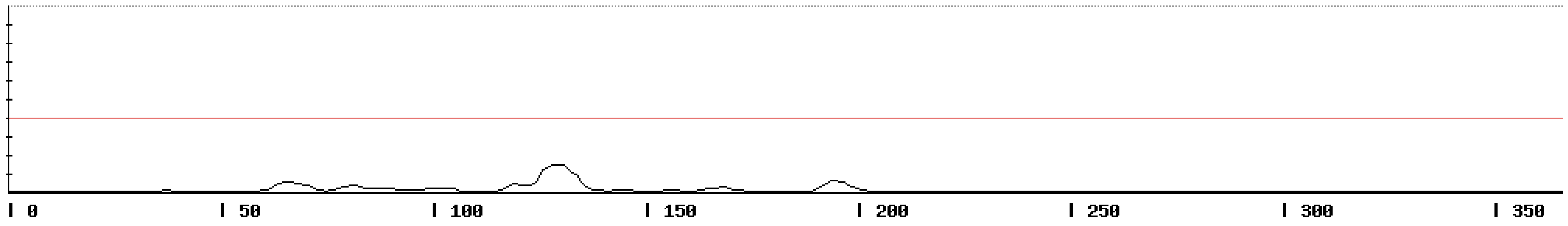

Predictions for Brassicaoleracea_XP_013607097.1

Viterbi Path	
Viterbi Path	
Posterior @ 0.4	
Posterior @ 0.4	
Posterior Graphic. Threshold Marked @ 0.4	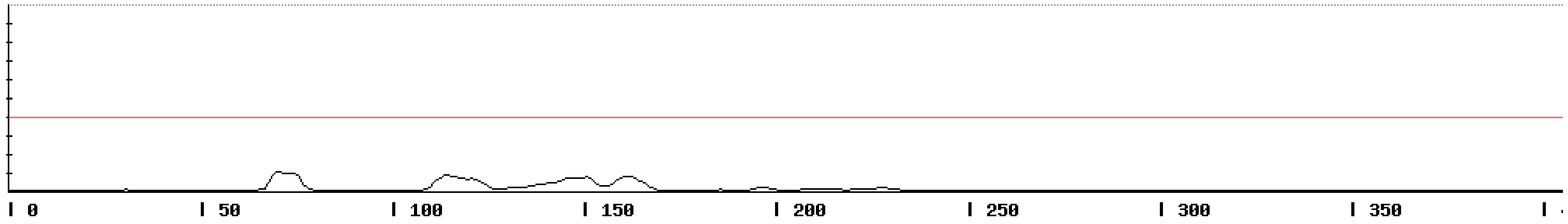

Predictions for Brassicaoleracea_XP_013607098.1

Viterbi Path	
Viterbi Path	
Posterior @ 0.4	
Posterior @ 0.4	
Posterior Graphic. Threshold Marked @ 0.4	

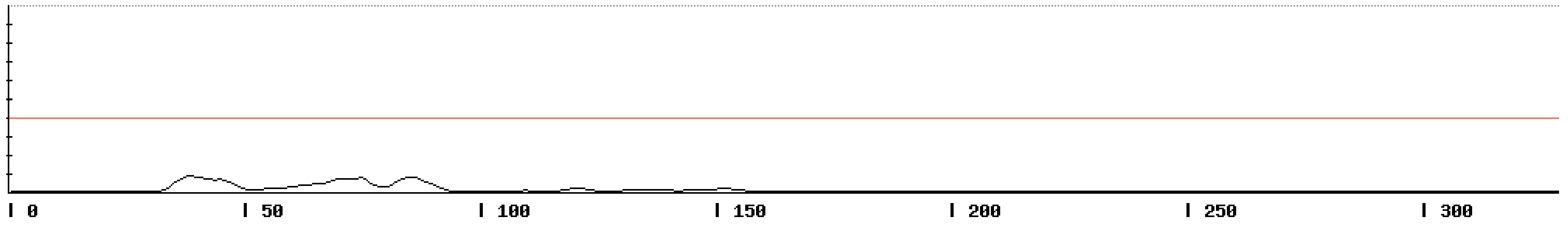

Predictions for Brassicaoleracea_XP_013607352.1

Viterbi Path	
Viterbi Path	
Posterior @ 0.4	126 - KR - 127
Posterior @ 0.4	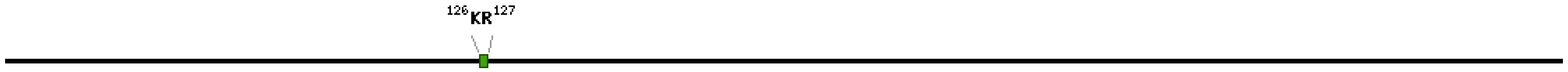
Posterior Graphic. Threshold Marked @ 0.4	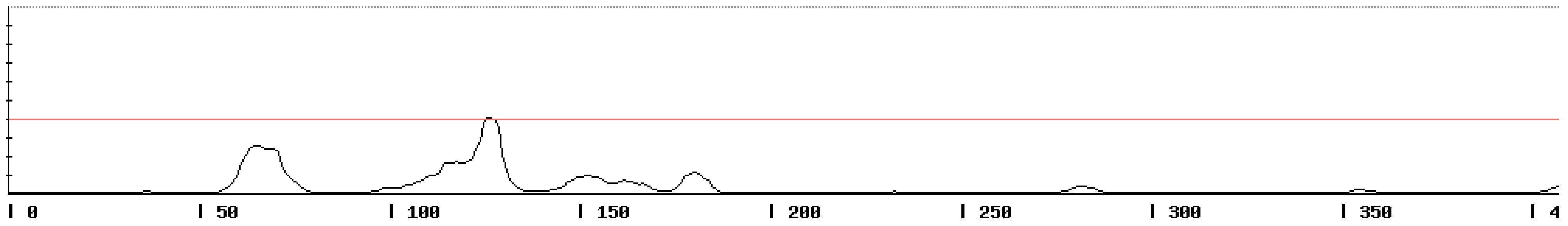

Predictions for Brassicaoleracea_XP_013607353.1

Viterbi Path	
Viterbi Path	
Posterior @ 0.4	126 - KR - 127
Posterior @ 0.4	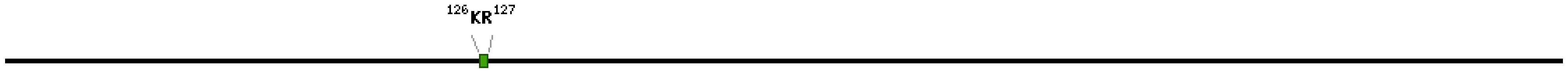

Posterior Graphic. Threshold Marked @ 0.4

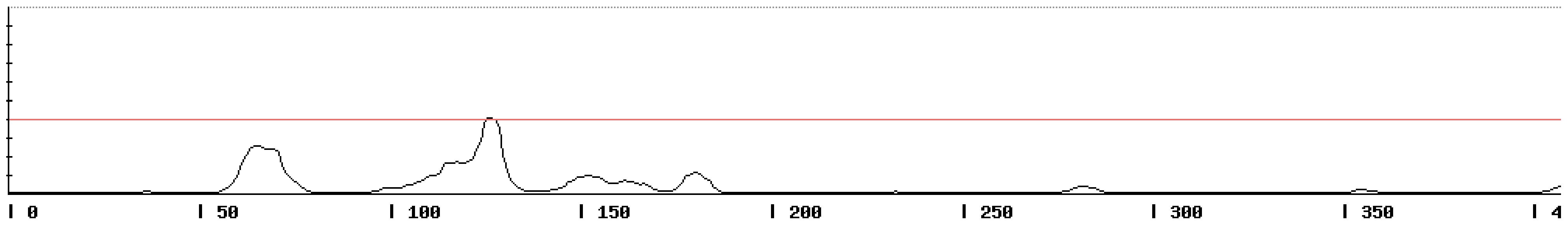

Predictions for Brassicaoleracea_XP_013607354.1

Viterbi Path

Viterbi Path

Posterior @ 0.4 126 - KR - 127

Posterior @ 0.4

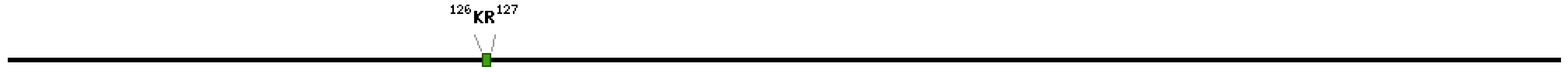

Posterior Graphic. Threshold Marked @ 0.4

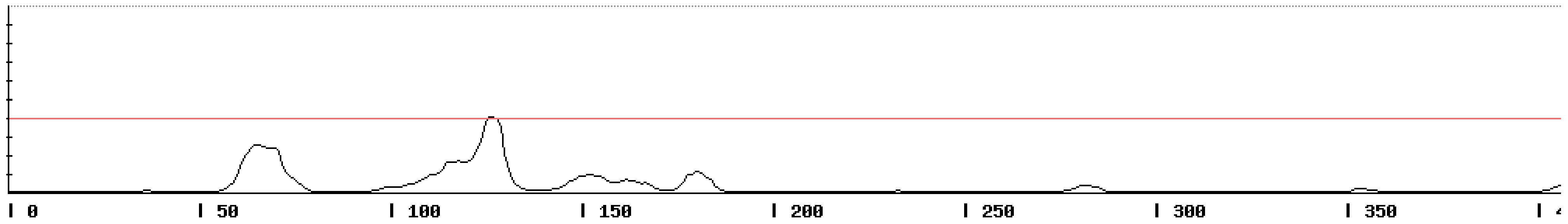

Predictions for Brassicaoleracea_XP_013607355.1

Viterbi Path

Viterbi Path

Posterior @ 0.4 126 - KR - 127

Posterior @ 0.4

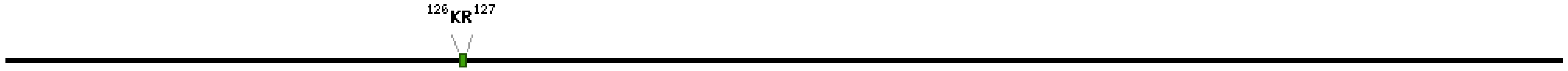

Posterior Graphic. Threshold Marked @ 0.4

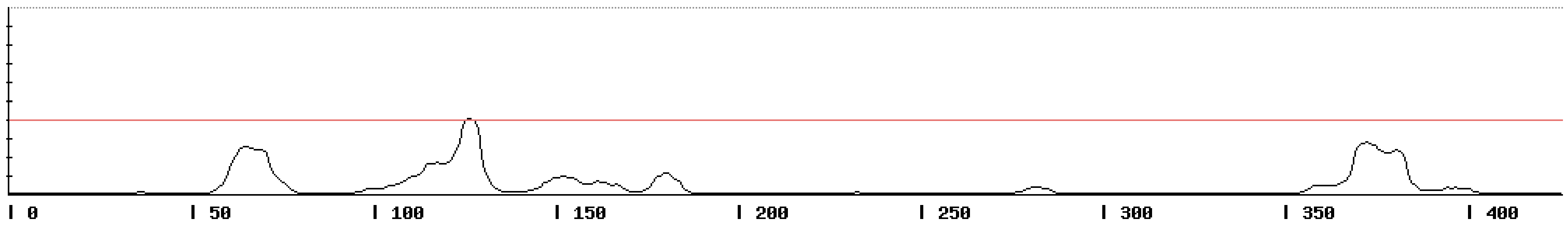

Predictions for Brassicaoleracea_XP_013607450.1

Viterbi Path

Viterbi Path

Posterior @ 0.4 72 - KSPKKSLTKRTTPSGFWKSTGKDRKVKEEKRRDG - 105

Posterior @ 0.4

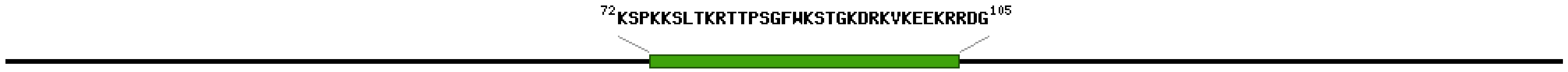

Posterior Graphic. Threshold Marked @ 0.4

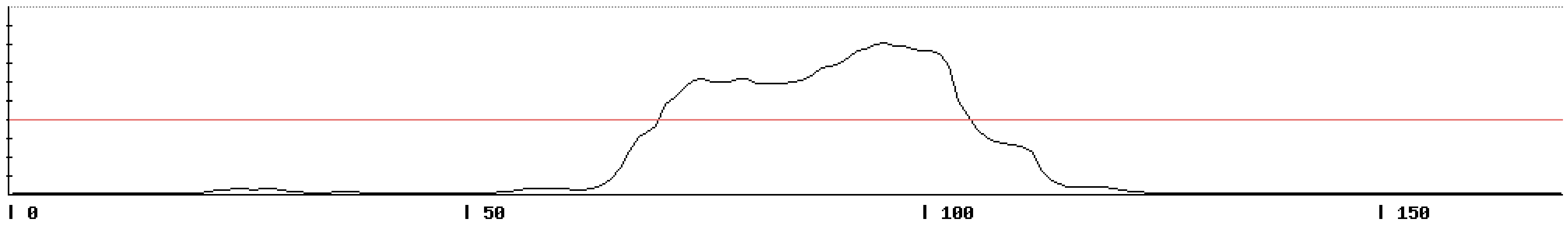

Predictions for Brassicaoleracea_XP_013607451.1

Viterbi Path

Viterbi Path

Posterior @ 0.4 — 76 - KKKGTKR - 82

Posterior @ 0.4

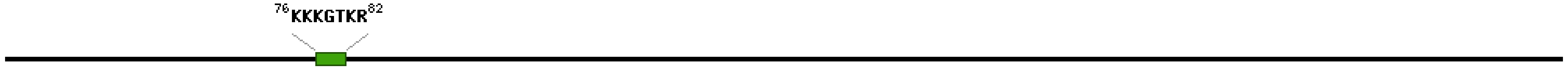

Posterior Graphic. Threshold Marked @ 0.4

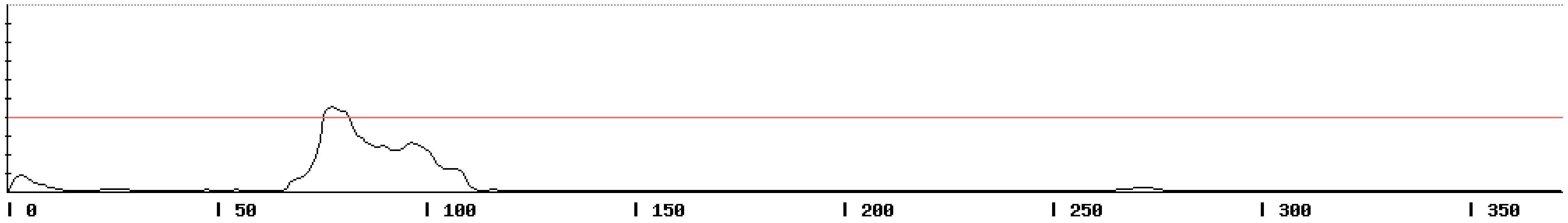

Predictions for Brassicaoleracea_XP_013607456.1

Viterbi Path

Viterbi Path

Posterior @ 0.4 — 65 - RRNKRPGKQKA - 75

Posterior @ 0.4

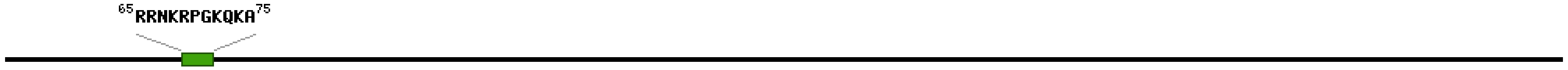

Posterior Graphic. Threshold Marked @ 0.4

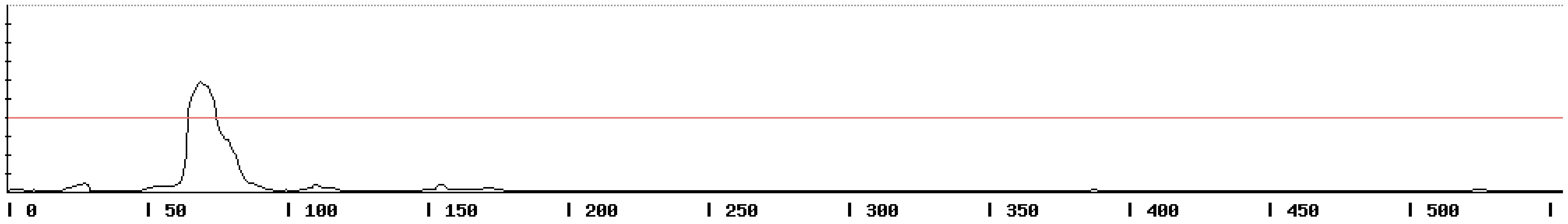

Predictions for Brassicaoleracea_XP_013607494.1

Viterbi Path

Viterbi Path

Posterior @ 0.4 | 23 - RTKIKKRRRETKTFTKKIKVK - 43

Posterior @ 0.4

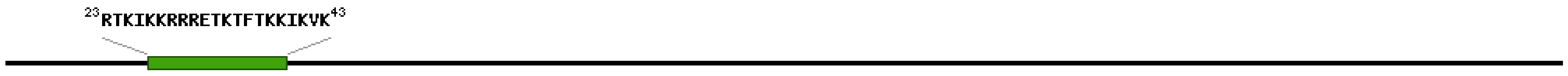

Posterior Graphic. Threshold Marked @ 0.4

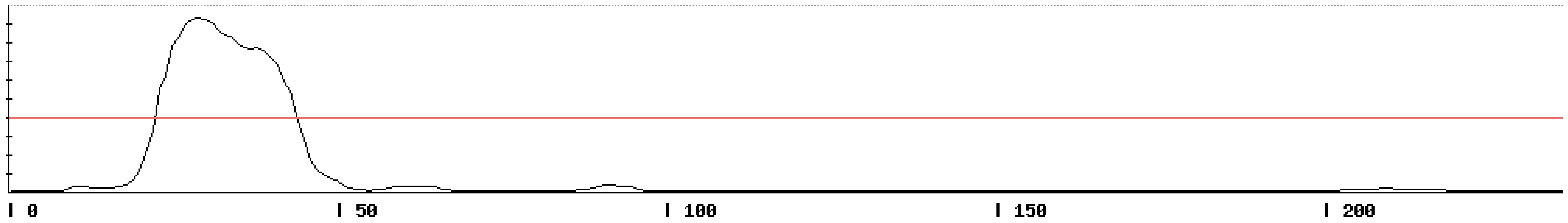

Predictions for Brassicaoleracea_XP_013608112.1

Viterbi Path

Viterbi Path

Posterior @ 0.4

Posterior @ 0.4

Posterior Graphic. Threshold Marked @ 0.4

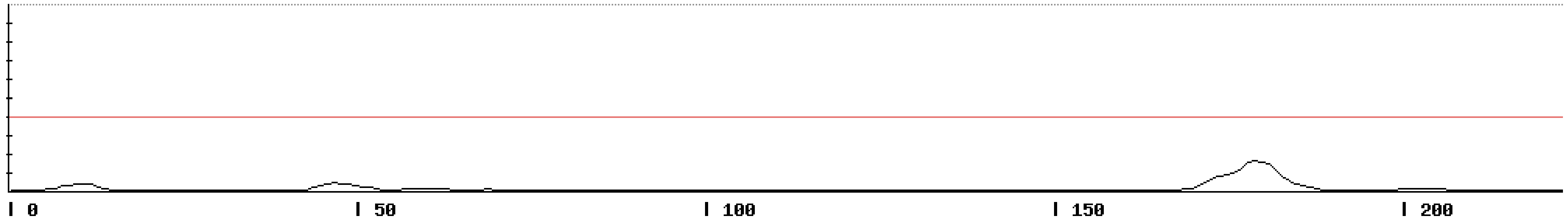

Predictions for Brassicaoleracea_XP_013608221.1

Viterbi Path

Viterbi Path

Posterior @ 0.4

Posterior @ 0.4

Posterior Graphic. Threshold Marked @ 0.4

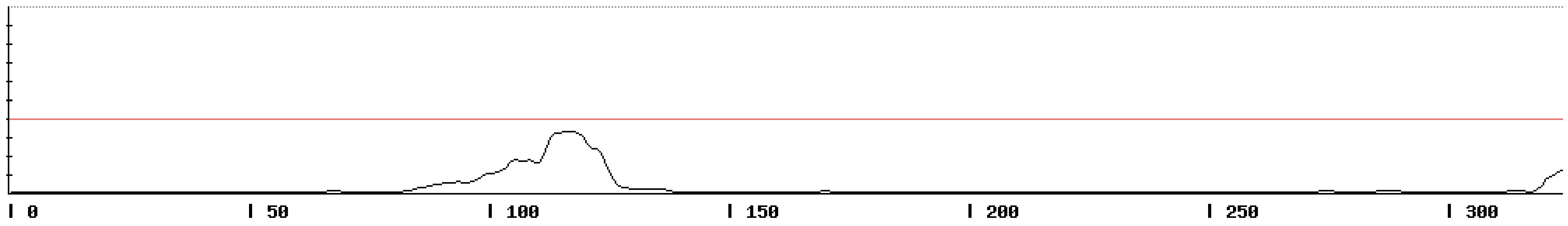

Predictions for Brassicaoleracea_XP_013608281.1

Viterbi Path

Viterbi Path

Posterior @ 0.4

Posterior @ 0.4

Posterior Graphic. Threshold Marked @ 0.4

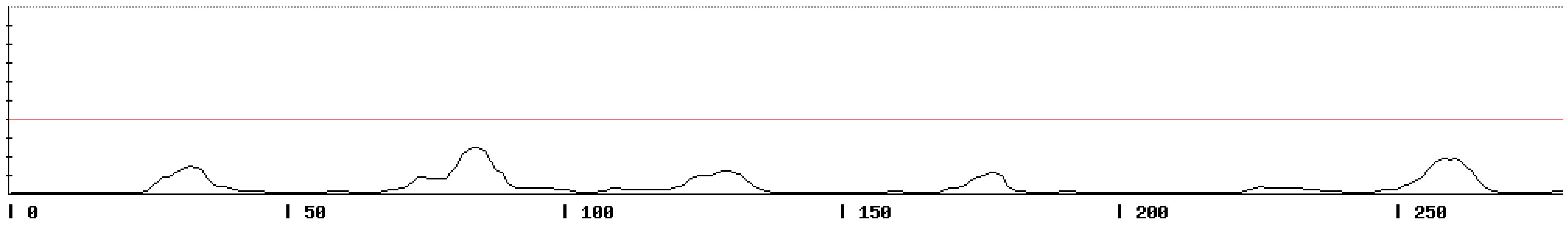

Predictions for Brassicaoleracea_XP_013608283.1	
Viterbi Path	
Viterbi Path	
Posterior @ 0.4	
Posterior @ 0.4	
Posterior Graphic. Threshold Marked @ 0.4	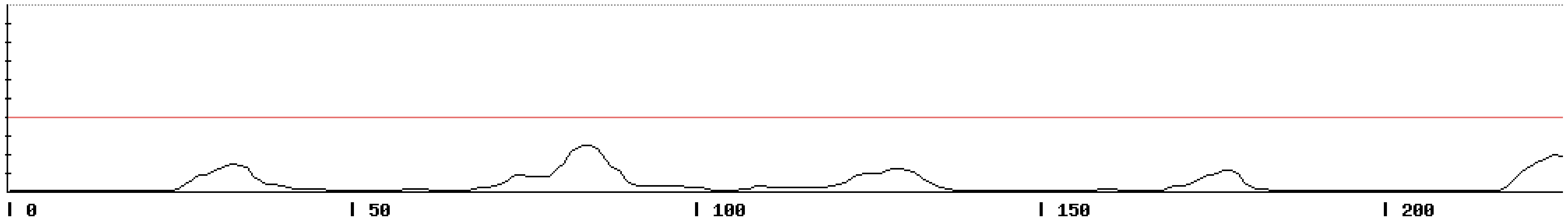

Predictions for Brassicaoleracea_XP_013608285.1	
Viterbi Path	
Viterbi Path	
Posterior @ 0.4	74 - KKKIGCGKKVKRKI - 87
Posterior @ 0.4	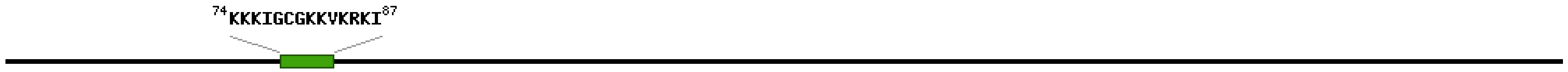
Posterior Graphic. Threshold Marked @ 0.4	

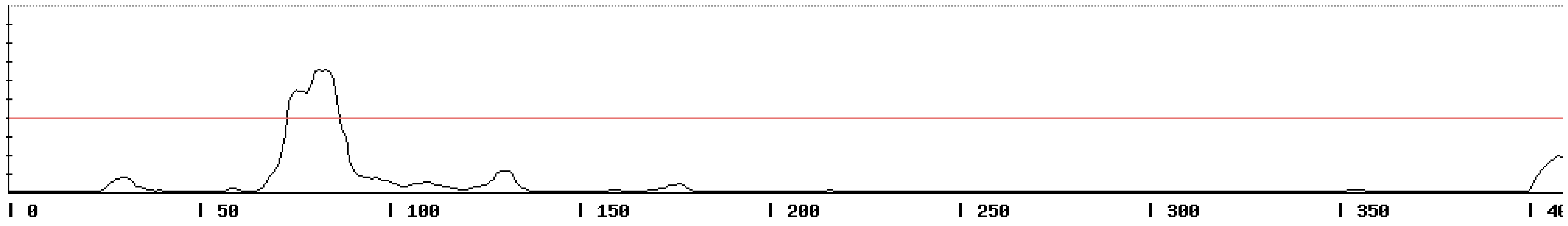

Predictions for Brassicaoleracea_XP_013608476.1

Viterbi Path

Viterbi Path

Posterior @ 0.4

Posterior @ 0.4

Posterior Graphic. Threshold Marked @ 0.4

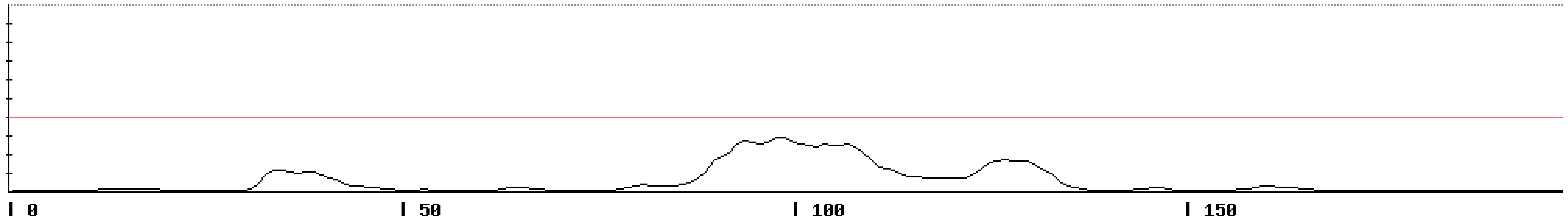

Predictions for Brassicaoleracea_XP_013608832.1

Viterbi Path

Viterbi Path

Posterior @ 0.4

Posterior @ 0.4

Posterior Graphic. Threshold Marked @ 0.4

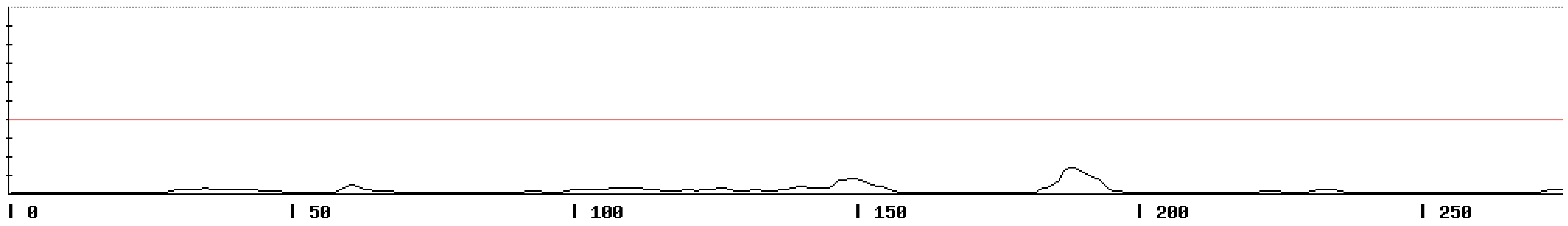

Predictions for Brassicaoleracea_XP_013609014.1

Viterbi Path

Viterbi Path

Posterior @ 0.4

Posterior @ 0.4

Posterior Graphic. Threshold Marked @ 0.4

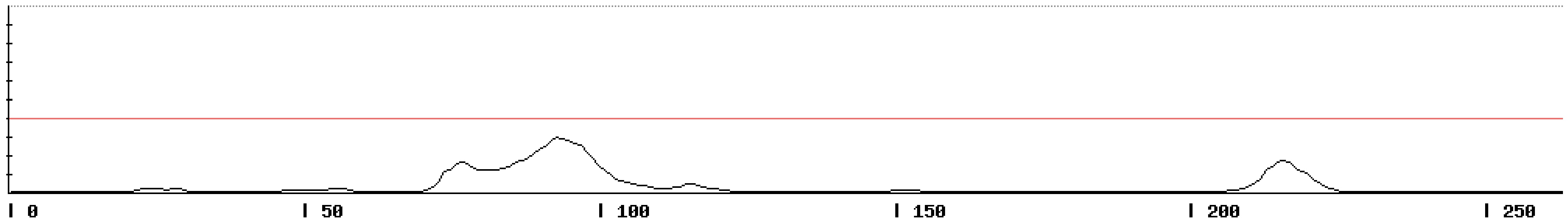

Predictions for Brassicaoleracea_XP_013609062.1

Viterbi Path

Viterbi Path

Posterior @ 0.4

Posterior @ 0.4

Posterior Graphic. Threshold Marked @ 0.4

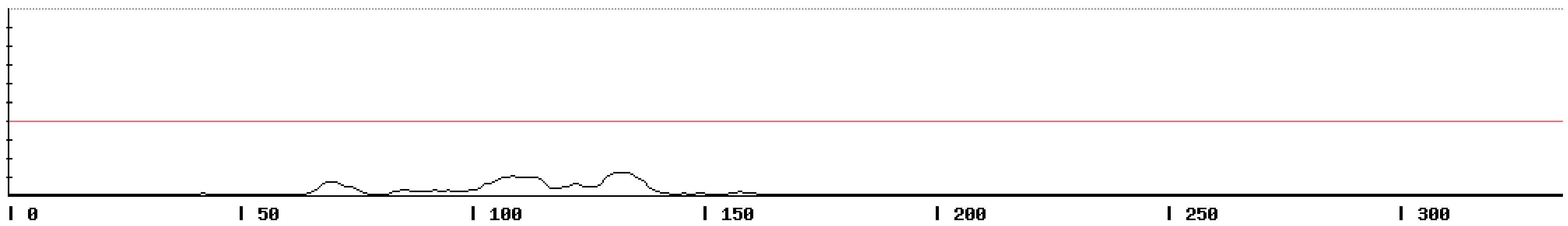

Predictions for Brassicaoleracea_XP_013609230.1

Viterbi Path

Viterbi Path

Posterior @ 0.4

Posterior @ 0.4

Posterior Graphic. Threshold Marked @ 0.4

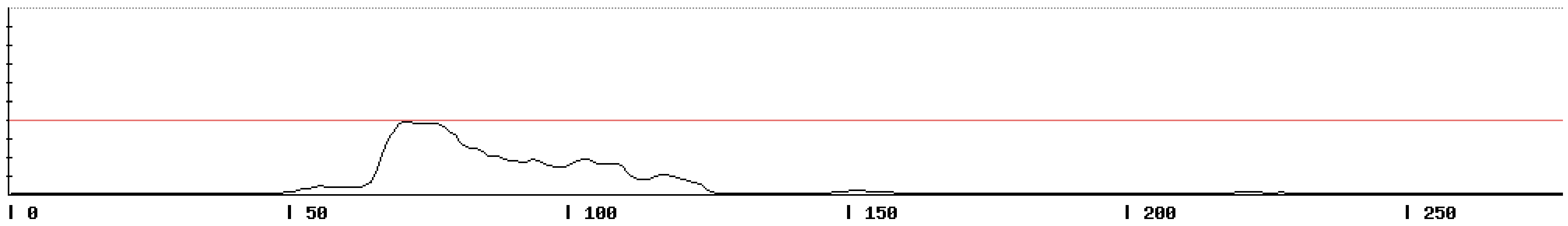

Predictions for Brassicaoleracea_XP_013609231.1

Viterbi Path

Viterbi Path

Posterior @ 0.4

Posterior @ 0.4

Posterior Graphic. Threshold Marked @ 0.4

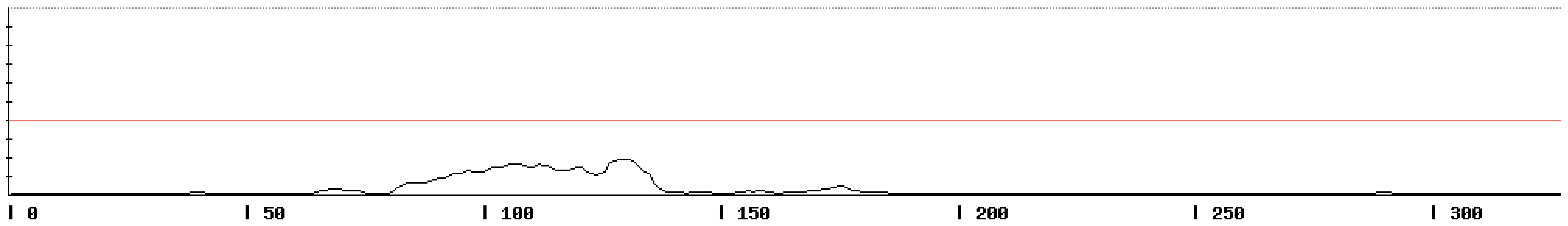

Predictions for Brassicaoleracea_XP_013609232.1

Viterbi Path

Viterbi Path

Posterior @ 0.4

Posterior @ 0.4

Posterior Graphic. Threshold Marked @ 0.4

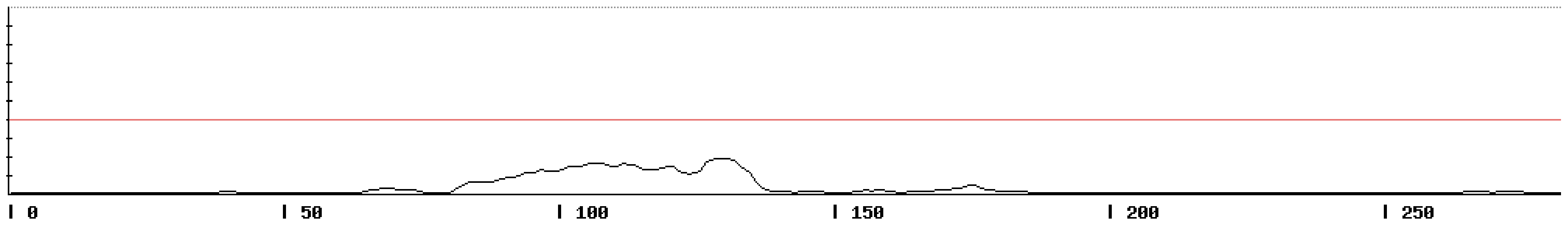

Predictions for Brassicaoleracea_XP_013609384.1

Viterbi Path

Viterbi Path

Posterior @ 0.4	
Posterior @ 0.4	
Posterior Graphic. Threshold Marked @ 0.4	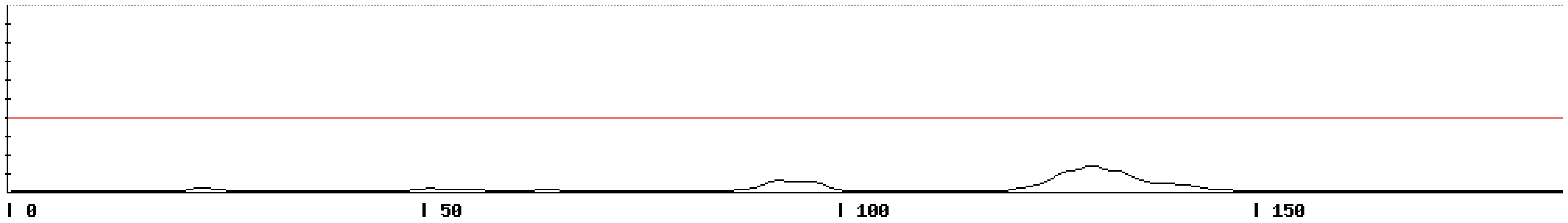

Predictions for Brassicaoleracea_XP_013609604.1

Viterbi Path	200 - RKASKGKRQRKSRGR - 214
Viterbi Path	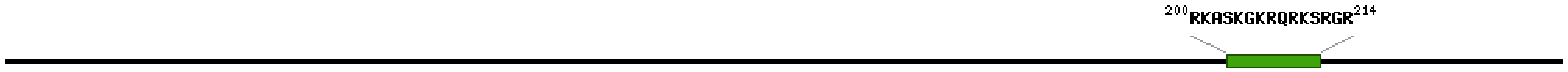
Posterior @ 0.4	115 - KKRR - 118<br>199 - ERKASKGKRQRKSRGR - 214
Posterior @ 0.4	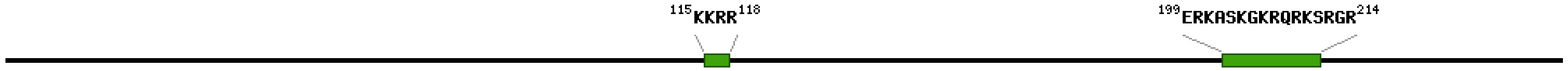
Posterior Graphic. Threshold Marked @ 0.4	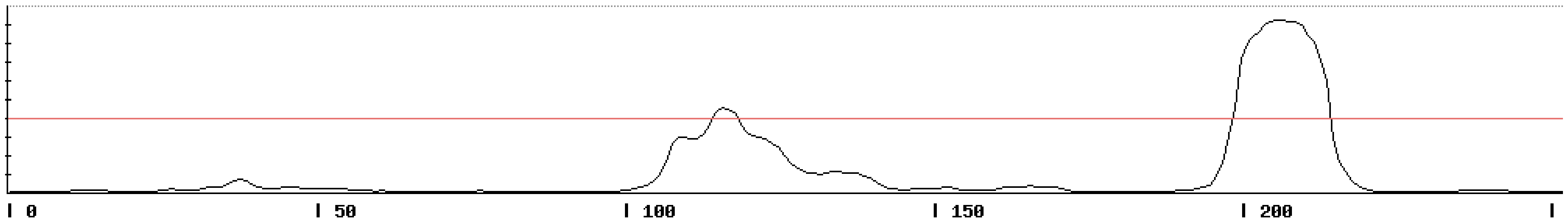

Predictions for Brassicaoleracea_XP_013609605.1	
Viterbi Path	199 - RKASKGKRQRKSRGR - 213
Viterbi Path	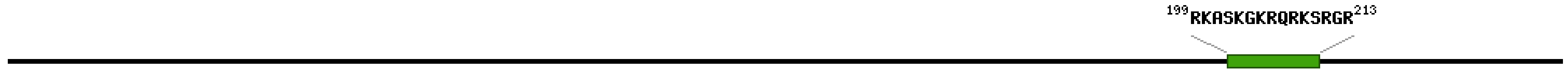
Posterior @ 0.4	115 - KKRR - 118
	198 - ERKASKGKRQRKSRGR - 213
Posterior @ 0.4	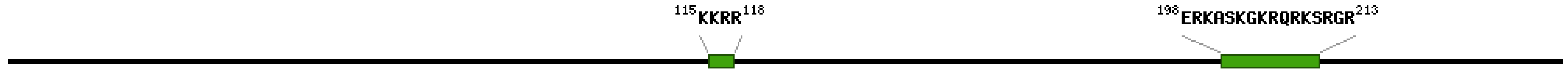
Posterior Graphic. Threshold Marked @ 0.4	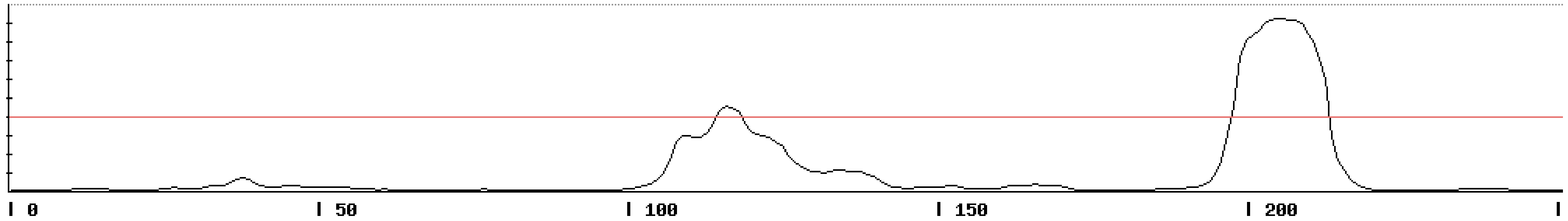

Predictions for Brassicaoleracea_XP_013609760.1	
Viterbi Path	
Viterbi Path	
Posterior @ 0.4	
Posterior @ 0.4	
Posterior Graphic. Threshold Marked @ 0.4	

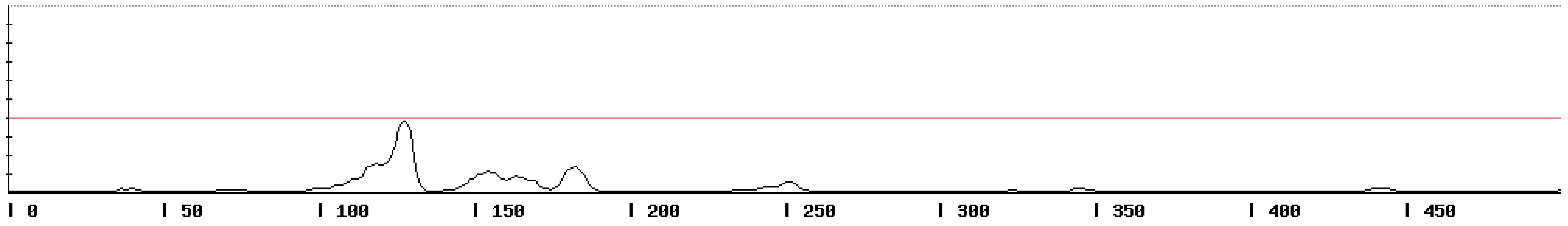

Predictions for Brassicaoleracea_XP_013609773.1

Viterbi Path

Viterbi Path

Posterior @ 0.4

Posterior @ 0.4

Posterior Graphic. Threshold Marked @ 0.4

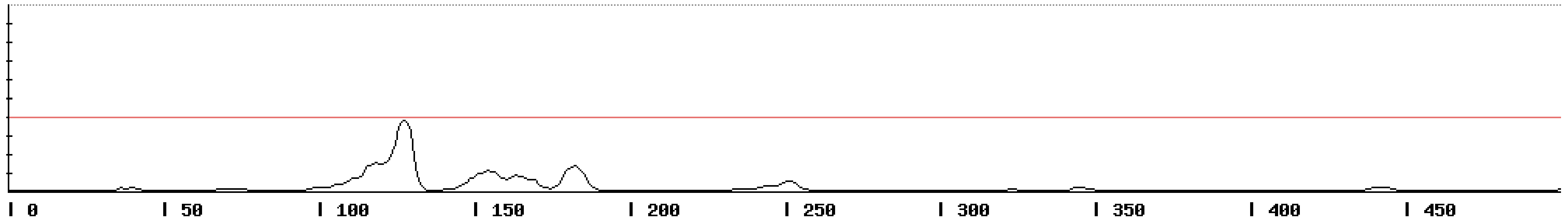

Predictions for Brassicaoleracea_XP_013609781.1

Viterbi Path

Viterbi Path

Posterior @ 0.4

Posterior @ 0.4

Posterior Graphic. Threshold Marked @ 0.4

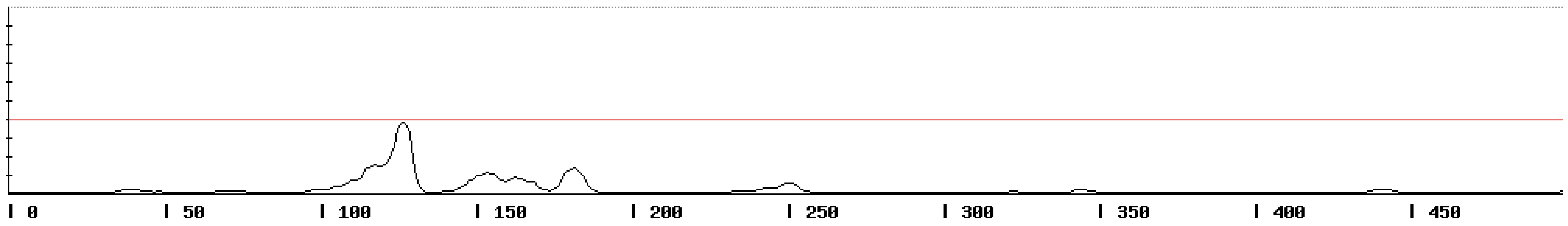

Predictions for Brassicaoleracea_XP_013609789.1

Viterbi Path

Viterbi Path

Posterior @ 0.4

Posterior @ 0.4

Posterior Graphic. Threshold Marked @ 0.4

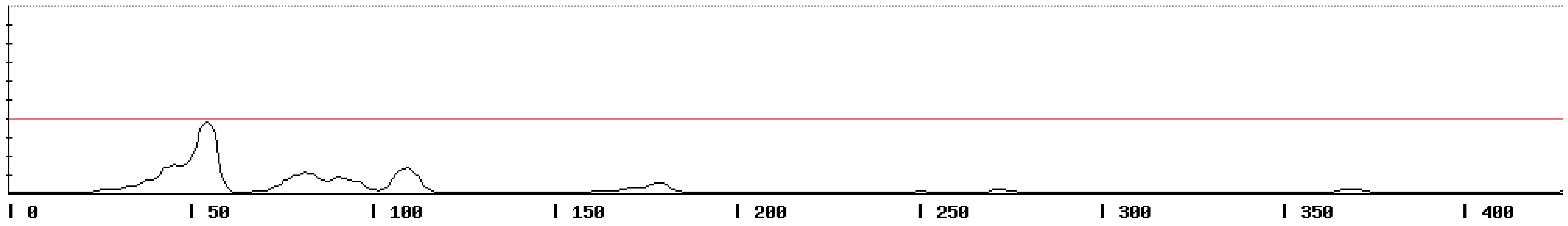

Predictions for Brassicaoleracea_XP_013609845.1

Viterbi Path

Viterbi Path

Posterior @ 0.4

Posterior @ 0.4

Posterior Graphic. Threshold Marked @ 0.4	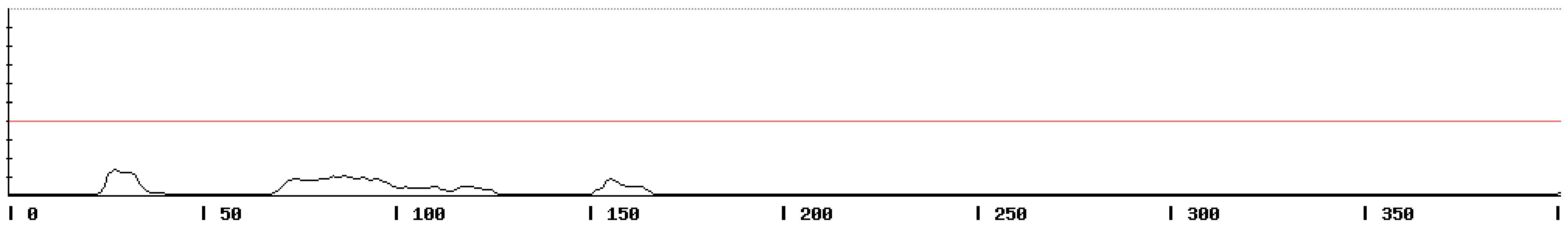

Predictions for Brassicaoleracea_XP_013609846.1

Viterbi Path	
Viterbi Path	
Posterior @ 0.4	
Posterior @ 0.4	
Posterior Graphic. Threshold Marked @ 0.4	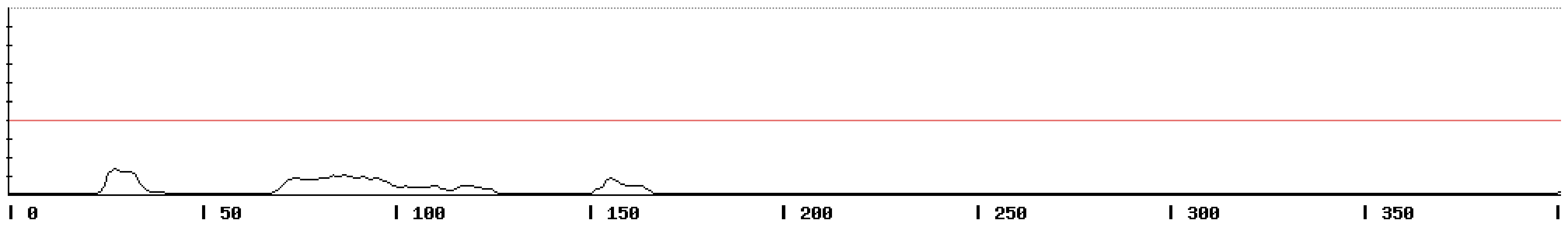

Predictions for Brassicaoleracea_XP_013609847.1

Viterbi Path	
Viterbi Path	

Posterior @ 0.4

Posterior @ 0.4

Posterior Graphic. Threshold Marked @ 0.4

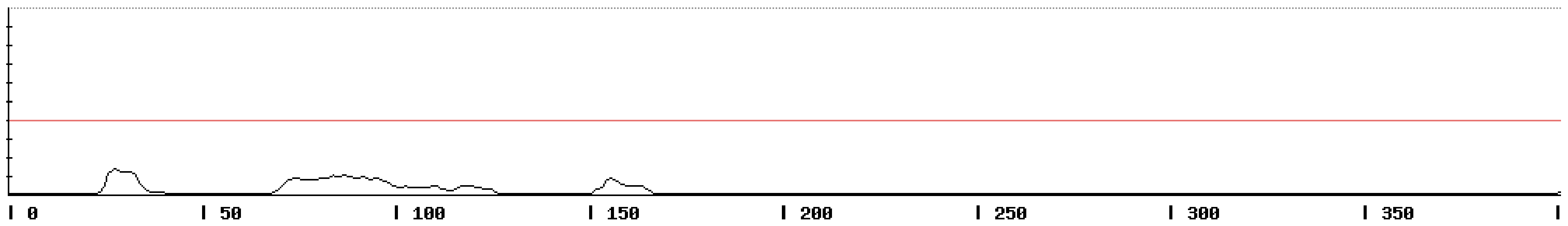

Predictions for Brassicaoleracea_XP_013609952.1

Viterbi Path

Viterbi Path

Posterior @ 0.4 — 138 - KRR - 140

Posterior @ 0.4

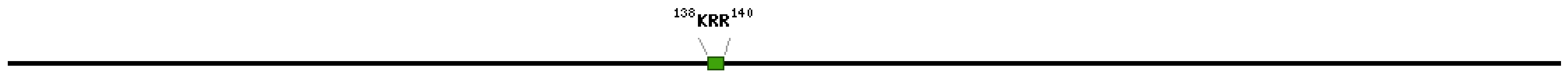

Posterior Graphic. Threshold Marked @ 0.4

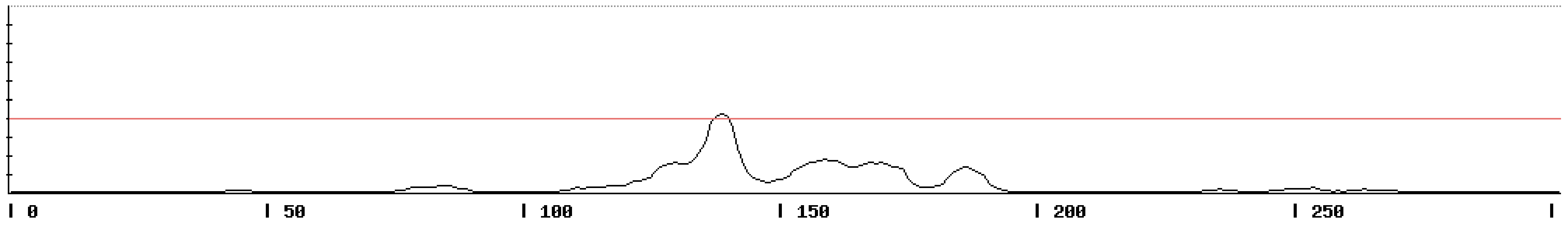

Predictions for Brassicaoleracea_XP_013610035.1

Viterbi Path

Viterbi Path

Posterior @ 0.4

Posterior @ 0.4

Posterior Graphic. Threshold Marked @ 0.4

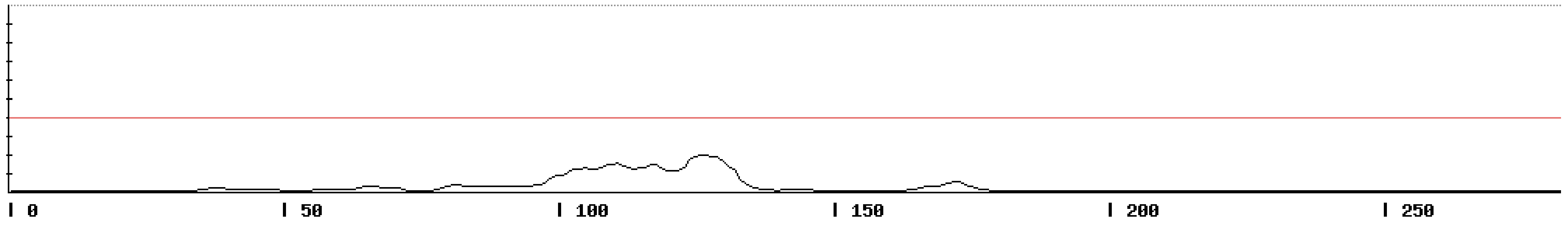

Predictions for Brassicaoleracea_XP_013610106.1

Viterbi Path

Viterbi Path

Posterior @ 0.4

Posterior @ 0.4

Posterior Graphic. Threshold Marked @ 0.4

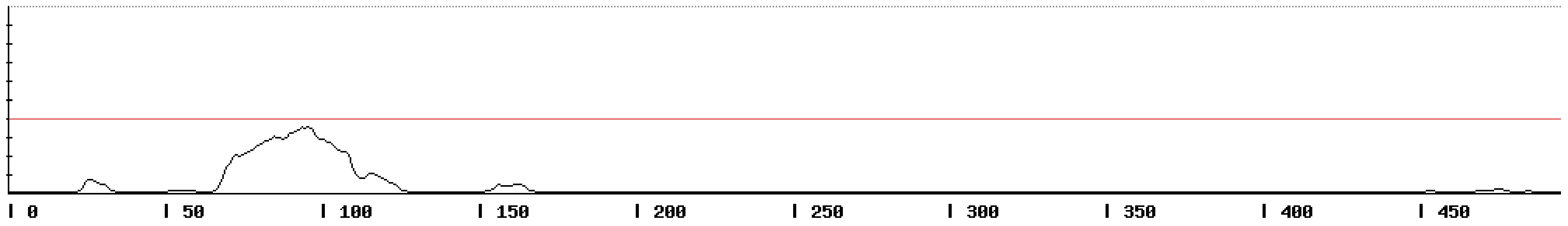

Predictions for Brassicaoleracea_XP_013610161.1

Viterbi Path	
Viterbi Path	
Posterior @ 0.4	
Posterior @ 0.4	
Posterior Graphic. Threshold Marked @ 0.4	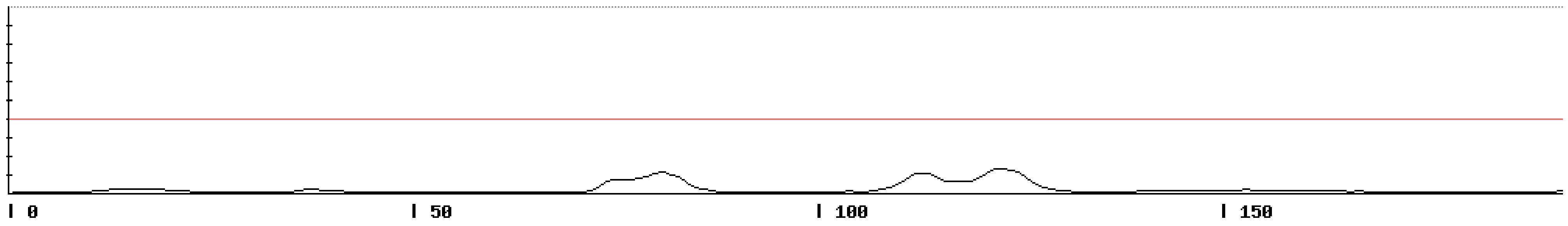

Predictions for Brassicaoleracea_XP_013610422.1

Viterbi Path	
Viterbi Path	
Posterior @ 0.4	
Posterior @ 0.4	
Posterior Graphic. Threshold Marked @ 0.4	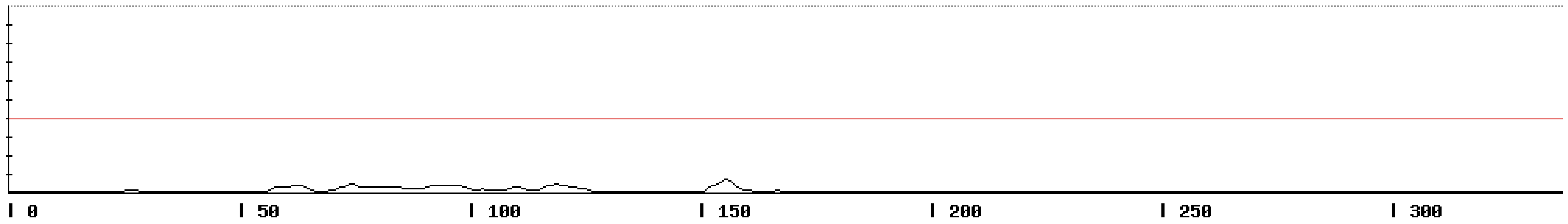

Predictions for Brassicaoleracea_XP_013610671.1

Viterbi Path	
Viterbi Path	
Posterior @ 0.4	114 - KKTKRAR - 120
Posterior @ 0.4	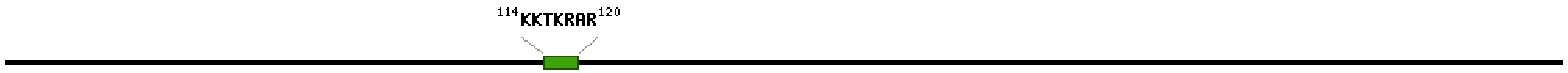
Posterior Graphic. Threshold Marked @ 0.4	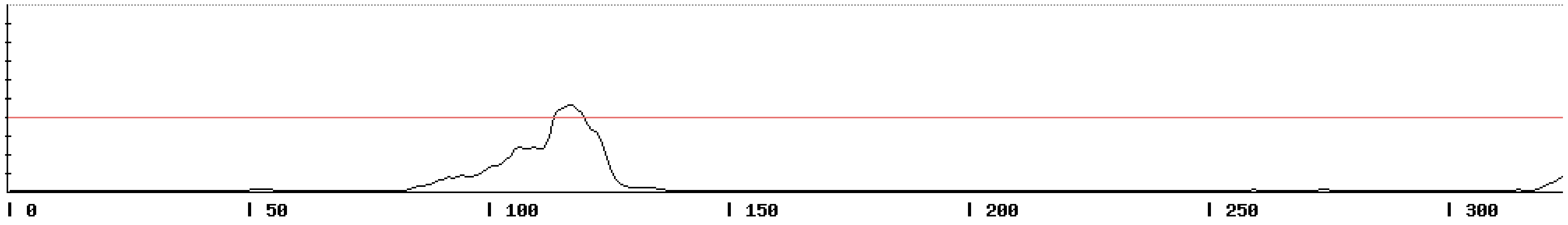

Predictions for Brassicaoleracea_XP_013610715.1

Viterbi Path	
Viterbi Path	
Posterior @ 0.4	
Posterior @ 0.4	
Posterior Graphic. Threshold Marked @ 0.4	

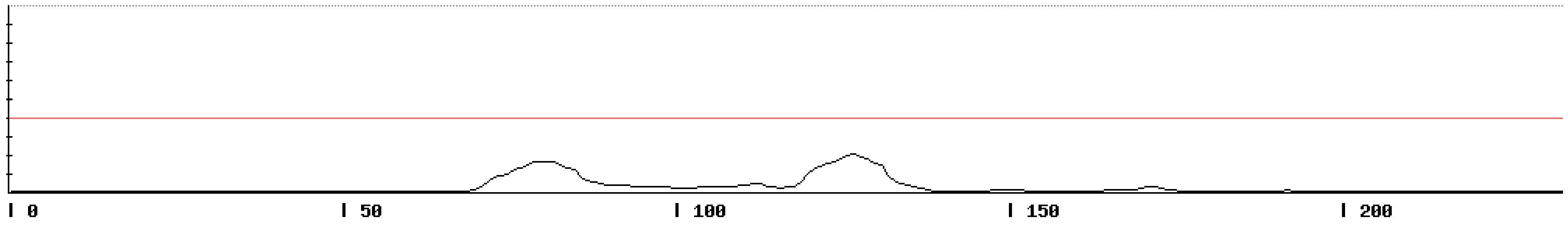

Predictions for Brassicaoleracea_XP_013611181.1

Viterbi Path

Viterbi Path

Posterior @ 0.4

Posterior @ 0.4

Posterior Graphic. Threshold Marked @ 0.4

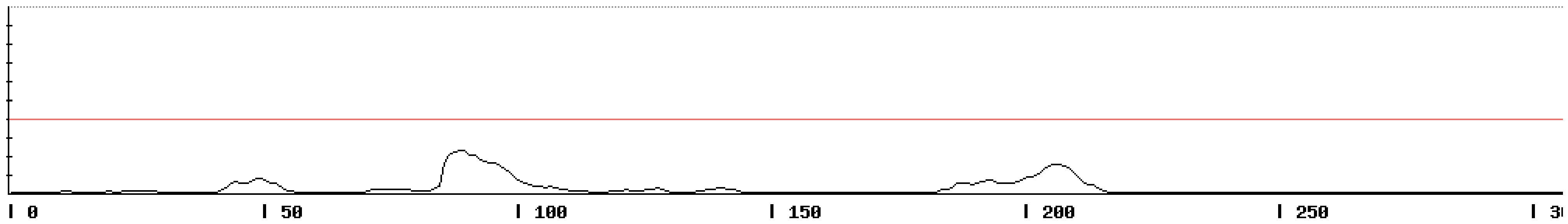

Predictions for Brassicaoleracea_XP_013611566.1

Viterbi Path

Viterbi Path

Posterior @ 0.4

Posterior @ 0.4

Posterior Graphic. Threshold Marked @ 0.4

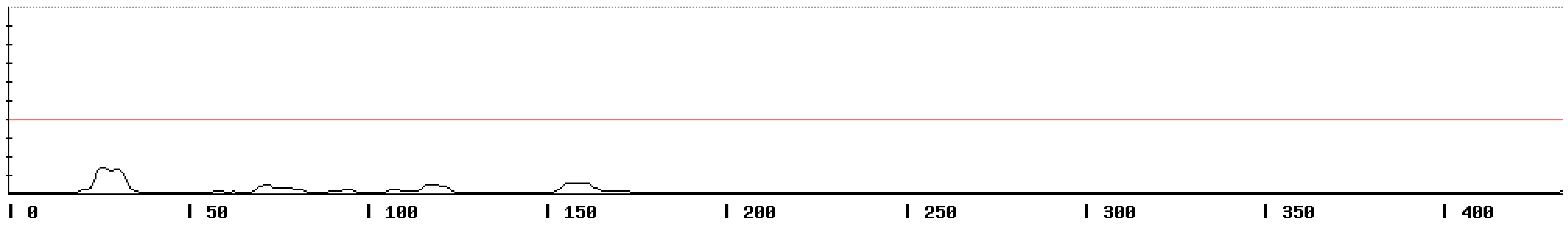

Predictions for Brassicaoleracea_XP_013611567.1

Viterbi Path

Viterbi Path

Posterior @ 0.4

Posterior @ 0.4

Posterior Graphic. Threshold Marked @ 0.4

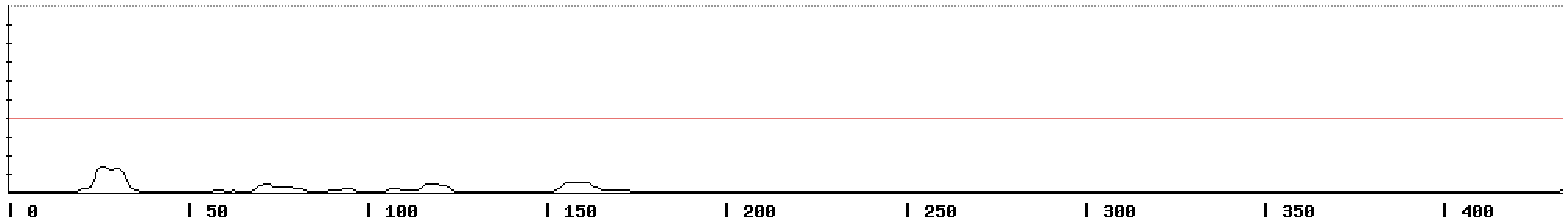

Predictions for Brassicaoleracea_XP_013612508.1

Viterbi Path

Viterbi Path

Posterior @ 0.4

Posterior @ 0.4

Posterior Graphic. Threshold Marked @ 0.4

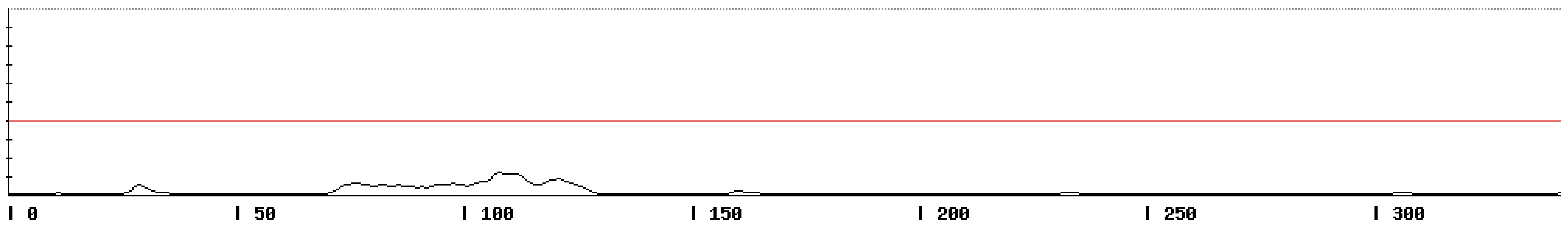

Predictions for Brassicaoleracea_XP_013612936.1

Viterbi Path

Viterbi Path

Posterior @ 0.4

Posterior @ 0.4

Posterior Graphic. Threshold Marked @ 0.4

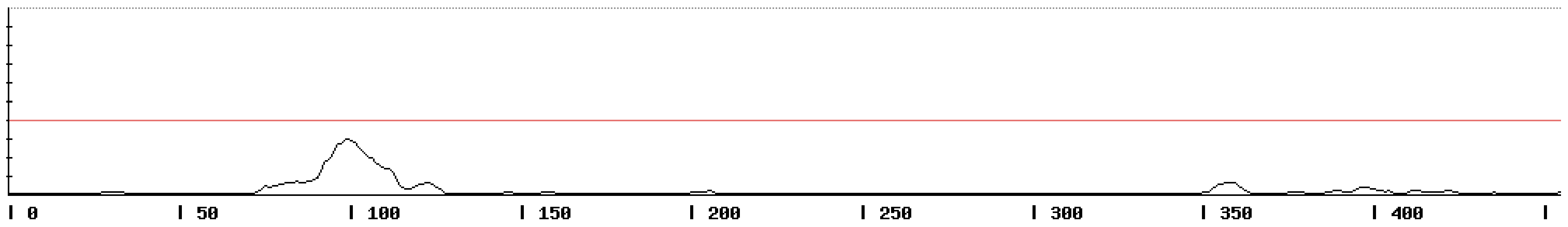

Predictions for Brassicaoleracea_XP_013612937.1

Viterbi Path

Viterbi Path

Posterior @ 0.4

Posterior @ 0.4

Posterior Graphic. Threshold Marked @ 0.4

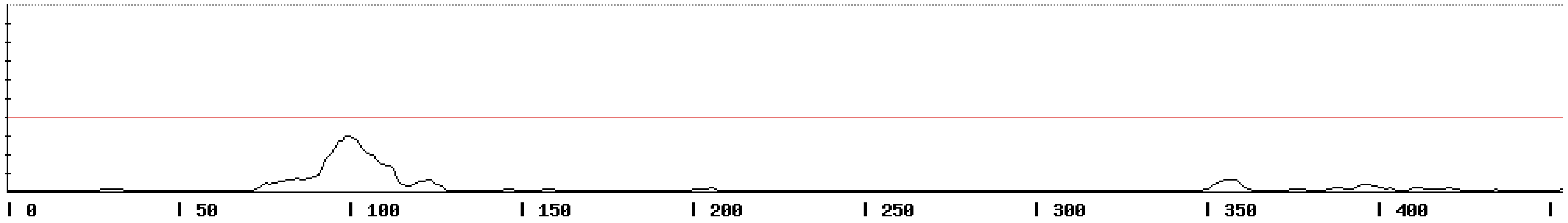

Predictions for Brassicaoleracea_XP_013613034.1

Viterbi Path

Viterbi Path

Posterior @ 0.4 — 98 - GKPK - 101

Posterior @ 0.4

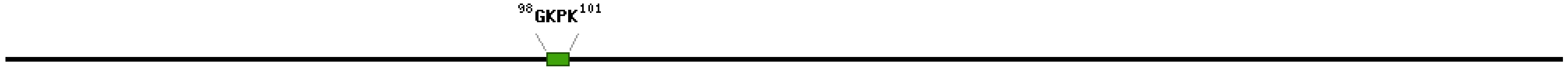

Posterior Graphic. Threshold Marked @ 0.4

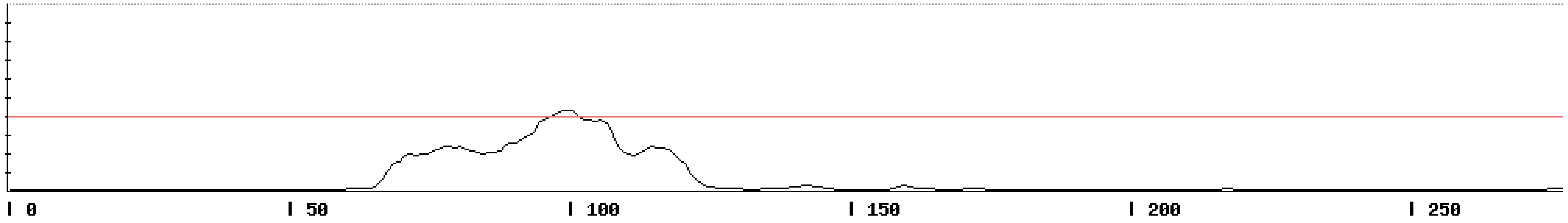

Predictions for Brassicaoleracea_XP_013613101.1

Viterbi Path

Viterbi Path

Posterior @ 0.4

Posterior @ 0.4

Posterior Graphic. Threshold Marked @ 0.4

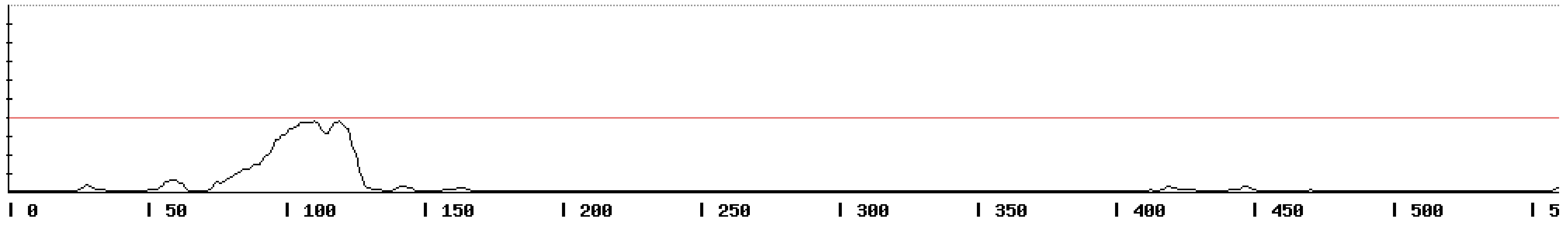

Predictions for Brassicaoleracea_XP_013613103.1

Viterbi Path

Viterbi Path

Posterior @ 0.4

Posterior @ 0.4

Posterior Graphic. Threshold Marked @ 0.4

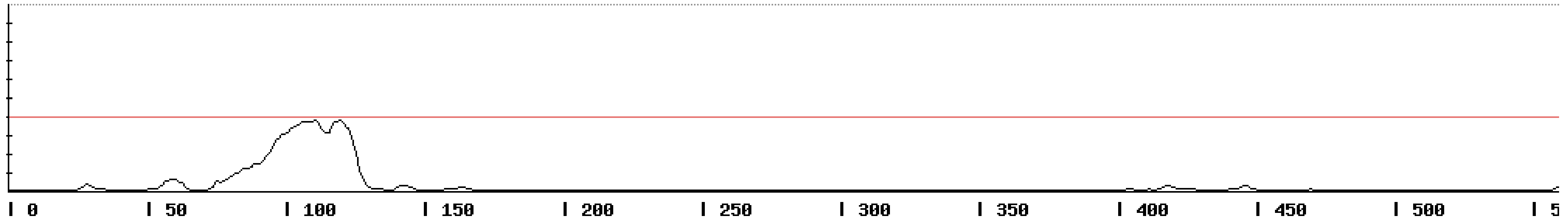

Predictions for Brassicaoleracea_XP_013613533.1

Viterbi Path

Viterbi Path

Posterior @ 0.4

Posterior @ 0.4

Posterior Graphic. Threshold Marked @ 0.4

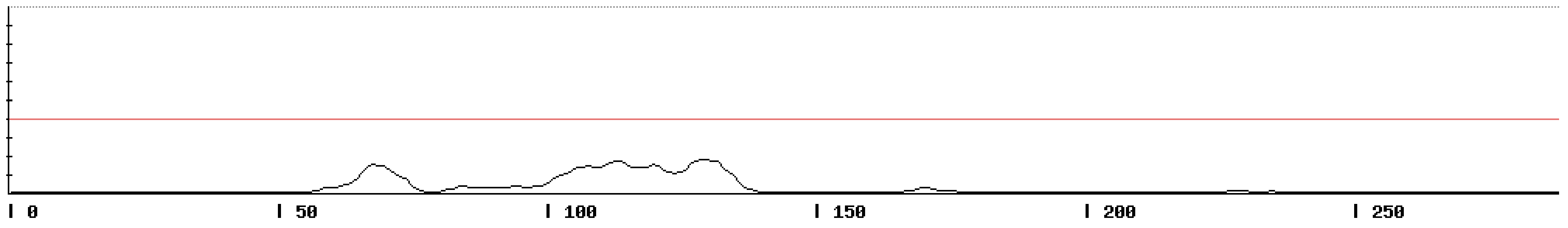

Predictions for Brassicaoleracea_XP_013614047.1

Viterbi Path

Viterbi Path

Posterior @ 0.4

Posterior @ 0.4

Posterior Graphic. Threshold Marked @ 0.4

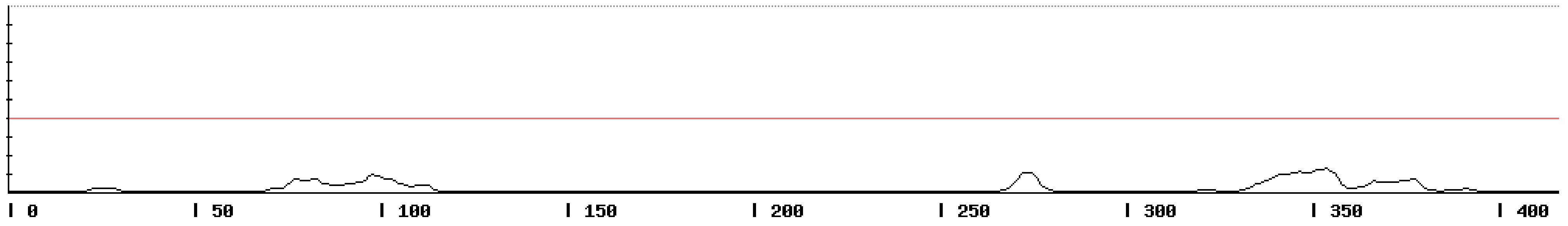

Predictions for Brassicaoleracea_XP_013614048.1	
Viterbi Path	
Viterbi Path	
Posterior @ 0.4	
Posterior @ 0.4	
Posterior Graphic. Threshold Marked @ 0.4	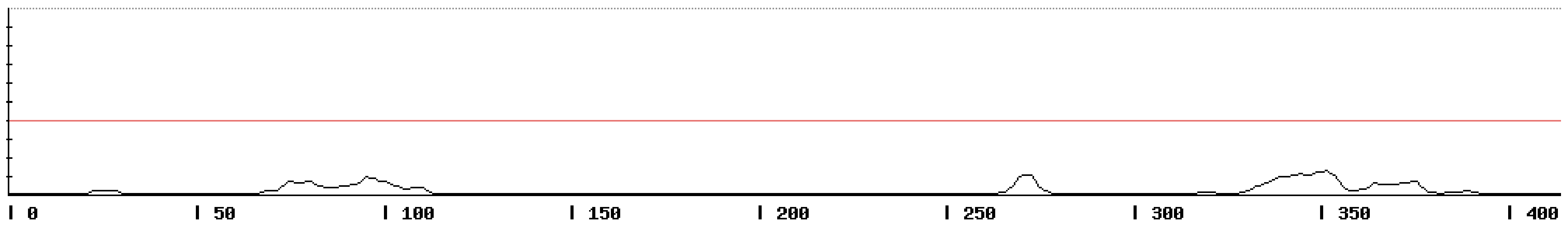

Predictions for Brassicaoleracea_XP_013614049.1	
Viterbi Path	
Viterbi Path	
Posterior @ 0.4	
Posterior @ 0.4	
Posterior Graphic. Threshold Marked @ 0.4	

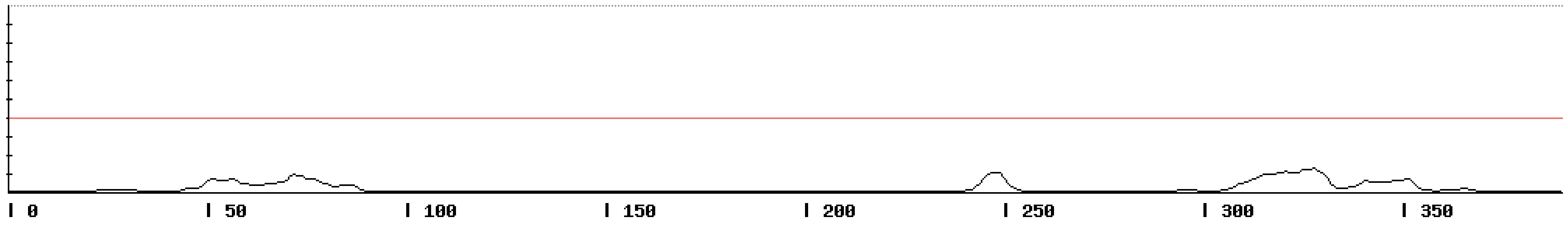

Predictions for Brassicaoleracea_XP_013614938.1

Viterbi Path

Viterbi Path

Posterior @ 0.4

Posterior @ 0.4

Posterior Graphic. Threshold Marked @ 0.4

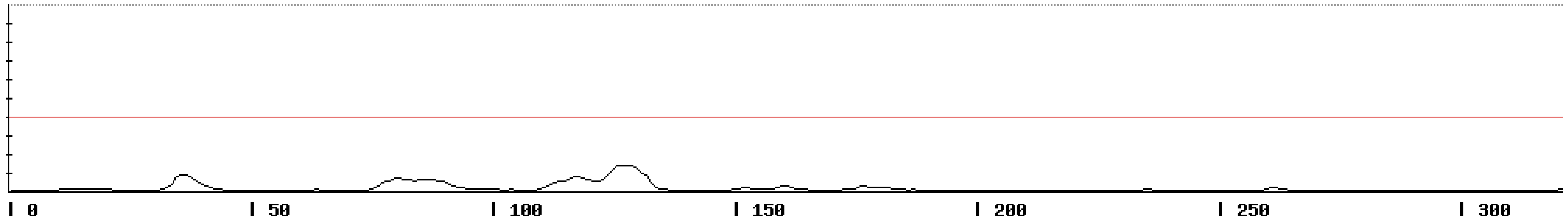

Predictions for Brassicaoleracea_XP_013614989.1

Viterbi Path

Viterbi Path

Posterior @ 0.4

Posterior @ 0.4

Posterior Graphic. Threshold Marked @ 0.4

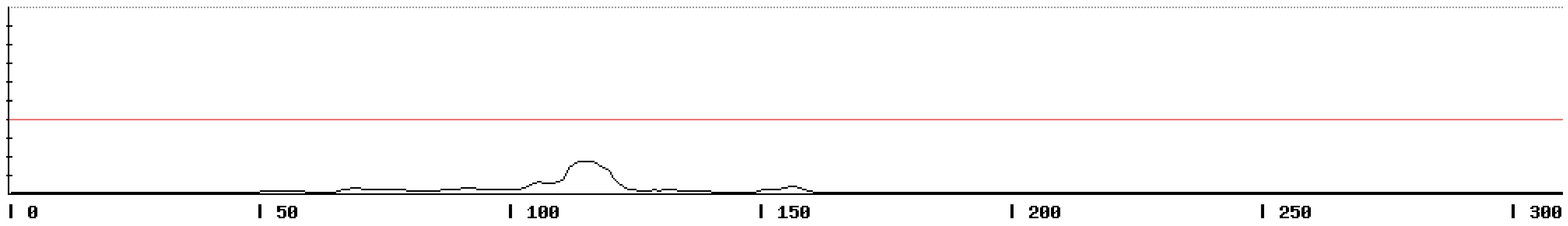

Predictions for Brassicaoleracea_XP_013615275.1

Viterbi Path

Viterbi Path

Posterior @ 0.4

Posterior @ 0.4

Posterior Graphic. Threshold Marked @ 0.4

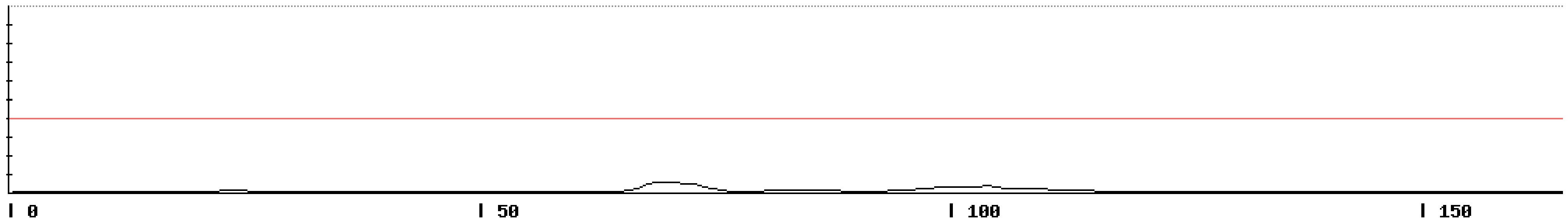

Predictions for Brassicaoleracea_XP_013615277.1

Viterbi Path

Viterbi Path

Posterior @ 0.4

Posterior @ 0.4

Posterior Graphic. Threshold Marked @ 0.4

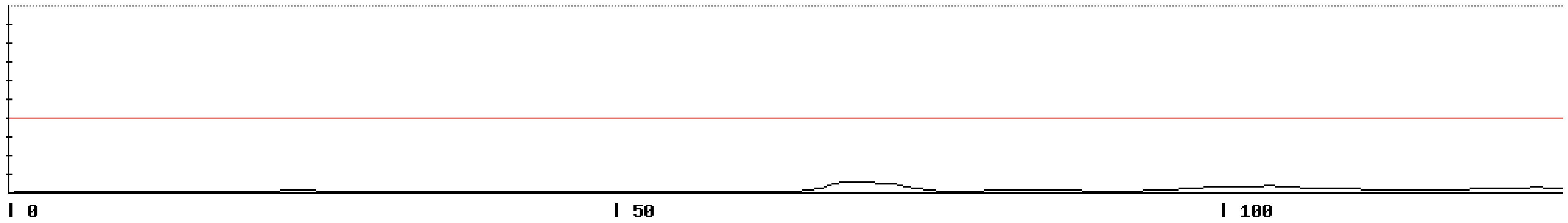

Predictions for Brassicaoleracea_XP_013615295.1

Viterbi Path

Viterbi Path

Posterior @ 0.4 — 98 - GKPK - 101

Posterior @ 0.4

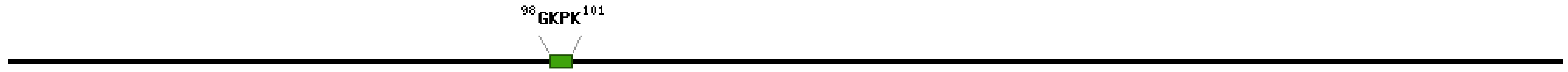

Posterior Graphic. Threshold Marked @ 0.4

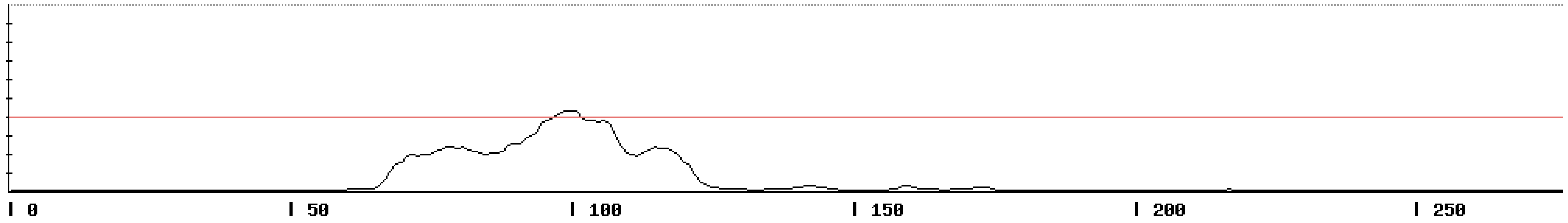

Predictions for Brassicaoleracea_XP_013615910.1

Viterbi Path

Viterbi Path

Posterior @ 0.4

Posterior @ 0.4

Posterior Graphic. Threshold Marked @ 0.4

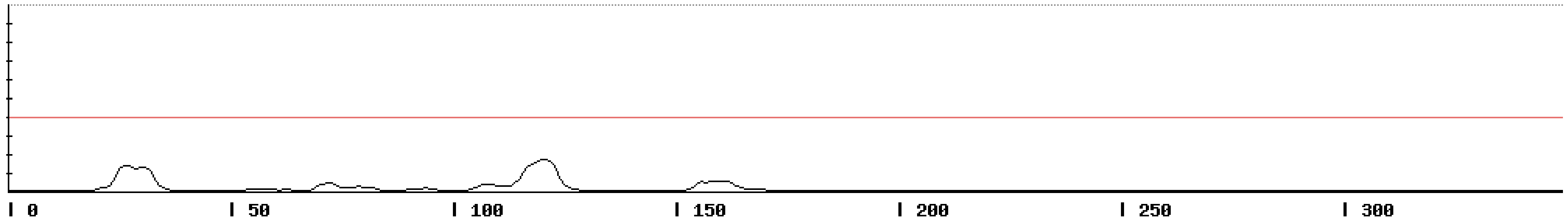

Predictions for Brassicaoleracea_XP_013615911.1

Viterbi Path

Viterbi Path

Posterior @ 0.4

Posterior @ 0.4

Posterior Graphic. Threshold Marked @ 0.4

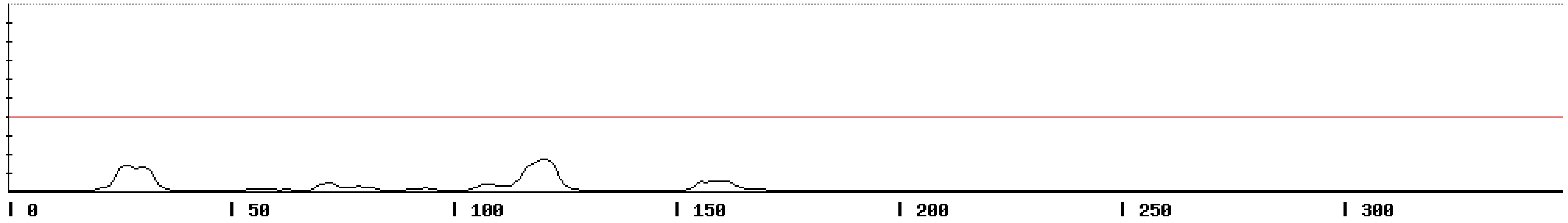

Predictions for Brassicaoleracea_XP_013616052.1

Viterbi Path

Viterbi Path

Posterior @ 0.4	98 - GKPK - 101
Posterior @ 0.4	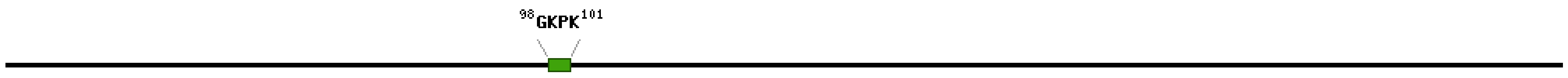
Posterior Graphic. Threshold Marked @ 0.4	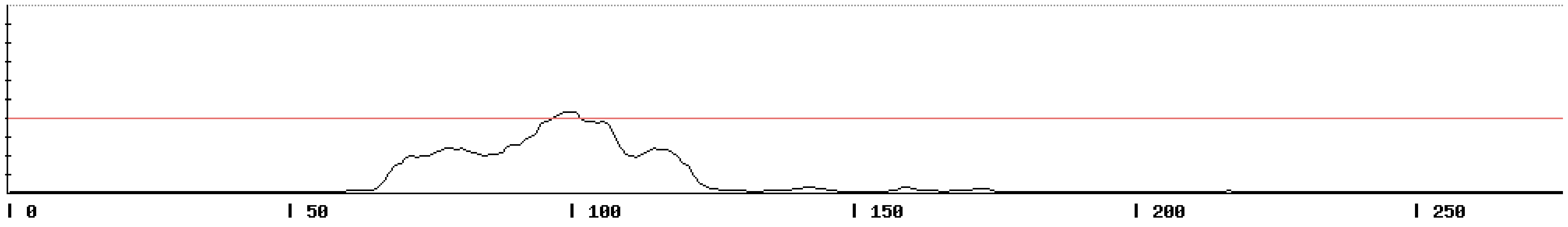

Predictions for Brassicaoleracea_XP_013616284.1

Viterbi Path	
Viterbi Path	
Posterior @ 0.4	
Posterior @ 0.4	
Posterior Graphic. Threshold Marked @ 0.4	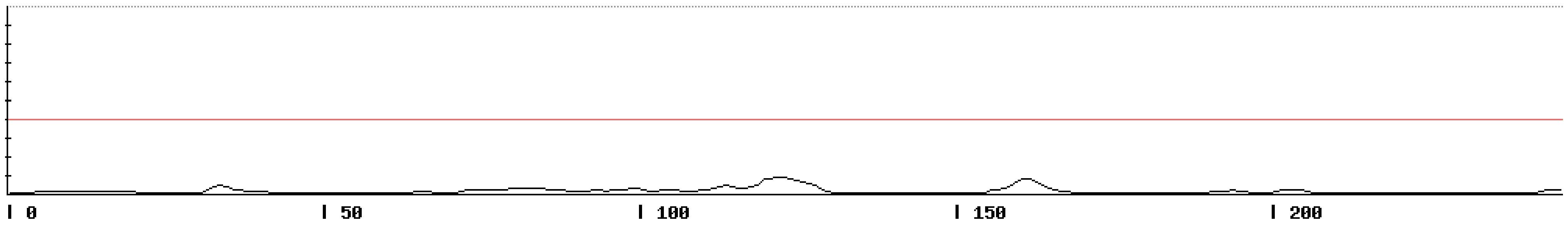

Predictions for Brassicaoleracea_XP_013616301.1

Viterbi Path

Viterbi Path

Posterior @ 0.4

Posterior @ 0.4

Posterior Graphic. Threshold Marked @ 0.4

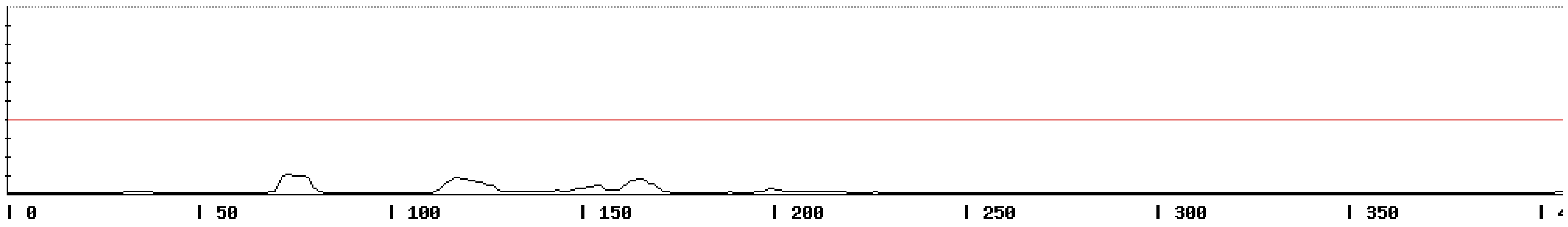

Predictions for Brassicaoleracea_XP_013616302.1

Viterbi Path

Viterbi Path

Posterior @ 0.4

Posterior @ 0.4

Posterior Graphic. Threshold Marked @ 0.4

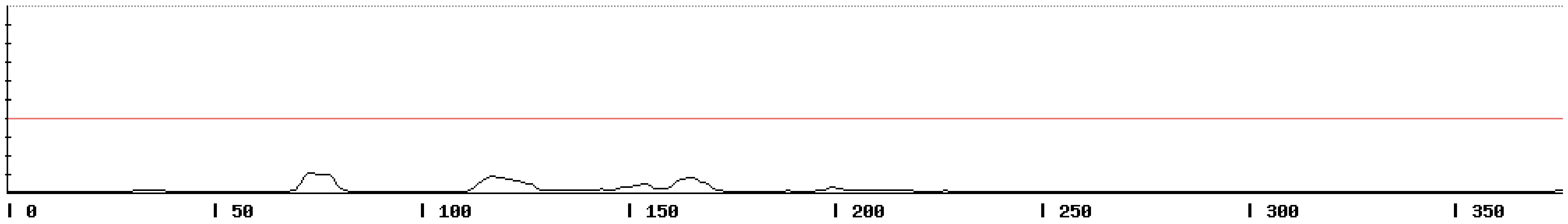

Predictions for Brassicaoleracea_XP_013616372.1	
Viterbi Path	
Viterbi Path	
Posterior @ 0.4	
Posterior @ 0.4	
Posterior Graphic. Threshold Marked @ 0.4	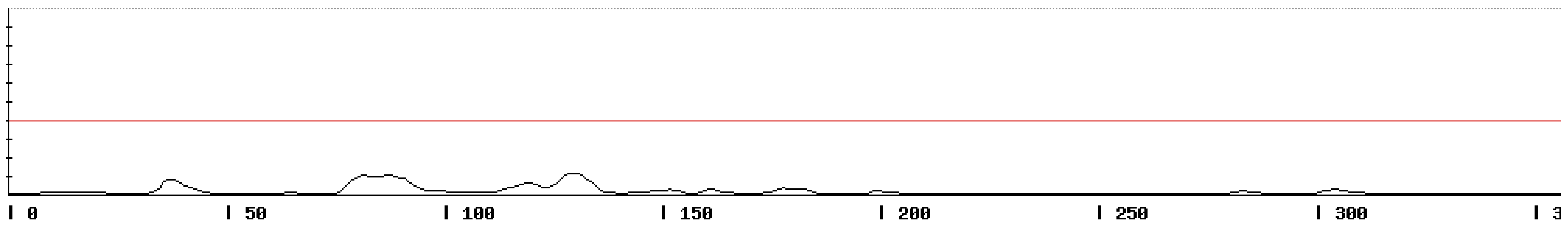

Predictions for Brassicaoleracea_XP_013616777.1	
Viterbi Path	
Viterbi Path	
Posterior @ 0.4	117 - GKKIKR - 122
Posterior @ 0.4	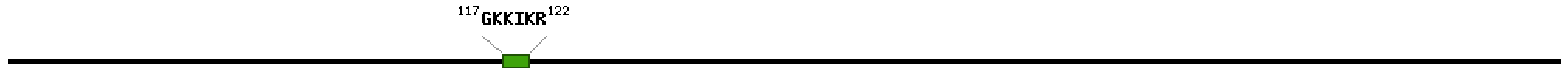
Posterior Graphic. Threshold Marked @ 0.4	

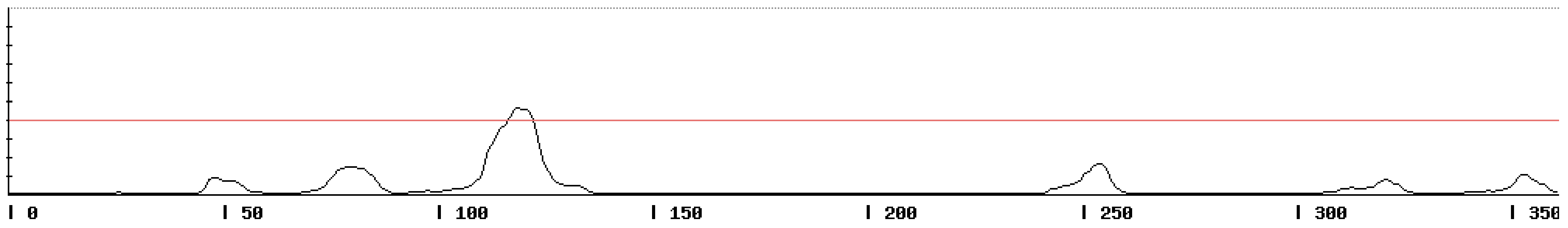

Predictions for Brassicaoleracea_XP_013616847.1

Viterbi Path

Viterbi Path

Posterior @ 0.4

Posterior @ 0.4

Posterior Graphic. Threshold Marked @ 0.4

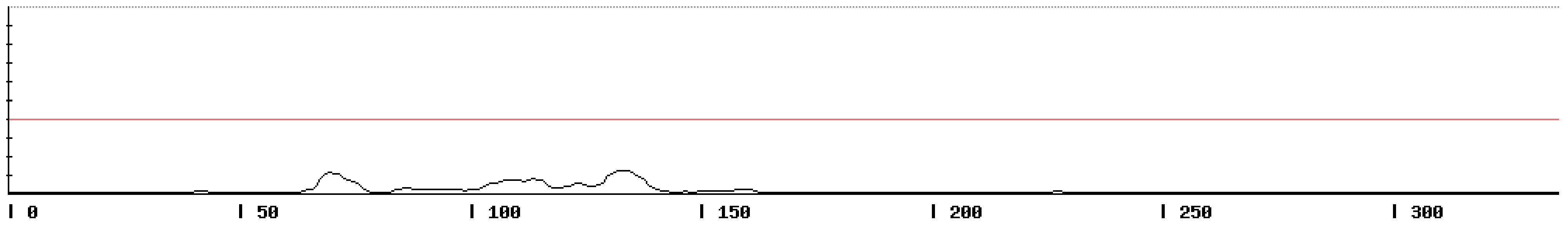

Predictions for Brassicaoleracea_XP_013616936.1

Viterbi Path

Viterbi Path

Posterior @ 0.4

105 - GRNSKRR - 111

Posterior @ 0.4

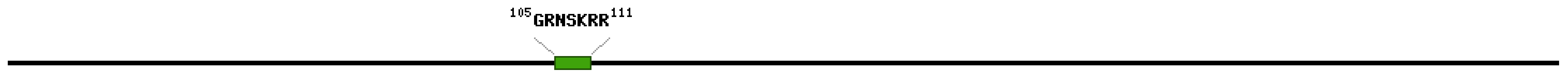

Posterior Graphic. Threshold Marked @ 0.4

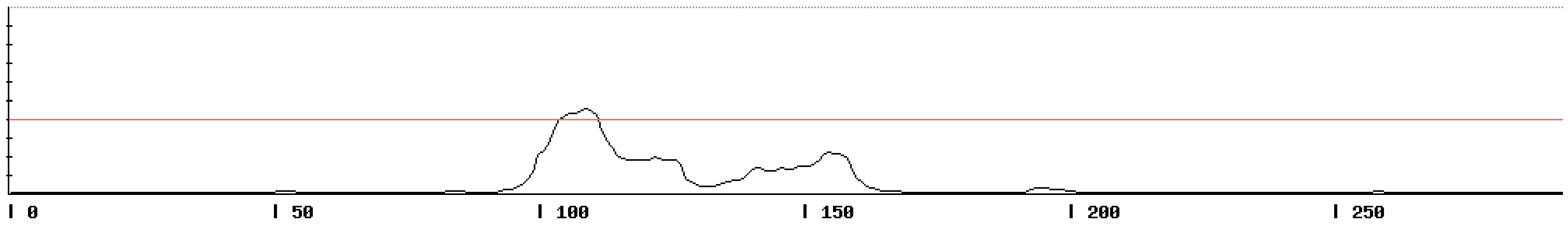

Predictions for Brassicaoleracea_XP_013616940.1

Viterbi Path

Viterbi Path

Posterior @ 0.4

Posterior @ 0.4

Posterior Graphic. Threshold Marked @ 0.4

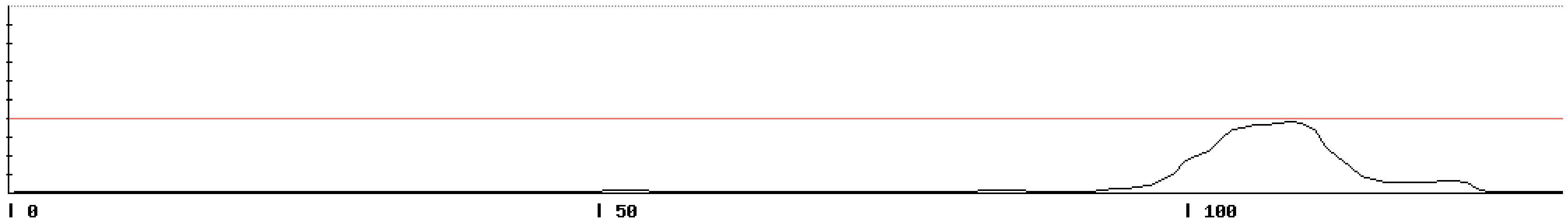

Predictions for Brassicaoleracea_XP_013616942.1

Viterbi Path

Viterbi Path

Posterior @ 0.4 | 42 - GRNSKRR - 48

Posterior @ 0.4

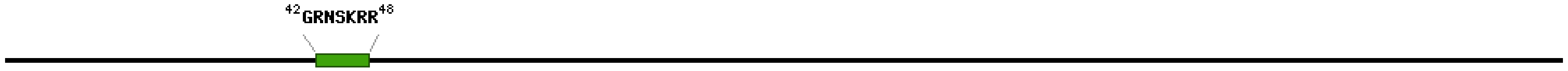

Posterior Graphic. Threshold Marked @ 0.4

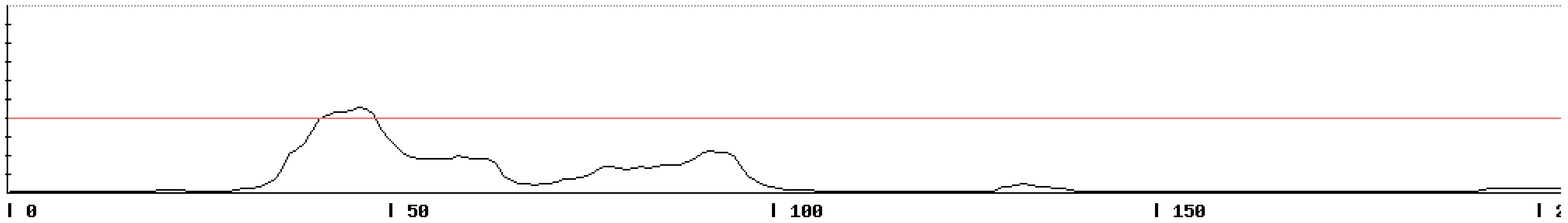

Predictions for Brassicaoleracea_XP_013616944.1

Viterbi Path

Viterbi Path

Posterior @ 0.4

117 - KKKTIIGKKQTLKF - 130

132 - K - 132

136 - NKRQK - 140

Posterior @ 0.4

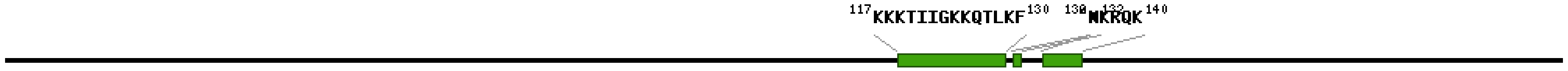

Posterior Graphic. Threshold Marked @ 0.4

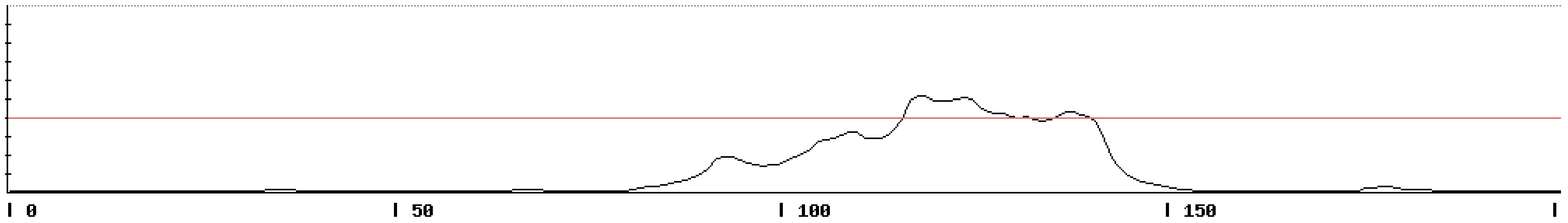

Predictions for Brassicaoleracea_XP_013617381.1

Viterbi Path

Viterbi Path

Posterior @ 0.4

Posterior @ 0.4

Posterior Graphic. Threshold Marked @ 0.4

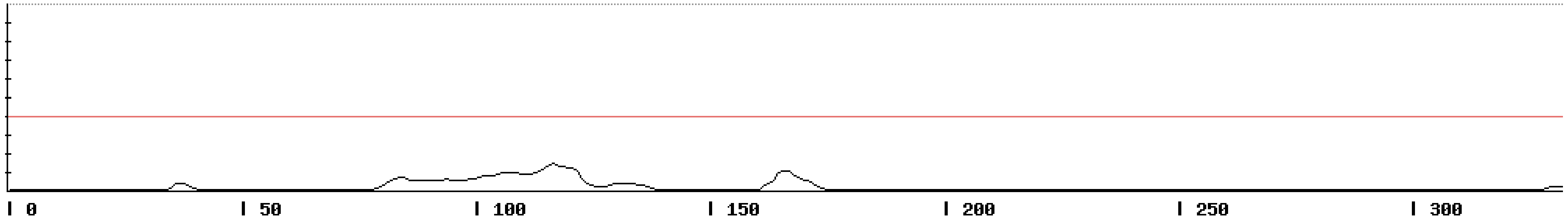

Predictions for Brassicaoleracea_XP_013617682.1

Viterbi Path

Viterbi Path

Posterior @ 0.4

Posterior @ 0.4

Posterior Graphic. Threshold Marked @ 0.4

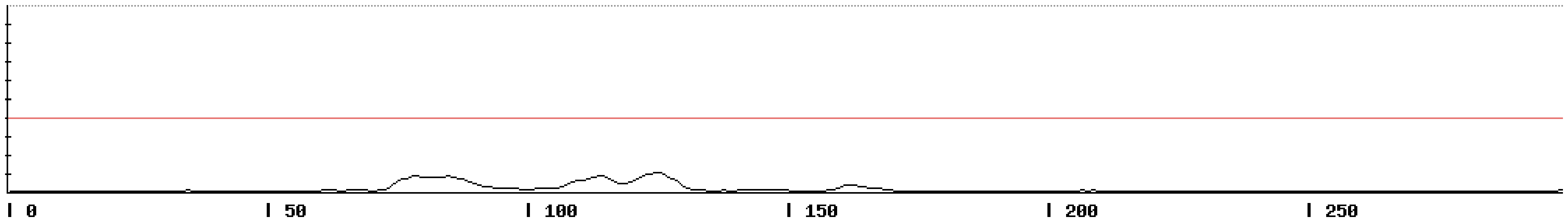

Predictions for Brassicaoleracea_XP_013617687.1

Viterbi Path

Viterbi Path

Posterior @ 0.4

Posterior @ 0.4

Posterior Graphic. Threshold Marked @ 0.4

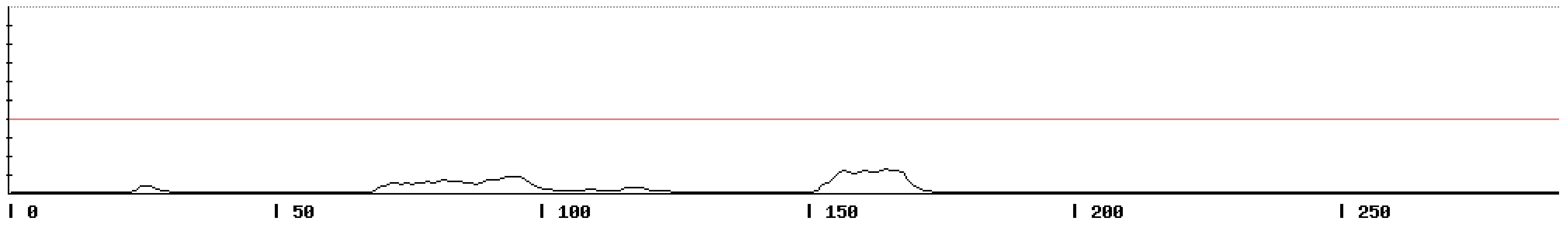

Predictions for Brassicaoleracea_XP_013618408.1

Viterbi Path

Viterbi Path

Posterior @ 0.4

98 - KPIGRPRPVGIKK - 110

Posterior @ 0.4

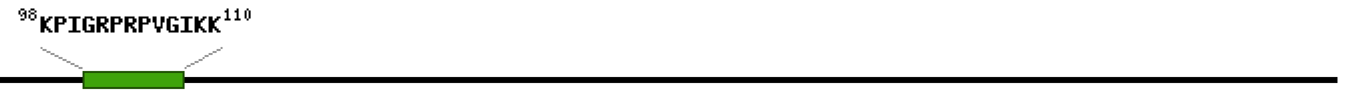

Posterior Graphic. Threshold Marked @ 0.4

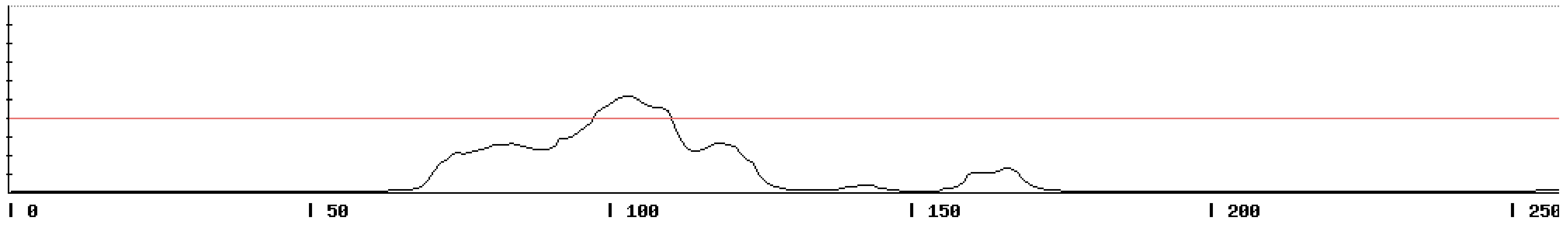

Predictions for Brassicaoleracea_XP_013618540.1	
Viterbi Path	
Viterbi Path	
Posterior @ 0.4	
Posterior @ 0.4	
Posterior Graphic. Threshold Marked @ 0.4	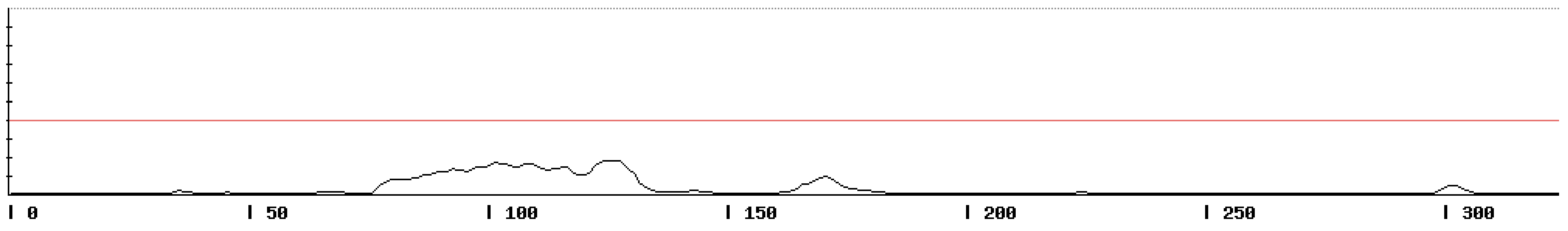

Predictions for Brassicaoleracea_XP_013618671.1	
Viterbi Path	
Viterbi Path	
Posterior @ 0.4	
Posterior @ 0.4	
Posterior Graphic. Threshold Marked @ 0.4	

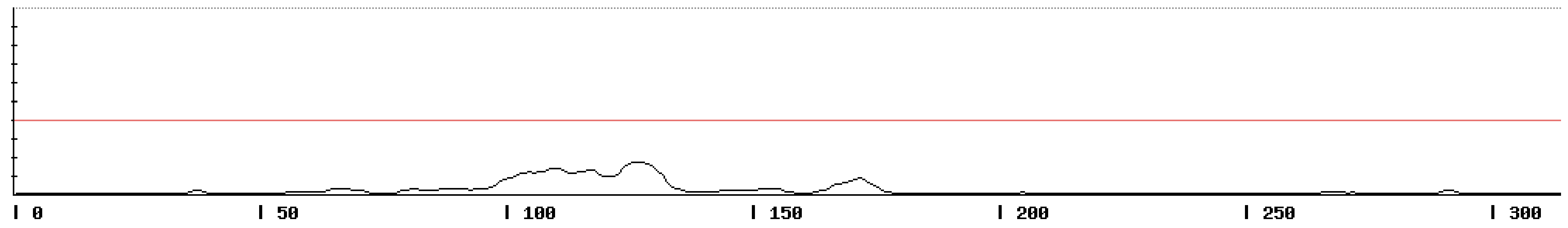

Predictions for Brassicaoleracea_XP_013618727.1

Viterbi Path	
Viterbi Path	
Posterior @ 0.4	585 - KKKK - 588
Posterior @ 0.4	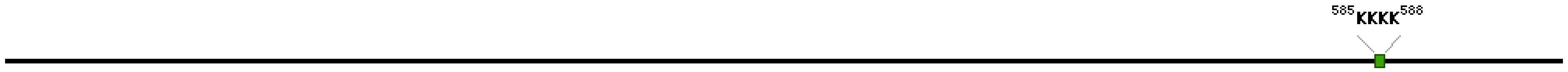
Posterior Graphic. Threshold Marked @ 0.4	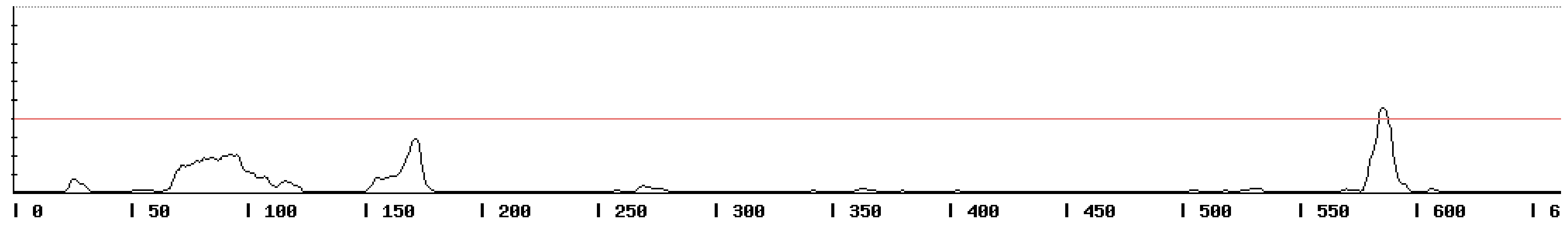

Predictions for Brassicaoleracea_XP_013618977.1

Viterbi Path	
Viterbi Path	
Posterior @ 0.4	
Posterior @ 0.4	

Posterior Graphic. Threshold Marked @ 0.4

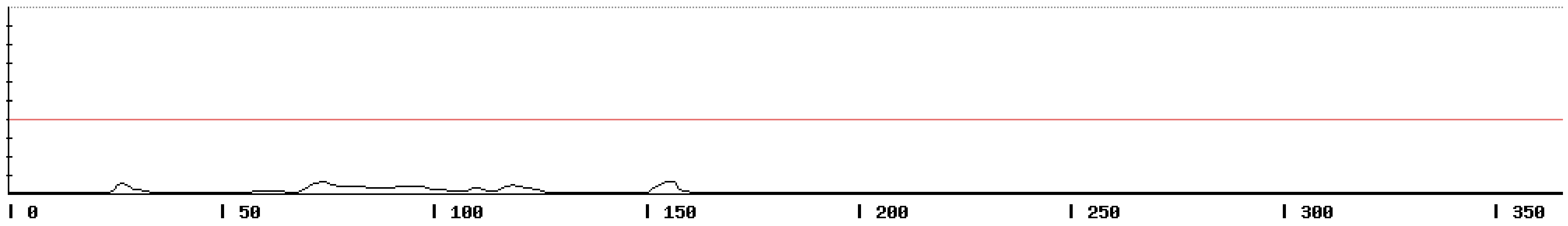

Predictions for Brassicaoleracea_XP_013618978.1

Viterbi Path

Viterbi Path

Posterior @ 0.4

Posterior @ 0.4

Posterior Graphic. Threshold Marked @ 0.4

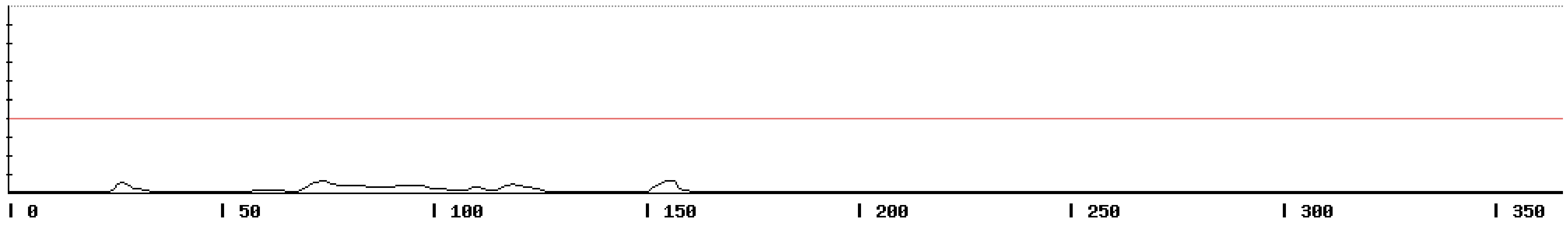

Predictions for Brassicaoleracea_XP_013618979.1

Viterbi Path

Viterbi Path

Posterior @ 0.4

Posterior @ 0.4

Posterior Graphic. Threshold Marked @ 0.4

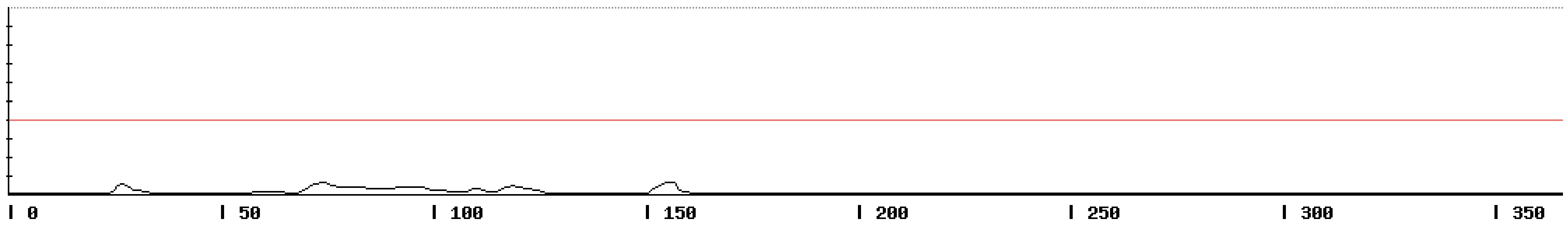

Predictions for Brassicaoleracea_XP_013618994.1

Viterbi Path

Viterbi Path

Posterior @ 0.4

Posterior @ 0.4

Posterior Graphic. Threshold Marked @ 0.4

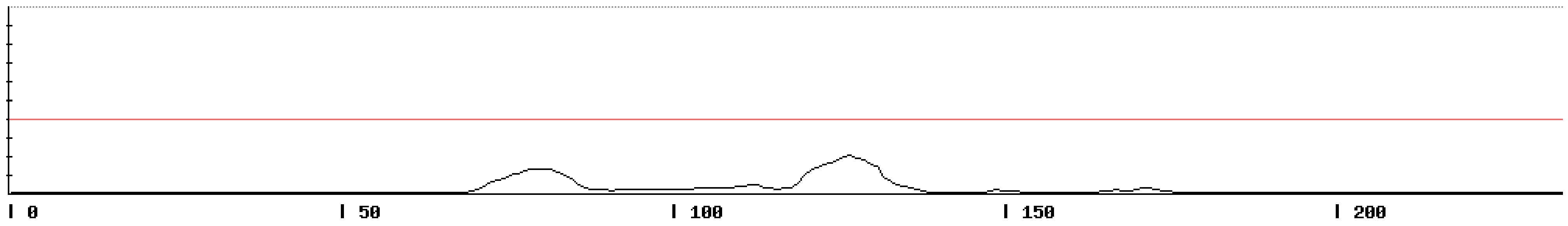

Predictions for Brassicaoleracea_XP_013619013.1

Viterbi Path

Viterbi Path

Posterior @ 0.4	
Posterior @ 0.4	
Posterior Graphic. Threshold Marked @ 0.4	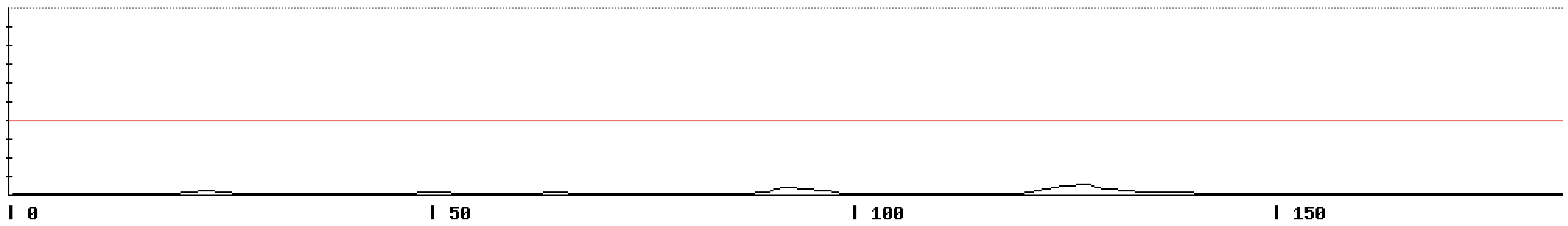

Predictions for Brassicaoleracea_XP_013619317.1	
Viterbi Path	
Viterbi Path	
Posterior @ 0.4	
Posterior @ 0.4	
Posterior Graphic. Threshold Marked @ 0.4	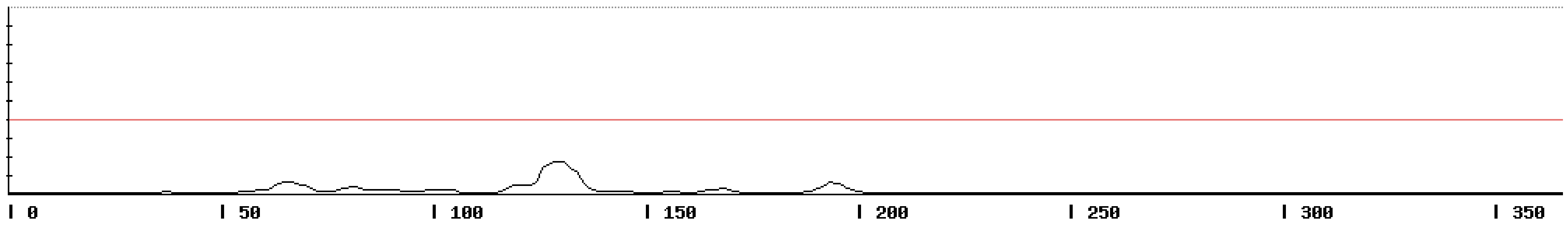

Predictions for Brassicaoleracea_XP_013619595.1	
Viterbi Path	
Viterbi Path	

Posterior @ 0.4

Posterior @ 0.4

Posterior Graphic. Threshold Marked @ 0.4

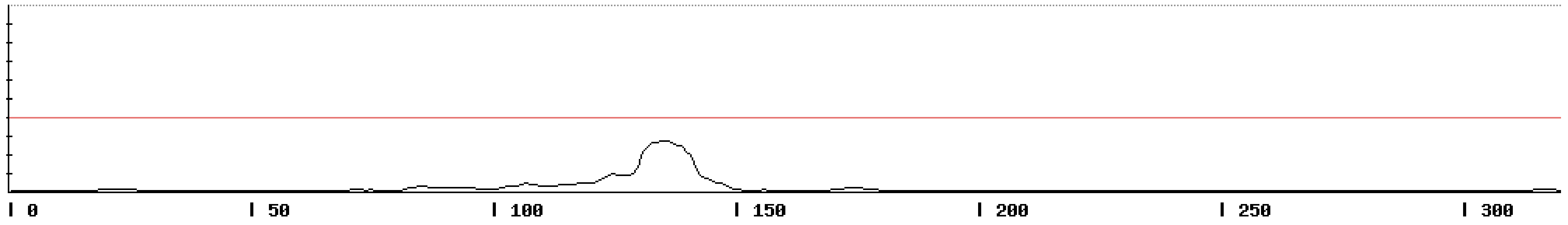

Predictions for Brassicaoleracea_XP_013619775.1

Viterbi Path

Viterbi Path

Posterior @ 0.4

Posterior @ 0.4

Posterior Graphic. Threshold Marked @ 0.4

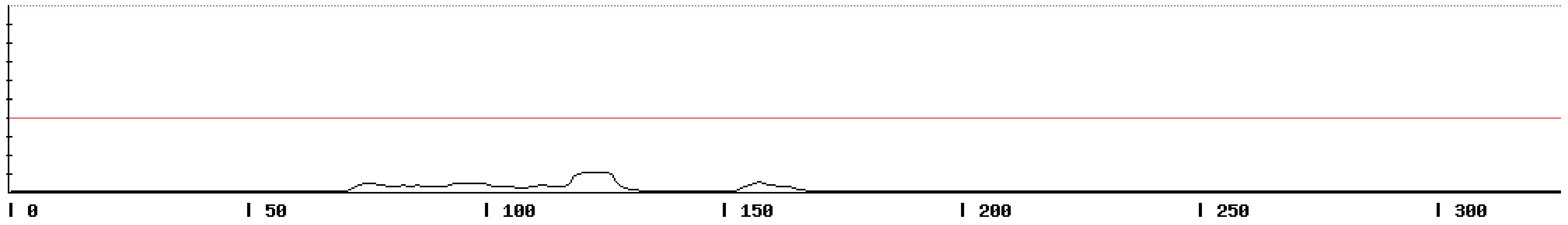

Predictions for Brassicaoleracea_XP_013619851.1

Viterbi Path	
Viterbi Path	
Posterior @ 0.4	
Posterior @ 0.4	
Posterior Graphic. Threshold Marked @ 0.4	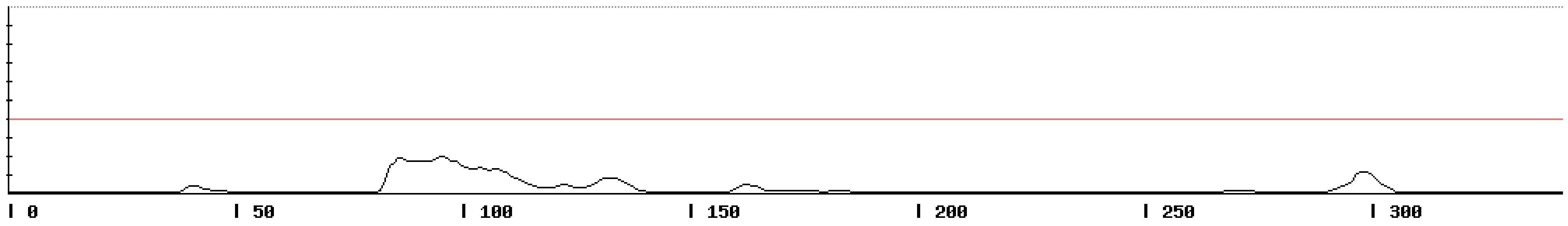

Predictions for Brassicaoleracea_XP_013620810.1

Viterbi Path	
Viterbi Path	
Posterior @ 0.4	
Posterior @ 0.4	
Posterior Graphic. Threshold Marked @ 0.4	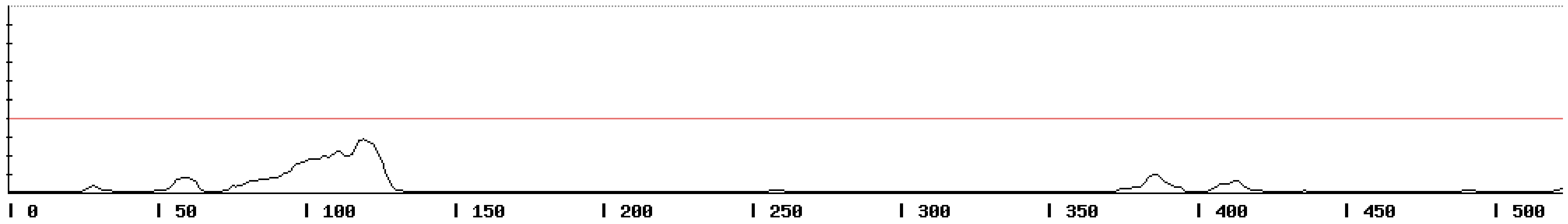

Predictions for Brassicaoleracea_XP_013620811.1

Viterbi Path	
Viterbi Path	
Posterior @ 0.4	
Posterior @ 0.4	
Posterior Graphic. Threshold Marked @ 0.4	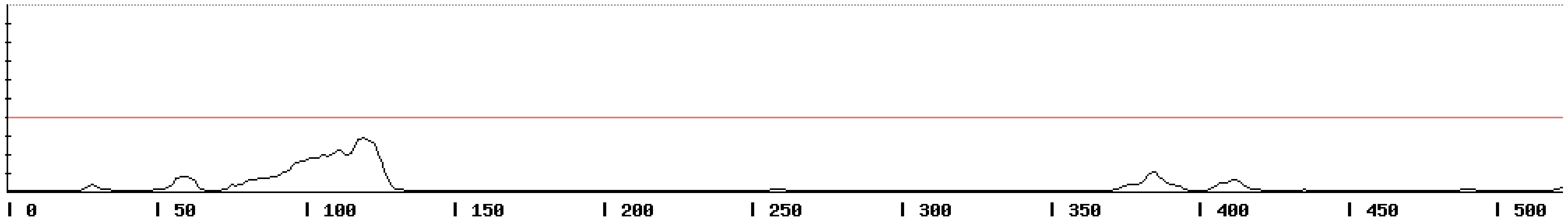

Predictions for Brassicaoleracea_XP_013620870.1

Viterbi Path	
Viterbi Path	
Posterior @ 0.4	99 - KP - 100
Posterior @ 0.4	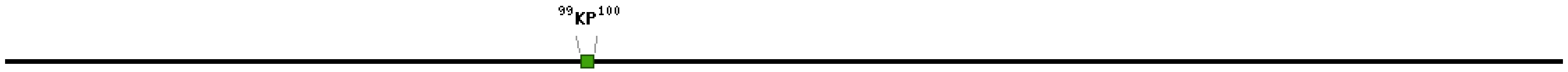
Posterior Graphic. Threshold Marked @ 0.4	

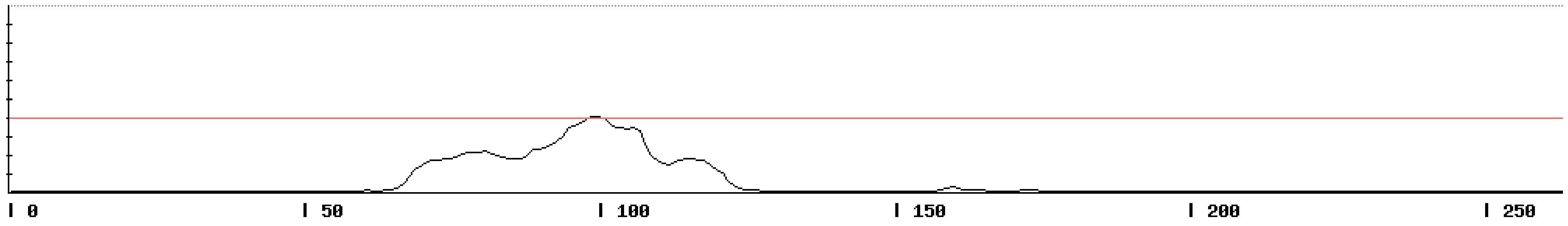

Predictions for Brassicaoleracea_XP_013621799.1

Viterbi Path	
Viterbi Path	
Posterior @ 0.4	
Posterior @ 0.4	
Posterior Graphic. Threshold Marked @ 0.4	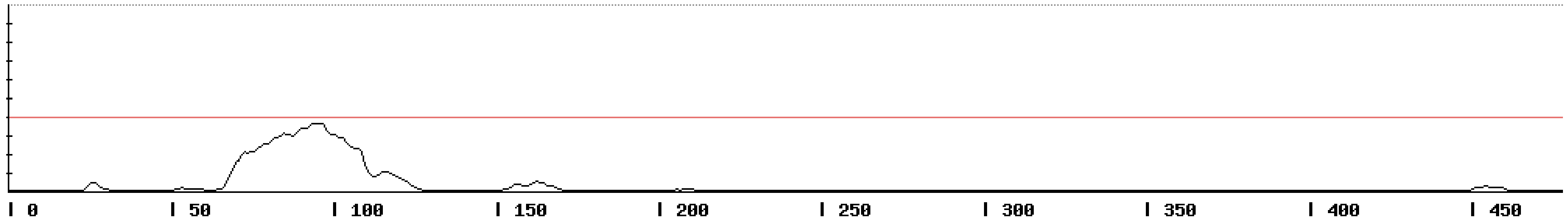

Predictions for Brassicaoleracea_XP_013621861.1

Viterbi Path	
Viterbi Path	
Posterior @ 0.4	
Posterior @ 0.4	

Posterior Graphic. Threshold Marked @ 0.4

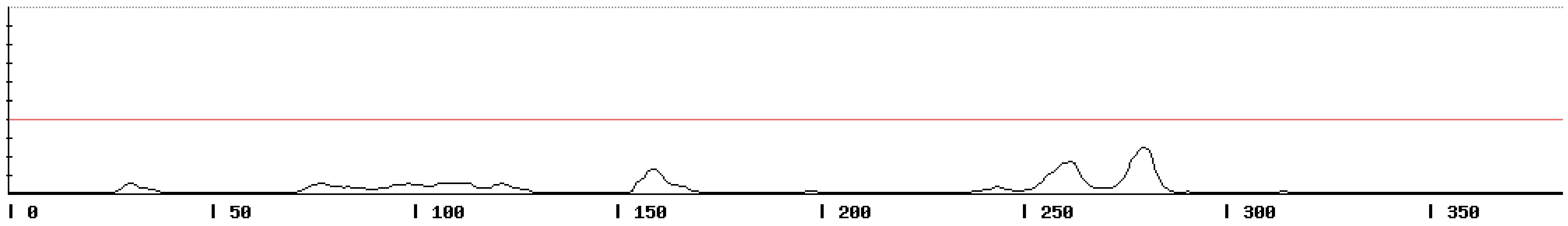

Predictions for Brassicaoleracea_XP_013622295.1

Viterbi Path

Viterbi Path

Posterior @ 0.4

Posterior @ 0.4

Posterior Graphic. Threshold Marked @ 0.4

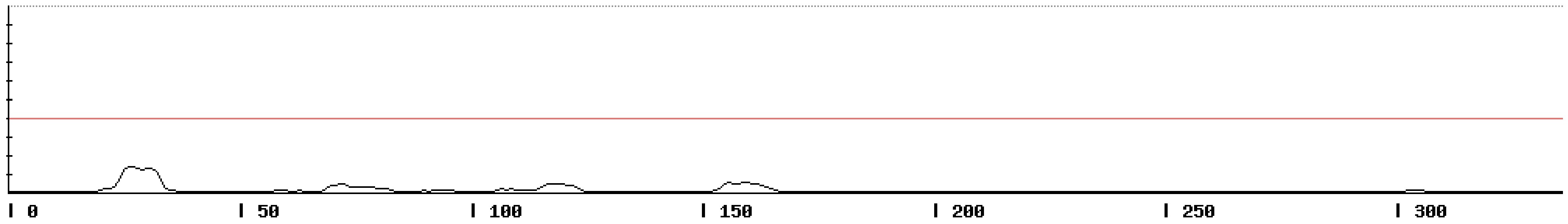

Predictions for Brassicaoleracea_XP_013622434.1

Viterbi Path

Viterbi Path

Posterior @ 0.4 98 - ATGKDRKIKSGK - 109

Posterior @ 0.4

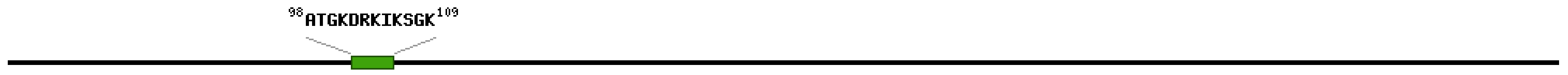

Posterior Graphic. Threshold Marked @ 0.4

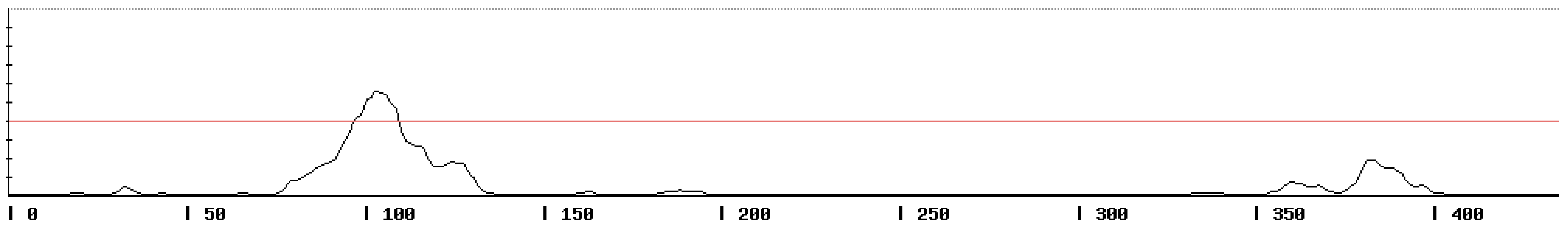

Predictions for Brassicaoleracea_XP_013622435.1

Viterbi Path

Viterbi Path

Posterior @ 0.4 98 - ATGKDRKIKSGK - 109

Posterior @ 0.4

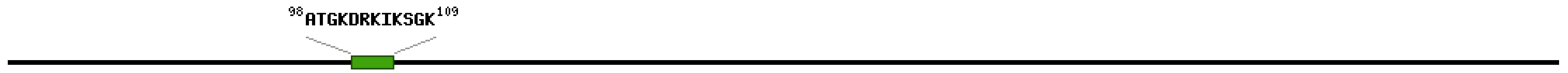

Posterior Graphic. Threshold Marked @ 0.4

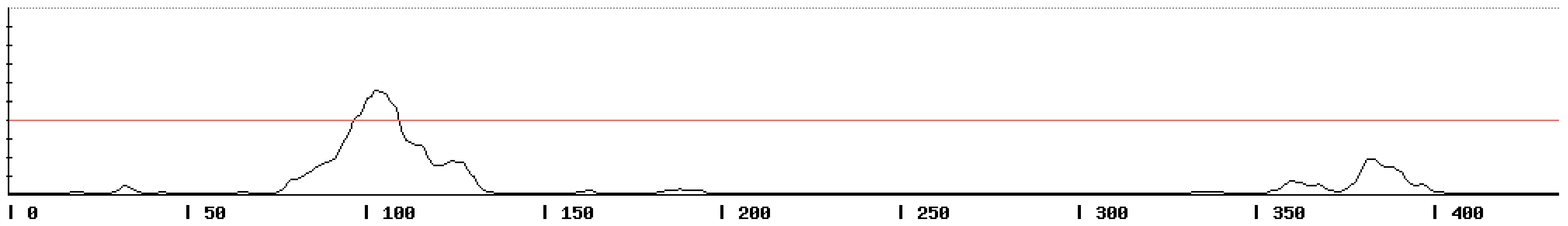

Predictions for Brassicaoleracea_XP_013622509.1

Viterbi Path

Viterbi Path

Posterior @ 0.4

Posterior @ 0.4

Posterior Graphic. Threshold Marked @ 0.4

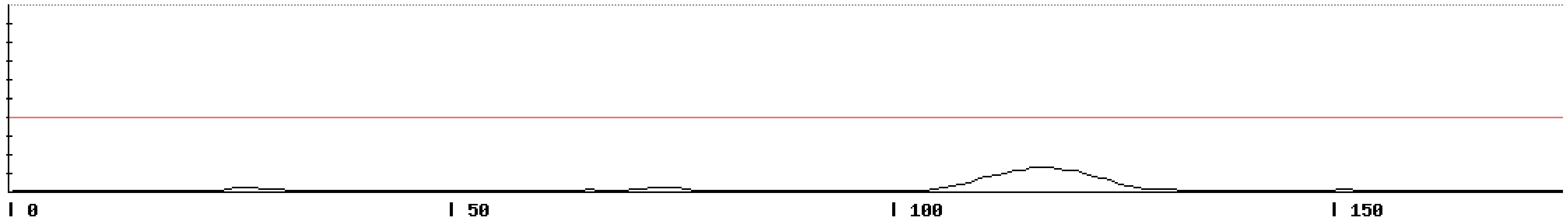

Predictions for Brassicaoleracea_XP_013622519.1

Viterbi Path

Viterbi Path

Posterior @ 0.4

Posterior @ 0.4

Posterior Graphic. Threshold Marked @ 0.4

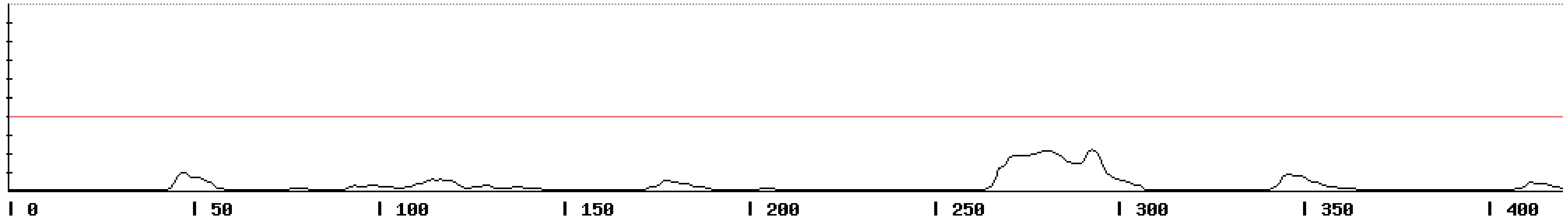

Predictions for Brassicaoleracea_XP_013622520.1

Viterbi Path

Viterbi Path

Posterior @ 0.4

Posterior @ 0.4

Posterior Graphic. Threshold Marked @ 0.4

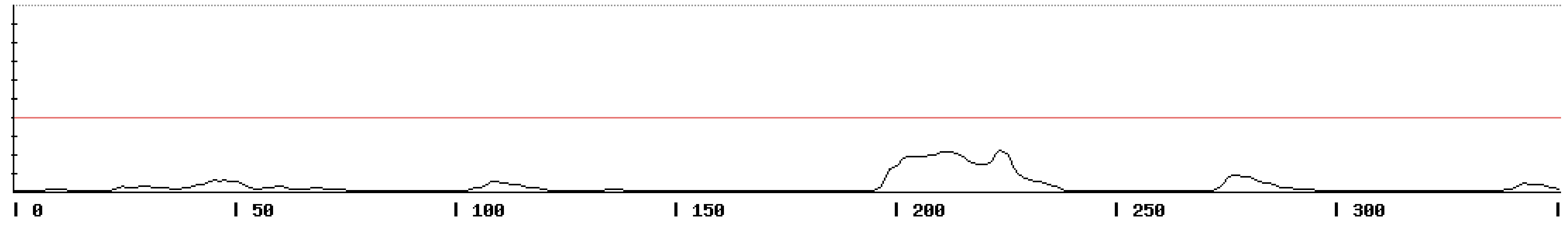

Predictions for Brassicaoleracea_XP_013623093.1

Viterbi Path

Viterbi Path

Posterior @ 0.4

Posterior @ 0.4

Posterior Graphic. Threshold Marked @ 0.4

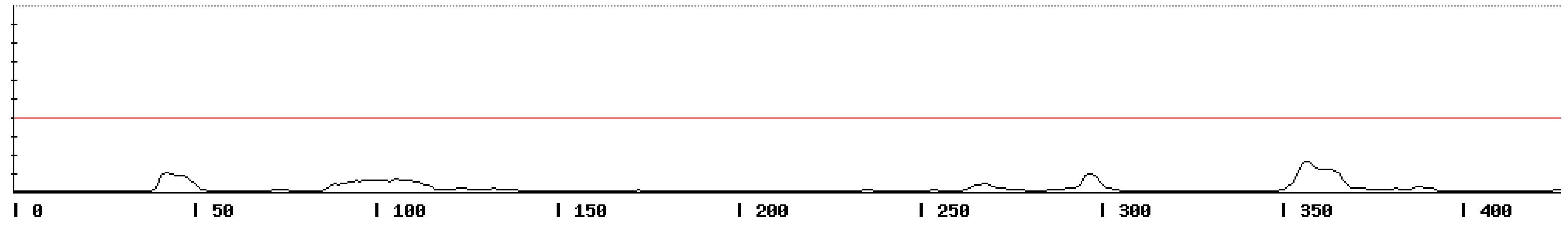

Predictions for Brassicaoleracea_XP_013624280.1

Viterbi Path	
Viterbi Path	
Posterior @ 0.4	121 - EKAEEKEKRRARDKR - 135
Posterior @ 0.4	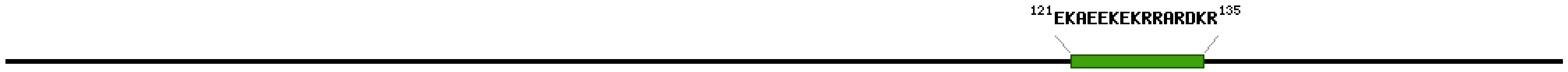
Posterior Graphic. Threshold Marked @ 0.4	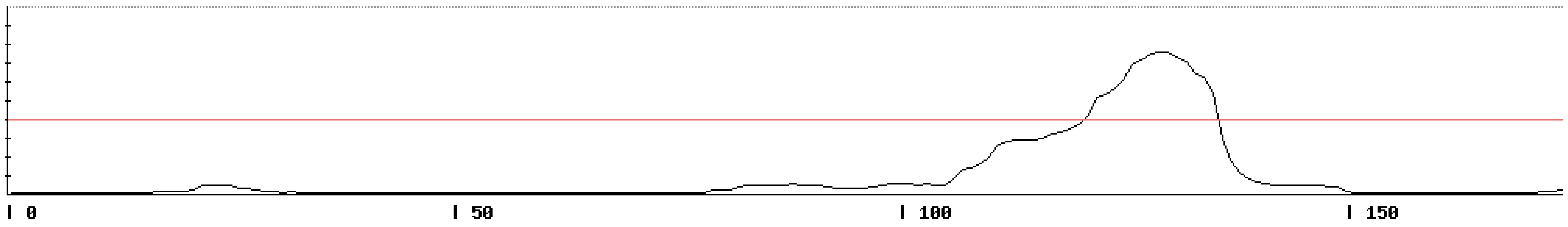

Predictions for Brassicaoleracea_XP_013624372.1

Viterbi Path	
Viterbi Path	
Posterior @ 0.4	
Posterior @ 0.4	
Posterior Graphic. Threshold Marked @ 0.4	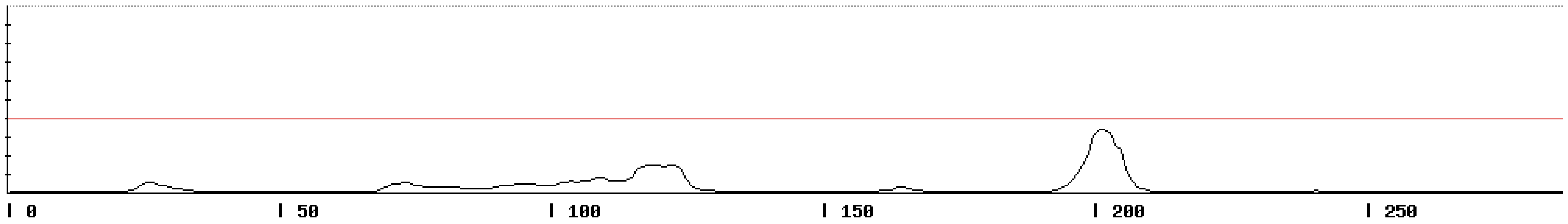

Predictions for Brassicaoleracea_XP_013625043.1	
Viterbi Path	
Viterbi Path	
Posterior @ 0.4	
Posterior @ 0.4	
Posterior Graphic. Threshold Marked @ 0.4	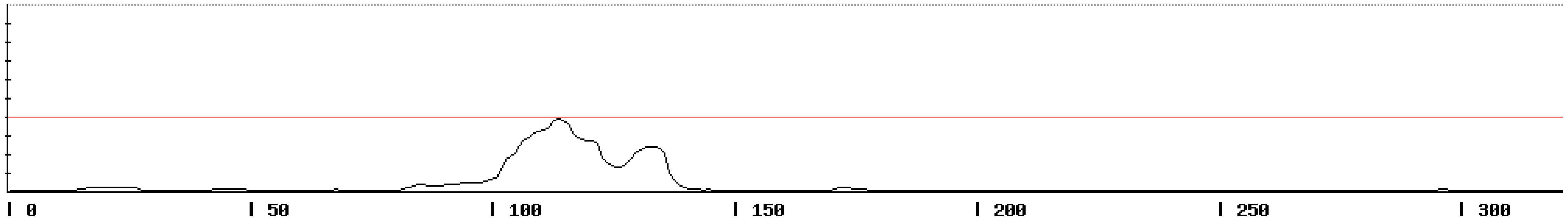

Predictions for Brassicaoleracea_XP_013625044.1	
Viterbi Path	
Viterbi Path	
Posterior @ 0.4	178 - KKKWK - 182
Posterior @ 0.4	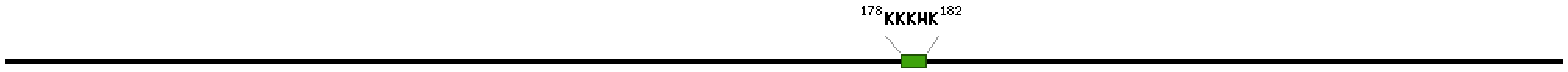
Posterior Graphic. Threshold Marked @ 0.4	

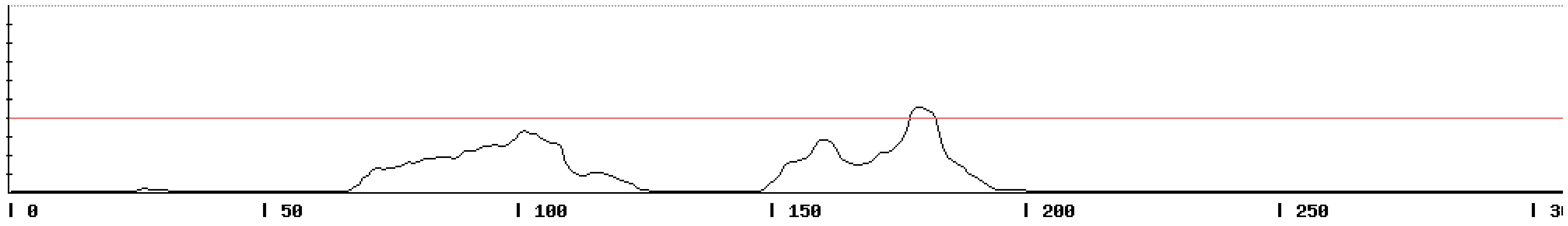

Predictions for Brassicaoleracea_XP_013625495.1

Viterbi Path

Viterbi Path

Posterior @ 0.4

Posterior @ 0.4

Posterior Graphic. Threshold Marked @ 0.4

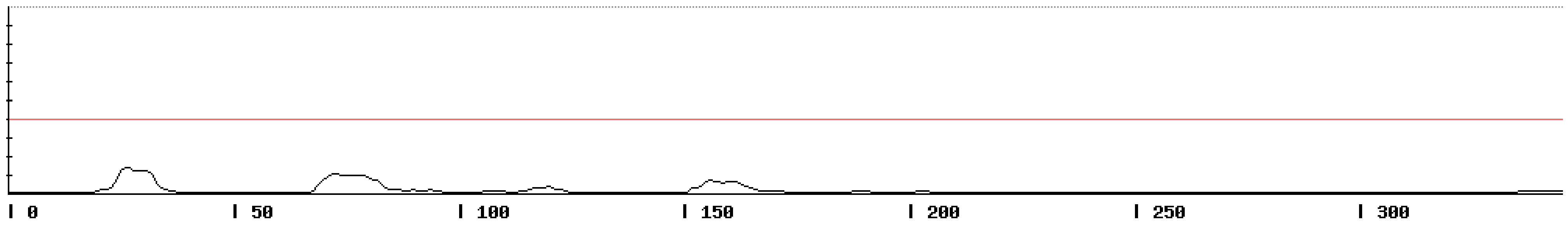

Predictions for Brassicaoleracea_XP_013625587.1

Viterbi Path

Viterbi Path

Posterior @ 0.4

Posterior @ 0.4

Posterior Graphic. Threshold Marked @ 0.4

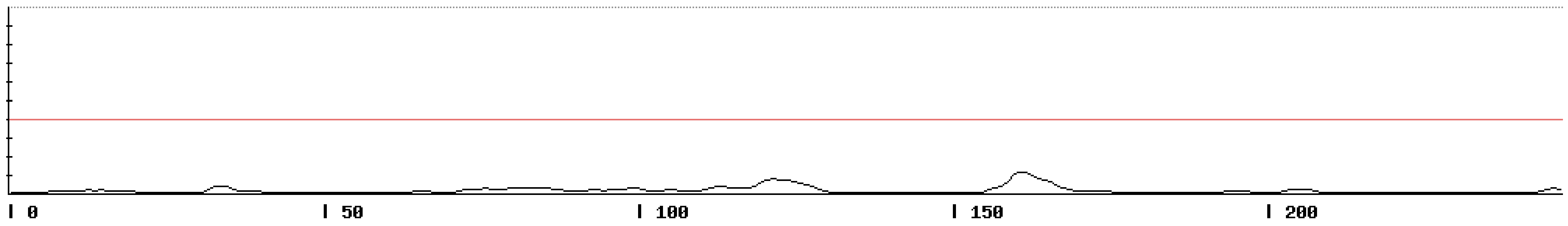

Predictions for Brassicaoleracea_XP_013625724.1

Viterbi Path

Viterbi Path

Posterior @ 0.4

Posterior @ 0.4

Posterior Graphic. Threshold Marked @ 0.4

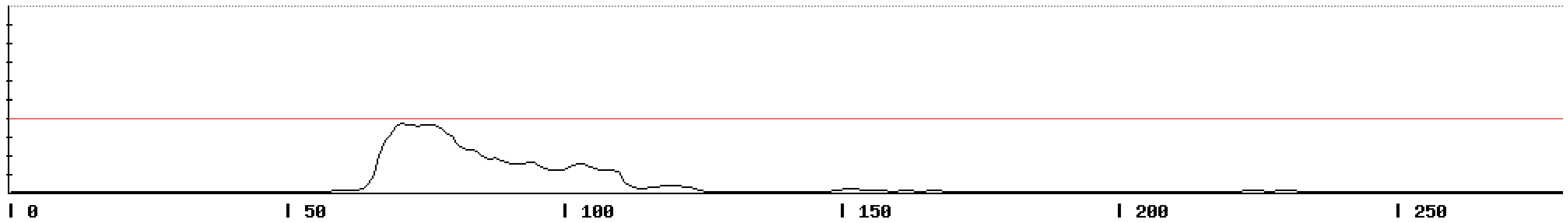

Predictions for Brassicaoleracea_XP_013625819.1

Viterbi Path

Viterbi Path

Posterior @ 0.4

Posterior @ 0.4

Posterior Graphic. Threshold Marked @ 0.4	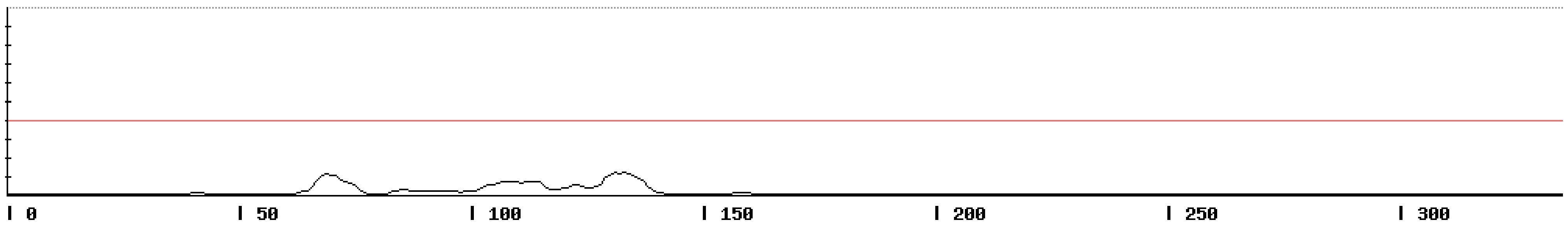

Predictions for Brassicaoleracea_XP_013626215.1

Viterbi Path	
Viterbi Path	
Posterior @ 0.4	176 - KSARKGSKPEK - 186
Posterior @ 0.4	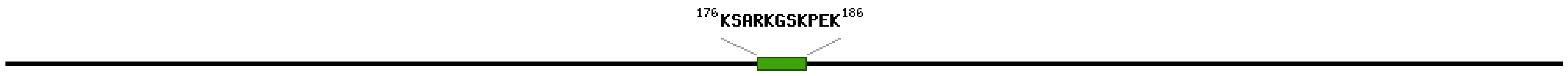
Posterior Graphic. Threshold Marked @ 0.4	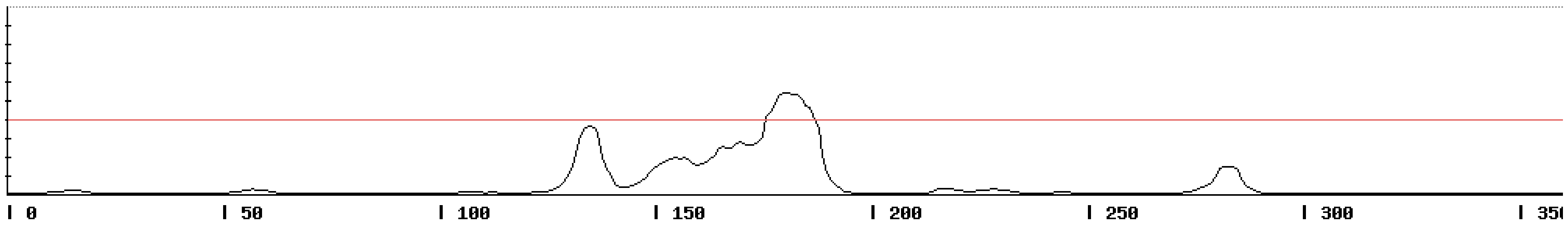

Predictions for Brassicaoleracea_XP_013626236.1

Viterbi Path	
Viterbi Path	

Posterior @ 0.4

Posterior @ 0.4

Posterior Graphic. Threshold Marked @ 0.4

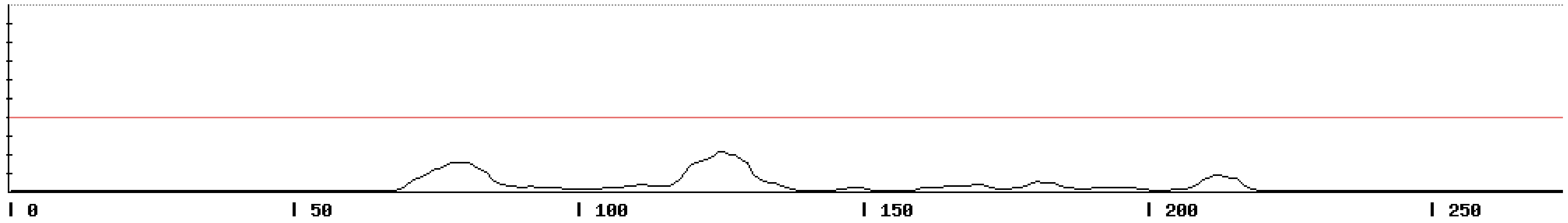

Predictions for Brassicaoleracea_XP_013626261.1

Viterbi Path

Viterbi Path

Posterior @ 0.4

Posterior @ 0.4

Posterior Graphic. Threshold Marked @ 0.4

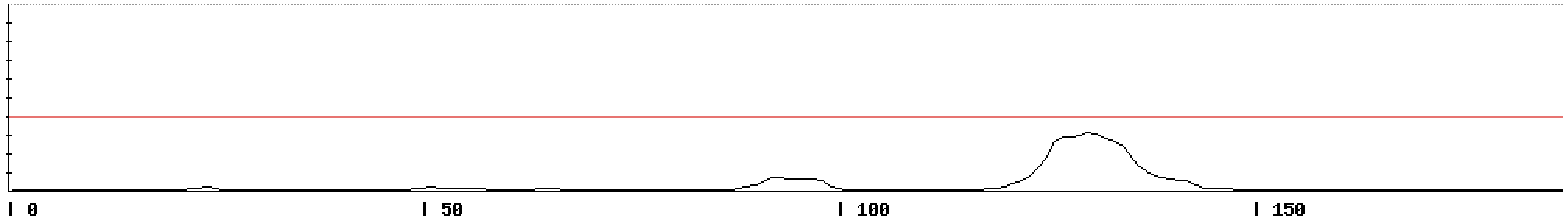

Predictions for Brassicaoleracea_XP_013626443.1

Viterbi Path

Viterbi Path

Posterior @ 0.4	
Posterior @ 0.4	
Posterior Graphic. Threshold Marked @ 0.4	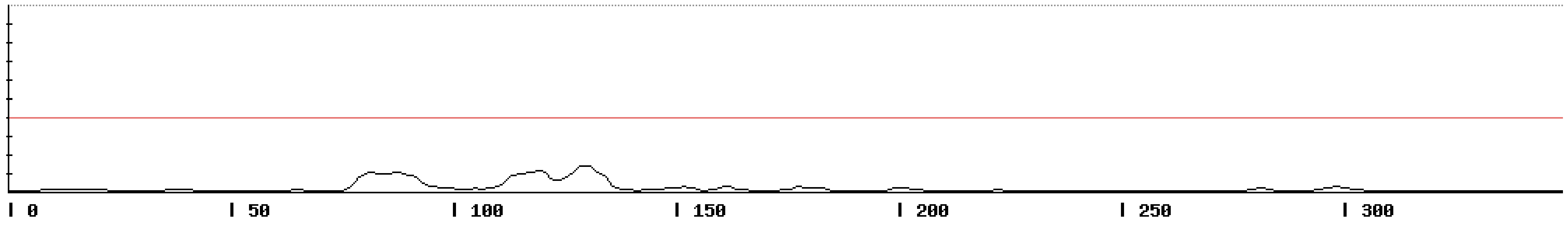

Predictions for Brassicaoleracea_XP_013626498.1

Viterbi Path	
Viterbi Path	
Posterior @ 0.4	
Posterior @ 0.4	
Posterior Graphic. Threshold Marked @ 0.4	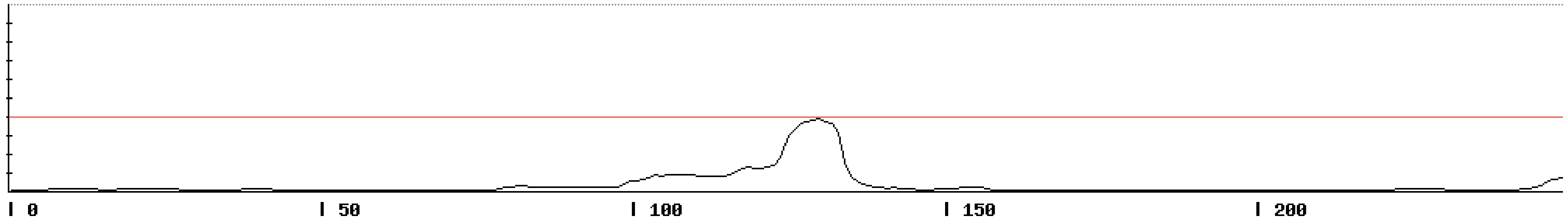

Predictions for Brassicaoleracea_XP_013626600.1

Viterbi Path

Viterbi Path

Posterior @ 0.4

Posterior @ 0.4

Posterior Graphic. Threshold Marked @ 0.4

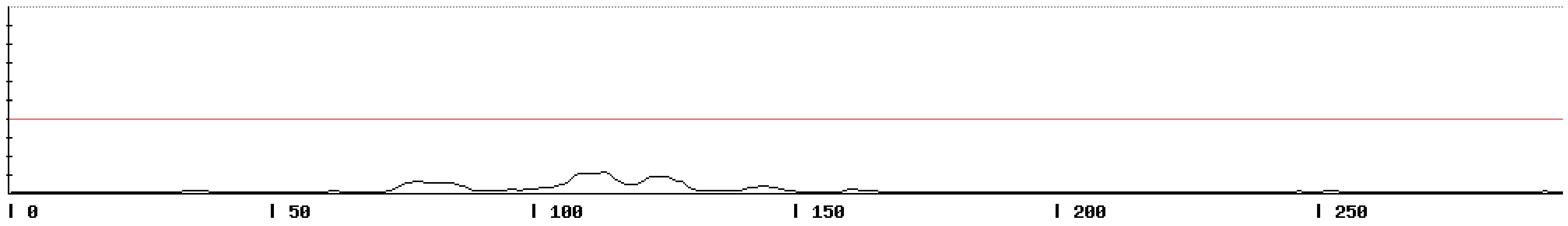

Predictions for Brassicaoleracea_XP_013626711.1

Viterbi Path

Viterbi Path

Posterior @ 0.4

Posterior @ 0.4

Posterior Graphic. Threshold Marked @ 0.4

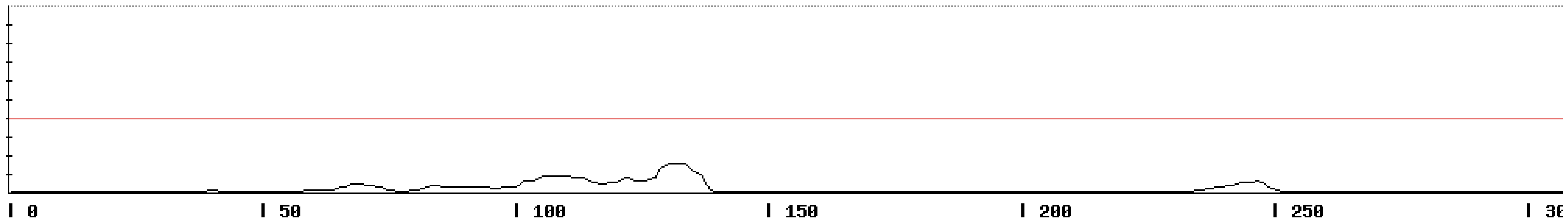

Predictions for Brassicaoleracea_XP_013626851.1

Viterbi Path

Viterbi Path

Posterior @ 0.4

Posterior @ 0.4

Posterior Graphic. Threshold Marked @ 0.4

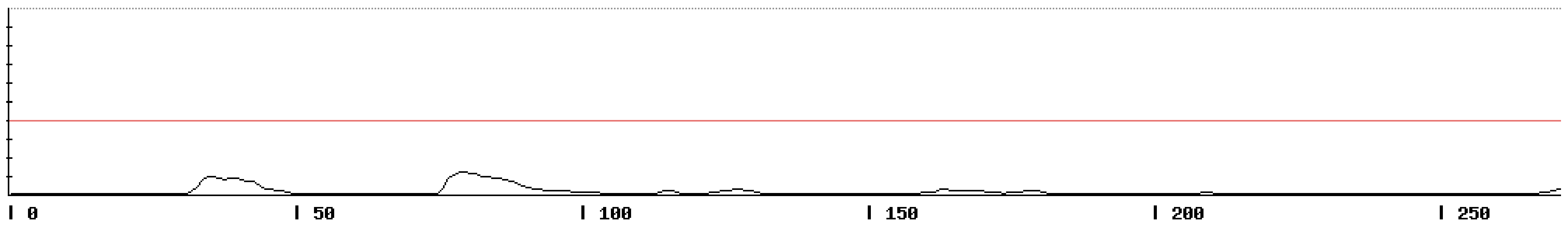

Predictions for Brassicaoleracea_XP_013626921.1

Viterbi Path

Viterbi Path

Posterior @ 0.4

Posterior @ 0.4

Posterior Graphic. Threshold Marked @ 0.4

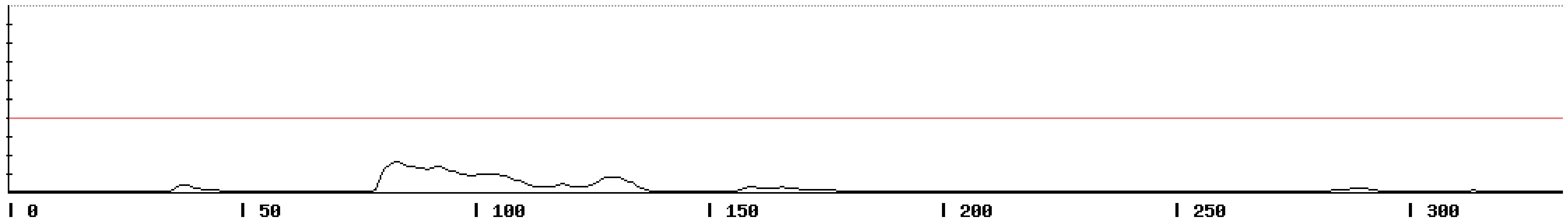

Predictions for Brassicaoleracea_XP_013626924.1

Viterbi Path

Viterbi Path

Posterior @ 0.4

Posterior @ 0.4

Posterior Graphic. Threshold Marked @ 0.4

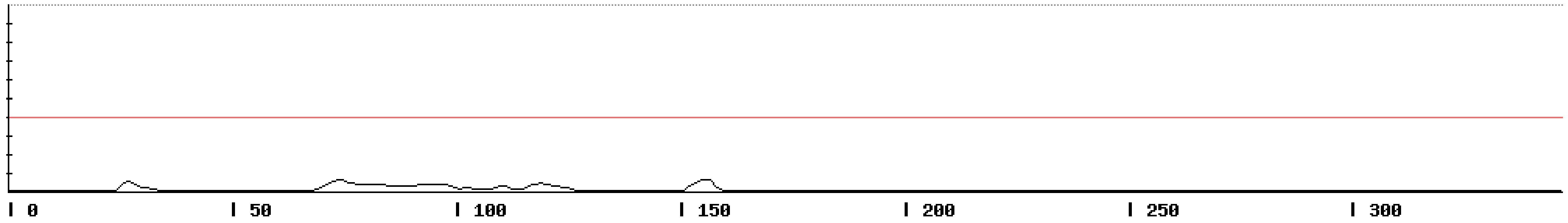

Predictions for Brassicaoleracea_XP_013626927.1

Viterbi Path

Viterbi Path

Posterior @ 0.4

Posterior @ 0.4

Posterior Graphic. Threshold Marked @ 0.4	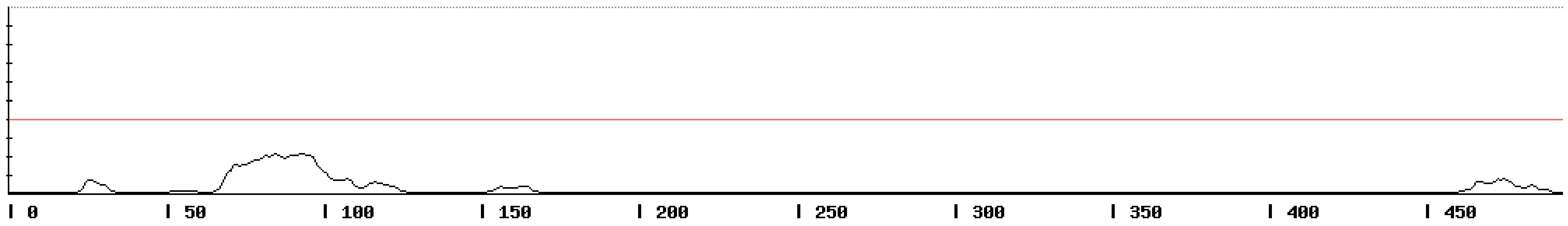

Predictions for Brassicaoleracea_XP_013626928.1

Viterbi Path	
Viterbi Path	
Posterior @ 0.4	
Posterior @ 0.4	
Posterior Graphic. Threshold Marked @ 0.4	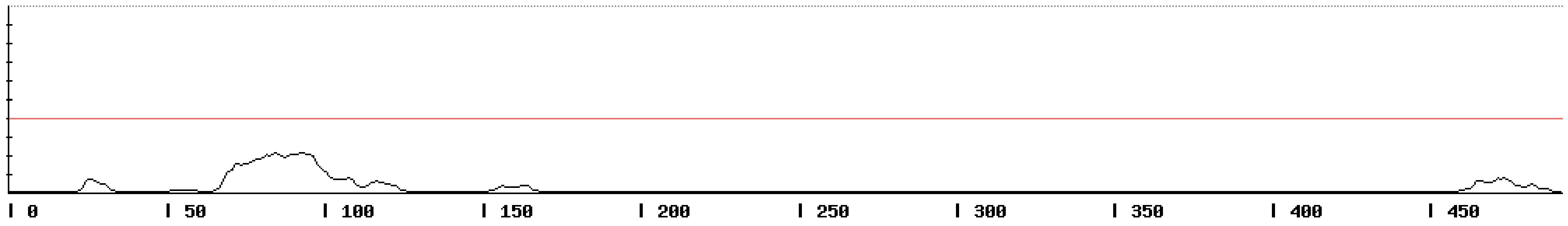

Predictions for Brassicaoleracea_XP_013627314.1

Viterbi Path	
Viterbi Path	
Posterior @ 0.4	
Posterior @ 0.4	

Posterior Graphic. Threshold Marked @ 0.4

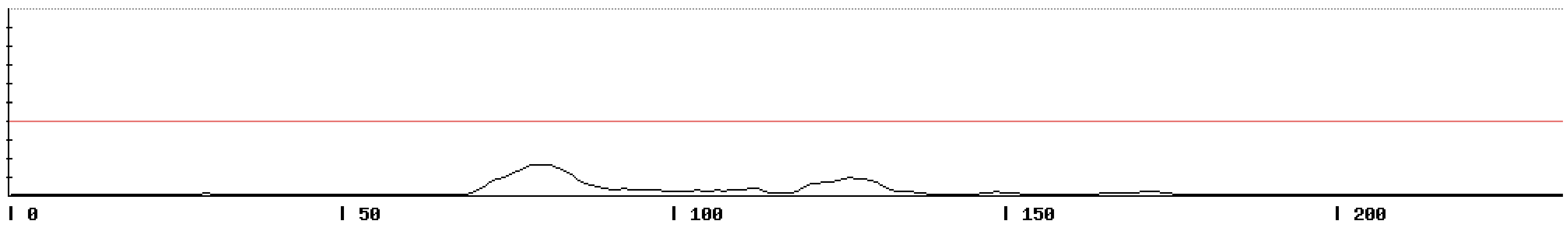

Predictions for Brassicaoleracea_XP_013627414.1

Viterbi Path

Viterbi Path

Posterior @ 0.4

Posterior @ 0.4

Posterior Graphic. Threshold Marked @ 0.4

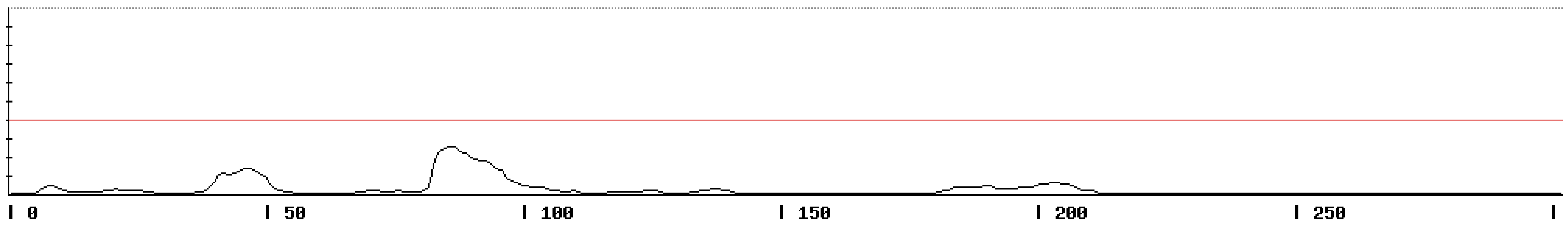

Predictions for Brassicaoleracea_XP_013627811.1

Viterbi Path

Viterbi Path

Posterior @ 0.4

Posterior @ 0.4

Posterior Graphic. Threshold Marked @ 0.4

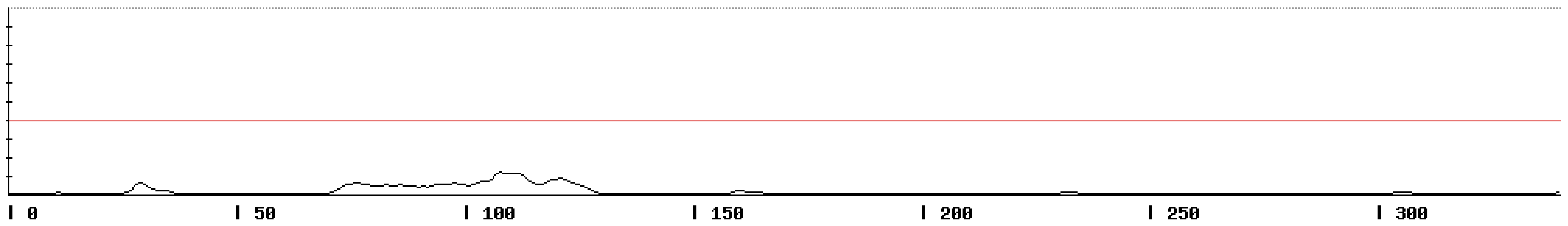

Predictions for Brassicaoleracea_XP_013628148.1

Viterbi Path

Viterbi Path

Posterior @ 0.4

Posterior @ 0.4

Posterior Graphic. Threshold Marked @ 0.4

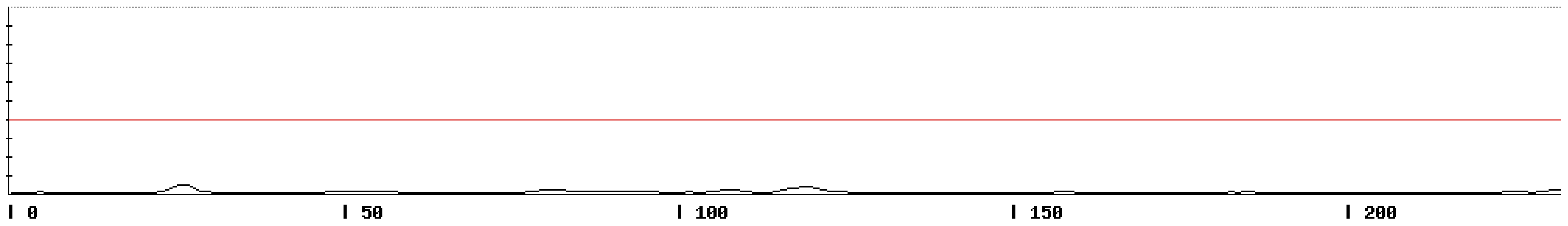

Predictions for Brassicaoleracea_XP_013628150.1

Viterbi Path

Viterbi Path

Posterior @ 0.4	
Posterior @ 0.4	
Posterior Graphic. Threshold Marked @ 0.4	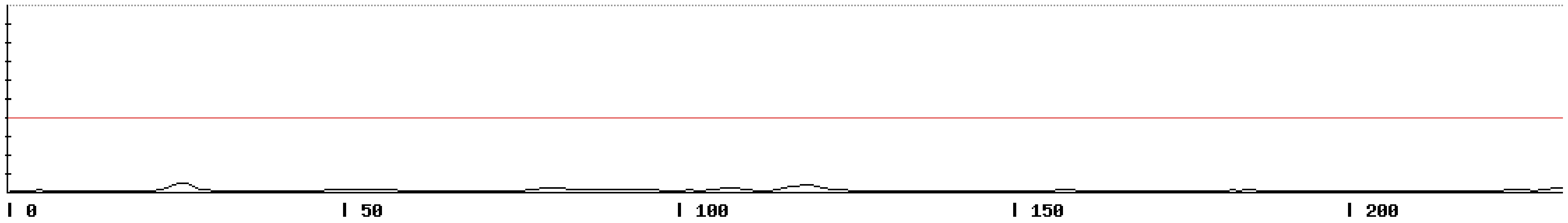

Predictions for Brassicaoleracea_XP_013628425.1	
Viterbi Path	160 - KRKEPKGRRQRKSRVR - 175
Viterbi Path	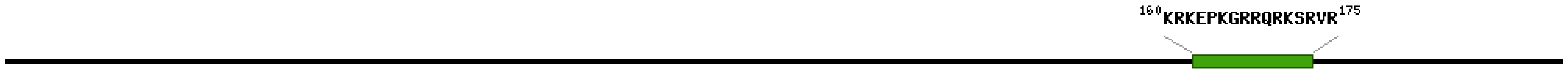
Posterior @ 0.4	159 - YKRKEPKGRRQRKSRVR - 175
Posterior @ 0.4	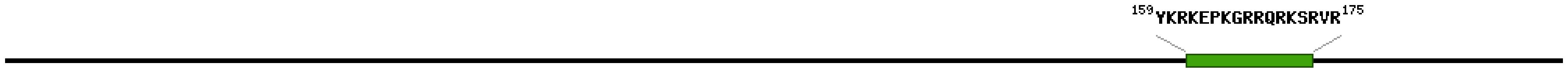
Posterior Graphic. Threshold Marked @ 0.4	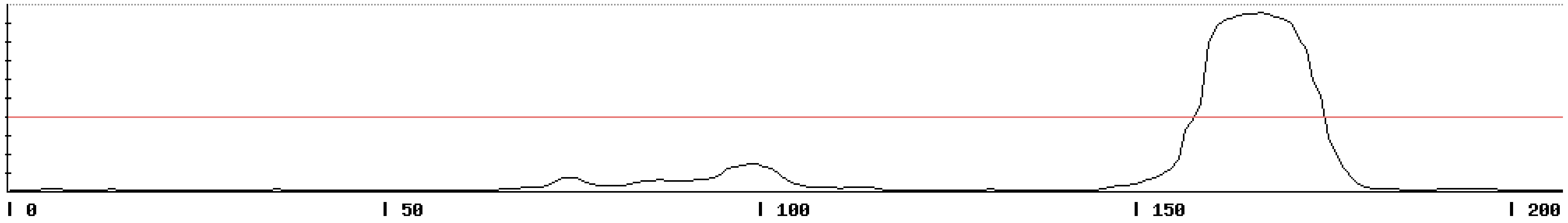

Predictions for Brassicaoleracea_XP_013628782.1

Viterbi Path

Viterbi Path

Posterior @ 0.4

Posterior @ 0.4

Posterior Graphic. Threshold Marked @ 0.4

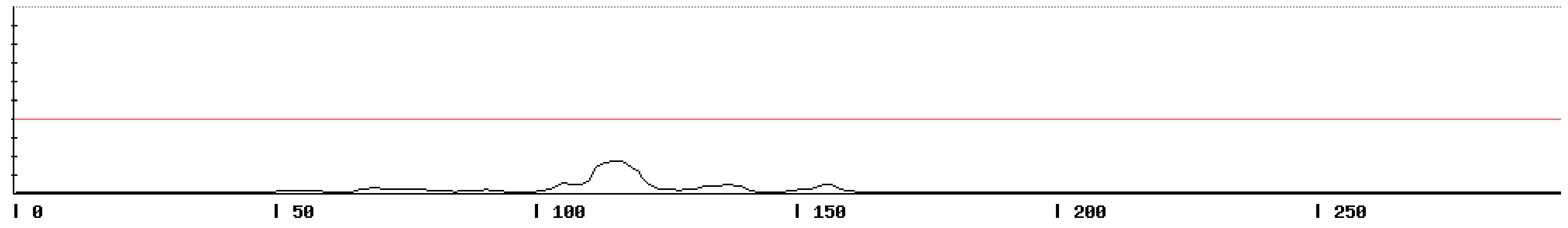

Predictions for Brassicaoleracea_XP_013628917.1

Viterbi Path

Viterbi Path

Posterior @ 0.4

Posterior @ 0.4

Posterior Graphic. Threshold Marked @ 0.4

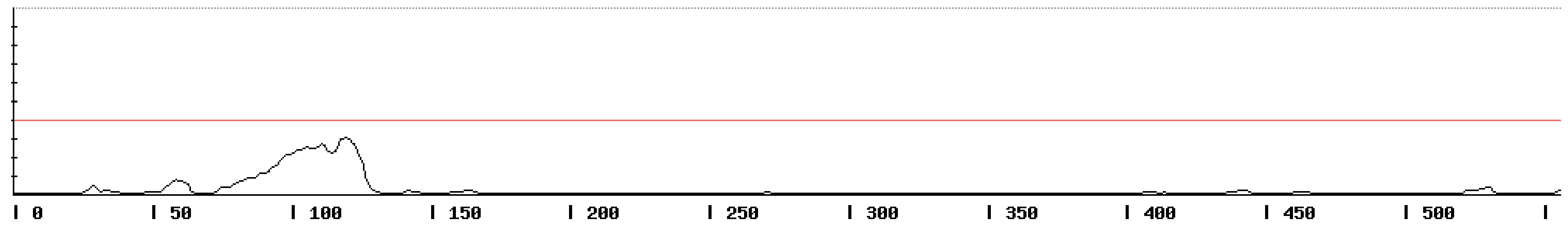

Predictions for Brassicaoleracea_XP_013628918.1

Viterbi Path	
Viterbi Path	
Posterior @ 0.4	
Posterior @ 0.4	
Posterior Graphic. Threshold Marked @ 0.4	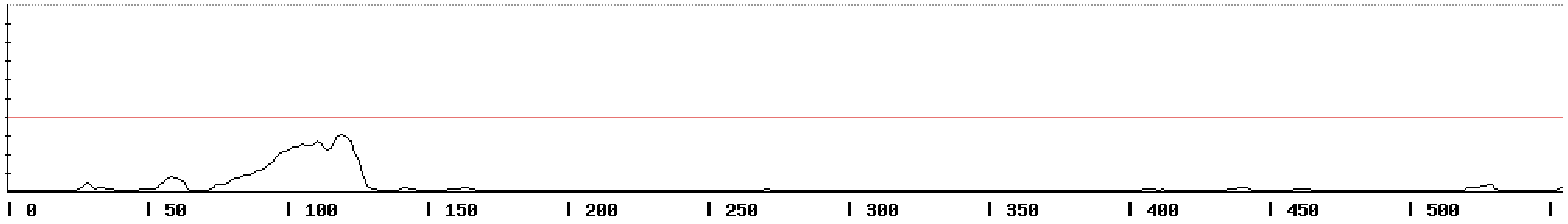

Predictions for Brassicaoleracea_XP_013629000.1

Viterbi Path	
Viterbi Path	
Posterior @ 0.4	95 - KPIGKPKTLGIKK - 107
Posterior @ 0.4	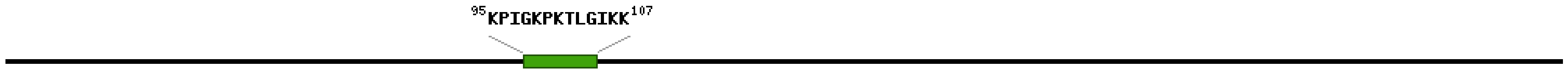
Posterior Graphic. Threshold Marked @ 0.4	

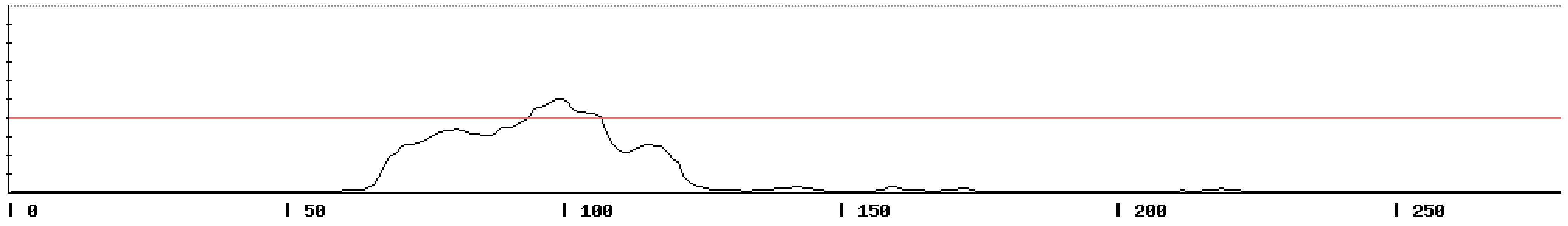

Predictions for Brassicaoleracea_XP_013630002.1

Viterbi Path

Viterbi Path

Posterior @ 0.4

Posterior @ 0.4

Posterior Graphic. Threshold Marked @ 0.4

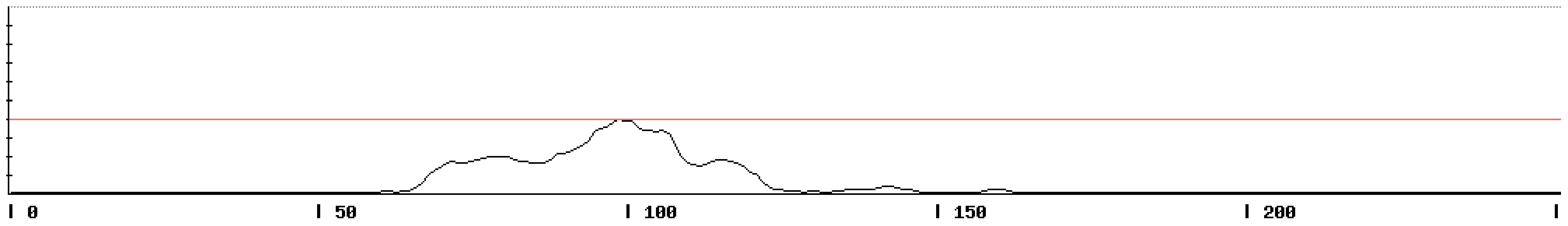

Predictions for Brassicaoleracea_XP_013630765.1

Viterbi Path

Viterbi Path

Posterior @ 0.4 — 121 - EKAEEKEKRRARDKR - 135

Posterior @ 0.4

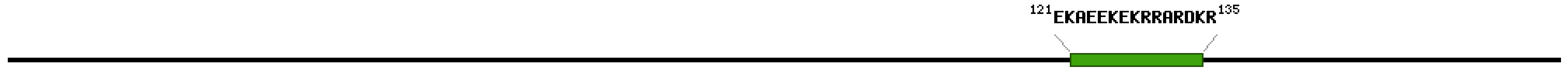

Posterior Graphic. Threshold Marked @ 0.4	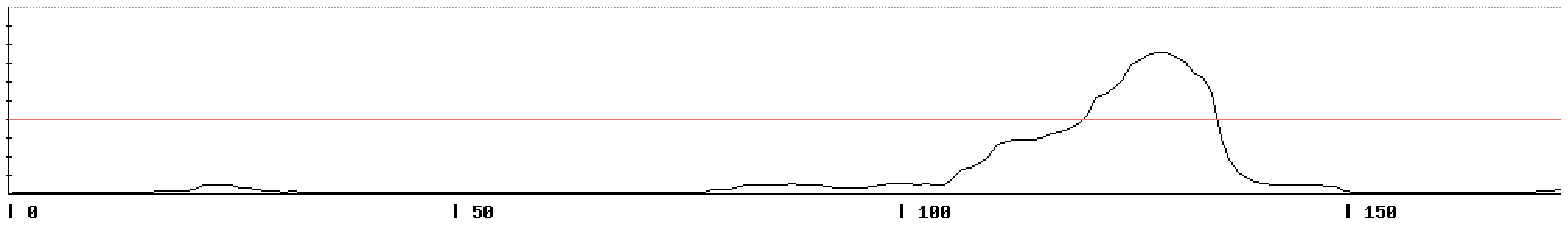

Predictions for Brassicaoleracea_XP_013630845.1	
Viterbi Path	
Viterbi Path	
Posterior @ 0.4	
Posterior @ 0.4	
Posterior Graphic. Threshold Marked @ 0.4	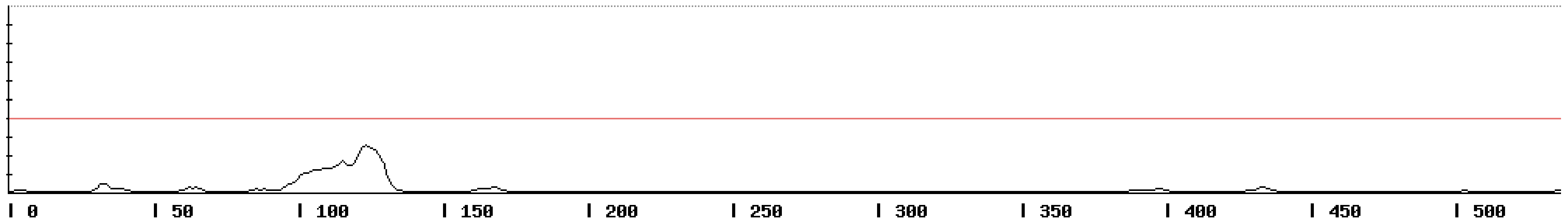

Predictions for Brassicaoleracea_XP_013632799.1	
Viterbi Path	
Viterbi Path	
Posterior @ 0.4	
Posterior @ 0.4	

Posterior Graphic. Threshold Marked @ 0.4

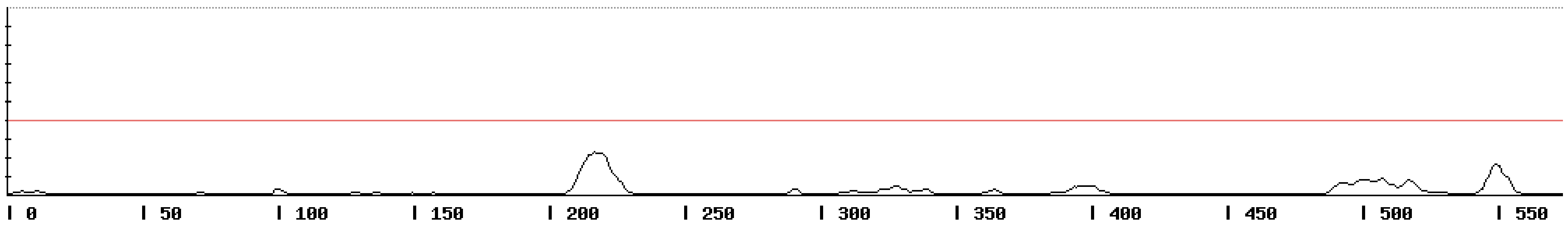

Predictions for Brassicaoleracea_XP_013634266.1

Viterbi Path

Viterbi Path

Posterior @ 0.4

197 - KDKKRK - 202

Posterior @ 0.4

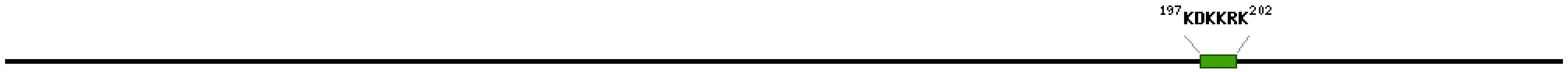

Posterior Graphic. Threshold Marked @ 0.4

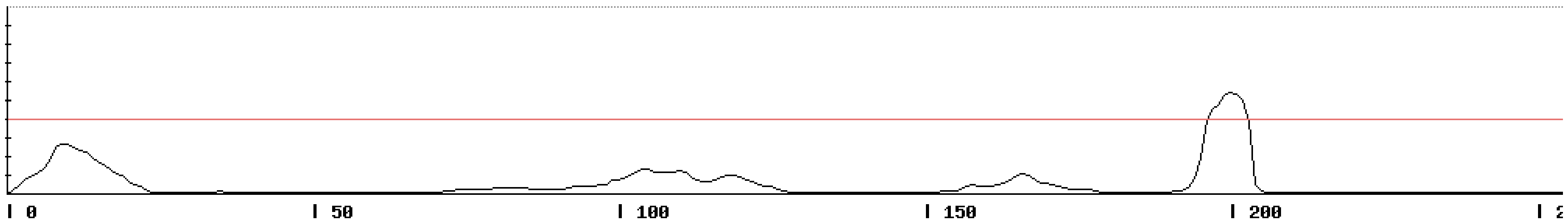

Predictions for Brassicaoleracea_XP_013634548.1

Viterbi Path

Viterbi Path

Posterior @ 0.4

Posterior @ 0.4

Posterior Graphic. Threshold Marked @ 0.4

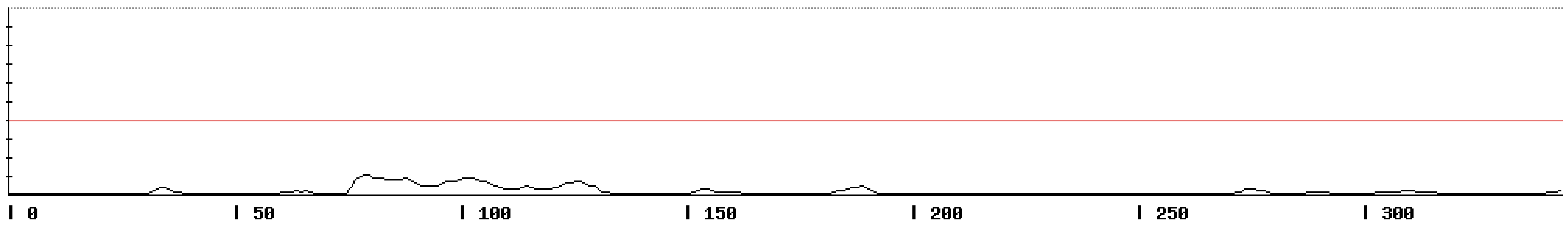

Predictions for Brassicaoleracea_XP_013634549.1

Viterbi Path

Viterbi Path

Posterior @ 0.4

Posterior @ 0.4

Posterior Graphic. Threshold Marked @ 0.4

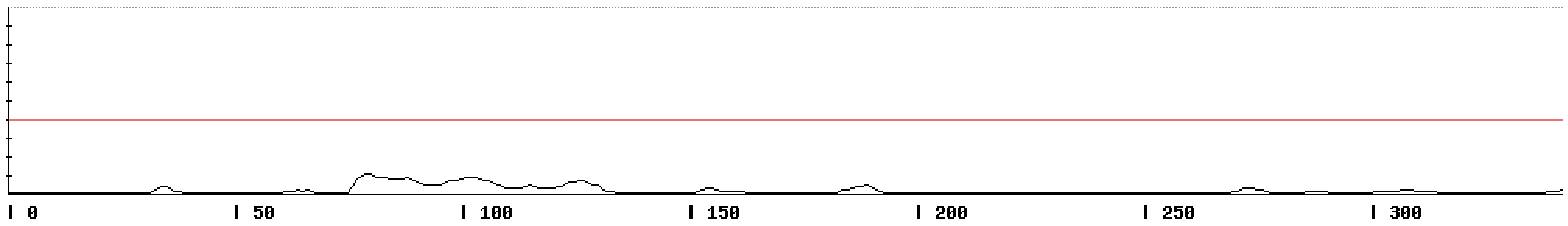

Predictions for Brassicaoleracea_XP_013634581.1

Viterbi Path 156 - KKRSNNNKKKKKEDEREKK - 174

Viterbi Path

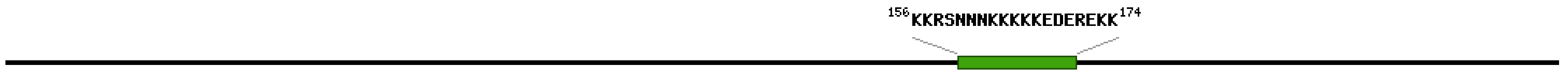

Posterior @ 0.4 | 156 - KKRSNNNKKKKKEDEREKK - 174

Posterior @ 0.4

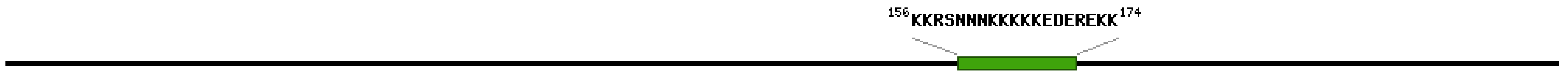

Posterior Graphic. Threshold Marked @ 0.4

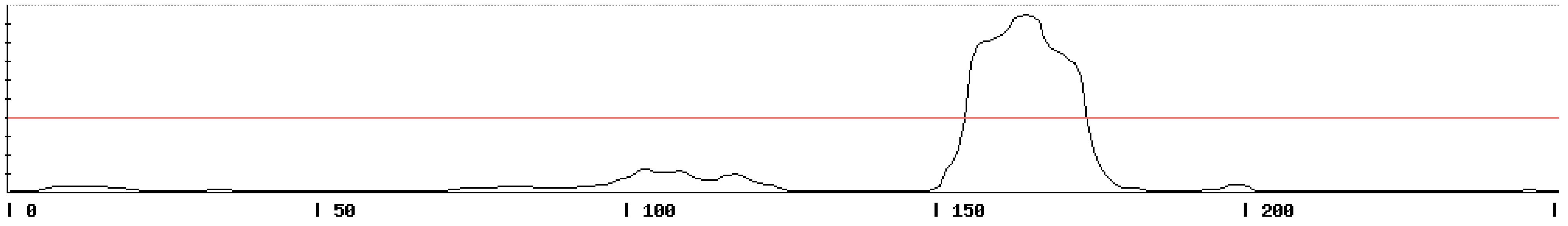

Predictions for Brassicaoleracea_XP_013634618.1

Viterbi Path

Viterbi Path

Posterior @ 0.4

Posterior @ 0.4

Posterior Graphic. Threshold Marked @ 0.4

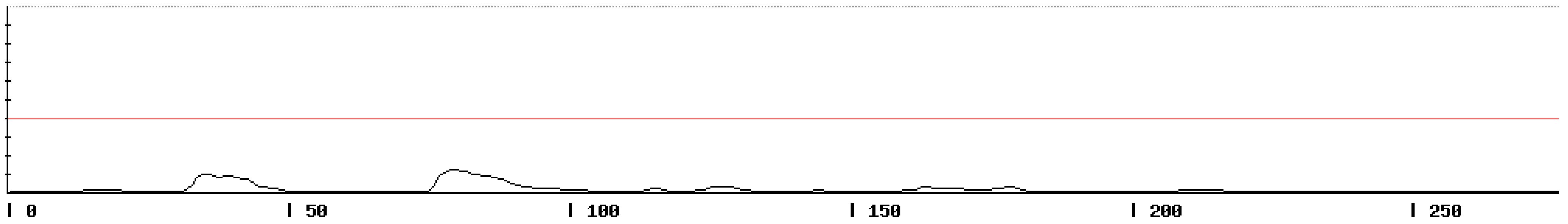

Predictions for Brassicaoleracea_XP_013634619.1

Viterbi Path

Viterbi Path

Posterior @ 0.4

Posterior @ 0.4

Posterior Graphic. Threshold Marked @ 0.4

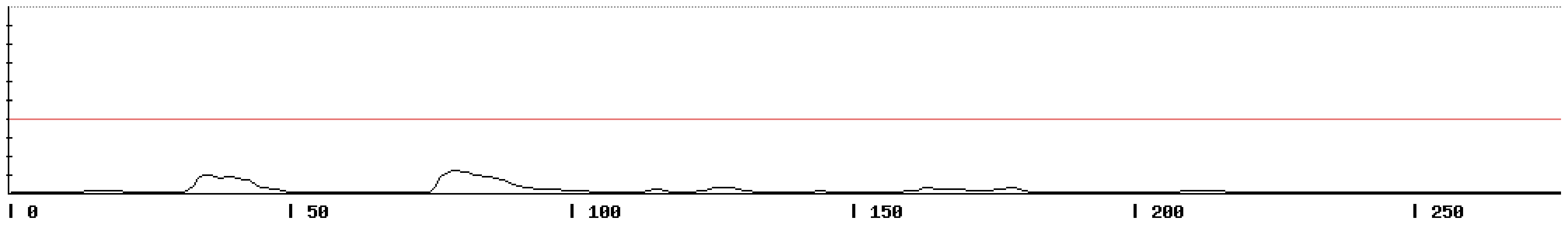

Predictions for Brassicaoleracea_XP_013634620.1

Viterbi Path

Viterbi Path

Posterior @ 0.4

Posterior @ 0.4

Posterior Graphic. Threshold Marked @ 0.4

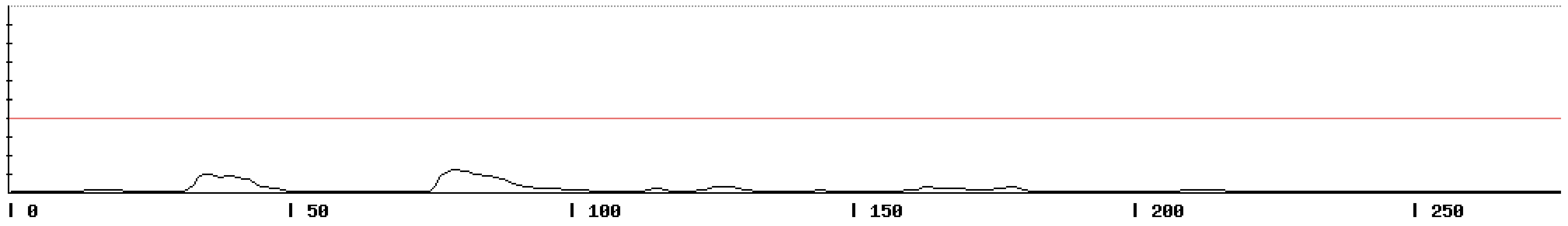

Predictions for Brassicaoleracea_XP_013634628.1	
Viterbi Path	
Viterbi Path	
Posterior @ 0.4	
Posterior @ 0.4	
Posterior Graphic. Threshold Marked @ 0.4	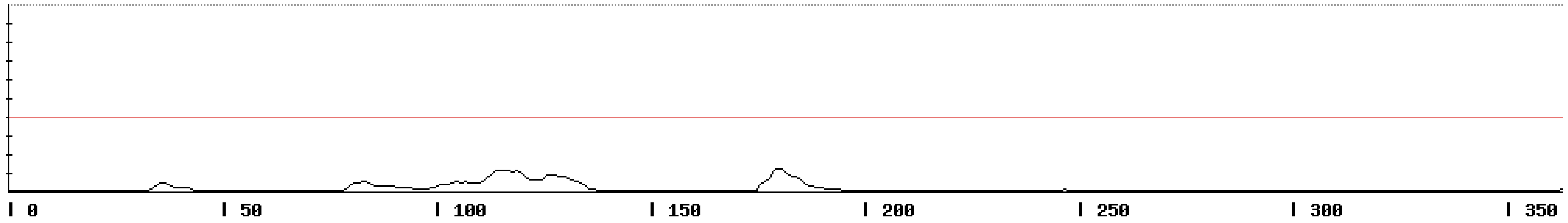

Predictions for Brassicaoleracea_XP_013634645.1	
Viterbi Path	
Viterbi Path	
Posterior @ 0.4	
Posterior @ 0.4	
Posterior Graphic. Threshold Marked @ 0.4	

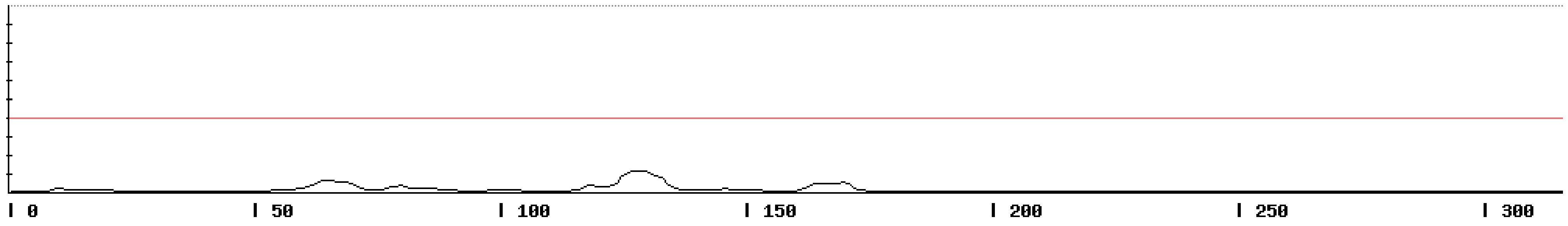

Predictions for Brassicaoleracea_XP_013634646.1

Viterbi Path

Viterbi Path

Posterior @ 0.4

Posterior @ 0.4

Posterior Graphic. Threshold Marked @ 0.4

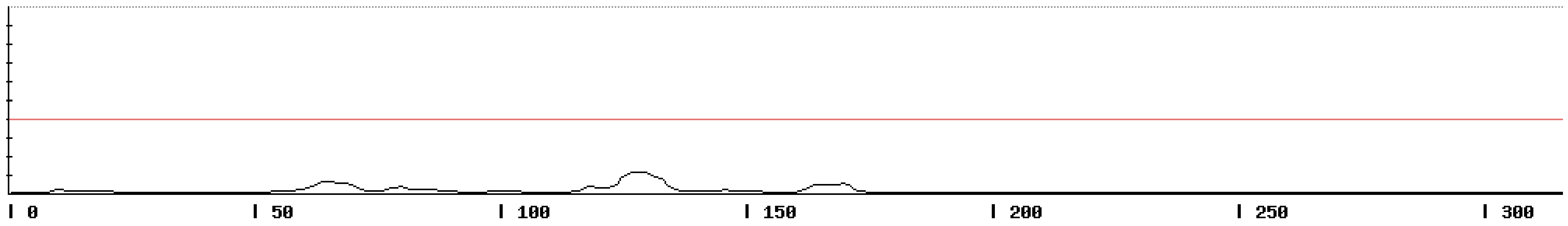

Predictions for Brassicaoleracea_XP_013634739.1

Viterbi Path

Viterbi Path

Posterior @ 0.4

Posterior @ 0.4

Posterior Graphic. Threshold Marked @ 0.4

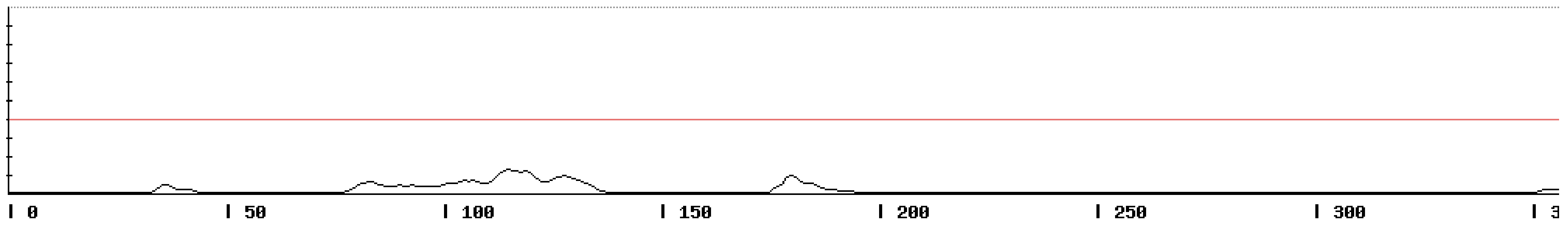

Predictions for Brassicaoleracea_XP_013634805.1

Viterbi Path

Viterbi Path

Posterior @ 0.4

Posterior @ 0.4

Posterior Graphic. Threshold Marked @ 0.4

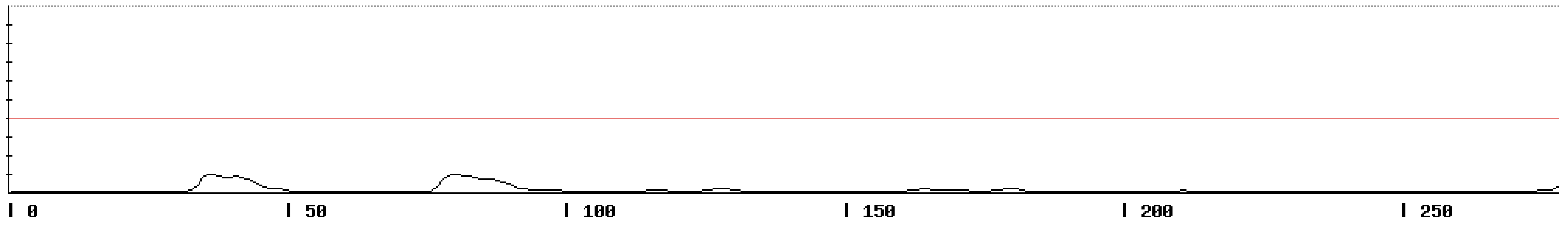

Predictions for Brassicaoleracea_XP_013635505.1

Viterbi Path

Viterbi Path

Posterior @ 0.4

Posterior @ 0.4

Posterior Graphic. Threshold Marked @ 0.4

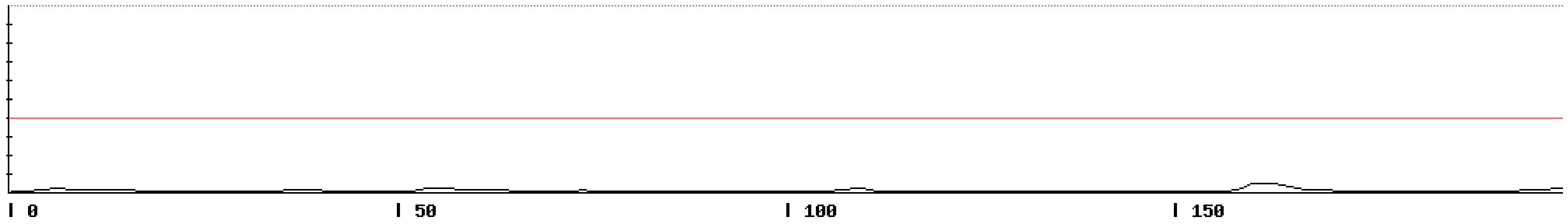

Predictions for Brassicaoleracea_XP_013636556.1

Viterbi Path

Viterbi Path

Posterior @ 0.4

Posterior @ 0.4

Posterior Graphic. Threshold Marked @ 0.4

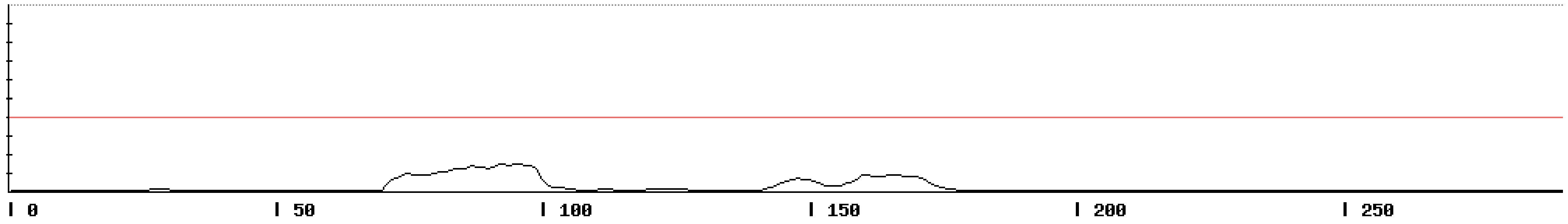

Predictions for Brassicaoleracea_XP_013637675.1

Viterbi Path

Viterbi Path

Posterior @ 0.4

Posterior @ 0.4

Posterior Graphic. Threshold Marked @ 0.4

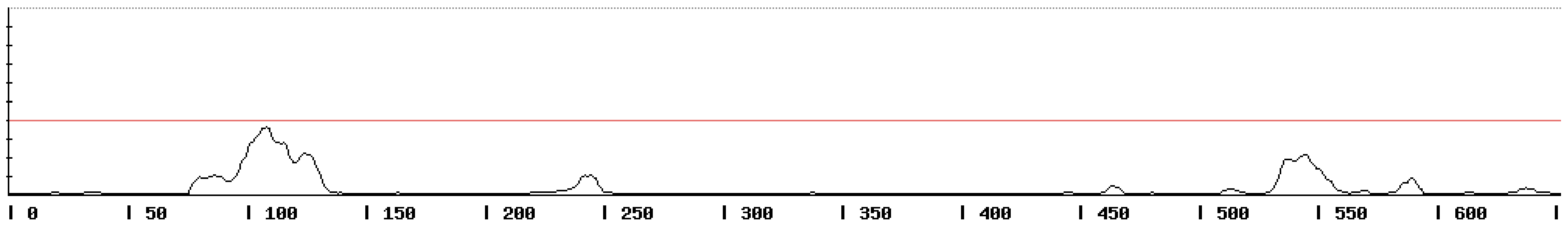

Predictions for Brassicaoleracea_XP_013637676.1

Viterbi Path

Viterbi Path

Posterior @ 0.4

Posterior @ 0.4

Posterior Graphic. Threshold Marked @ 0.4

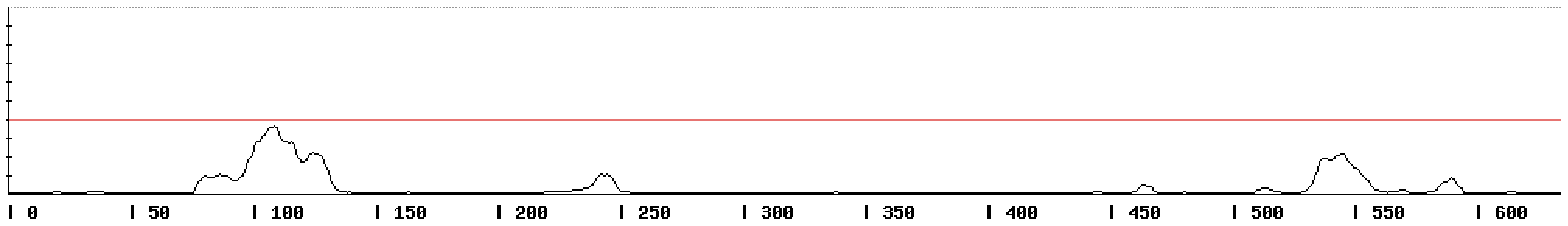

Predictions for Brassicaoleracea_XP_013638093.1

Viterbi Path

Viterbi Path

Posterior @ 0.4	138 - KRR - 140
Posterior @ 0.4	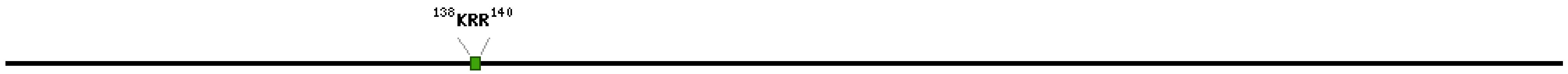
Posterior Graphic. Threshold Marked @ 0.4	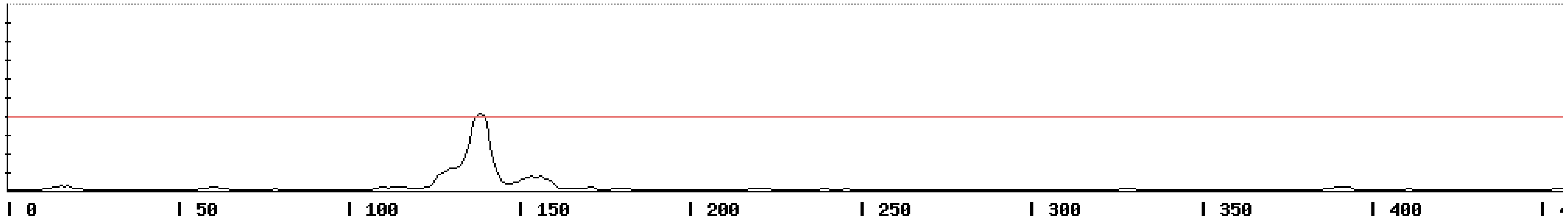

Predictions for Brassicaoleracea_XP_013638215.1

Viterbi Path	
Viterbi Path	
Posterior @ 0.4	
Posterior @ 0.4	
Posterior Graphic. Threshold Marked @ 0.4	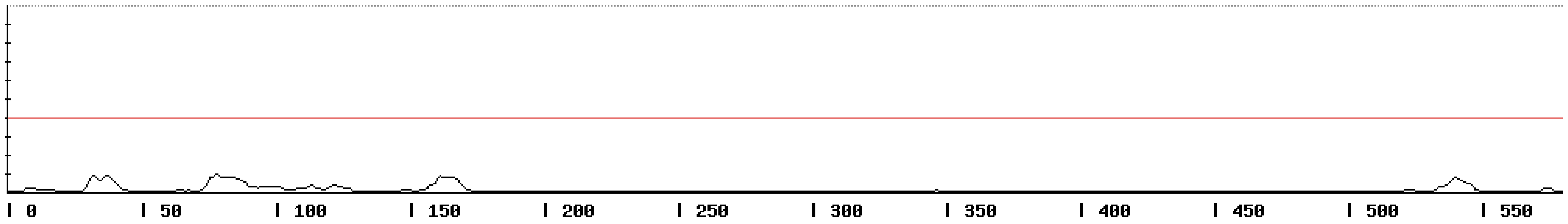

Predictions for Brassicaoleracea_XP_013638342.1

Viterbi Path

Viterbi Path

Posterior @ 0.4

Posterior @ 0.4

Posterior Graphic. Threshold Marked @ 0.4

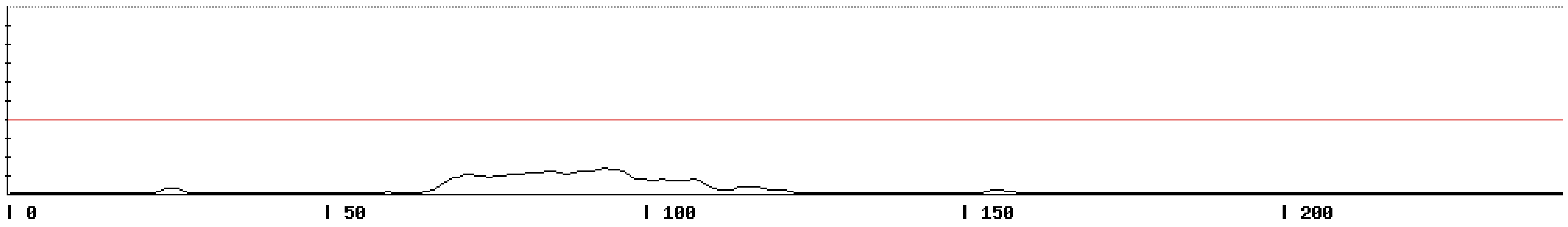

Predictions for Brassicaoleracea_XP_013638572.1

Viterbi Path

94 - KPIIRKKGGGGDHEKIGEKRVFVFKYSSKIPGGSKSKSKSKSKSKSK - 140

Viterbi Path

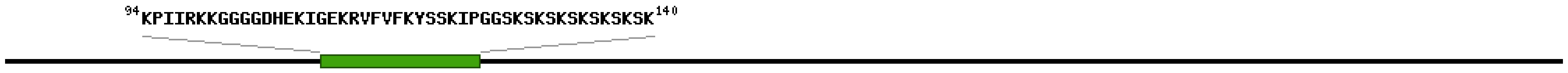

Posterior @ 0.4

94 - KPIIRKKGGGGDHEKIGEKRVFVFKYSSKIPGGSKSKSKSKSKSKSK - 140

Posterior @ 0.4

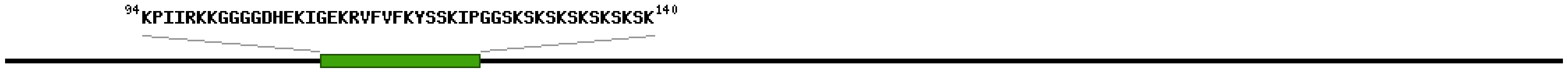

Posterior Graphic. Threshold Marked @ 0.4

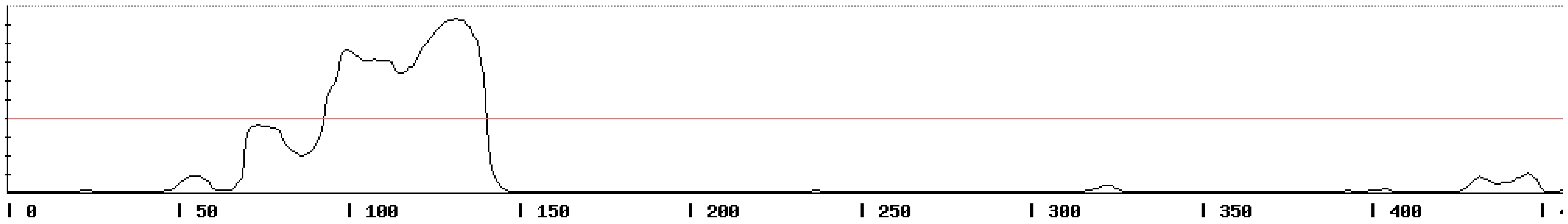

Predictions for Brassicaoleracea_XP_013638573.1	
Viterbi Path	94 - KPIIRKKGGGGDHEKIGEKRVFVFKYSSKIPGGSKSKSKSKSKSKSK - 140
Viterbi Path	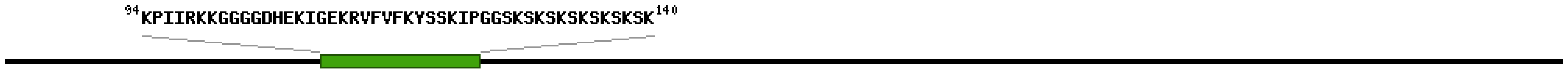
Posterior @ 0.4	94 - KPIIRKKGGGGDHEKIGEKRVFVFKYSSKIPGGSKSKSKSKSKSKSK - 140
Posterior @ 0.4	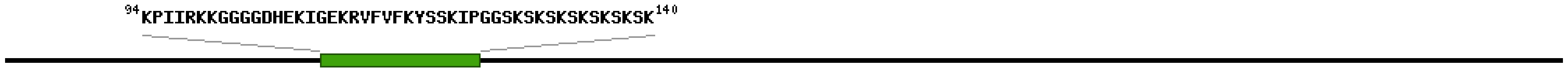
Posterior Graphic. Threshold Marked @ 0.4	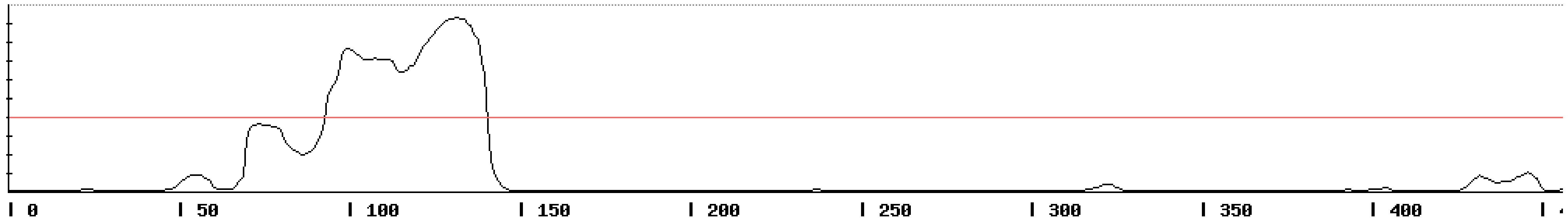

Predictions for Brassicaoleracea_XP_013638575.1	
Viterbi Path	94 - KPIIRKKGGGGDHEKIGEKRVFVFKYSSKIPGGSKSKSKSKSKSKSK - 140
Viterbi Path	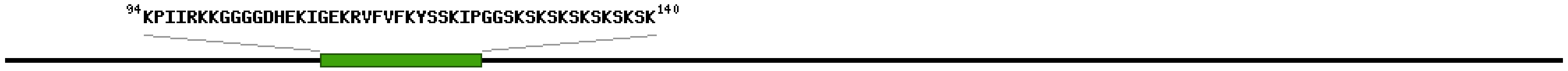
Posterior @ 0.4	94 - KPIIRKKGGGGDHEKIGEKRVFVFKYSSKIPGGSKSKSKSKSKSKSK - 140
Posterior @ 0.4	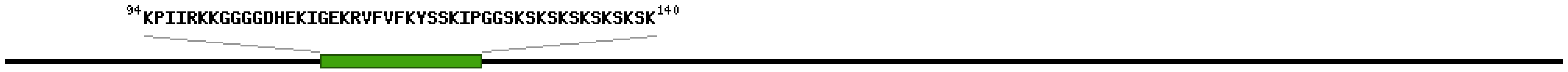
Posterior Graphic. Threshold Marked @ 0.4	

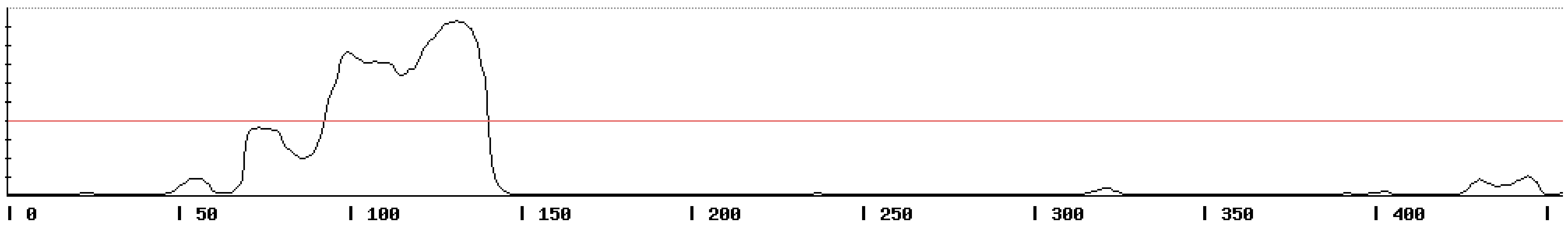

Predictions for Brassicaoleracea_XP_013638576.1	
Viterbi Path	94 - KPIIRKKGGGGDHEKIGEKRVFVFKYSSKIPGGSKSKSKSKSKSKSK - 140
Viterbi Path	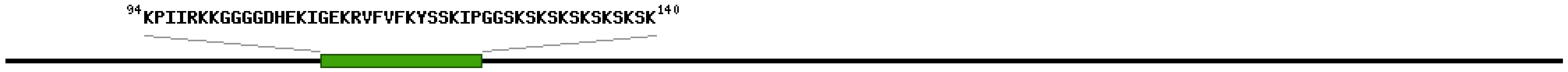
Posterior @ 0.4	94 - KPIIRKKGGGGDHEKIGEKRVFVFKYSSKIPGGSKSKSKSKSKSKSK - 140
Posterior @ 0.4	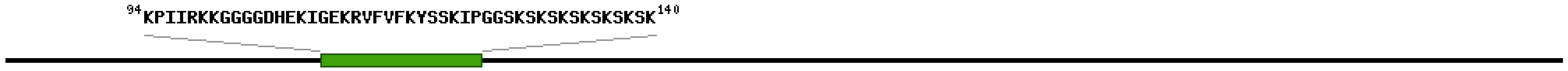
Posterior Graphic. Threshold Marked @ 0.4	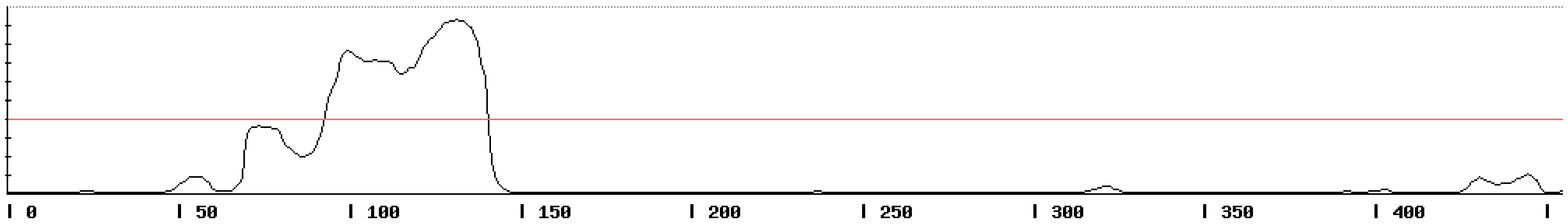

Predictions for Brassicaoleracea_XP_013638577.1	
Viterbi Path	94 - KPIIRKKGGGGDHEKIGEKRVFVFKSKSKSKSKSKSK - 130
Viterbi Path	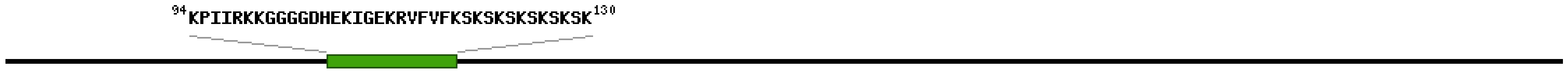
Posterior @ 0.4	93 - TKPIIRKKGGGGDHEKIGEKRVFVFKSKSKSKSKSKSK - 130
Posterior @ 0.4	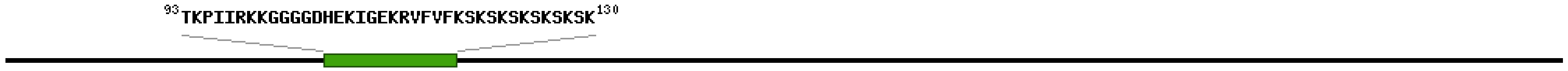

Posterior Graphic. Threshold Marked @ 0.4

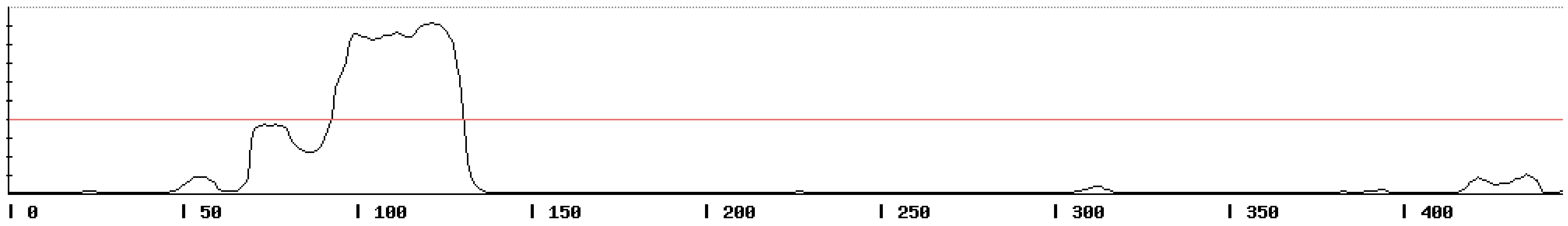

Predictions for Brassicaoleracea_XP_013638578.1

Viterbi Path: 94 - KPIIRKKGGGGDHEKIGEKRVFVFKYSSKIPGGSKSKSKSKSKSKSK - 140

Viterbi Path

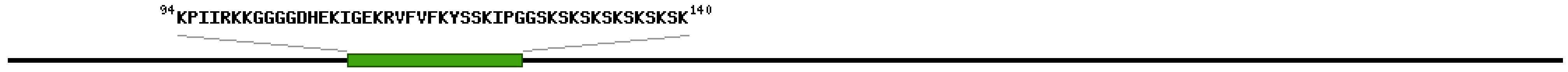

Posterior @ 0.4: 94 - KPIIRKKGGGGDHEKIGEKRVFVFKYSSKIPGGSKSKSKSKSKSKSK - 140

Posterior @ 0.4

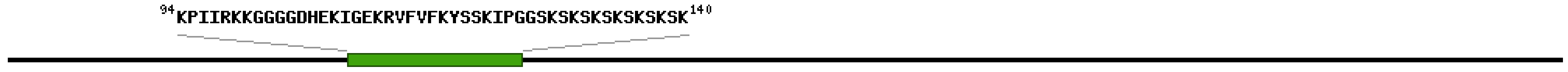

Posterior Graphic. Threshold Marked @ 0.4

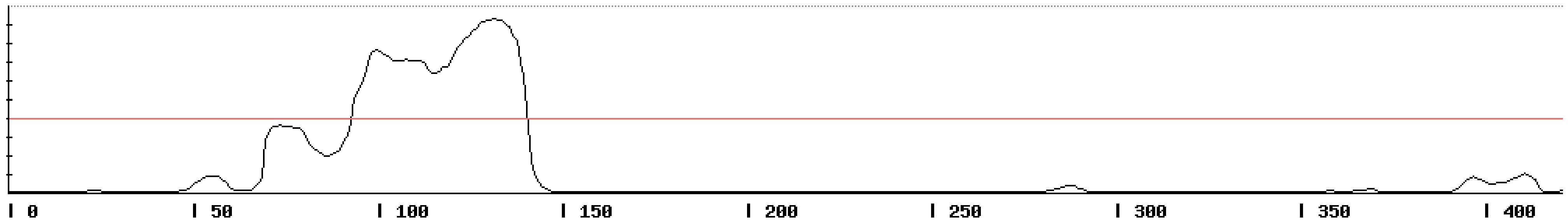

Predictions for Brassicaoleracea_XP_013638579.1

Viterbi Path

Viterbi Path

Posterior @ 0.4: 78 - SRKTK - 82

Posterior @ 0.4

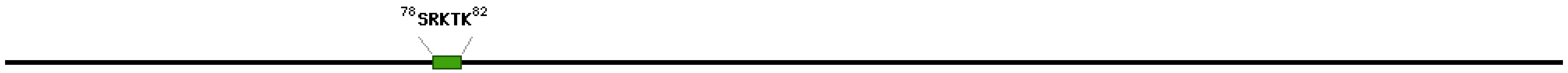

Posterior Graphic. Threshold Marked @ 0.4

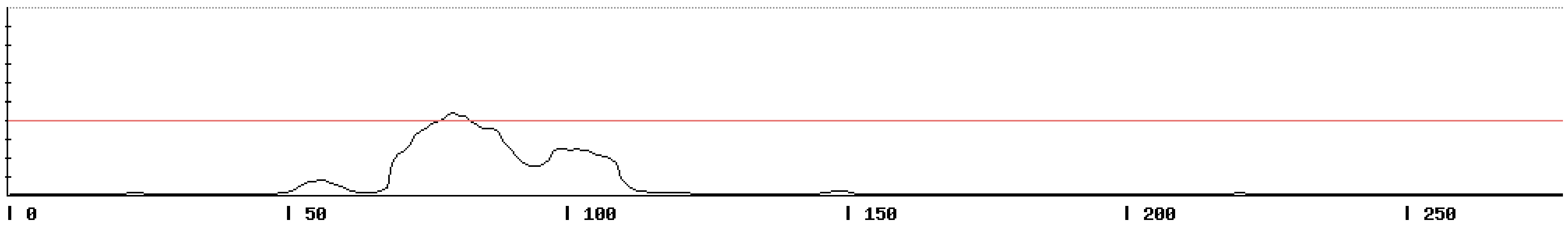

Predictions for Brassicaoleracea_XP_013638580.1

Viterbi Path	
Viterbi Path	
Posterior @ 0.4	78 - SRKTK - 82
Posterior @ 0.4	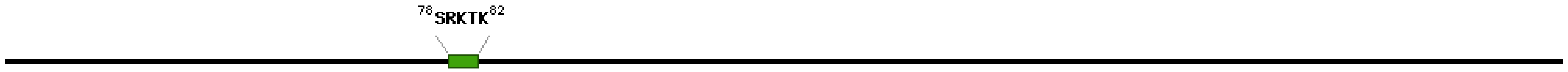
Posterior Graphic. Threshold Marked @ 0.4	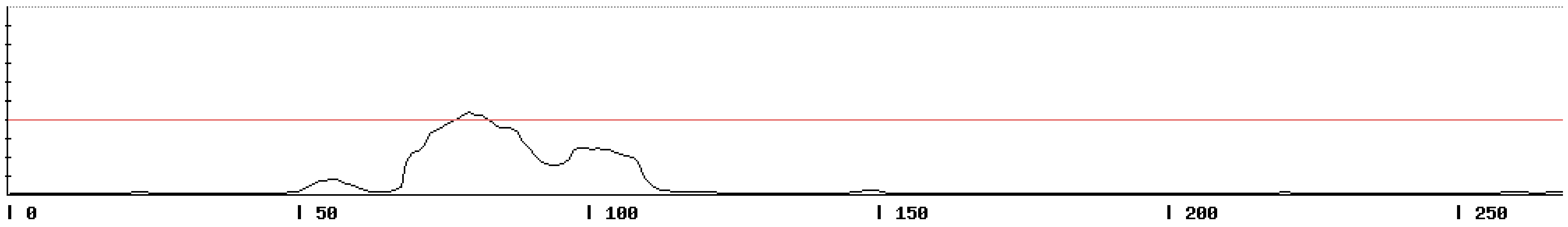

Predictions for Brassicaoleracea_XP_013638581.1

Viterbi Path	
Viterbi Path	

Posterior @ 0.4 — 78 - SRKTK - 82

Posterior @ 0.4

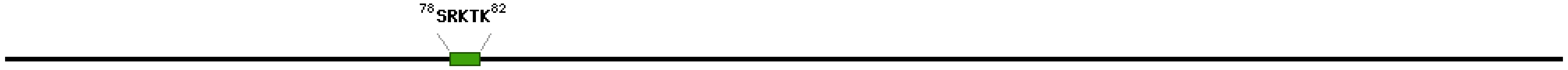

Posterior Graphic. Threshold Marked @ 0.4

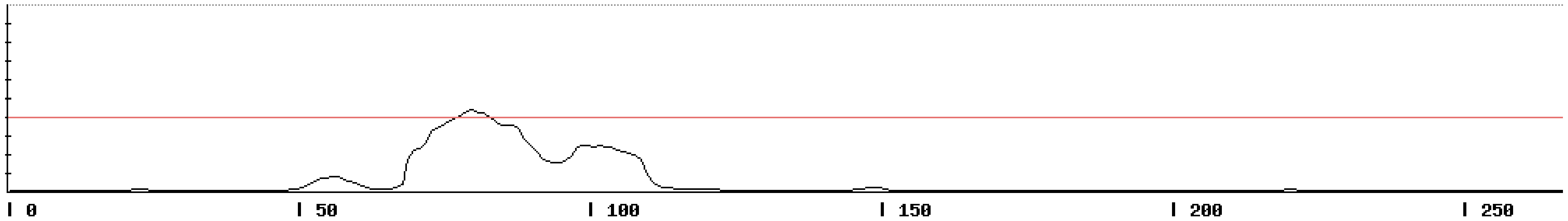

Predictions for Brassicaoleracea_XP_013638582.1

Viterbi Path

Viterbi Path

Posterior @ 0.4 — 78 - SRKTK - 82

Posterior @ 0.4

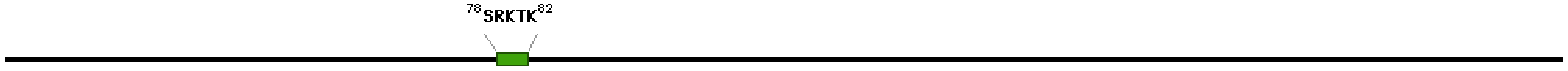

Posterior Graphic. Threshold Marked @ 0.4

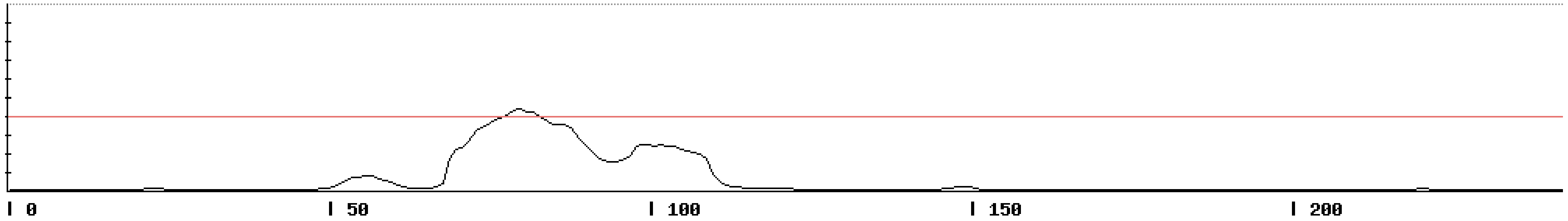

Predictions for Brassicaoleracea_XP_013638893.1

Viterbi Path

Viterbi Path

Posterior @ 0.4

Posterior @ 0.4

Posterior Graphic. Threshold Marked @ 0.4

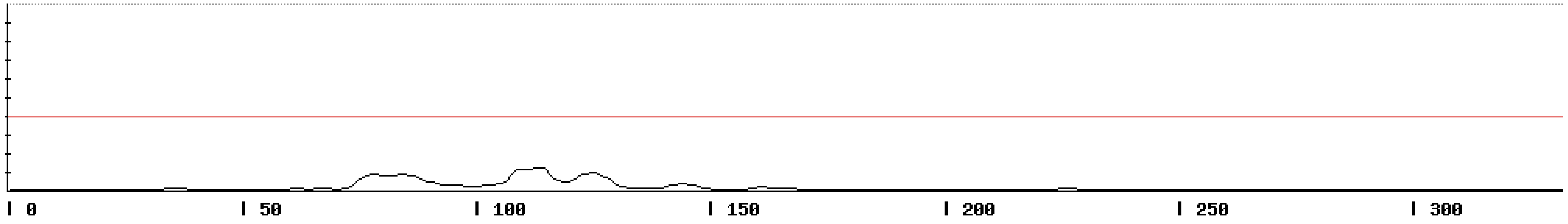

Predictions for Brassicaoleracea_XP_013638897.1

Viterbi Path

Viterbi Path

Posterior @ 0.4 — 152 - KRR - 154

Posterior @ 0.4

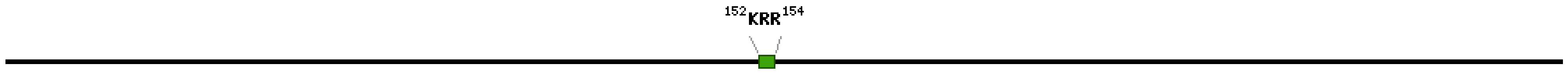

Posterior Graphic. Threshold Marked @ 0.4

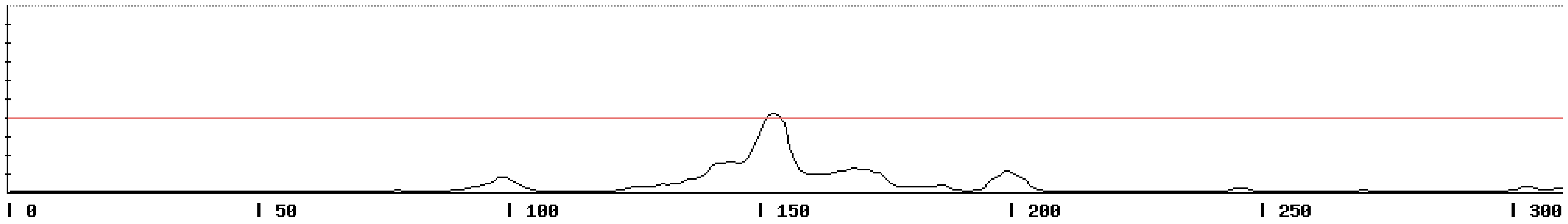

Predictions for Brassicaoleracea_XP_013638988.1

Viterbi Path

Viterbi Path

Posterior @ 0.4

Posterior @ 0.4

Posterior Graphic. Threshold Marked @ 0.4

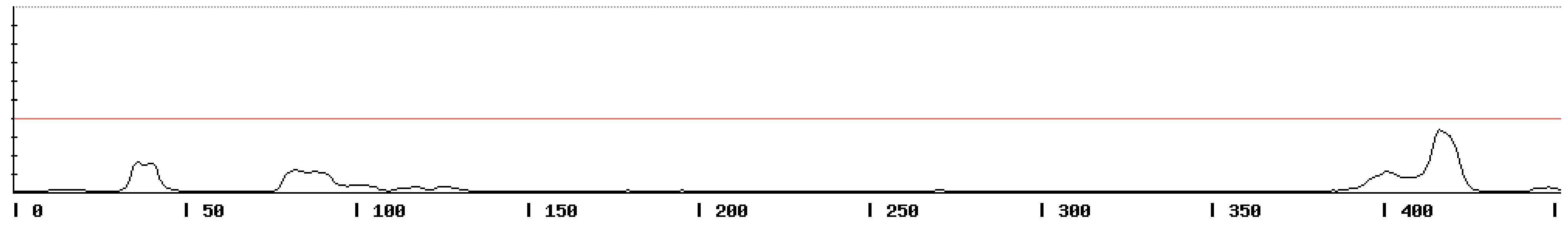

Predictions for Brassicaoleracea_XP_013638989.1

Viterbi Path

Viterbi Path

Posterior @ 0.4

Posterior @ 0.4

Posterior Graphic. Threshold Marked @ 0.4

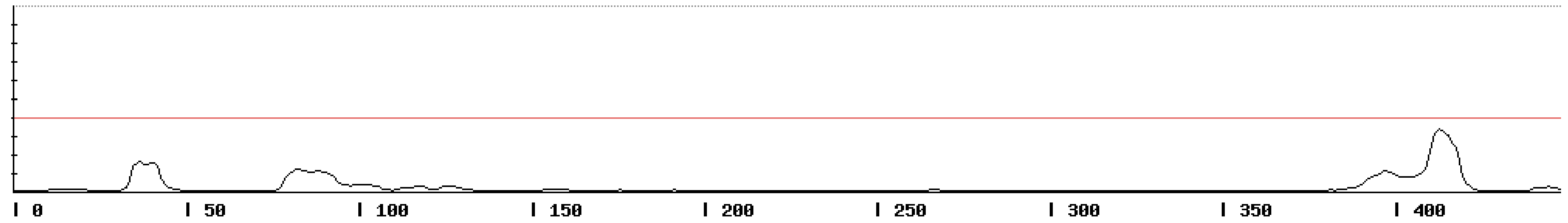

Predictions for Brassicaoleracea_XP_013639066.1	
Viterbi Path	
Viterbi Path	
Posterior @ 0.4	
Posterior @ 0.4	
Posterior Graphic. Threshold Marked @ 0.4	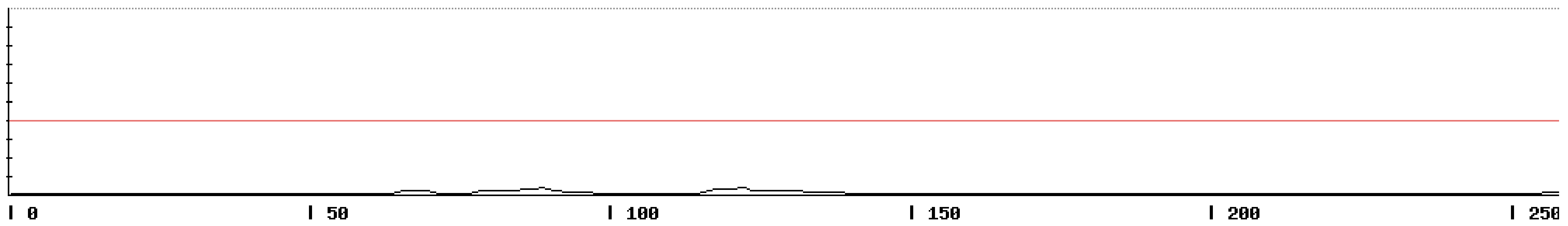

Predictions for Brassicaoleracea_XP_013639067.1	
Viterbi Path	
Viterbi Path	
Posterior @ 0.4	
Posterior @ 0.4	
Posterior Graphic. Threshold Marked @ 0.4	

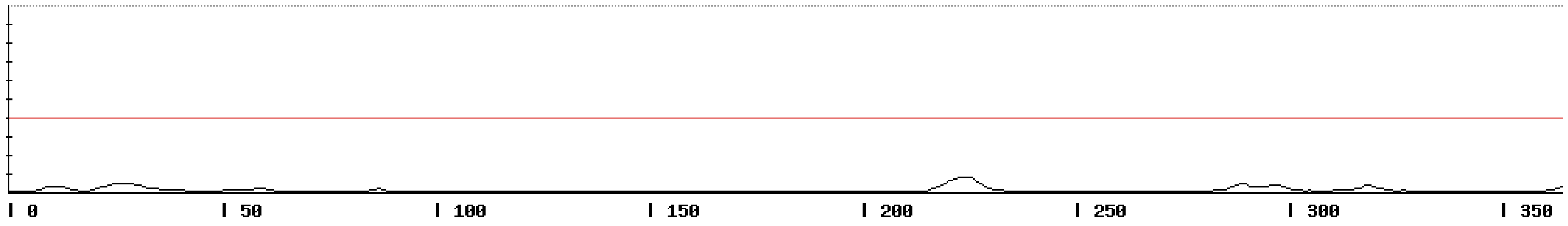

Predictions for Brassicaoleracea_XP_013639071.1	
Viterbi Path	
Viterbi Path	
Posterior @ 0.4	64 - VKKFGKLNRRKRASKTGH - 81
Posterior @ 0.4	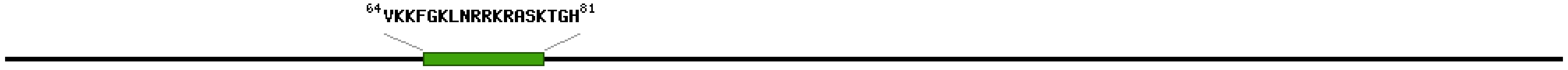
Posterior Graphic. Threshold Marked @ 0.4	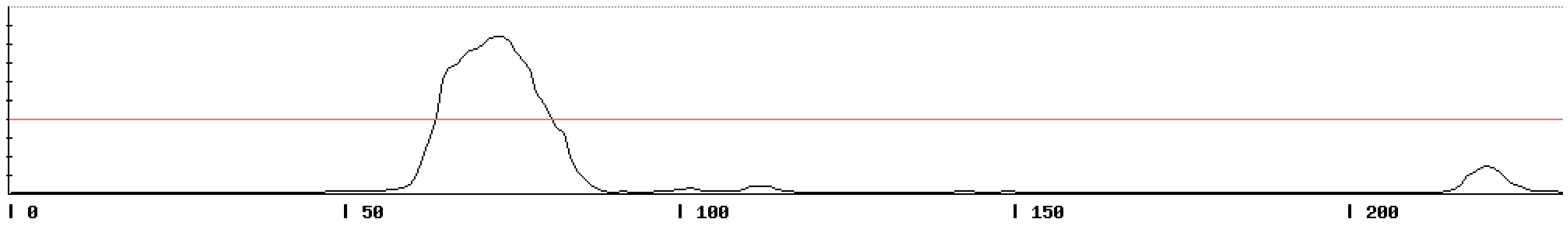

Predictions for Brassicaoleracea_XP_013639072.1	
Viterbi Path	
Viterbi Path	
Posterior @ 0.4	99 - RKRGNREKIGE - 109
Posterior @ 0.4	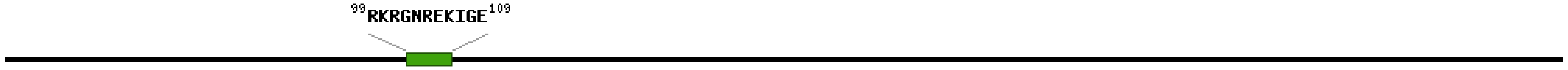

Posterior Graphic. Threshold Marked @ 0.4

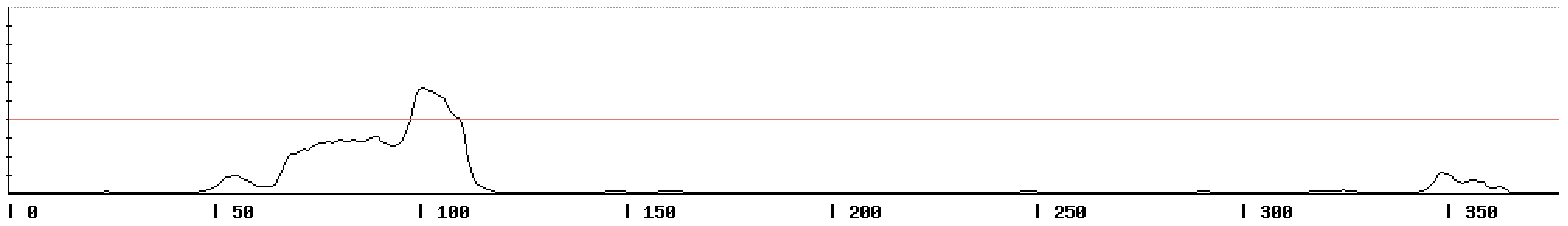

Predictions for Brassicaoleracea_XP_013639075.1

Viterbi Path	
Viterbi Path	
Posterior @ 0.4	85 - RKTK - 88
Posterior @ 0.4	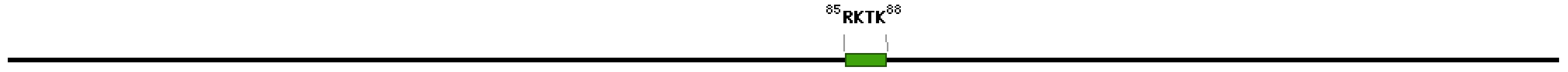
Posterior Graphic. Threshold Marked @ 0.4	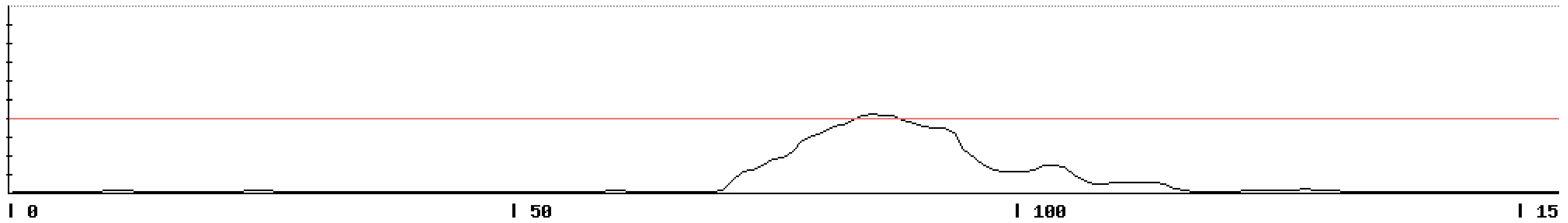

Predictions for Brassicaoleracea_XP_013639076.1

Viterbi Path	
Viterbi Path	
Posterior @ 0.4	
Posterior @ 0.4	

Posterior Graphic. Threshold Marked @ 0.4

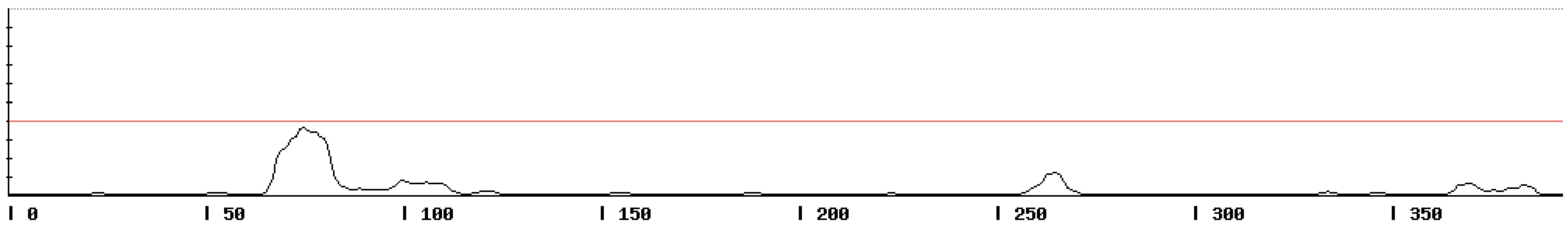

Predictions for Brassicaoleracea_XP_013639422.1

Viterbi Path

Viterbi Path

Posterior @ 0.4

Posterior @ 0.4

Posterior Graphic. Threshold Marked @ 0.4

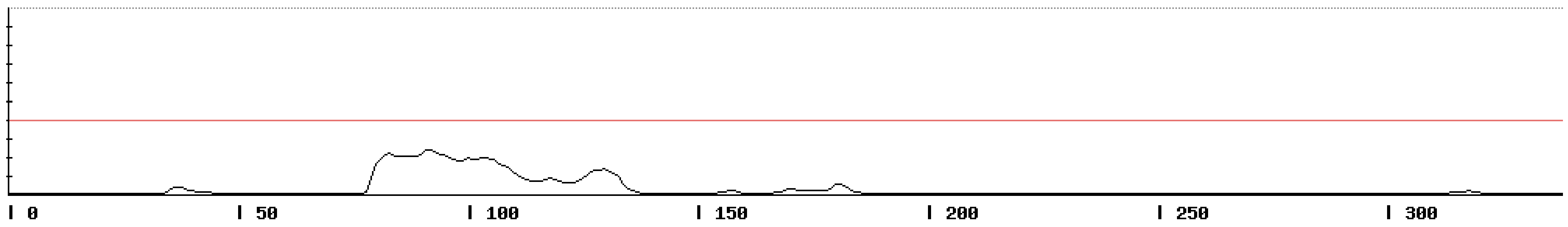

Predictions for Brassicaoleracea_XP_013639424.1

Viterbi Path

Viterbi Path

Posterior @ 0.4

Posterior @ 0.4

Posterior Graphic. Threshold Marked @ 0.4

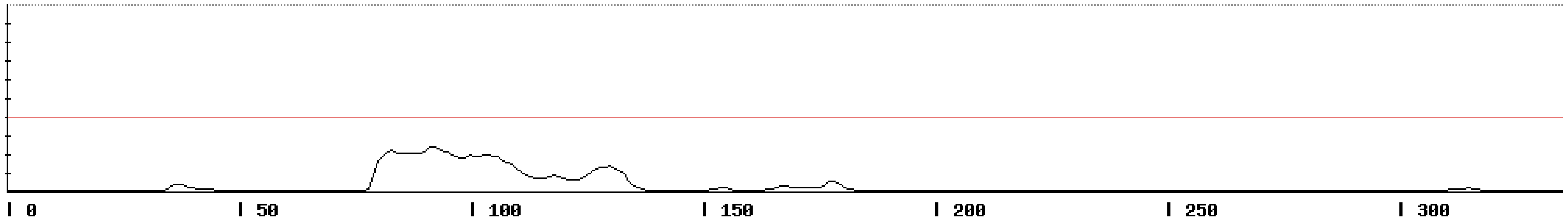

Predictions for Brassicaoleracea_XP_013639656.1

Viterbi Path

Viterbi Path

Posterior @ 0.4

Posterior @ 0.4

Posterior Graphic. Threshold Marked @ 0.4

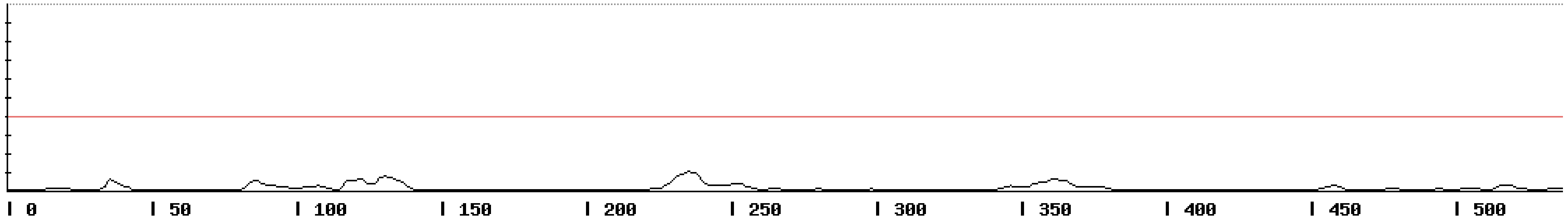

Predictions for Brassicaoleracea_XP_013639766.1

Viterbi Path

Viterbi Path

Posterior @ 0.4	
Posterior @ 0.4	
Posterior Graphic. Threshold Marked @ 0.4	

Revision r.9 - Last Updated on : February 24 2015 17:45:20.

alex.nguyenba@utoronto.ca

Intended to be used with Firefox

Viterbi Path

Posterior @ 0.4

Posterior @ 0.4

Posterior Graphic. Threshold Marked @ 0.4

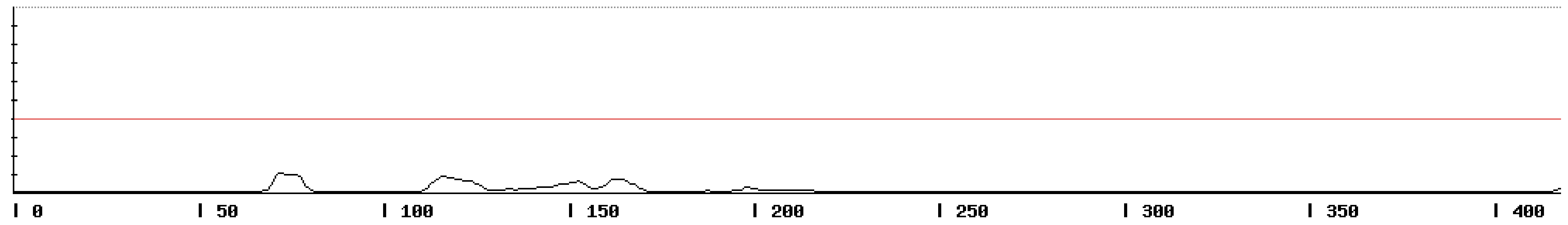

Predictions for Brassicarapa_XP_009101506.1

Viterbi Path

Viterbi Path

Posterior @ 0.4

Posterior @ 0.4

Posterior Graphic. Threshold Marked @ 0.4

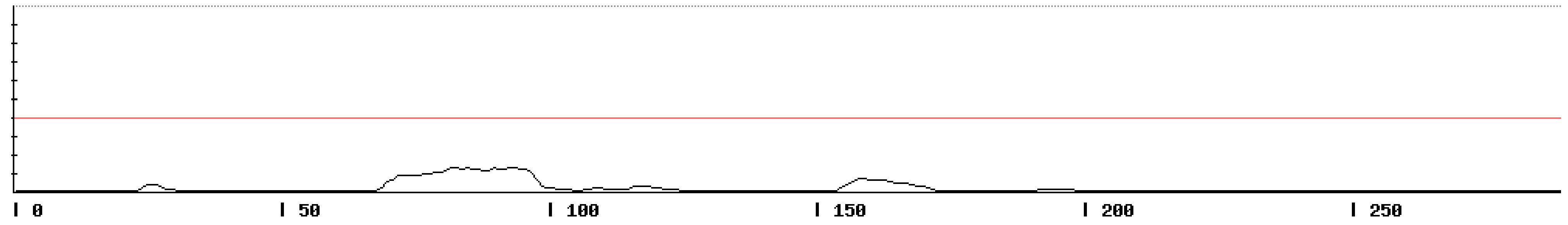

Predictions for Brassicarapa_XP_009102282.1

Viterbi Path	
Viterbi Path	
Posterior @ 0.4	274 - NKRKNKKKQVK - 284
Posterior @ 0.4	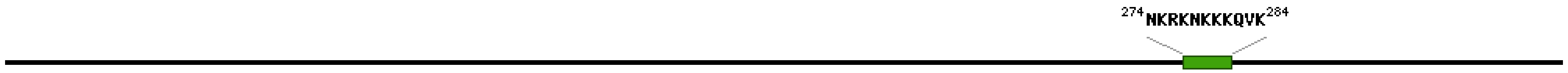
Posterior Graphic. Threshold Marked @ 0.4	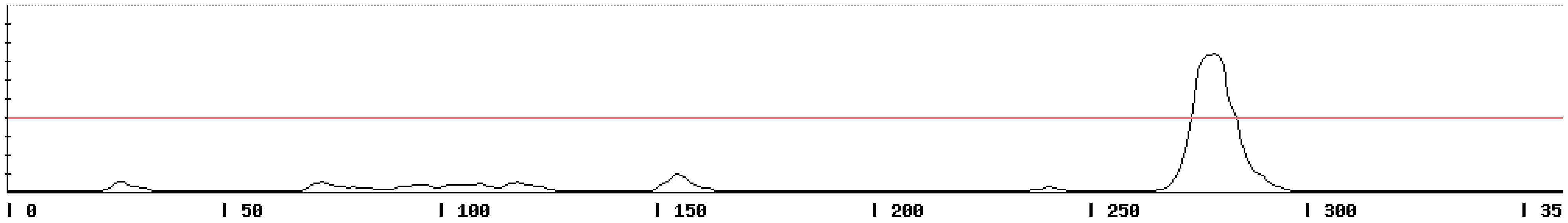

Predictions for Brassicarapa_XP_009102422.1

Viterbi Path	
Viterbi Path	
Posterior @ 0.4	
Posterior @ 0.4	
Posterior Graphic. Threshold Marked @ 0.4	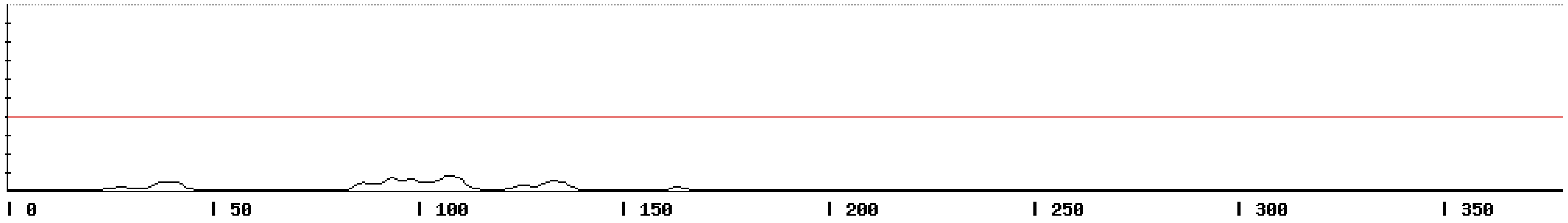

Predictions for Brassicarapa_XP_009102423.1	
Viterbi Path	
Viterbi Path	
Posterior @ 0.4	
Posterior @ 0.4	
Posterior Graphic. Threshold Marked @ 0.4	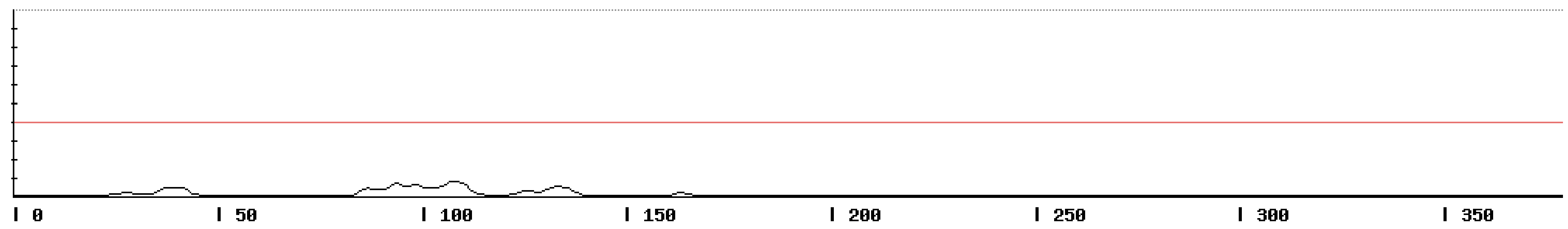

Predictions for Brassicarapa_XP_009102424.1	
Viterbi Path	
Viterbi Path	
Posterior @ 0.4	
Posterior @ 0.4	
Posterior Graphic. Threshold Marked @ 0.4	

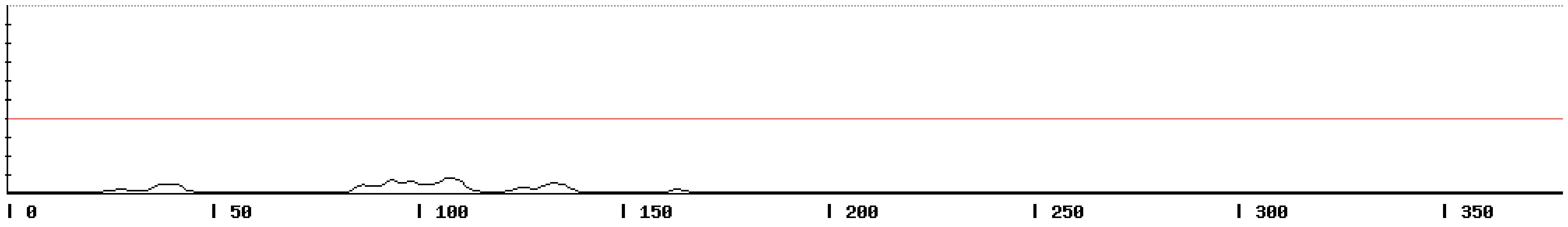

Predictions for Brassicarapa_XP_009102425.1

Viterbi Path

Viterbi Path

Posterior @ 0.4

Posterior @ 0.4

Posterior Graphic. Threshold Marked @ 0.4

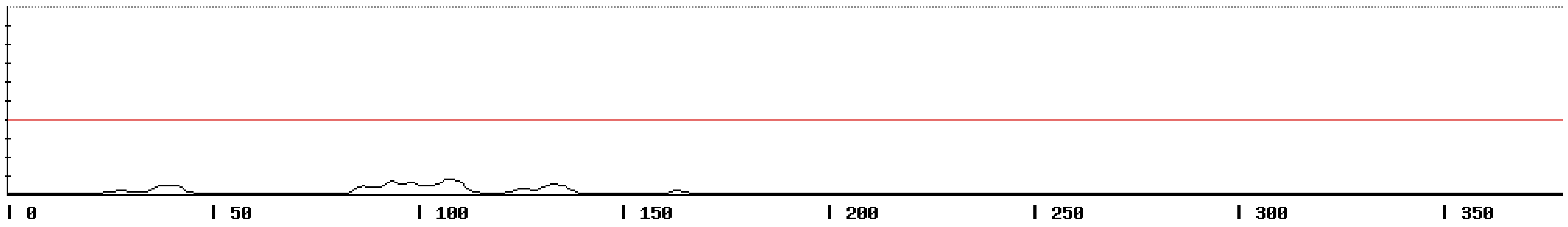

Predictions for Brassicarapa_XP_009102906.1

Viterbi Path

Viterbi Path

Posterior @ 0.4 | 160 - KRR - 162

Posterior @ 0.4

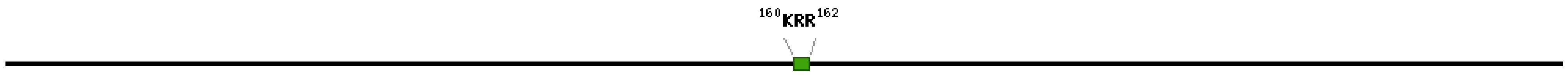

Posterior Graphic. Threshold Marked @ 0.4

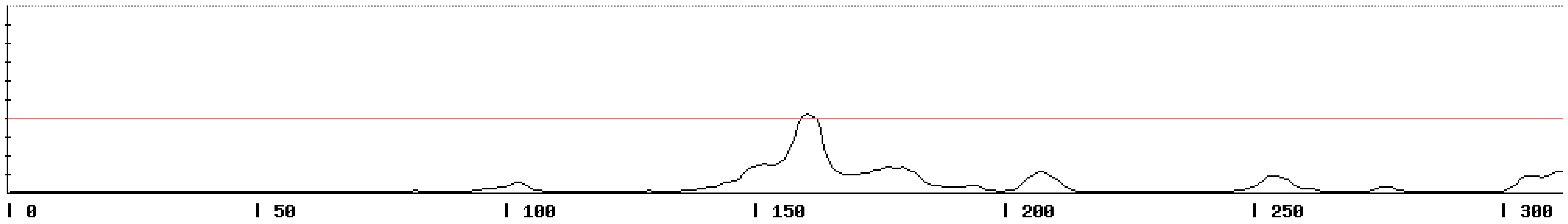

Predictions for Brassicarapa_XP_009102907.1

Viterbi Path	
Viterbi Path	
Posterior @ 0.4	156 - KRR - 158
Posterior @ 0.4	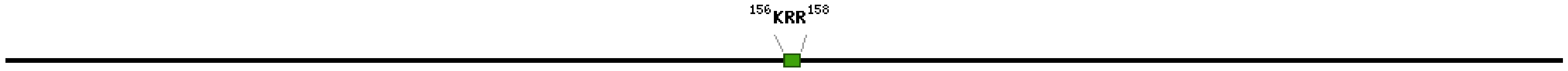
Posterior Graphic. Threshold Marked @ 0.4	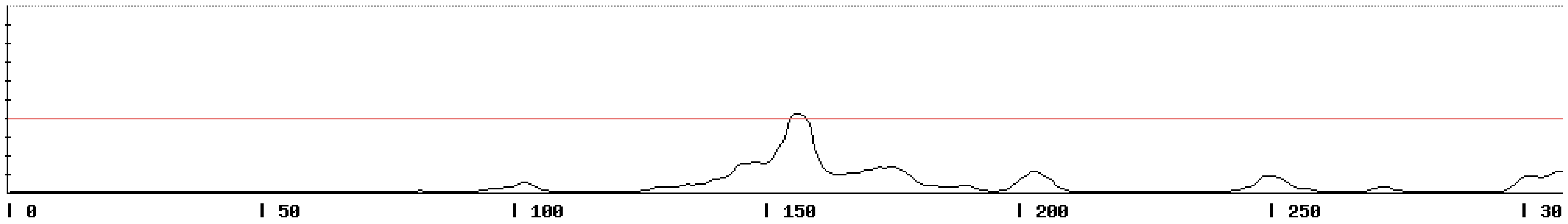

Predictions for Brassicarapa_XP_009102908.1

Viterbi Path	
Viterbi Path	
Posterior @ 0.4	160 - KRR - 162
Posterior @ 0.4	

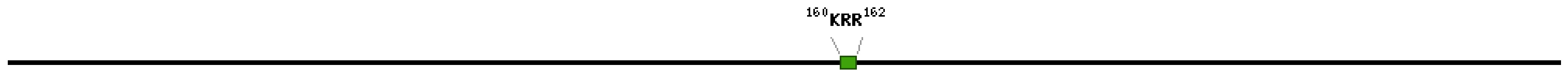

Posterior Graphic. Threshold Marked @ 0.4

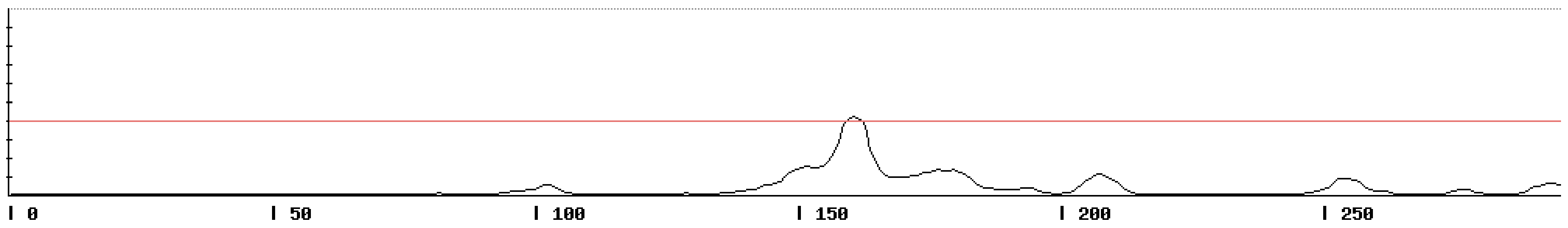

Predictions for Brassicarapa_XP_009103075.1

Viterbi Path

Viterbi Path

Posterior @ 0.4

Posterior @ 0.4

Posterior Graphic. Threshold Marked @ 0.4

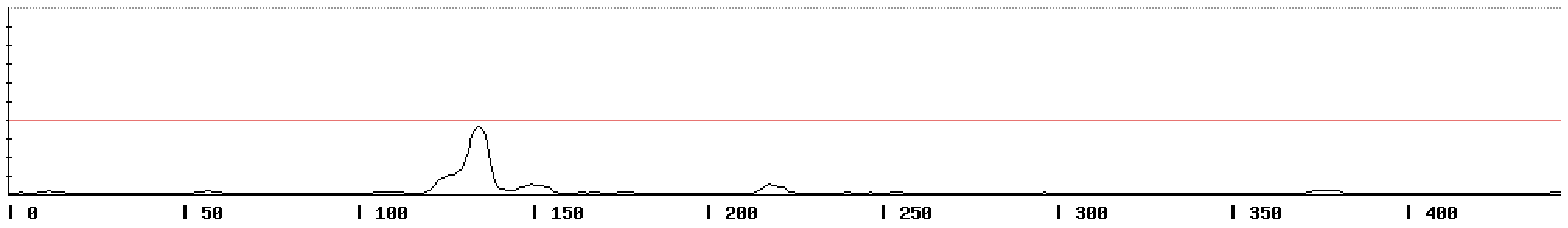

Predictions for Brassicarapa_XP_009103220.1

Viterbi Path

Viterbi Path

Posterior @ 0.4	35 - KRKIRRKKLR - 44
Posterior @ 0.4	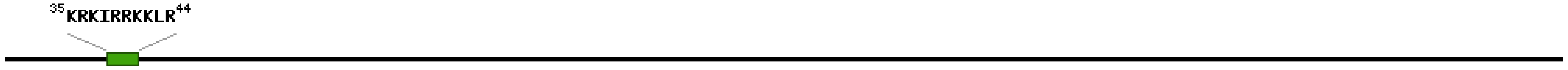
Posterior Graphic. Threshold Marked @ 0.4	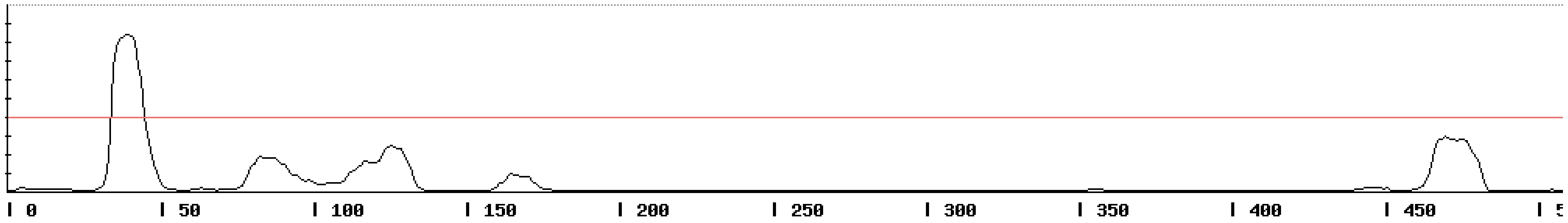

Predictions for Brassicarapa_XP_009103221.1

Viterbi Path	
Viterbi Path	
Posterior @ 0.4	35 - KRKIRRKKLR - 44
Posterior @ 0.4	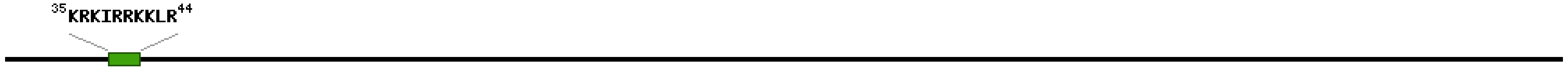
Posterior Graphic. Threshold Marked @ 0.4	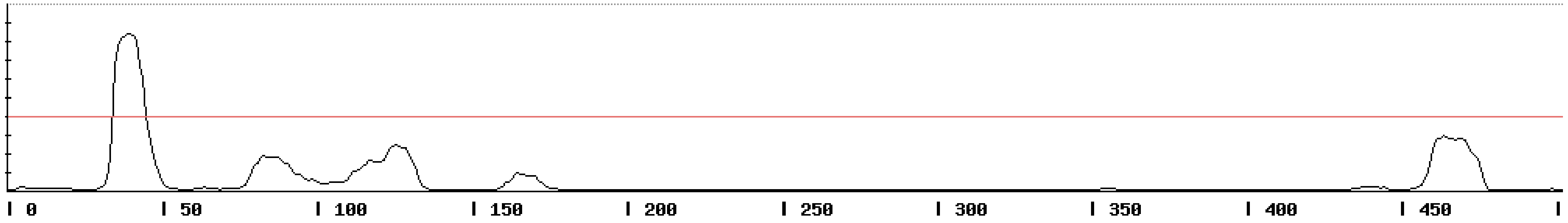

Predictions for Brassicarapa_XP_009103300.1

Viterbi Path	
Viterbi Path	

Posterior @ 0.4

Posterior @ 0.4

Posterior Graphic. Threshold Marked @ 0.4

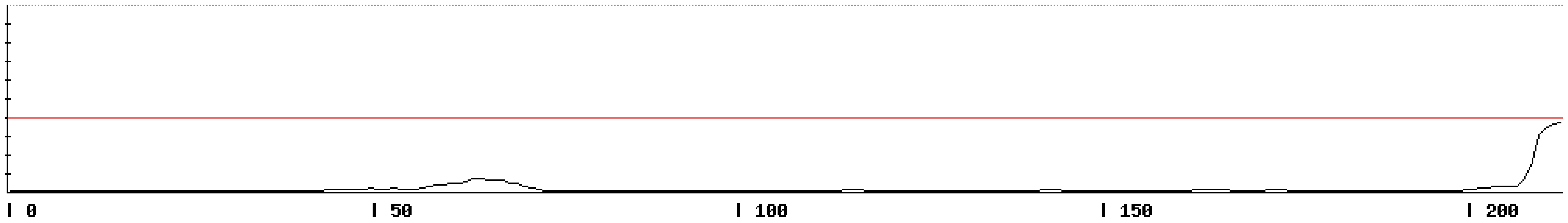

Predictions for Brassicarapa_XP_009103538.1

Viterbi Path

Viterbi Path

Posterior @ 0.4

Posterior @ 0.4

Posterior Graphic. Threshold Marked @ 0.4

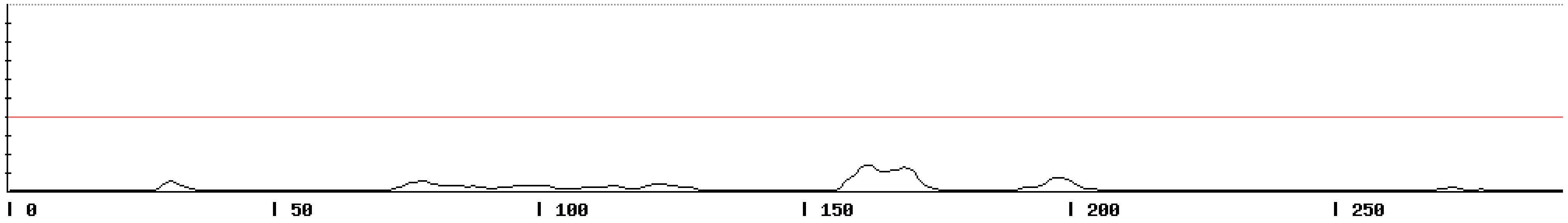

Predictions for Brassicarapa_XP_009103539.1

Viterbi Path	
Viterbi Path	
Posterior @ 0.4	
Posterior @ 0.4	
Posterior Graphic. Threshold Marked @ 0.4	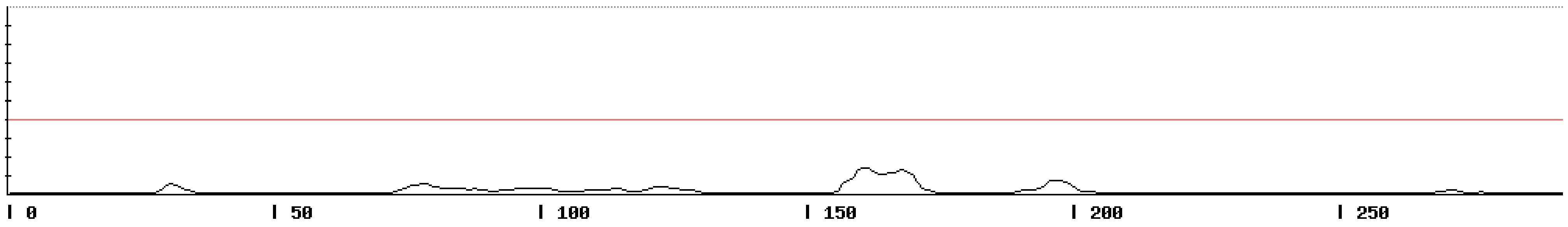

Predictions for Brassicarapa_XP_009103591.1

Viterbi Path	
Viterbi Path	
Posterior @ 0.4	
Posterior @ 0.4	
Posterior Graphic. Threshold Marked @ 0.4	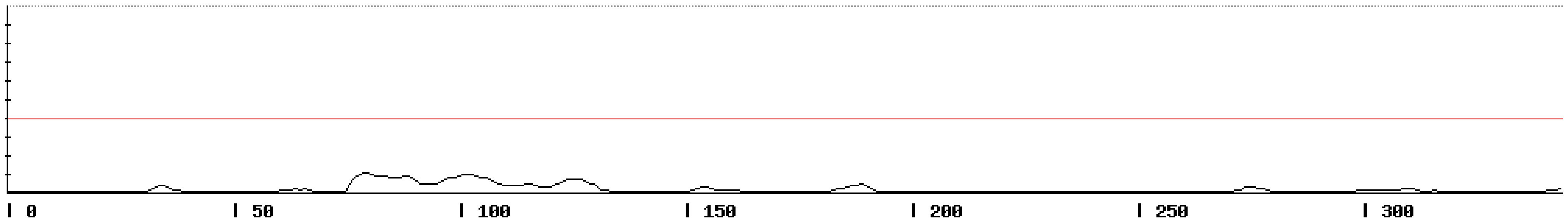

Predictions for Brassicarapa_XP_009103592.1	
Viterbi Path	
Viterbi Path	
Posterior @ 0.4	
Posterior @ 0.4	
Posterior Graphic. Threshold Marked @ 0.4	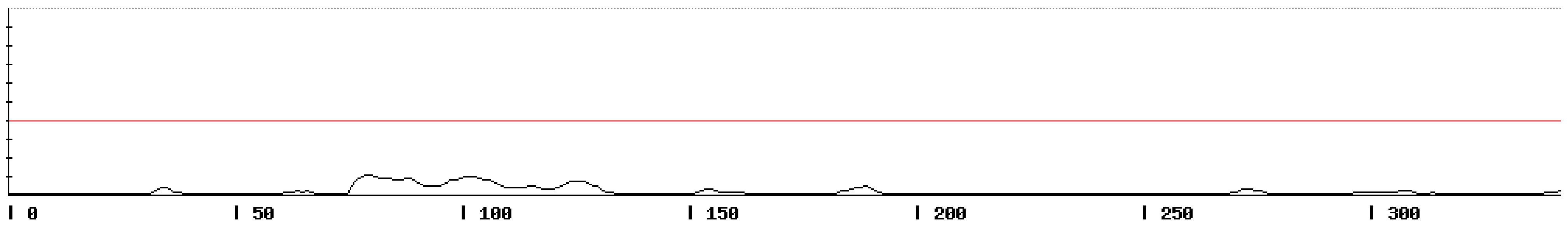

Predictions for Brassicarapa_XP_009103997.1	
Viterbi Path	
Viterbi Path	
Posterior @ 0.4	
Posterior @ 0.4	
Posterior Graphic. Threshold Marked @ 0.4	

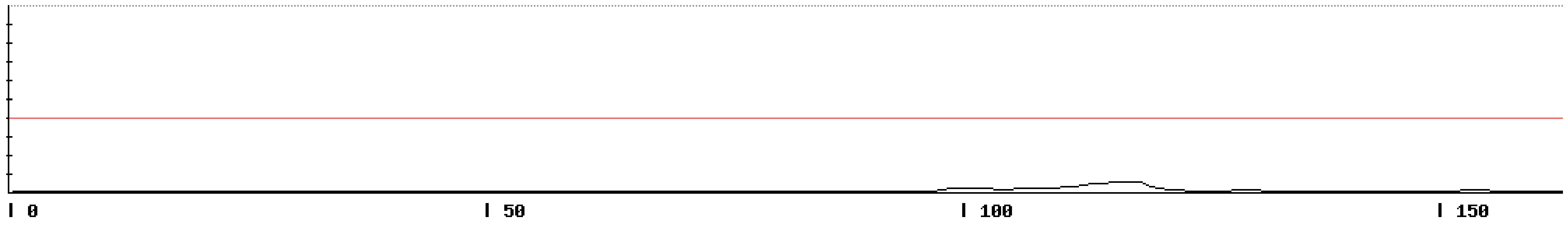

Predictions for Brassicarapa_XP_009104009.1

Viterbi Path	
Viterbi Path	
Posterior @ 0.4	
Posterior @ 0.4	
Posterior Graphic. Threshold Marked @ 0.4	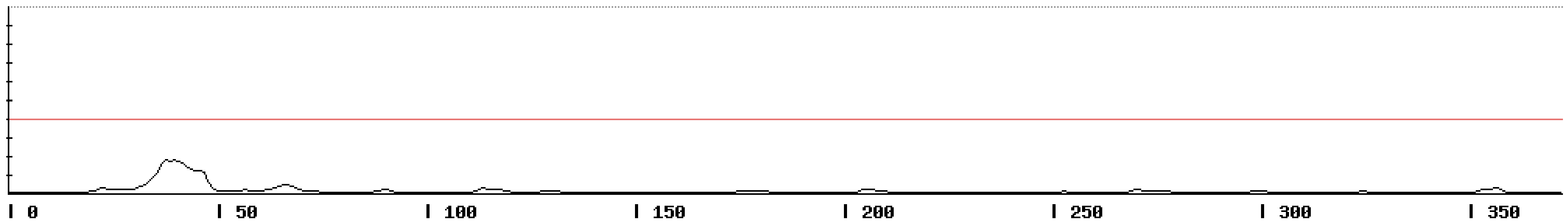

Predictions for Brassicarapa_XP_009104010.1

Viterbi Path	
Viterbi Path	
Posterior @ 0.4	
Posterior @ 0.4	

Posterior Graphic. Threshold Marked @ 0.4

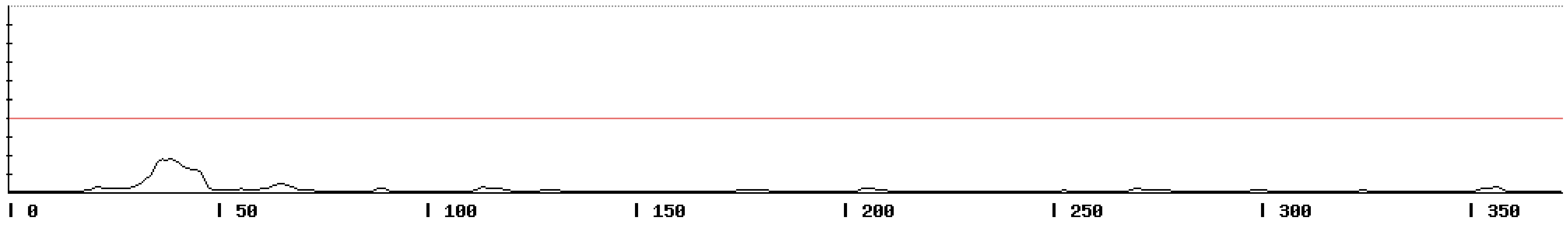

Predictions for Brassicarapa_XP_009104011.1

Viterbi Path	
Viterbi Path	
Posterior @ 0.4	
Posterior @ 0.4	
Posterior Graphic. Threshold Marked @ 0.4	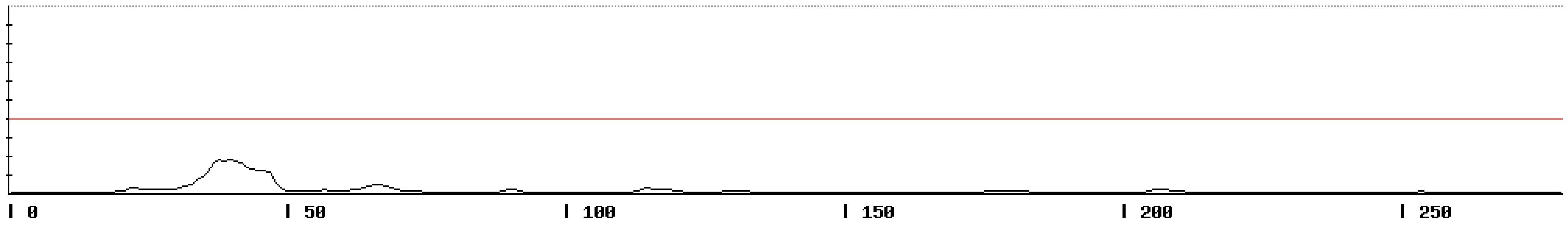

Predictions for Brassicarapa_XP_009104136.1

Viterbi Path	
Viterbi Path	
Posterior @ 0.4	100 - SKKMK - 104
Posterior @ 0.4	

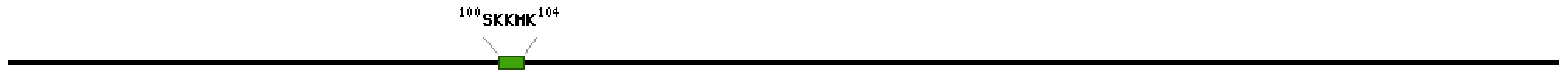

Posterior Graphic. Threshold Marked @ 0.4

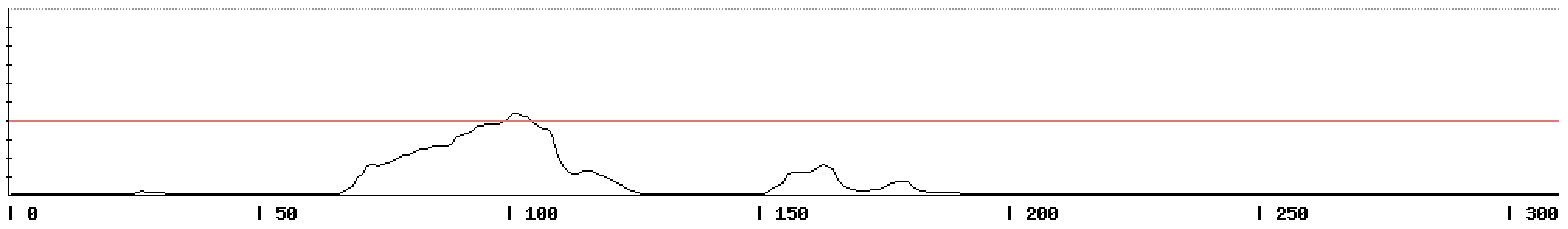

Predictions for Brassicarapa_XP_009104354.1

Viterbi Path

Viterbi Path

Posterior @ 0.4

Posterior @ 0.4

Posterior Graphic. Threshold Marked @ 0.4

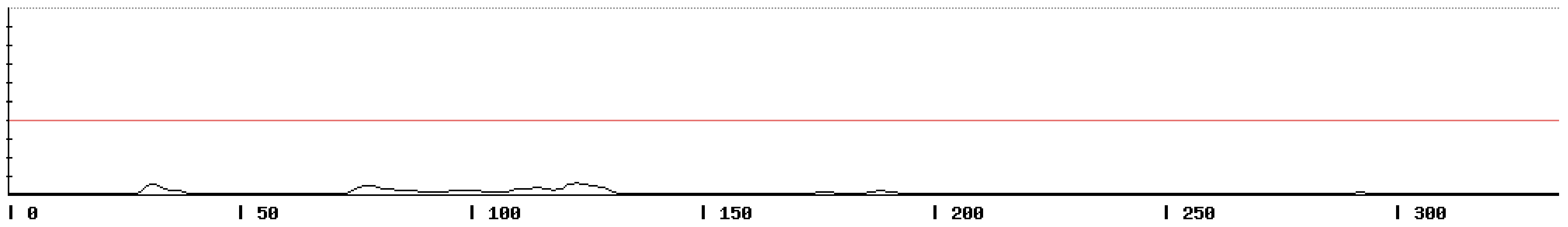

Predictions for Brassicarapa_XP_009104516.1

Viterbi Path

Viterbi Path

Posterior @ 0.4

Posterior @ 0.4

Posterior Graphic. Threshold Marked @ 0.4

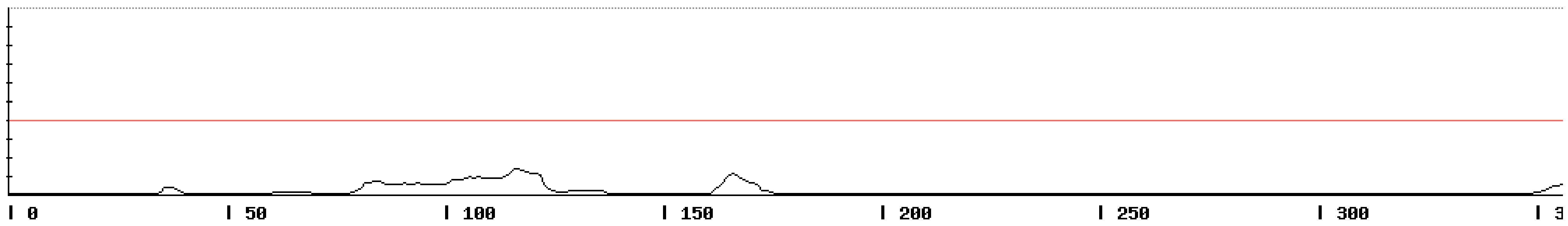

Predictions for Brassicarapa_XP_009104518.1

Viterbi Path

Viterbi Path

Posterior @ 0.4

Posterior @ 0.4

Posterior Graphic. Threshold Marked @ 0.4

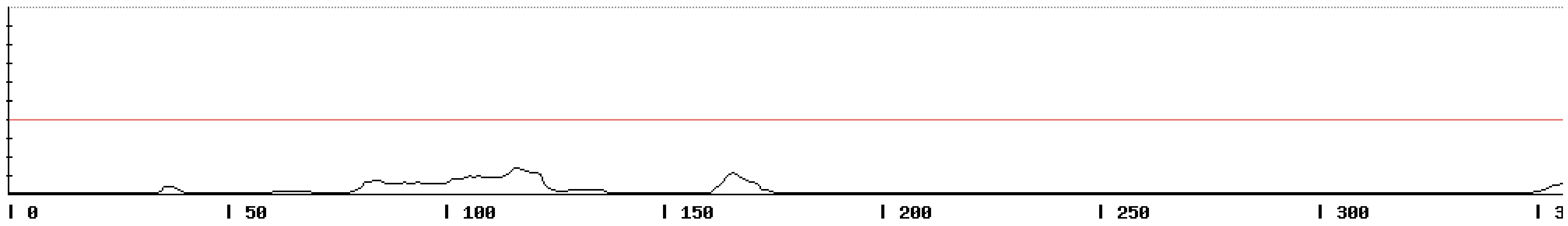

Predictions for Brassicarapa_XP_009104726.1

Viterbi Path

Viterbi Path

Posterior @ 0.4

Posterior @ 0.4

Posterior Graphic. Threshold Marked @ 0.4

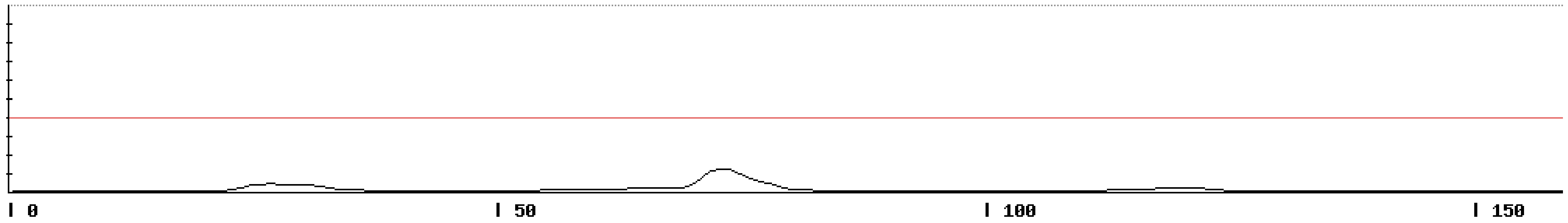

Predictions for Brassicarapa_XP_009105381.1

Viterbi Path

Viterbi Path

Posterior @ 0.4

Posterior @ 0.4

Posterior Graphic. Threshold Marked @ 0.4

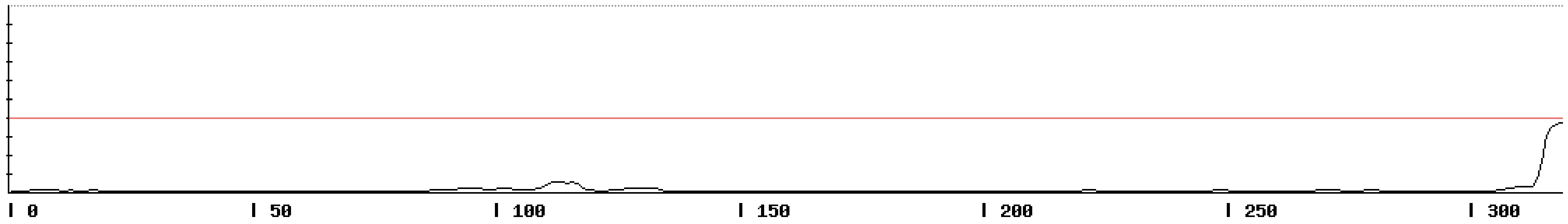

Predictions for Brassicarapa_XP_009105493.1

Viterbi Path	
Viterbi Path	
Posterior @ 0.4	
Posterior @ 0.4	
Posterior Graphic. Threshold Marked @ 0.4	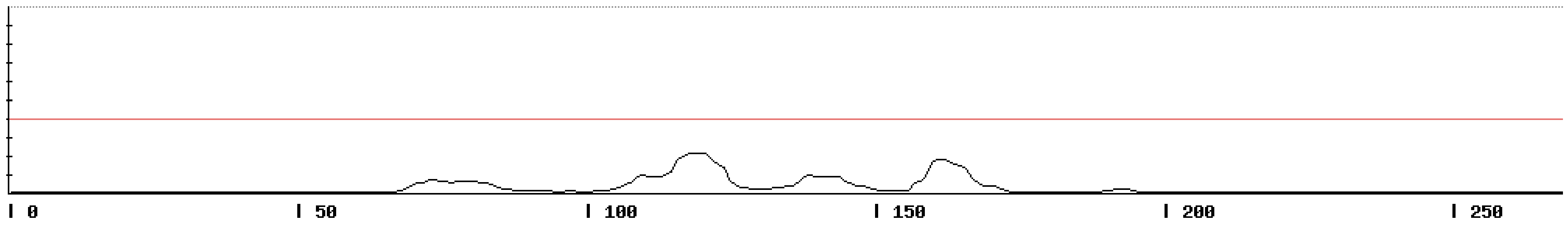

Predictions for Brassicarapa_XP_009105503.1

Viterbi Path	
Viterbi Path	
Posterior @ 0.4	109 - NEKKPKSFFKFSWEKAEEKEKRRARD - 134
Posterior @ 0.4	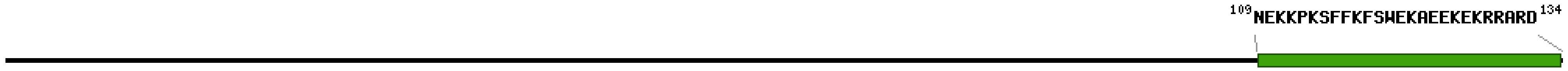
Posterior Graphic. Threshold Marked @ 0.4	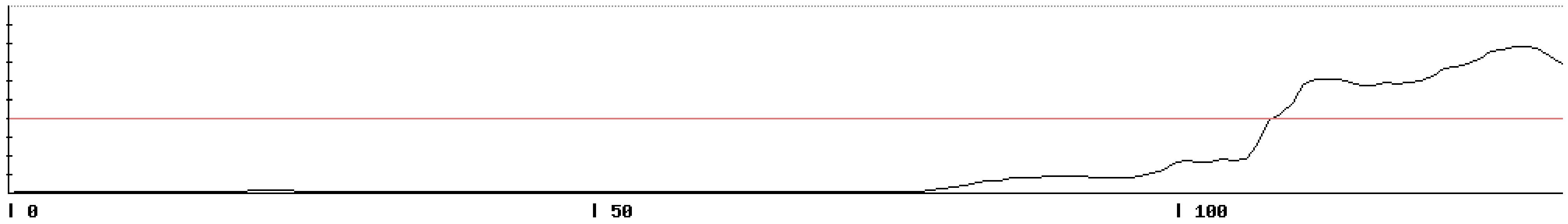

Predictions for Brassicarapa_XP_009105596.1	
Viterbi Path	
Viterbi Path	
Posterior @ 0.4	
Posterior @ 0.4	
Posterior Graphic. Threshold Marked @ 0.4	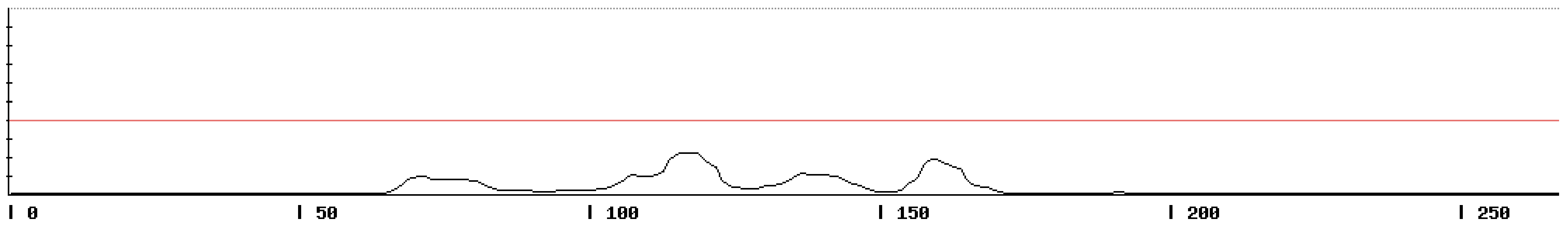

Predictions for Brassicarapa_XP_009105638.1	
Viterbi Path	
Viterbi Path	
Posterior @ 0.4	
Posterior @ 0.4	
Posterior Graphic. Threshold Marked @ 0.4	

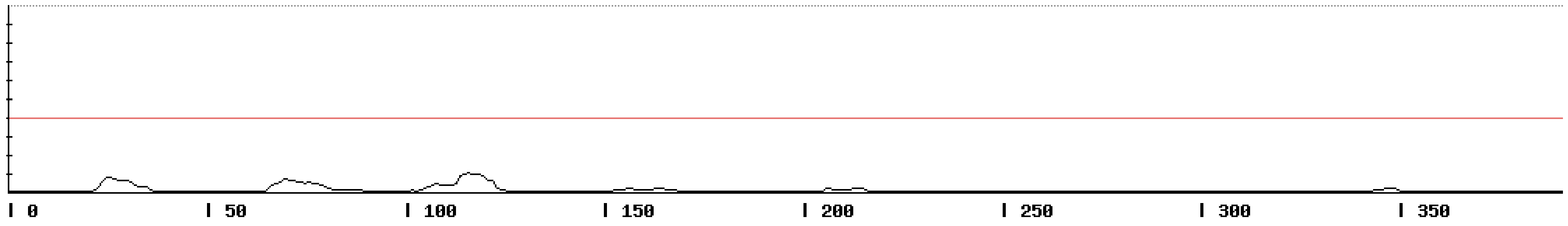

Predictions for Brassicarapa_XP_009105856.1

Viterbi Path

Viterbi Path

Posterior @ 0.4

Posterior @ 0.4

Posterior Graphic. Threshold Marked @ 0.4

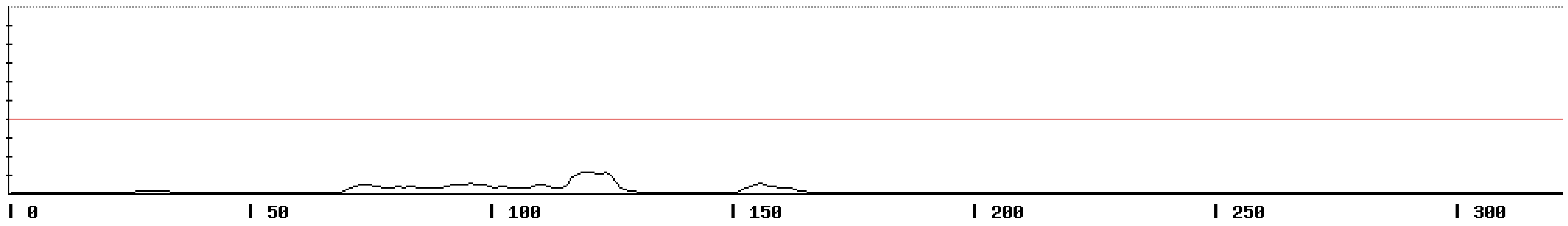

Predictions for Brassicarapa_XP_009105857.1

Viterbi Path

Viterbi Path

Posterior @ 0.4

Posterior @ 0.4

Posterior Graphic. Threshold Marked @ 0.4

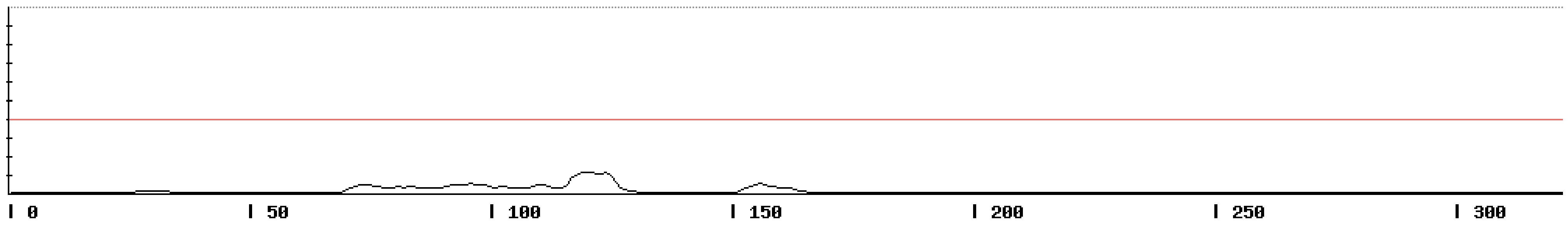

Predictions for Brassicarapa_XP_009106303.1

Viterbi Path	
Viterbi Path	
Posterior @ 0.4	
Posterior @ 0.4	
Posterior Graphic. Threshold Marked @ 0.4	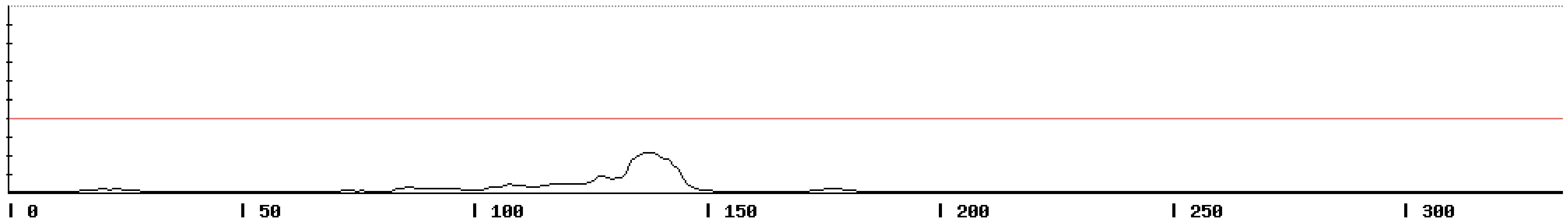

Predictions for Brassicarapa_XP_009106681.1

Viterbi Path	
Viterbi Path	
Posterior @ 0.4	
Posterior @ 0.4	

Posterior Graphic. Threshold Marked @ 0.4

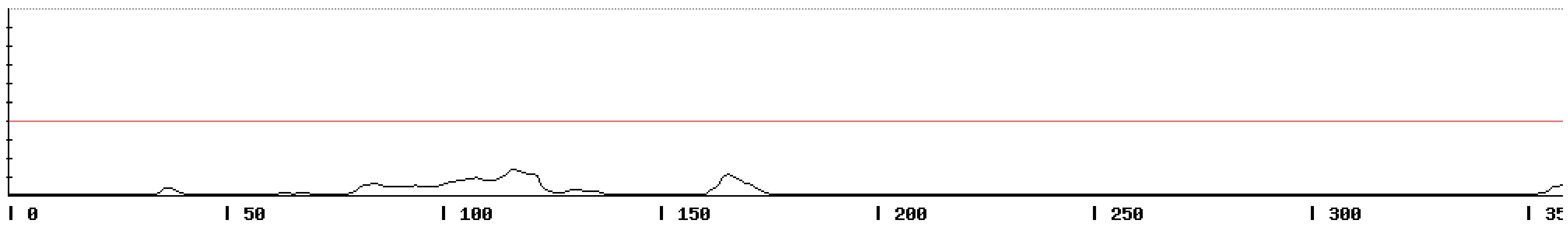

Predictions for Brassicarapa_XP_009106794.1

Viterbi Path

Viterbi Path

Posterior @ 0.4

Posterior @ 0.4

Posterior Graphic. Threshold Marked @ 0.4

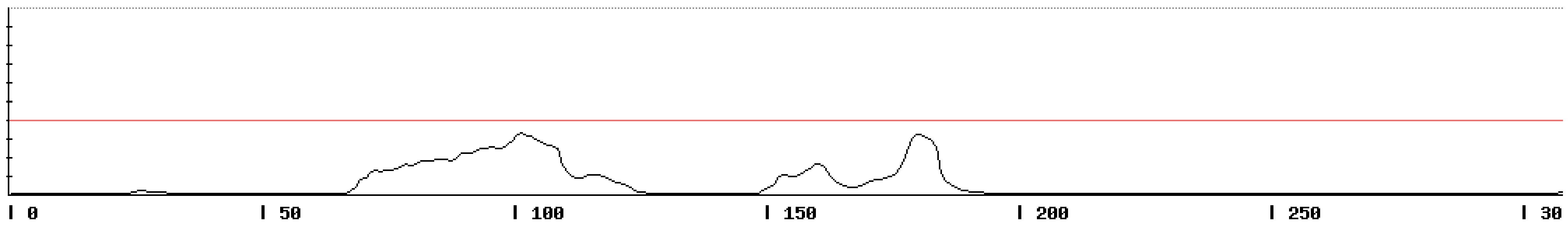

Predictions for Brassicarapa_XP_009106812.1

Viterbi Path

Viterbi Path

Posterior @ 0.4

Posterior @ 0.4

Posterior Graphic. Threshold Marked @ 0.4

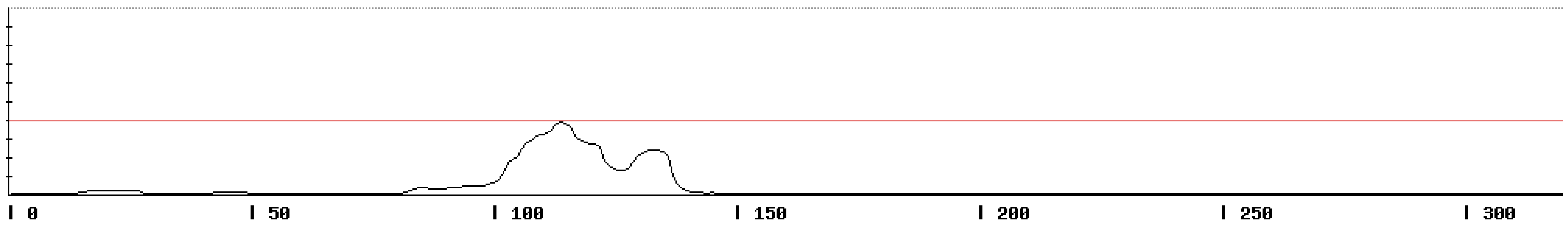

Predictions for Brassicarapa_XP_009107934.1

Viterbi Path

Viterbi Path

Posterior @ 0.4

Posterior @ 0.4

Posterior Graphic. Threshold Marked @ 0.4

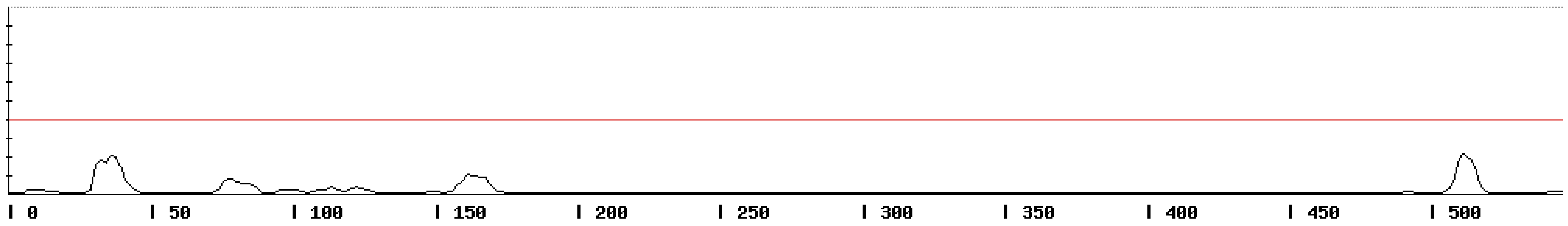

Predictions for Brassicarapa_XP_009107969.1

Viterbi Path

Viterbi Path

Posterior @ 0.4

Posterior @ 0.4

Posterior Graphic. Threshold Marked @ 0.4

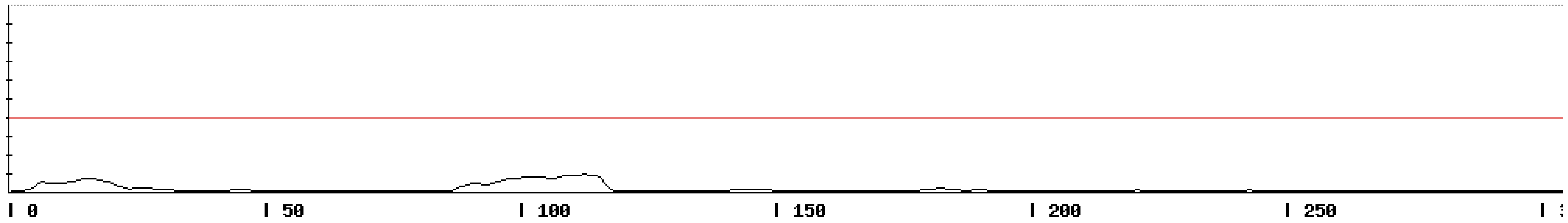

Predictions for Brassicarapa_XP_009107971.1

Viterbi Path

Viterbi Path

Posterior @ 0.4

Posterior @ 0.4

Posterior Graphic. Threshold Marked @ 0.4

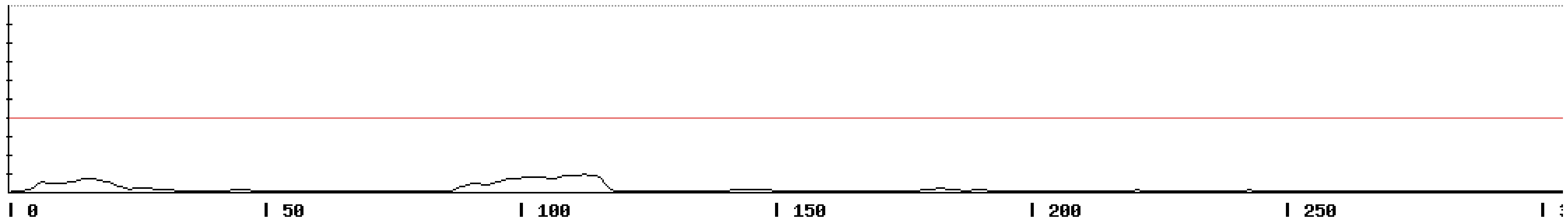

Predictions for Brassicarapa_XP_009107972.1

Viterbi Path	
Viterbi Path	
Posterior @ 0.4	
Posterior @ 0.4	
Posterior Graphic. Threshold Marked @ 0.4	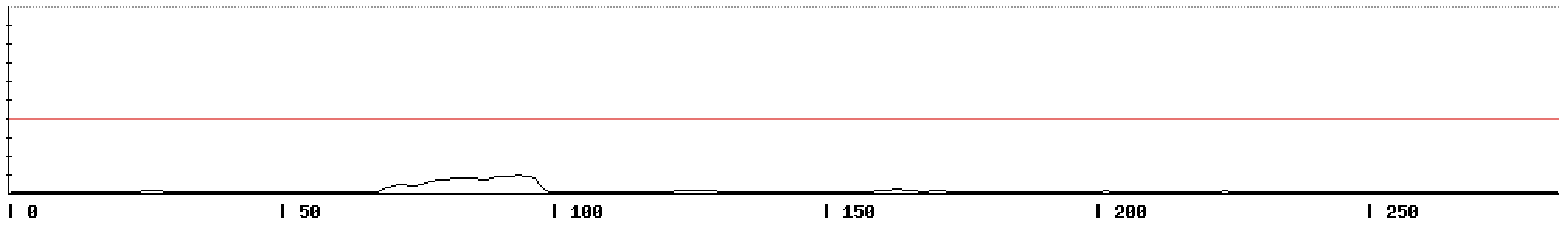

Predictions for Brassicarapa_XP_009109087.1

Viterbi Path	
Viterbi Path	
Posterior @ 0.4	
Posterior @ 0.4	
Posterior Graphic. Threshold Marked @ 0.4	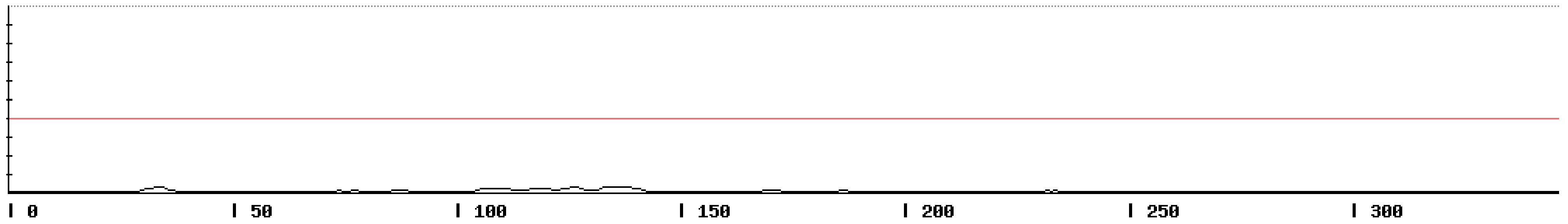

Predictions for Brassicarapa_XP_009110048.1

Viterbi Path	
Viterbi Path	
Posterior @ 0.4	
Posterior @ 0.4	
Posterior Graphic. Threshold Marked @ 0.4	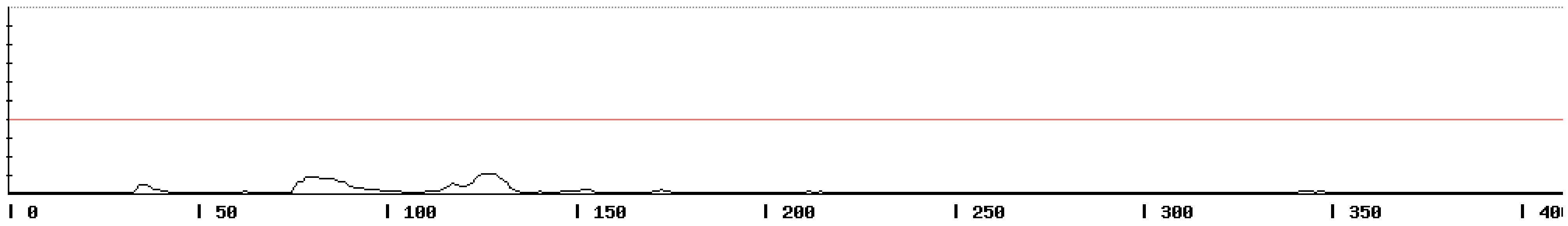

Predictions for Brassicarapa_XP_009110661.1

Viterbi Path	
Viterbi Path	
Posterior @ 0.4	
Posterior @ 0.4	
Posterior Graphic. Threshold Marked @ 0.4	

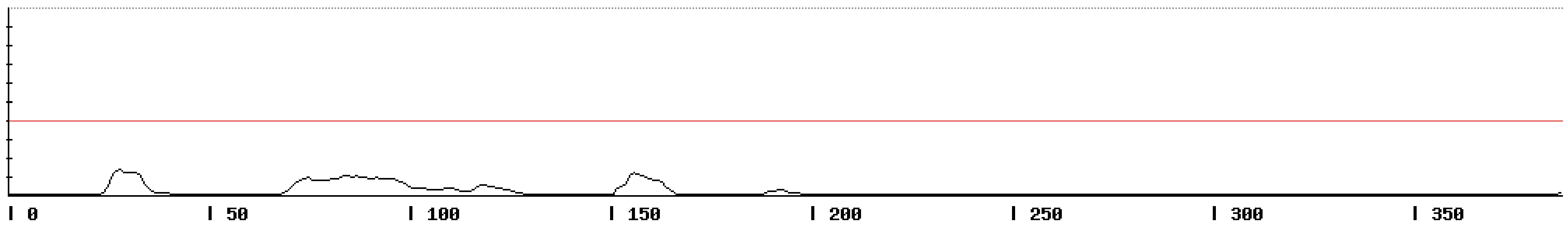

Predictions for Brassicarapa_XP_009110662.1	
Viterbi Path	
Viterbi Path	
Posterior @ 0.4	
Posterior @ 0.4	
Posterior Graphic. Threshold Marked @ 0.4	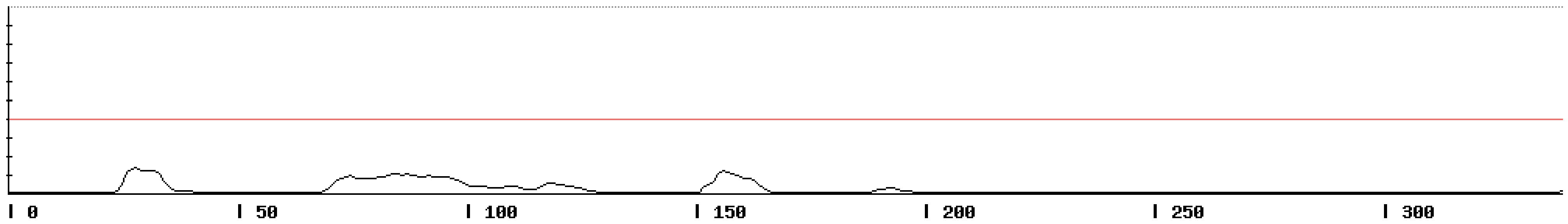

Predictions for Brassicarapa_XP_009110663.1	
Viterbi Path	
Viterbi Path	
Posterior @ 0.4	
Posterior @ 0.4	

Posterior Graphic. Threshold Marked @ 0.4

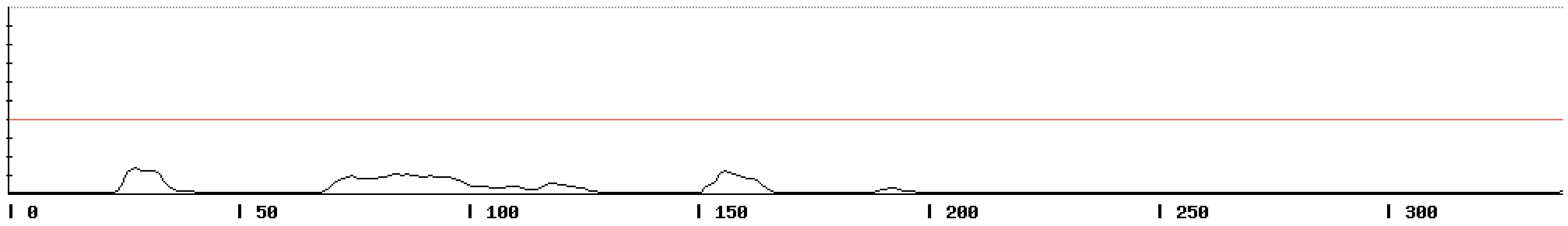

Predictions for Brassicarapa_XP_009111333.1

Viterbi Path

Viterbi Path

Posterior @ 0.4

Posterior @ 0.4

Posterior Graphic. Threshold Marked @ 0.4

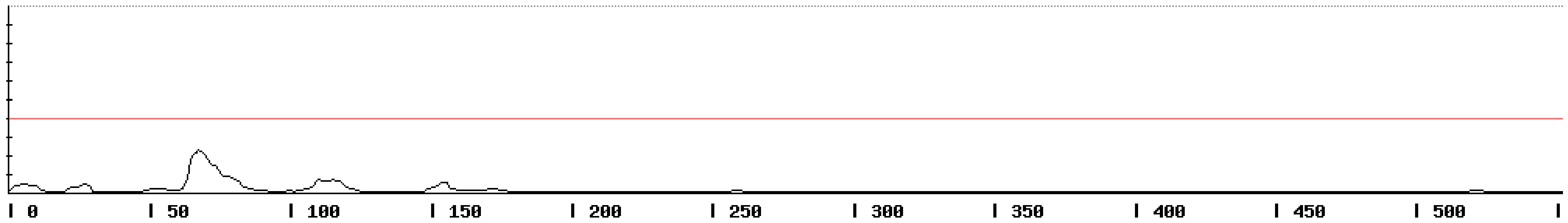

Predictions for Brassicarapa_XP_009111357.1

Viterbi Path

Viterbi Path

Posterior @ 0.4

75 - EKKKGTKRTTPSGFWKATGKDRIIRDKRGH - 104

389 - RNRKKP - 394

Posterior @ 0.4

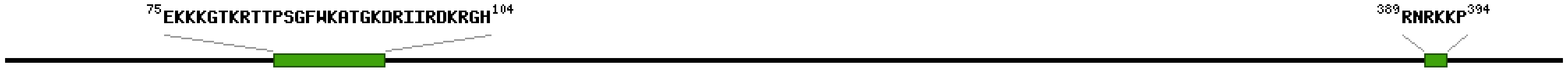

Posterior Graphic. Threshold Marked @ 0.4

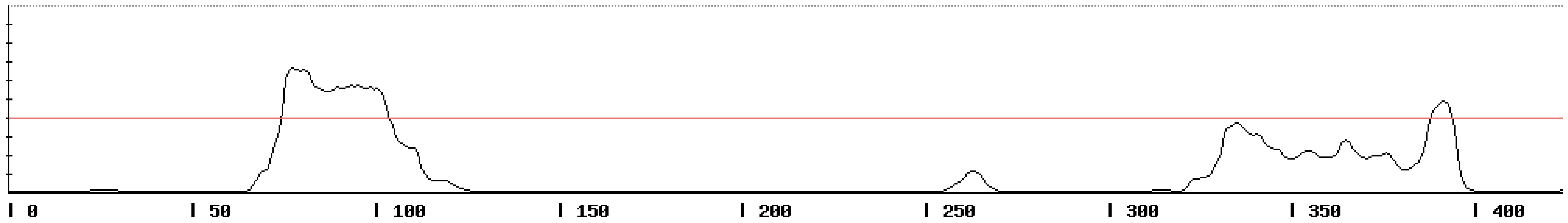

Predictions for Brassicarapa_XP_009111358.1

Viterbi Path

Viterbi Path

Posterior @ 0.4 — 72 - KSPKKSLTKRTTPSGFWKSTGKDRKIKEDKRRD - 104

Posterior @ 0.4

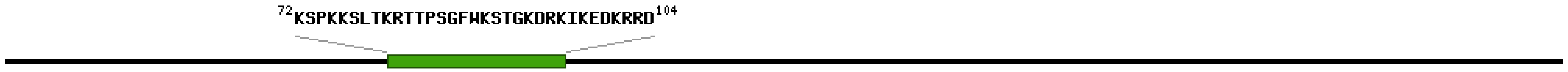

Posterior Graphic. Threshold Marked @ 0.4

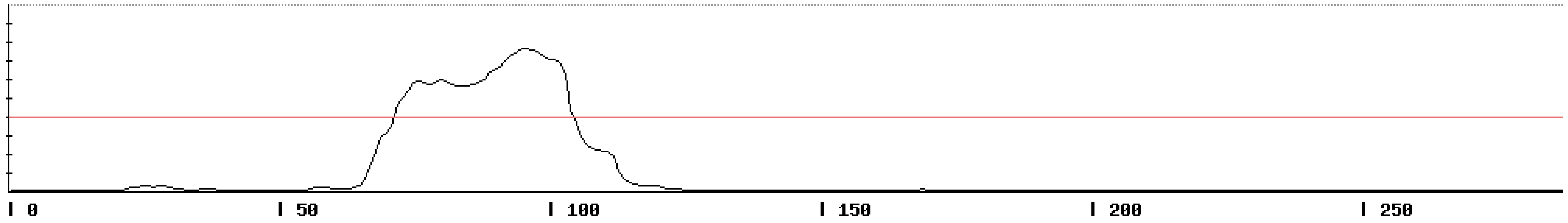

Predictions for Brassicarapa_XP_009111374.1

Viterbi Path

Viterbi Path

Posterior @ 0.4	77 - KKGTK - 81
Posterior @ 0.4	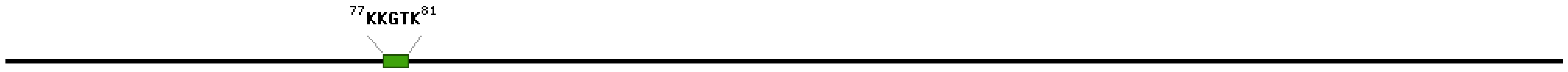
Posterior Graphic. Threshold Marked @ 0.4	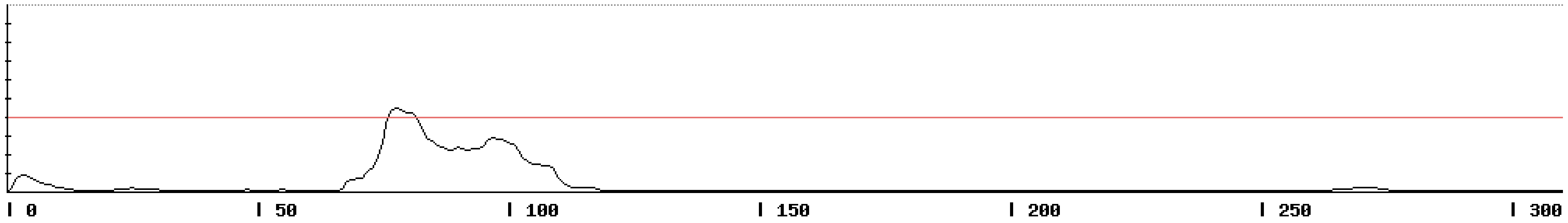

Predictions for Brassicarapa_XP_009111638.1

Viterbi Path	
Viterbi Path	
Posterior @ 0.4	
Posterior @ 0.4	
Posterior Graphic. Threshold Marked @ 0.4	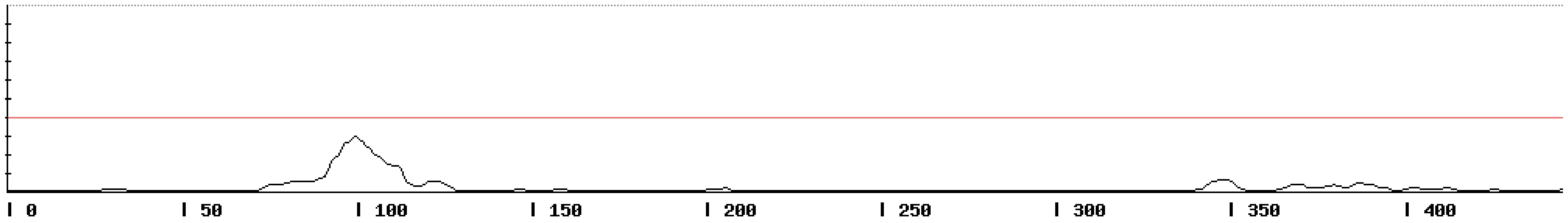

Predictions for Brassicarapa_XP_009111639.1

Viterbi Path	
Viterbi Path	

Posterior @ 0.4

Posterior @ 0.4

Posterior Graphic. Threshold Marked @ 0.4

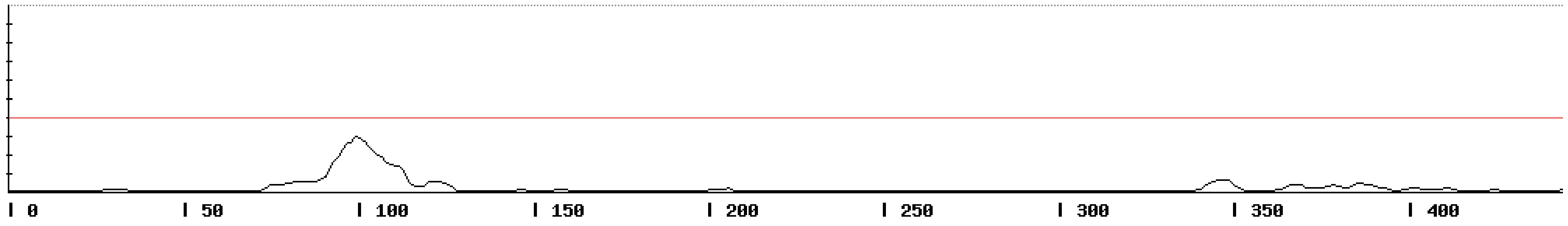

Predictions for Brassicarapa_XP_009111778.1

Viterbi Path

Viterbi Path

Posterior @ 0.4

Posterior @ 0.4

Posterior Graphic. Threshold Marked @ 0.4

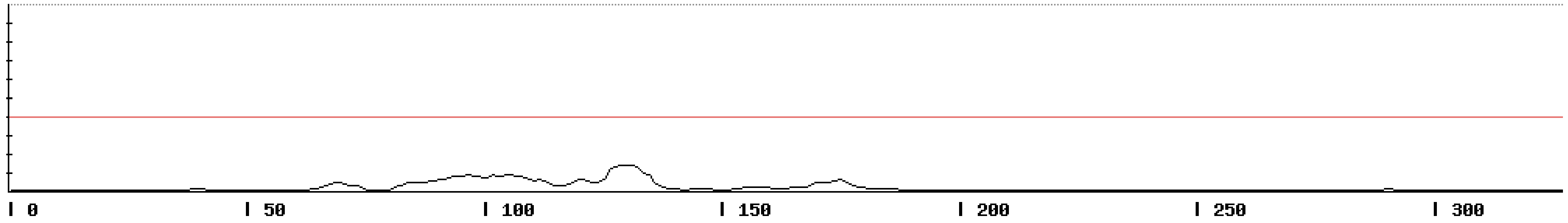

Predictions for Brassicarapa_XP_009112020.1

Viterbi Path

Viterbi Path

Posterior @ 0.4

Posterior @ 0.4

Posterior Graphic. Threshold Marked @ 0.4

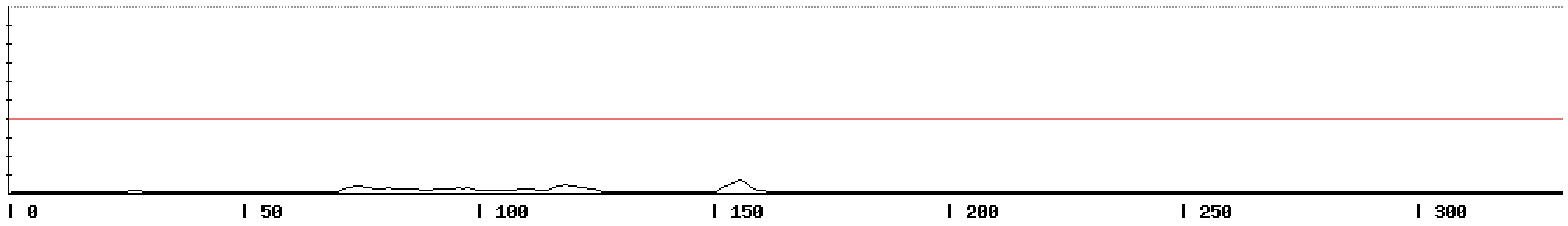

Predictions for Brassicarapa_XP_009112137.1

Viterbi Path

Viterbi Path

Posterior @ 0.4

Posterior @ 0.4

Posterior Graphic. Threshold Marked @ 0.4

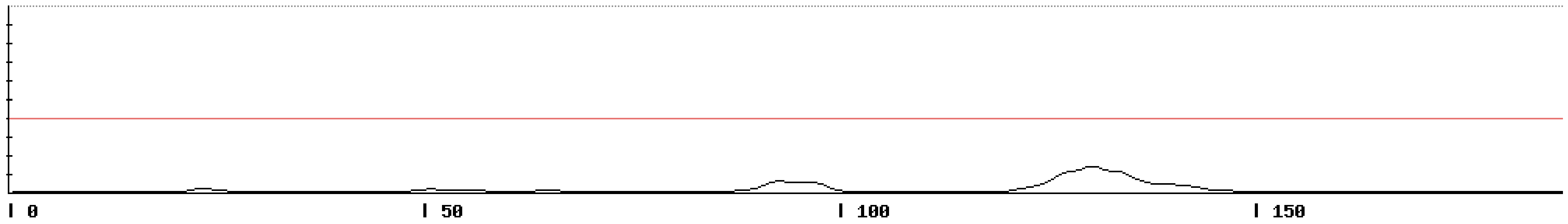

Predictions for Brassicarapa_XP_009112413.1

Viterbi Path

Viterbi Path

Posterior @ 0.4

Posterior @ 0.4

Posterior Graphic. Threshold Marked @ 0.4

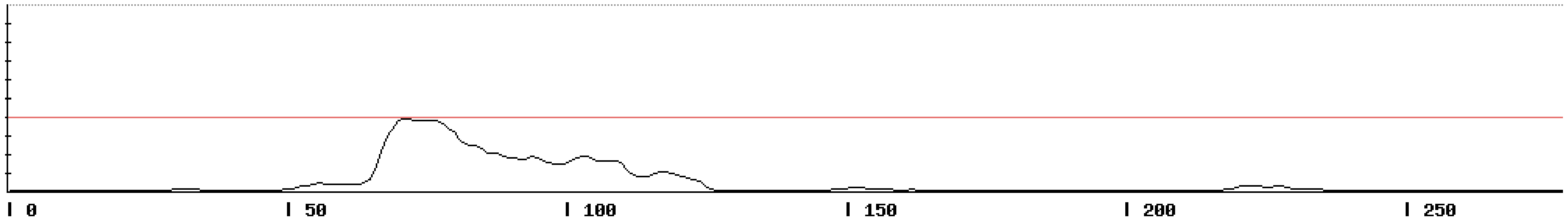

Predictions for Brassicarapa_XP_009112744.1

Viterbi Path

Viterbi Path

Posterior @ 0.4

Posterior @ 0.4

Posterior Graphic. Threshold Marked @ 0.4

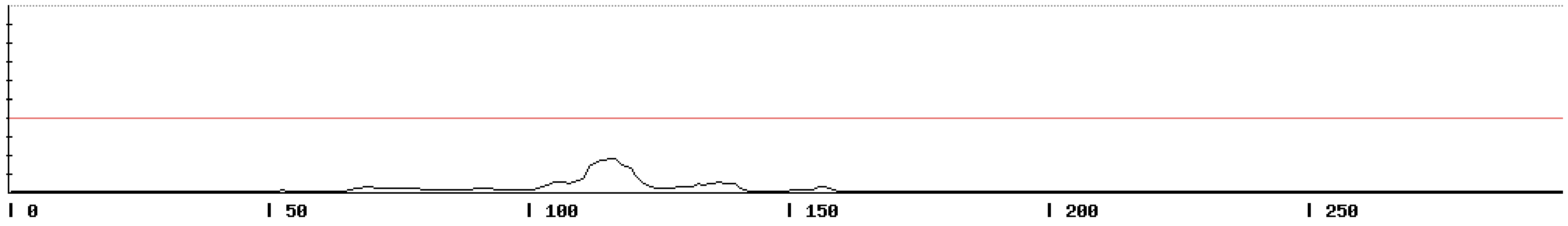

Predictions for Brassicarapa_XP_009113056.1

Viterbi Path	
Viterbi Path	
Posterior @ 0.4	
Posterior @ 0.4	
Posterior Graphic. Threshold Marked @ 0.4	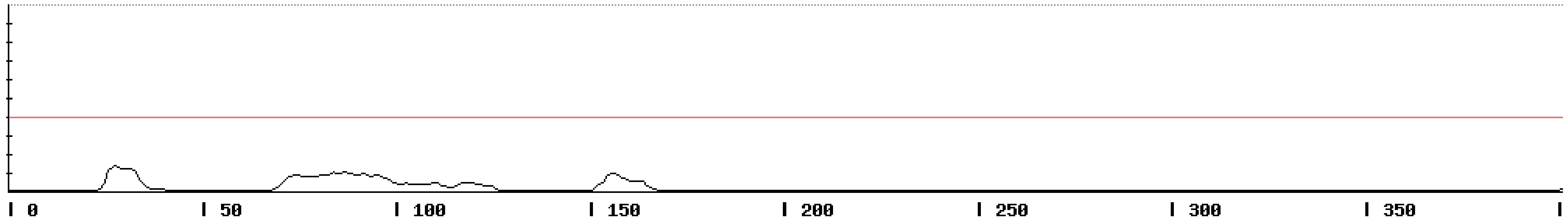

Predictions for Brassicarapa_XP_009113057.1

Viterbi Path	
Viterbi Path	
Posterior @ 0.4	
Posterior @ 0.4	

Posterior Graphic. Threshold Marked @ 0.4

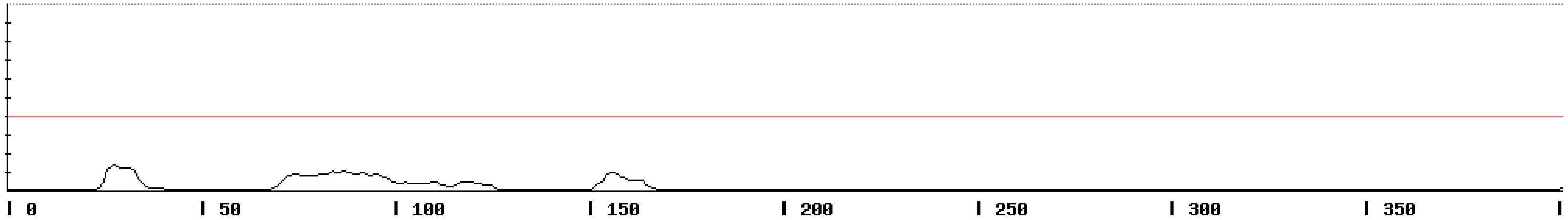

Predictions for Brassicarapa_XP_009113148.1

Viterbi Path

Viterbi Path

Posterior @ 0.4

Posterior @ 0.4

Posterior Graphic. Threshold Marked @ 0.4

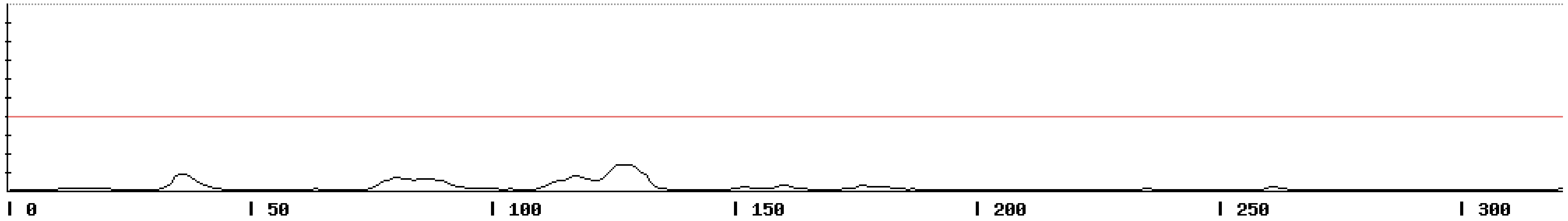

Predictions for Brassicarapa_XP_009113605.1

Viterbi Path

Viterbi Path

Posterior @ 0.4

Posterior @ 0.4

Posterior Graphic. Threshold Marked @ 0.4

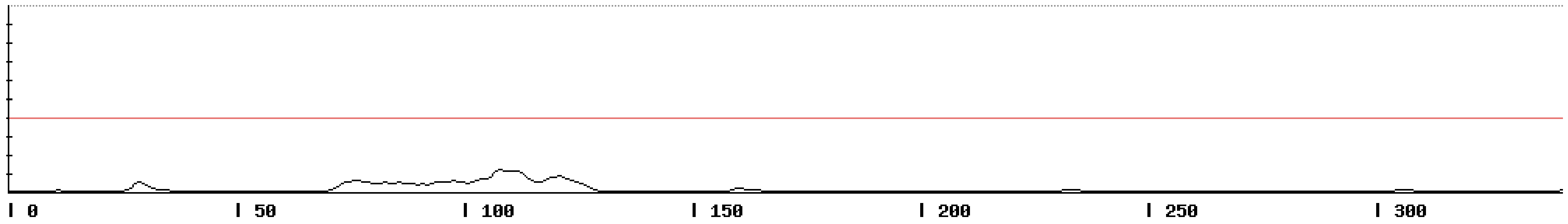

Predictions for Brassicarapa_XP_009113606.1

Viterbi Path

Viterbi Path

Posterior @ 0.4

Posterior @ 0.4

Posterior Graphic. Threshold Marked @ 0.4

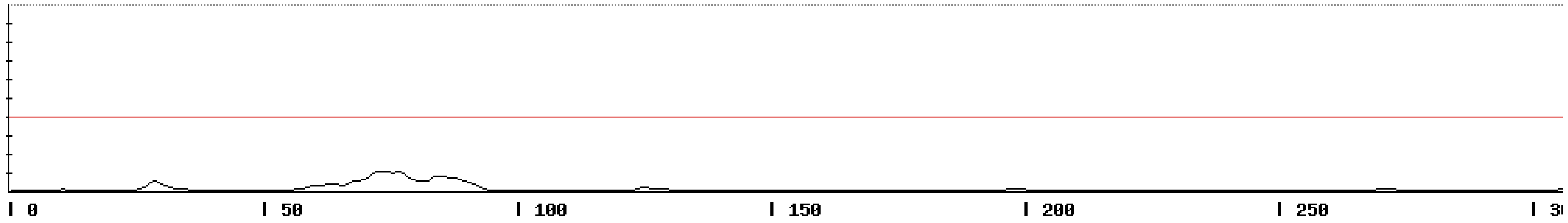

Predictions for Brassicarapa_XP_009113660.1

Viterbi Path

Viterbi Path

Posterior @ 0.4	152 - KRR - 154
Posterior @ 0.4	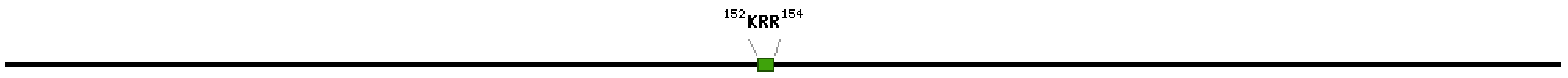
Posterior Graphic. Threshold Marked @ 0.4	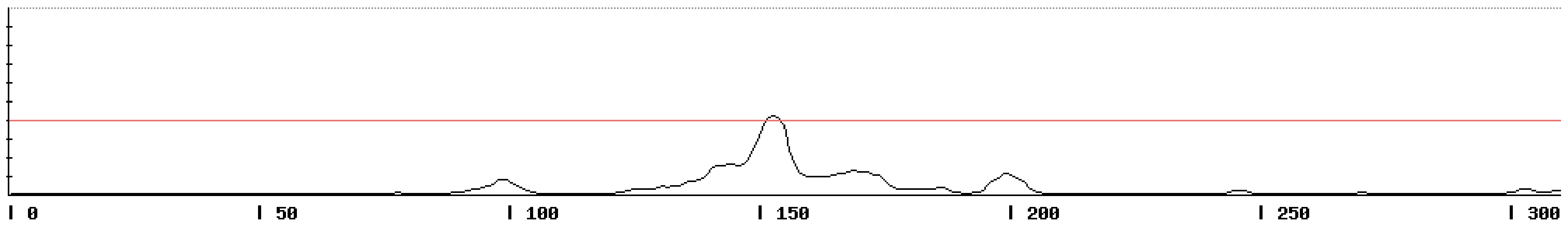

Predictions for Brassicarapa_XP_009113875.1

Viterbi Path	
Viterbi Path	
Posterior @ 0.4	
Posterior @ 0.4	
Posterior Graphic. Threshold Marked @ 0.4	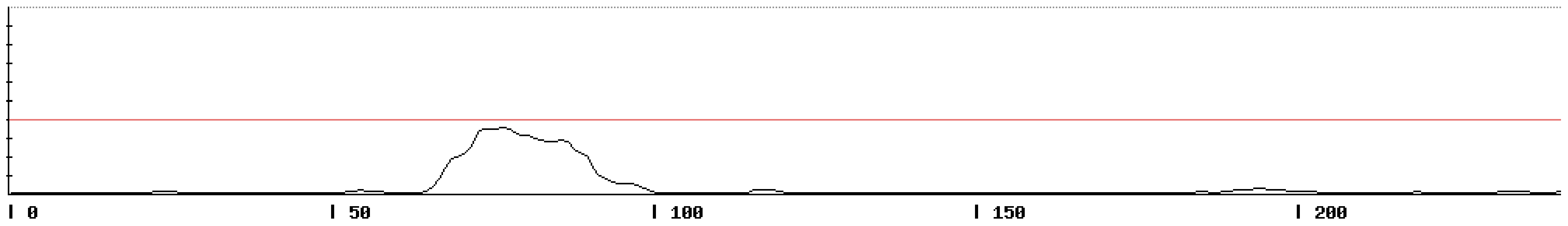

Predictions for Brassicarapa_XP_009114197.1

Viterbi Path	
Viterbi Path	

Posterior @ 0.4

Posterior @ 0.4

Posterior Graphic. Threshold Marked @ 0.4

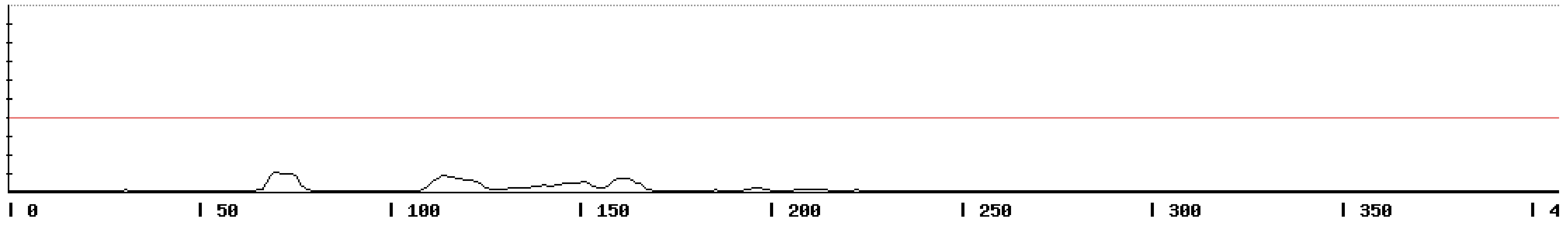

Predictions for Brassicarapa_XP_009114491.1

Viterbi Path

Viterbi Path

Posterior @ 0.4

Posterior @ 0.4

Posterior Graphic. Threshold Marked @ 0.4

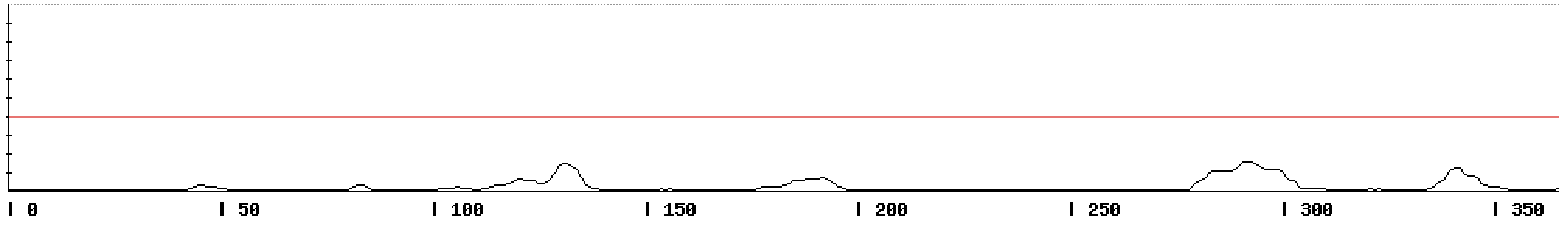

Predictions for Brassicarapa_XP_009114751.1

Viterbi Path

Viterbi Path

Posterior @ 0.4

Posterior @ 0.4

Posterior Graphic. Threshold Marked @ 0.4

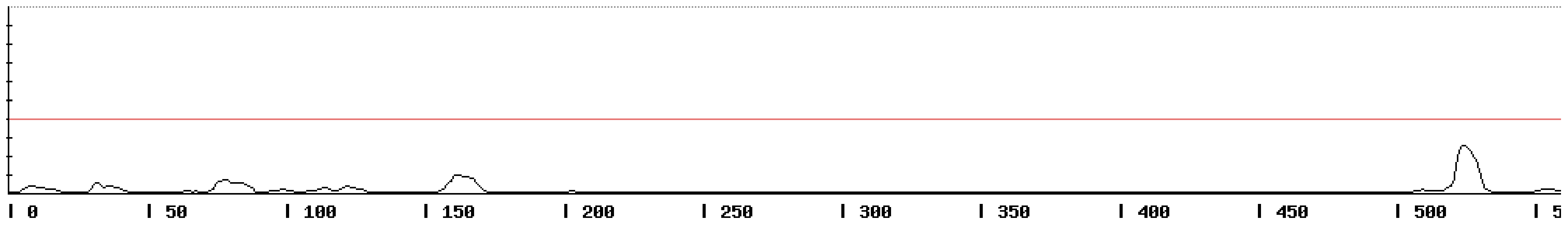

Predictions for Brassicarapa_XP_009114752.1

Viterbi Path

Viterbi Path

Posterior @ 0.4

Posterior @ 0.4

Posterior Graphic. Threshold Marked @ 0.4

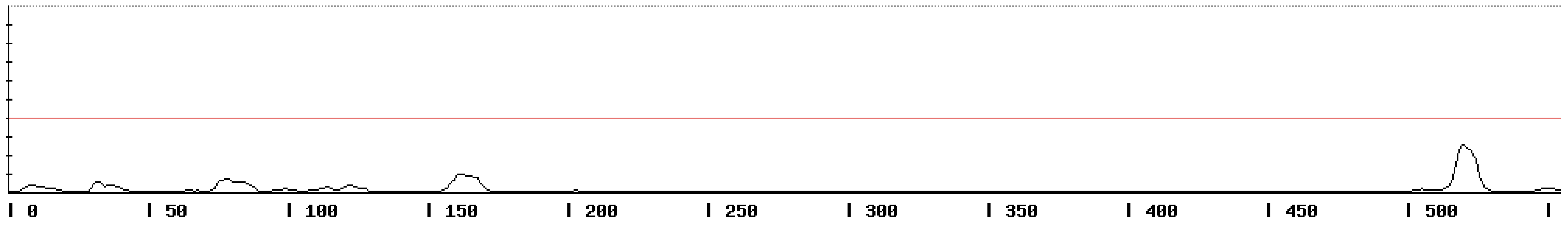

Predictions for Brassicarapa_XP_009114859.1

Viterbi Path

Viterbi Path

Posterior @ 0.4

Posterior @ 0.4

Posterior Graphic. Threshold Marked @ 0.4

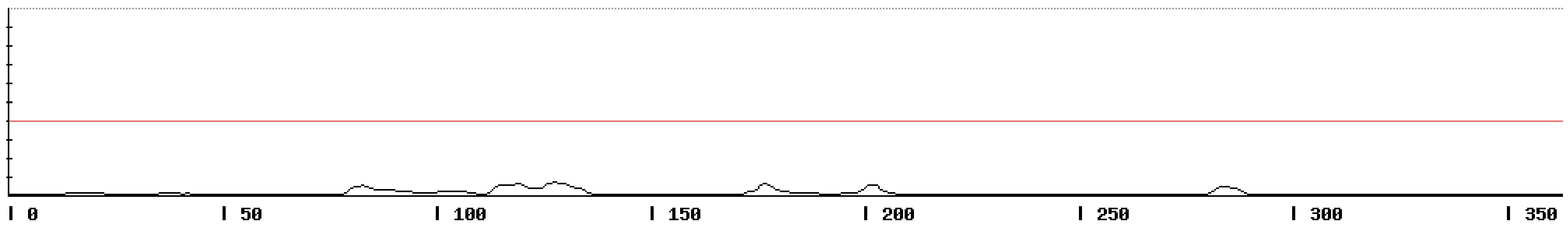

Predictions for Brassicarapa_XP_009114860.1

Viterbi Path

Viterbi Path

Posterior @ 0.4

Posterior @ 0.4

Posterior Graphic. Threshold Marked @ 0.4

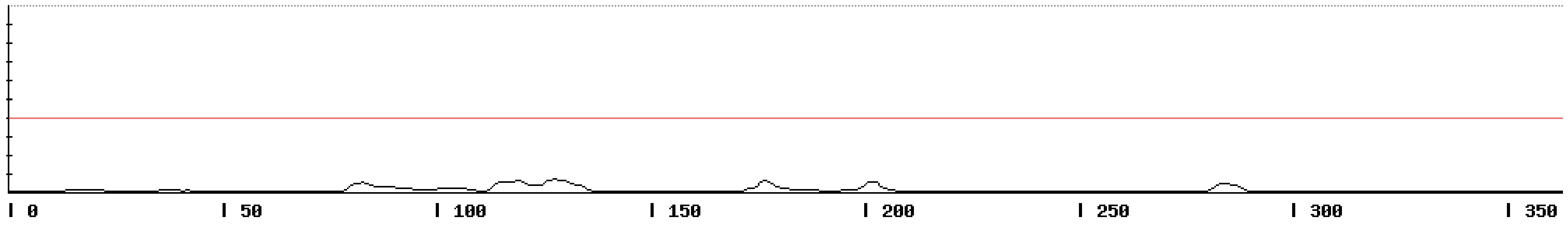

Predictions for Brassicarapa_XP_009114861.1

Viterbi Path

Viterbi Path

Posterior @ 0.4

Posterior @ 0.4

Posterior Graphic. Threshold Marked @ 0.4

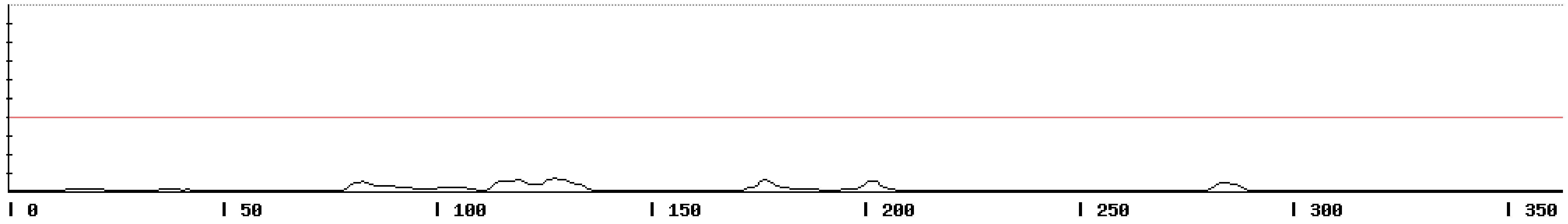

Predictions for Brassicarapa_XP_009115341.1

Viterbi Path

Viterbi Path

Posterior @ 0.4

137 - KRR - 139

Posterior @ 0.4

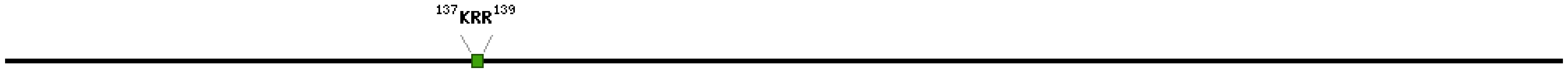

Posterior Graphic. Threshold Marked @ 0.4

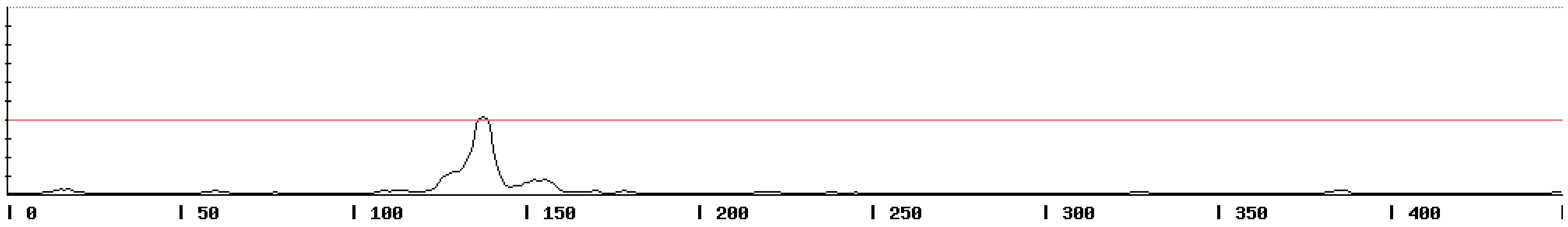

Predictions for Brassicarapa_XP_009115419.1

Viterbi Path

Viterbi Path

Posterior @ 0.4

Posterior @ 0.4

Posterior Graphic. Threshold Marked @ 0.4

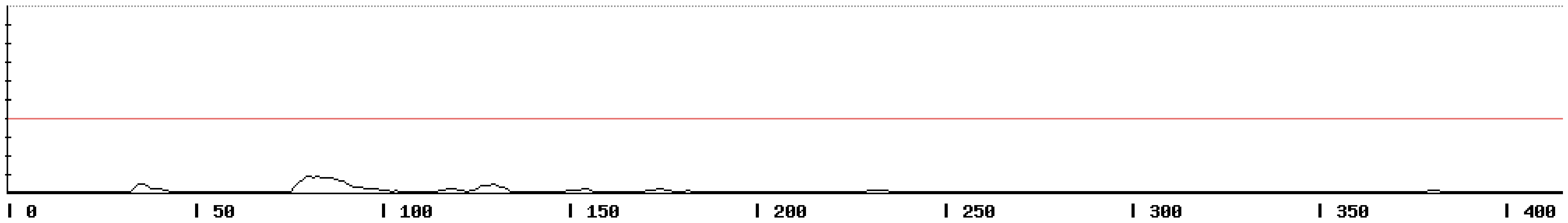

Predictions for Brassicarapa_XP_009115491.1

Viterbi Path

Viterbi Path

Posterior @ 0.4

Posterior @ 0.4

Posterior Graphic. Threshold Marked @ 0.4

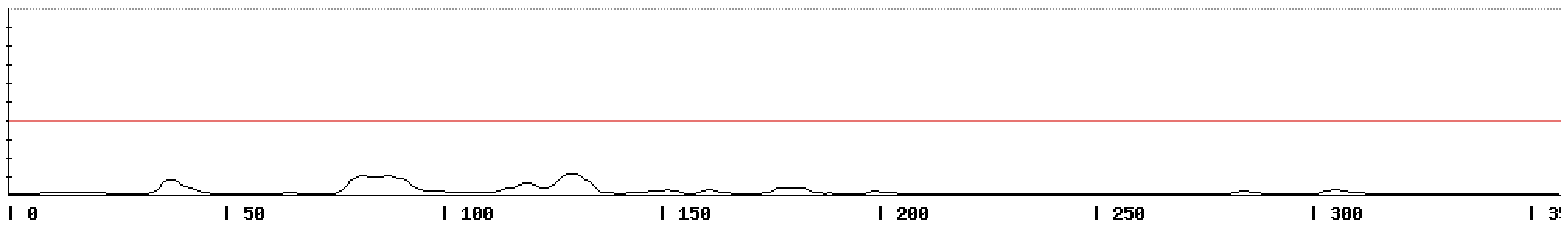

Predictions for Brassicarapa_XP_009115498.1

Viterbi Path

Viterbi Path

Posterior @ 0.4

Posterior @ 0.4

Posterior Graphic. Threshold Marked @ 0.4

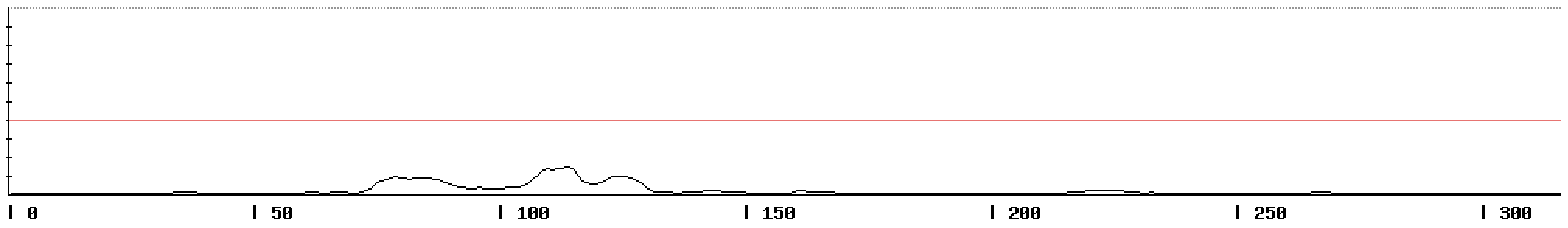

Predictions for Brassicarapa_XP_009116356.1

Viterbi Path

Viterbi Path

Posterior @ 0.4	339 - KKREKKK - 345
Posterior @ 0.4	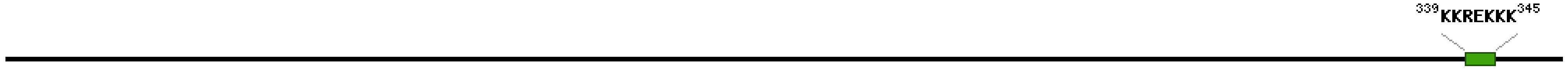
Posterior Graphic. Threshold Marked @ 0.4	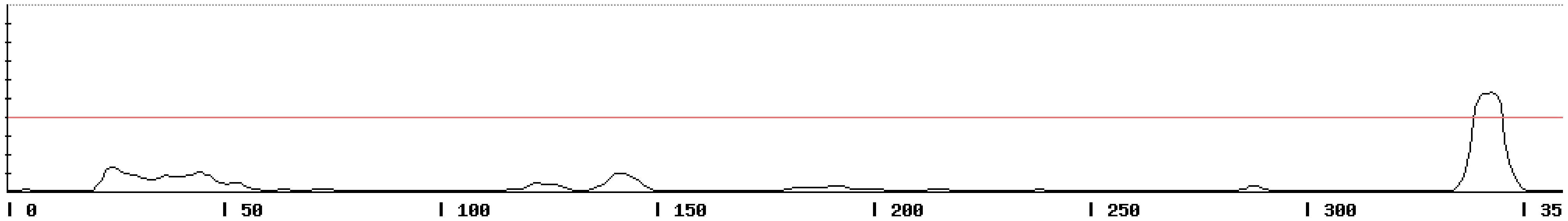

Predictions for Brassicarapa_XP_009116358.1

Viterbi Path	
Viterbi Path	
Posterior @ 0.4	338 - KKREKKK - 344
Posterior @ 0.4	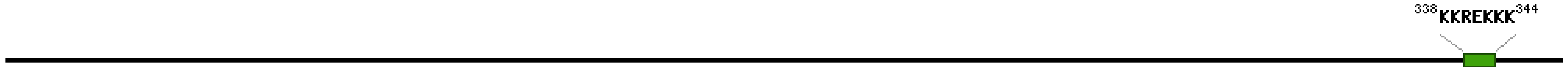
Posterior Graphic. Threshold Marked @ 0.4	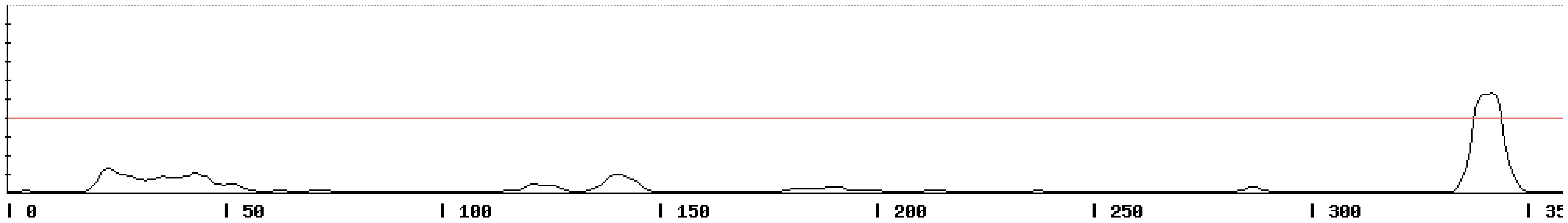

Predictions for Brassicarapa_XP_009116359.1

Viterbi Path	
Viterbi Path	

Posterior @ 0.4	338 - KKREKKK - 344
Posterior @ 0.4	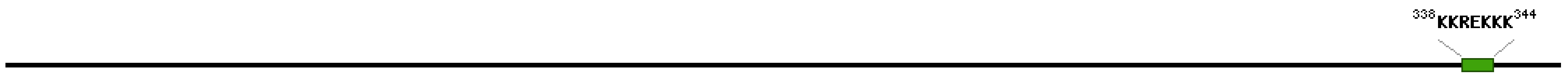
Posterior Graphic. Threshold Marked @ 0.4	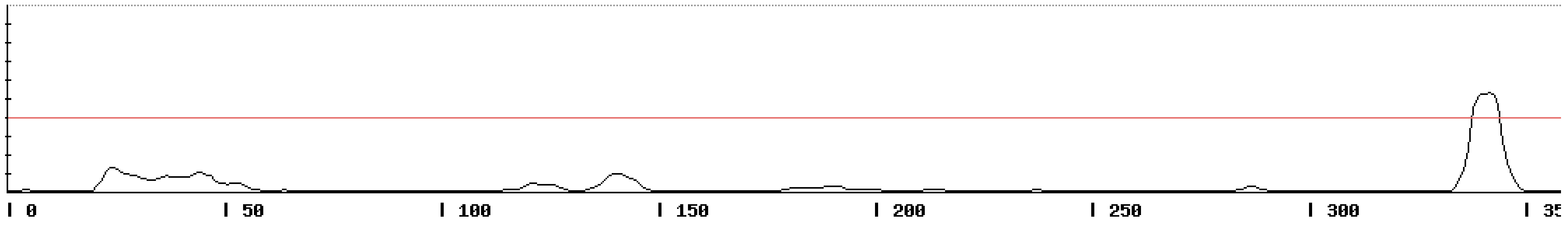

Predictions for Brassicarapa_XP_009116467.1

Viterbi Path	
Viterbi Path	
Posterior @ 0.4	
Posterior @ 0.4	
Posterior Graphic. Threshold Marked @ 0.4	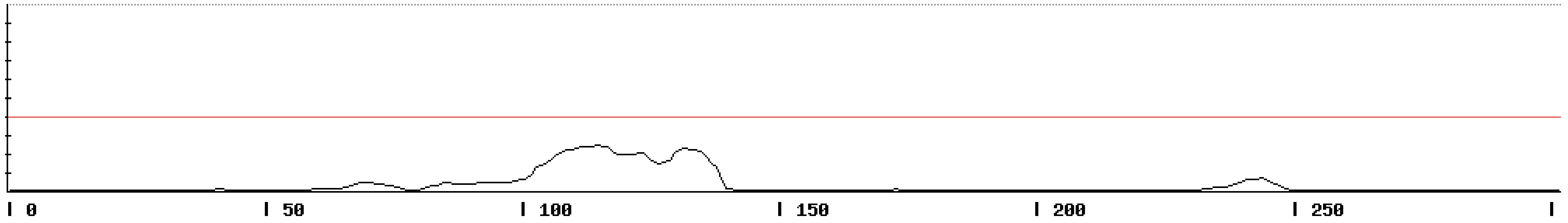

Predictions for Brassicarapa_XP_009116860.1

Viterbi Path

Viterbi Path

Posterior @ 0.4

Posterior @ 0.4

Posterior Graphic. Threshold Marked @ 0.4

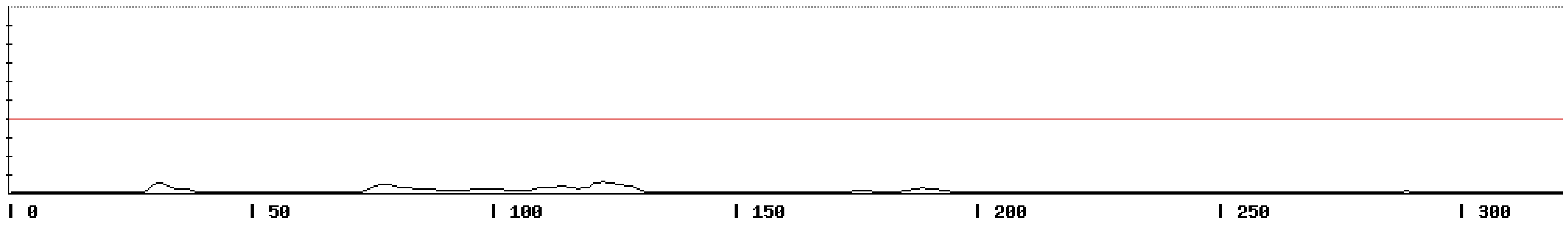

Predictions for Brassicarapa_XP_009117131.1

Viterbi Path

Viterbi Path

Posterior @ 0.4

Posterior @ 0.4

Posterior Graphic. Threshold Marked @ 0.4

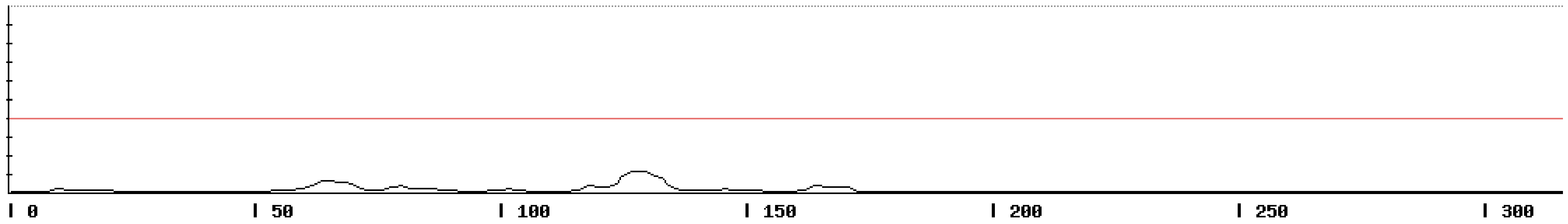

Predictions for Brassicarapa_XP_009117877.1	
Viterbi Path	
Viterbi Path	
Posterior @ 0.4	
Posterior @ 0.4	
Posterior Graphic. Threshold Marked @ 0.4	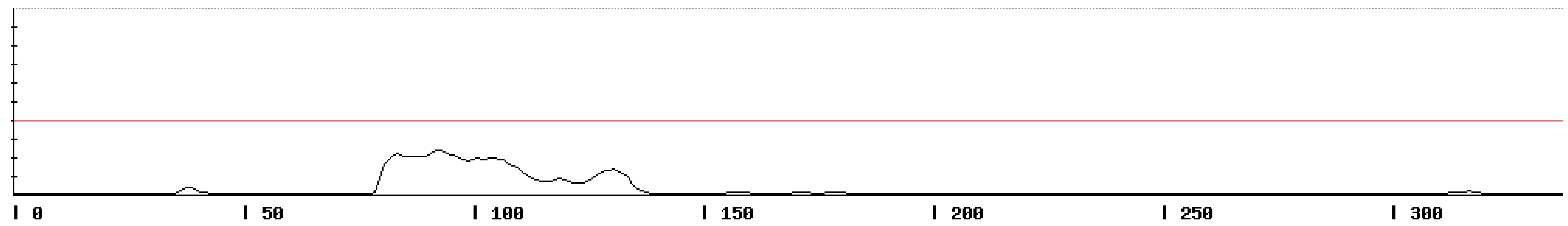

Predictions for Brassicarapa_XP_009118784.1	
Viterbi Path	
Viterbi Path	
Posterior @ 0.4	69 - RKESRYNRGERQKRKTKSGFWKKTGKTLPITRK - 101
Posterior @ 0.4	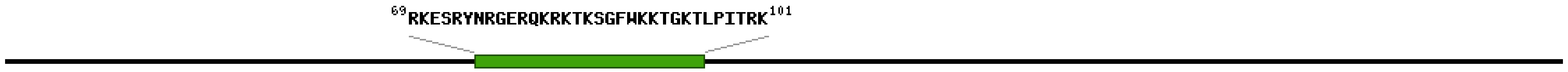
Posterior Graphic. Threshold Marked @ 0.4	

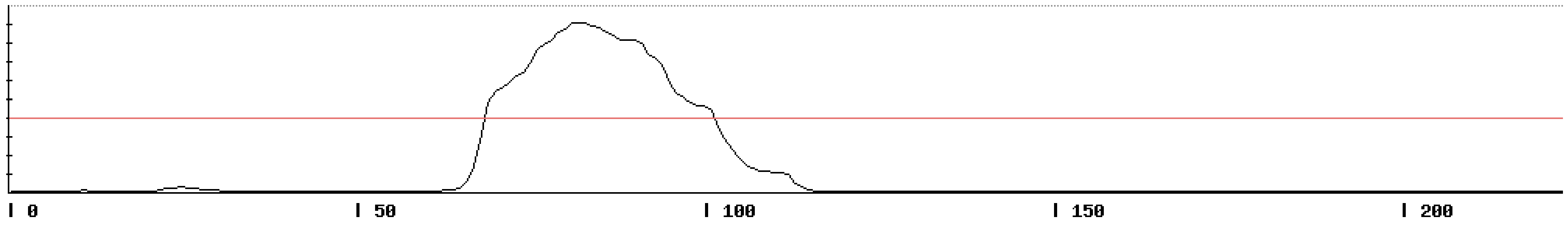

Predictions for Brassicarapa_XP_009118785.1

Viterbi Path	
Viterbi Path	
Posterior @ 0.4	69 - RKESRYNRGERQKRKTKSGFWKKTGKTLPITRK - 101
Posterior @ 0.4	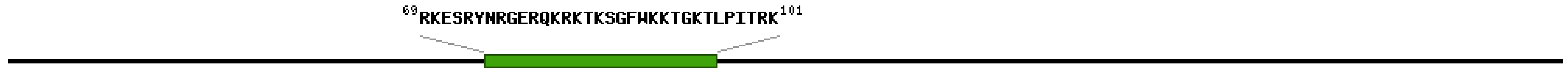
Posterior Graphic. Threshold Marked @ 0.4	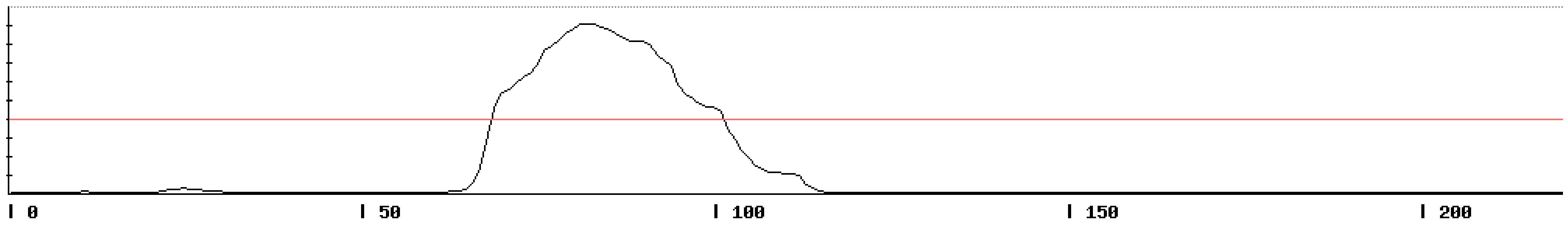

Predictions for Brassicarapa_XP_009119137.1

Viterbi Path	
Viterbi Path	
Posterior @ 0.4	
Posterior @ 0.4	

Posterior Graphic. Threshold Marked @ 0.4	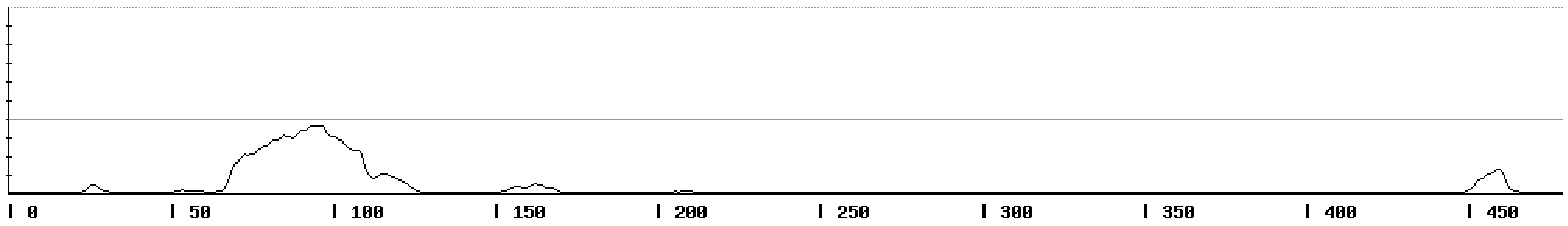

Predictions for Brassicarapa_XP_009119349.1

Viterbi Path	
Viterbi Path	
Posterior @ 0.4	
Posterior @ 0.4	
Posterior Graphic. Threshold Marked @ 0.4	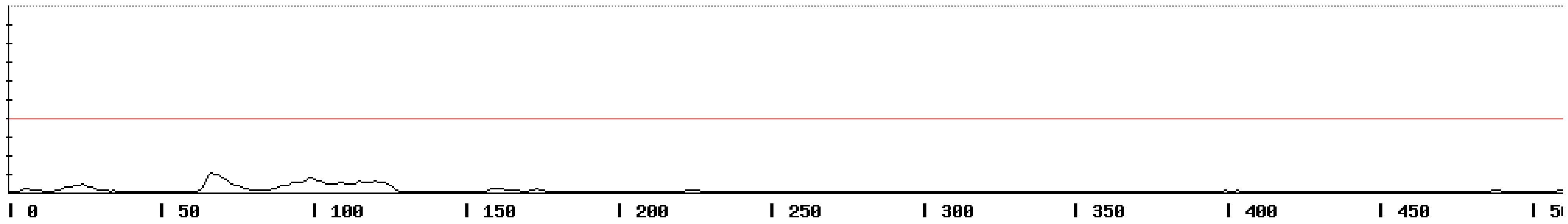

Predictions for Brassicarapa_XP_009119652.1

Viterbi Path	
Viterbi Path	
Posterior @ 0.4	99 - RKKGDHEKIGEKRVF - 113
	115 - FKYSSKIPGGSKSKSKSKSK - 134
Posterior @ 0.4	

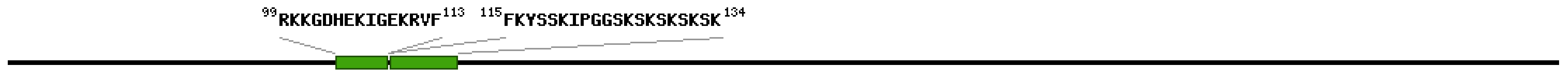

Posterior Graphic. Threshold Marked @ 0.4

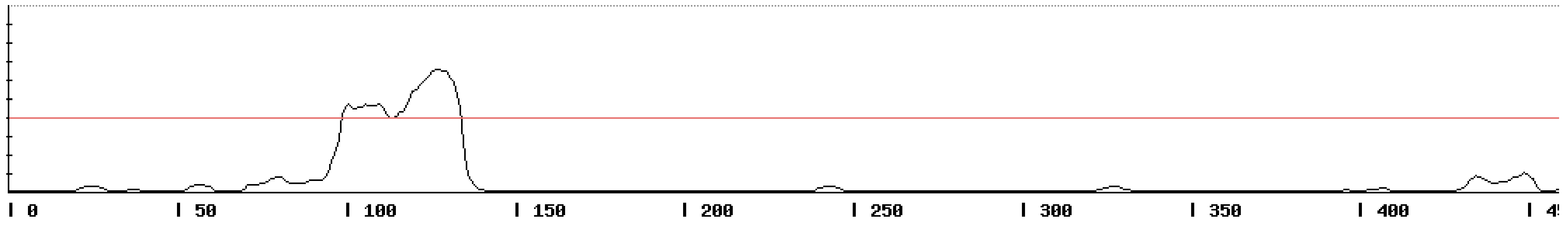

Predictions for Brassicarapa_XP_009119653.1

Viterbi Path

Viterbi Path

Posterior @ 0.4

98 - RKKGDHEKIGEKRVF - 112

114 - FKYSSKIPGGSKSKSKSKSK - 133

Posterior @ 0.4

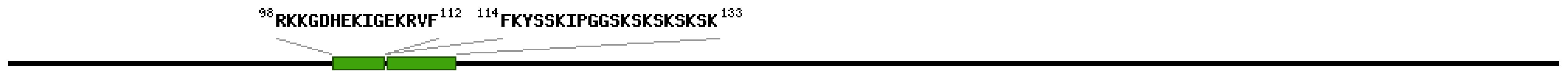

Posterior Graphic. Threshold Marked @ 0.4

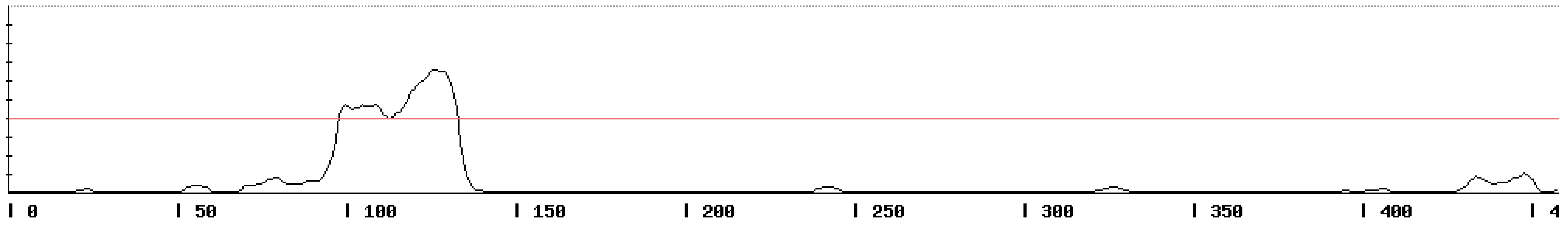

Predictions for Brassicarapa_XP_009119654.1

Viterbi Path

Viterbi Path

Posterior @ 0.4	99 - RKRGNREKIGE - 109
Posterior @ 0.4	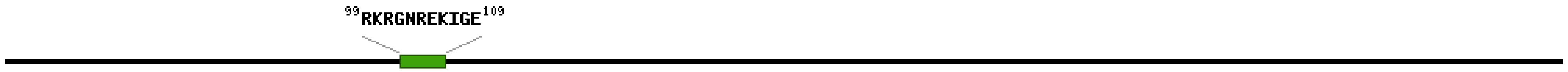
Posterior Graphic. Threshold Marked @ 0.4	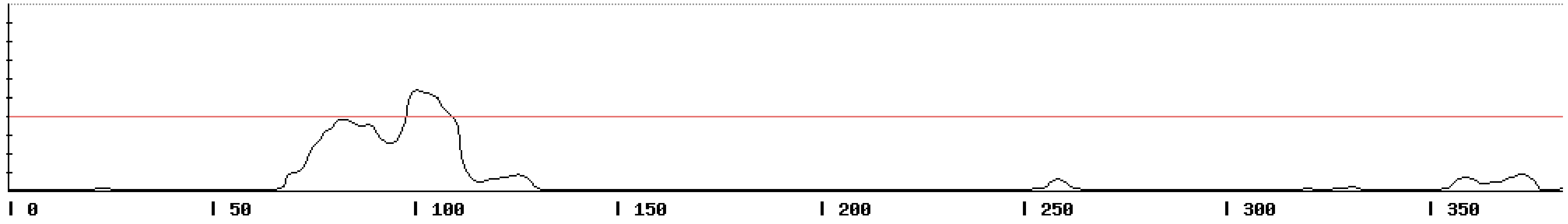

**Predictions for Brassicarapa_XP_009119656.1**	
Viterbi Path	68 - GRKEKRYNRGERQIRKTKSGFWKKTGVTMDIKRKRSAHREKIGEKR - 113
Viterbi Path	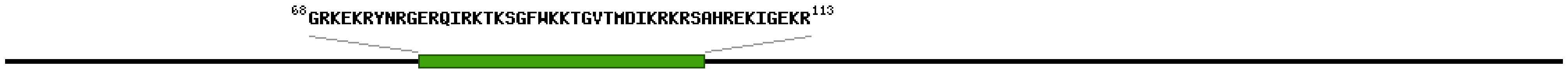
Posterior @ 0.4	68 - GRKEKRYNRGERQIRKTKSGFWKKTGVTMDIKRKRSAHREKIGEKR - 113
Posterior @ 0.4	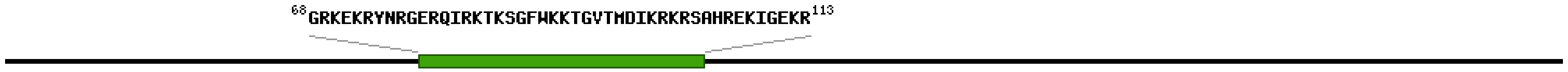
Posterior Graphic. Threshold Marked @ 0.4	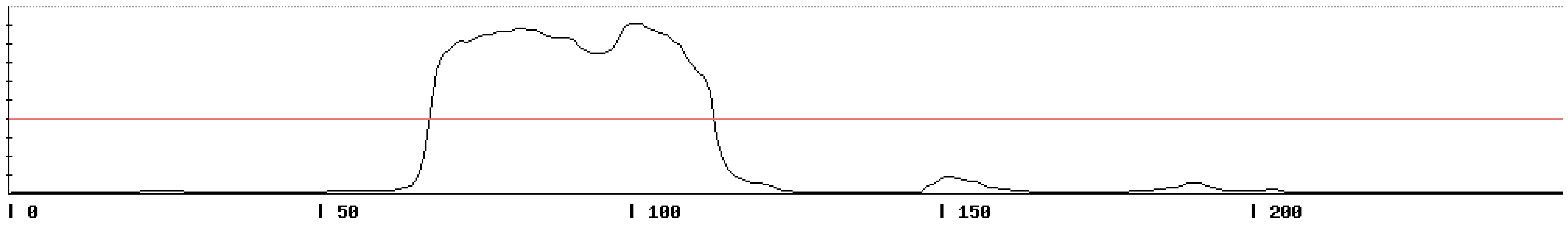

Predictions for Brassicarapa_XP_009119657.1

Viterbi Path	
Viterbi Path	
Posterior @ 0.4	74 - NRGERQSRKTRSGFWKKTGVTTEIIRKRGNREKIGEKR - 111
Posterior @ 0.4	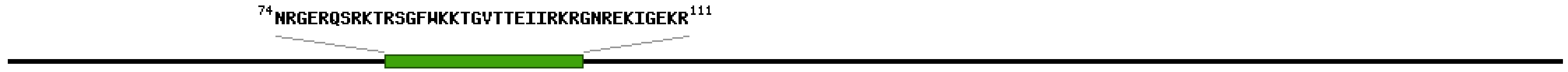
Posterior Graphic. Threshold Marked @ 0.4	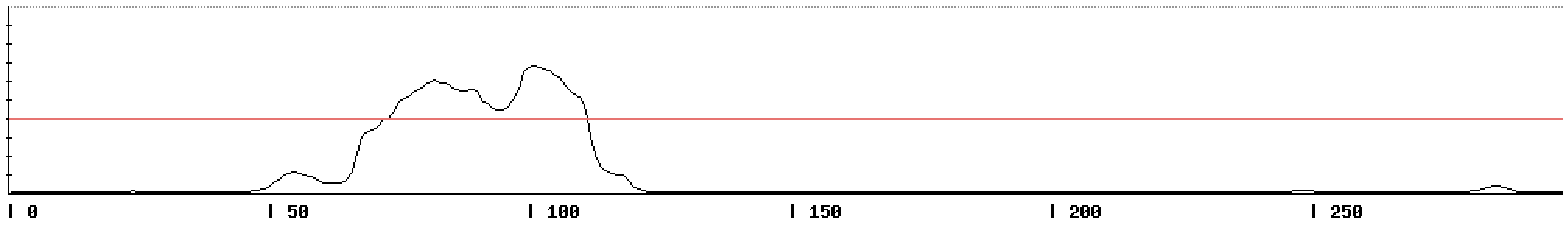

Predictions for Brassicarapa_XP_009119658.1

Viterbi Path	
Viterbi Path	
Posterior @ 0.4	
Posterior @ 0.4	
Posterior Graphic. Threshold Marked @ 0.4	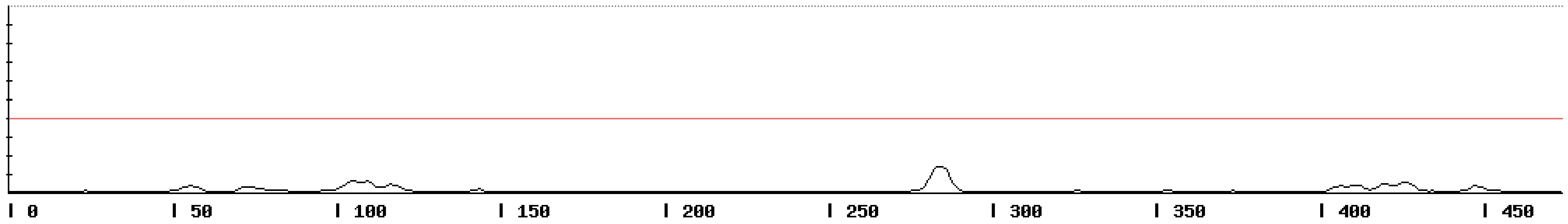

Predictions for Brassicarapa_XP_009119659.1

Viterbi Path	
Viterbi Path	
Posterior @ 0.4	
Posterior @ 0.4	
Posterior Graphic. Threshold Marked @ 0.4	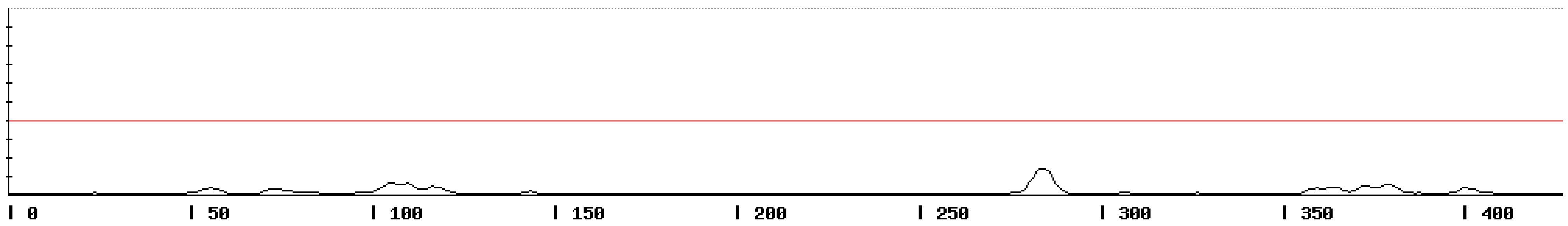

Predictions for Brassicarapa_XP_009119728.1

Viterbi Path	
Viterbi Path	
Posterior @ 0.4	
Posterior @ 0.4	
Posterior Graphic. Threshold Marked @ 0.4	

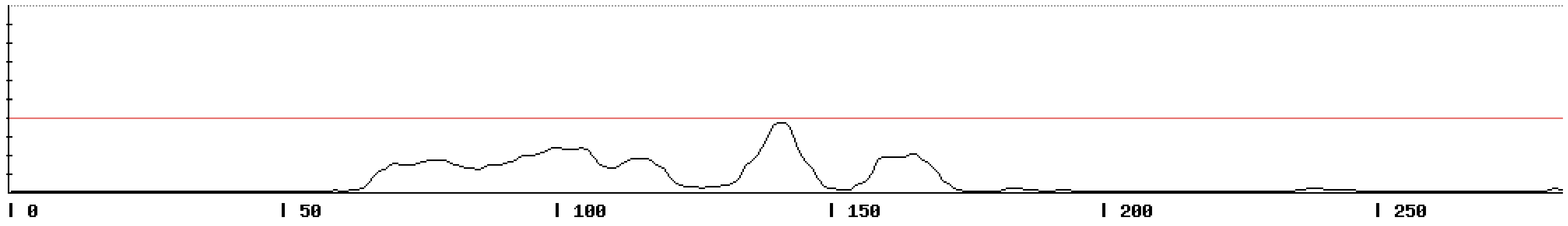

Predictions for Brassicarapa_XP_009119767.1

Viterbi Path	
Viterbi Path	
Posterior @ 0.4	94 - KKKDIKK - 100
Posterior @ 0.4	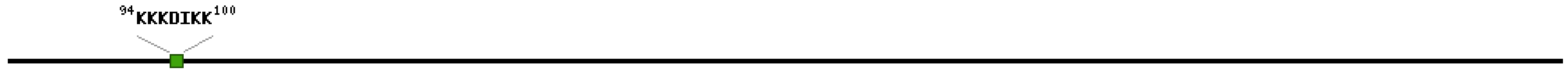
Posterior Graphic. Threshold Marked @ 0.4	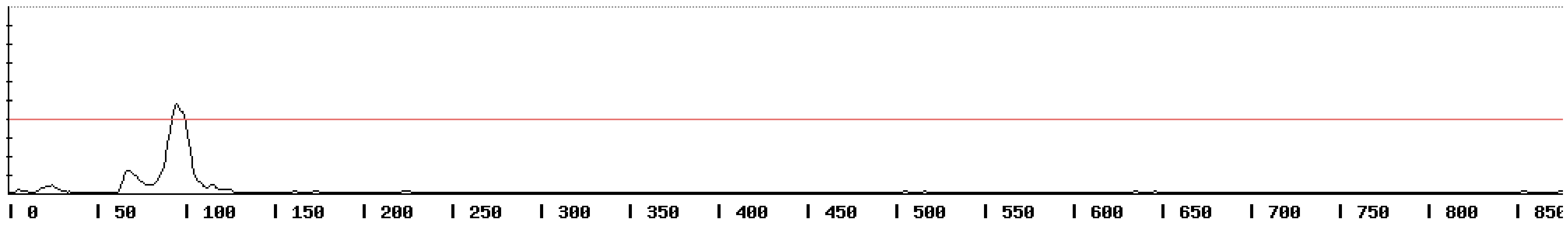

Predictions for Brassicarapa_XP_009119784.1

Viterbi Path	
Viterbi Path	
Posterior @ 0.4	100 - RKRGNREKIGE - 110
Posterior @ 0.4	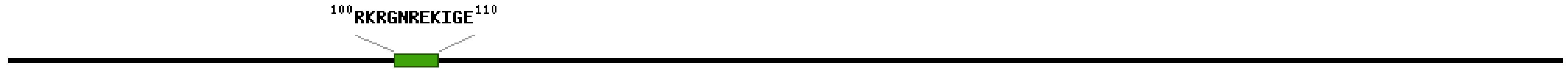

Posterior Graphic. Threshold Marked @ 0.4

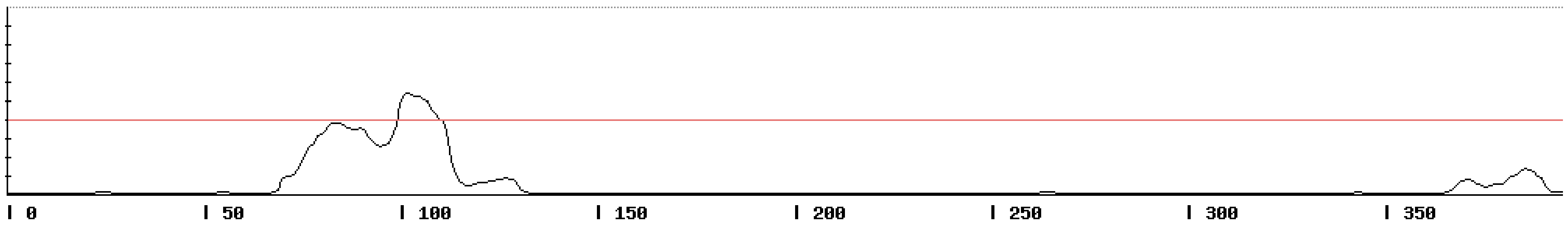

Predictions for Brassicarapa_XP_009119787.1

Viterbi Path	
Viterbi Path	
Posterior @ 0.4	86 - K - 86
Posterior @ 0.4	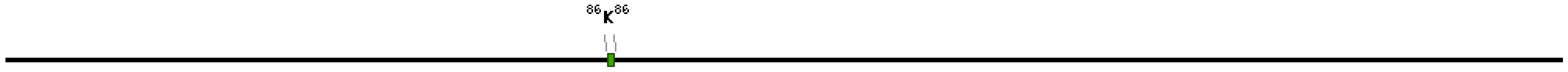
Posterior Graphic. Threshold Marked @ 0.4	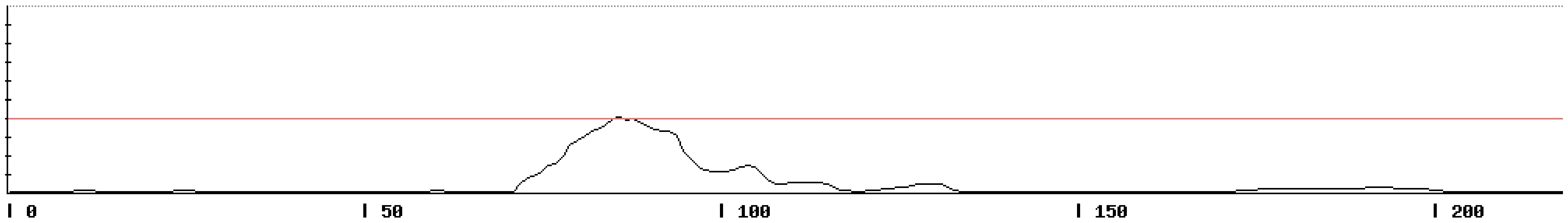

Predictions for Brassicarapa_XP_009119789.1

Viterbi Path	
Viterbi Path	
Posterior @ 0.4	99 - SRKTK - 103
Posterior @ 0.4	

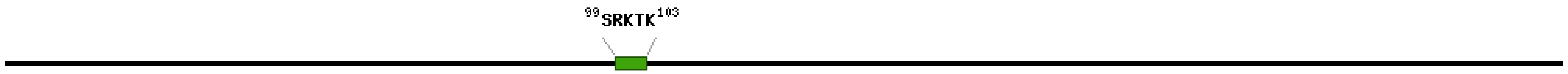

Posterior Graphic. Threshold Marked @ 0.4

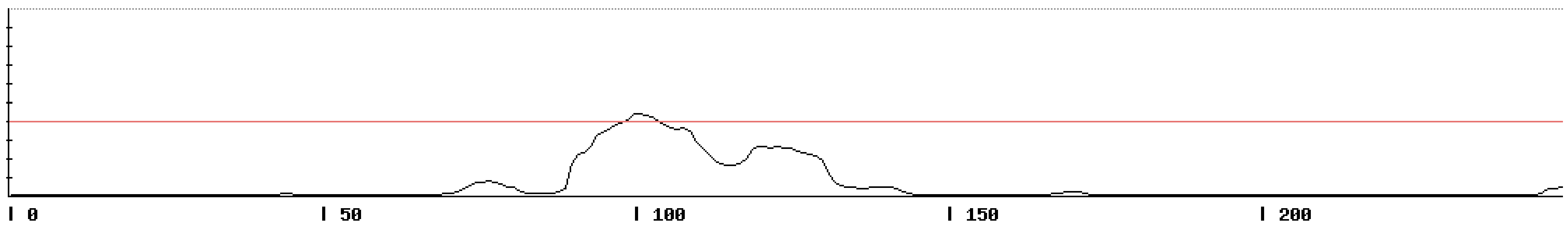

Predictions for Brassicarapa_XP_009119790.1

Viterbi Path

Viterbi Path

Posterior @ 0.4 — 68 - GKLNRRKRASKT - 79

Posterior @ 0.4

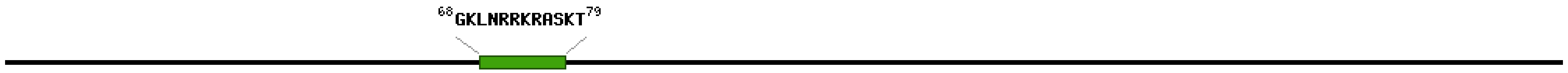

Posterior Graphic. Threshold Marked @ 0.4

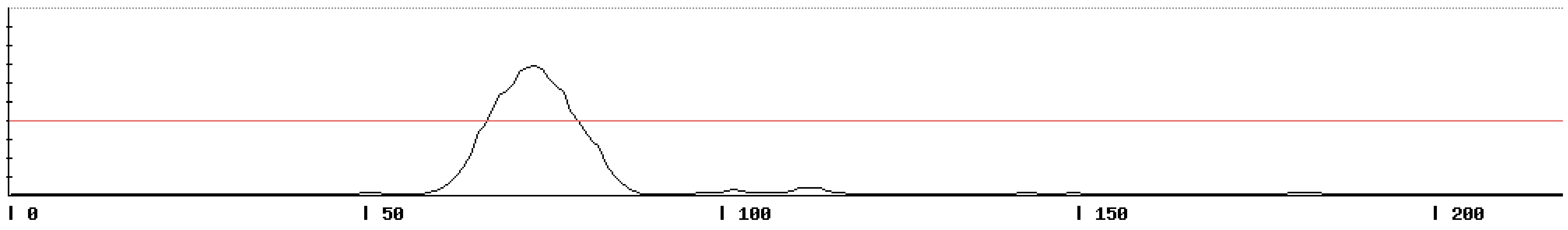

Predictions for Brassicarapa_XP_009119934.1

Viterbi Path

Viterbi Path

Posterior @ 0.4

Posterior @ 0.4

Posterior Graphic. Threshold Marked @ 0.4

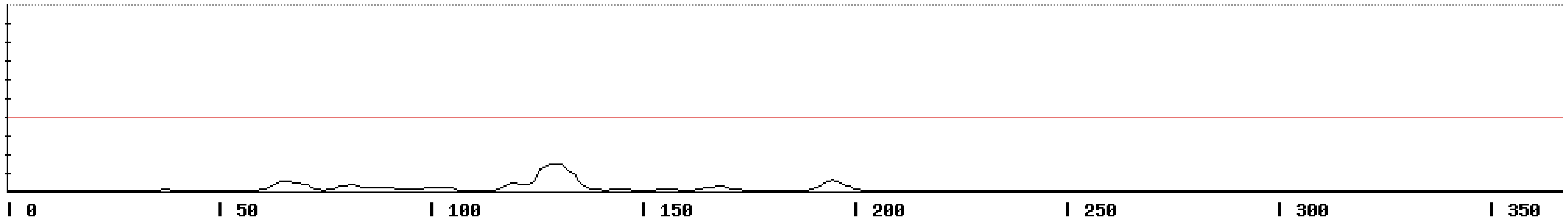

Predictions for Brassicarapa_XP_009120111.1

Viterbi Path

Viterbi Path

Posterior @ 0.4 113 - KKTKRGR - 119

Posterior @ 0.4

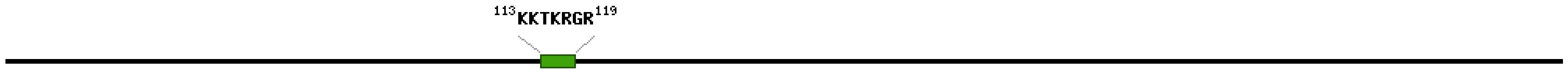

Posterior Graphic. Threshold Marked @ 0.4

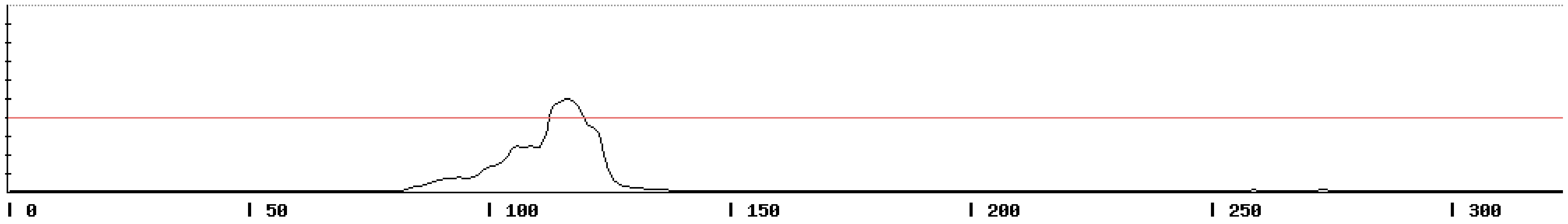

Predictions for Brassicarapa_XP_009120122.1

Viterbi Path

Viterbi Path

Posterior @ 0.4

Posterior @ 0.4

Posterior Graphic. Threshold Marked @ 0.4

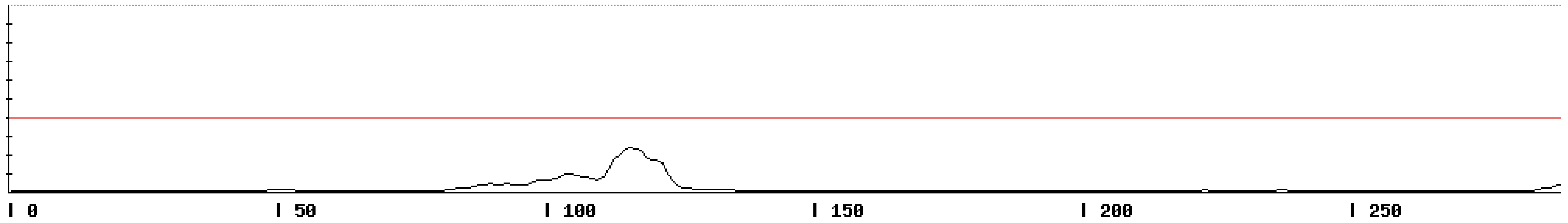

Predictions for Brassicarapa_XP_009120171.1

Viterbi Path

Viterbi Path

Posterior @ 0.4

127 - R - 127

Posterior @ 0.4

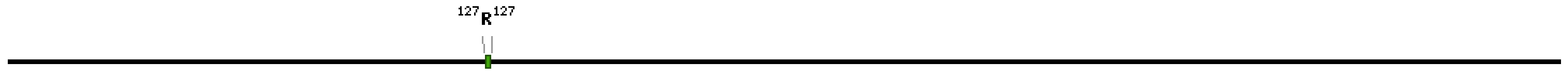

Posterior Graphic. Threshold Marked @ 0.4

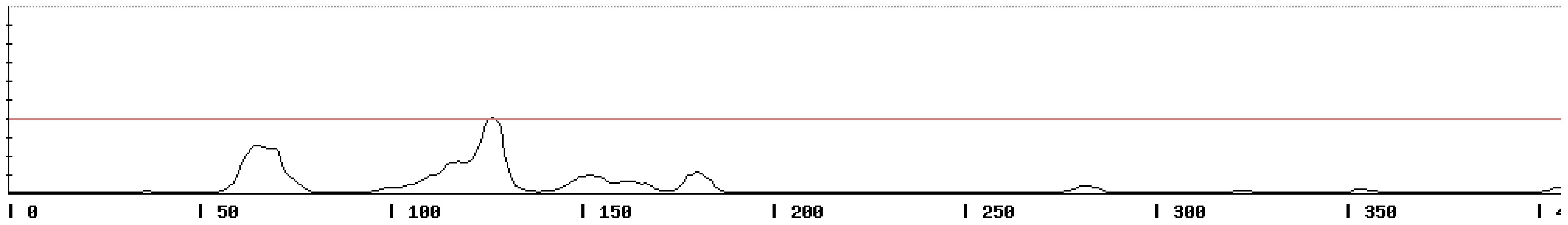

Predictions for Brassicarapa_XP_009120172.1

Viterbi Path

Viterbi Path

Posterior @ 0.4 127 - R - 127

Posterior @ 0.4

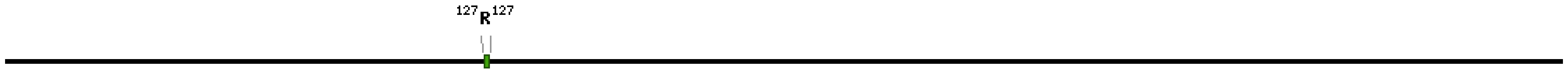

Posterior Graphic. Threshold Marked @ 0.4

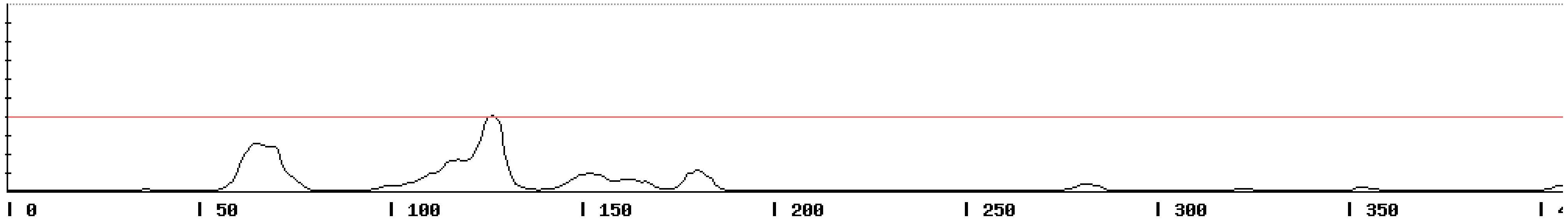

Predictions for Brassicarapa_XP_009120173.1

Viterbi Path

Viterbi Path

Posterior @ 0.4 127 - R - 127

Posterior @ 0.4

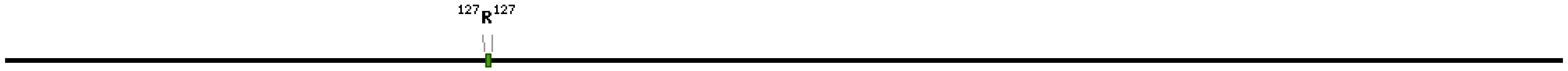

Posterior Graphic. Threshold Marked @ 0.4

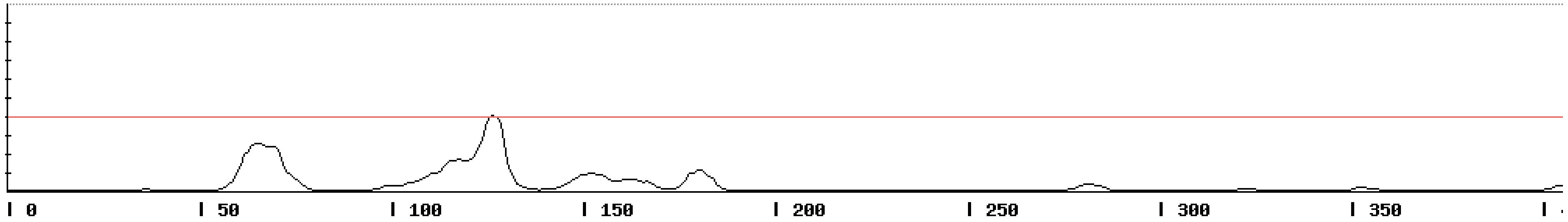

Predictions for Brassicarapa_XP_009120174.1

Viterbi Path	
Viterbi Path	
Posterior @ 0.4	127 - R - 127
	369 - RRGKFRSKSTAKRR - 382
Posterior @ 0.4	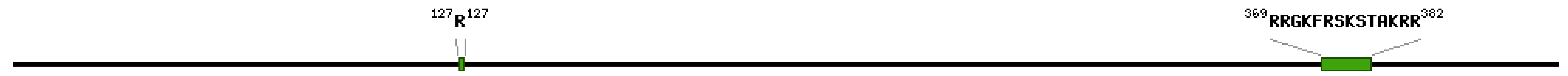
Posterior Graphic. Threshold Marked @ 0.4	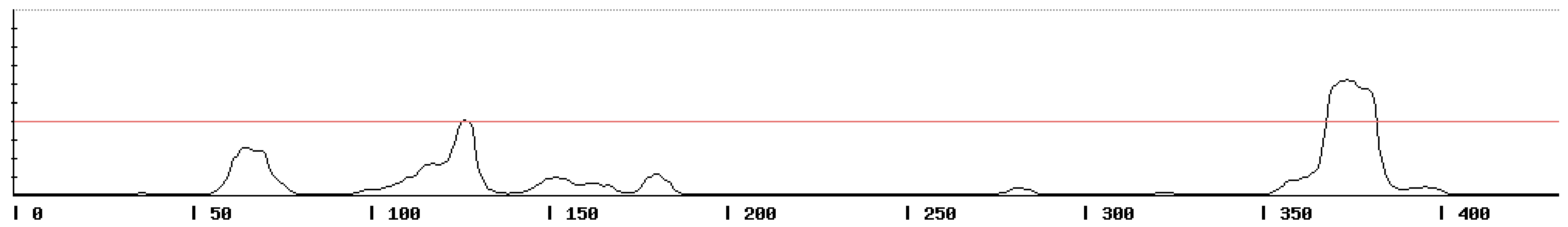

Predictions for Brassicarapa_XP_009120664.1

Viterbi Path	
Viterbi Path	
Posterior @ 0.4	
Posterior @ 0.4	
Posterior Graphic. Threshold Marked @ 0.4	

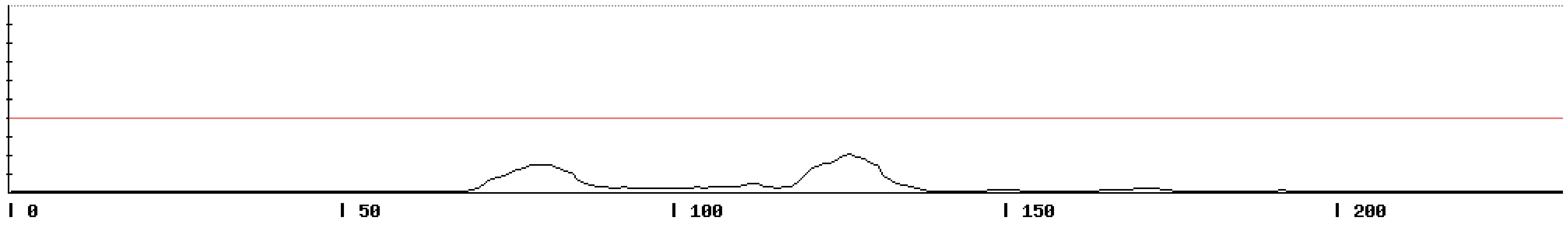

Predictions for Brassicarapa_XP_009120669.1

Viterbi Path	
Viterbi Path	
Posterior @ 0.4	
Posterior @ 0.4	
Posterior Graphic. Threshold Marked @ 0.4	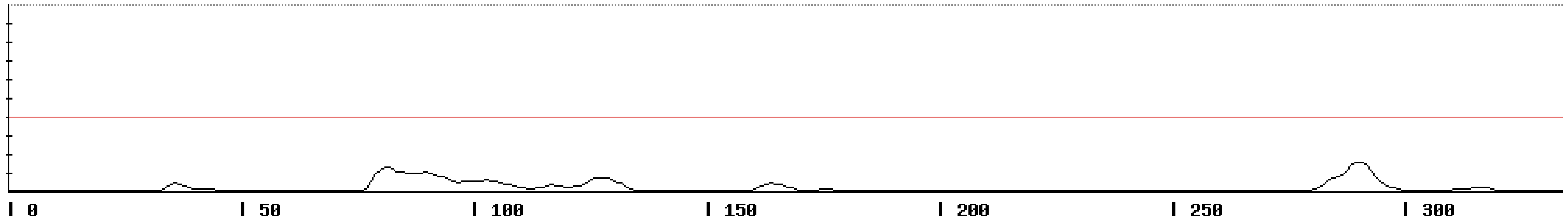

Predictions for Brassicarapa_XP_009120990.1

Viterbi Path	
Viterbi Path	
Posterior @ 0.4	
Posterior @ 0.4	

Posterior Graphic. Threshold Marked @ 0.4

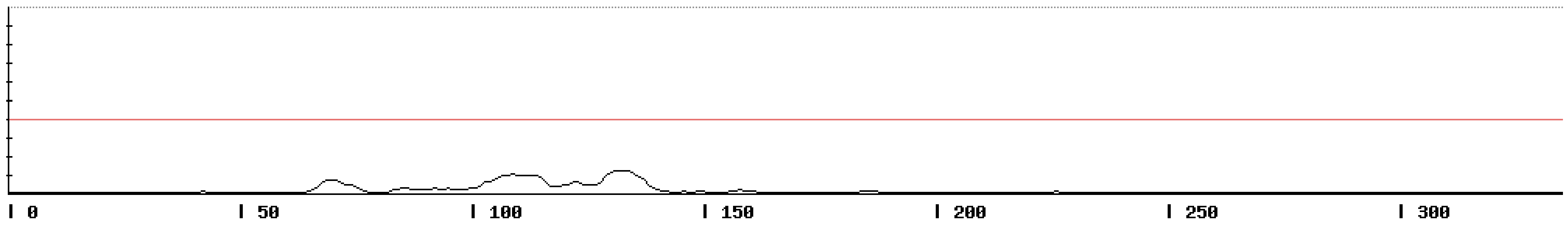

Predictions for Brassicarapa_XP_009121166.1

Viterbi Path

Viterbi Path

Posterior @ 0.4

Posterior @ 0.4

Posterior Graphic. Threshold Marked @ 0.4

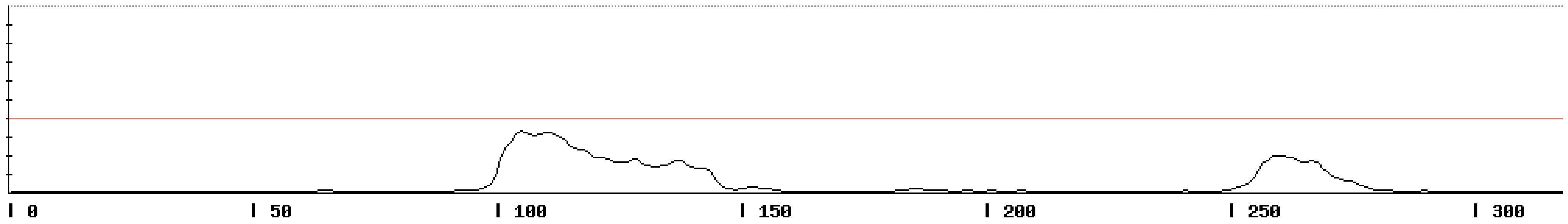

Predictions for Brassicarapa_XP_009121308.1

Viterbi Path

Viterbi Path

Posterior @ 0.4

Posterior @ 0.4

Posterior Graphic. Threshold Marked @ 0.4

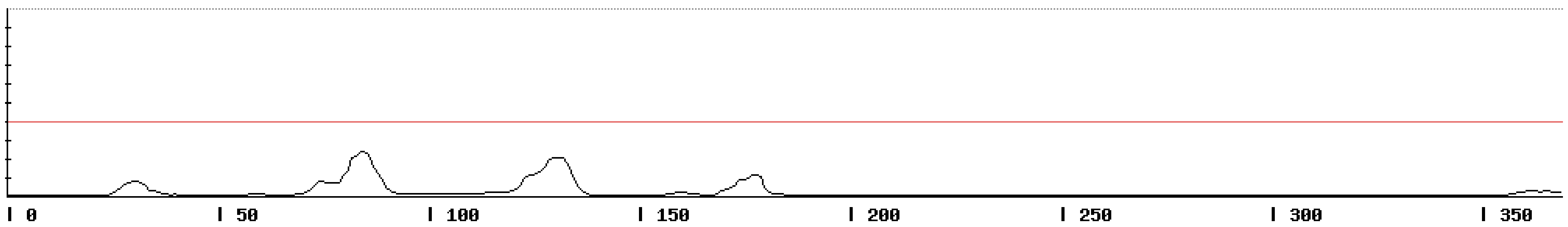

Predictions for Brassicarapa_XP_009121402.1

Viterbi Path	
Viterbi Path	
Posterior @ 0.4	
Posterior @ 0.4	
Posterior Graphic. Threshold Marked @ 0.4	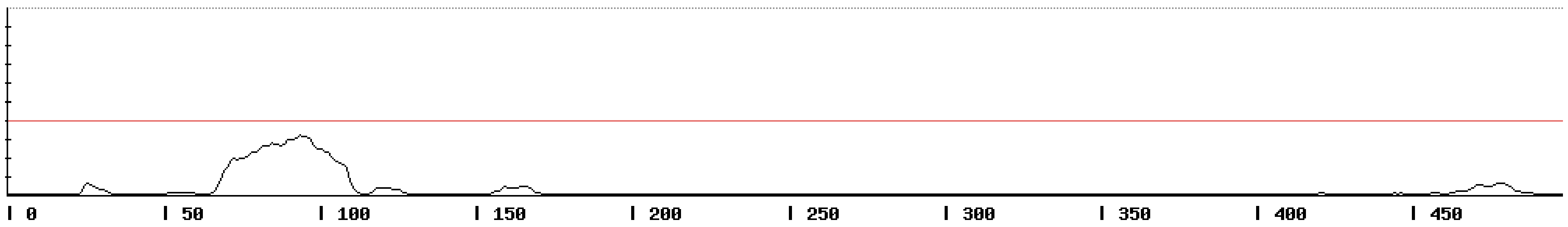

Predictions for Brassicarapa_XP_009121709.1

Viterbi Path	151 - RKASKGRRQRKSRGR - 165
Viterbi Path	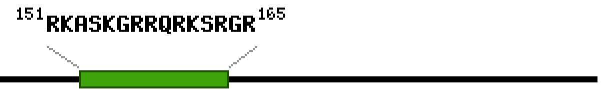

Posterior @ 0.4	151 - RKASKGRRQRKSRGR - 165
Posterior @ 0.4	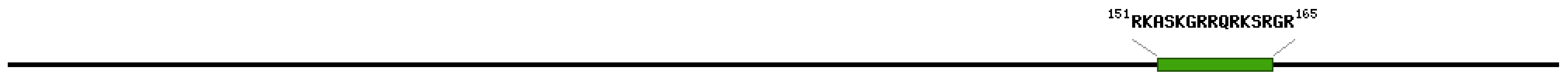
Posterior Graphic. Threshold Marked @ 0.4	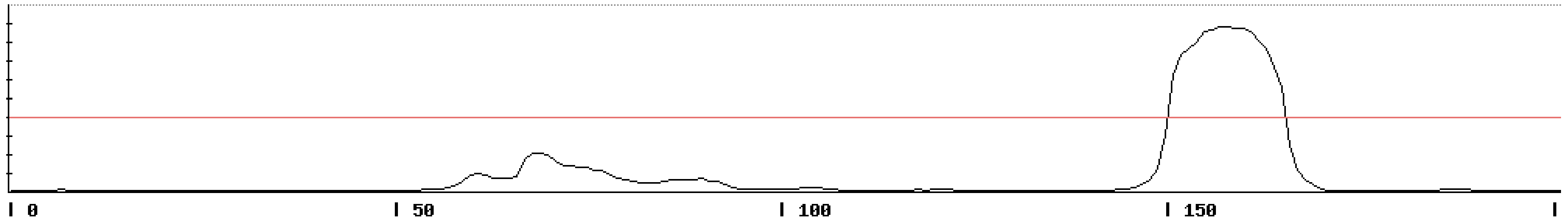

Predictions for Brassicarapa_XP_009121790.1

Viterbi Path	
Viterbi Path	
Posterior @ 0.4	
Posterior @ 0.4	
Posterior Graphic. Threshold Marked @ 0.4	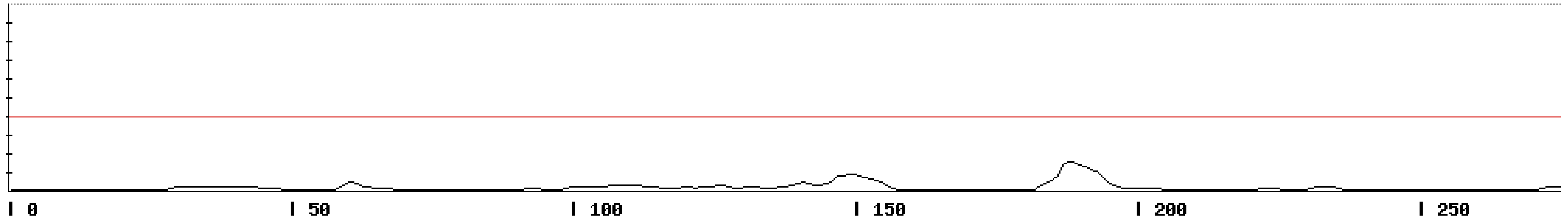

Predictions for Brassicarapa_XP_009121975.1

Viterbi Path	
Viterbi Path	

Posterior @ 0.4

Posterior @ 0.4

Posterior Graphic. Threshold Marked @ 0.4

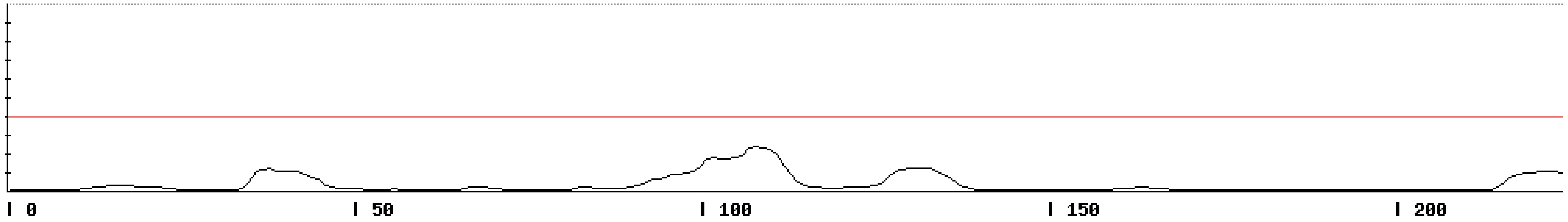

Predictions for Brassicarapa_XP_009121976.1

Viterbi Path

Viterbi Path

Posterior @ 0.4

Posterior @ 0.4

Posterior Graphic. Threshold Marked @ 0.4

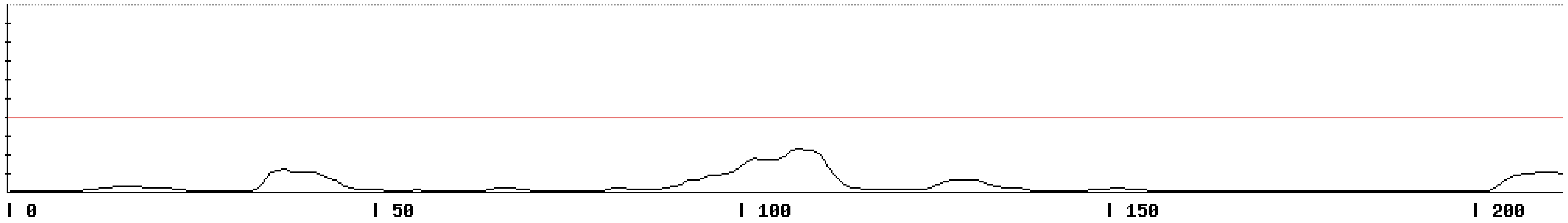

Predictions for Brassicarapa_XP_009121977.1

Viterbi Path

Viterbi Path

Posterior @ 0.4

Posterior @ 0.4

Posterior Graphic. Threshold Marked @ 0.4

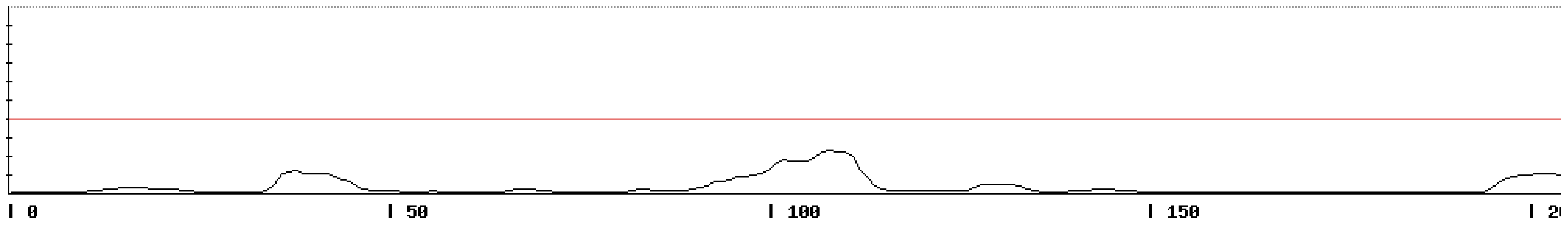

Predictions for Brassicarapa_XP_009122319.1

Viterbi Path

Viterbi Path

Posterior @ 0.4

Posterior @ 0.4

Posterior Graphic. Threshold Marked @ 0.4

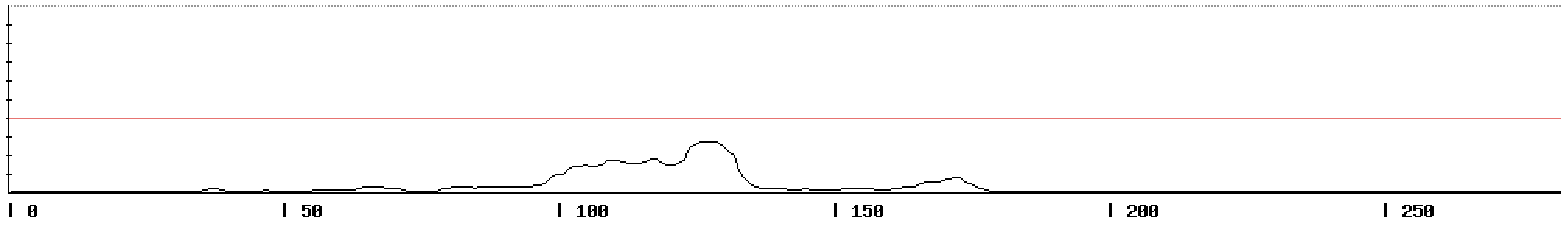

Predictions for Brassicarapa_XP_009122498.1	
Viterbi Path	
Viterbi Path	
Posterior @ 0.4	98 - GKPK - 101
Posterior @ 0.4	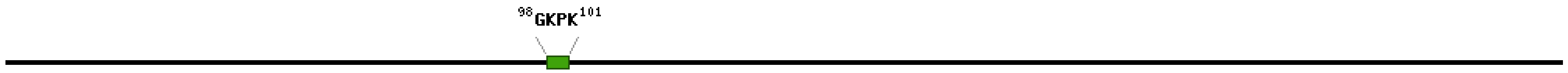
Posterior Graphic. Threshold Marked @ 0.4	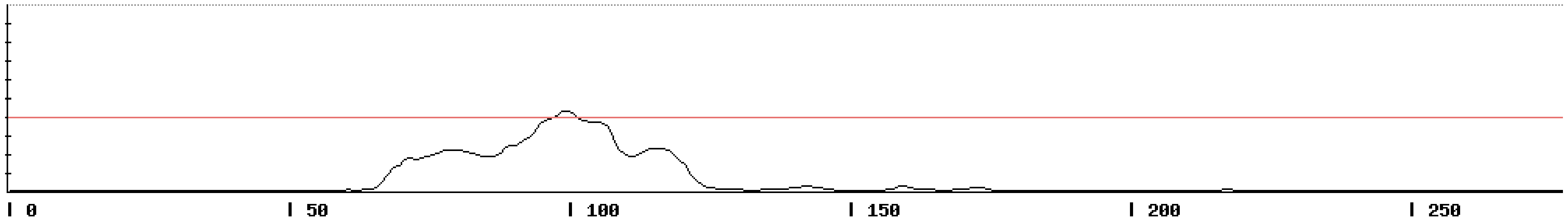

Predictions for Brassicarapa_XP_009122507.1	
Viterbi Path	
Viterbi Path	
Posterior @ 0.4	
Posterior @ 0.4	
Posterior Graphic. Threshold Marked @ 0.4	

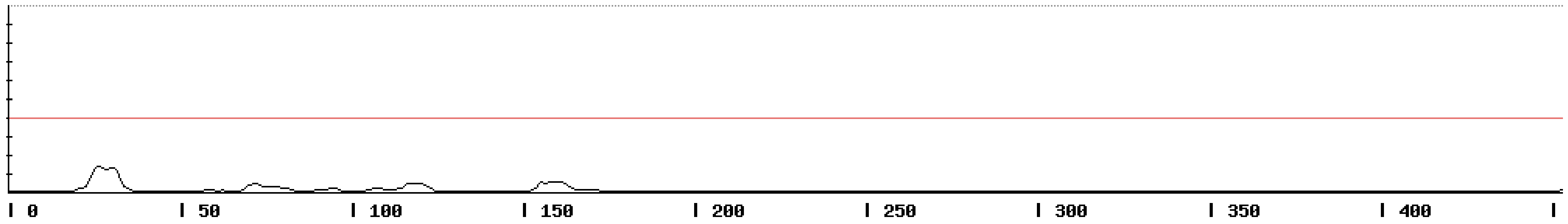

Predictions for Brassicarapa_XP_009122509.1

Viterbi Path	
Viterbi Path	
Posterior @ 0.4	
Posterior @ 0.4	
Posterior Graphic. Threshold Marked @ 0.4	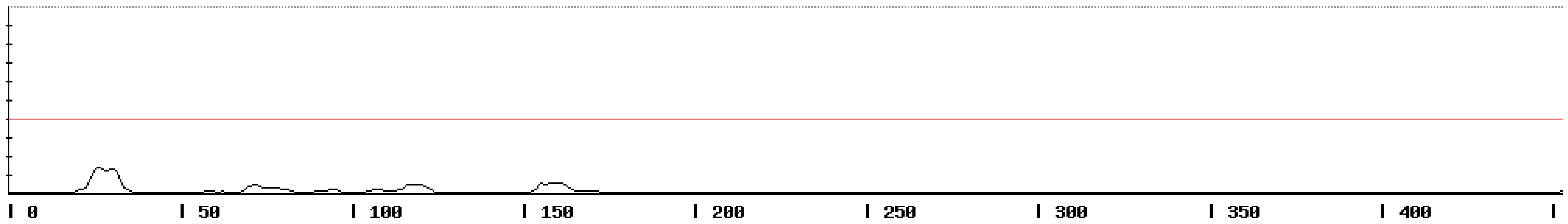

Predictions for Brassicarapa_XP_009122601.1

Viterbi Path	
Viterbi Path	
Posterior @ 0.4	
Posterior @ 0.4	

Posterior Graphic. Threshold Marked @ 0.4

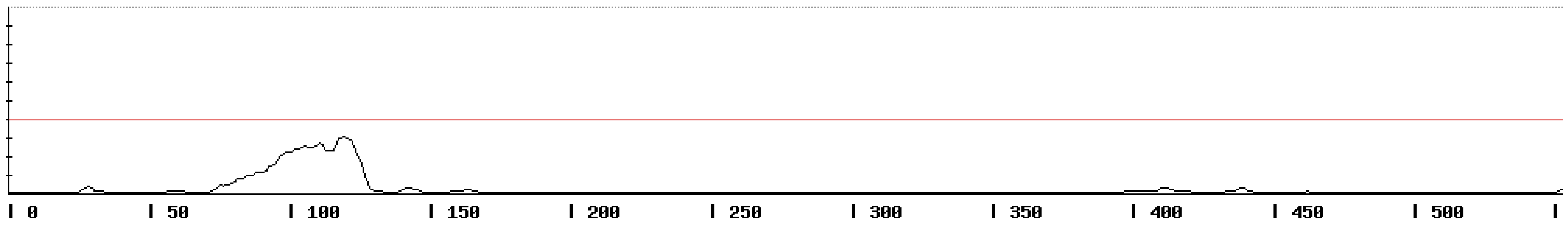

Predictions for Brassicarapa_XP_009122853.1

Viterbi Path

Viterbi Path

Posterior @ 0.4

Posterior @ 0.4

Posterior Graphic. Threshold Marked @ 0.4

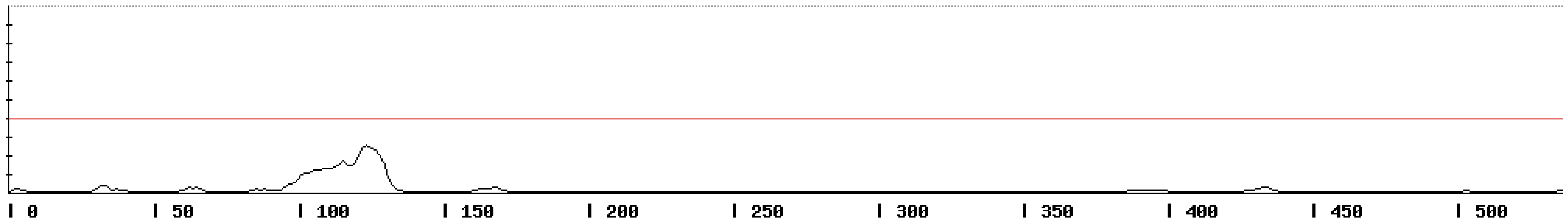

Predictions for Brassicarapa_XP_009122861.1

Viterbi Path

Viterbi Path

Posterior @ 0.4

Posterior @ 0.4

Posterior Graphic. Threshold Marked @ 0.4

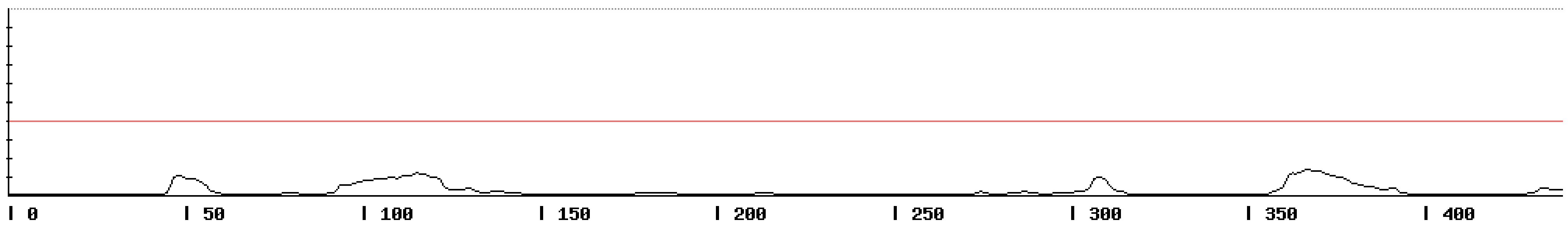

Predictions for Brassicarapa_XP_009122872.1

Viterbi Path

Viterbi Path

Posterior @ 0.4

Posterior @ 0.4

Posterior Graphic. Threshold Marked @ 0.4

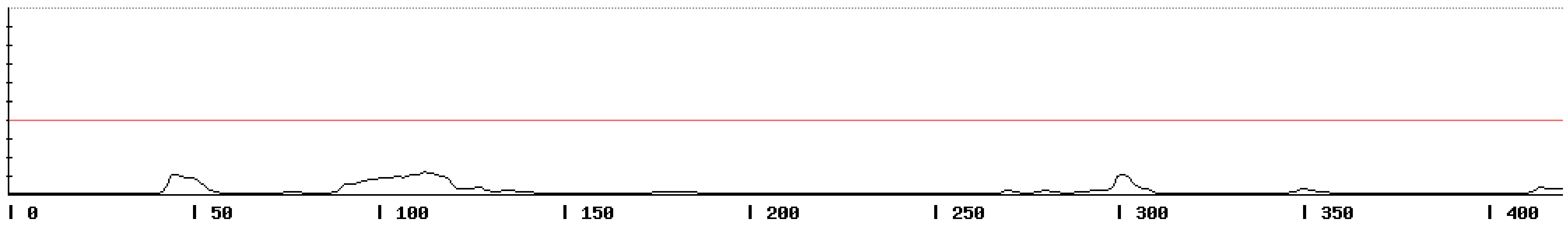

Predictions for Brassicarapa_XP_009124687.1

Viterbi Path

Viterbi Path

Posterior @ 0.4

Posterior @ 0.4

Posterior Graphic. Threshold Marked @ 0.4

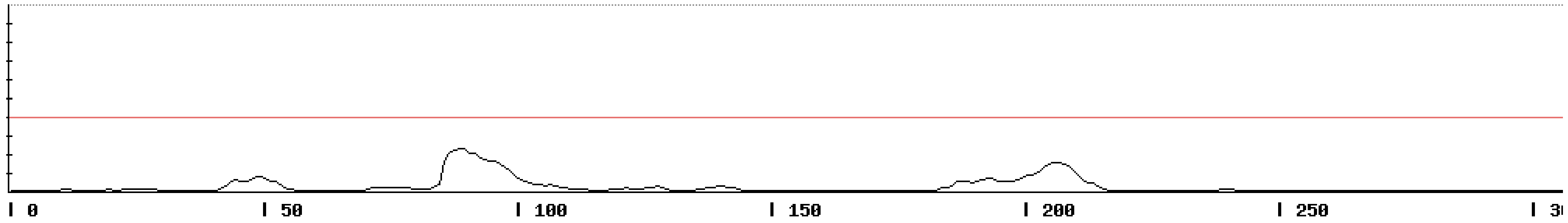

Predictions for Brassicarapa_XP_009125011.1

Viterbi Path

Viterbi Path

Posterior @ 0.4

Posterior @ 0.4

Posterior Graphic. Threshold Marked @ 0.4

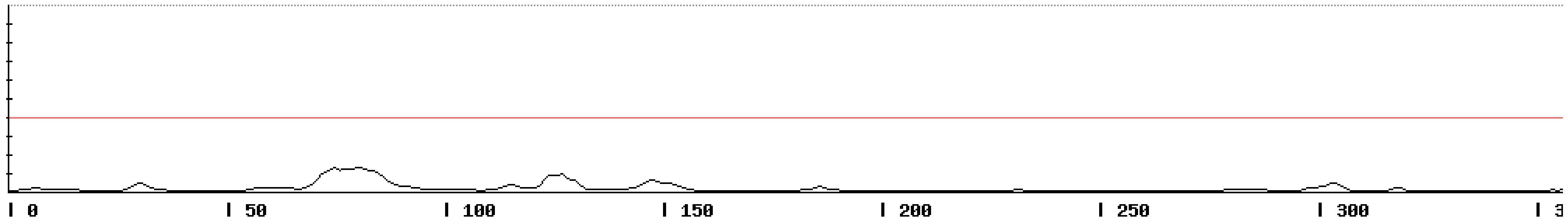

Predictions for Brassicarapa_XP_009125012.1

Viterbi Path

Viterbi Path

Posterior @ 0.4	
Posterior @ 0.4	
Posterior Graphic. Threshold Marked @ 0.4	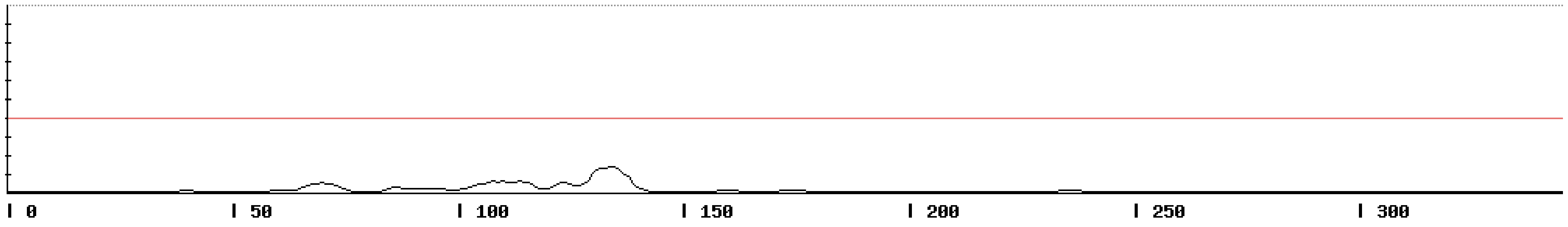

Predictions for Brassicarapa_XP_009125013.1

Viterbi Path	
Viterbi Path	
Posterior @ 0.4	
Posterior @ 0.4	
Posterior Graphic. Threshold Marked @ 0.4	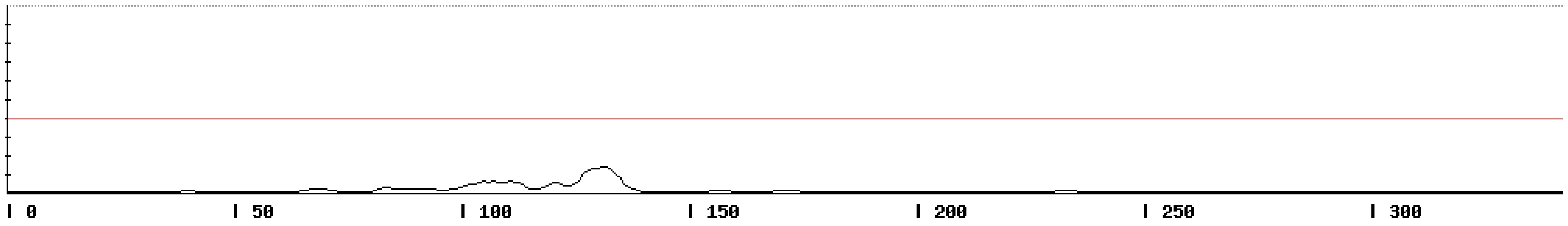

Predictions for Brassicarapa_XP_009125108.1

Viterbi Path

Viterbi Path

Posterior @ 0.4

Posterior @ 0.4

Posterior Graphic. Threshold Marked @ 0.4

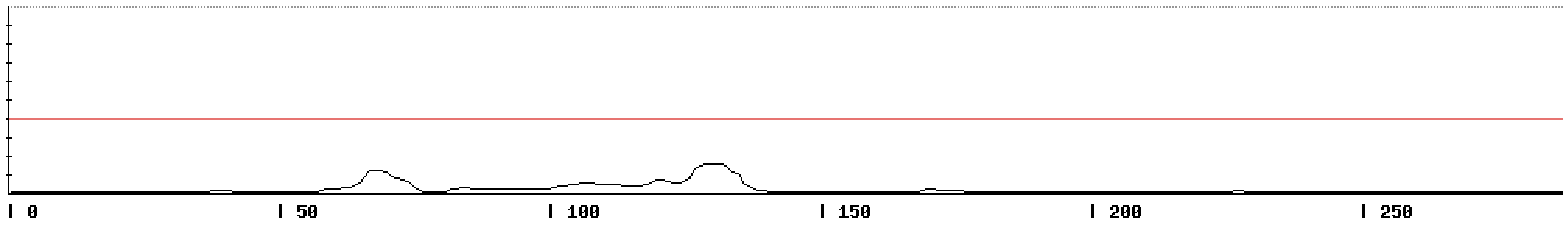

Predictions for Brassicarapa_XP_009125115.1

Viterbi Path

Viterbi Path

Posterior @ 0.4

Posterior @ 0.4

Posterior Graphic. Threshold Marked @ 0.4

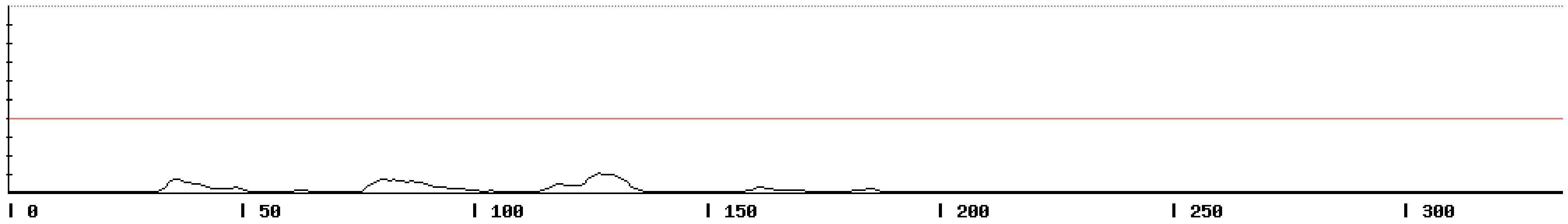

Predictions for Brassicarapa_XP_009125174.1

Viterbi Path

Viterbi Path

Posterior @ 0.4

Posterior @ 0.4

Posterior Graphic. Threshold Marked @ 0.4

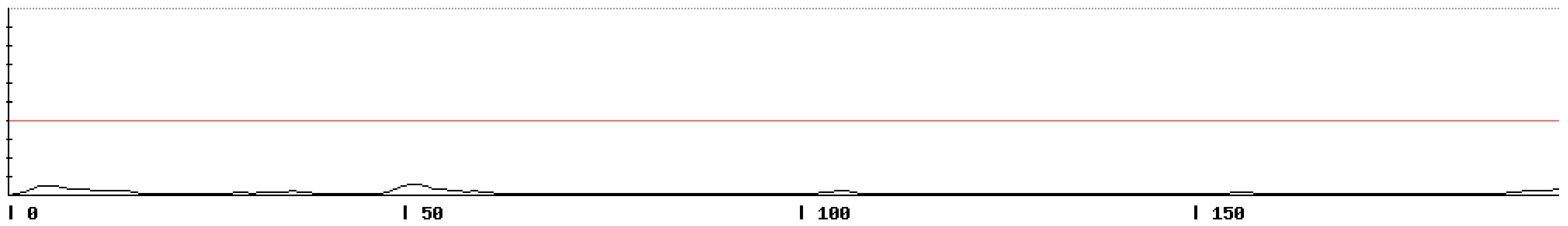

Predictions for Brassicarapa_XP_009125558.1

Viterbi Path

Viterbi Path

Posterior @ 0.4

Posterior @ 0.4

Posterior Graphic. Threshold Marked @ 0.4

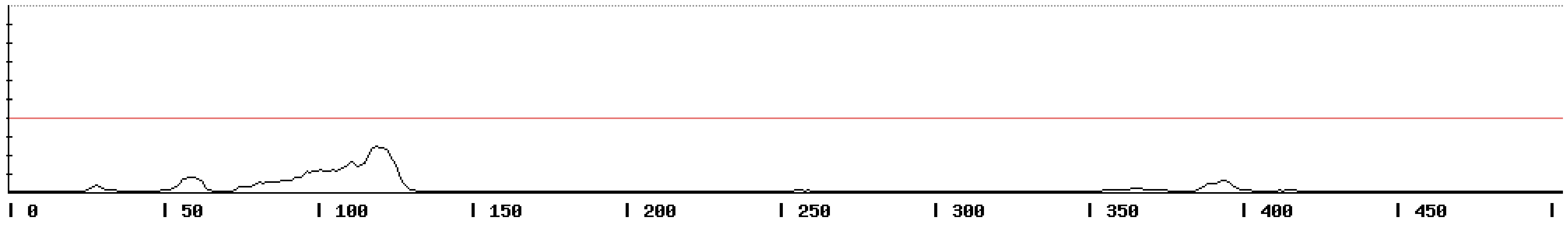

Predictions for Brassicarapa_XP_009125559.1

Viterbi Path

Viterbi Path

Posterior @ 0.4

Posterior @ 0.4

Posterior Graphic. Threshold Marked @ 0.4

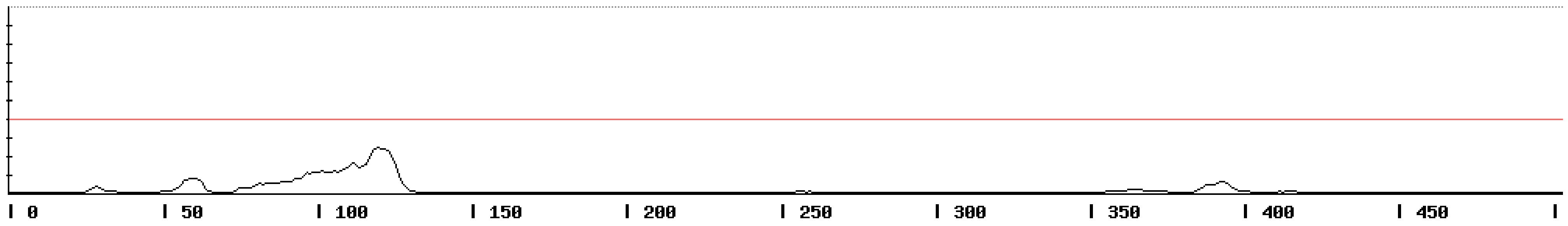

Predictions for Brassicarapa_XP_009125699.1

Viterbi Path

Viterbi Path

Posterior @ 0.4

Posterior @ 0.4

Posterior Graphic. Threshold Marked @ 0.4	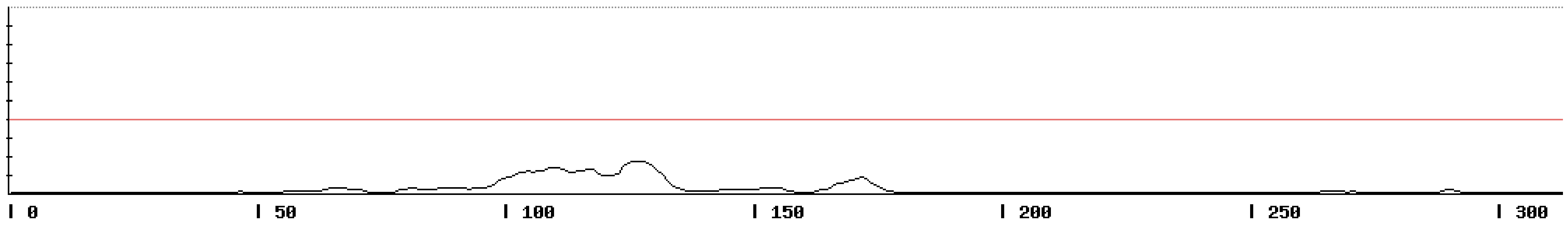

Predictions for Brassicarapa_XP_009125745.1	
Viterbi Path	
Viterbi Path	
Posterior @ 0.4	98 - GKPK - 101
Posterior @ 0.4	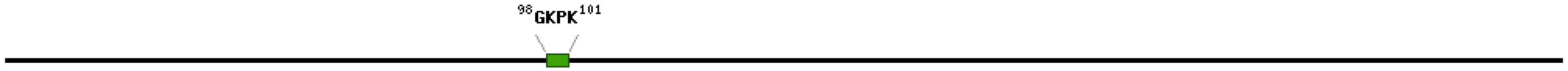
Posterior Graphic. Threshold Marked @ 0.4	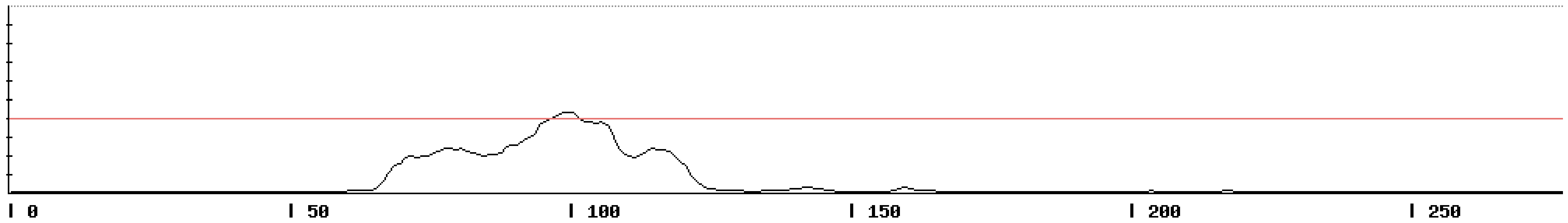

Predictions for Brassicarapa_XP_009125756.1	
Viterbi Path	
Viterbi Path	
Posterior @ 0.4	
Posterior @ 0.4	

Posterior Graphic. Threshold Marked @ 0.4

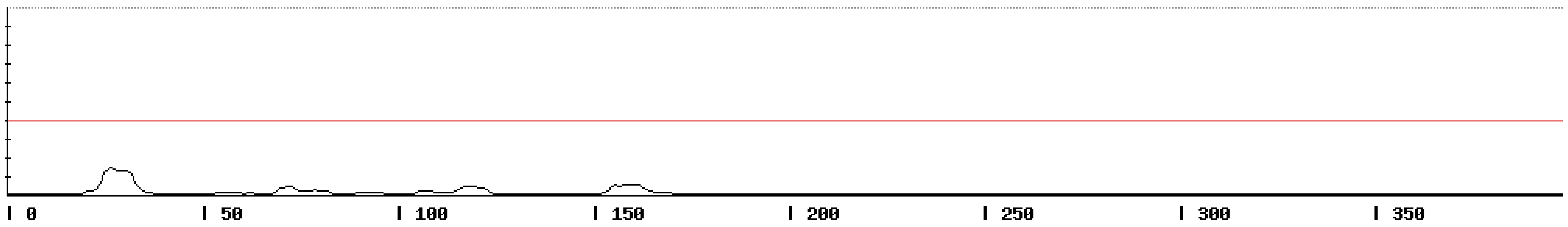

Predictions for Brassicarapa_XP_009125795.1

Viterbi Path

Viterbi Path

Posterior @ 0.4 | 105 - AKGNWRERGREKKIKRGG - 122

Posterior @ 0.4

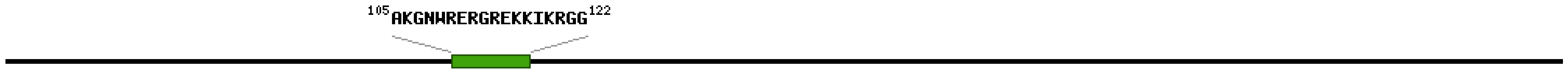

Posterior Graphic. Threshold Marked @ 0.4

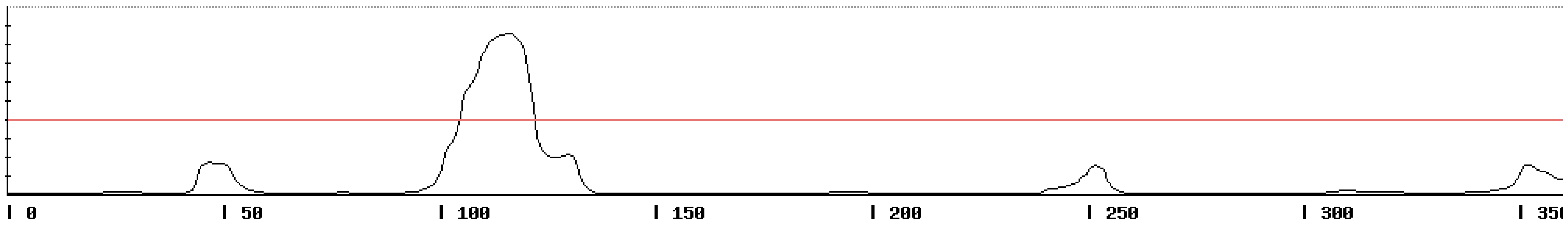

Predictions for Brassicarapa_XP_009125891.1

Viterbi Path

Viterbi Path

Posterior @ 0.4

Posterior @ 0.4

Posterior Graphic. Threshold Marked @ 0.4

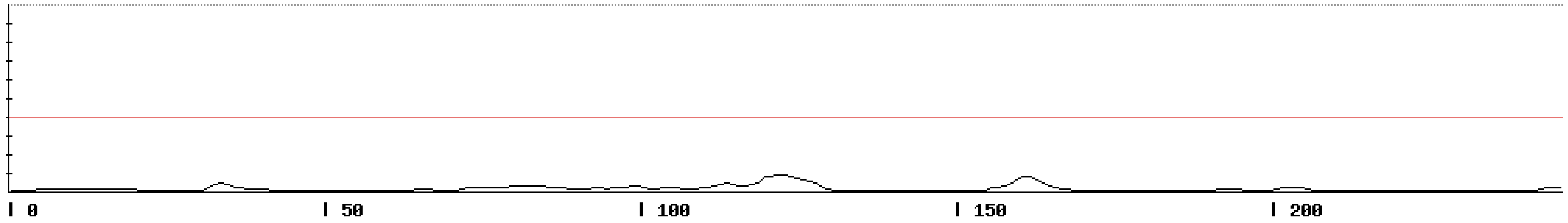

Predictions for Brassicarapa_XP_009126247.1

Viterbi Path

Viterbi Path

Posterior @ 0.4

Posterior @ 0.4

Posterior Graphic. Threshold Marked @ 0.4

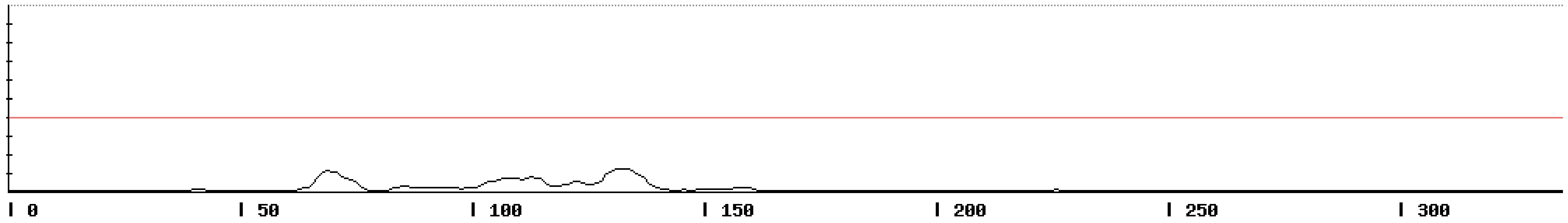

Predictions for Brassicarapa_XP_009126534.1

Viterbi Path

Viterbi Path

Posterior @ 0.4

Posterior @ 0.4

Posterior Graphic. Threshold Marked @ 0.4

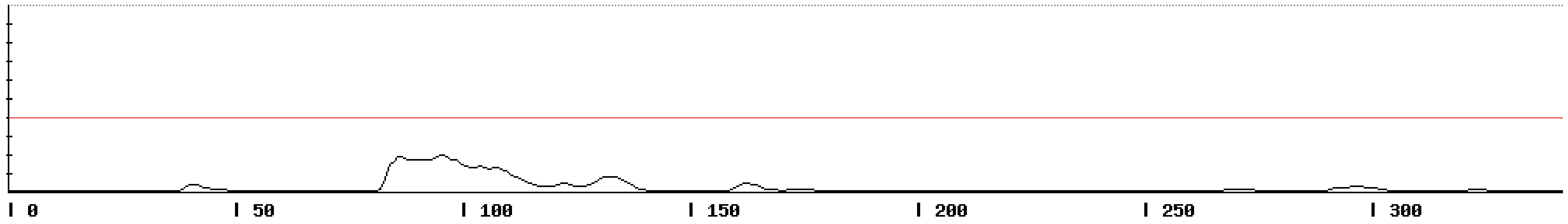

Predictions for Brassicarapa_XP_009126539.1

Viterbi Path

Viterbi Path

Posterior @ 0.4

Posterior @ 0.4

Posterior Graphic. Threshold Marked @ 0.4

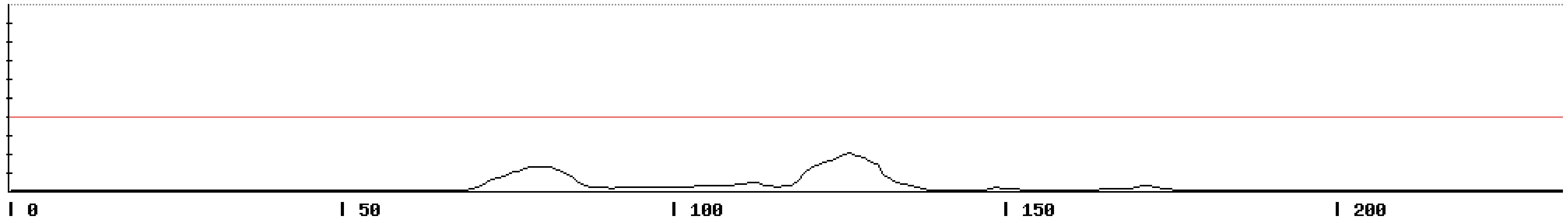

Predictions for Brassicarapa_XP_009126706.1

Viterbi Path

Viterbi Path

Posterior @ 0.4

103 - GRGRNSKRRIAGD - 115

119 - GSWKPNAKKYIEDEERKKTIIGKKQTLKFTKSDNNKRQK - 157

Posterior @ 0.4

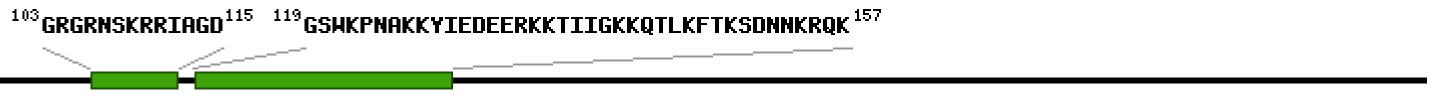

Posterior Graphic. Threshold Marked @ 0.4

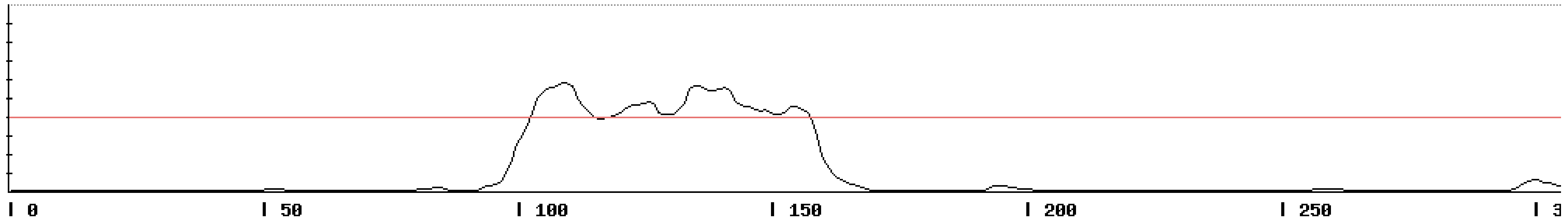

Predictions for Brassicarapa_XP_009126707.1

Viterbi Path

Viterbi Path

Posterior @ 0.4

103 - GRGRNSKRRIAGD - 115

119 - GSWKPNAKKYIEDEERKKTIIGKKQTLKFTKSDNNKRQK - 157

Posterior @ 0.4

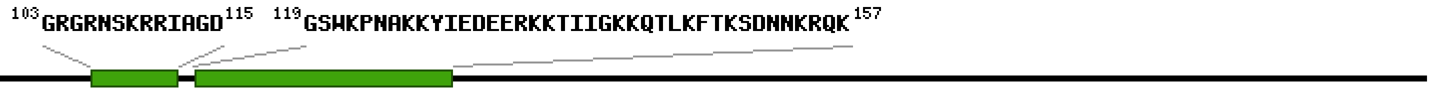

Posterior Graphic. Threshold Marked @ 0.4

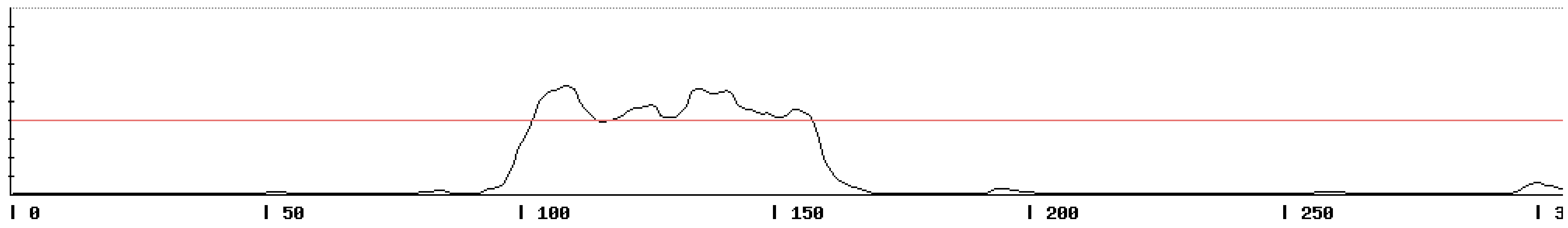

Predictions for Brassicarapa_XP_009127127.1

Viterbi Path

Viterbi Path

Posterior @ 0.4

Posterior @ 0.4

Posterior Graphic. Threshold Marked @ 0.4

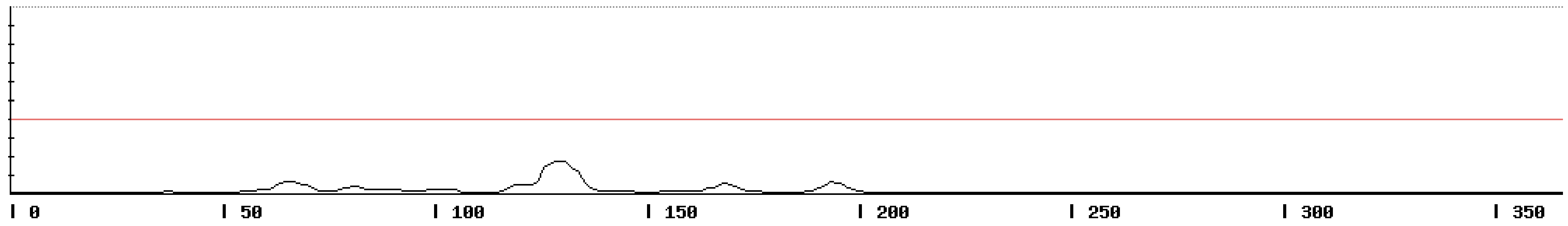

Predictions for Brassicarapa_XP_009127458.1

Viterbi Path

Viterbi Path

Posterior @ 0.4

Posterior @ 0.4

Posterior Graphic. Threshold Marked @ 0.4	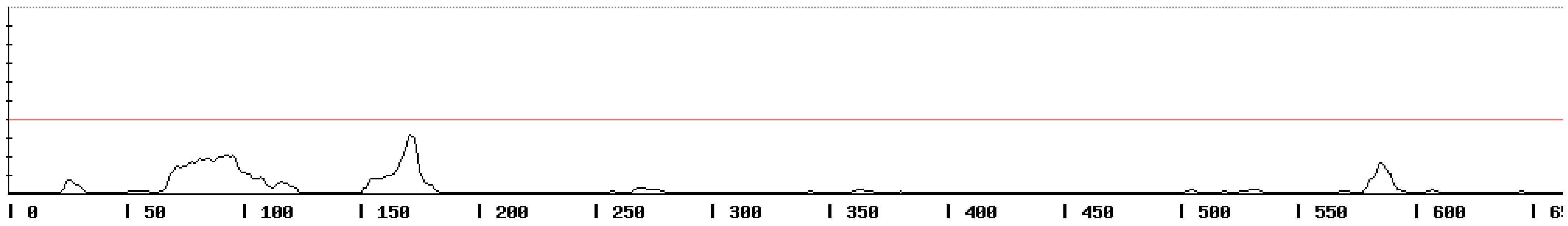

Predictions for Brassicarapa_XP_009127884.1	
Viterbi Path	
Viterbi Path	
Posterior @ 0.4	
Posterior @ 0.4	
Posterior Graphic. Threshold Marked @ 0.4	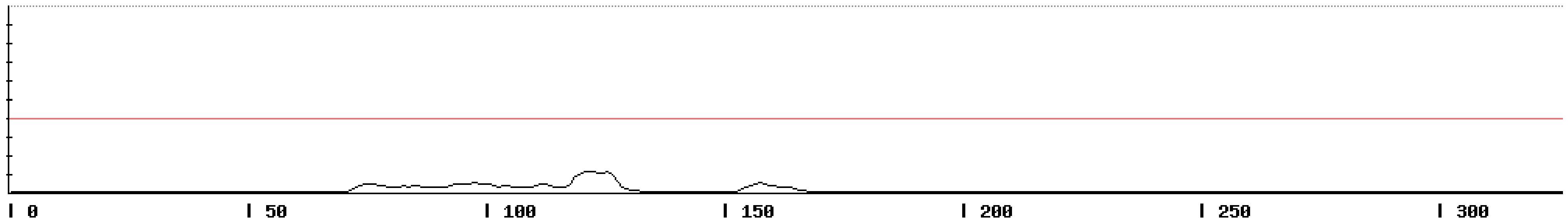

Predictions for Brassicarapa_XP_009127885.1	
Viterbi Path	
Viterbi Path	
Posterior @ 0.4	
Posterior @ 0.4	

Posterior Graphic. Threshold Marked @ 0.4

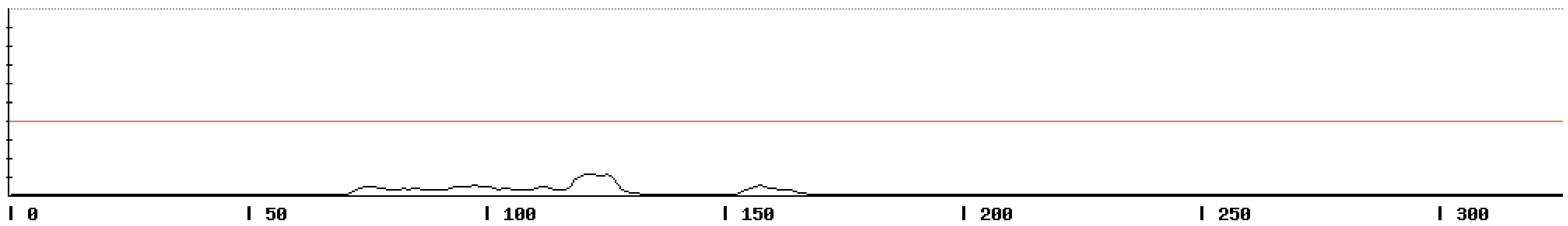

Predictions for Brassicarapa_XP_009128177.1

Viterbi Path

Viterbi Path

Posterior @ 0.4

Posterior @ 0.4

Posterior Graphic. Threshold Marked @ 0.4

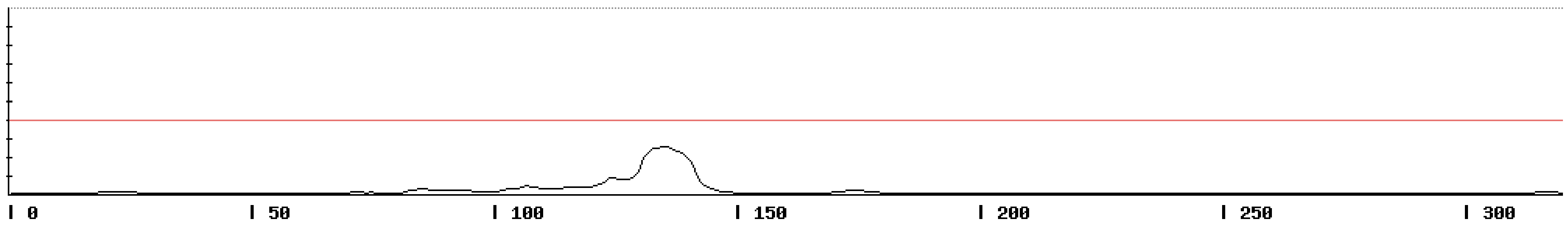

Predictions for Brassicarapa_XP_009128229.1

Viterbi Path

Viterbi Path

Posterior @ 0.4 | 98 - KPIGRPKPVGIKK - 110

Posterior @ 0.4

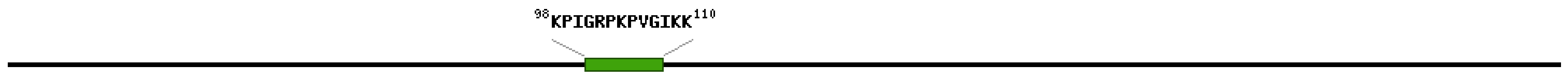

Posterior Graphic. Threshold Marked @ 0.4

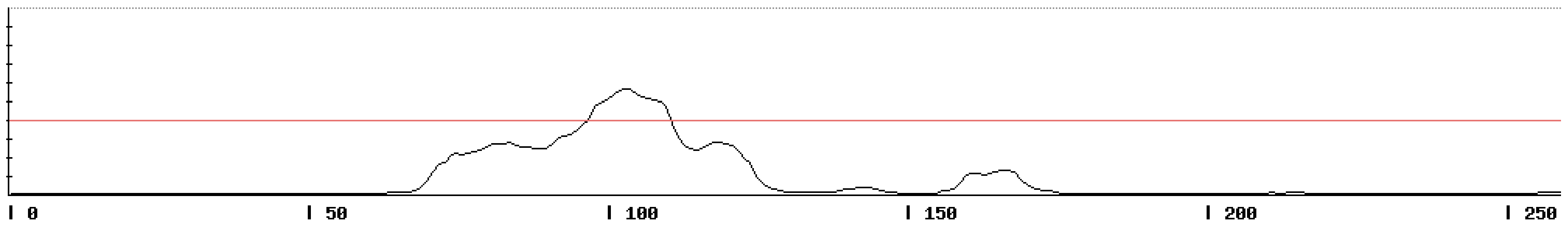

Predictions for Brassicarapa_XP_009128370.1

Viterbi Path

Viterbi Path

Posterior @ 0.4

Posterior @ 0.4

Posterior Graphic. Threshold Marked @ 0.4

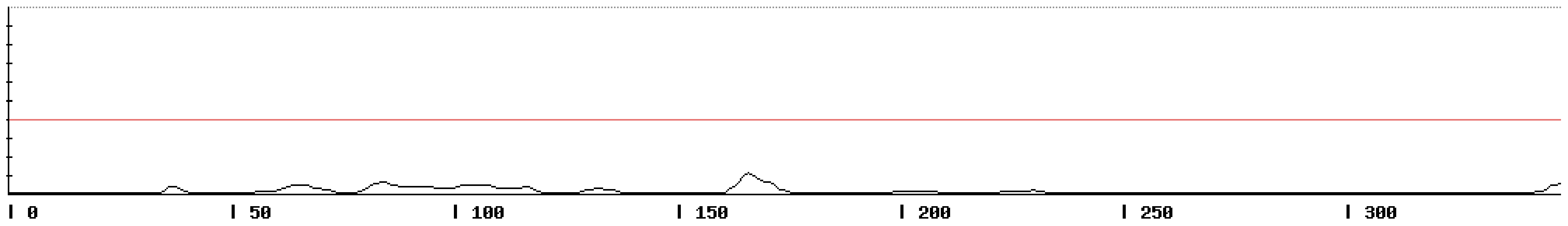

Predictions for Brassicarapa_XP_009128520.1

Viterbi Path

Viterbi Path

Posterior @ 0.4

Posterior @ 0.4

Posterior Graphic. Threshold Marked @ 0.4

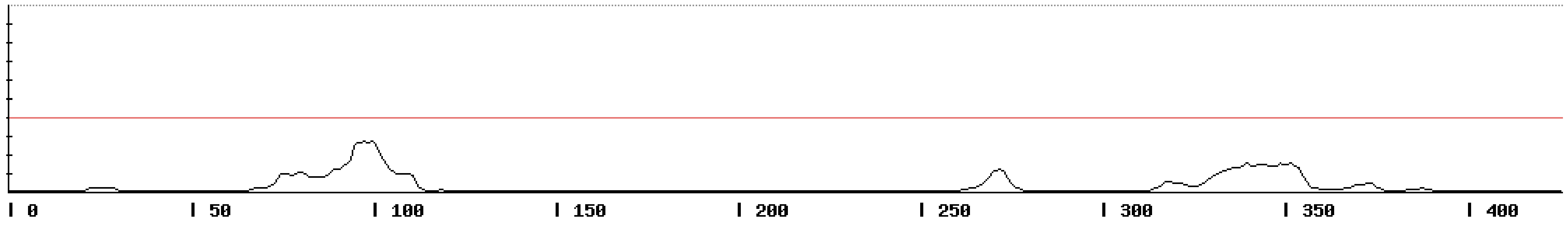

Predictions for Brassicarapa_XP_009128881.1

Viterbi Path

Viterbi Path

Posterior @ 0.4

Posterior @ 0.4

Posterior Graphic. Threshold Marked @ 0.4

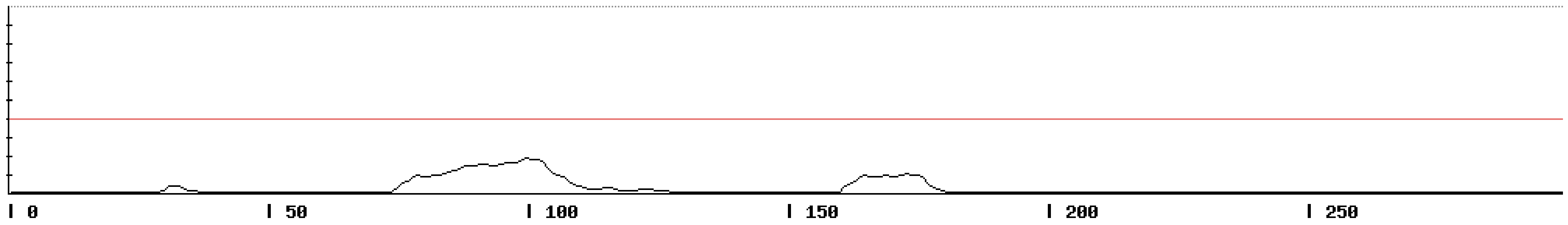

Predictions for Brassicarapa_XP_009128919.1

Viterbi Path

Viterbi Path

Posterior @ 0.4

Posterior @ 0.4

Posterior Graphic. Threshold Marked @ 0.4

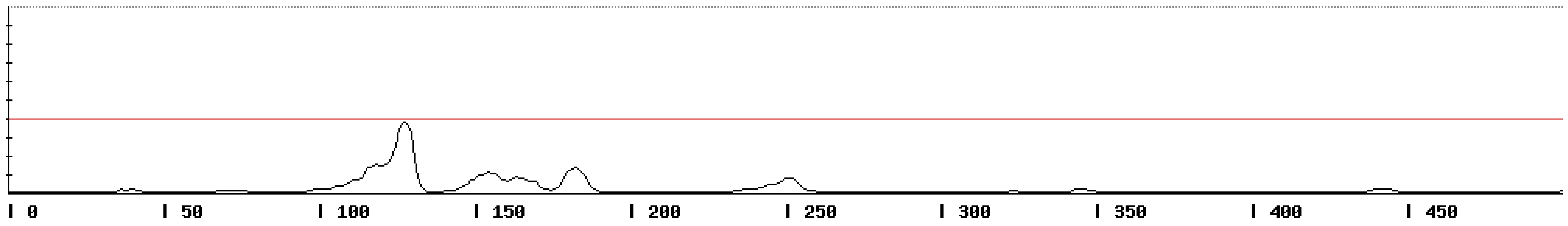

Predictions for Brassicarapa_XP_009128925.1

Viterbi Path

Viterbi Path

Posterior @ 0.4

Posterior @ 0.4

Posterior Graphic. Threshold Marked @ 0.4

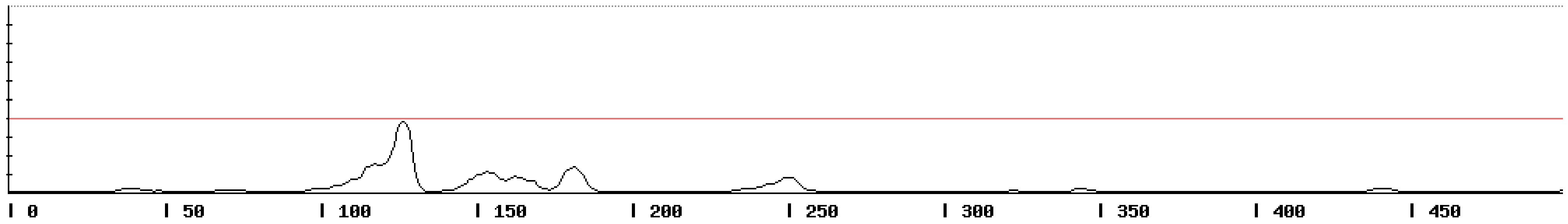

Predictions for Brassicarapa_XP_009128929.1	
Viterbi Path	
Viterbi Path	
Posterior @ 0.4	
Posterior @ 0.4	
Posterior Graphic. Threshold Marked @ 0.4	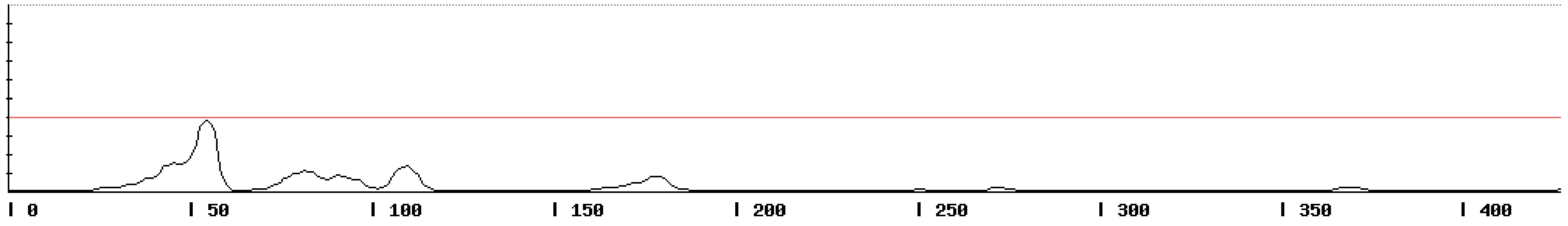

Predictions for Brassicarapa_XP_009129144.1	
Viterbi Path	
Viterbi Path	
Posterior @ 0.4	
Posterior @ 0.4	
Posterior Graphic. Threshold Marked @ 0.4	

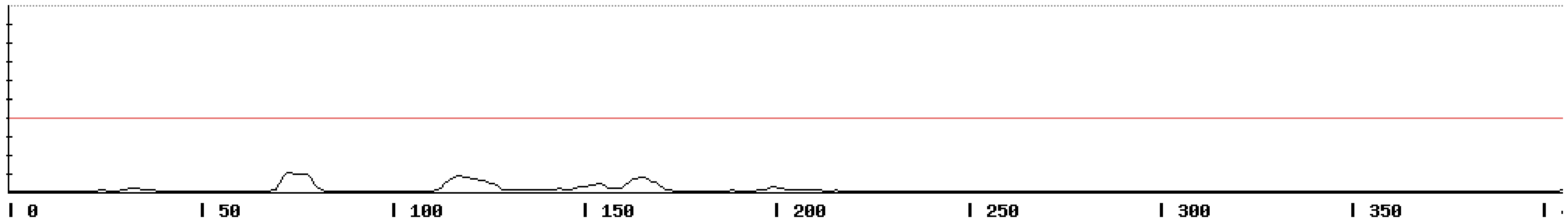

Predictions for Brassicarapa_XP_009129145.1

Viterbi Path	
Viterbi Path	
Posterior @ 0.4	
Posterior @ 0.4	
Posterior Graphic. Threshold Marked @ 0.4	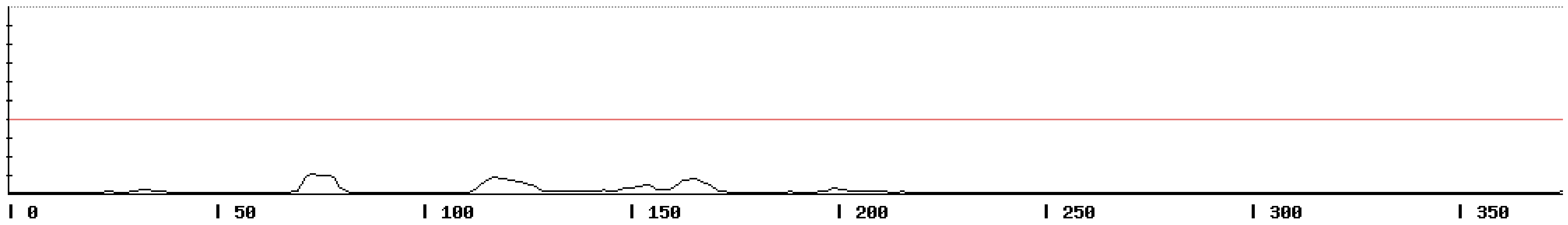

Predictions for Brassicarapa_XP_009129548.1

Viterbi Path	
Viterbi Path	
Posterior @ 0.4	
Posterior @ 0.4	

Posterior Graphic. Threshold Marked @ 0.4

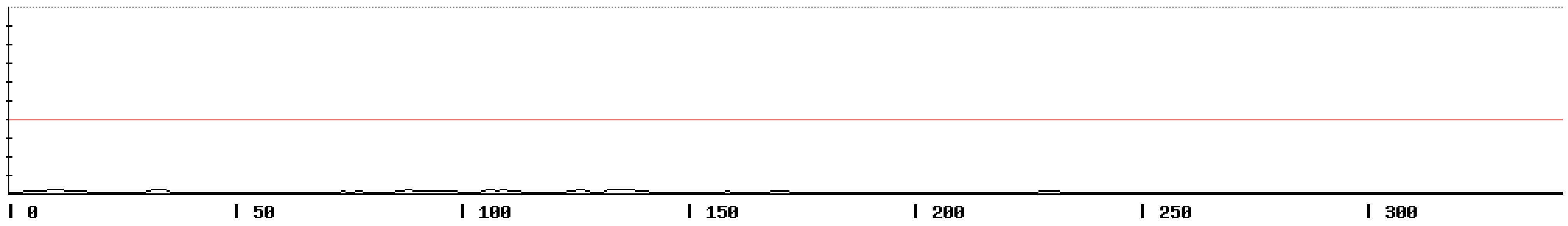

Predictions for Brassicarapa_XP_009129565.1

Viterbi Path

Viterbi Path

Posterior @ 0.4 — 137 - RKRRK - 141

Posterior @ 0.4

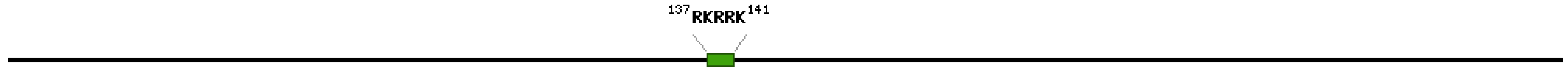

Posterior Graphic. Threshold Marked @ 0.4

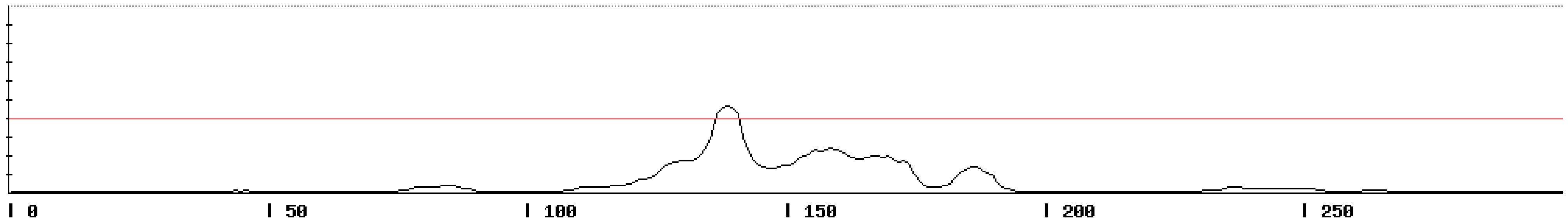

Predictions for Brassicarapa_XP_009130171.1

Viterbi Path

Viterbi Path

Posterior @ 0.4

Posterior @ 0.4

Posterior Graphic. Threshold Marked @ 0.4	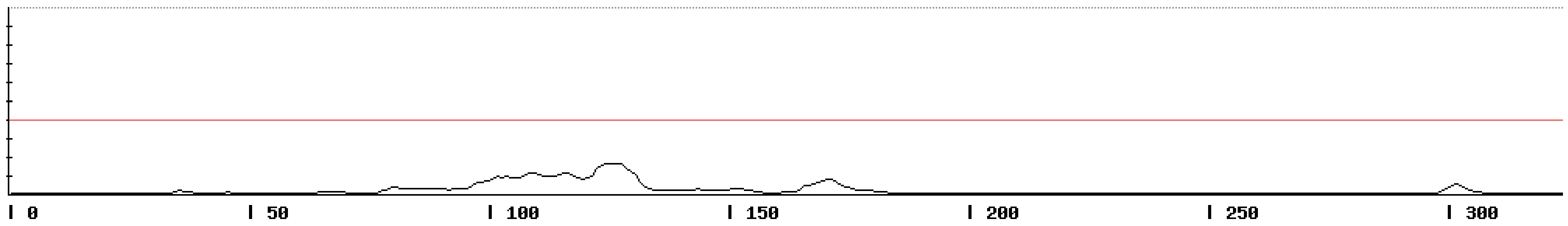

Predictions for Brassicarapa_XP_009130209.1

Viterbi Path	
Viterbi Path	
Posterior @ 0.4	
Posterior @ 0.4	
Posterior Graphic. Threshold Marked @ 0.4	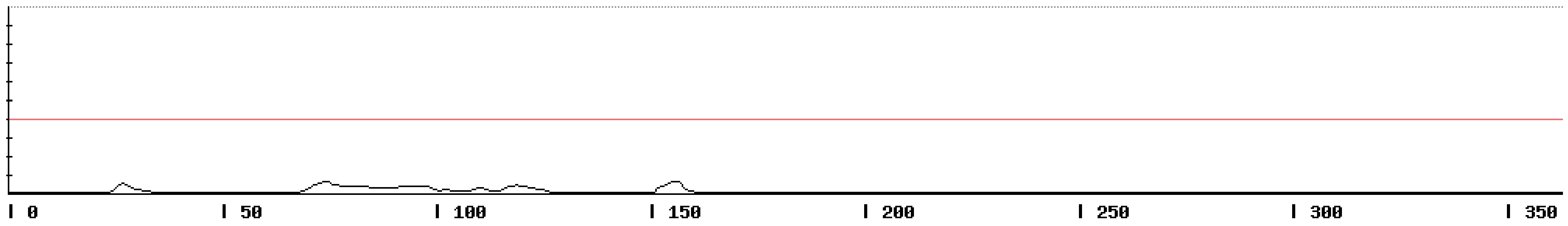

Predictions for Brassicarapa_XP_009130210.1

Viterbi Path	
Viterbi Path	

Posterior @ 0.4

Posterior @ 0.4

Posterior Graphic. Threshold Marked @ 0.4

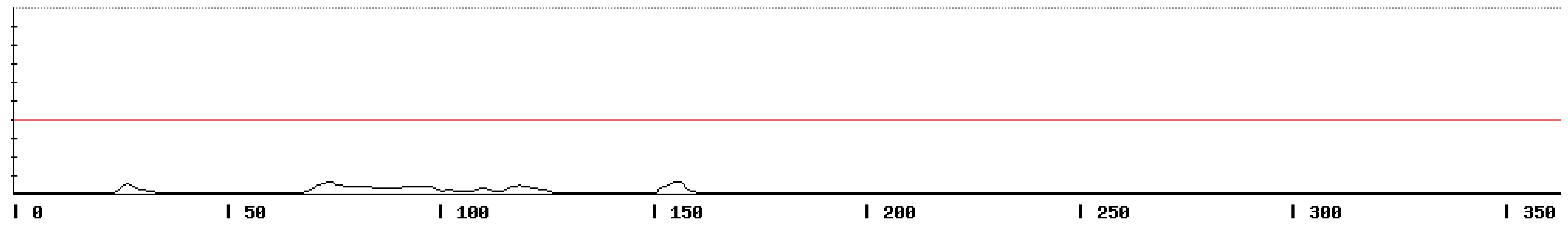

Predictions for Brassicarapa_XP_009130211.1

Viterbi Path

Viterbi Path

Posterior @ 0.4

Posterior @ 0.4

Posterior Graphic. Threshold Marked @ 0.4

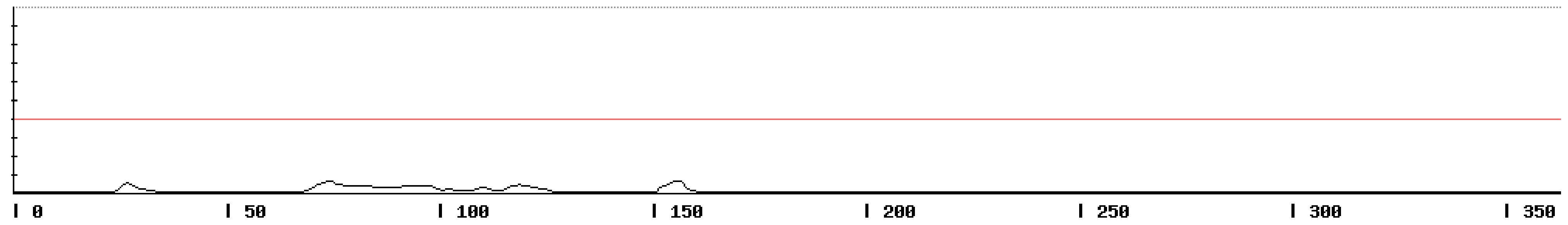

Predictions for Brassicarapa_XP_009130273.1

Viterbi Path

Viterbi Path

Posterior @ 0.4 | 99 - KP - 100

Posterior @ 0.4

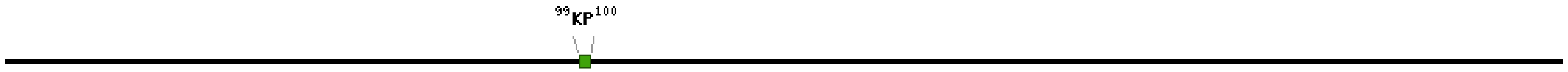

Posterior Graphic. Threshold Marked @ 0.4

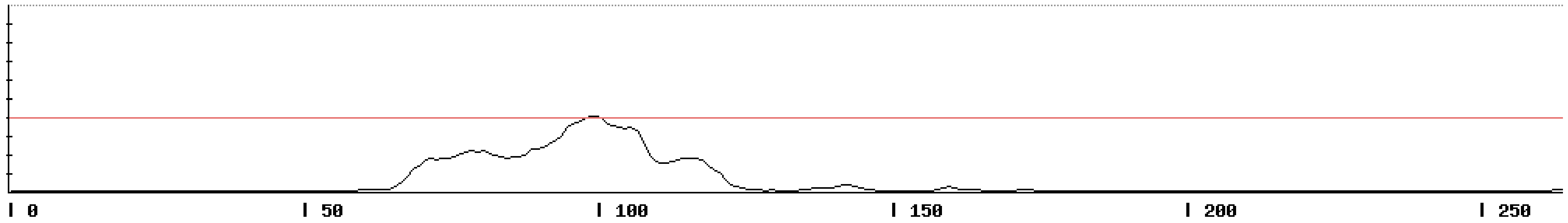

Predictions for Brassicarapa_XP_009130343.1

Viterbi Path

Viterbi Path

Posterior @ 0.4

Posterior @ 0.4

Posterior Graphic. Threshold Marked @ 0.4

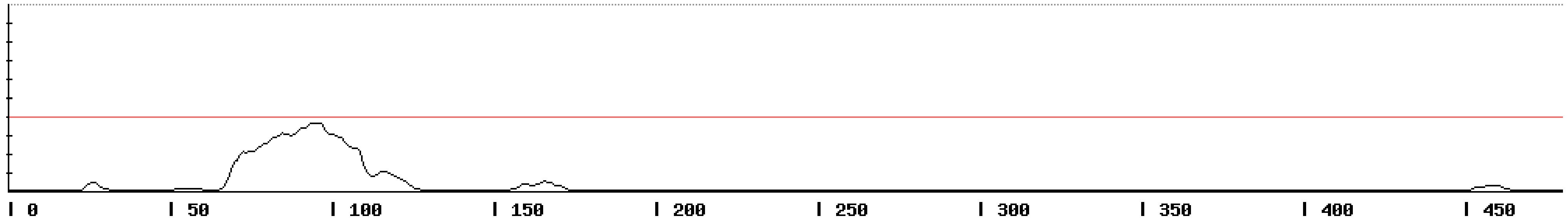

Predictions for Brassicarapa_XP_009130344.1

Viterbi Path

Viterbi Path

Posterior @ 0.4

Posterior @ 0.4

Posterior Graphic. Threshold Marked @ 0.4

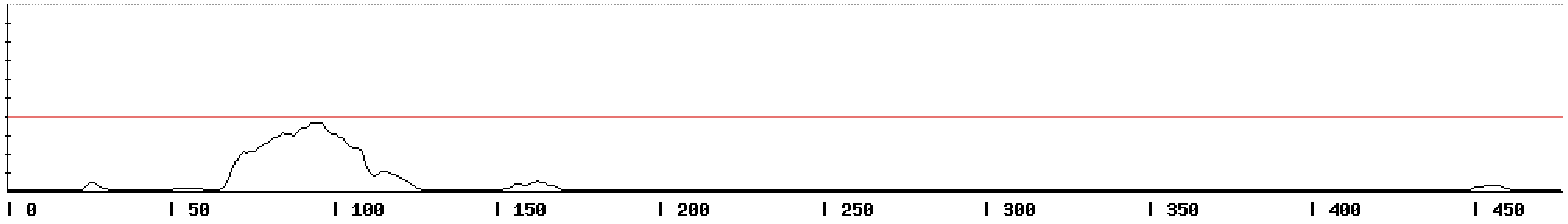

Predictions for Brassicarapa_XP_009130418.1

Viterbi Path

Viterbi Path

Posterior @ 0.4

Posterior @ 0.4

Posterior Graphic. Threshold Marked @ 0.4

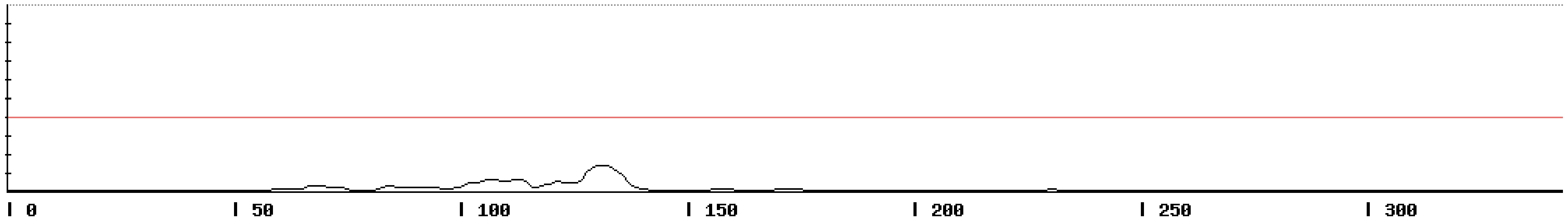

Predictions for Brassicarapa_XP_009130419.1	
Viterbi Path	
Viterbi Path	
Posterior @ 0.4	
Posterior @ 0.4	
Posterior Graphic. Threshold Marked @ 0.4	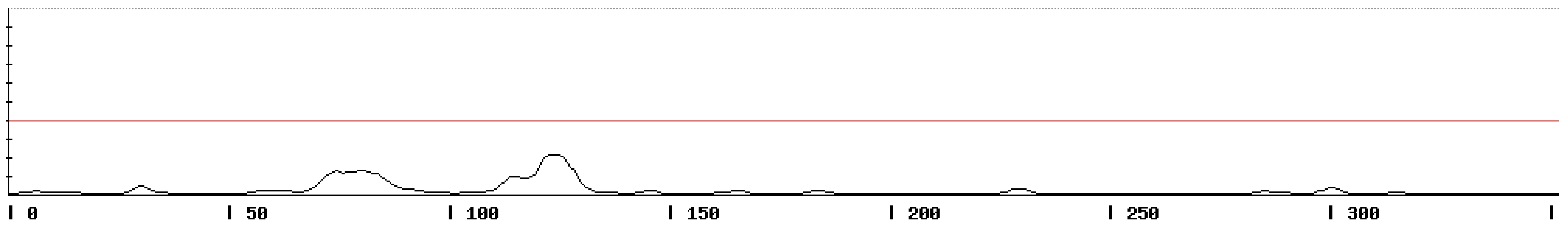

Predictions for Brassicarapa_XP_009130458.1	
Viterbi Path	
Viterbi Path	
Posterior @ 0.4	
Posterior @ 0.4	
Posterior Graphic. Threshold Marked @ 0.4	

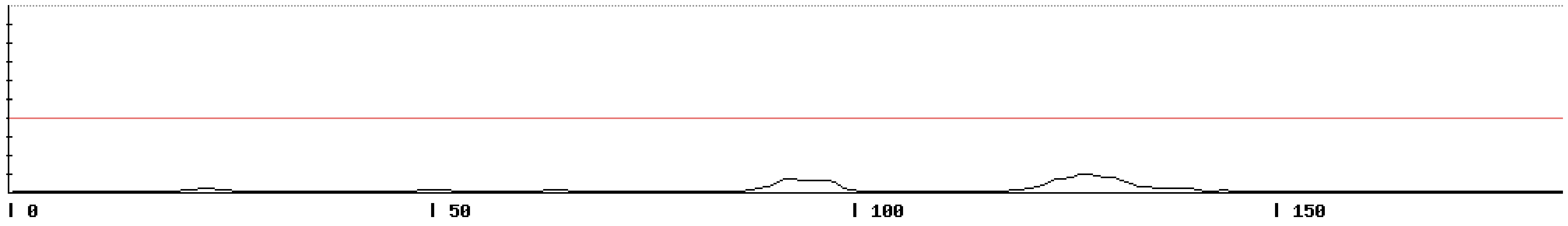

Predictions for Brassicarapa_XP_009130821.1

Viterbi Path

Viterbi Path

Posterior @ 0.4

Posterior @ 0.4

Posterior Graphic. Threshold Marked @ 0.4

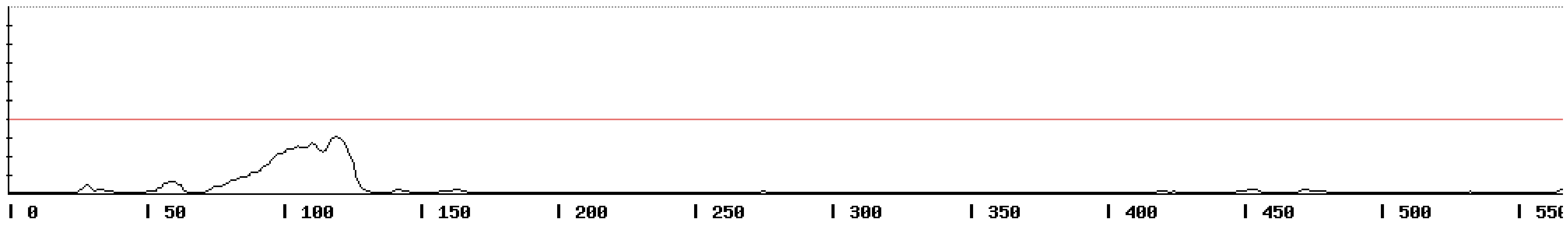

Predictions for Brassicarapa_XP_009130822.1

Viterbi Path

Viterbi Path

Posterior @ 0.4

Posterior @ 0.4

Posterior Graphic. Threshold Marked @ 0.4

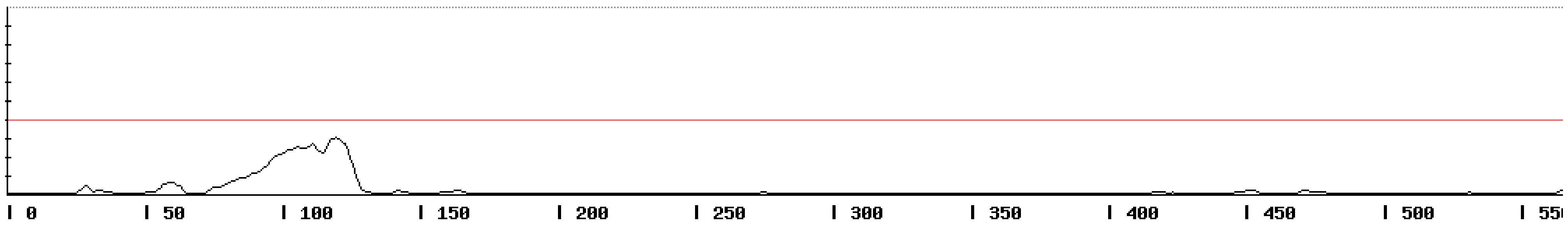

Predictions for Brassicarapa_XP_009131103.1

Viterbi Path

Viterbi Path

Posterior @ 0.4 — 98 - GKPK - 101

Posterior @ 0.4

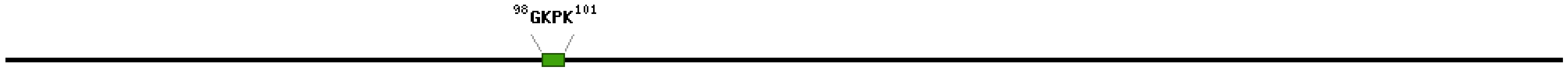

Posterior Graphic. Threshold Marked @ 0.4

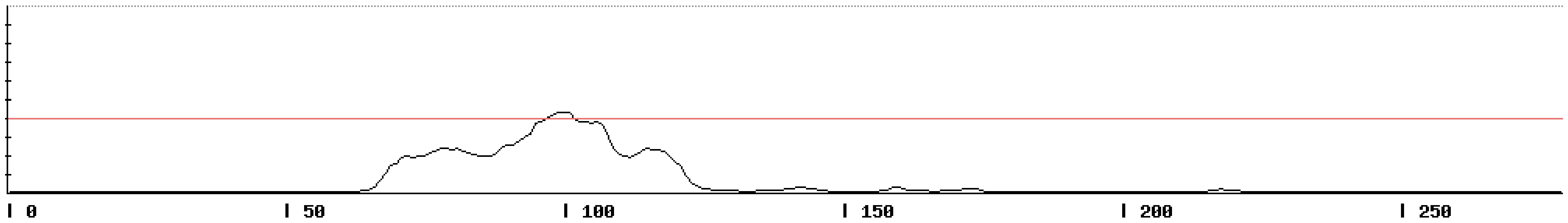

Predictions for Brassicarapa_XP_009131217.1

Viterbi Path

Viterbi Path

Posterior @ 0.4

Posterior @ 0.4

Posterior Graphic. Threshold Marked @ 0.4	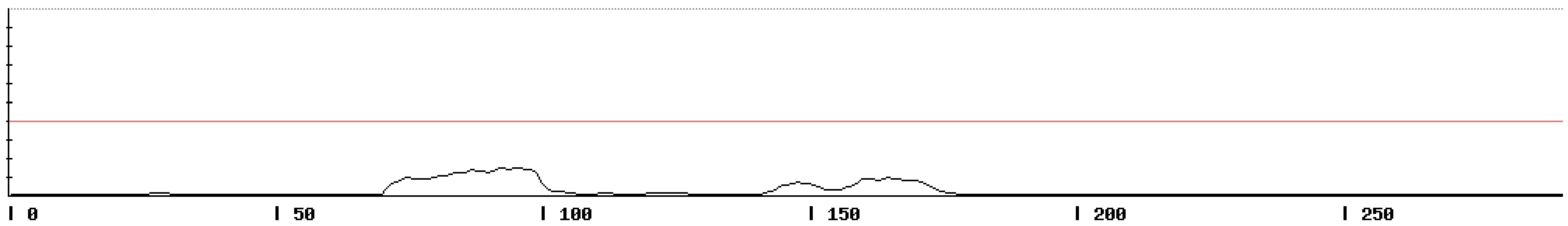

Predictions for Brassicarapa_XP_009131224.1

Viterbi Path	
Viterbi Path	
Posterior @ 0.4	
Posterior @ 0.4	
Posterior Graphic. Threshold Marked @ 0.4	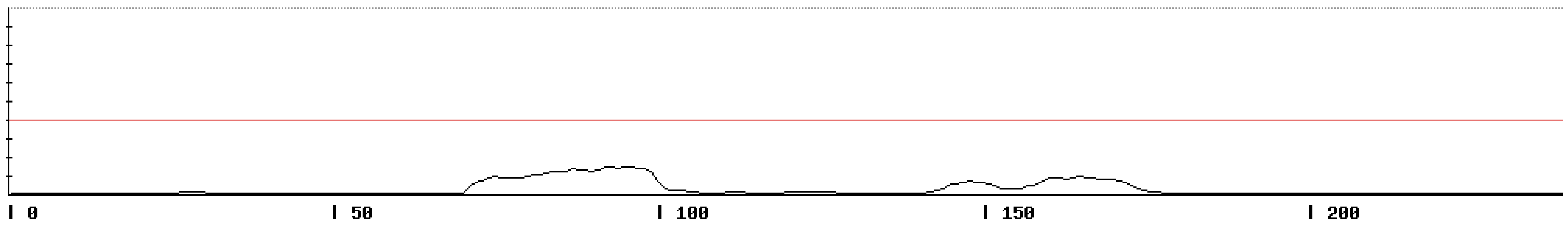

Predictions for Brassicarapa_XP_009131323.1

Viterbi Path	
Viterbi Path	

Posterior @ 0.4	
Posterior @ 0.4	
Posterior Graphic. Threshold Marked @ 0.4	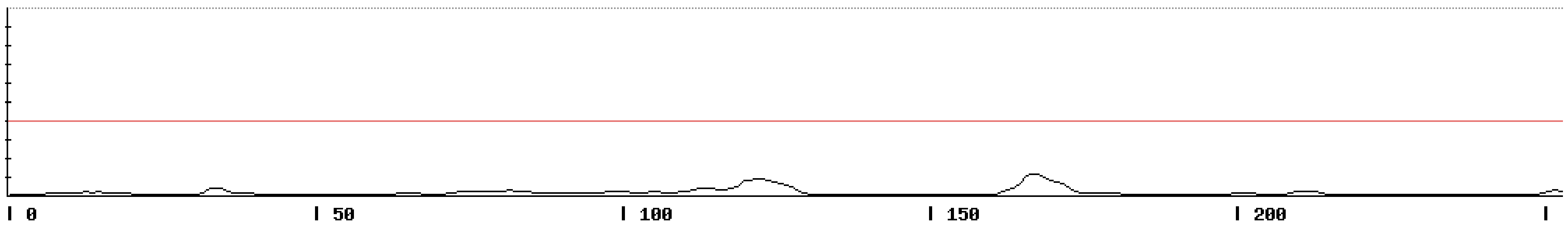

Predictions for Brassicarapa_XP_009131324.1	
Viterbi Path	
Viterbi Path	
Posterior @ 0.4	
Posterior @ 0.4	
Posterior Graphic. Threshold Marked @ 0.4	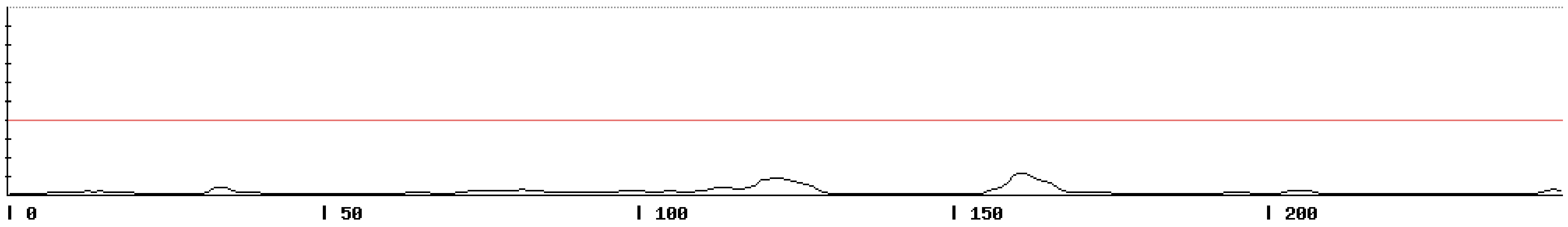

Predictions for Brassicarapa_XP_009131374.1	
Viterbi Path	160 - KRKEPKGRRQRKPRVR - 175
Viterbi Path	

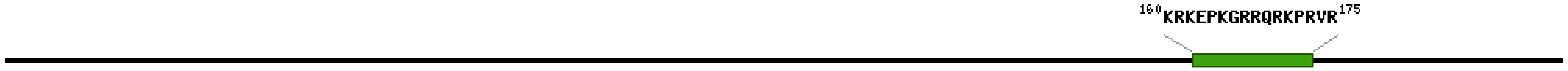

Posterior @ 0.4 | 159 - YKRKEPKGRRQRKPRVR - 175

Posterior @ 0.4

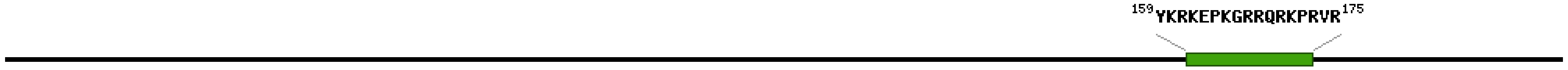

Posterior Graphic. Threshold Marked @ 0.4

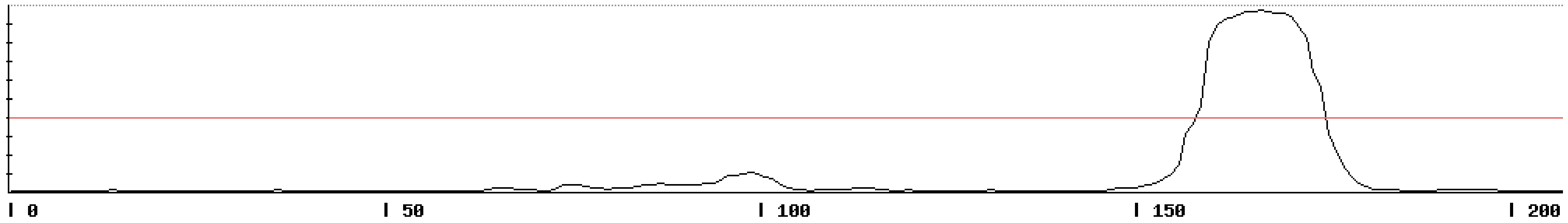

Predictions for Brassicarapa_XP_009131585.1

Viterbi Path

Viterbi Path

Posterior @ 0.4

Posterior @ 0.4

Posterior Graphic. Threshold Marked @ 0.4

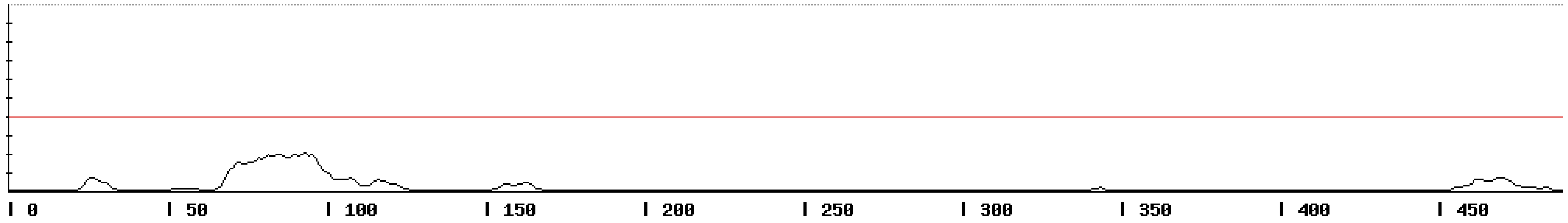

Predictions for Brassicarapa_XP_009131648.1

Viterbi Path

Viterbi Path

Posterior @ 0.4

Posterior @ 0.4

Posterior Graphic. Threshold Marked @ 0.4

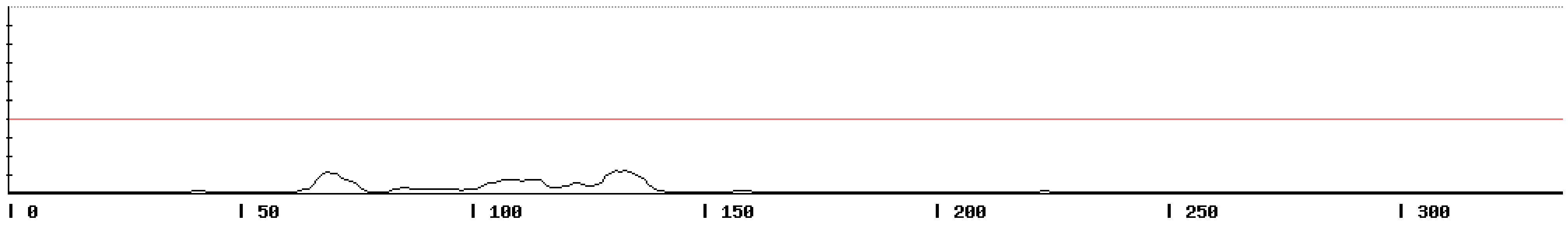

Predictions for Brassicarapa_XP_009131870.1

Viterbi Path

Viterbi Path

Posterior @ 0.4

Posterior @ 0.4

Posterior Graphic. Threshold Marked @ 0.4

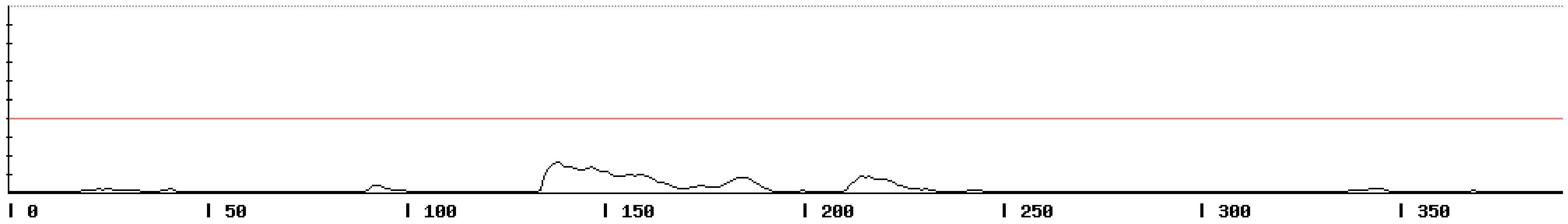

Predictions for Brassicarapa_XP_009131877.1

Viterbi Path	
Viterbi Path	
Posterior @ 0.4	
Posterior @ 0.4	
Posterior Graphic. Threshold Marked @ 0.4	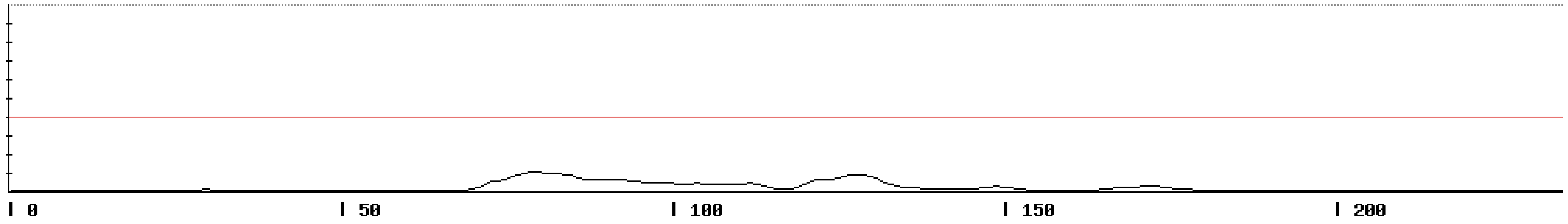

Predictions for Brassicarapa_XP_009132241.1

Viterbi Path	
Viterbi Path	
Posterior @ 0.4	8 - LVYKKKKKPWK - 18
	195 - AKKGLKPEK - 203
Posterior @ 0.4	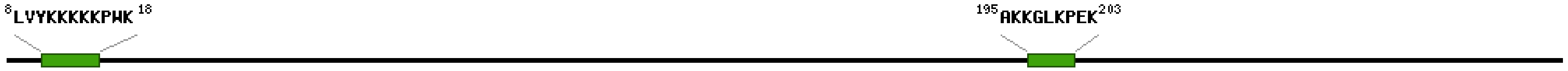
Posterior Graphic. Threshold Marked @ 0.4	

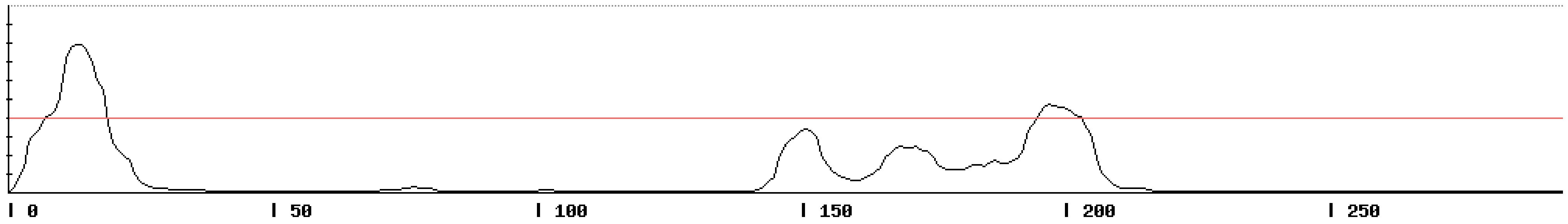

Predictions for Brassicarapa_XP_009133561.1	
Viterbi Path	
Viterbi Path	
Posterior @ 0.4	
Posterior @ 0.4	
Posterior Graphic. Threshold Marked @ 0.4	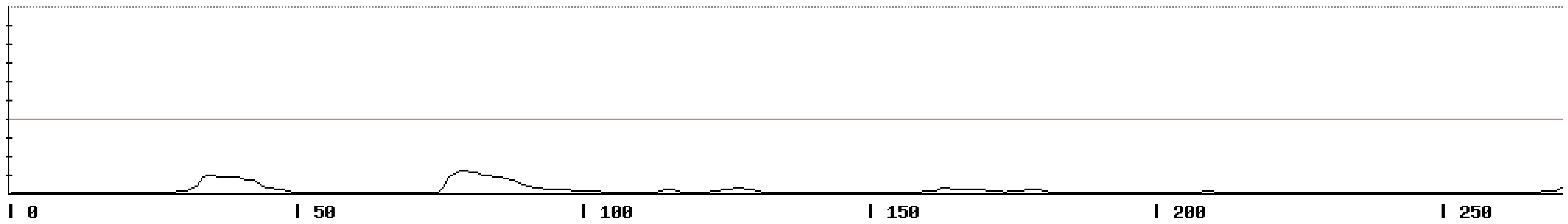

Predictions for Brassicarapa_XP_009134191.1	
Viterbi Path	
Viterbi Path	
Posterior @ 0.4	
Posterior @ 0.4	

Posterior Graphic. Threshold Marked @ 0.4

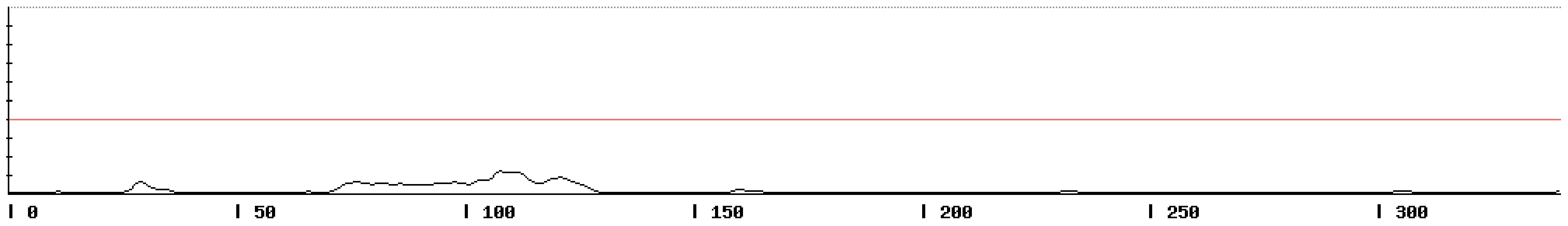

Predictions for Brassicarapa_XP_009134601.1

Viterbi Path

Viterbi Path

Posterior @ 0.4

180 - RKGSKPE - 186

Posterior @ 0.4

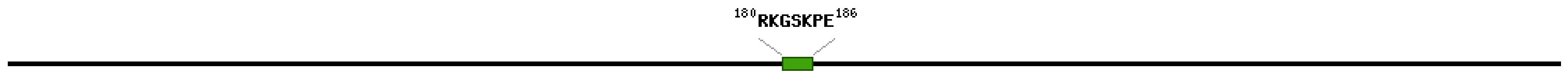

Posterior Graphic. Threshold Marked @ 0.4

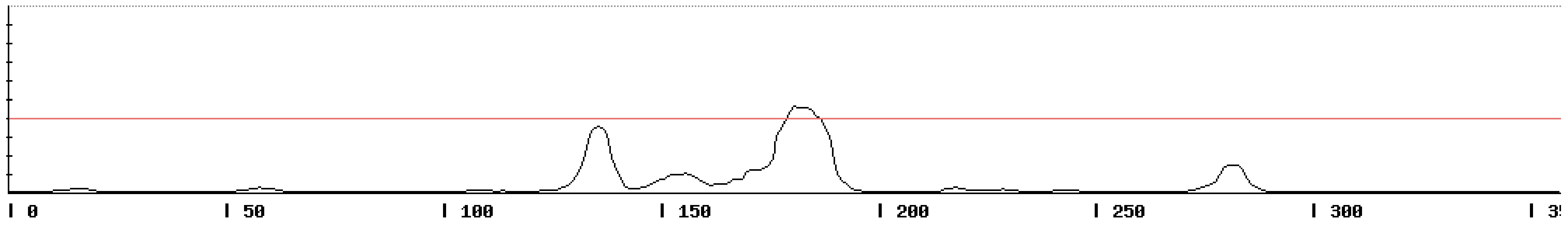

Predictions for Brassicarapa_XP_009135111.1

Viterbi Path

Viterbi Path

Posterior @ 0.4

Posterior @ 0.4

Posterior Graphic. Threshold Marked @ 0.4

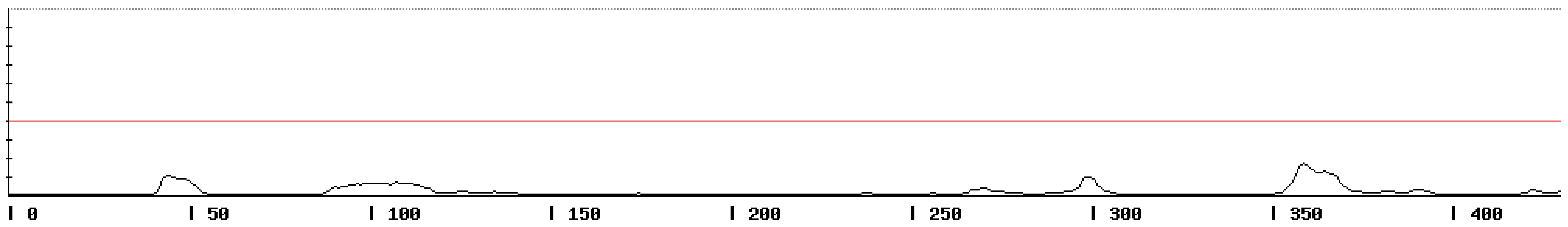

Predictions for Brassicarapa_XP_009135112.1

Viterbi Path

Viterbi Path

Posterior @ 0.4

Posterior @ 0.4

Posterior Graphic. Threshold Marked @ 0.4

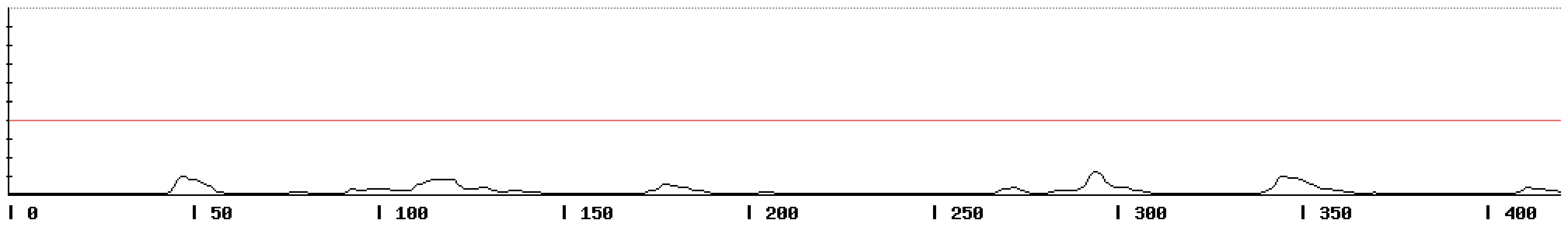

Predictions for Brassicarapa_XP_009135113.1

Viterbi Path

Viterbi Path

Posterior @ 0.4

Posterior @ 0.4

Posterior Graphic. Threshold Marked @ 0.4

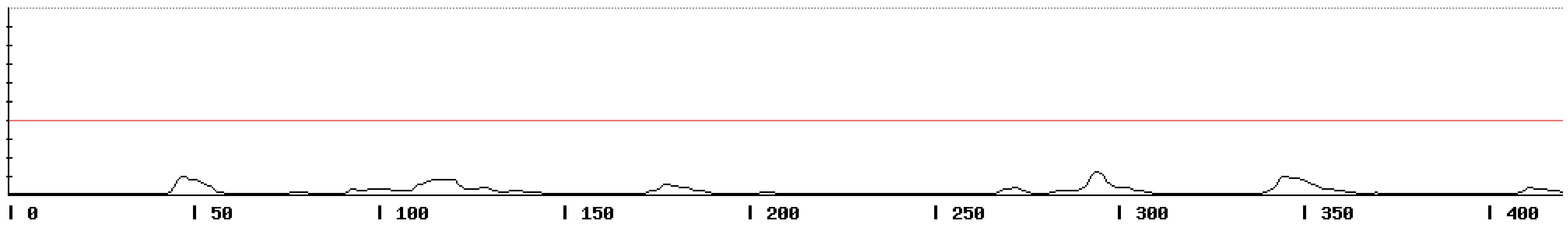

Predictions for Brassicarapa_XP_009135261.1

Viterbi Path

Viterbi Path

Posterior @ 0.4

Posterior @ 0.4

Posterior Graphic. Threshold Marked @ 0.4

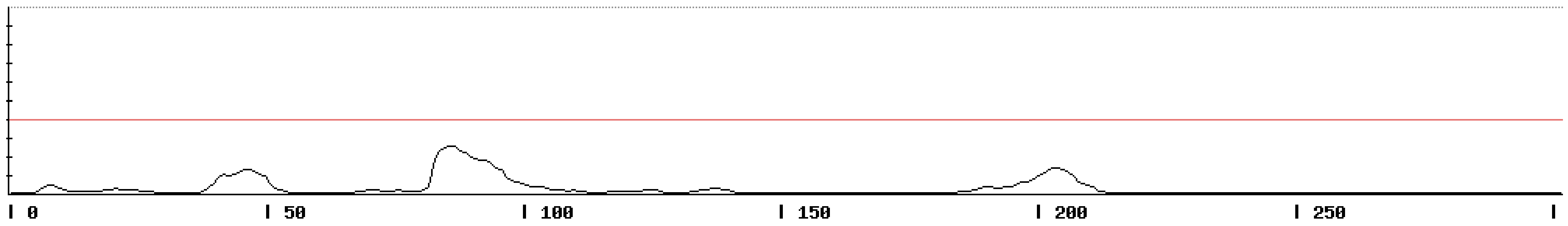

Predictions for Brassicarapa_XP_009135268.1

Viterbi Path

Viterbi Path

Posterior @ 0.4

Posterior @ 0.4

Posterior Graphic. Threshold Marked @ 0.4

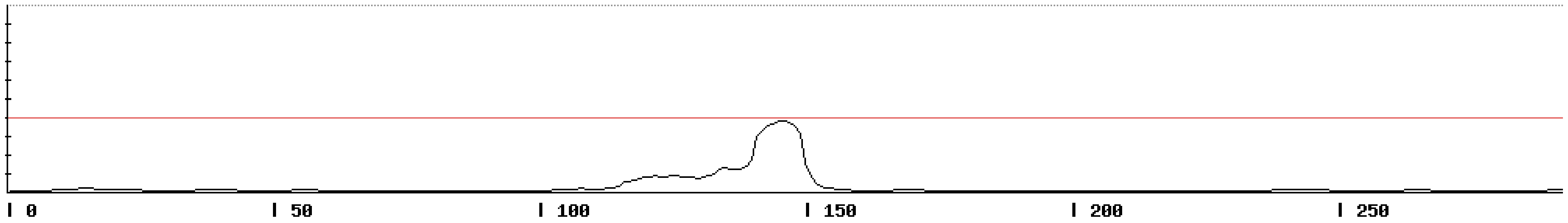

Predictions for Brassicarapa_XP_009135429.1

Viterbi Path

Viterbi Path

Posterior @ 0.4

Posterior @ 0.4

Posterior Graphic. Threshold Marked @ 0.4

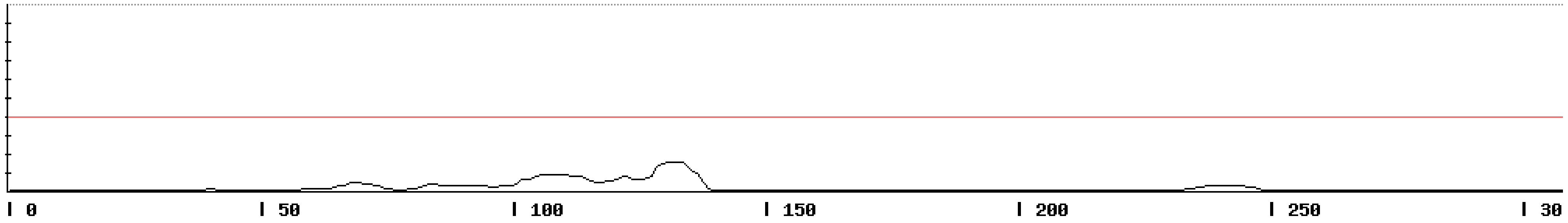

Predictions for Brassicarapa_XP_009135445.1

Viterbi Path	
Viterbi Path	
Posterior @ 0.4	
Posterior @ 0.4	
Posterior Graphic. Threshold Marked @ 0.4	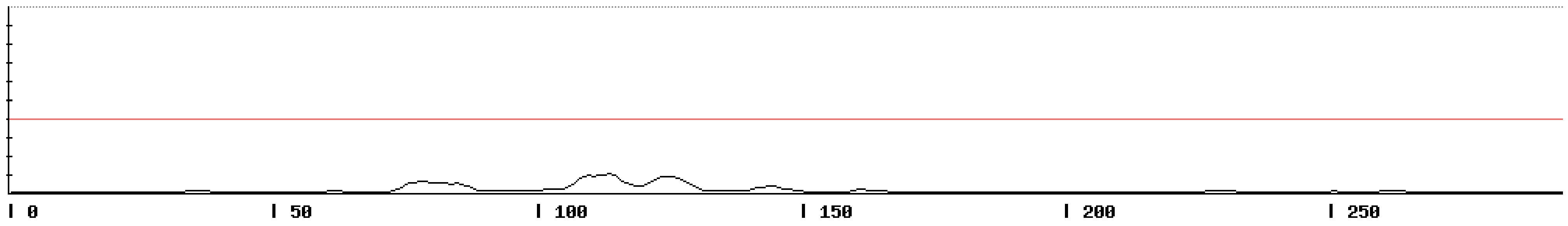

Predictions for Brassicarapa_XP_009135446.1	
Viterbi Path	
Viterbi Path	
Posterior @ 0.4	
Posterior @ 0.4	
Posterior Graphic. Threshold Marked @ 0.4	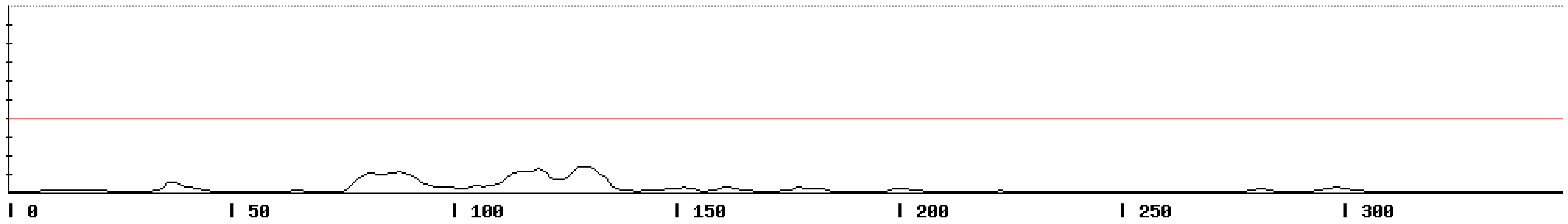

Predictions for Brassicarapa_XP_009135577.1

Viterbi Path	
Viterbi Path	
Posterior @ 0.4	
Posterior @ 0.4	
Posterior Graphic. Threshold Marked @ 0.4	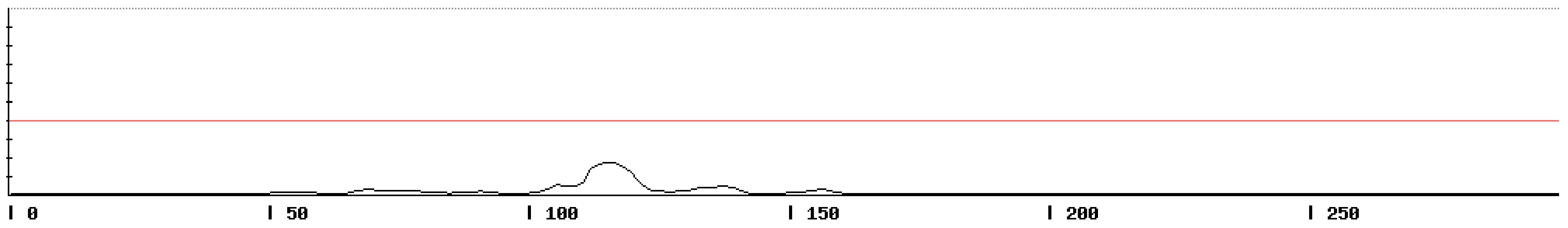

Predictions for Brassicarapa_XP_009136365.1

Viterbi Path	
Viterbi Path	
Posterior @ 0.4	
Posterior @ 0.4	
Posterior Graphic. Threshold Marked @ 0.4	

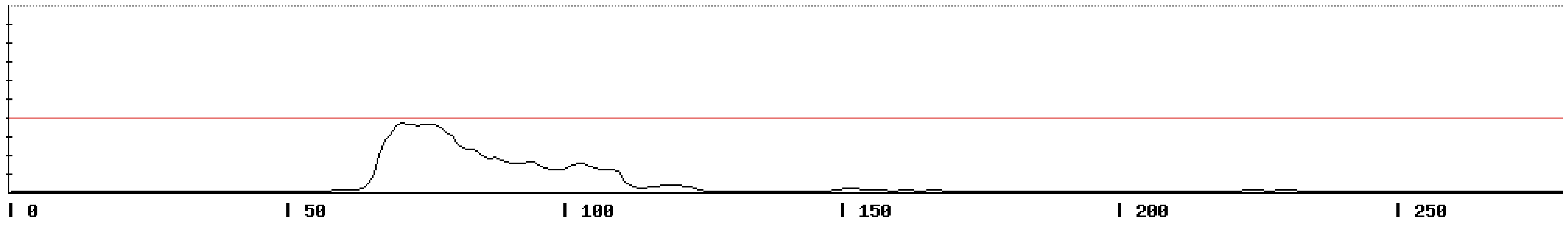

Predictions for Brassicarapa_XP_009136471.1

Viterbi Path

Viterbi Path

Posterior @ 0.4

Posterior @ 0.4

Posterior Graphic. Threshold Marked @ 0.4

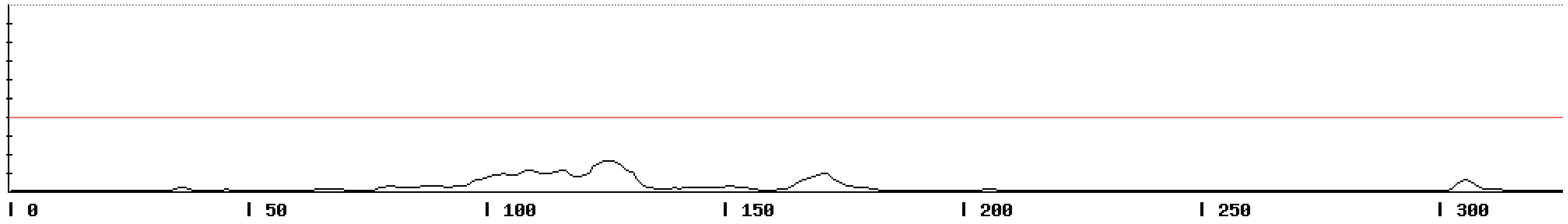

Predictions for Brassicarapa_XP_009136888.1

Viterbi Path

Viterbi Path

Posterior @ 0.4

Posterior @ 0.4

Posterior Graphic. Threshold Marked @ 0.4

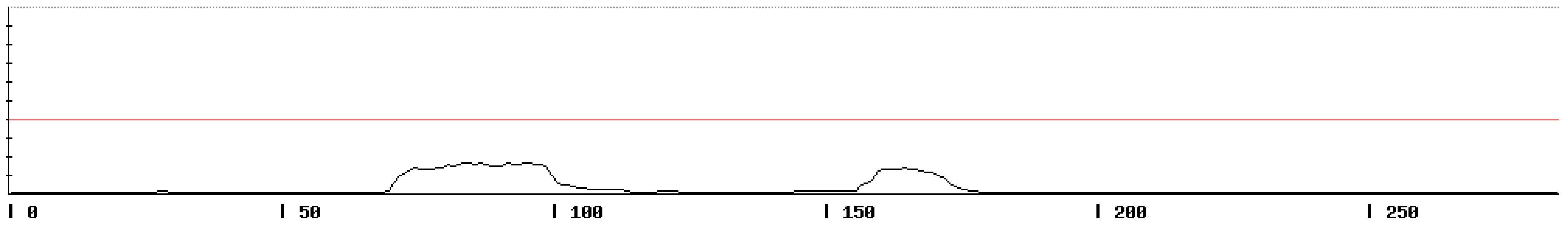

Predictions for Brassicarapa_XP_009137656.1

Viterbi Path

Viterbi Path

Posterior @ 0.4

Posterior @ 0.4

Posterior Graphic. Threshold Marked @ 0.4

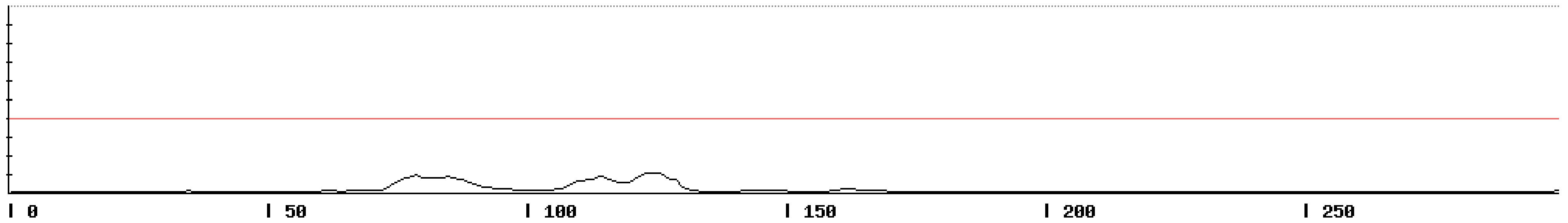

Predictions for Brassicarapa_XP_009137790.1

Viterbi Path

Viterbi Path

Posterior @ 0.4

Posterior @ 0.4

Posterior Graphic. Threshold Marked @ 0.4

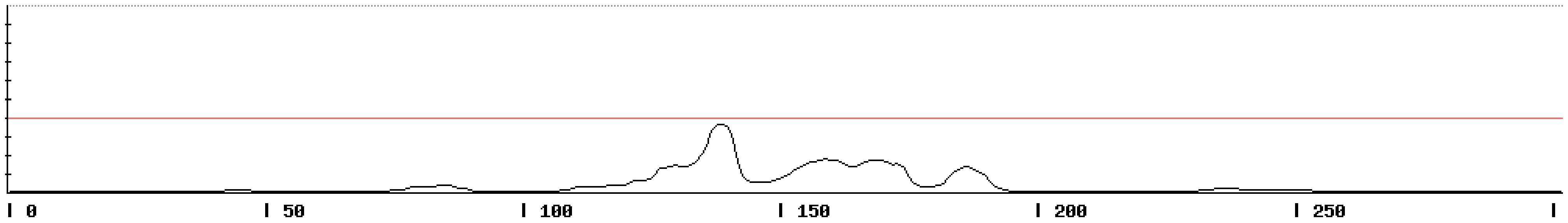

Predictions for Brassicarapa_XP_009137795.1

Viterbi Path

Viterbi Path

Posterior @ 0.4

Posterior @ 0.4

Posterior Graphic. Threshold Marked @ 0.4

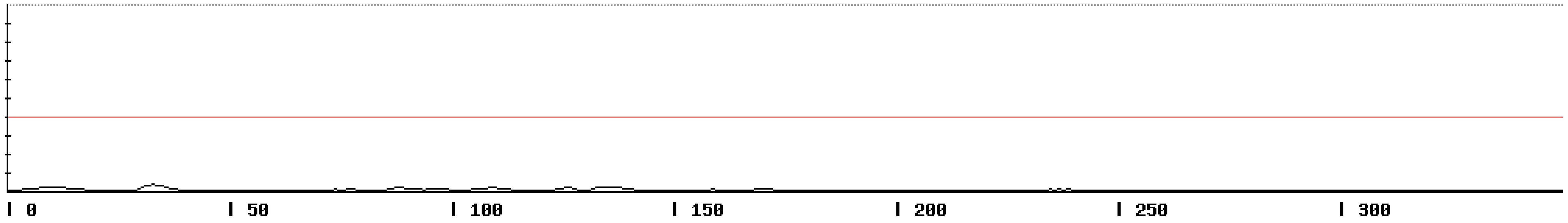

Predictions for Brassicarapa_XP_009137840.1

Viterbi Path

Viterbi Path

Posterior @ 0.4

Posterior @ 0.4

Posterior Graphic. Threshold Marked @ 0.4

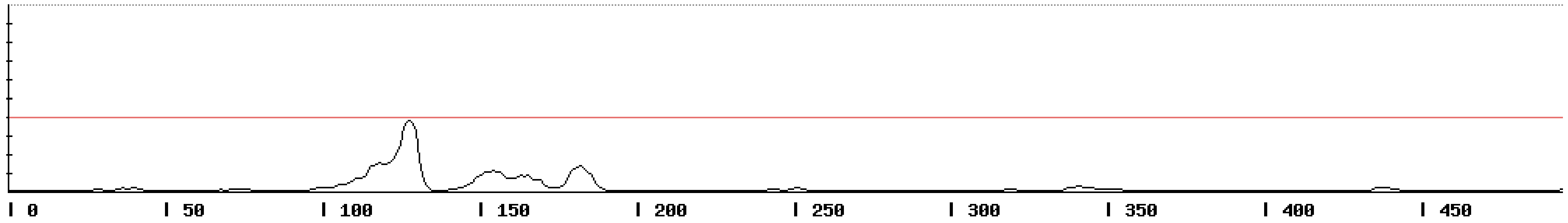

Predictions for Brassicarapa_XP_009137841.1

Viterbi Path

Viterbi Path

Posterior @ 0.4

Posterior @ 0.4

Posterior Graphic. Threshold Marked @ 0.4

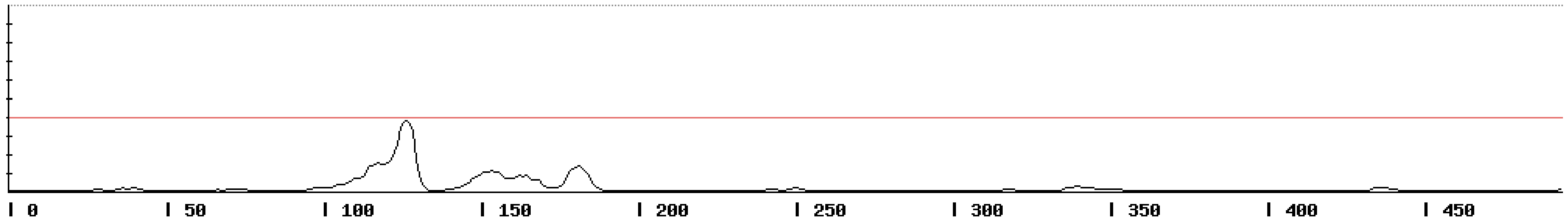

Predictions for Brassicarapa_XP_009138410.1

Viterbi Path

Viterbi Path

Posterior @ 0.4	279 - KSKDKKKK - 286
Posterior @ 0.4	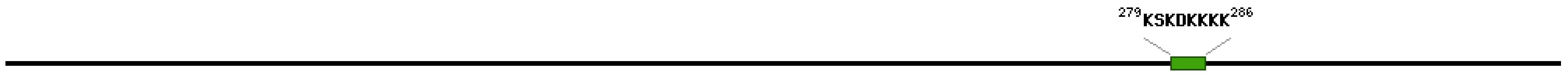
Posterior Graphic. Threshold Marked @ 0.4	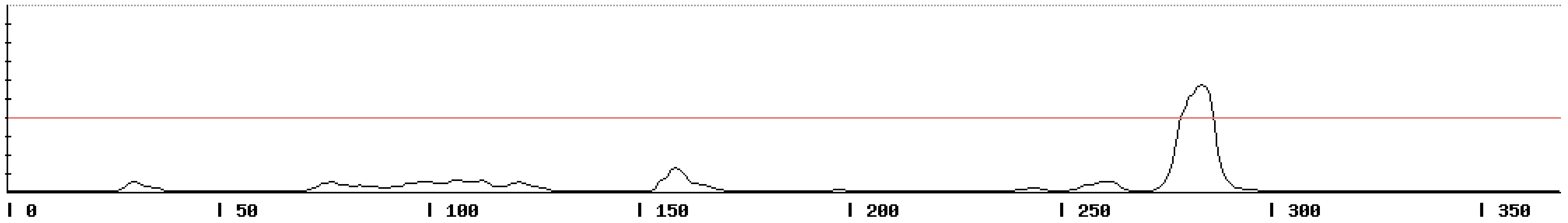

Predictions for Brassicarapa_XP_009138411.1

Viterbi Path	
Viterbi Path	
Posterior @ 0.4	279 - KSKDKKKK - 286
Posterior @ 0.4	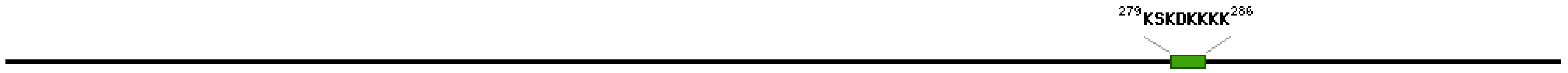
Posterior Graphic. Threshold Marked @ 0.4	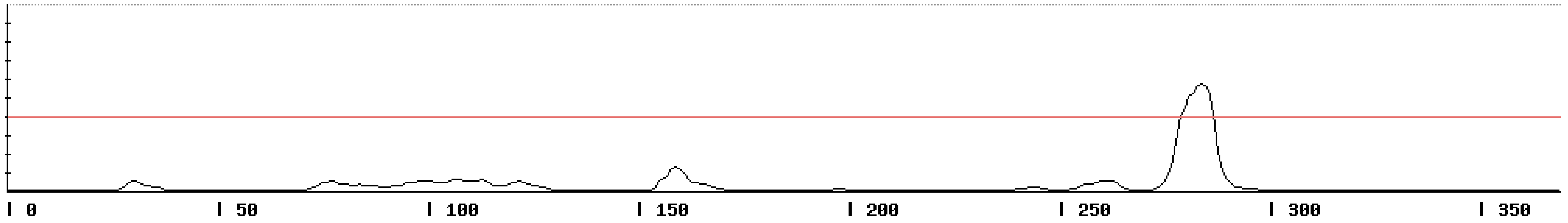

Predictions for Brassicarapa_XP_009139340.1

Viterbi Path 528 - KRKRKRKK - 535

Viterbi Path

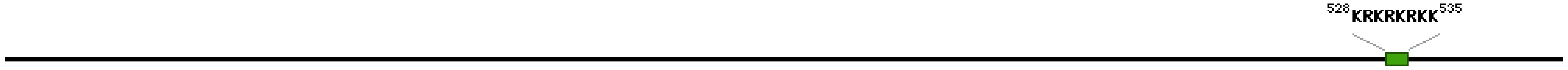

Posterior @ 0.4 526 - DEKRKRKRKKNEG - 538

Posterior @ 0.4

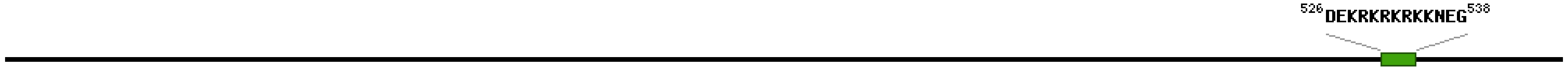

Posterior Graphic. Threshold Marked @ 0.4

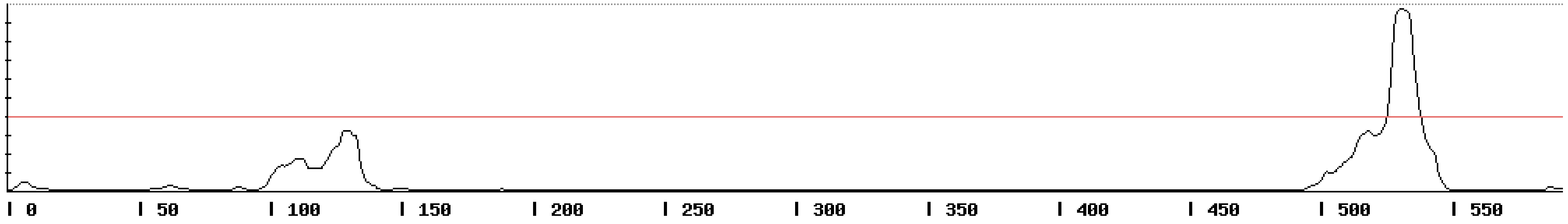

Predictions for Brassicarapa_XP_009139909.1

Viterbi Path

Viterbi Path

Posterior @ 0.4

Posterior @ 0.4

Posterior Graphic. Threshold Marked @ 0.4

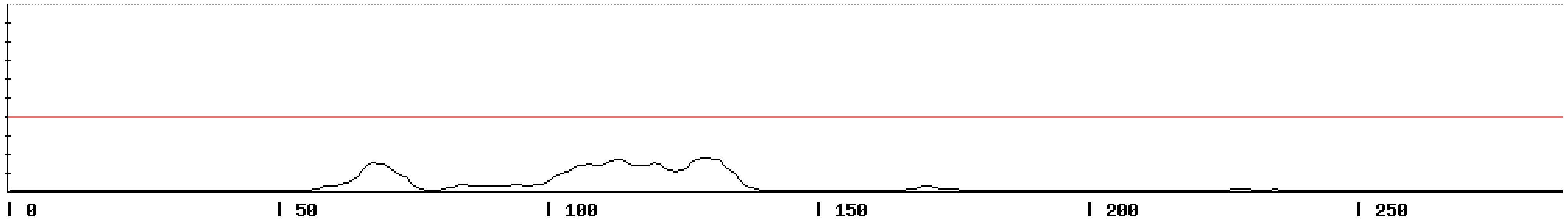

Predictions for Brassicarapa_XP_009140067.1

Viterbi Path	
Viterbi Path	
Posterior @ 0.4	
Posterior @ 0.4	
Posterior Graphic. Threshold Marked @ 0.4	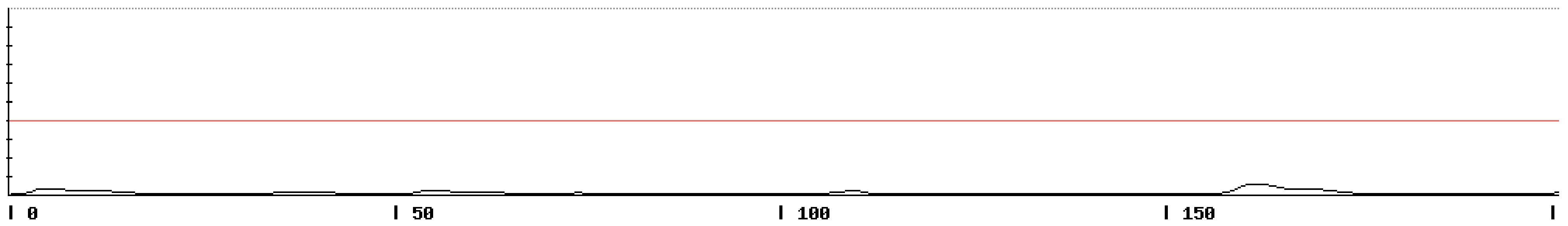

Predictions for Brassicarapa_XP_009140553.1

Viterbi Path	
Viterbi Path	
Posterior @ 0.4	
Posterior @ 0.4	
Posterior Graphic. Threshold Marked @ 0.4	

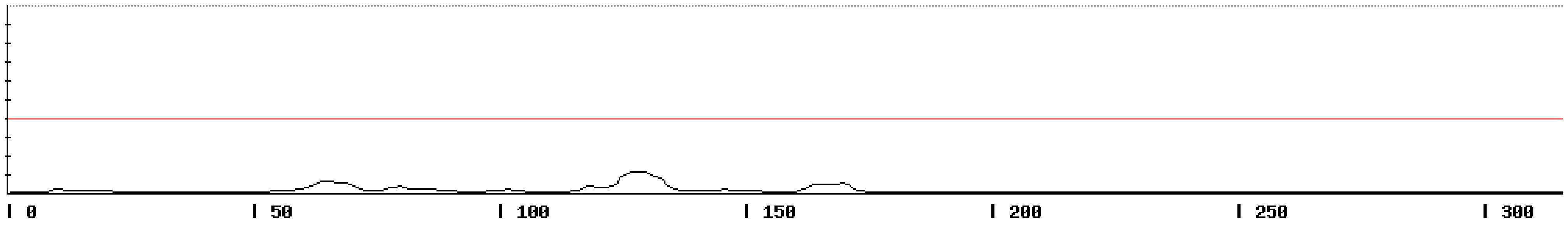

Predictions for Brassicarapa_XP_009140890.1

Viterbi Path	
Viterbi Path	
Posterior @ 0.4	
Posterior @ 0.4	
Posterior Graphic. Threshold Marked @ 0.4	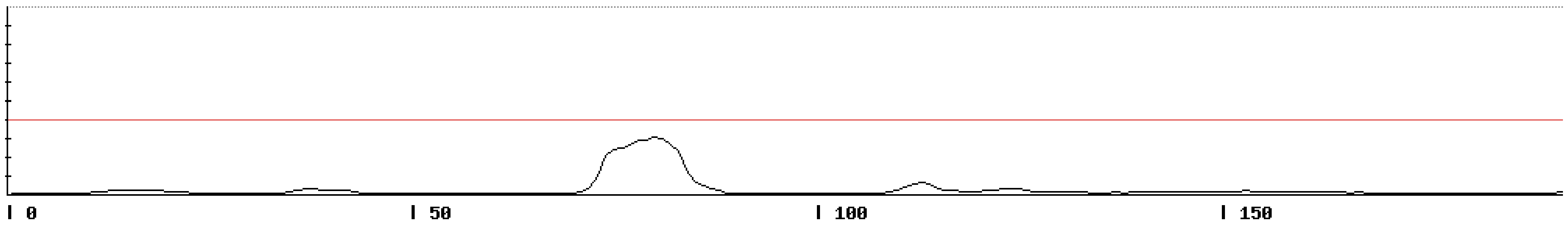

Predictions for Brassicarapa_XP_009141320.1

Viterbi Path	
Viterbi Path	
Posterior @ 0.4	197 - KDKKRK - 202
Posterior @ 0.4	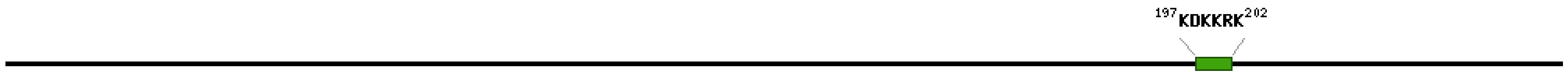

Posterior Graphic. Threshold Marked @ 0.4

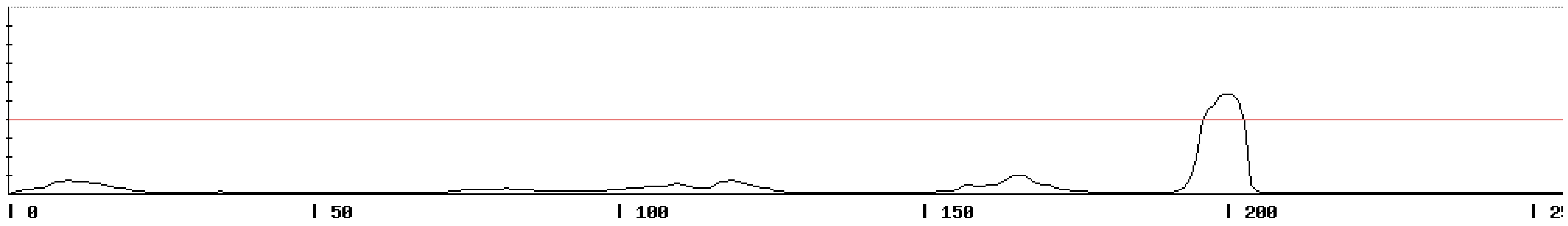

Predictions for Brassicarapa_XP_009142151.1

Viterbi Path

Viterbi Path

Posterior @ 0.4

Posterior @ 0.4

Posterior Graphic. Threshold Marked @ 0.4

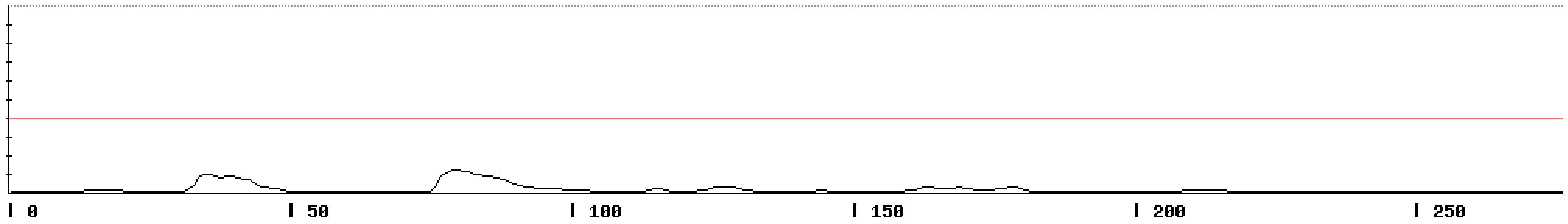

Predictions for Brassicarapa_XP_009142445.1

Viterbi Path

Viterbi Path

Posterior @ 0.4

Posterior @ 0.4

Posterior Graphic. Threshold Marked @ 0.4

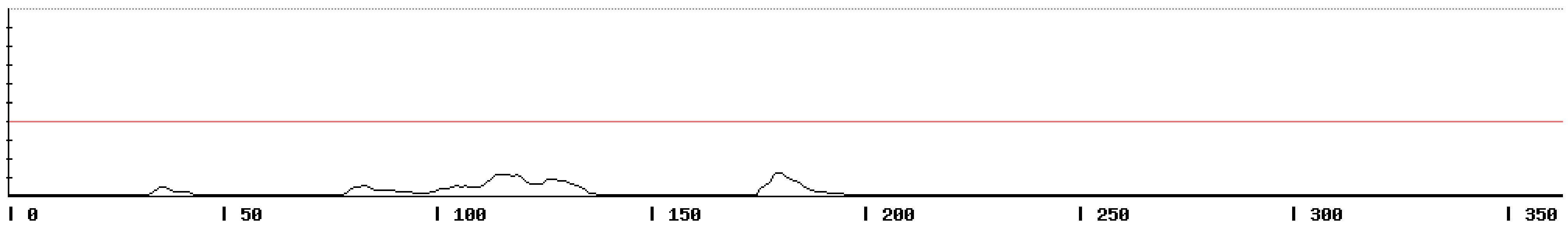

Predictions for Brassicarapa_XP_009142607.1

Viterbi Path

Viterbi Path

Posterior @ 0.4

Posterior @ 0.4

Posterior Graphic. Threshold Marked @ 0.4

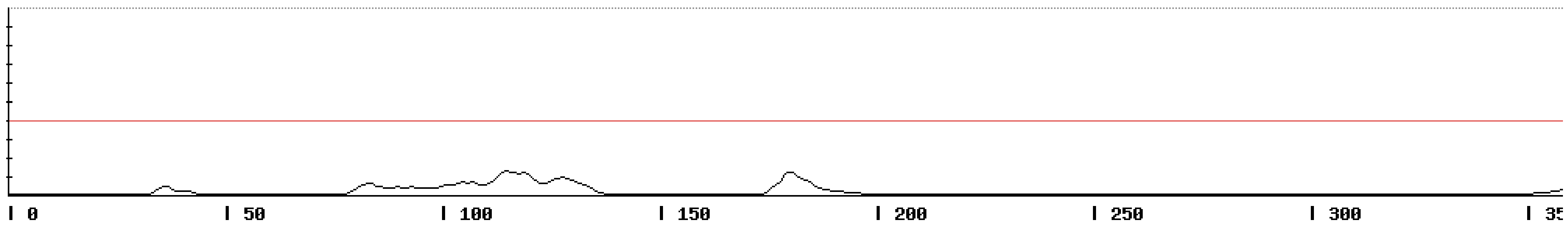

Predictions for Brassicarapa_XP_009142897.1

Viterbi Path

Viterbi Path

Posterior @ 0.4

Posterior @ 0.4

Posterior Graphic. Threshold Marked @ 0.4

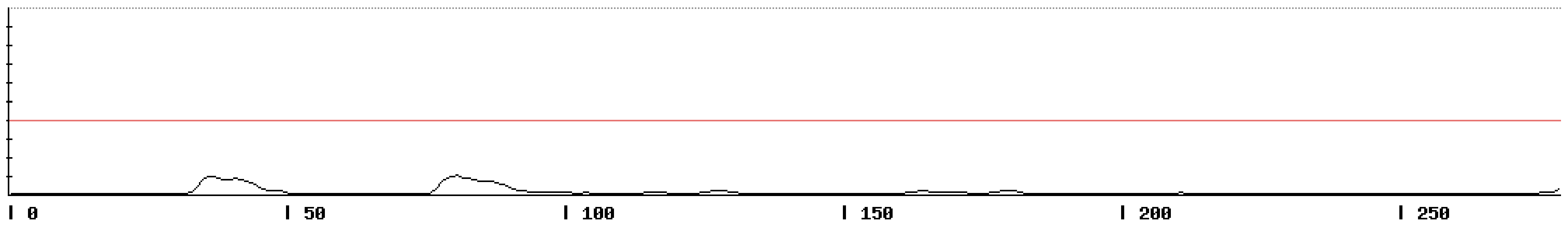

Predictions for Brassicarapa_XP_009143398.1

Viterbi Path

Viterbi Path

Posterior @ 0.4

Posterior @ 0.4

Posterior Graphic. Threshold Marked @ 0.4

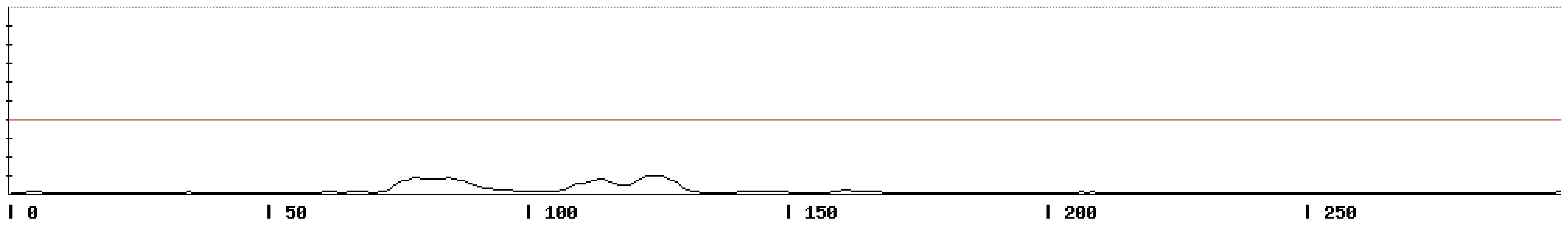

Predictions for Brassicarapa_XP_009143857.1

Viterbi Path

Viterbi Path

Posterior @ 0.4 — 156 - KKRSNNNNKKKKEDEREKK - 174

Posterior @ 0.4

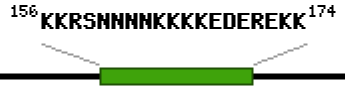

Posterior Graphic. Threshold Marked @ 0.4

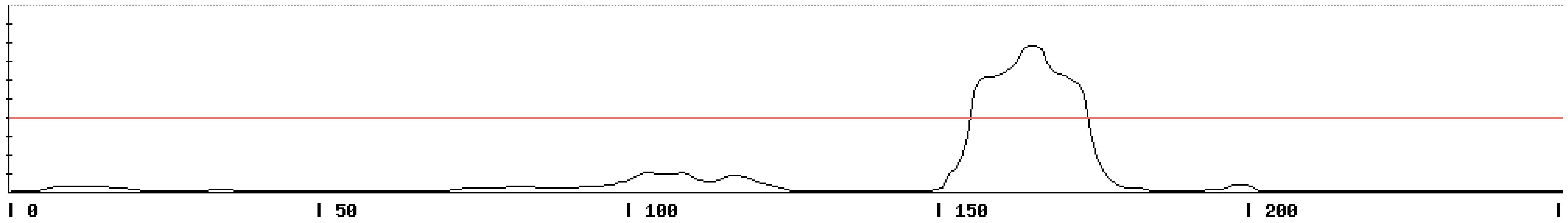

Predictions for Brassicarapa_XP_009144711.1

Viterbi Path

Viterbi Path

Posterior @ 0.4

Posterior @ 0.4

Posterior Graphic. Threshold Marked @ 0.4

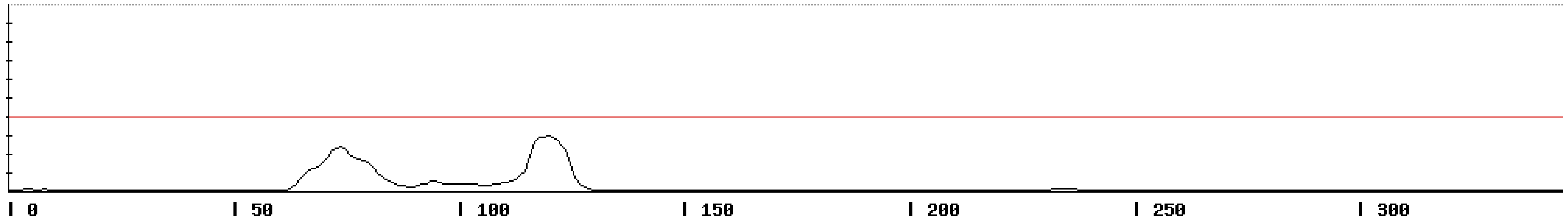

Predictions for Brassicarapa_XP_009145145.1

Viterbi Path

Viterbi Path

Posterior @ 0.4

Posterior @ 0.4

Posterior Graphic. Threshold Marked @ 0.4

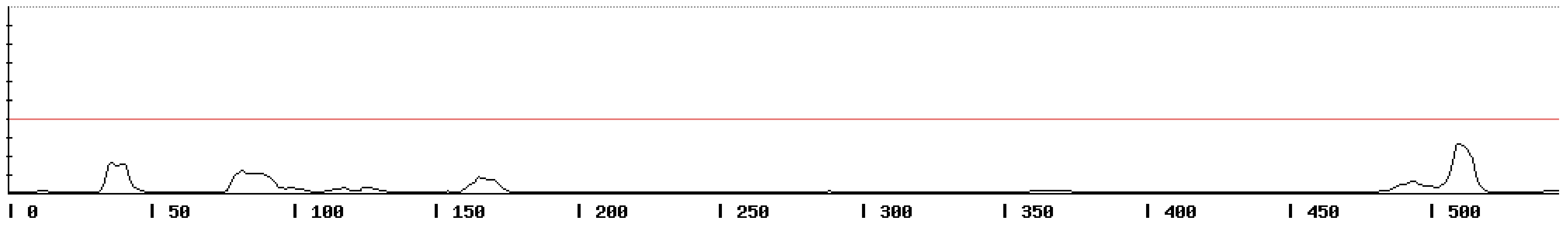

Predictions for Brassicarapa_XP_009145146.1

Viterbi Path

Viterbi Path

Posterior @ 0.4

Posterior @ 0.4

Posterior Graphic. Threshold Marked @ 0.4

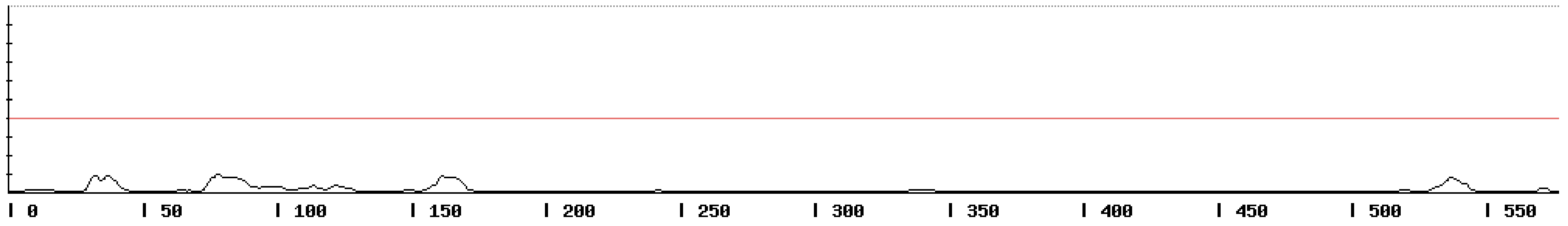

Predictions for Brassicarapa_XP_009145148.1	
Viterbi Path	
Viterbi Path	
Posterior @ 0.4	
Posterior @ 0.4	
Posterior Graphic. Threshold Marked @ 0.4	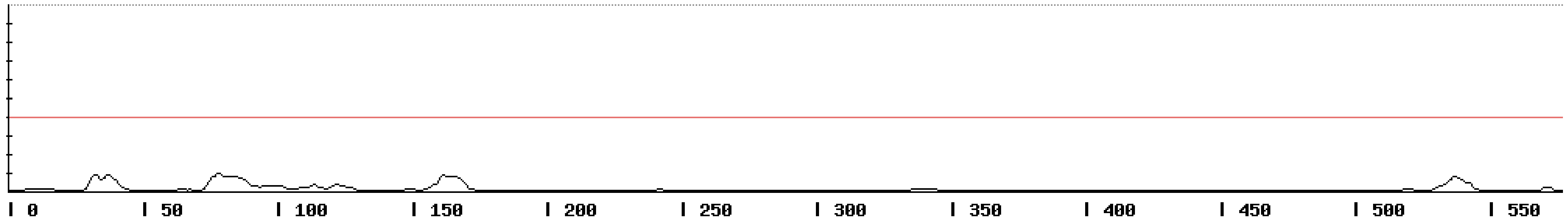

Predictions for Brassicarapa_XP_009145149.1	
Viterbi Path	
Viterbi Path	
Posterior @ 0.4	
Posterior @ 0.4	
Posterior Graphic. Threshold Marked @ 0.4	

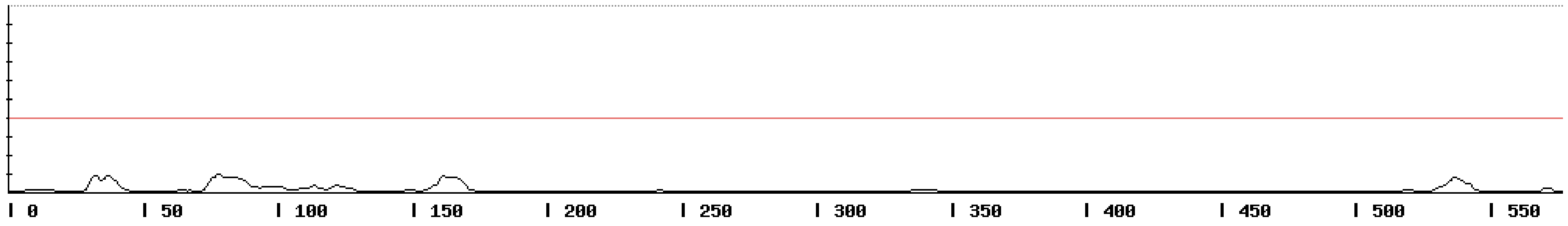

Predictions for Brassicarapa_XP_009145150.1

Viterbi Path

Viterbi Path

Posterior @ 0.4

Posterior @ 0.4

Posterior Graphic. Threshold Marked @ 0.4

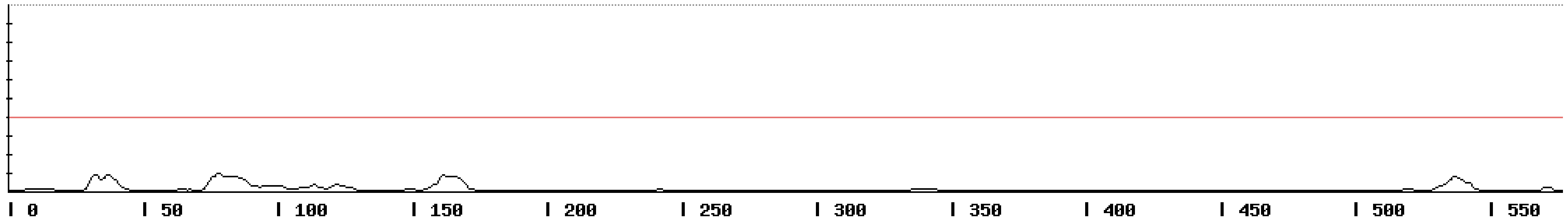

Predictions for Brassicarapa_XP_009145151.1

Viterbi Path

Viterbi Path

Posterior @ 0.4

Posterior @ 0.4

Posterior Graphic. Threshold Marked @ 0.4

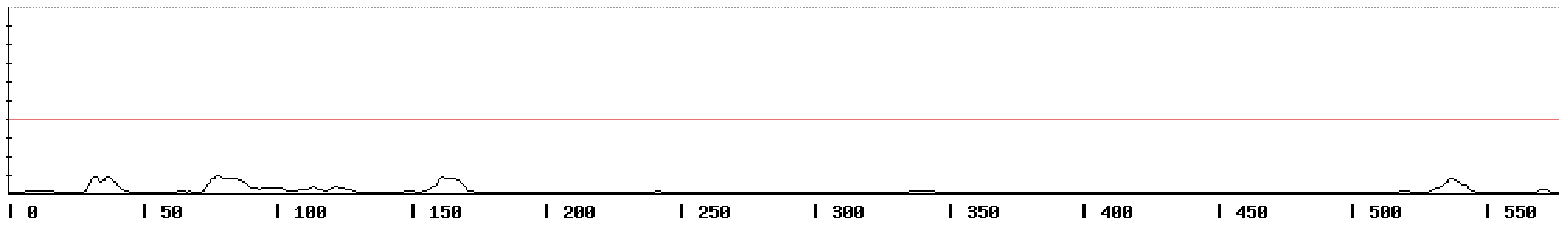

Predictions for Brassicarapa_XP_009145896.1

Viterbi Path

Viterbi Path

Posterior @ 0.4

Posterior @ 0.4

Posterior Graphic. Threshold Marked @ 0.4

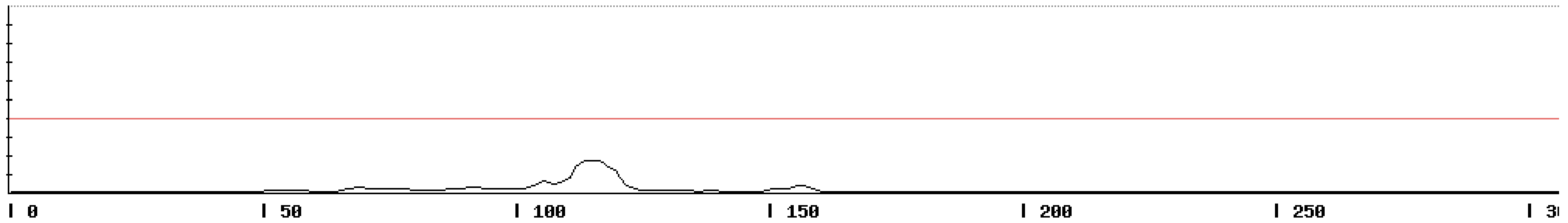

Predictions for Brassicarapa_XP_009145897.1

Viterbi Path

Viterbi Path

Posterior @ 0.4

Posterior @ 0.4

Posterior Graphic. Threshold Marked @ 0.4

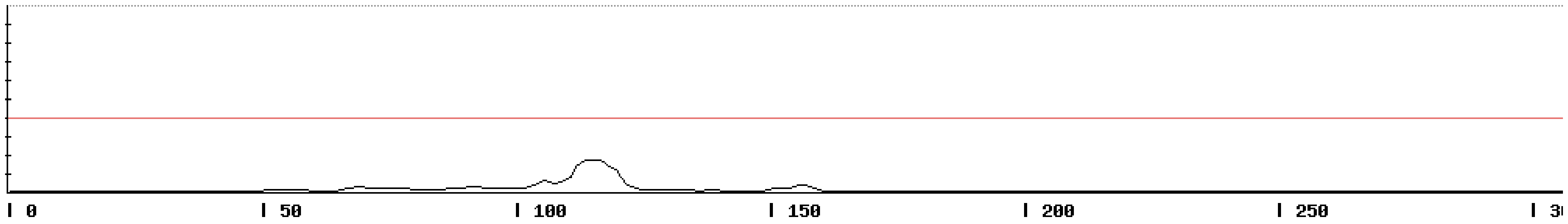

Predictions for Brassicarapa_XP_009145987.1

Viterbi Path

Viterbi Path

Posterior @ 0.4

Posterior @ 0.4

Posterior Graphic. Threshold Marked @ 0.4

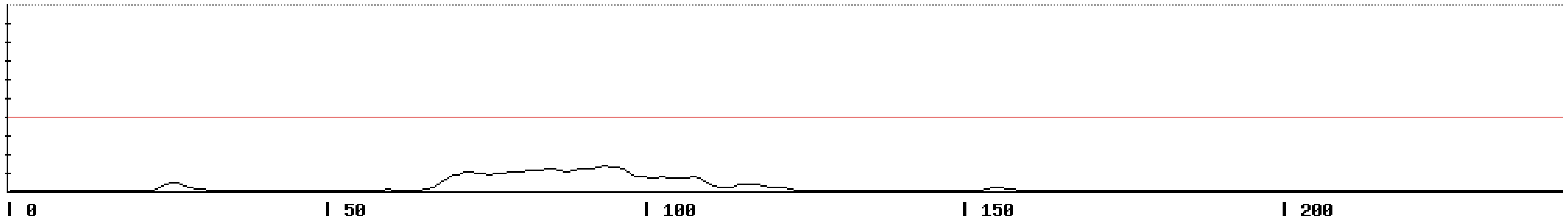

Predictions for Brassicarapa_XP_009146237.1

Viterbi Path

Viterbi Path

Posterior @ 0.4

Posterior @ 0.4

Posterior Graphic. Threshold Marked @ 0.4

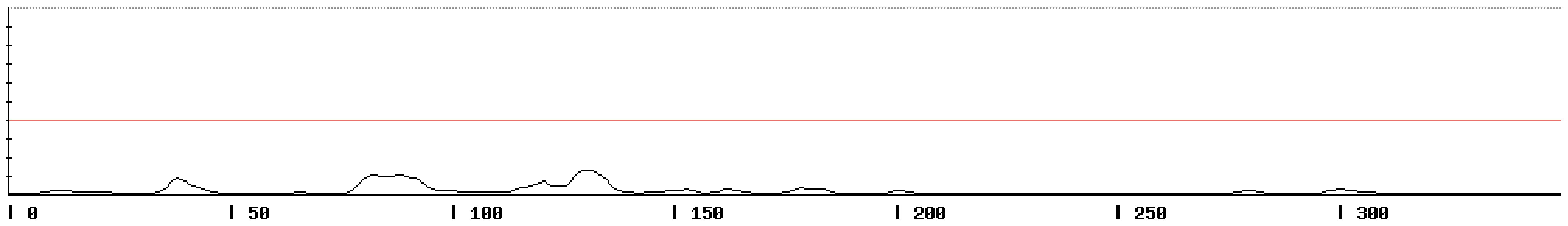

Predictions for Brassicarapa_XP_009146239.1

Viterbi Path

Viterbi Path

Posterior @ 0.4

Posterior @ 0.4

Posterior Graphic. Threshold Marked @ 0.4

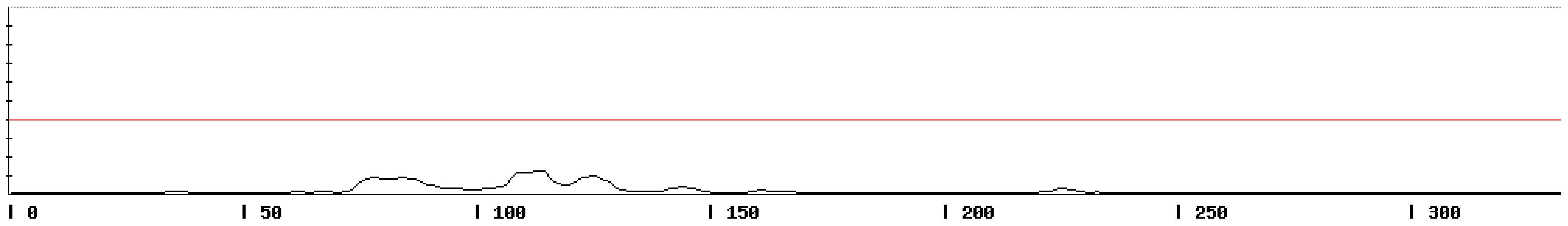

Predictions for Brassicarapa_XP_009146450.1

Viterbi Path

Viterbi Path

Posterior @ 0.4

Posterior @ 0.4

Posterior Graphic. Threshold Marked @ 0.4

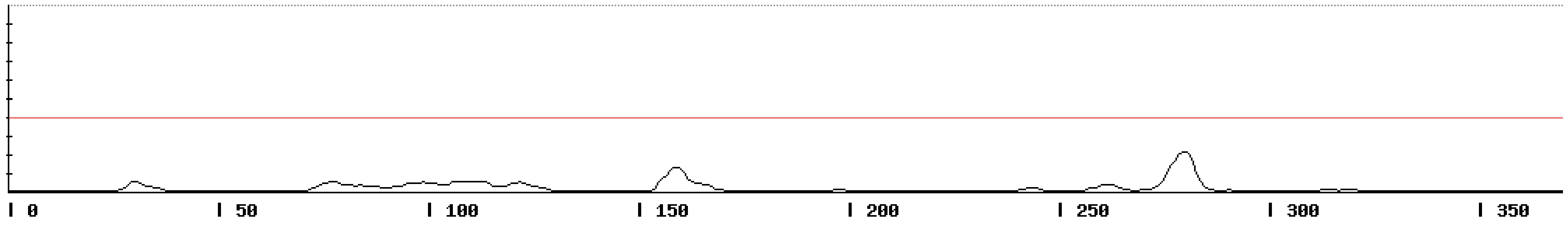

Predictions for Brassicarapa_XP_009146562.1

Viterbi Path

Viterbi Path

Posterior @ 0.4

Posterior @ 0.4

Posterior Graphic. Threshold Marked @ 0.4

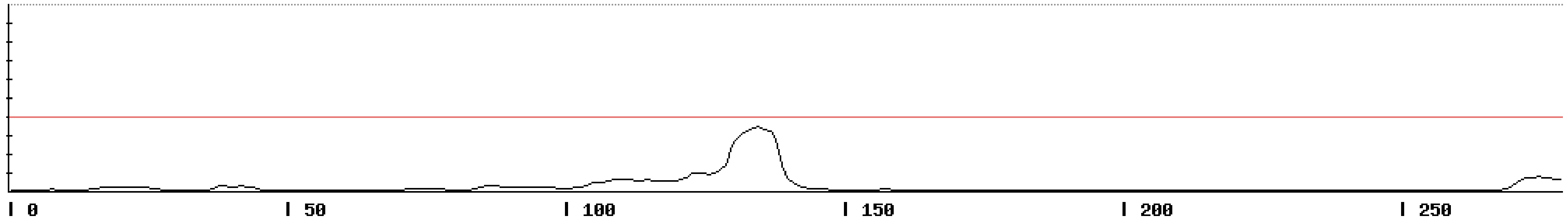

Predictions for Brassicarapa_XP_009146563.1

Viterbi Path

Viterbi Path

Posterior @ 0.4

Posterior @ 0.4

Posterior Graphic. Threshold Marked @ 0.4

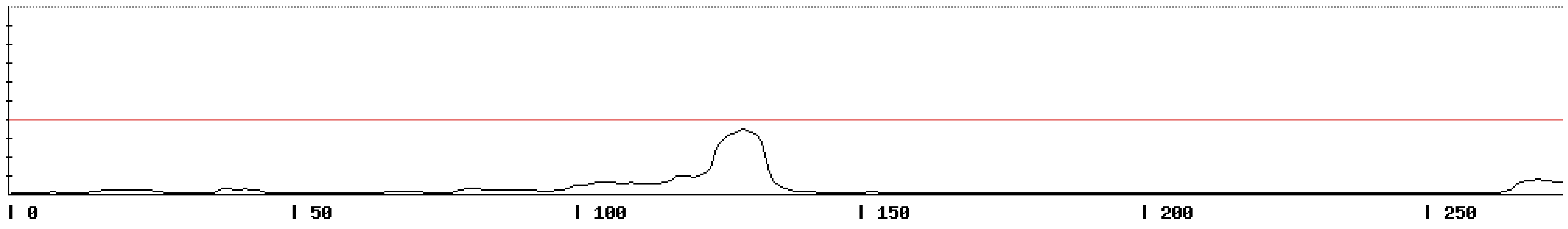

Predictions for Brassicarapa_XP_009146571.1

Viterbi Path

Viterbi Path

Posterior @ 0.4

Posterior @ 0.4

Posterior Graphic. Threshold Marked @ 0.4

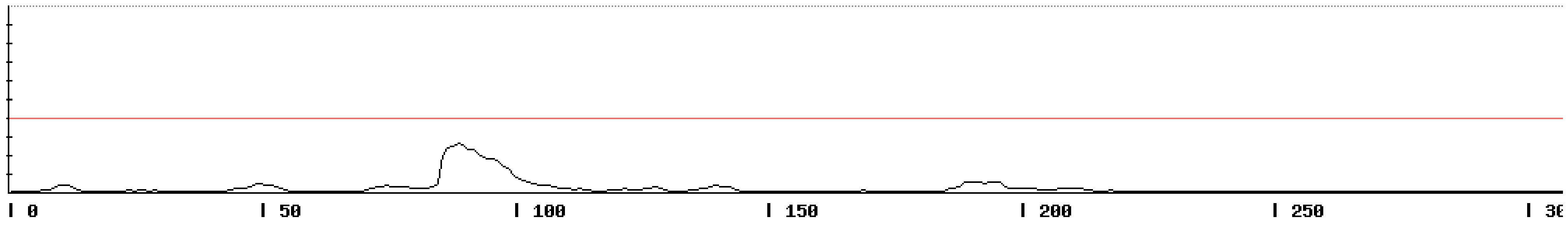

Predictions for Brassicarapa_XP_009146834.1

Viterbi Path

Viterbi Path

Posterior @ 0.4

Posterior @ 0.4

Posterior Graphic. Threshold Marked @ 0.4

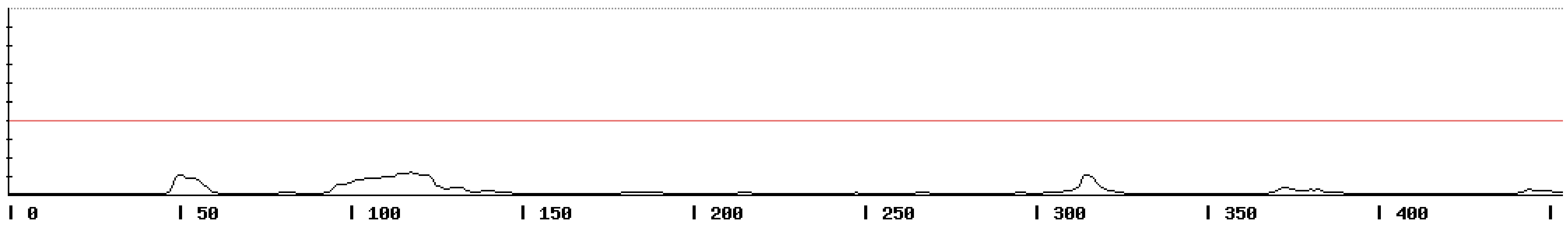

Predictions for Brassicarapa_XP_009146835.1

Viterbi Path

Viterbi Path

Posterior @ 0.4

Posterior @ 0.4

Posterior Graphic. Threshold Marked @ 0.4

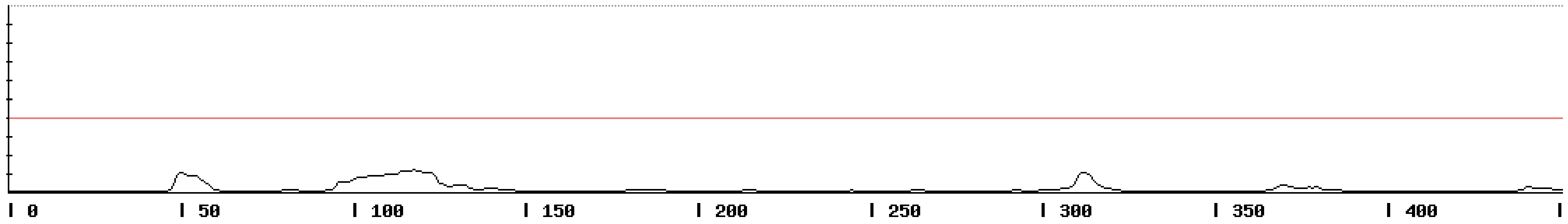

Predictions for Brassicarapa_XP_009147649.1	
Viterbi Path	
Viterbi Path	
Posterior @ 0.4	
Posterior @ 0.4	
Posterior Graphic. Threshold Marked @ 0.4	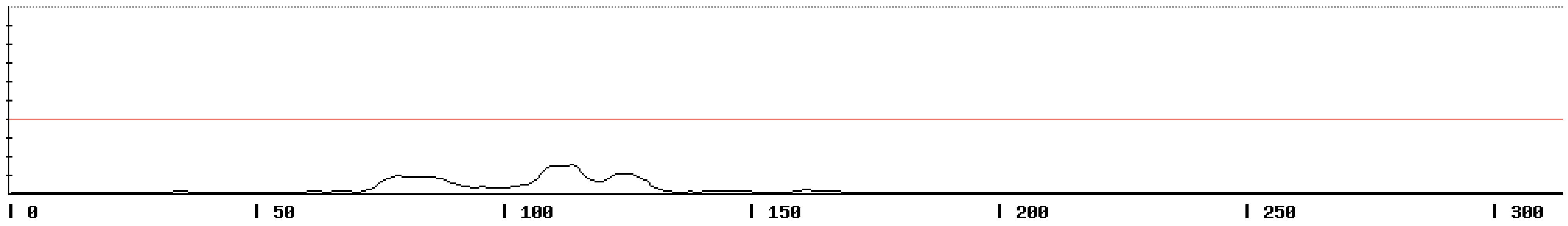

Predictions for Brassicarapa_XP_009147650.1	
Viterbi Path	
Viterbi Path	
Posterior @ 0.4	
Posterior @ 0.4	

Posterior Graphic. Threshold Marked @ 0.4

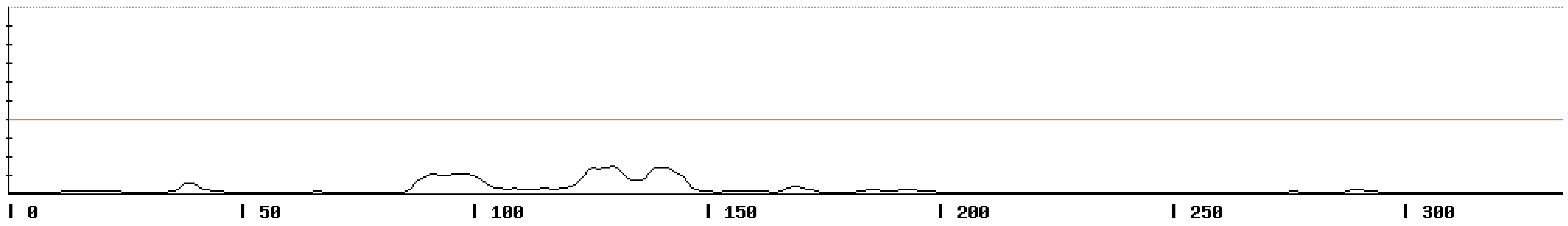

Predictions for Brassicarapa_XP_009147651.1

Viterbi Path

Viterbi Path

Posterior @ 0.4

Posterior @ 0.4

Posterior Graphic. Threshold Marked @ 0.4

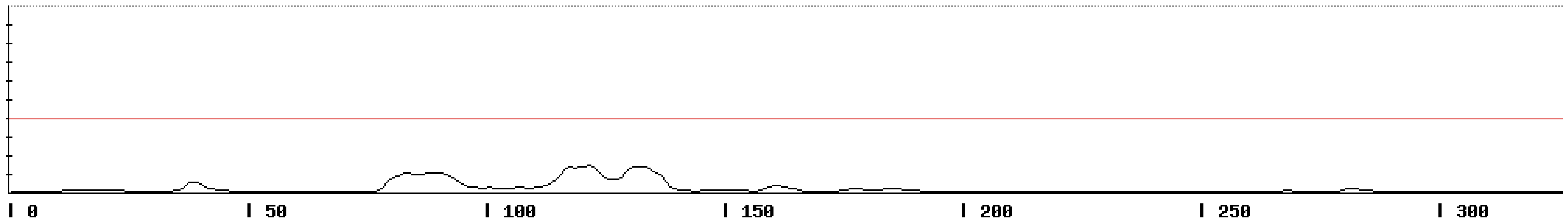

Predictions for Brassicarapa_XP_009148605.1

Viterbi Path

Viterbi Path

Posterior @ 0.4

Posterior @ 0.4

Posterior Graphic. Threshold Marked @ 0.4

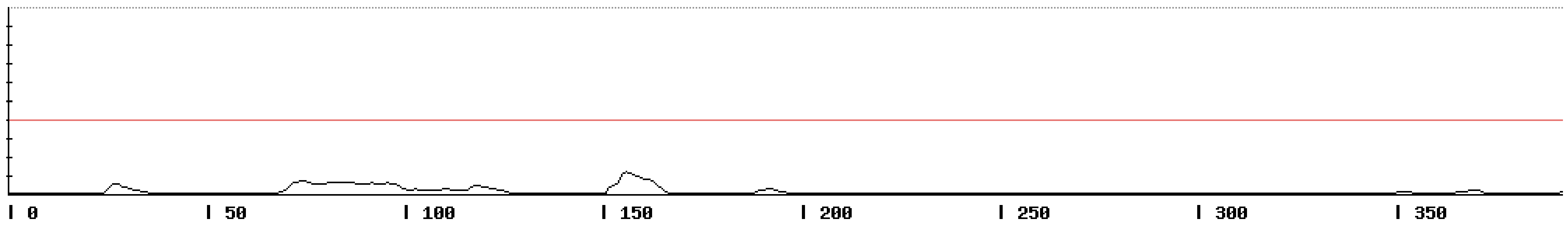

Predictions for Brassicarapa_XP_009148606.1

Viterbi Path

Viterbi Path

Posterior @ 0.4

Posterior @ 0.4

Posterior Graphic. Threshold Marked @ 0.4

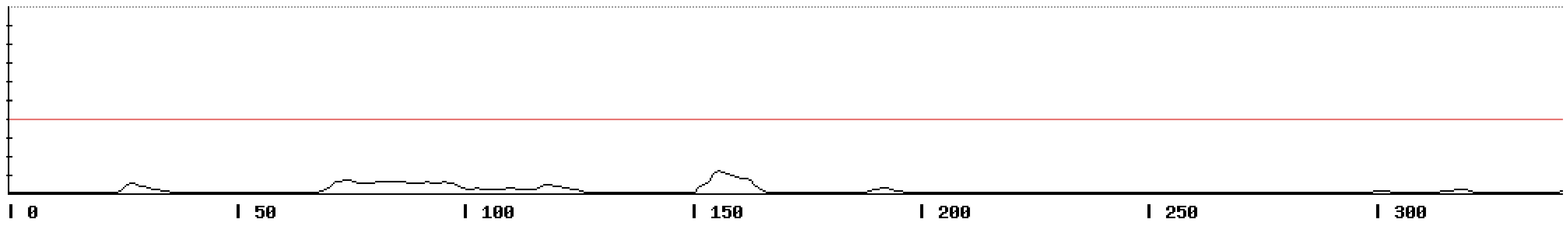

Predictions for Brassicarapa_XP_009149732.1

Viterbi Path

Viterbi Path

Posterior @ 0.4	104 - KDRKIKSGK - 112
	409 - KKGKPSK - 415
Posterior @ 0.4	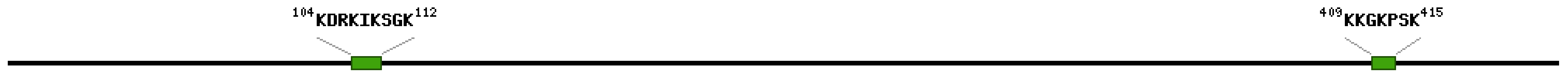
Posterior Graphic. Threshold Marked @ 0.4	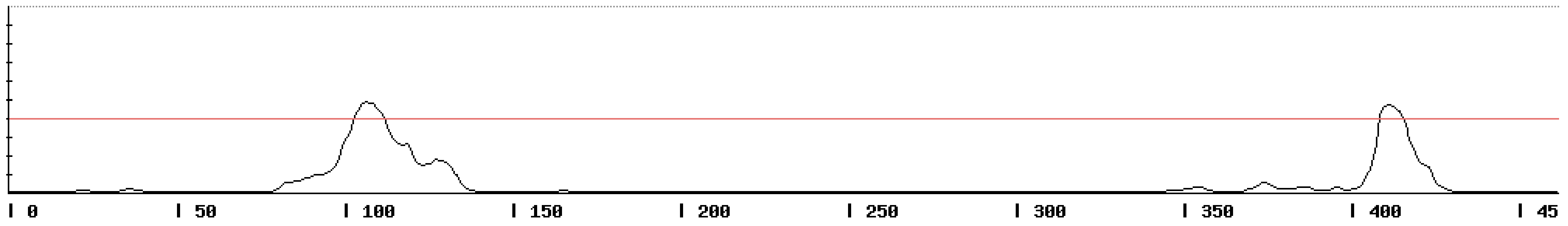

Predictions for Brassicarapa_XP_009150140.1

Viterbi Path	
Viterbi Path	
Posterior @ 0.4	98 - ATGKDRKIKSGK - 109
	374 - KAIKKGKLMRSKNR - 387
Posterior @ 0.4	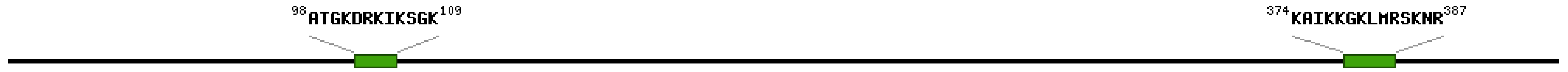
Posterior Graphic. Threshold Marked @ 0.4	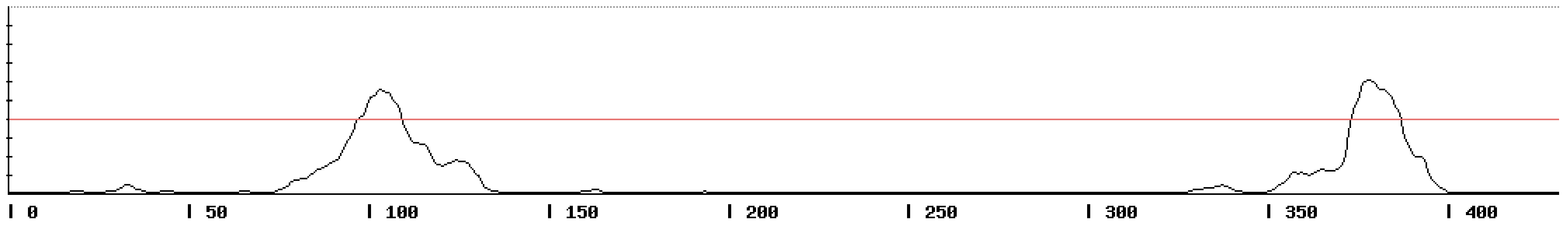

Predictions for Brassicarapa_XP_009150275.1

Viterbi Path	

Viterbi Path

Posterior @ 0.4 — 121 - KAPKGRKTKW - 130

Posterior @ 0.4

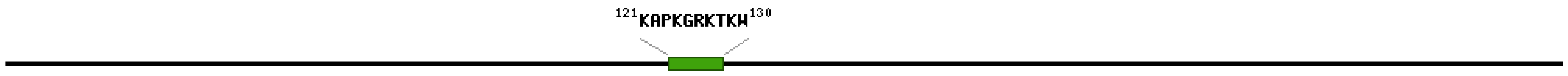

Posterior Graphic. Threshold Marked @ 0.4

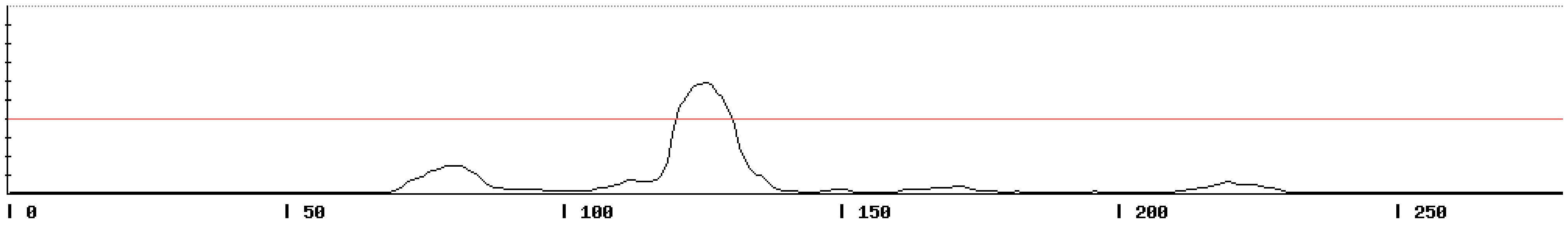

Predictions for Brassicarapa_XP_009150295.1

Viterbi Path

Viterbi Path

Posterior @ 0.4

Posterior @ 0.4

Posterior Graphic. Threshold Marked @ 0.4

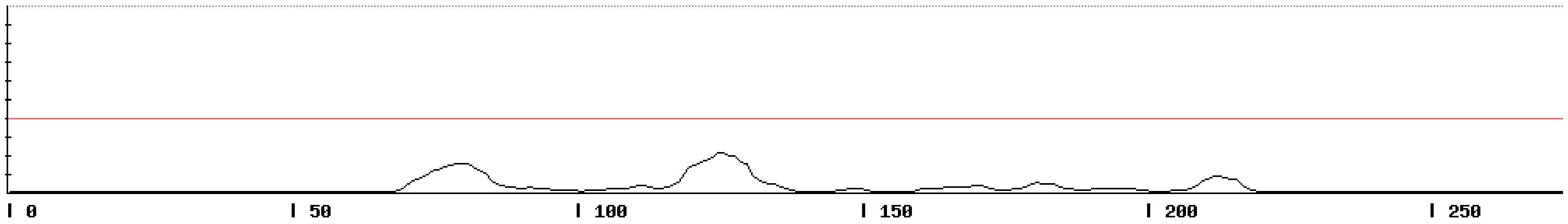

Predictions for Brassicarapa_XP_009150429.1

Viterbi Path

Viterbi Path

Posterior @ 0.4

Posterior @ 0.4

Posterior Graphic. Threshold Marked @ 0.4

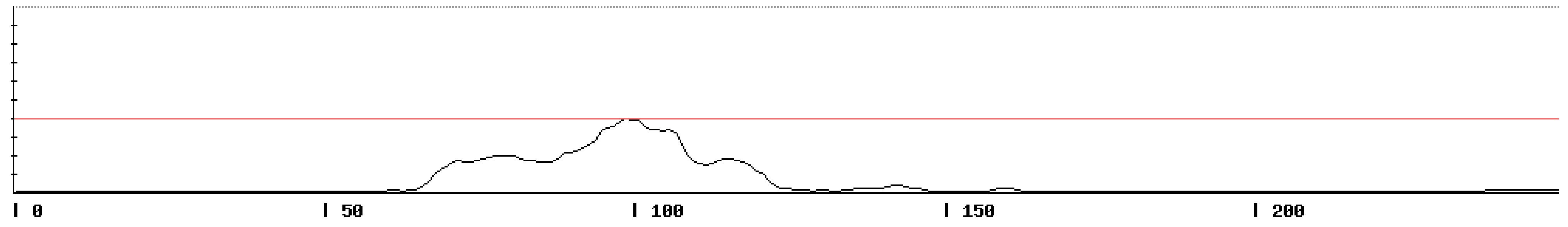

Predictions for Brassicarapa_XP_009150476.1

Viterbi Path

Viterbi Path

Posterior @ 0.4

Posterior @ 0.4

Posterior Graphic. Threshold Marked @ 0.4

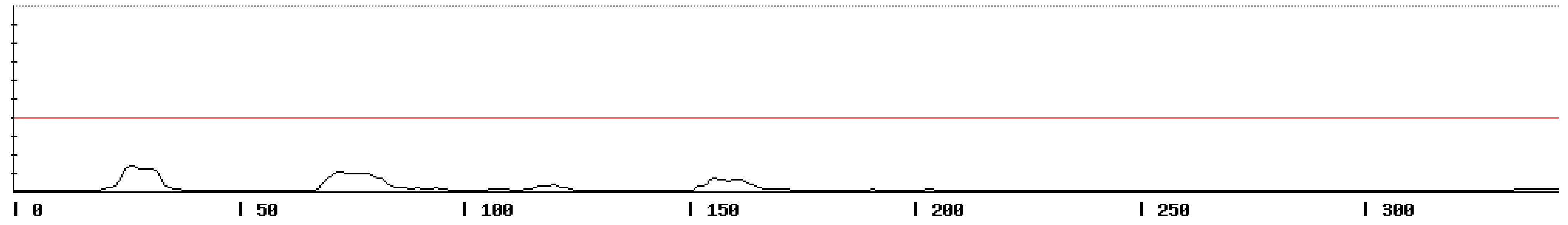

Predictions for Brassicarapa_XP_009150535.1	
Viterbi Path	
Viterbi Path	
Posterior @ 0.4	
Posterior @ 0.4	
Posterior Graphic. Threshold Marked @ 0.4	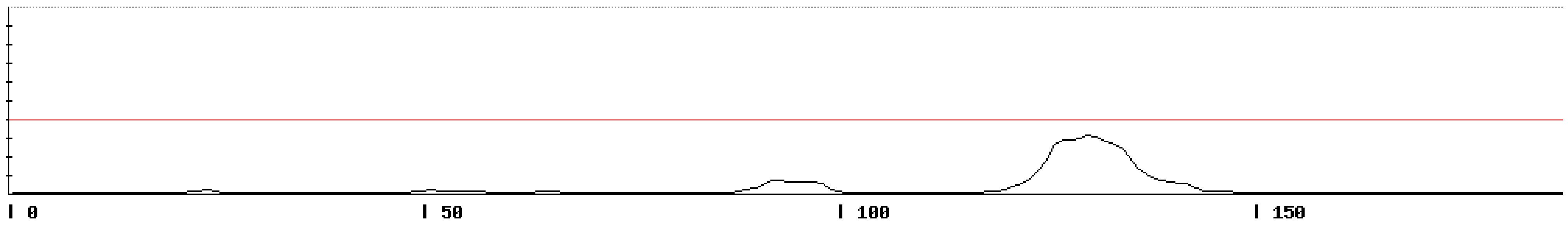

Predictions for Brassicarapa_XP_009150982.1	
Viterbi Path	
Viterbi Path	
Posterior @ 0.4	
Posterior @ 0.4	
Posterior Graphic. Threshold Marked @ 0.4	

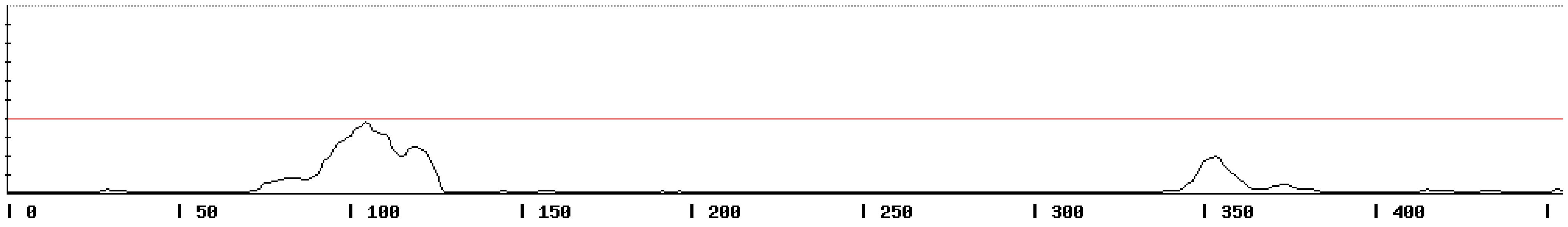

Predictions for Brassicarapa_XP_009150983.1	
Viterbi Path	
Viterbi Path	
Posterior @ 0.4	
Posterior @ 0.4	
Posterior Graphic. Threshold Marked @ 0.4	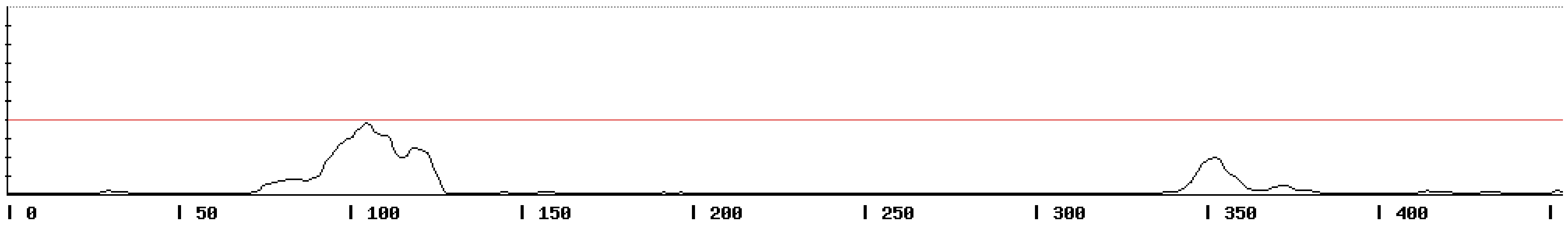

Predictions for Brassicarapa_XP_009151357.1	
Viterbi Path	
Viterbi Path	
Posterior @ 0.4	
Posterior @ 0.4	

Posterior Graphic. Threshold Marked @ 0.4	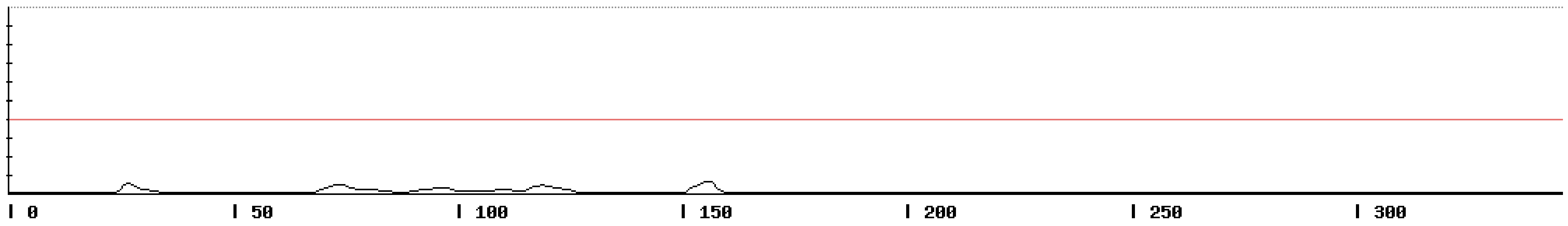

Predictions for Brassicarapa_XP_009151358.1

Viterbi Path	
Viterbi Path	
Posterior @ 0.4	
Posterior @ 0.4	
Posterior Graphic. Threshold Marked @ 0.4	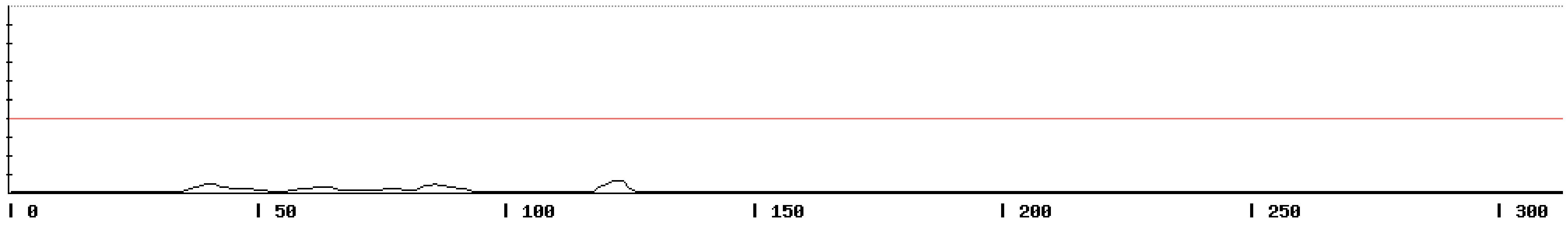

Predictions for Brassicarapa_XP_009151359.1

Viterbi Path	
Viterbi Path	
Posterior @ 0.4	
Posterior @ 0.4	

Posterior Graphic. Threshold Marked @ 0.4

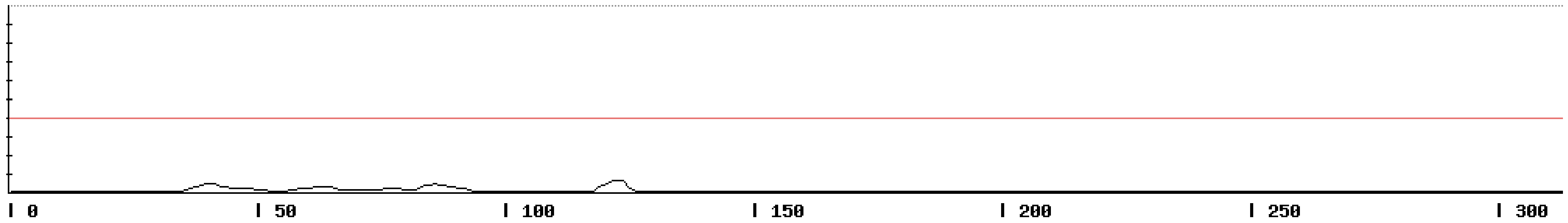

Predictions for Brassicarapa_XP_009151451.1

Viterbi Path

Viterbi Path

Posterior @ 0.4

Posterior @ 0.4

Posterior Graphic. Threshold Marked @ 0.4

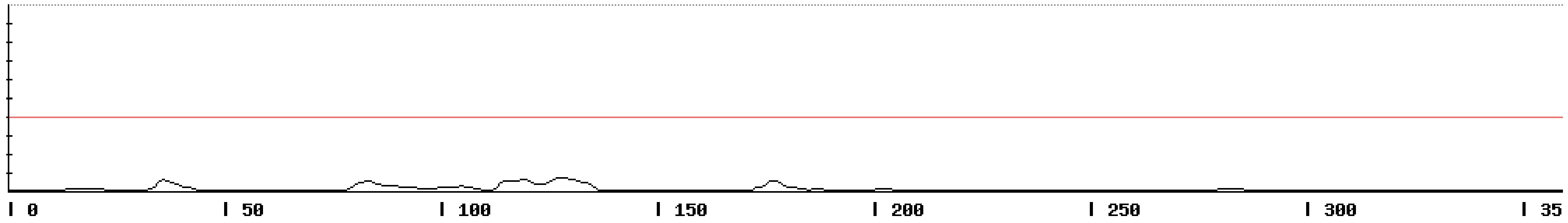

Predictions for Brassicarapa_XP_009151462.1

Viterbi Path

Viterbi Path

Posterior @ 0.4

Posterior @ 0.4

Posterior Graphic. Threshold Marked @ 0.4

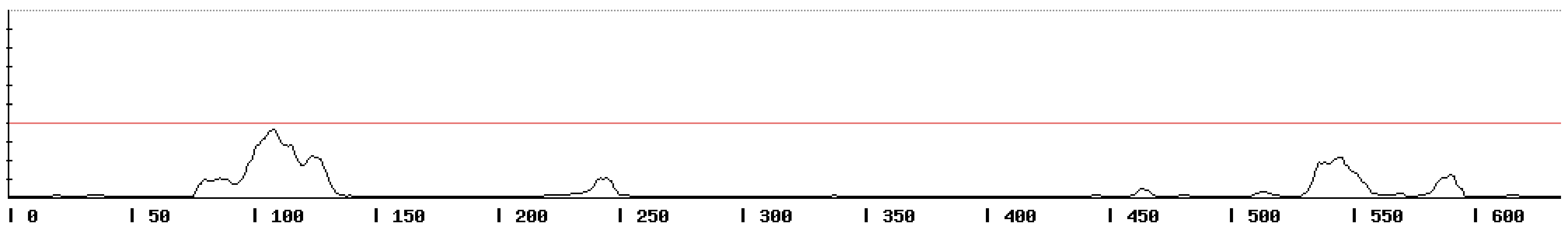

Revision r.9 - Last Updated on : February 24 2015 17:45:20.

alex.nguyenba@utoronto.ca

Intended to be used with Firefox

Viterbi Path

Posterior @ 0.4

Posterior @ 0.4

Posterior Graphic. Threshold Marked @ 0.4

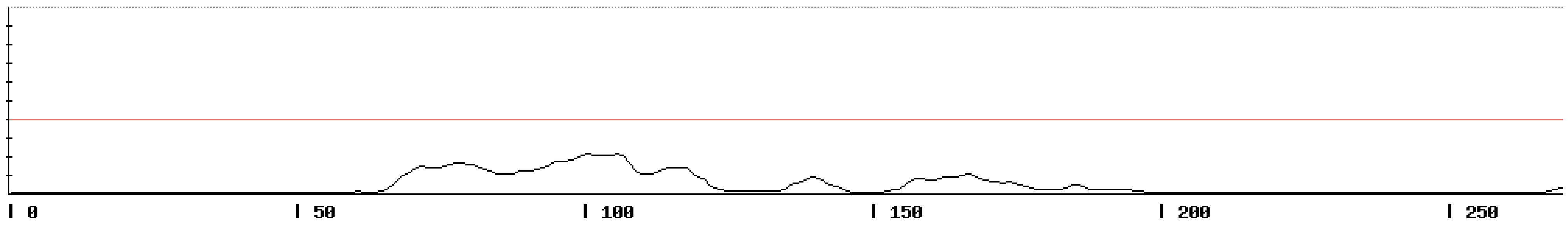

Predictions for Cajanuscajan_C.cajan_01440

Viterbi Path

Viterbi Path

Posterior @ 0.4

Posterior @ 0.4

Posterior Graphic. Threshold Marked @ 0.4

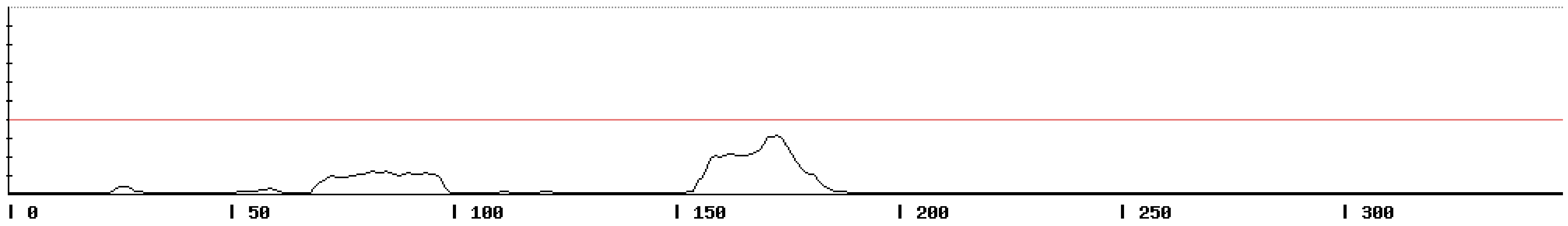

Predictions for Cajanuscajan_C.cajan_01567

Viterbi Path	
Viterbi Path	
Posterior @ 0.4	
Posterior @ 0.4	
Posterior Graphic. Threshold Marked @ 0.4	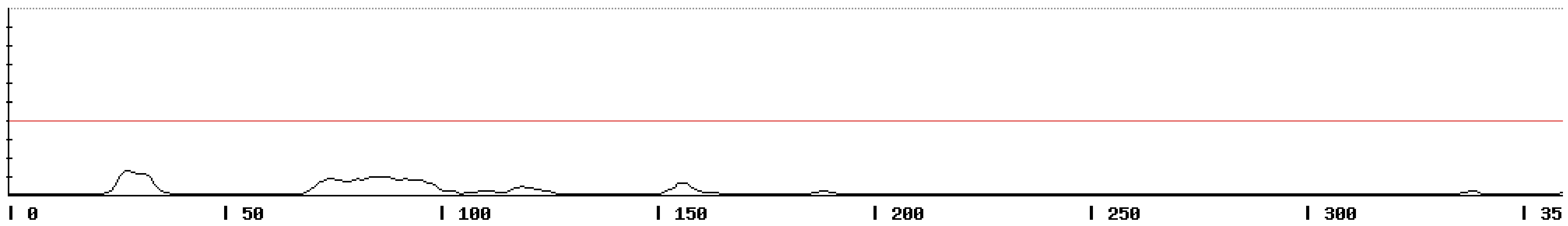

Predictions for Cajanuscajan_C.cajan_01726

Viterbi Path	
Viterbi Path	
Posterior @ 0.4	
Posterior @ 0.4	
Posterior Graphic. Threshold Marked @ 0.4	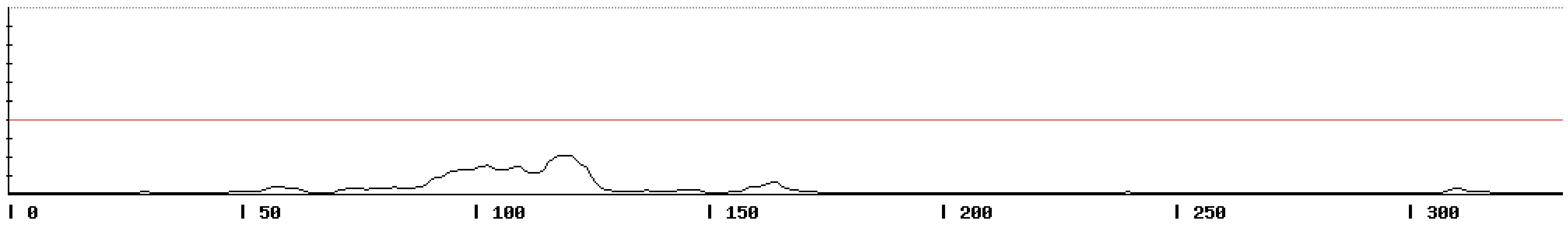

Predictions for Cajanuscajan_C.cajan_02029	
Viterbi Path	
Viterbi Path	
Posterior @ 0.4	
Posterior @ 0.4	
Posterior Graphic. Threshold Marked @ 0.4	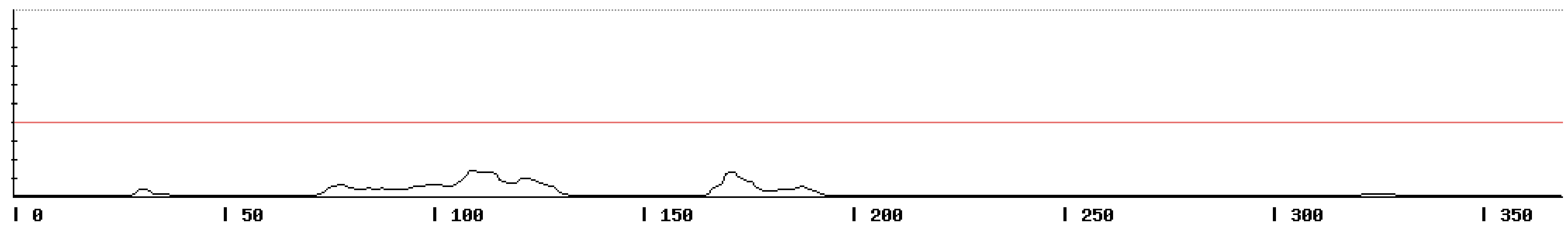

Predictions for Cajanuscajan_C.cajan_02050	
Viterbi Path	
Viterbi Path	
Posterior @ 0.4	
Posterior @ 0.4	
Posterior Graphic. Threshold Marked @ 0.4	

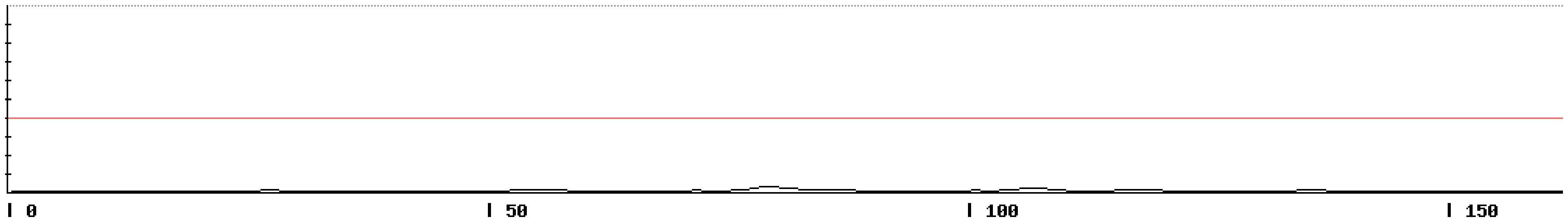

Predictions for Cajanuscajan_C.cajan_02051

Viterbi Path	
Viterbi Path	
Posterior @ 0.4	
Posterior @ 0.4	
Posterior Graphic. Threshold Marked @ 0.4	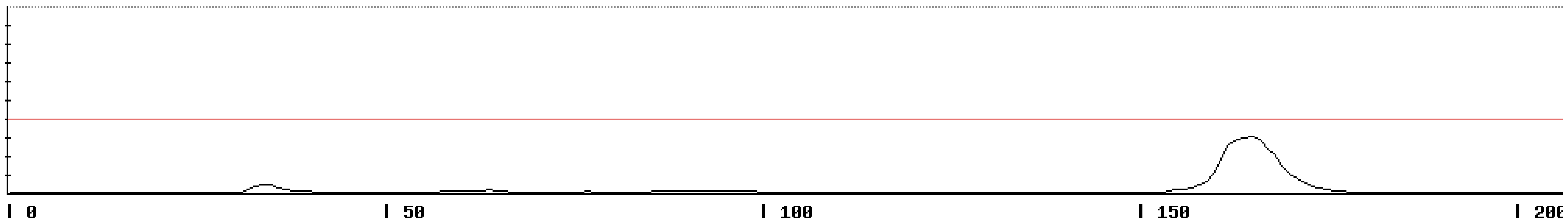

Predictions for Cajanuscajan_C.cajan_02052

Viterbi Path	
Viterbi Path	
Posterior @ 0.4	161 - GRRRKKARVSR - 171
Posterior @ 0.4	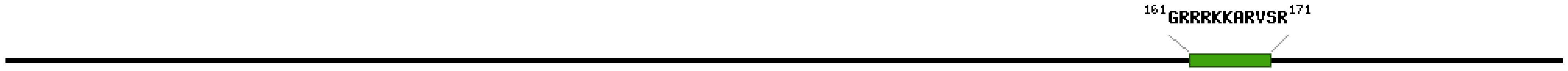

Posterior Graphic. Threshold Marked @ 0.4

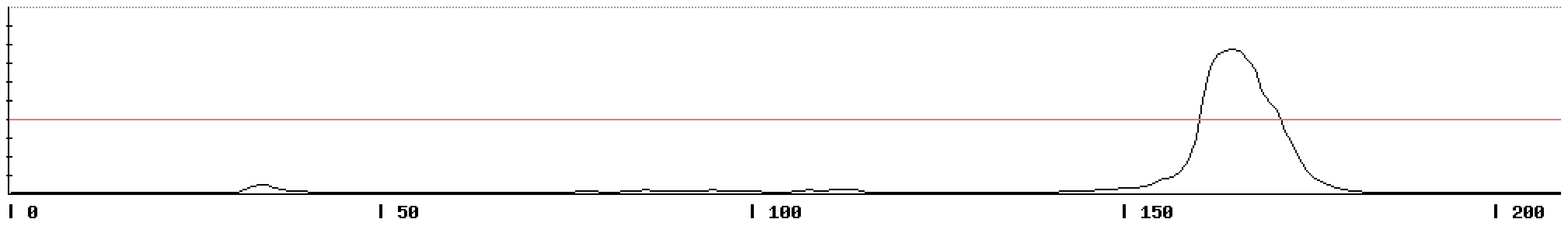

Predictions for Cajanuscajan_C.cajan_04121

Viterbi Path	
Viterbi Path	
Posterior @ 0.4	140 - RRRRK - 144

Posterior @ 0.4

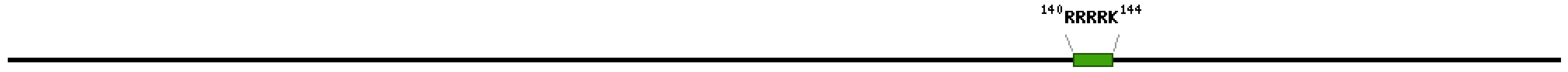

Posterior Graphic. Threshold Marked @ 0.4

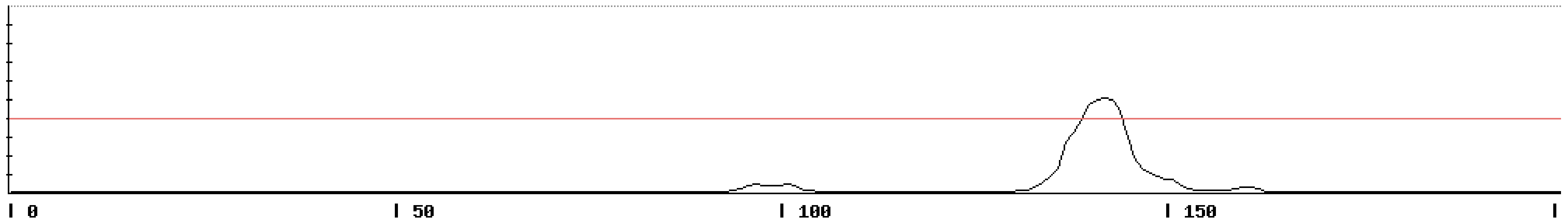

Predictions for Cajanuscajan_C.cajan_04140

Viterbi Path	
Viterbi Path	
Posterior @ 0.4	
Posterior @ 0.4	

Posterior Graphic. Threshold Marked @ 0.4

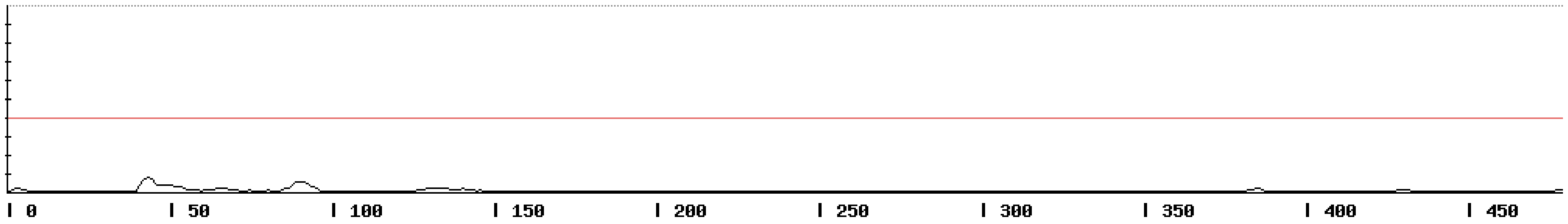

Predictions for Cajanuscajan_C.cajan_06648

Viterbi Path

Viterbi Path

Posterior @ 0.4

Posterior @ 0.4

Posterior Graphic. Threshold Marked @ 0.4

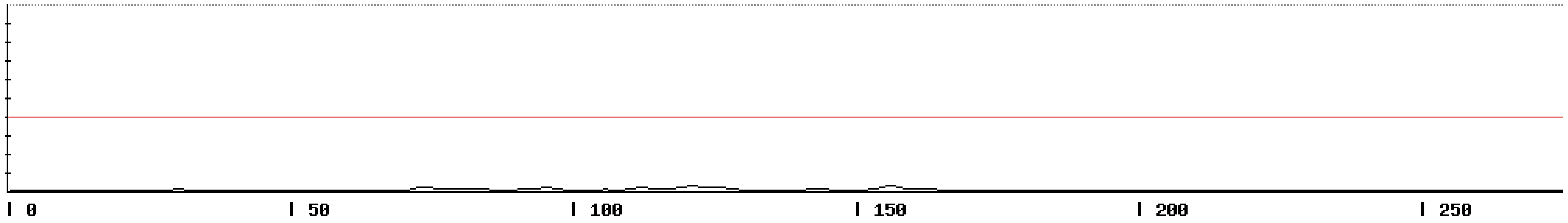

Predictions for Cajanuscajan_C.cajan_07217

Viterbi Path

Viterbi Path

Posterior @ 0.4

Posterior @ 0.4

Posterior Graphic. Threshold Marked @ 0.4

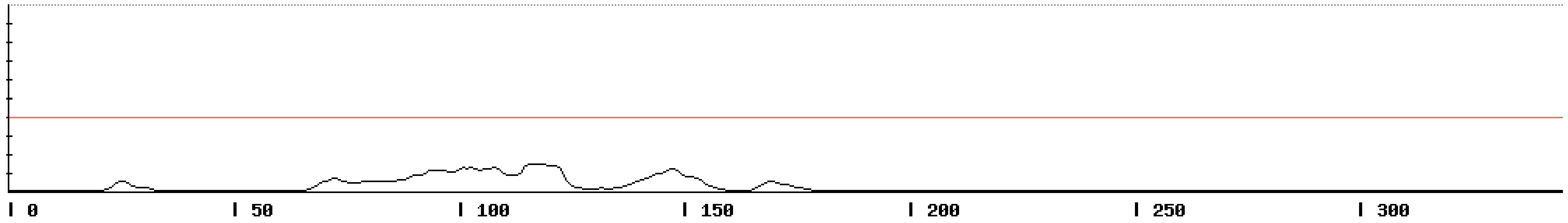

Predictions for Cajanuscajan_C.cajan_08648

Viterbi Path

Viterbi Path

Posterior @ 0.4

Posterior @ 0.4

Posterior Graphic. Threshold Marked @ 0.4

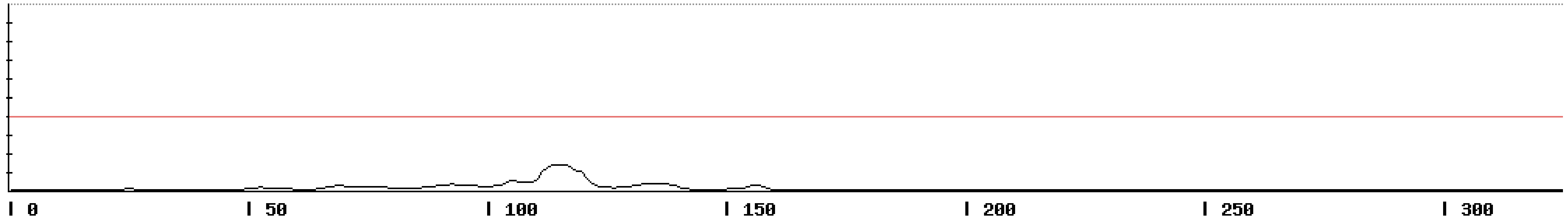

Predictions for Cajanuscajan_C.cajan_08704

Viterbi Path

Viterbi Path

Posterior @ 0.4

Posterior @ 0.4

Posterior Graphic. Threshold Marked @ 0.4

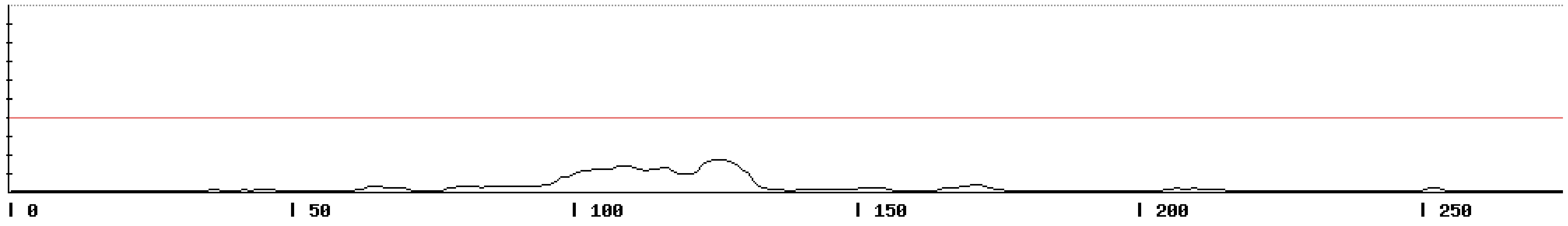

Predictions for Cajanuscajan_C.cajan_09117

Viterbi Path

Viterbi Path

Posterior @ 0.4

Posterior @ 0.4

Posterior Graphic. Threshold Marked @ 0.4

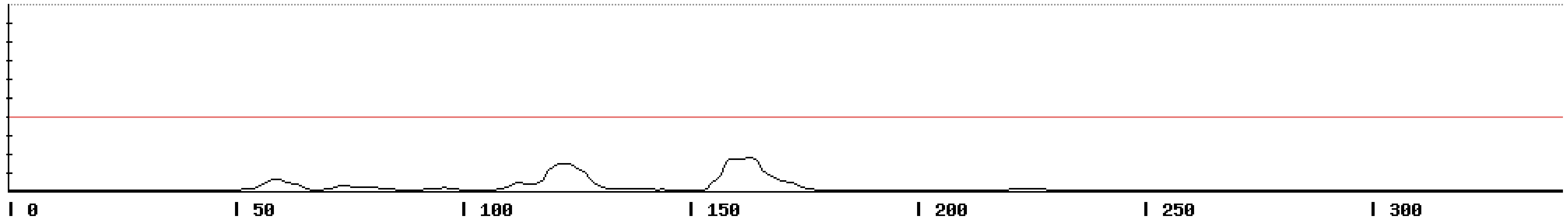

Predictions for Cajanuscajan_C.cajan_09817

Viterbi Path	
Viterbi Path	
Posterior @ 0.4	
Posterior @ 0.4	
Posterior Graphic. Threshold Marked @ 0.4	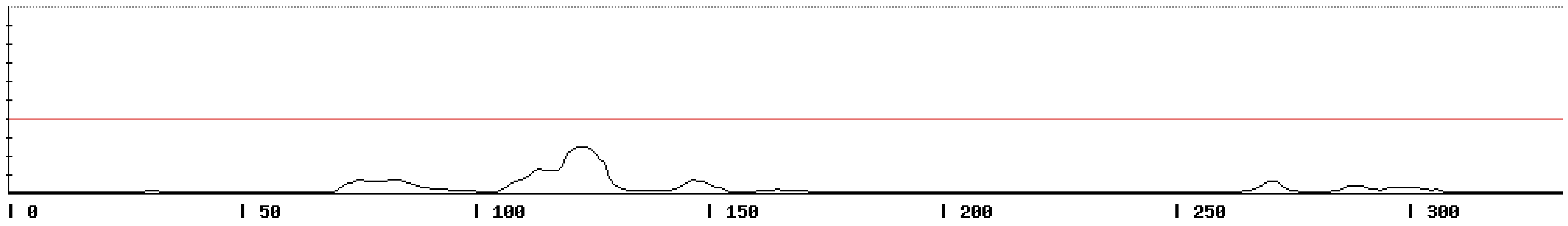

Predictions for Cajanuscajan_C.cajan_11824

Viterbi Path	
Viterbi Path	
Posterior @ 0.4	
Posterior @ 0.4	
Posterior Graphic. Threshold Marked @ 0.4	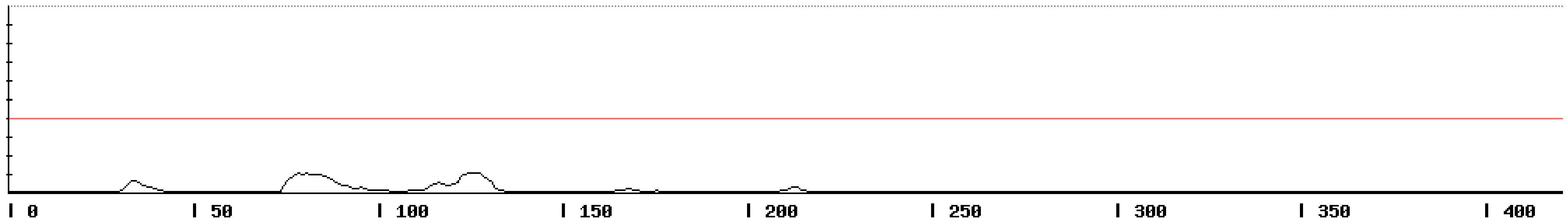

Predictions for Cajanuscajan_C.cajan_12803	
Viterbi Path	
Viterbi Path	
Posterior @ 0.4	
Posterior @ 0.4	
Posterior Graphic. Threshold Marked @ 0.4	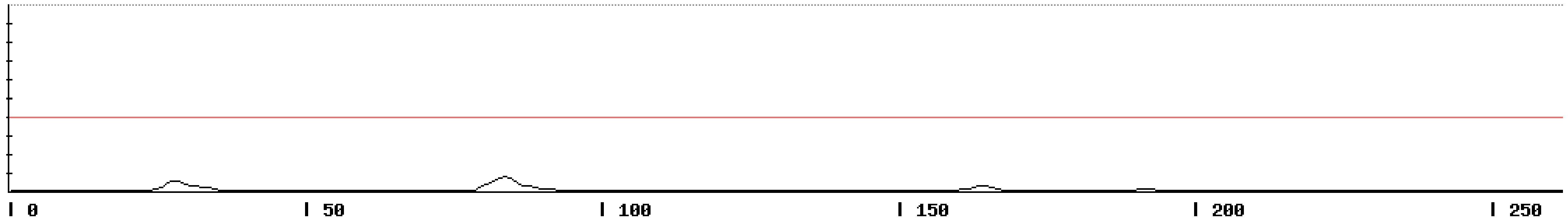

Predictions for Cajanuscajan_C.cajan_12845	
Viterbi Path	
Viterbi Path	
Posterior @ 0.4	
Posterior @ 0.4	
Posterior Graphic. Threshold Marked @ 0.4	

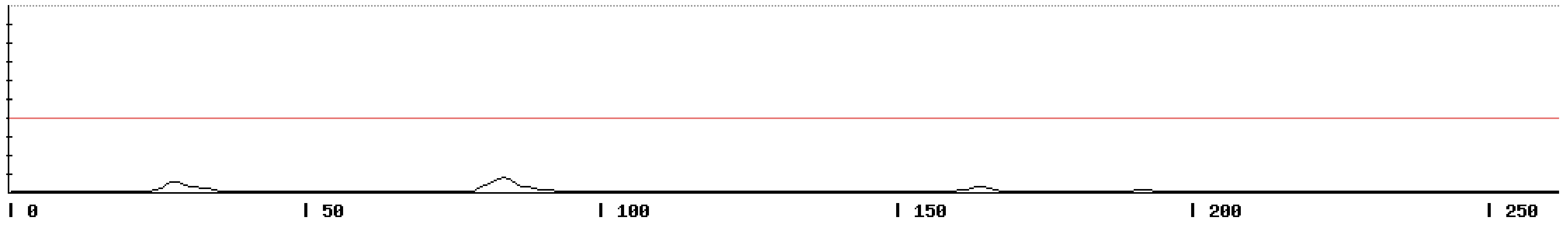

Predictions for Cajanuscajan_C.cajan_13790	
Viterbi Path	
Viterbi Path	
Posterior @ 0.4	127 - RKRRK - 131
Posterior @ 0.4	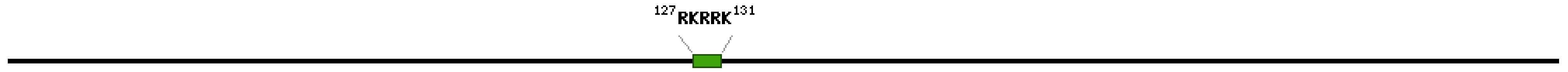
Posterior Graphic. Threshold Marked @ 0.4	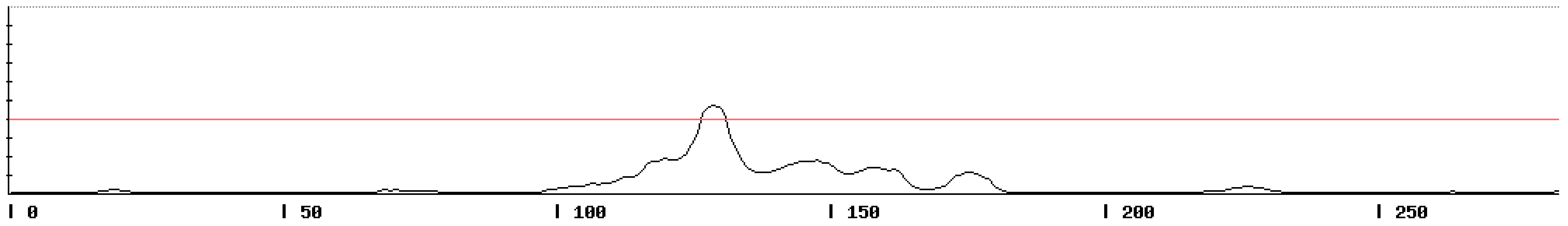

Predictions for Cajanuscajan_C.cajan_14029	
Viterbi Path	
Viterbi Path	
Posterior @ 0.4	
Posterior @ 0.4	

Posterior Graphic. Threshold Marked @ 0.4

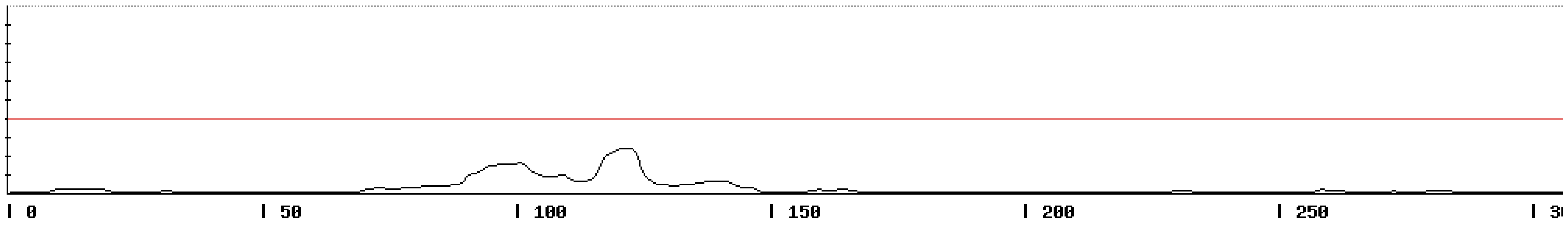

Predictions for Cajanuscajan_C.cajan_14854

Viterbi Path

Viterbi Path

Posterior @ 0.4

Posterior @ 0.4

Posterior Graphic. Threshold Marked @ 0.4

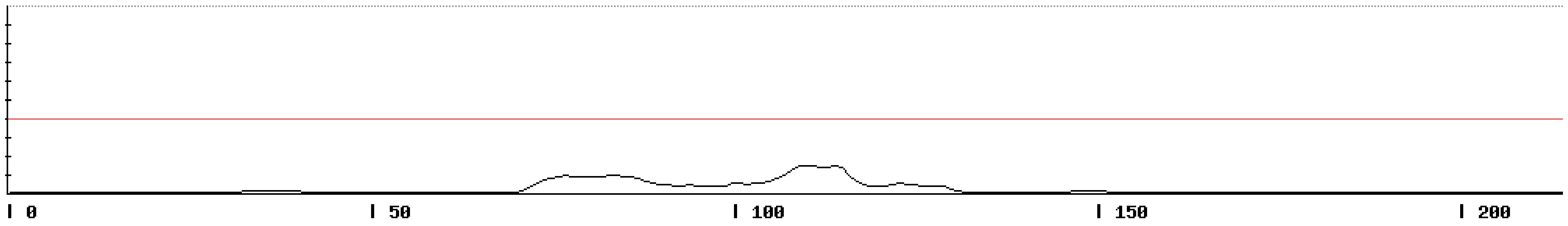

Predictions for Cajanuscajan_C.cajan_15439

Viterbi Path

Viterbi Path

Posterior @ 0.4

Posterior @ 0.4

Posterior Graphic. Threshold Marked @ 0.4

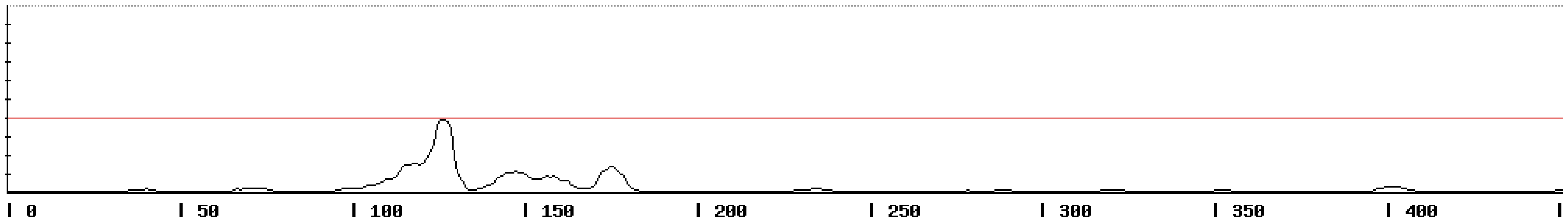

Predictions for Cajanuscajan_C.cajan_15622

Viterbi Path

Viterbi Path

Posterior @ 0.4

Posterior @ 0.4

Posterior Graphic. Threshold Marked @ 0.4

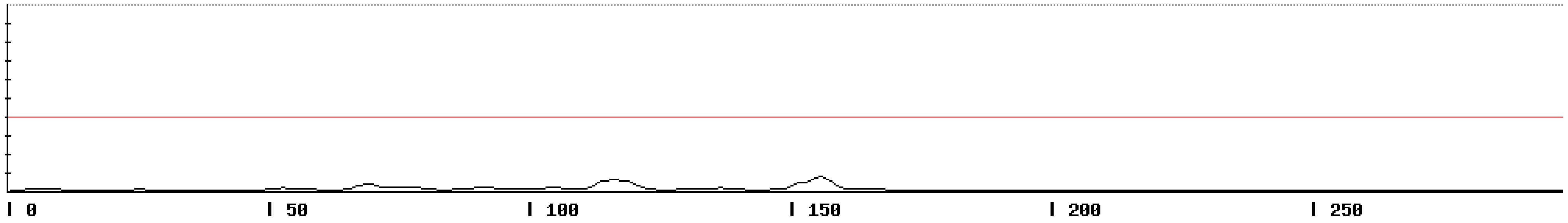

Predictions for Cajanuscajan_C.cajan_15737

Viterbi Path

Viterbi Path

Posterior @ 0.4	
Posterior @ 0.4	
Posterior Graphic. Threshold Marked @ 0.4	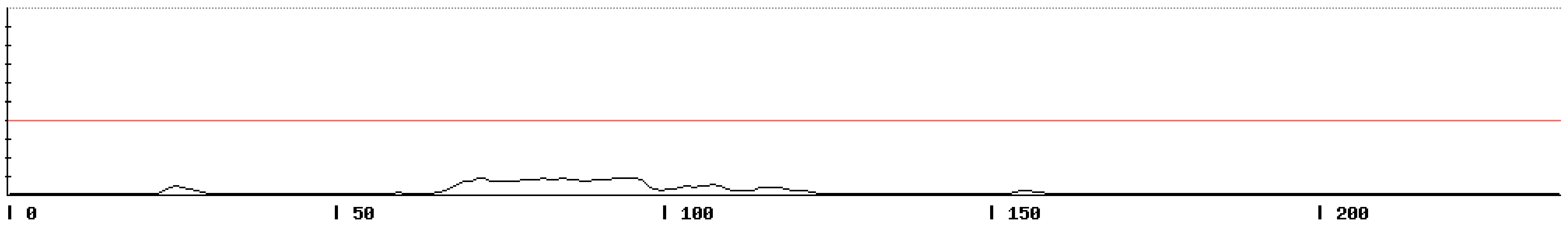

Predictions for Cajanuscajan_C.cajan_15921

Viterbi Path	
Viterbi Path	
Posterior @ 0.4	92 - GADKPIGKPKALGIKK - 107
Posterior @ 0.4	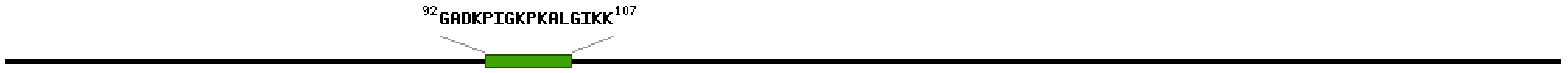
Posterior Graphic. Threshold Marked @ 0.4	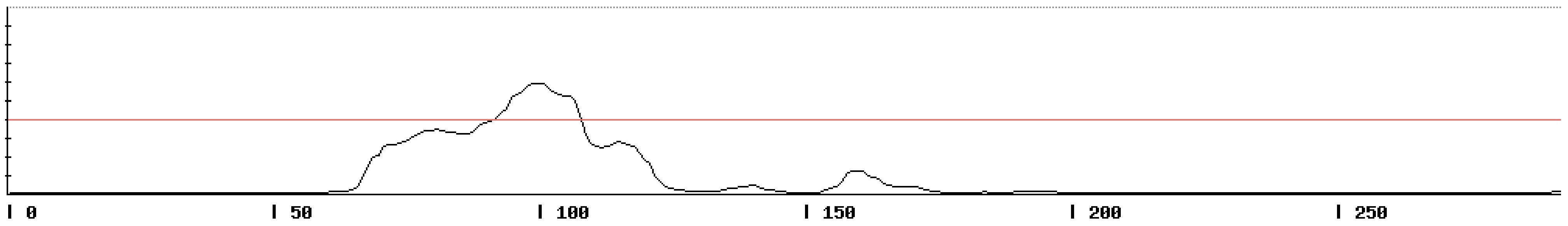

Predictions for Cajanuscajan_C.cajan_17016

Viterbi Path	
Viterbi Path	

Posterior @ 0.4

Posterior @ 0.4

Posterior Graphic. Threshold Marked @ 0.4

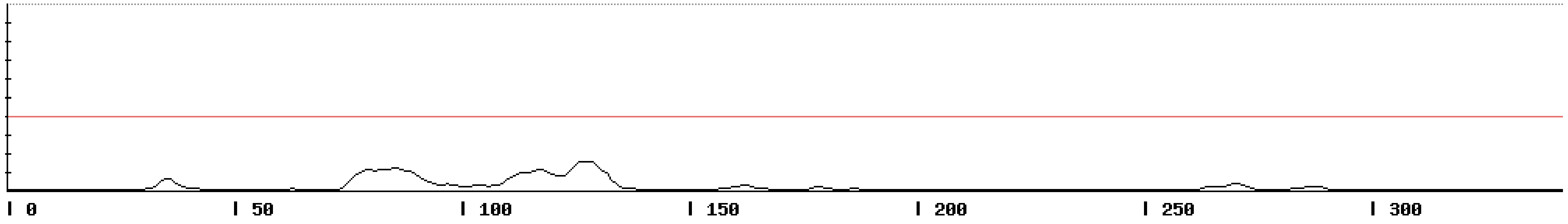

Predictions for Cajanuscajan_C.cajan_17157

Viterbi Path

Viterbi Path

Posterior @ 0.4

Posterior @ 0.4

Posterior Graphic. Threshold Marked @ 0.4

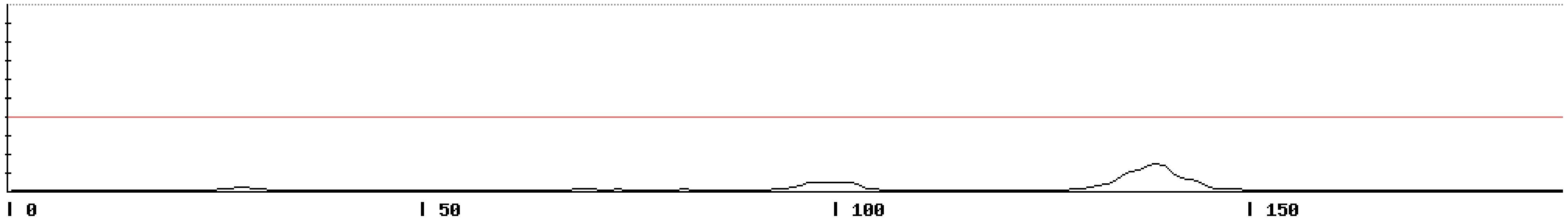

Predictions for Cajanuscajan_C.cajan_17807

Viterbi Path	
Viterbi Path	
Posterior @ 0.4	
Posterior @ 0.4	
Posterior Graphic. Threshold Marked @ 0.4	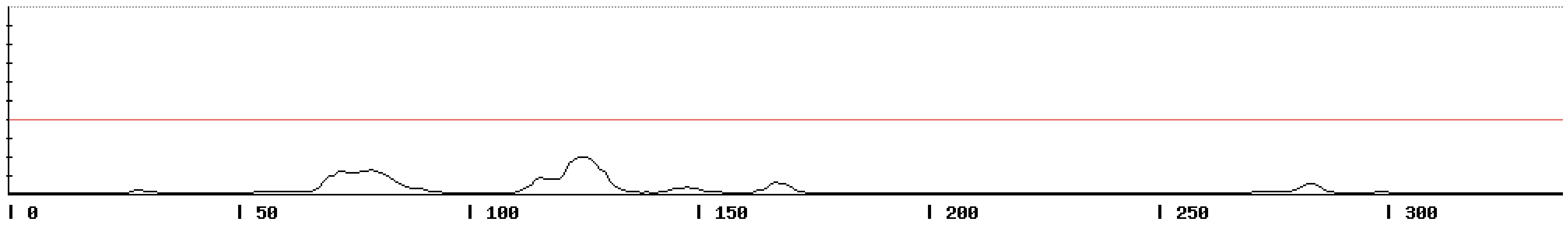

Predictions for Cajanuscajan_C.cajan_17814	
Viterbi Path	
Viterbi Path	
Posterior @ 0.4	
Posterior @ 0.4	
Posterior Graphic. Threshold Marked @ 0.4	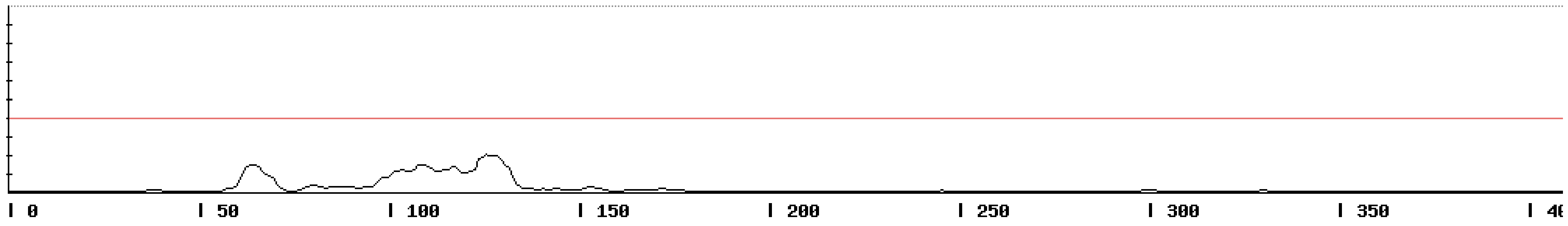

Predictions for Cajanuscajan_C.cajan_18402	
Viterbi Path	
Viterbi Path	
Posterior @ 0.4	
Posterior @ 0.4	
Posterior Graphic. Threshold Marked @ 0.4	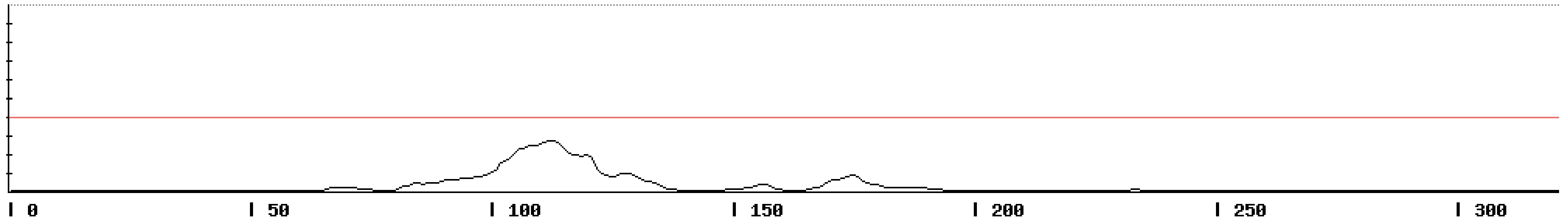

Predictions for Cajanuscajan_C.cajan_18734	
Viterbi Path	
Viterbi Path	
Posterior @ 0.4	
Posterior @ 0.4	
Posterior Graphic. Threshold Marked @ 0.4	

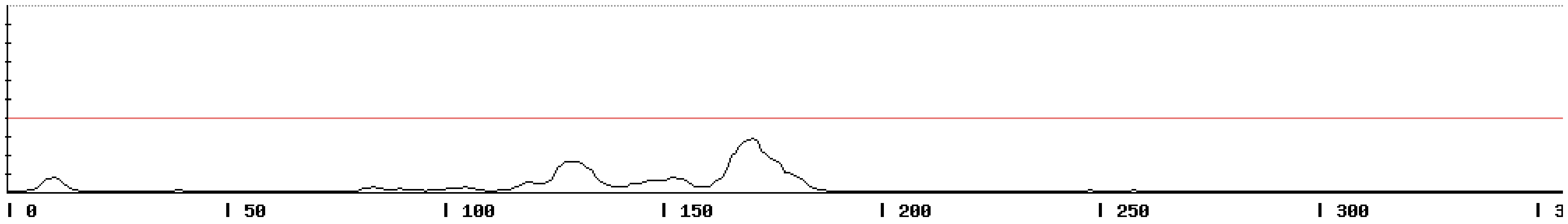

Predictions for Cajanuscajan_C.cajan_18948

Viterbi Path	
Viterbi Path	
Posterior @ 0.4	
Posterior @ 0.4	
Posterior Graphic. Threshold Marked @ 0.4	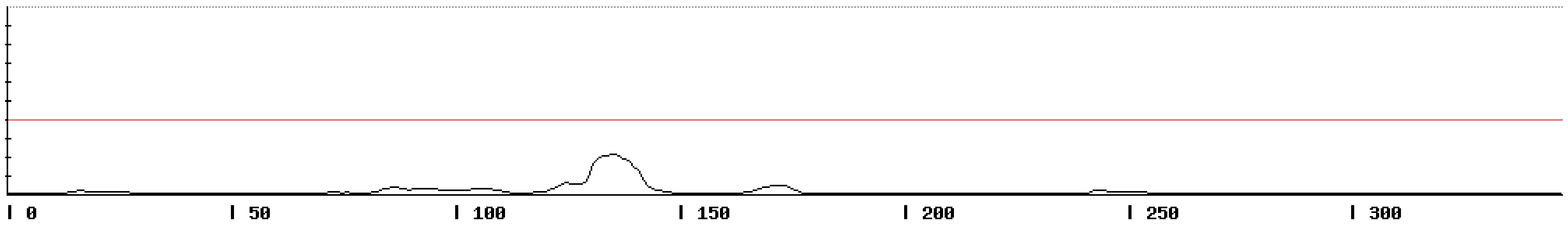

Predictions for Cajanuscajan_C.cajan_19098

Viterbi Path	
Viterbi Path	
Posterior @ 0.4	
Posterior @ 0.4	

Posterior Graphic. Threshold Marked @ 0.4

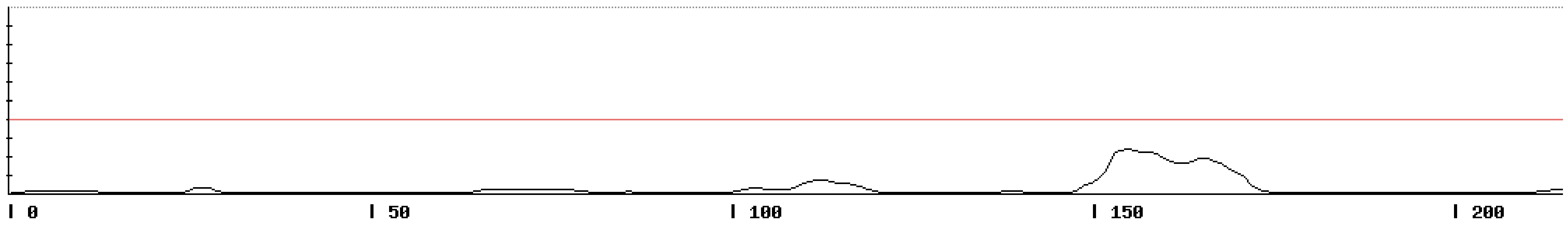

Predictions for Cajanuscajan_C.cajan_19195

Viterbi Path	
Viterbi Path	
Posterior @ 0.4	70 - FTKDRRKKCNRKTKAGFWKPTGKDREIKSRGSKNVIG - 106
Posterior @ 0.4	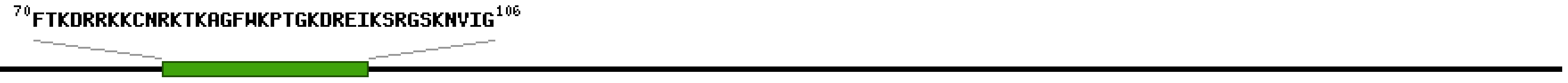
Posterior Graphic. Threshold Marked @ 0.4	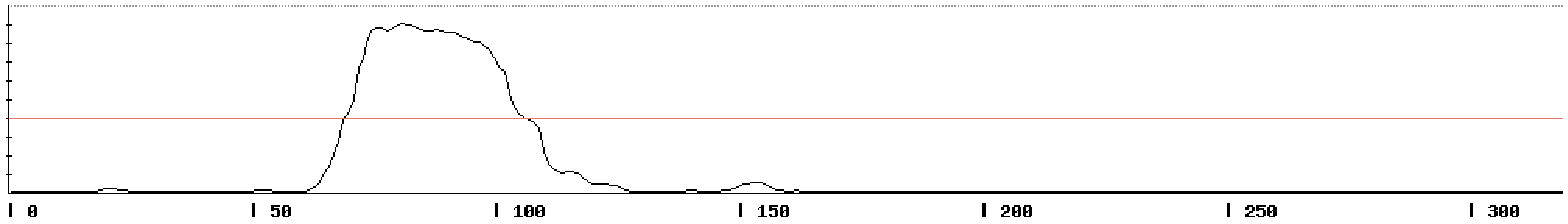

Predictions for Cajanuscajan_C.cajan_19966

Viterbi Path	
Viterbi Path	
Posterior @ 0.4	144 - RKRRK - 148
Posterior @ 0.4	

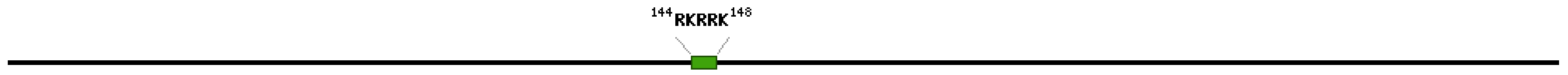

Posterior Graphic. Threshold Marked @ 0.4

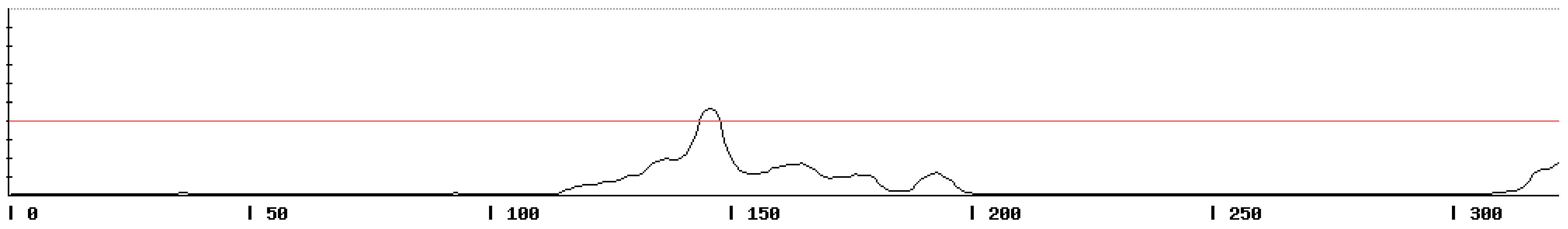

Predictions for Cajanuscajan_C.cajan_20225

Viterbi Path

Viterbi Path

Posterior @ 0.4

Posterior @ 0.4

Posterior Graphic. Threshold Marked @ 0.4

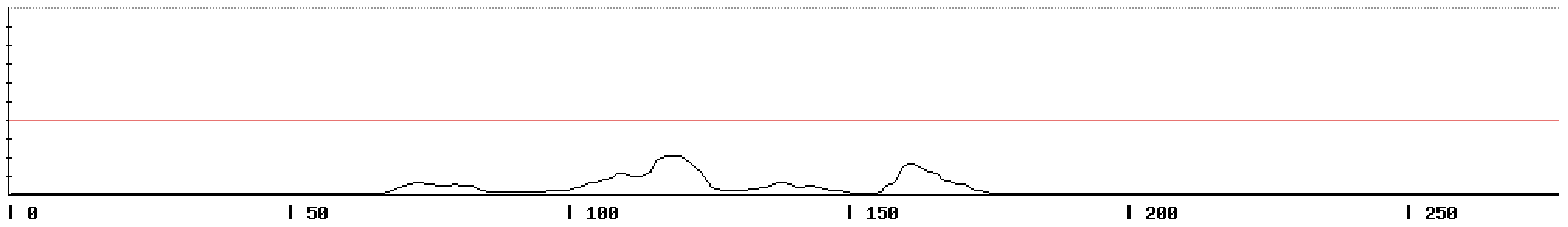

Predictions for Cajanuscajan_C.cajan_20892

Viterbi Path

Viterbi Path

Posterior @ 0.4	
Posterior @ 0.4	
Posterior Graphic. Threshold Marked @ 0.4	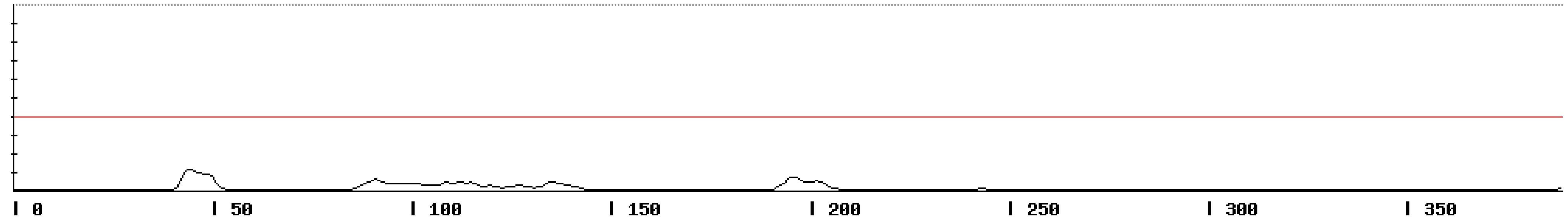

Predictions for Cajanuscajan_C.cajan_21061	
Viterbi Path	
Viterbi Path	
Posterior @ 0.4	
Posterior @ 0.4	
Posterior Graphic. Threshold Marked @ 0.4	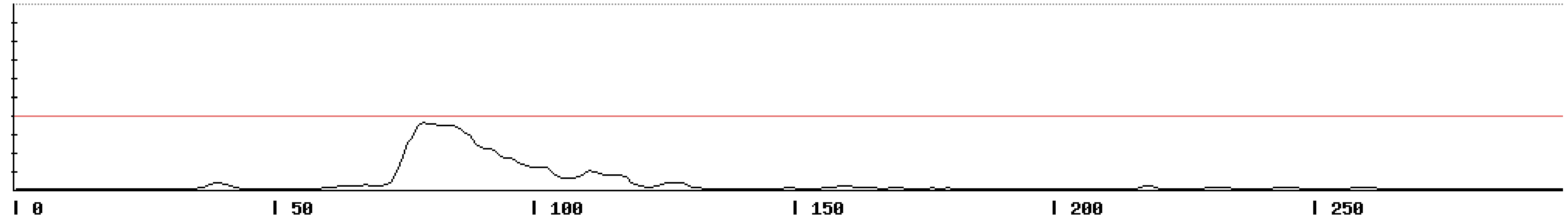

Predictions for Cajanuscajan_C.cajan_21708	
Viterbi Path	
Viterbi Path	

Posterior @ 0.4

Posterior @ 0.4

Posterior Graphic. Threshold Marked @ 0.4

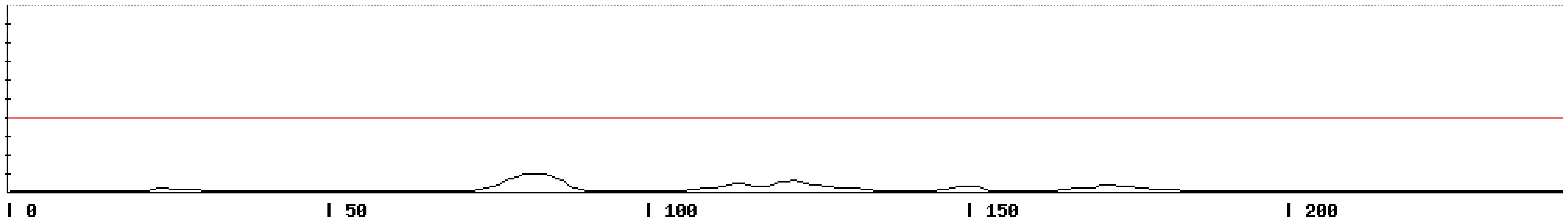

Predictions for Cajanuscajan_C.cajan_21774

Viterbi Path

Viterbi Path

Posterior @ 0.4

Posterior @ 0.4

Posterior Graphic. Threshold Marked @ 0.4

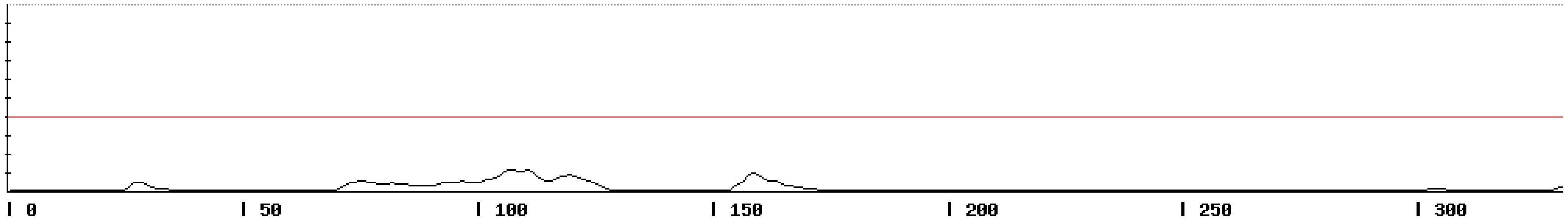

Predictions for Cajanuscajan_C.cajan_22429

Viterbi Path

Viterbi Path

Posterior @ 0.4

Posterior @ 0.4

Posterior Graphic. Threshold Marked @ 0.4

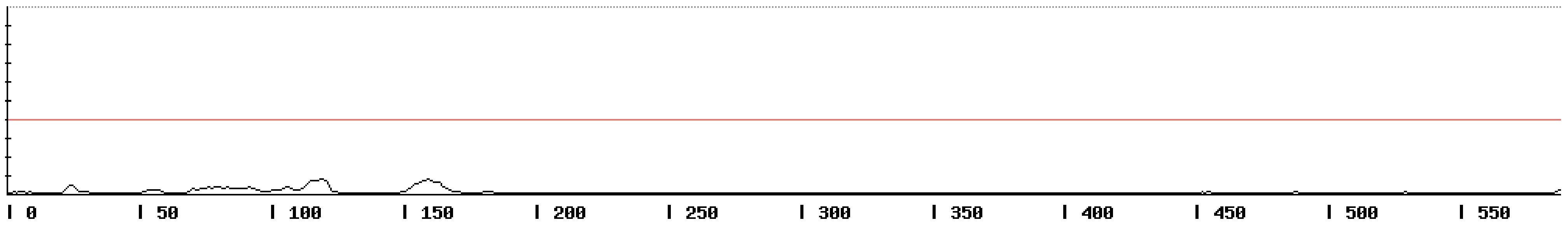

Predictions for Cajanuscajan_C.cajan_22430

Viterbi Path

Viterbi Path

Posterior @ 0.4

Posterior @ 0.4

Posterior Graphic. Threshold Marked @ 0.4

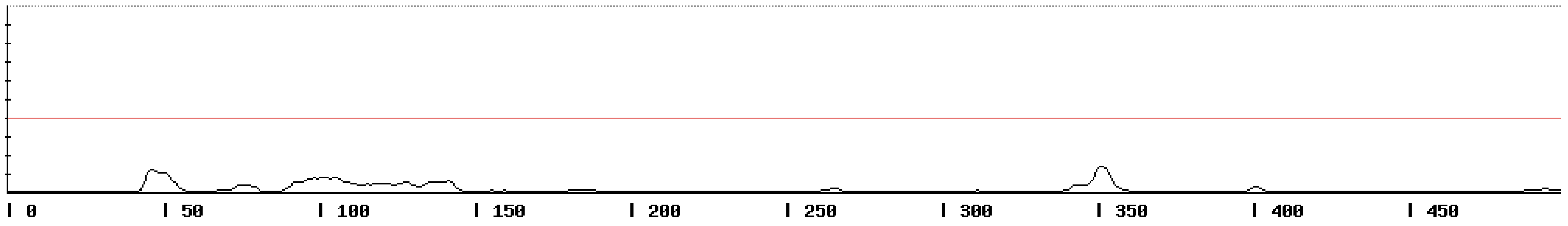

Predictions for Cajanuscajan_C.cajan_22489

Viterbi Path	
Viterbi Path	
Posterior @ 0.4	
Posterior @ 0.4	
Posterior Graphic. Threshold Marked @ 0.4	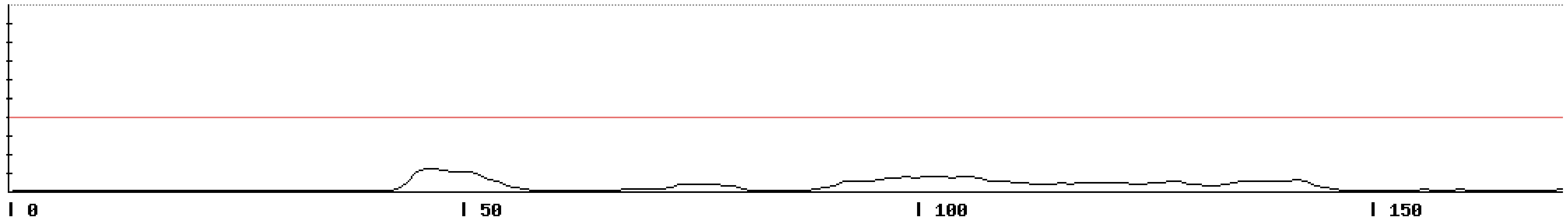

Predictions for Cajanuscajan_C.cajan_22852

Viterbi Path	
Viterbi Path	
Posterior @ 0.4	
Posterior @ 0.4	
Posterior Graphic. Threshold Marked @ 0.4	

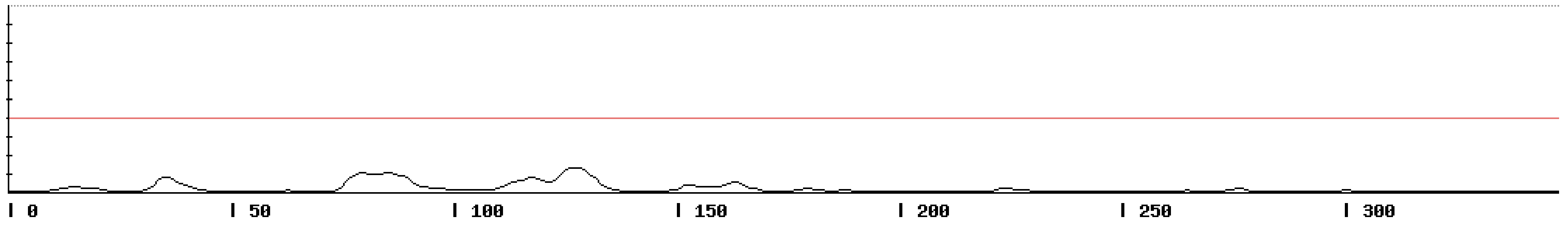

Predictions for Cajanuscajan_C.cajan_22870	
Viterbi Path	
Viterbi Path	
Posterior @ 0.4	
Posterior @ 0.4	
Posterior Graphic. Threshold Marked @ 0.4	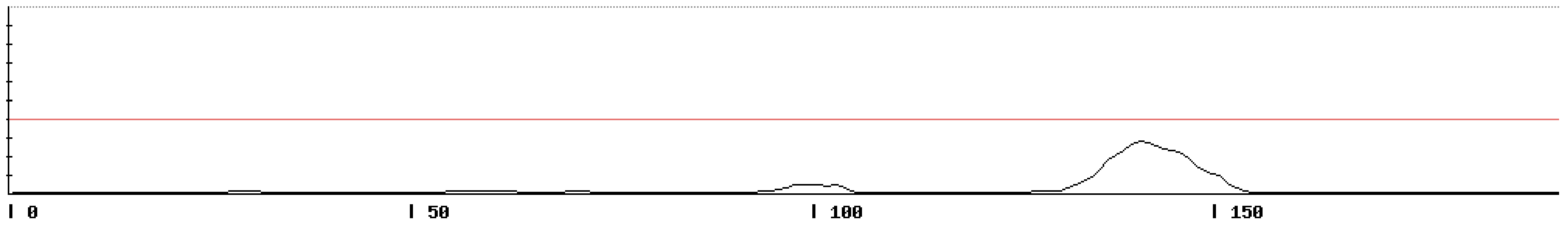

Predictions for Cajanuscajan_C.cajan_23161	
Viterbi Path	
Viterbi Path	
Posterior @ 0.4	
Posterior @ 0.4	

Posterior Graphic. Threshold Marked @ 0.4	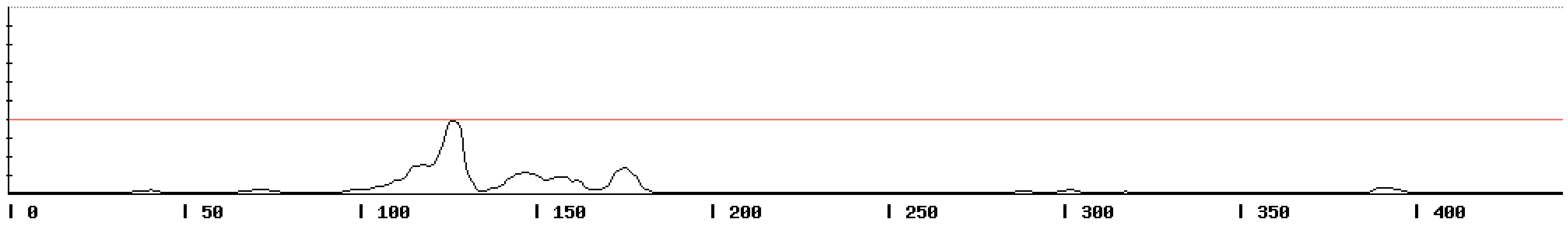

Predictions for Cajanuscajan_C.cajan_23518	
Viterbi Path	
Viterbi Path	
Posterior @ 0.4	
Posterior @ 0.4	
Posterior Graphic. Threshold Marked @ 0.4	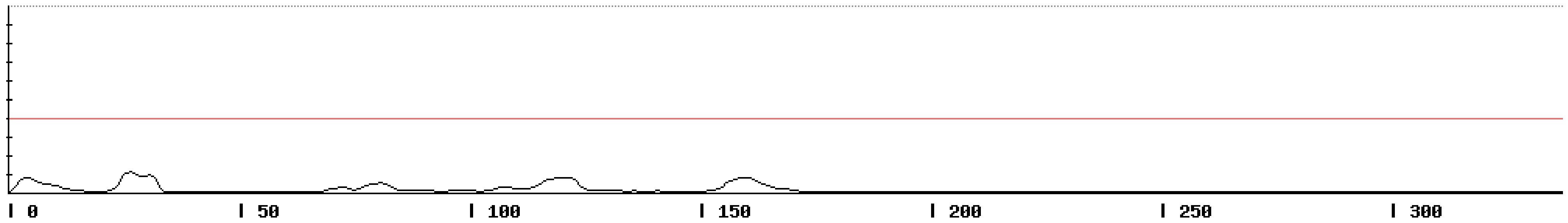

Predictions for Cajanuscajan_C.cajan_23529	
Viterbi Path	
Viterbi Path	
Posterior @ 0.4	
Posterior @ 0.4	

Posterior Graphic. Threshold Marked @ 0.4

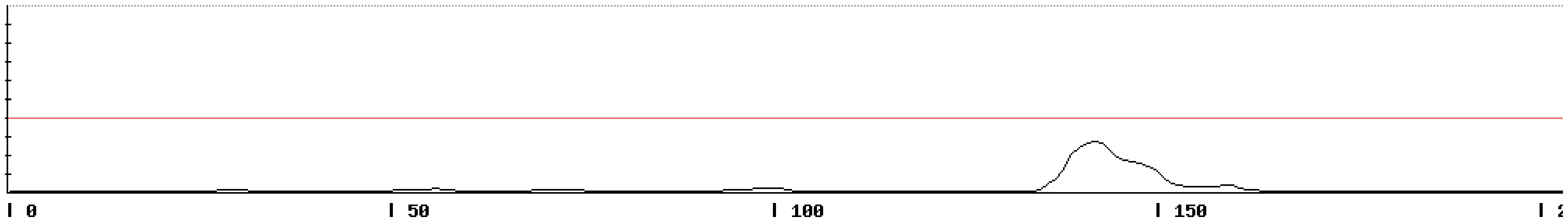

Predictions for Cajanuscajan_C.cajan_23928

Viterbi Path	
Viterbi Path	
Posterior @ 0.4	126 - RKRRK - 130
Posterior @ 0.4	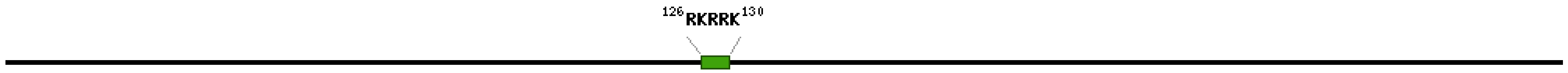

Posterior Graphic. Threshold Marked @ 0.4

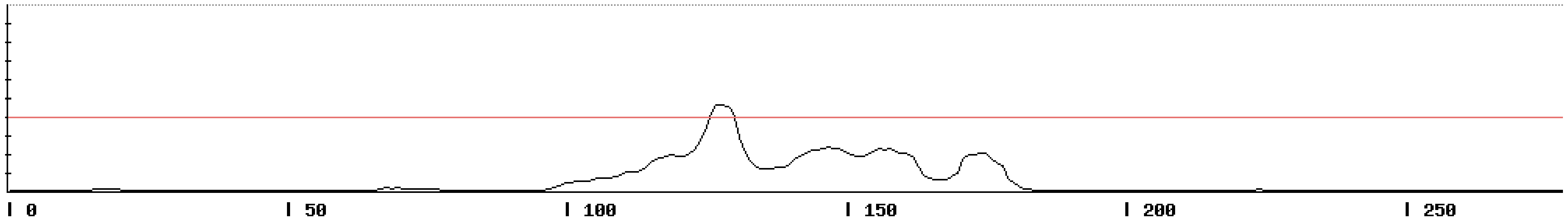

Predictions for Cajanuscajan_C.cajan_24591

Viterbi Path	
Viterbi Path	

Posterior @ 0.4

Posterior @ 0.4

Posterior Graphic. Threshold Marked @ 0.4

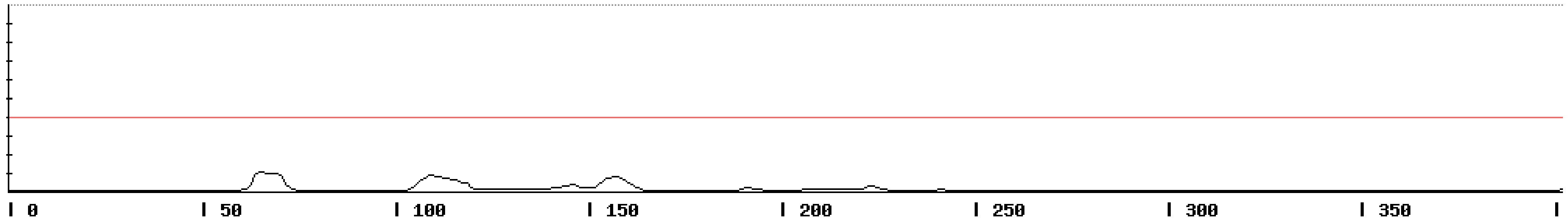

Predictions for Cajanuscajan_C.cajan_24657

Viterbi Path

Viterbi Path

Posterior @ 0.4

Posterior @ 0.4

Posterior Graphic. Threshold Marked @ 0.4

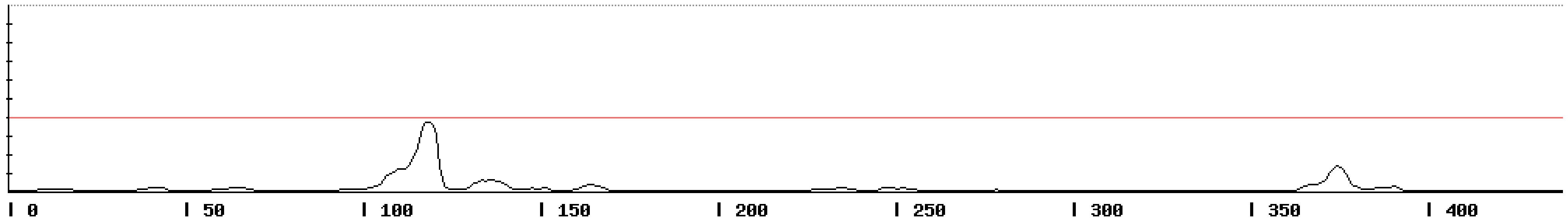

Predictions for Cajanuscajan_C.cajan_24755

Viterbi Path

Viterbi Path

Posterior @ 0.4	142 - KRR - 144
Posterior @ 0.4	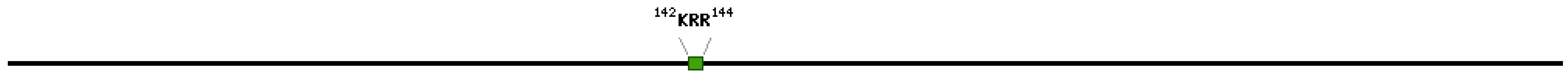
Posterior Graphic. Threshold Marked @ 0.4	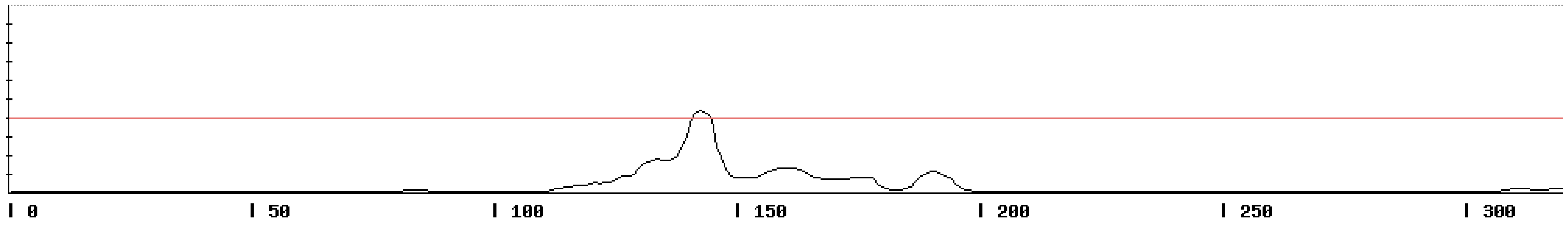

Predictions for Cajanuscajan_C.cajan_26125

Viterbi Path	
Viterbi Path	
Posterior @ 0.4	
Posterior @ 0.4	
Posterior Graphic. Threshold Marked @ 0.4	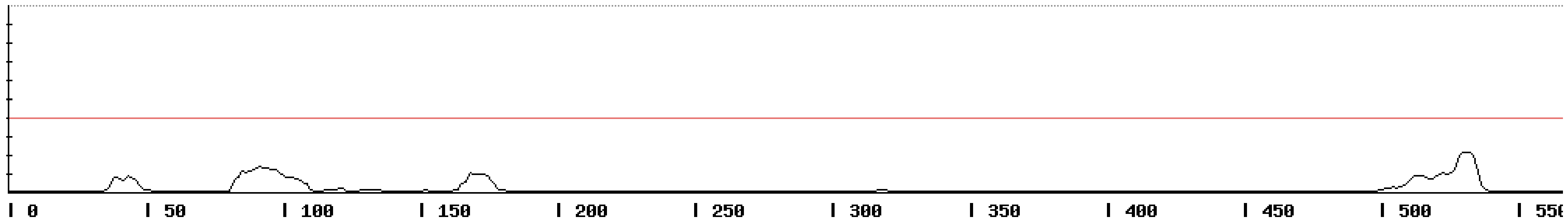

Predictions for Cajanuscajan_C.cajan_26764

Viterbi Path

Viterbi Path

Posterior @ 0.4

Posterior @ 0.4

Posterior Graphic. Threshold Marked @ 0.4

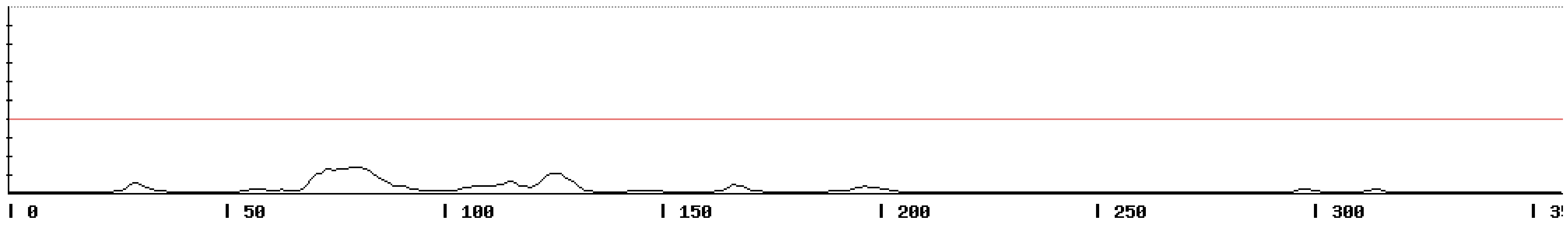

Predictions for Cajanuscajan_C.cajan_28603

Viterbi Path

Viterbi Path

Posterior @ 0.4

Posterior @ 0.4

Posterior Graphic. Threshold Marked @ 0.4

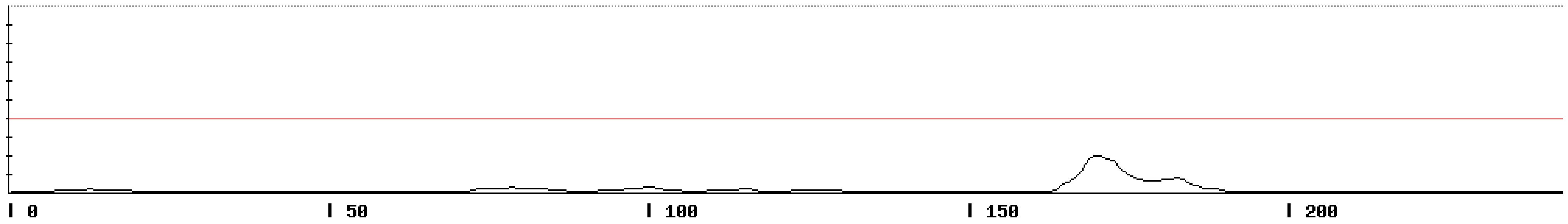

Predictions for Cajanuscajan_C.cajan_29425

Viterbi Path

Viterbi Path

Posterior @ 0.4

Posterior @ 0.4

Posterior Graphic. Threshold Marked @ 0.4

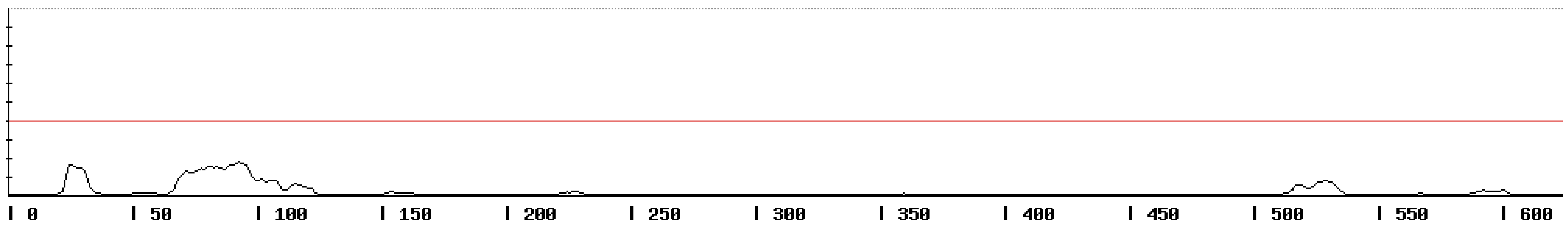

Predictions for Cajanuscajan_C.cajan_29427

Viterbi Path

Viterbi Path

Posterior @ 0.4

Posterior @ 0.4

Posterior Graphic. Threshold Marked @ 0.4

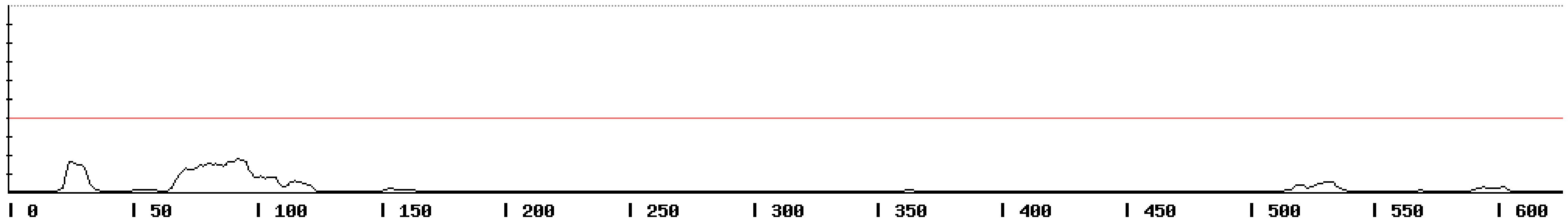

Predictions for Cajanuscajan_C.cajan_29871

Viterbi Path

Viterbi Path

Posterior @ 0.4 — 96 - ADKPIGKPKPVGIKKA - 111

Posterior @ 0.4

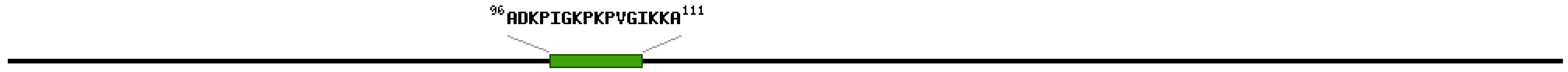

Posterior Graphic. Threshold Marked @ 0.4

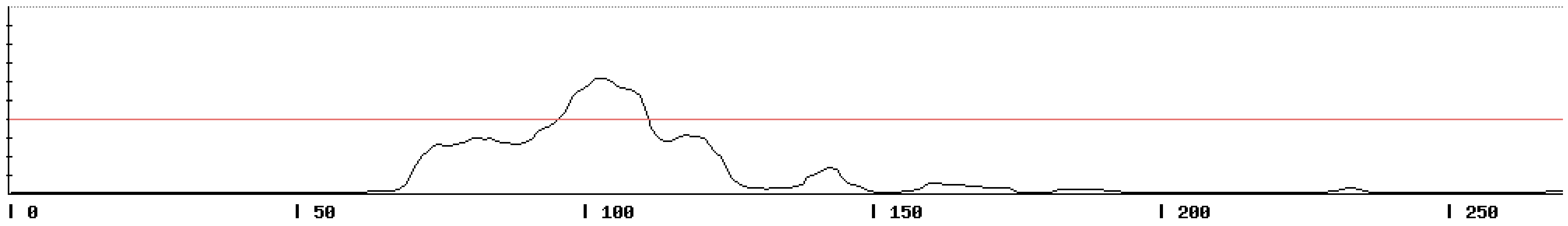

Predictions for Cajanuscajan_C.cajan_30472

Viterbi Path

Viterbi Path

Posterior @ 0.4

Posterior @ 0.4

Posterior Graphic. Threshold Marked @ 0.4	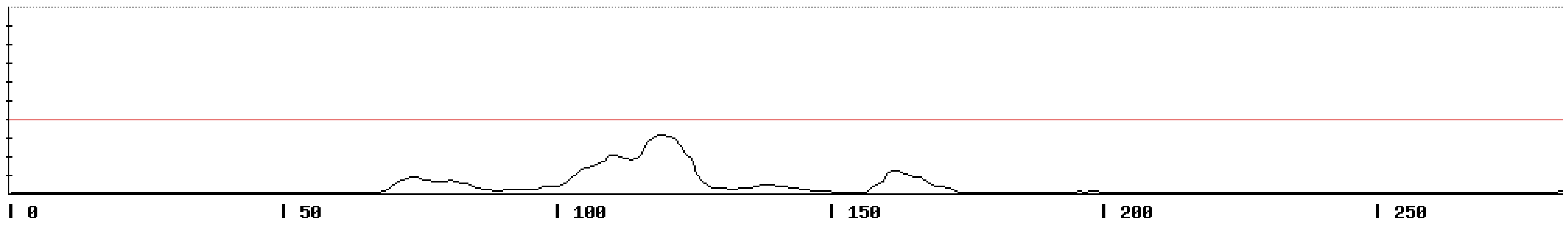

Predictions for Cajanuscajan_C.cajan_30485

Viterbi Path	
Viterbi Path	
Posterior @ 0.4	
Posterior @ 0.4	
Posterior Graphic. Threshold Marked @ 0.4	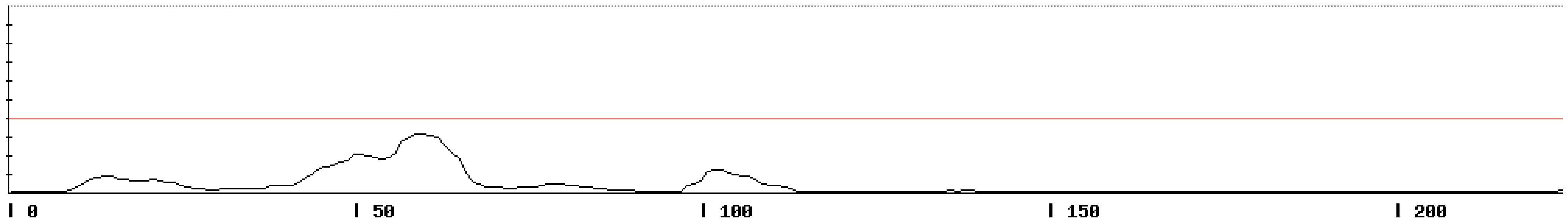

Predictions for Cajanuscajan_C.cajan_30687

Viterbi Path	
Viterbi Path	
Posterior @ 0.4	
Posterior @ 0.4	

Posterior Graphic. Threshold Marked @ 0.4

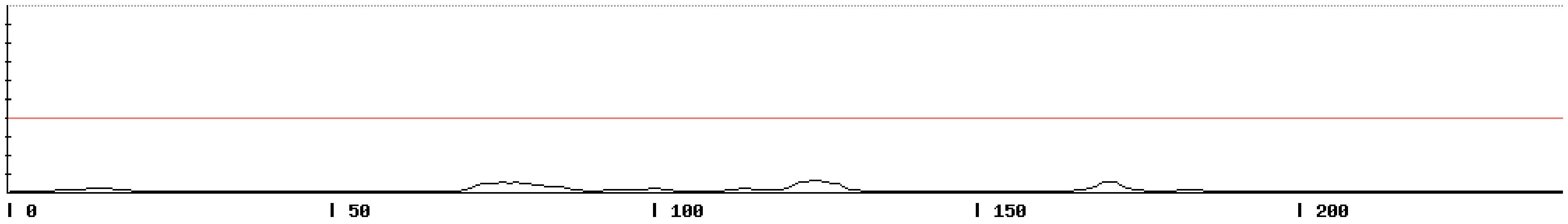

Predictions for Cajanuscajan_C.cajan_31437

Viterbi Path

Viterbi Path

Posterior @ 0.4 156 - RKRRK - 160

Posterior @ 0.4

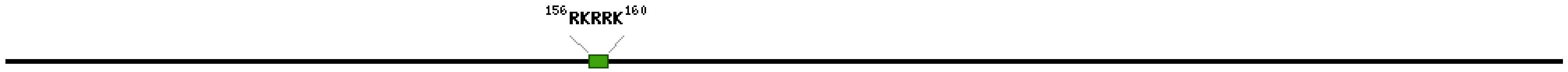

Posterior Graphic. Threshold Marked @ 0.4

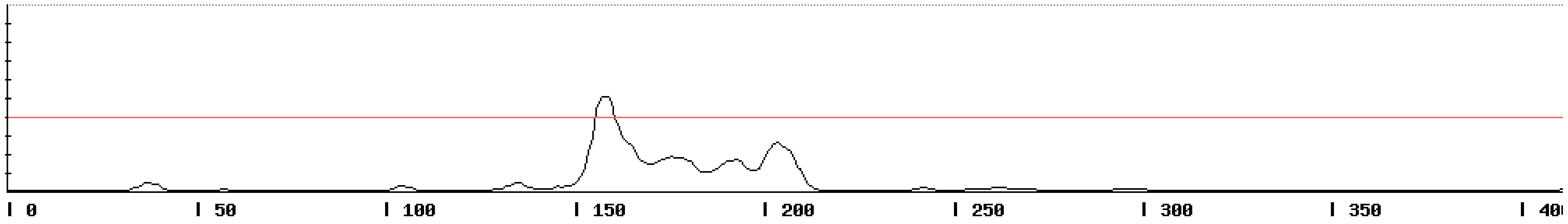

Predictions for Cajanuscajan_C.cajan_31741

Viterbi Path

Viterbi Path

Posterior @ 0.4 | 127 - GRK - 129

Posterior @ 0.4

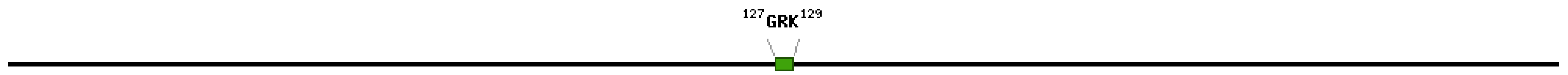

Posterior Graphic. Threshold Marked @ 0.4

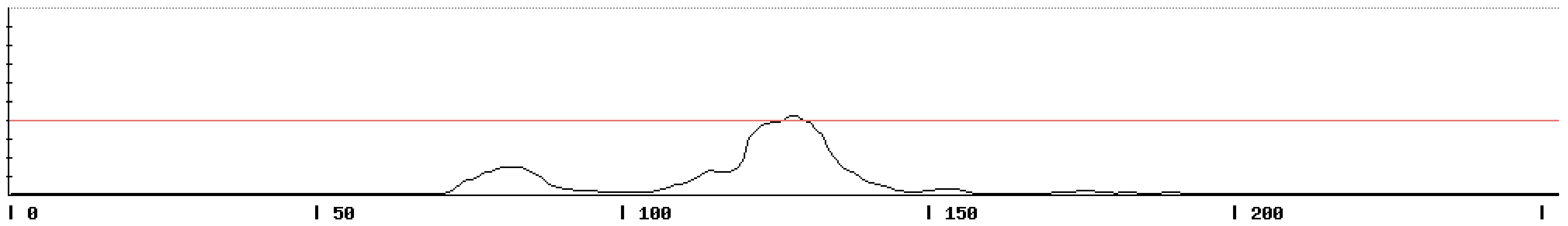

Predictions for Cajanuscajan_C.cajan_32331

Viterbi Path

Viterbi Path

Posterior @ 0.4

Posterior @ 0.4

Posterior Graphic. Threshold Marked @ 0.4

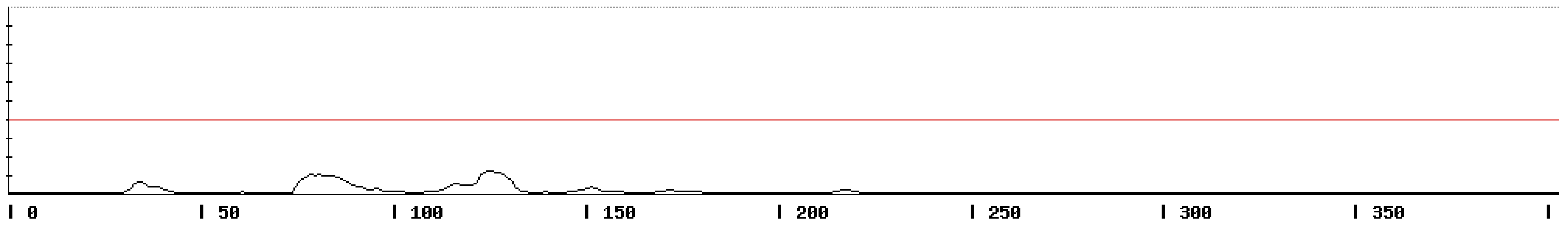

Predictions for Cajanuscajan_C.cajan_32993

Viterbi Path

Viterbi Path

Posterior @ 0.4

Posterior @ 0.4

Posterior Graphic. Threshold Marked @ 0.4

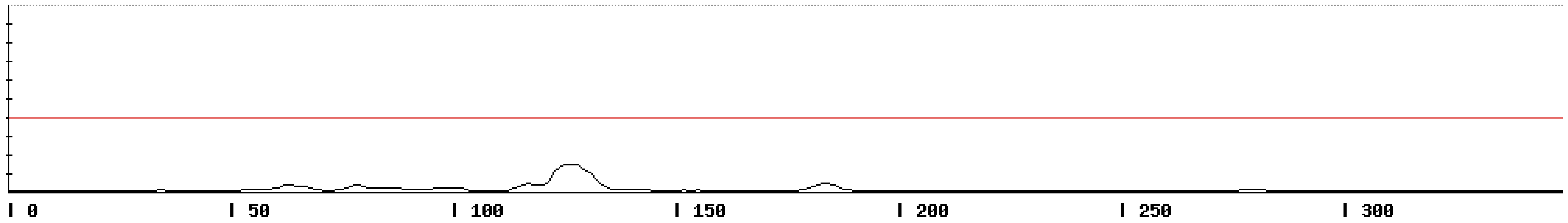

Predictions for Cajanuscajan_C.cajan_34115

Viterbi Path

Viterbi Path

Posterior @ 0.4

Posterior @ 0.4

Posterior Graphic. Threshold Marked @ 0.4

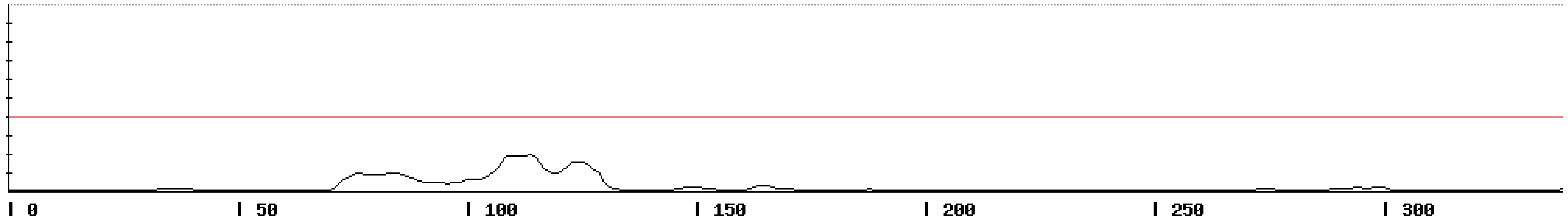

Predictions for Cajanuscajan_C.cajan_34162

Viterbi Path

Viterbi Path

Posterior @ 0.4

Posterior @ 0.4

Posterior Graphic. Threshold Marked @ 0.4

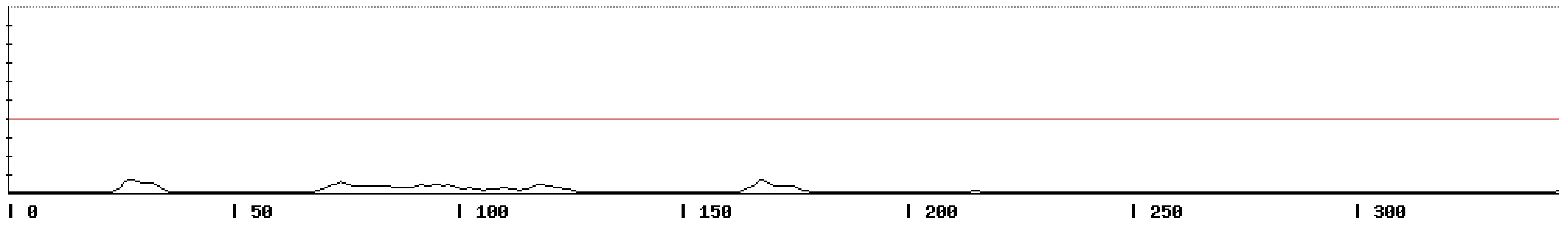

Predictions for Cajanuscajan_C.cajan_35648

Viterbi Path

Viterbi Path

Posterior @ 0.4

Posterior @ 0.4

Posterior Graphic. Threshold Marked @ 0.4

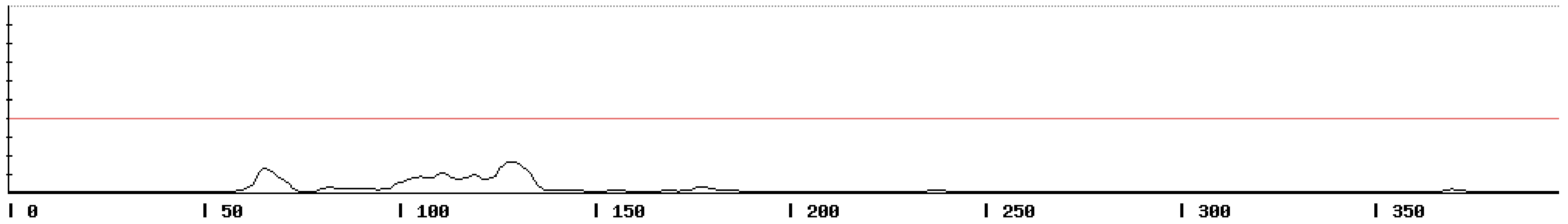

Predictions for Cajanuscajan_C.cajan_36091

Viterbi Path

Viterbi Path

Posterior @ 0.4

Posterior @ 0.4

Posterior Graphic. Threshold Marked @ 0.4

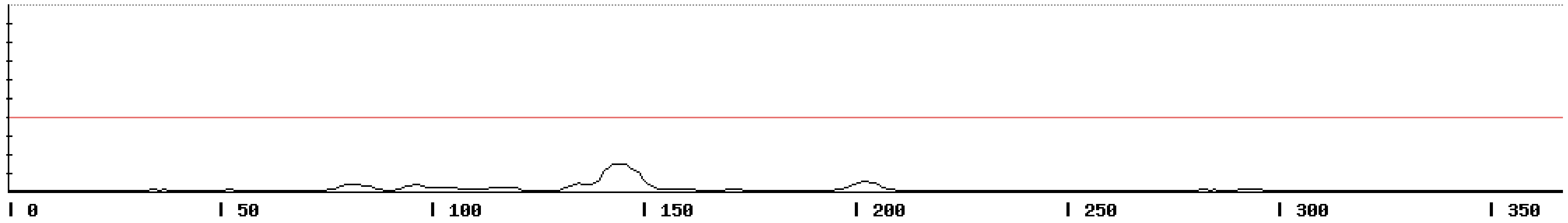

Predictions for Cajanuscajan_C.cajan_36992

Viterbi Path

Viterbi Path

Posterior @ 0.4

Posterior @ 0.4

Posterior Graphic. Threshold Marked @ 0.4

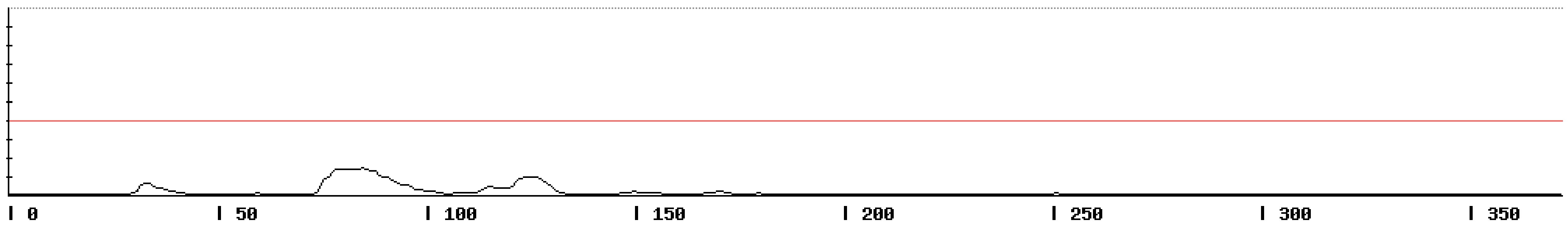

Predictions for Cajanuscajan_C.cajan_36997

Viterbi Path	
Viterbi Path	
Posterior @ 0.4	
Posterior @ 0.4	
Posterior Graphic. Threshold Marked @ 0.4	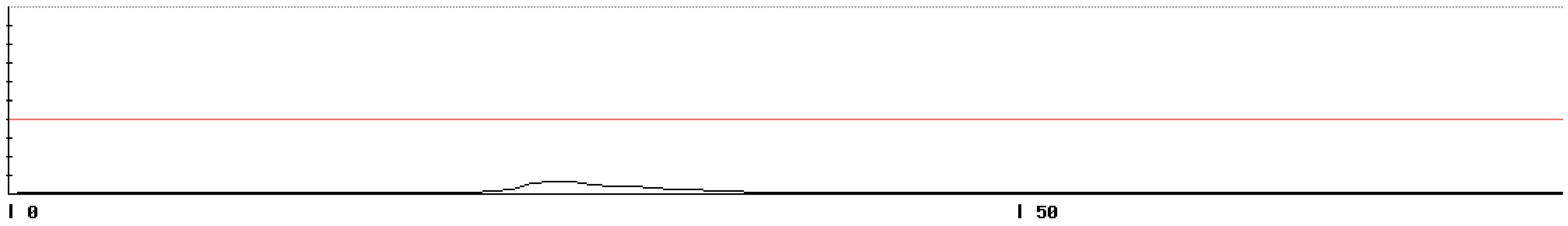

Predictions for Cajanuscajan_C.cajan_37341

Viterbi Path	
Viterbi Path	
Posterior @ 0.4	
Posterior @ 0.4	

Posterior Graphic. Threshold Marked @ 0.4

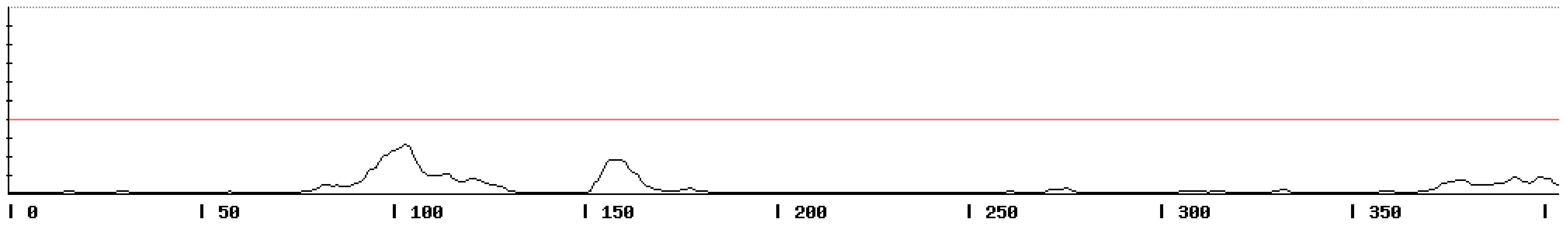

Predictions for Cajanuscajan_C.cajan_37342

Viterbi Path	
Viterbi Path	
Posterior @ 0.4	460 - RKRR - 463
Posterior @ 0.4	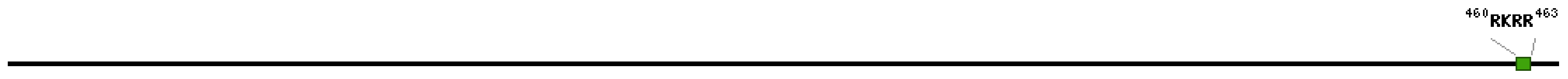

Posterior Graphic. Threshold Marked @ 0.4

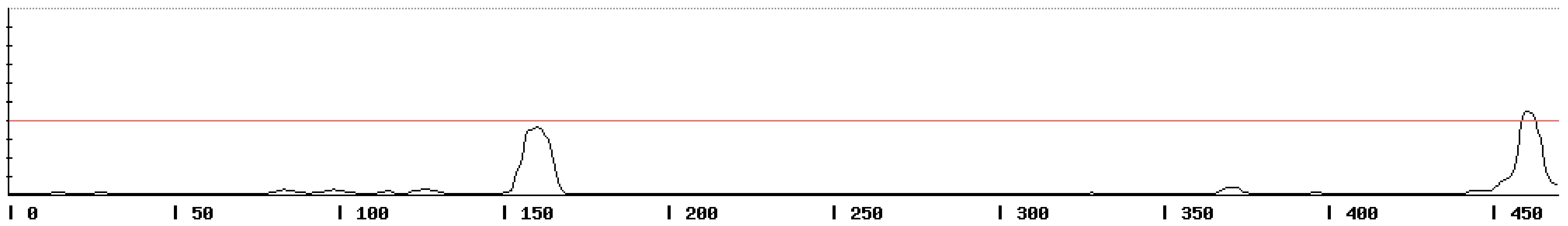

Predictions for Cajanuscajan_C.cajan_37971

Viterbi Path	
Viterbi Path	
Posterior @ 0.4	
Posterior @ 0.4	

Posterior Graphic. Threshold Marked @ 0.4	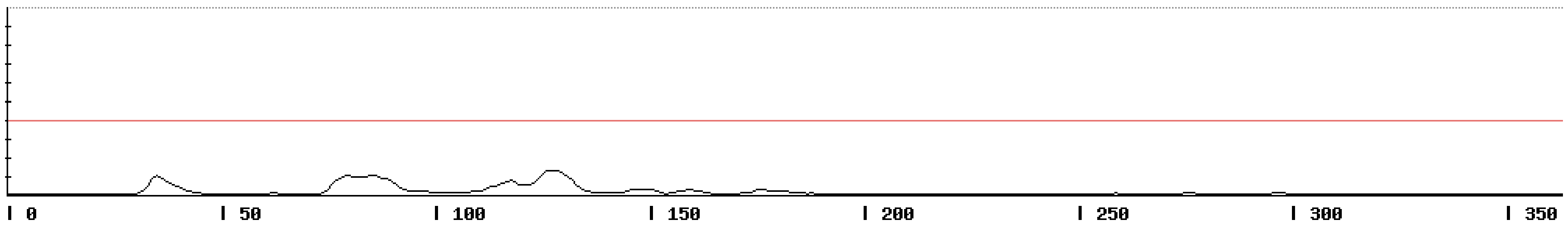

Predictions for Cajanuscajan_C.cajan_38151

Viterbi Path	
Viterbi Path	
Posterior @ 0.4	95 - KPIGKPKALGIKK - 107
Posterior @ 0.4	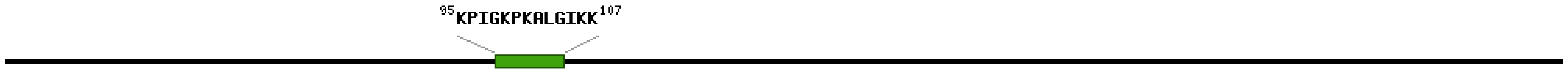
Posterior Graphic. Threshold Marked @ 0.4	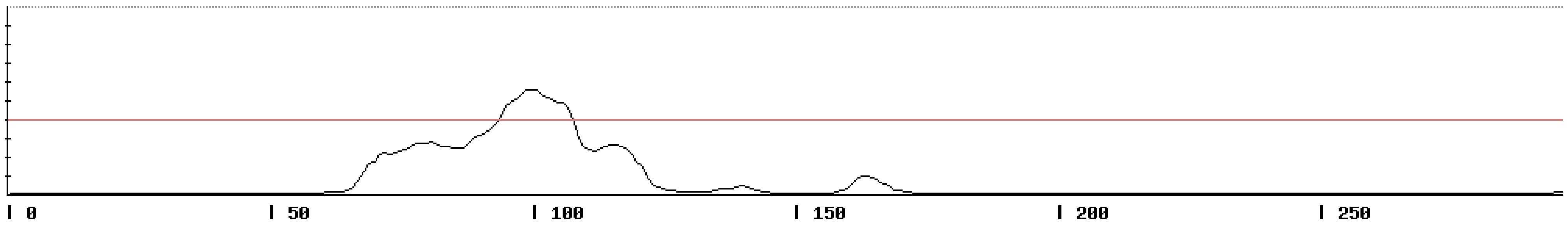

Predictions for Cajanuscajan_C.cajan_38763

Viterbi Path	
Viterbi Path	

Posterior @ 0.4

Posterior @ 0.4

Posterior Graphic. Threshold Marked @ 0.4

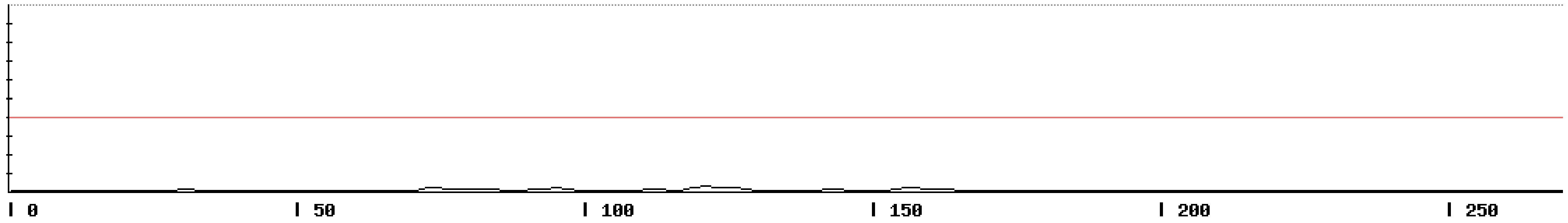

Predictions for Cajanuscajan_C.cajan_39141

Viterbi Path

Viterbi Path

Posterior @ 0.4

Posterior @ 0.4

Posterior Graphic. Threshold Marked @ 0.4

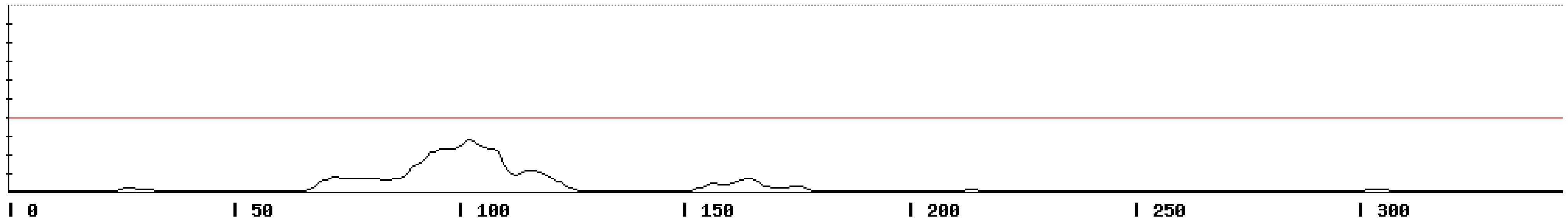

Predictions for Cajanuscajan_C.cajan_39689

Viterbi Path

Viterbi Path

Posterior @ 0.4

Posterior @ 0.4

Posterior Graphic. Threshold Marked @ 0.4

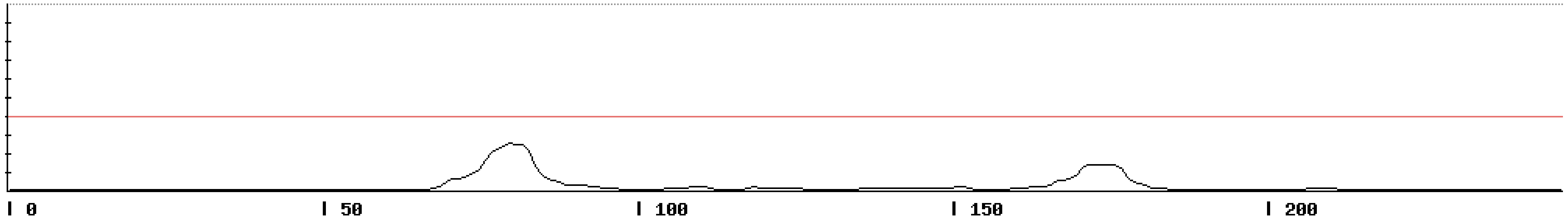

Predictions for Cajanuscajan_C.cajan_40054

Viterbi Path

Viterbi Path

Posterior @ 0.4

Posterior @ 0.4

Posterior Graphic. Threshold Marked @ 0.4

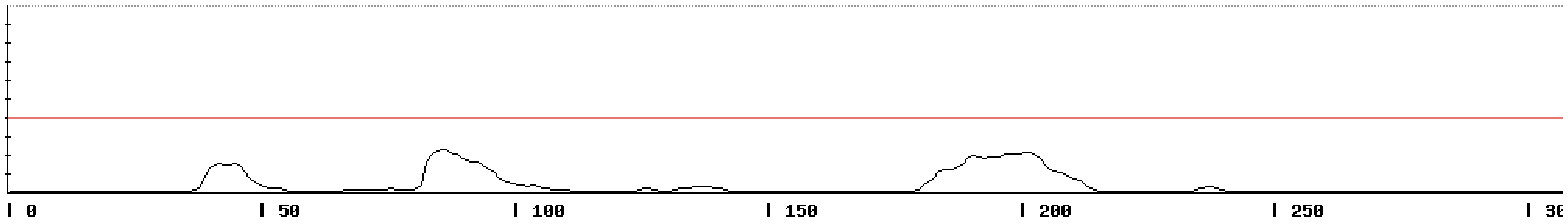

Predictions for Cajanuscajan_C.cajan_40311

Viterbi Path

Viterbi Path

Posterior @ 0.4

Posterior @ 0.4

Posterior Graphic. Threshold Marked @ 0.4

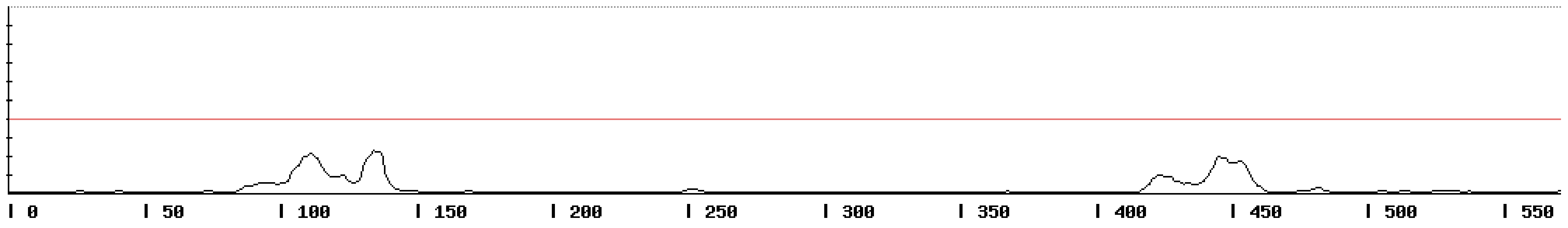

Predictions for Cajanuscajan_C.cajan_40755

Viterbi Path

Viterbi Path

Posterior @ 0.4

Posterior @ 0.4

Posterior Graphic. Threshold Marked @ 0.4

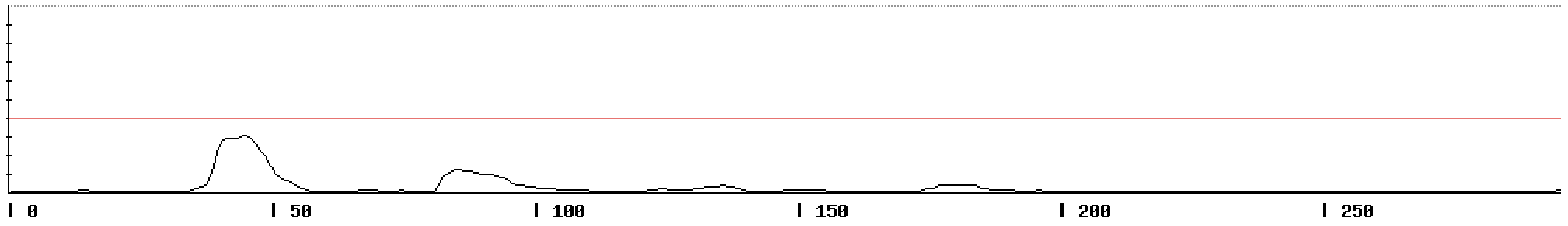

Predictions for Cajanuscajan_C.cajan_40877

Viterbi Path	
Viterbi Path	
Posterior @ 0.4	
Posterior @ 0.4	
Posterior Graphic. Threshold Marked @ 0.4	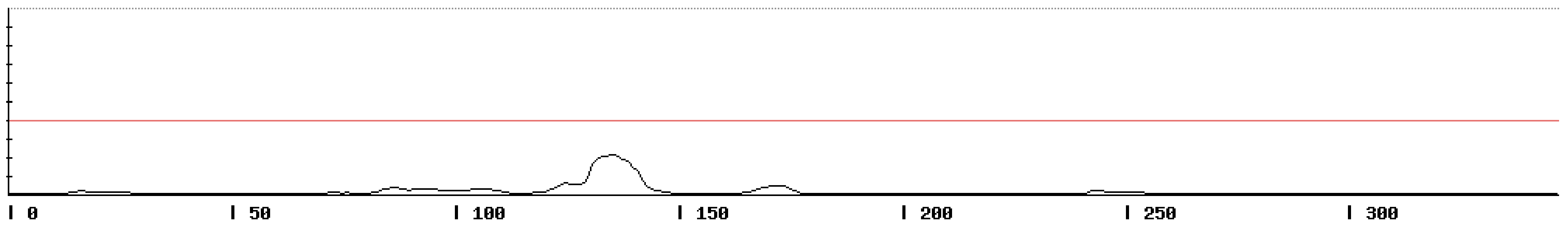

Predictions for Cajanuscajan_C.cajan_41044

Viterbi Path	
Viterbi Path	
Posterior @ 0.4	
Posterior @ 0.4	
Posterior Graphic. Threshold Marked @ 0.4	

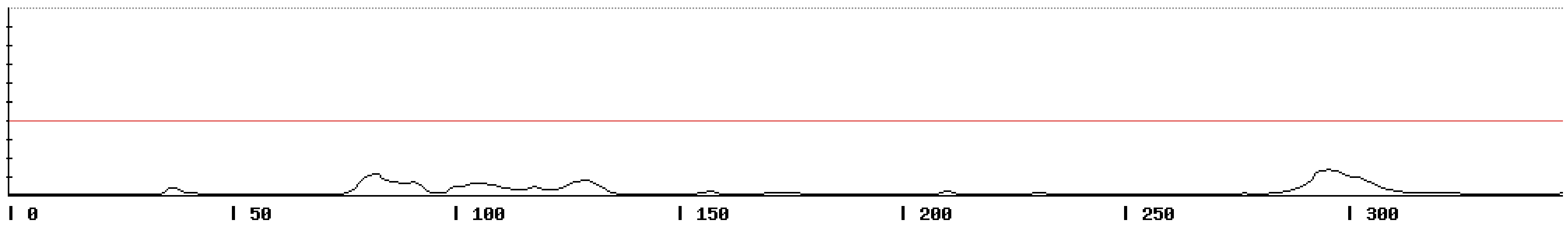

Predictions for Cajanuscajan_C.cajan_42082

Viterbi Path

Viterbi Path

Posterior @ 0.4

Posterior @ 0.4

Posterior Graphic. Threshold Marked @ 0.4

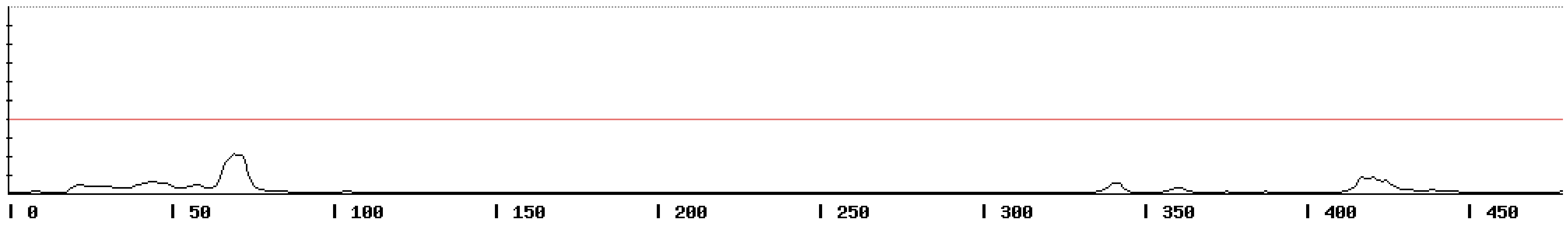

Predictions for Cajanuscajan_C.cajan_42309

Viterbi Path

Viterbi Path

Posterior @ 0.4

Posterior @ 0.4

Posterior Graphic. Threshold Marked @ 0.4

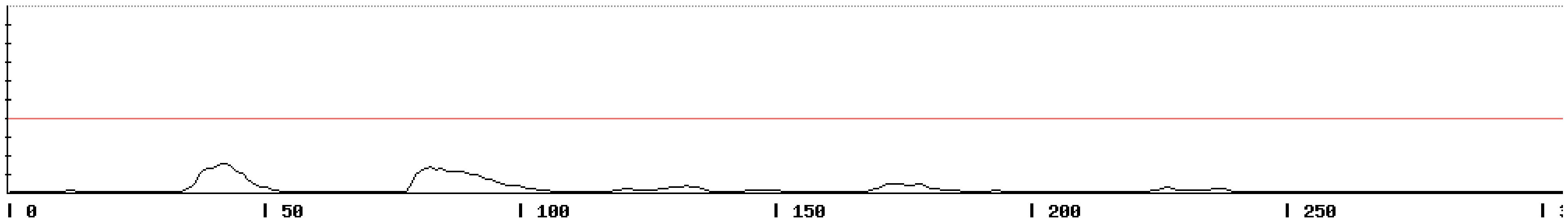

Predictions for Cajanuscajan_C.cajan_43030

Viterbi Path

Viterbi Path

Posterior @ 0.4

Posterior @ 0.4

Posterior Graphic. Threshold Marked @ 0.4

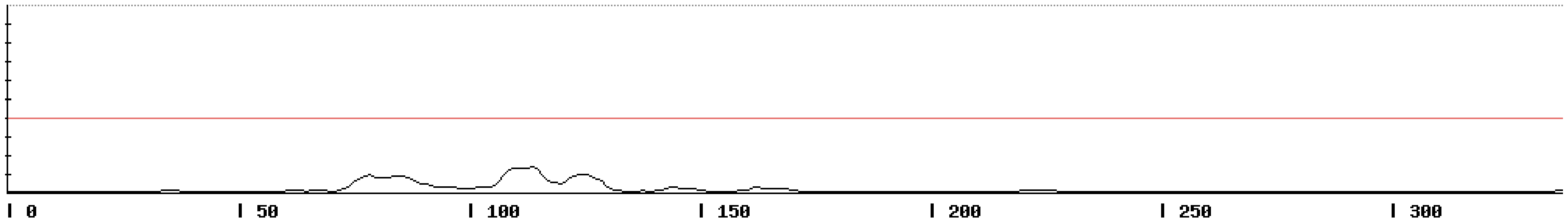

Predictions for Cajanuscajan_C.cajan_43785

Viterbi Path

Viterbi Path

Posterior @ 0.4

Posterior @ 0.4

Posterior Graphic. Threshold Marked @ 0.4	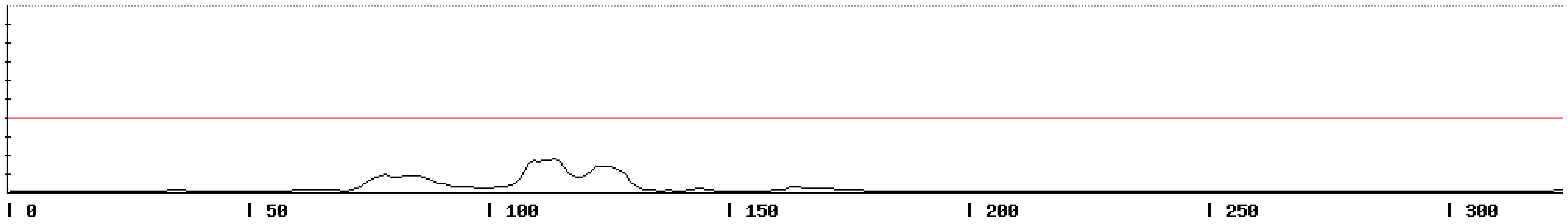

Predictions for Cajanuscajan_C.cajan_43786

Viterbi Path	
Viterbi Path	
Posterior @ 0.4	
Posterior @ 0.4	
Posterior Graphic. Threshold Marked @ 0.4	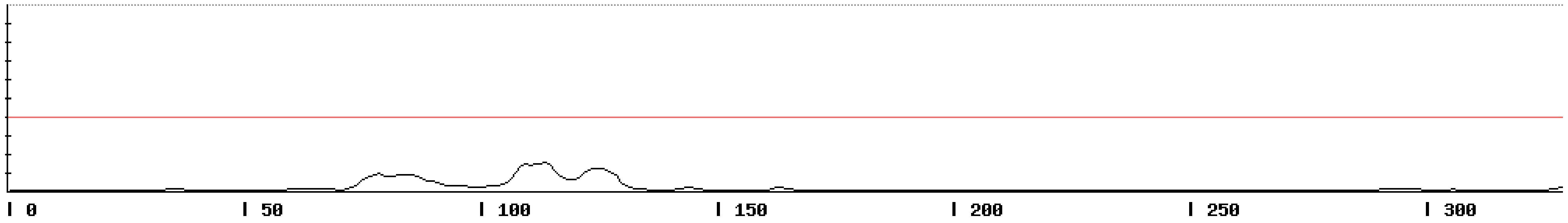

Predictions for Cajanuscajan_C.cajan_44182

Viterbi Path	
Viterbi Path	

Posterior @ 0.4

Posterior @ 0.4

Posterior Graphic. Threshold Marked @ 0.4

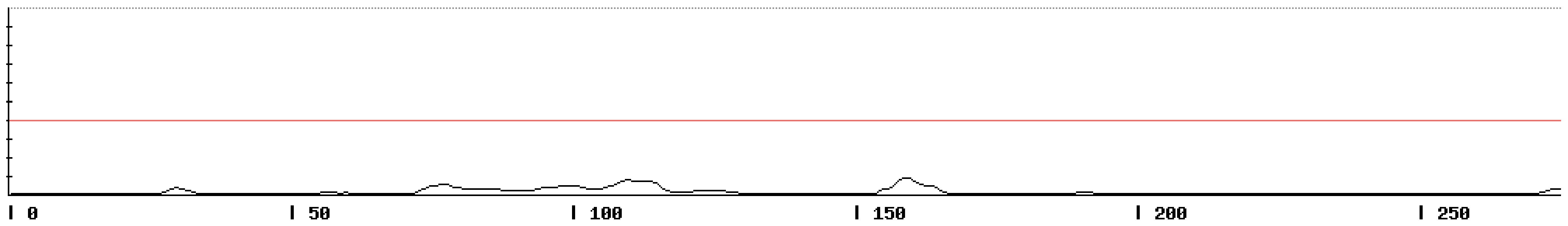

Predictions for Cajanuscajan_C.cajan_44515

Viterbi Path

Viterbi Path

Posterior @ 0.4

Posterior @ 0.4

Posterior Graphic. Threshold Marked @ 0.4

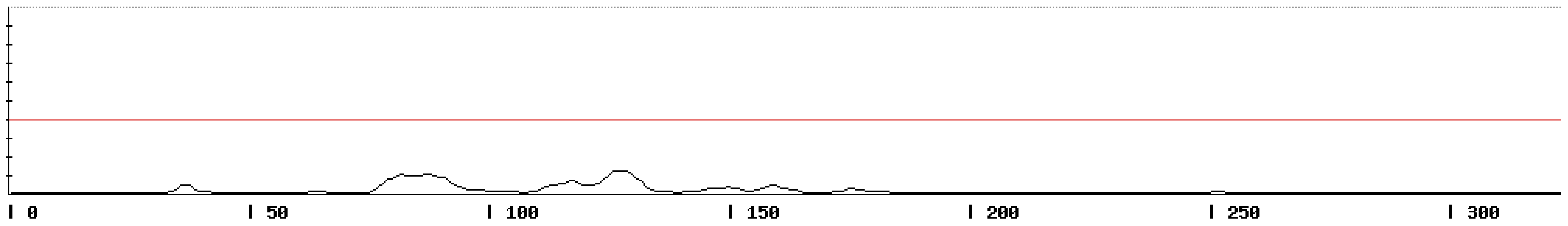

Predictions for Cajanuscajan_C.cajan_44516

Viterbi Path

Viterbi Path

Posterior @ 0.4

Posterior @ 0.4

Posterior Graphic. Threshold Marked @ 0.4

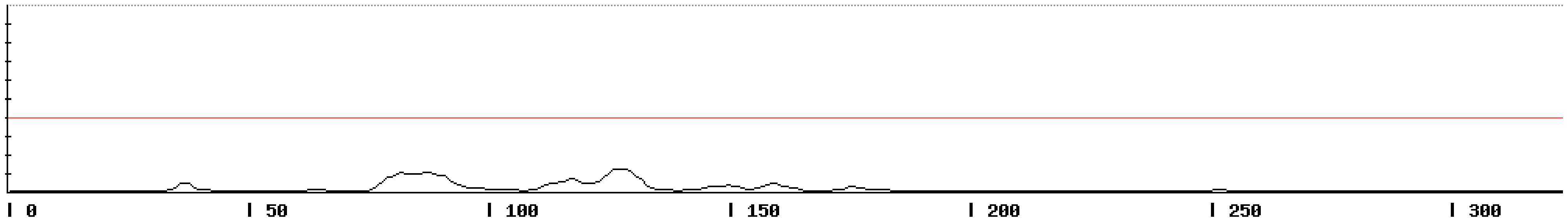

Predictions for Cajanuscajan_C.cajan_46578

Viterbi Path

Viterbi Path

Posterior @ 0.4

Posterior @ 0.4

Posterior Graphic. Threshold Marked @ 0.4

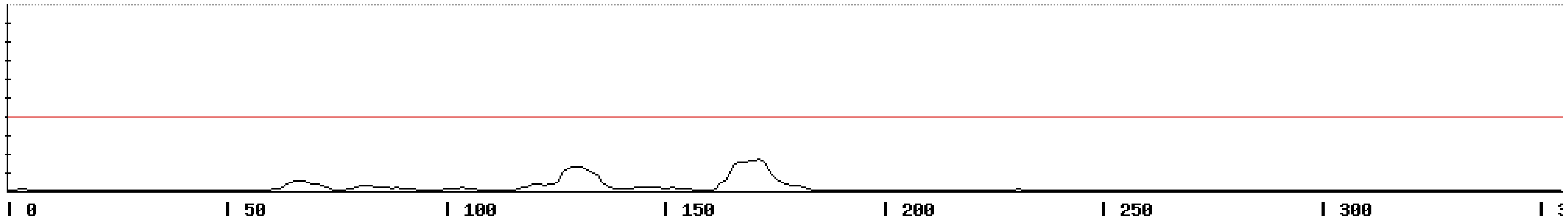

Predictions for Cajanuscajan_C.cajan_46953

Viterbi Path

Viterbi Path

Posterior @ 0.4 183 - R - 183

Posterior @ 0.4

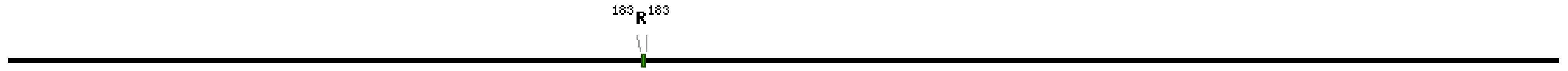

Posterior Graphic. Threshold Marked @ 0.4

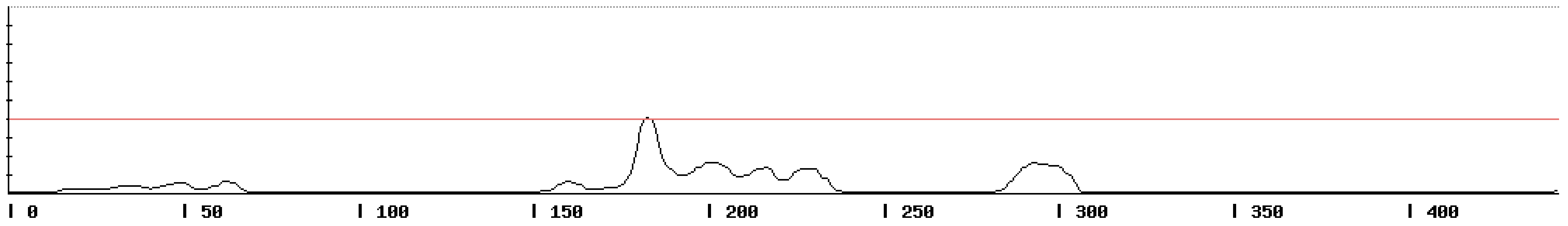

Predictions for Cajanuscajan_C.cajan_48342

Viterbi Path

Viterbi Path

Posterior @ 0.4

Posterior @ 0.4

Posterior Graphic. Threshold Marked @ 0.4

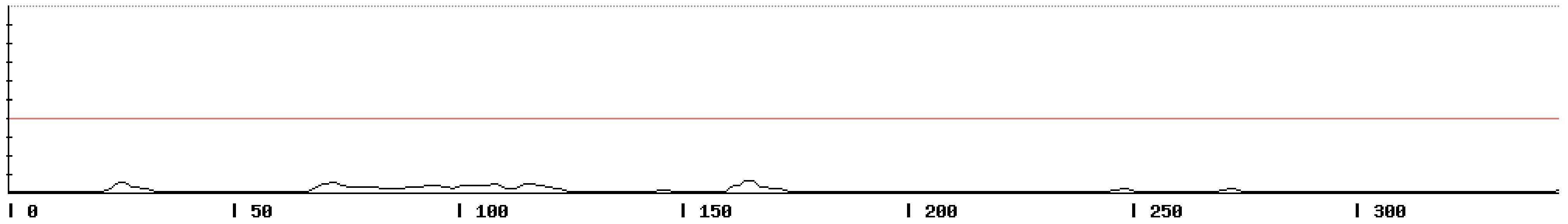

Predictions for Cajanuscajan_C.cajan_48539	
Viterbi Path	
Viterbi Path	
Posterior @ 0.4	
Posterior @ 0.4	
Posterior Graphic. Threshold Marked @ 0.4	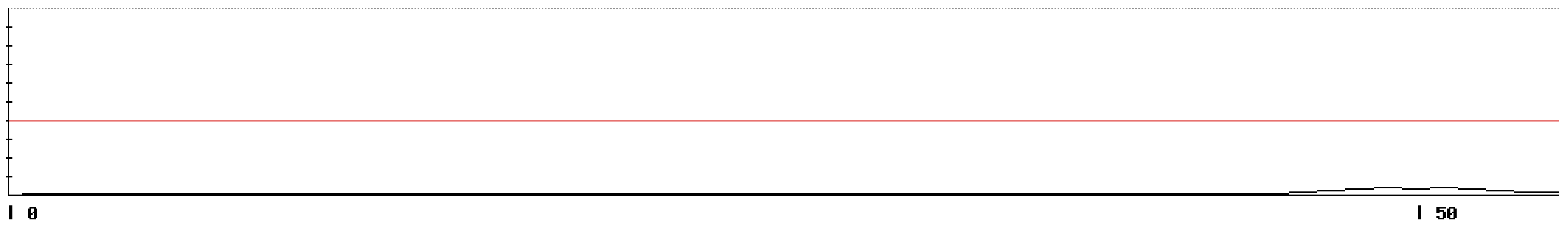

Revision r.9 - Last Updated on : February 24 2015 17:45:20. alex.nguyenba@utoronto.ca

Intended to be used with Firefox

Viterbi Path

Posterior @ 0.4

Posterior @ 0.4

Posterior Graphic. Threshold Marked @ 0.4

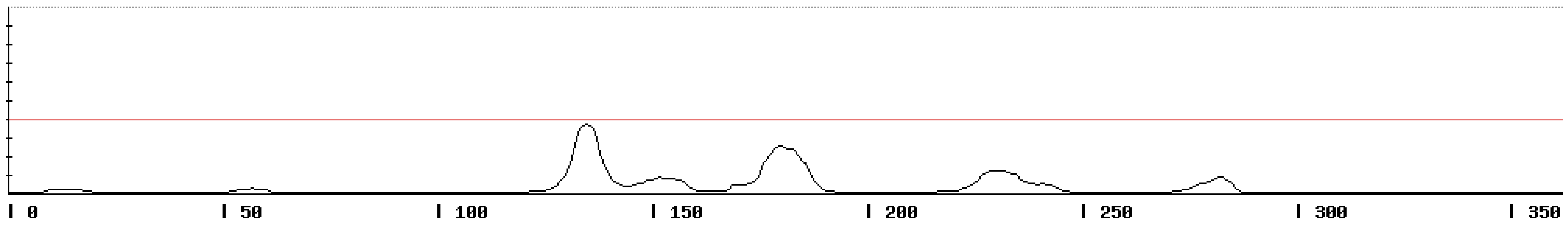

Predictions for Camelinasativa_Csa01g002880.1

Viterbi Path

Viterbi Path

Posterior @ 0.4

Posterior @ 0.4

Posterior Graphic. Threshold Marked @ 0.4

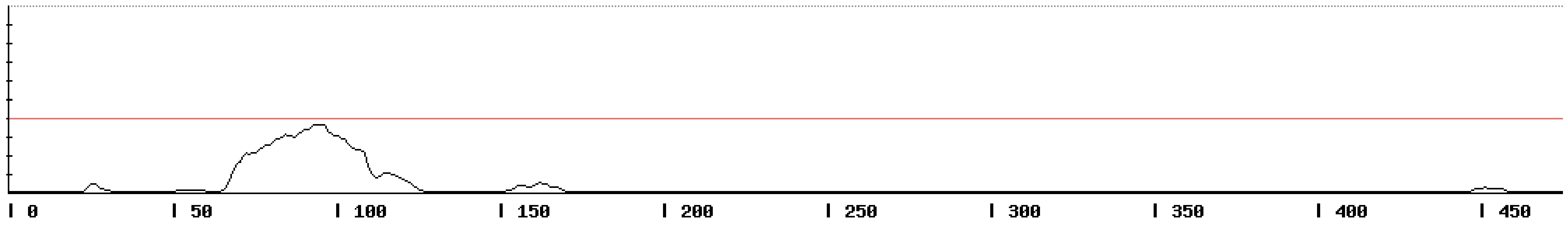

Predictions for Camelinasativa_Csa01g004850.1

Viterbi Path

Viterbi Path

Posterior @ 0.4

Posterior @ 0.4

Posterior Graphic. Threshold Marked @ 0.4

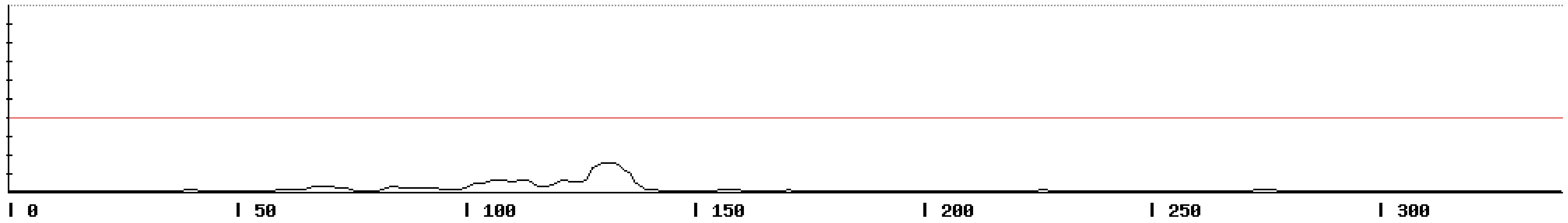

Predictions for Camelinasativa_Csa01g004860.1

Viterbi Path

Viterbi Path

Posterior @ 0.4

Posterior @ 0.4

Posterior Graphic. Threshold Marked @ 0.4

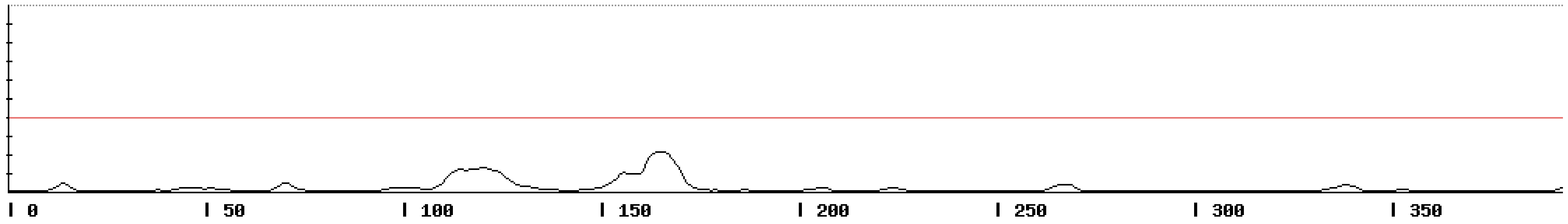

Predictions for Camelinasativa_Csa01g011360.1

Viterbi Path

Viterbi Path

Posterior @ 0.4

Posterior @ 0.4

Posterior Graphic. Threshold Marked @ 0.4

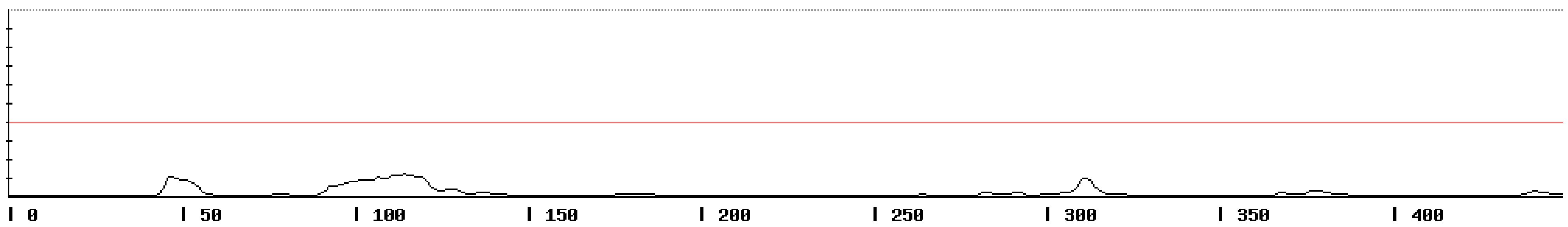

Predictions for Camelinasativa_Csa01g011370.1

Viterbi Path

Viterbi Path

Posterior @ 0.4

Posterior @ 0.4

Posterior Graphic. Threshold Marked @ 0.4

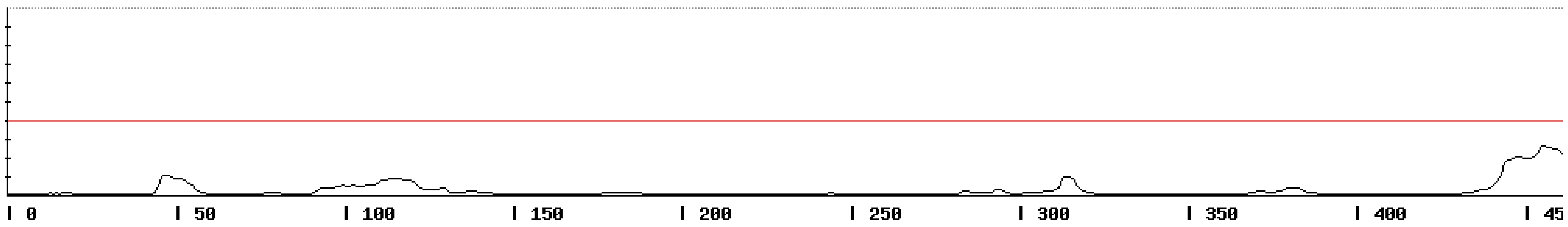

Predictions for Camelinasativa_Csa01g011380.1

Viterbi Path

Viterbi Path

Posterior @ 0.4

Posterior @ 0.4

Posterior Graphic. Threshold Marked @ 0.4

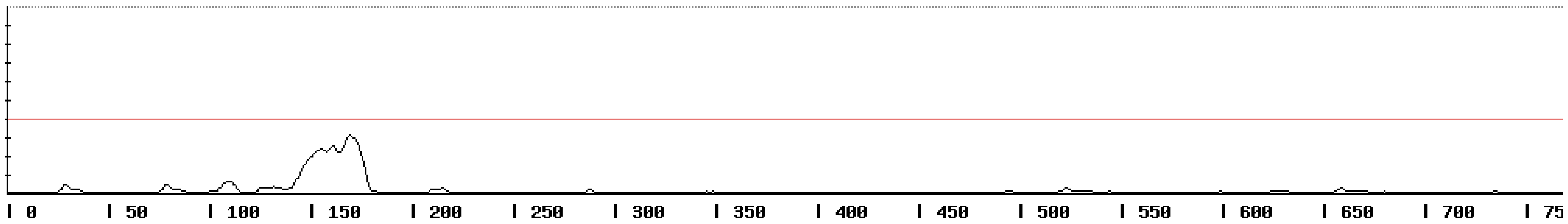

Predictions for Camelinasativa_Csa01g014020.1

Viterbi Path

Viterbi Path

Posterior @ 0.4

Posterior @ 0.4

Posterior Graphic. Threshold Marked @ 0.4

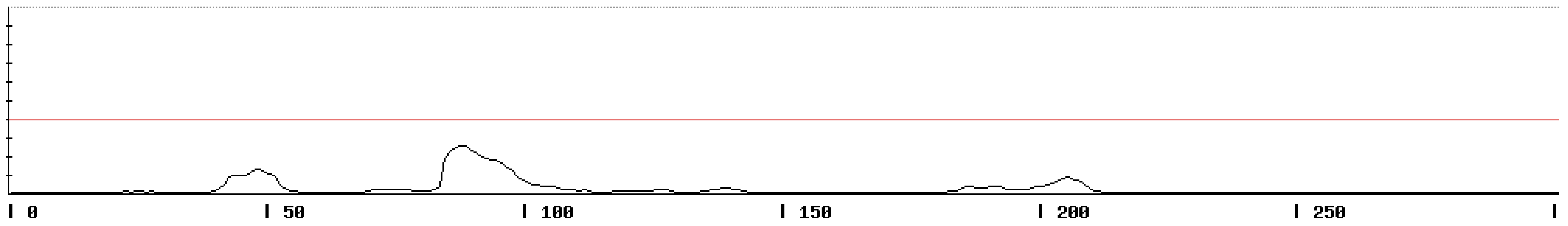

Predictions for Camelinasativa_Csa01g014150.1

Viterbi Path

Viterbi Path

Posterior @ 0.4

Posterior @ 0.4

Posterior Graphic. Threshold Marked @ 0.4

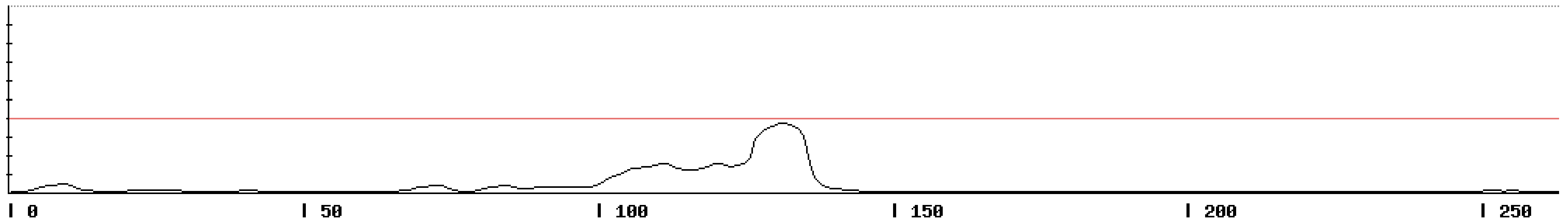

Predictions for Camelinasativa_Csa01g017710.1

Viterbi Path

Viterbi Path

Posterior @ 0.4

Posterior @ 0.4

Posterior Graphic. Threshold Marked @ 0.4

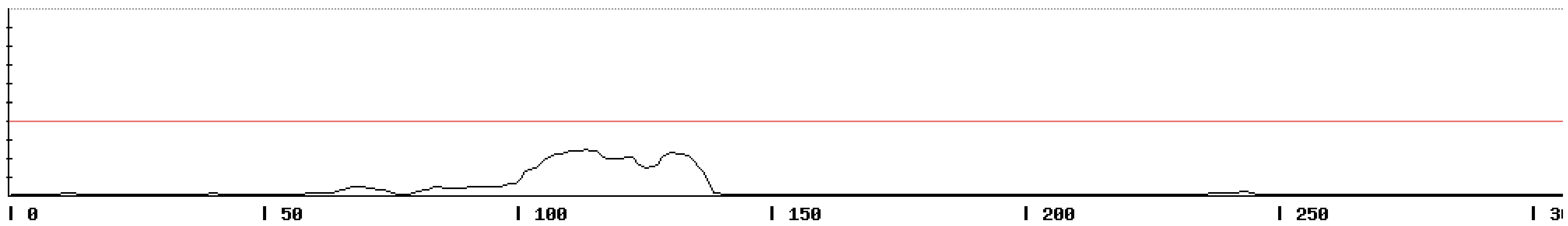

Predictions for Camelinasativa_Csa01g018090.1

Viterbi Path

Viterbi Path

Posterior @ 0.4

Posterior @ 0.4

Posterior Graphic. Threshold Marked @ 0.4

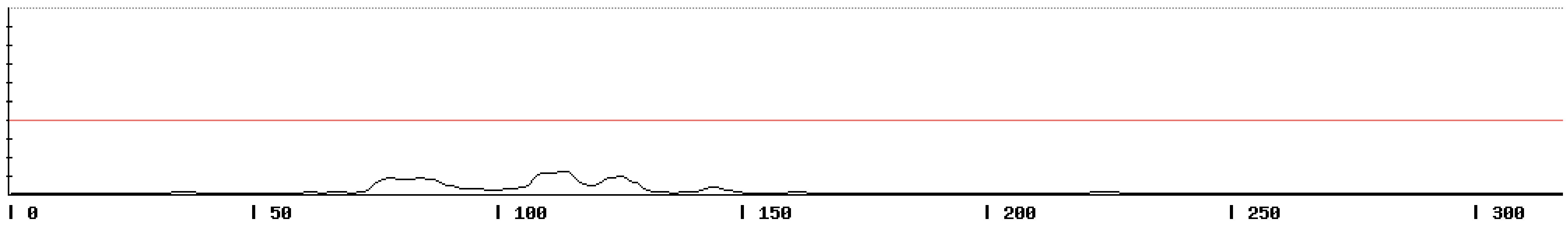

Predictions for Camelinasativa_Csa01g018100.1

Viterbi Path

Viterbi Path

Posterior @ 0.4

Posterior @ 0.4

Posterior Graphic. Threshold Marked @ 0.4

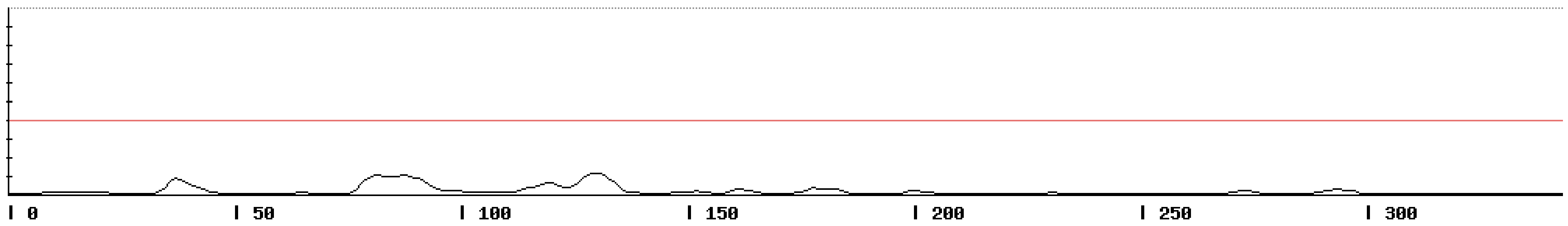

Predictions for Camelinasativa_Csa01g020430.1

Viterbi Path

Viterbi Path

Posterior @ 0.4

Posterior @ 0.4

Posterior Graphic. Threshold Marked @ 0.4

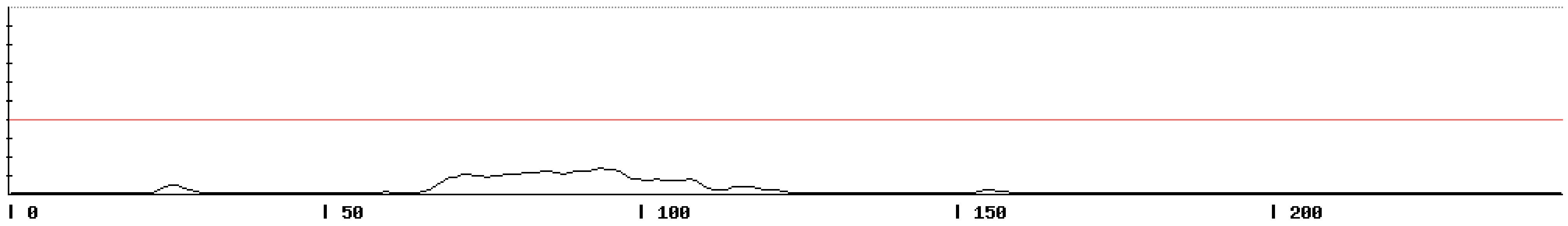

Predictions for Camelinasativa_Csa01g021240.1

Viterbi Path

Viterbi Path

Posterior @ 0.4

Posterior @ 0.4

Posterior Graphic. Threshold Marked @ 0.4

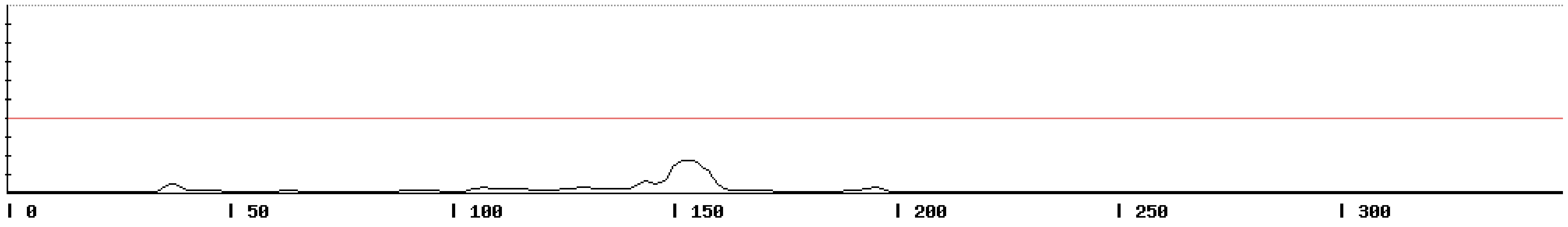

Predictions for Camelinasativa_Csa01g032870.1

Viterbi Path

Viterbi Path

Posterior @ 0.4

Posterior @ 0.4

Posterior Graphic. Threshold Marked @ 0.4

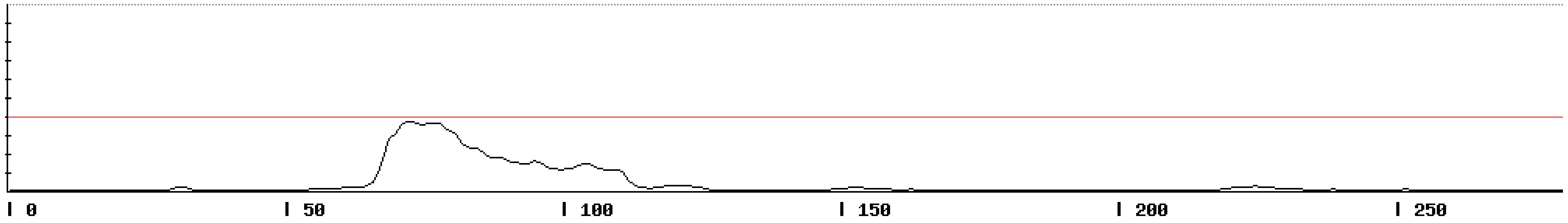

Predictions for Camelinasativa_Csa01g035930.1

Viterbi Path: 200 - KRMKAEKKEKLKKGKGVSK - 218

Viterbi Path

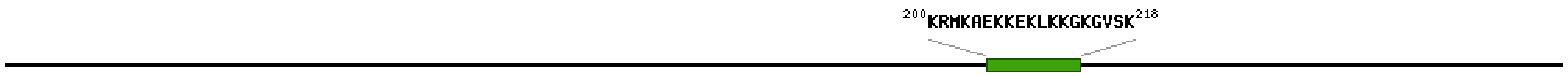

Posterior @ 0.4: 113 - KIGKSGKRQKR - 123

199 - VKRMKAEKKEKLKKGKGVSK - 218

Posterior @ 0.4

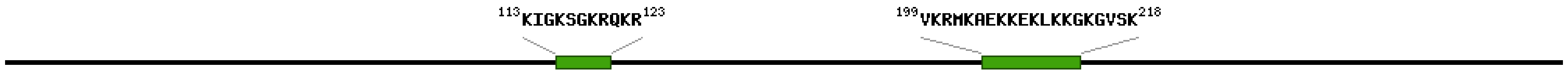

Posterior Graphic. Threshold Marked @ 0.4

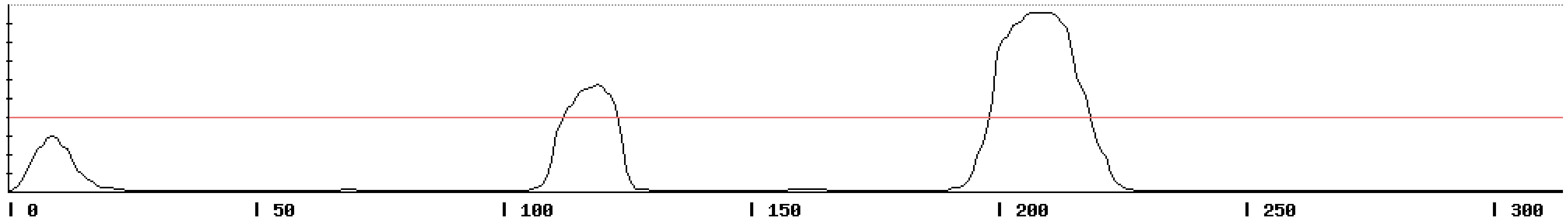

Predictions for Camelinasativa_Csa01g035940.1

Viterbi Path: 200 - KRMKAEKKEKLKKGKGVSK - 218

Viterbi Path

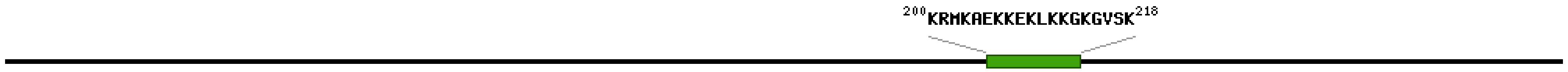

Posterior @ 0.4: 113 - KIGKSGKRQKR - 123

199 - VKRMKAEKKEKLKKGKGVSK - 218

Posterior @ 0.4

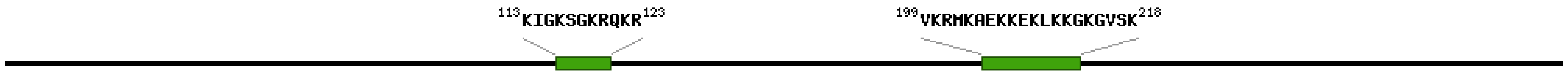

Posterior Graphic. Threshold Marked @ 0.4

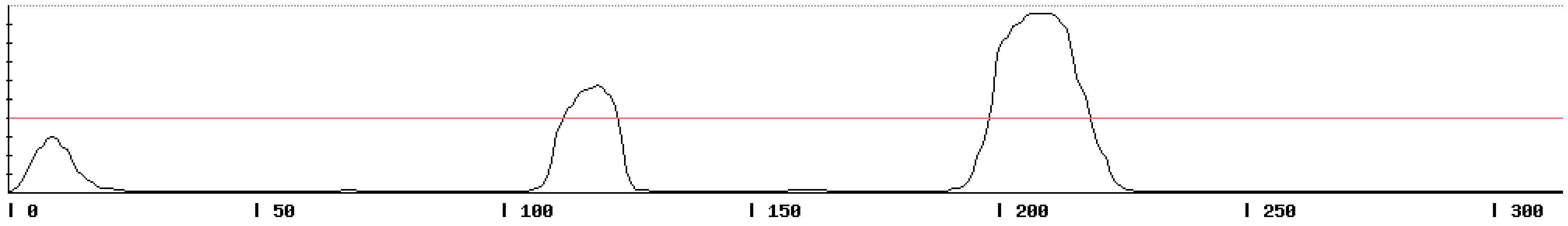

Predictions for Camelinasativa_Csa02g004650.1

Viterbi Path	
Viterbi Path	
Posterior @ 0.4	91 - ATGKDRKIKDKRGN - 104
Posterior @ 0.4	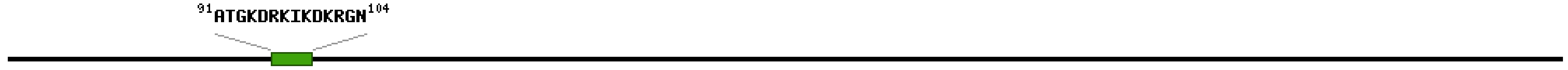
Posterior Graphic. Threshold Marked @ 0.4	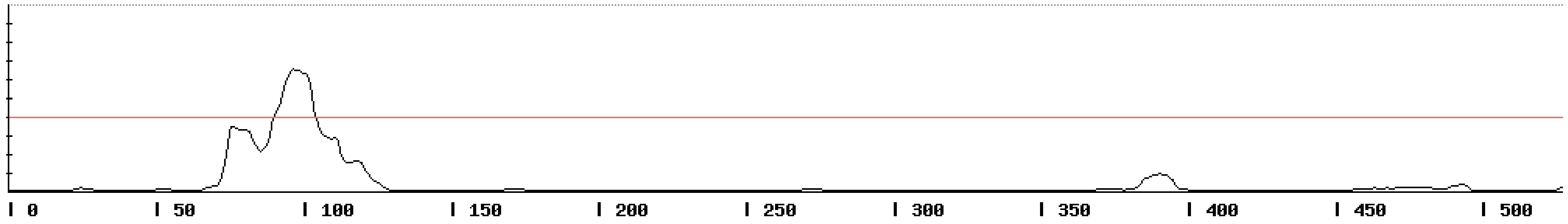

Predictions for Camelinasativa_Csa02g004670.1

Viterbi Path	
Viterbi Path	
Posterior @ 0.4	76 - KKKNAMKR - 83
	91 - KPTGKDRKIKDKRGNG - 106
Posterior @ 0.4	

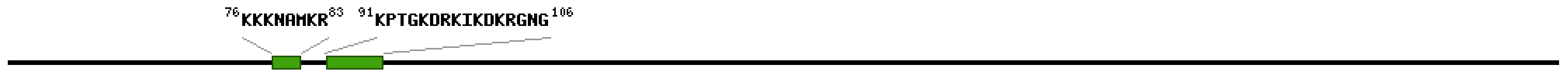

Posterior Graphic. Threshold Marked @ 0.4

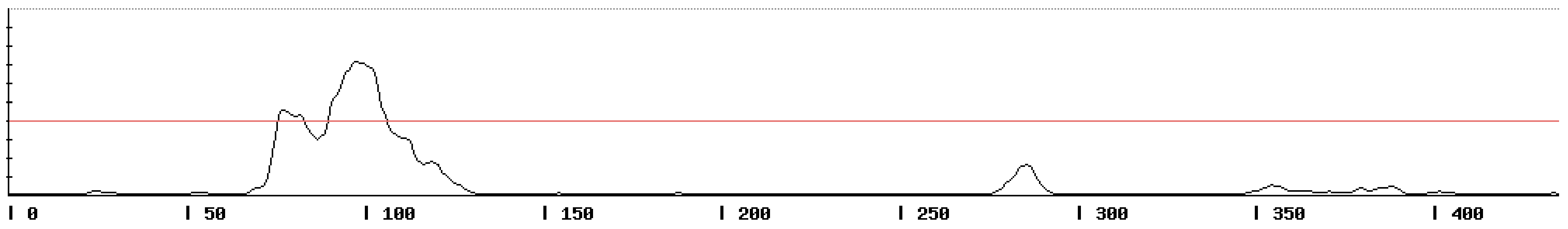

Predictions for Camelinasativa_Csa02g004860.1

Viterbi Path

Viterbi Path

Posterior @ 0.4

Posterior @ 0.4

Posterior Graphic. Threshold Marked @ 0.4

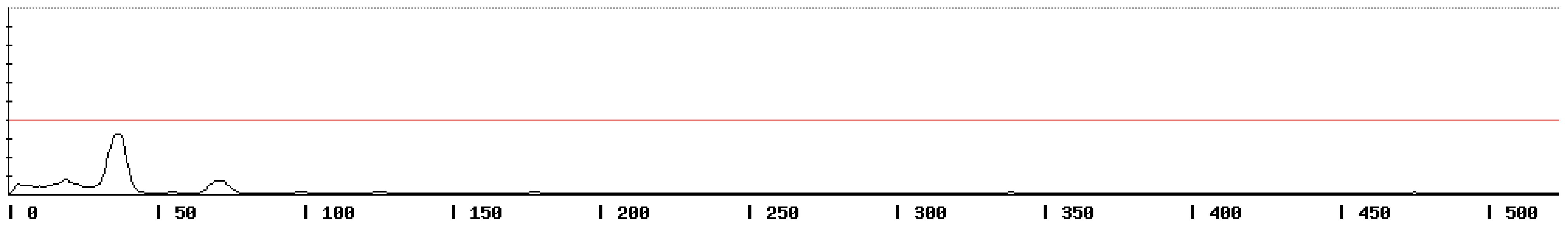

Predictions for Camelinasativa_Csa02g011430.1

Viterbi Path

Viterbi Path

Posterior @ 0.4	77 - K - 77
	92 - GVDRKIKDKRGNRGEI - 107
Posterior @ 0.4	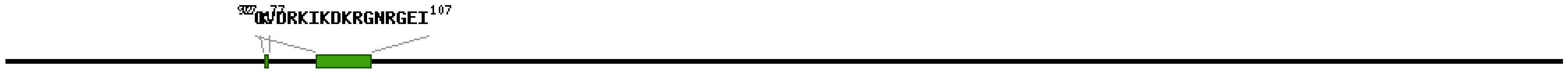
Posterior Graphic. Threshold Marked @ 0.4	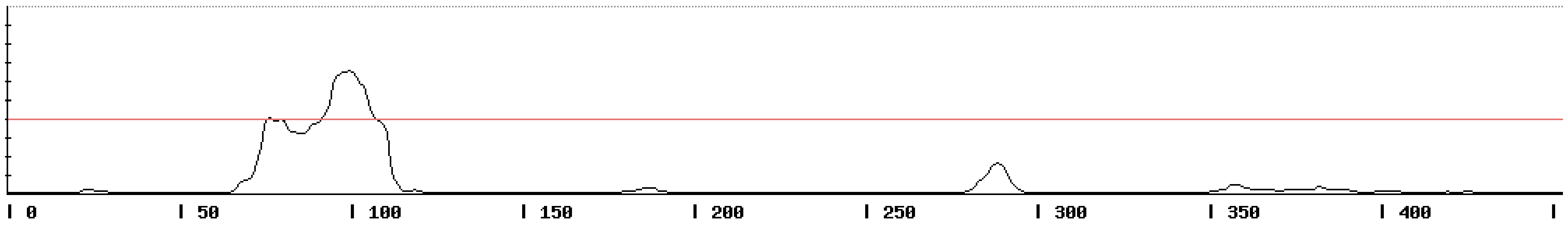

Predictions for Camelinasativa_Csa02g026430.1

Viterbi Path	
Viterbi Path	
Posterior @ 0.4	
Posterior @ 0.4	
Posterior Graphic. Threshold Marked @ 0.4	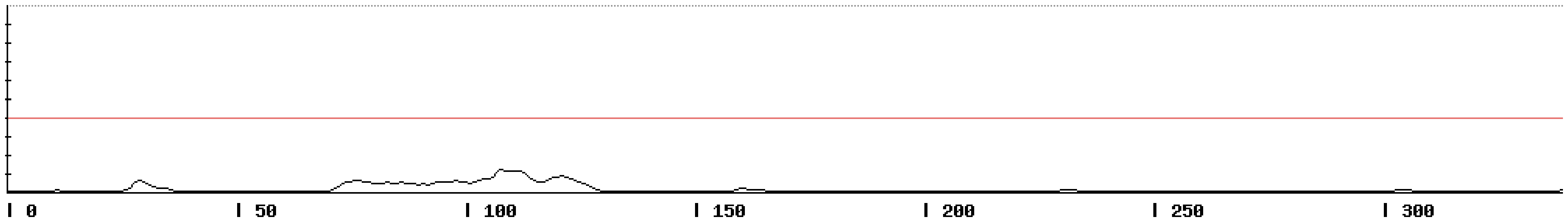

Predictions for Camelinasativa_Csa02g039410.1

Viterbi Path	
Viterbi Path	

Posterior @ 0.4

Posterior @ 0.4

Posterior Graphic. Threshold Marked @ 0.4

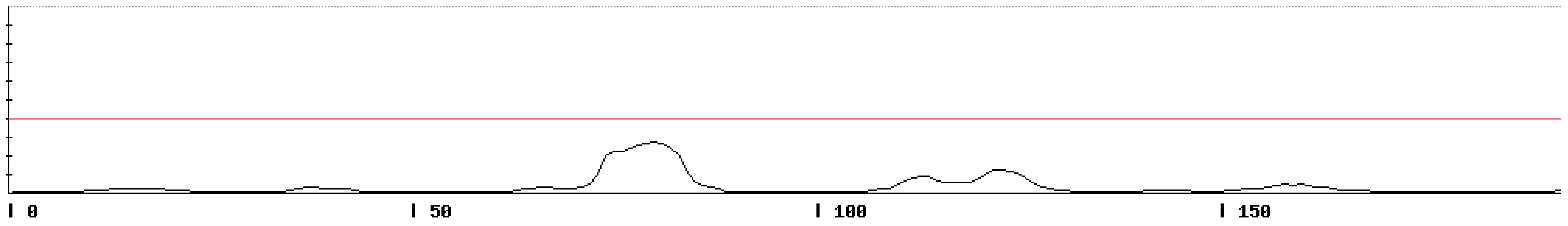

Predictions for Camelinasativa_Csa02g051570.1

Viterbi Path

Viterbi Path

Posterior @ 0.4

Posterior @ 0.4

Posterior Graphic. Threshold Marked @ 0.4

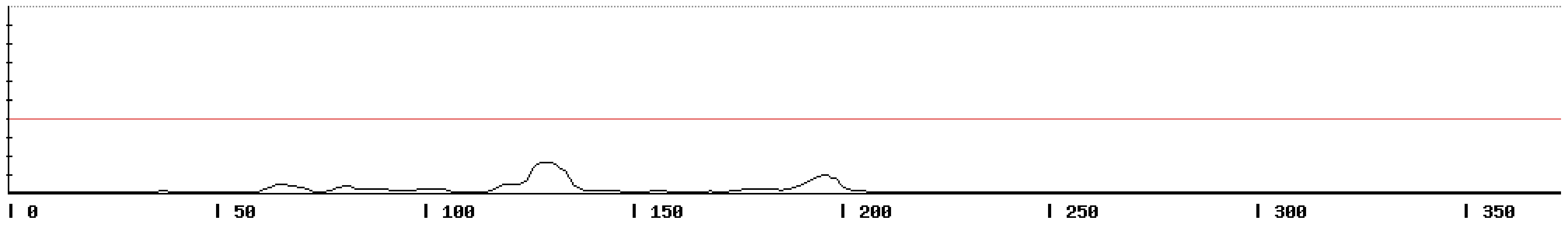

Predictions for Camelinasativa_Csa02g059890.1

Viterbi Path	
Viterbi Path	
Posterior @ 0.4	128 - R - 128
Posterior @ 0.4	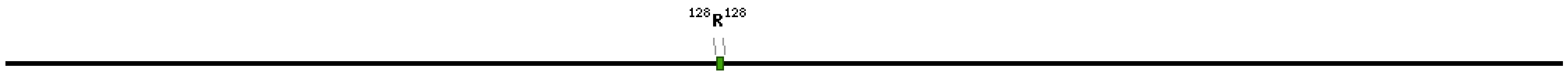
Posterior Graphic. Threshold Marked @ 0.4	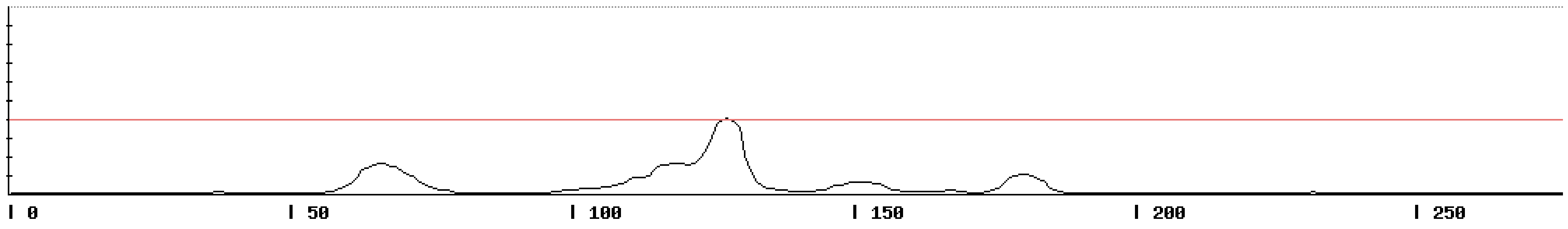

Predictions for Camelinasativa_Csa02g064730.1

Viterbi Path	178 - KIHKKKWSEKEKKKEKKKEEKKKEK - 202
	221 - KIHKKRNAKKKKEK - 234
Viterbi Path	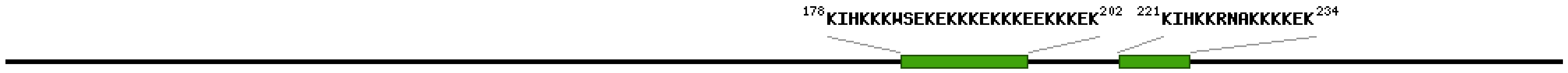
Posterior @ 0.4	132 - NKNKKQKR - 139
	178 - KIHKKKWSEKEKKKEKKKEEKKKEKE - 203
	221 - KIHKKRNAKKKKEK - 234
Posterior @ 0.4	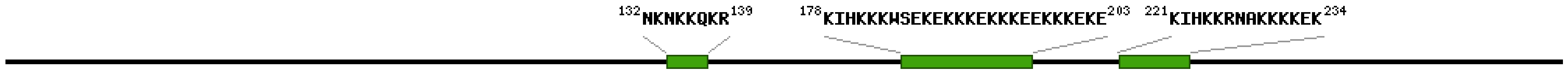
Posterior Graphic. Threshold Marked @ 0.4	

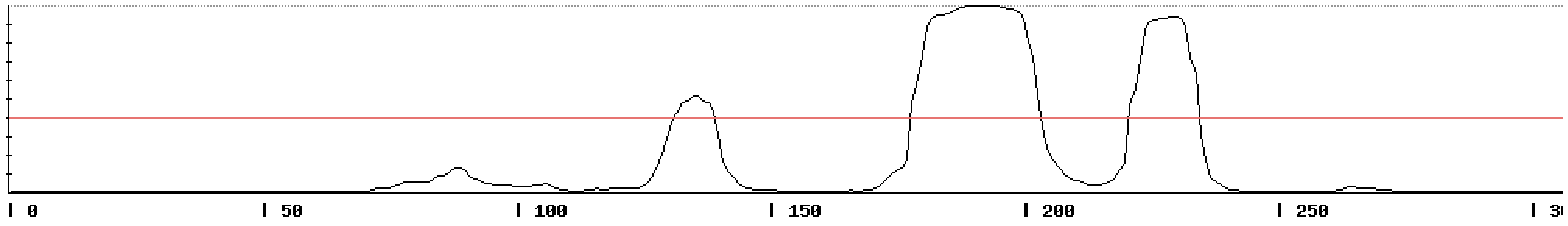

Predictions for Camelinasativa_Csa02g064950.1

Viterbi Path	178 - KIHKKKWSEKEKKKEKKKEEKEKEK - 202
Viterbi Path	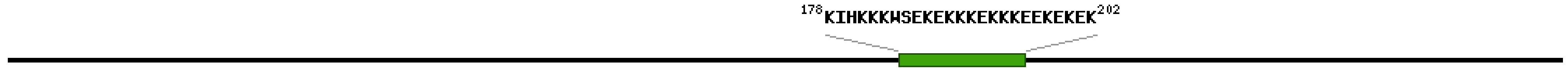
Posterior @ 0.4	132 - NKNKKQKR - 139
	178 - KIHKKKWSEKEKKKEKKKEEKEKEKE - 203
	221 - KIHKKKWSEKEKKKEK - 236
Posterior @ 0.4	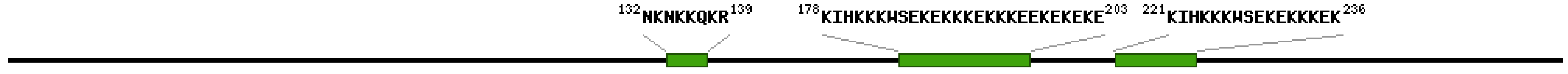
Posterior Graphic. Threshold Marked @ 0.4	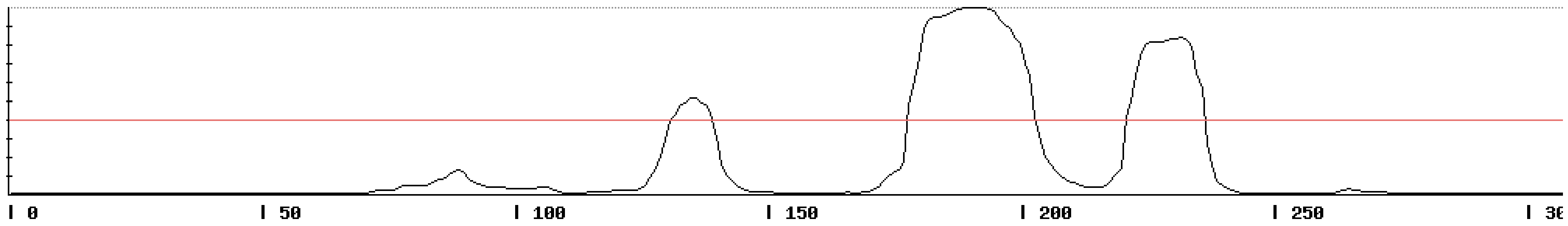

Predictions for Camelinasativa_Csa02g068390.1

Viterbi Path	
Viterbi Path	
Posterior @ 0.4	
Posterior @ 0.4	

Posterior Graphic. Threshold Marked @ 0.4

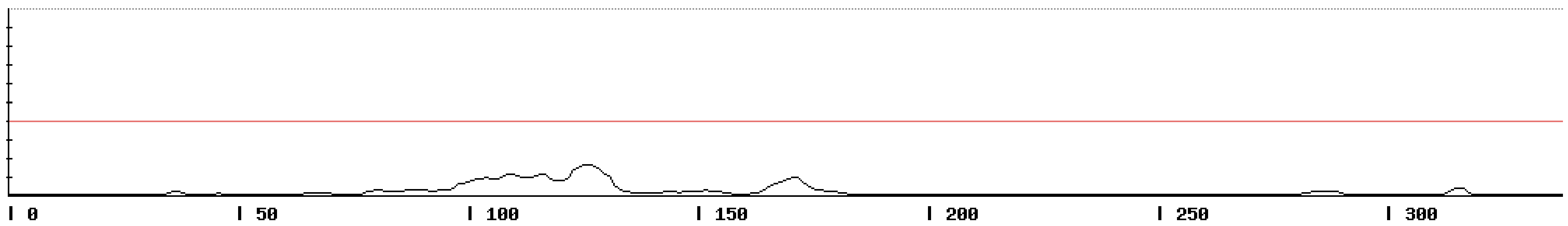

Predictions for Camelinasativa_Csa02g070520.1

Viterbi Path

Viterbi Path

Posterior @ 0.4

Posterior @ 0.4

Posterior Graphic. Threshold Marked @ 0.4

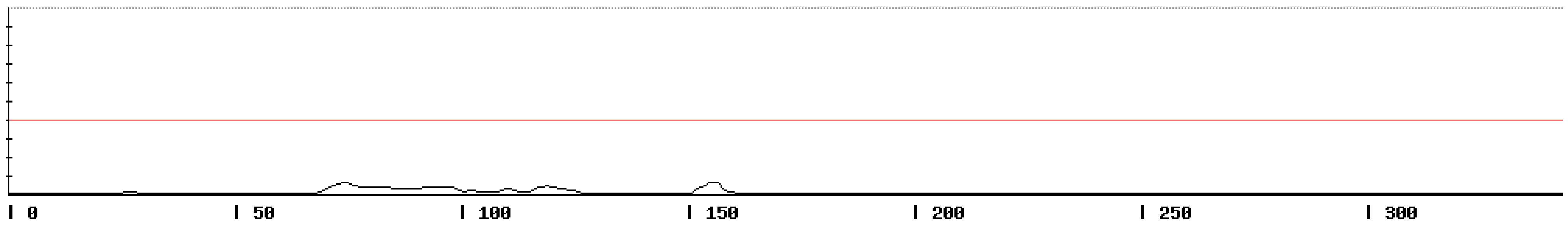

Predictions for Camelinasativa_Csa02g072480.1

Viterbi Path

Viterbi Path

Posterior @ 0.4 — 98 - GKPK - 101

Posterior @ 0.4

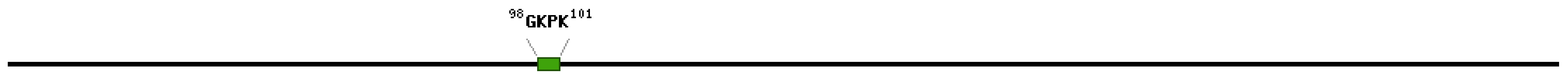

Posterior Graphic. Threshold Marked @ 0.4

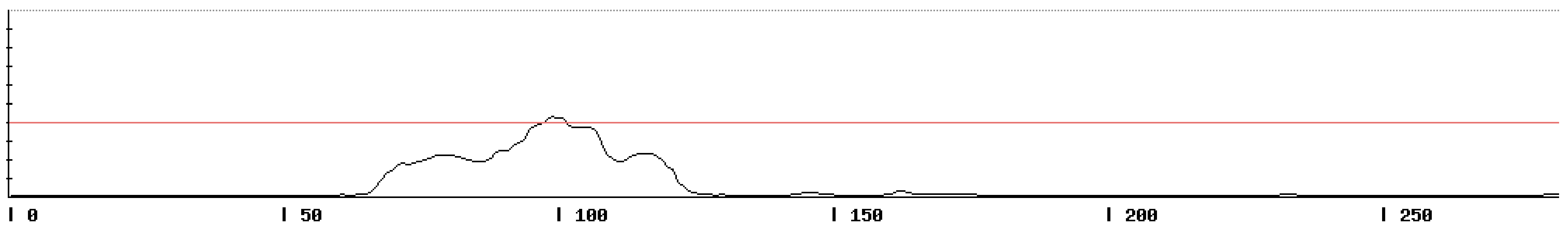

Predictions for Camelinasativa_Csa02g072750.1

Viterbi Path

Viterbi Path

Posterior @ 0.4

Posterior @ 0.4

Posterior Graphic. Threshold Marked @ 0.4

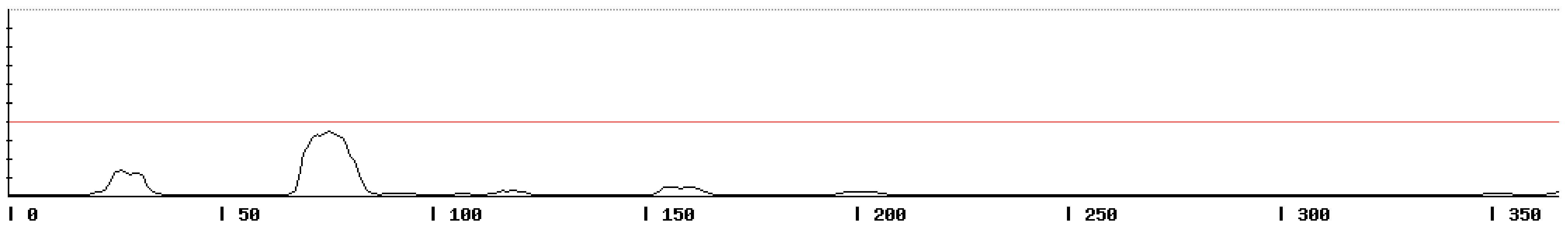

Predictions for Camelinasativa_Csa02g073300.1

Viterbi Path

Viterbi Path

Posterior @ 0.4

Posterior @ 0.4

Posterior Graphic. Threshold Marked @ 0.4

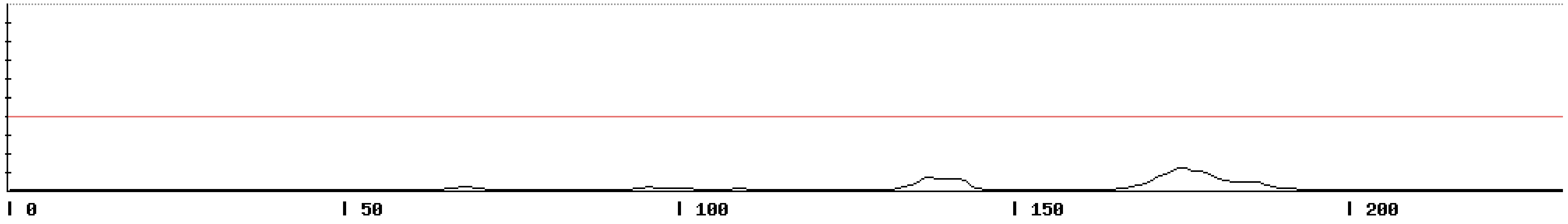

Predictions for Camelinasativa_Csa02g076500.1

Viterbi Path

Viterbi Path

Posterior @ 0.4

Posterior @ 0.4

Posterior Graphic. Threshold Marked @ 0.4

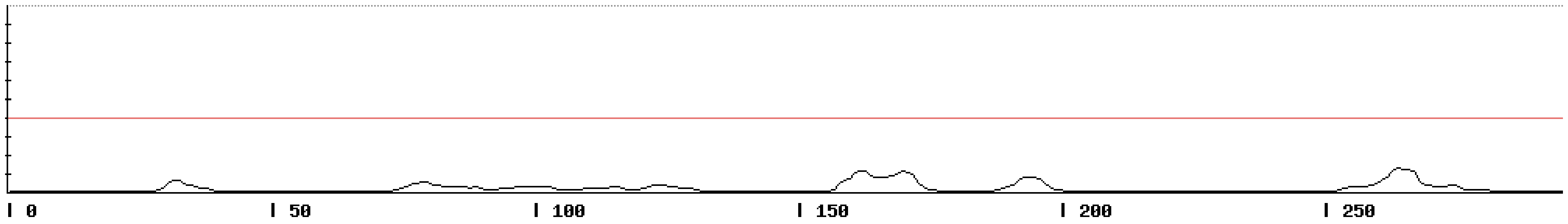

Predictions for Camelinasativa_Csa03g002220.1

Viterbi Path

Viterbi Path

Posterior @ 0.4

Posterior @ 0.4

Posterior Graphic. Threshold Marked @ 0.4

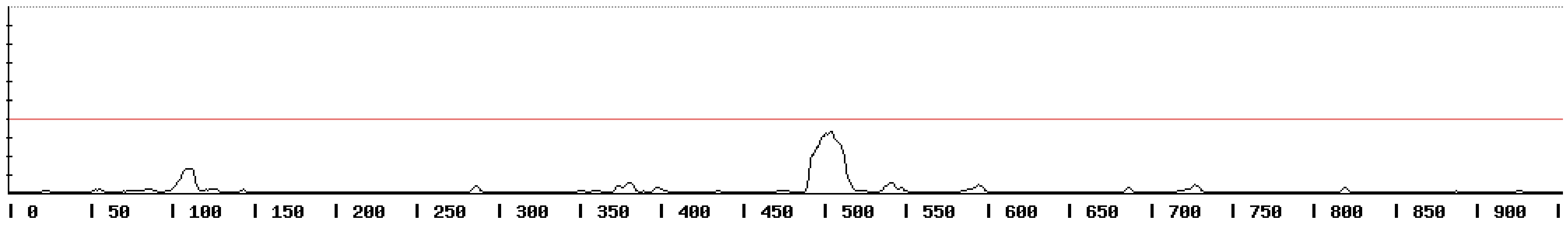

Predictions for Camelinasativa_Csa03g002230.1

Viterbi Path

Viterbi Path

Posterior @ 0.4

74 - TKSGYWKITGKPKAIIRNRVKIGAR - 98

496 - HKRRRKSKPKVVKA - 509

Posterior @ 0.4

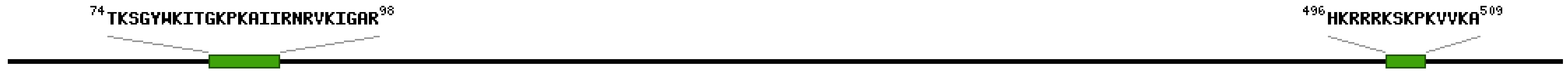

Posterior Graphic. Threshold Marked @ 0.4

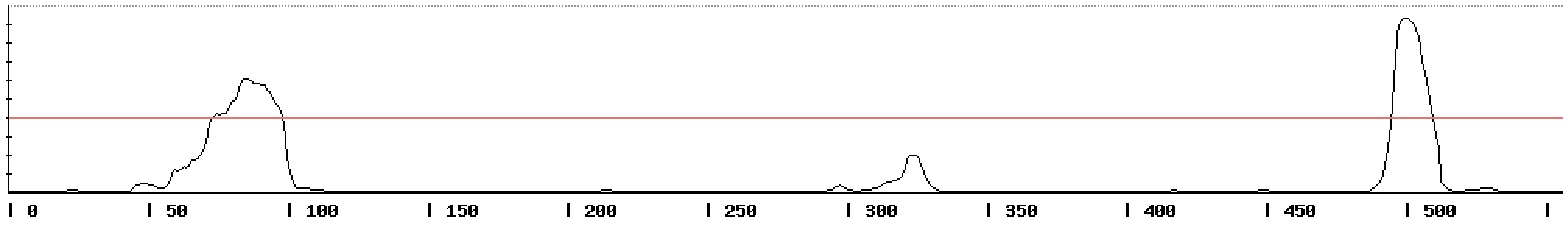

Predictions for Camelinasativa_Csa03g002250.1

Viterbi Path

Viterbi Path

Posterior @ 0.4

411 - IIRKRRGNREKIGEKRVLVFKHRGG - 435

651 - KKKRA - 655

Posterior @ 0.4

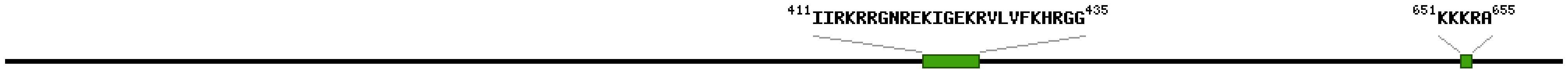

Posterior Graphic. Threshold Marked @ 0.4

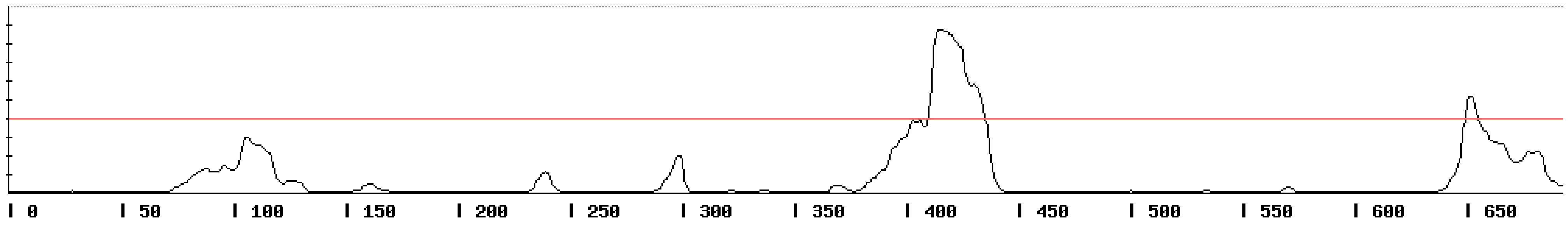

Predictions for Camelinasativa_Csa03g002570.1

Viterbi Path

Viterbi Path

Posterior @ 0.4

722 - RRKRRKQW - 729

Posterior @ 0.4

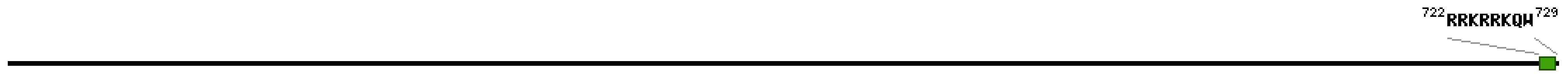

Posterior Graphic. Threshold Marked @ 0.4

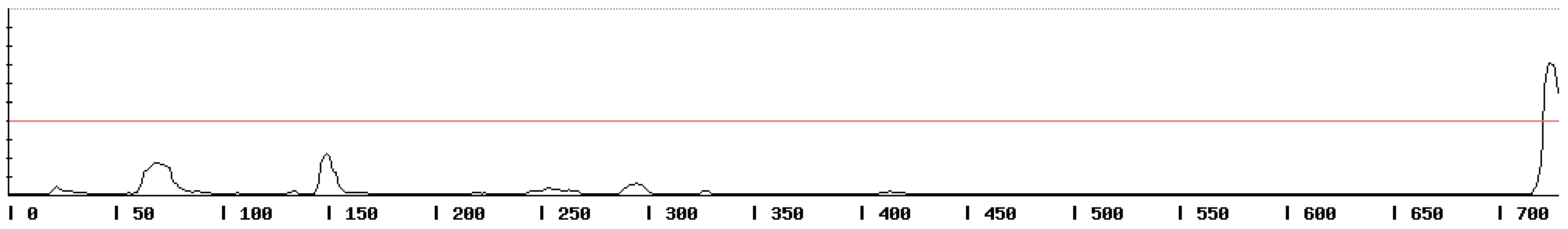

Predictions for Camelinasativa_Csa03g004610.1

Viterbi Path

Viterbi Path

Posterior @ 0.4

69 - PKFRDKRSSRSVPRRGGSKAGFWRSGVRK - 97

Posterior @ 0.4

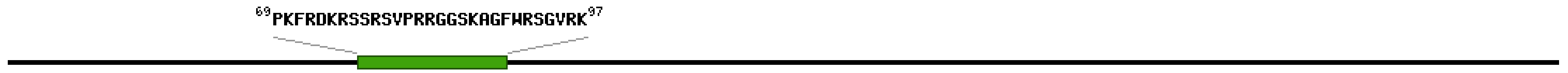

Posterior Graphic. Threshold Marked @ 0.4

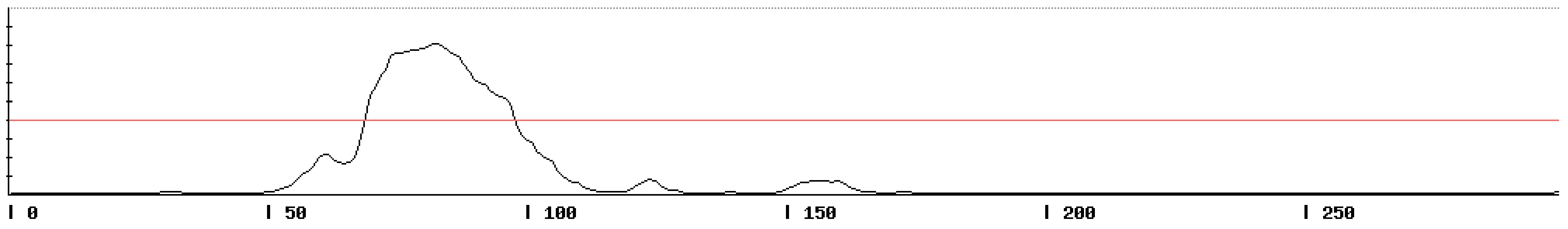

Predictions for Camelinasativa_Csa03g016020.1

Viterbi Path

Viterbi Path

Posterior @ 0.4

Posterior @ 0.4

Posterior Graphic. Threshold Marked @ 0.4

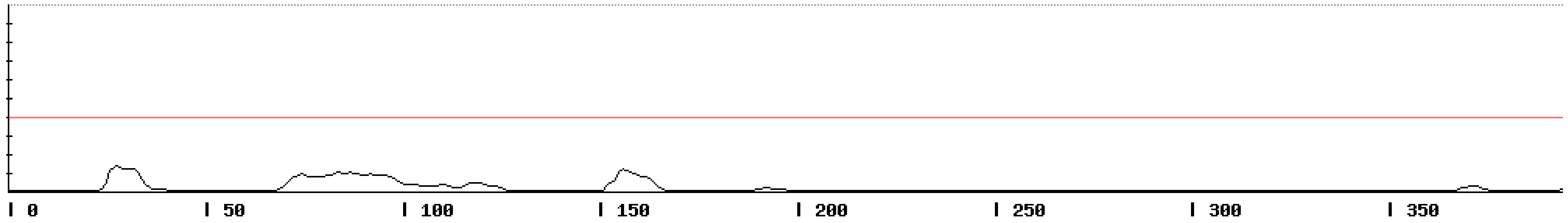

Predictions for Camelinasativa_Csa03g028620.1

Viterbi Path

Viterbi Path

Posterior @ 0.4

Posterior @ 0.4

Posterior Graphic. Threshold Marked @ 0.4

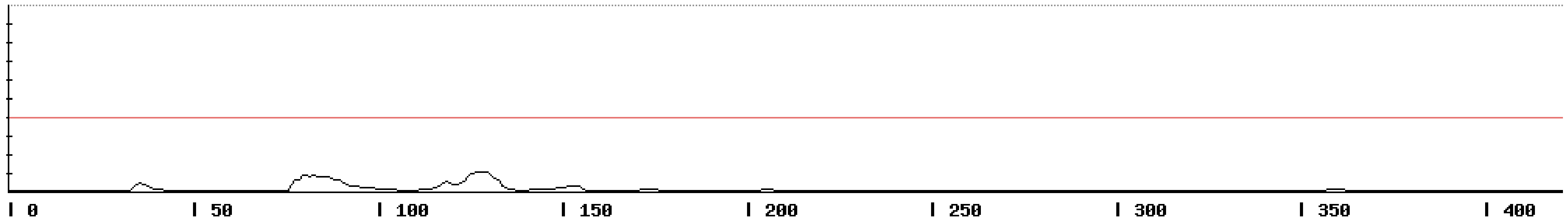

Predictions for Camelinasativa_Csa03g029490.1

Viterbi Path

Viterbi Path

Posterior @ 0.4	137 - KRR - 139
Posterior @ 0.4	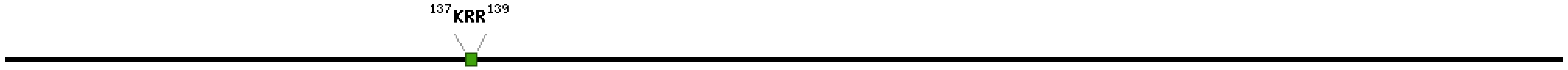
Posterior Graphic. Threshold Marked @ 0.4	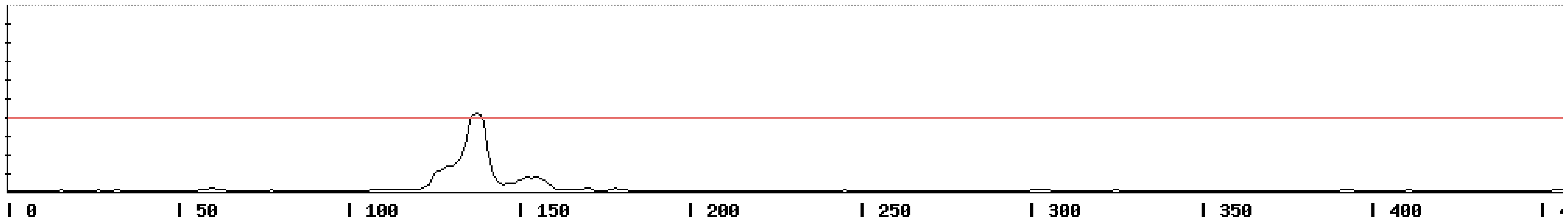

Predictions for Camelinasativa_Csa03g031810.1

Viterbi Path	
Viterbi Path	
Posterior @ 0.4	24 - KRR - 26<br>107 - KRR - 109
Posterior @ 0.4	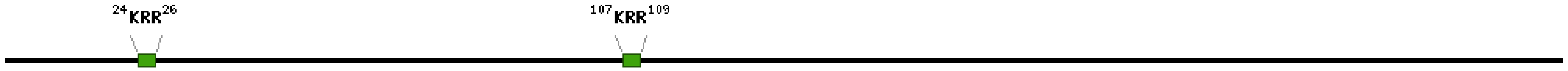
Posterior Graphic. Threshold Marked @ 0.4	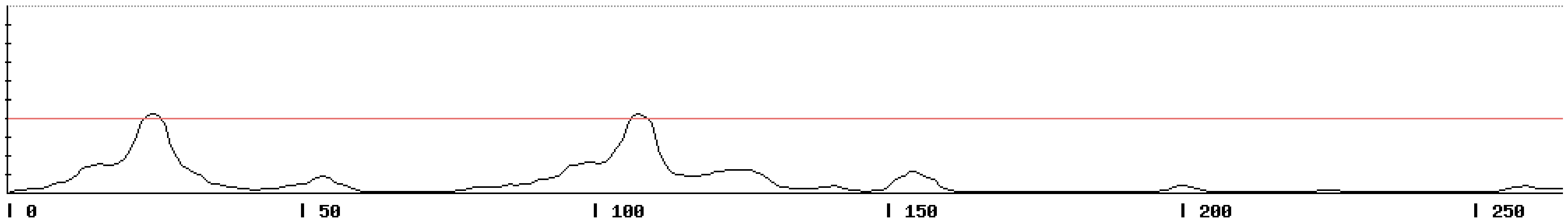

Predictions for Camelinasativa_Csa03g037070.1	
Viterbi Path	
Viterbi Path	
Posterior @ 0.4	
Posterior @ 0.4	
Posterior Graphic. Threshold Marked @ 0.4	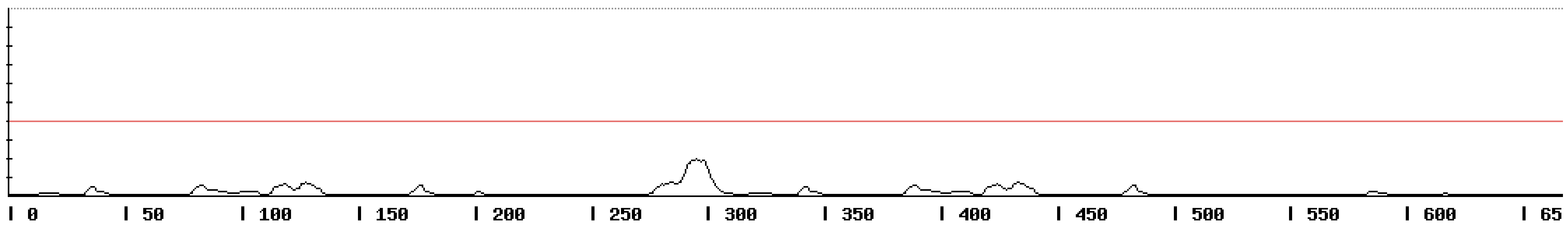

Predictions for Camelinasativa_Csa03g037360.1	
Viterbi Path	
Viterbi Path	
Posterior @ 0.4	20 - RPKKEK - 25
Posterior @ 0.4	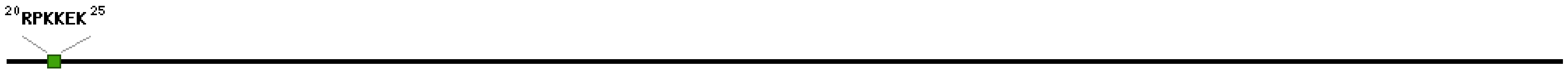
Posterior Graphic. Threshold Marked @ 0.4	

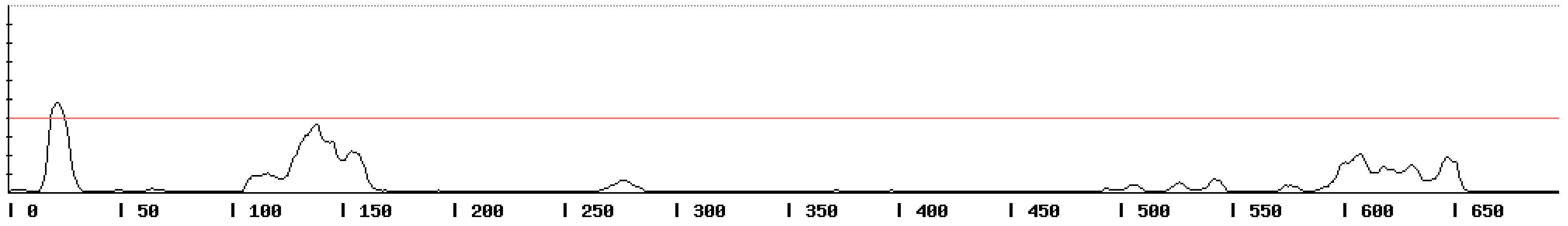

Predictions for Camelinasativa_Csa03g037530.1

Viterbi Path	
Viterbi Path	
Posterior @ 0.4	
Posterior @ 0.4	
Posterior Graphic. Threshold Marked @ 0.4	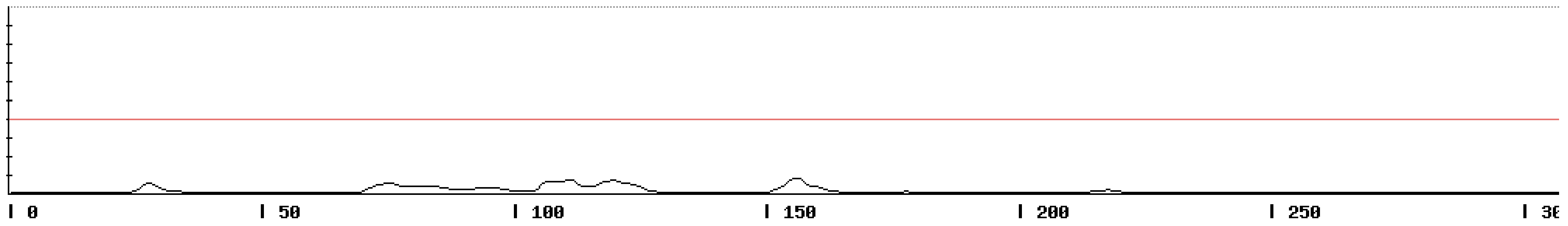

Predictions for Camelinasativa_Csa03g038330.1

Viterbi Path	
Viterbi Path	
Posterior @ 0.4	
Posterior @ 0.4	

Posterior Graphic. Threshold Marked @ 0.4

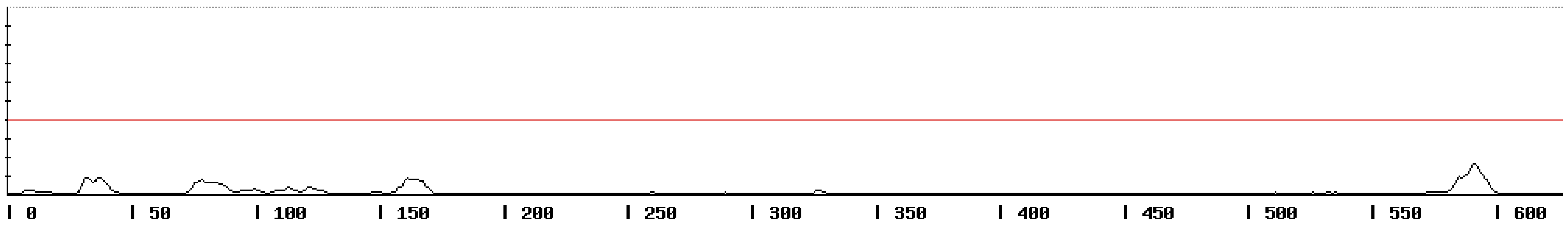

Predictions for Camelinasativa_Csa03g038340.1

Viterbi Path

Viterbi Path

Posterior @ 0.4

Posterior @ 0.4

Posterior Graphic. Threshold Marked @ 0.4

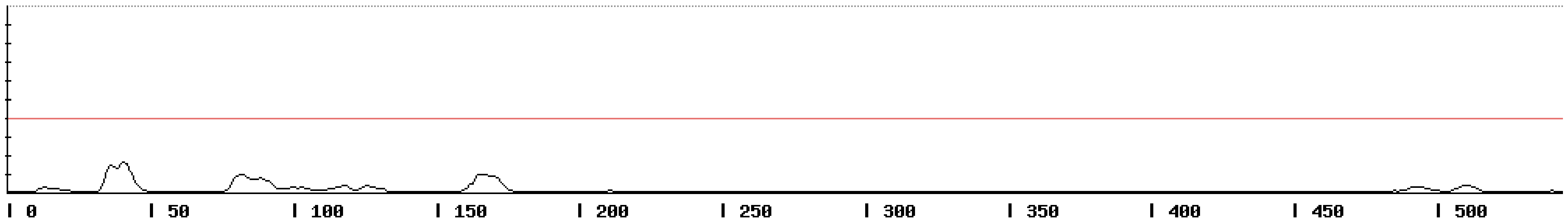

Predictions for Camelinasativa_Csa03g059990.1

Viterbi Path

Viterbi Path

Posterior @ 0.4

Posterior @ 0.4

Posterior Graphic. Threshold Marked @ 0.4	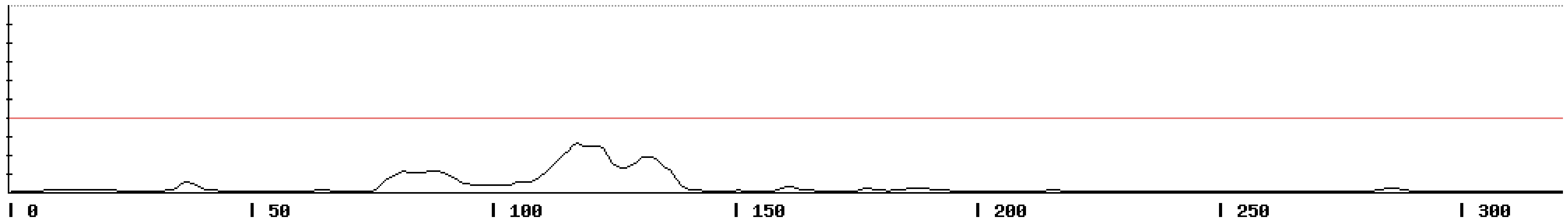

Predictions for Camelinasativa_Csa03g060000.1

Viterbi Path	
Viterbi Path	
Posterior @ 0.4	
Posterior @ 0.4	
Posterior Graphic. Threshold Marked @ 0.4	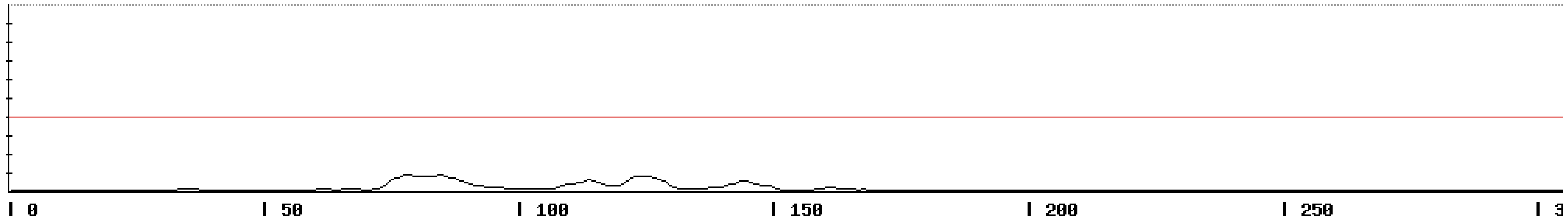

Predictions for Camelinasativa_Csa03g062660.1

Viterbi Path	
Viterbi Path	

Posterior @ 0.4

Posterior @ 0.4

Posterior Graphic. Threshold Marked @ 0.4

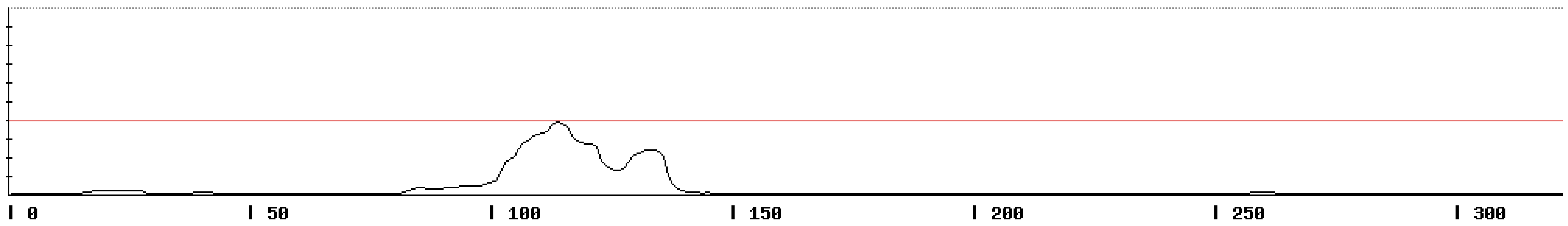

Predictions for Camelinasativa_Csa03g062870.1

Viterbi Path

Viterbi Path

Posterior @ 0.4

Posterior @ 0.4

Posterior Graphic. Threshold Marked @ 0.4

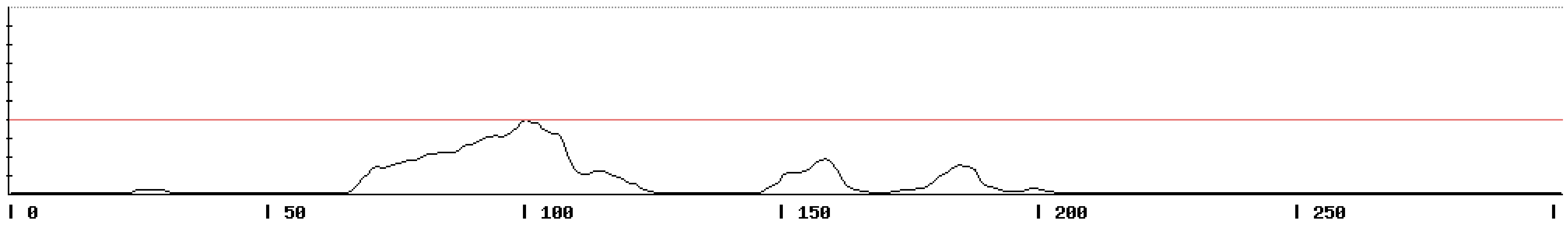

Predictions for Camelinasativa_Csa04g008000.1

Viterbi Path

Viterbi Path

Posterior @ 0.4

Posterior @ 0.4

Posterior Graphic. Threshold Marked @ 0.4

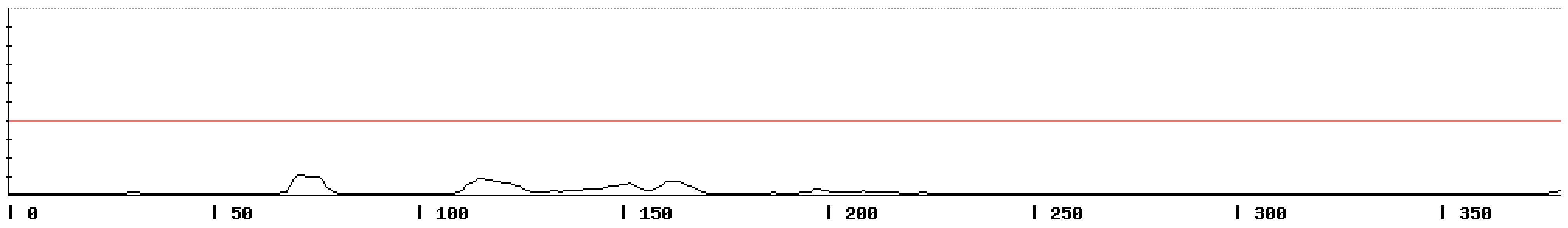

Predictions for Camelinasativa_Csa04g015120.1

Viterbi Path

Viterbi Path

Posterior @ 0.4

Posterior @ 0.4

Posterior Graphic. Threshold Marked @ 0.4

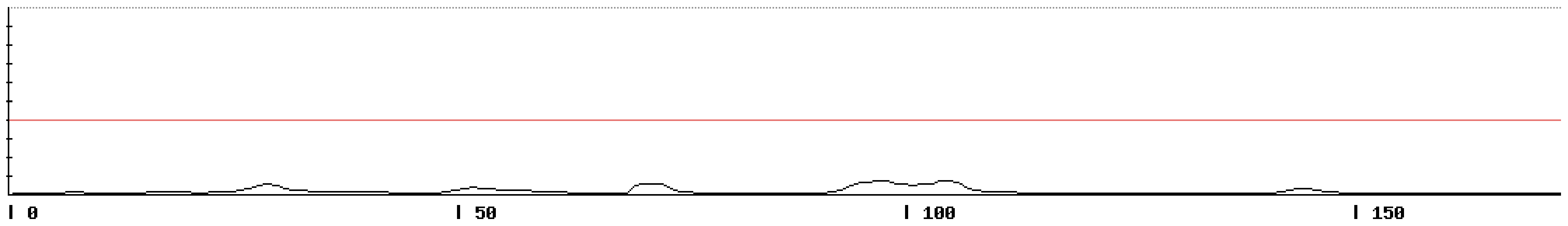

Predictions for Camelinasativa_Csa04g018350.1

Viterbi Path	
Viterbi Path	
Posterior @ 0.4	
Posterior @ 0.4	
Posterior Graphic. Threshold Marked @ 0.4	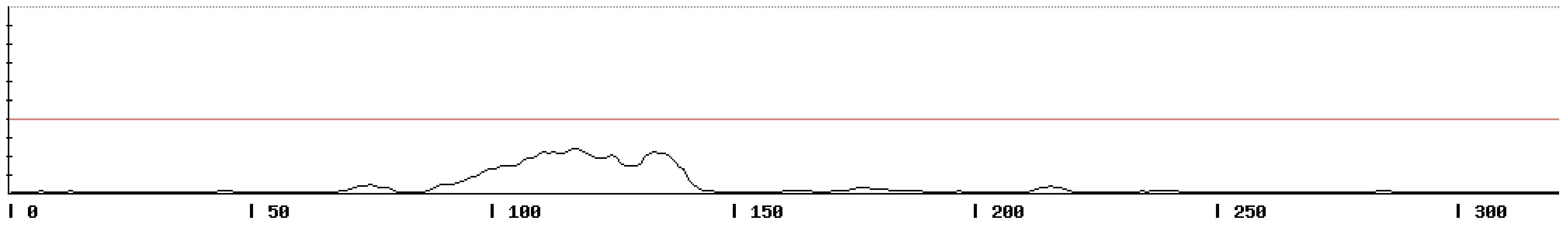

Predictions for Camelinasativa_Csa04g023980.1

Viterbi Path	
Viterbi Path	
Posterior @ 0.4	
Posterior @ 0.4	
Posterior Graphic. Threshold Marked @ 0.4	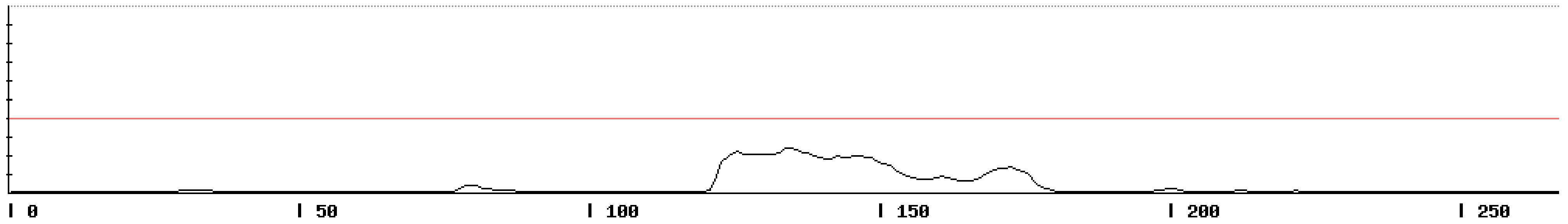

Predictions for Camelinasativa_Csa04g024130.1

Viterbi Path	
Viterbi Path	
Posterior @ 0.4	
Posterior @ 0.4	
Posterior Graphic. Threshold Marked @ 0.4	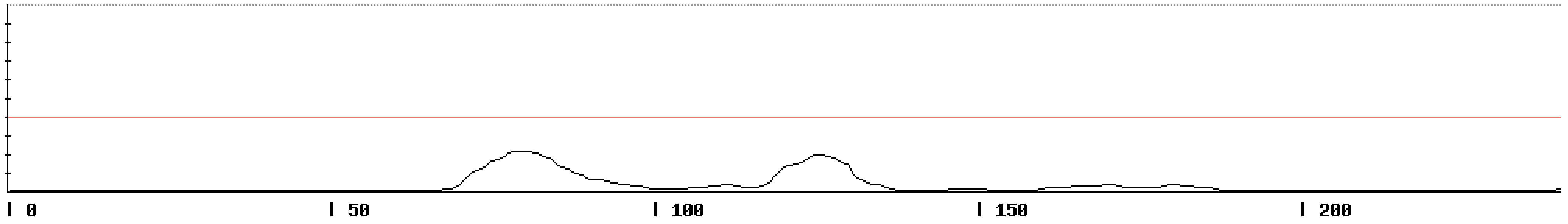

Predictions for Camelinasativa_Csa04g035000.1

Viterbi Path	
Viterbi Path	
Posterior @ 0.4	99 - TGKDRKIKNGKTKIIGVKR - 117
Posterior @ 0.4	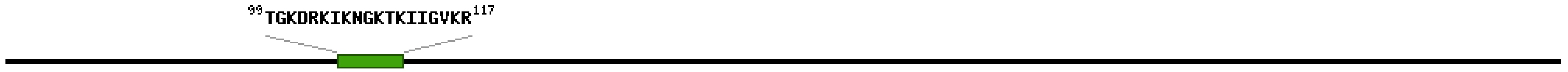
Posterior Graphic. Threshold Marked @ 0.4	

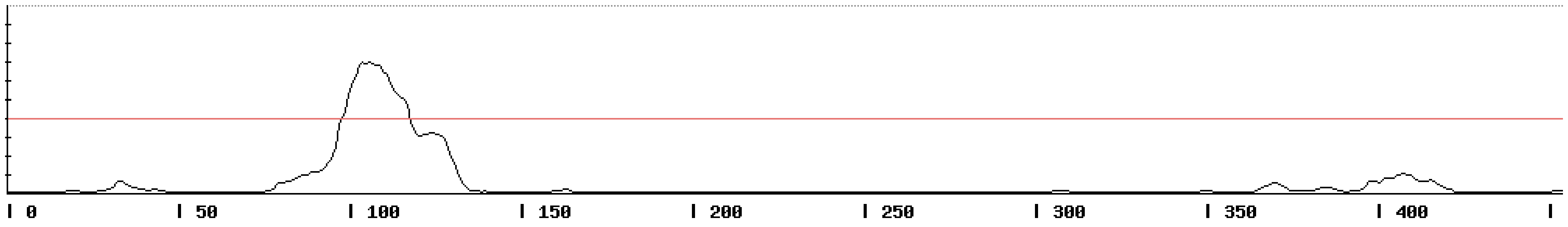

Predictions for Camelinasativa_Csa04g040470.1

Viterbi Path

Viterbi Path

Posterior @ 0.4

Posterior @ 0.4

Posterior Graphic. Threshold Marked @ 0.4

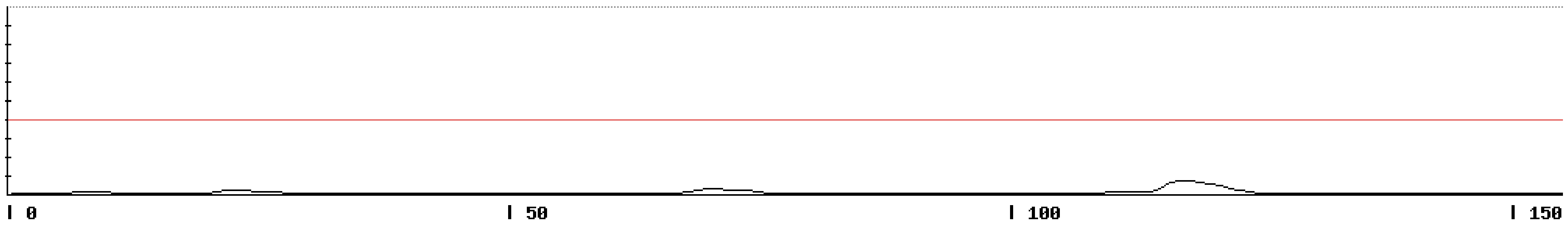

Predictions for Camelinasativa_Csa04g042950.1

Viterbi Path

Viterbi Path

Posterior @ 0.4 189 - KGAKKKK - 195

Posterior @ 0.4

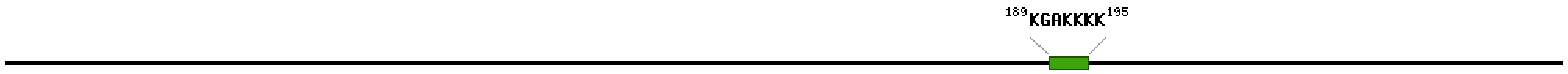

Posterior Graphic. Threshold Marked @ 0.4

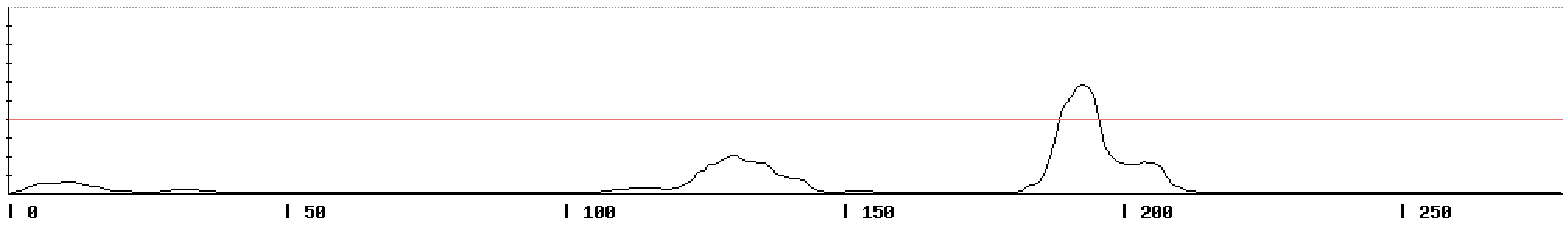

Predictions for Camelinasativa_Csa04g042970.1

Viterbi Path

Viterbi Path

Posterior @ 0.4 109 - KSKRNKTGKDGKRQKRDAKG - 128

Posterior @ 0.4

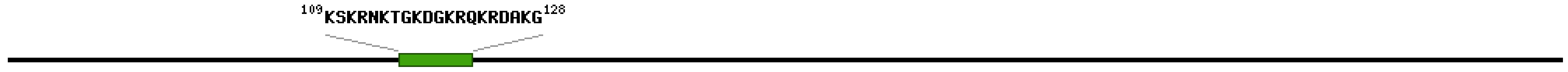

Posterior Graphic. Threshold Marked @ 0.4

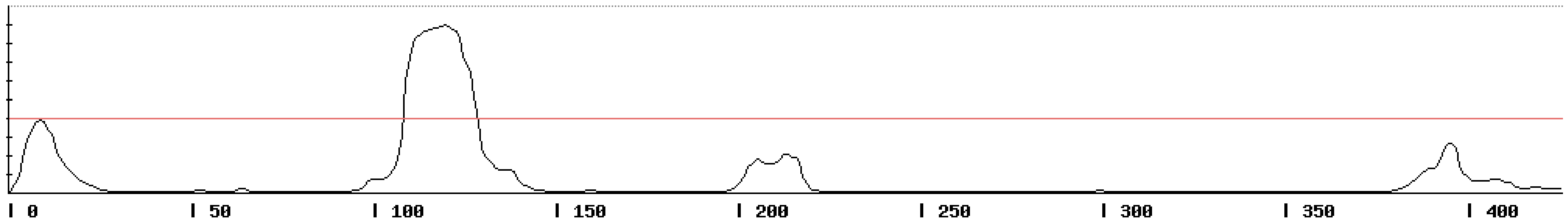

Predictions for Camelinasativa_Csa04g049120.1

Viterbi Path

Viterbi Path

Posterior @ 0.4 106 - SKRNKMGKGGKRQKRVDKG - 124

Posterior @ 0.4

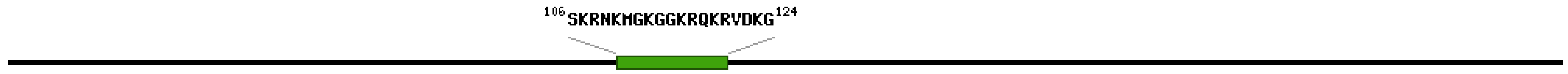

Posterior Graphic. Threshold Marked @ 0.4

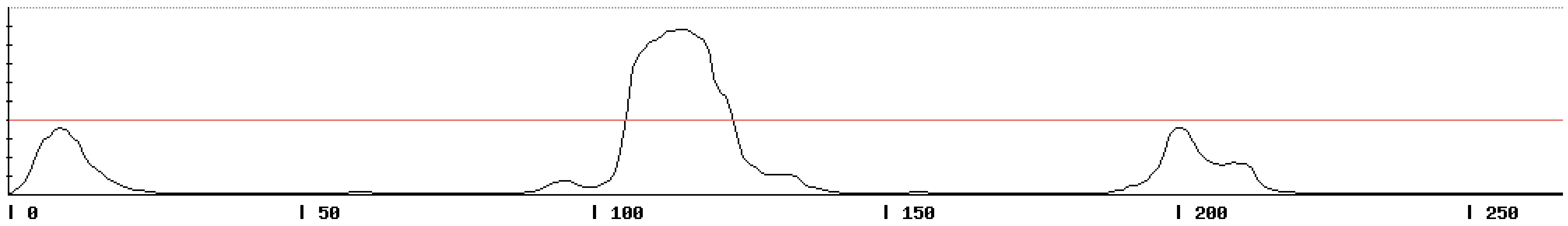

Predictions for Camelinasativa_Csa04g060600.1

Viterbi Path

Viterbi Path

Posterior @ 0.4

Posterior @ 0.4

Posterior Graphic. Threshold Marked @ 0.4

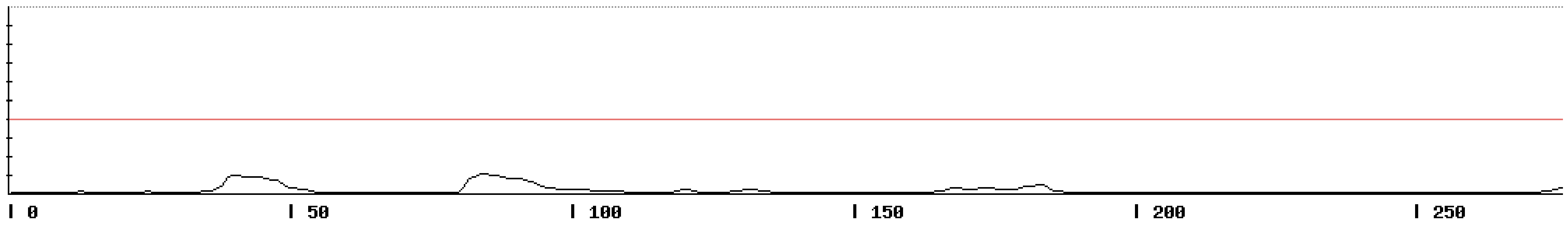

Predictions for Camelinasativa_Csa04g065900.1

Viterbi Path

Viterbi Path

Posterior @ 0.4

Posterior @ 0.4

Posterior Graphic. Threshold Marked @ 0.4

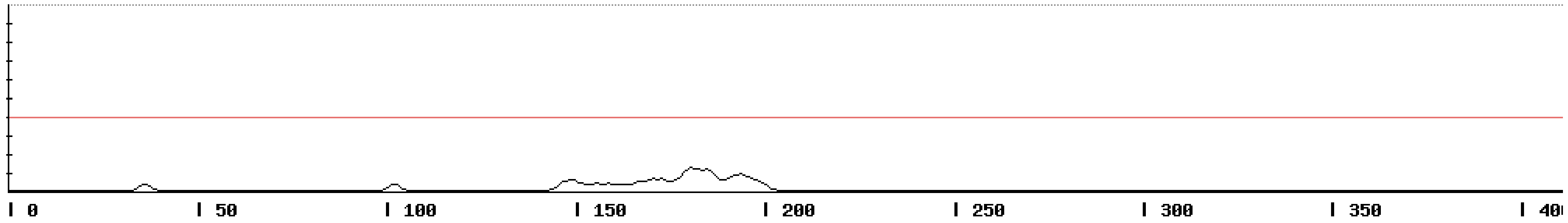

Predictions for Camelinasativa_Csa05g002390.1

Viterbi Path

Viterbi Path

Posterior @ 0.4

Posterior @ 0.4

Posterior Graphic. Threshold Marked @ 0.4

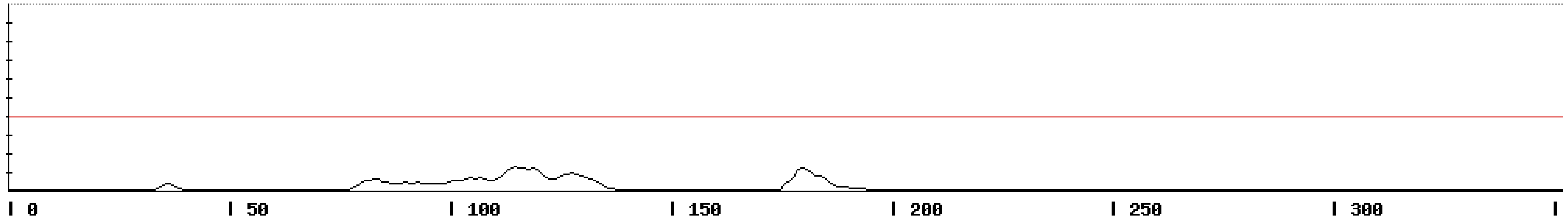

Predictions for Camelinasativa_Csa05g002930.1

Viterbi Path

Viterbi Path

Posterior @ 0.4

Posterior @ 0.4

Posterior Graphic. Threshold Marked @ 0.4

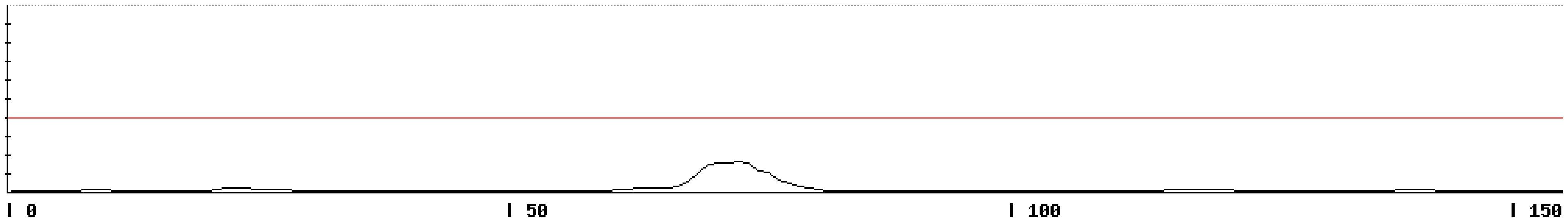

Predictions for Camelinasativa_Csa05g007460.1

Viterbi Path

Viterbi Path

Posterior @ 0.4

Posterior @ 0.4

Posterior Graphic. Threshold Marked @ 0.4

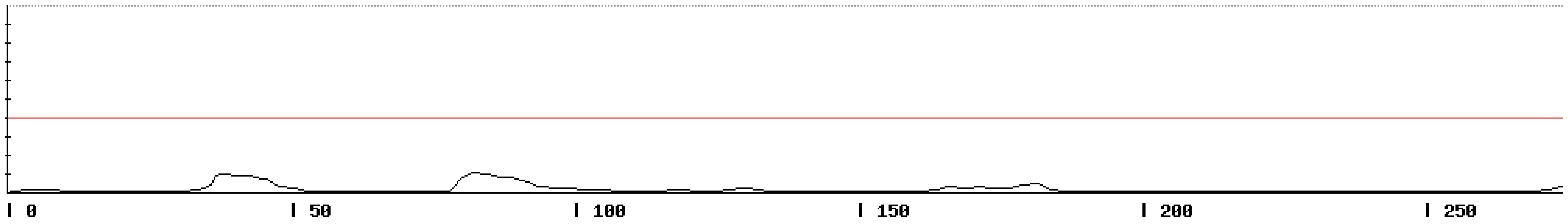

Predictions for Camelinasativa_Csa05g018490.1

Viterbi Path	
Viterbi Path	
Posterior @ 0.4	106 - SKRNKMGKGGKRQKRVDKGGYWHSK - 130
Posterior @ 0.4	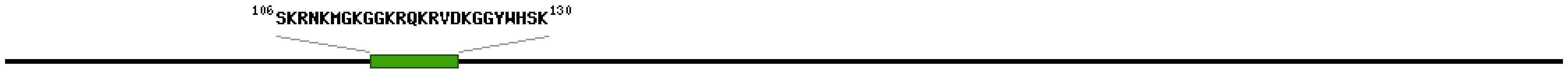
Posterior Graphic. Threshold Marked @ 0.4	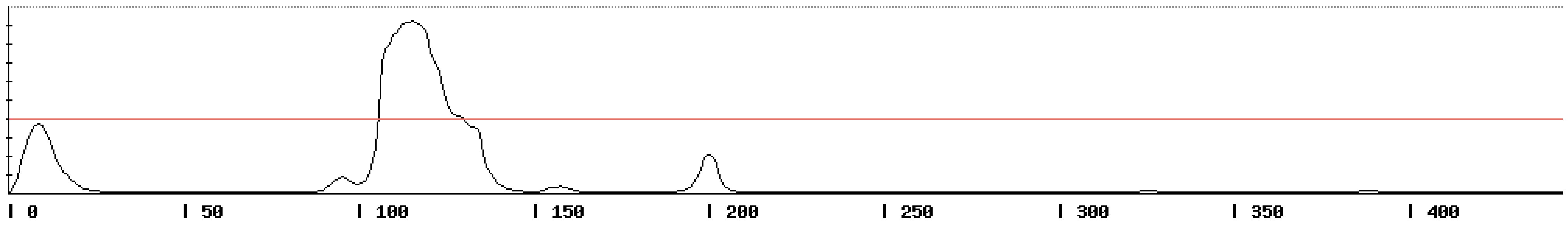

Predictions for Camelinasativa_Csa05g022930.1

Viterbi Path	
Viterbi Path	
Posterior @ 0.4	188 - RKDKKNKIRRRR - 199
Posterior @ 0.4	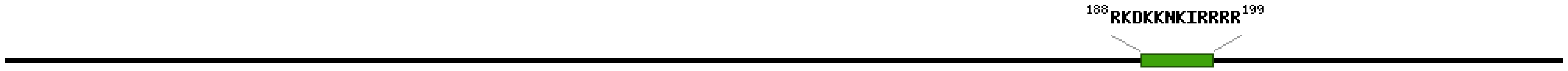
Posterior Graphic. Threshold Marked @ 0.4	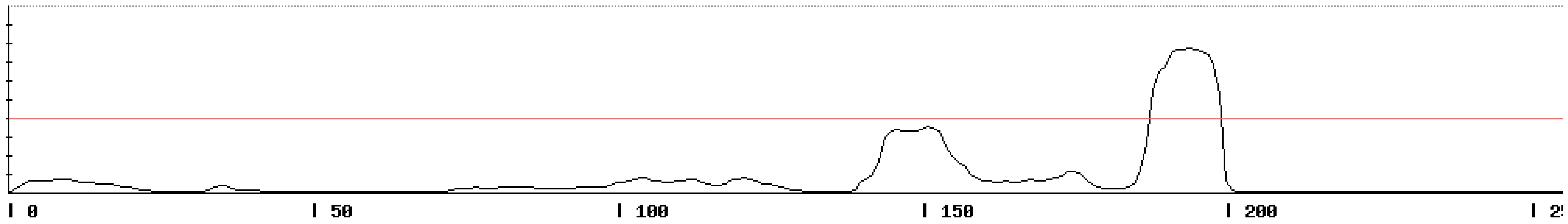

Predictions for Camelinasativa_Csa05g041670.1

Viterbi Path

Viterbi Path

Posterior @ 0.4

Posterior @ 0.4

Posterior Graphic. Threshold Marked @ 0.4

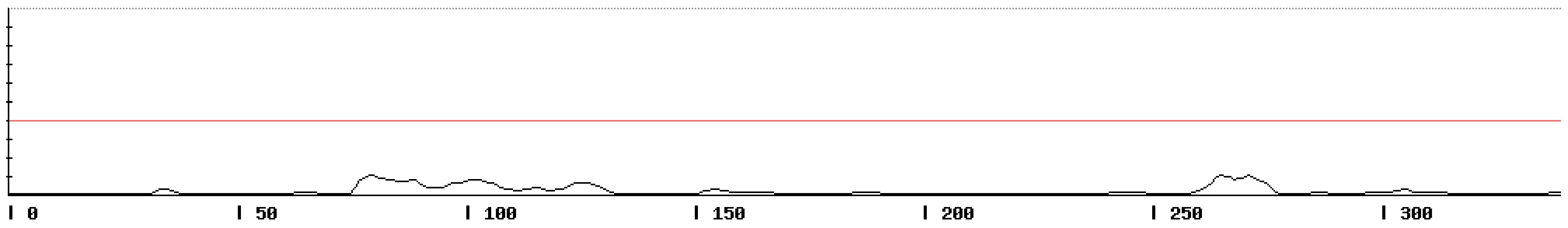

Predictions for Camelinasativa_Csa05g042090.1

Viterbi Path

Viterbi Path

Posterior @ 0.4 | 119 - SKKNKMGKGGKKQKR - 133

Posterior @ 0.4

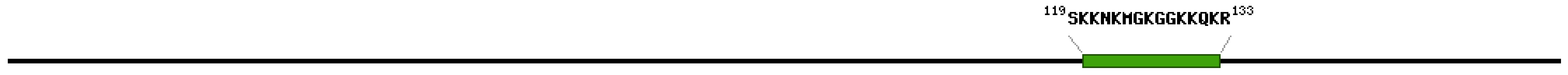

Posterior Graphic. Threshold Marked @ 0.4

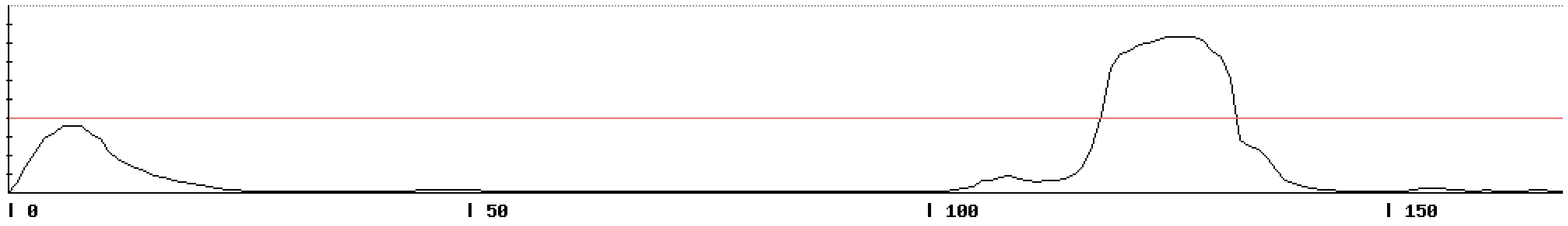

Predictions for Camelinasativa_Csa05g042120.1

Viterbi Path

Viterbi Path

Posterior @ 0.4

9 - R - 9

121 - SKKNKMGKGGKKQKR - 135

Posterior @ 0.4

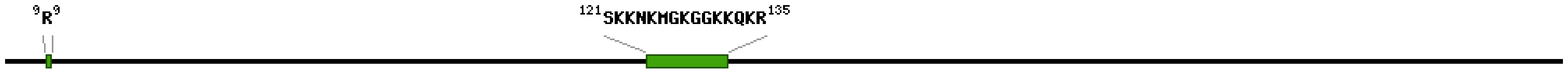

Posterior Graphic. Threshold Marked @ 0.4

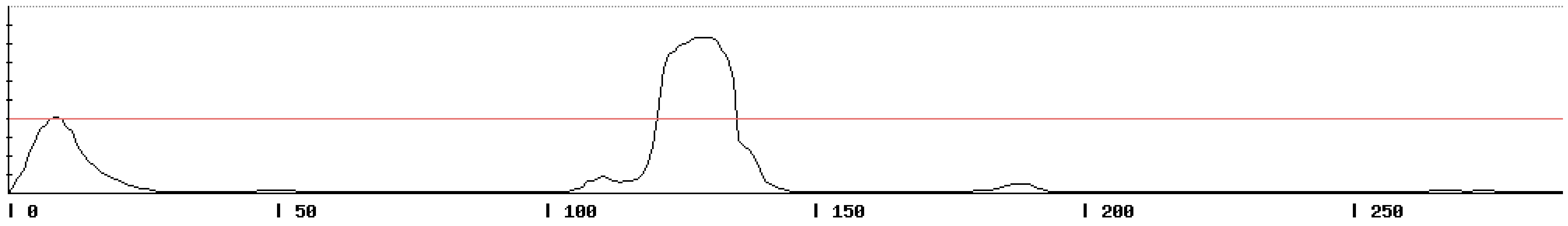

Predictions for Camelinasativa_Csa05g047220.1

Viterbi Path

Viterbi Path

Posterior @ 0.4

Posterior @ 0.4

Posterior Graphic. Threshold Marked @ 0.4

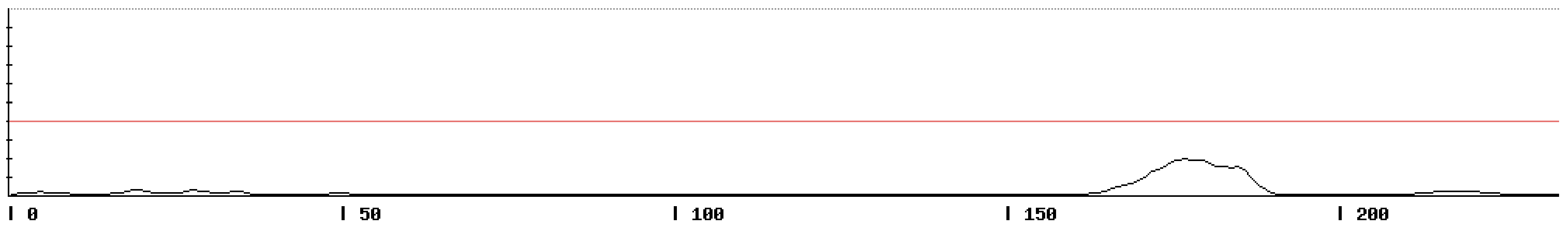

Predictions for Camelinasativa_Csa05g047230.1

Viterbi Path

Viterbi Path

Posterior @ 0.4 — 128 - KRKPR - 132

Posterior @ 0.4

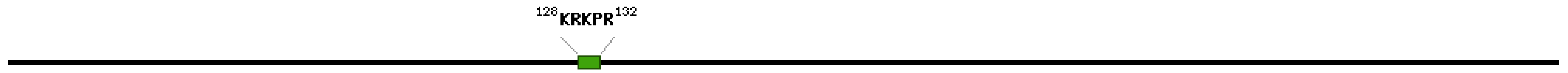

Posterior Graphic. Threshold Marked @ 0.4

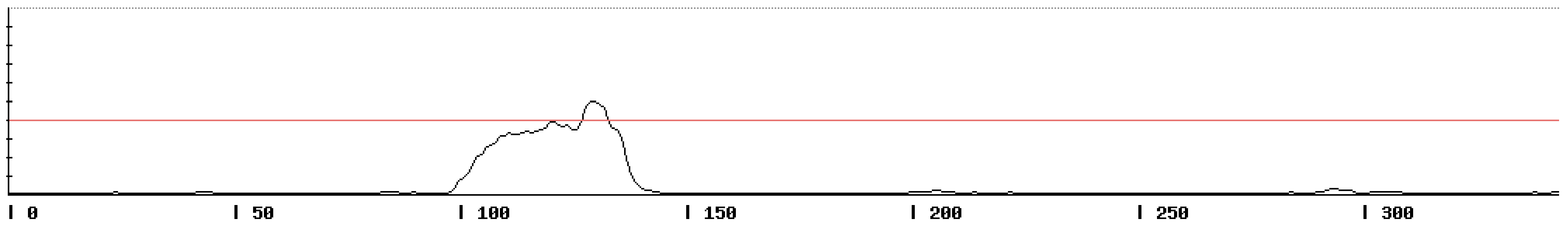

Predictions for Camelinasativa_Csa05g047250.1

Viterbi Path

Viterbi Path

Posterior @ 0.4 — 93 - KEKPKSMFKFWEKEKRRVRDKR - 114

Posterior @ 0.4

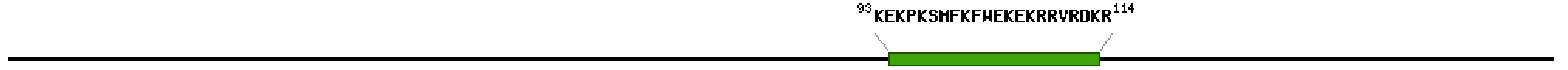

Posterior Graphic. Threshold Marked @ 0.4

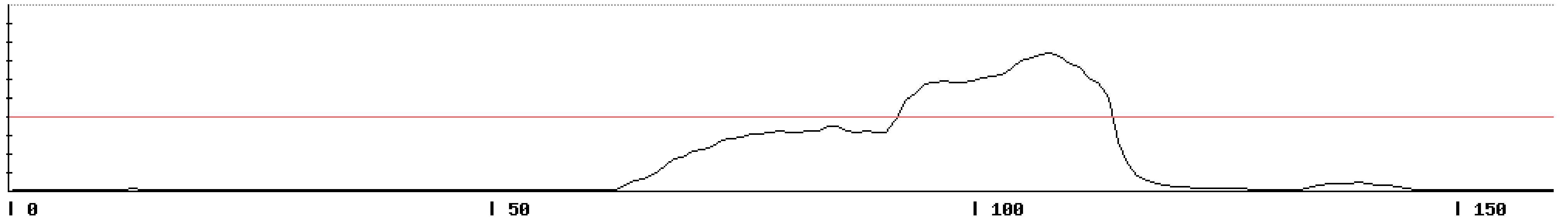

Predictions for Camelinasativa_Csa05g070860.1

Viterbi Path

Viterbi Path

Posterior @ 0.4 — 5 - GKKQKR - 10

Posterior @ 0.4

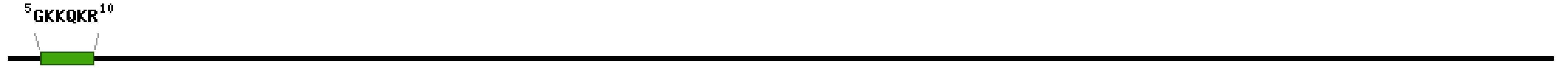

Posterior Graphic. Threshold Marked @ 0.4

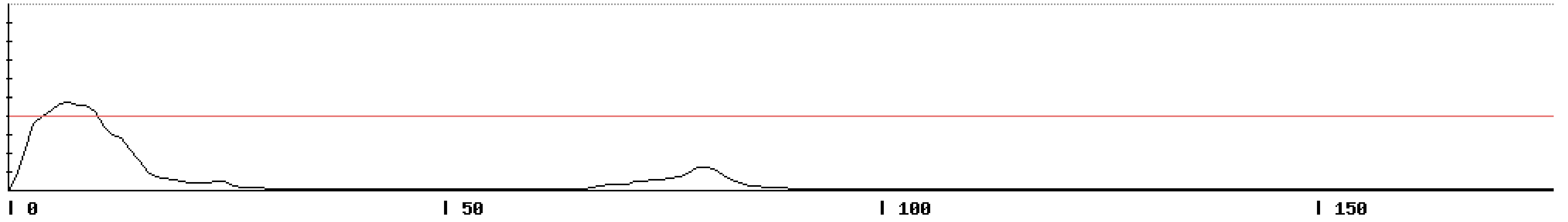

Predictions for Camelinasativa_Csa05g073600.1

Viterbi Path

Viterbi Path

Posterior @ 0.4	585 - RRKIWKKEKGR - 595
Posterior @ 0.4	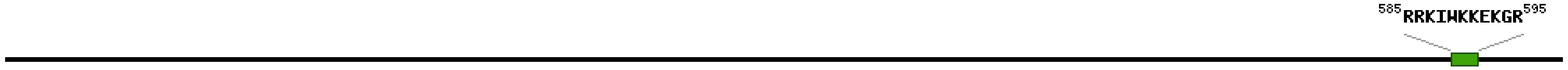
Posterior Graphic. Threshold Marked @ 0.4	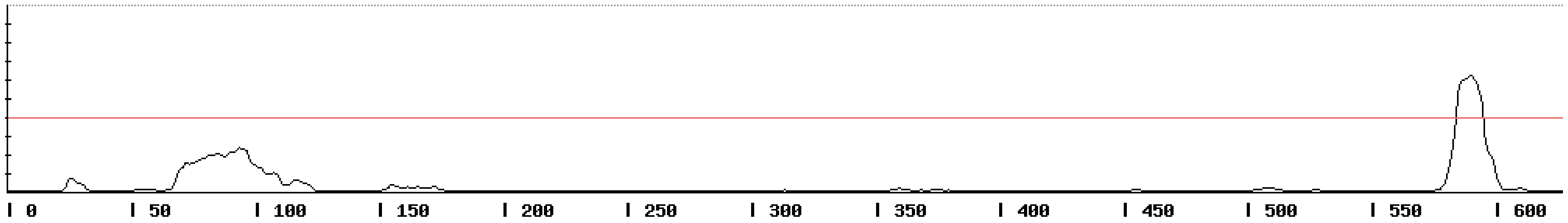

Predictions for Camelinasativa_Csa05g086200.1

Viterbi Path	
Viterbi Path	
Posterior @ 0.4	
Posterior @ 0.4	
Posterior Graphic. Threshold Marked @ 0.4	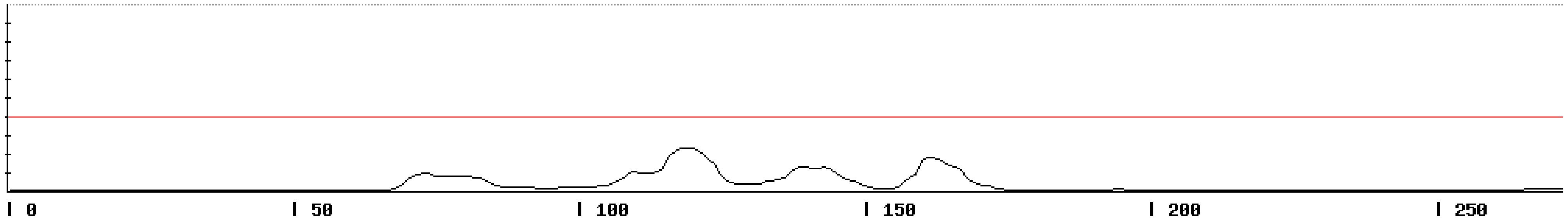

Predictions for Camelinasativa_Csa05g086390.1

Viterbi Path

Viterbi Path

Posterior @ 0.4

Posterior @ 0.4

Posterior Graphic. Threshold Marked @ 0.4

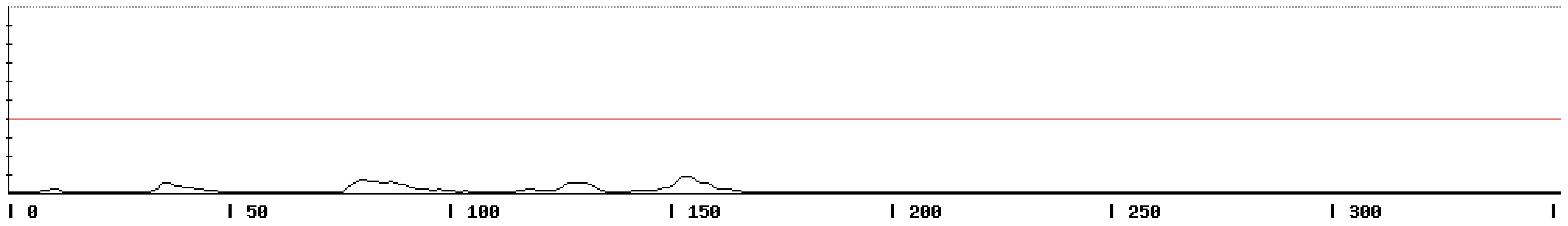

Predictions for Camelinasativa_Csa05g094110.1

Viterbi Path

Viterbi Path

Posterior @ 0.4

Posterior @ 0.4

Posterior Graphic. Threshold Marked @ 0.4

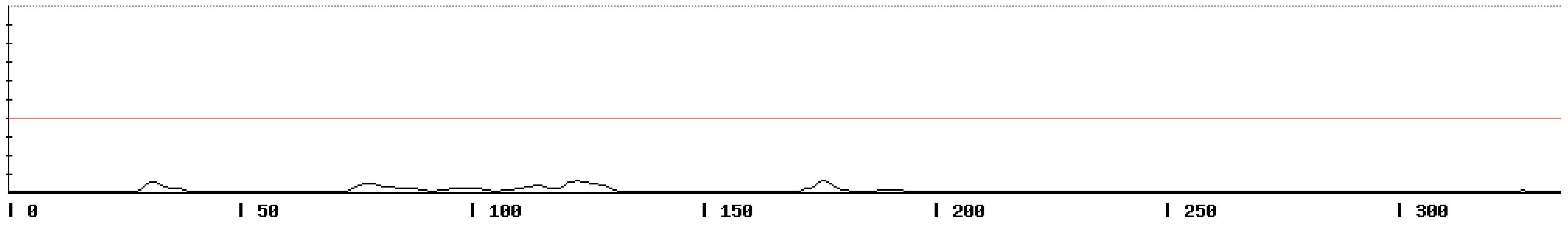

Predictions for Camelinasativa_Csa06g002590.1	
Viterbi Path	
Viterbi Path	
Posterior @ 0.4	
Posterior @ 0.4	
Posterior Graphic. Threshold Marked @ 0.4	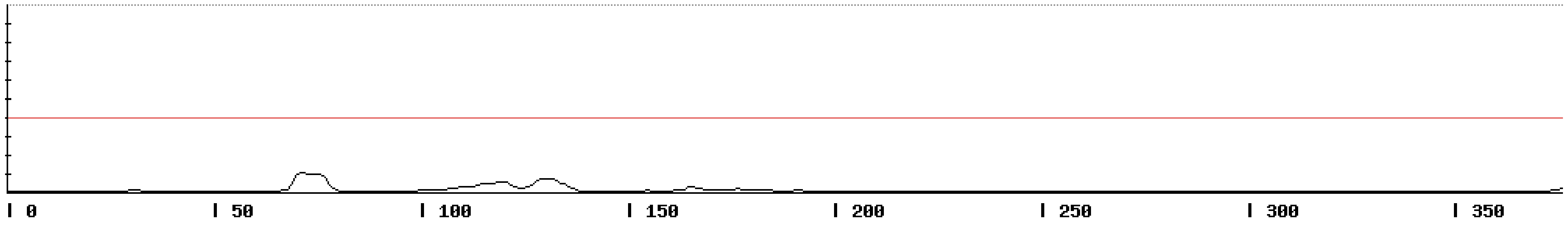

Predictions for Camelinasativa_Csa06g006850.1	
Viterbi Path	
Viterbi Path	
Posterior @ 0.4	
Posterior @ 0.4	
Posterior Graphic. Threshold Marked @ 0.4	

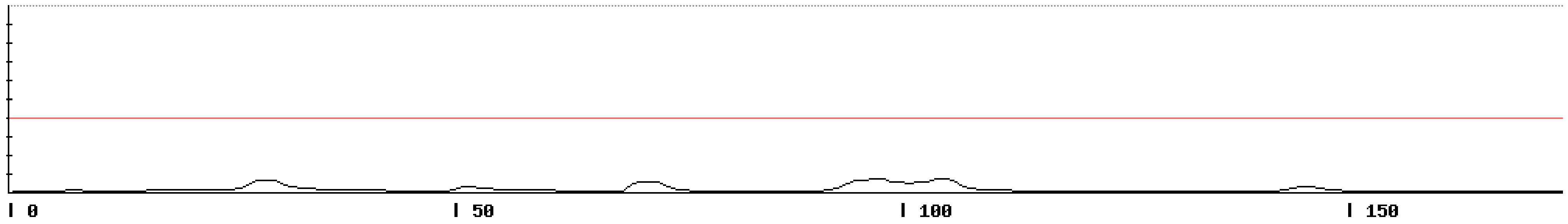

Predictions for Camelinasativa_Csa06g007190.1	
Viterbi Path	
Viterbi Path	
Posterior @ 0.4	
Posterior @ 0.4	
Posterior Graphic. Threshold Marked @ 0.4	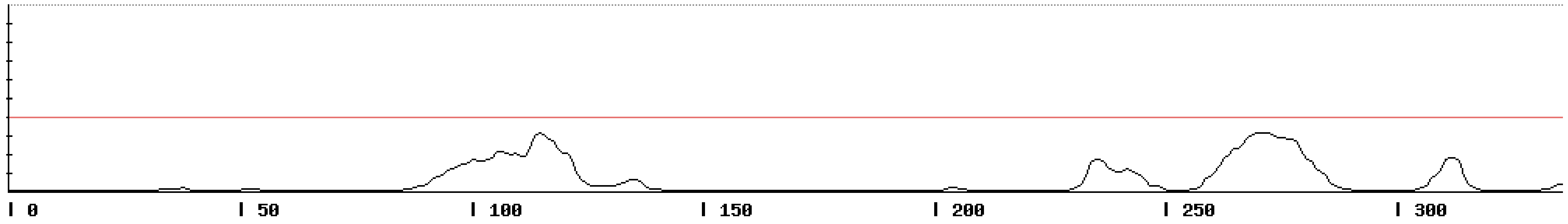

Predictions for Camelinasativa_Csa06g011390.1	
Viterbi Path	
Viterbi Path	
Posterior @ 0.4	
Posterior @ 0.4	

Posterior Graphic. Threshold Marked @ 0.4

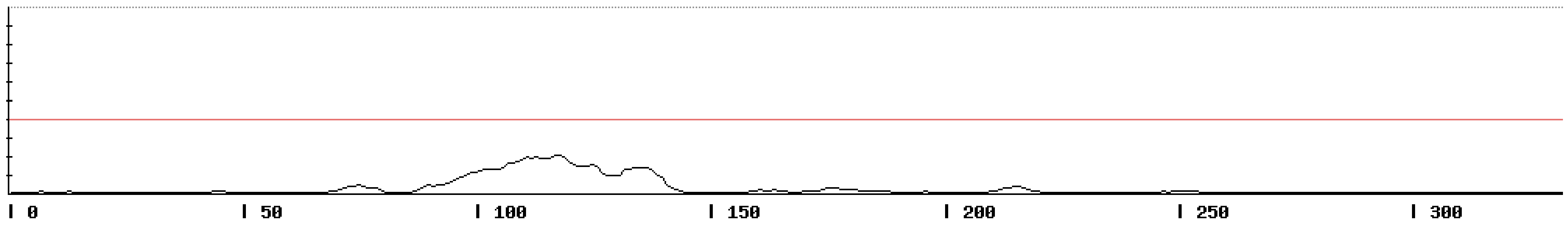

Predictions for Camelinasativa_Csa06g016930.1

Viterbi Path

Viterbi Path

Posterior @ 0.4

Posterior @ 0.4

Posterior Graphic. Threshold Marked @ 0.4

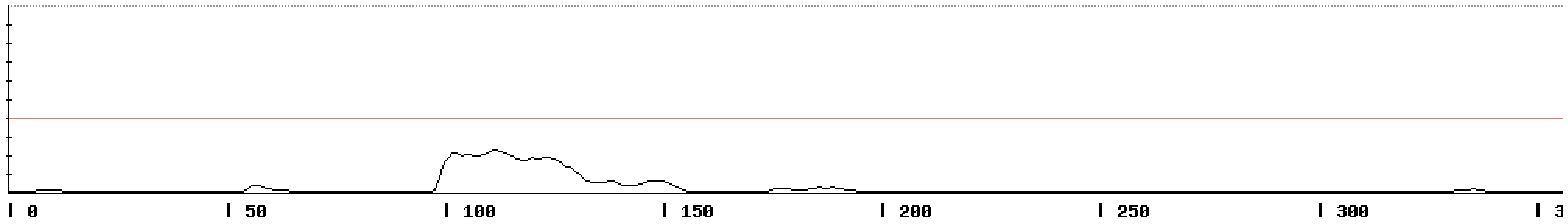

Predictions for Camelinasativa_Csa06g017030.1

Viterbi Path

Viterbi Path

Posterior @ 0.4

Posterior @ 0.4

Posterior Graphic. Threshold Marked @ 0.4

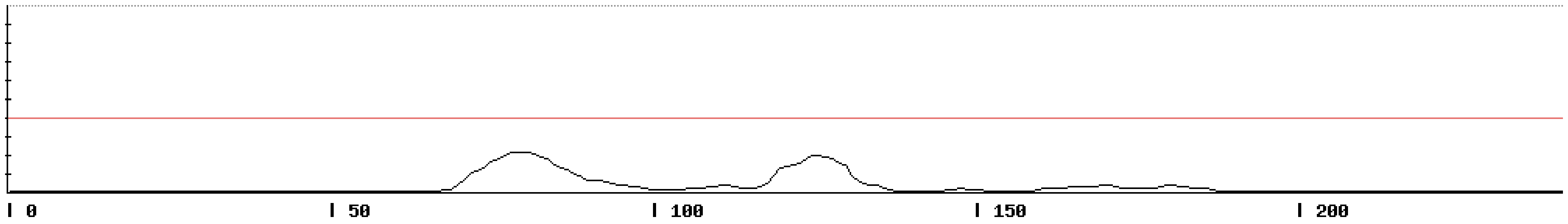

Predictions for Camelinasativa_Csa06g023540.1

Viterbi Path

Viterbi Path

Posterior @ 0.4

99 - TGKDRKIKNGKTKIIGVKR - 117

Posterior @ 0.4

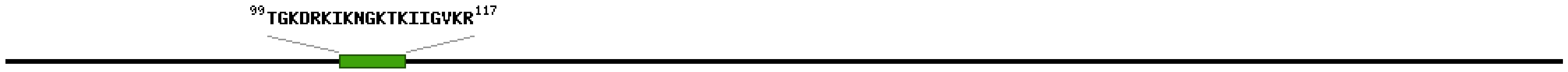

Posterior Graphic. Threshold Marked @ 0.4

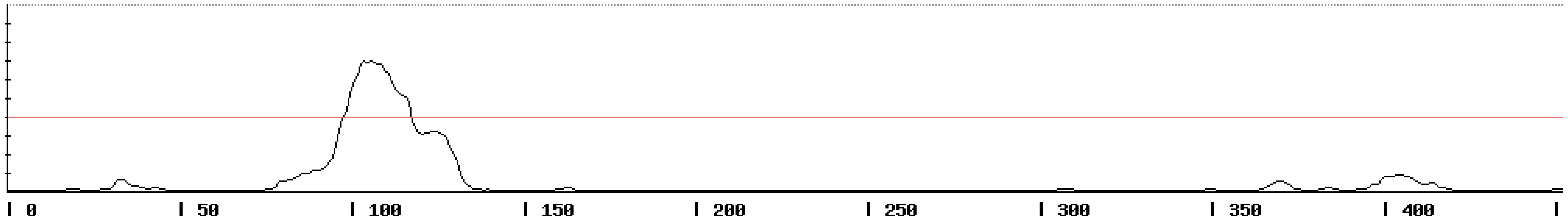

Predictions for Camelinasativa_Csa06g031260.1

Viterbi Path

Viterbi Path

Posterior @ 0.4	114 - KMGKDGKRQKRVAK - 127
Posterior @ 0.4	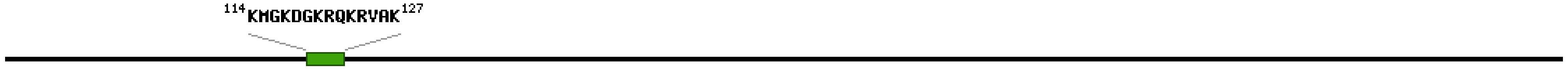
Posterior Graphic. Threshold Marked @ 0.4	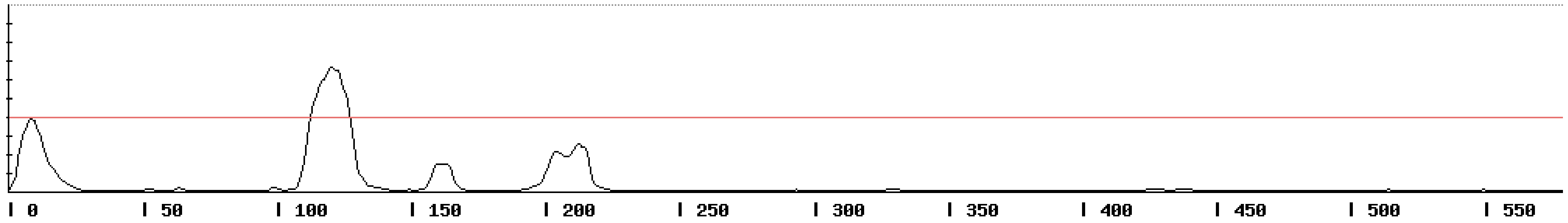

Predictions for Camelinasativa_Csa06g031280.1

Viterbi Path	123 - KKNKMGKGGKKQKRGDKGG - 141
Viterbi Path	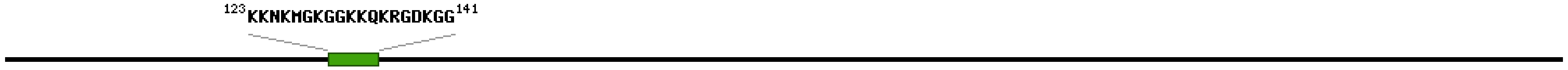
Posterior @ 0.4	122 - SKKNKMGKGGKKQKRGDKG - 140
Posterior @ 0.4	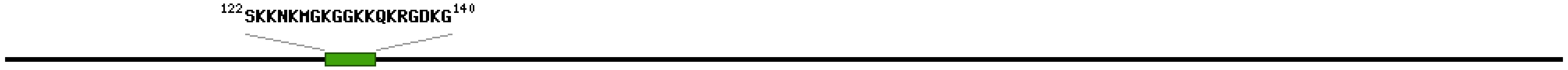
Posterior Graphic. Threshold Marked @ 0.4	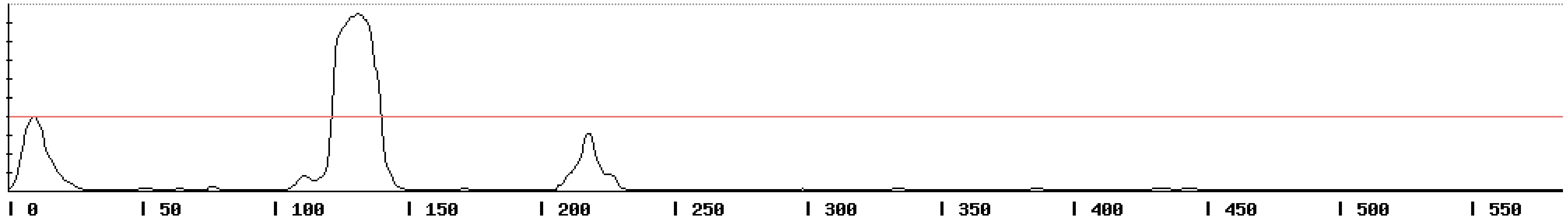

Predictions for Camelinasativa_Csa06g038290.1

Viterbi Path	
Viterbi Path	

Posterior @ 0.4	111 - KRNKMGKDGKRQKRVDK - 127
	203 - KKKKAE - 208
	214 - KQKK - 217
Posterior @ 0.4	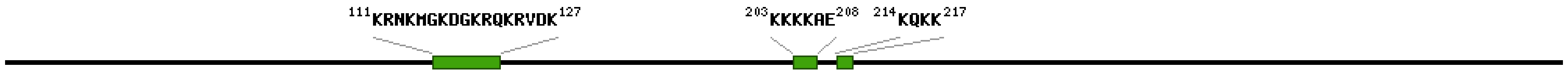
Posterior Graphic. Threshold Marked @ 0.4	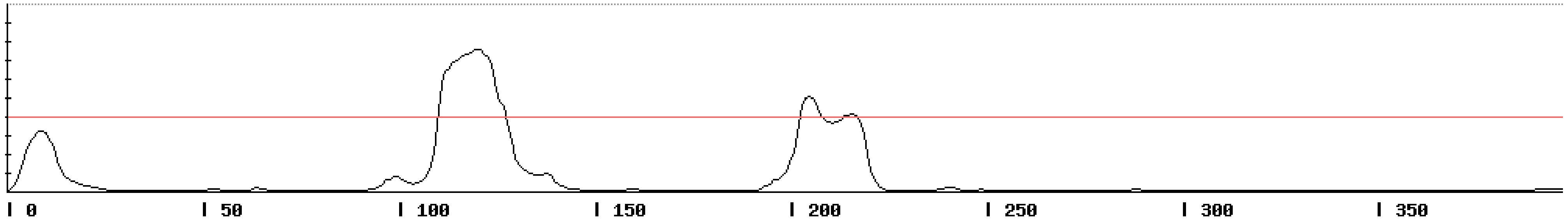

**Predictions for Camelinasativa_Csa06g049190.1**	
Viterbi Path	
Viterbi Path	
Posterior @ 0.4	
Posterior @ 0.4	
Posterior Graphic. Threshold Marked @ 0.4	

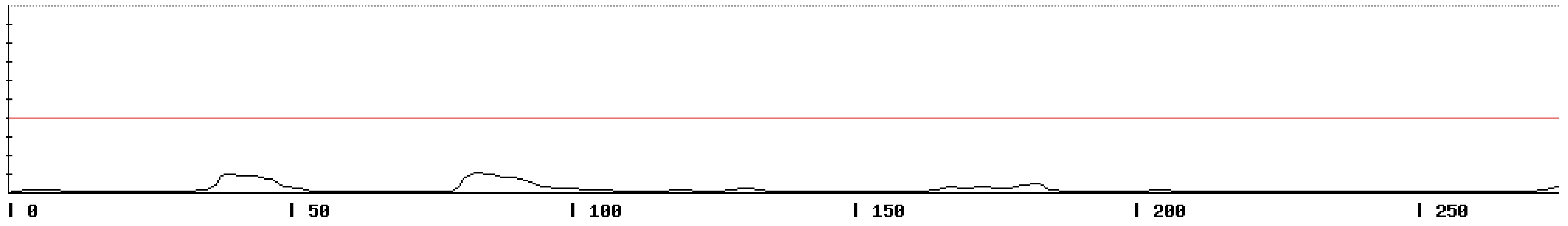

Predictions for Camelinasativa_Csa06g053280.1

Viterbi Path

Viterbi Path

Posterior @ 0.4

Posterior @ 0.4

Posterior Graphic. Threshold Marked @ 0.4

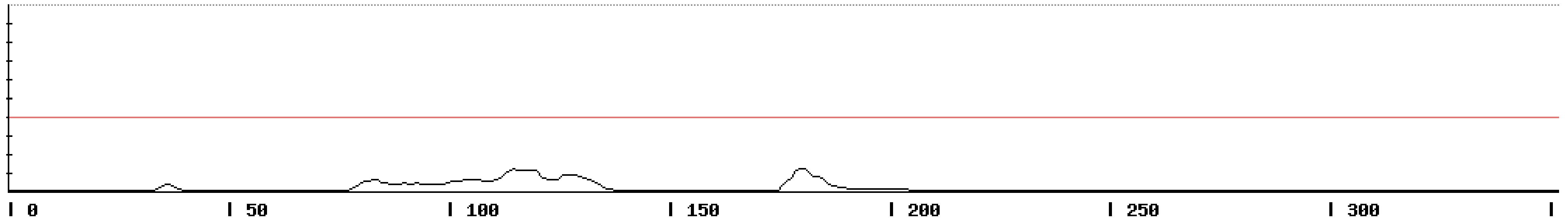

Predictions for Camelinasativa_Csa07482s010.1

Viterbi Path

Viterbi Path

Posterior @ 0.4

3 - KLGKGGGRGGRQKKRGS - 19

Posterior @ 0.4

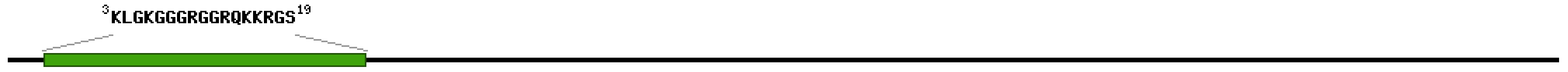

Posterior Graphic. Threshold Marked @ 0.4

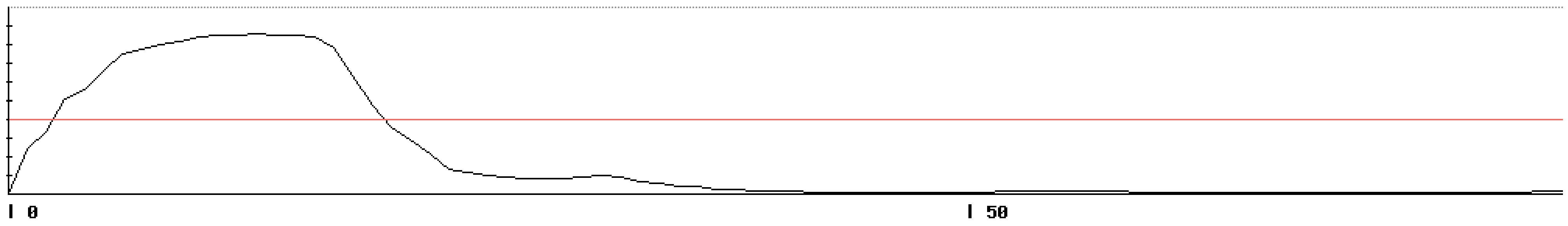

Predictions for Camelinasativa_Csa07g002690.1

Viterbi Path

Viterbi Path

Posterior @ 0.4

Posterior @ 0.4

Posterior Graphic. Threshold Marked @ 0.4

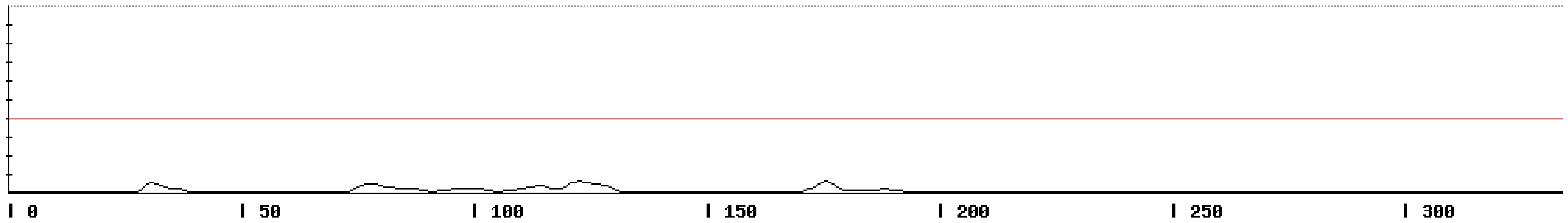

Predictions for Camelinasativa_Csa07g007900.1

Viterbi Path

Viterbi Path

Posterior @ 0.4

Posterior @ 0.4

Posterior Graphic. Threshold Marked @ 0.4

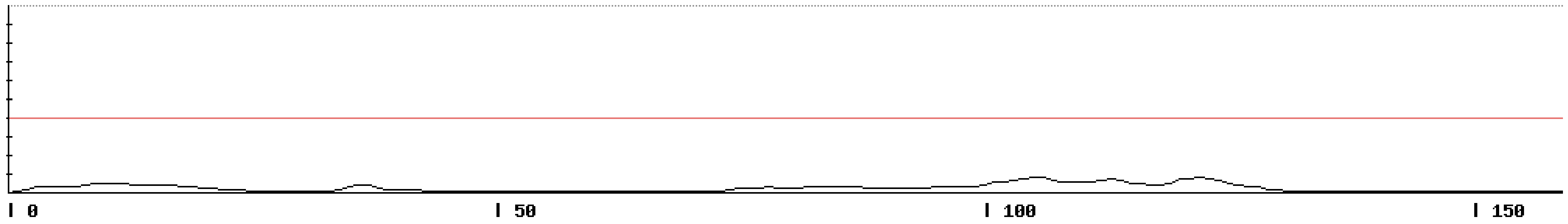

Predictions for Camelinasativa_Csa07g036200.1

Viterbi Path

Viterbi Path

Posterior @ 0.4

Posterior @ 0.4

Posterior Graphic. Threshold Marked @ 0.4

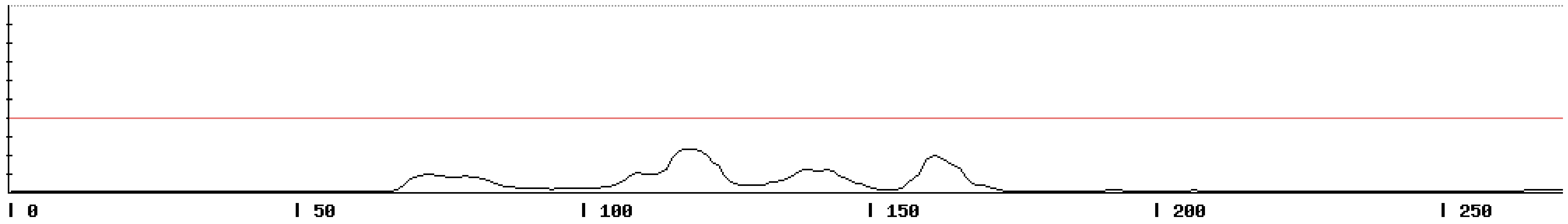

Predictions for Camelinasativa_Csa07g036390.1

Viterbi Path

Viterbi Path

Posterior @ 0.4

Posterior @ 0.4

Posterior Graphic. Threshold Marked @ 0.4

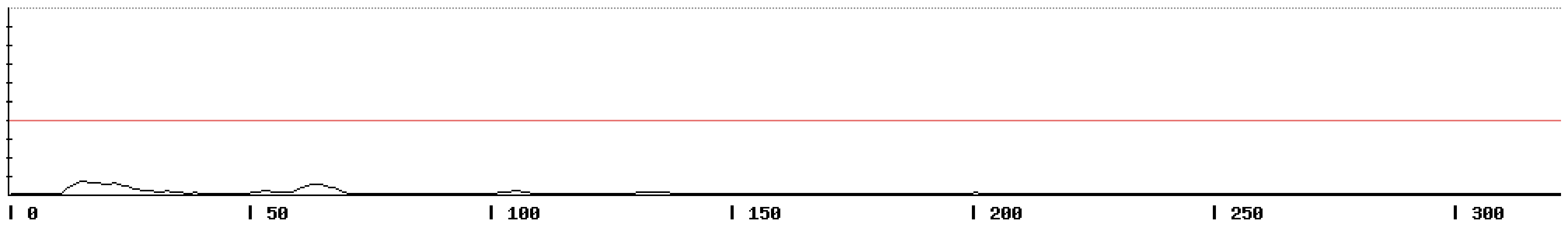

Predictions for Camelinasativa_Csa07g038870.1

Viterbi Path

Viterbi Path

Posterior @ 0.4

Posterior @ 0.4

Posterior Graphic. Threshold Marked @ 0.4

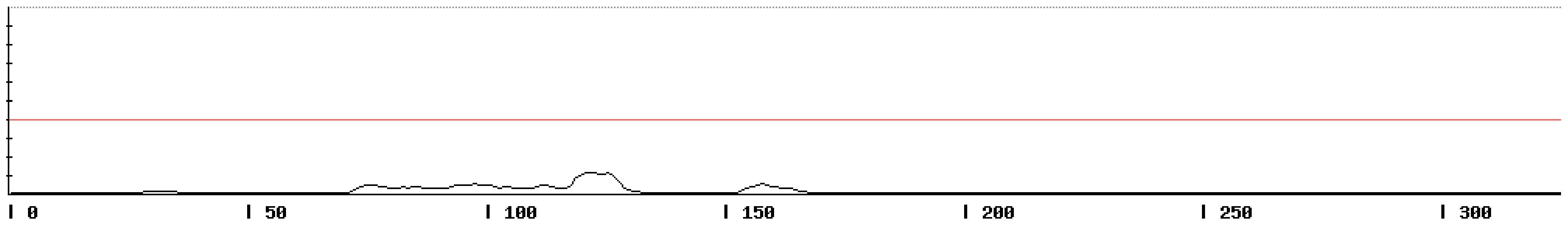

Predictions for Camelinasativa_Csa07g047980.1

Viterbi Path

Viterbi Path

Posterior @ 0.4

Posterior @ 0.4

Posterior Graphic. Threshold Marked @ 0.4

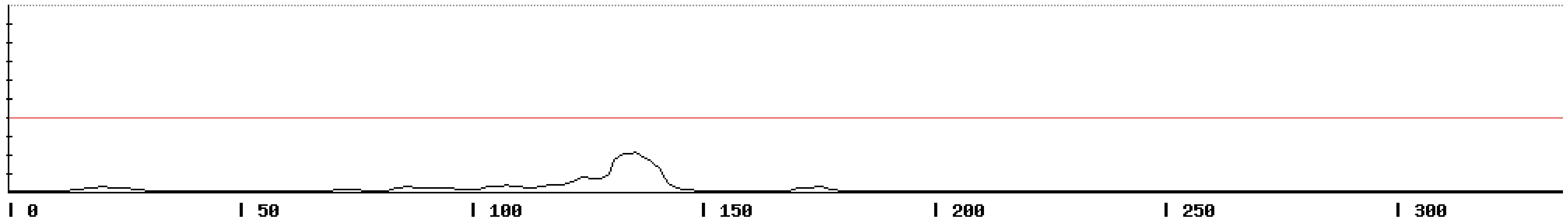

Predictions for Camelinasativa_Csa07g050100.1

Viterbi Path

Viterbi Path

Posterior @ 0.4

Posterior @ 0.4

Posterior Graphic. Threshold Marked @ 0.4

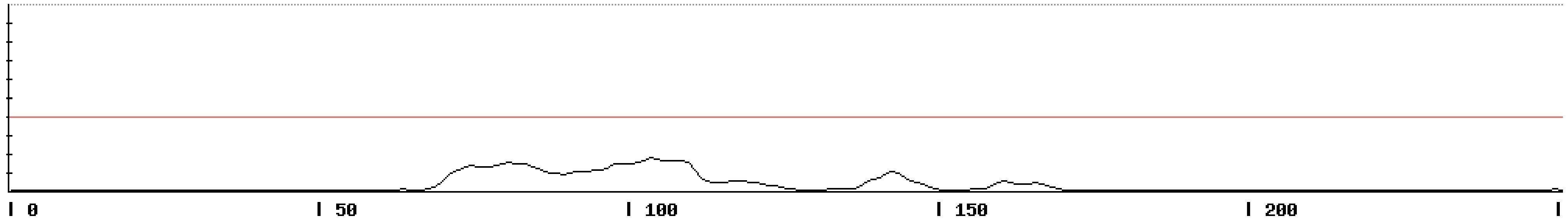

Predictions for Camelinasativa_Csa07g054410.1

Viterbi Path

Viterbi Path

Posterior @ 0.4

Posterior @ 0.4

Posterior Graphic. Threshold Marked @ 0.4

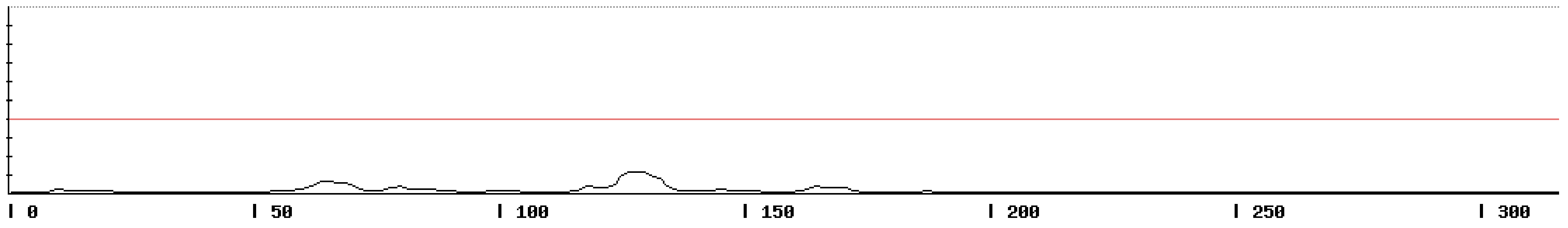

Predictions for Camelinasativa_Csa07g058710.1

Viterbi Path

Viterbi Path

Posterior @ 0.4

Posterior @ 0.4

Posterior Graphic. Threshold Marked @ 0.4

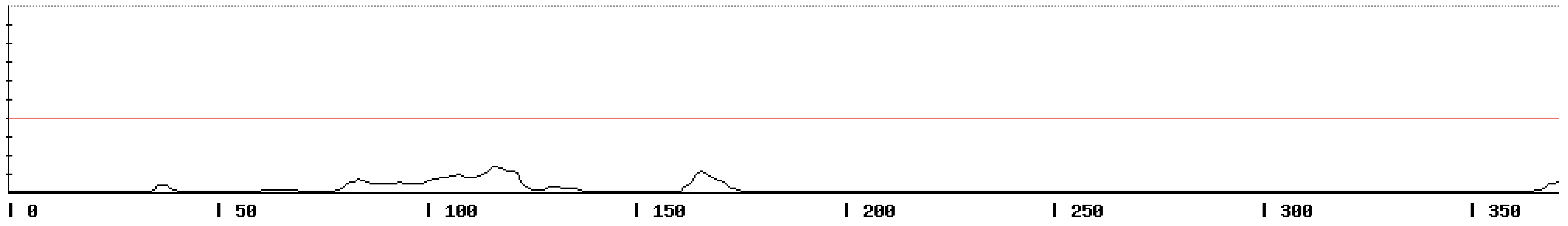

Predictions for Camelinasativa_Csa07g060910.1	
Viterbi Path	
Viterbi Path	
Posterior @ 0.4	
Posterior @ 0.4	
Posterior Graphic. Threshold Marked @ 0.4	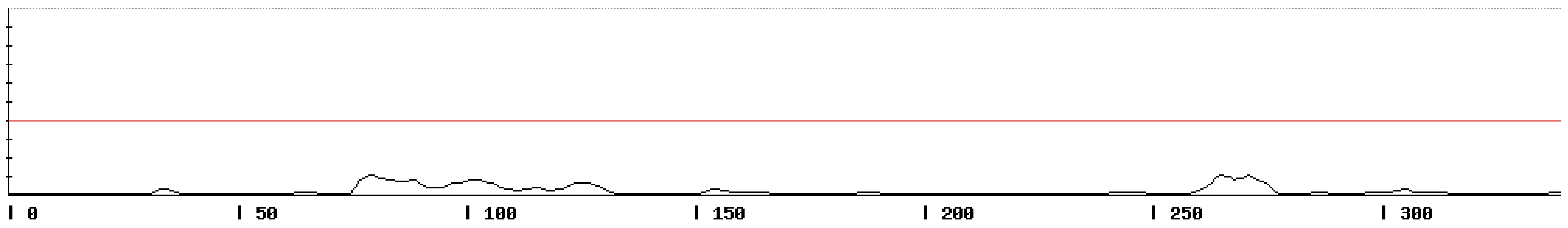

Predictions for Camelinasativa_Csa07g061560.1	
Viterbi Path	
Viterbi Path	
Posterior @ 0.4	116 - KEKPKSMFKFWEKEKRRVRDKR - 137
Posterior @ 0.4	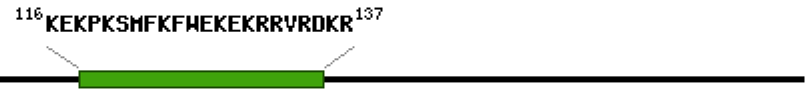
Posterior Graphic. Threshold Marked @ 0.4	

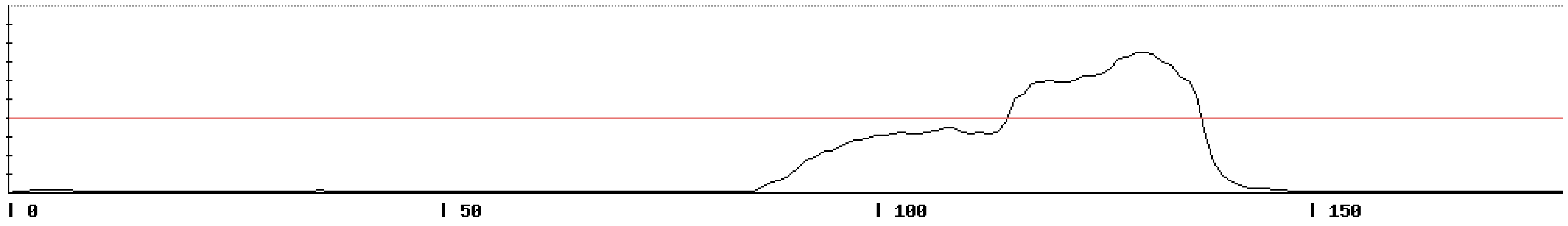

Predictions for Camelinasativa_Csa07g061590.1

Viterbi Path	
Viterbi Path	
Posterior @ 0.4	
Posterior @ 0.4	
Posterior Graphic. Threshold Marked @ 0.4	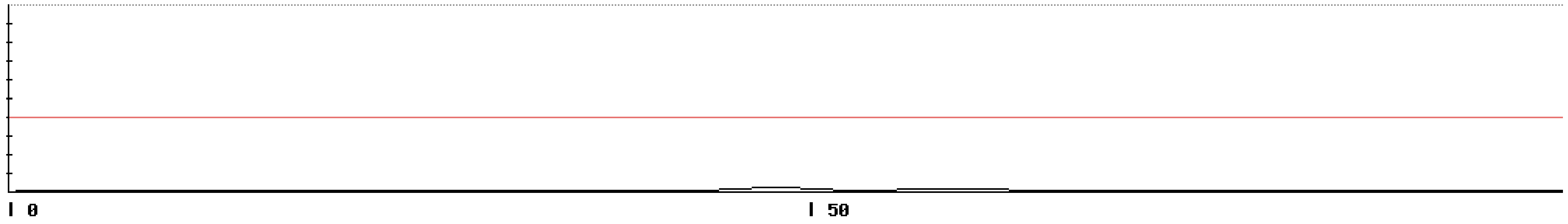

Predictions for Camelinasativa_Csa07g061600.1

Viterbi Path	
Viterbi Path	
Posterior @ 0.4	
Posterior @ 0.4	

Posterior Graphic. Threshold Marked @ 0.4

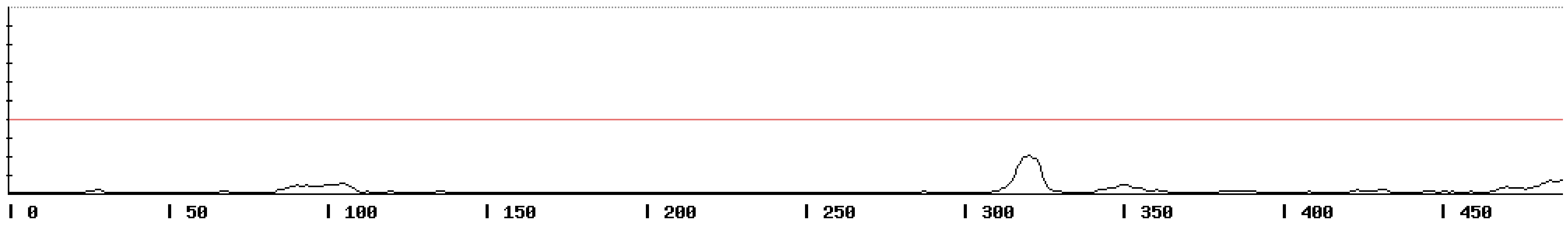

Predictions for Camelinasativa_Csa07g062260.1

Viterbi Path

Viterbi Path

Posterior @ 0.4

Posterior @ 0.4

Posterior Graphic. Threshold Marked @ 0.4

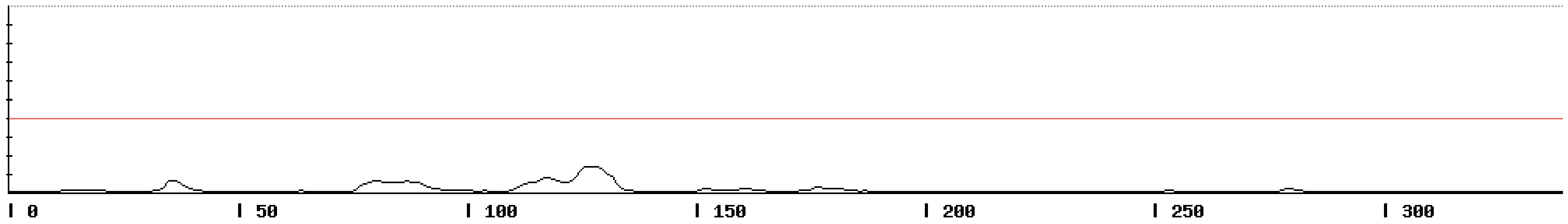

Predictions for Camelinasativa_Csa07g064020.1

Viterbi Path

Viterbi Path

Posterior @ 0.4

Posterior @ 0.4

Posterior Graphic. Threshold Marked @ 0.4

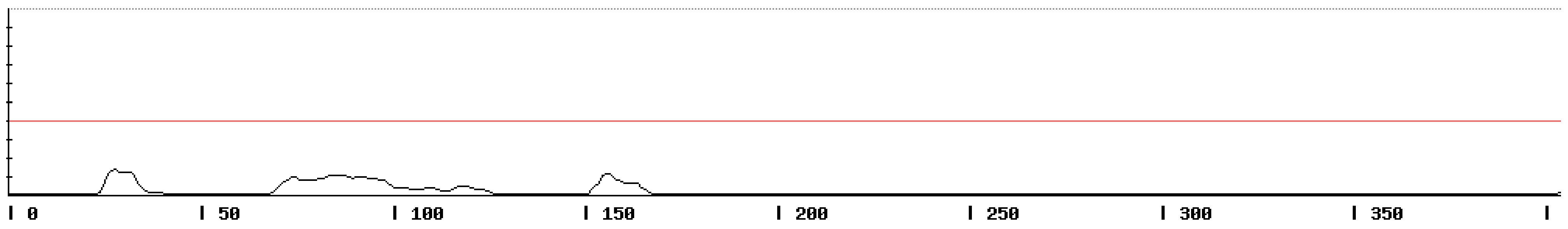

Predictions for Camelinasativa_Csa08945s010.1

Viterbi Path

Viterbi Path

Posterior @ 0.4

Posterior @ 0.4

Posterior Graphic. Threshold Marked @ 0.4

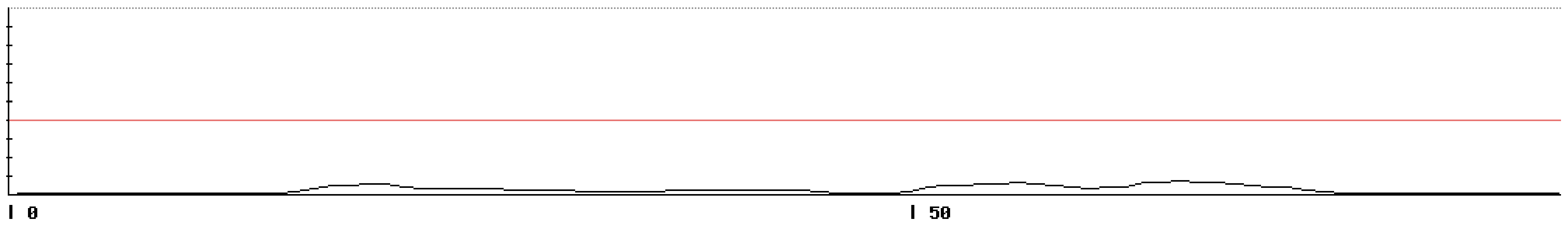

Predictions for Camelinasativa_Csa08g005060.1

Viterbi Path

Viterbi Path

Posterior @ 0.4

Posterior @ 0.4

Posterior Graphic. Threshold Marked @ 0.4

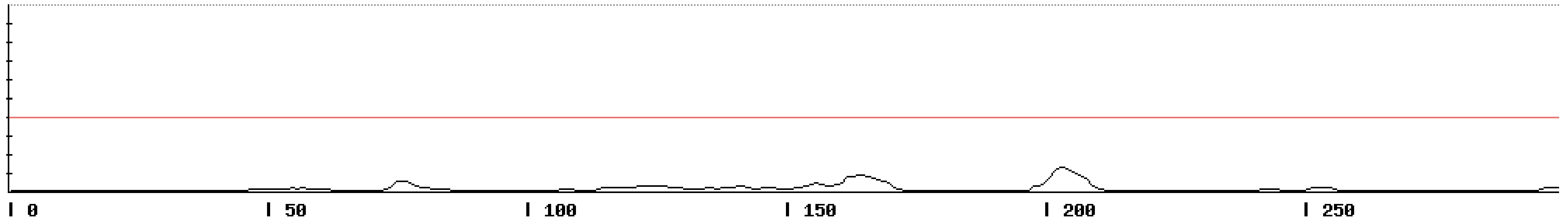

Predictions for Camelinasativa_Csa08g006010.1

Viterbi Path

Viterbi Path

Posterior @ 0.4

98 - RKGSKARKQRKSR - 110

284 - RKGSKARKQRKSR - 296

Posterior @ 0.4

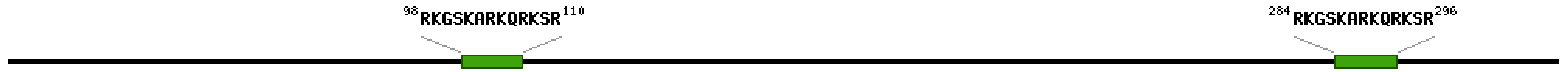

Posterior Graphic. Threshold Marked @ 0.4

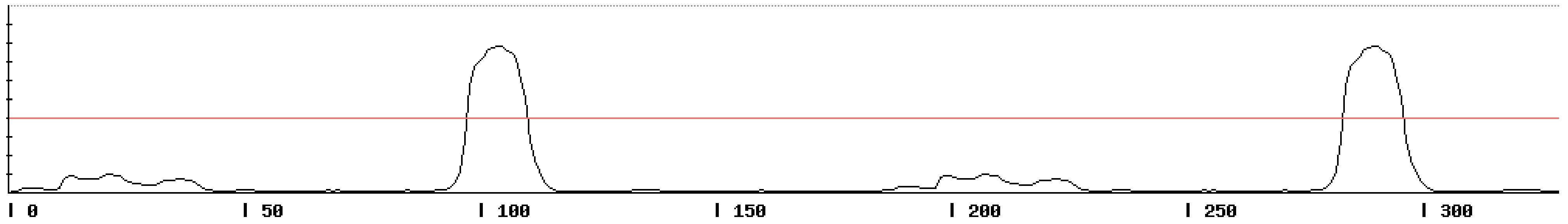

Predictions for Camelinasativa_Csa08g006520.1

Viterbi Path

Viterbi Path

Posterior @ 0.4

Posterior @ 0.4

Posterior Graphic. Threshold Marked @ 0.4

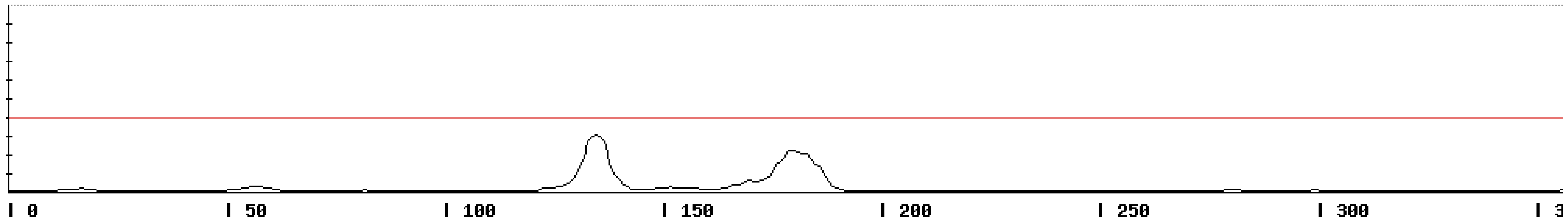

Predictions for Camelinasativa_Csa08g009420.1

Viterbi Path

Viterbi Path

Posterior @ 0.4

Posterior @ 0.4

Posterior Graphic. Threshold Marked @ 0.4

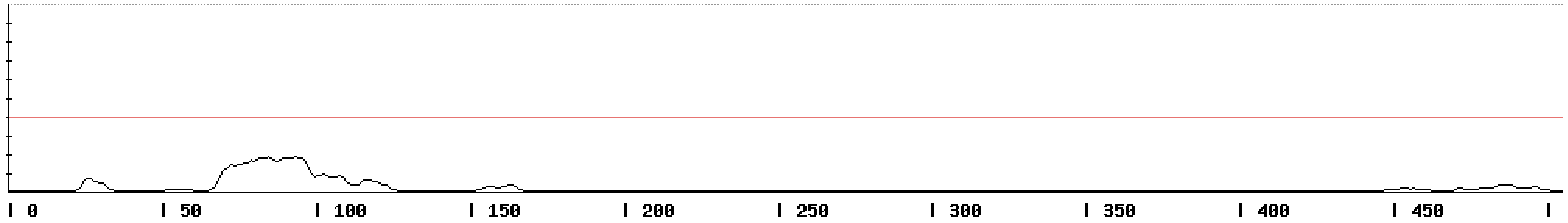

Predictions for Camelinasativa_Csa08g010450.1

Viterbi Path	
Viterbi Path	
Posterior @ 0.4	
Posterior @ 0.4	
Posterior Graphic. Threshold Marked @ 0.4	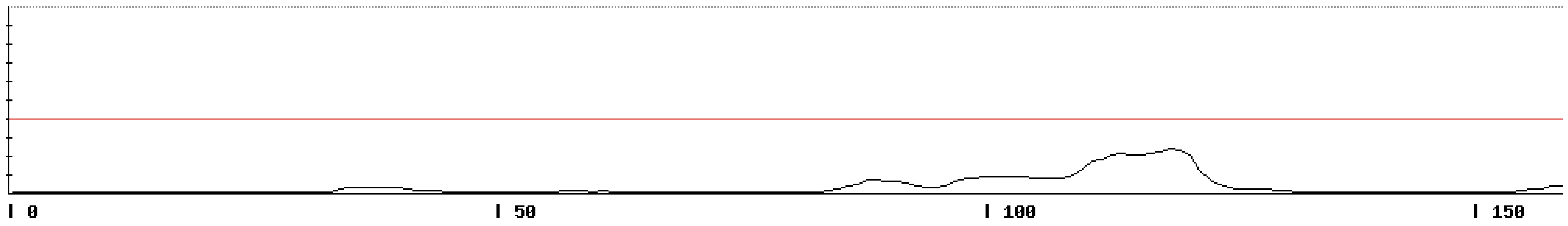

Predictions for Camelinasativa_Csa08g010510.1

Viterbi Path	
Viterbi Path	
Posterior @ 0.4	
Posterior @ 0.4	
Posterior Graphic. Threshold Marked @ 0.4	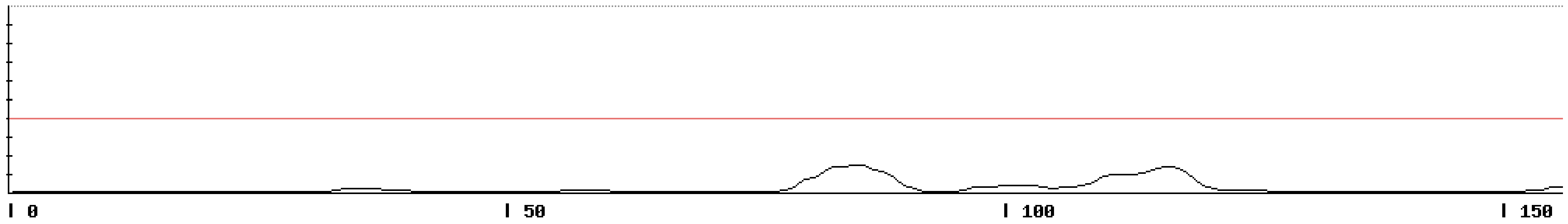

Predictions for Camelinasativa_Csa08g010750.1

Viterbi Path

Viterbi Path

Posterior @ 0.4

Posterior @ 0.4

Posterior Graphic. Threshold Marked @ 0.4

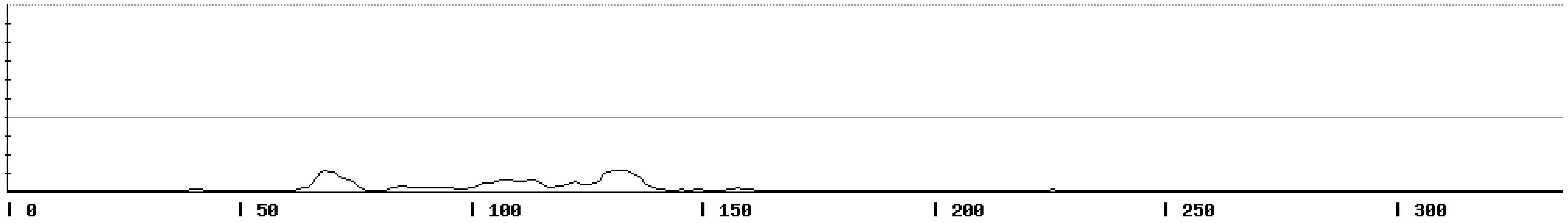

Predictions for Camelinasativa_Csa08g015450.1

Viterbi Path

Viterbi Path

Posterior @ 0.4

Posterior @ 0.4

Posterior Graphic. Threshold Marked @ 0.4

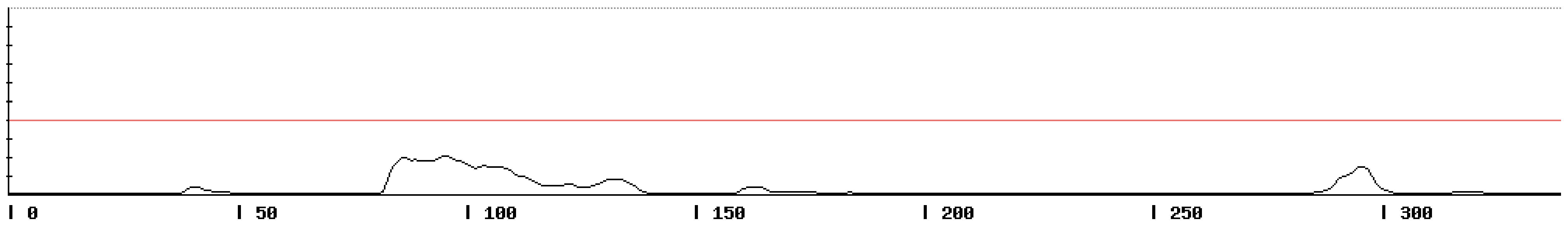

Predictions for Camelinasativa_Csa08g015570.1

Viterbi Path

Viterbi Path

Posterior @ 0.4

Posterior @ 0.4

Posterior Graphic. Threshold Marked @ 0.4

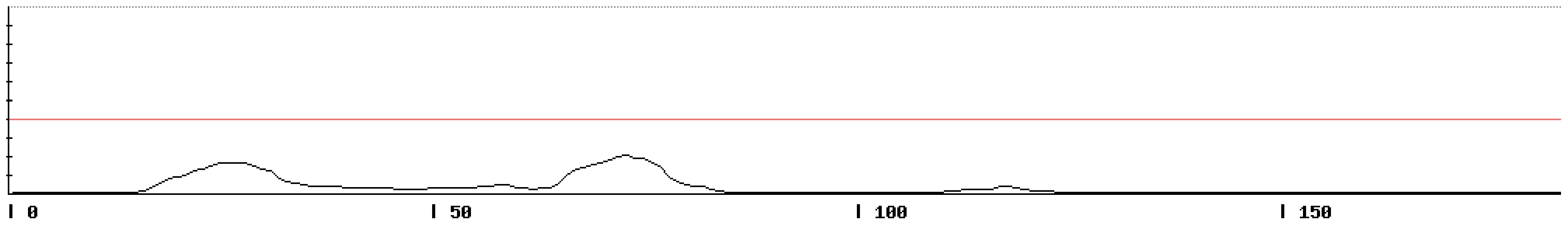

Predictions for Camelinasativa_Csa08g020160.1

Viterbi Path

Viterbi Path

Posterior @ 0.4 — 95 - TGKDRKIKSGKTKII - 109

Posterior @ 0.4

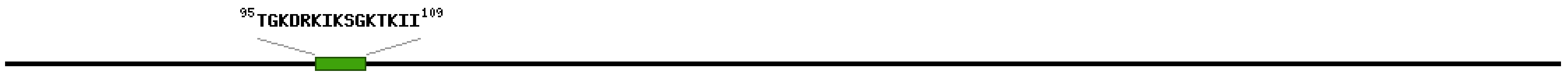

Posterior Graphic. Threshold Marked @ 0.4	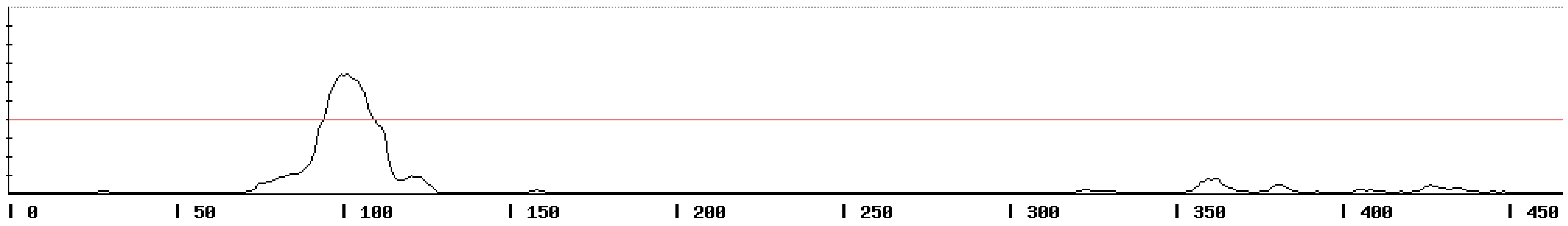

Predictions for Camelinasativa_Csa08g040330.1	
Viterbi Path	
Viterbi Path	
Posterior @ 0.4	
Posterior @ 0.4	
Posterior Graphic. Threshold Marked @ 0.4	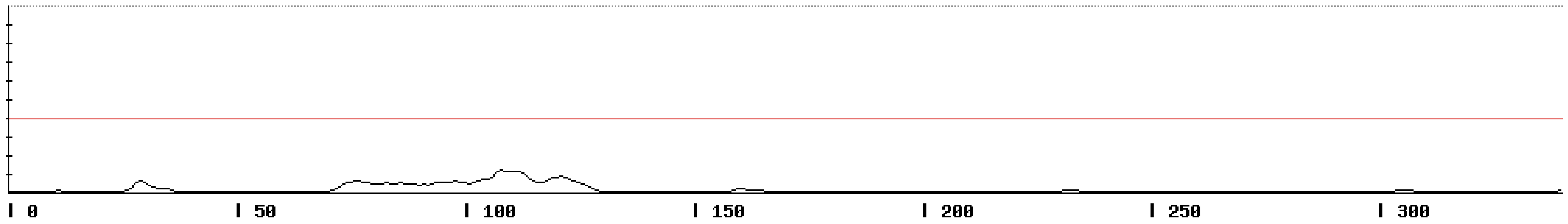

Predictions for Camelinasativa_Csa08g052780.1	
Viterbi Path	
Viterbi Path	
Posterior @ 0.4	
Posterior @ 0.4	

Posterior Graphic. Threshold Marked @ 0.4	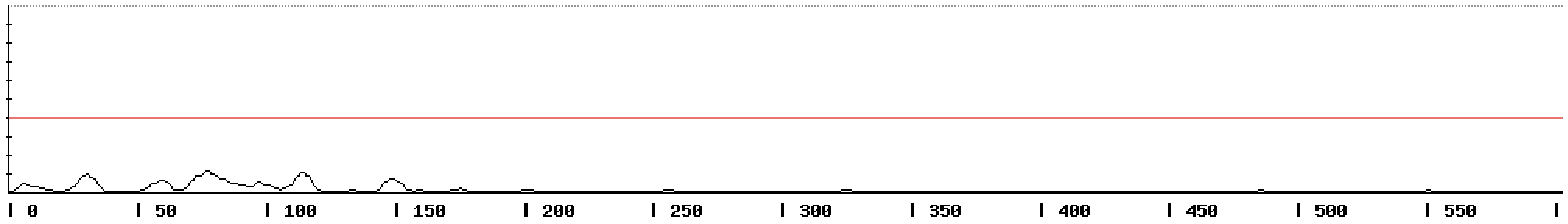

Predictions for Camelinasativa_Csa08g052950.1

Viterbi Path	
Viterbi Path	
Posterior @ 0.4	77 - K - 77
	92 - GVDRKIKDKRGNRGEIG - 108
Posterior @ 0.4	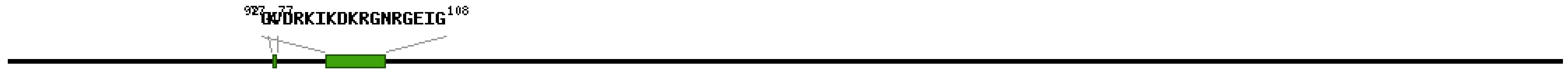
Posterior Graphic. Threshold Marked @ 0.4	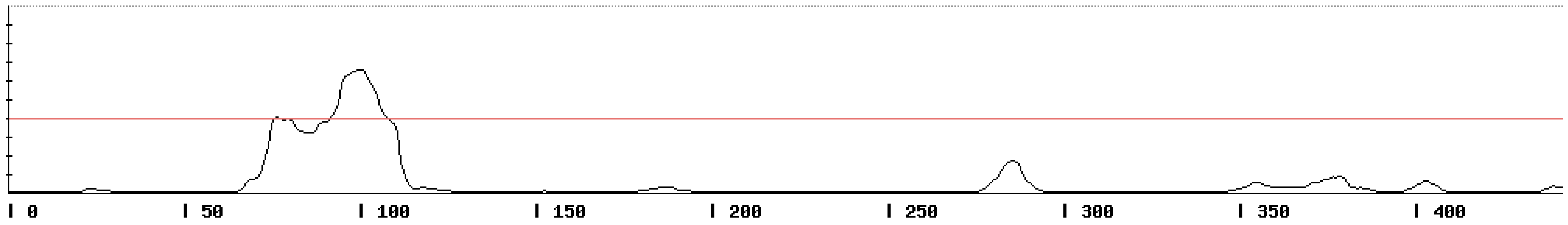

Predictions for Camelinasativa_Csa08g055310.1

Viterbi Path	
Viterbi Path	

Posterior @ 0.4	
Posterior @ 0.4	
Posterior Graphic. Threshold Marked @ 0.4	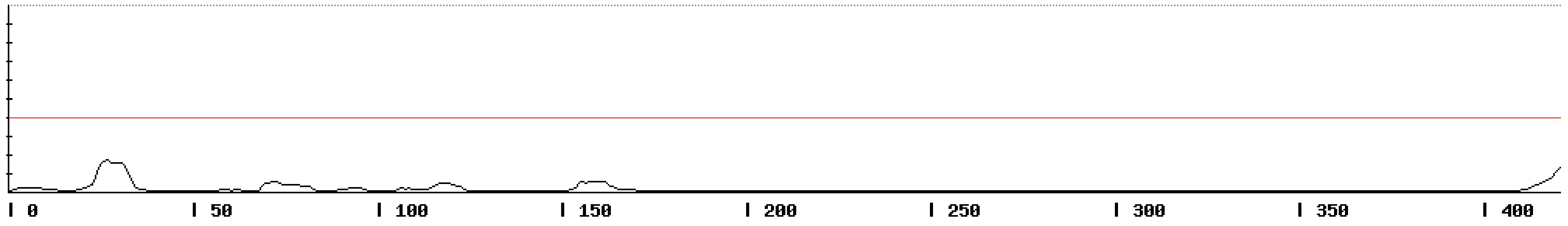

Predictions for Camelinasativa_Csa08g055440.1

Viterbi Path	
Viterbi Path	
Posterior @ 0.4	98 - GKPK - 101
Posterior @ 0.4	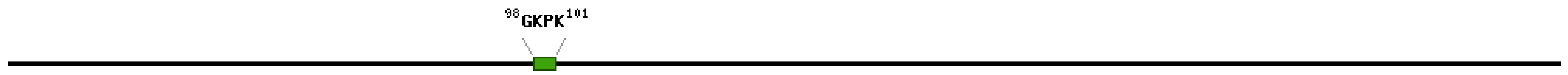
Posterior Graphic. Threshold Marked @ 0.4	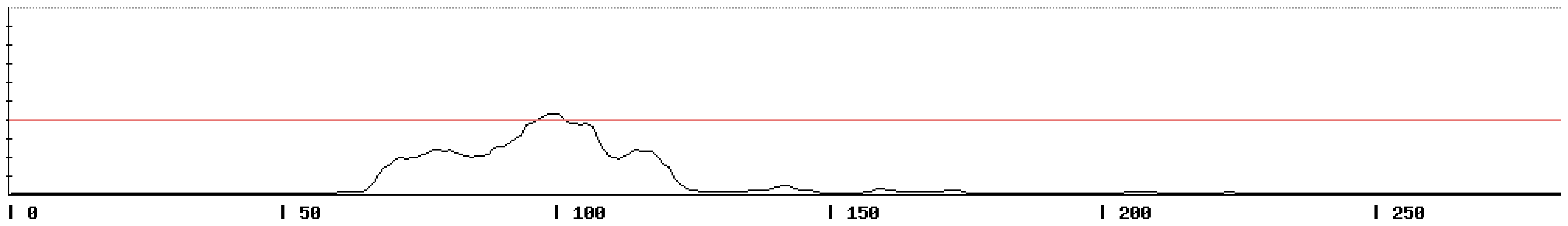

Predictions for Camelinasativa_Csa08g056700.1

Viterbi Path

Viterbi Path

Posterior @ 0.4

Posterior @ 0.4

Posterior Graphic. Threshold Marked @ 0.4

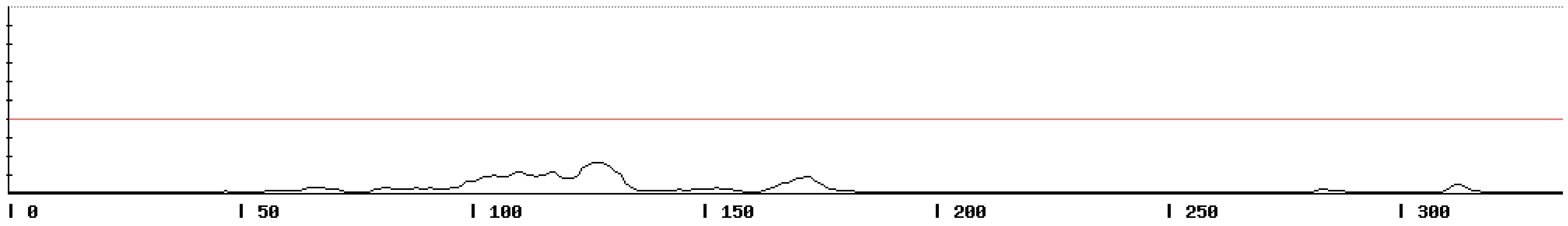

Predictions for Camelinasativa_Csa08g056710.1

Viterbi Path

Viterbi Path

Posterior @ 0.4

Posterior @ 0.4

Posterior Graphic. Threshold Marked @ 0.4

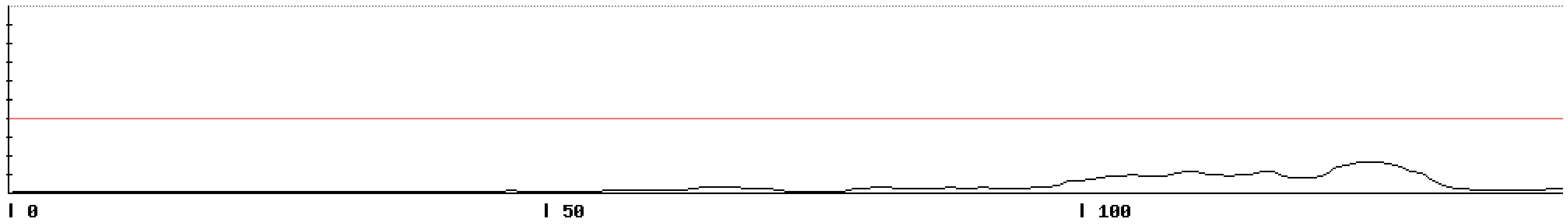

Predictions for Camelinasativa_Csa08g060070.1	
Viterbi Path	
Viterbi Path	
Posterior @ 0.4	
Posterior @ 0.4	
Posterior Graphic. Threshold Marked @ 0.4	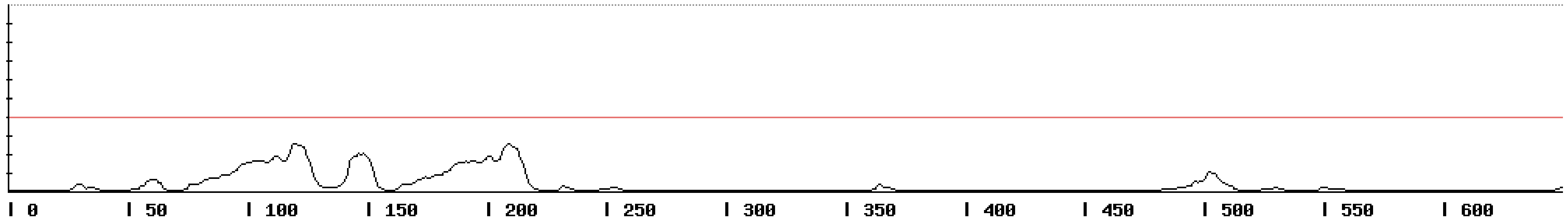

Predictions for Camelinasativa_Csa08g060080.1	
Viterbi Path	
Viterbi Path	
Posterior @ 0.4	
Posterior @ 0.4	
Posterior Graphic. Threshold Marked @ 0.4	

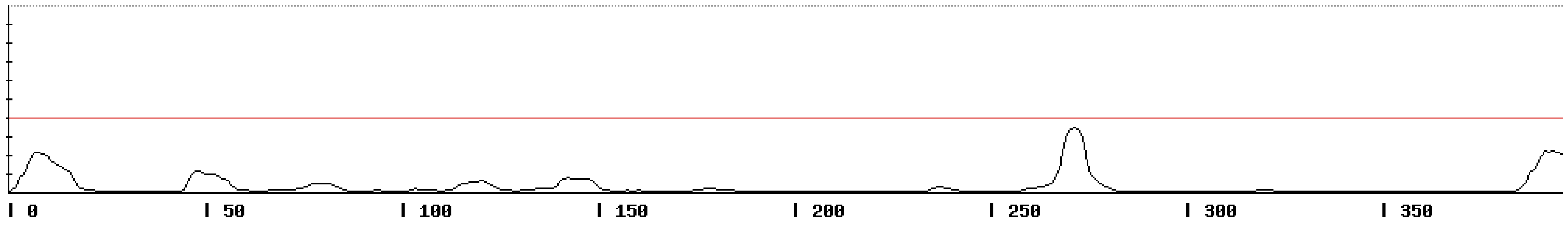

Predictions for Camelinasativa_Csa09g004780.1

Viterbi Path

Viterbi Path

Posterior @ 0.4

Posterior @ 0.4

Posterior Graphic. Threshold Marked @ 0.4

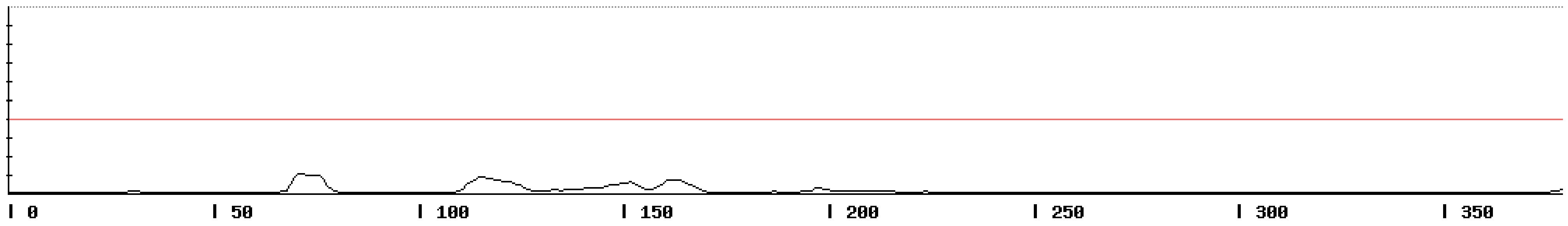

Predictions for Camelinasativa_Csa09g013080.1

Viterbi Path

Viterbi Path

Posterior @ 0.4

Posterior @ 0.4

Posterior Graphic. Threshold Marked @ 0.4

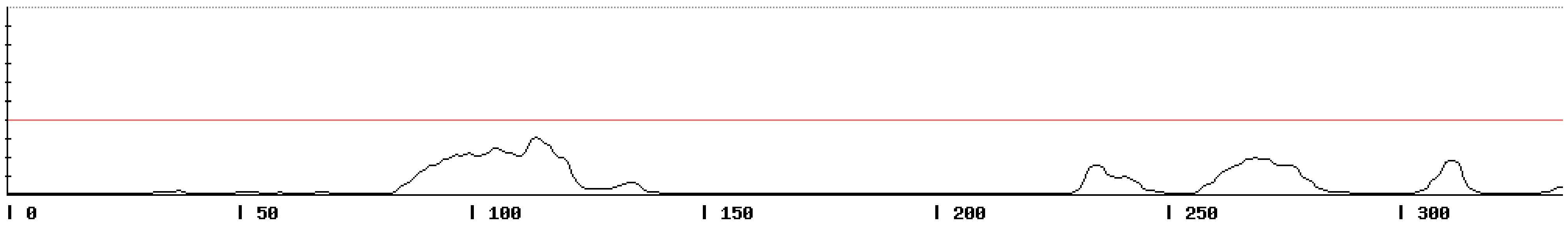

Predictions for Camelinasativa_Csa09g022510.1

Viterbi Path

Viterbi Path

Posterior @ 0.4

Posterior @ 0.4

Posterior Graphic. Threshold Marked @ 0.4

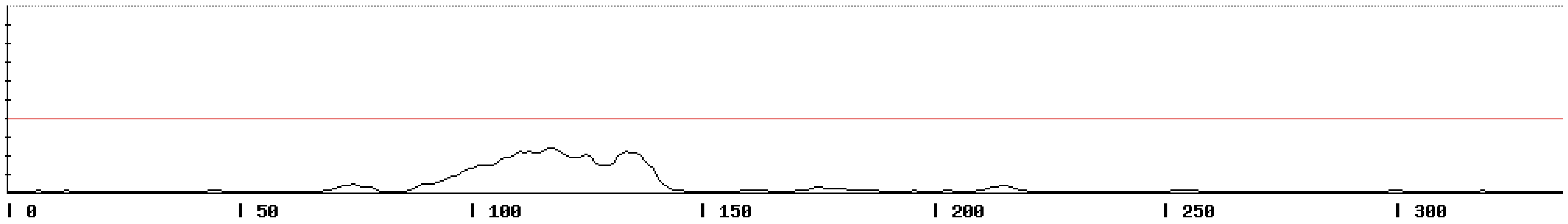

Predictions for Camelinasativa_Csa09g034120.1

Viterbi Path

Viterbi Path

Posterior @ 0.4

Posterior @ 0.4

Posterior Graphic. Threshold Marked @ 0.4

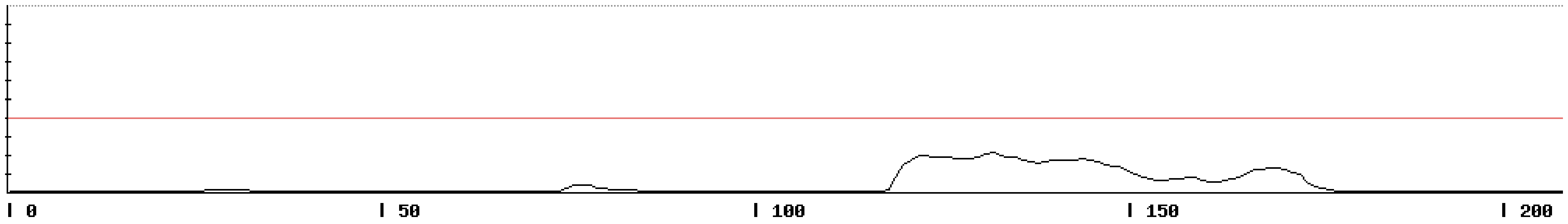

Predictions for Camelinasativa_Csa09g034210.1

Viterbi Path

Viterbi Path

Posterior @ 0.4

Posterior @ 0.4

Posterior Graphic. Threshold Marked @ 0.4

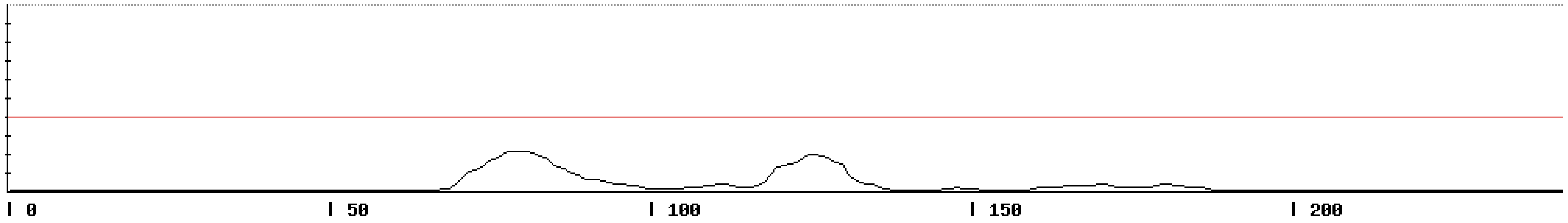

Predictions for Camelinasativa_Csa09g052140.1

Viterbi Path

Viterbi Path

Posterior @ 0.4	99 - TGKDRKIKNGKTKIIGVKR - 117
Posterior @ 0.4	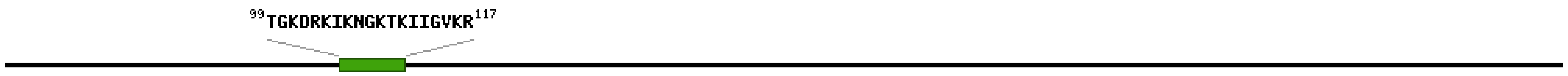
Posterior Graphic. Threshold Marked @ 0.4	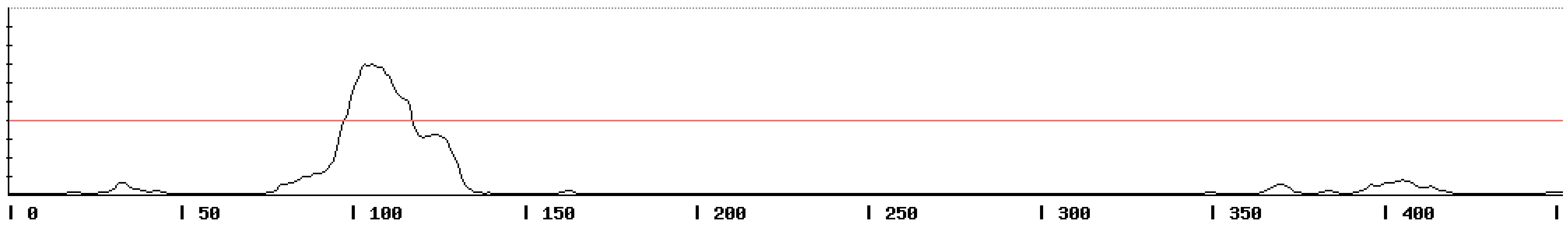

Predictions for Camelinasativa_Csa09g067970.1

Viterbi Path	
Viterbi Path	
Posterior @ 0.4	110 - SKKNKMGKGGKKQKRYDK - 127
	208 - KSSQKADRKKKKLKEKLLKEASK - 230
Posterior @ 0.4	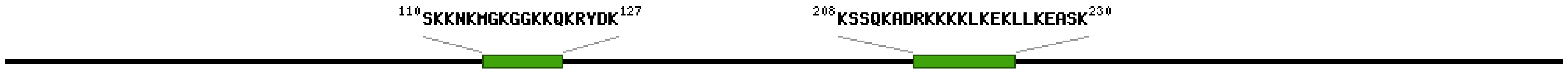
Posterior Graphic. Threshold Marked @ 0.4	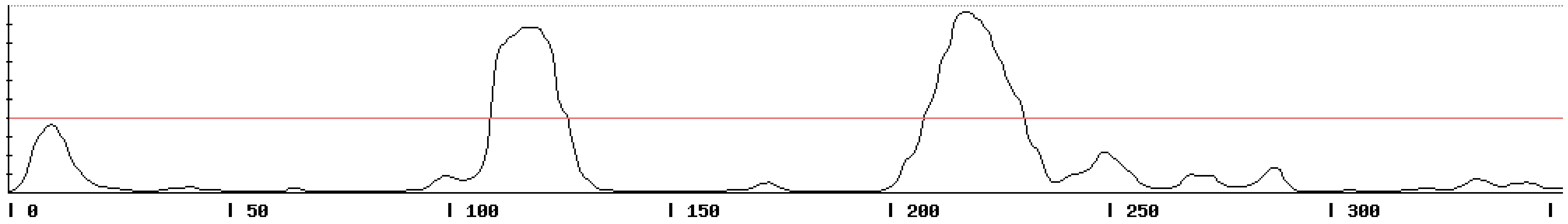

Predictions for Camelinasativa_Csa09g068810.1

Viterbi Path	
Viterbi Path	

Posterior @ 0.4	111 - SKRNKMGKDGKRQKRDAKGGYWK - 133
Posterior @ 0.4	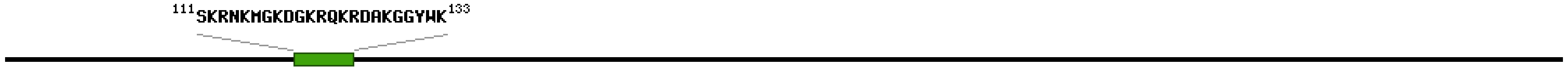
Posterior Graphic. Threshold Marked @ 0.4	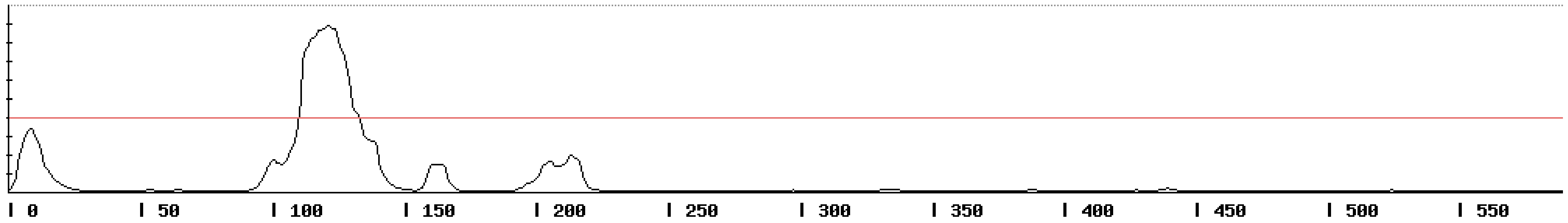

Predictions for Camelinasativa_Csa09g068820.1

Viterbi Path	
Viterbi Path	
Posterior @ 0.4	
Posterior @ 0.4	
Posterior Graphic. Threshold Marked @ 0.4	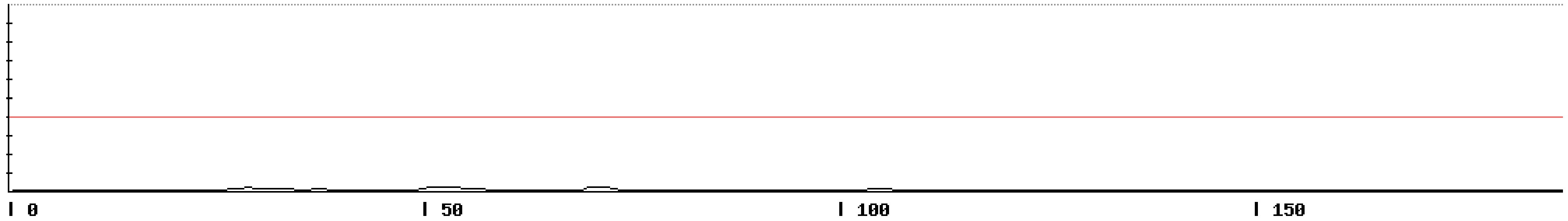

Predictions for Camelinasativa_Csa09g068830.1

Viterbi Path

Viterbi Path

Posterior @ 0.4

115 - RTKLGKGGGRGGRQKKRGS - 133

208 - PRGAKKKKKS - 217

Posterior @ 0.4

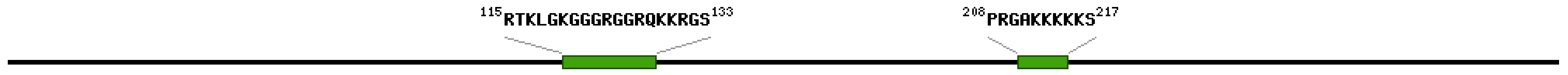

Posterior Graphic. Threshold Marked @ 0.4

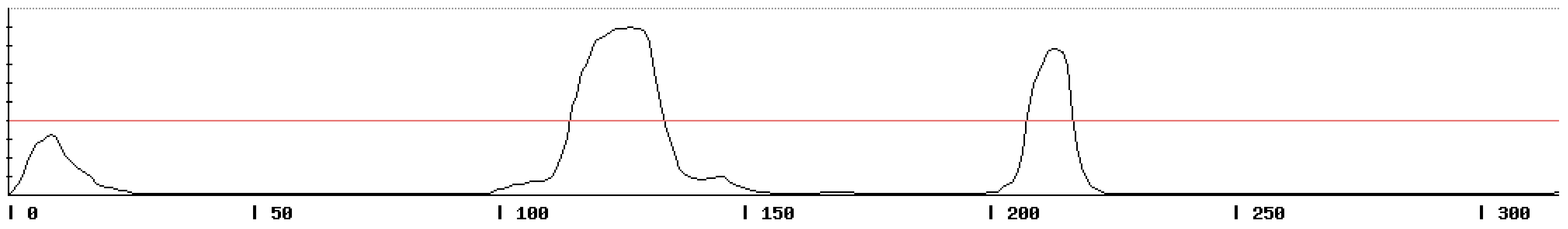

Predictions for Camelinasativa_Csa09g068840.1

Viterbi Path

Viterbi Path

Posterior @ 0.4

110 - KRNKMGKDGKRQKRDAKG - 127

Posterior @ 0.4

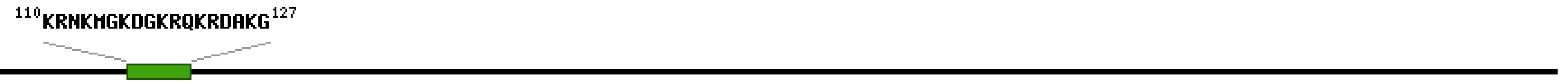

Posterior Graphic. Threshold Marked @ 0.4

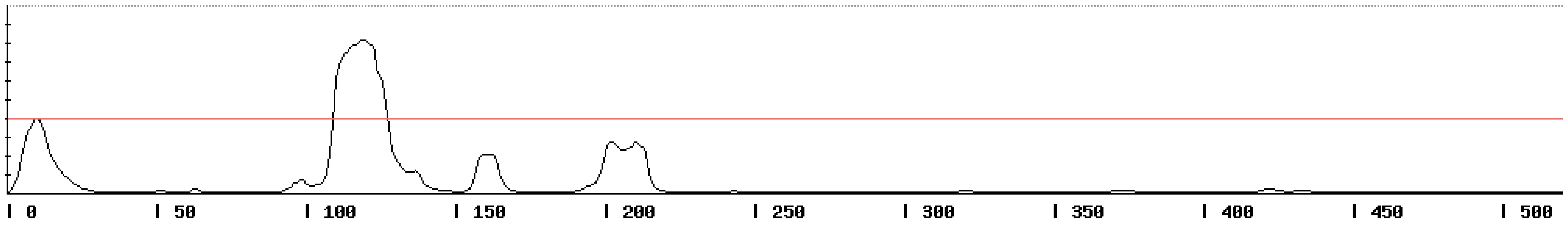

Predictions for Camelinasativa_Csa09g068850.1

Viterbi Path	
Viterbi Path	
Posterior @ 0.4	128 - GRSLKRQKR - 136
	209 - KGAKKKKA - 216
Posterior @ 0.4	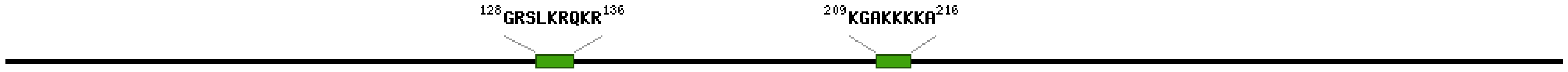
Posterior Graphic. Threshold Marked @ 0.4	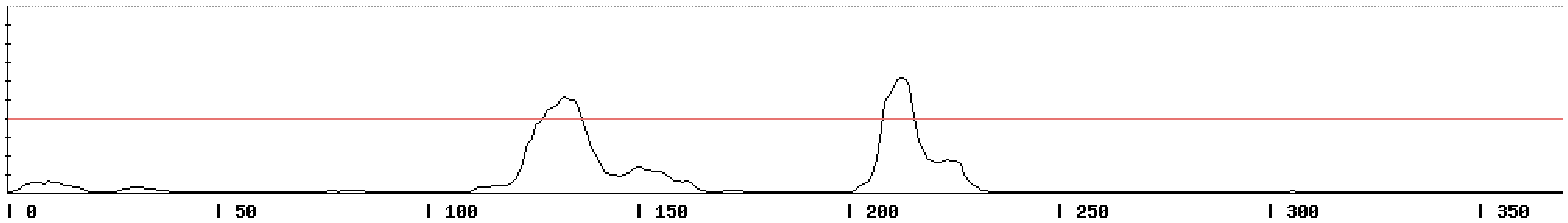

Predictions for Camelinasativa_Csa09g068880.1

Viterbi Path	
Viterbi Path	
Posterior @ 0.4	110 - NKLGKDGKRQKRVAK - 124
Posterior @ 0.4	

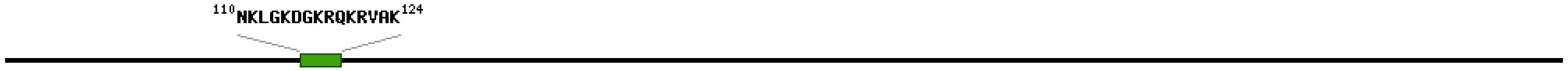

Posterior Graphic. Threshold Marked @ 0.4

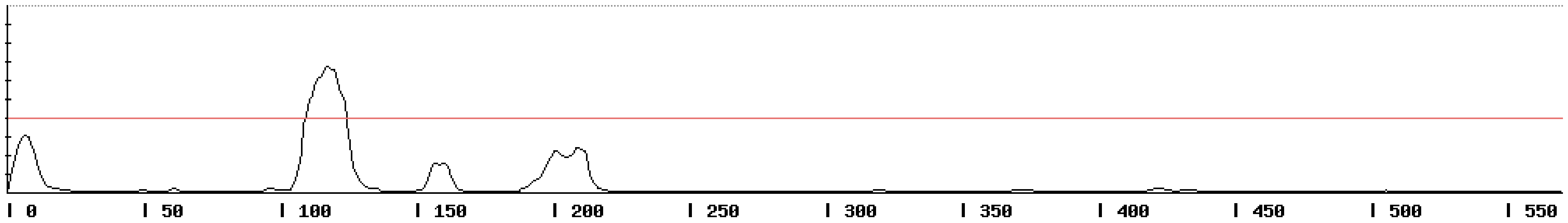

Predictions for Camelinasativa_Csa09g068890.1

Viterbi Path

Viterbi Path

Posterior @ 0.4

115 - RTKLGKGGGRGGRQKKRGS - 133

208 - PRGAKKKKKS - 217

Posterior @ 0.4

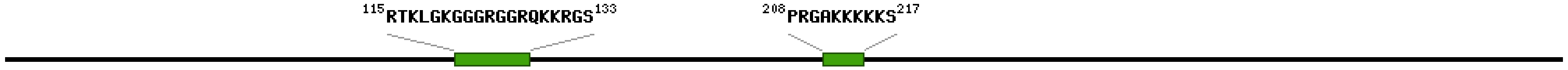

Posterior Graphic. Threshold Marked @ 0.4

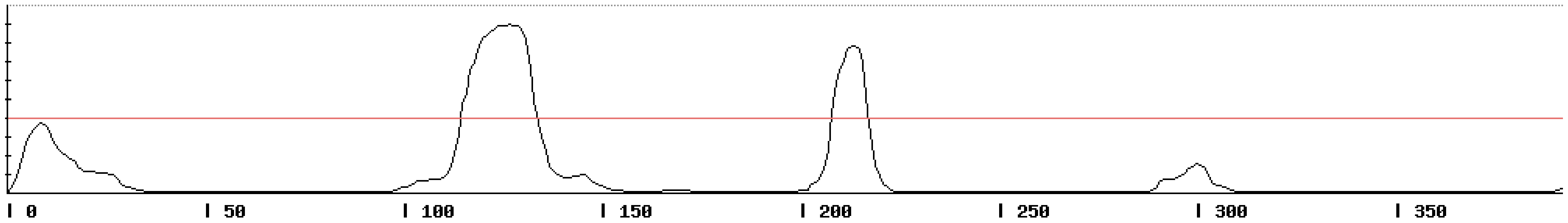

Predictions for Camelinasativa_Csa09g068910.1

Viterbi Path

Viterbi Path

Posterior @ 0.4 | 111 - KRNKMGKDGKRQTR - 124

Posterior @ 0.4

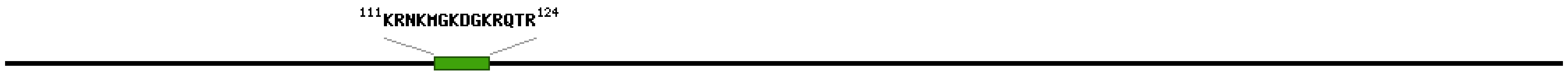

Posterior Graphic. Threshold Marked @ 0.4

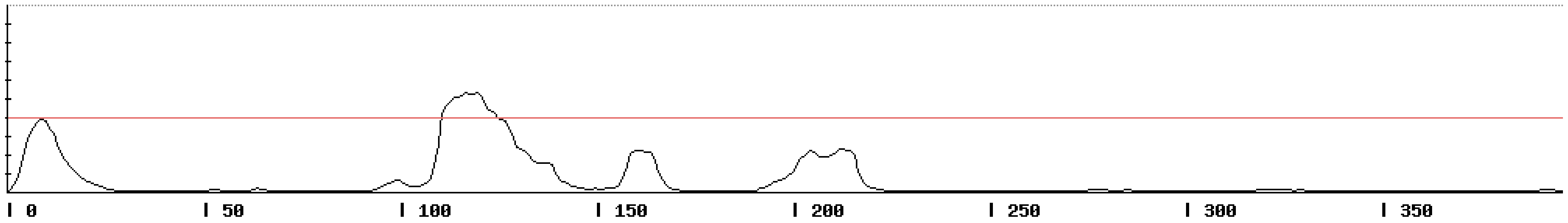

Predictions for Camelinasativa_Csa09g069310.1

Viterbi Path

Viterbi Path

Posterior @ 0.4 | 110 - SKRNKIGKDGKRQKRDAKGGYWK - 132

Posterior @ 0.4

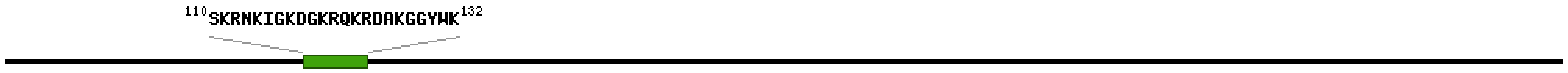

Posterior Graphic. Threshold Marked @ 0.4

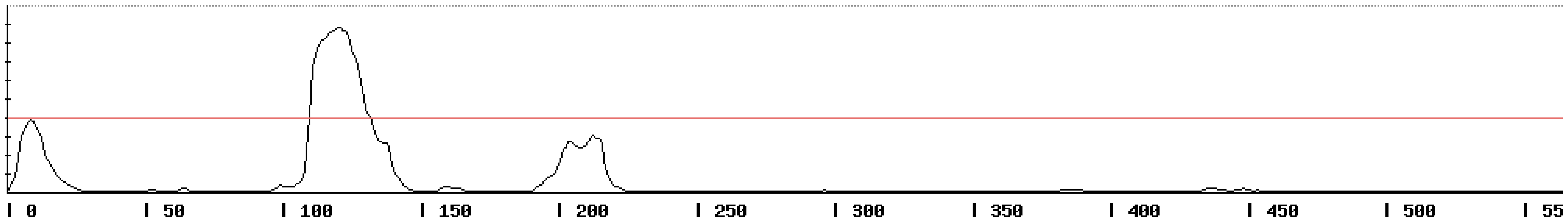

Predictions for Camelinasativa_Csa09g070900.1

Viterbi Path	
Viterbi Path	
Posterior @ 0.4	69 - GAKKKK - 74
Posterior @ 0.4	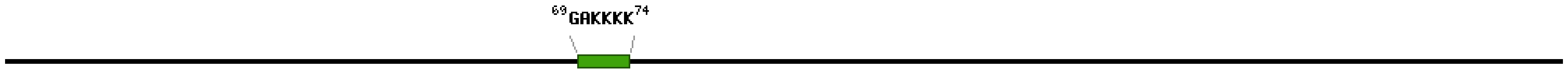
Posterior Graphic. Threshold Marked @ 0.4	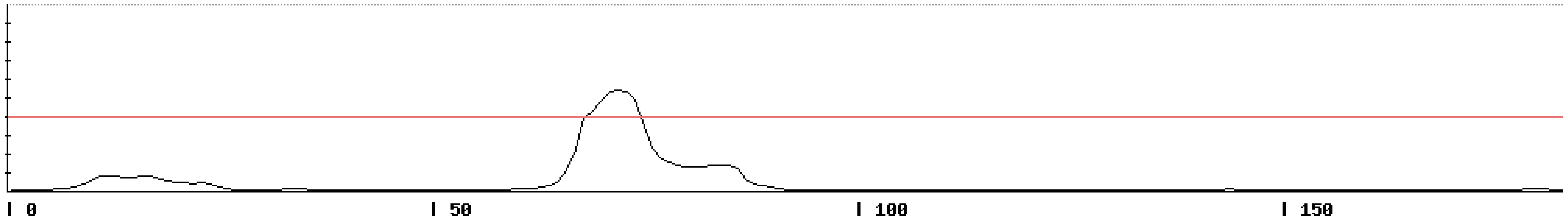

Predictions for Camelinasativa_Csa09g075270.1

Viterbi Path	
Viterbi Path	
Posterior @ 0.4	
Posterior @ 0.4	
Posterior Graphic. Threshold Marked @ 0.4	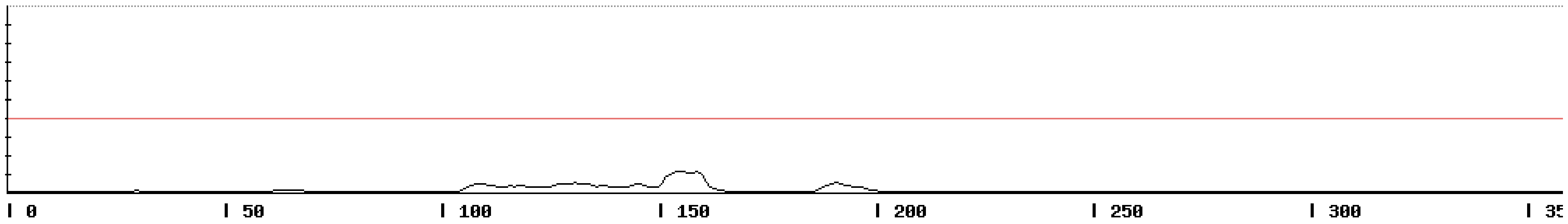

Predictions for Camelinasativa_Csa09g081150.1

Viterbi Path	
Viterbi Path	
Posterior @ 0.4	
Posterior @ 0.4	
Posterior Graphic. Threshold Marked @ 0.4	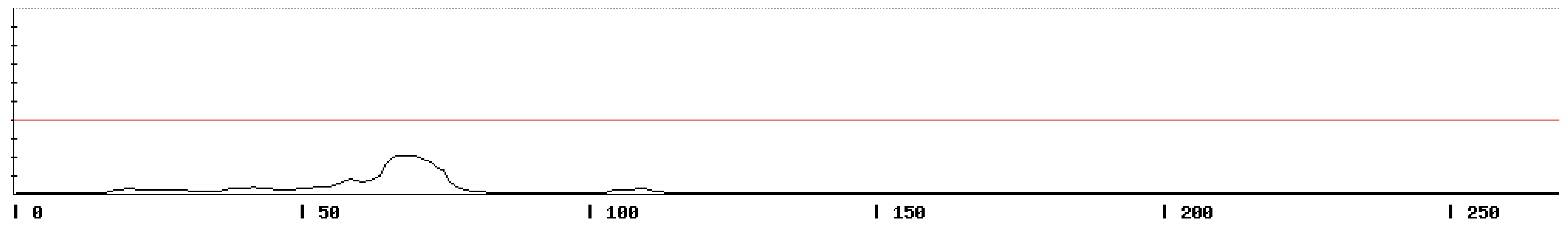

Predictions for Camelinasativa_Csa09g081160.1

Viterbi Path	
Viterbi Path	
Posterior @ 0.4	
Posterior @ 0.4	
Posterior Graphic. Threshold Marked @ 0.4	

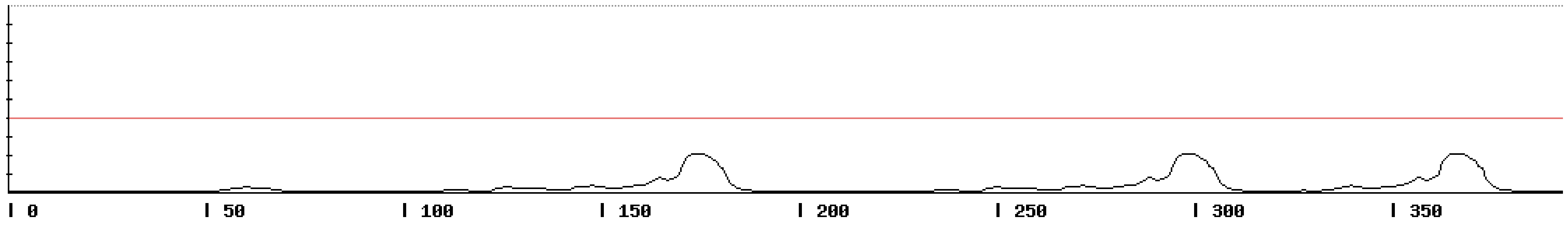

Predictions for Camelinasativa_Csa09g084210.1	
Viterbi Path	
Viterbi Path	
Posterior @ 0.4	98 - KPIGRPKPVGIK - 109
Posterior @ 0.4	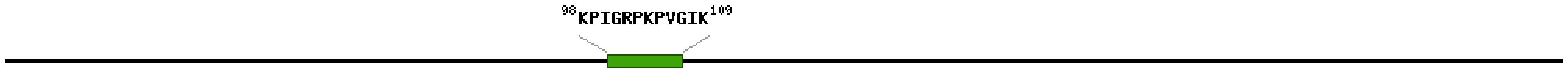
Posterior Graphic. Threshold Marked @ 0.4	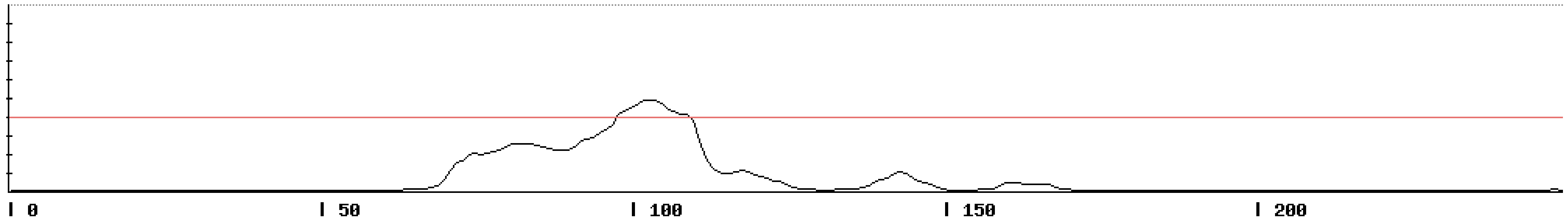

Predictions for Camelinasativa_Csa09g088620.1	
Viterbi Path	
Viterbi Path	
Posterior @ 0.4	
Posterior @ 0.4	

Posterior Graphic. Threshold Marked @ 0.4

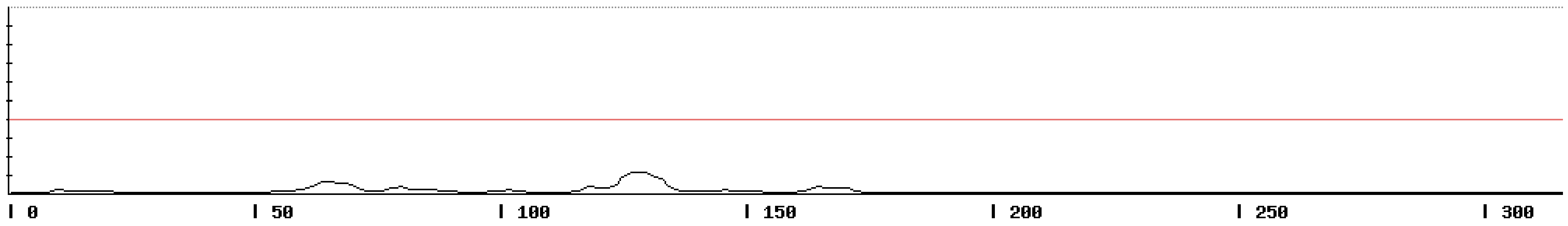

Predictions for Camelinasativa_Csa09g093850.1

Viterbi Path

Viterbi Path

Posterior @ 0.4

Posterior @ 0.4

Posterior Graphic. Threshold Marked @ 0.4

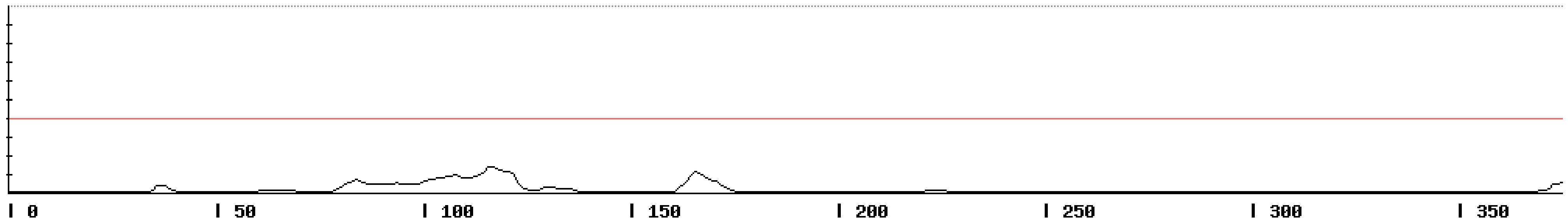

Predictions for Camelinasativa_Csa09g095630.1

Viterbi Path

Viterbi Path

Posterior @ 0.4

Posterior @ 0.4

Posterior Graphic. Threshold Marked @ 0.4

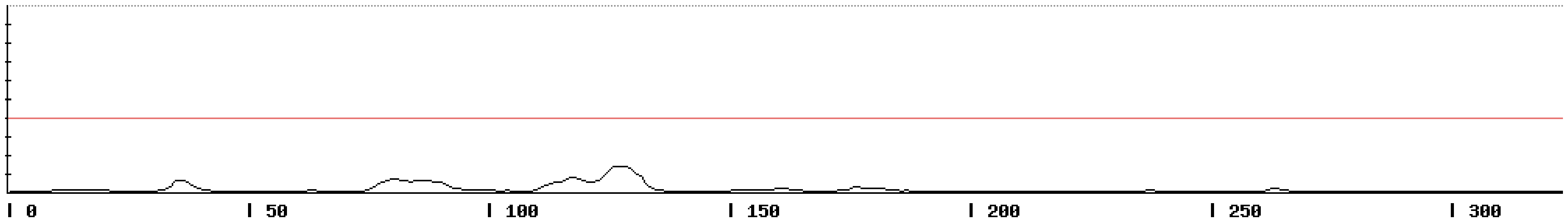

Predictions for Camelinasativa_Csa09g097350.1

Viterbi Path

Viterbi Path

Posterior @ 0.4

Posterior @ 0.4

Posterior Graphic. Threshold Marked @ 0.4

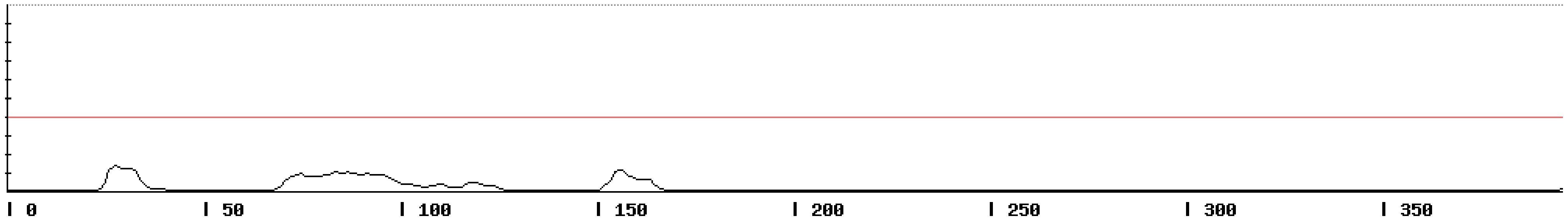

Predictions for Camelinasativa_Csa10g004970.1

Viterbi Path

Viterbi Path

Posterior @ 0.4 | 282 - KDKKKKK - 288

Posterior @ 0.4

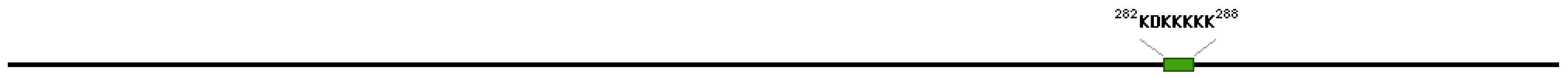

Posterior Graphic. Threshold Marked @ 0.4

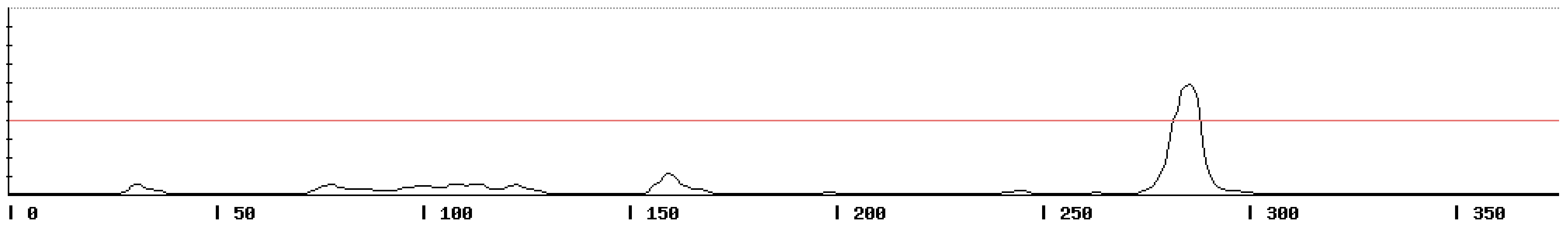

Predictions for Camelinasativa_Csa10g006570.1

Viterbi Path

Viterbi Path

Posterior @ 0.4 | 101 - IKSKK - 105
489 - RRKRRREGKQ - 498

Posterior @ 0.4

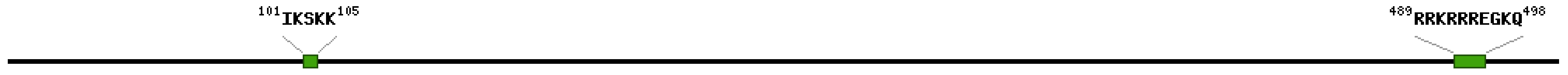

Posterior Graphic. Threshold Marked @ 0.4

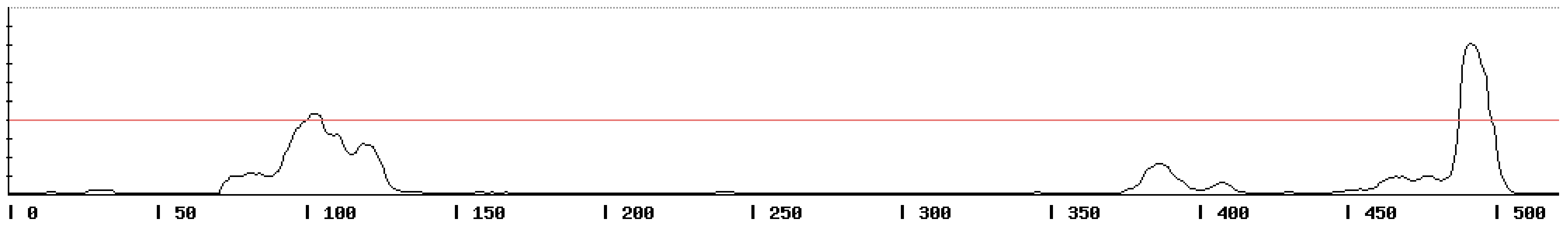

Predictions for Camelinasativa_Csa10g013350.1

Viterbi Path

Viterbi Path

Posterior @ 0.4

Posterior @ 0.4

Posterior Graphic. Threshold Marked @ 0.4

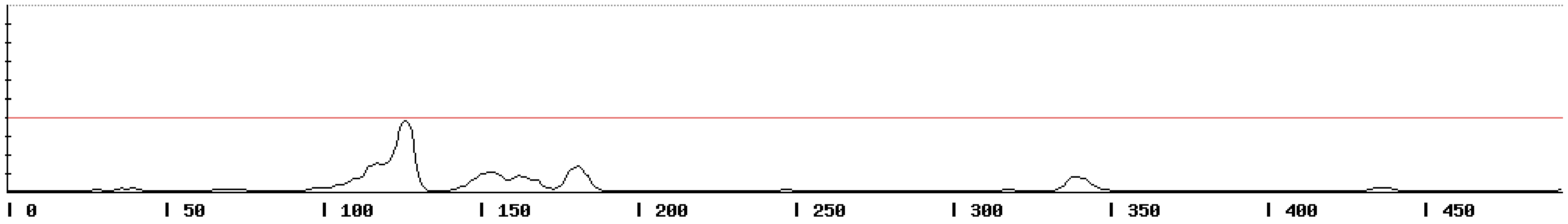

Predictions for Camelinasativa_Csa10g014090.1

Viterbi Path

Viterbi Path

Posterior @ 0.4

Posterior @ 0.4

Posterior Graphic. Threshold Marked @ 0.4

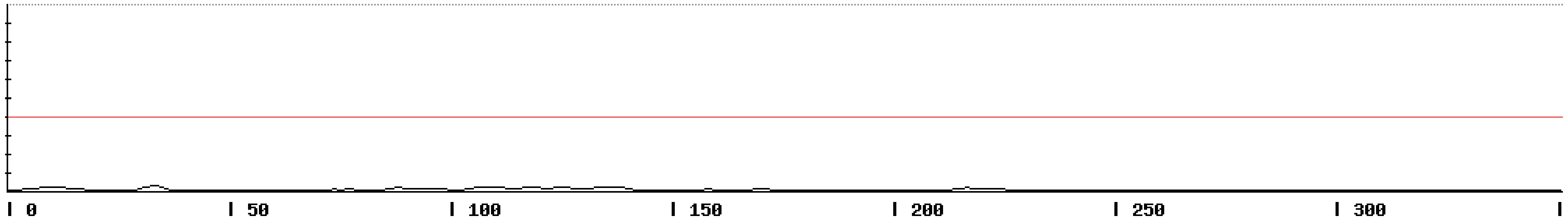

Predictions for Camelinasativa_Csa10g014120.1

Viterbi Path	
Viterbi Path	
Posterior @ 0.4	137 - RKRRK - 141
Posterior @ 0.4	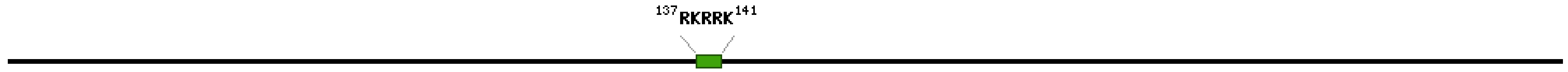
Posterior Graphic. Threshold Marked @ 0.4	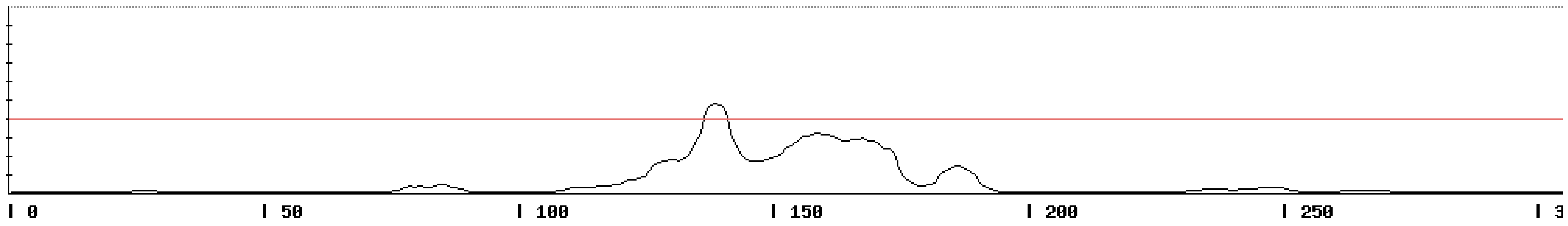

Predictions for Camelinasativa_Csa10g015440.1

Viterbi Path	
Viterbi Path	
Posterior @ 0.4	
Posterior @ 0.4	
Posterior Graphic. Threshold Marked @ 0.4	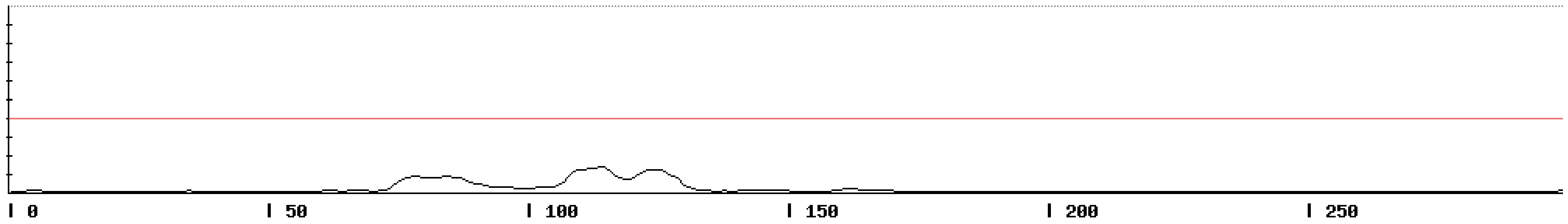

Predictions for Camelinasativa_Csa10g027250.1

Viterbi Path

Viterbi Path

Posterior @ 0.4

Posterior @ 0.4

Posterior Graphic. Threshold Marked @ 0.4

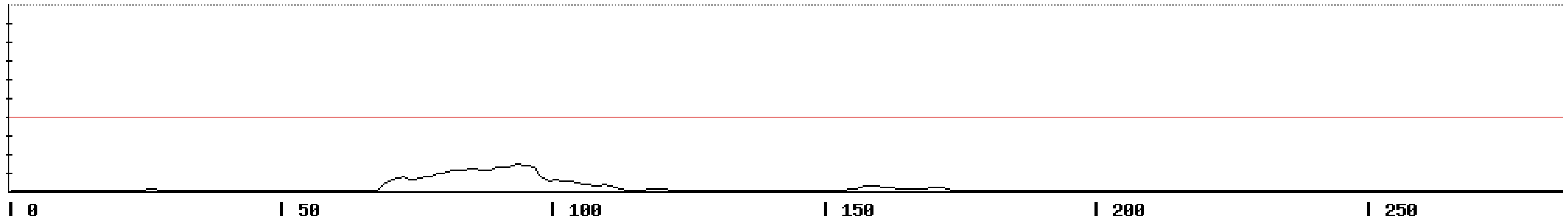

Predictions for Camelinasativa_Csa10g042450.1

Viterbi Path

Viterbi Path

Posterior @ 0.4

Posterior @ 0.4

Posterior Graphic. Threshold Marked @ 0.4

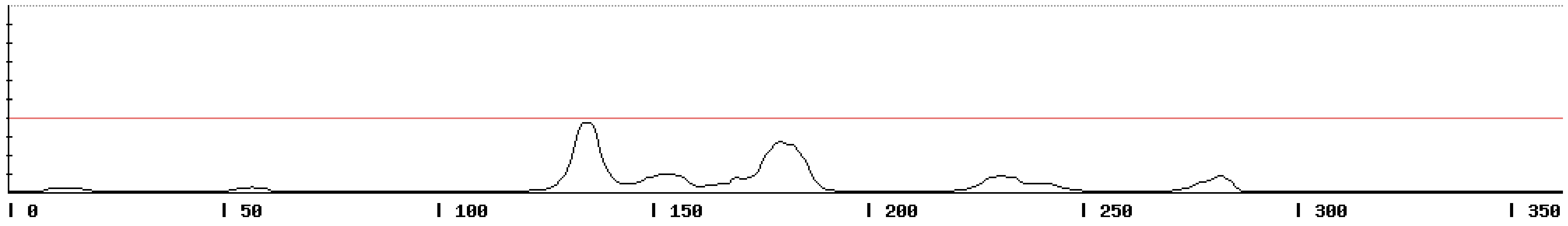

Predictions for Camelinasativa_Csa10g046660.1	
Viterbi Path	
Viterbi Path	
Posterior @ 0.4	76 - ARKYNRGPRTKRVTPHGRWGVKLKEGGGSKKKEF - 109
Posterior @ 0.4	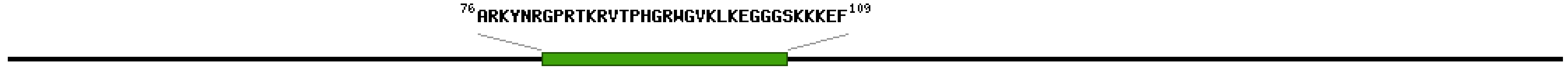
Posterior Graphic. Threshold Marked @ 0.4	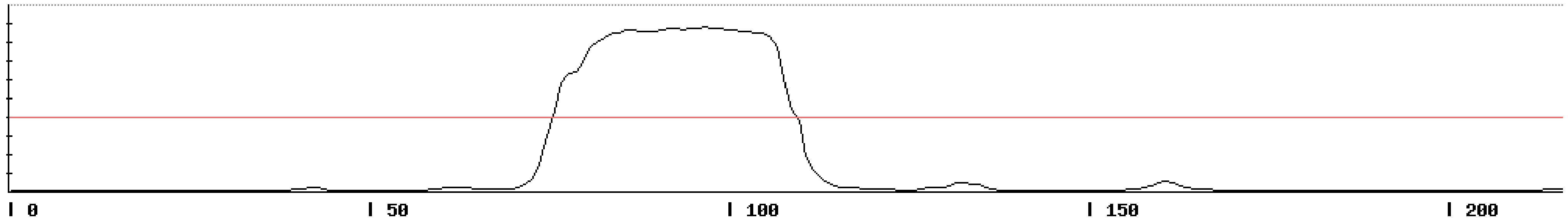

Predictions for Camelinasativa_Csa10g046670.1	
Viterbi Path	
Viterbi Path	
Posterior @ 0.4	
Posterior @ 0.4	

Posterior Graphic. Threshold Marked @ 0.4

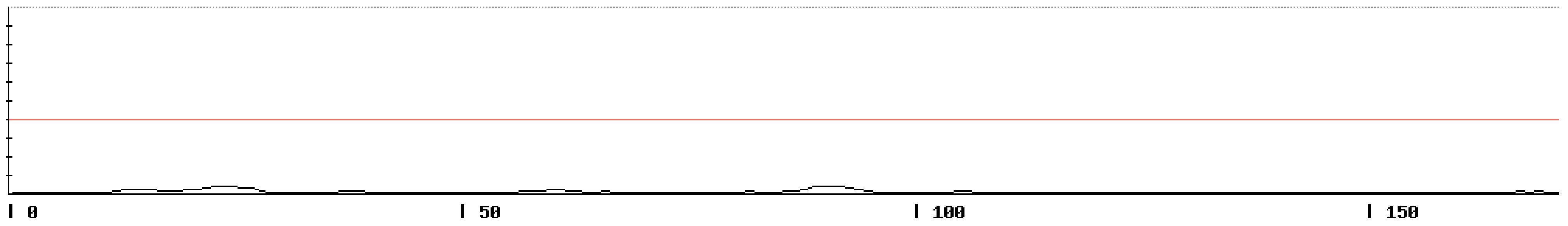

Predictions for Camelinasativa_Csa10g046680.1

Viterbi Path

Viterbi Path

Posterior @ 0.4

Posterior @ 0.4

Posterior Graphic. Threshold Marked @ 0.4

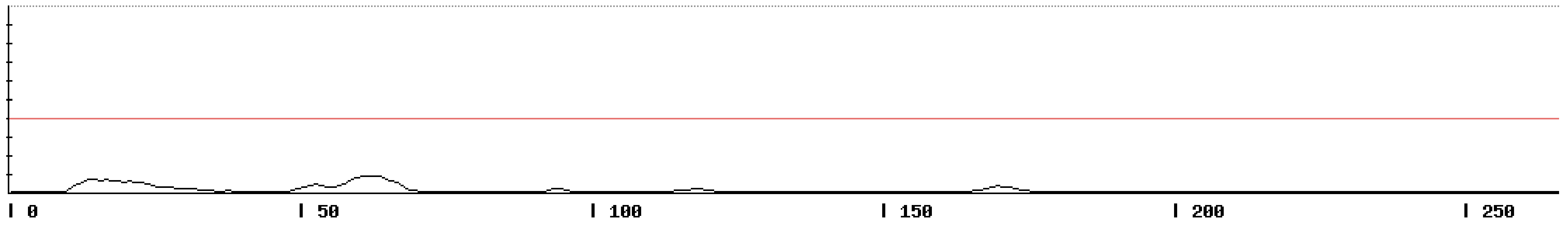

Predictions for Camelinasativa_Csa10g046940.1

Viterbi Path

Viterbi Path

Posterior @ 0.4

Posterior @ 0.4

Posterior Graphic. Threshold Marked @ 0.4	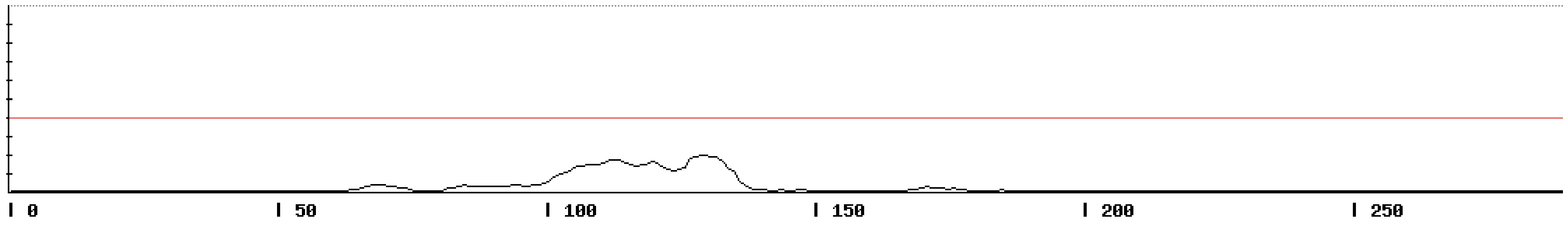

Predictions for Camelinasativa_Csa10g049630.1

Viterbi Path	
Viterbi Path	
Posterior @ 0.4	
Posterior @ 0.4	
Posterior Graphic. Threshold Marked @ 0.4	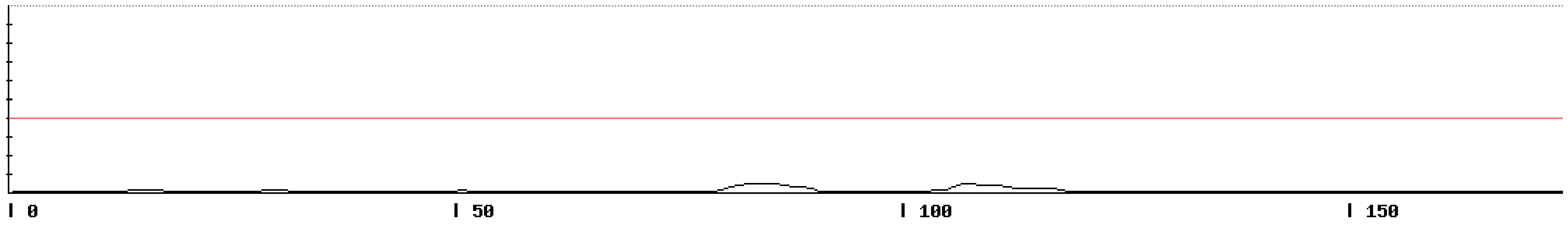

Predictions for Camelinasativa_Csa11g005520.1

Viterbi Path	
Viterbi Path	

Posterior @ 0.4	282 - KDKKKKK - 288
Posterior @ 0.4	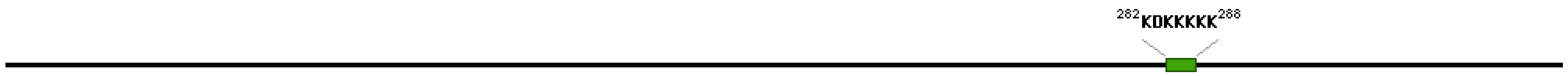
Posterior Graphic. Threshold Marked @ 0.4	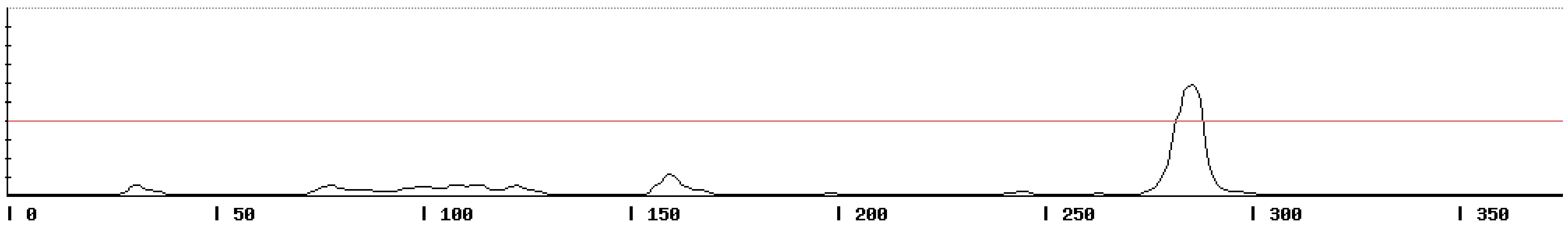

Predictions for Camelinasativa_Csa11g006210.1

Viterbi Path	
Viterbi Path	
Posterior @ 0.4	79 - IKSKK - 83<br>470 - RRRRREGK - 477
Posterior @ 0.4	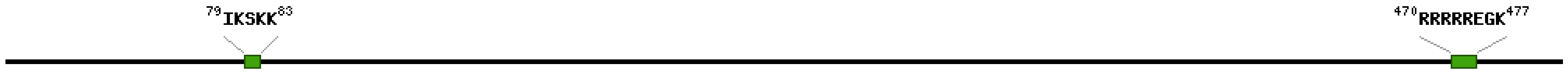
Posterior Graphic. Threshold Marked @ 0.4	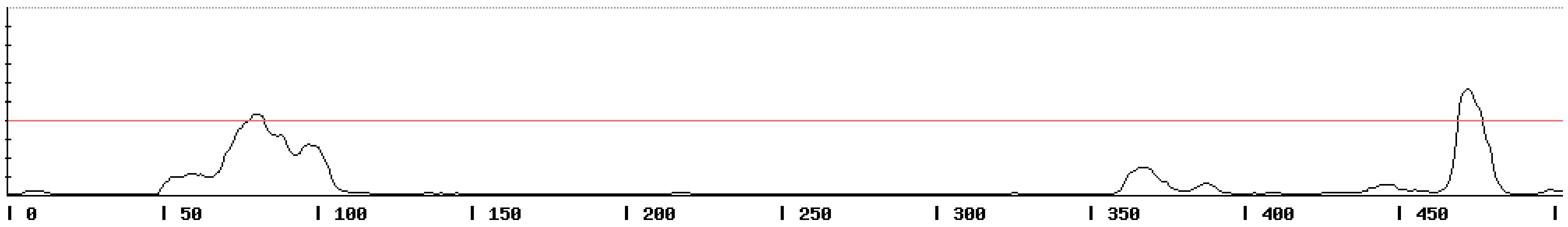

Predictions for Camelinasativa_Csa11g014290.1

Viterbi Path	
Viterbi Path	

Posterior @ 0.4

Posterior @ 0.4

Posterior Graphic. Threshold Marked @ 0.4

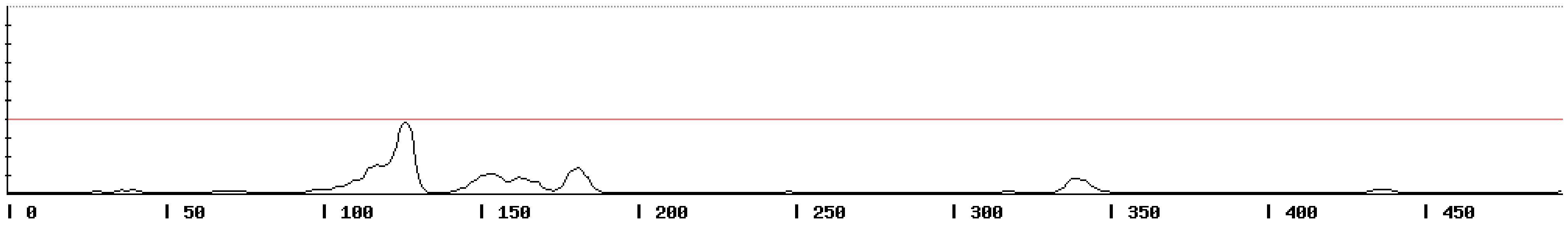

Predictions for Camelinasativa_Csa11g015230.1

Viterbi Path

Viterbi Path

Posterior @ 0.4

Posterior @ 0.4

Posterior Graphic. Threshold Marked @ 0.4

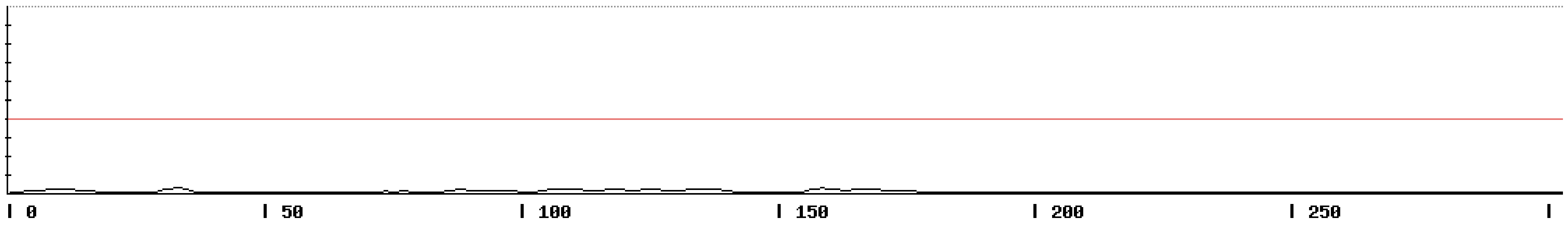

Predictions for Camelinasativa_Csa11g015260.1

Viterbi Path

Viterbi Path

Posterior @ 0.4 137 - RKRRK - 141

Posterior @ 0.4

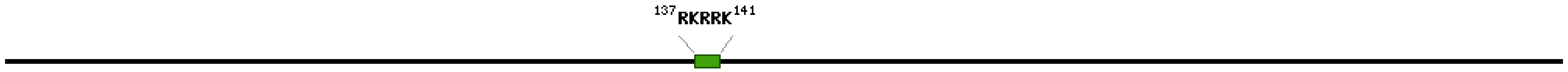

Posterior Graphic. Threshold Marked @ 0.4

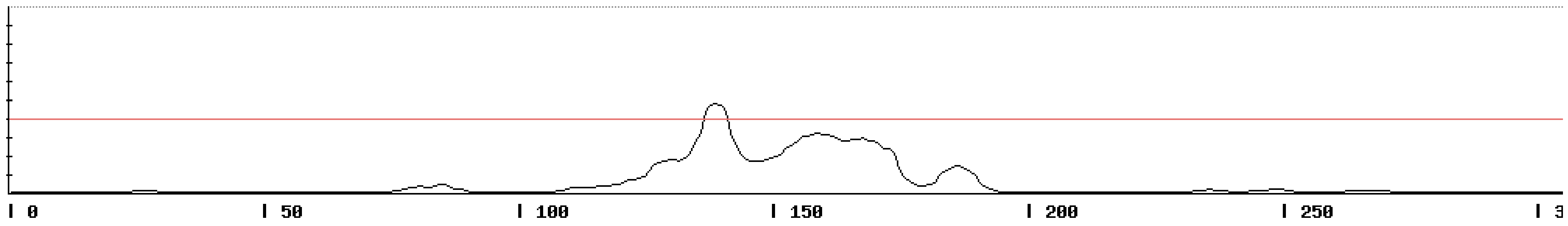

Predictions for Camelinasativa_Csa11g016750.1

Viterbi Path

Viterbi Path

Posterior @ 0.4

Posterior @ 0.4

Posterior Graphic. Threshold Marked @ 0.4

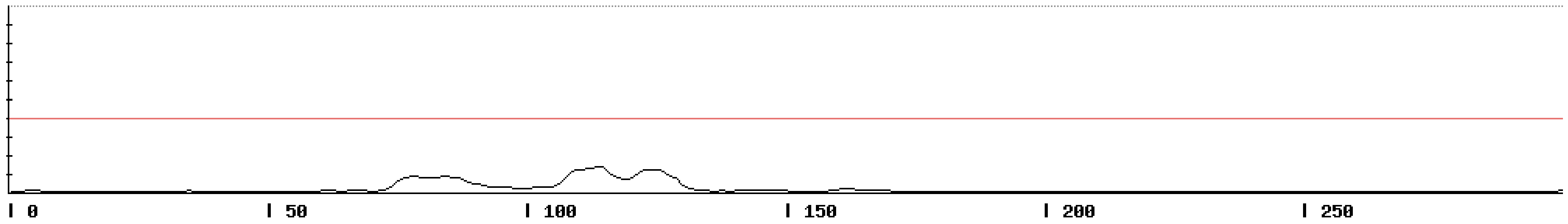

Predictions for Camelinasativa_Csa11g055650.1	
Viterbi Path	
Viterbi Path	
Posterior @ 0.4	
Posterior @ 0.4	
Posterior Graphic. Threshold Marked @ 0.4	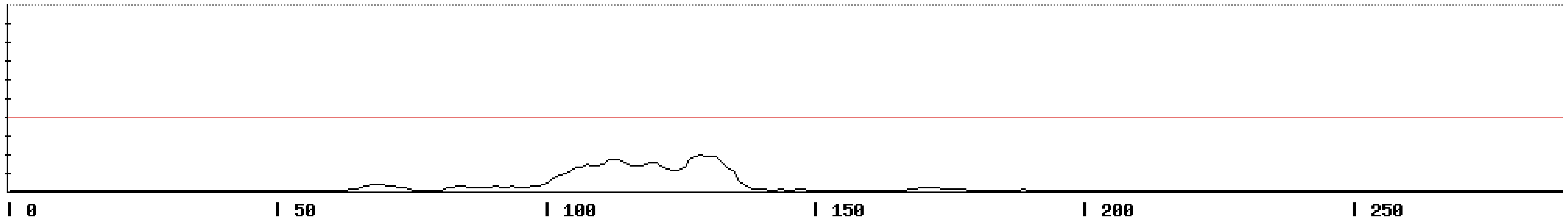

Predictions for Camelinasativa_Csa11g055880.1	
Viterbi Path	
Viterbi Path	
Posterior @ 0.4	
Posterior @ 0.4	
Posterior Graphic. Threshold Marked @ 0.4	

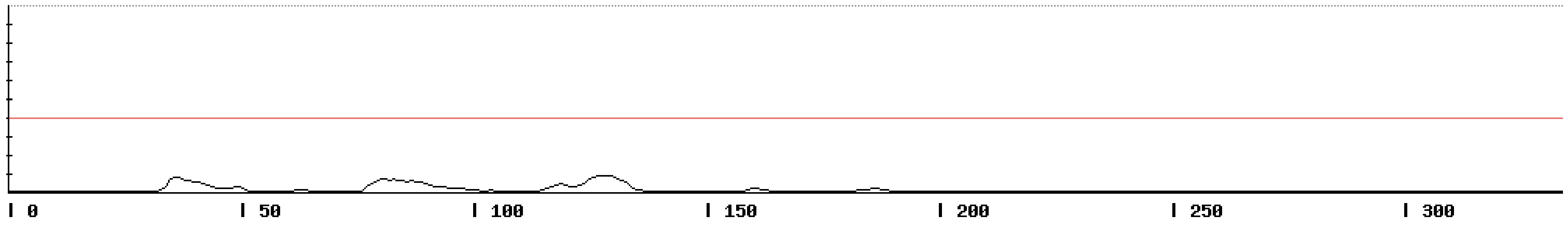

Predictions for Camelinasativa_Csa11g059640.1

Viterbi Path	
Viterbi Path	
Posterior @ 0.4	
Posterior @ 0.4	
Posterior Graphic. Threshold Marked @ 0.4	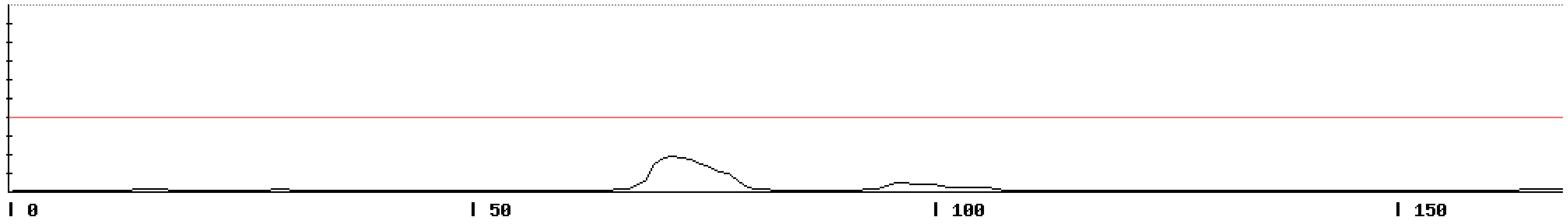

Predictions for Camelinasativa_Csa11g065390.1

Viterbi Path	
Viterbi Path	
Posterior @ 0.4	
Posterior @ 0.4	

Posterior Graphic. Threshold Marked @ 0.4	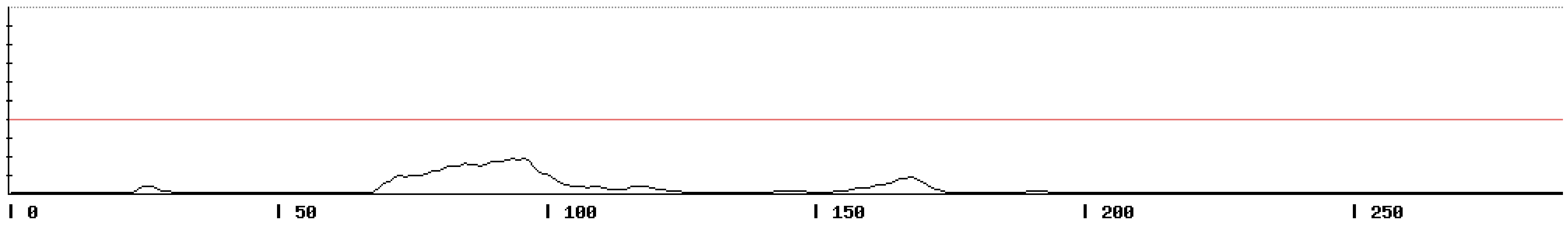

Predictions for Camelinasativa_Csa11g082140.1

Viterbi Path	
Viterbi Path	
Posterior @ 0.4	
Posterior @ 0.4	
Posterior Graphic. Threshold Marked @ 0.4	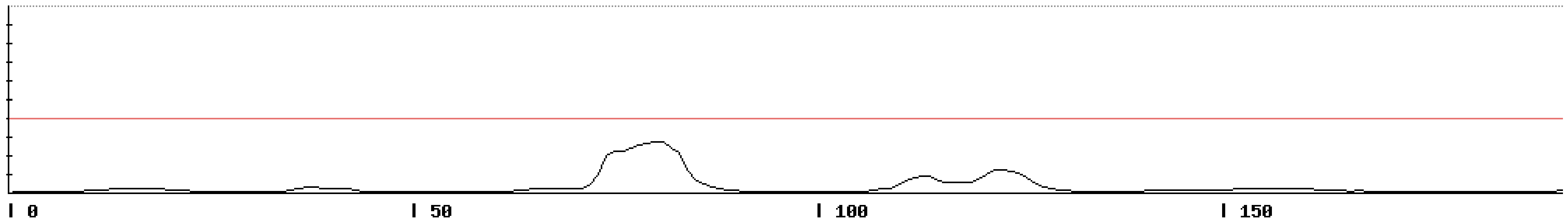

Predictions for Camelinasativa_Csa11g085880.1

Viterbi Path	
Viterbi Path	
Posterior @ 0.4	
Posterior @ 0.4	

Posterior Graphic. Threshold Marked @ 0.4	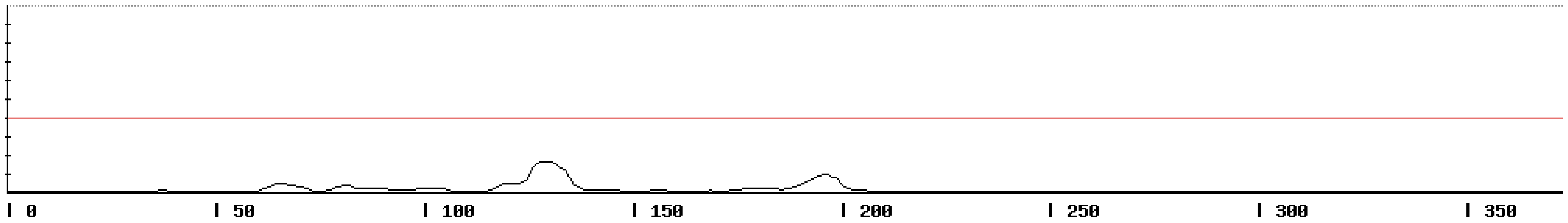

Predictions for Camelinasativa_Csa11g090050.1

Viterbi Path	
Viterbi Path	
Posterior @ 0.4	127 - KR - 128
Posterior @ 0.4	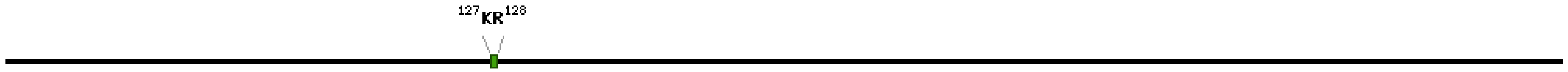
Posterior Graphic. Threshold Marked @ 0.4	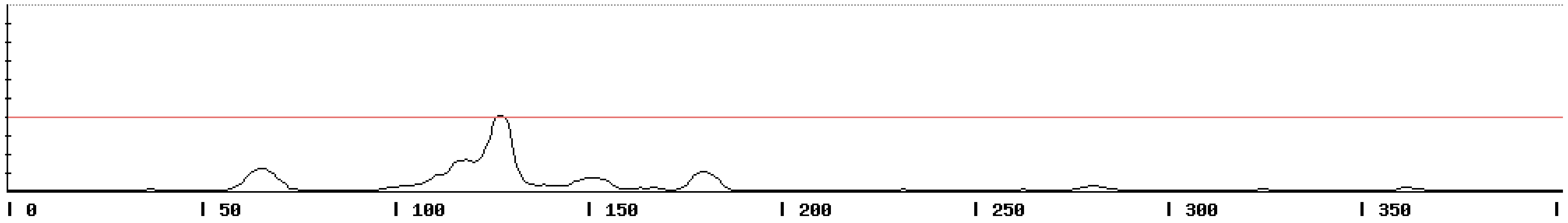

Predictions for Camelinasativa_Csa11g092840.1

Viterbi Path	172 - KIHKKKWSEKKKKKEKENDHHEEDKLDQELVVPPKIHKKRNFKKKKEK - 219
Viterbi Path	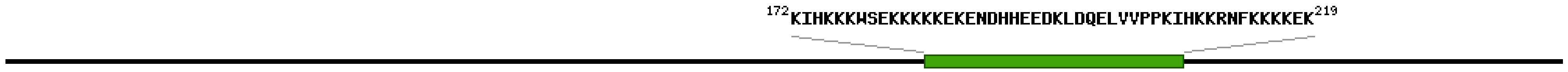

Posterior @ 0.4	131 - KNKKQKR - 137
	172 - KIHKKKWSEKKKKKEKE - 188
	204 - PPKIHKKRNFKKKKEKD - 220

Posterior @ 0.4

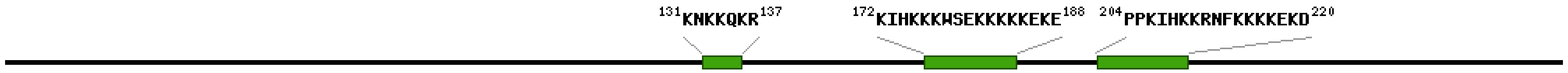

Posterior Graphic. Threshold Marked @ 0.4

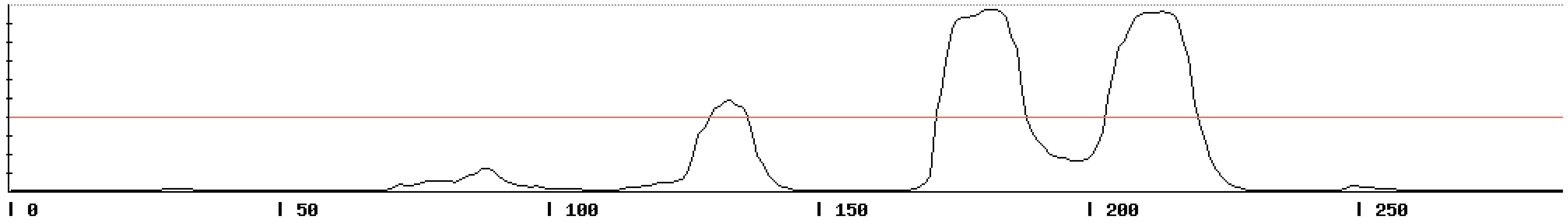

Predictions for Camelinasativa_Csa11g093080.1

Viterbi Path	154 - KIHKKKWSEKKKKKEKKK - 171
	191 - KVHKKRNSKKKKEK - 204

Viterbi Path

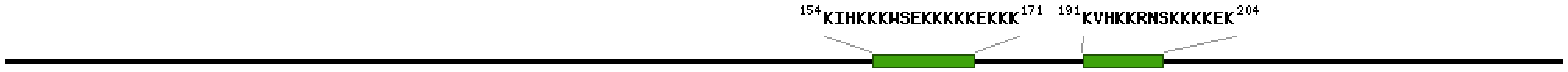

Posterior @ 0.4	131 - KNKNKKHRR - 139
	154 - KIHKKKWSEKKKKKEKKKDHH - 174
	191 - KVHKKRNSKKKKEKD - 205

Posterior @ 0.4

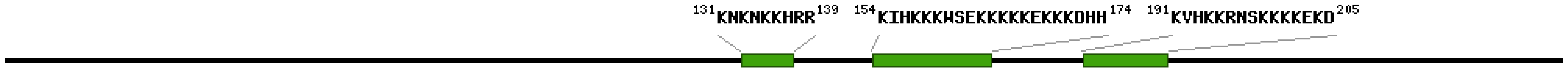

Posterior Graphic. Threshold Marked @ 0.4

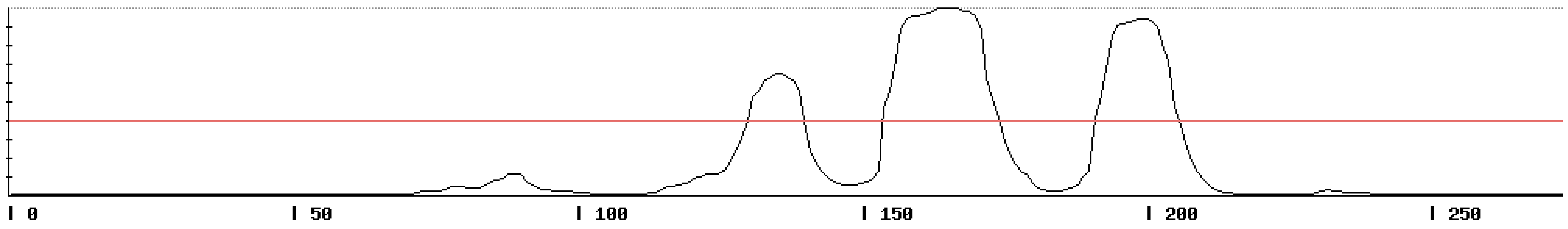

Predictions for Camelinasativa_Csa11g097230.1

Viterbi Path

Viterbi Path

Posterior @ 0.4

Posterior @ 0.4

Posterior Graphic. Threshold Marked @ 0.4

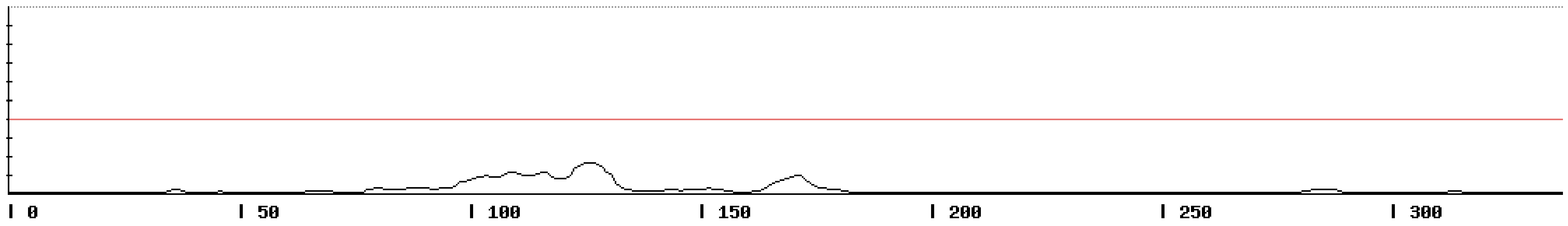

Predictions for Camelinasativa_Csa11g099250.1

Viterbi Path

Viterbi Path

Posterior @ 0.4

Posterior @ 0.4

Posterior Graphic. Threshold Marked @ 0.4

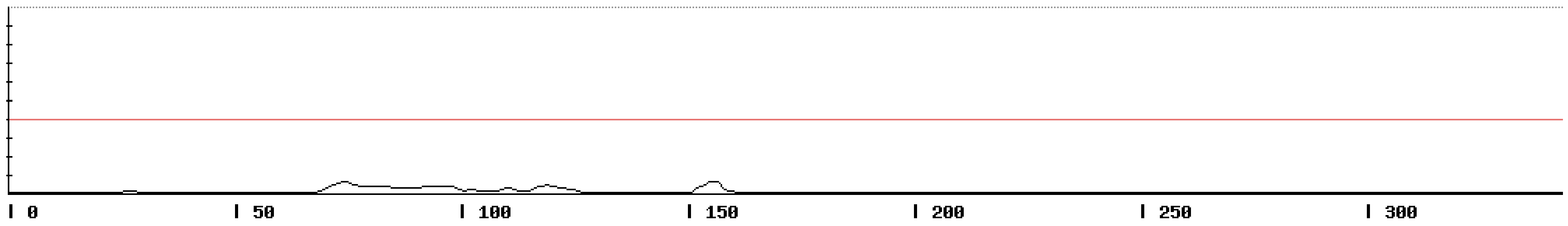

Predictions for Camelinasativa_Csa11g100860.1

Viterbi Path	
Viterbi Path	
Posterior @ 0.4	98 - GKPK - 101
Posterior @ 0.4	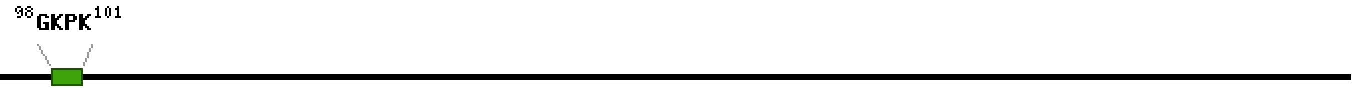
Posterior Graphic. Threshold Marked @ 0.4	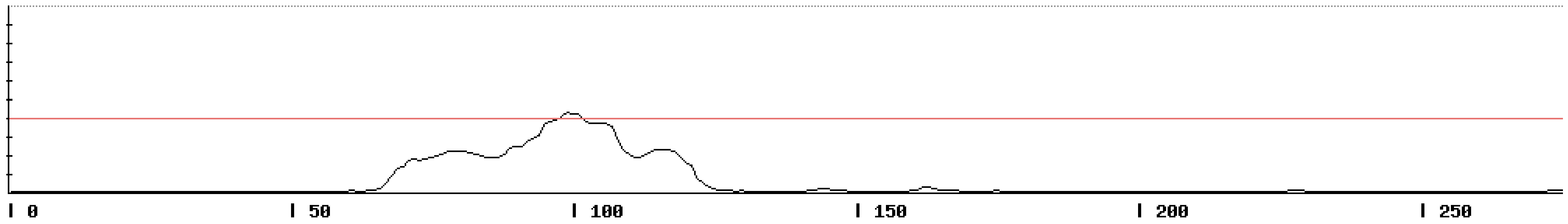

Predictions for Camelinasativa_Csa11g101160.1

Viterbi Path	
Viterbi Path	
Posterior @ 0.4	
Posterior @ 0.4	

Posterior Graphic. Threshold Marked @ 0.4

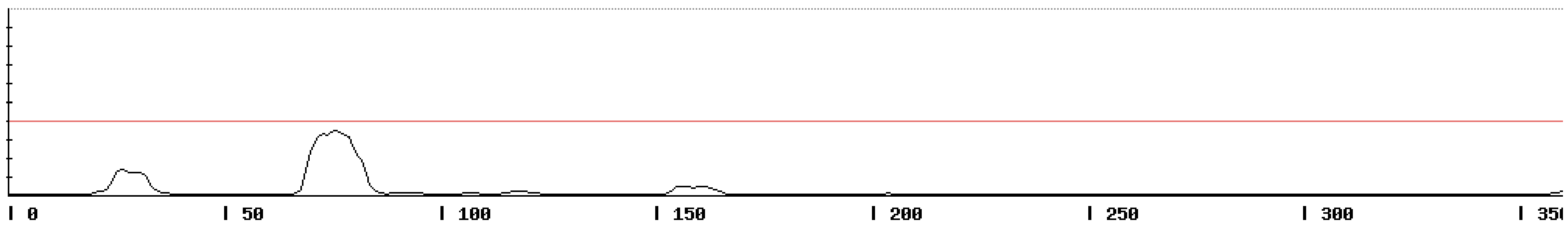

Predictions for Camelinasativa_Csa11g101660.1

Viterbi Path

Viterbi Path

Posterior @ 0.4

Posterior @ 0.4

Posterior Graphic. Threshold Marked @ 0.4

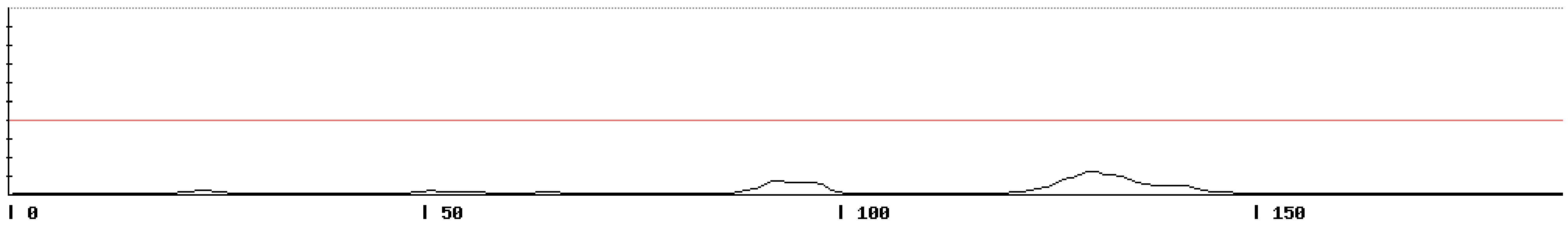

Predictions for Camelinasativa_Csa11g104660.1

Viterbi Path

Viterbi Path

Posterior @ 0.4

Posterior @ 0.4

Posterior Graphic. Threshold Marked @ 0.4

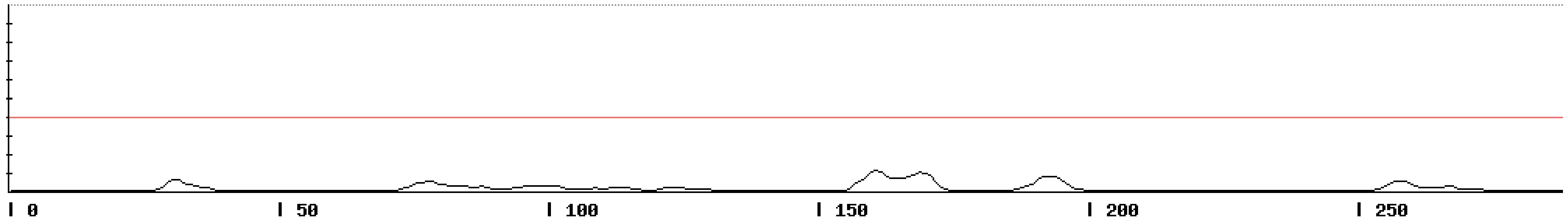

Predictions for Camelinasativa_Csa12g006210.1

Viterbi Path

Viterbi Path

Posterior @ 0.4 | 282 - KDKKKKK - 288

Posterior @ 0.4

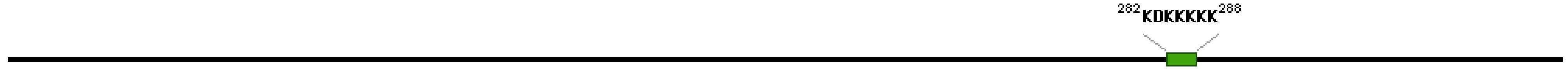

Posterior Graphic. Threshold Marked @ 0.4

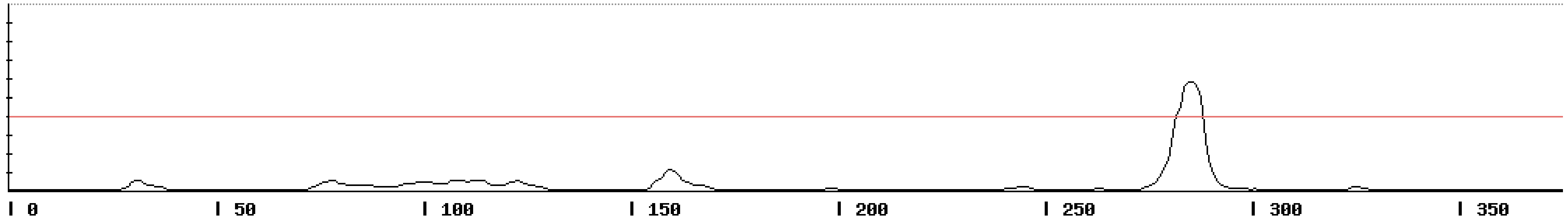

Predictions for Camelinasativa_Csa12g007970.1

Viterbi Path

Viterbi Path

Posterior @ 0.4	101 - IKSKK - 105
Posterior @ 0.4	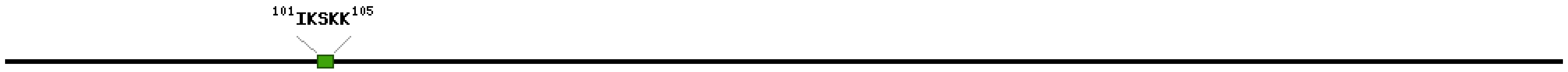
Posterior Graphic. Threshold Marked @ 0.4	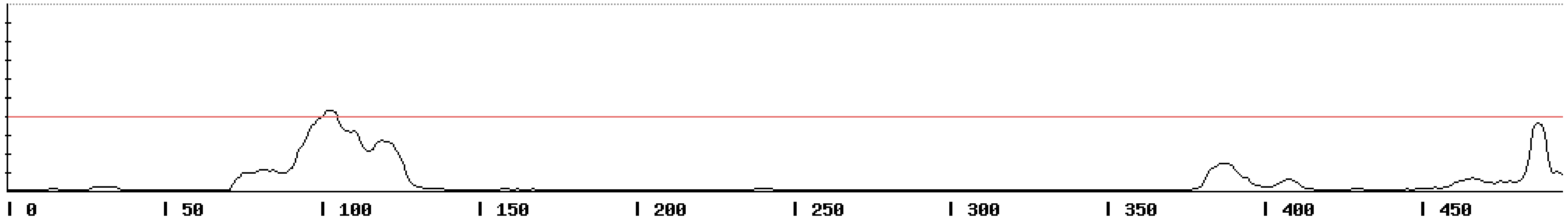

Predictions for Camelinasativa_Csa12g019250.1

Viterbi Path	
Viterbi Path	
Posterior @ 0.4	
Posterior @ 0.4	
Posterior Graphic. Threshold Marked @ 0.4	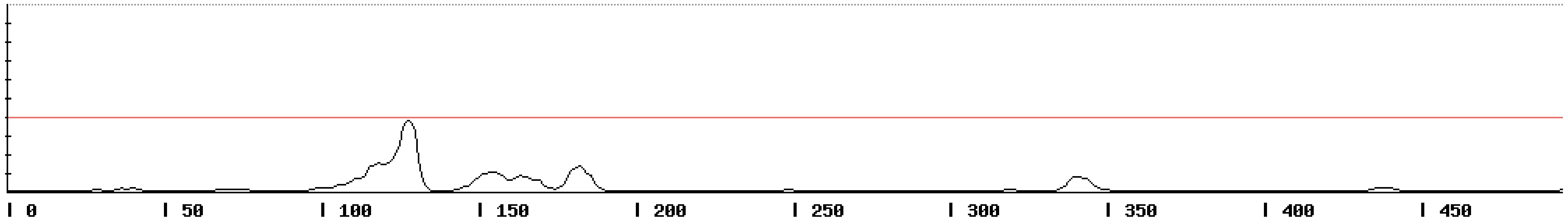

Predictions for Camelinasativa_Csa12g021980.1

Viterbi Path	
Viterbi Path	
Posterior @ 0.4	
Posterior @ 0.4	
Posterior Graphic. Threshold Marked @ 0.4	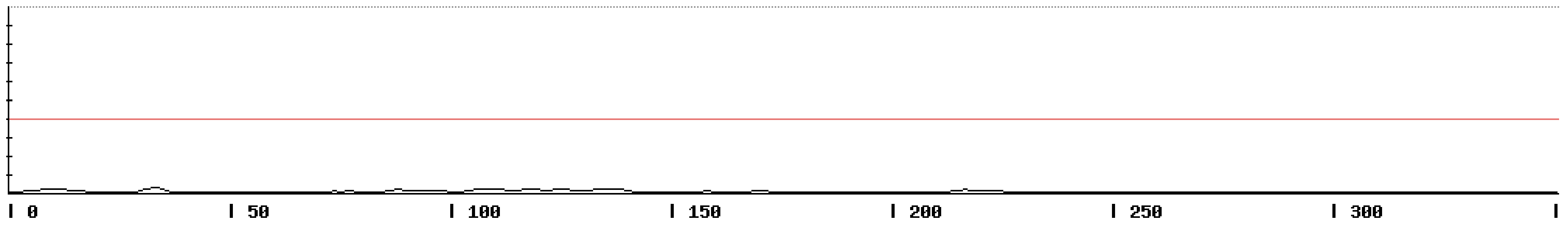

Predictions for Camelinasativa_Csa12g022020.1

Viterbi Path	
Viterbi Path	
Posterior @ 0.4	137 - RKRRK - 141
Posterior @ 0.4	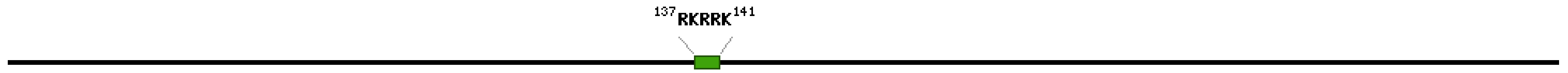
Posterior Graphic. Threshold Marked @ 0.4	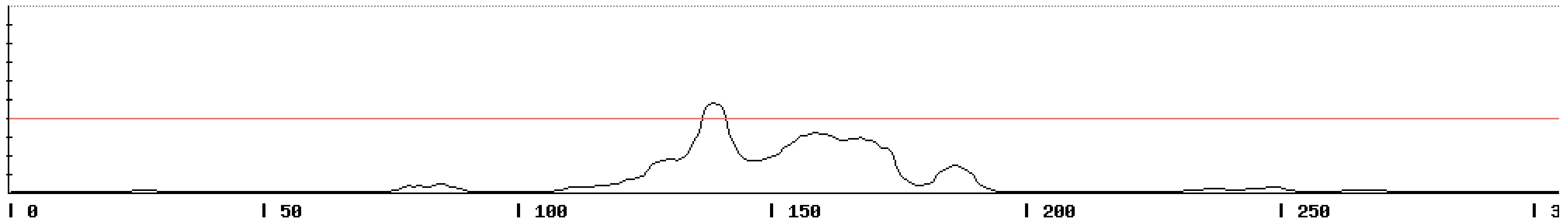

Predictions for Camelinasativa_Csa12g023460.1	
Viterbi Path	
Viterbi Path	
Posterior @ 0.4	
Posterior @ 0.4	
Posterior Graphic. Threshold Marked @ 0.4	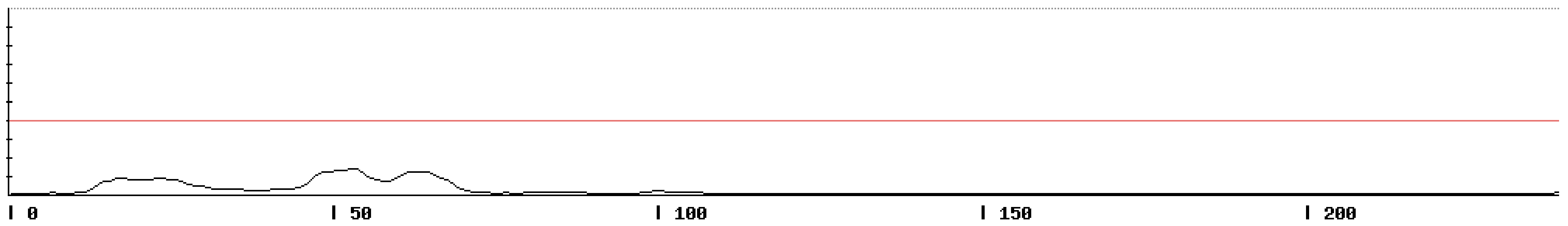

Predictions for Camelinasativa_Csa12g045480.1	
Viterbi Path	
Viterbi Path	
Posterior @ 0.4	
Posterior @ 0.4	
Posterior Graphic. Threshold Marked @ 0.4	

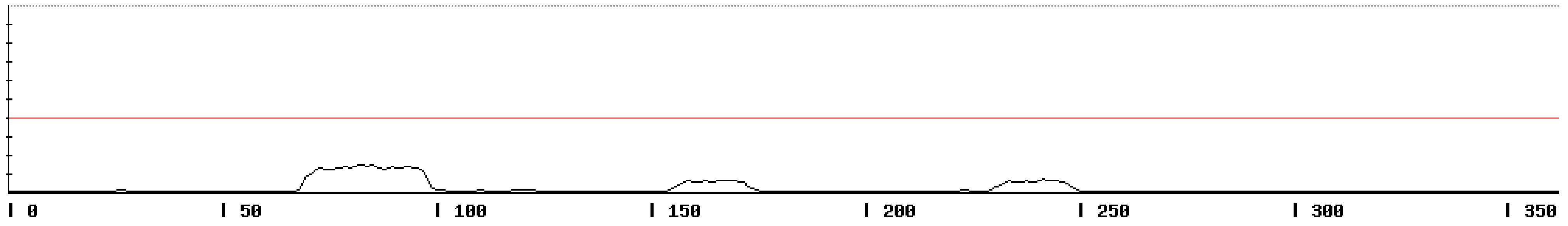

Predictions for Camelinasativa_Csa12g077490.1

Viterbi Path	
Viterbi Path	
Posterior @ 0.4	
Posterior @ 0.4	
Posterior Graphic. Threshold Marked @ 0.4	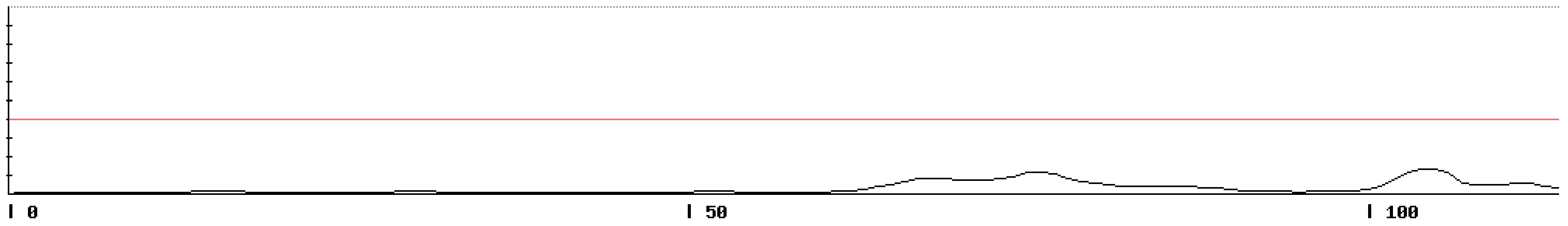

Predictions for Camelinasativa_Csa12g081560.1

Viterbi Path	
Viterbi Path	
Posterior @ 0.4	
Posterior @ 0.4	

Posterior Graphic. Threshold Marked @ 0.4

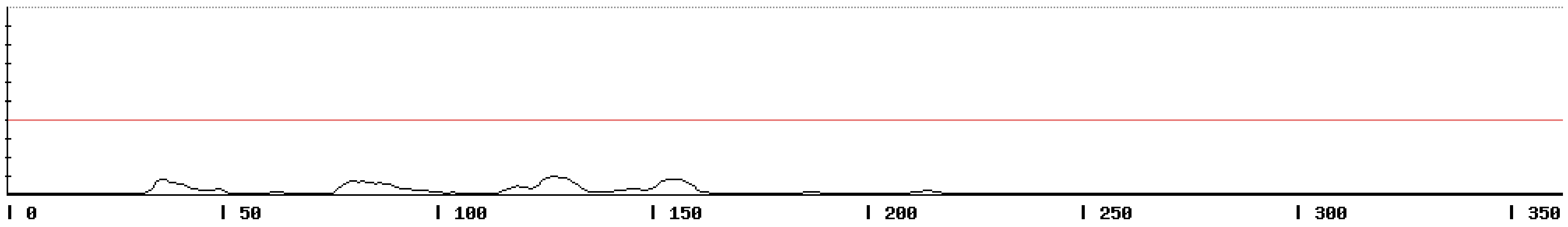

Predictions for Camelinasativa_Csa12g081820.1

Viterbi Path

Viterbi Path

Posterior @ 0.4

Posterior @ 0.4

Posterior Graphic. Threshold Marked @ 0.4

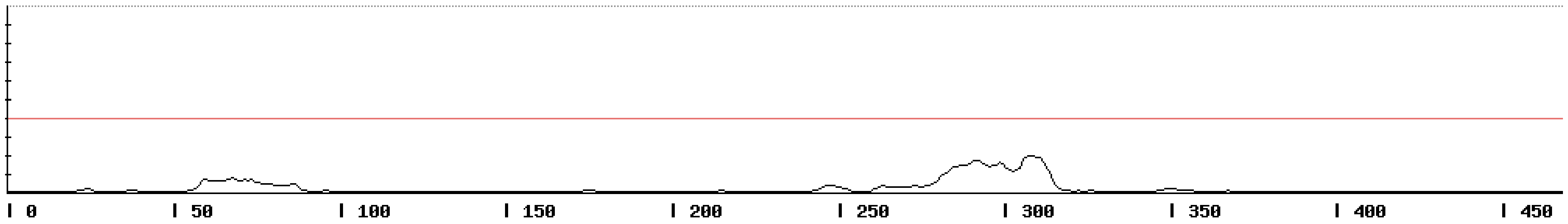

Predictions for Camelinasativa_Csa13g006400.1

Viterbi Path

Viterbi Path

Posterior @ 0.4

Posterior @ 0.4

Posterior Graphic. Threshold Marked @ 0.4

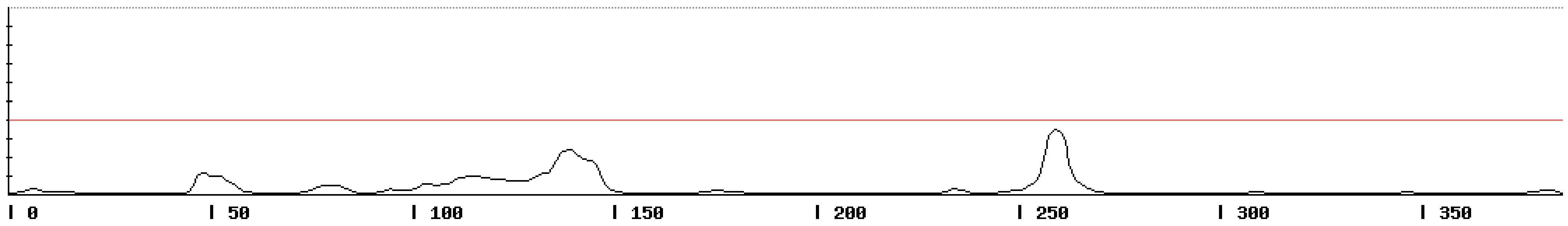

Predictions for Camelinasativa_Csa13g006410.1

Viterbi Path

Viterbi Path

Posterior @ 0.4

Posterior @ 0.4

Posterior Graphic. Threshold Marked @ 0.4

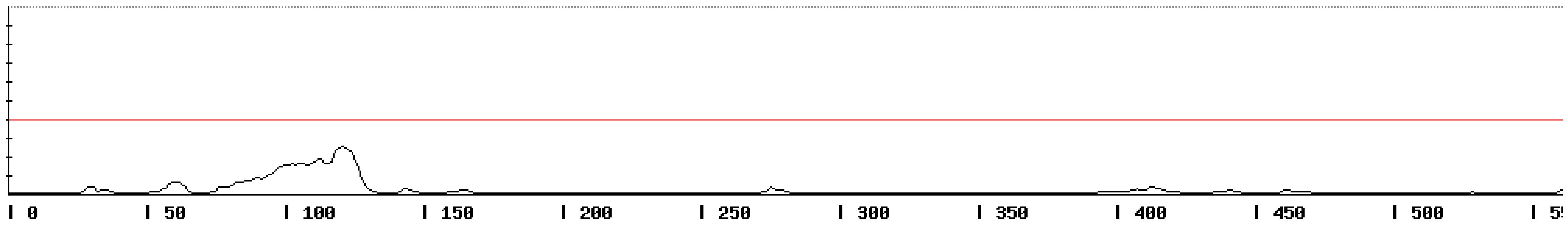

Predictions for Camelinasativa_Csa13g009710.1

Viterbi Path

Viterbi Path

Posterior @ 0.4

Posterior @ 0.4

Posterior Graphic. Threshold Marked @ 0.4

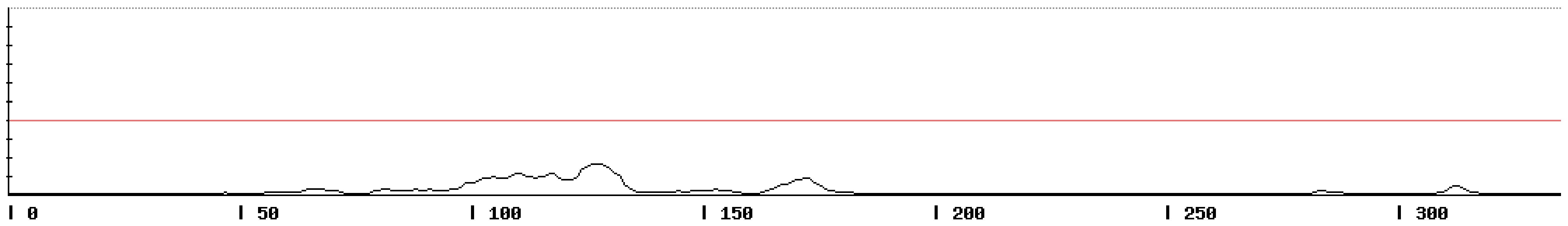

Predictions for Camelinasativa_Csa13g010890.1

Viterbi Path

Viterbi Path

Posterior @ 0.4 | 44 - GKPK - 47

Posterior @ 0.4

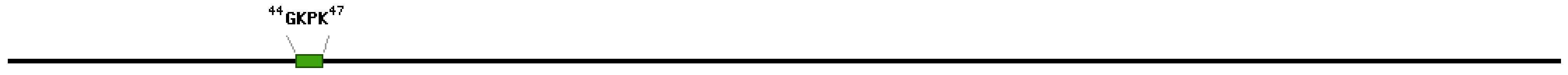

Posterior Graphic. Threshold Marked @ 0.4

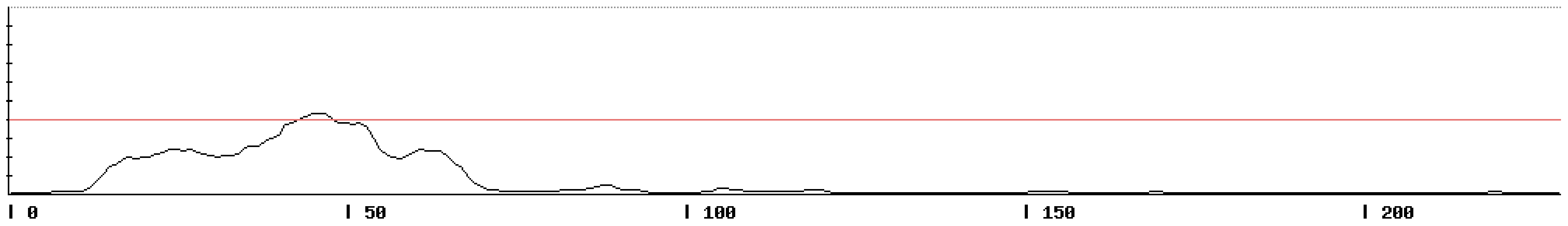

Predictions for Camelinasativa_Csa13g011010.1

Viterbi Path

Viterbi Path

Posterior @ 0.4	
Posterior @ 0.4	
Posterior Graphic. Threshold Marked @ 0.4	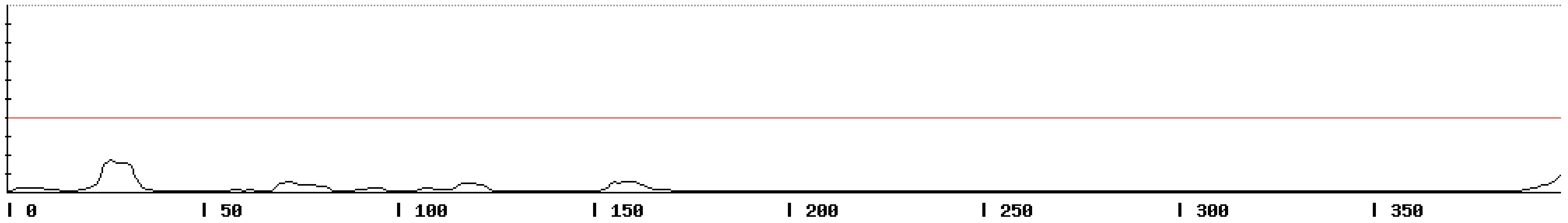

Predictions for Camelinasativa_Csa13g015790.1	
Viterbi Path	
Viterbi Path	
Posterior @ 0.4	
Posterior @ 0.4	
Posterior Graphic. Threshold Marked @ 0.4	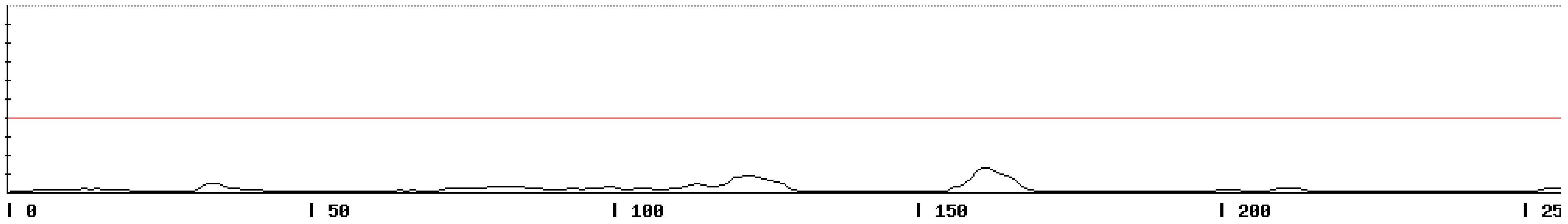

Predictions for Camelinasativa_Csa13g016770.1

Viterbi Path	
Viterbi Path	
Posterior @ 0.4	160 - RKGSKARKQRKSR - 172
Posterior @ 0.4	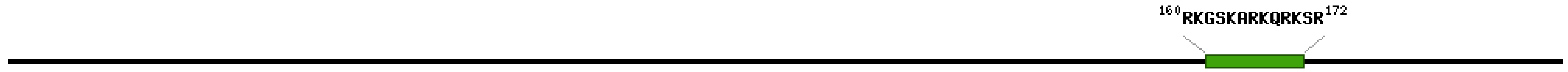
Posterior Graphic. Threshold Marked @ 0.4	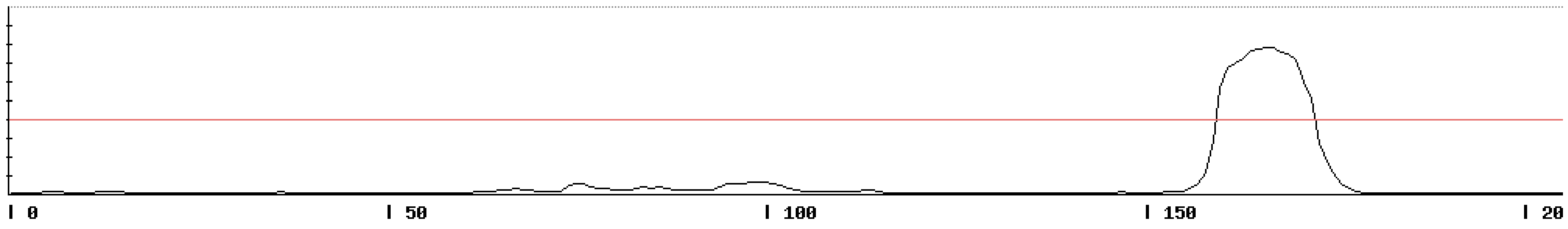

Predictions for Camelinasativa_Csa13g017310.1

Viterbi Path	
Viterbi Path	
Posterior @ 0.4	
Posterior @ 0.4	
Posterior Graphic. Threshold Marked @ 0.4	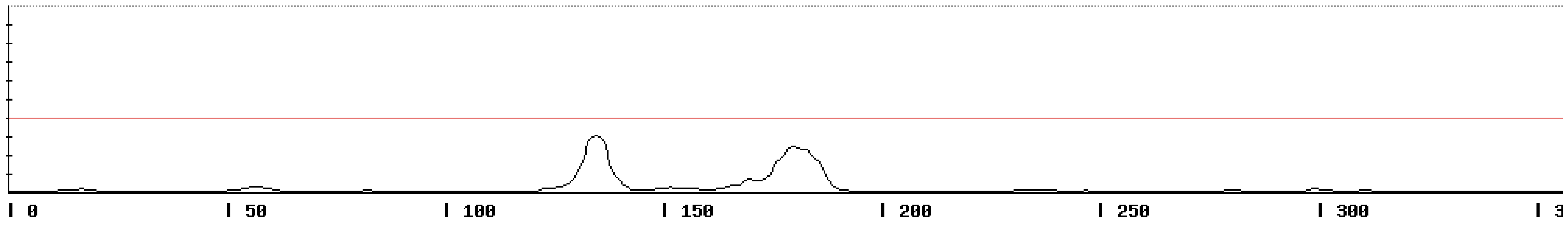

Predictions for Camelinasativa_Csa13g020020.1

Viterbi Path

Viterbi Path

Posterior @ 0.4

Posterior @ 0.4

Posterior Graphic. Threshold Marked @ 0.4

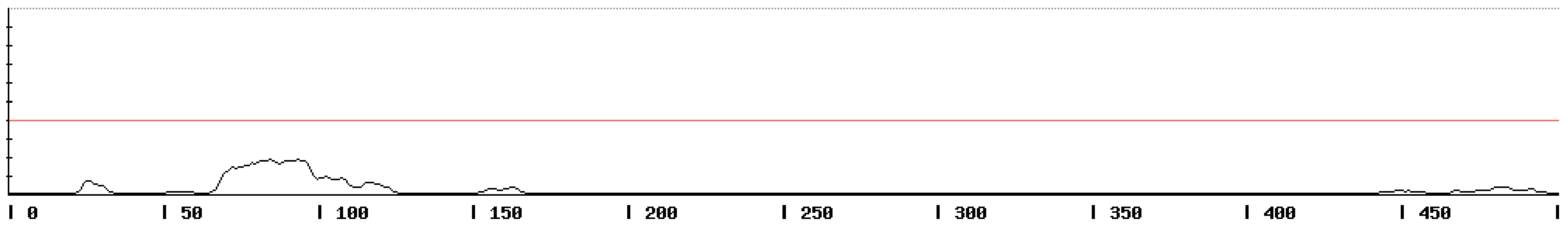

Predictions for Camelinasativa_Csa13g020920.1

Viterbi Path

Viterbi Path

Posterior @ 0.4

Posterior @ 0.4

Posterior Graphic. Threshold Marked @ 0.4

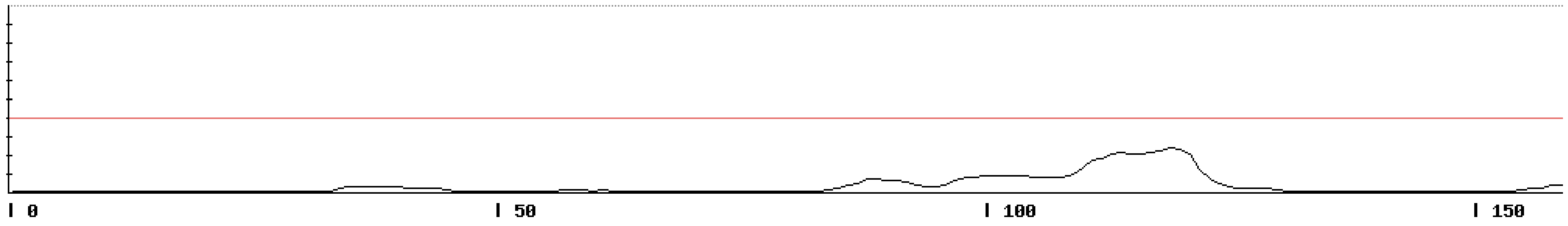

Predictions for Camelinasativa_Csa13g020970.1

Viterbi Path

Viterbi Path

Posterior @ 0.4

Posterior @ 0.4

Posterior Graphic. Threshold Marked @ 0.4

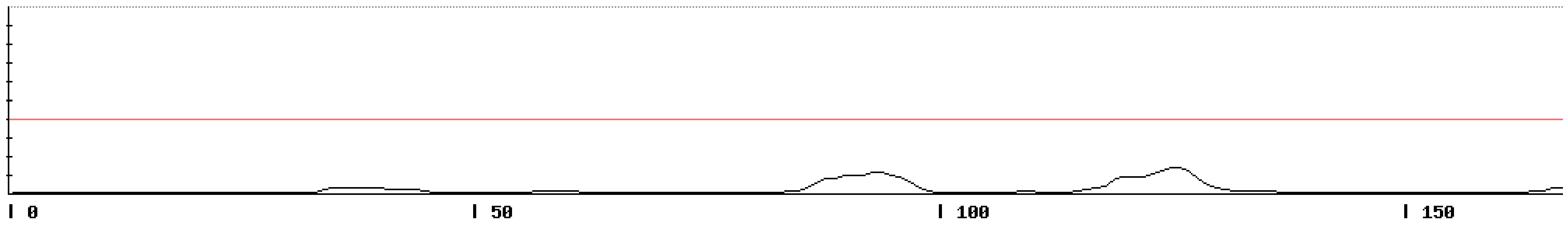

Predictions for Camelinasativa_Csa13g021170.1

Viterbi Path

Viterbi Path

Posterior @ 0.4

Posterior @ 0.4

Posterior Graphic. Threshold Marked @ 0.4

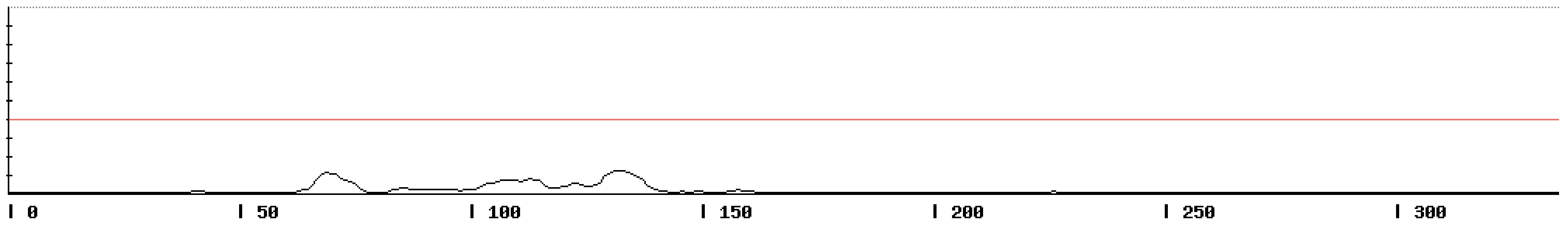

Predictions for Camelinasativa_Csa13g021200.1

Viterbi Path

Viterbi Path

Posterior @ 0.4 86 - K - 86

Posterior @ 0.4

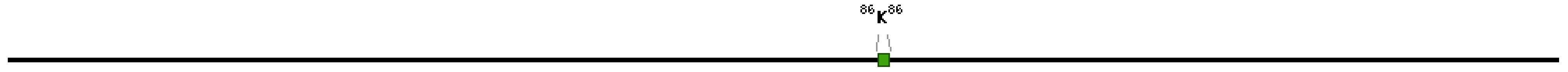

Posterior Graphic. Threshold Marked @ 0.4

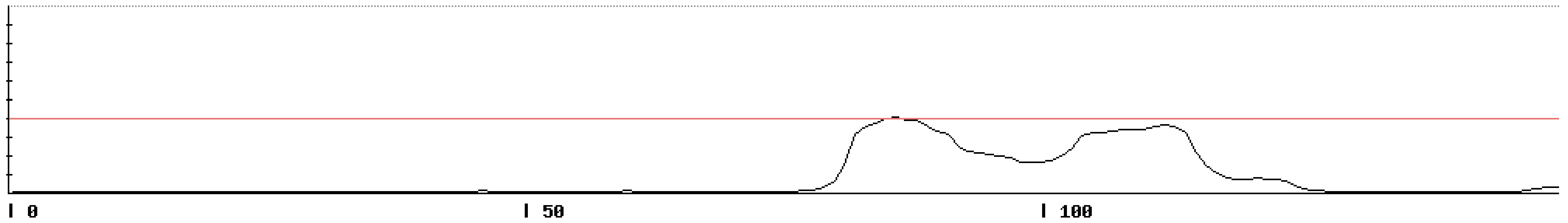

Predictions for Camelinasativa_Csa13g025730.1

Viterbi Path

Viterbi Path

Posterior @ 0.4

Posterior @ 0.4

Posterior Graphic. Threshold Marked @ 0.4

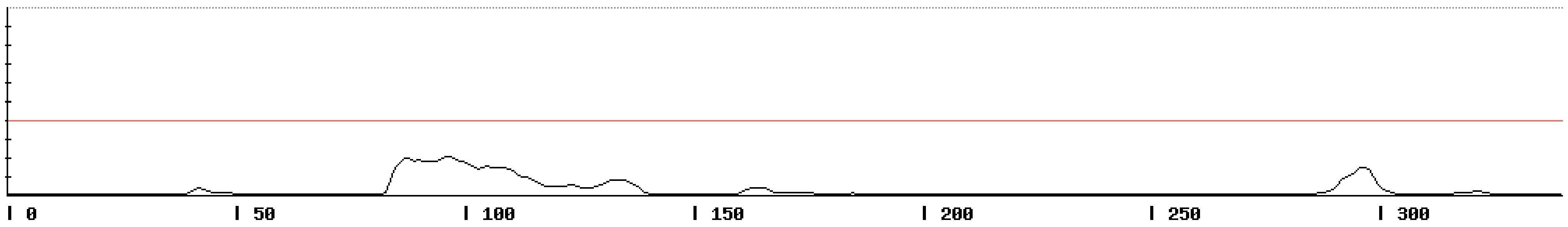

Predictions for Camelinasativa_Csa13g025850.1

Viterbi Path

Viterbi Path

Posterior @ 0.4

Posterior @ 0.4

Posterior Graphic. Threshold Marked @ 0.4

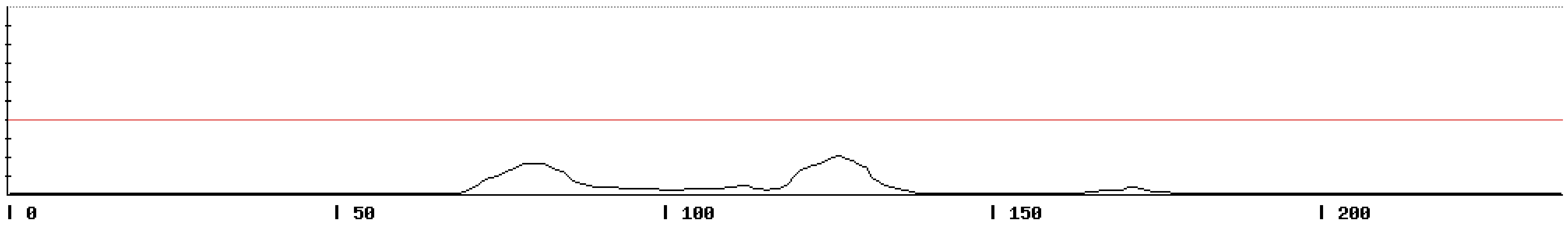

Predictions for Camelinasativa_Csa13g030320.1

Viterbi Path

Viterbi Path

Posterior @ 0.4	95 - TGKDRKIKSGKTKII - 109
Posterior @ 0.4	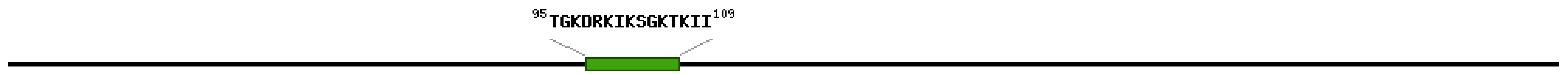
Posterior Graphic. Threshold Marked @ 0.4	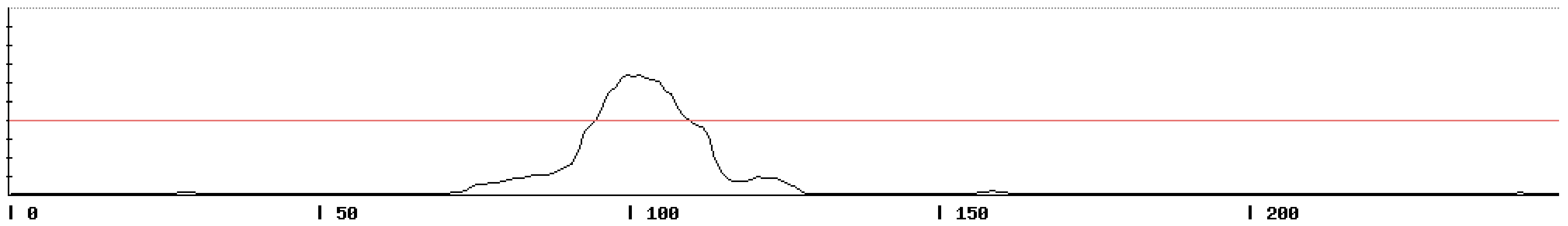

Predictions for Camelinasativa_Csa13g047490.1

Viterbi Path	
Viterbi Path	
Posterior @ 0.4	
Posterior @ 0.4	
Posterior Graphic. Threshold Marked @ 0.4	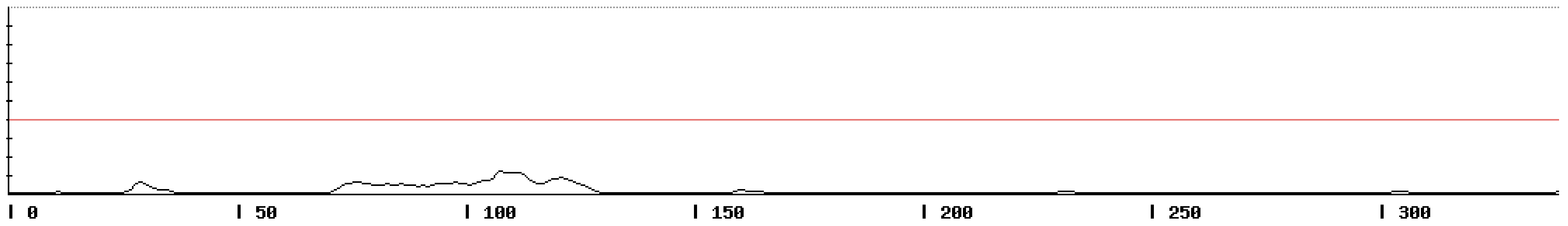

Predictions for Camelinasativa_Csa13g055570.1

Viterbi Path	
Viterbi Path	

Posterior @ 0.4

Posterior @ 0.4

Posterior Graphic. Threshold Marked @ 0.4

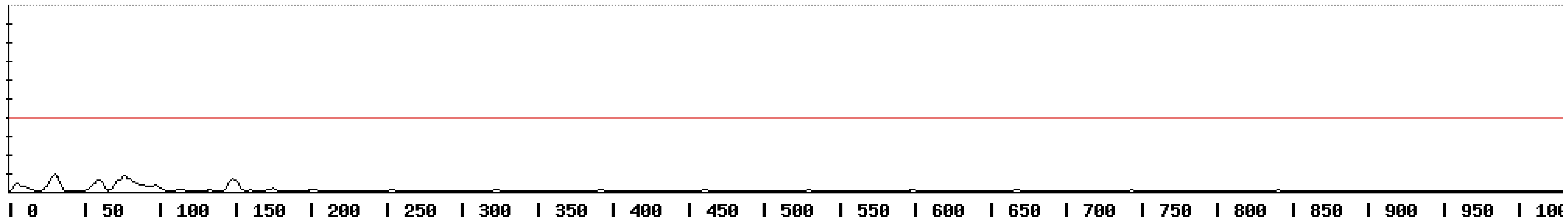

Predictions for Camelinasativa_Csa13g055730.1

Viterbi Path

Viterbi Path

Posterior @ 0.4

75 - KKKNAMKR - 82

90 - KPTGKDRKIKDKRG - 103

Posterior @ 0.4

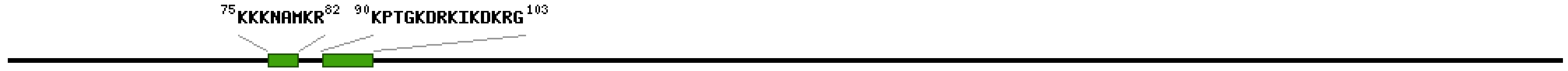

Posterior Graphic. Threshold Marked @ 0.4

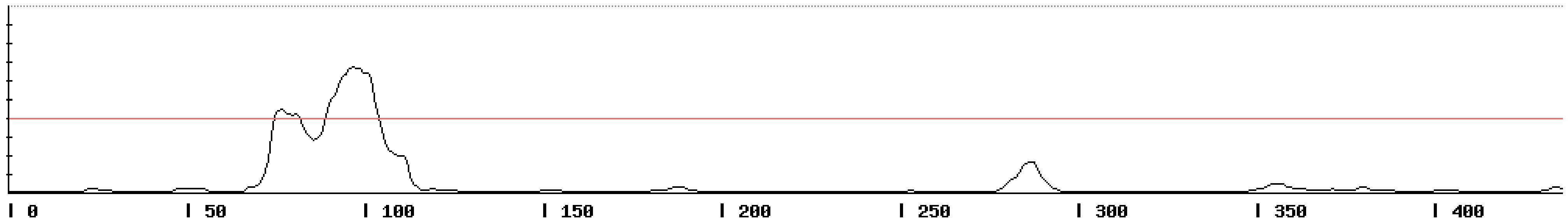

Predictions for Camelinasativa_Csa14g002180.1

Viterbi Path

Viterbi Path

Posterior @ 0.4

Posterior @ 0.4

Posterior Graphic. Threshold Marked @ 0.4

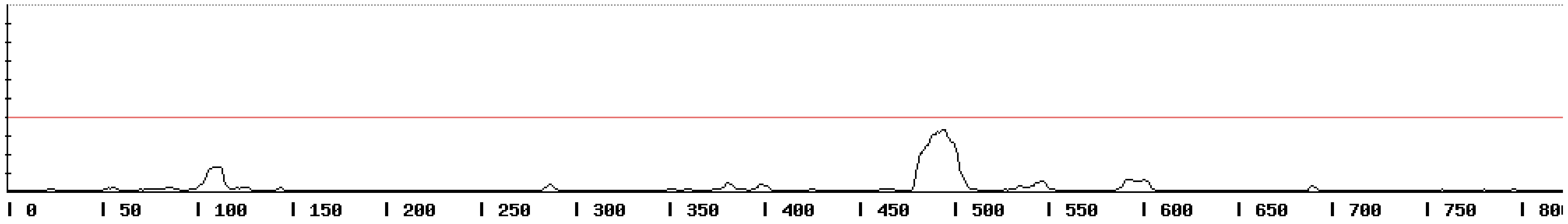

Predictions for Camelinasativa_Csa14g002190.1

Viterbi Path

Viterbi Path

Posterior @ 0.4

98 - WKITGKPKSIIRNRVKIGA - 116

Posterior @ 0.4

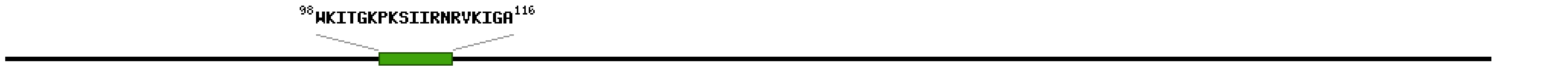

Posterior Graphic. Threshold Marked @ 0.4

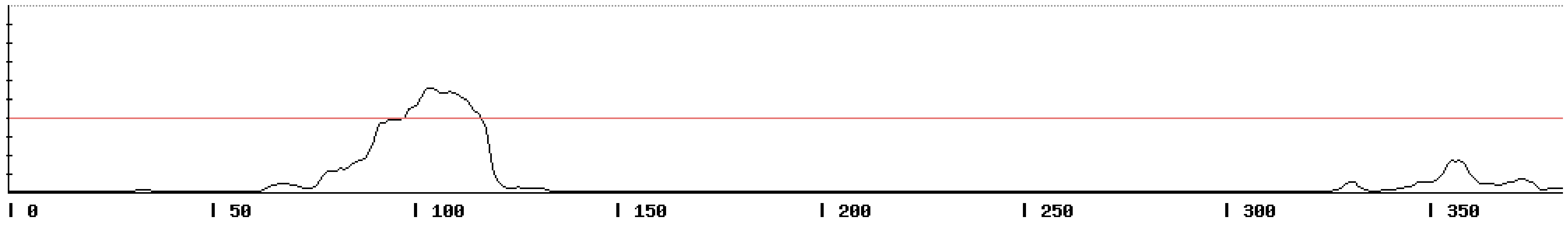

Predictions for Camelinasativa_Csa14g002230.1

Viterbi Path	
Viterbi Path	
Posterior @ 0.4	105 - KRKSGNREK - 113
Posterior @ 0.4	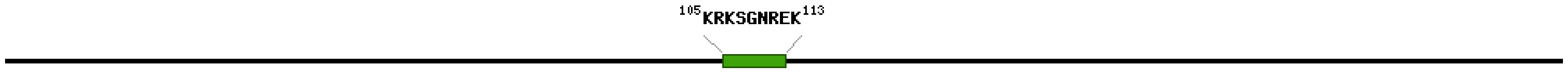
Posterior Graphic. Threshold Marked @ 0.4	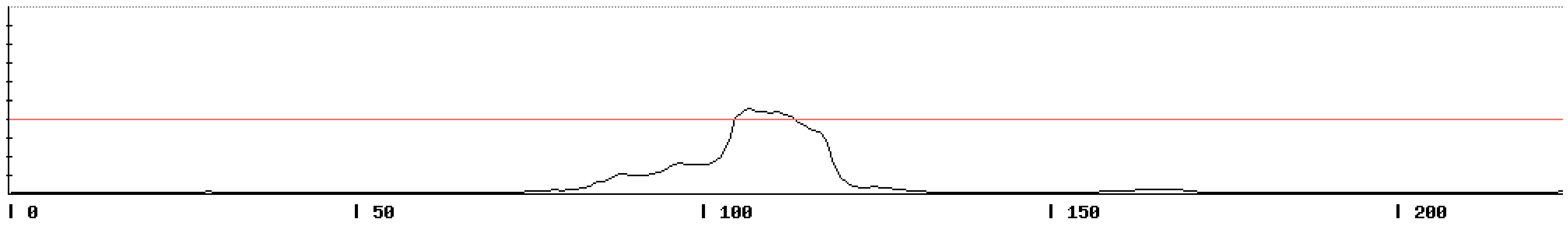

Predictions for Camelinasativa_Csa14g002590.1

Viterbi Path	
Viterbi Path	
Posterior @ 0.4	66 - TLKARKTPNRKV - 77
	579 - RRKRRK - 584
Posterior @ 0.4	

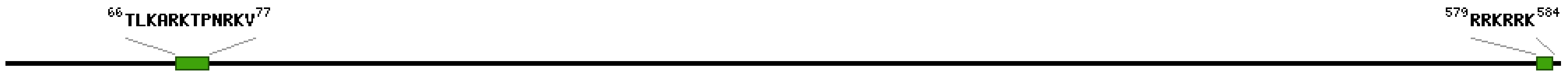

Posterior Graphic. Threshold Marked @ 0.4

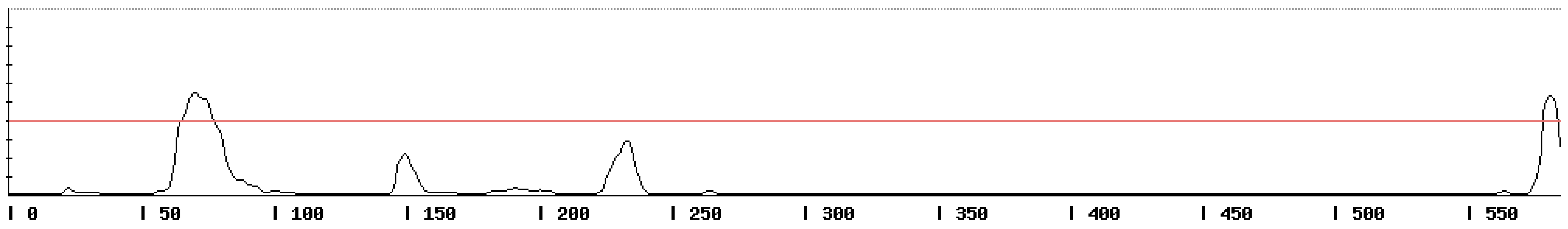

Predictions for Camelinasativa_Csa14g004560.1

Viterbi Path	
Viterbi Path	
Posterior @ 0.4	70 - KFRDKRPSRSVPGRGGSKAGVWRSGVTKRNIK - 101
Posterior @ 0.4	

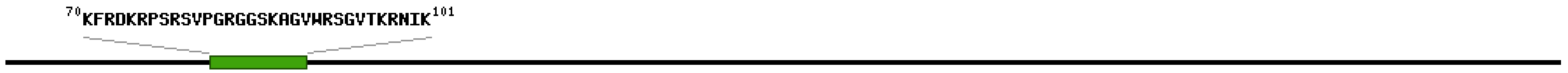

Posterior Graphic. Threshold Marked @ 0.4

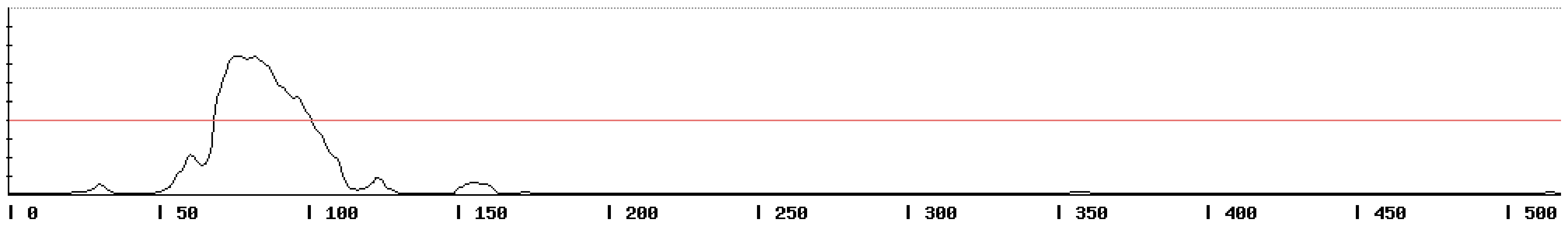

Predictions for Camelinasativa_Csa14g015200.1

Viterbi Path

Viterbi Path

Posterior @ 0.4

Posterior @ 0.4

Posterior Graphic. Threshold Marked @ 0.4

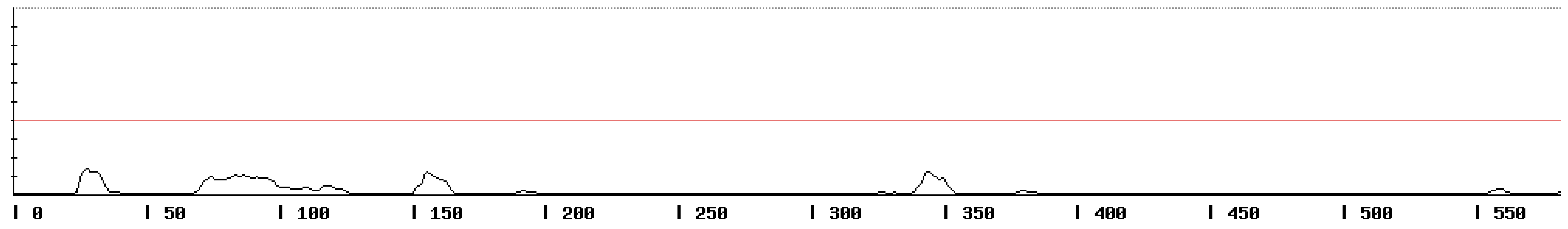

Predictions for Camelinasativa_Csa14g031790.1

Viterbi Path

Viterbi Path

Posterior @ 0.4

Posterior @ 0.4

Posterior Graphic. Threshold Marked @ 0.4

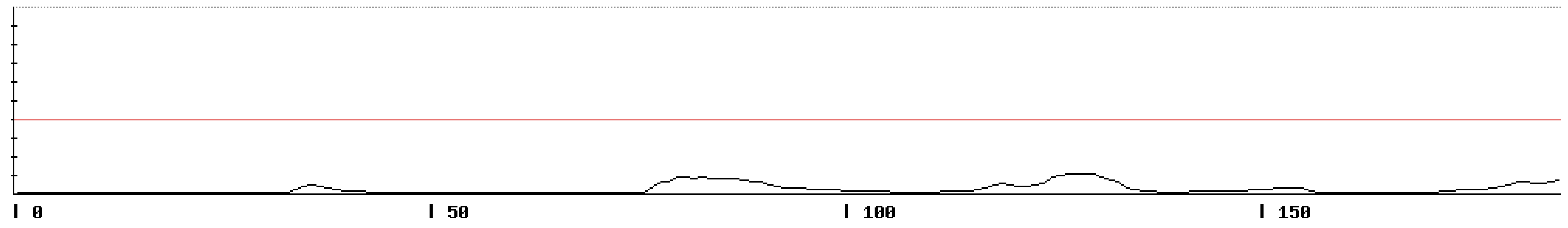

Predictions for Camelinasativa_Csa14g032780.1

Viterbi Path

Viterbi Path

Posterior @ 0.4 | 137 - KRR - 139

Posterior @ 0.4

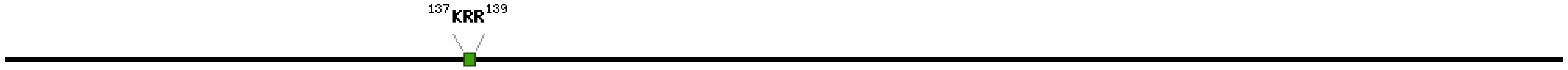

Posterior Graphic. Threshold Marked @ 0.4

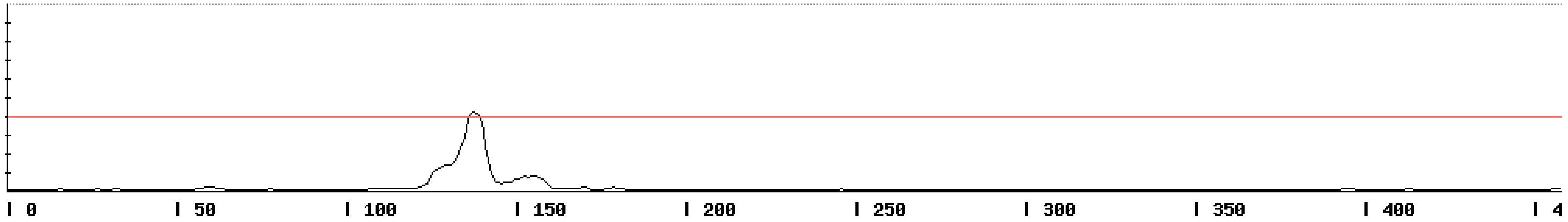

Predictions for Camelinasativa_Csa14g036150.1

Viterbi Path

Viterbi Path

Posterior @ 0.4

Posterior @ 0.4

Posterior Graphic. Threshold Marked @ 0.4

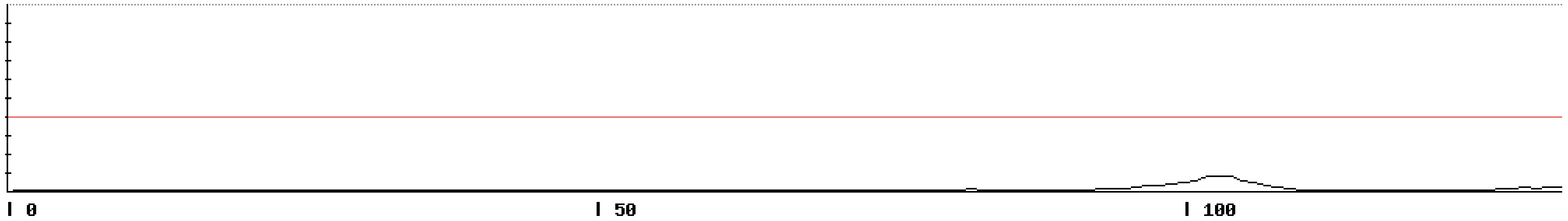

Predictions for Camelinasativa_Csa14g042520.1

Viterbi Path

Viterbi Path

Posterior @ 0.4

Posterior @ 0.4

Posterior Graphic. Threshold Marked @ 0.4

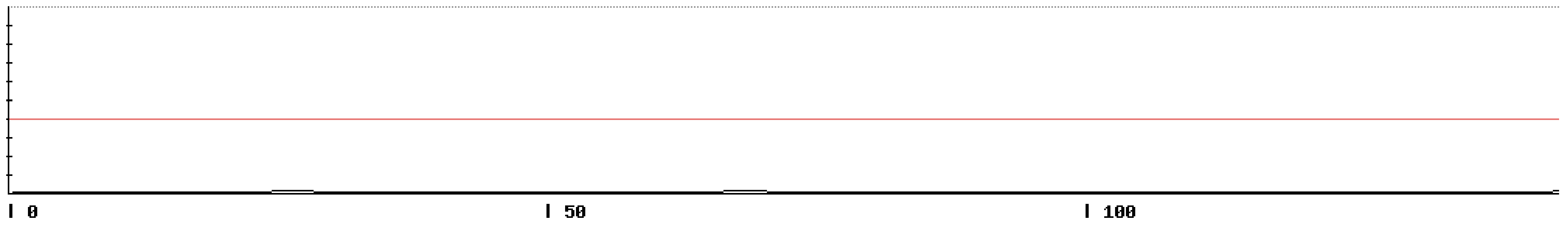

Predictions for Camelinasativa_Csa14g042630.1

Viterbi Path

Viterbi Path

Posterior @ 0.4

70 - KRKIRRKKLR - 79

514 - KRKKSGGNNDKKNKNK - 529

Posterior @ 0.4

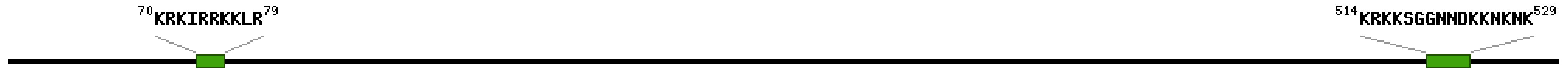

Posterior Graphic. Threshold Marked @ 0.4

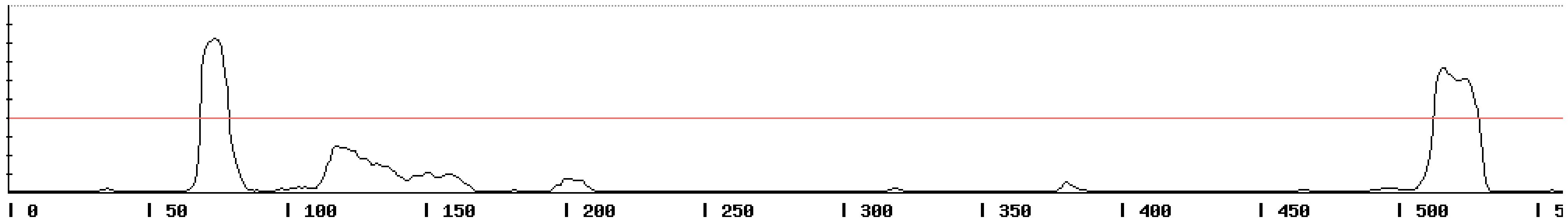

Predictions for Camelinasativa_Csa14g042710.1	
Viterbi Path	
Viterbi Path	
Posterior @ 0.4	
Posterior @ 0.4	
Posterior Graphic. Threshold Marked @ 0.4	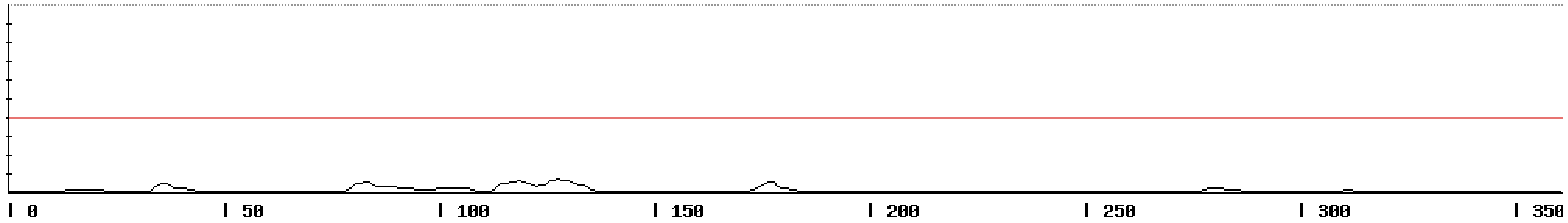

Predictions for Camelinasativa_Csa14g043230.1	
Viterbi Path	
Viterbi Path	
Posterior @ 0.4	
Posterior @ 0.4	

Posterior Graphic. Threshold Marked @ 0.4

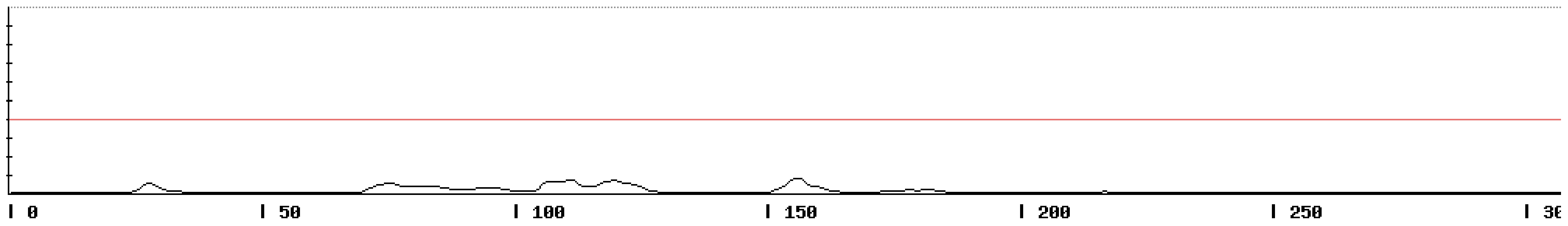

Predictions for Camelinasativa_Csa14g044030.1

Viterbi Path

Viterbi Path

Posterior @ 0.4

Posterior @ 0.4

Posterior Graphic. Threshold Marked @ 0.4

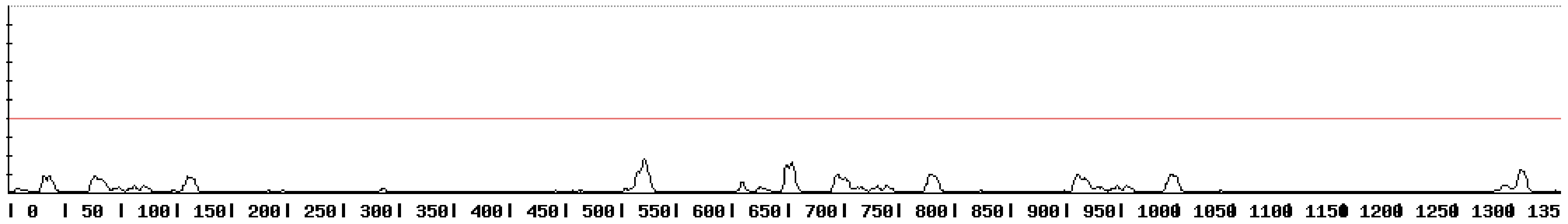

Predictions for Camelinasativa_Csa14g063200.1

Viterbi Path

Viterbi Path

Posterior @ 0.4

Posterior @ 0.4

Posterior Graphic. Threshold Marked @ 0.4

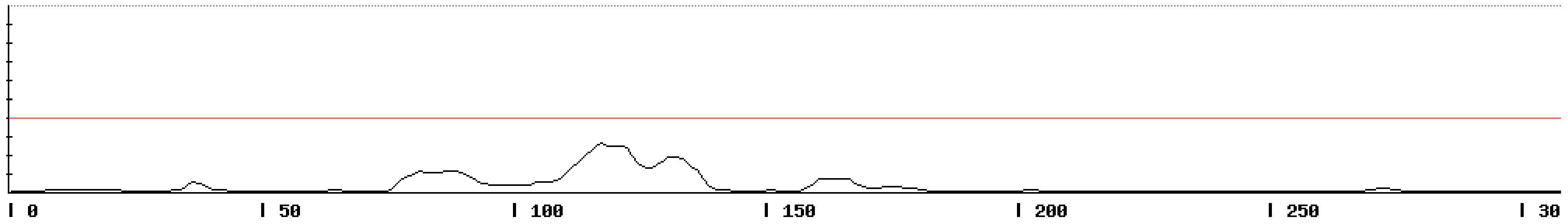

Predictions for Camelinasativa_Csa14g063210.1

Viterbi Path

Viterbi Path

Posterior @ 0.4

Posterior @ 0.4

Posterior Graphic. Threshold Marked @ 0.4

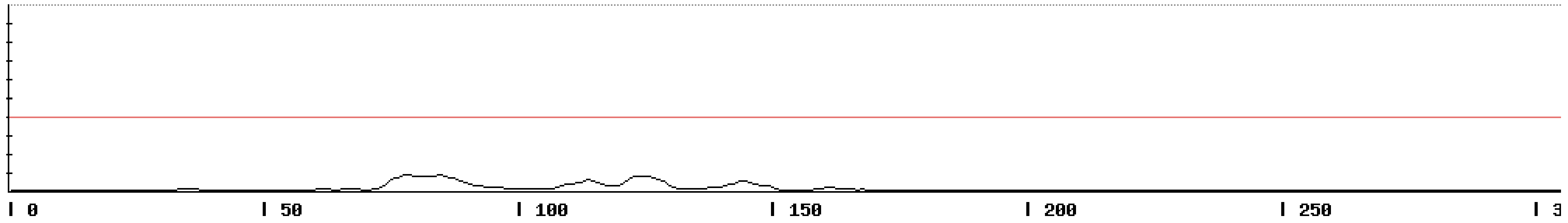

Predictions for Camelinasativa_Csa14g067910.1

Viterbi Path

Viterbi Path

Posterior @ 0.4

Posterior @ 0.4

Posterior Graphic. Threshold Marked @ 0.4

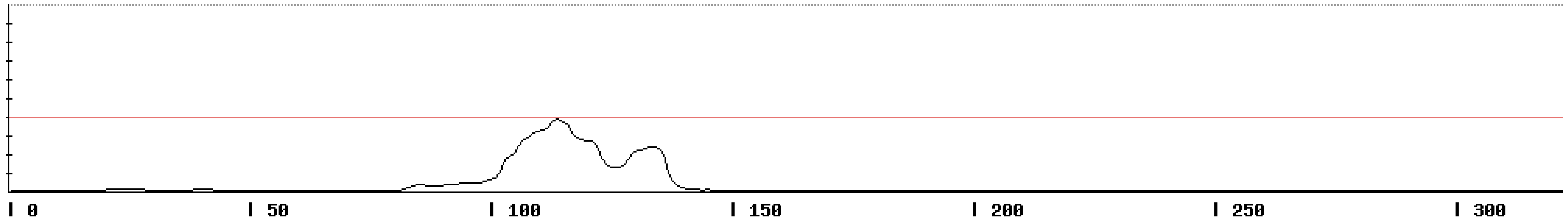

Predictions for Camelinasativa_Csa14g069120.1

Viterbi Path

Viterbi Path

Posterior @ 0.4

Posterior @ 0.4

Posterior Graphic. Threshold Marked @ 0.4

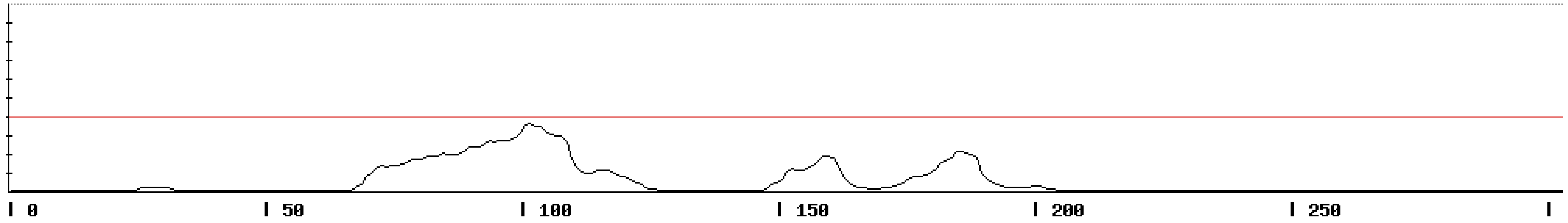

Predictions for Camelinasativa_Csa15g002950.1

Viterbi Path

Viterbi Path

Posterior @ 0.4

Posterior @ 0.4

Posterior Graphic. Threshold Marked @ 0.4

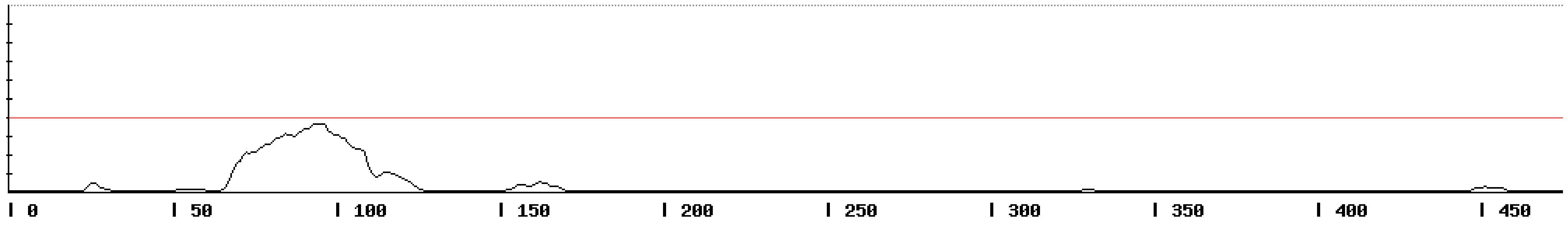

Predictions for Camelinasativa_Csa15g003940.1

Viterbi Path

Viterbi Path

Posterior @ 0.4

Posterior @ 0.4

Posterior Graphic. Threshold Marked @ 0.4

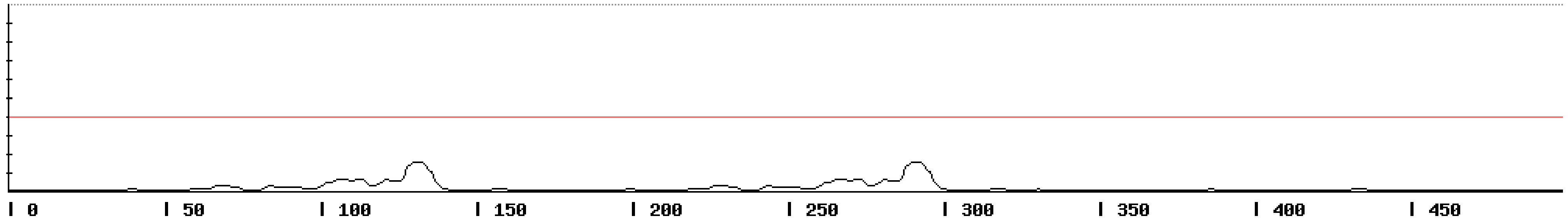

Predictions for Camelinasativa_Csa15g003950.1

Viterbi Path	
Viterbi Path	
Posterior @ 0.4	
Posterior @ 0.4	
Posterior Graphic. Threshold Marked @ 0.4	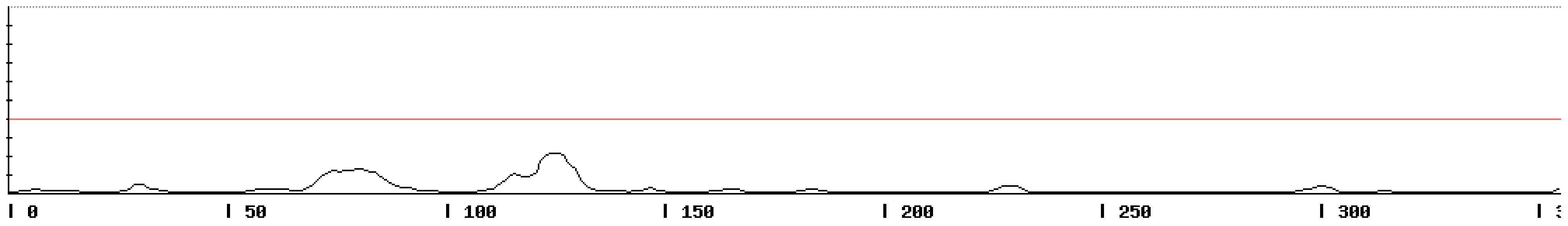

Predictions for Camelinasativa_Csa15g013970.1

Viterbi Path	
Viterbi Path	
Posterior @ 0.4	
Posterior @ 0.4	
Posterior Graphic. Threshold Marked @ 0.4	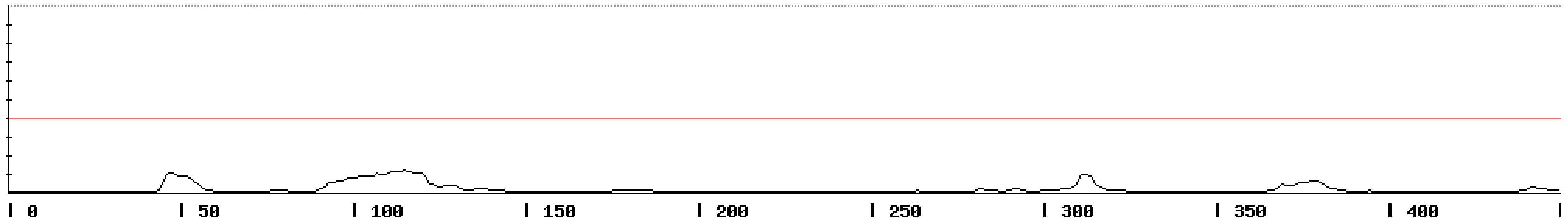

Predictions for Camelinasativa_Csa15g013990.1	
Viterbi Path	
Viterbi Path	
Posterior @ 0.4	
Posterior @ 0.4	
Posterior Graphic. Threshold Marked @ 0.4	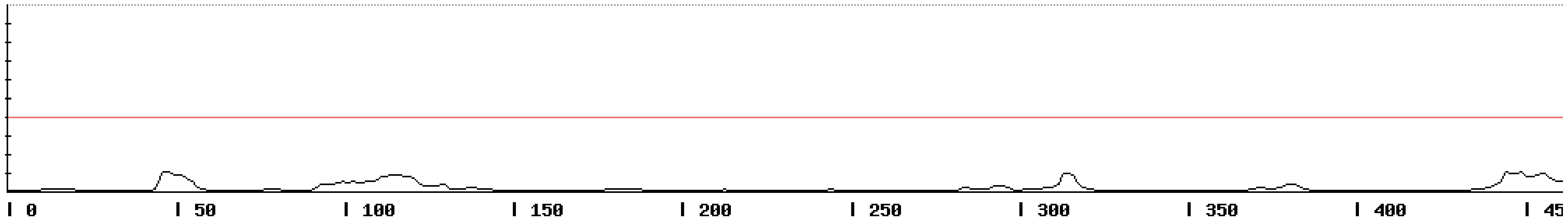

Predictions for Camelinasativa_Csa15g014000.1	
Viterbi Path	
Viterbi Path	
Posterior @ 0.4	
Posterior @ 0.4	
Posterior Graphic. Threshold Marked @ 0.4	

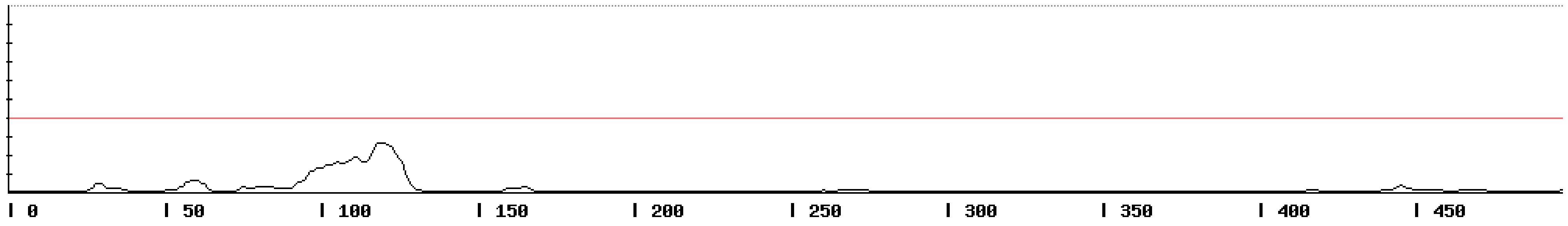

Predictions for Camelinasativa_Csa15g016850.1

Viterbi Path

Viterbi Path

Posterior @ 0.4

Posterior @ 0.4

Posterior Graphic. Threshold Marked @ 0.4

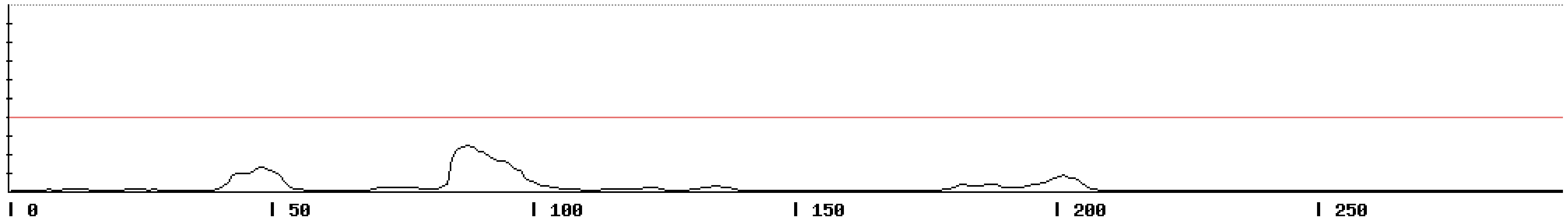

Predictions for Camelinasativa_Csa15g016960.1

Viterbi Path

Viterbi Path

Posterior @ 0.4

Posterior @ 0.4

Posterior Graphic. Threshold Marked @ 0.4

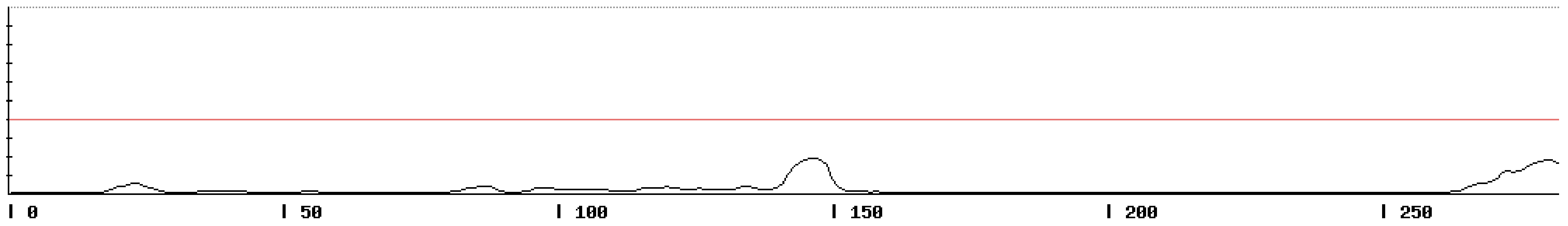

Predictions for Camelinasativa_Csa15g019650.1

Viterbi Path

Viterbi Path

Posterior @ 0.4

Posterior @ 0.4

Posterior Graphic. Threshold Marked @ 0.4

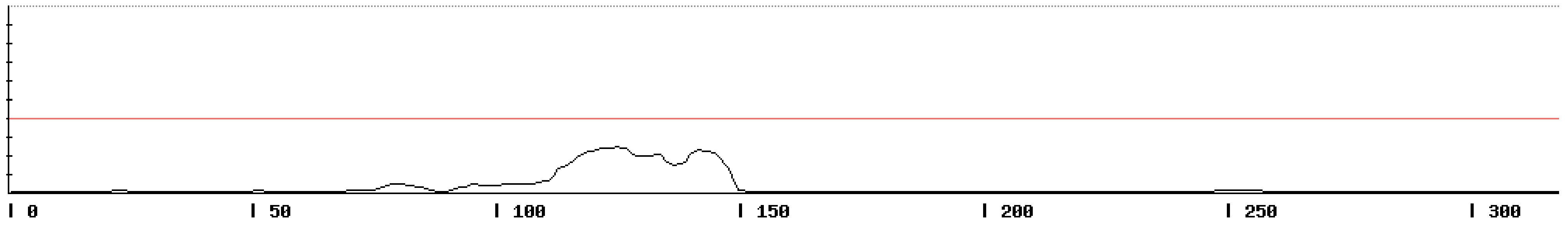

Predictions for Camelinasativa_Csa15g020040.1

Viterbi Path

Viterbi Path

Posterior @ 0.4

Posterior @ 0.4

Posterior Graphic. Threshold Marked @ 0.4

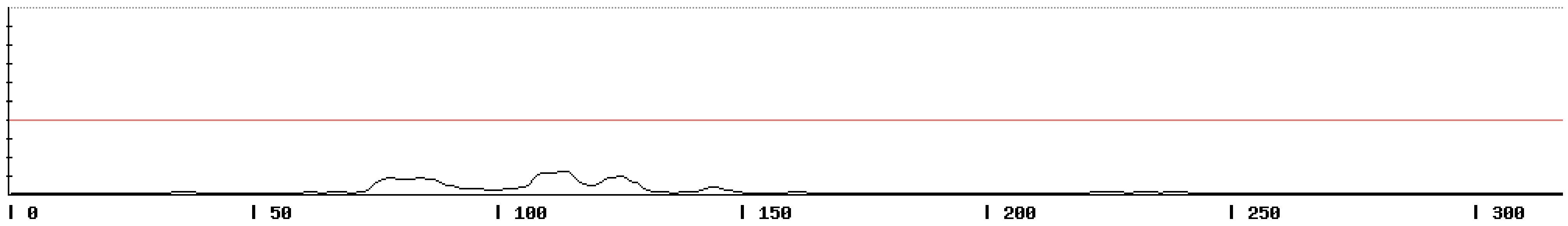

Predictions for Camelinasativa_Csa15g020050.1

Viterbi Path

Viterbi Path

Posterior @ 0.4

Posterior @ 0.4

Posterior Graphic. Threshold Marked @ 0.4

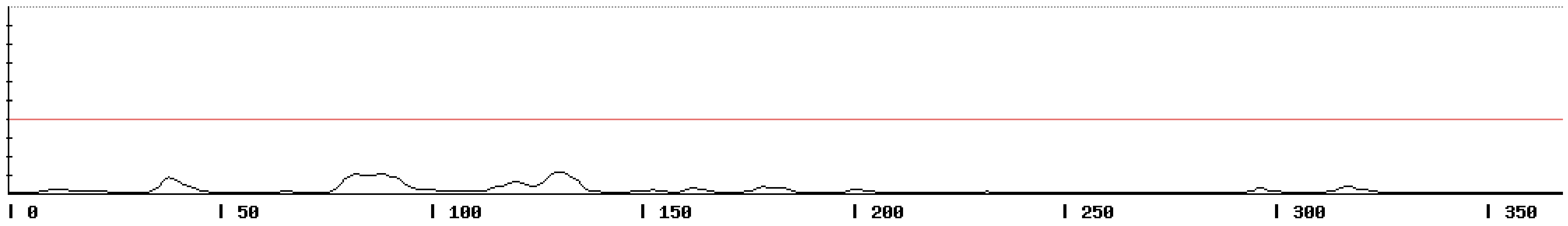

Predictions for Camelinasativa_Csa15g023980.1

Viterbi Path

Viterbi Path

Posterior @ 0.4

Posterior @ 0.4

Posterior Graphic. Threshold Marked @ 0.4

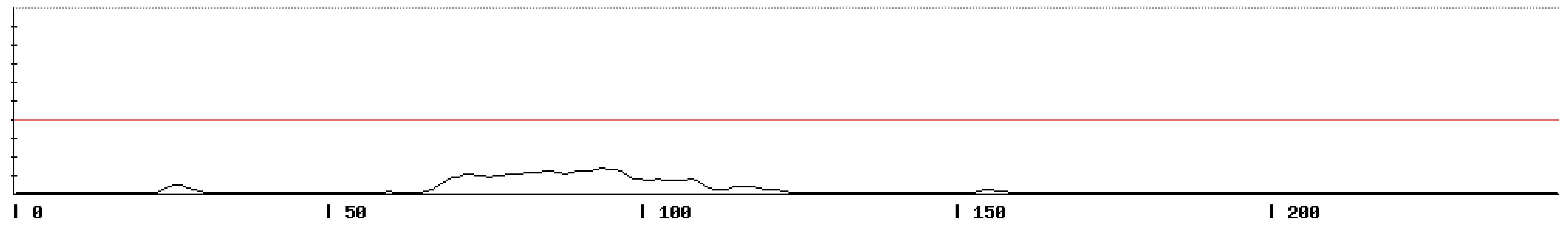

Predictions for Camelinasativa_Csa15g024970.1

Viterbi Path

Viterbi Path

Posterior @ 0.4 175 - KRARQRRKHK - 184

Posterior @ 0.4

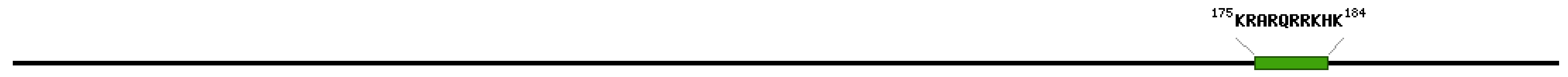

Posterior Graphic. Threshold Marked @ 0.4

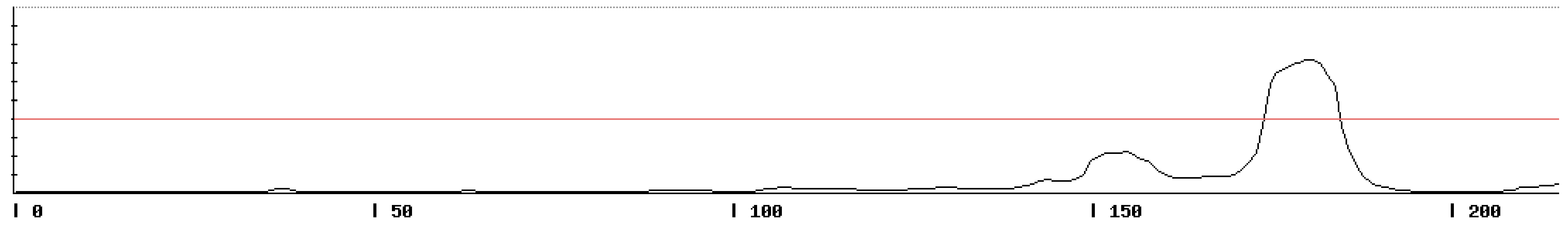

Predictions for Camelinasativa_Csa15g071340.1

Viterbi Path 72 - KRMKAEKKEKLKKGKGVSK - 90

Viterbi Path

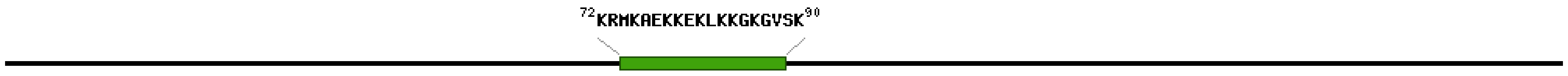

Posterior @ 0.4	71 - VKRMKAEKKEKLKKGKGVSK - 90
Posterior @ 0.4	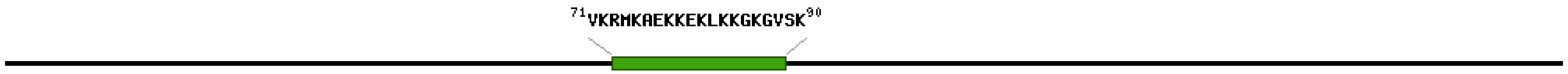
Posterior Graphic. Threshold Marked @ 0.4	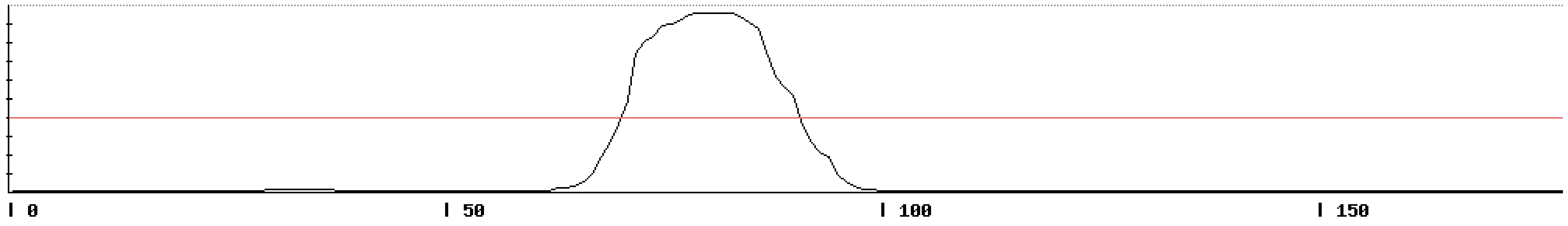

Predictions for Camelinasativa_Csa15g074090.1

Viterbi Path	
Viterbi Path	
Posterior @ 0.4	
Posterior @ 0.4	
Posterior Graphic. Threshold Marked @ 0.4	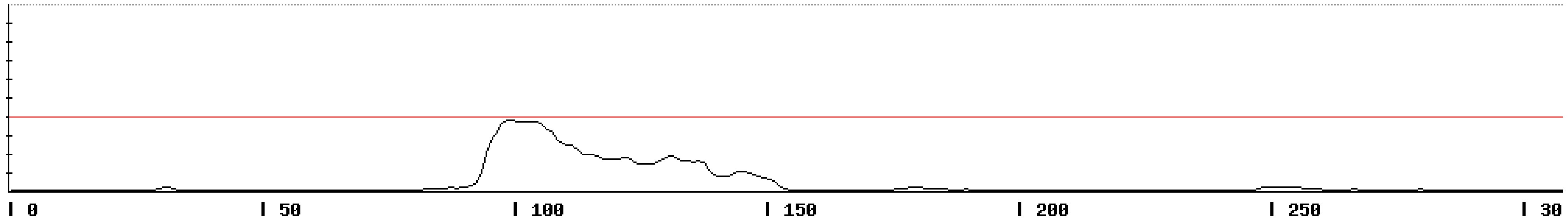

Predictions for Camelinasativa_Csa16729s010.1

Viterbi Path

Viterbi Path

Posterior @ 0.4

Posterior @ 0.4

Posterior Graphic. Threshold Marked @ 0.4

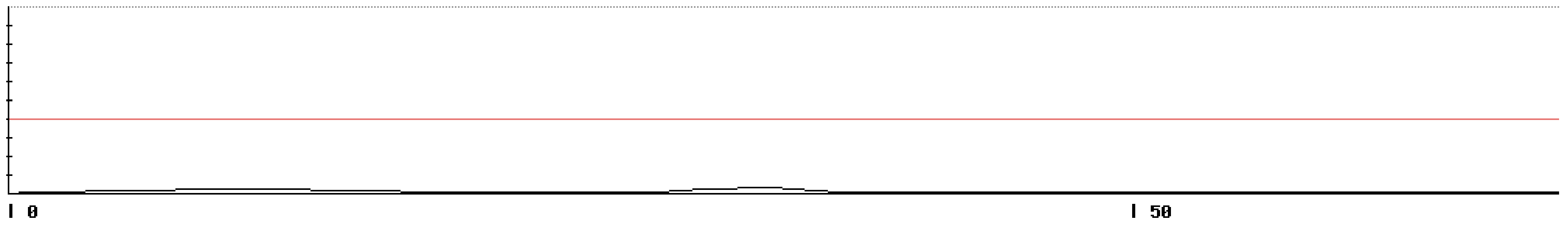

Predictions for Camelinasativa_Csa16g002750.1

Viterbi Path

Viterbi Path

Posterior @ 0.4

Posterior @ 0.4

Posterior Graphic. Threshold Marked @ 0.4

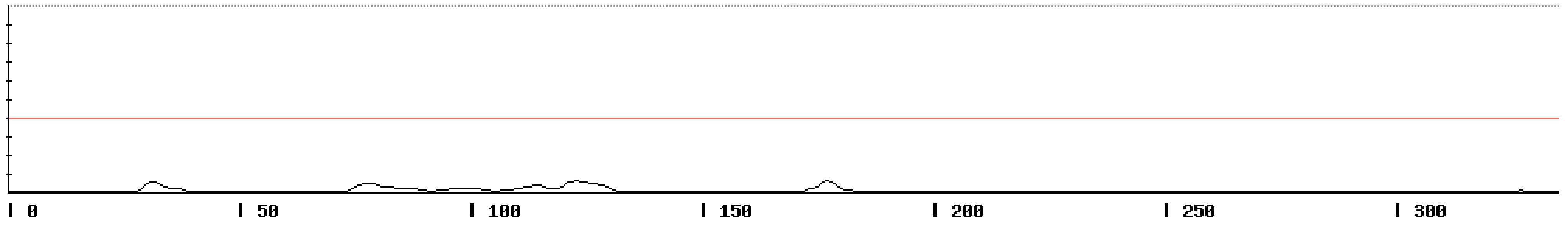

Predictions for Camelinasativa_Csa16g007760.1

Viterbi Path	
Viterbi Path	
Posterior @ 0.4	
Posterior @ 0.4	
Posterior Graphic. Threshold Marked @ 0.4	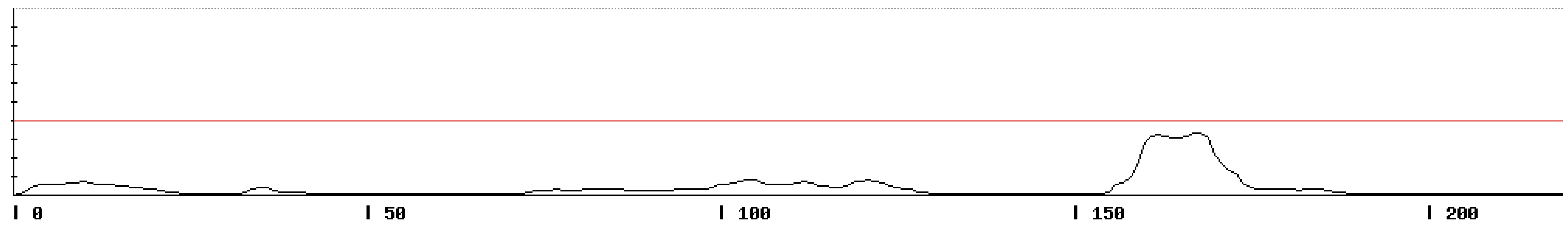

Predictions for Camelinasativa_Csa16g023250.1

Viterbi Path	
Viterbi Path	
Posterior @ 0.4	580 - RRKIWKKEKGR - 590
Posterior @ 0.4	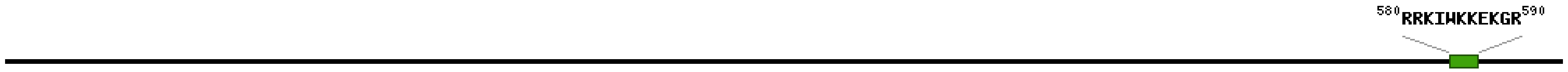
Posterior Graphic. Threshold Marked @ 0.4	

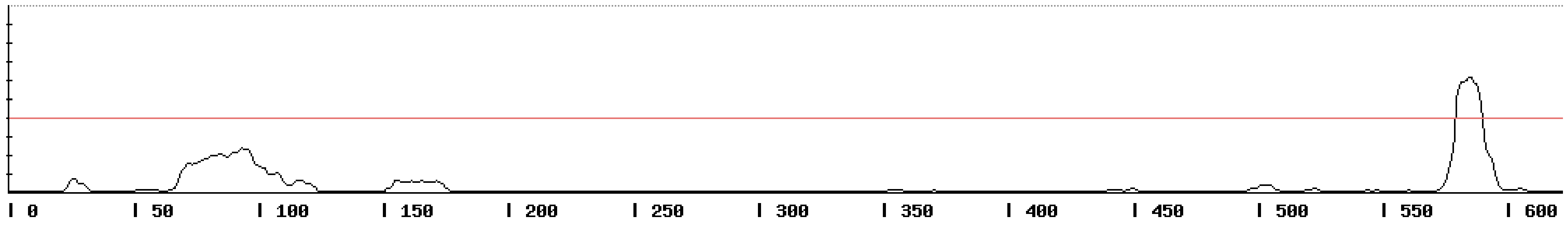

Predictions for Camelinasativa_Csa16g030850.1

Viterbi Path	
Viterbi Path	
Posterior @ 0.4	
Posterior @ 0.4	
Posterior Graphic. Threshold Marked @ 0.4	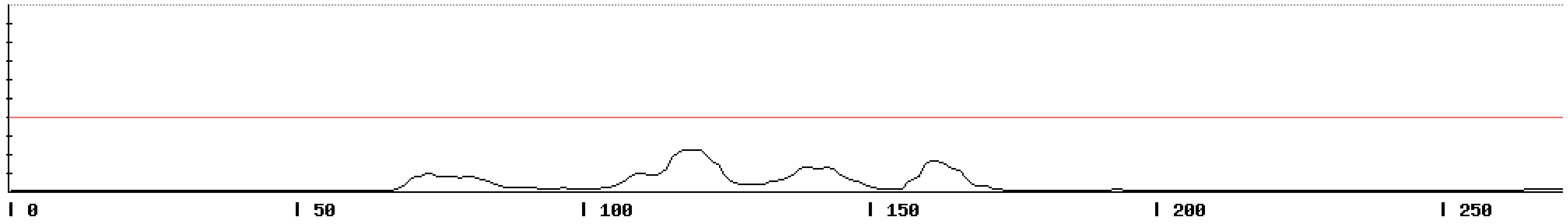

Predictions for Camelinasativa_Csa16g031130.1

Viterbi Path	
Viterbi Path	
Posterior @ 0.4	
Posterior @ 0.4	

Posterior Graphic. Threshold Marked @ 0.4

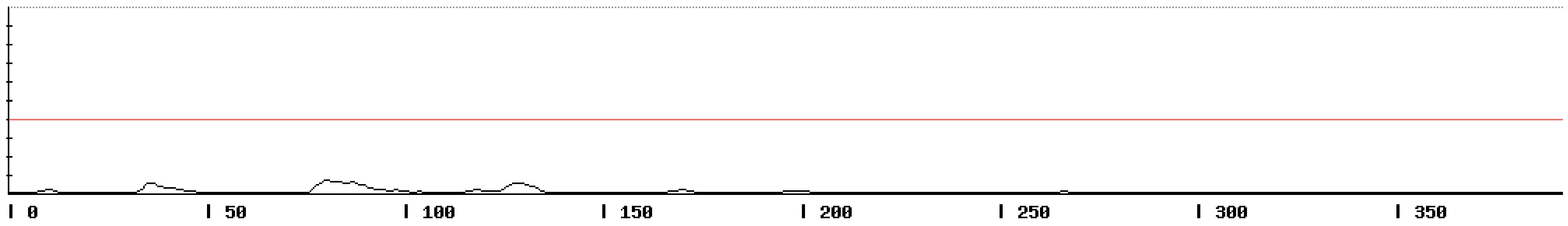

Predictions for Camelinasativa_Csa16g034580.1

Viterbi Path

Viterbi Path

Posterior @ 0.4

Posterior @ 0.4

Posterior Graphic. Threshold Marked @ 0.4

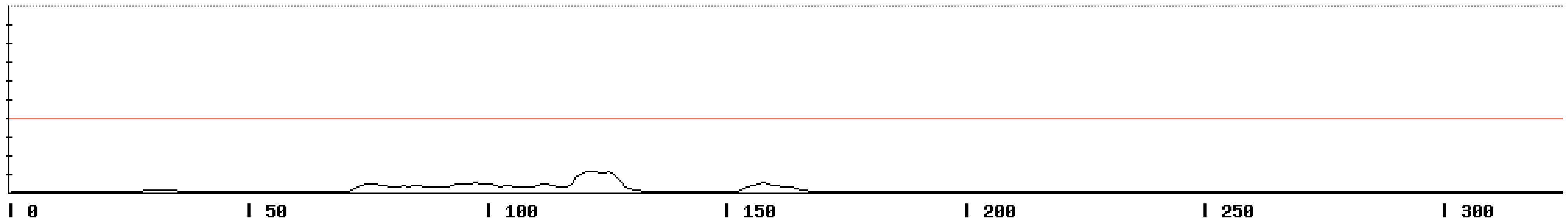

Predictions for Camelinasativa_Csa16g040560.1

Viterbi Path

Viterbi Path

Posterior @ 0.4

Posterior @ 0.4

Posterior Graphic. Threshold Marked @ 0.4

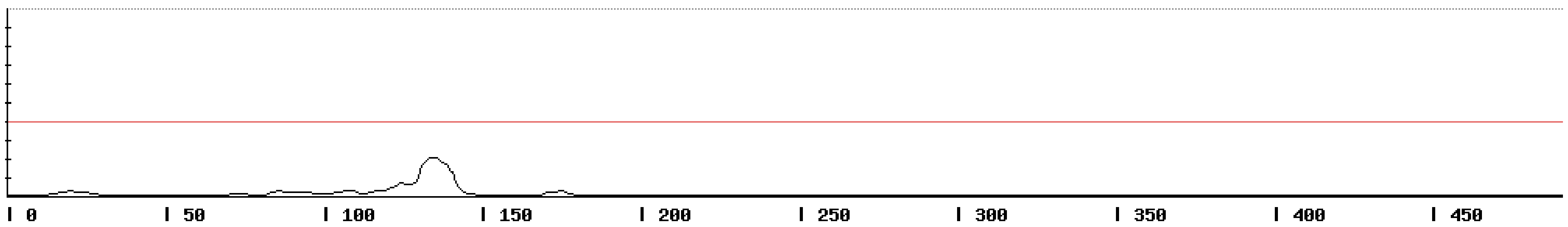

Predictions for Camelinasativa_Csa16g041630.1

Viterbi Path

Viterbi Path

Posterior @ 0.4

98 - KPIGRPKPVGIK - 109

Posterior @ 0.4

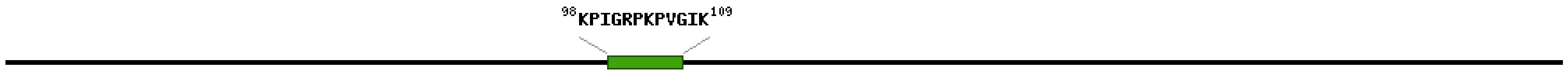

Posterior Graphic. Threshold Marked @ 0.4

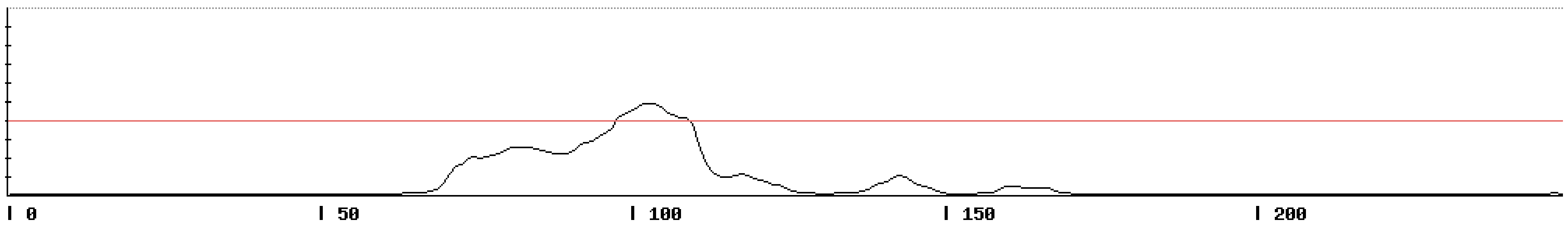

Predictions for Camelinasativa_Csa16g046090.1

Viterbi Path

Viterbi Path

Posterior @ 0.4

Posterior @ 0.4

Posterior Graphic. Threshold Marked @ 0.4

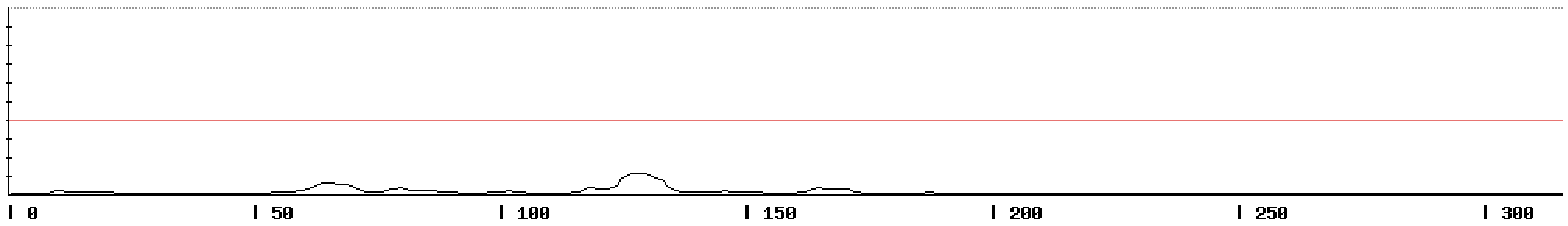

Predictions for Camelinasativa_Csa16g049230.1

Viterbi Path

Viterbi Path

Posterior @ 0.4

Posterior @ 0.4

Posterior Graphic. Threshold Marked @ 0.4

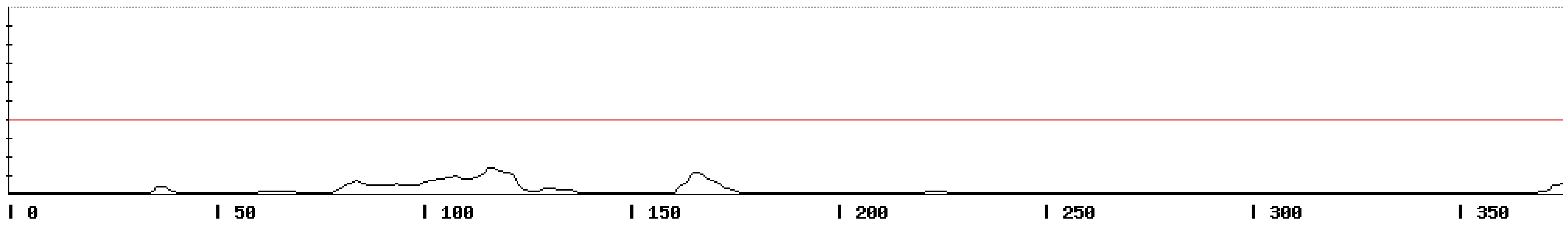

Predictions for Camelinasativa_Csa16g051500.1

Viterbi Path

Viterbi Path

Posterior @ 0.4	
Posterior @ 0.4	
Posterior Graphic. Threshold Marked @ 0.4	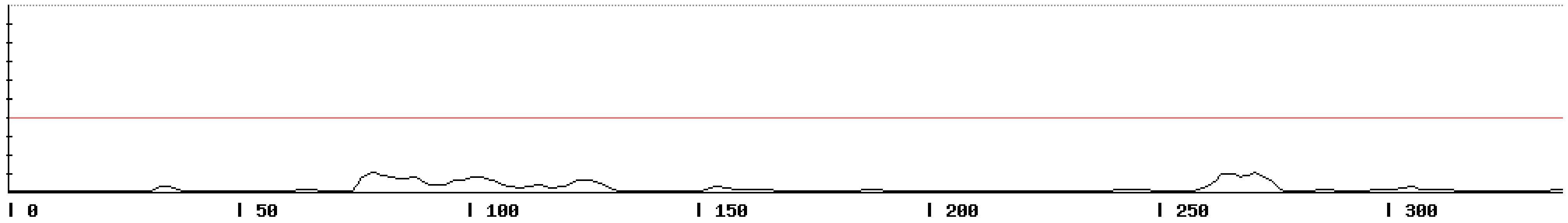

Predictions for Camelinasativa_Csa16g052230.1	
Viterbi Path	
Viterbi Path	
Posterior @ 0.4	116 - KEKPKSMFKFWEKEKRRVRDKR - 137
Posterior @ 0.4	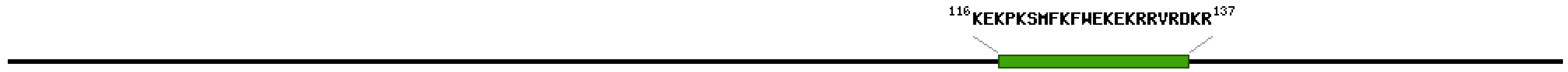
Posterior Graphic. Threshold Marked @ 0.4	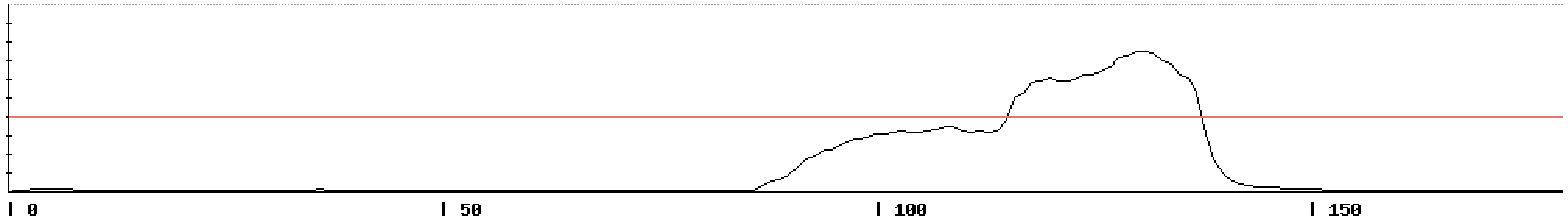

Predictions for Camelinasativa_Csa16g052260.1

Viterbi Path

Viterbi Path

Posterior @ 0.4

568 - KRKPR - 572

Posterior @ 0.4

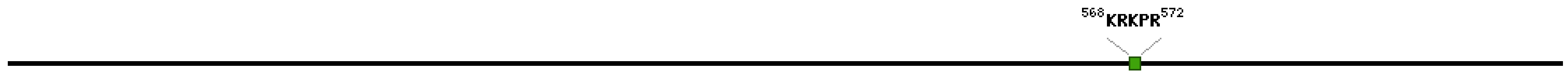

Posterior Graphic. Threshold Marked @ 0.4

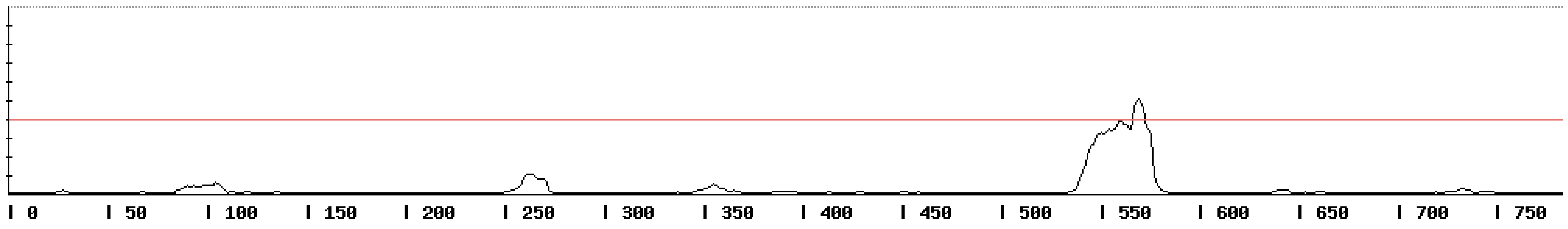

Predictions for Camelinasativa_Csa16g053010.1

Viterbi Path

Viterbi Path

Posterior @ 0.4

Posterior @ 0.4

Posterior Graphic. Threshold Marked @ 0.4

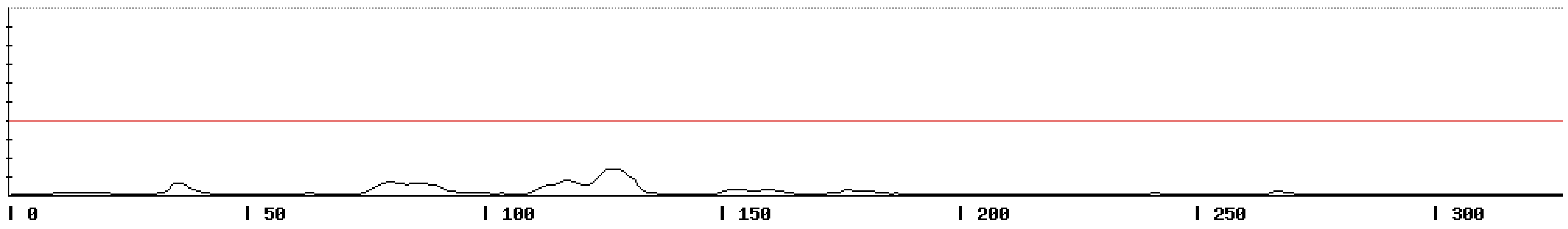

Predictions for Camelinasativa_Csa16g055400.1

Viterbi Path

Viterbi Path

Posterior @ 0.4

Posterior @ 0.4

Posterior Graphic. Threshold Marked @ 0.4

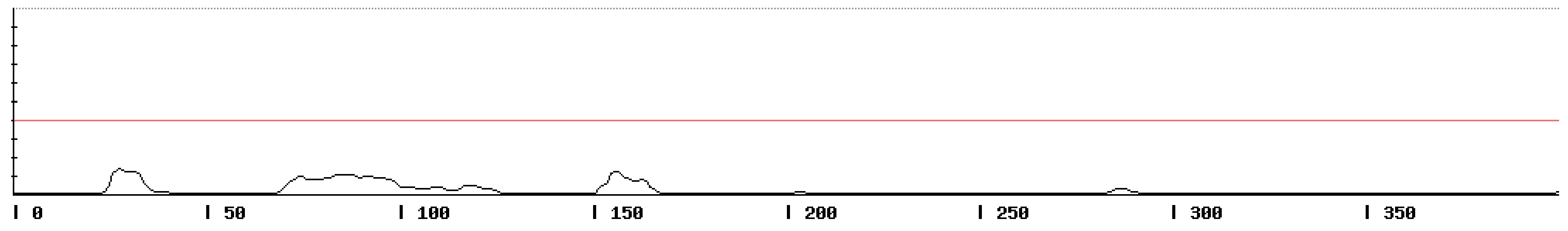

Predictions for Camelinasativa_Csa17g001000.1

Viterbi Path

Viterbi Path

Posterior @ 0.4

81 - RTTKSGVWKKTGKTMSIIRKRRGNNEKIGEKRVLVFKHRG - 120

346 - KKKRA - 350

Posterior @ 0.4

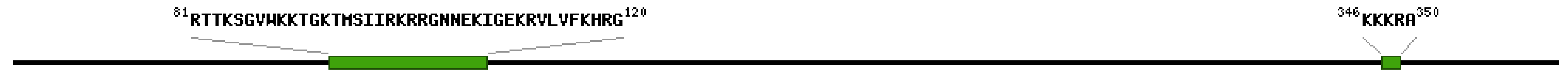

Posterior Graphic. Threshold Marked @ 0.4

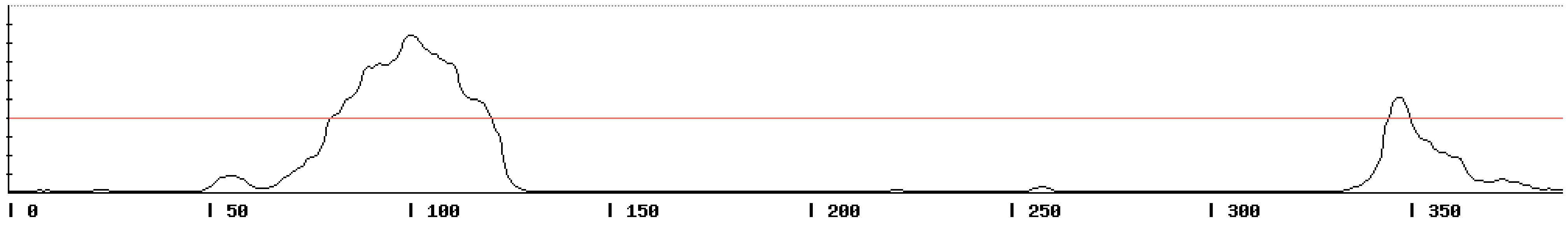

Predictions for Camelinasativa_Csa17g001010.1

Viterbi Path	
Viterbi Path	
Posterior @ 0.4	
Posterior @ 0.4	
Posterior Graphic. Threshold Marked @ 0.4	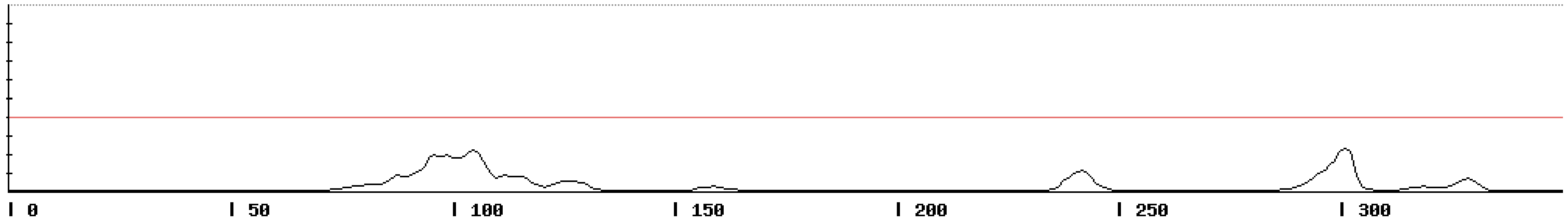

Predictions for Camelinasativa_Csa17g001330.1

Viterbi Path	
Viterbi Path	
Posterior @ 0.4	66 - TLKARKTPNRKV - 77
	684 - RRKRRK - 689
Posterior @ 0.4	

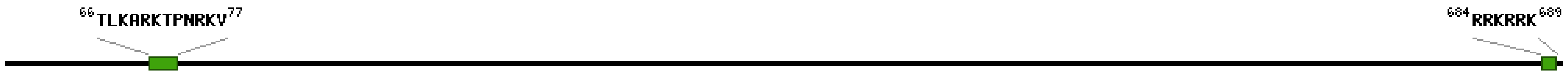

Posterior Graphic. Threshold Marked @ 0.4

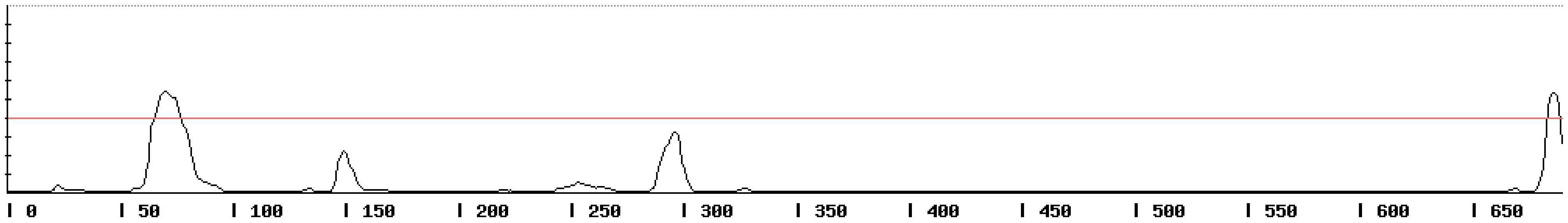

Predictions for Camelinasativa_Csa17g001470.1

Viterbi Path

Viterbi Path

Posterior @ 0.4

Posterior @ 0.4

Posterior Graphic. Threshold Marked @ 0.4

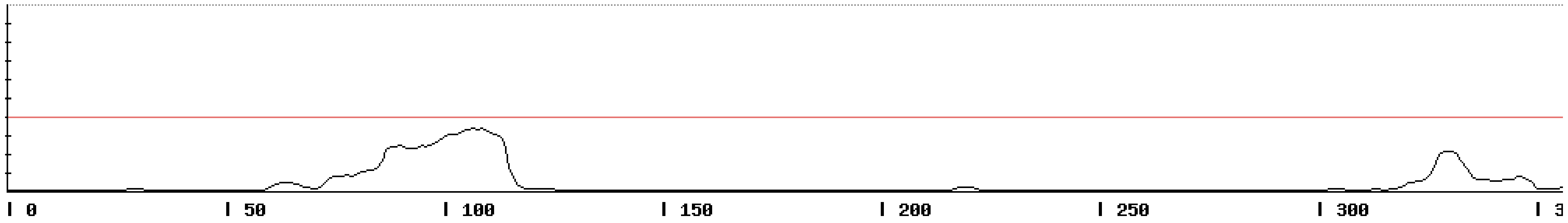

Predictions for Camelinasativa_Csa17g001480.1

Viterbi Path

Viterbi Path

Posterior @ 0.4	1021 - RLKKSKERSKKK - 1032
Posterior @ 0.4	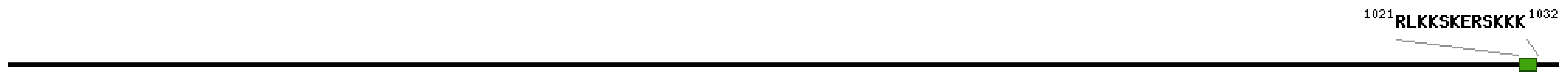
Posterior Graphic. Threshold Marked @ 0.4	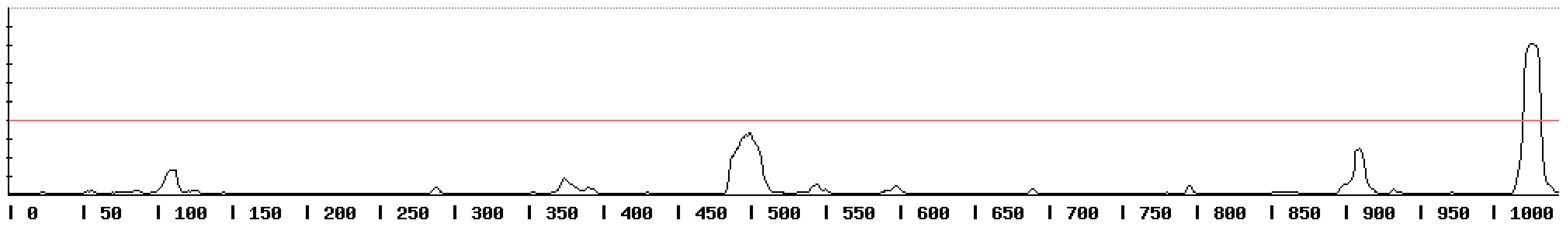

Predictions for Camelinasativa_Csa17g002230.1

Viterbi Path	
Viterbi Path	
Posterior @ 0.4	
Posterior @ 0.4	
Posterior Graphic. Threshold Marked @ 0.4	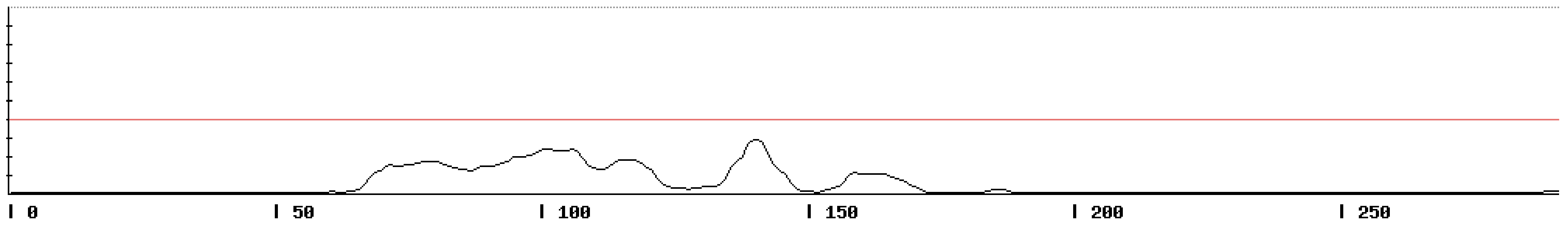

Predictions for Camelinasativa_Csa17g005560.1

Viterbi Path	
Viterbi Path	

Posterior @ 0.4	71 - RDKRSSRSVPGRGGSK - 86
Posterior @ 0.4	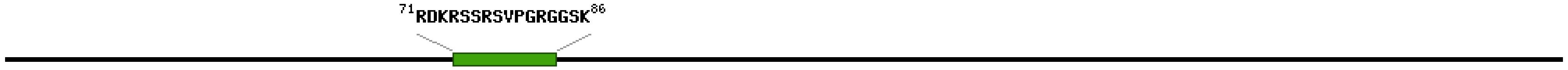
Posterior Graphic. Threshold Marked @ 0.4	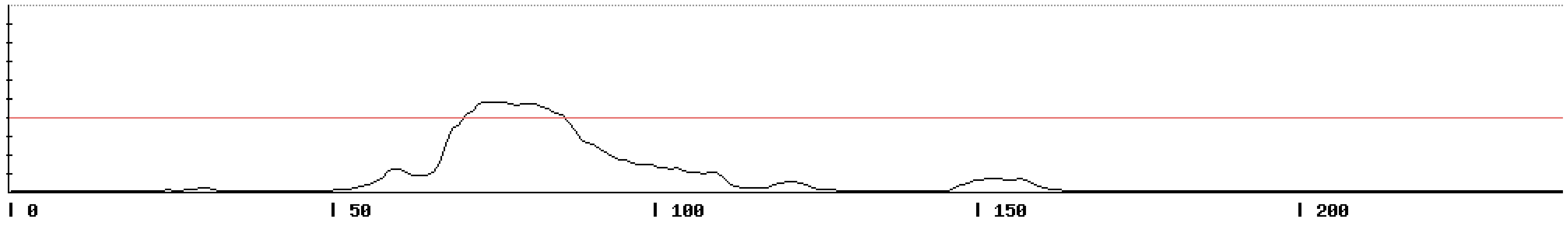

Predictions for Camelinasativa_Csa17g016480.1

Viterbi Path	
Viterbi Path	
Posterior @ 0.4	
Posterior @ 0.4	
Posterior Graphic. Threshold Marked @ 0.4	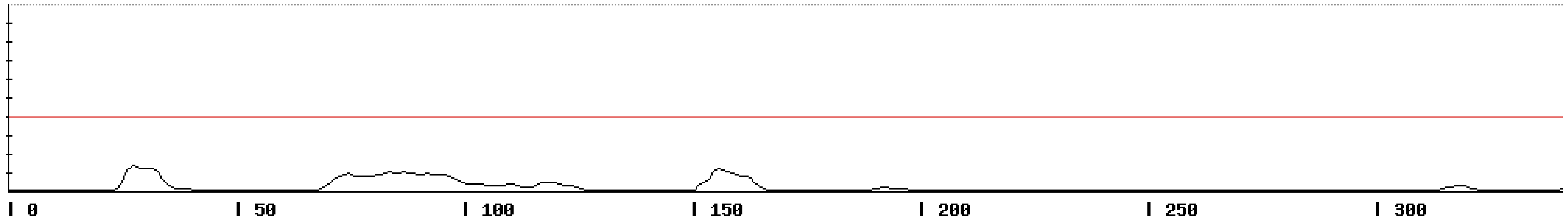

Predictions for Camelinasativa_Csa17g033960.1

Viterbi Path	
Viterbi Path	
Posterior @ 0.4	
Posterior @ 0.4	
Posterior Graphic. Threshold Marked @ 0.4	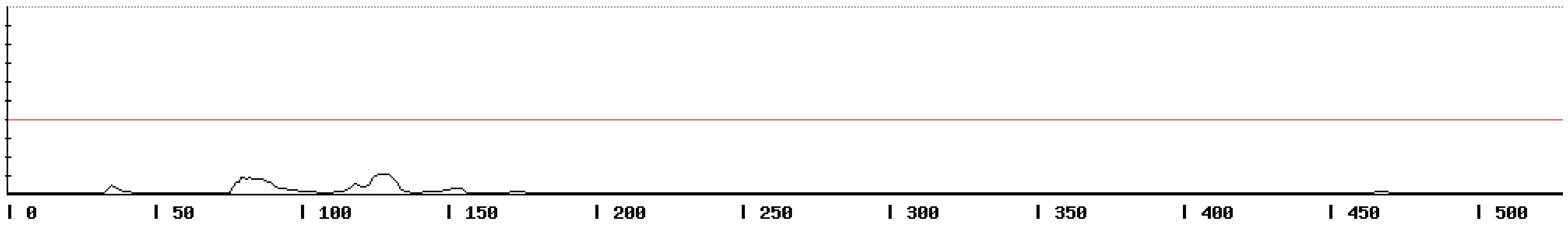

**Predictions for Camelinasativa_Csa17g034890.1**	
Viterbi Path	
Viterbi Path	
Posterior @ 0.4	137 - KRR - 139
Posterior @ 0.4	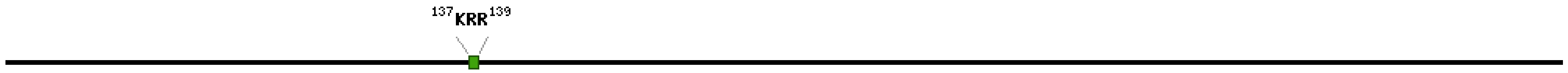
Posterior Graphic. Threshold Marked @ 0.4	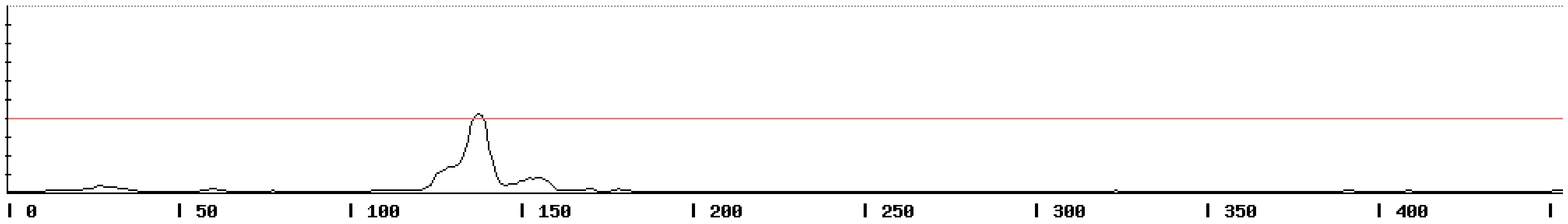

Predictions for Camelinasativa_Csa17g039350.1	
Viterbi Path	
Viterbi Path	
Posterior @ 0.4	94 - KRR - 96
Posterior @ 0.4	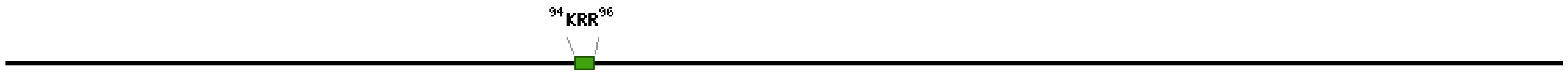
Posterior Graphic. Threshold Marked @ 0.4	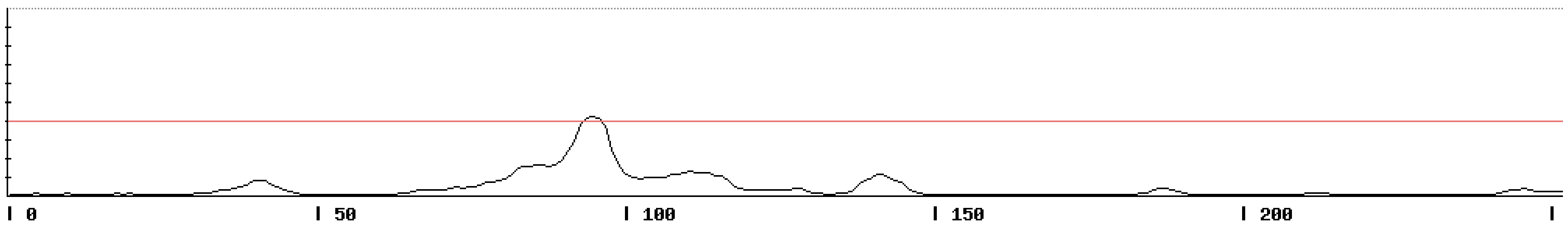

Predictions for Camelinasativa_Csa17g054170.1	
Viterbi Path	
Viterbi Path	
Posterior @ 0.4	
Posterior @ 0.4	
Posterior Graphic. Threshold Marked @ 0.4	

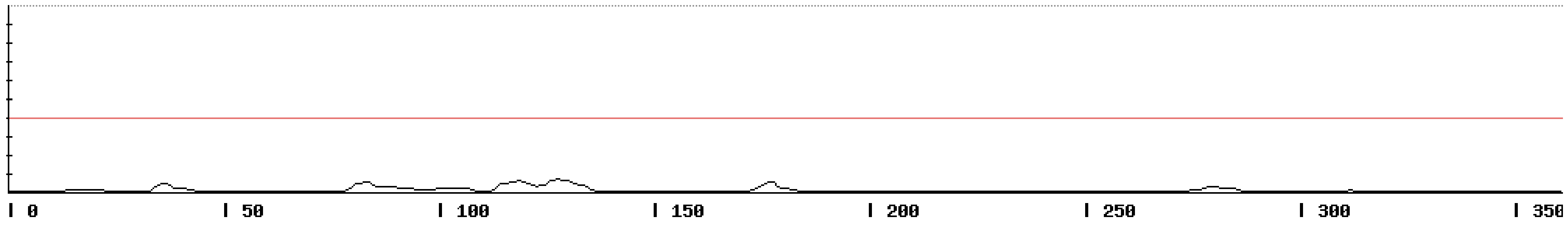

Predictions for Camelinasativa_Csa17g054250.1

Viterbi Path	
Viterbi Path	
Posterior @ 0.4	29 - KRKIRRKKLR - 38
	473 - KRKKSGGNNDKKNKNK - 488
Posterior @ 0.4	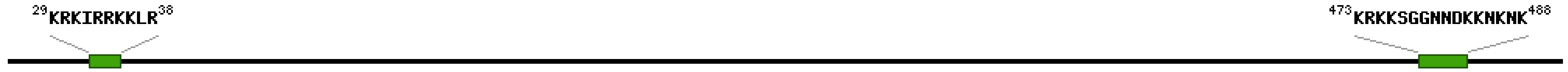
Posterior Graphic. Threshold Marked @ 0.4	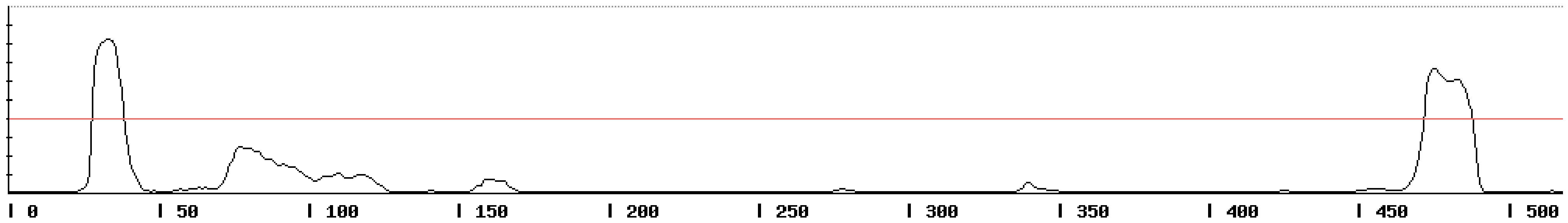

Predictions for Camelinasativa_Csa17g055470.1

Viterbi Path	
Viterbi Path	
Posterior @ 0.4	
Posterior @ 0.4	

Posterior Graphic. Threshold Marked @ 0.4

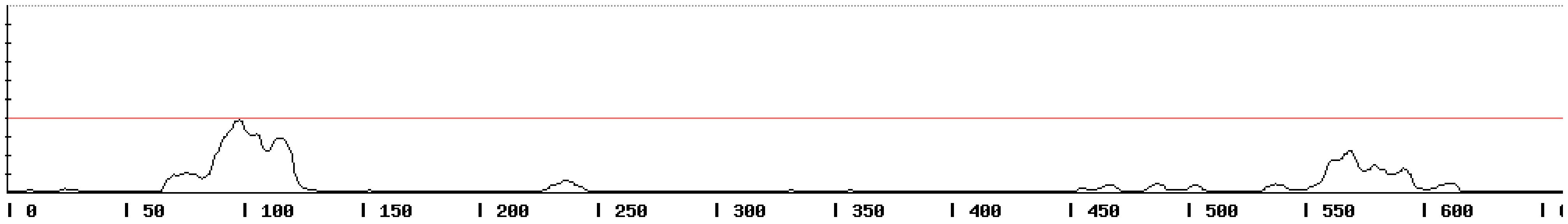

Predictions for Camelinasativa_Csa17g055620.1

Viterbi Path

Viterbi Path

Posterior @ 0.4

Posterior @ 0.4

Posterior Graphic. Threshold Marked @ 0.4

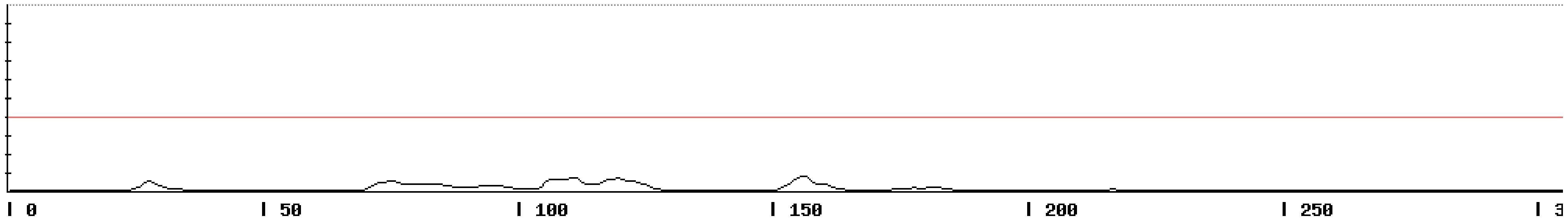

Predictions for Camelinasativa_Csa17g057040.1

Viterbi Path

Viterbi Path

Posterior @ 0.4	
Posterior @ 0.4	
Posterior Graphic. Threshold Marked @ 0.4	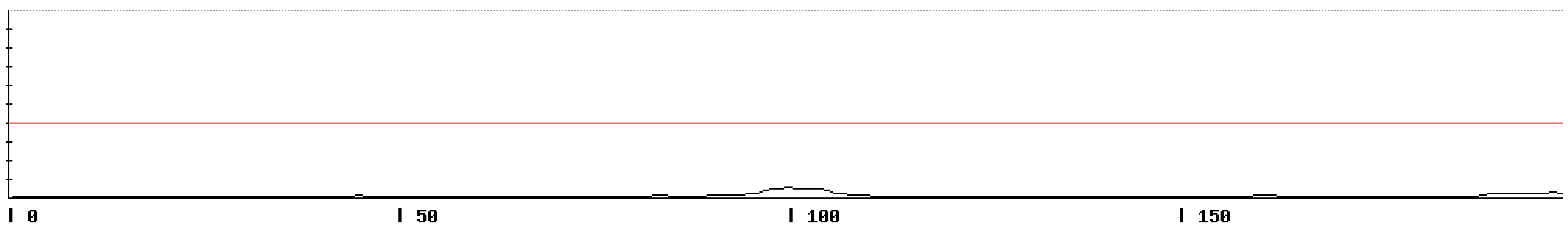

Predictions for Camelinasativa_Csa17g059460.1

Viterbi Path	
Viterbi Path	
Posterior @ 0.4	
Posterior @ 0.4	
Posterior Graphic. Threshold Marked @ 0.4	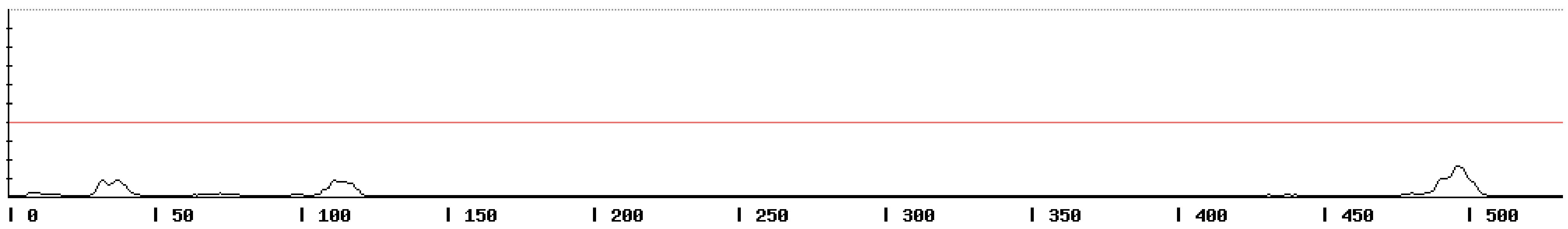

Predictions for Camelinasativa_Csa17g059480.1

Viterbi Path	
Viterbi Path	

Posterior @ 0.4

Posterior @ 0.4

Posterior Graphic. Threshold Marked @ 0.4

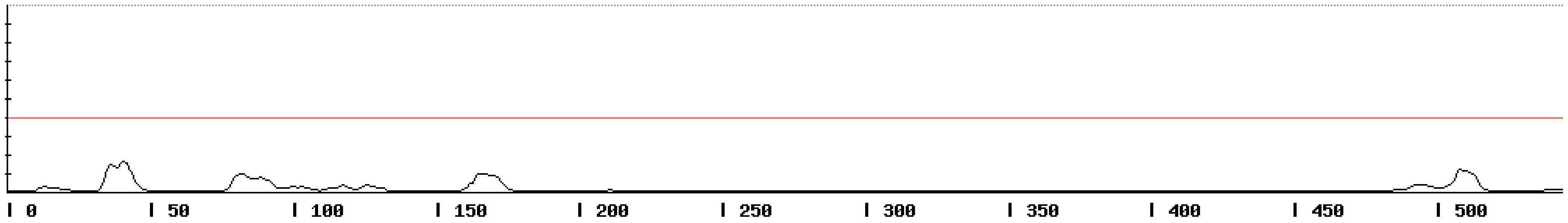

Predictions for Camelinasativa_Csa17g062260.1

Viterbi Path

Viterbi Path

Posterior @ 0.4 — 109 - SKRNKMGKDGKRQKRDAKG - 127

Posterior @ 0.4

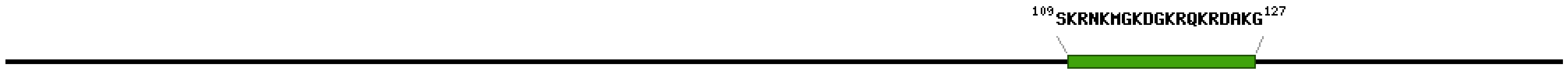

Posterior Graphic. Threshold Marked @ 0.4

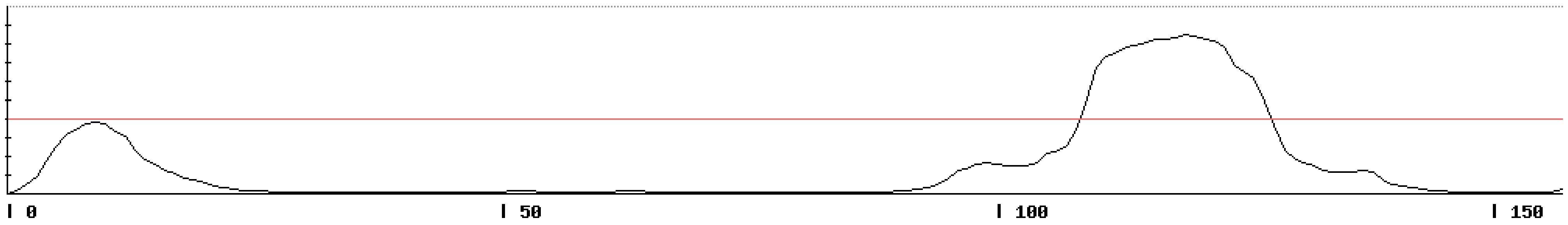

Predictions for Camelinasativa_Csa17g093060.1

Viterbi Path	22 - KKKKKKK - 28
Viterbi Path	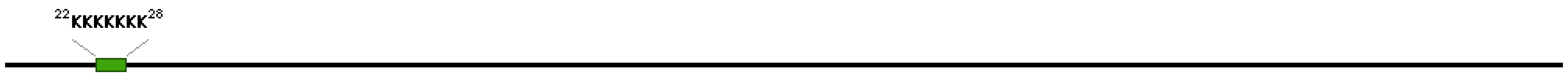
Posterior @ 0.4	21 - SKKKKKKKESL - 31
Posterior @ 0.4	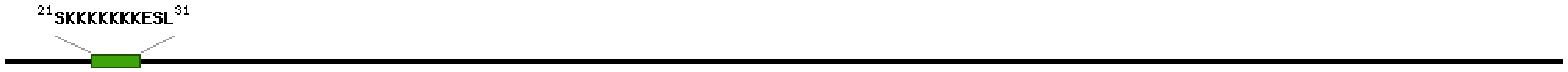
Posterior Graphic. Threshold Marked @ 0.4	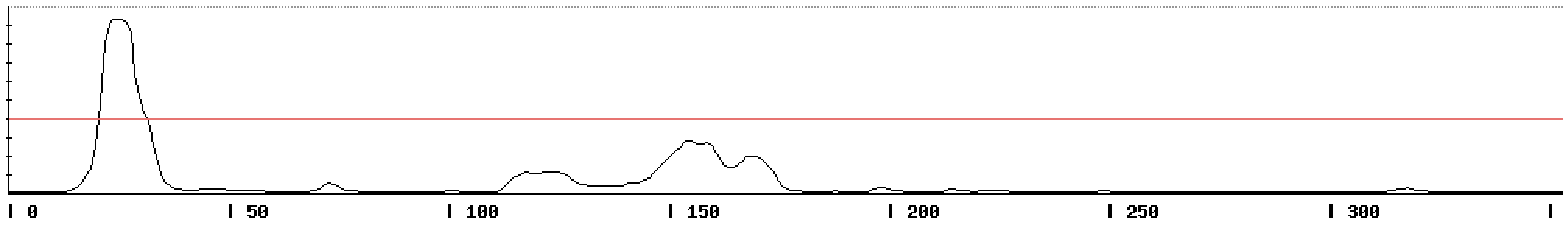

Predictions for Camelinasativa_Csa17g093080.1

Viterbi Path	
Viterbi Path	
Posterior @ 0.4	
Posterior @ 0.4	
Posterior Graphic. Threshold Marked @ 0.4	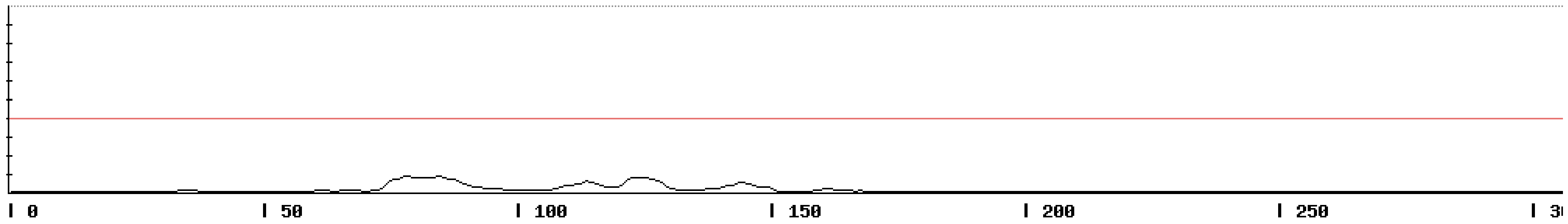

Predictions for Camelinasativa_Csa17g095950.1	
Viterbi Path	
Viterbi Path	
Posterior @ 0.4	
Posterior @ 0.4	
Posterior Graphic. Threshold Marked @ 0.4	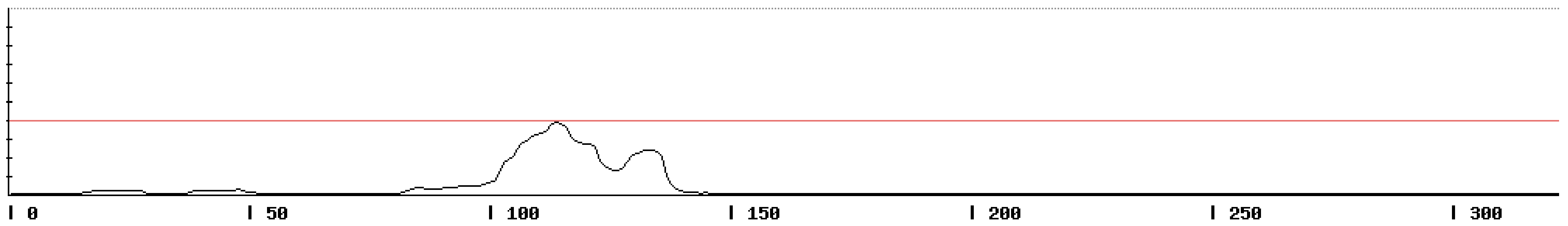

Predictions for Camelinasativa_Csa17g097090.1	
Viterbi Path	
Viterbi Path	
Posterior @ 0.4	
Posterior @ 0.4	
Posterior Graphic. Threshold Marked @ 0.4	

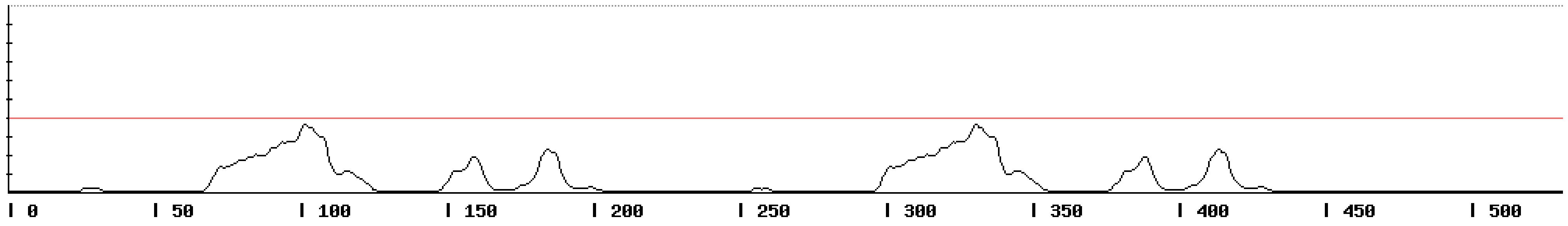

Predictions for Camelinasativa_Csa18g003450.1	
Viterbi Path	
Viterbi Path	
Posterior @ 0.4	
Posterior @ 0.4	
Posterior Graphic. Threshold Marked @ 0.4	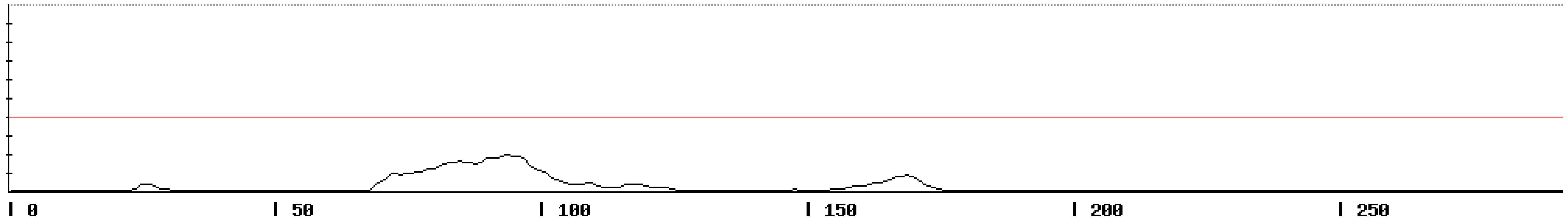

Predictions for Camelinasativa_Csa18g021500.1	
Viterbi Path	
Viterbi Path	
Posterior @ 0.4	
Posterior @ 0.4	

Posterior Graphic. Threshold Marked @ 0.4

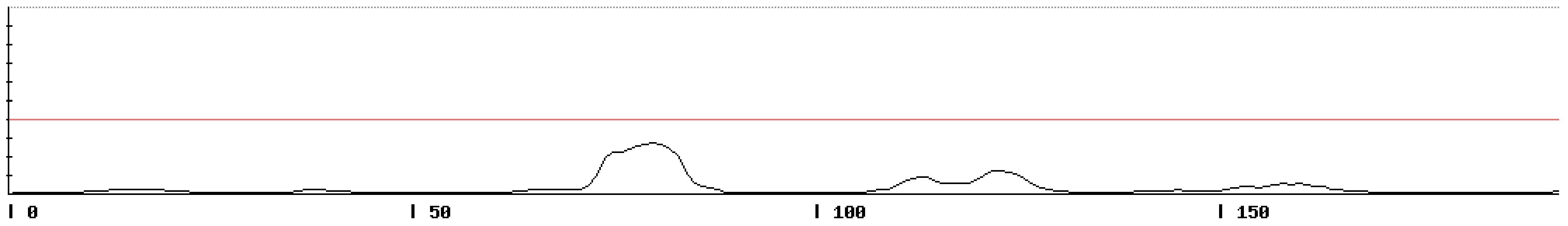

Predictions for Camelinasativa_Csa18g025220.1

Viterbi Path

Viterbi Path

Posterior @ 0.4

Posterior @ 0.4

Posterior Graphic. Threshold Marked @ 0.4

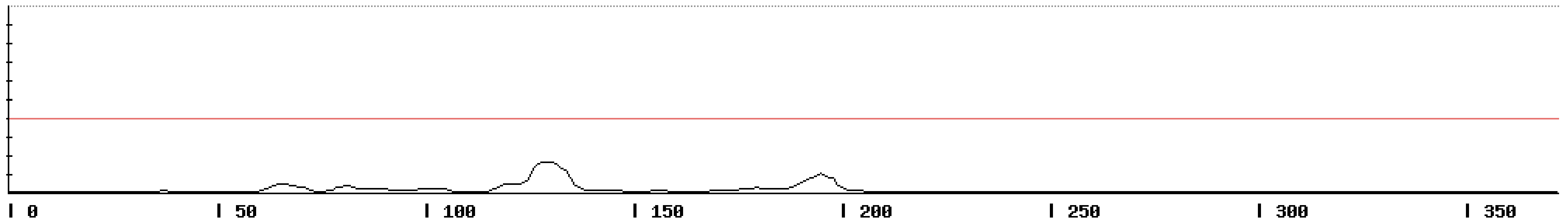

Predictions for Camelinasativa_Csa18g029530.1

Viterbi Path

Viterbi Path

Posterior @ 0.4

Posterior @ 0.4

Posterior Graphic. Threshold Marked @ 0.4

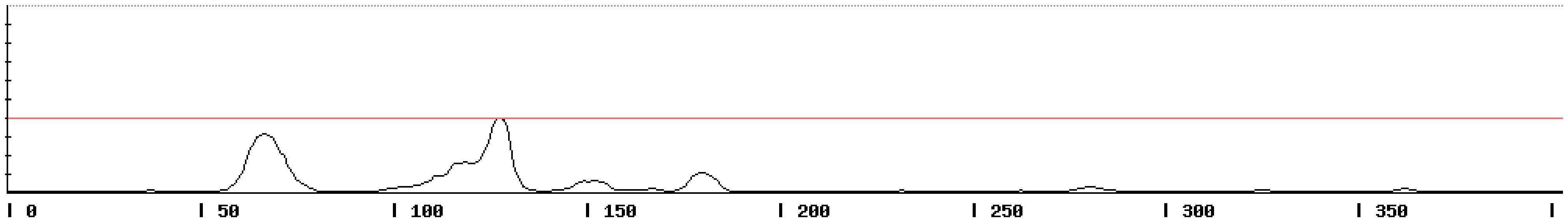

Predictions for Camelinasativa_Csa18g032320.1

Viterbi Path: 171 - KIHKKKWSEKKKEKEKENDHHVEDKLDQELVVPRKIHKKRNSKKKKEK - 218

Viterbi Path

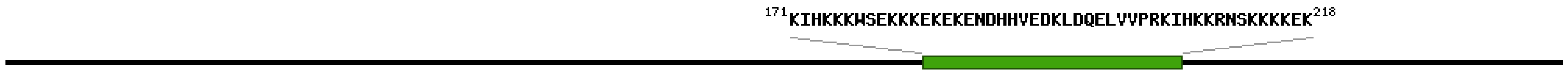

Posterior @ 0.4:

128 - KNKNKKQKR - 136

172 - IHKKKWSEKKKEKEKE - 187

203 - PRKIHKKRNSKKKKEKD - 219

Posterior @ 0.4

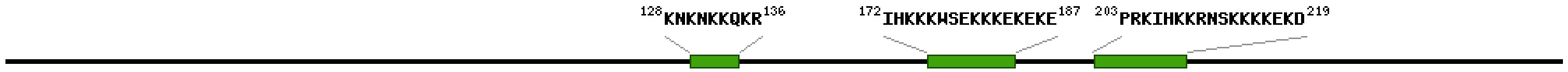

Posterior Graphic. Threshold Marked @ 0.4

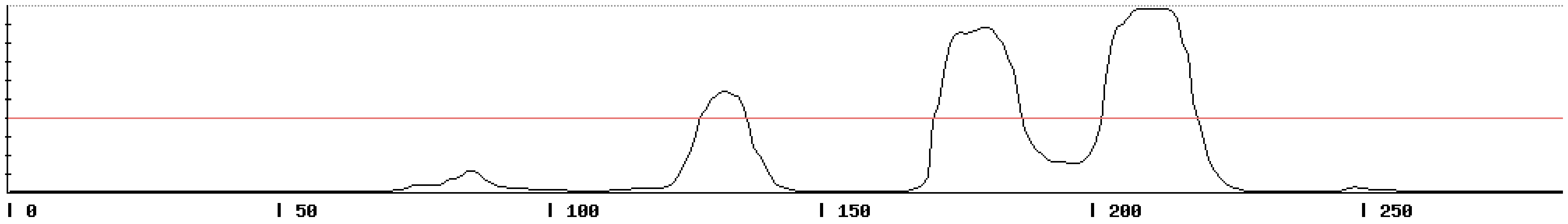

Predictions for Camelinasativa_Csa18g032550.1

Viterbi Path:

178 - KIHKKKWSEKKEKKKEEKKKEK - 199

218 - KIHKKRNSKKKKEK - 231

Viterbi Path	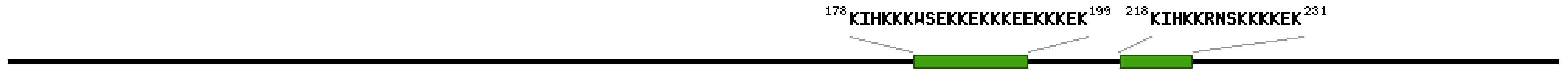
Posterior @ 0.4	132 - NKNKKQKR - 139
	178 - KIHKKKWSEKKEKKKEEKKKEKE - 200
	218 - KIHKKRNSKKKKEK - 231
Posterior @ 0.4	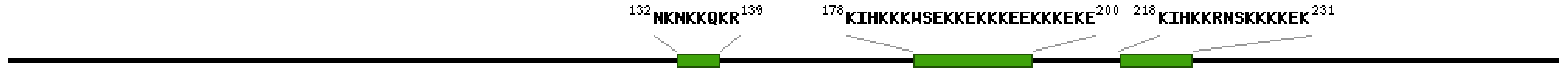
Posterior Graphic. Threshold Marked @ 0.4	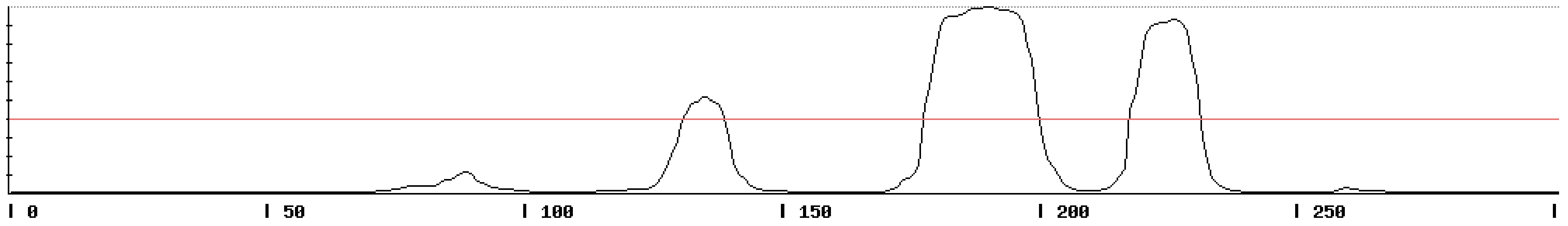

Predictions for Camelinasativa_Csa18g034760.1

Viterbi Path	
Viterbi Path	
Posterior @ 0.4	
Posterior @ 0.4	
Posterior Graphic. Threshold Marked @ 0.4	

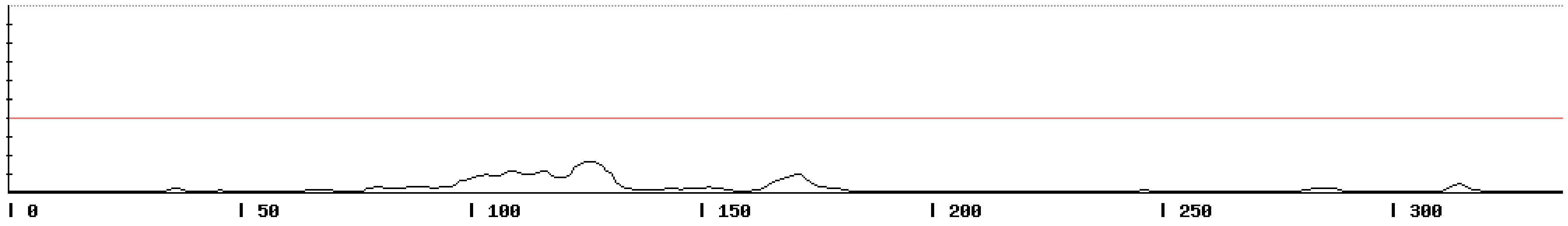

Predictions for Camelinasativa_Csa18g035850.1

Viterbi Path	
Viterbi Path	
Posterior @ 0.4	
Posterior @ 0.4	
Posterior Graphic. Threshold Marked @ 0.4	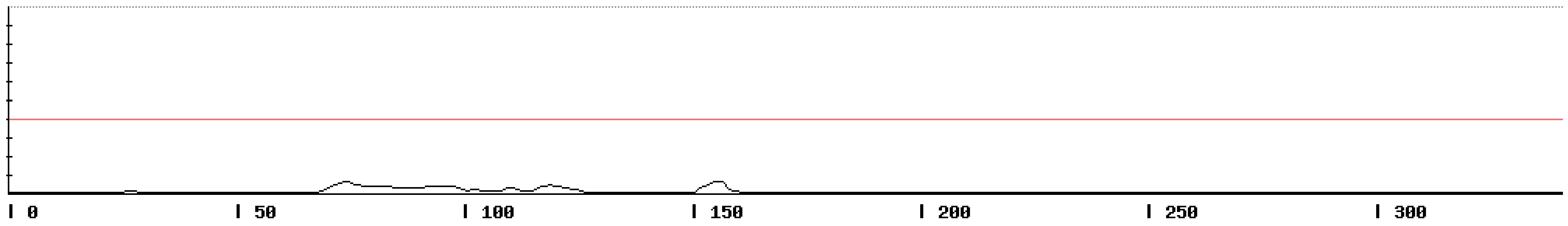

Predictions for Camelinasativa_Csa18g037380.1

Viterbi Path	
Viterbi Path	
Posterior @ 0.4	98 - GKPK - 101
Posterior @ 0.4	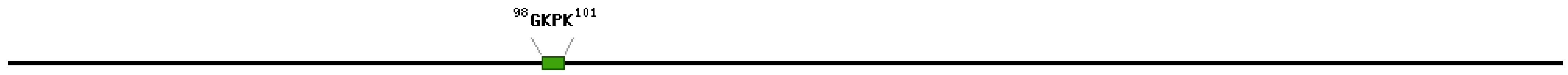

Posterior Graphic. Threshold Marked @ 0.4

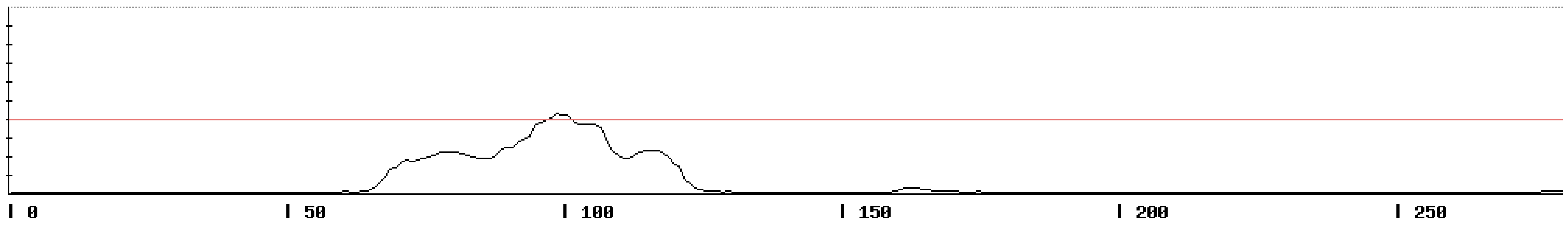

Predictions for Camelinasativa_Csa18g037670.1

Viterbi Path

Viterbi Path

Posterior @ 0.4

Posterior @ 0.4

Posterior Graphic. Threshold Marked @ 0.4

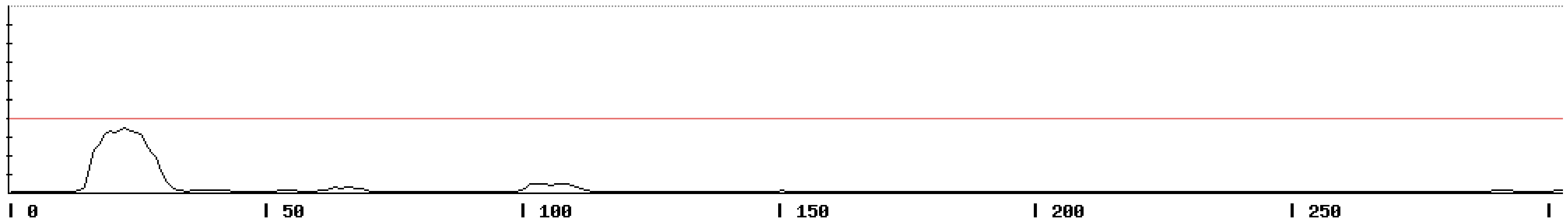

Predictions for Camelinasativa_Csa18g038180.1

Viterbi Path

Viterbi Path

Posterior @ 0.4

Posterior @ 0.4

Posterior Graphic. Threshold Marked @ 0.4

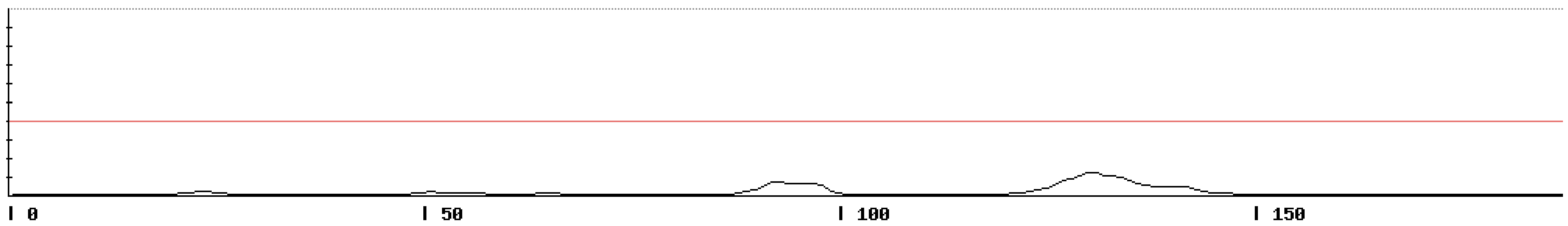

Predictions for Camelinasativa_Csa18g042250.1

Viterbi Path

Viterbi Path

Posterior @ 0.4

Posterior @ 0.4

Posterior Graphic. Threshold Marked @ 0.4

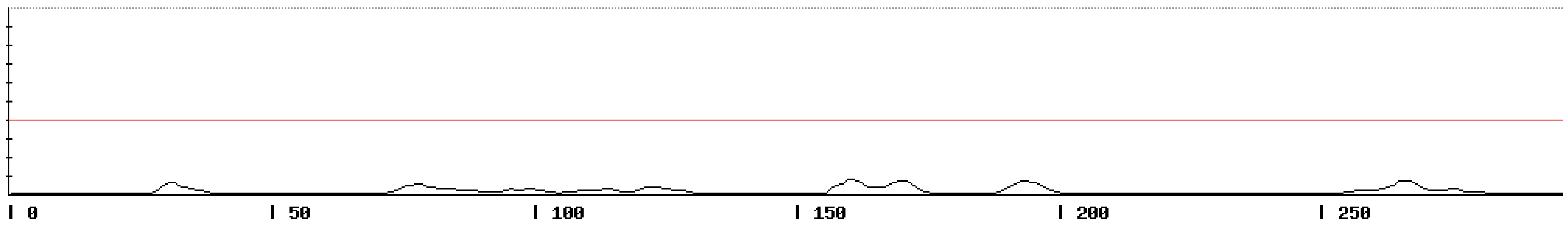

Predictions for Camelinasativa_Csa19g001330.1

Viterbi Path

Viterbi Path

Posterior @ 0.4

Posterior @ 0.4

Posterior Graphic. Threshold Marked @ 0.4

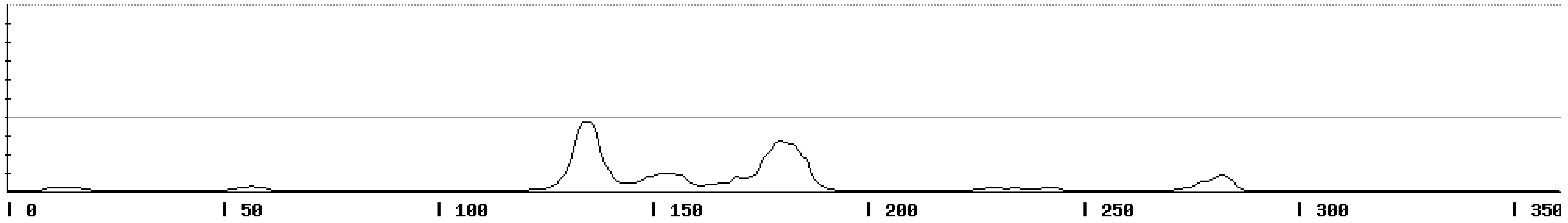

Predictions for Camelinasativa_Csa19g005320.1

Viterbi Path

Viterbi Path

Posterior @ 0.4

Posterior @ 0.4

Posterior Graphic. Threshold Marked @ 0.4

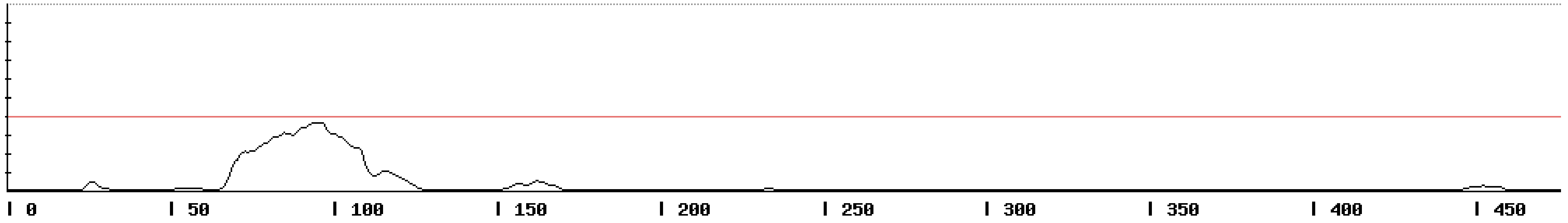

Predictions for Camelinasativa_Csa19g006270.1

Viterbi Path

Viterbi Path

Posterior @ 0.4	
Posterior @ 0.4	
Posterior Graphic. Threshold Marked @ 0.4	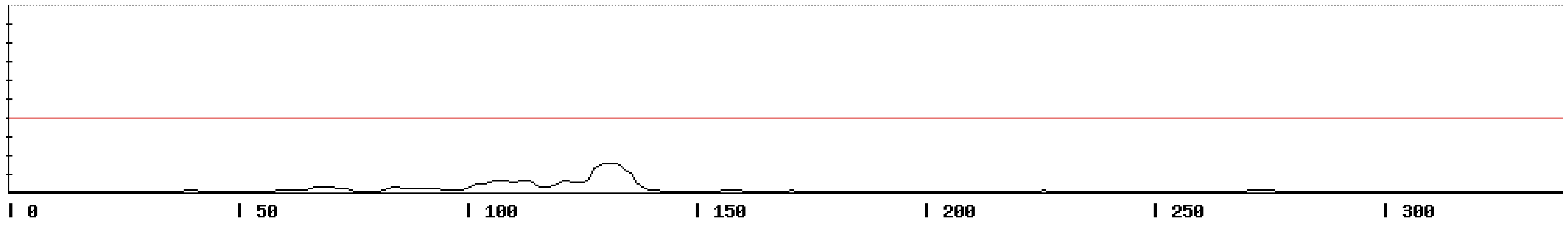

Predictions for Camelinasativa_Csa19g006280.1

Viterbi Path	
Viterbi Path	
Posterior @ 0.4	
Posterior @ 0.4	
Posterior Graphic. Threshold Marked @ 0.4	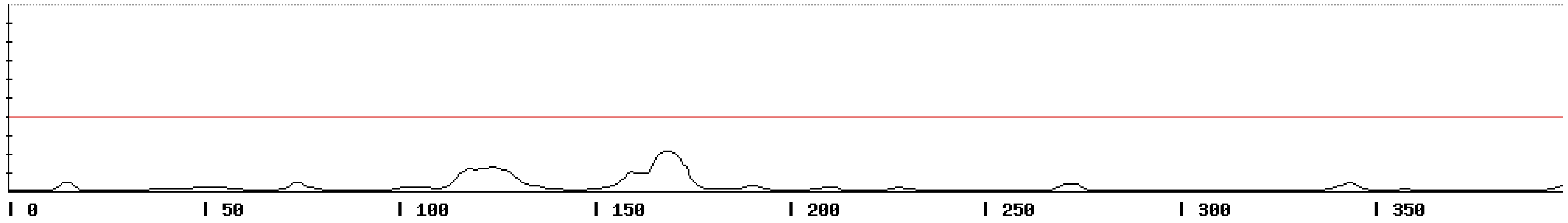

Predictions for Camelinasativa_Csa19g014110.1

Viterbi Path

Viterbi Path

Posterior @ 0.4

Posterior @ 0.4

Posterior Graphic. Threshold Marked @ 0.4

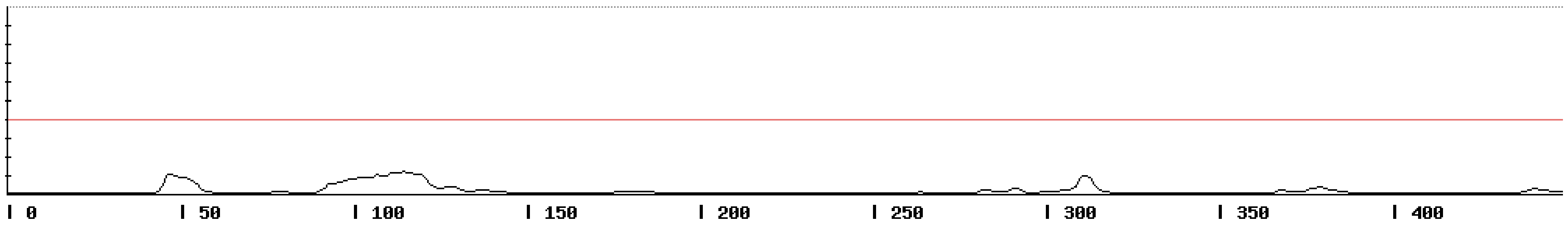

Predictions for Camelinasativa_Csa19g014120.1

Viterbi Path

Viterbi Path

Posterior @ 0.4

Posterior @ 0.4

Posterior Graphic. Threshold Marked @ 0.4

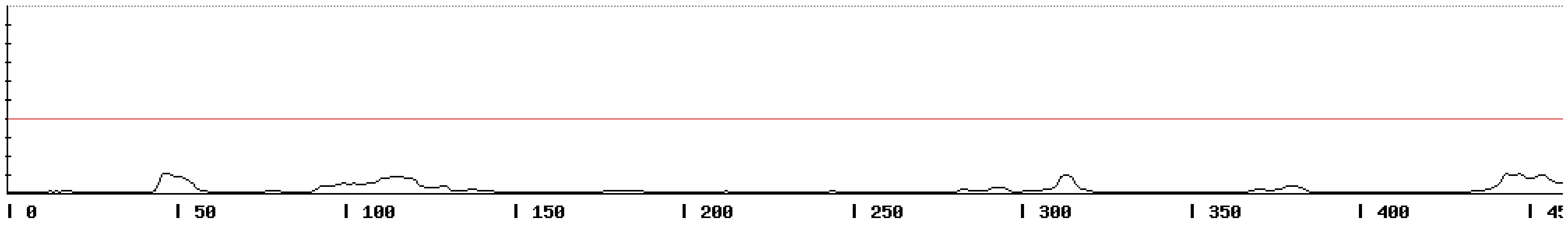

Predictions for Camelinasativa_Csa19g014130.1

Viterbi Path

Viterbi Path

Posterior @ 0.4

Posterior @ 0.4

Posterior Graphic. Threshold Marked @ 0.4

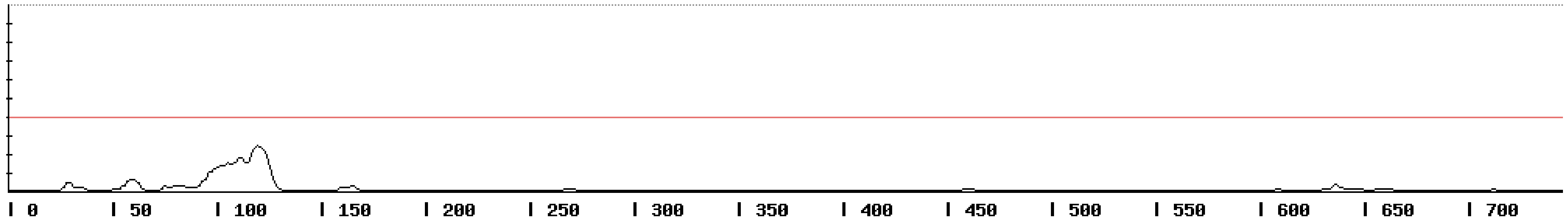

Predictions for Camelinasativa_Csa19g017110.1

Viterbi Path

Viterbi Path

Posterior @ 0.4

Posterior @ 0.4

Posterior Graphic. Threshold Marked @ 0.4

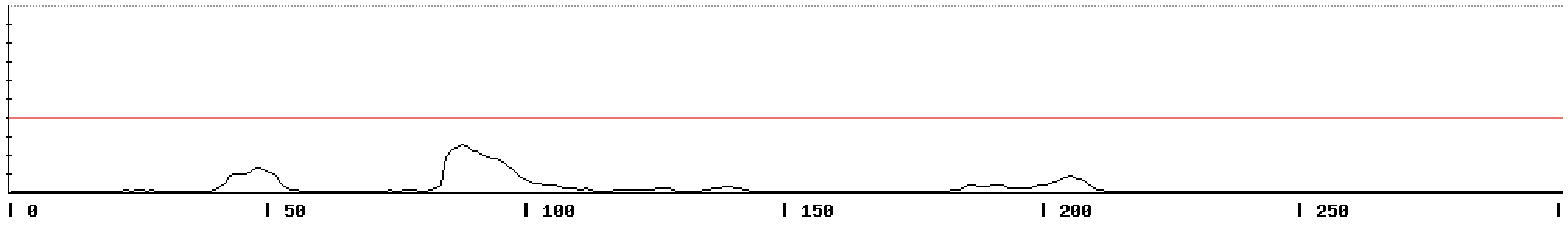

Predictions for Camelinasativa_Csa19g017210.1	
Viterbi Path	
Viterbi Path	
Posterior @ 0.4	
Posterior @ 0.4	
Posterior Graphic. Threshold Marked @ 0.4	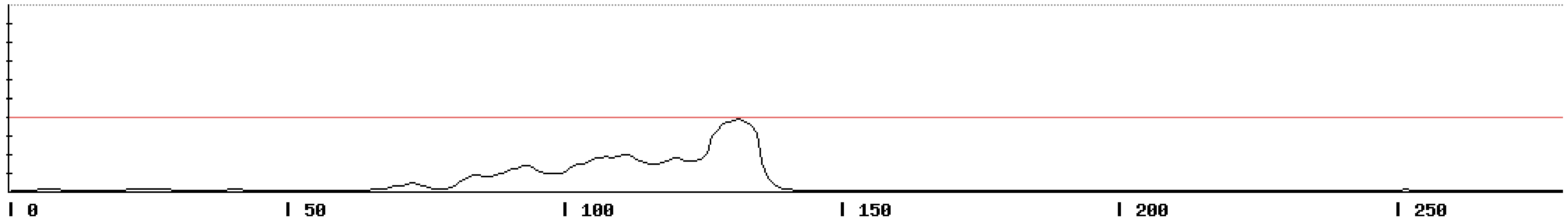

Predictions for Camelinasativa_Csa19g021860.1	
Viterbi Path	
Viterbi Path	
Posterior @ 0.4	
Posterior @ 0.4	

Posterior Graphic. Threshold Marked @ 0.4

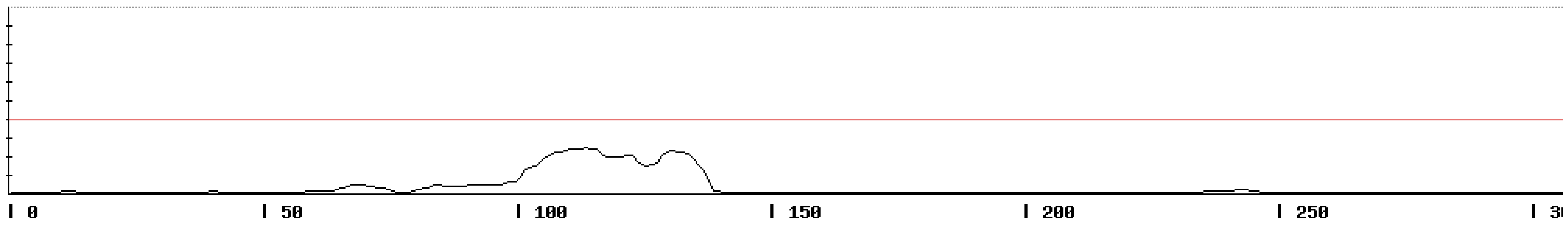

Predictions for Camelinasativa_Csa19g022270.1

Viterbi Path

Viterbi Path

Posterior @ 0.4

Posterior @ 0.4

Posterior Graphic. Threshold Marked @ 0.4

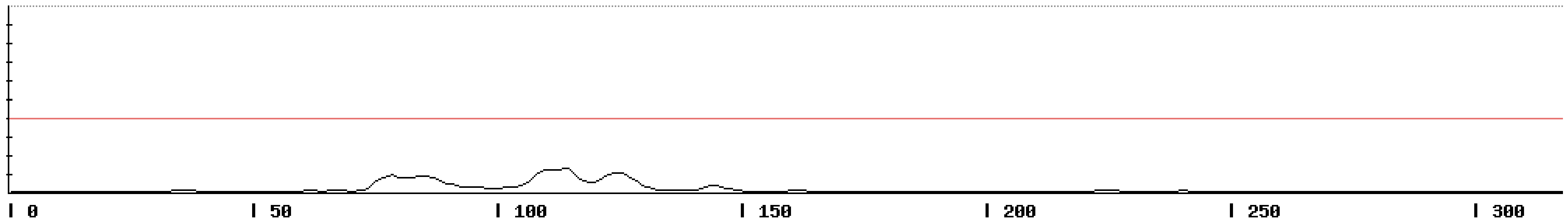

Predictions for Camelinasativa_Csa19g022280.1

Viterbi Path

Viterbi Path

Posterior @ 0.4

Posterior @ 0.4

Posterior Graphic. Threshold Marked @ 0.4

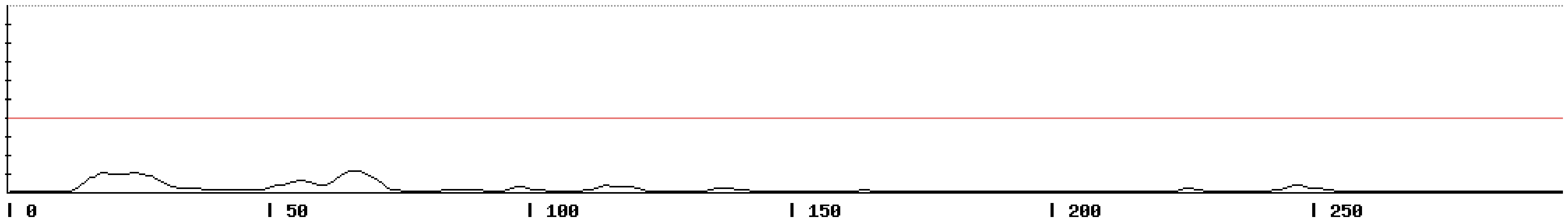

Predictions for Camelinasativa_Csa19g024880.1

Viterbi Path

Viterbi Path

Posterior @ 0.4

Posterior @ 0.4

Posterior Graphic. Threshold Marked @ 0.4

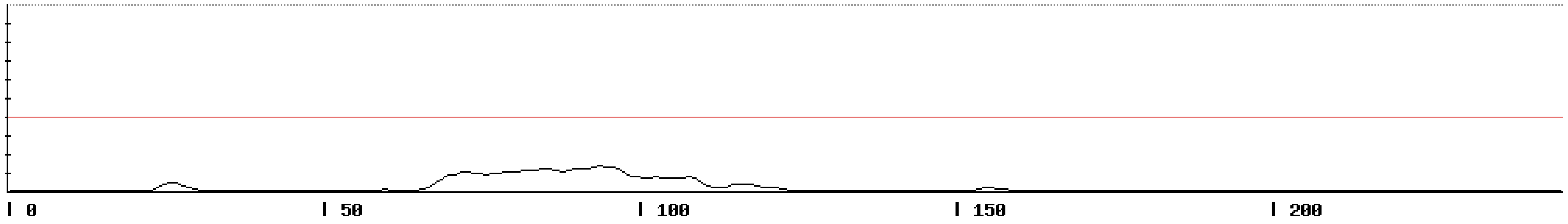

Predictions for Camelinasativa_Csa19g025790.1

Viterbi Path

Viterbi Path

Posterior @ 0.4

Posterior @ 0.4

Posterior Graphic. Threshold Marked @ 0.4

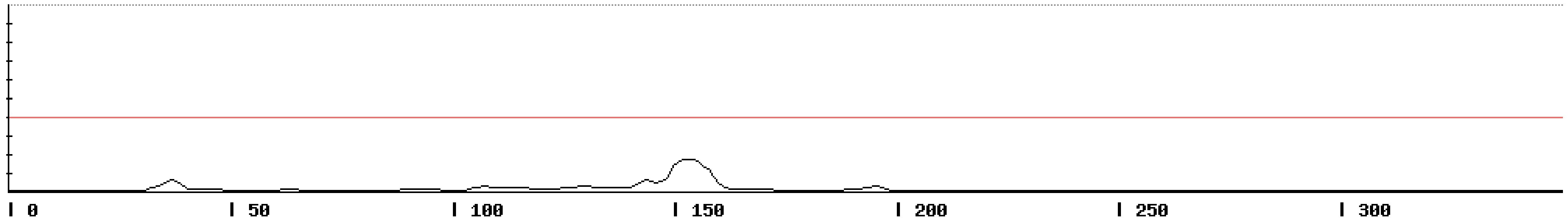

Predictions for Camelinasativa_Csa19g046490.1

Viterbi Path	153 - KRMKAEKKEKLKKGKGVSK - 171
Viterbi Path	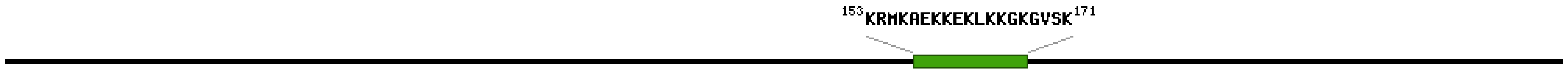
Posterior @ 0.4	152 - VKRMKAEKKEKLKKGKGVSK - 171
Posterior @ 0.4	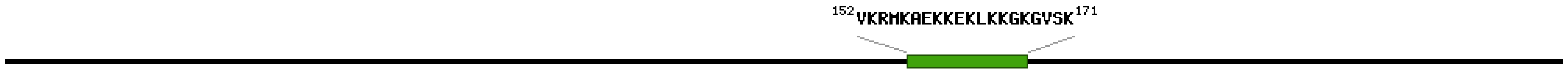
Posterior Graphic. Threshold Marked @ 0.4	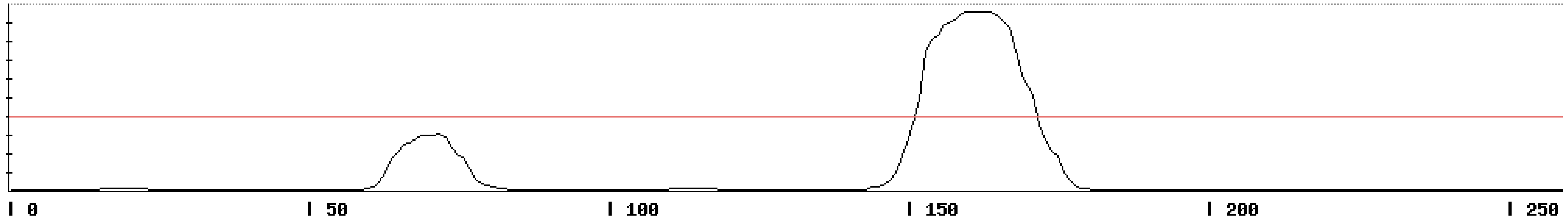

Predictions for Camelinasativa_Csa19g053810.1

Viterbi Path

Viterbi Path

Posterior @ 0.4

Posterior @ 0.4

Posterior Graphic. Threshold Marked @ 0.4

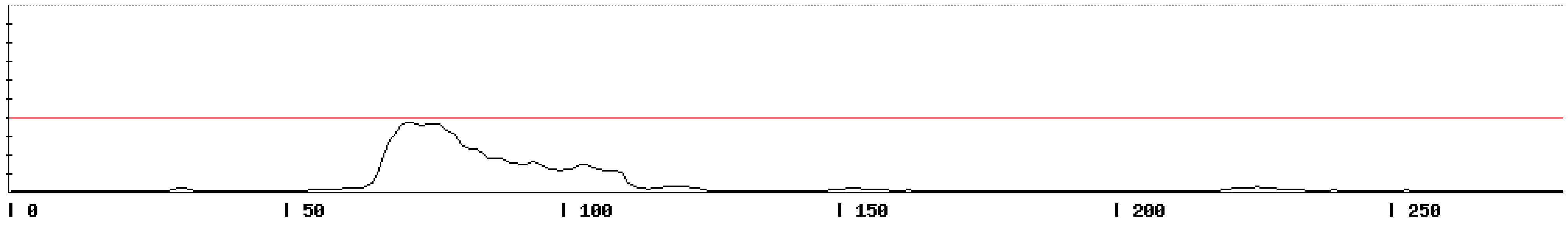

Predictions for Camelinasativa_Csa20g005600.1

Viterbi Path

Viterbi Path

Posterior @ 0.4

Posterior @ 0.4

Posterior Graphic. Threshold Marked @ 0.4

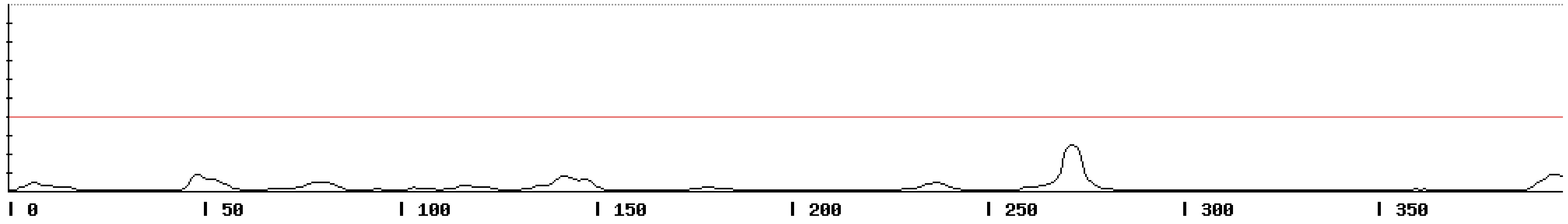

Predictions for Camelinasativa_Csa20g005610.1

Viterbi Path	
Viterbi Path	
Posterior @ 0.4	
Posterior @ 0.4	
Posterior Graphic. Threshold Marked @ 0.4	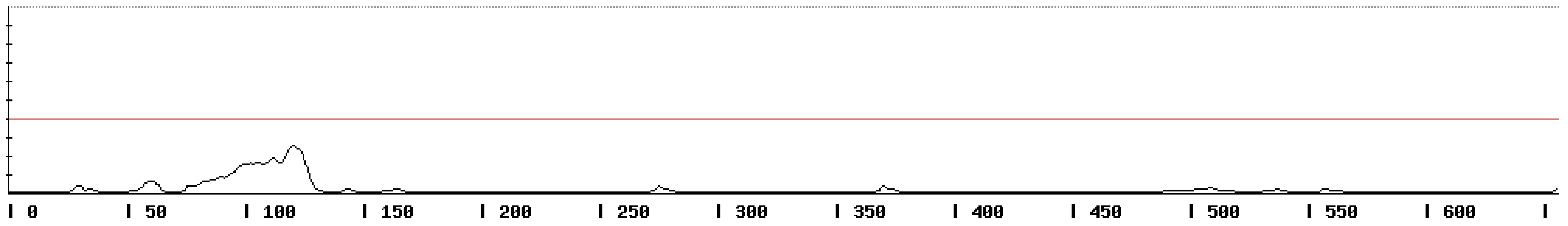

Predictions for Camelinasativa_Csa20g010110.1

Viterbi Path	
Viterbi Path	
Posterior @ 0.4	
Posterior @ 0.4	
Posterior Graphic. Threshold Marked @ 0.4	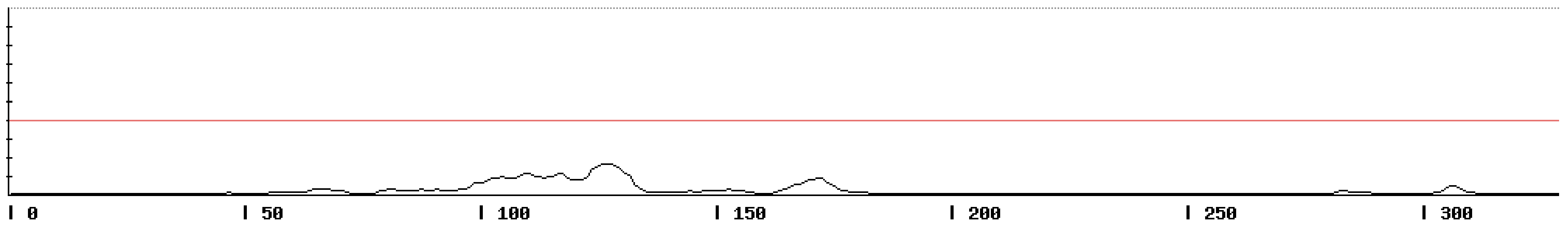

Predictions for Camelinasativa_Csa20g012360.1	
Viterbi Path	
Viterbi Path	
Posterior @ 0.4	98 - GKPK - 101
Posterior @ 0.4	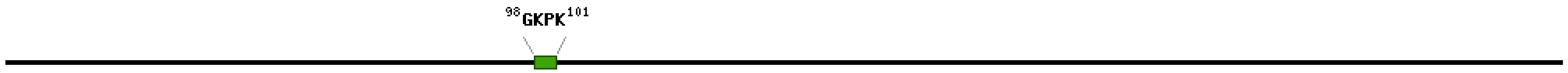
Posterior Graphic. Threshold Marked @ 0.4	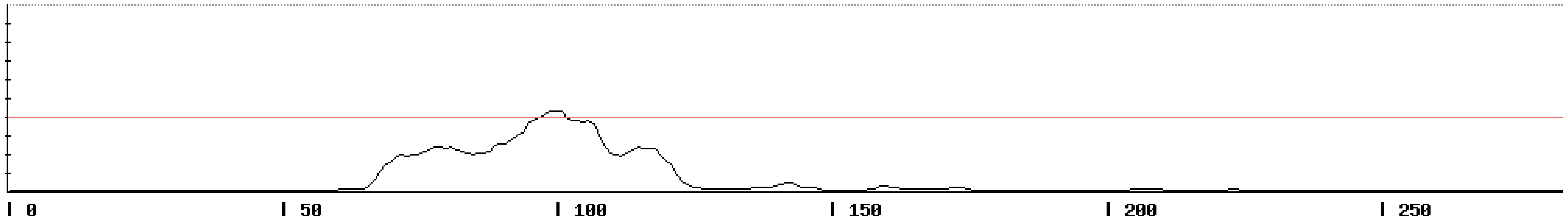

Predictions for Camelinasativa_Csa20g012490.1	
Viterbi Path	
Viterbi Path	
Posterior @ 0.4	
Posterior @ 0.4	
Posterior Graphic. Threshold Marked @ 0.4	

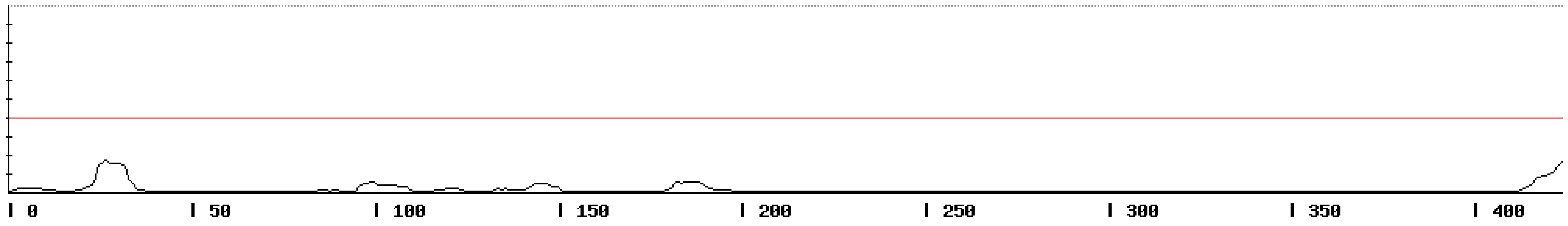

Predictions for Camelinasativa_Csa20g018530.1

Viterbi Path

Viterbi Path

Posterior @ 0.4

Posterior @ 0.4

Posterior Graphic. Threshold Marked @ 0.4

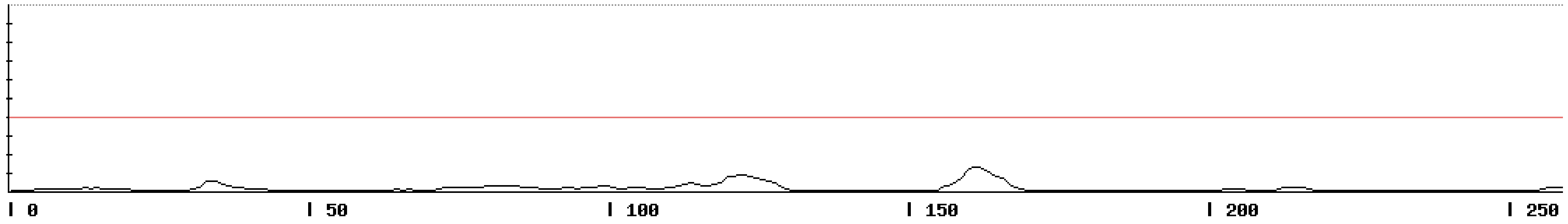

Predictions for Camelinasativa_Csa20g020460.1

Viterbi Path

Viterbi Path

Posterior @ 0.4

18 - KKERKGRR - 25

187 - RKGTKARKQRKSR - 199

Posterior @ 0.4

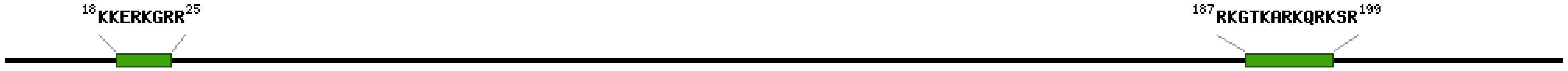

Posterior Graphic. Threshold Marked @ 0.4

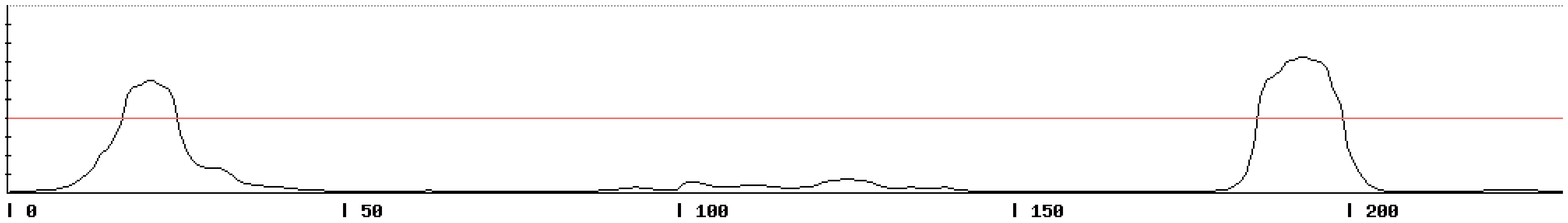

Predictions for Camelinasativa_Csa20g021030.1

Viterbi Path

Viterbi Path

Posterior @ 0.4

Posterior @ 0.4

Posterior Graphic. Threshold Marked @ 0.4

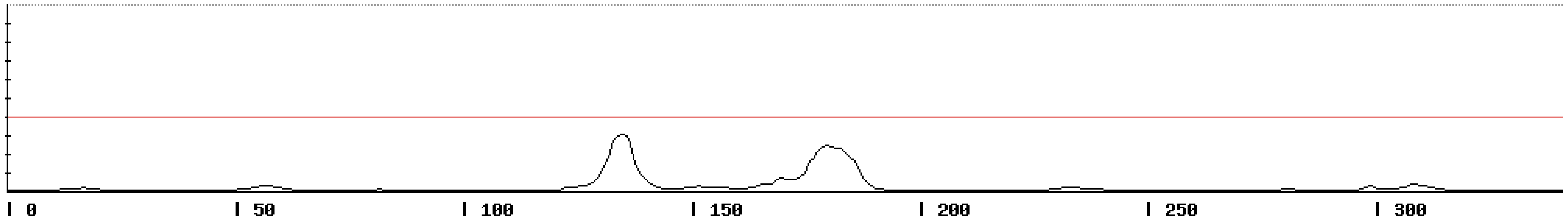

Predictions for Camelinasativa_Csa20g023680.1

Viterbi Path

Viterbi Path

Posterior @ 0.4

Posterior @ 0.4

Posterior Graphic. Threshold Marked @ 0.4

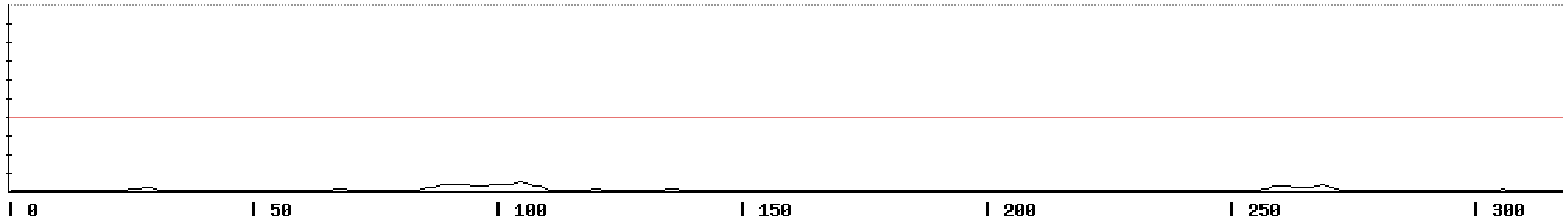

Predictions for Camelinasativa_Csa20g025340.1

Viterbi Path

Viterbi Path

Posterior @ 0.4

Posterior @ 0.4

Posterior Graphic. Threshold Marked @ 0.4

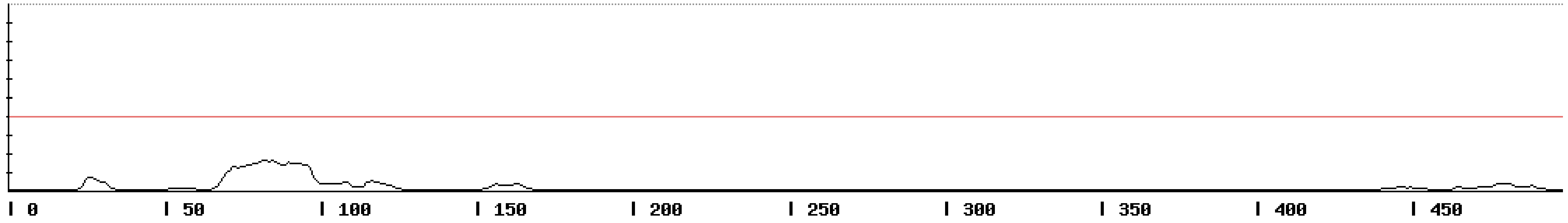

Predictions for Camelinasativa_Csa20g026310.1

Viterbi Path

Viterbi Path

Posterior @ 0.4	
Posterior @ 0.4	
Posterior Graphic. Threshold Marked @ 0.4	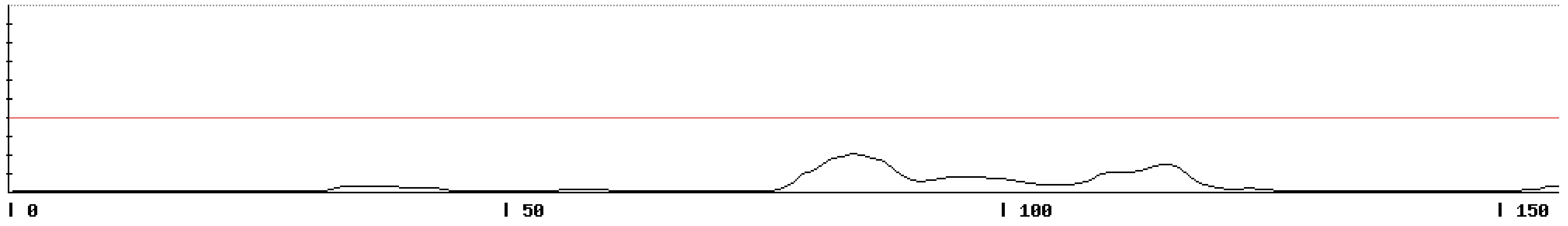

Predictions for Camelinasativa_Csa20g026350.1

Viterbi Path	
Viterbi Path	
Posterior @ 0.4	
Posterior @ 0.4	
Posterior Graphic. Threshold Marked @ 0.4	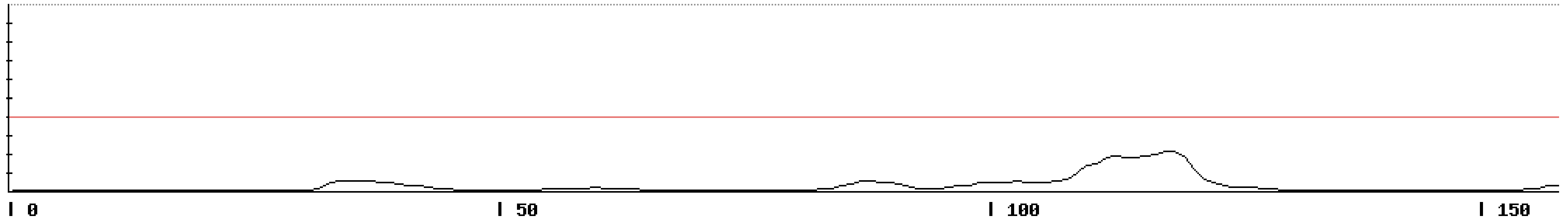

Predictions for Camelinasativa_Csa20g026400.1

Viterbi Path	
Viterbi Path	
Posterior @ 0.4	
Posterior @ 0.4	
Posterior Graphic. Threshold Marked @ 0.4	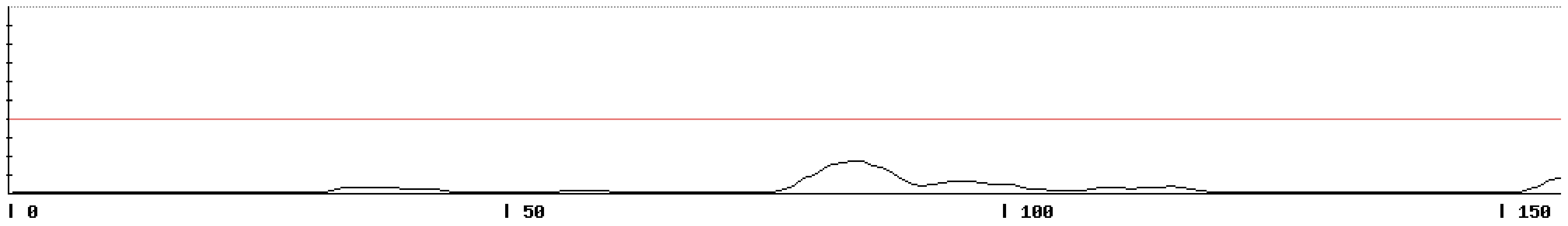

Predictions for Camelinasativa_Csa20g026650.1

Viterbi Path	
Viterbi Path	
Posterior @ 0.4	
Posterior @ 0.4	
Posterior Graphic. Threshold Marked @ 0.4	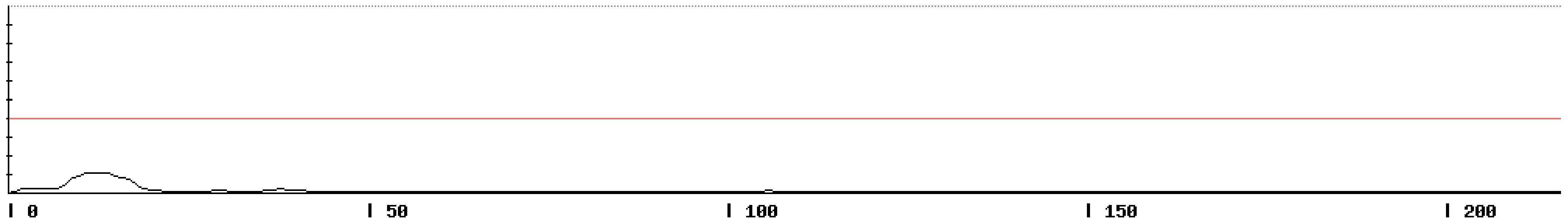

Predictions for Camelinasativa_Csa20g026680.1	
Viterbi Path	
Viterbi Path	
Posterior @ 0.4	55 - RK - 56
Posterior @ 0.4	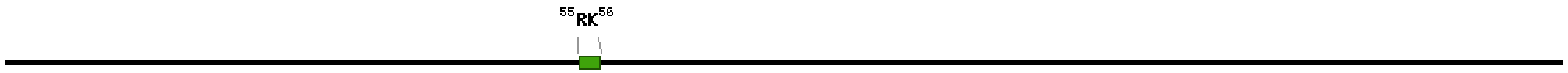
Posterior Graphic. Threshold Marked @ 0.4	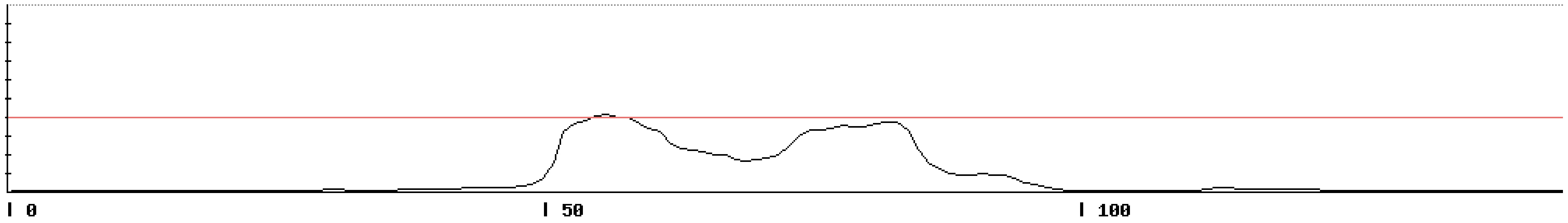

Predictions for Camelinasativa_Csa20g031750.1	
Viterbi Path	
Viterbi Path	
Posterior @ 0.4	189 - KGAKKKKA - 196
Posterior @ 0.4	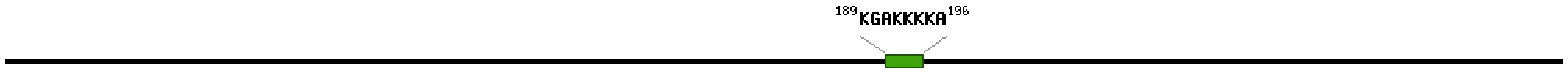
Posterior Graphic. Threshold Marked @ 0.4	

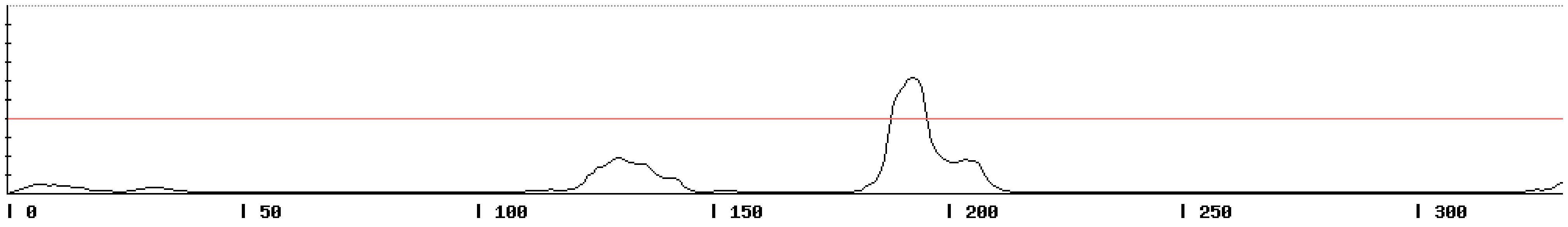

Predictions for Camelinasativa_Csa20g031760.1

Viterbi Path

Viterbi Path

Posterior @ 0.4

110 - KRNKMGKDGKRQKRDAKG - 127

202 - QKKAEEEEKKKQKK - 215

Posterior @ 0.4

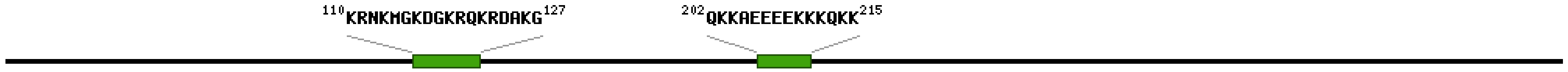

Posterior Graphic. Threshold Marked @ 0.4

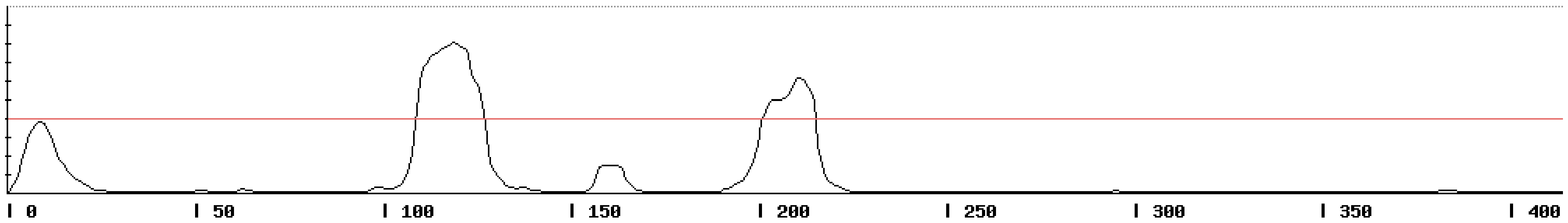

Predictions for Camelinasativa_Csa20g035850.1

Viterbi Path

Viterbi Path

Posterior @ 0.4

Posterior @ 0.4

Posterior Graphic. Threshold Marked @ 0.4

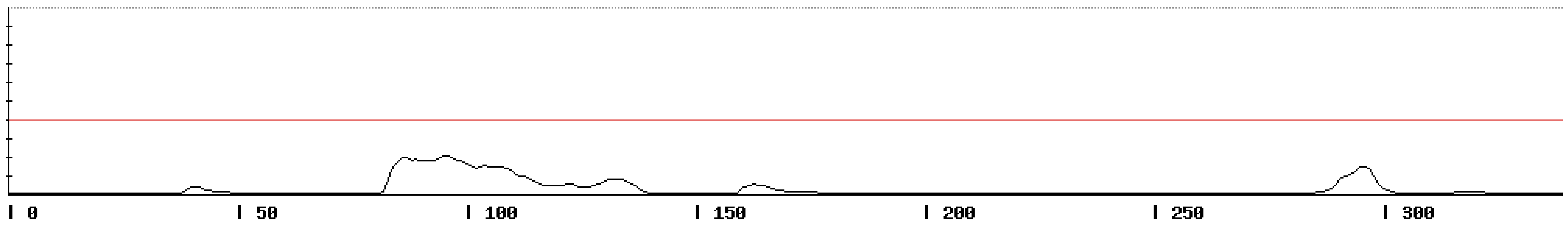

Predictions for Camelinasativa_Csa20g036010.1

Viterbi Path

Viterbi Path

Posterior @ 0.4

Posterior @ 0.4

Posterior Graphic. Threshold Marked @ 0.4

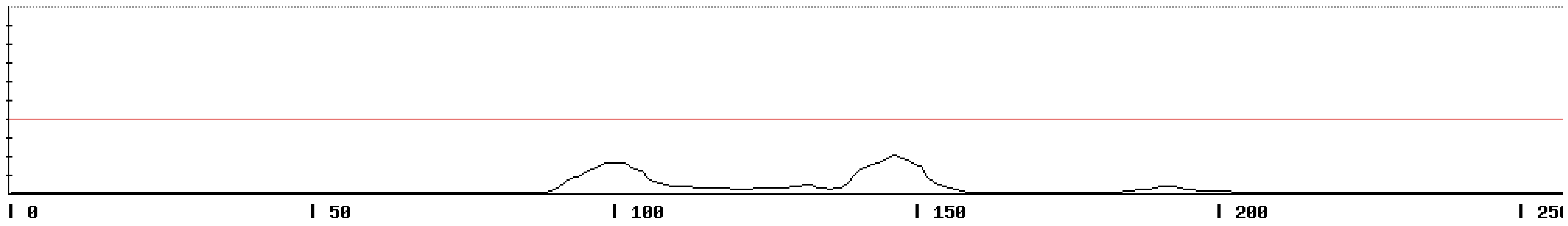

Predictions for Camelinasativa_Csa20g042750.1

Viterbi Path

Viterbi Path

Posterior @ 0.4 95 - TGKDRKIKSGKTKII - 109

Posterior @ 0.4

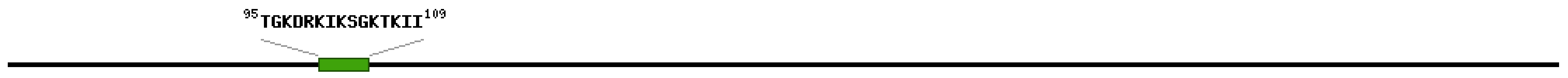

Posterior Graphic. Threshold Marked @ 0.4

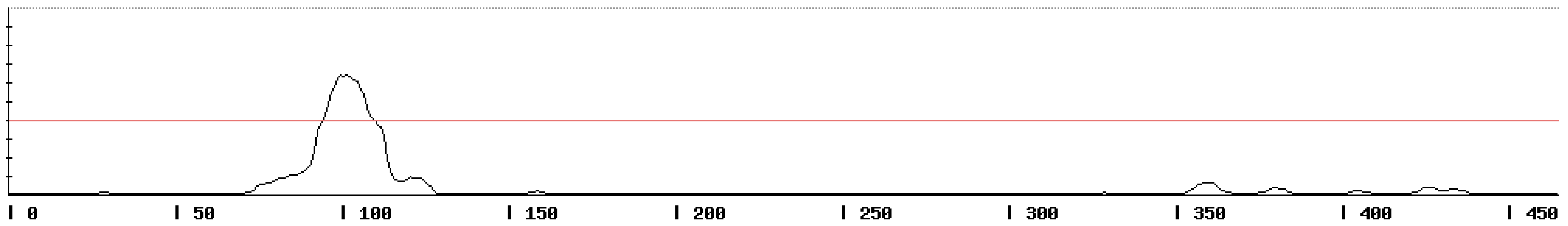

Predictions for Camelinasativa_Csa20g057380.1

Viterbi Path

Viterbi Path

Posterior @ 0.4 2 - KKKRISRWLKITTRKSNK - 19

Posterior @ 0.4

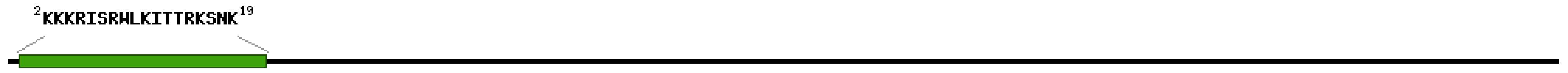

Posterior Graphic. Threshold Marked @ 0.4

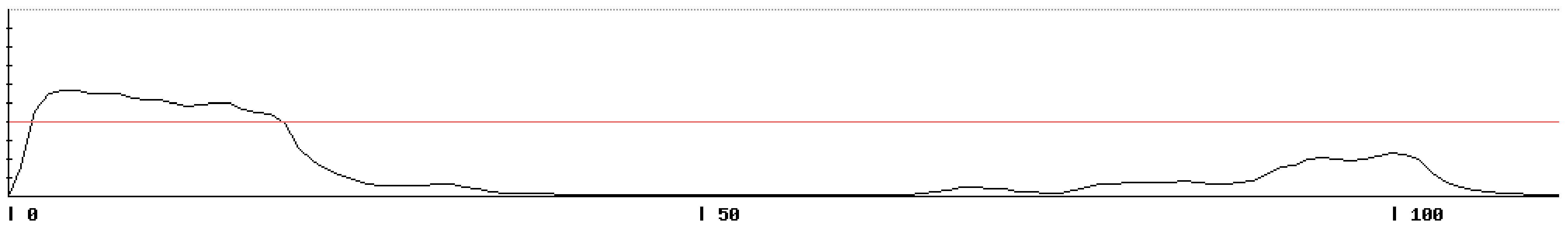

Predictions for Camelinasativa_Csa20g080730.1

Viterbi Path

Viterbi Path

Posterior @ 0.4	
Posterior @ 0.4	
Posterior Graphic. Threshold Marked @ 0.4	

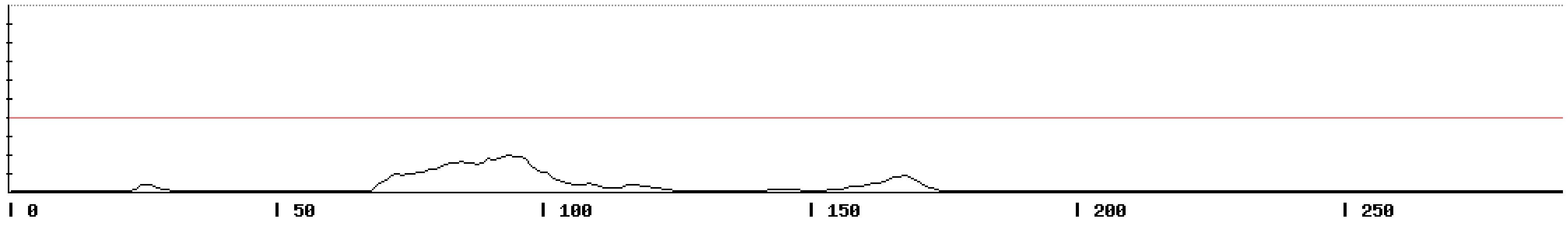

Predictions for Camelinasativa_Csa26370s010.1

alex.nguyenba@utoronto.ca

Intended to be used with Firefox

Viterbi Path

Posterior @ 0.4

Posterior @ 0.4

Posterior Graphic. Threshold Marked @ 0.4

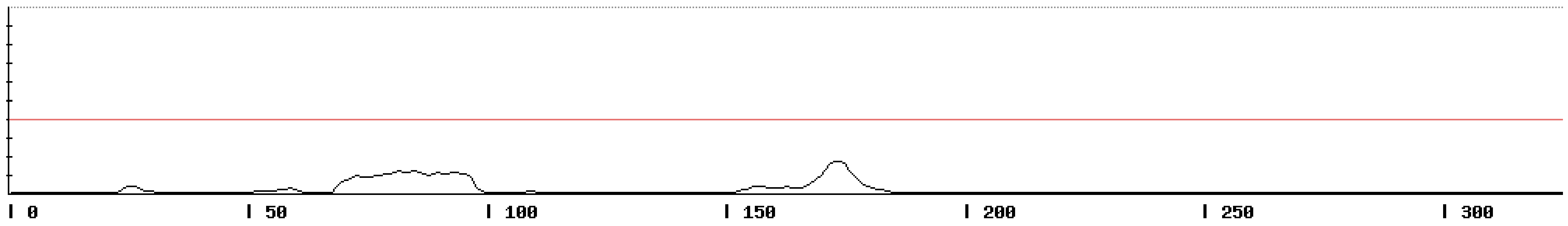

Predictions for Cannabissativa_PK00547.1

Viterbi Path

Viterbi Path

Posterior @ 0.4

Posterior @ 0.4

Posterior Graphic. Threshold Marked @ 0.4

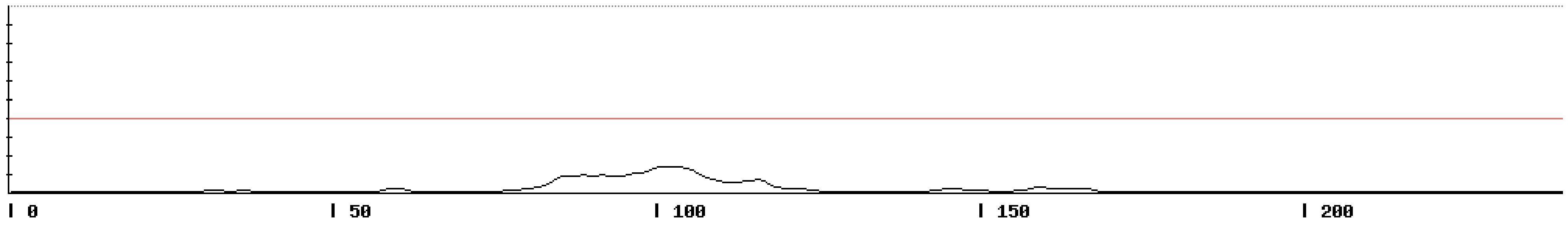

Predictions for Cannabissativa_PK01070.1

Viterbi Path

Viterbi Path

Posterior @ 0.4

Posterior @ 0.4

Posterior Graphic. Threshold Marked @ 0.4

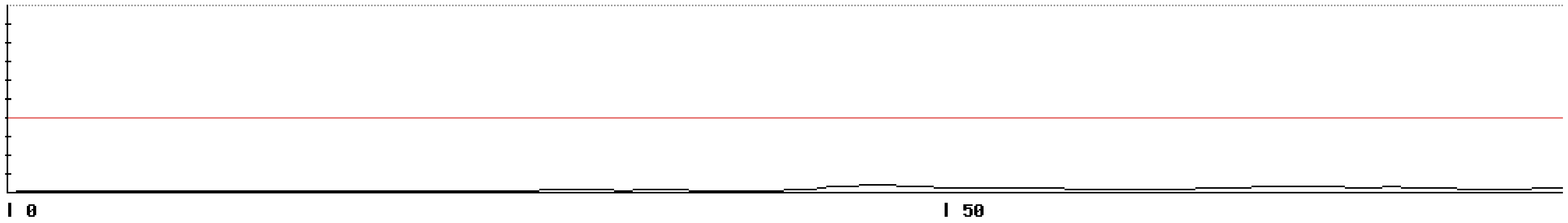

Predictions for Cannabissativa_PK01104.1

Viterbi Path

Viterbi Path

Posterior @ 0.4

Posterior @ 0.4

Posterior Graphic. Threshold Marked @ 0.4

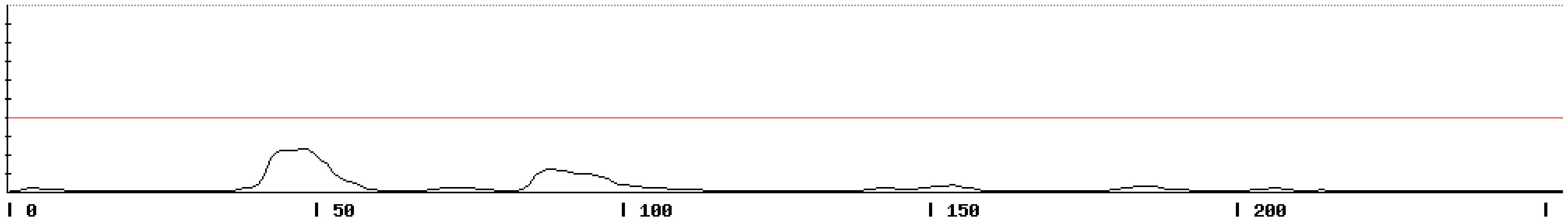

Predictions for Cannabissativa_PK02019.1

Viterbi Path

Viterbi Path

Posterior @ 0.4

Posterior @ 0.4

Posterior Graphic. Threshold Marked @ 0.4

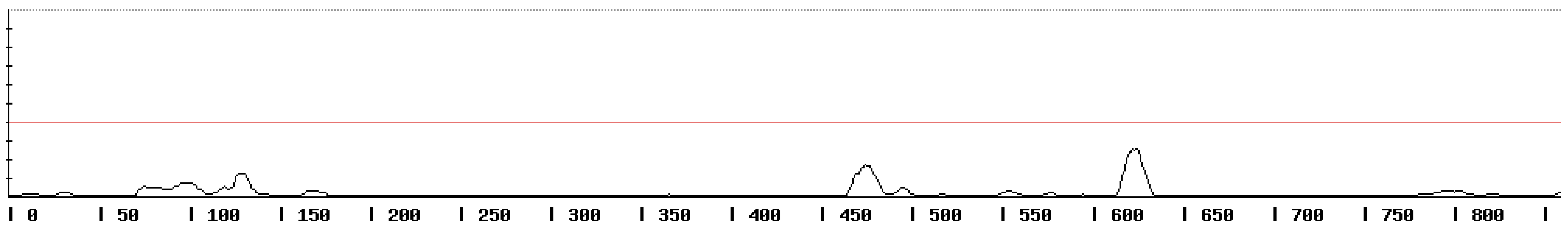

Predictions for Cannabissativa_PK02366.1

Viterbi Path

Viterbi Path

Posterior @ 0.4

Posterior @ 0.4

Posterior Graphic. Threshold Marked @ 0.4

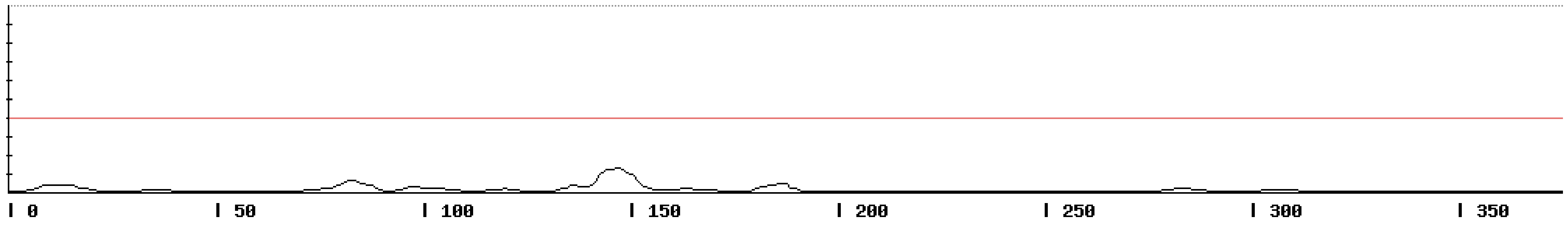

Predictions for Cannabissativa_PK02804.1

Viterbi Path

Viterbi Path

Posterior @ 0.4

Posterior @ 0.4

Posterior Graphic. Threshold Marked @ 0.4

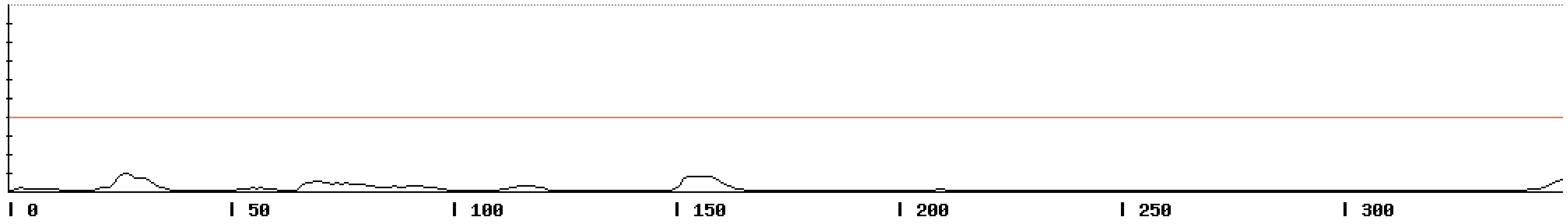

Predictions for Cannabissativa_PK03341.1

Viterbi Path

Viterbi Path

Posterior @ 0.4

Posterior @ 0.4

Posterior Graphic. Threshold Marked @ 0.4

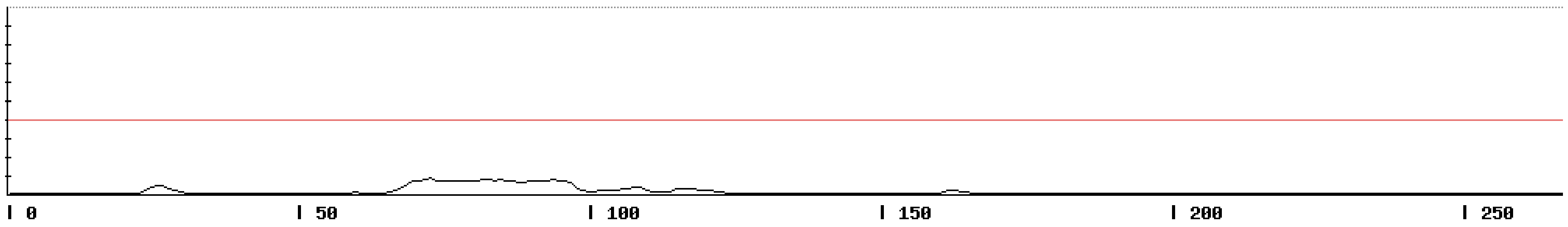

Predictions for Cannabissativa_PK04961.1

Viterbi Path

Viterbi Path

Posterior @ 0.4

Posterior @ 0.4

Posterior Graphic. Threshold Marked @ 0.4

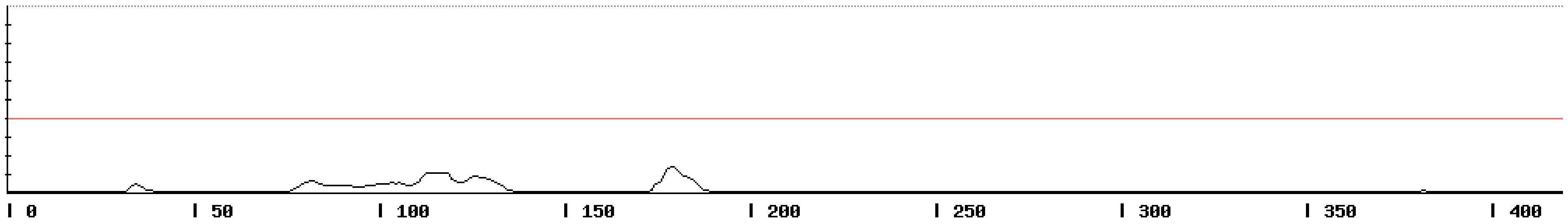

Predictions for Cannabissativa_PK05686.1

Viterbi Path

Viterbi Path

Posterior @ 0.4

Posterior @ 0.4

Posterior Graphic. Threshold Marked @ 0.4

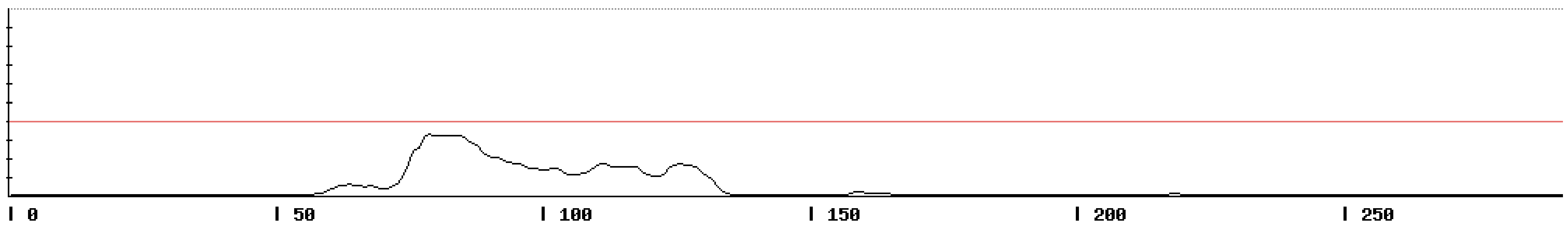

Predictions for Cannabissativa_PK06287.1

Viterbi Path

Viterbi Path

Posterior @ 0.4

Posterior @ 0.4

Posterior Graphic. Threshold Marked @ 0.4

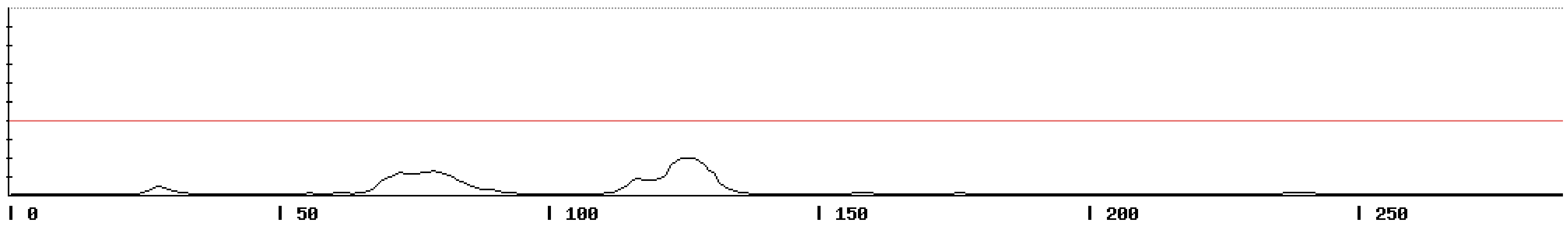

Predictions for Cannabissativa_PK06477.1

Viterbi Path

Viterbi Path

Posterior @ 0.4

Posterior @ 0.4

Posterior Graphic. Threshold Marked @ 0.4

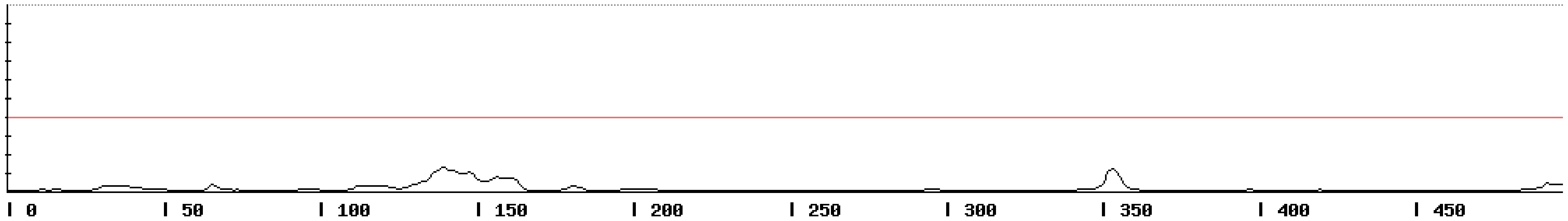

Predictions for Cannabissativa_PK06766.1

Viterbi Path	384 - KKEAHGRRKKLRKK - 397

Viterbi Path

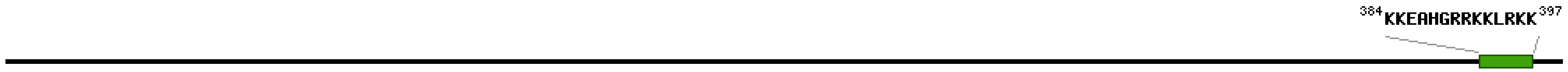

Posterior @ 0.4	384 - KKEAHGRRKKLRKK - 397

Posterior @ 0.4

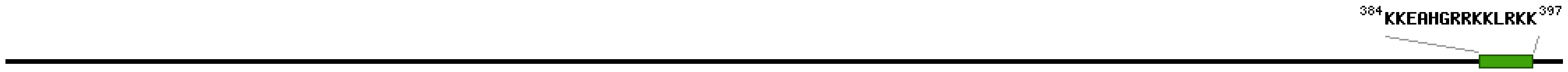

Posterior Graphic. Threshold Marked @ 0.4

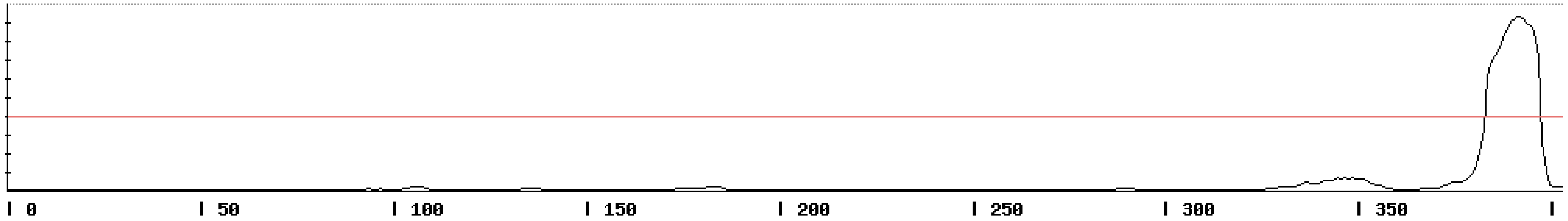

Predictions for Cannabissativa_PK07042.1

Viterbi Path

Viterbi Path

Posterior @ 0.4

Posterior @ 0.4

Posterior Graphic. Threshold Marked @ 0.4

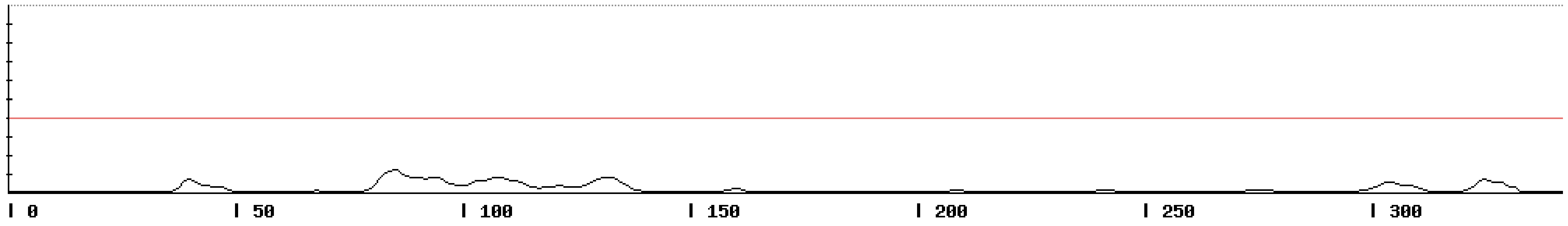

Predictions for Cannabissativa_PK08180.1

Viterbi Path

Viterbi Path

Posterior @ 0.4

Posterior @ 0.4

Posterior Graphic. Threshold Marked @ 0.4

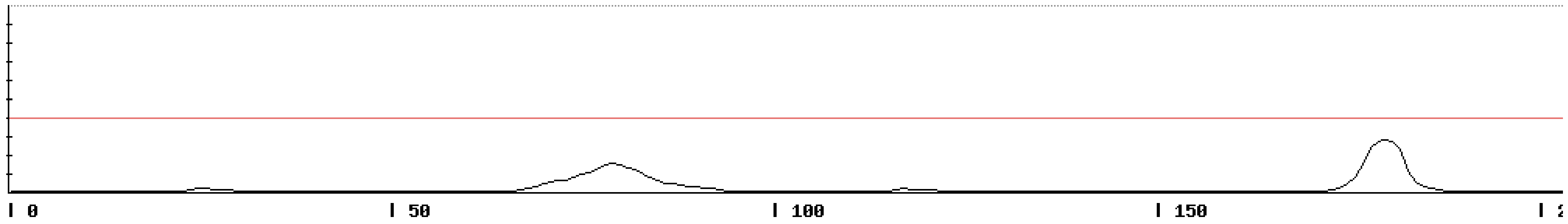

Predictions for Cannabissativa_PK08629.1

Viterbi Path

Viterbi Path

Posterior @ 0.4

Posterior @ 0.4

Posterior Graphic. Threshold Marked @ 0.4

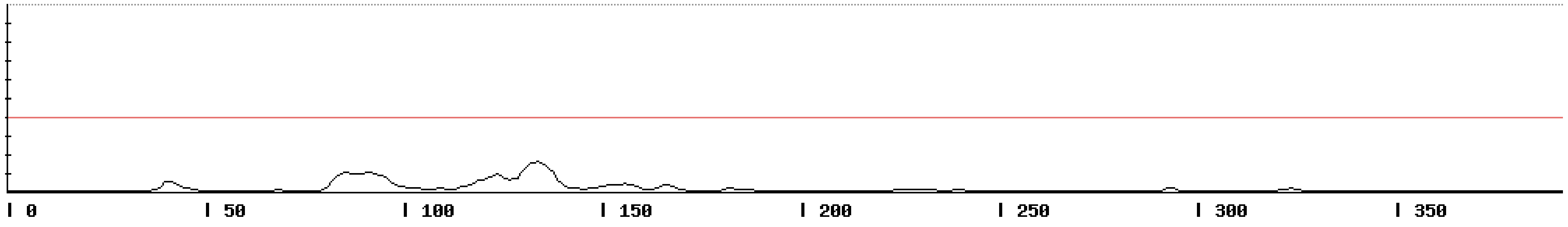

Predictions for Cannabissativa_PK10271.1

Viterbi Path

Viterbi Path

Posterior @ 0.4

Posterior @ 0.4

Posterior Graphic. Threshold Marked @ 0.4

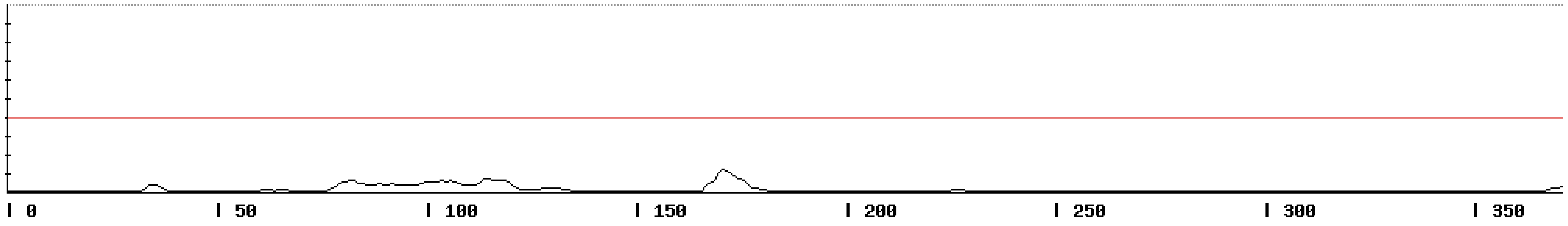

Predictions for Cannabissativa_PK11173.1	
Viterbi Path	
Viterbi Path	
Posterior @ 0.4	
Posterior @ 0.4	
Posterior Graphic. Threshold Marked @ 0.4	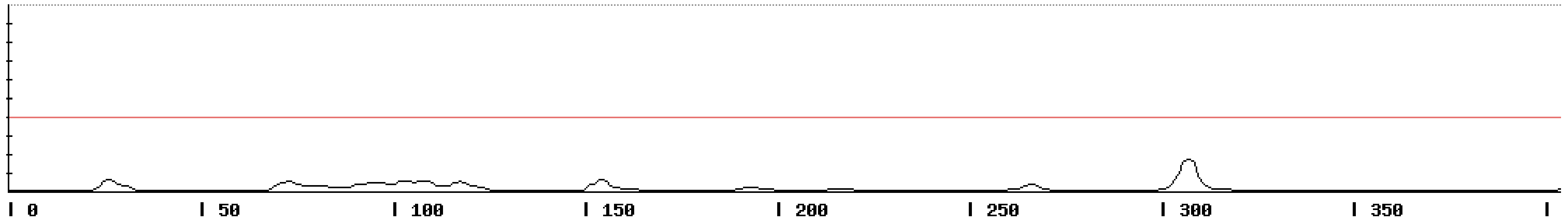

Predictions for Cannabissativa_PK11281.7	
Viterbi Path	
Viterbi Path	
Posterior @ 0.4	
Posterior @ 0.4	
Posterior Graphic. Threshold Marked @ 0.4	

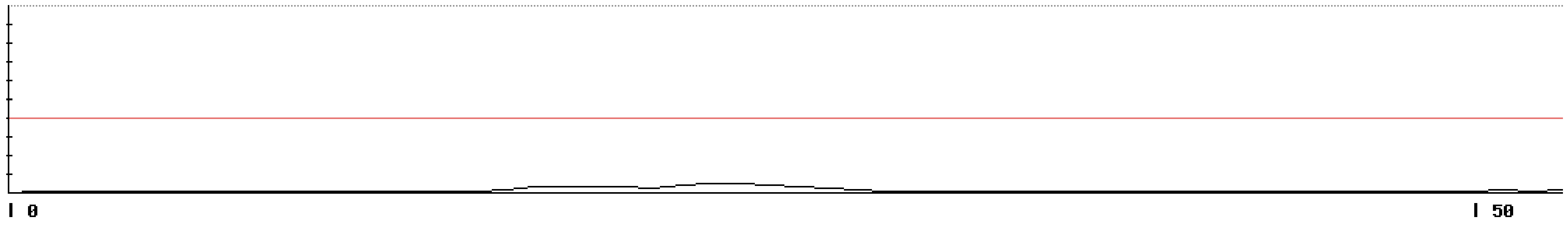

Predictions for Cannabissativa_PK11467.1

Viterbi Path	
Viterbi Path	
Posterior @ 0.4	
Posterior @ 0.4	
Posterior Graphic. Threshold Marked @ 0.4	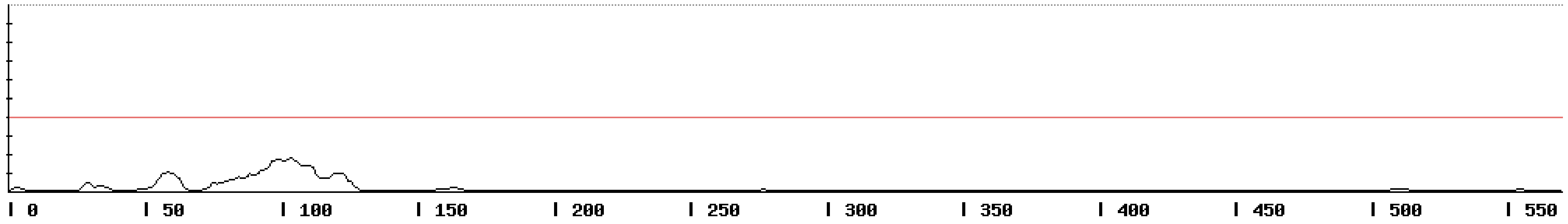

Predictions for Cannabissativa_PK11990.1

Viterbi Path	
Viterbi Path	
Posterior @ 0.4	173 - R - 173
Posterior @ 0.4	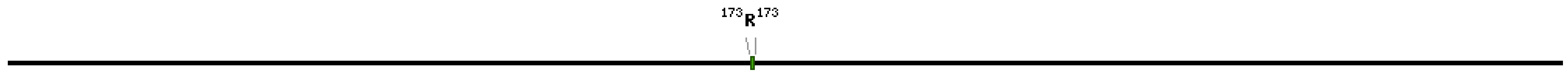

Posterior Graphic. Threshold Marked @ 0.4

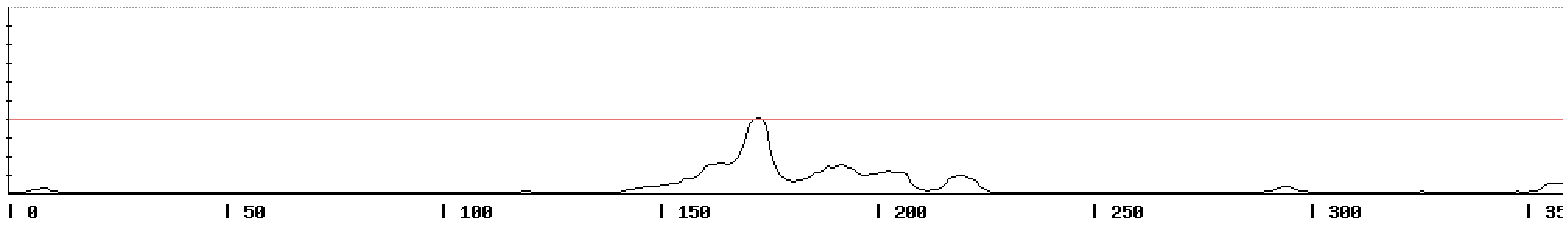

Predictions for Cannabissativa_PK12461.1

Viterbi Path

Viterbi Path

Posterior @ 0.4

Posterior @ 0.4

Posterior Graphic. Threshold Marked @ 0.4

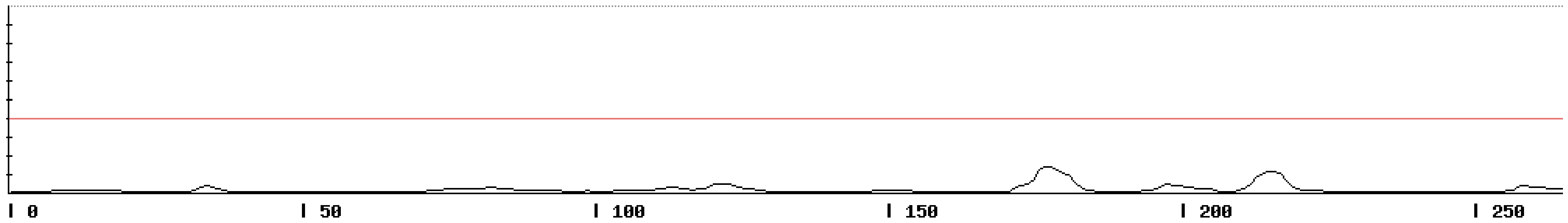

Predictions for Cannabissativa_PK12901.1

Viterbi Path

Viterbi Path

Posterior @ 0.4

Posterior @ 0.4

Posterior Graphic. Threshold Marked @ 0.4

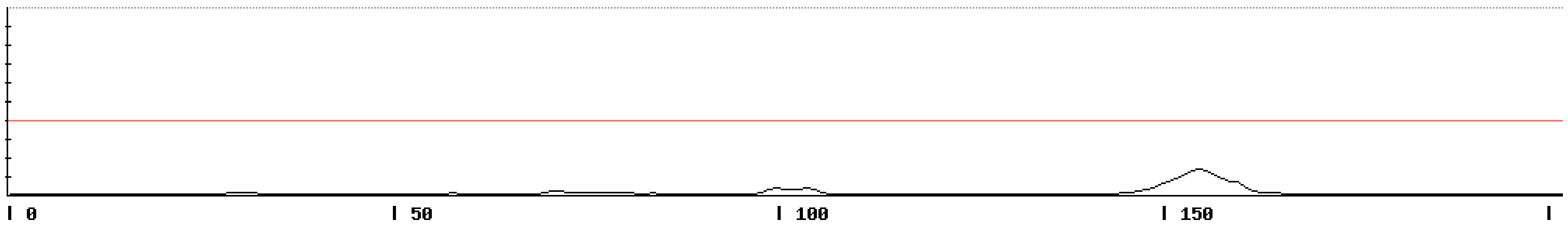

Predictions for Cannabissativa_PK13251.1

Viterbi Path

Viterbi Path

Posterior @ 0.4

Posterior @ 0.4

Posterior Graphic. Threshold Marked @ 0.4

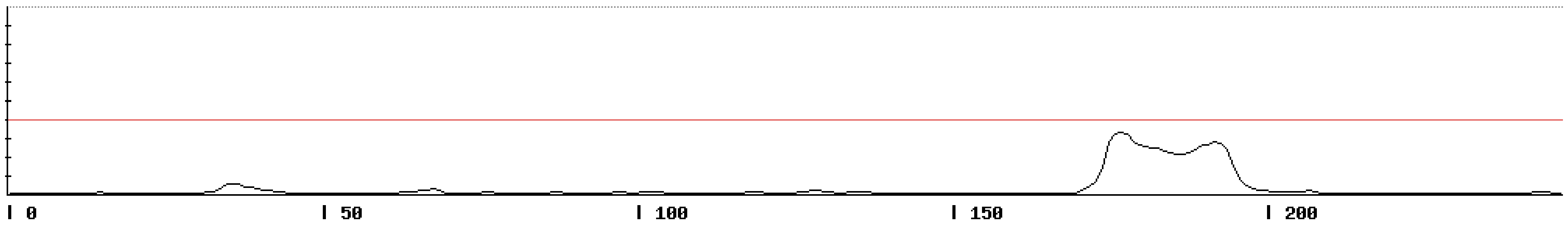

Predictions for Cannabissativa_PK13860.1

Viterbi Path

Viterbi Path

Posterior @ 0.4

Posterior @ 0.4

Posterior Graphic. Threshold Marked @ 0.4

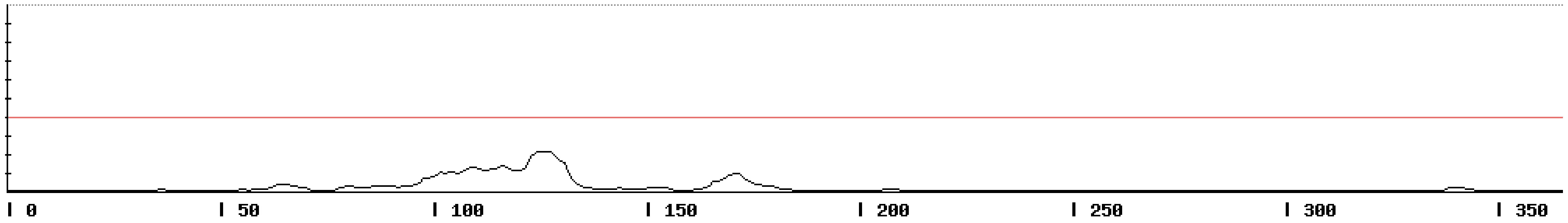

Predictions for Cannabissativa_PK13964.1

Viterbi Path

Viterbi Path

Posterior @ 0.4

Posterior @ 0.4

Posterior Graphic. Threshold Marked @ 0.4

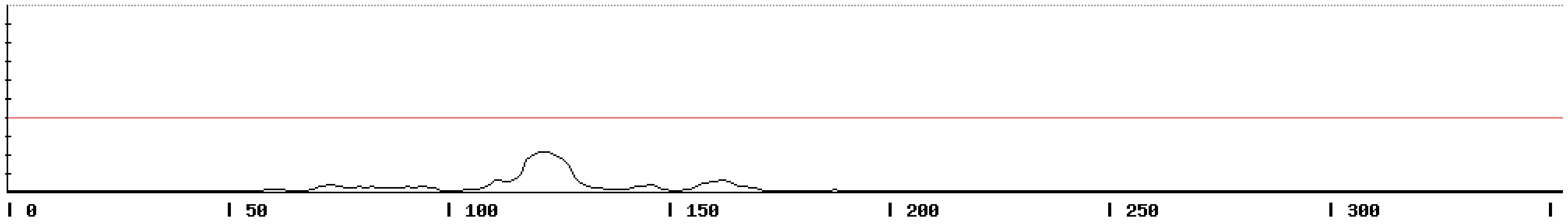

Predictions for Cannabissativa_PK15363.1

Viterbi Path

Viterbi Path

Posterior @ 0.4

Posterior @ 0.4

Posterior Graphic. Threshold Marked @ 0.4

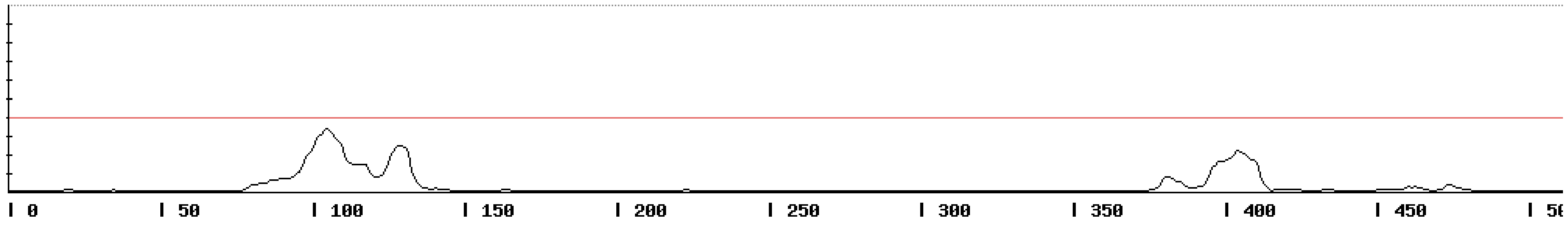

Predictions for Cannabissativa_PK17141.1

Viterbi Path

Viterbi Path

Posterior @ 0.4

Posterior @ 0.4

Posterior Graphic. Threshold Marked @ 0.4

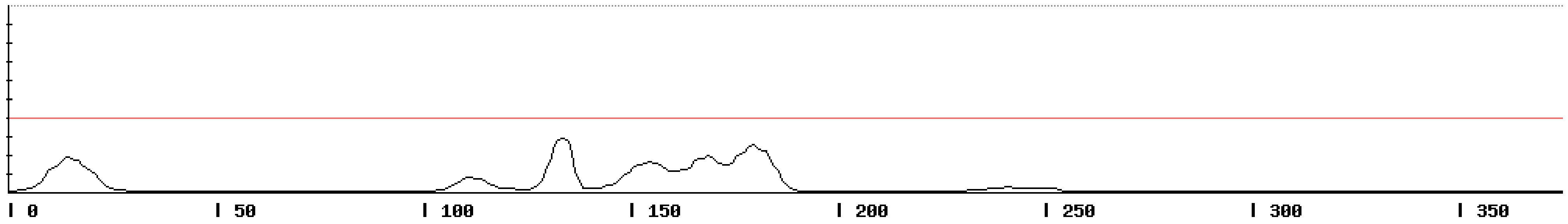

Predictions for Cannabissativa_PK17295.1

Viterbi Path

Viterbi Path

Posterior @ 0.4

Posterior @ 0.4

Posterior Graphic. Threshold Marked @ 0.4

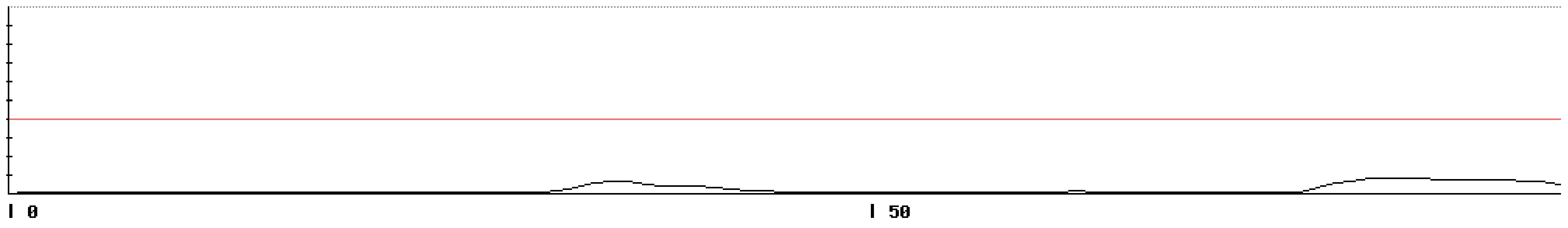

Predictions for Cannabissativa_PK18158.1

Viterbi Path

Viterbi Path

Posterior @ 0.4

Posterior @ 0.4

Posterior Graphic. Threshold Marked @ 0.4

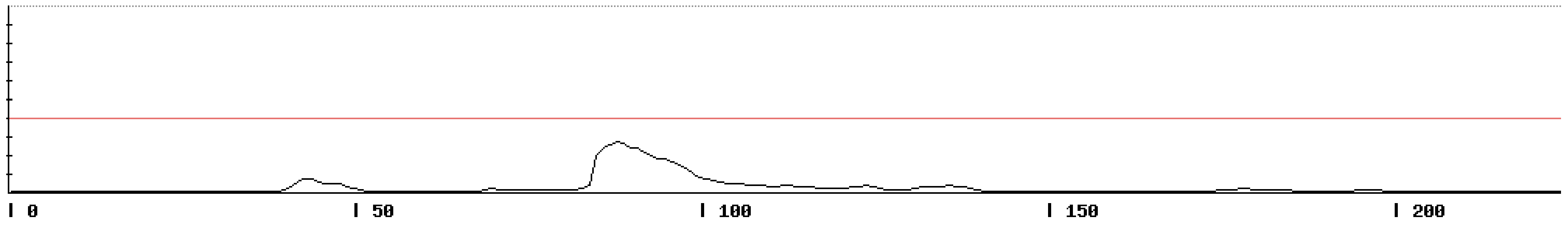

Predictions for Cannabissativa_PK18821.1	
Viterbi Path	
Viterbi Path	
Posterior @ 0.4	89 - RTTKTGFWKKTGKERKTKGCNGRKR - 113
Posterior @ 0.4	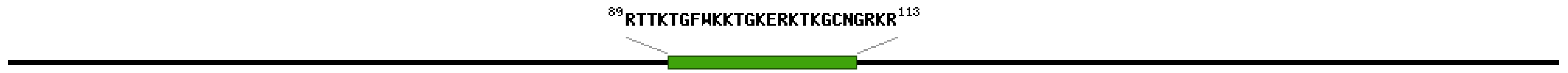
Posterior Graphic. Threshold Marked @ 0.4	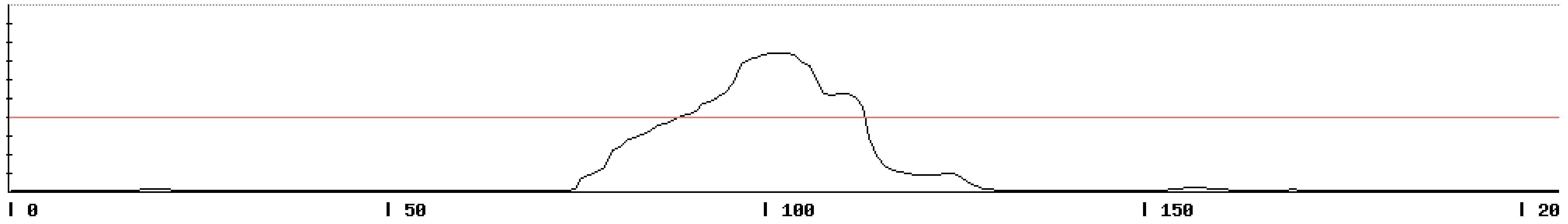

Predictions for Cannabissativa_PK19051.1	
Viterbi Path	
Viterbi Path	
Posterior @ 0.4	
Posterior @ 0.4	
Posterior Graphic. Threshold Marked @ 0.4	

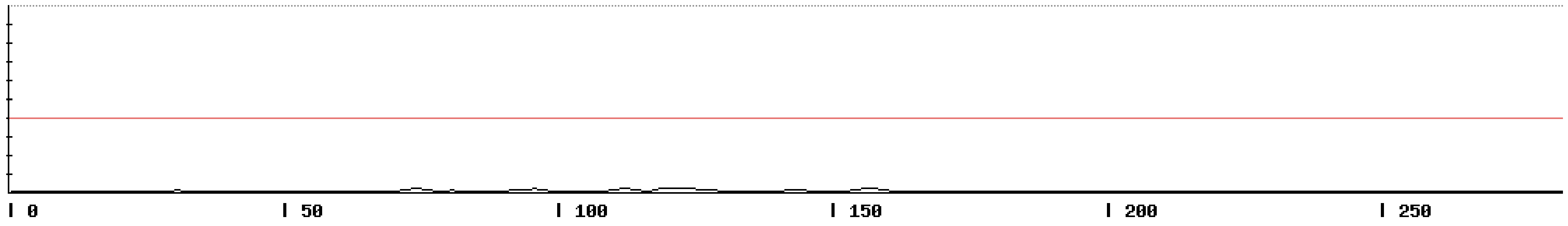

Predictions for Cannabissativa_PK19059.1

Viterbi Path

Viterbi Path

Posterior @ 0.4

Posterior @ 0.4

Posterior Graphic. Threshold Marked @ 0.4

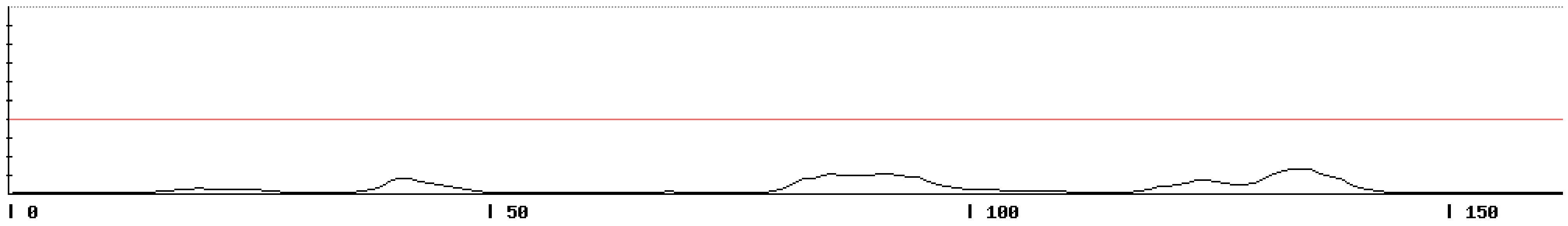

Predictions for Cannabissativa_PK19195.1

Viterbi Path

Viterbi Path

Posterior @ 0.4

Posterior @ 0.4

Posterior Graphic. Threshold Marked @ 0.4

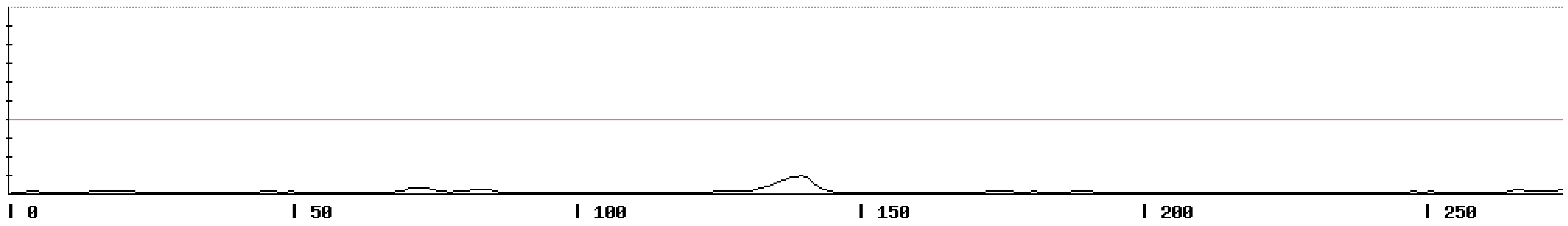

Predictions for Cannabissativa_PK19314.1

Viterbi Path

Viterbi Path

Posterior @ 0.4

Posterior @ 0.4

Posterior Graphic. Threshold Marked @ 0.4

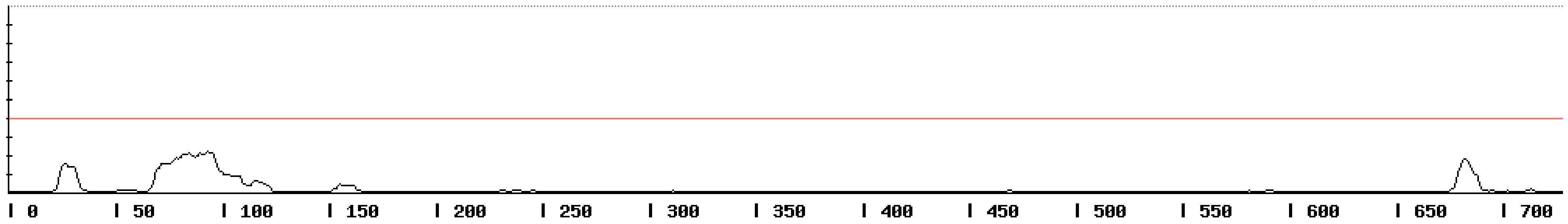

Predictions for Cannabissativa_PK20135.1

Viterbi Path

Viterbi Path

Posterior @ 0.4

Posterior @ 0.4

Posterior Graphic. Threshold Marked @ 0.4

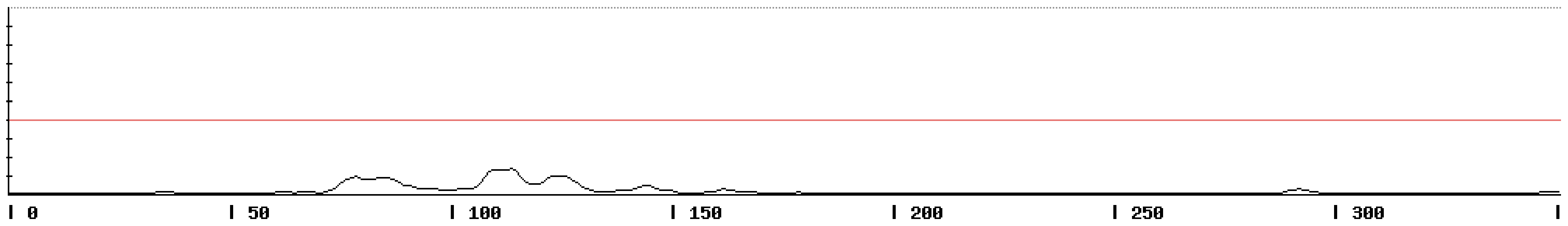

Predictions for Cannabissativa_PK20262.1

Viterbi Path

Viterbi Path

Posterior @ 0.4

Posterior @ 0.4

Posterior Graphic. Threshold Marked @ 0.4

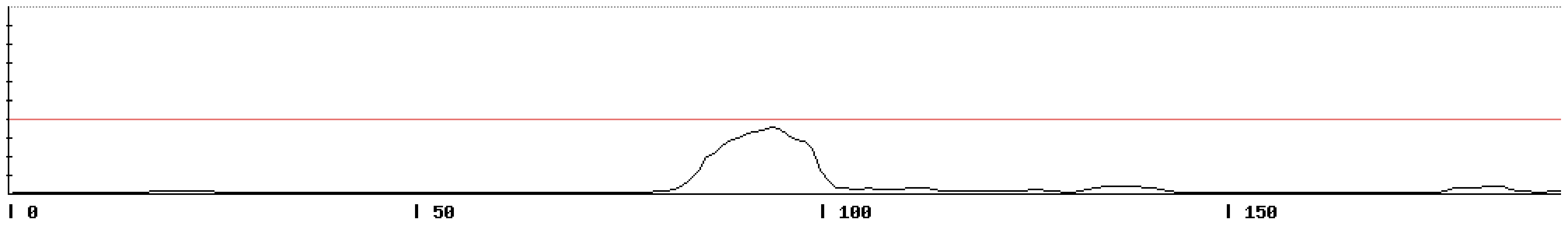

Predictions for Cannabissativa_PK22182.1

Viterbi Path

Viterbi Path

Posterior @ 0.4

Posterior @ 0.4

Posterior Graphic. Threshold Marked @ 0.4

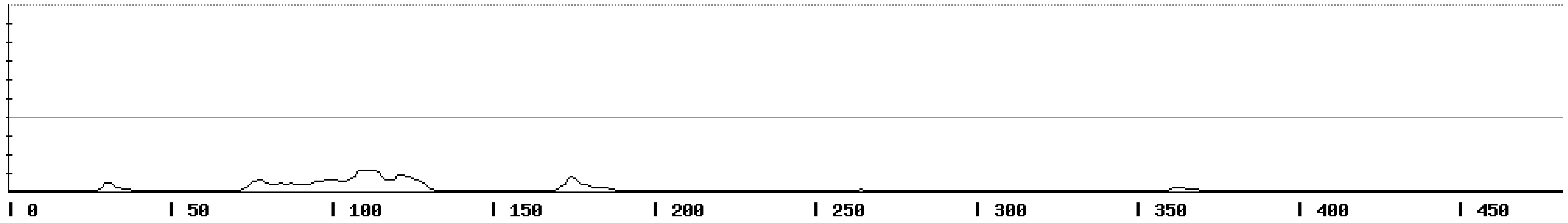

Predictions for Cannabissativa_PK22221.1

Viterbi Path

Viterbi Path

Posterior @ 0.4

Posterior @ 0.4

Posterior Graphic. Threshold Marked @ 0.4

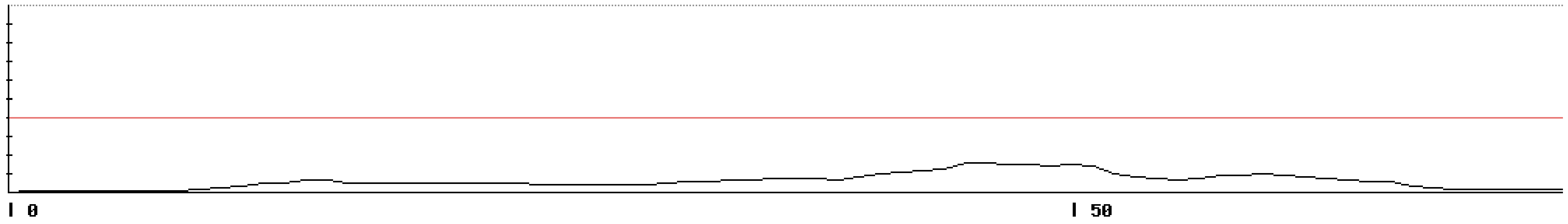

Predictions for Cannabissativa_PK22704.1

Viterbi Path

Viterbi Path

Posterior @ 0.4

Posterior @ 0.4

Posterior Graphic. Threshold Marked @ 0.4

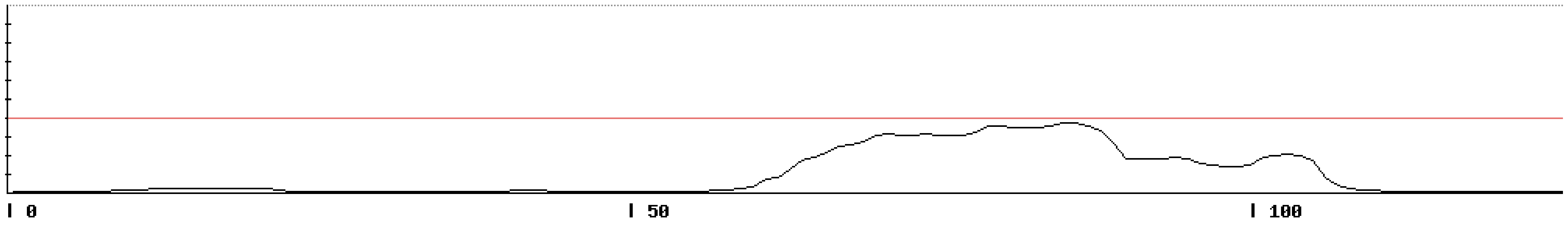

Predictions for Cannabissativa_PK23050.1

Viterbi Path

Viterbi Path

Posterior @ 0.4 147 - SRKRGRKSDKQKPT - 160

Posterior @ 0.4

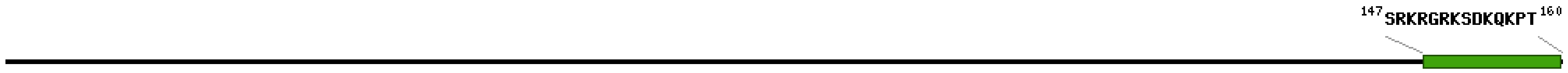

Posterior Graphic. Threshold Marked @ 0.4

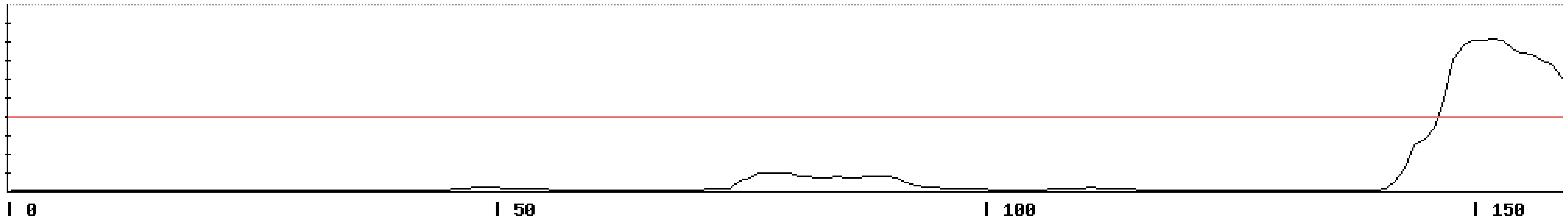

Predictions for Cannabissativa_PK23416.1

Viterbi Path

Viterbi Path

Posterior @ 0.4

Posterior @ 0.4

Posterior Graphic. Threshold Marked @ 0.4

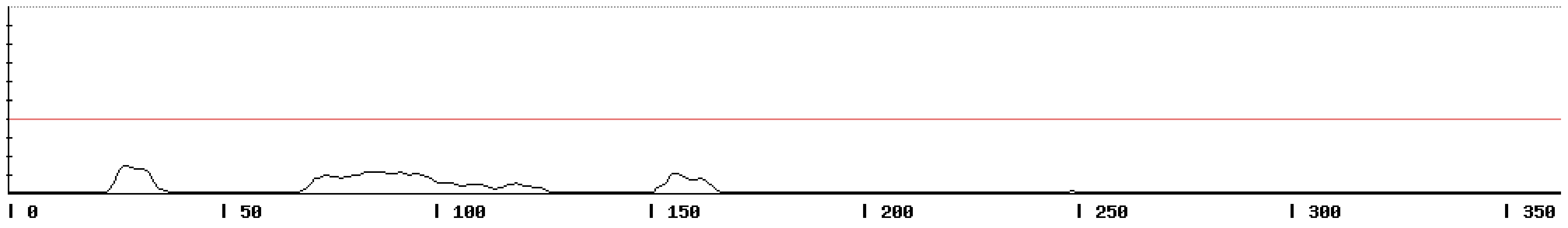

Predictions for Cannabissativa_PK23481.1

Viterbi Path

Viterbi Path

Posterior @ 0.4

Posterior @ 0.4

Posterior Graphic. Threshold Marked @ 0.4

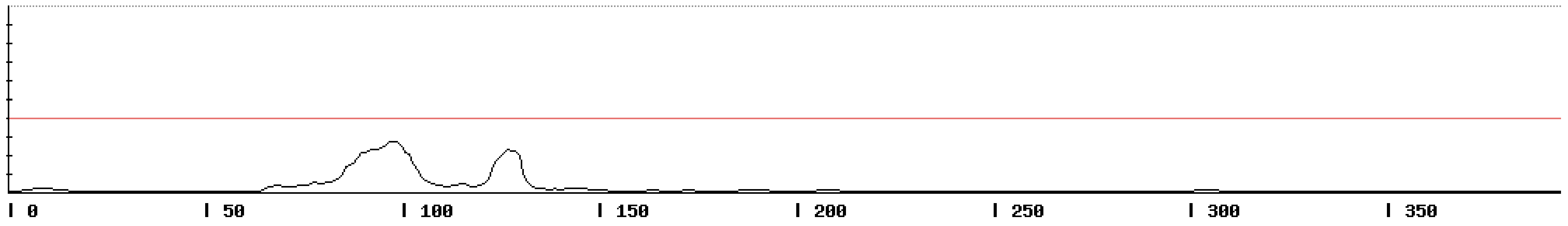

Predictions for Cannabissativa_PK23488.1	
Viterbi Path	
Viterbi Path	
Posterior @ 0.4	
Posterior @ 0.4	
Posterior Graphic. Threshold Marked @ 0.4	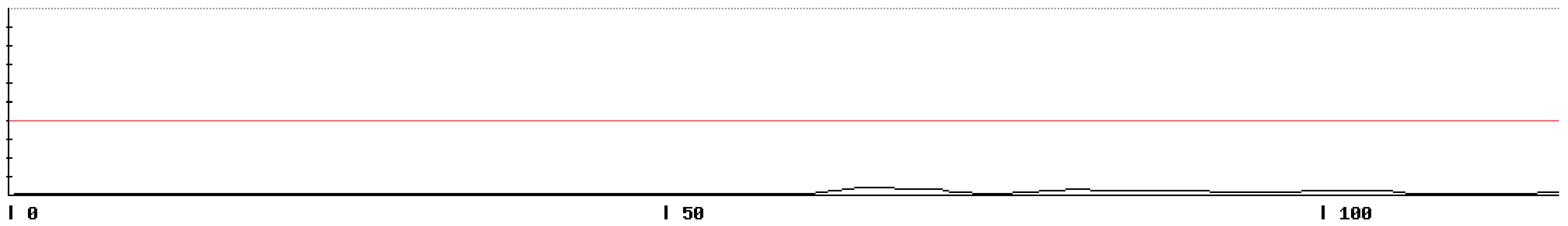

Predictions for Cannabissativa_PK24240.1	
Viterbi Path	
Viterbi Path	
Posterior @ 0.4	
Posterior @ 0.4	
Posterior Graphic. Threshold Marked @ 0.4	

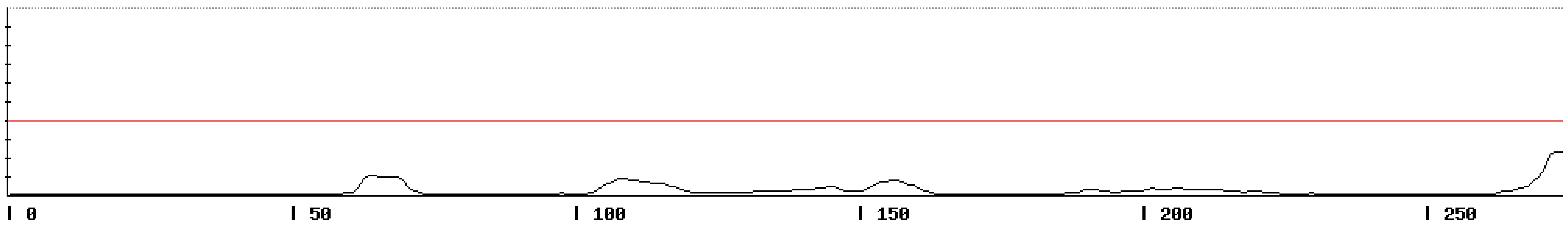

Predictions for Cannabissativa_PK24620.1

Viterbi Path	
Viterbi Path	
Posterior @ 0.4	81 - NRKTKTG - 87
	93 - GRGKK - 97
	151 - KLVSRKRGRKS - 161

Posterior @ 0.4

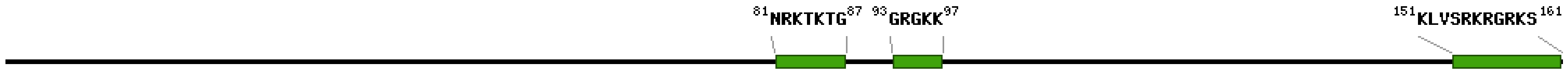

Posterior Graphic. Threshold Marked @ 0.4

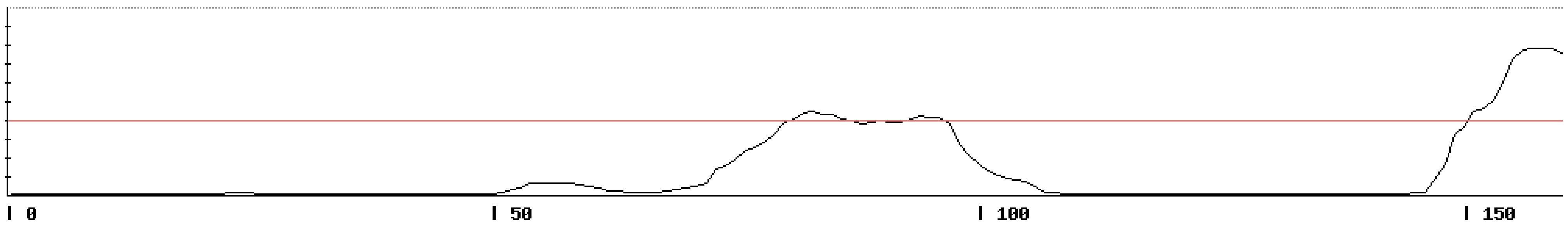

Predictions for Cannabissativa_PK25119.1

Viterbi Path	
Viterbi Path	
Posterior @ 0.4	
Posterior @ 0.4	

Posterior Graphic. Threshold Marked @ 0.4

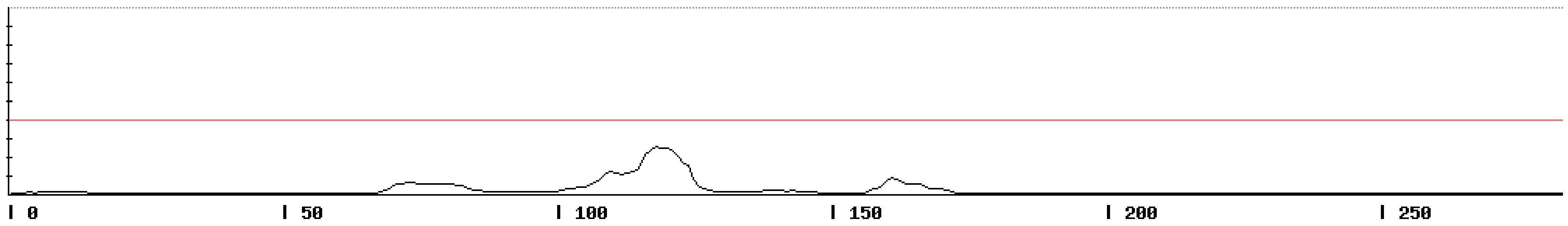

Predictions for Cannabissativa_PK25206.1

Viterbi Path

Viterbi Path

Posterior @ 0.4

Posterior @ 0.4

Posterior Graphic. Threshold Marked @ 0.4

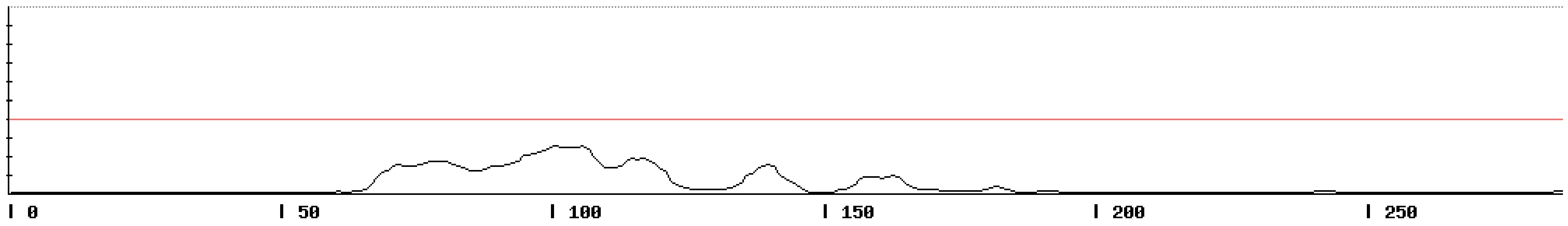

Predictions for Cannabissativa_PK25436.1

Viterbi Path

Viterbi Path

Posterior @ 0.4	153 - RRRK - 156
Posterior @ 0.4	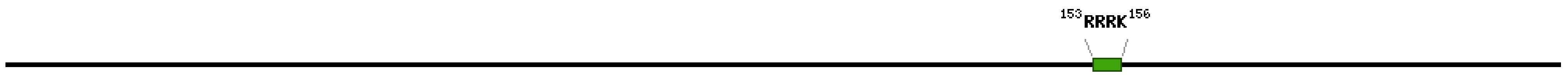
Posterior Graphic. Threshold Marked @ 0.4	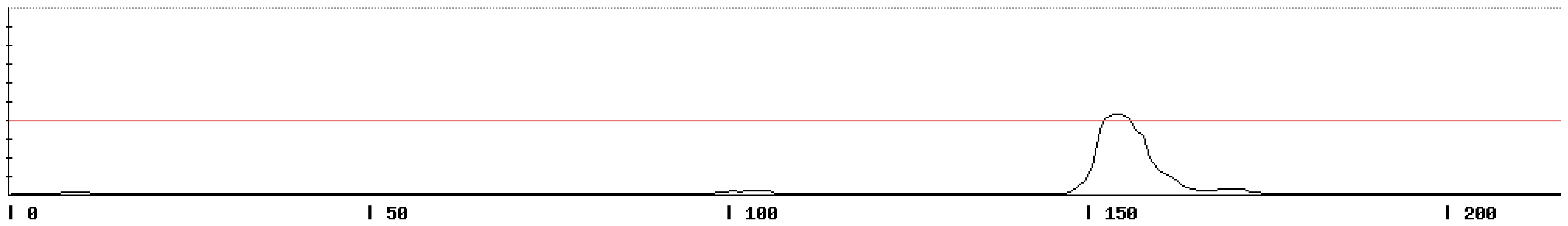

Predictions for Cannabissativa_PK25812.1

Viterbi Path	
Viterbi Path	
Posterior @ 0.4	138 - R - 138
Posterior @ 0.4	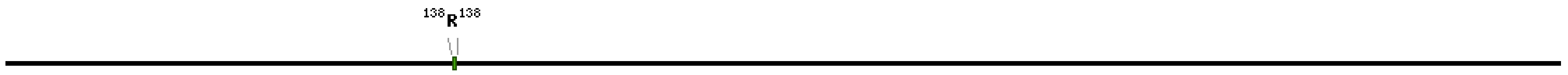
Posterior Graphic. Threshold Marked @ 0.4	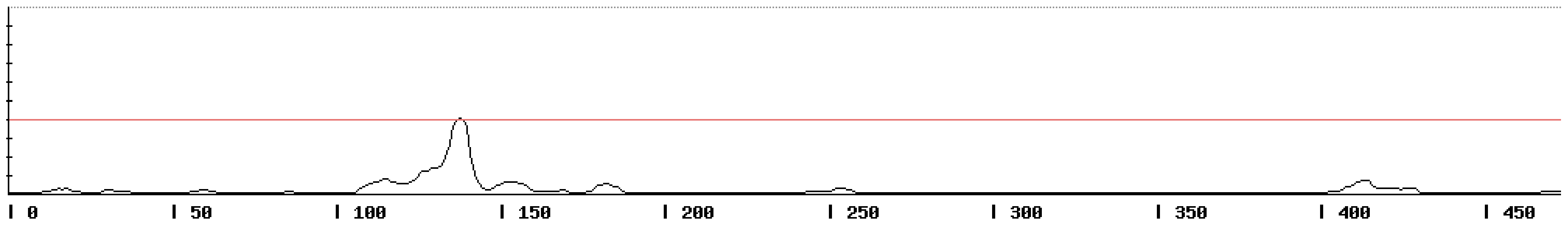

Predictions for Cannabissativa_PK26157.1

Viterbi Path	
Viterbi Path	

Posterior @ 0.4 — 95 - ADKPIGKPKPLGIKKA - 110

Posterior @ 0.4

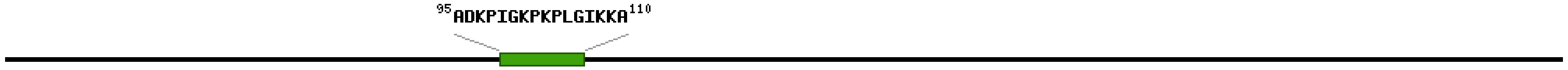

Posterior Graphic. Threshold Marked @ 0.4

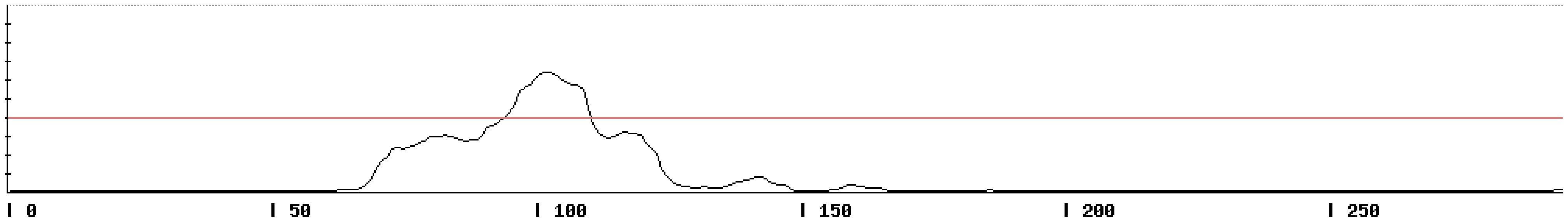

Predictions for Cannabissativa_PK26618.1

Viterbi Path

Viterbi Path

Posterior @ 0.4

Posterior @ 0.4

Posterior Graphic. Threshold Marked @ 0.4

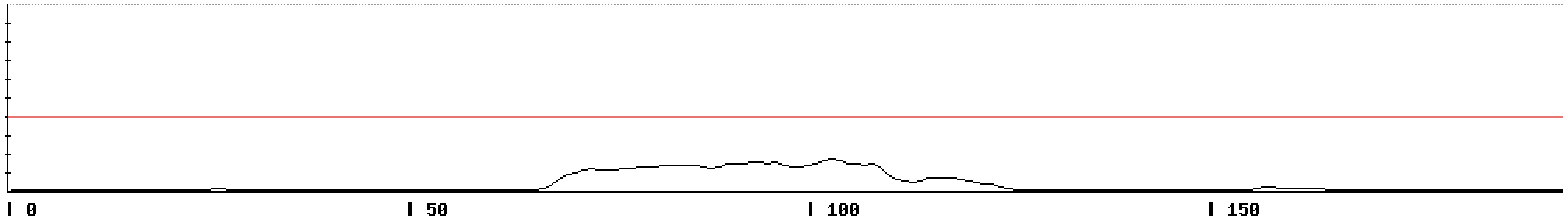

Predictions for Cannabissativa_PK26741.1

Viterbi Path

Viterbi Path

Posterior @ 0.4

Posterior @ 0.4

Posterior Graphic. Threshold Marked @ 0.4

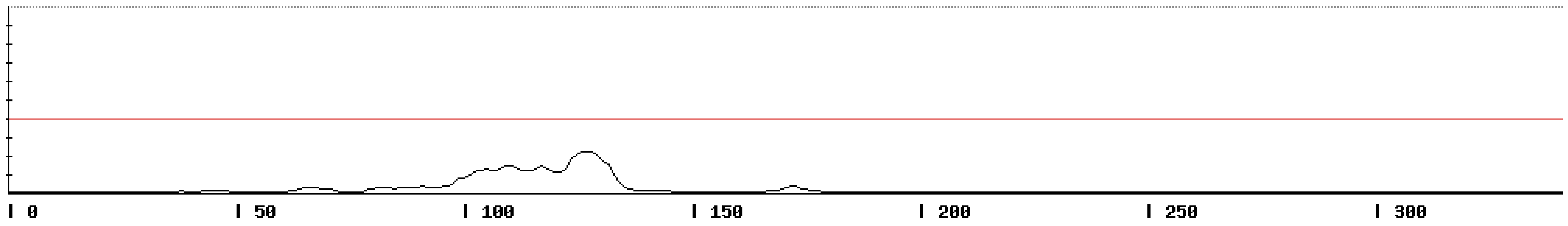

Predictions for Cannabissativa_PK27085.1

Viterbi Path

Viterbi Path

Posterior @ 0.4

Posterior @ 0.4

Posterior Graphic. Threshold Marked @ 0.4

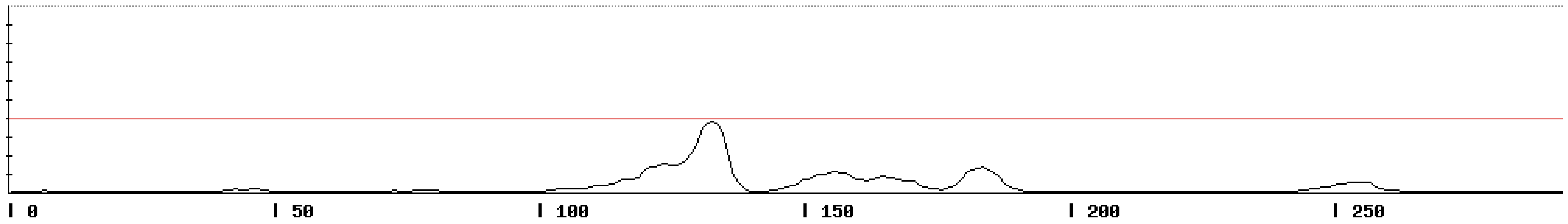

Predictions for Cannabissativa_PK27925.1	
Viterbi Path	
Viterbi Path	
Posterior @ 0.4	
Posterior @ 0.4	
Posterior Graphic. Threshold Marked @ 0.4	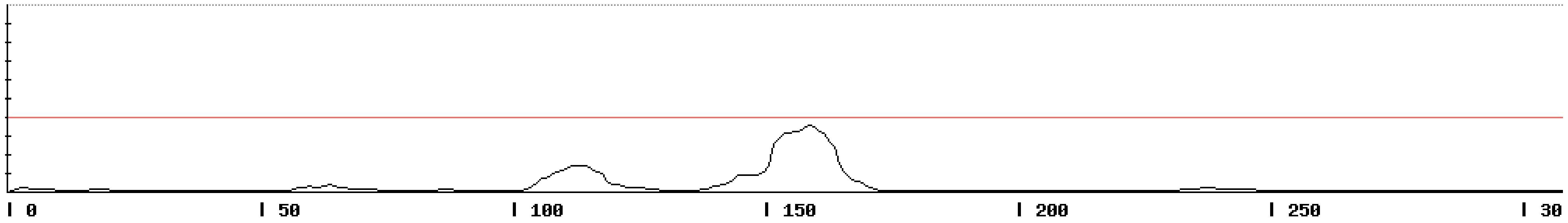

Predictions for Cannabissativa_PK28347.1	
Viterbi Path	
Viterbi Path	
Posterior @ 0.4	118 - RKRRK - 122
Posterior @ 0.4	

Revision r.9 - Last Updated on : February 24 2015 17:45:20.

alex.nguyenba@utoronto.ca

Intended to be used with Firefox

Viterbi Path

Posterior @ 0.4

Posterior @ 0.4

Posterior Graphic. Threshold Marked @ 0.4

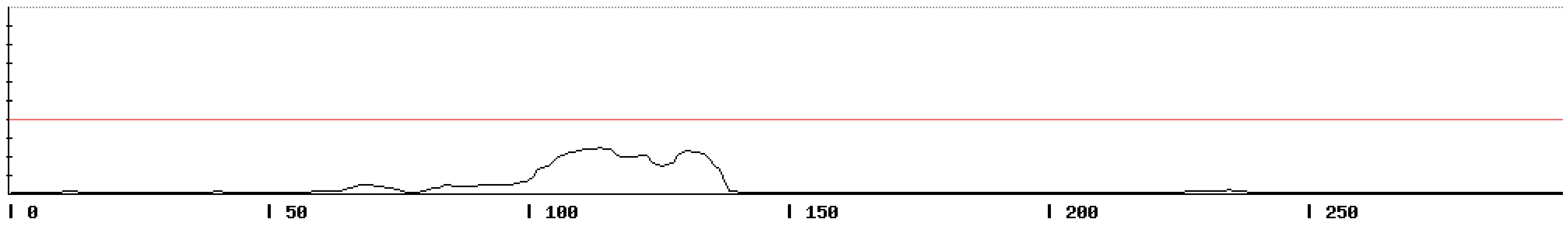

Predictions for Capsellagrandiflora_Cagra.0007s0097

Viterbi Path

Viterbi Path

Posterior @ 0.4

Posterior @ 0.4

Posterior Graphic. Threshold Marked @ 0.4

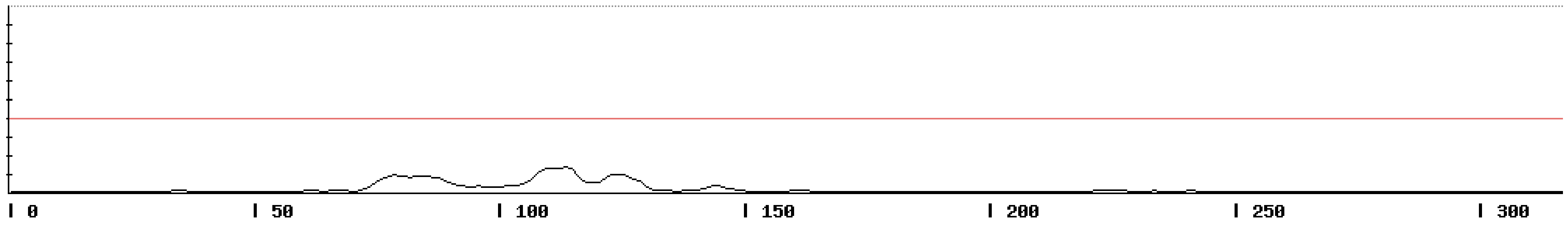

Predictions for Capsellagrandiflora_Cagra.0050s0024

Viterbi Path	
Viterbi Path	
Posterior @ 0.4	
Posterior @ 0.4	
Posterior Graphic. Threshold Marked @ 0.4	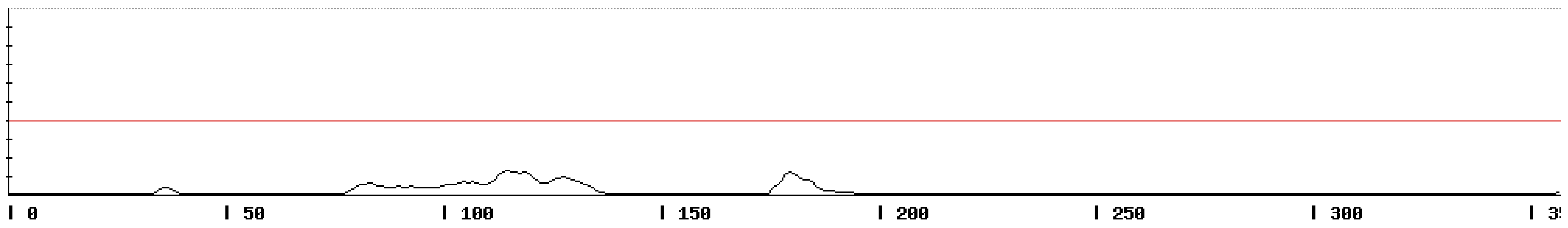

Predictions for Capsellagrandiflora_Cagra.0096s0087

Viterbi Path	
Viterbi Path	
Posterior @ 0.4	98 - KPIGRPKPVGIK - 109
Posterior @ 0.4	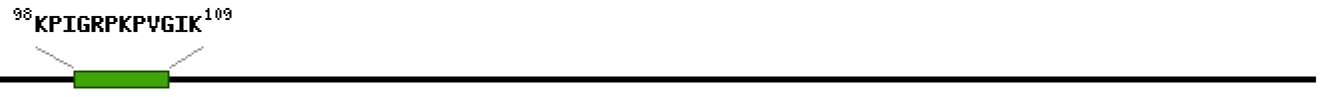
Posterior Graphic. Threshold Marked @ 0.4	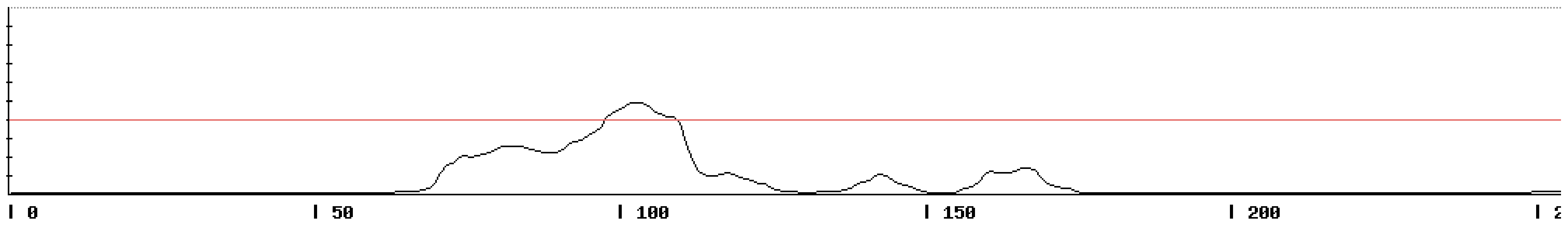

Predictions for Capsellagrandiflora_Cagra.0202s0012	
Viterbi Path	
Viterbi Path	
Posterior @ 0.4	
Posterior @ 0.4	
Posterior Graphic. Threshold Marked @ 0.4	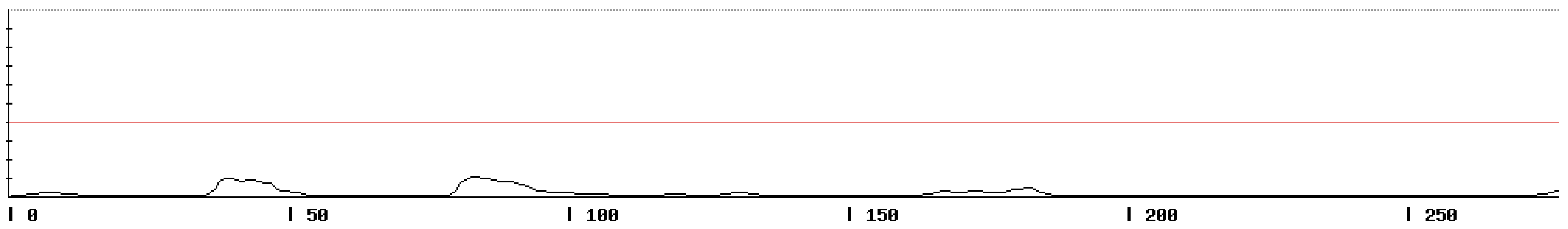

Predictions for Capsellagrandiflora_Cagra.0248s0059	
Viterbi Path	
Viterbi Path	
Posterior @ 0.4	98 - GKPK - 101
Posterior @ 0.4	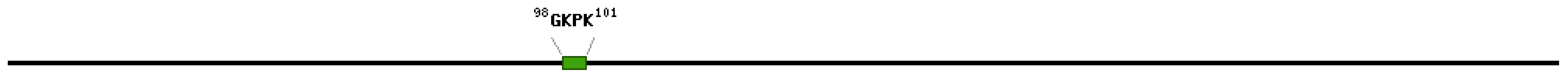
Posterior Graphic. Threshold Marked @ 0.4	

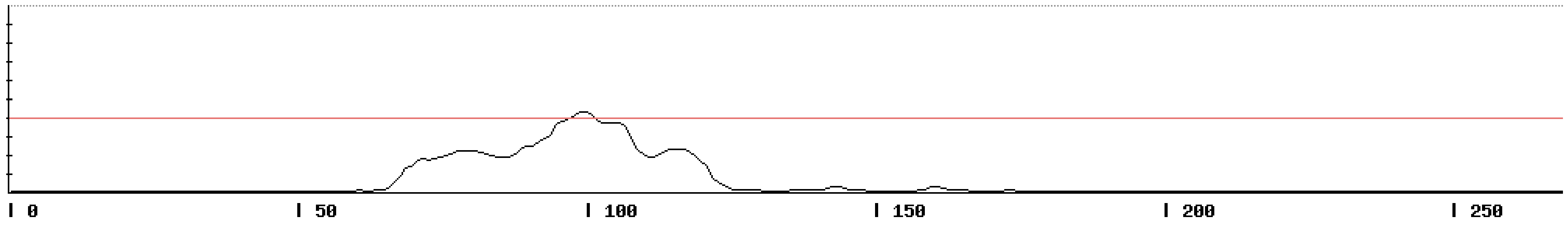

Predictions for Capsellagrandiflora_Cagra.0248s0087

Viterbi Path

Viterbi Path

Posterior @ 0.4

Posterior @ 0.4

Posterior Graphic. Threshold Marked @ 0.4

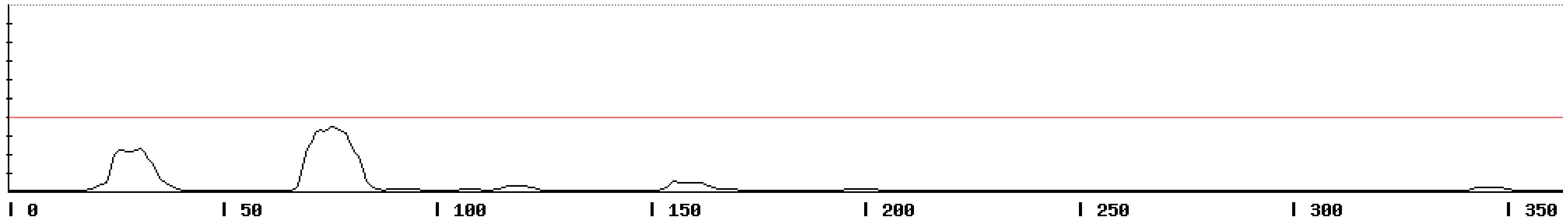

Predictions for Capsellagrandiflora_Cagra.0248s0135

Viterbi Path

Viterbi Path

Posterior @ 0.4

Posterior @ 0.4

Posterior Graphic. Threshold Marked @ 0.4

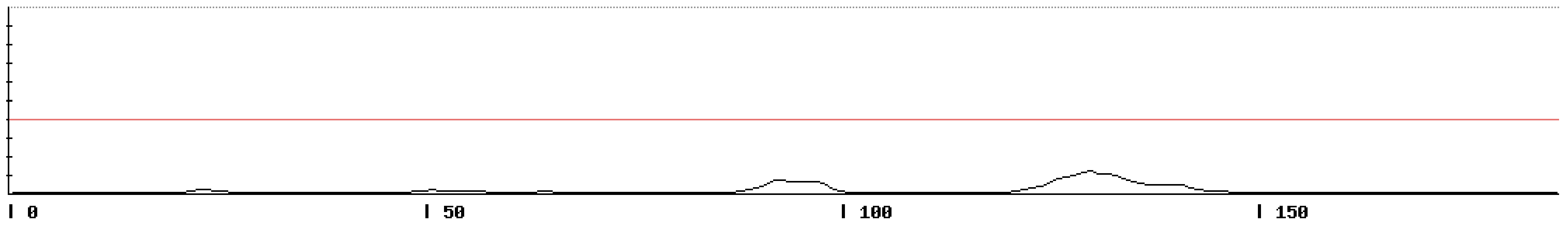

Predictions for Capsellagrandiflora_Cagra.0301s0034

Viterbi Path: 202 - RKADKKKRRRR - 212

Viterbi Path

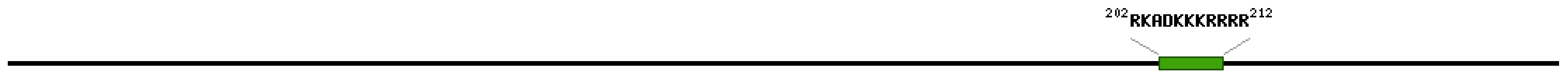

Posterior @ 0.4: 202 - RKADKKKRRRR - 212

Posterior @ 0.4

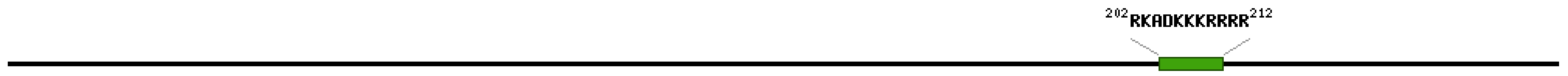

Posterior Graphic. Threshold Marked @ 0.4

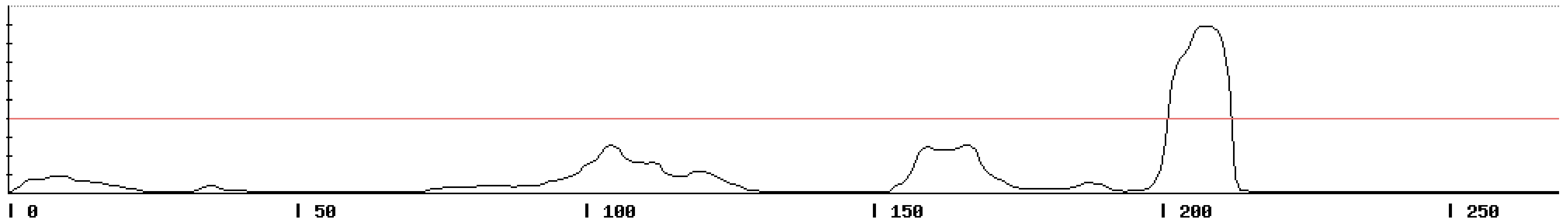

Predictions for Capsellagrandiflora_Cagra.0334s0001

Viterbi Path

Viterbi Path

Posterior @ 0.4: 65 - DRKIKDKRGNRGE - 77

Posterior @ 0.4

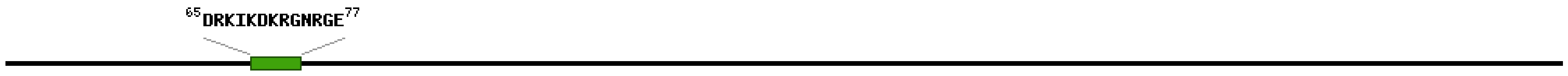

Posterior Graphic. Threshold Marked @ 0.4

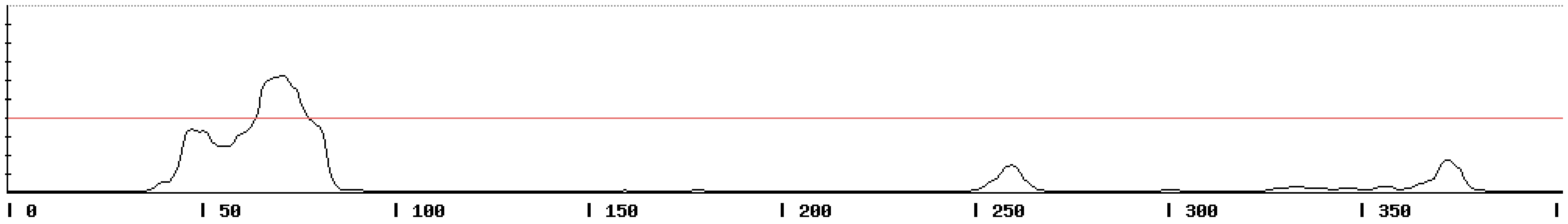

Predictions for Capsellagrandiflora_Cagra.0483s0052

Viterbi Path

Viterbi Path

Posterior @ 0.4

Posterior @ 0.4

Posterior Graphic. Threshold Marked @ 0.4

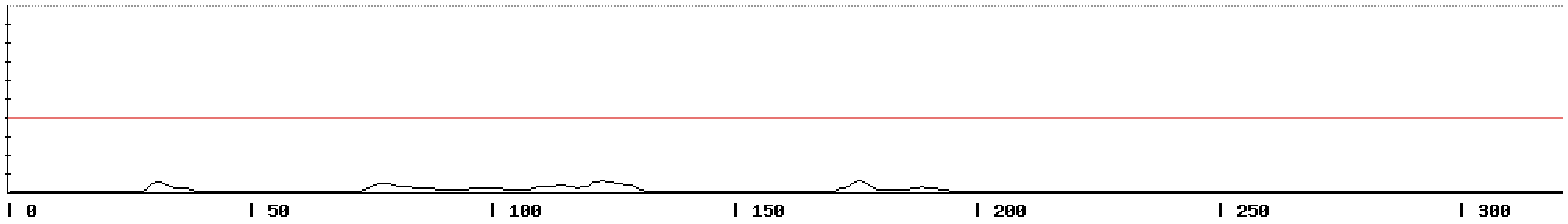

Predictions for Capsellagrandiflora_Cagra.0534s0046

Viterbi Path

Viterbi Path

Posterior @ 0.4

Posterior @ 0.4

Posterior Graphic. Threshold Marked @ 0.4

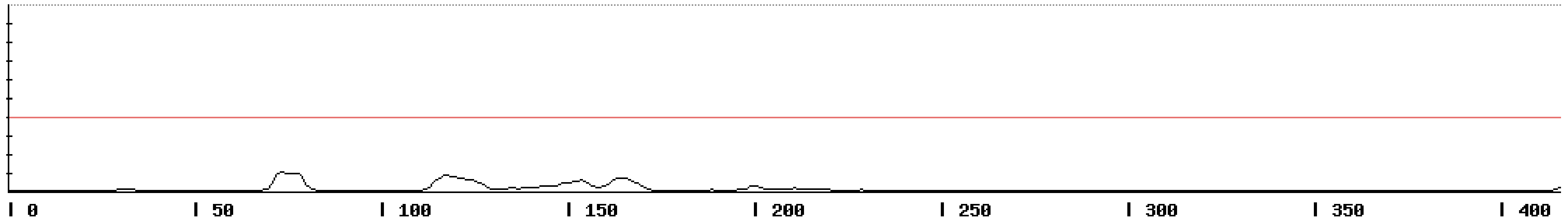

Predictions for Capsellagrandiflora_Cagra.0542s0037

Viterbi Path

Viterbi Path

Posterior @ 0.4

Posterior @ 0.4

Posterior Graphic. Threshold Marked @ 0.4

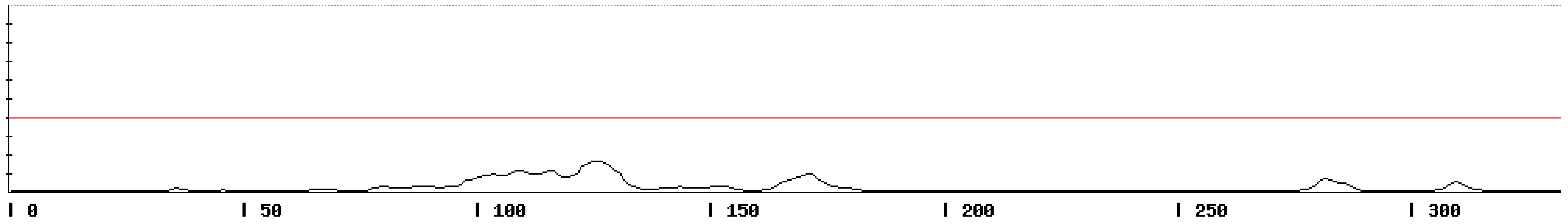

Predictions for Capsellagrandiflora_Cagra.0568s0135

Viterbi Path

Viterbi Path

Posterior @ 0.4

Posterior @ 0.4

Posterior Graphic. Threshold Marked @ 0.4

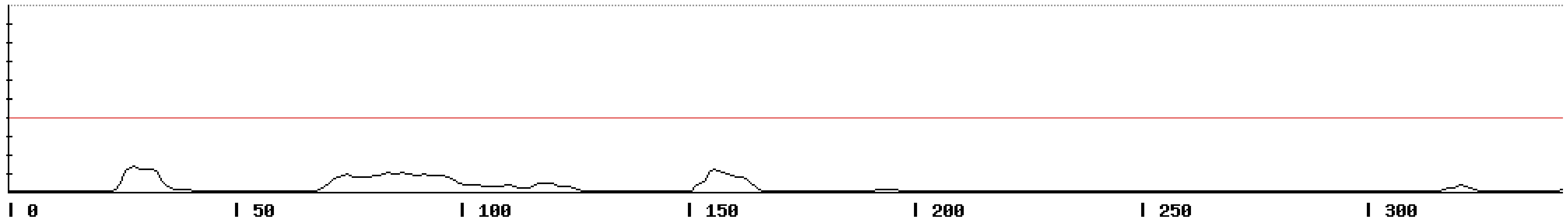

Predictions for Capsellagrandiflora_Cagra.0569s0030

Viterbi Path

Viterbi Path

Posterior @ 0.4

Posterior @ 0.4

Posterior Graphic. Threshold Marked @ 0.4

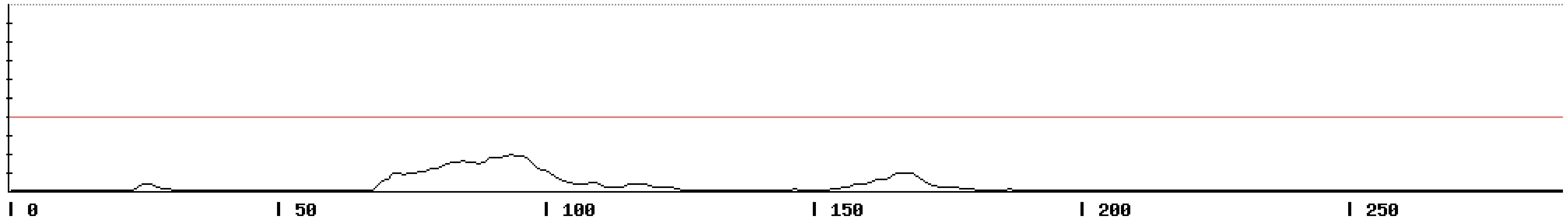

Predictions for Capsellagrandiflora_Cagra.0570s0027

Viterbi Path

Viterbi Path

Posterior @ 0.4

Posterior @ 0.4

Posterior Graphic. Threshold Marked @ 0.4

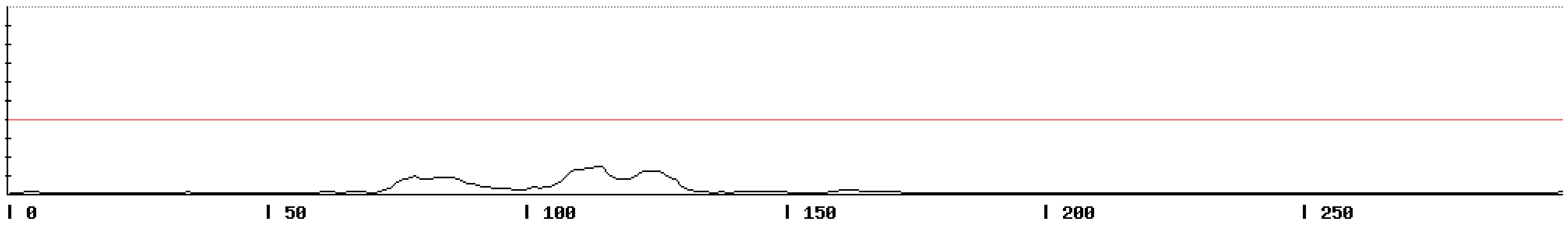

Predictions for Capsellagrandiflora_Cagra.0596s0010

Viterbi Path

Viterbi Path

Posterior @ 0.4

Posterior @ 0.4

Posterior Graphic. Threshold Marked @ 0.4

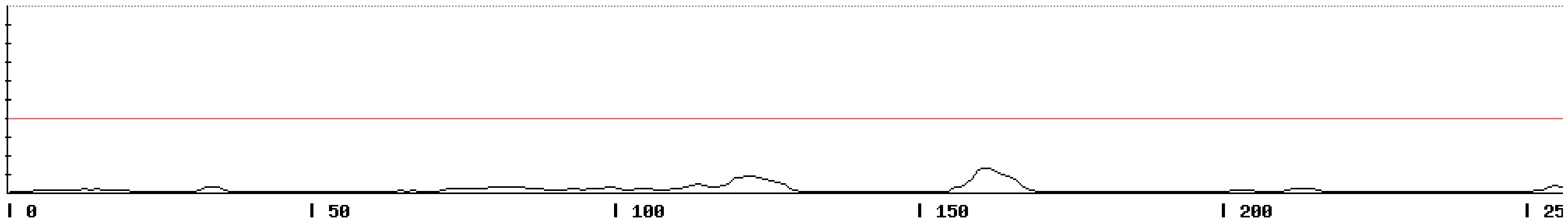

Predictions for Capsellagrandiflora_Cagra.0799s0114	
Viterbi Path	
Viterbi Path	
Posterior @ 0.4	
Posterior @ 0.4	
Posterior Graphic. Threshold Marked @ 0.4	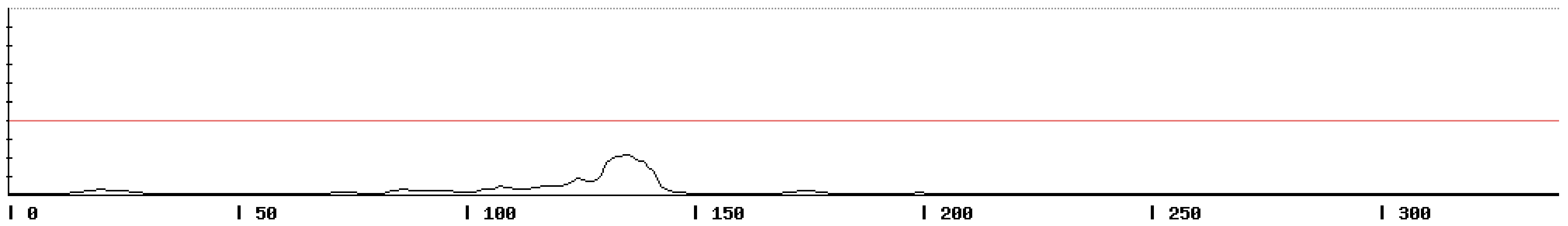

Predictions for Capsellagrandiflora_Cagra.0805s0005	
Viterbi Path	
Viterbi Path	
Posterior @ 0.4	
Posterior @ 0.4	
Posterior Graphic. Threshold Marked @ 0.4	

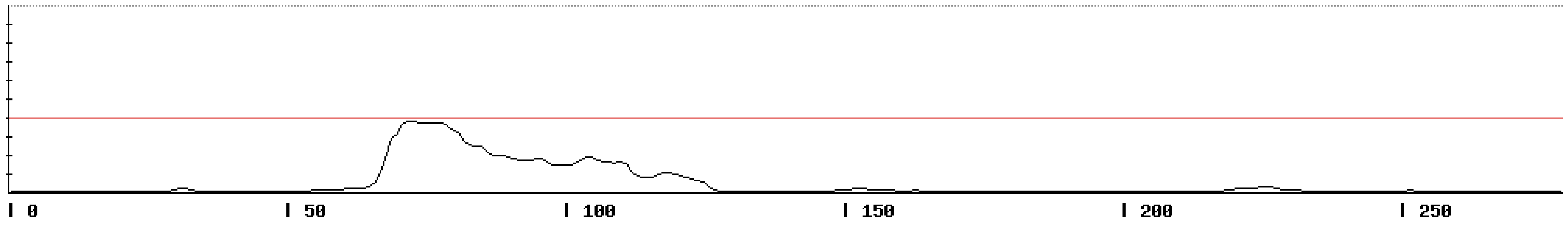

Predictions for Capsellagrandiflora_Cagra.0834s0027

Viterbi Path

Viterbi Path

Posterior @ 0.4

Posterior @ 0.4

Posterior Graphic. Threshold Marked @ 0.4

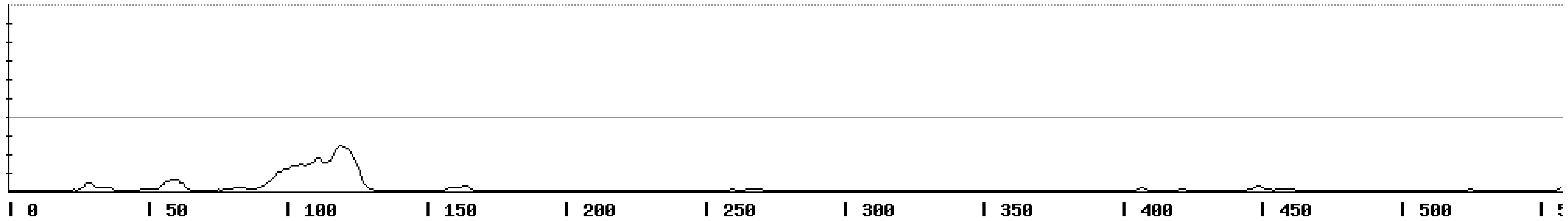

Predictions for Capsellagrandiflora_Cagra.0834s0028

Viterbi Path

Viterbi Path

Posterior @ 0.4

Posterior @ 0.4

Posterior Graphic. Threshold Marked @ 0.4

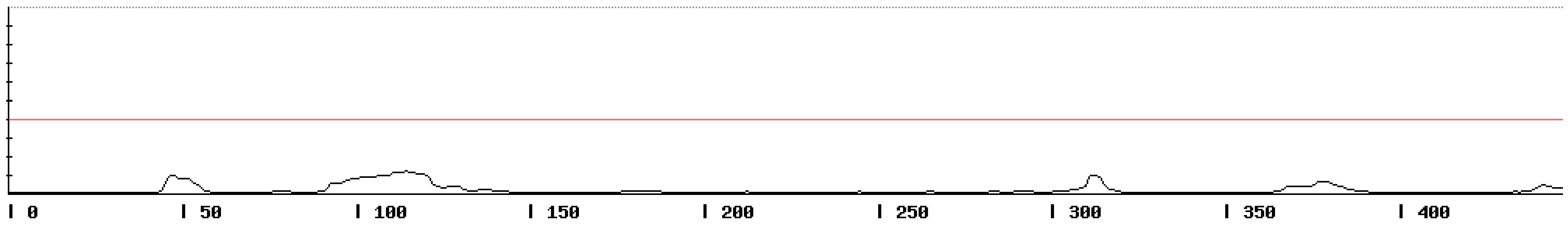

Predictions for Capsellagrandiflora_Cagra.0834s0029

Viterbi Path

Viterbi Path

Posterior @ 0.4

Posterior @ 0.4

Posterior Graphic. Threshold Marked @ 0.4

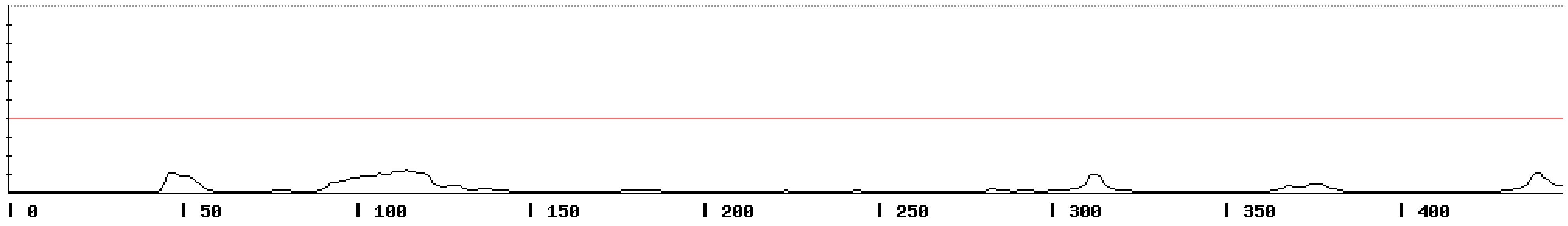

Predictions for Capsellagrandiflora_Cagra.0846s0004

Viterbi Path

Viterbi Path

Posterior @ 0.4

Posterior @ 0.4

Posterior Graphic. Threshold Marked @ 0.4

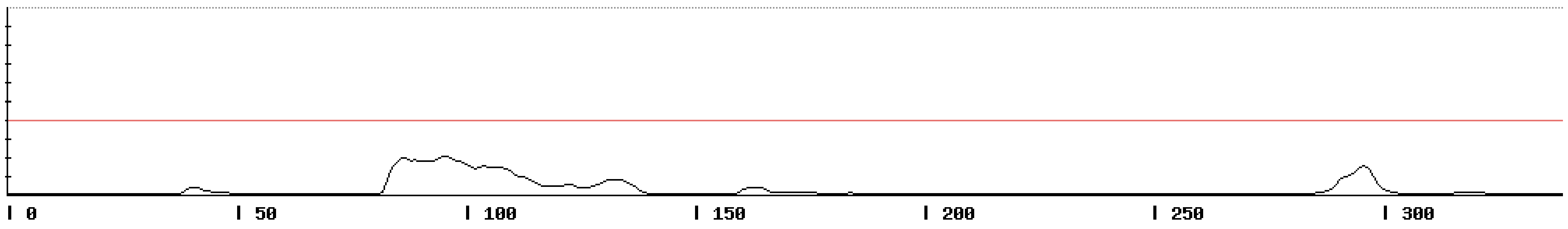

Predictions for Capsellagrandiflora_Cagra.0846s0014

Viterbi Path

Viterbi Path

Posterior @ 0.4

Posterior @ 0.4

Posterior Graphic. Threshold Marked @ 0.4

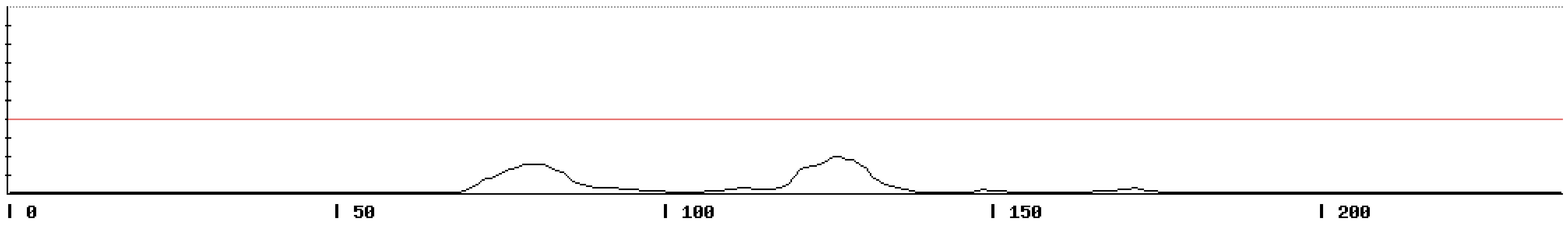

Predictions for Capsellagrandiflora_Cagra.0887s0008

Viterbi Path

Viterbi Path

Posterior @ 0.4	
Posterior @ 0.4	
Posterior Graphic. Threshold Marked @ 0.4	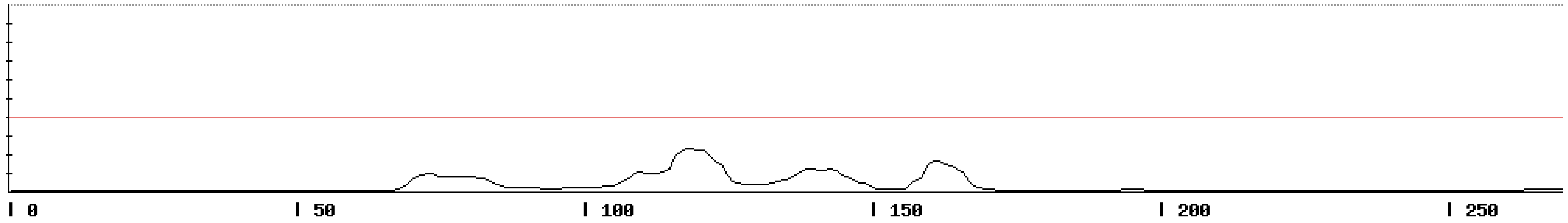

Predictions for Capsellagrandiflora_Cagra.0897s0030

Viterbi Path	
Viterbi Path	
Posterior @ 0.4	41 - PRTKRHRNKPRRTVPPRGGS - 60
Posterior @ 0.4	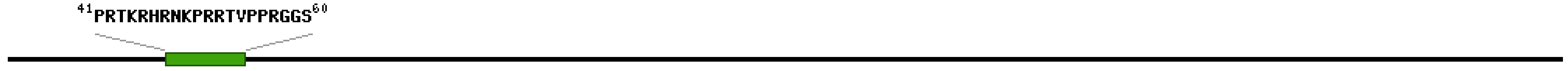
Posterior Graphic. Threshold Marked @ 0.4	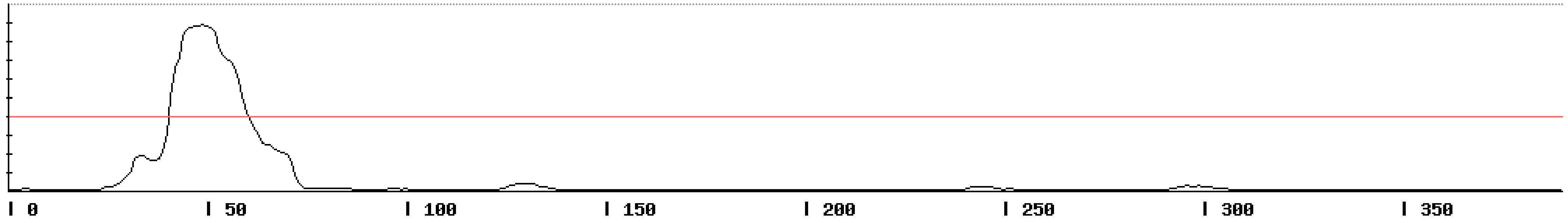

Predictions for Capsellagrandiflora_Cagra.0906s0001

Viterbi Path	
Viterbi Path	

Posterior @ 0.4

Posterior @ 0.4

Posterior Graphic. Threshold Marked @ 0.4

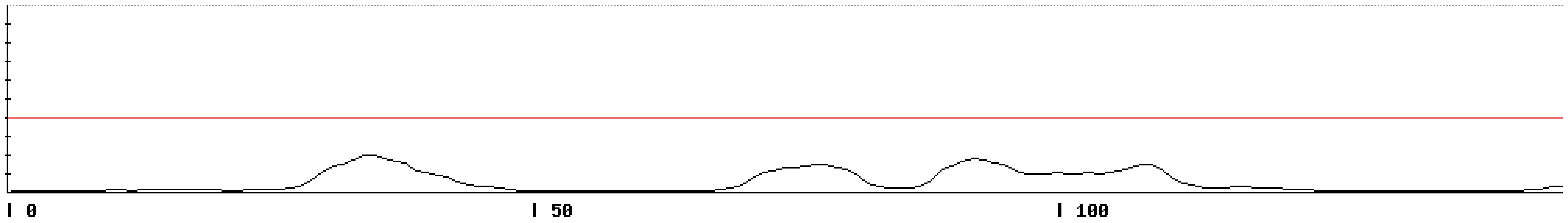

Predictions for Capsellagrandiflora_Cagra.0906s0022

Viterbi Path

Viterbi Path

Posterior @ 0.4

Posterior @ 0.4

Posterior Graphic. Threshold Marked @ 0.4

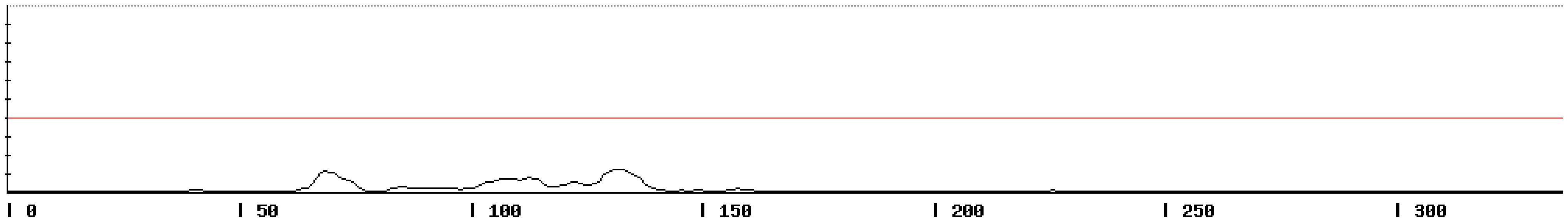

Predictions for Capsellagrandiflora_Cagra.0925s0069

Viterbi Path

Viterbi Path

Posterior @ 0.4 — 203 - KAAKKRKAE - 211

Posterior @ 0.4

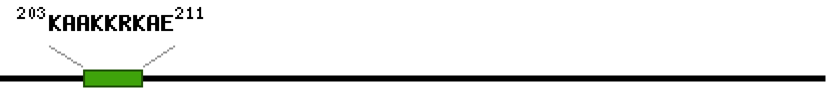

Posterior Graphic. Threshold Marked @ 0.4

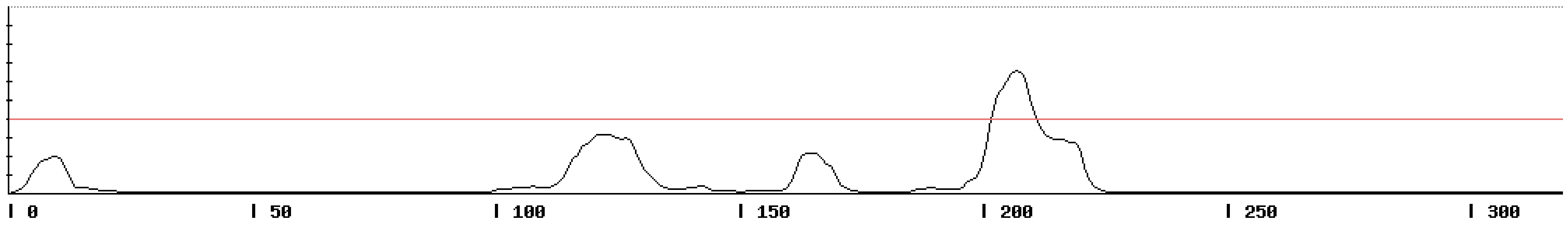

Predictions for Capsellagrandiflora_Cagra.0946s0024

Viterbi Path

Viterbi Path

Posterior @ 0.4

Posterior @ 0.4

Posterior Graphic. Threshold Marked @ 0.4

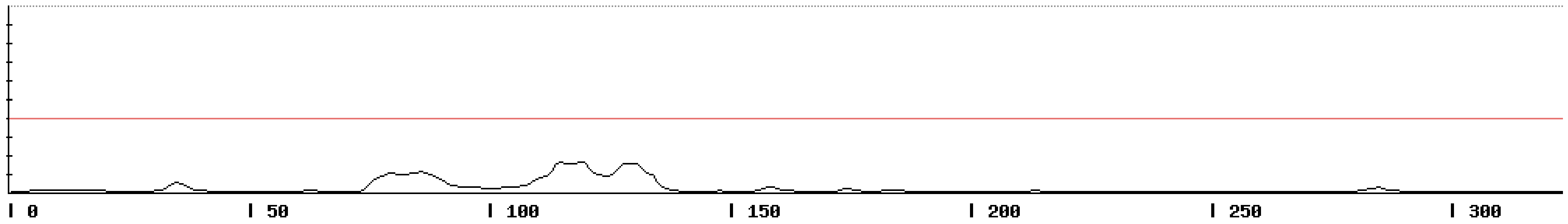

Predictions for Capsellagrandiflora_Cagra.0946s0025

Viterbi Path

Viterbi Path

Posterior @ 0.4

Posterior @ 0.4

Posterior Graphic. Threshold Marked @ 0.4

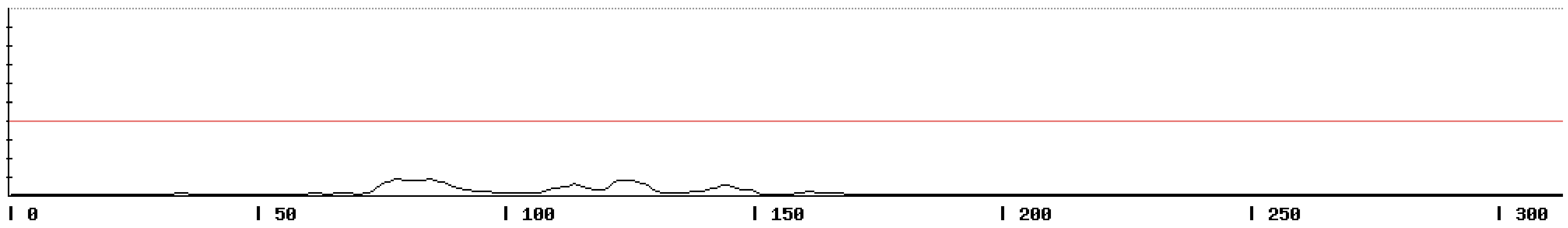

Predictions for Capsellagrandiflora_Cagra.10427s0040

Viterbi Path

Viterbi Path

Posterior @ 0.4

Posterior @ 0.4

Posterior Graphic. Threshold Marked @ 0.4

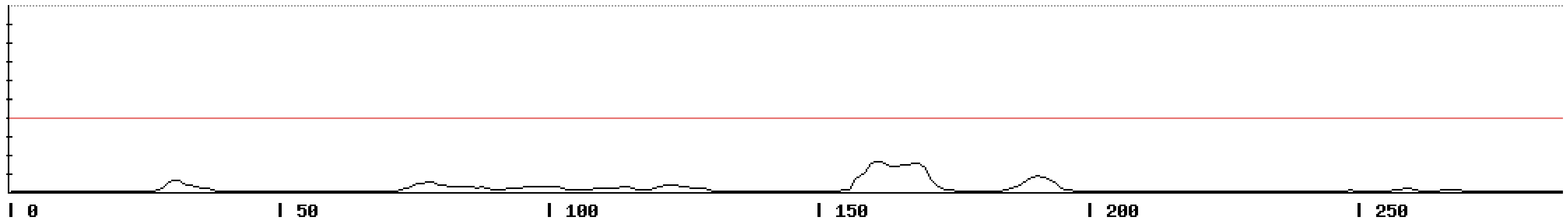

**Predictions for Capsellagrandiflora_Cagra.11446s0003**	
Viterbi Path	
Viterbi Path	
Posterior @ 0.4	
Posterior @ 0.4	
Posterior Graphic. Threshold Marked @ 0.4	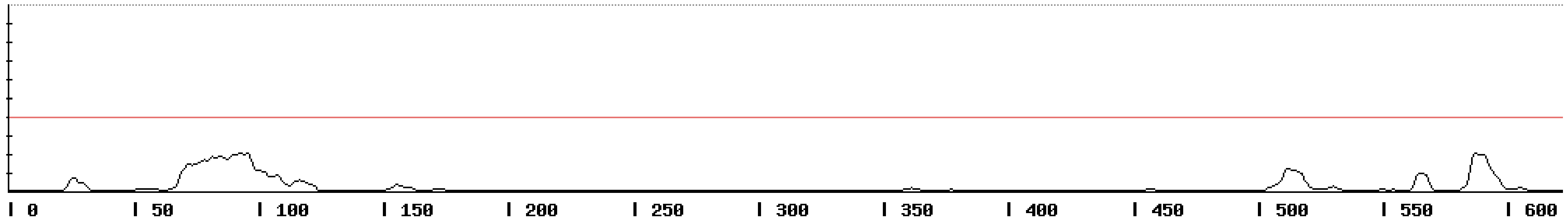

**Predictions for Capsellagrandiflora_Cagra.1150s0013**	
Viterbi Path	
Viterbi Path	
Posterior @ 0.4	
Posterior @ 0.4	

Posterior Graphic. Threshold Marked @ 0.4

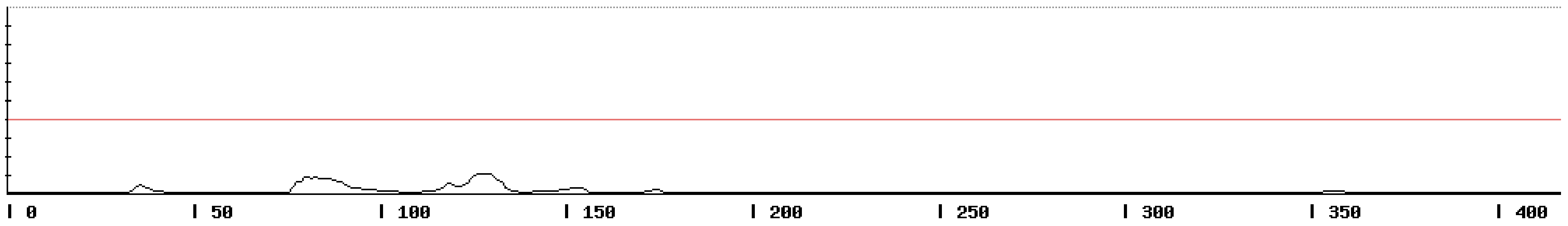

Predictions for Capsellagrandiflora_Cagra.11546s0003

Viterbi Path

Viterbi Path

Posterior @ 0.4

137 - KRR - 139

Posterior @ 0.4

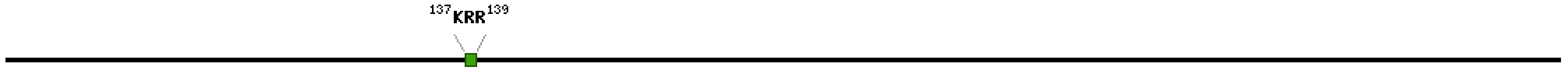

Posterior Graphic. Threshold Marked @ 0.4

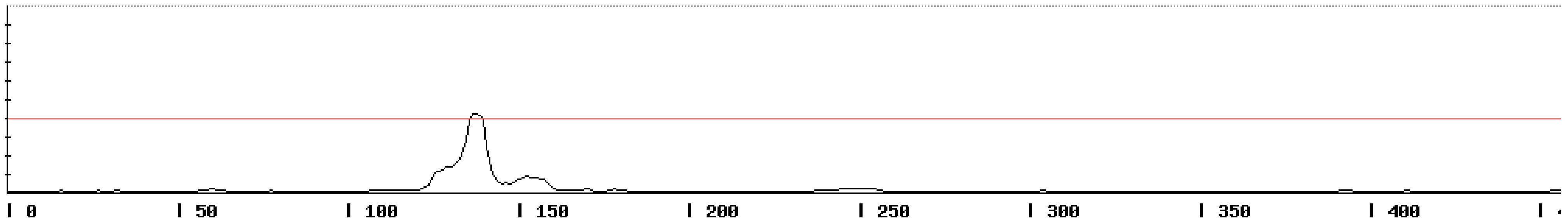

Predictions for Capsellagrandiflora_Cagra.1225s0038

Viterbi Path

Viterbi Path

Posterior @ 0.4

Posterior @ 0.4

Posterior Graphic. Threshold Marked @ 0.4

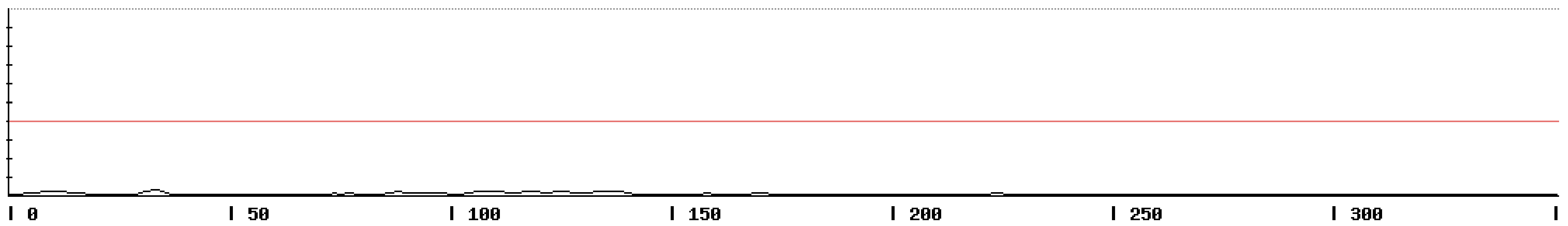

Predictions for Capsellagrandiflora_Cagra.12529s0001

Viterbi Path

Viterbi Path

Posterior @ 0.4

Posterior @ 0.4

Posterior Graphic. Threshold Marked @ 0.4

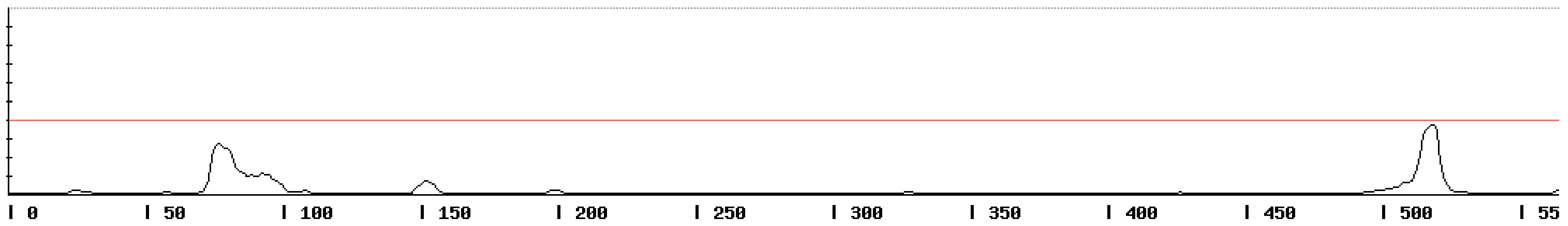

Predictions for Capsellagrandiflora_Cagra.12686s0005

Viterbi Path

Viterbi Path

Posterior @ 0.4

Posterior @ 0.4

Posterior Graphic. Threshold Marked @ 0.4

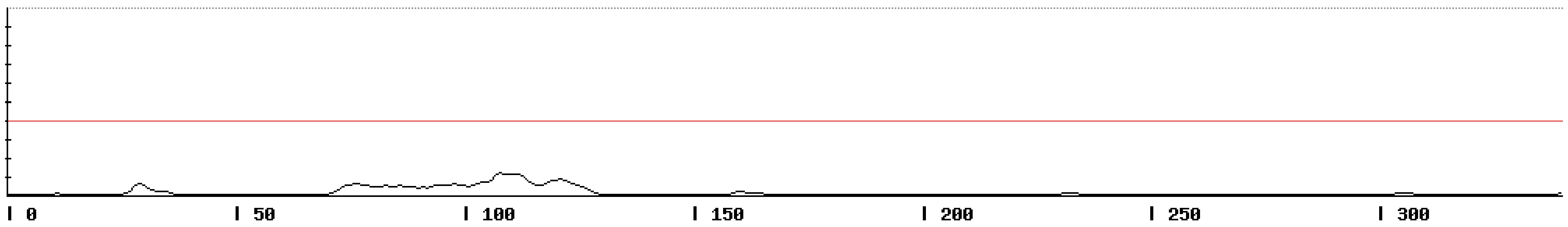

Predictions for Capsellagrandiflora_Cagra.1301s0009

Viterbi Path

Viterbi Path

Posterior @ 0.4

76 - KK - 77

83 - VKRKPRE - 89

Posterior @ 0.4

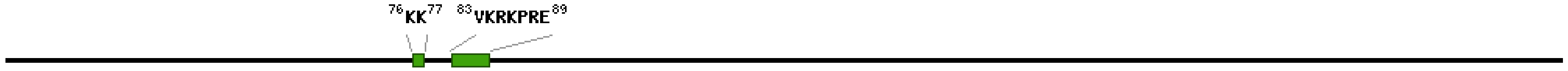

Posterior Graphic. Threshold Marked @ 0.4

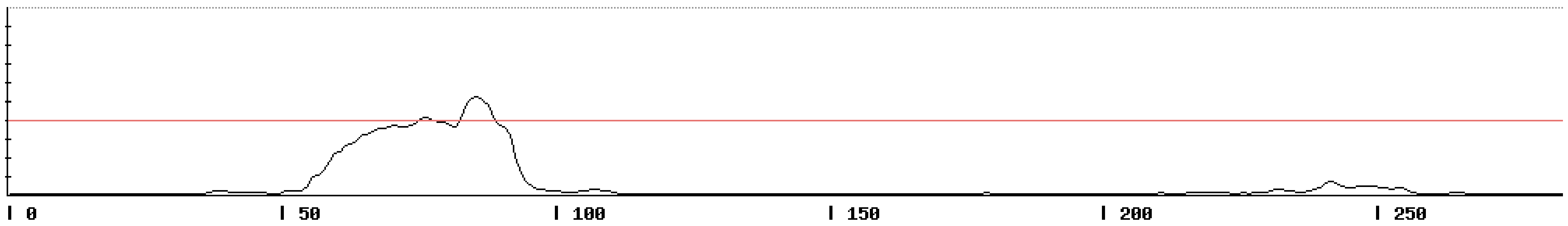

Predictions for Capsellagrandiflora_Cagra.1301s0010

Viterbi Path

Viterbi Path

Posterior @ 0.4	
Posterior @ 0.4	
Posterior Graphic. Threshold Marked @ 0.4	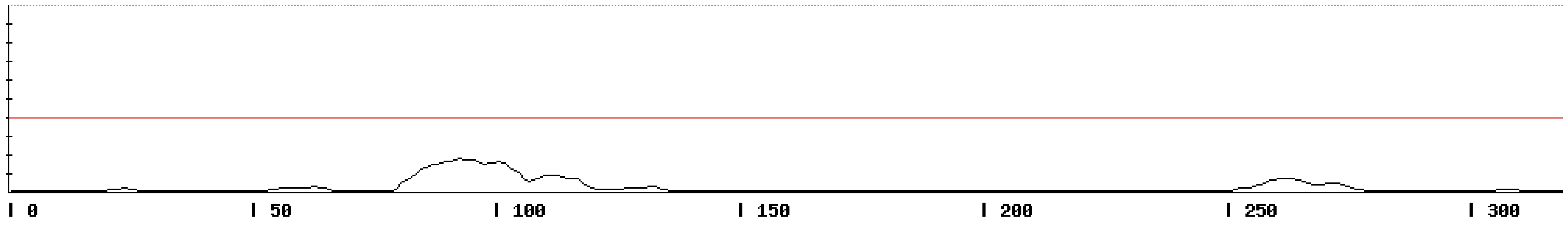

Predictions for Capsellagrandiflora_Cagra.1310s0016	
Viterbi Path	
Viterbi Path	
Posterior @ 0.4	
Posterior @ 0.4	
Posterior Graphic. Threshold Marked @ 0.4	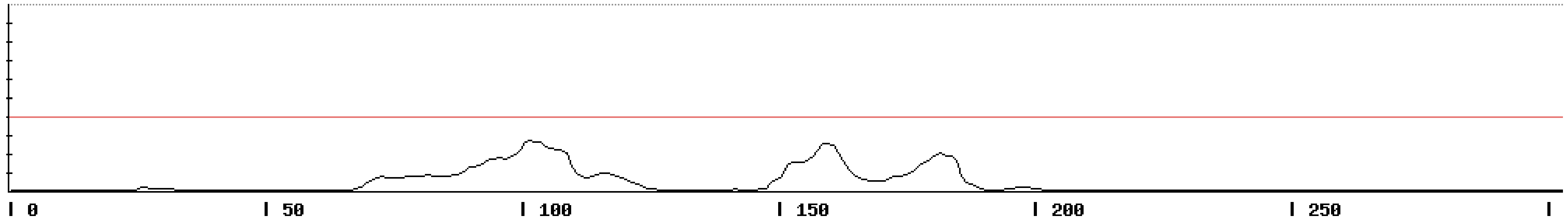

Predictions for Capsellagrandiflora_Cagra.1365s0094

Viterbi Path

Viterbi Path

Posterior @ 0.4

Posterior @ 0.4

Posterior Graphic. Threshold Marked @ 0.4

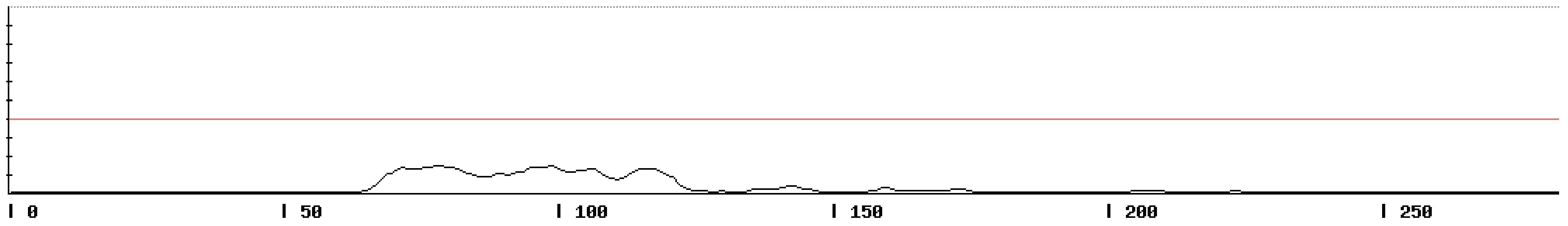

Predictions for Capsellagrandiflora_Cagra.1365s0106

Viterbi Path

Viterbi Path

Posterior @ 0.4

Posterior @ 0.4

Posterior Graphic. Threshold Marked @ 0.4

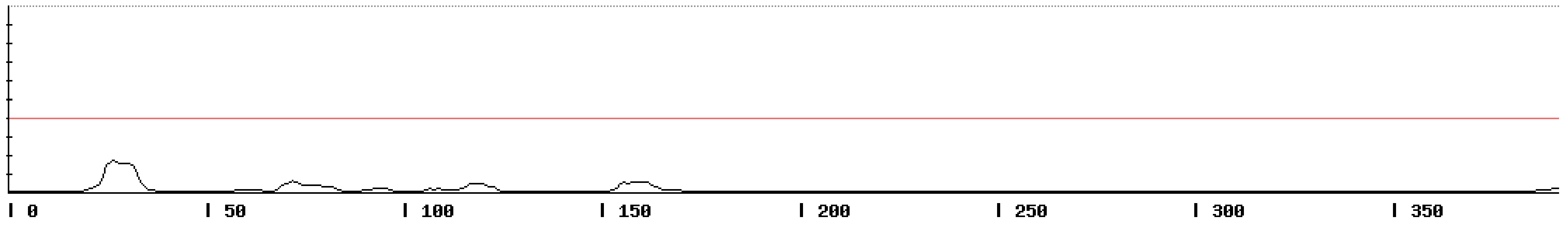

Predictions for Capsellagrandiflora_Cagra.1378s0012	
Viterbi Path	
Viterbi Path	
Posterior @ 0.4	
Posterior @ 0.4	
Posterior Graphic. Threshold Marked @ 0.4	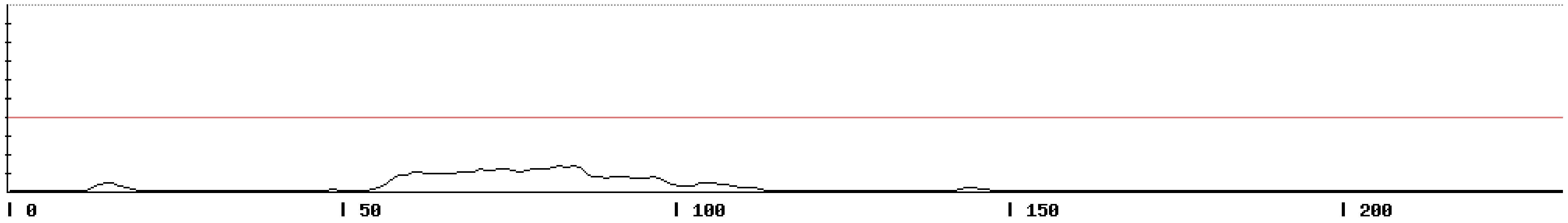

Predictions for Capsellagrandiflora_Cagra.1443s0001	
Viterbi Path	
Viterbi Path	
Posterior @ 0.4	
Posterior @ 0.4	
Posterior Graphic. Threshold Marked @ 0.4	

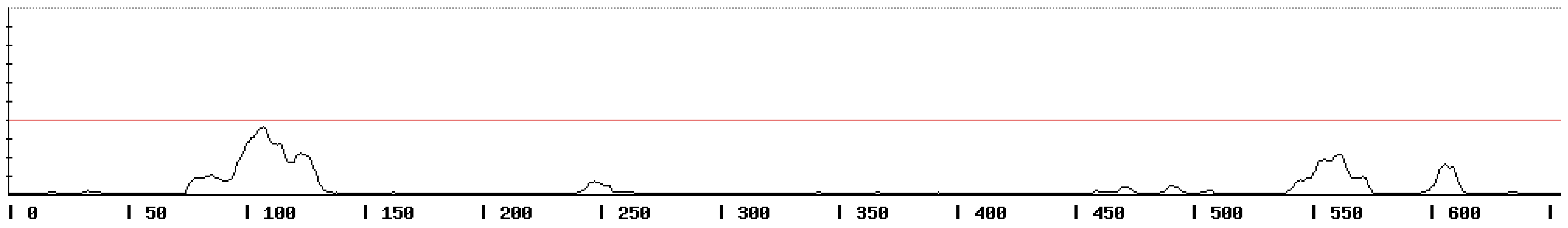

Predictions for Capsellagrandiflora_Cagra.1443s0015

Viterbi Path

Viterbi Path

Posterior @ 0.4

30 - KRKIRRKKLR - 39

485 - NRKKSGRKNDKKNNKSK - 501

Posterior @ 0.4

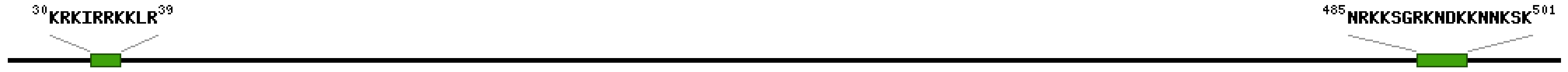

Posterior Graphic. Threshold Marked @ 0.4

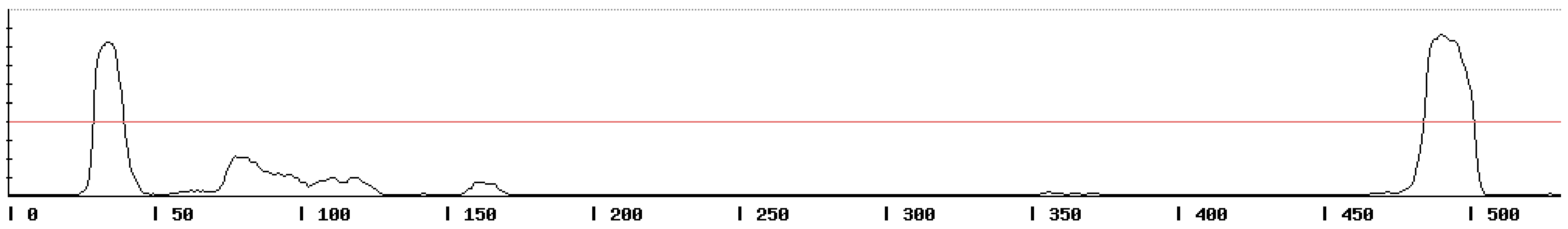

Predictions for Capsellagrandiflora_Cagra.14513s0001

Viterbi Path

Viterbi Path

Posterior @ 0.4

Posterior @ 0.4

Posterior Graphic. Threshold Marked @ 0.4

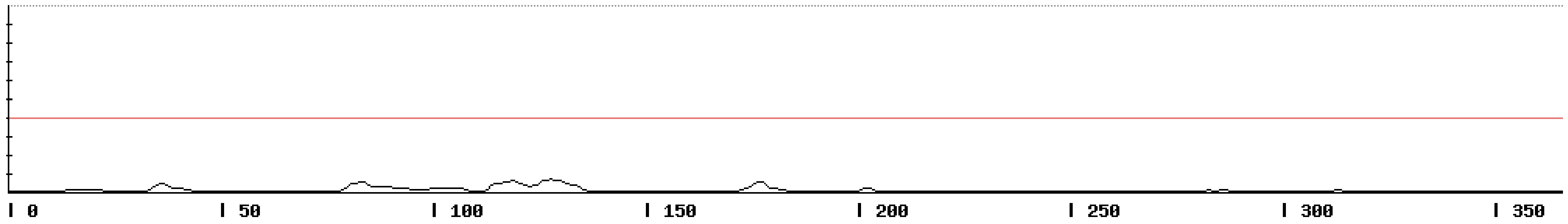

Predictions for Capsellagrandiflora_Cagra.1670s0031

Viterbi Path

Viterbi Path

Posterior @ 0.4

Posterior @ 0.4

Posterior Graphic. Threshold Marked @ 0.4

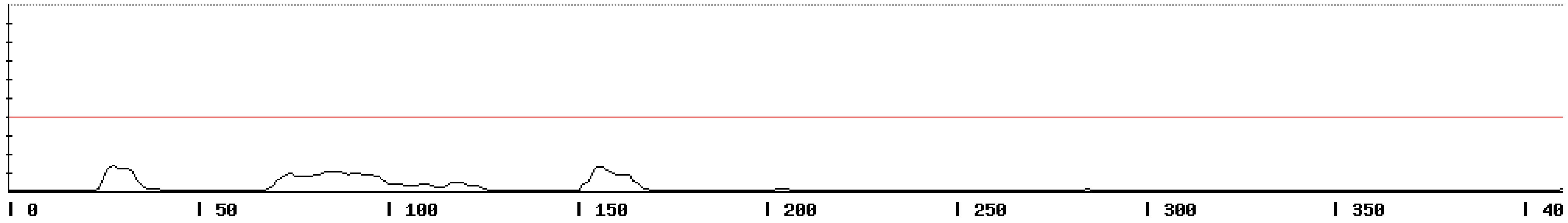

Predictions for Capsellagrandiflora_Cagra.1681s0009

Viterbi Path

Viterbi Path

Posterior @ 0.4

Posterior @ 0.4

Posterior Graphic. Threshold Marked @ 0.4

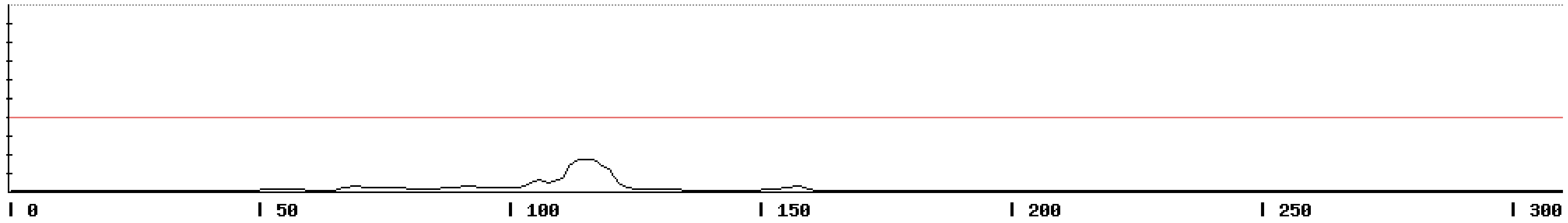

Predictions for Capsellagrandiflora_Cagra.1725s0032

Viterbi Path

Viterbi Path

Posterior @ 0.4

Posterior @ 0.4

Posterior Graphic. Threshold Marked @ 0.4

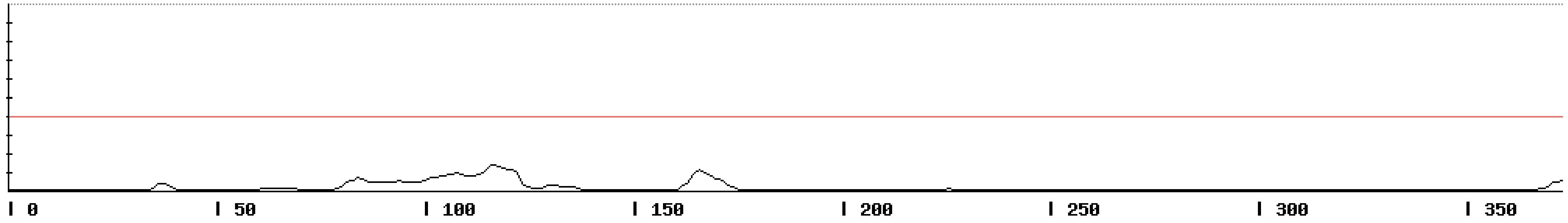

Predictions for Capsellagrandiflora_Cagra.1822s0012

Viterbi Path

Viterbi Path

Posterior @ 0.4	156 - KRR - 158
Posterior @ 0.4	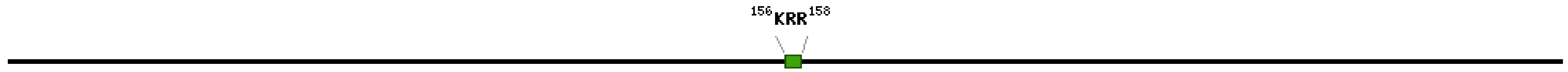
Posterior Graphic. Threshold Marked @ 0.4	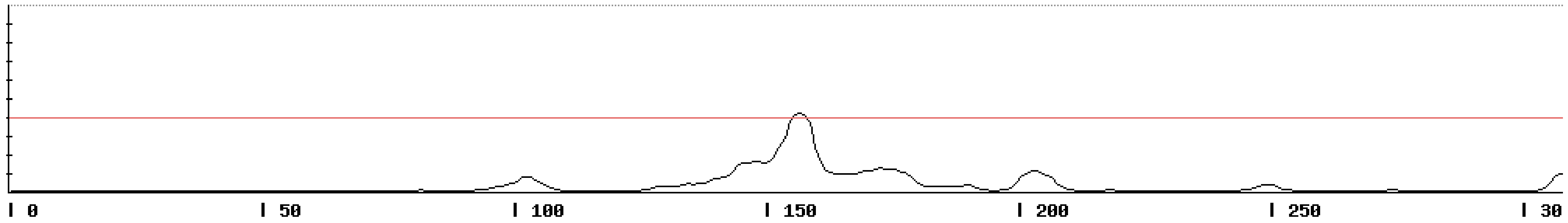

Predictions for Capsellagrandiflora_Cagra.1838s0033

Viterbi Path	
Viterbi Path	
Posterior @ 0.4	
Posterior @ 0.4	
Posterior Graphic. Threshold Marked @ 0.4	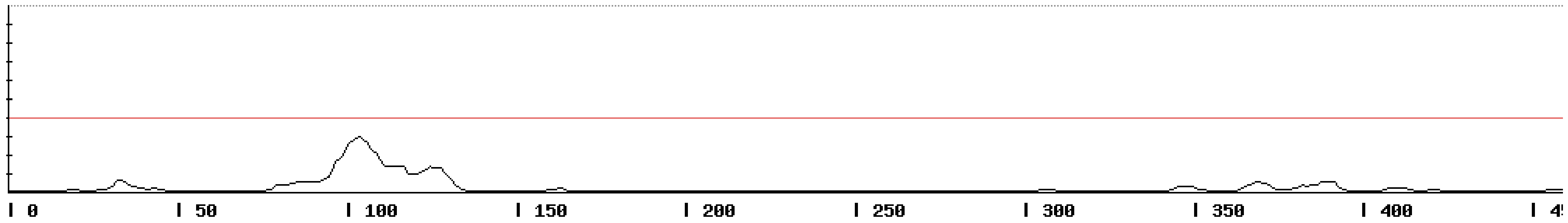

Predictions for Capsellagrandiflora_Cagra.1968s0008

Viterbi Path

Viterbi Path

Posterior @ 0.4

Posterior @ 0.4

Posterior Graphic. Threshold Marked @ 0.4

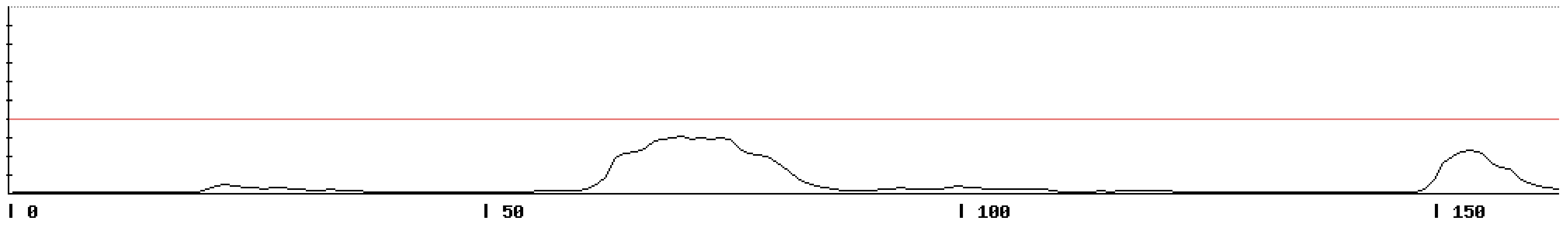

Predictions for Capsellagrandiflora_Cagra.1968s0009

Viterbi Path

Viterbi Path

Posterior @ 0.4

Posterior @ 0.4

Posterior Graphic. Threshold Marked @ 0.4

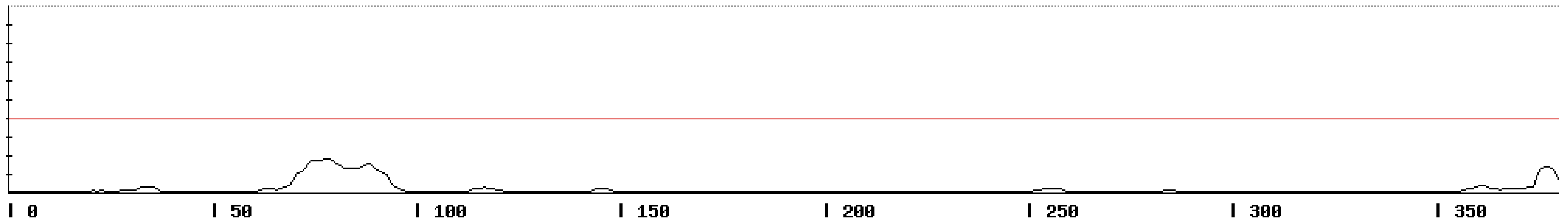

Predictions for Capsellagrandiflora_Cagra.1968s0040	
Viterbi Path	
Viterbi Path	
Posterior @ 0.4	
Posterior @ 0.4	
Posterior Graphic. Threshold Marked @ 0.4	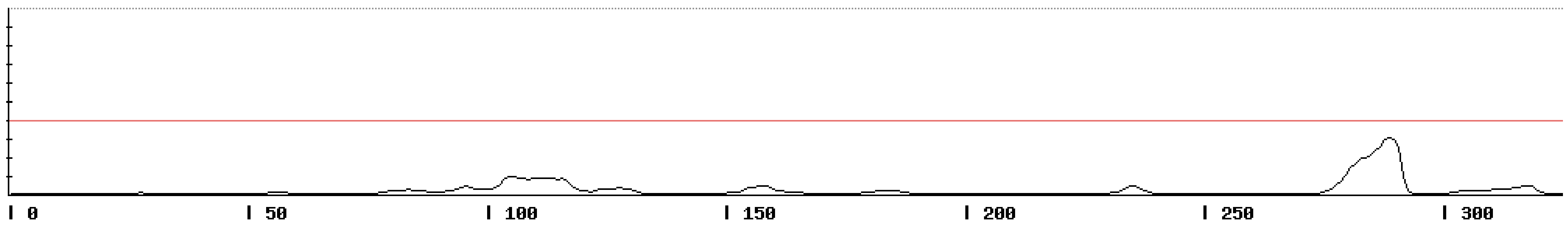

Predictions for Capsellagrandiflora_Cagra.1968s0041	
Viterbi Path	
Viterbi Path	
Posterior @ 0.4	81 - KYDKGERQIRKTKSGFWKITGKPKDIMRNRVKIGEK - 116
Posterior @ 0.4	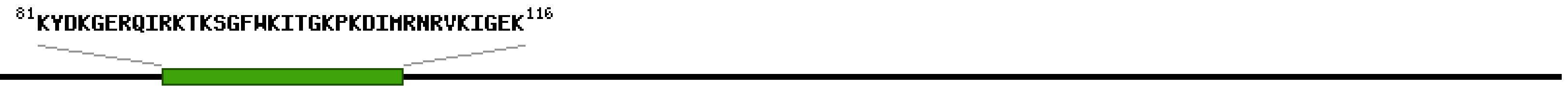
Posterior Graphic. Threshold Marked @ 0.4	

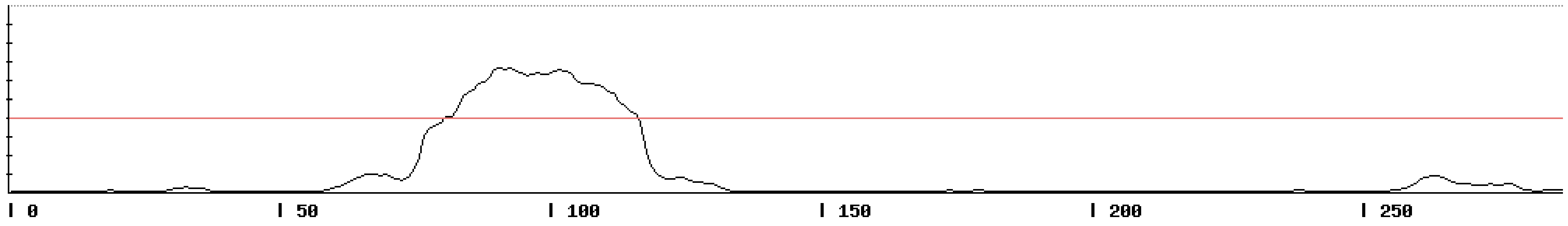

Predictions for Capsellagrandiflora_Cagra.1968s0043

Viterbi Path

Viterbi Path

Posterior @ 0.4

Posterior @ 0.4

Posterior Graphic. Threshold Marked @ 0.4

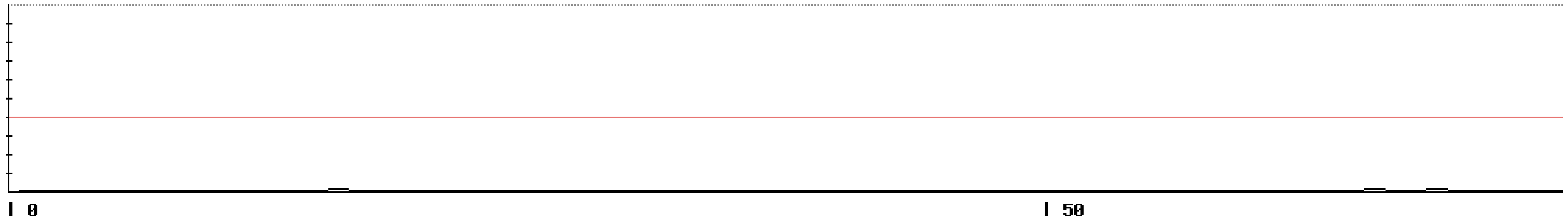

Predictions for Capsellagrandiflora_Cagra.1968s0044

Viterbi Path

Viterbi Path

Posterior @ 0.4

Posterior @ 0.4

Posterior Graphic. Threshold Marked @ 0.4	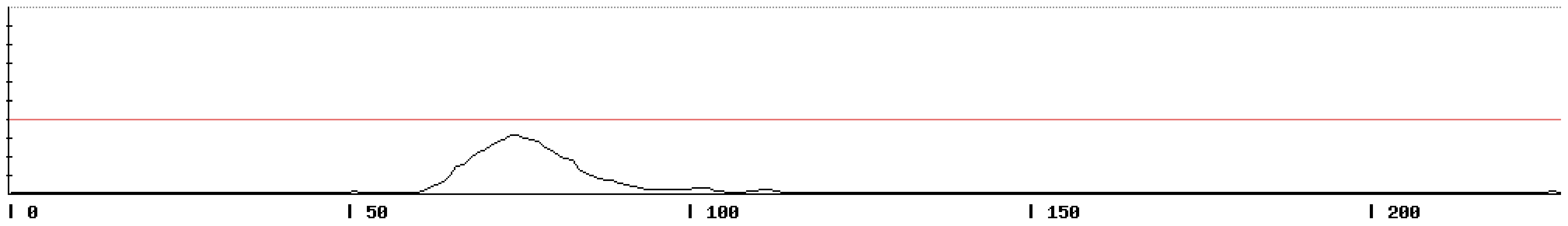

Predictions for Capsellagrandiflora_Cagra.1968s0045	
Viterbi Path	
Viterbi Path	
Posterior @ 0.4	
Posterior @ 0.4	
Posterior Graphic. Threshold Marked @ 0.4	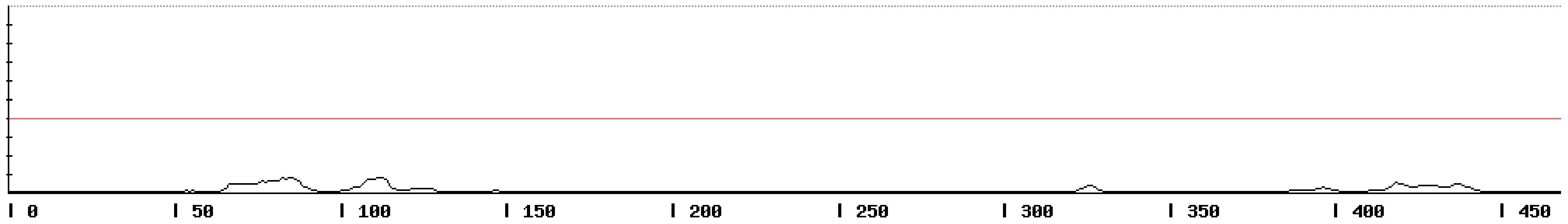

Predictions for Capsellagrandiflora_Cagra.1968s0046	
Viterbi Path	
Viterbi Path	
Posterior @ 0.4	
Posterior @ 0.4	

Posterior Graphic. Threshold Marked @ 0.4

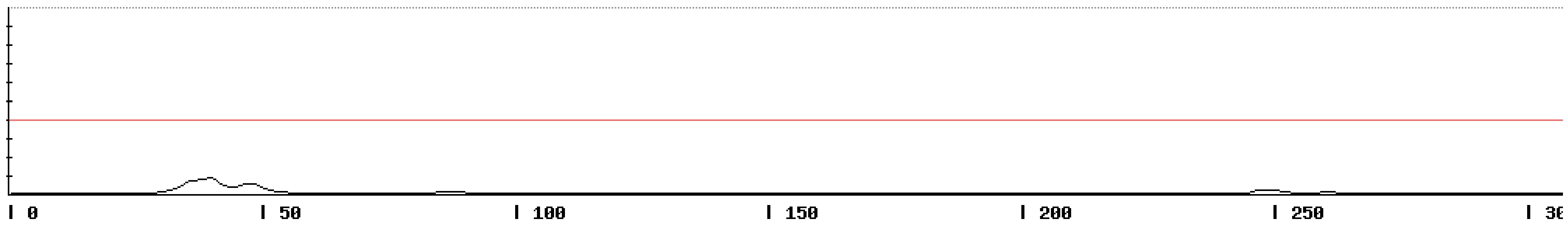

Predictions for Capsellagrandiflora_Cagra.1968s0112

Viterbi Path

Viterbi Path

Posterior @ 0.4

Posterior @ 0.4

Posterior Graphic. Threshold Marked @ 0.4

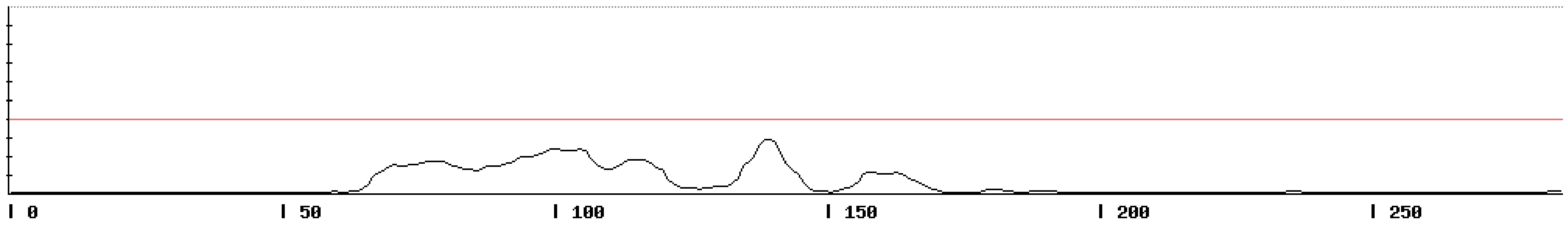

Predictions for Capsellagrandiflora_Cagra.2026s0001

Viterbi Path

Viterbi Path

Posterior @ 0.4

Posterior @ 0.4

Posterior Graphic. Threshold Marked @ 0.4

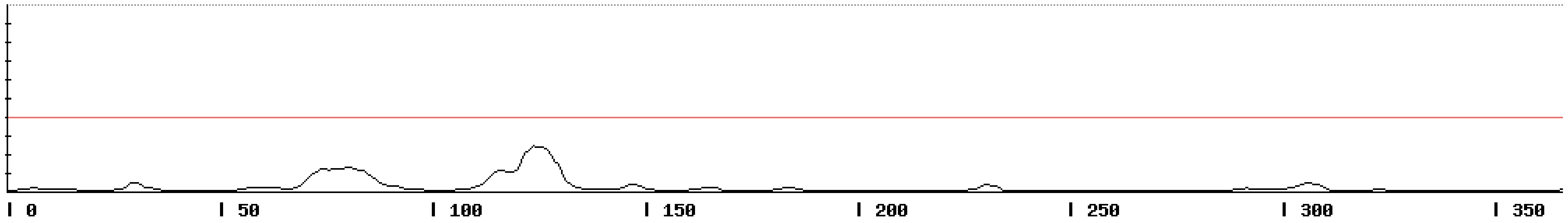

Predictions for Capsellagrandiflora_Cagra.2026s0002

Viterbi Path

Viterbi Path

Posterior @ 0.4

Posterior @ 0.4

Posterior Graphic. Threshold Marked @ 0.4

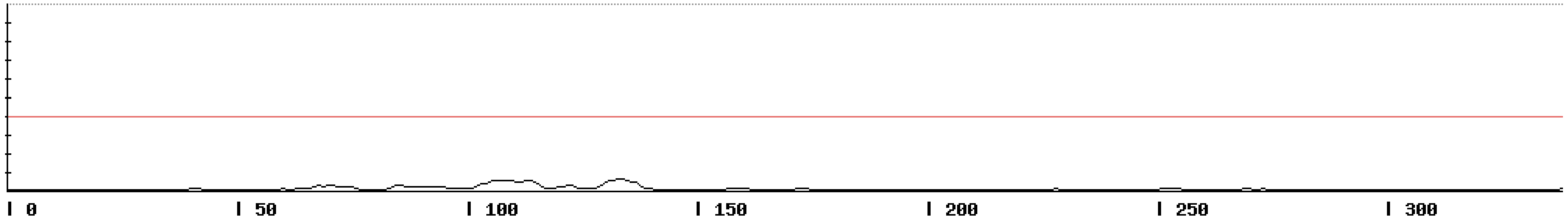

Predictions for Capsellagrandiflora_Cagra.2098s0066

Viterbi Path

Viterbi Path

Posterior @ 0.4

Posterior @ 0.4

Posterior Graphic. Threshold Marked @ 0.4

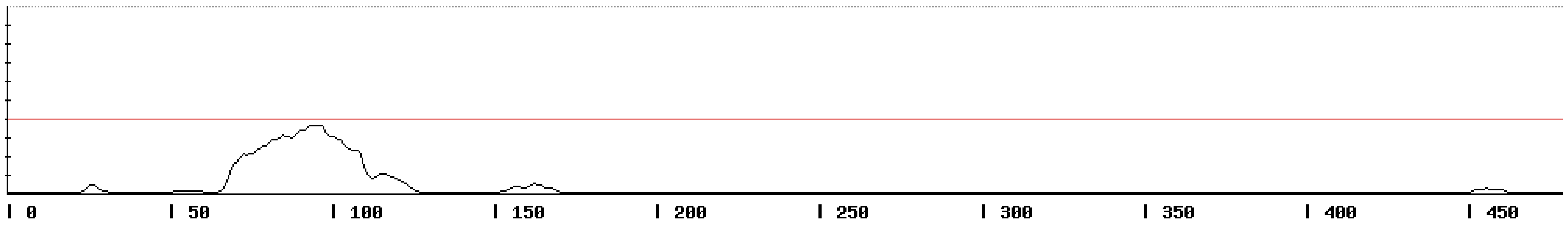

Predictions for Capsellagrandiflora_Cagra.21346s0001

Viterbi Path

Viterbi Path

Posterior @ 0.4

99 - TGKDRKIKSGKTKII - 113

Posterior @ 0.4

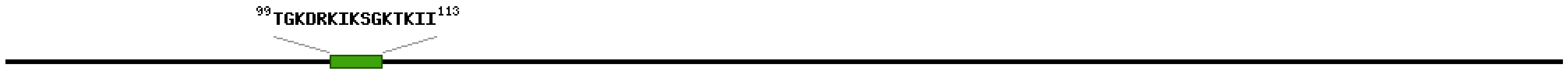

Posterior Graphic. Threshold Marked @ 0.4

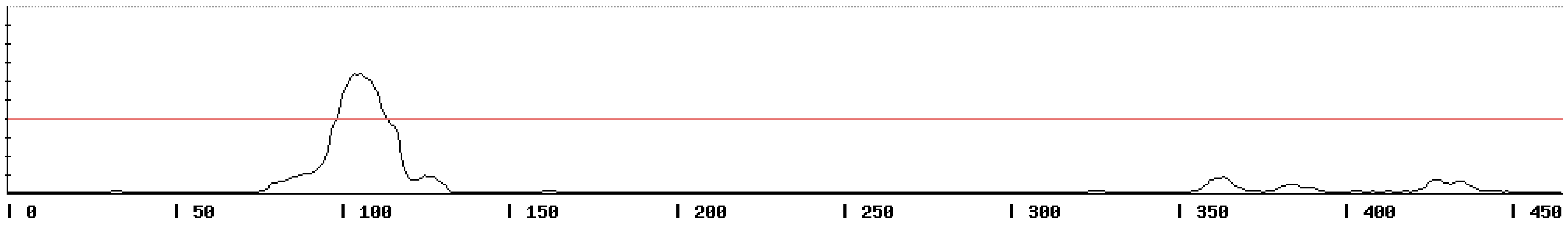

Predictions for Capsellagrandiflora_Cagra.2192s0006

Viterbi Path

Viterbi Path

Posterior @ 0.4

Posterior @ 0.4

Posterior Graphic. Threshold Marked @ 0.4

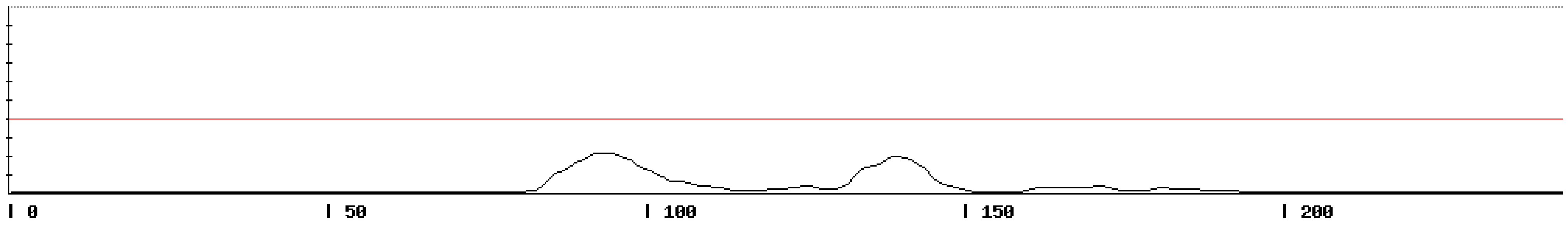

Predictions for Capsellagrandiflora_Cagra.2204s0002

Viterbi Path

Viterbi Path

Posterior @ 0.4

113 - KKGISGKKHKR - 123

206 - KALSKAKKGKGKLKE - 220

Posterior @ 0.4

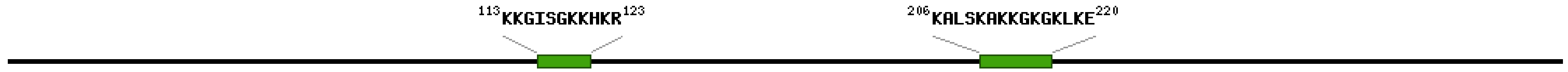

Posterior Graphic. Threshold Marked @ 0.4

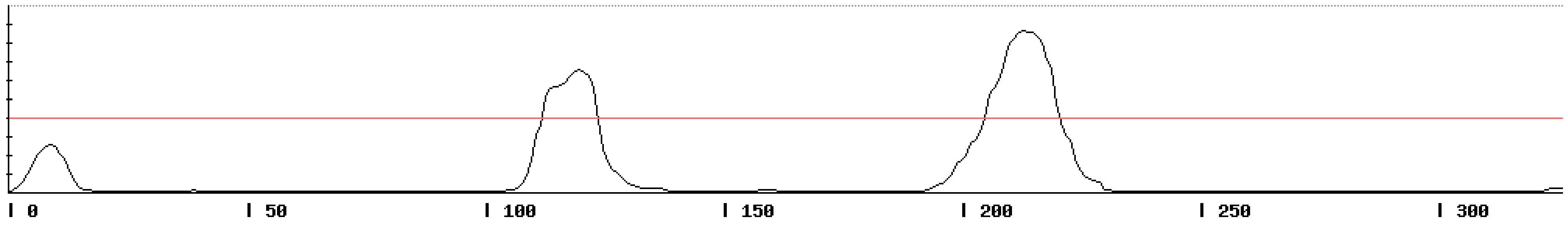

Predictions for Capsellagrandiflora_Cagra.2213s0001	
Viterbi Path	
Viterbi Path	
Posterior @ 0.4	
Posterior @ 0.4	
Posterior Graphic. Threshold Marked @ 0.4	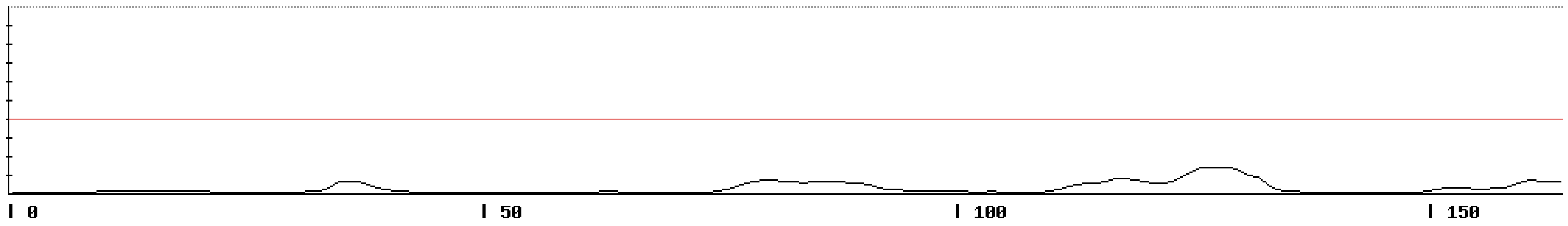

Predictions for Capsellagrandiflora_Cagra.2350s0100	
Viterbi Path	
Viterbi Path	
Posterior @ 0.4	101 - IKSKK - 105
	491 - SRKKRREGKRWK - 502
Posterior @ 0.4	

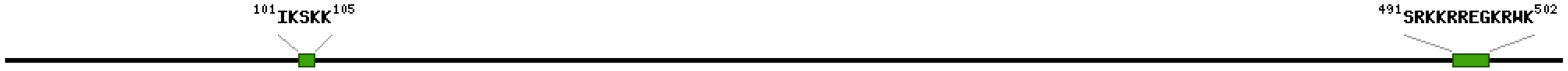

Posterior Graphic. Threshold Marked @ 0.4

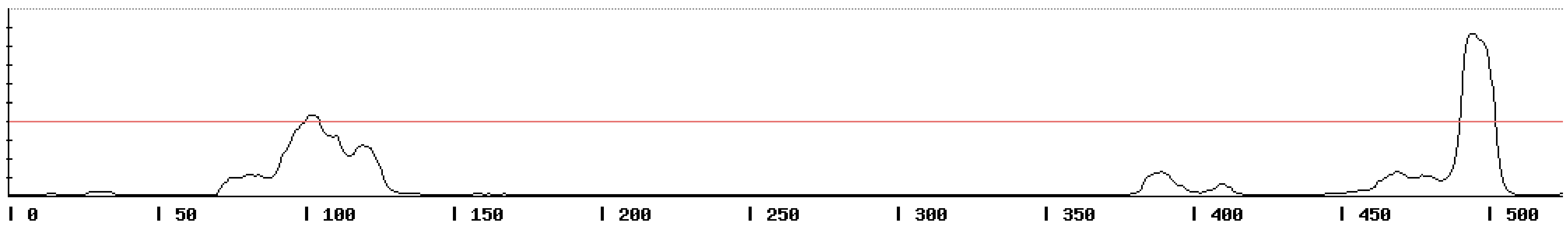

Predictions for Capsellagrandiflora_Cagra.27691s0001

Viterbi Path

Viterbi Path

Posterior @ 0.4

Posterior @ 0.4

Posterior Graphic. Threshold Marked @ 0.4

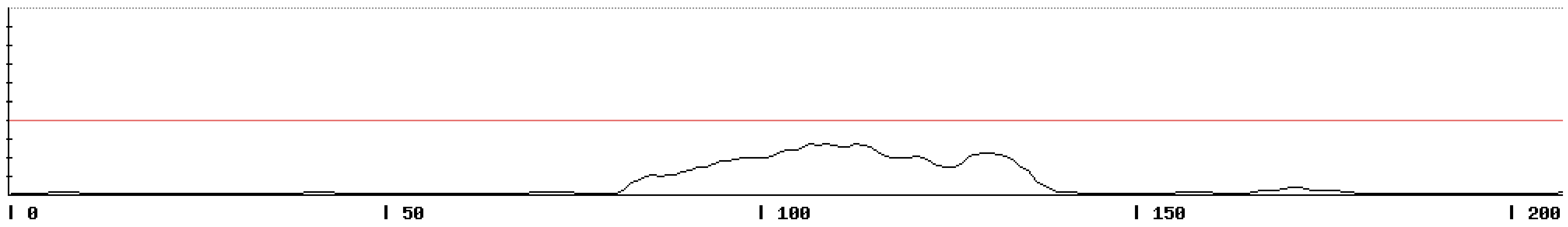

Predictions for Capsellagrandiflora_Cagra.27949s0001

Viterbi Path

Viterbi Path

Posterior @ 0.4

Posterior @ 0.4

Posterior Graphic. Threshold Marked @ 0.4

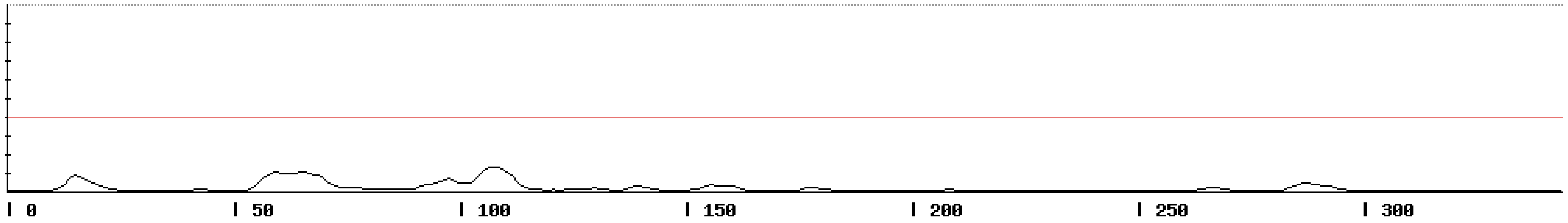

Predictions for Capsellagrandiflora_Cagra.2925s0018

Viterbi Path

Viterbi Path

Posterior @ 0.4

Posterior @ 0.4

Posterior Graphic. Threshold Marked @ 0.4

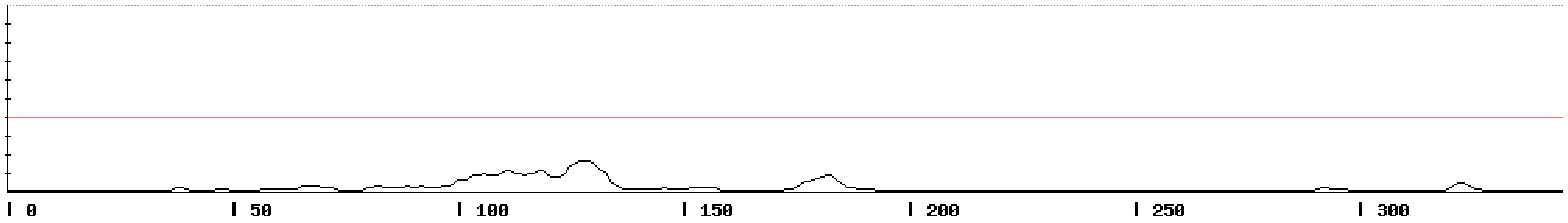

Predictions for Capsellagrandiflora_Cagra.3125s0012

Viterbi Path

Viterbi Path

Posterior @ 0.4

Posterior @ 0.4

Posterior Graphic. Threshold Marked @ 0.4

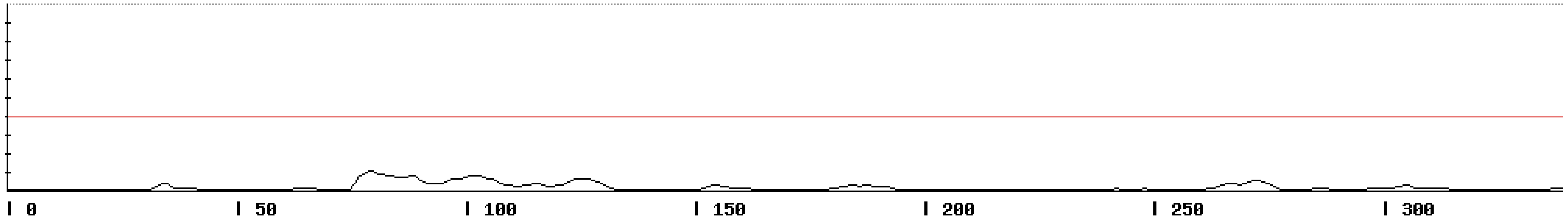

Predictions for Capsellagrandiflora_Cagra.3356s0092

Viterbi Path

Viterbi Path

Posterior @ 0.4

Posterior @ 0.4

Posterior Graphic. Threshold Marked @ 0.4

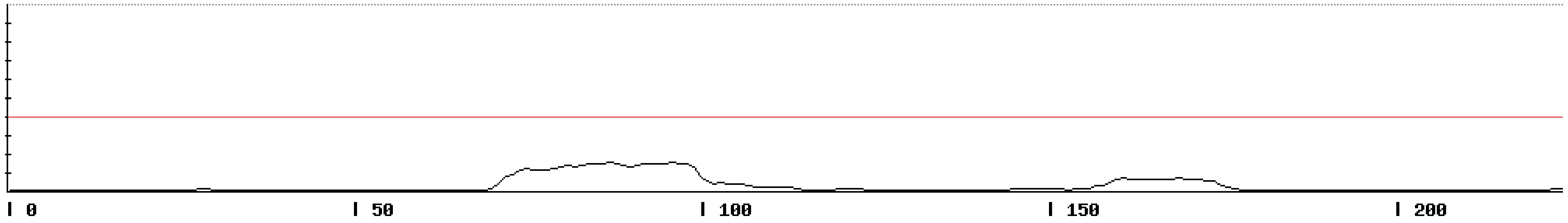

Predictions for Capsellagrandiflora_Cagra.3375s0004

Viterbi Path

Viterbi Path

Posterior @ 0.4

Posterior @ 0.4

Posterior Graphic. Threshold Marked @ 0.4

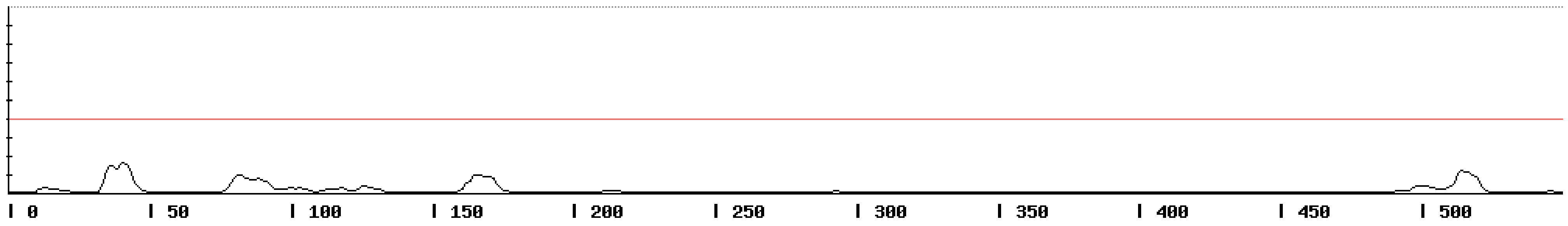

Predictions for Capsellagrandiflora_Cagra.3375s0006

Viterbi Path

Viterbi Path

Posterior @ 0.4

Posterior @ 0.4

Posterior Graphic. Threshold Marked @ 0.4

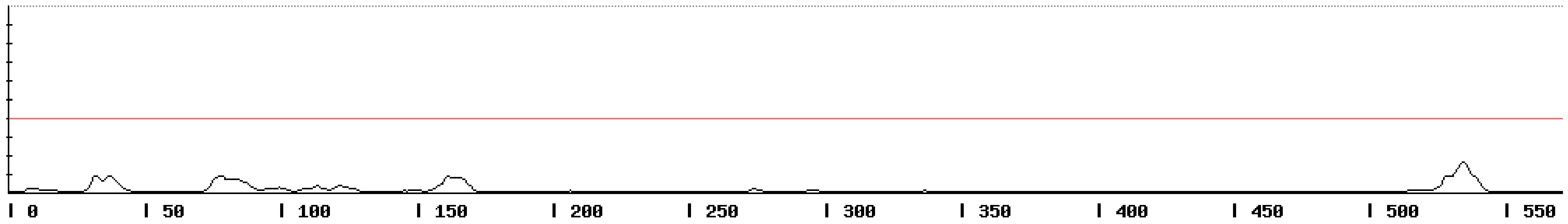

Predictions for Capsellagrandiflora_Cagra.3545s0001

Viterbi Path

Viterbi Path

Posterior @ 0.4

Posterior @ 0.4

Posterior Graphic. Threshold Marked @ 0.4

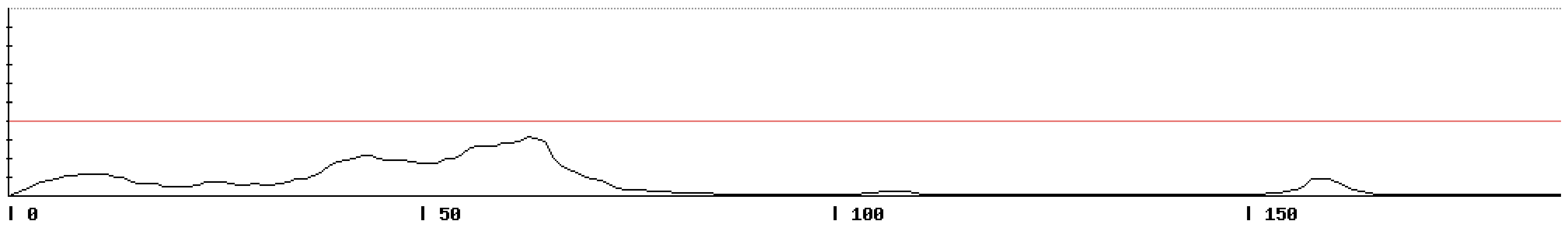

Predictions for Capsellagrandiflora_Cagra.4003s0027

Viterbi Path

Viterbi Path

Posterior @ 0.4

Posterior @ 0.4

Posterior Graphic. Threshold Marked @ 0.4

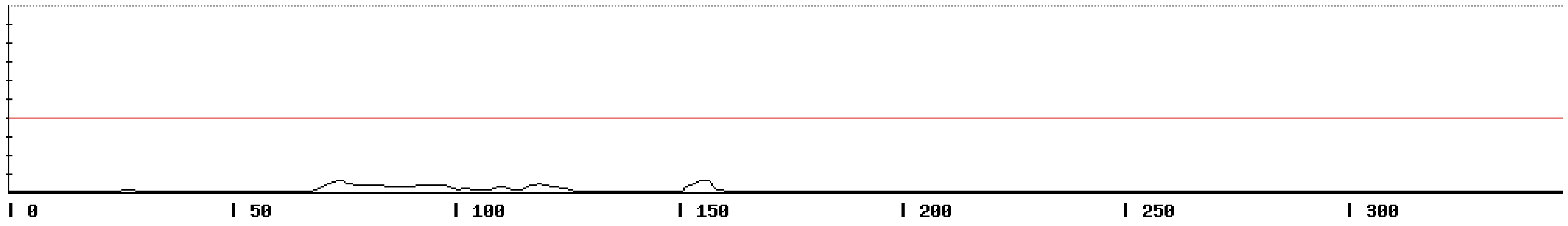

Predictions for Capsellagrandiflora_Cagra.4006s0034

Viterbi Path

Viterbi Path

Posterior @ 0.4

Posterior @ 0.4

Posterior Graphic. Threshold Marked @ 0.4

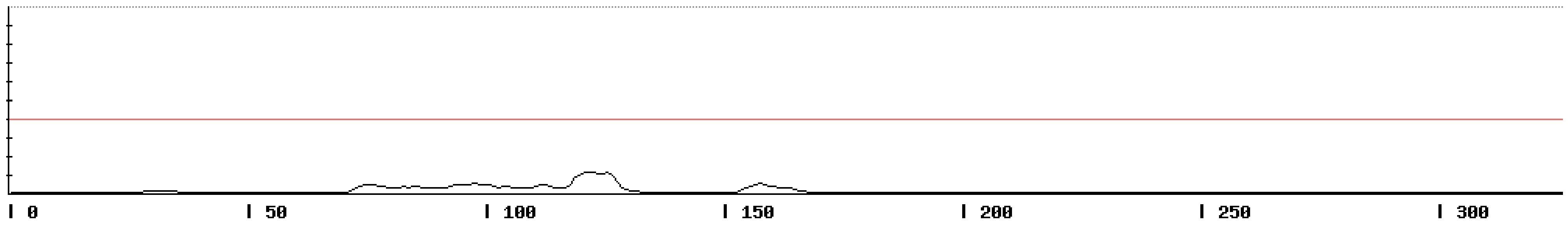

Predictions for Capsellagrandiflora_Cagra.4091s0015

Viterbi Path

Viterbi Path

Posterior @ 0.4

Posterior @ 0.4

Posterior Graphic. Threshold Marked @ 0.4

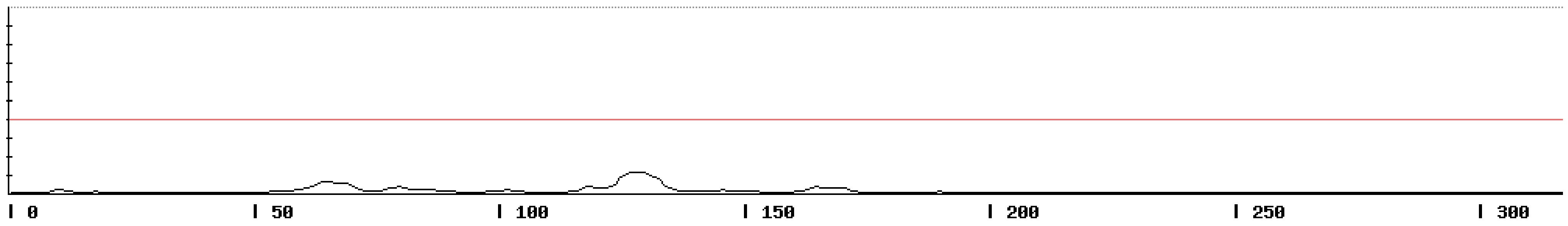

Predictions for Capsellagrandiflora_Cagra.4344s0001

Viterbi Path

Viterbi Path

Posterior @ 0.4

Posterior @ 0.4

Posterior Graphic. Threshold Marked @ 0.4

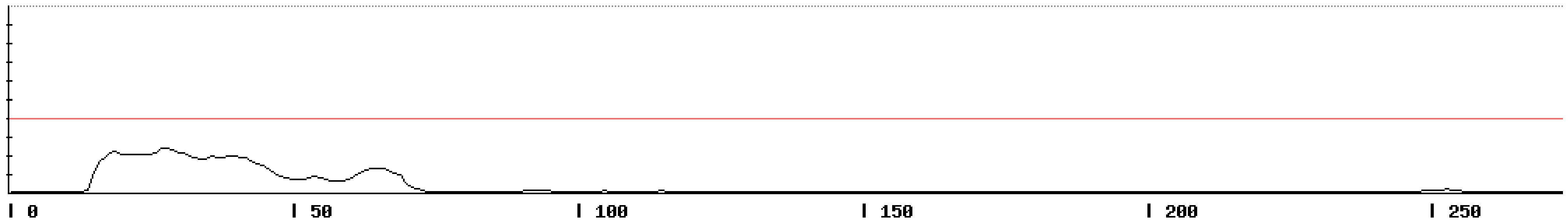

Predictions for Capsellagrandiflora_Cagra.4622s0005

Viterbi Path

Viterbi Path

Posterior @ 0.4

Posterior @ 0.4

Posterior Graphic. Threshold Marked @ 0.4	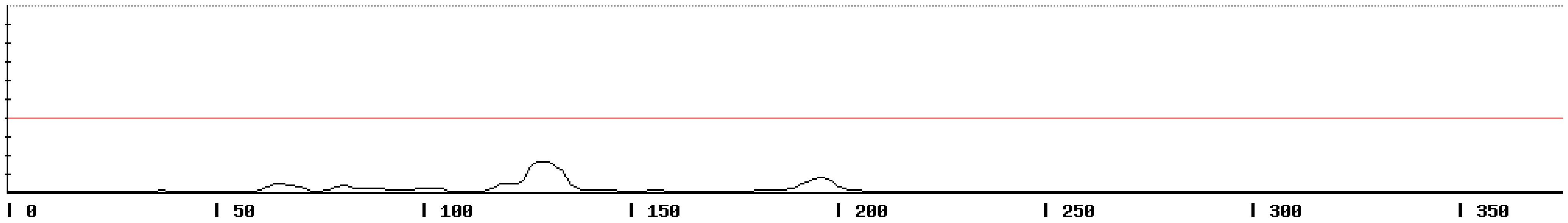

Predictions for Capsellagrandiflora_Cagra.4734s0006	
Viterbi Path	
Viterbi Path	
Posterior @ 0.4	
Posterior @ 0.4	
Posterior Graphic. Threshold Marked @ 0.4	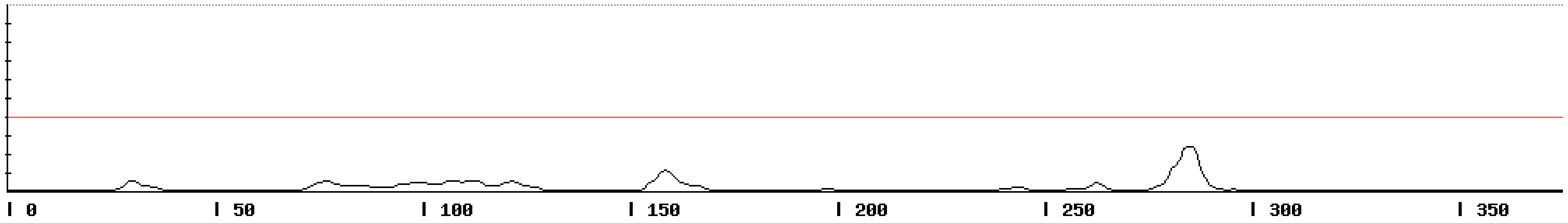

Predictions for Capsellagrandiflora_Cagra.4849s0008	
Viterbi Path	
Viterbi Path	

Posterior @ 0.4	
Posterior @ 0.4	
Posterior Graphic. Threshold Marked @ 0.4	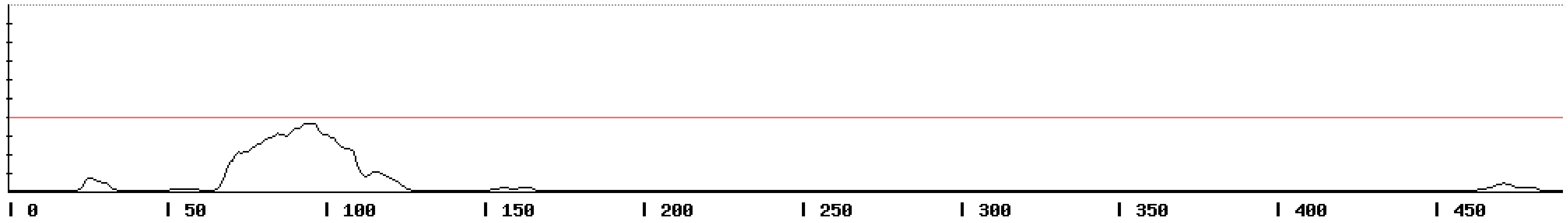

Predictions for Capsellagrandiflora_Cagra.5641s0055

Viterbi Path	
Viterbi Path	
Posterior @ 0.4	
Posterior @ 0.4	
Posterior Graphic. Threshold Marked @ 0.4	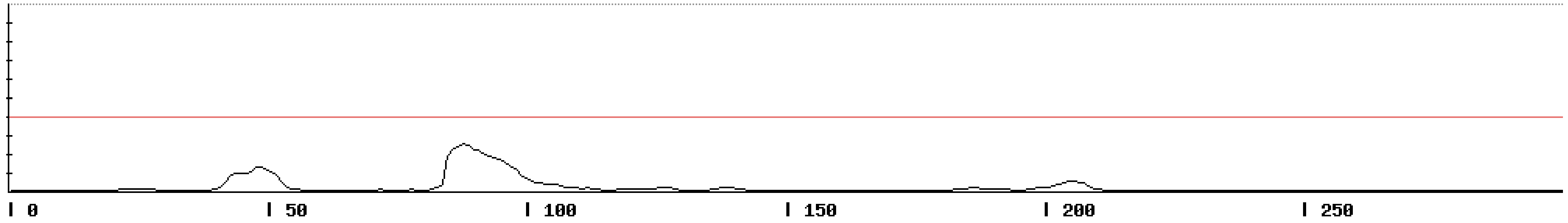

Predictions for Capsellagrandiflora_Cagra.5641s0060

Viterbi Path	
Viterbi Path	

Posterior @ 0.4

Posterior @ 0.4

Posterior Graphic. Threshold Marked @ 0.4

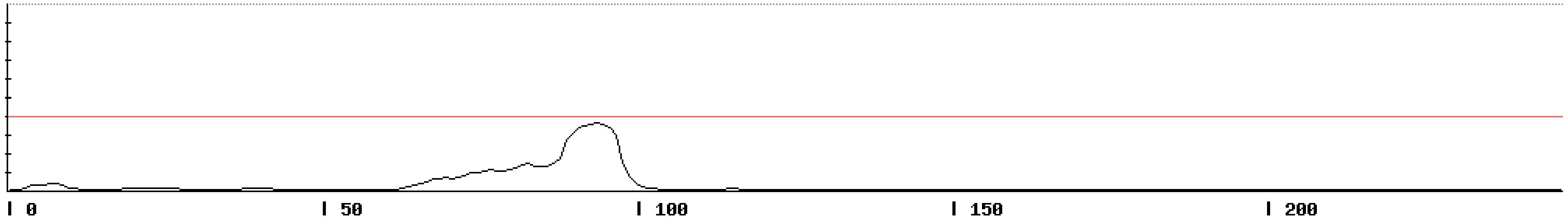

Predictions for Capsellagrandiflora_Cagra.5905s0003

Viterbi Path

Viterbi Path

Posterior @ 0.4

Posterior @ 0.4

Posterior Graphic. Threshold Marked @ 0.4

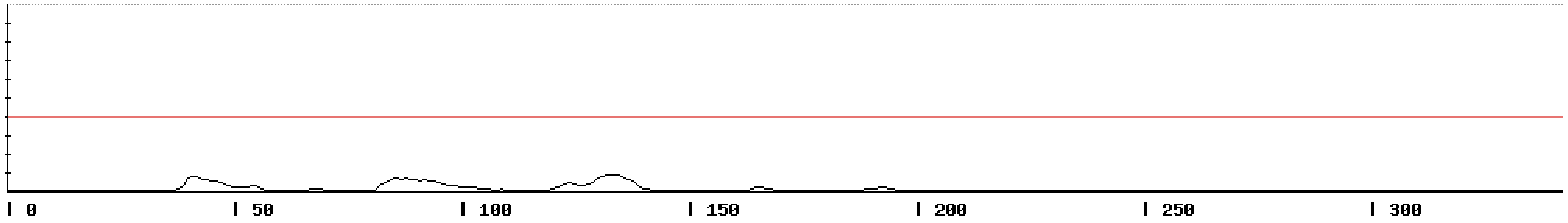

Predictions for Capsellagrandiflora_Cagra.6133s0030

Viterbi Path

Viterbi Path

Posterior @ 0.4

Posterior @ 0.4

Posterior Graphic. Threshold Marked @ 0.4

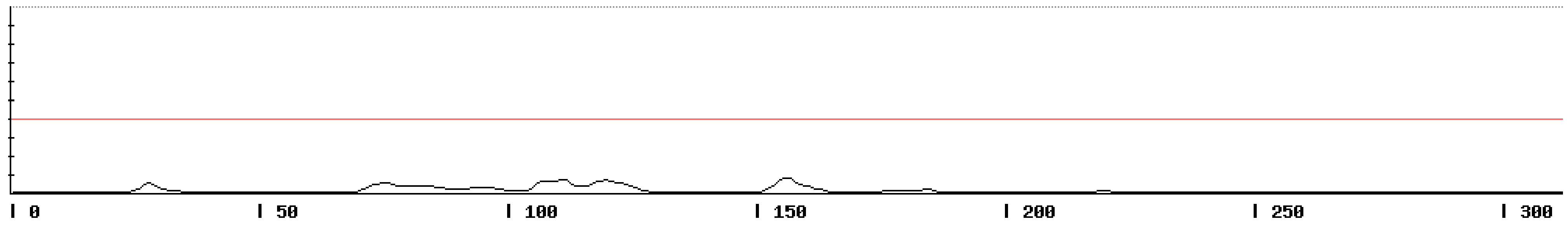

Predictions for Capsellagrandiflora_Cagra.6372s0006

Viterbi Path

Viterbi Path

Posterior @ 0.4

Posterior @ 0.4

Posterior Graphic. Threshold Marked @ 0.4

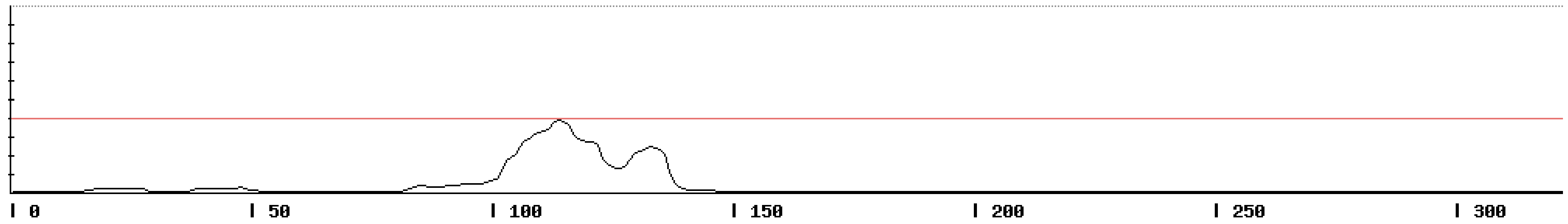

Predictions for Capsellagrandiflora_Cagra.6981s0001	
Viterbi Path	
Viterbi Path	
Posterior @ 0.4	
Posterior @ 0.4	
Posterior Graphic. Threshold Marked @ 0.4	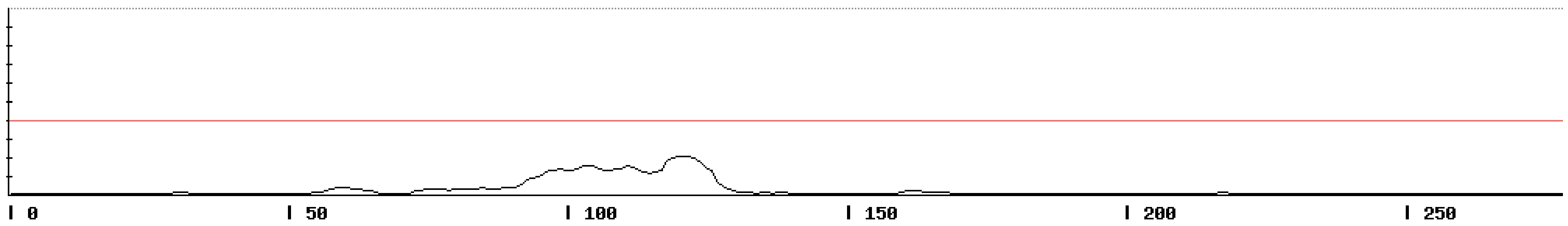

Predictions for Capsellagrandiflora_Cagra.7393s0016	
Viterbi Path	
Viterbi Path	
Posterior @ 0.4	
Posterior @ 0.4	
Posterior Graphic. Threshold Marked @ 0.4	

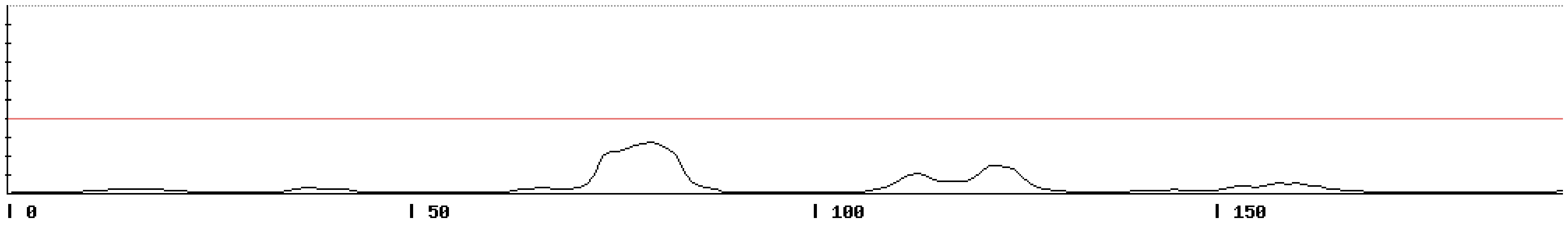

Predictions for Capsellagrandiflora_Cagra.7865s0013	
Viterbi Path	
Viterbi Path	
Posterior @ 0.4	
Posterior @ 0.4	
Posterior Graphic. Threshold Marked @ 0.4	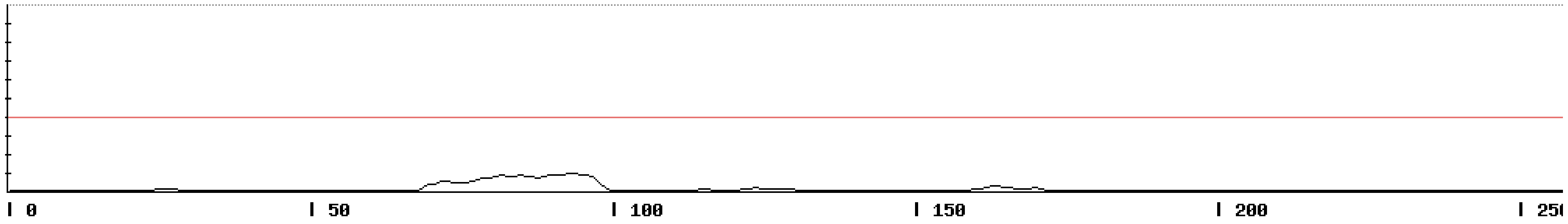

Predictions for Capsellagrandiflora_Cagra.8367s0003	
Viterbi Path	
Viterbi Path	
Posterior @ 0.4	
Posterior @ 0.4	

Viterbi Path

Posterior @ 0.4

Posterior @ 0.4

Posterior Graphic. Threshold Marked @ 0.4

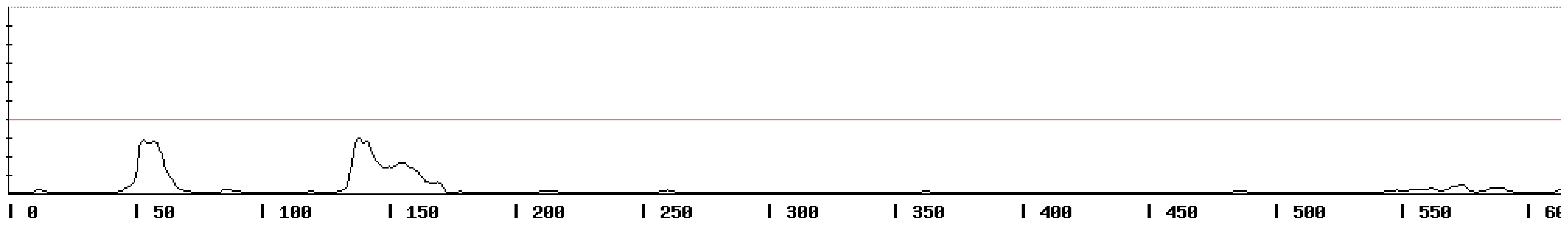

Predictions for Capsellarubella_Carubv10000500m

Viterbi Path

Viterbi Path

Posterior @ 0.4

Posterior @ 0.4

Posterior Graphic. Threshold Marked @ 0.4

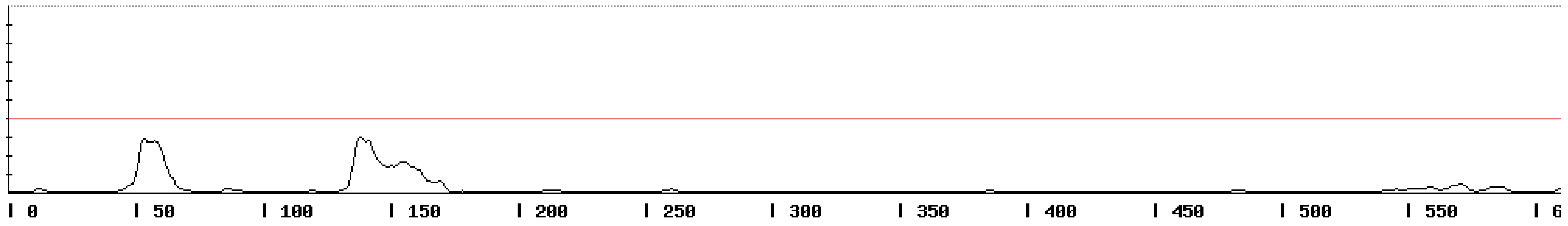

Predictions for Capsellarubella_Carubv10000892m

Viterbi Path	
Viterbi Path	
Posterior @ 0.4	99 - TGKDRKIKSGKTKII - 113
Posterior @ 0.4	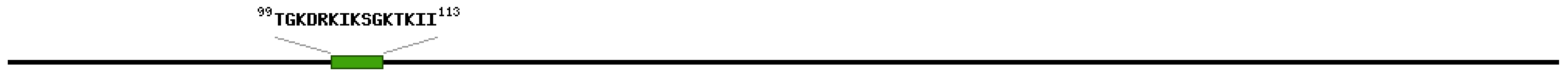
Posterior Graphic. Threshold Marked @ 0.4	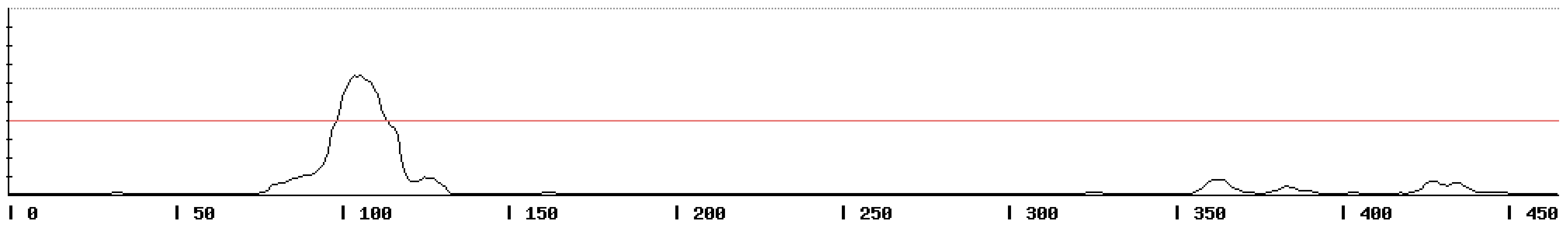

Predictions for Capsellarubella_Carubv10001072m

Viterbi Path	
Viterbi Path	
Posterior @ 0.4	99 - TGKDRKIKSGKTKII - 113
Posterior @ 0.4	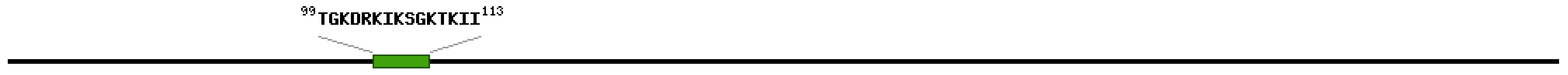
Posterior Graphic. Threshold Marked @ 0.4	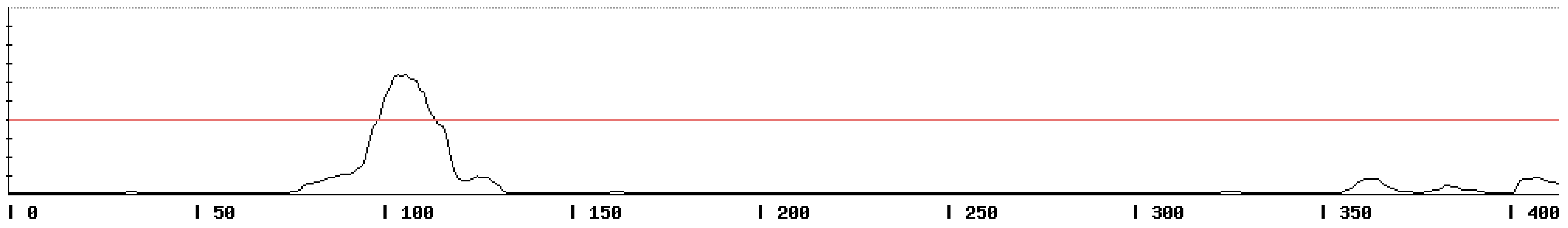

Predictions for Capsellarubella_Carubv10001139m	
Viterbi Path	
Viterbi Path	
Posterior @ 0.4	
Posterior @ 0.4	
Posterior Graphic. Threshold Marked @ 0.4	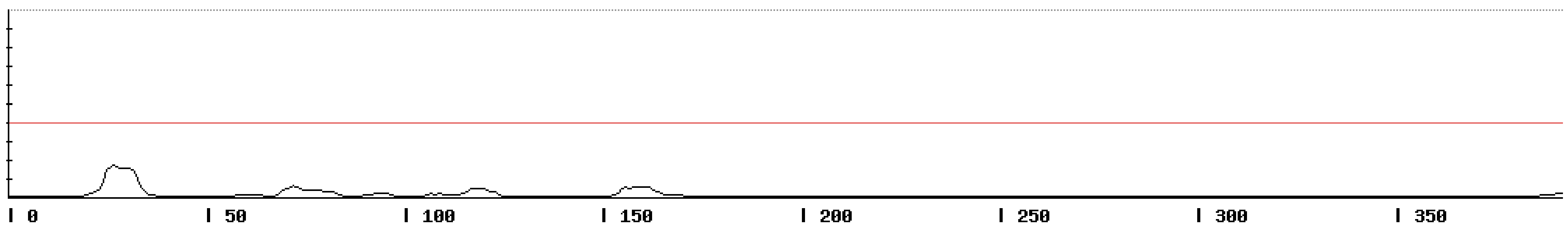

Predictions for Capsellarubella_Carubv10001357m	
Viterbi Path	
Viterbi Path	
Posterior @ 0.4	
Posterior @ 0.4	
Posterior Graphic. Threshold Marked @ 0.4	

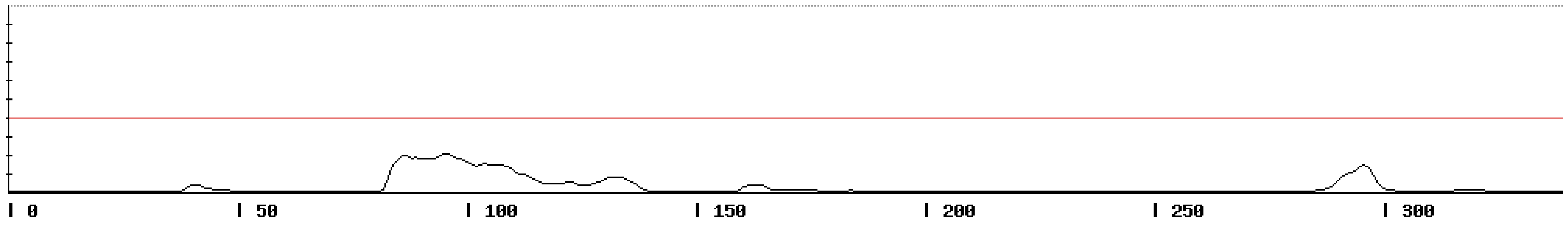

Predictions for Capsellarubella_Carubv10001372m

Viterbi Path	
Viterbi Path	
Posterior @ 0.4	
Posterior @ 0.4	
Posterior Graphic. Threshold Marked @ 0.4	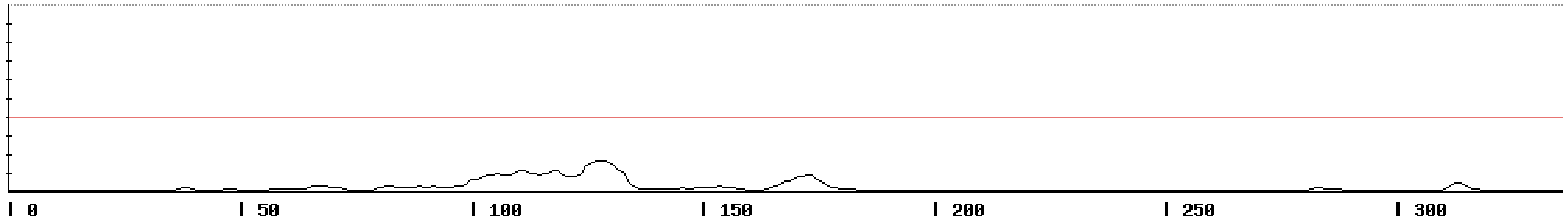

Predictions for Capsellarubella_Carubv10001373m

Viterbi Path	
Viterbi Path	
Posterior @ 0.4	
Posterior @ 0.4	

Posterior Graphic. Threshold Marked @ 0.4

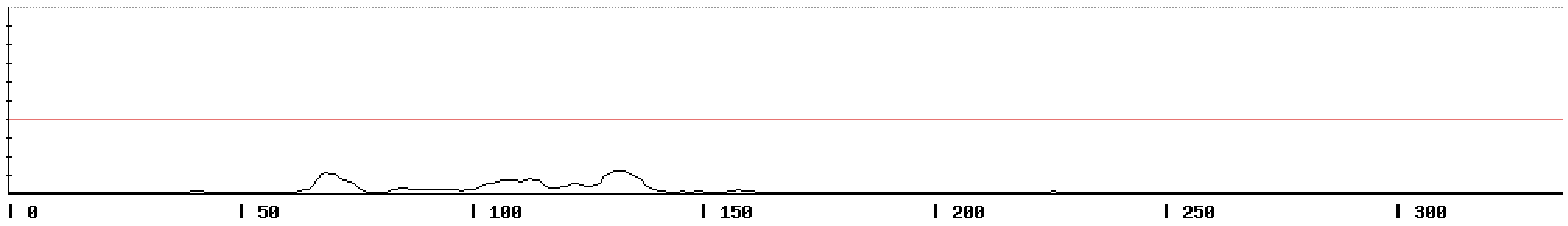

Predictions for Capsellarubella_Carubv10001688m

Viterbi Path

47 - RRPNFKKKEKKRRRRRRRR - 65

Viterbi Path

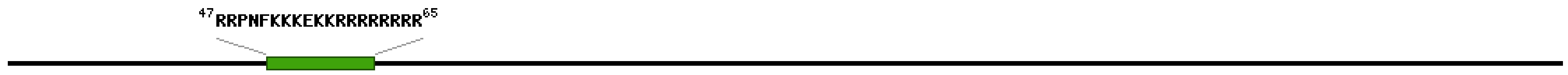

Posterior @ 0.4

47 - RRPNFKKKEKKRRRRRRRRE - 66

227 - RKGSKARKQRKSR - 239

Posterior @ 0.4

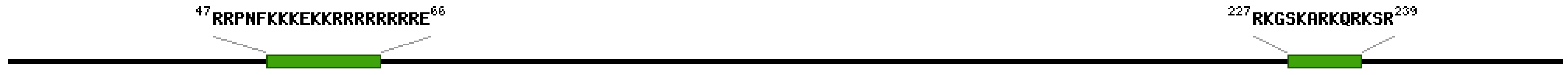

Posterior Graphic. Threshold Marked @ 0.4

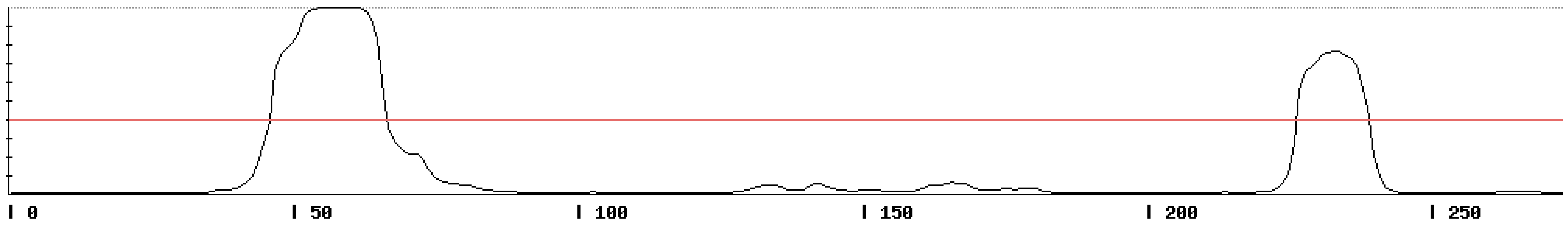

Predictions for Capsellarubella_Carubv10001788m

Viterbi Path

Viterbi Path

Posterior @ 0.4

Posterior @ 0.4

Posterior Graphic. Threshold Marked @ 0.4

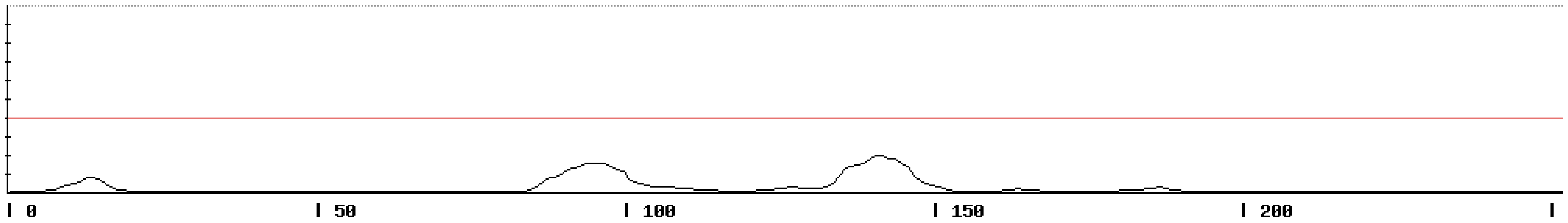

Predictions for Capsellarubella_Carubv10002548m

Viterbi Path

Viterbi Path

Posterior @ 0.4

Posterior @ 0.4

Posterior Graphic. Threshold Marked @ 0.4

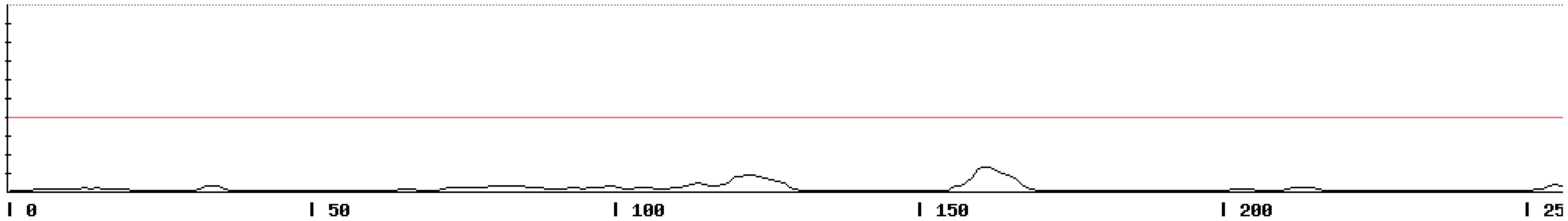

Predictions for Capsellarubella_Carubv10002665m

Viterbi Path

Viterbi Path

Posterior @ 0.4

Posterior @ 0.4

Posterior Graphic. Threshold Marked @ 0.4

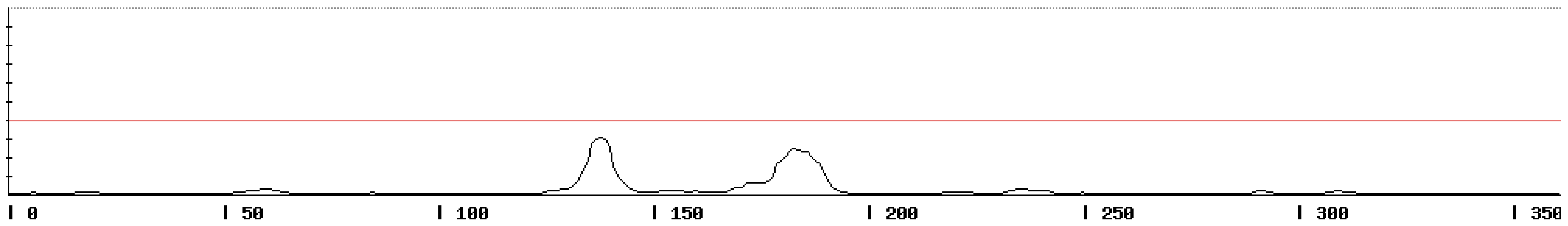

Predictions for Capsellarubella_Carubv10002791m

Viterbi Path

Viterbi Path

Posterior @ 0.4

Posterior @ 0.4

Posterior Graphic. Threshold Marked @ 0.4

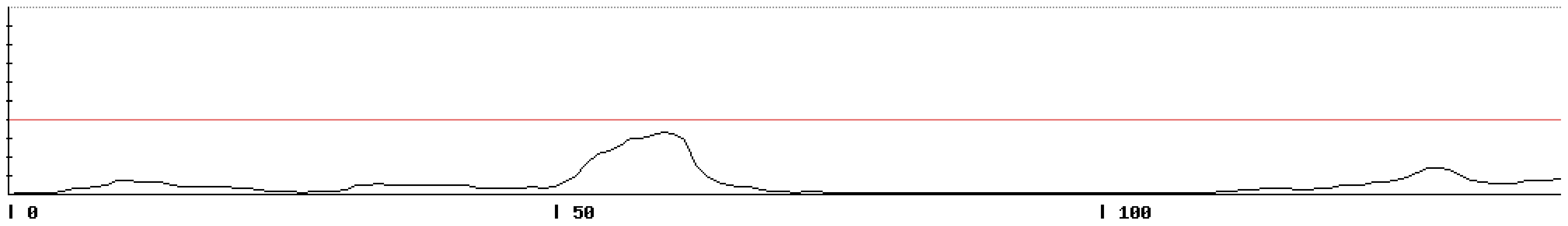

Predictions for Capsellarubella_Carubv10003054m

Viterbi Path

Viterbi Path

Posterior @ 0.4

Posterior @ 0.4

Posterior Graphic. Threshold Marked @ 0.4

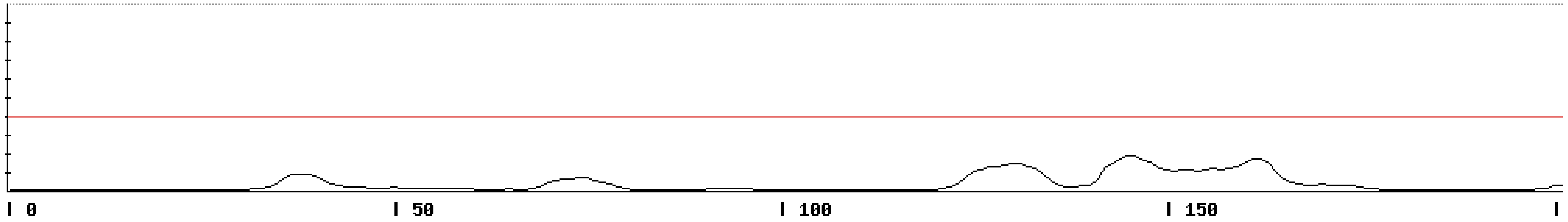

Predictions for Capsellarubella_Carubv10003254m

Viterbi Path

Viterbi Path

Posterior @ 0.4

Posterior @ 0.4

Posterior Graphic. Threshold Marked @ 0.4

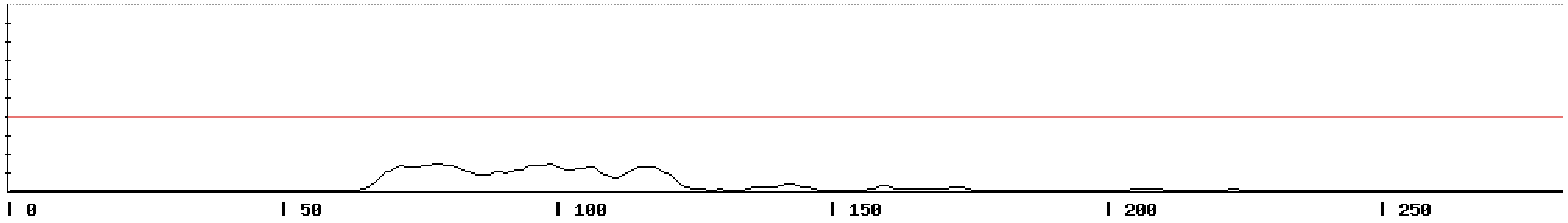

Predictions for Capsellarubella_Carubv10003260m

Viterbi Path	
Viterbi Path	
Posterior @ 0.4	94 - DRKIKDKRGNRGE - 106
Posterior @ 0.4	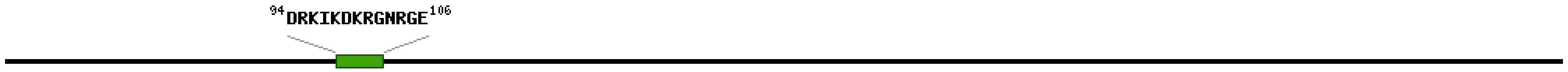
Posterior Graphic. Threshold Marked @ 0.4	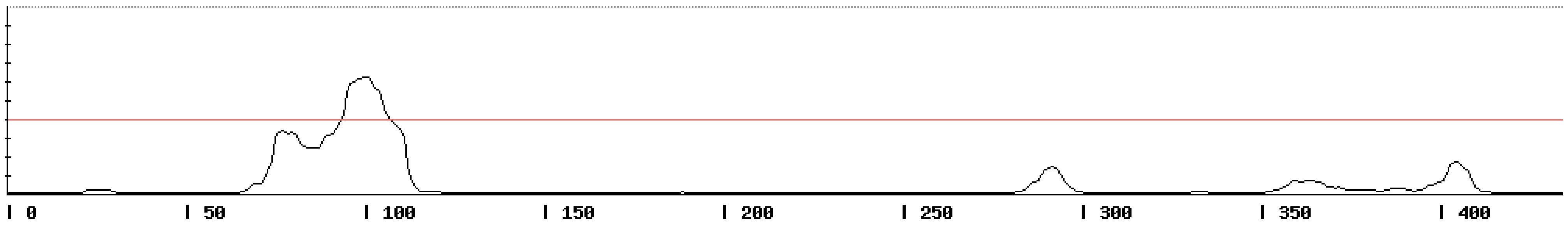

Predictions for Capsellarubella_Carubv10003671m

Viterbi Path	
Viterbi Path	
Posterior @ 0.4	
Posterior @ 0.4	
Posterior Graphic. Threshold Marked @ 0.4	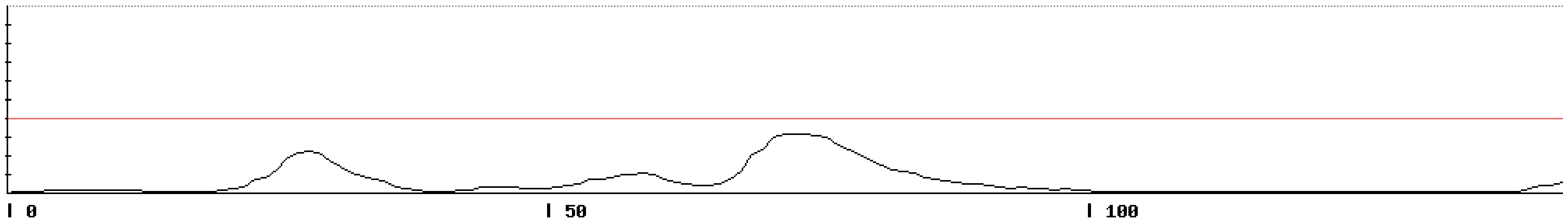

Predictions for Capsellarubella_Carubv10003768m	
Viterbi Path	
Viterbi Path	
Posterior @ 0.4	131 - RERKSKLK - 138
Posterior @ 0.4	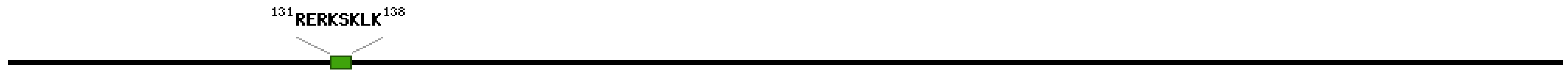
Posterior Graphic. Threshold Marked @ 0.4	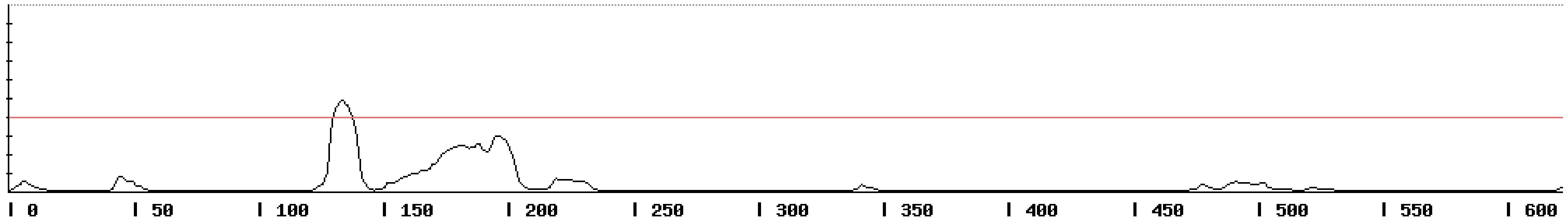

Predictions for Capsellarubella_Carubv10003817m	
Viterbi Path	132 - KRMKDNEKEKVKKGKGKLKEKVK - 154
Viterbi Path	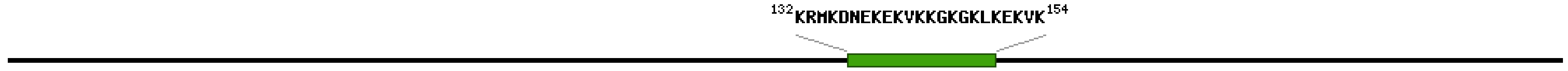
Posterior @ 0.4	62 - KKGISGKKHKL - 72
	80 - GAKKIERYRARKPH - 93
	132 - KRMKDNEKEKVKKGKGKLKEKVK - 154
Posterior @ 0.4	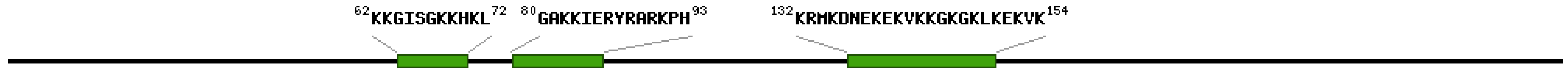
Posterior Graphic. Threshold Marked @ 0.4	

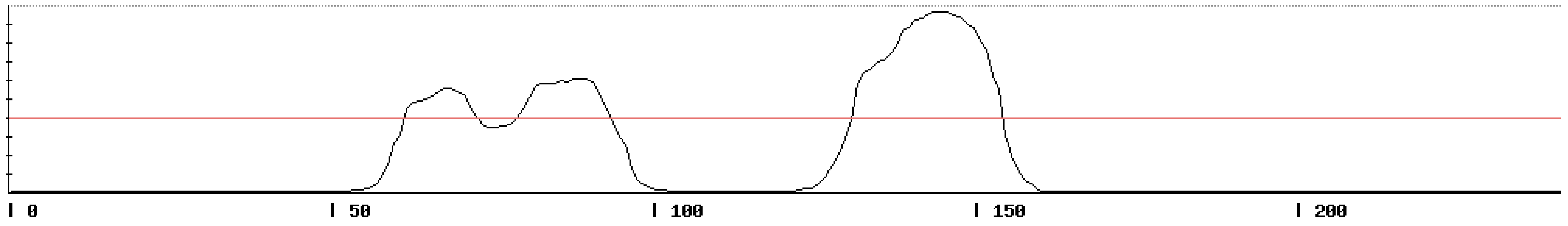

Predictions for Capsellarubella_Carubv10003869m

Viterbi Path	
Viterbi Path	
Posterior @ 0.4	
Posterior @ 0.4	
Posterior Graphic. Threshold Marked @ 0.4	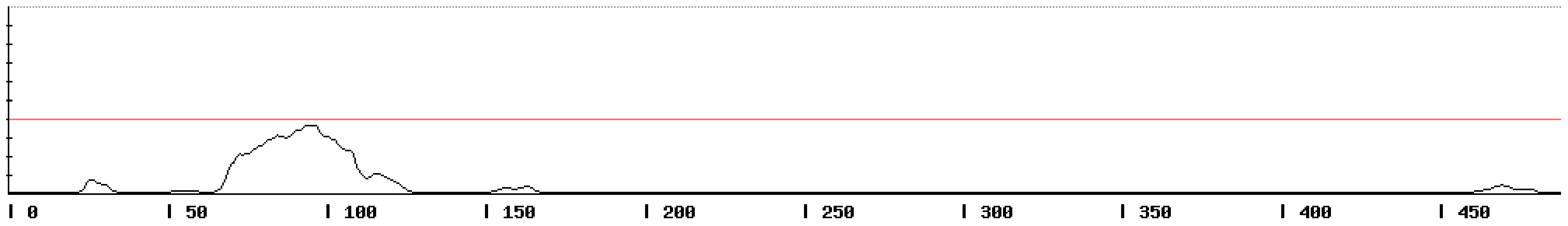

Predictions for Capsellarubella_Carubv10003879m

Viterbi Path	
Viterbi Path	
Posterior @ 0.4	
Posterior @ 0.4	

Posterior Graphic. Threshold Marked @ 0.4

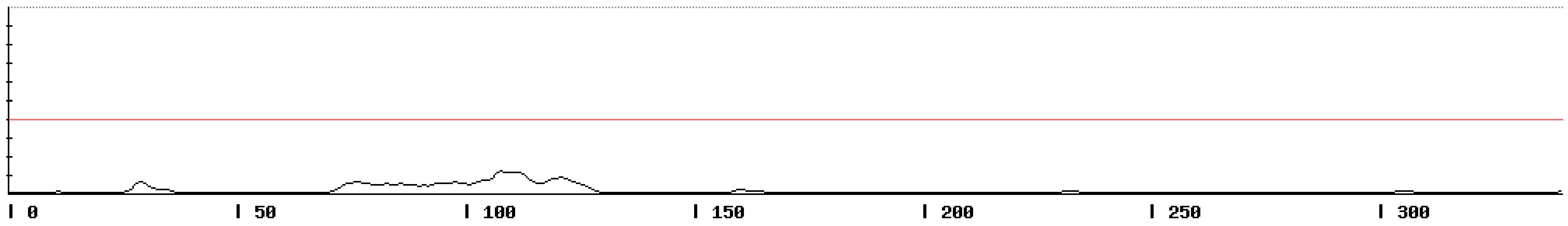

Predictions for Capsellarubella_Carubv10004602m

Viterbi Path

Viterbi Path

Posterior @ 0.4

Posterior @ 0.4

Posterior Graphic. Threshold Marked @ 0.4

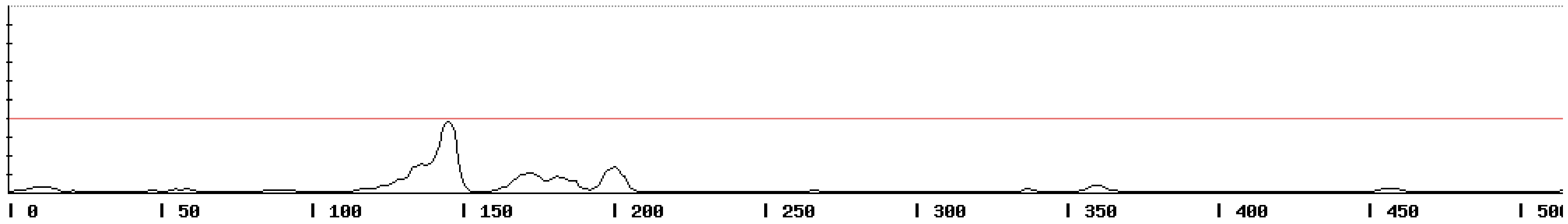

Predictions for Capsellarubella_Carubv10004997m

Viterbi Path

Viterbi Path

Posterior @ 0.4

Posterior @ 0.4

Posterior Graphic. Threshold Marked @ 0.4

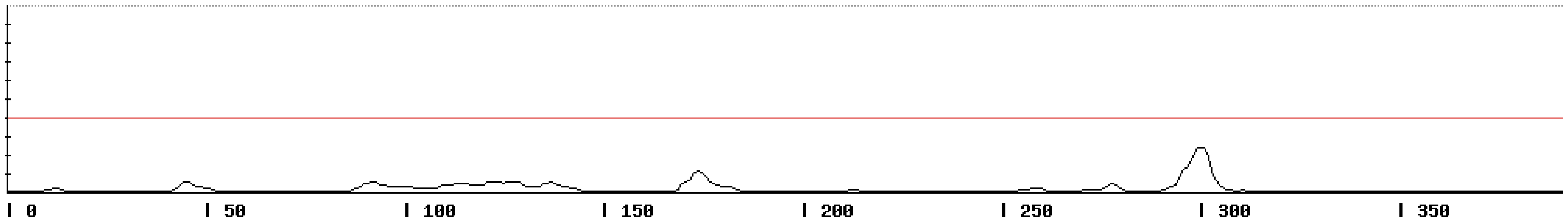

Predictions for Capsellarubella_Carubv10005139m

Viterbi Path

Viterbi Path

Posterior @ 0.4

Posterior @ 0.4

Posterior Graphic. Threshold Marked @ 0.4

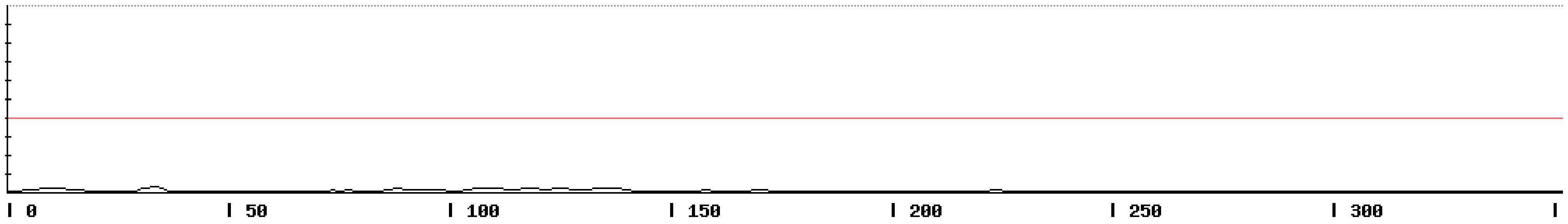

Predictions for Capsellarubella_Carubv10005201m

Viterbi Path

Viterbi Path

Posterior @ 0.4

Posterior @ 0.4

Posterior Graphic. Threshold Marked @ 0.4

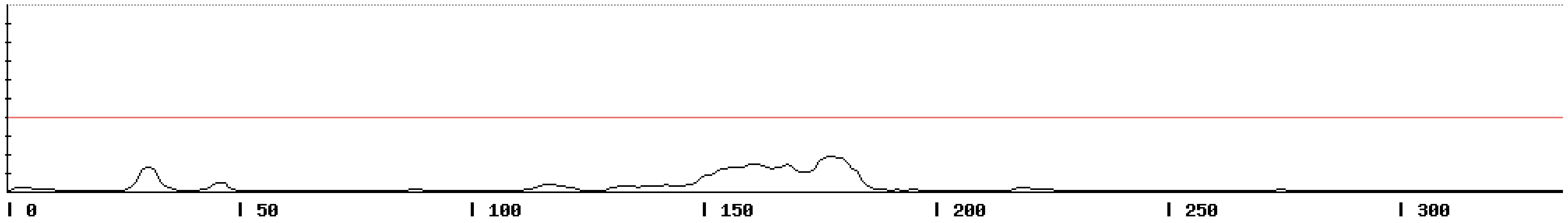

Predictions for Capsellarubella_Carubv10005324m

Viterbi Path

Viterbi Path

Posterior @ 0.4 — 137 - RKRRK - 141

Posterior @ 0.4

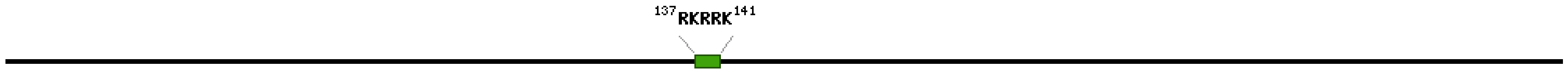

Posterior Graphic. Threshold Marked @ 0.4

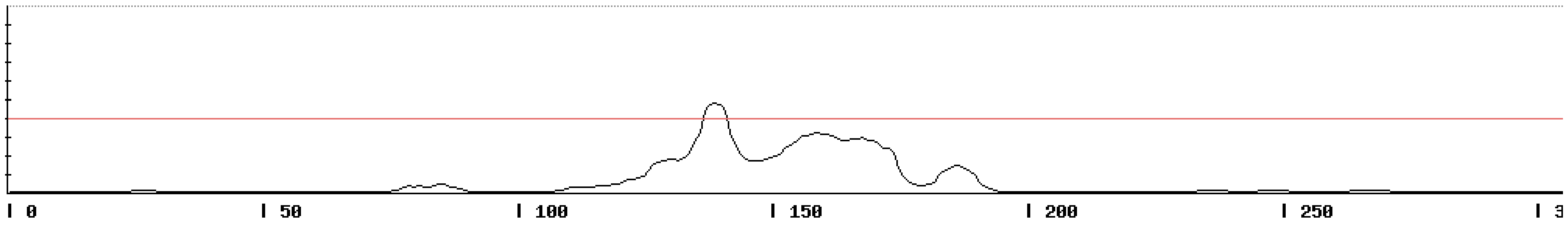

Predictions for Capsellarubella_Carubv10005353m

Viterbi Path

Viterbi Path

Posterior @ 0.4	
Posterior @ 0.4	
Posterior Graphic. Threshold Marked @ 0.4	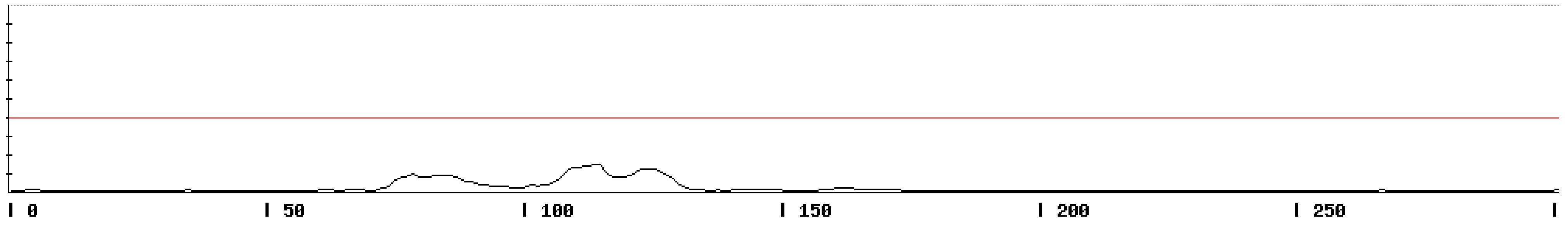

Predictions for Capsellarubella_Carubv10005574m

Viterbi Path	
Viterbi Path	
Posterior @ 0.4	
Posterior @ 0.4	
Posterior Graphic. Threshold Marked @ 0.4	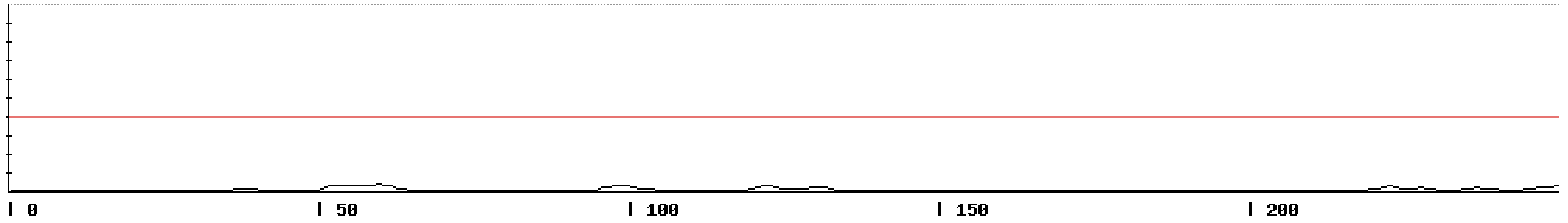

Predictions for Capsellarubella_Carubv10006906m

Viterbi Path

Viterbi Path

Posterior @ 0.4

Posterior @ 0.4

Posterior Graphic. Threshold Marked @ 0.4

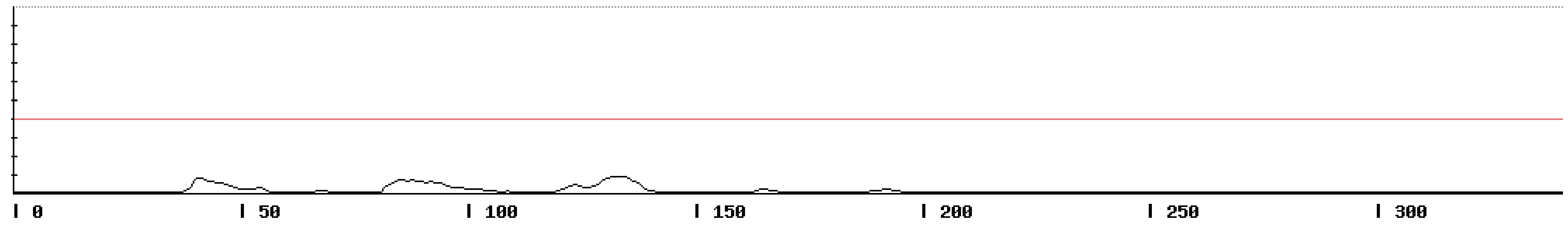

Predictions for Capsellarubella_Carubv10007390m

Viterbi Path

Viterbi Path

Posterior @ 0.4

Posterior @ 0.4

Posterior Graphic. Threshold Marked @ 0.4

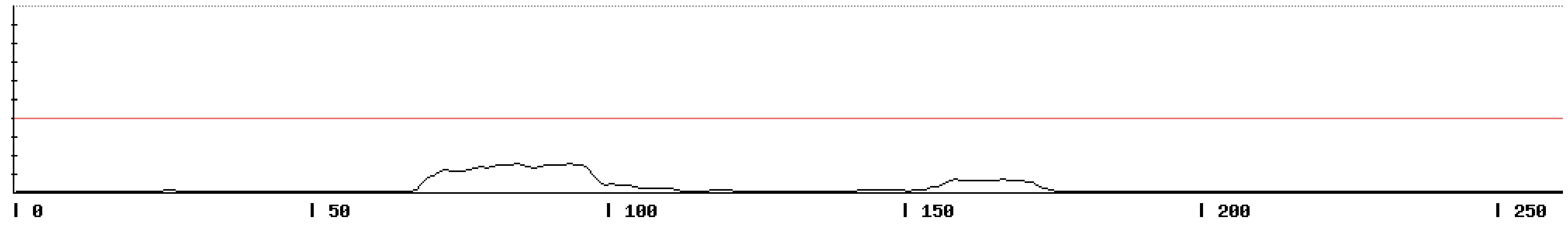

Predictions for Capsellarubella_Carubv10007613m

Viterbi Path	
Viterbi Path	
Posterior @ 0.4	101 - IKSKK - 105
	491 - SRKKRREGKKWK - 502
Posterior @ 0.4	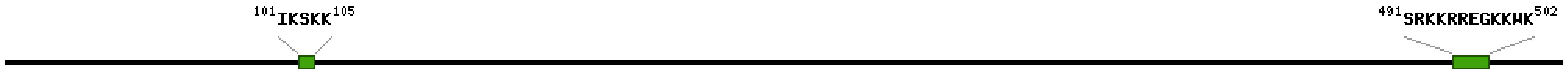
Posterior Graphic. Threshold Marked @ 0.4	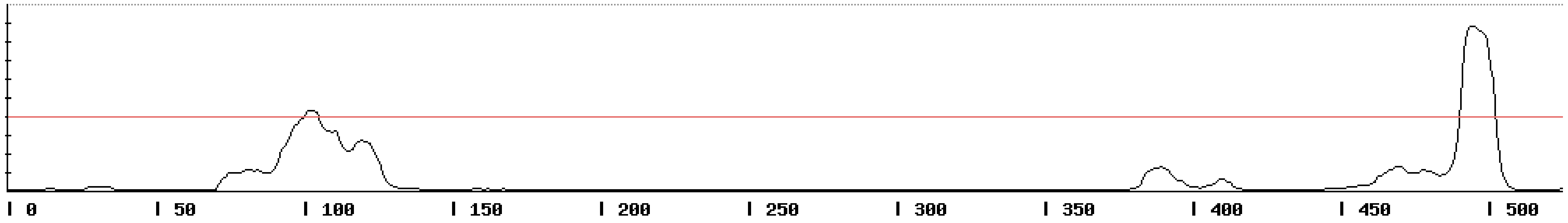

Predictions for Capsellarubella_Carubv10008035m

Viterbi Path	
Viterbi Path	
Posterior @ 0.4	
Posterior @ 0.4	
Posterior Graphic. Threshold Marked @ 0.4	

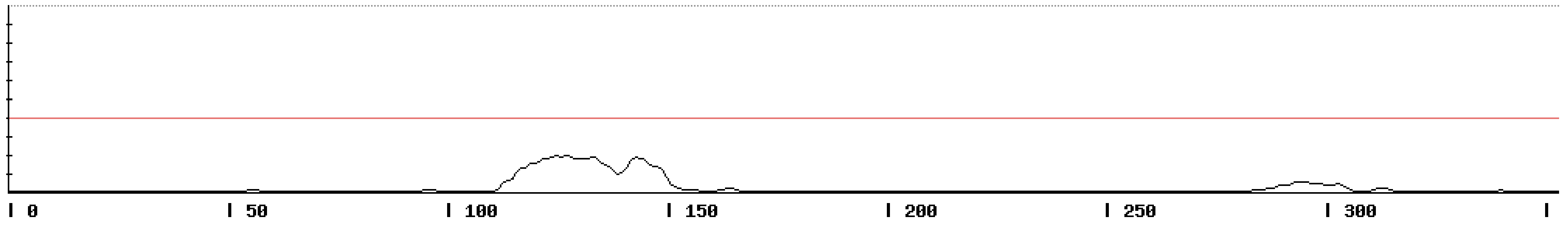

Predictions for Capsellarubella_Carubv10008036m

Viterbi Path

Viterbi Path

Posterior @ 0.4

Posterior @ 0.4

Posterior Graphic. Threshold Marked @ 0.4

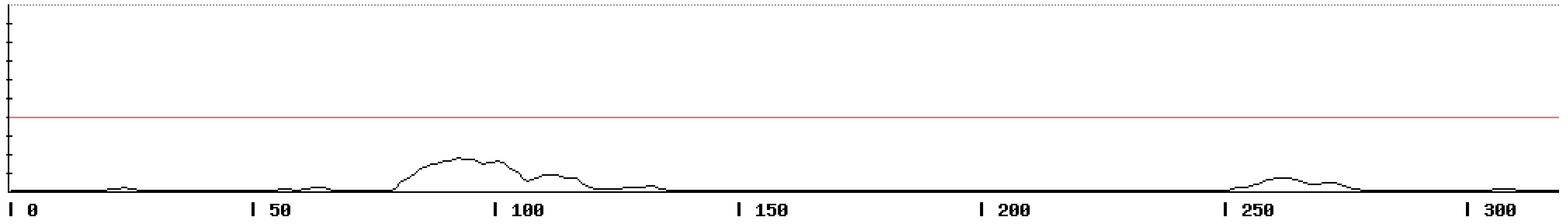

Predictions for Capsellarubella_Carubv10008529m

Viterbi Path

Viterbi Path

Posterior @ 0.4

Posterior @ 0.4

Posterior Graphic. Threshold Marked @ 0.4

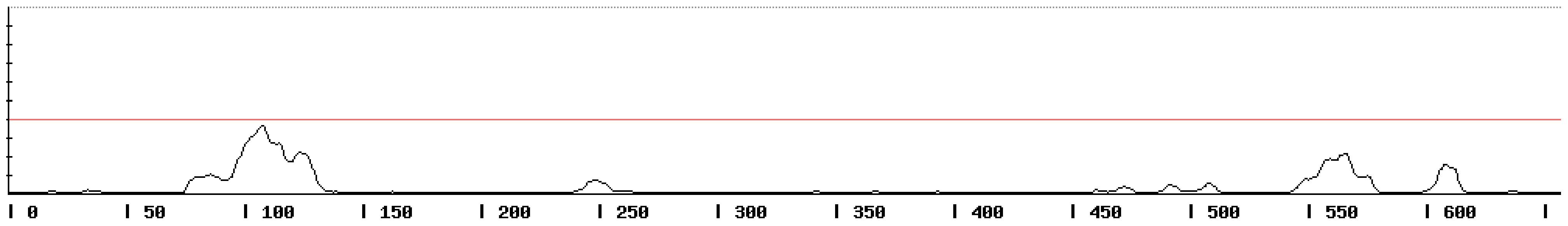

Predictions for Capsellarubella_Carubv10008531m

Viterbi Path

Viterbi Path

Posterior @ 0.4

Posterior @ 0.4

Posterior Graphic. Threshold Marked @ 0.4

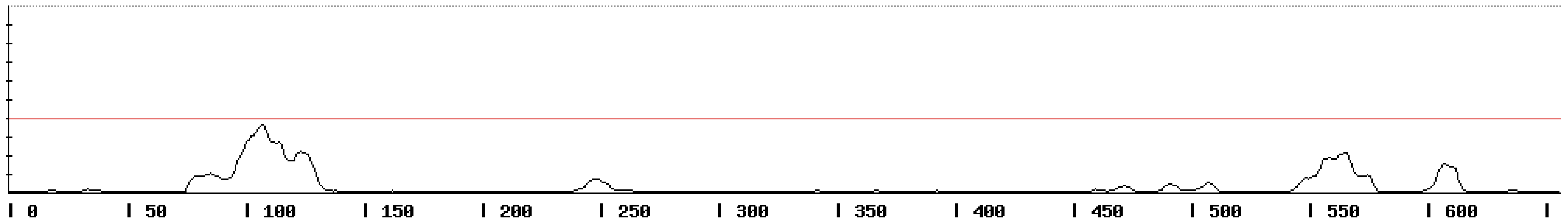

Predictions for Capsellarubella_Carubv10008533m

Viterbi Path

Viterbi Path

Posterior @ 0.4

Posterior @ 0.4

Posterior Graphic. Threshold Marked @ 0.4

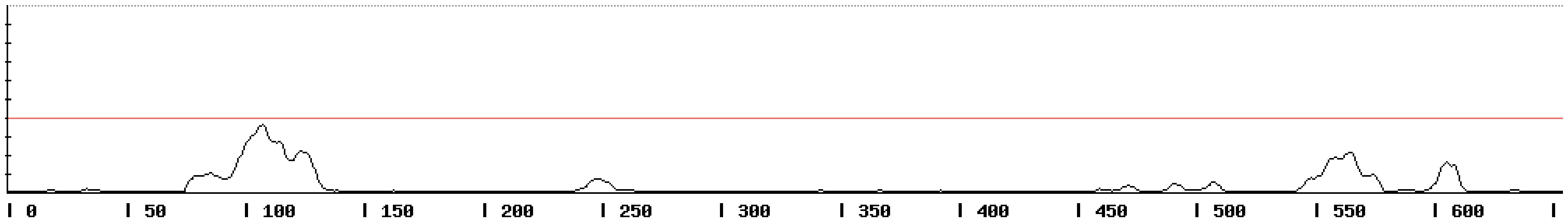

Predictions for Capsellarubella_Carubv10008706m

Viterbi Path

Viterbi Path

Posterior @ 0.4

Posterior @ 0.4

Posterior Graphic. Threshold Marked @ 0.4

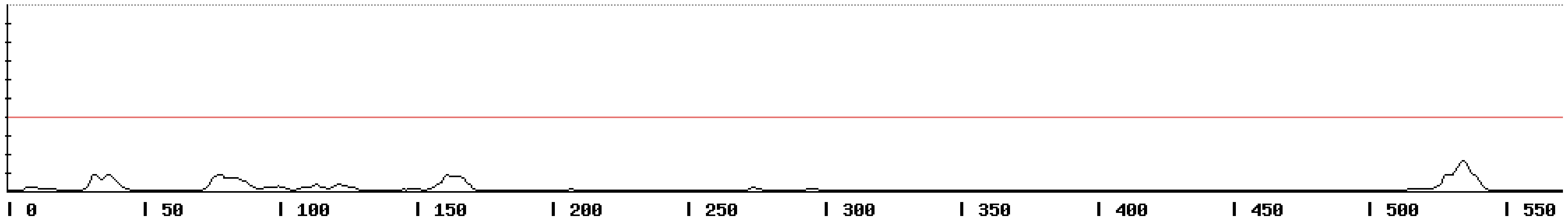

Predictions for Capsellarubella_Carubv10008761m

Viterbi Path

Viterbi Path

Posterior @ 0.4

Posterior @ 0.4

Posterior Graphic. Threshold Marked @ 0.4

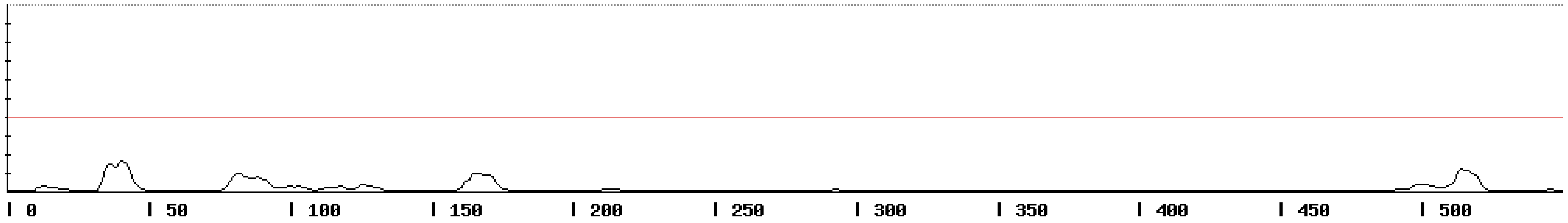

Predictions for Capsellarubella_Carubv10008817m

Viterbi Path

Viterbi Path

Posterior @ 0.4

30 - KRKIRRKKLR - 39

486 - RKKSGGKTDKKNNKSK - 501

Posterior @ 0.4

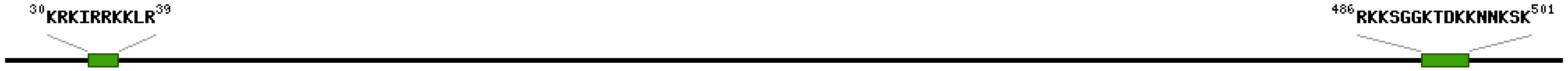

Posterior Graphic. Threshold Marked @ 0.4

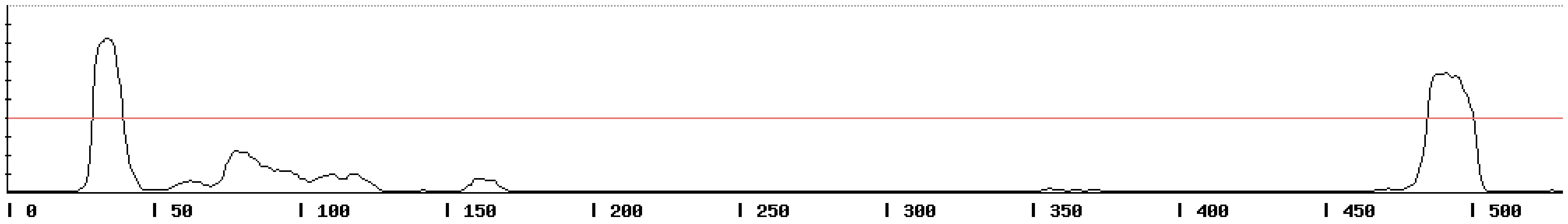

Predictions for Capsellarubella_Carubv10008886m

Viterbi Path

Viterbi Path

Posterior @ 0.4	
Posterior @ 0.4	
Posterior Graphic. Threshold Marked @ 0.4	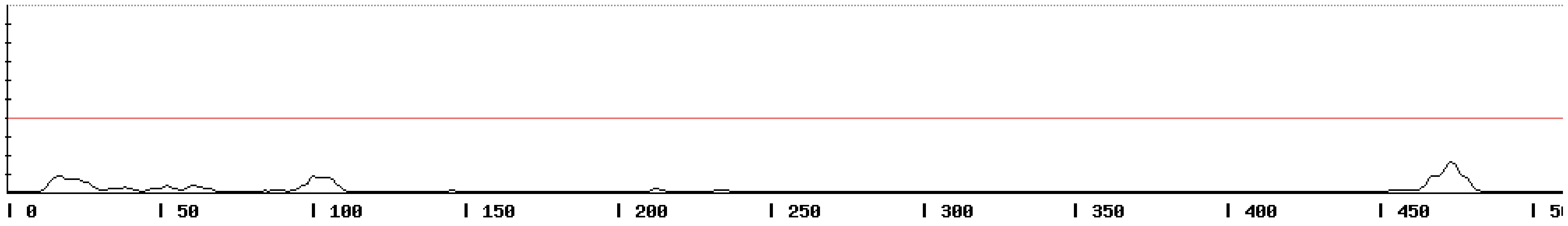

Predictions for Capsellarubella_Carubv10009049m	
Viterbi Path	
Viterbi Path	
Posterior @ 0.4	
Posterior @ 0.4	
Posterior Graphic. Threshold Marked @ 0.4	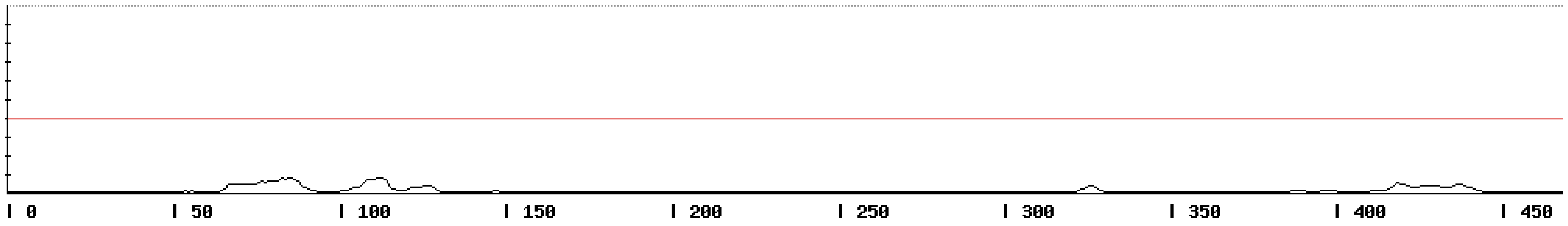

Predictions for Capsellarubella_Carubv10009087m

Viterbi Path	
Viterbi Path	
Posterior @ 0.4	137 - KRR - 139
Posterior @ 0.4	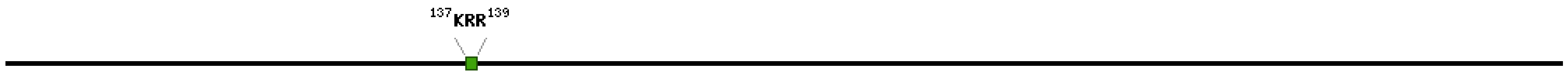
Posterior Graphic. Threshold Marked @ 0.4	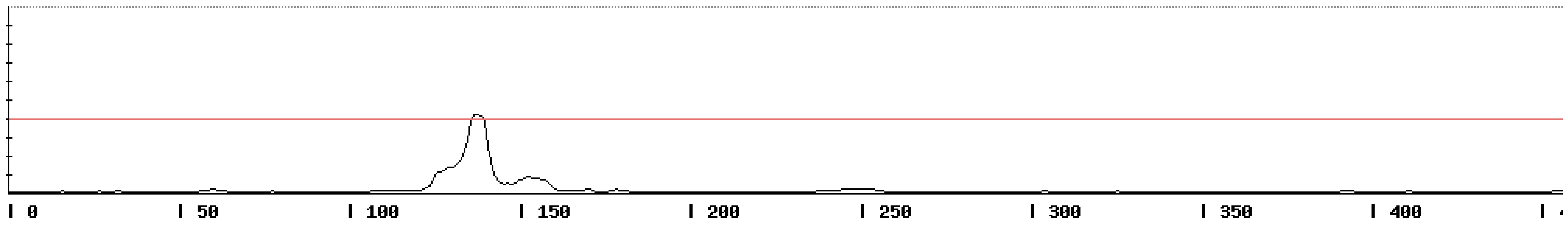

Predictions for Capsellarubella_Carubv10009366m

Viterbi Path	
Viterbi Path	
Posterior @ 0.4	
Posterior @ 0.4	
Posterior Graphic. Threshold Marked @ 0.4	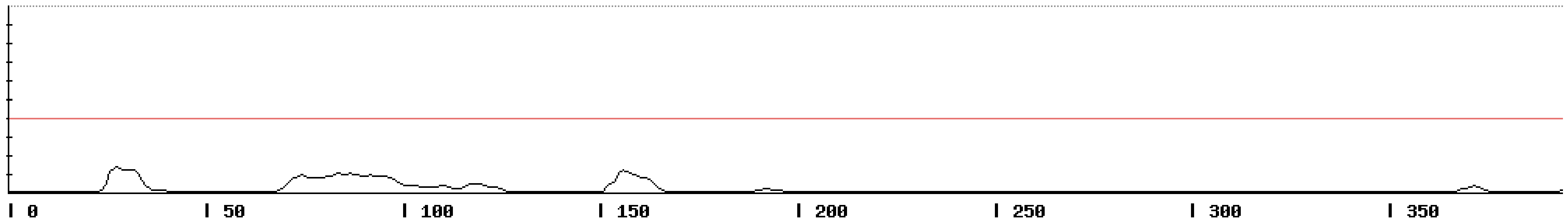

Predictions for Capsellarubella_Carubv10009390m	
Viterbi Path	
Viterbi Path	
Posterior @ 0.4	82 - NKYDKGERQIRKTKSGFWKITGKPKAIMRNRVKIGEKR - 119
Posterior @ 0.4	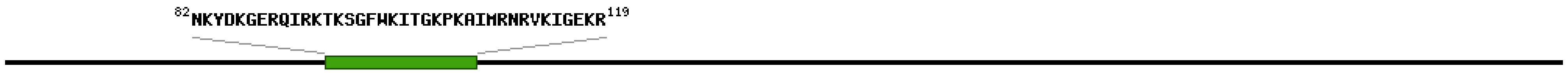
Posterior Graphic. Threshold Marked @ 0.4	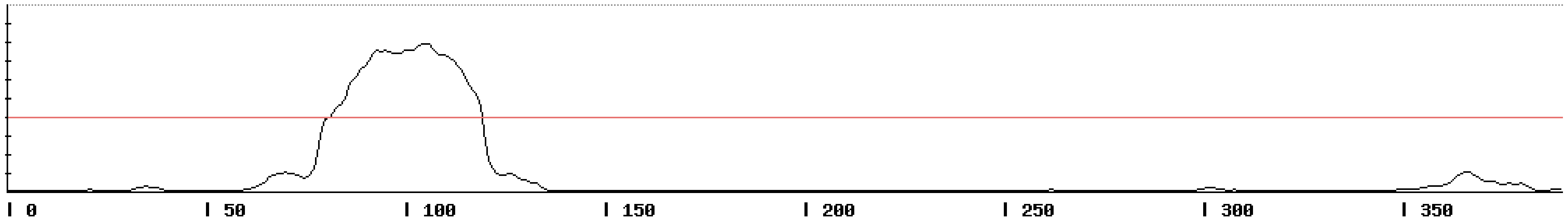

Predictions for Capsellarubella_Carubv10009451m	
Viterbi Path	
Viterbi Path	
Posterior @ 0.4	
Posterior @ 0.4	
Posterior Graphic. Threshold Marked @ 0.4	

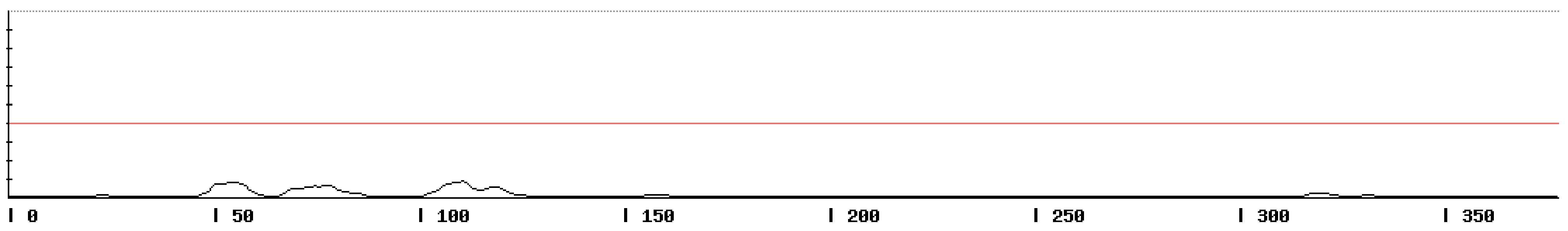

Predictions for Capsellarubella_Carubv10009524m

Viterbi Path	19 - RRPKKKKKSR - 28
Viterbi Path	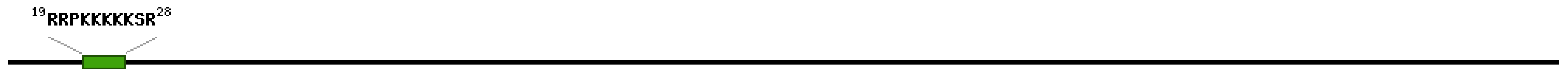
Posterior @ 0.4	18 - SRRPKKKKKSRI - 29
Posterior @ 0.4	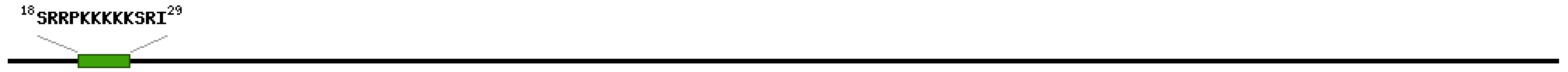
Posterior Graphic. Threshold Marked @ 0.4	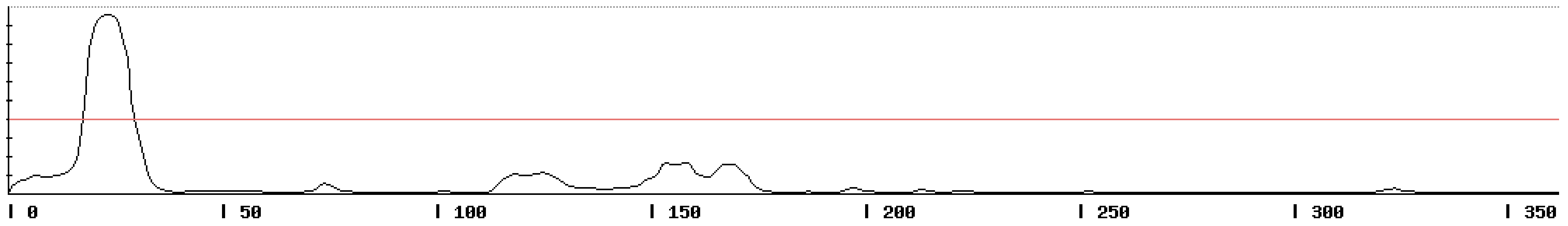

Predictions for Capsellarubella_Carubv10009638m

Viterbi Path	
Viterbi Path	
Posterior @ 0.4	15 - REREREVSKRR - 25<br>182 - KRR - 184
Posterior @ 0.4	

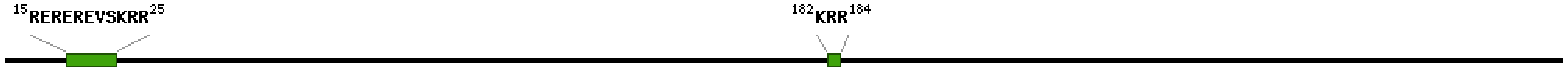

Posterior Graphic. Threshold Marked @ 0.4

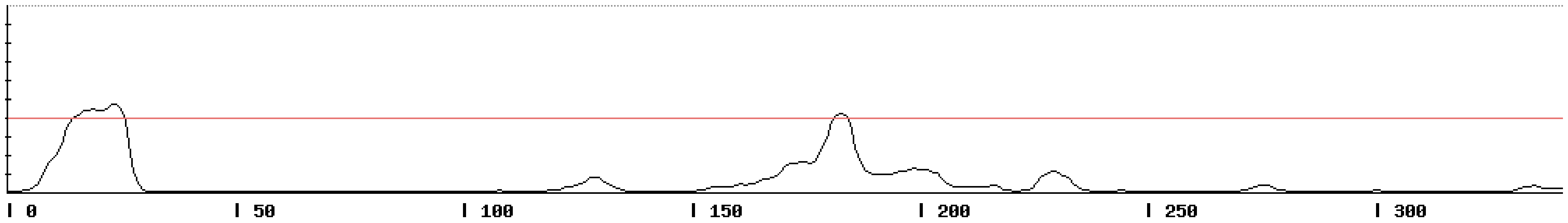

Predictions for Capsellarubella_Carubv10009640m

Viterbi Path

Viterbi Path

Posterior @ 0.4

Posterior @ 0.4

Posterior Graphic. Threshold Marked @ 0.4

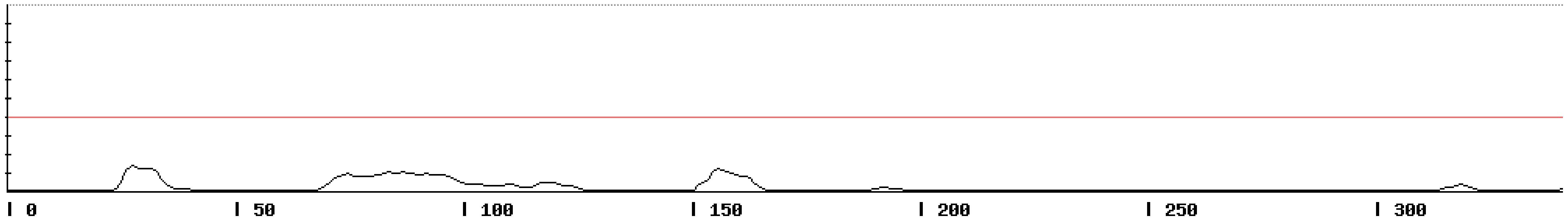

Predictions for Capsellarubella_Carubv10009727m

Viterbi Path

Viterbi Path

Posterior @ 0.4	
Posterior @ 0.4	
Posterior Graphic. Threshold Marked @ 0.4	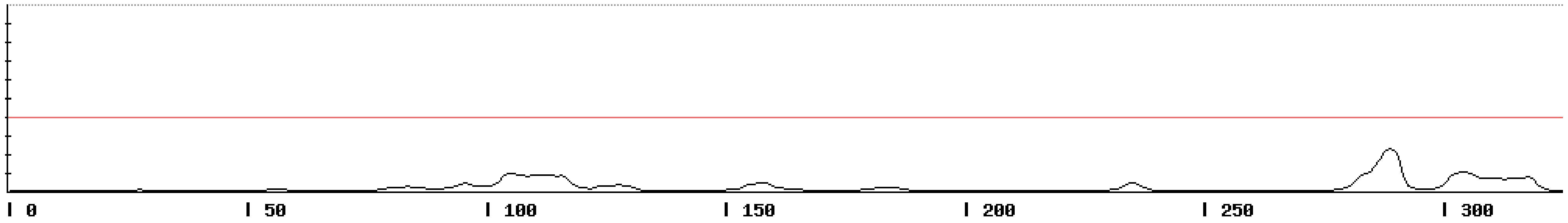

Predictions for Capsellarubella_Carubv10009816m

Viterbi Path	
Viterbi Path	
Posterior @ 0.4	
Posterior @ 0.4	
Posterior Graphic. Threshold Marked @ 0.4	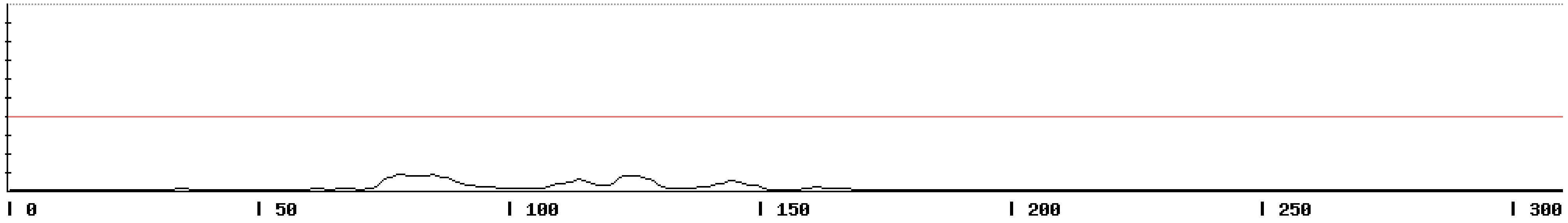

Predictions for Capsellarubella_Carubv10009870m

Viterbi Path	
Viterbi Path	

Posterior @ 0.4

Posterior @ 0.4

Posterior Graphic. Threshold Marked @ 0.4

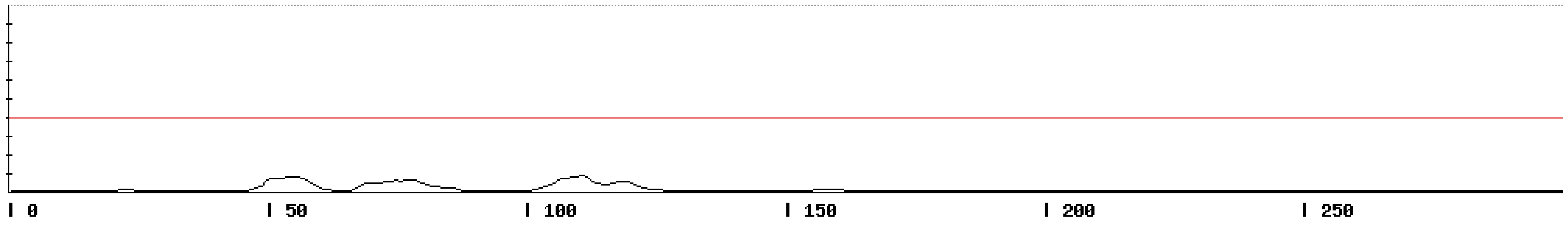

Predictions for Capsellarubella_Carubv10009926m

Viterbi Path

Viterbi Path

Posterior @ 0.4

Posterior @ 0.4

Posterior Graphic. Threshold Marked @ 0.4

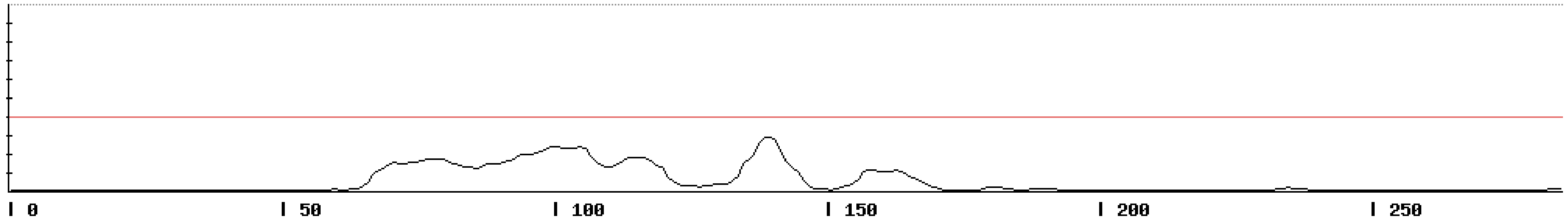

Predictions for Capsellarubella_Carubv10010920m

Viterbi Path

Viterbi Path

Posterior @ 0.4

Posterior @ 0.4

Posterior Graphic. Threshold Marked @ 0.4

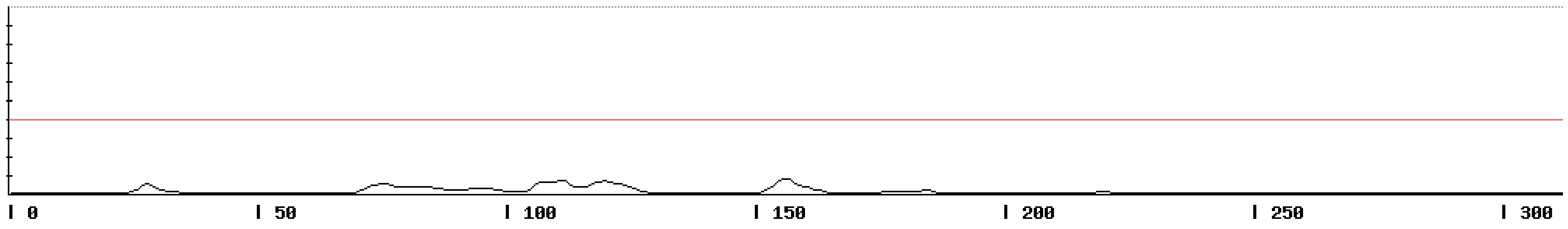

Predictions for Capsellarubella_Carubv10011081m

Viterbi Path

Viterbi Path

Posterior @ 0.4

Posterior @ 0.4

Posterior Graphic. Threshold Marked @ 0.4

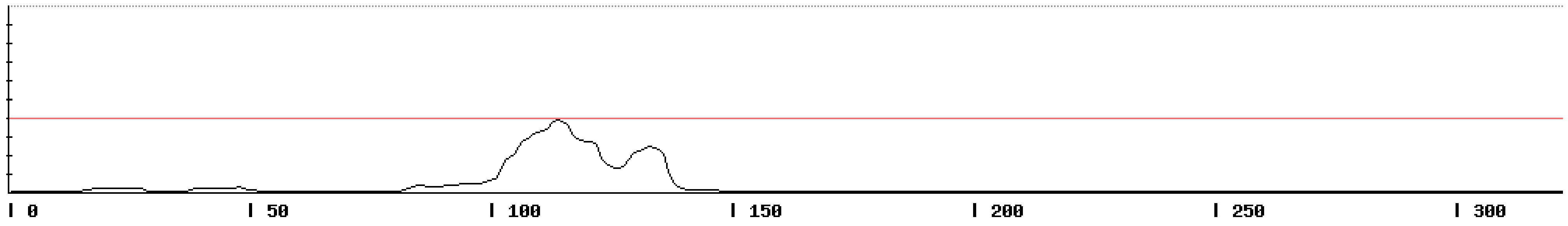

Predictions for Capsellarubella_Carubv10011108m

Viterbi Path

Viterbi Path

Posterior @ 0.4

Posterior @ 0.4

Posterior Graphic. Threshold Marked @ 0.4

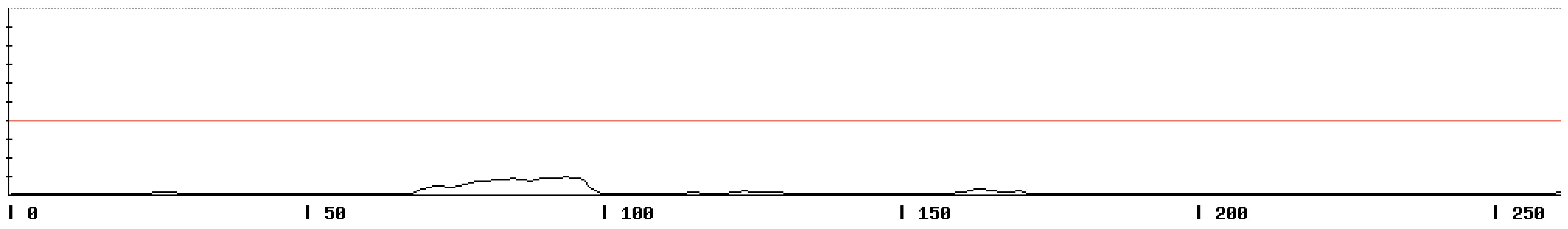

Predictions for Capsellarubella_Carubv10011253m

Viterbi Path

Viterbi Path

Posterior @ 0.4

Posterior @ 0.4

Posterior Graphic. Threshold Marked @ 0.4

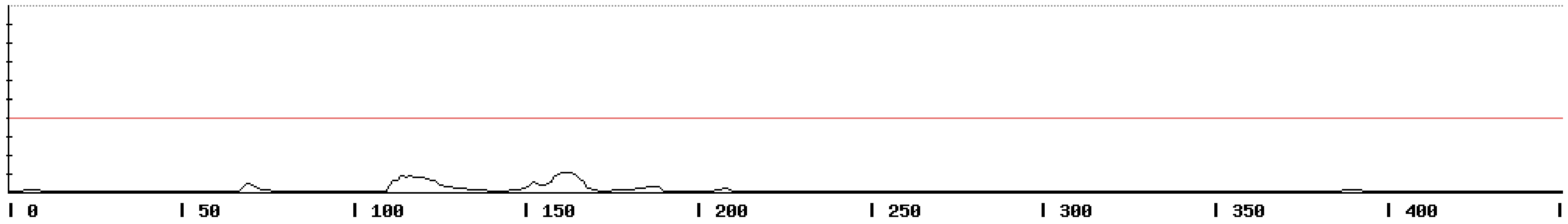

Predictions for Capsellarubella_Carubv10011623m

Viterbi Path

Viterbi Path

Posterior @ 0.4

Posterior @ 0.4

Posterior Graphic. Threshold Marked @ 0.4

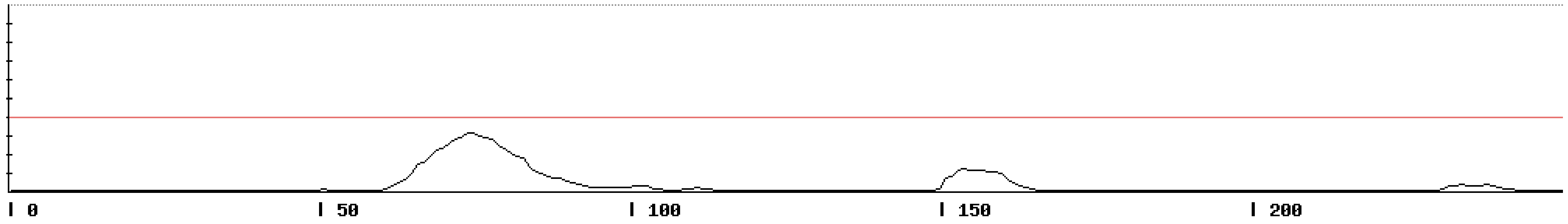

Predictions for Capsellarubella_Carubv10011900m

Viterbi Path

Viterbi Path

Posterior @ 0.4

Posterior @ 0.4

Posterior Graphic. Threshold Marked @ 0.4

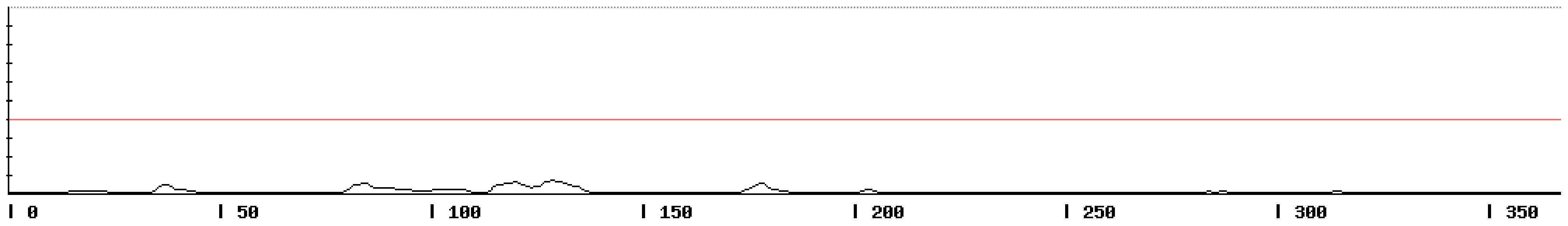

Predictions for Capsellarubella_Carubv10011902m

Viterbi Path

Viterbi Path

Posterior @ 0.4

Posterior @ 0.4

Posterior Graphic. Threshold Marked @ 0.4

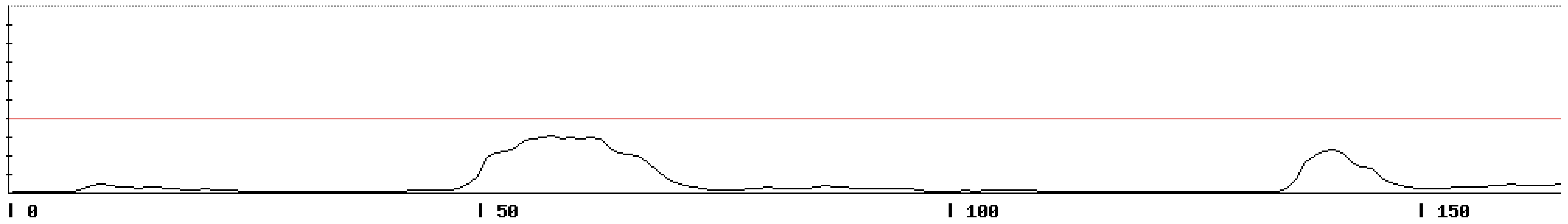

Predictions for Capsellarubella_Carubv10012163m

Viterbi Path

Viterbi Path

Posterior @ 0.4

Posterior @ 0.4

Posterior Graphic. Threshold Marked @ 0.4

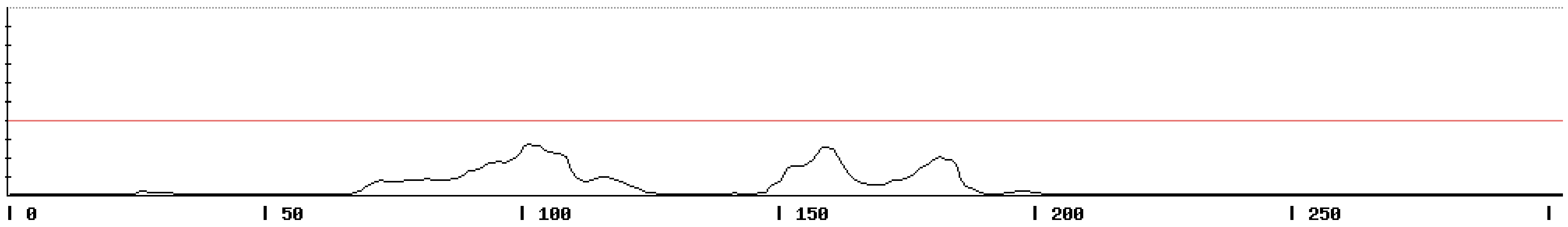

Predictions for Capsellarubella_Carubv10012206m

Viterbi Path

Viterbi Path

Posterior @ 0.4 74 - PRTKRHRNKPRRTVPPRGGS - 93

Posterior @ 0.4

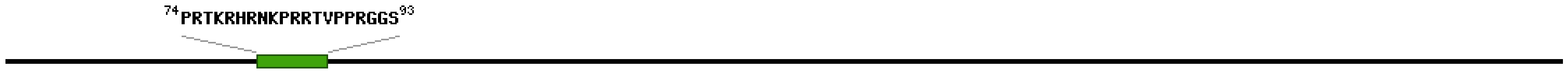

Posterior Graphic. Threshold Marked @ 0.4

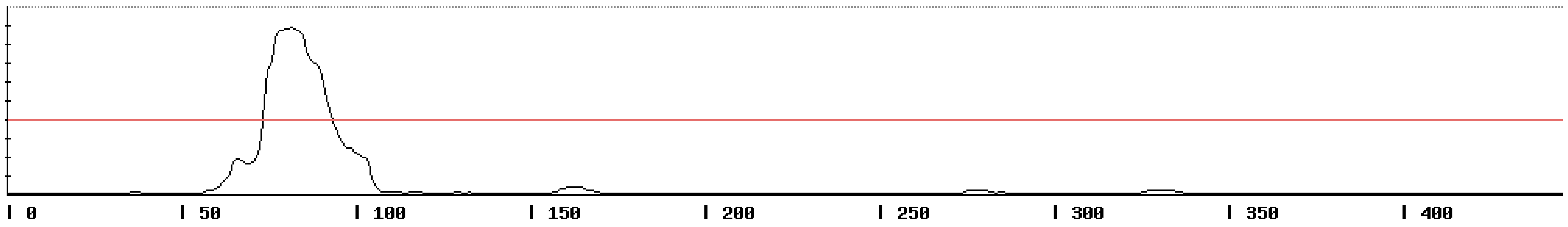

Predictions for Capsellarubella_Carubv10013370m

Viterbi Path

Viterbi Path

Posterior @ 0.4

Posterior @ 0.4

Posterior Graphic. Threshold Marked @ 0.4

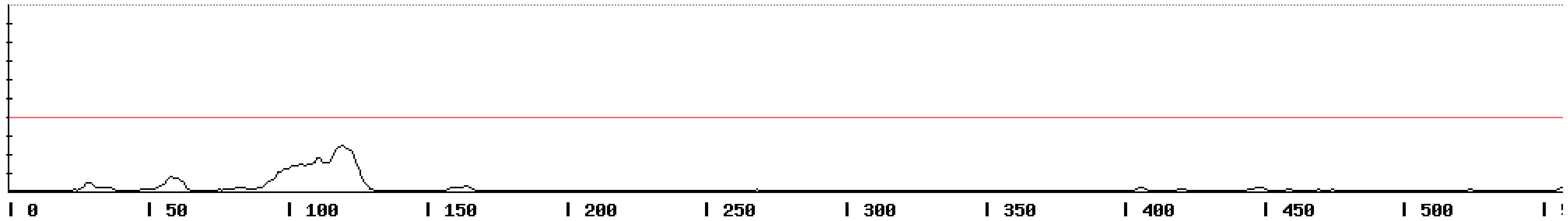

Predictions for Capsellarubella_Carubv10013564m

Viterbi Path

Viterbi Path

Posterior @ 0.4

Posterior @ 0.4

Posterior Graphic. Threshold Marked @ 0.4

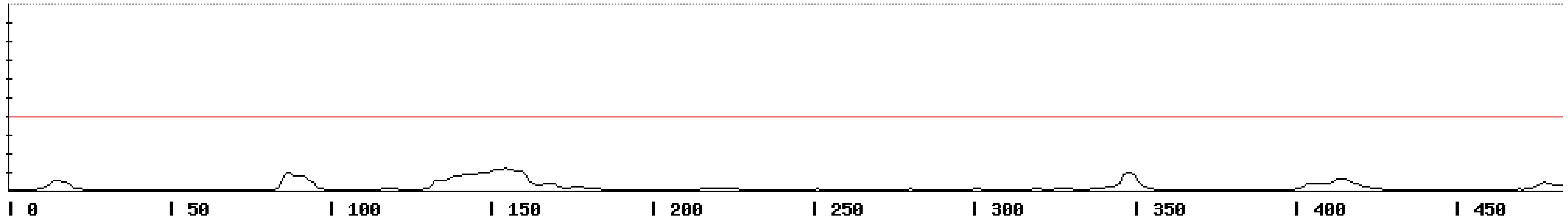

Predictions for Capsellarubella_Carubv10013584m

Viterbi Path

Viterbi Path

Posterior @ 0.4

Posterior @ 0.4

Posterior Graphic. Threshold Marked @ 0.4

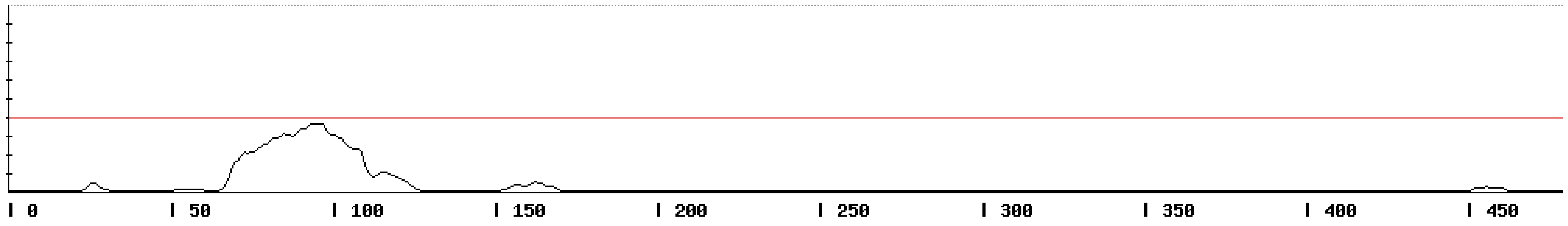

Predictions for Capsellarubella_Carubv10013694m

Viterbi Path

Viterbi Path

Posterior @ 0.4

Posterior @ 0.4

Posterior Graphic. Threshold Marked @ 0.4

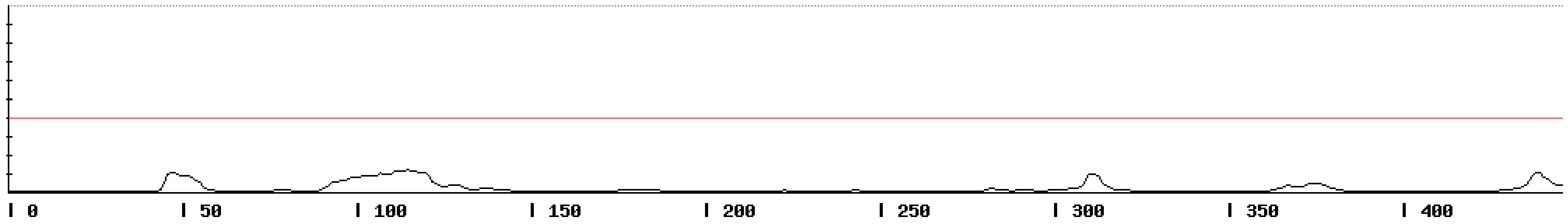

Predictions for Capsellarubella_Carubv10014000m

Viterbi Path

Viterbi Path

Posterior @ 0.4

Posterior @ 0.4

Posterior Graphic. Threshold Marked @ 0.4

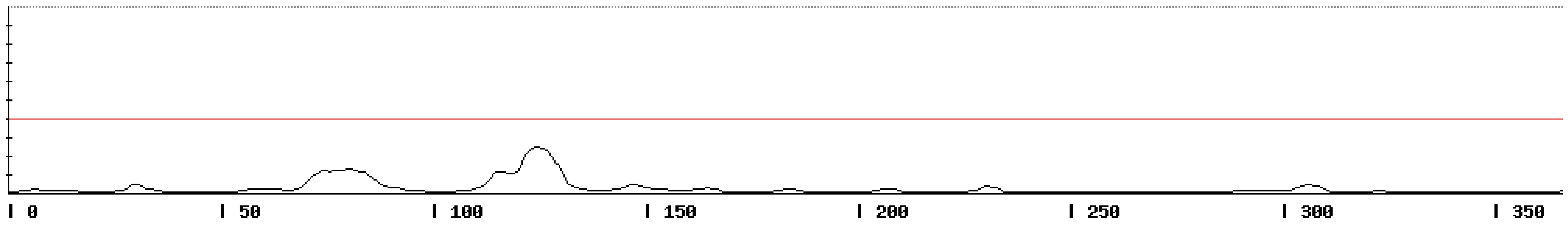

Predictions for Capsellarubella_Carubv10014004m

Viterbi Path

Viterbi Path

Posterior @ 0.4

Posterior @ 0.4

Posterior Graphic. Threshold Marked @ 0.4

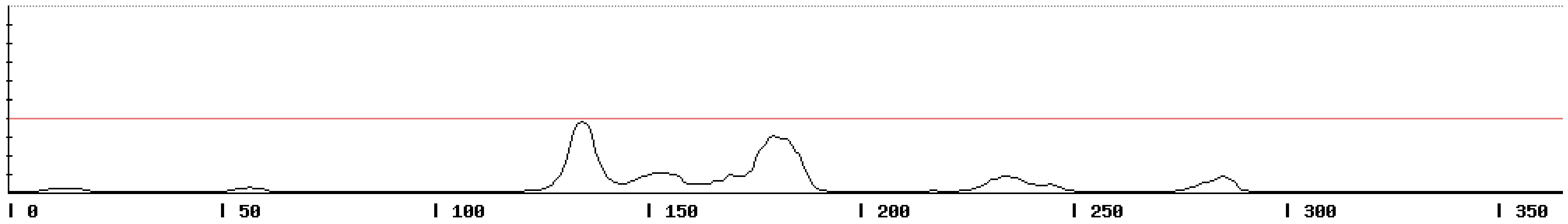

Predictions for Capsellarubella_Carubv10014008m	
Viterbi Path	
Viterbi Path	
Posterior @ 0.4	
Posterior @ 0.4	
Posterior Graphic. Threshold Marked @ 0.4	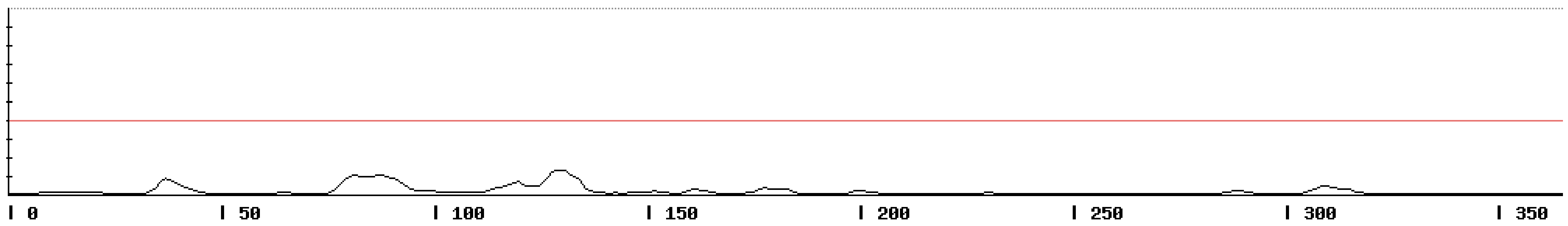

Predictions for Capsellarubella_Carubv10014118m	
Viterbi Path	
Viterbi Path	
Posterior @ 0.4	
Posterior @ 0.4	
Posterior Graphic. Threshold Marked @ 0.4	

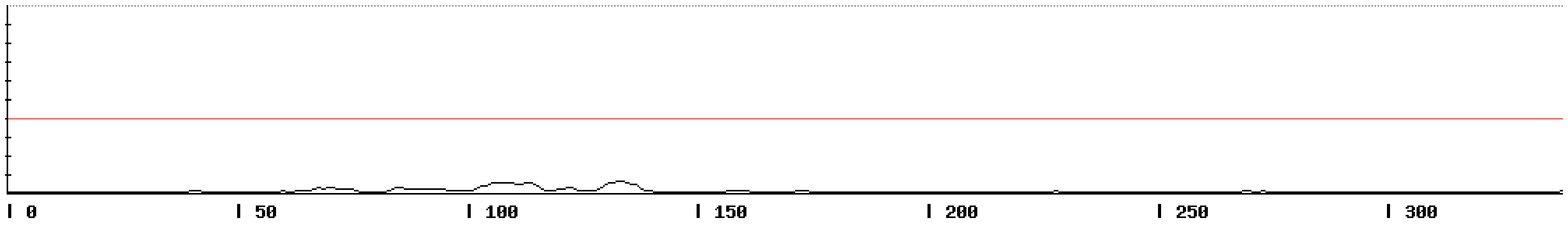

Predictions for Capsellarubella_Carubv10014211m	
Viterbi Path	
Viterbi Path	
Posterior @ 0.4	
Posterior @ 0.4	
Posterior Graphic. Threshold Marked @ 0.4	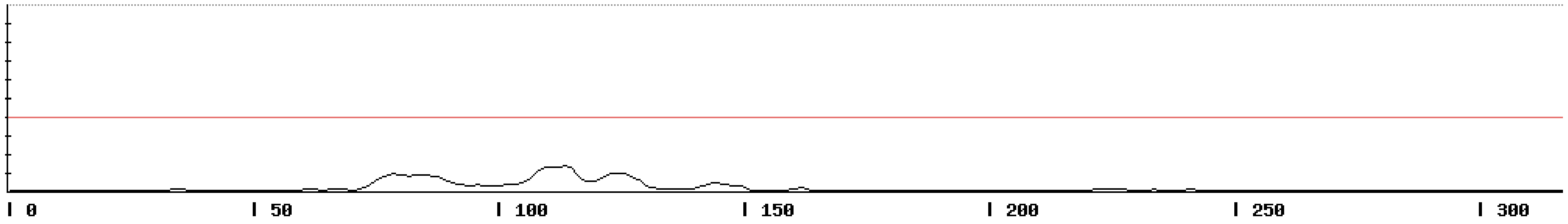

Predictions for Capsellarubella_Carubv10014270m	
Viterbi Path	
Viterbi Path	
Posterior @ 0.4	
Posterior @ 0.4	

Posterior Graphic. Threshold Marked @ 0.4

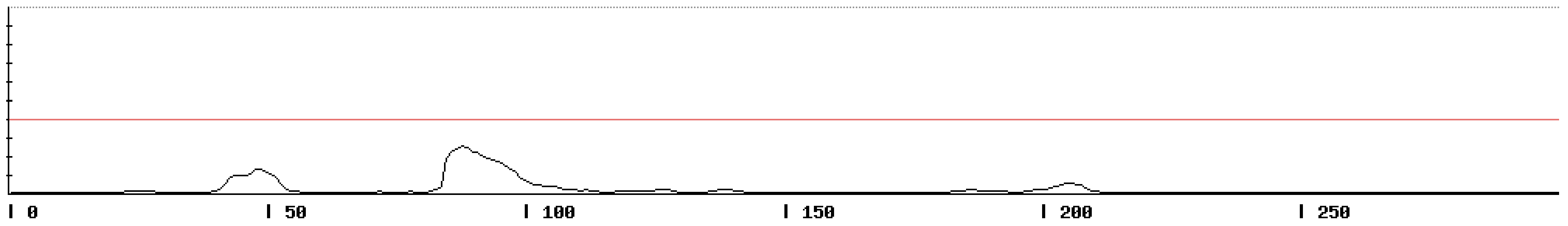

Predictions for Capsellarubella_Carubv10014272m

Viterbi Path

Viterbi Path

Posterior @ 0.4

Posterior @ 0.4

Posterior Graphic. Threshold Marked @ 0.4

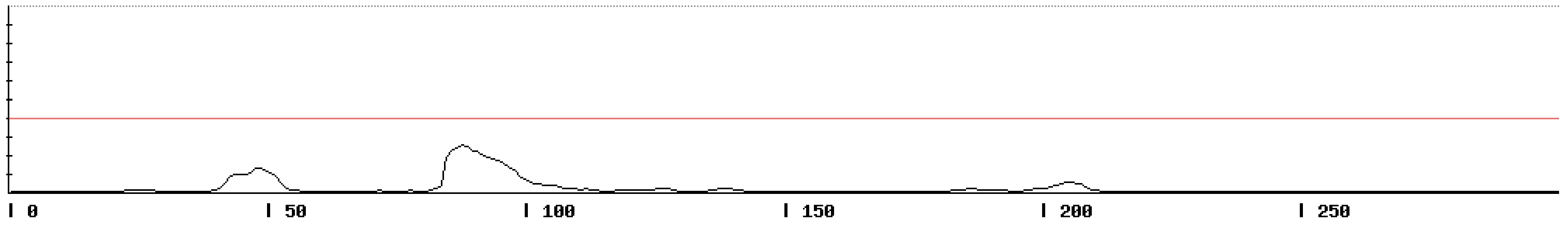

Predictions for Capsellarubella_Carubv10014276m

Viterbi Path

Viterbi Path

Posterior @ 0.4

Posterior @ 0.4

Posterior Graphic. Threshold Marked @ 0.4	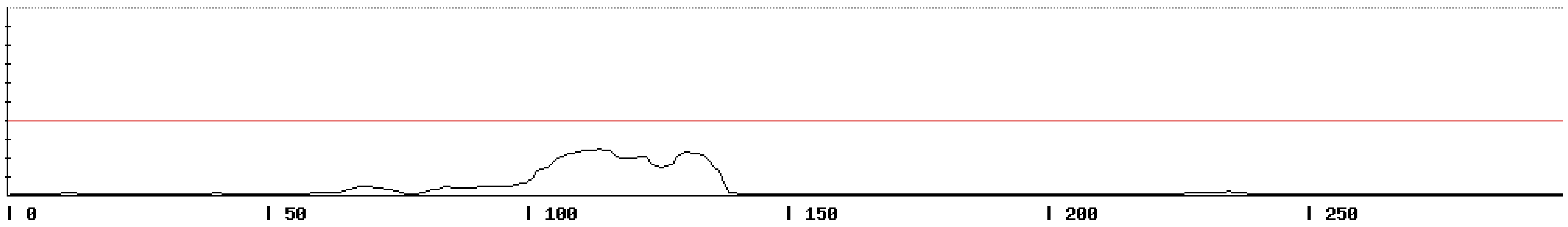

Predictions for Capsellarubella_Carubv10014350m	
Viterbi Path	
Viterbi Path	
Posterior @ 0.4	
Posterior @ 0.4	
Posterior Graphic. Threshold Marked @ 0.4	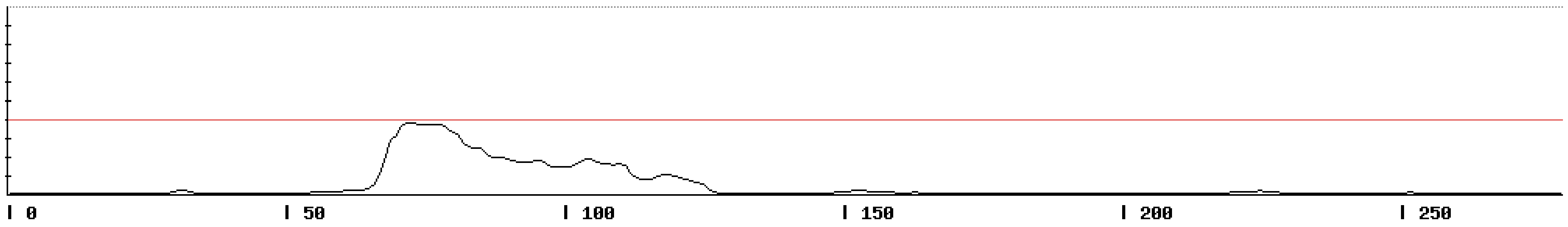

Predictions for Capsellarubella_Carubv10014426m	
Viterbi Path	
Viterbi Path	

Posterior @ 0.4

Posterior @ 0.4

Posterior Graphic. Threshold Marked @ 0.4

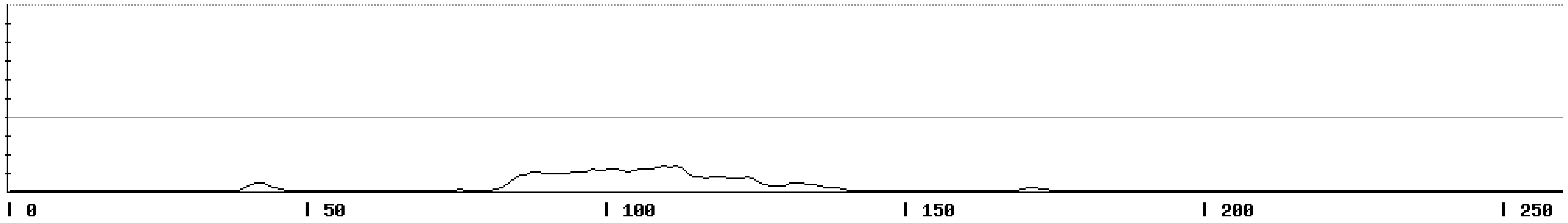

Predictions for Capsellarubella_Carubv10015289m

Viterbi Path

Viterbi Path

Posterior @ 0.4

114 - KKGISGKKHKR - 124

207 - KAMSKAKKGKGKLKV - 221

Posterior @ 0.4

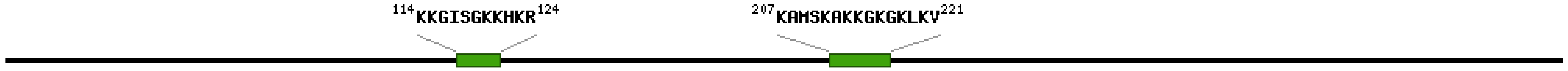

Posterior Graphic. Threshold Marked @ 0.4

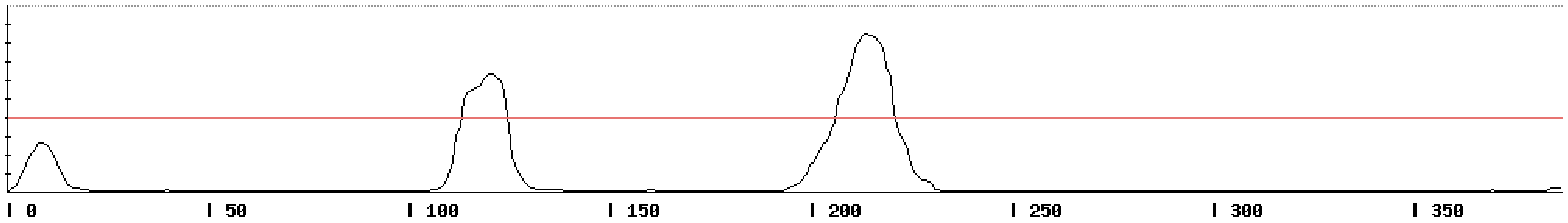

Predictions for Capsellarubella_Carubv10015990m

Viterbi Path

Viterbi Path

Posterior @ 0.4

Posterior @ 0.4

Posterior Graphic. Threshold Marked @ 0.4

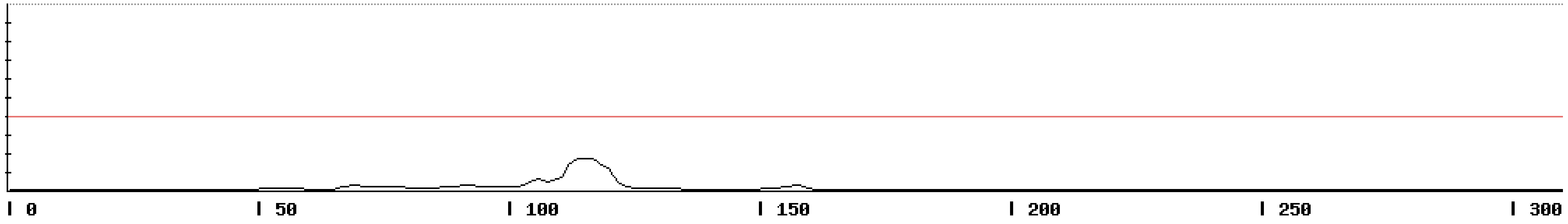

Predictions for Capsellarubella_Carubv10017199m

Viterbi Path

Viterbi Path

Posterior @ 0.4

Posterior @ 0.4

Posterior Graphic. Threshold Marked @ 0.4

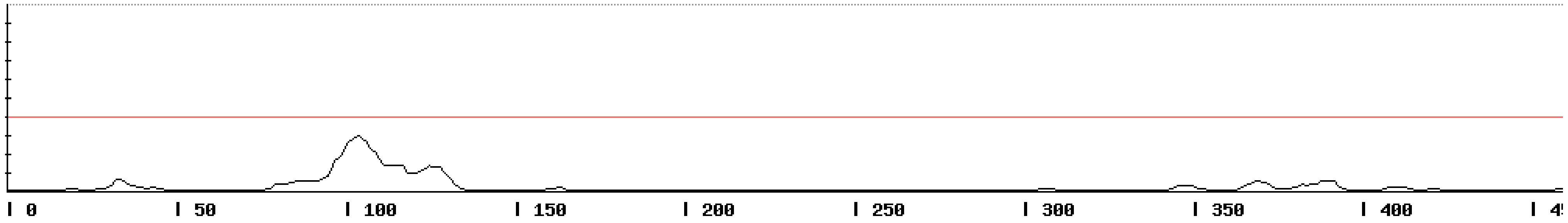

Predictions for Capsellarubella_Carubv10017334m

Viterbi Path

Viterbi Path

Posterior @ 0.4

Posterior @ 0.4

Posterior Graphic. Threshold Marked @ 0.4

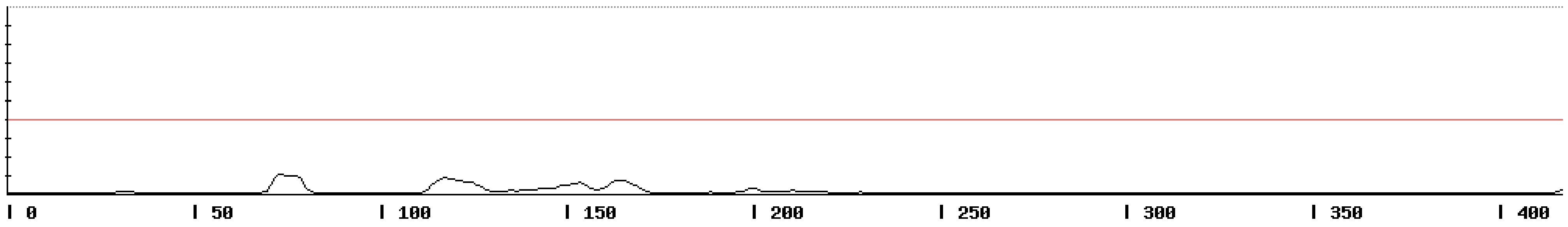

Predictions for Capsellarubella_Carubv10017737m

Viterbi Path

Viterbi Path

Posterior @ 0.4

Posterior @ 0.4

Posterior Graphic. Threshold Marked @ 0.4

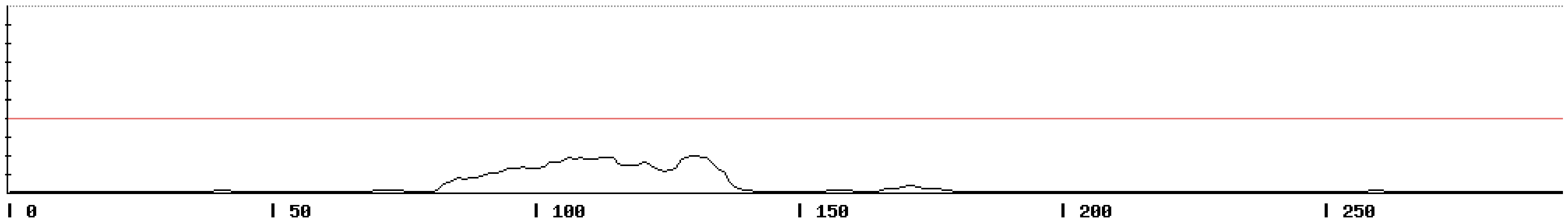

Predictions for Capsellarubella_Carubv10017870m	
Viterbi Path	
Viterbi Path	
Posterior @ 0.4	
Posterior @ 0.4	
Posterior Graphic. Threshold Marked @ 0.4	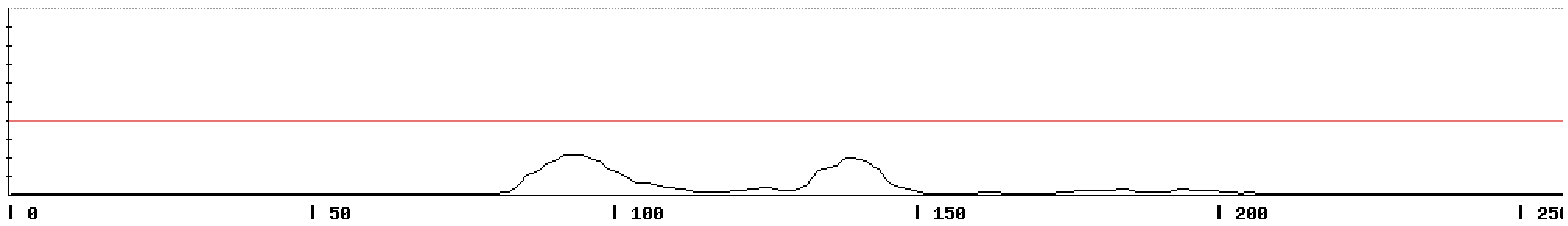

Predictions for Capsellarubella_Carubv10017972m	
Viterbi Path	
Viterbi Path	
Posterior @ 0.4	
Posterior @ 0.4	
Posterior Graphic. Threshold Marked @ 0.4	

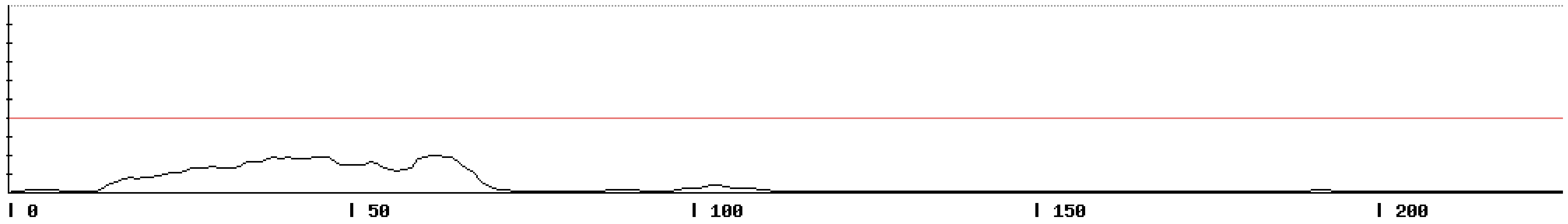

Predictions for Capsellarubella_Carubv10018723m

Viterbi Path	
Viterbi Path	
Posterior @ 0.4	
Posterior @ 0.4	
Posterior Graphic. Threshold Marked @ 0.4	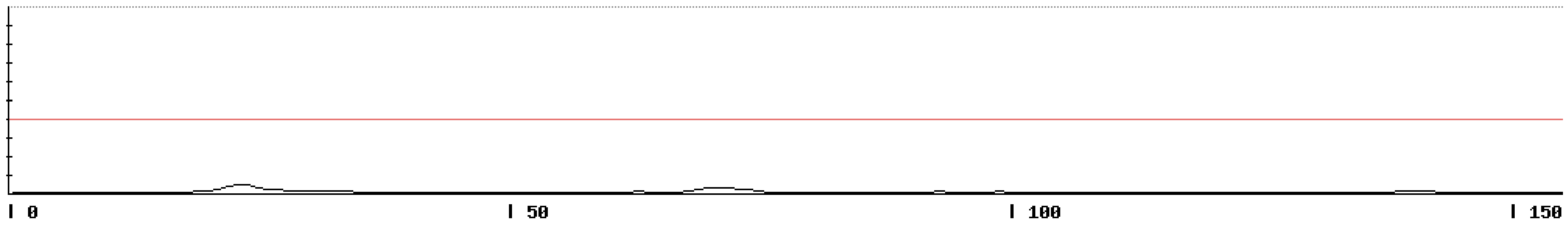

Predictions for Capsellarubella_Carubv10019084m

Viterbi Path	
Viterbi Path	
Posterior @ 0.4	
Posterior @ 0.4	

Posterior Graphic. Threshold Marked @ 0.4

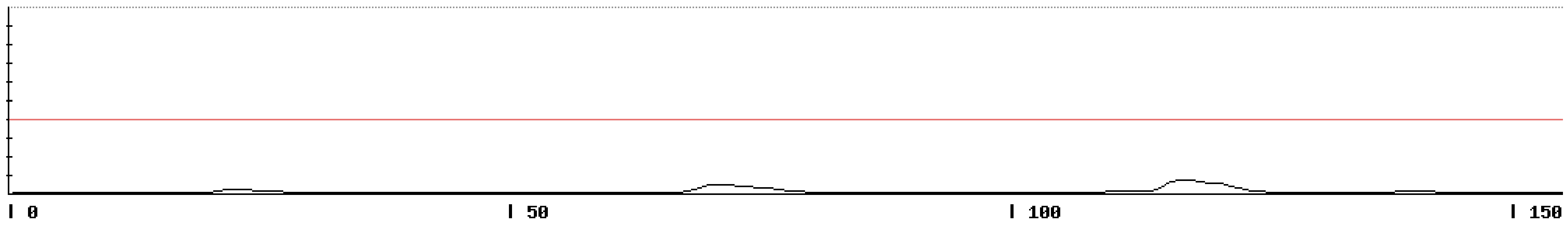

Predictions for Capsellarubella_Carubv10019187m

Viterbi Path

Viterbi Path

Posterior @ 0.4

Posterior @ 0.4

Posterior Graphic. Threshold Marked @ 0.4

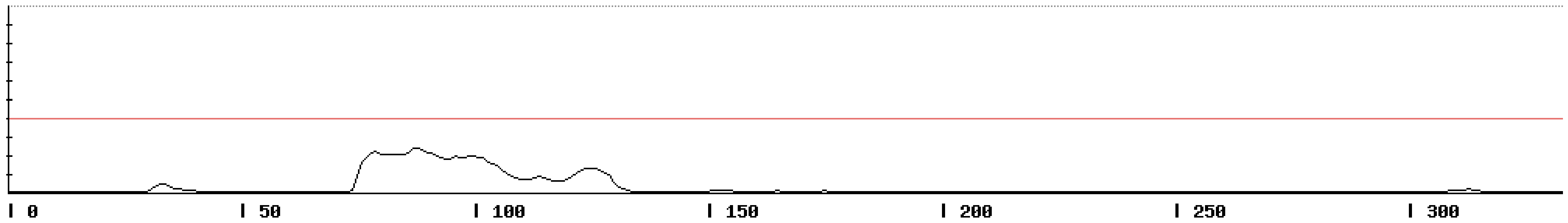

Predictions for Capsellarubella_Carubv10019223m

Viterbi Path

Viterbi Path

Posterior @ 0.4

Posterior @ 0.4

Posterior Graphic. Threshold Marked @ 0.4

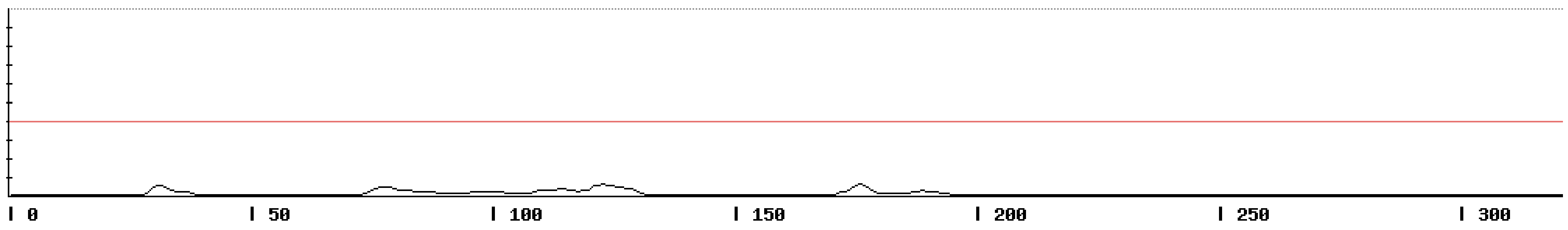

Predictions for Capsellarubella_Carubv10019452m

Viterbi Path

189 - RKKKKTEETENERKKKKALRNK - 210

Viterbi Path

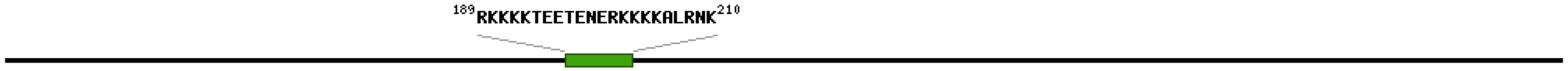

Posterior @ 0.4

187 - NERKKKKTEETENERKKKKALRNK - 210

Posterior @ 0.4

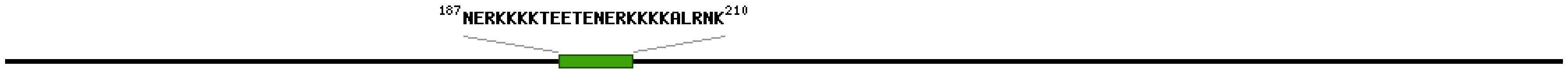

Posterior Graphic. Threshold Marked @ 0.4

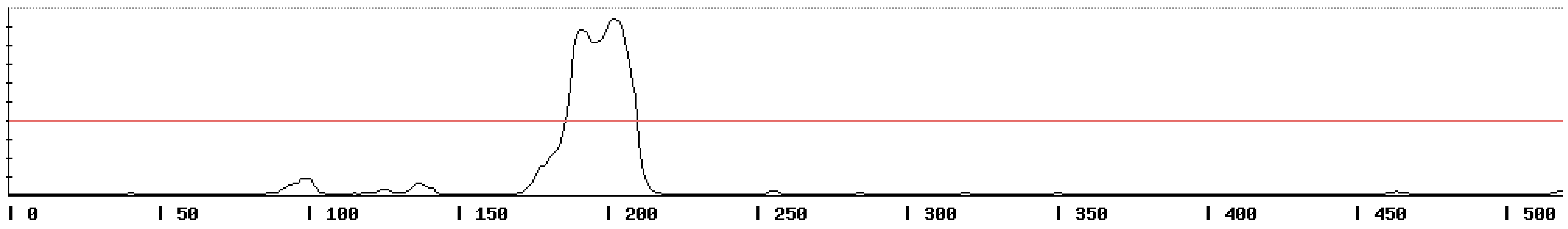

Predictions for Capsellarubella_Carubv10020504m

Viterbi Path

Viterbi Path

Posterior @ 0.4

Posterior @ 0.4

Posterior Graphic. Threshold Marked @ 0.4

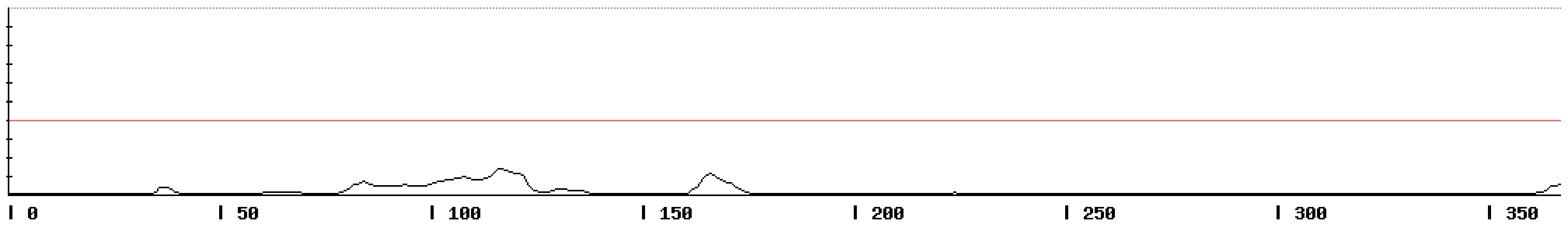

Predictions for Capsellarubella_Carubv10020645m

Viterbi Path

Viterbi Path

Posterior @ 0.4

Posterior @ 0.4

Posterior Graphic. Threshold Marked @ 0.4

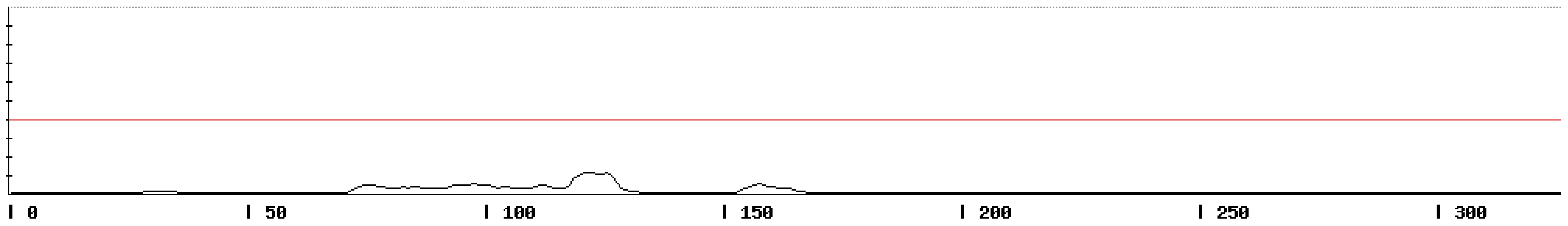

Predictions for Capsellarubella_Carubv10020776m

Viterbi Path

Viterbi Path

Posterior @ 0.4	
Posterior @ 0.4	
Posterior Graphic. Threshold Marked @ 0.4	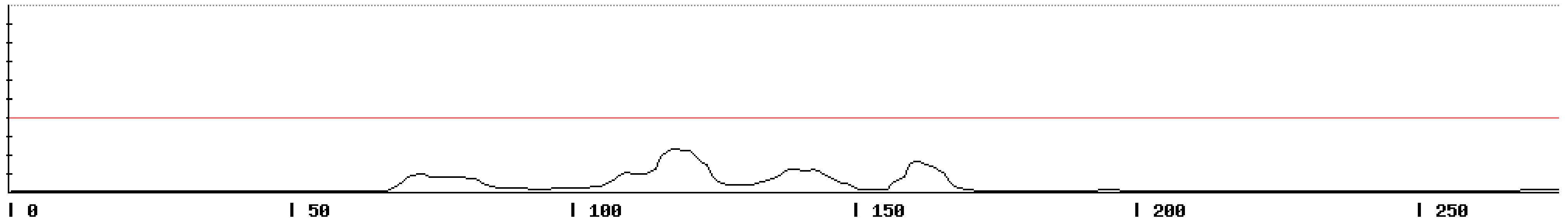

Predictions for Capsellarubella_Carubv10020834m

Viterbi Path	
Viterbi Path	
Posterior @ 0.4	98 - KPIGRPKPVGIK - 109
Posterior @ 0.4	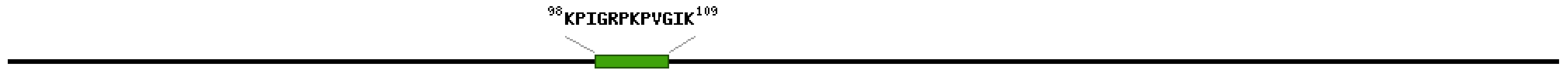
Posterior Graphic. Threshold Marked @ 0.4	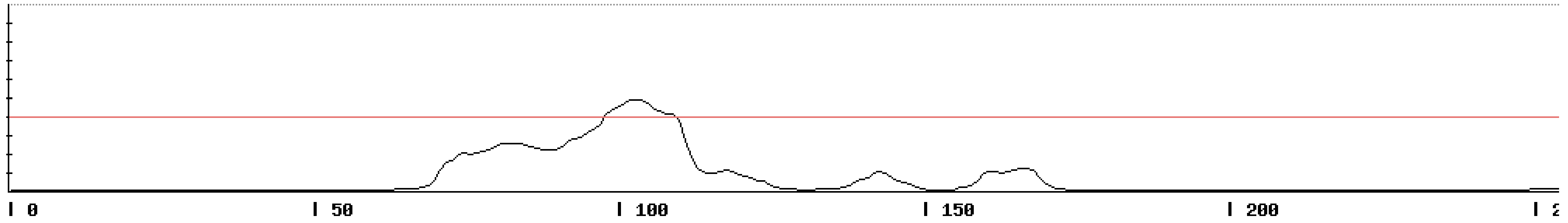

Predictions for Capsellarubella_Carubv10021319m

Viterbi Path	
Viterbi Path	
Posterior @ 0.4	115 - RKP - 117
Posterior @ 0.4	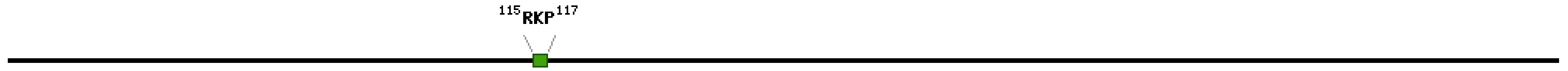
Posterior Graphic. Threshold Marked @ 0.4	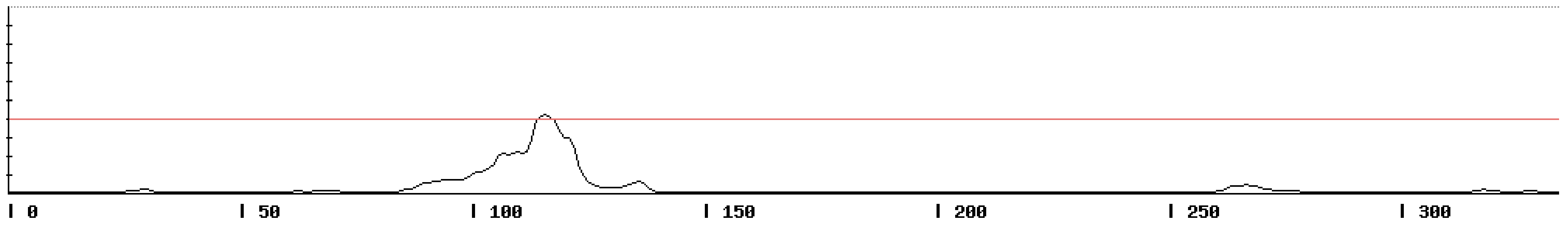

Predictions for Capsellarubella_Carubv10021371m

Viterbi Path	
Viterbi Path	
Posterior @ 0.4	
Posterior @ 0.4	
Posterior Graphic. Threshold Marked @ 0.4	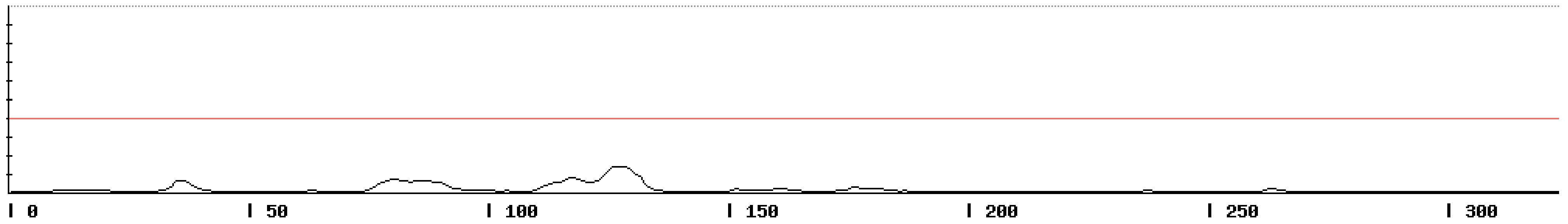

Predictions for Capsellarubella_Carubv10021514m

Viterbi Path

Viterbi Path

Posterior @ 0.4

Posterior @ 0.4

Posterior Graphic. Threshold Marked @ 0.4

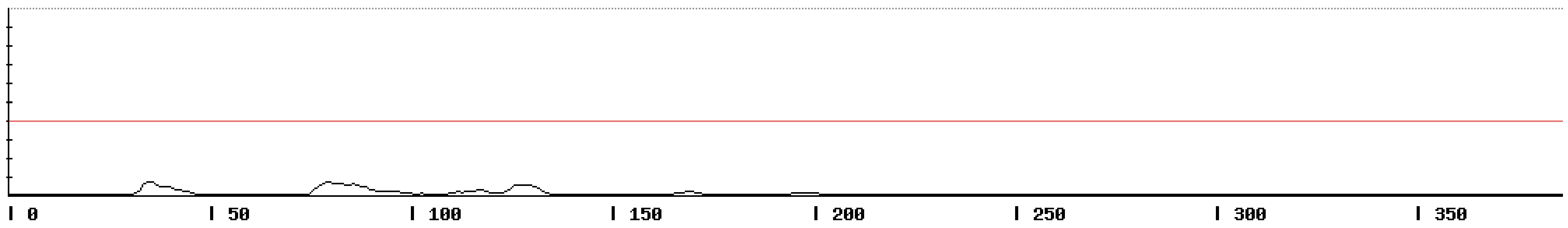

Predictions for Capsellarubella_Carubv10021788m

Viterbi Path

Viterbi Path

Posterior @ 0.4

Posterior @ 0.4

Posterior Graphic. Threshold Marked @ 0.4

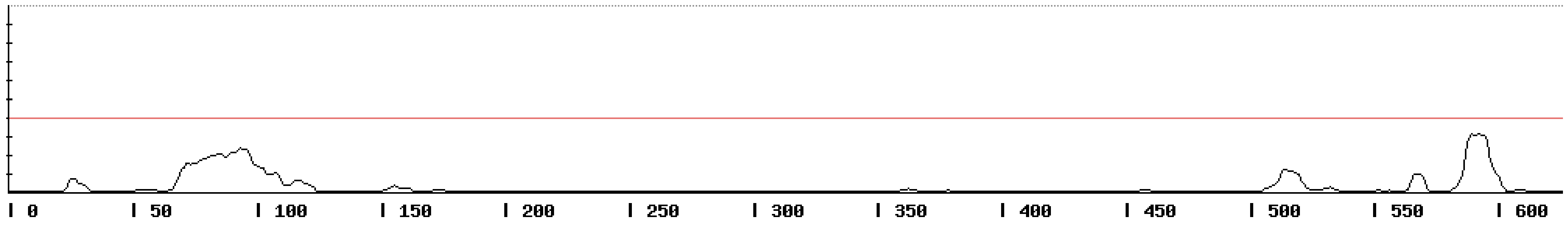

Predictions for Capsellarubella_Carubv10021818m

Viterbi Path	
Viterbi Path	
Posterior @ 0.4	
Posterior @ 0.4	
Posterior Graphic. Threshold Marked @ 0.4	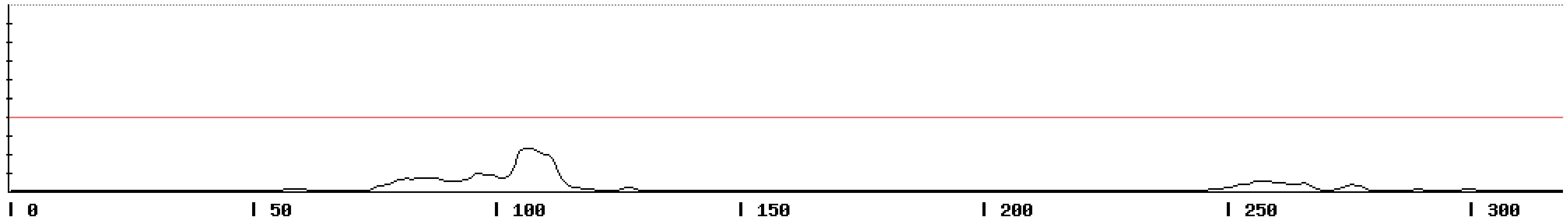

Predictions for Capsellarubella_Carubv10022010m

Viterbi Path	
Viterbi Path	
Posterior @ 0.4	121 - KEKRRVRDK - 129
Posterior @ 0.4	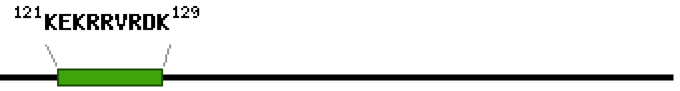

Posterior Graphic. Threshold Marked @ 0.4

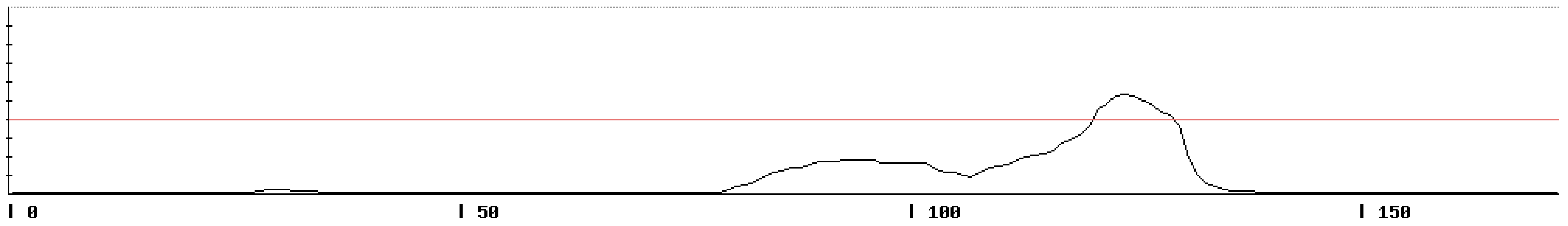

Predictions for Capsellarubella_Carubv10022061m

Viterbi Path

Viterbi Path

Posterior @ 0.4

Posterior @ 0.4

Posterior Graphic. Threshold Marked @ 0.4

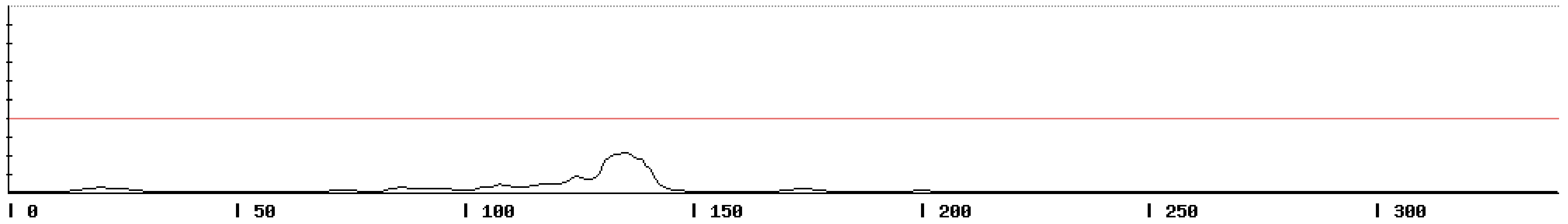

Predictions for Capsellarubella_Carubv10022099m

Viterbi Path

Viterbi Path

Posterior @ 0.4

Posterior @ 0.4

Posterior Graphic. Threshold Marked @ 0.4

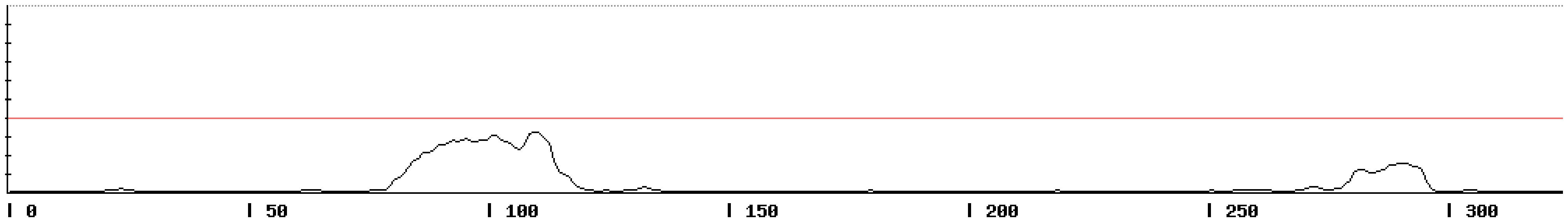

Predictions for Capsellarubella_Carubv10022302m

Viterbi Path

173 - KKKKAKEEESEKQKKAEEEESEKKKKALKKK - 203

Viterbi Path

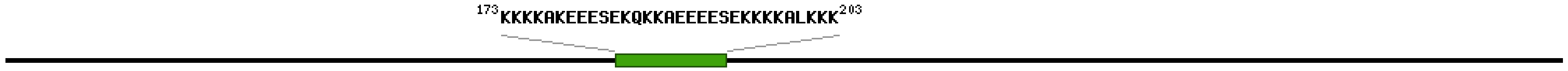

Posterior @ 0.4

89 - RNKKKKGKGGINQKRGG - 105

172 - YKKKKAKEEESEKQKKAEEEESEKKKKALKKKVE - 205

Posterior @ 0.4

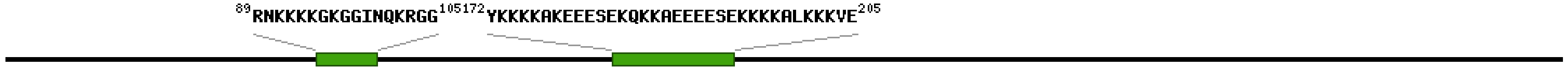

Posterior Graphic. Threshold Marked @ 0.4

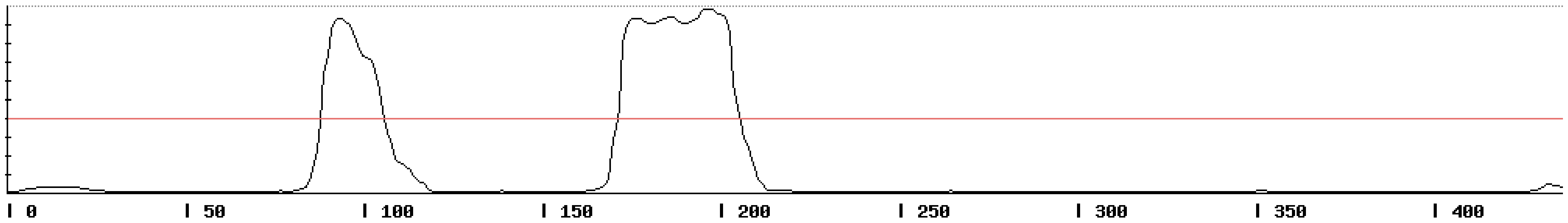

Predictions for Capsellarubella_Carubv10022351m

Viterbi Path

Viterbi Path

Posterior @ 0.4	
Posterior @ 0.4	
Posterior Graphic. Threshold Marked @ 0.4	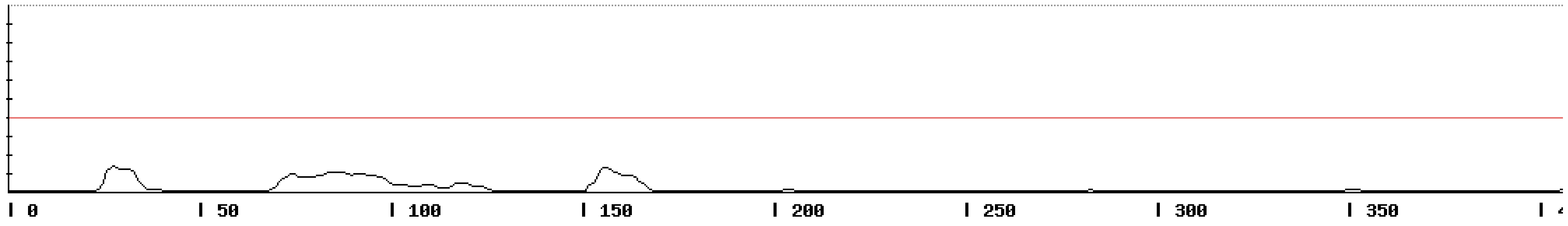

Predictions for Capsellarubella_Carubv10022440m

Viterbi Path	154 - KKKKTEEEENEKKKKVPKKKK - 174
Viterbi Path	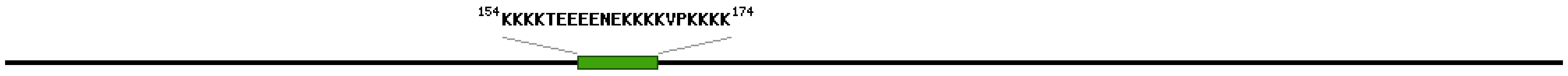
Posterior @ 0.4	64 - RNKKKKGKGGINQKR - 78
	153 - TKKKKTEEEENEKKKKVPKKKKV - 175
Posterior @ 0.4	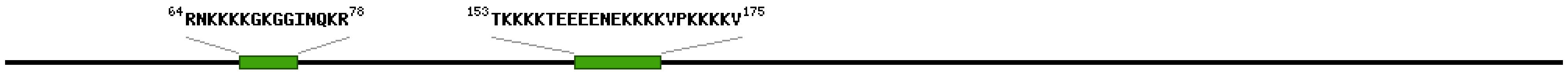
Posterior Graphic. Threshold Marked @ 0.4	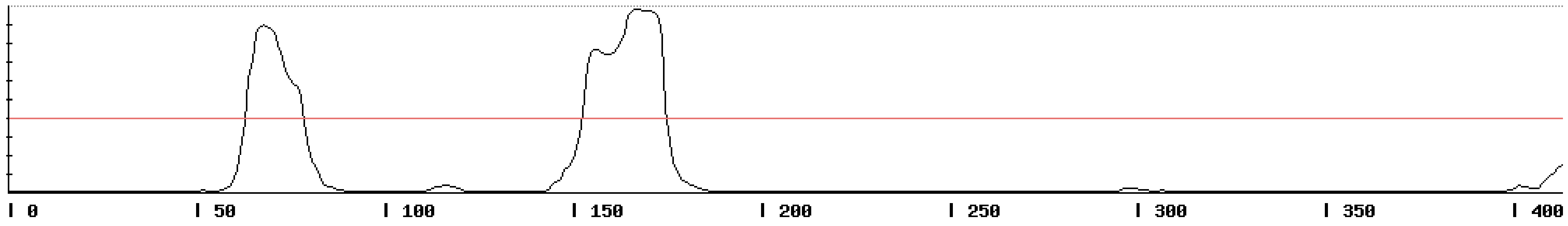

Predictions for Capsellarubella_Carubv10023503m

Viterbi Path	
Viterbi Path	
Posterior @ 0.4	
Posterior @ 0.4	
Posterior Graphic. Threshold Marked @ 0.4	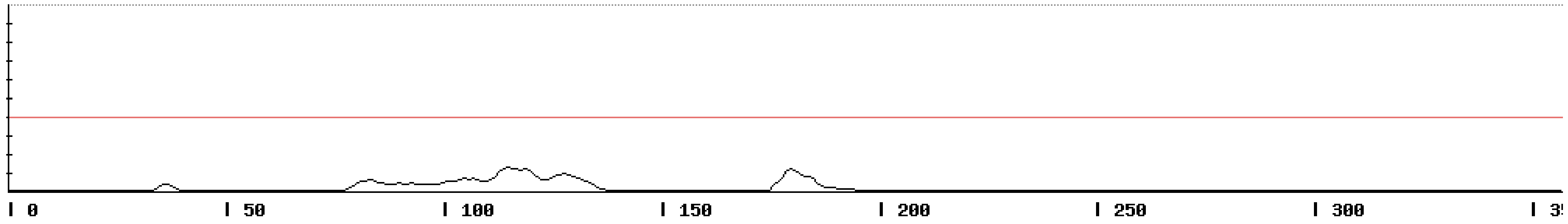

Predictions for Capsellarubella_Carubv10023568m

Viterbi Path	
Viterbi Path	
Posterior @ 0.4	
Posterior @ 0.4	
Posterior Graphic. Threshold Marked @ 0.4	

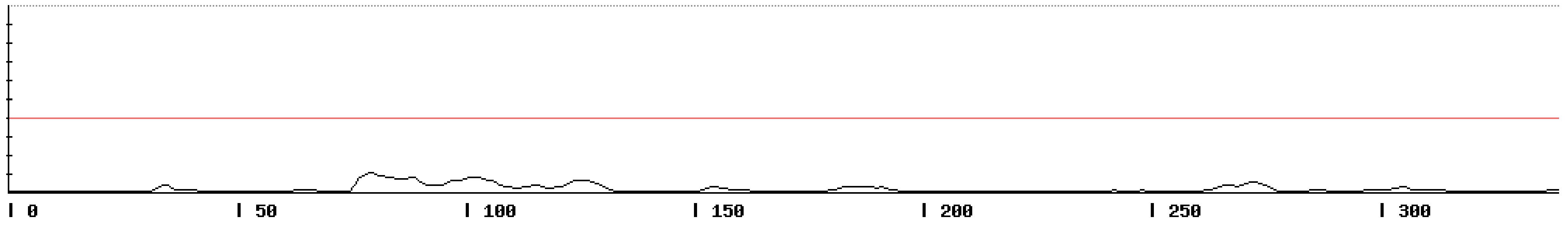

Predictions for Capsellarubella_Carubv10023730m

Viterbi Path	
Viterbi Path	
Posterior @ 0.4	
Posterior @ 0.4	
Posterior Graphic. Threshold Marked @ 0.4	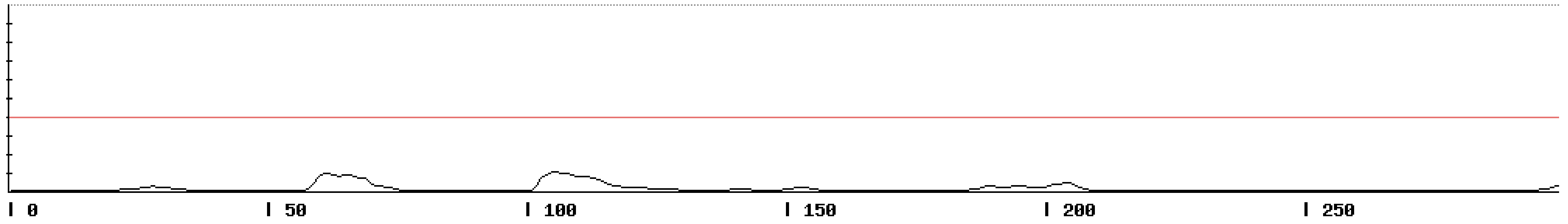

Predictions for Capsellarubella_Carubv10023833m

Viterbi Path	204 - RKEDKKKRRRRR - 215
Viterbi Path	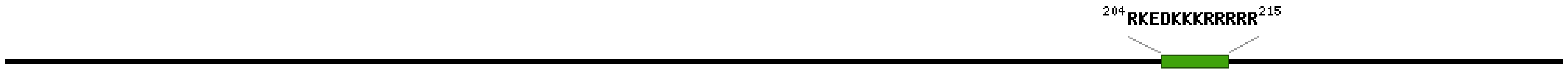
Posterior @ 0.4	204 - RKEDKKKRRRRR - 215
Posterior @ 0.4	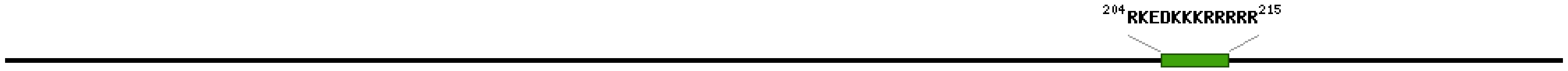

Posterior Graphic. Threshold Marked @ 0.4	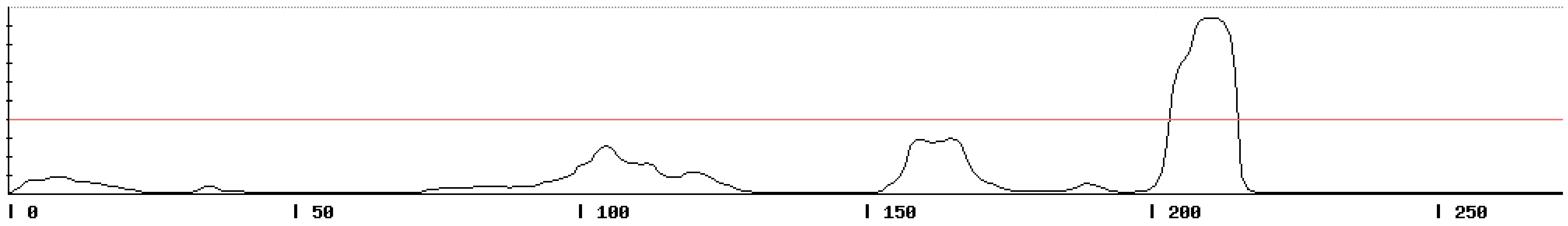

Predictions for Capsellarubella_Carubv10025508m

Viterbi Path	
Viterbi Path	
Posterior @ 0.4	
Posterior @ 0.4	
Posterior Graphic. Threshold Marked @ 0.4	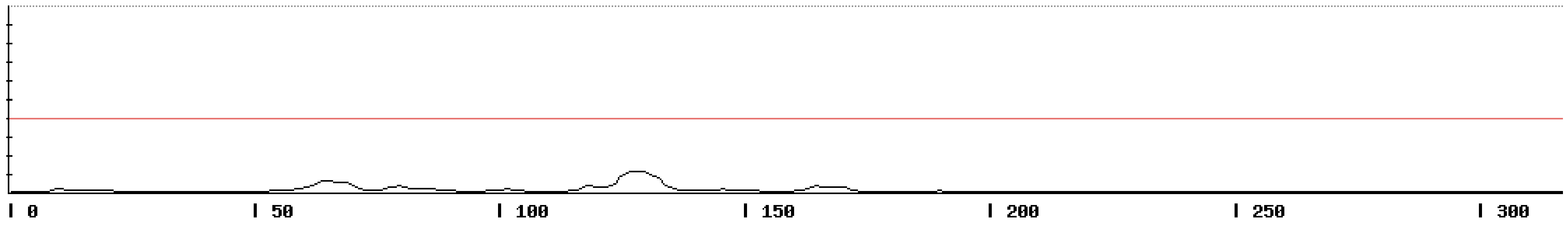

Predictions for Capsellarubella_Carubv10026643m

Viterbi Path	
Viterbi Path	
Posterior @ 0.4	
Posterior @ 0.4	

Posterior Graphic. Threshold Marked @ 0.4

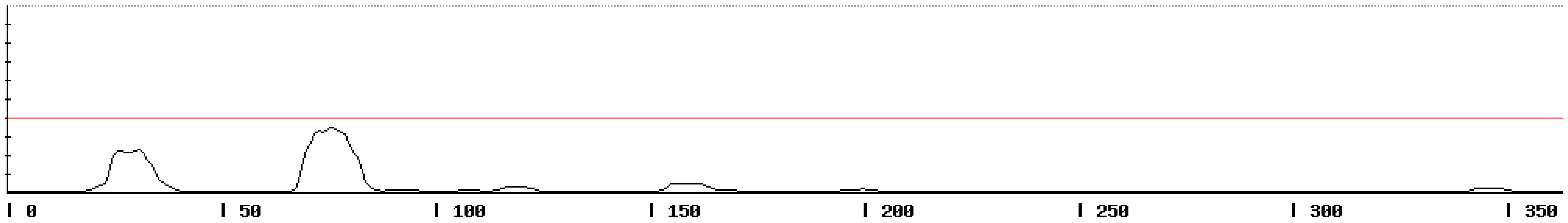

Predictions for Capsellarubella_Carubv10026697m

Viterbi Path

Viterbi Path

Posterior @ 0.4

Posterior @ 0.4

Posterior Graphic. Threshold Marked @ 0.4

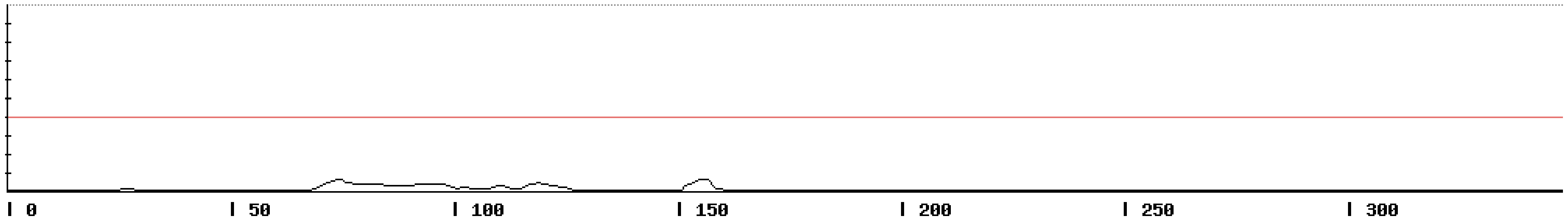

Predictions for Capsellarubella_Carubv10026752m

Viterbi Path

Viterbi Path

Posterior @ 0.4

Posterior @ 0.4

Posterior Graphic. Threshold Marked @ 0.4

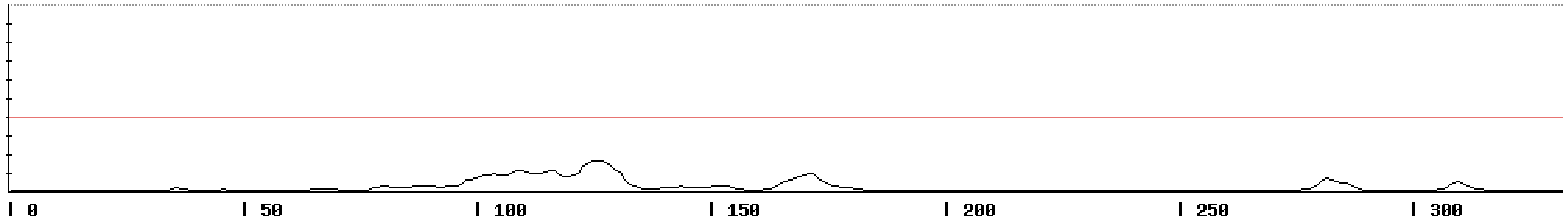

Predictions for Capsellarubella_Carubv10026898m

Viterbi Path

Viterbi Path

Posterior @ 0.4

Posterior @ 0.4

Posterior Graphic. Threshold Marked @ 0.4

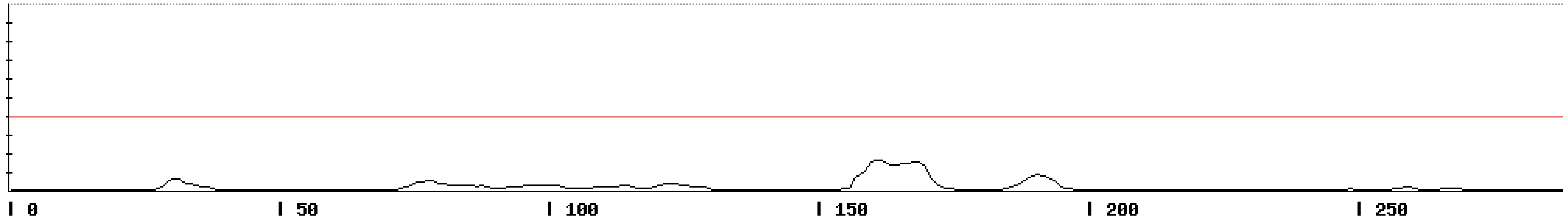

Predictions for Capsellarubella_Carubv10026953m

Viterbi Path

Viterbi Path

Posterior @ 0.4	98 - GKPK - 101
Posterior @ 0.4	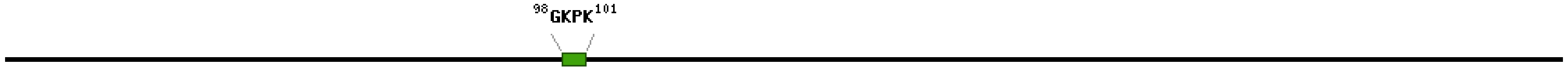
Posterior Graphic. Threshold Marked @ 0.4	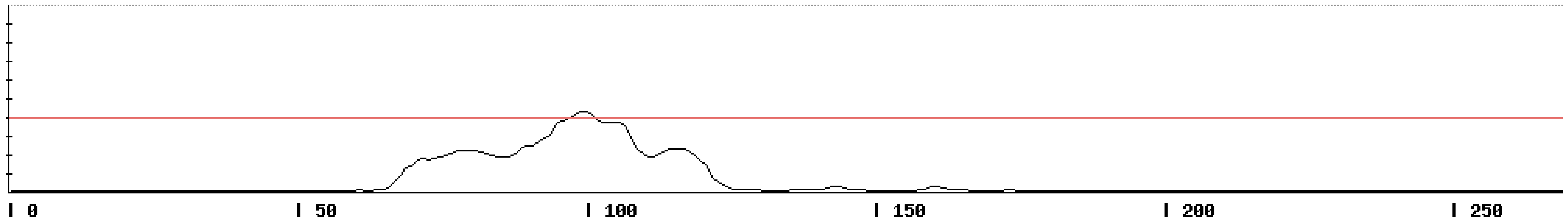

Predictions for Capsellarubella_Carubv10027180m

Viterbi Path	
Viterbi Path	
Posterior @ 0.4	
Posterior @ 0.4	
Posterior Graphic. Threshold Marked @ 0.4	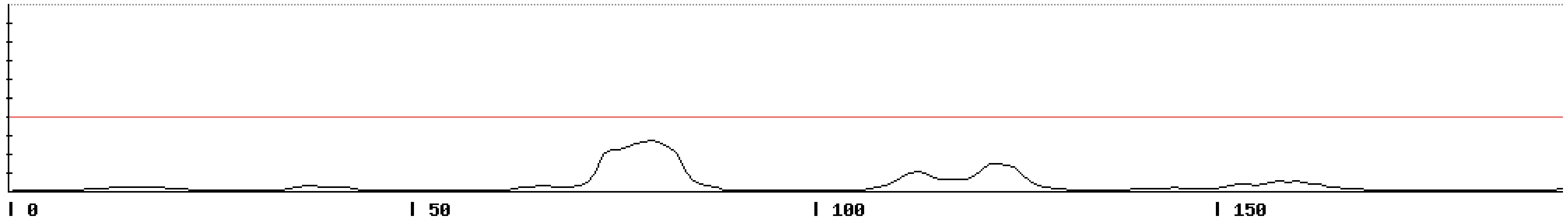

Predictions for Capsellarubella_Carubv10027198m

Viterbi Path

Viterbi Path

Posterior @ 0.4

Posterior @ 0.4

Posterior Graphic. Threshold Marked @ 0.4

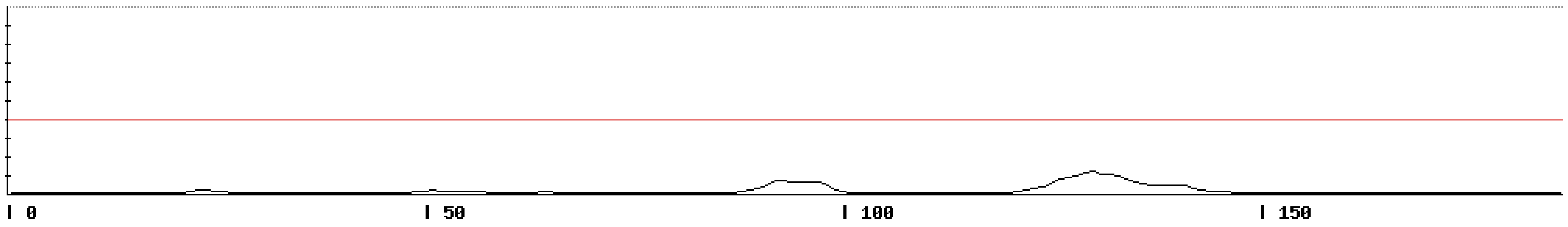

Predictions for Capsellarubella_Carubv10027934m

Viterbi Path

210 - KKHTKRKSKKKK - 221

Viterbi Path

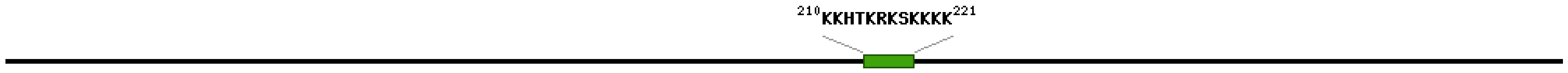

Posterior @ 0.4

130 - PRKSKKHKRGD - 140

176 - IHKKKWCKKKGKGKE - 190

210 - KKHTKRKSKKKKE - 222

Posterior @ 0.4

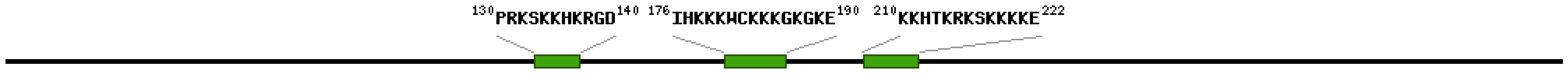

Posterior Graphic. Threshold Marked @ 0.4

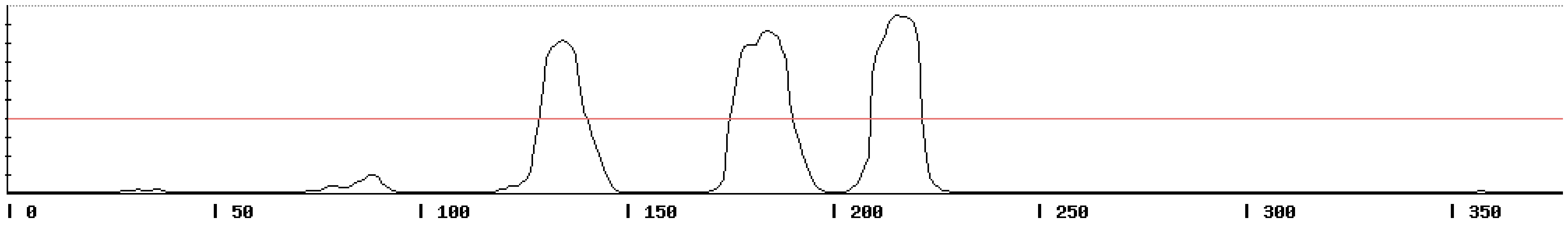

Predictions for Capsellarubella_Carubv10028131m

Viterbi Path	
Viterbi Path	
Posterior @ 0.4	128 - KR - 129
Posterior @ 0.4	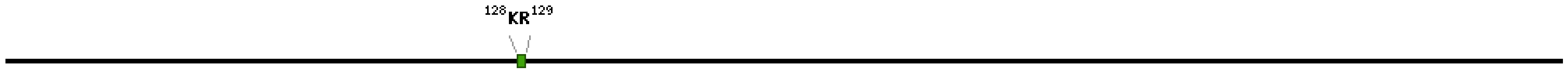
Posterior Graphic. Threshold Marked @ 0.4	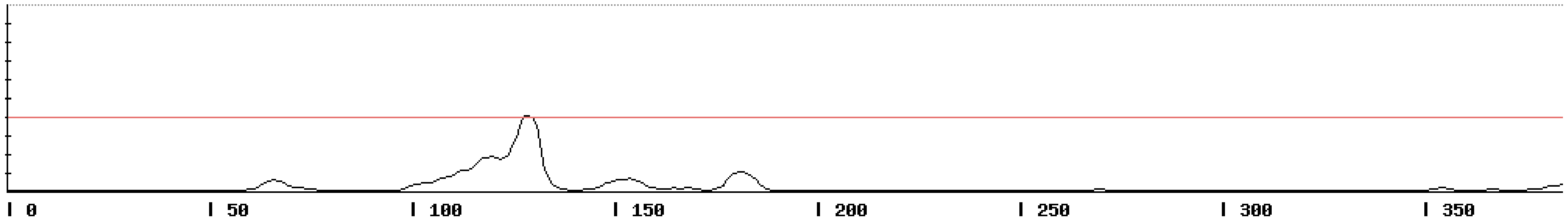

Predictions for Capsellarubella_Carubv10028284m

Viterbi Path	
Viterbi Path	
Posterior @ 0.4	
Posterior @ 0.4	

Viterbi Path	
Posterior @ 0.4	96 - KAIGRPKPMGIKK - 108
Posterior @ 0.4	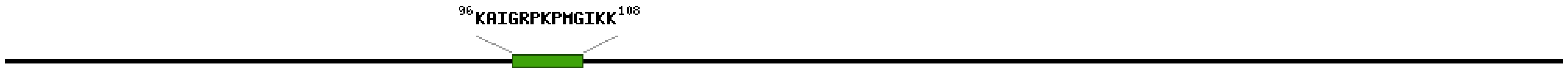
Posterior Graphic. Threshold Marked @ 0.4	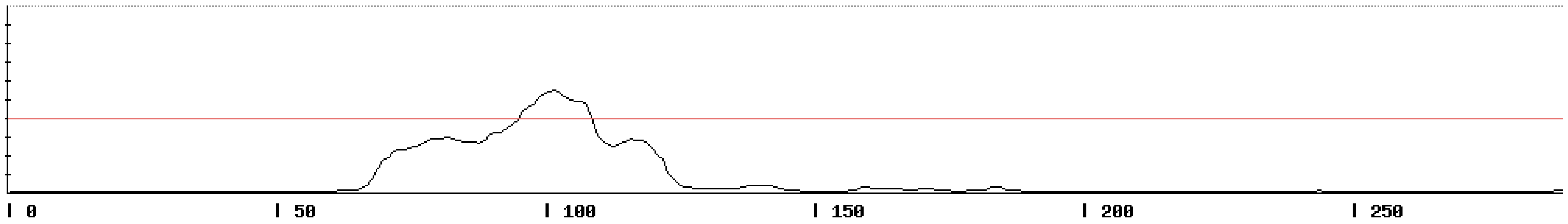

Predictions for Capsicumannuum_CA00g46270

Viterbi Path	
Viterbi Path	
Posterior @ 0.4	
Posterior @ 0.4	
Posterior Graphic. Threshold Marked @ 0.4	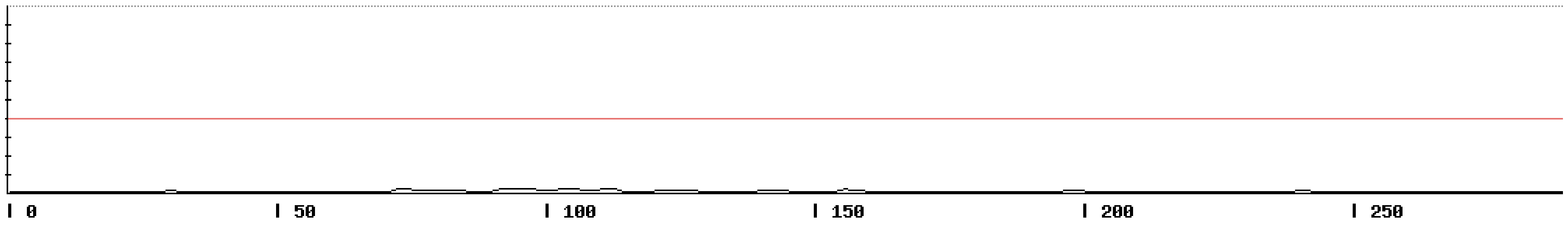

Predictions for Capsicumannuum_CA00g54690

Viterbi Path	
Viterbi Path	
Posterior @ 0.4	187 - KKKKK - 191
Posterior @ 0.4	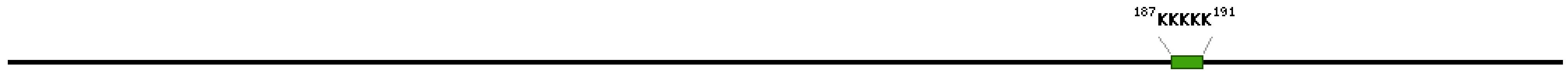
Posterior Graphic. Threshold Marked @ 0.4	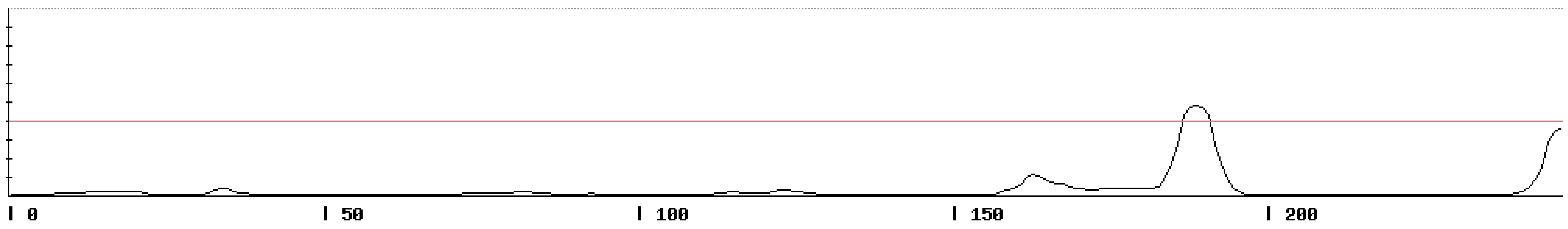

Predictions for Capsicumannuum_CA00g66680

Viterbi Path	
Viterbi Path	
Posterior @ 0.4	
Posterior @ 0.4	
Posterior Graphic. Threshold Marked @ 0.4	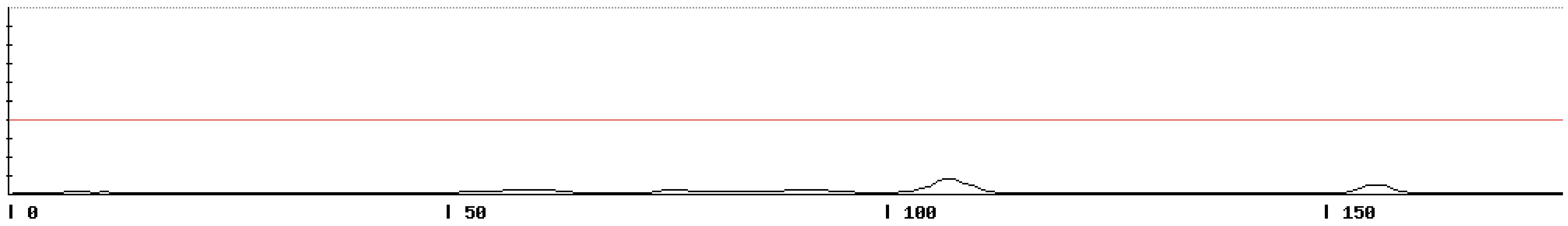

Predictions for Capsicumannuum_CA00g67620	
Viterbi Path	
Viterbi Path	
Posterior @ 0.4	73 - KAKRFTRK - 80
Posterior @ 0.4	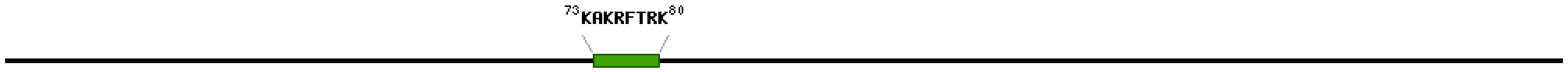
Posterior Graphic. Threshold Marked @ 0.4	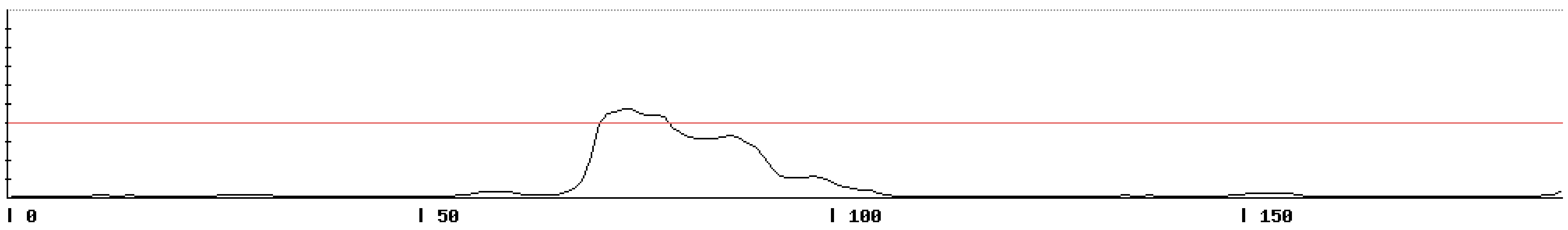

Predictions for Capsicumannuum_CA00g73220	
Viterbi Path	
Viterbi Path	
Posterior @ 0.4	
Posterior @ 0.4	
Posterior Graphic. Threshold Marked @ 0.4	

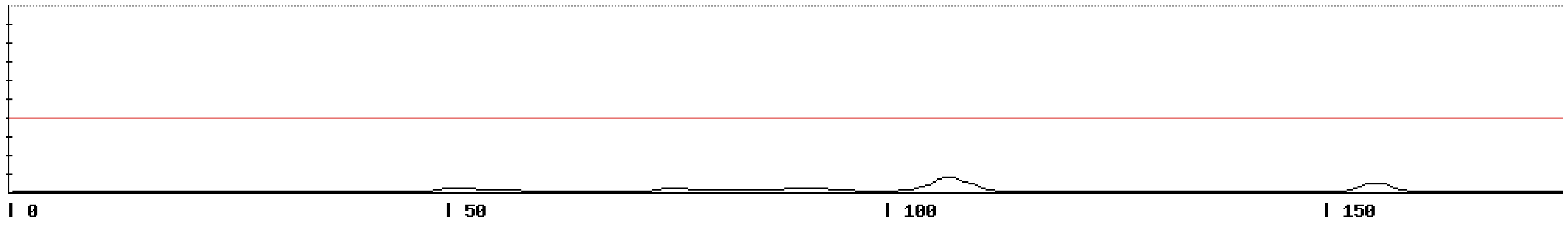

Predictions for Capsicumannuum_CA00g77790

Viterbi Path	
Viterbi Path	
Posterior @ 0.4	
Posterior @ 0.4	
Posterior Graphic. Threshold Marked @ 0.4	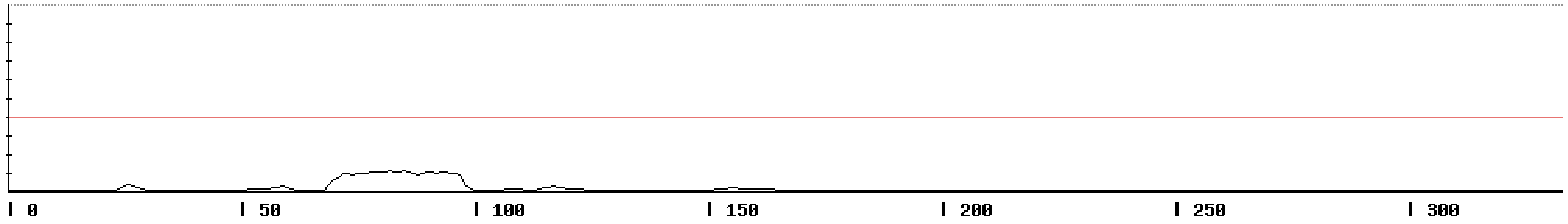

Predictions for Capsicumannuum_CA00g84200

Viterbi Path	
Viterbi Path	
Posterior @ 0.4	127 - RKRRK - 131
	144 - HKTGKTRPVVFKNKVKGHKK - 163
Posterior @ 0.4	

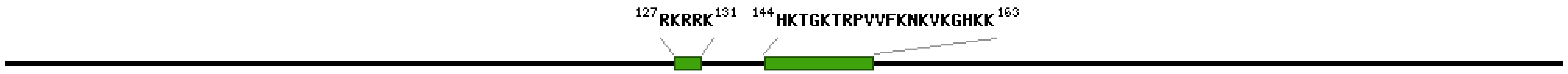

Posterior Graphic. Threshold Marked @ 0.4

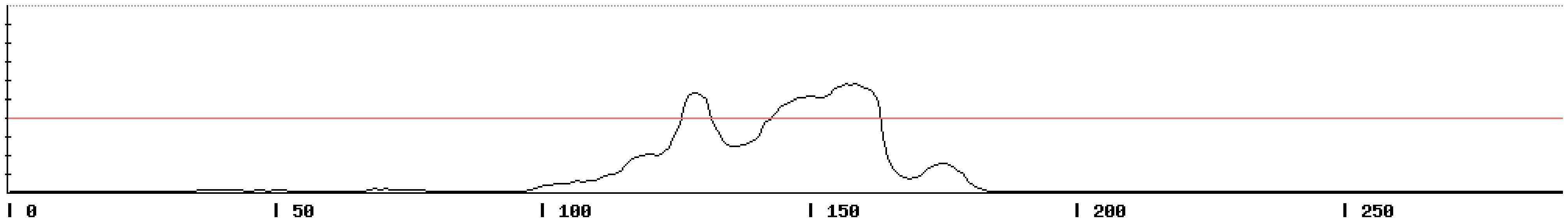

Predictions for Capsicumannuum_CA00g86400

Viterbi Path

Viterbi Path

Posterior @ 0.4

Posterior @ 0.4

Posterior Graphic. Threshold Marked @ 0.4

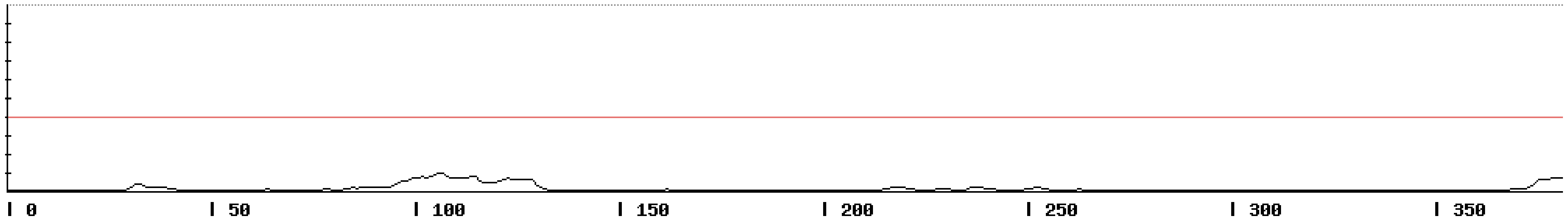

Predictions for Capsicumannuum_CA00g86410

Viterbi Path

Viterbi Path

Posterior @ 0.4

Posterior @ 0.4

Posterior Graphic. Threshold Marked @ 0.4

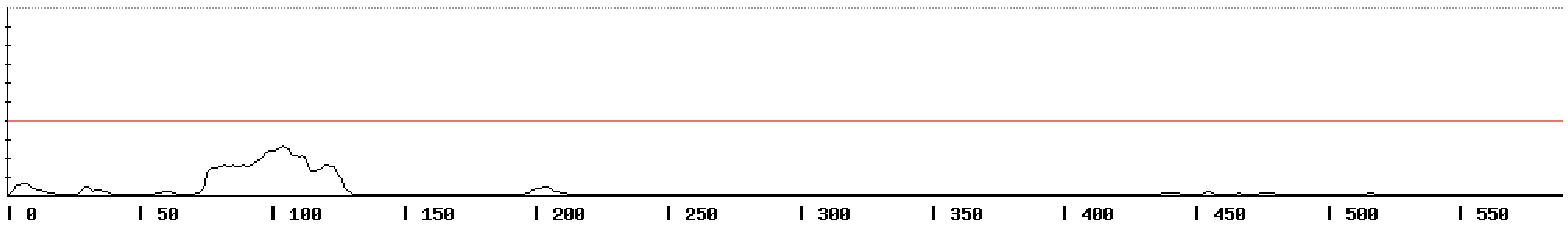

Predictions for Capsicumannuum_CA00g89750

Viterbi Path

Viterbi Path

Posterior @ 0.4

Posterior @ 0.4

Posterior Graphic. Threshold Marked @ 0.4

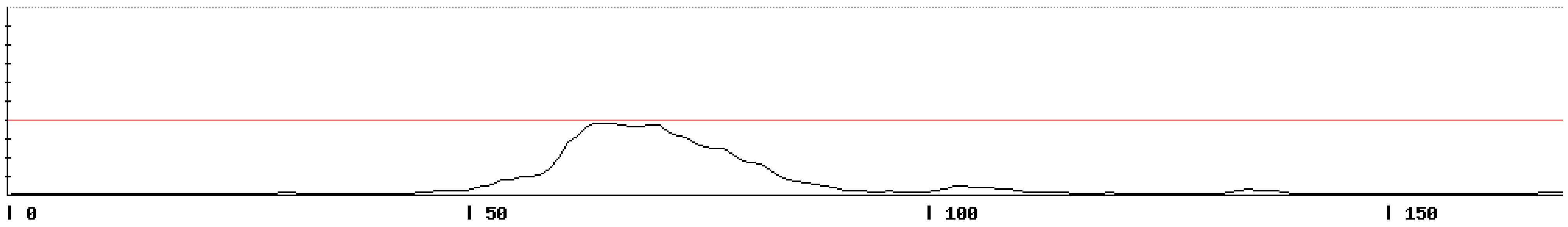

Predictions for Capsicumannuum_CA00g89790

Viterbi Path

Viterbi Path

Posterior @ 0.4	
Posterior @ 0.4	
Posterior Graphic. Threshold Marked @ 0.4	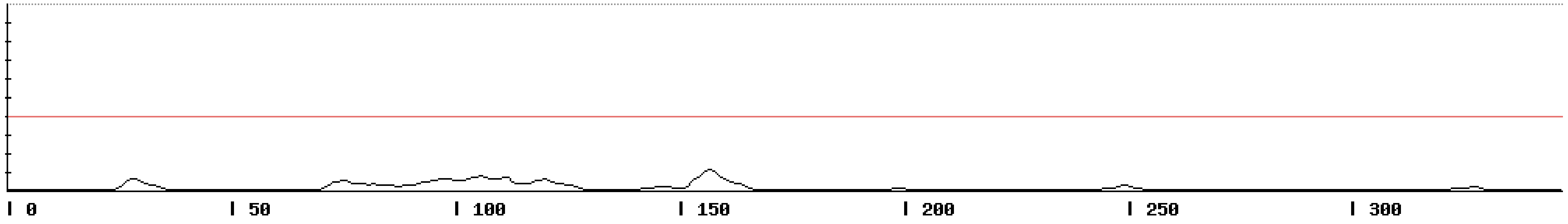

Predictions for Capsicumannuum_CA01g04740

Viterbi Path	
Viterbi Path	
Posterior @ 0.4	
Posterior @ 0.4	
Posterior Graphic. Threshold Marked @ 0.4	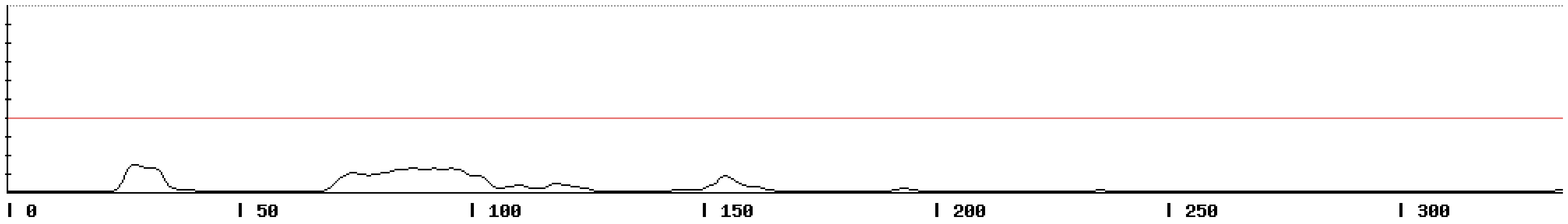

Predictions for Capsicumannuum_CA01g07550

Viterbi Path

Viterbi Path

Posterior @ 0.4

72 - HKNKIKKKKKSLWDDKKKDR - 91

Posterior @ 0.4

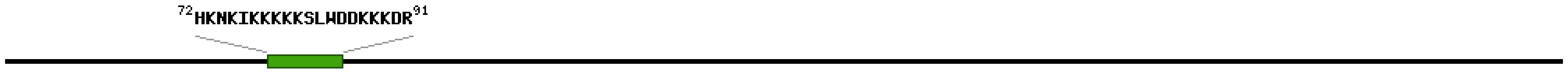

Posterior Graphic. Threshold Marked @ 0.4

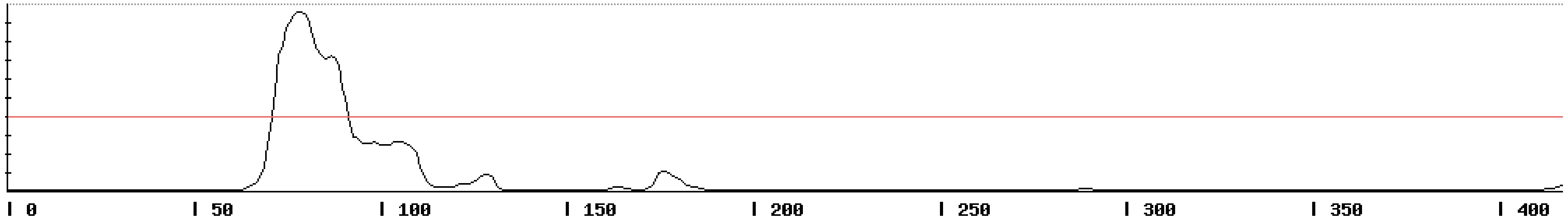

Predictions for Capsicumannuum_CA01g09650

Viterbi Path

Viterbi Path

Posterior @ 0.4

Posterior @ 0.4

Posterior Graphic. Threshold Marked @ 0.4

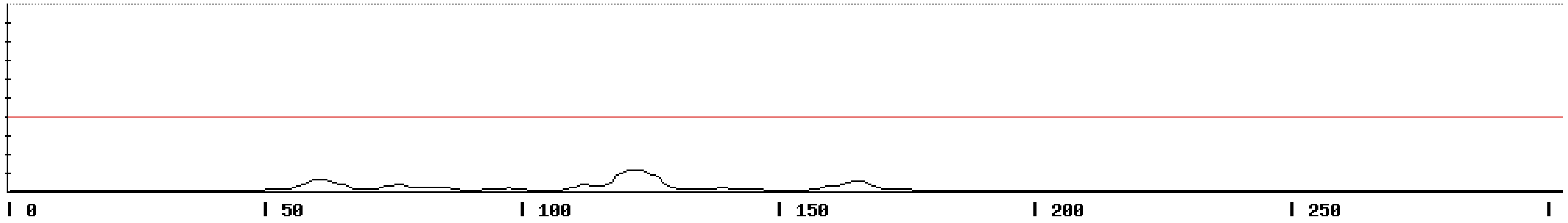

Predictions for Capsicumannuum_CA01g11960	
Viterbi Path	
Viterbi Path	
Posterior @ 0.4	58 - KKKTR - 62
Posterior @ 0.4	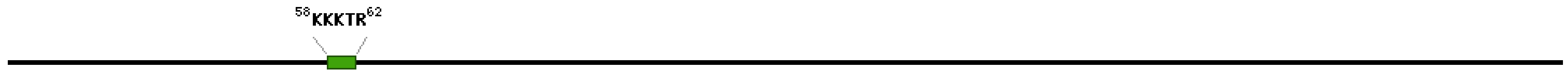
Posterior Graphic. Threshold Marked @ 0.4	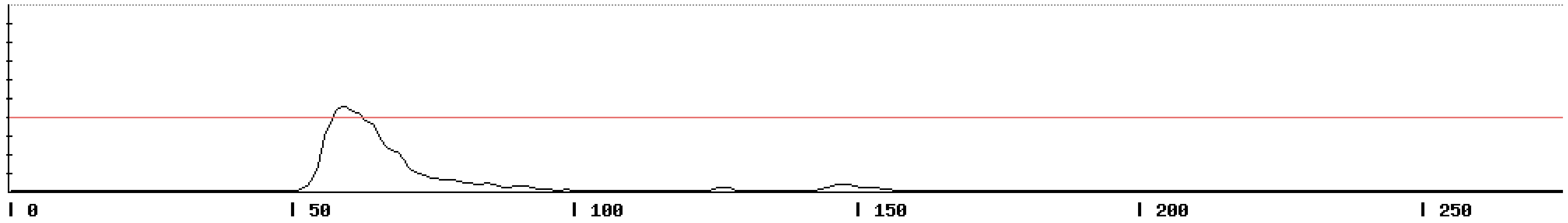

Predictions for Capsicumannuum_CA01g17160	
Viterbi Path	
Viterbi Path	
Posterior @ 0.4	
Posterior @ 0.4	
Posterior Graphic. Threshold Marked @ 0.4	

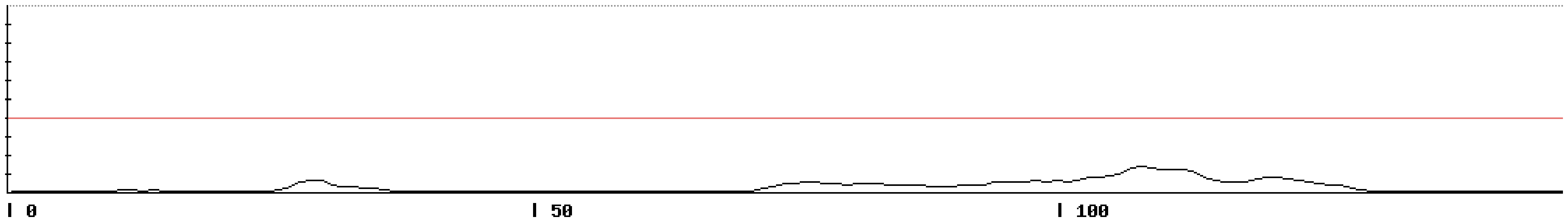

Predictions for Capsicumannuum_CA01g26660

Viterbi Path	
Viterbi Path	
Posterior @ 0.4	80 - KKKK - 83
Posterior @ 0.4	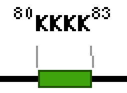
Posterior Graphic. Threshold Marked @ 0.4	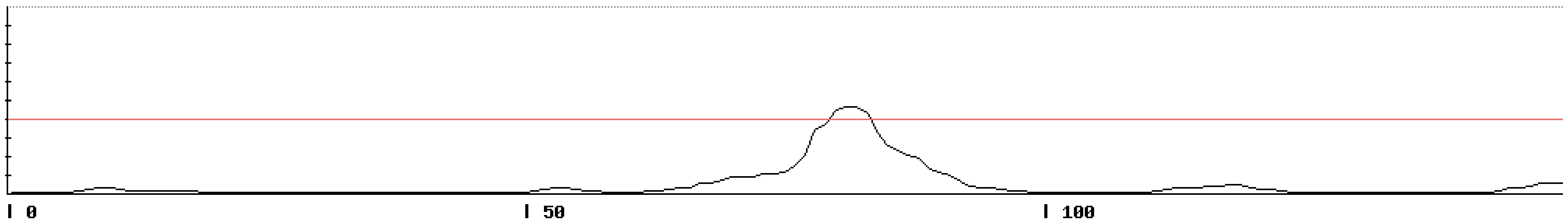

Predictions for Capsicumannuum_CA01g30550

Viterbi Path	
Viterbi Path	
Posterior @ 0.4	
Posterior @ 0.4	

Posterior Graphic. Threshold Marked @ 0.4

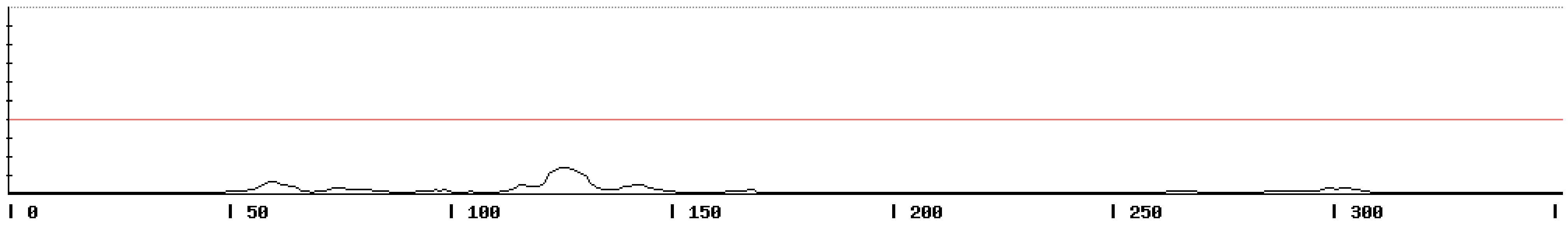

Predictions for Capsicumannuum_CA01g34750

Viterbi Path

Viterbi Path

Posterior @ 0.4 — 104 - IYGKRKRIGMRK - 115

Posterior @ 0.4

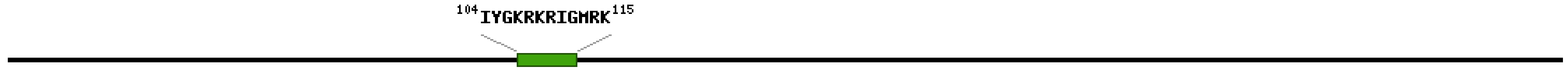

Posterior Graphic. Threshold Marked @ 0.4

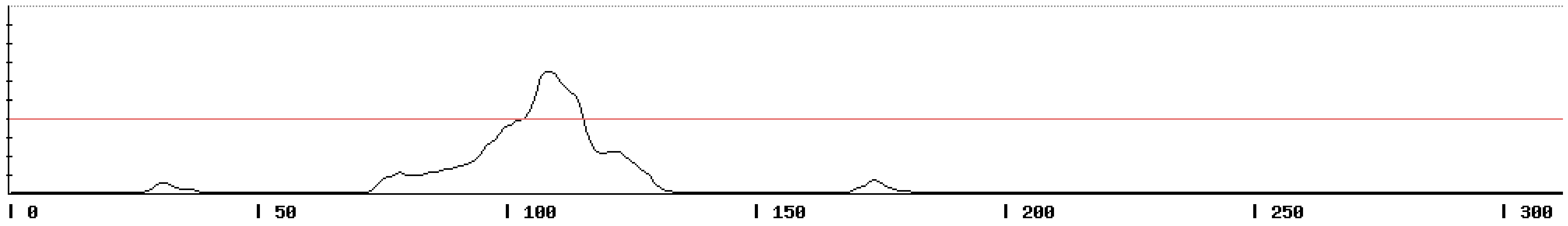

Predictions for Capsicumannuum_CA02g03060

Viterbi Path

Viterbi Path

Posterior @ 0.4 — 101 - RKHGPKGKPNRTTR - 114

Posterior @ 0.4

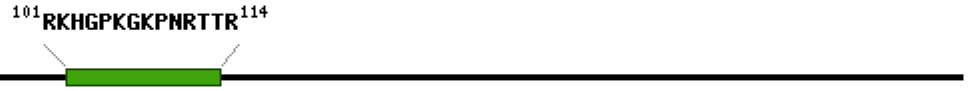

Posterior Graphic. Threshold Marked @ 0.4

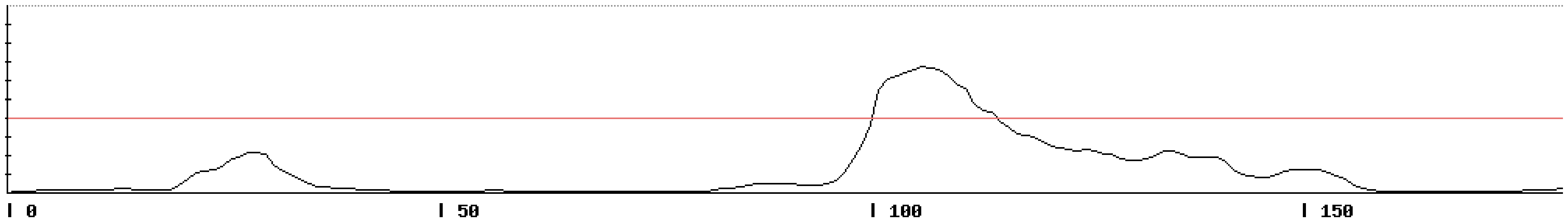

Predictions for Capsicumannuum_CA02g04820

Viterbi Path

Viterbi Path

Posterior @ 0.4

Posterior @ 0.4

Posterior Graphic. Threshold Marked @ 0.4

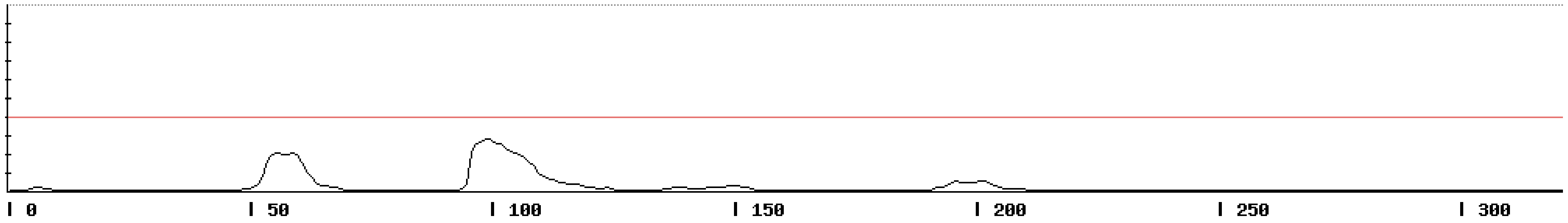

Predictions for Capsicumannuum_CA02g05470

Viterbi Path

Viterbi Path

Posterior @ 0.4

Posterior @ 0.4

Posterior Graphic. Threshold Marked @ 0.4

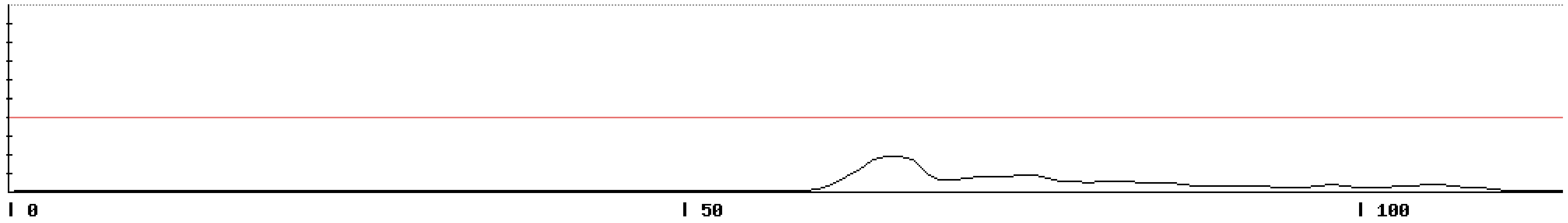

Predictions for Capsicumannuum_CA02g06450

Viterbi Path

Viterbi Path

Posterior @ 0.4

Posterior @ 0.4

Posterior Graphic. Threshold Marked @ 0.4

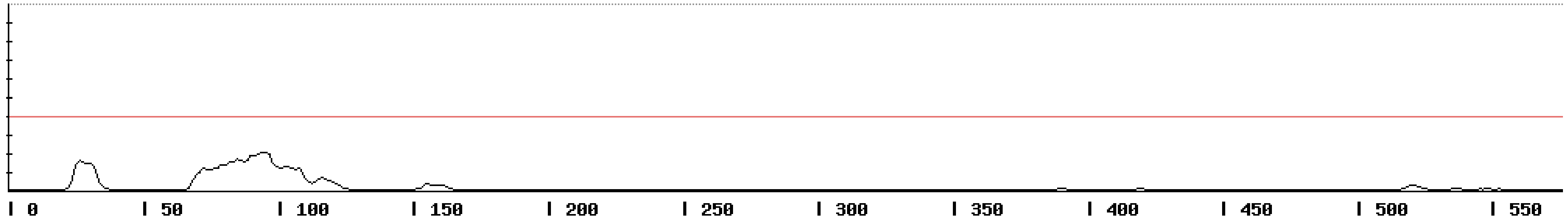

Predictions for Capsicumannuum_CA02g15520

Viterbi Path

Viterbi Path

Posterior @ 0.4

Posterior @ 0.4

Posterior Graphic. Threshold Marked @ 0.4

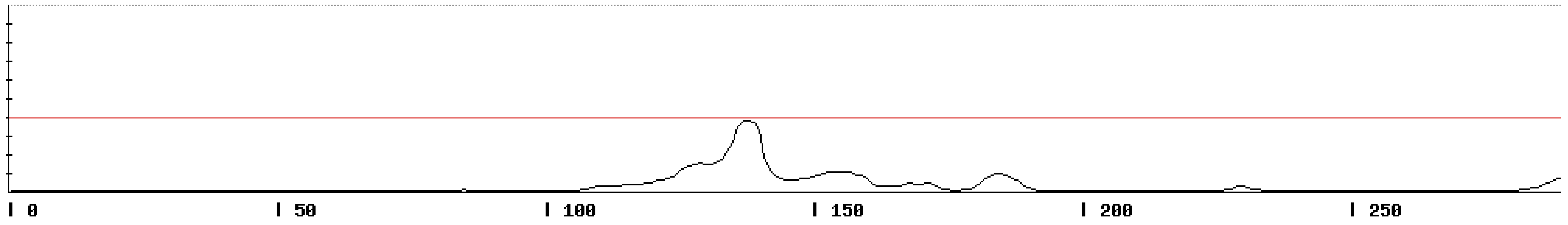

Predictions for Capsicumannuum_CA02g17910

Viterbi Path

Viterbi Path

Posterior @ 0.4

Posterior @ 0.4

Posterior Graphic. Threshold Marked @ 0.4

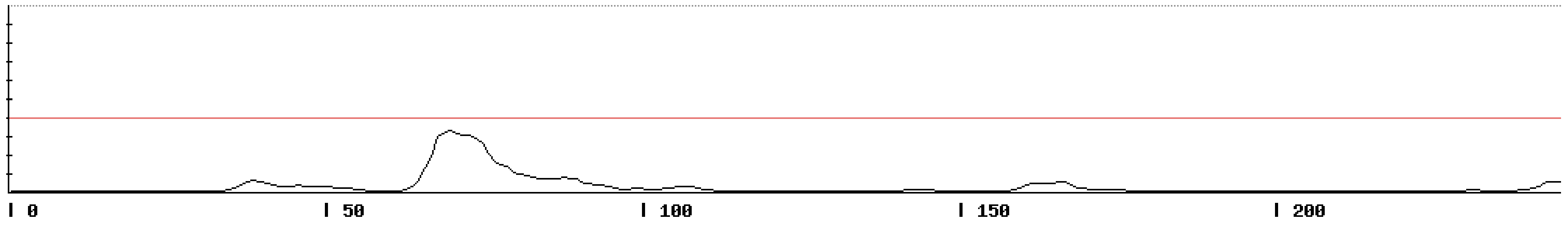

Predictions for Capsicumannuum_CA02g20290

Viterbi Path

Viterbi Path

Posterior @ 0.4

Posterior @ 0.4

Posterior Graphic. Threshold Marked @ 0.4

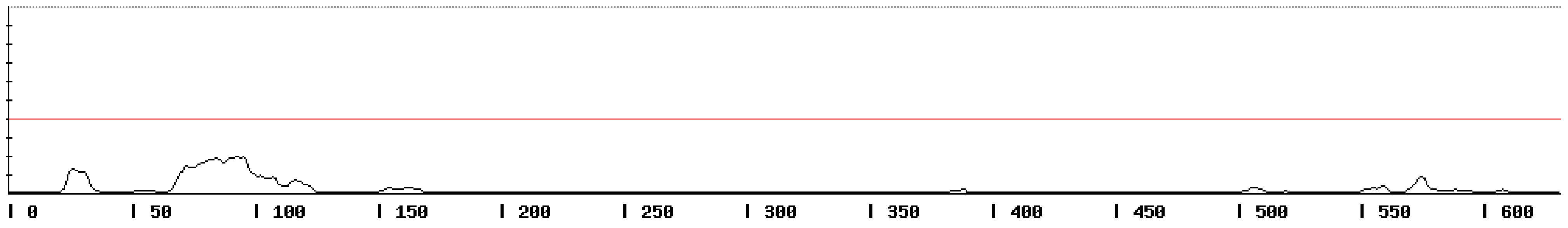

Predictions for Capsicumannuum_CA02g22730

Viterbi Path

Viterbi Path

Posterior @ 0.4

Posterior @ 0.4

Posterior Graphic. Threshold Marked @ 0.4

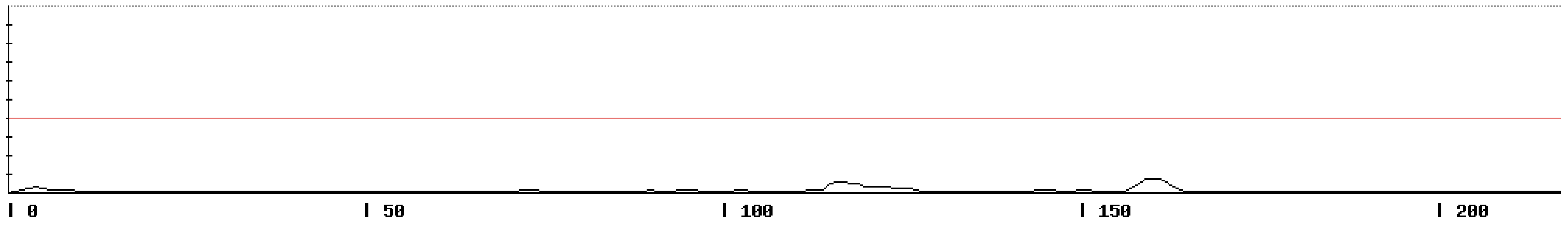

Predictions for Capsicumannuum_CA02g28070

Viterbi Path

Viterbi Path

Posterior @ 0.4

Posterior @ 0.4

Posterior Graphic. Threshold Marked @ 0.4

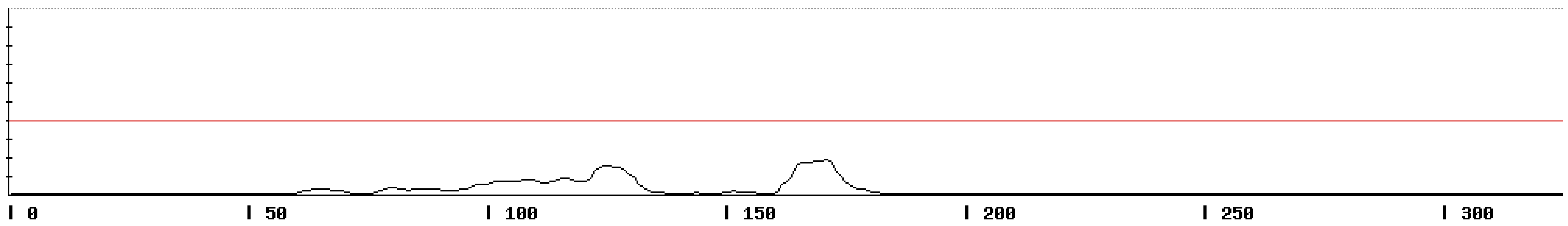

Predictions for Capsicumannuum_CA02g28290

Viterbi Path

Viterbi Path

Posterior @ 0.4

Posterior @ 0.4

Posterior Graphic. Threshold Marked @ 0.4

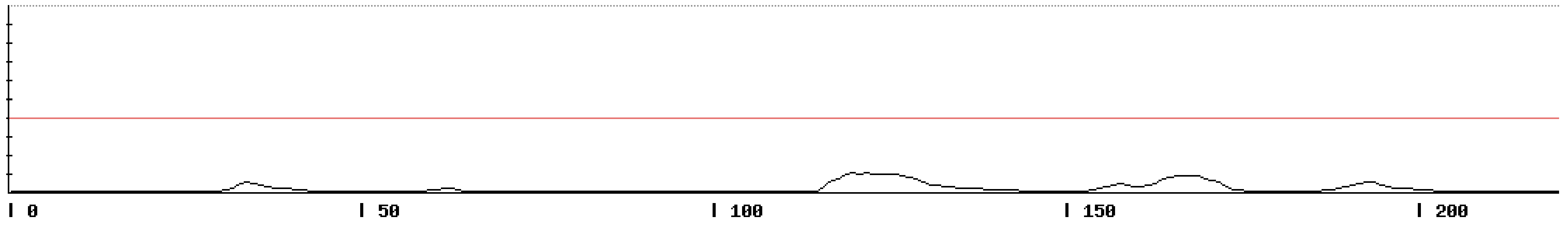

Predictions for Capsicumannuum_CA02g30090

Viterbi Path	
Viterbi Path	
Posterior @ 0.4	131 - GKRKRRKI - 138
Posterior @ 0.4	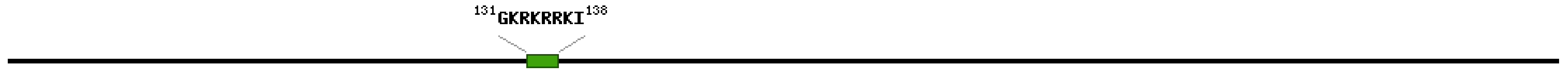
Posterior Graphic. Threshold Marked @ 0.4	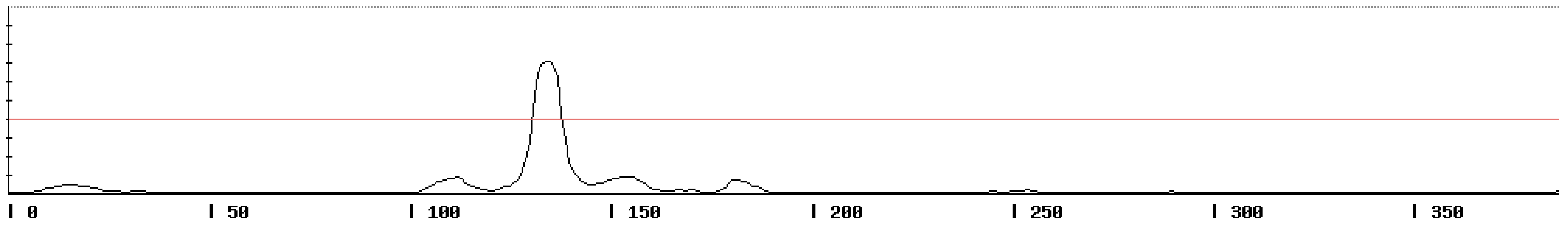

Predictions for Capsicumannuum_CA03g14470

Viterbi Path	
Viterbi Path	
Posterior @ 0.4	
Posterior @ 0.4	

Posterior Graphic. Threshold Marked @ 0.4

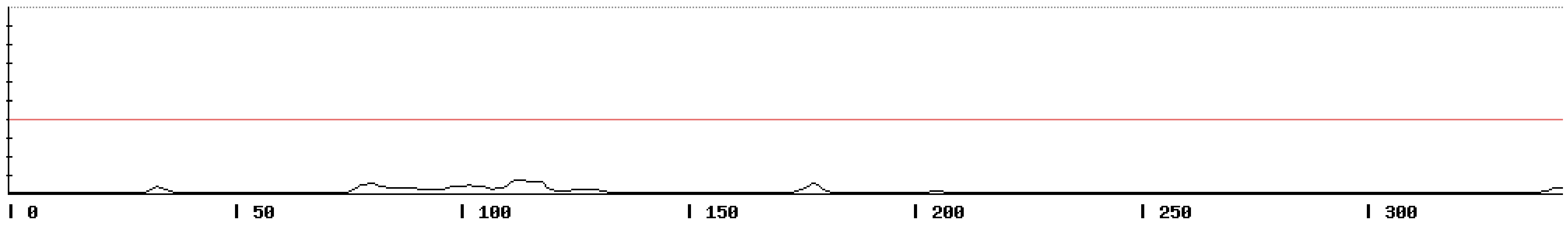

Predictions for Capsicumannuum_CA03g15410

Viterbi Path

Viterbi Path

Posterior @ 0.4

Posterior @ 0.4

Posterior Graphic. Threshold Marked @ 0.4

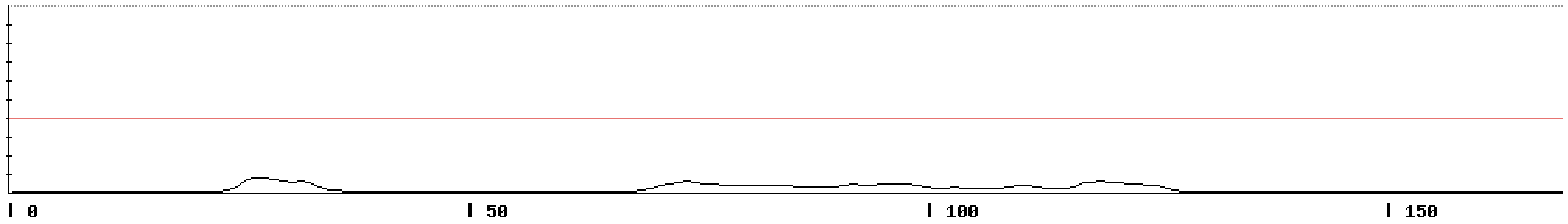

Predictions for Capsicumannuum_CA03g21000

Viterbi Path

Viterbi Path

Posterior @ 0.4

Posterior @ 0.4

Posterior Graphic. Threshold Marked @ 0.4

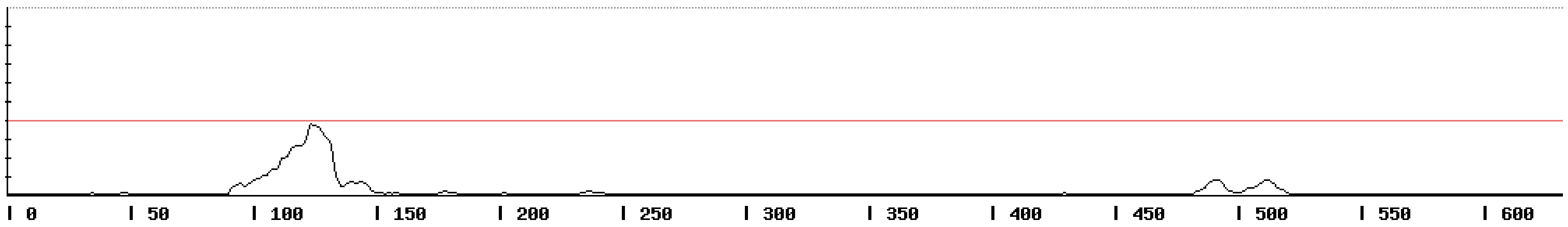

Predictions for Capsicumannuum_CA03g22350

Viterbi Path

Viterbi Path

Posterior @ 0.4

Posterior @ 0.4

Posterior Graphic. Threshold Marked @ 0.4

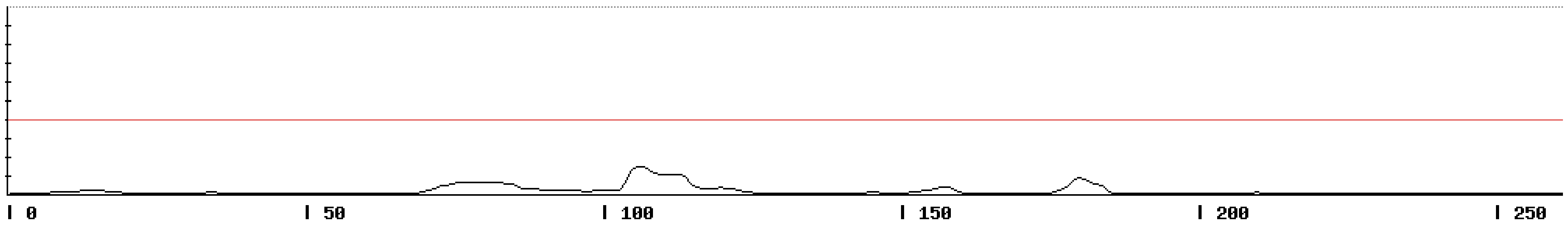

Predictions for Capsicumannuum_CA03g23090

Viterbi Path

Viterbi Path

Posterior @ 0.4

Posterior @ 0.4

Posterior Graphic. Threshold Marked @ 0.4

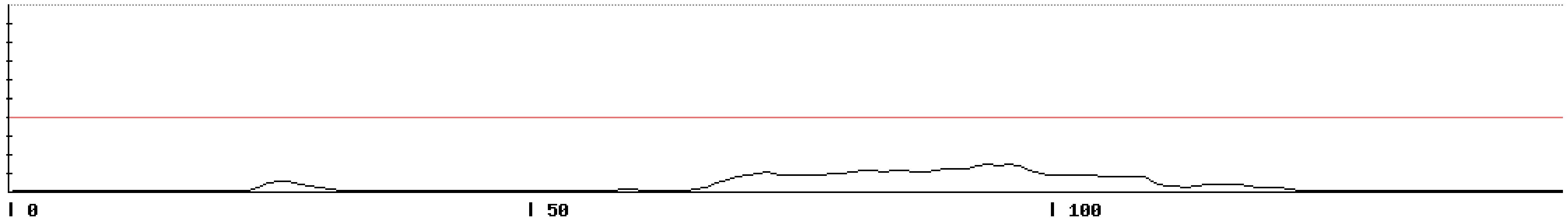

Predictions for Capsicumannuum_CA03g27130

Viterbi Path

Viterbi Path

Posterior @ 0.4 — 62 - KKRKKSDQ - 69

Posterior @ 0.4

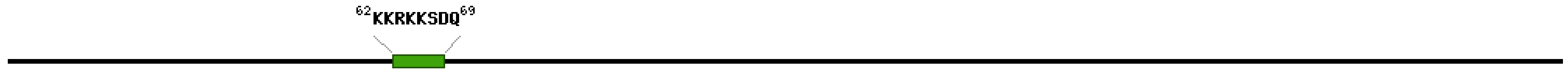

Posterior Graphic. Threshold Marked @ 0.4

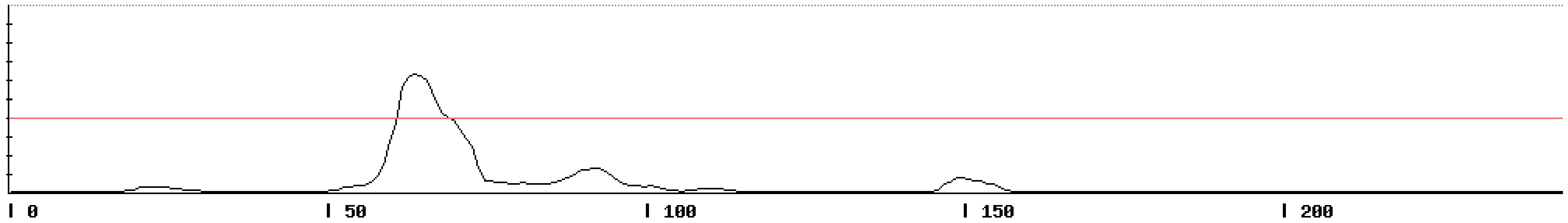

Predictions for Capsicumannuum_CA03g27300

Viterbi Path

Viterbi Path

Posterior @ 0.4	62 - KKRKK - 66
Posterior @ 0.4	
Posterior Graphic. Threshold Marked @ 0.4	

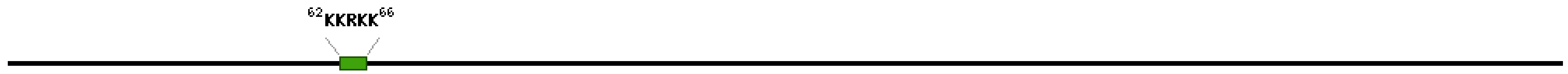

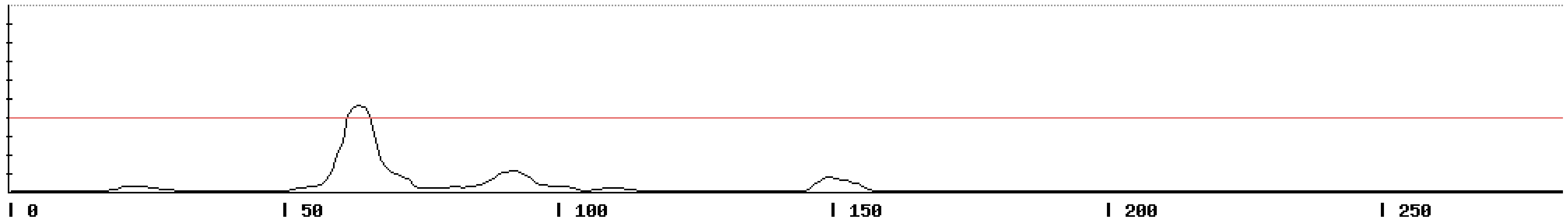

Predictions for Capsicumannuum_CA03g28100

Viterbi Path	
Viterbi Path	
Posterior @ 0.4	
Posterior @ 0.4	
Posterior Graphic. Threshold Marked @ 0.4	

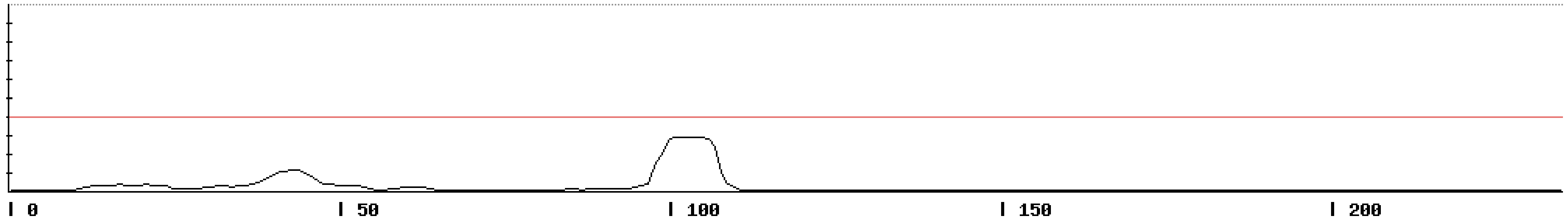

Predictions for Capsicumannuum_CA04g14740

Viterbi Path

Viterbi Path

Posterior @ 0.4

Posterior @ 0.4

Posterior Graphic. Threshold Marked @ 0.4

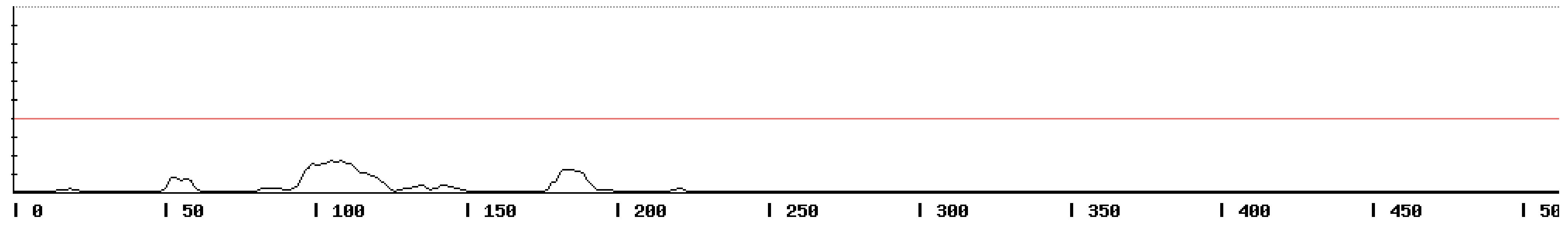

Predictions for Capsicumannuum_CA04g20070

Viterbi Path

Viterbi Path

Posterior @ 0.4

Posterior @ 0.4

Posterior Graphic. Threshold Marked @ 0.4

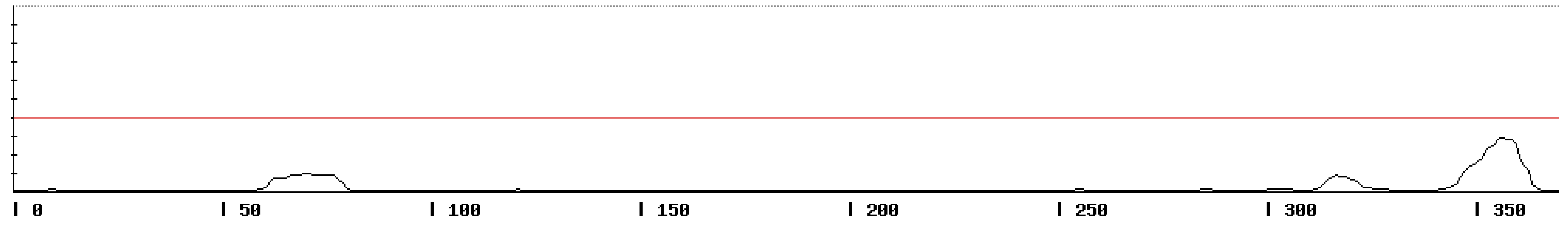

Predictions for Capsicumannuum_CA04g23340

Viterbi Path

Viterbi Path

Posterior @ 0.4

Posterior @ 0.4

Posterior Graphic. Threshold Marked @ 0.4

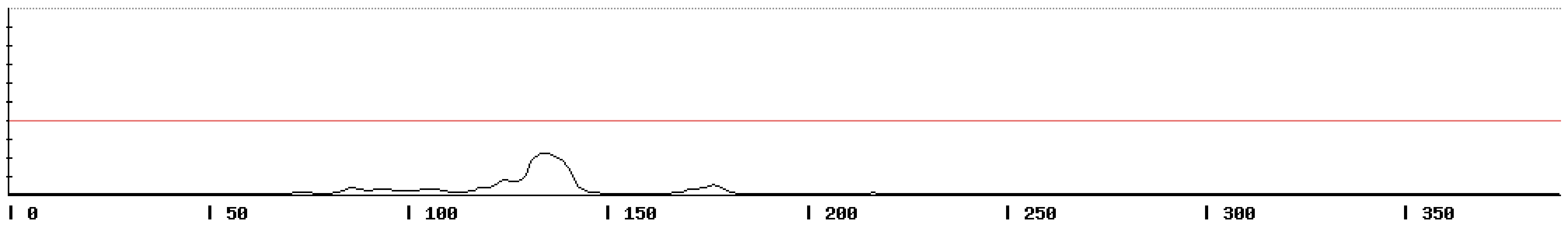

Predictions for Capsicumannuum_CA05g04410

Viterbi Path

Viterbi Path

Posterior @ 0.4

Posterior @ 0.4

Posterior Graphic. Threshold Marked @ 0.4

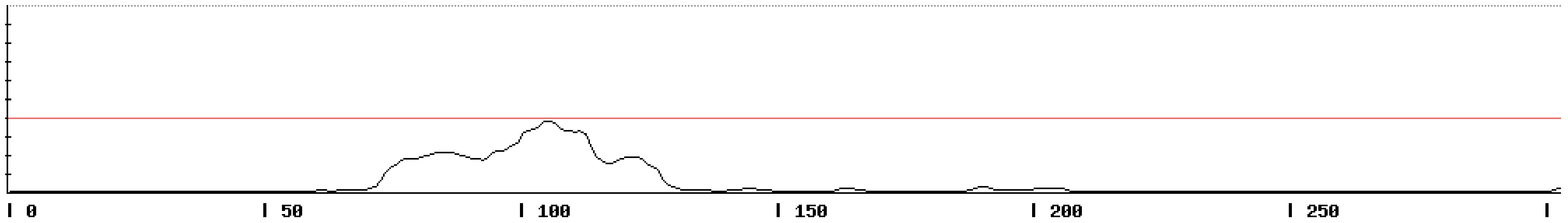

Predictions for Capsicumannuum_CA05g09500

Viterbi Path

Viterbi Path

Posterior @ 0.4

Posterior @ 0.4

Posterior Graphic. Threshold Marked @ 0.4

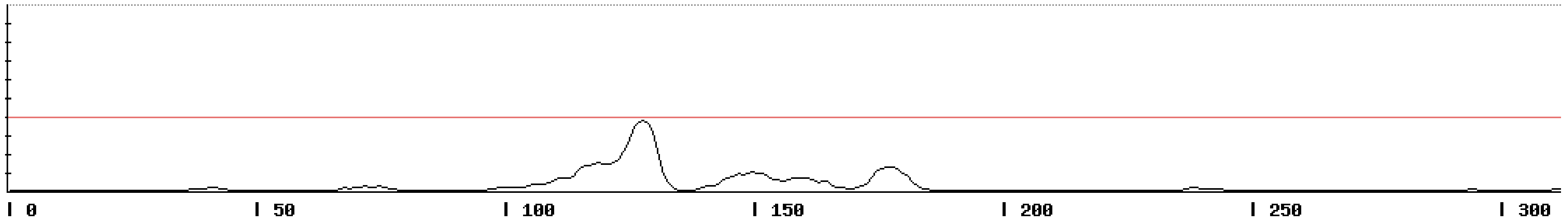

Predictions for Capsicumannuum_CA05g09600

Viterbi Path

Viterbi Path

Posterior @ 0.4

Posterior @ 0.4

Posterior Graphic. Threshold Marked @ 0.4

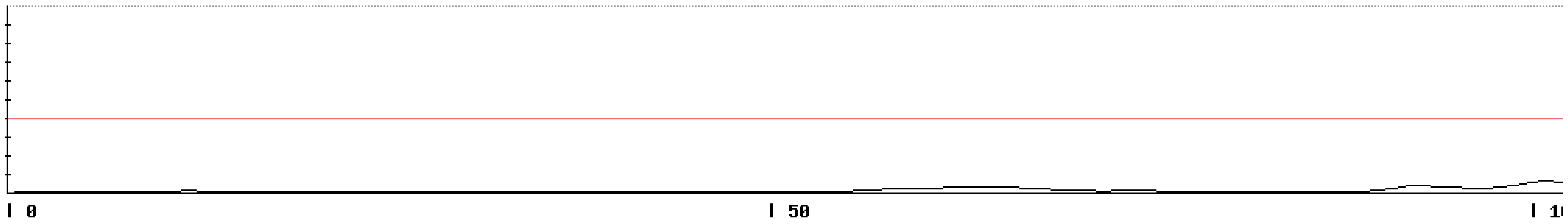

Predictions for Capsicumannuum_CA06g01680

Viterbi Path

Viterbi Path

Posterior @ 0.4

Posterior @ 0.4

Posterior Graphic. Threshold Marked @ 0.4

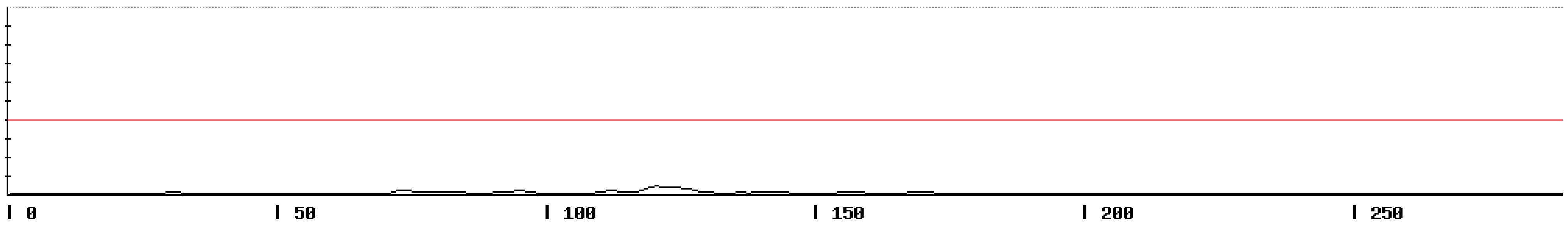

Predictions for Capsicumannuum_CA06g02260

Viterbi Path

Viterbi Path

Posterior @ 0.4

Posterior @ 0.4

Posterior Graphic. Threshold Marked @ 0.4

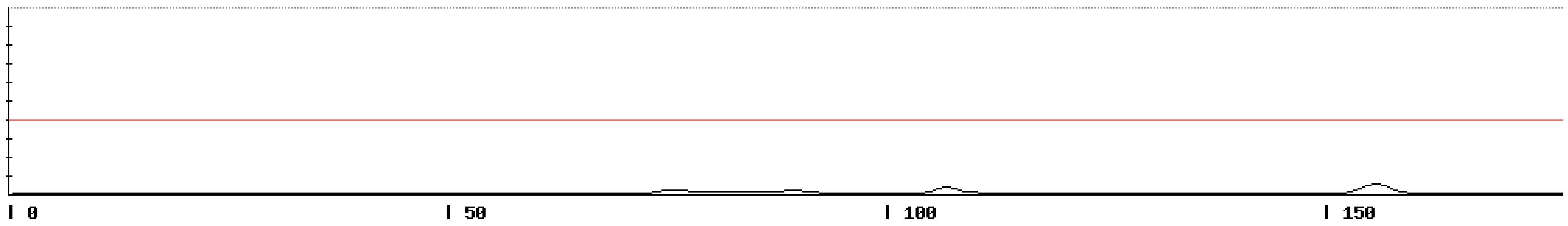

Predictions for Capsicumannuum_CA06g06410

Viterbi Path

Viterbi Path

Posterior @ 0.4

Posterior @ 0.4

Posterior Graphic. Threshold Marked @ 0.4

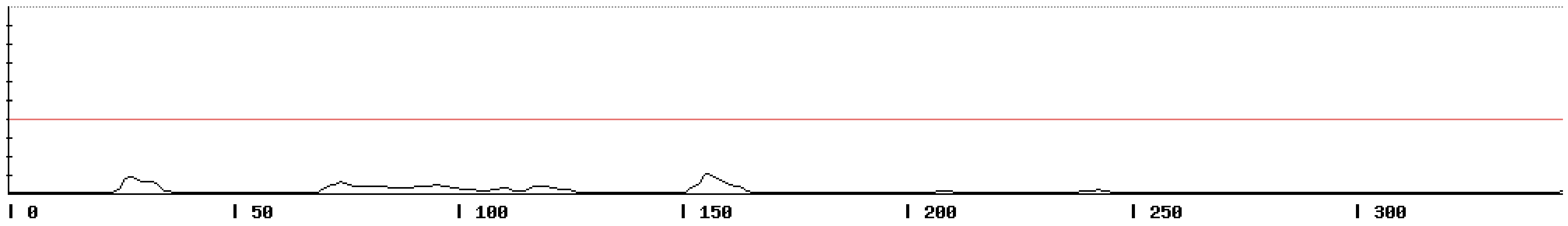

Predictions for Capsicumannuum_CA06g11310

Viterbi Path

Viterbi Path

Posterior @ 0.4

Posterior @ 0.4

Posterior Graphic. Threshold Marked @ 0.4

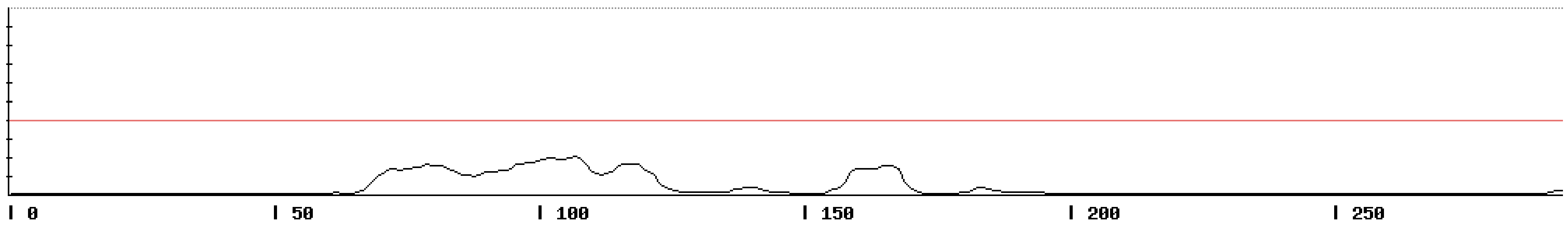

Predictions for Capsicumannuum_CA06g13110

Viterbi Path

Viterbi Path

Posterior @ 0.4

Posterior @ 0.4

Posterior Graphic. Threshold Marked @ 0.4

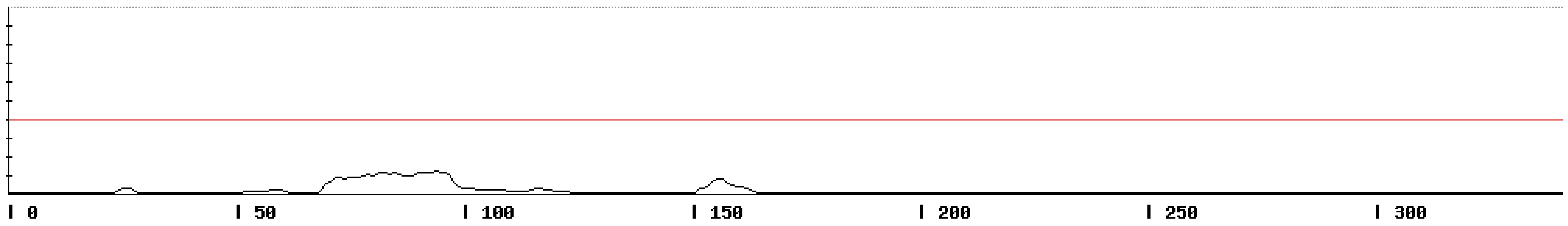

Predictions for Capsicumannuum_CA06g14050

Viterbi Path

Viterbi Path

Posterior @ 0.4

Posterior @ 0.4

Posterior Graphic. Threshold Marked @ 0.4

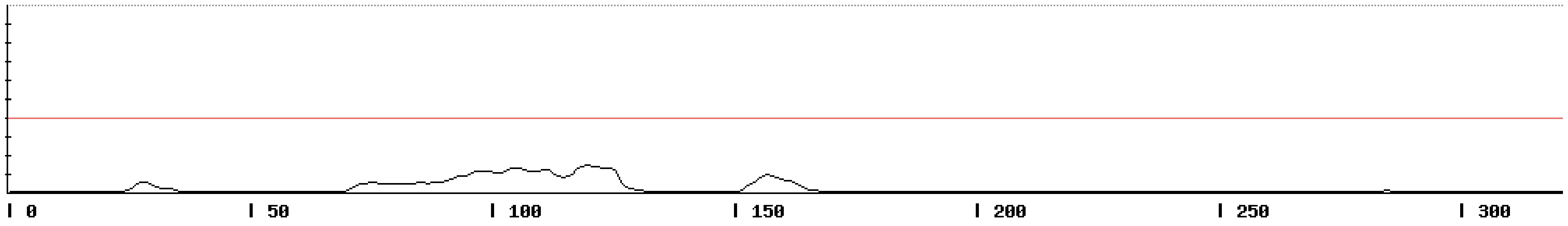

Predictions for Capsicumannuum_CA06g14960

Viterbi Path

Viterbi Path

Posterior @ 0.4

Posterior @ 0.4

Posterior Graphic. Threshold Marked @ 0.4

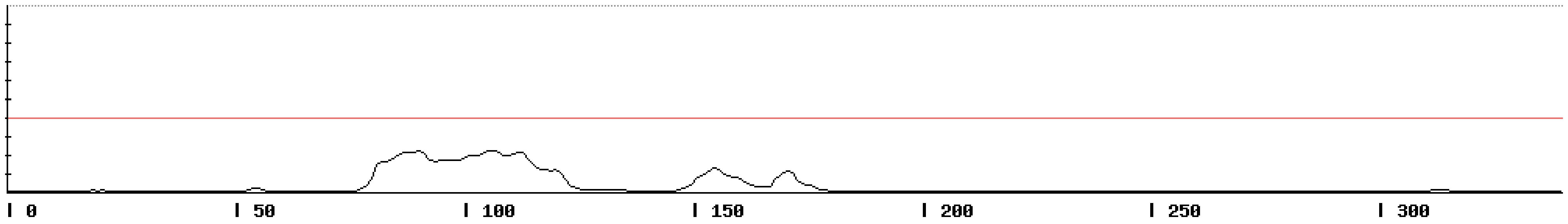

Predictions for Capsicumannuum_CA06g15350

Viterbi Path

Viterbi Path

Posterior @ 0.4

74 - HKNKIKKKNKSLAGDAKKDR - 93

178 - RKSPK - 182

Posterior @ 0.4

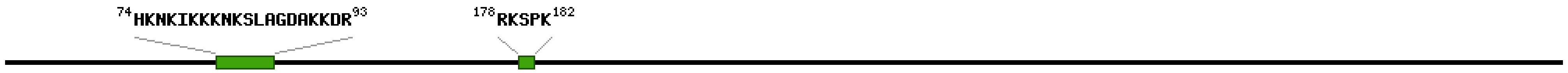

Posterior Graphic. Threshold Marked @ 0.4

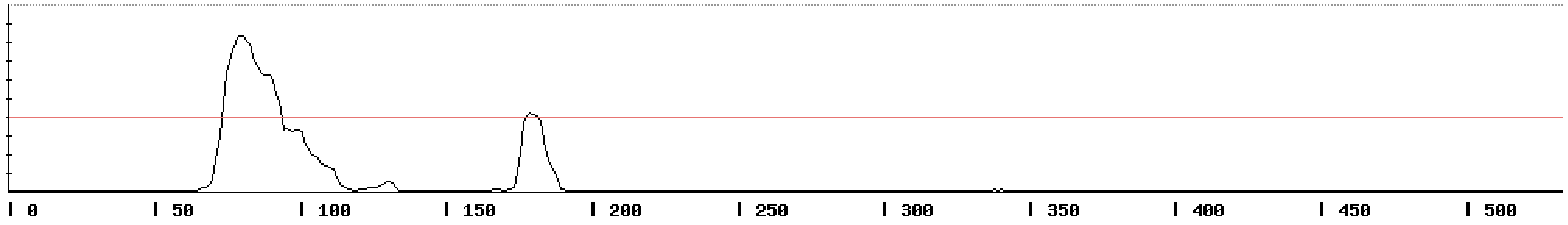

Predictions for Capsicumannuum_CA06g17290

Viterbi Path

Viterbi Path

Posterior @ 0.4

Posterior @ 0.4

Posterior Graphic. Threshold Marked @ 0.4

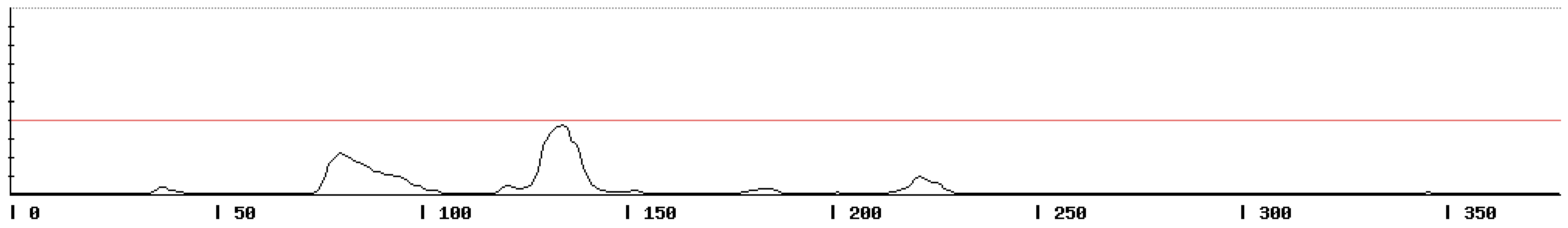

Predictions for Capsicumannuum_CA06g17870

Viterbi Path	
Viterbi Path	
Posterior @ 0.4	
Posterior @ 0.4	
Posterior Graphic. Threshold Marked @ 0.4	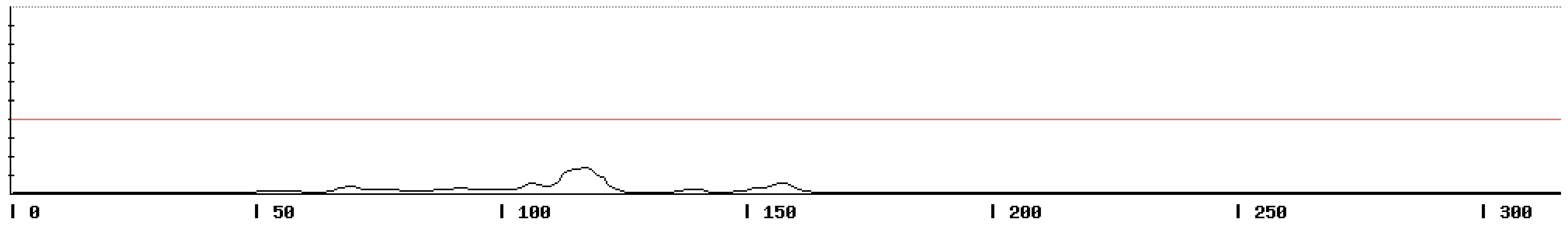

Predictions for Capsicumannuum_CA06g18770

Viterbi Path	
Viterbi Path	
Posterior @ 0.4	
Posterior @ 0.4	

Posterior Graphic. Threshold Marked @ 0.4

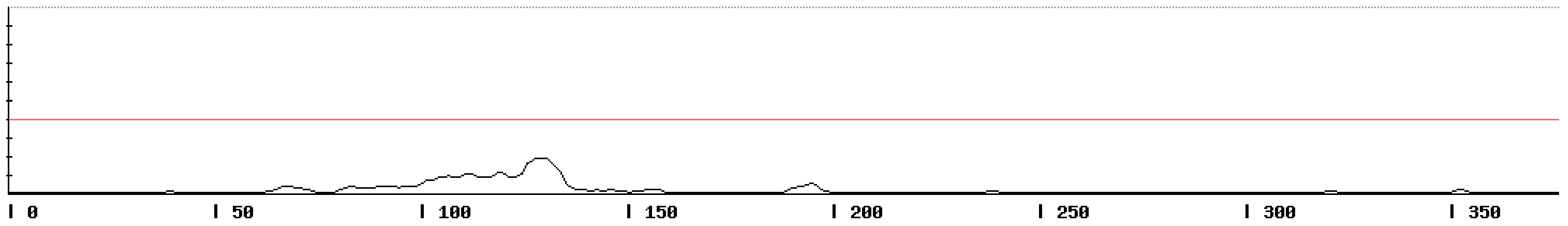

Predictions for Capsicumannuum_CA06g21590

Viterbi Path

Viterbi Path

Posterior @ 0.4

113 - RKGAKIGM - 120

Posterior @ 0.4

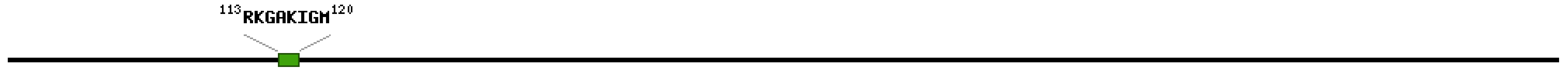

Posterior Graphic. Threshold Marked @ 0.4

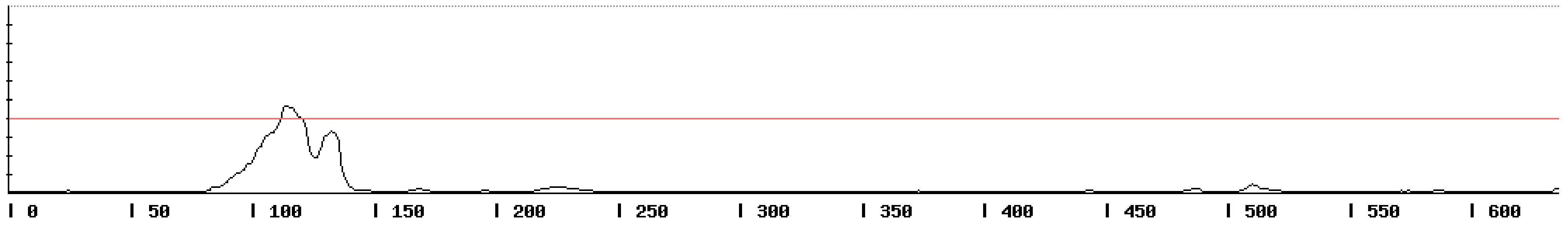

Predictions for Capsicumannuum_CA06g22780

Viterbi Path

Viterbi Path

Posterior @ 0.4

Posterior @ 0.4

Posterior Graphic. Threshold Marked @ 0.4

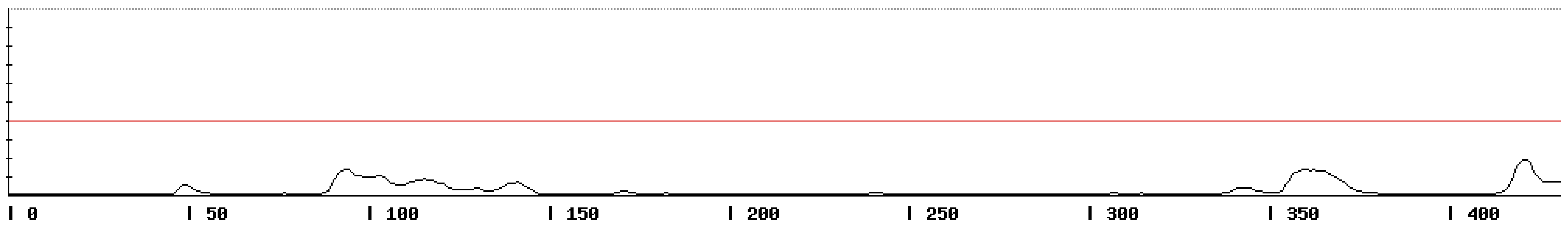

Predictions for Capsicumannuum_CA06g25420

Viterbi Path

Viterbi Path

Posterior @ 0.4

Posterior @ 0.4

Posterior Graphic. Threshold Marked @ 0.4

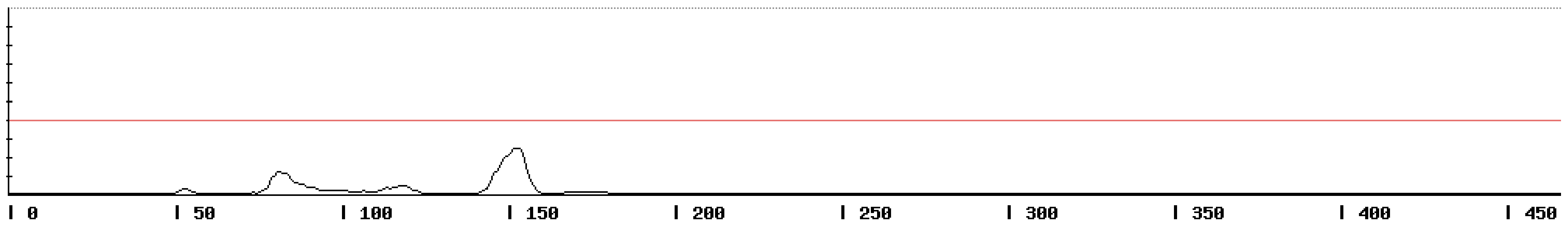

Predictions for Capsicumannuum_CA06g27940

Viterbi Path

Viterbi Path

Posterior @ 0.4

Posterior @ 0.4

Posterior Graphic. Threshold Marked @ 0.4

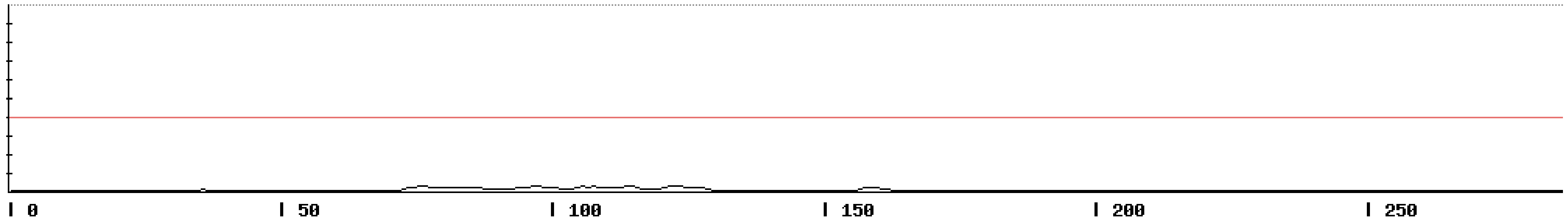

Predictions for Capsicumannuum_CA07g00500

Viterbi Path

Viterbi Path

Posterior @ 0.4

Posterior @ 0.4

Posterior Graphic. Threshold Marked @ 0.4

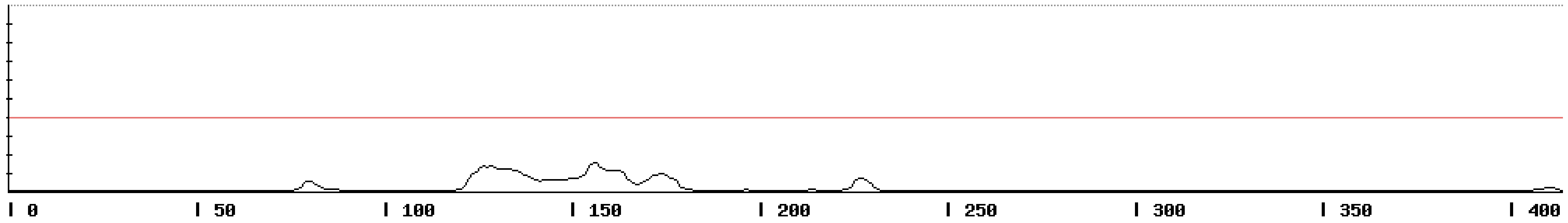

Predictions for Capsicumannuum_CA07g02120

Viterbi Path

Viterbi Path

Posterior @ 0.4

Posterior @ 0.4

Posterior Graphic. Threshold Marked @ 0.4

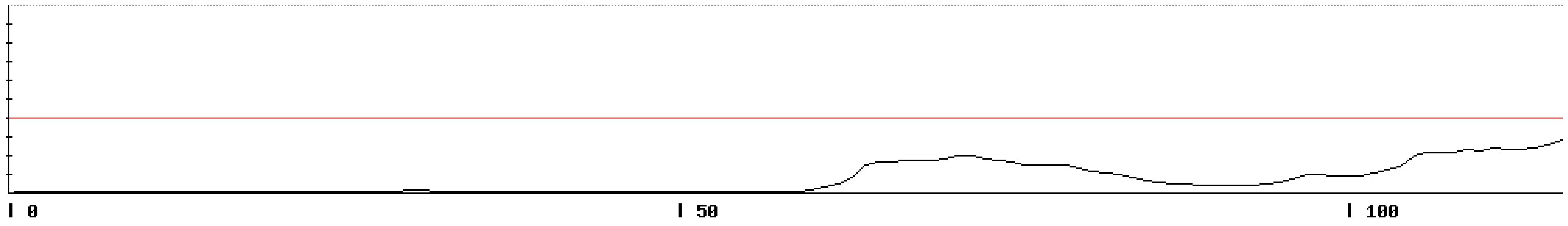

Predictions for Capsicumannuum_CA07g03900

Viterbi Path

Viterbi Path

Posterior @ 0.4

Posterior @ 0.4

Posterior Graphic. Threshold Marked @ 0.4

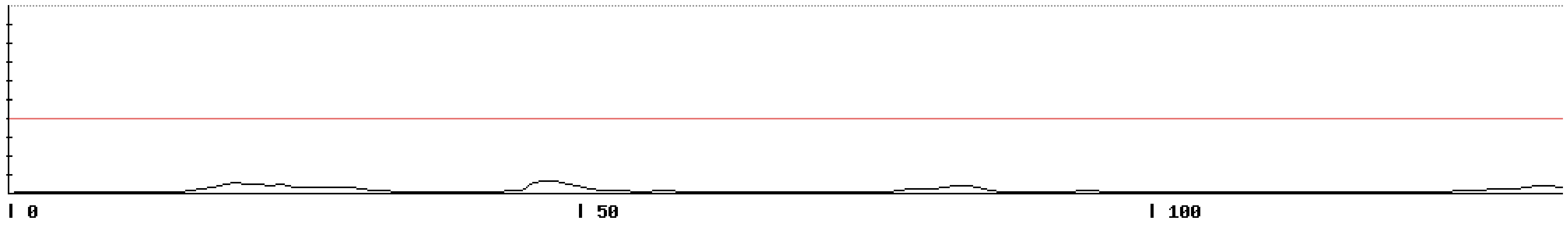

Predictions for Capsicumannuum_CA07g16790

Viterbi Path

Viterbi Path

Posterior @ 0.4

Posterior @ 0.4

Posterior Graphic. Threshold Marked @ 0.4

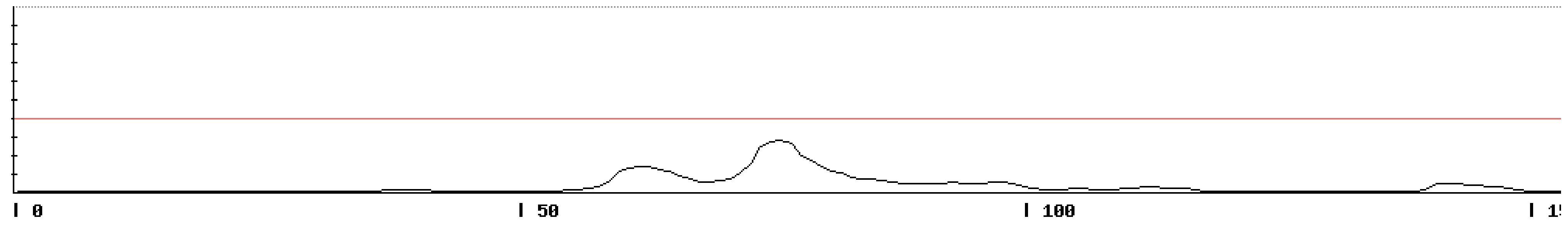

Predictions for Capsicumannuum_CA07g17460

Viterbi Path

Viterbi Path

Posterior @ 0.4

Posterior @ 0.4

Posterior Graphic. Threshold Marked @ 0.4

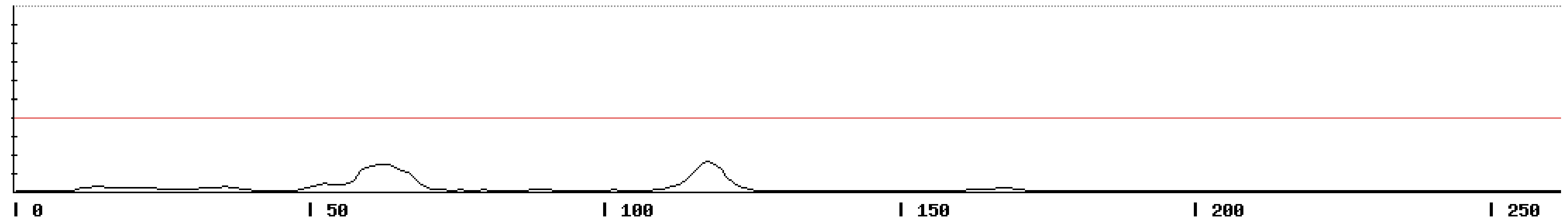

Predictions for Capsicumannuum_CA07g18020

Viterbi Path

Viterbi Path

Posterior @ 0.4

Posterior @ 0.4

Posterior Graphic. Threshold Marked @ 0.4

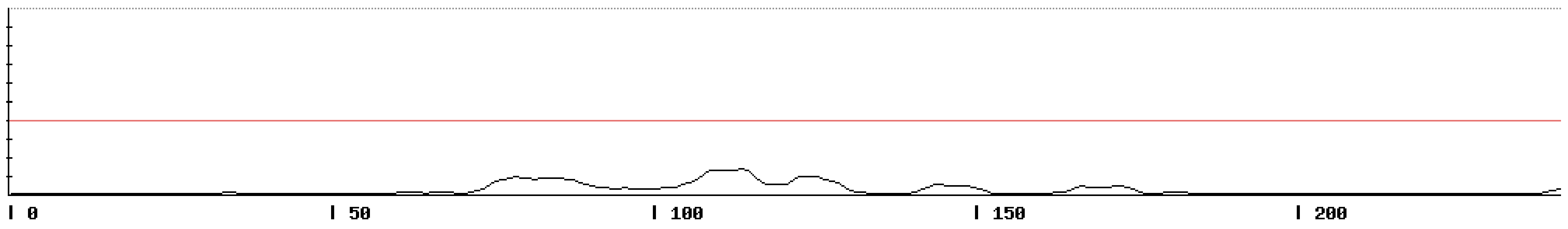

Predictions for Capsicumannuum_CA07g18030

Viterbi Path

Viterbi Path

Posterior @ 0.4

Posterior @ 0.4

Posterior Graphic. Threshold Marked @ 0.4

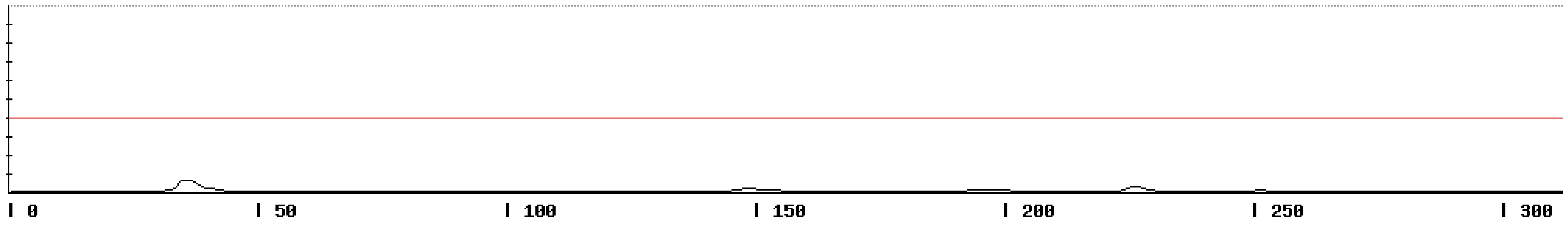

Predictions for Capsicumannuum_CA07g21320	
Viterbi Path	
Viterbi Path	
Posterior @ 0.4	
Posterior @ 0.4	
Posterior Graphic. Threshold Marked @ 0.4	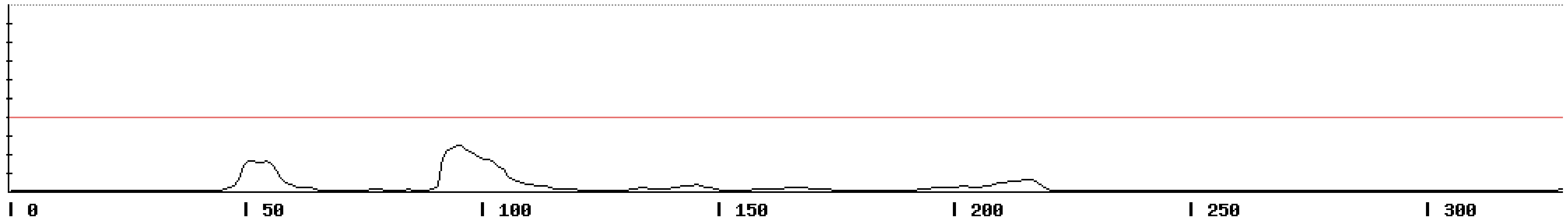

Predictions for Capsicumannuum_CA07g21470	
Viterbi Path	
Viterbi Path	
Posterior @ 0.4	
Posterior @ 0.4	

Posterior Graphic. Threshold Marked @ 0.4

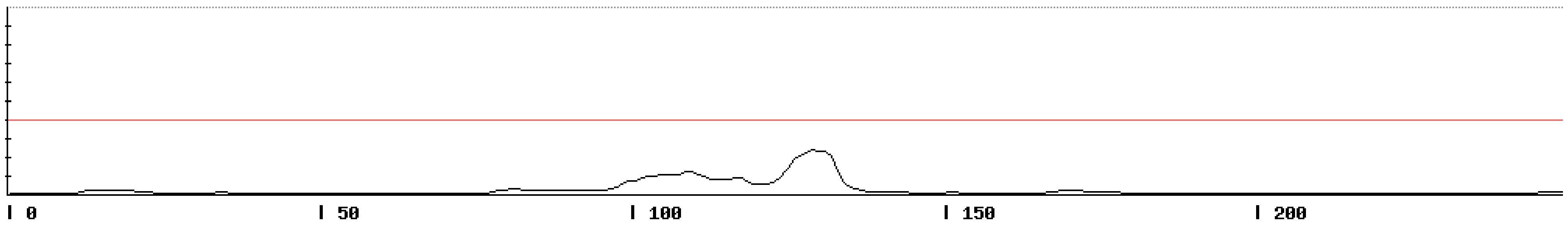

Predictions for Capsicumannuum_CA08g06780

Viterbi Path	
Viterbi Path	
Posterior @ 0.4	73 - KKK - 75
	156 - KIKKKKKND - 164
Posterior @ 0.4	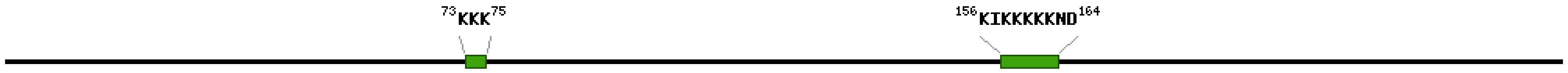
Posterior Graphic. Threshold Marked @ 0.4	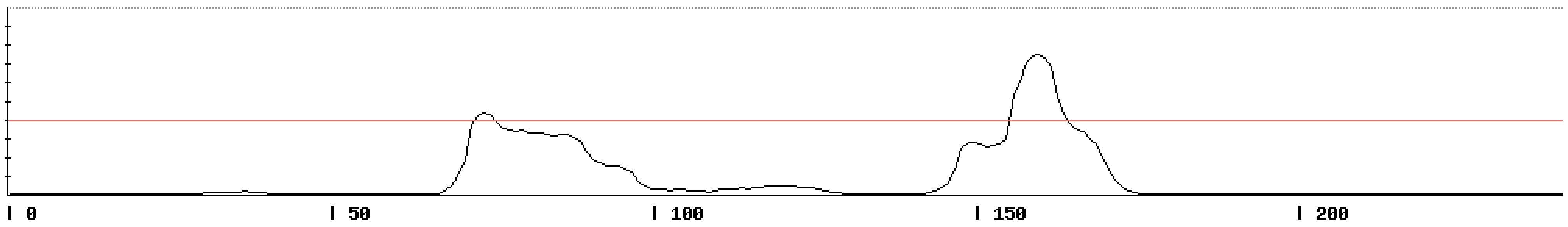

Predictions for Capsicumannuum_CA08g06790

Viterbi Path	
Viterbi Path	
Posterior @ 0.4	72 - KKKKADSIRTERSAGKG - 88
	156 - KKKK - 159

Posterior @ 0.4

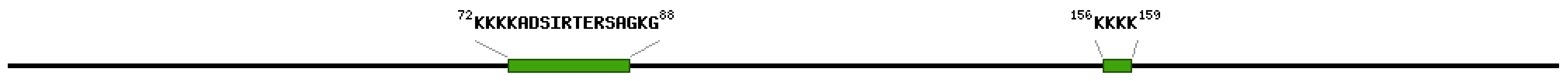

Posterior Graphic. Threshold Marked @ 0.4

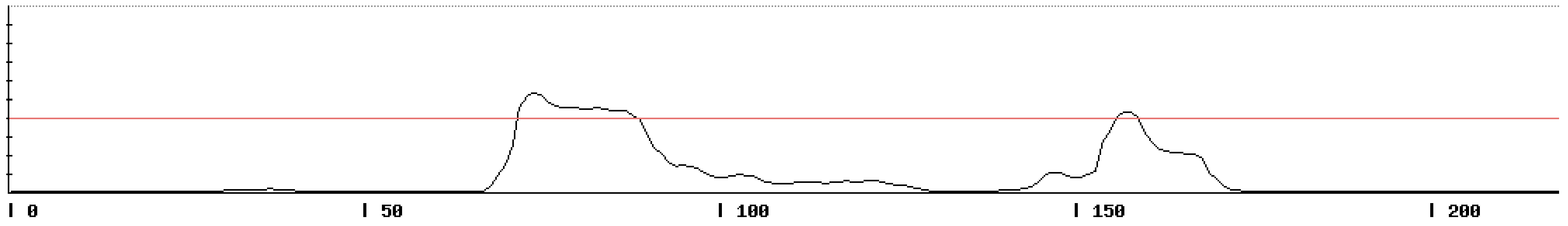

Predictions for Capsicumannuum_CA08g13660

Viterbi Path

Viterbi Path

Posterior @ 0.4

Posterior @ 0.4

Posterior Graphic. Threshold Marked @ 0.4

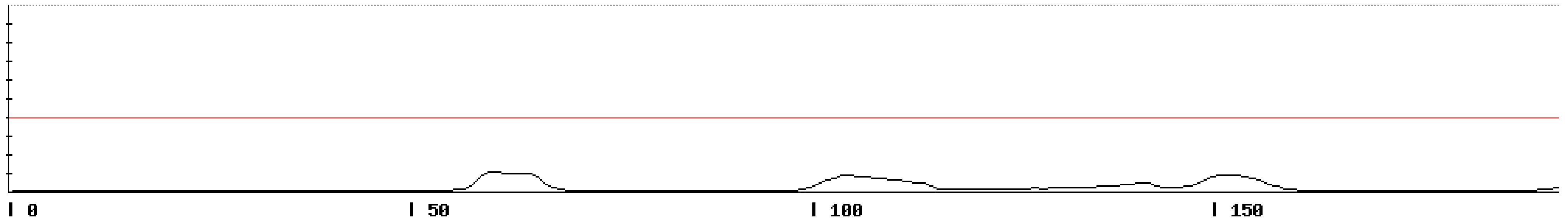

Predictions for Capsicumannuum_CA08g15460

Viterbi Path

Viterbi Path

Posterior @ 0.4

Posterior @ 0.4

Posterior Graphic. Threshold Marked @ 0.4

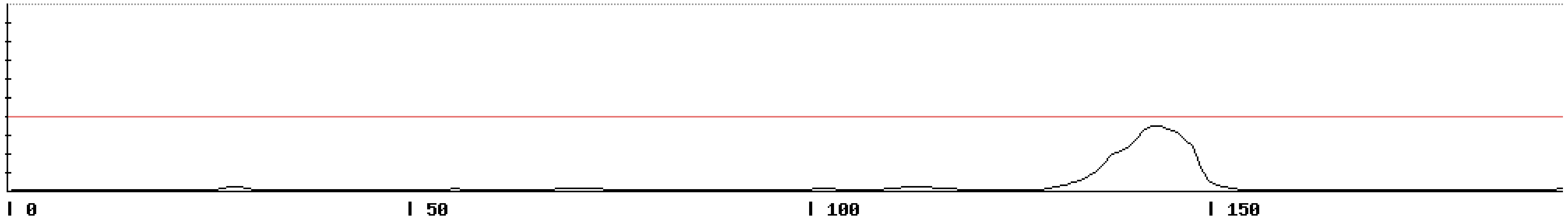

Predictions for Capsicumannuum_CA09g00160

Viterbi Path

Viterbi Path

Posterior @ 0.4

Posterior @ 0.4

Posterior Graphic. Threshold Marked @ 0.4

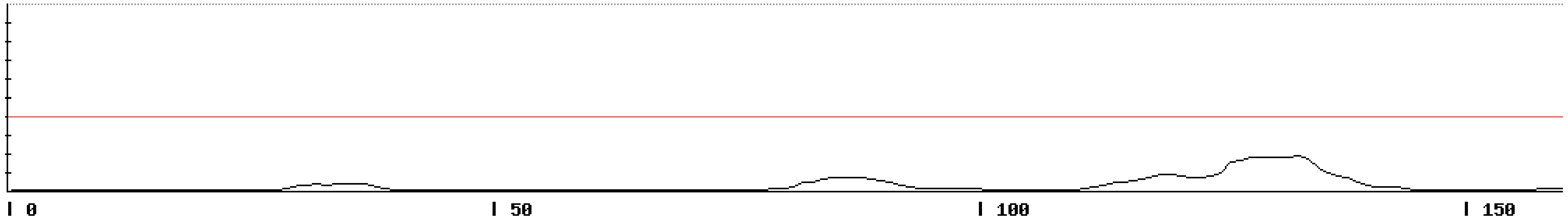

Predictions for Capsicumannuum_CA09g12970

Viterbi Path

Viterbi Path

Posterior @ 0.4

Posterior @ 0.4

Posterior Graphic. Threshold Marked @ 0.4

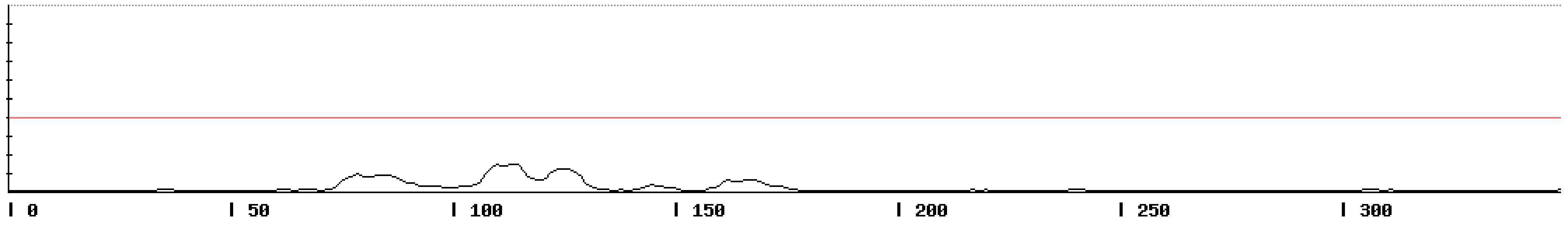

Predictions for Capsicumannuum_CA09g18600

Viterbi Path

Viterbi Path

Posterior @ 0.4

Posterior @ 0.4

Posterior Graphic. Threshold Marked @ 0.4

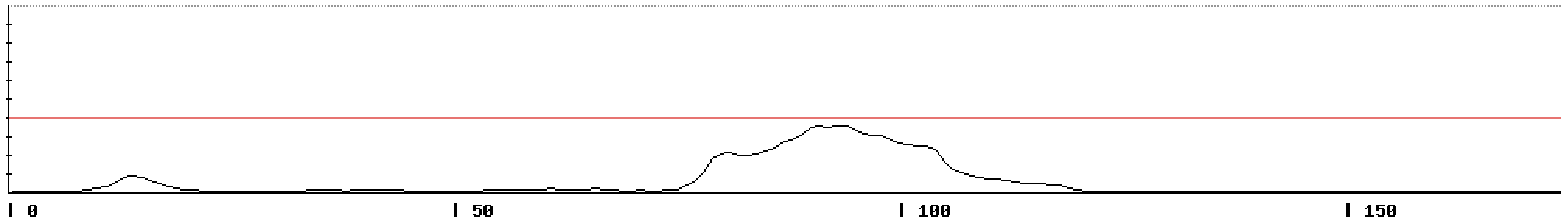

Predictions for Capsicumannuum_CA10g00460

Viterbi Path

Viterbi Path

Posterior @ 0.4

Posterior @ 0.4

Posterior Graphic. Threshold Marked @ 0.4

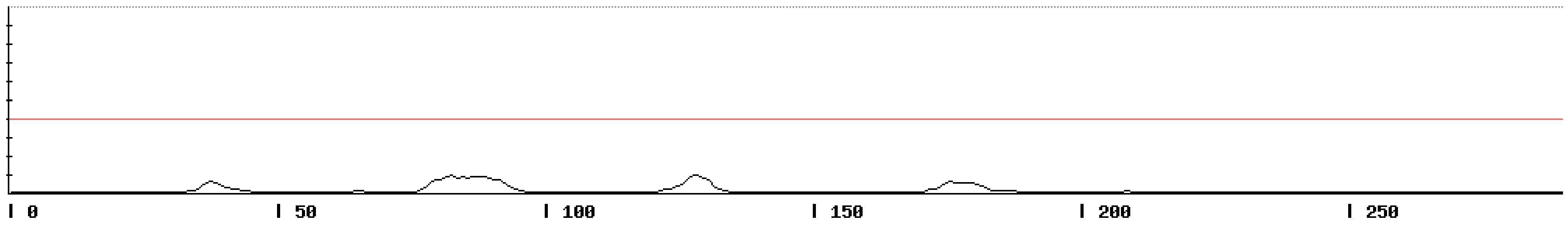

Predictions for Capsicumannuum_CA10g09910

Viterbi Path

Viterbi Path

Posterior @ 0.4

Posterior @ 0.4

Posterior Graphic. Threshold Marked @ 0.4

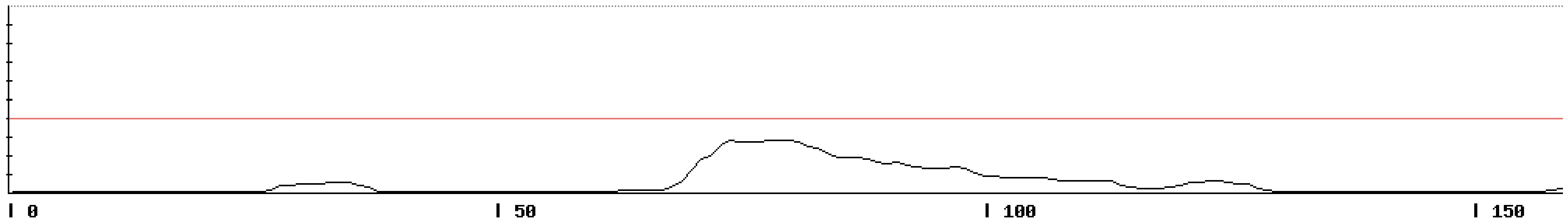

Predictions for Capsicumannuum_CA10g13700

Viterbi Path

Viterbi Path

Posterior @ 0.4

Posterior @ 0.4

Posterior Graphic. Threshold Marked @ 0.4

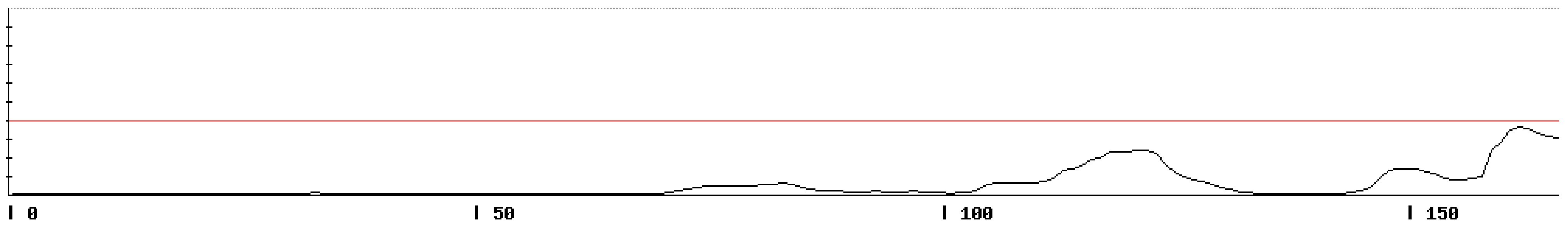

Predictions for Capsicumannuum_CA10g13720

Viterbi Path

Viterbi Path

Posterior @ 0.4

Posterior @ 0.4

Posterior Graphic. Threshold Marked @ 0.4

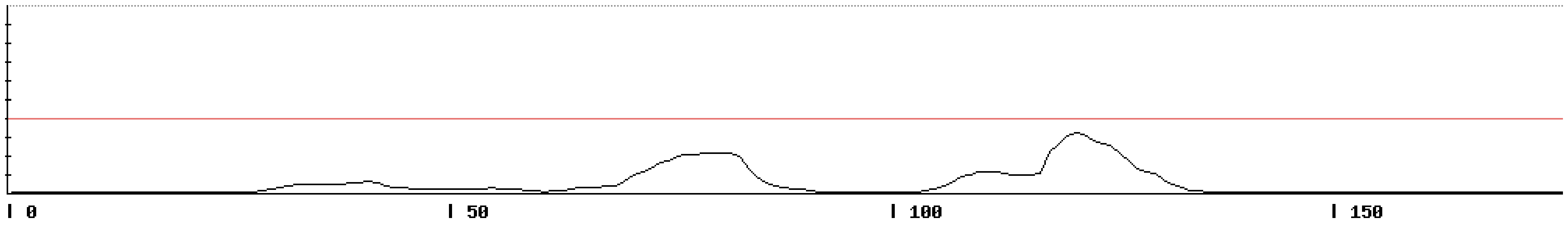

Predictions for Capsicumannuum_CA10g20530

Viterbi Path

Viterbi Path

Posterior @ 0.4 68 - KLKKKKSDGRRF - 79

Posterior @ 0.4

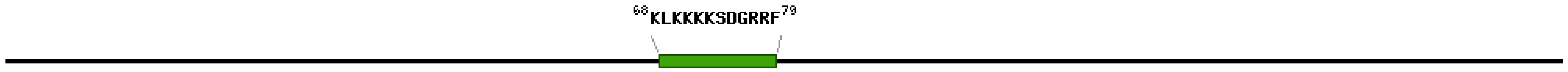

Posterior Graphic. Threshold Marked @ 0.4

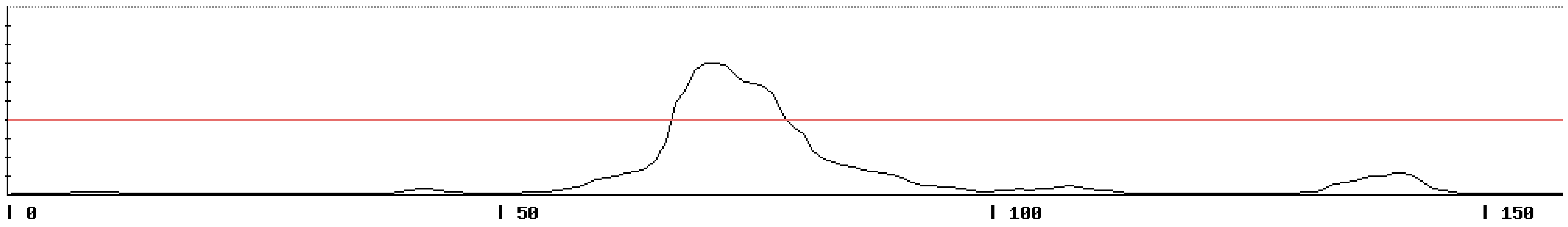

Predictions for Capsicumannuum_CA10g20640

Viterbi Path

Viterbi Path

Posterior @ 0.4 67 - KLKKKKSDGRRF - 78

Posterior @ 0.4

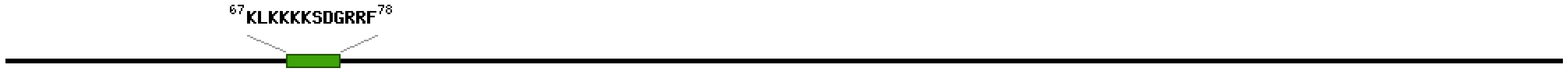

Posterior Graphic. Threshold Marked @ 0.4

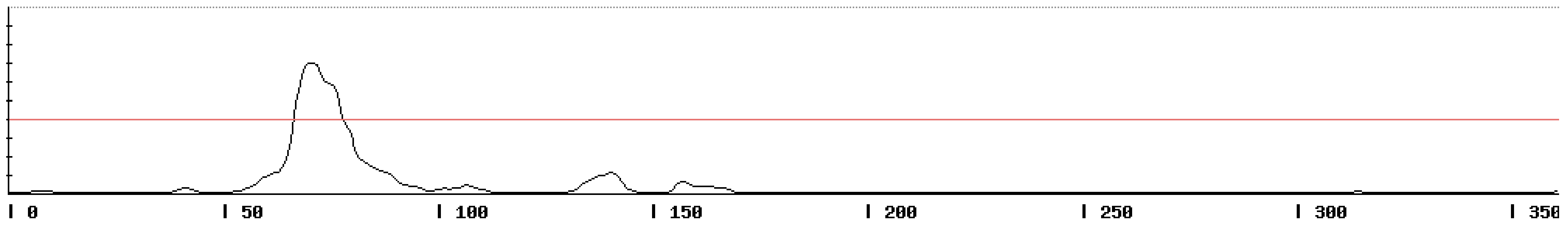

Predictions for Capsicumannuum_CA11g01550

Viterbi Path

Viterbi Path

Posterior @ 0.4 168 - RKRRK - 172

Posterior @ 0.4

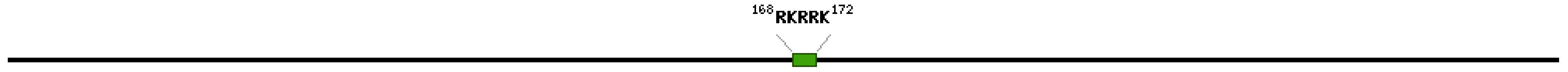

Posterior Graphic. Threshold Marked @ 0.4

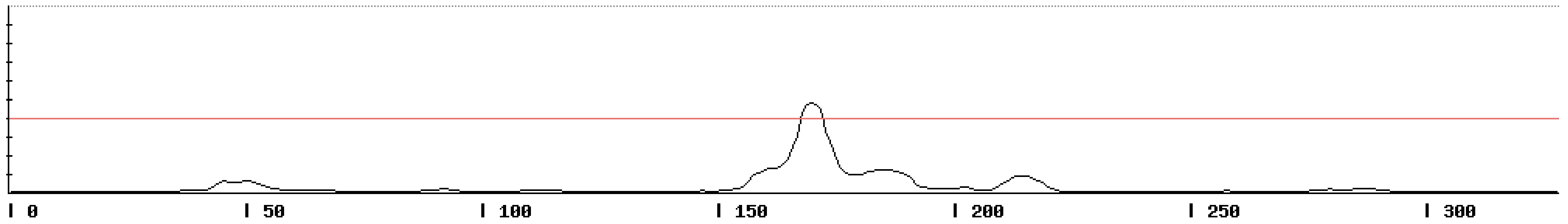

Predictions for Capsicumannuum_CA11g04440

Viterbi Path

Viterbi Path

Posterior @ 0.4

Posterior @ 0.4

Posterior Graphic. Threshold Marked @ 0.4

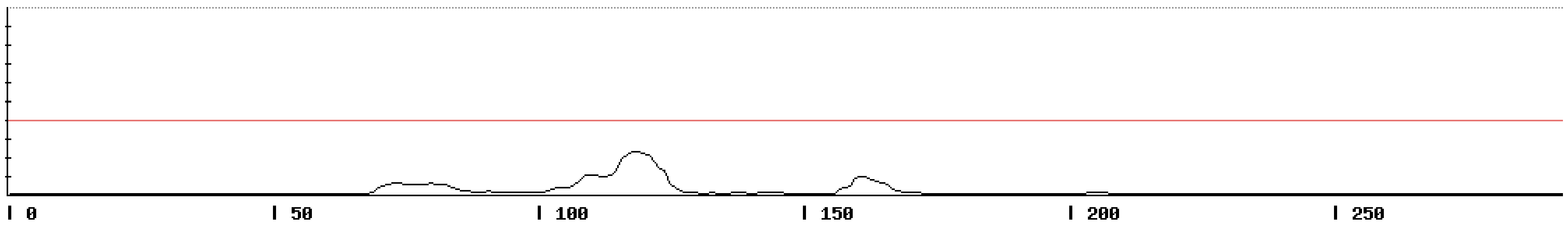

Predictions for Capsicumannuum_CA11g04580

Viterbi Path

Viterbi Path

Posterior @ 0.4

Posterior @ 0.4

Posterior Graphic. Threshold Marked @ 0.4

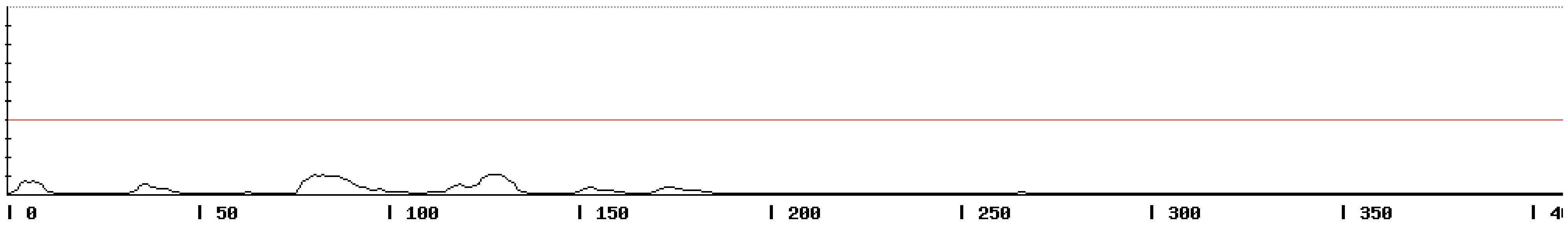

Predictions for Capsicumannuum_CA11g08290

Viterbi Path

Viterbi Path

Posterior @ 0.4

Posterior @ 0.4

Posterior Graphic. Threshold Marked @ 0.4

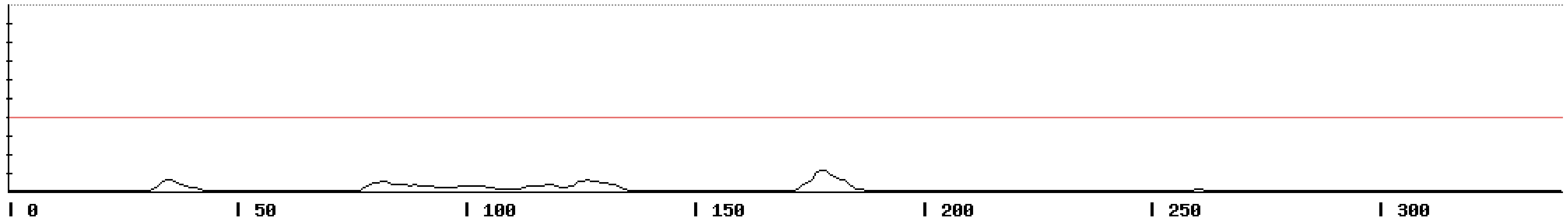

Predictions for Capsicumannuum_CA11g12260

Viterbi Path

Viterbi Path

Posterior @ 0.4

Posterior @ 0.4

Posterior Graphic. Threshold Marked @ 0.4

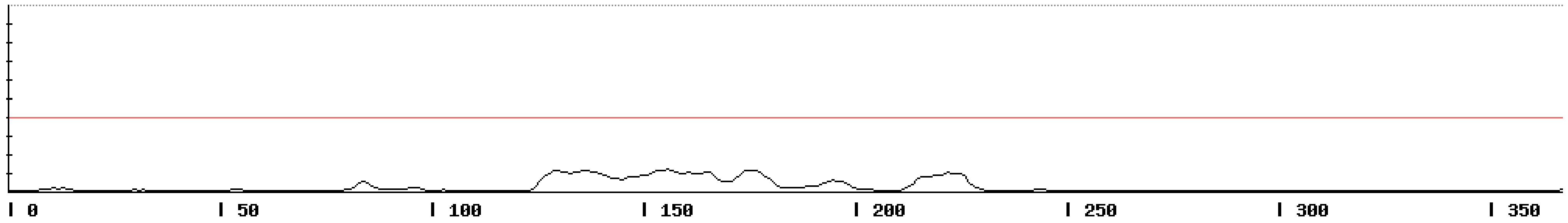

Predictions for Capsicumannuum_CA11g16490

Viterbi Path

Viterbi Path

Posterior @ 0.4 | 340 - RRLKLQTEKPPRRVKRSEPHEAIKKNS - 366

Posterior @ 0.4

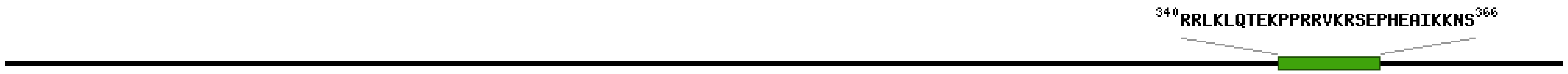

Posterior Graphic. Threshold Marked @ 0.4

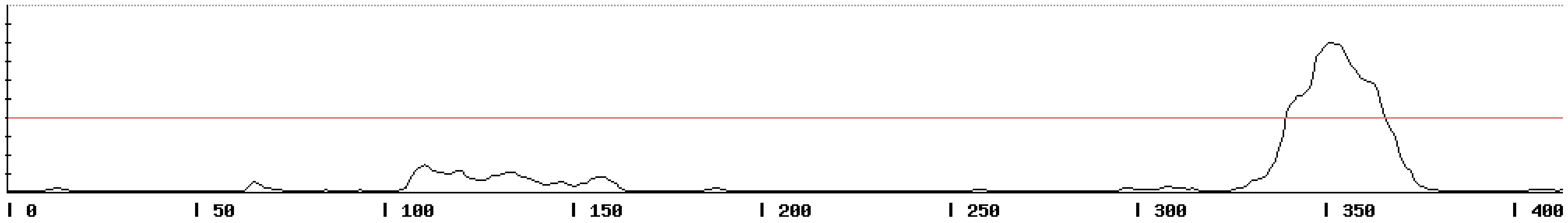

Predictions for Capsicumannuum_CA12g00080

Viterbi Path

Viterbi Path

Posterior @ 0.4

Posterior @ 0.4

Posterior Graphic. Threshold Marked @ 0.4

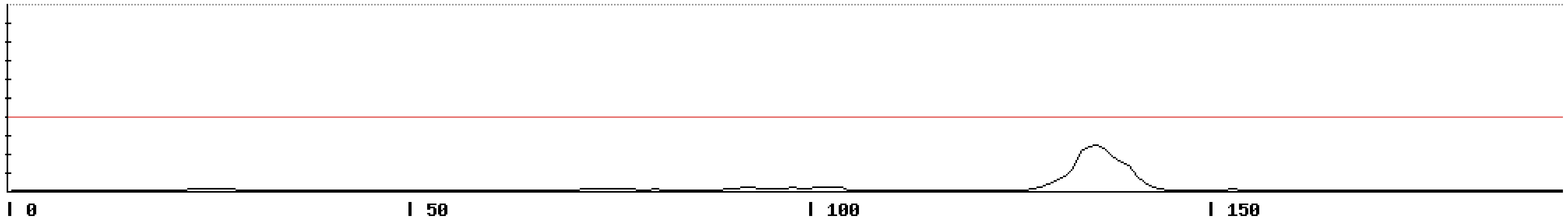

Predictions for Capsicumannuum_CA12g04950

Viterbi Path

Viterbi Path

Posterior @ 0.4

Posterior @ 0.4

Posterior Graphic. Threshold Marked @ 0.4

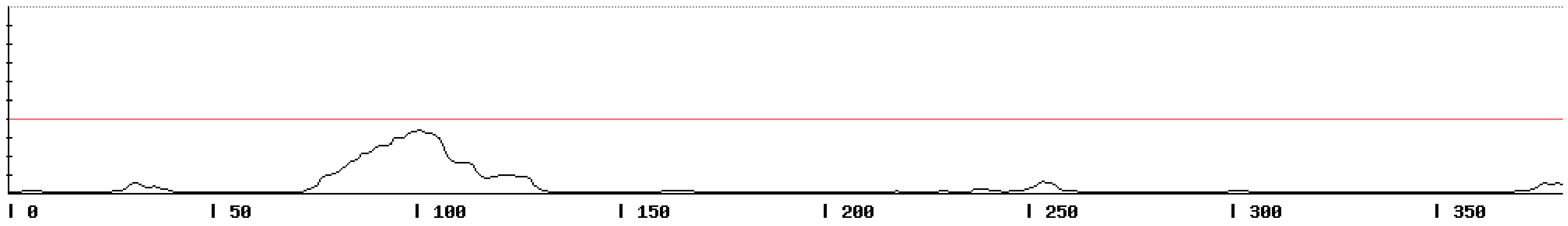

Predictions for Capsicumannuum_CA12g04970

Viterbi Path

Viterbi Path

Posterior @ 0.4

Posterior @ 0.4

Posterior Graphic. Threshold Marked @ 0.4

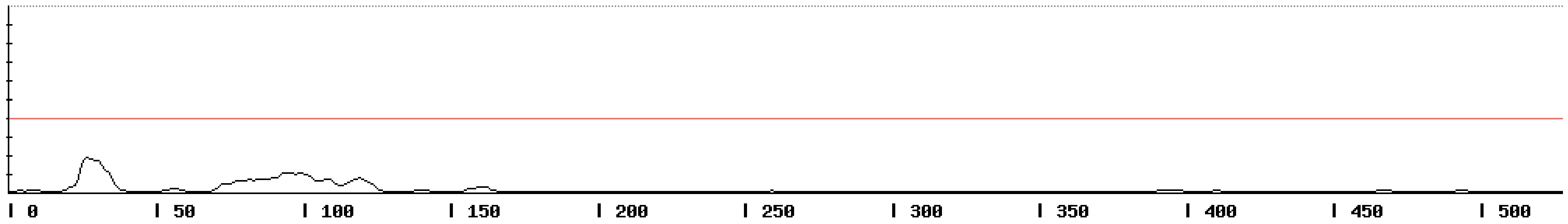

Predictions for Capsicumannuum_CA12g05890	
Viterbi Path	
Viterbi Path	
Posterior @ 0.4	142 - VKKRIKG - 148
Posterior @ 0.4	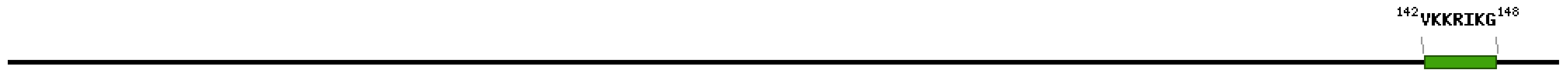
Posterior Graphic. Threshold Marked @ 0.4	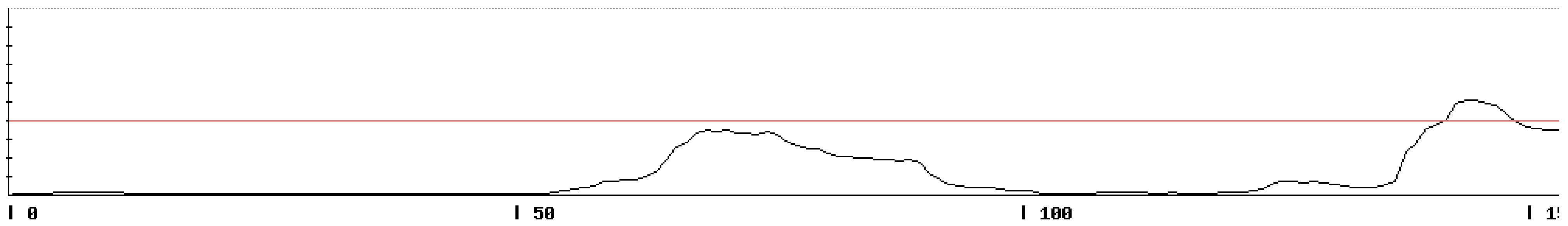

Predictions for Capsicumannuum_CA12g05910	
Viterbi Path	
Viterbi Path	
Posterior @ 0.4	146 - KIKEKKKNEKKNK - 158
Posterior @ 0.4	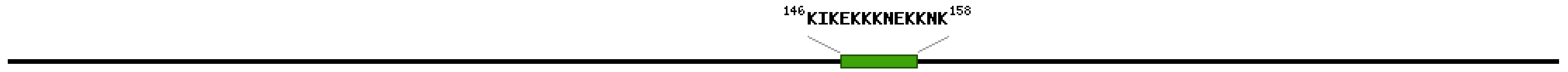
Posterior Graphic. Threshold Marked @ 0.4	

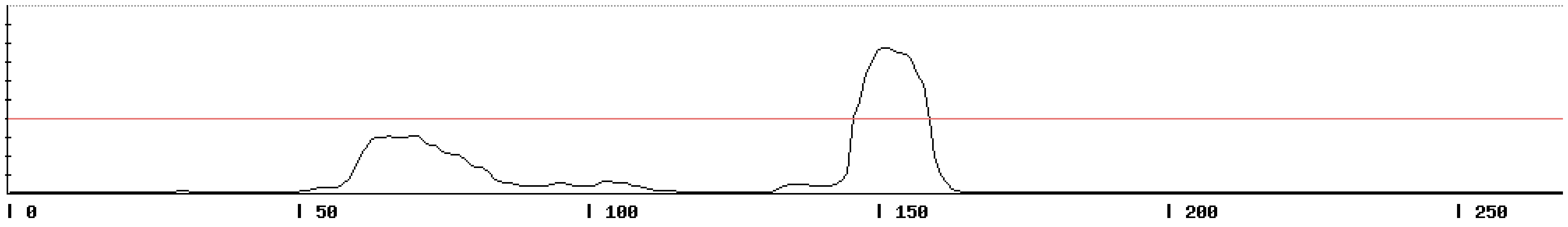

Predictions for Capsicumannuum_CA12g06210

Viterbi Path	
Viterbi Path	
Posterior @ 0.4	60 - SRLKRLKNSTKKFSRTVRKG - 79
	147 - IKEKRKNEKKNK - 158
Posterior @ 0.4	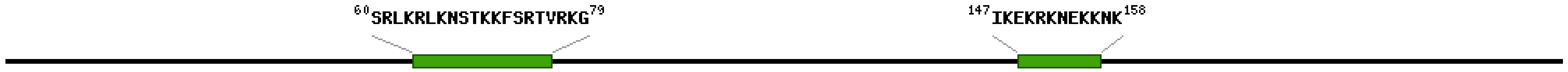
Posterior Graphic. Threshold Marked @ 0.4	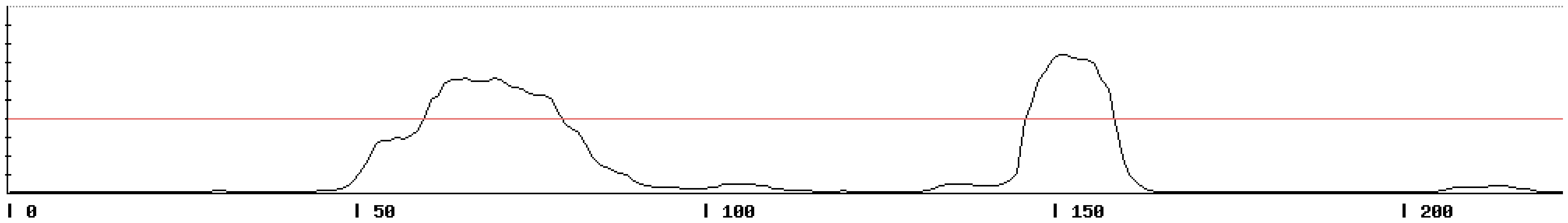

Predictions for Capsicumannuum_CA12g07920

Viterbi Path	
Viterbi Path	
Posterior @ 0.4	
Posterior @ 0.4	

Viterbi Path

Posterior @ 0.4

Posterior @ 0.4

Posterior Graphic. Threshold Marked @ 0.4

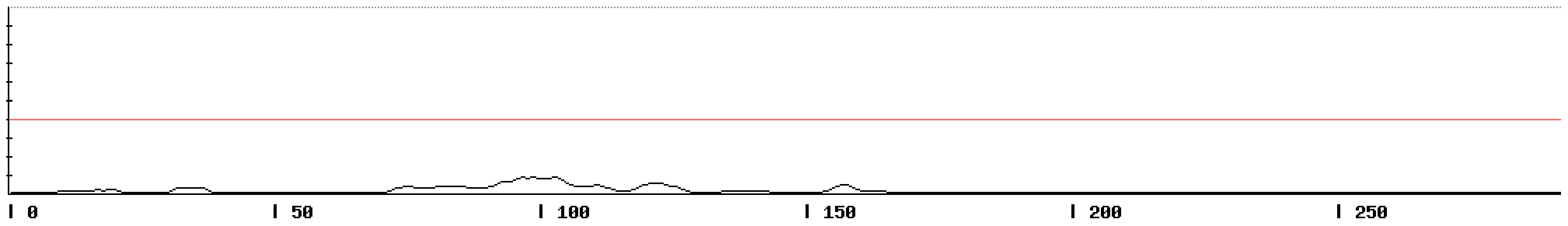

Predictions for Caricapapaya_evm.model.supercontig_104.98

Viterbi Path

Viterbi Path

Posterior @ 0.4

Posterior @ 0.4

Posterior Graphic. Threshold Marked @ 0.4

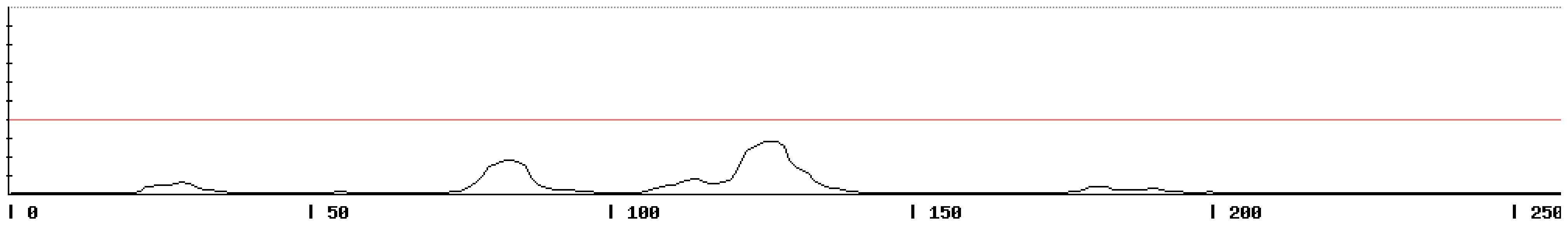

Predictions for Caricapapaya_evm.model.supercontig_106.80

Viterbi Path

Viterbi Path

Posterior @ 0.4 | 138 - KKKKK - 142

Posterior @ 0.4

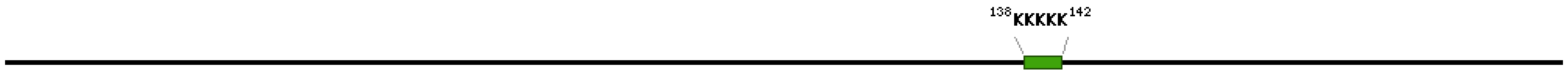

Posterior Graphic. Threshold Marked @ 0.4

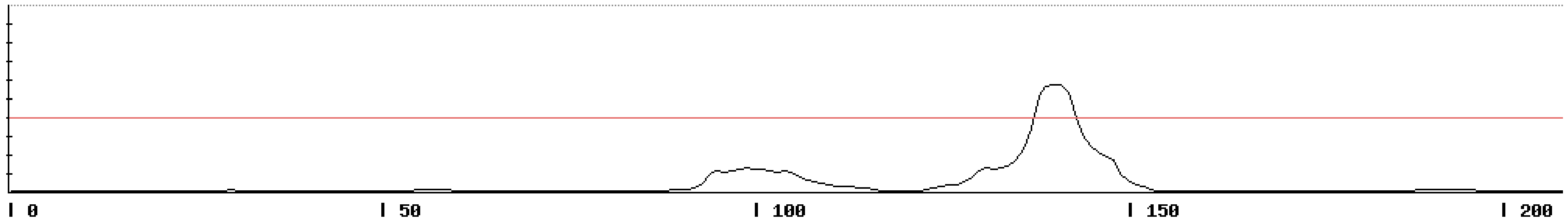

Predictions for Caricapapaya_evm.model.supercontig_1.108

Viterbi Path

Viterbi Path

Posterior @ 0.4 | 96 - KGKKRMASSSSK - 107

Posterior @ 0.4

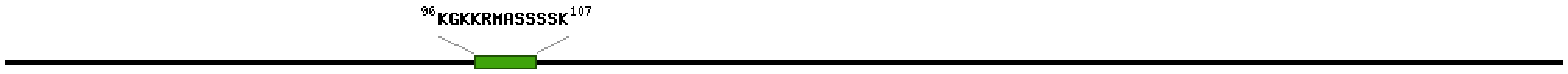

Posterior Graphic. Threshold Marked @ 0.4

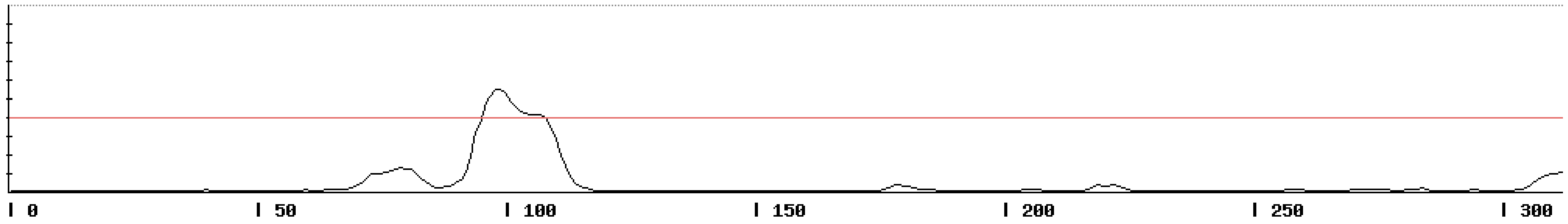

Predictions for Caricapapaya_evm.model.supercontig_111.22

Viterbi Path	
Viterbi Path	
Posterior @ 0.4	
Posterior @ 0.4	
Posterior Graphic. Threshold Marked @ 0.4	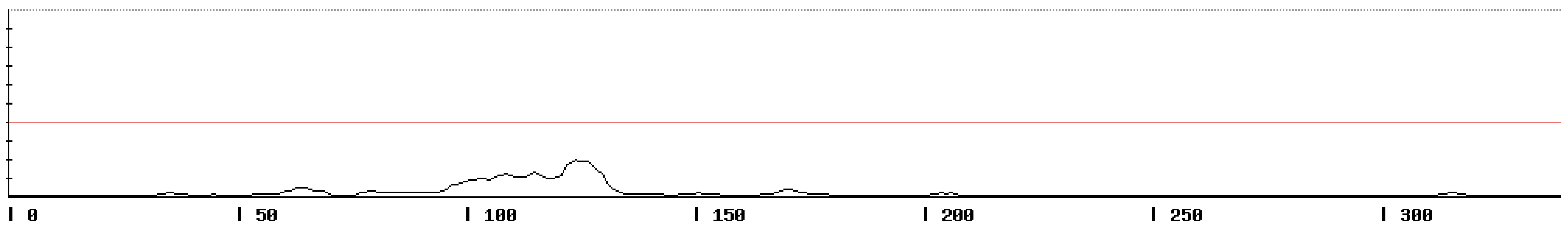

Predictions for Caricapapaya_evm.model.supercontig_113.19

Viterbi Path	
Viterbi Path	
Posterior @ 0.4	117 - RRGKVIGRKKT - 127<br>135 - DPSKKRKE - 142
Posterior @ 0.4	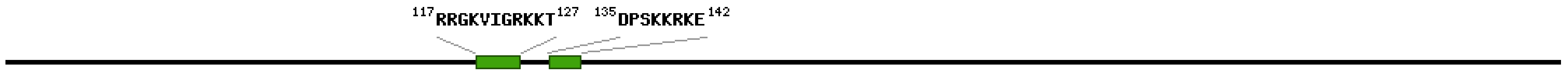
Posterior Graphic. Threshold Marked @ 0.4	

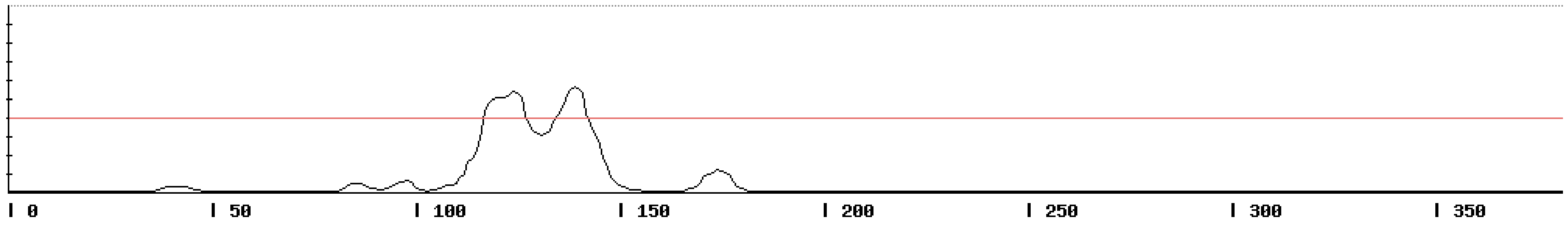

Predictions for Caricapapaya_evm.model.supercontig_113.36	
Viterbi Path	
Viterbi Path	
Posterior @ 0.4	
Posterior @ 0.4	
Posterior Graphic. Threshold Marked @ 0.4	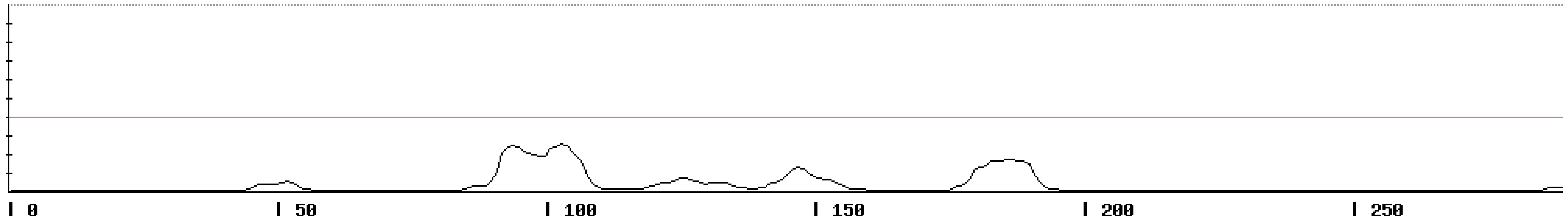

Predictions for Caricapapaya_evm.model.supercontig_1.141	
Viterbi Path	
Viterbi Path	
Posterior @ 0.4	
Posterior @ 0.4	

Posterior Graphic. Threshold Marked @ 0.4	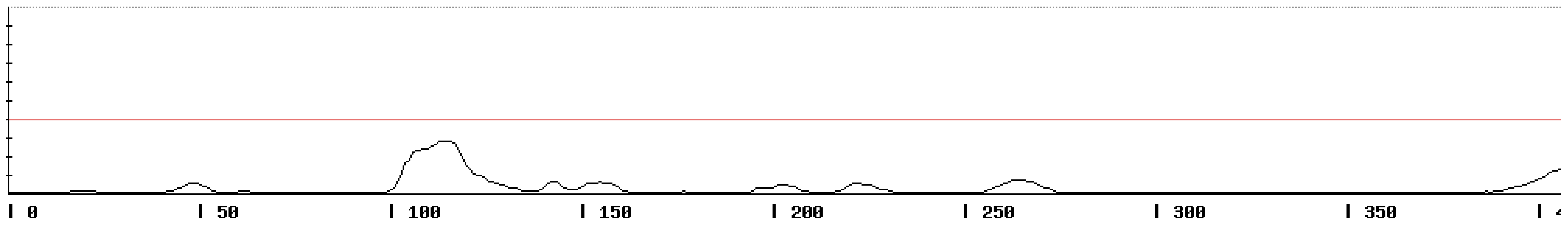

Predictions for Caricapapaya_evm.model.supercontig_11.52

Viterbi Path	
Viterbi Path	
Posterior @ 0.4	
Posterior @ 0.4	
Posterior Graphic. Threshold Marked @ 0.4	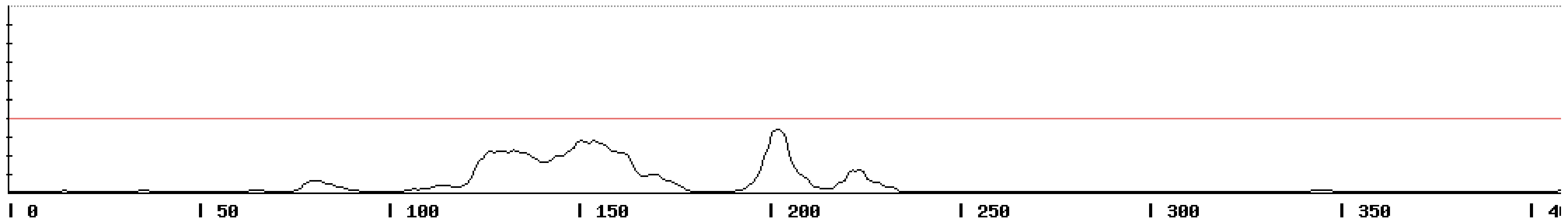

Predictions for Caricapapaya_evm.model.supercontig_118.26

Viterbi Path	
Viterbi Path	
Posterior @ 0.4	
Posterior @ 0.4	

Posterior Graphic. Threshold Marked @ 0.4

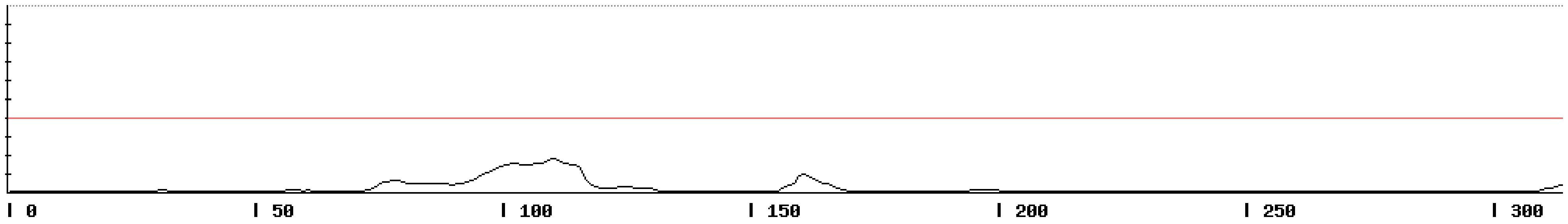

Predictions for Caricapapaya_evm.model.supercontig_12.156

Viterbi Path

Viterbi Path

Posterior @ 0.4: 63 - KKKSRSR - 69

Posterior @ 0.4

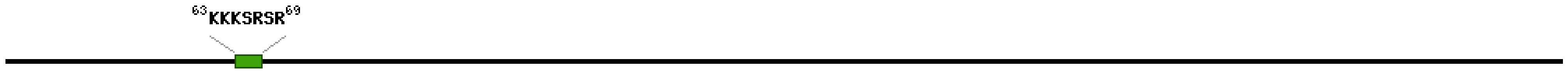

Posterior Graphic. Threshold Marked @ 0.4

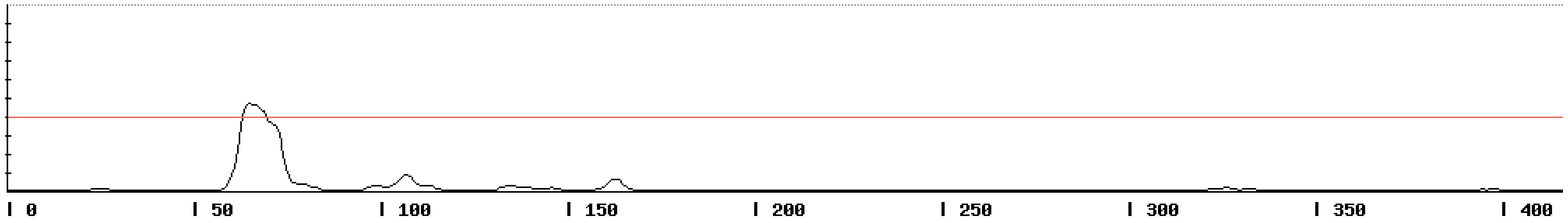

Predictions for Caricapapaya_evm.model.supercontig_128.20

Viterbi Path

Viterbi Path

Posterior @ 0.4

Posterior @ 0.4

Posterior Graphic. Threshold Marked @ 0.4

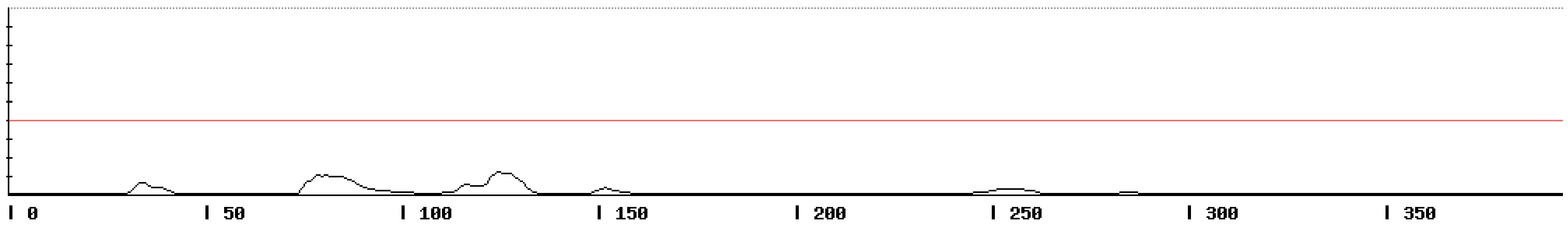

Predictions for Caricapapaya_evm.model.supercontig_131.88

Viterbi Path

Viterbi Path

Posterior @ 0.4

Posterior @ 0.4

Posterior Graphic. Threshold Marked @ 0.4

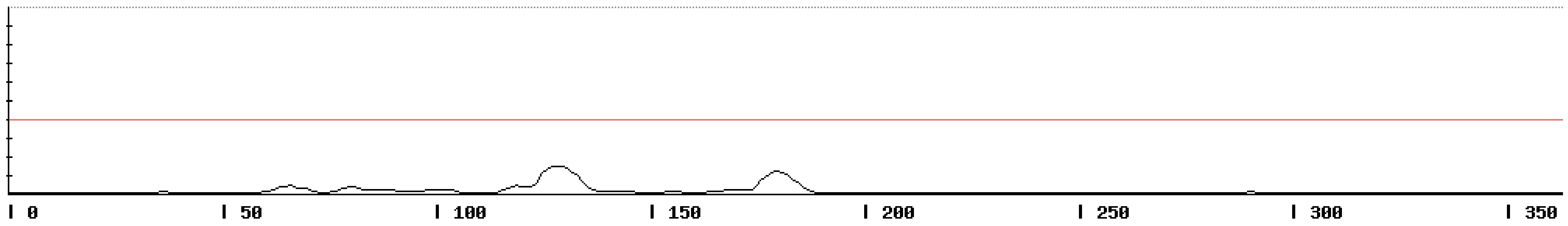

Predictions for Caricapapaya_evm.model.supercontig_14.101

Viterbi Path

Viterbi Path

Posterior @ 0.4

Posterior @ 0.4

Posterior Graphic. Threshold Marked @ 0.4

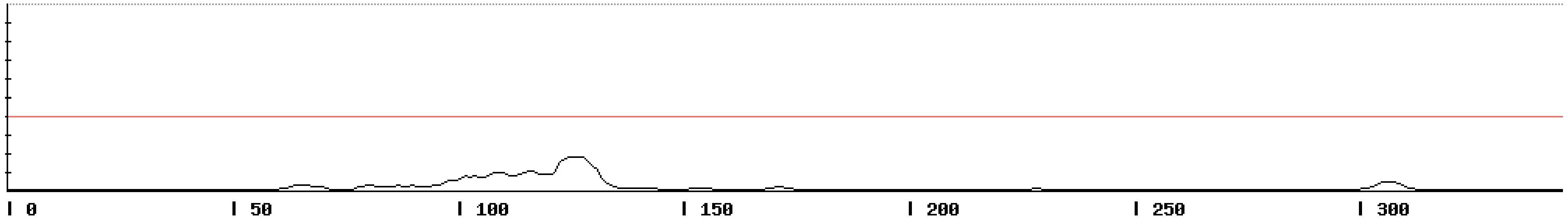

Predictions for Caricapapaya_evm.model.supercontig_14.2

Viterbi Path

Viterbi Path

Posterior @ 0.4

Posterior @ 0.4

Posterior Graphic. Threshold Marked @ 0.4

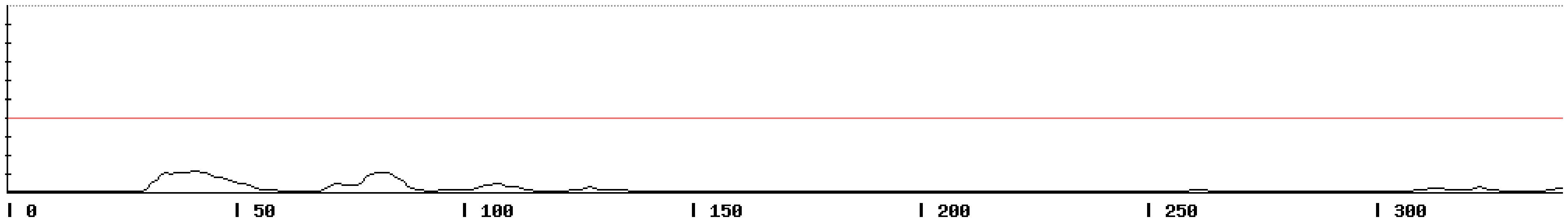

Predictions for Caricapapaya_evm.model.supercontig_14.213

Viterbi Path

Viterbi Path

Posterior @ 0.4

Posterior @ 0.4

Posterior Graphic. Threshold Marked @ 0.4

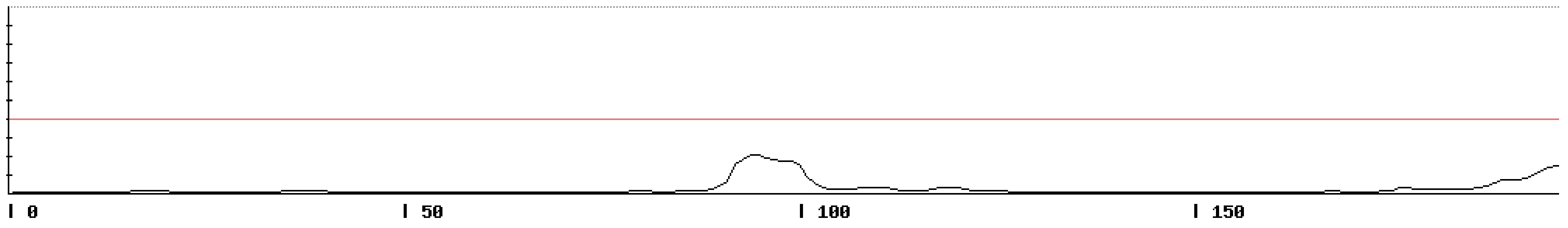

Predictions for Caricapapaya_evm.model.supercontig_1.48

Viterbi Path

Viterbi Path

Posterior @ 0.4

Posterior @ 0.4

Posterior Graphic. Threshold Marked @ 0.4

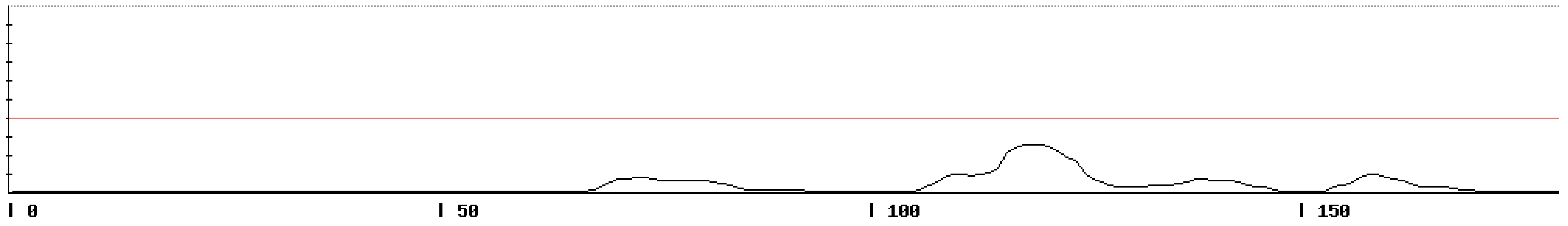

Predictions for Caricapapaya_evm.model.supercontig_165.12	
Viterbi Path	
Viterbi Path	
Posterior @ 0.4	
Posterior @ 0.4	
Posterior Graphic. Threshold Marked @ 0.4	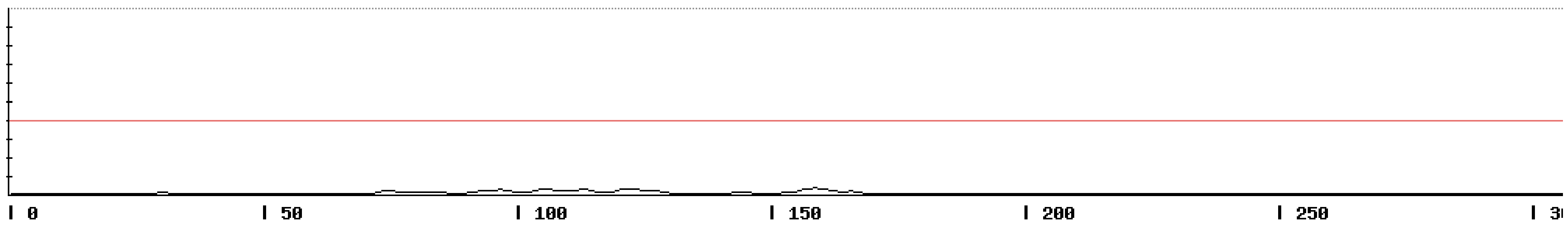

Predictions for Caricapapaya_evm.model.supercontig_17.29	
Viterbi Path	
Viterbi Path	
Posterior @ 0.4	
Posterior @ 0.4	
Posterior Graphic. Threshold Marked @ 0.4	

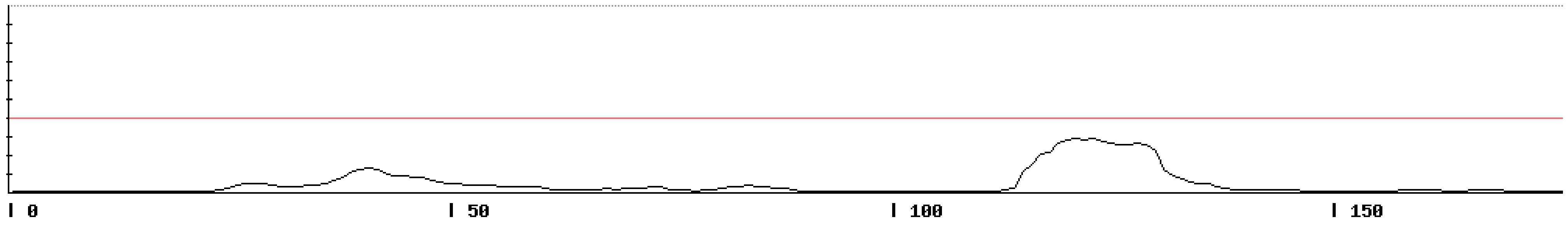

Predictions for Caricapapaya_evm.model.supercontig_178.32

Viterbi Path

Viterbi Path

Posterior @ 0.4

Posterior @ 0.4

Posterior Graphic. Threshold Marked @ 0.4

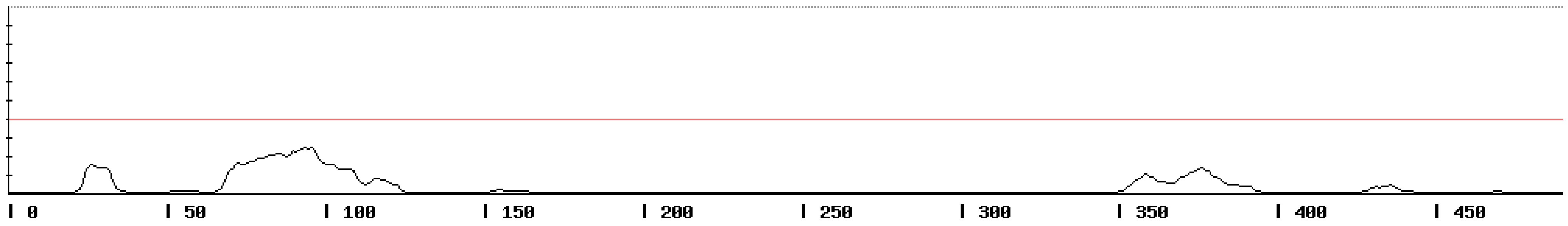

Predictions for Caricapapaya_evm.model.supercontig_192.19

Viterbi Path

Viterbi Path

Posterior @ 0.4

Posterior @ 0.4

Posterior Graphic. Threshold Marked @ 0.4	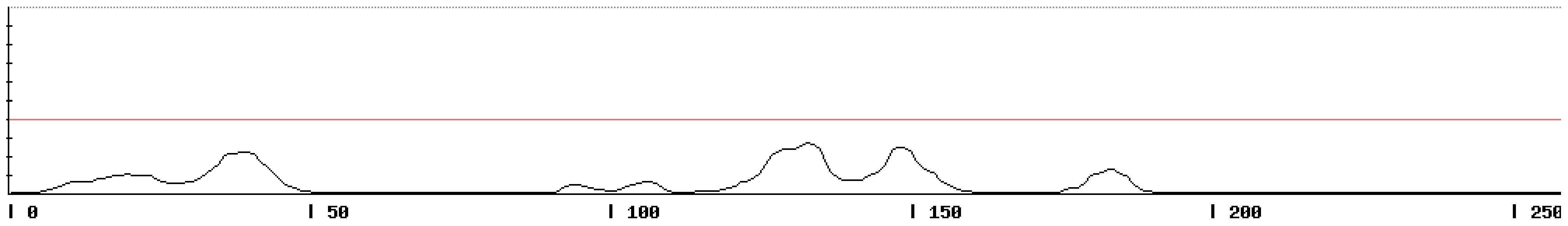

Predictions for Caricapapaya_evm.model.supercontig_195.14

Viterbi Path	
Viterbi Path	
Posterior @ 0.4	
Posterior @ 0.4	
Posterior Graphic. Threshold Marked @ 0.4	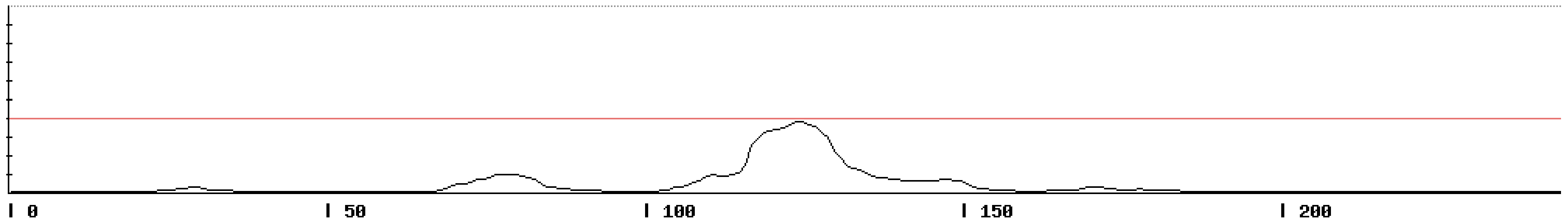

Predictions for Caricapapaya_evm.model.supercontig_195.35

Viterbi Path	
Viterbi Path	
Posterior @ 0.4	
Posterior @ 0.4	

Posterior Graphic. Threshold Marked @ 0.4

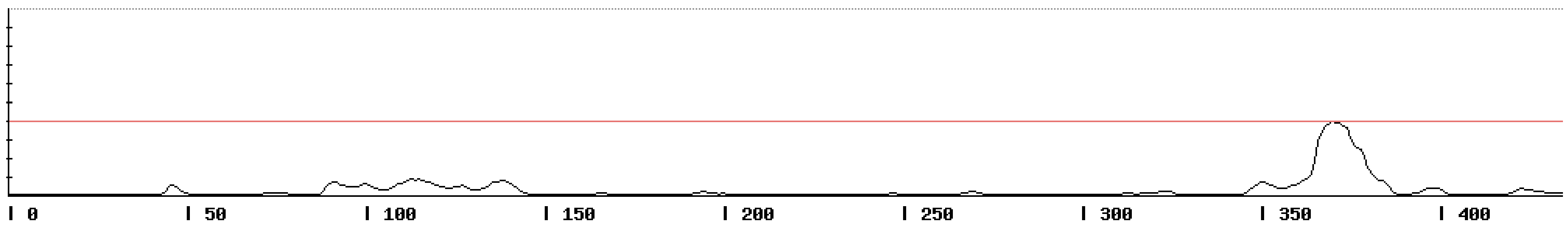

Predictions for Caricapapaya_evm.model.supercontig_200.10

Viterbi Path

Viterbi Path

Posterior @ 0.4

113 - KKKKK - 117

Posterior @ 0.4

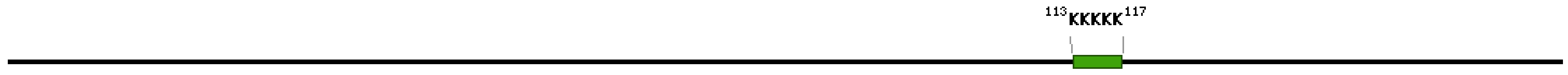

Posterior Graphic. Threshold Marked @ 0.4

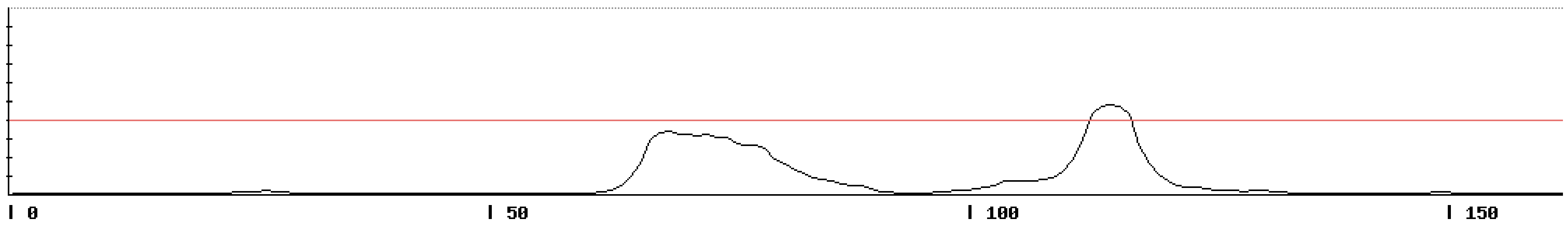

Predictions for Caricapapaya_evm.model.supercontig_202.7

Viterbi Path

Viterbi Path

Posterior @ 0.4

Posterior @ 0.4

Posterior Graphic. Threshold Marked @ 0.4

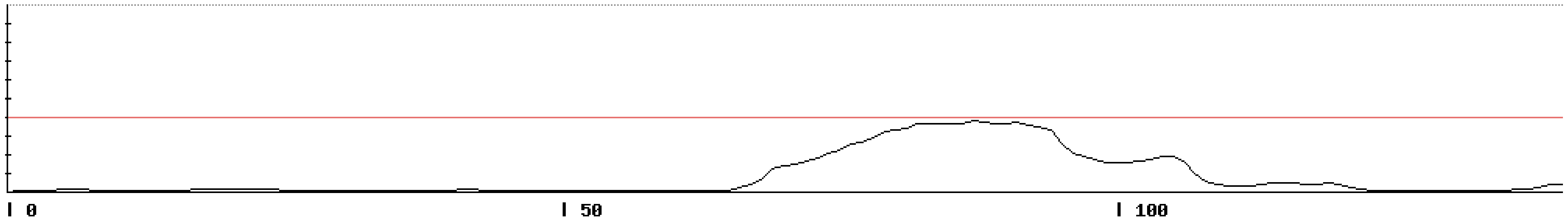

Predictions for Caricapapaya_evm.model.supercontig_21.173

Viterbi Path

Viterbi Path

Posterior @ 0.4

Posterior @ 0.4

Posterior Graphic. Threshold Marked @ 0.4

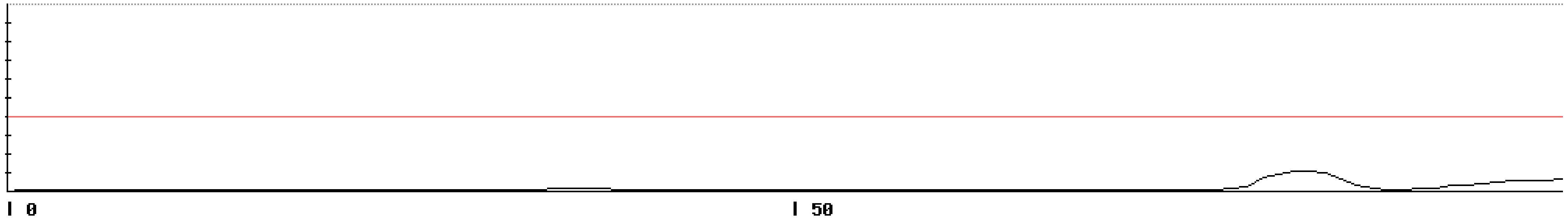

Predictions for Caricapapaya_evm.model.supercontig_244.7

Viterbi Path

Viterbi Path

Posterior @ 0.4	
Posterior @ 0.4	
Posterior Graphic. Threshold Marked @ 0.4	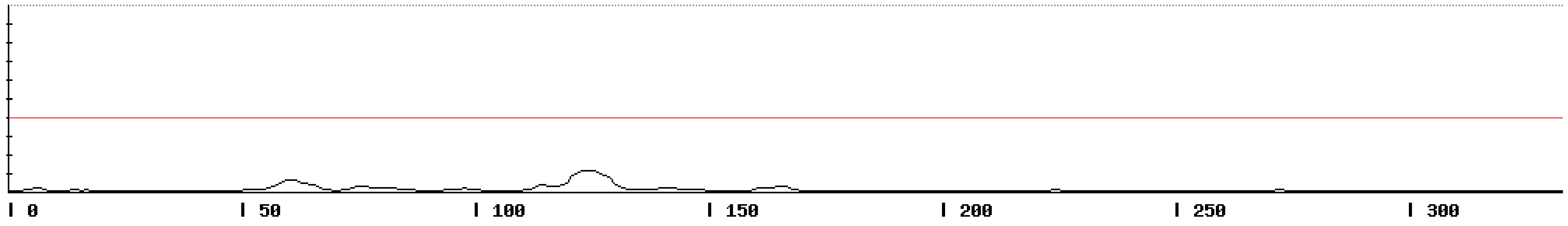

Predictions for Caricapapaya_evm.model.supercontig_2586.1

Viterbi Path	
Viterbi Path	
Posterior @ 0.4	135 - RKRRK - 139
Posterior @ 0.4	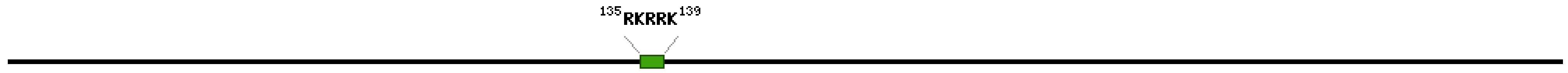
Posterior Graphic. Threshold Marked @ 0.4	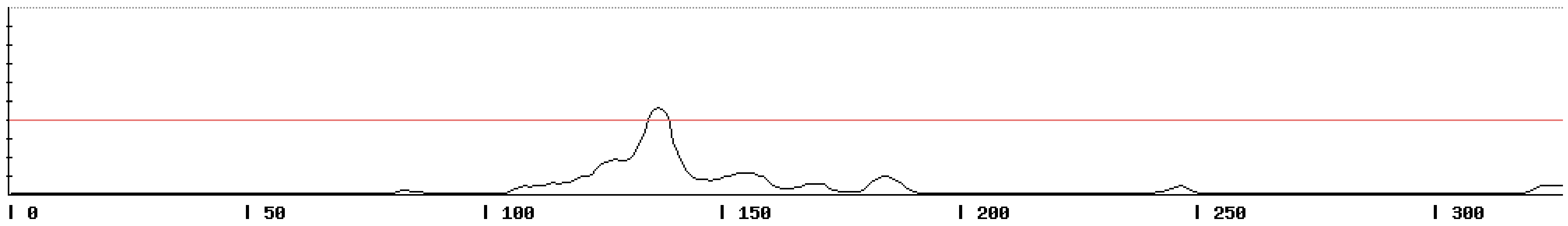

Predictions for Caricapapaya_evm.model.supercontig_26.135

Viterbi Path

Viterbi Path

Posterior @ 0.4

Posterior @ 0.4

Posterior Graphic. Threshold Marked @ 0.4

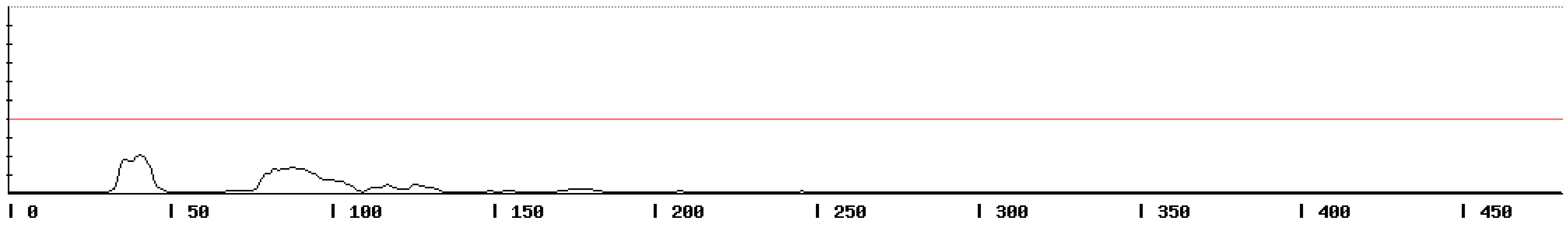

Predictions for Caricapapaya_evm.model.supercontig_27.81

Viterbi Path

Viterbi Path

Posterior @ 0.4

Posterior @ 0.4

Posterior Graphic. Threshold Marked @ 0.4

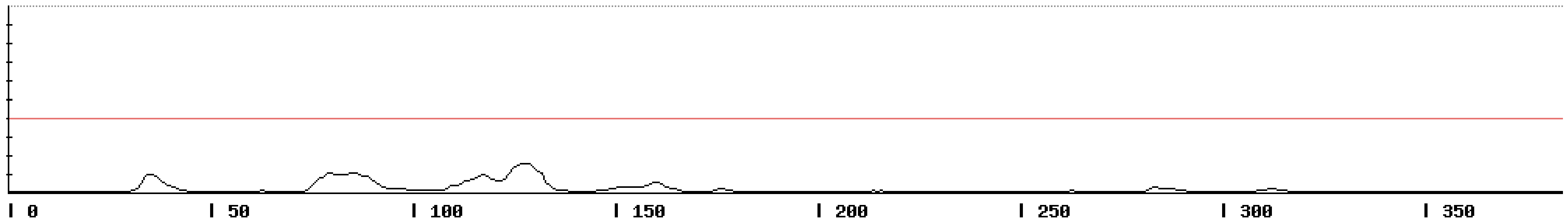

Predictions for Caricapapaya_evm.model.supercontig_286.12

Viterbi Path

Viterbi Path

Posterior @ 0.4

Posterior @ 0.4

Posterior Graphic. Threshold Marked @ 0.4

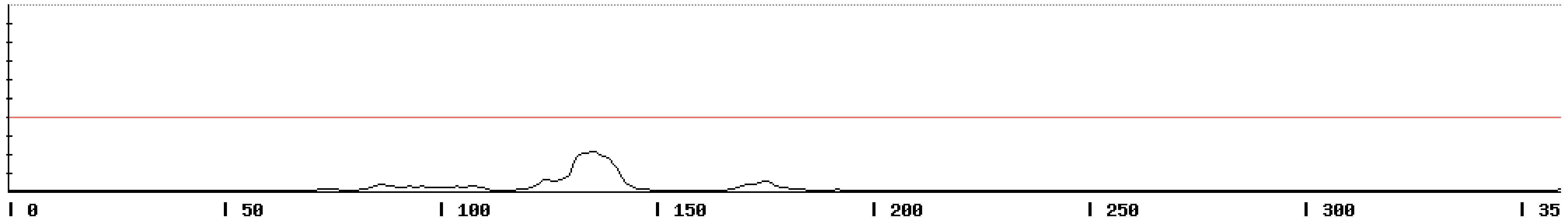

Predictions for Caricapapaya_evm.model.supercontig_29.118

Viterbi Path

Viterbi Path

Posterior @ 0.4

Posterior @ 0.4

Posterior Graphic. Threshold Marked @ 0.4

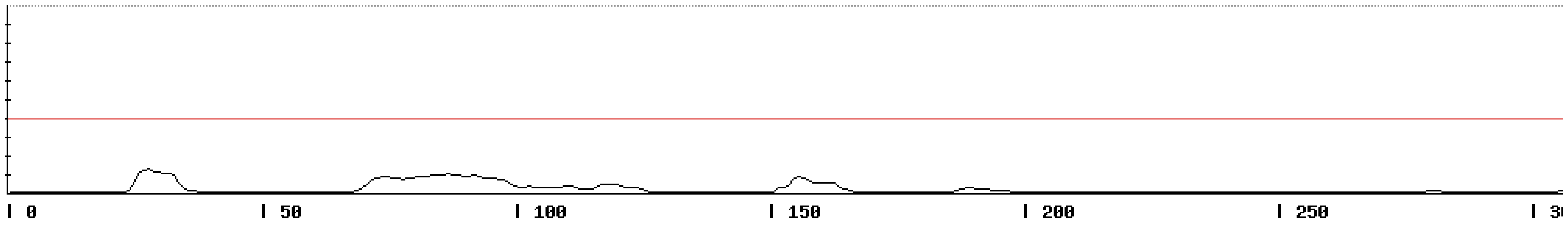

Predictions for Caricapapaya_evm.model.supercontig_3.170

Viterbi Path

Viterbi Path

Posterior @ 0.4

Posterior @ 0.4

Posterior Graphic. Threshold Marked @ 0.4

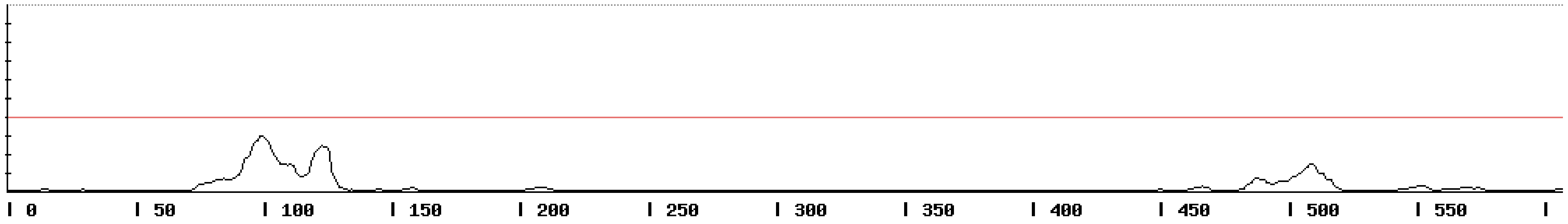

Predictions for Caricapapaya_evm.model.supercontig_320.2

Viterbi Path

Viterbi Path

Posterior @ 0.4

Posterior @ 0.4

Posterior Graphic. Threshold Marked @ 0.4	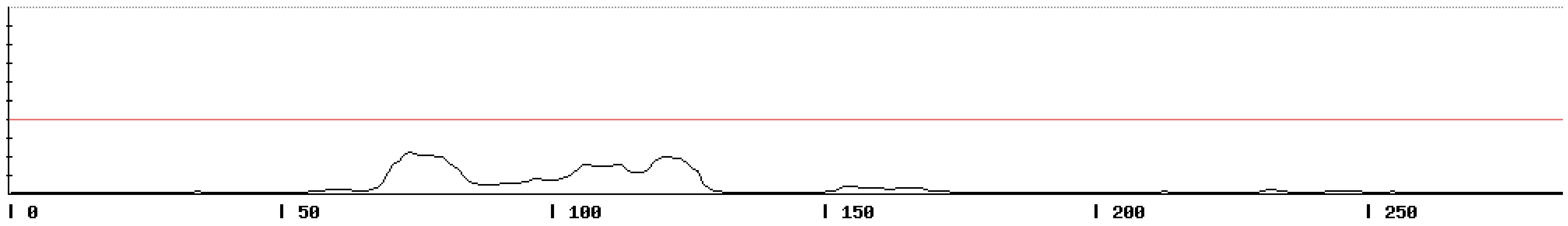

Predictions for Caricapapaya_evm.model.supercontig_3.311	
Viterbi Path	
Viterbi Path	
Posterior @ 0.4	
Posterior @ 0.4	
Posterior Graphic. Threshold Marked @ 0.4	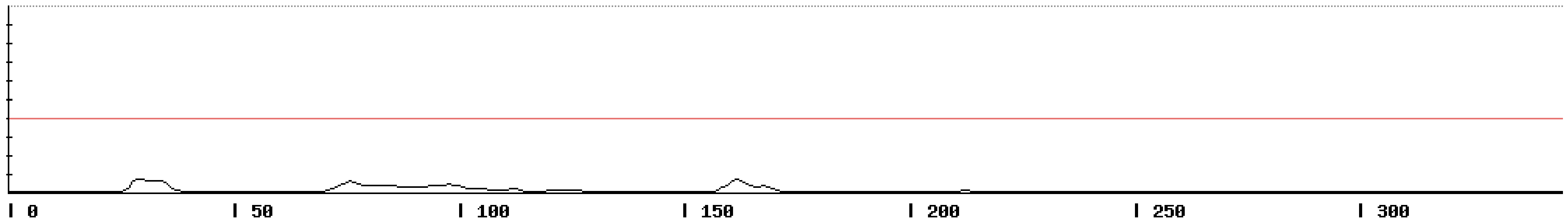

Predictions for Caricapapaya_evm.model.supercontig_33.206	
Viterbi Path	
Viterbi Path	
Posterior @ 0.4	
Posterior @ 0.4	

Posterior Graphic. Threshold Marked @ 0.4

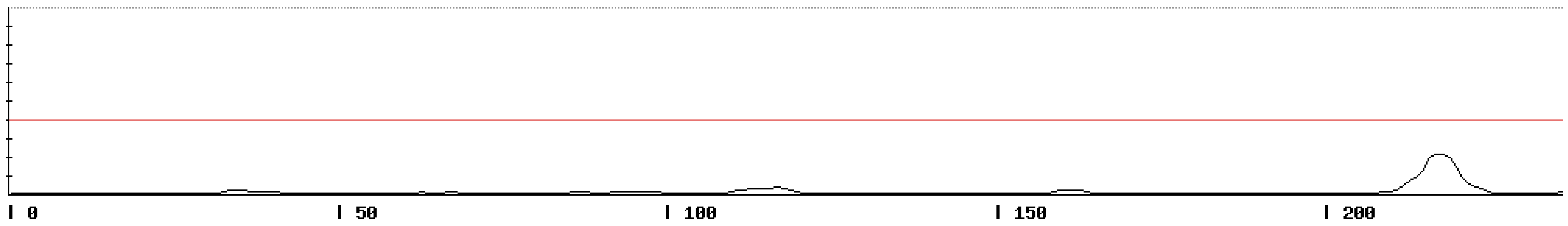

Predictions for Caricapapaya_evm.model.supercontig_33.207

Viterbi Path

Viterbi Path

Posterior @ 0.4

Posterior @ 0.4

Posterior Graphic. Threshold Marked @ 0.4

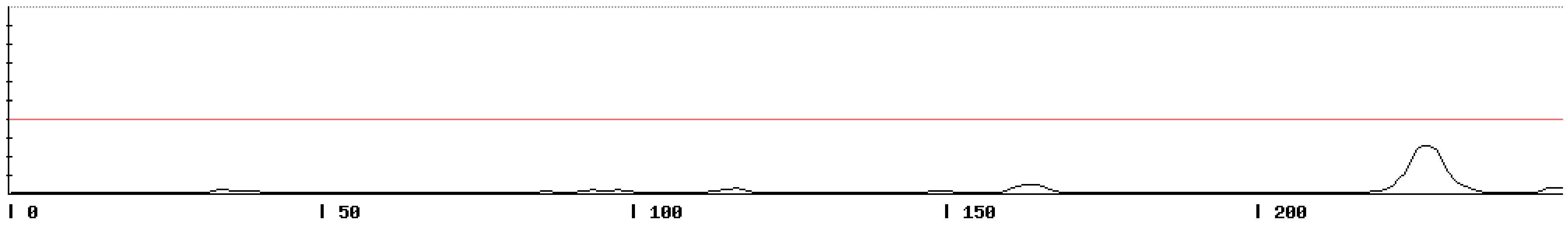

Predictions for Caricapapaya_evm.model.supercontig_3.509

Viterbi Path

Viterbi Path

Posterior @ 0.4

Posterior @ 0.4

Posterior Graphic. Threshold Marked @ 0.4

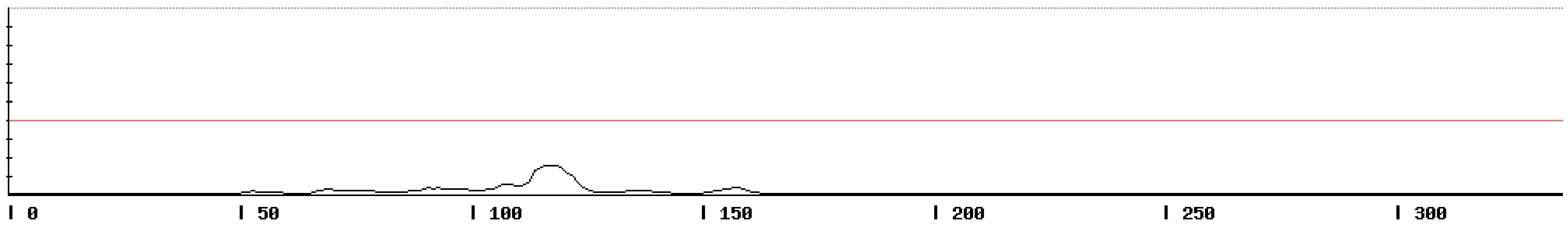

Predictions for Caricapapaya_evm.model.supercontig_37.112

Viterbi Path

Viterbi Path

Posterior @ 0.4

Posterior @ 0.4

Posterior Graphic. Threshold Marked @ 0.4

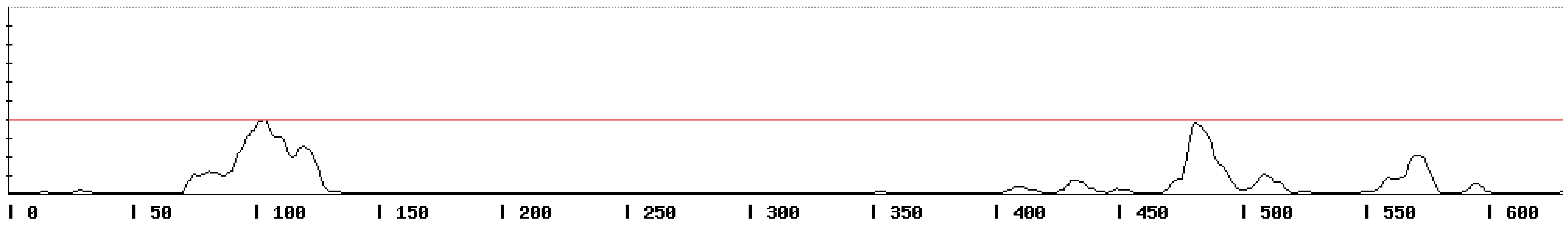

Predictions for Caricapapaya_evm.model.supercontig_37.28

Viterbi Path

Viterbi Path

Posterior @ 0.4	
Posterior @ 0.4	
Posterior Graphic. Threshold Marked @ 0.4	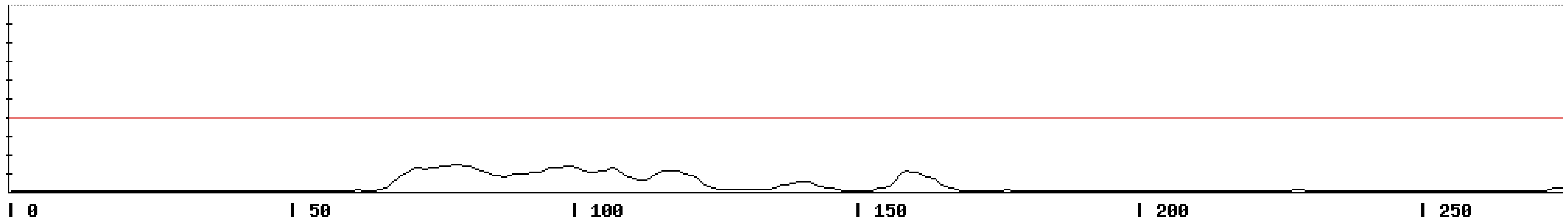

Predictions for Caricapapaya_evm.model.supercontig_37.89

Viterbi Path	
Viterbi Path	
Posterior @ 0.4	
Posterior @ 0.4	
Posterior Graphic. Threshold Marked @ 0.4	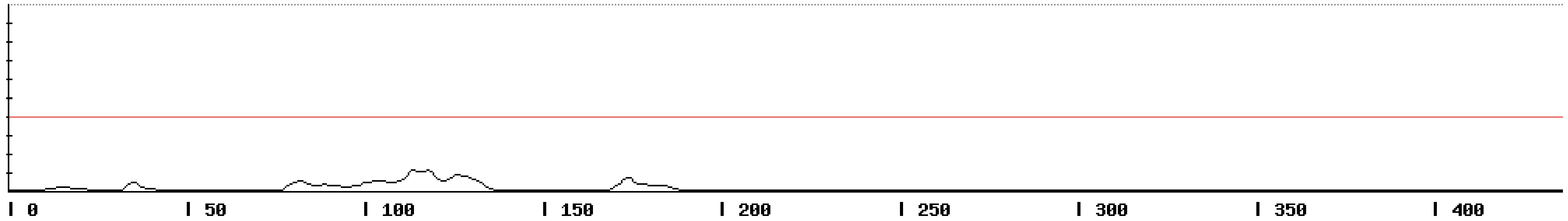

Predictions for Caricapapaya_evm.model.supercontig_38.43

Viterbi Path

Viterbi Path

Posterior @ 0.4

Posterior @ 0.4

Posterior Graphic. Threshold Marked @ 0.4

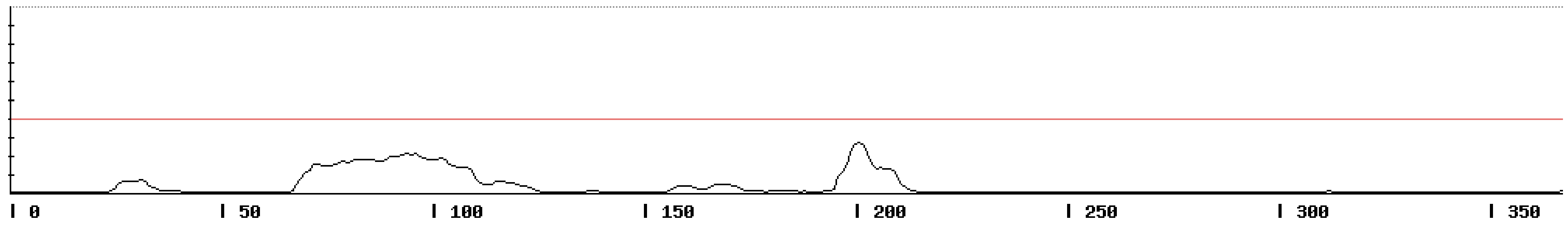

Predictions for Caricapapaya_evm.model.supercontig_4056.1

Viterbi Path

Viterbi Path

Posterior @ 0.4

Posterior @ 0.4

Posterior Graphic. Threshold Marked @ 0.4

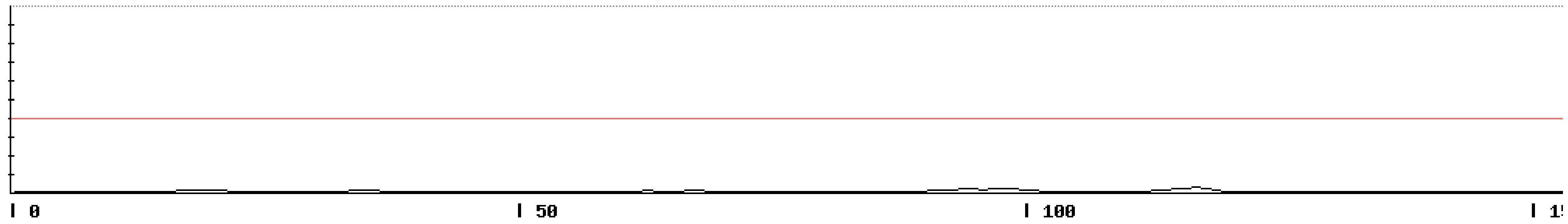

Predictions for Caricapapaya_evm.model.supercontig_4.138	
Viterbi Path	
Viterbi Path	
Posterior @ 0.4	
Posterior @ 0.4	
Posterior Graphic. Threshold Marked @ 0.4	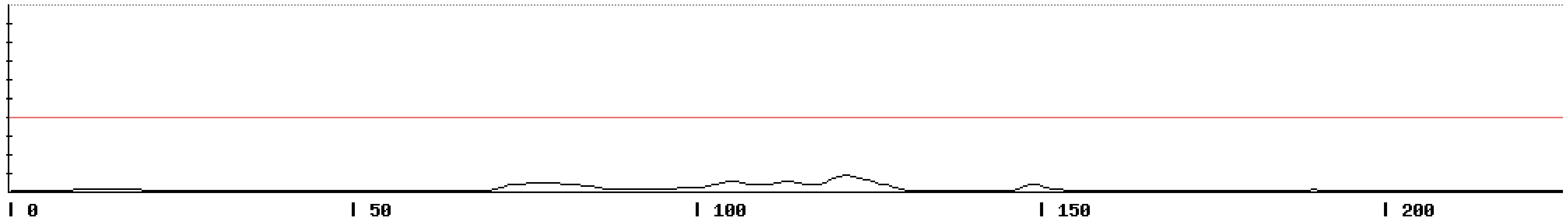

Predictions for Caricapapaya_evm.model.supercontig_42.100	
Viterbi Path	
Viterbi Path	
Posterior @ 0.4	37 - KKKKKN - 42
Posterior @ 0.4	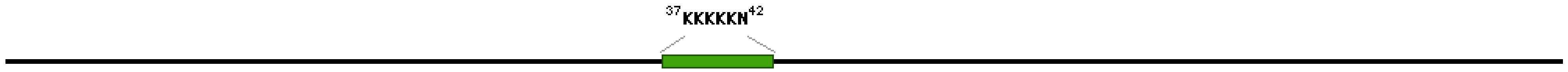
Posterior Graphic. Threshold Marked @ 0.4	

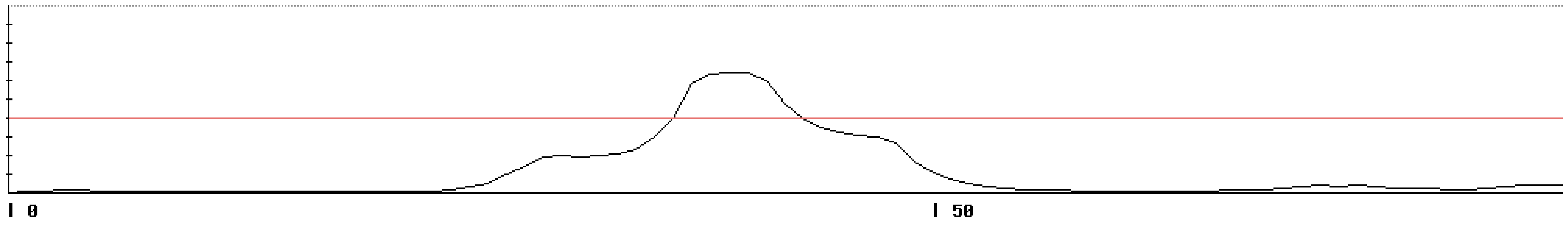

Predictions for Caricapapaya_evm.model.supercontig_42.145

Viterbi Path

Viterbi Path

Posterior @ 0.4

Posterior @ 0.4

Posterior Graphic. Threshold Marked @ 0.4

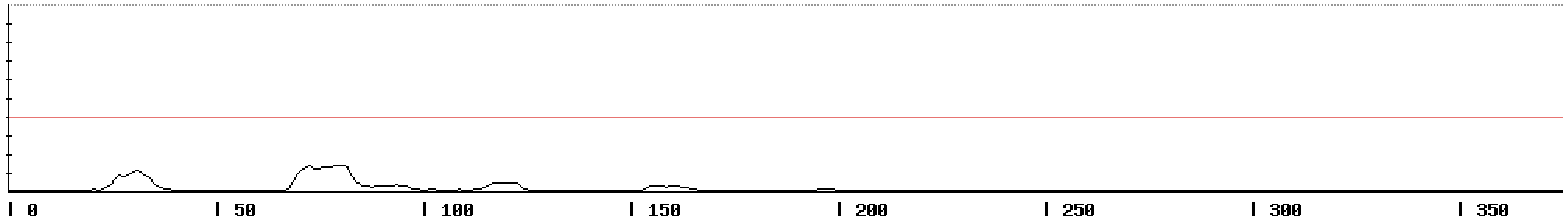

Predictions for Caricapapaya_evm.model.supercontig_42.167

Viterbi Path

Viterbi Path

Posterior @ 0.4

Posterior @ 0.4

Posterior Graphic. Threshold Marked @ 0.4

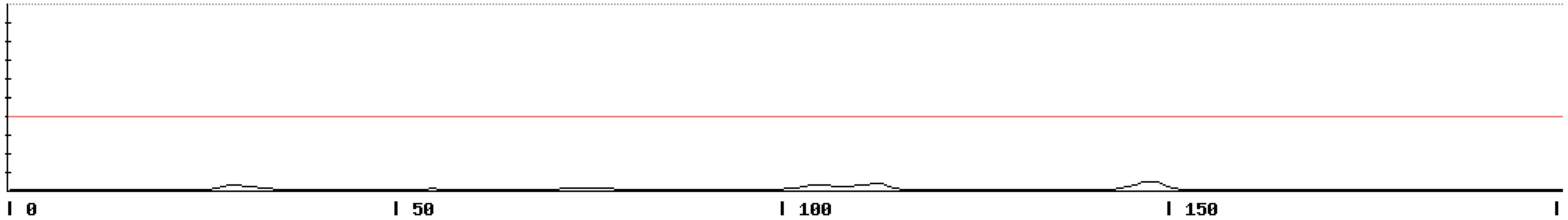

Predictions for Caricapapaya_evm.model.supercontig_435.1

Viterbi Path	
Viterbi Path	
Posterior @ 0.4	
Posterior @ 0.4	
Posterior Graphic. Threshold Marked @ 0.4	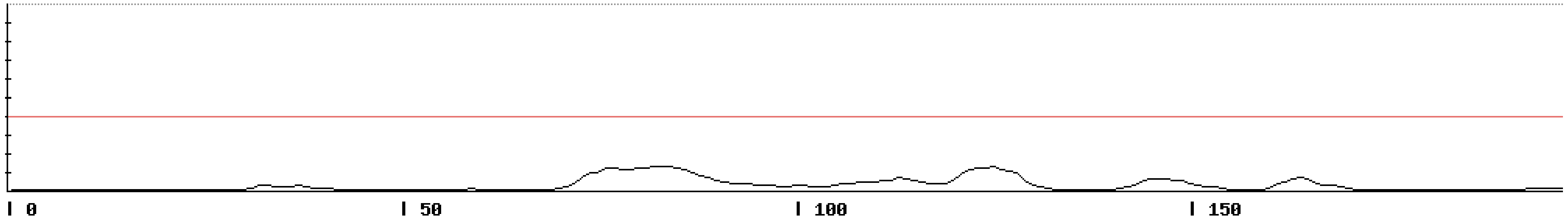

Predictions for Caricapapaya_evm.model.supercontig_53.116

Viterbi Path	
Viterbi Path	
Posterior @ 0.4	148 - RKRRK - 152
Posterior @ 0.4	

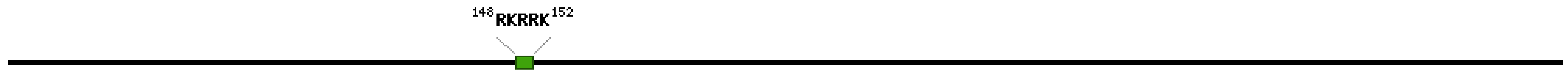

Posterior Graphic. Threshold Marked @ 0.4

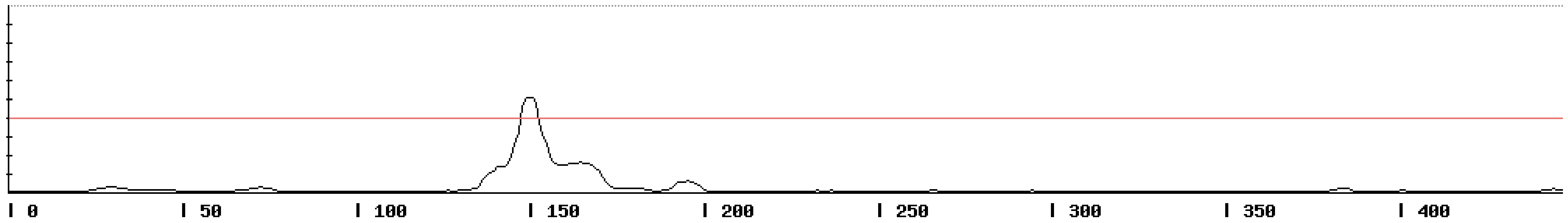

Predictions for Caricapapaya_evm.model.supercontig_53.164

Viterbi Path

Viterbi Path

Posterior @ 0.4

Posterior @ 0.4

Posterior Graphic. Threshold Marked @ 0.4

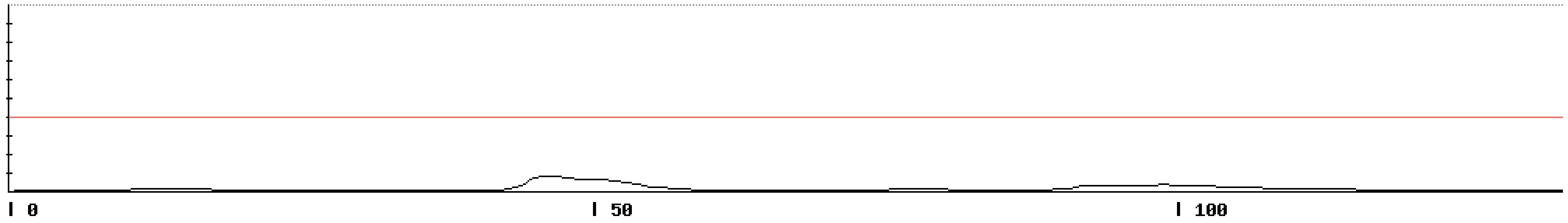

Predictions for Caricapapaya_evm.model.supercontig_566.2

Viterbi Path

Viterbi Path

Posterior @ 0.4	19 - AKRQKVQKWRSPKS - 32
Posterior @ 0.4	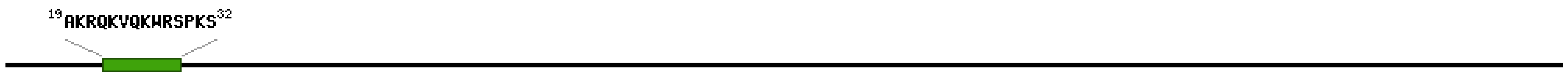
Posterior Graphic. Threshold Marked @ 0.4	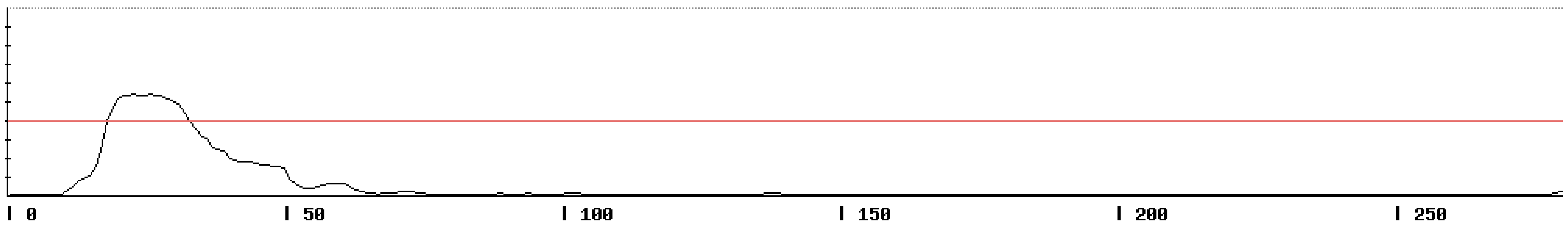

Predictions for Caricapapaya_evm.model.supercontig_566.3	
Viterbi Path	
Viterbi Path	
Posterior @ 0.4	
Posterior @ 0.4	
Posterior Graphic. Threshold Marked @ 0.4	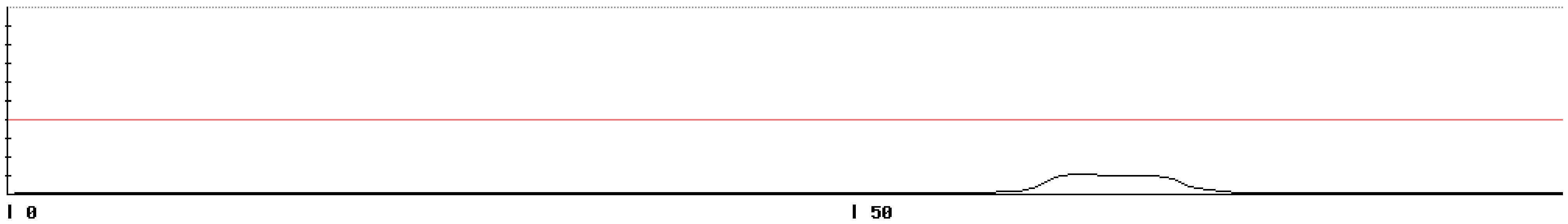

Predictions for Caricapapaya_evm.model.supercontig_57.70	
Viterbi Path	
Viterbi Path	

Posterior @ 0.4

Posterior @ 0.4

Posterior Graphic. Threshold Marked @ 0.4

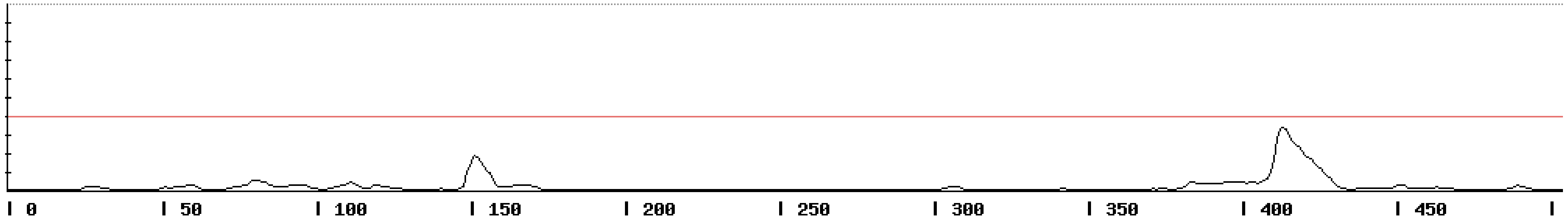

Predictions for Caricapapaya_evm.model.supercontig_594.1

Viterbi Path

Viterbi Path

Posterior @ 0.4

Posterior @ 0.4

Posterior Graphic. Threshold Marked @ 0.4

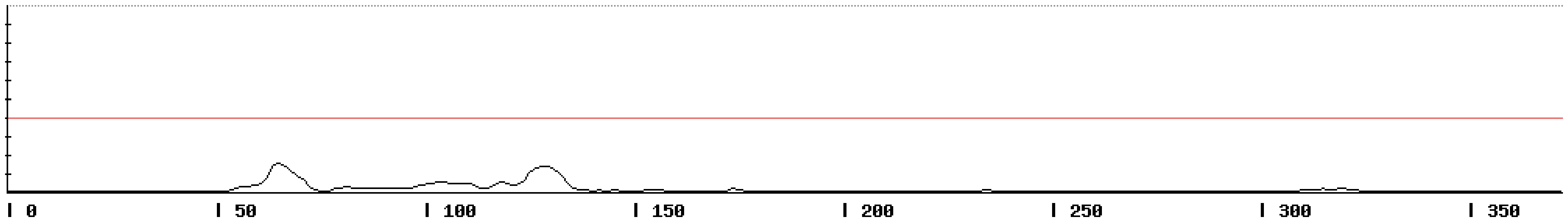

Predictions for Caricapapaya_evm.model.supercontig_6.226

Viterbi Path

Viterbi Path

Posterior @ 0.4

Posterior @ 0.4

Posterior Graphic. Threshold Marked @ 0.4

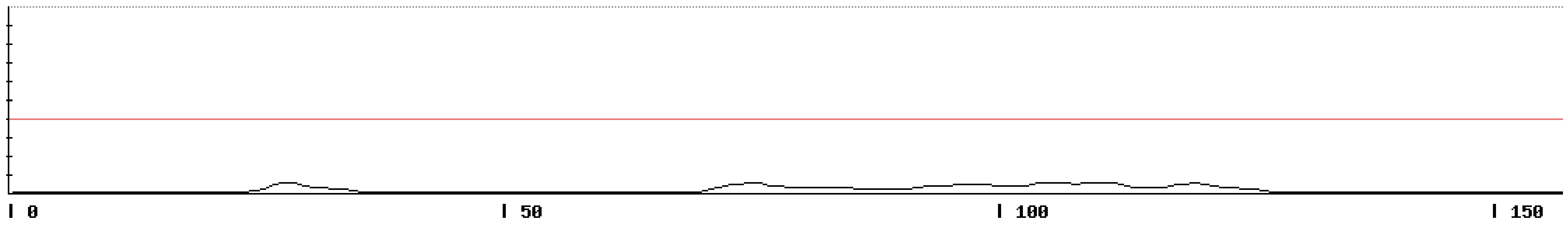

Predictions for Caricapapaya_evm.model.supercontig_63.29

Viterbi Path

Viterbi Path

Posterior @ 0.4

Posterior @ 0.4

Posterior Graphic. Threshold Marked @ 0.4

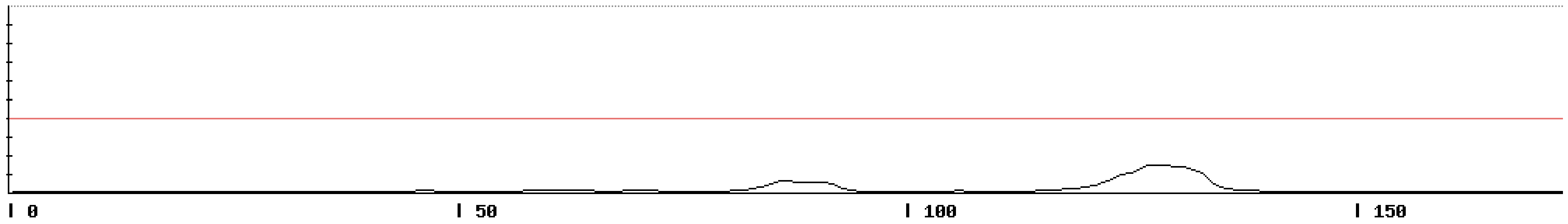

Predictions for Caricapapaya_evm.model.supercontig_64.115

Viterbi Path	
Viterbi Path	
Posterior @ 0.4	
Posterior @ 0.4	
Posterior Graphic. Threshold Marked @ 0.4	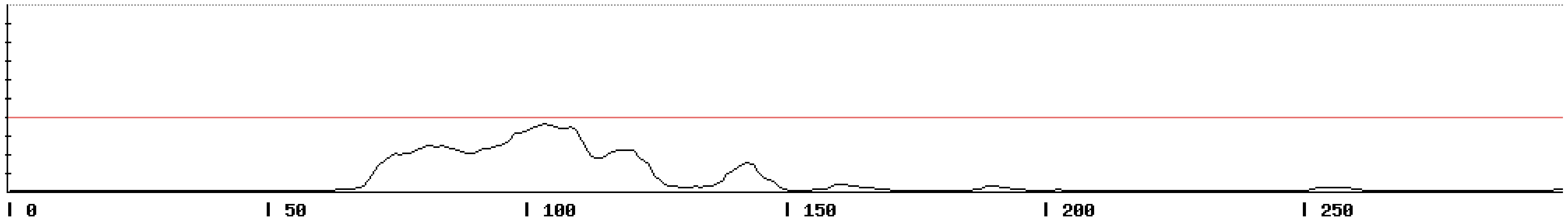

Predictions for Caricapapaya_evm.model.supercontig_64.53

Viterbi Path	
Viterbi Path	
Posterior @ 0.4	
Posterior @ 0.4	
Posterior Graphic. Threshold Marked @ 0.4	

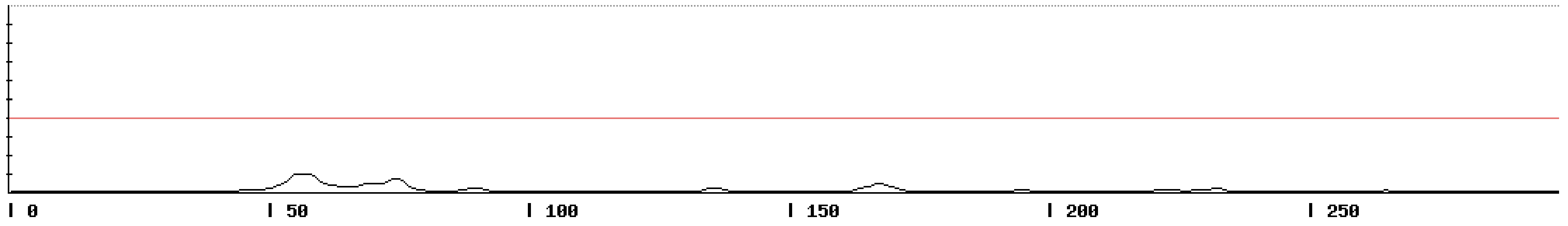

Predictions for Caricapapaya_evm.model.supercontig_67.60

Viterbi Path	
Viterbi Path	
Posterior @ 0.4	
Posterior @ 0.4	
Posterior Graphic. Threshold Marked @ 0.4	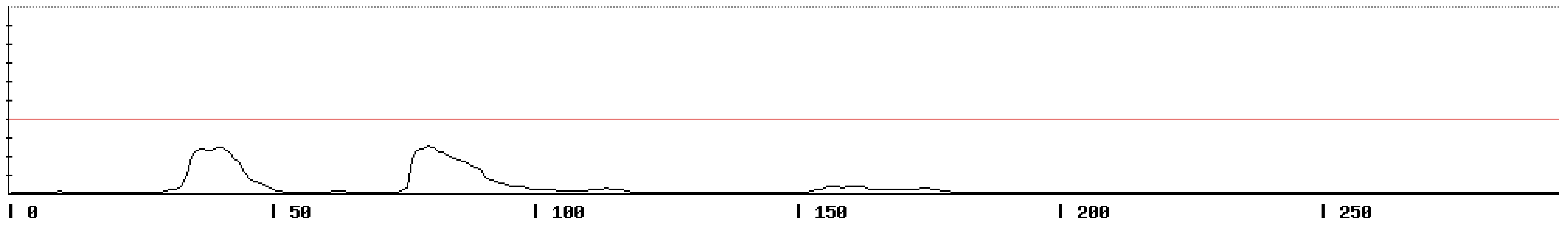

Predictions for Caricapapaya_evm.model.supercontig_70.104

Viterbi Path	
Viterbi Path	
Posterior @ 0.4	119 - SKKKKKKTSH - 128
	170 - RKRKARRS - 177
Posterior @ 0.4	

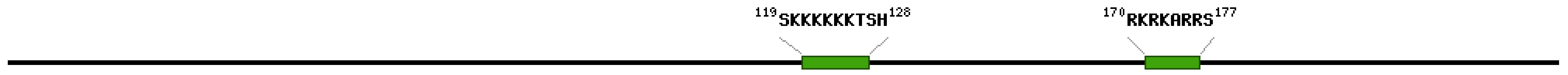

Posterior Graphic. Threshold Marked @ 0.4

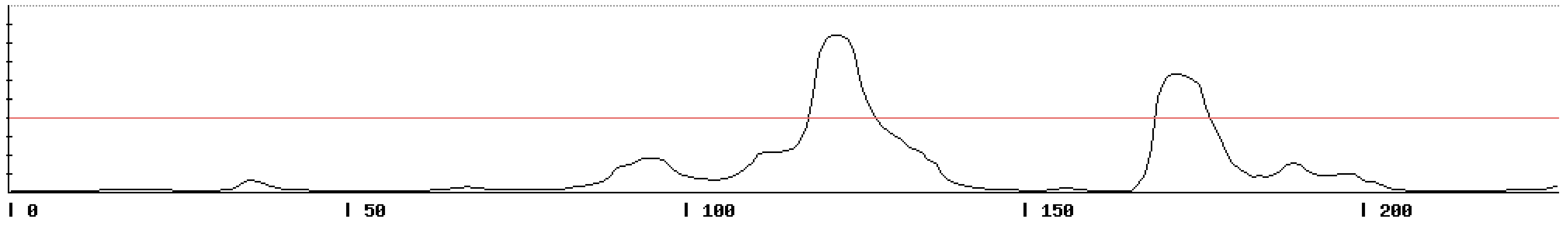

Predictions for Caricapapaya_evm.model.supercontig_78.40

Viterbi Path

Viterbi Path

Posterior @ 0.4

Posterior @ 0.4

Posterior Graphic. Threshold Marked @ 0.4

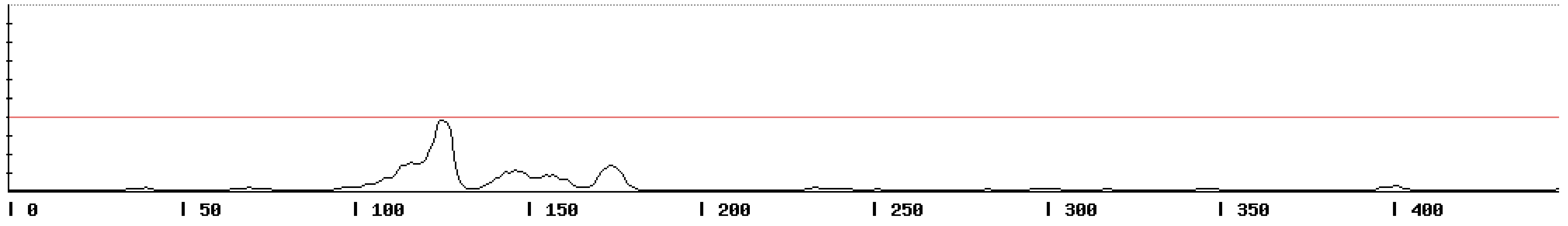

Predictions for Caricapapaya_evm.model.supercontig_80.14

Viterbi Path 133 - KKGVREREIRRARERERERERKKRKKDENKRIRSR - 167

Viterbi Path

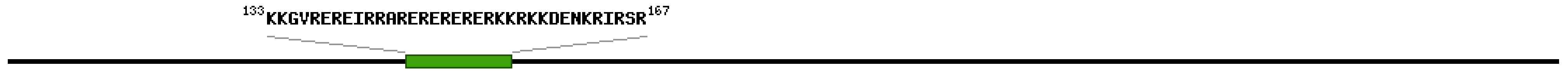

Posterior @ 0.4	133 - KKGVREREIRRARERERERERKKRKKDENKRIRSR - 167
	242 - K - 242
Posterior @ 0.4	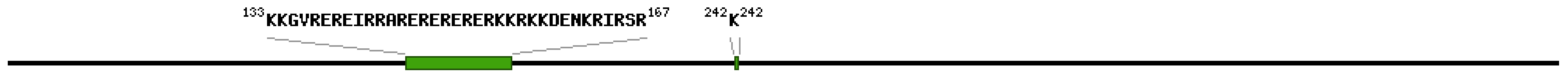
Posterior Graphic. Threshold Marked @ 0.4	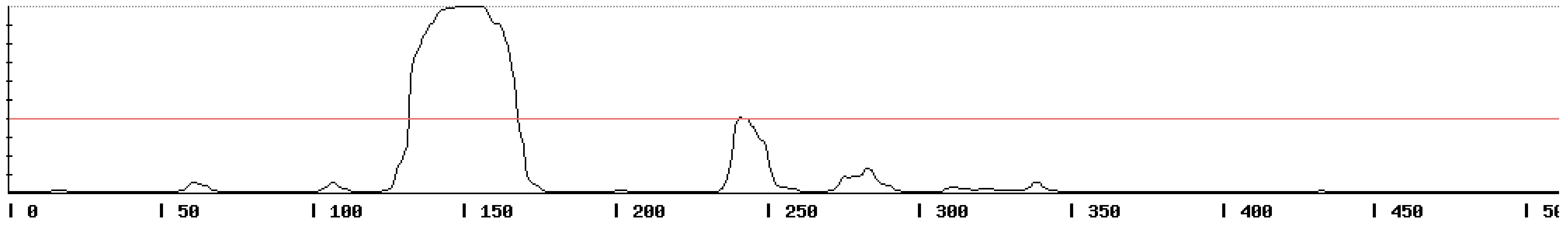

Predictions for Caricapapaya_evm.model.supercontig_80.18

Viterbi Path	
Viterbi Path	
Posterior @ 0.4	65 - K - 65
Posterior @ 0.4	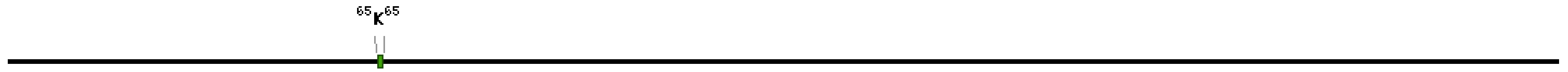
Posterior Graphic. Threshold Marked @ 0.4	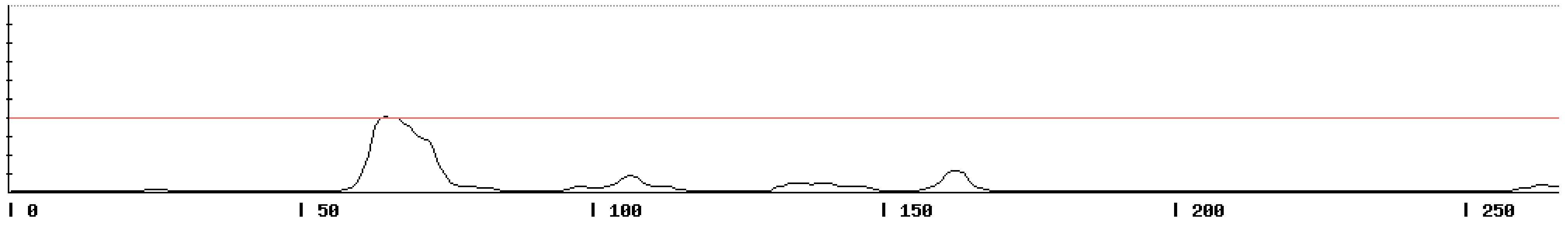

Predictions for Caricapapaya_evm.model.supercontig_80.19

Viterbi Path	327 - RRREAAGGGRRRRRKNVLREKK - 348
Viterbi Path	

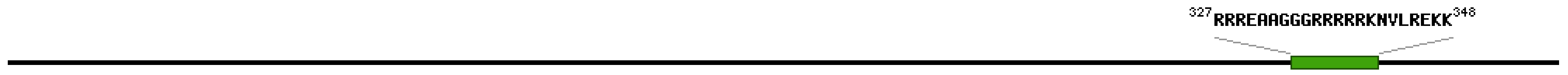

Posterior @ 0.4

65 - K - 65

327 - RRREAAGGGRRRRRKNVLREKKTDR - 351

Posterior @ 0.4

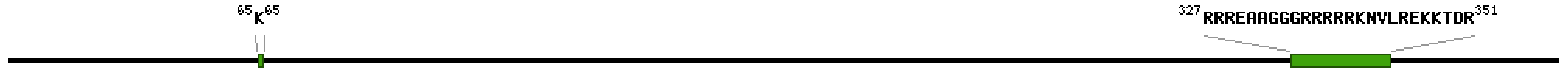

Posterior Graphic. Threshold Marked @ 0.4

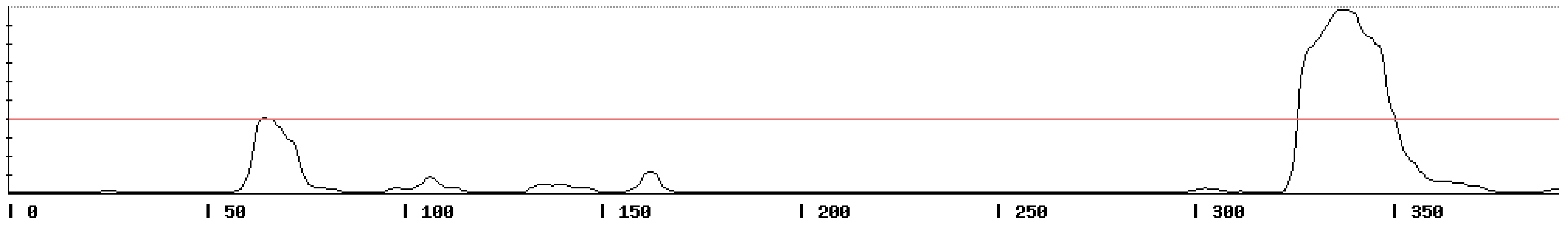

Predictions for Caricapapaya_evm.model.supercontig_80.20

Viterbi Path

Viterbi Path

Posterior @ 0.4

63 - KKKSRSR - 69

Posterior @ 0.4

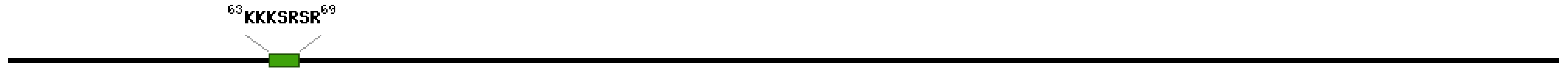

Posterior Graphic. Threshold Marked @ 0.4

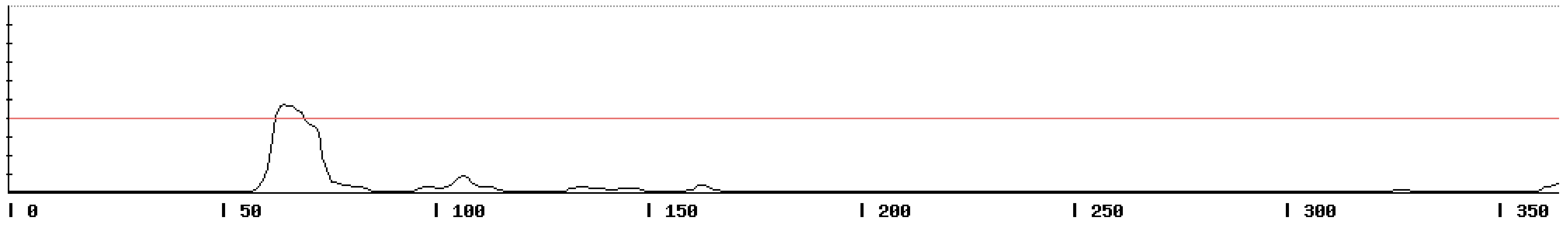

Predictions for Caricapapaya_evm.model.supercontig_80.21

Viterbi Path	
Viterbi Path	
Posterior @ 0.4	65 - K - 65
Posterior @ 0.4	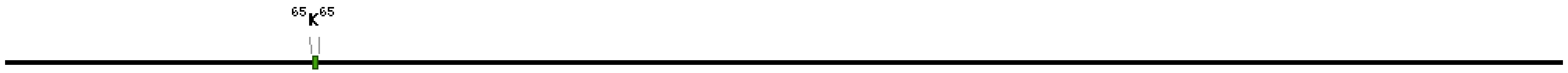
Posterior Graphic. Threshold Marked @ 0.4	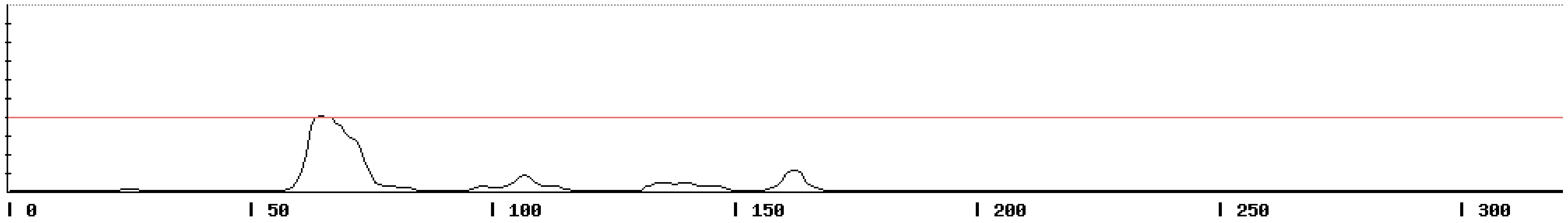

Predictions for Caricapapaya_evm.model.supercontig_80.23

Viterbi Path	
Viterbi Path	
Posterior @ 0.4	63 - KKKSRSR - 69
Posterior @ 0.4	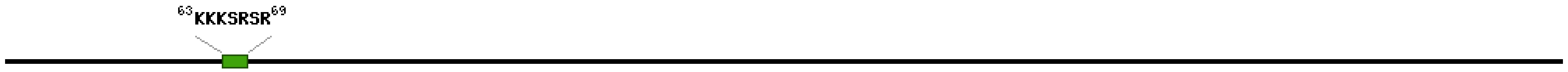
Posterior Graphic. Threshold Marked @ 0.4	

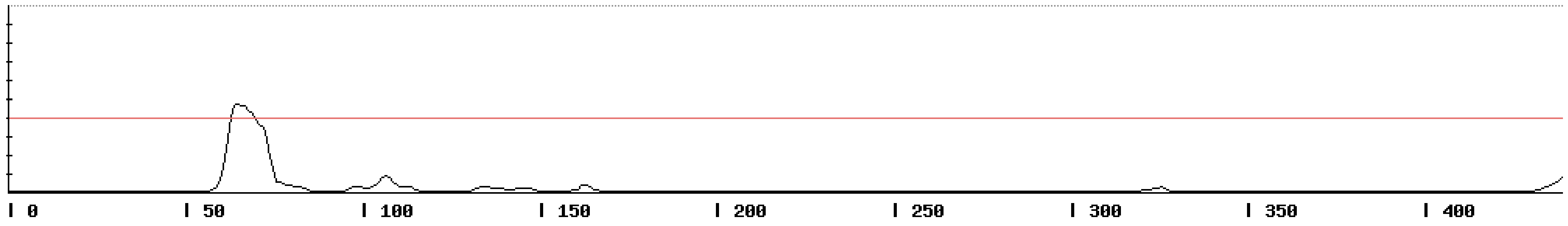

Predictions for Caricapapaya_evm.model.supercontig_80.24

Viterbi Path	327 - RRREAAGGGRRRRRKNVLREKK - 348
Viterbi Path	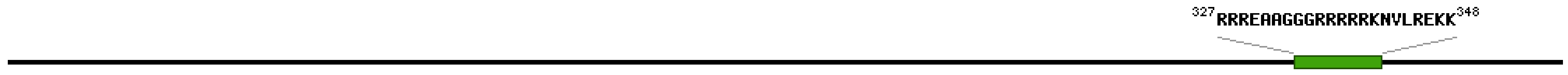
Posterior @ 0.4	65 - K - 65<br>327 - RRREAAGGGRRRRRKNVLREKKTDR - 351
Posterior @ 0.4	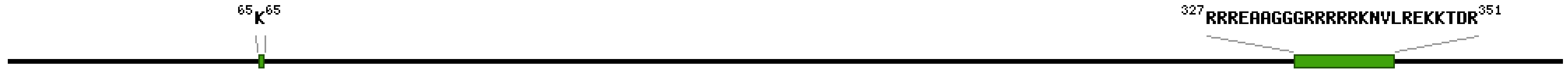
Posterior Graphic. Threshold Marked @ 0.4	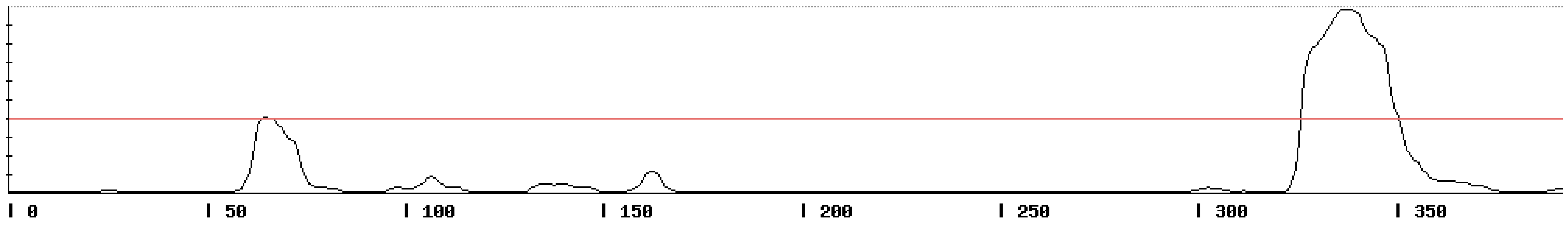

Predictions for Caricapapaya_evm.model.supercontig_80.93

Viterbi Path	
Viterbi Path	
Posterior @ 0.4	
Posterior @ 0.4	

Posterior Graphic. Threshold Marked @ 0.4

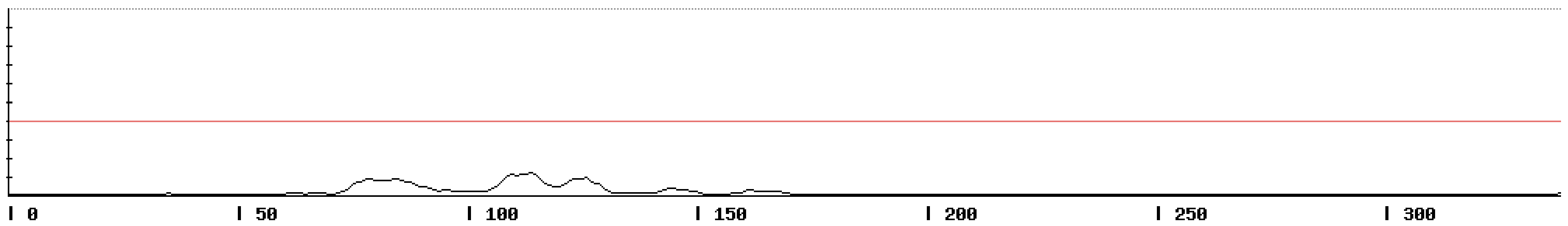

Predictions for Caricapapaya_evm.model.supercontig_80.94

Viterbi Path

Viterbi Path

Posterior @ 0.4

Posterior @ 0.4

Posterior Graphic. Threshold Marked @ 0.4

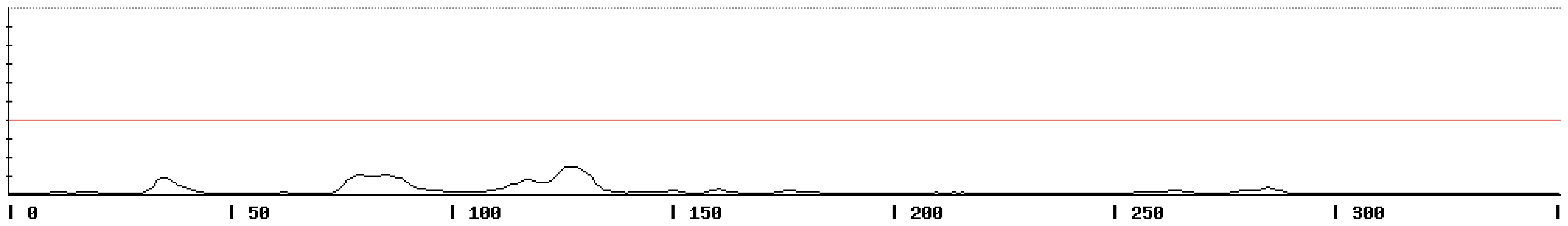

Predictions for Caricapapaya_evm.model.supercontig_83.99

Viterbi Path

Viterbi Path

Posterior @ 0.4 146 - GTRKRRKVHSDSEGGETRWHKTGKTRPVFIGGKAKGYK - 183

Posterior @ 0.4

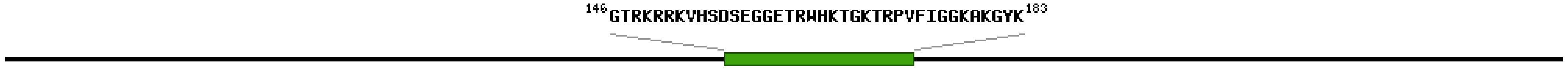

Posterior Graphic. Threshold Marked @ 0.4

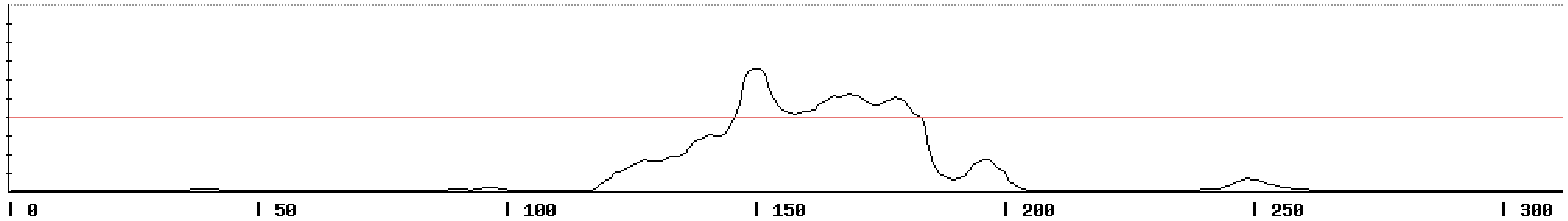

Predictions for Caricapapaya_evm.model.supercontig_84.41

Viterbi Path

Viterbi Path

Posterior @ 0.4 112 - RDKKYPKGNRPSRKAG - 127

Posterior @ 0.4

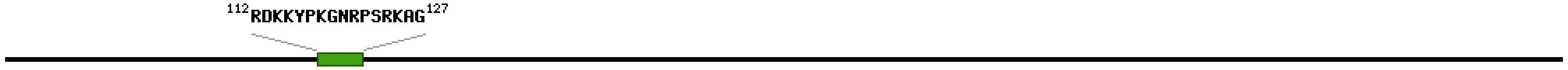

Posterior Graphic. Threshold Marked @ 0.4

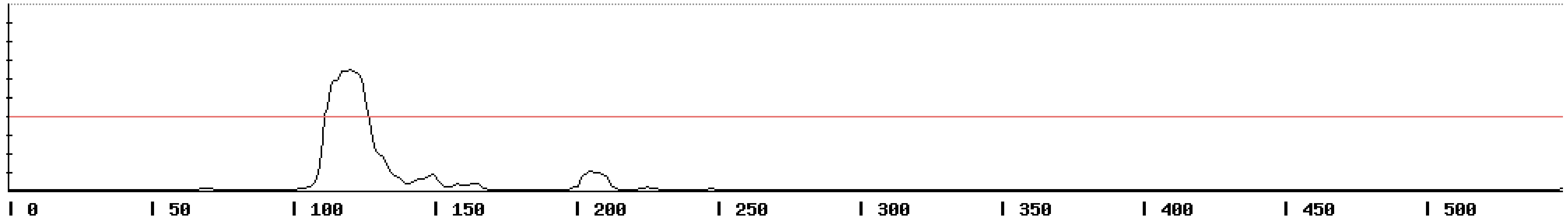

Predictions for Caricapapaya_evm.model.supercontig_90.52

Viterbi Path

Viterbi Path

Posterior @ 0.4

Posterior @ 0.4

Posterior Graphic. Threshold Marked @ 0.4

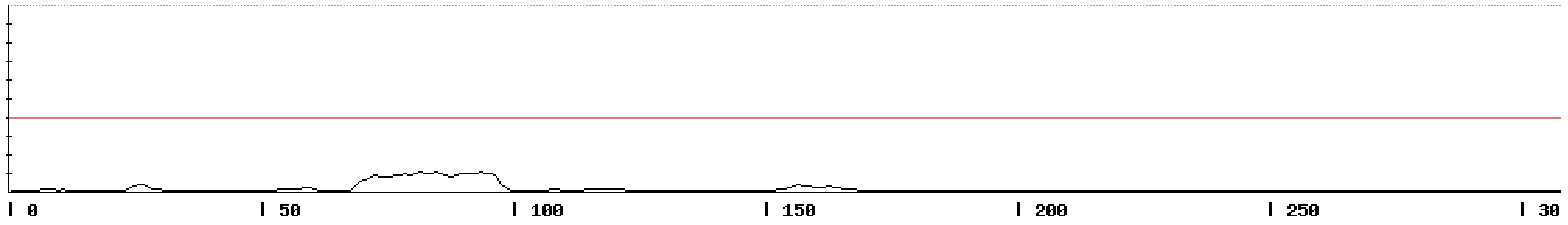

Predictions for Caricapapaya_evm.model.supercontig_94.23

Viterbi Path

Viterbi Path

Posterior @ 0.4

Posterior @ 0.4

Posterior Graphic. Threshold Marked @ 0.4

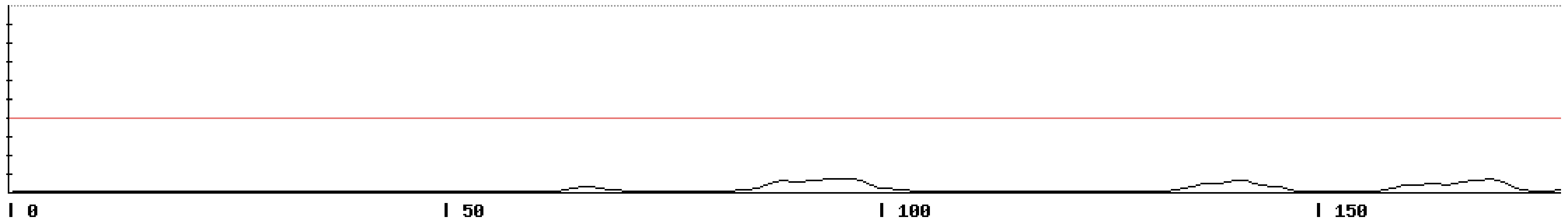

Predictions for Caricapapaya_evm.model.supercontig_94.54

Viterbi Path	
Viterbi Path	
Posterior @ 0.4	
Posterior @ 0.4	
Posterior Graphic. Threshold Marked @ 0.4	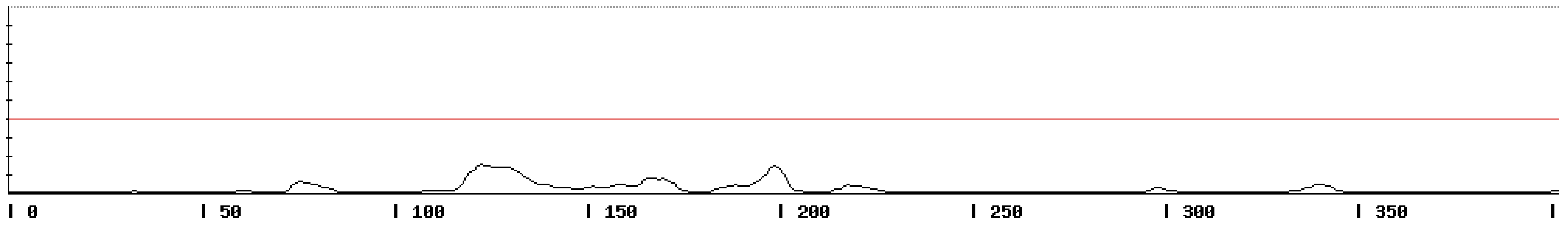

Predictions for Caricapapaya_evm.model.supercontig_99.63

Viterbi Path	
Viterbi Path	
Posterior @ 0.4	
Posterior @ 0.4	
Posterior Graphic. Threshold Marked @ 0.4	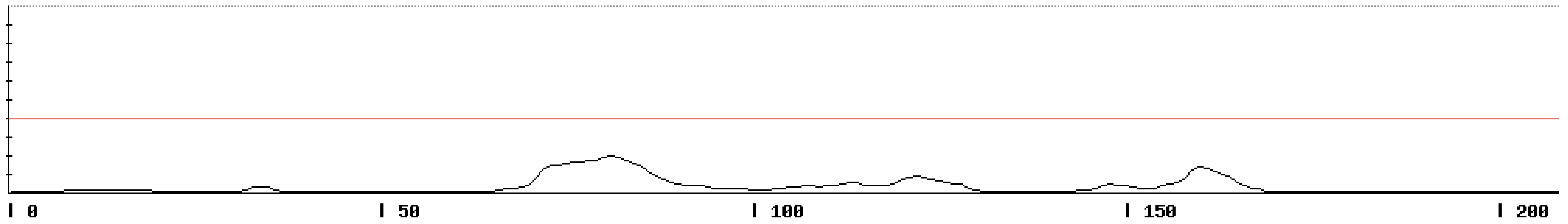

Predictions for Caricapapaya_evm.TU.contig_29561.1

Viterbi Path	
Viterbi Path	
Posterior @ 0.4	
Posterior @ 0.4	
Posterior Graphic. Threshold Marked @ 0.4	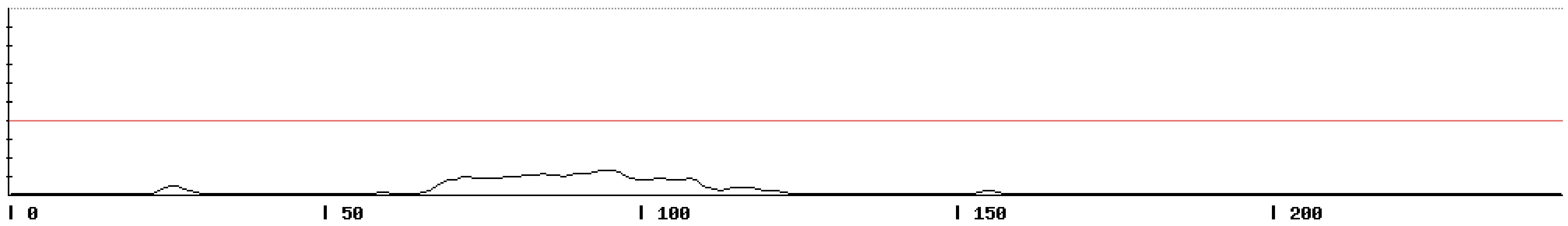

Predictions for Caricapapaya_evm.TU.contig_30558.1

Viterbi Path	
Viterbi Path	
Posterior @ 0.4	
Posterior @ 0.4	
Posterior Graphic. Threshold Marked @ 0.4	

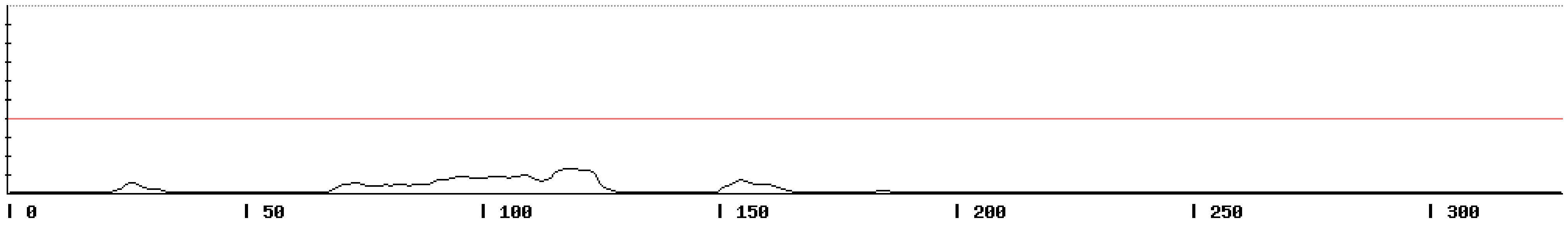

Predictions for Caricapapaya_evm.TU.contig_33298.1	
Viterbi Path	
Viterbi Path	
Posterior @ 0.4	
Posterior @ 0.4	
Posterior Graphic. Threshold Marked @ 0.4	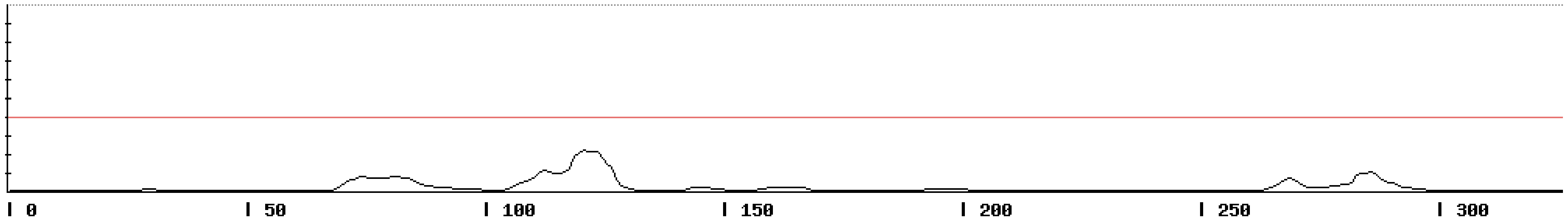

Predictions for Caricapapaya_evm.TU.contig_37010.1	
Viterbi Path	
Viterbi Path	
Posterior @ 0.4	
Posterior @ 0.4	

Viterbi Path	
Posterior @ 0.4	
Posterior @ 0.4	
Posterior Graphic. Threshold Marked @ 0.4	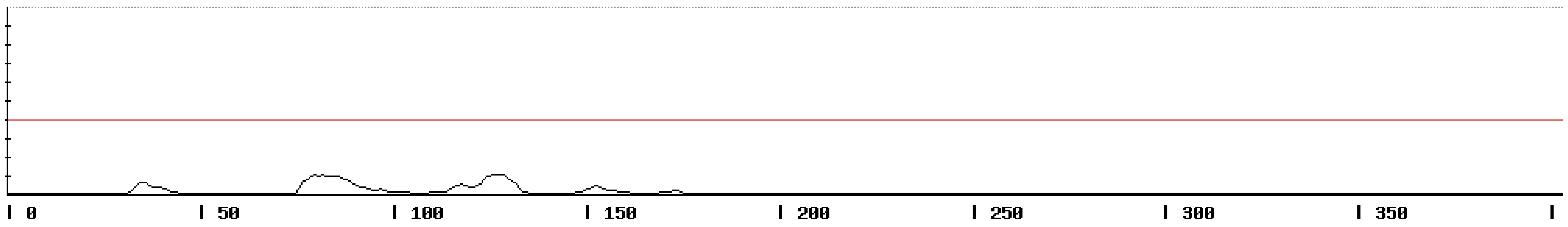

Predictions for Castaneamollissima_scaffold00498-abinit-gene-0.4d00498

Viterbi Path	
Viterbi Path	
Posterior @ 0.4	
Posterior @ 0.4	
Posterior Graphic. Threshold Marked @ 0.4	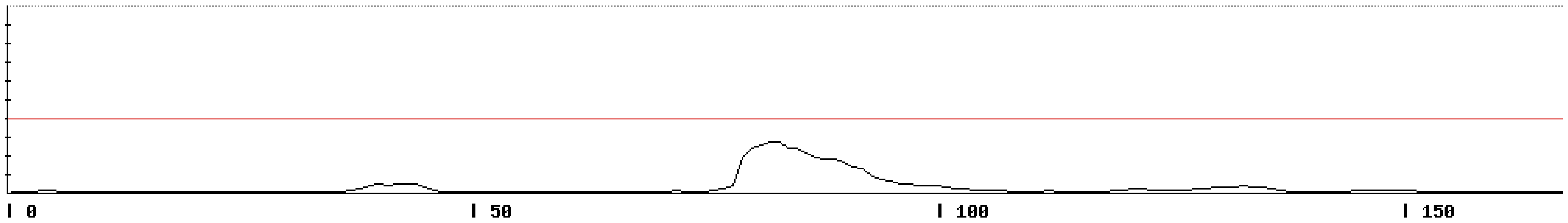

Predictions for Castaneamollissima_scaffold00534-abinit-gene-0.10d00534

Viterbi Path

Viterbi Path

Posterior @ 0.4

Posterior @ 0.4

Posterior Graphic. Threshold Marked @ 0.4

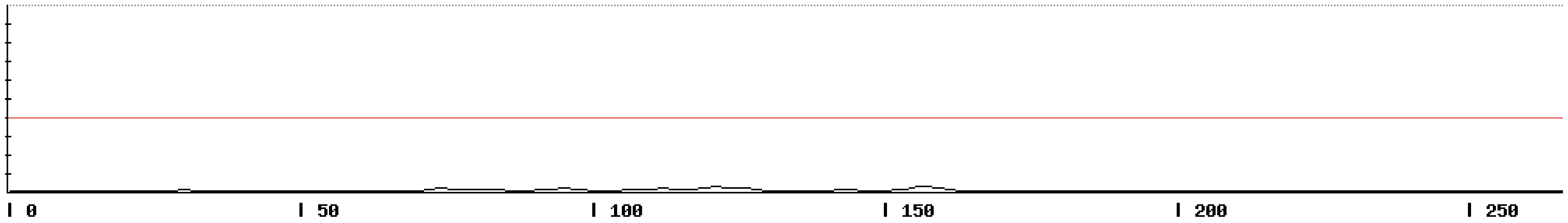

Predictions for Castaneamollissima_scaffold00906-abinit-gene-0.7d00906

Viterbi Path

Viterbi Path

Posterior @ 0.4

Posterior @ 0.4

Posterior Graphic. Threshold Marked @ 0.4

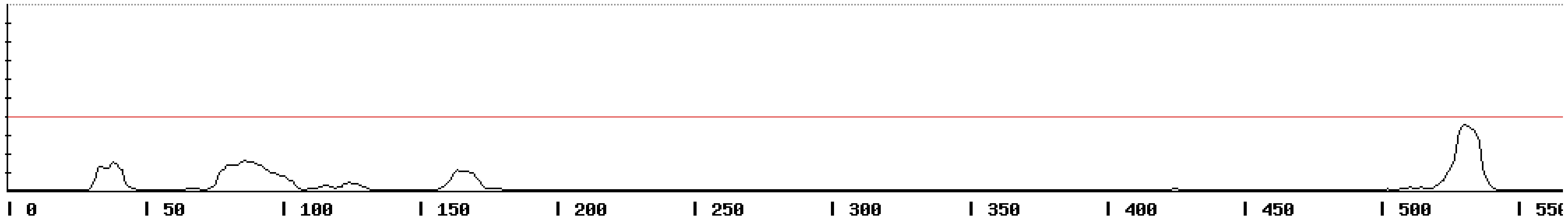

Predictions for Castaneamollissima_scaffold01826-abinit-gene-0.0d01826	
Viterbi Path	
Viterbi Path	
Posterior @ 0.4	126 - RKRRK - 130
Posterior @ 0.4	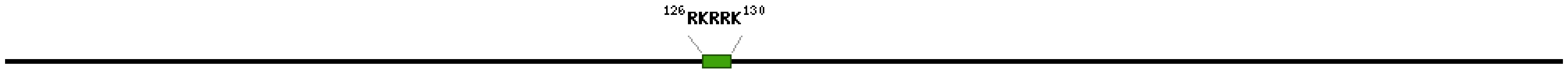
Posterior Graphic. Threshold Marked @ 0.4	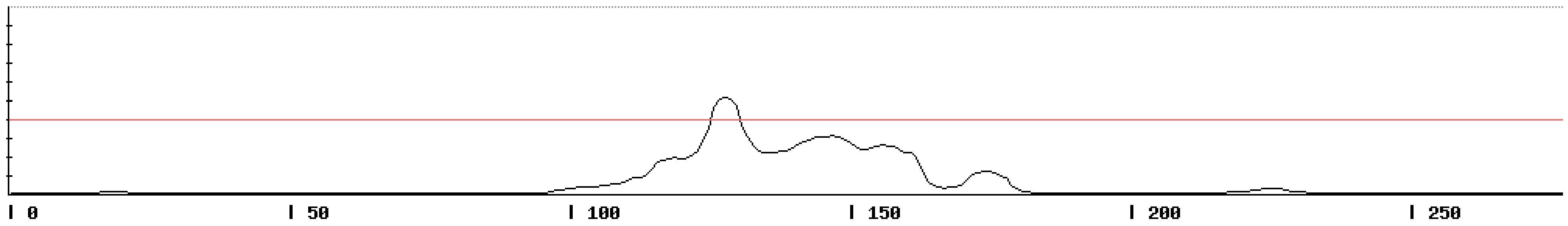

Predictions for Castaneamollissima_scaffold02790-abinit-gene-0.8d02790	
Viterbi Path	
Viterbi Path	
Posterior @ 0.4	
Posterior @ 0.4	
Posterior Graphic. Threshold Marked @ 0.4	

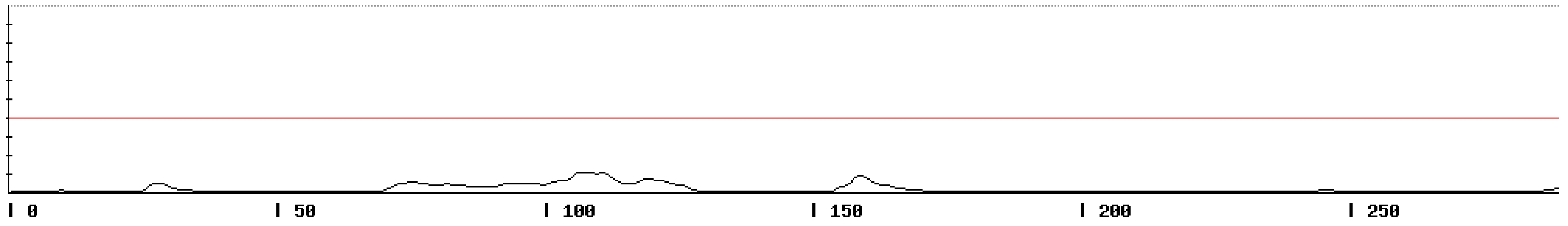

Predictions for Castaneamollissima_scaffold02867-abinit-gene-0.1d02867

Viterbi Path	
Viterbi Path	
Posterior @ 0.4	
Posterior @ 0.4	
Posterior Graphic. Threshold Marked @ 0.4	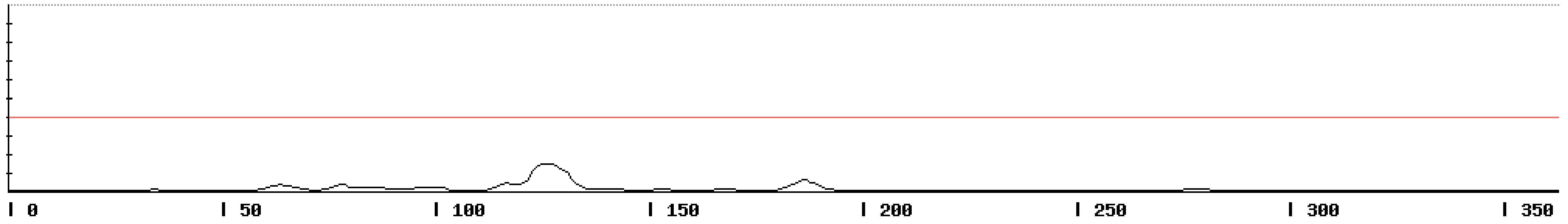

Predictions for Castaneamollissima_scaffold03666-abinit-gene-0.5d03666

Viterbi Path	545 - KKERKRKRKEHNDERK - 560
Viterbi Path	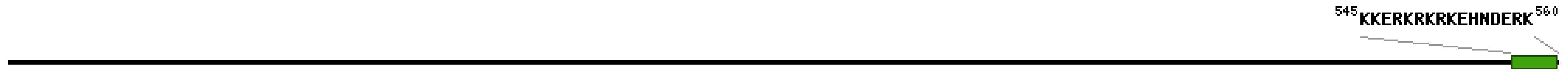
Posterior @ 0.4	543 - SNKKERKRKRKEHNDERK - 560
Posterior @ 0.4	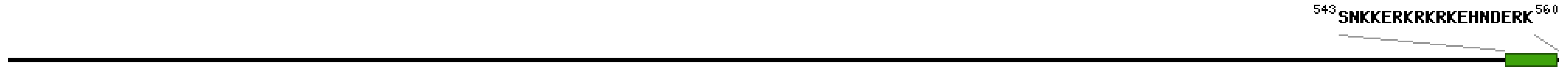

Posterior Graphic. Threshold Marked @ 0.4

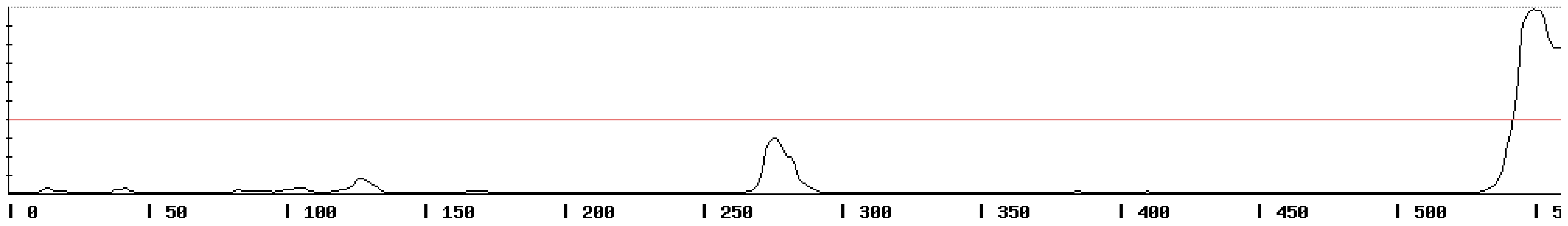

Predictions for Castaneamollissima_scaffold04550-abinit-gene-0.6d04550

Viterbi Path

Viterbi Path

Posterior @ 0.4

Posterior @ 0.4

Posterior Graphic. Threshold Marked @ 0.4

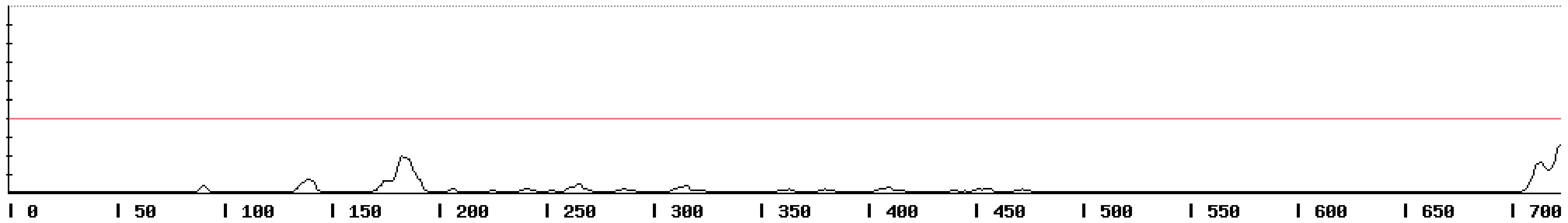

Predictions for Castaneamollissima_scaffold10669-abinit-gene-0.0d10669

Viterbi Path

Viterbi Path

Posterior @ 0.4

Posterior @ 0.4

Posterior Graphic. Threshold Marked @ 0.4

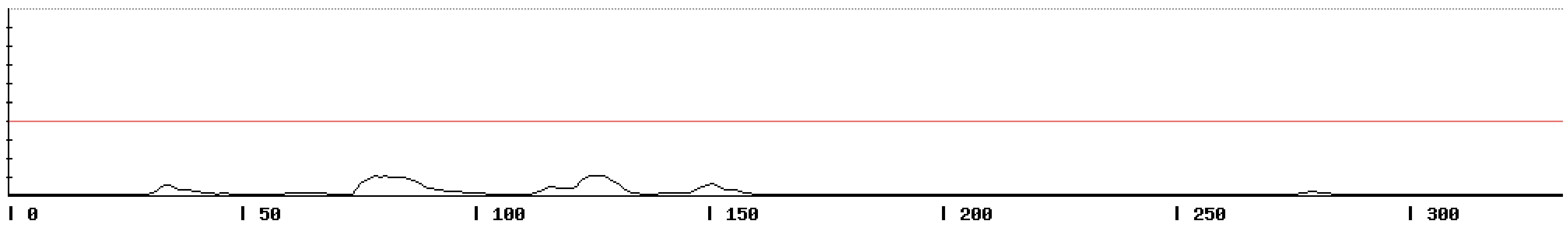

Predictions for Castaneamollissima_scaffold12148-abinit-gene-0.2d12148

Viterbi Path

Viterbi Path

Posterior @ 0.4

Posterior @ 0.4

Posterior Graphic. Threshold Marked @ 0.4

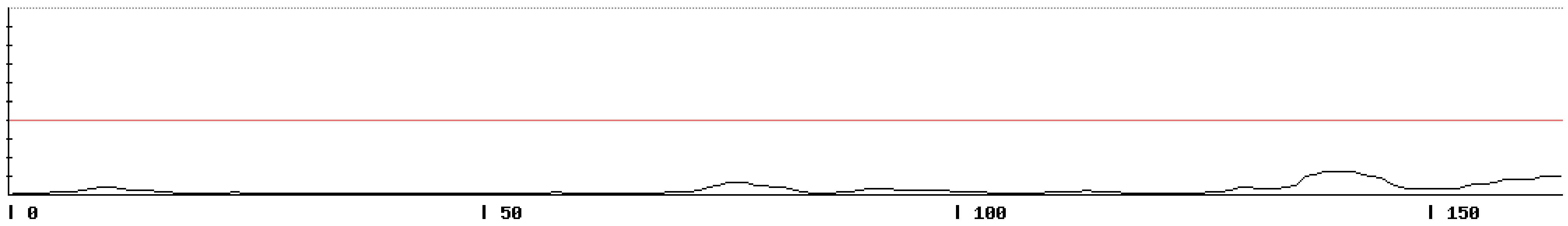

Predictions for Castaneamollissima_scaffold13692-abinit-gene-0.0d13692

Viterbi Path

Viterbi Path

Posterior @ 0.4

Posterior @ 0.4

Posterior Graphic. Threshold Marked @ 0.4

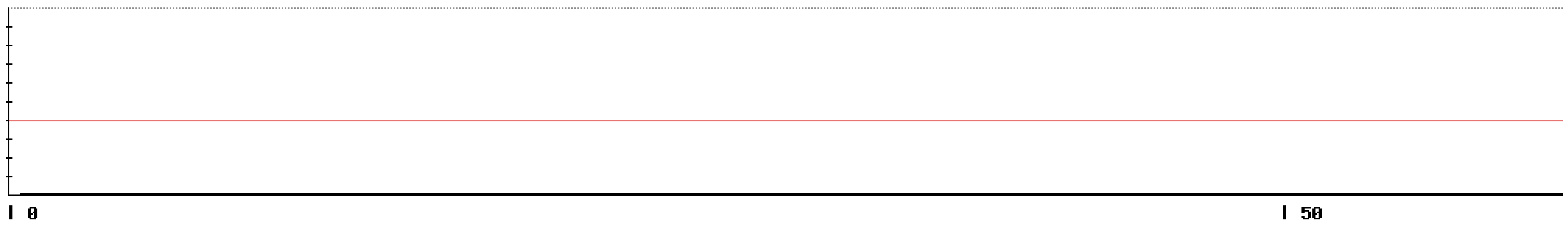

Predictions for Castaneamollissima_scaffold00002-augustus-gene-1.34

Viterbi Path

Viterbi Path

Posterior @ 0.4

Posterior @ 0.4

Posterior Graphic. Threshold Marked @ 0.4

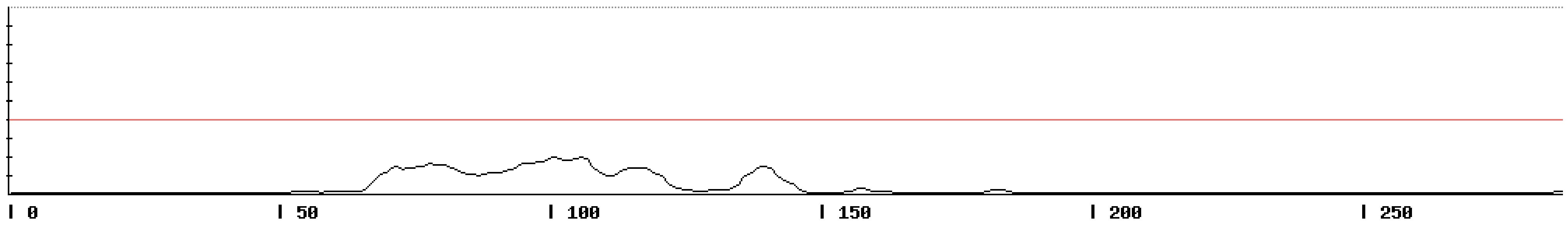

Predictions for Castaneamollissima_scaffold00005-augustus-gene-0.28

Viterbi Path

Viterbi Path

Posterior @ 0.4

Posterior @ 0.4

Posterior Graphic. Threshold Marked @ 0.4

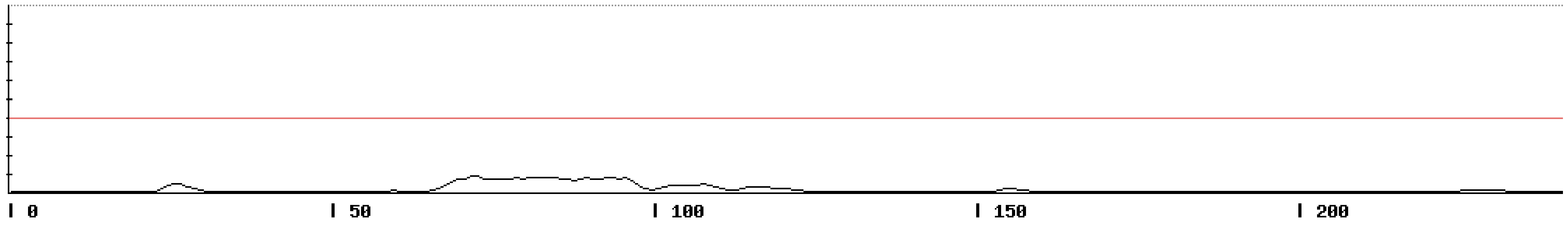

Predictions for Castaneamollissima_scaffold00018-augustus-gene-0.30

Viterbi Path

Viterbi Path

Posterior @ 0.4

Posterior @ 0.4

Posterior Graphic. Threshold Marked @ 0.4

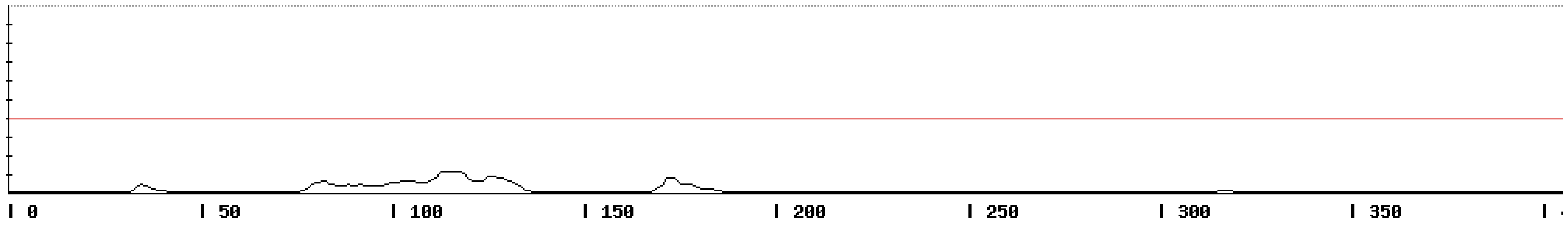

Predictions for Castaneamollissima_scaffold00057-snap-gene-0.53

Viterbi Path

Viterbi Path

Posterior @ 0.4

Posterior @ 0.4

Posterior Graphic. Threshold Marked @ 0.4

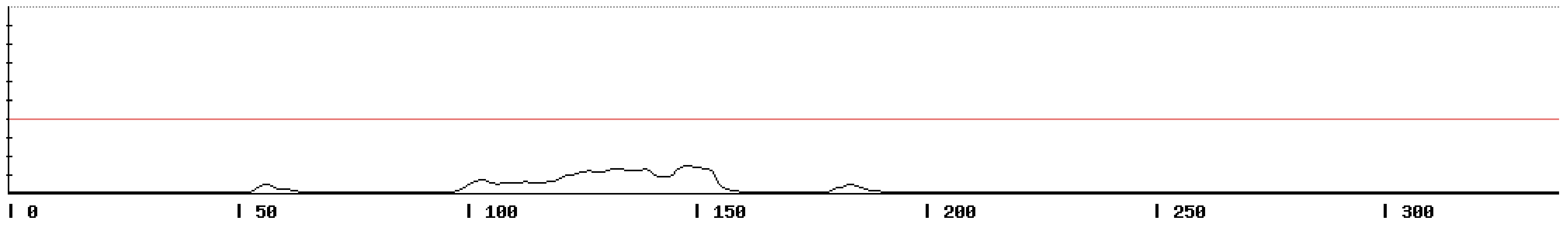

Predictions for Castaneamollissima_scaffold00345-augustus-gene-0.38

Viterbi Path

Viterbi Path

Posterior @ 0.4

Posterior @ 0.4

Posterior Graphic. Threshold Marked @ 0.4

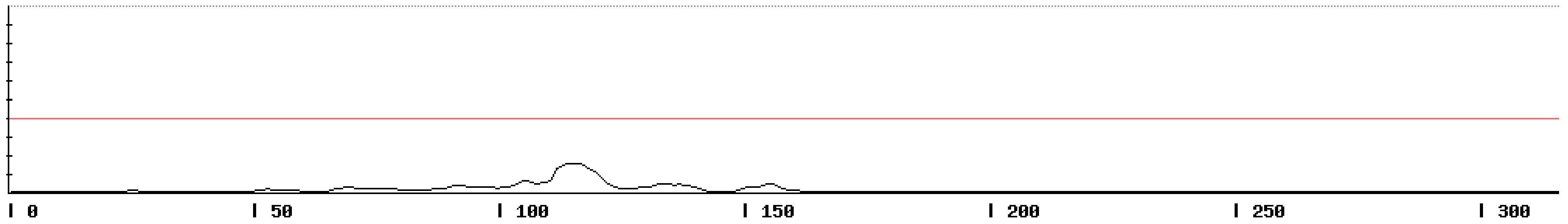

Predictions for Castaneamollissima_scaffold00745-snap-gene-0.42	
Viterbi Path	
Viterbi Path	
Posterior @ 0.4	
Posterior @ 0.4	
Posterior Graphic. Threshold Marked @ 0.4	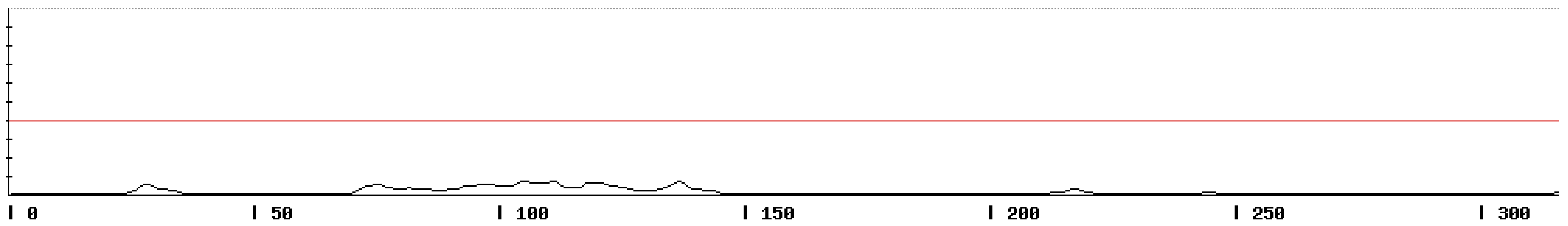

Predictions for Castaneamollissima_scaffold00830-augustus-gene-0.42	
Viterbi Path	
Viterbi Path	
Posterior @ 0.4	93 - ADKPIGKPKPLGIKKA - 108
Posterior @ 0.4	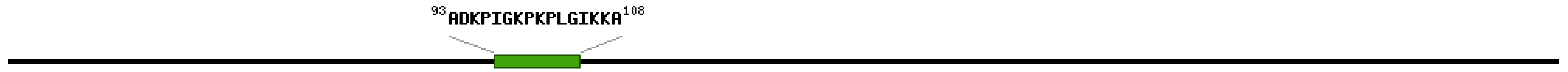
Posterior Graphic. Threshold Marked @ 0.4	

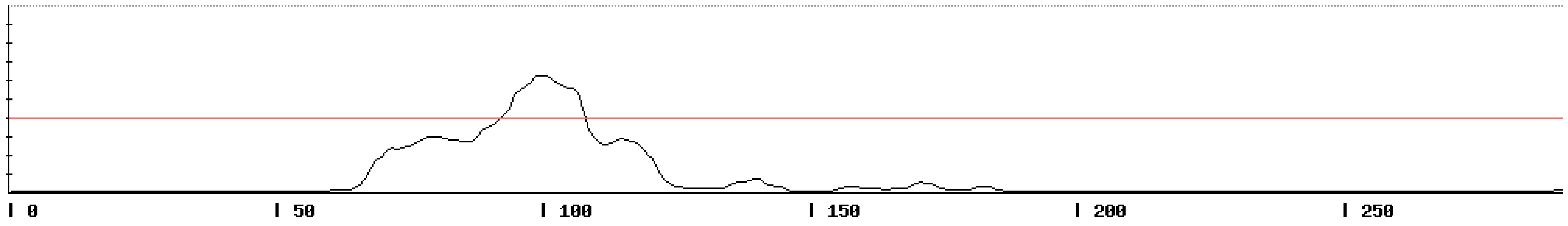

Predictions for Castaneamollissima_scaffold00860-augustus-gene-0.36

Viterbi Path	
Viterbi Path	
Posterior @ 0.4	
Posterior @ 0.4	
Posterior Graphic. Threshold Marked @ 0.4	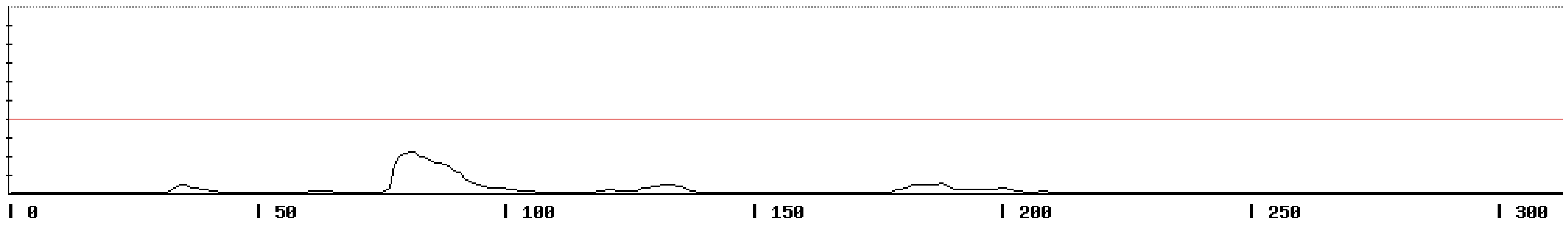

Predictions for Castaneamollissima_scaffold00873-snap-gene-0.35

Viterbi Path	
Viterbi Path	
Posterior @ 0.4	
Posterior @ 0.4	

Posterior Graphic. Threshold Marked @ 0.4

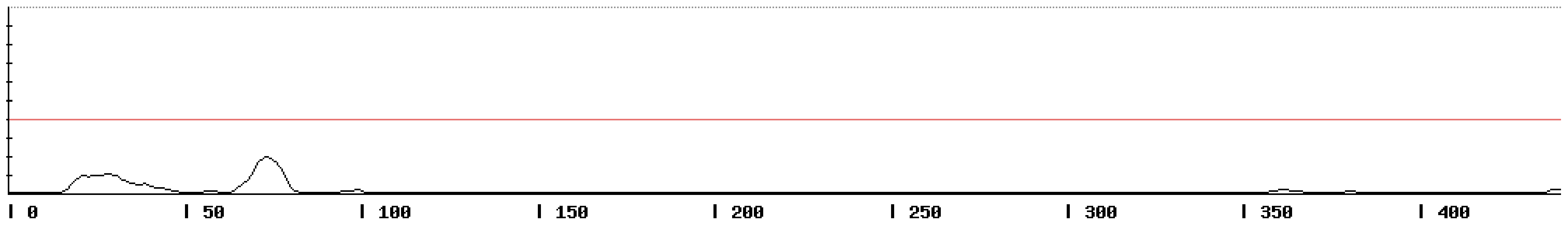

Predictions for Castaneamollissima_scaffold00890-augustus-gene-0.33

Viterbi Path

Viterbi Path

Posterior @ 0.4

Posterior @ 0.4

Posterior Graphic. Threshold Marked @ 0.4

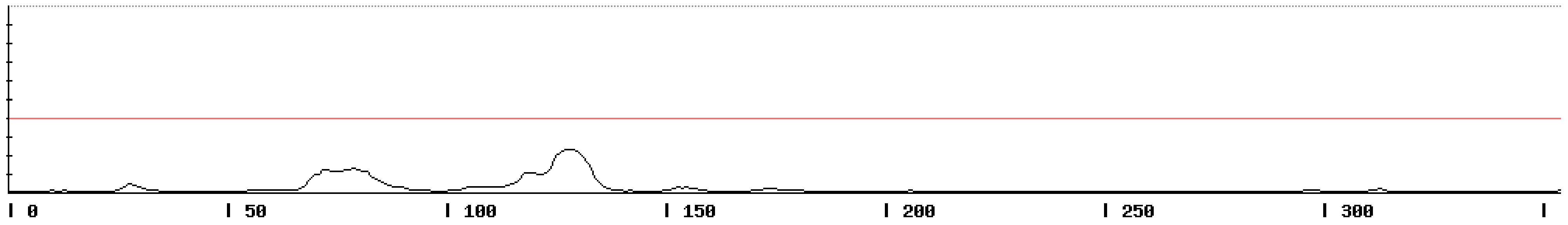

Predictions for Castaneamollissima_scaffold00983-snap-gene-0.43

Viterbi Path

Viterbi Path

Posterior @ 0.4

Posterior @ 0.4

Posterior Graphic. Threshold Marked @ 0.4

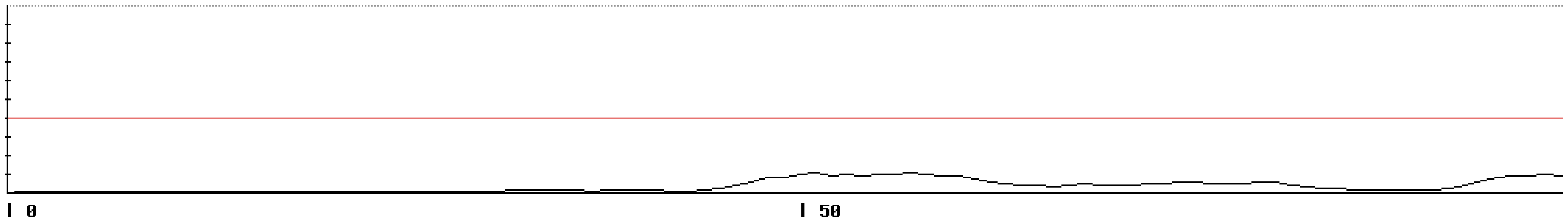

Predictions for Castaneamollissima_scaffold01061-augustus-gene-0.30

Viterbi Path

Viterbi Path

Posterior @ 0.4

Posterior @ 0.4

Posterior Graphic. Threshold Marked @ 0.4

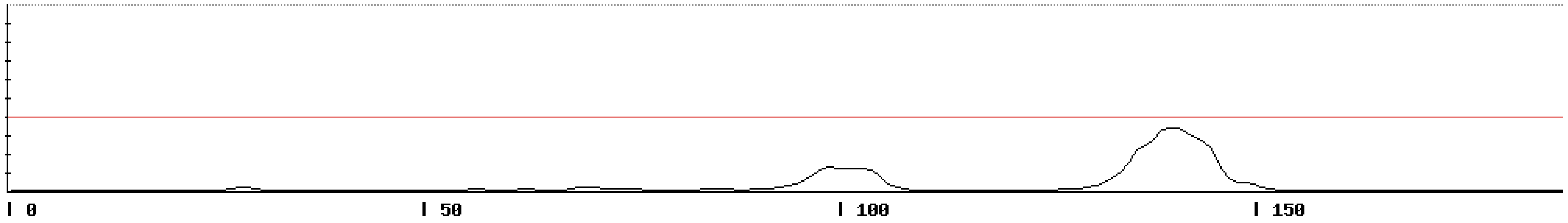

Predictions for Castaneamollissima_scaffold01636-snap-gene-0.46

Viterbi Path

Viterbi Path

Posterior @ 0.4	61 - KNEKEEKKK - 69
	128 - RKIK - 131
Posterior @ 0.4	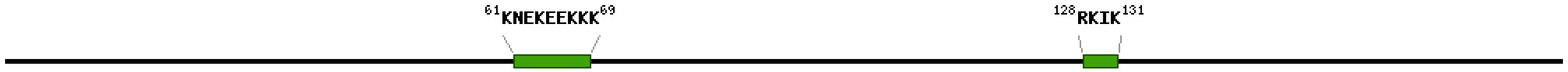
Posterior Graphic. Threshold Marked @ 0.4	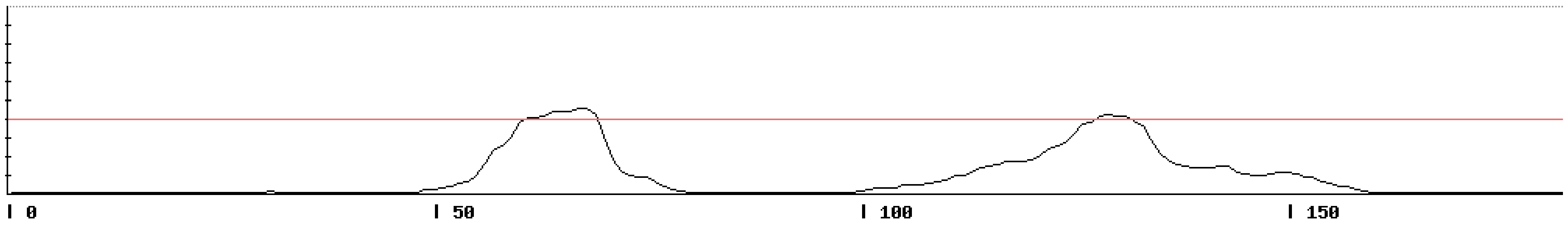

Predictions for Castaneamollissima_scaffold01655-augustus-gene-0.20

Viterbi Path	
Viterbi Path	
Posterior @ 0.4	
Posterior @ 0.4	
Posterior Graphic. Threshold Marked @ 0.4	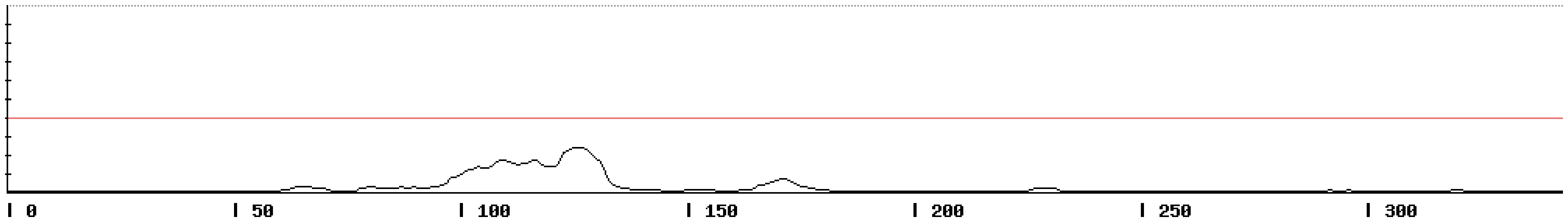

Predictions for Castaneamollissima_scaffold01684-snap-gene-0.33

Viterbi Path	
Viterbi Path	

Posterior @ 0.4

Posterior @ 0.4

Posterior Graphic. Threshold Marked @ 0.4

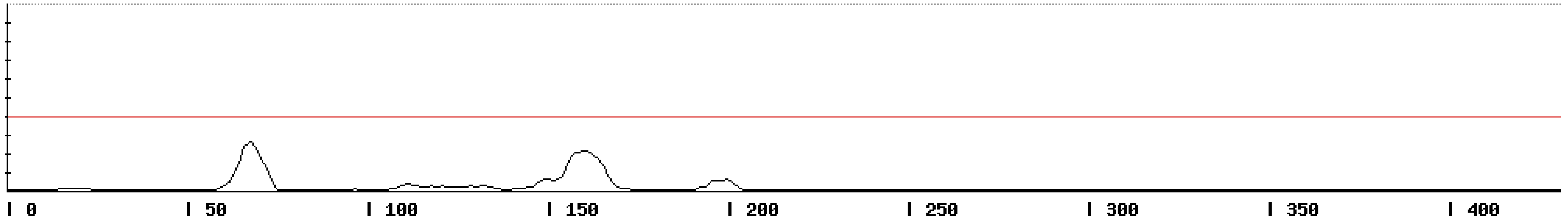

Predictions for Castaneamollissima_scaffold01789-snap-gene-0.35

Viterbi Path

Viterbi Path

Posterior @ 0.4 — 99 - RIRSRRKTKNGYWKRNGK - 116

Posterior @ 0.4

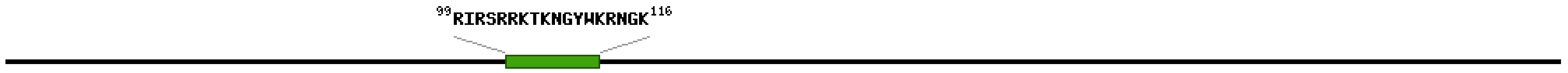

Posterior Graphic. Threshold Marked @ 0.4

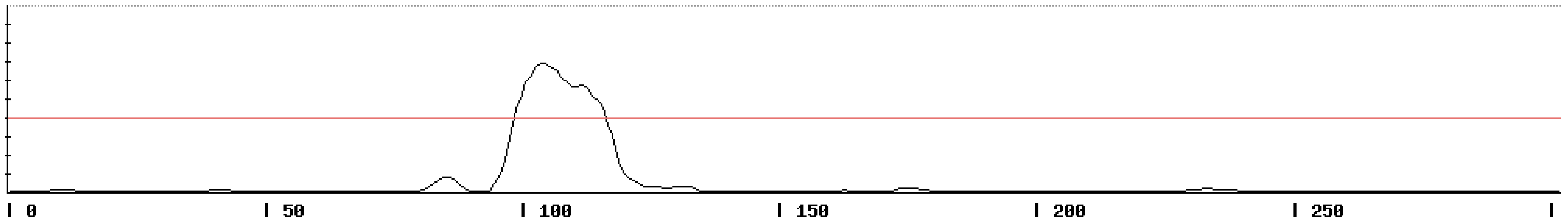

Predictions for Castaneamollissima_scaffold02051-augustus-gene-0.10

Viterbi Path

Viterbi Path

Posterior @ 0.4

Posterior @ 0.4

Posterior Graphic. Threshold Marked @ 0.4

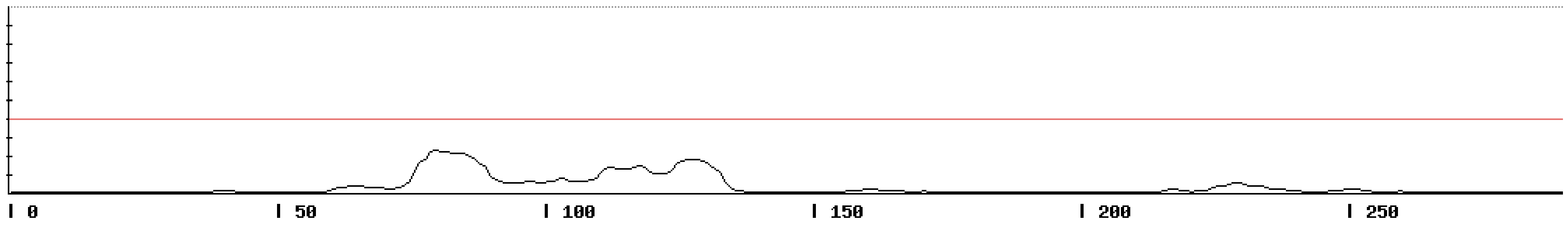

Predictions for Castaneamollissima_scaffold02306-augustus-gene-0.21

Viterbi Path

Viterbi Path

Posterior @ 0.4

Posterior @ 0.4

Posterior Graphic. Threshold Marked @ 0.4

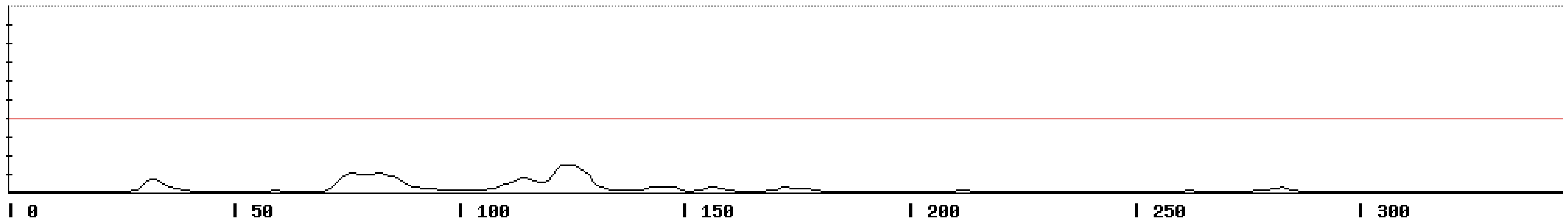

Predictions for Castaneamollissima_scaffold02433-augustus-gene-0.7

Viterbi Path

Viterbi Path

Posterior @ 0.4

Posterior @ 0.4

Posterior Graphic. Threshold Marked @ 0.4

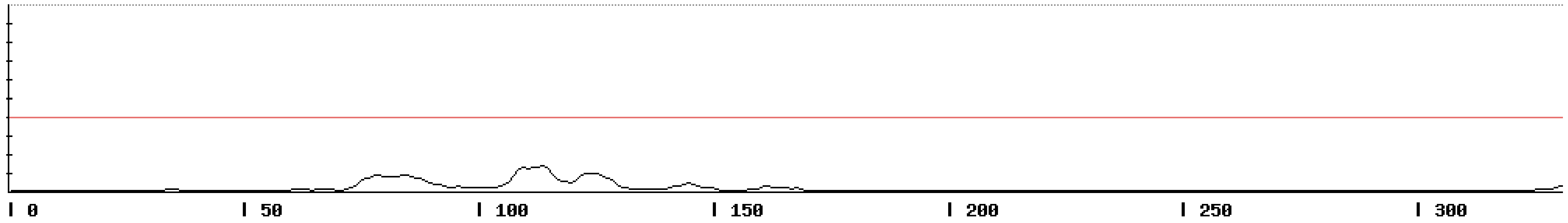

Predictions for Castaneamollissima_scaffold02656-augustus-gene-0.15

Viterbi Path

Viterbi Path

Posterior @ 0.4

Posterior @ 0.4

Posterior Graphic. Threshold Marked @ 0.4

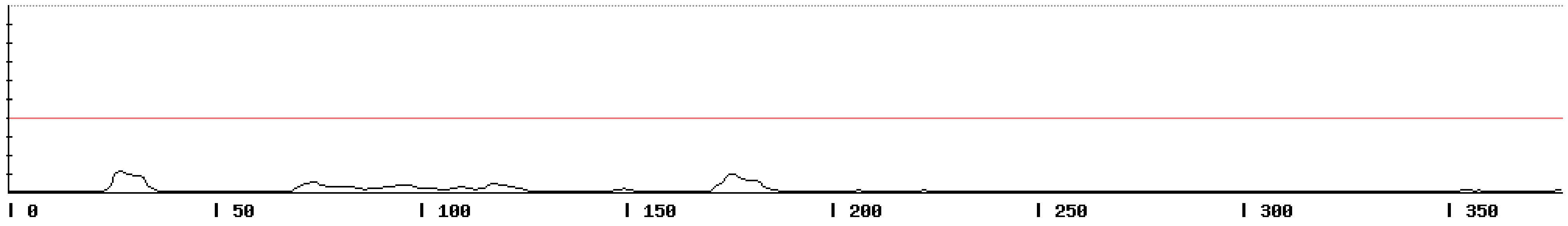

Predictions for Castaneamollissima_scaffold02751-augustus-gene-0.20

Viterbi Path	
Viterbi Path	
Posterior @ 0.4	
Posterior @ 0.4	
Posterior Graphic. Threshold Marked @ 0.4	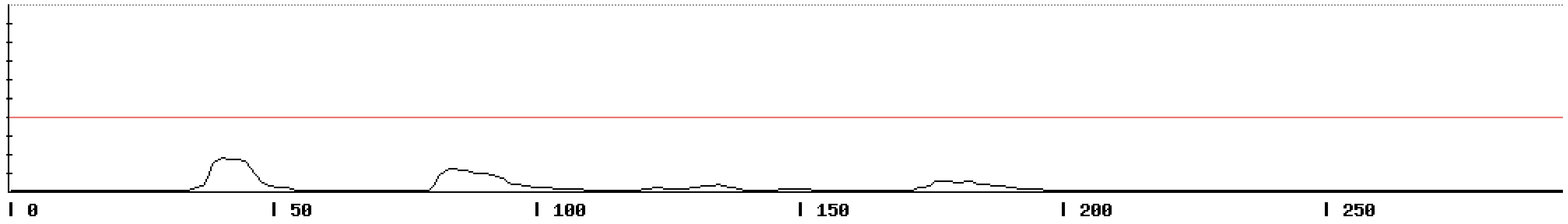

Predictions for Castaneamollissima_scaffold02784-augustus-gene-0.30

Viterbi Path	
Viterbi Path	
Posterior @ 0.4	
Posterior @ 0.4	

Posterior Graphic. Threshold Marked @ 0.4

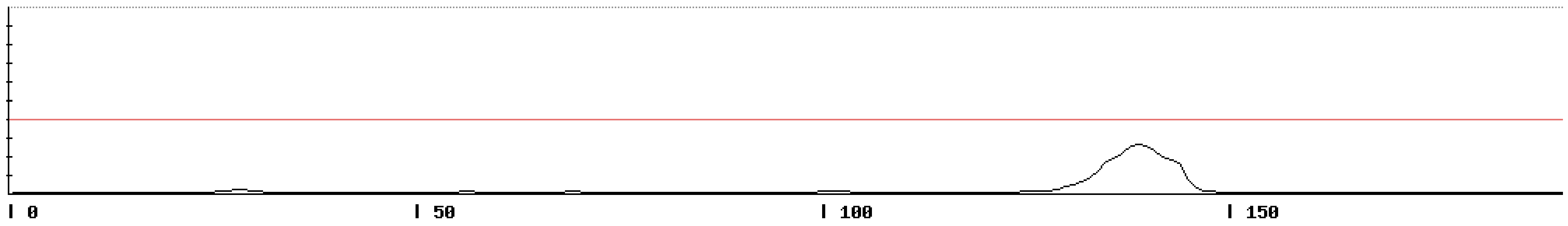

Predictions for Castaneamollissima_scaffold02784-augustus-gene-0.30

Viterbi Path

Viterbi Path

Posterior @ 0.4

Posterior @ 0.4

Posterior Graphic. Threshold Marked @ 0.4

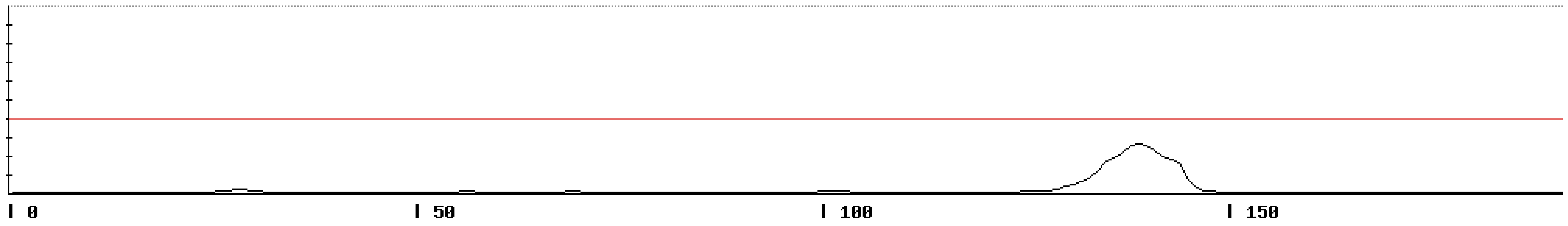

Predictions for Castaneamollissima_scaffold02799-augustus-gene-0.20

Viterbi Path

Viterbi Path

Posterior @ 0.4

Posterior @ 0.4

Posterior Graphic. Threshold Marked @ 0.4

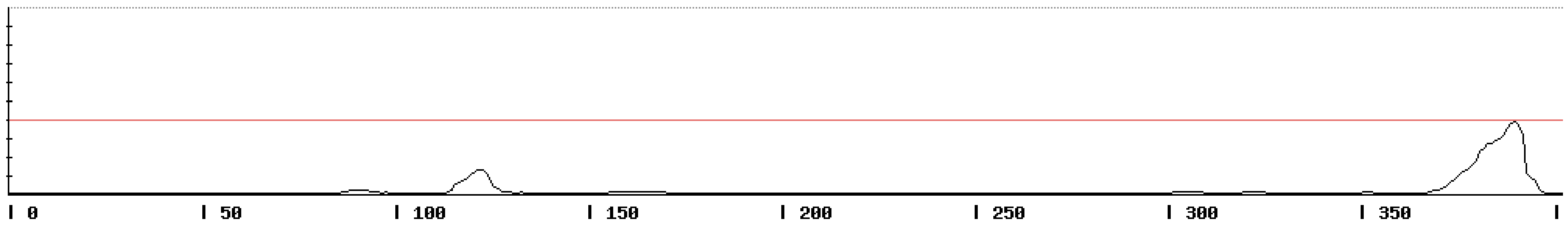

Predictions for Castaneamollissima_scaffold02824-augustus-gene-0.20

Viterbi Path

Viterbi Path

Posterior @ 0.4

Posterior @ 0.4

Posterior Graphic. Threshold Marked @ 0.4

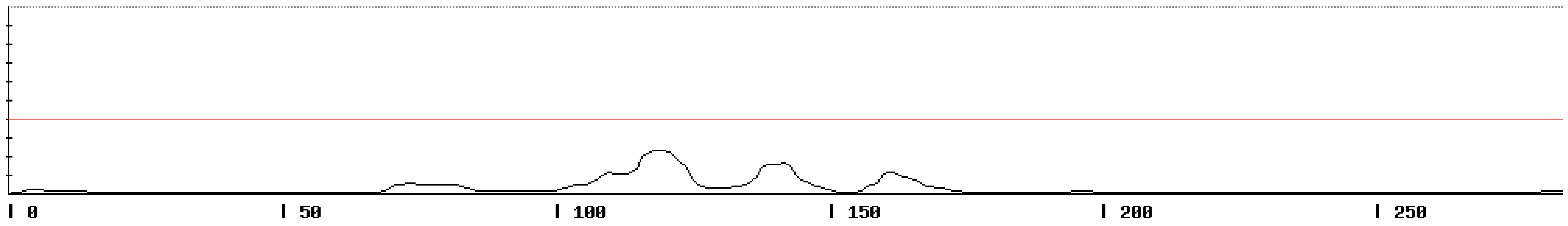

Predictions for Castaneamollissima_scaffold02829-snap-gene-0.27

Viterbi Path

Viterbi Path

Posterior @ 0.4

Posterior @ 0.4

Posterior Graphic. Threshold Marked @ 0.4

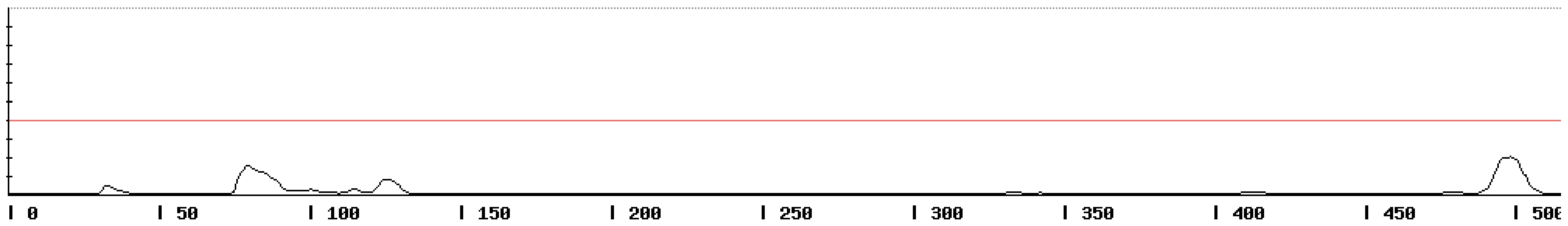

Predictions for Castaneamollissima_scaffold02959-augustus-gene-0.14

Viterbi Path

Viterbi Path

Posterior @ 0.4

Posterior @ 0.4

Posterior Graphic. Threshold Marked @ 0.4

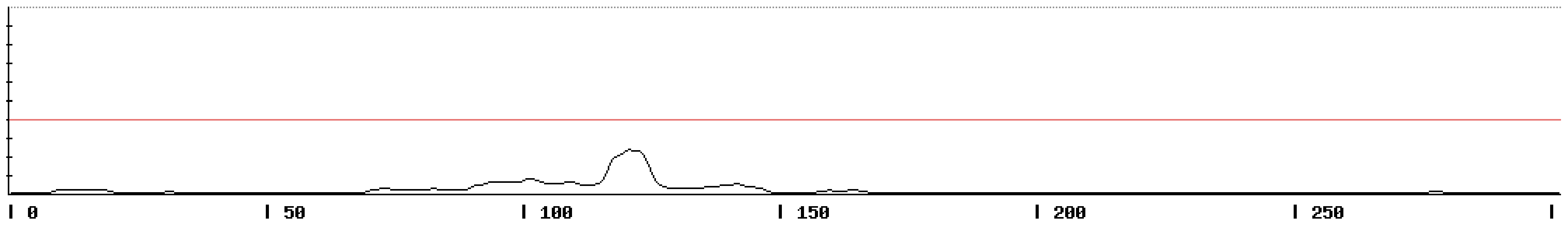

Predictions for Castaneamollissima_scaffold03016-augustus-gene-0.16

Viterbi Path

Viterbi Path

Posterior @ 0.4

Posterior @ 0.4

Posterior Graphic. Threshold Marked @ 0.4

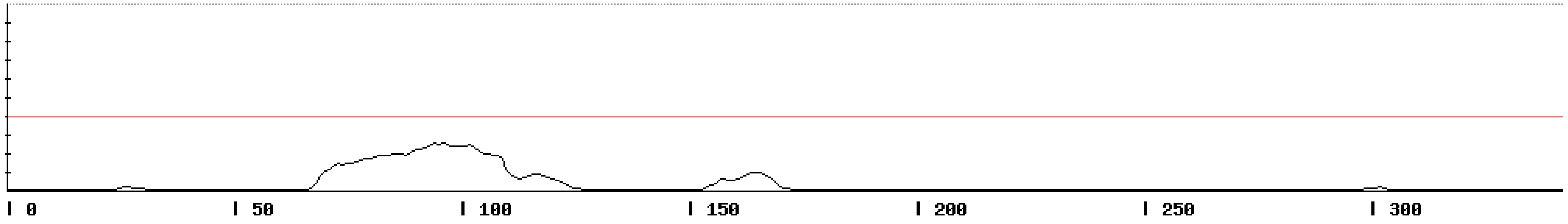

Predictions for Castaneamollissima_scaffold03032-augustus-gene-0.24

Viterbi Path

Viterbi Path

Posterior @ 0.4

Posterior @ 0.4

Posterior Graphic. Threshold Marked @ 0.4

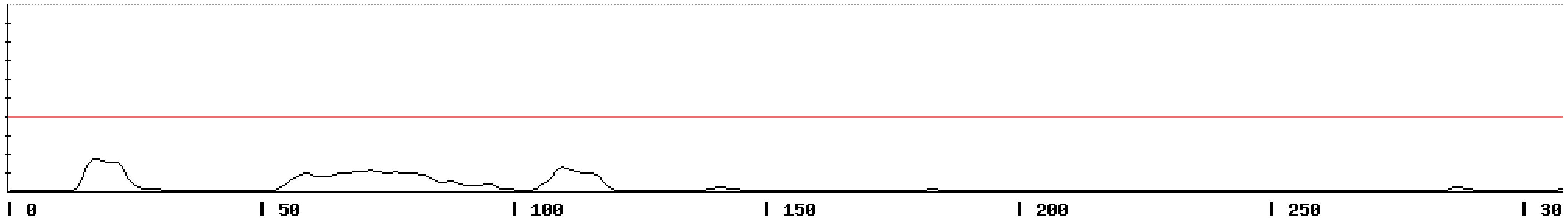

Predictions for Castaneamollissima_scaffold03547-augustus-gene-0.16

Viterbi Path

Viterbi Path

Posterior @ 0.4

Posterior @ 0.4

Posterior Graphic. Threshold Marked @ 0.4

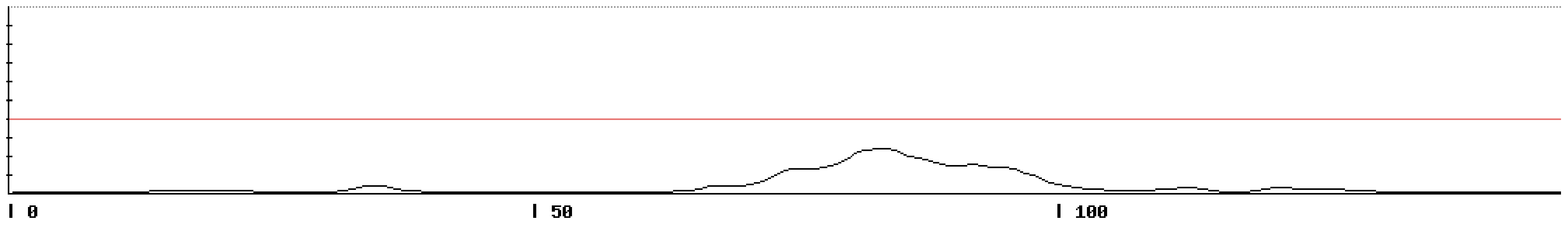

Predictions for Castaneamollissima_scaffold03703-augustus-gene-0.17

Viterbi Path

Viterbi Path

Posterior @ 0.4

Posterior @ 0.4

Posterior Graphic. Threshold Marked @ 0.4

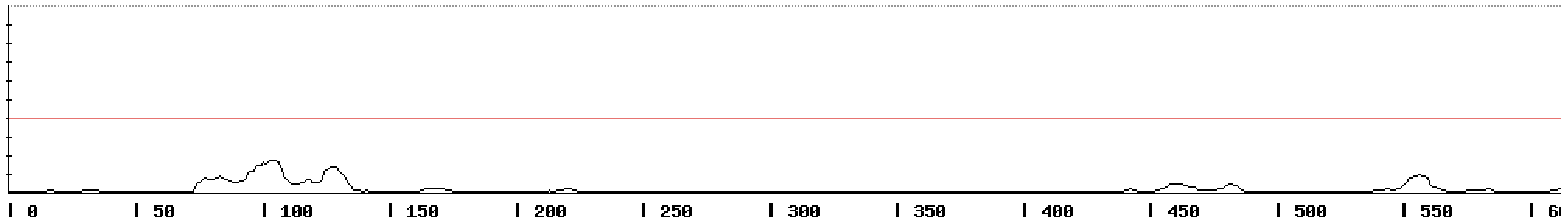

Predictions for Castaneamollissima_scaffold03973-snap-gene-0.12	
Viterbi Path	
Viterbi Path	
Posterior @ 0.4	
Posterior @ 0.4	
Posterior Graphic. Threshold Marked @ 0.4	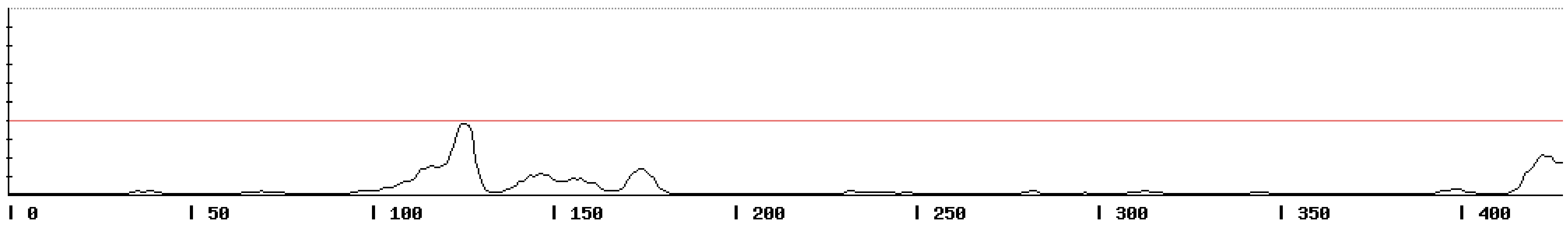

Predictions for Castaneamollissima_scaffold04069-snap-gene-0.18	
Viterbi Path	
Viterbi Path	
Posterior @ 0.4	129 - RGKR - 132
Posterior @ 0.4	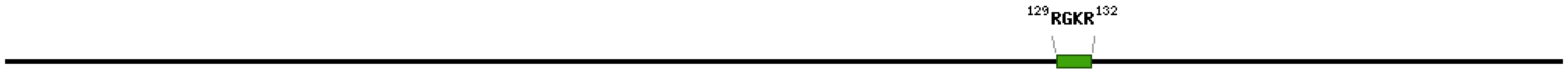
Posterior Graphic. Threshold Marked @ 0.4	

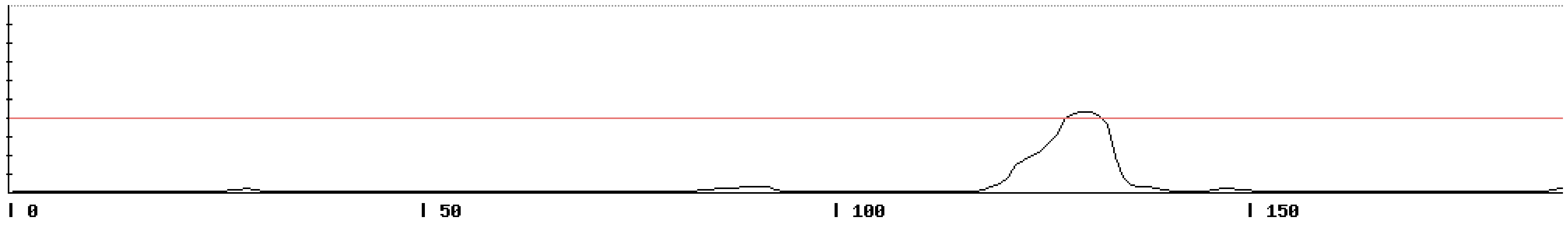

Predictions for Castaneamollissima_scaffold04103-augustus-gene-0.16

Viterbi Path	
Viterbi Path	
Posterior @ 0.4	
Posterior @ 0.4	
Posterior Graphic. Threshold Marked @ 0.4	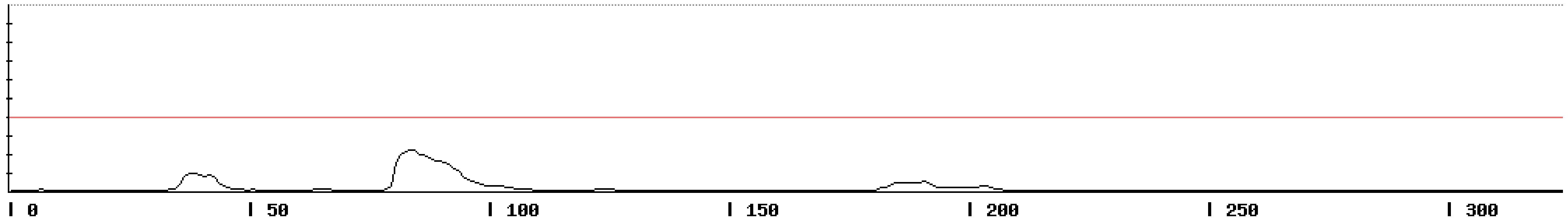

Predictions for Castaneamollissima_scaffold04107-augustus-gene-0.9

Viterbi Path	
Viterbi Path	
Posterior @ 0.4	
Posterior @ 0.4	

Posterior Graphic. Threshold Marked @ 0.4

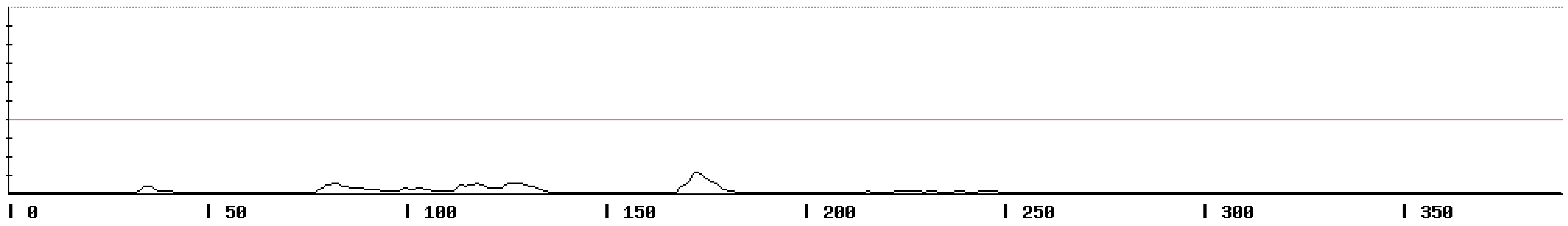

Predictions for Castaneamollissima_scaffold04312-snap-gene-0.20	
Viterbi Path	
Viterbi Path	
Posterior @ 0.4	
Posterior @ 0.4	
Posterior Graphic. Threshold Marked @ 0.4	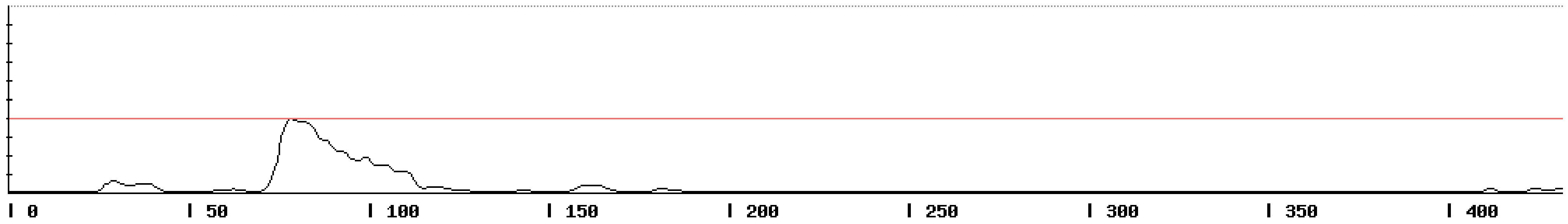

Predictions for Castaneamollissima_scaffold04312-snap-gene-0.21	
Viterbi Path	
Viterbi Path	
Posterior @ 0.4	
Posterior @ 0.4	

Posterior Graphic. Threshold Marked @ 0.4

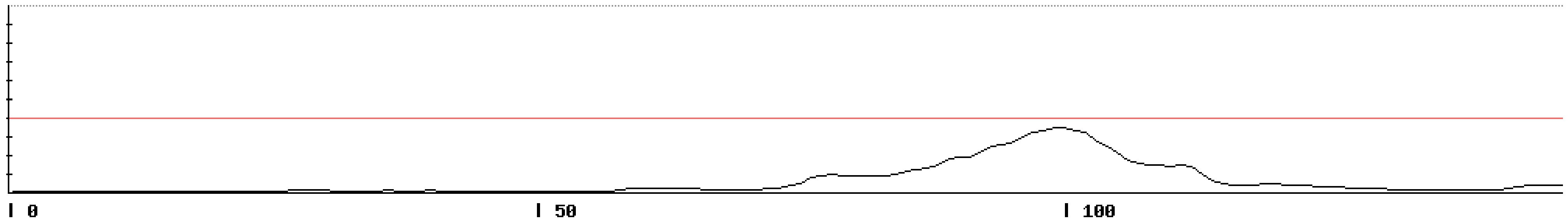

Predictions for Castaneamollissima_scaffold04399-augustus-gene-0.11

Viterbi Path

Viterbi Path

Posterior @ 0.4

Posterior @ 0.4

Posterior Graphic. Threshold Marked @ 0.4

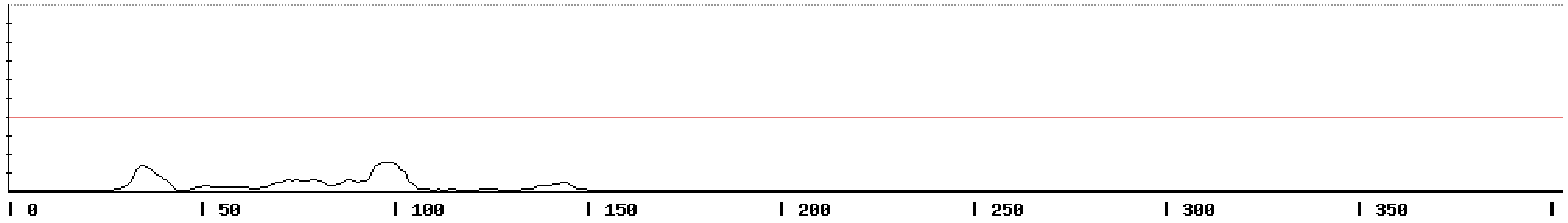

Predictions for Castaneamollissima_scaffold04399-augustus-gene-0.11

Viterbi Path

Viterbi Path

Posterior @ 0.4

Posterior @ 0.4

Posterior Graphic. Threshold Marked @ 0.4

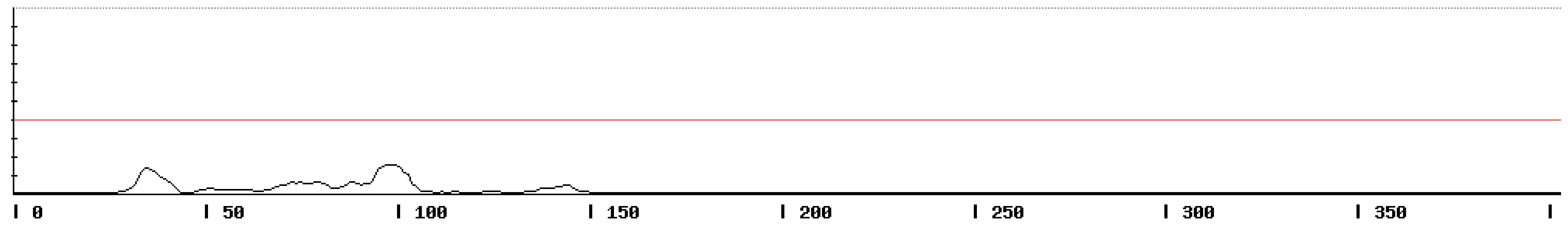

Predictions for Castaneamollissima_scaffold04550-snap-gene-0.24

Viterbi Path

Viterbi Path

Posterior @ 0.4 — 431 - KKIK - 434

Posterior @ 0.4

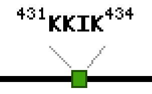

Posterior Graphic. Threshold Marked @ 0.4

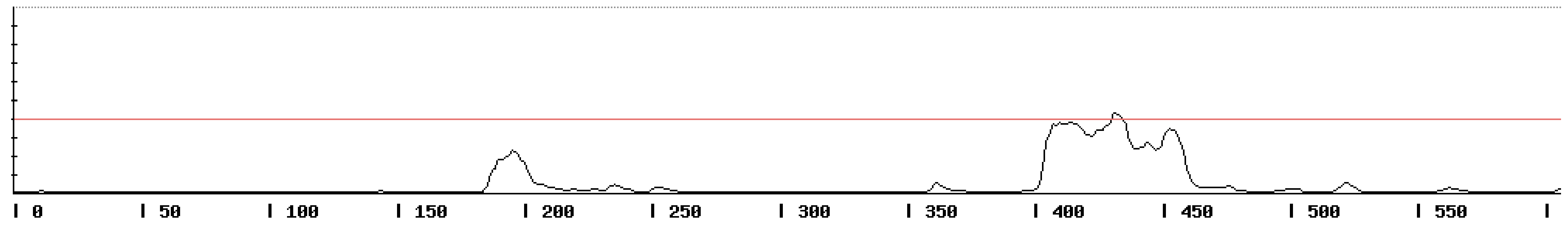

Predictions for Castaneamollissima_scaffold04550-snap-gene-0.25

Viterbi Path

Viterbi Path

Posterior @ 0.4	171 - RKQKYQNRKRPNR - 183
Posterior @ 0.4	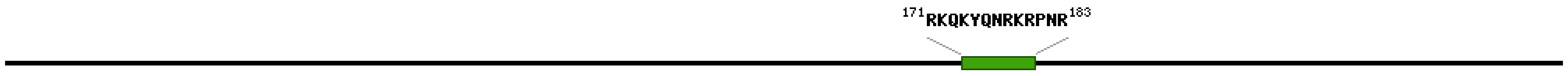
Posterior Graphic. Threshold Marked @ 0.4	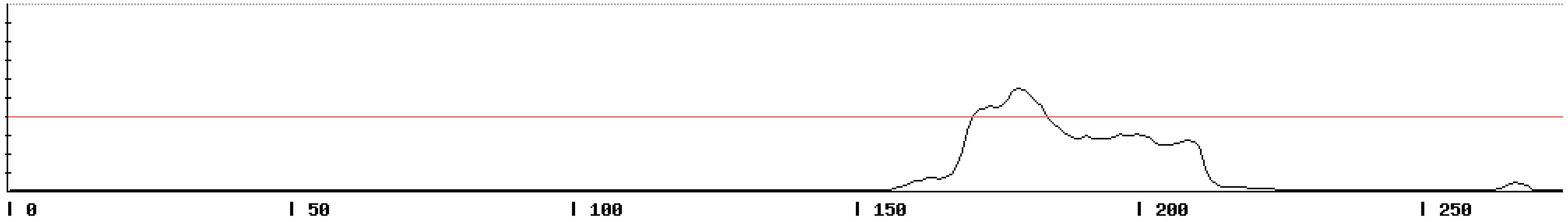

Predictions for Castaneamollissima_scaffold04583-augustus-gene-0.13

Viterbi Path	
Viterbi Path	
Posterior @ 0.4	
Posterior @ 0.4	
Posterior Graphic. Threshold Marked @ 0.4	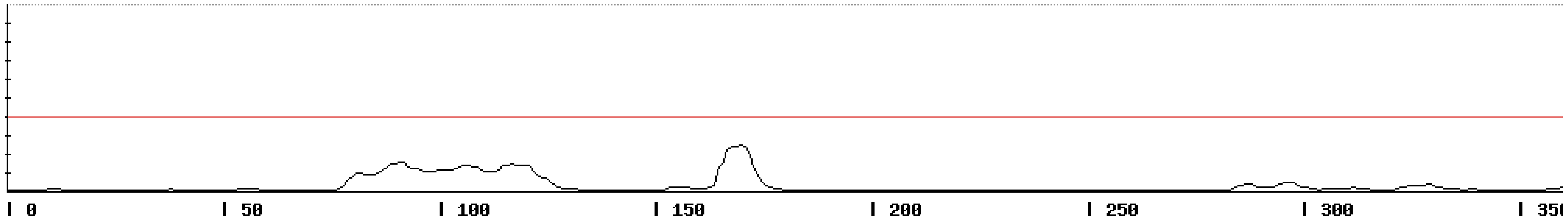

Predictions for Castaneamollissima_scaffold04981-augustus-gene-0.2

Viterbi Path	
Viterbi Path	
Posterior @ 0.4	
Posterior @ 0.4	
Posterior Graphic. Threshold Marked @ 0.4	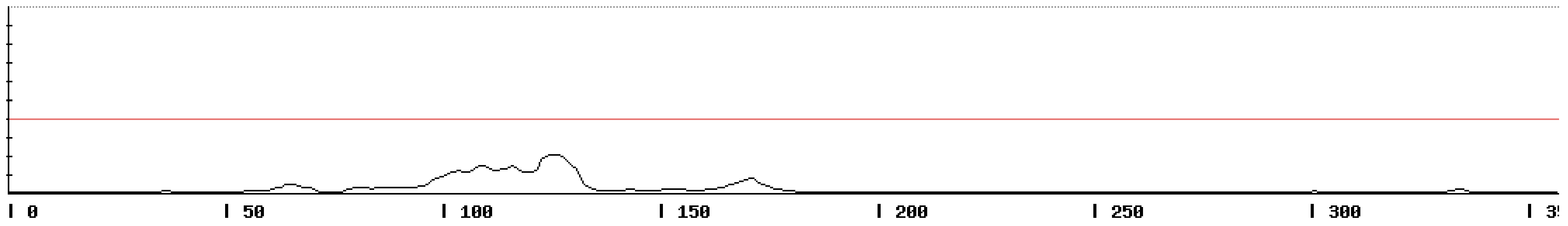

Predictions for Castaneamollissima_scaffold05403-augustus-gene-0.18

Viterbi Path	
Viterbi Path	
Posterior @ 0.4	116 - QKGKGRNRKTEKGHWKVTGNDREIKSKGKVIGMKKTLVFHIGRSRK - 161
Posterior @ 0.4	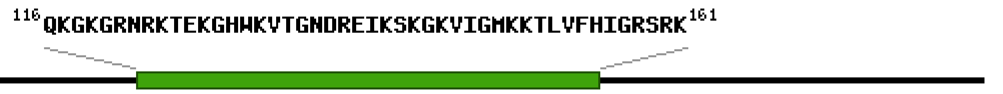
Posterior Graphic. Threshold Marked @ 0.4	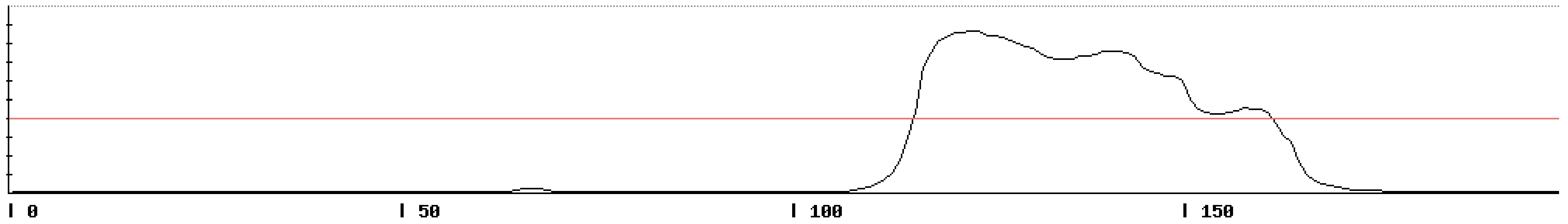

Predictions for Castaneamollissima_scaffold05469-snap-gene-0.9	
Viterbi Path	
Viterbi Path	
Posterior @ 0.4	
Posterior @ 0.4	
Posterior Graphic. Threshold Marked @ 0.4	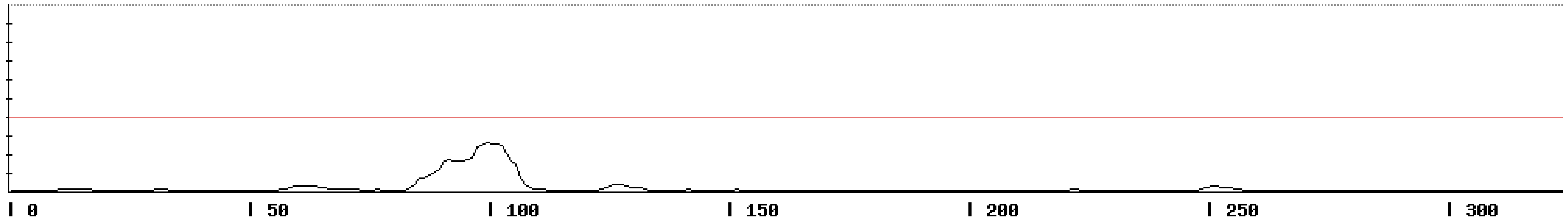

Predictions for Castaneamollissima_scaffold06450-augustus-gene-0.17	
Viterbi Path	
Viterbi Path	
Posterior @ 0.4	
Posterior @ 0.4	
Posterior Graphic. Threshold Marked @ 0.4	

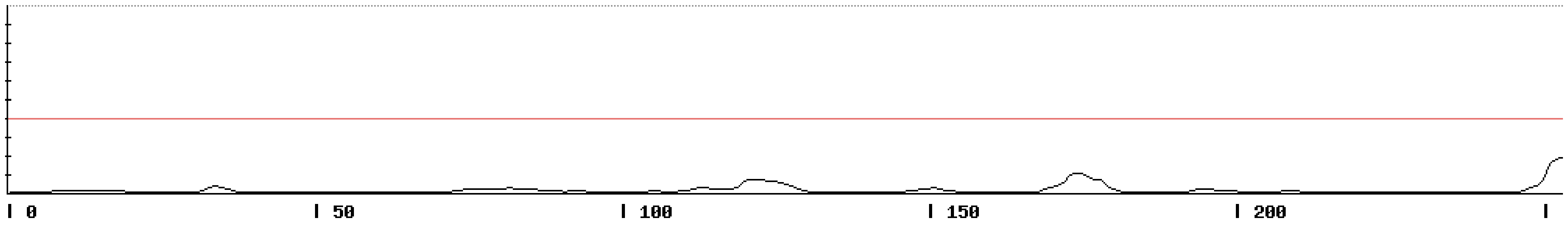

Predictions for Castaneamollissima_scaffold06646-augustus-gene-0.12	
Viterbi Path	
Viterbi Path	
Posterior @ 0.4	35 - KRR - 37
Posterior @ 0.4	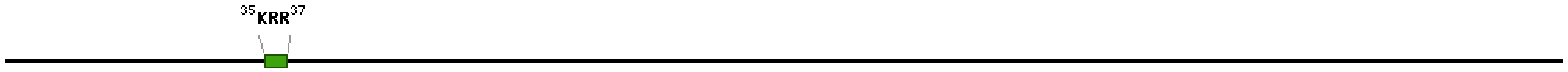
Posterior Graphic. Threshold Marked @ 0.4	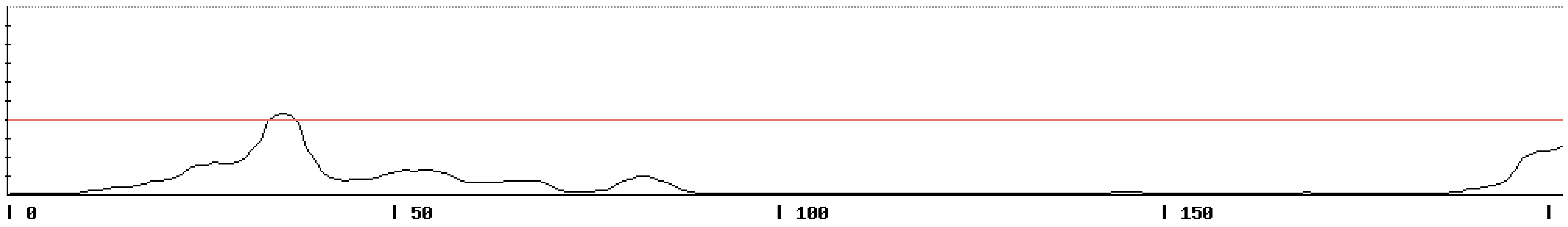

Predictions for Castaneamollissima_scaffold07067-augustus-gene-0.6	
Viterbi Path	
Viterbi Path	
Posterior @ 0.4	104 - PKKRKRDQKQERVSRKTK - 121
	618 - PKKRKRGQK - 626
Posterior @ 0.4	

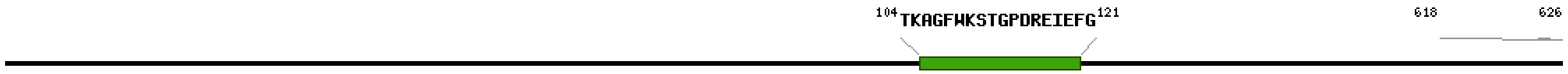

Posterior Graphic. Threshold Marked @ 0.4	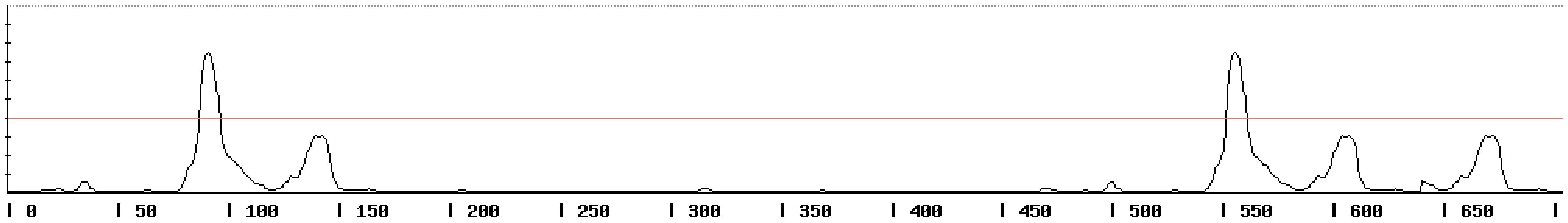

Predictions for Castaneamollissima_scaffold07067-augustus-gene-0.6

Viterbi Path	
Viterbi Path	
Posterior @ 0.4	82 - PKKRKRDQKQERVSRKTK - 99
	553 - PKKRKRGQK - 561
Posterior @ 0.4	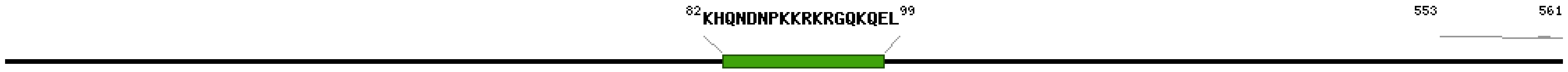
Posterior Graphic. Threshold Marked @ 0.4	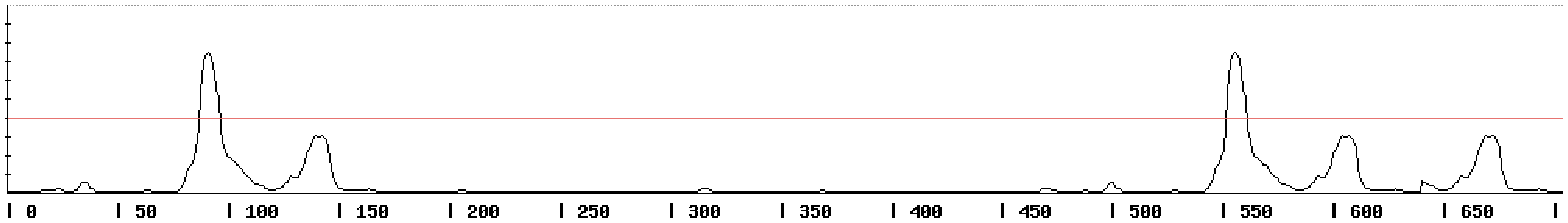

Predictions for Castaneamollissima_scaffold07067-augustus-gene-0.6

Viterbi Path	
Viterbi Path	

Posterior @ 0.4	88 - PKKRKRGQK - 96
Posterior @ 0.4	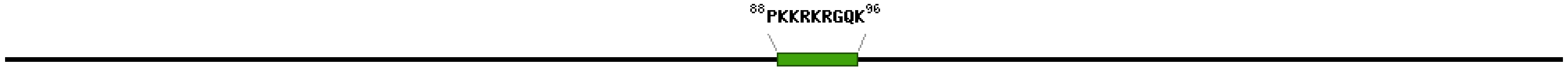
Posterior Graphic. Threshold Marked @ 0.4	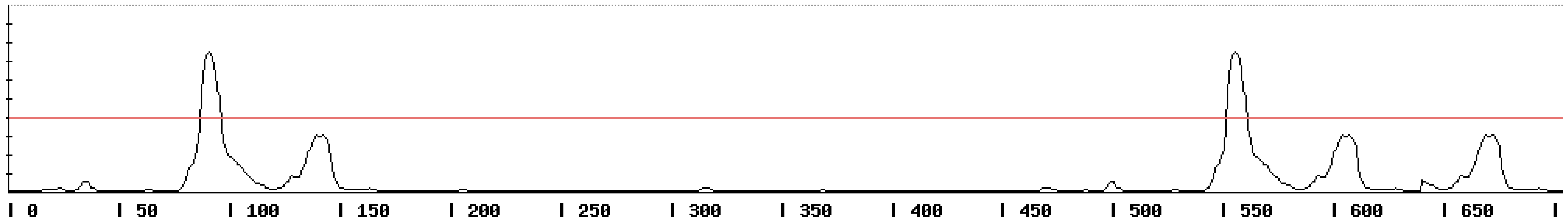

Predictions for Castaneamollissima_scaffold07459-snap-gene-0.9

Viterbi Path	
Viterbi Path	
Posterior @ 0.4	99 - KDREIKSKK - 107
Posterior @ 0.4	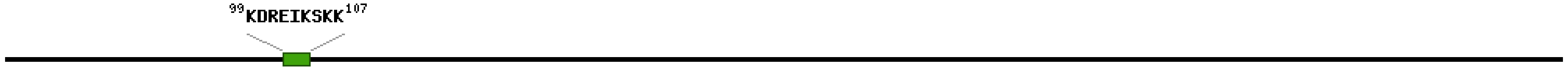
Posterior Graphic. Threshold Marked @ 0.4	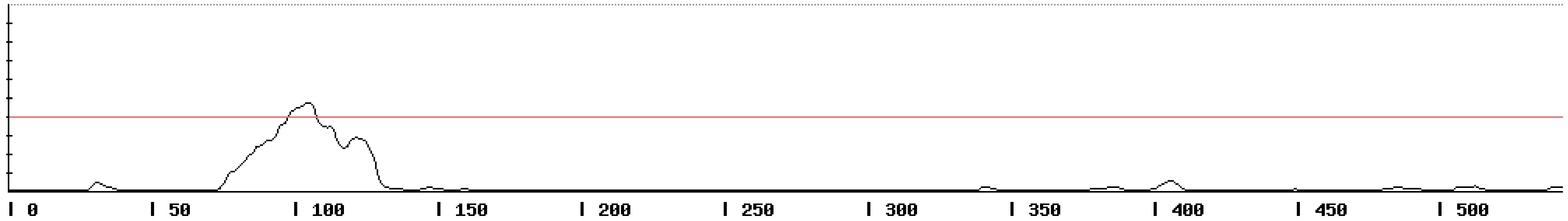

Predictions for Castaneamollissima_scaffold08043-augustus-gene-0.8

Viterbi Path

Viterbi Path

Posterior @ 0.4

Posterior @ 0.4

Posterior Graphic. Threshold Marked @ 0.4

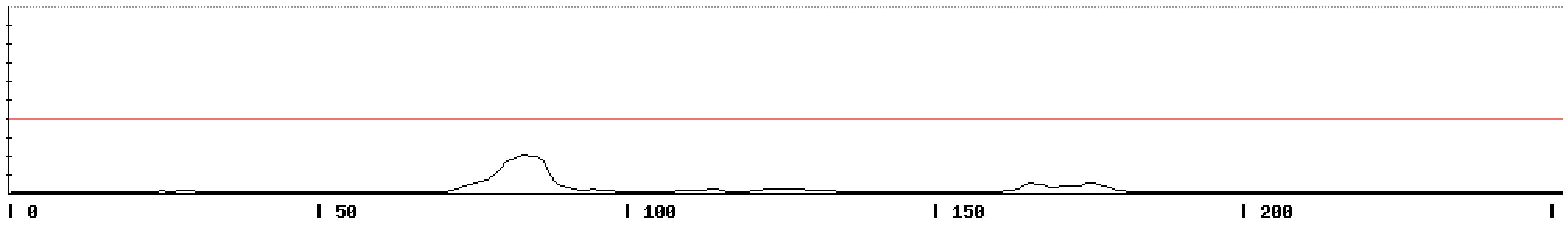

Predictions for Castaneamollissima_scaffold08137-snap-gene-0.8

Viterbi Path

Viterbi Path

Posterior @ 0.4

88 - RSRANRATKRGYWK - 101

Posterior @ 0.4

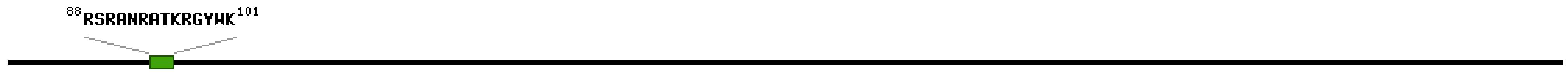

Posterior Graphic. Threshold Marked @ 0.4

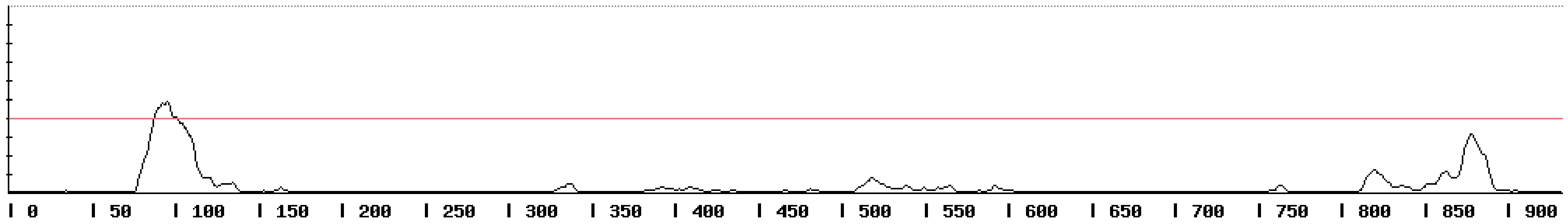

Predictions for Castaneamollissima_scaffold08188-augustus-gene-0.7	
Viterbi Path	
Viterbi Path	
Posterior @ 0.4	
Posterior @ 0.4	
Posterior Graphic. Threshold Marked @ 0.4	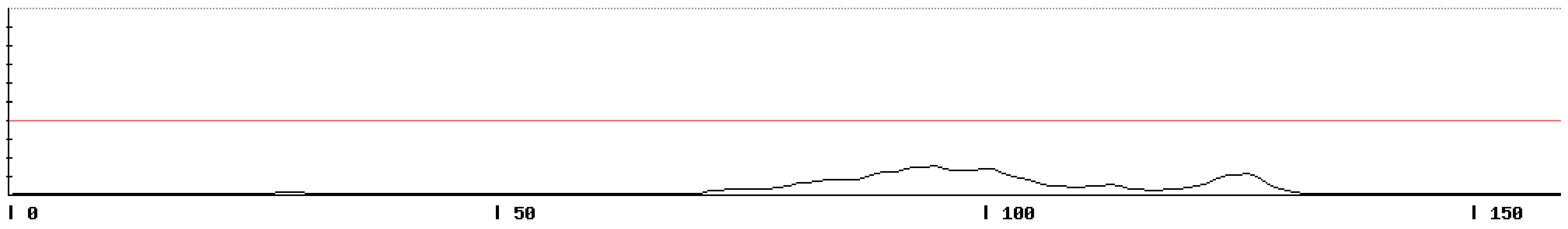

Predictions for Castaneamollissima_scaffold08294-augustus-gene-0.4	
Viterbi Path	
Viterbi Path	
Posterior @ 0.4	
Posterior @ 0.4	
Posterior Graphic. Threshold Marked @ 0.4	

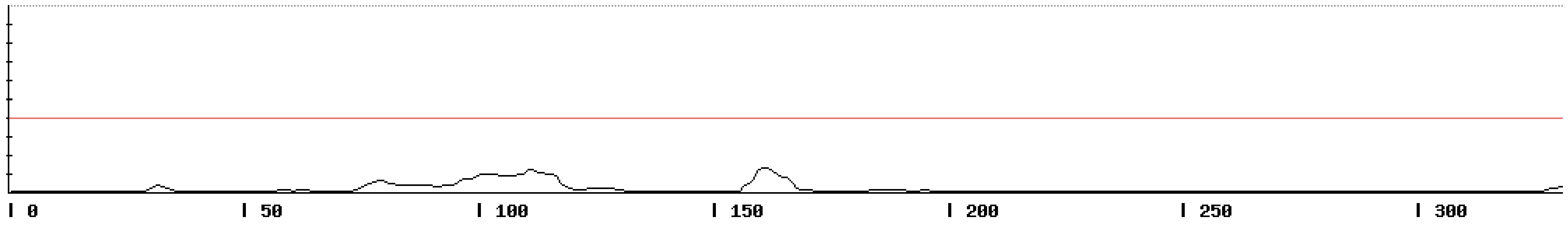

Predictions for Castaneamollissima_scaffold08297-augustus-gene-0.5	
Viterbi Path	
Viterbi Path	
Posterior @ 0.4	
Posterior @ 0.4	
Posterior Graphic. Threshold Marked @ 0.4	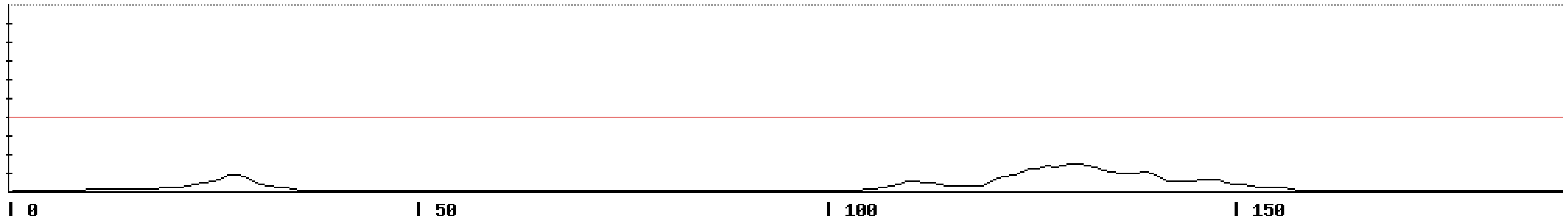

Predictions for Castaneamollissima_scaffold08573-augustus-gene-0.8	
Viterbi Path	
Viterbi Path	
Posterior @ 0.4	517 - KERKRKR - 523
Posterior @ 0.4	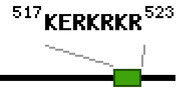

Posterior Graphic. Threshold Marked @ 0.4

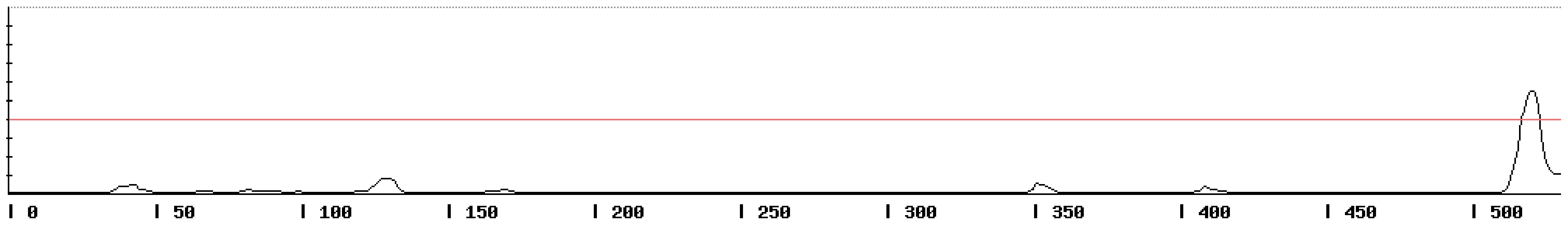

Predictions for Castaneamollissima_scaffold09059-snap-gene-0.18

Viterbi Path	
Viterbi Path	
Posterior @ 0.4	133 - RKRRK - 137
Posterior @ 0.4	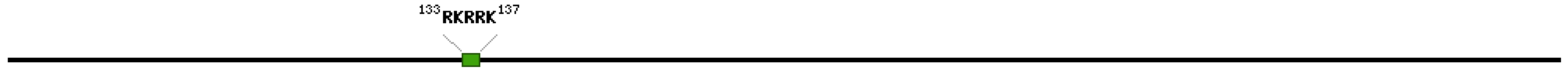
Posterior Graphic. Threshold Marked @ 0.4	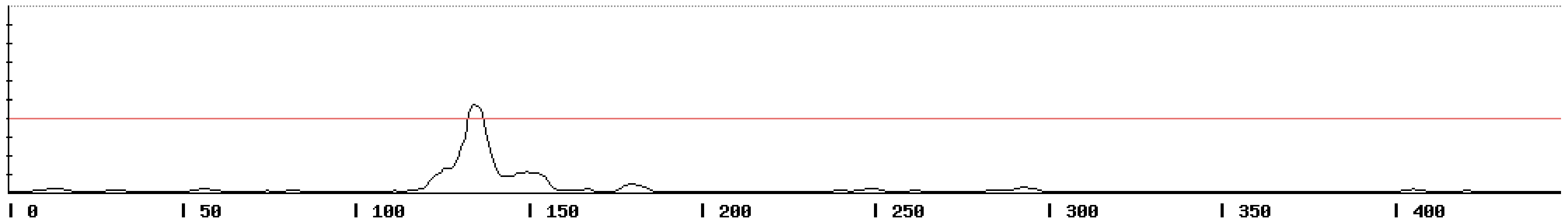

Predictions for Castaneamollissima_scaffold09484-snap-gene-0.17

Viterbi Path	
Viterbi Path	
Posterior @ 0.4	98 - KDRKIKAK - 105
Posterior @ 0.4	

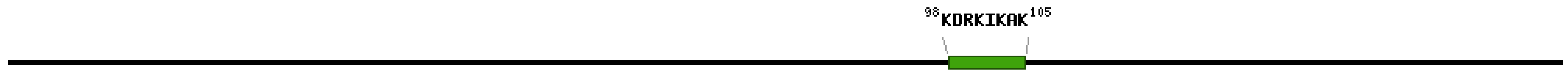

Posterior Graphic. Threshold Marked @ 0.4

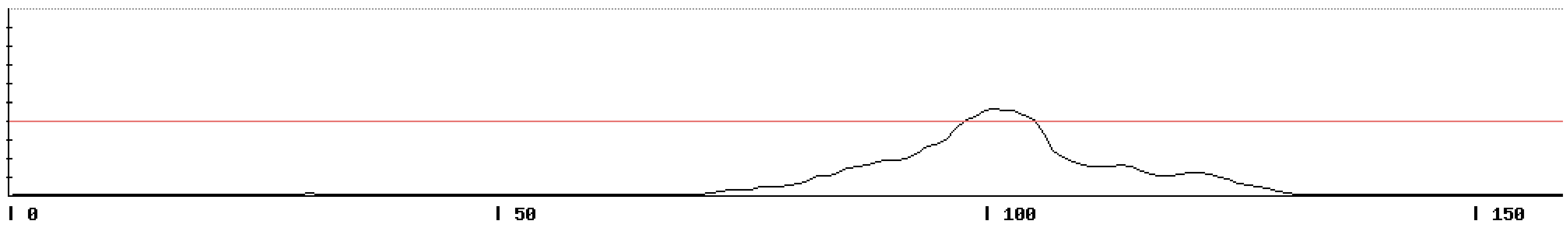

Predictions for Castaneamollissima_scaffold09786-augustus-gene-0.7	
Viterbi Path	
Viterbi Path	
Posterior @ 0.4	
Posterior @ 0.4	
Posterior Graphic. Threshold Marked @ 0.4	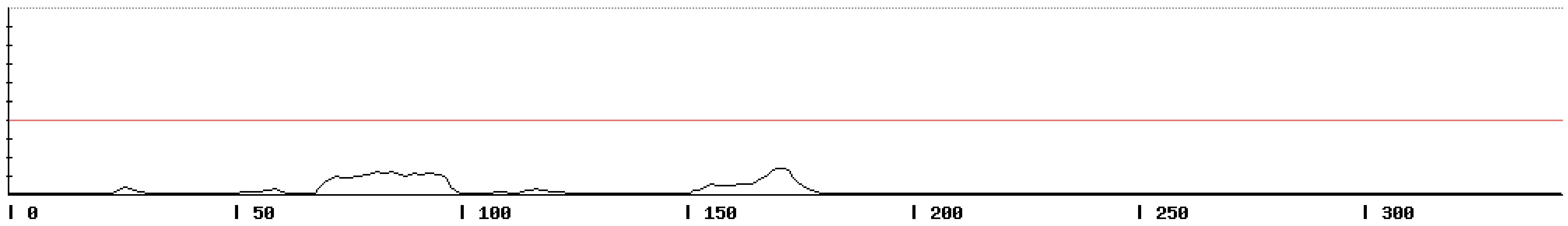

Predictions for Castaneamollissima_scaffold09973-snap-gene-0.10	
Viterbi Path	
Viterbi Path	

Posterior @ 0.4	
Posterior @ 0.4	
Posterior Graphic. Threshold Marked @ 0.4	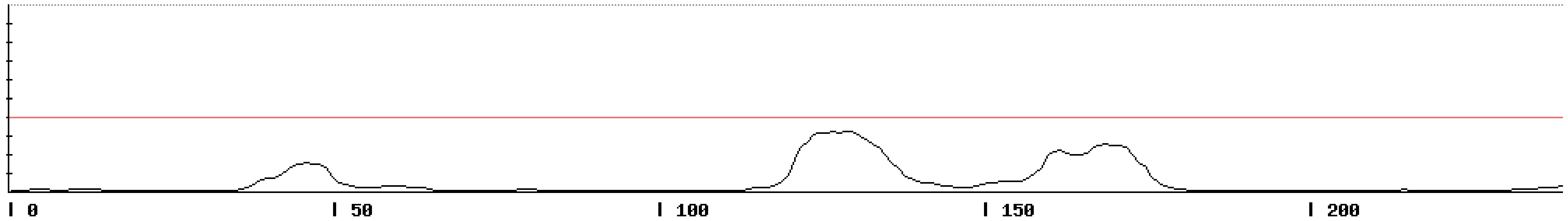

Predictions for Castaneamollissima_scaffold09973-snap-gene-0.11

Viterbi Path	
Viterbi Path	
Posterior @ 0.4	16 - KRAKERKYPNGSRPKR - 31
	412 - KRKHTKGKSK - 421
Posterior @ 0.4	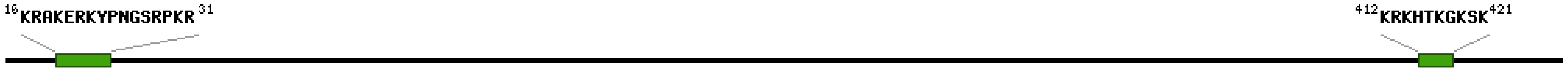
Posterior Graphic. Threshold Marked @ 0.4	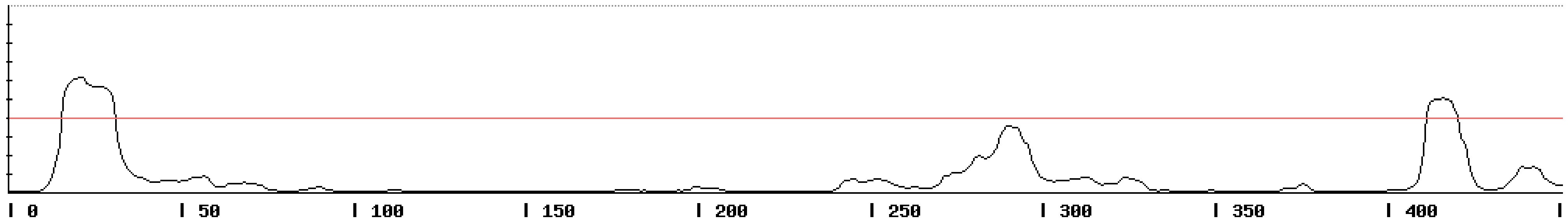

Predictions for Castaneamollissima_scaffold10692-snap-gene-0.12

Viterbi Path	
Viterbi Path	

Posterior @ 0.4	82 - QKKRKR - 87
Posterior @ 0.4	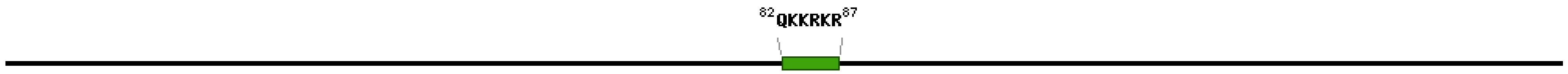
Posterior Graphic. Threshold Marked @ 0.4	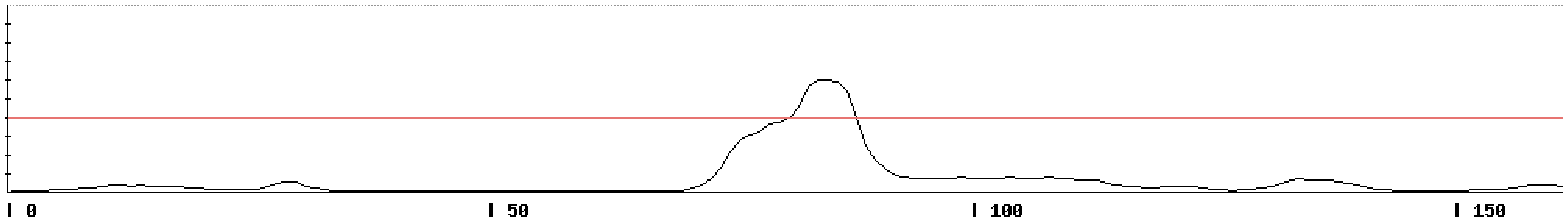

Predictions for Castaneamollissima_scaffold12065-snap-gene-0.6-mRNA

Viterbi Path	
Viterbi Path	
Posterior @ 0.4	
Posterior @ 0.4	
Posterior Graphic. Threshold Marked @ 0.4	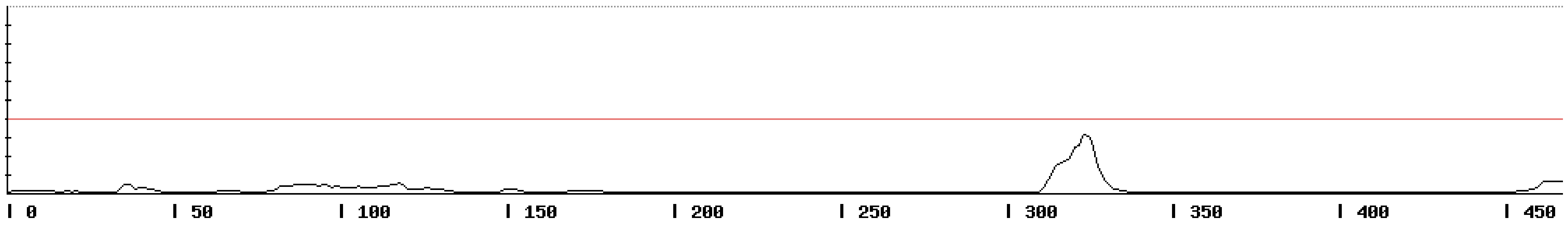

Predictions for Castaneamollissima_scaffold13586-augustus-gene-0.8

Viterbi Path

Viterbi Path

Posterior @ 0.4 82 - PKKRKRDQKQERVSRKTKAG - 101

Posterior @ 0.4

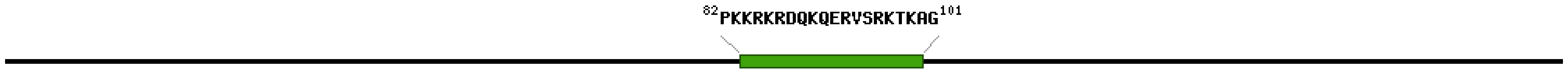

Posterior Graphic. Threshold Marked @ 0.4

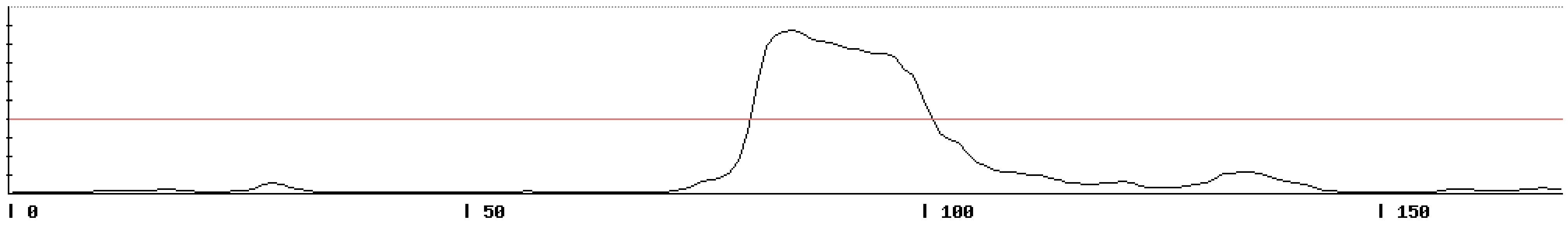

Predictions for Castaneamollissima_scaffold13838-snap-gene-0.5

Viterbi Path

Viterbi Path

Posterior @ 0.4

Posterior @ 0.4

Posterior Graphic. Threshold Marked @ 0.4

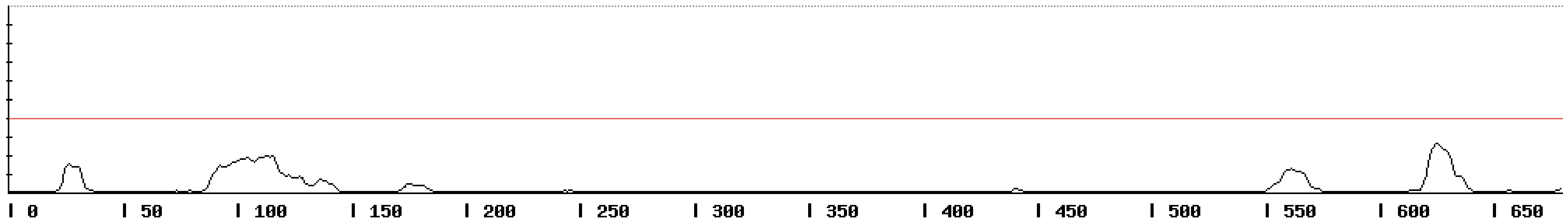

Predictions for Castaneamollissima_scaffold14402-augustus-gene-0.4

Viterbi Path

Viterbi Path

Posterior @ 0.4

Posterior @ 0.4

Posterior Graphic. Threshold Marked @ 0.4

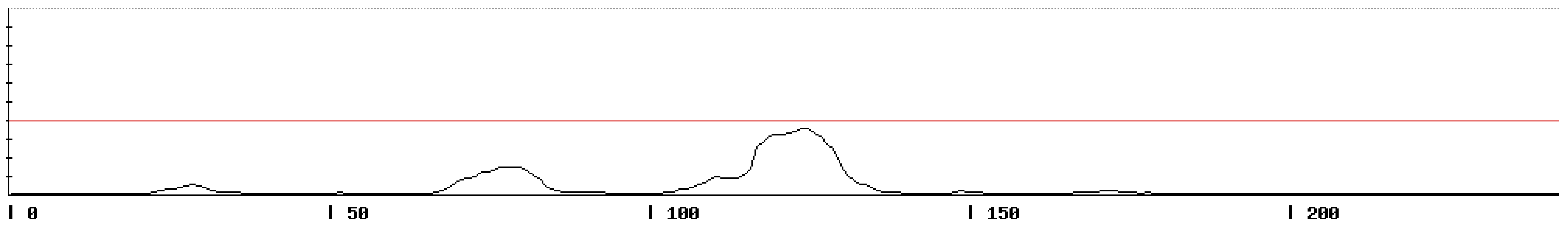

Predictions for Castaneamollissima_scaffold14907-augustus-gene-0.3

Viterbi Path

Viterbi Path

Posterior @ 0.4

Posterior @ 0.4

Posterior Graphic. Threshold Marked @ 0.4

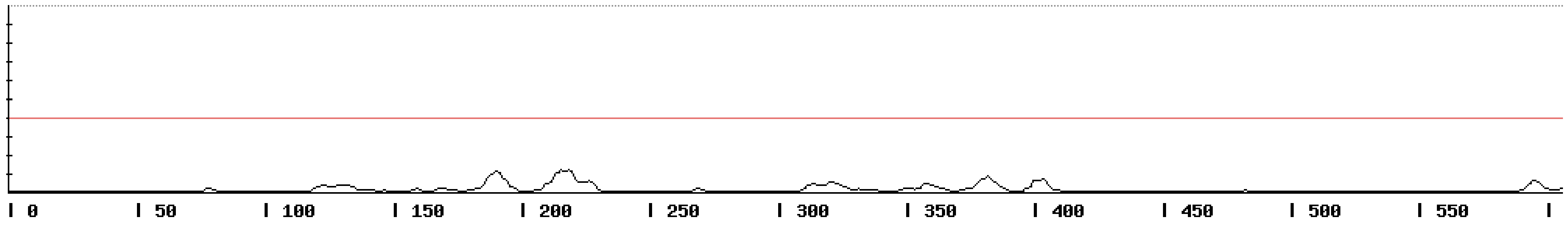

Predictions for Castaneamollissima_scaffold15610-augustus-gene-0.4

Viterbi Path

Viterbi Path

Posterior @ 0.4

Posterior @ 0.4

Posterior Graphic. Threshold Marked @ 0.4

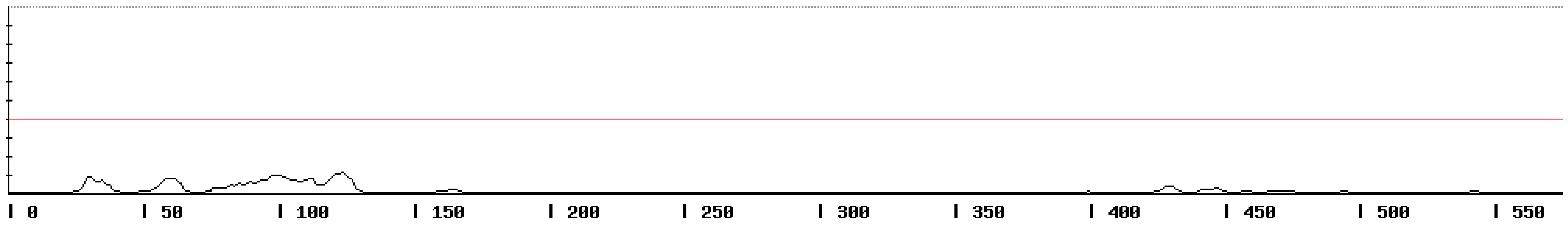

Predictions for Castaneamollissima_scaffold16081-augustus-gene-0.6

Viterbi Path

Viterbi Path

Posterior @ 0.4 82 - PKKRKRDQKQERVNRKTKAG - 101

Posterior @ 0.4

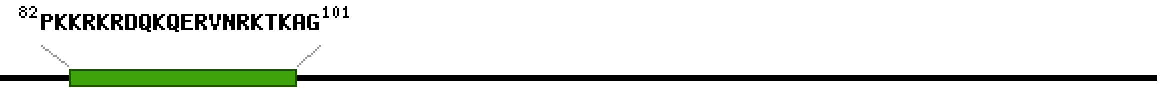

Posterior Graphic. Threshold Marked @ 0.4

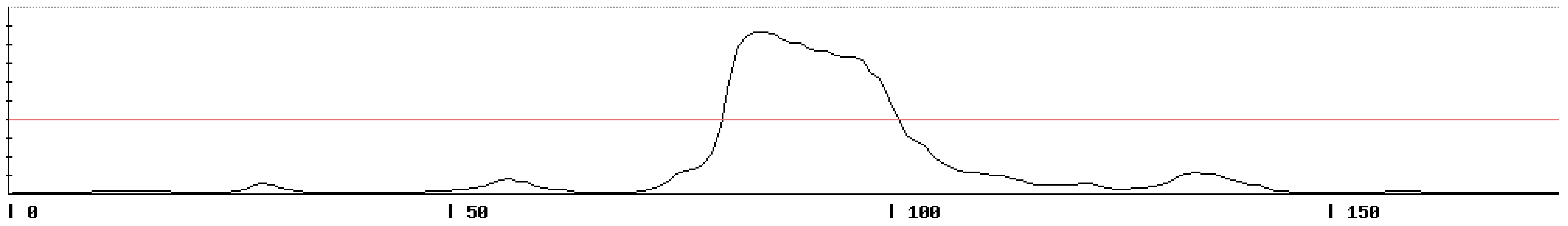

Predictions for Castaneamollissima_scaffold16464-augustus-gene-0.2

Viterbi Path

Viterbi Path

Posterior @ 0.4

Posterior @ 0.4

Posterior Graphic. Threshold Marked @ 0.4

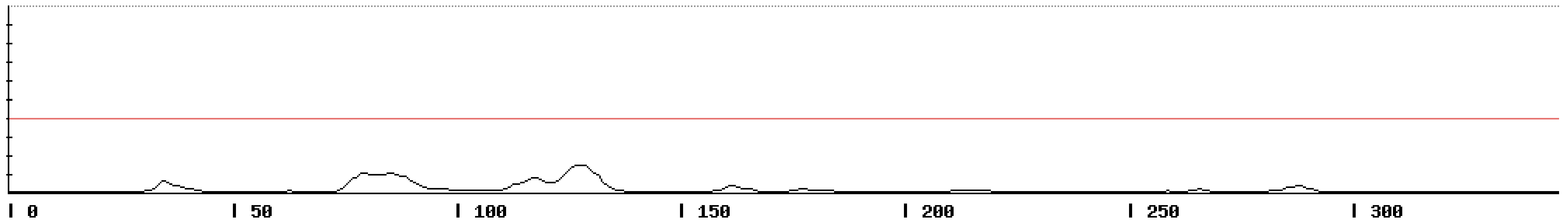

Predictions for Castaneamollissima_scaffold17927-snap-gene-0.8

Viterbi Path

Viterbi Path

Posterior @ 0.4 81 - SKKRKRGQK - 89

Posterior @ 0.4

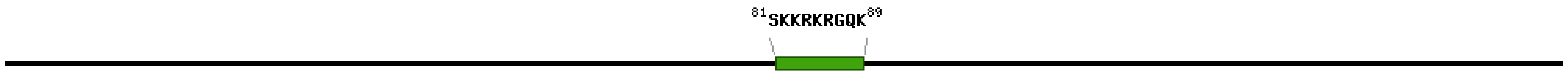

Posterior Graphic. Threshold Marked @ 0.4

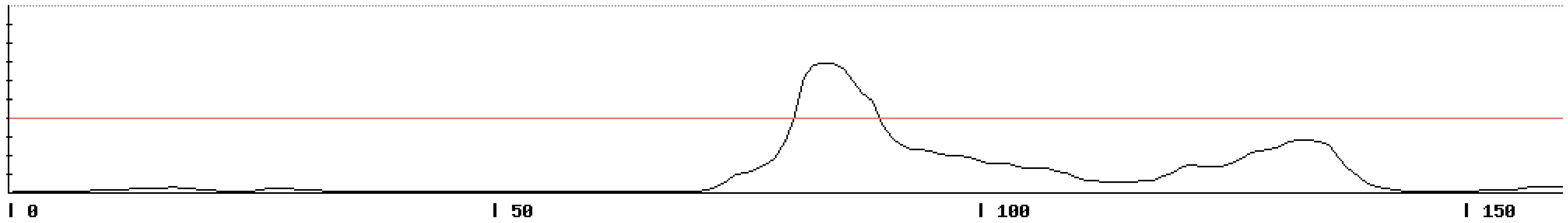

Predictions for Castaneamollissima_scaffold22384-snap-gene-0.5

Viterbi Path

Viterbi Path

Posterior @ 0.4

Posterior @ 0.4

Posterior Graphic. Threshold Marked @ 0.4

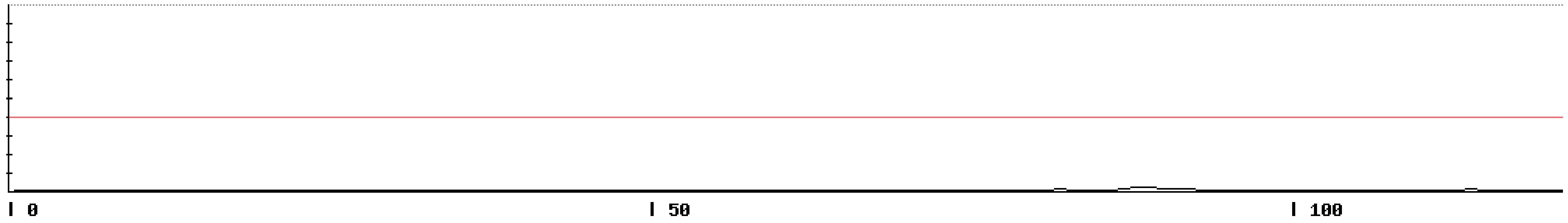

Predictions for Castaneamollissima_scaffold23714-snap-gene-0.3

Viterbi Path

Viterbi Path

Posterior @ 0.4 141 - KRR - 143

Posterior @ 0.4

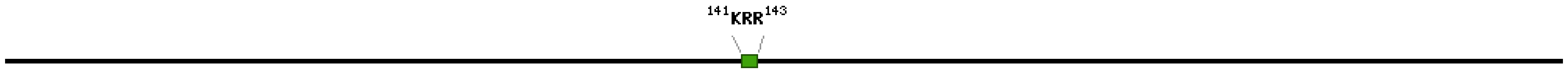

Posterior Graphic. Threshold Marked @ 0.4

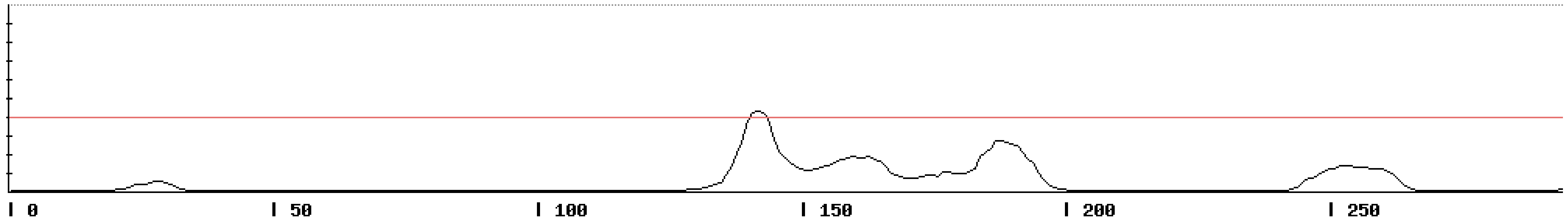

Predictions for Castaneamollissima_scaffold08137-abinit-gene-0.4

Viterbi Path

Viterbi Path

Posterior @ 0.4

Posterior @ 0.4

Posterior Graphic. Threshold Marked @ 0.4

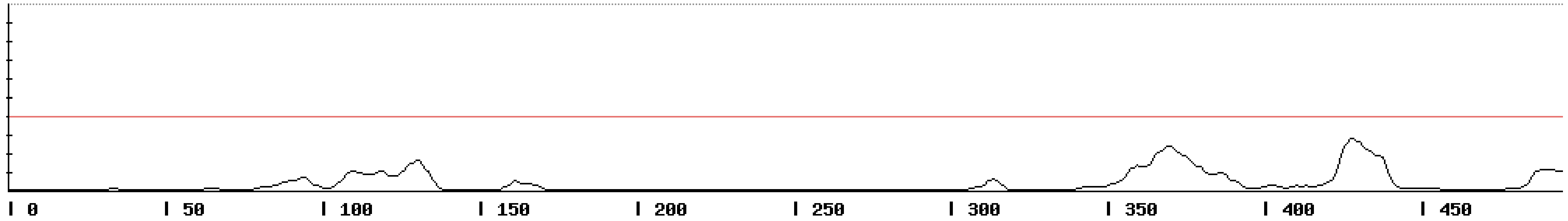

Predictions for Castaneamollissima_scaffold08253-abinit-gene-0.10

Viterbi Path

Viterbi Path

Posterior @ 0.4

Posterior @ 0.4

Posterior Graphic. Threshold Marked @ 0.4

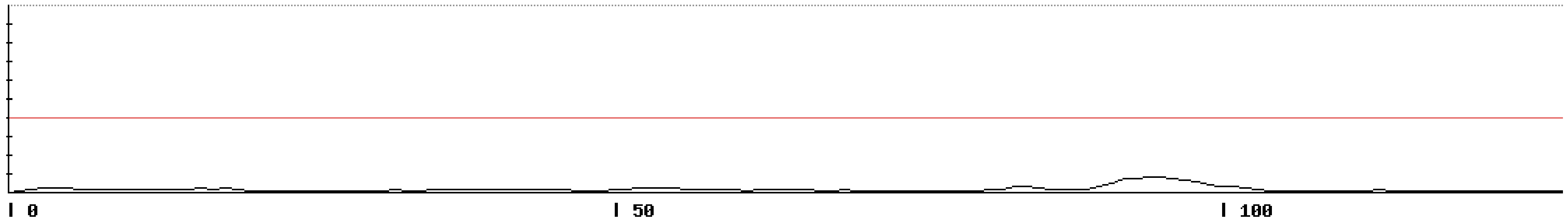

Predictions for Castaneamollissima_scaffold08322-abinit-gene-0.9

Viterbi Path

Viterbi Path

Posterior @ 0.4

Posterior @ 0.4

Posterior Graphic. Threshold Marked @ 0.4

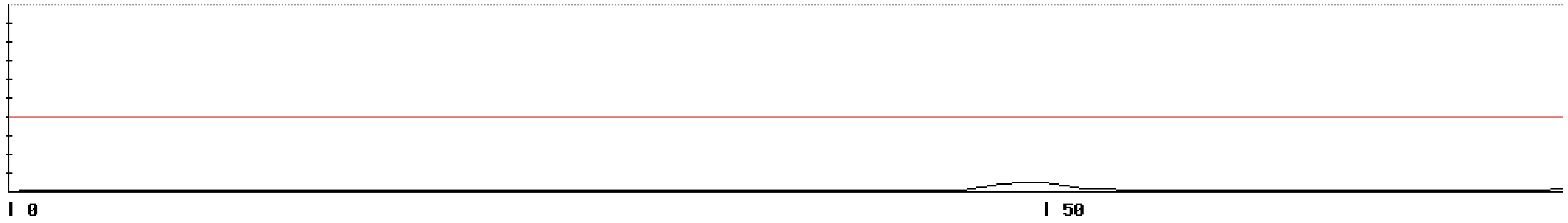

Predictions for Castaneamollissima_scaffold12800-abinit-gene-0.4

Viterbi Path	
Viterbi Path	
Posterior @ 0.4	
Posterior @ 0.4	
Posterior Graphic. Threshold Marked @ 0.4	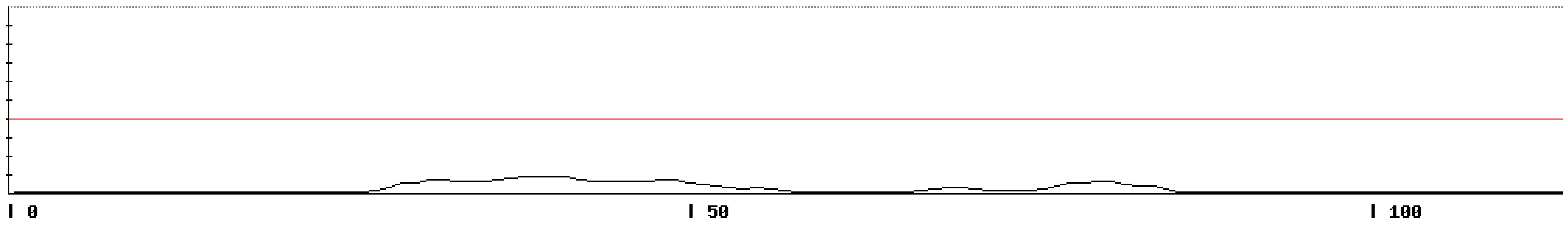

Predictions for Castaneamollissima_scaffold13705-abinit-gene-0.5

Viterbi Path	
Viterbi Path	
Posterior @ 0.4	
Posterior @ 0.4	

Revision r.9 - Last Updated on : February 24 2015 17:45:20. alex.nguyenba@utoronto.ca

Intended to be used with Firefox

Viterbi Path

Posterior @ 0.4

Posterior @ 0.4

Posterior Graphic. Threshold Marked @ 0.4

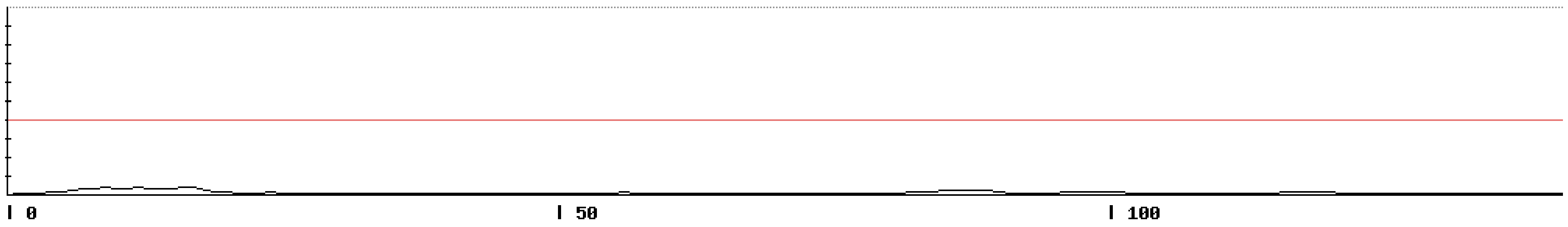

Predictions for Catharanthusroseus_cra_locus_10450_iso_4

Viterbi Path

Viterbi Path

Posterior @ 0.4

Posterior @ 0.4

Posterior Graphic. Threshold Marked @ 0.4

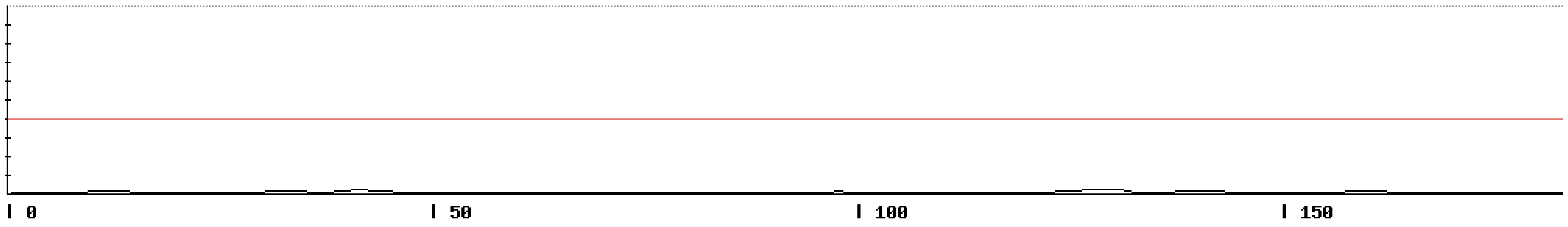

Predictions for Catharanthusroseus_cra_locus_10904_iso_1

Viterbi Path	
Viterbi Path	
Posterior @ 0.4	164 - KIKKRAKKGRNS - 175
Posterior @ 0.4	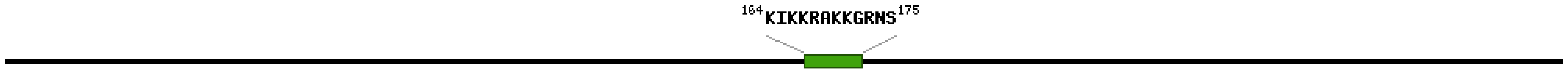
Posterior Graphic. Threshold Marked @ 0.4	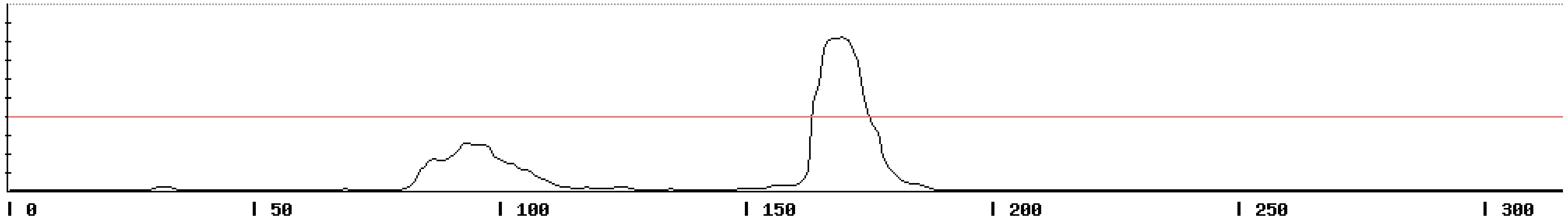

Predictions for Catharanthusroseus_cra_locus_10904_iso_2

Viterbi Path	
Viterbi Path	
Posterior @ 0.4	164 - KIKKRAKKGRNS - 175
Posterior @ 0.4	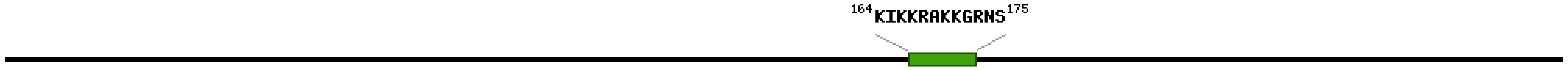
Posterior Graphic. Threshold Marked @ 0.4	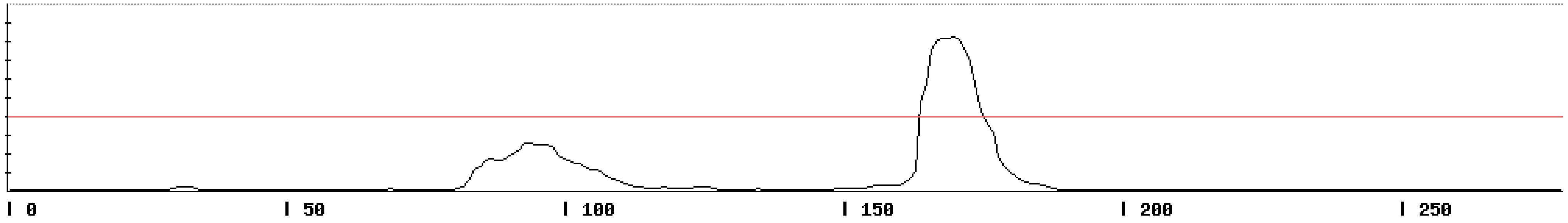

Predictions for Catharanthusroseus_cra_locus_10952_iso_1	
Viterbi Path	
Viterbi Path	
Posterior @ 0.4	
Posterior @ 0.4	
Posterior Graphic. Threshold Marked @ 0.4	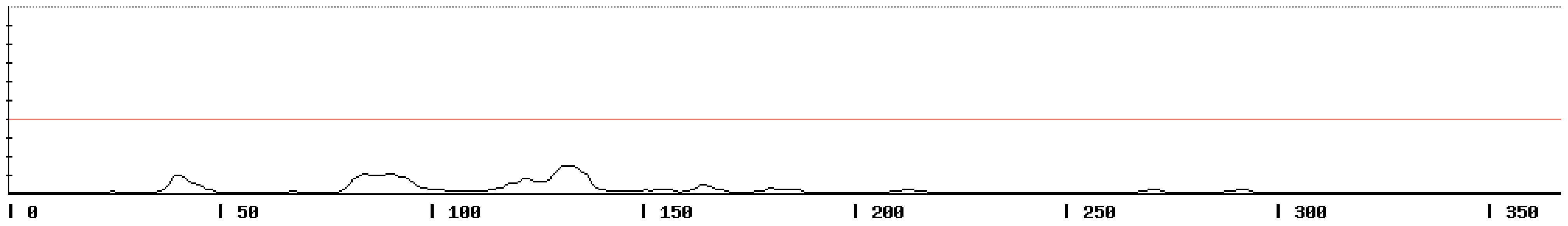

Predictions for Catharanthusroseus_cra_locus_10952_iso_2	
Viterbi Path	
Viterbi Path	
Posterior @ 0.4	
Posterior @ 0.4	
Posterior Graphic. Threshold Marked @ 0.4	

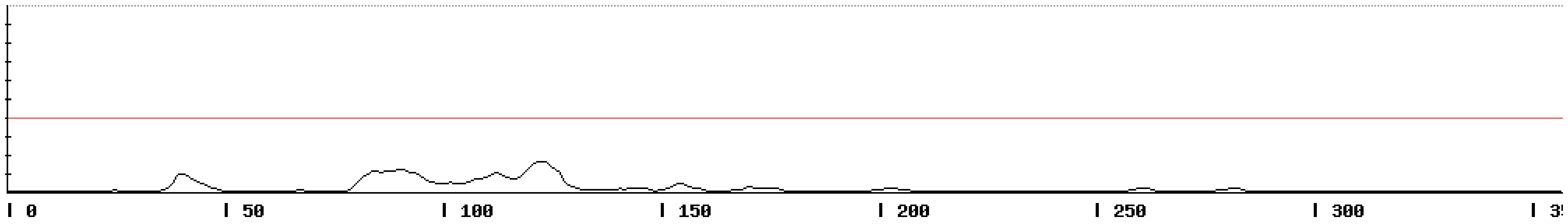

Predictions for Catharanthusroseus_cra_locus_112403_iso_1

Viterbi Path	
Viterbi Path	
Posterior @ 0.4	
Posterior @ 0.4	
Posterior Graphic. Threshold Marked @ 0.4	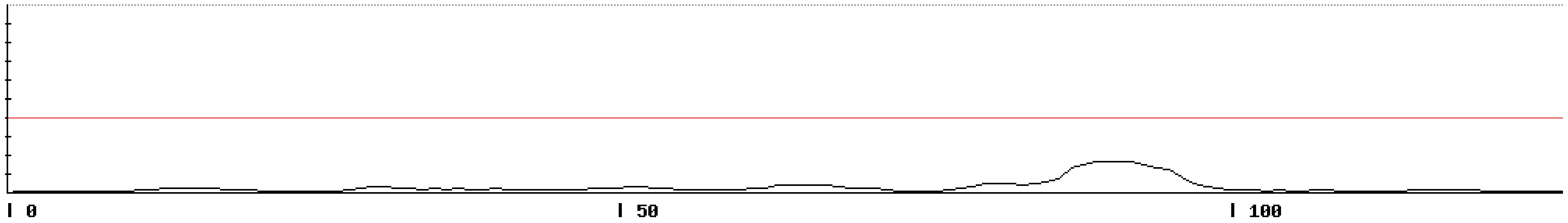

Predictions for Catharanthusroseus_cra_locus_112403_iso_2

Viterbi Path	
Viterbi Path	
Posterior @ 0.4	
Posterior @ 0.4	

Posterior Graphic. Threshold Marked @ 0.4

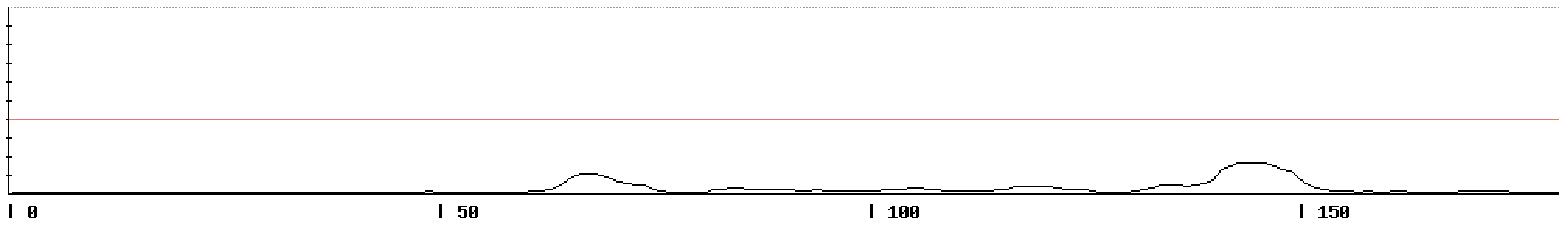

Predictions for Catharanthusroseus_cra_locus_112403_iso_3

Viterbi Path

Viterbi Path

Posterior @ 0.4

Posterior @ 0.4

Posterior Graphic. Threshold Marked @ 0.4

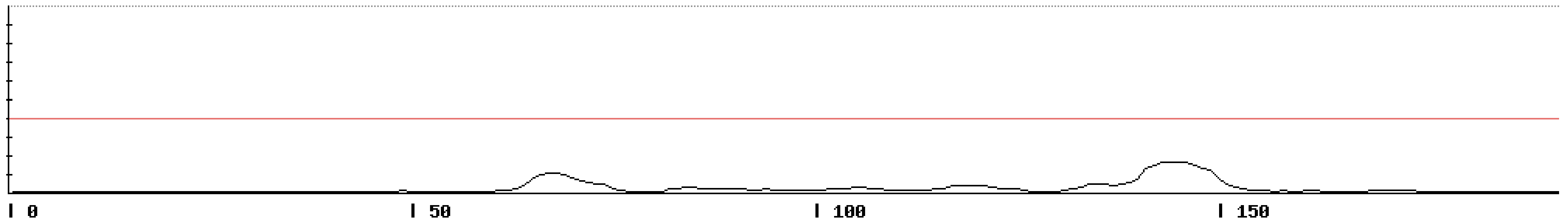

Predictions for Catharanthusroseus_cra_locus_112403_iso_4

Viterbi Path

Viterbi Path

Posterior @ 0.4

Posterior @ 0.4

Posterior Graphic. Threshold Marked @ 0.4

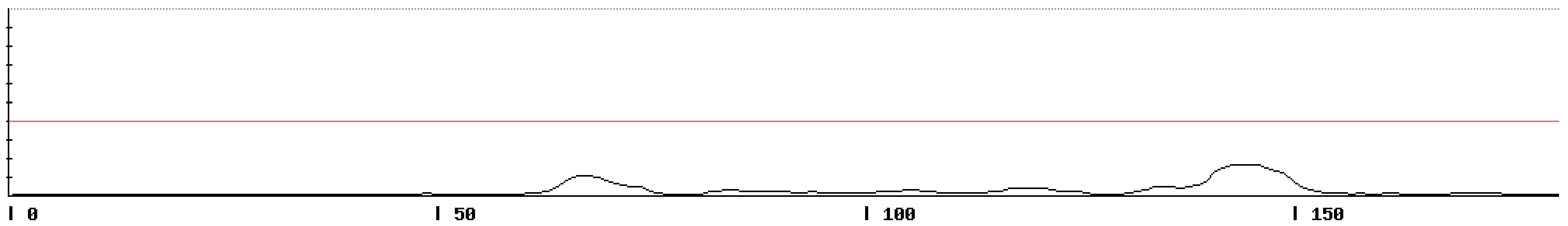

Predictions for Catharanthusroseus_cra_locus_11431_iso_1

Viterbi Path	
Viterbi Path	
Posterior @ 0.4	157 - RRKKRKTK - 164
Posterior @ 0.4	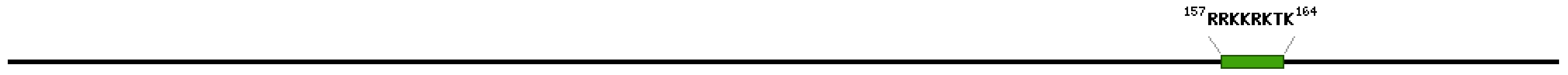
Posterior Graphic. Threshold Marked @ 0.4	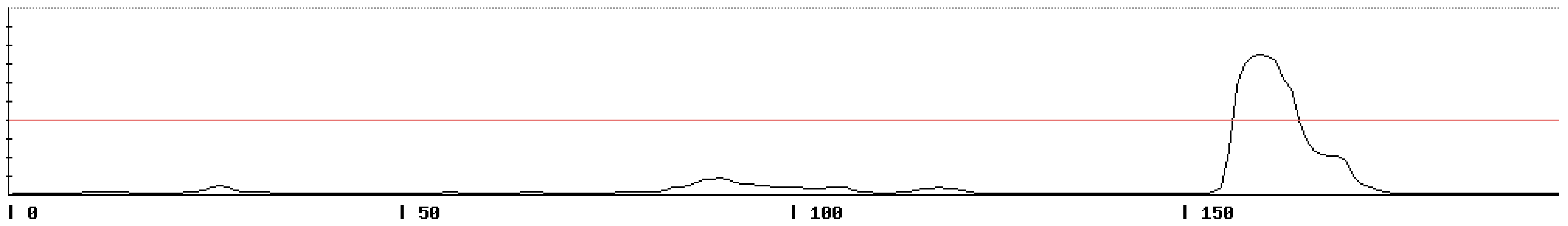

Predictions for Catharanthusroseus_cra_locus_12026_iso_1

Viterbi Path	
Viterbi Path	

Posterior @ 0.4	
Posterior @ 0.4	
Posterior Graphic. Threshold Marked @ 0.4	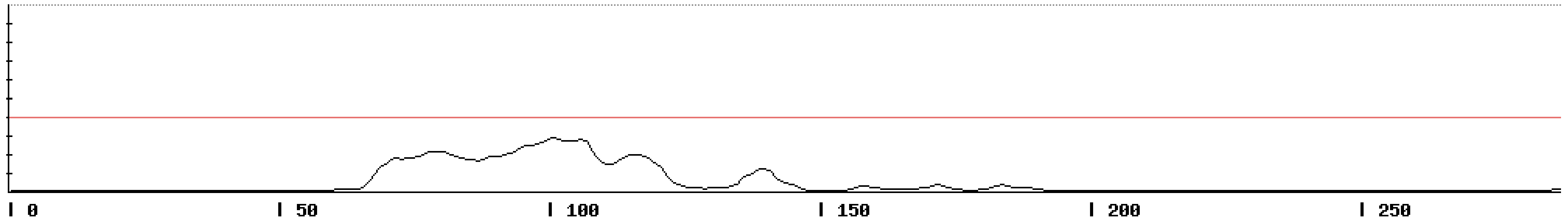

Predictions for Catharanthusroseus_cra_locus_12026_iso_3	
Viterbi Path	
Viterbi Path	
Posterior @ 0.4	
Posterior @ 0.4	
Posterior Graphic. Threshold Marked @ 0.4	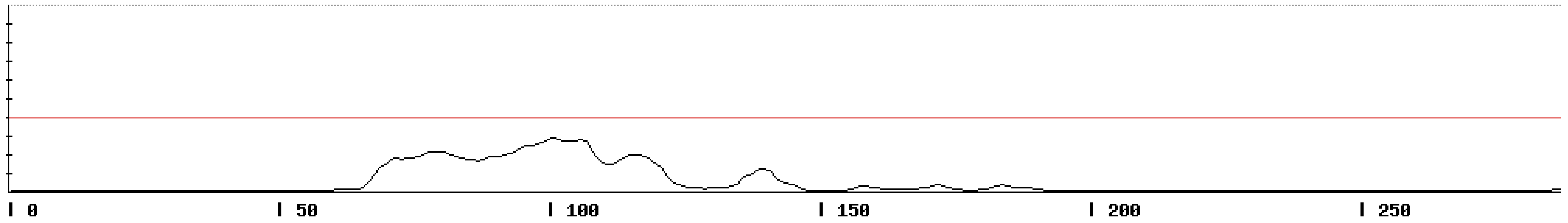

Predictions for Catharanthusroseus_cra_locus_12026_iso_4	
Viterbi Path	
Viterbi Path	

Posterior @ 0.4

Posterior @ 0.4

Posterior Graphic. Threshold Marked @ 0.4

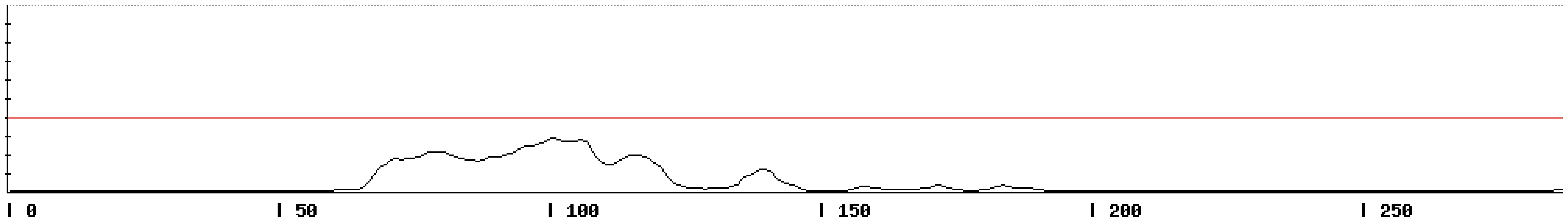

Predictions for Catharanthusroseus_cra_locus_12026_iso_5

Viterbi Path

Viterbi Path

Posterior @ 0.4

Posterior @ 0.4

Posterior Graphic. Threshold Marked @ 0.4

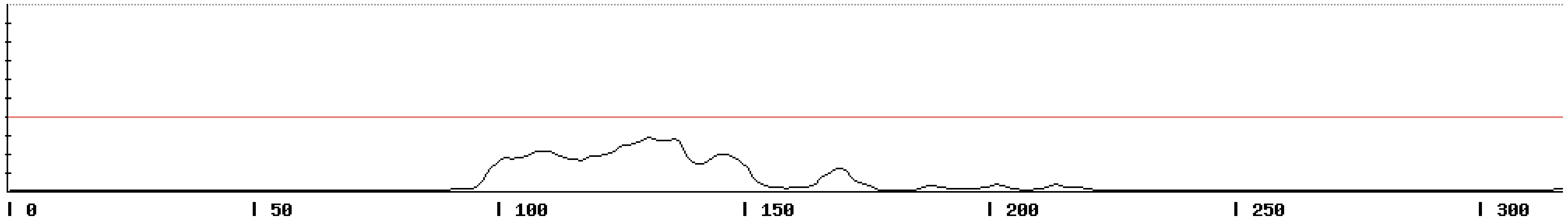

Predictions for Catharanthusroseus_cra_locus_12026_iso_6

Viterbi Path	
Viterbi Path	
Posterior @ 0.4	
Posterior @ 0.4	
Posterior Graphic. Threshold Marked @ 0.4	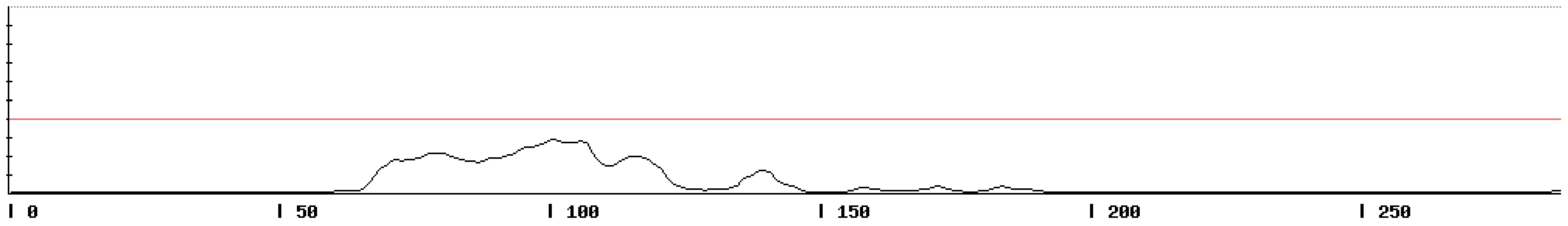

Predictions for Catharanthusroseus_cra_locus_12235_iso_1

Viterbi Path	
Viterbi Path	
Posterior @ 0.4	355 - RLRLRRPK - 362
Posterior @ 0.4	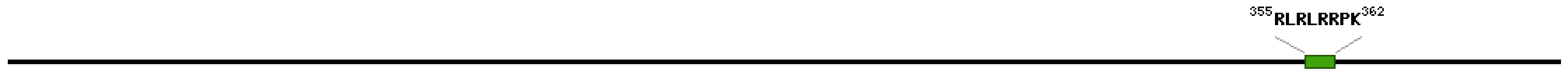
Posterior Graphic. Threshold Marked @ 0.4	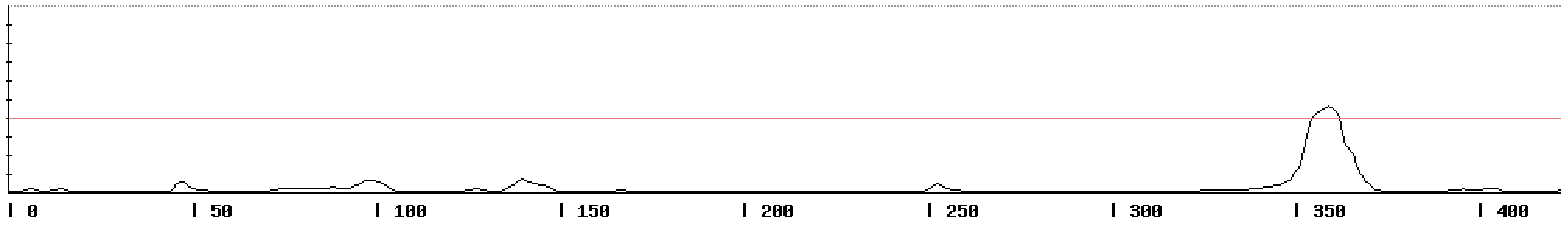

Predictions for Catharanthusroseus_cra_locus_12235_iso_3	
Viterbi Path	
Viterbi Path	
Posterior @ 0.4	278 - RLRLRRPK - 285
Posterior @ 0.4	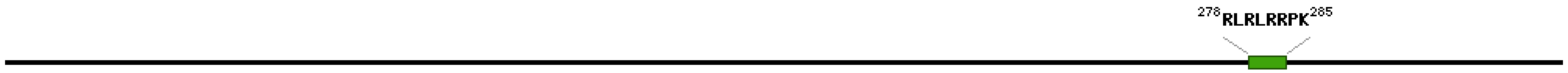
Posterior Graphic. Threshold Marked @ 0.4	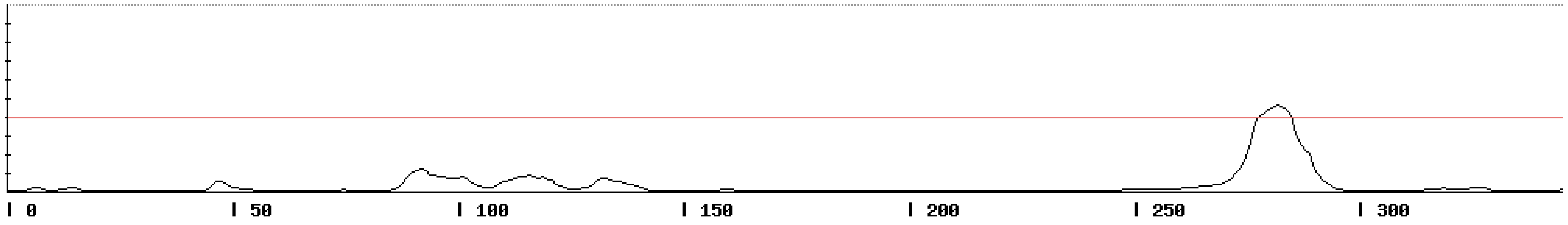

Predictions for Catharanthusroseus_cra_locus_12235_iso_4	
Viterbi Path	
Viterbi Path	
Posterior @ 0.4	285 - RLRLRRPK - 292
Posterior @ 0.4	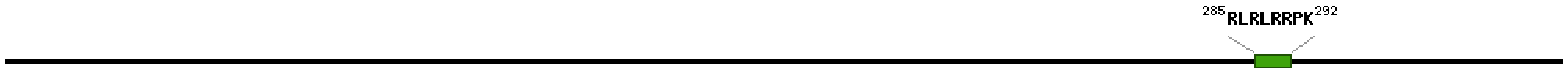
Posterior Graphic. Threshold Marked @ 0.4	

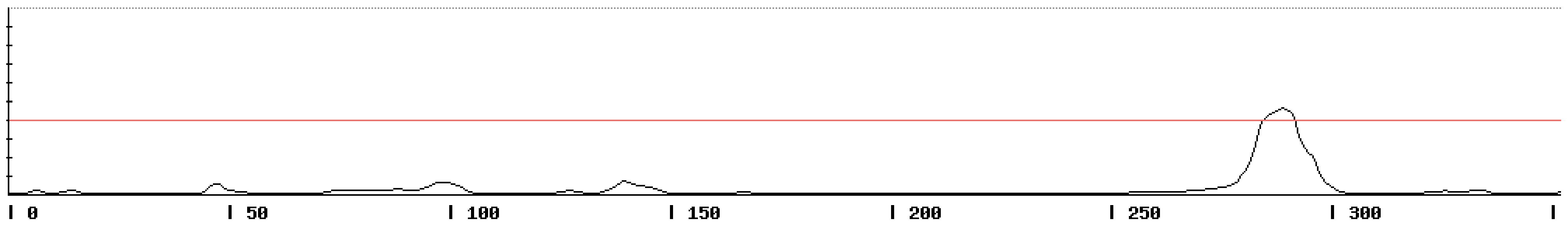

Predictions for Catharanthusroseus_cra_locus_12712_iso_1

Viterbi Path

Viterbi Path

Posterior @ 0.4

Posterior @ 0.4

Posterior Graphic. Threshold Marked @ 0.4

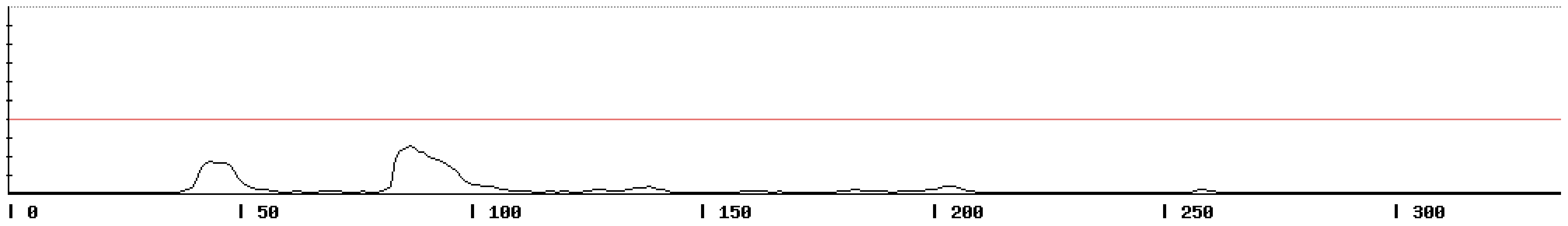

Predictions for Catharanthusroseus_cra_locus_12712_iso_2

Viterbi Path

Viterbi Path

Posterior @ 0.4

Posterior @ 0.4

Posterior Graphic. Threshold Marked @ 0.4	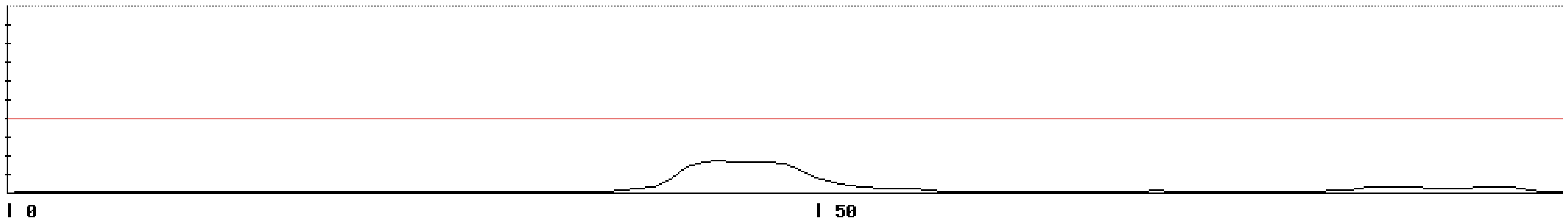

Predictions for Catharanthusroseus_cra_locus_12712_iso_3

Viterbi Path	
Viterbi Path	
Posterior @ 0.4	
Posterior @ 0.4	
Posterior Graphic. Threshold Marked @ 0.4	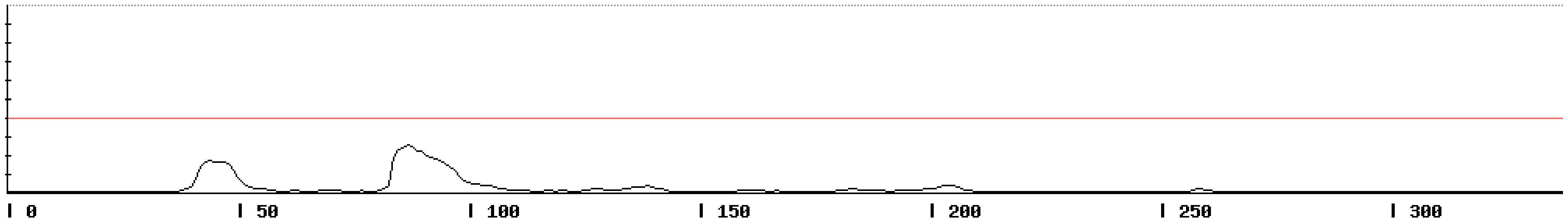

Predictions for Catharanthusroseus_cra_locus_1291_iso_2

Viterbi Path	
Viterbi Path	
Posterior @ 0.4	
Posterior @ 0.4	

Posterior Graphic. Threshold Marked @ 0.4

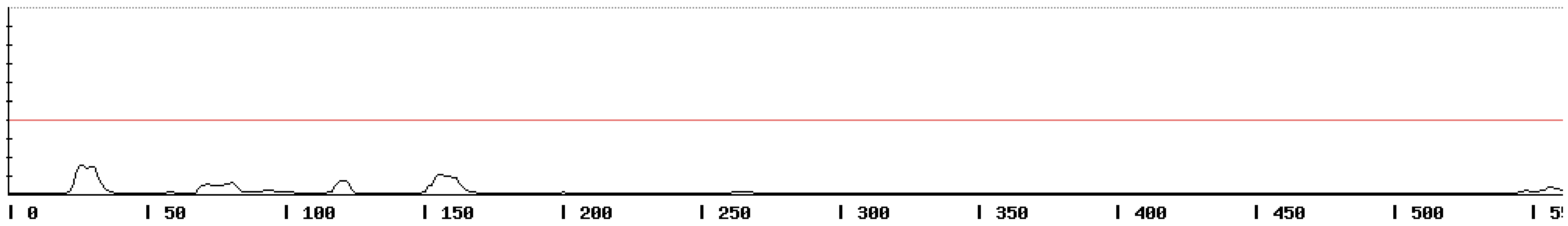

Predictions for Catharanthusroseus_cra_locus_1291_iso_3

Viterbi Path

Viterbi Path

Posterior @ 0.4

Posterior @ 0.4

Posterior Graphic. Threshold Marked @ 0.4

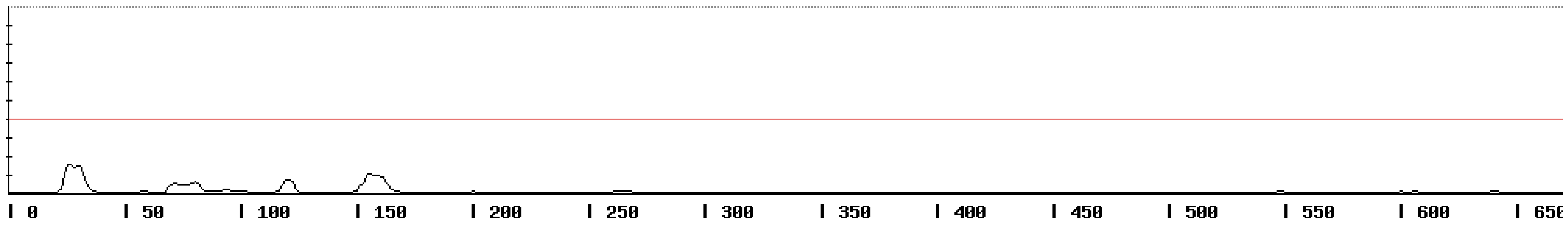

Predictions for Catharanthusroseus_cra_locus_12960_iso_1

Viterbi Path

Viterbi Path

Posterior @ 0.4

Posterior @ 0.4

Posterior Graphic. Threshold Marked @ 0.4

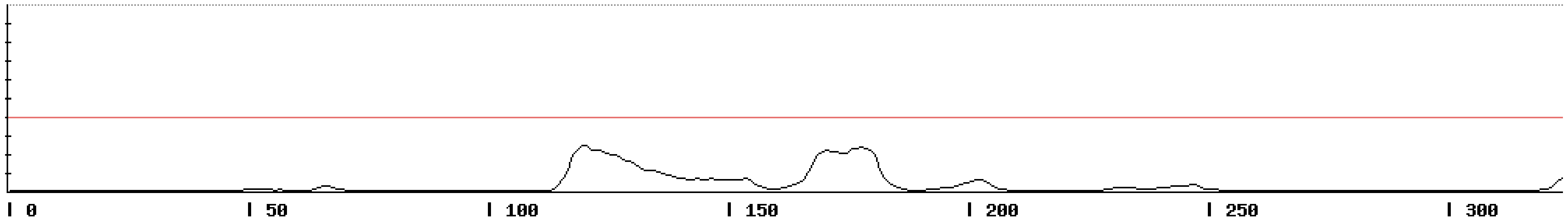

Predictions for Catharanthusroseus_cra_locus_12983_iso_1

Viterbi Path

Viterbi Path

Posterior @ 0.4

Posterior @ 0.4

Posterior Graphic. Threshold Marked @ 0.4

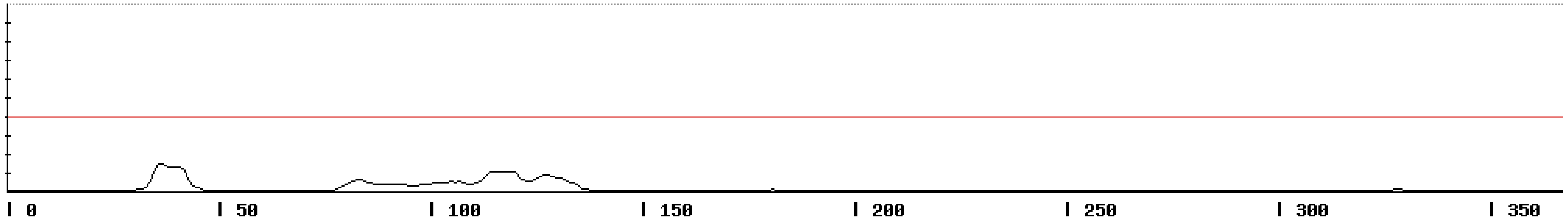

Predictions for Catharanthusroseus_cra_locus_13006_iso_2

Viterbi Path

Viterbi Path

Posterior @ 0.4	431 - GKKKVMKNKRIKK - 443
Posterior @ 0.4	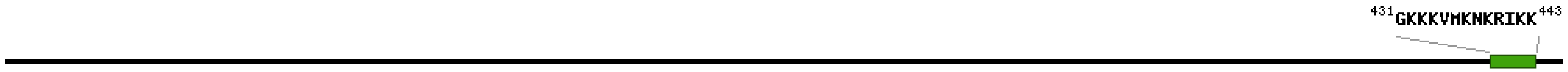
Posterior Graphic. Threshold Marked @ 0.4	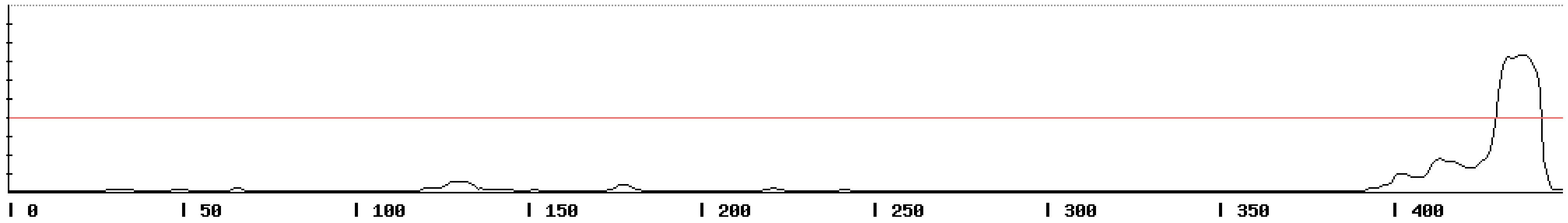

Predictions for Catharanthusroseus_cra_locus_13006_iso_4

Viterbi Path	
Viterbi Path	
Posterior @ 0.4	
Posterior @ 0.4	
Posterior Graphic. Threshold Marked @ 0.4	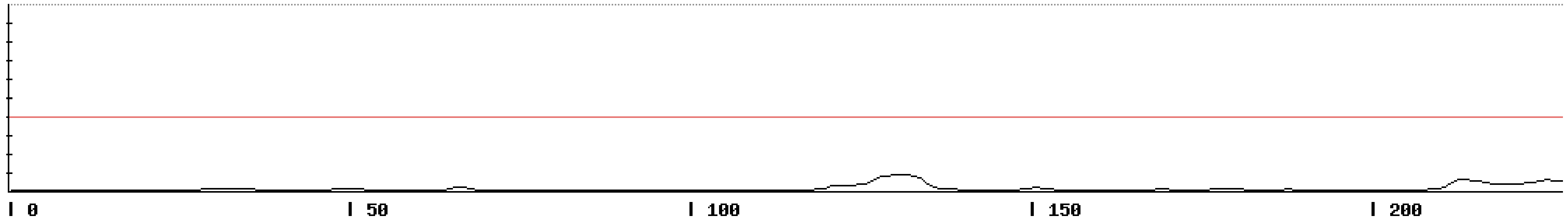

Predictions for Catharanthusroseus_cra_locus_13006_iso_5

Viterbi Path	
Viterbi Path	
Posterior @ 0.4	
Posterior @ 0.4	
Posterior Graphic. Threshold Marked @ 0.4	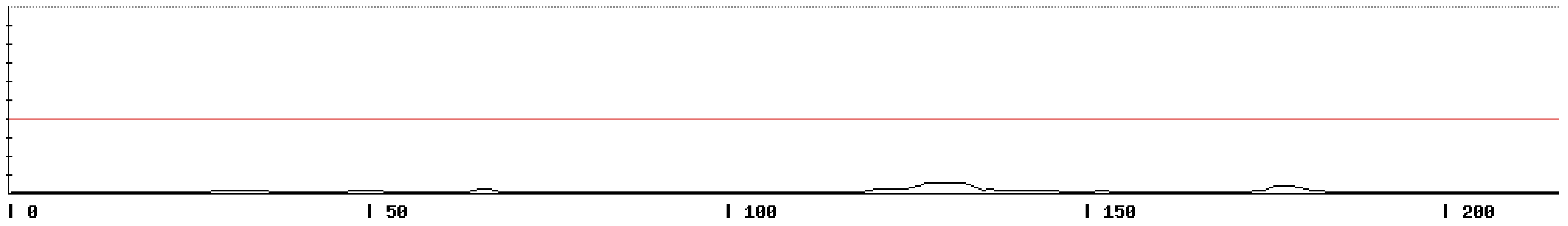

Predictions for Catharanthusroseus_cra_locus_13006_iso_6

Viterbi Path	
Viterbi Path	
Posterior @ 0.4	360 - GKKKVMKNKRIKK - 372
Posterior @ 0.4	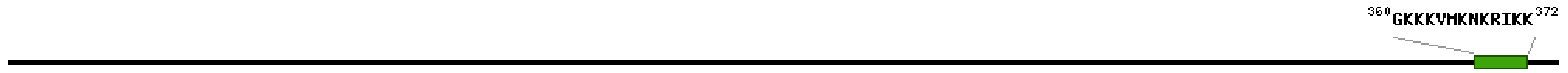
Posterior Graphic. Threshold Marked @ 0.4	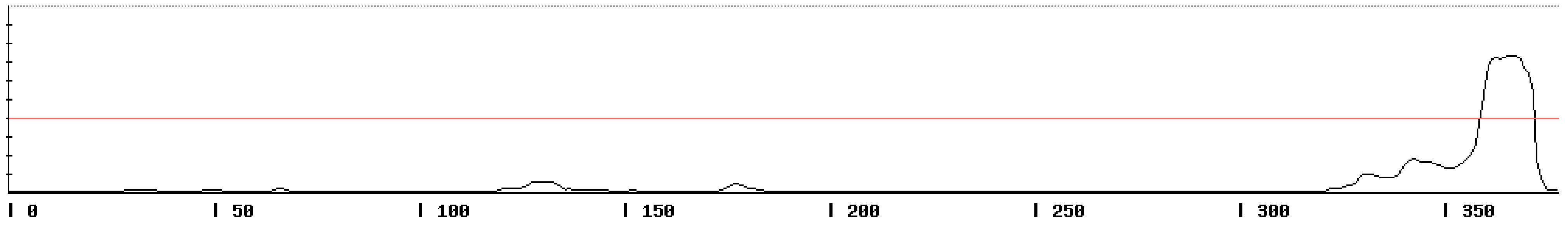

Predictions for Catharanthusroseus_cra_locus_13047_iso_1

Viterbi Path

Viterbi Path

Posterior @ 0.4

Posterior @ 0.4

Posterior Graphic. Threshold Marked @ 0.4

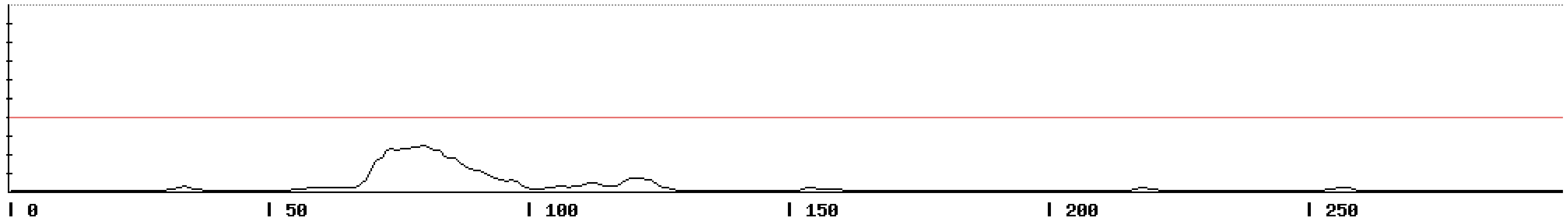

Predictions for Catharanthusroseus_cra_locus_13047_iso_2

Viterbi Path

Viterbi Path

Posterior @ 0.4

Posterior @ 0.4

Posterior Graphic. Threshold Marked @ 0.4

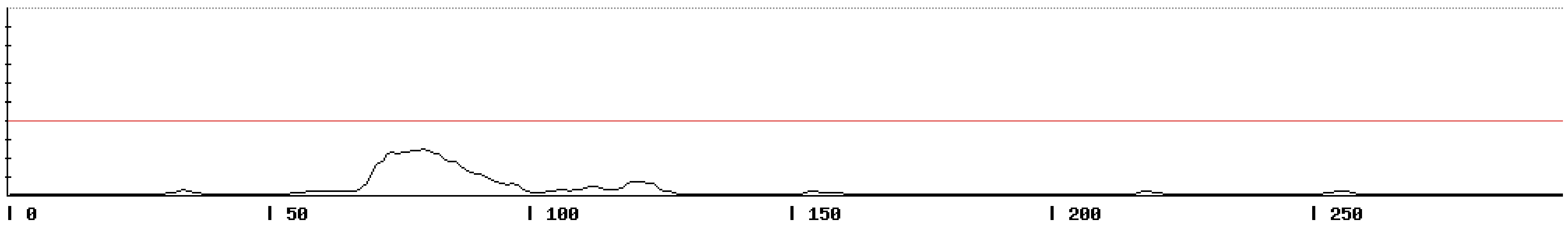

Predictions for Catharanthusroseus_cra_locus_1368_iso_1

Viterbi Path

Viterbi Path

Posterior @ 0.4

Posterior @ 0.4

Posterior Graphic. Threshold Marked @ 0.4

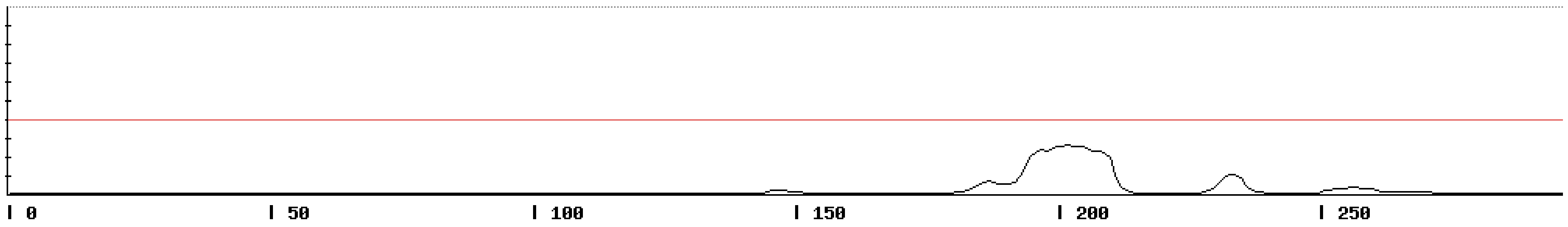

Predictions for Catharanthusroseus_cra_locus_1368_iso_3

Viterbi Path

Viterbi Path

Posterior @ 0.4

2 - RDRRRRQRNGYNRDRRPRGRQREPR - 26

Posterior @ 0.4

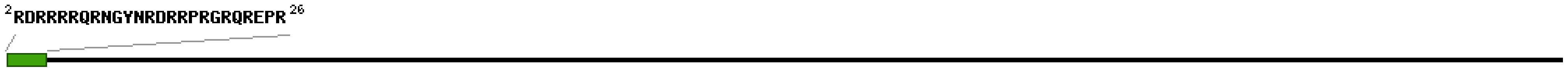

Posterior Graphic. Threshold Marked @ 0.4

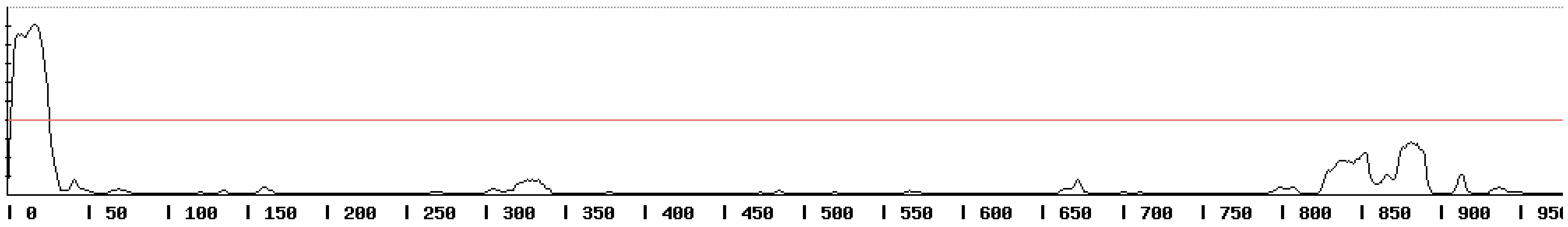

Predictions for Catharanthusroseus_cra_locus_1368_iso_4

Viterbi Path

Viterbi Path

Posterior @ 0.4

2 - RDRRRRQRNGYNRDRRPRGRQREPR - 26

Posterior @ 0.4

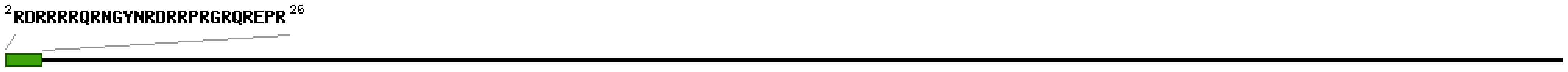

Posterior Graphic. Threshold Marked @ 0.4

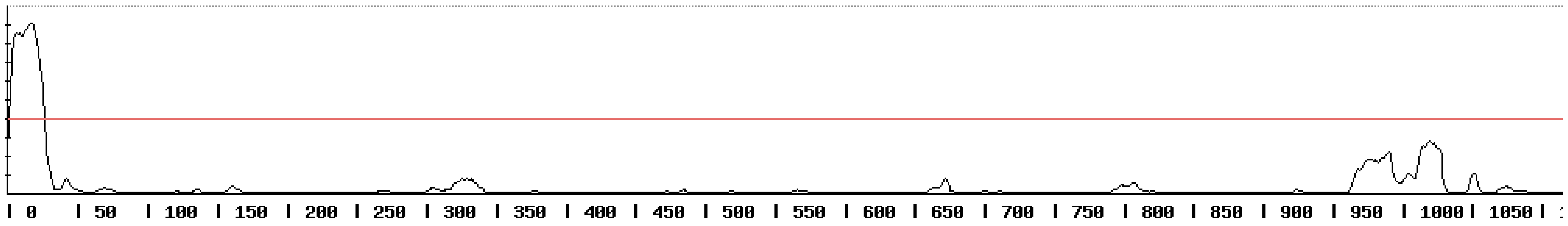

Predictions for Catharanthusroseus_cra_locus_1368_iso_6

Viterbi Path

Viterbi Path

Posterior @ 0.4

Posterior @ 0.4

Posterior Graphic. Threshold Marked @ 0.4

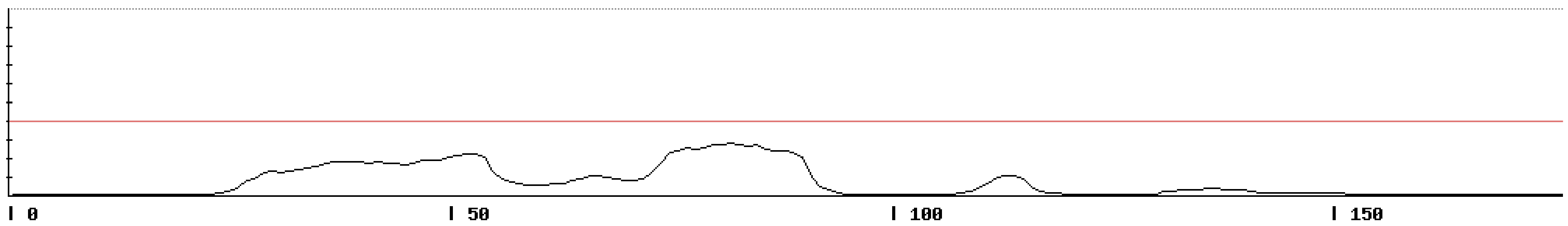

Predictions for Catharanthusroseus_cra_locus_1368_iso_8

Viterbi Path

Viterbi Path

Posterior @ 0.4

Posterior @ 0.4

Posterior Graphic. Threshold Marked @ 0.4

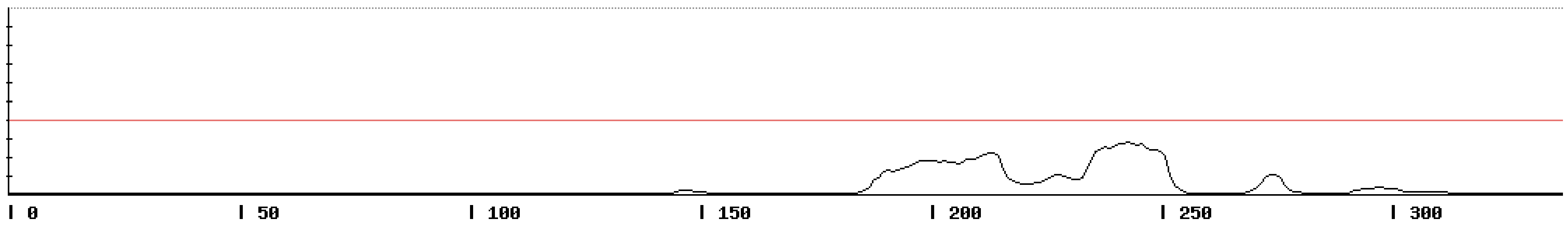

Predictions for Catharanthusroseus_cra_locus_15344_iso_1

Viterbi Path

Viterbi Path

Posterior @ 0.4

Posterior @ 0.4

Posterior Graphic. Threshold Marked @ 0.4

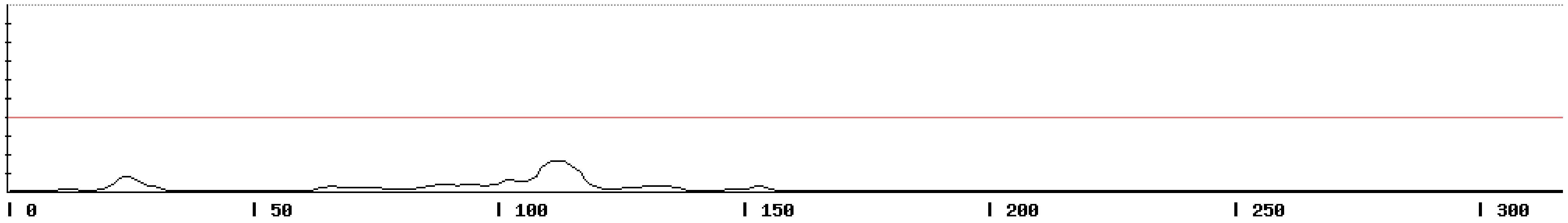

Predictions for Catharanthusroseus_cra_locus_15356_iso_1

Viterbi Path

Viterbi Path

Posterior @ 0.4

150 - HKTGKTRPVIVKGKVKGYK - 168

Posterior @ 0.4

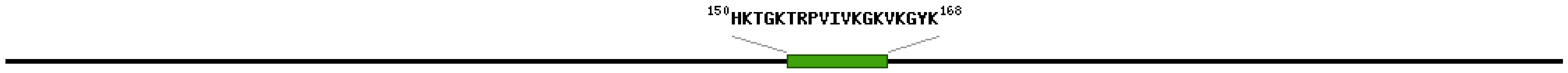

Posterior Graphic. Threshold Marked @ 0.4

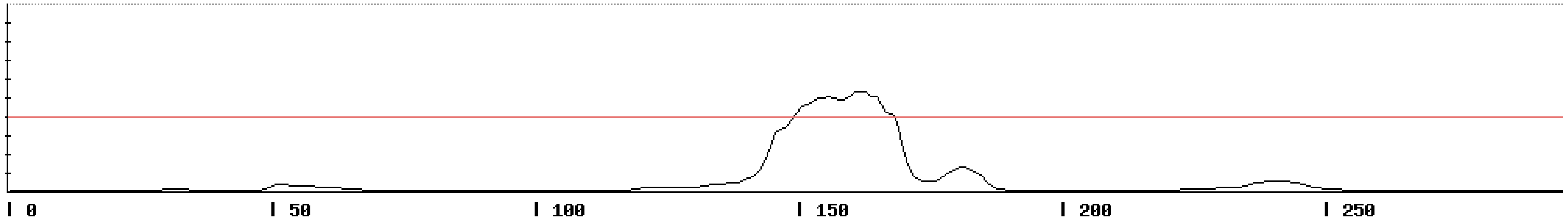

Predictions for Catharanthusroseus_cra_locus_16332_iso_1

Viterbi Path

Viterbi Path

Posterior @ 0.4

Posterior @ 0.4

Posterior Graphic. Threshold Marked @ 0.4

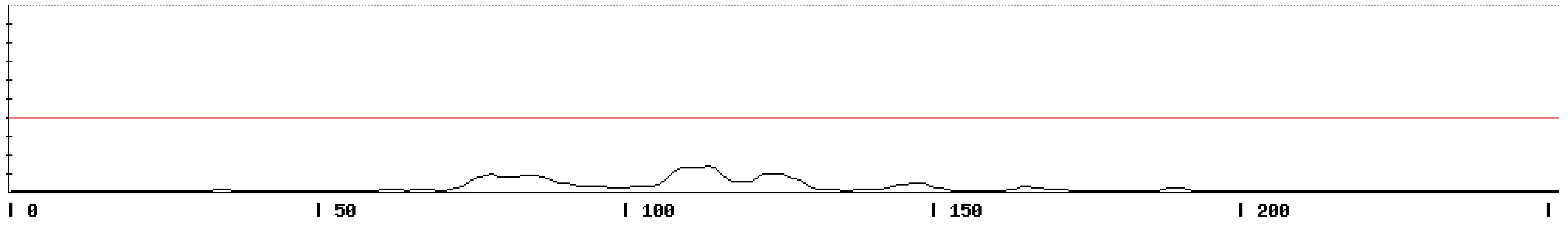

Predictions for Catharanthusroseus_cra_locus_16332_iso_2

Viterbi Path

Viterbi Path

Posterior @ 0.4

Posterior @ 0.4

Posterior Graphic. Threshold Marked @ 0.4

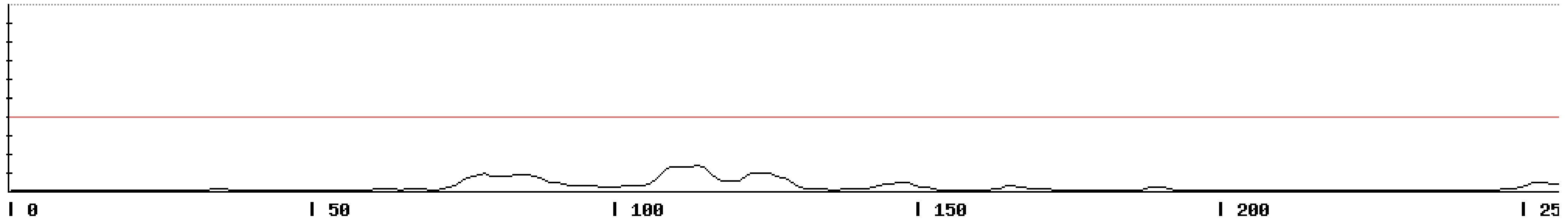

Predictions for Catharanthusroseus_cra_locus_16332_iso_3

Viterbi Path

Viterbi Path

Posterior @ 0.4

Posterior @ 0.4

Posterior Graphic. Threshold Marked @ 0.4

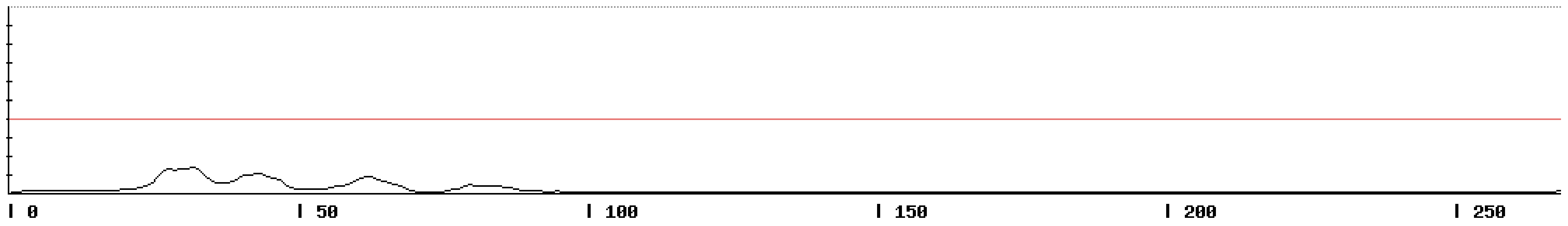

Predictions for Catharanthusroseus_cra_locus_16332_iso_4

Viterbi Path

Viterbi Path

Posterior @ 0.4

Posterior @ 0.4

Posterior Graphic. Threshold Marked @ 0.4

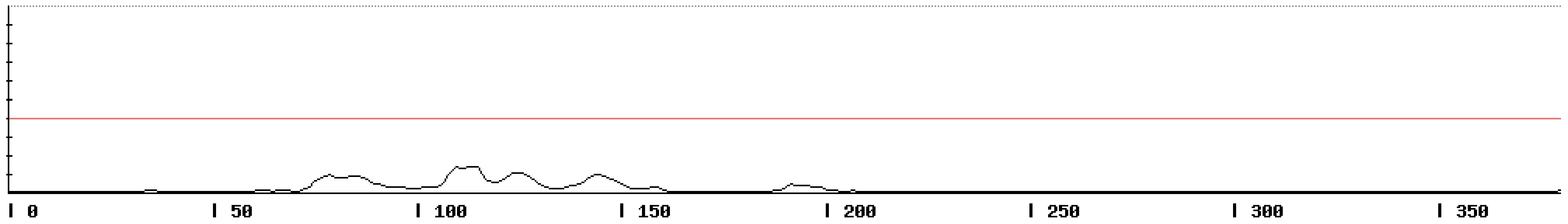

Predictions for Catharanthusroseus_cra_locus_16332_iso_5	
Viterbi Path	
Viterbi Path	
Posterior @ 0.4	
Posterior @ 0.4	
Posterior Graphic. Threshold Marked @ 0.4	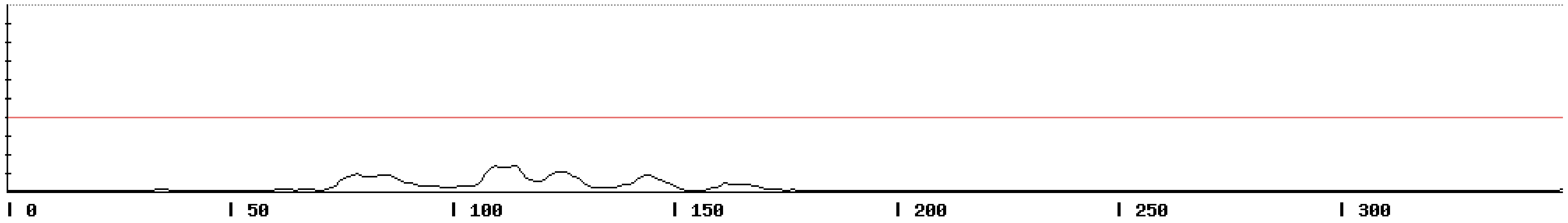

Predictions for Catharanthusroseus_cra_locus_16366_iso_1	
Viterbi Path	
Viterbi Path	
Posterior @ 0.4	127 - RIIRERGRRRAGSGYWKKRGKVKDI - 151
Posterior @ 0.4	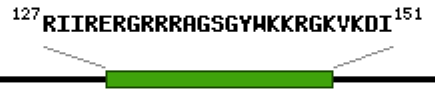
Posterior Graphic. Threshold Marked @ 0.4	

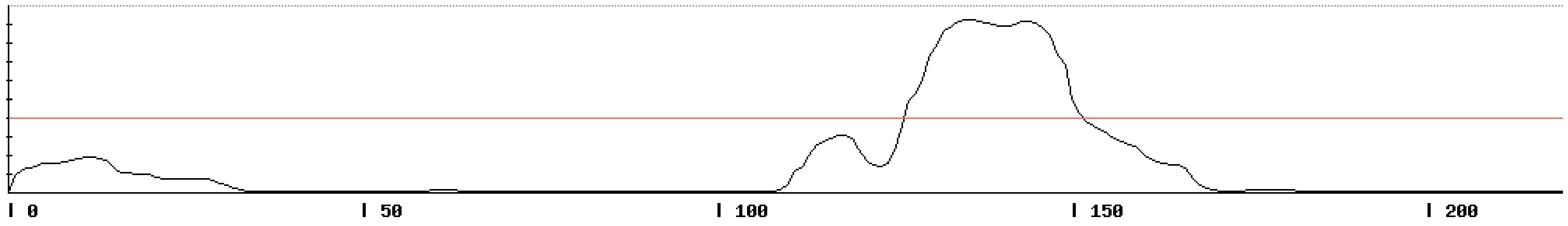

Predictions for Catharanthusroseus_cra_locus_16366_iso_2

Viterbi Path

Viterbi Path

Posterior @ 0.4: 127 - RIIRERGRRRAGSGYWKKRGKVKDI - 151

Posterior @ 0.4

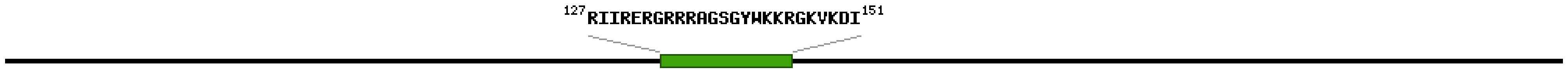

Posterior Graphic. Threshold Marked @ 0.4

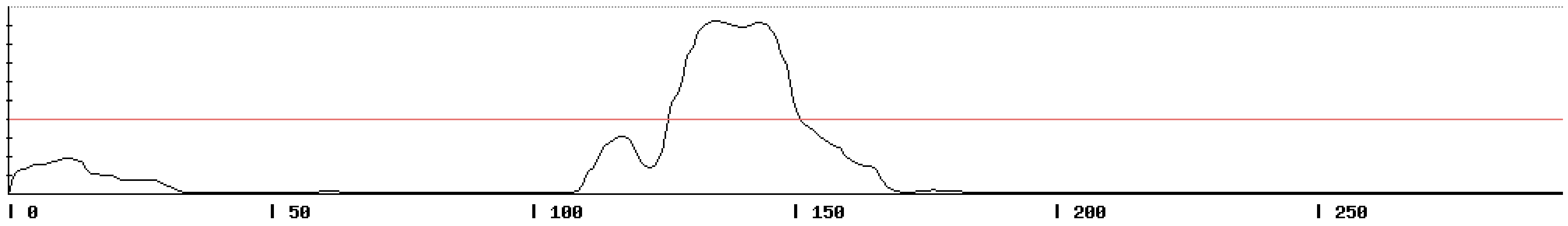

Predictions for Catharanthusroseus_cra_locus_16366_iso_5

Viterbi Path

Viterbi Path

Posterior @ 0.4: 127 - RIIRERGRRRAGSGYWKKRGKVKDI - 151

Posterior @ 0.4

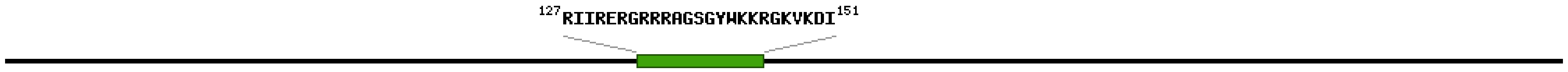

Posterior Graphic. Threshold Marked @ 0.4

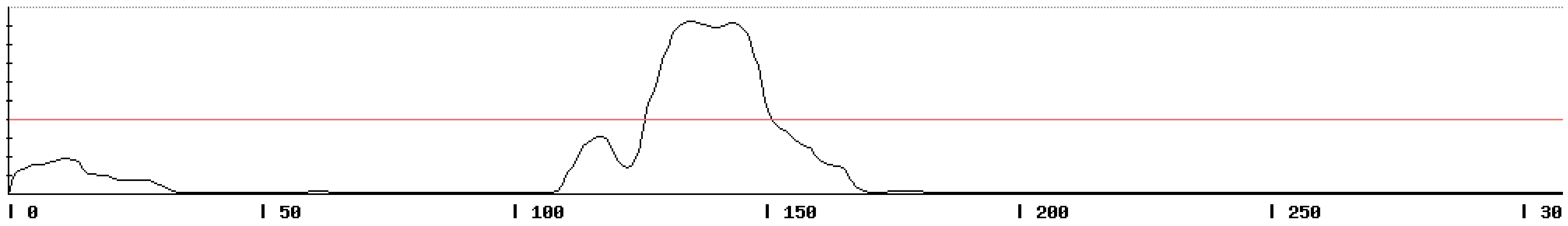

Predictions for Catharanthusroseus_cra_locus_16413_iso_1

Viterbi Path

Viterbi Path

Posterior @ 0.4

Posterior @ 0.4

Posterior Graphic. Threshold Marked @ 0.4

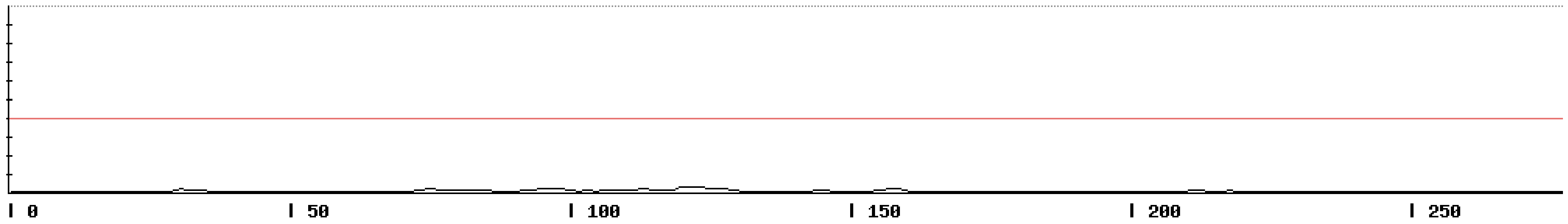

Predictions for Catharanthusroseus_cra_locus_16413_iso_2

Viterbi Path

Viterbi Path

Posterior @ 0.4

Posterior @ 0.4

Posterior Graphic. Threshold Marked @ 0.4

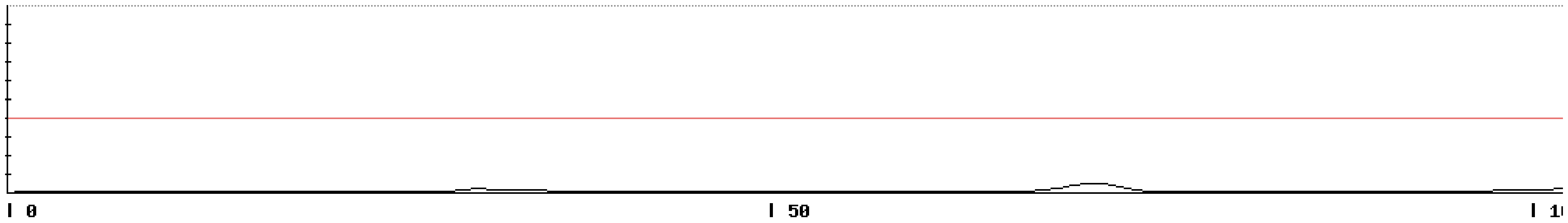

Predictions for Catharanthusroseus_cra_locus_17418_iso_1

Viterbi Path	
Viterbi Path	
Posterior @ 0.4	134 - RR - 135
Posterior @ 0.4	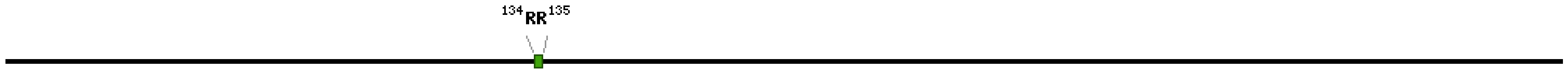
Posterior Graphic. Threshold Marked @ 0.4	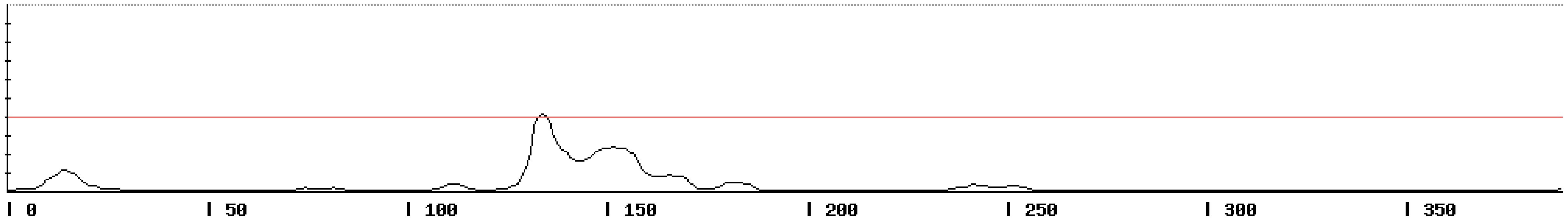

Predictions for Catharanthusroseus_cra_locus_18519_iso_1

Viterbi Path	
Viterbi Path	

Posterior @ 0.4

Posterior @ 0.4

Posterior Graphic. Threshold Marked @ 0.4

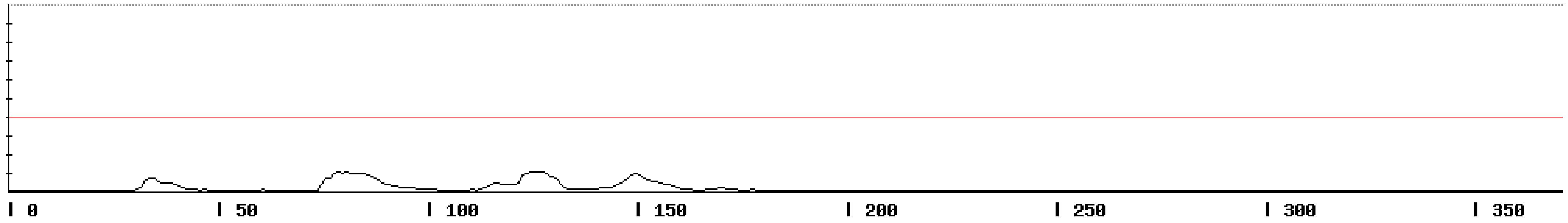

Predictions for Catharanthusroseus_cra_locus_18775_iso_1

Viterbi Path

Viterbi Path

Posterior @ 0.4

Posterior @ 0.4

Posterior Graphic. Threshold Marked @ 0.4

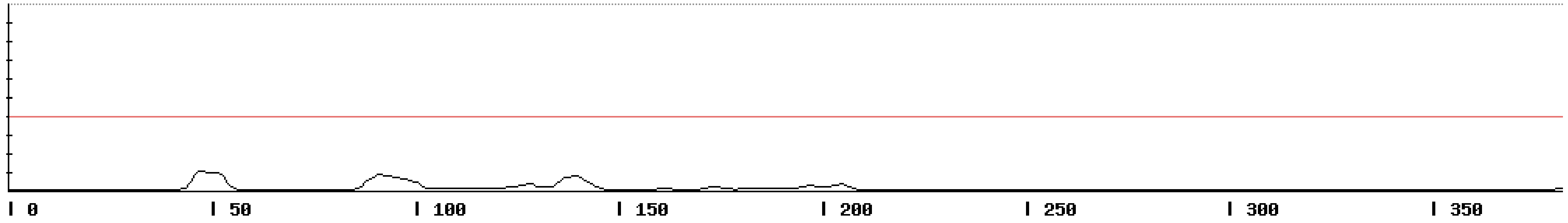

Predictions for Catharanthusroseus_cra_locus_19122_iso_1

Viterbi Path

Viterbi Path

Posterior @ 0.4

Posterior @ 0.4

Posterior Graphic. Threshold Marked @ 0.4

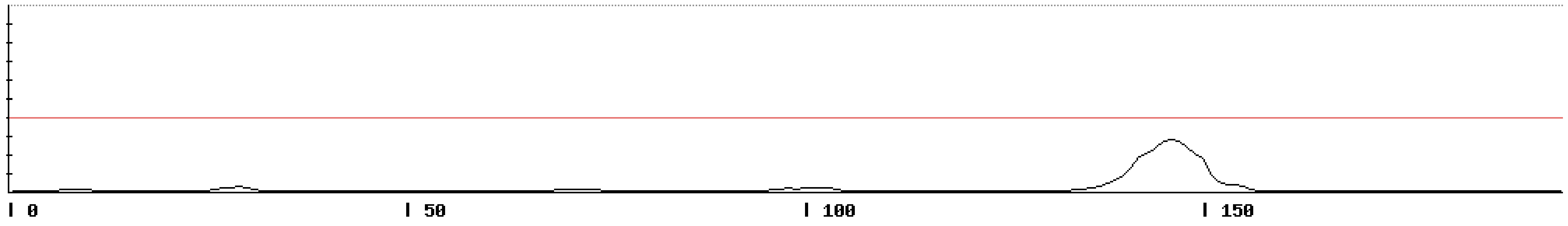

Predictions for Catharanthusroseus_cra_locus_19122_iso_2

Viterbi Path

Viterbi Path

Posterior @ 0.4

Posterior @ 0.4

Posterior Graphic. Threshold Marked @ 0.4

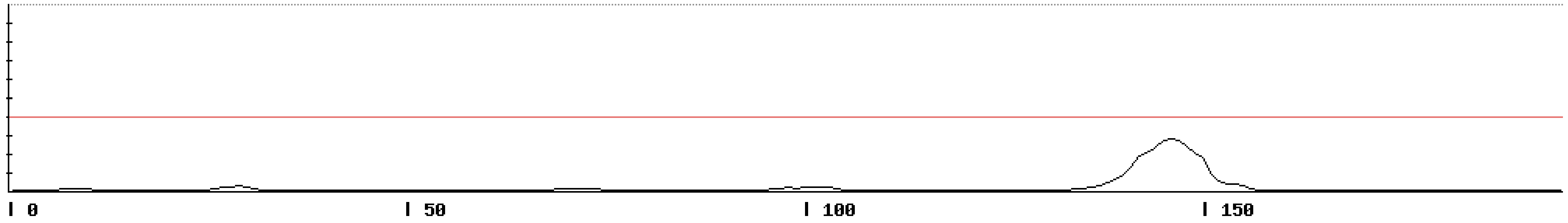

Predictions for Catharanthusroseus_cra_locus_19122_iso_3

Viterbi Path

Viterbi Path

Posterior @ 0.4

Posterior @ 0.4

Posterior Graphic. Threshold Marked @ 0.4

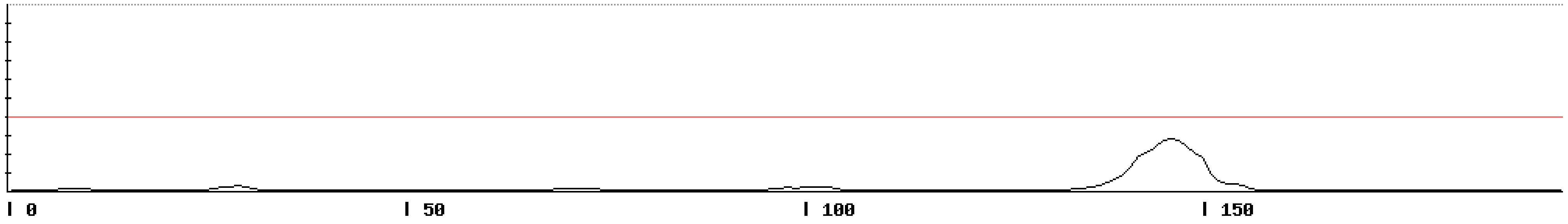

Predictions for Catharanthusroseus_cra_locus_19122_iso_4

Viterbi Path

Viterbi Path

Posterior @ 0.4

Posterior @ 0.4

Posterior Graphic. Threshold Marked @ 0.4

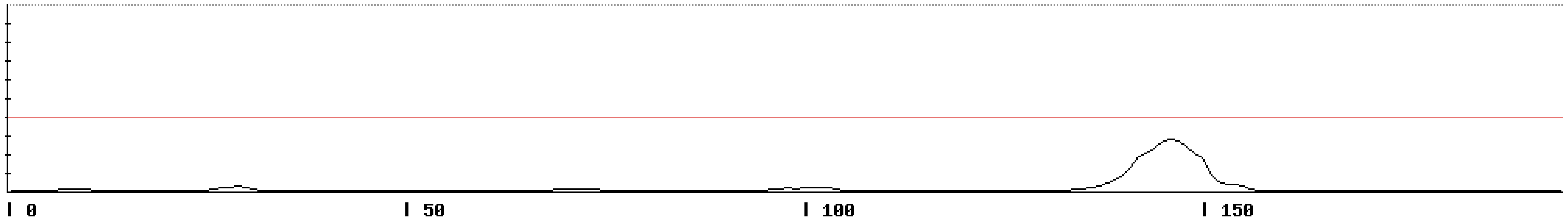

Predictions for Catharanthusroseus_cra_locus_19122_iso_5	
Viterbi Path	
Viterbi Path	
Posterior @ 0.4	
Posterior @ 0.4	
Posterior Graphic. Threshold Marked @ 0.4	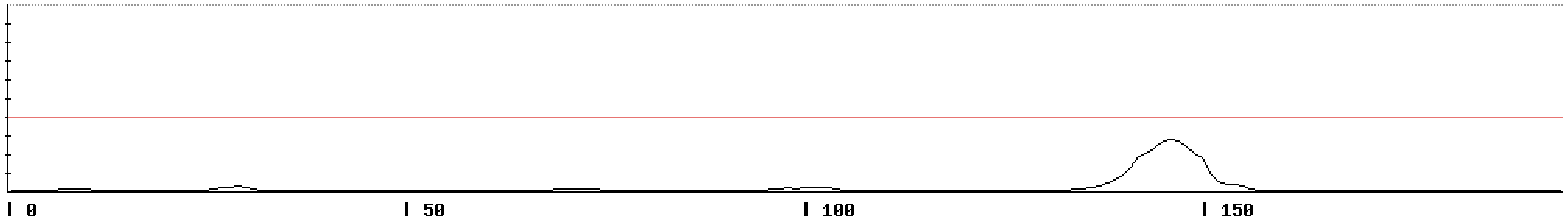

Predictions for Catharanthusroseus_cra_locus_21223_iso_1	
Viterbi Path	
Viterbi Path	
Posterior @ 0.4	
Posterior @ 0.4	
Posterior Graphic. Threshold Marked @ 0.4	

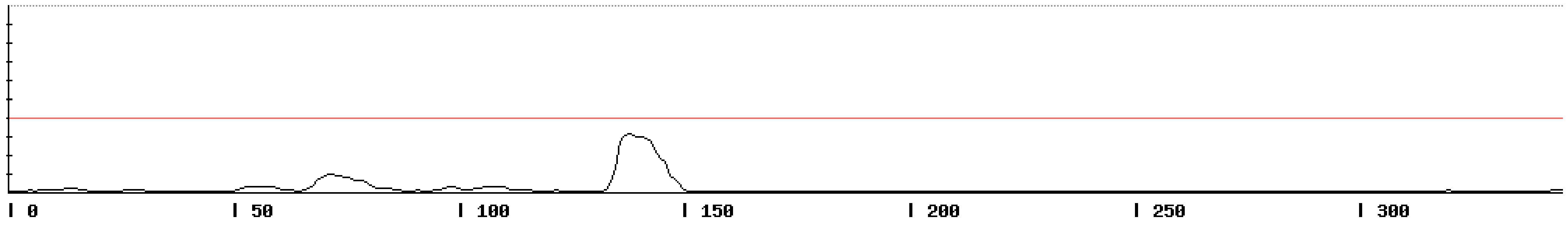

Predictions for Catharanthusroseus_cra_locus_21223_iso_2	
Viterbi Path	
Viterbi Path	
Posterior @ 0.4	
Posterior @ 0.4	
Posterior Graphic. Threshold Marked @ 0.4	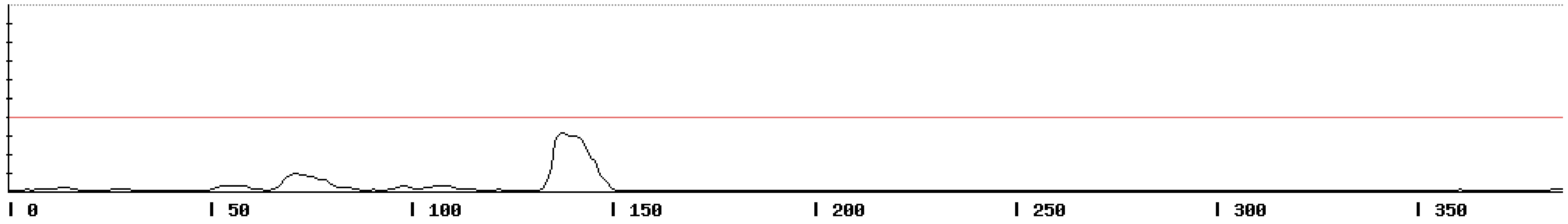

Predictions for Catharanthusroseus_cra_locus_2128_iso_10	
Viterbi Path	
Viterbi Path	
Posterior @ 0.4	
Posterior @ 0.4	

Posterior Graphic. Threshold Marked @ 0.4

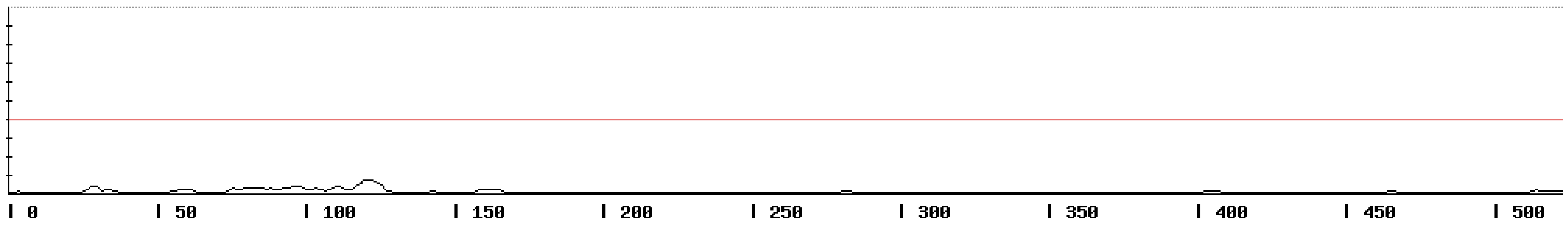

Predictions for Catharanthusroseus_cra_locus_2128_iso_1

Viterbi Path

Viterbi Path

Posterior @ 0.4

Posterior @ 0.4

Posterior Graphic. Threshold Marked @ 0.4

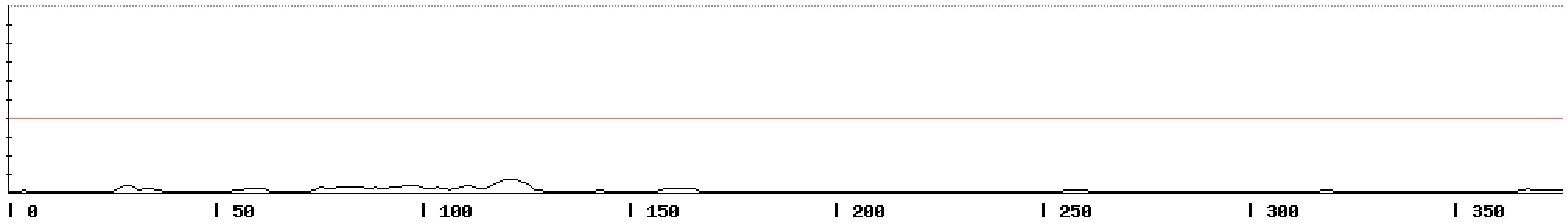

Predictions for Catharanthusroseus_cra_locus_2128_iso_5

Viterbi Path

Viterbi Path

Posterior @ 0.4

Posterior @ 0.4

Posterior Graphic. Threshold Marked @ 0.4	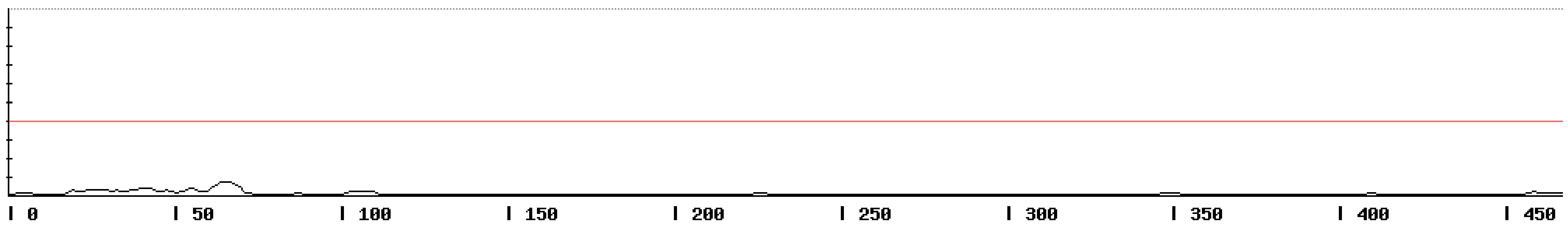

Predictions for Catharanthusroseus_cra_locus_2128_iso_6

Viterbi Path	
Viterbi Path	
Posterior @ 0.4	
Posterior @ 0.4	
Posterior Graphic. Threshold Marked @ 0.4	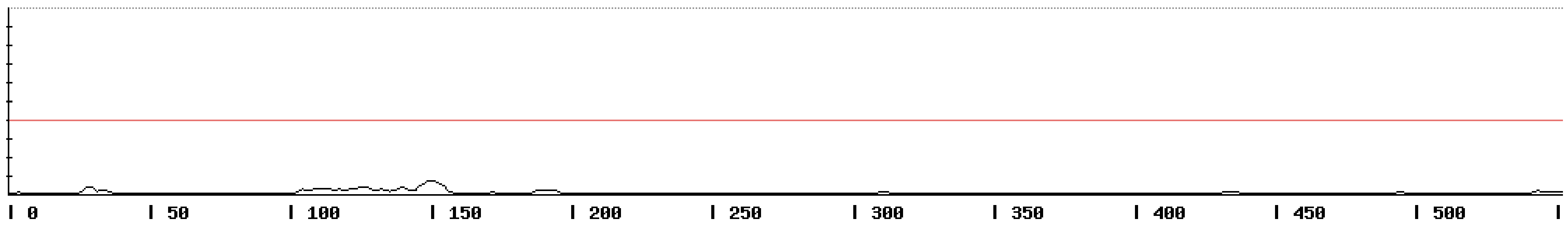

Predictions for Catharanthusroseus_cra_locus_21743_iso_1

Viterbi Path	
Viterbi Path	

Posterior @ 0.4	
Posterior @ 0.4	
Posterior Graphic. Threshold Marked @ 0.4	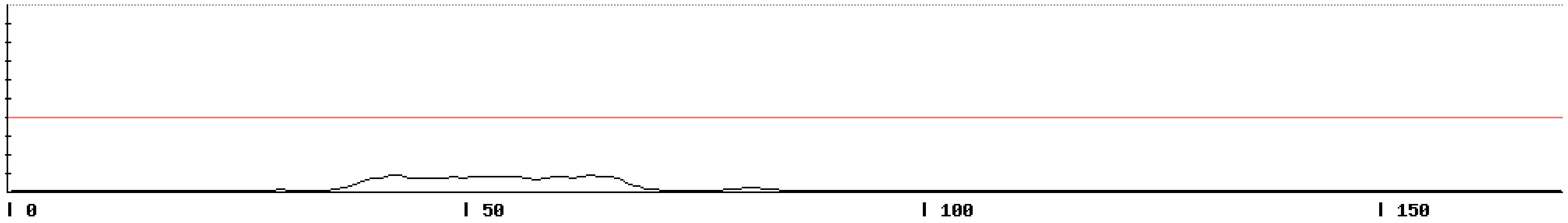

Predictions for Catharanthusroseus_cra_locus_21743_iso_2	
Viterbi Path	
Viterbi Path	
Posterior @ 0.4	
Posterior @ 0.4	
Posterior Graphic. Threshold Marked @ 0.4	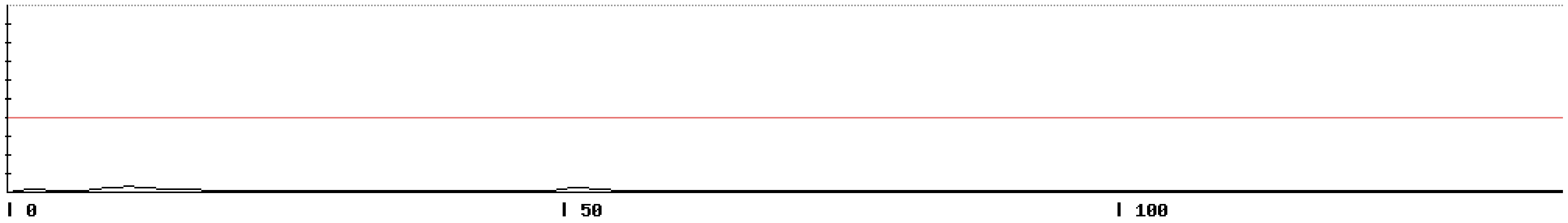

Predictions for Catharanthusroseus_cra_locus_22400_iso_1	
Viterbi Path	
Viterbi Path	

Posterior @ 0.4

Posterior @ 0.4

Posterior Graphic. Threshold Marked @ 0.4

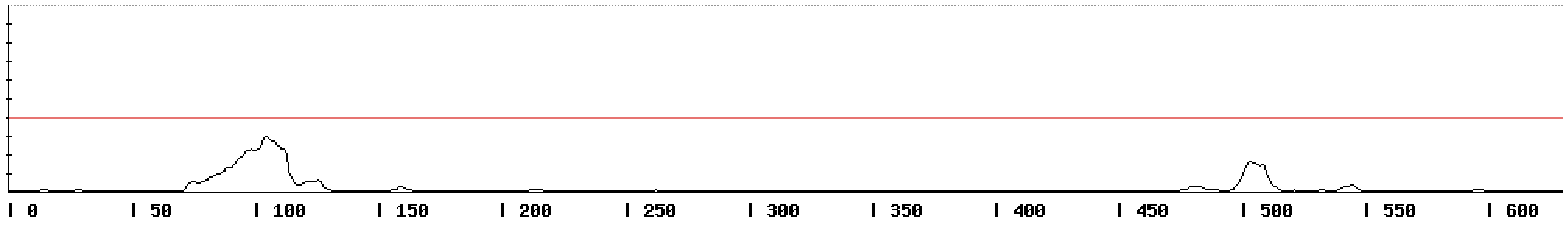

Predictions for Catharanthusroseus_cra_locus_22400_iso_2

Viterbi Path

Viterbi Path

Posterior @ 0.4

Posterior @ 0.4

Posterior Graphic. Threshold Marked @ 0.4

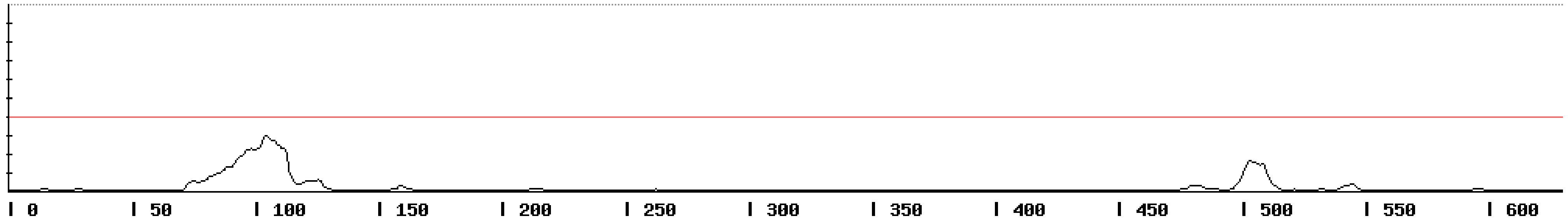

Predictions for Catharanthusroseus_cra_locus_22400_iso_3

Viterbi Path	
Viterbi Path	
Posterior @ 0.4	
Posterior @ 0.4	
Posterior Graphic. Threshold Marked @ 0.4	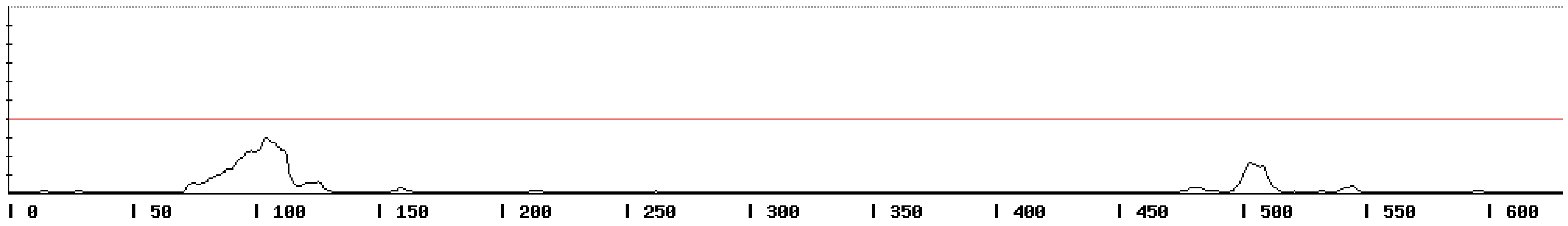

Predictions for Catharanthusroseus_cra_locus_22400_iso_4

Viterbi Path	
Viterbi Path	
Posterior @ 0.4	
Posterior @ 0.4	
Posterior Graphic. Threshold Marked @ 0.4	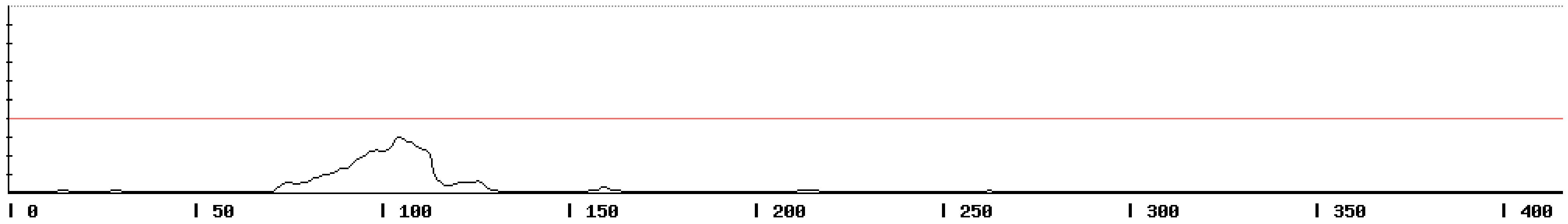

Predictions for Catharanthusroseus_cra_locus_22400_iso_5

Viterbi Path	
Viterbi Path	
Posterior @ 0.4	
Posterior @ 0.4	
Posterior Graphic. Threshold Marked @ 0.4	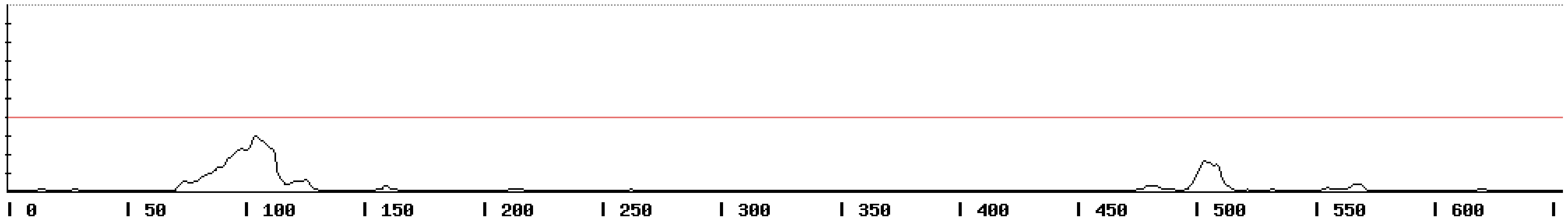

Predictions for Catharanthusroseus_cra_locus_22400_iso_6

Viterbi Path	
Viterbi Path	
Posterior @ 0.4	
Posterior @ 0.4	
Posterior Graphic. Threshold Marked @ 0.4	

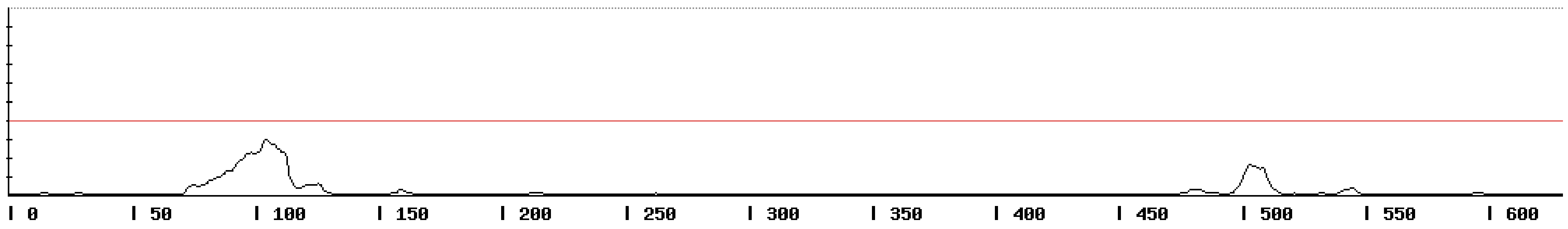

Predictions for Catharanthusroseus_cra_locus_2287_iso_2

Viterbi Path	
Viterbi Path	
Posterior @ 0.4	
Posterior @ 0.4	
Posterior Graphic. Threshold Marked @ 0.4	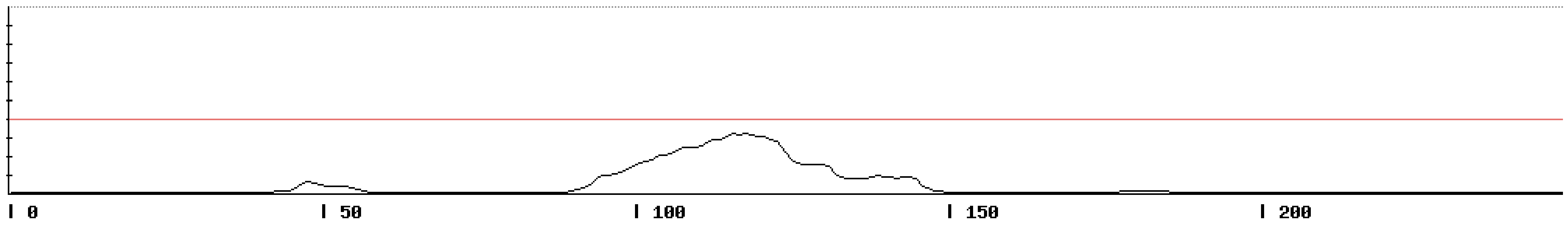

Predictions for Catharanthusroseus_cra_locus_2287_iso_4

Viterbi Path	
Viterbi Path	
Posterior @ 0.4	
Posterior @ 0.4	

Posterior Graphic. Threshold Marked @ 0.4	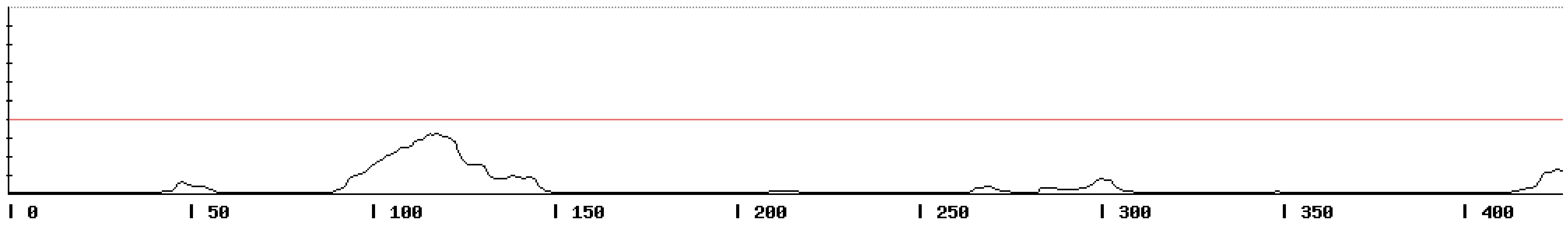

Predictions for Catharanthusroseus_cra_locus_2287_iso_5	
Viterbi Path	
Viterbi Path	
Posterior @ 0.4	
Posterior @ 0.4	
Posterior Graphic. Threshold Marked @ 0.4	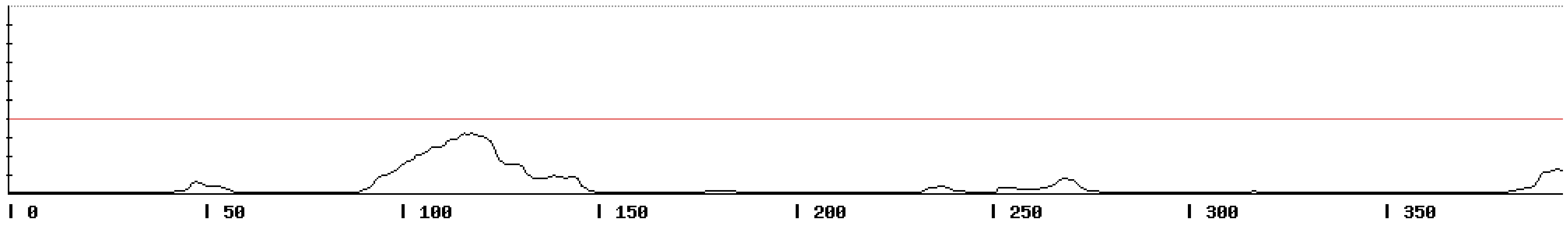

Predictions for Catharanthusroseus_cra_locus_23250_iso_1	
Viterbi Path	
Viterbi Path	
Posterior @ 0.4	
Posterior @ 0.4	

Posterior Graphic. Threshold Marked @ 0.4

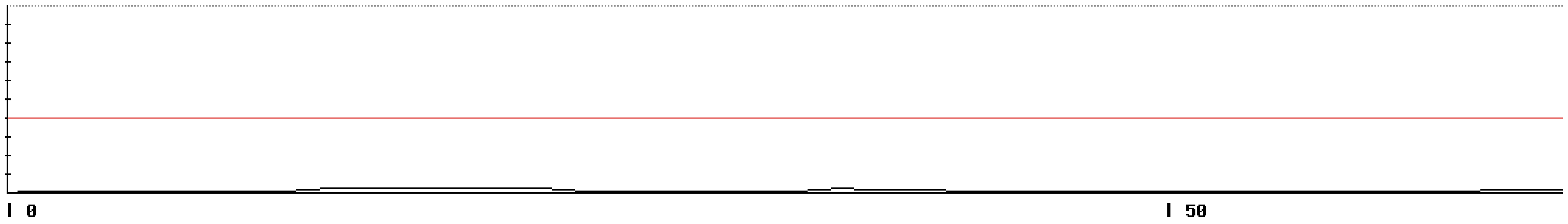

Predictions for Catharanthusroseus_cra_locus_23250_iso_2

Viterbi Path

Viterbi Path

Posterior @ 0.4

Posterior @ 0.4

Posterior Graphic. Threshold Marked @ 0.4

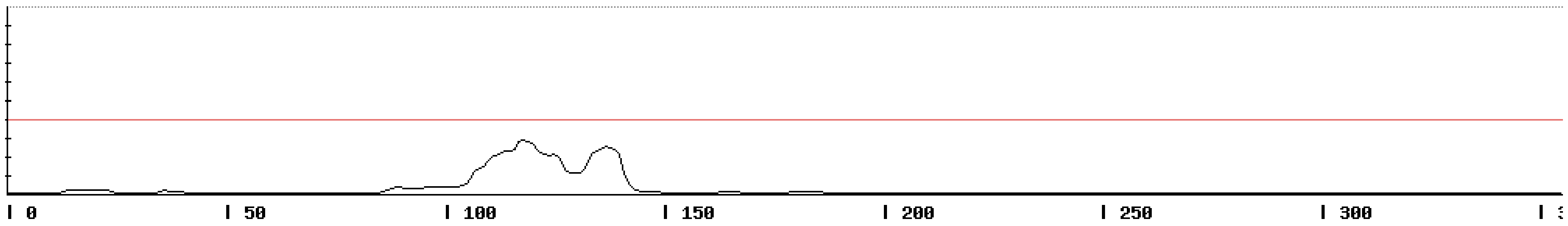

Predictions for Catharanthusroseus_cra_locus_23496_iso_1

Viterbi Path

Viterbi Path

Posterior @ 0.4	
Posterior @ 0.4	
Posterior Graphic. Threshold Marked @ 0.4	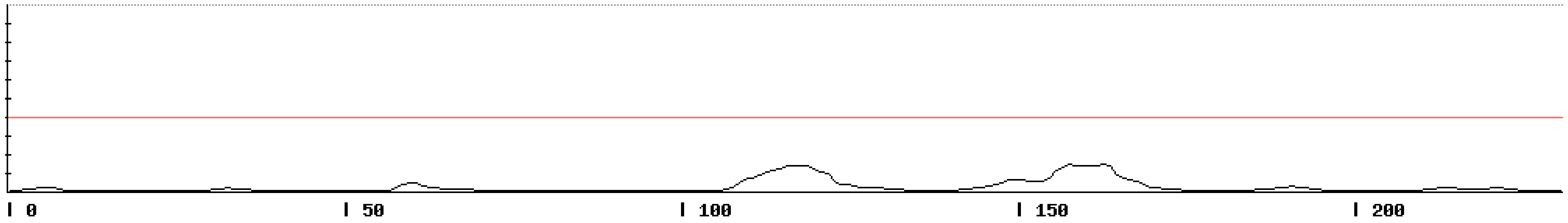

Predictions for Catharanthusroseus_cra_locus_24682_iso_1

Viterbi Path	
Viterbi Path	
Posterior @ 0.4	
Posterior @ 0.4	
Posterior Graphic. Threshold Marked @ 0.4	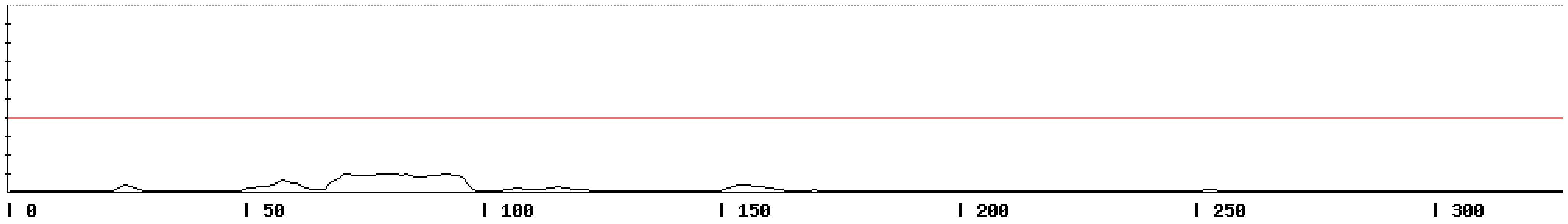

Predictions for Catharanthusroseus_cra_locus_24682_iso_2

Viterbi Path	
Viterbi Path	

Posterior @ 0.4

Posterior @ 0.4

Posterior Graphic. Threshold Marked @ 0.4

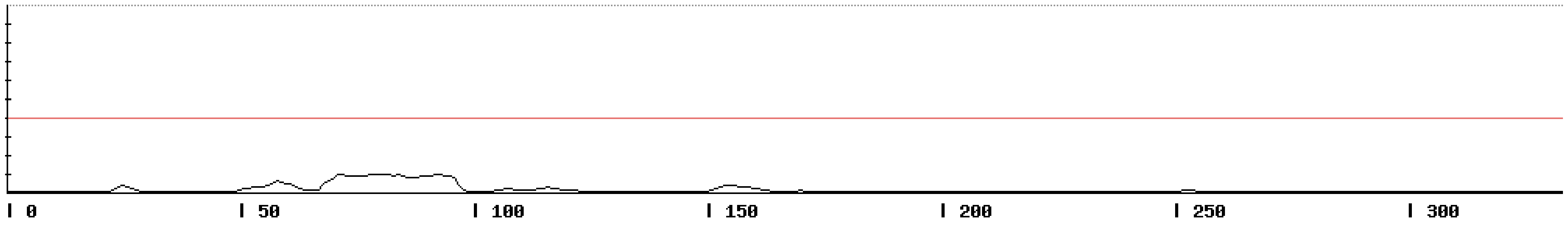

Predictions for Catharanthusroseus_cra_locus_2614_iso_1

Viterbi Path

Viterbi Path

Posterior @ 0.4

Posterior @ 0.4

Posterior Graphic. Threshold Marked @ 0.4

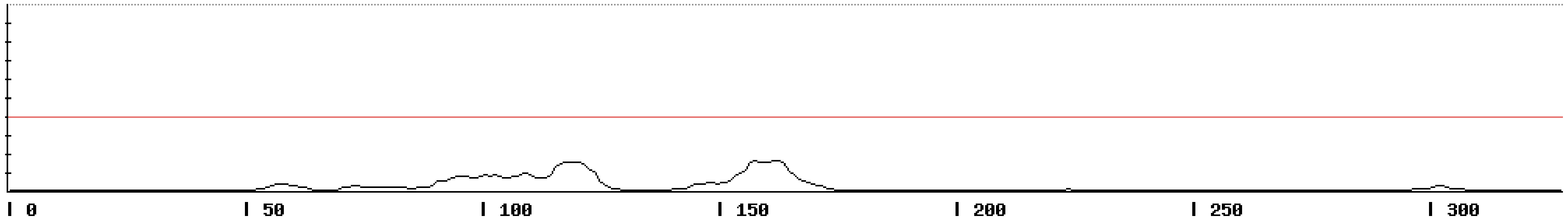

Predictions for Catharanthusroseus_cra_locus_2614_iso_2

Viterbi Path	
Viterbi Path	
Posterior @ 0.4	
Posterior @ 0.4	
Posterior Graphic. Threshold Marked @ 0.4	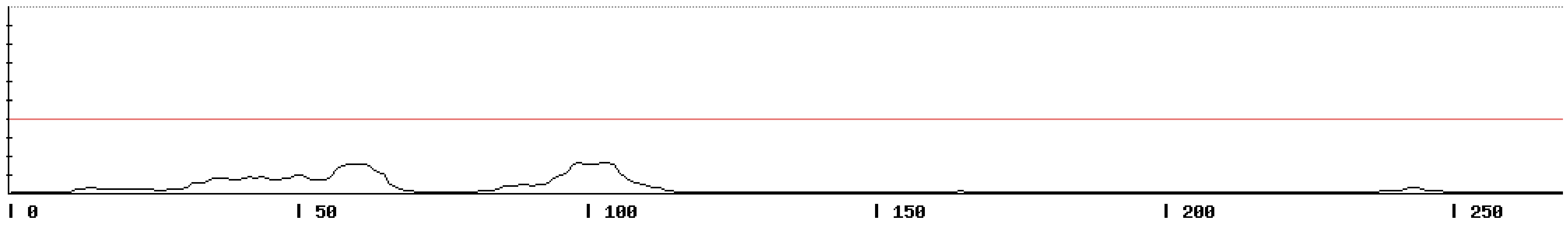

Predictions for Catharanthusroseus_cra_locus_2614_iso_3

Viterbi Path	
Viterbi Path	
Posterior @ 0.4	
Posterior @ 0.4	
Posterior Graphic. Threshold Marked @ 0.4	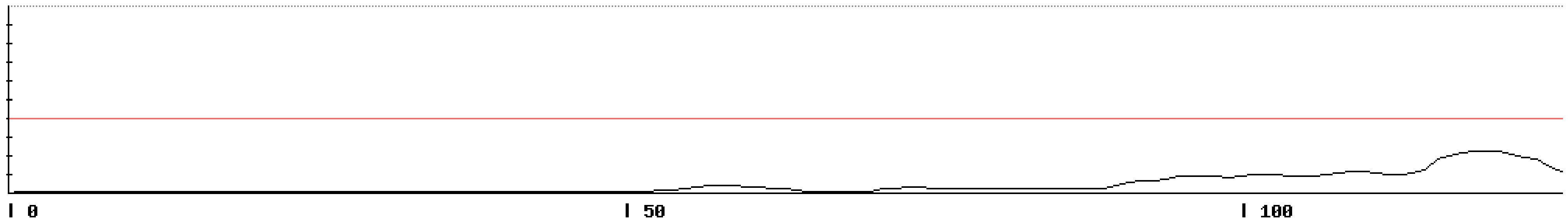

Predictions for Catharanthusroseus_cra_locus_2614_iso_5

Viterbi Path

Viterbi Path

Posterior @ 0.4

Posterior @ 0.4

Posterior Graphic. Threshold Marked @ 0.4

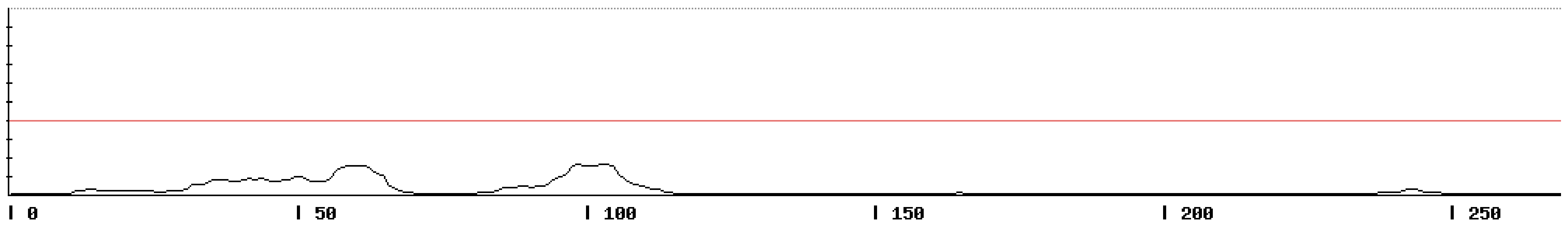

Predictions for Catharanthusroseus_cra_locus_2614_iso_7

Viterbi Path

Viterbi Path

Posterior @ 0.4

Posterior @ 0.4

Posterior Graphic. Threshold Marked @ 0.4

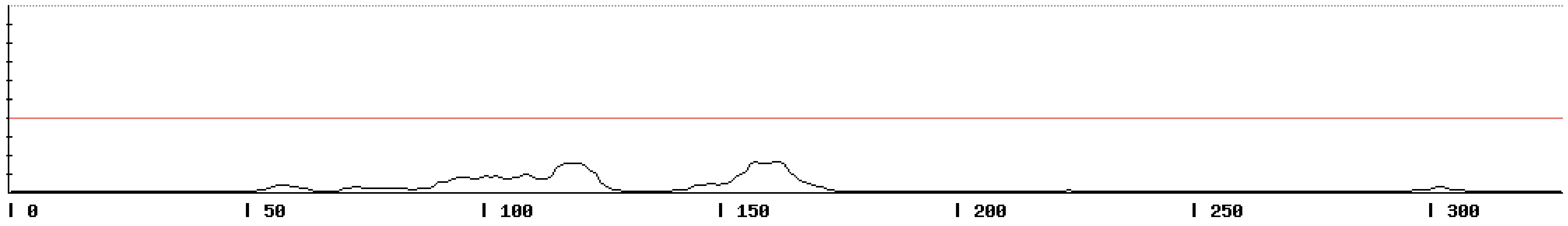

Predictions for Catharanthusroseus_cra_locus_26911_iso_1

Viterbi Path

Viterbi Path

Posterior @ 0.4

Posterior @ 0.4

Posterior Graphic. Threshold Marked @ 0.4

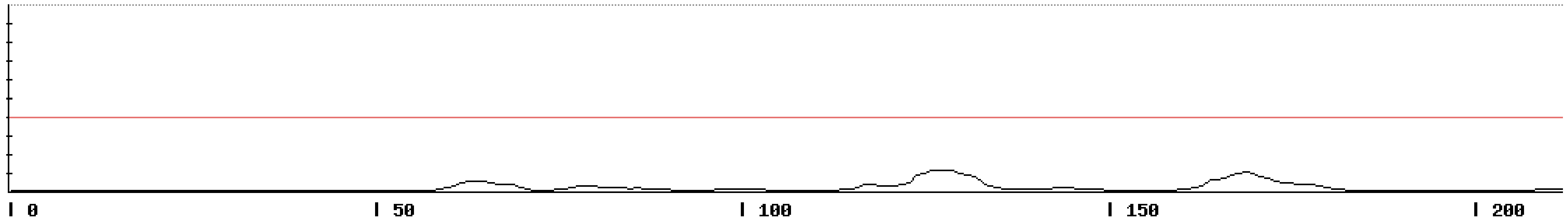

Predictions for Catharanthusroseus_cra_locus_290_iso_1

Viterbi Path

Viterbi Path

Posterior @ 0.4 — 39 - ADKPIGKPKPLGIKKA - 54

Posterior @ 0.4

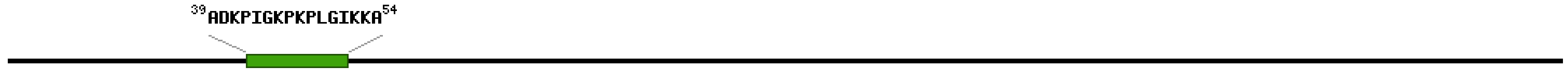

Posterior Graphic. Threshold Marked @ 0.4

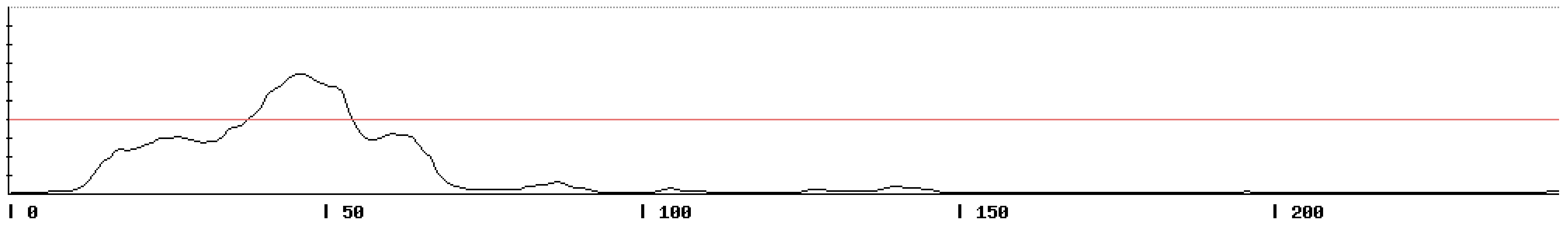

Predictions for Catharanthusroseus_cra_locus_290_iso_2

Viterbi Path

Viterbi Path

Posterior @ 0.4: 154 - ADKPIGKPKPLGIKKA - 169

Posterior @ 0.4

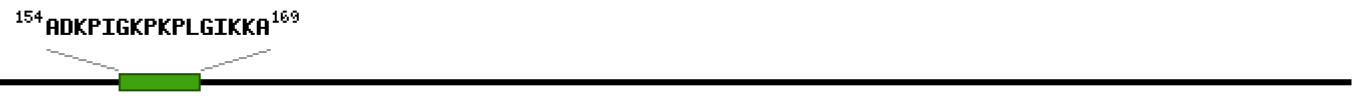

Posterior Graphic. Threshold Marked @ 0.4

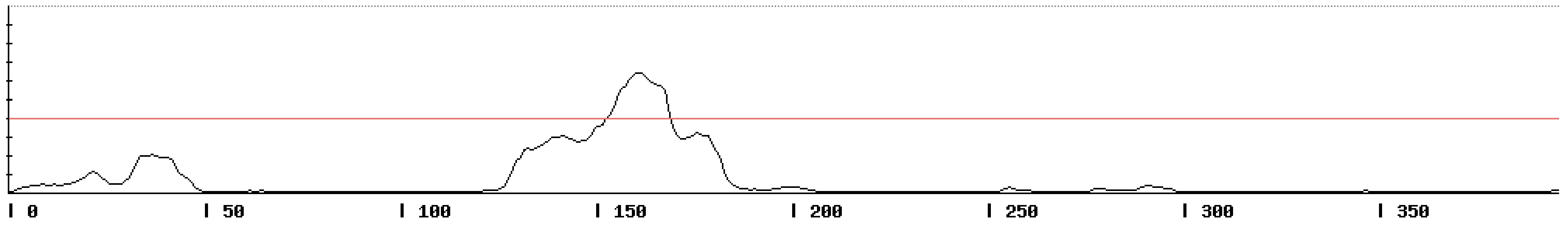

Predictions for Catharanthusroseus_cra_locus_290_iso_3

Viterbi Path

Viterbi Path

Posterior @ 0.4: 154 - ADKPIGKPKPLGIKKA - 169

Posterior @ 0.4

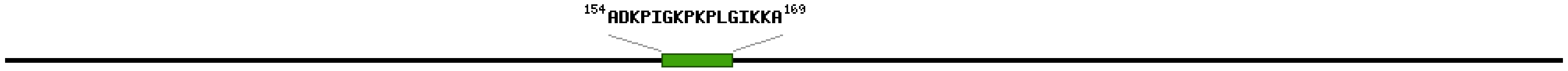

Posterior Graphic. Threshold Marked @ 0.4

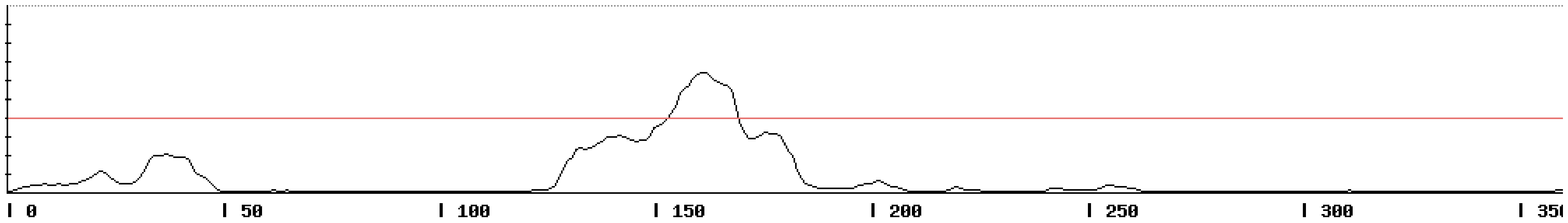

Predictions for Catharanthusroseus_cra_locus_31747_iso_1

Viterbi Path

Viterbi Path

Posterior @ 0.4

Posterior @ 0.4

Posterior Graphic. Threshold Marked @ 0.4

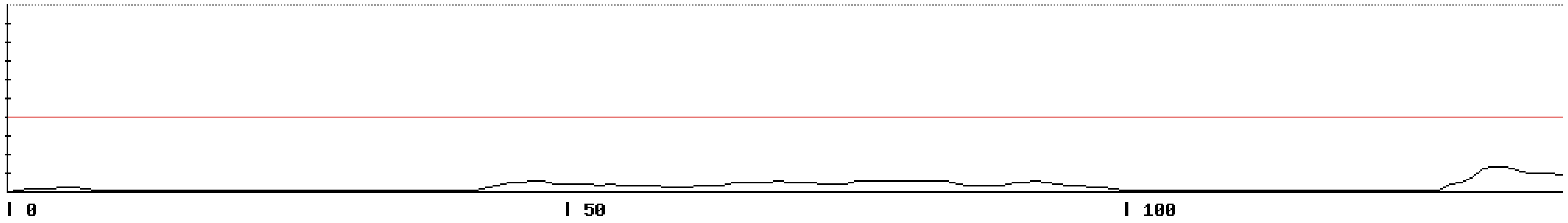

Predictions for Catharanthusroseus_cra_locus_31747_iso_2

Viterbi Path

Viterbi Path

Posterior @ 0.4

Posterior @ 0.4

Posterior Graphic. Threshold Marked @ 0.4

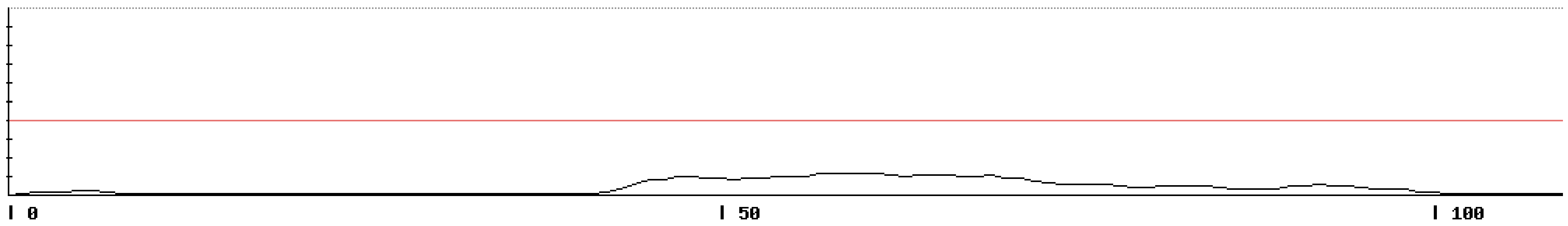

Predictions for Catharanthusroseus_cra_locus_31747_iso_3

Viterbi Path

Viterbi Path

Posterior @ 0.4

Posterior @ 0.4

Posterior Graphic. Threshold Marked @ 0.4

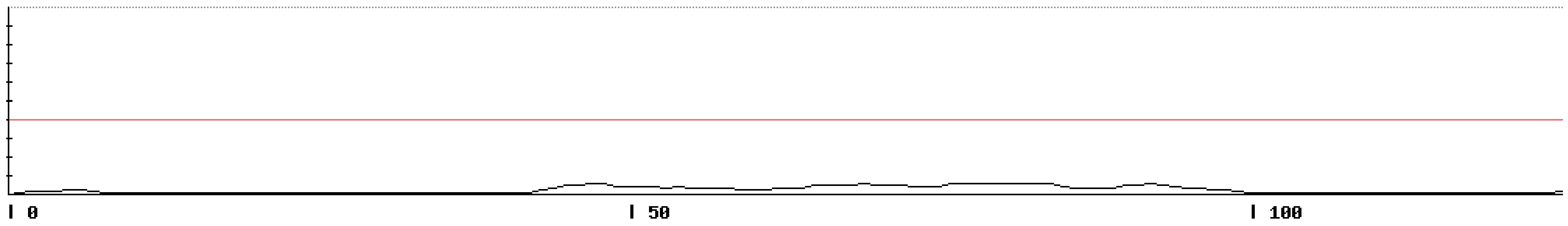

Predictions for Catharanthusroseus_cra_locus_35120_iso_1

Viterbi Path

Viterbi Path

Posterior @ 0.4	
Posterior @ 0.4	
Posterior Graphic. Threshold Marked @ 0.4	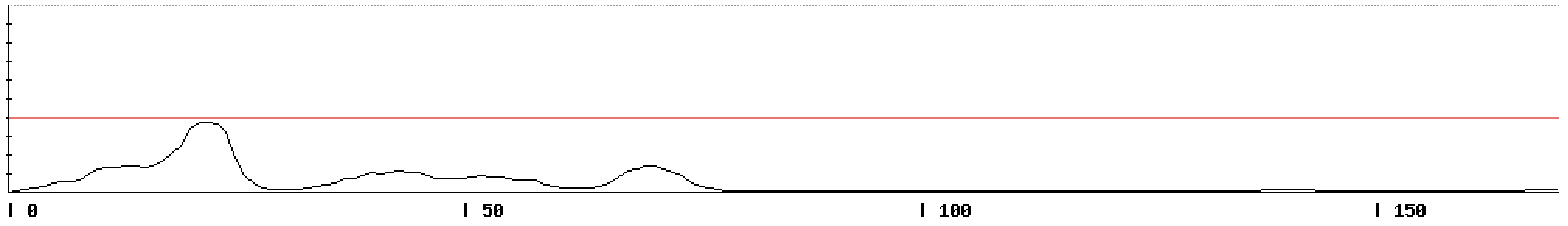

Predictions for Catharanthusroseus_cra_locus_35121_iso_1

Viterbi Path	
Viterbi Path	
Posterior @ 0.4	
Posterior @ 0.4	
Posterior Graphic. Threshold Marked @ 0.4	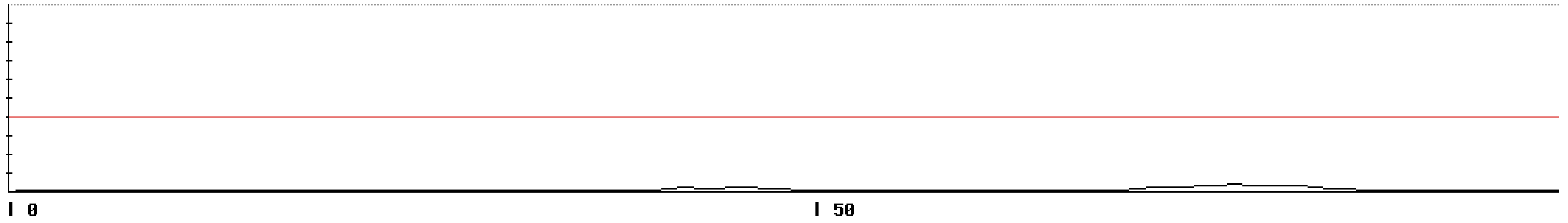

Predictions for Catharanthusroseus_cra_locus_4016_iso_1

Viterbi Path

Viterbi Path

Posterior @ 0.4

Posterior @ 0.4

Posterior Graphic. Threshold Marked @ 0.4

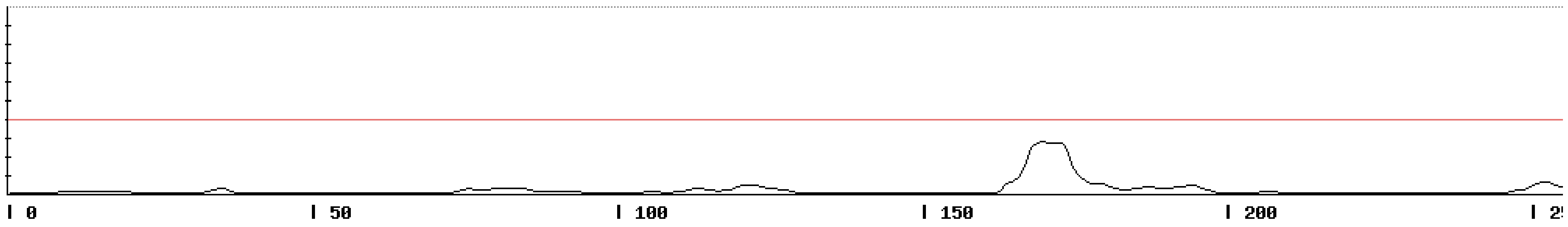

Predictions for Catharanthusroseus_cra_locus_4016_iso_3

Viterbi Path

Viterbi Path

Posterior @ 0.4

Posterior @ 0.4

Posterior Graphic. Threshold Marked @ 0.4

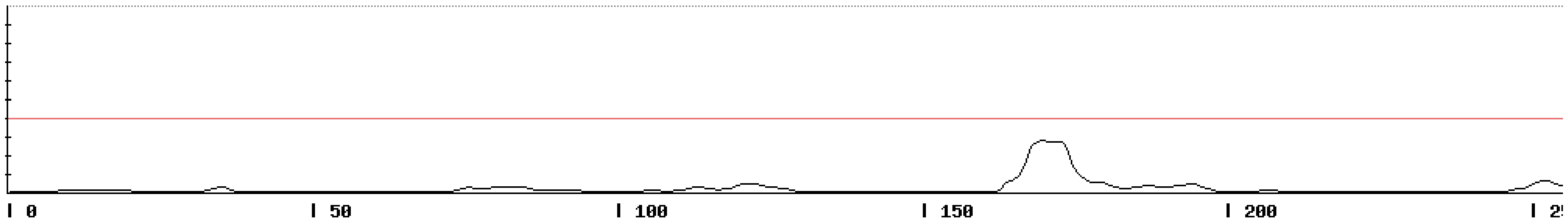

Predictions for Catharanthusroseus_cra_locus_5081_iso_1	
Viterbi Path	
Viterbi Path	
Posterior @ 0.4	
Posterior @ 0.4	
Posterior Graphic. Threshold Marked @ 0.4	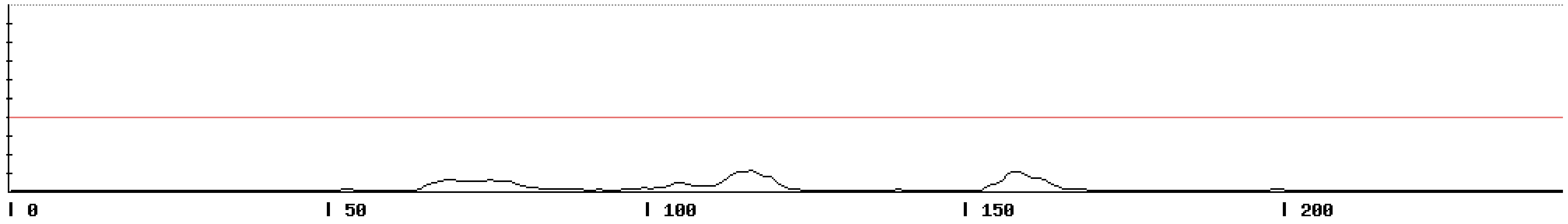

Predictions for Catharanthusroseus_cra_locus_5081_iso_2	
Viterbi Path	
Viterbi Path	
Posterior @ 0.4	
Posterior @ 0.4	
Posterior Graphic. Threshold Marked @ 0.4	

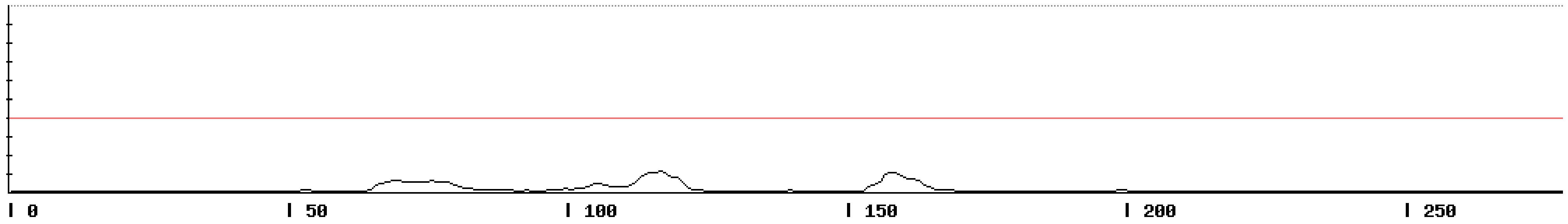

Predictions for Catharanthusroseus_cra_locus_5081_iso_3

Viterbi Path	
Viterbi Path	
Posterior @ 0.4	
Posterior @ 0.4	
Posterior Graphic. Threshold Marked @ 0.4	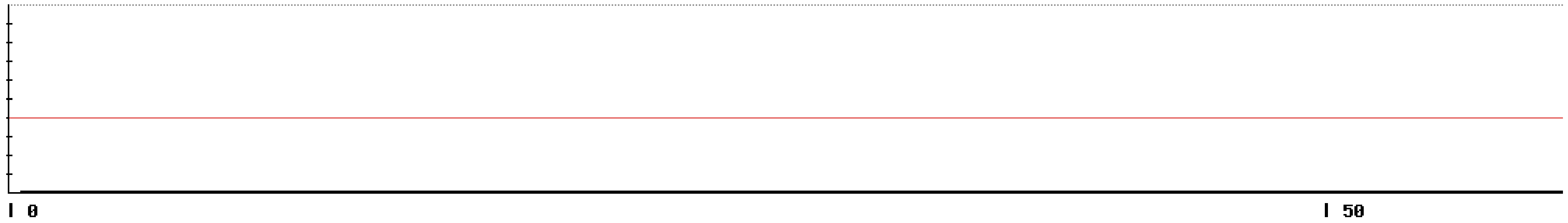

Predictions for Catharanthusroseus_cra_locus_5081_iso_4

Viterbi Path	
Viterbi Path	
Posterior @ 0.4	
Posterior @ 0.4	

Posterior Graphic. Threshold Marked @ 0.4

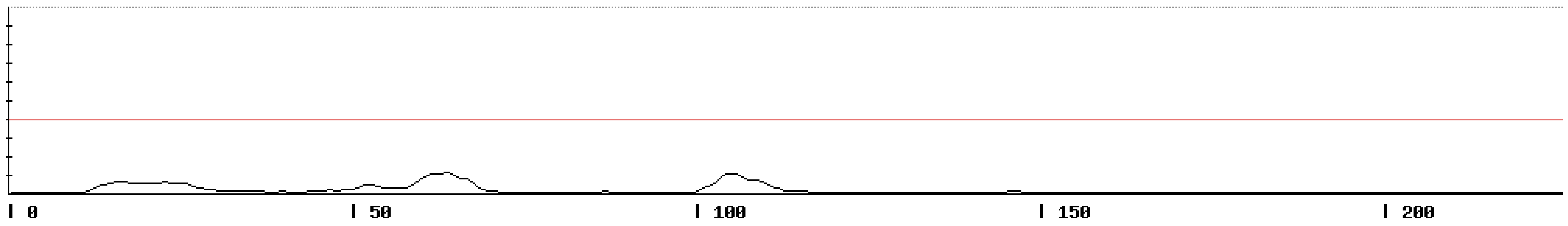

Predictions for Catharanthusroseus_cra_locus_5081_iso_5

Viterbi Path

Viterbi Path

Posterior @ 0.4

Posterior @ 0.4

Posterior Graphic. Threshold Marked @ 0.4

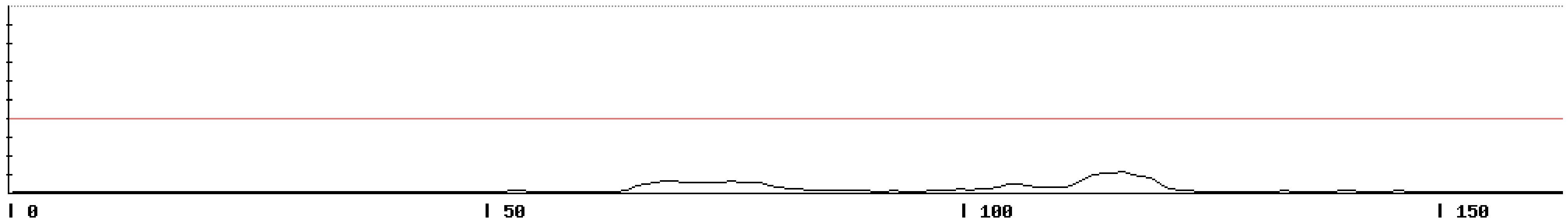

Predictions for Catharanthusroseus_cra_locus_5081_iso_6

Viterbi Path

Viterbi Path

Posterior @ 0.4

Posterior @ 0.4

Posterior Graphic. Threshold Marked @ 0.4

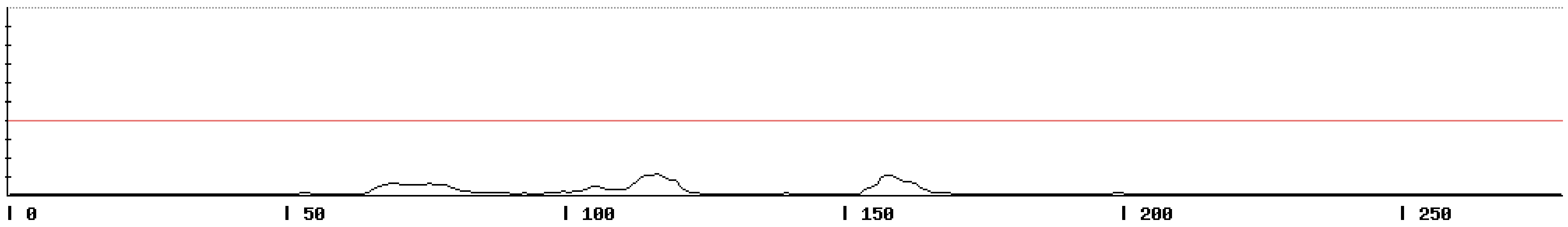

Predictions for Catharanthusroseus_cra_locus_5366_iso_3

Viterbi Path

Viterbi Path

Posterior @ 0.4

Posterior @ 0.4

Posterior Graphic. Threshold Marked @ 0.4

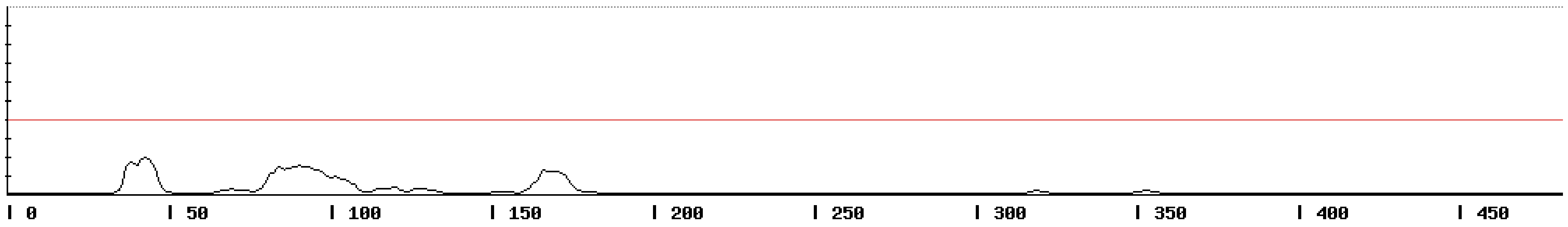

Predictions for Catharanthusroseus_cra_locus_5366_iso_4

Viterbi Path

Viterbi Path

Posterior @ 0.4

Posterior @ 0.4

Posterior Graphic. Threshold Marked @ 0.4

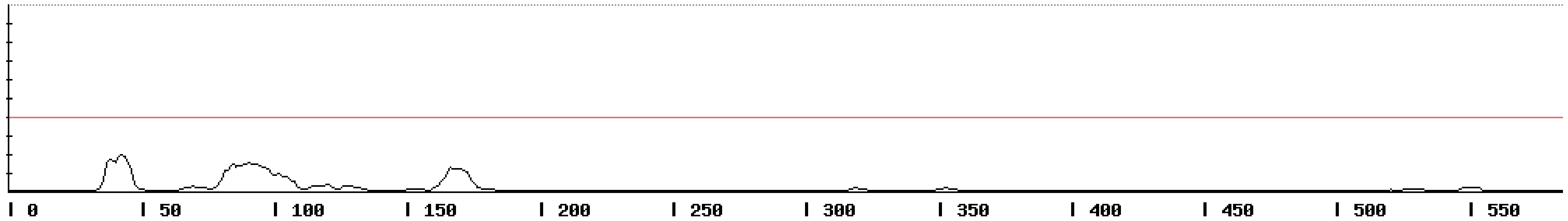

Predictions for Catharanthusroseus_cra_locus_5600_iso_1

Viterbi Path

Viterbi Path

Posterior @ 0.4

170 - RKRRK - 174

Posterior @ 0.4

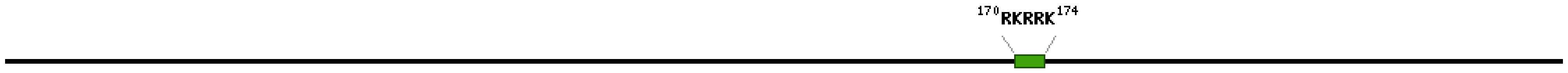

Posterior Graphic. Threshold Marked @ 0.4

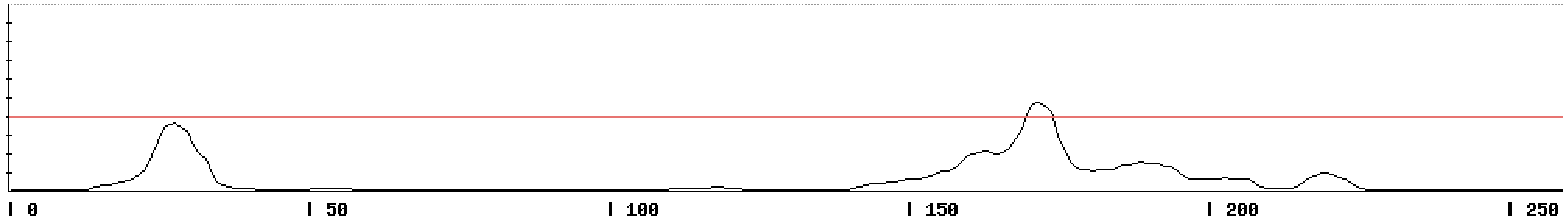

Predictions for Catharanthusroseus_cra_locus_5600_iso_2

Viterbi Path

Viterbi Path

Posterior @ 0.4	49 - RKRRK - 53
Posterior @ 0.4	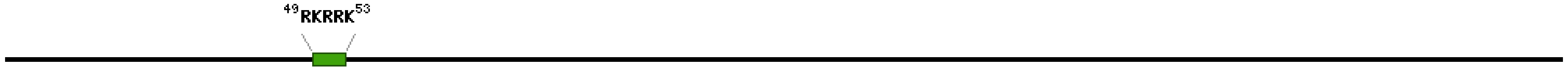
Posterior Graphic. Threshold Marked @ 0.4	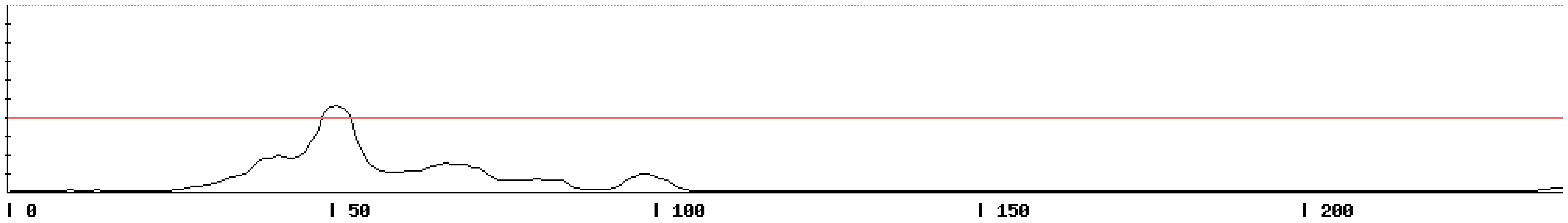

Predictions for Catharanthusroseus_cra_locus_5600_iso_3

Viterbi Path	
Viterbi Path	
Posterior @ 0.4	139 - RKRRK - 143
Posterior @ 0.4	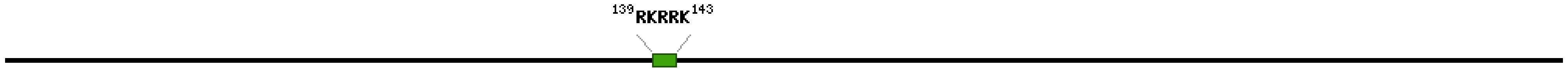
Posterior Graphic. Threshold Marked @ 0.4	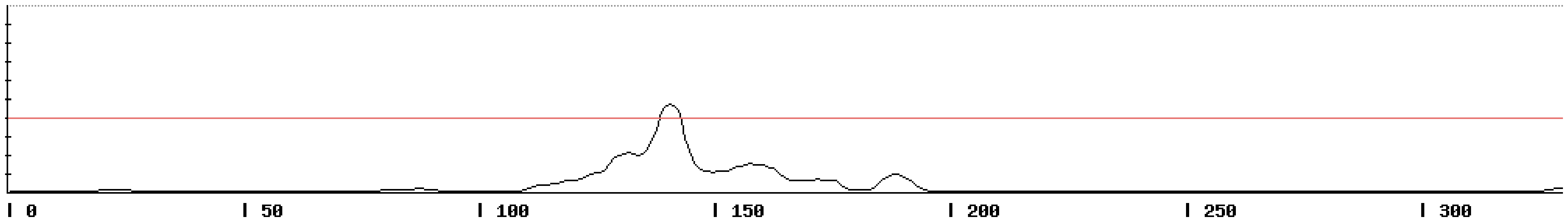

Predictions for Catharanthusroseus_cra_locus_5600_iso_4

Viterbi Path

Viterbi Path

Posterior @ 0.4

170 - RKRRK - 174

Posterior @ 0.4

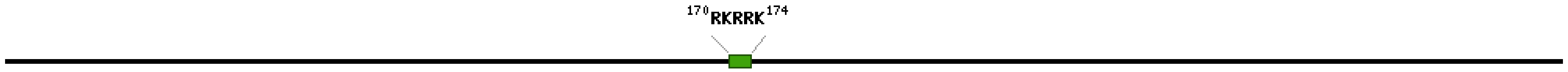

Posterior Graphic. Threshold Marked @ 0.4

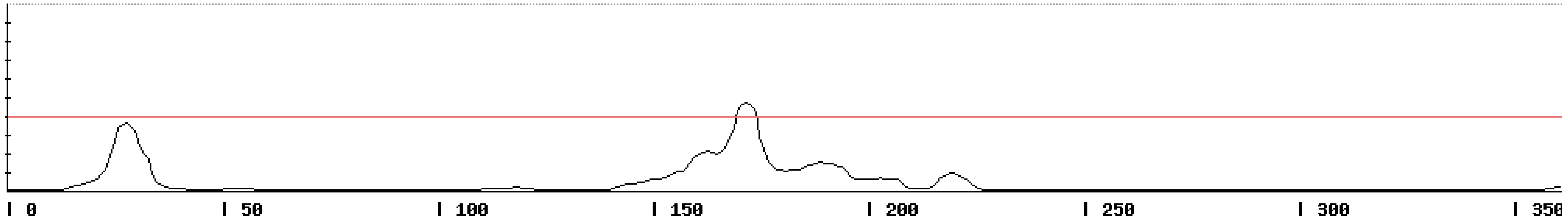

Predictions for Catharanthusroseus_cra_locus_61901_iso_1

Viterbi Path

Viterbi Path

Posterior @ 0.4

Posterior @ 0.4

Posterior Graphic. Threshold Marked @ 0.4

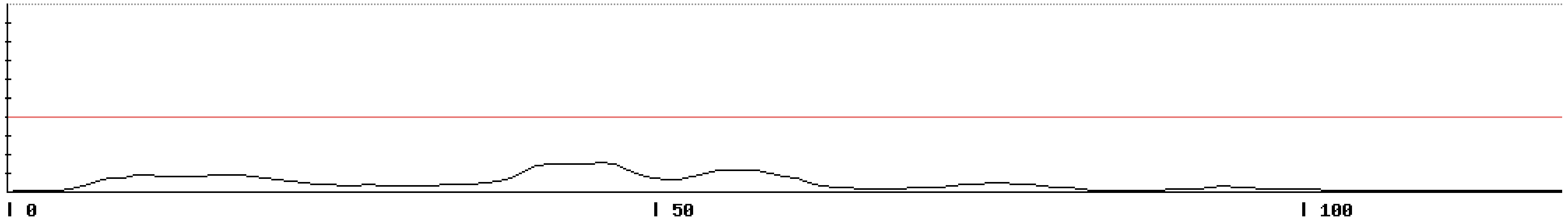

Predictions for Catharanthusroseus_cra_locus_6334_iso_1	
Viterbi Path	
Viterbi Path	
Posterior @ 0.4	136 - RKRRK - 140
Posterior @ 0.4	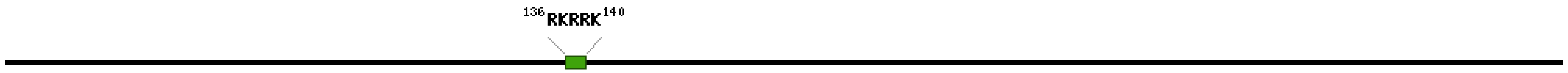
Posterior Graphic. Threshold Marked @ 0.4	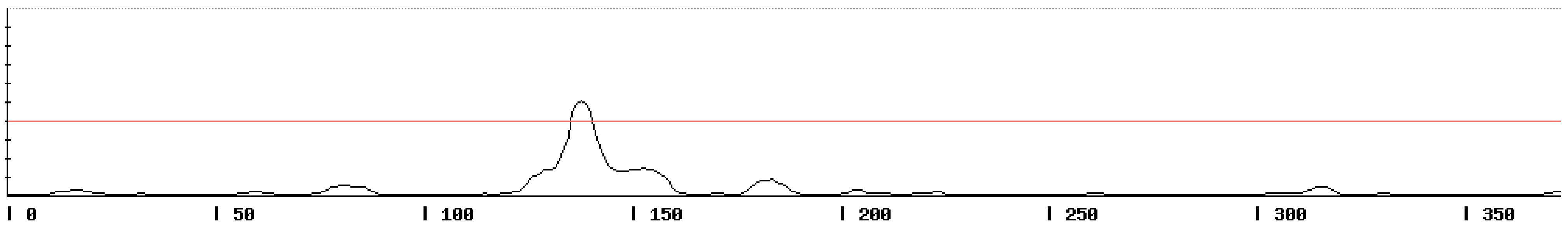

Predictions for Catharanthusroseus_cra_locus_6334_iso_2	
Viterbi Path	
Viterbi Path	
Posterior @ 0.4	136 - RKRRK - 140
Posterior @ 0.4	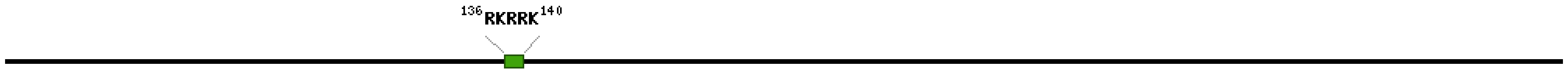
Posterior Graphic. Threshold Marked @ 0.4	

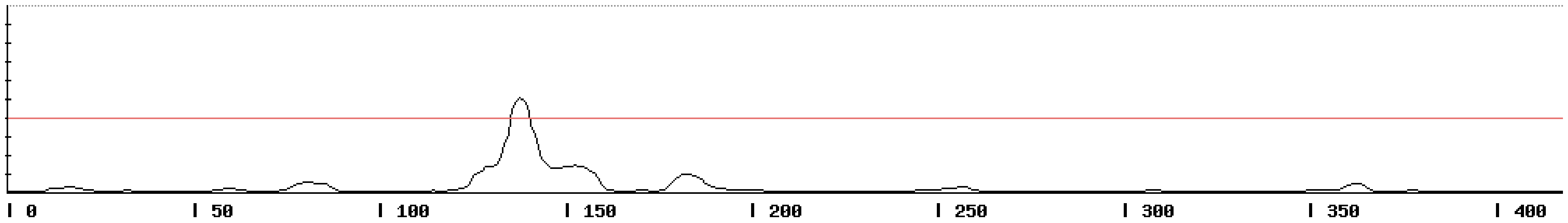

Predictions for Catharanthusroseus_cra_locus_6334_iso_3

Viterbi Path

Viterbi Path

Posterior @ 0.4

136 - RKRRK - 140

301 - R - 301

Posterior @ 0.4

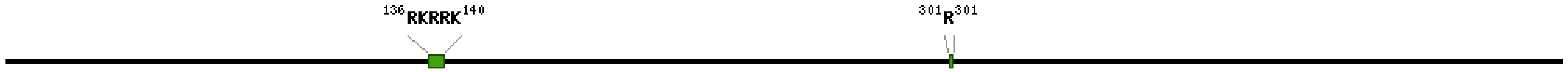

Posterior Graphic. Threshold Marked @ 0.4

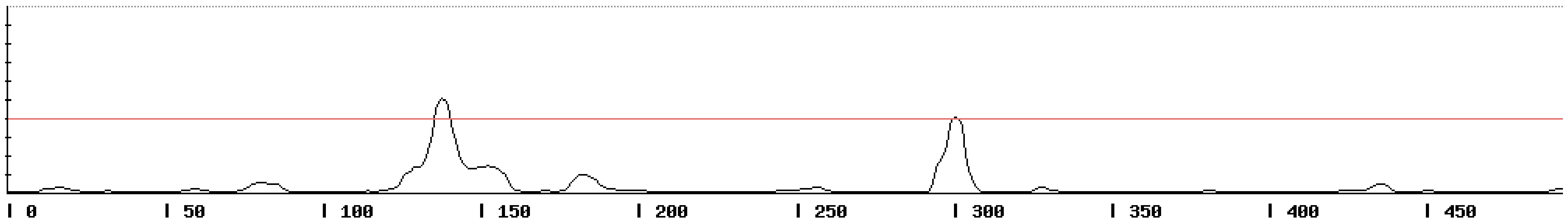

Predictions for Catharanthusroseus_cra_locus_6334_iso_4

Viterbi Path

Viterbi Path

Posterior @ 0.4

136 - RKRRK - 140

Posterior @ 0.4

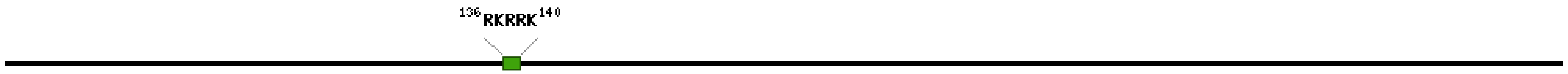

Posterior Graphic. Threshold Marked @ 0.4

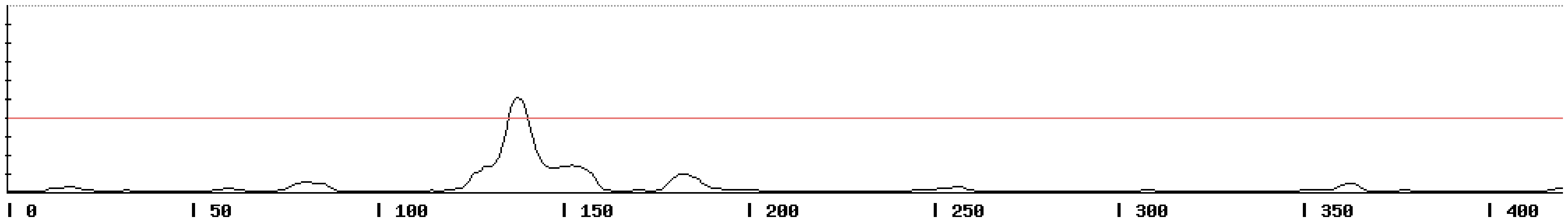

Predictions for Catharanthusroseus_cra_locus_63860_iso_1

Viterbi Path

Viterbi Path

Posterior @ 0.4

Posterior @ 0.4

Posterior Graphic. Threshold Marked @ 0.4

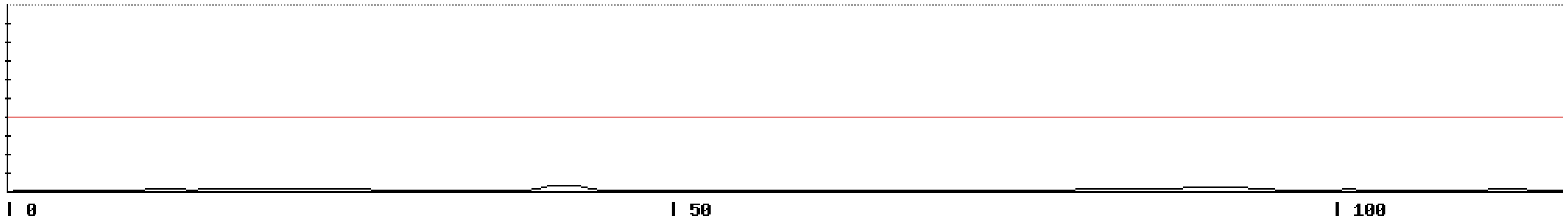

Predictions for Catharanthusroseus_cra_locus_6720_iso_3

Viterbi Path

Viterbi Path

Posterior @ 0.4

Posterior @ 0.4

Posterior Graphic. Threshold Marked @ 0.4

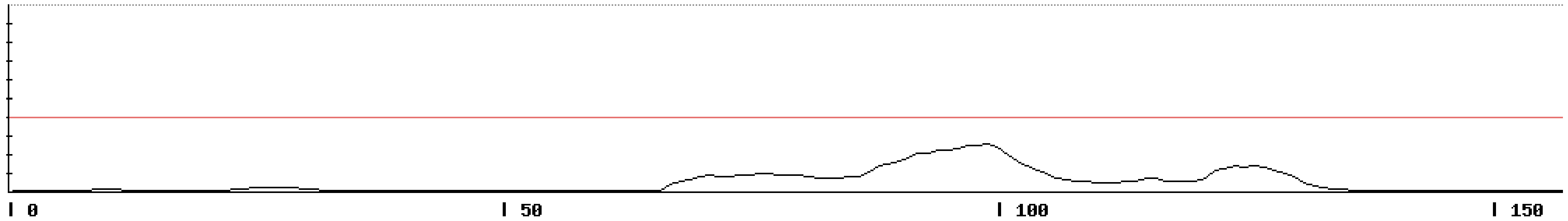

Predictions for Catharanthusroseus_cra_locus_6720_iso_4

Viterbi Path

Viterbi Path

Posterior @ 0.4

Posterior @ 0.4

Posterior Graphic. Threshold Marked @ 0.4

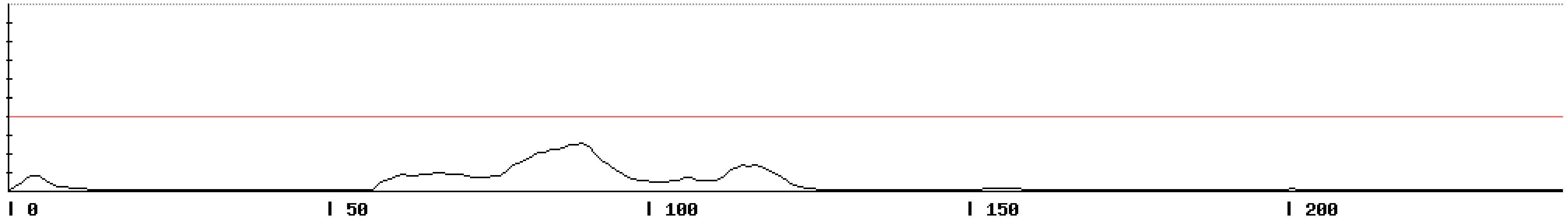

Predictions for Catharanthusroseus_cra_locus_6720_iso_7

Viterbi Path

Viterbi Path

Posterior @ 0.4

Posterior @ 0.4

Posterior Graphic. Threshold Marked @ 0.4

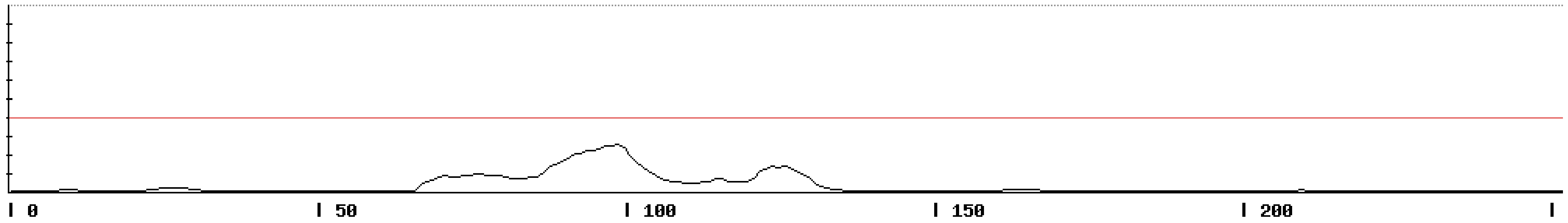

Predictions for Catharanthusroseus_cra_locus_7294_iso_2

Viterbi Path

Viterbi Path

Posterior @ 0.4

Posterior @ 0.4

Posterior Graphic. Threshold Marked @ 0.4

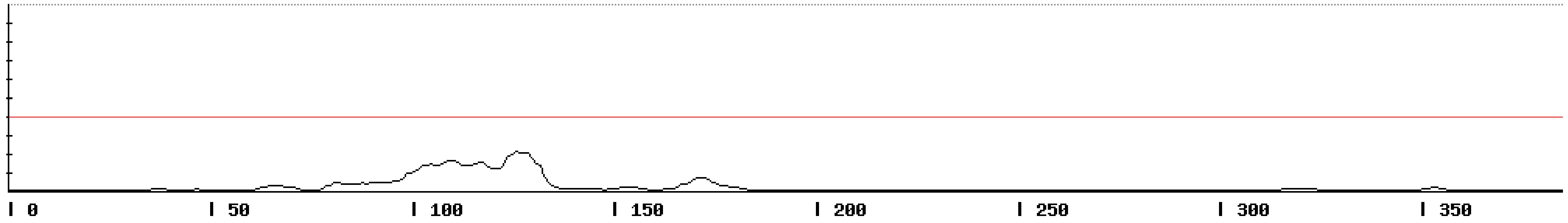

Predictions for Catharanthusroseus_cra_locus_7294_iso_3

Viterbi Path	
Viterbi Path	
Posterior @ 0.4	
Posterior @ 0.4	
Posterior Graphic. Threshold Marked @ 0.4	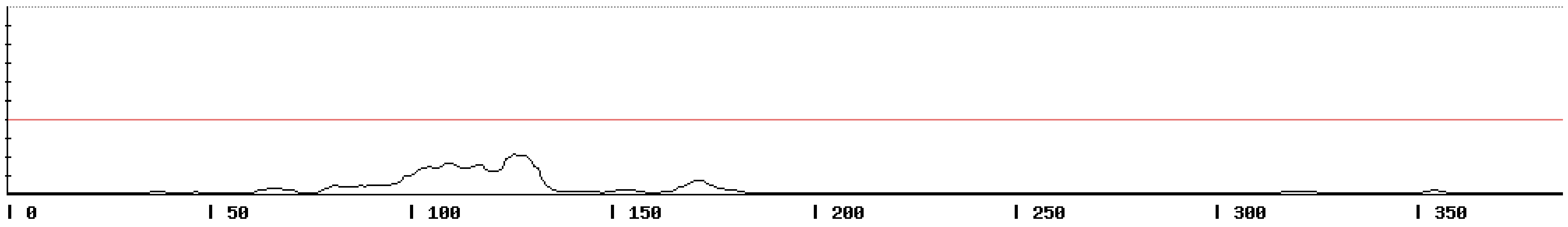

Predictions for Catharanthusroseus_cra_locus_84210_iso_1

Viterbi Path	
Viterbi Path	
Posterior @ 0.4	
Posterior @ 0.4	
Posterior Graphic. Threshold Marked @ 0.4	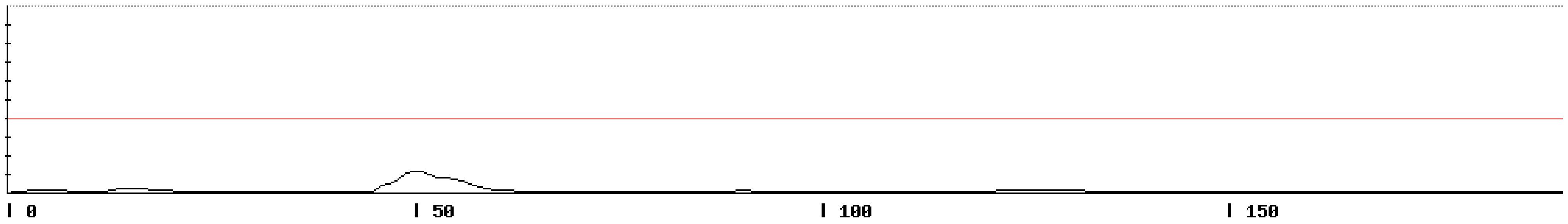

Predictions for Catharanthusroseus_cra_locus_9229_iso_1

Viterbi Path

Viterbi Path

Posterior @ 0.4

Posterior @ 0.4

Posterior Graphic. Threshold Marked @ 0.4

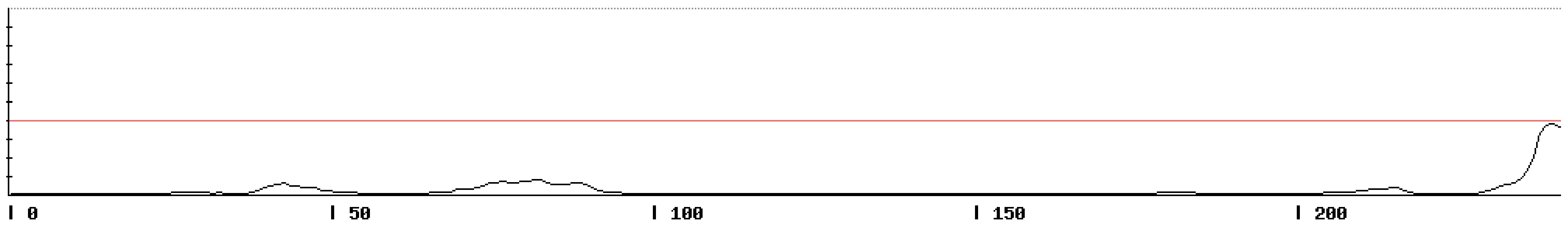

Predictions for Catharanthusroseus_cra_locus_9524_iso_1

Viterbi Path

Viterbi Path

Posterior @ 0.4

Posterior @ 0.4

Posterior Graphic. Threshold Marked @ 0.4

Revision r.9 - Last Updated on : February 24 2015 17:45:20.

alex.nguyenba@utoronto.ca

Intended to be used with Firefox

Viterbi Path

Posterior @ 0.4

Posterior @ 0.4

Posterior Graphic. Threshold Marked @ 0.4

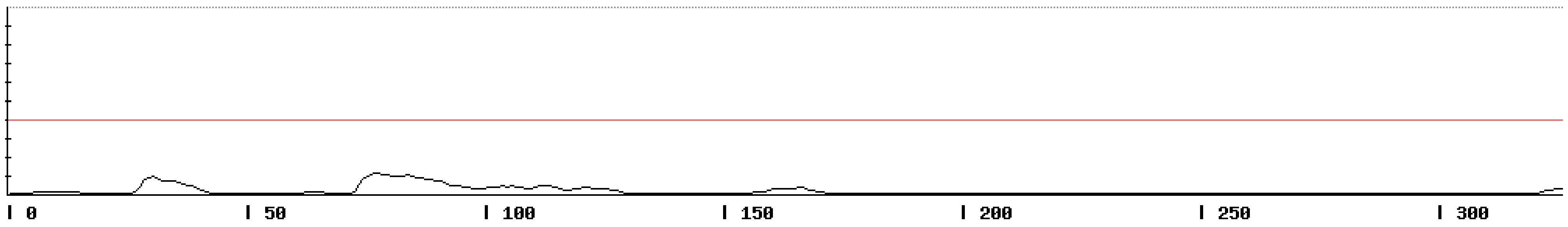

Predictions for Chenopodiumquinoa_AUR62000493

Viterbi Path

Viterbi Path

Posterior @ 0.4

Posterior @ 0.4

Posterior Graphic. Threshold Marked @ 0.4

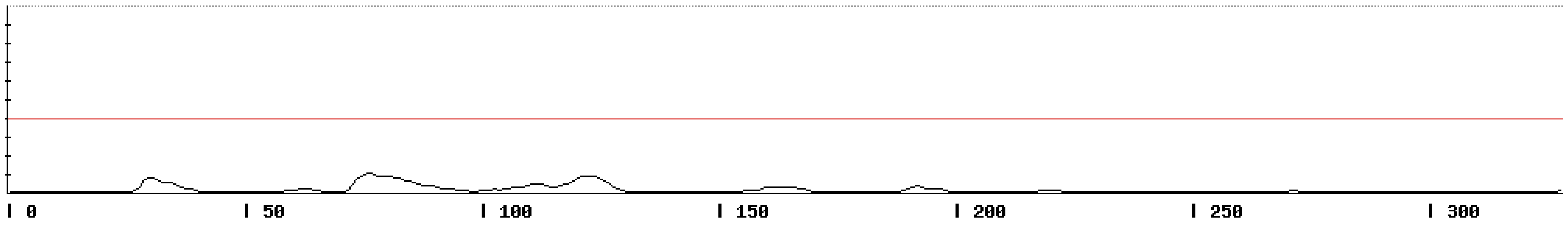

Predictions for Chenopodiumquinoa_AUR62000536

Viterbi Path

Viterbi Path

Posterior @ 0.4

44 - GKPK - 47

Posterior @ 0.4

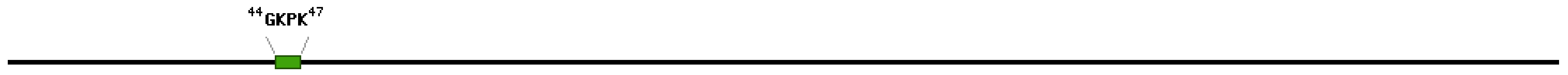

Posterior Graphic. Threshold Marked @ 0.4

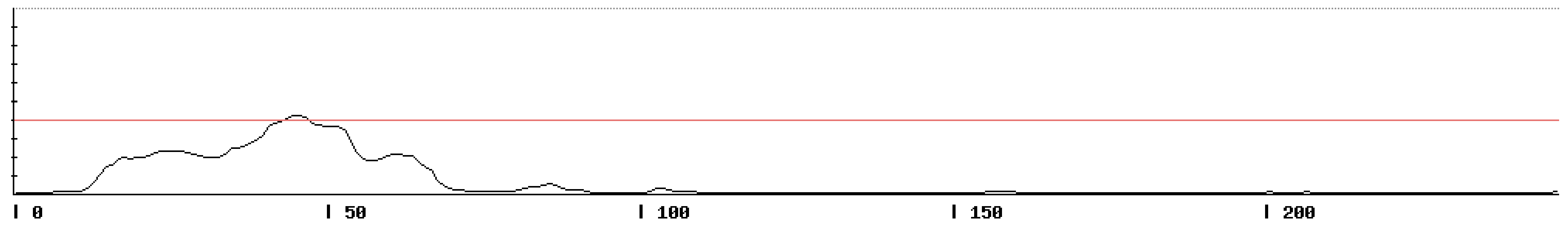

Predictions for Chenopodiumquinoa_AUR62000898

Viterbi Path

Viterbi Path

Posterior @ 0.4

96 - GRPKRSASNGKW - 107

145 - NHKKKSKKTLSHKKK - 159

204 - KKENKGKGRKKSKNS - 218

Posterior @ 0.4

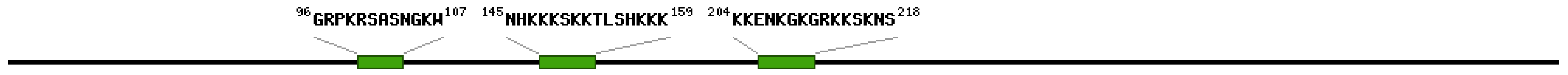

Posterior Graphic. Threshold Marked @ 0.4

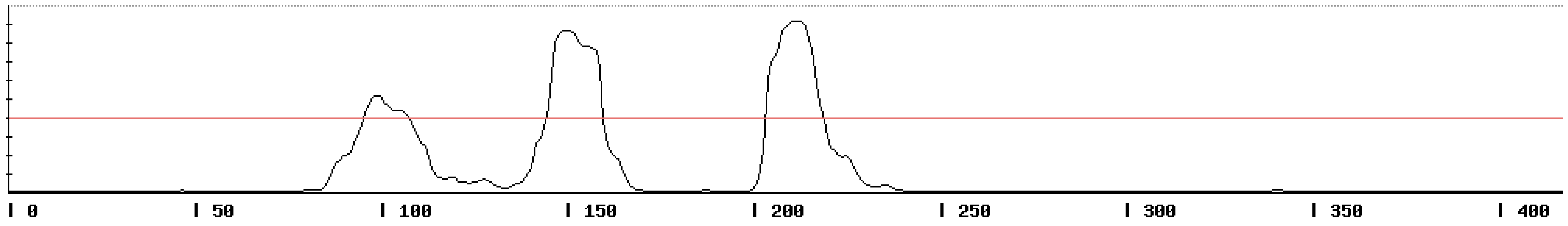

Predictions for Chenopodiumquinoa_AUR62001365

Viterbi Path	
Viterbi Path	
Posterior @ 0.4	
Posterior @ 0.4	
Posterior Graphic. Threshold Marked @ 0.4	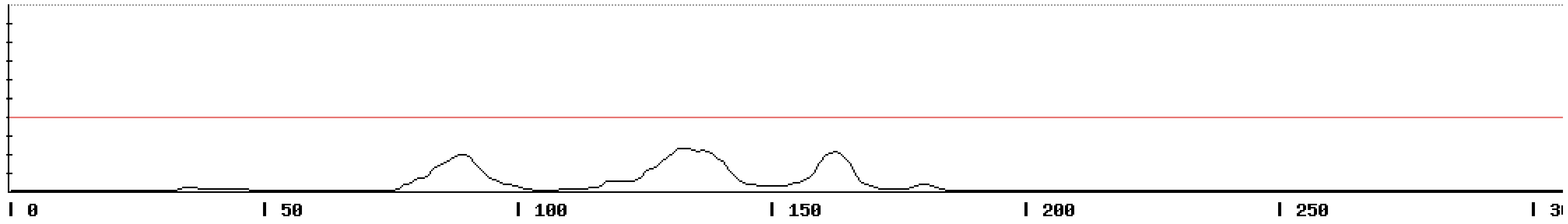

Predictions for Chenopodiumquinoa_AUR62001522

Viterbi Path	
Viterbi Path	
Posterior @ 0.4	115 - PIGRKTK - 121
	263 - SIKRKK - 268
Posterior @ 0.4	

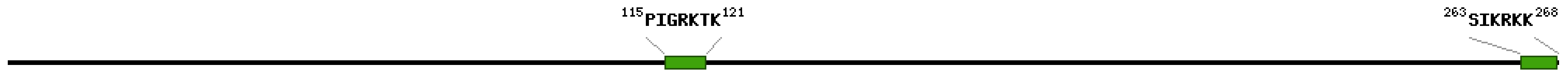

Posterior Graphic. Threshold Marked @ 0.4

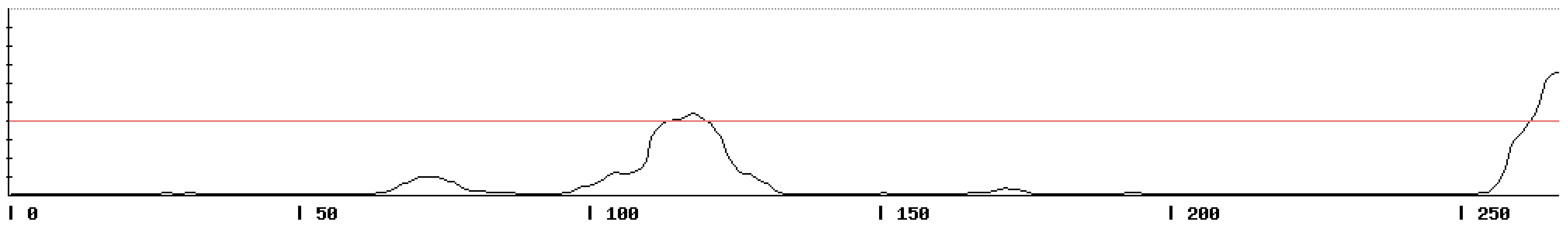

Predictions for Chenopodiumquinoa_AUR62001531

Viterbi Path

Viterbi Path

Posterior @ 0.4 255 - NRRLRLRKLRRLSPRK - 270

Posterior @ 0.4

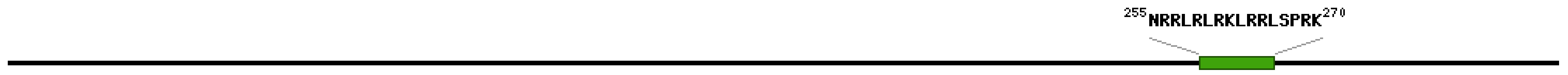

Posterior Graphic. Threshold Marked @ 0.4

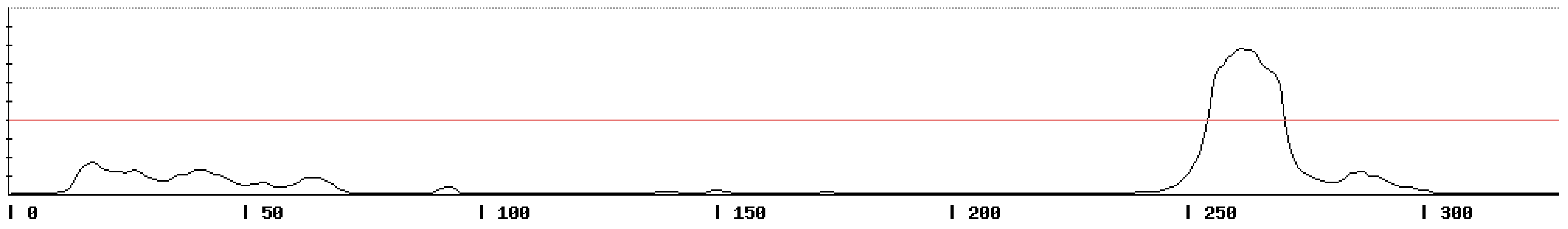

Predictions for Chenopodiumquinoa_AUR62002191

Viterbi Path

Viterbi Path

Posterior @ 0.4	
Posterior @ 0.4	
Posterior Graphic. Threshold Marked @ 0.4	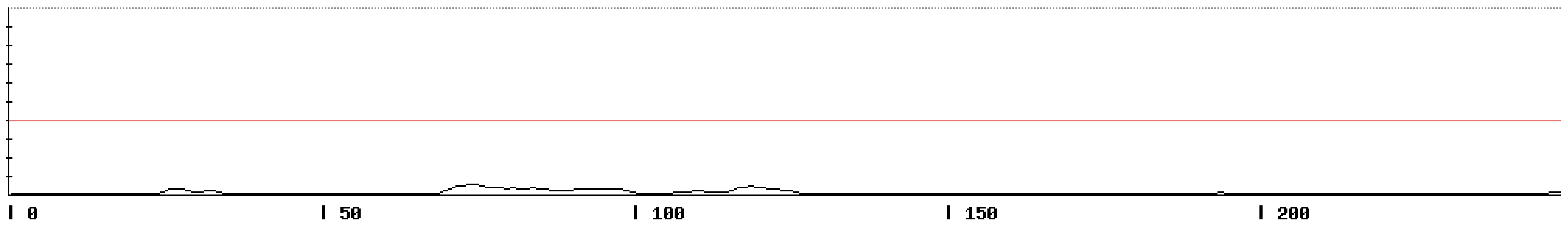

Predictions for Chenopodiumquinoa_AUR62003400	
Viterbi Path	
Viterbi Path	
Posterior @ 0.4	
Posterior @ 0.4	
Posterior Graphic. Threshold Marked @ 0.4	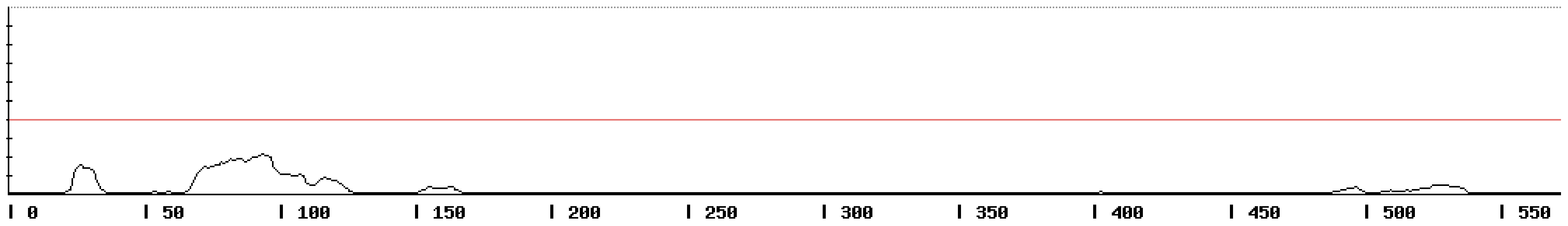

Predictions for Chenopodiumquinoa_AUR62003583	
Viterbi Path	
Viterbi Path	

Posterior @ 0.4

Posterior @ 0.4

Posterior Graphic. Threshold Marked @ 0.4

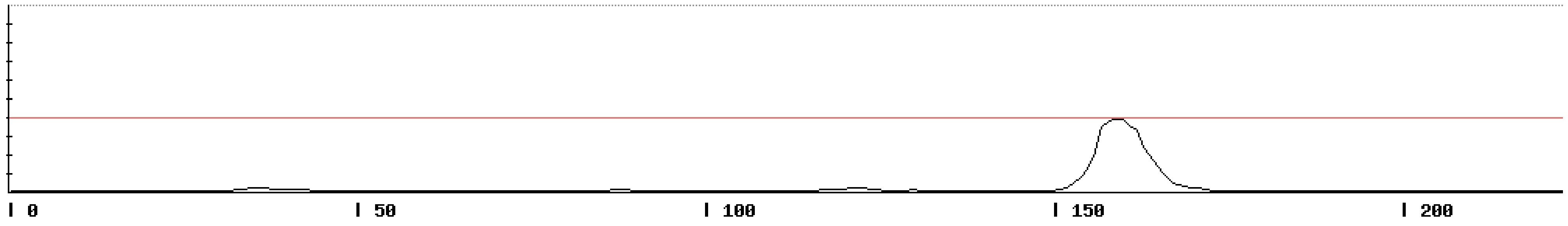

Predictions for Chenopodiumquinoa_AUR62004554

Viterbi Path

Viterbi Path

Posterior @ 0.4

Posterior @ 0.4

Posterior Graphic. Threshold Marked @ 0.4

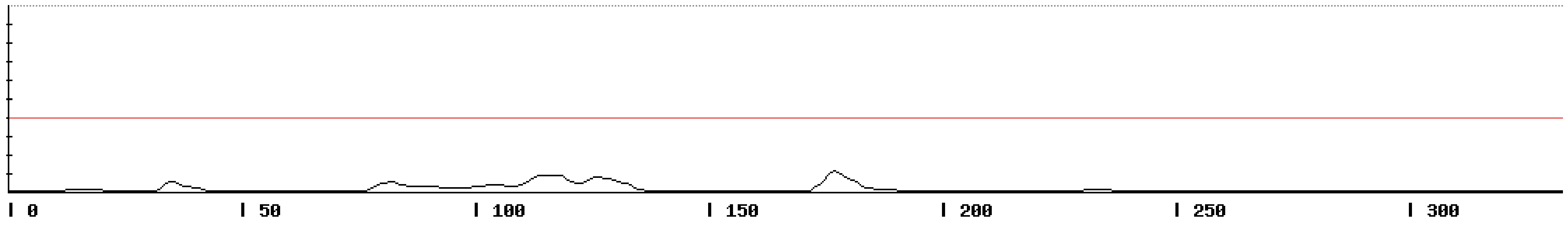

Predictions for Chenopodiumquinoa_AUR62004648

Viterbi Path	
Viterbi Path	
Posterior @ 0.4	
Posterior @ 0.4	
Posterior Graphic. Threshold Marked @ 0.4	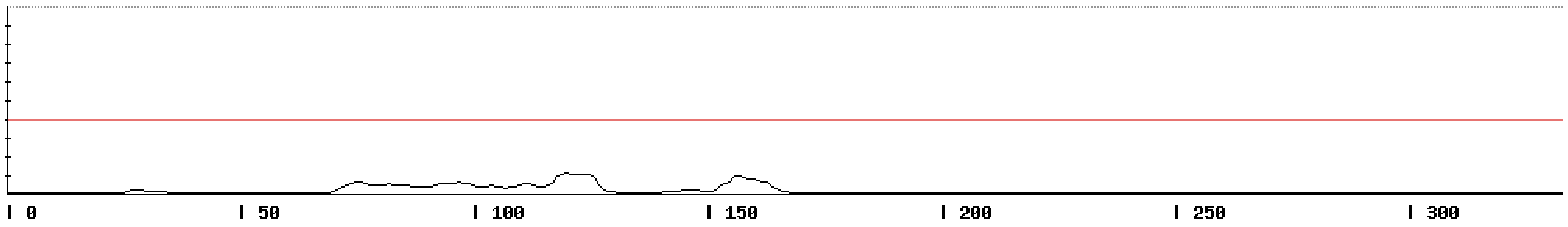

Predictions for Chenopodiumquinoa_AUR62004773

Viterbi Path	
Viterbi Path	
Posterior @ 0.4	
Posterior @ 0.4	
Posterior Graphic. Threshold Marked @ 0.4	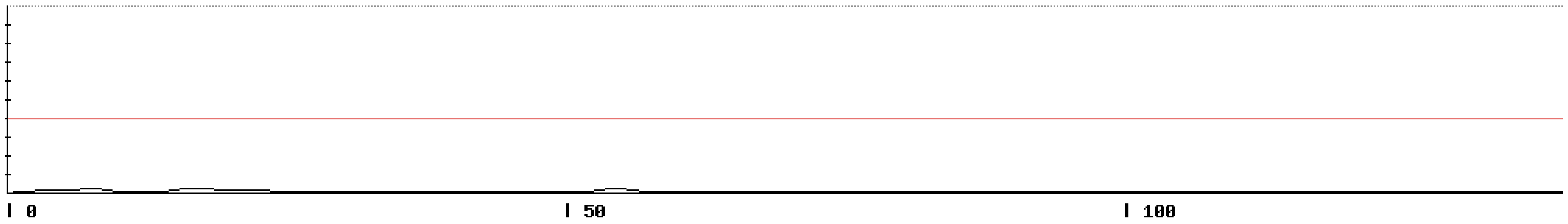

Predictions for Chenopodiumquinoa_AUR62004778

Viterbi Path	
Viterbi Path	
Posterior @ 0.4	
Posterior @ 0.4	
Posterior Graphic. Threshold Marked @ 0.4	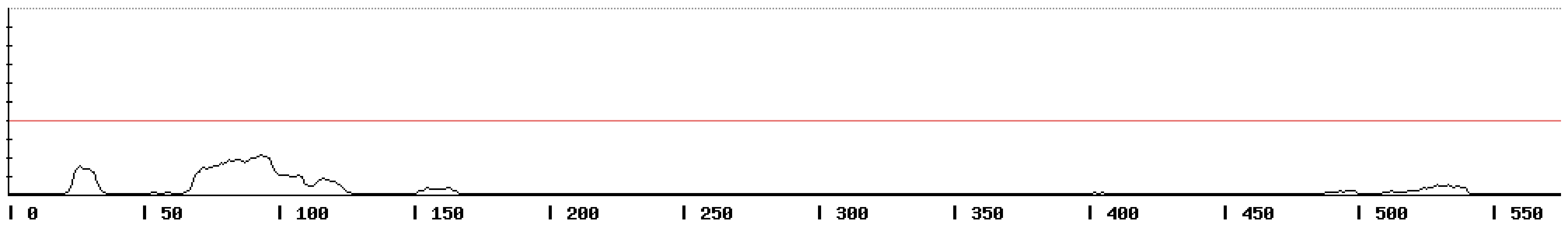

Predictions for Chenopodiumquinoa_AUR62005686

Viterbi Path	
Viterbi Path	
Posterior @ 0.4	
Posterior @ 0.4	
Posterior Graphic. Threshold Marked @ 0.4	

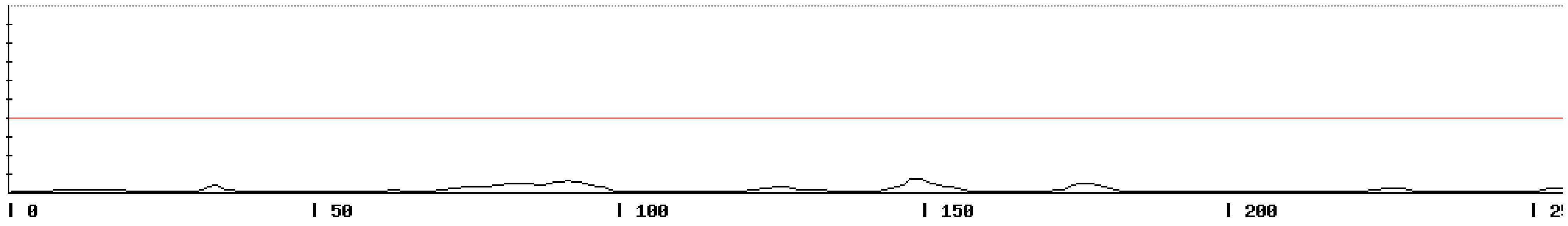

Predictions for Chenopodiumquinoa_AUR62005772

Viterbi Path

Viterbi Path

Posterior @ 0.4

Posterior @ 0.4

Posterior Graphic. Threshold Marked @ 0.4

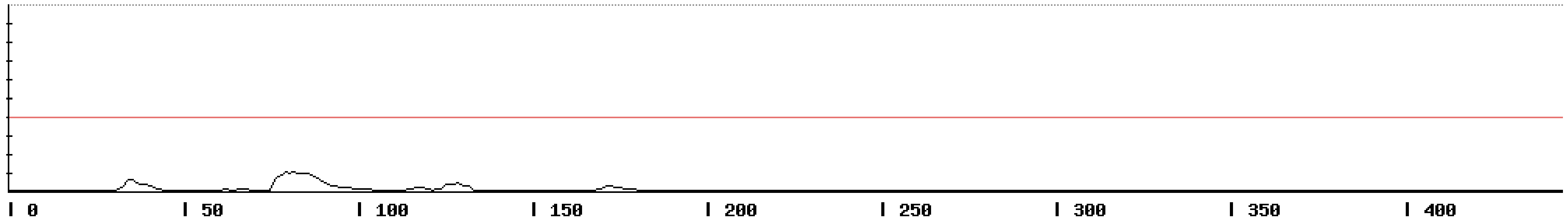

Predictions for Chenopodiumquinoa_AUR62005821

Viterbi Path

Viterbi Path

Posterior @ 0.4

Posterior @ 0.4

Posterior Graphic. Threshold Marked @ 0.4

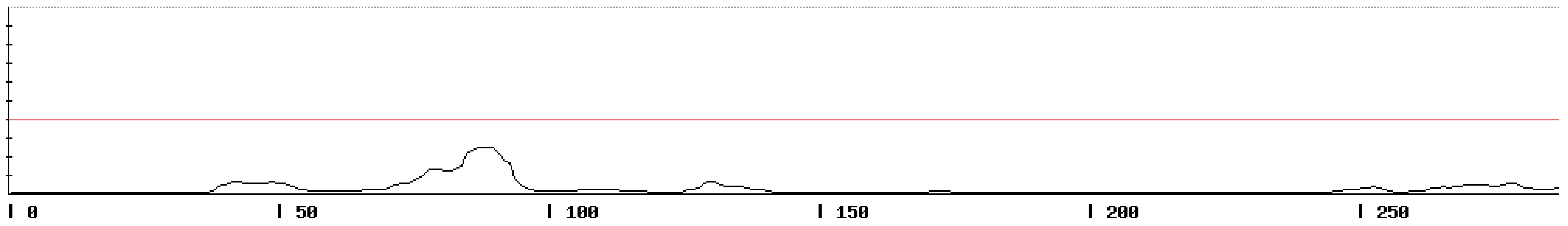

Predictions for Chenopodiumquinoa_AUR62005966

Viterbi Path

Viterbi Path

Posterior @ 0.4

103 - IKQGKK - 108

Posterior @ 0.4

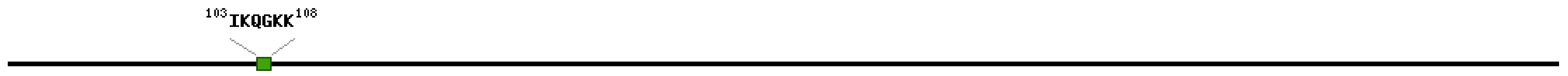

Posterior Graphic. Threshold Marked @ 0.4

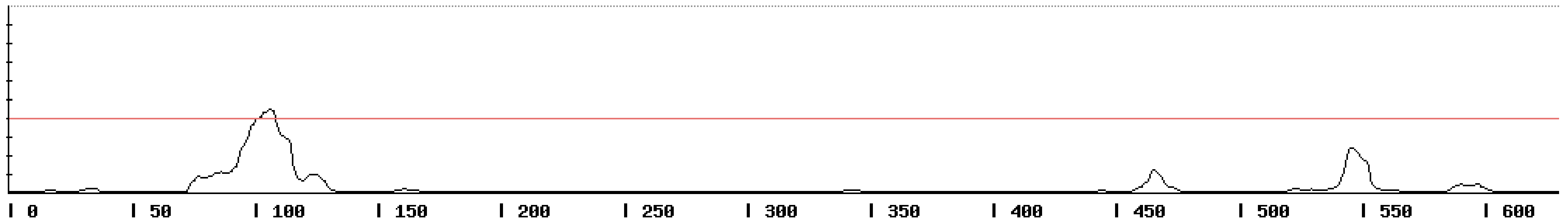

Predictions for Chenopodiumquinoa_AUR62006223

Viterbi Path

Viterbi Path

Posterior @ 0.4

Posterior @ 0.4

Posterior Graphic. Threshold Marked @ 0.4

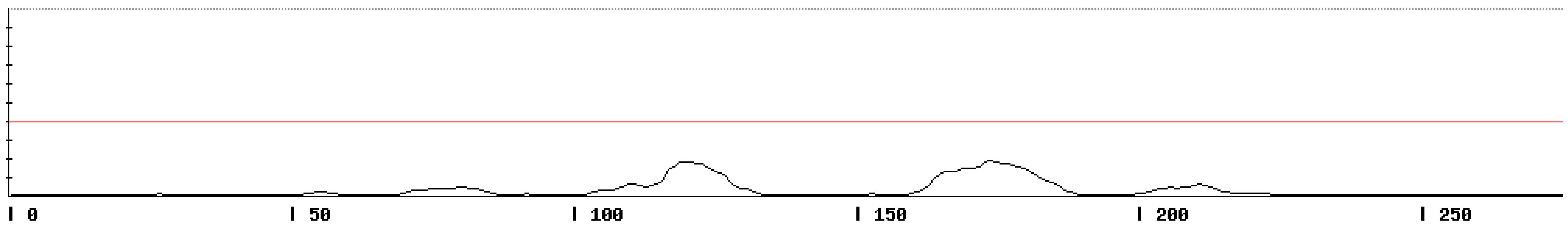

Predictions for Chenopodiumquinoa_AUR62006407

Viterbi Path

Viterbi Path

Posterior @ 0.4

Posterior @ 0.4

Posterior Graphic. Threshold Marked @ 0.4

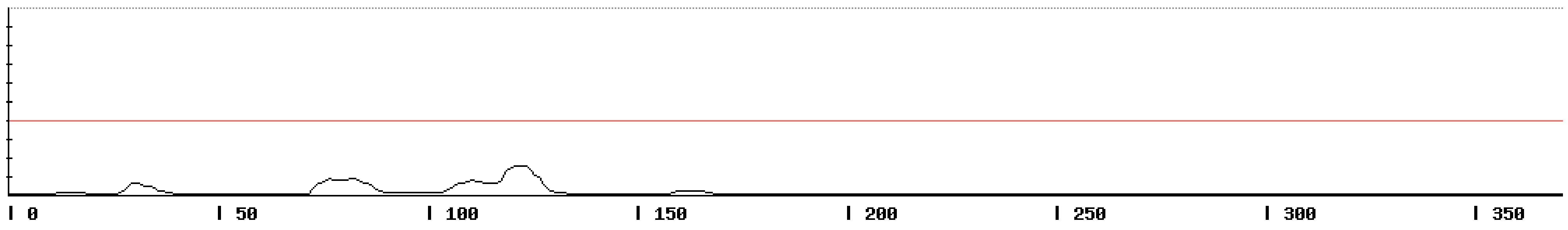

Predictions for Chenopodiumquinoa_AUR62006836

Viterbi Path

Viterbi Path

Posterior @ 0.4

Posterior @ 0.4

Posterior Graphic. Threshold Marked @ 0.4

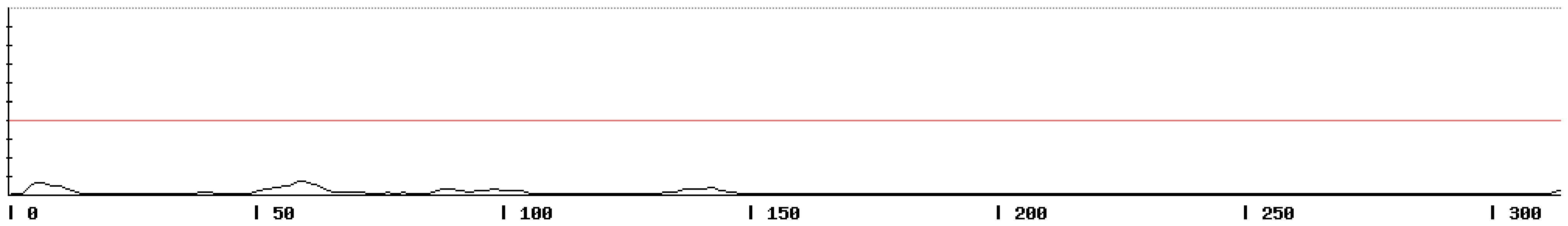

Predictions for Chenopodiumquinoa_AUR62006837

Viterbi Path

Viterbi Path

Posterior @ 0.4

Posterior @ 0.4

Posterior Graphic. Threshold Marked @ 0.4

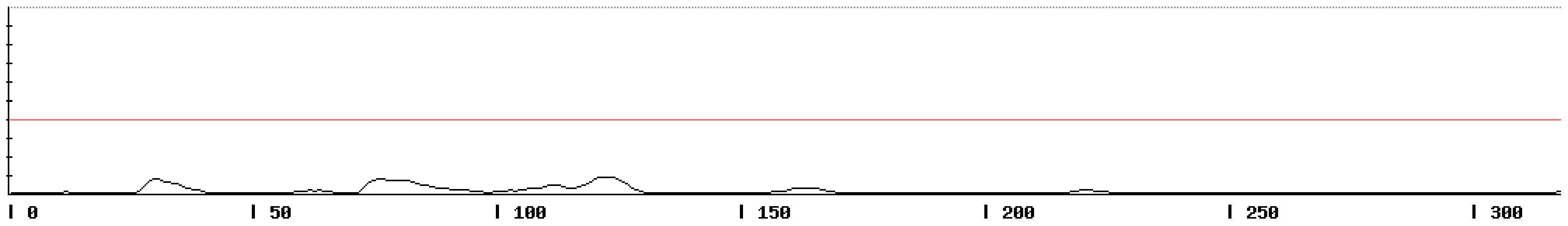

Predictions for Chenopodiumquinoa_AUR62006885

Viterbi Path

Viterbi Path

Posterior @ 0.4	125 - GKPK - 128
Posterior @ 0.4	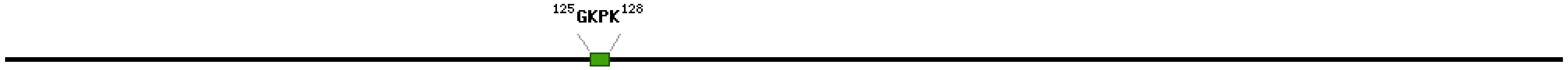
Posterior Graphic. Threshold Marked @ 0.4	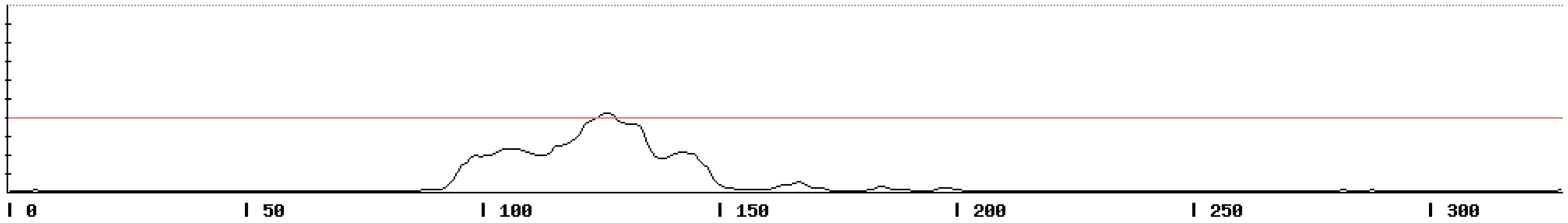

Predictions for Chenopodiumquinoa_AUR62007071

Viterbi Path	
Viterbi Path	
Posterior @ 0.4	
Posterior @ 0.4	
Posterior Graphic. Threshold Marked @ 0.4	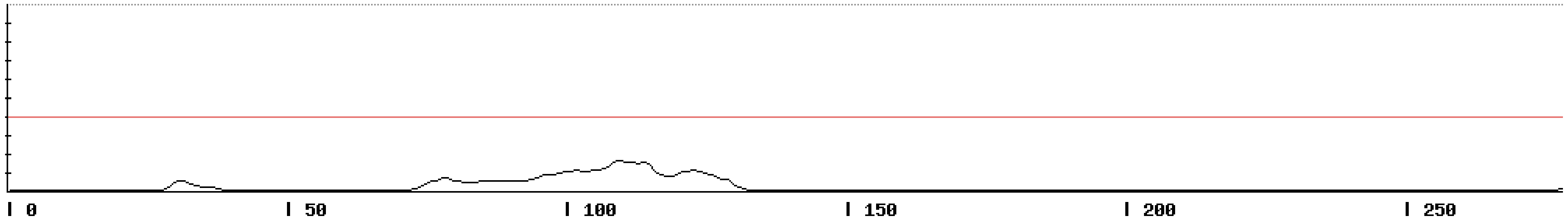

Predictions for Chenopodiumquinoa_AUR62007247

Viterbi Path

Viterbi Path

Posterior @ 0.4

Posterior @ 0.4

Posterior Graphic. Threshold Marked @ 0.4

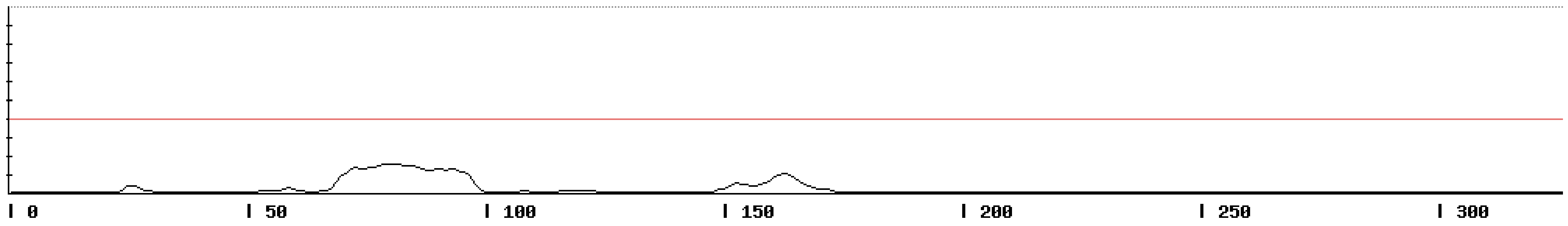

Predictions for Chenopodiumquinoa_AUR62007469

Viterbi Path

Viterbi Path

Posterior @ 0.4

Posterior @ 0.4

Posterior Graphic. Threshold Marked @ 0.4

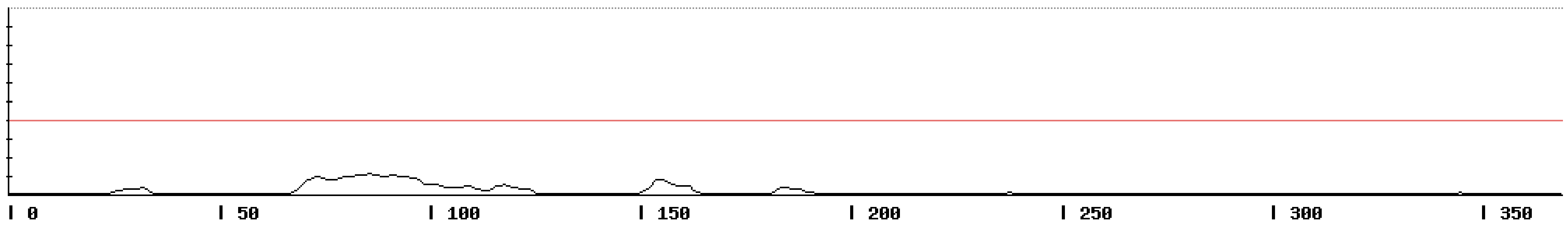

Predictions for Chenopodiumquinoa_AUR62008438	
Viterbi Path	
Viterbi Path	
Posterior @ 0.4	
Posterior @ 0.4	
Posterior Graphic. Threshold Marked @ 0.4	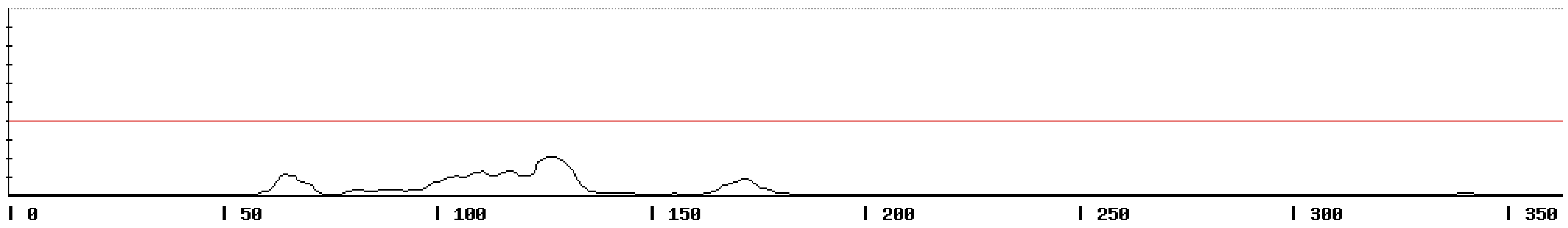

Predictions for Chenopodiumquinoa_AUR62010080	
Viterbi Path	
Viterbi Path	
Posterior @ 0.4	
Posterior @ 0.4	
Posterior Graphic. Threshold Marked @ 0.4	

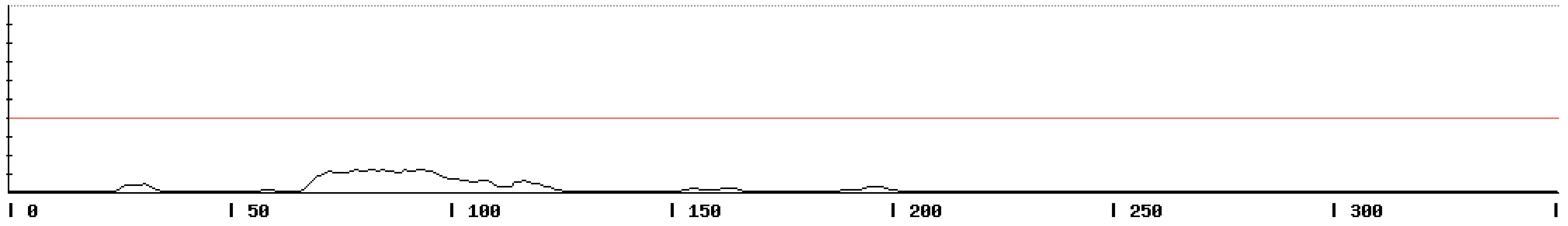

Predictions for Chenopodiumquinoa_AUR62011638

Viterbi Path

Viterbi Path

Posterior @ 0.4 — 79 - SRNRKYKNGKRPDR - 92

Posterior @ 0.4

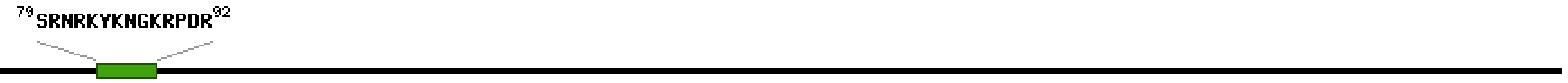

Posterior Graphic. Threshold Marked @ 0.4

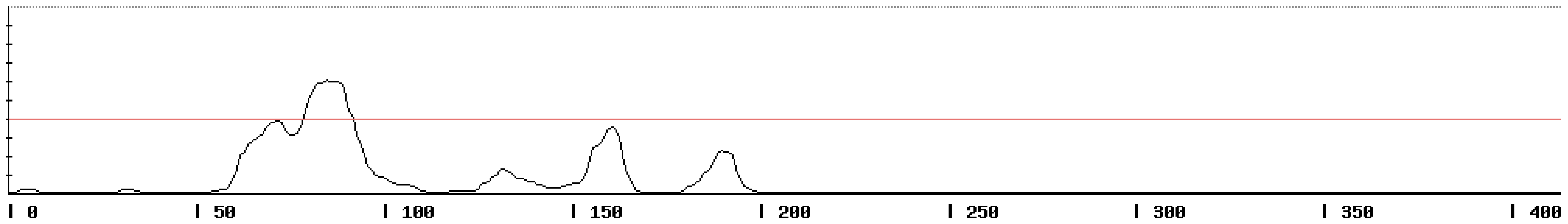

Predictions for Chenopodiumquinoa_AUR62011853

Viterbi Path

Viterbi Path

Posterior @ 0.4

Posterior @ 0.4

Posterior Graphic. Threshold Marked @ 0.4

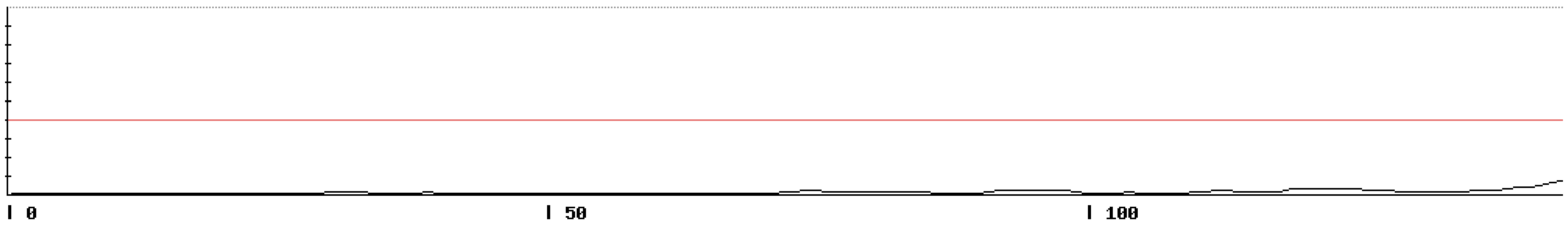

Predictions for Chenopodiumquinoa_AUR62012948

Viterbi Path

Viterbi Path

Posterior @ 0.4

Posterior @ 0.4

Posterior Graphic. Threshold Marked @ 0.4

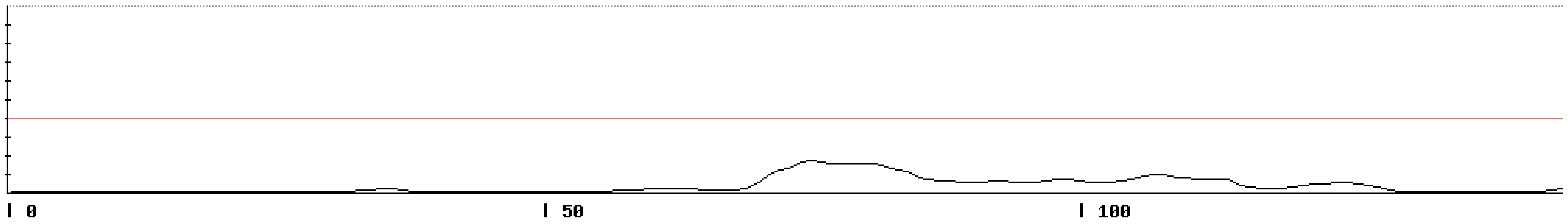

Predictions for Chenopodiumquinoa_AUR62013074

Viterbi Path

Viterbi Path

Posterior @ 0.4

Posterior @ 0.4

Posterior Graphic. Threshold Marked @ 0.4

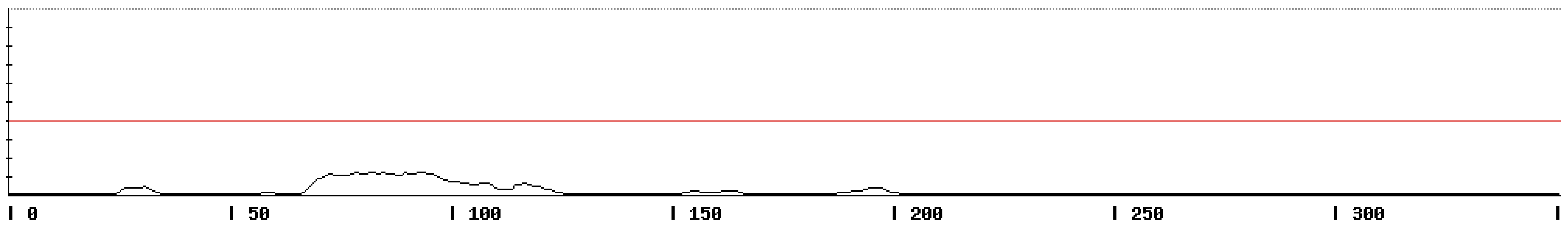

Predictions for Chenopodiumquinoa_AUR62013541

Viterbi Path

Viterbi Path

Posterior @ 0.4

Posterior @ 0.4

Posterior Graphic. Threshold Marked @ 0.4

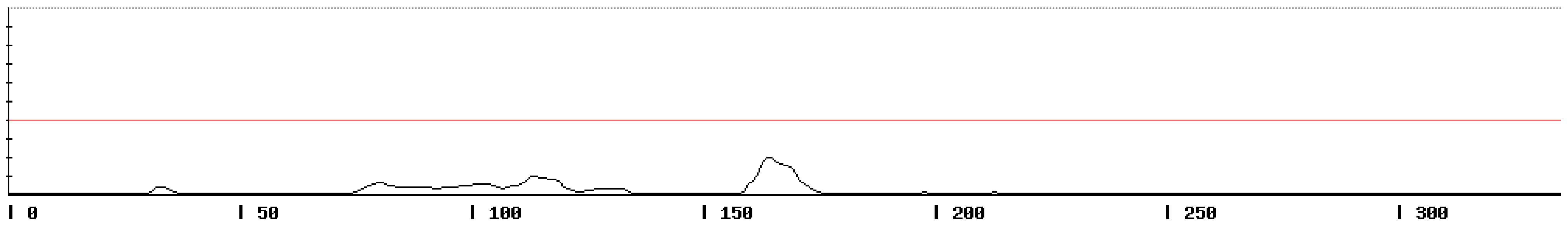

Predictions for Chenopodiumquinoa_AUR62014768

Viterbi Path

Viterbi Path

Posterior @ 0.4

Posterior @ 0.4

Posterior Graphic. Threshold Marked @ 0.4

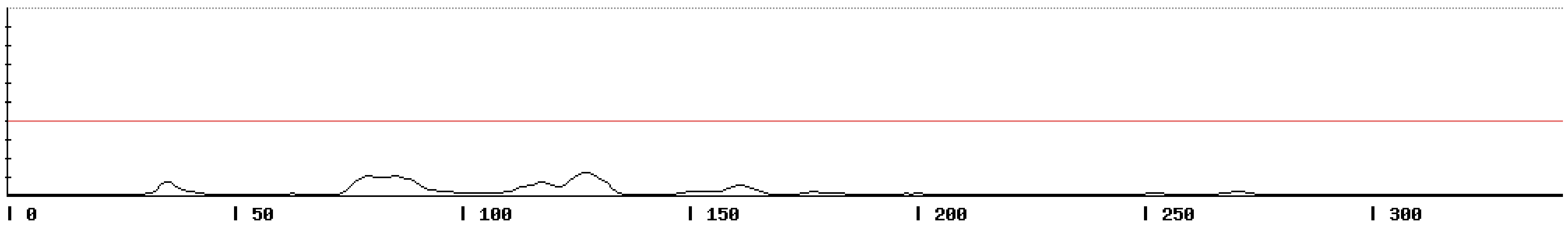

Predictions for Chenopodiumquinoa_AUR62015520

Viterbi Path

Viterbi Path

Posterior @ 0.4

Posterior @ 0.4

Posterior Graphic. Threshold Marked @ 0.4

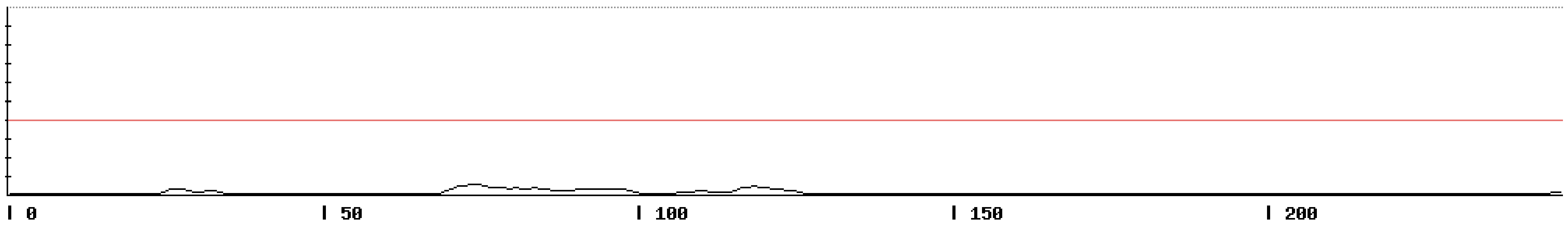

Predictions for Chenopodiumquinoa_AUR62015629

Viterbi Path

Viterbi Path

Posterior @ 0.4

Posterior @ 0.4

Posterior Graphic. Threshold Marked @ 0.4

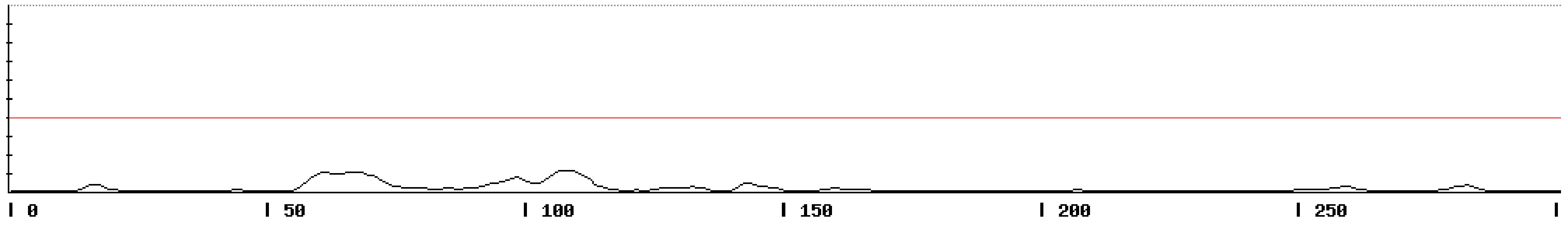

Predictions for Chenopodiumquinoa_AUR62015630

Viterbi Path

Viterbi Path

Posterior @ 0.4

Posterior @ 0.4

Posterior Graphic. Threshold Marked @ 0.4

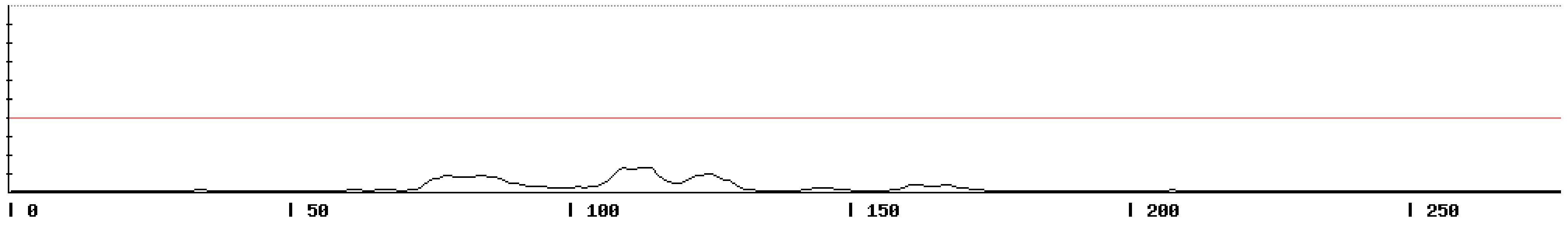

Predictions for Chenopodiumquinoa_AUR62016200

Viterbi Path

Viterbi Path

Posterior @ 0.4

Posterior @ 0.4

Posterior Graphic. Threshold Marked @ 0.4

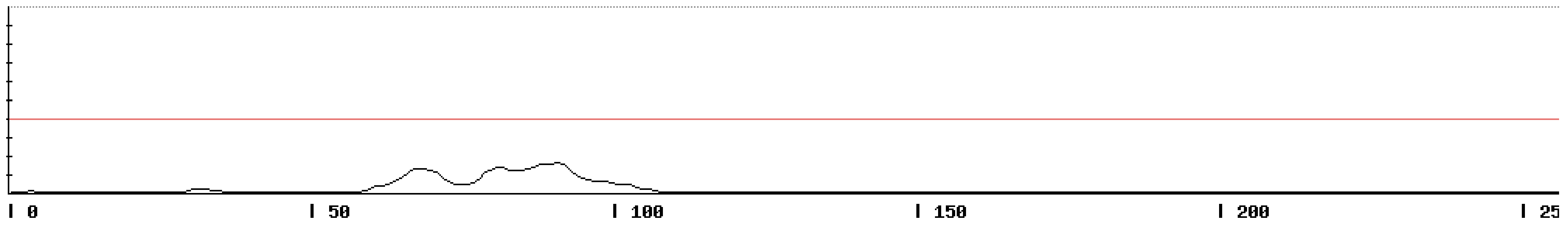

Predictions for Chenopodiumquinoa_AUR62016213

Viterbi Path

Viterbi Path

Posterior @ 0.4

80 - RNRKYKNGKRP - 90

Posterior @ 0.4

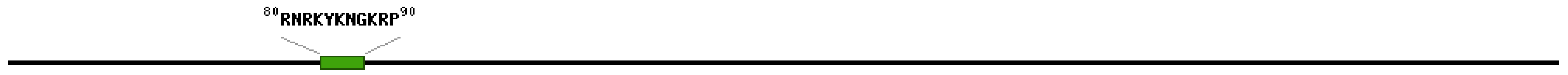

Posterior Graphic. Threshold Marked @ 0.4

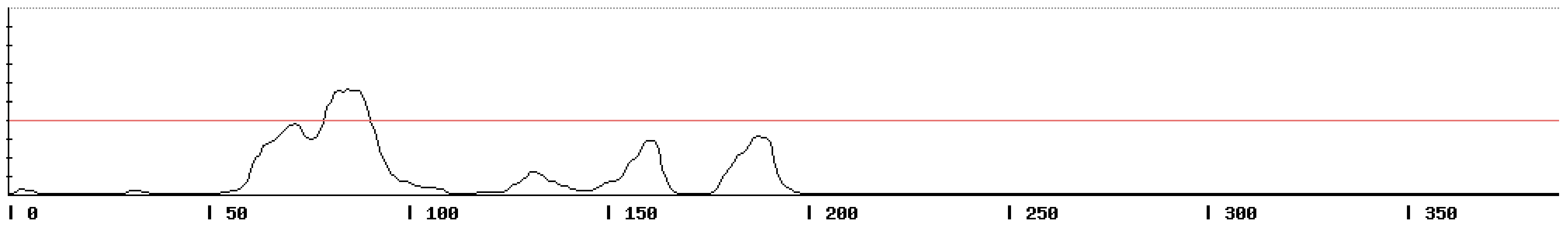

Predictions for Chenopodiumquinoa_AUR62016954

Viterbi Path	
Viterbi Path	
Posterior @ 0.4	
Posterior @ 0.4	
Posterior Graphic. Threshold Marked @ 0.4	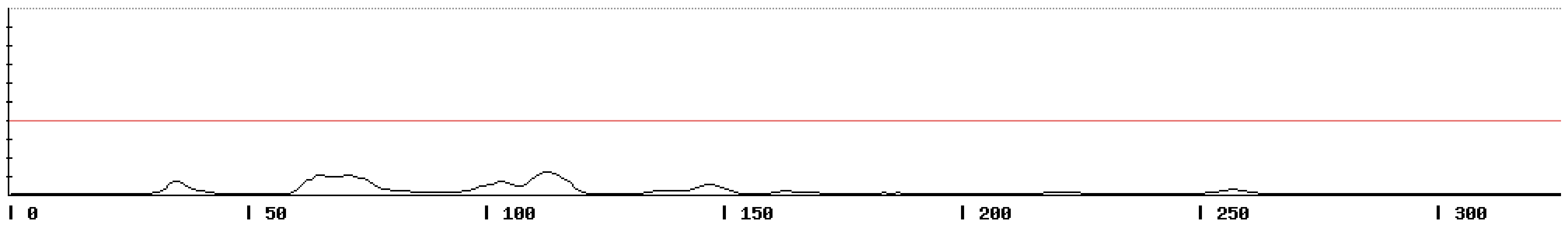

Predictions for Chenopodiumquinoa_AUR62017681

Viterbi Path	
Viterbi Path	
Posterior @ 0.4	
Posterior @ 0.4	
Posterior Graphic. Threshold Marked @ 0.4	

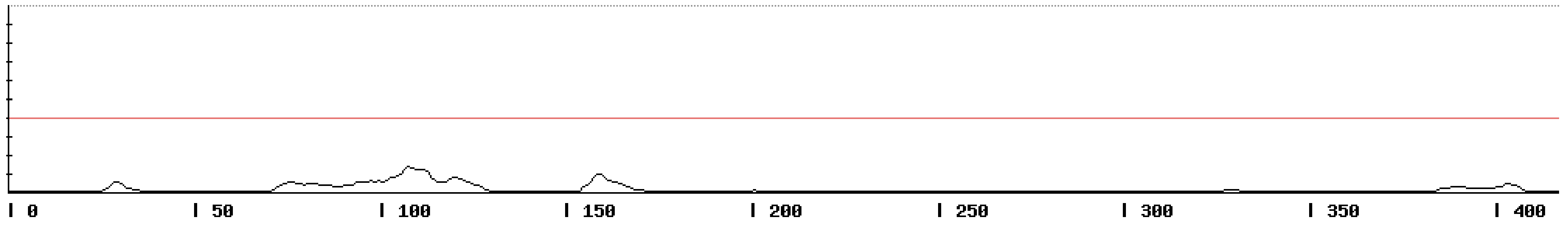

Predictions for Chenopodiumquinoa_AUR62017896	
Viterbi Path	
Viterbi Path	
Posterior @ 0.4	
Posterior @ 0.4	
Posterior Graphic. Threshold Marked @ 0.4	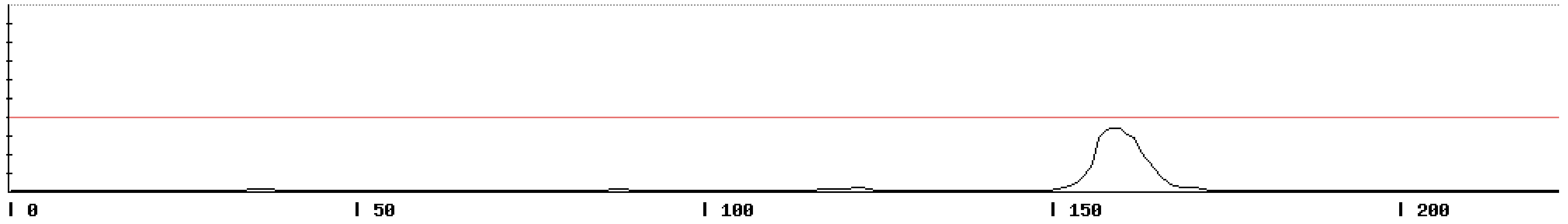

Predictions for Chenopodiumquinoa_AUR62018102	
Viterbi Path	
Viterbi Path	
Posterior @ 0.4	
Posterior @ 0.4	

Posterior Graphic. Threshold Marked @ 0.4

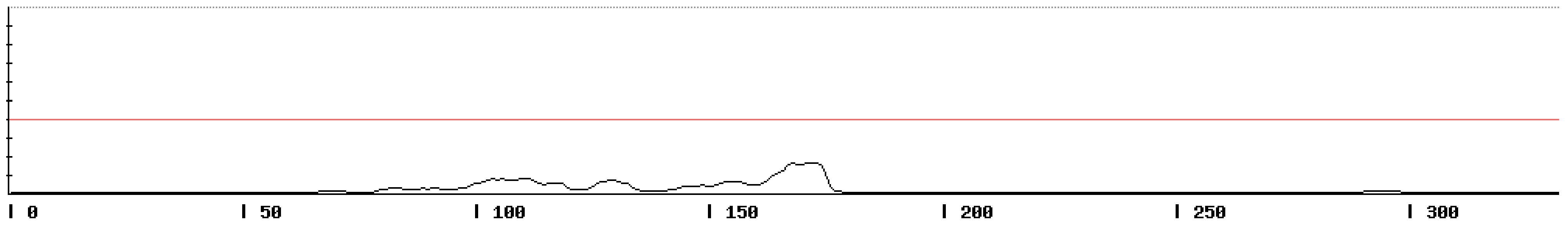

Predictions for Chenopodiumquinoa_AUR62018752

Viterbi Path

Viterbi Path

Posterior @ 0.4

Posterior @ 0.4

Posterior Graphic. Threshold Marked @ 0.4

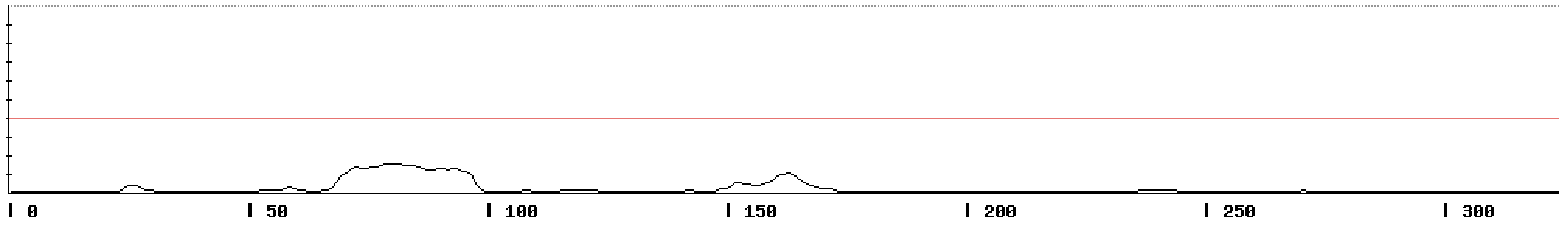

Predictions for Chenopodiumquinoa_AUR62019477

Viterbi Path

Viterbi Path

Posterior @ 0.4

Posterior @ 0.4

Posterior Graphic. Threshold Marked @ 0.4

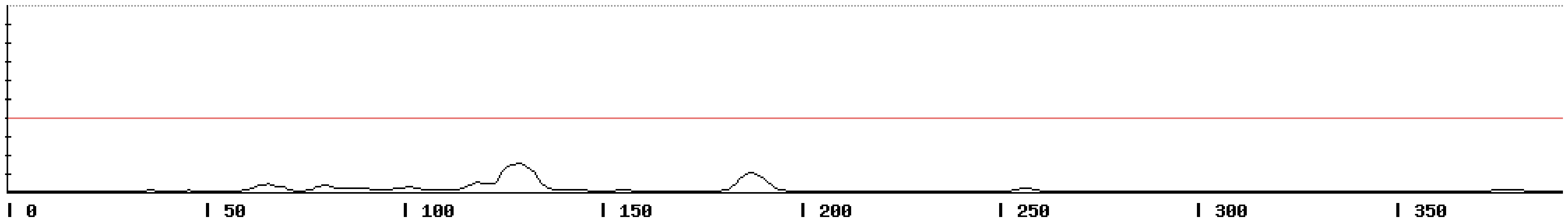

Predictions for Chenopodiumquinoa_AUR62019936

Viterbi Path

Viterbi Path

Posterior @ 0.4

Posterior @ 0.4

Posterior Graphic. Threshold Marked @ 0.4

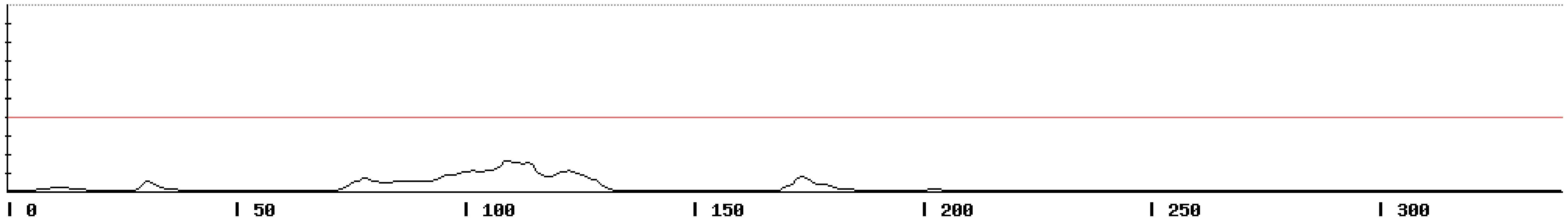

Predictions for Chenopodiumquinoa_AUR62020251

Viterbi Path

Viterbi Path

Posterior @ 0.4 | 354 - NRRIRLRKLRRLGPRKE - 370

Posterior @ 0.4

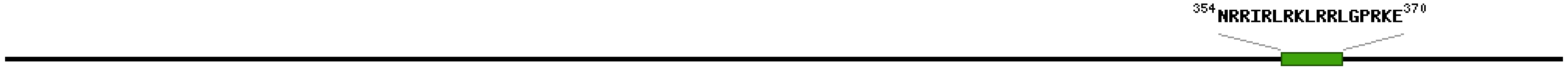

Posterior Graphic. Threshold Marked @ 0.4

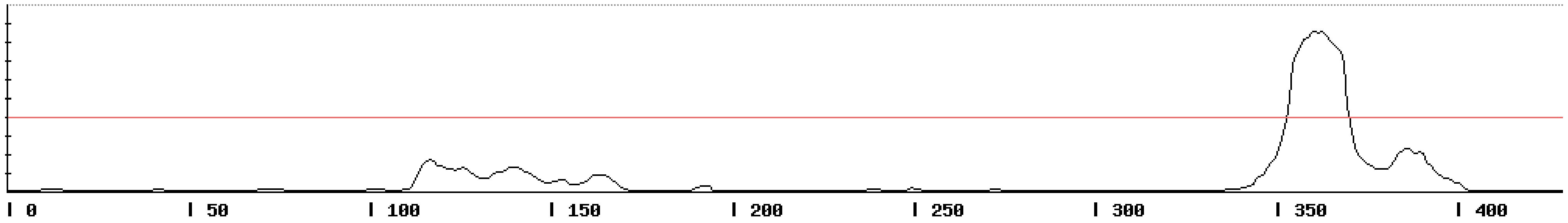

Predictions for Chenopodiumquinoa_AUR62020261

Viterbi Path

Viterbi Path

Posterior @ 0.4 | 118 - PIGRKT - 123

Posterior @ 0.4

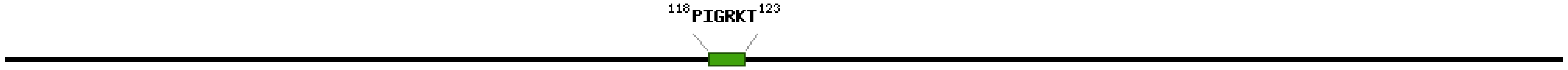

Posterior Graphic. Threshold Marked @ 0.4

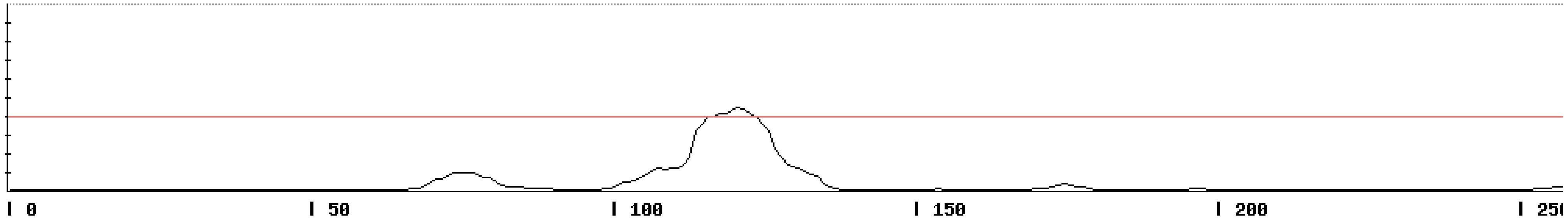

Predictions for Chenopodiumquinoa_AUR62020634

Viterbi Path

Viterbi Path

Posterior @ 0.4

Posterior @ 0.4

Posterior Graphic. Threshold Marked @ 0.4

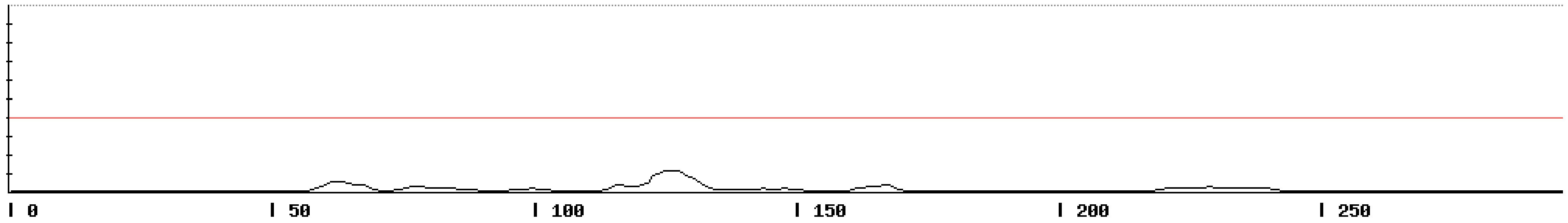

Predictions for Chenopodiumquinoa_AUR62021621

Viterbi Path

Viterbi Path

Posterior @ 0.4

Posterior @ 0.4

Posterior Graphic. Threshold Marked @ 0.4

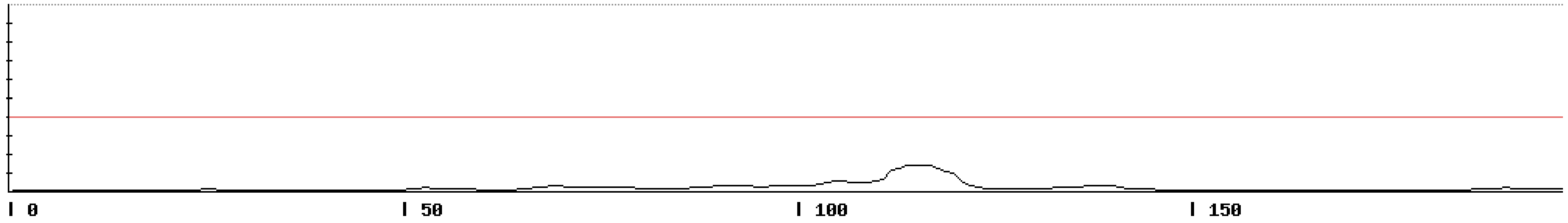

Predictions for Chenopodiumquinoa_AUR62021652

Viterbi Path

Viterbi Path

Posterior @ 0.4

Posterior @ 0.4

Posterior Graphic. Threshold Marked @ 0.4

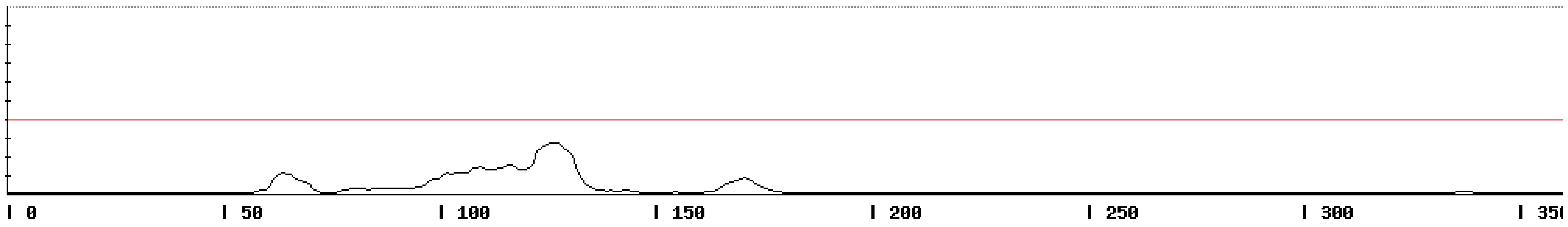

Predictions for Chenopodiumquinoa_AUR62022486

Viterbi Path

Viterbi Path

Posterior @ 0.4

110 - WKRKGR - 115

Posterior @ 0.4

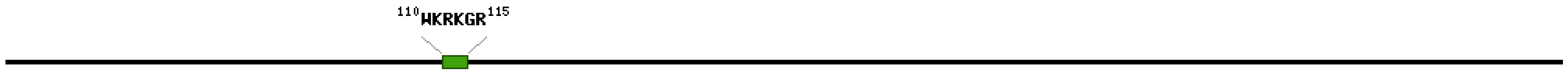

Posterior Graphic. Threshold Marked @ 0.4

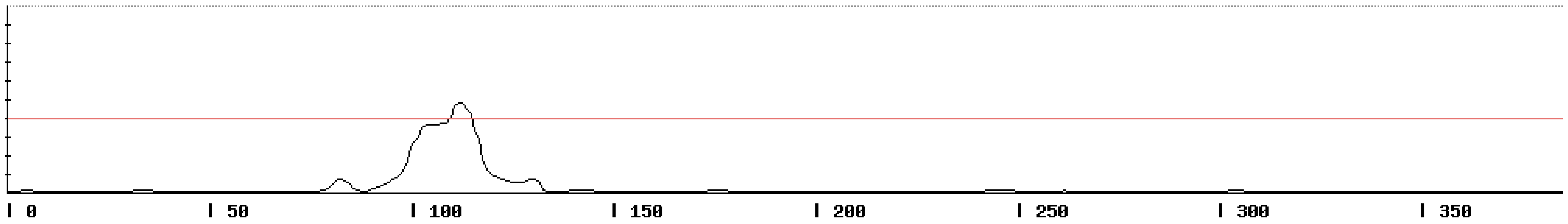

Predictions for Chenopodiumquinoa_AUR62022669

Viterbi Path	
Viterbi Path	
Posterior @ 0.4	
Posterior @ 0.4	
Posterior Graphic. Threshold Marked @ 0.4	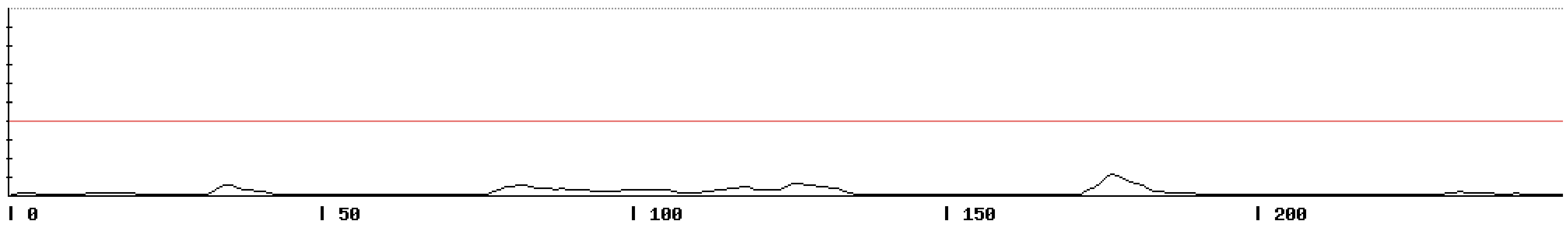

Predictions for Chenopodiumquinoa_AUR62022690

Viterbi Path	
Viterbi Path	
Posterior @ 0.4	
Posterior @ 0.4	
Posterior Graphic. Threshold Marked @ 0.4	

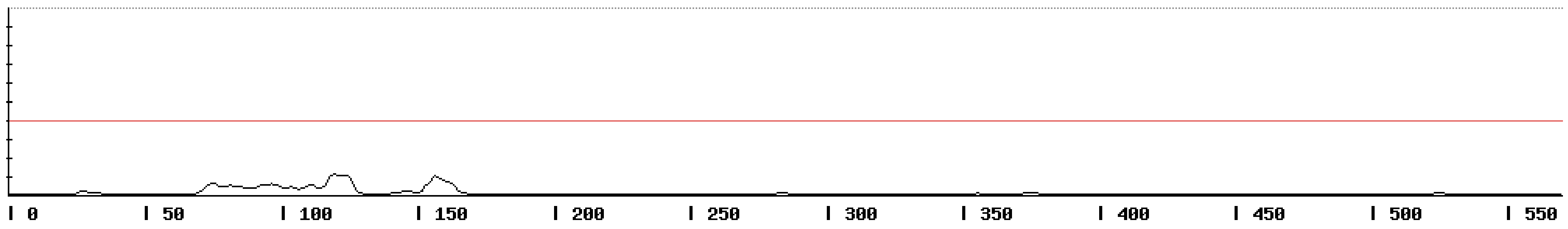

Predictions for Chenopodiumquinoa_AUR62024101	
Viterbi Path	
Viterbi Path	
Posterior @ 0.4	
Posterior @ 0.4	
Posterior Graphic. Threshold Marked @ 0.4	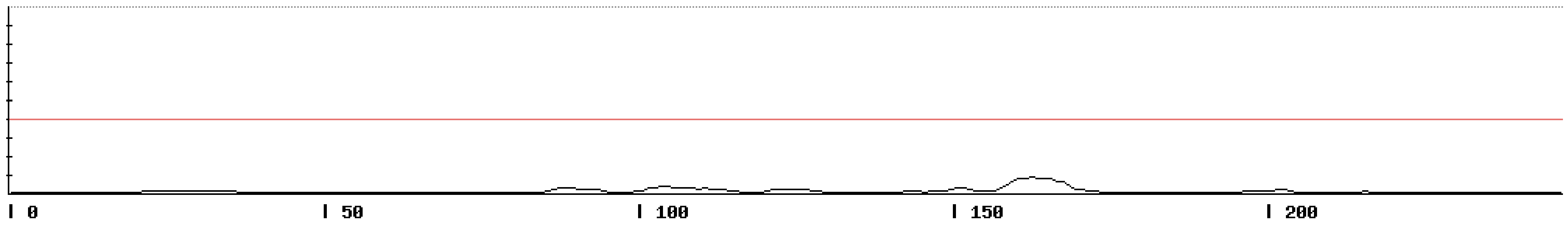

Predictions for Chenopodiumquinoa_AUR62024698	
Viterbi Path	
Viterbi Path	
Posterior @ 0.4	
Posterior @ 0.4	

Posterior Graphic. Threshold Marked @ 0.4

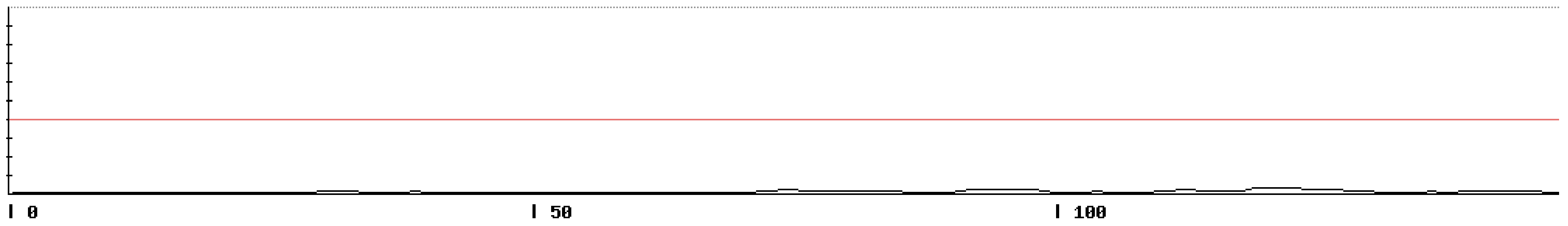

Predictions for Chenopodiumquinoa_AUR62025580

Viterbi Path

Viterbi Path

Posterior @ 0.4

158 - KPKRRK - 163

Posterior @ 0.4

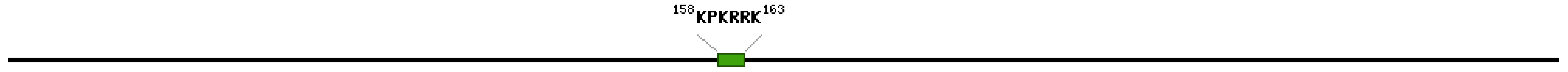

Posterior Graphic. Threshold Marked @ 0.4

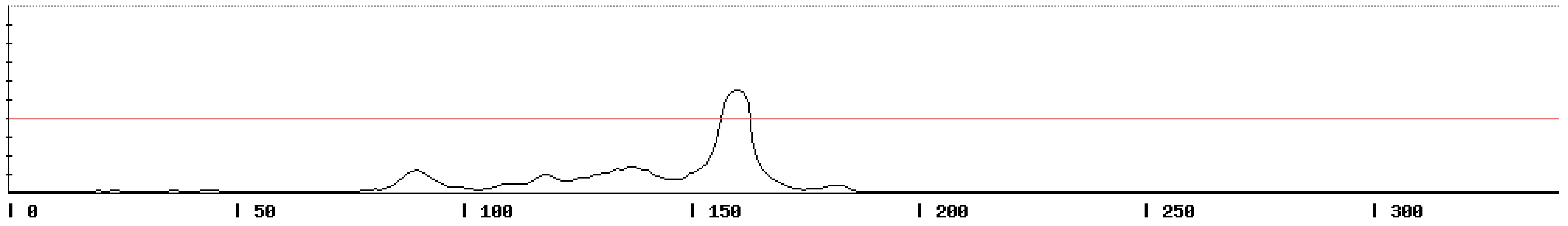

Predictions for Chenopodiumquinoa_AUR62025636

Viterbi Path

Viterbi Path

Posterior @ 0.4

Posterior @ 0.4

Posterior Graphic. Threshold Marked @ 0.4

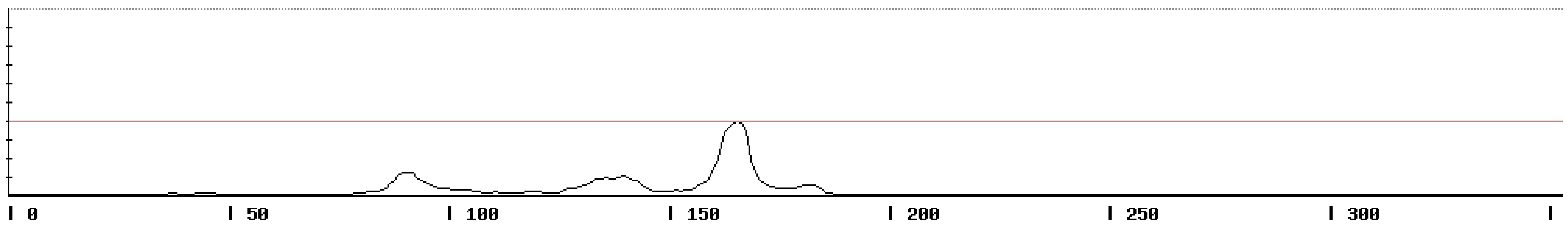

Predictions for Chenopodiumquinoa_AUR62026741

Viterbi Path

Viterbi Path

Posterior @ 0.4

Posterior @ 0.4

Posterior Graphic. Threshold Marked @ 0.4

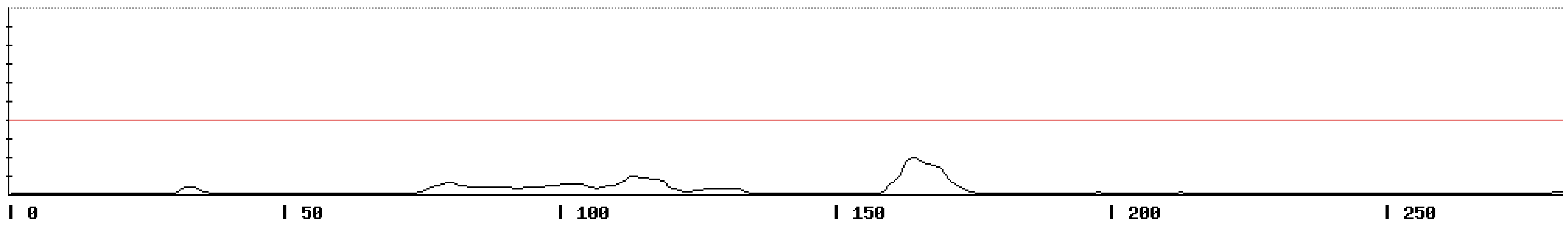

Predictions for Chenopodiumquinoa_AUR62026904

Viterbi Path

Viterbi Path

Posterior @ 0.4

Posterior @ 0.4

Posterior Graphic. Threshold Marked @ 0.4

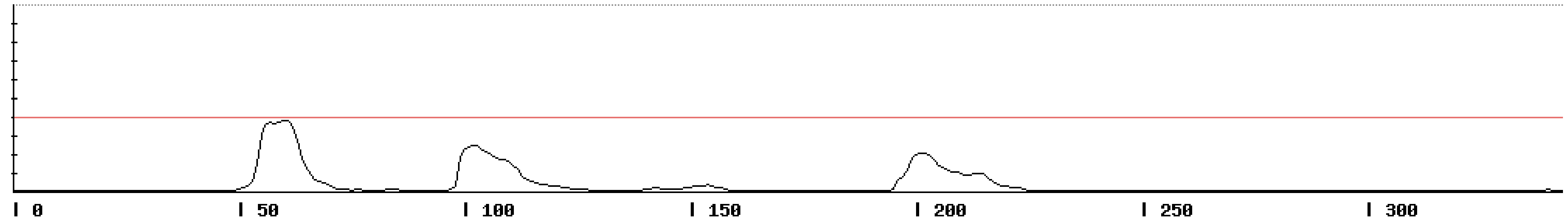

Predictions for Chenopodiumquinoa_AUR62027152

Viterbi Path

Viterbi Path

Posterior @ 0.4

Posterior @ 0.4

Posterior Graphic. Threshold Marked @ 0.4

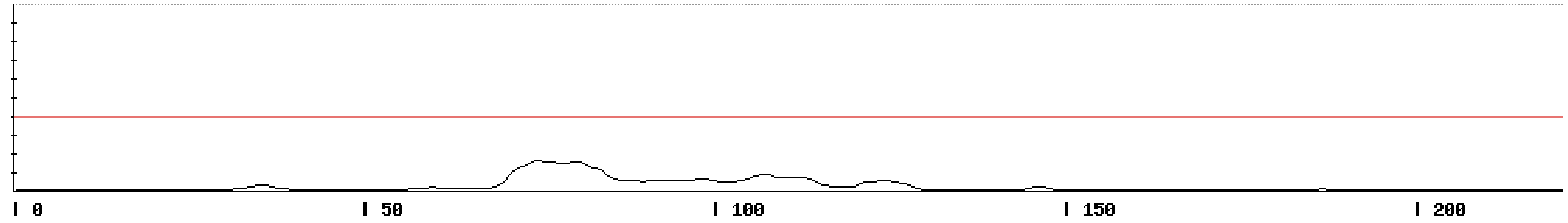

Predictions for Chenopodiumquinoa_AUR62027390

Viterbi Path

Viterbi Path

Posterior @ 0.4	
Posterior @ 0.4	
Posterior Graphic. Threshold Marked @ 0.4	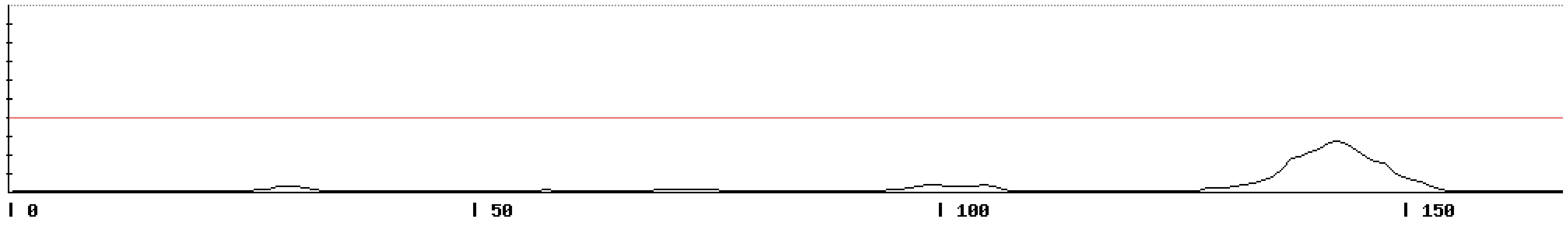

Predictions for Chenopodiumquinoa_AUR62028103

Viterbi Path	
Viterbi Path	
Posterior @ 0.4	97 - TGKDRSIKKVKKGLIGMK - 114
Posterior @ 0.4	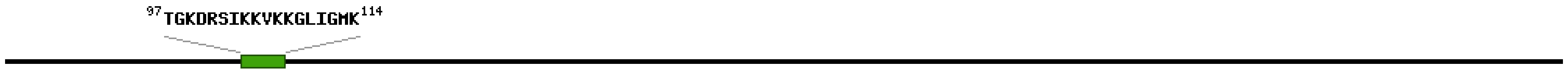
Posterior Graphic. Threshold Marked @ 0.4	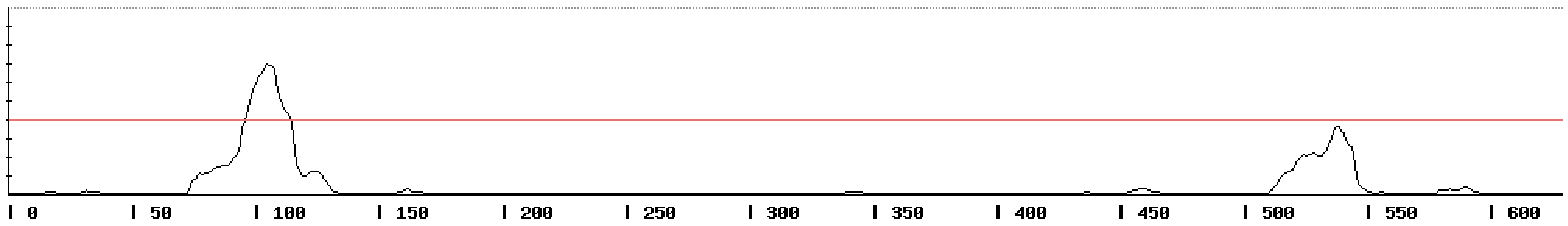

Predictions for Chenopodiumquinoa_AUR62028872

Viterbi Path

Viterbi Path

Posterior @ 0.4

Posterior @ 0.4

Posterior Graphic. Threshold Marked @ 0.4

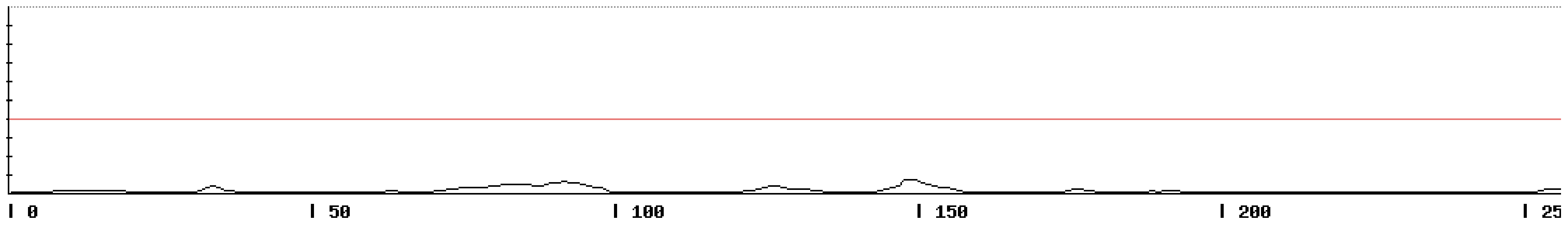

Predictions for Chenopodiumquinoa_AUR62029057

Viterbi Path

Viterbi Path

Posterior @ 0.4

Posterior @ 0.4

Posterior Graphic. Threshold Marked @ 0.4

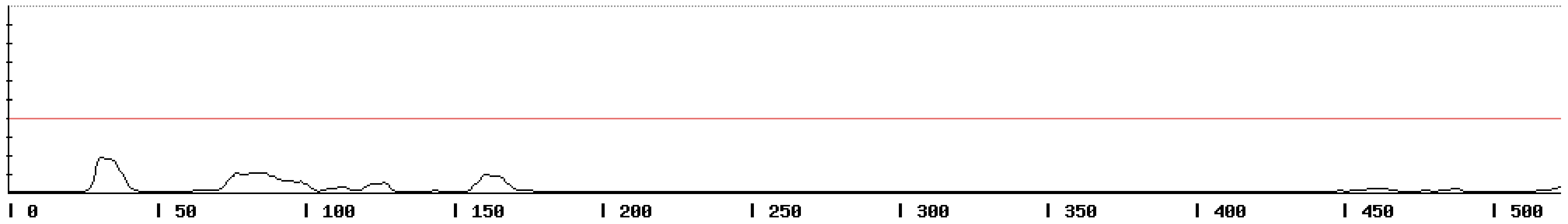

Predictions for Chenopodiumquinoa_AUR62029330	
Viterbi Path	
Viterbi Path	
Posterior @ 0.4	
Posterior @ 0.4	
Posterior Graphic. Threshold Marked @ 0.4	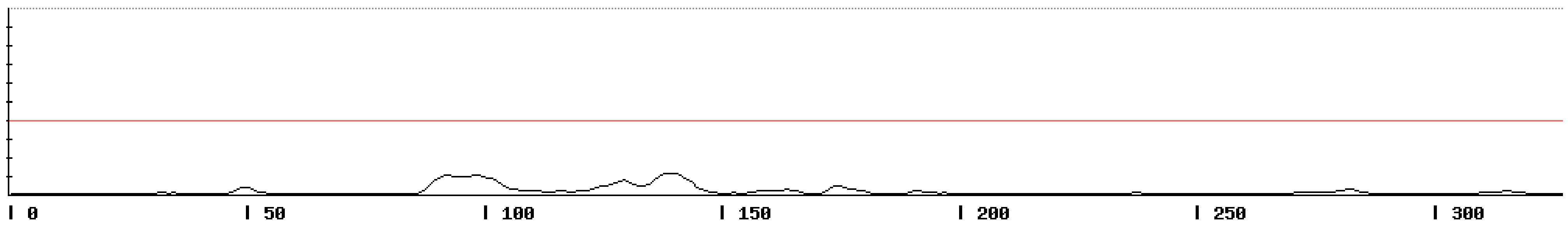

Predictions for Chenopodiumquinoa_AUR62029331	
Viterbi Path	
Viterbi Path	
Posterior @ 0.4	
Posterior @ 0.4	
Posterior Graphic. Threshold Marked @ 0.4	

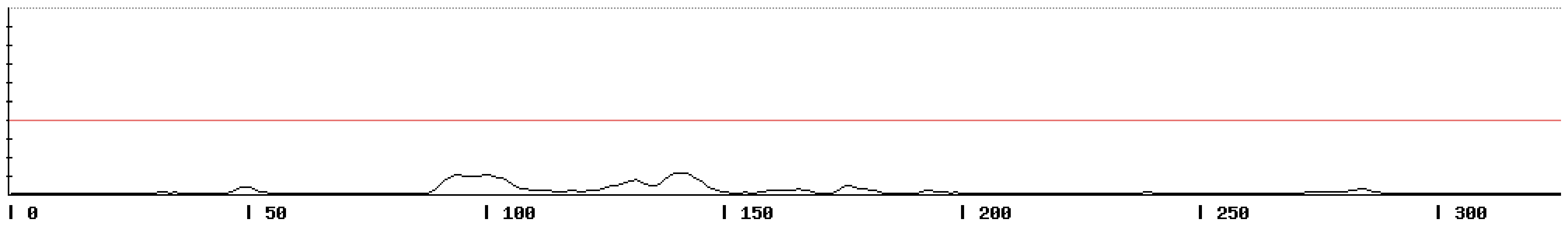

Predictions for Chenopodiumquinoa_AUR62029344

Viterbi Path

Viterbi Path

Posterior @ 0.4

Posterior @ 0.4

Posterior Graphic. Threshold Marked @ 0.4

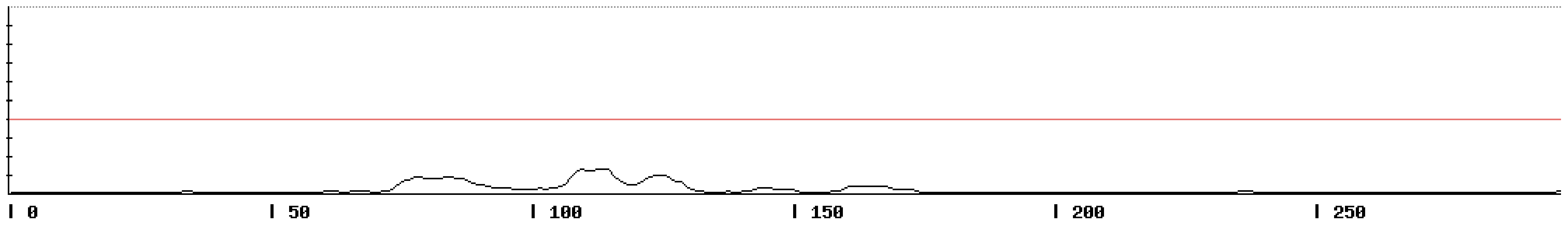

Predictions for Chenopodiumquinoa_AUR62029543

Viterbi Path: 269 - GKGERQMRAKGDKKEGDVAIGEKGRGHRKINILKRENKGGKEGKMLSGGKR - 319

Viterbi Path

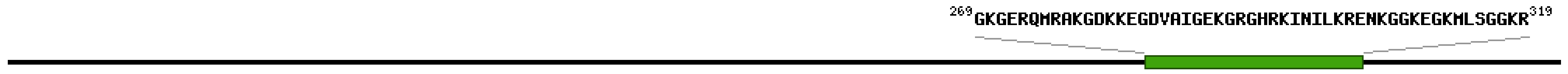

Posterior @ 0.4:

72 - KKGGRHKKRNQN - 83

158 - ASPKKKSRID - 167

175 - KK - 176

270 - KGERQMRAKGDKKEGDVAIGEKGRGHRKINILKRENKGGKEGKMLSGGKR - 319

Posterior @ 0.4

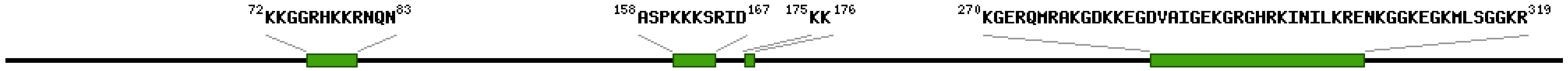

Posterior Graphic. Threshold Marked @ 0.4

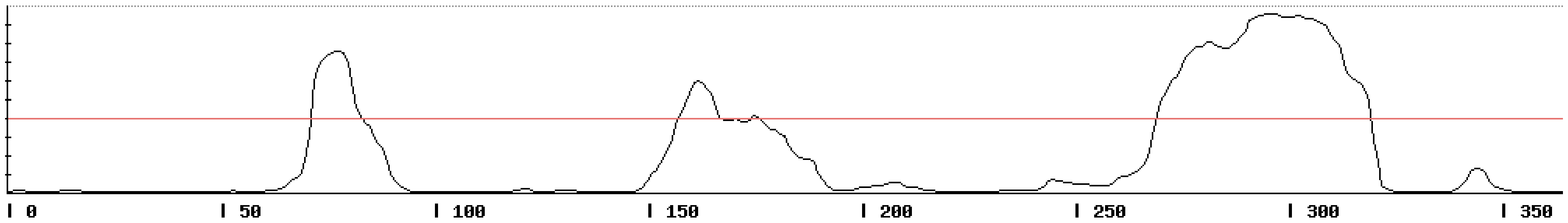

Predictions for Chenopodiumquinoa_AUR62029755

Viterbi Path

Viterbi Path

Posterior @ 0.4

Posterior @ 0.4

Posterior Graphic. Threshold Marked @ 0.4

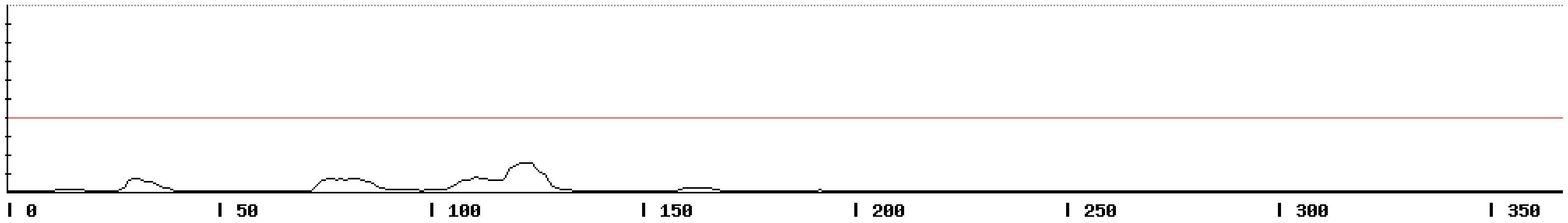

Predictions for Chenopodiumquinoa_AUR62031305

Viterbi Path

Viterbi Path

Posterior @ 0.4

Posterior @ 0.4

Posterior Graphic. Threshold Marked @ 0.4

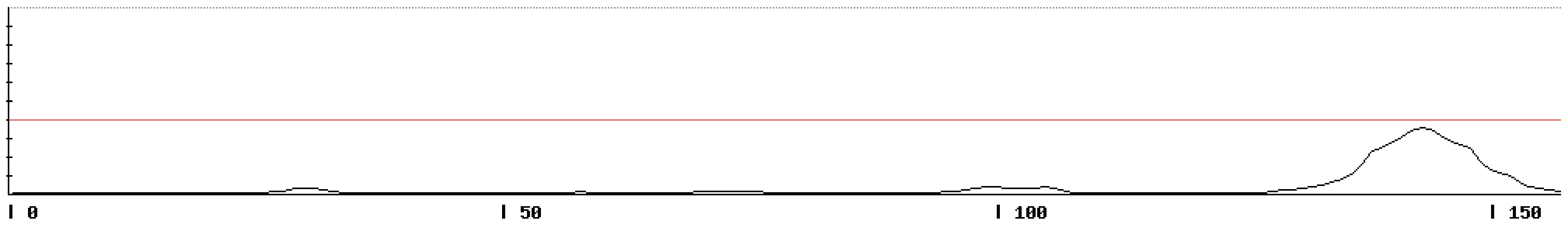

Predictions for Chenopodiumquinoa_AUR62032488

Viterbi Path

Viterbi Path

Posterior @ 0.4

Posterior @ 0.4

Posterior Graphic. Threshold Marked @ 0.4

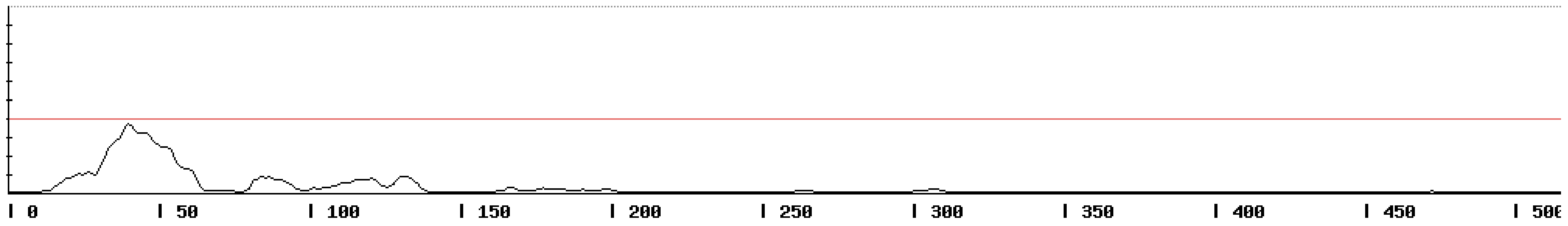

Predictions for Chenopodiumquinoa_AUR62032816

Viterbi Path

Viterbi Path

Posterior @ 0.4

Posterior @ 0.4

Posterior Graphic. Threshold Marked @ 0.4

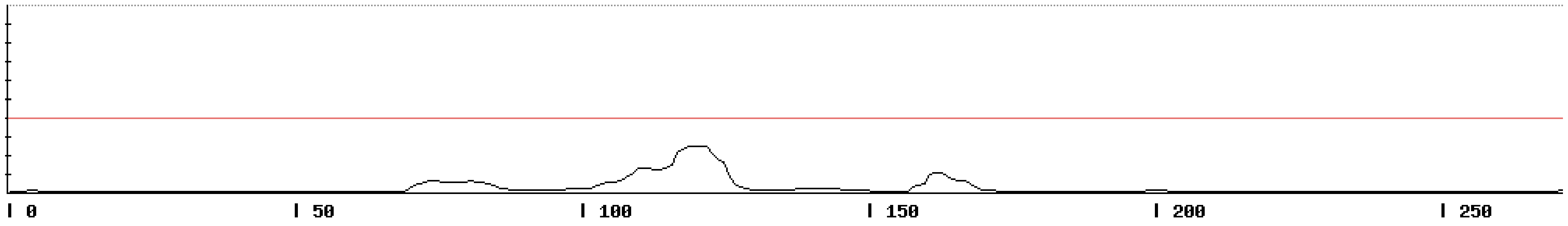

Predictions for Chenopodiumquinoa_AUR62033952

Viterbi Path

Viterbi Path

Posterior @ 0.4

Posterior @ 0.4

Posterior Graphic. Threshold Marked @ 0.4

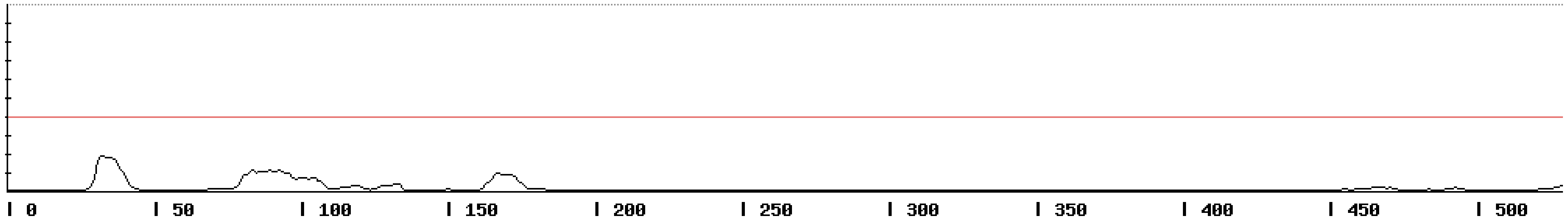

Predictions for Chenopodiumquinoa_AUR62034400

Viterbi Path	
Viterbi Path	
Posterior @ 0.4	
Posterior @ 0.4	
Posterior Graphic. Threshold Marked @ 0.4	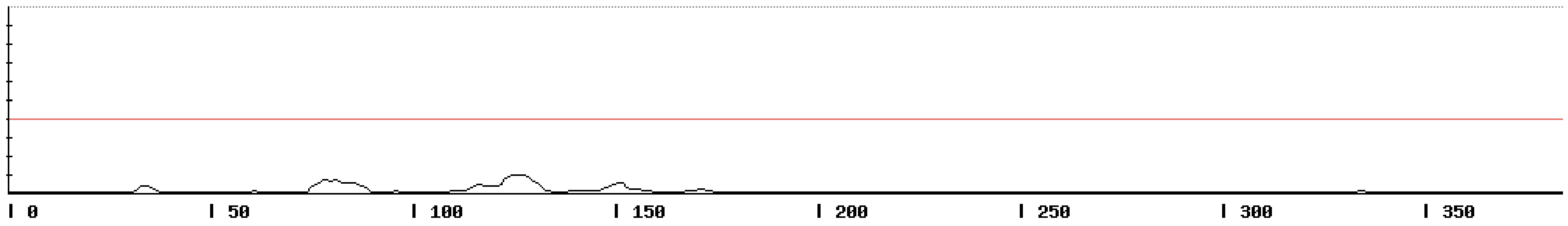

Predictions for Chenopodiumquinoa_AUR62034435

Viterbi Path	
Viterbi Path	
Posterior @ 0.4	
Posterior @ 0.4	
Posterior Graphic. Threshold Marked @ 0.4	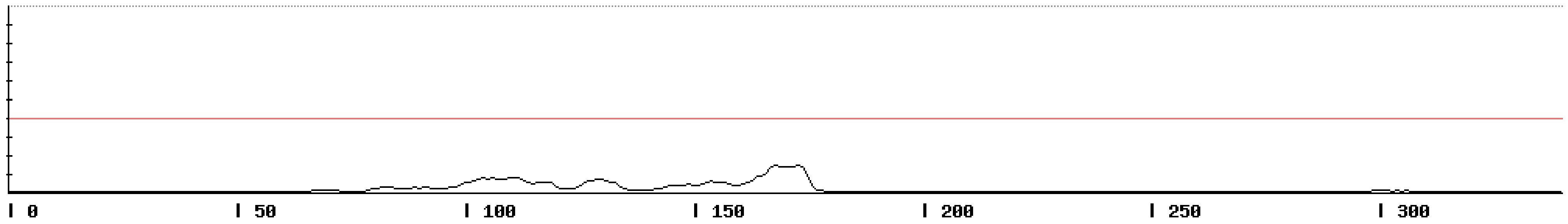

Predictions for Chenopodiumquinoa_AUR62034813	
Viterbi Path	
Viterbi Path	
Posterior @ 0.4	72 - KKGGRHKKRNQ - 82
Posterior @ 0.4	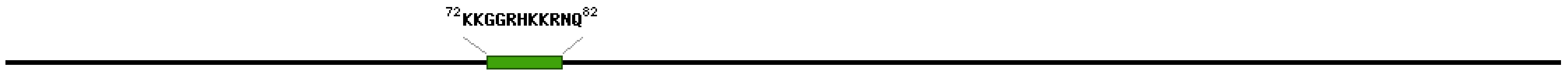
Posterior Graphic. Threshold Marked @ 0.4	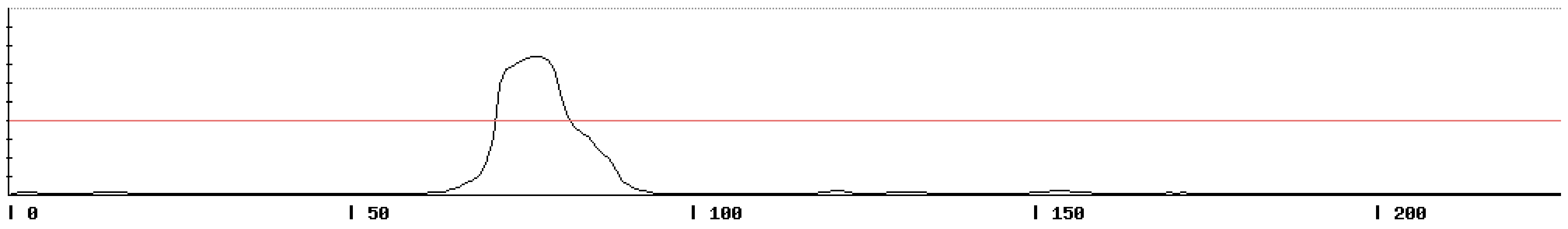

Predictions for Chenopodiumquinoa_AUR62035165	
Viterbi Path	
Viterbi Path	
Posterior @ 0.4	
Posterior @ 0.4	
Posterior Graphic. Threshold Marked @ 0.4	

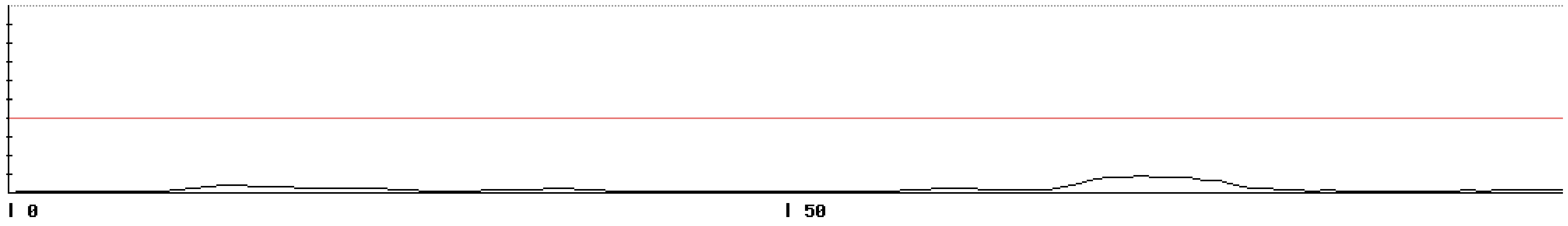

Predictions for Chenopodiumquinoa_AUR62035514

Viterbi Path

Viterbi Path

Posterior @ 0.4

Posterior @ 0.4

Posterior Graphic. Threshold Marked @ 0.4

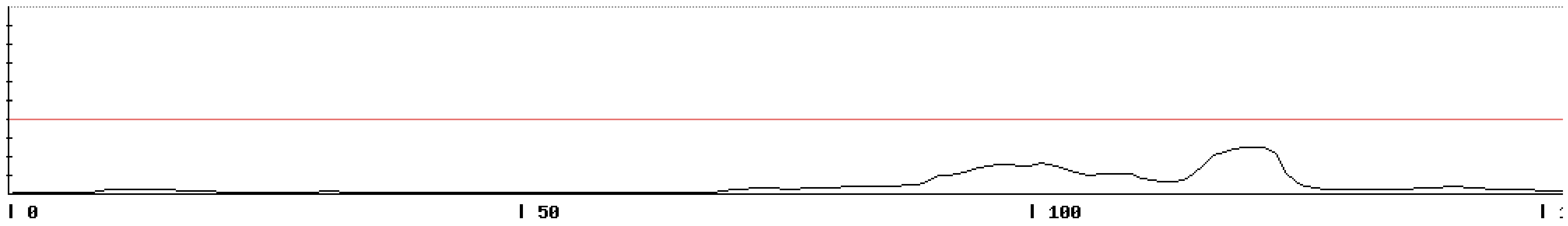

Predictions for Chenopodiumquinoa_AUR62036158

Viterbi Path

Viterbi Path

Posterior @ 0.4

Posterior @ 0.4

Posterior Graphic. Threshold Marked @ 0.4

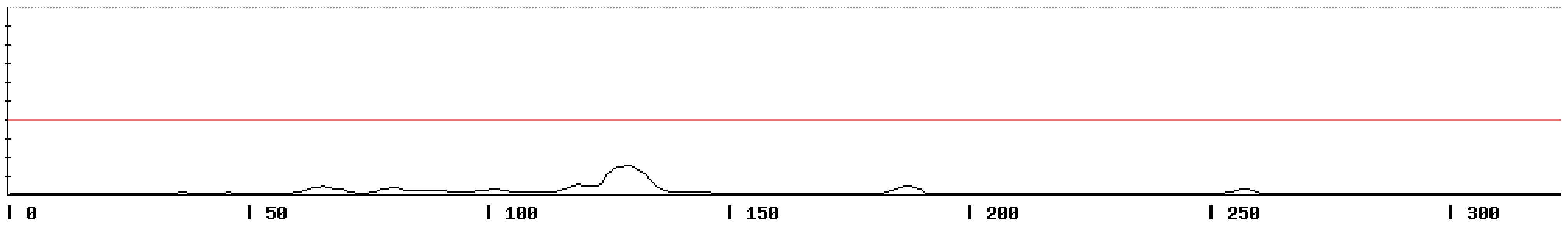

Predictions for Chenopodiumquinoa_AUR62036626

Viterbi Path	
Viterbi Path	
Posterior @ 0.4	110 - WKRKGR - 115
Posterior @ 0.4	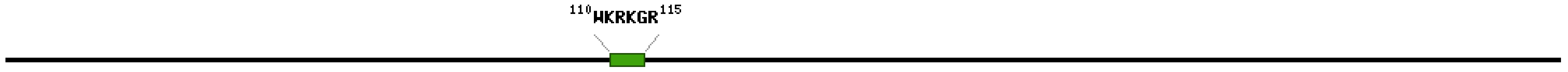
Posterior Graphic. Threshold Marked @ 0.4	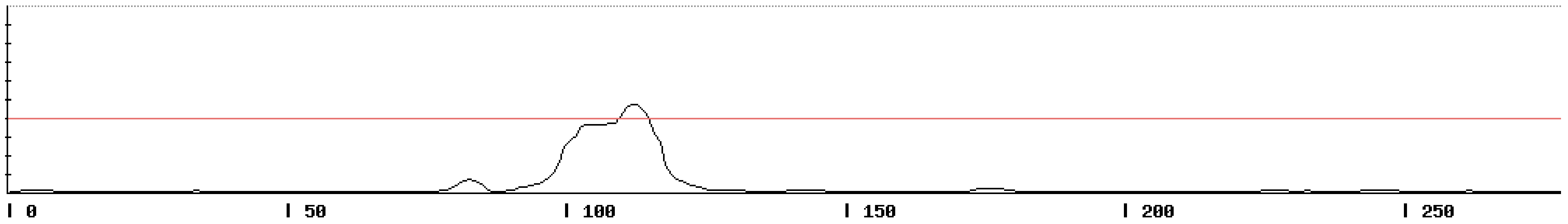

Predictions for Chenopodiumquinoa_AUR62036991

Viterbi Path	
Viterbi Path	
Posterior @ 0.4	
Posterior @ 0.4	

Posterior Graphic. Threshold Marked @ 0.4	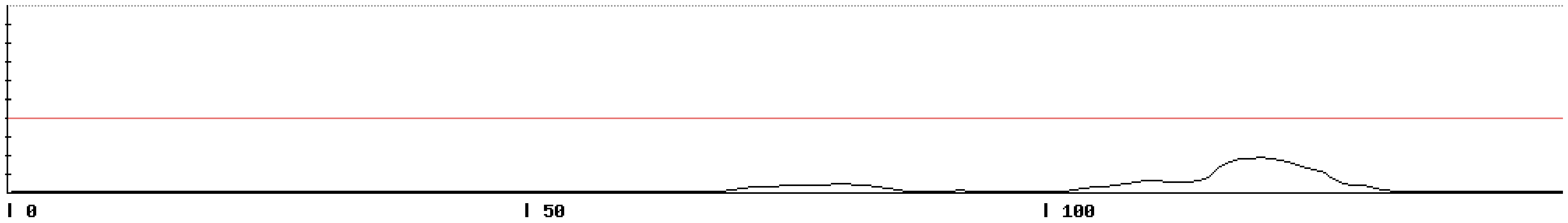

Predictions for Chenopodiumquinoa_AUR62037478	
Viterbi Path	
Viterbi Path	
Posterior @ 0.4	
Posterior @ 0.4	
Posterior Graphic. Threshold Marked @ 0.4	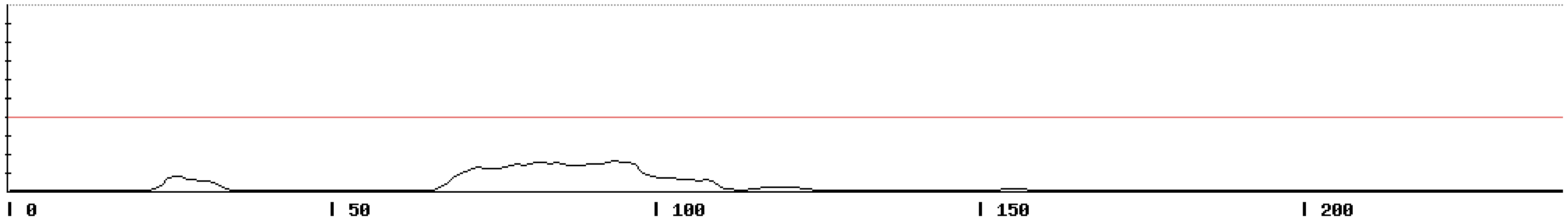

Predictions for Chenopodiumquinoa_AUR62038748	
Viterbi Path	
Viterbi Path	

Posterior @ 0.4

Posterior @ 0.4

Posterior Graphic. Threshold Marked @ 0.4

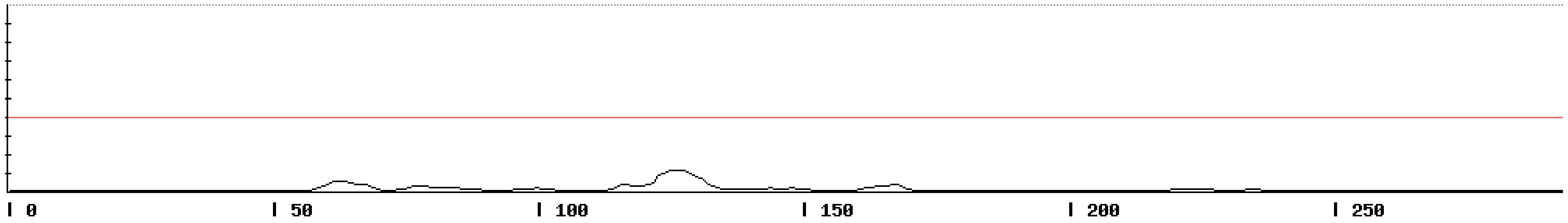

Predictions for Chenopodiumquinoa_AUR62039815

Viterbi Path

Viterbi Path

Posterior @ 0.4

Posterior @ 0.4

Posterior Graphic. Threshold Marked @ 0.4

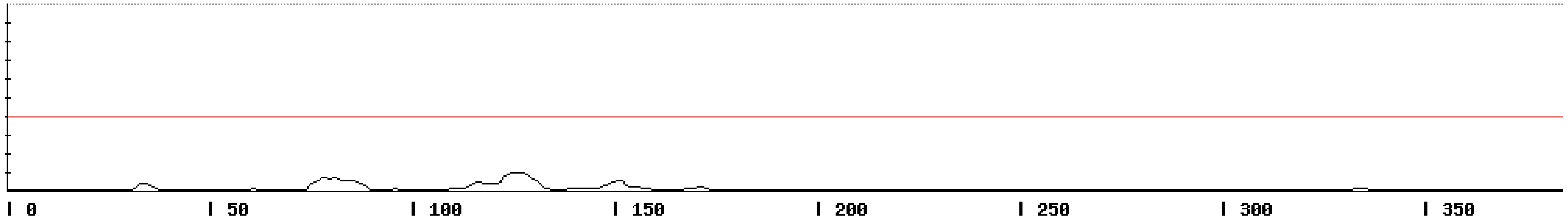

Predictions for Chenopodiumquinoa_AUR62039927

Viterbi Path

Viterbi Path

Posterior @ 0.4

Posterior @ 0.4

Posterior Graphic. Threshold Marked @ 0.4

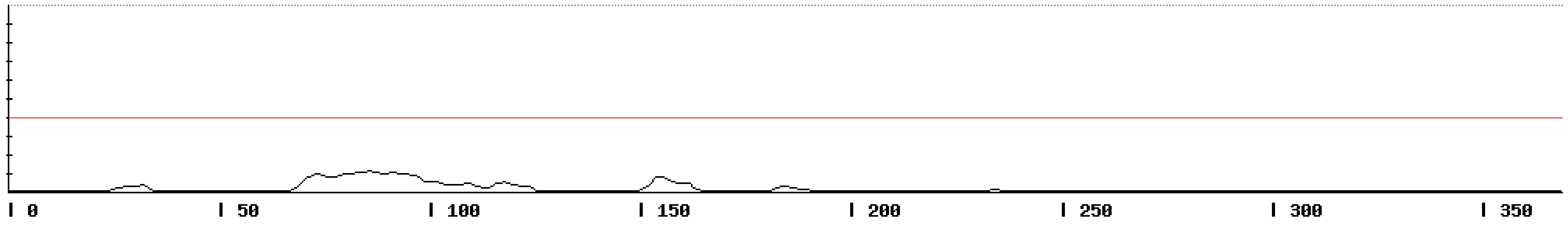

Predictions for Chenopodiumquinoa_AUR62040120

Viterbi Path

Viterbi Path

Posterior @ 0.4

Posterior @ 0.4

Posterior Graphic. Threshold Marked @ 0.4

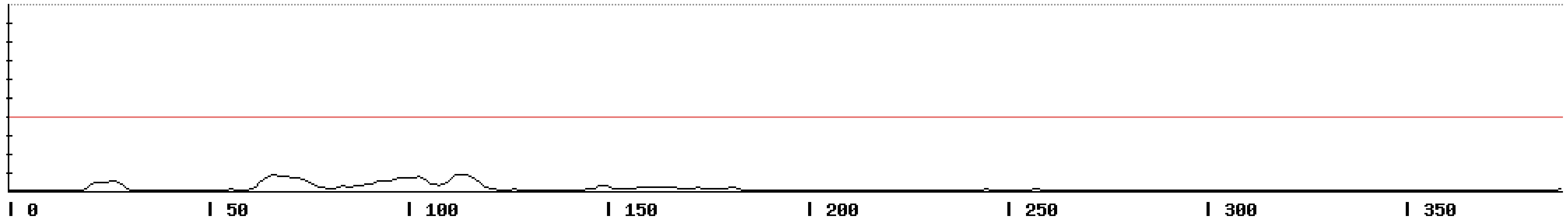

Predictions for Chenopodiumquinoa_AUR62040889

Viterbi Path

Viterbi Path

Posterior @ 0.4

Posterior @ 0.4

Posterior Graphic. Threshold Marked @ 0.4

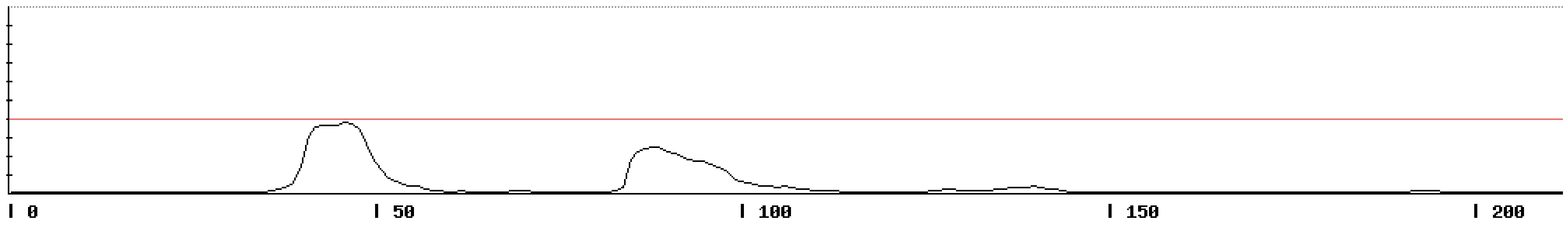

Predictions for Chenopodiumquinoa_AUR62041525

Viterbi Path

Viterbi Path

Posterior @ 0.4

Posterior @ 0.4

Posterior Graphic. Threshold Marked @ 0.4

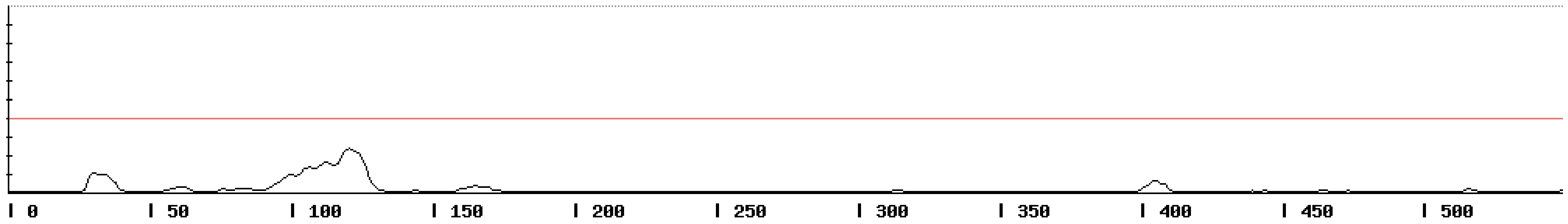

Predictions for Chenopodiumquinoa_AUR62041539	
Viterbi Path	
Viterbi Path	
Posterior @ 0.4	
Posterior @ 0.4	
Posterior Graphic. Threshold Marked @ 0.4	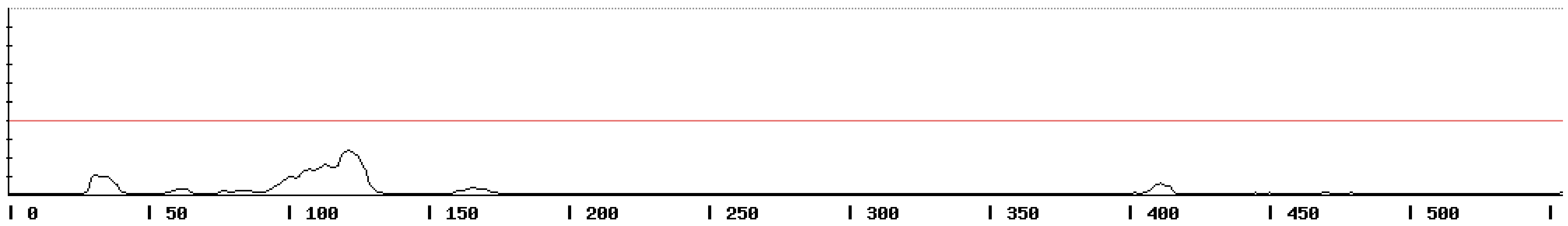

Predictions for Chenopodiumquinoa_AUR62043235	
Viterbi Path	
Viterbi Path	
Posterior @ 0.4	
Posterior @ 0.4	
Posterior Graphic. Threshold Marked @ 0.4	

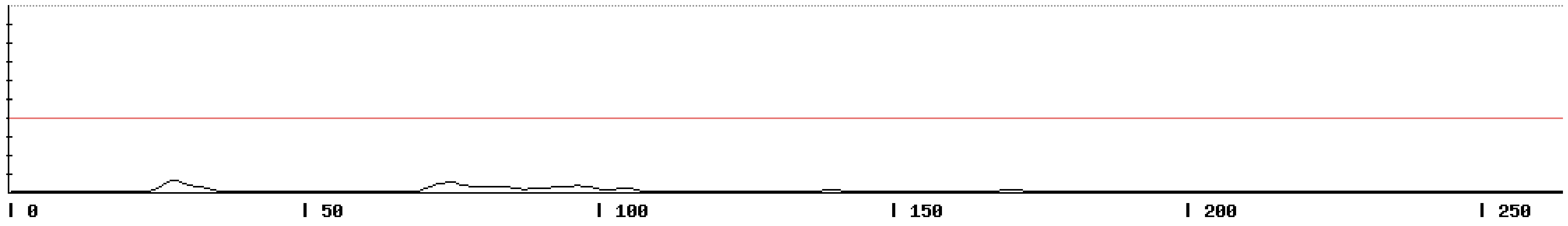

Predictions for Chenopodiumquinoa_AUR62043491

Viterbi Path

Viterbi Path

Posterior @ 0.4

Posterior @ 0.4

Posterior Graphic. Threshold Marked @ 0.4

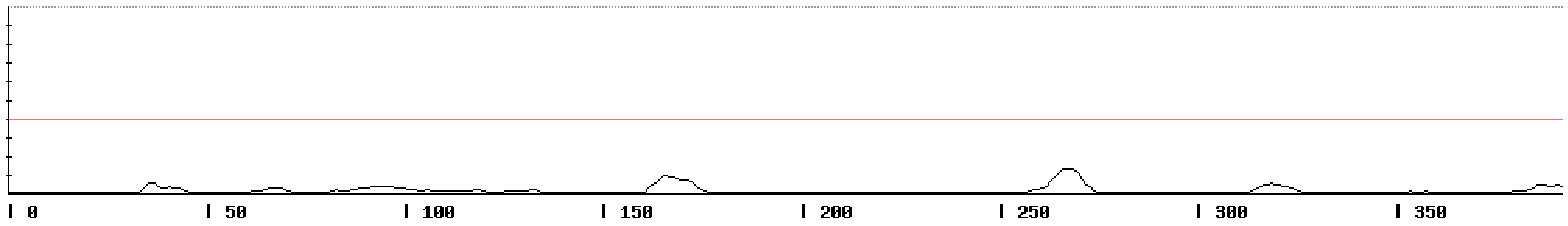

Predictions for Chenopodiumquinoa_AUR62043497

Viterbi Path

Viterbi Path

Posterior @ 0.4

Posterior @ 0.4

Posterior Graphic. Threshold Marked @ 0.4

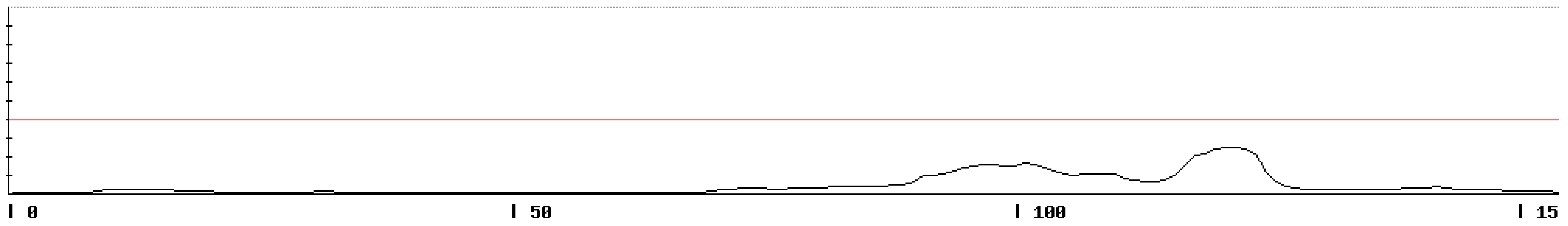

Predictions for Chenopodiumquinoa_AUR62043713

Viterbi Path	
Viterbi Path	
Posterior @ 0.4	
Posterior @ 0.4	
Posterior Graphic. Threshold Marked @ 0.4	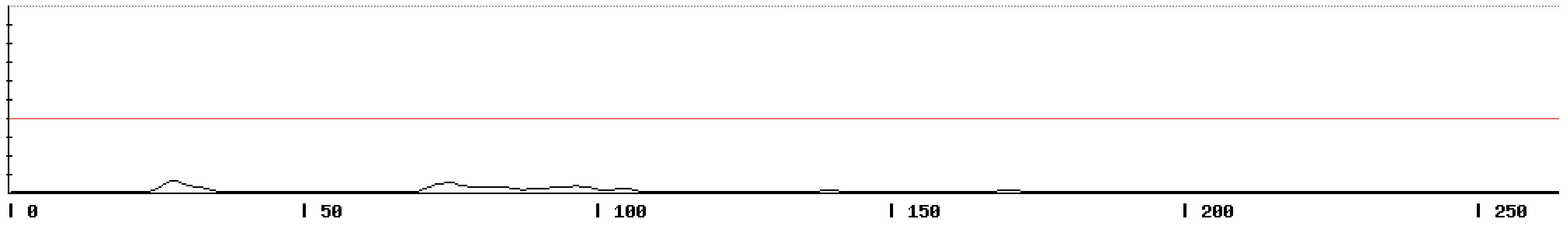

Predictions for Chenopodiumquinoa_AUR62043741

Viterbi Path	
Viterbi Path	
Posterior @ 0.4	
Posterior @ 0.4	

Viterbi Path	
Posterior @ 0.4	
Posterior @ 0.4	
Posterior Graphic. Threshold Marked @ 0.4	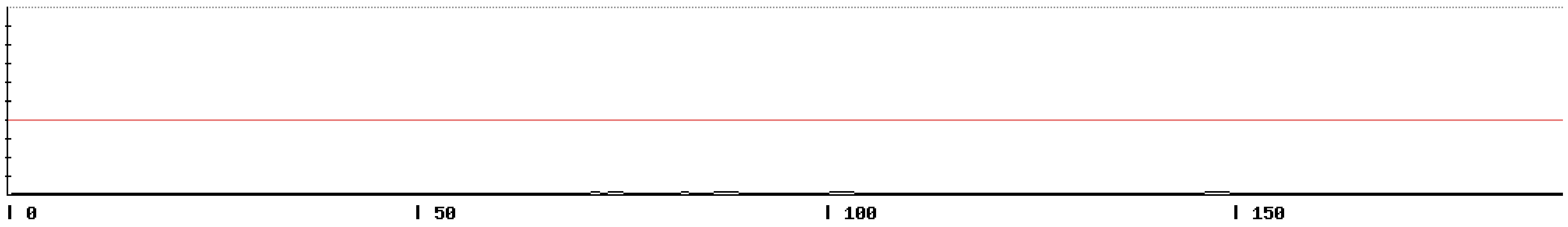

Predictions for Cicerarietinum_NP_001266090.1

Viterbi Path	
Viterbi Path	
Posterior @ 0.4	157 - KKRGGAKNGGGR - 168
Posterior @ 0.4	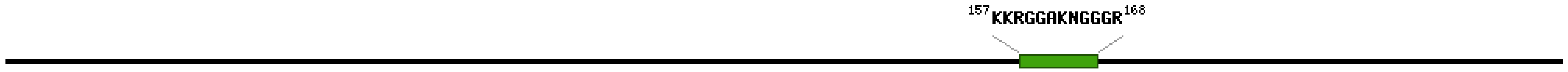
Posterior Graphic. Threshold Marked @ 0.4	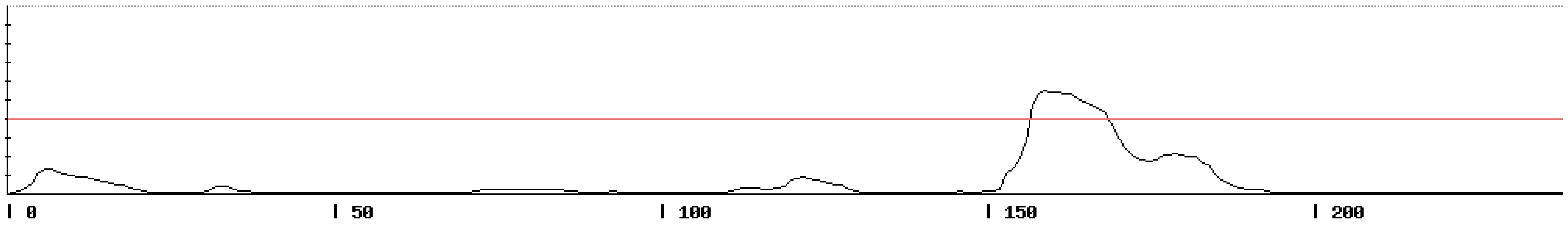

Predictions for Cicerarietinum_XP_004485547.1

Viterbi Path

Viterbi Path

Posterior @ 0.4

Posterior @ 0.4

Posterior Graphic. Threshold Marked @ 0.4

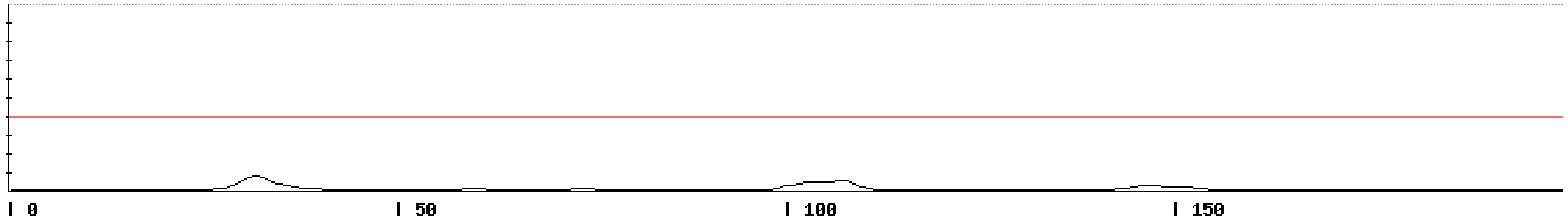

Predictions for Cicerarietinum_XP_004487133.1

Viterbi Path

Viterbi Path

Posterior @ 0.4

Posterior @ 0.4

Posterior Graphic. Threshold Marked @ 0.4

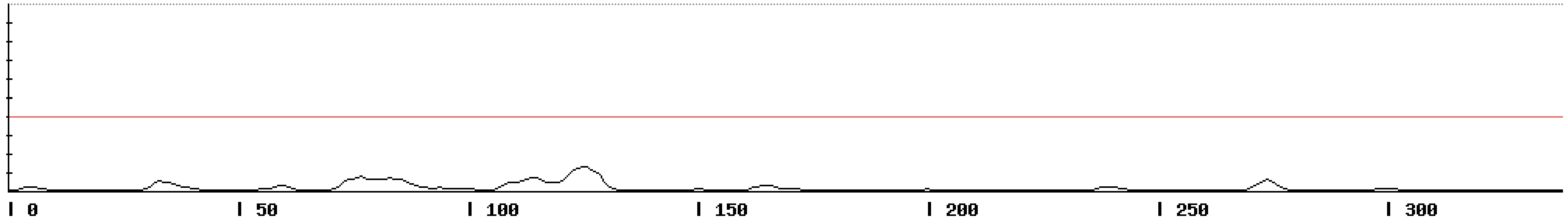

Predictions for Cicerarietinum_XP_004487194.1

Viterbi Path	
Viterbi Path	
Posterior @ 0.4	127 - RKRR - 130
Posterior @ 0.4	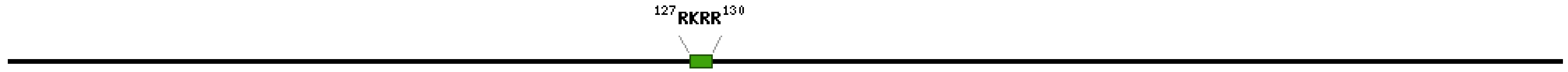
Posterior Graphic. Threshold Marked @ 0.4	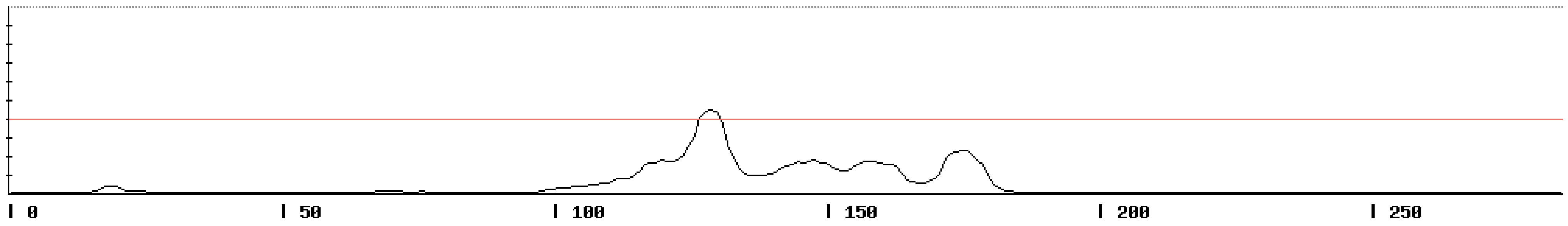

Predictions for Cicerarietinum_XP_004487678.1

Viterbi Path	
Viterbi Path	
Posterior @ 0.4	
Posterior @ 0.4	
Posterior Graphic. Threshold Marked @ 0.4	

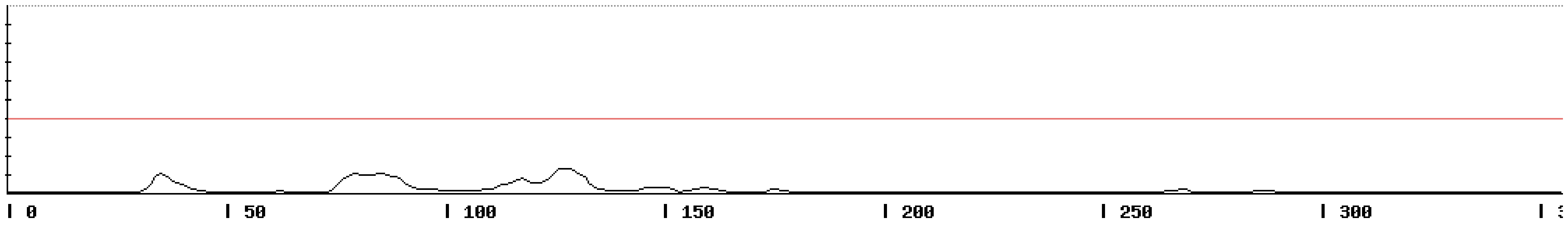

Predictions for Cicerarietinum_XP_004487679.1

Viterbi Path

Viterbi Path

Posterior @ 0.4

Posterior @ 0.4

Posterior Graphic. Threshold Marked @ 0.4

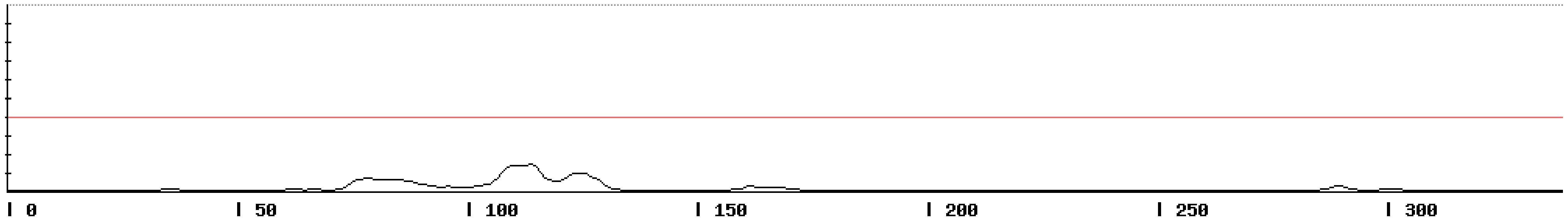

Predictions for Cicerarietinum_XP_004488556.1

Viterbi Path

Viterbi Path

Posterior @ 0.4

Posterior @ 0.4

Posterior Graphic. Threshold Marked @ 0.4

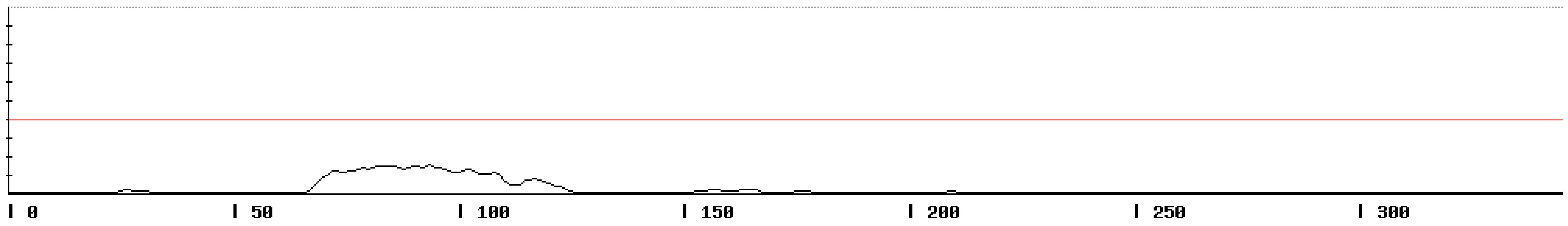

Predictions for Cicerarietinum_XP_004488594.1

Viterbi Path

Viterbi Path

Posterior @ 0.4

Posterior @ 0.4

Posterior Graphic. Threshold Marked @ 0.4

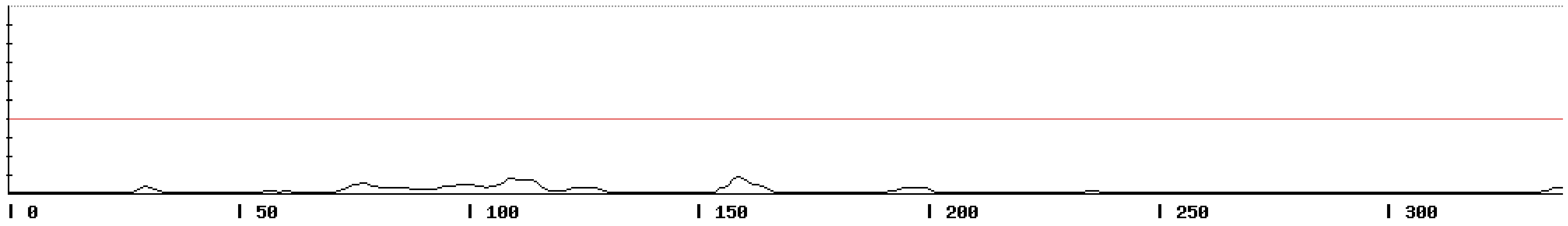

Predictions for Cicerarietinum_XP_004488746.1

Viterbi Path

Viterbi Path

Posterior @ 0.4

Posterior @ 0.4

Posterior Graphic. Threshold Marked @ 0.4

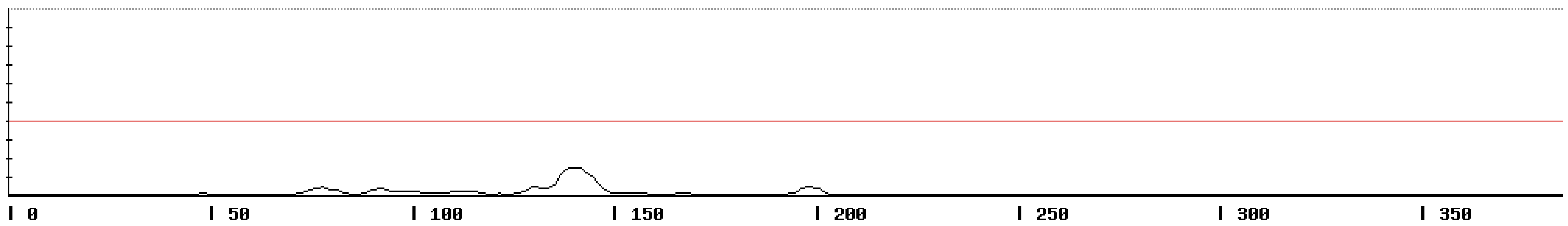

Predictions for Cicerarietinum_XP_004488808.1

Viterbi Path

Viterbi Path

Posterior @ 0.4

Posterior @ 0.4

Posterior Graphic. Threshold Marked @ 0.4

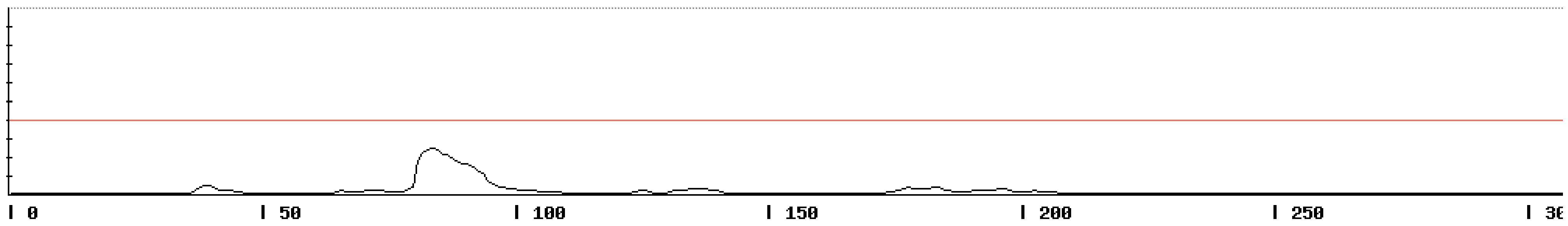

Predictions for Cicerarietinum_XP_004488843.1

Viterbi Path

Viterbi Path

Posterior @ 0.4

Posterior @ 0.4

Posterior Graphic. Threshold Marked @ 0.4

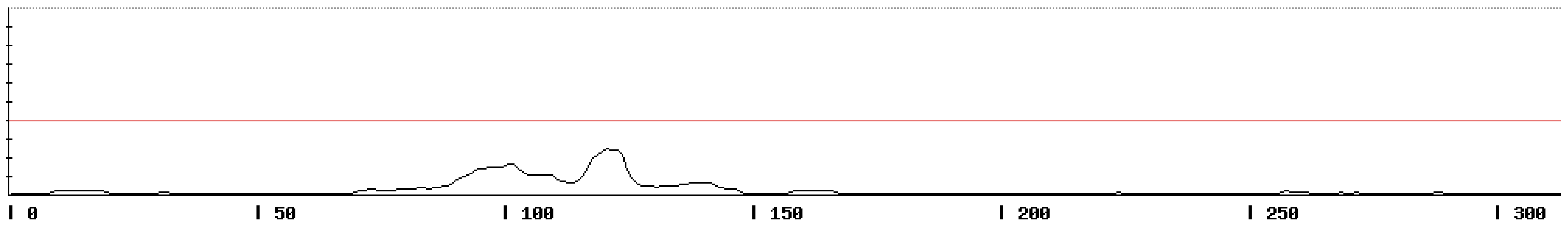

Predictions for Cicerarietinum_XP_004489410.1

Viterbi Path

Viterbi Path

Posterior @ 0.4

Posterior @ 0.4

Posterior Graphic. Threshold Marked @ 0.4

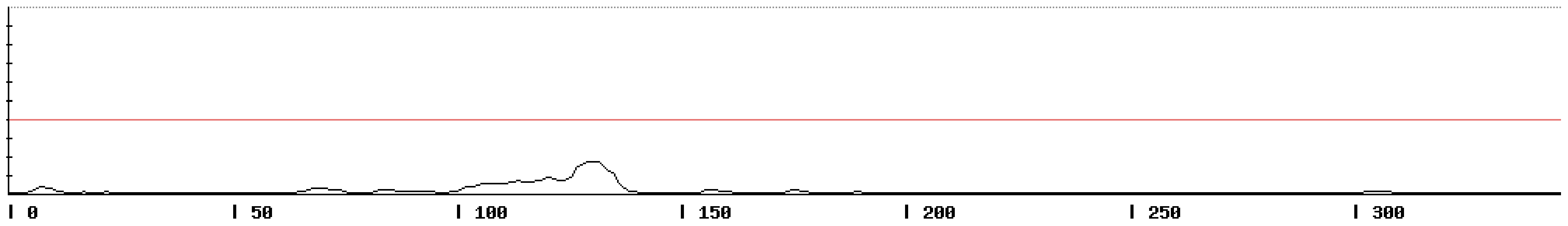

Predictions for Cicerarietinum_XP_004489492.1

Viterbi Path

Viterbi Path

Posterior @ 0.4

Posterior @ 0.4

Posterior Graphic. Threshold Marked @ 0.4

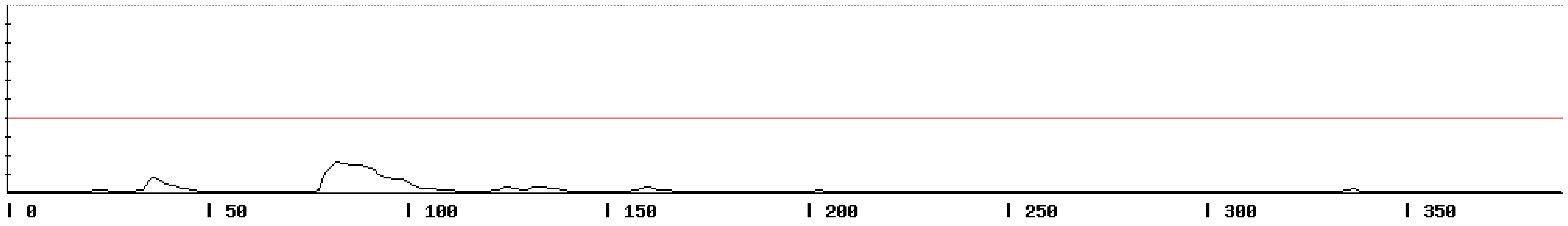

Predictions for Cicerarietinum_XP_004489720.1

Viterbi Path

Viterbi Path

Posterior @ 0.4

Posterior @ 0.4

Posterior Graphic. Threshold Marked @ 0.4

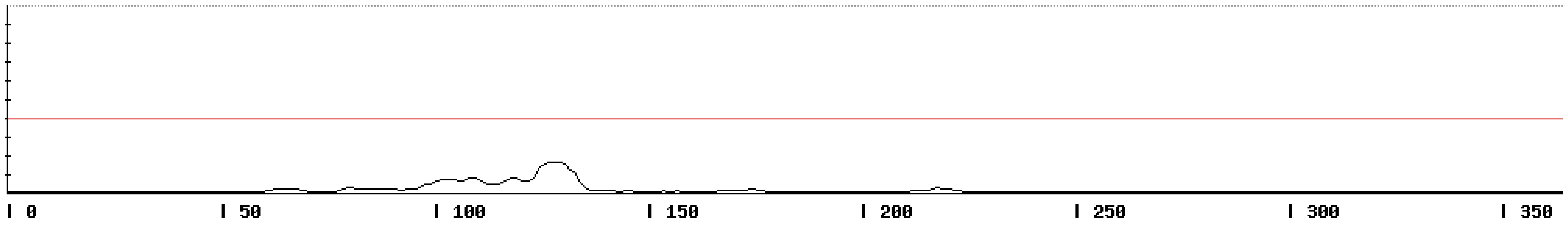

Predictions for Cicerarietinum_XP_004490046.1

Viterbi Path

Viterbi Path

Posterior @ 0.4

Posterior @ 0.4

Posterior Graphic. Threshold Marked @ 0.4

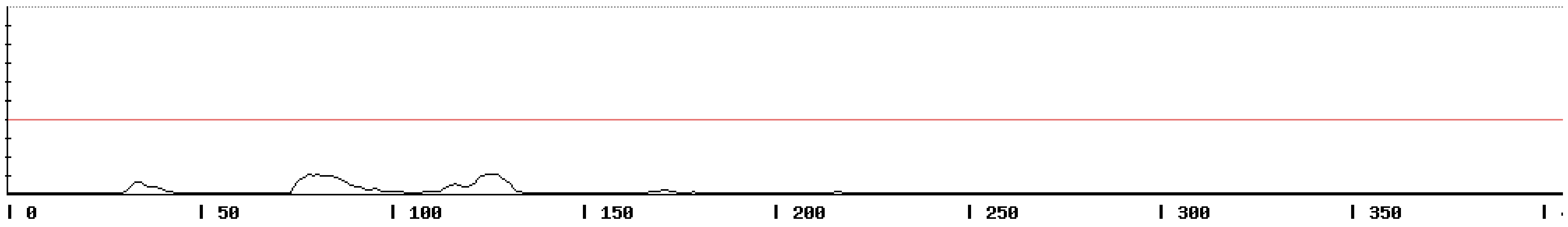

Predictions for Cicerarietinum_XP_004490093.1

Viterbi Path

Viterbi Path

Posterior @ 0.4

Posterior @ 0.4

Posterior Graphic. Threshold Marked @ 0.4

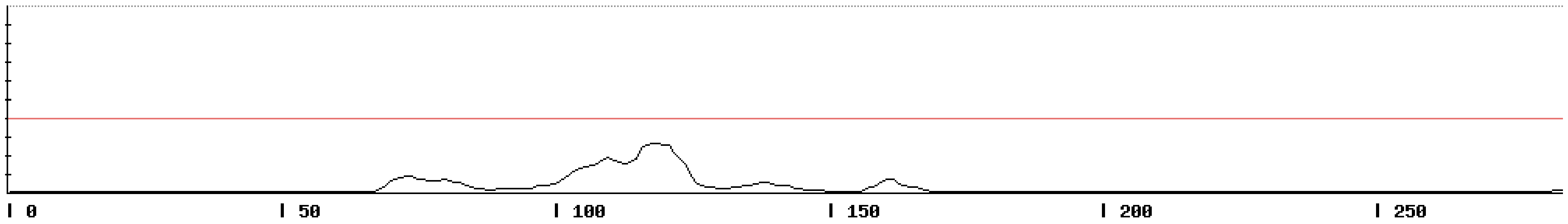

Predictions for Cicerarietinum_XP_004490175.1	
Viterbi Path	
Viterbi Path	
Posterior @ 0.4	157 - RKNVKKNPVSTRNRIAKNANRRLQRNNTSMGNTGRGRKRKK - 197
Posterior @ 0.4	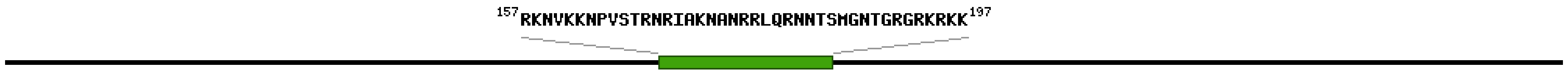
Posterior Graphic. Threshold Marked @ 0.4	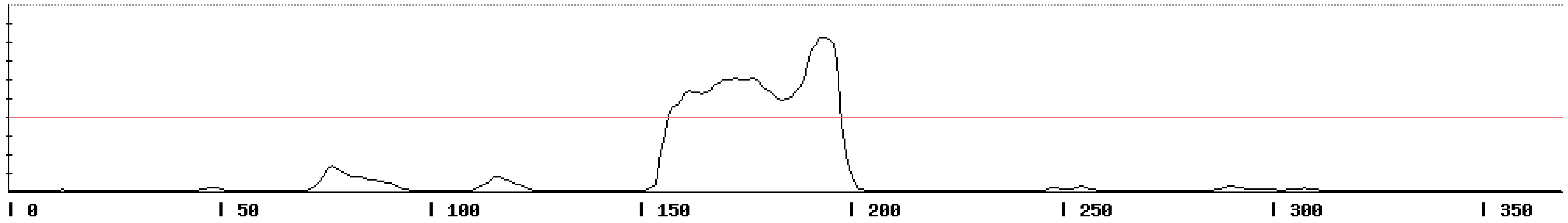

Predictions for Cicerarietinum_XP_004490275.1	
Viterbi Path	
Viterbi Path	
Posterior @ 0.4	137 - KRR - 139
Posterior @ 0.4	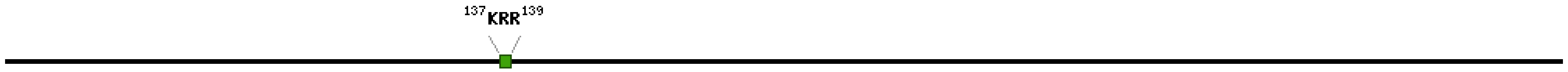
Posterior Graphic. Threshold Marked @ 0.4	

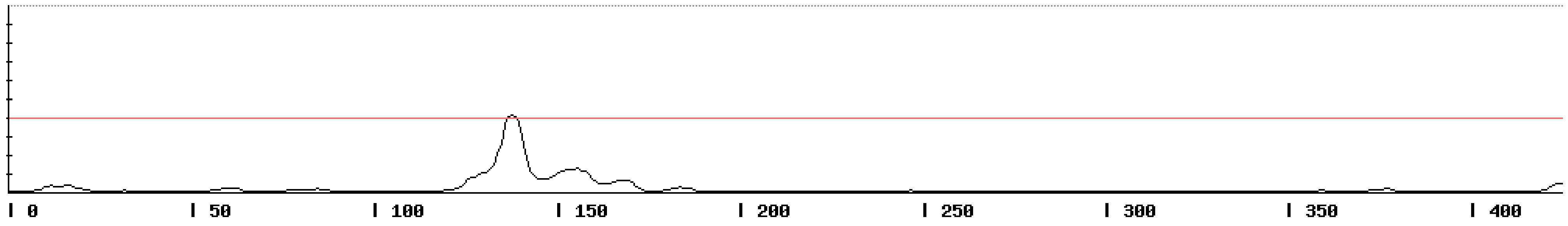

Predictions for Cicerarietinum_XP_004490776.1

Viterbi Path

Viterbi Path

Posterior @ 0.4

Posterior @ 0.4

Posterior Graphic. Threshold Marked @ 0.4

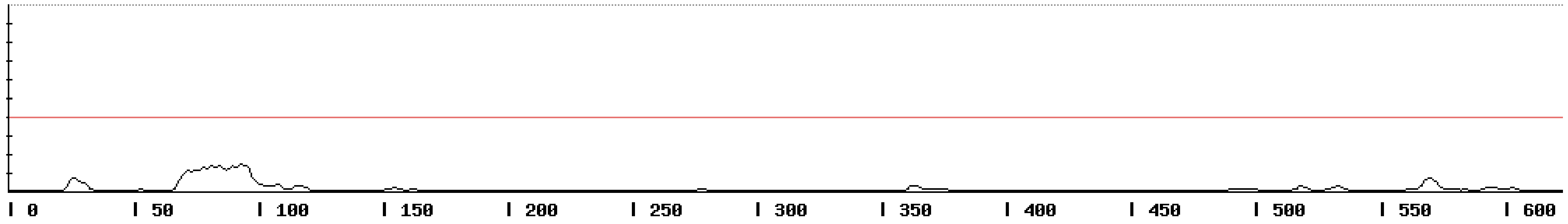

Predictions for Cicerarietinum_XP_004491313.1

Viterbi Path

Viterbi Path

Posterior @ 0.4

Posterior @ 0.4

Posterior Graphic. Threshold Marked @ 0.4

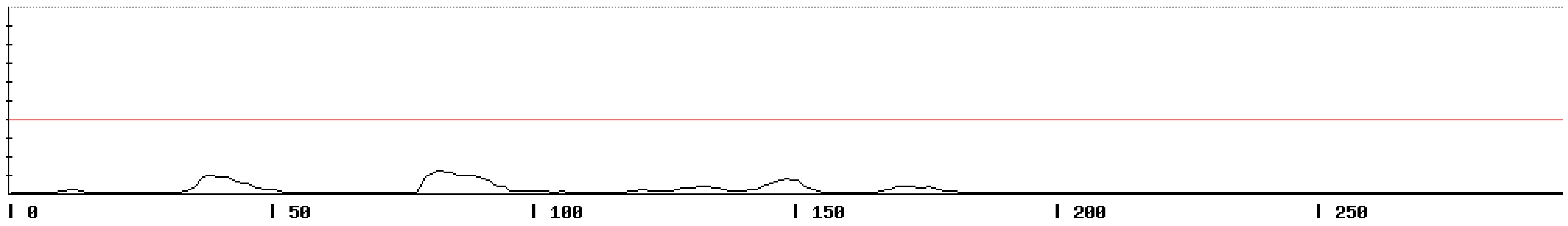

Predictions for Cicerarietinum_XP_004491830.1

Viterbi Path

Viterbi Path

Posterior @ 0.4

Posterior @ 0.4

Posterior Graphic. Threshold Marked @ 0.4

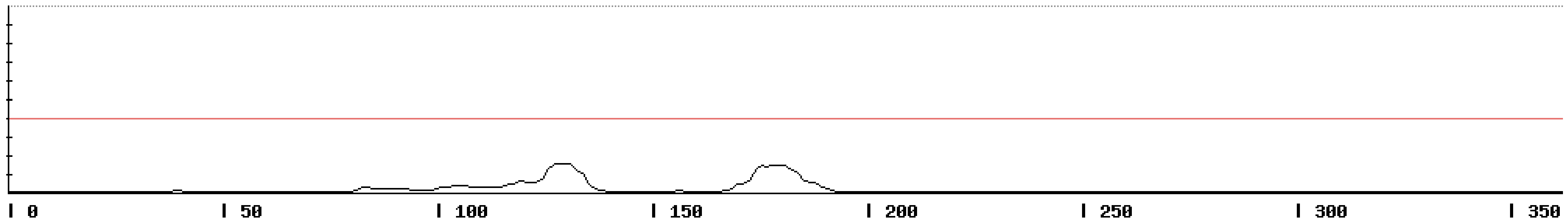

Predictions for Cicerarietinum_XP_004491831.1

Viterbi Path

Viterbi Path

Posterior @ 0.4

Posterior @ 0.4

Posterior Graphic. Threshold Marked @ 0.4

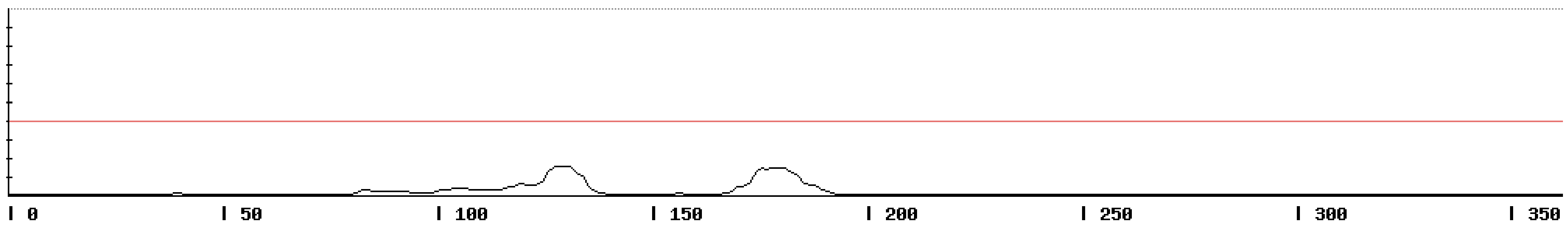

Predictions for Cicerarietinum_XP_004492996.1

Viterbi Path

Viterbi Path

Posterior @ 0.4

Posterior @ 0.4

Posterior Graphic. Threshold Marked @ 0.4

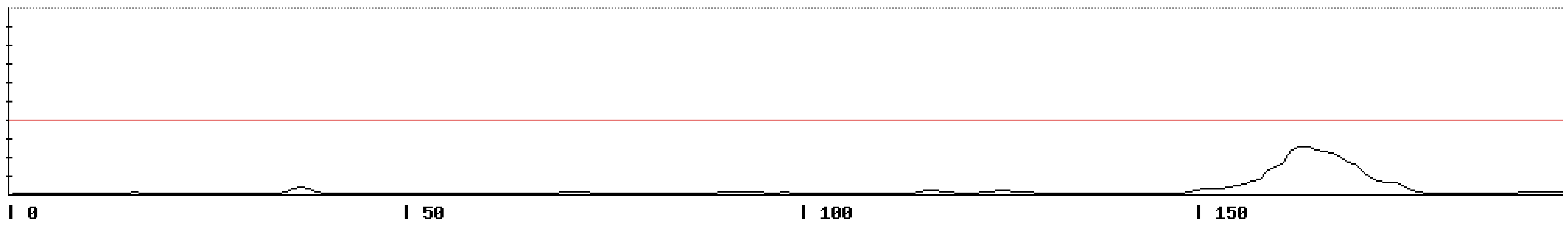

Predictions for Cicerarietinum_XP_004493136.1

Viterbi Path

Viterbi Path

Posterior @ 0.4

Posterior @ 0.4

Posterior Graphic. Threshold Marked @ 0.4

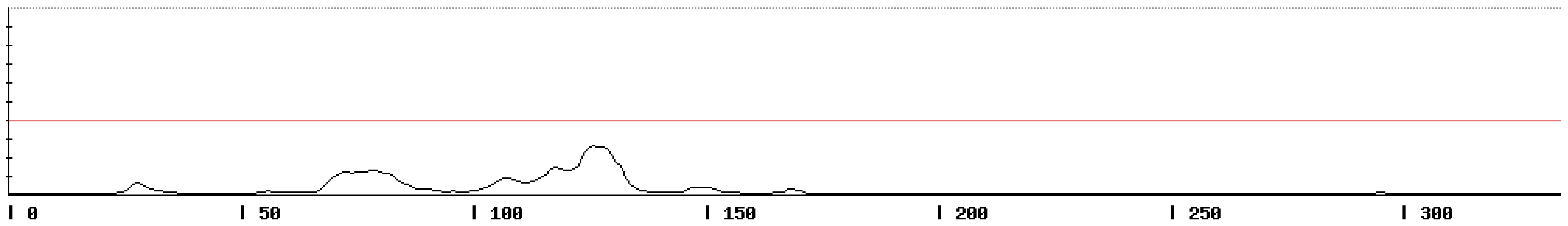

Predictions for Cicerarietinum_XP_004493138.1

Viterbi Path

Viterbi Path

Posterior @ 0.4

Posterior @ 0.4

Posterior Graphic. Threshold Marked @ 0.4

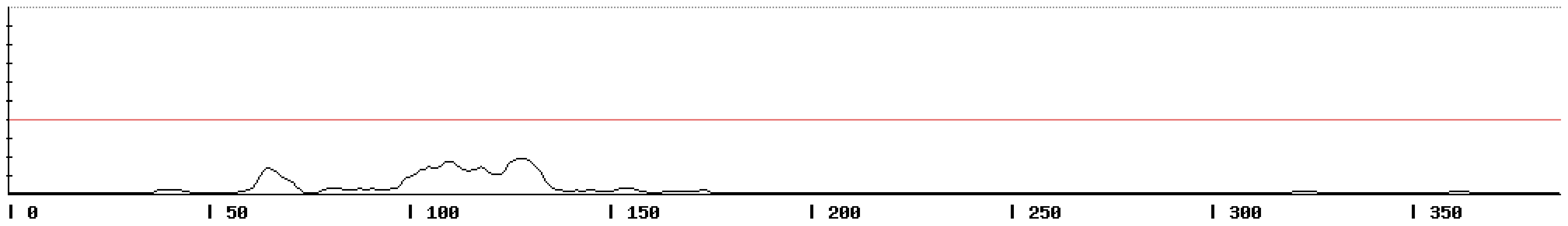

Predictions for Cicerarietinum_XP_004493788.1

Viterbi Path

Viterbi Path

Posterior @ 0.4

Posterior @ 0.4

Posterior Graphic. Threshold Marked @ 0.4

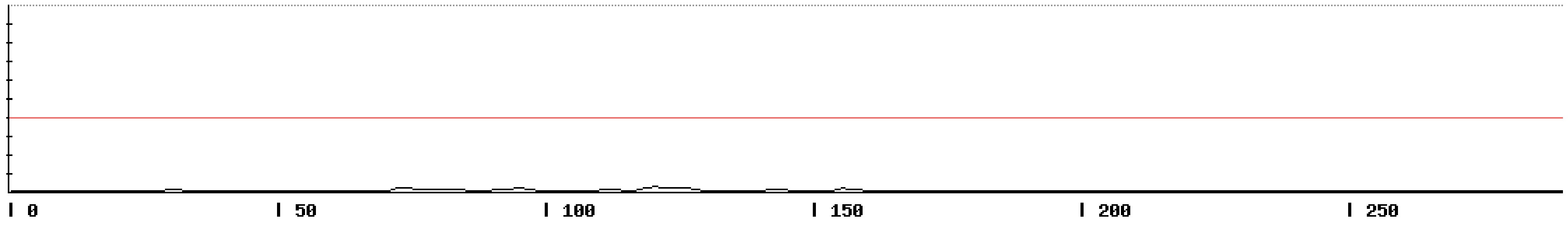

Predictions for Cicerarietinum_XP_004493961.2

Viterbi Path

Viterbi Path

Posterior @ 0.4

Posterior @ 0.4

Posterior Graphic. Threshold Marked @ 0.4

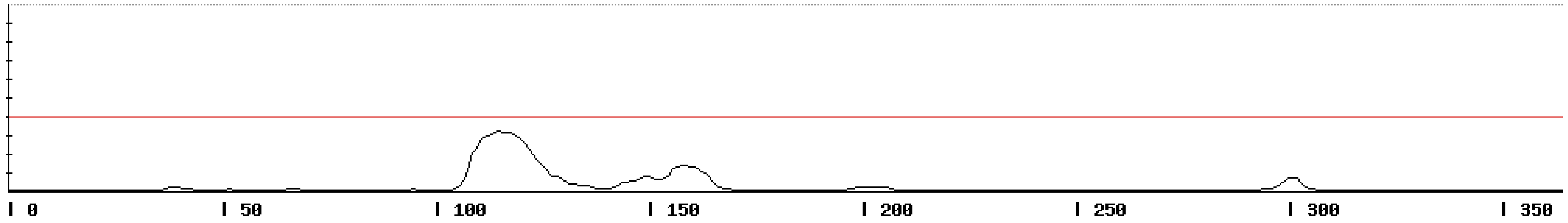

Predictions for Cicerarietinum_XP_004494211.1

Viterbi Path	
Viterbi Path	
Posterior @ 0.4	
Posterior @ 0.4	
Posterior Graphic. Threshold Marked @ 0.4	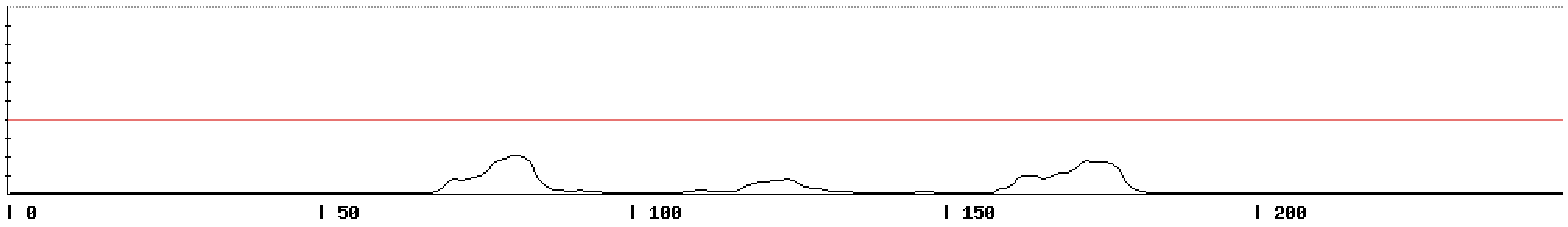

Predictions for Cicerarietinum_XP_004496131.1

Viterbi Path	
Viterbi Path	
Posterior @ 0.4	
Posterior @ 0.4	
Posterior Graphic. Threshold Marked @ 0.4	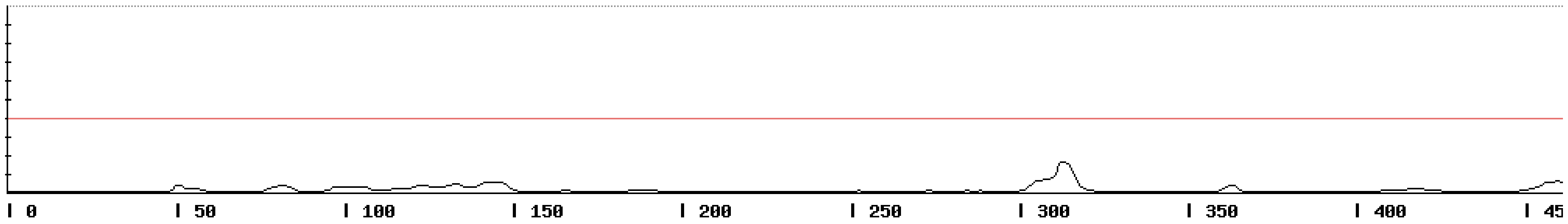

Predictions for Cicerarietinum_XP_004496133.1

Viterbi Path

Viterbi Path

Posterior @ 0.4

Posterior @ 0.4

Posterior Graphic. Threshold Marked @ 0.4

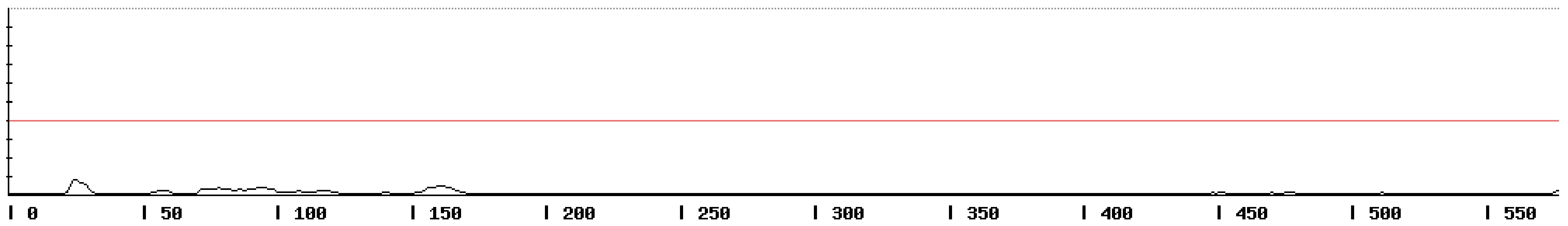

Predictions for Cicerarietinum_XP_004496134.1

Viterbi Path

Viterbi Path

Posterior @ 0.4

Posterior @ 0.4

Posterior Graphic. Threshold Marked @ 0.4

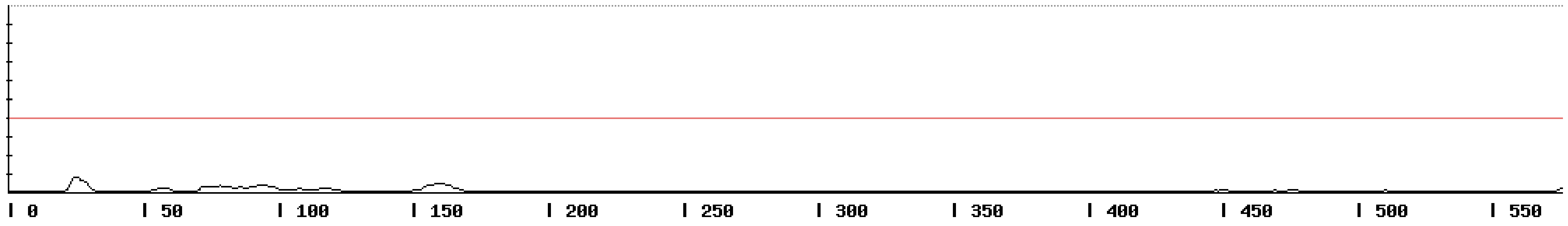

Predictions for Cicerarietinum_XP_004496367.1

Viterbi Path	
Viterbi Path	
Posterior @ 0.4	
Posterior @ 0.4	
Posterior Graphic. Threshold Marked @ 0.4	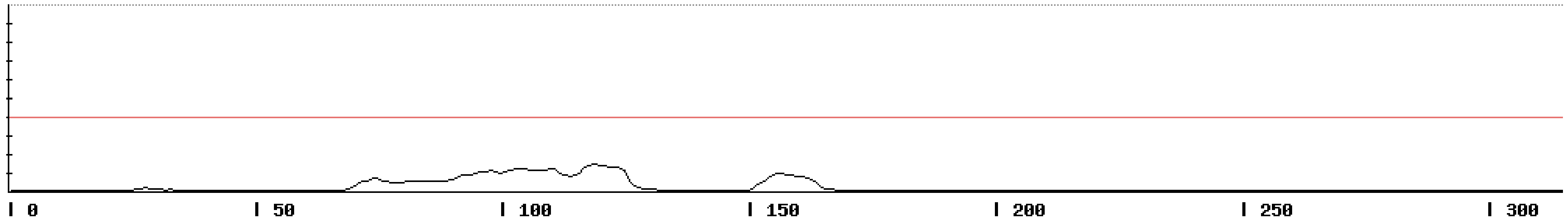

Predictions for Cicerarietinum_XP_004496404.1

Viterbi Path	
Viterbi Path	
Posterior @ 0.4	
Posterior @ 0.4	

Posterior Graphic. Threshold Marked @ 0.4

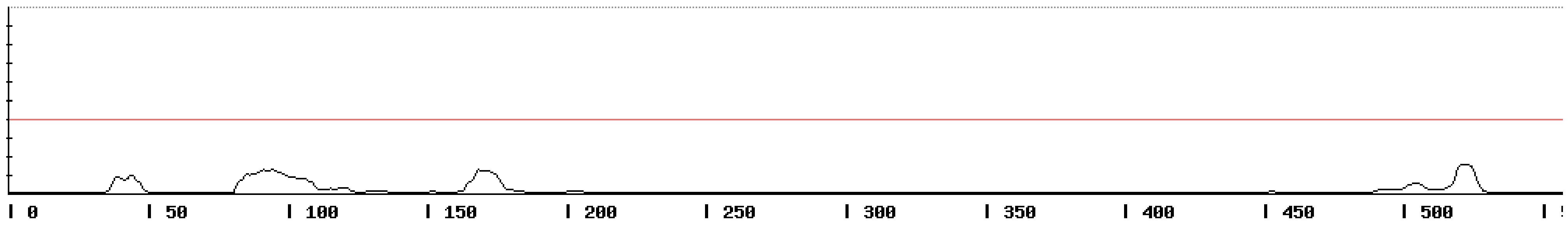

Predictions for Cicerarietinum_XP_004498547.1

Viterbi Path	
Viterbi Path	
Posterior @ 0.4	
Posterior @ 0.4	
Posterior Graphic. Threshold Marked @ 0.4	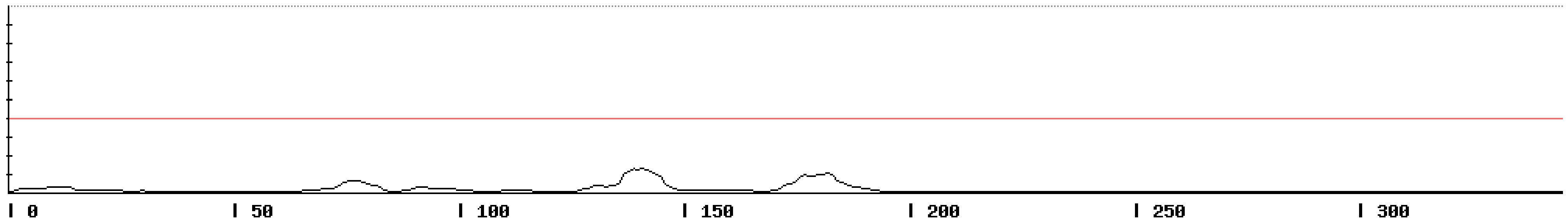

Predictions for Cicerarietinum_XP_004499353.2

Viterbi Path	
Viterbi Path	
Posterior @ 0.4	
Posterior @ 0.4	

Posterior Graphic. Threshold Marked @ 0.4

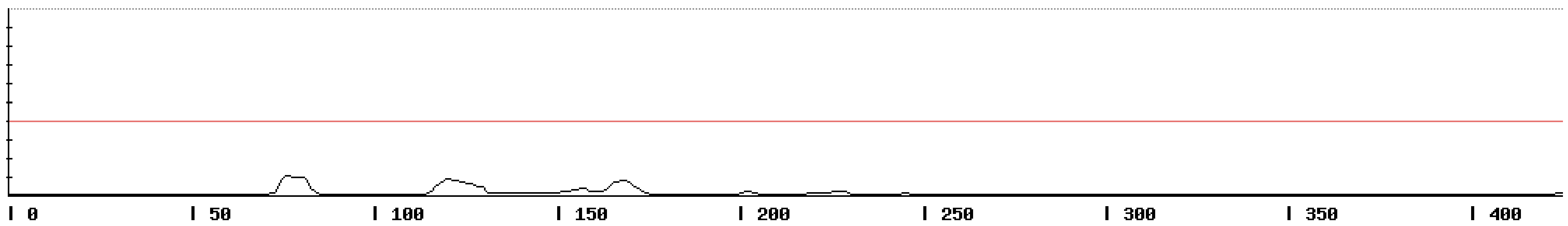

Predictions for Cicerarietinum_XP_004499395.1

Viterbi Path

Viterbi Path

Posterior @ 0.4 — 133 - RKRRKIHHHRG - 143

Posterior @ 0.4

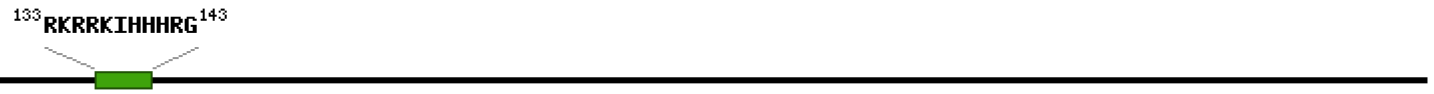

Posterior Graphic. Threshold Marked @ 0.4

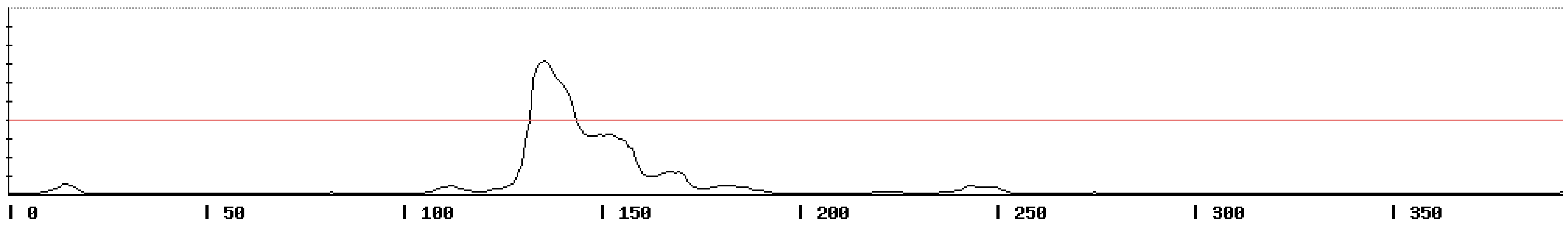

Predictions for Cicerarietinum_XP_004500832.1

Viterbi Path

Viterbi Path

Posterior @ 0.4

Posterior @ 0.4

Posterior Graphic. Threshold Marked @ 0.4

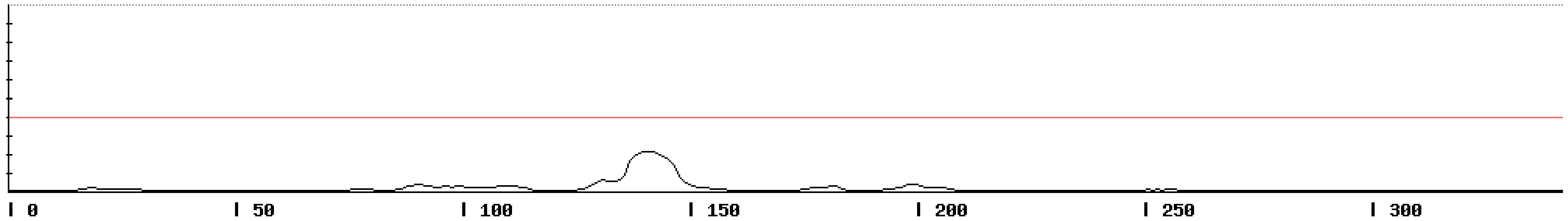

Predictions for Cicerarietinum_XP_004501729.1

Viterbi Path

Viterbi Path

Posterior @ 0.4

Posterior @ 0.4

Posterior Graphic. Threshold Marked @ 0.4

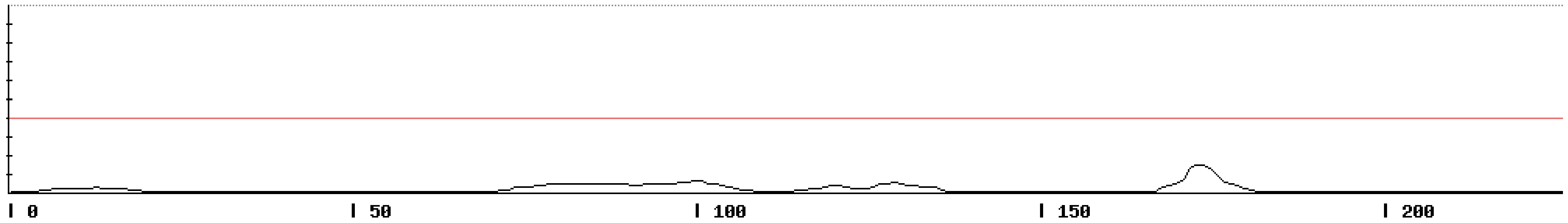

Predictions for Cicerarietinum_XP_004502479.1

Viterbi Path

Viterbi Path

Posterior @ 0.4	
Posterior @ 0.4	
Posterior Graphic. Threshold Marked @ 0.4	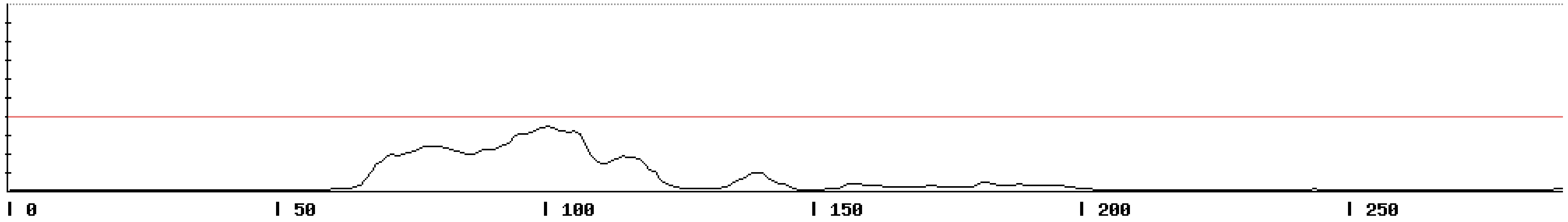

Predictions for Cicerarietinum_XP_004502779.1	
Viterbi Path	
Viterbi Path	
Posterior @ 0.4	
Posterior @ 0.4	
Posterior Graphic. Threshold Marked @ 0.4	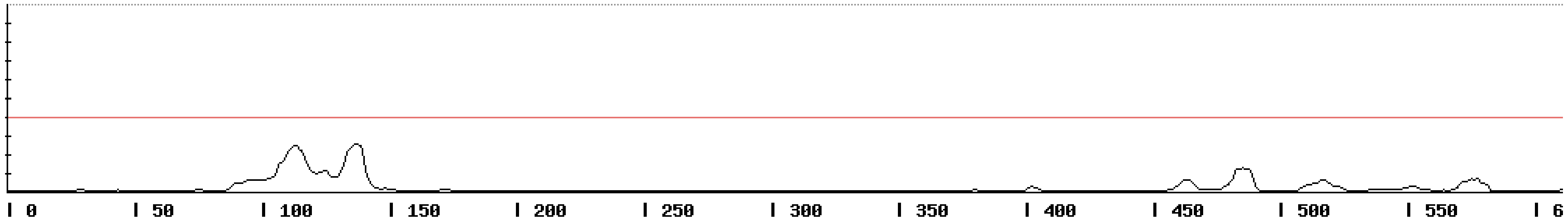

Predictions for Cicerarietinum_XP_004502931.1

Viterbi Path

Viterbi Path

Posterior @ 0.4

Posterior @ 0.4

Posterior Graphic. Threshold Marked @ 0.4

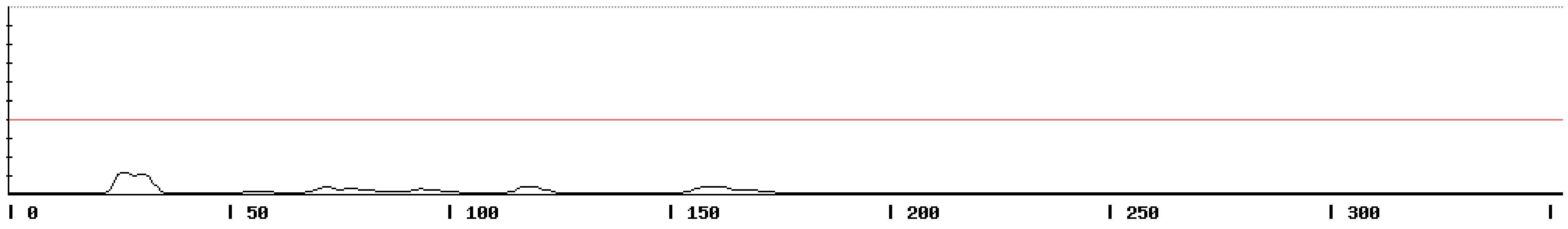

Predictions for Cicerarietinum_XP_004502932.1

Viterbi Path

Viterbi Path

Posterior @ 0.4

Posterior @ 0.4

Posterior Graphic. Threshold Marked @ 0.4

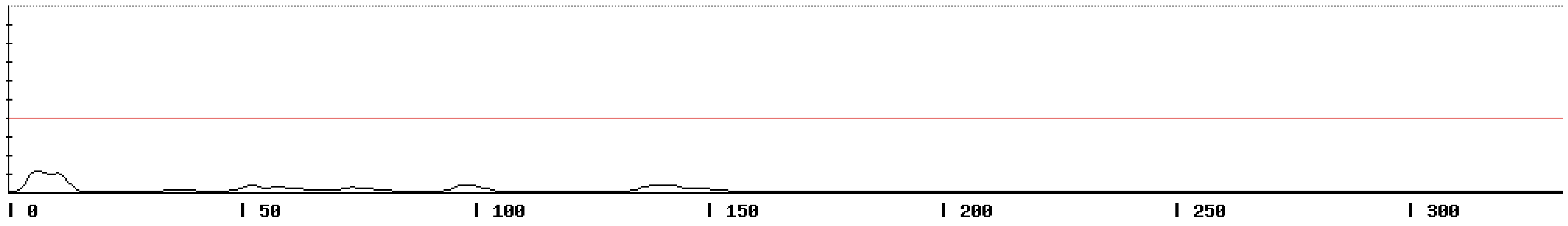

Predictions for Cicerarietinum_XP_004502933.1	
Viterbi Path	
Viterbi Path	
Posterior @ 0.4	
Posterior @ 0.4	
Posterior Graphic. Threshold Marked @ 0.4	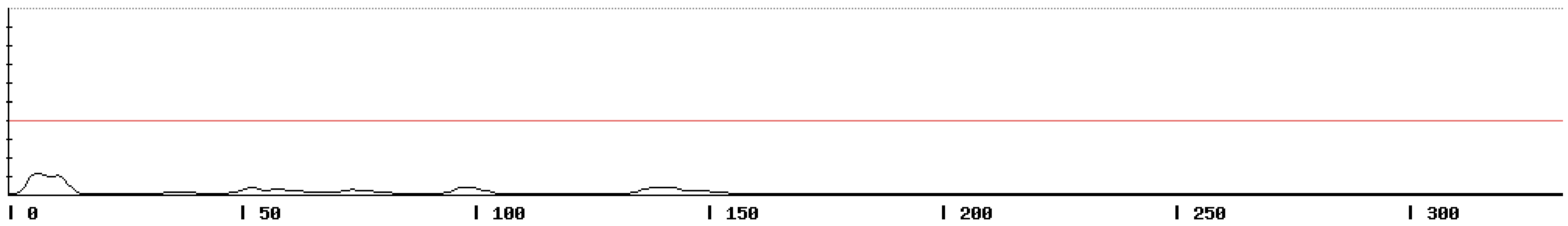

Predictions for Cicerarietinum_XP_004502989.1	
Viterbi Path	
Viterbi Path	
Posterior @ 0.4	93 - ADKPIGKPKAMGIKK - 107
Posterior @ 0.4	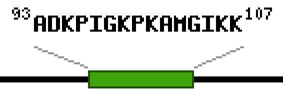
Posterior Graphic. Threshold Marked @ 0.4	

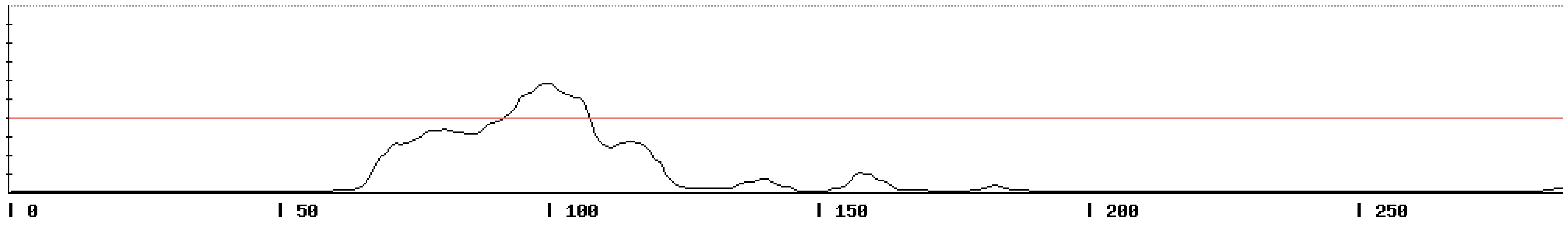

Predictions for Cicerarietinum_XP_004503093.1	
Viterbi Path	
Viterbi Path	
Posterior @ 0.4	
Posterior @ 0.4	
Posterior Graphic. Threshold Marked @ 0.4	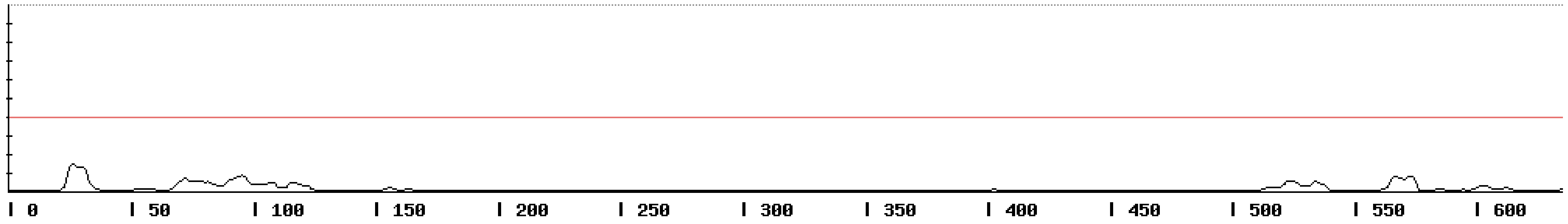

Predictions for Cicerarietinum_XP_004503507.1	
Viterbi Path	
Viterbi Path	
Posterior @ 0.4	
Posterior @ 0.4	

Posterior Graphic. Threshold Marked @ 0.4

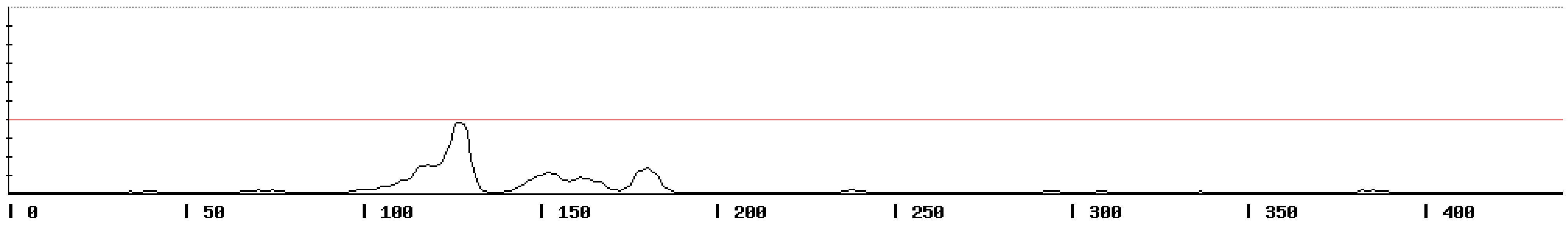

Predictions for Cicerarietinum_XP_004503781.1

Viterbi Path

Viterbi Path

Posterior @ 0.4

Posterior @ 0.4

Posterior Graphic. Threshold Marked @ 0.4

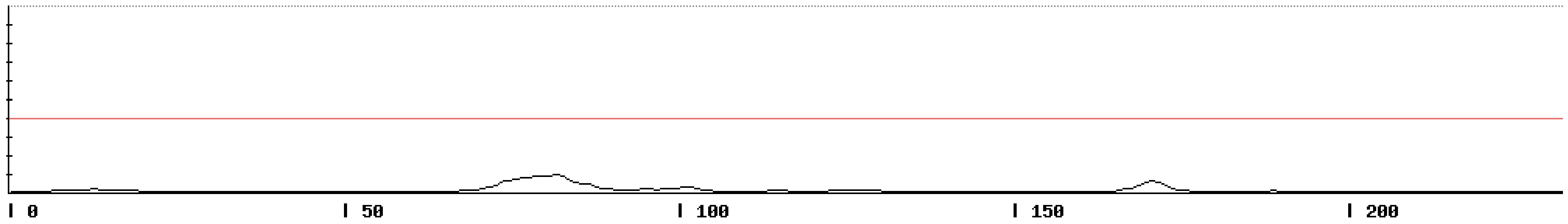

Predictions for Cicerarietinum_XP_004503901.1

Viterbi Path

Viterbi Path

Posterior @ 0.4 — 93 - ADKPIGKPKPLGIKKA - 108

Posterior @ 0.4

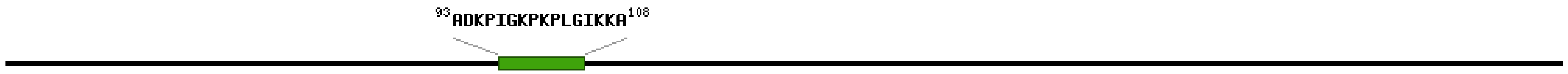

Posterior Graphic. Threshold Marked @ 0.4

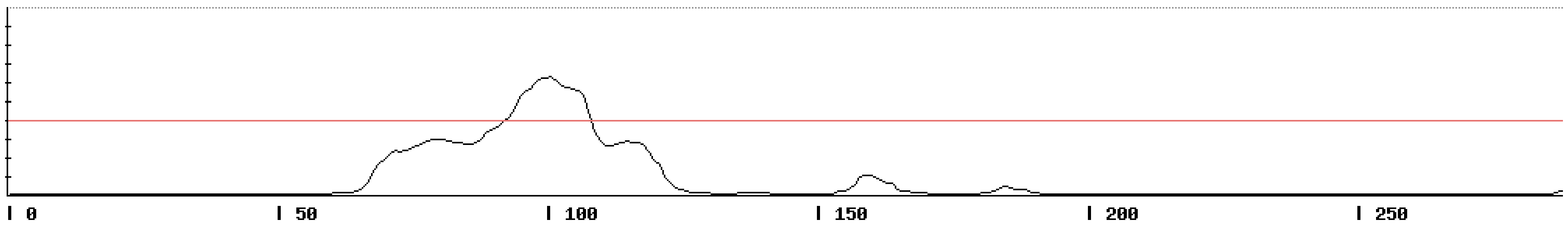

Predictions for Cicerarietinum_XP_004504766.1

Viterbi Path

Viterbi Path

Posterior @ 0.4

Posterior @ 0.4

Posterior Graphic. Threshold Marked @ 0.4

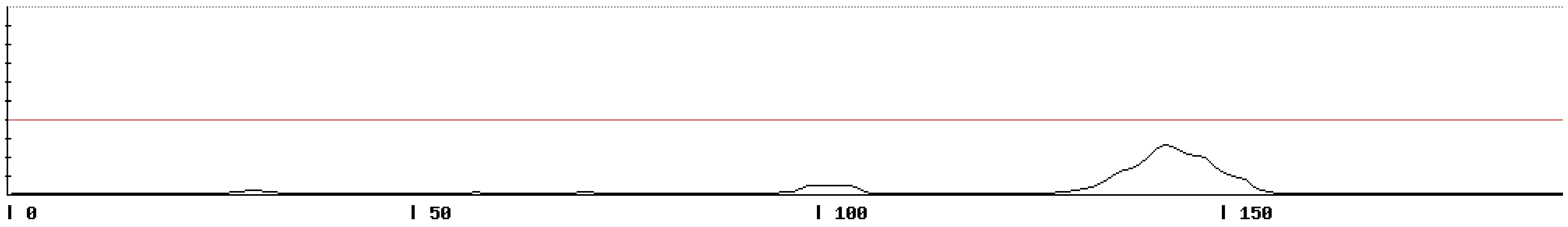

Predictions for Cicerarietinum_XP_004505006.1

Viterbi Path

Viterbi Path

Posterior @ 0.4

Posterior @ 0.4

Posterior Graphic. Threshold Marked @ 0.4

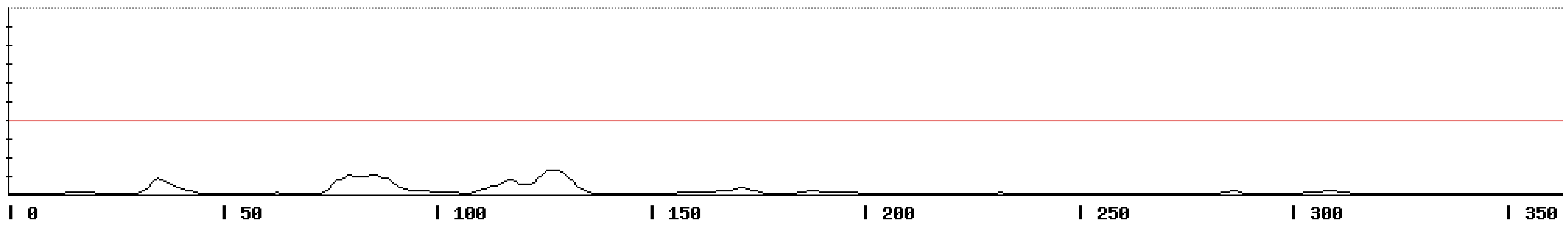

Predictions for Cicerarietinum_XP_004505102.1

Viterbi Path

Viterbi Path

Posterior @ 0.4

Posterior @ 0.4

Posterior Graphic. Threshold Marked @ 0.4

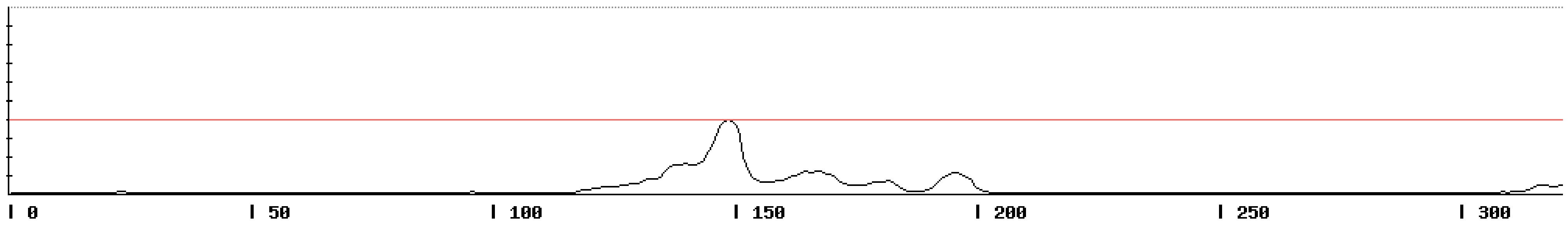

Predictions for Cicerarietinum_XP_004505536.1

Viterbi Path

Viterbi Path

Posterior @ 0.4 | 131 - RKRRK - 135

Posterior @ 0.4

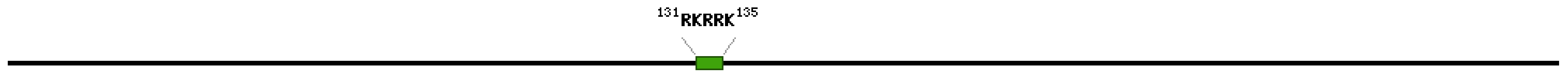

Posterior Graphic. Threshold Marked @ 0.4

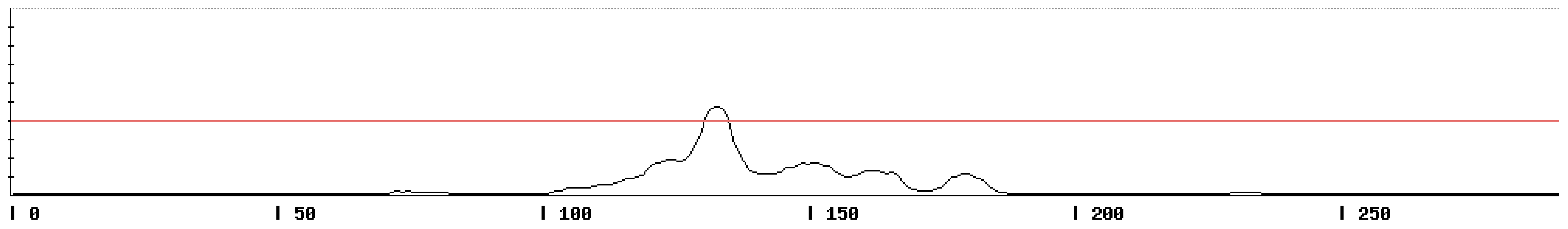

Predictions for Cicerarietinum_XP_004505672.1

Viterbi Path

Viterbi Path

Posterior @ 0.4

Posterior @ 0.4

Posterior Graphic. Threshold Marked @ 0.4

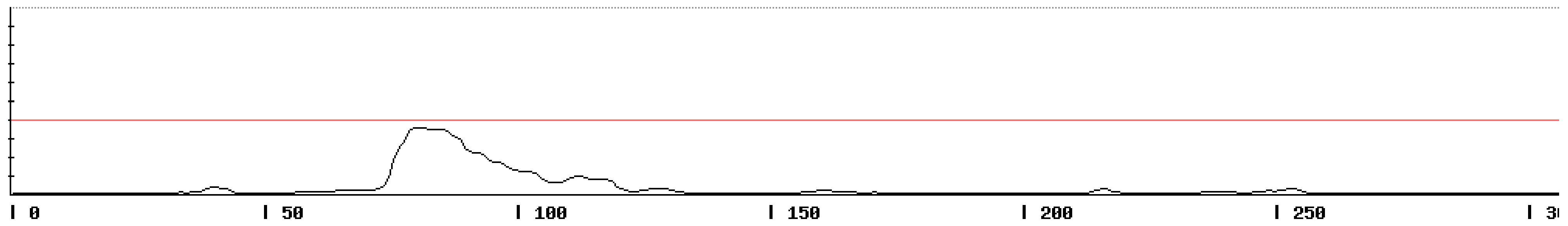

Predictions for Cicerarietinum_XP_004506197.1

Viterbi Path	
Viterbi Path	
Posterior @ 0.4	
Posterior @ 0.4	
Posterior Graphic. Threshold Marked @ 0.4	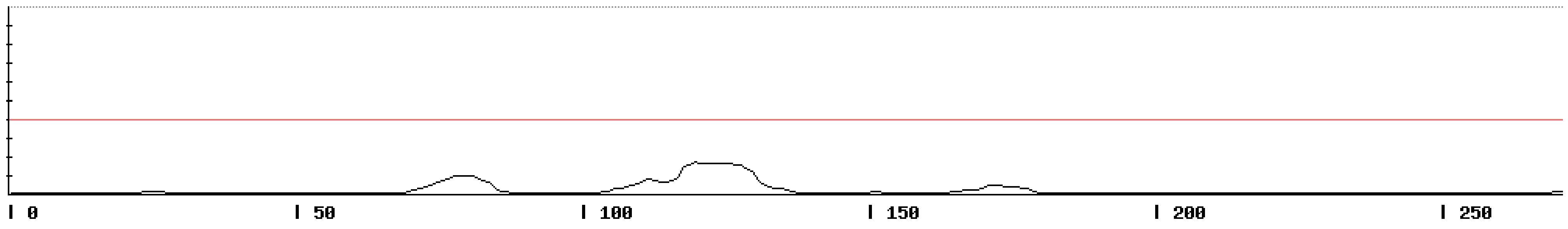

Predictions for Cicerarietinum_XP_004506361.1

Viterbi Path	
Viterbi Path	
Posterior @ 0.4	
Posterior @ 0.4	
Posterior Graphic. Threshold Marked @ 0.4	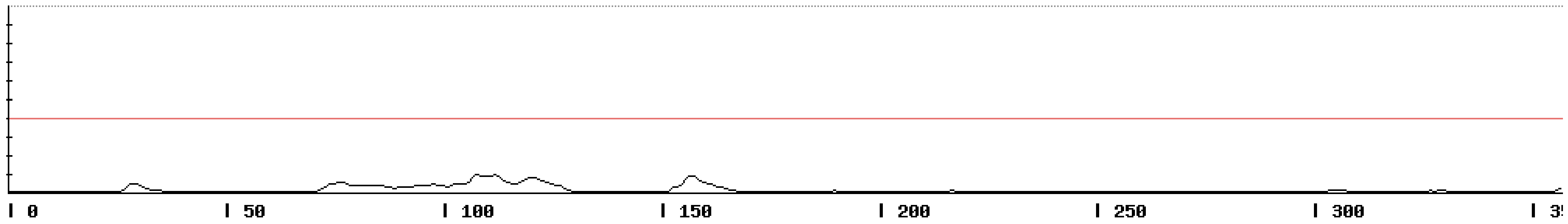

Predictions for Cicerarietinum_XP_004508725.1	
Viterbi Path	
Viterbi Path	
Posterior @ 0.4	
Posterior @ 0.4	
Posterior Graphic. Threshold Marked @ 0.4	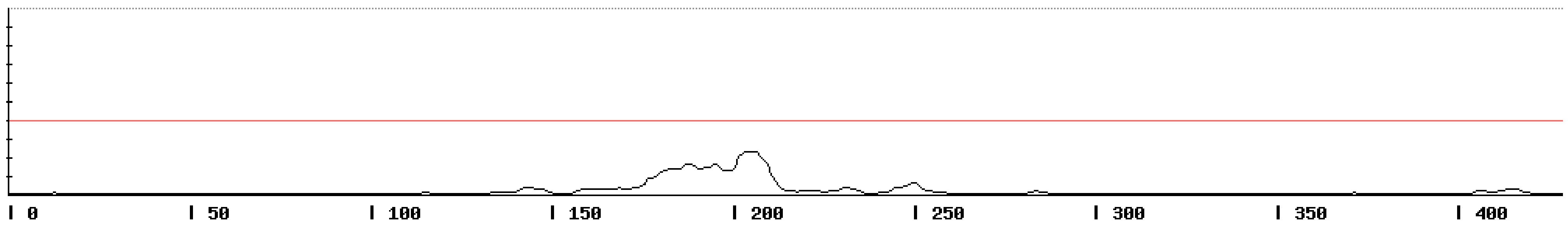

Predictions for Cicerarietinum_XP_004509014.1	
Viterbi Path	
Viterbi Path	
Posterior @ 0.4	
Posterior @ 0.4	
Posterior Graphic. Threshold Marked @ 0.4	

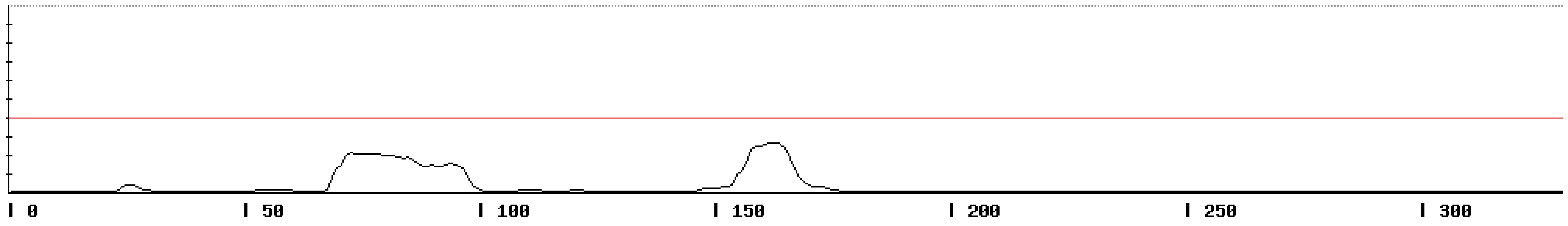

Predictions for Cicerarietinum_XP_004509344.1

Viterbi Path

Viterbi Path

Posterior @ 0.4

Posterior @ 0.4

Posterior Graphic. Threshold Marked @ 0.4

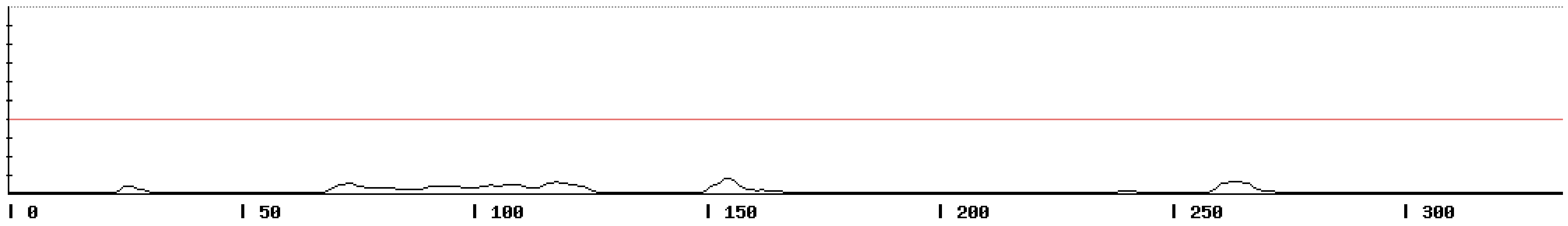

Predictions for Cicerarietinum_XP_004510270.1

Viterbi Path

Viterbi Path

Posterior @ 0.4

Posterior @ 0.4

Posterior Graphic. Threshold Marked @ 0.4

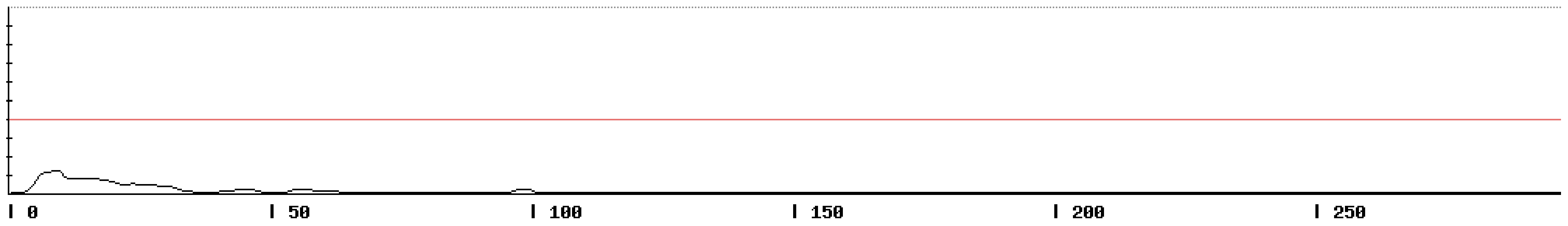

Predictions for Cicerarietinum_XP_004511662.1

Viterbi Path

Viterbi Path

Posterior @ 0.4

Posterior @ 0.4

Posterior Graphic. Threshold Marked @ 0.4

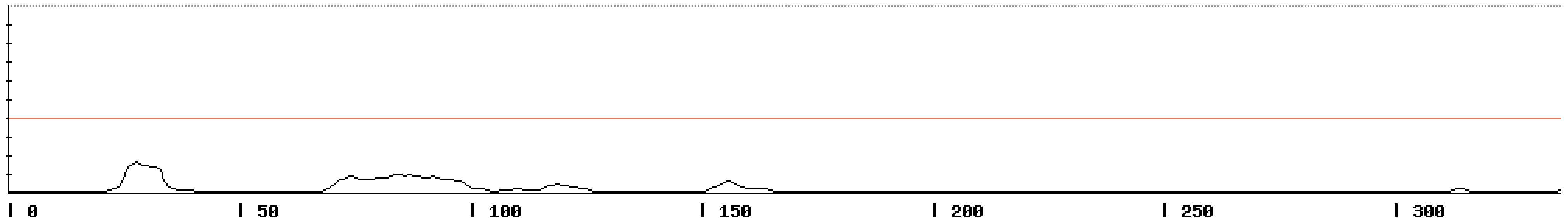

Predictions for Cicerarietinum_XP_004511779.1

Viterbi Path

Viterbi Path

Posterior @ 0.4

Posterior @ 0.4

Posterior Graphic. Threshold Marked @ 0.4

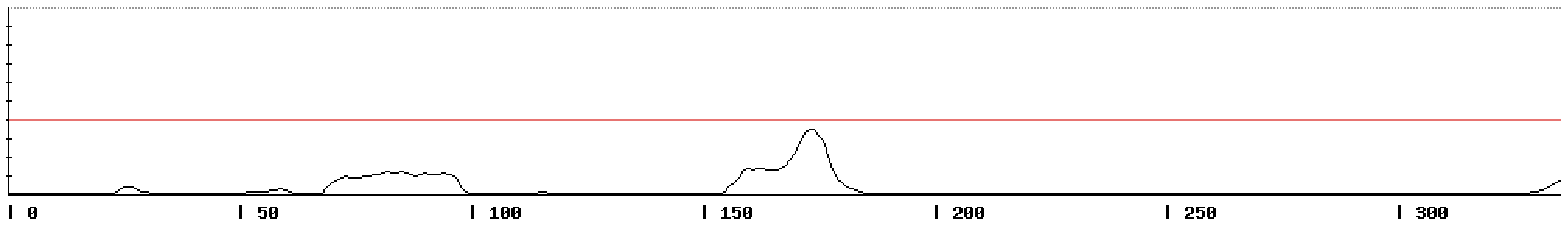

Predictions for Cicerarietinum_XP_004512121.1

Viterbi Path

Viterbi Path

Posterior @ 0.4

Posterior @ 0.4

Posterior Graphic. Threshold Marked @ 0.4

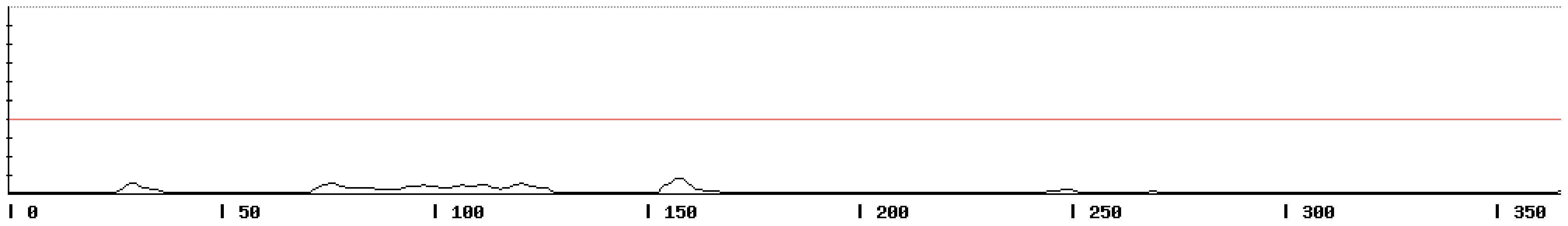

Predictions for Cicerarietinum_XP_004512922.1

Viterbi Path

Viterbi Path

Posterior @ 0.4

Posterior @ 0.4

Posterior Graphic. Threshold Marked @ 0.4

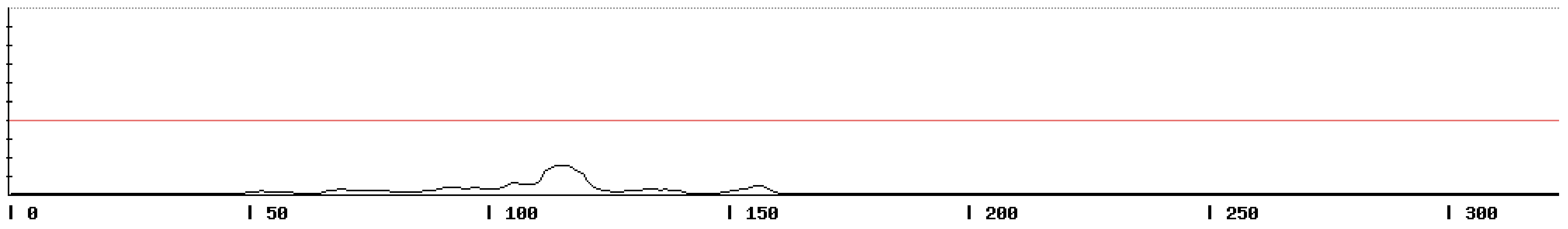

Predictions for Cicerarietinum_XP_004513071.1

Viterbi Path

Viterbi Path

Posterior @ 0.4

Posterior @ 0.4

Posterior Graphic. Threshold Marked @ 0.4

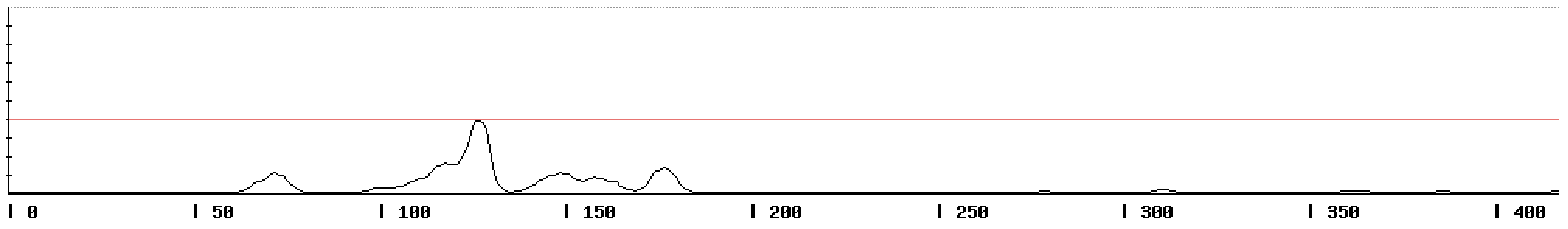

Predictions for Cicerarietinum_XP_004513072.1

Viterbi Path

Viterbi Path

Posterior @ 0.4

Posterior @ 0.4

Posterior Graphic. Threshold Marked @ 0.4

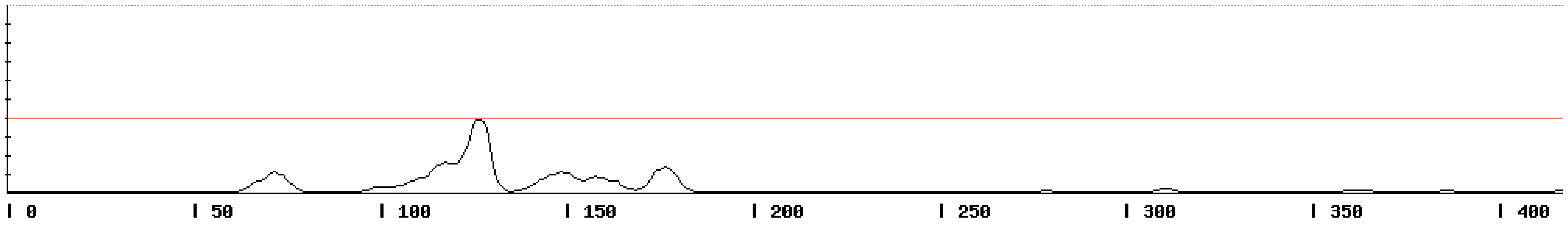

Predictions for Cicerarietinum_XP_004513361.2

Viterbi Path

Viterbi Path

Posterior @ 0.4

71 - KRNRKYPKGERPDR - 84

173 - KKR - 175

Posterior @ 0.4

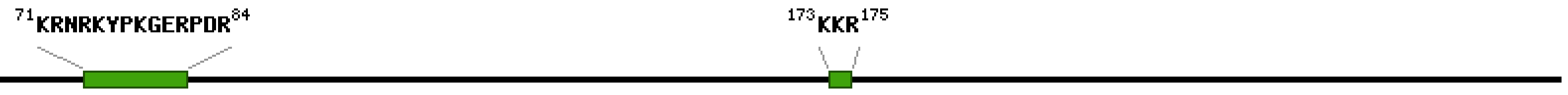

Posterior Graphic. Threshold Marked @ 0.4

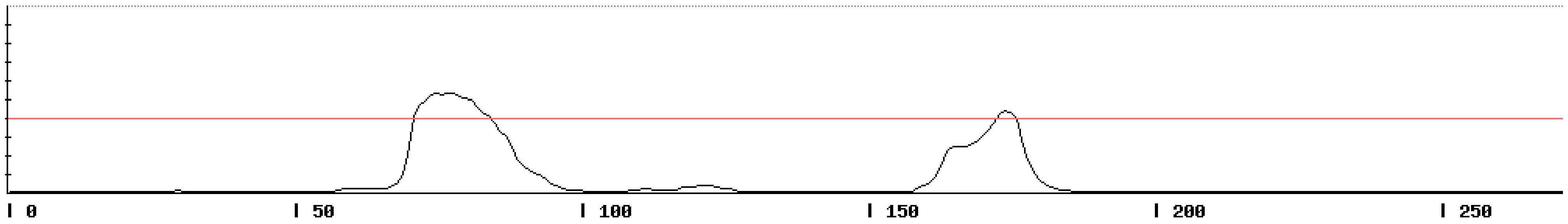

Predictions for Cicerarietinum_XP_004514021.1	
Viterbi Path	
Viterbi Path	
Posterior @ 0.4	112 - DRKIK - 116<br>133 - GRAPKGK - 139
Posterior @ 0.4	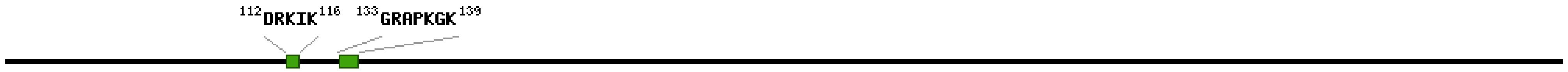
Posterior Graphic. Threshold Marked @ 0.4	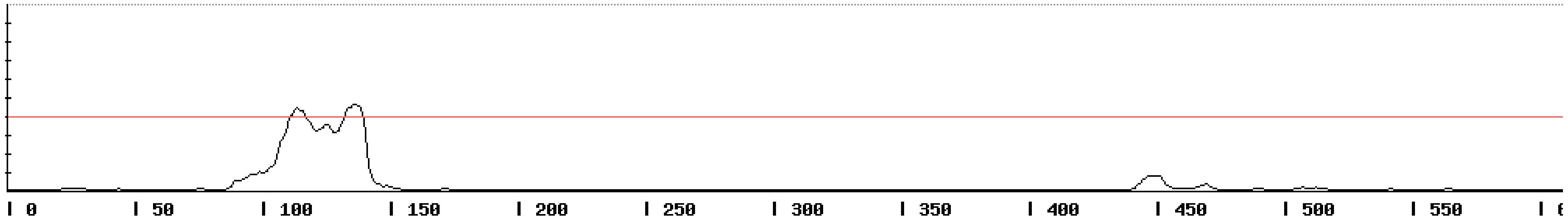

Predictions for Cicerarietinum_XP_004514350.1	
Viterbi Path	
Viterbi Path	
Posterior @ 0.4	
Posterior @ 0.4	
Posterior Graphic. Threshold Marked @ 0.4	

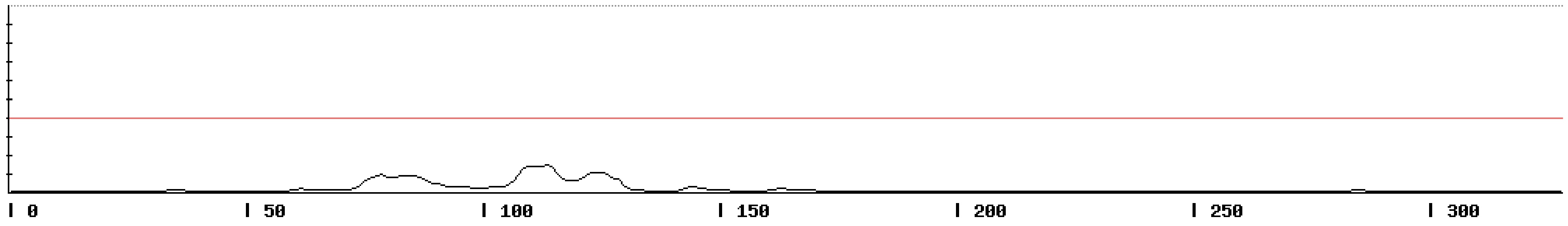

Predictions for Cicerarietinum_XP_004514529.1

Viterbi Path	
Viterbi Path	
Posterior @ 0.4	
Posterior @ 0.4	
Posterior Graphic. Threshold Marked @ 0.4	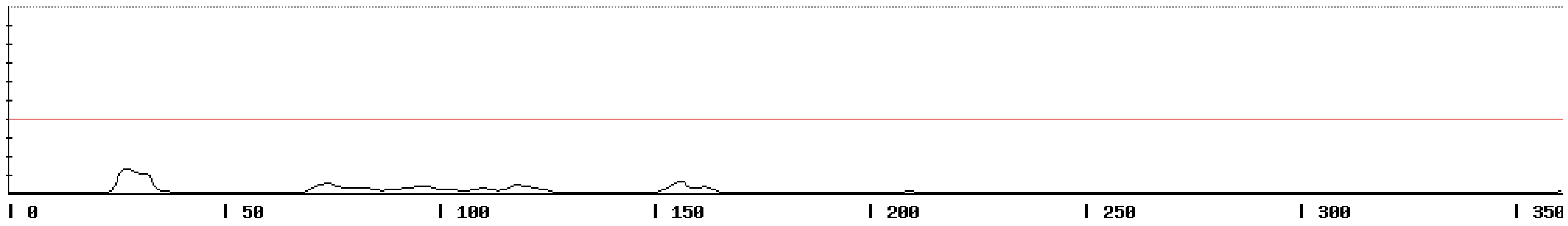

Predictions for Cicerarietinum_XP_004514929.1

Viterbi Path	
Viterbi Path	
Posterior @ 0.4	
Posterior @ 0.4	

Posterior Graphic. Threshold Marked @ 0.4

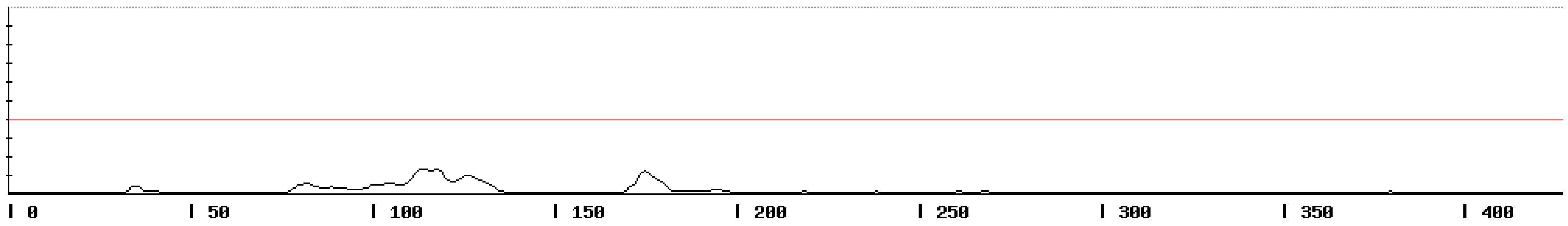

Predictions for Cicerarietinum_XP_004514930.1

Viterbi Path

Viterbi Path

Posterior @ 0.4

Posterior @ 0.4

Posterior Graphic. Threshold Marked @ 0.4

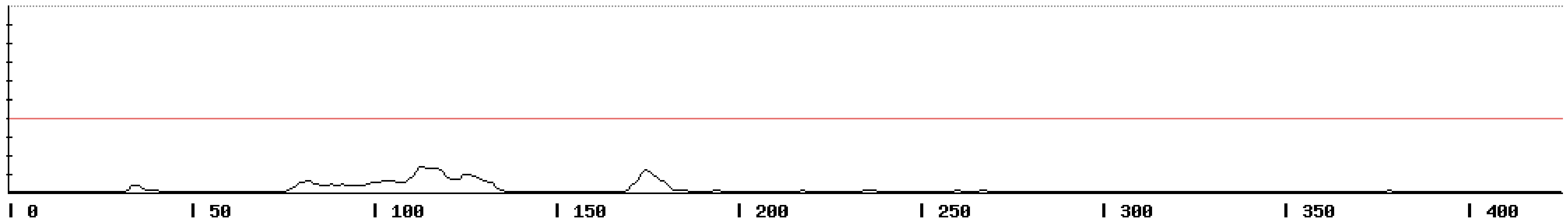

Predictions for Cicerarietinum_XP_004515256.1

Viterbi Path

Viterbi Path

Posterior @ 0.4

Posterior @ 0.4

Posterior Graphic. Threshold Marked @ 0.4

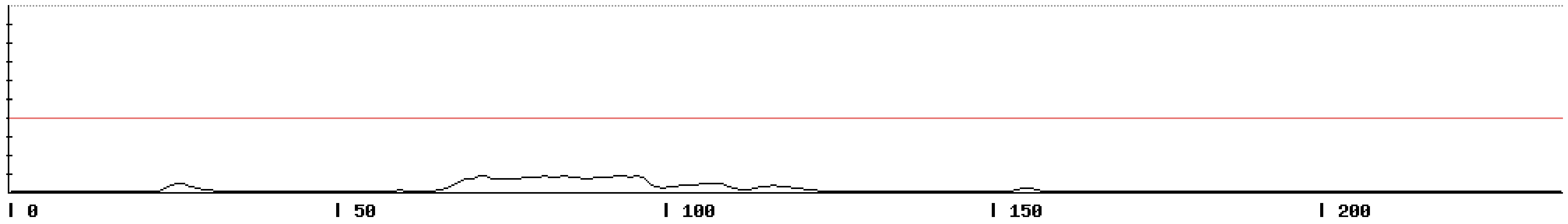

Predictions for Cicerarietinum_XP_004515741.1

Viterbi Path

Viterbi Path

Posterior @ 0.4

Posterior @ 0.4

Posterior Graphic. Threshold Marked @ 0.4

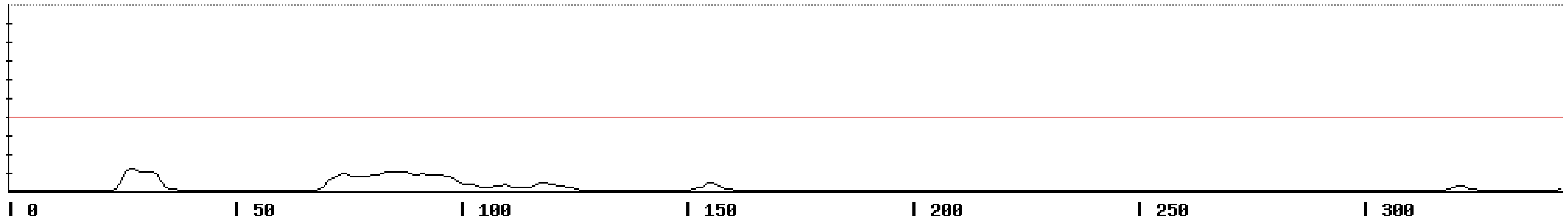

Predictions for Cicerarietinum_XP_004516088.1

Viterbi Path

Viterbi Path

Posterior @ 0.4

Posterior @ 0.4

Posterior Graphic. Threshold Marked @ 0.4

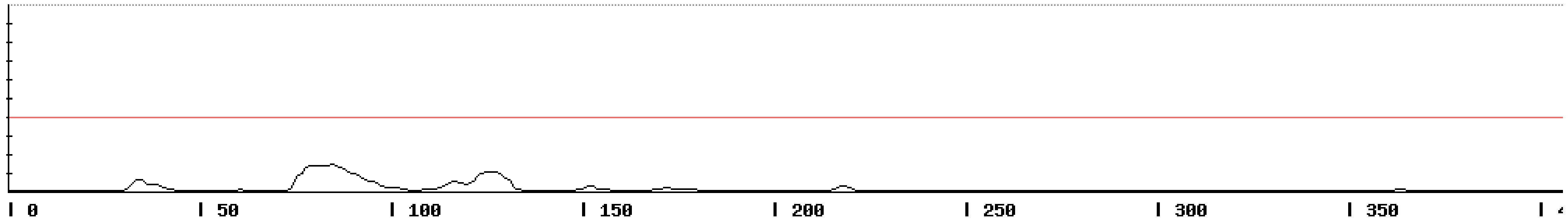

Predictions for Cicerarietinum_XP_012567502.1

Viterbi Path

Viterbi Path

Posterior @ 0.4

Posterior @ 0.4

Posterior Graphic. Threshold Marked @ 0.4

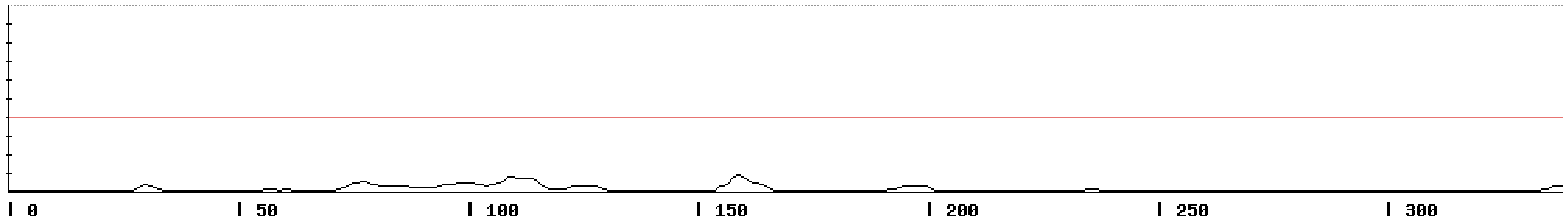

Predictions for Cicerarietinum_XP_012568084.1

Viterbi Path

Viterbi Path

Posterior @ 0.4

Posterior @ 0.4

Posterior Graphic. Threshold Marked @ 0.4

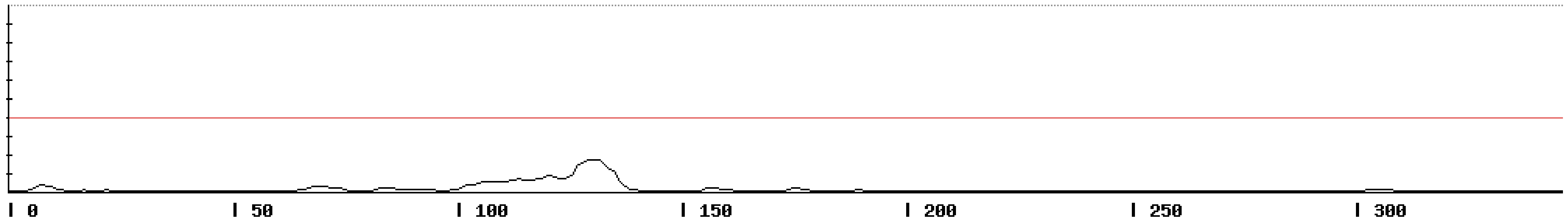

Predictions for Cicerarietinum_XP_012568792.1

Viterbi Path

Viterbi Path

Posterior @ 0.4

Posterior @ 0.4

Posterior Graphic. Threshold Marked @ 0.4

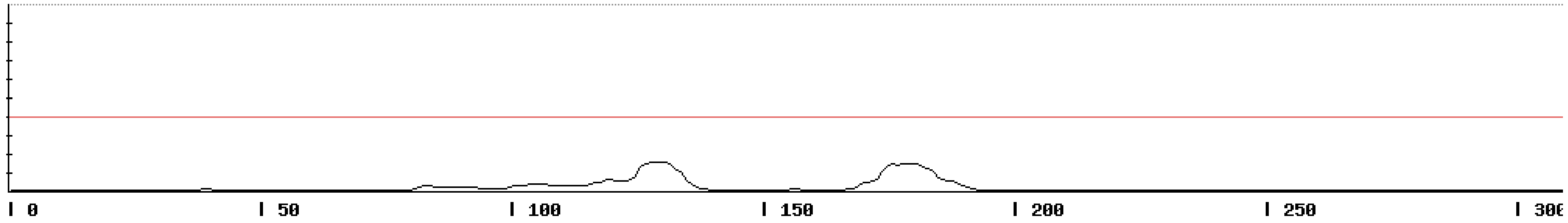

Predictions for Cicerarietinum_XP_012570459.1

Viterbi Path

Viterbi Path

Posterior @ 0.4 — 156 - RKNNIRNKIKMPRKSTRTKNVGQHRRRKII - 185

Posterior @ 0.4

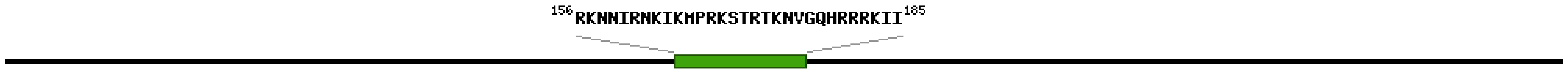

Posterior Graphic. Threshold Marked @ 0.4

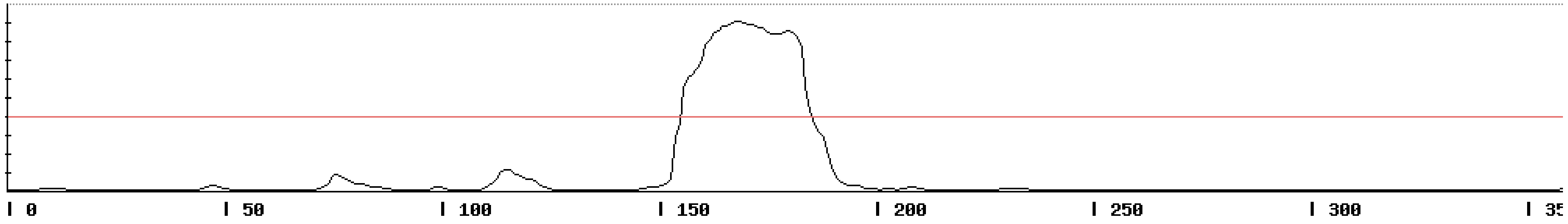

Predictions for Cicerarietinum_XP_012571554.1

Viterbi Path

Viterbi Path

Posterior @ 0.4 — 145 - KRR - 147

Posterior @ 0.4

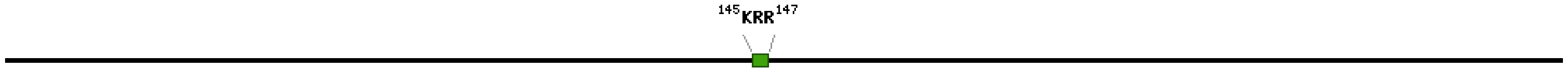

Posterior Graphic. Threshold Marked @ 0.4

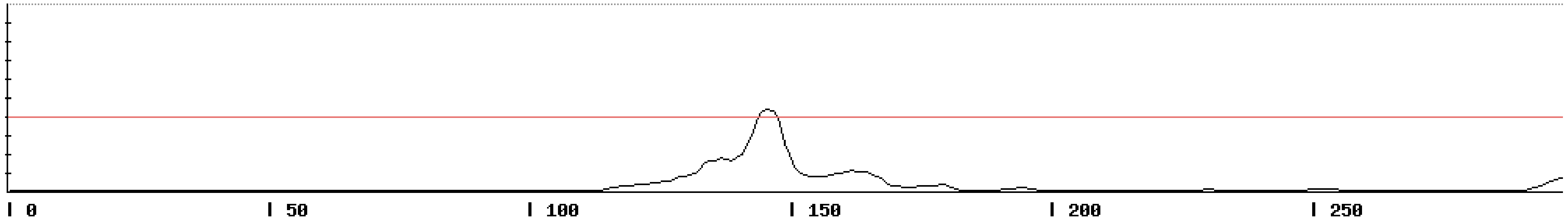

Predictions for Cicerarietinum_XP_012571948.1

Viterbi Path

Viterbi Path

Posterior @ 0.4

Posterior @ 0.4

Posterior Graphic. Threshold Marked @ 0.4

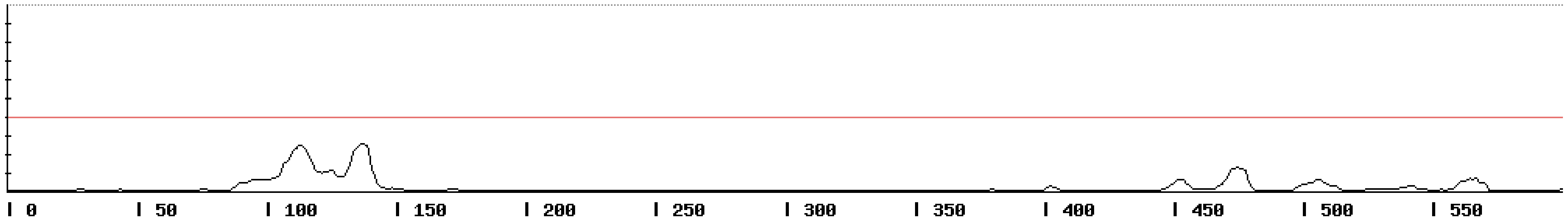

Predictions for Cicerarietinum_XP_012572103.1

Viterbi Path

Viterbi Path

Posterior @ 0.4

Posterior @ 0.4

Posterior Graphic. Threshold Marked @ 0.4

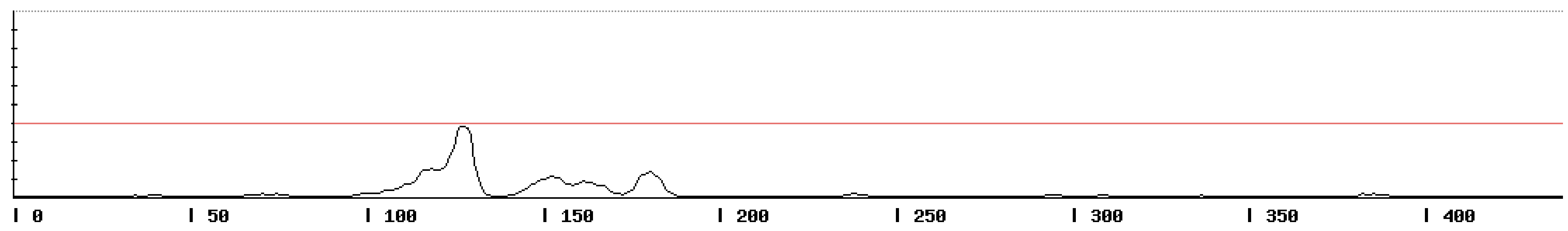

Predictions for Cicerarietinum_XP_012572104.1

Viterbi Path

Viterbi Path

Posterior @ 0.4

Posterior @ 0.4

Posterior Graphic. Threshold Marked @ 0.4

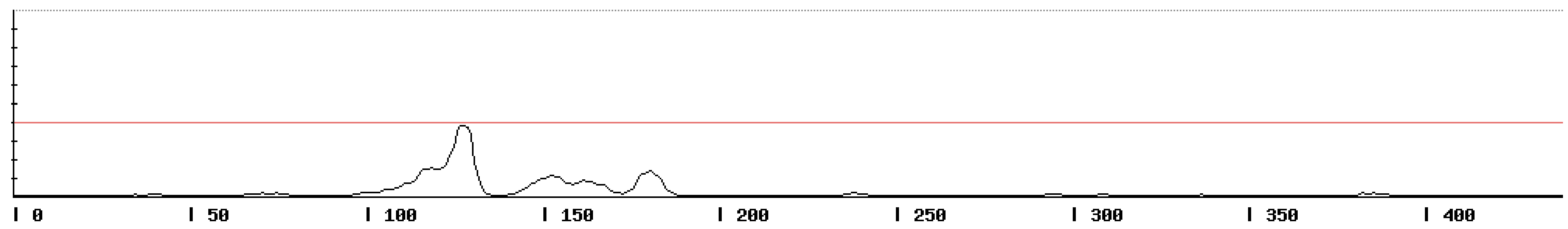

Predictions for Cicerarietinum_XP_012572105.1

Viterbi Path

Viterbi Path

Posterior @ 0.4

Posterior @ 0.4

Posterior Graphic. Threshold Marked @ 0.4	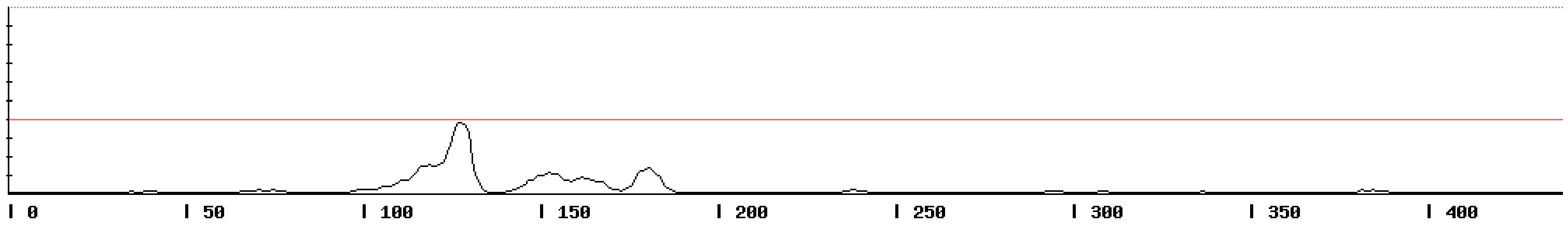

Predictions for Cicerarietinum_XP_012572255.1	
Viterbi Path	
Viterbi Path	
Posterior @ 0.4	
Posterior @ 0.4	
Posterior Graphic. Threshold Marked @ 0.4	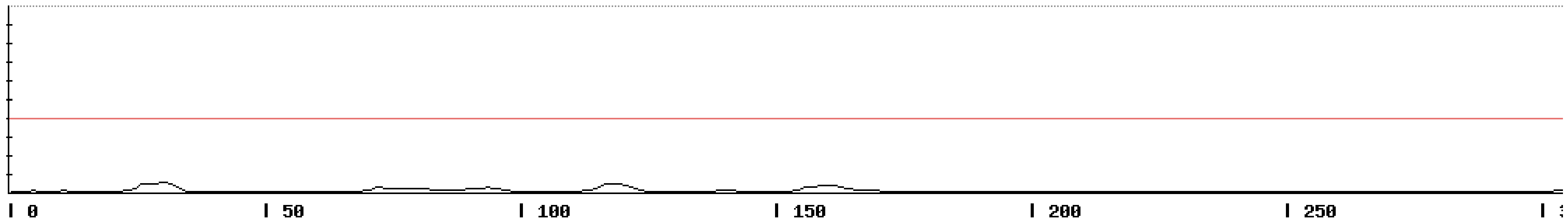

Predictions for Cicerarietinum_XP_012572610.1	
Viterbi Path	
Viterbi Path	
Posterior @ 0.4	
Posterior @ 0.4	

Posterior Graphic. Threshold Marked @ 0.4

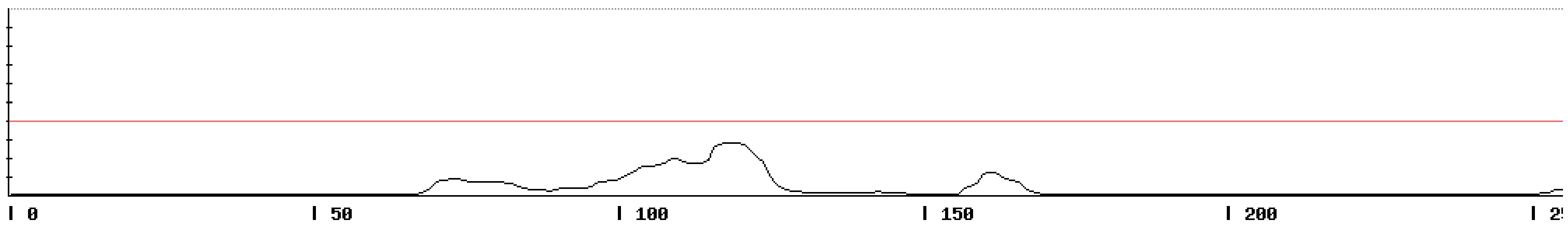

Predictions for Cicerarietinum_XP_012572681.1

Viterbi Path

Viterbi Path

Posterior @ 0.4

Posterior @ 0.4

Posterior Graphic. Threshold Marked @ 0.4

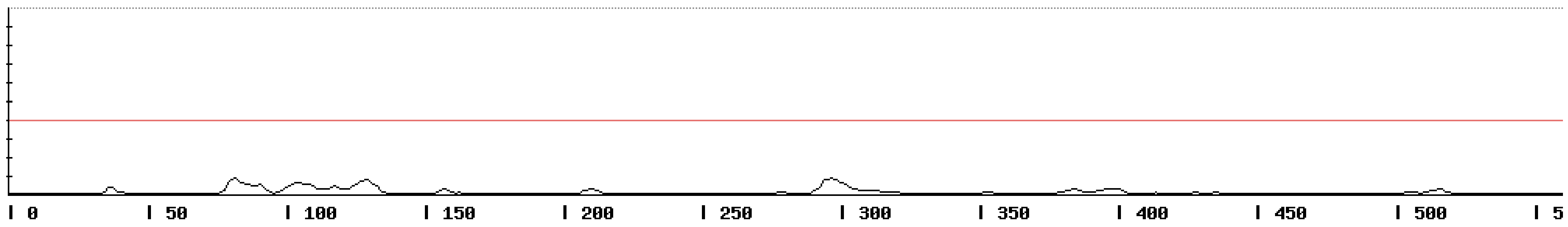

Predictions for Cicerarietinum_XP_012574126.1

Viterbi Path

Viterbi Path

Posterior @ 0.4

Posterior @ 0.4

Posterior Graphic. Threshold Marked @ 0.4

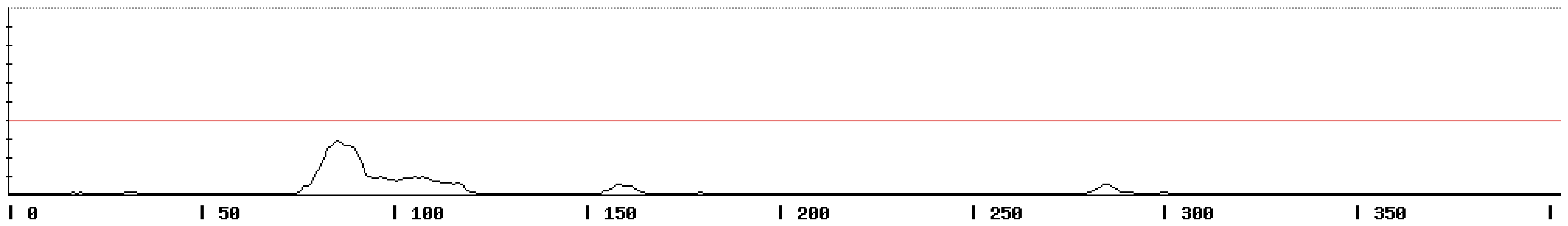

Predictions for Cicerarietinum_XP_012574127.1

Viterbi Path

Viterbi Path

Posterior @ 0.4

Posterior @ 0.4

Posterior Graphic. Threshold Marked @ 0.4

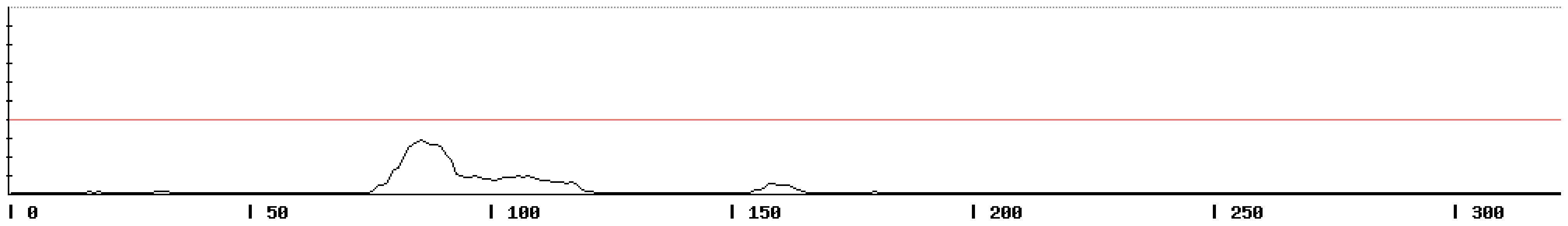

Predictions for Cicerarietinum_XP_012574420.1

Viterbi Path

Viterbi Path

Posterior @ 0.4	
Posterior @ 0.4	
Posterior Graphic. Threshold Marked @ 0.4	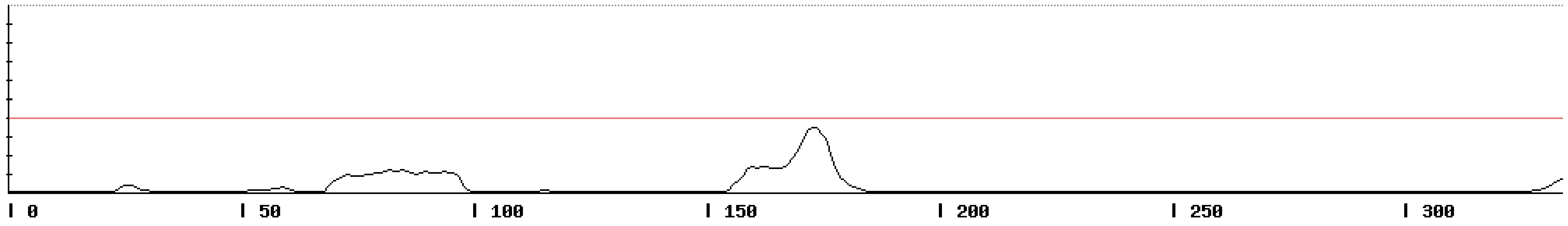

Predictions for Cicerarietinum_XP_012574421.1

Viterbi Path	
Viterbi Path	
Posterior @ 0.4	
Posterior @ 0.4	
Posterior Graphic. Threshold Marked @ 0.4	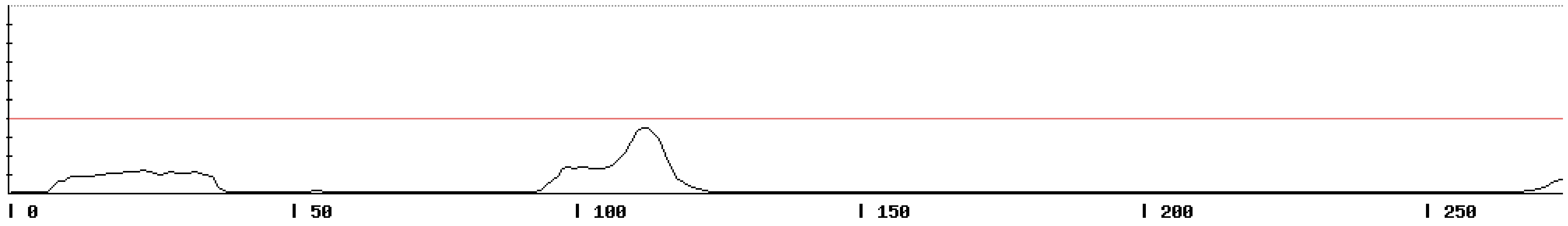

Predictions for Cicerarietinum_XP_012574450.1

Viterbi Path

Viterbi Path

Posterior @ 0.4

Posterior @ 0.4

Posterior Graphic. Threshold Marked @ 0.4

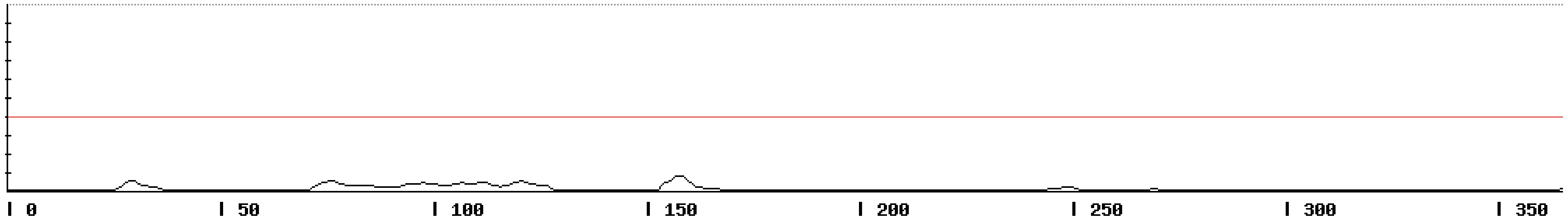

Predictions for Cicerarietinum_XP_012574451.1

Viterbi Path

Viterbi Path

Posterior @ 0.4

Posterior @ 0.4

Posterior Graphic. Threshold Marked @ 0.4

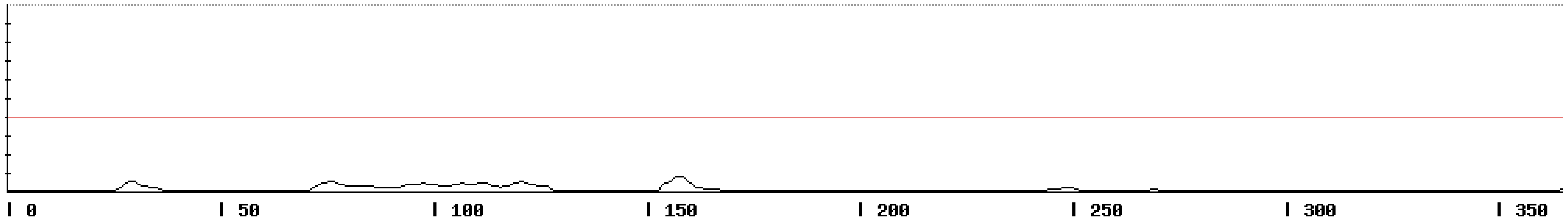

Predictions for Cicerarietinum_XP_012574502.1	
Viterbi Path	
Viterbi Path	
Posterior @ 0.4	
Posterior @ 0.4	
Posterior Graphic. Threshold Marked @ 0.4	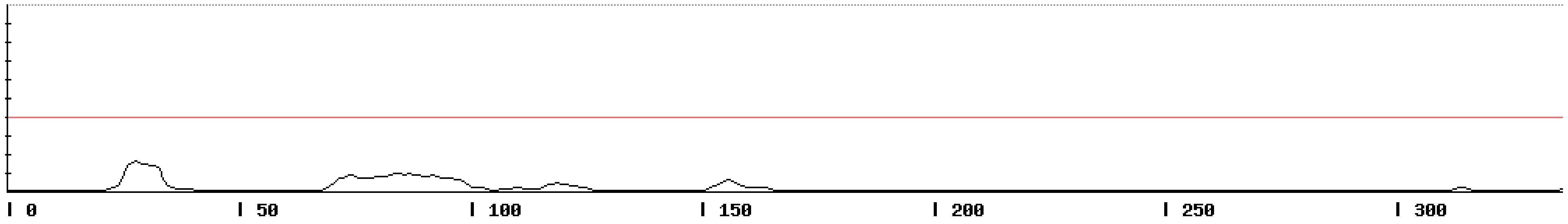

Predictions for Cicerarietinum_XP_012575345.1	
Viterbi Path	
Viterbi Path	
Posterior @ 0.4	67 - KRNRKYPKGERPDR - 80
Posterior @ 0.4	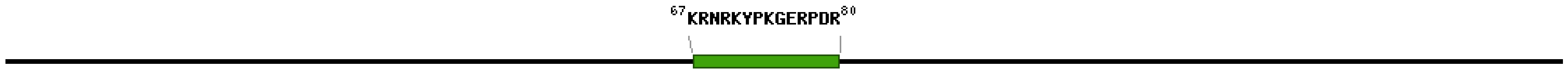

Viterbi Path

Posterior @ 0.4

Posterior @ 0.4

Posterior Graphic. Threshold Marked @ 0.4

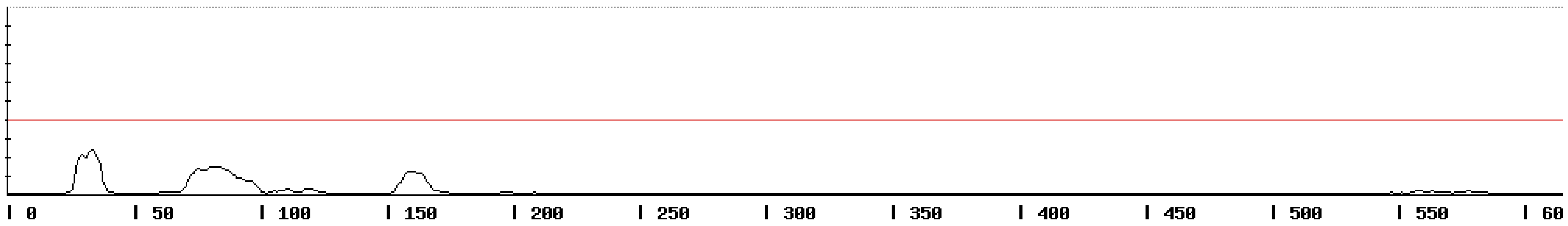

Predictions for Citrusclementina_Ciclev10000870m

Viterbi Path

Viterbi Path

Posterior @ 0.4

Posterior @ 0.4

Posterior Graphic. Threshold Marked @ 0.4

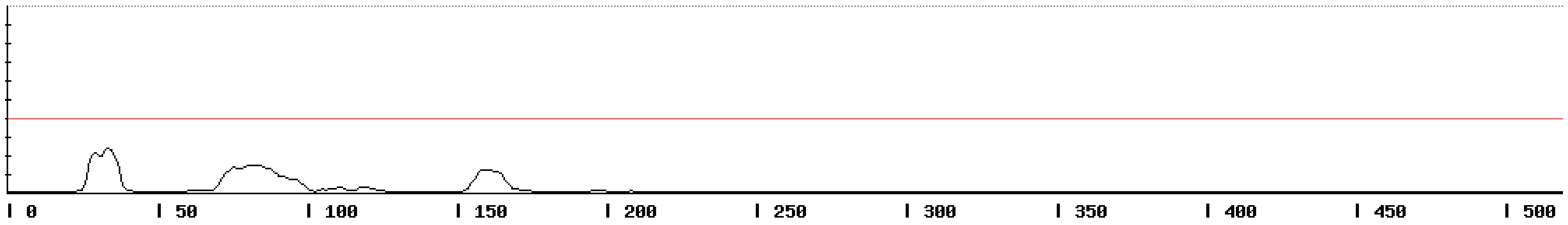

Predictions for Citrusclementina_Ciclev10000936m

Viterbi Path

Viterbi Path

Posterior @ 0.4

Posterior @ 0.4

Posterior Graphic. Threshold Marked @ 0.4

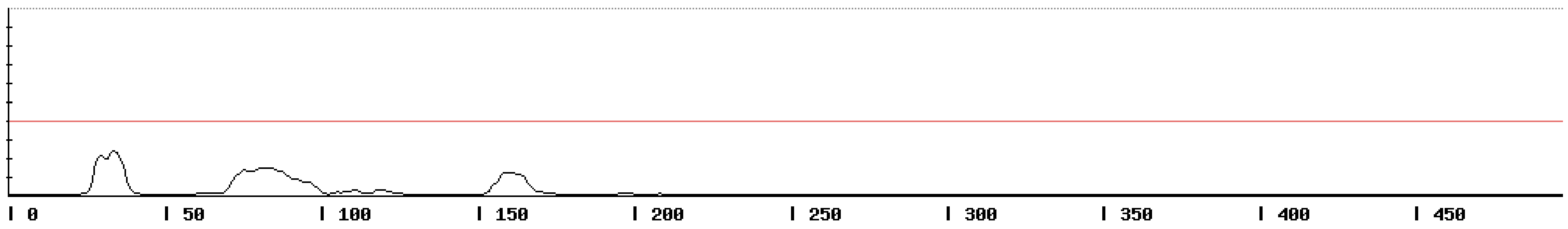

Predictions for Citrusclementina_Ciclev10001199m

Viterbi Path

Viterbi Path

Posterior @ 0.4

Posterior @ 0.4

Posterior Graphic. Threshold Marked @ 0.4

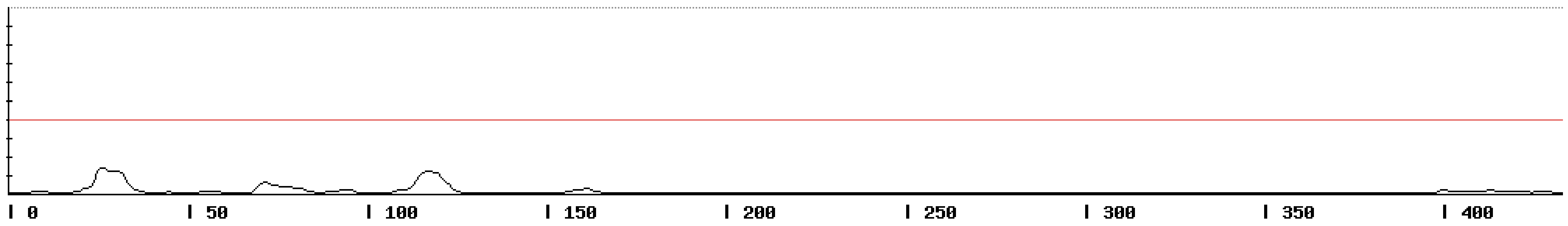

Predictions for Citrusclementina_Ciclev10001200m

Viterbi Path

Viterbi Path

Posterior @ 0.4

Posterior @ 0.4

Posterior Graphic. Threshold Marked @ 0.4

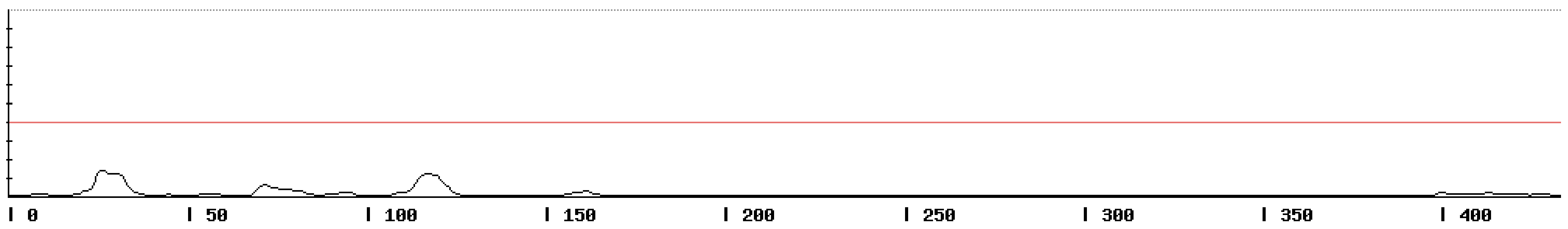

Predictions for Citrusclementina_Ciclev10001201m

Viterbi Path

Viterbi Path

Posterior @ 0.4

Posterior @ 0.4

Posterior Graphic. Threshold Marked @ 0.4

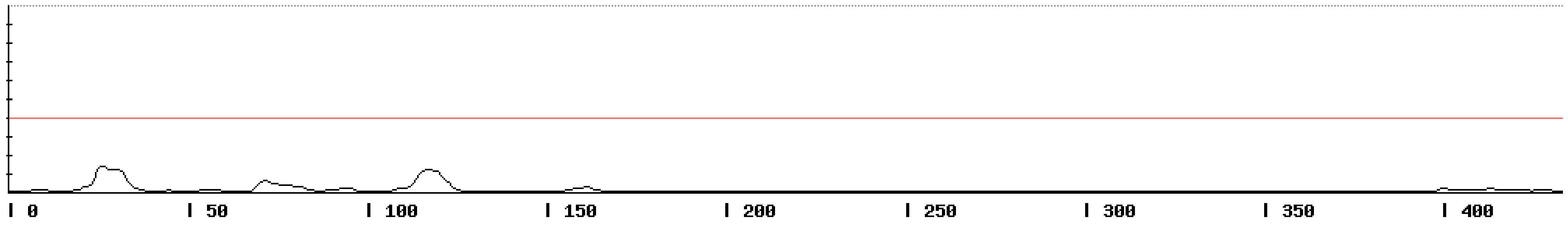

Predictions for Citrusclementina_Ciclev10001204m

Viterbi Path	
Viterbi Path	
Posterior @ 0.4	
Posterior @ 0.4	
Posterior Graphic. Threshold Marked @ 0.4	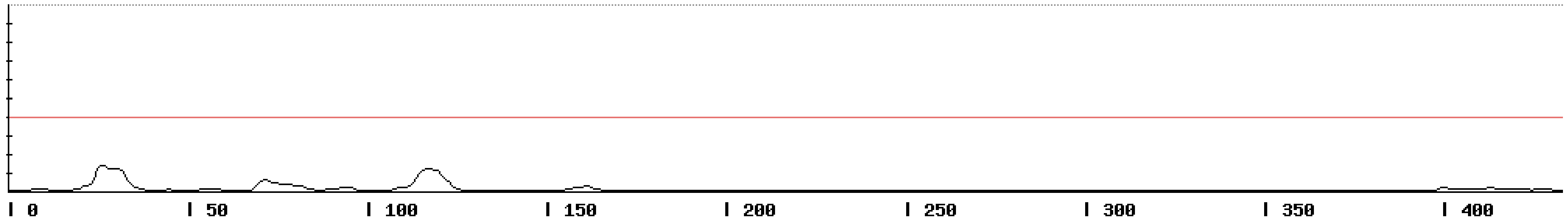

Predictions for Citrusclementina_Ciclev10001205m

Viterbi Path	
Viterbi Path	
Posterior @ 0.4	
Posterior @ 0.4	

Posterior Graphic. Threshold Marked @ 0.4

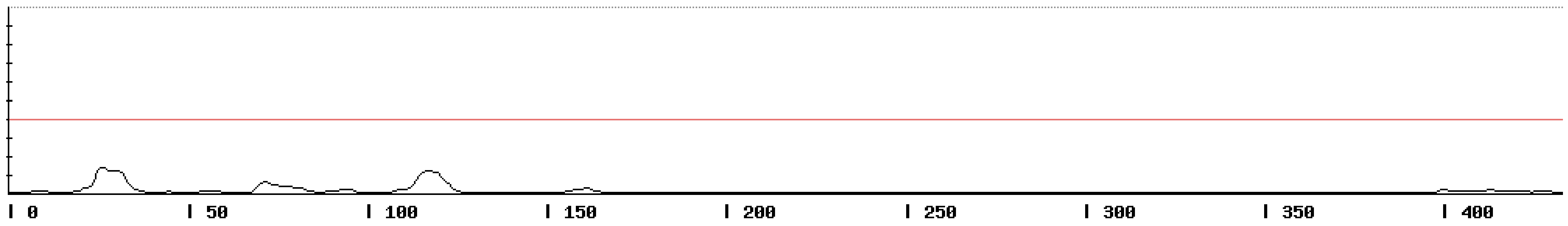

Predictions for Citrusclementina_Ciclev10001206m

Viterbi Path

Viterbi Path

Posterior @ 0.4

Posterior @ 0.4

Posterior Graphic. Threshold Marked @ 0.4

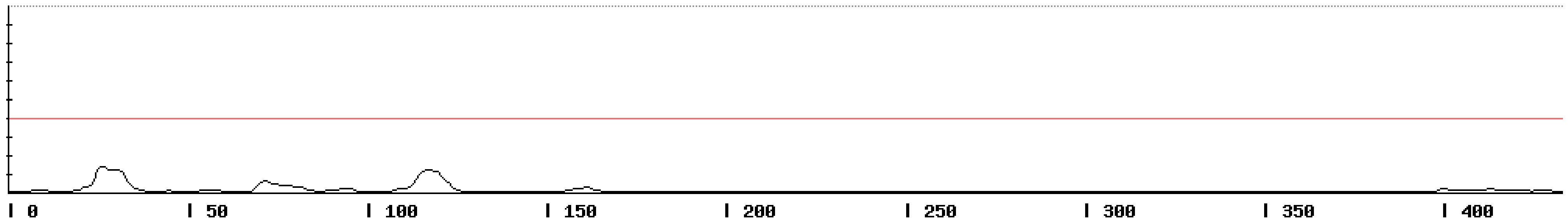

Predictions for Citrusclementina_Ciclev10001210m

Viterbi Path

Viterbi Path

Posterior @ 0.4

Posterior @ 0.4

Posterior Graphic. Threshold Marked @ 0.4

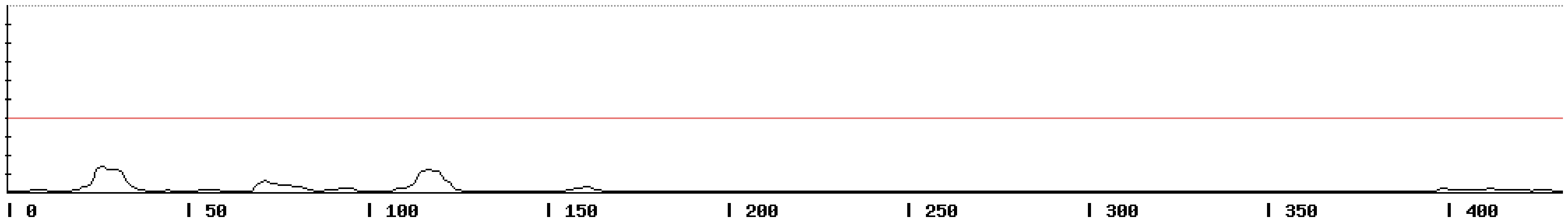

Predictions for Citrusclementina_Ciclev10001346m

Viterbi Path

Viterbi Path

Posterior @ 0.4

Posterior @ 0.4

Posterior Graphic. Threshold Marked @ 0.4

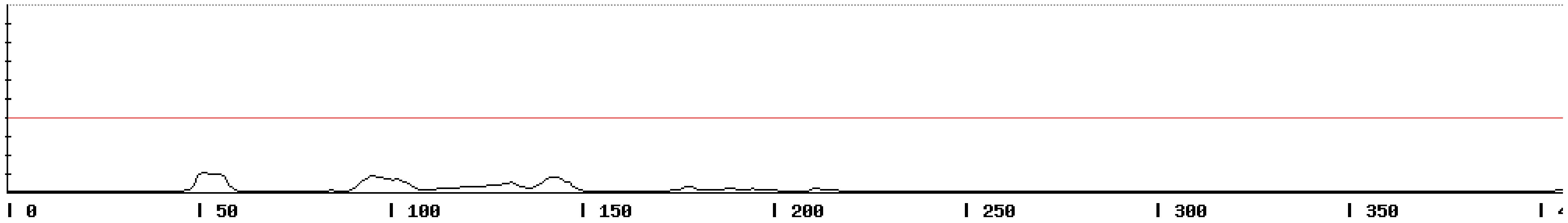

Predictions for Citrusclementina_Ciclev10001389m

Viterbi Path

Viterbi Path

Posterior @ 0.4	376 - GKKAAVGRMKKLKRK - 390
Posterior @ 0.4	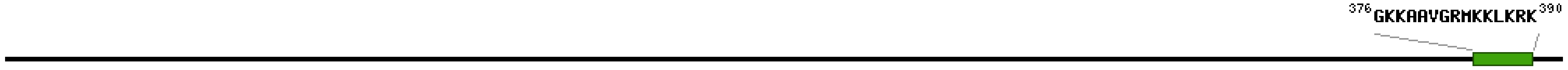
Posterior Graphic. Threshold Marked @ 0.4	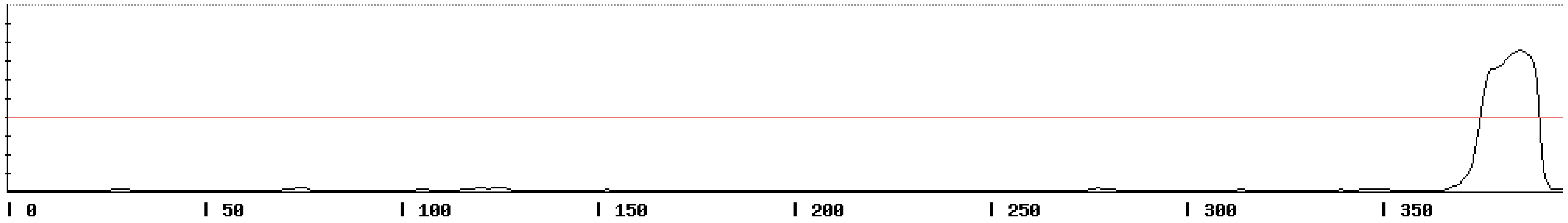

Predictions for Citrusclementina_Ciclev10001401m

Viterbi Path	
Viterbi Path	
Posterior @ 0.4	
Posterior @ 0.4	
Posterior Graphic. Threshold Marked @ 0.4	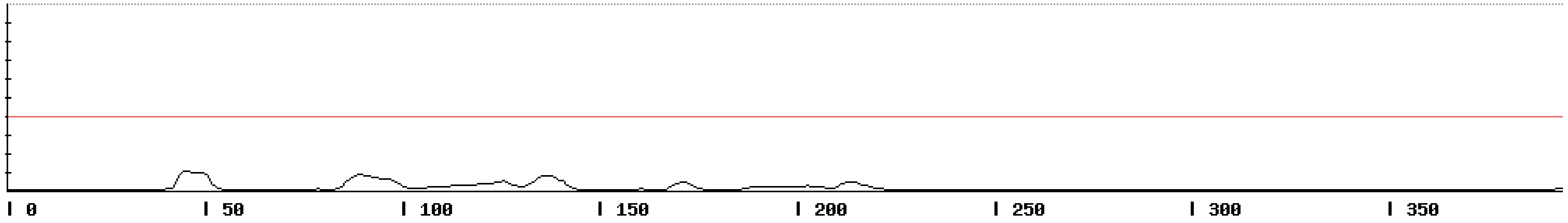

Predictions for Citrusclementina_Ciclev10001402m

Viterbi Path	
Viterbi Path	

Posterior @ 0.4

Posterior @ 0.4

Posterior Graphic. Threshold Marked @ 0.4

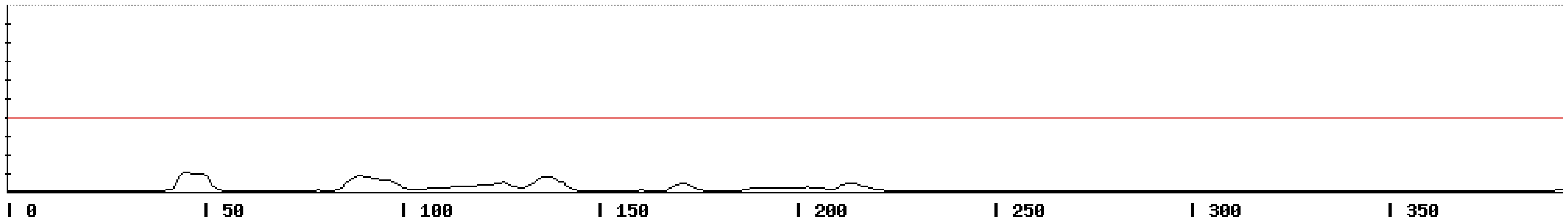

Predictions for Citrusclementina_Ciclev10001403m

Viterbi Path

Viterbi Path

Posterior @ 0.4

Posterior @ 0.4

Posterior Graphic. Threshold Marked @ 0.4

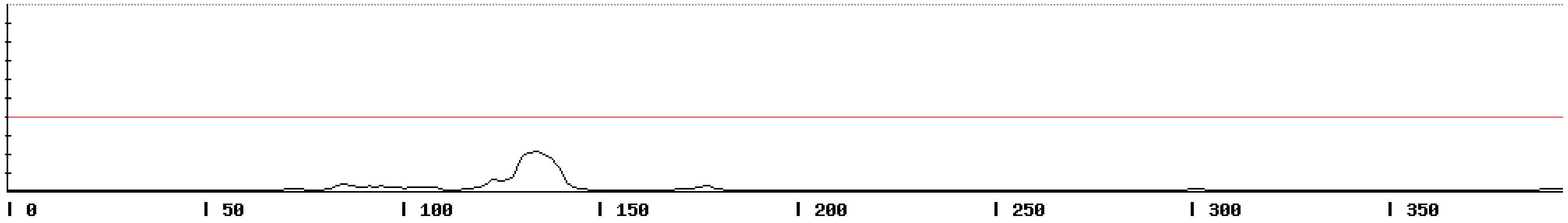

Predictions for Citrusclementina_Ciclev10001433m

Viterbi Path	
Viterbi Path	
Posterior @ 0.4	94 - QKKYKKGSIRERKAKGGFWK - 113
Posterior @ 0.4	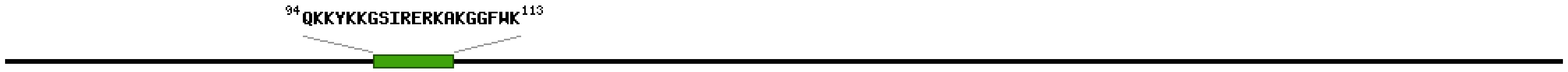
Posterior Graphic. Threshold Marked @ 0.4	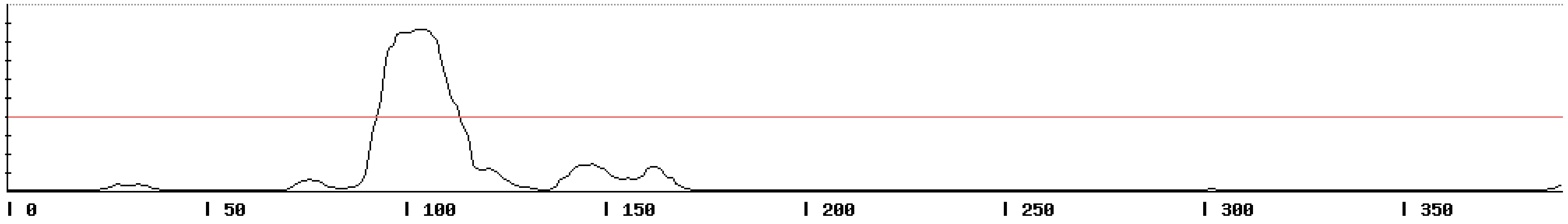

Predictions for Citrusclementina_Ciclev10001471m

Viterbi Path	
Viterbi Path	
Posterior @ 0.4	365 - GKKAAVGRMKKLKRK - 379
Posterior @ 0.4	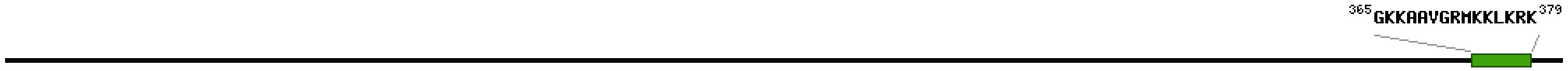
Posterior Graphic. Threshold Marked @ 0.4	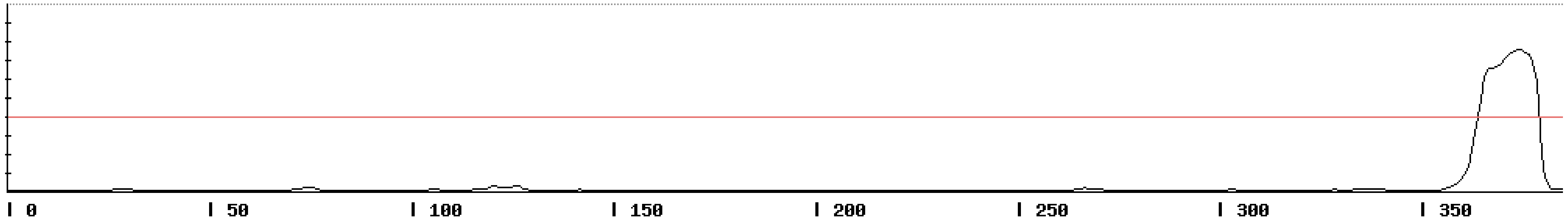

Predictions for Citrusclementina_Ciclev10001473m	
Viterbi Path	
Viterbi Path	
Posterior @ 0.4	365 - GKKAAVGRMKKLKRK - 379
Posterior @ 0.4	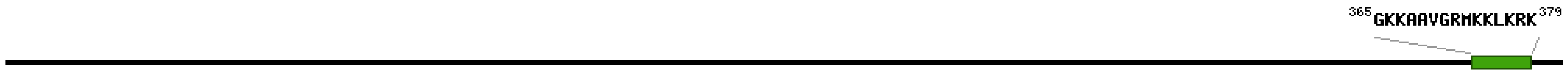
Posterior Graphic. Threshold Marked @ 0.4	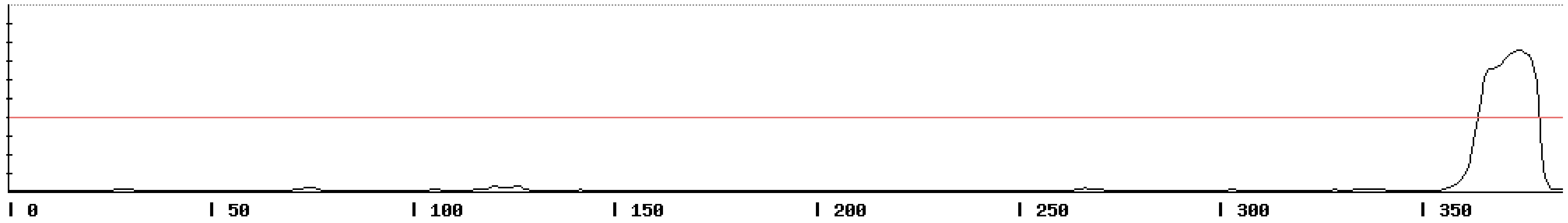

Predictions for Citrusclementina_Ciclev10001956m	
Viterbi Path	
Viterbi Path	
Posterior @ 0.4	97 - KPIGKPKTLGIK - 108
Posterior @ 0.4	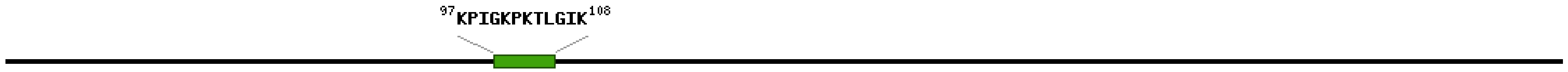
Posterior Graphic. Threshold Marked @ 0.4	

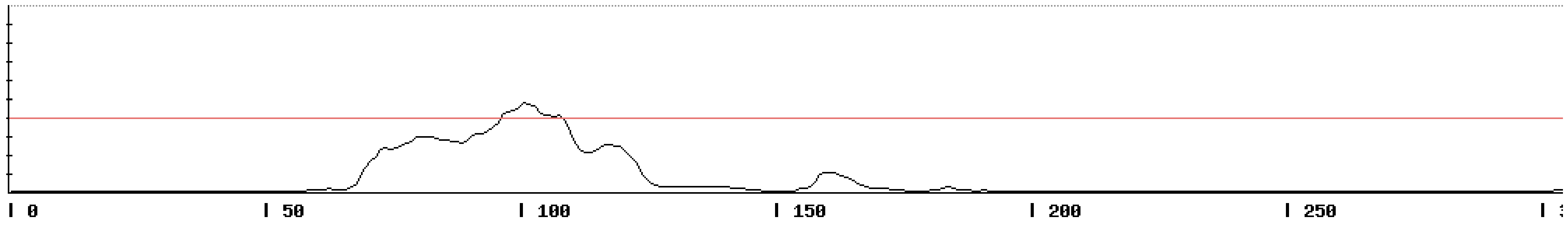

Predictions for Citrusclementina_Ciclev10001976m	
Viterbi Path	
Viterbi Path	
Posterior @ 0.4	
Posterior @ 0.4	
Posterior Graphic. Threshold Marked @ 0.4	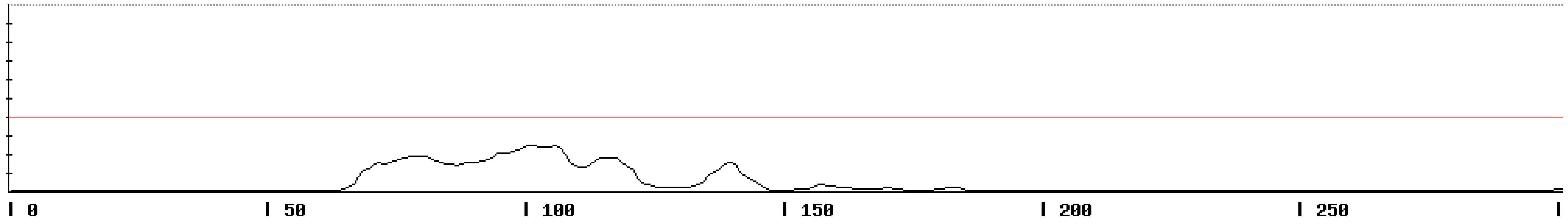

Predictions for Citrusclementina_Ciclev10002070m	
Viterbi Path	
Viterbi Path	
Posterior @ 0.4	
Posterior @ 0.4	

Posterior Graphic. Threshold Marked @ 0.4	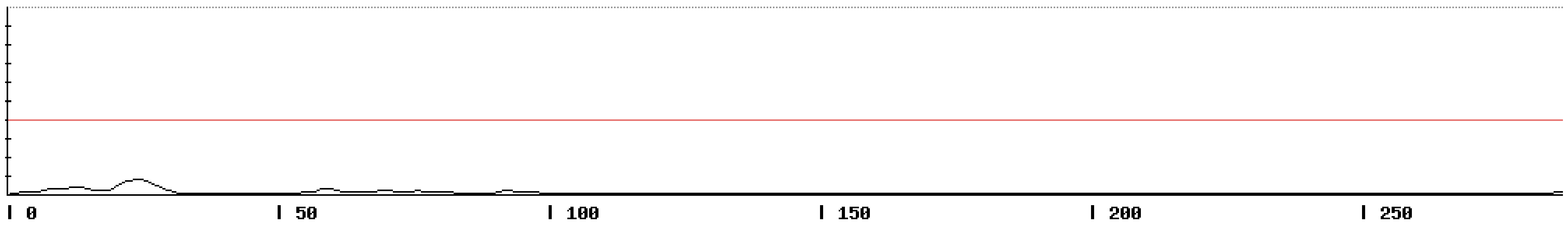

Predictions for Citrusclementina_Ciclev10002094m

Viterbi Path	
Viterbi Path	
Posterior @ 0.4	
Posterior @ 0.4	
Posterior Graphic. Threshold Marked @ 0.4	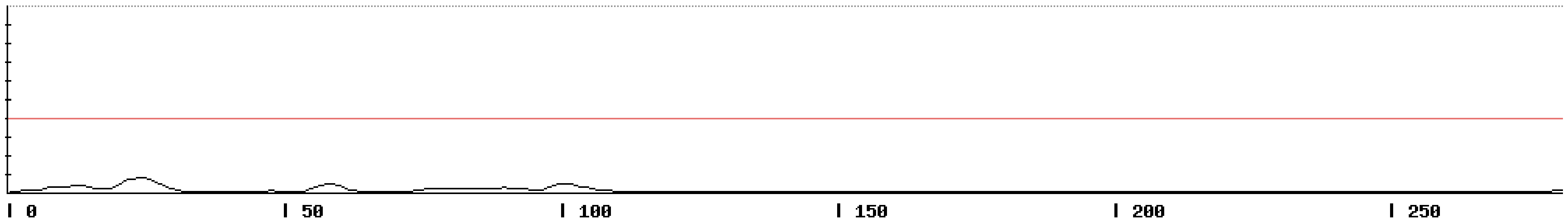

Predictions for Citrusclementina_Ciclev10002273m

Viterbi Path	
Viterbi Path	
Posterior @ 0.4	41 - KPIGKPKTLGIK - 52
Posterior @ 0.4	

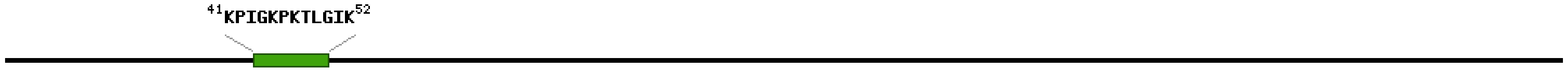

Posterior Graphic. Threshold Marked @ 0.4

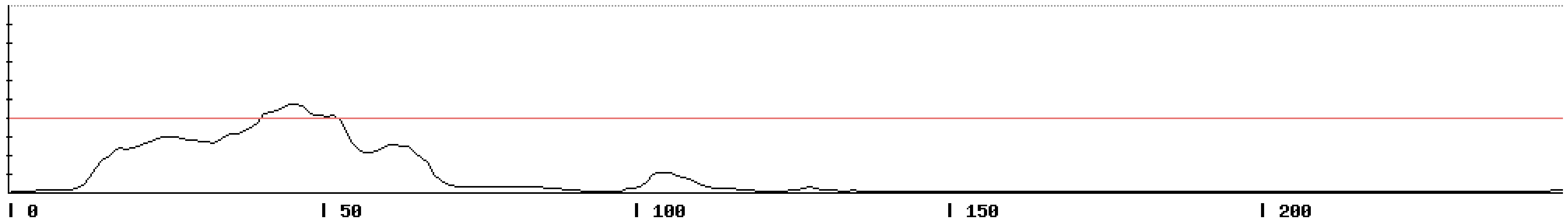

Predictions for Citrusclementina_Ciclev10002277m

Viterbi Path

Viterbi Path

Posterior @ 0.4

41 - KPIGKPKTLGIK - 52

Posterior @ 0.4

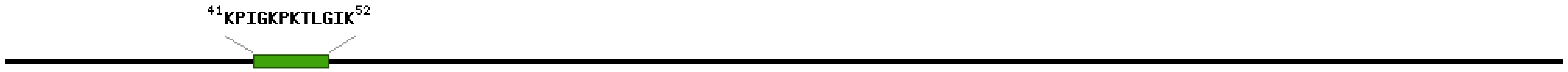

Posterior Graphic. Threshold Marked @ 0.4

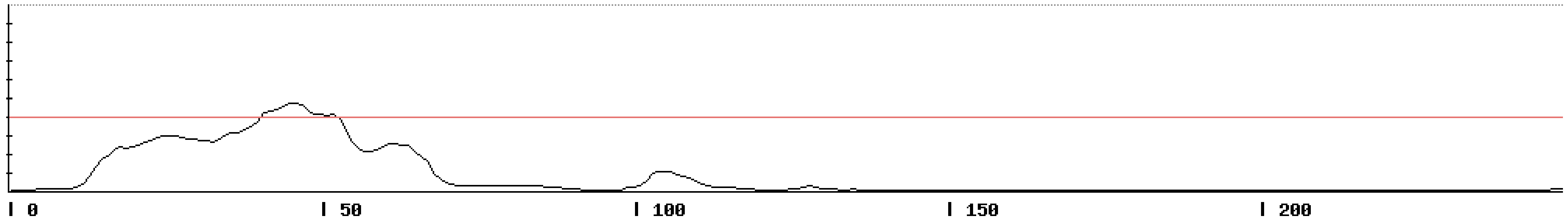

Predictions for Citrusclementina_Ciclev10002538m

Viterbi Path

Viterbi Path

Posterior @ 0.4

Posterior @ 0.4

Posterior Graphic. Threshold Marked @ 0.4

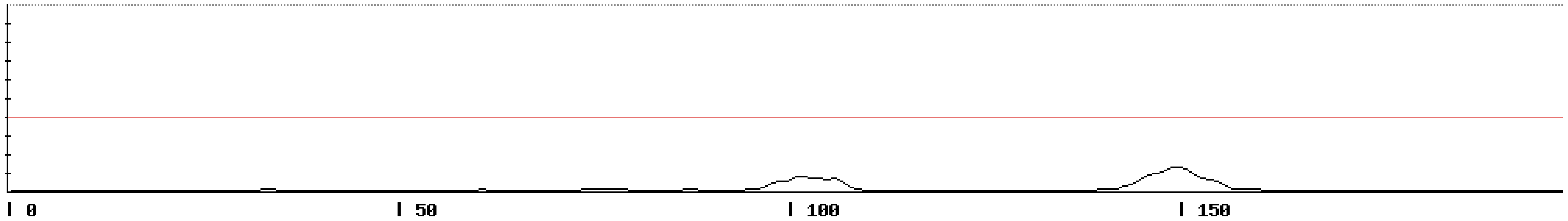

Predictions for Citrusclementina_Ciclev10003255m

Viterbi Path

Viterbi Path

Posterior @ 0.4

Posterior @ 0.4

Posterior Graphic. Threshold Marked @ 0.4

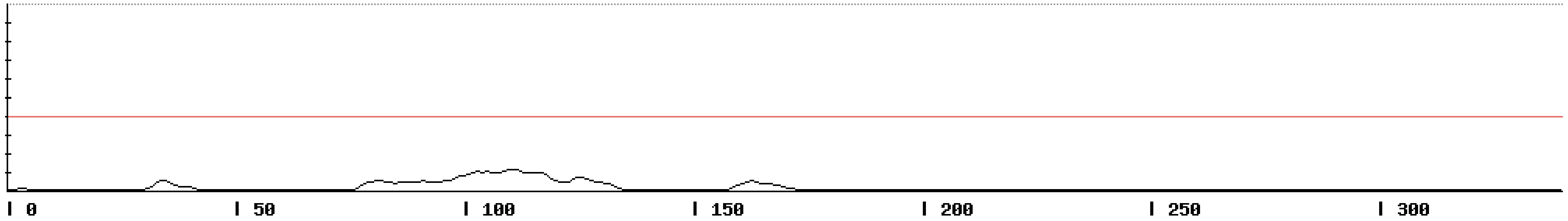

Predictions for Citrusclementina_Ciclev10003273m

Viterbi Path

Viterbi Path

Posterior @ 0.4	73 - KKAGKKRISRVAGKGTWHGESSAKEIVDSKKKNVVIGFKKTFKFRIKGKK - 122
	146 - IKKKKPKA - 153
Posterior @ 0.4	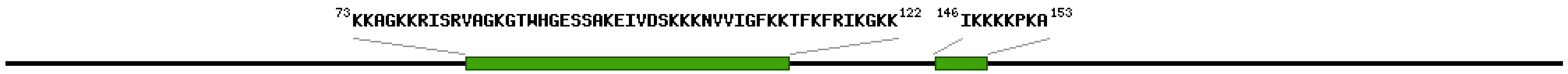
Posterior Graphic. Threshold Marked @ 0.4	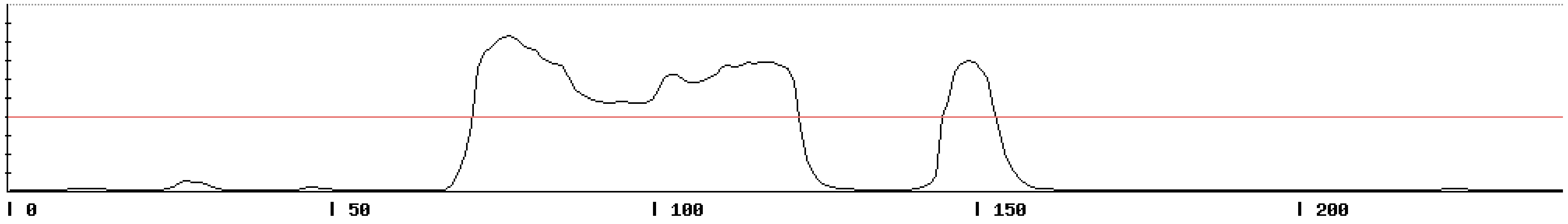

Predictions for Citrusclementina_Ciclev10003379m

Viterbi Path	
Viterbi Path	
Posterior @ 0.4	
Posterior @ 0.4	
Posterior Graphic. Threshold Marked @ 0.4	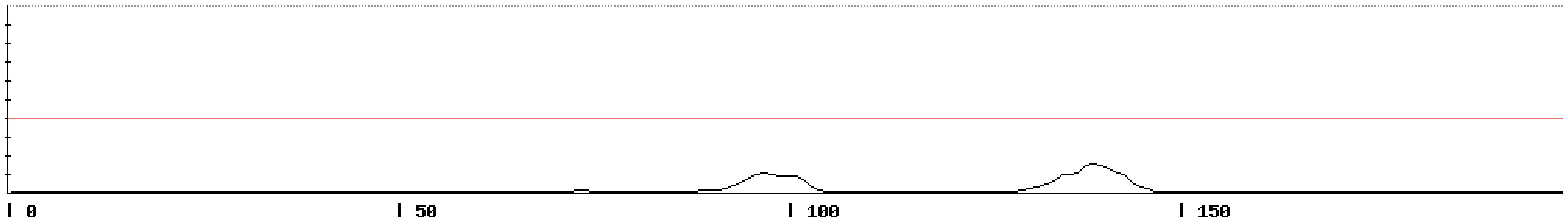

Predictions for Citrusclementina_Ciclev10003814m

Viterbi Path	
Viterbi Path	
Posterior @ 0.4	18 - K - 18
	121 - RKGKNGYWRRR - 131
Posterior @ 0.4	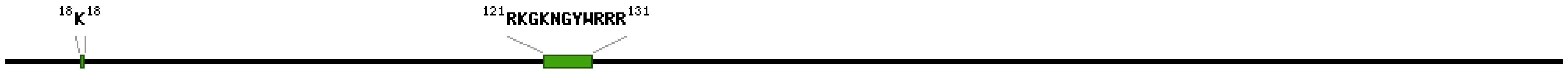
Posterior Graphic. Threshold Marked @ 0.4	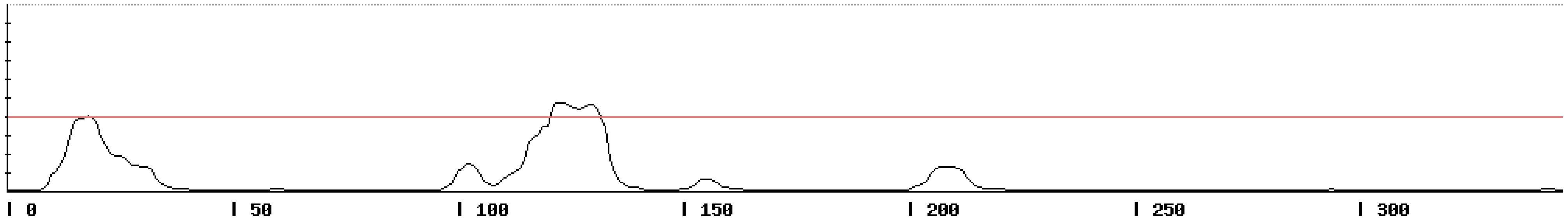

Predictions for Citrusclementina_Ciclev10005260m

Viterbi Path	
Viterbi Path	
Posterior @ 0.4	
Posterior @ 0.4	
Posterior Graphic. Threshold Marked @ 0.4	

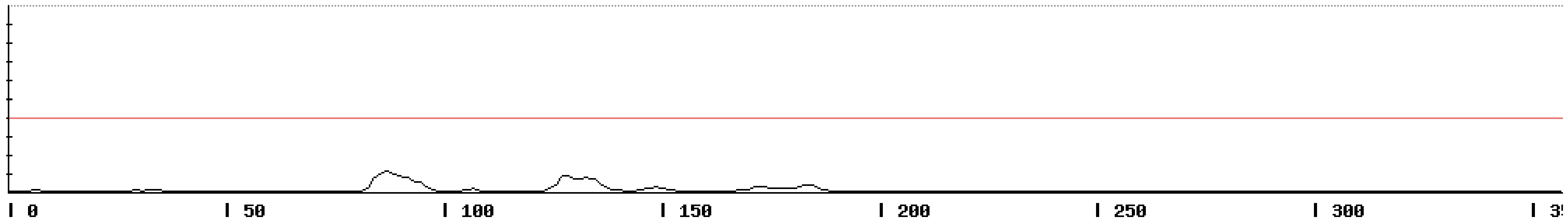

Predictions for Citrusclementina_Ciclev10005341m

Viterbi Path

Viterbi Path

Posterior @ 0.4

Posterior @ 0.4

Posterior Graphic. Threshold Marked @ 0.4

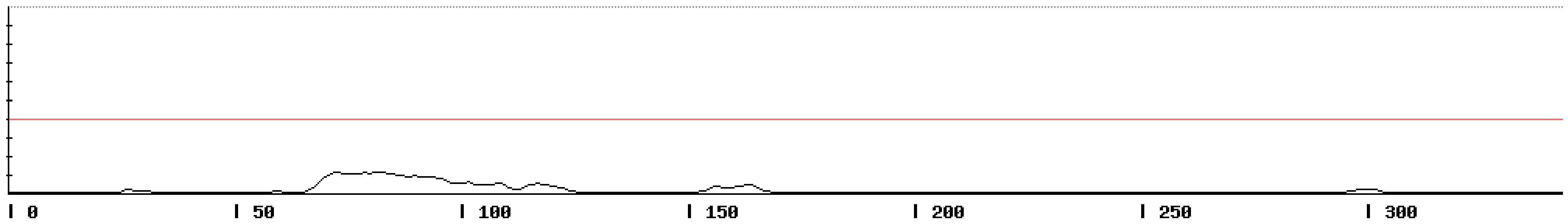

Predictions for Citrusclementina_Ciclev10005465m

Viterbi Path

Viterbi Path

Posterior @ 0.4

Posterior @ 0.4

Posterior Graphic. Threshold Marked @ 0.4	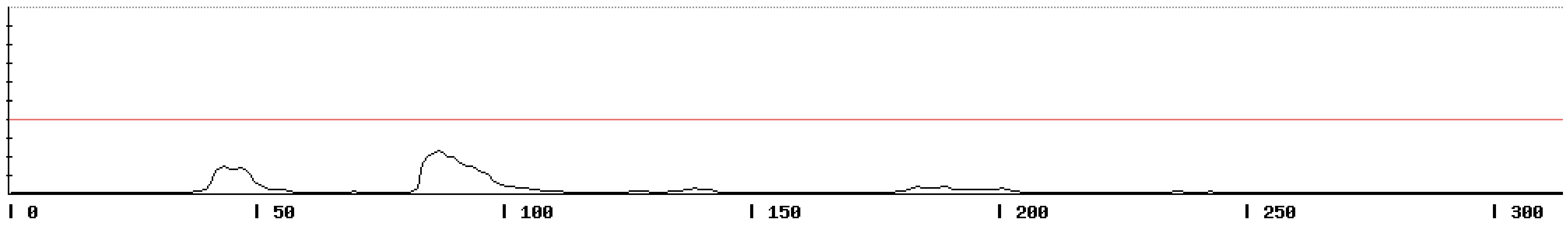

Predictions for Citrusclementina_Ciclev10006623m

Viterbi Path	
Viterbi Path	
Posterior @ 0.4	
Posterior @ 0.4	
Posterior Graphic. Threshold Marked @ 0.4	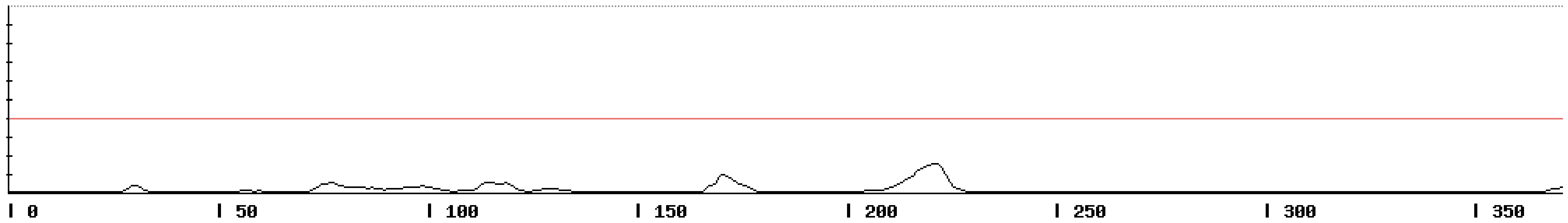

Predictions for Citrusclementina_Ciclev10006625m

Viterbi Path	
Viterbi Path	
Posterior @ 0.4	38 - RRR - 40
Posterior @ 0.4	

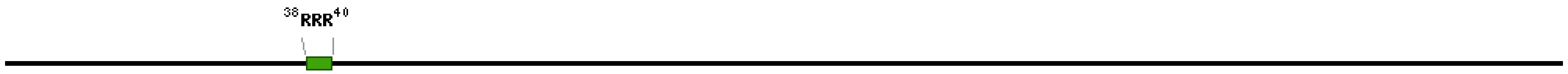

Posterior Graphic. Threshold Marked @ 0.4

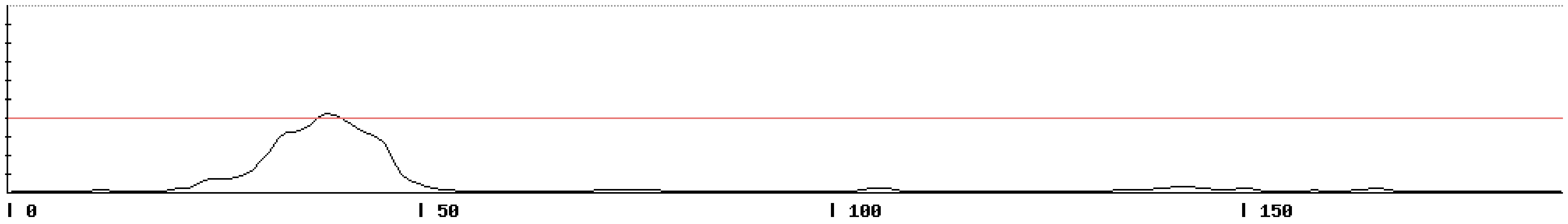

Predictions for Citrusclementina_Ciclev10006695m

Viterbi Path

Viterbi Path

Posterior @ 0.4

Posterior @ 0.4

Posterior Graphic. Threshold Marked @ 0.4

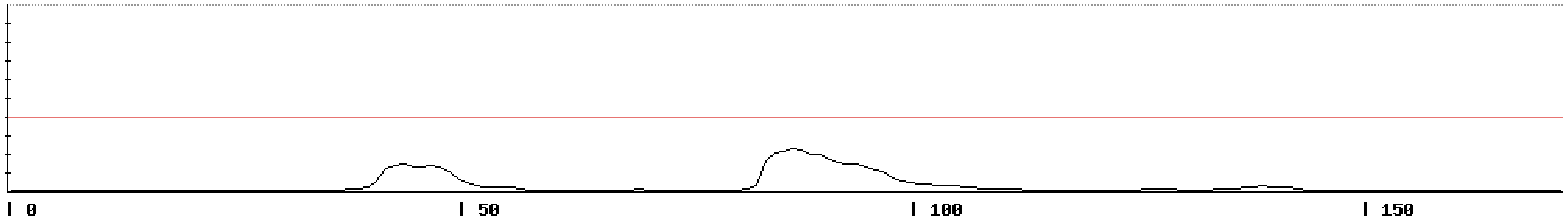

Predictions for Citrusclementina_Ciclev10006869m

Viterbi Path

Viterbi Path

Posterior @ 0.4

Posterior @ 0.4

Posterior Graphic. Threshold Marked @ 0.4

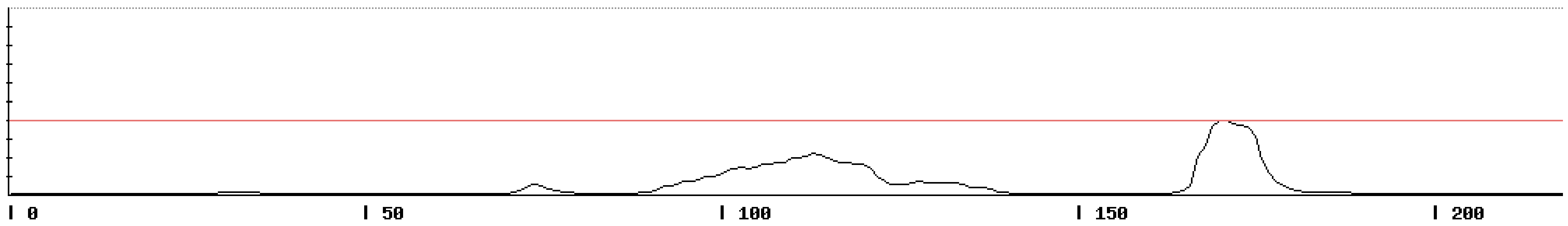

Predictions for Citrusclementina_Ciclev10006985m

Viterbi Path

Viterbi Path

Posterior @ 0.4

Posterior @ 0.4

Posterior Graphic. Threshold Marked @ 0.4

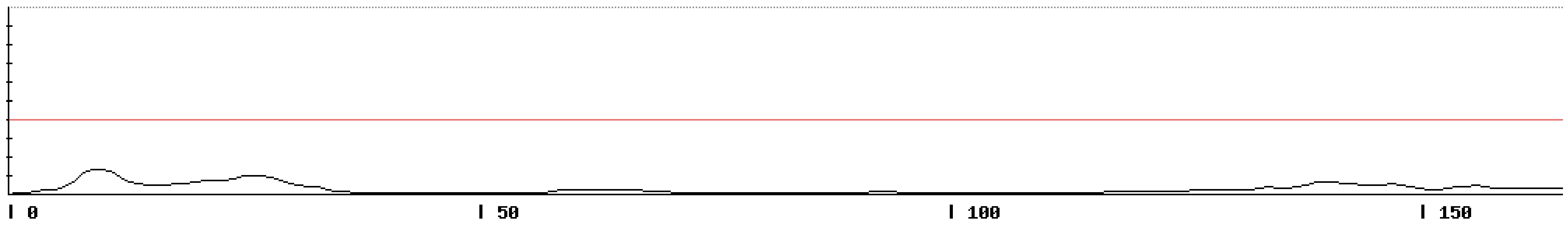

Predictions for Citrusclementina_Ciclev10006988m

Viterbi Path

Viterbi Path

Posterior @ 0.4

Posterior @ 0.4

Posterior Graphic. Threshold Marked @ 0.4

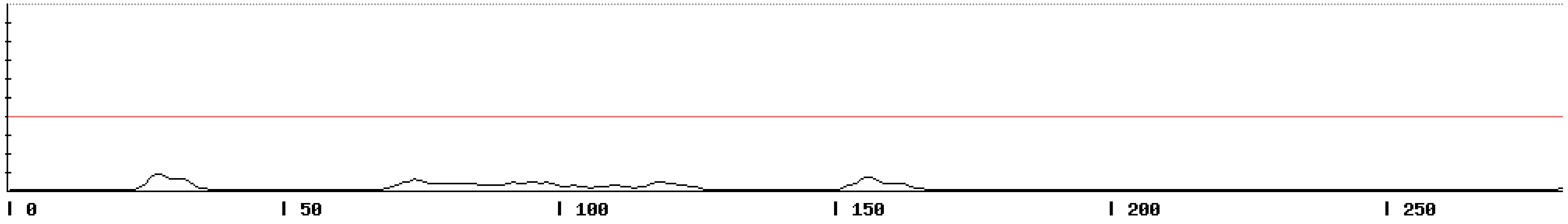

Predictions for Citrusclementina_Ciclev10007172m

Viterbi Path

Viterbi Path

Posterior @ 0.4

Posterior @ 0.4

Posterior Graphic. Threshold Marked @ 0.4

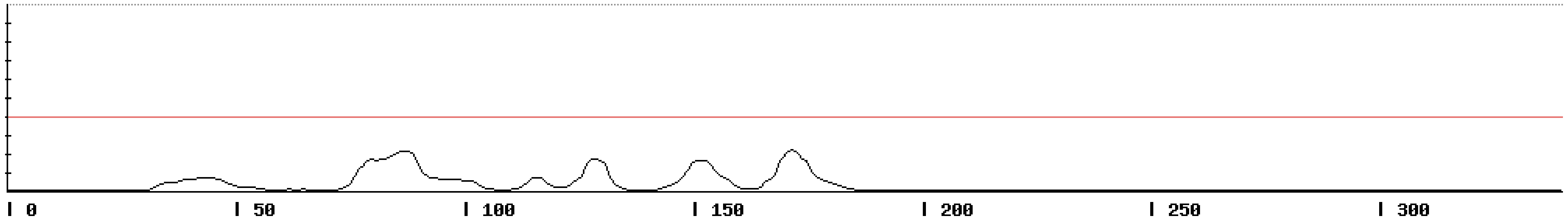

Predictions for Citrusclementina_Ciclev10008619m

Viterbi Path

Viterbi Path

Posterior @ 0.4

Posterior @ 0.4

Posterior Graphic. Threshold Marked @ 0.4

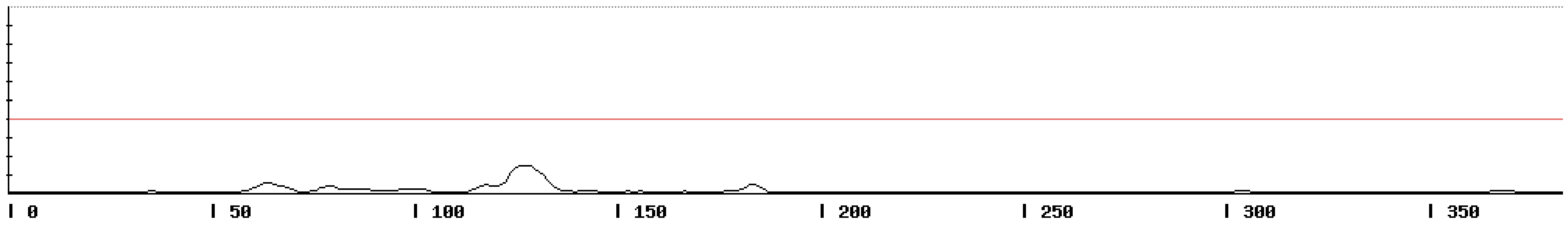

Predictions for Citrusclementina_Ciclev10008628m

Viterbi Path

Viterbi Path

Posterior @ 0.4

Posterior @ 0.4

Posterior Graphic. Threshold Marked @ 0.4

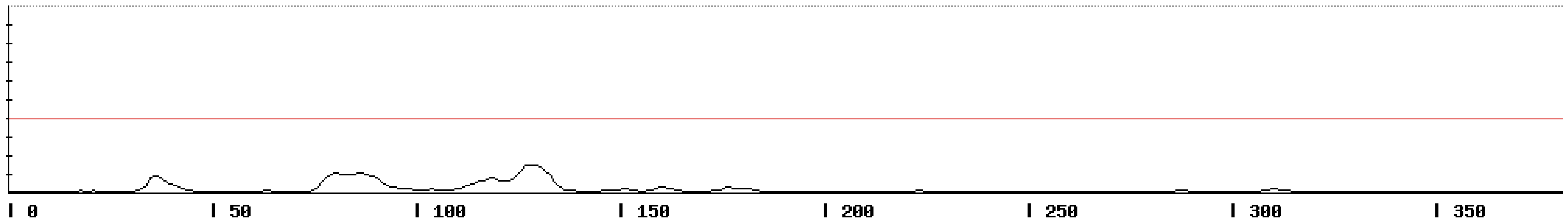

Predictions for Citrusclementina_Ciclev10008812m

Viterbi Path

Viterbi Path

Posterior @ 0.4

Posterior @ 0.4

Posterior Graphic. Threshold Marked @ 0.4

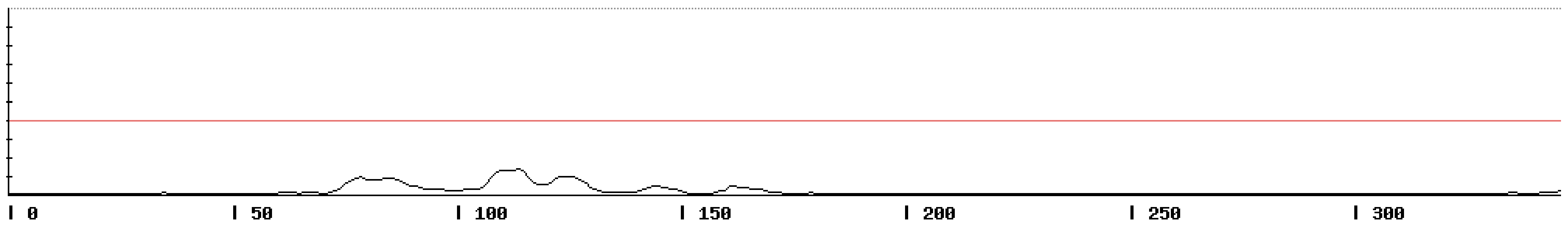

Predictions for Citrusclementina_Ciclev10008885m

Viterbi Path

Viterbi Path

Posterior @ 0.4

Posterior @ 0.4

Posterior Graphic. Threshold Marked @ 0.4

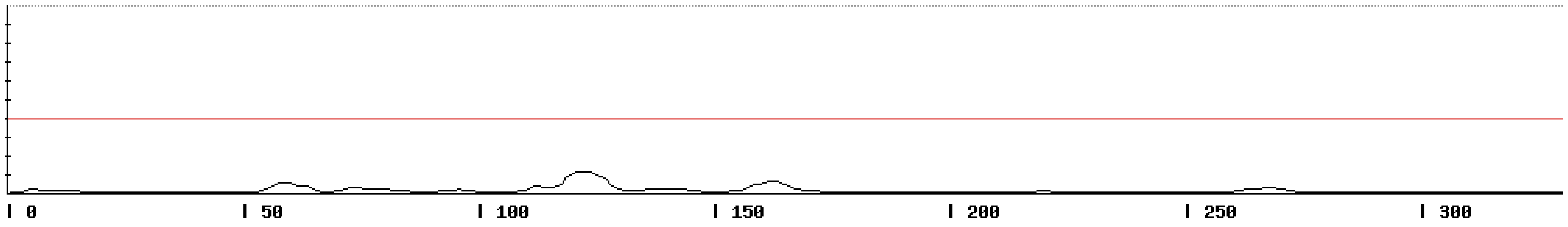

Predictions for Citrusclementina_Ciclev10010221m

Viterbi Path

Viterbi Path

Posterior @ 0.4 — 29 - KGKR - 32

Posterior @ 0.4

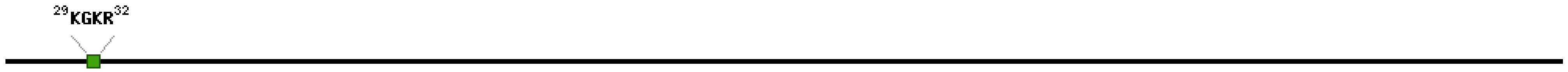

Posterior Graphic. Threshold Marked @ 0.4

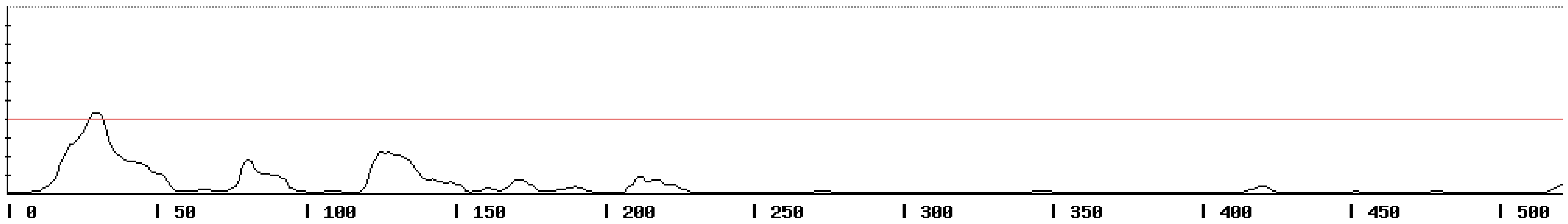

Predictions for Citrusclementina_Ciclev10010524m

Viterbi Path

Viterbi Path

Posterior @ 0.4

Posterior @ 0.4

Posterior Graphic. Threshold Marked @ 0.4

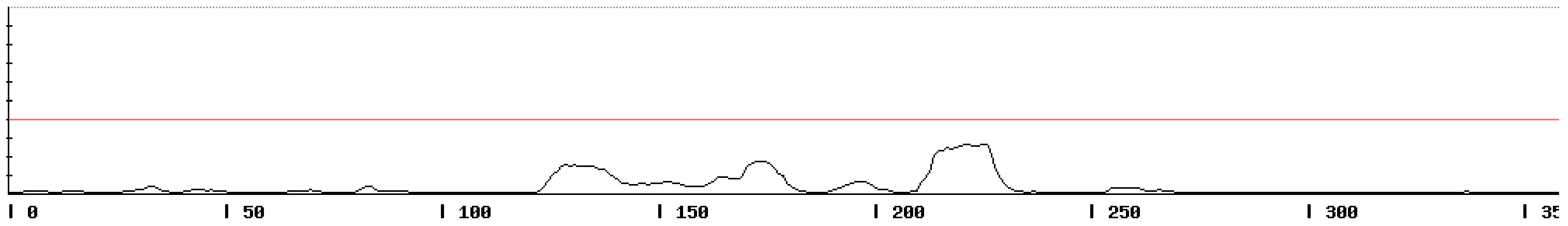

**Predictions for Citrusclementina_Ciclev10010545m**	
Viterbi Path	
Viterbi Path	
Posterior @ 0.4	
Posterior @ 0.4	
Posterior Graphic. Threshold Marked @ 0.4	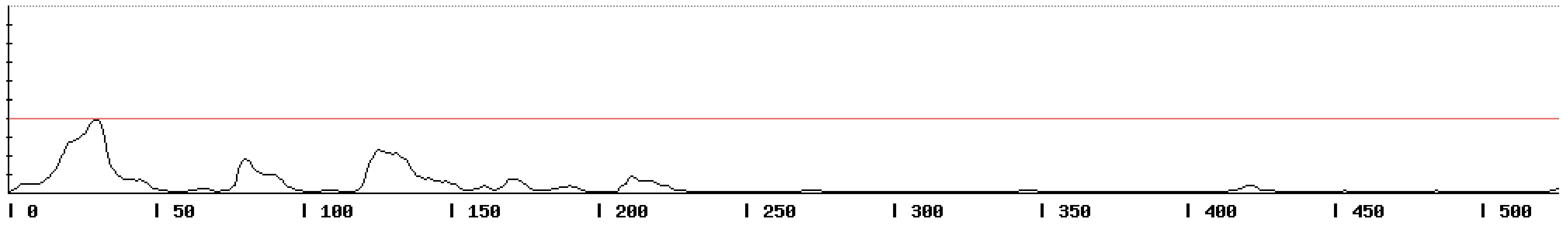

**Predictions for Citrusclementina_Ciclev10010568m**	
Viterbi Path	
Viterbi Path	
Posterior @ 0.4	68 - KRKWNKKKKS - 77
Posterior @ 0.4	

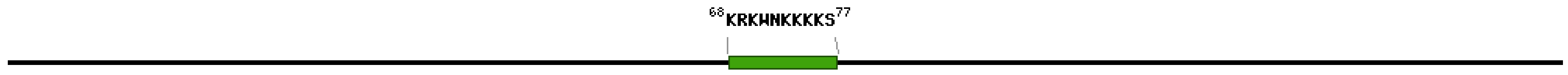

Posterior Graphic. Threshold Marked @ 0.4

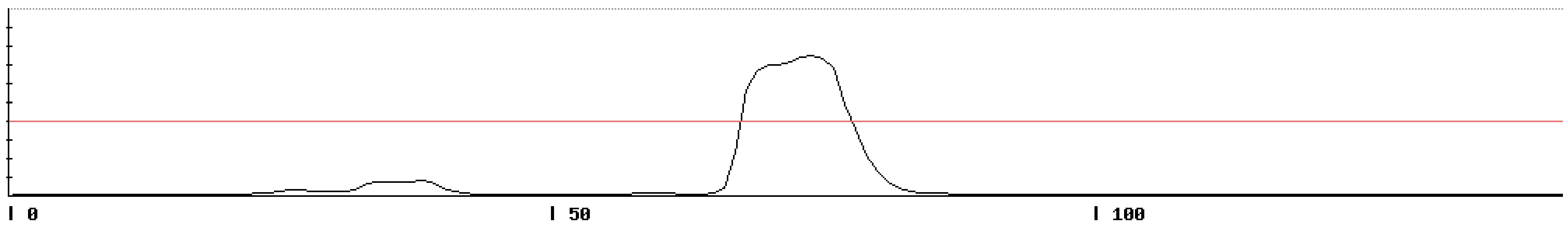

Predictions for Citrusclementina_Ciclev10010579m

Viterbi Path

Viterbi Path

Posterior @ 0.4

Posterior @ 0.4

Posterior Graphic. Threshold Marked @ 0.4

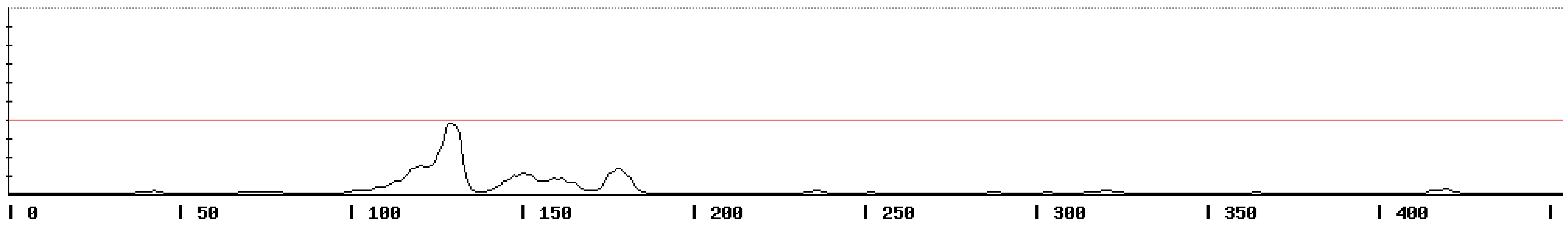

Predictions for Citrusclementina_Ciclev10010632m

Viterbi Path

Viterbi Path

Posterior @ 0.4

Posterior @ 0.4

Posterior Graphic. Threshold Marked @ 0.4

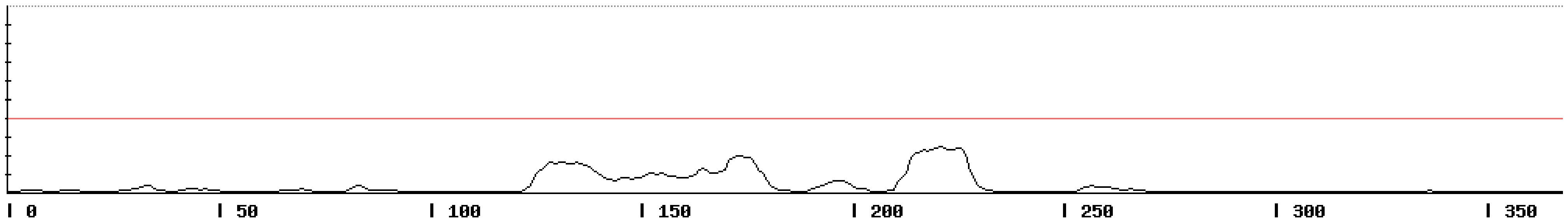

Predictions for Citrusclementina_Ciclev10011293m

Viterbi Path

Viterbi Path

Posterior @ 0.4

Posterior @ 0.4

Posterior Graphic. Threshold Marked @ 0.4

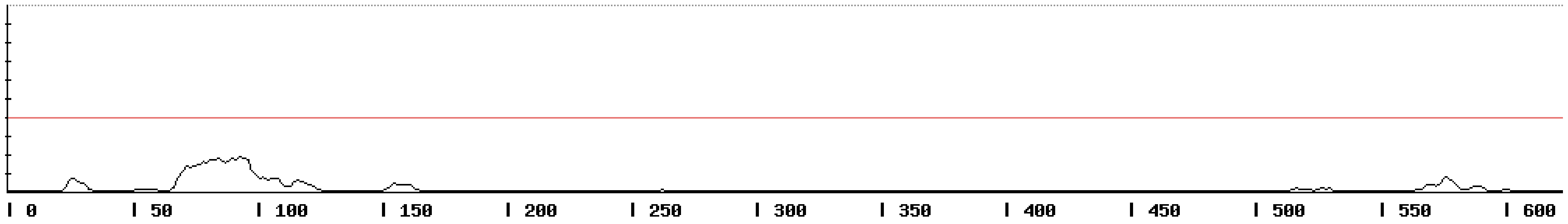

Predictions for Citrusclementina_Ciclev10011891m

Viterbi Path

Viterbi Path

Posterior @ 0.4

Posterior @ 0.4

Posterior Graphic. Threshold Marked @ 0.4

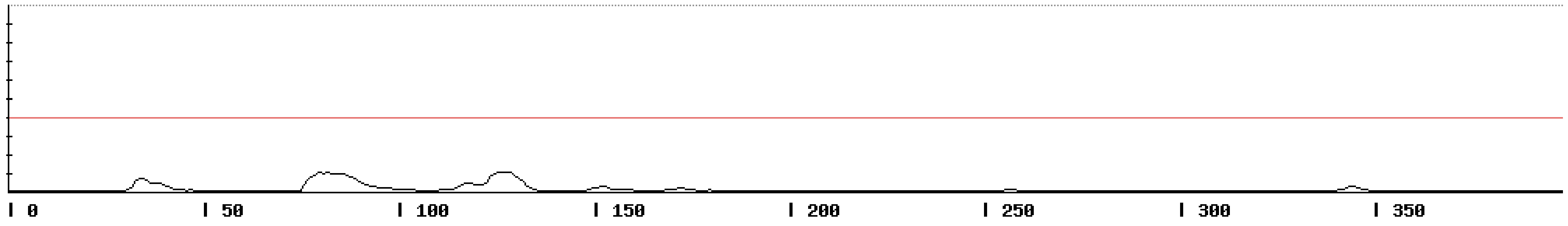

Predictions for Citrusclementina_Ciclev10012093m

Viterbi Path

Viterbi Path

Posterior @ 0.4

Posterior @ 0.4

Posterior Graphic. Threshold Marked @ 0.4

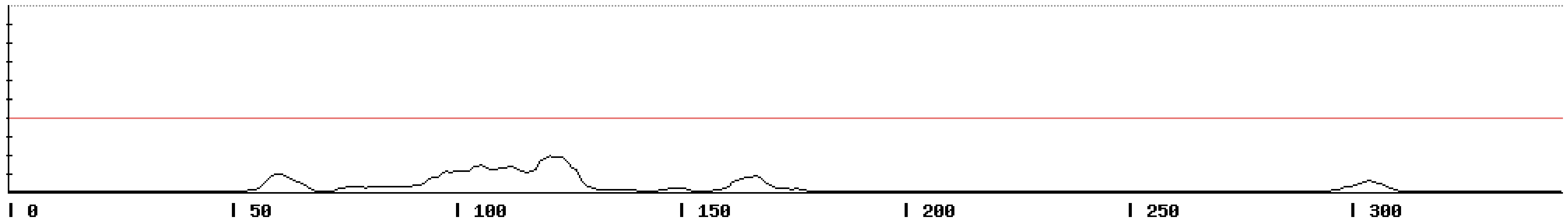

Predictions for Citrusclementina_Ciclev10012468m

Viterbi Path	
Viterbi Path	
Posterior @ 0.4	
Posterior @ 0.4	
Posterior Graphic. Threshold Marked @ 0.4	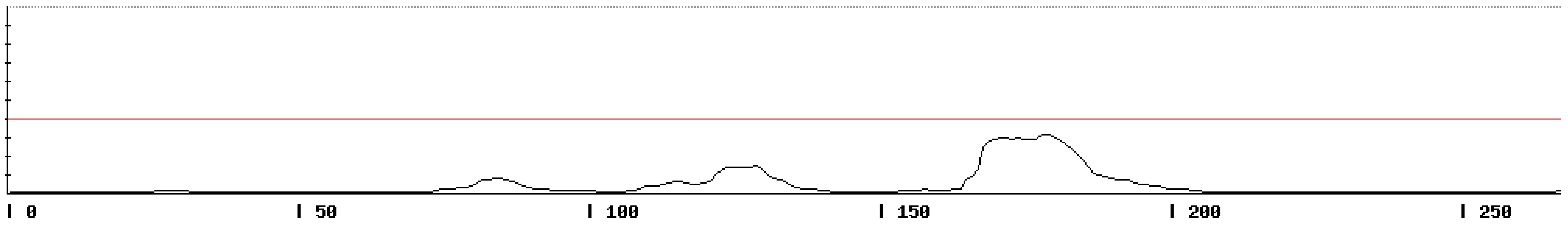

Predictions for Citrusclementina_Ciclev10013622m	
Viterbi Path	
Viterbi Path	
Posterior @ 0.4	104 - SRNSRKKIGSKKRFRYEKDK - 123
Posterior @ 0.4	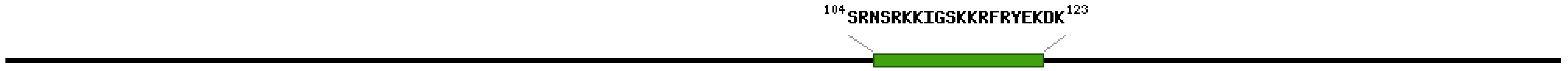
Posterior Graphic. Threshold Marked @ 0.4	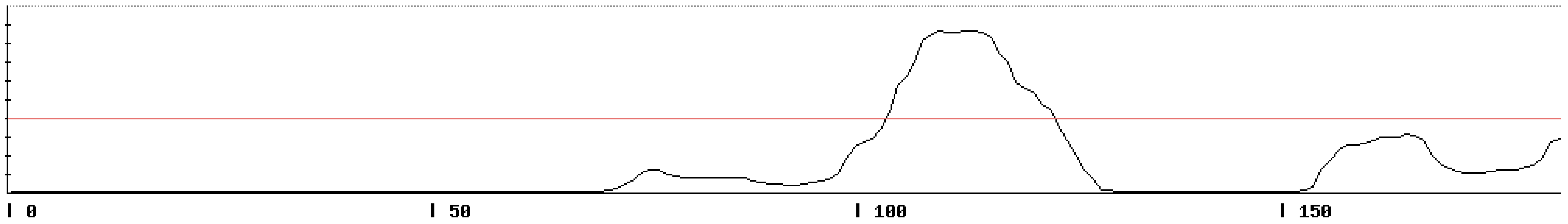

Predictions for Citrusclementina_Ciclev10013626m	
Viterbi Path	
Viterbi Path	
Posterior @ 0.4	163 - KNGGRSVGIKDSRKKIK - 179
Posterior @ 0.4	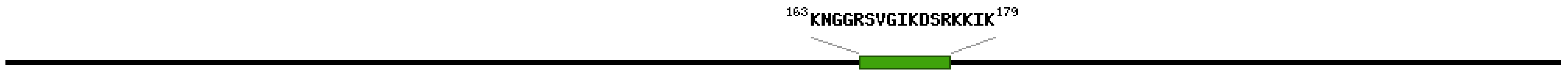
Posterior Graphic. Threshold Marked @ 0.4	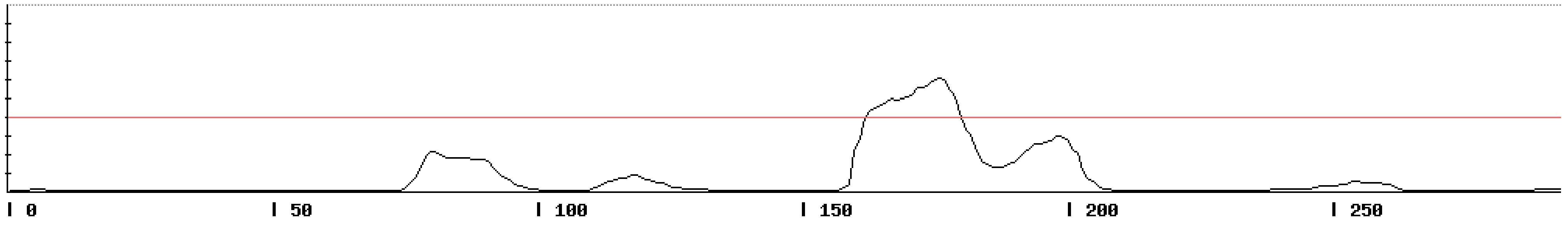

Predictions for Citrusclementina_Ciclev10013691m	
Viterbi Path	108 - RKSKKKIGSKKRFR - 121
Viterbi Path	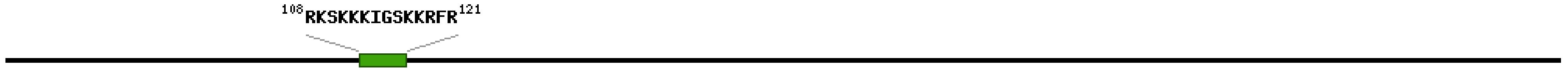
Posterior @ 0.4	103 - KAVVSRKSKKKIGSKKRFRYEKDK - 126<br>161 - KNGEPAAGGQKNSRKRRK - 178
Posterior @ 0.4	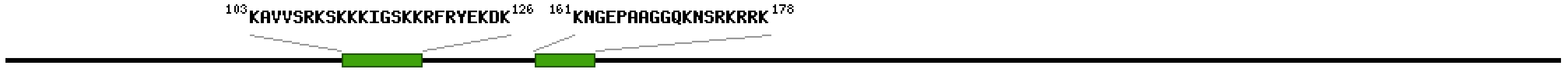
Posterior Graphic. Threshold Marked @ 0.4	

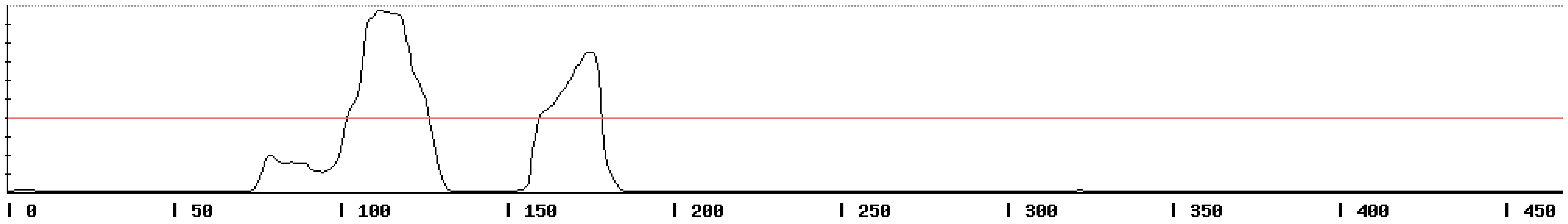

Predictions for Citrusclementina_Ciclev10013694m

Viterbi Path

Viterbi Path

Posterior @ 0.4

Posterior @ 0.4

Posterior Graphic. Threshold Marked @ 0.4

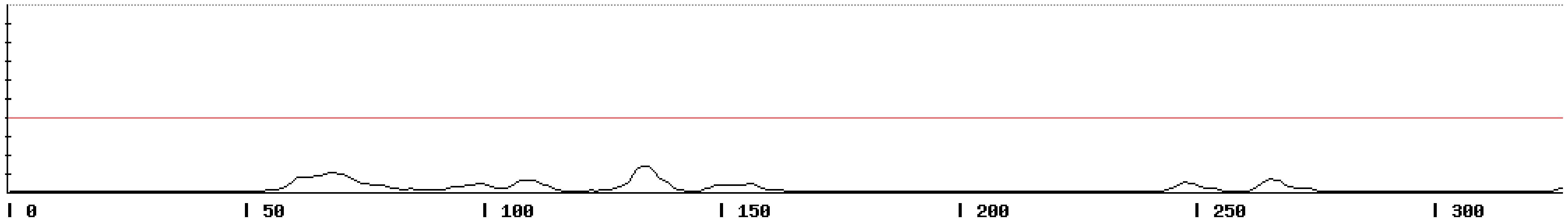

Predictions for Citrusclementina_Ciclev10013730m

Viterbi Path

Viterbi Path

Posterior @ 0.4

163 - KNGGRSVGIKDSRKKIK - 179

Posterior @ 0.4

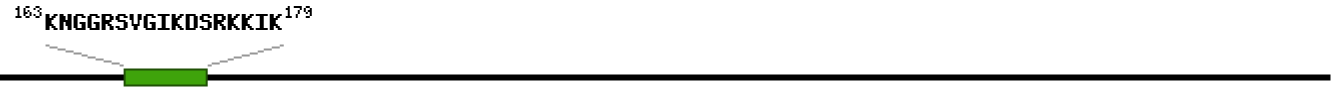

Posterior Graphic. Threshold Marked @ 0.4

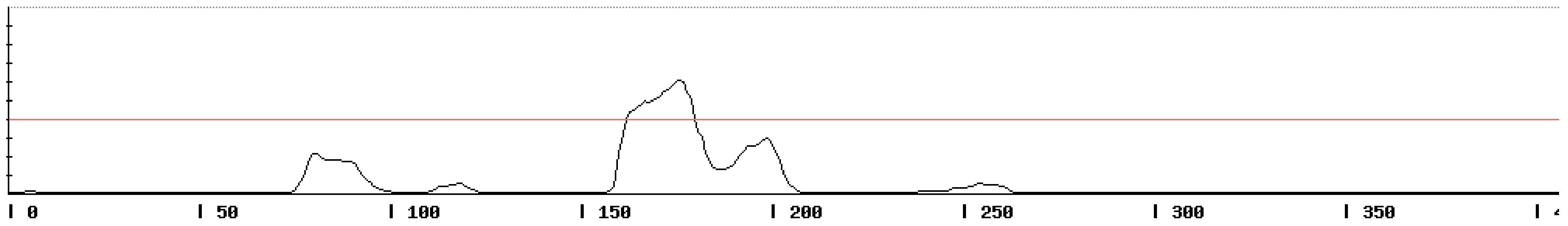

Predictions for Citrusclementina_Ciclev10013879m

Viterbi Path

108 - RKSKKKIGSKKRFR - 121

Viterbi Path

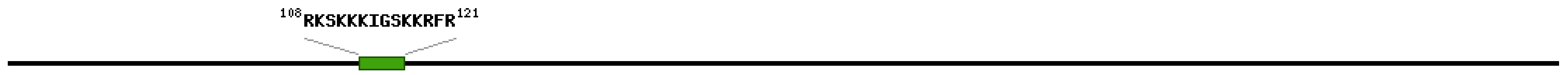

Posterior @ 0.4

103 - KAVVSRKSKKKIGSKKRFRYEKDK - 126

161 - KNGEPAAGGQKNSRKRRK - 178

Posterior @ 0.4

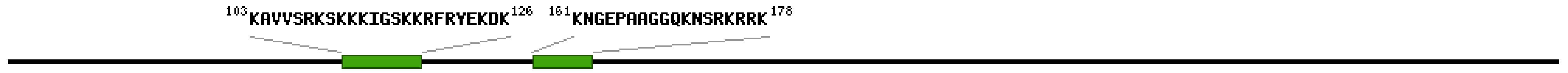

Posterior Graphic. Threshold Marked @ 0.4

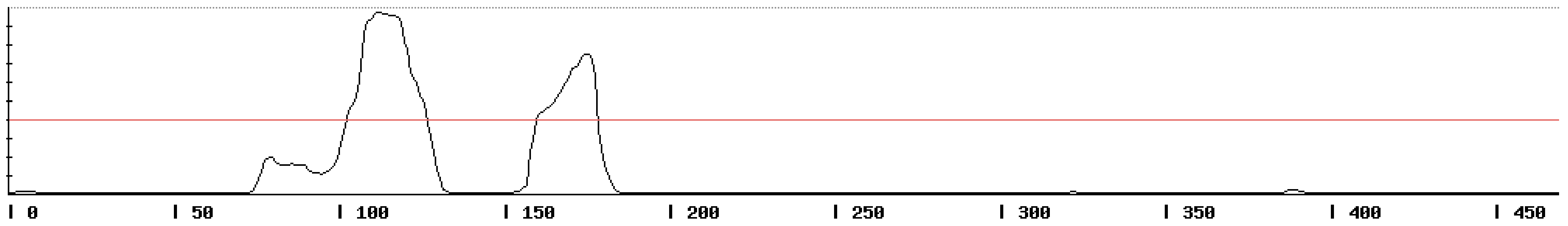

Predictions for Citrusclementina_Ciclev10015507m

Viterbi Path

Viterbi Path

Posterior @ 0.4

Posterior @ 0.4

Posterior Graphic. Threshold Marked @ 0.4	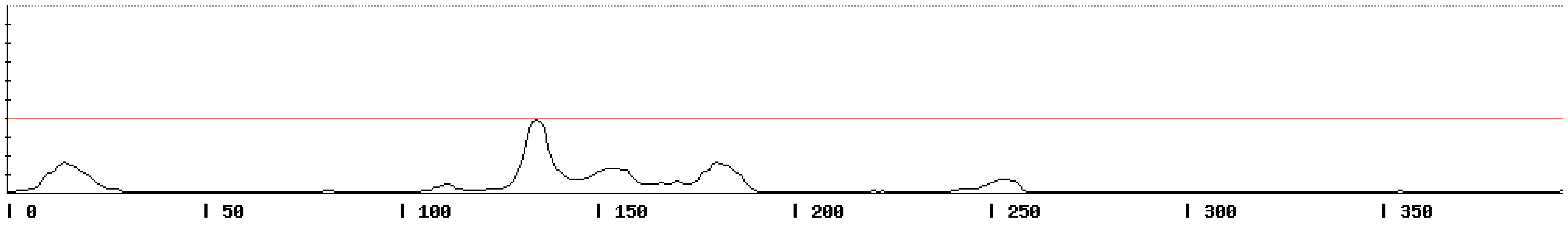

**Predictions for Citrusclementina_Ciclev10015688m**	
Viterbi Path	
Viterbi Path	
Posterior @ 0.4	
Posterior @ 0.4	
Posterior Graphic. Threshold Marked @ 0.4	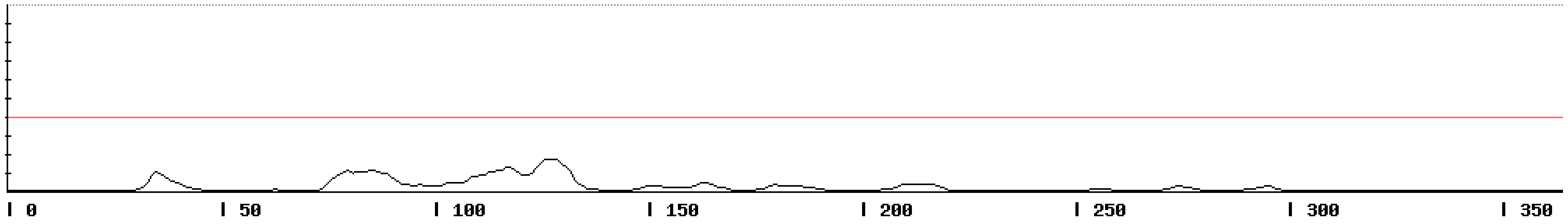

**Predictions for Citrusclementina_Ciclev10015976m**	
Viterbi Path	
Viterbi Path	

Posterior @ 0.4 | 151 - KRR - 153

Posterior @ 0.4

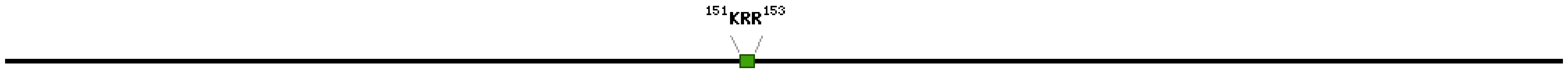

Posterior Graphic. Threshold Marked @ 0.4

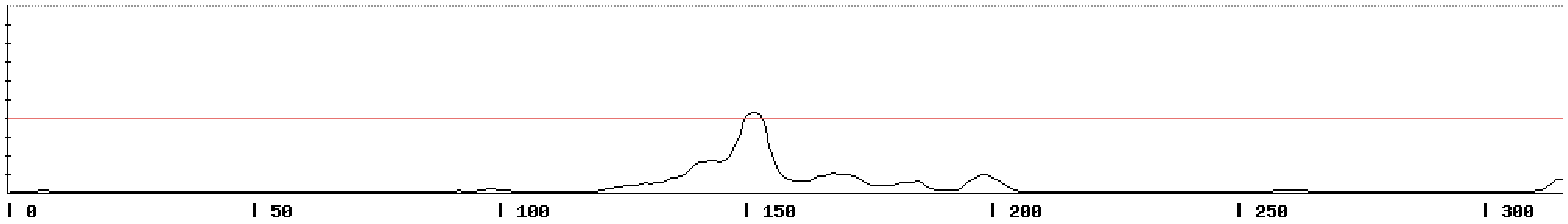

Predictions for Citrusclementina_Ciclev10016434m

Viterbi Path

Viterbi Path

Posterior @ 0.4 | 171 - QRKKKPKKRG - 180

Posterior @ 0.4

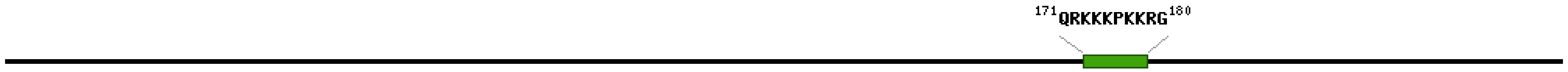

Posterior Graphic. Threshold Marked @ 0.4

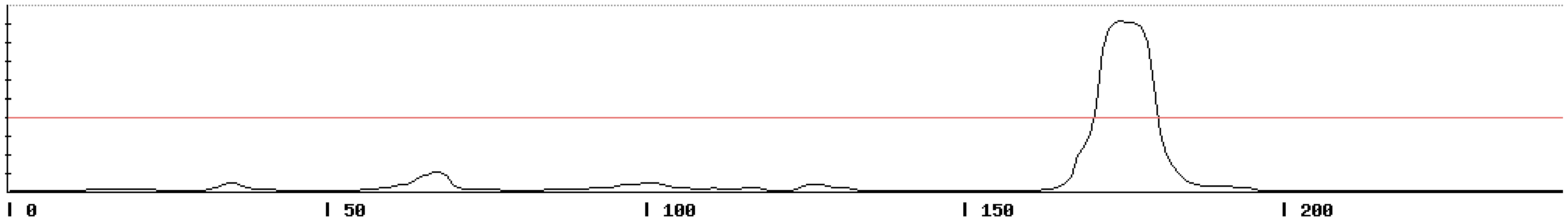

Predictions for Citrusclementina_Ciclev10016877m

Viterbi Path

Viterbi Path

Posterior @ 0.4 | 108 - QRKKKPKKRG - 117

Posterior @ 0.4

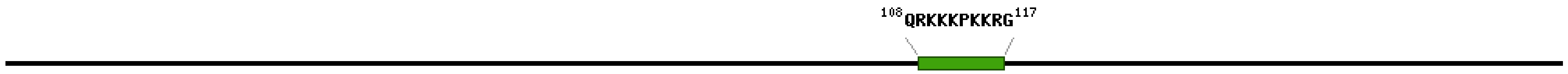

Posterior Graphic. Threshold Marked @ 0.4

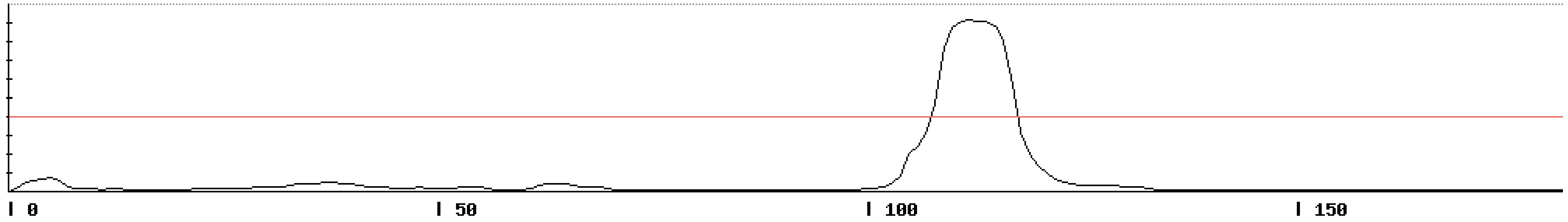

Predictions for Citrusclementina_Ciclev10017618m

Viterbi Path

Viterbi Path

Posterior @ 0.4

Posterior @ 0.4

Posterior Graphic. Threshold Marked @ 0.4

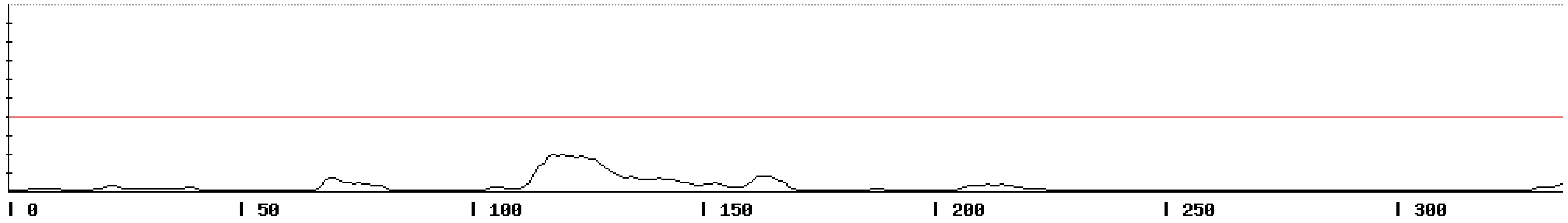

Predictions for Citrusclementina_Ciclev10017822m

Viterbi Path

Viterbi Path

Posterior @ 0.4 | 154 - RKRRK - 158

Posterior @ 0.4

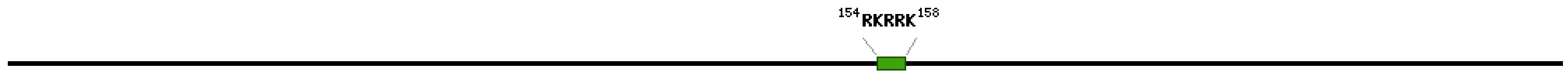

Posterior Graphic. Threshold Marked @ 0.4

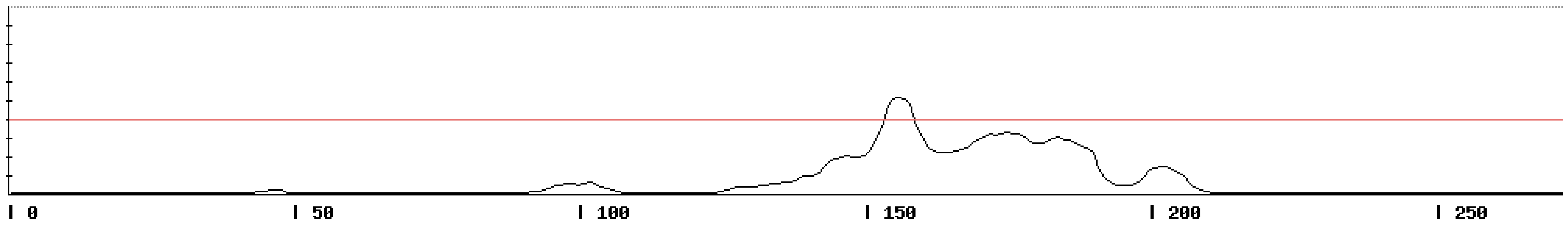

Predictions for Citrusclementina_Ciclev10019282m

Viterbi Path

Viterbi Path

Posterior @ 0.4

Posterior @ 0.4

Posterior Graphic. Threshold Marked @ 0.4

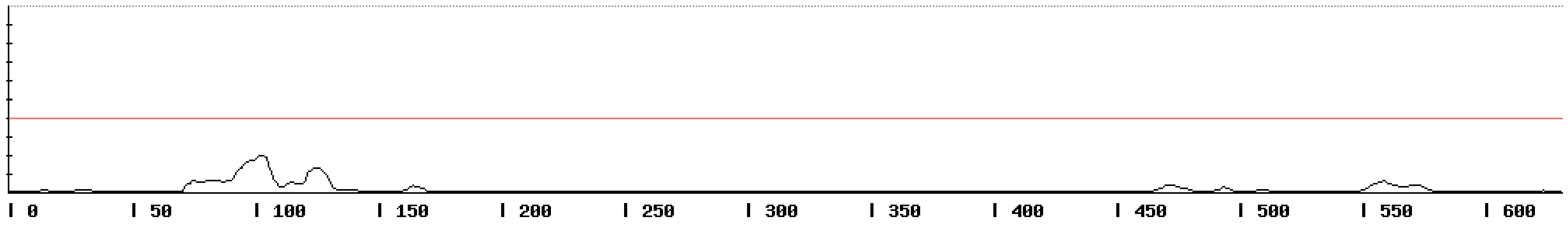

Predictions for Citrusclementina_Ciclev10019368m

Viterbi Path	
Viterbi Path	
Posterior @ 0.4	99 - RK - 100
Posterior @ 0.4	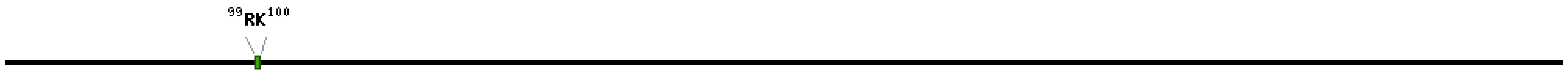
Posterior Graphic. Threshold Marked @ 0.4	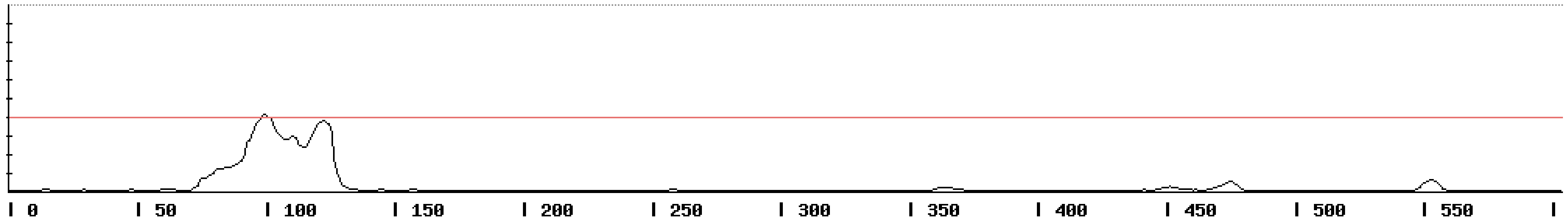

Predictions for Citrusclementina_Ciclev10019378m

Viterbi Path	
Viterbi Path	
Posterior @ 0.4	99 - RK - 100
Posterior @ 0.4	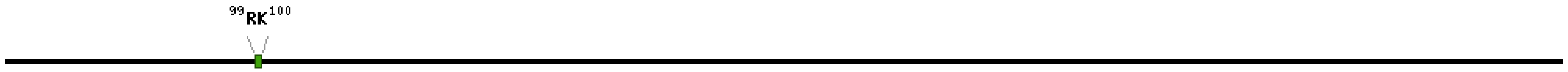
Posterior Graphic. Threshold Marked @ 0.4	

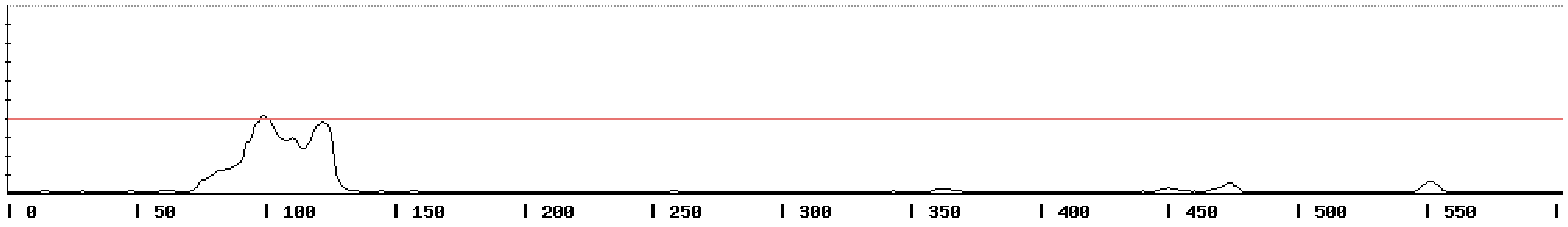

Predictions for Citrusclementina_Ciclev10019533m

Viterbi Path	
Viterbi Path	
Posterior @ 0.4	124 - KKKSG - 128
Posterior @ 0.4	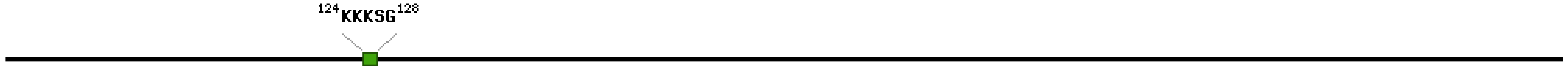
Posterior Graphic. Threshold Marked @ 0.4	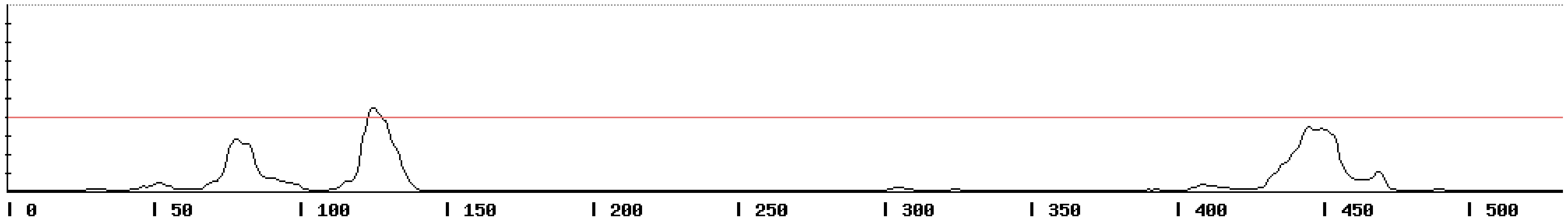

Predictions for Citrusclementina_Ciclev10019769m

Viterbi Path	
Viterbi Path	
Posterior @ 0.4	99 - RK - 100
Posterior @ 0.4	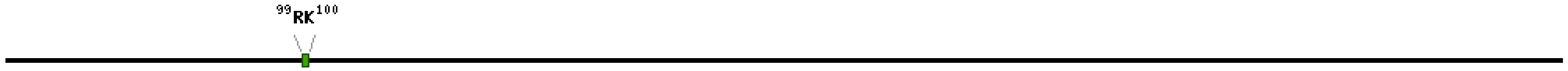

Posterior Graphic. Threshold Marked @ 0.4

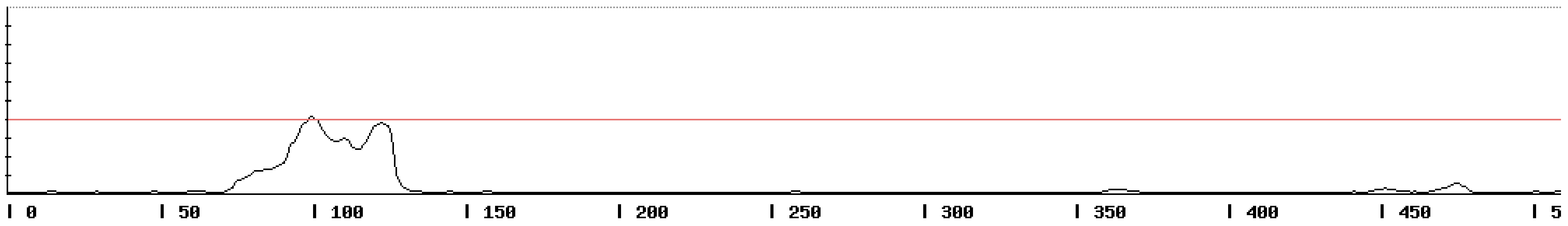

Predictions for Citrusclementina_Ciclev10019781m

Viterbi Path

Viterbi Path

Posterior @ 0.4 99 - RK - 100

Posterior @ 0.4

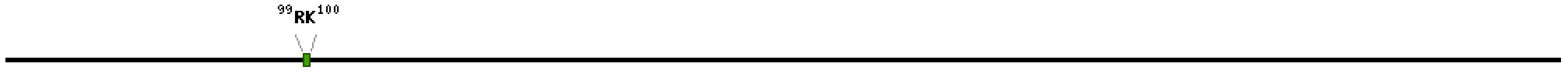

Posterior Graphic. Threshold Marked @ 0.4

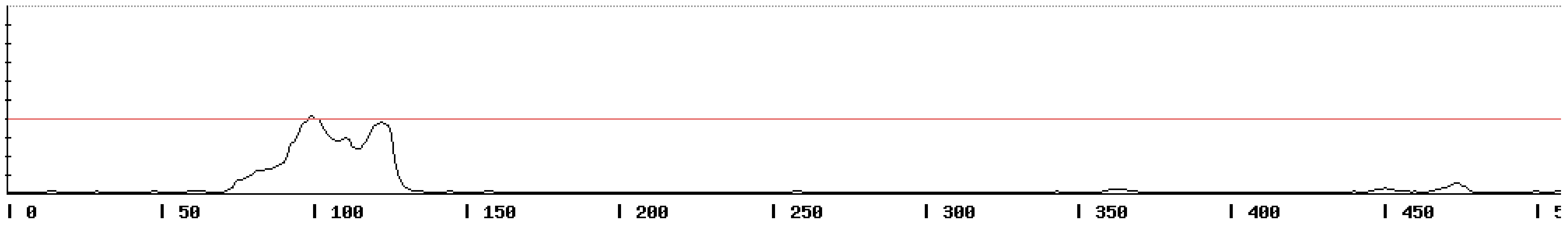

Predictions for Citrusclementina_Ciclev10019845m

Viterbi Path

Viterbi Path

Posterior @ 0.4

Posterior @ 0.4

Posterior Graphic. Threshold Marked @ 0.4

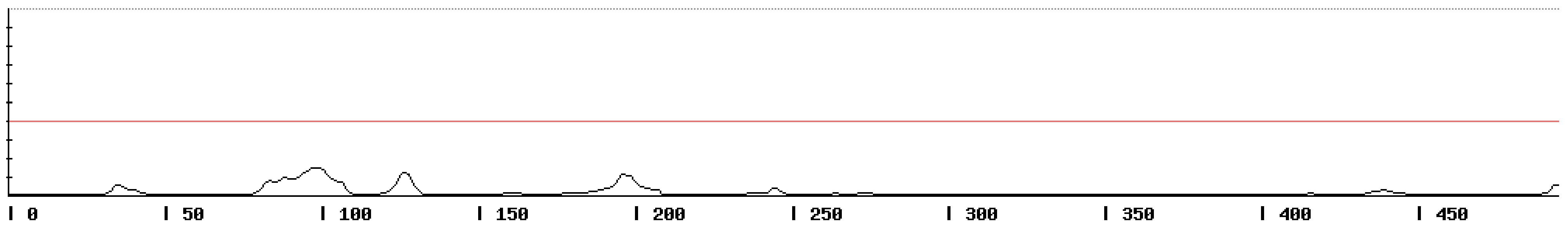

Predictions for Citrusclementina_Ciclev10020480m

Viterbi Path

Viterbi Path

Posterior @ 0.4

Posterior @ 0.4

Posterior Graphic. Threshold Marked @ 0.4

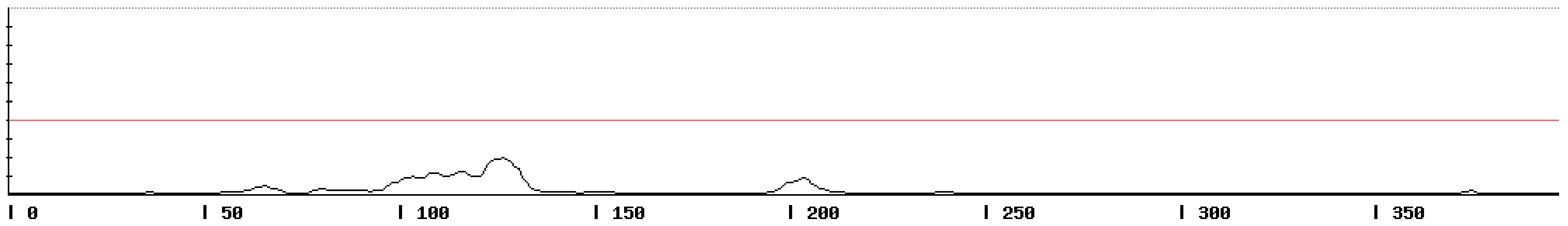

Predictions for Citrusclementina_Ciclev10020549m

Viterbi Path

Viterbi Path

Posterior @ 0.4

Posterior @ 0.4

Posterior Graphic. Threshold Marked @ 0.4

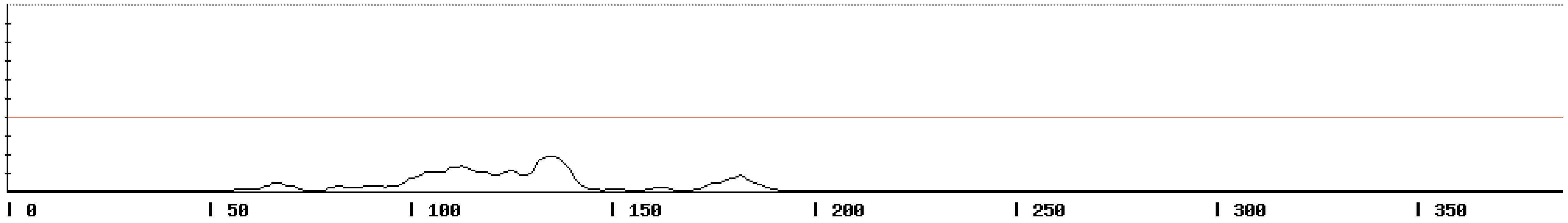

Predictions for Citrusclementina_Ciclev10020717m

Viterbi Path

Viterbi Path

Posterior @ 0.4

Posterior @ 0.4

Posterior Graphic. Threshold Marked @ 0.4

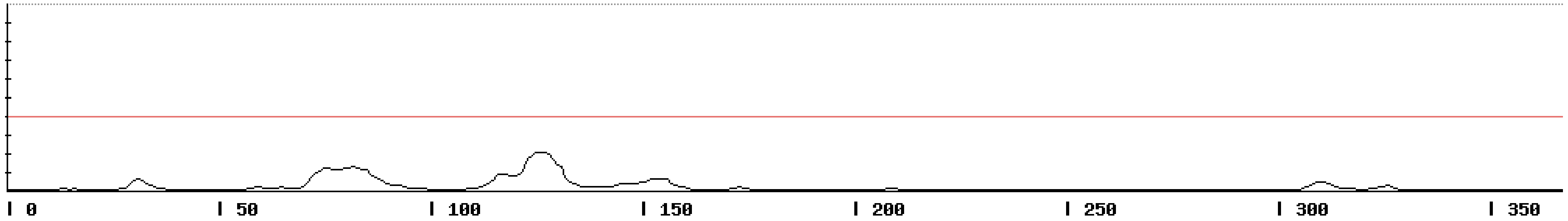

Predictions for Citrusclementina_Ciclev10020734m

Viterbi Path

Viterbi Path

Posterior @ 0.4

Posterior @ 0.4

Posterior Graphic. Threshold Marked @ 0.4

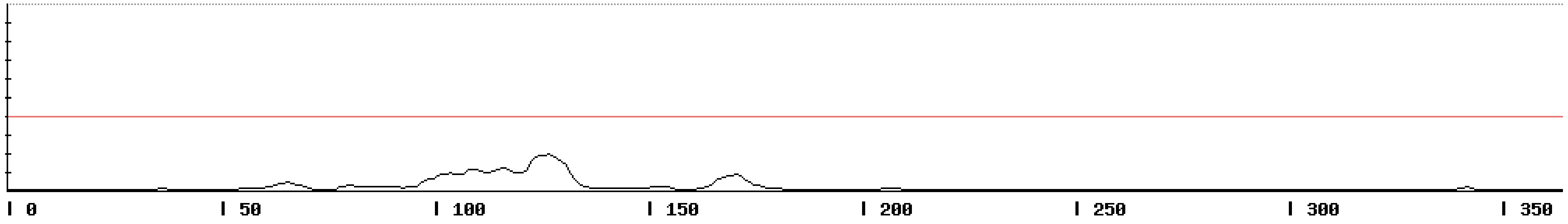

Predictions for Citrusclementina_Ciclev10021117m

Viterbi Path

Viterbi Path

Posterior @ 0.4 — 125 - GRAPKGKK - 132

Posterior @ 0.4

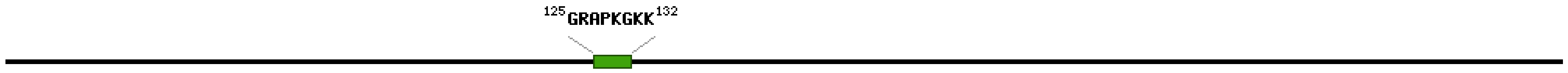

Posterior Graphic. Threshold Marked @ 0.4

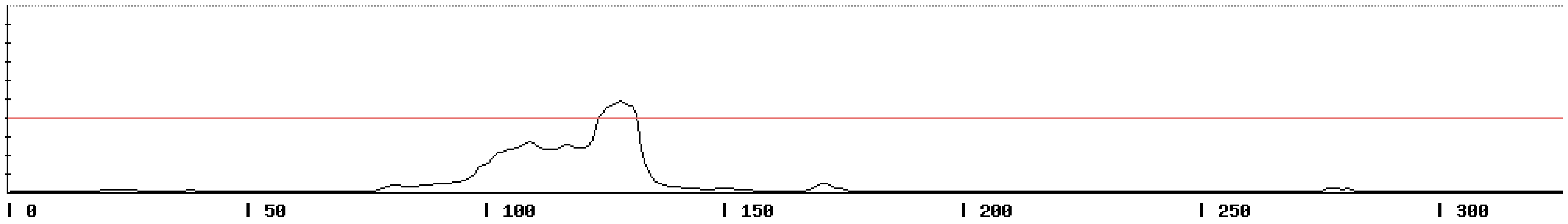

Predictions for Citrusclementina_Ciclev10021141m

Viterbi Path

Viterbi Path

Posterior @ 0.4

Posterior @ 0.4

Posterior Graphic. Threshold Marked @ 0.4

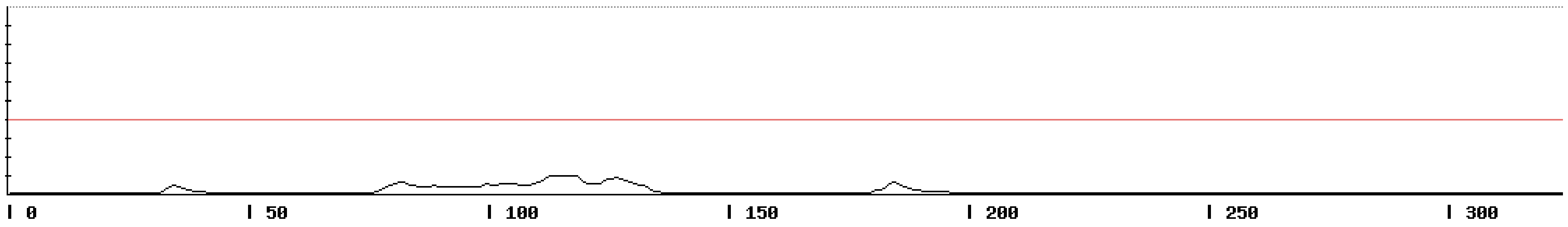

Predictions for Citrusclementina_Ciclev10021155m

Viterbi Path

Viterbi Path

Posterior @ 0.4

125 - GRAPKGKK - 132

Posterior @ 0.4

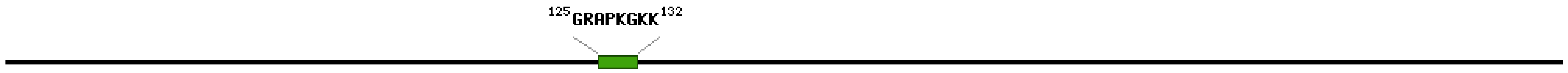

Posterior Graphic. Threshold Marked @ 0.4

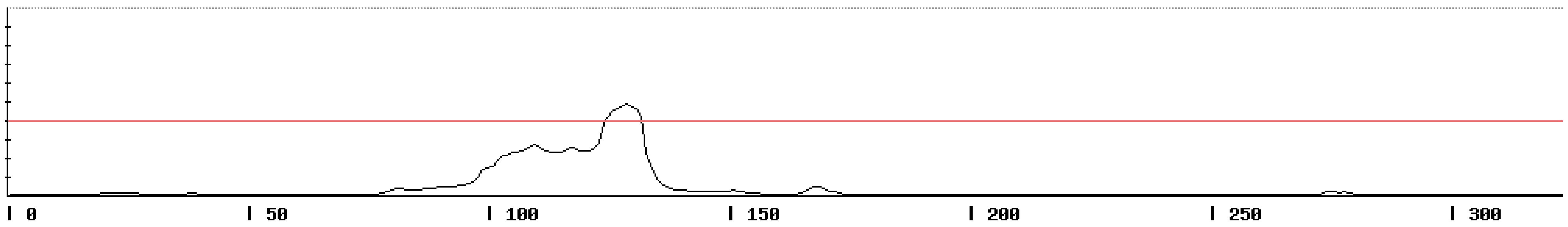

Predictions for Citrusclementina_Ciclev10021312m

Viterbi Path

Viterbi Path

Posterior @ 0.4

Posterior @ 0.4

Posterior Graphic. Threshold Marked @ 0.4

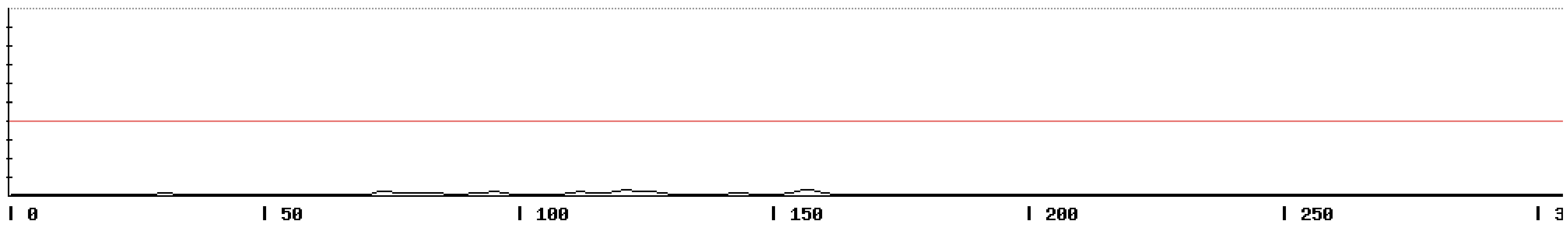

Predictions for Citrusclementina_Ciclev10022564m

Viterbi Path

Viterbi Path

Posterior @ 0.4

Posterior @ 0.4

Posterior Graphic. Threshold Marked @ 0.4

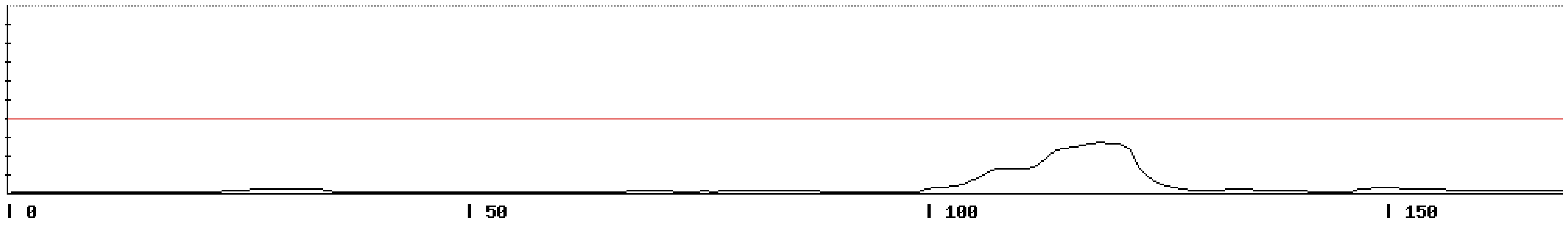

Predictions for Citrusclementina_Ciclev10022859m	
Viterbi Path	
Viterbi Path	
Posterior @ 0.4	
Posterior @ 0.4	
Posterior Graphic. Threshold Marked @ 0.4	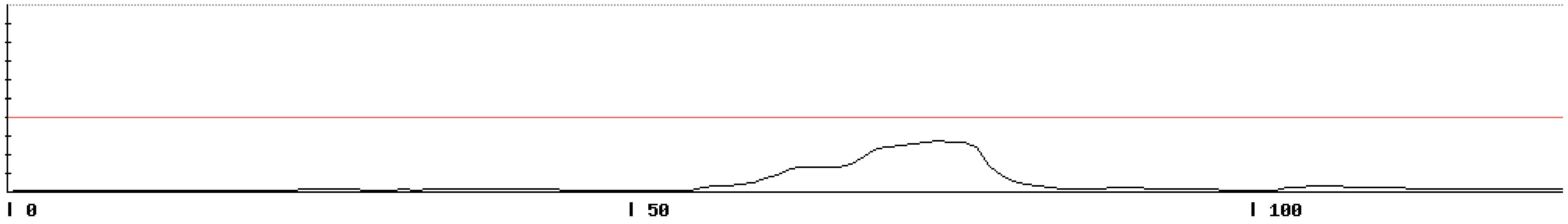

Predictions for Citrusclementina_Ciclev10023292m	
Viterbi Path	
Viterbi Path	
Posterior @ 0.4	98 - KSREKIDSKKR - 108
Posterior @ 0.4	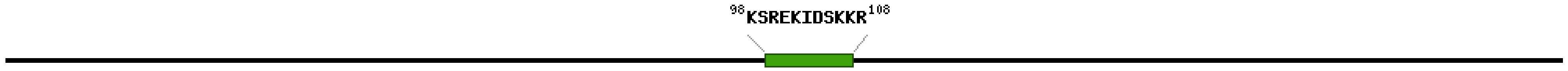

Posterior Graphic. Threshold Marked @ 0.4

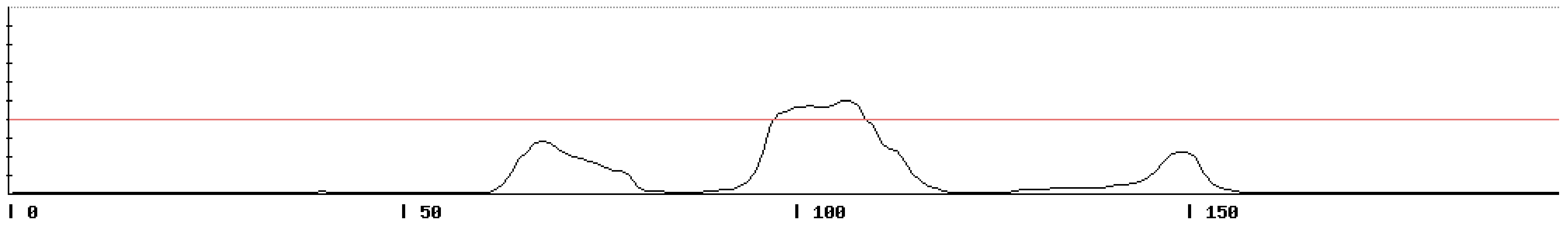

Predictions for Citrusclementina_Ciclev10023312m

Viterbi Path

Viterbi Path

Posterior @ 0.4

Posterior @ 0.4

Posterior Graphic. Threshold Marked @ 0.4

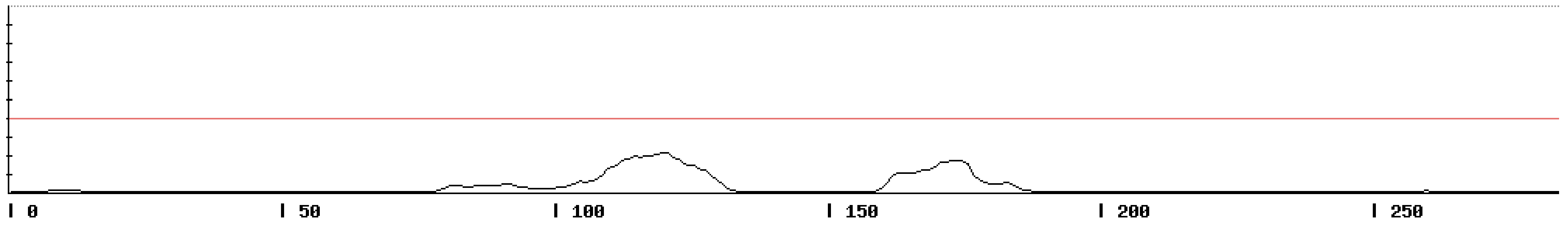

Predictions for Citrusclementina_Ciclev10023578m

Viterbi Path

Viterbi Path

Posterior @ 0.4

Posterior @ 0.4

Posterior Graphic. Threshold Marked @ 0.4

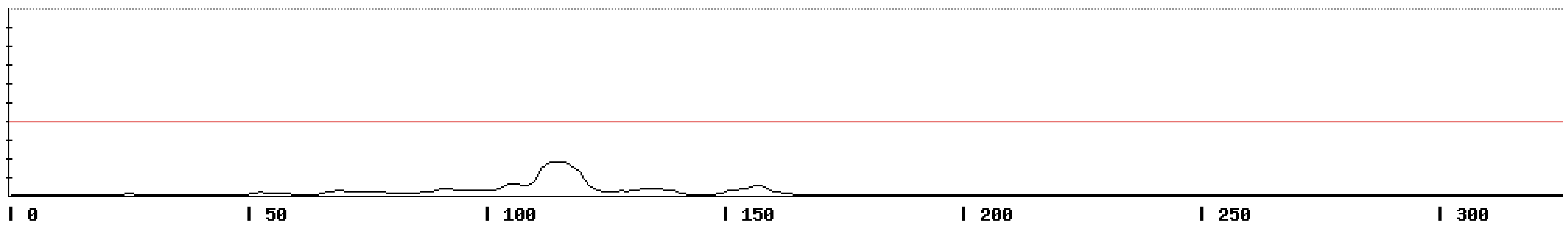

Predictions for Citrusclementina_Ciclev10023601m

Viterbi Path

Viterbi Path

Posterior @ 0.4

Posterior @ 0.4

Posterior Graphic. Threshold Marked @ 0.4

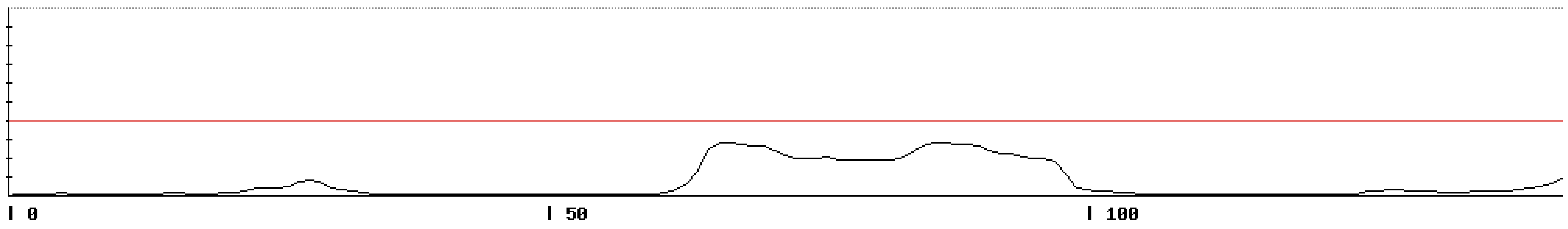

Predictions for Citrusclementina_Ciclev10023783m

Viterbi Path

Viterbi Path

Posterior @ 0.4	55 - MRKKNMAKKIPRKQKET - 71
Posterior @ 0.4	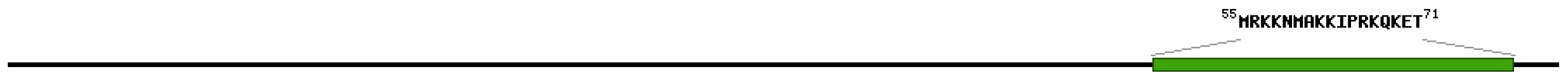
Posterior Graphic. Threshold Marked @ 0.4	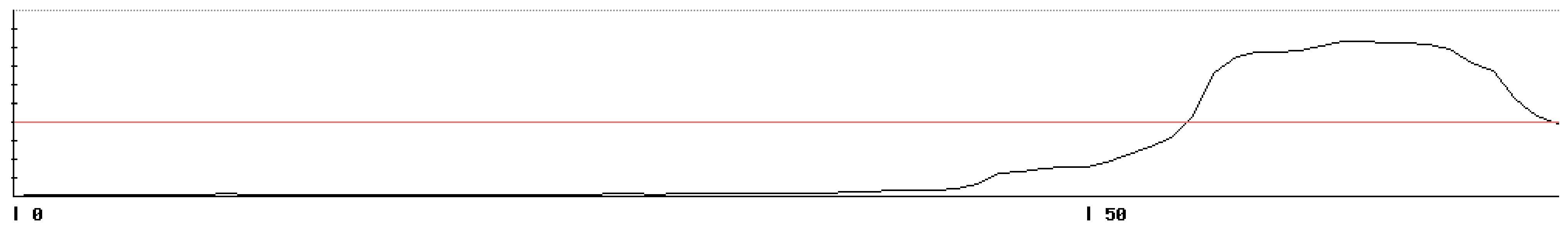

Predictions for Citrusclementina_Ciclev10024035m

Viterbi Path	
Viterbi Path	
Posterior @ 0.4	
Posterior @ 0.4	
Posterior Graphic. Threshold Marked @ 0.4	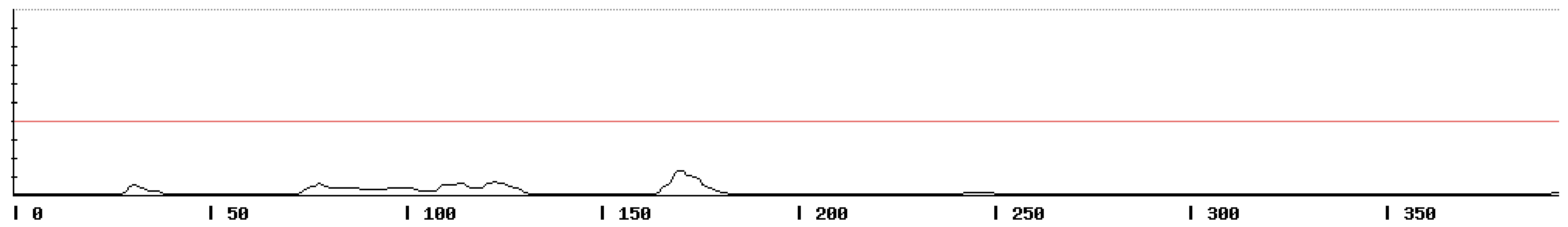

Predictions for Citrusclementina_Ciclev10024200m

Viterbi Path	117 - RIKKNGEPRQPGRKIQGKRESRA - 139
Viterbi Path	

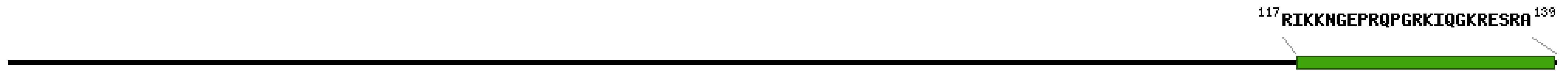

Posterior @ 0.4

67 - RNSRKKIGFKKRFRYEKD - 84

118 - IKKNGEPRQPGRKIQGKRESRA - 139

Posterior @ 0.4

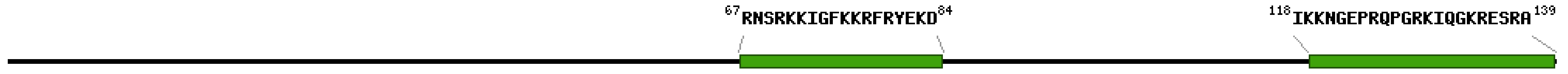

Posterior Graphic. Threshold Marked @ 0.4

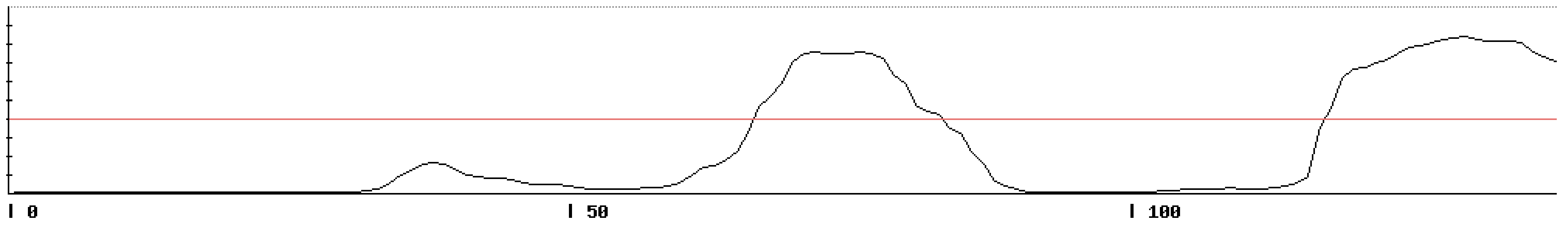

Predictions for Citrusclementina_Ciclev10024218m

Viterbi Path

Viterbi Path

Posterior @ 0.4

Posterior @ 0.4

Posterior Graphic. Threshold Marked @ 0.4

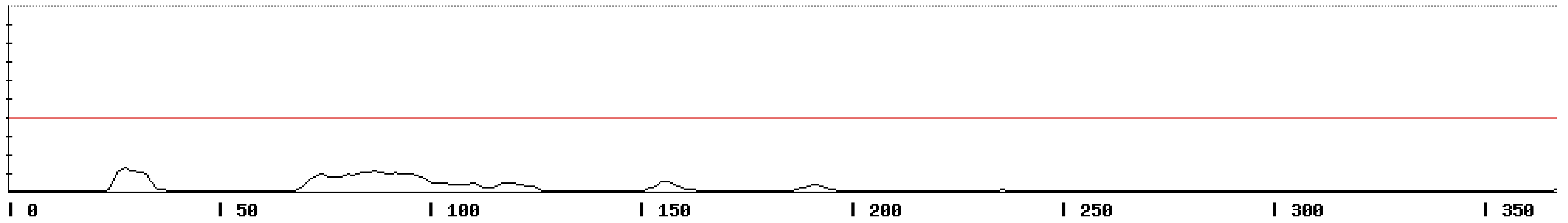

Predictions for Citrusclementina_Ciclev10024520m

Viterbi Path

Viterbi Path

Posterior @ 0.4

Posterior @ 0.4

Posterior Graphic. Threshold Marked @ 0.4

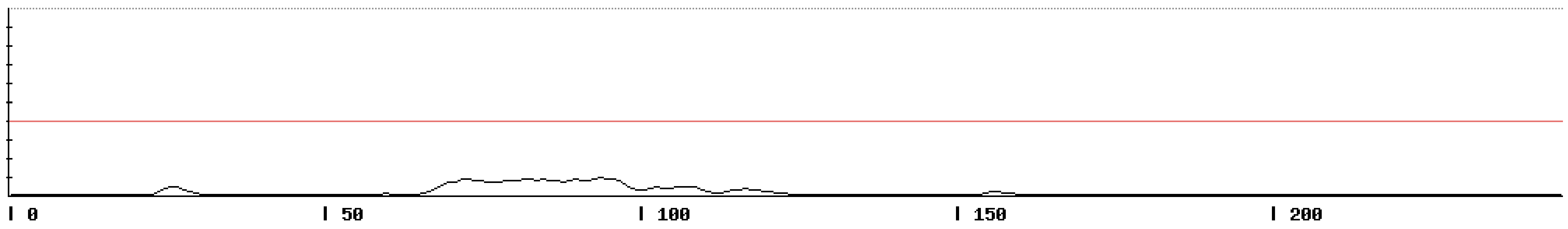

Predictions for Citrusclementina_Ciclev10024588m

Viterbi Path

Viterbi Path

Posterior @ 0.4

Posterior @ 0.4

Posterior Graphic. Threshold Marked @ 0.4

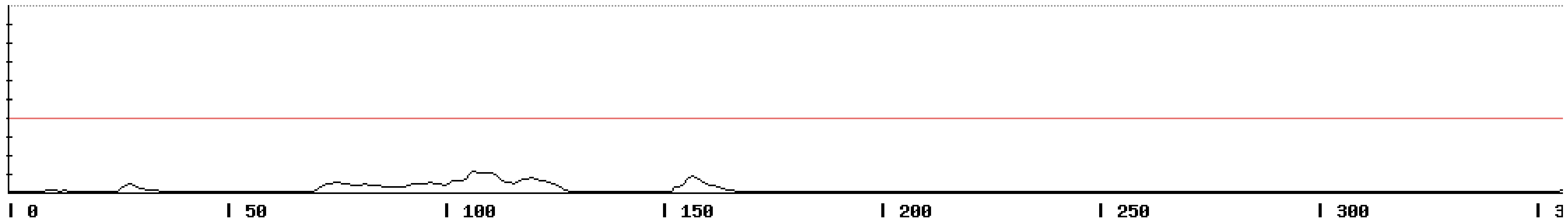

Predictions for Ciclev10026114m

Viterbi Path	
Viterbi Path	
Posterior @ 0.4	
Posterior @ 0.4	
Posterior Graphic. Threshold Marked @ 0.4	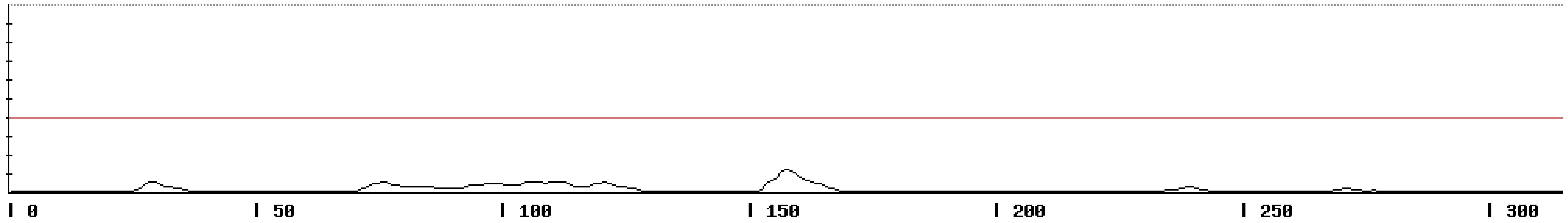

Predictions for Citrusclementina_Ciclev10026981m

Viterbi Path	
Viterbi Path	
Posterior @ 0.4	67 - SRKKIDSKKR - 76
Posterior @ 0.4	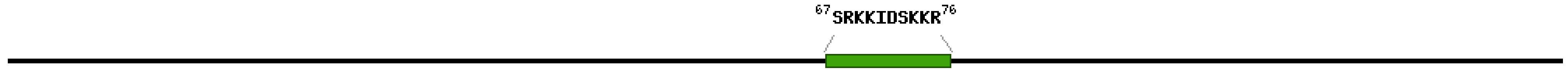

Posterior Graphic. Threshold Marked @ 0.4

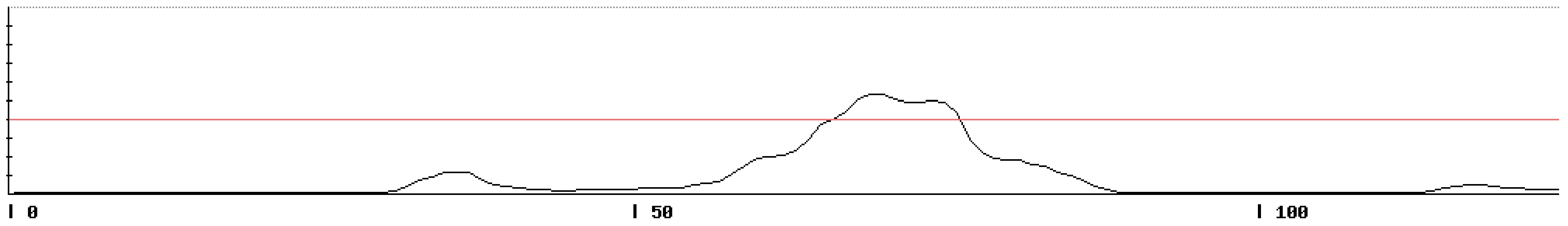

Predictions for Citrusclementina_Ciclev10028618m

Viterbi Path

Viterbi Path

Posterior @ 0.4

308 - RIKLRK - 313

Posterior @ 0.4

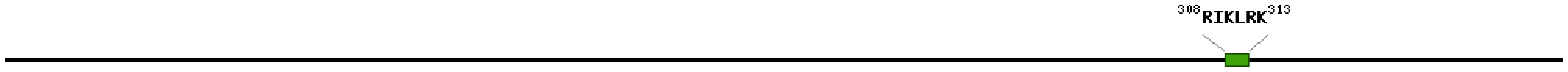

Posterior Graphic. Threshold Marked @ 0.4

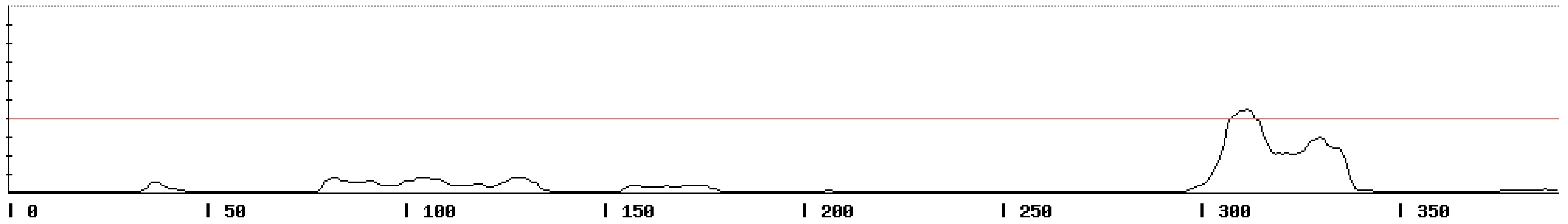

Predictions for Citrusclementina_Ciclev10029007m

Viterbi Path

Viterbi Path

Posterior @ 0.4

Posterior @ 0.4

Posterior Graphic. Threshold Marked @ 0.4

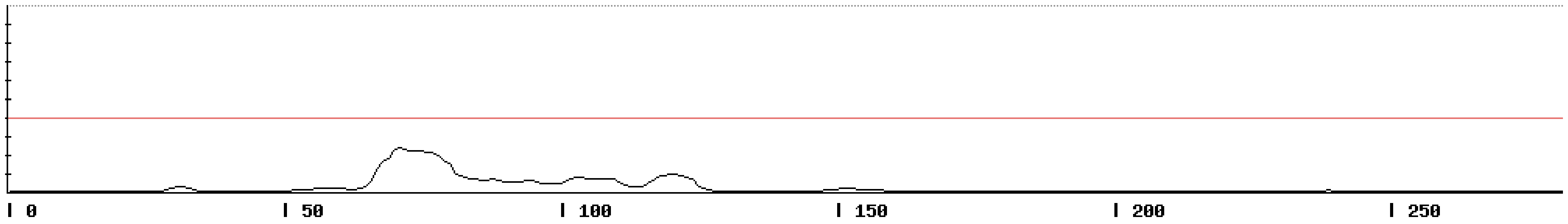

Predictions for Citrusclementina_Ciclev10029032m

Viterbi Path

Viterbi Path

Posterior @ 0.4 124 - R - 124

Posterior @ 0.4

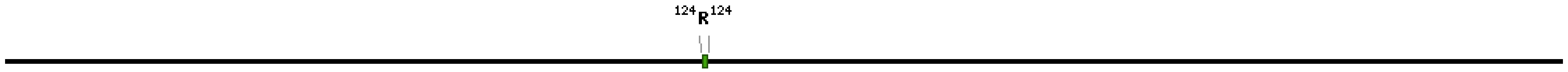

Posterior Graphic. Threshold Marked @ 0.4

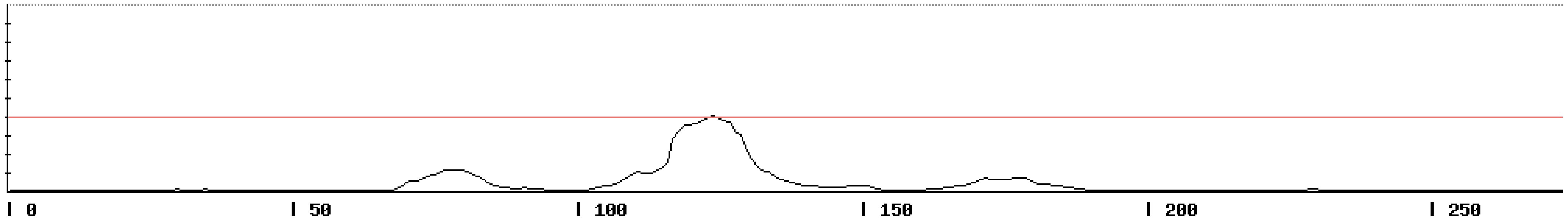

Predictions for Citrusclementina_Ciclev10029131m

Viterbi Path

Viterbi Path

Posterior @ 0.4 | 100 - R - 100

Posterior @ 0.4

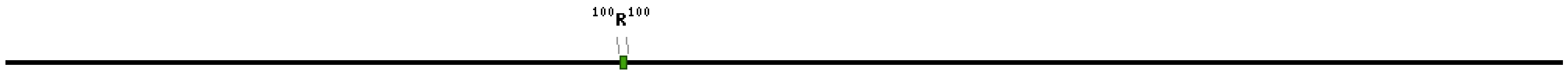

Posterior Graphic. Threshold Marked @ 0.4

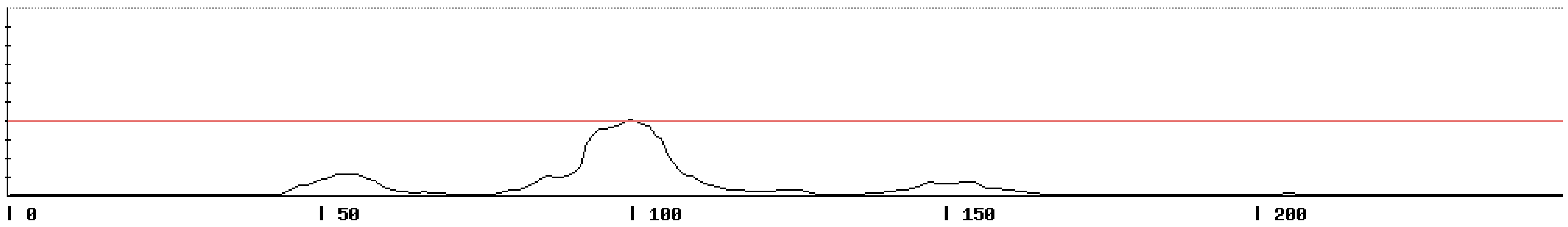

Predictions for Citrusclementina_Ciclev10030252m

Viterbi Path

Viterbi Path

Posterior @ 0.4

Posterior @ 0.4

Posterior Graphic. Threshold Marked @ 0.4

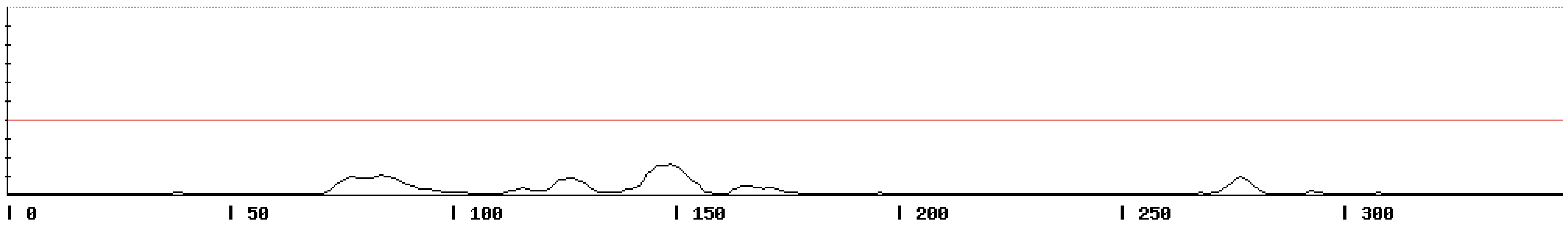

Predictions for Citrusclementina_Ciclev10031100m

Viterbi Path

Viterbi Path

Posterior @ 0.4	549 - RKRREGPFKRT - 559
Posterior @ 0.4	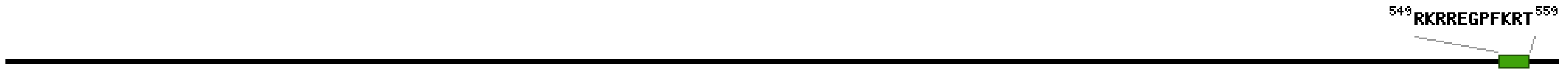
Posterior Graphic. Threshold Marked @ 0.4	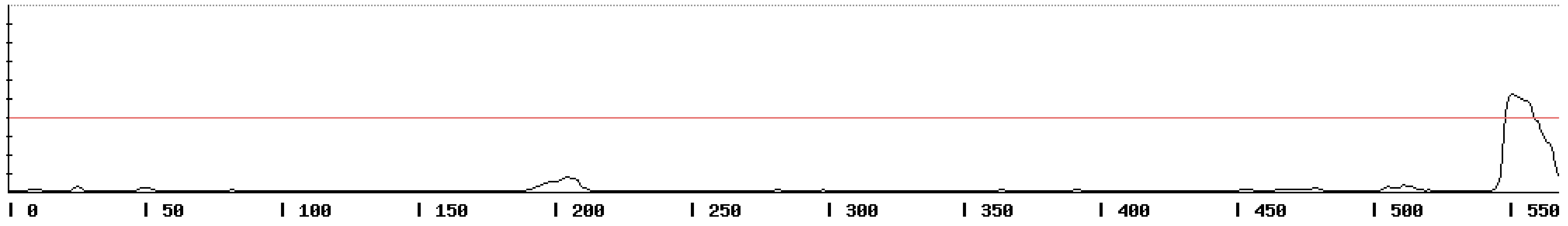

Predictions for Citrusclementina_Ciclev10031147m

Viterbi Path	
Viterbi Path	
Posterior @ 0.4	
Posterior @ 0.4	
Posterior Graphic. Threshold Marked @ 0.4	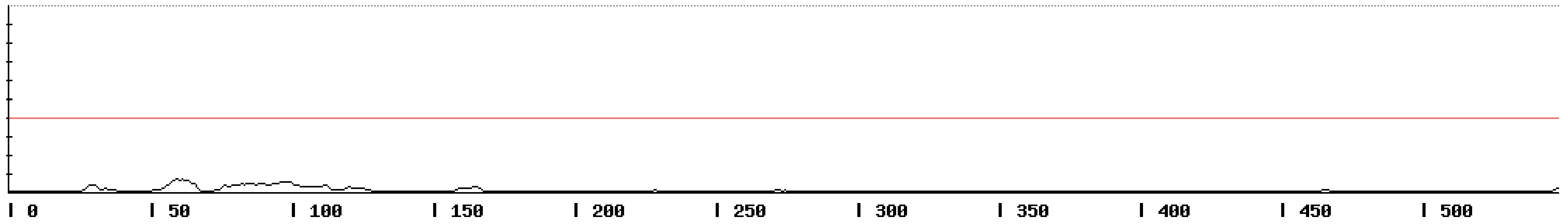

Predictions for Citrusclementina_Ciclev10031150m

Viterbi Path

Viterbi Path

Posterior @ 0.4

Posterior @ 0.4

Posterior Graphic. Threshold Marked @ 0.4

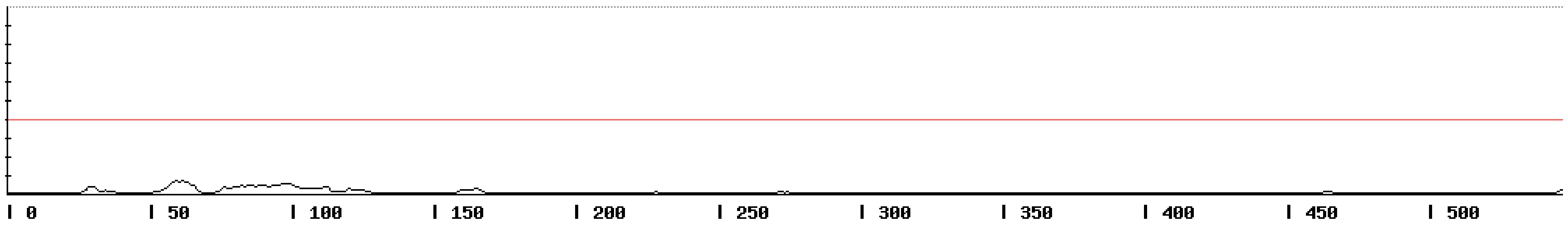

Predictions for Citrusclementina_Ciclev10031406m

Viterbi Path

Viterbi Path

Posterior @ 0.4

Posterior @ 0.4

Posterior Graphic. Threshold Marked @ 0.4

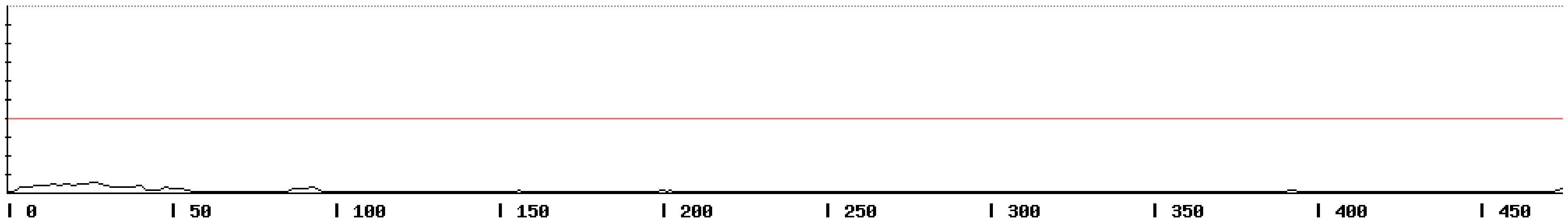

Predictions for Citrusclementina_Ciclev10031598m	
Viterbi Path	
Viterbi Path	
Posterior @ 0.4	137 - KRR - 139
Posterior @ 0.4	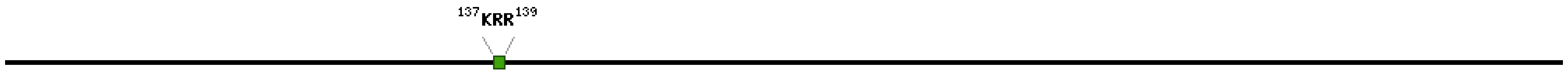
Posterior Graphic. Threshold Marked @ 0.4	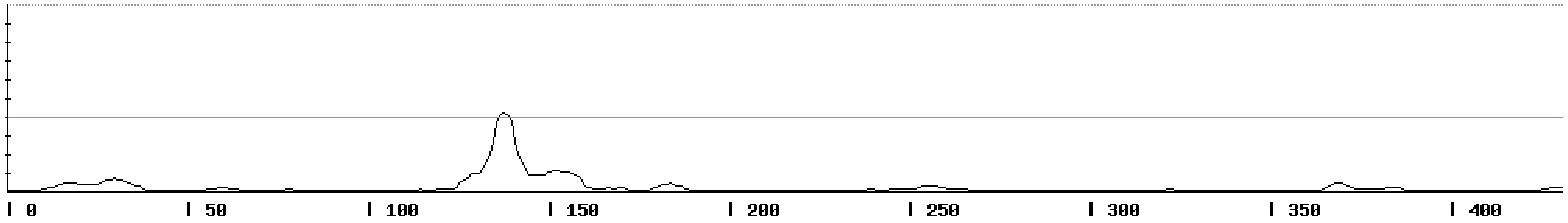

Predictions for Citrusclementina_Ciclev10031606m	
Viterbi Path	
Viterbi Path	
Posterior @ 0.4	
Posterior @ 0.4	
Posterior Graphic. Threshold Marked @ 0.4	

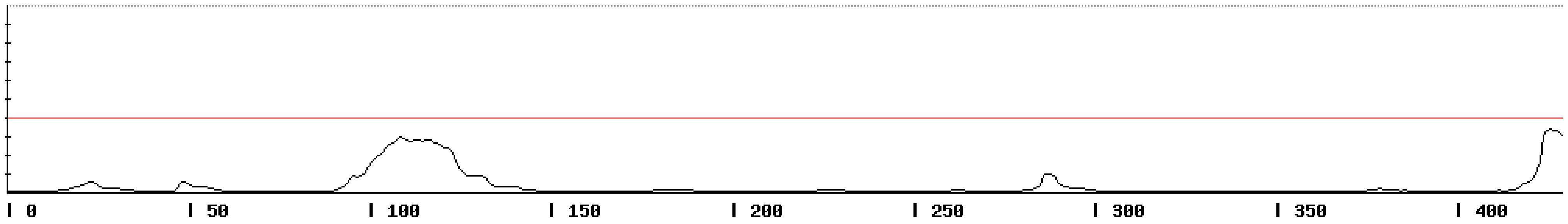

Predictions for Citrusclementina_Ciclev10031623m

Viterbi Path

Viterbi Path

Posterior @ 0.4

Posterior @ 0.4

Posterior Graphic. Threshold Marked @ 0.4

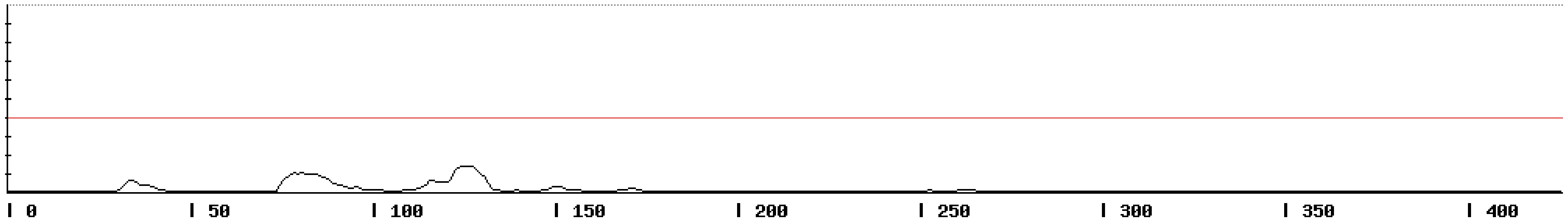

Predictions for Citrusclementina_Ciclev10031966m

Viterbi Path

Viterbi Path

Posterior @ 0.4

Posterior @ 0.4

Posterior Graphic. Threshold Marked @ 0.4	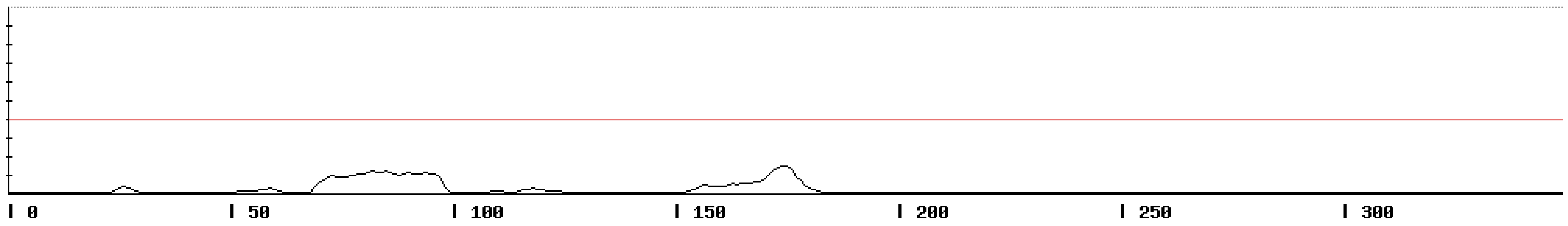

Predictions for Citrusclementina_Ciclev10032203m

Viterbi Path	
Viterbi Path	
Posterior @ 0.4	
Posterior @ 0.4	
Posterior Graphic. Threshold Marked @ 0.4	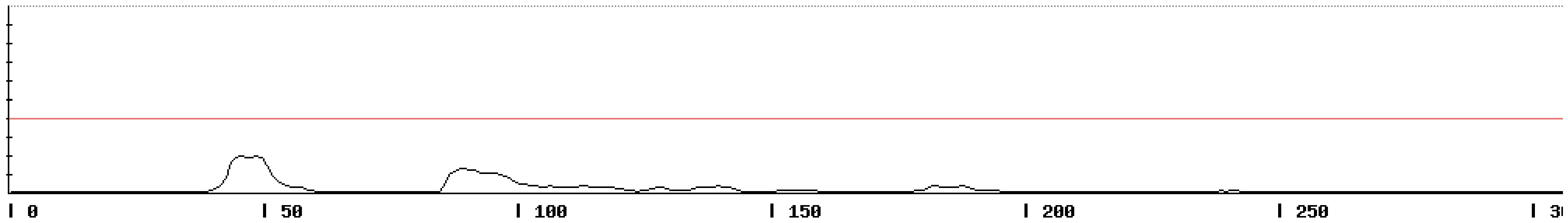

Predictions for Citrusclementina_Ciclev10032211m

Viterbi Path	
Viterbi Path	
Posterior @ 0.4	
Posterior @ 0.4	

Posterior Graphic. Threshold Marked @ 0.4

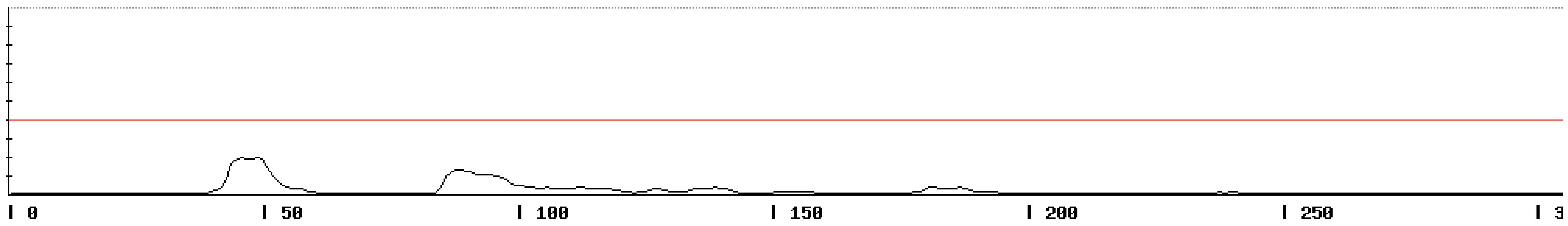

Predictions for Citrusclementina_Ciclev10032304m

Viterbi Path

Viterbi Path

Posterior @ 0.4

Posterior @ 0.4

Posterior Graphic. Threshold Marked @ 0.4

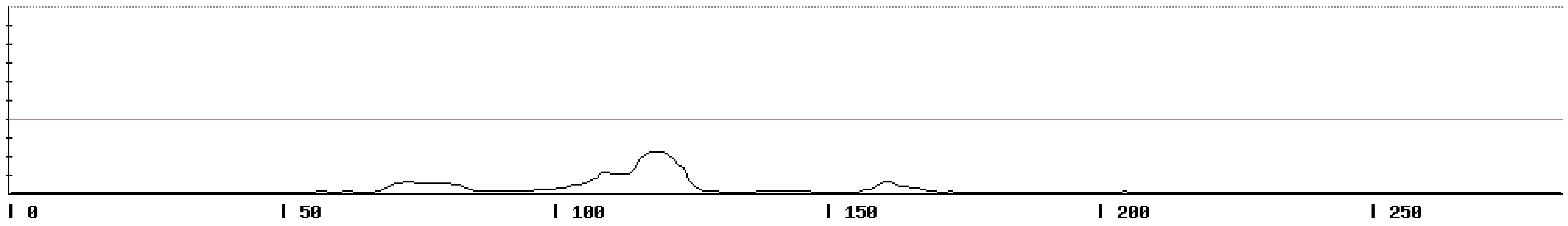

Predictions for Citrusclementina_Ciclev10032547m

Viterbi Path

Viterbi Path

Posterior @ 0.4

Posterior @ 0.4

Posterior Graphic. Threshold Marked @ 0.4

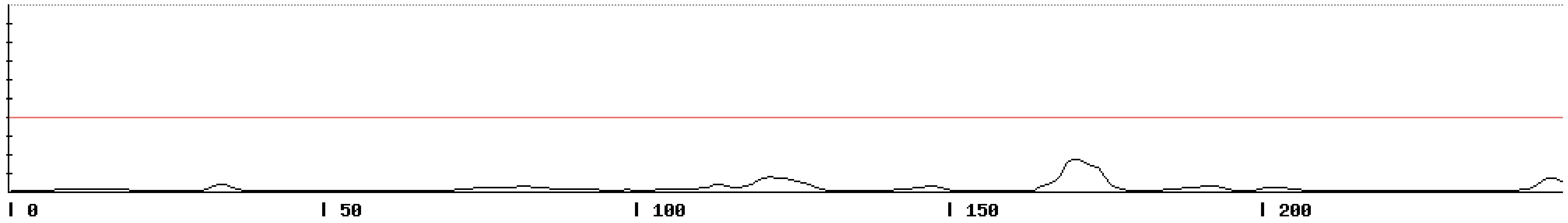

Predictions for Citrusclementina_Ciclev10032627m

Viterbi Path

Viterbi Path

Posterior @ 0.4

Posterior @ 0.4

Posterior Graphic. Threshold Marked @ 0.4

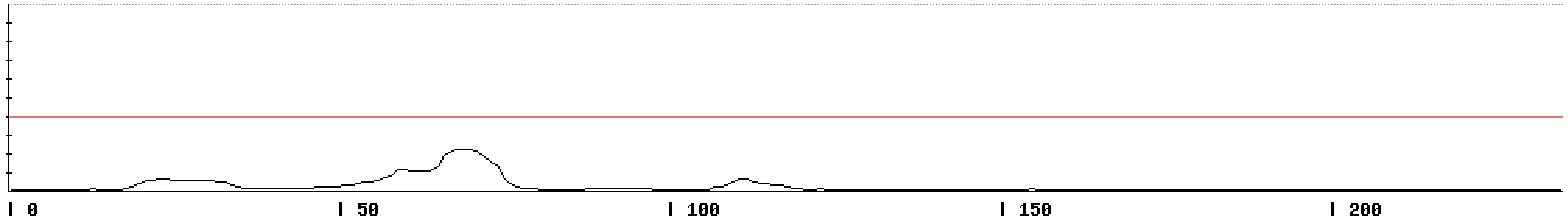

Predictions for Citrusclementina_Ciclev10032776m

Viterbi Path

Viterbi Path

Posterior @ 0.4

Posterior @ 0.4

Posterior Graphic. Threshold Marked @ 0.4

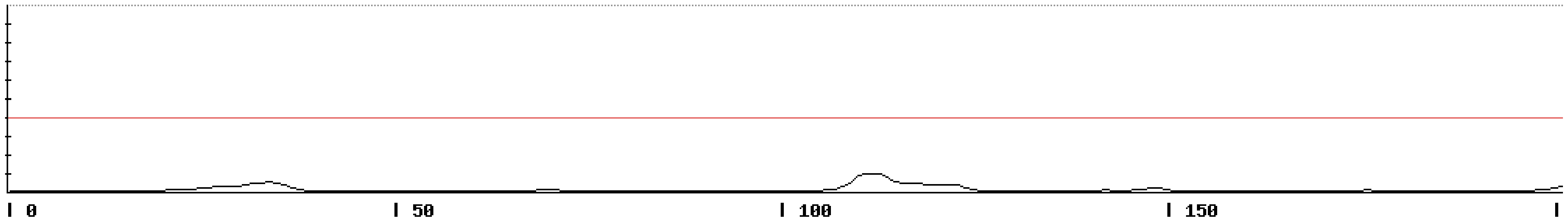

Predictions for Ciclev10032817m

Viterbi Path: 165 - KIHHKKESGNKRSEAGKKKNLPVRKKTKAC - 194

Viterbi Path

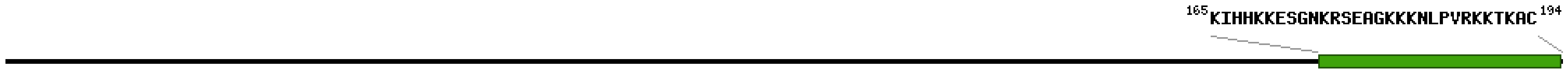

Posterior @ 0.4: 167 - HHKKESGNKRSEAGKKKNLPVRKKTKA - 193

Posterior @ 0.4

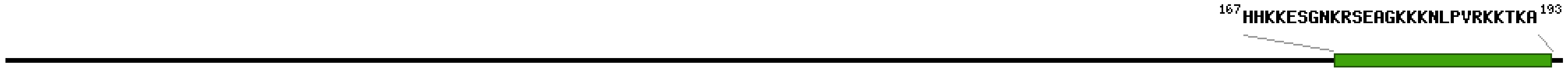

Posterior Graphic. Threshold Marked @ 0.4

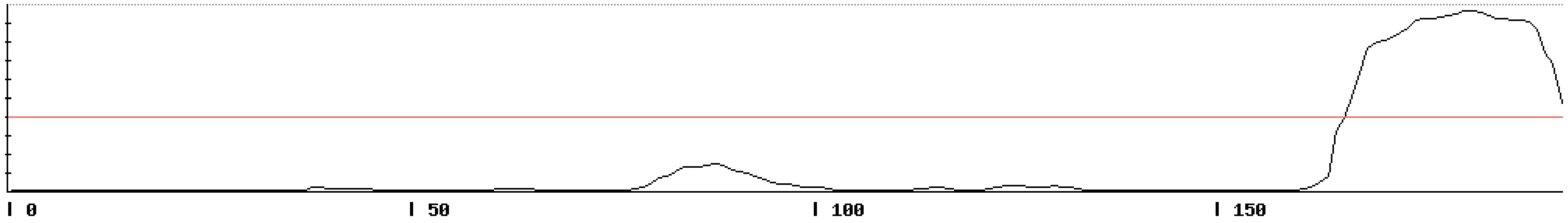

Predictions for Ciclev10032865m

Viterbi Path

Viterbi Path

Posterior @ 0.4

Posterior @ 0.4

Posterior Graphic. Threshold Marked @ 0.4

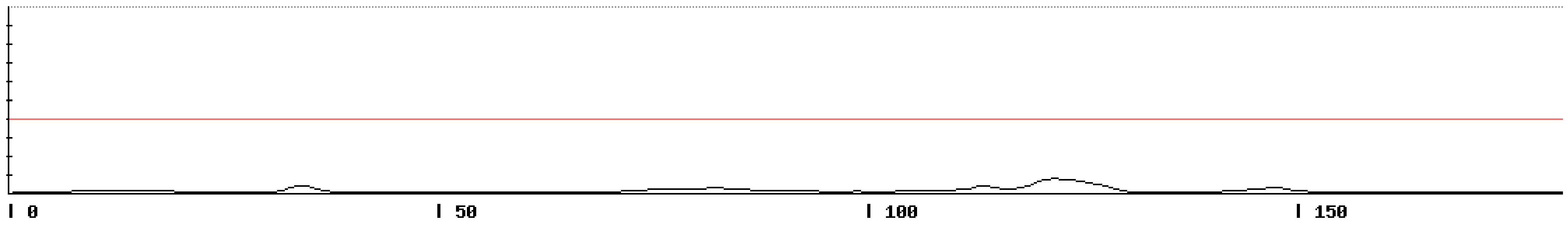

Predictions for Citrusclementina_Ciclev10033346m

Viterbi Path

Viterbi Path

Posterior @ 0.4

Posterior @ 0.4

Posterior Graphic. Threshold Marked @ 0.4

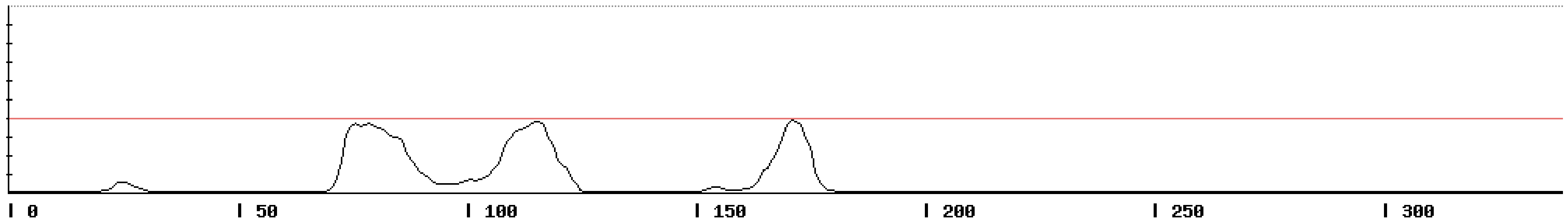

Predictions for Citrusclementina_Ciclev10033389m	
Viterbi Path	
Viterbi Path	
Posterior @ 0.4	
Posterior @ 0.4	
Posterior Graphic. Threshold Marked @ 0.4	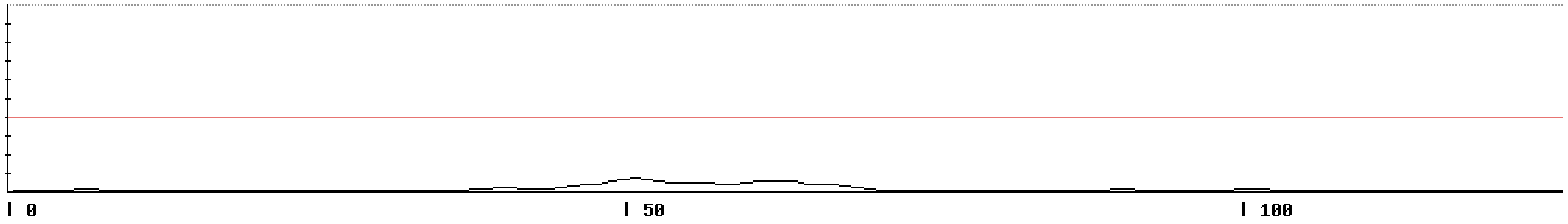

Predictions for Citrusclementina_Ciclev10033415m	
Viterbi Path	
Viterbi Path	
Posterior @ 0.4	
Posterior @ 0.4	
Posterior Graphic. Threshold Marked @ 0.4	

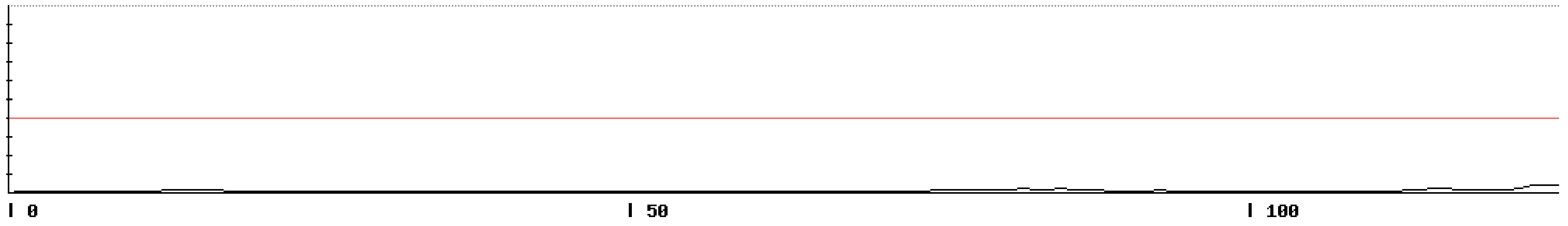

Predictions for Citrusclementina_Ciclev10033436m

Viterbi Path

Viterbi Path

Posterior @ 0.4

77 - KKKSSKGSR - 85

174 - KRK - 176

Posterior @ 0.4

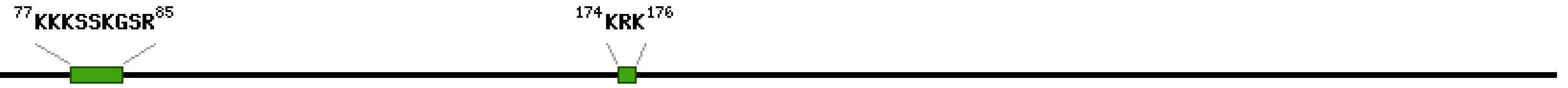

Posterior Graphic. Threshold Marked @ 0.4

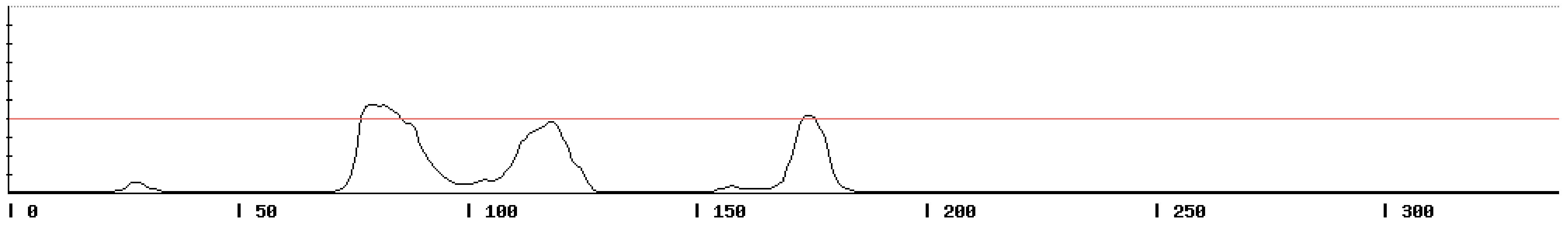

Predictions for Citrusclementina_Ciclev10033459m

Viterbi Path

Viterbi Path

Posterior @ 0.4

78 - KKSSK - 82

Posterior @ 0.4

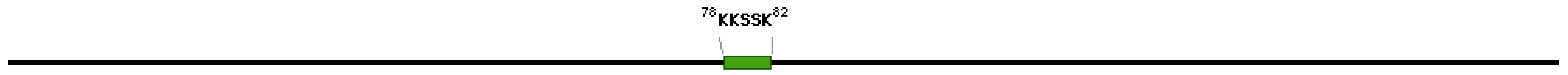

Posterior Graphic. Threshold Marked @ 0.4

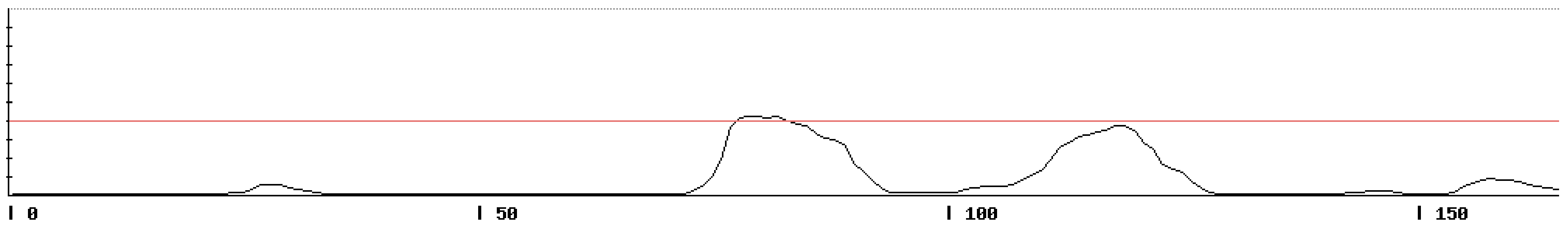

Predictions for Citrusclementina_Ciclev10033526m

Viterbi Path

Viterbi Path

Posterior @ 0.4

77 - KKKSSKGSR - 85

173 - KKRK - 176

Posterior @ 0.4

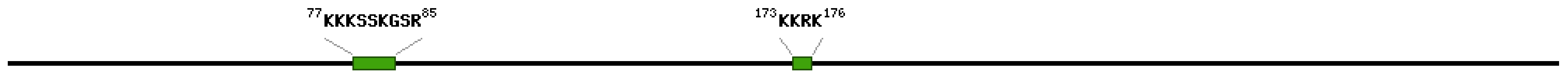

Posterior Graphic. Threshold Marked @ 0.4

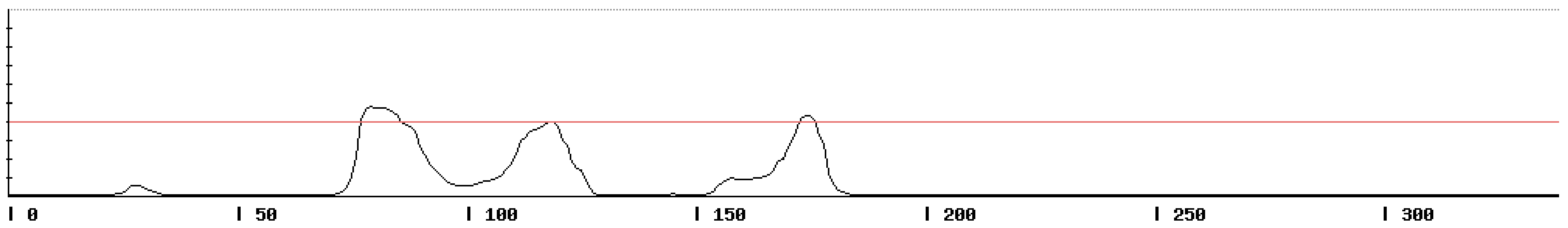

Predictions for Citrusclementina_Ciclev10033536m

Viterbi Path

Viterbi Path

Posterior @ 0.4

77 - KKKSSKGSR - 85

173 - KKRK - 176

Posterior @ 0.4

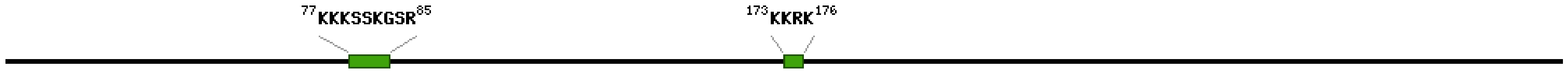

Posterior Graphic. Threshold Marked @ 0.4

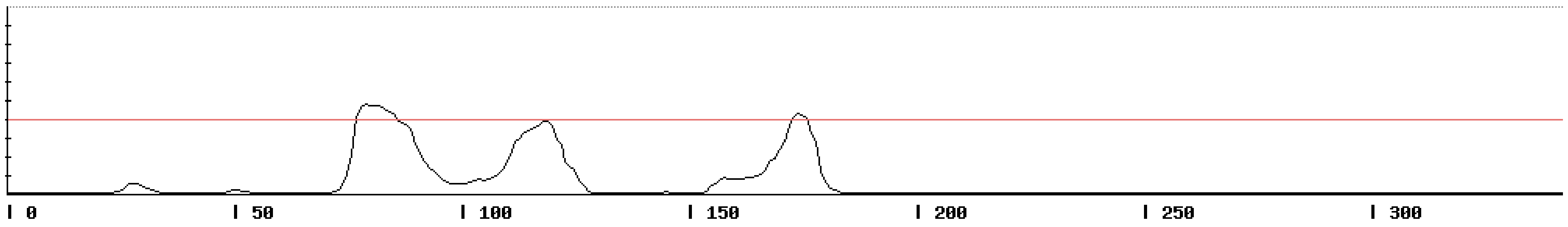

Predictions for Citrusclementina_Ciclev10033541m

Viterbi Path

Viterbi Path

Posterior @ 0.4

77 - KKKSSKGSR - 85

174 - KR - 175

Posterior @ 0.4

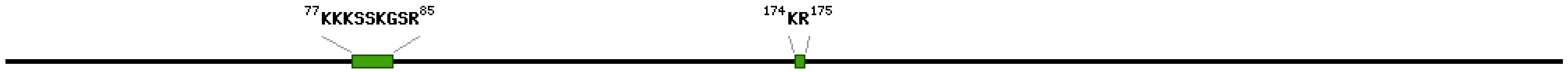

Posterior Graphic. Threshold Marked @ 0.4

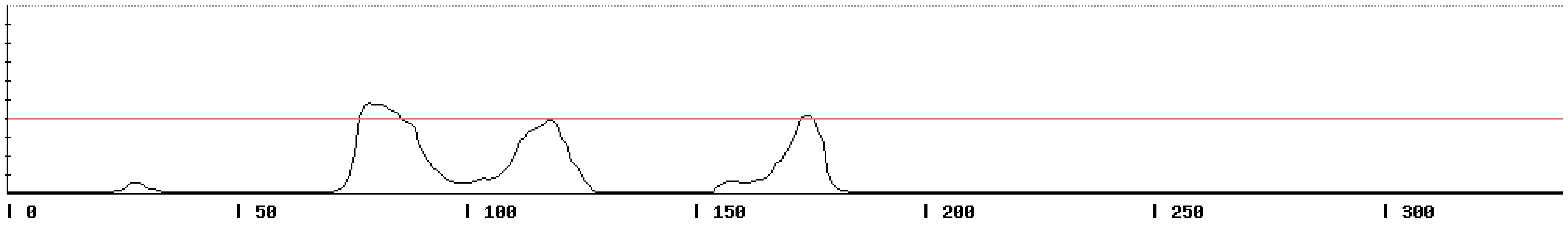

Predictions for Citrusclementina_Ciclev10033719m

Viterbi Path	
Viterbi Path	
Posterior @ 0.4	78 - KKSSKGSR - 85
	113 - IKIGSKKRFR - 122
	173 - KKRK - 176
Posterior @ 0.4	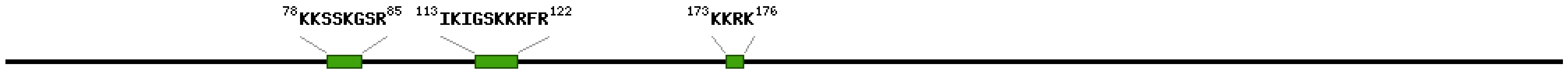
Posterior Graphic. Threshold Marked @ 0.4	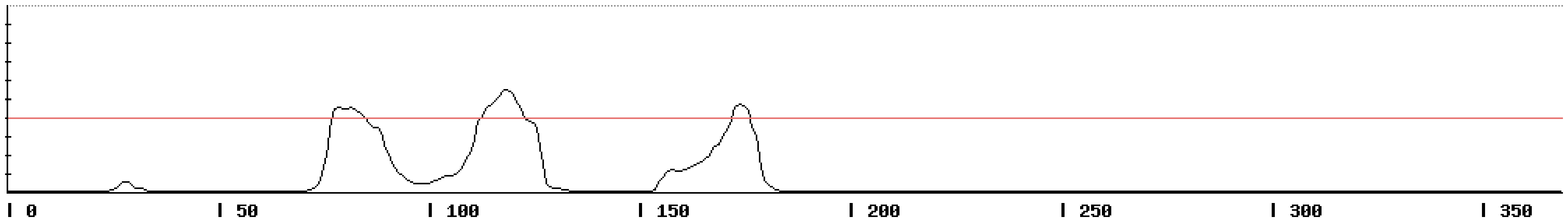

Predictions for Citrusclementina_Ciclev10033744m

Viterbi Path	
Viterbi Path	
Posterior @ 0.4	65 - KKKSSKGSR - 73
	161 - KKRK - 164

Viterbi Path

Posterior @ 0.4 — 99 - RK - 100

Posterior @ 0.4

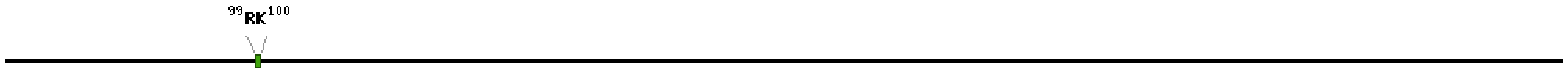

Posterior Graphic. Threshold Marked @ 0.4

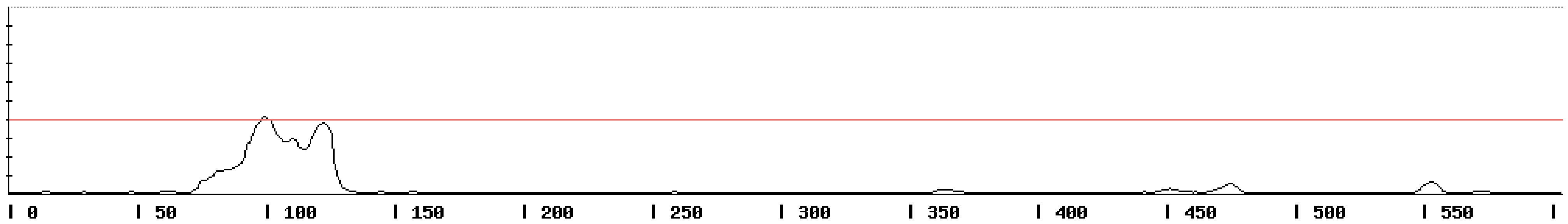

Predictions for Citrussinensis_orange1.1g007432m

Viterbi Path

Viterbi Path

Posterior @ 0.4 — 99 - RK - 100

Posterior @ 0.4

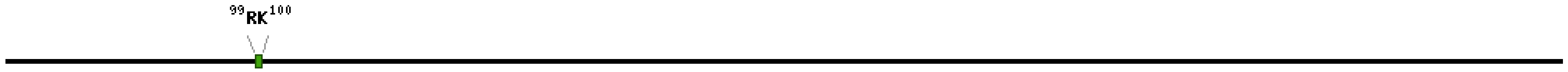

Posterior Graphic. Threshold Marked @ 0.4

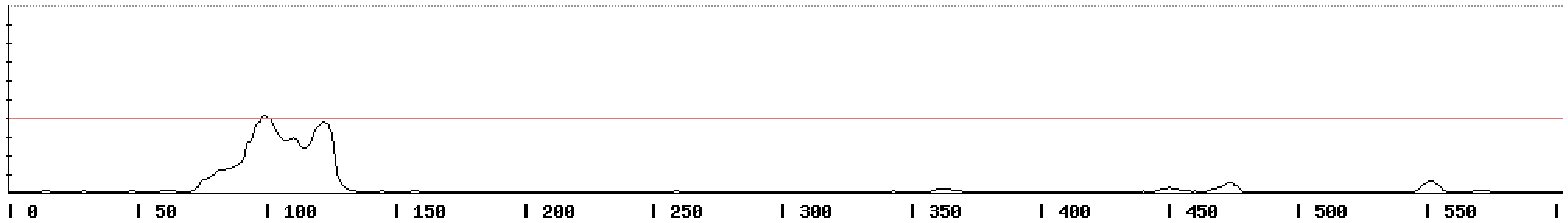

Predictions for Citrussinensis_orange1.1g007598m

Viterbi Path	
Viterbi Path	
Posterior @ 0.4	
Posterior @ 0.4	
Posterior Graphic. Threshold Marked @ 0.4	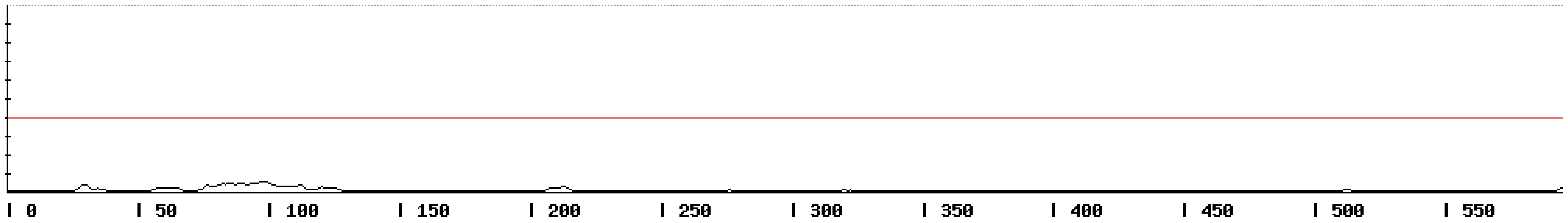

Predictions for Citrussinensis_orange1.1g007938m

Viterbi Path	
Viterbi Path	
Posterior @ 0.4	
Posterior @ 0.4	
Posterior Graphic. Threshold Marked @ 0.4	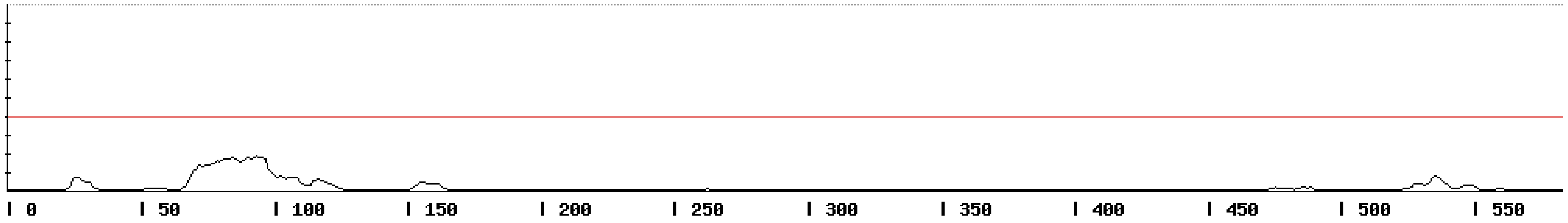

Predictions for Citrussinensis_orange1.1g008125m	
Viterbi Path	
Viterbi Path	
Posterior @ 0.4	
Posterior @ 0.4	
Posterior Graphic. Threshold Marked @ 0.4	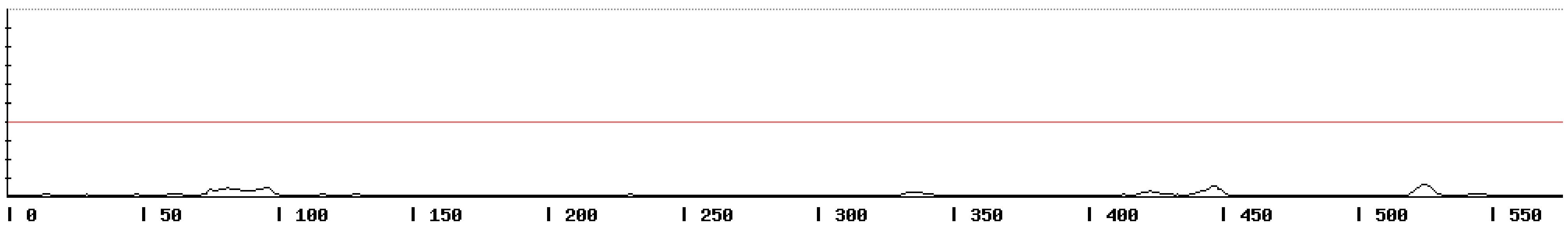

Predictions for Citrussinensis_orange1.1g008335m	
Viterbi Path	
Viterbi Path	
Posterior @ 0.4	549 - RKRREGPFKRT - 559
Posterior @ 0.4	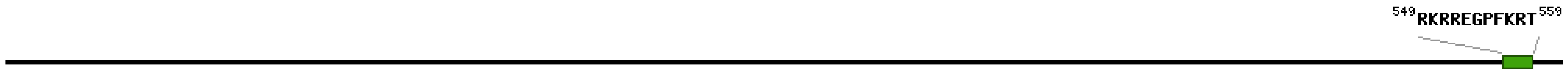
Posterior Graphic. Threshold Marked @ 0.4	

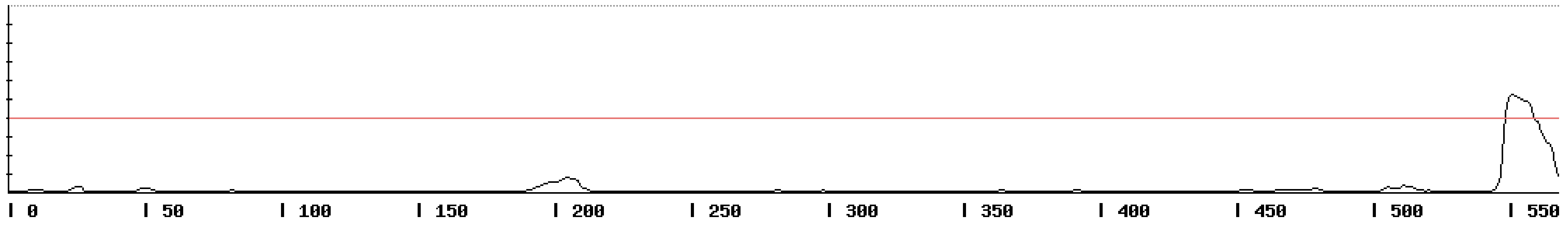

Predictions for Citrussinensis_orange1.1g008830m

Viterbi Path	
Viterbi Path	
Posterior @ 0.4	46 - RK - 47
Posterior @ 0.4	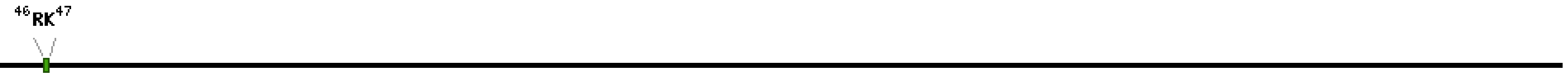
Posterior Graphic. Threshold Marked @ 0.4	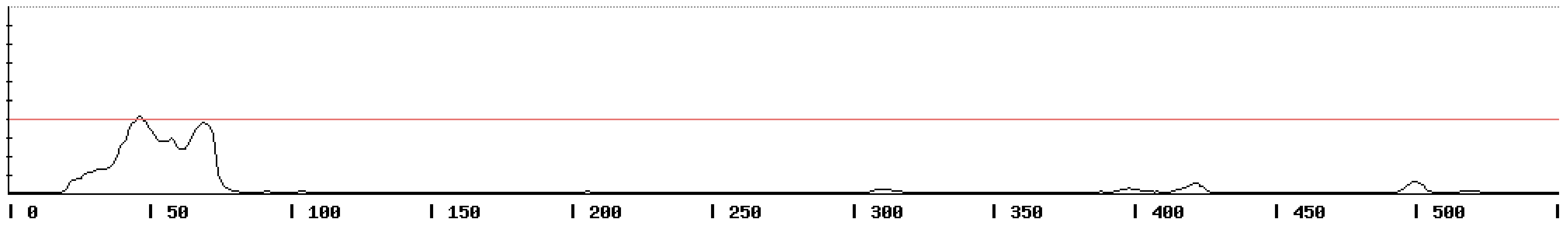

Predictions for Citrussinensis_orange1.1g008907m

Viterbi Path	
Viterbi Path	
Posterior @ 0.4	
Posterior @ 0.4	

Posterior Graphic. Threshold Marked @ 0.4

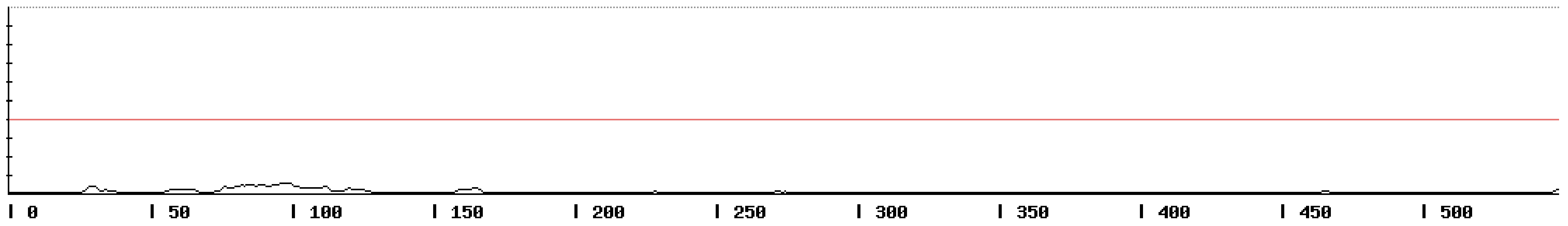

Predictions for Citrussinensis_orange1.1g008921m

Viterbi Path

Viterbi Path

Posterior @ 0.4

Posterior @ 0.4

Posterior Graphic. Threshold Marked @ 0.4

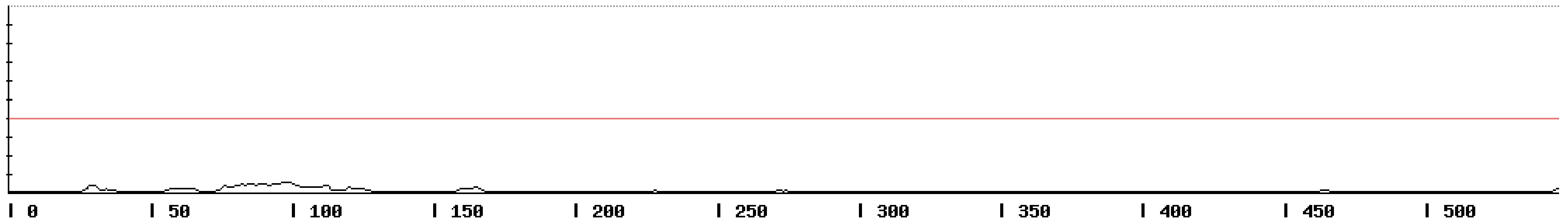

Predictions for Citrussinensis_orange1.1g009388m

Viterbi Path

Viterbi Path

Posterior @ 0.4

Posterior @ 0.4

Posterior Graphic. Threshold Marked @ 0.4

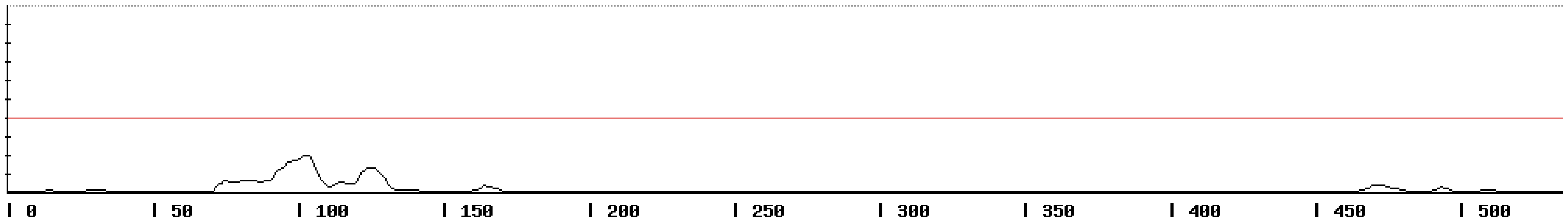

Predictions for Citrussinensis_orange1.1g009389m

Viterbi Path

Viterbi Path

Posterior @ 0.4

Posterior @ 0.4

Posterior Graphic. Threshold Marked @ 0.4

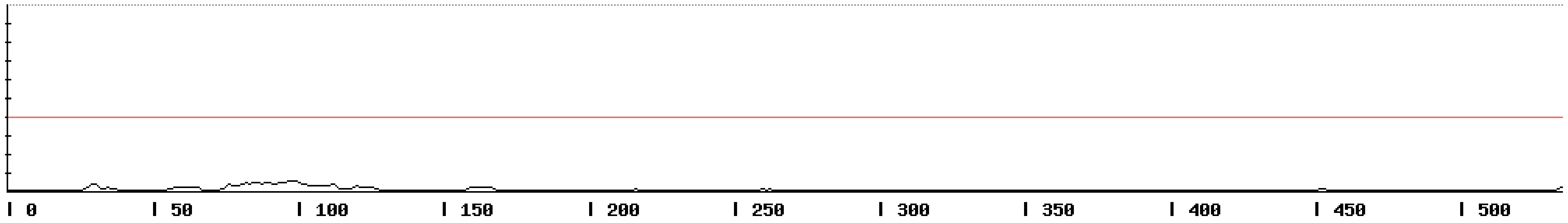

Predictions for Citrussinensis_orange1.1g009697m

Viterbi Path

Viterbi Path

Posterior @ 0.4

Posterior @ 0.4

Posterior Graphic. Threshold Marked @ 0.4

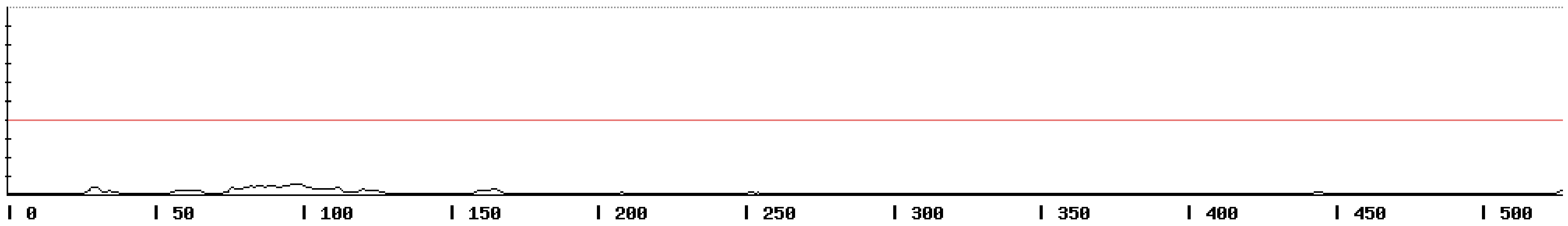

Predictions for Citrussinensis_orange1.1g011830m

Viterbi Path

Viterbi Path

Posterior @ 0.4

Posterior @ 0.4

Posterior Graphic. Threshold Marked @ 0.4

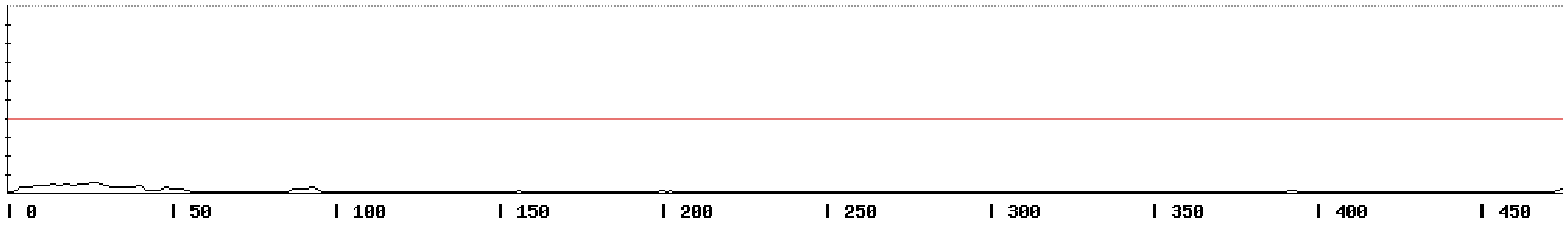

Predictions for Citrussinensis_orange1.1g012118m

Viterbi Path

Viterbi Path

Posterior @ 0.4	
Posterior @ 0.4	
Posterior Graphic. Threshold Marked @ 0.4	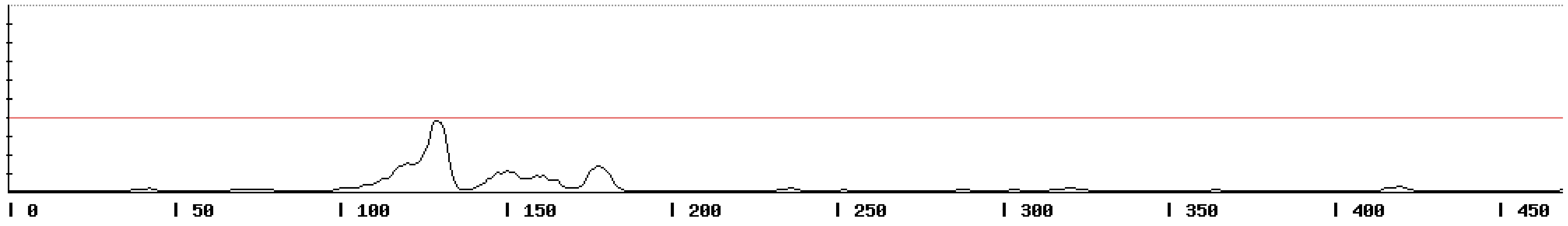

Predictions for Citrussinensis_orange1.1g012155m	
Viterbi Path	
Viterbi Path	
Posterior @ 0.4	
Posterior @ 0.4	
Posterior Graphic. Threshold Marked @ 0.4	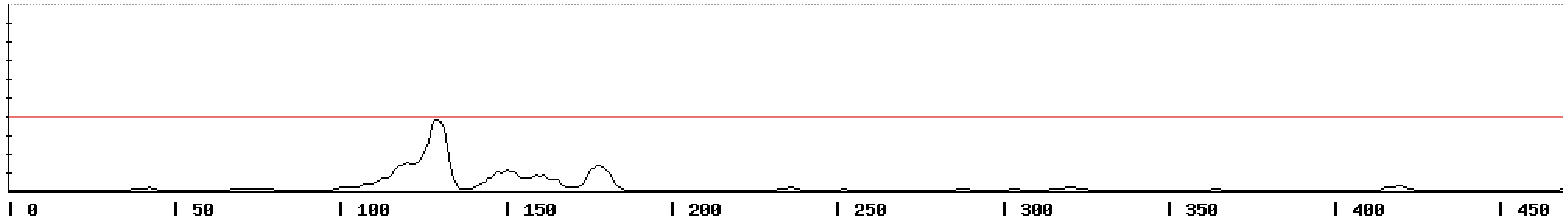

Predictions for Citrussinensis_orange1.1g013027m

Viterbi Path	
Viterbi Path	
Posterior @ 0.4	150 - KKKSG - 154
Posterior @ 0.4	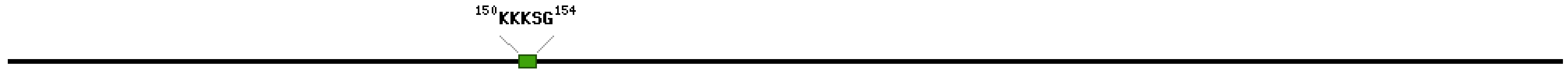
Posterior Graphic. Threshold Marked @ 0.4	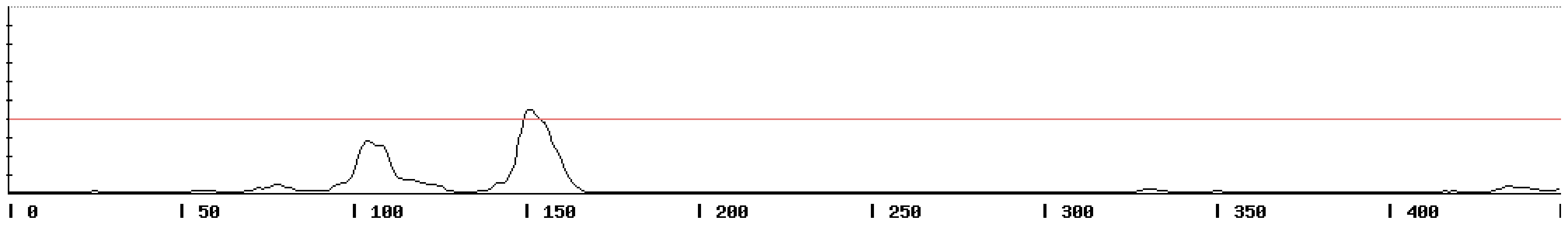

Predictions for Citrussinensis_orange1.1g013077m

Viterbi Path	
Viterbi Path	
Posterior @ 0.4	150 - KKS - 152
Posterior @ 0.4	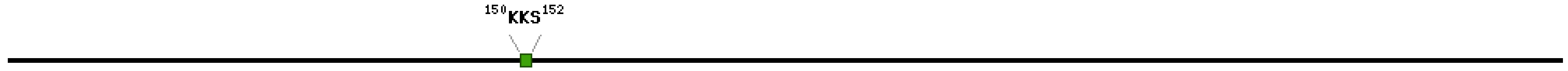
Posterior Graphic. Threshold Marked @ 0.4	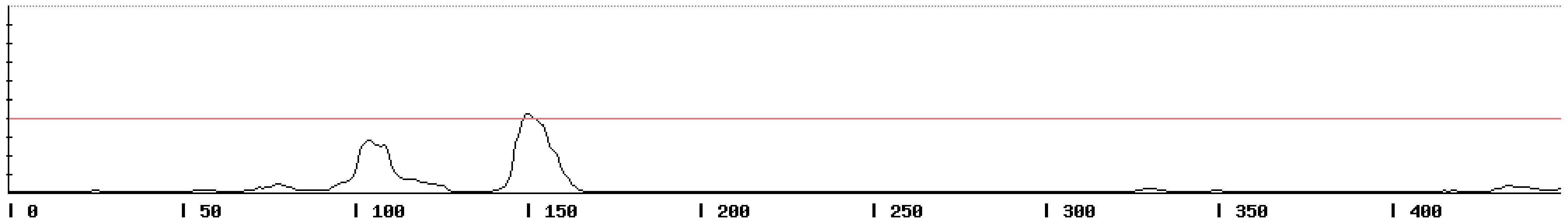

Predictions for Citrussinensis_orange1.1g013960m	
Viterbi Path	
Viterbi Path	
Posterior @ 0.4	
Posterior @ 0.4	
Posterior Graphic. Threshold Marked @ 0.4	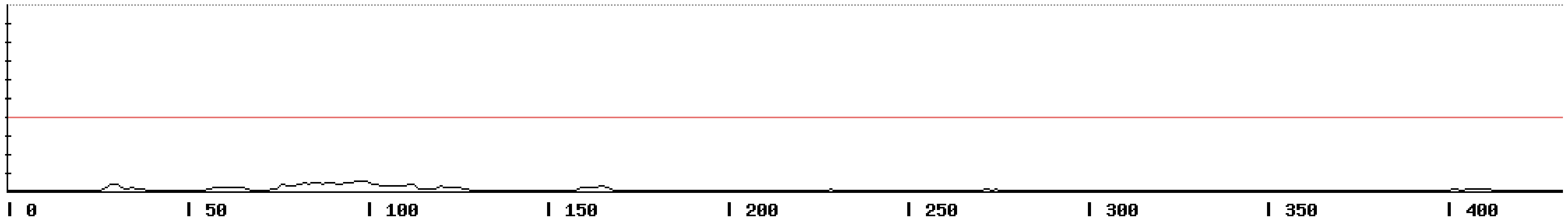

Predictions for Citrussinensis_orange1.1g014130m	
Viterbi Path	
Viterbi Path	
Posterior @ 0.4	
Posterior @ 0.4	
Posterior Graphic. Threshold Marked @ 0.4	

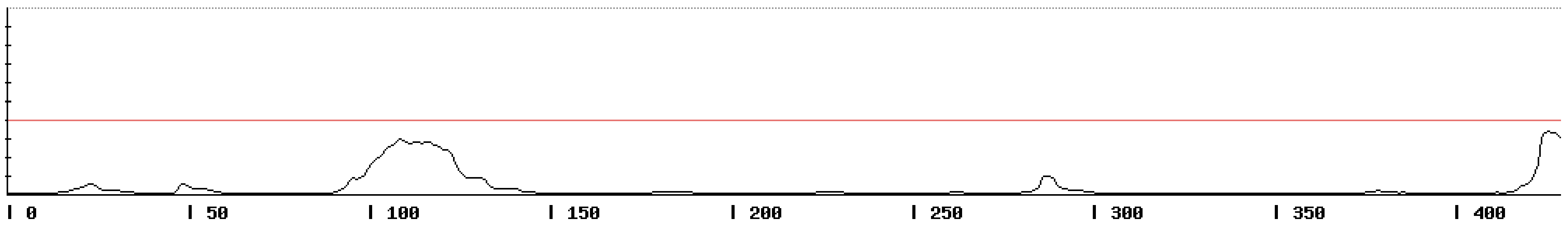

Predictions for Citrussinensis_orange1.1g014260m

Viterbi Path

Viterbi Path

Posterior @ 0.4

Posterior @ 0.4

Posterior Graphic. Threshold Marked @ 0.4

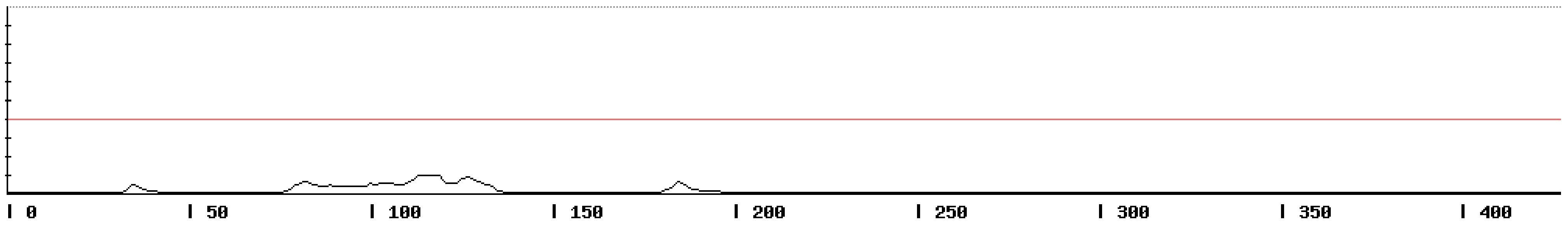

Predictions for Citrussinensis_orange1.1g014310m

Viterbi Path

Viterbi Path

Posterior @ 0.4 137 - KRR - 139

Posterior @ 0.4

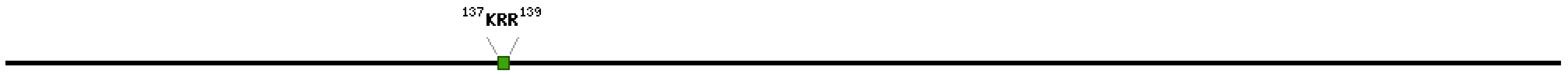

Posterior Graphic. Threshold Marked @ 0.4

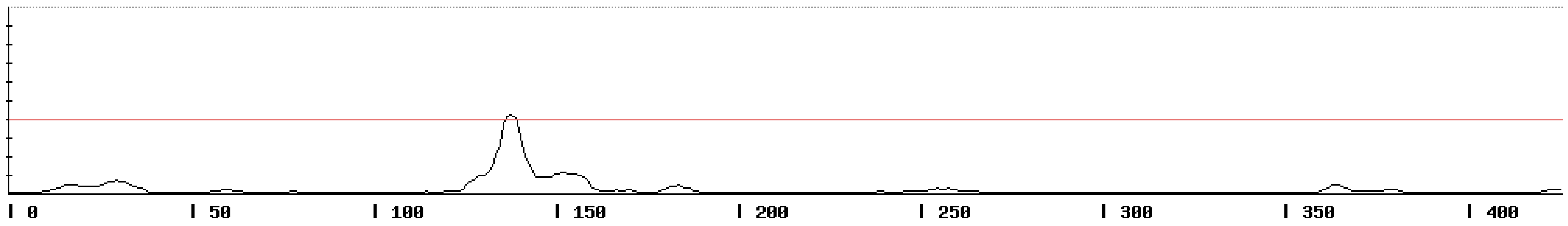

Predictions for Citrussinensis_orange1.1g014445m

Viterbi Path

Viterbi Path

Posterior @ 0.4 — 137 - KRR - 139

Posterior @ 0.4

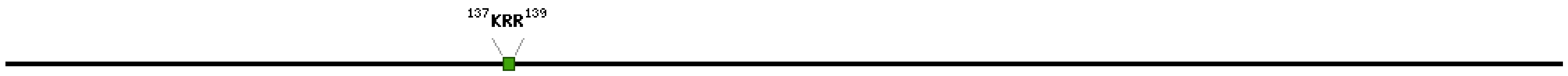

Posterior Graphic. Threshold Marked @ 0.4

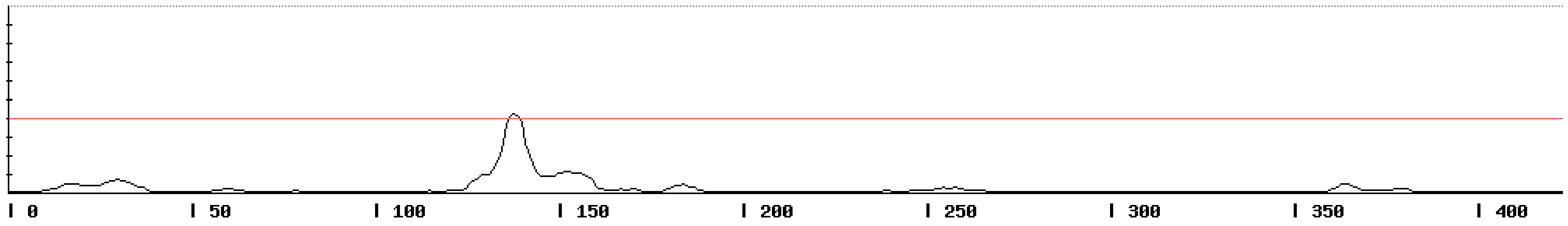

Predictions for Citrussinensis_orange1.1g015081m

Viterbi Path

Viterbi Path

Posterior @ 0.4

Posterior @ 0.4

Posterior Graphic. Threshold Marked @ 0.4	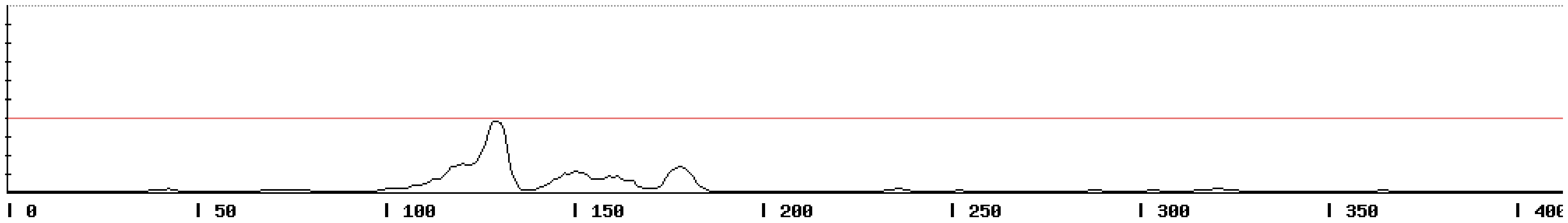

Predictions for Citrussinensis_orange1.1g015575m	
Viterbi Path	
Viterbi Path	
Posterior @ 0.4	147 - FRRRNKKKRG - 156
Posterior @ 0.4	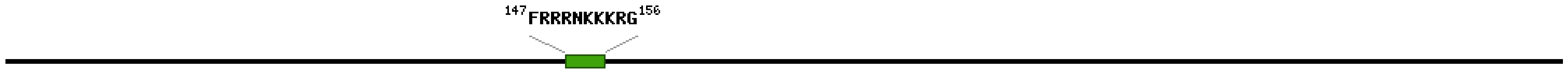
Posterior Graphic. Threshold Marked @ 0.4	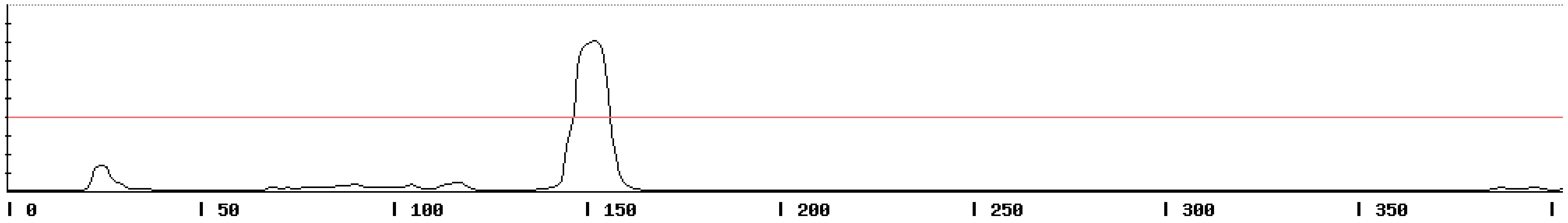

Predictions for Citrussinensis_orange1.1g015827m	
Viterbi Path	
Viterbi Path	

Posterior @ 0.4

Posterior @ 0.4

Posterior Graphic. Threshold Marked @ 0.4

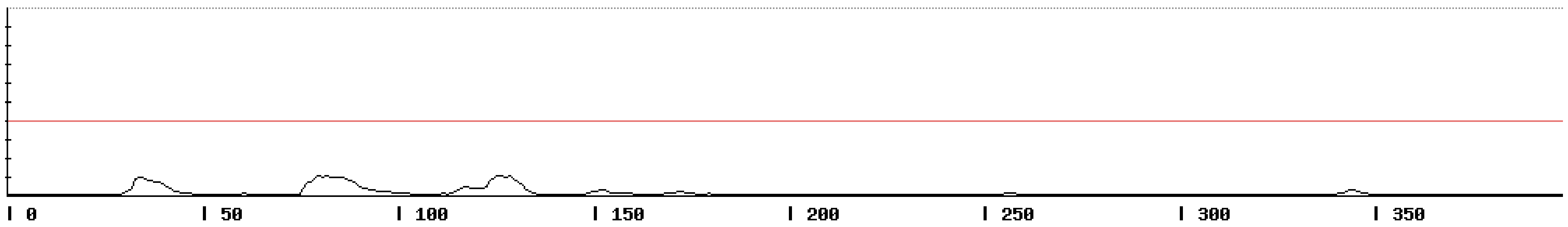

Predictions for Citrussinensis_orange1.1g015853m

Viterbi Path

Viterbi Path

Posterior @ 0.4

Posterior @ 0.4

Posterior Graphic. Threshold Marked @ 0.4

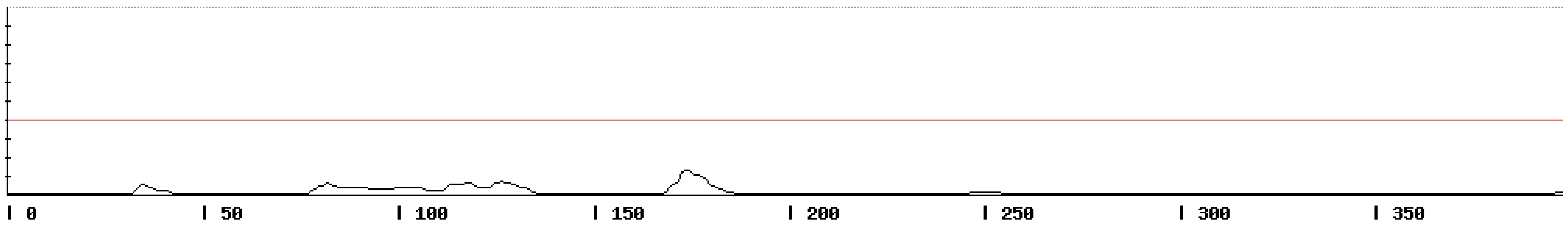

Predictions for Citrussinensis_orange1.1g015924m

Viterbi Path

Viterbi Path

Posterior @ 0.4

Posterior @ 0.4

Posterior Graphic. Threshold Marked @ 0.4

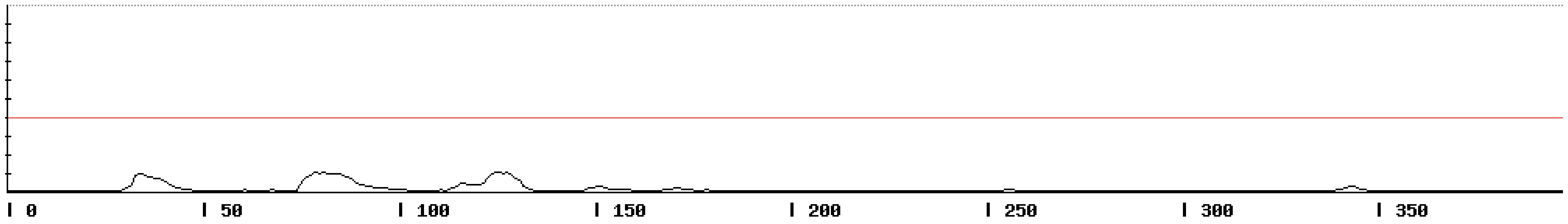

Predictions for Citrussinensis_orange1.1g015998m

Viterbi Path

Viterbi Path

Posterior @ 0.4

Posterior @ 0.4

Posterior Graphic. Threshold Marked @ 0.4

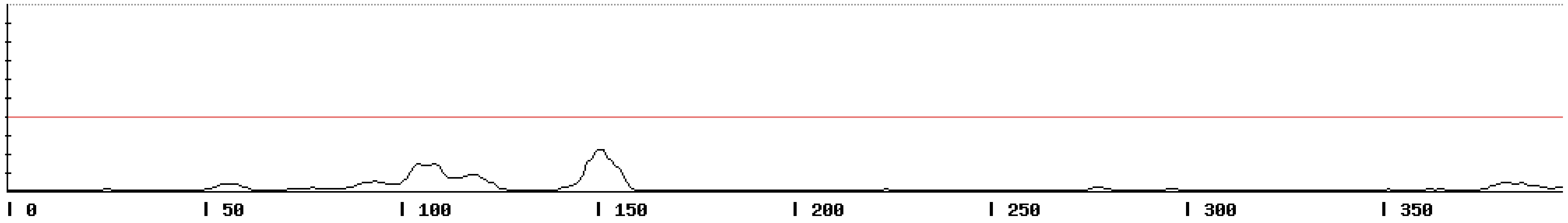

Predictions for Citrussinensis_orange1.1g016086m

Viterbi Path

Viterbi Path

Posterior @ 0.4

Posterior @ 0.4

Posterior Graphic. Threshold Marked @ 0.4

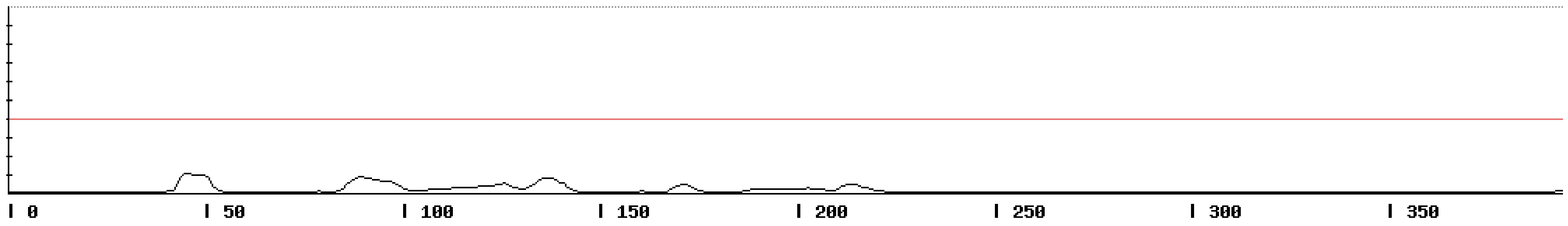

Predictions for Citrussinensis_orange1.1g016095m

Viterbi Path

Viterbi Path

Posterior @ 0.4

Posterior @ 0.4

Posterior Graphic. Threshold Marked @ 0.4

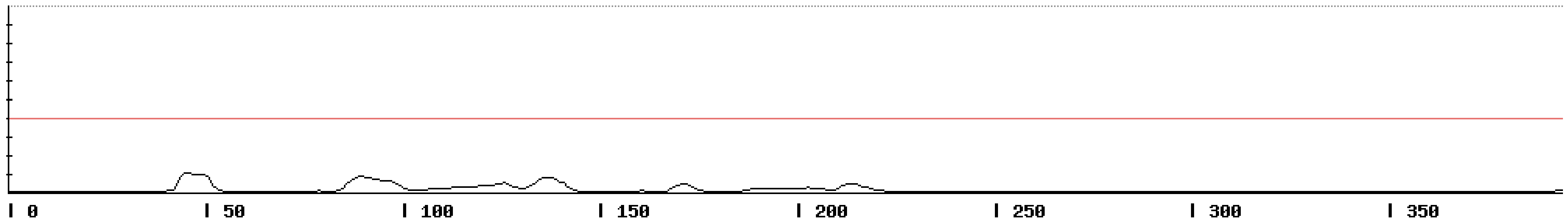

Predictions for Citrussinensis_orange1.1g016181m	
Viterbi Path	
Viterbi Path	
Posterior @ 0.4	94 - QKKYKKGSIRERKAKGGFWK - 113
Posterior @ 0.4	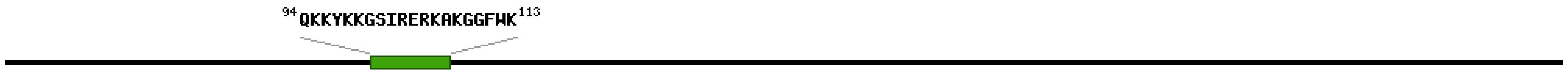
Posterior Graphic. Threshold Marked @ 0.4	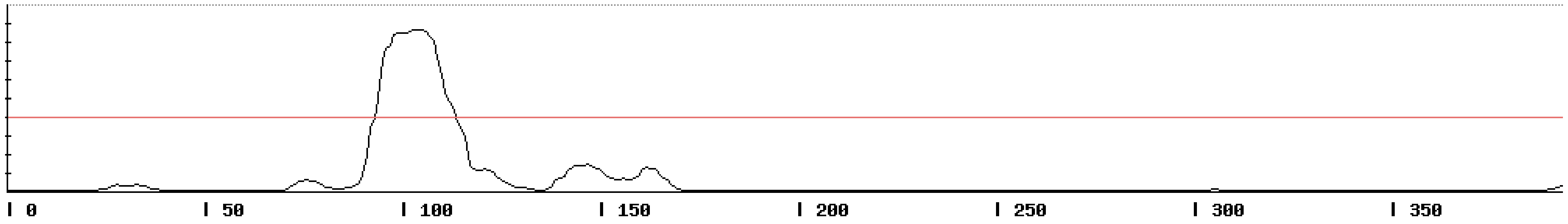

Predictions for Citrussinensis_orange1.1g016283m	
Viterbi Path	
Viterbi Path	
Posterior @ 0.4	
Posterior @ 0.4	
Posterior Graphic. Threshold Marked @ 0.4	

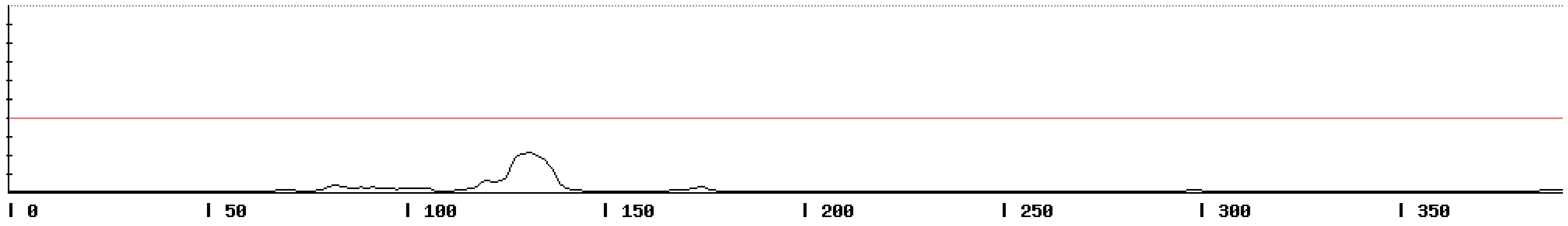

Predictions for Citrussinensis_orange1.1g016337m

Viterbi Path	
Viterbi Path	
Posterior @ 0.4	94 - QKKYKKGSIRERKAKGGFWK - 113
Posterior @ 0.4	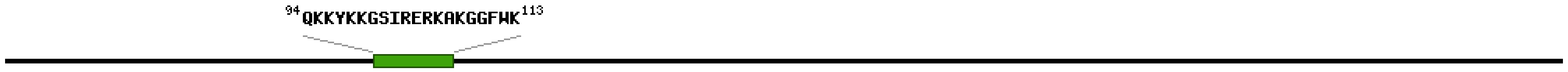
Posterior Graphic. Threshold Marked @ 0.4	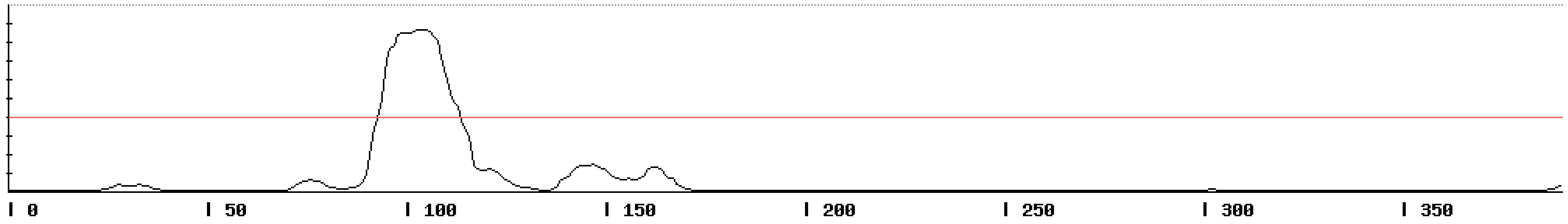

Predictions for Citrussinensis_orange1.1g016435m

Viterbi Path	150 - RKREKKTLGVVSTKRKREKTLGVVSSKRKRDKK - 182
Viterbi Path	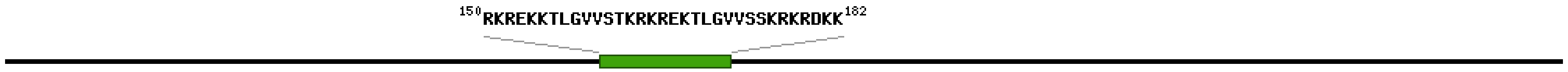
Posterior @ 0.4	75 - KRVHRRTKEGYWKKTGRGS - 93
	149 - ERKREKKTLGVVSTKRKREKTLGVVSSKRKRDKKL - 183
Posterior @ 0.4	

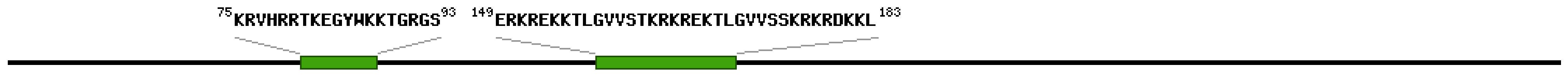

Posterior Graphic. Threshold Marked @ 0.4

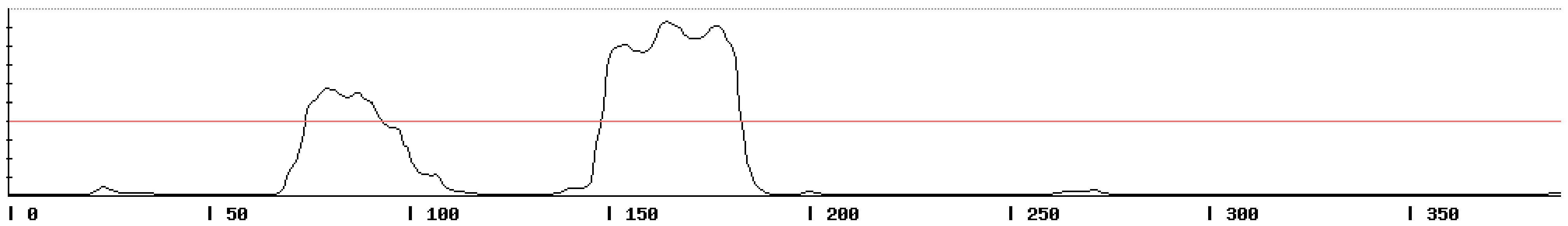

Predictions for Citrussinensis_orange1.1g016542m

Viterbi Path

Viterbi Path

Posterior @ 0.4

Posterior @ 0.4

Posterior Graphic. Threshold Marked @ 0.4

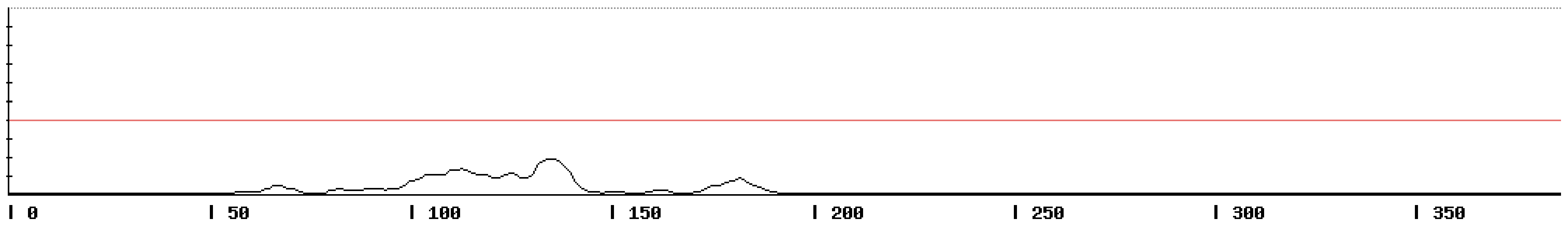

Predictions for Citrussinensis_orange1.1g016791m

Viterbi Path

Viterbi Path

Posterior @ 0.4

Posterior @ 0.4

Posterior Graphic. Threshold Marked @ 0.4

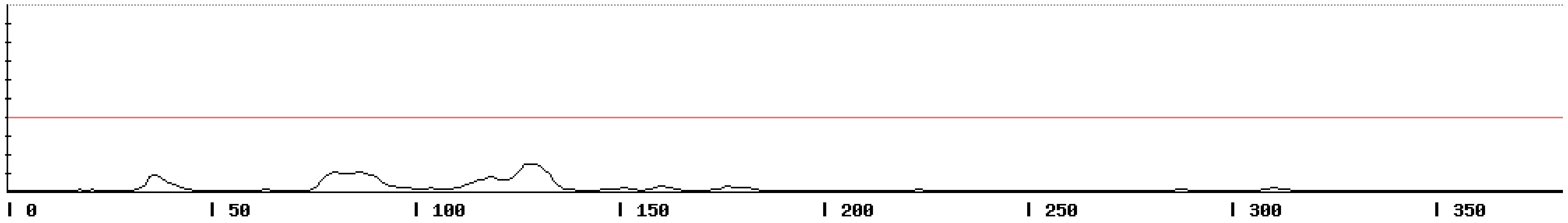

Predictions for Citrussinensis_orange1.1g017142m

Viterbi Path

150 - RKREKKTLGVVSTKRKREKKTLGVVSTKRKREKK - 183

Viterbi Path

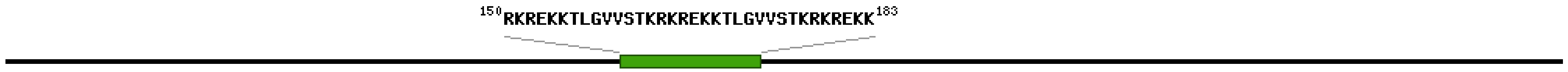

Posterior @ 0.4

73 - ESKRVRRRTKEGYWKKTGRGSNIKRKYKT - 101

149 - ERKREKKTLGVVSTKRKREKKTLGVVSTKRKREKKL - 184

Posterior @ 0.4

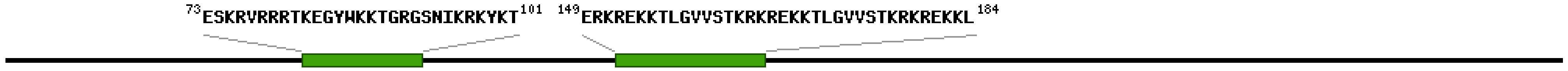

Posterior Graphic. Threshold Marked @ 0.4

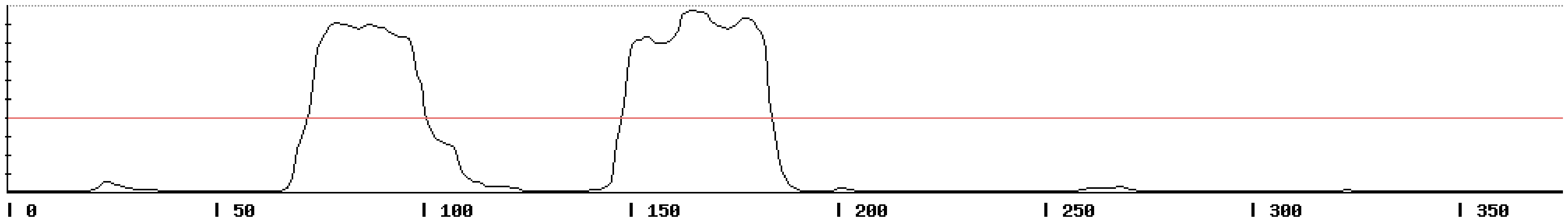

Predictions for Citrussinensis_orange1.1g017473m

Viterbi Path

Viterbi Path

Posterior @ 0.4 | 147 - FRRRNKKKRG - 156

Posterior @ 0.4

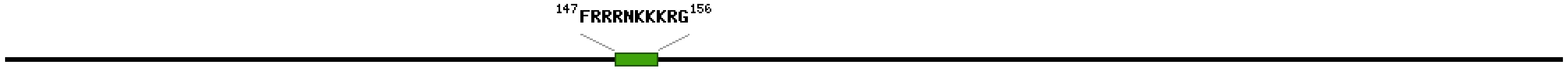

Posterior Graphic. Threshold Marked @ 0.4

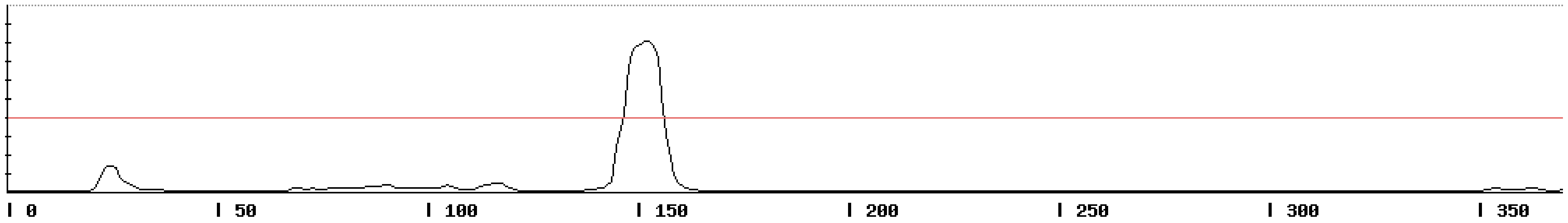

Predictions for Citrussinensis_orange1.1g017665m

Viterbi Path

Viterbi Path

Posterior @ 0.4

Posterior @ 0.4

Posterior Graphic. Threshold Marked @ 0.4

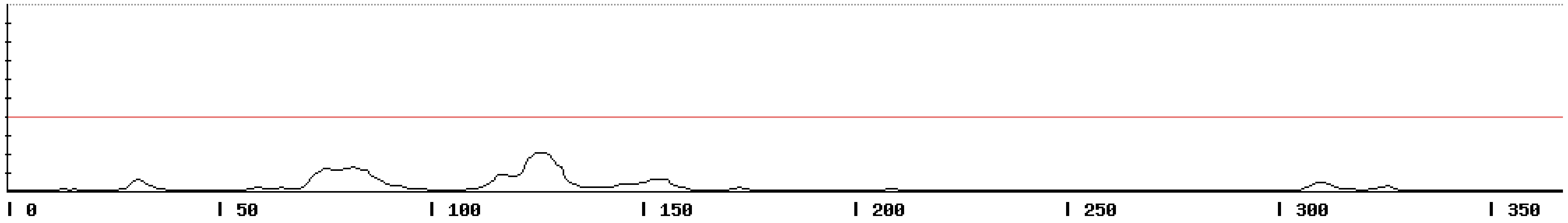

Predictions for Citrussinensis_orange1.1g017827m

Viterbi Path

Viterbi Path

Posterior @ 0.4

Posterior @ 0.4

Posterior Graphic. Threshold Marked @ 0.4

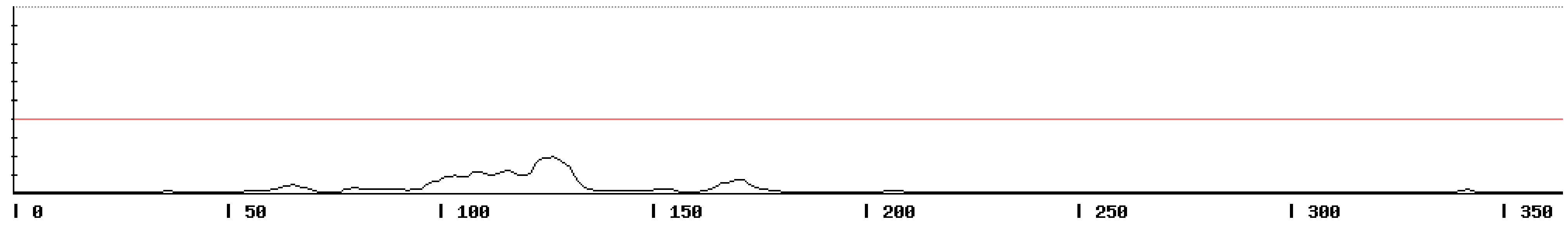

Predictions for Citrussinensis_orange1.1g017854m

Viterbi Path

Viterbi Path

Posterior @ 0.4

Posterior @ 0.4

Posterior Graphic. Threshold Marked @ 0.4

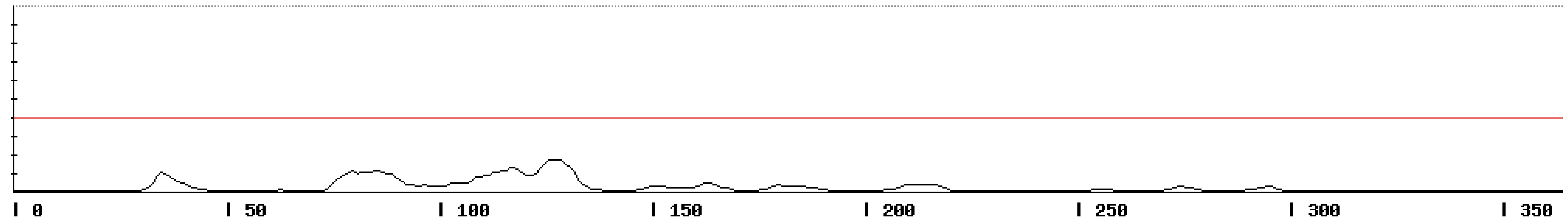

Predictions for Citrussinensis_orange1.1g018596m

Viterbi Path

Viterbi Path

Posterior @ 0.4

Posterior @ 0.4

Posterior Graphic. Threshold Marked @ 0.4

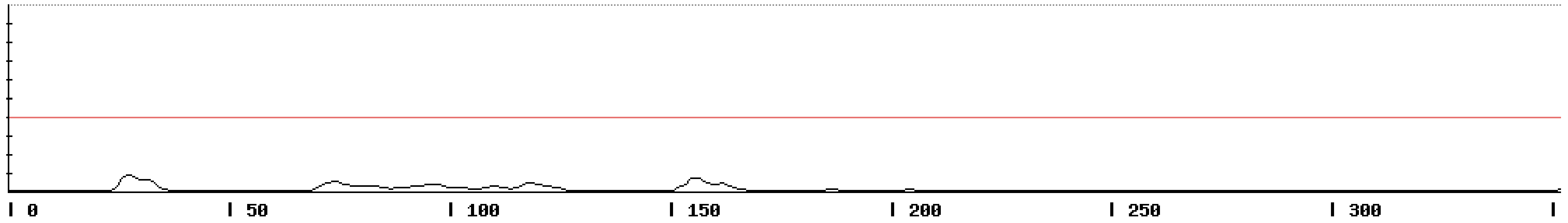

Predictions for Citrussinensis_orange1.1g018835m

Viterbi Path

Viterbi Path

Posterior @ 0.4

Posterior @ 0.4

Posterior Graphic. Threshold Marked @ 0.4

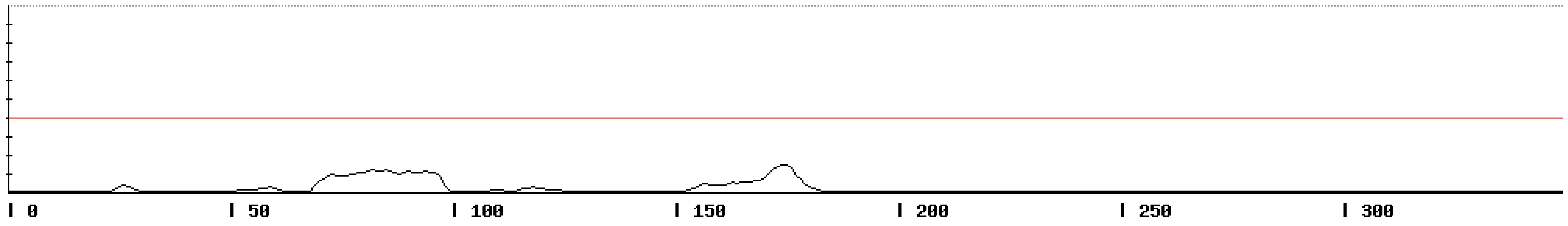

Predictions for Citrussinensis_orange1.1g018950m

Viterbi Path	
Viterbi Path	
Posterior @ 0.4	
Posterior @ 0.4	
Posterior Graphic. Threshold Marked @ 0.4	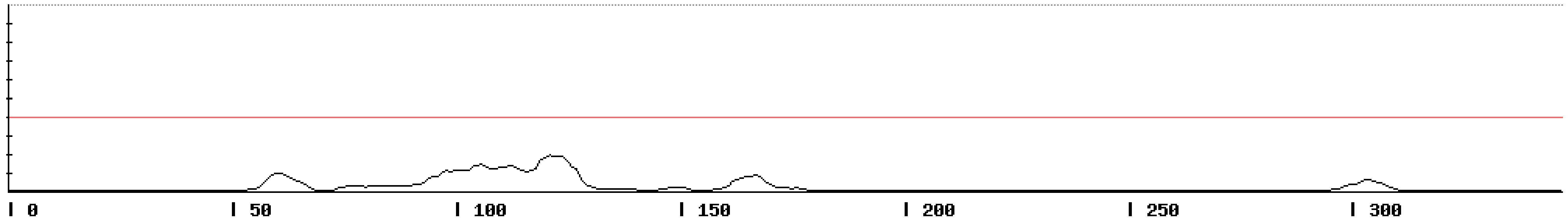

Predictions for Citrussinensis_orange1.1g019011m

Viterbi Path	
Viterbi Path	
Posterior @ 0.4	
Posterior @ 0.4	

Posterior Graphic. Threshold Marked @ 0.4

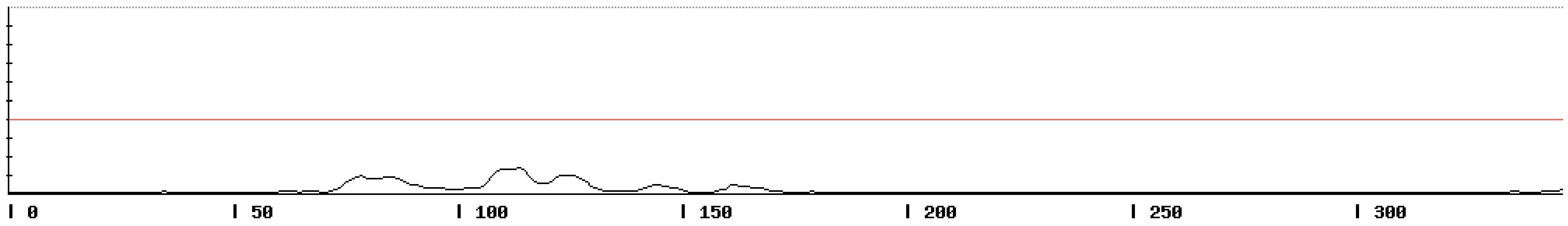

Predictions for Citrussinensis_orange1.1g019821m

Viterbi Path

Viterbi Path

Posterior @ 0.4

Posterior @ 0.4

Posterior Graphic. Threshold Marked @ 0.4

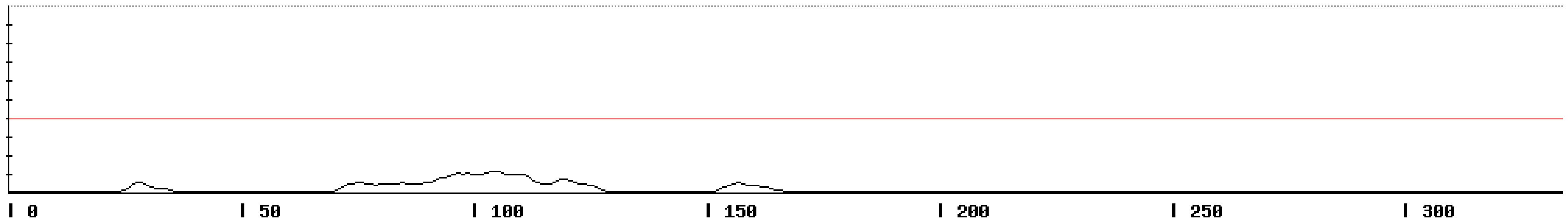

Predictions for Citrussinensis_orange1.1g020354m

Viterbi Path

Viterbi Path

Posterior @ 0.4

Posterior @ 0.4

Posterior Graphic. Threshold Marked @ 0.4

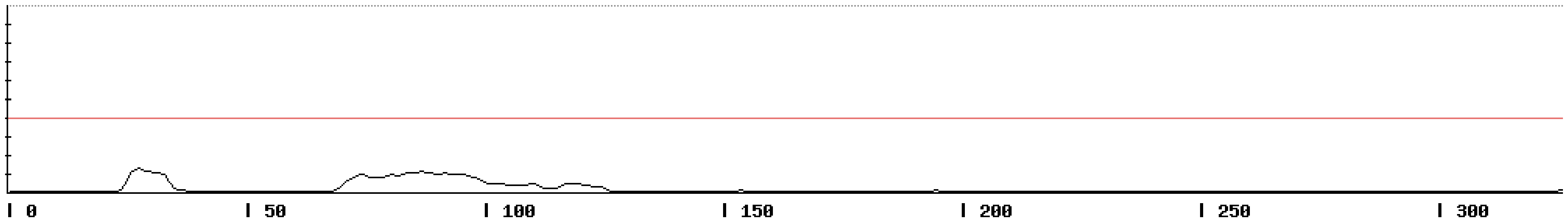

Predictions for Citrussinensis_orange1.1g021045m

Viterbi Path: 150 - RKREKKTLGVVSTKRKREKKTLGVVSTKRKREKK - 183

Viterbi Path

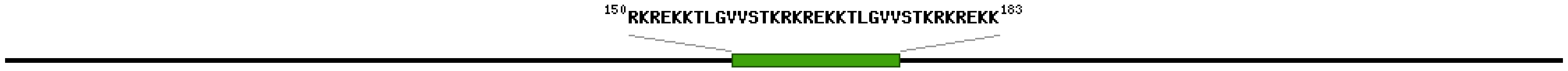

Posterior @ 0.4

73 - ESKRVRRRTKEGYWKKTGRGSNIKRKYKT - 101

149 - ERKREKKTLGVVSTKRKREKKTLGVVSTKRKREKKL - 184

Posterior @ 0.4

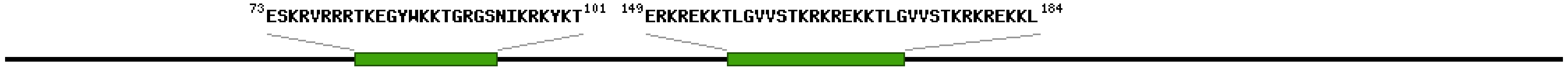

Posterior Graphic. Threshold Marked @ 0.4

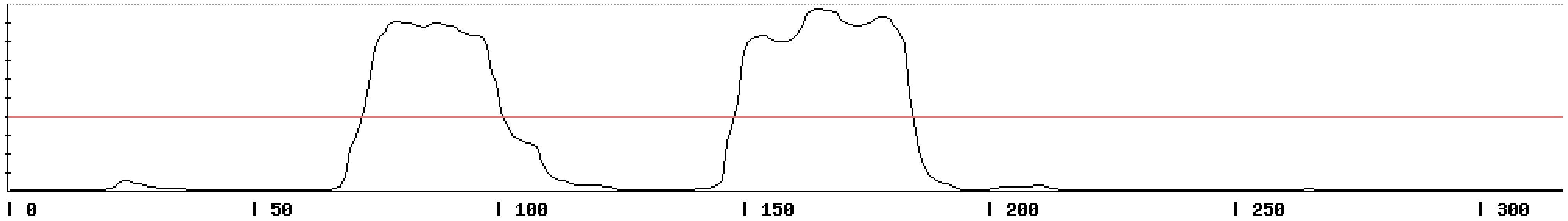

Predictions for Citrussinensis_orange1.1g021172m

Viterbi Path

Viterbi Path

Posterior @ 0.4

Posterior @ 0.4

Posterior Graphic. Threshold Marked @ 0.4

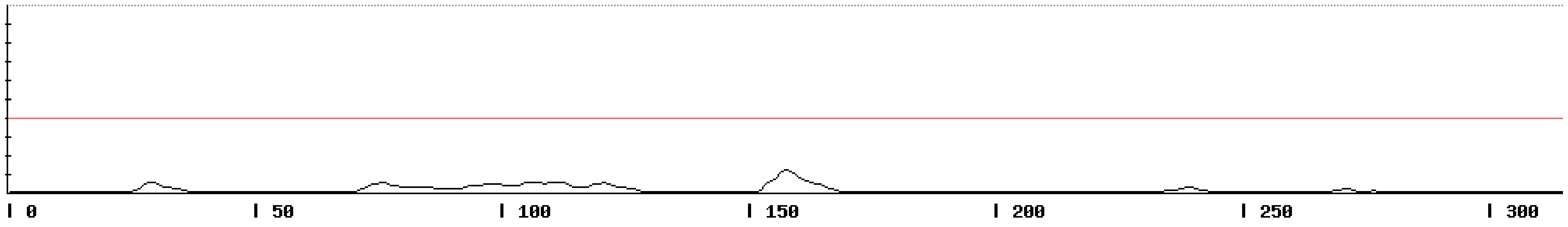

Predictions for Citrussinensis_orange1.1g021187m

Viterbi Path

Viterbi Path

Posterior @ 0.4

Posterior @ 0.4

Posterior Graphic. Threshold Marked @ 0.4

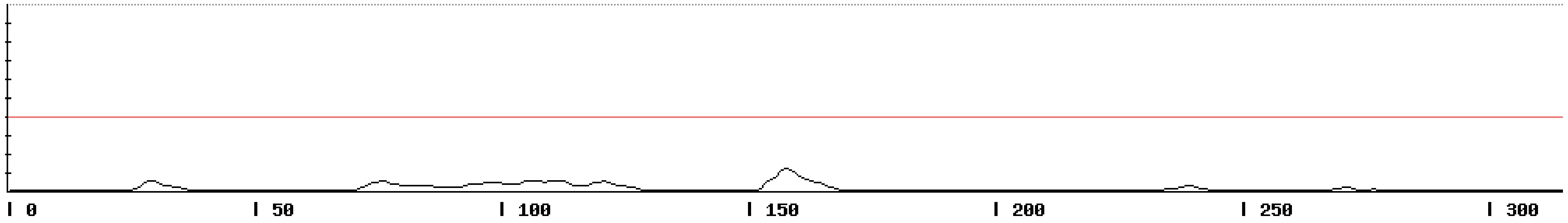

Predictions for Citrussinensis_orange1.1g021353m

Viterbi Path

Viterbi Path

Posterior @ 0.4

Posterior @ 0.4

Posterior Graphic. Threshold Marked @ 0.4

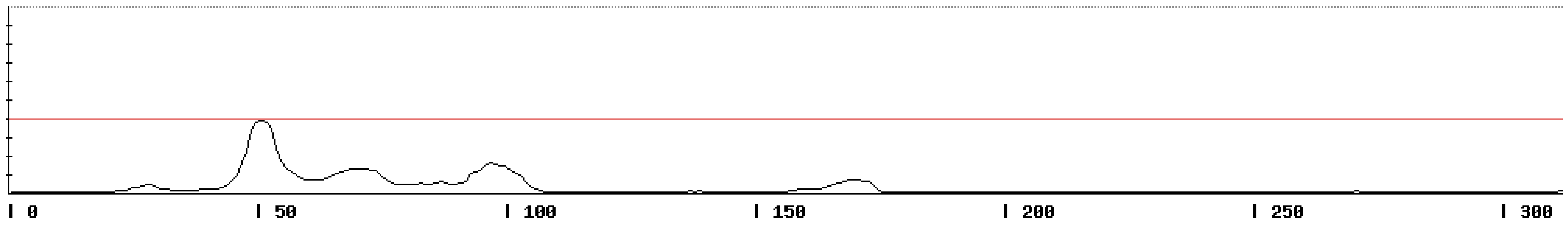

Predictions for Citrussinensis_orange1.1g021635m

Viterbi Path

Viterbi Path

Posterior @ 0.4

150 - RKRRK - 154

Posterior @ 0.4

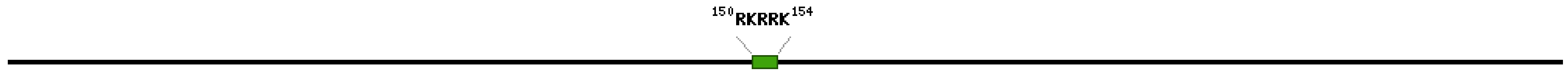

Posterior Graphic. Threshold Marked @ 0.4

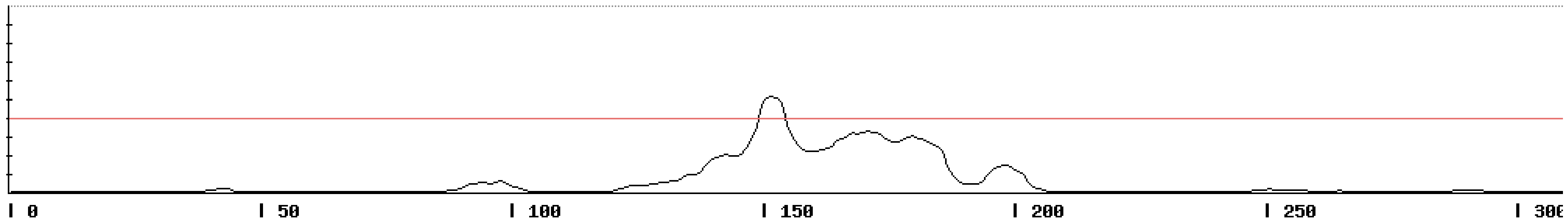

Predictions for Citrussinensis_orange1.1g021772m

Viterbi Path	
Viterbi Path	
Posterior @ 0.4	71 - AKSKRANRRTKEGYWKKTGNGSKIKRK - 97
Posterior @ 0.4	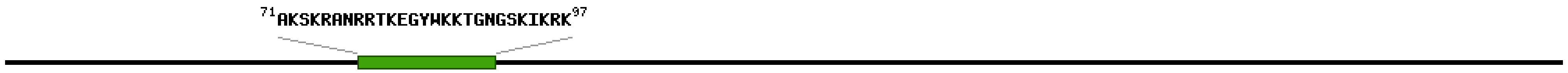
Posterior Graphic. Threshold Marked @ 0.4	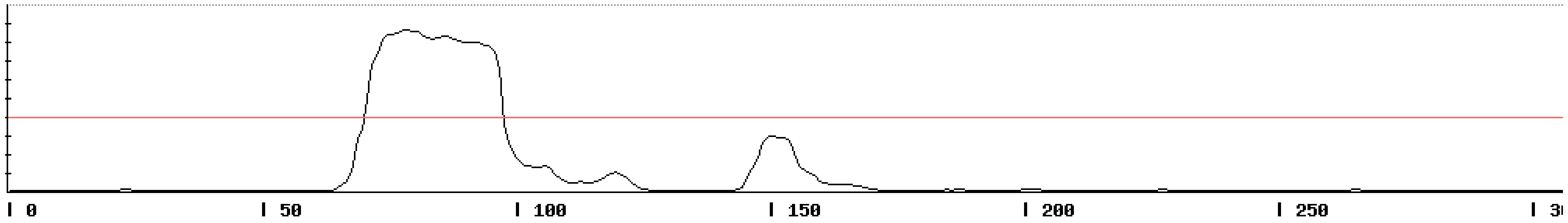

Predictions for Citrussinensis_orange1.1g021785m

Viterbi Path	
Viterbi Path	
Posterior @ 0.4	
Posterior @ 0.4	
Posterior Graphic. Threshold Marked @ 0.4	

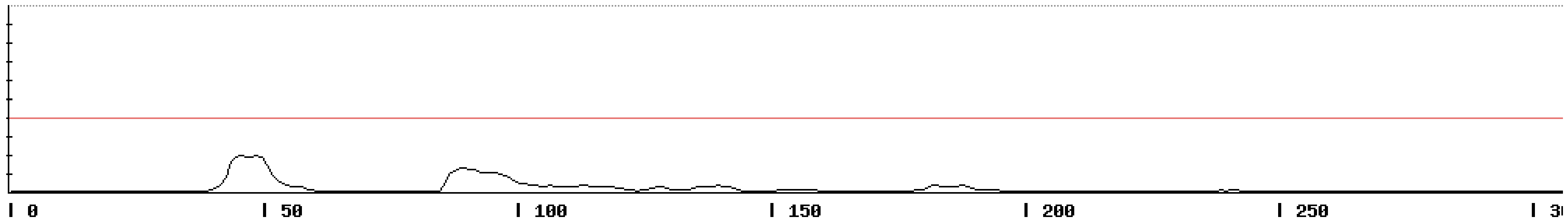

Predictions for Citrussinensis_orange1.1g021877m

Viterbi Path

Viterbi Path

Posterior @ 0.4

Posterior @ 0.4

Posterior Graphic. Threshold Marked @ 0.4

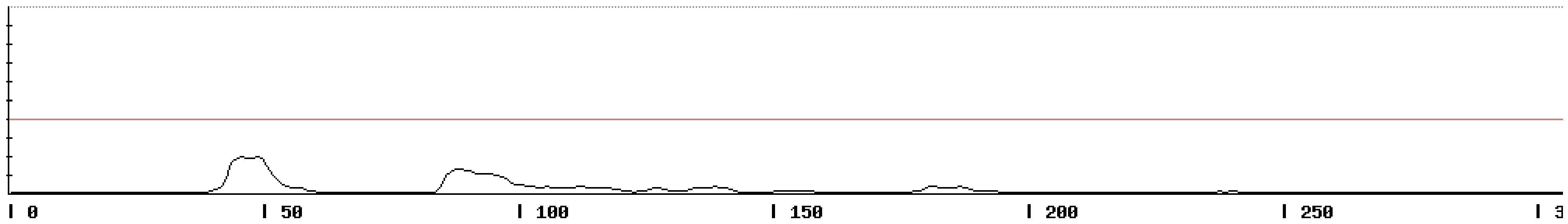

Predictions for Citrussinensis_orange1.1g021882m

Viterbi Path

Viterbi Path

Posterior @ 0.4

Posterior @ 0.4

Posterior Graphic. Threshold Marked @ 0.4

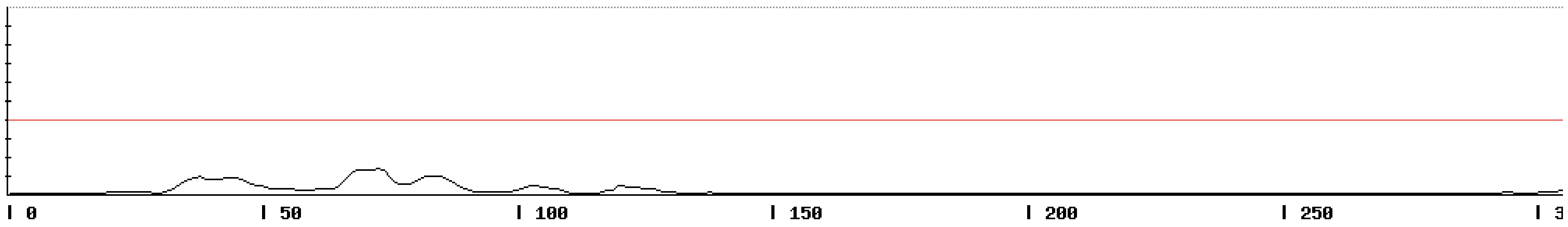

Predictions for Citrussinensis_orange1.1g021910m

Viterbi Path

Viterbi Path

Posterior @ 0.4 — 97 - KPIGKPKTLGIK - 108

Posterior @ 0.4

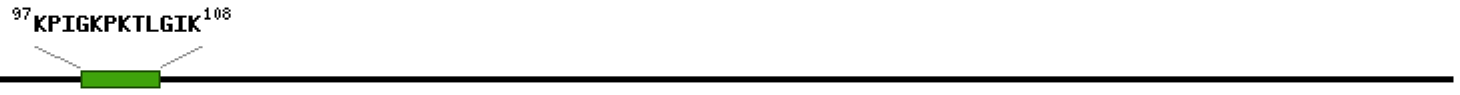

Posterior Graphic. Threshold Marked @ 0.4

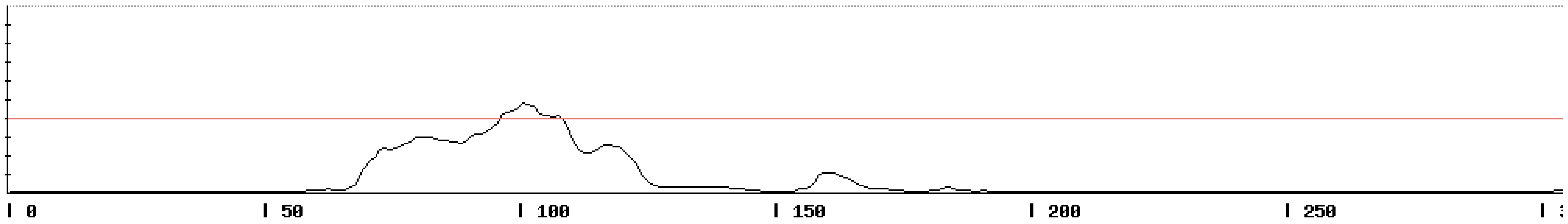

Predictions for Citrussinensis_orange1.1g022711m

Viterbi Path

Viterbi Path

Posterior @ 0.4 — 101 - RKGKNGYWRRR - 111

Posterior @ 0.4

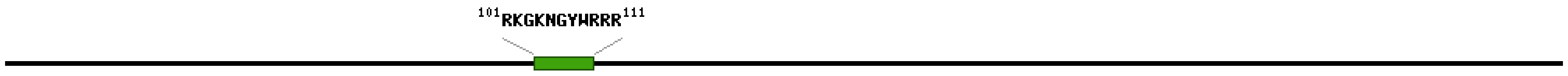

Posterior Graphic. Threshold Marked @ 0.4

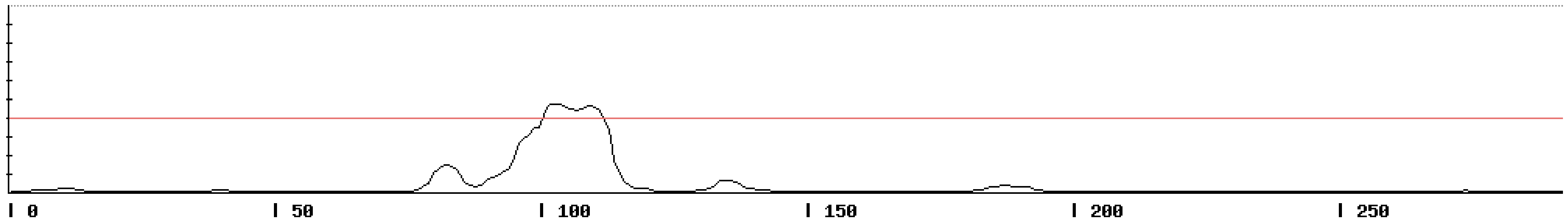

Predictions for Citrussinensis_orange1.1g023190m

Viterbi Path

Viterbi Path

Posterior @ 0.4

Posterior @ 0.4

Posterior Graphic. Threshold Marked @ 0.4

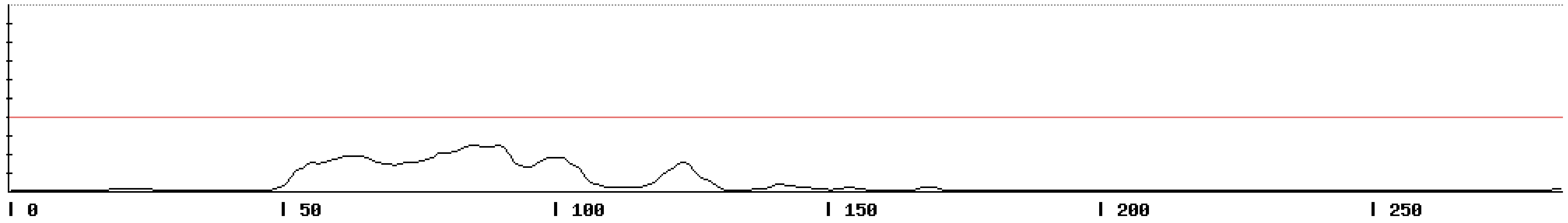

Predictions for Citrussinensis_orange1.1g023197m

Viterbi Path

Viterbi Path

Posterior @ 0.4

Posterior @ 0.4

Posterior Graphic. Threshold Marked @ 0.4

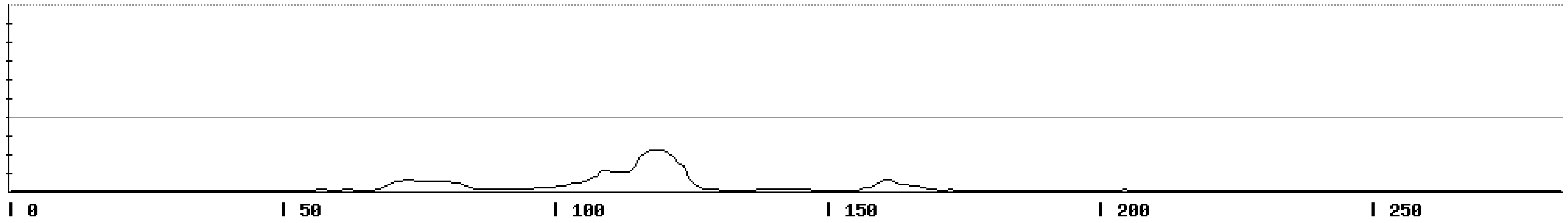

Predictions for Citrussinensis_orange1.1g023419m

Viterbi Path

Viterbi Path

Posterior @ 0.4

Posterior @ 0.4

Posterior Graphic. Threshold Marked @ 0.4

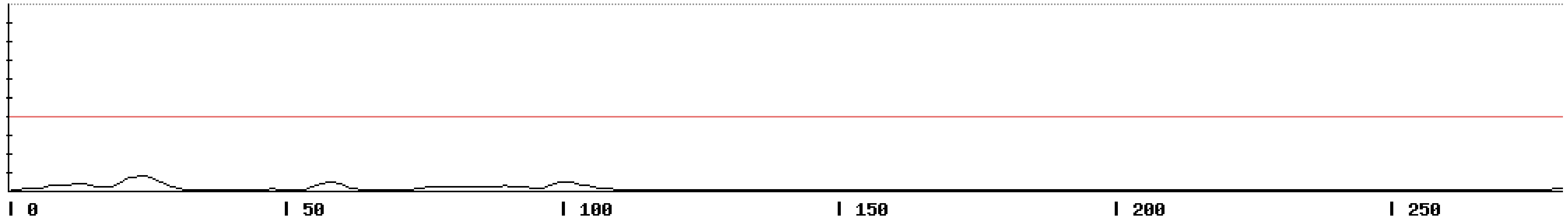

Predictions for Citrussinensis_orange1.1g023427m

Viterbi Path

Viterbi Path

Posterior @ 0.4

Posterior @ 0.4

Posterior Graphic. Threshold Marked @ 0.4

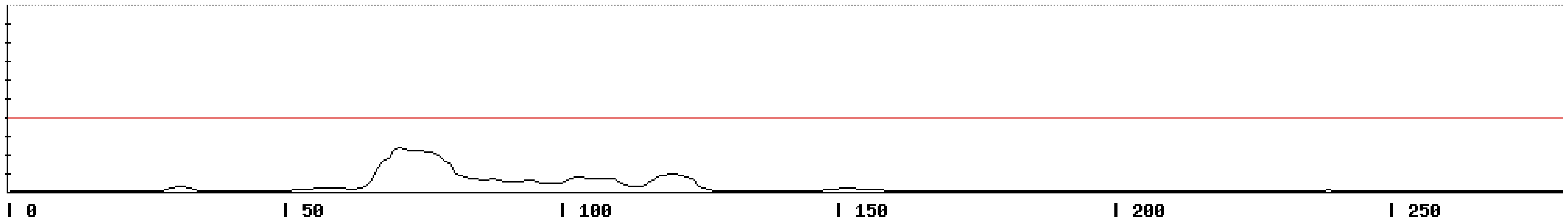

Predictions for Citrussinensis_orange1.1g023821m

Viterbi Path

Viterbi Path

Posterior @ 0.4

Posterior @ 0.4

Posterior Graphic. Threshold Marked @ 0.4

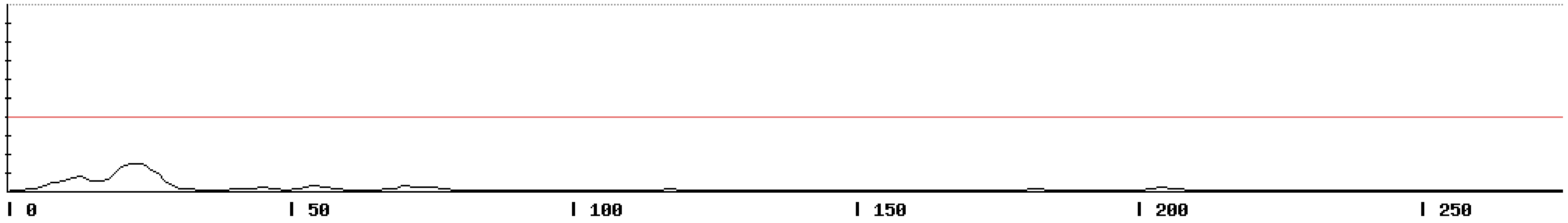

Predictions for Citrussinensis_orange1.1g023996m

Viterbi Path

Viterbi Path

Posterior @ 0.4 — 124 - R - 124

Posterior @ 0.4

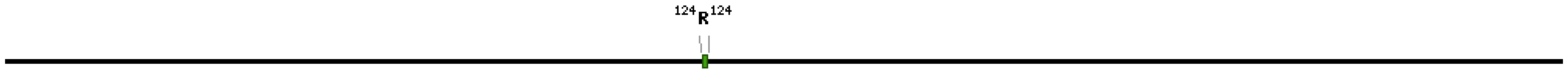

Posterior Graphic. Threshold Marked @ 0.4

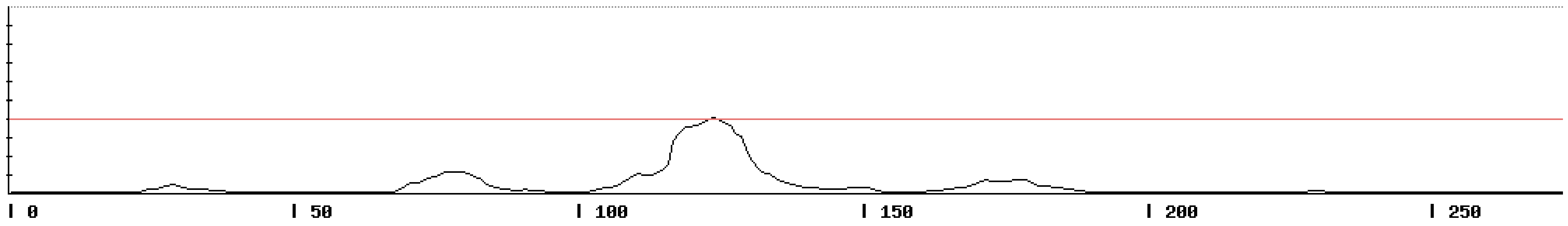

Predictions for Citrussinensis_orange1.1g025053m

Viterbi Path

Viterbi Path

Posterior @ 0.4 — 98 - RKRRK - 102

Posterior @ 0.4

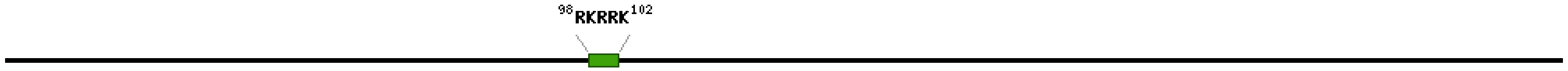

Posterior Graphic. Threshold Marked @ 0.4

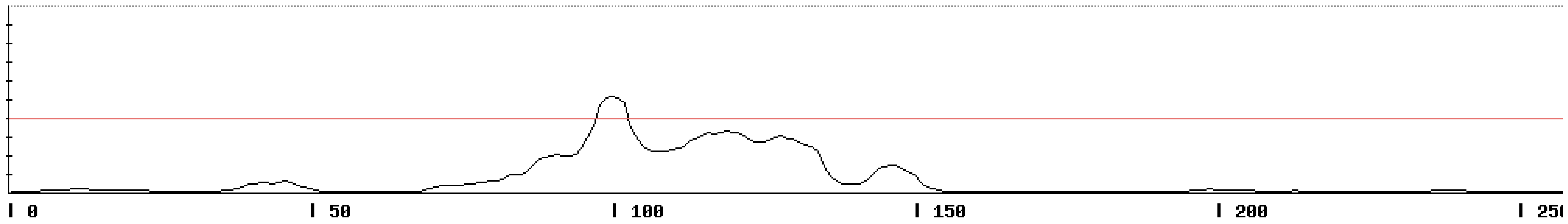

Predictions for Citrussinensis_orange1.1g025156m	
Viterbi Path	
Viterbi Path	
Posterior @ 0.4	
Posterior @ 0.4	
Posterior Graphic. Threshold Marked @ 0.4	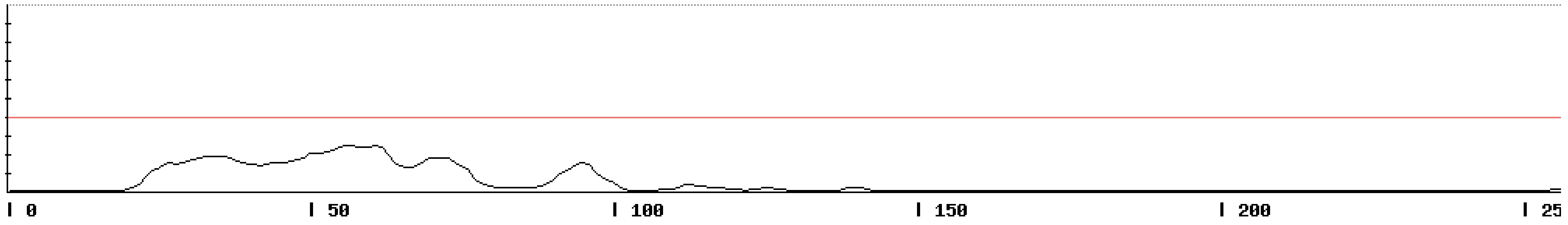

Predictions for Citrussinensis_orange1.1g025570m	
Viterbi Path	
Viterbi Path	
Posterior @ 0.4	100 - R - 100
Posterior @ 0.4	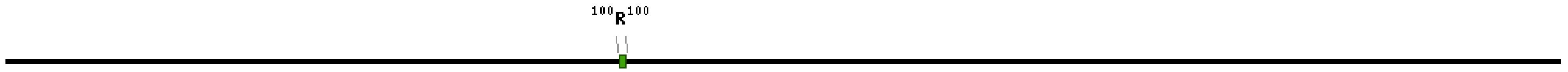
Posterior Graphic. Threshold Marked @ 0.4	

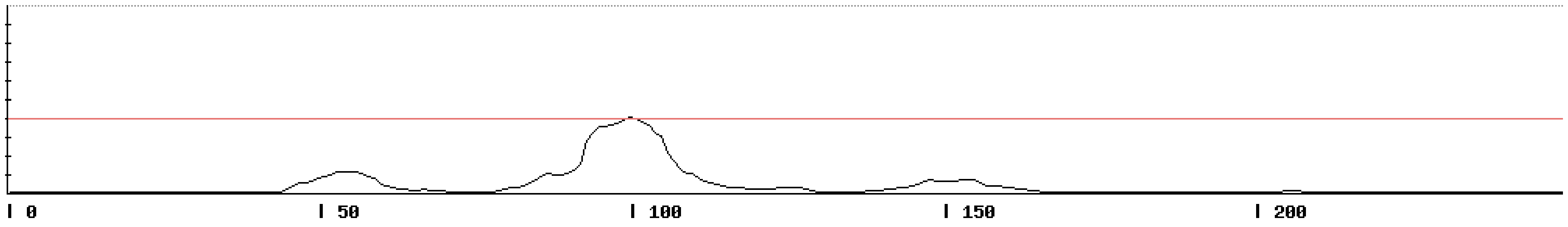

Predictions for Citrussinensis_orange1.1g025667m

Viterbi Path

Viterbi Path

Posterior @ 0.4 — 41 - KPIGKPKTLGIK - 52

Posterior @ 0.4

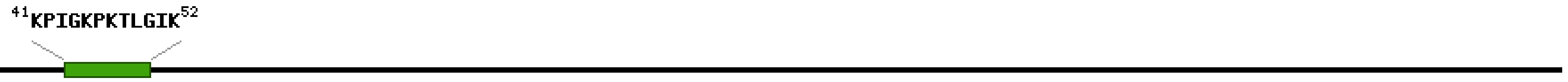

Posterior Graphic. Threshold Marked @ 0.4

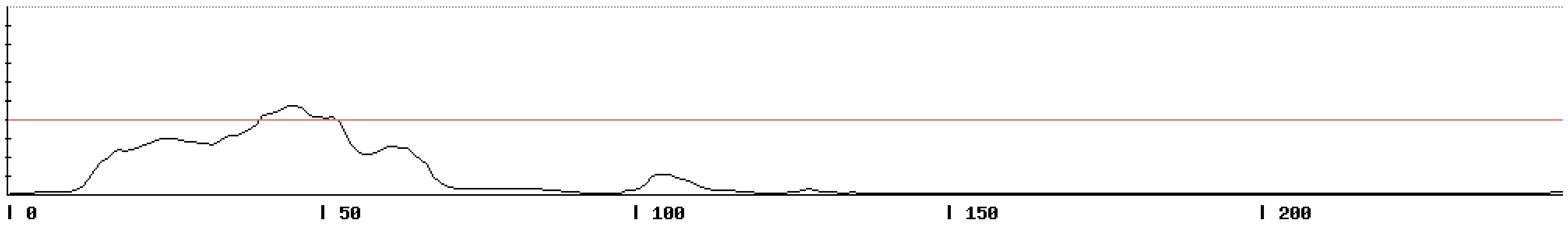

Predictions for Citrussinensis_orange1.1g025990m

Viterbi Path

Viterbi Path

Posterior @ 0.4 — 171 - QRKKKPKKRG - 180

Posterior @ 0.4

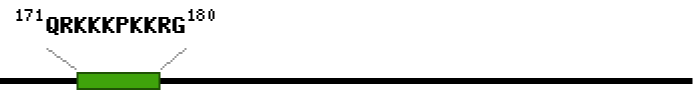

Posterior Graphic. Threshold Marked @ 0.4	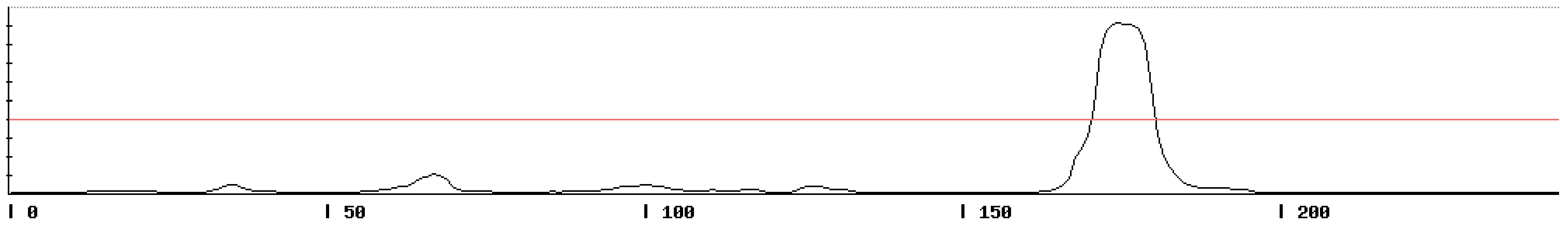

Predictions for Citrussinensis_orange1.1g026592m	
Viterbi Path	
Viterbi Path	
Posterior @ 0.4	
Posterior @ 0.4	
Posterior Graphic. Threshold Marked @ 0.4	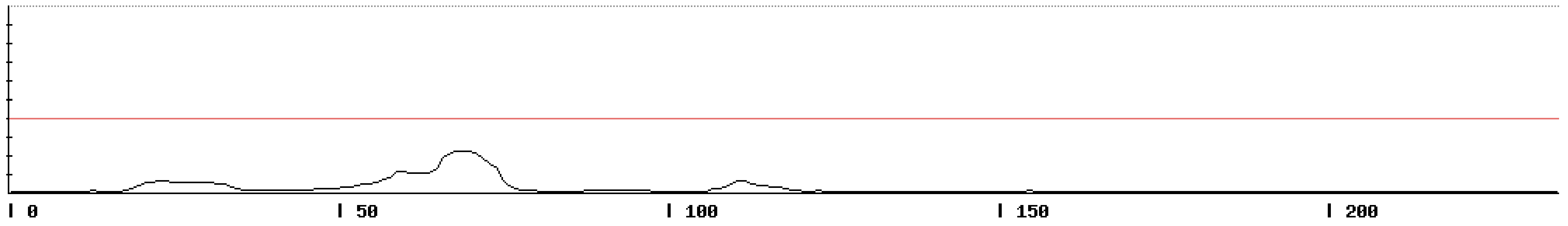

Predictions for Citrussinensis_orange1.1g027074m	
Viterbi Path	
Viterbi Path	
Posterior @ 0.4	
Posterior @ 0.4	

Posterior Graphic. Threshold Marked @ 0.4

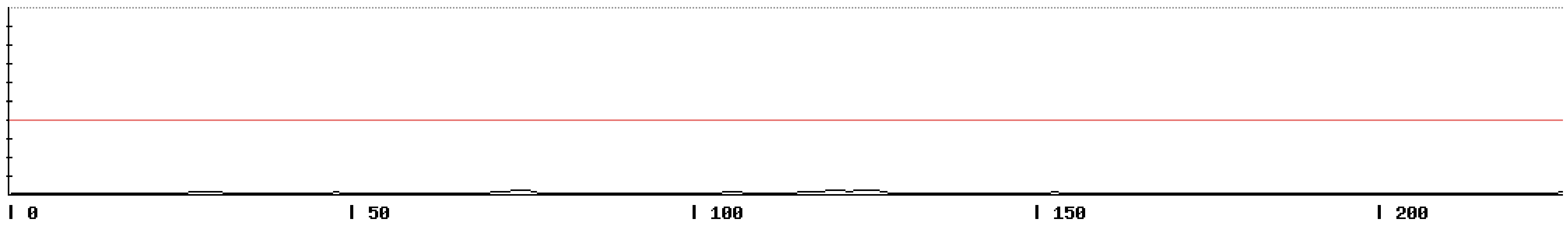

Predictions for Citrussinensis_orange1.1g027859m

Viterbi Path

Viterbi Path

Posterior @ 0.4

Posterior @ 0.4

Posterior Graphic. Threshold Marked @ 0.4

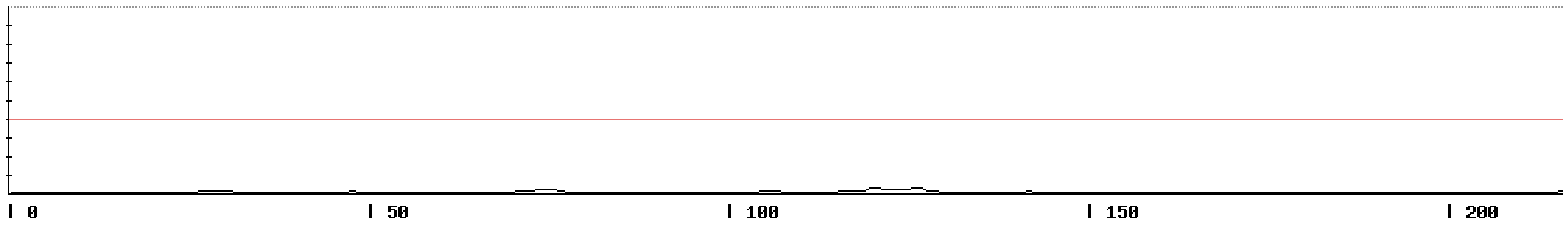

Predictions for Citrussinensis_orange1.1g027874m

Viterbi Path

Viterbi Path

Posterior @ 0.4

Posterior @ 0.4

Posterior Graphic. Threshold Marked @ 0.4

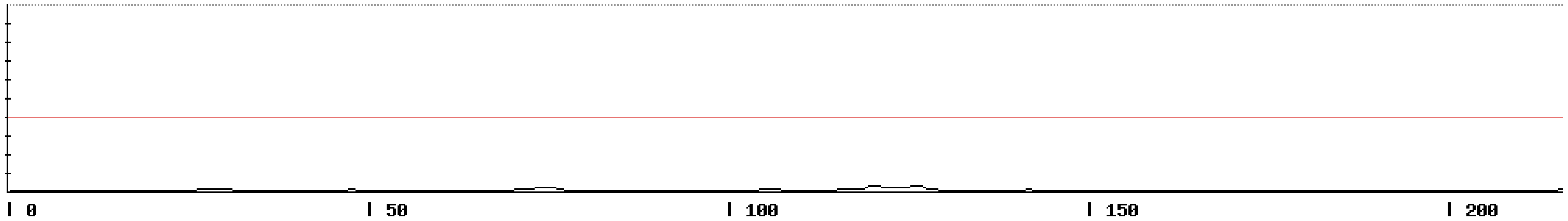

Predictions for Citrussinensis_orange1.1g027916m

Viterbi Path

Viterbi Path

Posterior @ 0.4

Posterior @ 0.4

Posterior Graphic. Threshold Marked @ 0.4

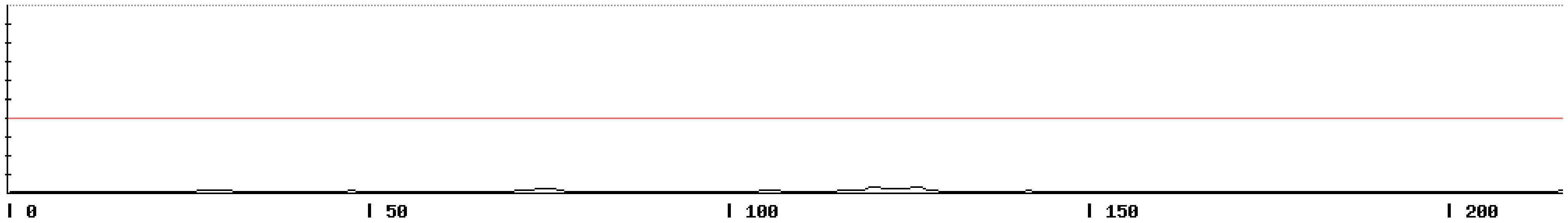

Predictions for Citrussinensis_orange1.1g028803m

Viterbi Path

Viterbi Path

Posterior @ 0.4

Posterior @ 0.4

Posterior Graphic. Threshold Marked @ 0.4

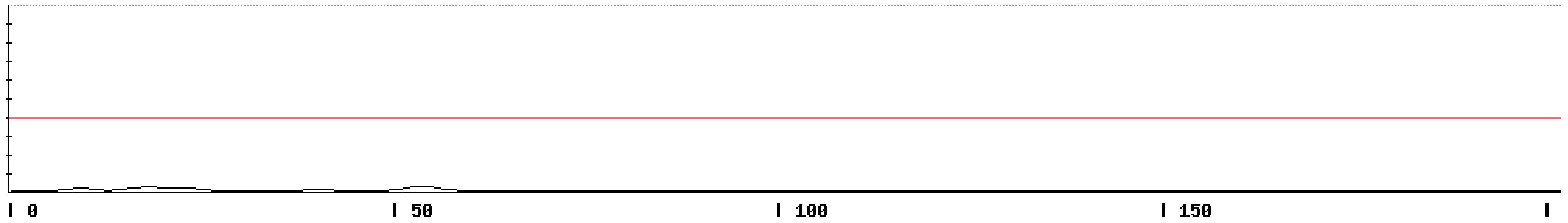

Predictions for Citrussinensis_orange1.1g029016m

Viterbi Path

Viterbi Path

Posterior @ 0.4

Posterior @ 0.4

Posterior Graphic. Threshold Marked @ 0.4

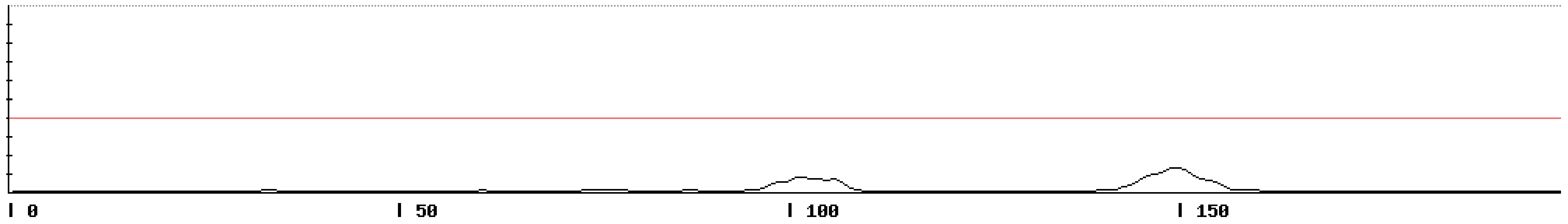

Predictions for Citrussinensis_orange1.1g029281m

Viterbi Path

Viterbi Path

Posterior @ 0.4

Posterior @ 0.4

Posterior Graphic. Threshold Marked @ 0.4

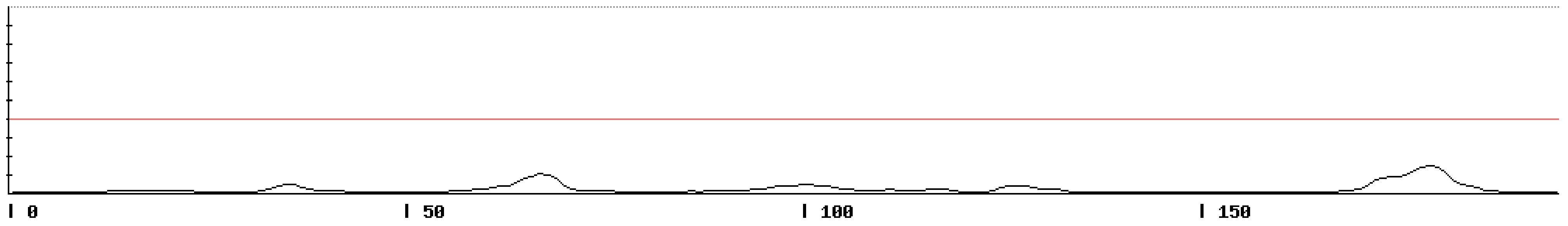

Predictions for Citrussinensis_orange1.1g030079m

Viterbi Path

Viterbi Path

Posterior @ 0.4

Posterior @ 0.4

Posterior Graphic. Threshold Marked @ 0.4

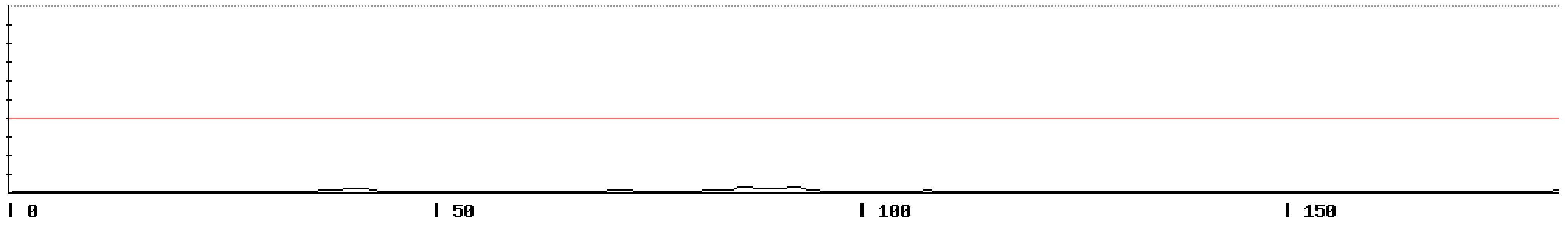

Predictions for Citrussinensis_orange1.1g030168m	
Viterbi Path	
Viterbi Path	
Posterior @ 0.4	108 - QRKKKPKKRG - 117
Posterior @ 0.4	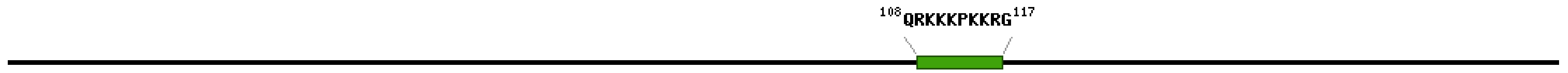
Posterior Graphic. Threshold Marked @ 0.4	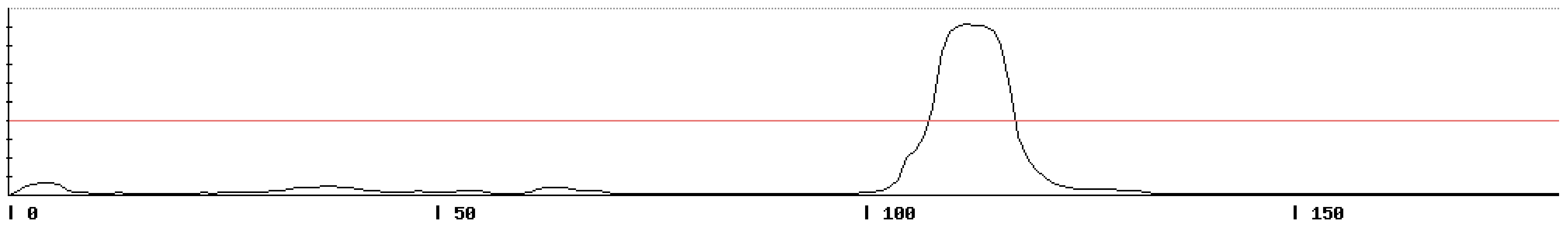

Predictions for Citrussinensis_orange1.1g030306m	
Viterbi Path	
Viterbi Path	
Posterior @ 0.4	
Posterior @ 0.4	
Posterior Graphic. Threshold Marked @ 0.4	

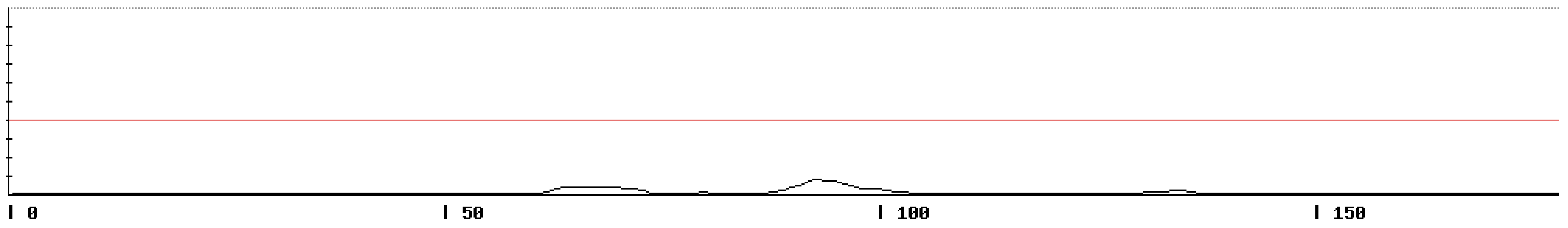

Predictions for Citrussinensis_orange1.1g030727m

Viterbi Path	
Viterbi Path	
Posterior @ 0.4	
Posterior @ 0.4	
Posterior Graphic. Threshold Marked @ 0.4	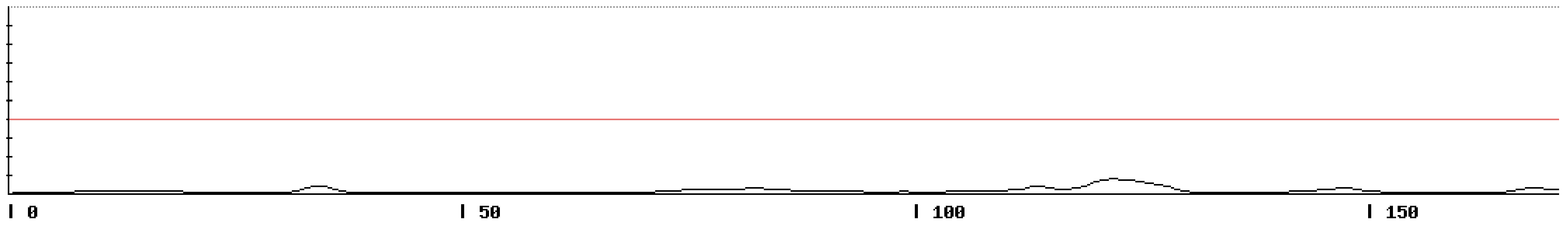

Predictions for Citrussinensis_orange1.1g030909m

Viterbi Path	
Viterbi Path	
Posterior @ 0.4	103 - GRAPKGKK - 110
Posterior @ 0.4	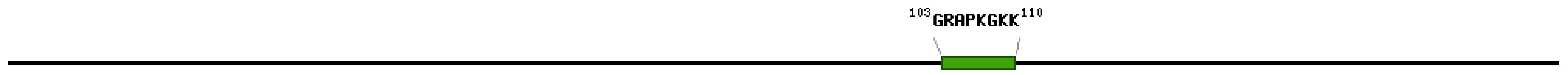

Posterior Graphic. Threshold Marked @ 0.4

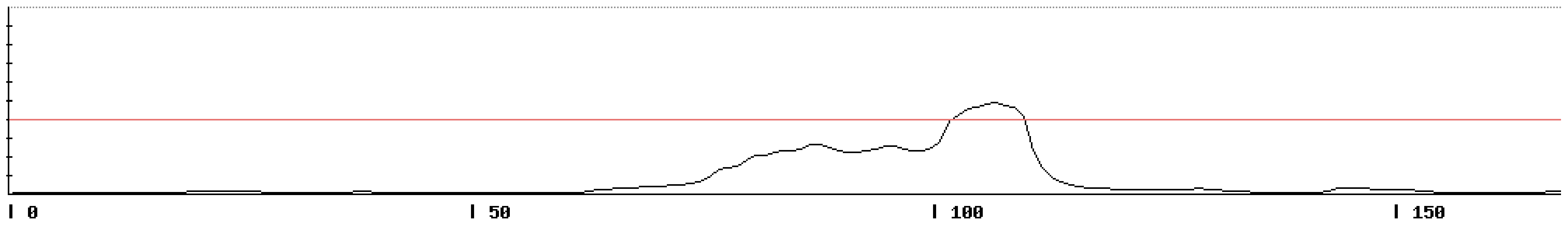

Predictions for Citrussinensis_orange1.1g031030m

Viterbi Path

Viterbi Path

Posterior @ 0.4

Posterior @ 0.4

Posterior Graphic. Threshold Marked @ 0.4

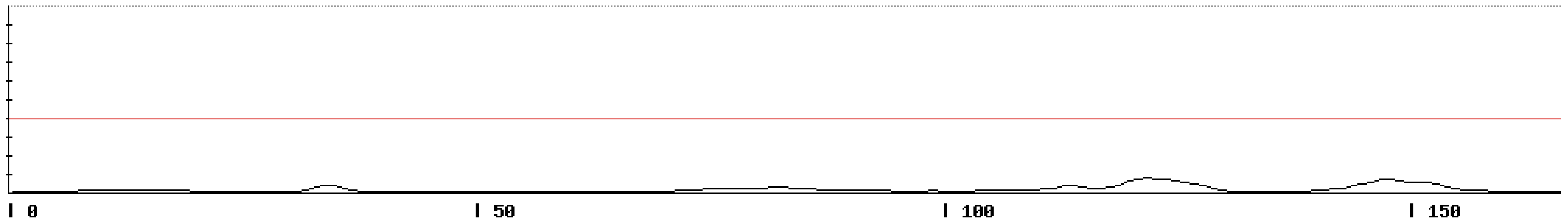

Predictions for Citrussinensis_orange1.1g031672m

Viterbi Path

Viterbi Path

Posterior @ 0.4

Posterior @ 0.4

Posterior Graphic. Threshold Marked @ 0.4

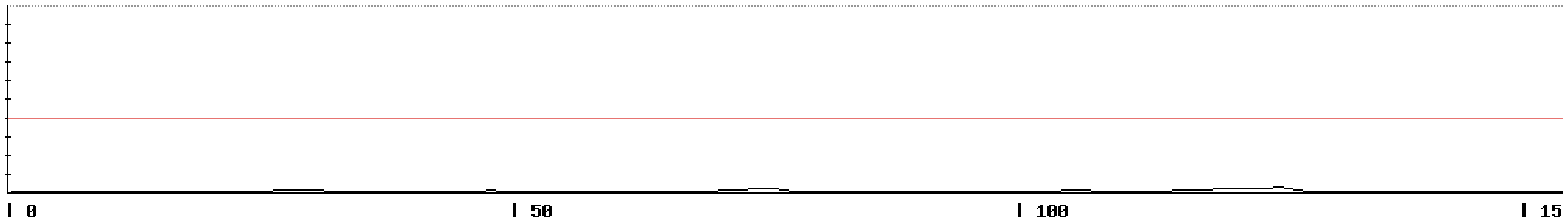

Predictions for Citrussinensis_orange1.1g031844m

Viterbi Path

Viterbi Path

Posterior @ 0.4

Posterior @ 0.4

Posterior Graphic. Threshold Marked @ 0.4

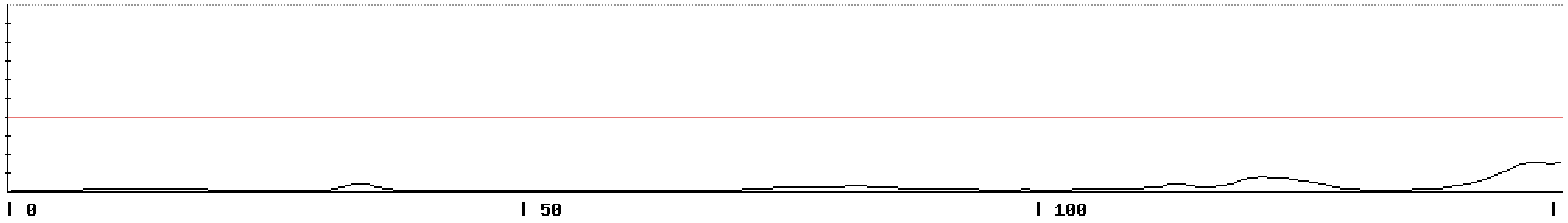

Predictions for Citrussinensis_orange1.1g031935m

Viterbi Path

Viterbi Path

Posterior @ 0.4

Posterior @ 0.4

Posterior Graphic. Threshold Marked @ 0.4

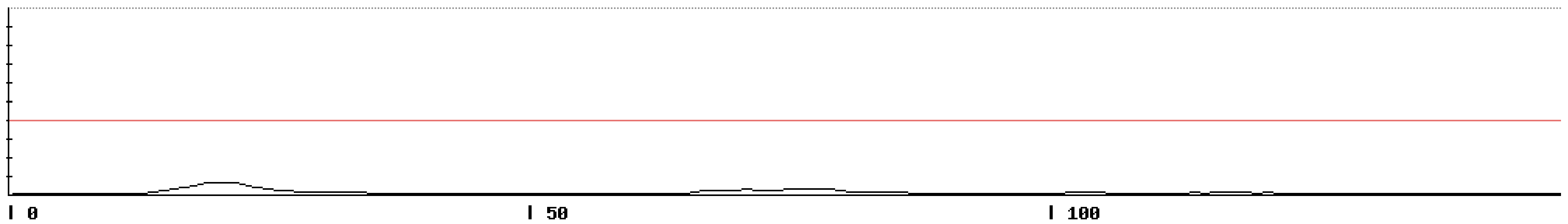

Predictions for Citrussinensis_orange1.1g032247m

Viterbi Path

Viterbi Path

Posterior @ 0.4

Posterior @ 0.4

Posterior Graphic. Threshold Marked @ 0.4

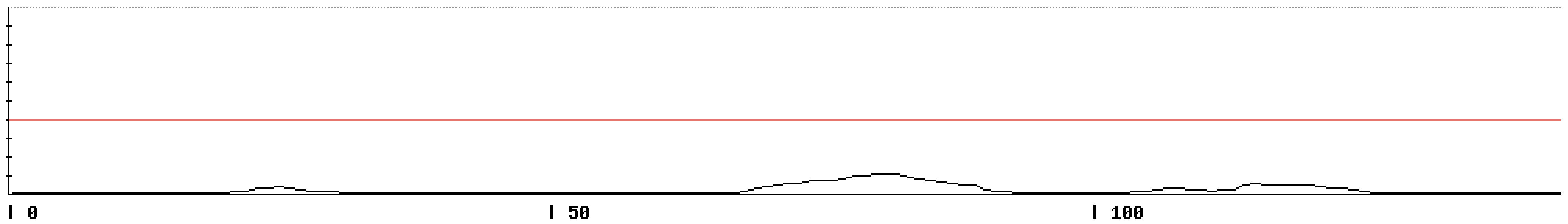

Predictions for Citrussinensis_orange1.1g032371m

Viterbi Path

Viterbi Path

Posterior @ 0.4	
Posterior @ 0.4	
Posterior Graphic. Threshold Marked @ 0.4	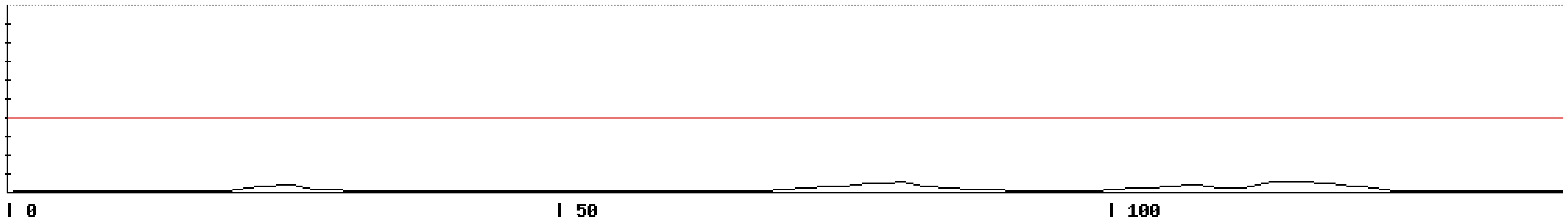

Predictions for Citrussinensis_orange1.1g032472m

Viterbi Path	
Viterbi Path	
Posterior @ 0.4	72 - KSKQAHRRTKSGYWKVTGKGS - 92<br>94 - IKR - 96
Posterior @ 0.4	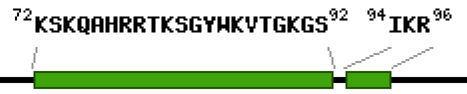
Posterior Graphic. Threshold Marked @ 0.4	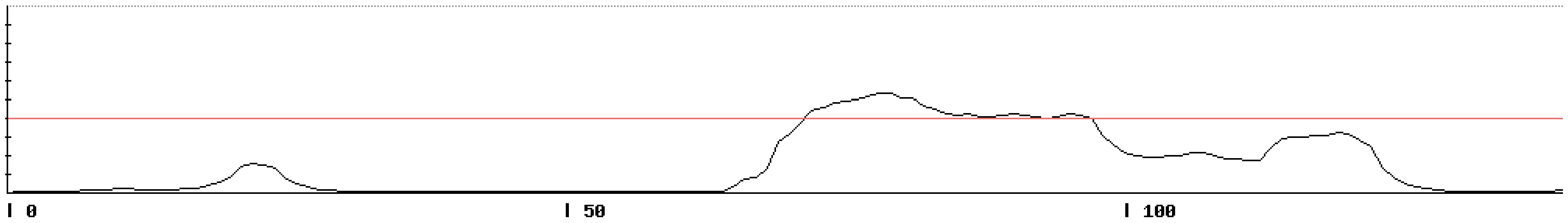

Predictions for Citrussinensis_orange1.1g033258m	
Viterbi Path	
Viterbi Path	
Posterior @ 0.4	
Posterior @ 0.4	
Posterior Graphic. Threshold Marked @ 0.4	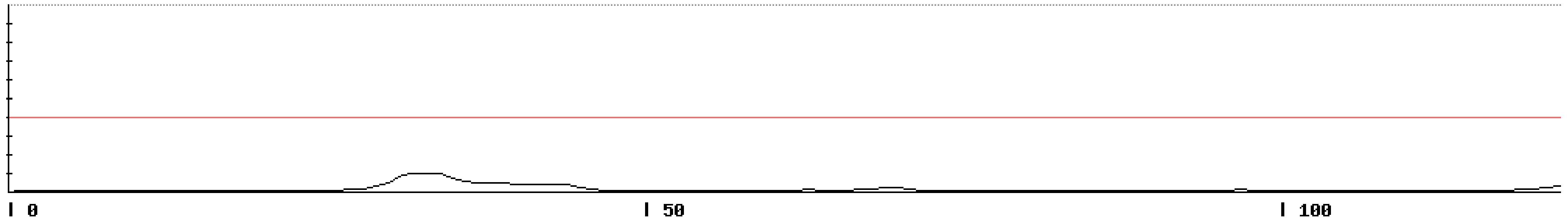

Predictions for Citrussinensis_orange1.1g035726m	
Viterbi Path	
Viterbi Path	
Posterior @ 0.4	
Posterior @ 0.4	
Posterior Graphic. Threshold Marked @ 0.4	

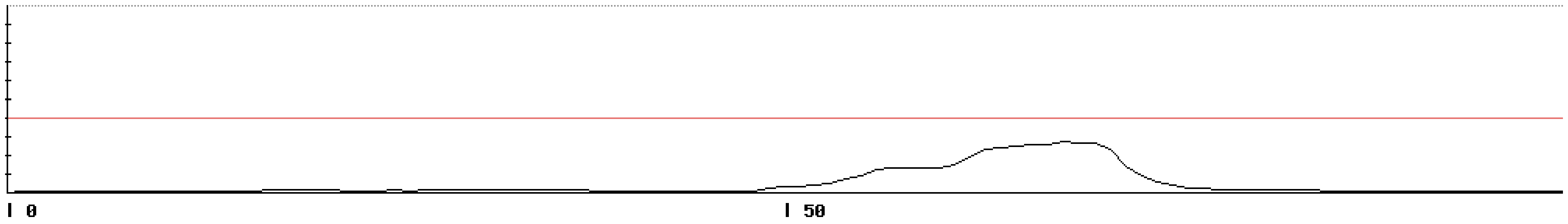

Predictions for Citrussinensis_orange1.1g036036m

Viterbi Path

Viterbi Path

Posterior @ 0.4

Posterior @ 0.4

Posterior Graphic. Threshold Marked @ 0.4

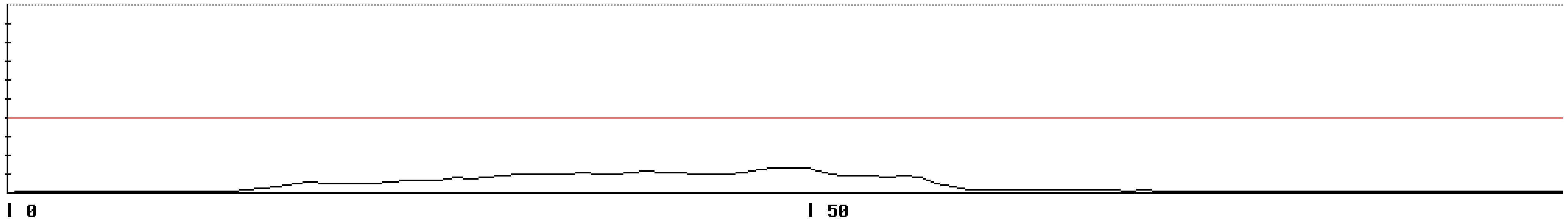

Predictions for Citrussinensis_orange1.1g036240m

Viterbi Path

Viterbi Path

Posterior @ 0.4

Posterior @ 0.4

Posterior Graphic. Threshold Marked @ 0.4

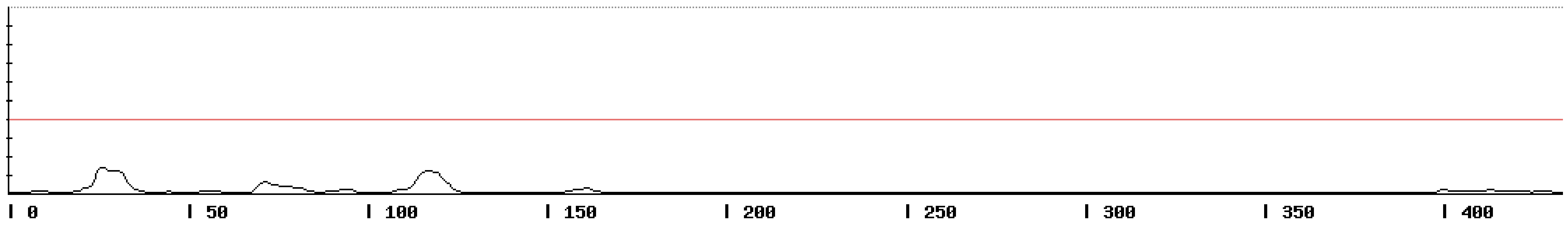

Predictions for Citrussinensis_orange1.1g036376m

Viterbi Path

Viterbi Path

Posterior @ 0.4

Posterior @ 0.4

Posterior Graphic. Threshold Marked @ 0.4

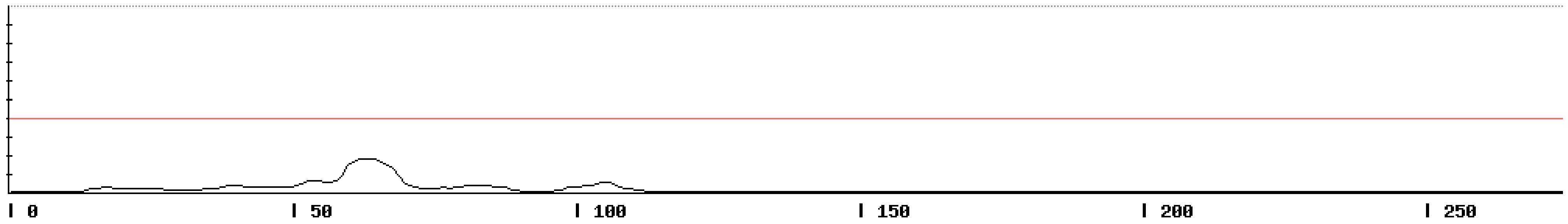

Predictions for Citrussinensis_orange1.1g036898m

Viterbi Path

Viterbi Path

Posterior @ 0.4

Posterior @ 0.4

Posterior Graphic. Threshold Marked @ 0.4

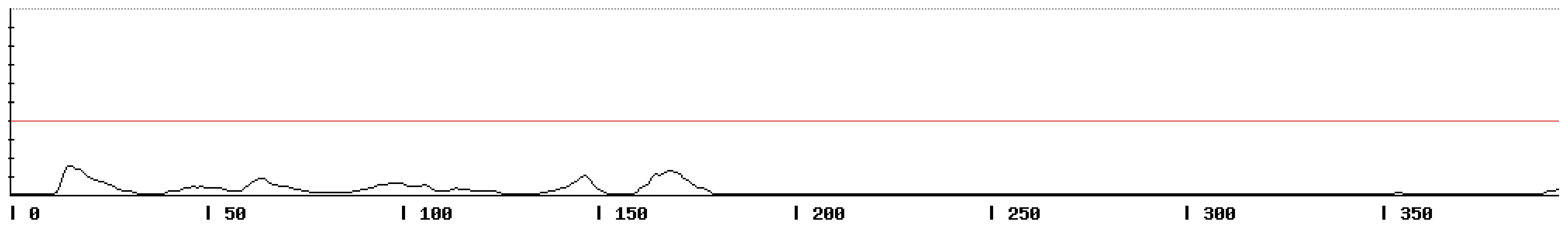

Predictions for Citrussinensis_orange1.1g036998m

Viterbi Path

Viterbi Path

Posterior @ 0.4

Posterior @ 0.4

Posterior Graphic. Threshold Marked @ 0.4

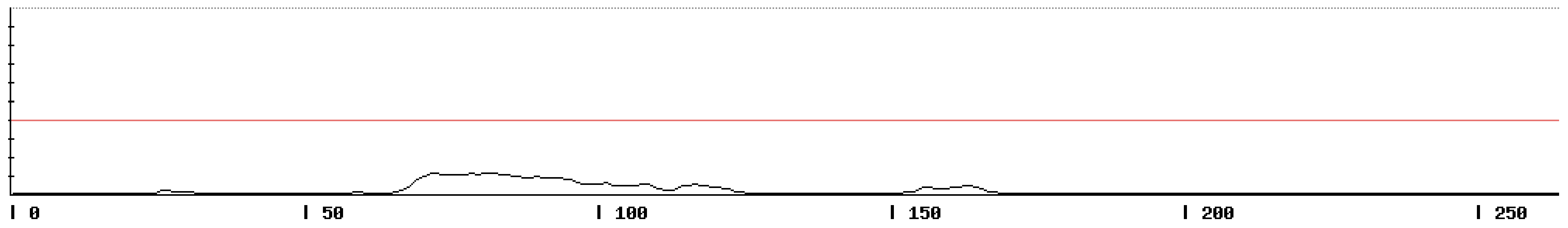

Predictions for Citrussinensis_orange1.1g037150m

Viterbi Path

Viterbi Path

Posterior @ 0.4

Posterior @ 0.4

Posterior Graphic. Threshold Marked @ 0.4

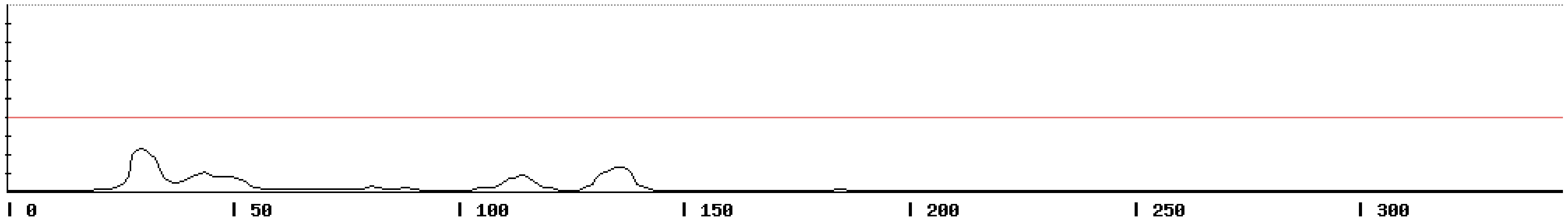

Predictions for Citrussinensis_orange1.1g037198m

Viterbi Path

Viterbi Path

Posterior @ 0.4 — 182 - GGKRSSGSKRKL - 193

Posterior @ 0.4

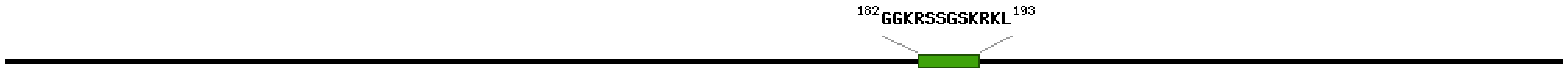

Posterior Graphic. Threshold Marked @ 0.4

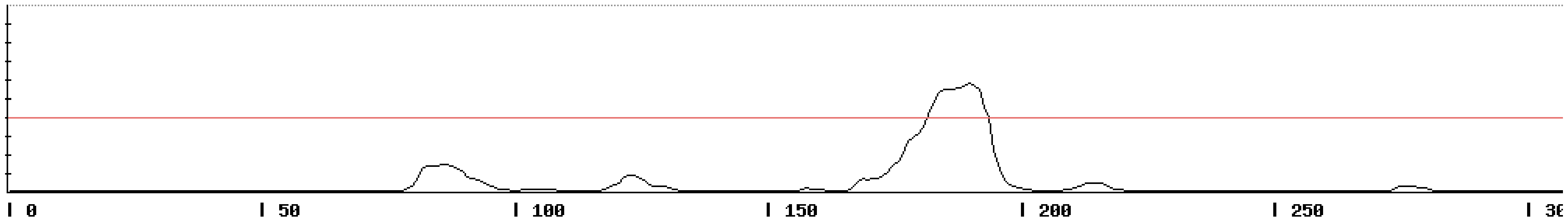

Predictions for Citrussinensis_orange1.1g037220m

Viterbi Path

Viterbi Path

Posterior @ 0.4	73 - KSNRAHRRTKAGHGKIT - 89
Posterior @ 0.4	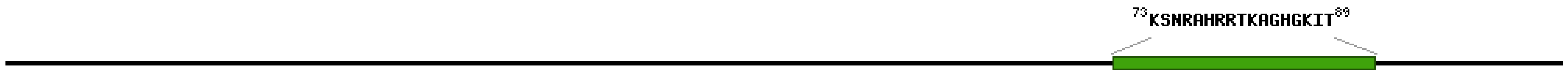
Posterior Graphic. Threshold Marked @ 0.4	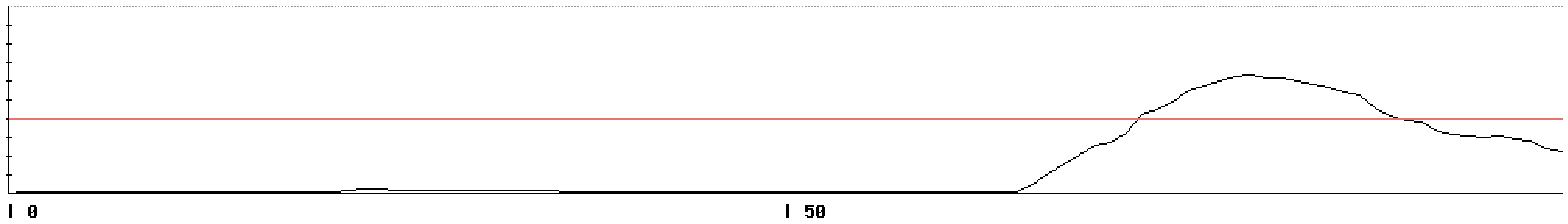

Predictions for Citrussinensis_orange1.1g037309m

Viterbi Path	
Viterbi Path	
Posterior @ 0.4	
Posterior @ 0.4	
Posterior Graphic. Threshold Marked @ 0.4	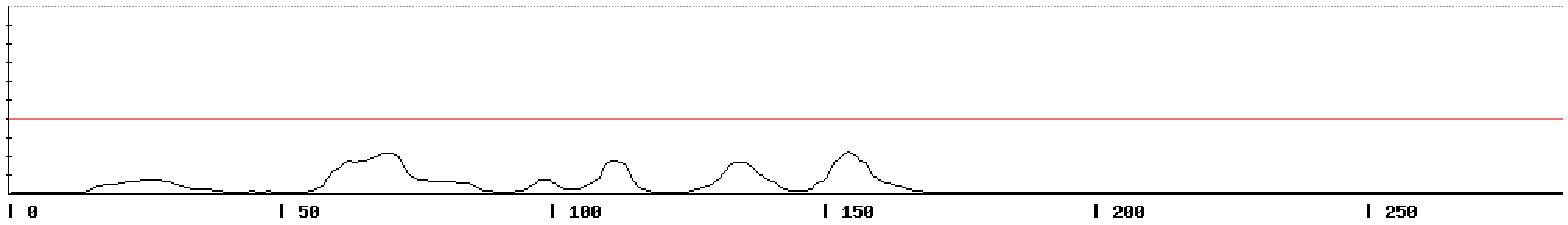

Predictions for Citrussinensis_orange1.1g037457m

Viterbi Path

Viterbi Path

Posterior @ 0.4

Posterior @ 0.4

Posterior Graphic. Threshold Marked @ 0.4

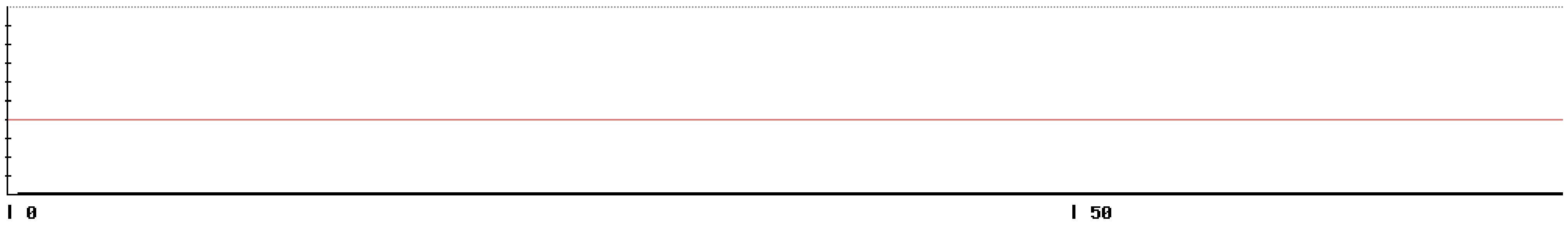

Predictions for Citrussinensis_orange1.1g037591m

Viterbi Path

Viterbi Path

Posterior @ 0.4

Posterior @ 0.4

Posterior Graphic. Threshold Marked @ 0.4

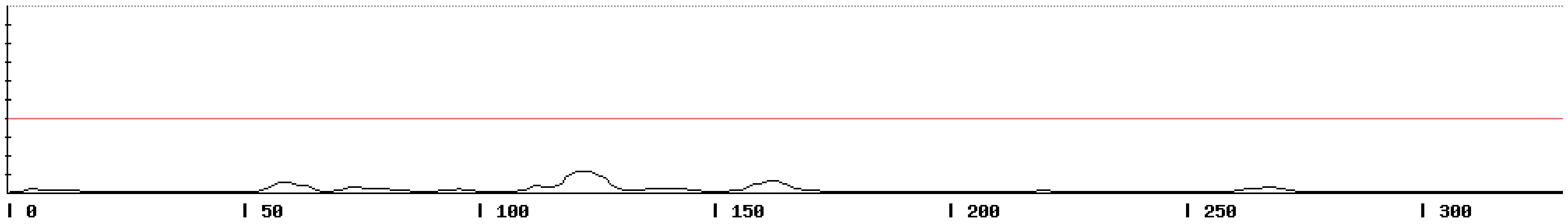

Predictions for Citrussinensis_orange1.1g037709m

Viterbi Path: 65 - KPKYRGAKRKRSGQQQHRKTKSGTWKKTGSGSEMKRK - 101

Viterbi Path

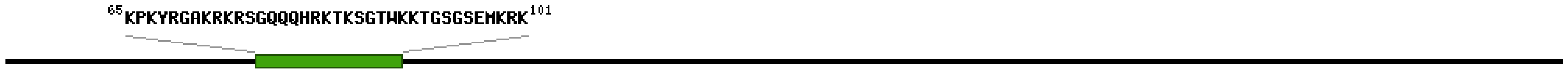

Posterior @ 0.4: 65 - KPKYRGAKRKRSGQQQHRKTKSGTWKKTGSGSEMKRK - 101

Posterior @ 0.4

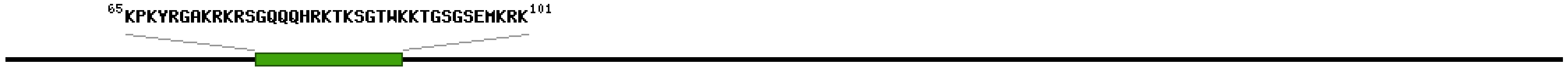

Posterior Graphic. Threshold Marked @ 0.4

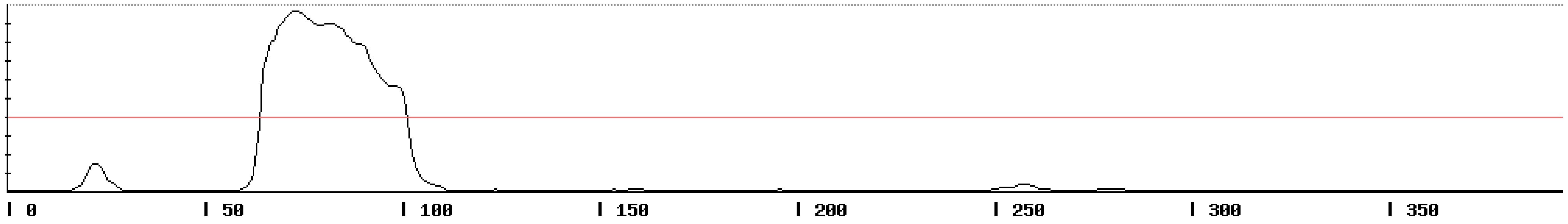

Predictions for Citrussinensis_orange1.1g038703m

Viterbi Path: 170 - KIHHKKESGNKRSEAGKKKNLPVRKKTKAC - 199

Viterbi Path

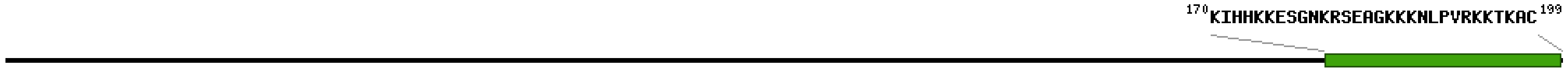

Posterior @ 0.4: 172 - HHKKESGNKRSEAGKKKNLPVRKKTKA - 198

Posterior @ 0.4

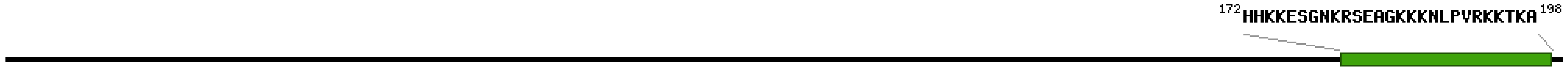

Posterior Graphic. Threshold Marked @ 0.4

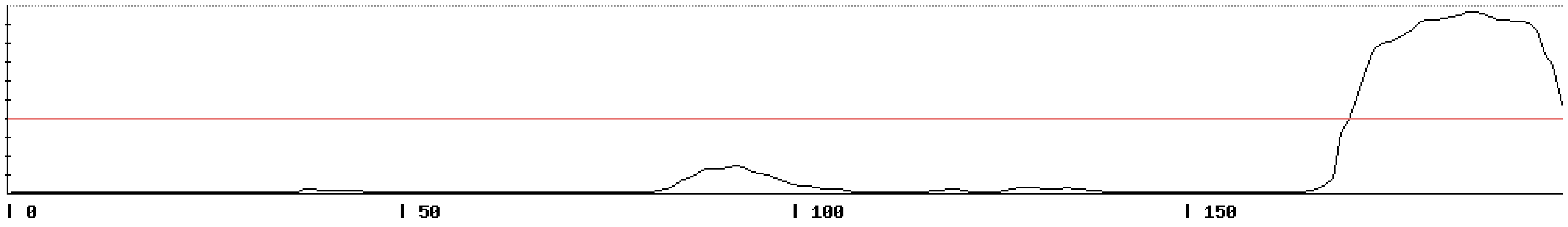

Predictions for Citrussinensis_orange1.1g038778m	
Viterbi Path	
Viterbi Path	
Posterior @ 0.4	73 - KSTRAHRRTNAG - 84
	86 - WKVTGRGSKIKTRNGLSGTKK - 106
	115 - PASKKA - 120
Posterior @ 0.4	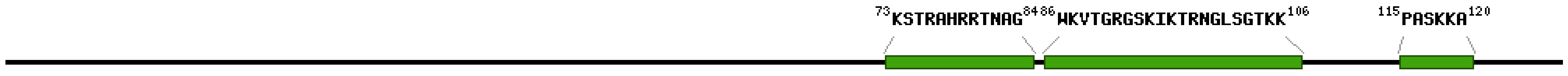
Posterior Graphic. Threshold Marked @ 0.4	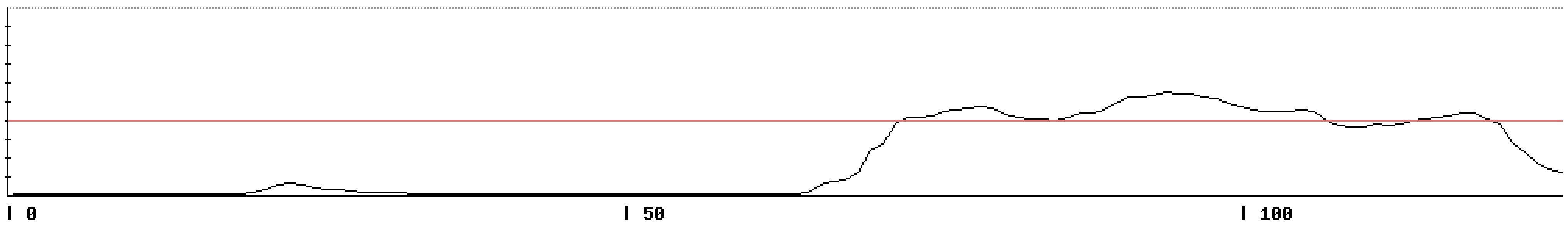

Predictions for Citrussinensis_orange1.1g038876m	
Viterbi Path	
Viterbi Path	
Posterior @ 0.4	78 - KK - 79
	81 - SK - 82

113 - IKIGSKKRFR - 122

Posterior @ 0.4

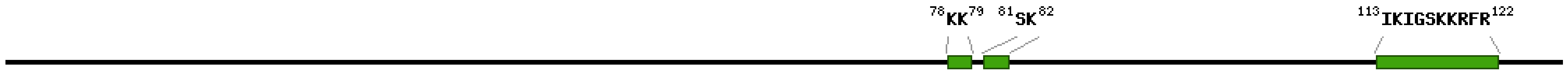

Posterior Graphic. Threshold Marked @ 0.4

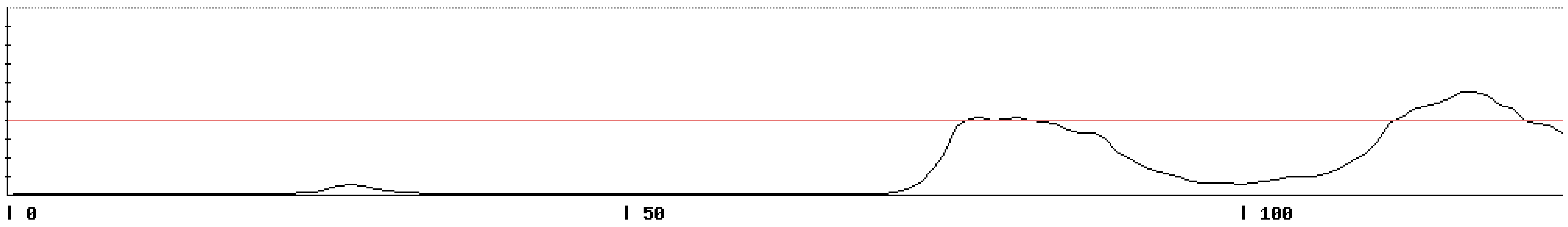

Predictions for Citrussinensis_orange1.1g038974m

Viterbi Path

Viterbi Path

Posterior @ 0.4

Posterior @ 0.4

Posterior Graphic. Threshold Marked @ 0.4

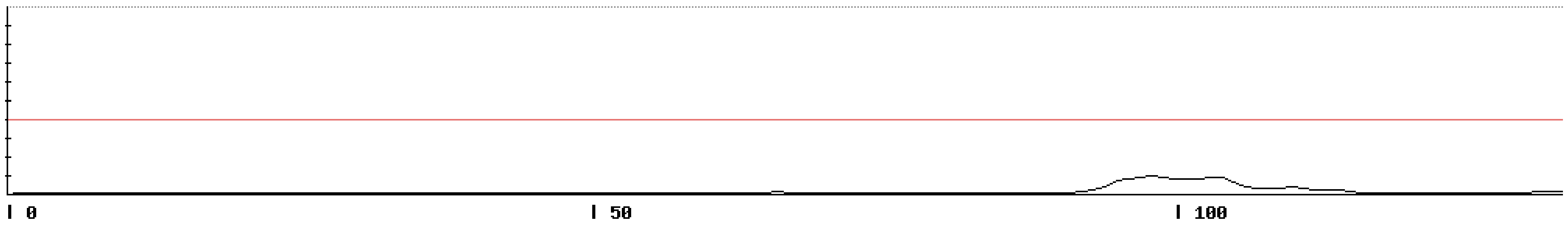

Predictions for Citrussinensis_orange1.1g039532m

Viterbi Path

Viterbi Path

Posterior @ 0.4

Posterior @ 0.4

Posterior Graphic. Threshold Marked @ 0.4

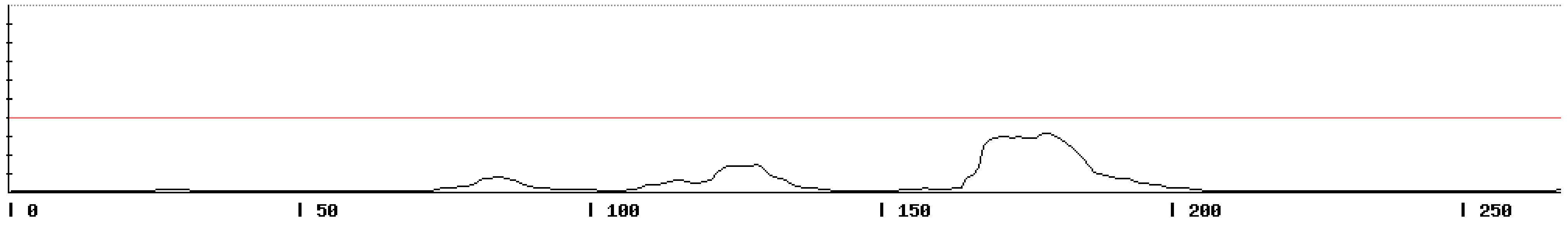

Predictions for Citrussinensis_orange1.1g039886m

Viterbi Path

Viterbi Path

Posterior @ 0.4

Posterior @ 0.4

Posterior Graphic. Threshold Marked @ 0.4

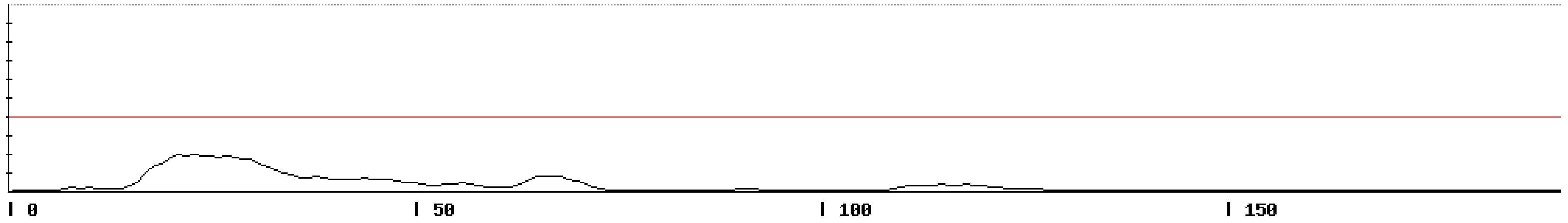

Predictions for Citrussinensis_orange1.1g040419m

Viterbi Path	
Viterbi Path	
Posterior @ 0.4	92 - SKIKR - 96<br>149 - RKREKKMLGVVSTKRKR - 165
Posterior @ 0.4	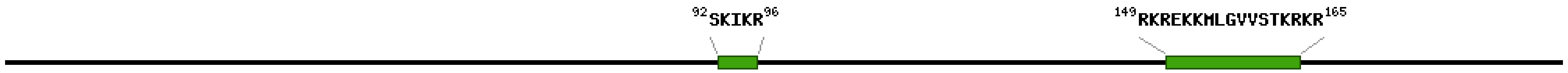
Posterior Graphic. Threshold Marked @ 0.4	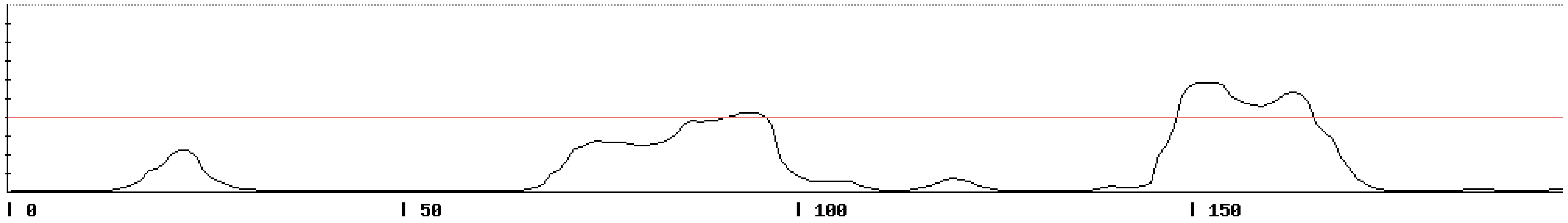

Predictions for Citrussinensis_orange1.1g040553m

Viterbi Path	
Viterbi Path	
Posterior @ 0.4	
Posterior @ 0.4	
Posterior Graphic. Threshold Marked @ 0.4	

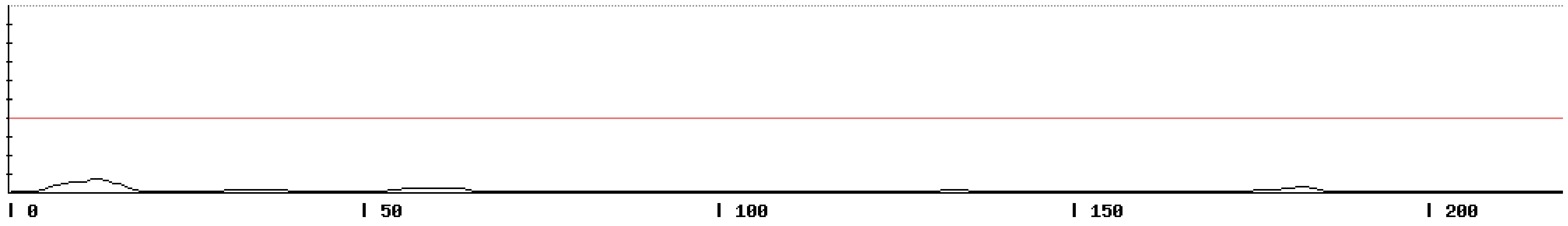

Predictions for Citrussinensis_orange1.1g040617m

Viterbi Path

Viterbi Path

Posterior @ 0.4

Posterior @ 0.4

Posterior Graphic. Threshold Marked @ 0.4

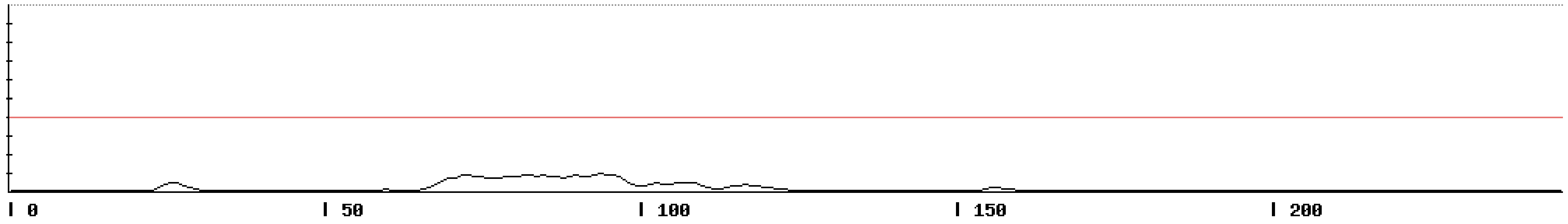

Predictions for Citrussinensis_orange1.1g040747m

Viterbi Path

Viterbi Path

Posterior @ 0.4

Posterior @ 0.4

Posterior Graphic. Threshold Marked @ 0.4

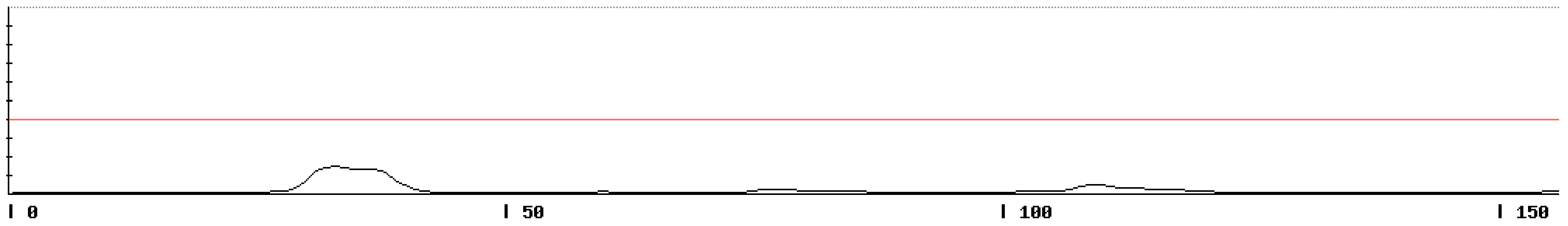

Predictions for Citrussinensis_orange1.1g041697m

Viterbi Path

Viterbi Path

Posterior @ 0.4 | 77 - KKKSSKGSRI - 86

Posterior @ 0.4

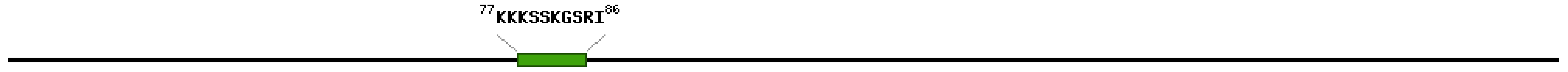

Posterior Graphic. Threshold Marked @ 0.4

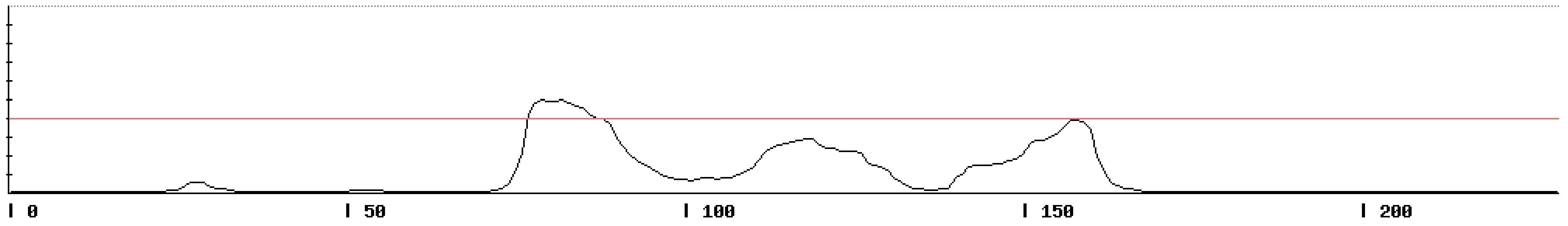

Predictions for Citrussinensis_orange1.1g042311m

Viterbi Path

Viterbi Path

Posterior @ 0.4

Posterior @ 0.4

Posterior Graphic. Threshold Marked @ 0.4

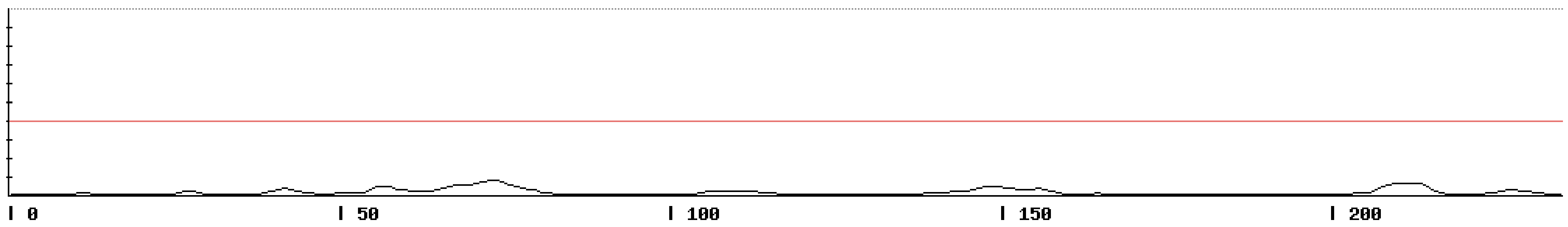

Predictions for Citrussinensis_orange1.1g042376m

Viterbi Path

Viterbi Path

Posterior @ 0.4

Posterior @ 0.4

Posterior Graphic. Threshold Marked @ 0.4

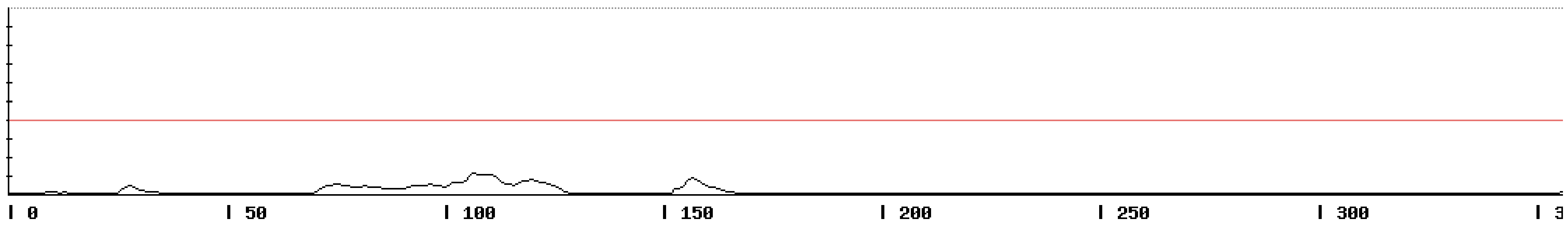

Predictions for Citrussinensis_orange1.1g042614m

Viterbi Path

Viterbi Path

Posterior @ 0.4

Posterior @ 0.4

Posterior Graphic. Threshold Marked @ 0.4

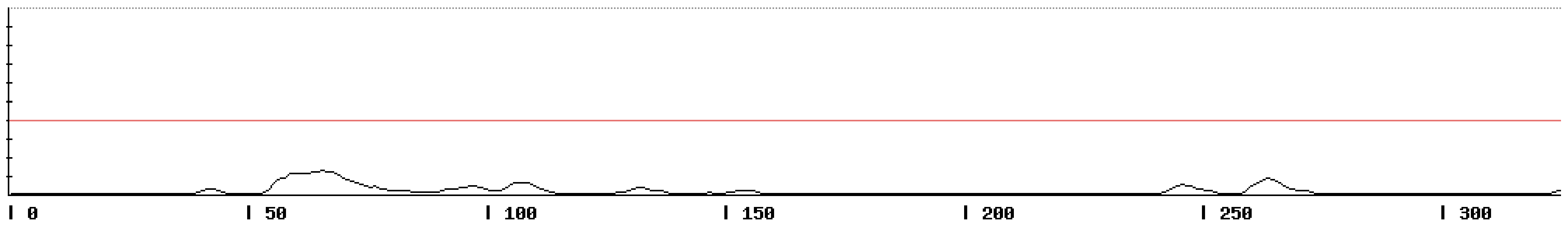

Predictions for Citrussinensis_orange1.1g042765m

Viterbi Path

Viterbi Path

Posterior @ 0.4

100 - KKSSKG - 105

136 - KIGSKKRFR - 144

Posterior @ 0.4

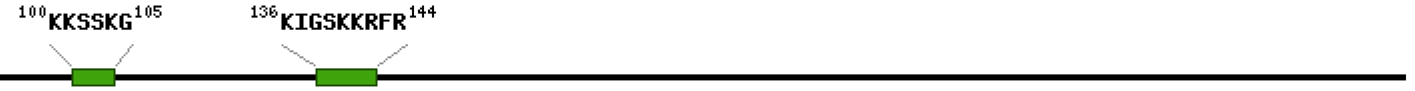

Posterior Graphic. Threshold Marked @ 0.4

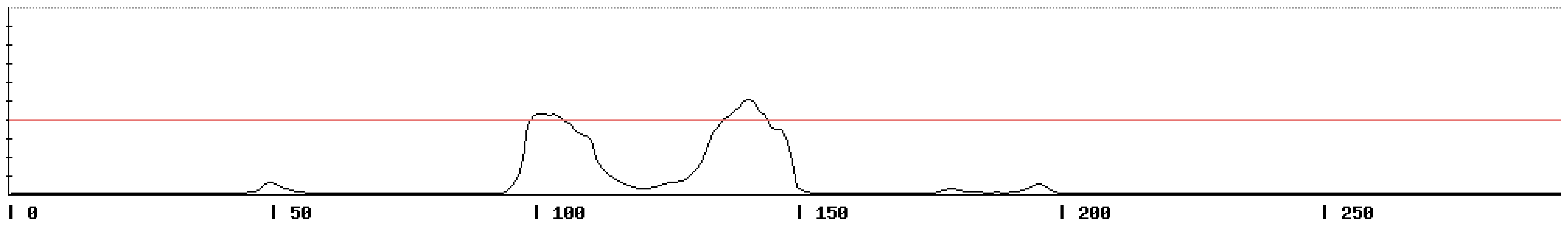

Predictions for Citrussinensis_orange1.1g043175m

Viterbi Path

Viterbi Path

Posterior @ 0.4

Posterior @ 0.4

Posterior Graphic. Threshold Marked @ 0.4

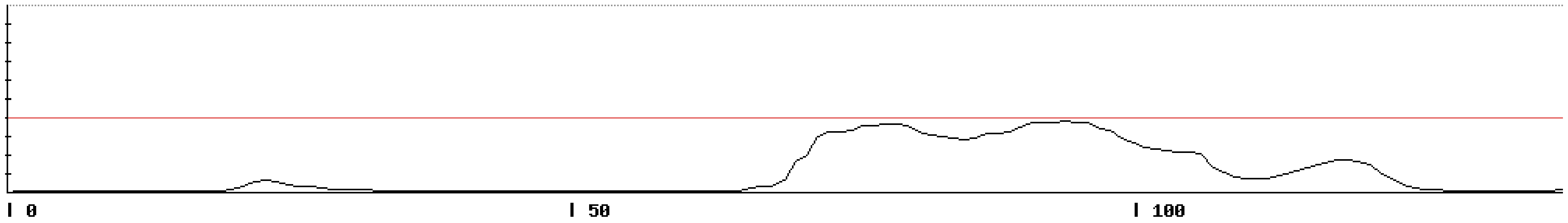

Predictions for Citrussinensis_orange1.1g043513m

Viterbi Path

Viterbi Path

Posterior @ 0.4

Posterior @ 0.4

Posterior Graphic. Threshold Marked @ 0.4

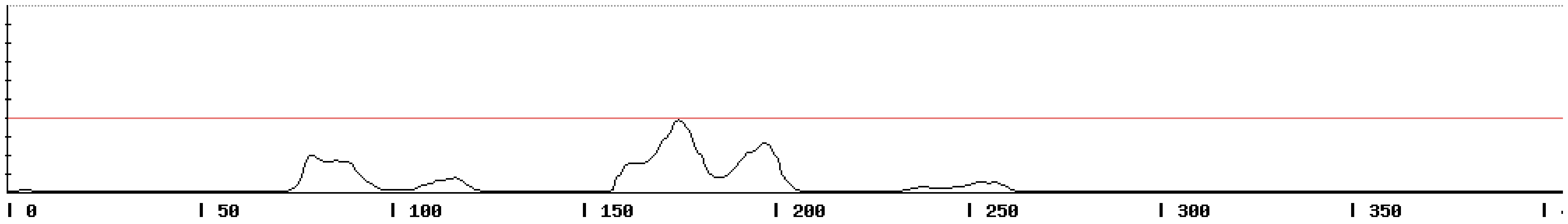

Predictions for Citrussinensis_orange1.1g043911m

Viterbi Path

Viterbi Path

Posterior @ 0.4

Posterior @ 0.4

Posterior Graphic. Threshold Marked @ 0.4

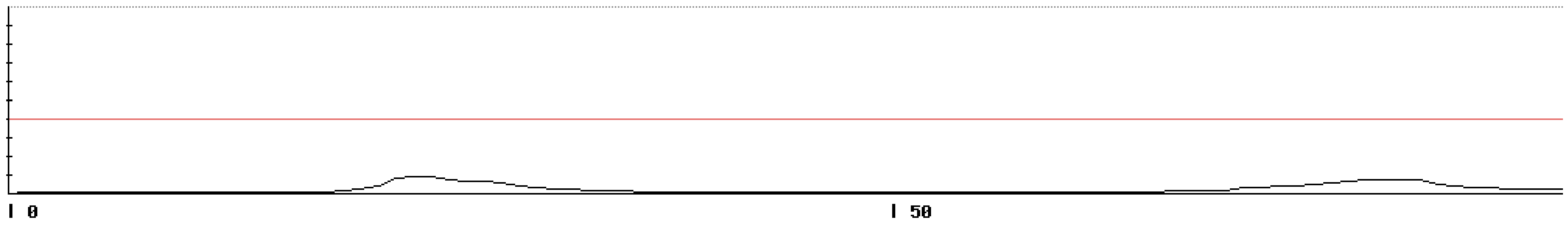

Predictions for Citrussinensis_orange1.1g044381m

Viterbi Path

Viterbi Path

Posterior @ 0.4

Posterior @ 0.4

Posterior Graphic. Threshold Marked @ 0.4

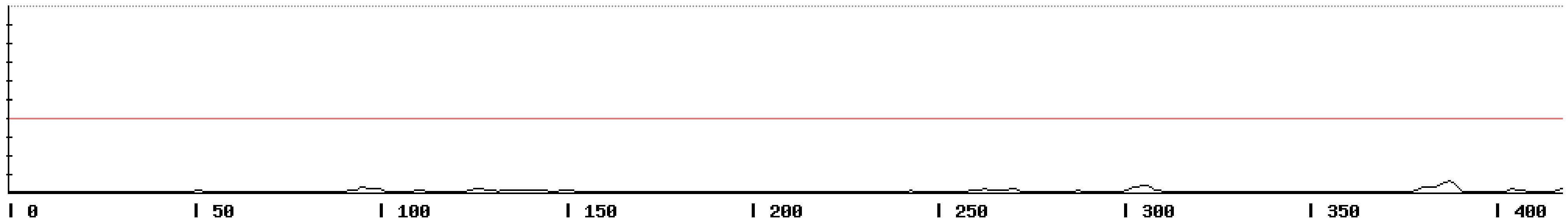

Predictions for Citrussinensis_orange1.1g044940m	
Viterbi Path	
Viterbi Path	
Posterior @ 0.4	
Posterior @ 0.4	
Posterior Graphic. Threshold Marked @ 0.4	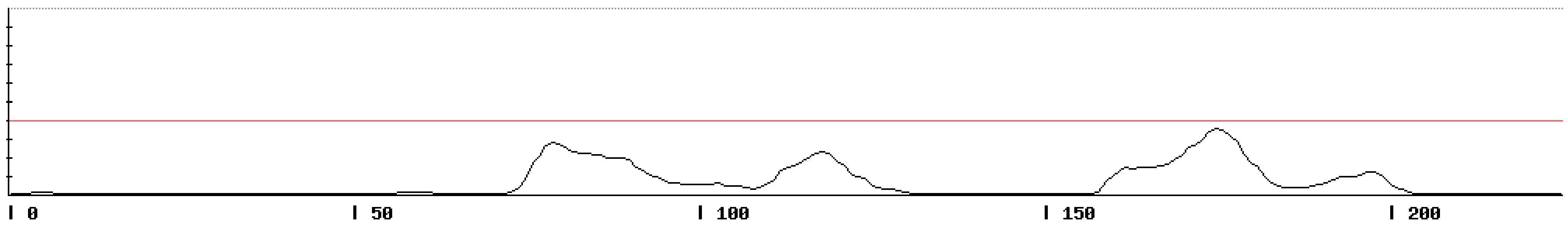

Predictions for Citrussinensis_orange1.1g045278m	
Viterbi Path	148 - RKRNKKKKRG - 157
Viterbi Path	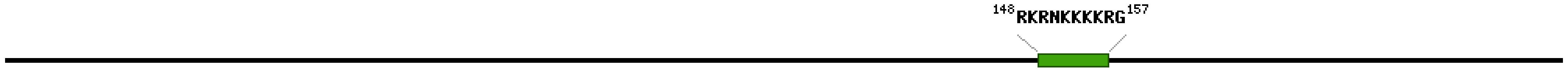
Posterior @ 0.4	76 - RAHRRTKAGH - 85<br>148 - RKRNKKKKRG - 157
Posterior @ 0.4	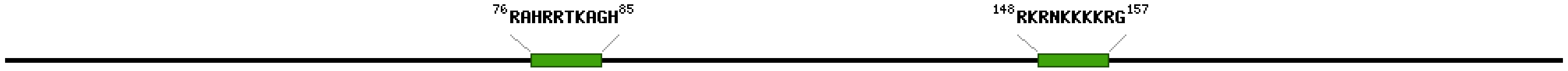
Posterior Graphic. Threshold Marked @ 0.4	

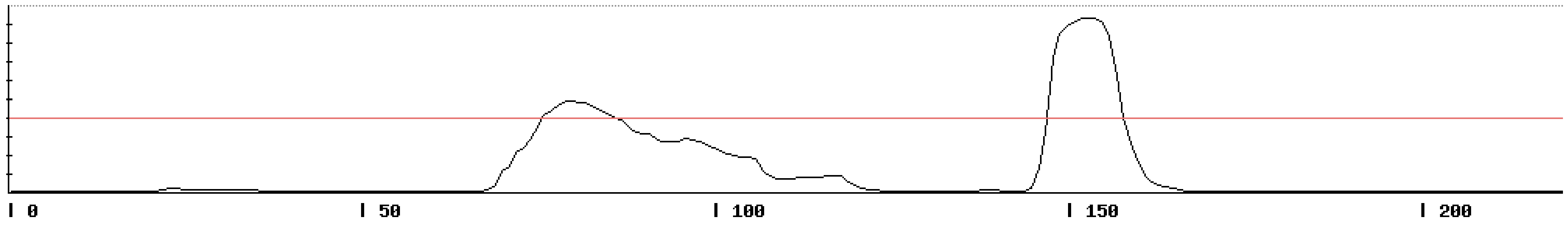

Predictions for Citrussinensis_orange1.1g045641m

Viterbi Path	
Viterbi Path	
Posterior @ 0.4	
Posterior @ 0.4	
Posterior Graphic. Threshold Marked @ 0.4	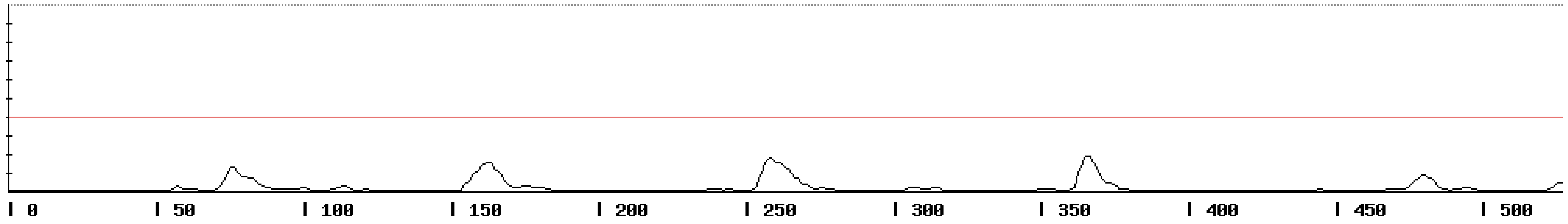

Predictions for Citrussinensis_orange1.1g045647m

Viterbi Path	
Viterbi Path	
Posterior @ 0.4	107 - SRNSRKKIGSKKRFR - 121
	161 - KNGEPAAAGQKTPRKKRK - 178
Posterior @ 0.4	

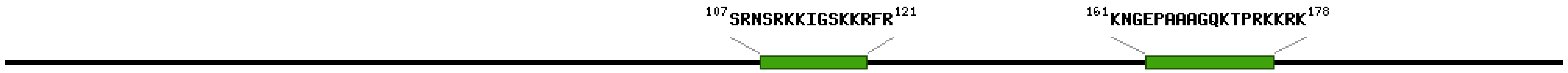

Posterior Graphic. Threshold Marked @ 0.4

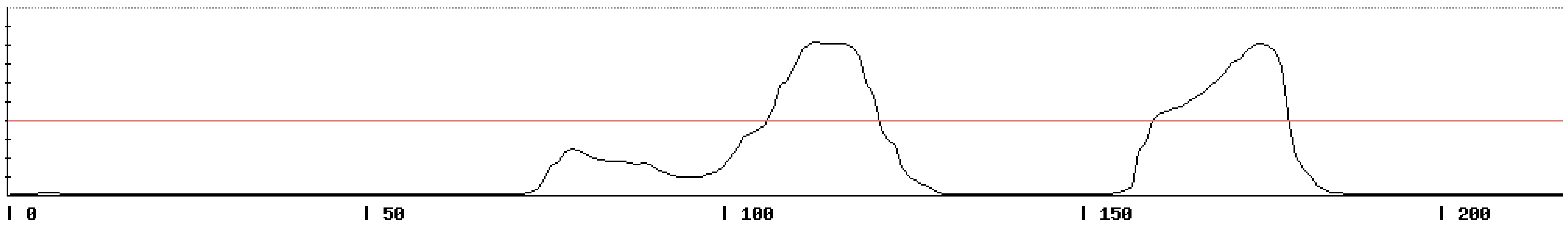

Predictions for Citrussinensis_orange1.1g045827m

Viterbi Path

Viterbi Path

Posterior @ 0.4

Posterior @ 0.4

Posterior Graphic. Threshold Marked @ 0.4

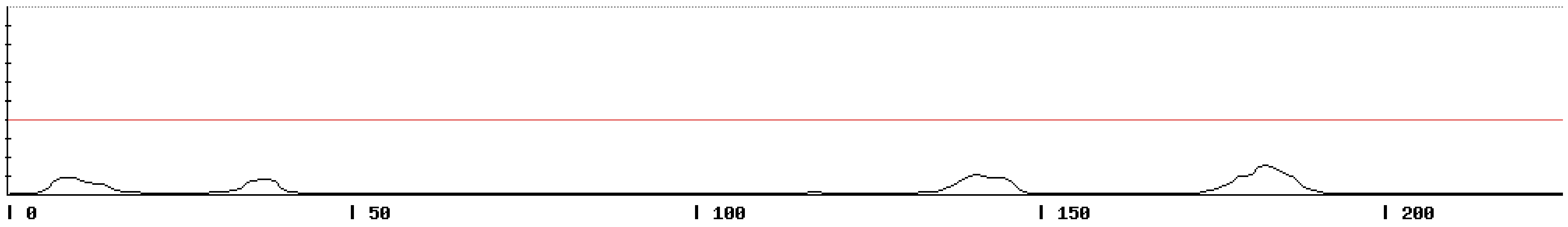

Predictions for Citrussinensis_orange1.1g045934m

Viterbi Path

Viterbi Path

Posterior @ 0.4

Posterior @ 0.4

Posterior Graphic. Threshold Marked @ 0.4

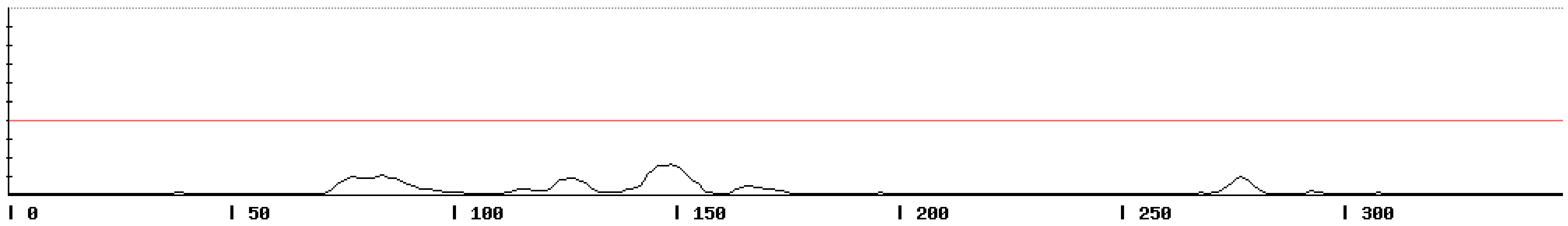

Predictions for Citrussinensis_orange1.1g046003m

Viterbi Path

Viterbi Path

Posterior @ 0.4

77 - KKKSSKGSRIDR - 88

112 - KIKIGSKKRFRY - 123

173 - K - 173

Posterior @ 0.4

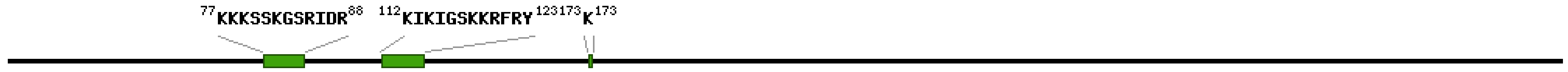

Posterior Graphic. Threshold Marked @ 0.4

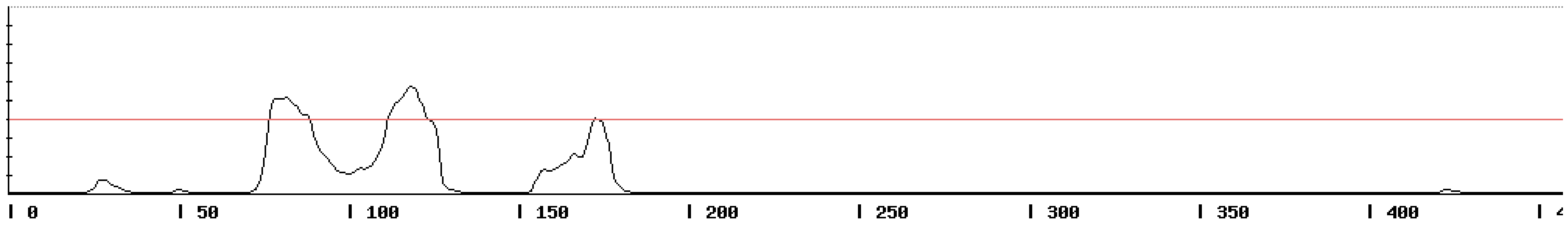

Predictions for Citrussinensis_orange1.1g046218m

Viterbi Path

Viterbi Path

Posterior @ 0.4 — 83 - PKGTRPNR - 90

Posterior @ 0.4

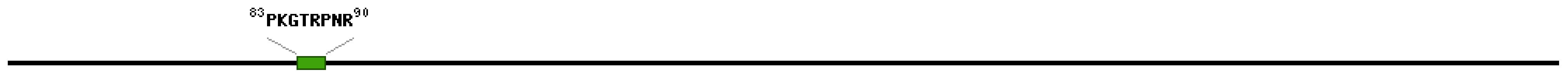

Posterior Graphic. Threshold Marked @ 0.4

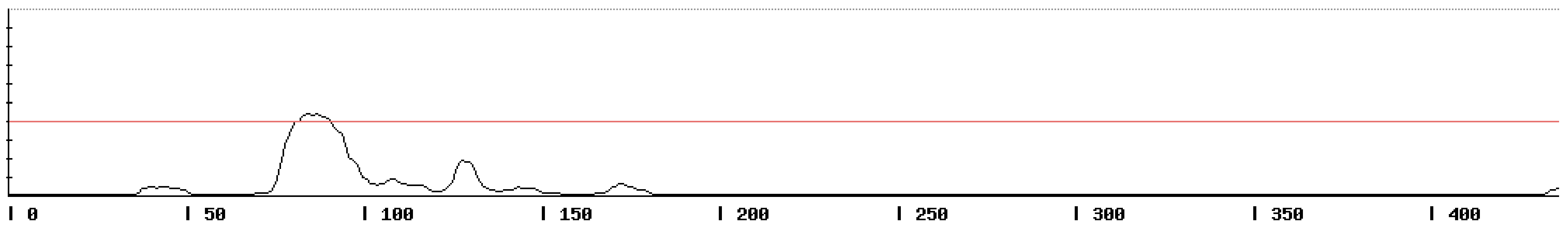

Predictions for Citrussinensis_orange1.1g046437m

Viterbi Path

Viterbi Path

Posterior @ 0.4 — 29 - KGKR - 32

Posterior @ 0.4

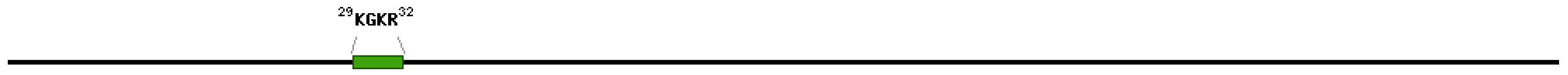

Posterior Graphic. Threshold Marked @ 0.4

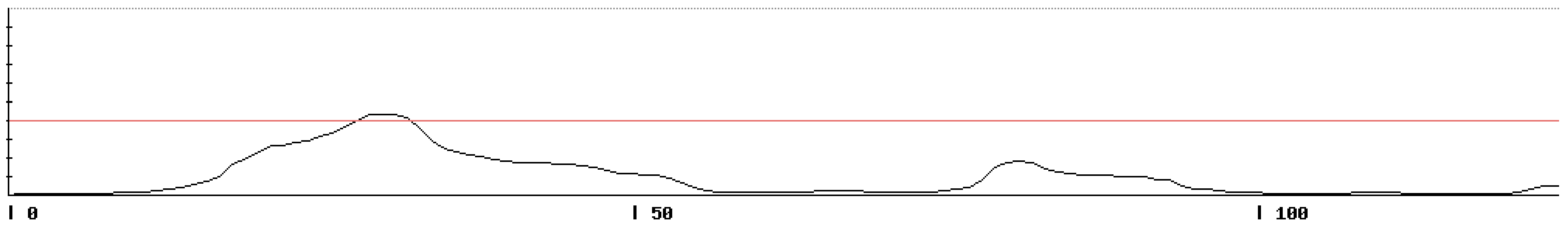

Predictions for Citrussinensis_orange1.1g046523m

Viterbi Path

Viterbi Path

Posterior @ 0.4

Posterior @ 0.4

Posterior Graphic. Threshold Marked @ 0.4

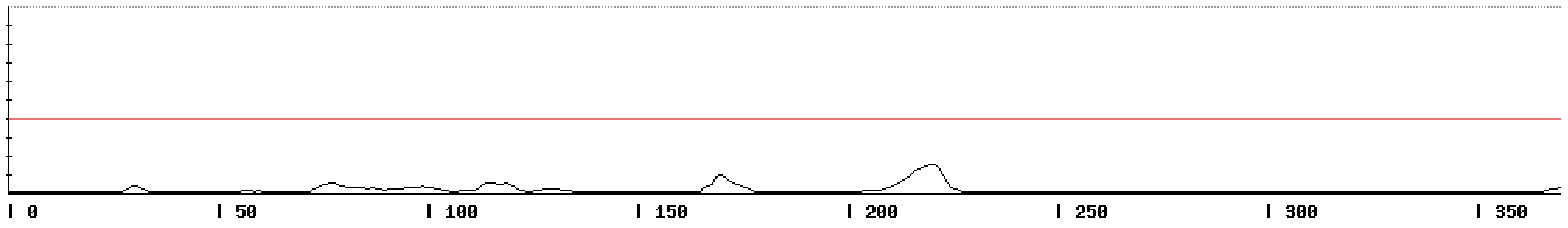

Predictions for Citrussinensis_orange1.1g046632m

Viterbi Path

Viterbi Path

Posterior @ 0.4

Posterior @ 0.4

Posterior Graphic. Threshold Marked @ 0.4

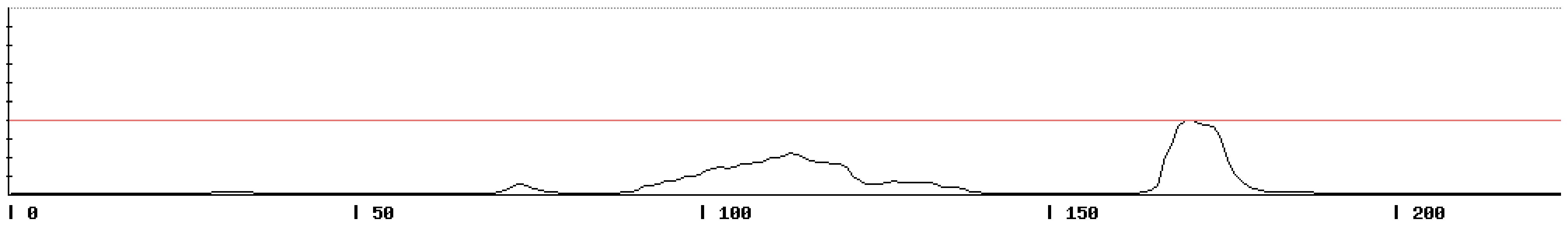

Predictions for Citrussinensis_orange1.1g046940m	
Viterbi Path	
Viterbi Path	
Posterior @ 0.4	107 - KRR - 109
Posterior @ 0.4	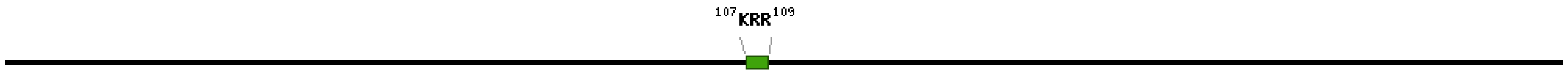
Posterior Graphic. Threshold Marked @ 0.4	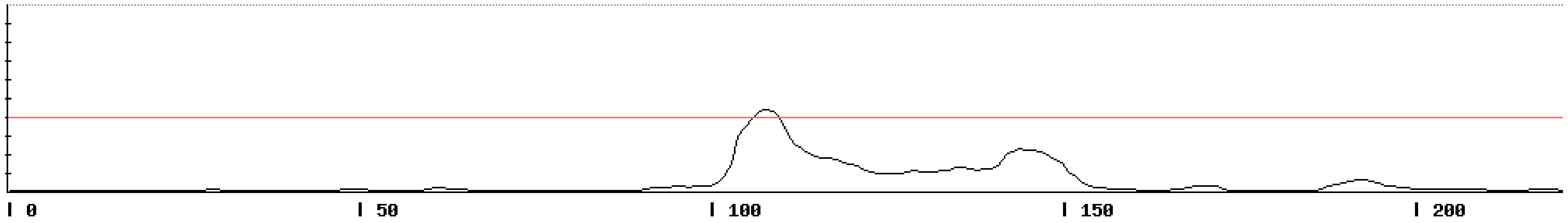

Predictions for Citrussinensis_orange1.1g046948m	
Viterbi Path	
Viterbi Path	
Posterior @ 0.4	
Posterior @ 0.4	
Posterior Graphic. Threshold Marked @ 0.4	

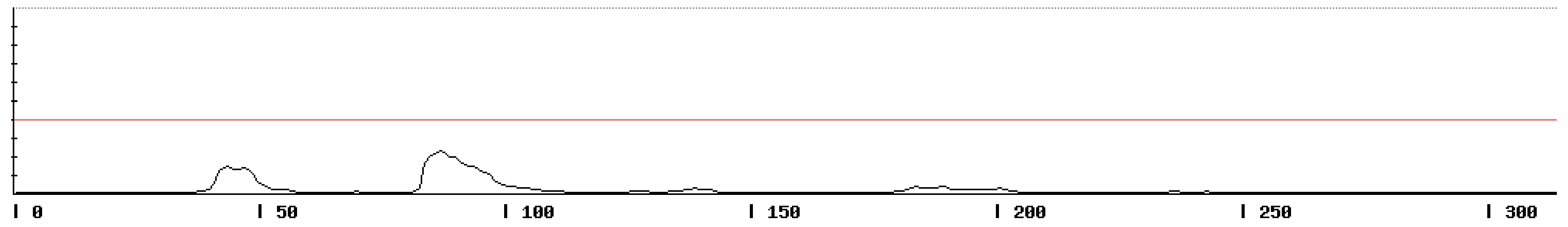

Predictions for Citrussinensis_orange1.1g046996m

Viterbi Path

Viterbi Path

Posterior @ 0.4

Posterior @ 0.4

Posterior Graphic. Threshold Marked @ 0.4

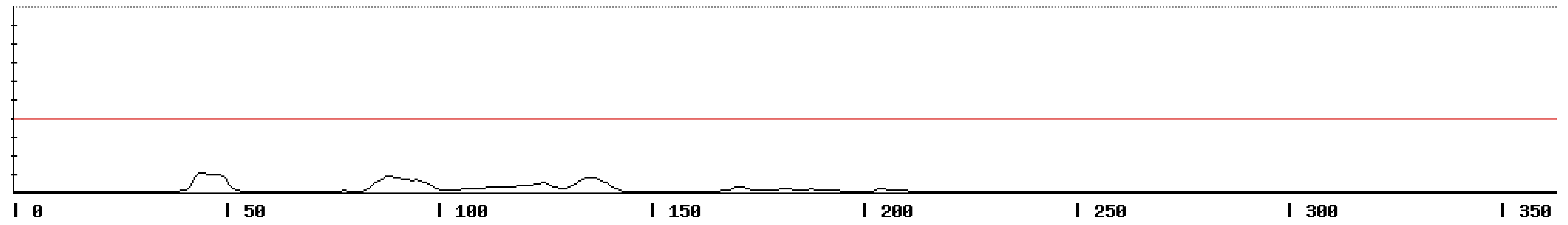

Predictions for Citrussinensis_orange1.1g047398m

Viterbi Path

Viterbi Path

Posterior @ 0.4

Posterior @ 0.4

Posterior Graphic. Threshold Marked @ 0.4

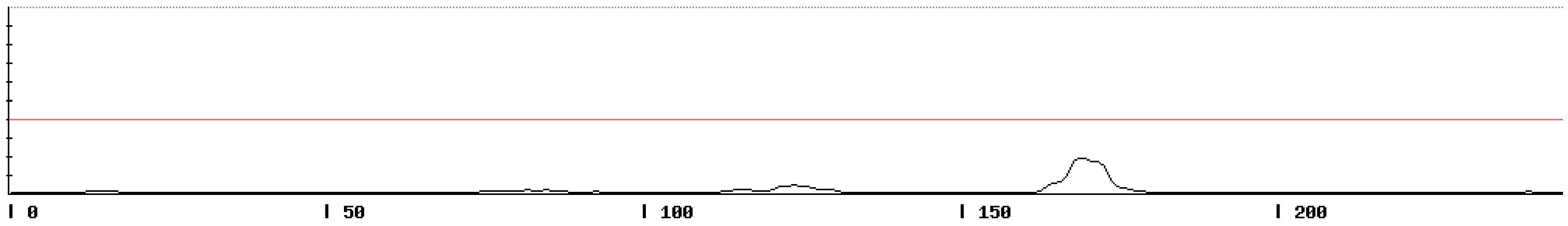

Predictions for Citrussinensis_orange1.1g047610m

Viterbi Path	
Viterbi Path	
Posterior @ 0.4	
Posterior @ 0.4	
Posterior Graphic. Threshold Marked @ 0.4	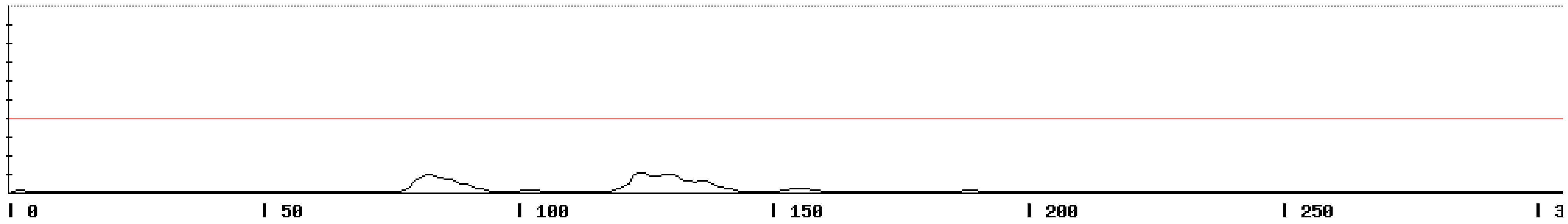

Predictions for Citrussinensis_orange1.1g047611m

Viterbi Path	
Viterbi Path	
Posterior @ 0.4	53 - RRR - 55
Posterior @ 0.4	

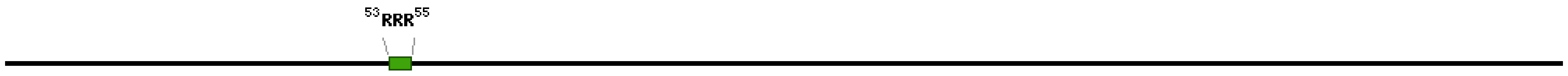

Posterior Graphic. Threshold Marked @ 0.4

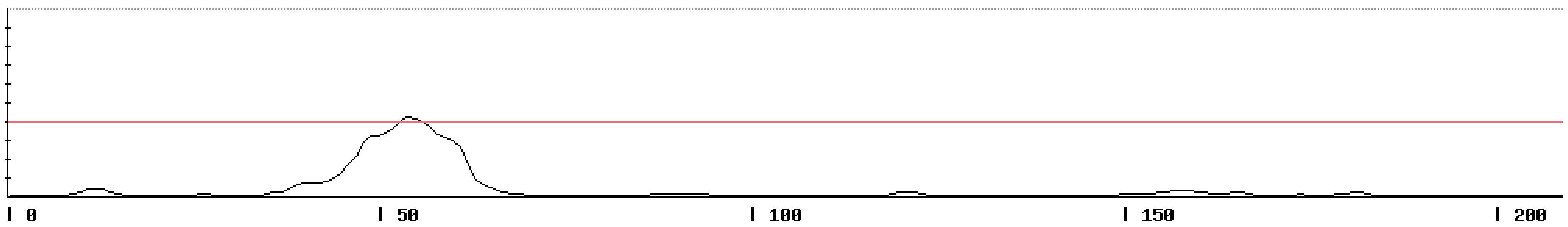

Predictions for Citrussinensis_orange1.1g047622m

Viterbi Path	108 - RKSKKKIGSKKRFR - 121
Viterbi Path	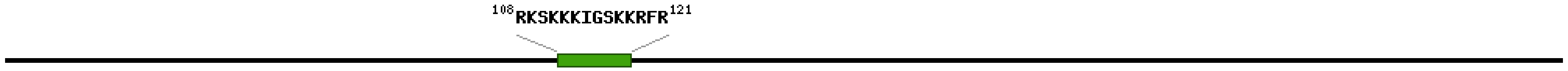
Posterior @ 0.4	103 - KAVVSRKSKKKIGSKKRFRYEKDK - 126
Posterior @ 0.4	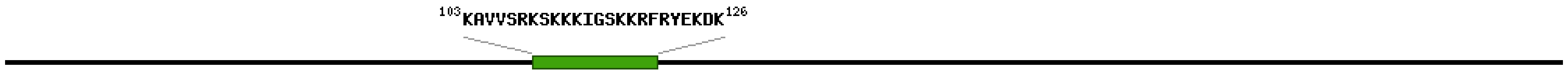
Posterior Graphic. Threshold Marked @ 0.4	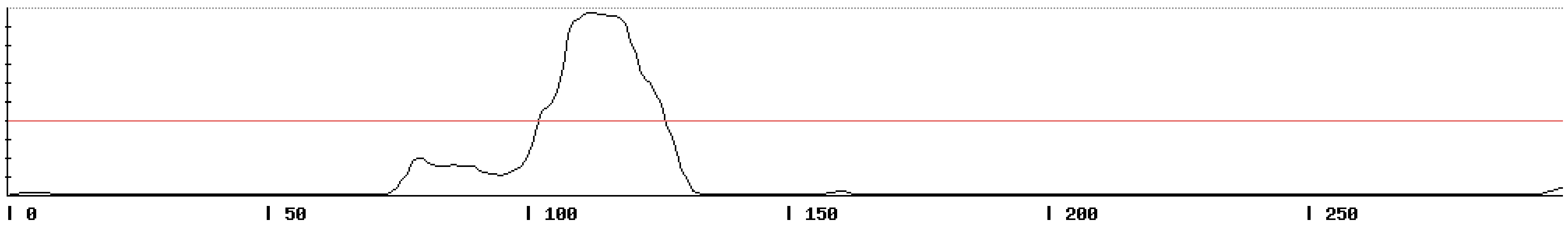

Predictions for Citrussinensis_orange1.1g047639m

Viterbi Path	
Viterbi Path	

Posterior @ 0.4	
Posterior @ 0.4	
Posterior Graphic. Threshold Marked @ 0.4	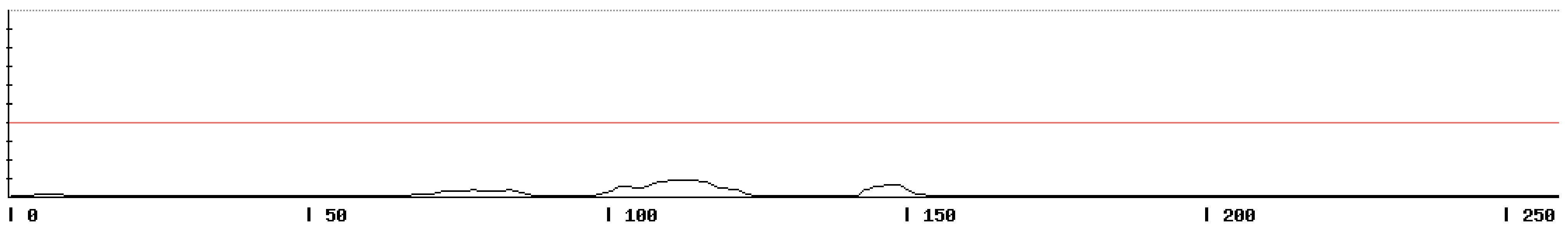

Predictions for Citrussinensis_orange1.1g047710m

Viterbi Path	
Viterbi Path	
Posterior @ 0.4	
Posterior @ 0.4	
Posterior Graphic. Threshold Marked @ 0.4	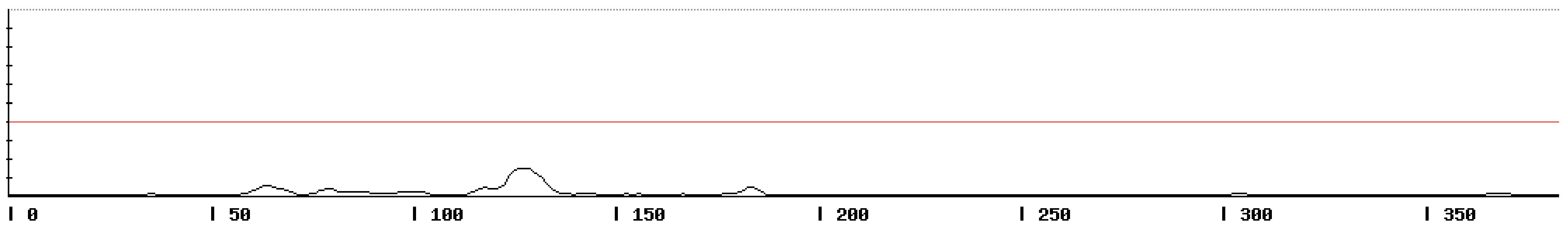

Predictions for Citrussinensis_orange1.1g047852m

Viterbi Path	
Viterbi Path	

Viterbi Path

Posterior @ 0.6

Posterior @ 0.6

Posterior Graphic. Threshold Marked @ 0.6

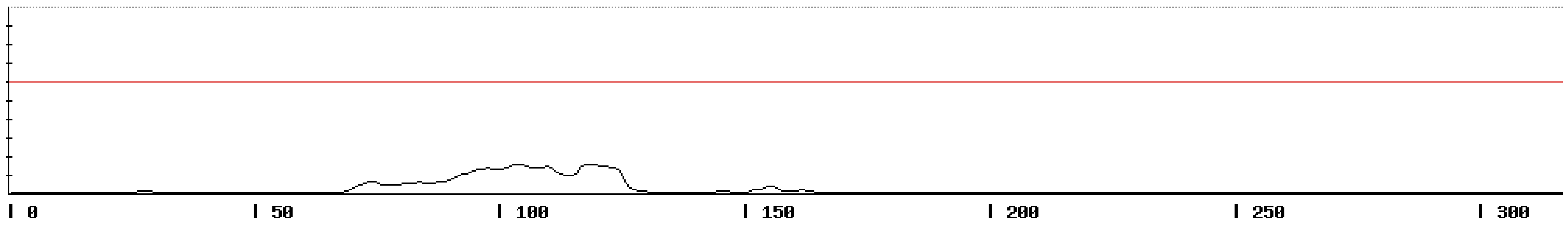

Predictions for Coffeacanephora_Cc00_g08030

Viterbi Path

Viterbi Path

Posterior @ 0.6

Posterior @ 0.6

Posterior Graphic. Threshold Marked @ 0.6

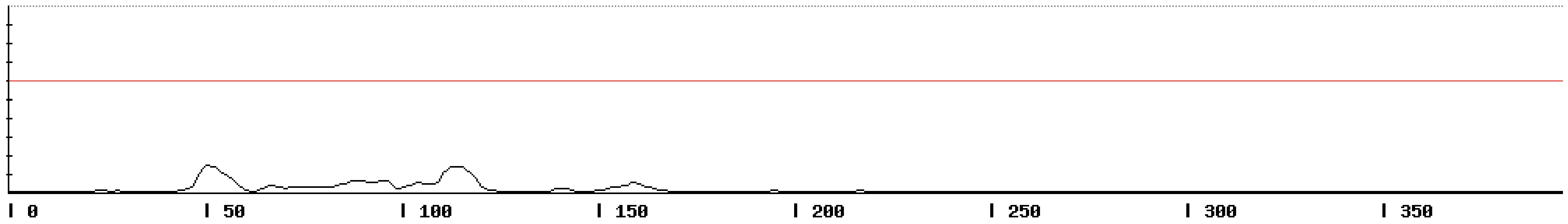

Predictions for Coffeacanephora_Cc00_g08040

Viterbi Path

Viterbi Path

Posterior @ 0.6

Posterior @ 0.6

Posterior Graphic. Threshold Marked @ 0.6

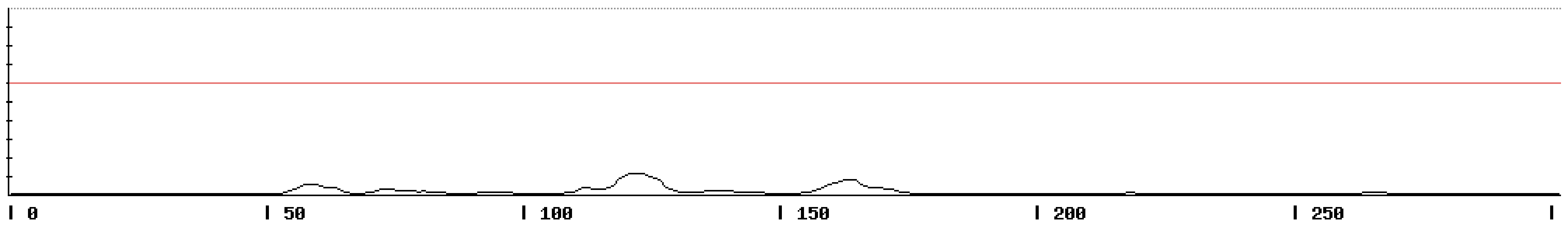

Predictions for Coffeacanephora_Cc00_g09070

Viterbi Path

Viterbi Path

Posterior @ 0.6

Posterior @ 0.6

Posterior Graphic. Threshold Marked @ 0.6

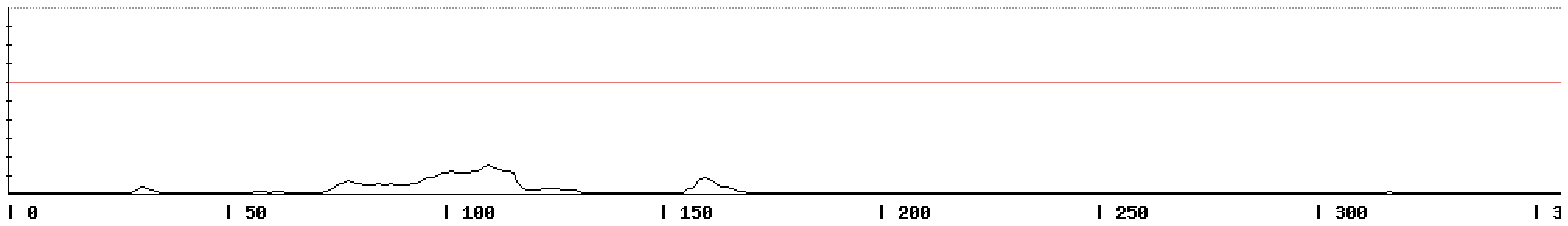

Predictions for Coffeacanephora_Cc00_g14270

Viterbi Path

Viterbi Path

Posterior @ 0.6

Posterior @ 0.6

Posterior Graphic. Threshold Marked @ 0.6

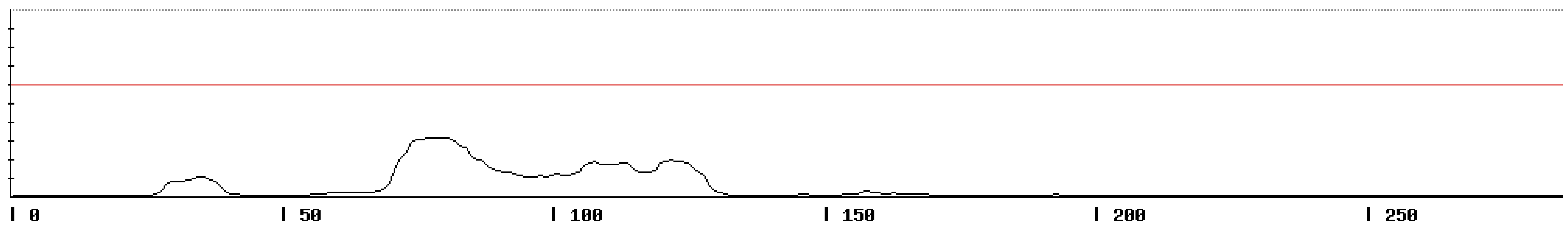

Predictions for Coffeacanephora_Cc00_g14290

Viterbi Path

Viterbi Path

Posterior @ 0.6

Posterior @ 0.6

Posterior Graphic. Threshold Marked @ 0.6

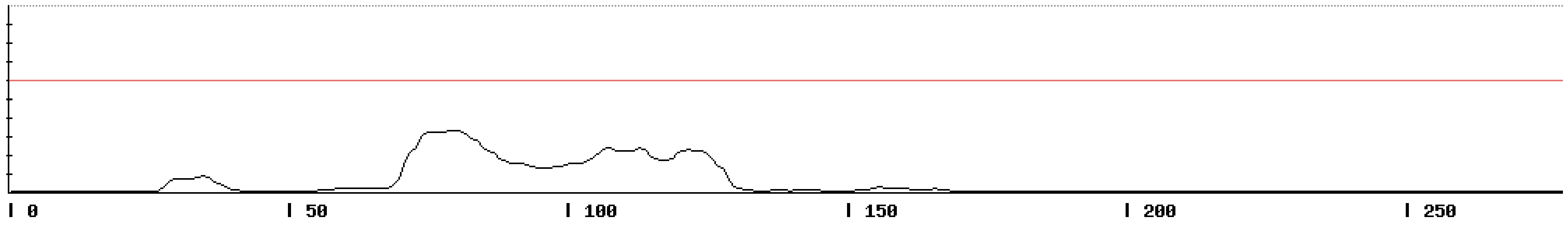

Predictions for Coffeacanephora_Cc00_g26010

Viterbi Path	
Viterbi Path	
Posterior @ 0.6	
Posterior @ 0.6	
Posterior Graphic. Threshold Marked @ 0.6	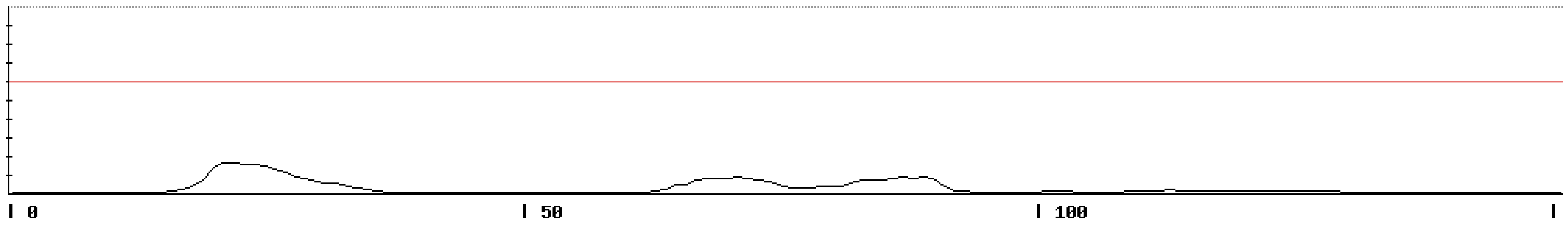

Predictions for Coffeacanephora_Cc00_g30840

Viterbi Path	
Viterbi Path	
Posterior @ 0.6	
Posterior @ 0.6	

Posterior Graphic. Threshold Marked @ 0.6

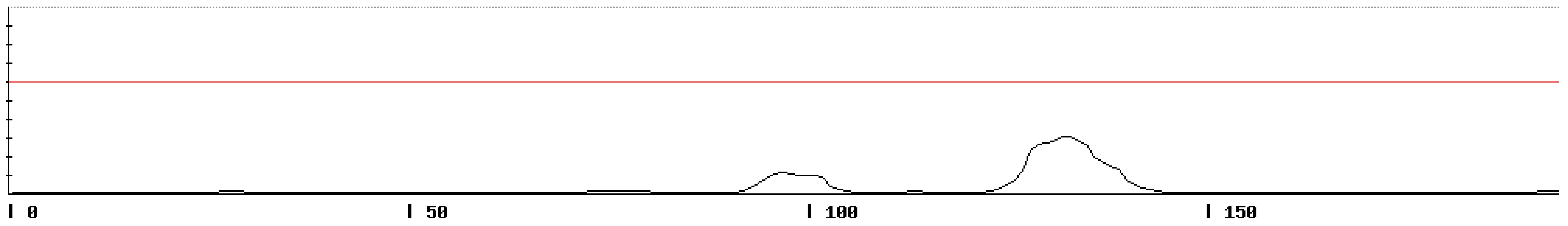

Predictions for Coffeacanephora_Cc01_g04540

Viterbi Path

Viterbi Path

Posterior @ 0.6

Posterior @ 0.6

Posterior Graphic. Threshold Marked @ 0.6

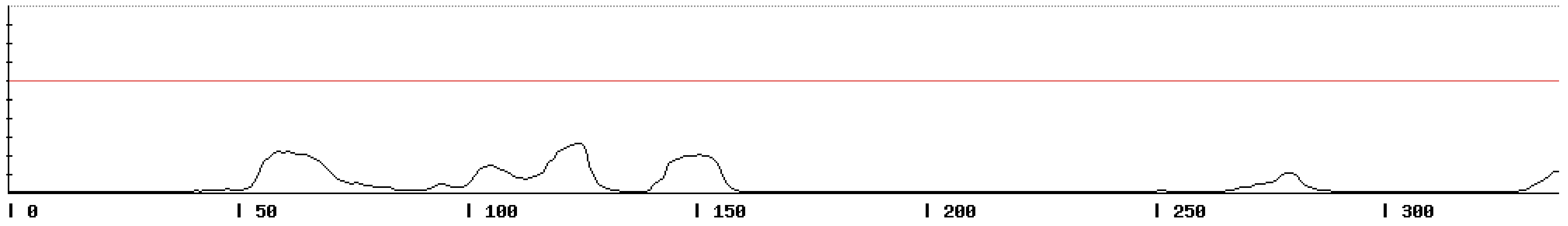

Predictions for Coffeacanephora_Cc01_g04560

Viterbi Path

Viterbi Path

Posterior @ 0.6

Posterior @ 0.6

Posterior Graphic. Threshold Marked @ 0.6

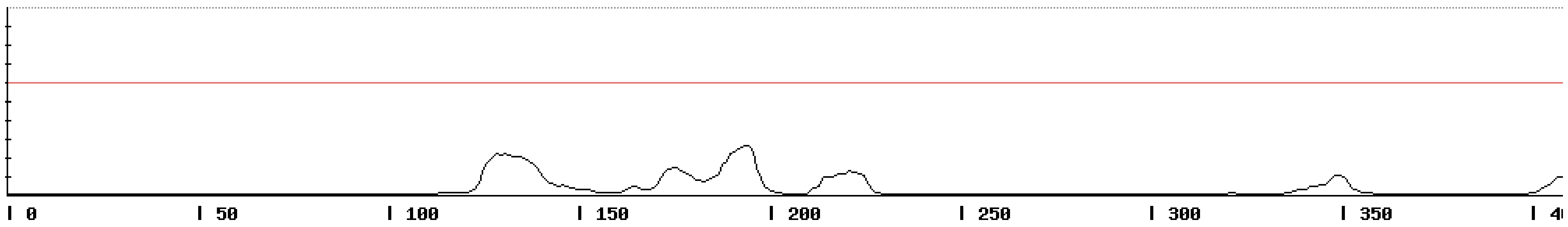

Predictions for Coffeacanephora_Cc01_g04590

Viterbi Path

Viterbi Path

Posterior @ 0.6

Posterior @ 0.6

Posterior Graphic. Threshold Marked @ 0.6

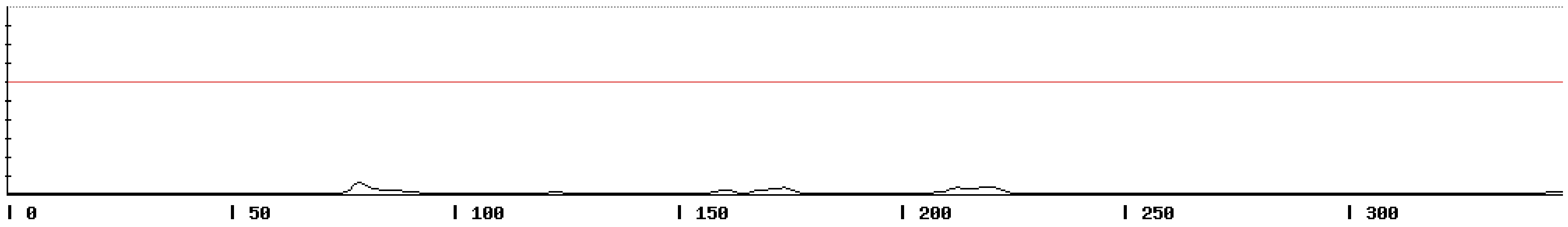

Predictions for Coffeacanephora_Cc01_g08720

Viterbi Path

Viterbi Path

Posterior @ 0.6

Posterior @ 0.6

Posterior Graphic. Threshold Marked @ 0.6

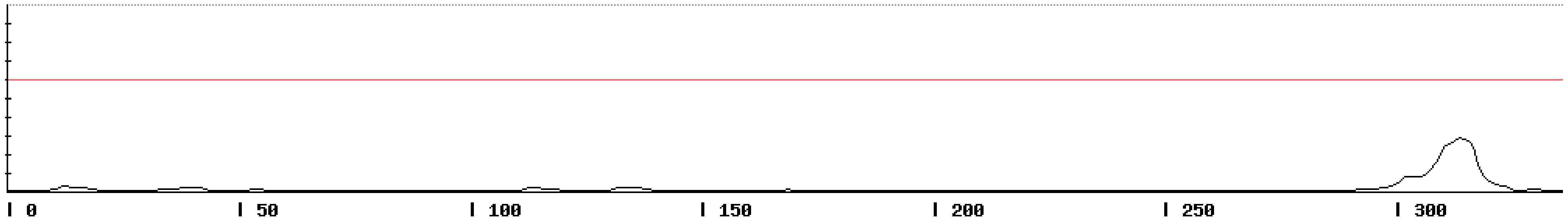

Predictions for Coffeacanephora_Cc01_g08730

Viterbi Path

Viterbi Path

Posterior @ 0.6

Posterior @ 0.6

Posterior Graphic. Threshold Marked @ 0.6

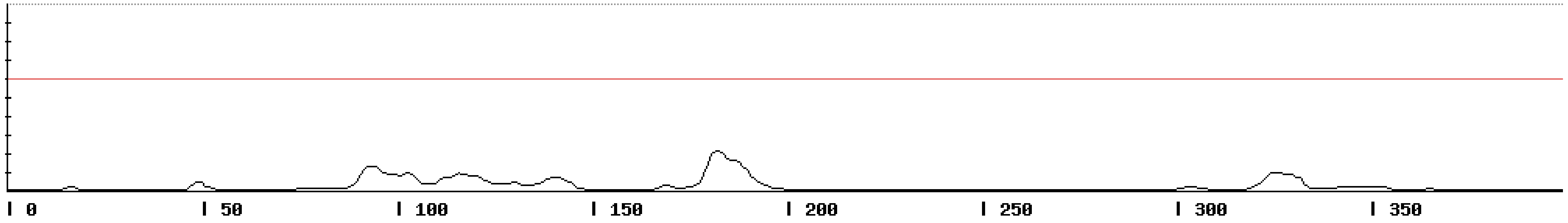

Predictions for Coffeacanephora_Cc01_g08900

Viterbi Path

Viterbi Path

Posterior @ 0.6

Posterior @ 0.6

Posterior Graphic. Threshold Marked @ 0.6

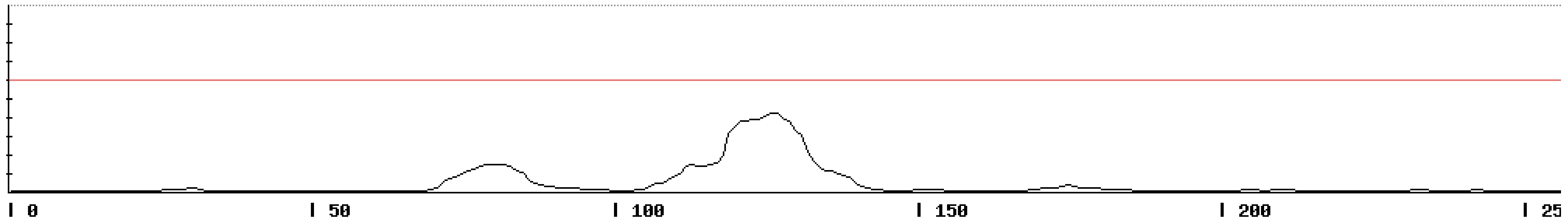

Predictions for Coffeacanephora_Cc01_g11320

Viterbi Path

Viterbi Path

Posterior @ 0.6

Posterior @ 0.6

Posterior Graphic. Threshold Marked @ 0.6

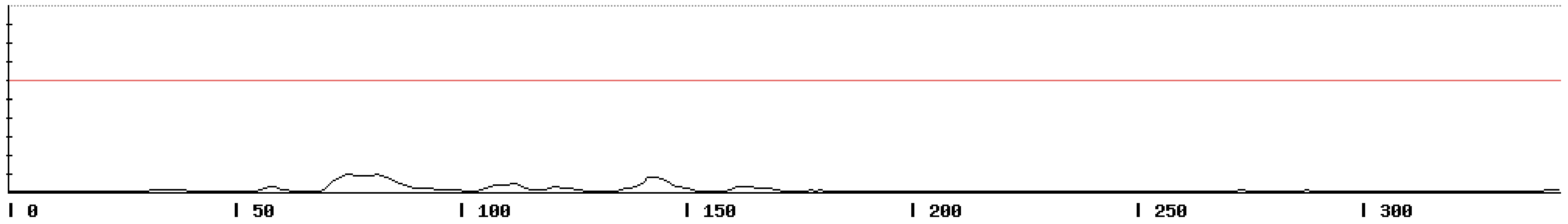

Predictions for Coffeacanephora_Cc01_g11350

Viterbi Path

Viterbi Path

Posterior @ 0.6

Posterior @ 0.6

Posterior Graphic. Threshold Marked @ 0.6

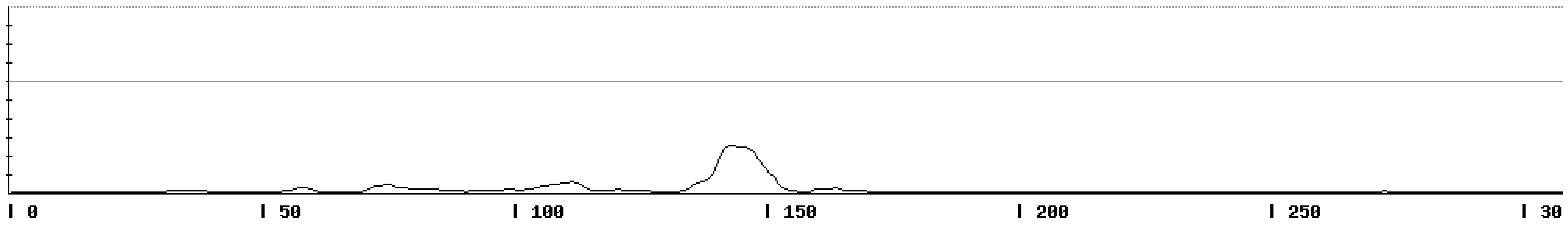

Predictions for Coffeacanephora_Cc01_g18220

Viterbi Path

Viterbi Path

Posterior @ 0.6

Posterior @ 0.6

Posterior Graphic. Threshold Marked @ 0.6

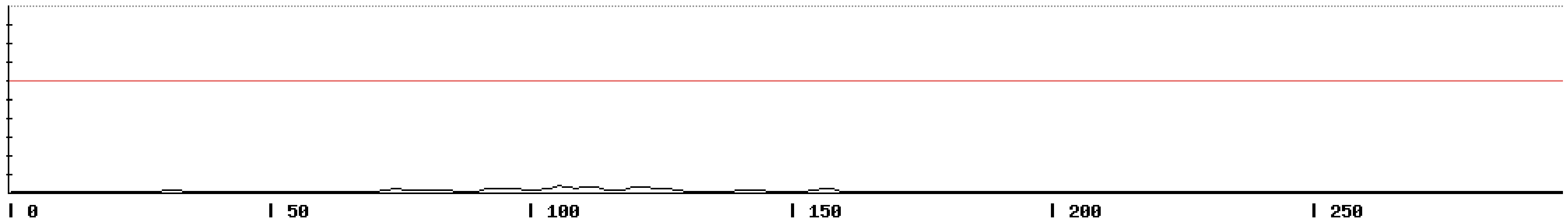

Predictions for Coffeacanephora_Cc01_g19370	
Viterbi Path	
Viterbi Path	
Posterior @ 0.6	
Posterior @ 0.6	
Posterior Graphic. Threshold Marked @ 0.6	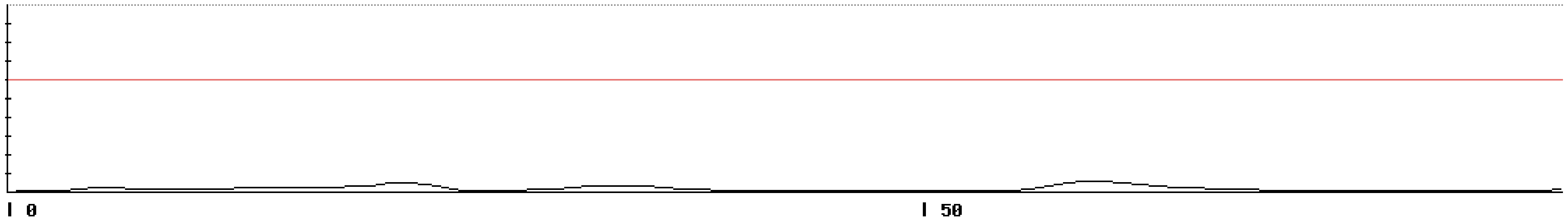

Predictions for Coffeacanephora_Cc02_g00700	
Viterbi Path	
Viterbi Path	
Posterior @ 0.6	100 - RNIRARKGTK - 109
Posterior @ 0.6	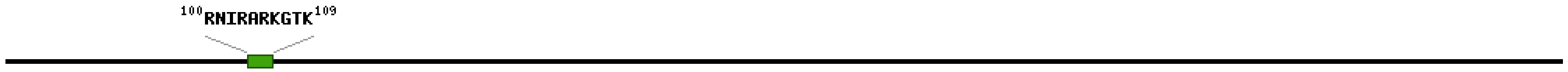
Posterior Graphic. Threshold Marked @ 0.6	

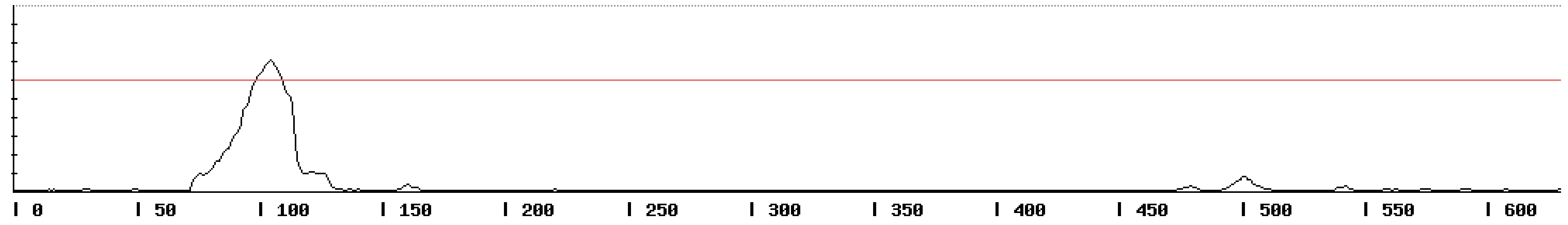

Predictions for Coffeacanephora_Cc02_g04160

Viterbi Path

Viterbi Path

Posterior @ 0.6

Posterior @ 0.6

Posterior Graphic. Threshold Marked @ 0.6

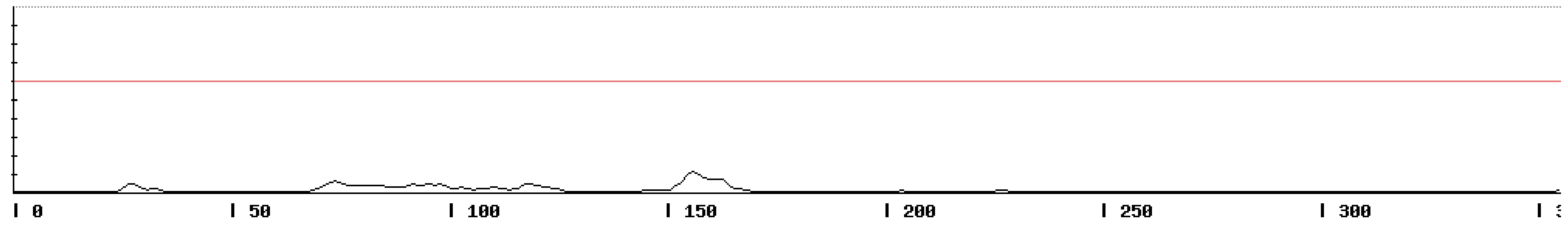

Predictions for Coffeacanephora_Cc02_g10630

Viterbi Path

Viterbi Path

Posterior @ 0.6

Posterior @ 0.6

Posterior Graphic. Threshold Marked @ 0.6

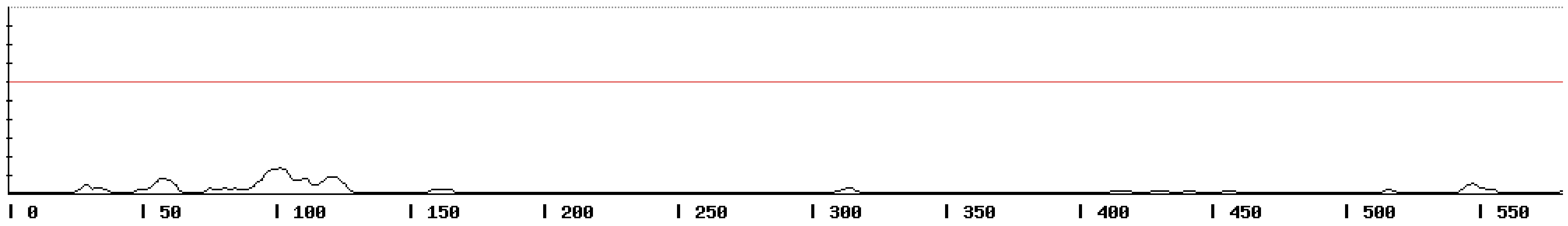

Predictions for Coffeacanephora_Cc02_g10640

Viterbi Path

Viterbi Path

Posterior @ 0.6

Posterior @ 0.6

Posterior Graphic. Threshold Marked @ 0.6

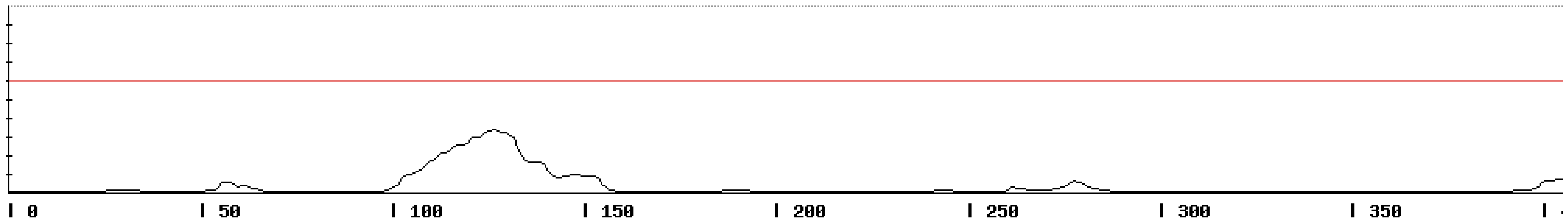

Predictions for Coffeacanephora_Cc02_g13910

Viterbi Path

Viterbi Path

Posterior @ 0.6

Posterior @ 0.6

Posterior Graphic. Threshold Marked @ 0.6

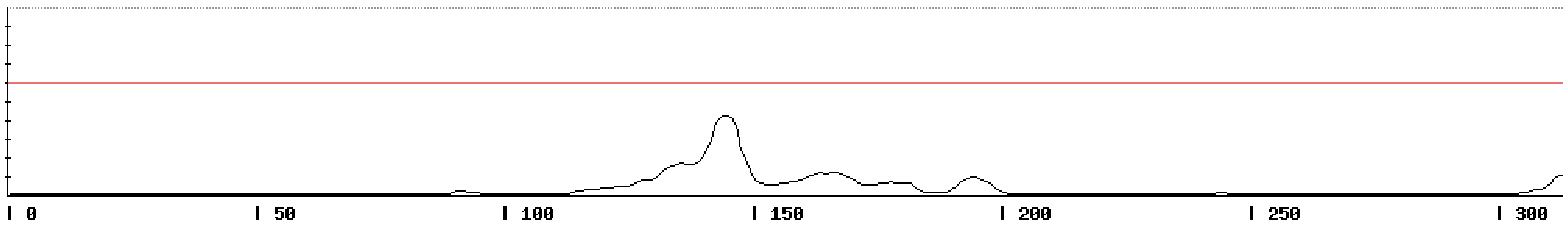

Predictions for Coffeacanephora_Cc02_g14010

Viterbi Path

Viterbi Path

Posterior @ 0.6

Posterior @ 0.6

Posterior Graphic. Threshold Marked @ 0.6

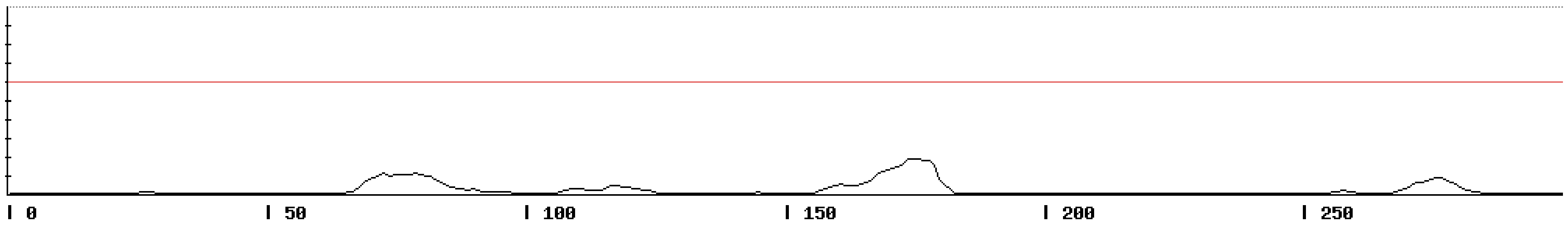

Predictions for Coffeacanephora_Cc02_g21890

Viterbi Path

Viterbi Path

Posterior @ 0.6

Posterior @ 0.6

Posterior Graphic. Threshold Marked @ 0.6

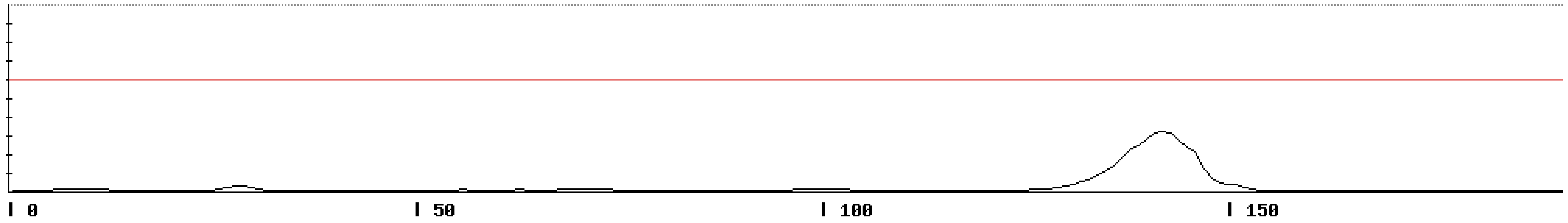

Predictions for Coffeacanephora_Cc02_g23810

Viterbi Path

Viterbi Path

Posterior @ 0.6

Posterior @ 0.6

Posterior Graphic. Threshold Marked @ 0.6

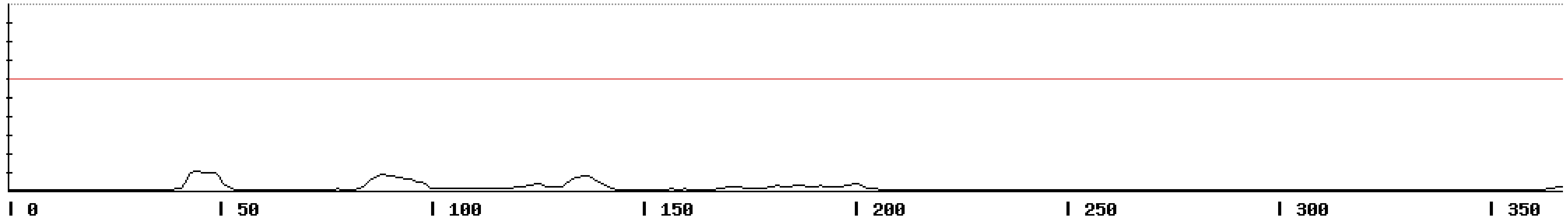

Predictions for Coffeacanephora_Cc02_g33930

Viterbi Path

Viterbi Path

Posterior @ 0.6

Posterior @ 0.6

Posterior Graphic. Threshold Marked @ 0.6

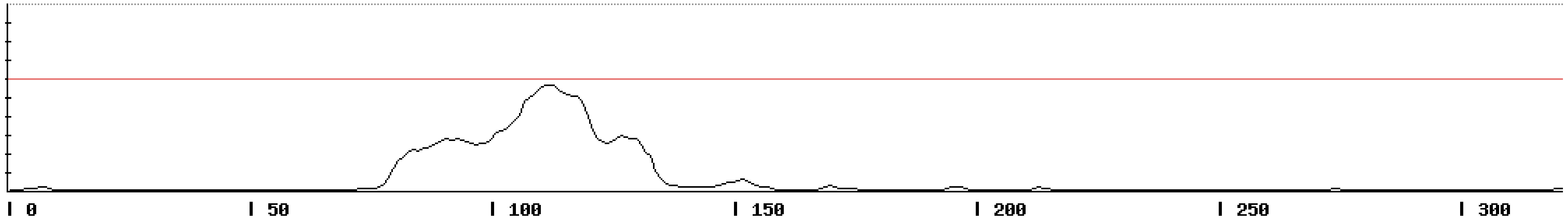

Predictions for Coffeacanephora_Cc02_g39920

Viterbi Path

Viterbi Path

Posterior @ 0.6

Posterior @ 0.6

Posterior Graphic. Threshold Marked @ 0.6

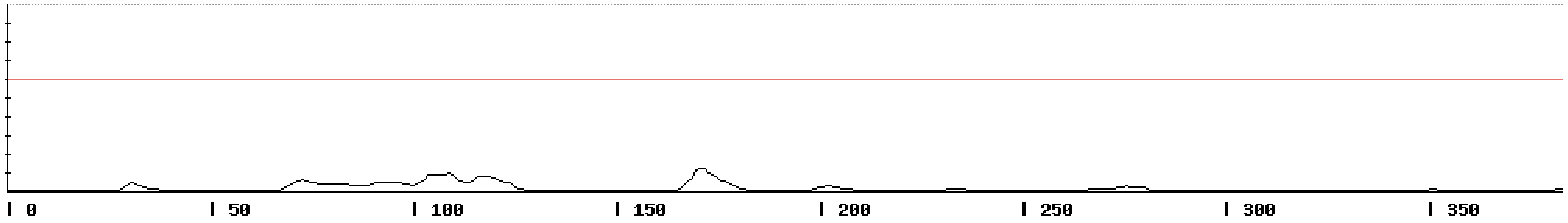

Predictions for Coffeacanephora_Cc04_g07090

Viterbi Path

Viterbi Path

Posterior @ 0.6

Posterior @ 0.6

Posterior Graphic. Threshold Marked @ 0.6

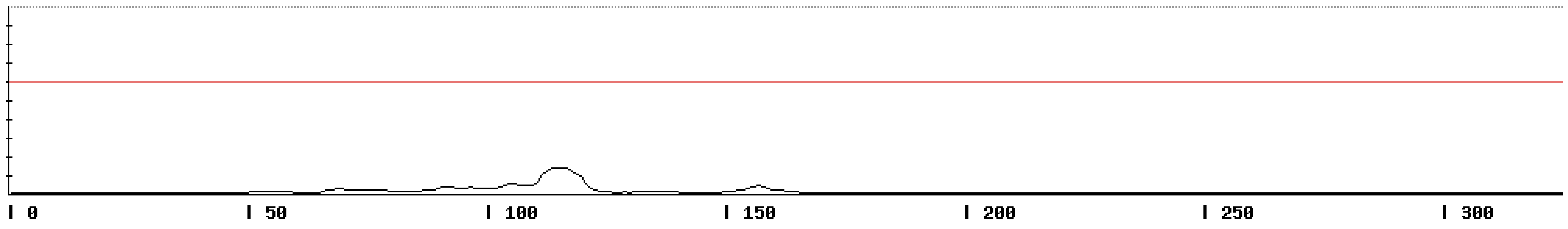

Predictions for Coffeacanephora_Cc04_g08930

Viterbi Path

Viterbi Path

Posterior @ 0.6

Posterior @ 0.6

Posterior Graphic. Threshold Marked @ 0.6

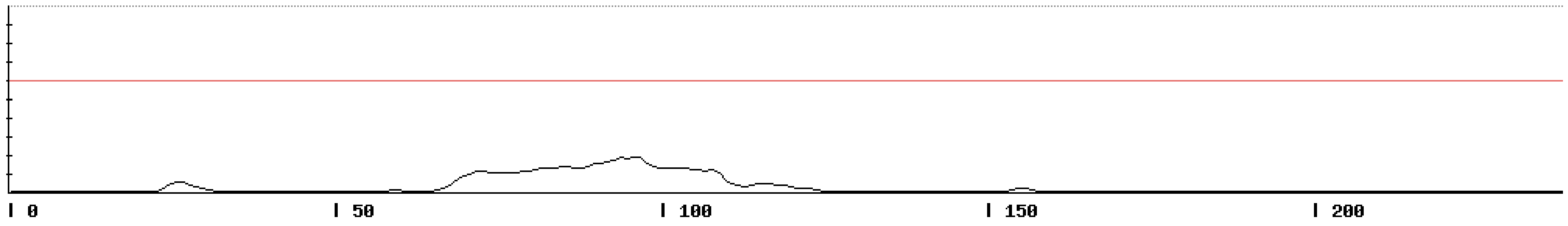

Predictions for Coffeacanephora_Cc04_g09840	
Viterbi Path	
Viterbi Path	
Posterior @ 0.6	
Posterior @ 0.6	
Posterior Graphic. Threshold Marked @ 0.6	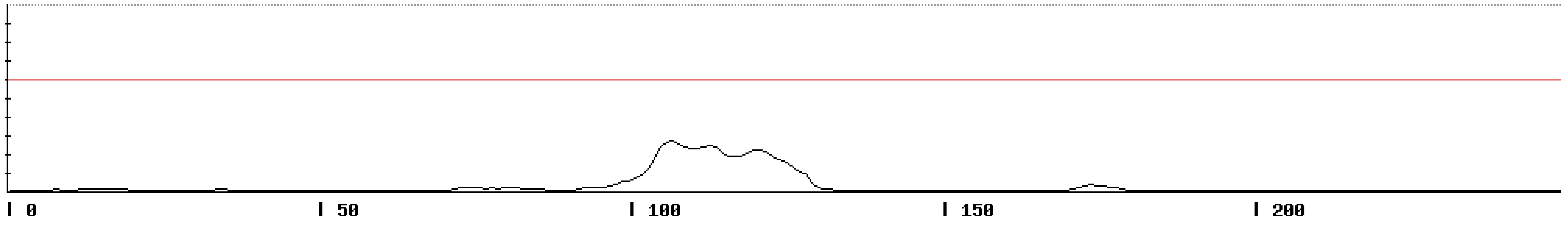

Predictions for Coffeacanephora_Cc04_g16290	
Viterbi Path	
Viterbi Path	
Posterior @ 0.6	
Posterior @ 0.6	
Posterior Graphic. Threshold Marked @ 0.6	

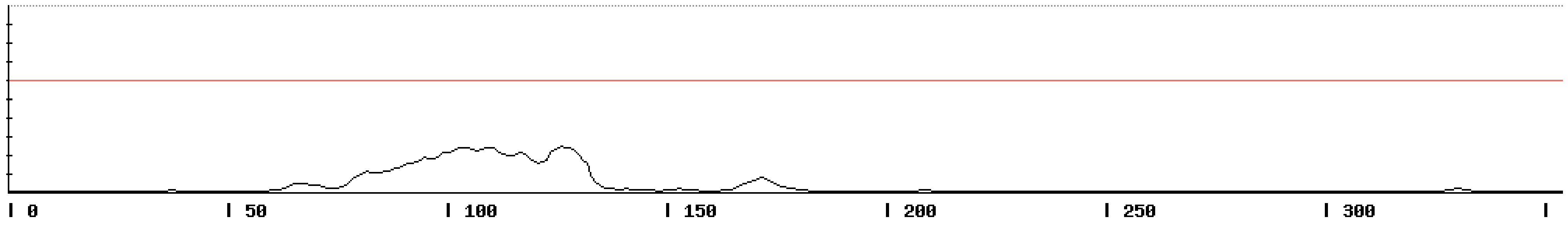

Predictions for Coffeacanephora_Cc05_g01340

Viterbi Path

Viterbi Path

Posterior @ 0.6

Posterior @ 0.6

Posterior Graphic. Threshold Marked @ 0.6

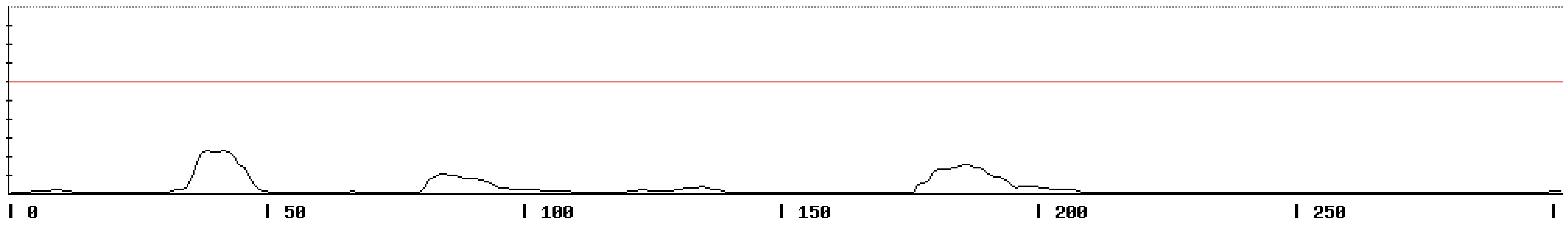

Predictions for Coffeacanephora_Cc05_g07770

Viterbi Path

Viterbi Path

Posterior @ 0.6

Posterior @ 0.6

Posterior Graphic. Threshold Marked @ 0.6	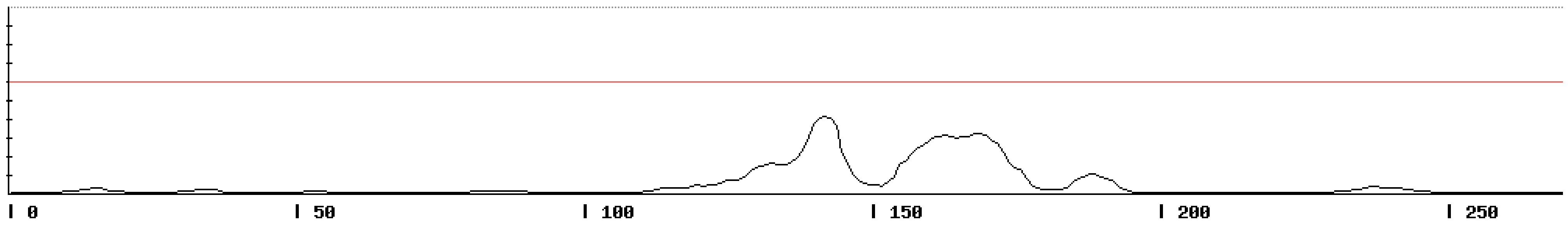

Predictions for Coffeacanephora_Cc05_g11720

Viterbi Path	
Viterbi Path	
Posterior @ 0.6	
Posterior @ 0.6	
Posterior Graphic. Threshold Marked @ 0.6	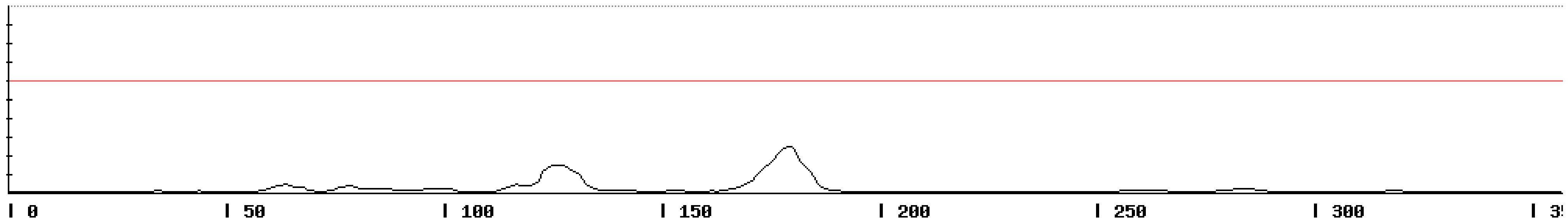

Predictions for Coffeacanephora_Cc05_g12500

Viterbi Path	
Viterbi Path	
Posterior @ 0.6	
Posterior @ 0.6	

Posterior Graphic. Threshold Marked @ 0.6

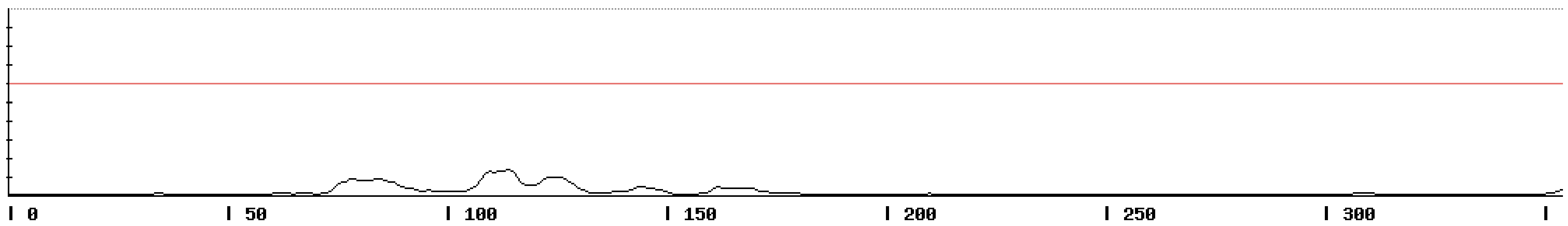

Predictions for Coffeacanephora_Cc05_g12510

Viterbi Path

Viterbi Path

Posterior @ 0.6

Posterior @ 0.6

Posterior Graphic. Threshold Marked @ 0.6

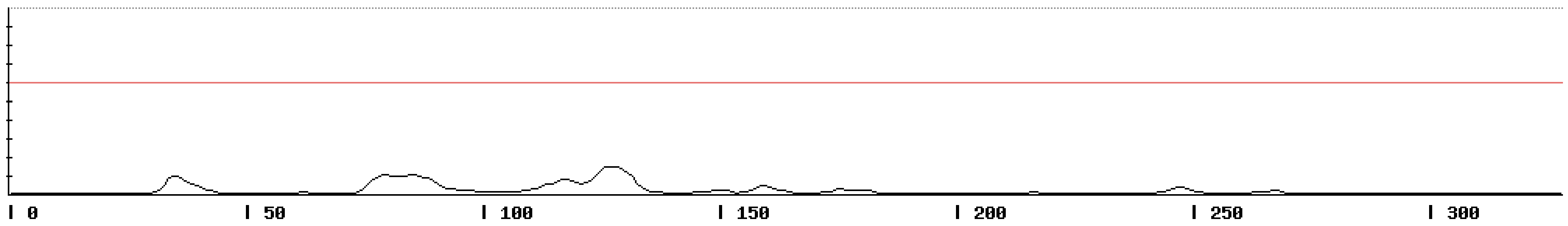

Predictions for Coffeacanephora_Cc05_g15960

Viterbi Path

Viterbi Path

Posterior @ 0.6

Posterior @ 0.6

Posterior Graphic. Threshold Marked @ 0.6

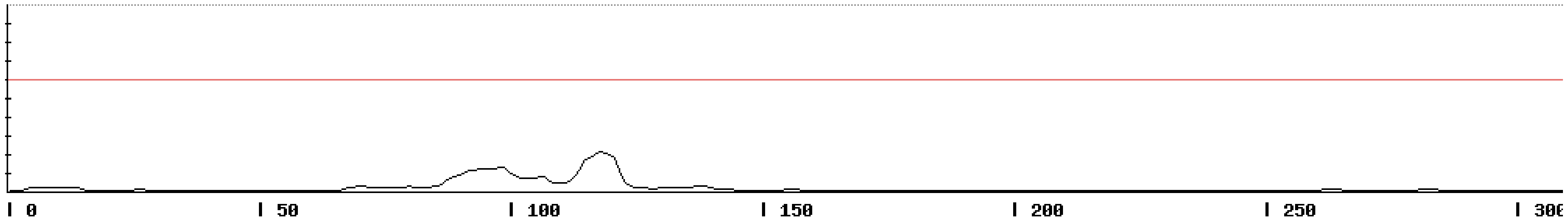

Predictions for Coffeacanephora_Cc05_g16190

Viterbi Path

Viterbi Path

Posterior @ 0.6

Posterior @ 0.6

Posterior Graphic. Threshold Marked @ 0.6

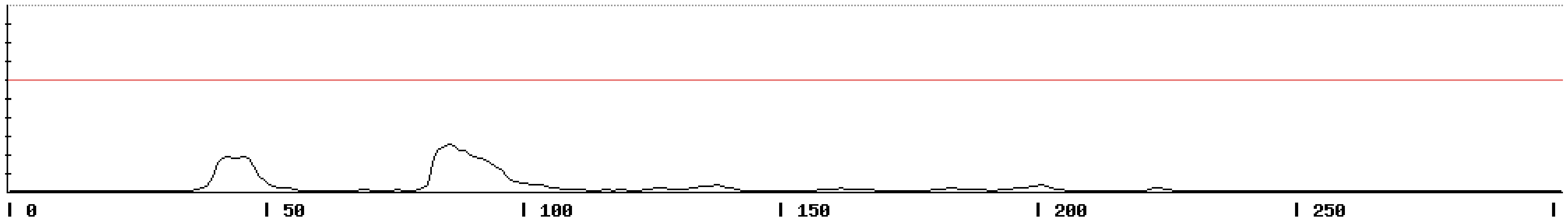

Predictions for Coffeacanephora_Cc06_g03790

Viterbi Path

Viterbi Path

Posterior @ 0.6

Posterior @ 0.6

Posterior Graphic. Threshold Marked @ 0.6

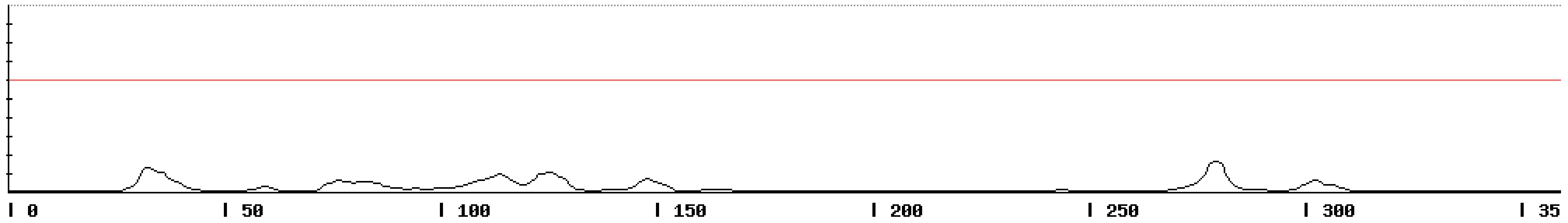

Predictions for Coffeacanephora_Cc06_g06140

Viterbi Path

Viterbi Path

Posterior @ 0.6

Posterior @ 0.6

Posterior Graphic. Threshold Marked @ 0.6

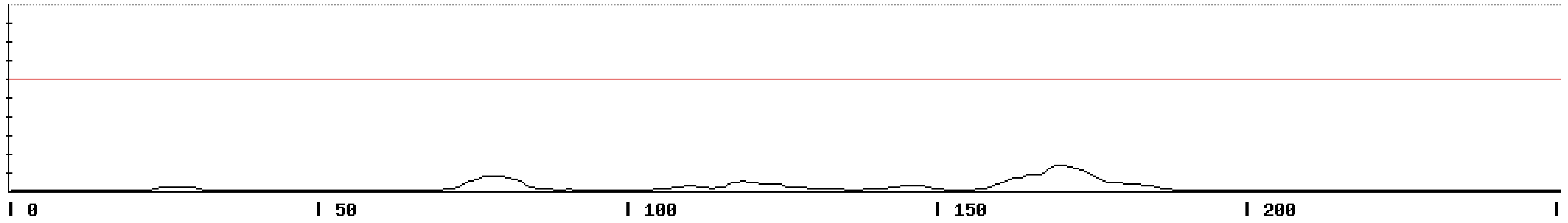

Predictions for Coffeacanephora_Cc06_g10730

Viterbi Path

Viterbi Path

Posterior @ 0.6

Posterior @ 0.6

Posterior Graphic. Threshold Marked @ 0.6

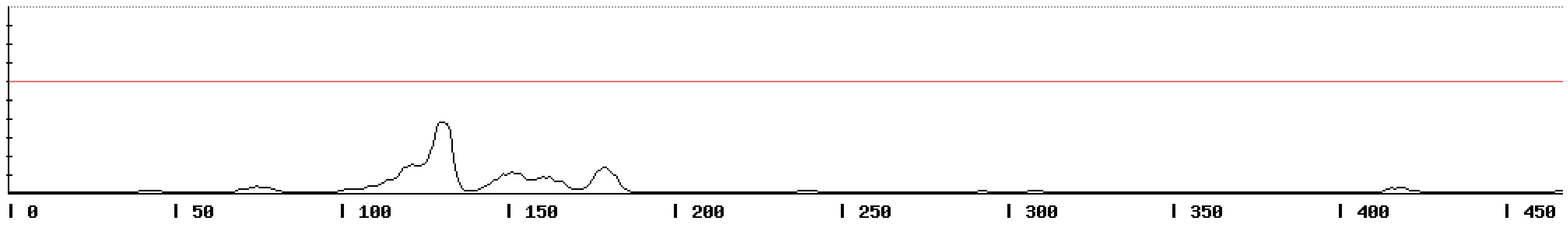

Predictions for Coffeacanephora_Cc07_g01550

Viterbi Path

Viterbi Path

Posterior @ 0.6

Posterior @ 0.6

Posterior Graphic. Threshold Marked @ 0.6

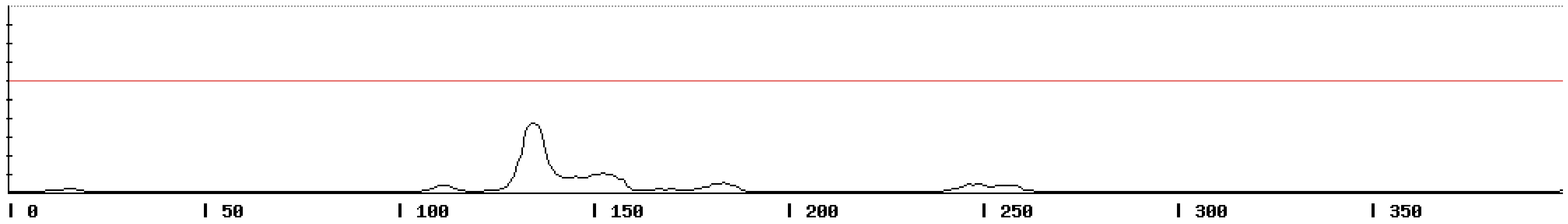

Predictions for Coffeacanephora_Cc07_g03320	
Viterbi Path	
Viterbi Path	
Posterior @ 0.6	
Posterior @ 0.6	
Posterior Graphic. Threshold Marked @ 0.6	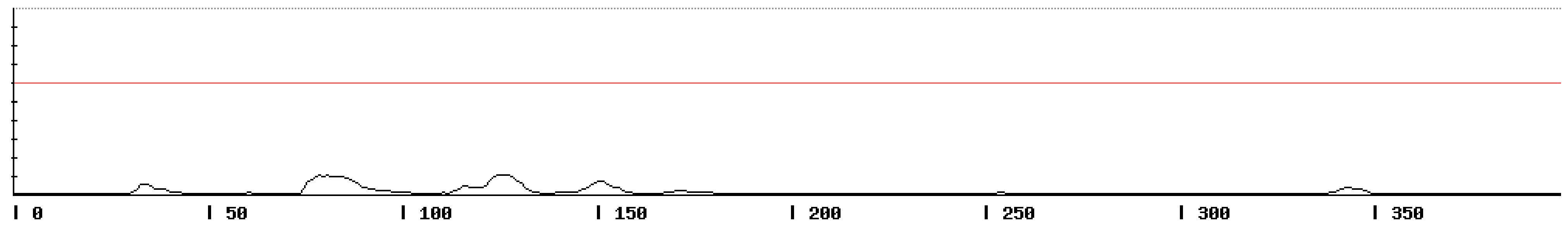

Predictions for Coffeacanephora_Cc07_g03570	
Viterbi Path	
Viterbi Path	
Posterior @ 0.6	
Posterior @ 0.6	
Posterior Graphic. Threshold Marked @ 0.6	

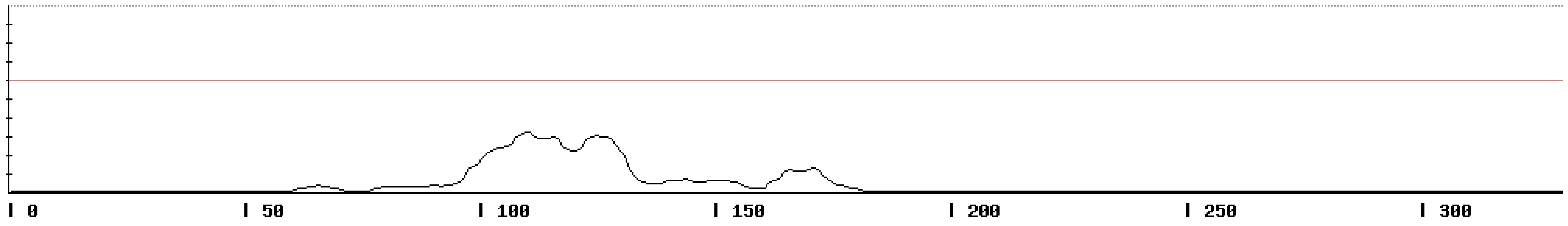

Predictions for Coffeacanephora_Cc07_g10520

Viterbi Path

Viterbi Path

Posterior @ 0.6

Posterior @ 0.6

Posterior Graphic. Threshold Marked @ 0.6

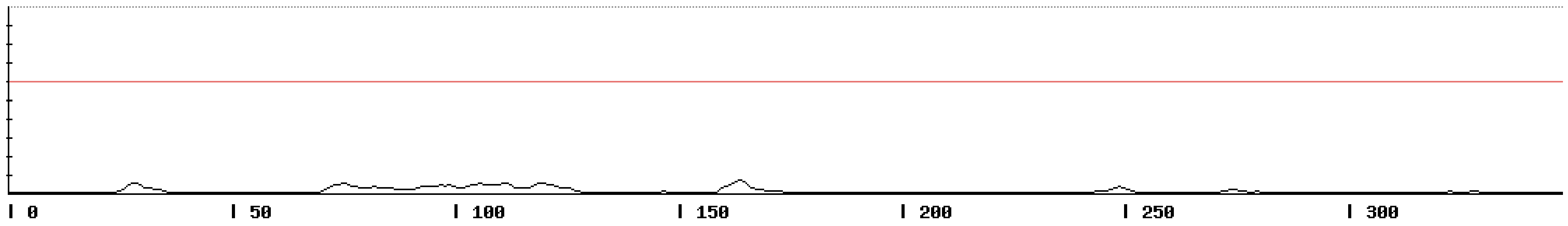

Predictions for Coffeacanephora_Cc07_g12760

Viterbi Path

Viterbi Path

Posterior @ 0.6

Posterior @ 0.6

Posterior Graphic. Threshold Marked @ 0.6

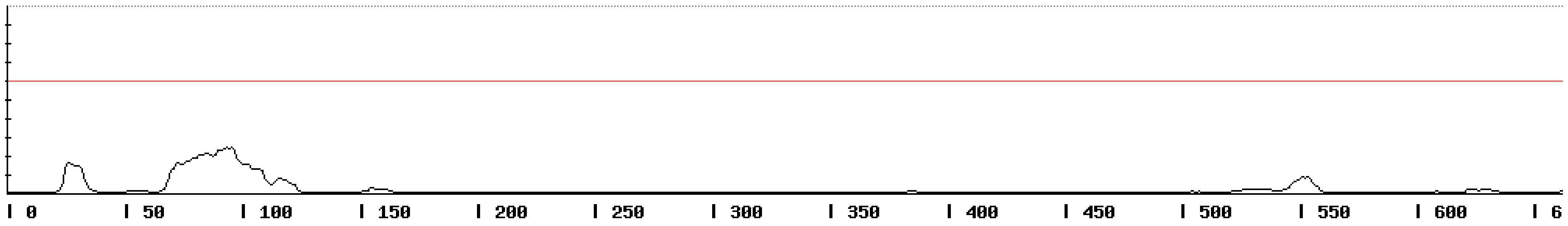

Predictions for Coffeacanephora_Cc07_g18360

Viterbi Path

Viterbi Path

Posterior @ 0.6

Posterior @ 0.6

Posterior Graphic. Threshold Marked @ 0.6

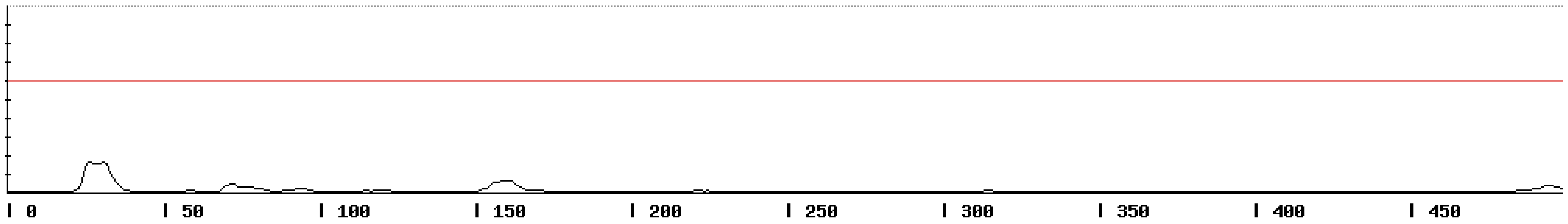

Predictions for Coffeacanephora_Cc08_g02440

Viterbi Path

Viterbi Path

Posterior @ 0.6

Posterior @ 0.6

Posterior Graphic. Threshold Marked @ 0.6

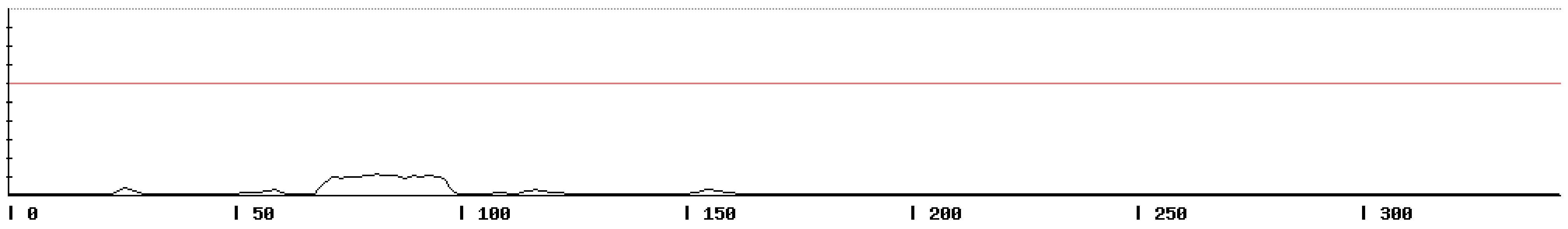

Predictions for Coffeacanephora_Cc08_g12970

Viterbi Path

Viterbi Path

Posterior @ 0.6

Posterior @ 0.6

Posterior Graphic. Threshold Marked @ 0.6

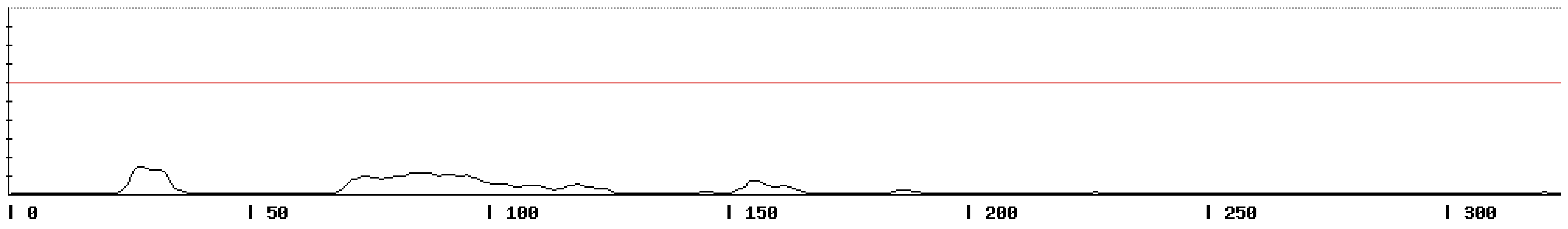

Predictions for Coffeacanephora_Cc08_g16900

Viterbi Path

Viterbi Path

Posterior @ 0.6

Posterior @ 0.6

Posterior Graphic. Threshold Marked @ 0.6

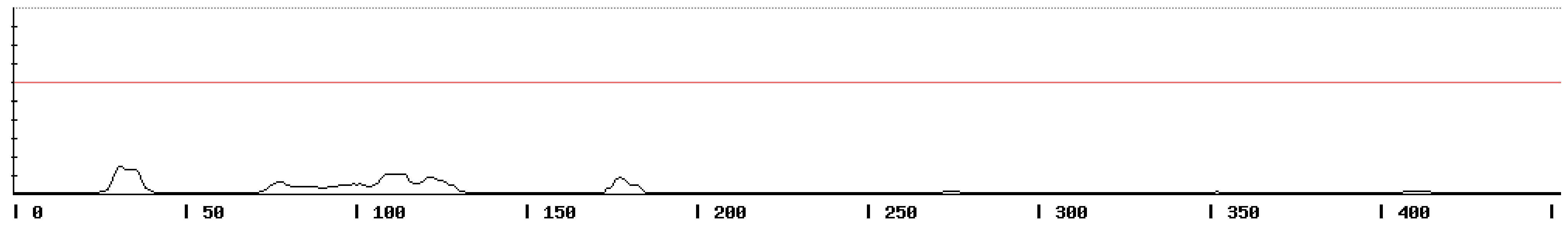

Predictions for Coffeacanephora_Cc08_g17070

Viterbi Path

Viterbi Path

Posterior @ 0.6

Posterior @ 0.6

Posterior Graphic. Threshold Marked @ 0.6

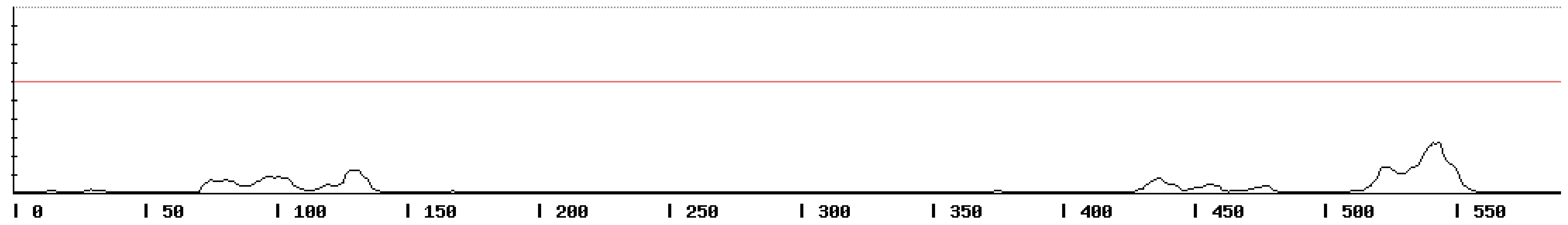

Predictions for Coffeacanephora_Cc09_g10560

Viterbi Path

Viterbi Path

Posterior @ 0.6 | 74 - DRKRGSGGGGRPNRTTKRGFWK - 95

Posterior @ 0.6

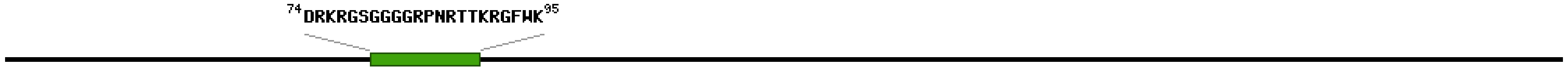

Posterior Graphic. Threshold Marked @ 0.6

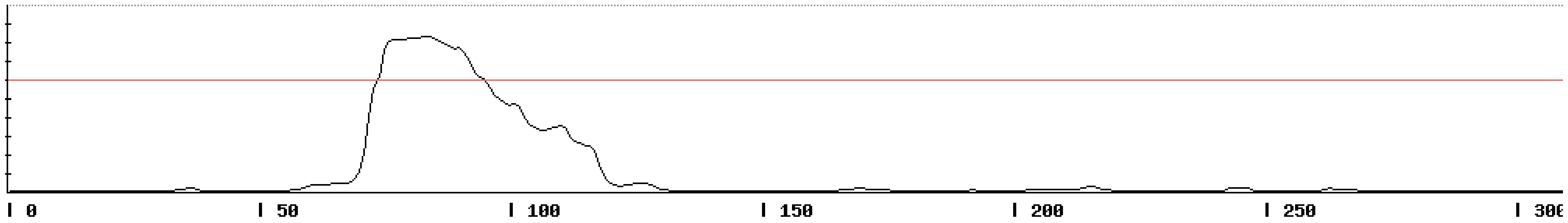

Predictions for Coffeacanephora_Cc10_g03330

Viterbi Path

Viterbi Path

Posterior @ 0.6

Posterior @ 0.6

Posterior Graphic. Threshold Marked @ 0.6

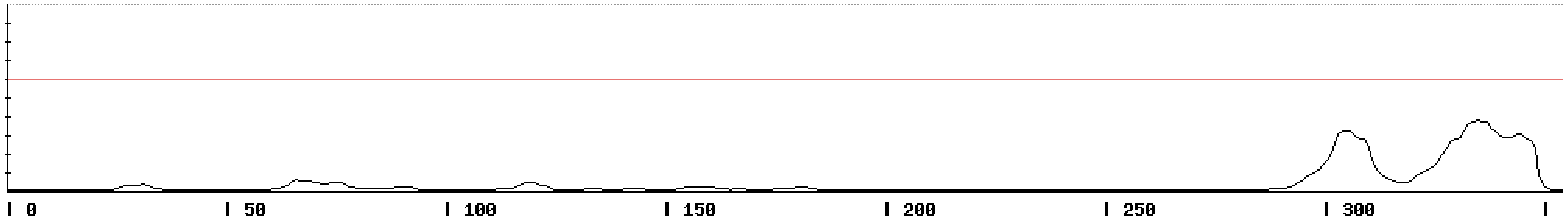

Predictions for Coffeacanephora_Cc10_g04680

Viterbi Path

Viterbi Path

Posterior @ 0.6 — 90 - RERGRRRVAGGGYWKKRGR - 108

Posterior @ 0.6

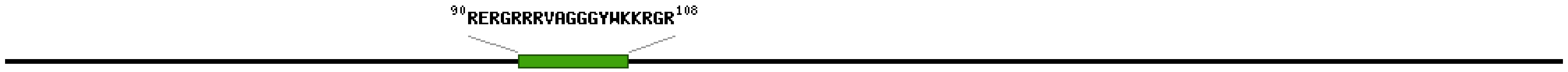

Posterior Graphic. Threshold Marked @ 0.6

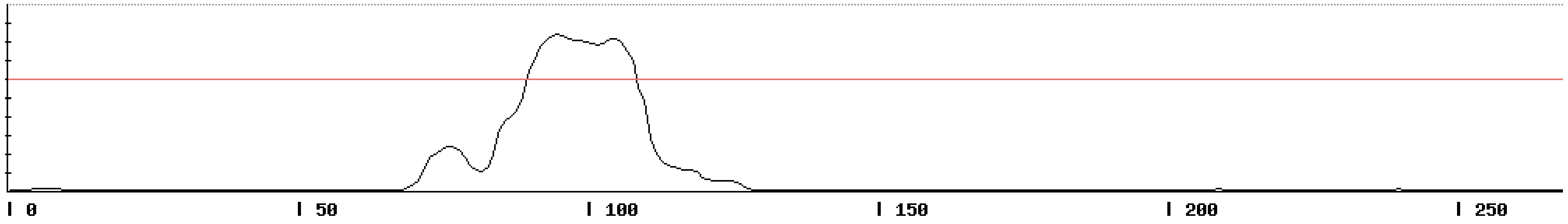

Predictions for Coffeacanephora_Cc10_g06320

Viterbi Path

Viterbi Path

Posterior @ 0.6

Posterior @ 0.6

Posterior Graphic. Threshold Marked @ 0.6

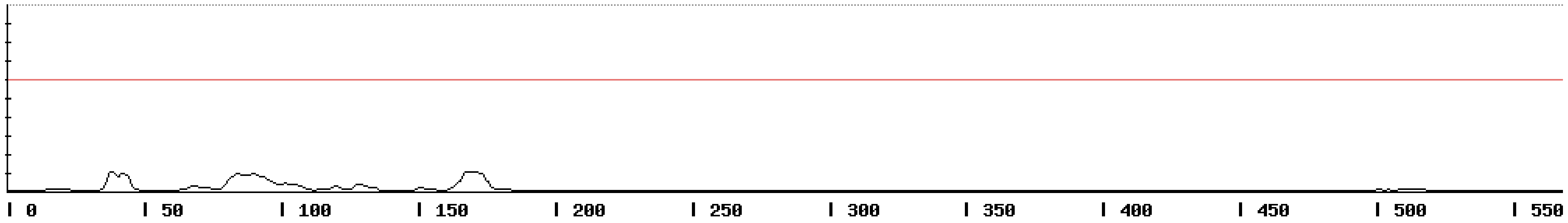

Predictions for Coffeacanephora_Cc10_g11470	
Viterbi Path	
Viterbi Path	
Posterior @ 0.6	
Posterior @ 0.6	
Posterior Graphic. Threshold Marked @ 0.6	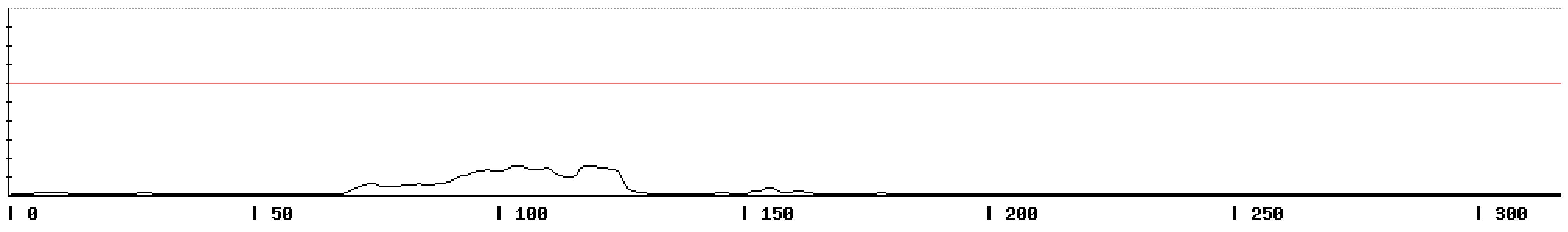

Predictions for Coffeacanephora_Cc10_g12200	
Viterbi Path	
Viterbi Path	
Posterior @ 0.6	
Posterior @ 0.6	
Posterior Graphic. Threshold Marked @ 0.6	

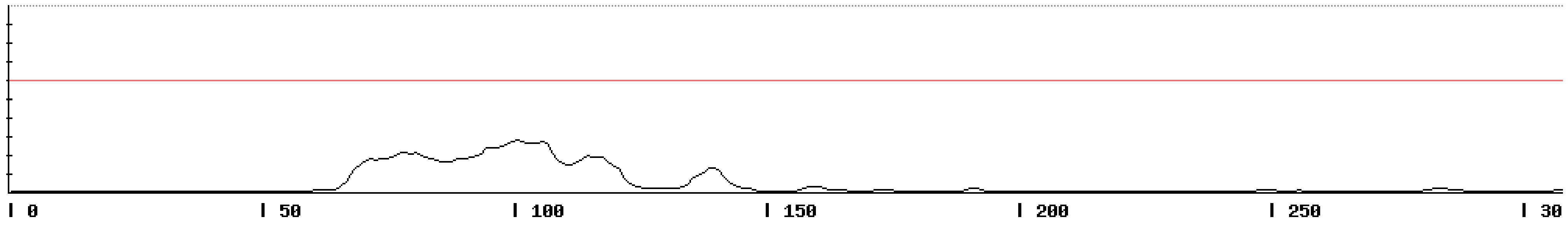

Predictions for Coffeacanephora_Cc10_g16190	
Viterbi Path	
Viterbi Path	
Posterior @ 0.6	
Posterior @ 0.6	
Posterior Graphic. Threshold Marked @ 0.6	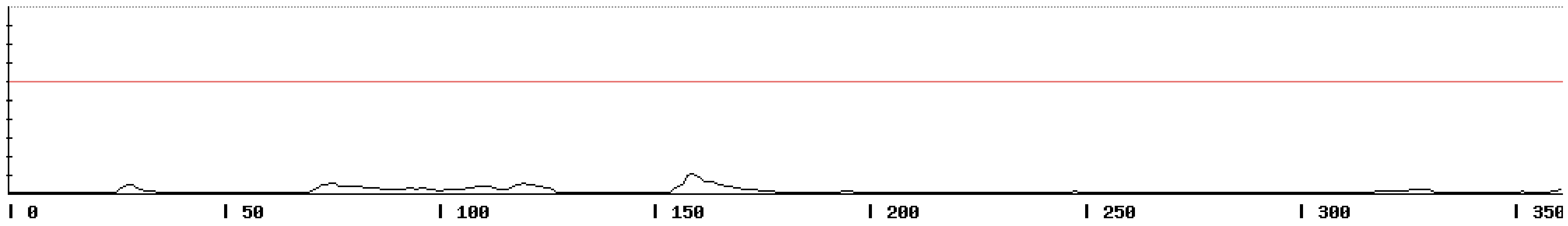

Predictions for Coffeacanephora_Cc11_g10610	
Viterbi Path	
Viterbi Path	
Posterior @ 0.6	

Revision r.9 - Last Updated on : February 24 2015 17:45:20. alex.nguyenba@utoronto.ca

Intended to be used with Firefox

Viterbi Path	
Posterior @ 0.4	
Posterior @ 0.4	
Posterior Graphic. Threshold Marked @ 0.4	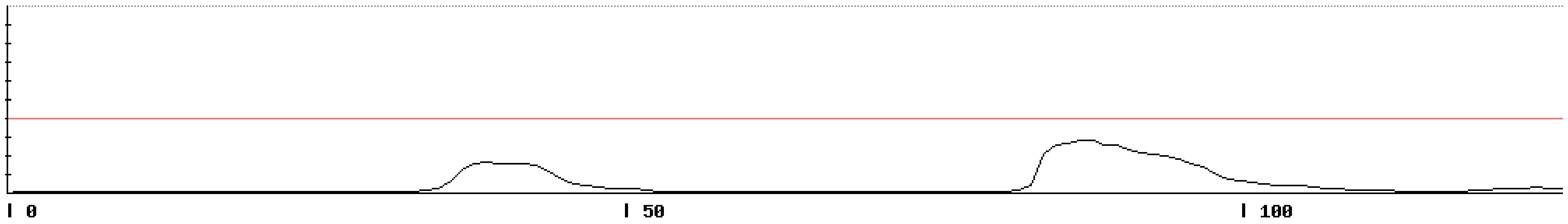

Predictions for Cucumismelo_MELO3C001959P1

Viterbi Path	
Viterbi Path	
Posterior @ 0.4	
Posterior @ 0.4	
Posterior Graphic. Threshold Marked @ 0.4	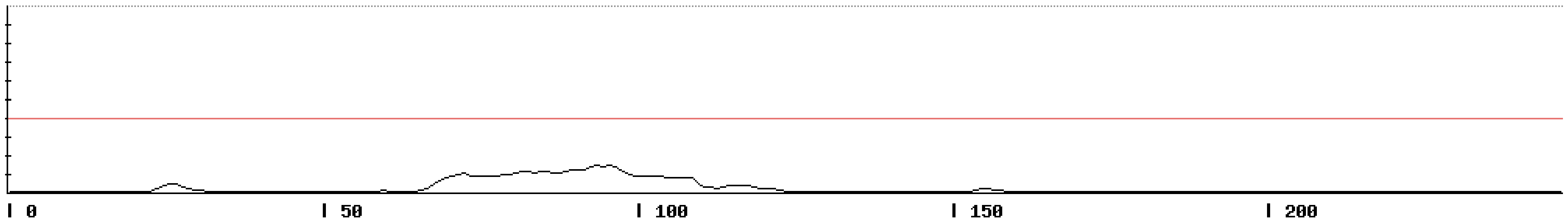

Predictions for Cucumismelo_MELO3C001996P1

Viterbi Path	
Viterbi Path	
Posterior @ 0.4	
Posterior @ 0.4	
Posterior Graphic. Threshold Marked @ 0.4	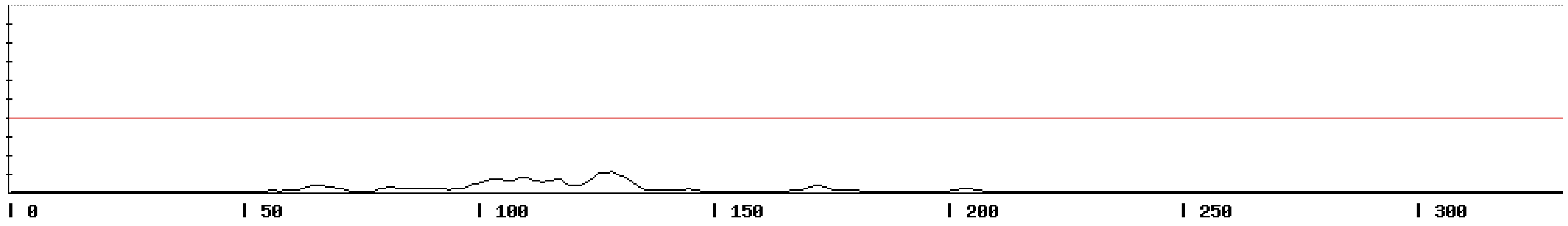

Predictions for Cucumismelo_MELO3C002573P1

Viterbi Path	
Viterbi Path	
Posterior @ 0.4	
Posterior @ 0.4	
Posterior Graphic. Threshold Marked @ 0.4	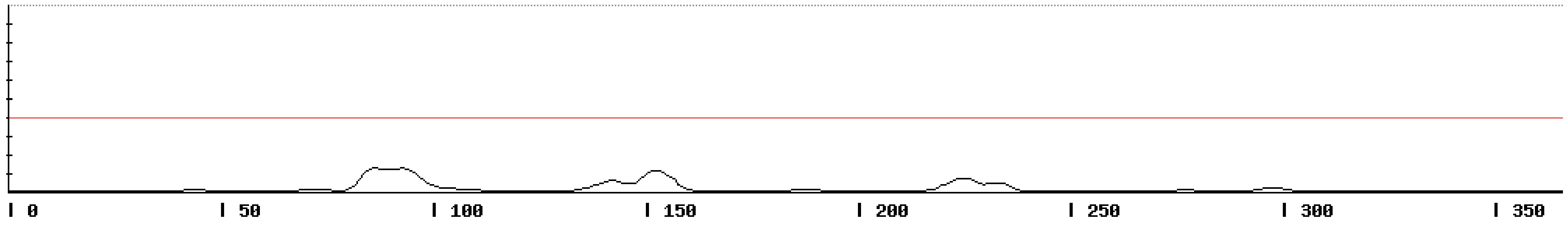

Predictions for Cucumismelo_MELO3C002628P1

Viterbi Path

Viterbi Path

Posterior @ 0.4

132 - RKRR - 135

Posterior @ 0.4

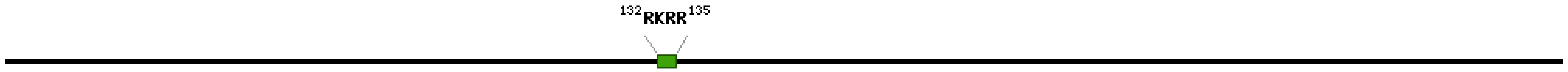

Posterior Graphic. Threshold Marked @ 0.4

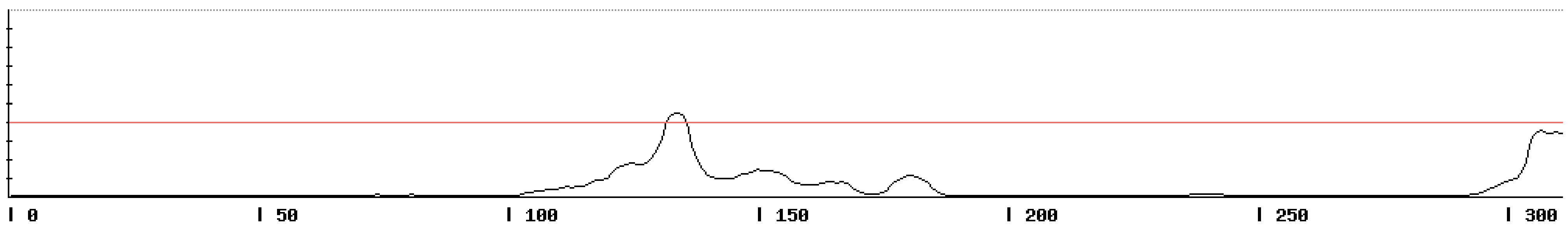

Predictions for Cucumismelo_MELO3C003390P1

Viterbi Path

Viterbi Path

Posterior @ 0.4

Posterior @ 0.4

Posterior Graphic. Threshold Marked @ 0.4

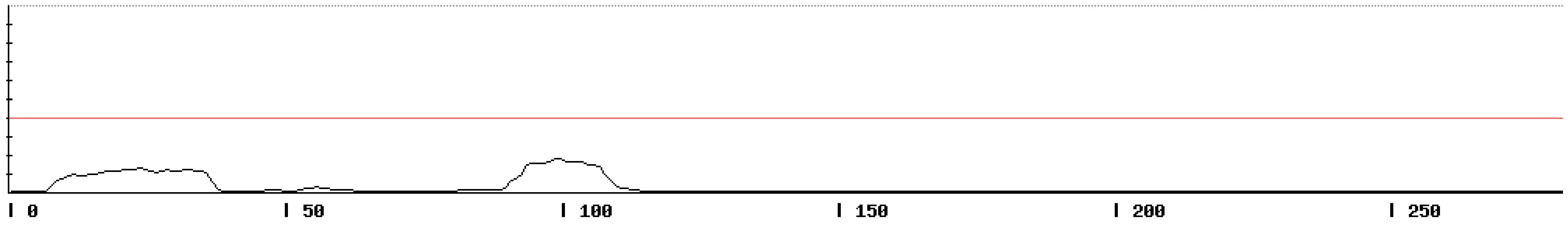

Predictions for Cucumismelo_MELO3C003390P2

Viterbi Path

Viterbi Path

Posterior @ 0.4

Posterior @ 0.4

Posterior Graphic. Threshold Marked @ 0.4

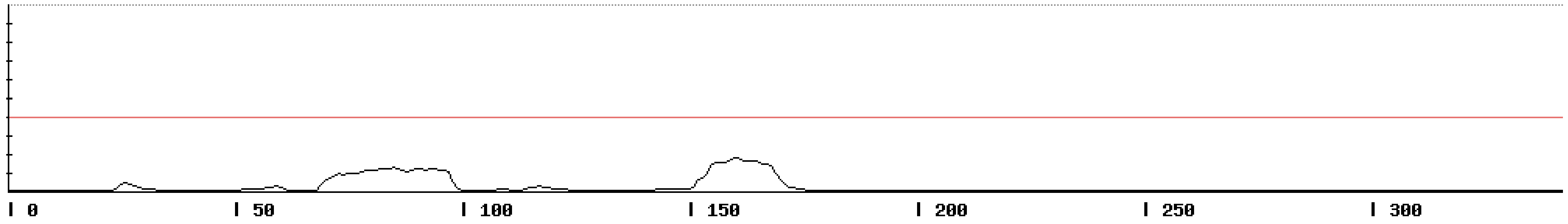

Predictions for Cucumismelo_MELO3C004604P1

Viterbi Path

Viterbi Path

Posterior @ 0.4

Posterior @ 0.4

Posterior Graphic. Threshold Marked @ 0.4

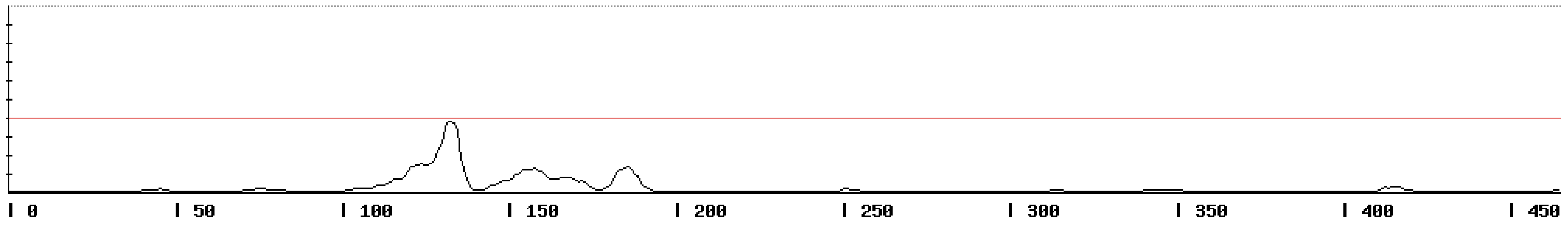

Predictions for Cucumismelo_MELO3C004694P1

Viterbi Path

Viterbi Path

Posterior @ 0.4

Posterior @ 0.4

Posterior Graphic. Threshold Marked @ 0.4

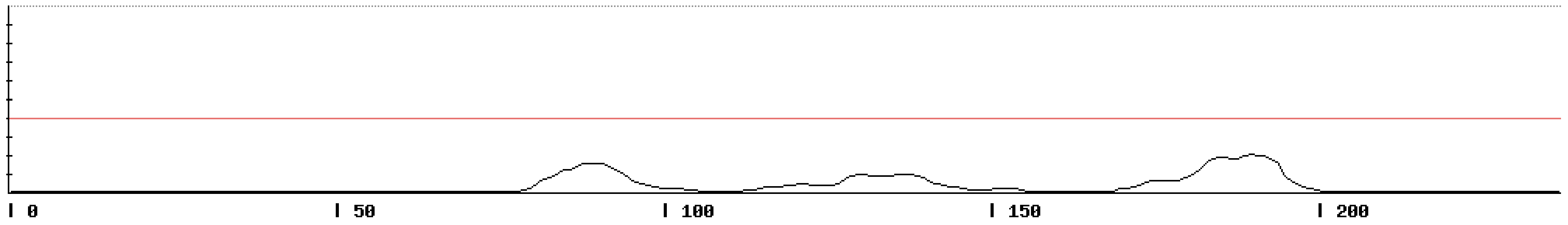

Predictions for Cucumismelo_MELO3C005563P1

Viterbi Path

Viterbi Path

Posterior @ 0.4

Posterior @ 0.4

Posterior Graphic. Threshold Marked @ 0.4

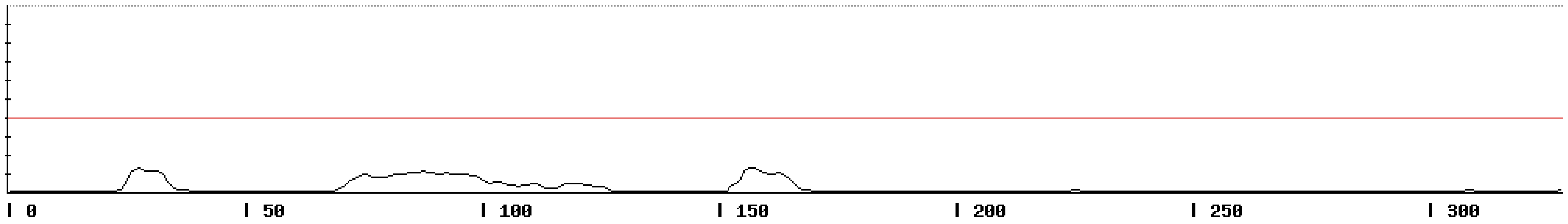

Predictions for Cucumismelo_MELO3C005791P1

Viterbi Path

Viterbi Path

Posterior @ 0.4

Posterior @ 0.4

Posterior Graphic. Threshold Marked @ 0.4

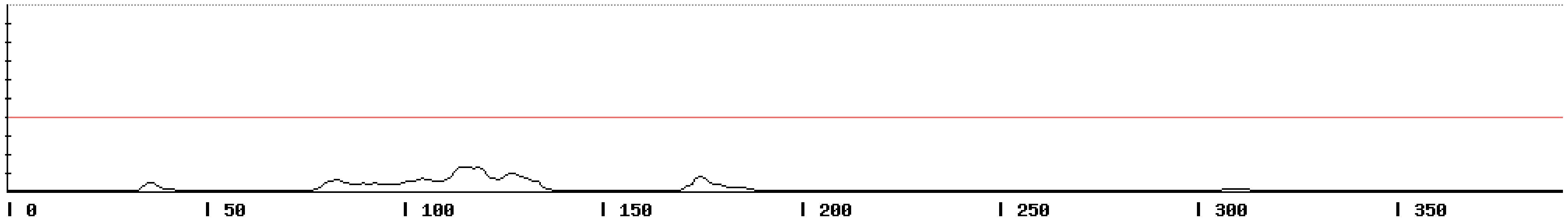

Predictions for Cucumismelo_MELO3C006814P1

Viterbi Path

Viterbi Path

Posterior @ 0.4	
Posterior @ 0.4	
Posterior Graphic. Threshold Marked @ 0.4	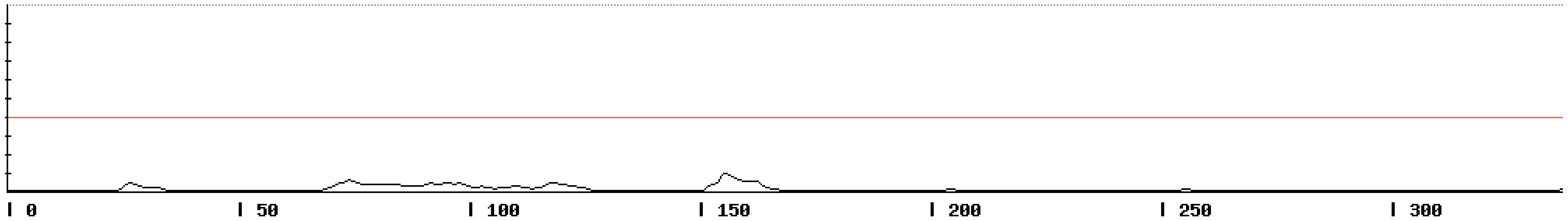

Predictions for Cucumismelo_MELO3C007255P1

Viterbi Path	
Viterbi Path	
Posterior @ 0.4	108 - GTRKRRKVK - 116
Posterior @ 0.4	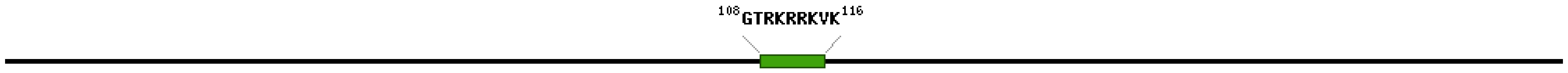
Posterior Graphic. Threshold Marked @ 0.4	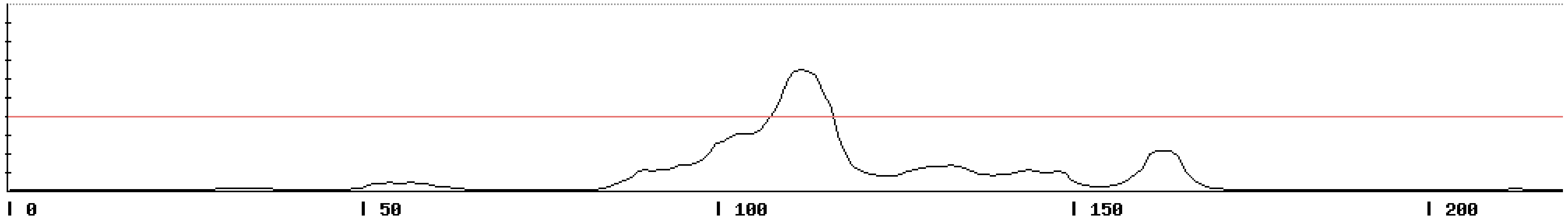

Predictions for Cucumismelo_MELO3C007888P1

Viterbi Path	
Viterbi Path	

Posterior @ 0.4

Posterior @ 0.4

Posterior Graphic. Threshold Marked @ 0.4

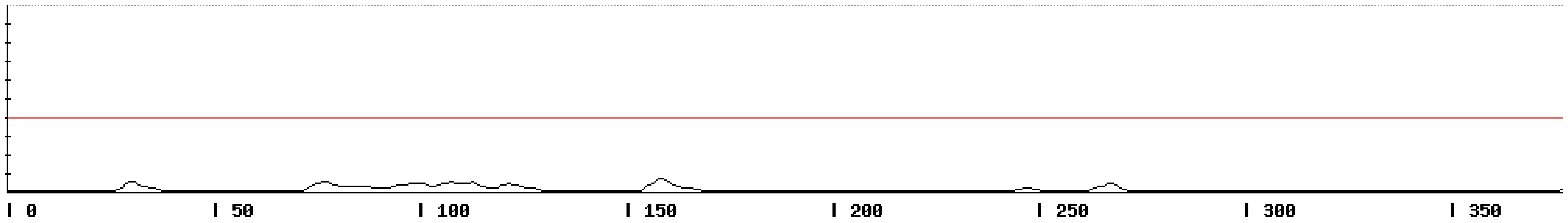

Predictions for Cucumismelo_MELO3C008056P1

Viterbi Path

Viterbi Path

Posterior @ 0.4

Posterior @ 0.4

Posterior Graphic. Threshold Marked @ 0.4

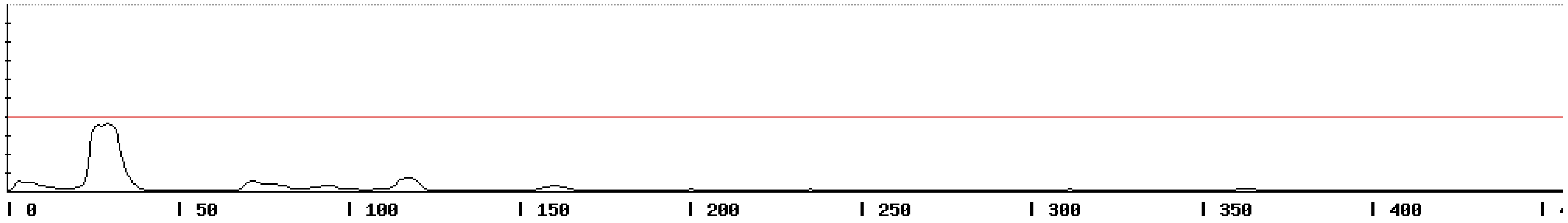

Predictions for Cucumismelo_MELO3C008534P1

Viterbi Path

Viterbi Path

Posterior @ 0.4

Posterior @ 0.4

Posterior Graphic. Threshold Marked @ 0.4

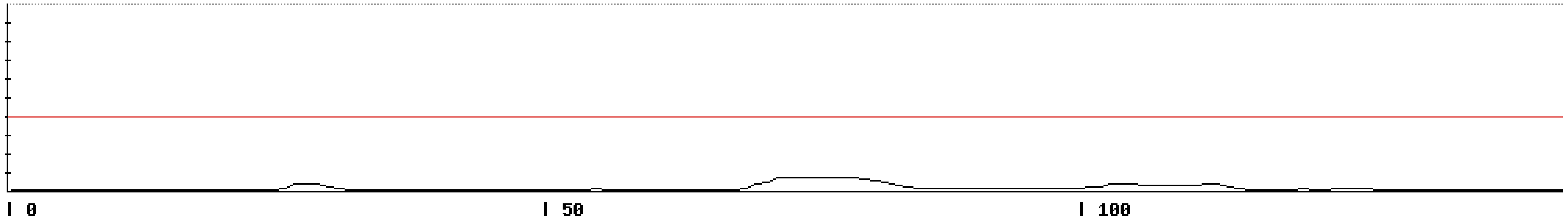

Predictions for Cucumismelo_MELO3C008790P1

Viterbi Path

Viterbi Path

Posterior @ 0.4

Posterior @ 0.4

Posterior Graphic. Threshold Marked @ 0.4

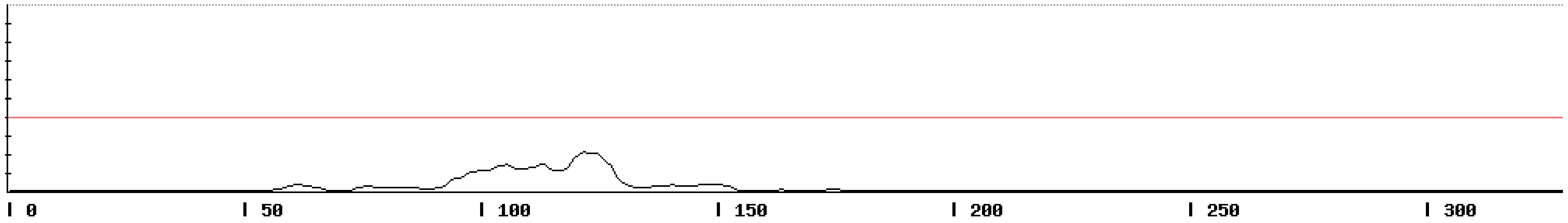

Predictions for Cucumismelo_MELO3C009236P1

Viterbi Path

Viterbi Path

Posterior @ 0.4

70 - KKKKSRIIRK - 79

Posterior @ 0.4

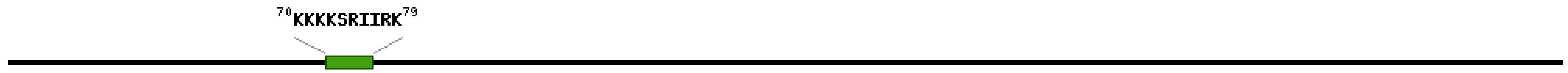

Posterior Graphic. Threshold Marked @ 0.4

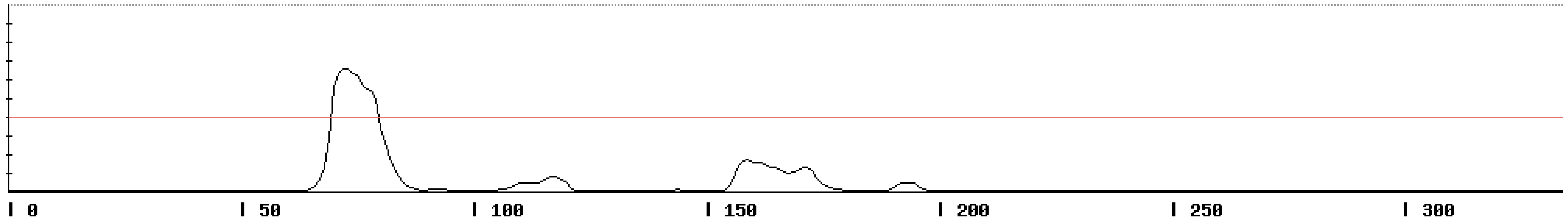

Predictions for Cucumismelo_MELO3C009855P1

Viterbi Path

Viterbi Path

Posterior @ 0.4

Posterior @ 0.4

Posterior Graphic. Threshold Marked @ 0.4

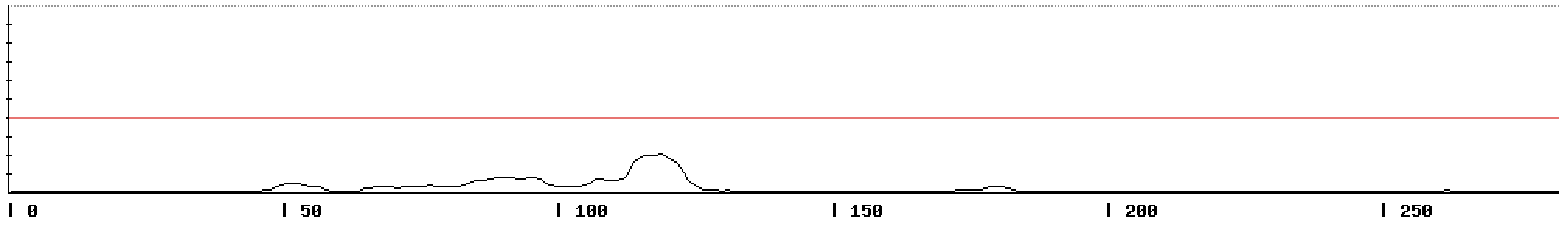

Predictions for Cucumismelo_MELO3C009980P1

Viterbi Path: 167 - RIRKNERAKRKLKVQRELKQPNKKR - 191

Viterbi Path

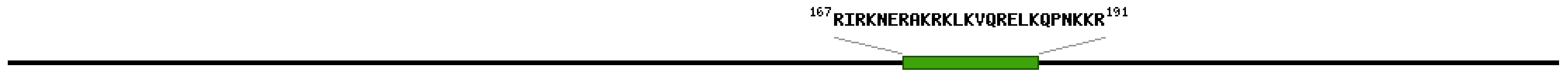

Posterior @ 0.4: 167 - RIRKNERAKRKLKVQRELKQPNKKRI - 192

Posterior @ 0.4

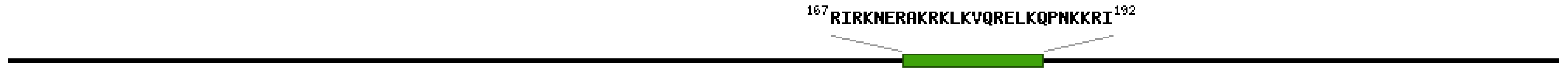

Posterior Graphic. Threshold Marked @ 0.4

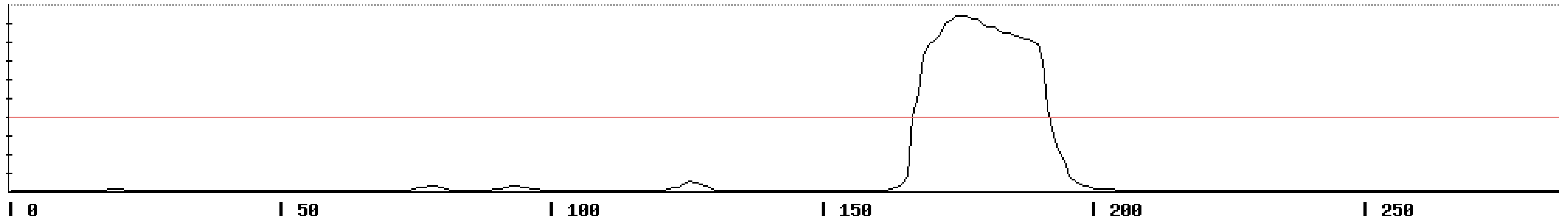

Predictions for Cucumismelo_MELO3C010501P1

Viterbi Path:

Viterbi Path

Posterior @ 0.4: 95 - KPIGRPKPV - 103

Posterior @ 0.4

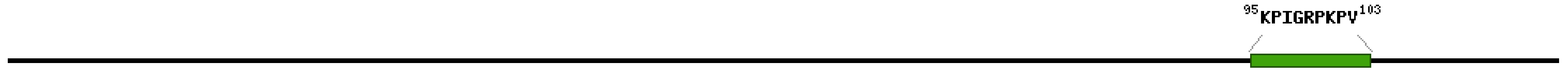

Posterior Graphic. Threshold Marked @ 0.4

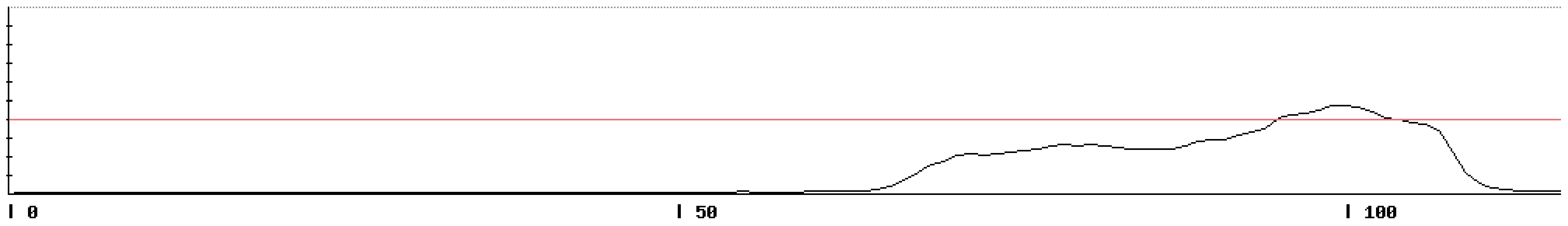

Predictions for Cucumismelo_MELO3C010555P1

Viterbi Path

Viterbi Path

Posterior @ 0.4

Posterior @ 0.4

Posterior Graphic. Threshold Marked @ 0.4

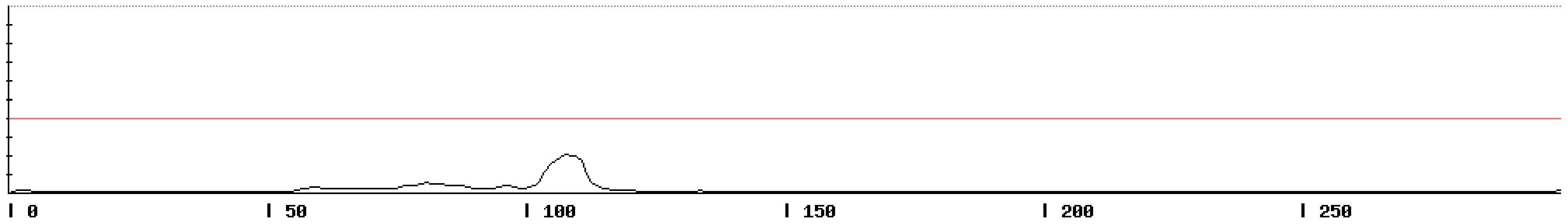

Predictions for Cucumismelo_MELO3C010632P1

Viterbi Path

Viterbi Path

Posterior @ 0.4

Posterior @ 0.4

Posterior Graphic. Threshold Marked @ 0.4

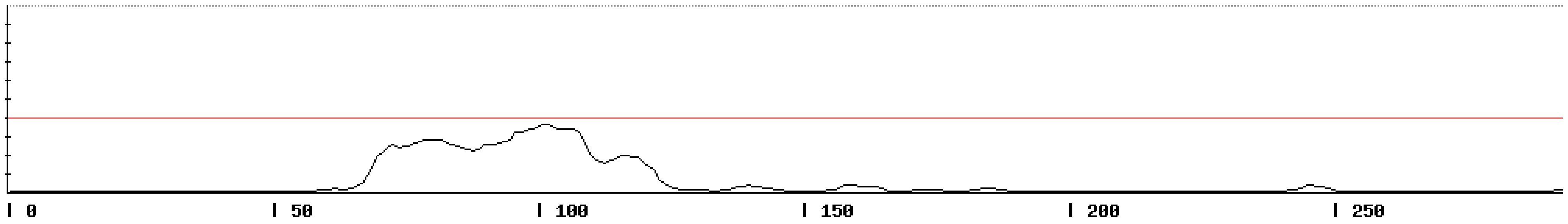

Predictions for Cucumismelo_MELO3C010923P1

Viterbi Path: 369 - GGKKKKKKAVGRMKK - 383

Viterbi Path

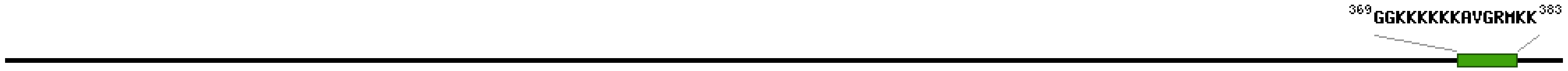

Posterior @ 0.4: 369 - GGKKKKKKAVGRMKKI - 384

Posterior @ 0.4

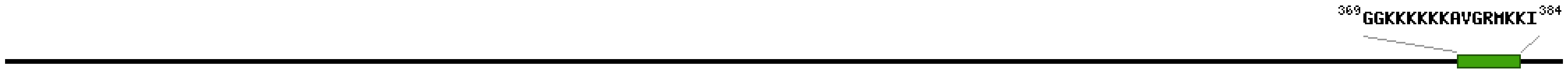

Posterior Graphic. Threshold Marked @ 0.4

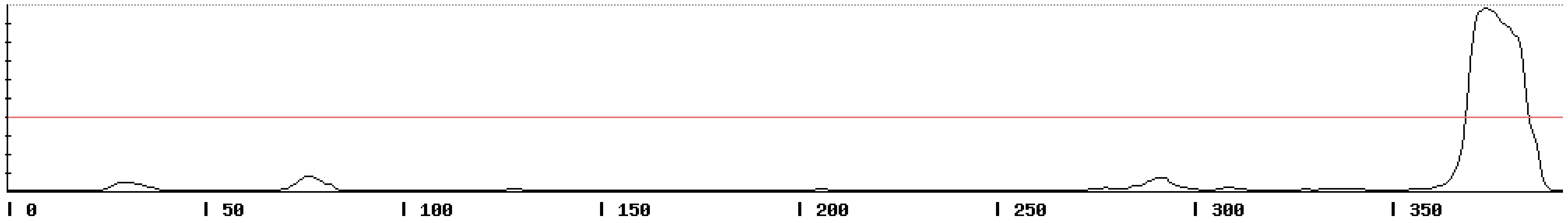

Predictions for Cucumismelo_MELO3C010923P2

Viterbi Path

Viterbi Path

Posterior @ 0.4

Posterior @ 0.4

Posterior Graphic. Threshold Marked @ 0.4

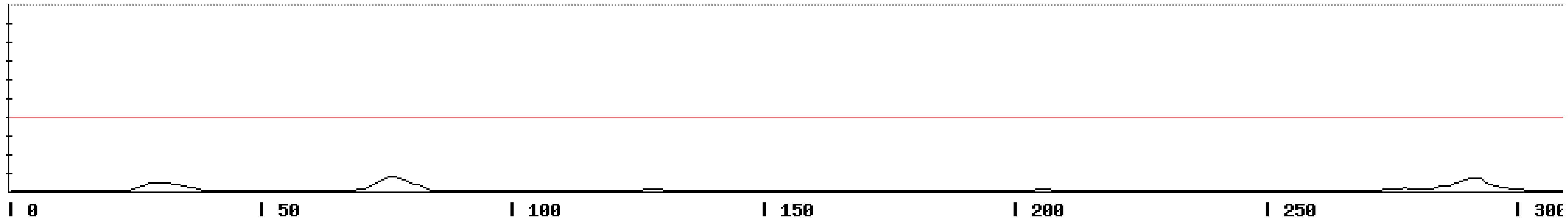

Predictions for Cucumismelo_MELO3C011164P1

Viterbi Path

Viterbi Path

Posterior @ 0.4

Posterior @ 0.4

Posterior Graphic. Threshold Marked @ 0.4

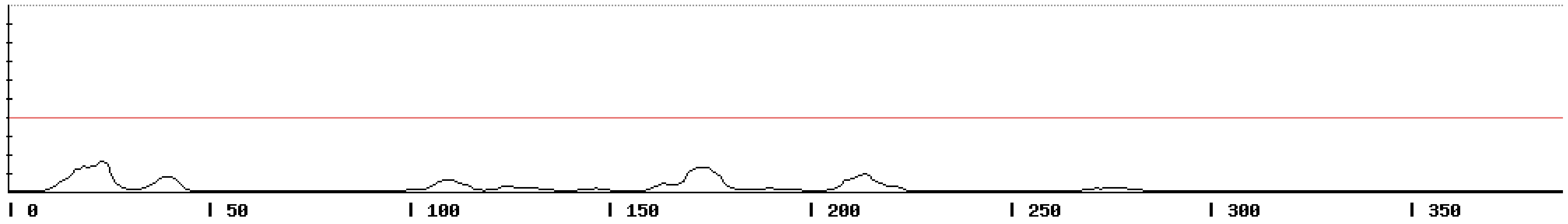

Predictions for Cucumismelo_MELO3C012091P1

Viterbi Path

Viterbi Path

Posterior @ 0.4

Posterior @ 0.4

Posterior Graphic. Threshold Marked @ 0.4

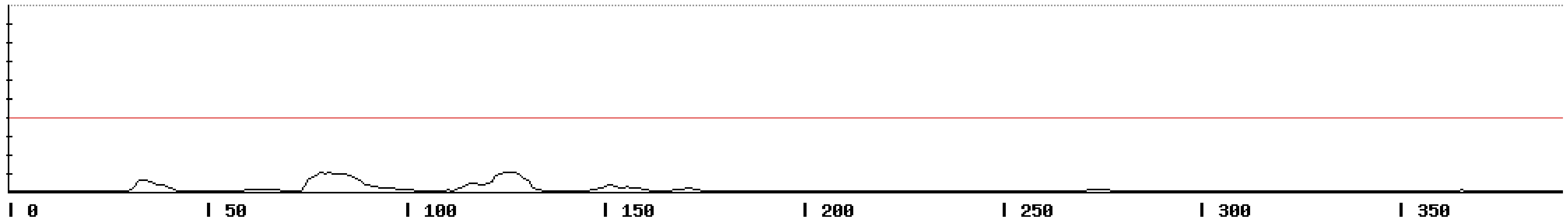

Predictions for Cucumismelo_MELO3C012114P1

Viterbi Path

Viterbi Path

Posterior @ 0.4

Posterior @ 0.4

Posterior Graphic. Threshold Marked @ 0.4

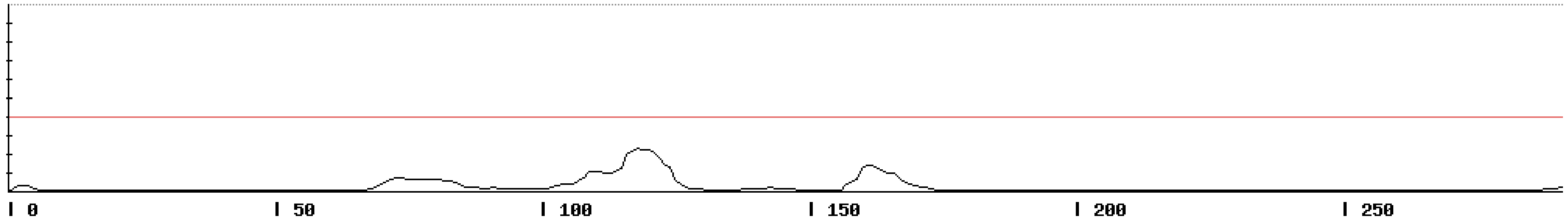

Predictions for Cucumismelo_MELO3C012215P1

Viterbi Path	
Viterbi Path	
Posterior @ 0.4	
Posterior @ 0.4	
Posterior Graphic. Threshold Marked @ 0.4	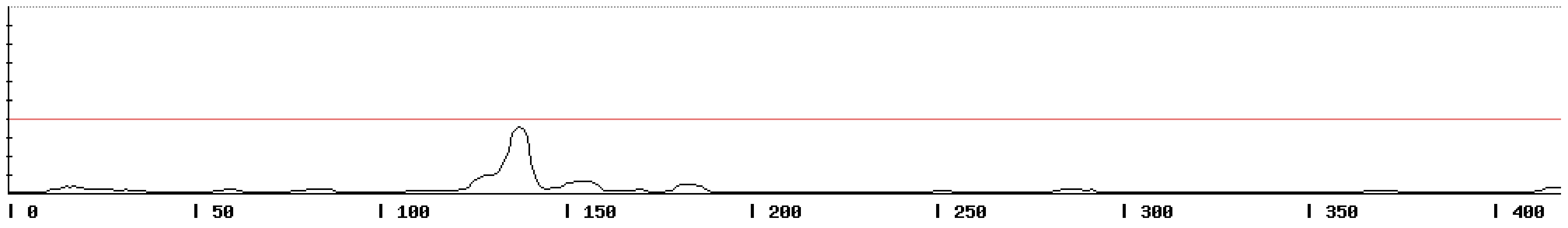

Predictions for Cucumismelo_MELO3C012390P1

Viterbi Path	
Viterbi Path	
Posterior @ 0.4	
Posterior @ 0.4	
Posterior Graphic. Threshold Marked @ 0.4	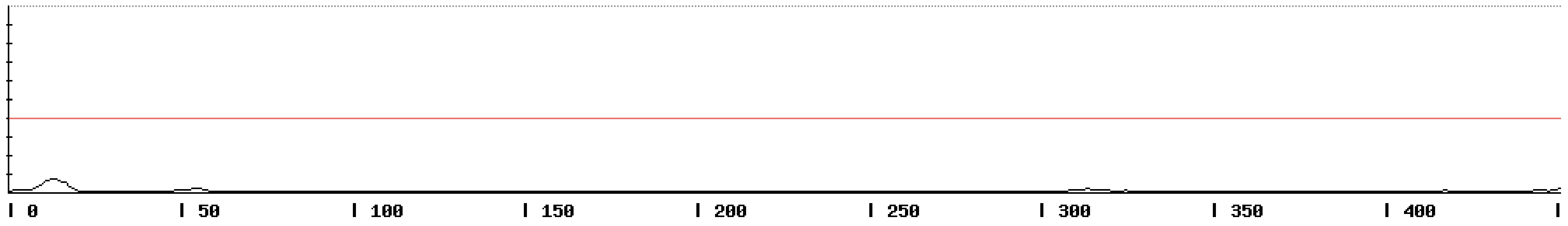

Predictions for Cucumismelo_MELO3C012390P2	
Viterbi Path	
Viterbi Path	
Posterior @ 0.4	
Posterior @ 0.4	
Posterior Graphic. Threshold Marked @ 0.4	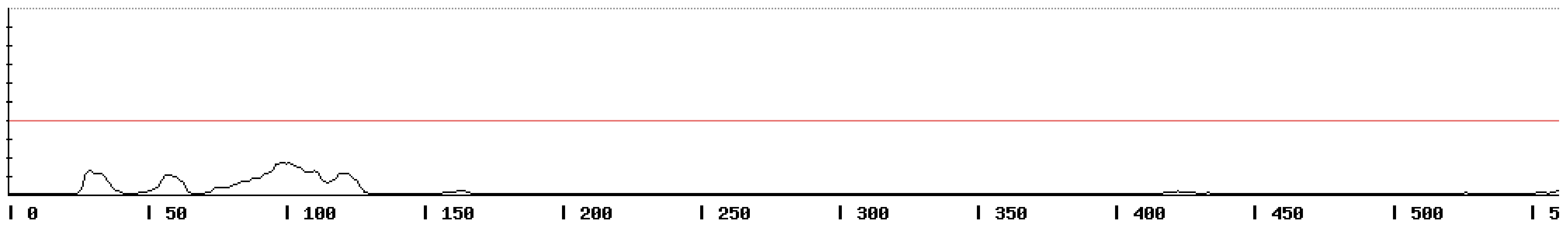

Predictions for Cucumismelo_MELO3C012391P1	
Viterbi Path	
Viterbi Path	
Posterior @ 0.4	
Posterior @ 0.4	
Posterior Graphic. Threshold Marked @ 0.4	

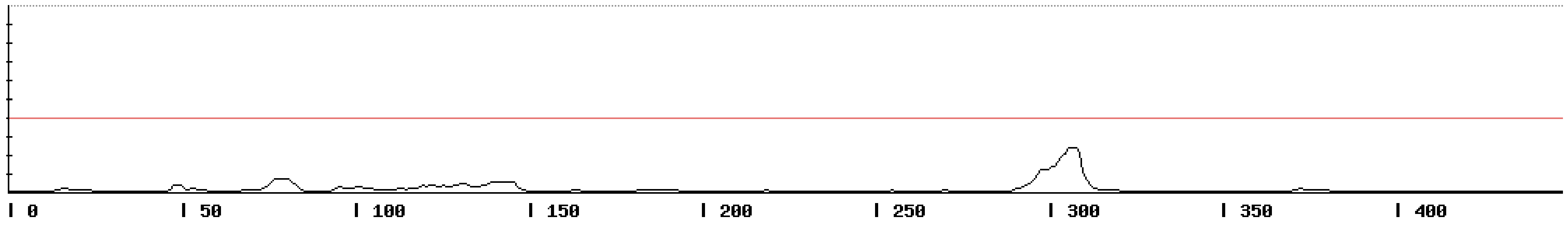

Predictions for Cucumismelo_MELO3C012391P3

Viterbi Path

Viterbi Path

Posterior @ 0.4

Posterior @ 0.4

Posterior Graphic. Threshold Marked @ 0.4

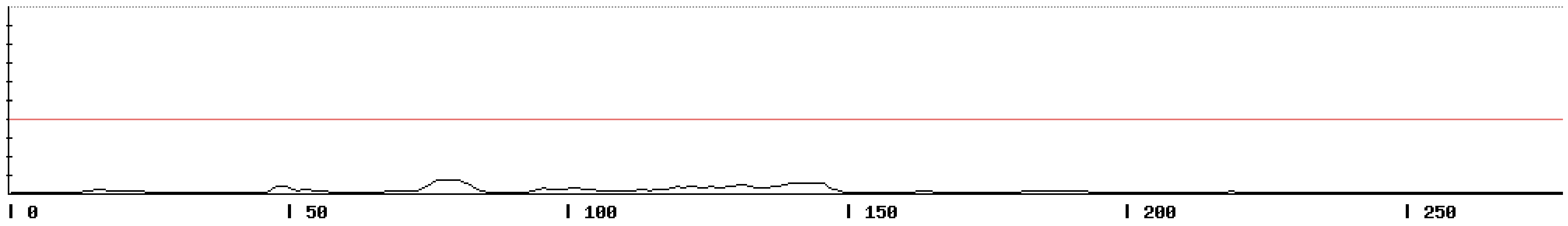

Predictions for Cucumismelo_MELO3C012573P1

Viterbi Path

Viterbi Path

Posterior @ 0.4

Posterior @ 0.4

Posterior Graphic. Threshold Marked @ 0.4	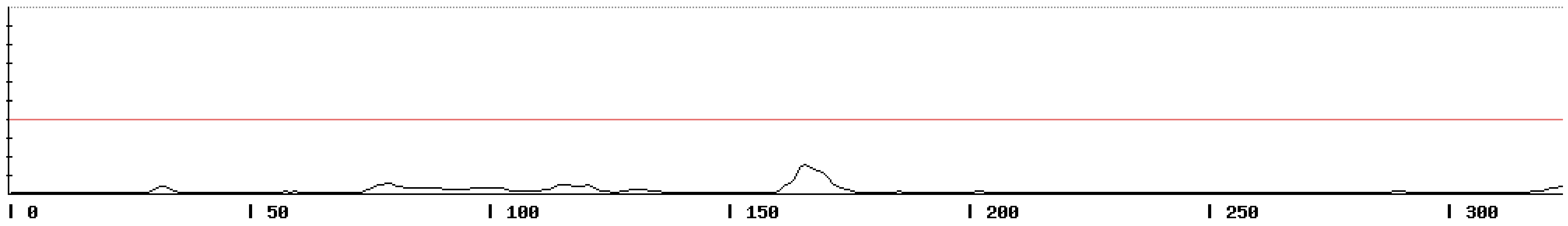

Predictions for Cucumismelo_MELO3C012873P1

Viterbi Path	
Viterbi Path	
Posterior @ 0.4	
Posterior @ 0.4	
Posterior Graphic. Threshold Marked @ 0.4	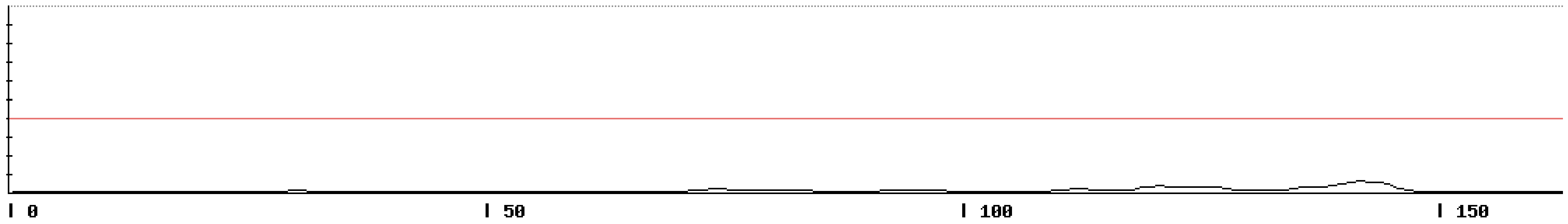

Predictions for Cucumismelo_MELO3C013173P1

Viterbi Path	
Viterbi Path	
Posterior @ 0.4	527 - K - 527
Posterior @ 0.4	

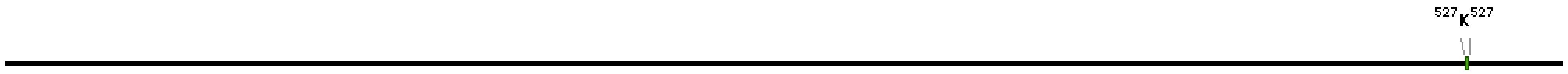

Posterior Graphic. Threshold Marked @ 0.4

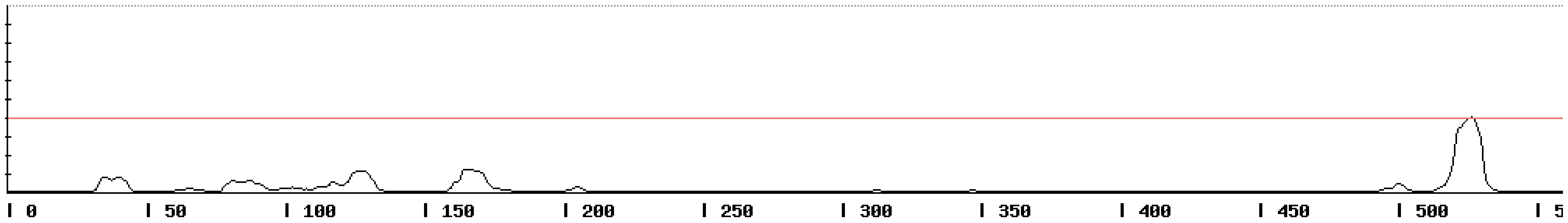

Predictions for Cucumismelo_MELO3C013287P1

Viterbi Path

Viterbi Path

Posterior @ 0.4

Posterior @ 0.4

Posterior Graphic. Threshold Marked @ 0.4

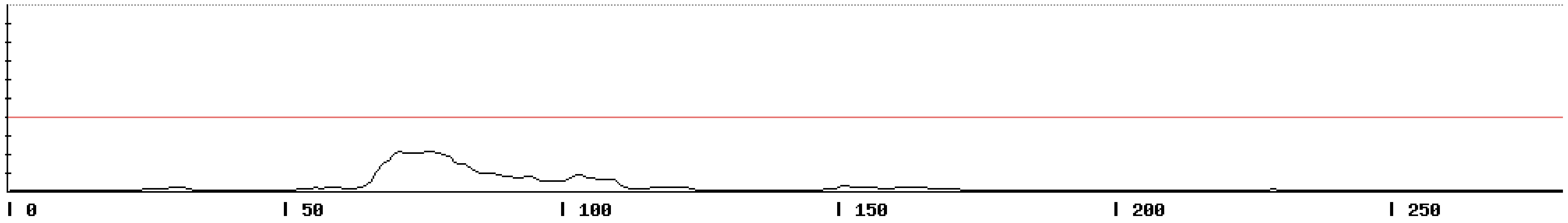

Predictions for Cucumismelo_MELO3C013641P1

Viterbi Path

Viterbi Path

Posterior @ 0.4

Posterior @ 0.4

Posterior Graphic. Threshold Marked @ 0.4

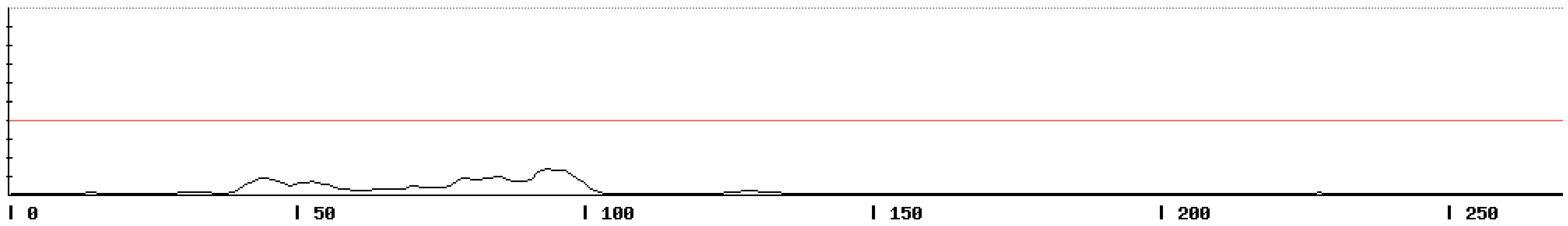

Predictions for Cucumismelo_MELO3C013641P2

Viterbi Path

Viterbi Path

Posterior @ 0.4

Posterior @ 0.4

Posterior Graphic. Threshold Marked @ 0.4

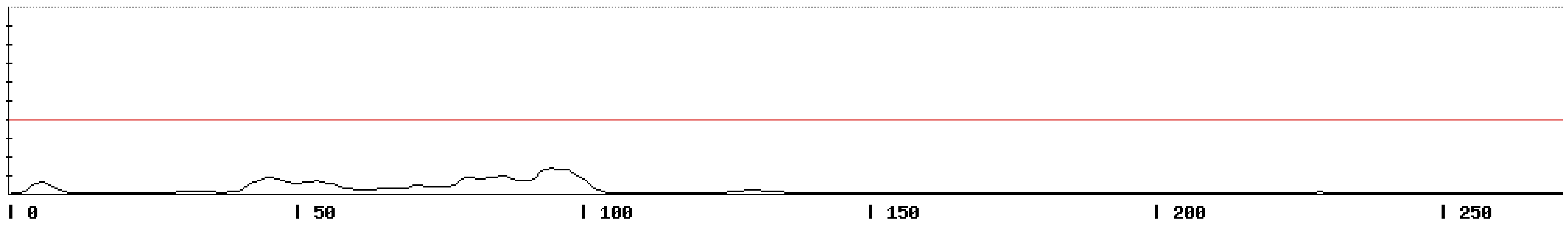

Predictions for Cucumismelo_MELO3C013971P1

Viterbi Path

Viterbi Path

Posterior @ 0.4	
Posterior @ 0.4	
Posterior Graphic. Threshold Marked @ 0.4	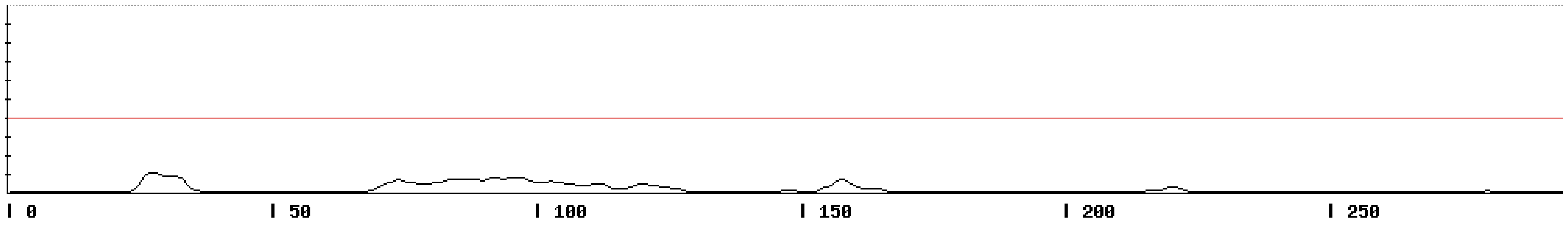

Predictions for Cucumismelo_MELO3C014141P1	
Viterbi Path	
Viterbi Path	
Posterior @ 0.4	166 - QRKKRKKRQIR - 176
Posterior @ 0.4	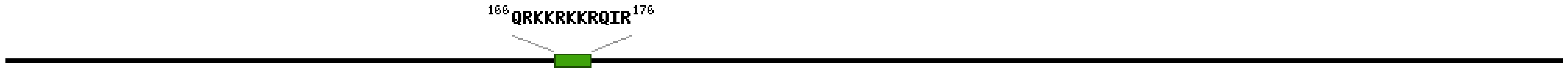
Posterior Graphic. Threshold Marked @ 0.4	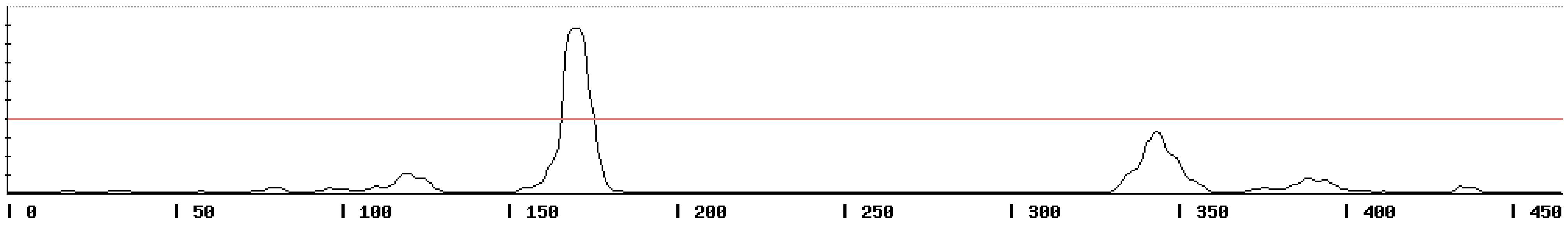

Predictions for Cucumismelo_MELO3C014505P1

Viterbi Path

Viterbi Path

Posterior @ 0.4

Posterior @ 0.4

Posterior Graphic. Threshold Marked @ 0.4

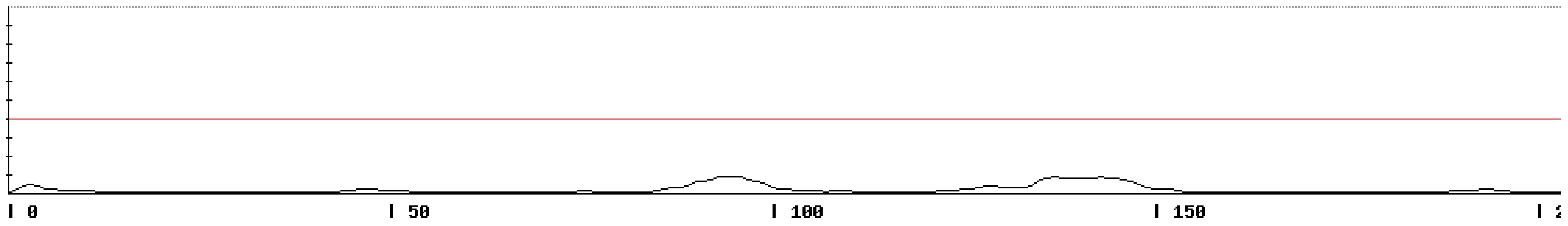

Predictions for Cucumismelo_MELO3C014509P1

Viterbi Path

Viterbi Path

Posterior @ 0.4

Posterior @ 0.4

Posterior Graphic. Threshold Marked @ 0.4

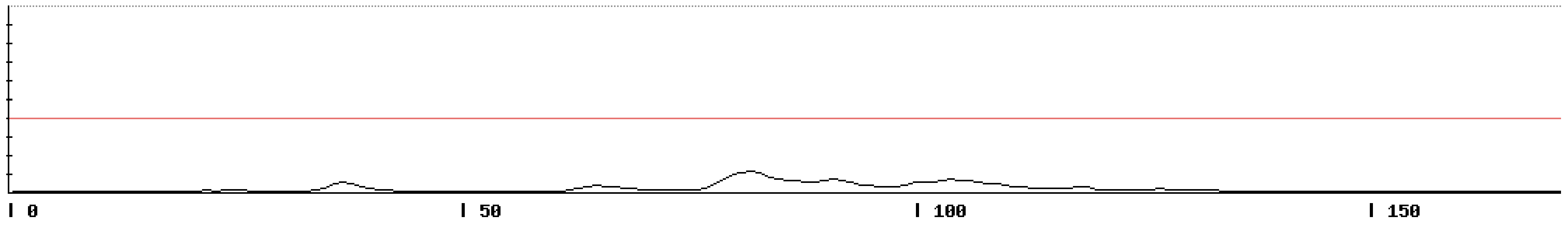

Predictions for Cucumismelo_MELO3C014922P1	
Viterbi Path	
Viterbi Path	
Posterior @ 0.4	163 - KRRRPKRQGVEE - 174
Posterior @ 0.4	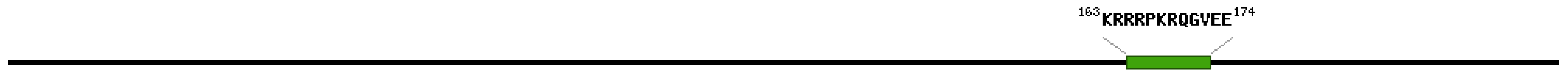
Posterior Graphic. Threshold Marked @ 0.4	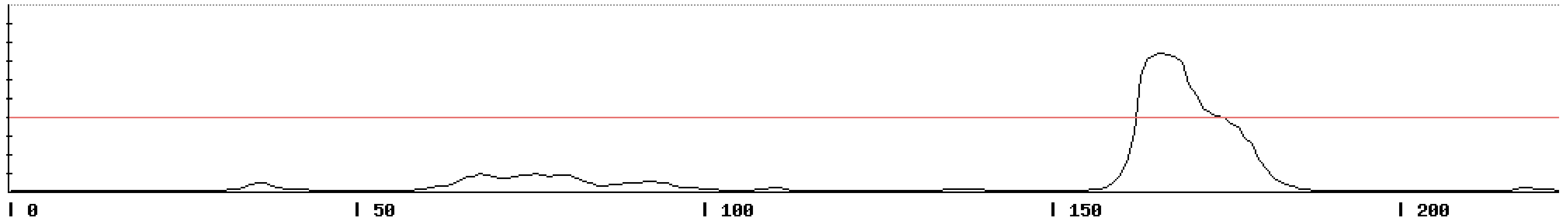

Predictions for Cucumismelo_MELO3C014922P2	
Viterbi Path	
Viterbi Path	
Posterior @ 0.4	
Posterior @ 0.4	
Posterior Graphic. Threshold Marked @ 0.4	

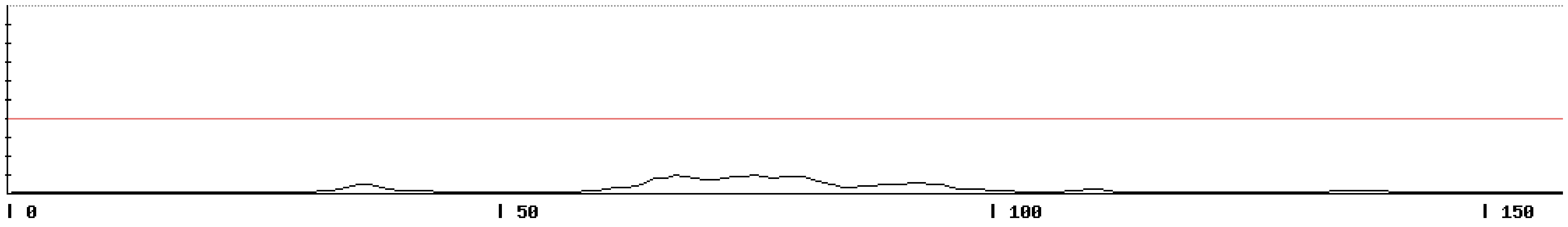

Predictions for Cucumismelo_MELO3C015355P1

Viterbi Path

Viterbi Path

Posterior @ 0.4

85 - RRSKRATGTGYWKSTGKDRKIRARGTNKLIGTKK - 118

Posterior @ 0.4

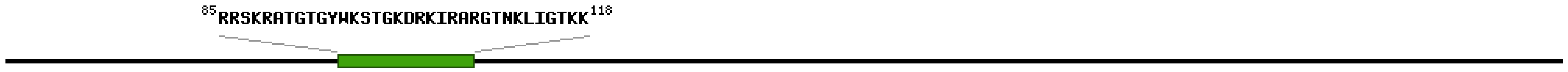

Posterior Graphic. Threshold Marked @ 0.4

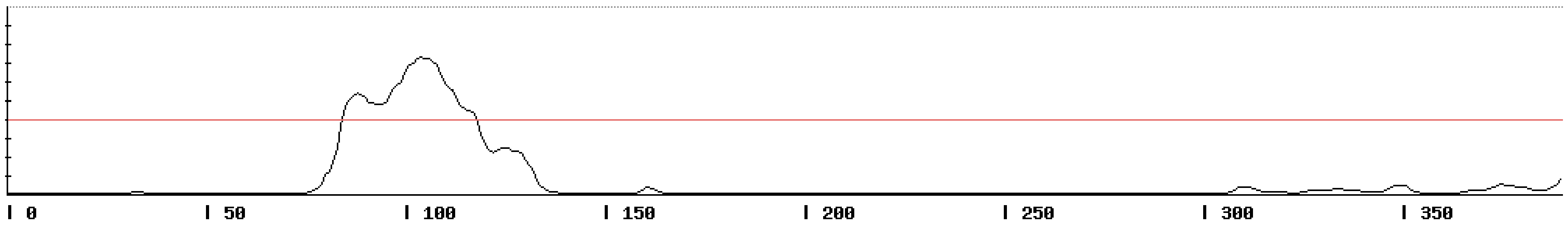

Predictions for Cucumismelo_MELO3C015357P1

Viterbi Path

Viterbi Path

Posterior @ 0.4

Posterior @ 0.4

Posterior Graphic. Threshold Marked @ 0.4

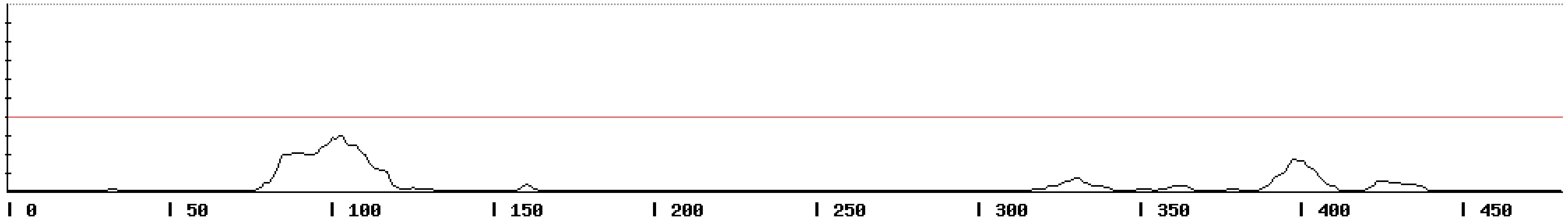

Predictions for Cucumismelo_MELO3C015427P1

Viterbi Path

Viterbi Path

Posterior @ 0.4

Posterior @ 0.4

Posterior Graphic. Threshold Marked @ 0.4

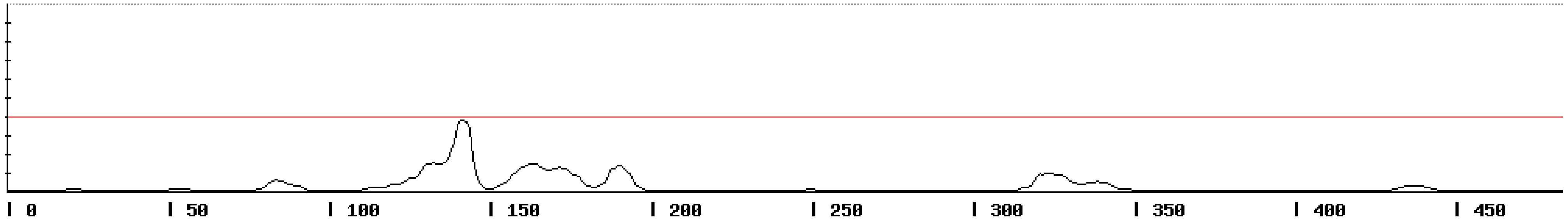

Predictions for Cucumismelo_MELO3C016444P1

Viterbi Path

Viterbi Path

Posterior @ 0.4

Posterior @ 0.4

Posterior Graphic. Threshold Marked @ 0.4	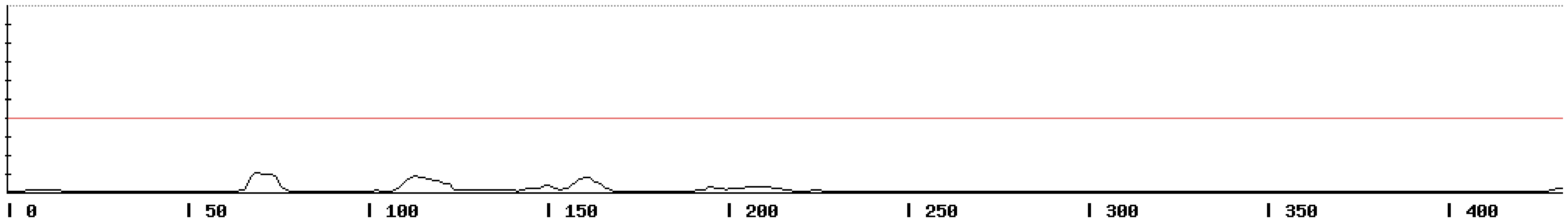

Predictions for Cucumismelo_MELO3C016536P1	
Viterbi Path	
Viterbi Path	
Posterior @ 0.4	
Posterior @ 0.4	
Posterior Graphic. Threshold Marked @ 0.4	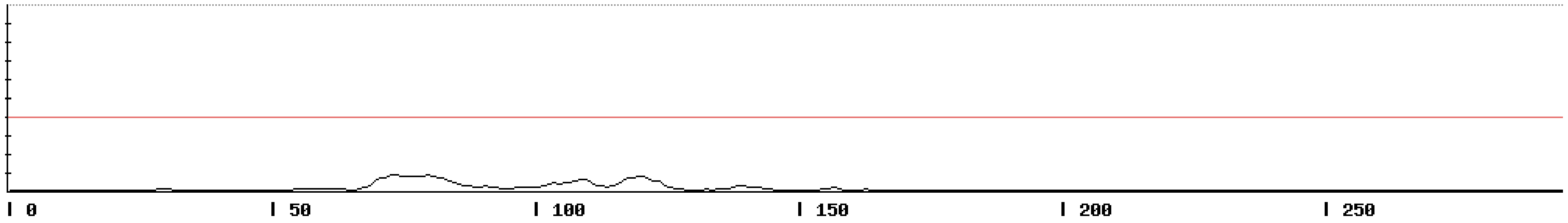

Predictions for Cucumismelo_MELO3C016540P1	
Viterbi Path	
Viterbi Path	

Posterior @ 0.4	
Posterior @ 0.4	
Posterior Graphic. Threshold Marked @ 0.4	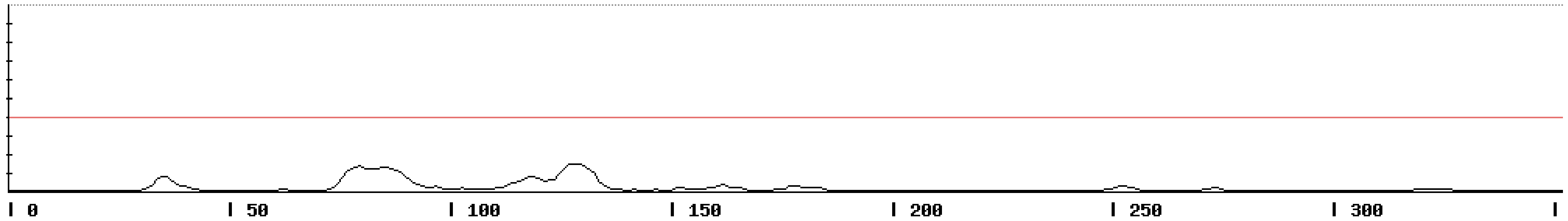

Predictions for Cucumismelo_MELO3C016767P1

Viterbi Path	
Viterbi Path	
Posterior @ 0.4	110 - RRKGR - 114
Posterior @ 0.4	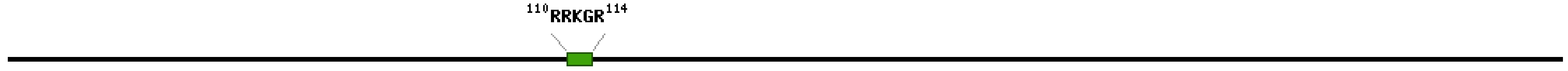
Posterior Graphic. Threshold Marked @ 0.4	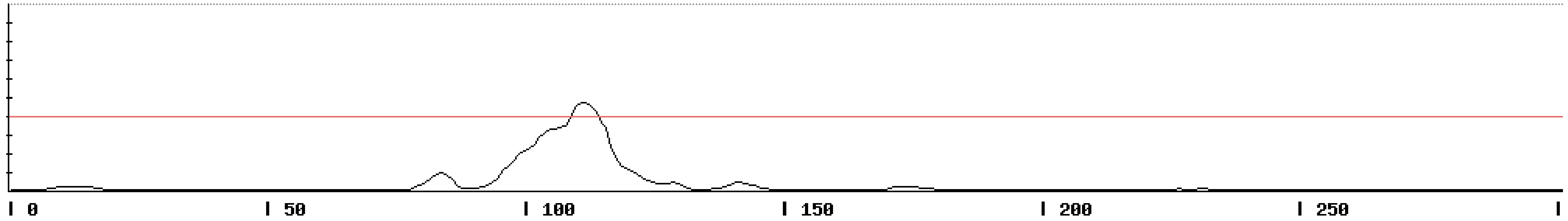

Predictions for Cucumismelo_MELO3C017052P1

Viterbi Path	
Viterbi Path	

Posterior @ 0.4

Posterior @ 0.4

Posterior Graphic. Threshold Marked @ 0.4

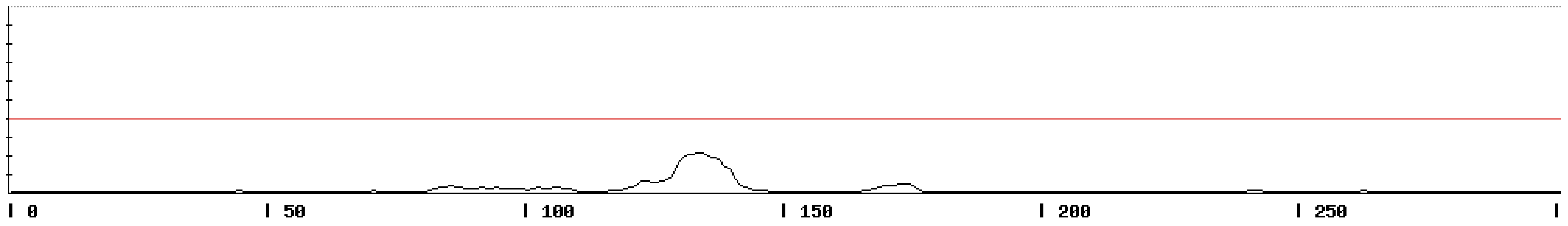

Predictions for Cucumismelo_MELO3C017185P1

Viterbi Path

Viterbi Path

Posterior @ 0.4

Posterior @ 0.4

Posterior Graphic. Threshold Marked @ 0.4

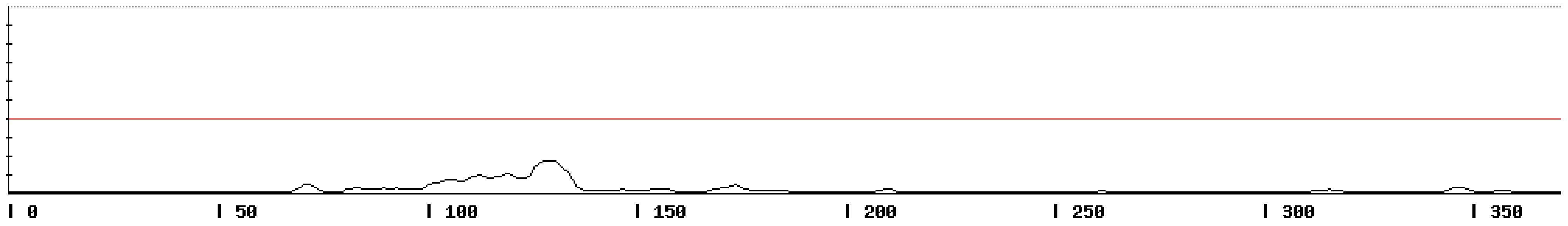

Predictions for Cucumismelo_MELO3C017308P1

Viterbi Path

Viterbi Path

Posterior @ 0.4

Posterior @ 0.4

Posterior Graphic. Threshold Marked @ 0.4

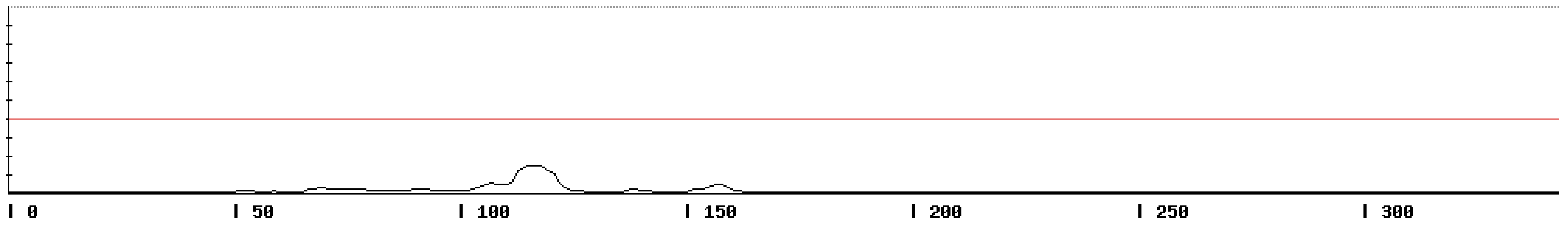

Predictions for Cucumismelo_MELO3C017754P1

Viterbi Path

Viterbi Path

Posterior @ 0.4

Posterior @ 0.4

Posterior Graphic. Threshold Marked @ 0.4

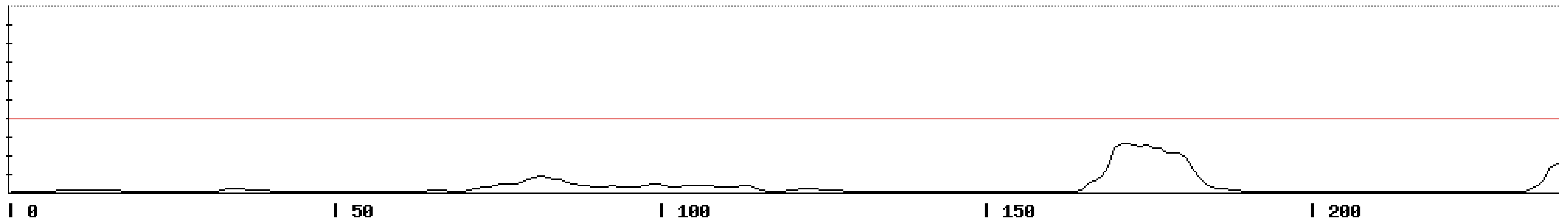

Predictions for Cucumismelo_MELO3C018237P1	
Viterbi Path	
Viterbi Path	
Posterior @ 0.4	
Posterior @ 0.4	
Posterior Graphic. Threshold Marked @ 0.4	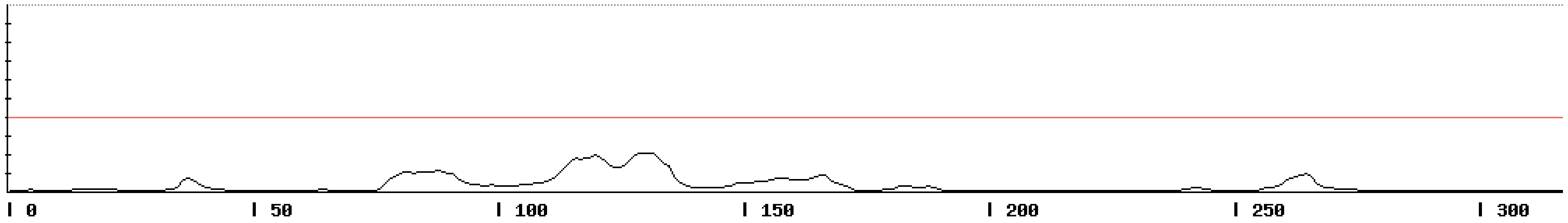

Predictions for Cucumismelo_MELO3C018242P1	
Viterbi Path	
Viterbi Path	
Posterior @ 0.4	
Posterior @ 0.4	
Posterior Graphic. Threshold Marked @ 0.4	

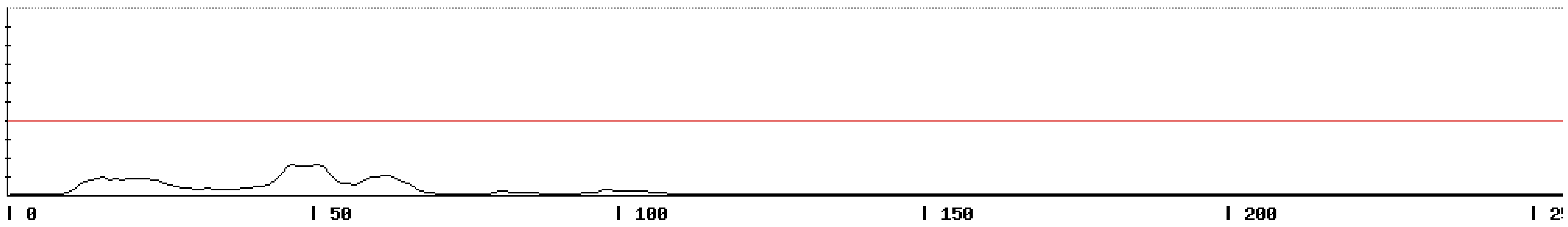

Predictions for Cucumismelo_MELO3C018242P2	
Viterbi Path	
Viterbi Path	
Posterior @ 0.4	
Posterior @ 0.4	
Posterior Graphic. Threshold Marked @ 0.4	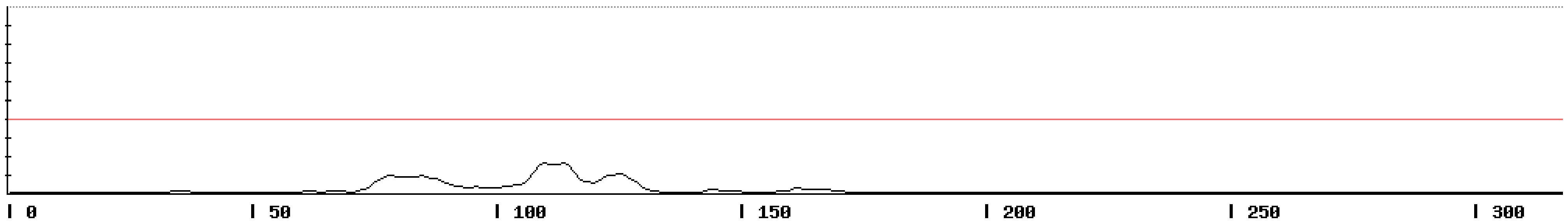

Predictions for Cucumismelo_MELO3C018675P1	
Viterbi Path	
Viterbi Path	
Posterior @ 0.4	143 - SRRRGRPKR - 151
Posterior @ 0.4	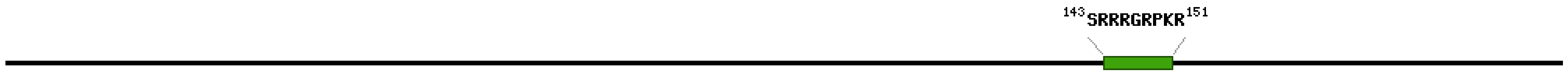

Posterior Graphic. Threshold Marked @ 0.4	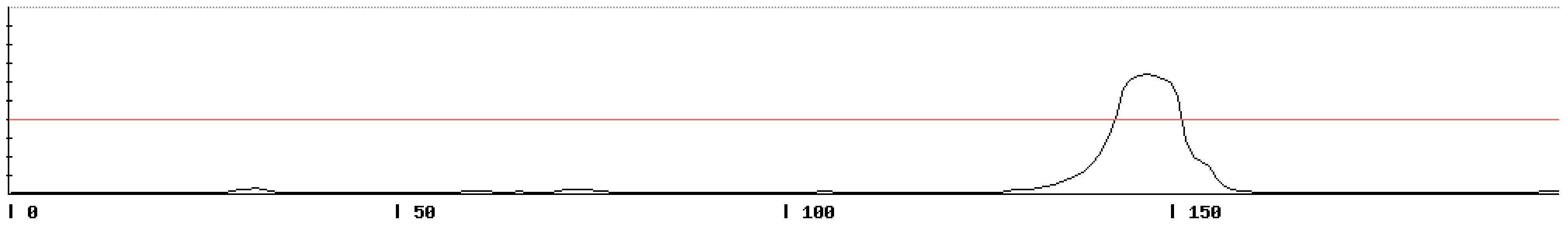

Predictions for Cucumismelo_MELO3C019332P1

Viterbi Path	
Viterbi Path	
Posterior @ 0.4	127 - RKRRK - 131
Posterior @ 0.4	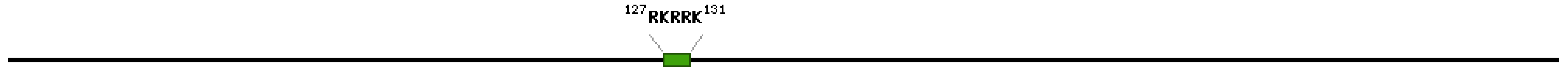
Posterior Graphic. Threshold Marked @ 0.4	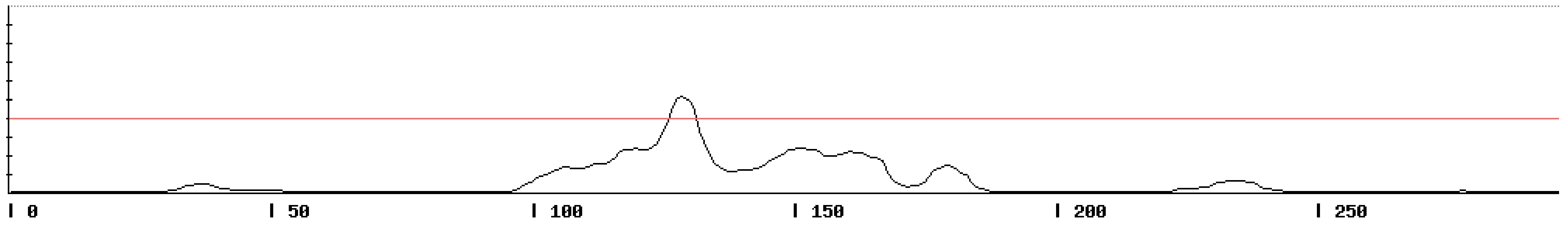

Predictions for Cucumismelo_MELO3C019401P1

Viterbi Path	
Viterbi Path	
Posterior @ 0.4	
Posterior @ 0.4	

Posterior Graphic. Threshold Marked @ 0.4	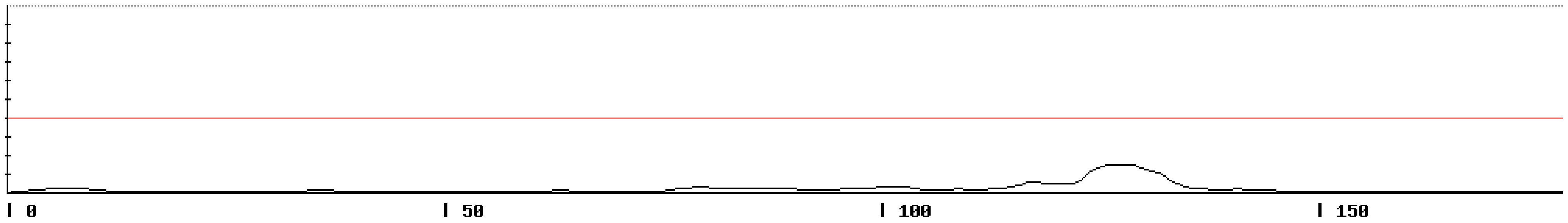

Predictions for Cucumismelo_MELO3C019620P1

Viterbi Path	
Viterbi Path	
Posterior @ 0.4	
Posterior @ 0.4	
Posterior Graphic. Threshold Marked @ 0.4	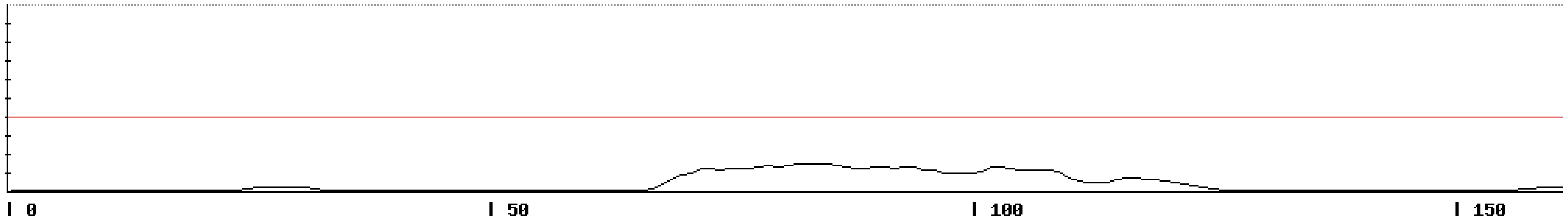

Predictions for Cucumismelo_MELO3C019663P1

Viterbi Path	
Viterbi Path	

Posterior @ 0.4	
Posterior @ 0.4	
Posterior Graphic. Threshold Marked @ 0.4	

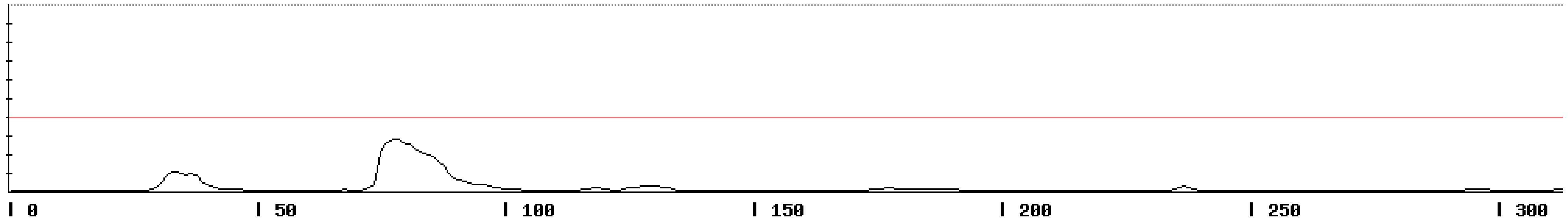

Predictions for Cucumismelo_MELO3C019665P1

Viterbi Path	
Viterbi Path	
Posterior @ 0.4	256 - KKKKMKR - 262
Posterior @ 0.4	
Posterior Graphic. Threshold Marked @ 0.4	

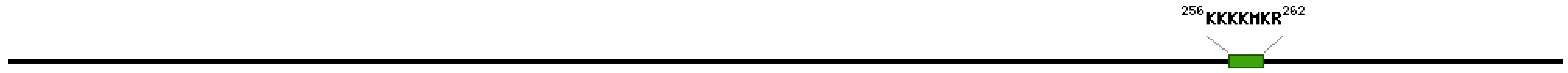

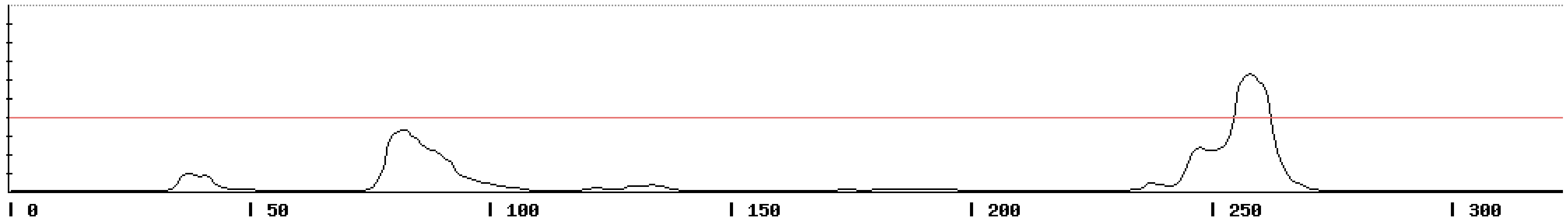

Predictions for Cucumismelo_MELO3C019666P1

Viterbi Path	
Viterbi Path	

Posterior @ 0.4

Posterior @ 0.4

Posterior Graphic. Threshold Marked @ 0.4

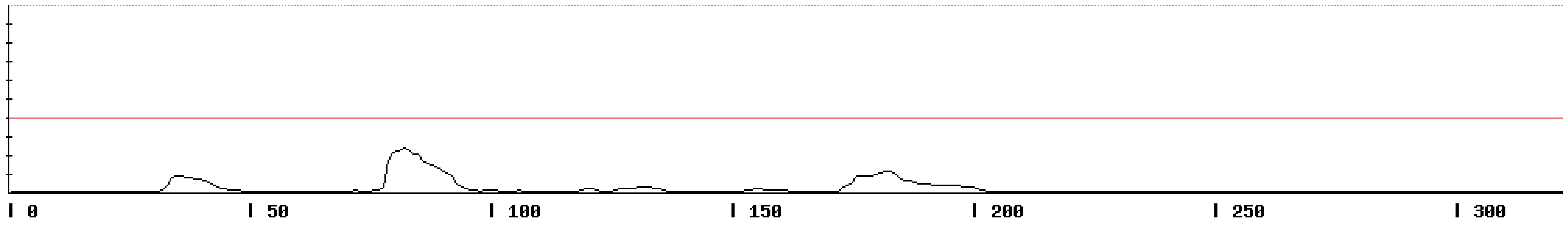

Predictions for Cucumismelo_MELO3C019845P1

Viterbi Path

Viterbi Path

Posterior @ 0.4

Posterior @ 0.4

Posterior Graphic. Threshold Marked @ 0.4

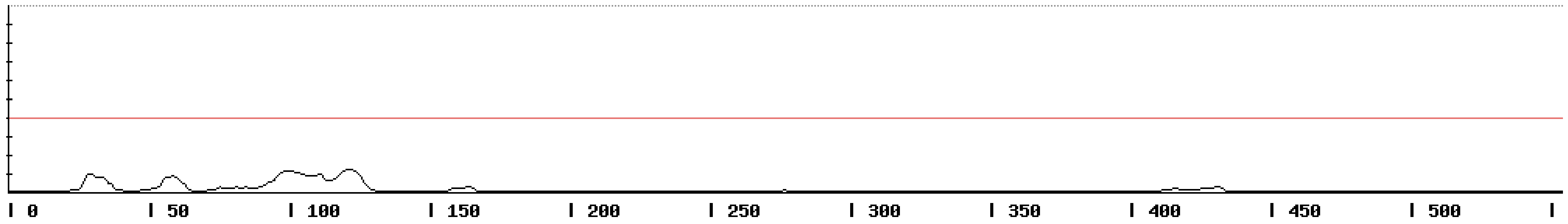

Predictions for Cucumismelo_MELO3C019845P2

Viterbi Path

Viterbi Path

Posterior @ 0.4

Posterior @ 0.4

Posterior Graphic. Threshold Marked @ 0.4

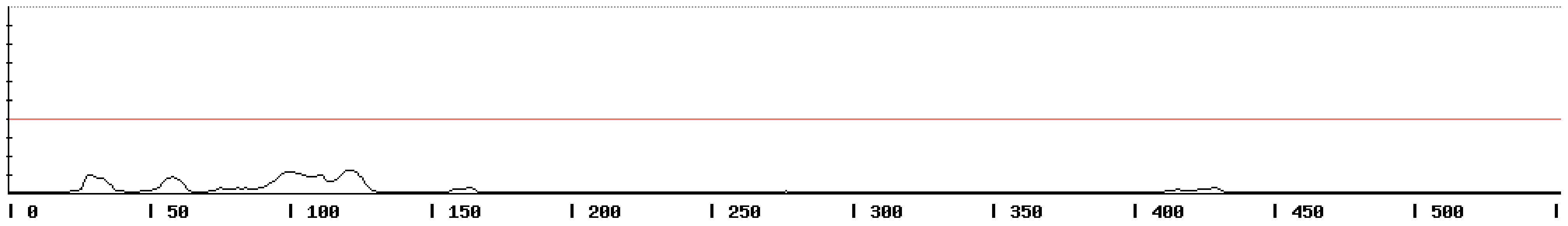

Predictions for Cucumismelo_MELO3C019954P1

Viterbi Path

Viterbi Path

Posterior @ 0.4

Posterior @ 0.4

Posterior Graphic. Threshold Marked @ 0.4

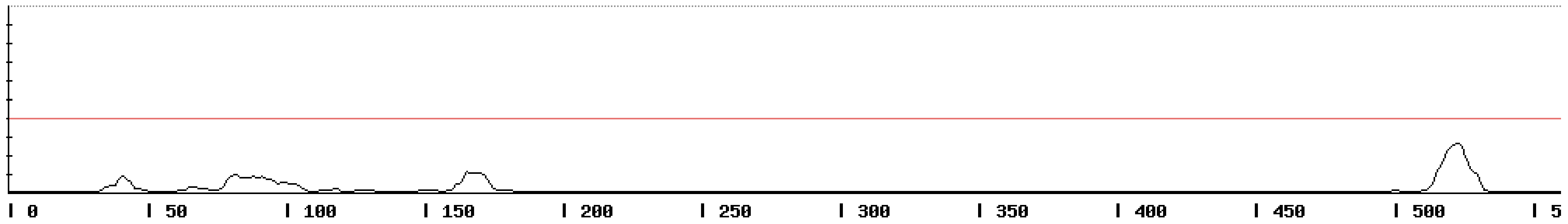

Predictions for Cucumismelo_MELO3C019954P2	
Viterbi Path	
Viterbi Path	
Posterior @ 0.4	
Posterior @ 0.4	
Posterior Graphic. Threshold Marked @ 0.4	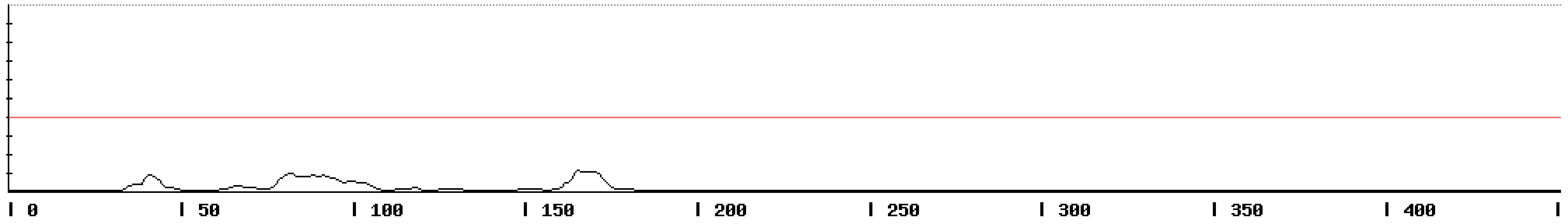

Predictions for Cucumismelo_MELO3C021131P1	
Viterbi Path	
Viterbi Path	
Posterior @ 0.4	
Posterior @ 0.4	
Posterior Graphic. Threshold Marked @ 0.4	

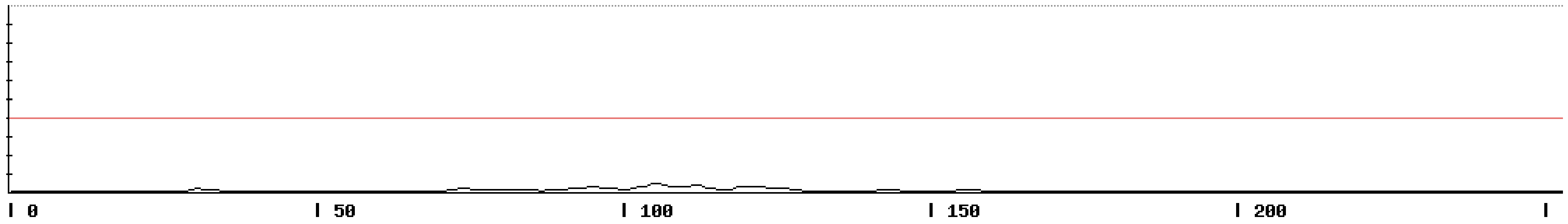

Predictions for Cucumismelo_MELO3C021587P1

Viterbi Path

Viterbi Path

Posterior @ 0.4

Posterior @ 0.4

Posterior Graphic. Threshold Marked @ 0.4

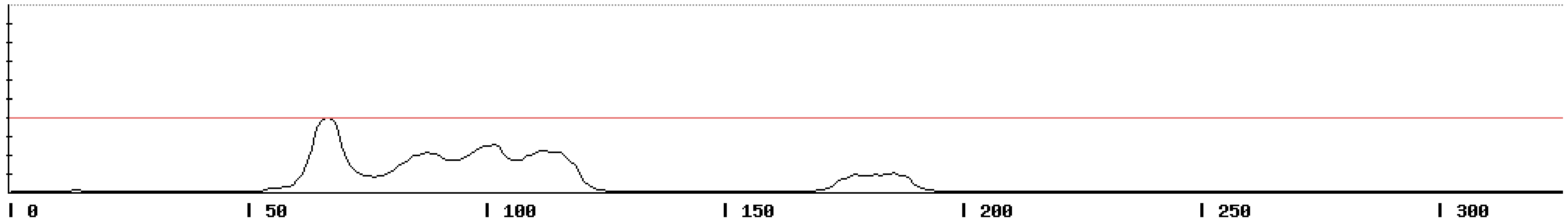

Predictions for Cucumismelo_MELO3C022002P1

Viterbi Path

Viterbi Path

Posterior @ 0.4

116 - RGKKLVG - 122

124 - K - 124

Posterior @ 0.4

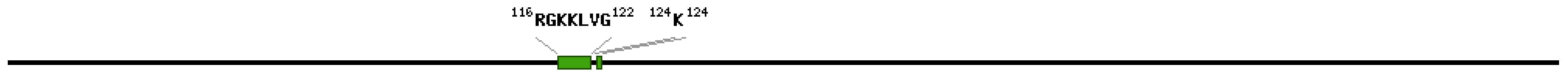

Posterior Graphic. Threshold Marked @ 0.4

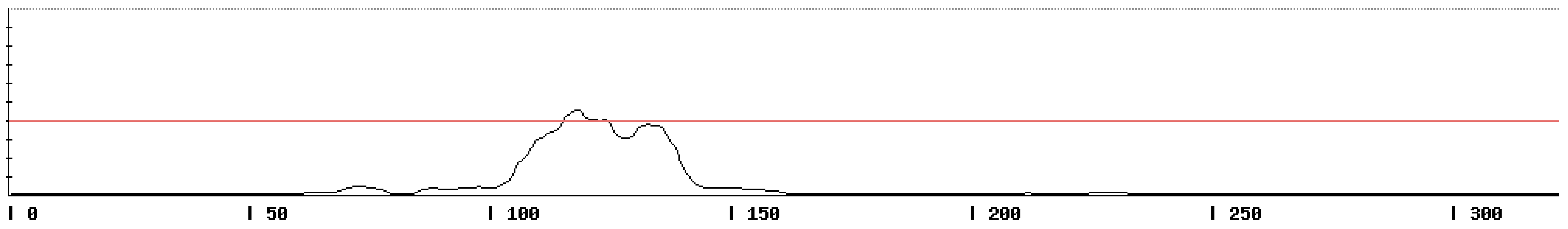

Predictions for Cucumismelo_MELO3C022117P1

Viterbi Path

Viterbi Path

Posterior @ 0.4

Posterior @ 0.4

Posterior Graphic. Threshold Marked @ 0.4

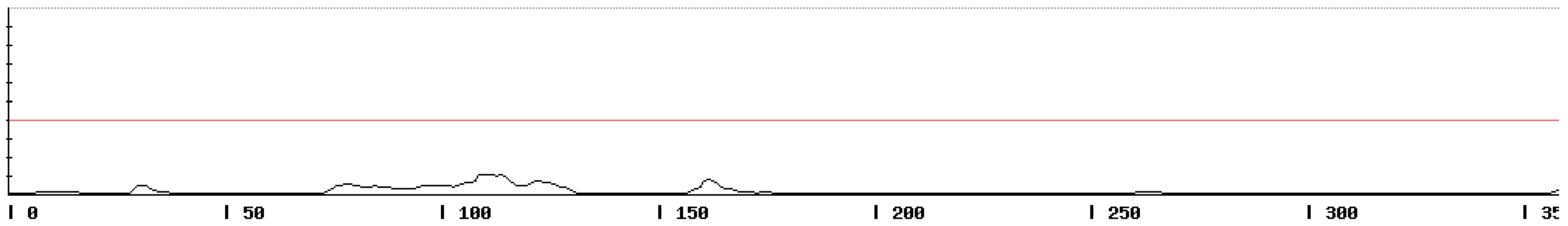

Predictions for Cucumismelo_MELO3C022254P1

Viterbi Path

Viterbi Path

Posterior @ 0.4

Posterior @ 0.4

Posterior Graphic. Threshold Marked @ 0.4

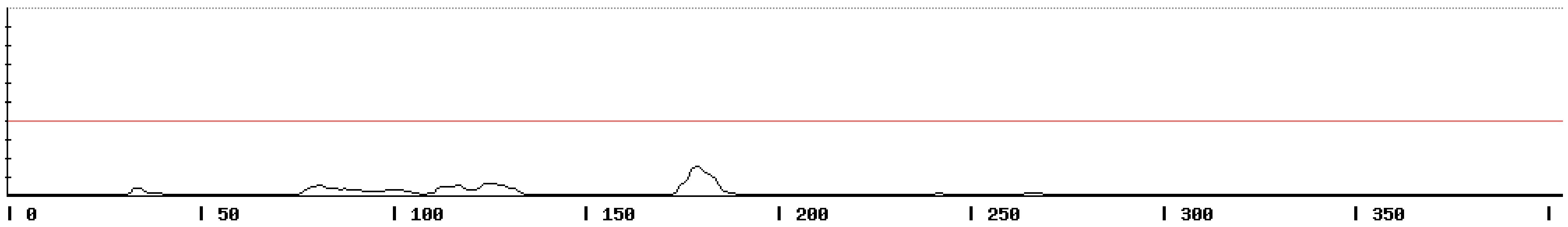

Predictions for Cucumismelo_MELO3C022342P1

Viterbi Path

Viterbi Path

Posterior @ 0.4

Posterior @ 0.4

Posterior Graphic. Threshold Marked @ 0.4

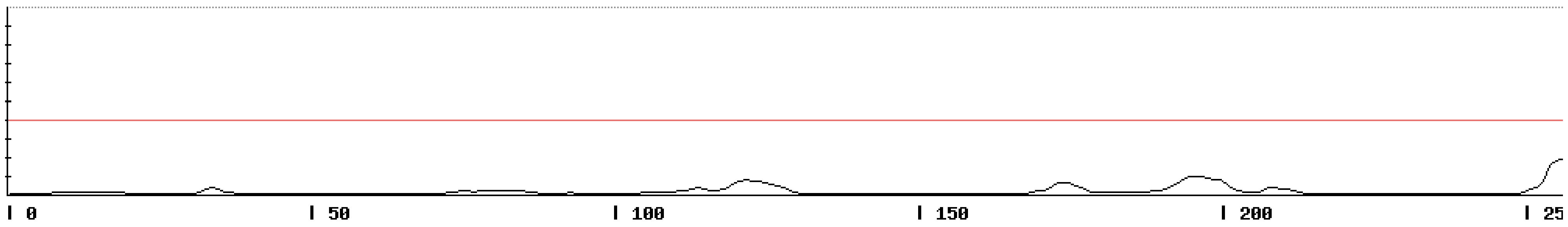

Predictions for Cucumismelo_MELO3C022962P1

Viterbi Path

Viterbi Path

Posterior @ 0.4

Posterior @ 0.4

Posterior Graphic. Threshold Marked @ 0.4

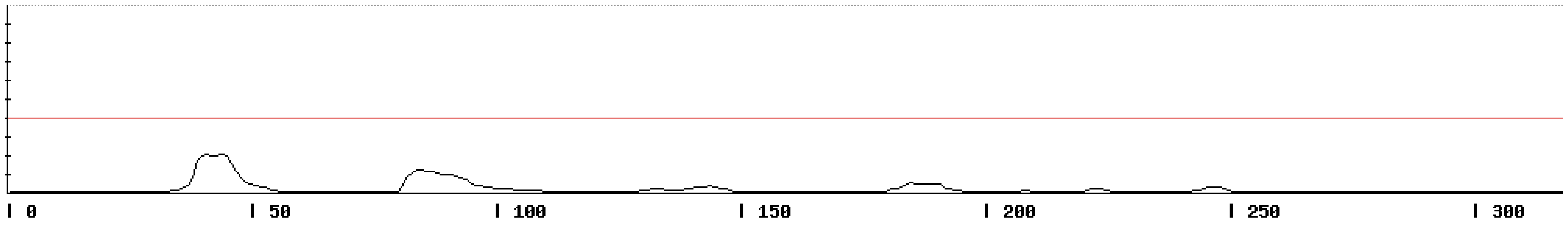

Predictions for Cucumismelo_MELO3C023195P1

Viterbi Path

Viterbi Path

Posterior @ 0.4

Posterior @ 0.4

Posterior Graphic. Threshold Marked @ 0.4

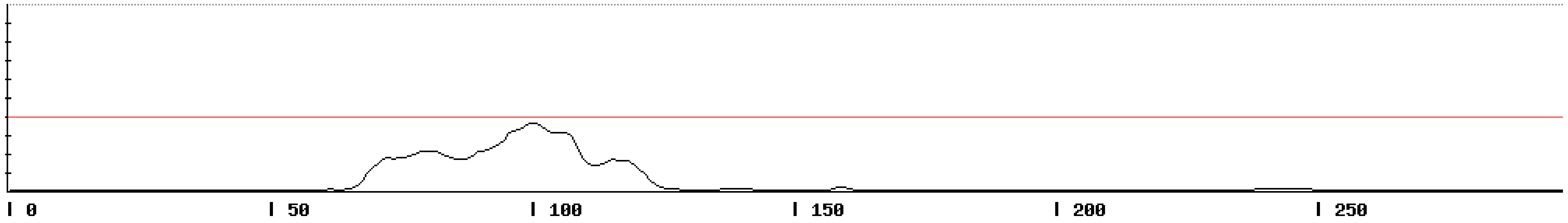

Predictions for Cucumismelo_MELO3C023230P1

Viterbi Path

Viterbi Path

Posterior @ 0.4

Posterior @ 0.4

Posterior Graphic. Threshold Marked @ 0.4

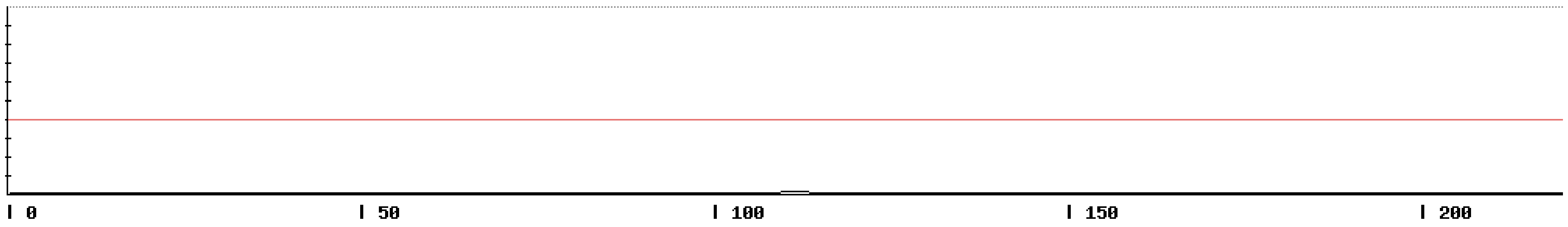

Predictions for Cucumismelo_MELO3C024115P1

Viterbi Path

Viterbi Path

Posterior @ 0.4

Posterior @ 0.4

Posterior Graphic. Threshold Marked @ 0.4

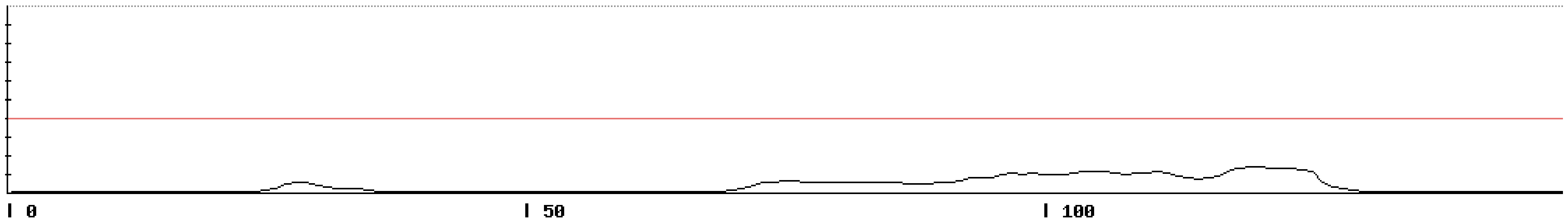

Predictions for Cucumismelo_MELO3C024560P1

Viterbi Path

Viterbi Path

Posterior @ 0.4

Posterior @ 0.4

Posterior Graphic. Threshold Marked @ 0.4

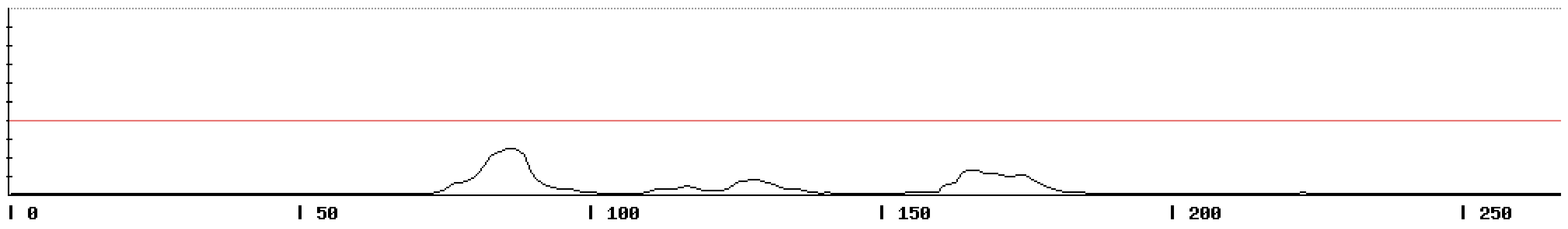

Predictions for Cucumismelo_MELO3C024561P1

Viterbi Path

Viterbi Path

Posterior @ 0.4

Posterior @ 0.4

Posterior Graphic. Threshold Marked @ 0.4

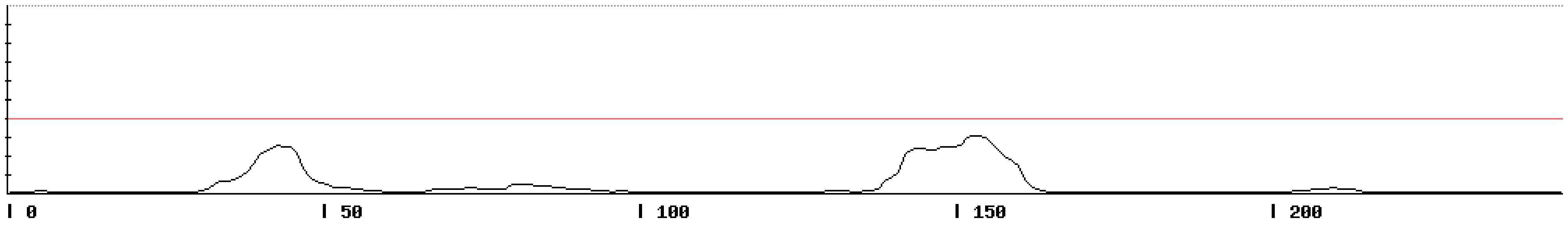

Predictions for Cucumismelo_MELO3C024865P1

Viterbi Path	
Viterbi Path	
Posterior @ 0.4	84 - KR - 85
	90 - GYWKATGKDRKIKSGTKLIGMKKTLVFYKGRAPKGKR - 126
Posterior @ 0.4	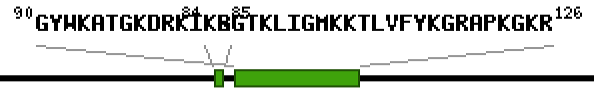
Posterior Graphic. Threshold Marked @ 0.4	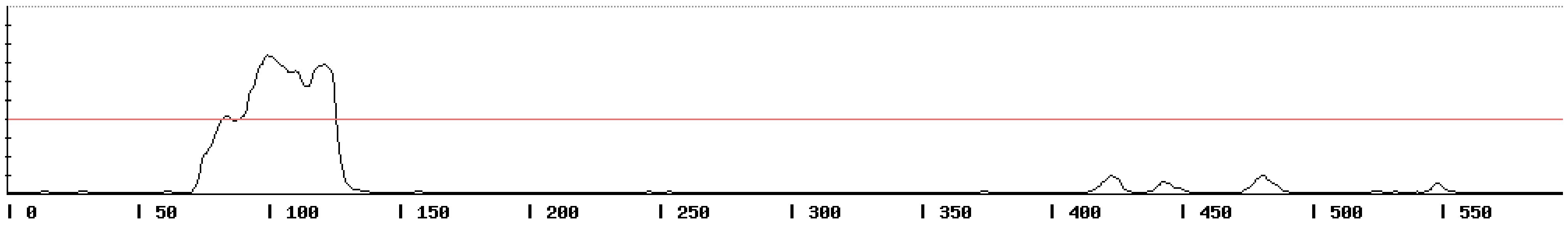

Predictions for Cucumismelo_MELO3C025099P1

Viterbi Path	
Viterbi Path	
Posterior @ 0.4	622 - KGKKKG - 627
Posterior @ 0.4	

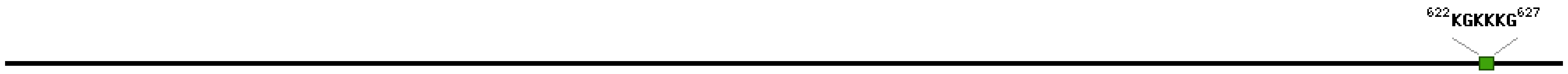

Posterior Graphic. Threshold Marked @ 0.4

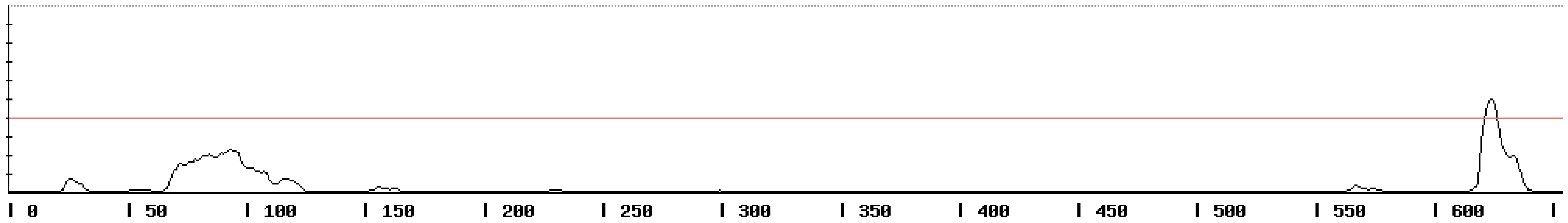

Predictions for Cucumismelo_MELO3C025584P2

Viterbi Path

Viterbi Path

Posterior @ 0.4 280 - NRRIRLKKREATGGKKL - 296

Posterior @ 0.4

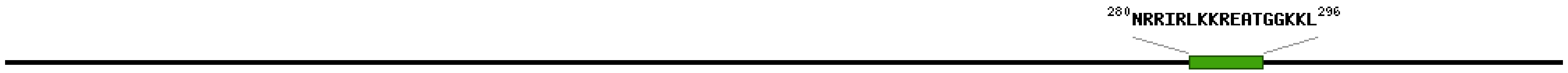

Posterior Graphic. Threshold Marked @ 0.4

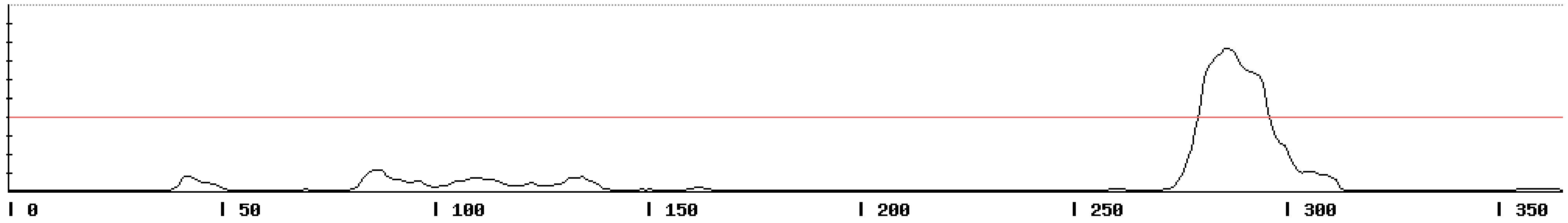

Predictions for Cucumismelo_MELO3C025584P3

Viterbi Path

Viterbi Path

Posterior @ 0.4

Posterior @ 0.4

Posterior Graphic. Threshold Marked @ 0.4

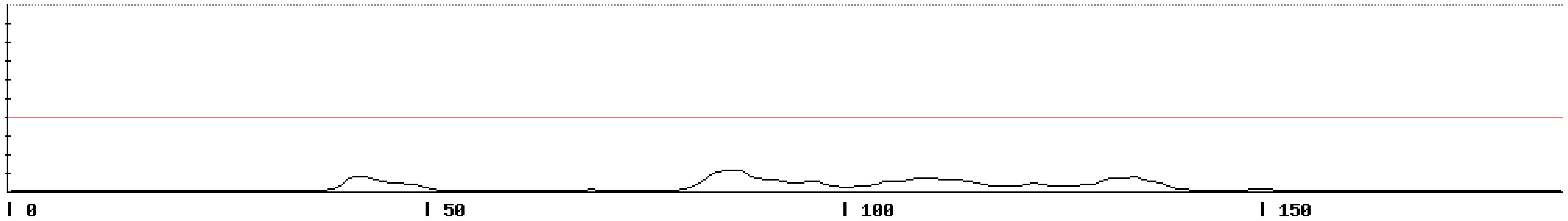

Predictions for Cucumismelo_MELO3C025611P1

Viterbi Path

Viterbi Path

Posterior @ 0.4

Posterior @ 0.4

Posterior Graphic. Threshold Marked @ 0.4

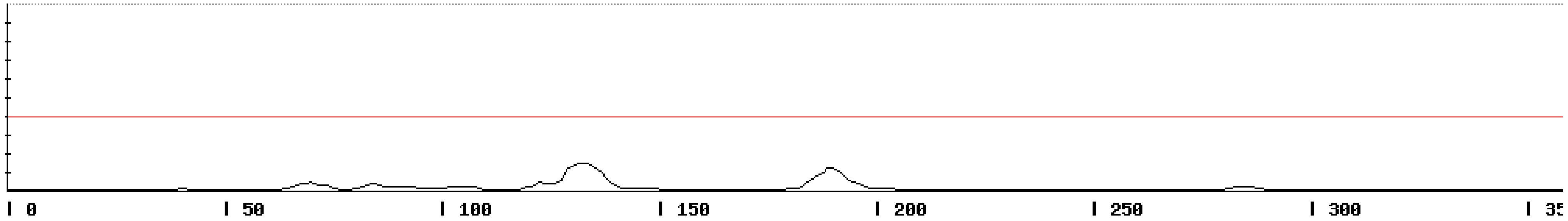

Predictions for Cucumismelo_MELO3C026180P1

Viterbi Path

Viterbi Path

Posterior @ 0.4

Posterior @ 0.4

Posterior Graphic. Threshold Marked @ 0.4

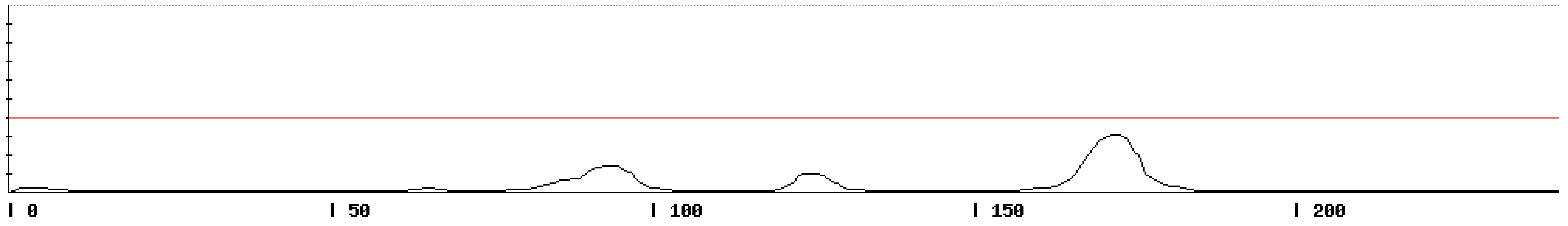

Predictions for Cucumismelo_MELO3C026251P1

Viterbi Path

Viterbi Path

Posterior @ 0.4

42 - KKRKSRIIRK - 51

128 - RKKILKQHKRAPCTRKEHLIPKRKR - 152

Posterior @ 0.4

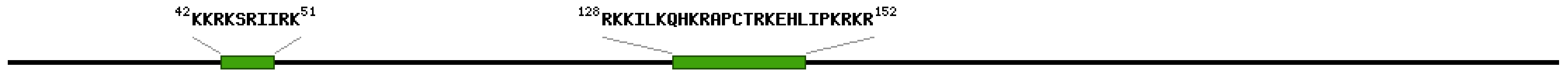

Posterior Graphic. Threshold Marked @ 0.4

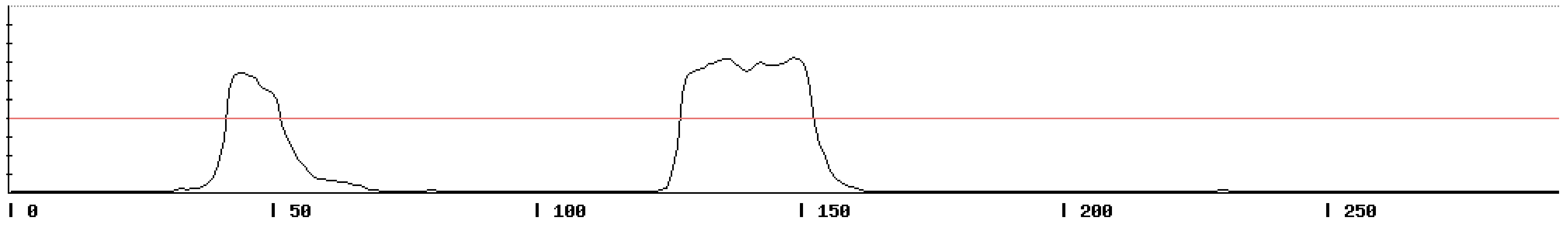

Predictions for Cucumismelo_MELO3C026521P1	
Viterbi Path	
Viterbi Path	
Posterior @ 0.4	
Posterior @ 0.4	
Posterior Graphic. Threshold Marked @ 0.4	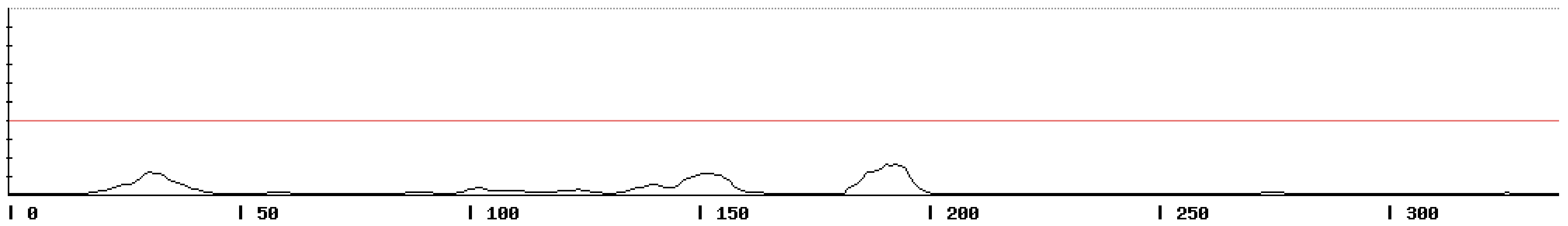

Predictions for Cucumismelo_MELO3C026521P2	
Viterbi Path	
Viterbi Path	
Posterior @ 0.4	
Posterior @ 0.4	
Posterior Graphic. Threshold Marked @ 0.4	

Revision r.9 - Last Updated on : February 24 2015 17:45:20. alex.nguyenba@utoronto.ca

Intended to be used with Firefox

Viterbi Path

Posterior @ 0.4

Posterior @ 0.4

Posterior Graphic. Threshold Marked @ 0.4

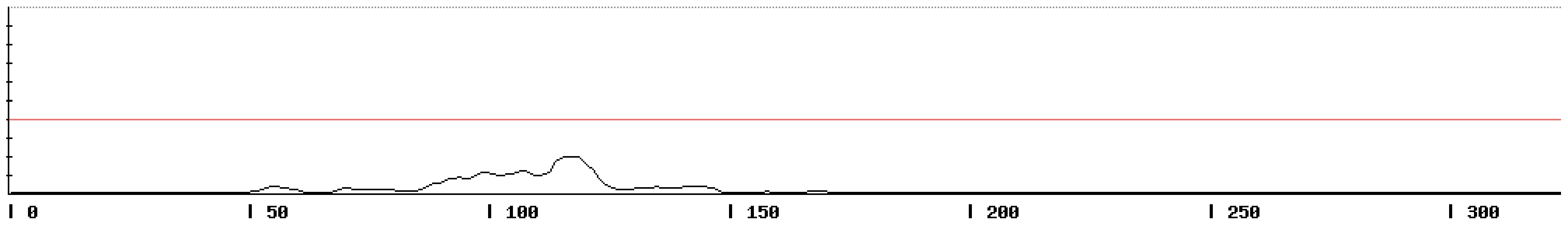

Predictions for Cucumissativus_Cucsa.011210.1

Viterbi Path

Viterbi Path

Posterior @ 0.4

Posterior @ 0.4

Posterior Graphic. Threshold Marked @ 0.4

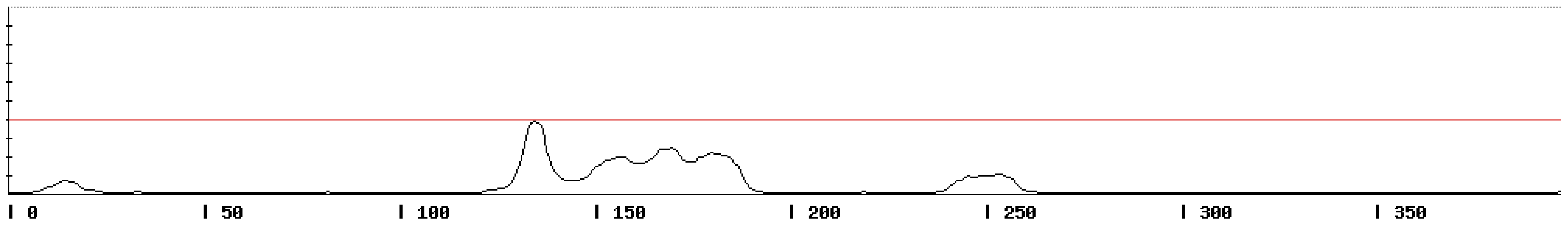

Predictions for Cucumissativus_Cucsa.032500.1

Viterbi Path

Viterbi Path

Posterior @ 0.4

Posterior @ 0.4

Posterior Graphic. Threshold Marked @ 0.4

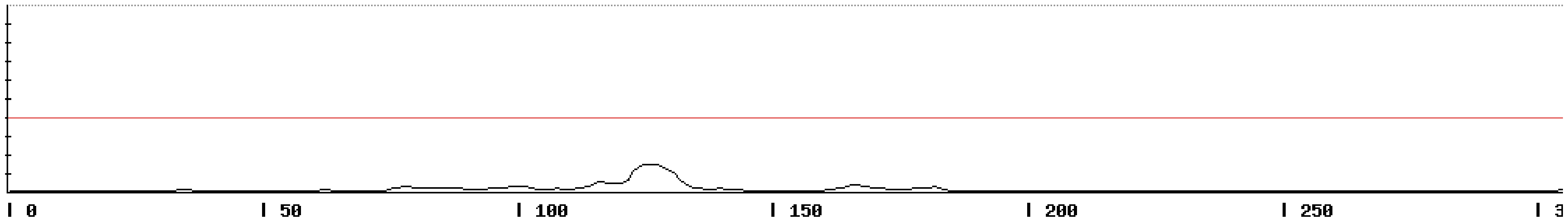

Predictions for Cucumissativus_Cucsa.040380.1

Viterbi Path

Viterbi Path

Posterior @ 0.4

Posterior @ 0.4

Posterior Graphic. Threshold Marked @ 0.4

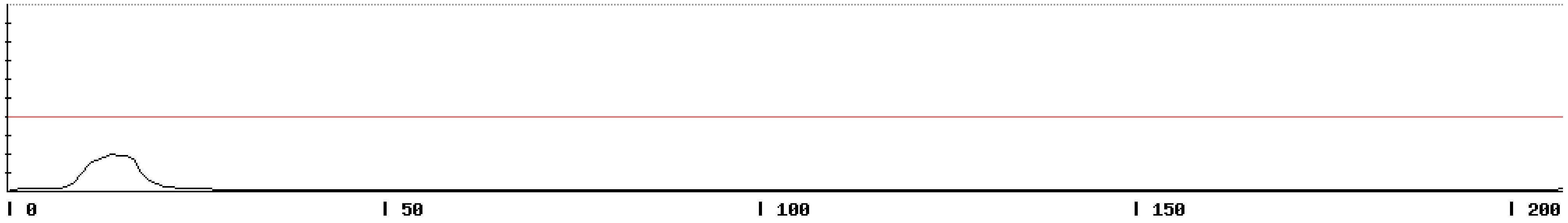

Predictions for Cucumissativus_Cucsa.042390.1	
Viterbi Path	
Viterbi Path	
Posterior @ 0.4	168 - RKKSKKR - 174
Posterior @ 0.4	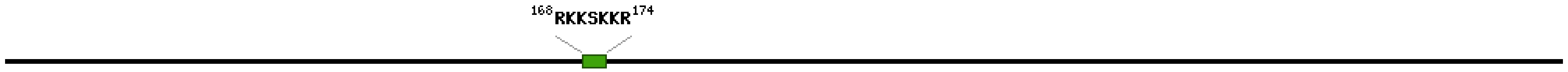
Posterior Graphic. Threshold Marked @ 0.4	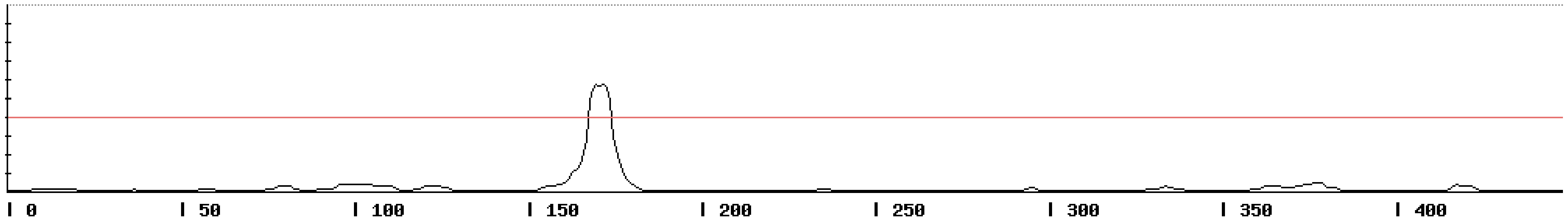

Predictions for Cucumissativus_Cucsa.043420.1	
Viterbi Path	
Viterbi Path	
Posterior @ 0.4	
Posterior @ 0.4	
Posterior Graphic. Threshold Marked @ 0.4	

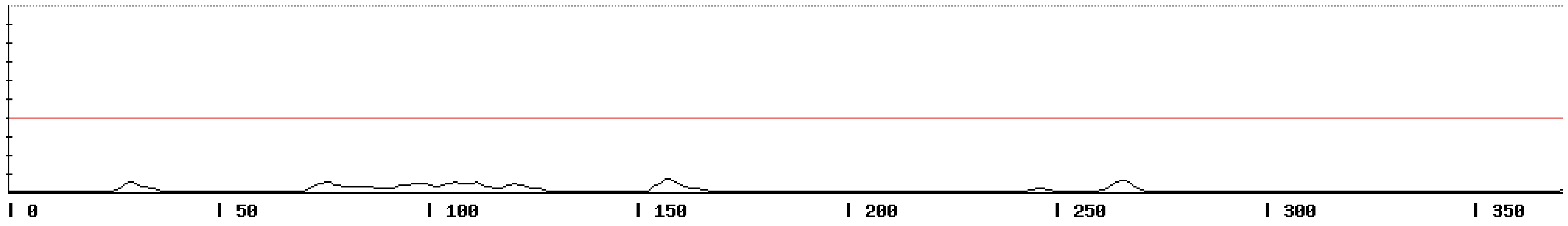

Predictions for Cucumissativus_Cucsa.046530.1

Viterbi Path

Viterbi Path

Posterior @ 0.4 — 108 - GTRKRRKV - 115

Posterior @ 0.4

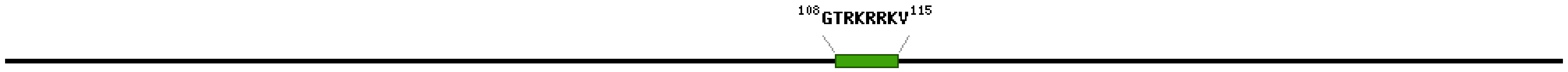

Posterior Graphic. Threshold Marked @ 0.4

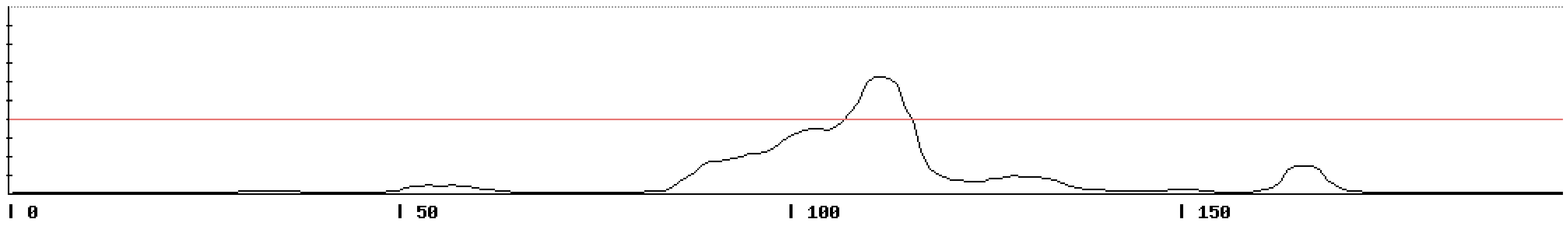

Predictions for Cucumissativus_Cucsa.053890.1

Viterbi Path

Viterbi Path

Posterior @ 0.4 — 164 - KRRRPKRQG - 172

Posterior @ 0.4

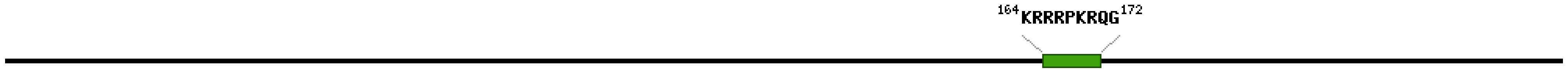

Posterior Graphic. Threshold Marked @ 0.4

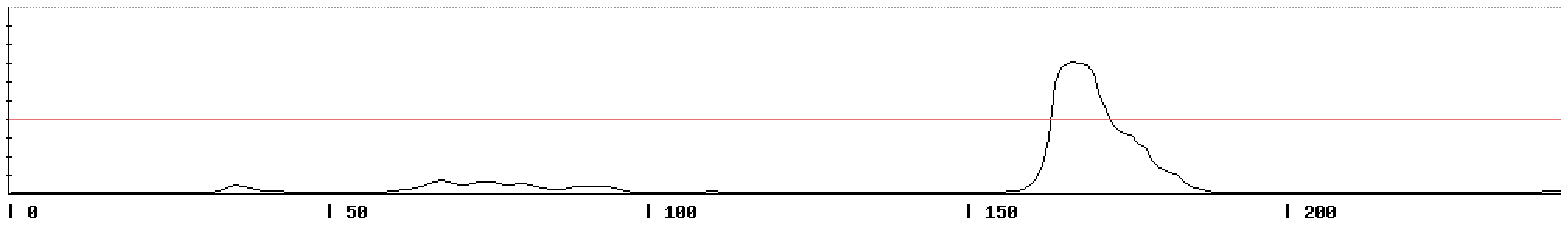

Predictions for Cucumissativus_Cucsa.058780.1

Viterbi Path

Viterbi Path

Posterior @ 0.4

Posterior @ 0.4

Posterior Graphic. Threshold Marked @ 0.4

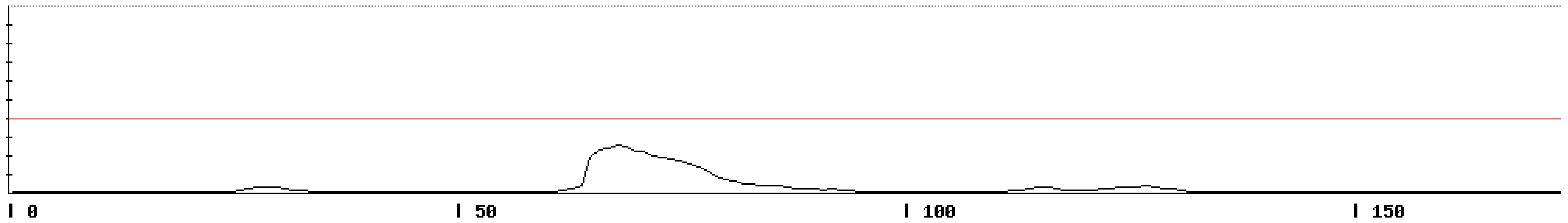

Predictions for Cucumissativus_Cucsa.060600.1

Viterbi Path

Viterbi Path

Posterior @ 0.4

Posterior @ 0.4

Posterior Graphic. Threshold Marked @ 0.4

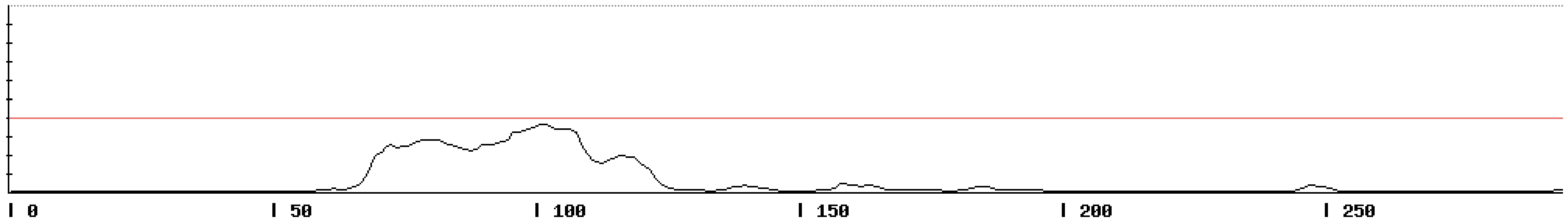

Predictions for Cucumissativus_Cucsa.074600.1

Viterbi Path

Viterbi Path

Posterior @ 0.4

Posterior @ 0.4

Posterior Graphic. Threshold Marked @ 0.4

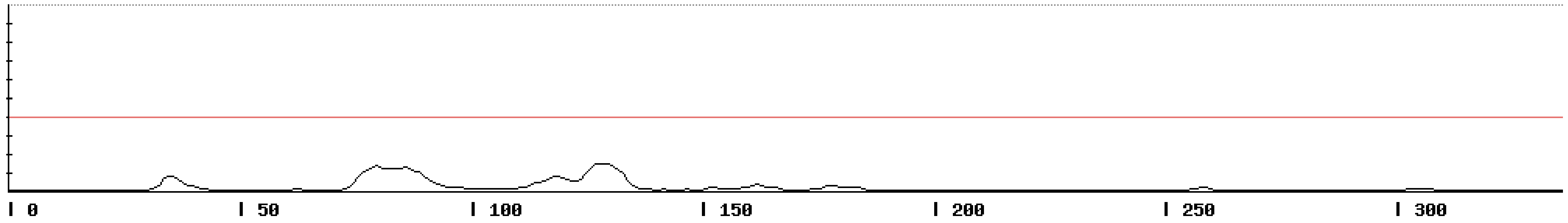

Predictions for Cucumissativus_Cucsa.074610.1

Viterbi Path

Viterbi Path

Posterior @ 0.4

Posterior @ 0.4

Posterior Graphic. Threshold Marked @ 0.4

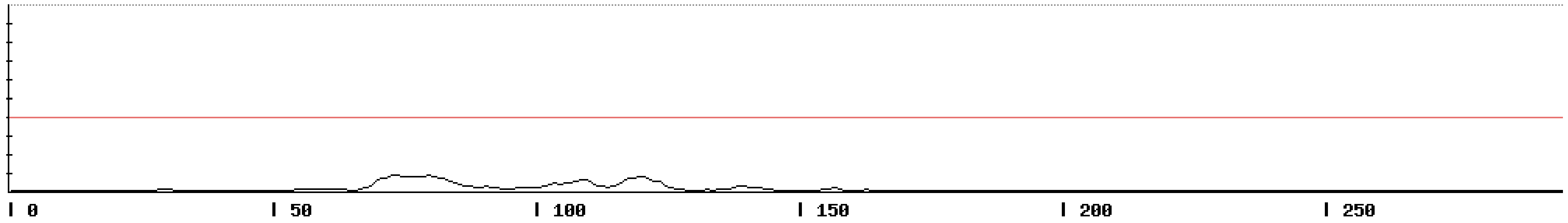

Predictions for Cucumissativus_Cucsa.077390.1

Viterbi Path

Viterbi Path

Posterior @ 0.4

Posterior @ 0.4

Posterior Graphic. Threshold Marked @ 0.4

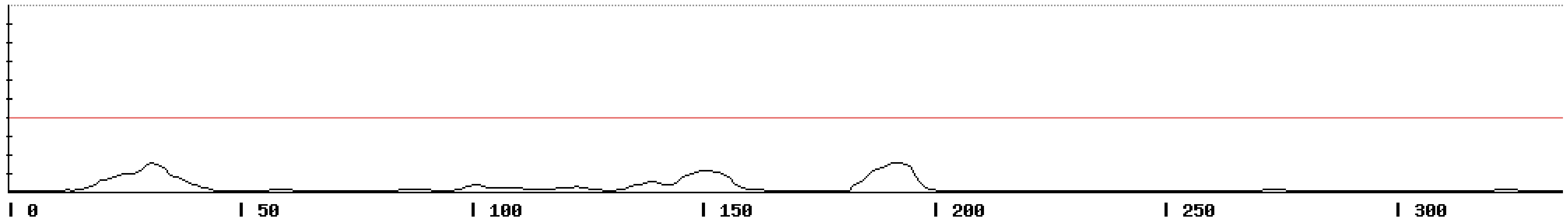

Predictions for Cucumissativus_Cucsa.077940.1

Viterbi Path

Viterbi Path

Posterior @ 0.4

Posterior @ 0.4

Posterior Graphic. Threshold Marked @ 0.4

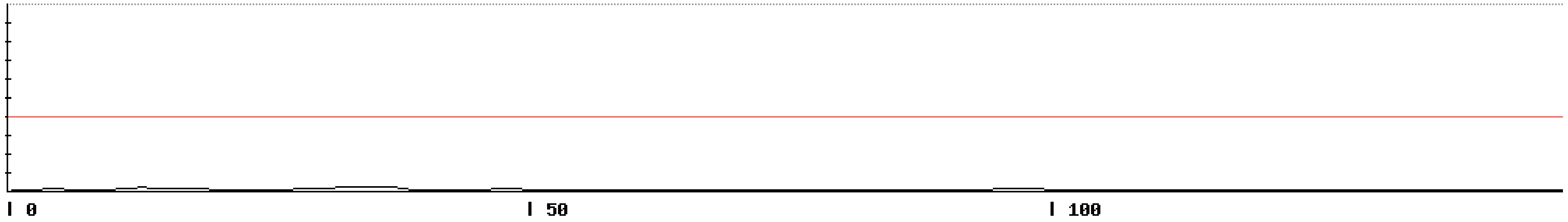

Predictions for Cucumissativus_Cucsa.081320.1

Viterbi Path

Viterbi Path

Posterior @ 0.4 — 72 - KKSR - 75

Posterior @ 0.4

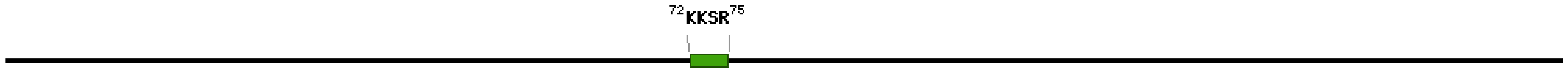

Posterior Graphic. Threshold Marked @ 0.4

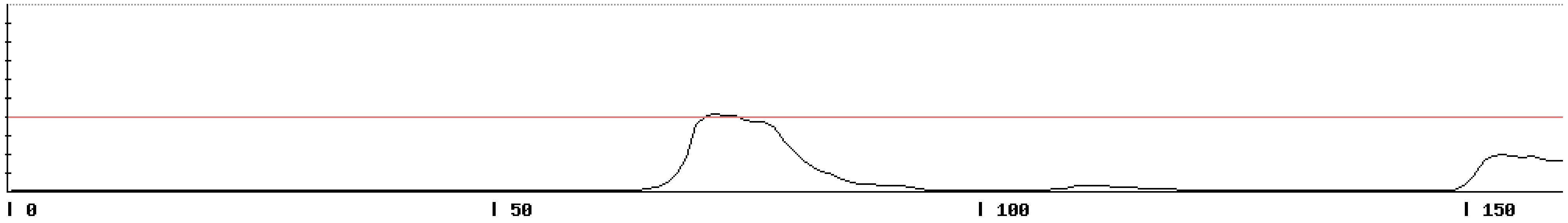

Predictions for Cucumissativus_Cucsa.083530.1

Viterbi Path

Viterbi Path

Posterior @ 0.4

Posterior @ 0.4

Posterior Graphic. Threshold Marked @ 0.4

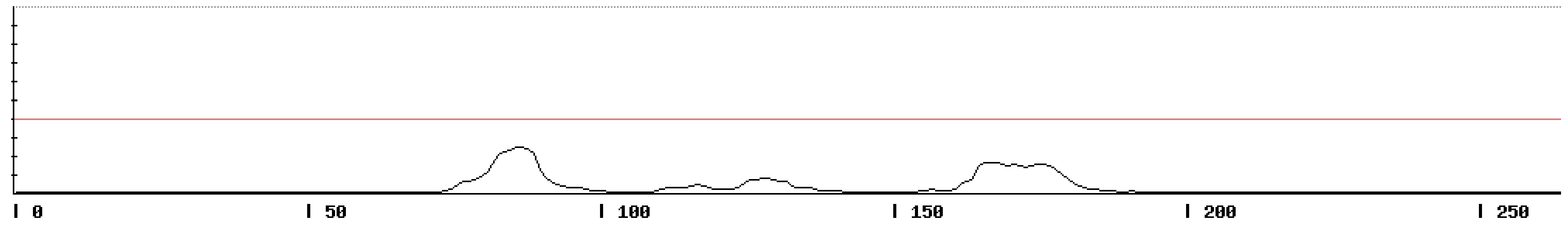

Predictions for Cucumissativus_Cucsa.083540.1

Viterbi Path

Viterbi Path

Posterior @ 0.4

Posterior @ 0.4

Posterior Graphic. Threshold Marked @ 0.4

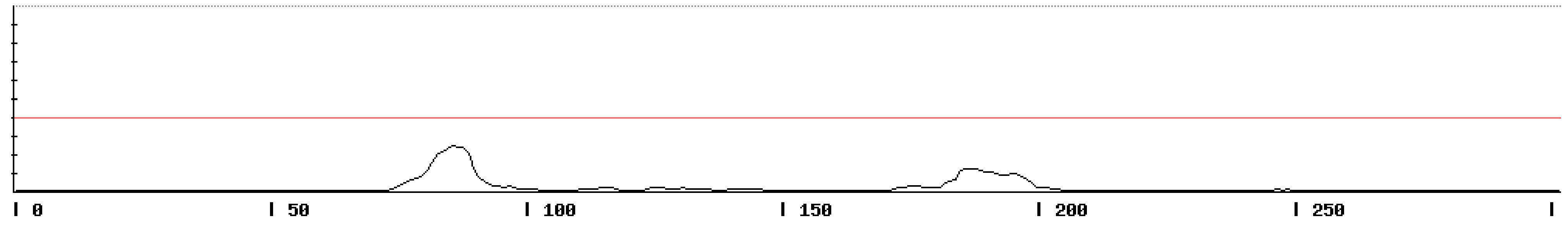

Predictions for Cucumissativus_Cucsa.087480.1

Viterbi Path

Viterbi Path

Posterior @ 0.4

88 - TRKRRKV - 94

Posterior @ 0.4

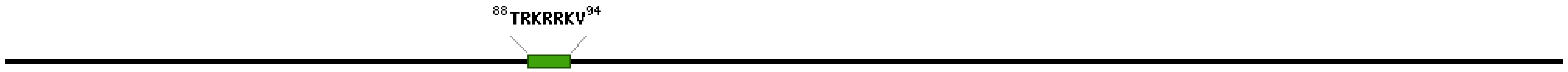

Posterior Graphic. Threshold Marked @ 0.4

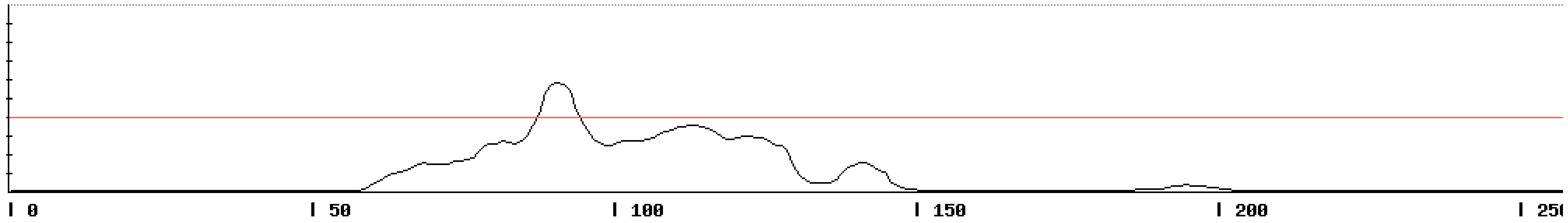

Predictions for Cucumissativus_Cucsa.095210.1

Viterbi Path

Viterbi Path

Posterior @ 0.4

170 - KKRSTRERDEKKK - 182

Posterior @ 0.4

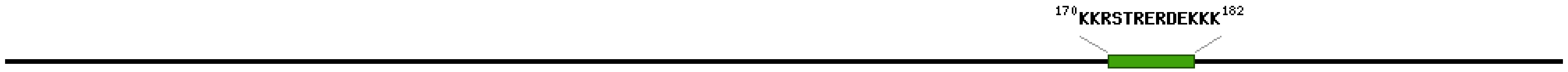

Posterior Graphic. Threshold Marked @ 0.4

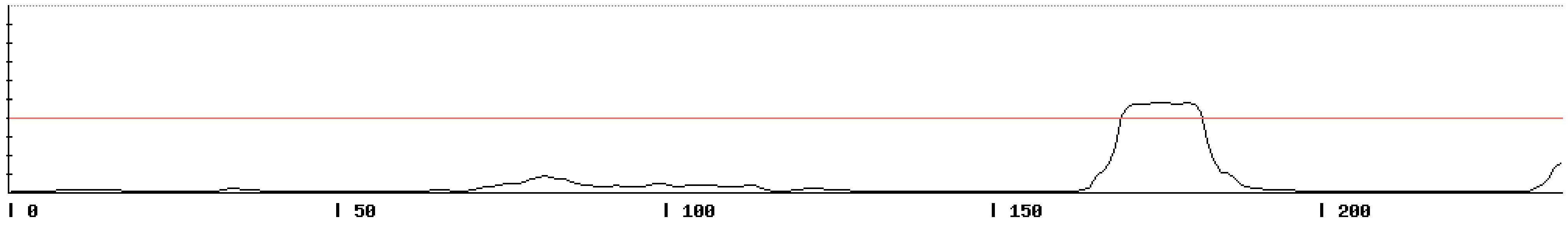

Predictions for Cucumissativus_Cucsa.099390.1

Viterbi Path	
Viterbi Path	
Posterior @ 0.4	76 - GRPNR - 80
Posterior @ 0.4	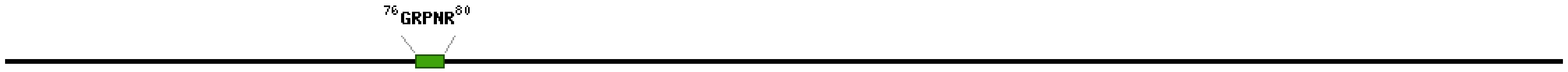
Posterior Graphic. Threshold Marked @ 0.4	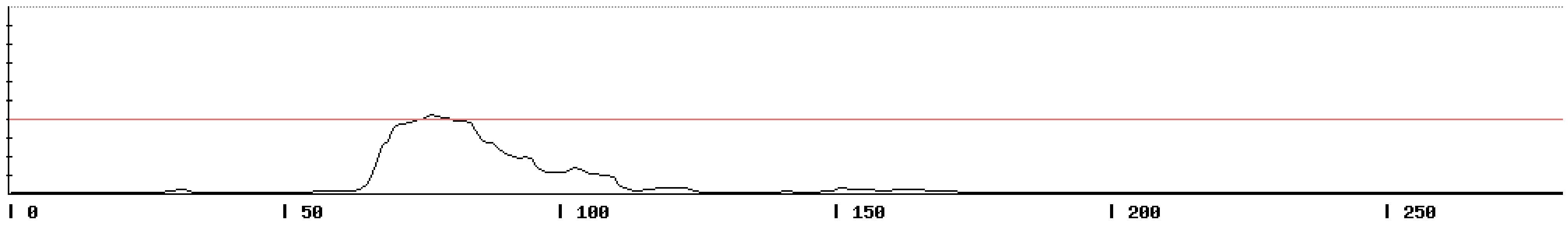

Predictions for Cucumissativus_Cucsa.100920.1

Viterbi Path	
Viterbi Path	
Posterior @ 0.4	
Posterior @ 0.4	

Posterior Graphic. Threshold Marked @ 0.4

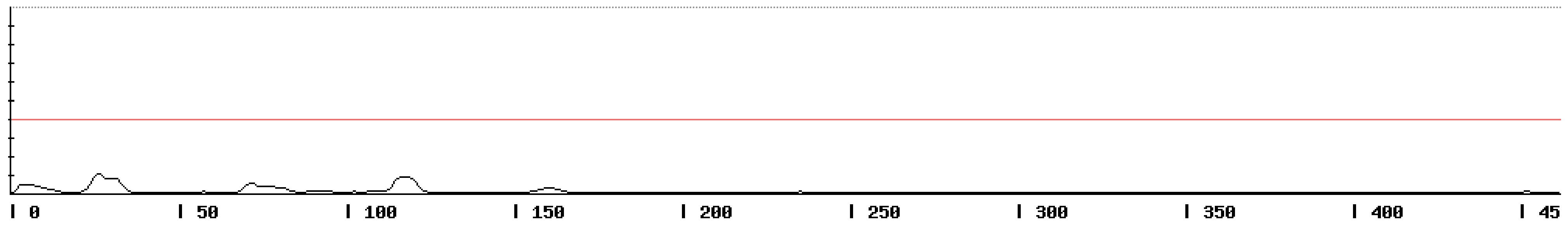

Predictions for Cucumissativus_Cucsa.112500.1

Viterbi Path

Viterbi Path

Posterior @ 0.4

Posterior @ 0.4

Posterior Graphic. Threshold Marked @ 0.4

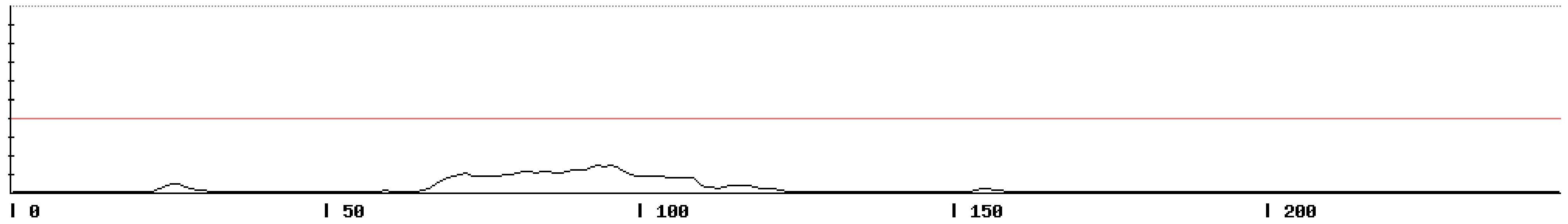

Predictions for Cucumissativus_Cucsa.112890.1

Viterbi Path

Viterbi Path

Posterior @ 0.4

Posterior @ 0.4

Posterior Graphic. Threshold Marked @ 0.4

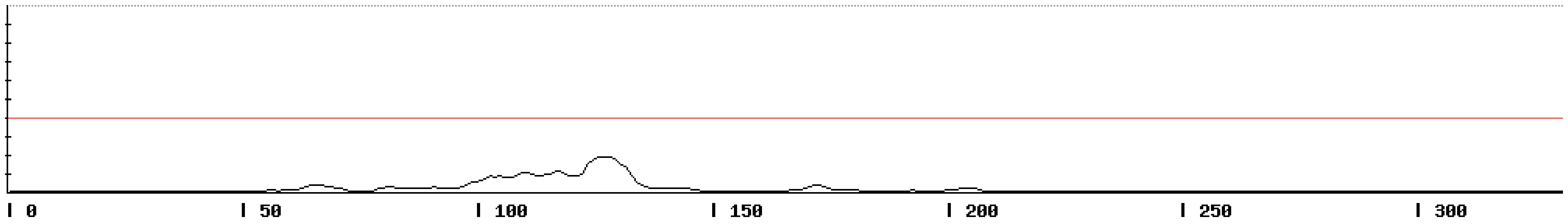

Predictions for Cucumissativus_Cucsa.116800.1

Viterbi Path

Viterbi Path

Posterior @ 0.4

Posterior @ 0.4

Posterior Graphic. Threshold Marked @ 0.4

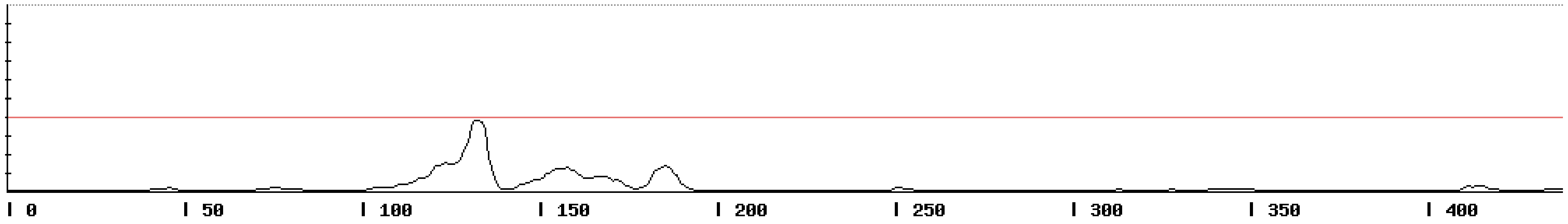

Predictions for Cucumissativus_Cucsa.118320.1

Viterbi Path

Viterbi Path

Posterior @ 0.4

Posterior @ 0.4

Posterior Graphic. Threshold Marked @ 0.4

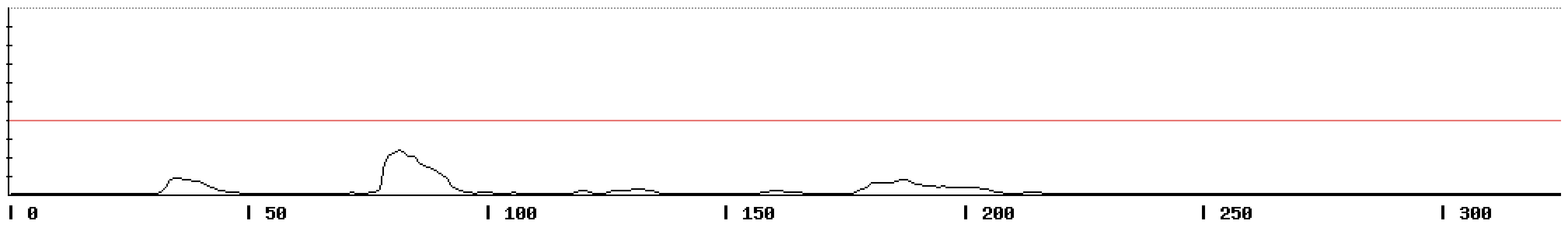

Predictions for Cucumissativus_Cucsa.118330.1

Viterbi Path

Viterbi Path

Posterior @ 0.4

Posterior @ 0.4

Posterior Graphic. Threshold Marked @ 0.4

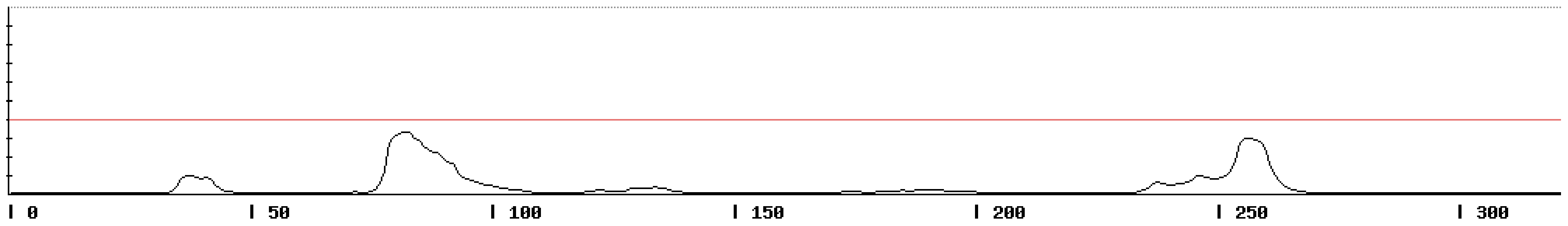

Predictions for Cucumissativus_Cucsa.118340.1

Viterbi Path

Viterbi Path

Posterior @ 0.4

Posterior @ 0.4

Posterior Graphic. Threshold Marked @ 0.4

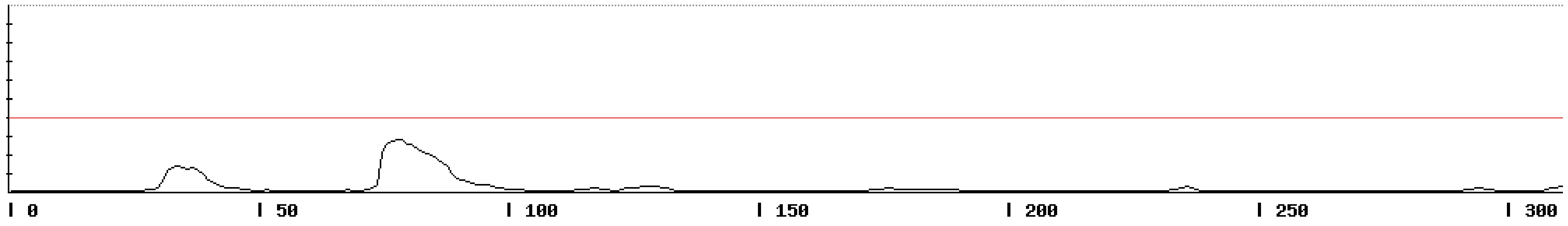

Predictions for Cucumissativus_Cucsa.118620.1

Viterbi Path

Viterbi Path

Posterior @ 0.4

Posterior @ 0.4

Posterior Graphic. Threshold Marked @ 0.4

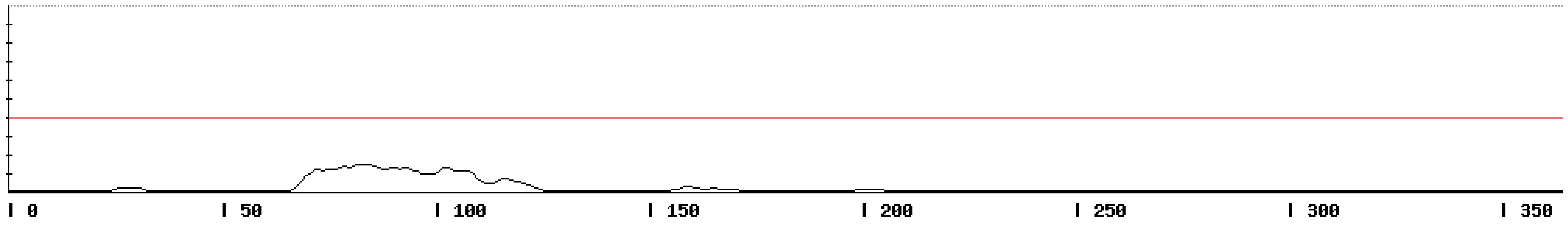

Predictions for Cucumissativus_Cucsa.120870.1

Viterbi Path	
Viterbi Path	
Posterior @ 0.4	
Posterior @ 0.4	
Posterior Graphic. Threshold Marked @ 0.4	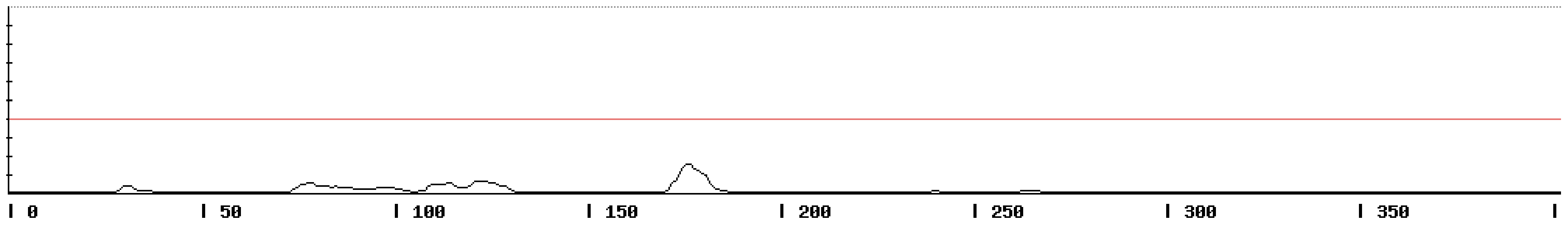

Predictions for Cucumissativus_Cucsa.122890.1

Viterbi Path	
Viterbi Path	
Posterior @ 0.4	
Posterior @ 0.4	
Posterior Graphic. Threshold Marked @ 0.4	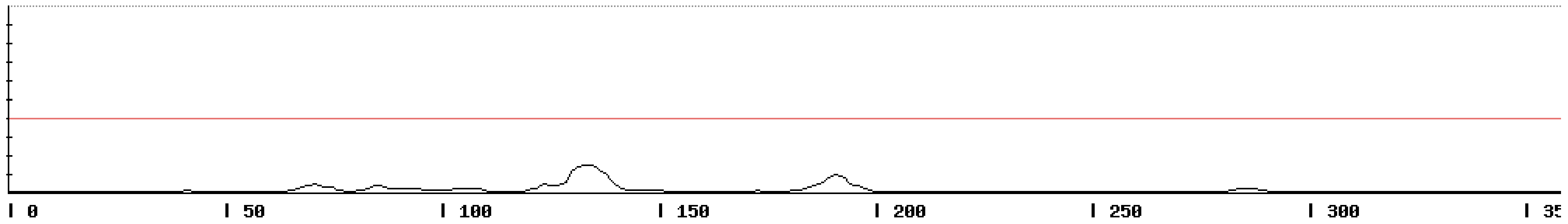

Predictions for Cucumissativus_Cucsa.125330.1	
Viterbi Path	
Viterbi Path	
Posterior @ 0.4	95 - KPIGRPKAVGIKK - 107
Posterior @ 0.4	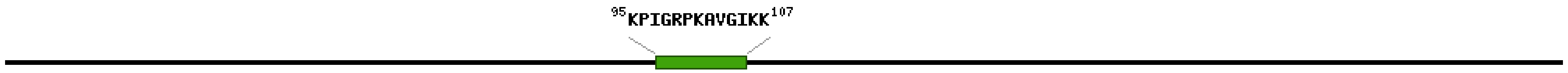
Posterior Graphic. Threshold Marked @ 0.4	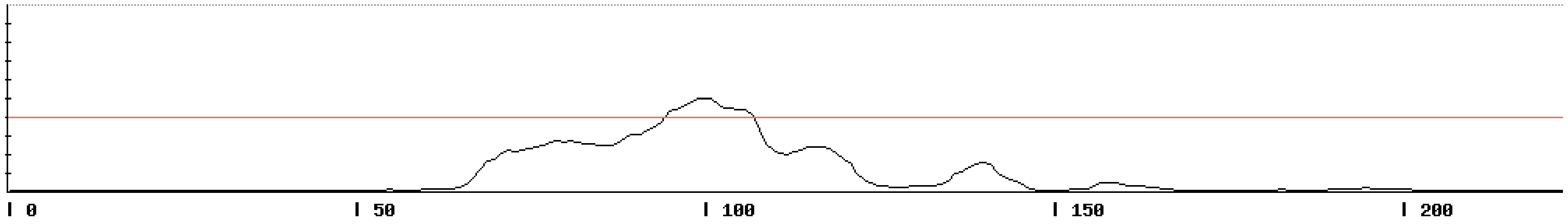

Predictions for Cucumissativus_Cucsa.126450.1	
Viterbi Path	
Viterbi Path	
Posterior @ 0.4	
Posterior @ 0.4	
Posterior Graphic. Threshold Marked @ 0.4	

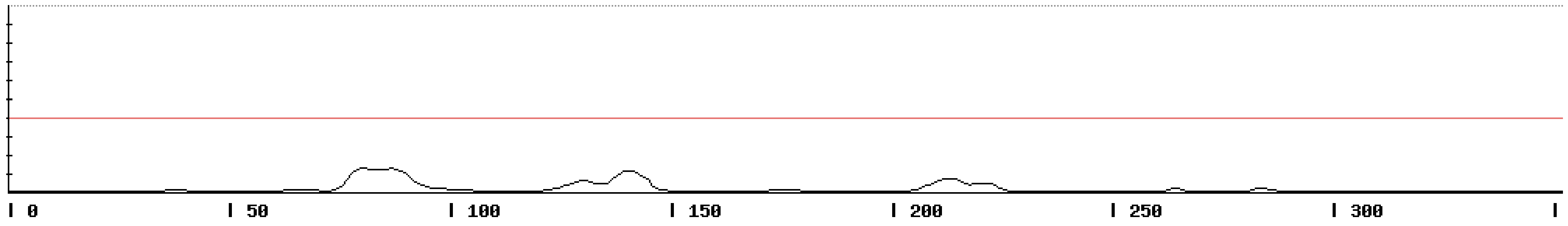

Predictions for Cucumissativus_Cucsa.126960.1

Viterbi Path	
Viterbi Path	
Posterior @ 0.4	131 - RKRRK - 135
Posterior @ 0.4	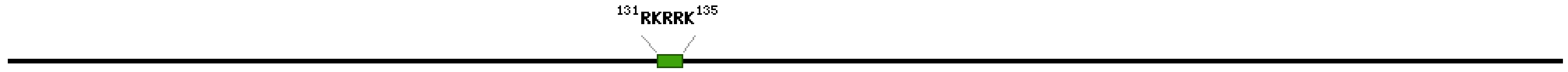
Posterior Graphic. Threshold Marked @ 0.4	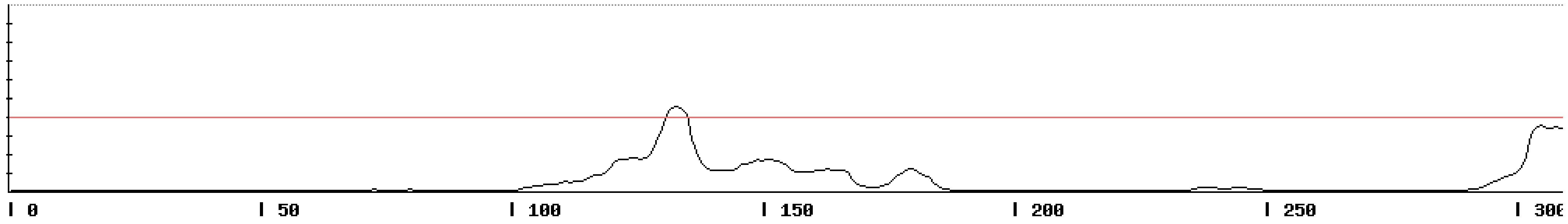

Predictions for Cucumissativus_Cucsa.150740.1

Viterbi Path	
Viterbi Path	
Posterior @ 0.4	
Posterior @ 0.4	

Posterior Graphic. Threshold Marked @ 0.4

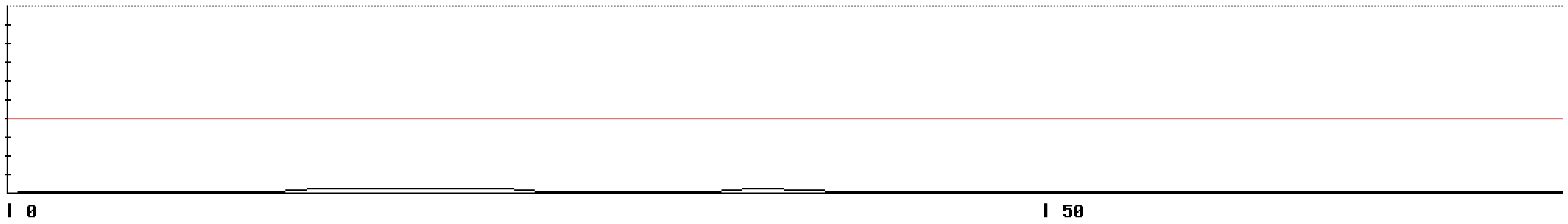

Predictions for Cucumissativus_Cucsa.157080.1

Viterbi Path

Viterbi Path

Posterior @ 0.4

Posterior @ 0.4

Posterior Graphic. Threshold Marked @ 0.4

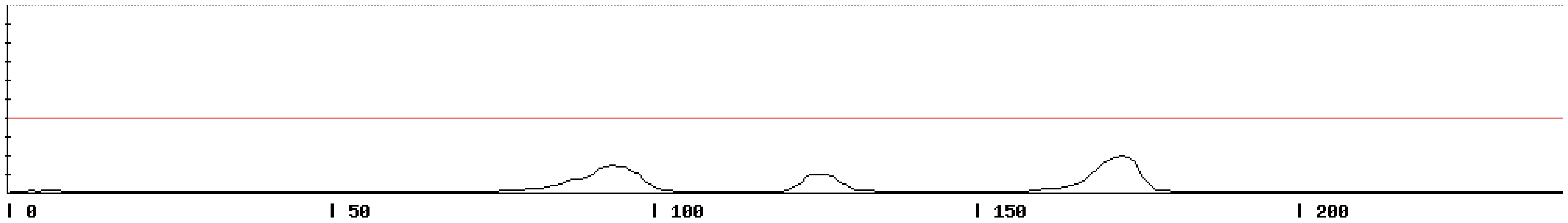

Predictions for Cucumissativus_Cucsa.158180.1

Viterbi Path

Viterbi Path

Posterior @ 0.4

119 - RGKKLVGMK - 127

Posterior @ 0.4

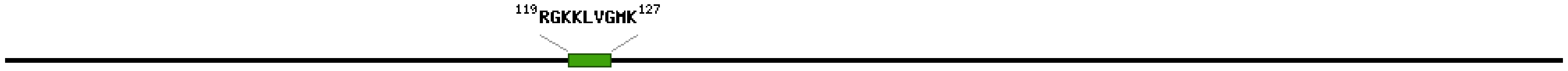

Posterior Graphic. Threshold Marked @ 0.4

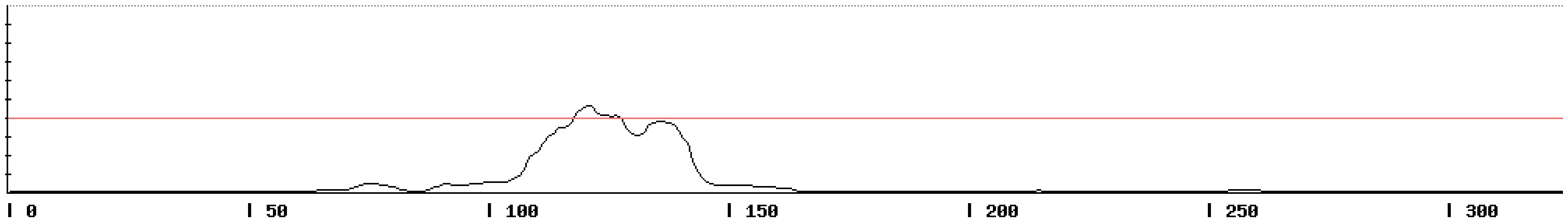

Predictions for Cucumissativus_Cucsa.161510.1

Viterbi Path

Viterbi Path

Posterior @ 0.4

Posterior @ 0.4

Posterior Graphic. Threshold Marked @ 0.4

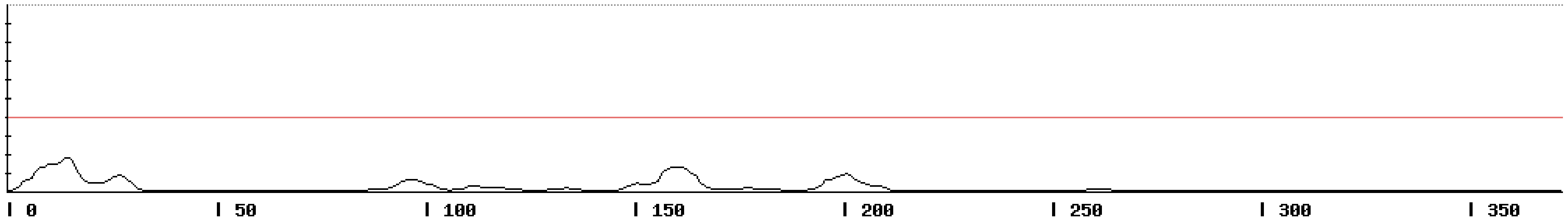

Predictions for Cucumissativus_Cucsa.163680.1

Viterbi Path

Viterbi Path

Posterior @ 0.4

Posterior @ 0.4

Posterior Graphic. Threshold Marked @ 0.4

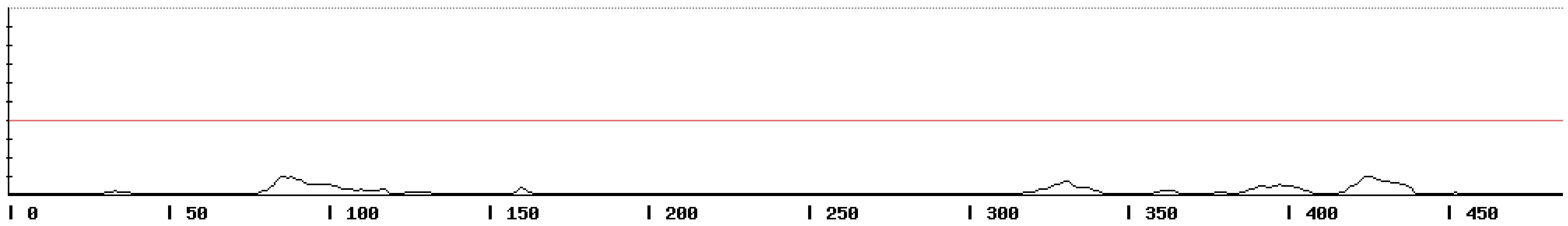

Predictions for Cucumissativus_Cucsa.171660.1

Viterbi Path

Viterbi Path

Posterior @ 0.4

Posterior @ 0.4

Posterior Graphic. Threshold Marked @ 0.4

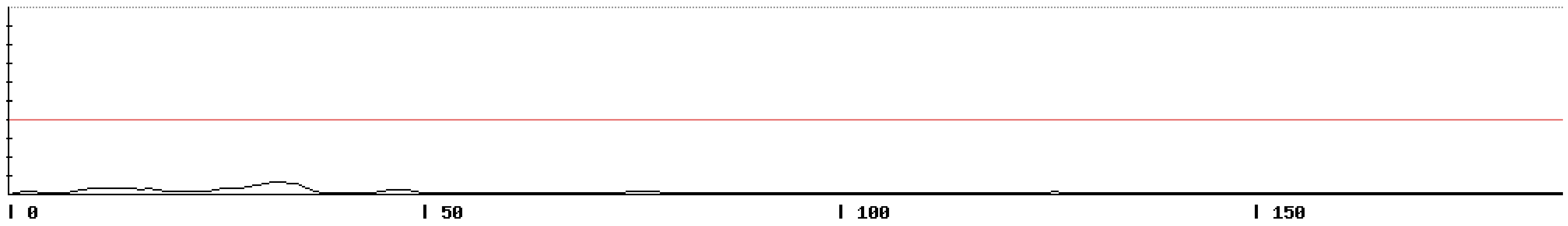

Predictions for Cucumissativus_Cucsa.173670.1

Viterbi Path

Viterbi Path

Posterior @ 0.4	623 - KK - 624
Posterior @ 0.4	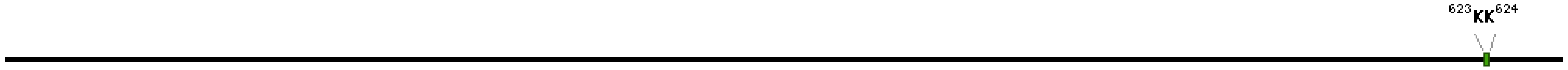
Posterior Graphic. Threshold Marked @ 0.4	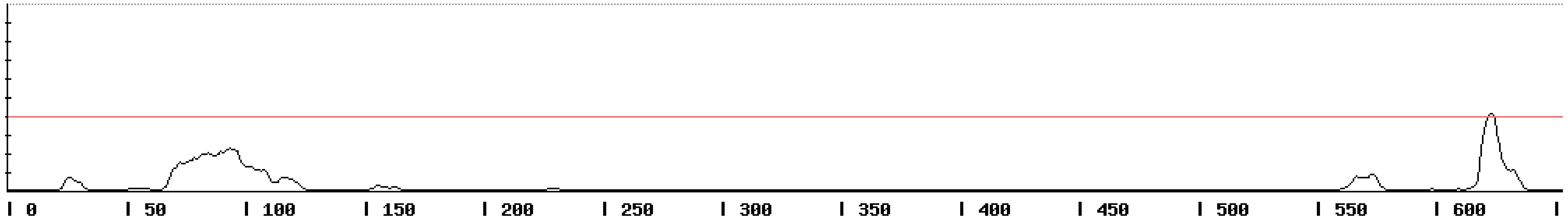

Predictions for Cucumissativus_Cucsa.175050.1

Viterbi Path	
Viterbi Path	
Posterior @ 0.4	
Posterior @ 0.4	
Posterior Graphic. Threshold Marked @ 0.4	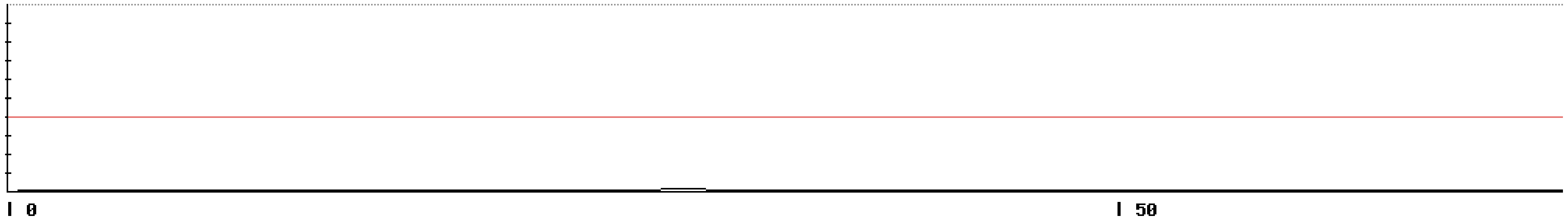

Predictions for Cucumissativus_Cucsa.175670.1

Viterbi Path	
Viterbi Path	
Posterior @ 0.4	73 - K - 73
Posterior @ 0.4	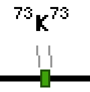
Posterior Graphic. Threshold Marked @ 0.4	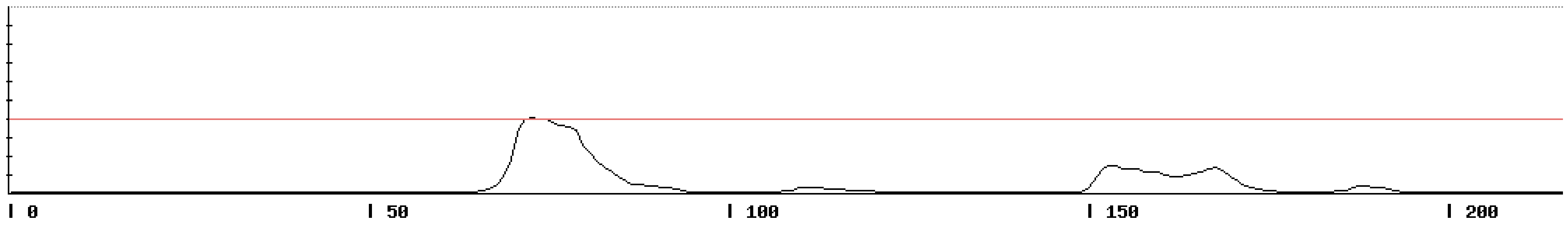

Predictions for Cucumissativus_Cucsa.185760.1

Viterbi Path	
Viterbi Path	
Posterior @ 0.4	
Posterior @ 0.4	
Posterior Graphic. Threshold Marked @ 0.4	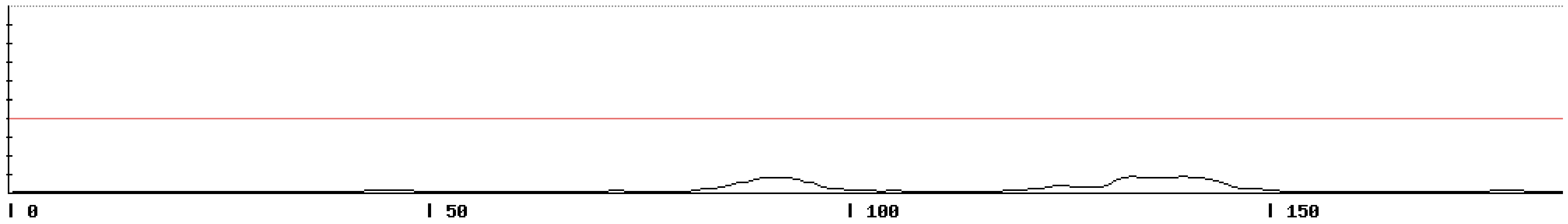

Predictions for Cucumissativus_Cucsa.185800.1

Viterbi Path

Viterbi Path

Posterior @ 0.4

Posterior @ 0.4

Posterior Graphic. Threshold Marked @ 0.4

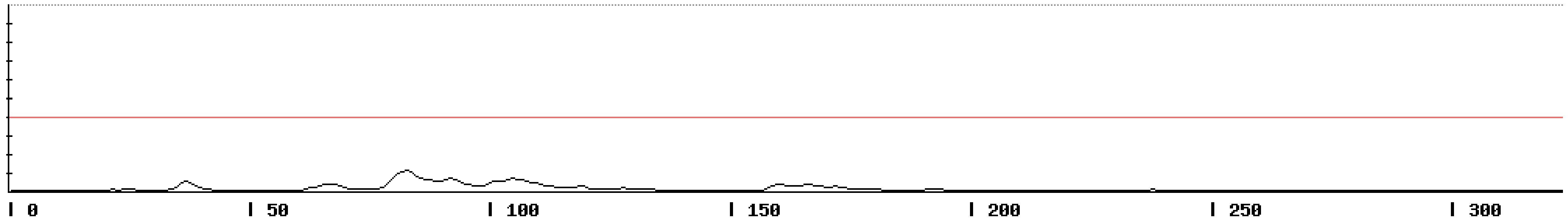

Predictions for Cucumissativus_Cucsa.186150.1

Viterbi Path

Viterbi Path

Posterior @ 0.4

Posterior @ 0.4

Posterior Graphic. Threshold Marked @ 0.4

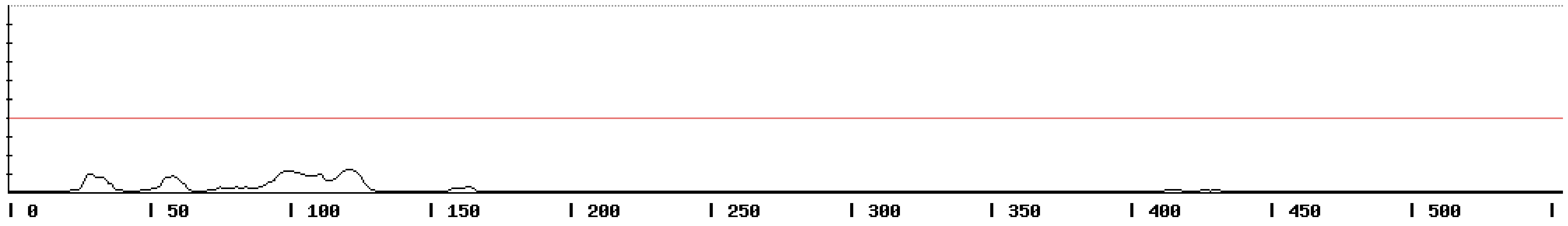

Predictions for Cucumissativus_Cucsa.193460.1	
Viterbi Path	
Viterbi Path	
Posterior @ 0.4	
Posterior @ 0.4	
Posterior Graphic. Threshold Marked @ 0.4	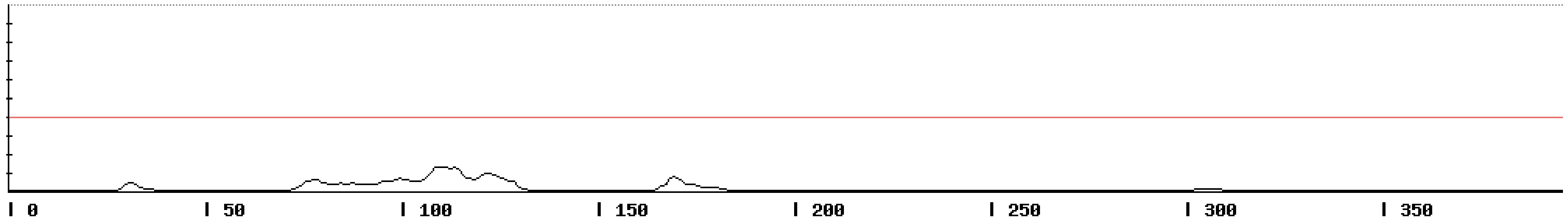

Predictions for Cucumissativus_Cucsa.197830.1	
Viterbi Path	
Viterbi Path	
Posterior @ 0.4	
Posterior @ 0.4	

Posterior Graphic. Threshold Marked @ 0.4	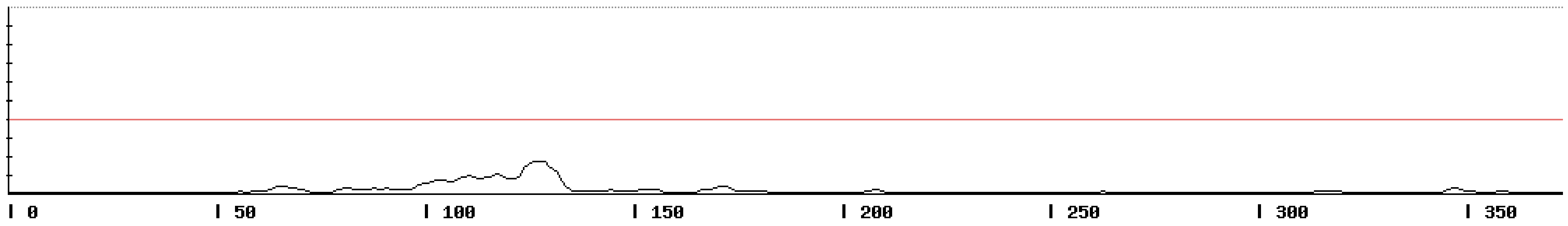

Predictions for Cucumissativus_Cucsa.199090.1

Viterbi Path	
Viterbi Path	
Posterior @ 0.4	
Posterior @ 0.4	
Posterior Graphic. Threshold Marked @ 0.4	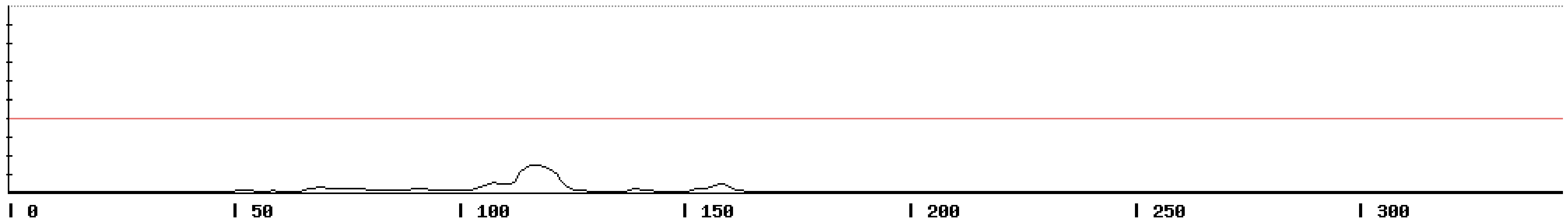

Predictions for Cucumissativus_Cucsa.228100.1

Viterbi Path	
Viterbi Path	
Posterior @ 0.4	
Posterior @ 0.4	

Posterior Graphic. Threshold Marked @ 0.4

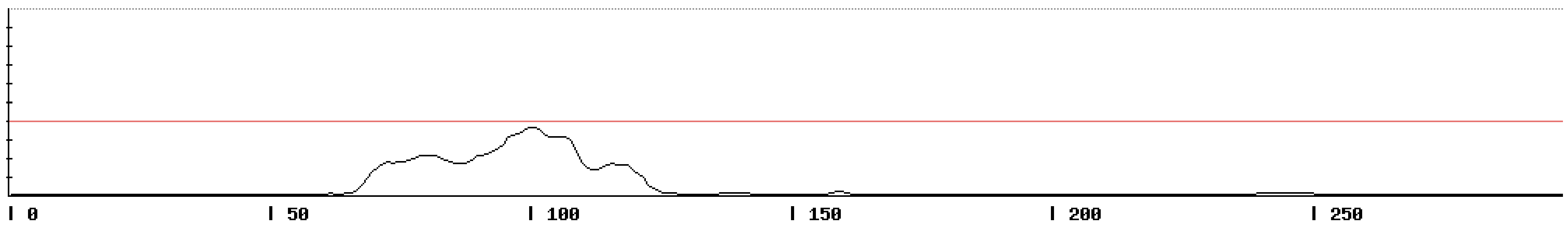

Predictions for Cucumissativus_Cucsa.228480.1

Viterbi Path

Viterbi Path

Posterior @ 0.4

Posterior @ 0.4

Posterior Graphic. Threshold Marked @ 0.4

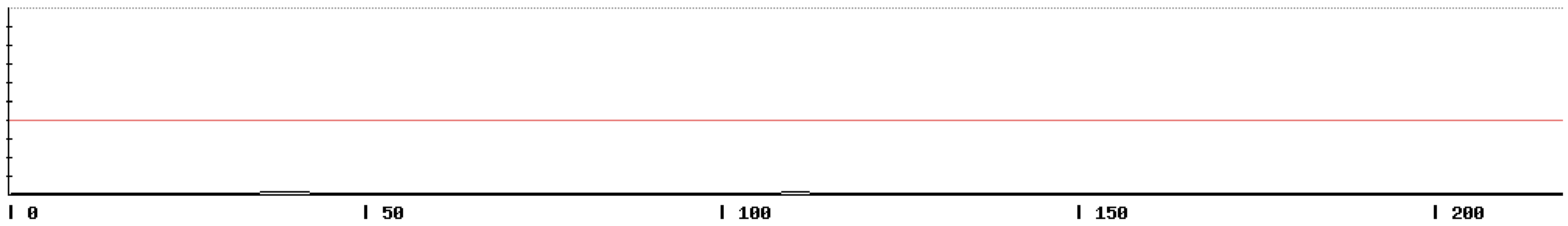

Predictions for Cucumissativus_Cucsa.232360.1

Viterbi Path 370 - GGKKKKKKAVGRMKKIQKK - 388

Viterbi Path

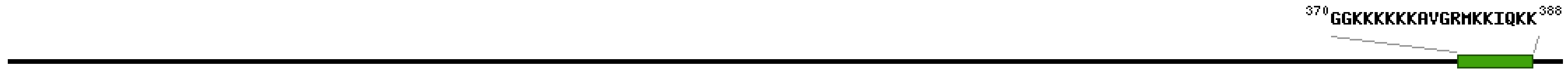

Posterior @ 0.4	370 - GGKKKKKKAVGRMKKIQKK - 388
Posterior @ 0.4	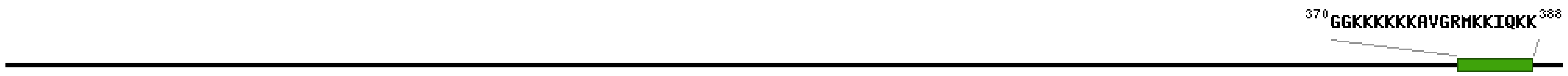
Posterior Graphic. Threshold Marked @ 0.4	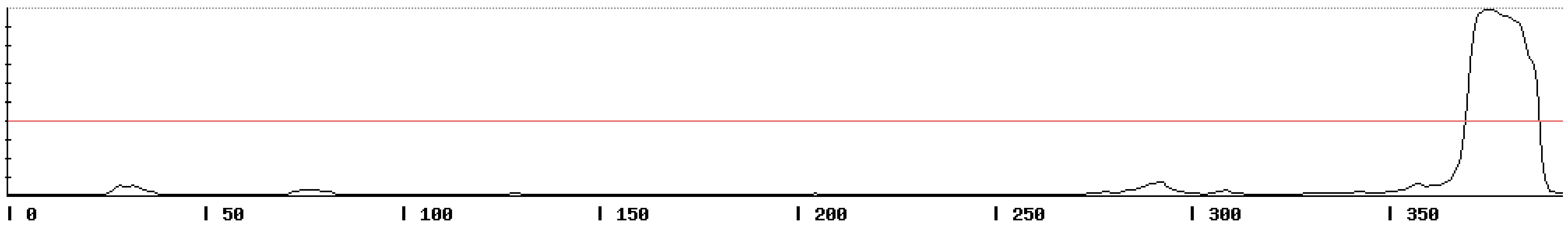

Predictions for Cucumissativus_Cucsa.236170.1

Viterbi Path	
Viterbi Path	
Posterior @ 0.4	
Posterior @ 0.4	
Posterior Graphic. Threshold Marked @ 0.4	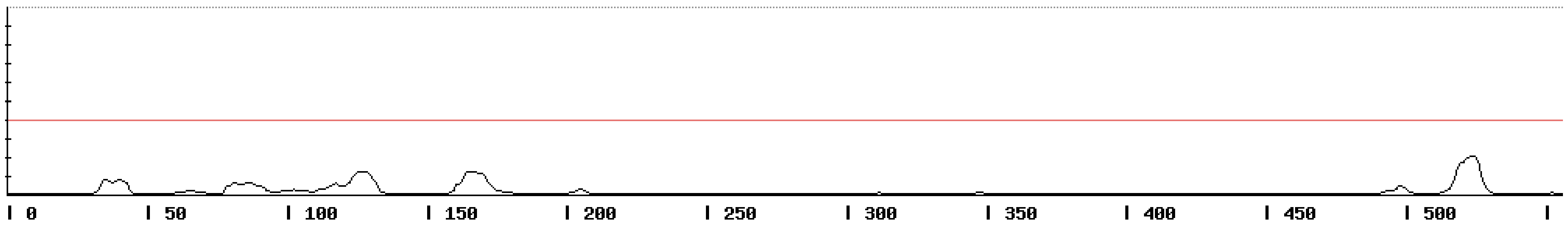

Predictions for Cucumissativus_Cucsa.237820.1

Viterbi Path	
Viterbi Path	

Posterior @ 0.4

Posterior @ 0.4

Posterior Graphic. Threshold Marked @ 0.4

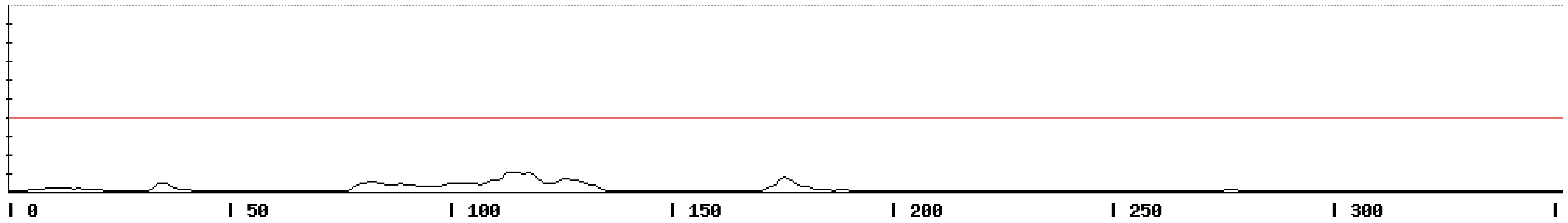

Predictions for Cucumissativus_Cucsa.239650.1

Viterbi Path

Viterbi Path

Posterior @ 0.4

Posterior @ 0.4

Posterior Graphic. Threshold Marked @ 0.4

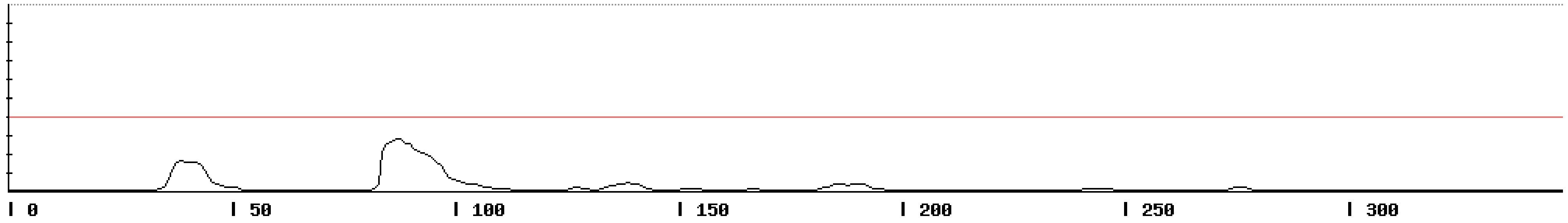

Predictions for Cucumissativus_Cucsa.240210.1

Viterbi Path

Viterbi Path

Posterior @ 0.4

Posterior @ 0.4

Posterior Graphic. Threshold Marked @ 0.4

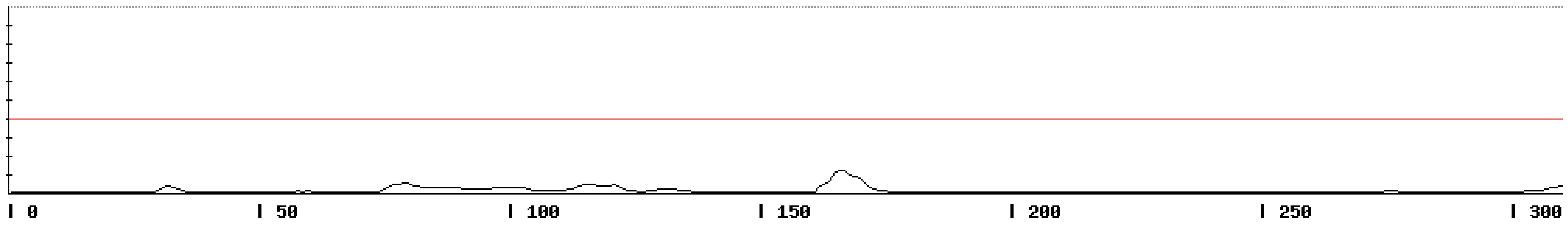

Predictions for Cucumissativus_Cucsa.240650.1

Viterbi Path

155 - KKSKEKKHKNIKKLKKK - 171

Viterbi Path

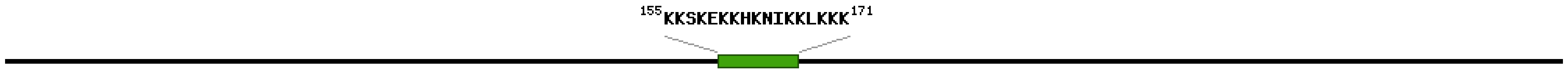

Posterior @ 0.4

154 - IKKSKEKKHKNIKKLKKKI - 172

Posterior @ 0.4

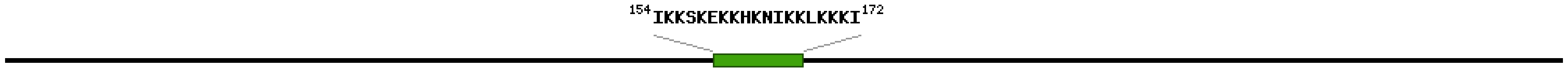

Posterior Graphic. Threshold Marked @ 0.4

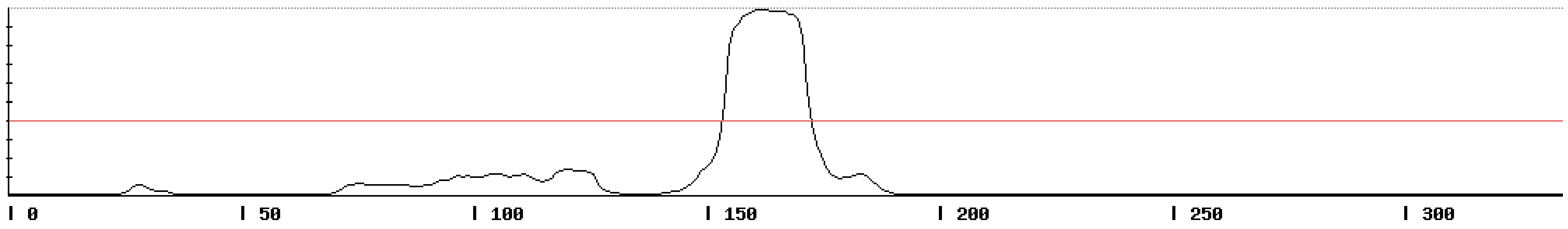

Predictions for Cucumissativus_Cucsa.249390.1

Viterbi Path	
Viterbi Path	
Posterior @ 0.4	63 - KKKKSRIVRKV - 73
Posterior @ 0.4	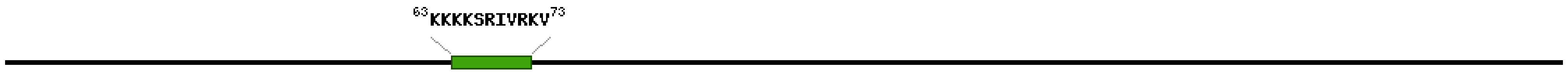
Posterior Graphic. Threshold Marked @ 0.4	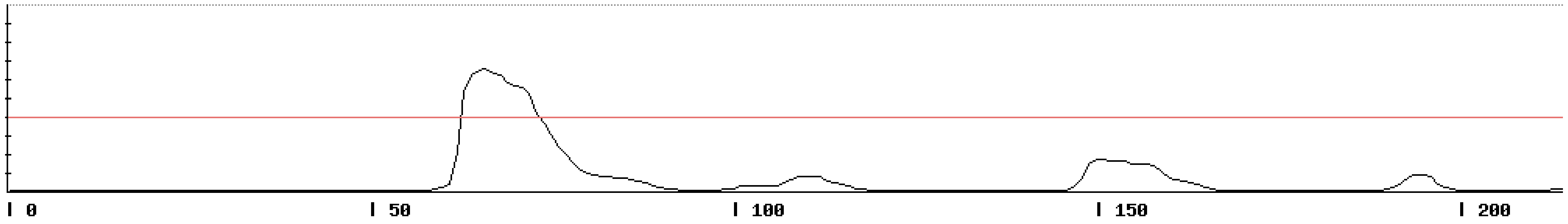

Predictions for Cucumissativus_Cucsa.251620.1

Viterbi Path	
Viterbi Path	
Posterior @ 0.4	
Posterior @ 0.4	
Posterior Graphic. Threshold Marked @ 0.4	

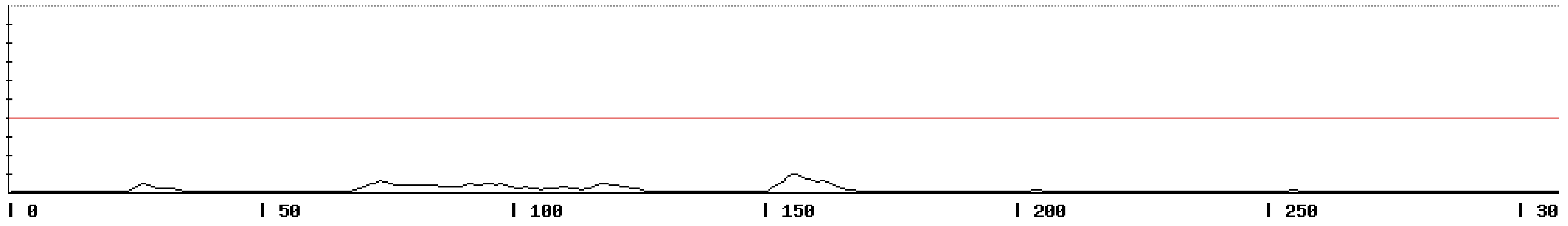

Predictions for Cucumissativus_Cucsa.260400.1

Viterbi Path

Viterbi Path

Posterior @ 0.4

Posterior @ 0.4

Posterior Graphic. Threshold Marked @ 0.4

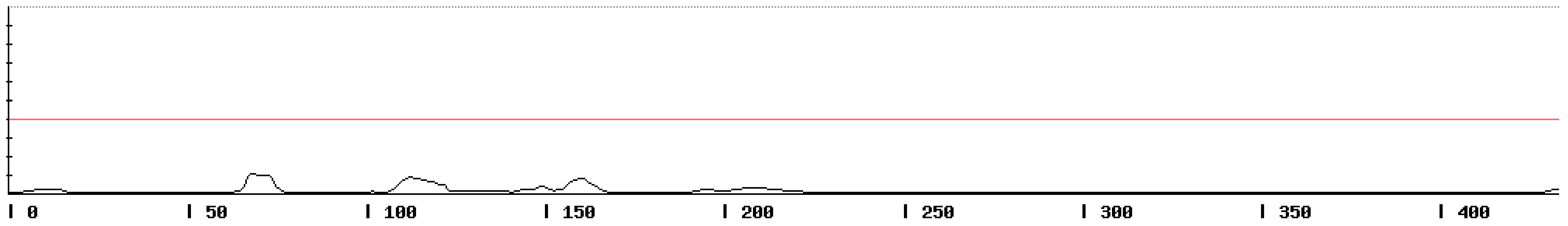

Predictions for Cucumissativus_Cucsa.265620.1

Viterbi Path

Viterbi Path

Posterior @ 0.4

Posterior @ 0.4

Posterior Graphic. Threshold Marked @ 0.4

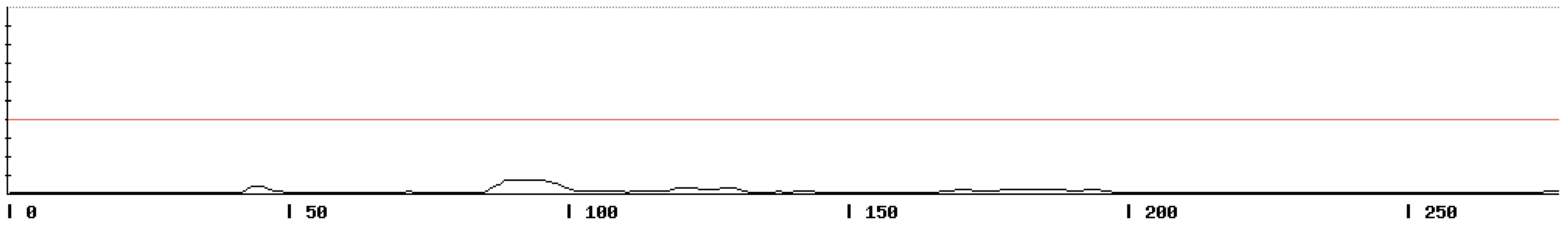

Predictions for Cucumissativus_Cucsa.272370.1

Viterbi Path

Viterbi Path

Posterior @ 0.4

Posterior @ 0.4

Posterior Graphic. Threshold Marked @ 0.4

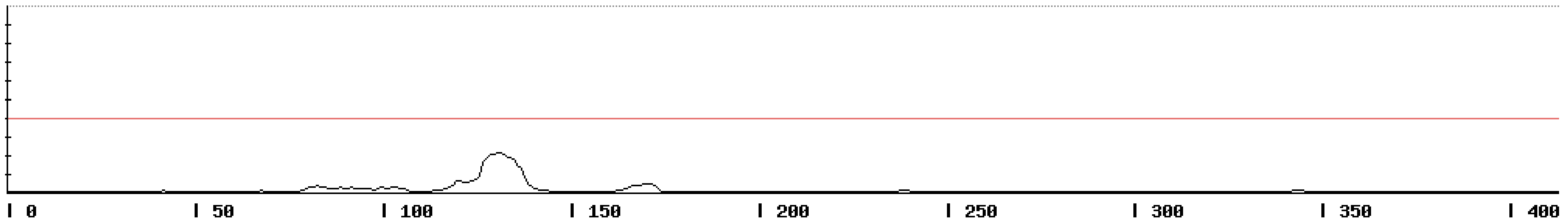

Predictions for Cucumissativus_Cucsa.273280.1

Viterbi Path

Viterbi Path

Posterior @ 0.4

84 - KRA - 86

90 - GYWKATGKDRKIKSGTKLIGMKKTLVFYKGRAPKGKR - 126

Posterior @ 0.4

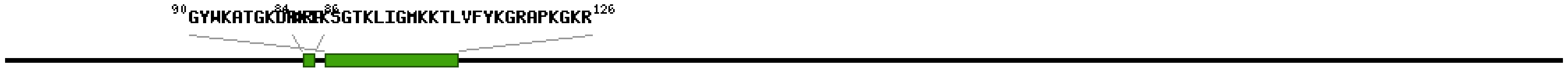

Posterior Graphic. Threshold Marked @ 0.4

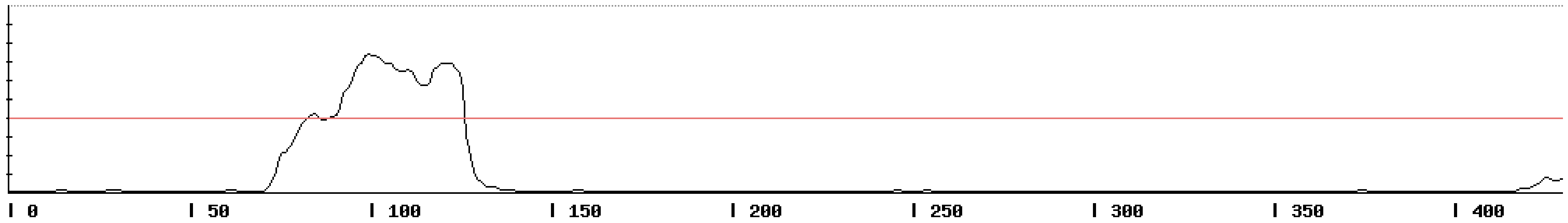

Predictions for Cucumissativus_Cucsa.283900.1

Viterbi Path

Viterbi Path

Posterior @ 0.4

Posterior @ 0.4

Posterior Graphic. Threshold Marked @ 0.4

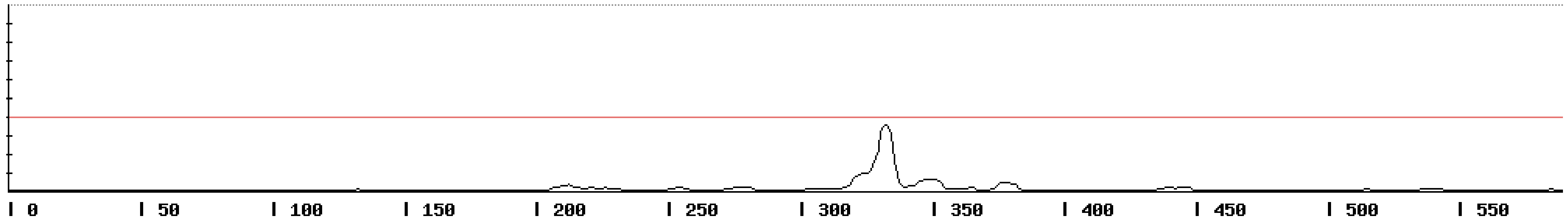

Predictions for Cucumissativus_Cucsa.284890.1

Viterbi Path

Viterbi Path

Posterior @ 0.4

Posterior @ 0.4

Posterior Graphic. Threshold Marked @ 0.4

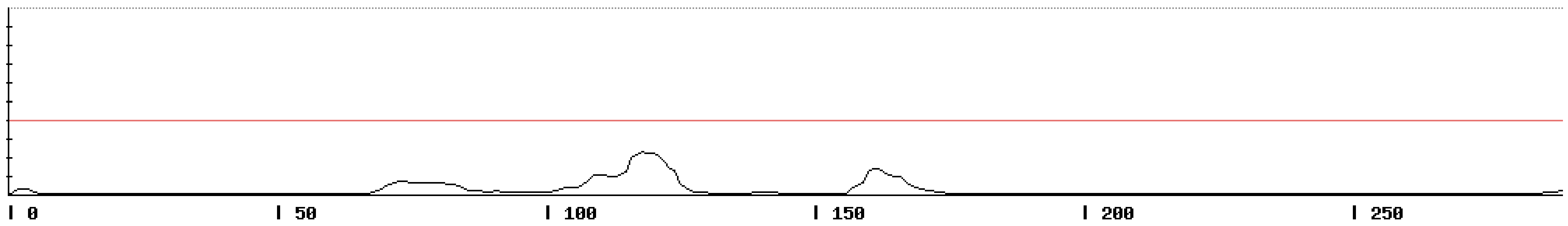

Predictions for Cucumissativus_Cucsa.285120.1

Viterbi Path

Viterbi Path

Posterior @ 0.4

Posterior @ 0.4

Posterior Graphic. Threshold Marked @ 0.4

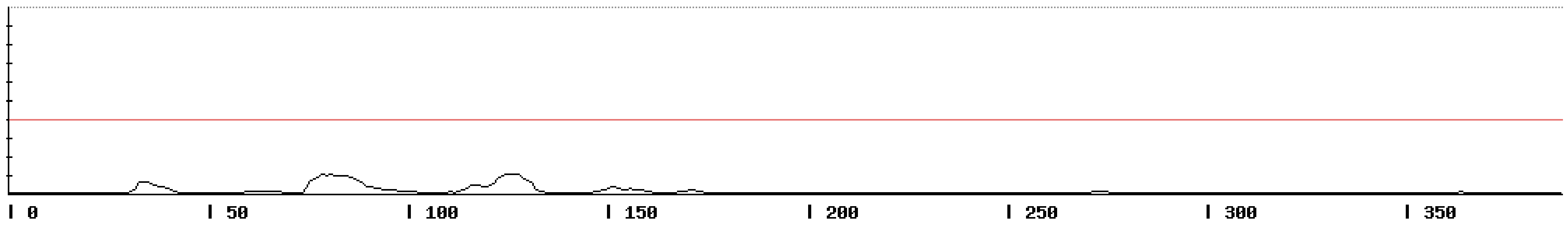

Predictions for Cucumissativus_Cucsa.288680.1

Viterbi Path

Viterbi Path

Posterior @ 0.4

Posterior @ 0.4

Posterior Graphic. Threshold Marked @ 0.4

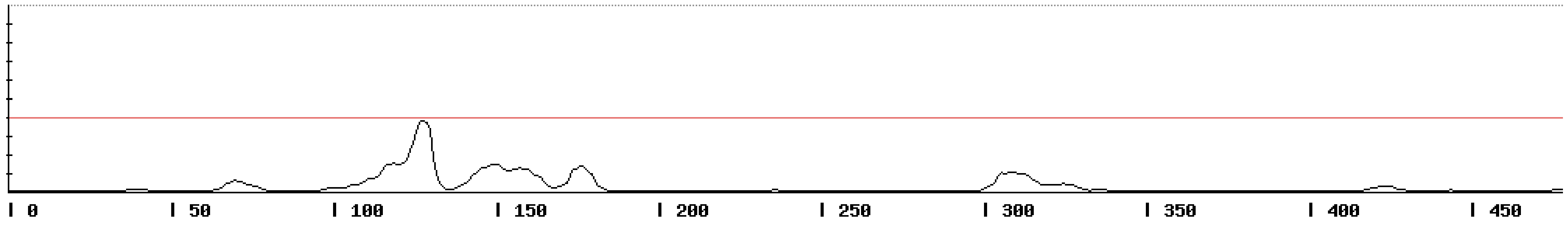

Predictions for Cucumissativus_Cucsa.302120.1

Viterbi Path

Viterbi Path

Posterior @ 0.4

Posterior @ 0.4

Posterior Graphic. Threshold Marked @ 0.4

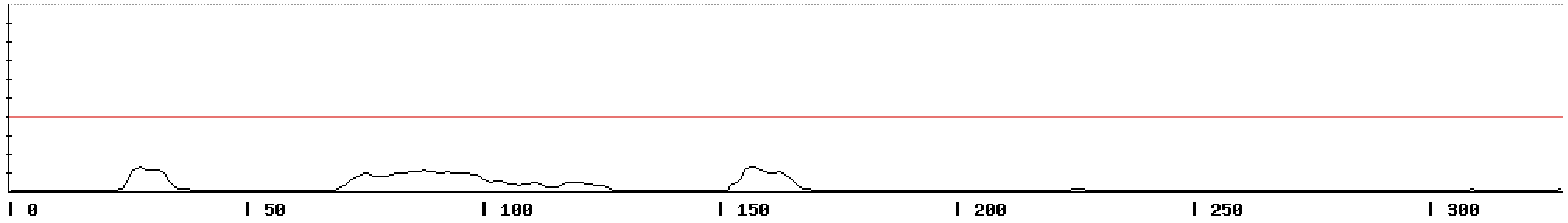

Predictions for Cucumissativus_Cucsa.308760.1

Viterbi Path

Viterbi Path

Posterior @ 0.4

Posterior @ 0.4

Posterior Graphic. Threshold Marked @ 0.4

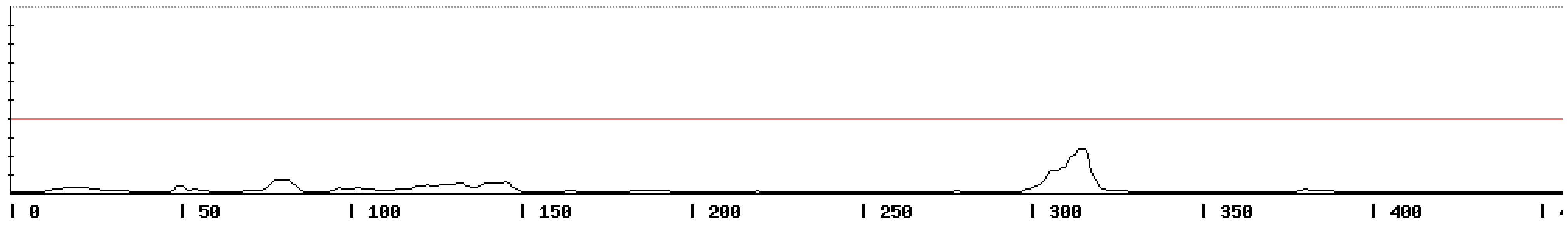

Predictions for Cucumissativus_Cucsa.308770.1

Viterbi Path

Viterbi Path

Posterior @ 0.4

Posterior @ 0.4

Posterior Graphic. Threshold Marked @ 0.4

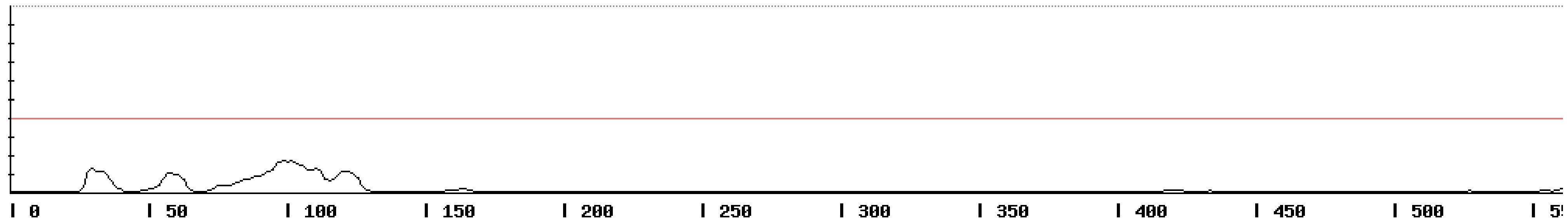

Predictions for Cucumissativus_Cucsa.313350.1

Viterbi Path

Viterbi Path

Posterior @ 0.4: 162 - KKK - 164

Posterior @ 0.4

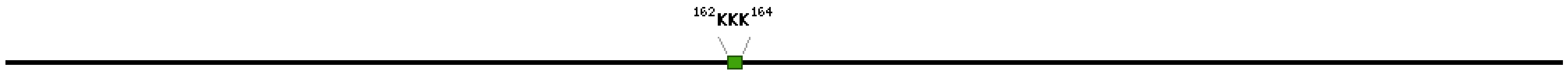

Posterior Graphic. Threshold Marked @ 0.4

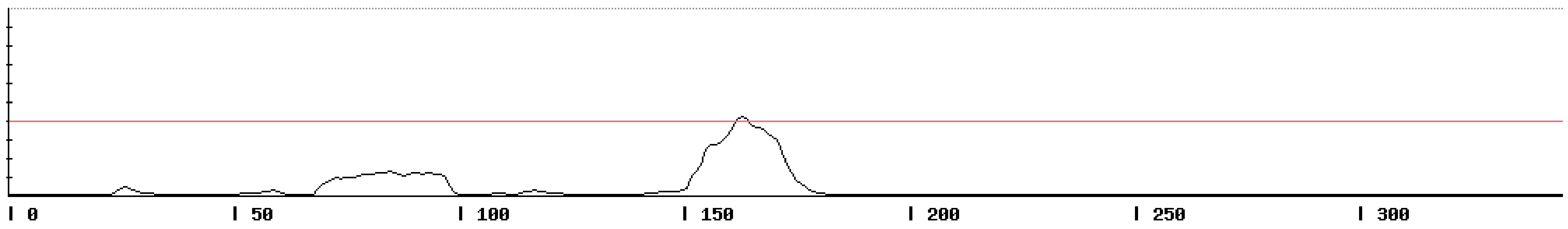

Predictions for Cucumissativus_Cucsa.319680.1

Viterbi Path

Viterbi Path

Posterior @ 0.4

Posterior @ 0.4

Posterior Graphic. Threshold Marked @ 0.4

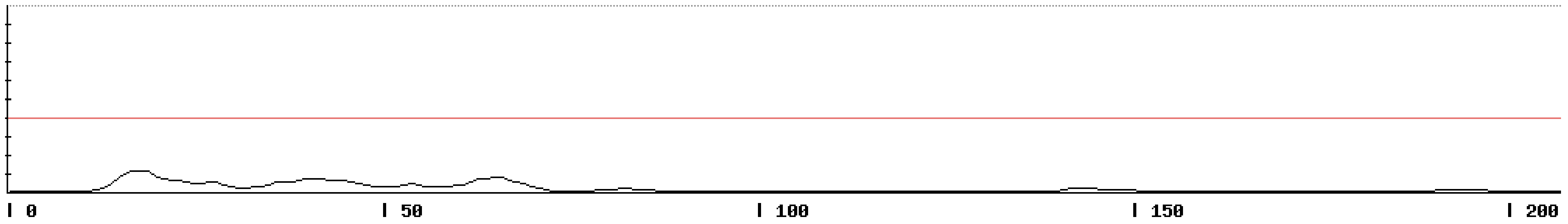

Predictions for Cucumissativus_Cucsa.319840.1

Viterbi Path	
Viterbi Path	
Posterior @ 0.4	184 - RKNADSKRKGK - 194
Posterior @ 0.4	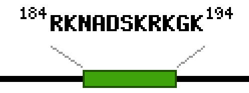
Posterior Graphic. Threshold Marked @ 0.4	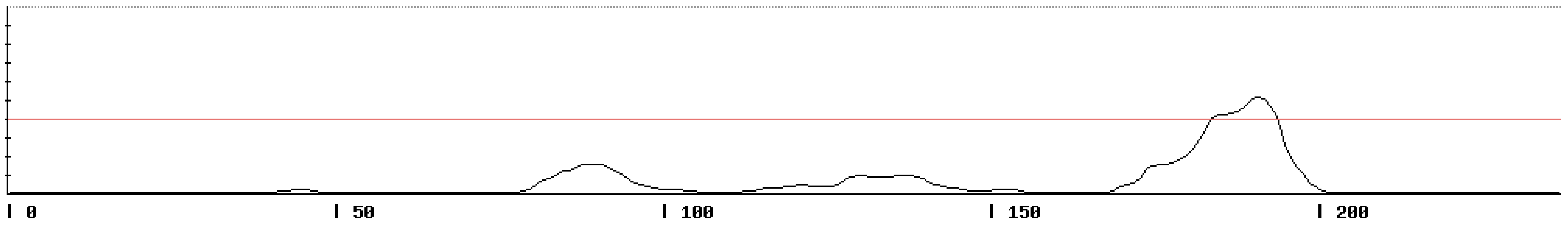

Predictions for Cucumissativus_Cucsa.320590.1

Viterbi Path	
Viterbi Path	
Posterior @ 0.4	
Posterior @ 0.4	

Posterior Graphic. Threshold Marked @ 0.4

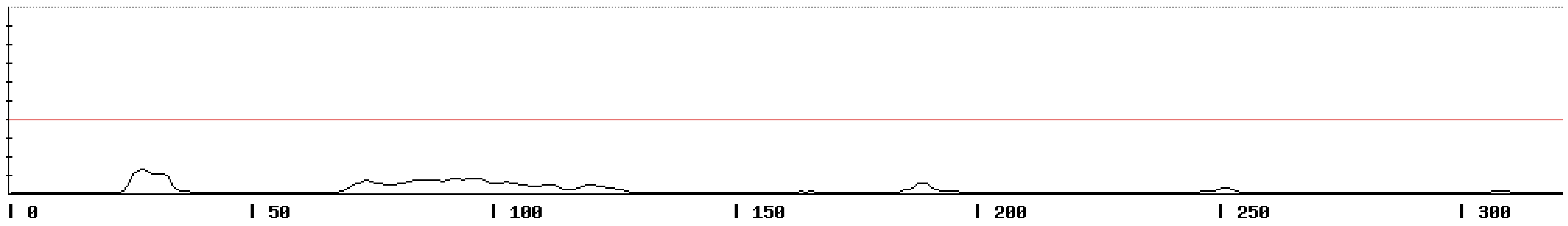

Predictions for Cucumissativus_Cucsa.338700.1

Viterbi Path

Viterbi Path

Posterior @ 0.4

Posterior @ 0.4

Posterior Graphic. Threshold Marked @ 0.4

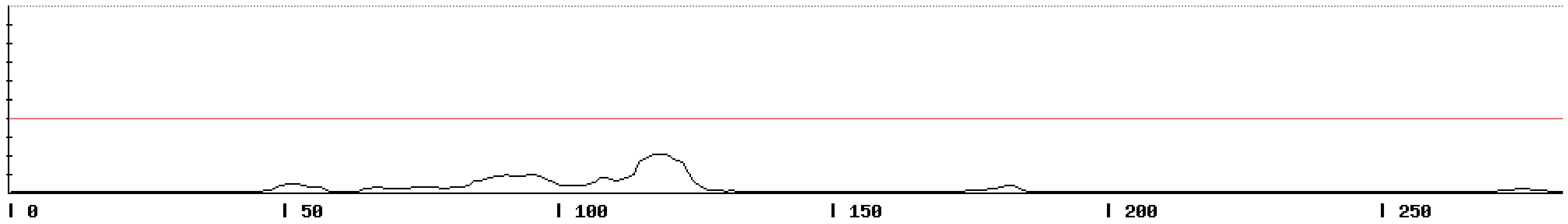

Predictions for Cucumissativus_Cucsa.341790.1

Viterbi Path

Viterbi Path

Posterior @ 0.4

Posterior @ 0.4

Posterior Graphic. Threshold Marked @ 0.4

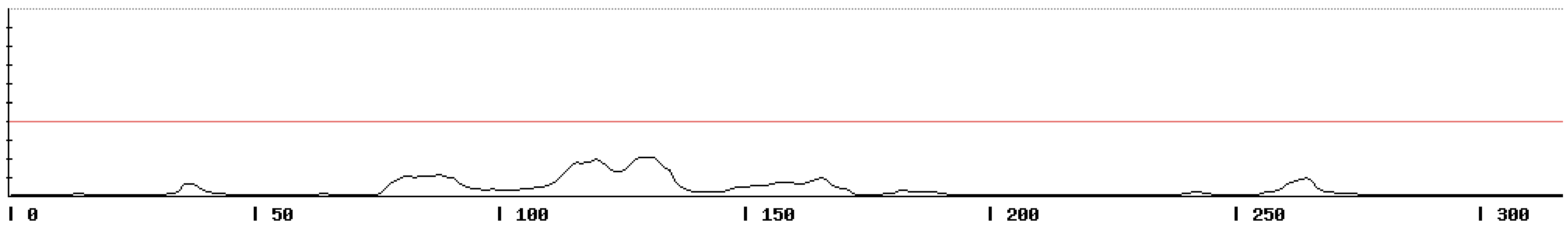

Predictions for Cucumissativus_Cucsa.341800.1

Viterbi Path

Viterbi Path

Posterior @ 0.4

Posterior @ 0.4

Posterior Graphic. Threshold Marked @ 0.4

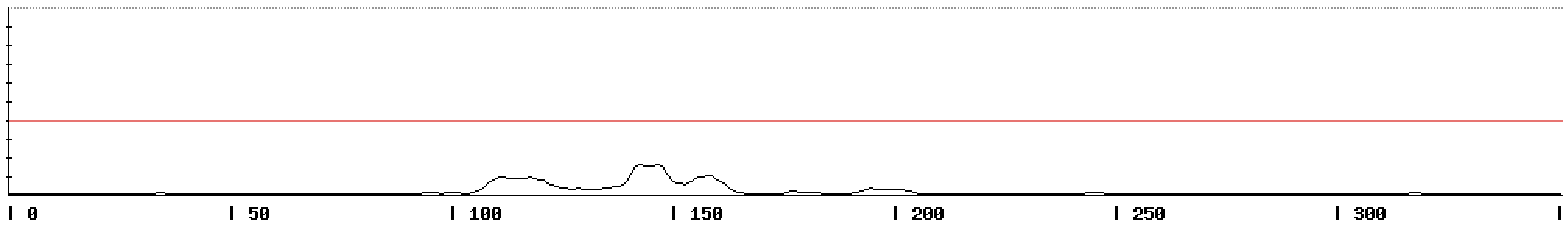

Predictions for Cucumissativus_Cucsa.345830.1

Viterbi Path

Viterbi Path

Posterior @ 0.4	146 - RRRGRPKR - 153
Posterior @ 0.4	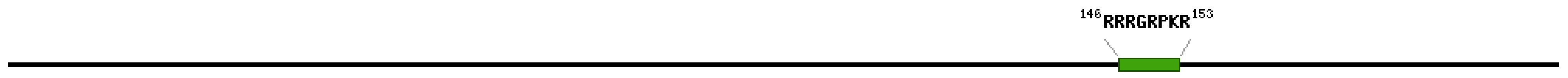
Posterior Graphic. Threshold Marked @ 0.4	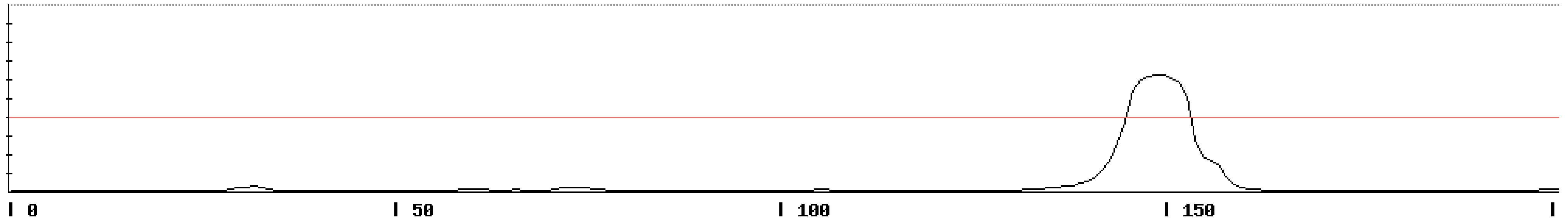

Predictions for Cucumissativus_Cucsa.349370.1

Viterbi Path	
Viterbi Path	
Posterior @ 0.4	
Posterior @ 0.4	
Posterior Graphic. Threshold Marked @ 0.4	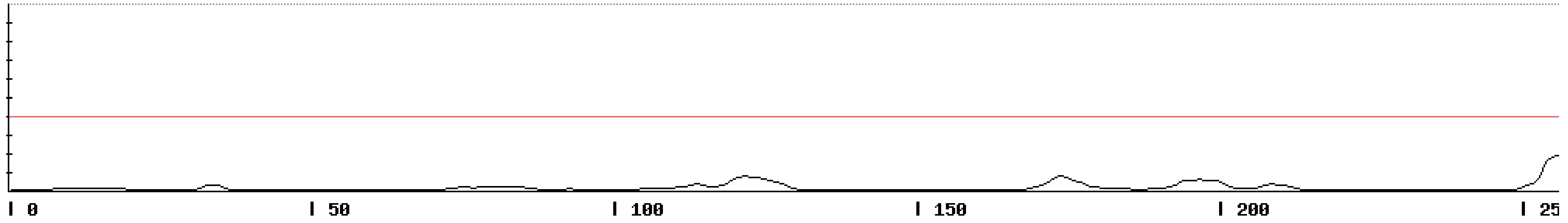

Predictions for Cucumissativus_Cucsa.352340.1

Viterbi Path	
Viterbi Path	

Posterior @ 0.4	108 - WRRKGKVKEVTGR - 120
Posterior @ 0.4	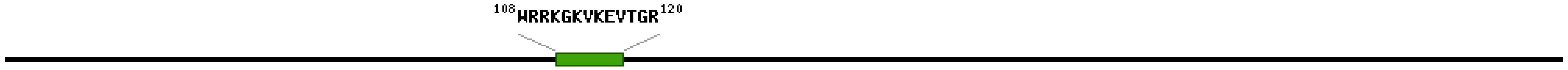
Posterior Graphic. Threshold Marked @ 0.4	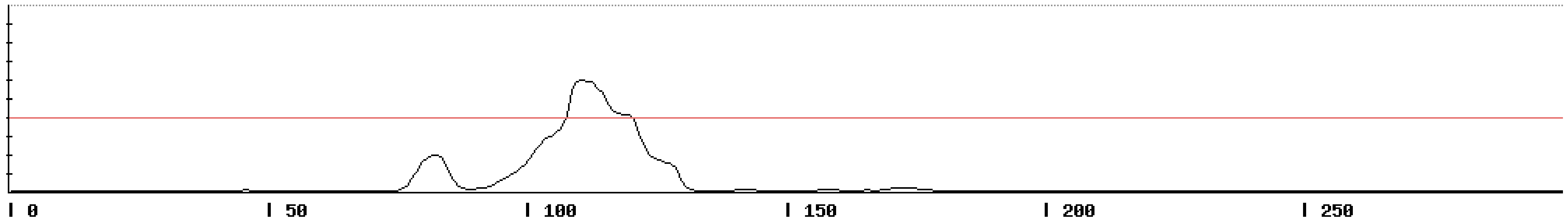

Predictions for Cucumissativus_Cucsa.364130.1

Viterbi Path	
Viterbi Path	
Posterior @ 0.4	
Posterior @ 0.4	
Posterior Graphic. Threshold Marked @ 0.4	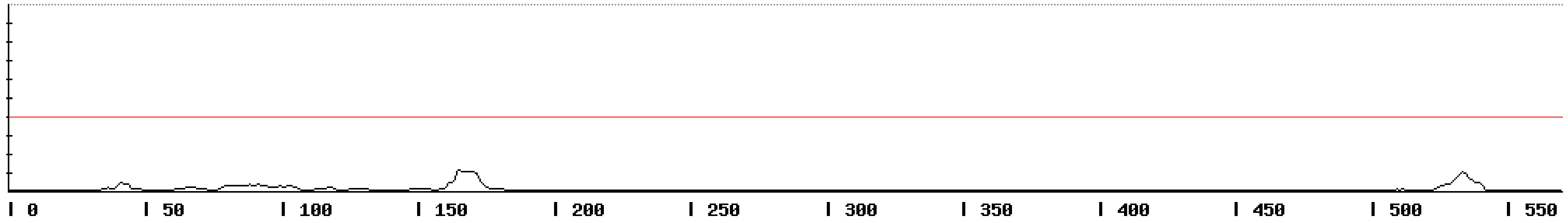

Predictions for Cucumissativus_Cucsa.370520.1

Viterbi Path	
Viterbi Path	
Posterior @ 0.4	63 - KKKKSRVVRK - 72
Posterior @ 0.4	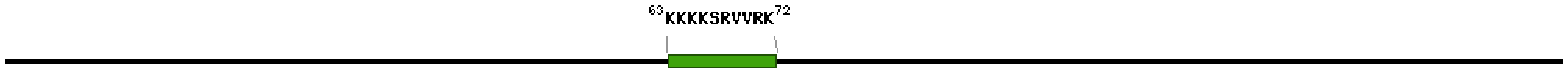
Posterior Graphic. Threshold Marked @ 0.4	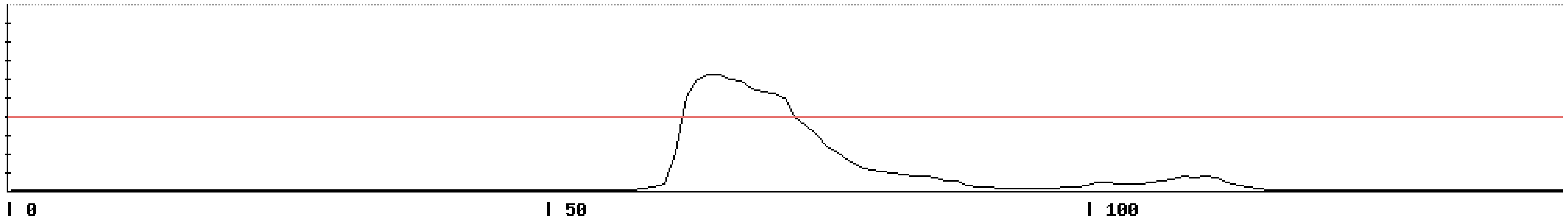

Revision r.9 - Last Updated on : February 24 2015 17:45:20.

alex.nguyenba@utoronto.ca

Intended to be used with Firefox

Viterbi Path	
Posterior @ 0.4	
Posterior @ 0.4	
Posterior Graphic. Threshold Marked @ 0.4	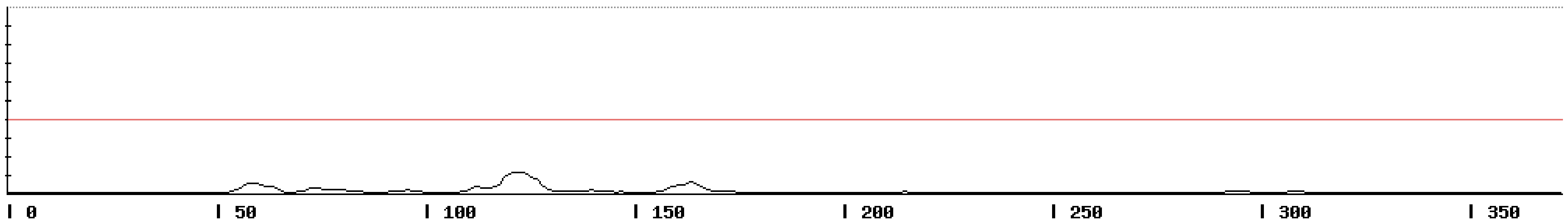

Predictions for Daucuscarota_DCAR_000935

Viterbi Path	
Viterbi Path	
Posterior @ 0.4	
Posterior @ 0.4	
Posterior Graphic. Threshold Marked @ 0.4	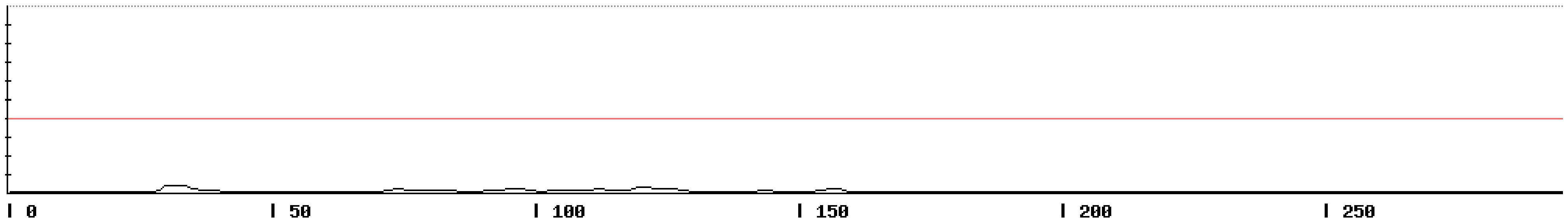

Predictions for Daucuscarota_DCAR_001346

Viterbi Path

Viterbi Path

Posterior @ 0.4

Posterior @ 0.4

Posterior Graphic. Threshold Marked @ 0.4

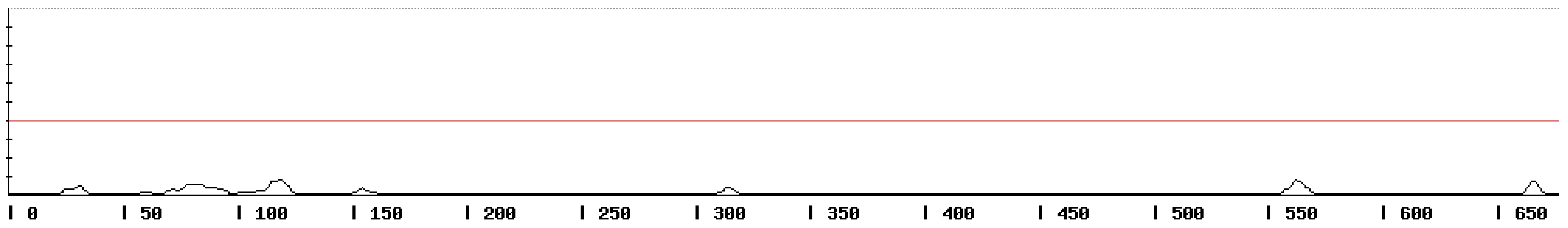

Predictions for Daucuscarota_DCAR_001479

Viterbi Path

Viterbi Path

Posterior @ 0.4

Posterior @ 0.4

Posterior Graphic. Threshold Marked @ 0.4

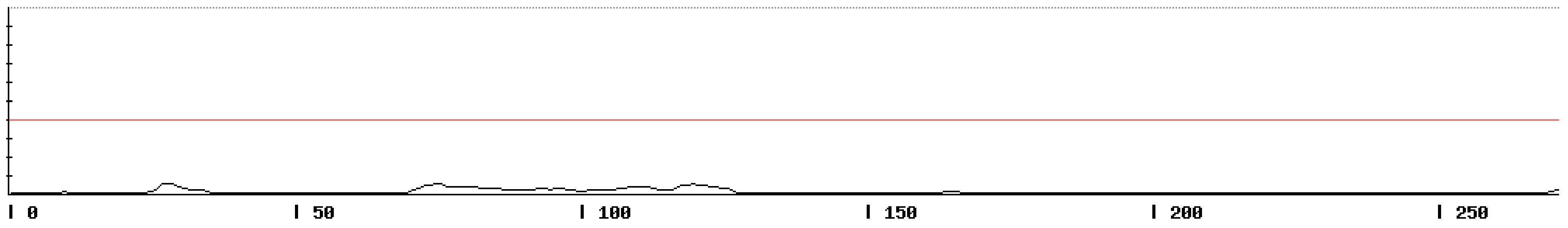

Predictions for Daucuscarota_DCAR_001804

Viterbi Path

Viterbi Path

Posterior @ 0.4

Posterior @ 0.4

Posterior Graphic. Threshold Marked @ 0.4

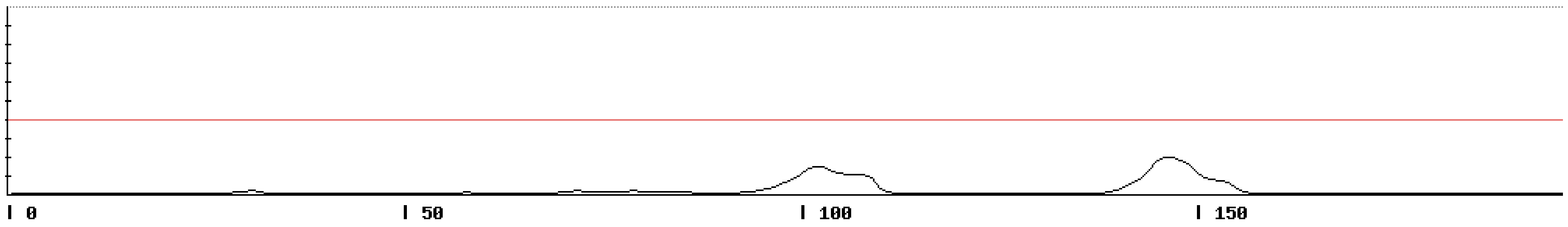

Predictions for Daucuscarota_DCAR_001871

Viterbi Path

Viterbi Path

Posterior @ 0.4

Posterior @ 0.4

Posterior Graphic. Threshold Marked @ 0.4

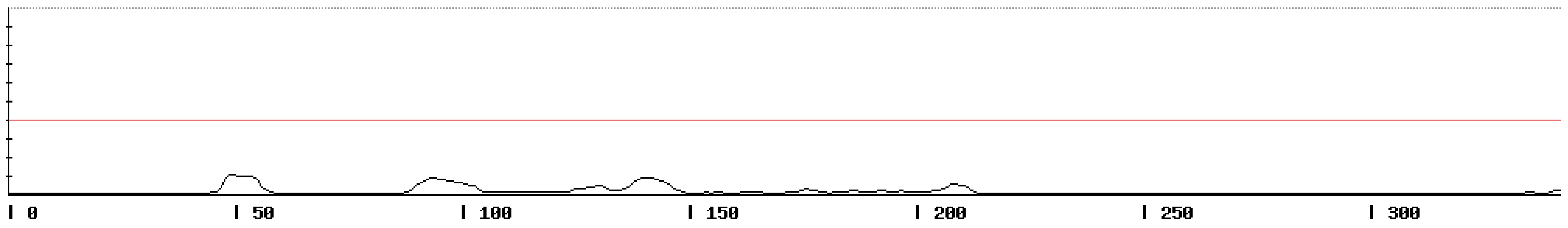

Predictions for Daucuscarota_DCAR_002069

Viterbi Path

Viterbi Path

Posterior @ 0.4

Posterior @ 0.4

Posterior Graphic. Threshold Marked @ 0.4

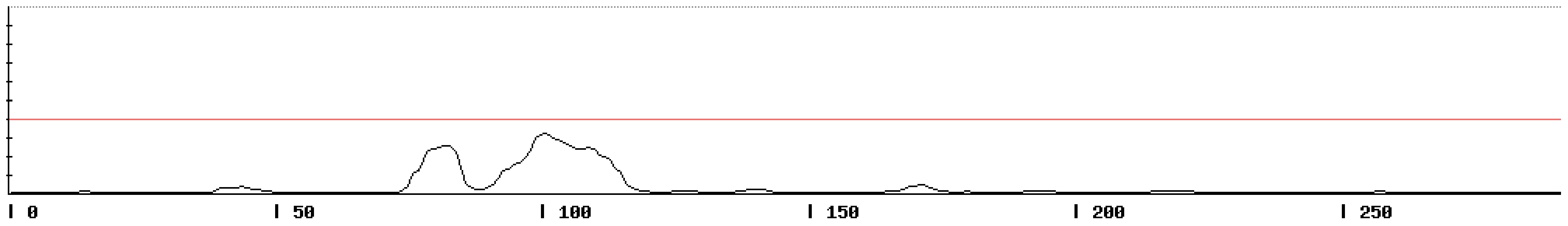

Predictions for Daucuscarota_DCAR_002079

Viterbi Path

Viterbi Path

Posterior @ 0.4

Posterior @ 0.4

Posterior Graphic. Threshold Marked @ 0.4

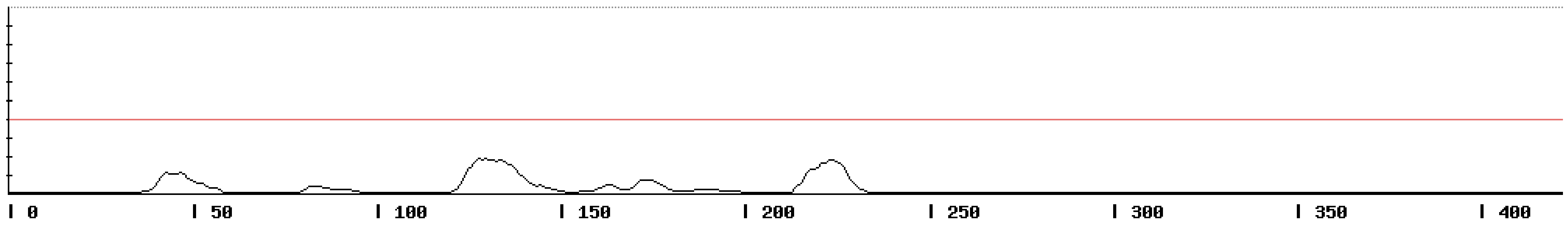

Predictions for Daucuscarota_DCAR_003260

Viterbi Path

Viterbi Path

Posterior @ 0.4

Posterior @ 0.4

Posterior Graphic. Threshold Marked @ 0.4

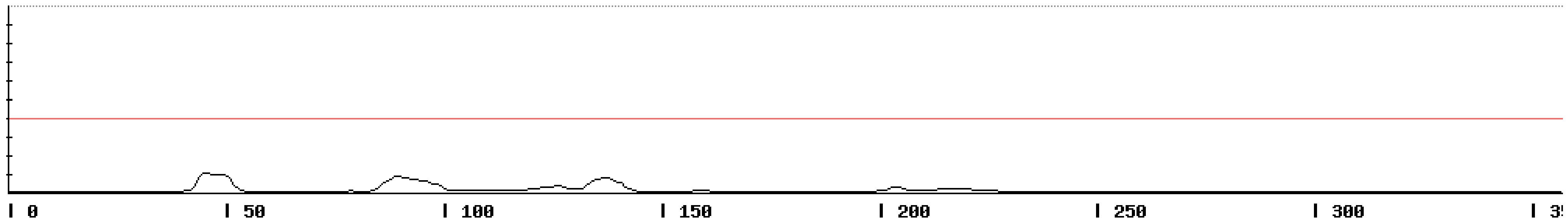

Predictions for Daucuscarota_DCAR_003409

Viterbi Path

Viterbi Path

Posterior @ 0.4

Posterior @ 0.4

Posterior Graphic. Threshold Marked @ 0.4

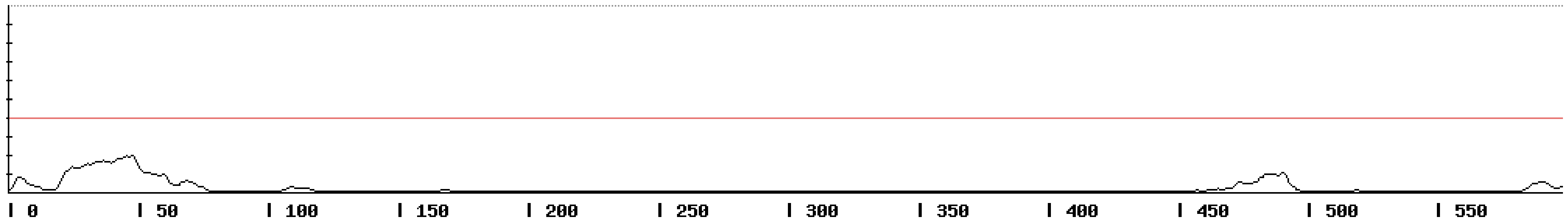

Predictions for Daucuscarota_DCAR_004918

Viterbi Path

Viterbi Path

Posterior @ 0.4

Posterior @ 0.4

Posterior Graphic. Threshold Marked @ 0.4

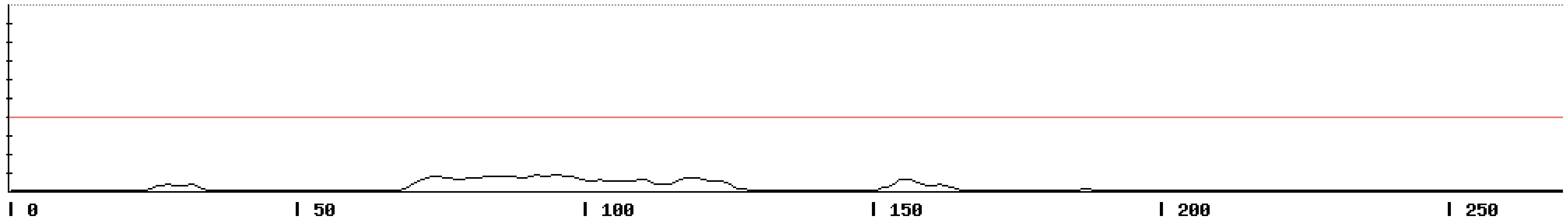

Predictions for Daucuscarota_DCAR_005363

Viterbi Path

Viterbi Path

Posterior @ 0.4

Posterior @ 0.4

Posterior Graphic. Threshold Marked @ 0.4

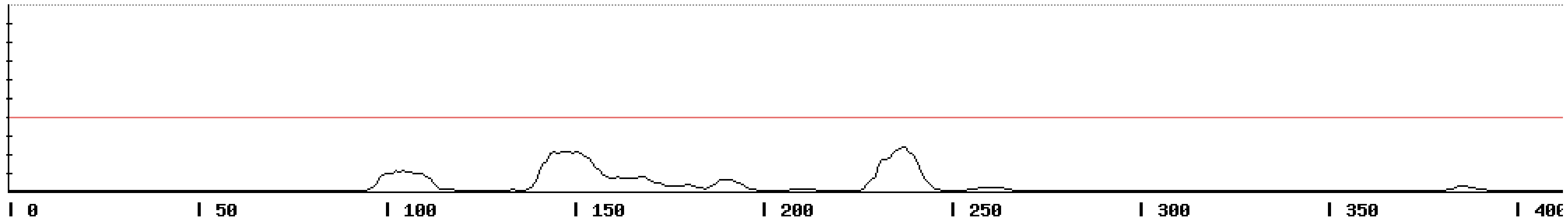

Predictions for Daucuscarota_DCAR_005684

Viterbi Path

Viterbi Path

Posterior @ 0.4

Posterior @ 0.4

Posterior Graphic. Threshold Marked @ 0.4

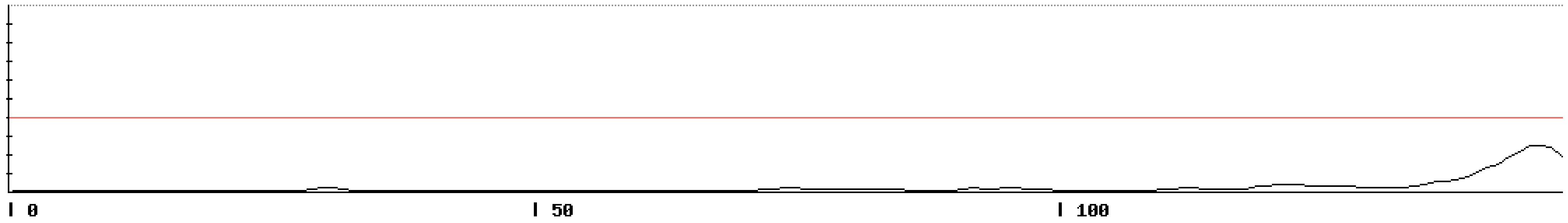

Predictions for Daucuscarota_DCAR_006054

Viterbi Path

Viterbi Path

Posterior @ 0.4

Posterior @ 0.4

Posterior Graphic. Threshold Marked @ 0.4

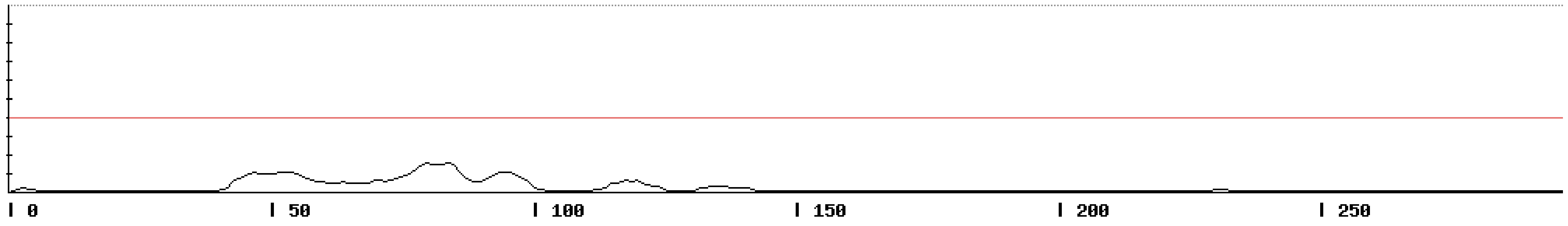

Predictions for Daucuscarota_DCAR_006174

Viterbi Path

Viterbi Path

Posterior @ 0.4

Posterior @ 0.4

Posterior Graphic. Threshold Marked @ 0.4

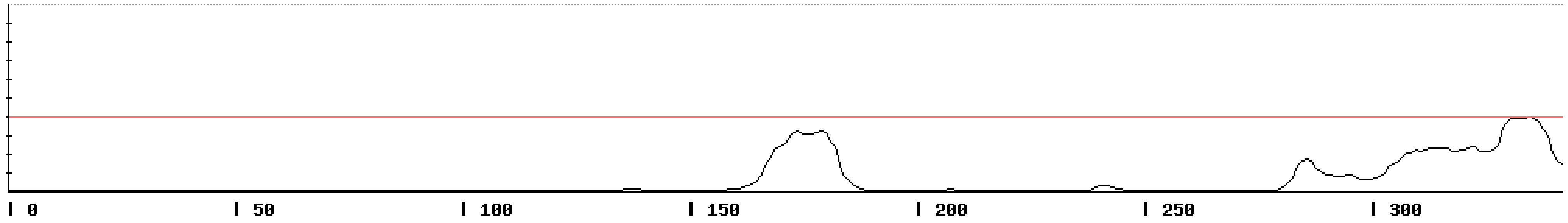

Predictions for Daucuscarota_DCAR_006295

Viterbi Path

Viterbi Path

Posterior @ 0.4

Posterior @ 0.4

Posterior Graphic. Threshold Marked @ 0.4

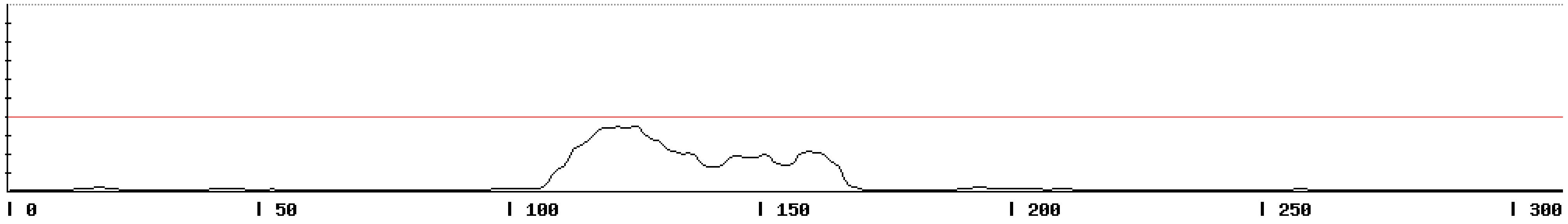

Predictions for Daucuscarota_DCAR_006394

Viterbi Path

Viterbi Path

Posterior @ 0.4

Posterior @ 0.4

Posterior Graphic. Threshold Marked @ 0.4

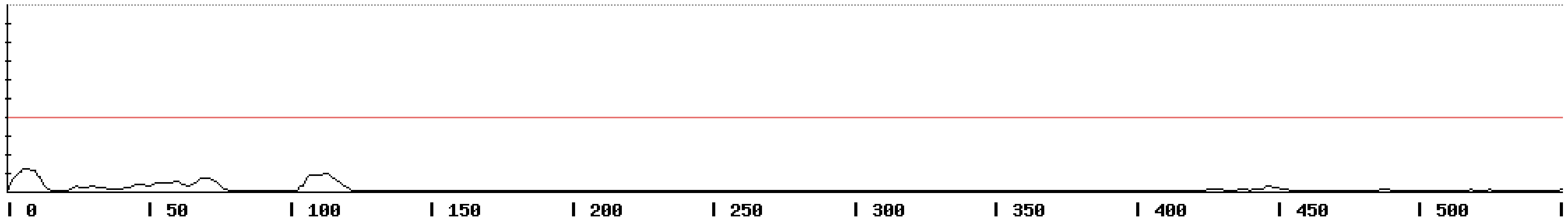

Predictions for Daucuscarota_DCAR_006395	
Viterbi Path	
Viterbi Path	
Posterior @ 0.4	
Posterior @ 0.4	
Posterior Graphic. Threshold Marked @ 0.4	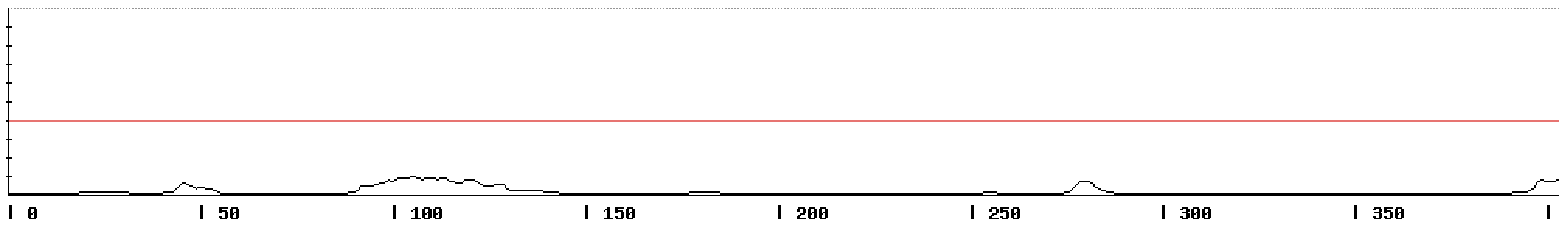

Predictions for Daucuscarota_DCAR_006562	
Viterbi Path	
Viterbi Path	
Posterior @ 0.4	
Posterior @ 0.4	
Posterior Graphic. Threshold Marked @ 0.4	

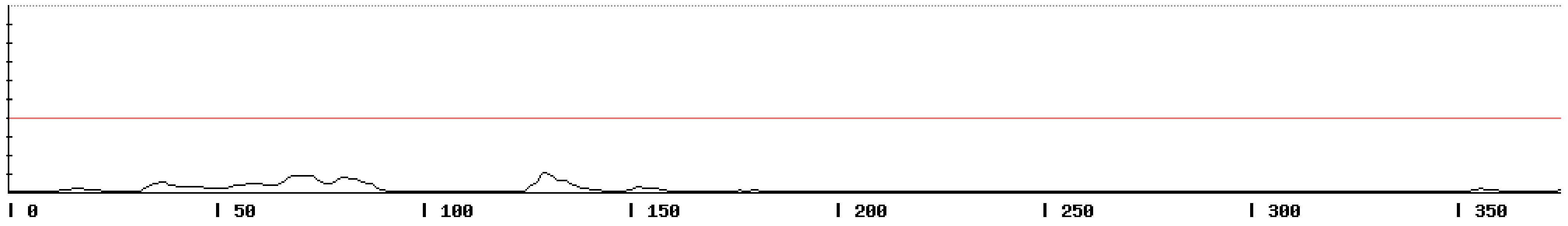

Predictions for Daucuscarota_DCAR_007150	
Viterbi Path	
Viterbi Path	
Posterior @ 0.4	
Posterior @ 0.4	
Posterior Graphic. Threshold Marked @ 0.4	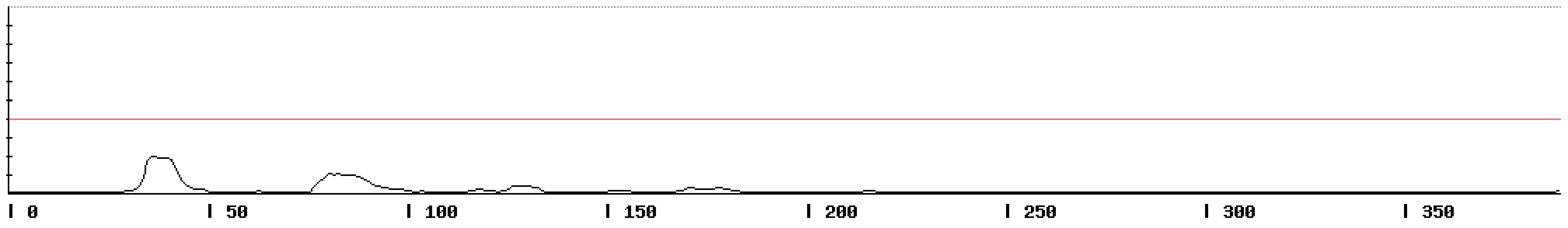

Predictions for Daucuscarota_DCAR_009603	
Viterbi Path	
Viterbi Path	
Posterior @ 0.4	
Posterior @ 0.4	

Posterior Graphic. Threshold Marked @ 0.4	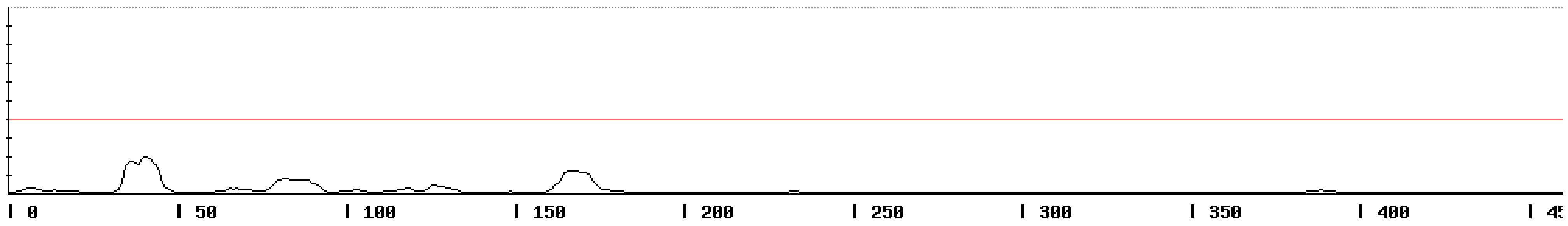

Predictions for Daucuscarota_DCAR_009635	
Viterbi Path	
Viterbi Path	
Posterior @ 0.4	
Posterior @ 0.4	
Posterior Graphic. Threshold Marked @ 0.4	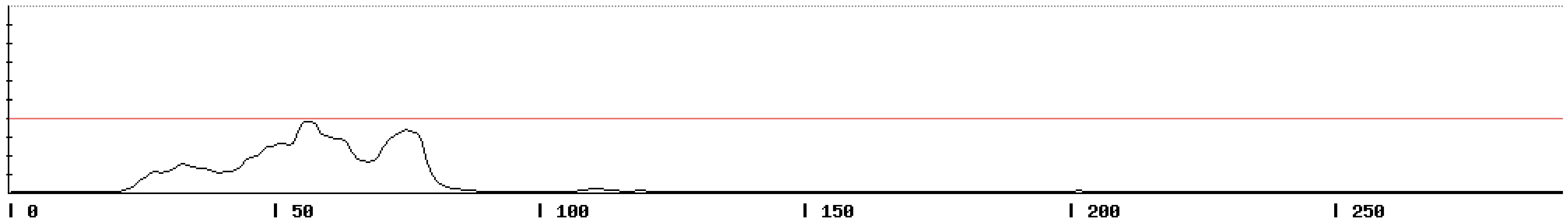

Predictions for Daucuscarota_DCAR_009670	
Viterbi Path	
Viterbi Path	
Posterior @ 0.4	
Posterior @ 0.4	

Posterior Graphic. Threshold Marked @ 0.4	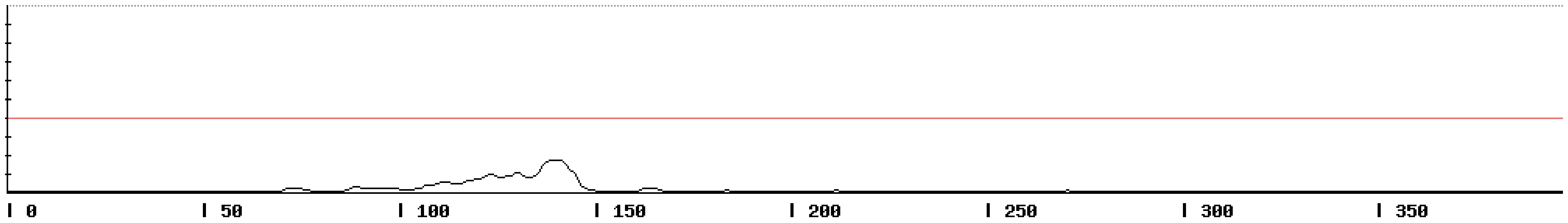

Predictions for Daucuscarota_DCAR_009852	
Viterbi Path	
Viterbi Path	
Posterior @ 0.4	
Posterior @ 0.4	
Posterior Graphic. Threshold Marked @ 0.4	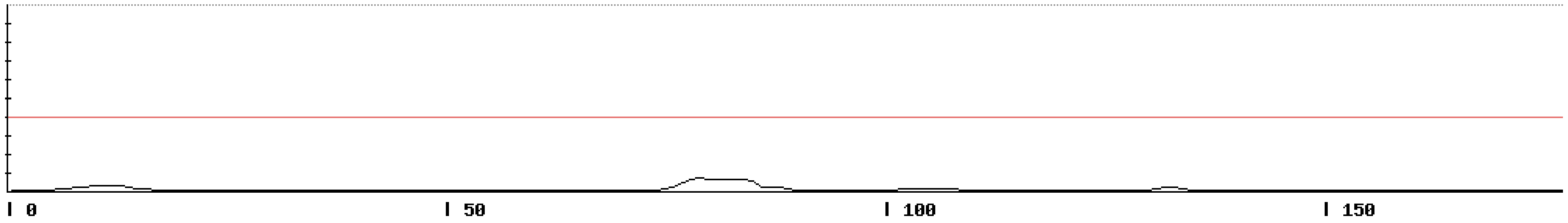

Predictions for Daucuscarota_DCAR_009973	
Viterbi Path	
Viterbi Path	

Posterior @ 0.4

Posterior @ 0.4

Posterior Graphic. Threshold Marked @ 0.4

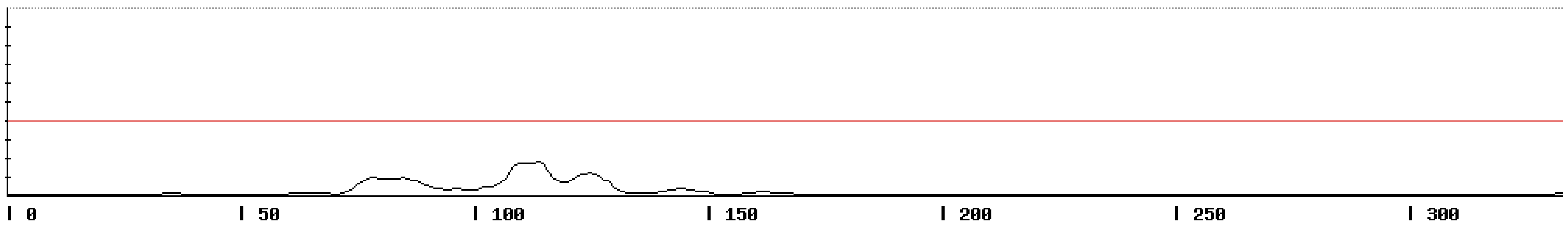

Predictions for Daucuscarota_DCAR_009975

Viterbi Path

Viterbi Path

Posterior @ 0.4

Posterior @ 0.4

Posterior Graphic. Threshold Marked @ 0.4

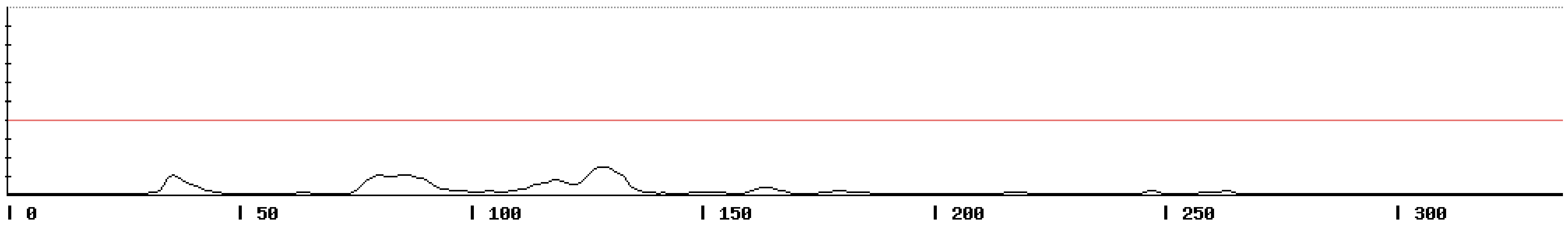

Predictions for Daucuscarota_DCAR_010211

Viterbi Path

Viterbi Path

Posterior @ 0.4

Posterior @ 0.4

Posterior Graphic. Threshold Marked @ 0.4

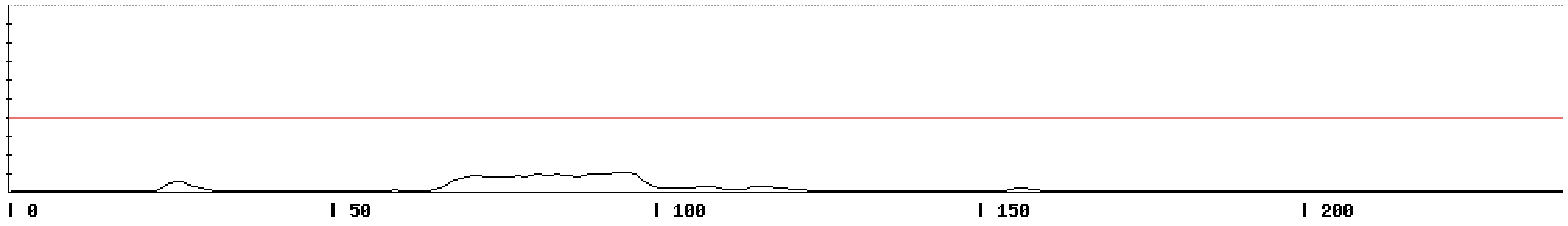

Predictions for Daucuscarota_DCAR_010568

Viterbi Path

Viterbi Path

Posterior @ 0.4

Posterior @ 0.4

Posterior Graphic. Threshold Marked @ 0.4

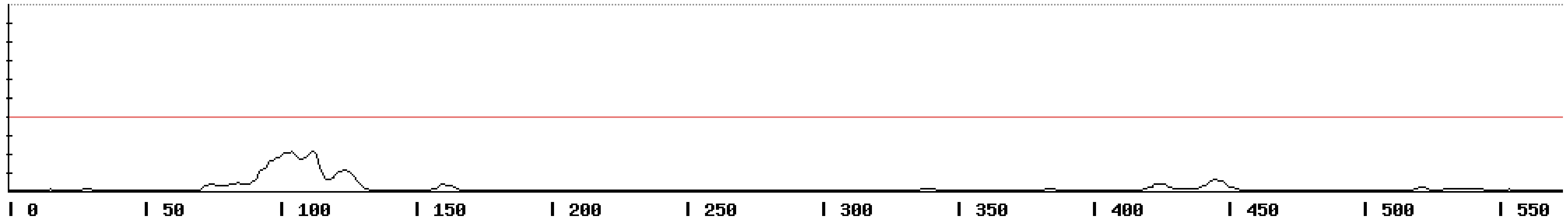

Predictions for Daucuscarota_DCAR_011084

Viterbi Path	
Viterbi Path	
Posterior @ 0.4	
Posterior @ 0.4	
Posterior Graphic. Threshold Marked @ 0.4	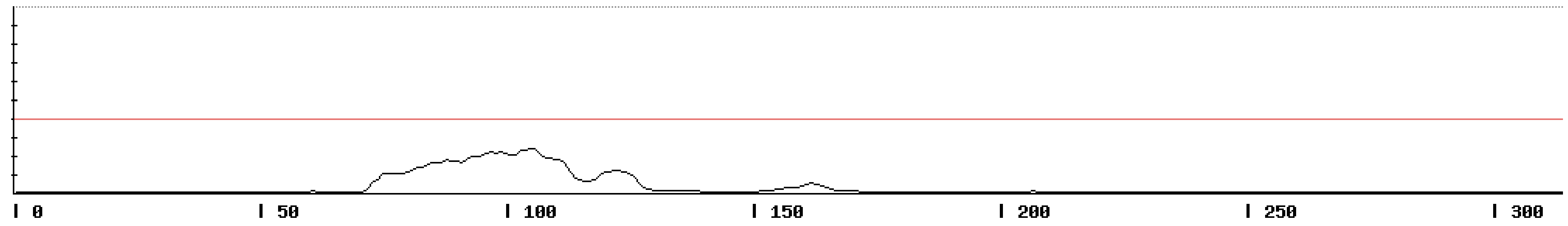

Predictions for Daucuscarota_DCAR_011133

Viterbi Path	
Viterbi Path	
Posterior @ 0.4	
Posterior @ 0.4	
Posterior Graphic. Threshold Marked @ 0.4	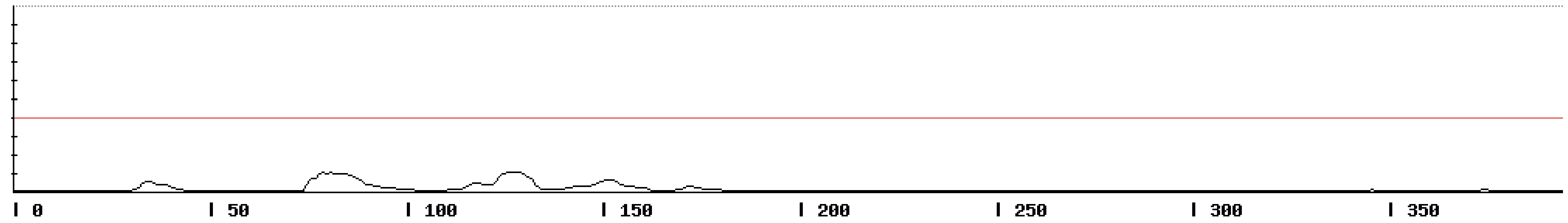

Predictions for Daucuscarota_DCAR_011258

Viterbi Path	
Viterbi Path	
Posterior @ 0.4	118 - SGKRKRRKIQS - 128
Posterior @ 0.4	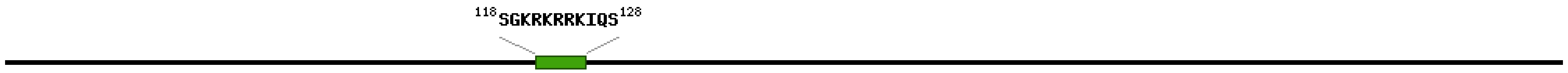
Posterior Graphic. Threshold Marked @ 0.4	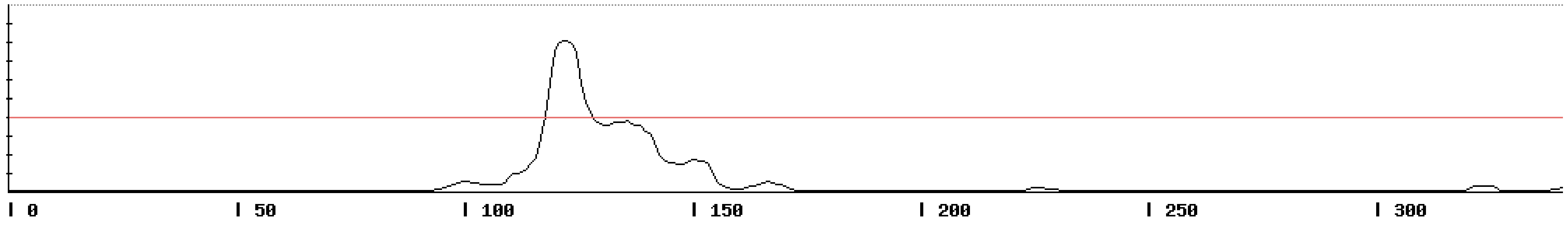

Predictions for Daucuscarota_DCAR_011259

Viterbi Path	
Viterbi Path	
Posterior @ 0.4	47 - GKRKRRKIQS - 56
Posterior @ 0.4	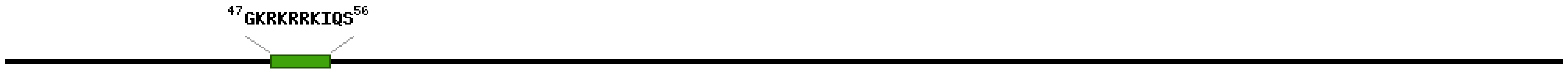
Posterior Graphic. Threshold Marked @ 0.4	

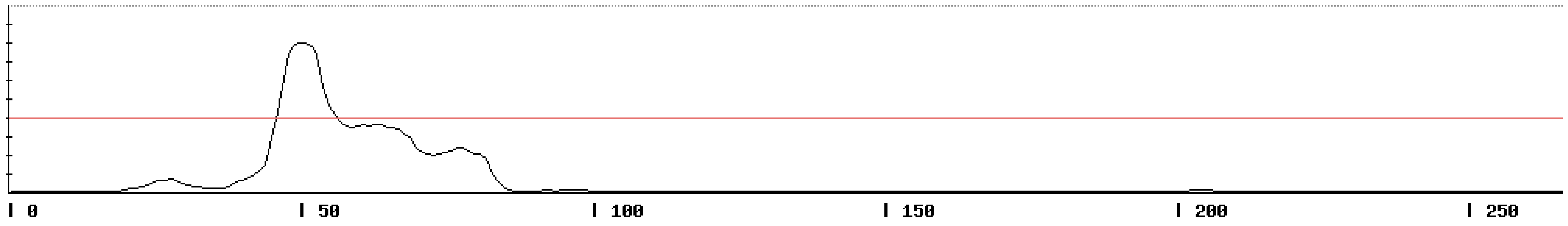

Predictions for Daucuscarota_DCAR_012021

Viterbi Path

Viterbi Path

Posterior @ 0.4

Posterior @ 0.4

Posterior Graphic. Threshold Marked @ 0.4

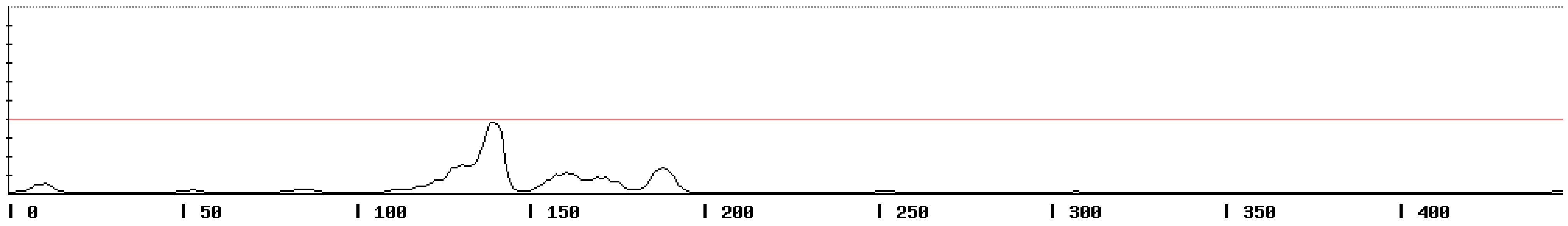

Predictions for Daucuscarota_DCAR_012209

Viterbi Path

Viterbi Path

Posterior @ 0.4

Posterior @ 0.4

Posterior Graphic. Threshold Marked @ 0.4	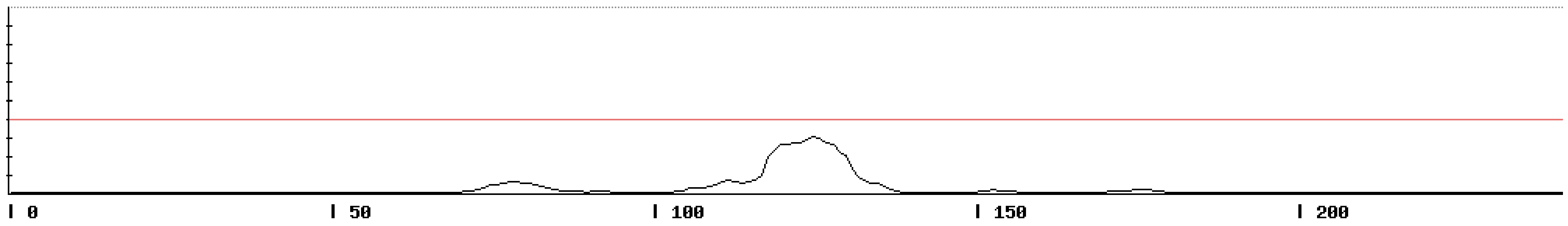

Predictions for Daucuscarota_DCAR_012553	
Viterbi Path	
Viterbi Path	
Posterior @ 0.4	
Posterior @ 0.4	
Posterior Graphic. Threshold Marked @ 0.4	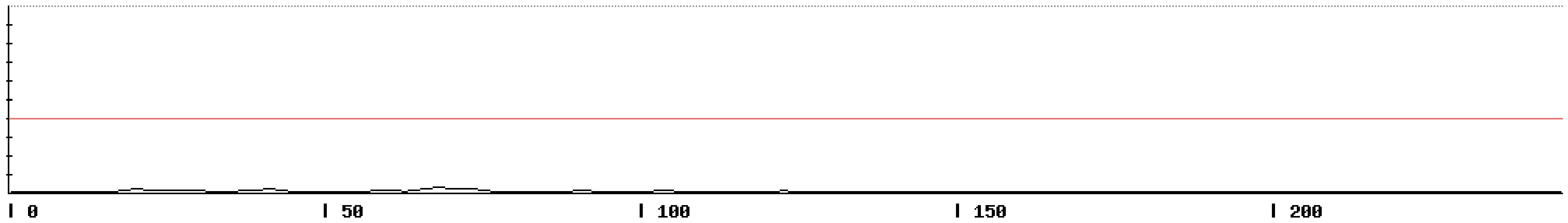

Predictions for Daucuscarota_DCAR_012554	
Viterbi Path	
Viterbi Path	
Posterior @ 0.4	
Posterior @ 0.4	

Posterior Graphic. Threshold Marked @ 0.4

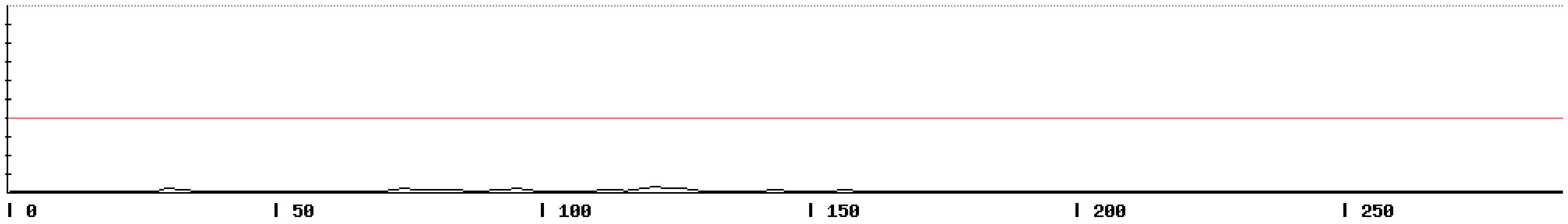

Predictions for Daucuscarota_DCAR_012555

Viterbi Path

Viterbi Path

Posterior @ 0.4

Posterior @ 0.4

Posterior Graphic. Threshold Marked @ 0.4

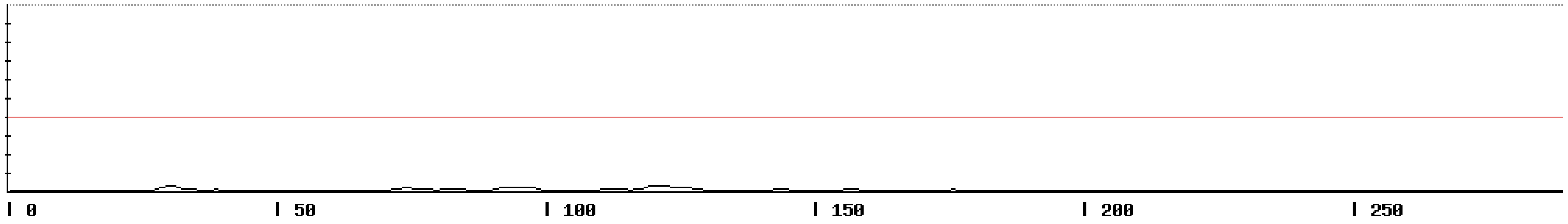

Predictions for Daucuscarota_DCAR_012697

Viterbi Path

Viterbi Path

Posterior @ 0.4

Posterior @ 0.4

Posterior Graphic. Threshold Marked @ 0.4

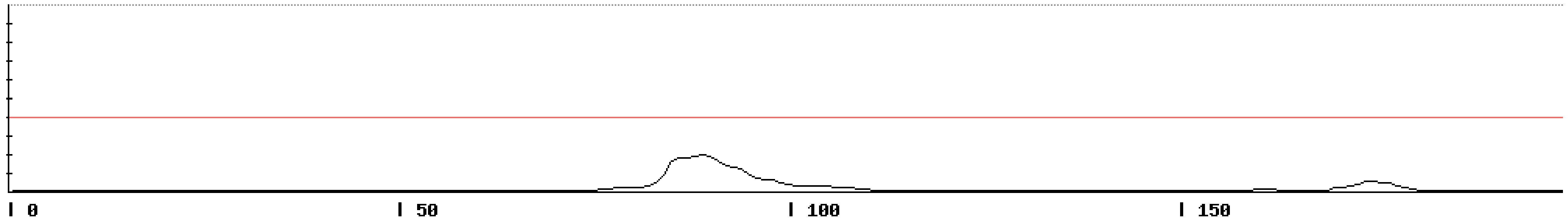

Predictions for Daucuscarota_DCAR_012752

Viterbi Path

Viterbi Path

Posterior @ 0.4 298 - KRKEKKG - 304

Posterior @ 0.4

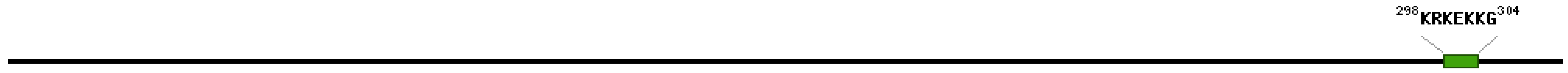

Posterior Graphic. Threshold Marked @ 0.4

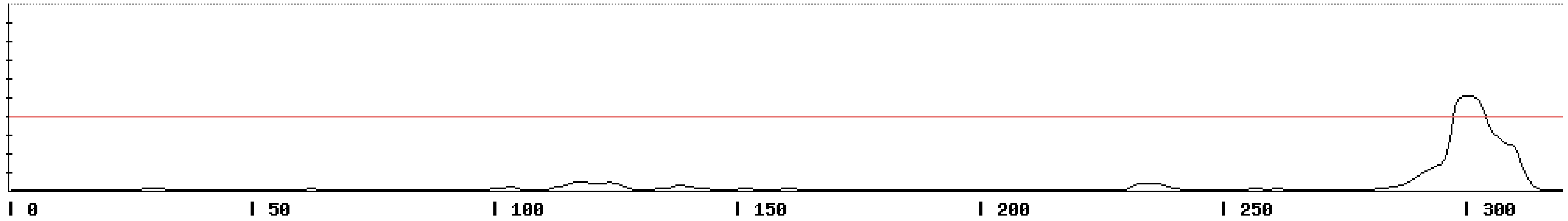

Predictions for Daucuscarota_DCAR_013082

Viterbi Path

Viterbi Path

Posterior @ 0.4

Posterior @ 0.4

Posterior Graphic. Threshold Marked @ 0.4

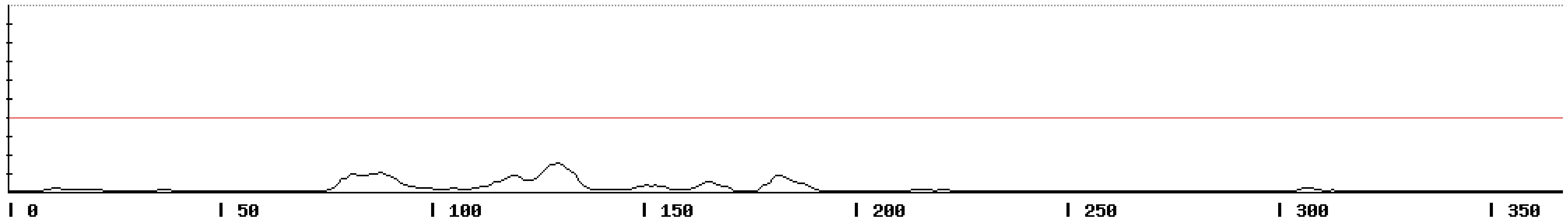

Predictions for Daucuscarota_DCAR_013122

Viterbi Path

Viterbi Path

Posterior @ 0.4

Posterior @ 0.4

Posterior Graphic. Threshold Marked @ 0.4

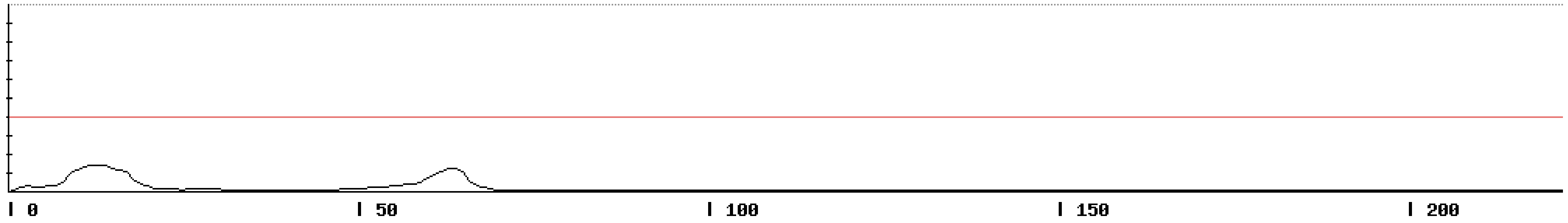

Predictions for Daucuscarota_DCAR_013176

Viterbi Path

Viterbi Path

Posterior @ 0.4

Posterior @ 0.4

Posterior Graphic. Threshold Marked @ 0.4

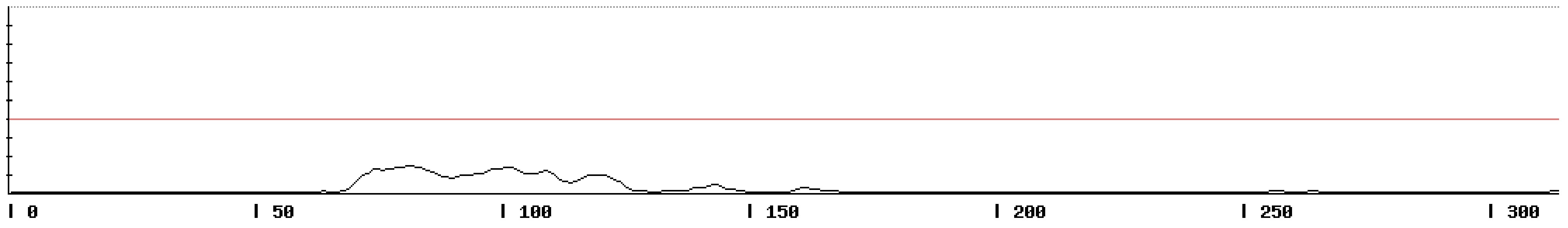

Predictions for Daucuscarota_DCAR_013698

Viterbi Path

Viterbi Path

Posterior @ 0.4

Posterior @ 0.4

Posterior Graphic. Threshold Marked @ 0.4

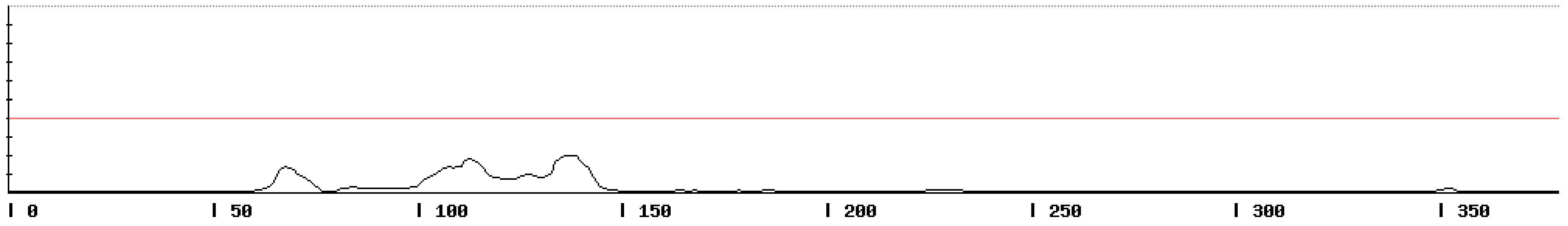

Predictions for Daucuscarota_DCAR_013734	
Viterbi Path	
Viterbi Path	
Posterior @ 0.4	
Posterior @ 0.4	
Posterior Graphic. Threshold Marked @ 0.4	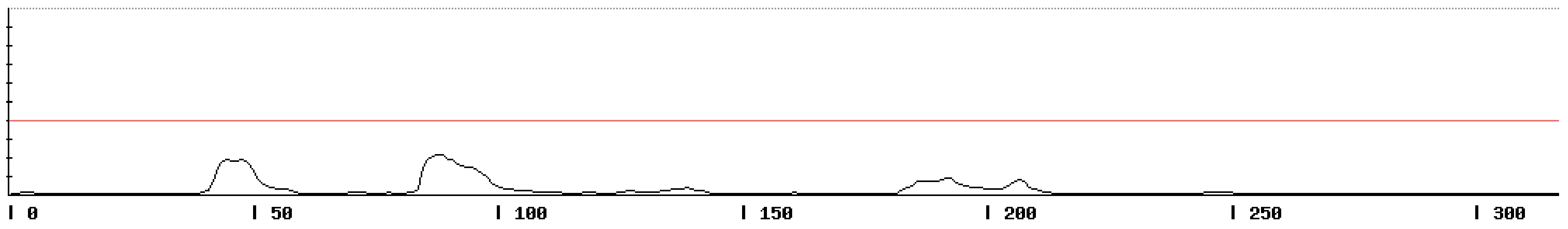

Predictions for Daucuscarota_DCAR_013939	
Viterbi Path	
Viterbi Path	
Posterior @ 0.4	
Posterior @ 0.4	
Posterior Graphic. Threshold Marked @ 0.4	

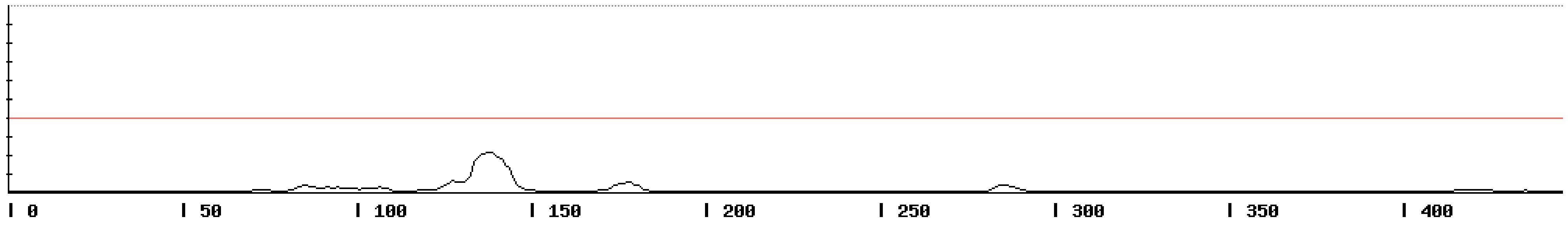

Predictions for Daucuscarota_DCAR_014143

Viterbi Path	
Viterbi Path	
Posterior @ 0.4	
Posterior @ 0.4	
Posterior Graphic. Threshold Marked @ 0.4	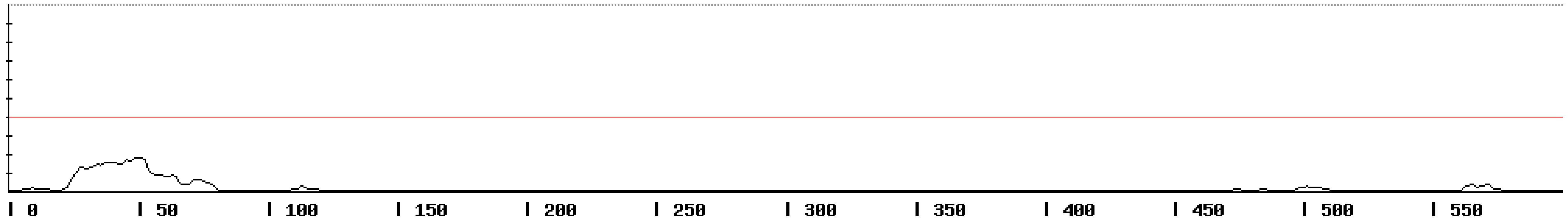

Predictions for Daucuscarota_DCAR_014340

Viterbi Path	
Viterbi Path	
Posterior @ 0.4	
Posterior @ 0.4	

Posterior Graphic. Threshold Marked @ 0.4	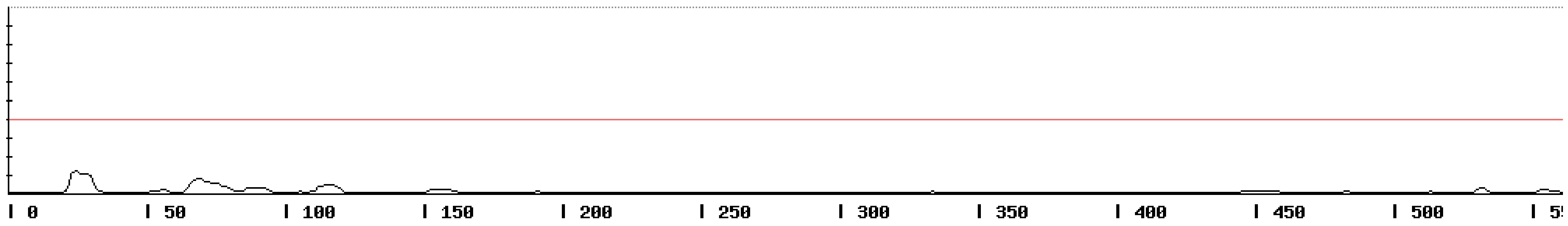

Predictions for Daucuscarota_DCAR_014487

Viterbi Path	162 - RRRRGKKHQVKKTRR - 176
Viterbi Path	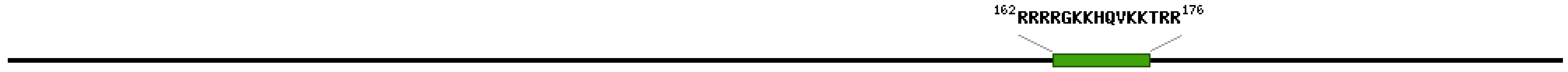
Posterior @ 0.4	162 - RRRRGKKHQVKKTRR - 176
Posterior @ 0.4	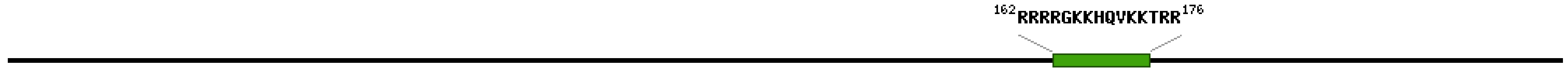
Posterior Graphic. Threshold Marked @ 0.4	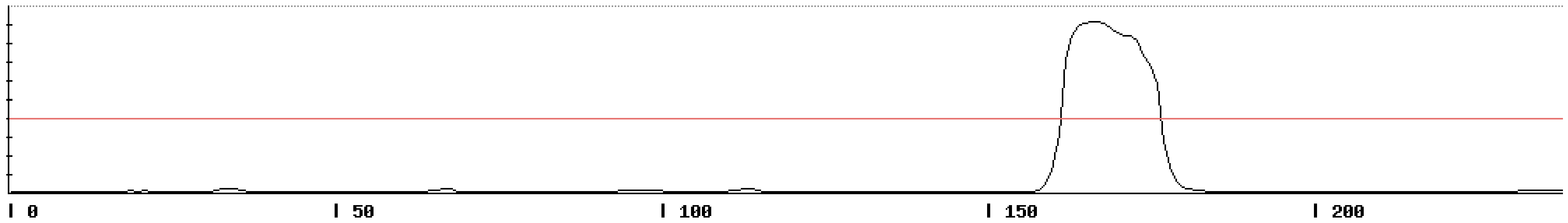

Predictions for Daucuscarota_DCAR_014567

Viterbi Path	
Viterbi Path	
Posterior @ 0.4	
Posterior @ 0.4	

Posterior Graphic. Threshold Marked @ 0.4

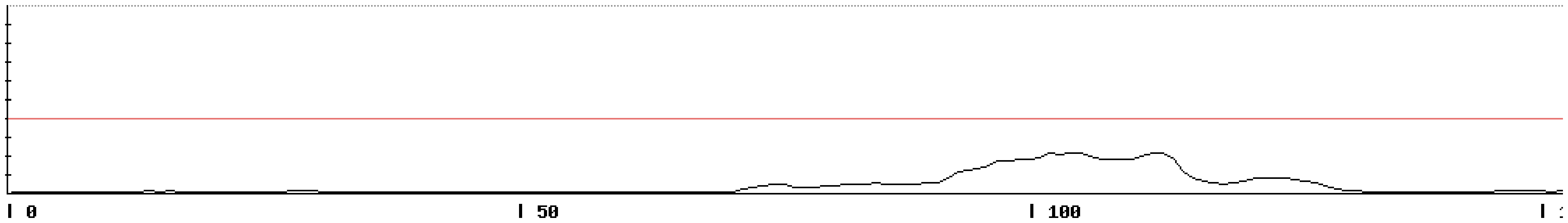

Predictions for Daucuscarota_DCAR_014625

Viterbi Path

Viterbi Path

Posterior @ 0.4

Posterior @ 0.4

Posterior Graphic. Threshold Marked @ 0.4

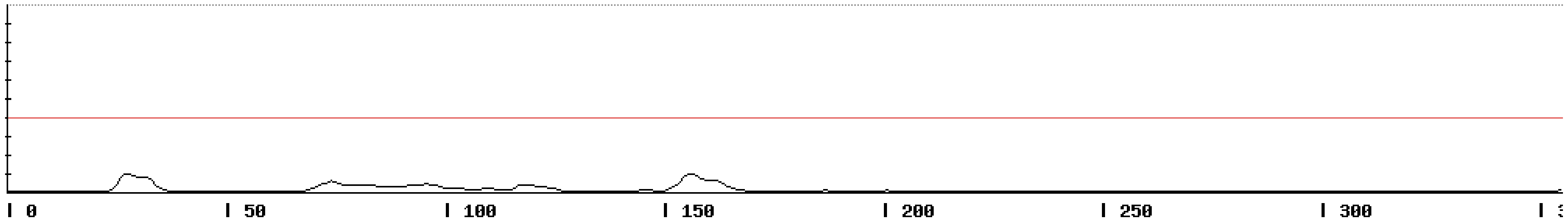

Predictions for Daucuscarota_DCAR_015676

Viterbi Path

Viterbi Path

Posterior @ 0.4	
Posterior @ 0.4	
Posterior Graphic. Threshold Marked @ 0.4	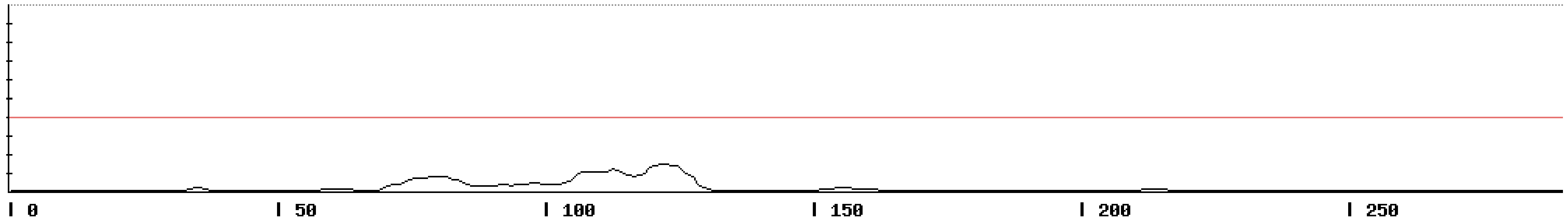

Predictions for Daucuscarota_DCAR_015694

Viterbi Path	
Viterbi Path	
Posterior @ 0.4	310 - SRKGNKPR - 317
Posterior @ 0.4	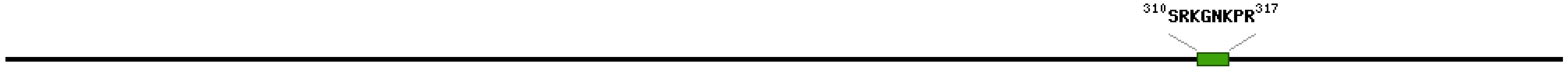
Posterior Graphic. Threshold Marked @ 0.4	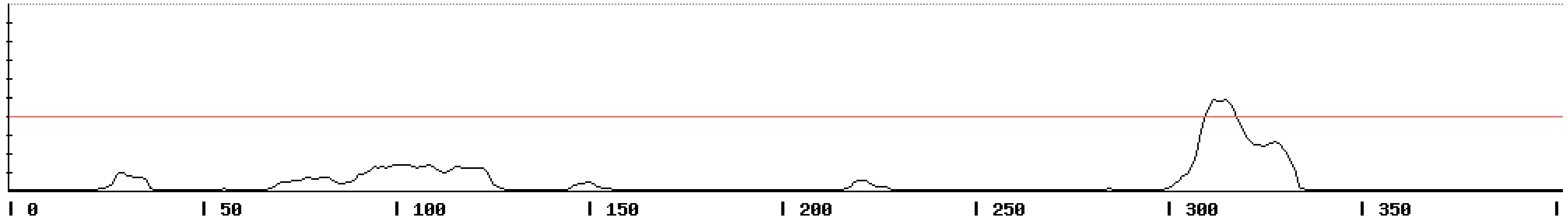

Predictions for Daucuscarota_DCAR_016571

Viterbi Path	
Viterbi Path	

Posterior @ 0.4

Posterior @ 0.4

Posterior Graphic. Threshold Marked @ 0.4

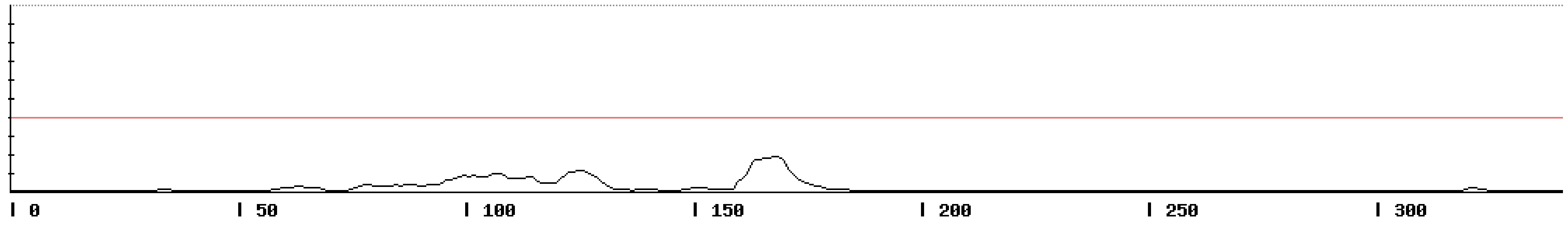

Predictions for Daucuscarota_DCAR_016661

Viterbi Path

Viterbi Path

Posterior @ 0.4

Posterior @ 0.4

Posterior Graphic. Threshold Marked @ 0.4

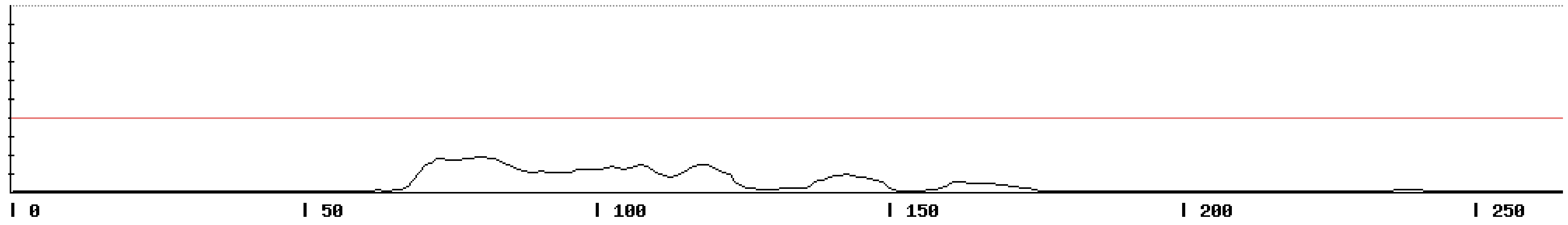

Predictions for Daucuscarota_DCAR_016795

Viterbi Path

Viterbi Path

Posterior @ 0.4

Posterior @ 0.4

Posterior Graphic. Threshold Marked @ 0.4

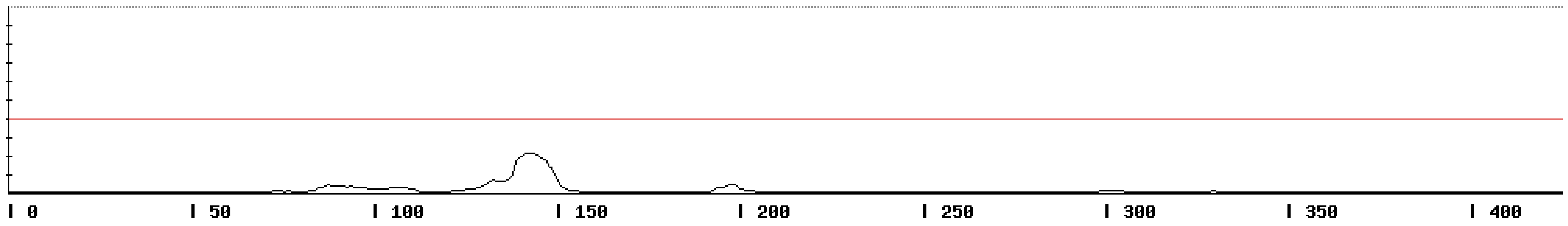

Predictions for Daucuscarota_DCAR_016827

Viterbi Path

Viterbi Path

Posterior @ 0.4

Posterior @ 0.4

Posterior Graphic. Threshold Marked @ 0.4

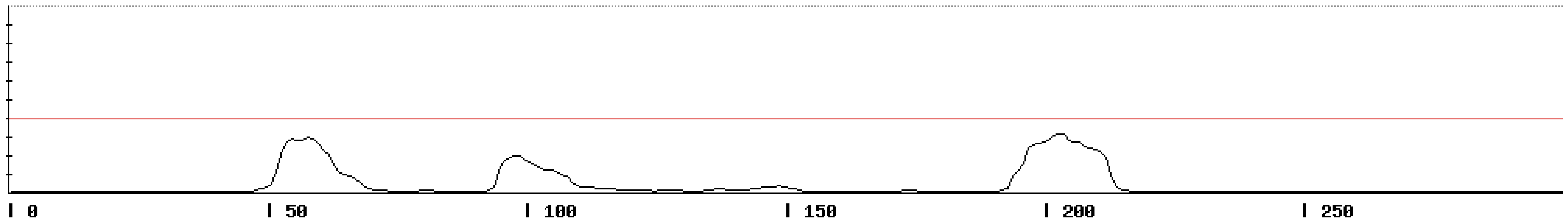

Predictions for Daucuscarota_DCAR_016852	
Viterbi Path	
Viterbi Path	
Posterior @ 0.4	
Posterior @ 0.4	
Posterior Graphic. Threshold Marked @ 0.4	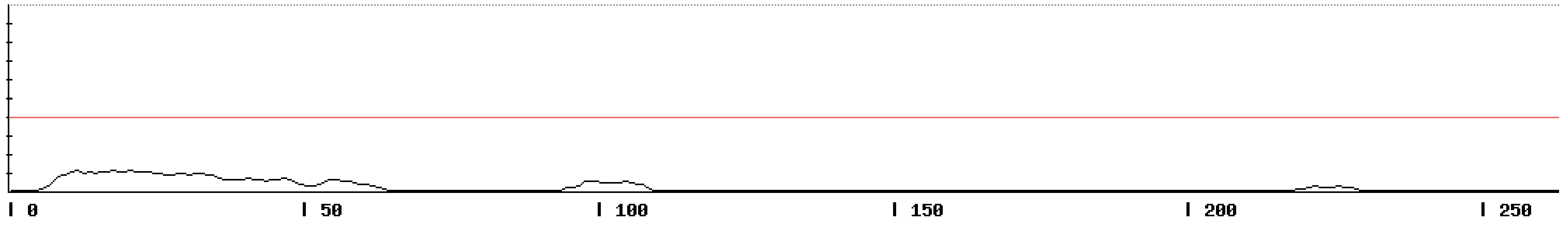

Predictions for Daucuscarota_DCAR_016856	
Viterbi Path	
Viterbi Path	
Posterior @ 0.4	
Posterior @ 0.4	
Posterior Graphic. Threshold Marked @ 0.4	

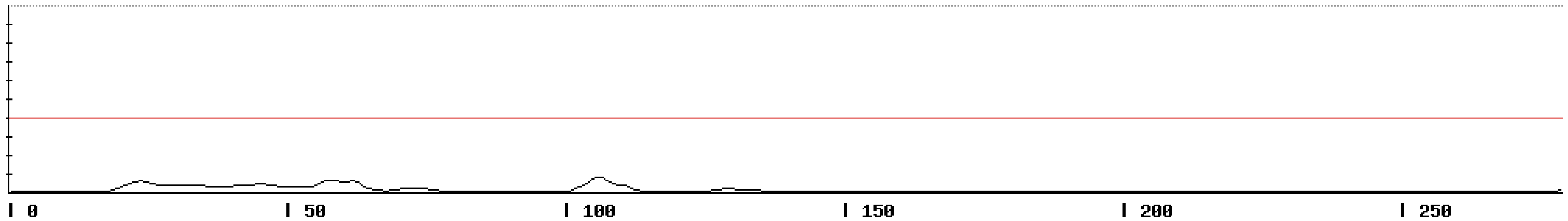

Predictions for Daucuscarota_DCAR_016858

Viterbi Path	
Viterbi Path	
Posterior @ 0.4	
Posterior @ 0.4	
Posterior Graphic. Threshold Marked @ 0.4	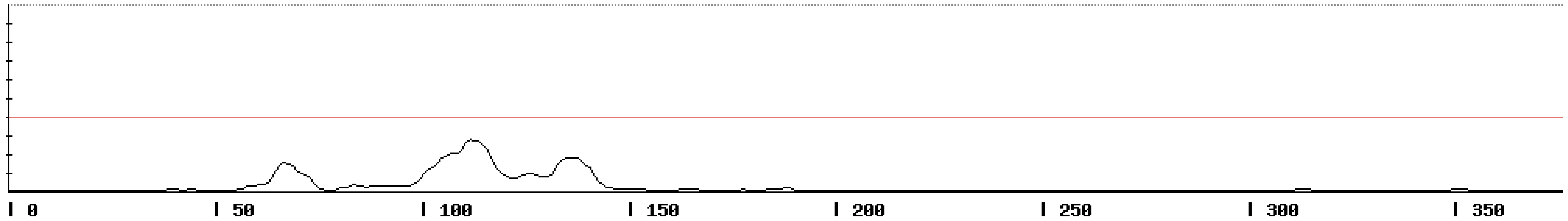

Predictions for Daucuscarota_DCAR_017156

Viterbi Path	
Viterbi Path	
Posterior @ 0.4	
Posterior @ 0.4	

Posterior Graphic. Threshold Marked @ 0.4

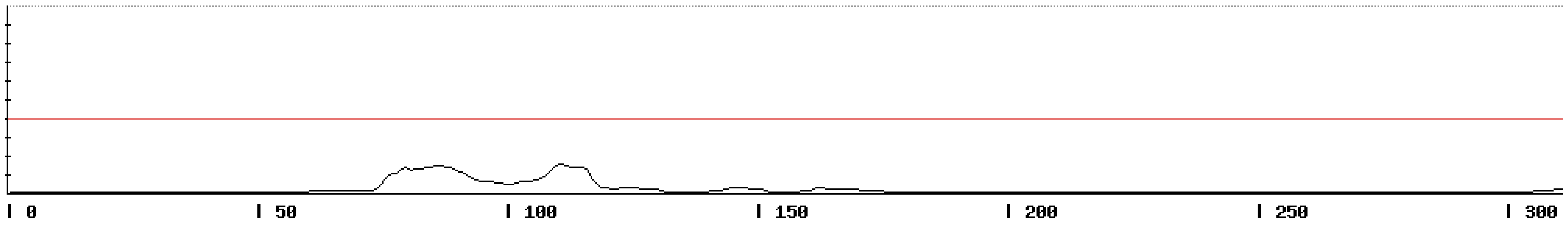

Predictions for Daucuscarota_DCAR_017344

Viterbi Path

Viterbi Path

Posterior @ 0.4

Posterior @ 0.4

Posterior Graphic. Threshold Marked @ 0.4

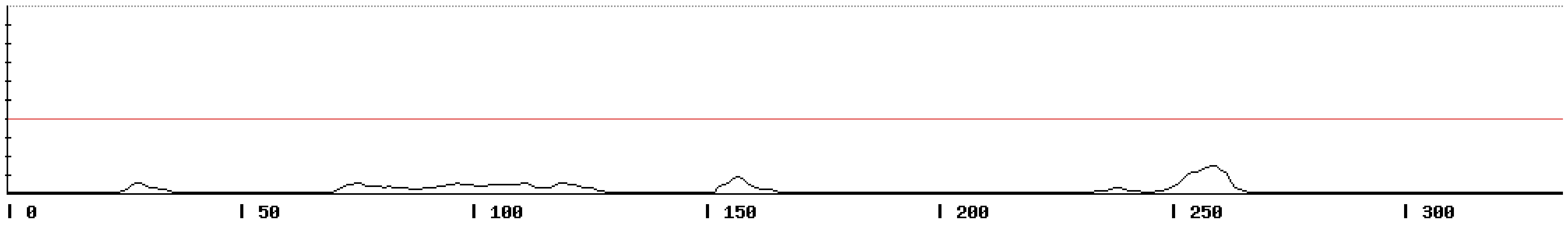

Predictions for Daucuscarota_DCAR_017967

Viterbi Path

Viterbi Path

Posterior @ 0.4

Posterior @ 0.4

Posterior Graphic. Threshold Marked @ 0.4

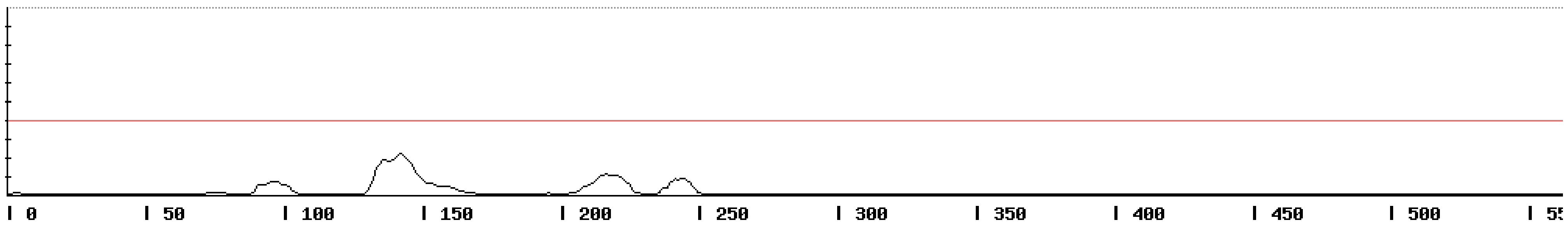

Predictions for Daucuscarota_DCAR_018104

Viterbi Path

Viterbi Path

Posterior @ 0.4

Posterior @ 0.4

Posterior Graphic. Threshold Marked @ 0.4

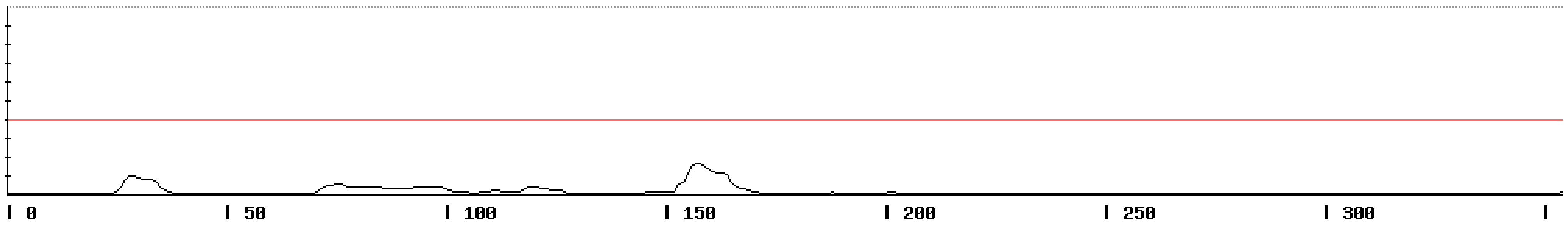

Predictions for Daucuscarota_DCAR_019076

Viterbi Path

Viterbi Path

Posterior @ 0.4

Posterior @ 0.4

Posterior Graphic. Threshold Marked @ 0.4

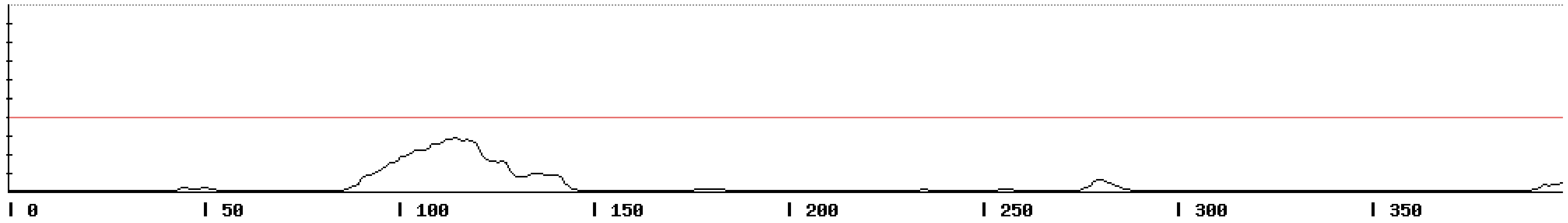

Predictions for Daucuscarota_DCAR_019077

Viterbi Path

Viterbi Path

Posterior @ 0.4

Posterior @ 0.4

Posterior Graphic. Threshold Marked @ 0.4

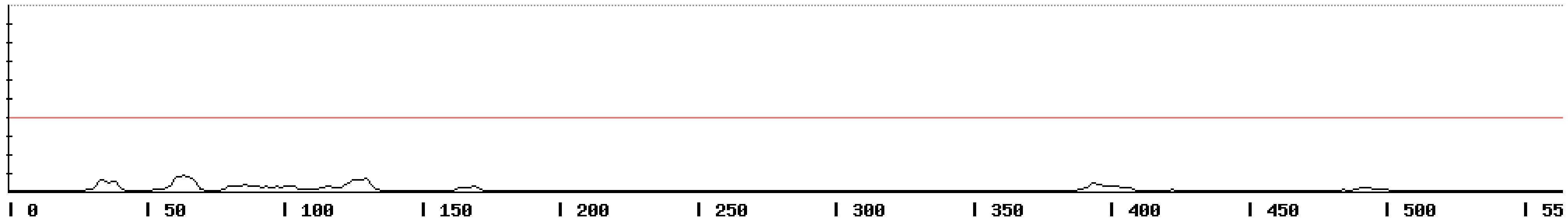

Predictions for Daucuscarota_DCAR_019571

Viterbi Path

Viterbi Path

Posterior @ 0.4

Posterior @ 0.4

Posterior Graphic. Threshold Marked @ 0.4

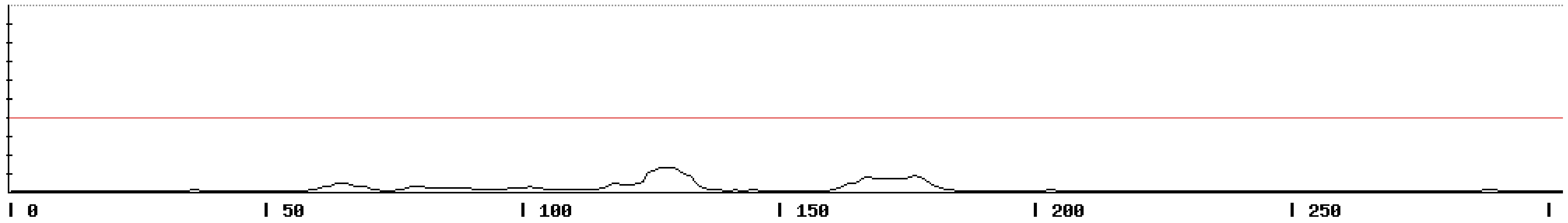

Predictions for Daucuscarota_DCAR_019896

Viterbi Path

Viterbi Path

Posterior @ 0.4

136 - RKGG - 139

Posterior @ 0.4

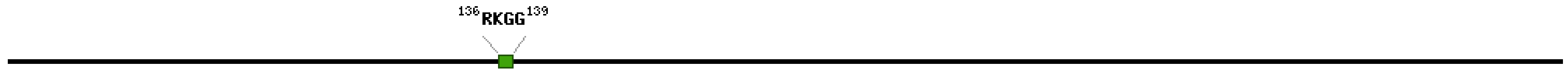

Posterior Graphic. Threshold Marked @ 0.4

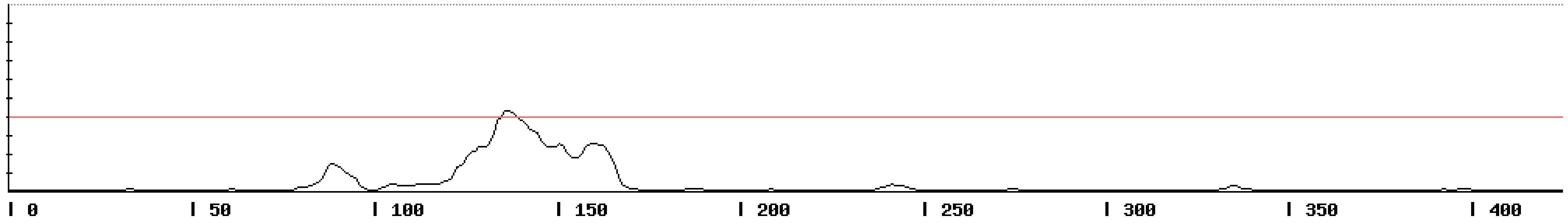

Predictions for Daucuscarota_DCAR_019979

Viterbi Path	
Viterbi Path	
Posterior @ 0.4	
Posterior @ 0.4	
Posterior Graphic. Threshold Marked @ 0.4	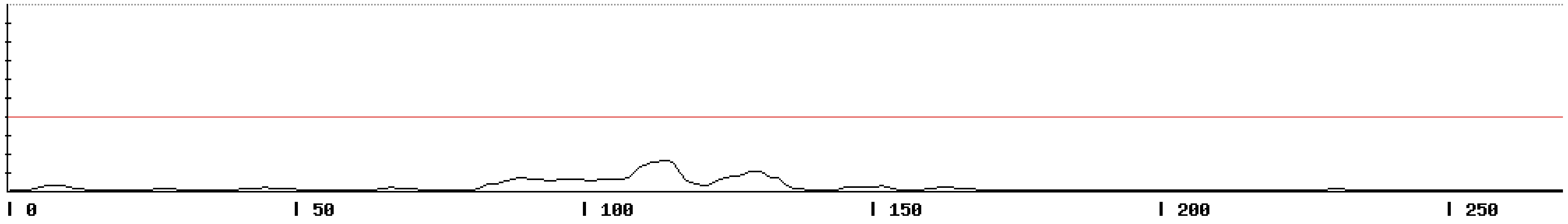

Predictions for Daucuscarota_DCAR_019984

Viterbi Path	
Viterbi Path	
Posterior @ 0.4	
Posterior @ 0.4	
Posterior Graphic. Threshold Marked @ 0.4	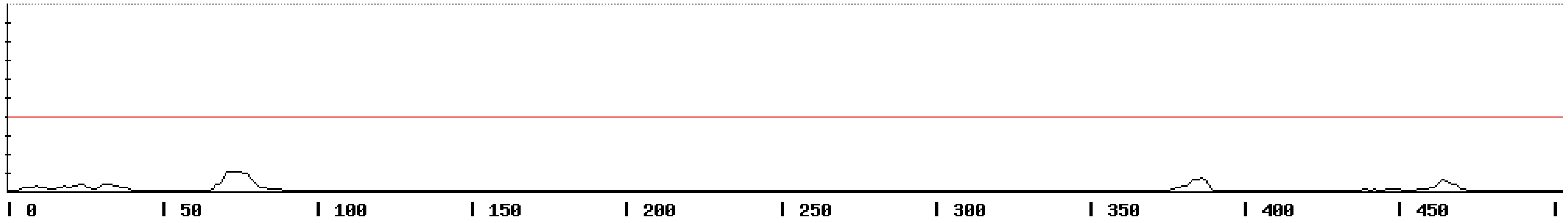

Predictions for Daucuscarota_DCAR_020036	
Viterbi Path	
Viterbi Path	
Posterior @ 0.4	
Posterior @ 0.4	
Posterior Graphic. Threshold Marked @ 0.4	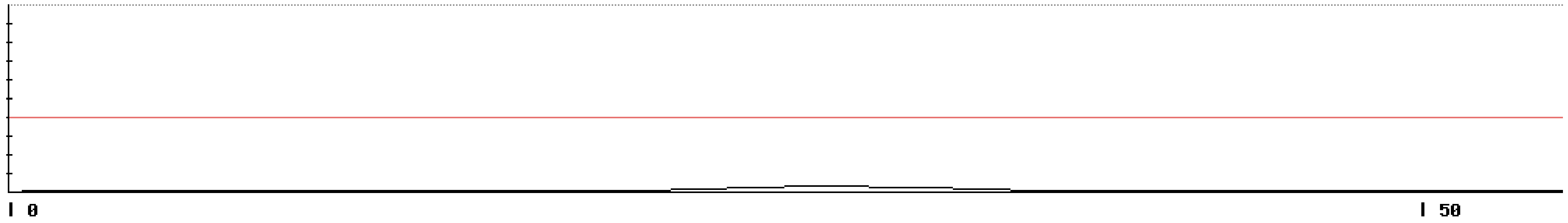

Predictions for Daucuscarota_DCAR_020385	
Viterbi Path	
Viterbi Path	
Posterior @ 0.4	
Posterior @ 0.4	
Posterior Graphic. Threshold Marked @ 0.4	

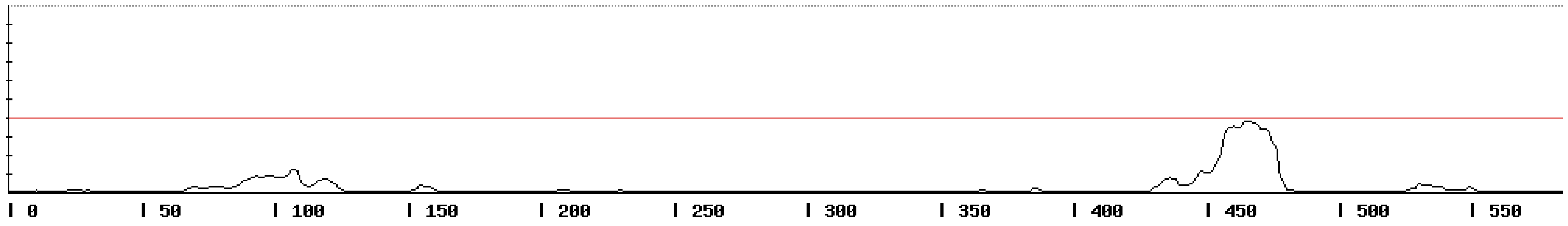

Predictions for Daucuscarota_DCAR_020996

Viterbi Path	
Viterbi Path	
Posterior @ 0.4	
Posterior @ 0.4	
Posterior Graphic. Threshold Marked @ 0.4	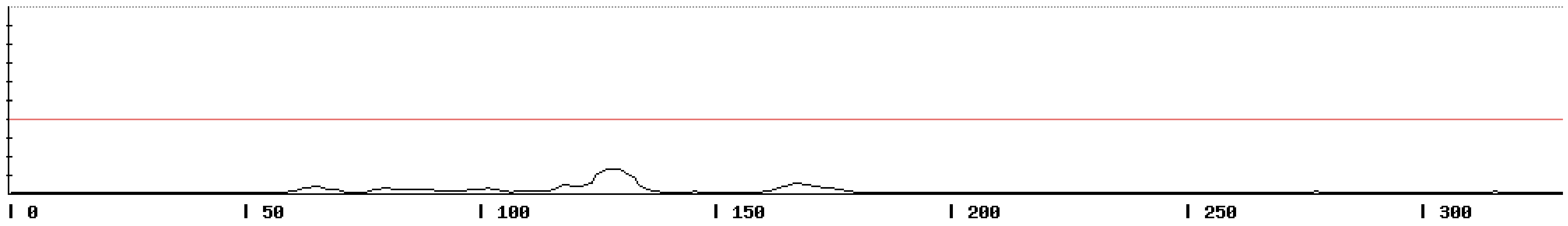

Predictions for Daucuscarota_DCAR_021266

Viterbi Path	
Viterbi Path	
Posterior @ 0.4	142 - RKRRK - 146
Posterior @ 0.4	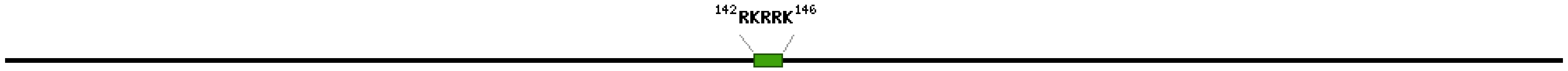

Posterior Graphic. Threshold Marked @ 0.4

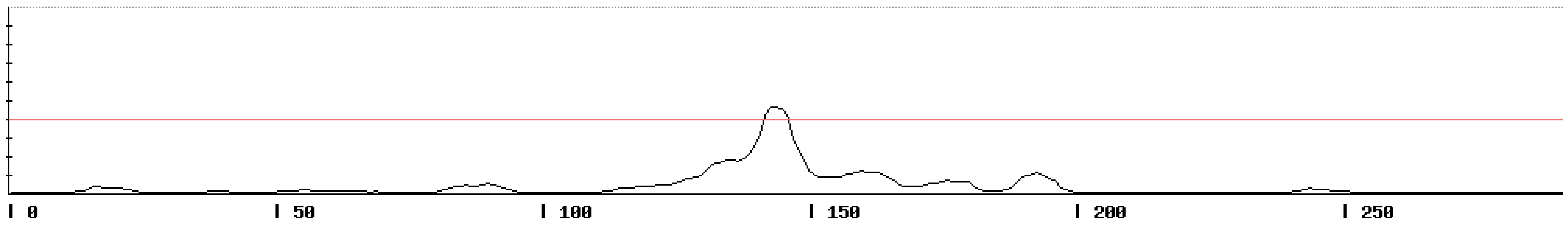

Predictions for Daucuscarota_DCAR_021419

Viterbi Path

Viterbi Path

Posterior @ 0.4

Posterior @ 0.4

Posterior Graphic. Threshold Marked @ 0.4

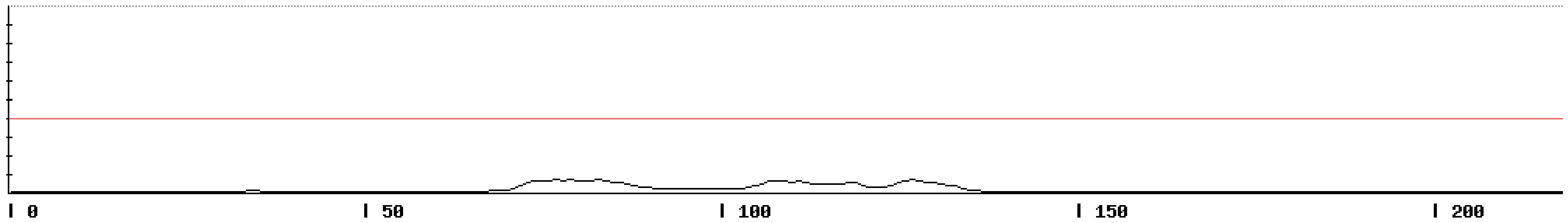

Predictions for Daucuscarota_DCAR_021729

Viterbi Path: 505 - RGKSMRSKVETRMRKESGKTLREIRRSKKLRKK - 537

Viterbi Path

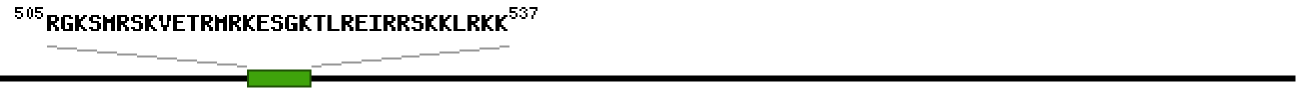

Posterior @ 0.4:

133 - RKRRK - 137

505 - RGKSMRSKVETRMRKESGKTLREIRRSKKLRKKMM - 539

549 - LRRAKKKV - 556

Posterior @ 0.4

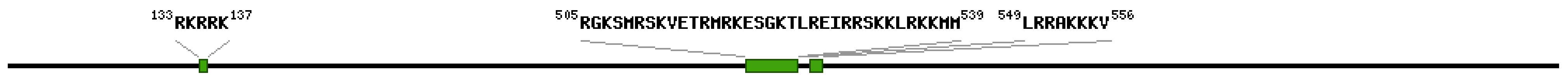

Posterior Graphic. Threshold Marked @ 0.4

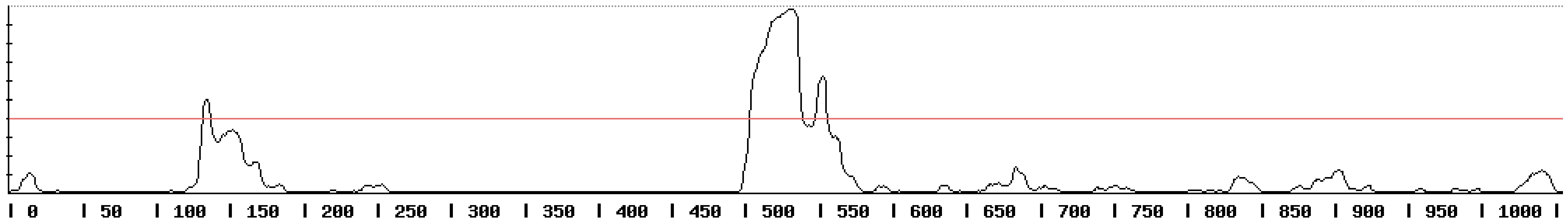

Predictions for Daucuscarota_DCAR_021811

Viterbi Path

Viterbi Path

Posterior @ 0.4

Posterior @ 0.4

Posterior Graphic. Threshold Marked @ 0.4

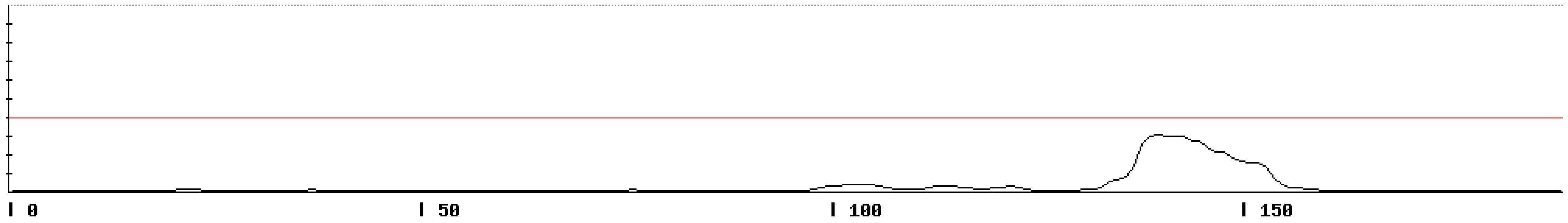

Predictions for Daucuscarota_DCAR_021838

Viterbi Path

Viterbi Path

Posterior @ 0.4

Posterior @ 0.4

Posterior Graphic. Threshold Marked @ 0.4

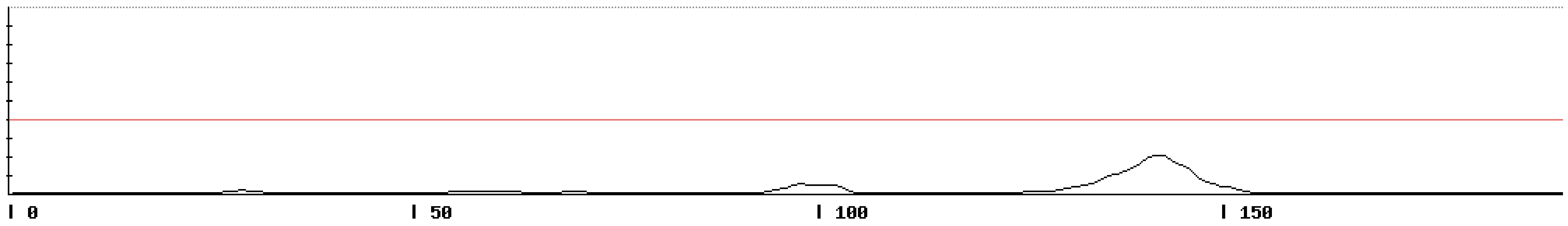

Predictions for Daucuscarota_DCAR_022323

Viterbi Path

Viterbi Path

Posterior @ 0.4

Posterior @ 0.4

Posterior Graphic. Threshold Marked @ 0.4

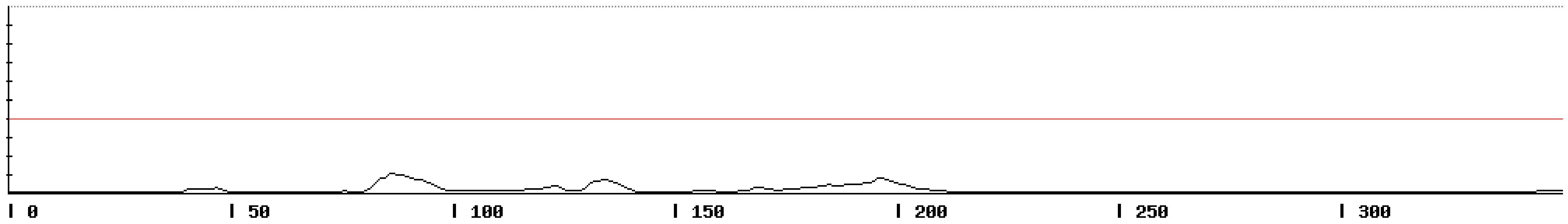

Predictions for Daucuscarota_DCAR_022387

Viterbi Path

Viterbi Path

Posterior @ 0.4

Posterior @ 0.4

Posterior Graphic. Threshold Marked @ 0.4

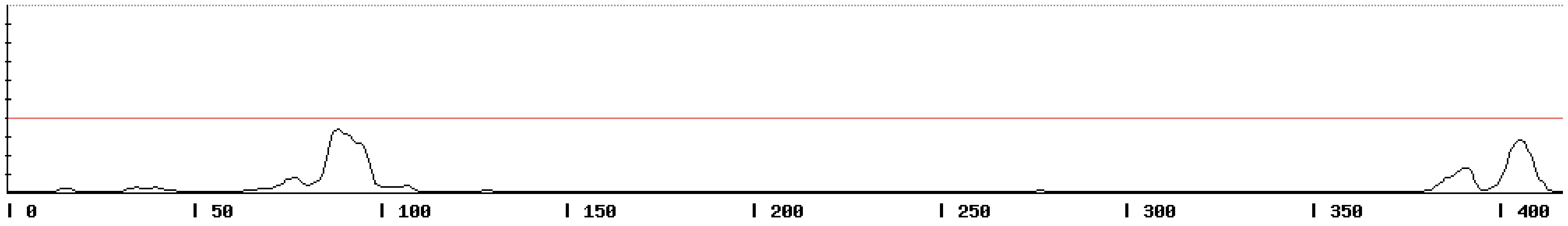

Predictions for Daucuscarota_DCAR_022389

Viterbi Path

Viterbi Path

Posterior @ 0.4

Posterior @ 0.4

Posterior Graphic. Threshold Marked @ 0.4

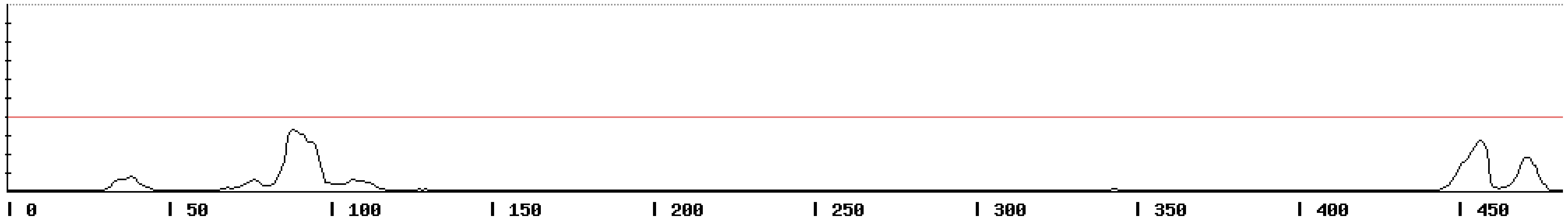

Predictions for Daucuscarota_DCAR_022479

Viterbi Path	
Viterbi Path	
Posterior @ 0.4	
Posterior @ 0.4	
Posterior Graphic. Threshold Marked @ 0.4	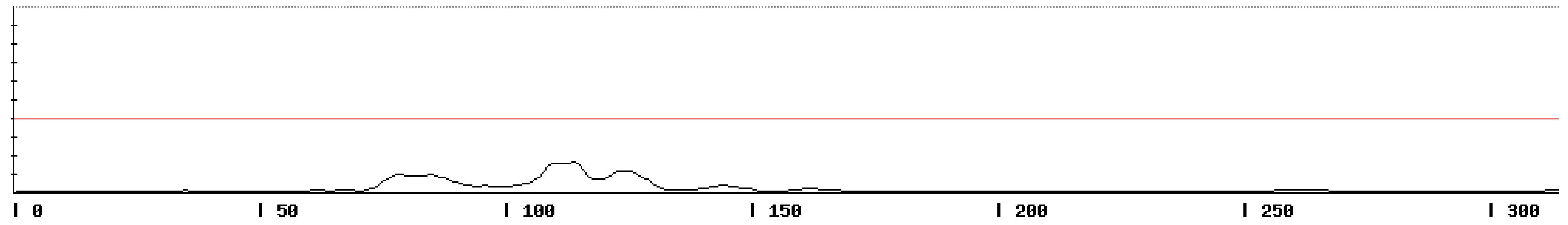

Predictions for Daucuscarota_DCAR_022480

Viterbi Path	
Viterbi Path	
Posterior @ 0.4	
Posterior @ 0.4	
Posterior Graphic. Threshold Marked @ 0.4	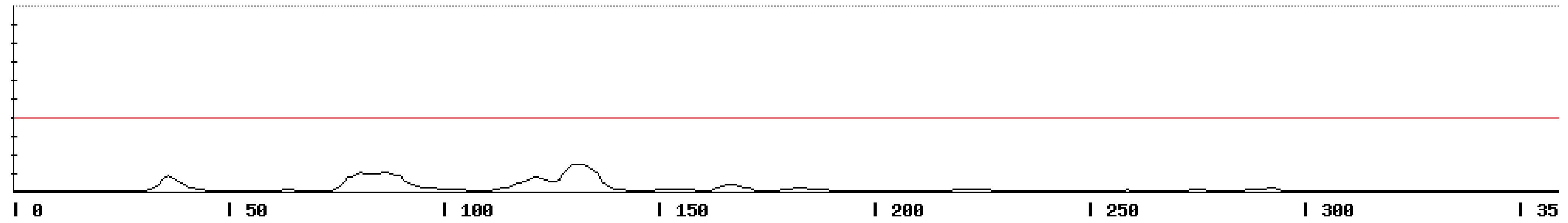

Predictions for Daucuscarota_DCAR_023688

Viterbi Path	
Viterbi Path	
Posterior @ 0.4	136 - RKRRK - 140
Posterior @ 0.4	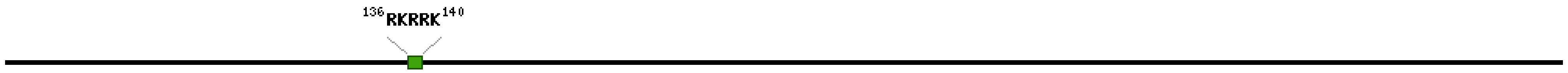
Posterior Graphic. Threshold Marked @ 0.4	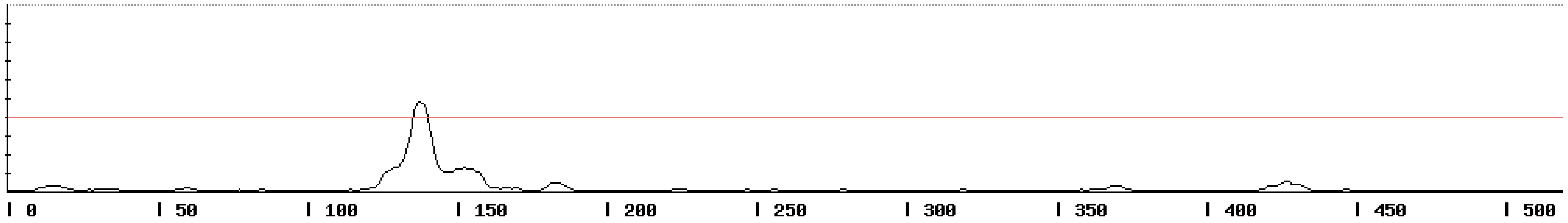

Predictions for Daucuscarota_DCAR_023696

Viterbi Path	
Viterbi Path	
Posterior @ 0.4	
Posterior @ 0.4	
Posterior Graphic. Threshold Marked @ 0.4	

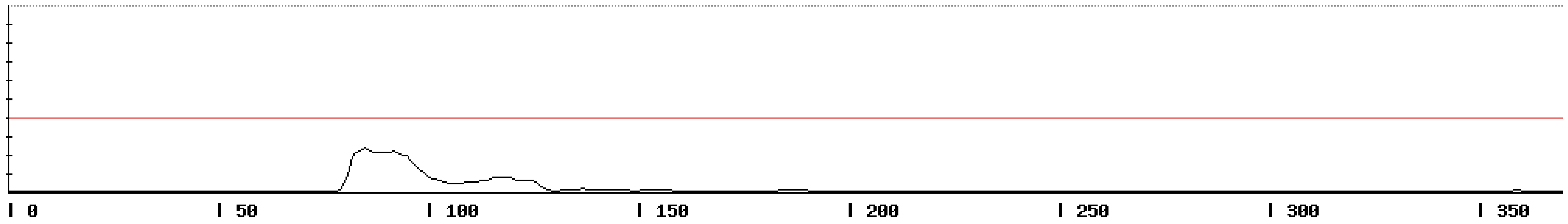

Predictions for Daucuscarota_DCAR_024642

Viterbi Path	
Viterbi Path	
Posterior @ 0.4	
Posterior @ 0.4	
Posterior Graphic. Threshold Marked @ 0.4	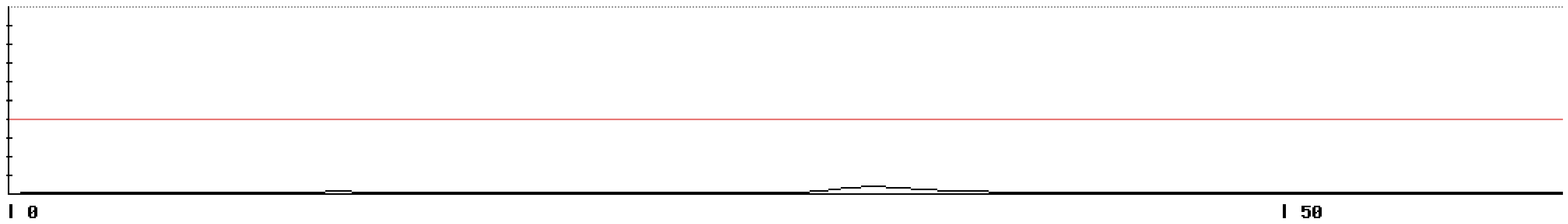

Predictions for Daucuscarota_DCAR_025382

Viterbi Path	
Viterbi Path	
Posterior @ 0.4	
Posterior @ 0.4	

Posterior Graphic. Threshold Marked @ 0.4

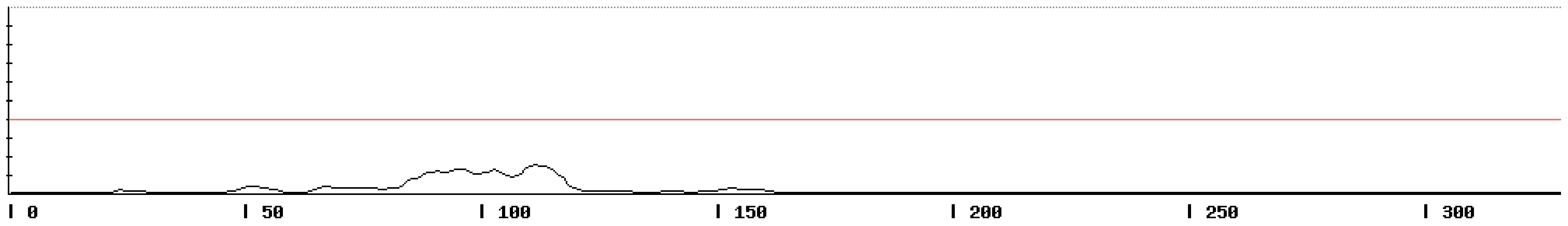

Predictions for Daucuscarota_DCAR_026063	
Viterbi Path	
Viterbi Path	
Posterior @ 0.4	
Posterior @ 0.4	
Posterior Graphic. Threshold Marked @ 0.4	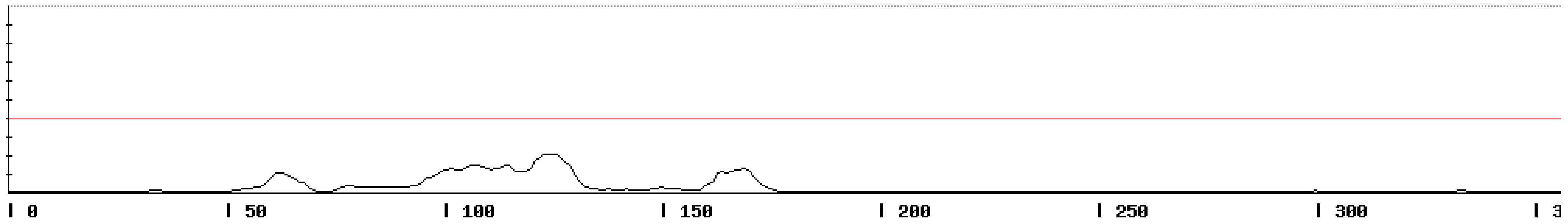

Predictions for Daucuscarota_DCAR_026711	
Viterbi Path	
Viterbi Path	
Posterior @ 0.4	
Posterior @ 0.4	

Posterior Graphic. Threshold Marked @ 0.4

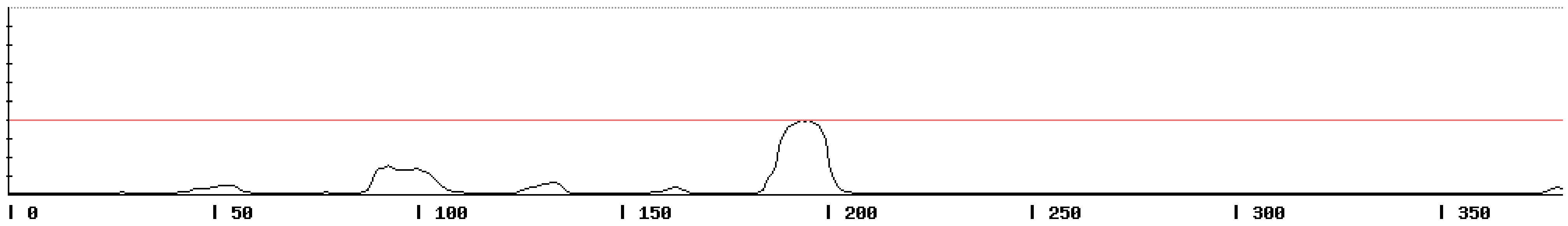

Predictions for Daucuscarota_DCAR_026712

Viterbi Path

Viterbi Path

Posterior @ 0.4

Posterior @ 0.4

Posterior Graphic. Threshold Marked @ 0.4

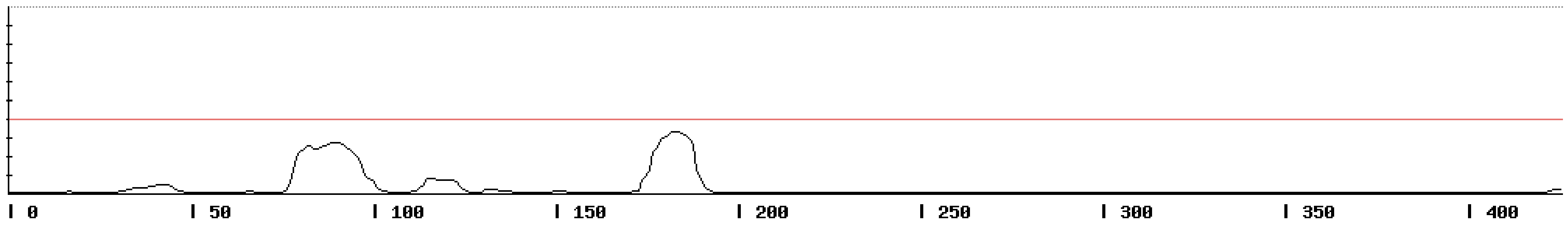

Predictions for Daucuscarota_DCAR_027411

Viterbi Path

Viterbi Path

Posterior @ 0.4	
Posterior @ 0.4	
Posterior Graphic. Threshold Marked @ 0.4	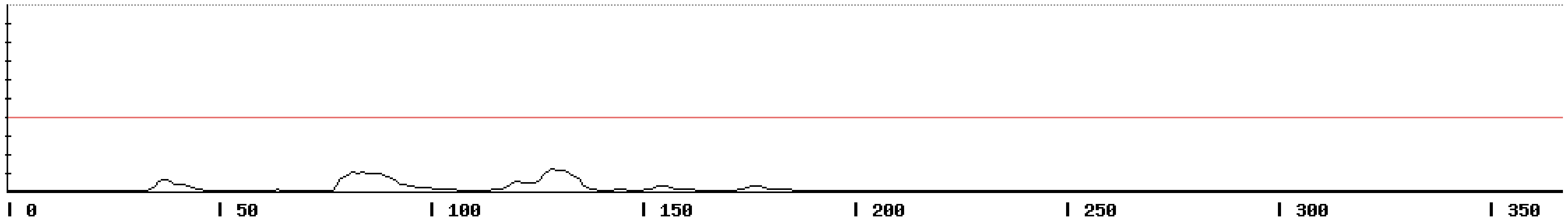

Predictions for Daucuscarota_DCAR_027802

Viterbi Path	
Viterbi Path	
Posterior @ 0.4	
Posterior @ 0.4	
Posterior Graphic. Threshold Marked @ 0.4	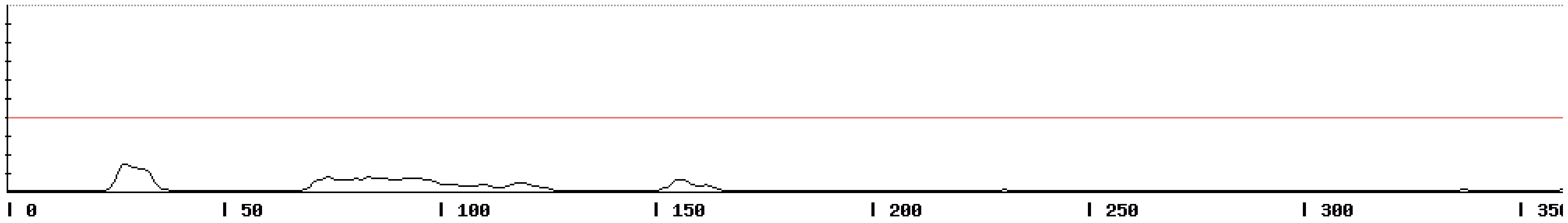

Predictions for Daucuscarota_DCAR_028042

Viterbi Path	
Viterbi Path	

Posterior @ 0.4 — 126 - KRR - 128

Posterior @ 0.4

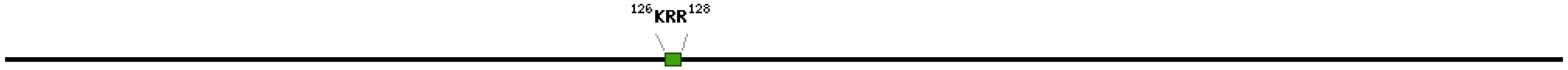

Posterior Graphic. Threshold Marked @ 0.4

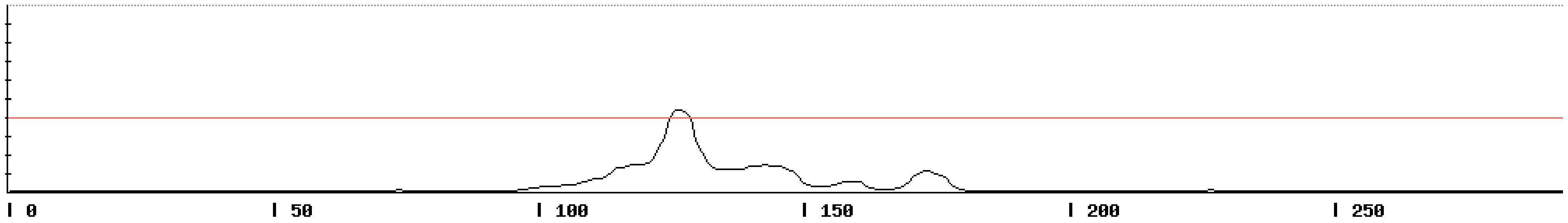

Predictions for Daucuscarota_DCAR_028641

Viterbi Path

Viterbi Path

Posterior @ 0.4

Posterior @ 0.4

Posterior Graphic. Threshold Marked @ 0.4

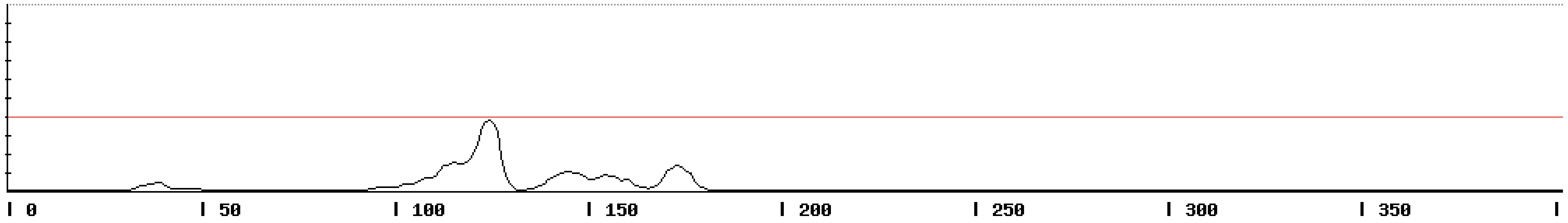

Predictions for Daucuscarota_DCAR_030911

Viterbi Path

Viterbi Path

Posterior @ 0.4

Posterior @ 0.4

Posterior Graphic. Threshold Marked @ 0.4

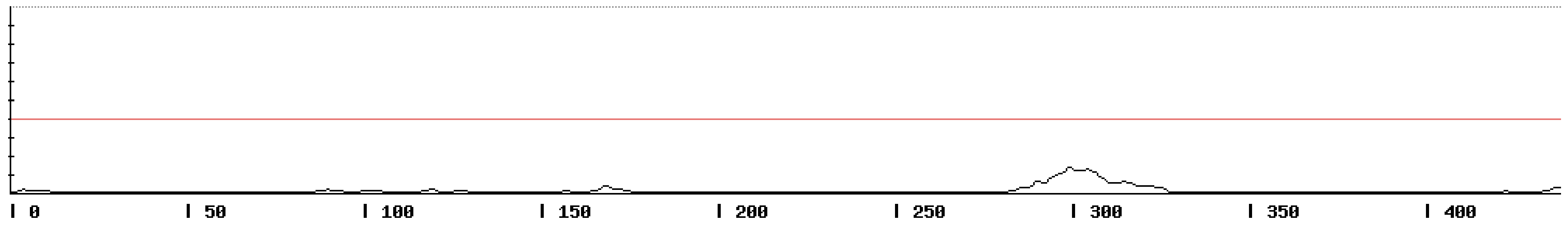

Predictions for Daucuscarota_DCAR_031001

Viterbi Path

Viterbi Path

Posterior @ 0.4

Posterior @ 0.4

Posterior Graphic. Threshold Marked @ 0.4

Revision r.9 - Last Updated on : February 24 2015 17:45:20.

alex.nguyenba@utoronto.ca

Intended to be used with Firefox

Viterbi Path

Posterior @ 0.4

Posterior @ 0.4

Posterior Graphic. Threshold Marked @ 0.4

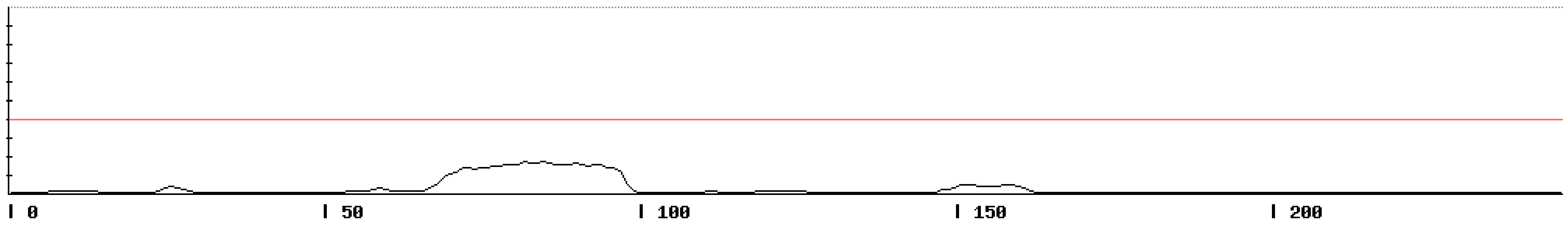

Predictions for Dianthuscaryophyllus_Dca12235.1

Viterbi Path

Viterbi Path

Posterior @ 0.4

Posterior @ 0.4

Posterior Graphic. Threshold Marked @ 0.4

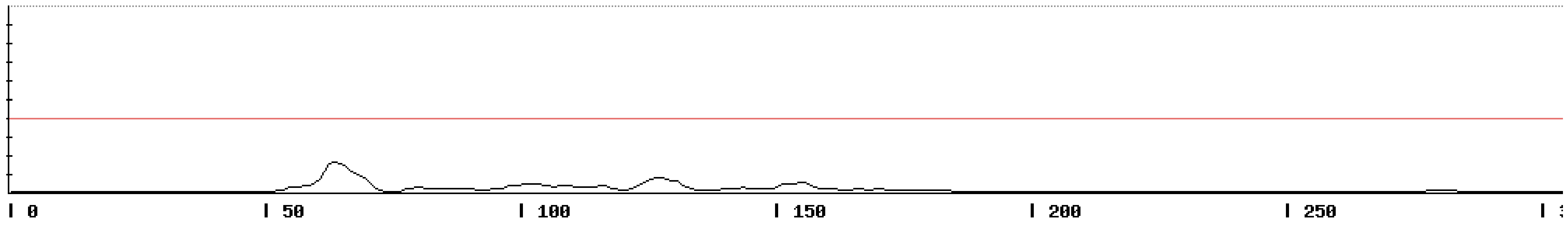

Predictions for Dianthuscaryophyllus_Dca12464.1

Viterbi Path	
Viterbi Path	
Posterior @ 0.4	205 - R - 205
Posterior @ 0.4	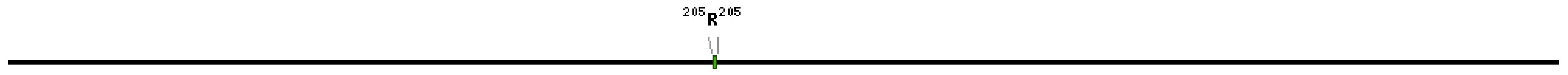
Posterior Graphic. Threshold Marked @ 0.4	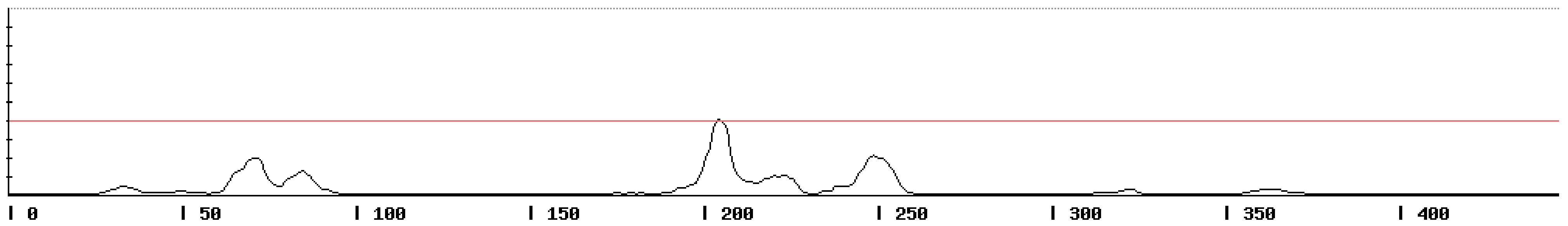

Predictions for Dianthuscaryophyllus_Dca13008.1

Viterbi Path	
Viterbi Path	
Posterior @ 0.4	
Posterior @ 0.4	
Posterior Graphic. Threshold Marked @ 0.4	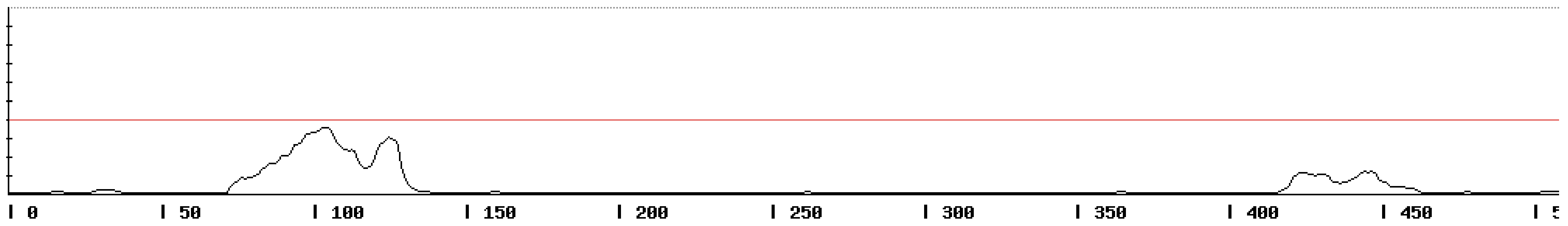

Predictions for Dianthuscaryophyllus_Dca13547.1

Viterbi Path

Viterbi Path

Posterior @ 0.4: 434 - KGKKIAHGKMKMLKKK - 449

Posterior @ 0.4

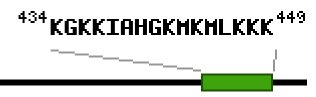

Posterior Graphic. Threshold Marked @ 0.4

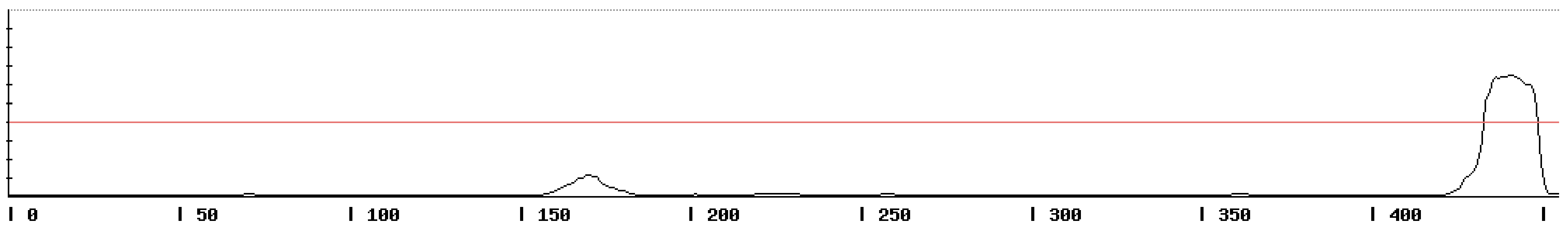

Predictions for Dianthuscaryophyllus_Dca13740.1

Viterbi Path

Viterbi Path

Posterior @ 0.4

Posterior @ 0.4

Posterior Graphic. Threshold Marked @ 0.4

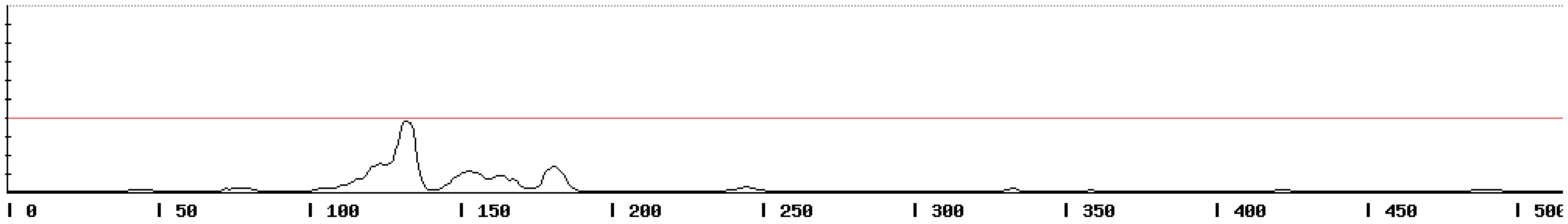

Predictions for Dianthuscaryophyllus_Dca17969.1

Viterbi Path	
Viterbi Path	
Posterior @ 0.4	100 - GKPK - 103
Posterior @ 0.4	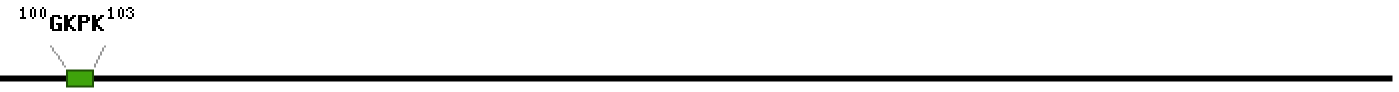
Posterior Graphic. Threshold Marked @ 0.4	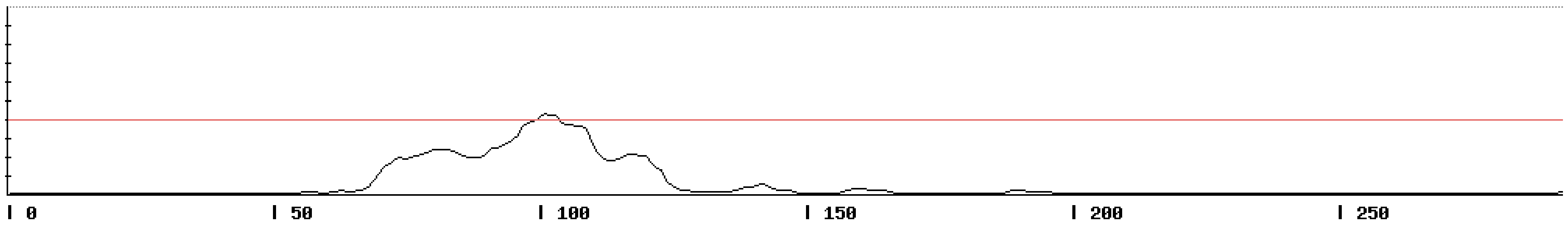

Predictions for Dianthuscaryophyllus_Dca1834.1

Viterbi Path	
Viterbi Path	
Posterior @ 0.4	
Posterior @ 0.4	

Posterior Graphic. Threshold Marked @ 0.4

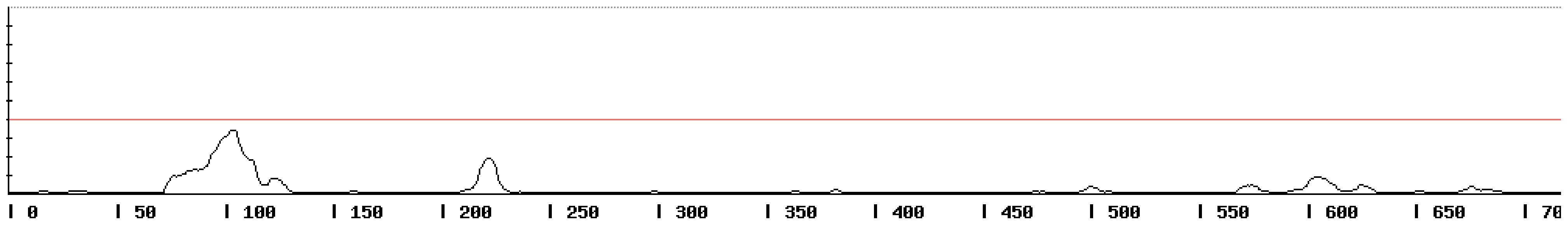

Predictions for Dianthuscaryophyllus_Dca20822.1

Viterbi Path

Viterbi Path

Posterior @ 0.4

254 - NRRIRLRKLRRVSPRK - 269

Posterior @ 0.4

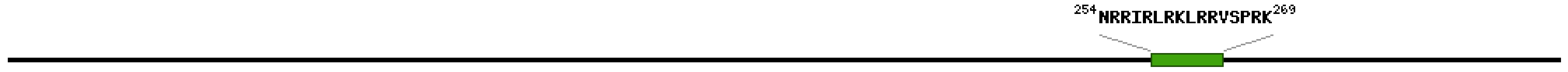

Posterior Graphic. Threshold Marked @ 0.4

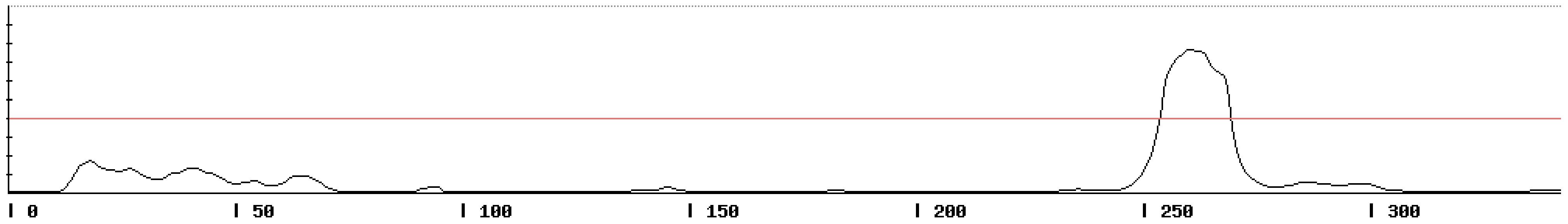

Predictions for Dianthuscaryophyllus_Dca21465.1

Viterbi Path

Viterbi Path

Posterior @ 0.4

Posterior @ 0.4

Posterior Graphic. Threshold Marked @ 0.4

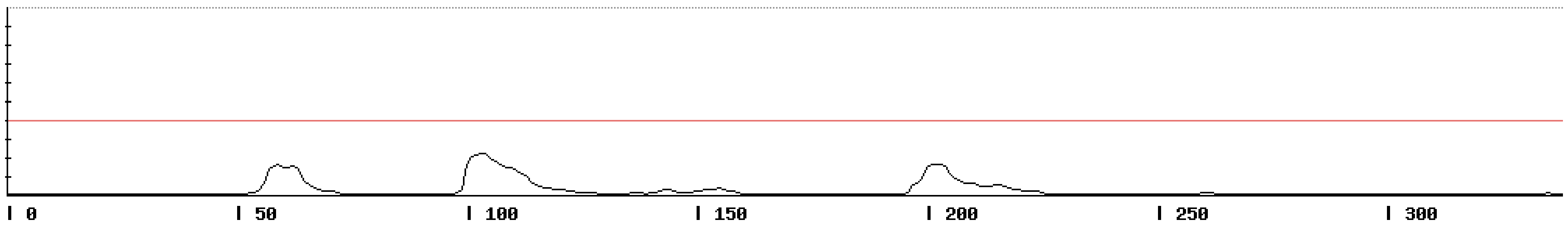

Predictions for Dianthuscaryophyllus_Dca21608.1

Viterbi Path

Viterbi Path

Posterior @ 0.4

Posterior @ 0.4

Posterior Graphic. Threshold Marked @ 0.4

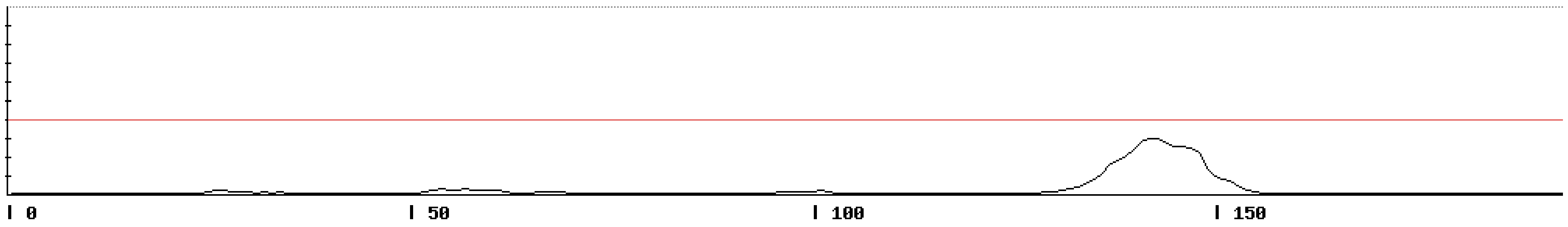

Predictions for Dianthuscaryophyllus_Dca21650.1

Viterbi Path

Viterbi Path

Posterior @ 0.4

Posterior @ 0.4

Posterior Graphic. Threshold Marked @ 0.4

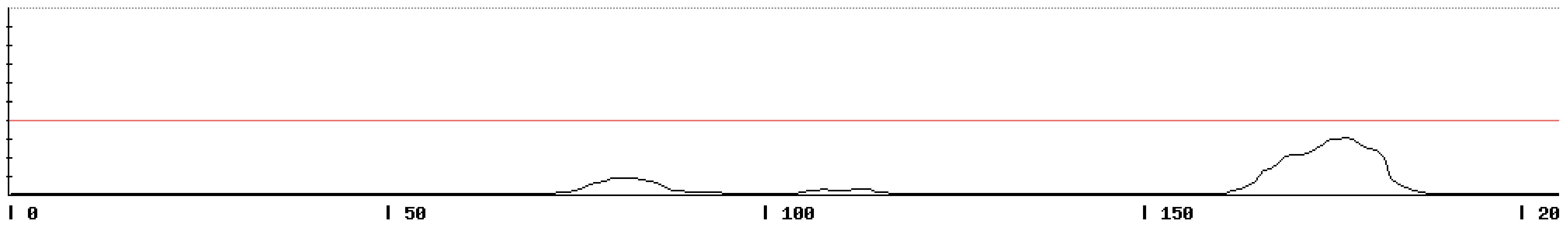

Predictions for Dianthuscaryophyllus_Dca21702.1

Viterbi Path

Viterbi Path

Posterior @ 0.4

Posterior @ 0.4

Posterior Graphic. Threshold Marked @ 0.4

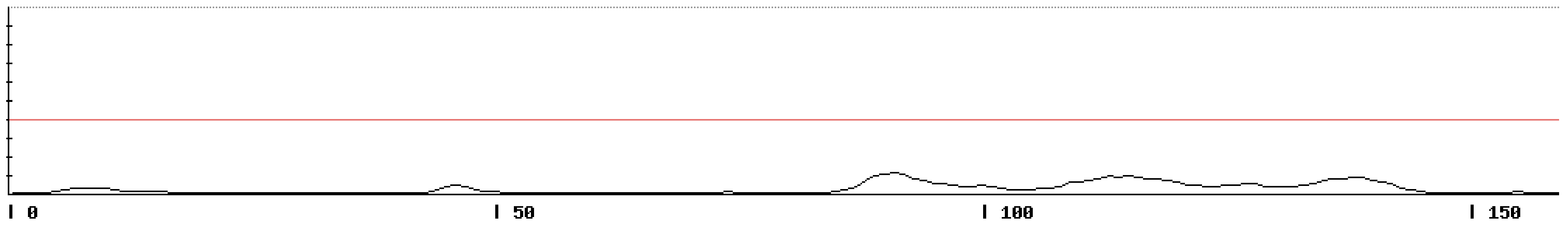

Predictions for Dianthuscaryophyllus_Dca21771.1

Viterbi Path

Viterbi Path

Posterior @ 0.4 — 85 - KLKKKKKNHERVI - 97

Posterior @ 0.4

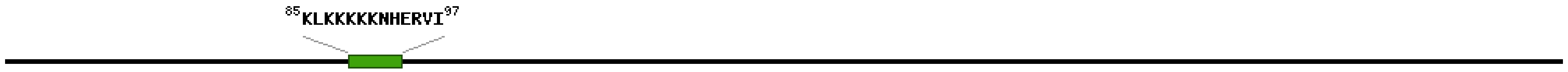

Posterior Graphic. Threshold Marked @ 0.4

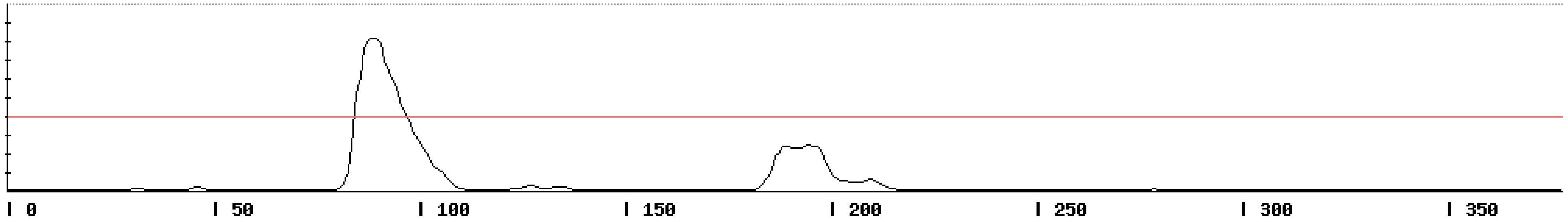

Predictions for Dianthuscaryophyllus_Dca22412.1

Viterbi Path

Viterbi Path

Posterior @ 0.4

Posterior @ 0.4

Posterior Graphic. Threshold Marked @ 0.4

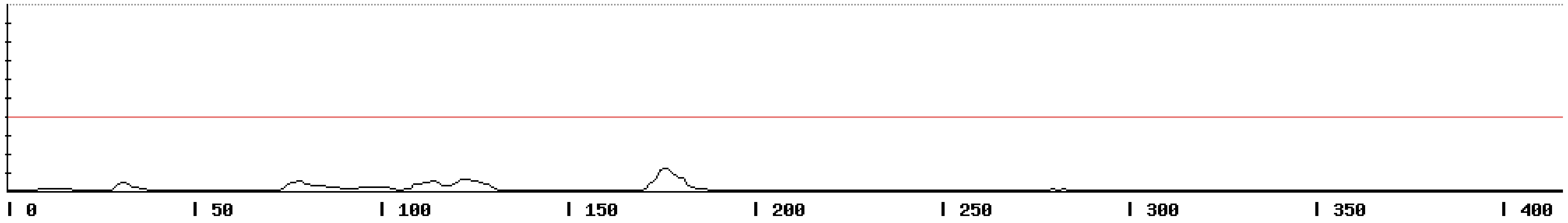

Predictions for Dianthuscaryophyllus_Dca25155.1

Viterbi Path

Viterbi Path

Posterior @ 0.4

Posterior @ 0.4

Posterior Graphic. Threshold Marked @ 0.4

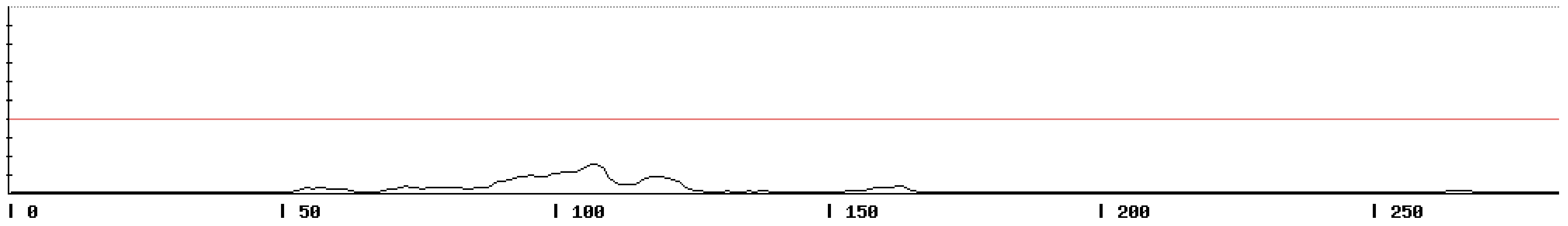

Predictions for Dianthuscaryophyllus_Dca25159.1

Viterbi Path

Viterbi Path

Posterior @ 0.4

Posterior @ 0.4

Posterior Graphic. Threshold Marked @ 0.4

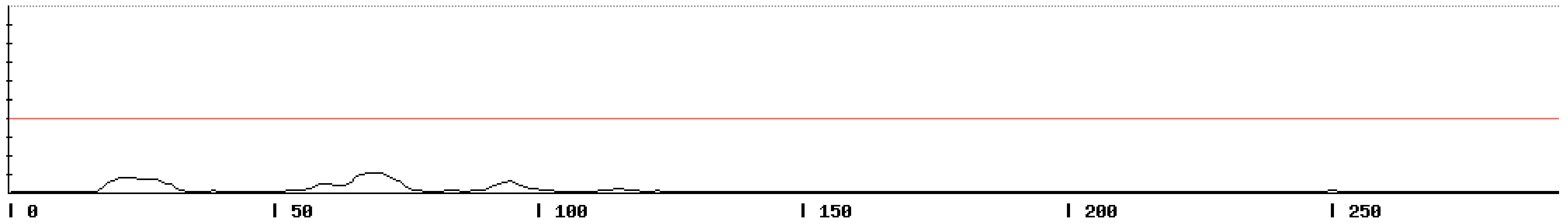

Predictions for Dianthuscaryophyllus_Dca27021.1	
Viterbi Path	
Viterbi Path	
Posterior @ 0.4	
Posterior @ 0.4	
Posterior Graphic. Threshold Marked @ 0.4	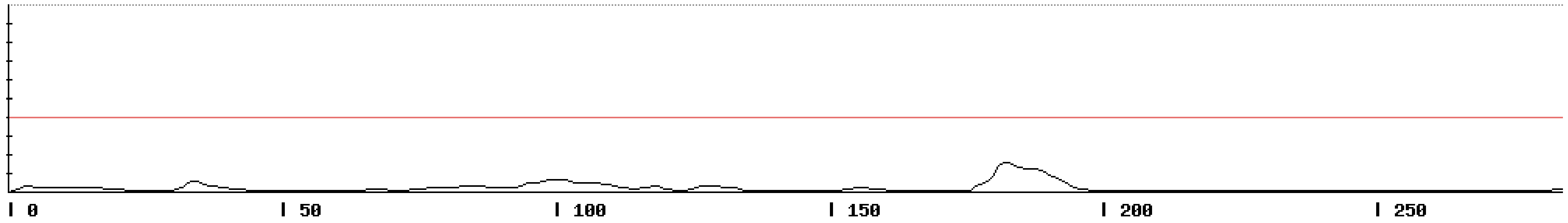

Predictions for Dianthuscaryophyllus_Dca27100.1	
Viterbi Path	
Viterbi Path	
Posterior @ 0.4	
Posterior @ 0.4	
Posterior Graphic. Threshold Marked @ 0.4	

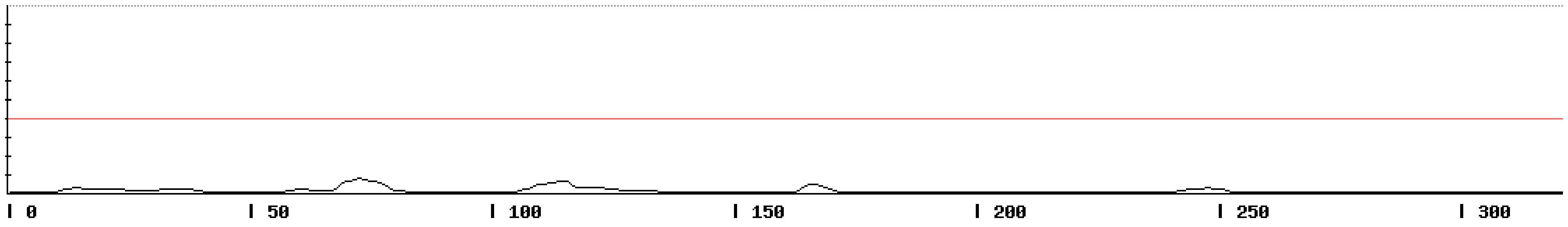

Predictions for Dianthuscaryophyllus_Dca28783.1

Viterbi Path

Viterbi Path

Posterior @ 0.4

Posterior @ 0.4

Posterior Graphic. Threshold Marked @ 0.4

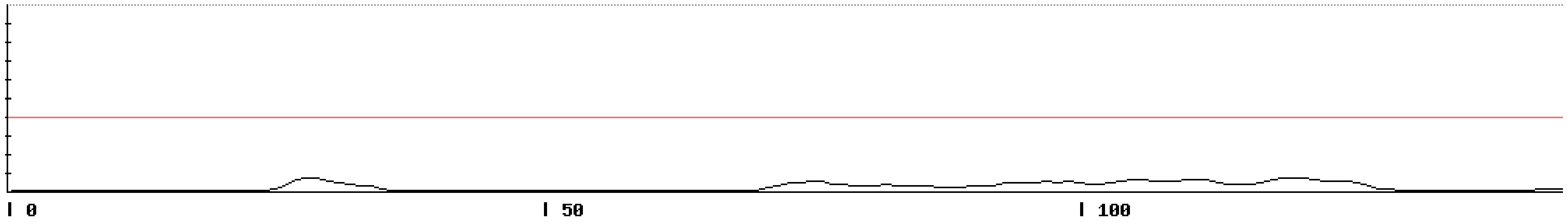

Predictions for Dianthuscaryophyllus_Dca29384.1

Viterbi Path

Viterbi Path

Posterior @ 0.4

Posterior @ 0.4

Posterior Graphic. Threshold Marked @ 0.4

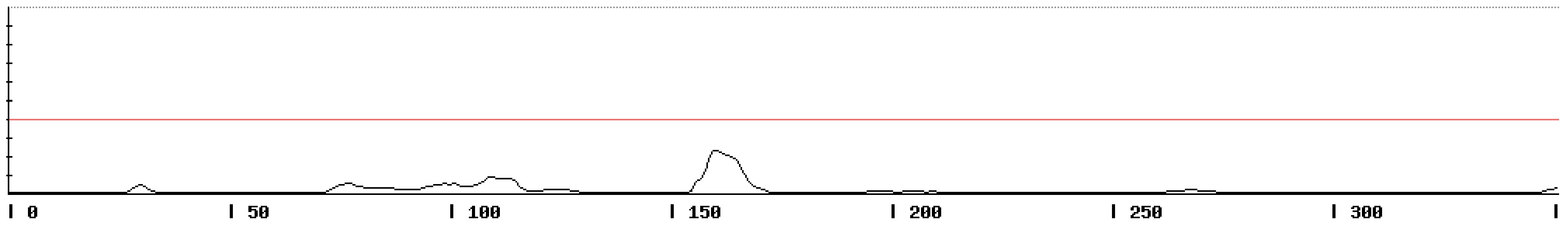

Predictions for Dianthuscaryophyllus_Dca29398.1

Viterbi Path

Viterbi Path

Posterior @ 0.4

Posterior @ 0.4

Posterior Graphic. Threshold Marked @ 0.4

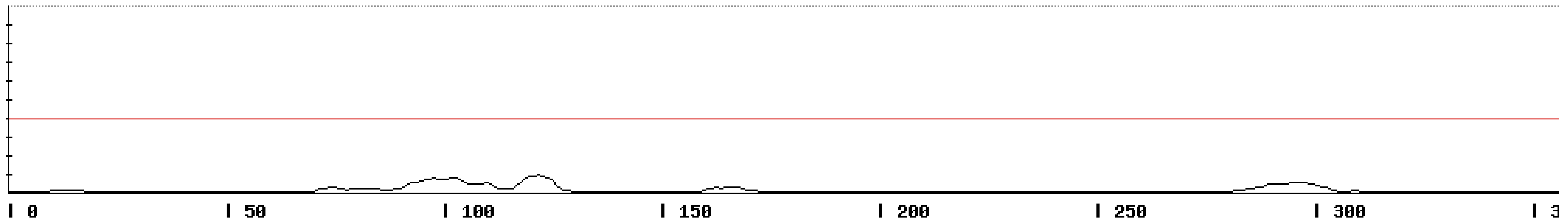

Predictions for Dianthuscaryophyllus_Dca29406.1

Viterbi Path

Viterbi Path

Posterior @ 0.4

Posterior @ 0.4

Posterior Graphic. Threshold Marked @ 0.4

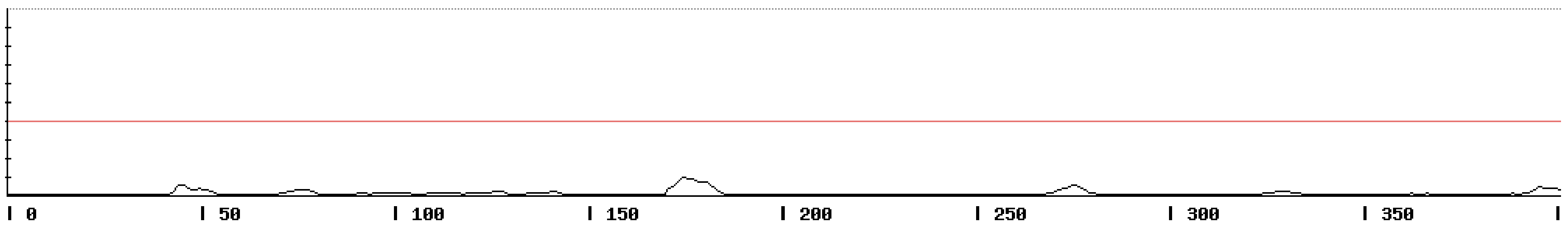

Predictions for Dianthuscaryophyllus_Dca29495.1

Viterbi Path

Viterbi Path

Posterior @ 0.4

100 - DKPIGKPKPVAIKK - 113

Posterior @ 0.4

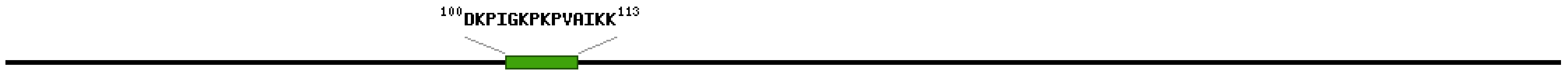

Posterior Graphic. Threshold Marked @ 0.4

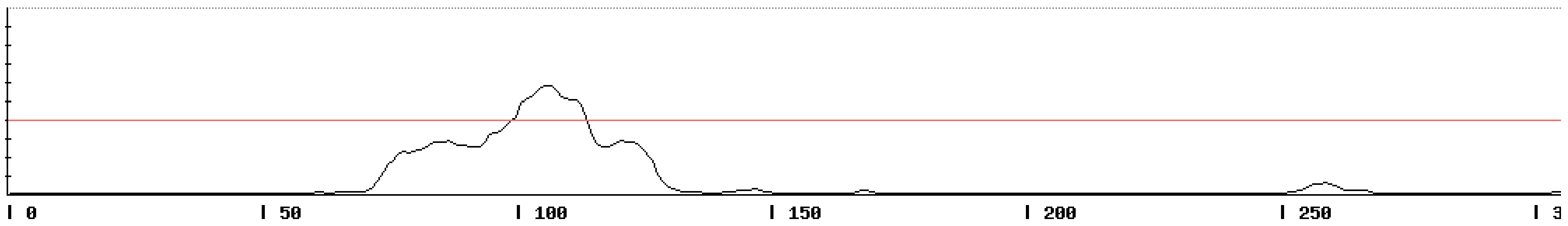

Predictions for Dianthuscaryophyllus_Dca30054.1

Viterbi Path

Viterbi Path

Posterior @ 0.4

Posterior @ 0.4

Posterior Graphic. Threshold Marked @ 0.4

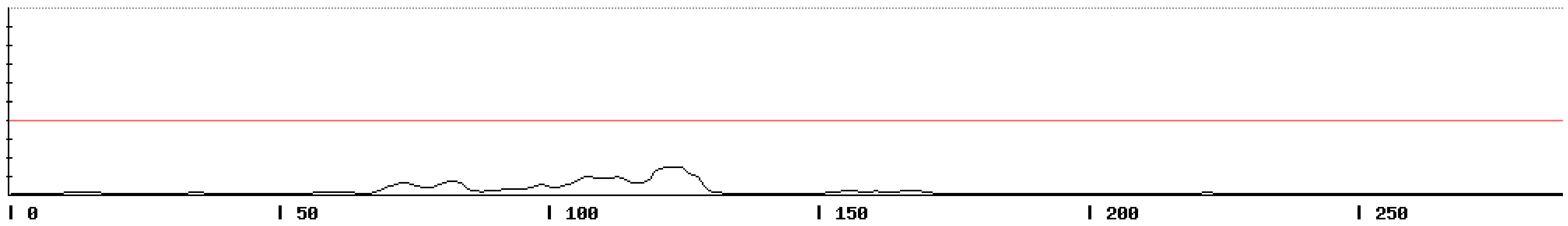

Predictions for Dianthuscaryophyllus_Dca30668.1

Viterbi Path

Viterbi Path

Posterior @ 0.4

Posterior @ 0.4

Posterior Graphic. Threshold Marked @ 0.4

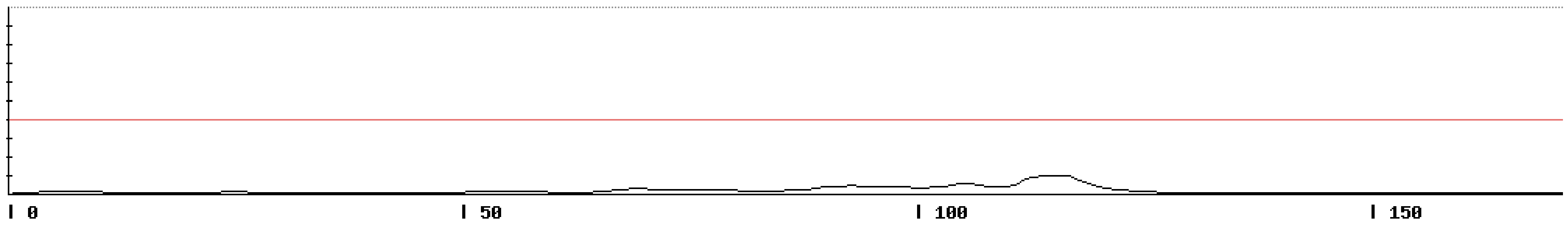

Predictions for Dianthuscaryophyllus_Dca30753.1

Viterbi Path

Viterbi Path

Posterior @ 0.4

Posterior @ 0.4

Posterior Graphic. Threshold Marked @ 0.4

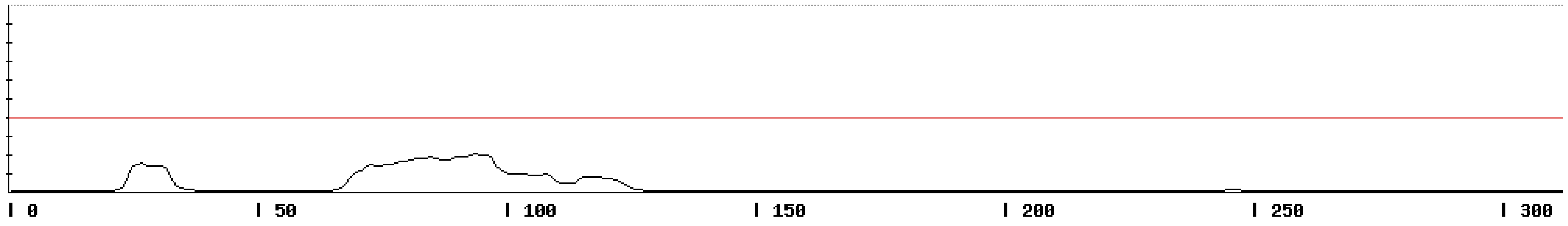

Predictions for Dianthuscaryophyllus_Dca30941.1

Viterbi Path

Viterbi Path

Posterior @ 0.4

Posterior @ 0.4

Posterior Graphic. Threshold Marked @ 0.4

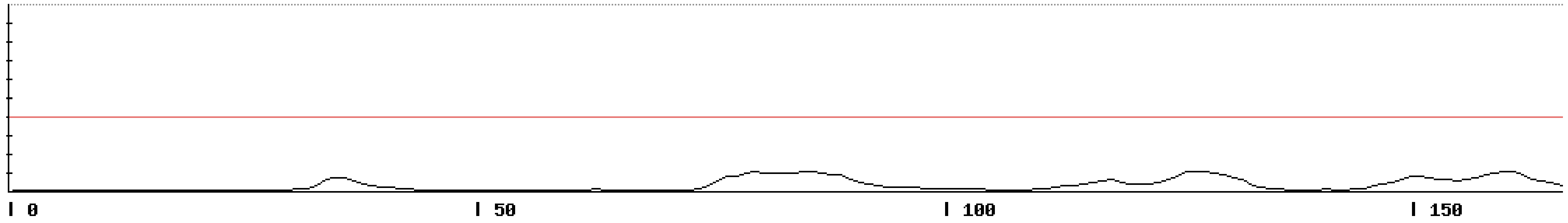

Predictions for Dianthuscaryophyllus_Dca31341.1

Viterbi Path	
Viterbi Path	
Posterior @ 0.4	
Posterior @ 0.4	
Posterior Graphic. Threshold Marked @ 0.4	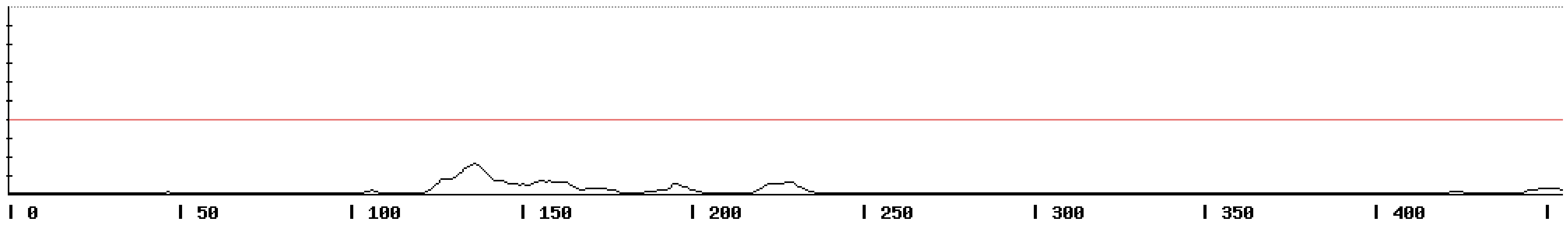

Predictions for Dianthuscaryophyllus_Dca31737.1

Viterbi Path	
Viterbi Path	
Posterior @ 0.4	
Posterior @ 0.4	
Posterior Graphic. Threshold Marked @ 0.4	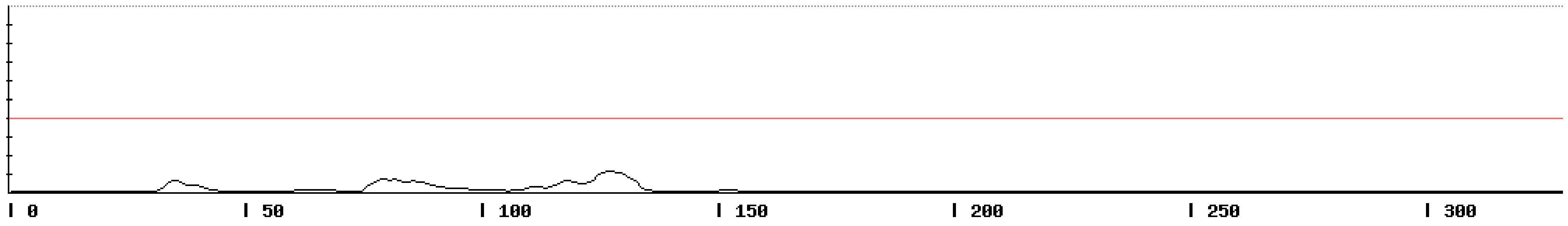

Predictions for Dianthuscaryophyllus_Dca31870.1

Viterbi Path

Viterbi Path

Posterior @ 0.4

Posterior @ 0.4

Posterior Graphic. Threshold Marked @ 0.4

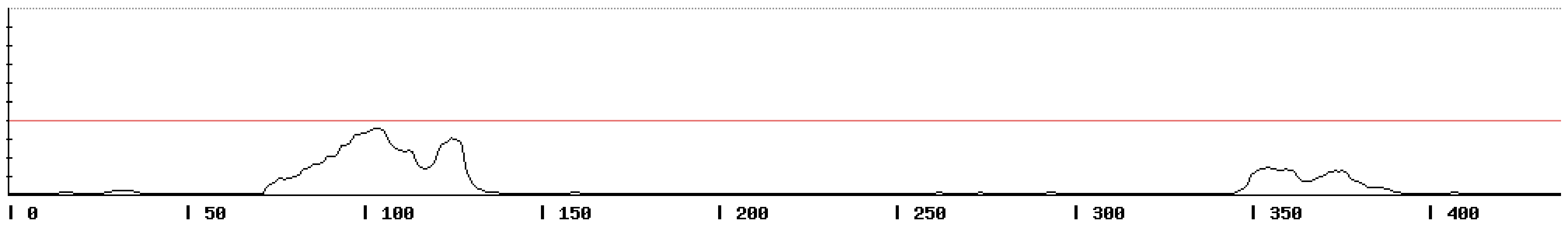

Predictions for Dianthuscaryophyllus_Dca34636.1

Viterbi Path

Viterbi Path

Posterior @ 0.4

Posterior @ 0.4

Posterior Graphic. Threshold Marked @ 0.4

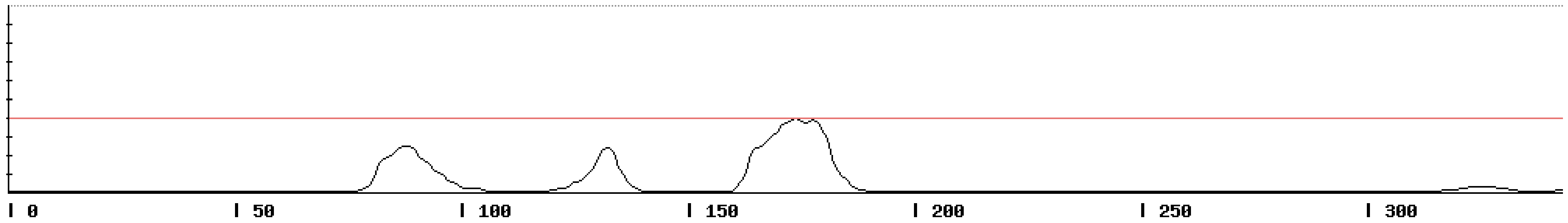

Predictions for Dianthuscaryophyllus_Dca35574.1

Viterbi Path	
Viterbi Path	
Posterior @ 0.4	
Posterior @ 0.4	
Posterior Graphic. Threshold Marked @ 0.4	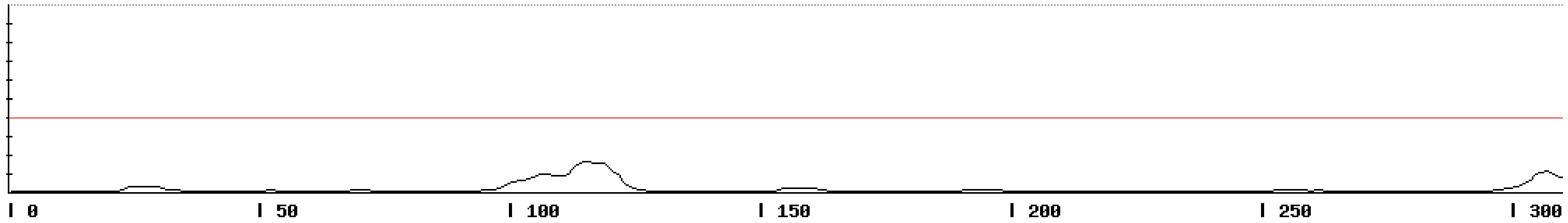

Predictions for Dianthuscaryophyllus_Dca37363.1

Viterbi Path	
Viterbi Path	
Posterior @ 0.4	
Posterior @ 0.4	

Posterior Graphic. Threshold Marked @ 0.4

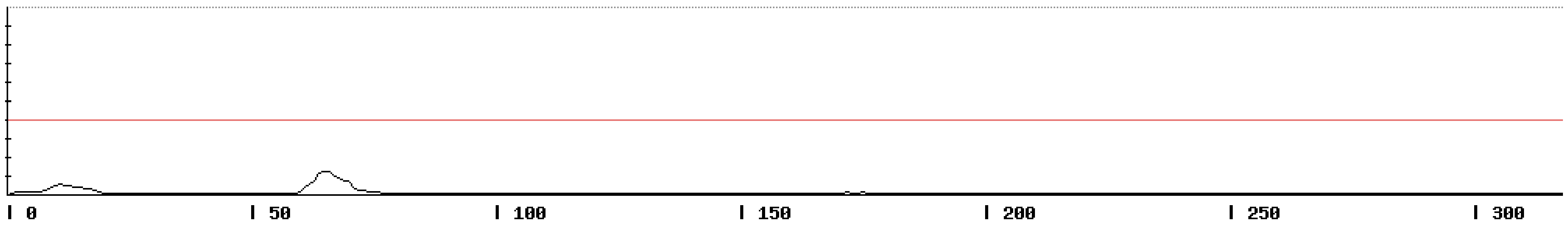

Predictions for Dianthuscaryophyllus_Dca38095.1

Viterbi Path

Viterbi Path

Posterior @ 0.4

Posterior @ 0.4

Posterior Graphic. Threshold Marked @ 0.4

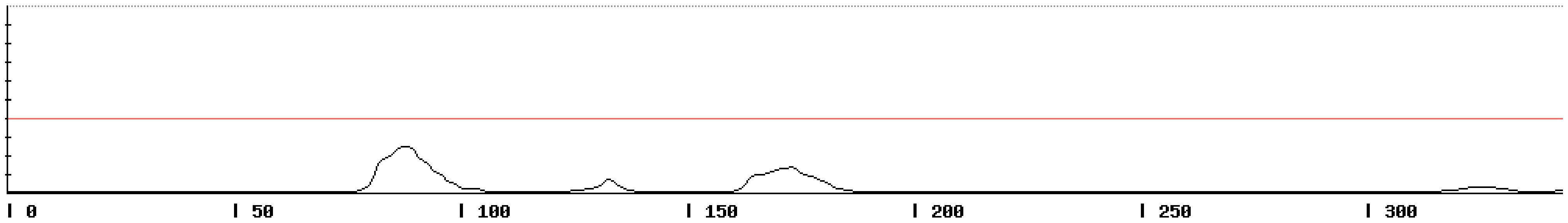

Predictions for Dianthuscaryophyllus_Dca39411.1

Viterbi Path

Viterbi Path

Posterior @ 0.4

Posterior @ 0.4

Posterior Graphic. Threshold Marked @ 0.4

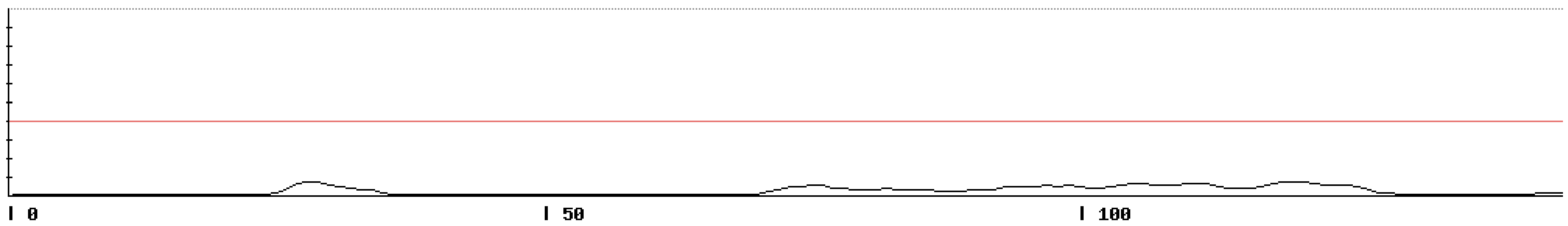

Predictions for Dianthuscaryophyllus_Dca3955.1

Viterbi Path

Viterbi Path

Posterior @ 0.4 27 - RKRR - 30

Posterior @ 0.4

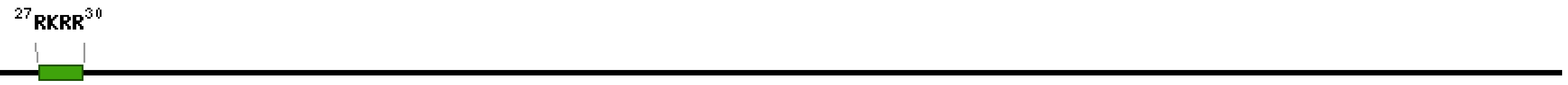

Posterior Graphic. Threshold Marked @ 0.4

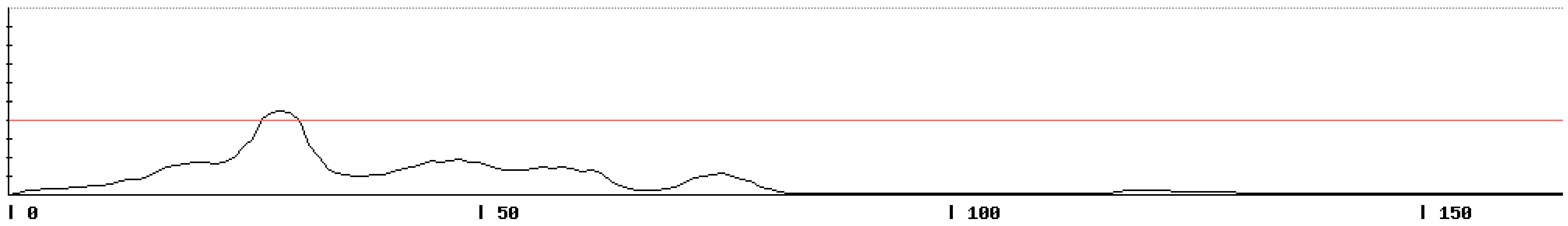

Predictions for Dianthuscaryophyllus_Dca39946.1

Viterbi Path

Viterbi Path

Posterior @ 0.4

Posterior @ 0.4

Posterior Graphic. Threshold Marked @ 0.4

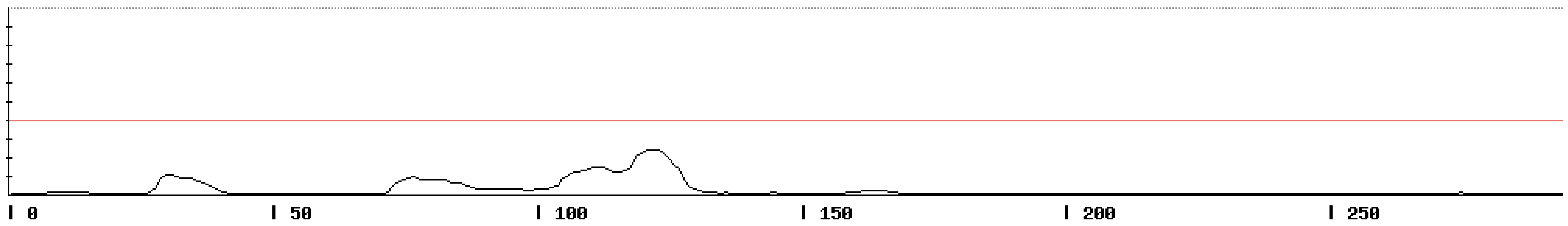

Predictions for Dianthuscaryophyllus_Dca4023.1

Viterbi Path

Viterbi Path

Posterior @ 0.4

Posterior @ 0.4

Posterior Graphic. Threshold Marked @ 0.4

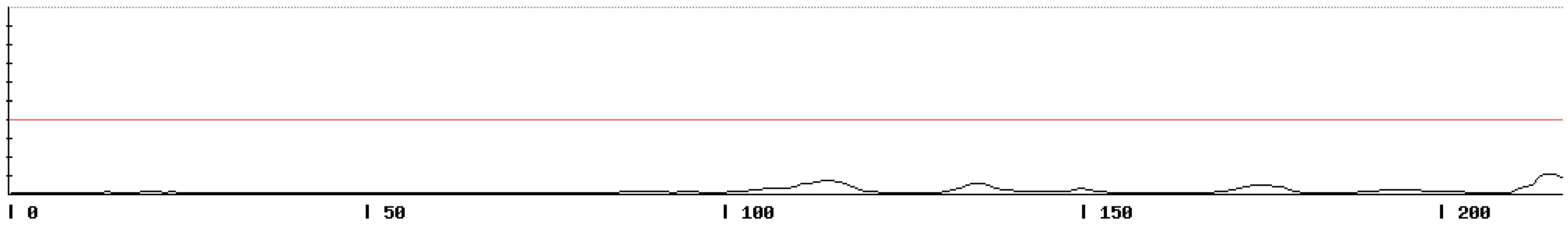

Predictions for Dianthuscaryophyllus_Dca41801.1

Viterbi Path

Viterbi Path

Posterior @ 0.4

Posterior @ 0.4

Posterior Graphic. Threshold Marked @ 0.4

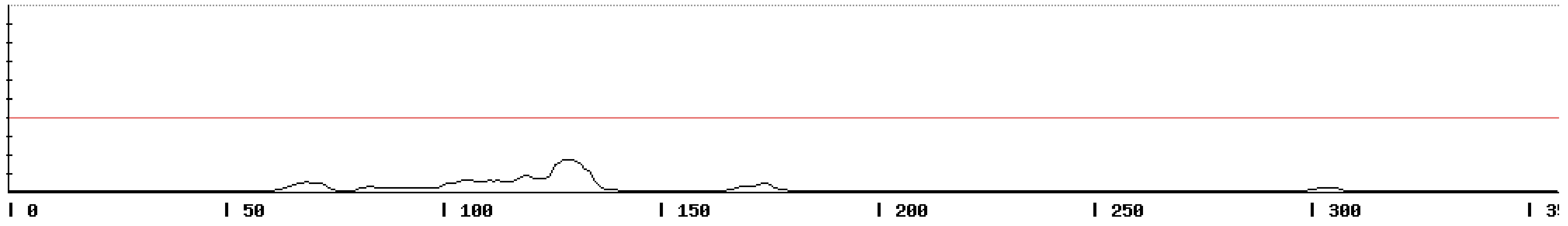

Predictions for Dianthuscaryophyllus_Dca44840.1

Viterbi Path

Viterbi Path

Posterior @ 0.4 238 - K - 238

Posterior @ 0.4

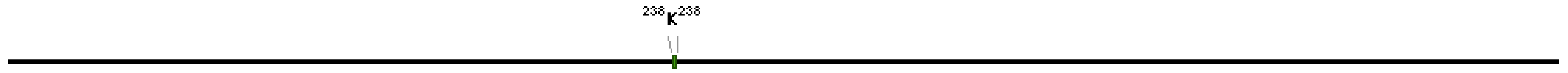

Posterior Graphic. Threshold Marked @ 0.4

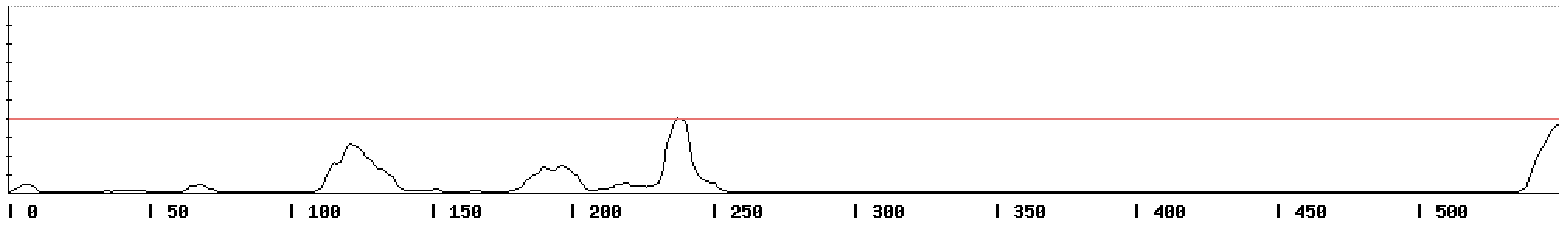

Predictions for Dianthuscaryophyllus_Dca44899.1

Viterbi Path

Viterbi Path

Posterior @ 0.4

Posterior @ 0.4

Posterior Graphic. Threshold Marked @ 0.4

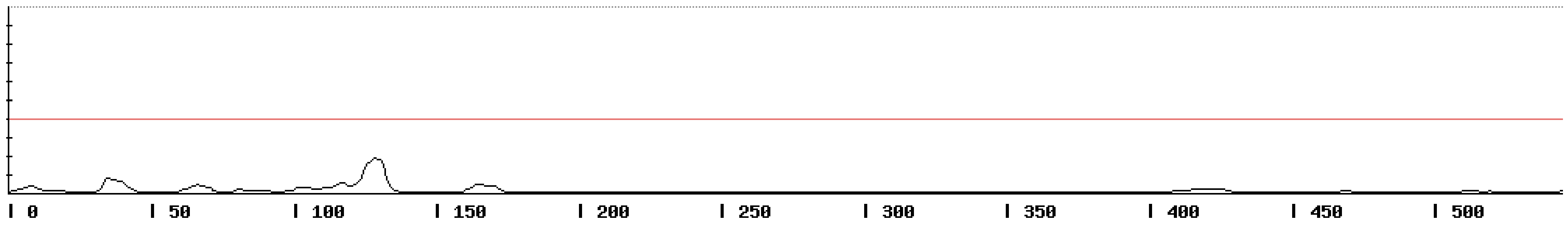

Predictions for Dianthuscaryophyllus_Dca45711.1

Viterbi Path

Viterbi Path

Posterior @ 0.4

Posterior @ 0.4

Posterior Graphic. Threshold Marked @ 0.4

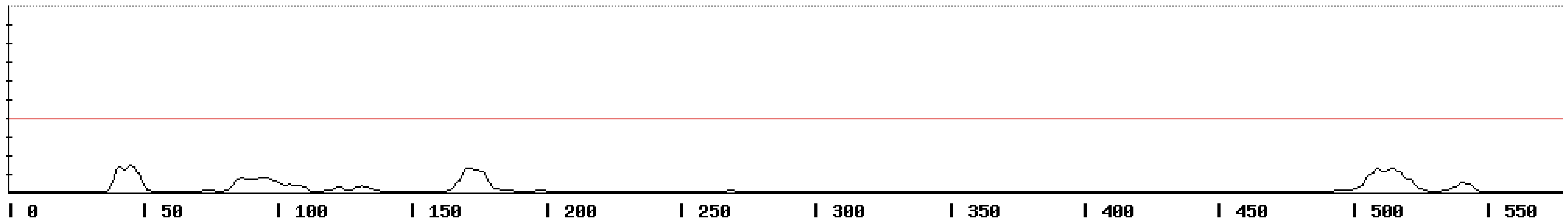

Predictions for Dianthuscaryophyllus_Dca45788.1	
Viterbi Path	
Viterbi Path	
Posterior @ 0.4	
Posterior @ 0.4	
Posterior Graphic. Threshold Marked @ 0.4	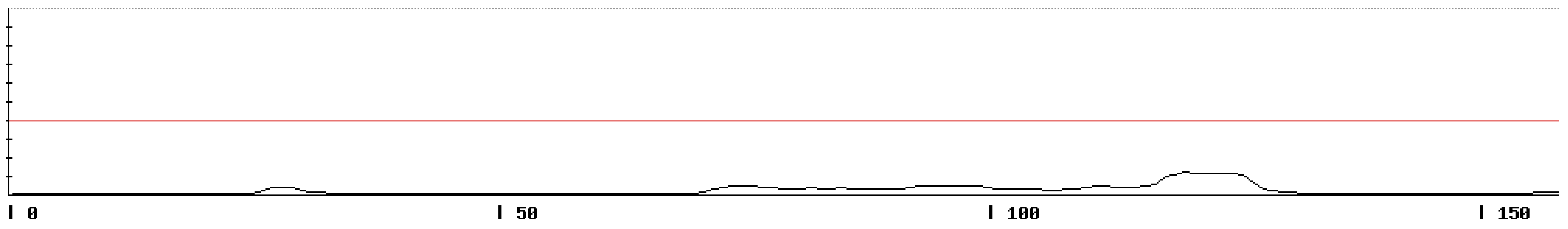

Predictions for Dianthuscaryophyllus_Dca47145.1	
Viterbi Path	
Viterbi Path	
Posterior @ 0.4	
Posterior @ 0.4	
Posterior Graphic. Threshold Marked @ 0.4	

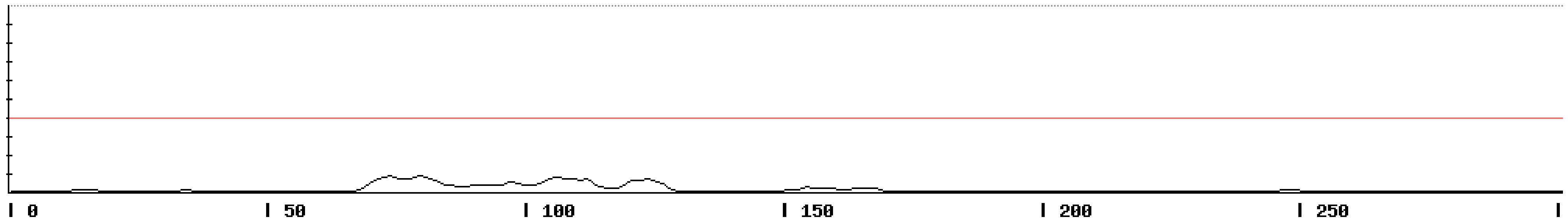

Predictions for Dianthuscaryophyllus_Dca47256.1

Viterbi Path

Viterbi Path

Posterior @ 0.4

Posterior @ 0.4

Posterior Graphic. Threshold Marked @ 0.4

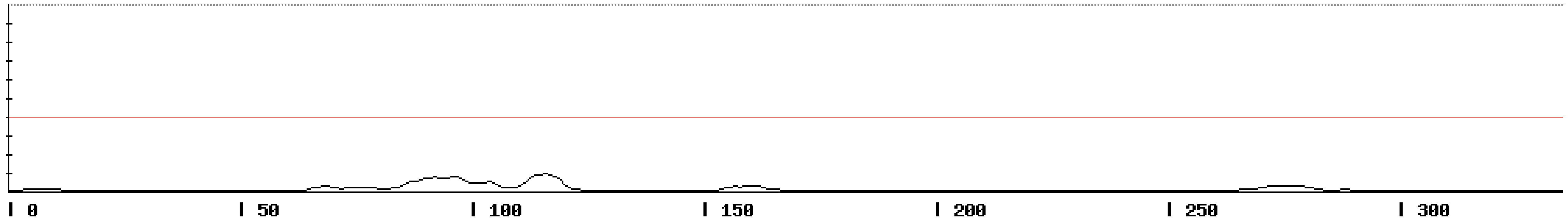

Predictions for Dianthuscaryophyllus_Dca48635.1

Viterbi Path

Viterbi Path

Posterior @ 0.4

Posterior @ 0.4

Posterior Graphic. Threshold Marked @ 0.4

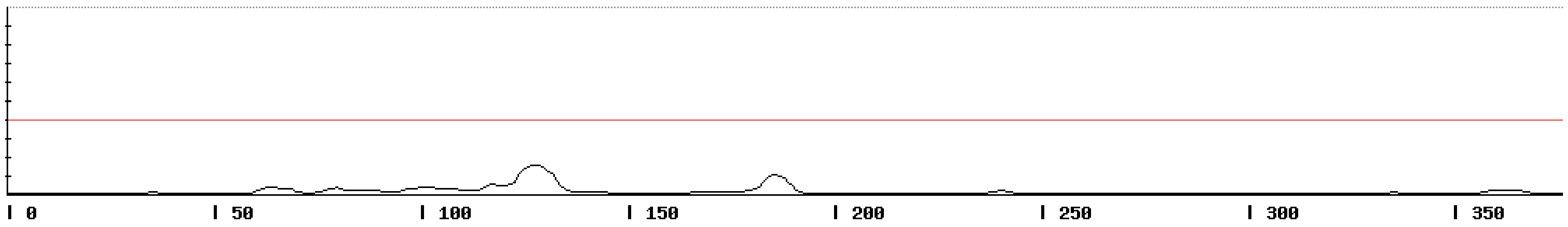

Predictions for Dianthuscaryophyllus_Dca48700.1

Viterbi Path

Viterbi Path

Posterior @ 0.4

Posterior @ 0.4

Posterior Graphic. Threshold Marked @ 0.4

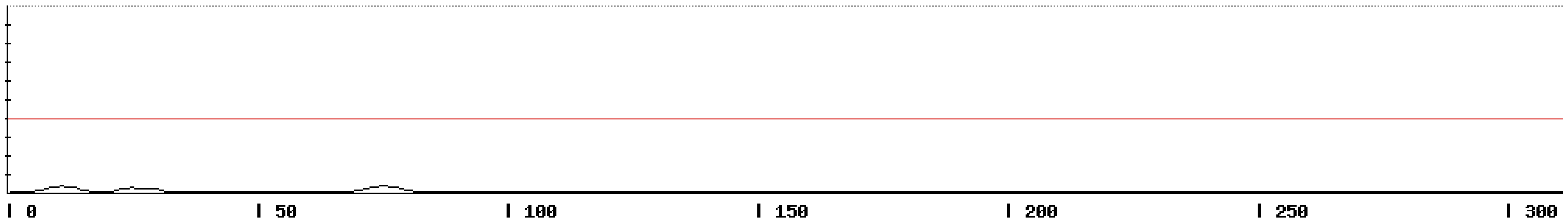

Predictions for Dianthuscaryophyllus_Dca49116.1

Viterbi Path

Viterbi Path

Posterior @ 0.4

Posterior @ 0.4

Posterior Graphic. Threshold Marked @ 0.4

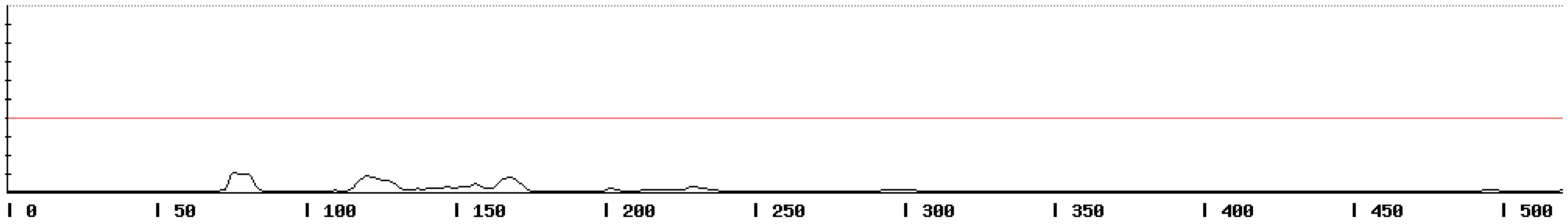

Predictions for Dianthuscaryophyllus_Dca4987.1

Viterbi Path	
Viterbi Path	
Posterior @ 0.4	126 - SRPKAKR - 132
Posterior @ 0.4	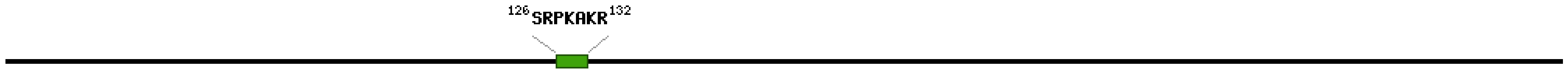
Posterior Graphic. Threshold Marked @ 0.4	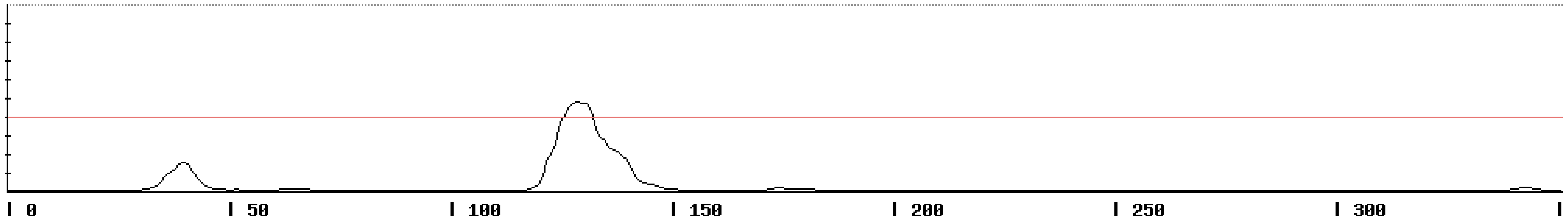

Predictions for Dianthuscaryophyllus_Dca50076.1

Viterbi Path	
Viterbi Path	

Posterior @ 0.4	
Posterior @ 0.4	
Posterior Graphic. Threshold Marked @ 0.4	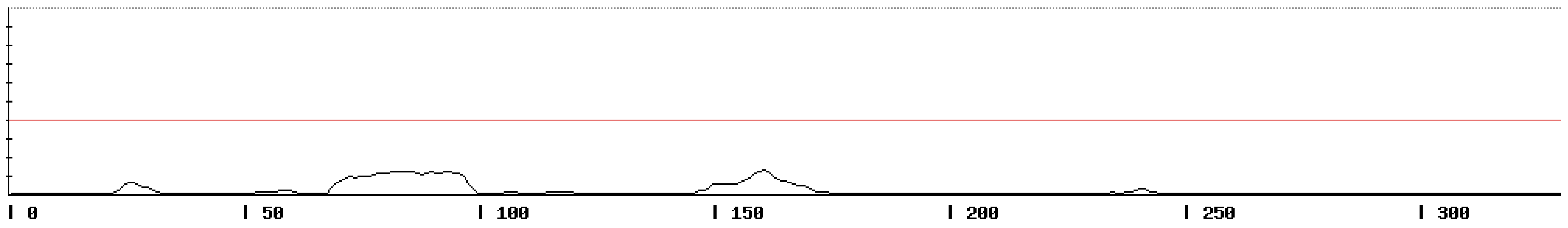

Predictions for Dianthuscaryophyllus_Dca50793.1	
Viterbi Path	
Viterbi Path	
Posterior @ 0.4	
Posterior @ 0.4	
Posterior Graphic. Threshold Marked @ 0.4	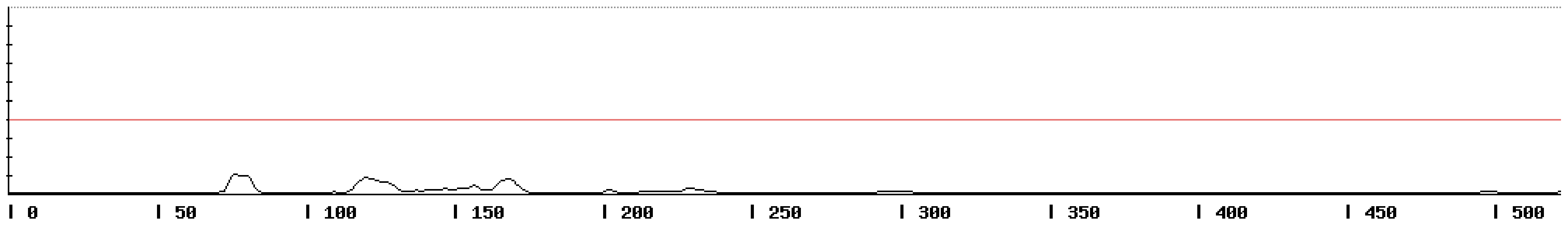

Predictions for Dianthuscaryophyllus_Dca50873.1	
Viterbi Path	
Viterbi Path	

Posterior @ 0.4

Posterior @ 0.4

Posterior Graphic. Threshold Marked @ 0.4

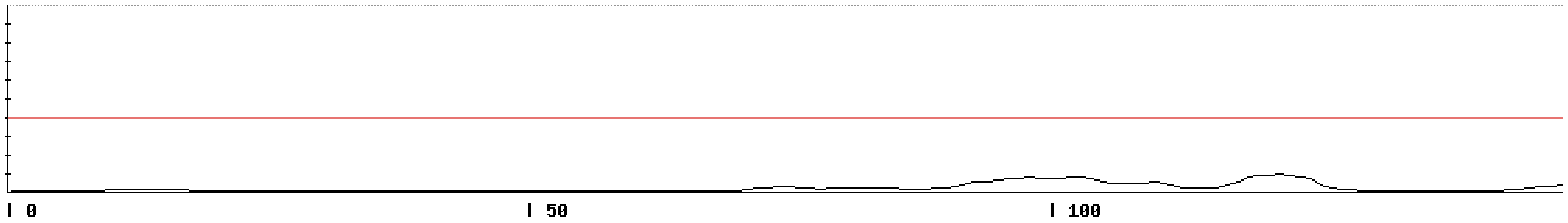

Predictions for Dianthuscaryophyllus_Dca51137.1

Viterbi Path

Viterbi Path

Posterior @ 0.4

Posterior @ 0.4

Posterior Graphic. Threshold Marked @ 0.4

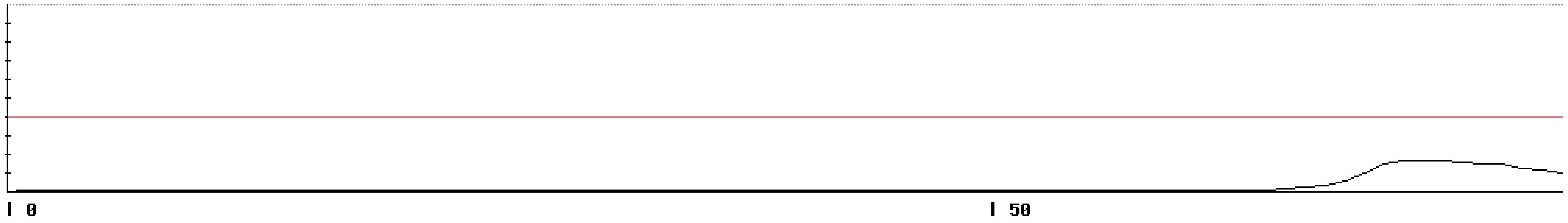

Predictions for Dianthuscaryophyllus_Dca51258.1

Viterbi Path

Viterbi Path

Posterior @ 0.4

Posterior @ 0.4

Posterior Graphic. Threshold Marked @ 0.4

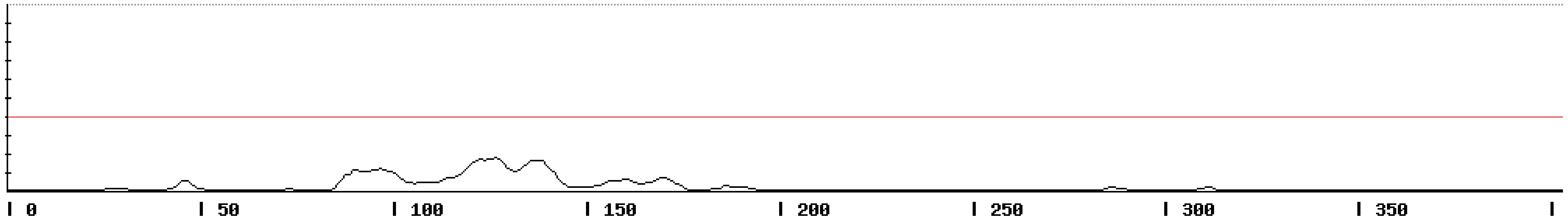

Predictions for Dianthuscaryophyllus_Dca51261.1

Viterbi Path

Viterbi Path

Posterior @ 0.4

Posterior @ 0.4

Posterior Graphic. Threshold Marked @ 0.4

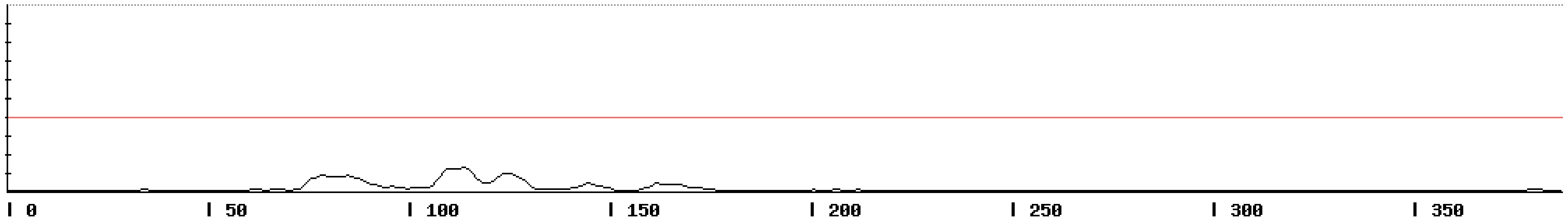

Predictions for Dianthuscaryophyllus_Dca51723.1

Viterbi Path

Viterbi Path

Posterior @ 0.4

Posterior @ 0.4

Posterior Graphic. Threshold Marked @ 0.4

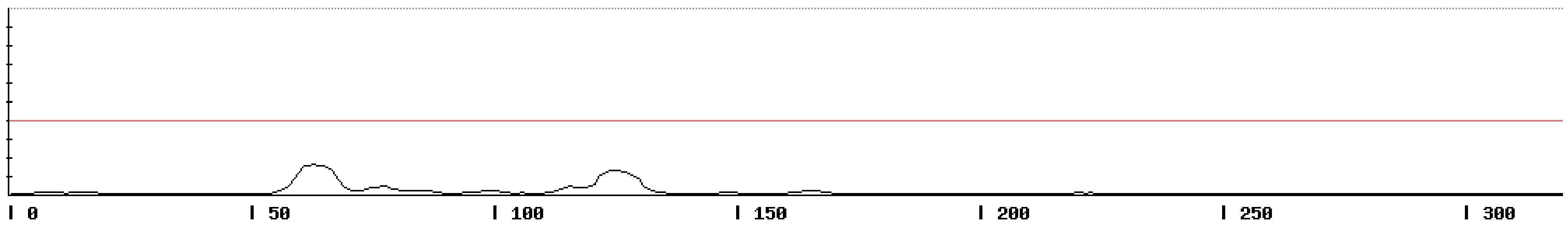

Predictions for Dianthuscaryophyllus_Dca52337.1

Viterbi Path

Viterbi Path

Posterior @ 0.4

Posterior @ 0.4

Posterior Graphic. Threshold Marked @ 0.4

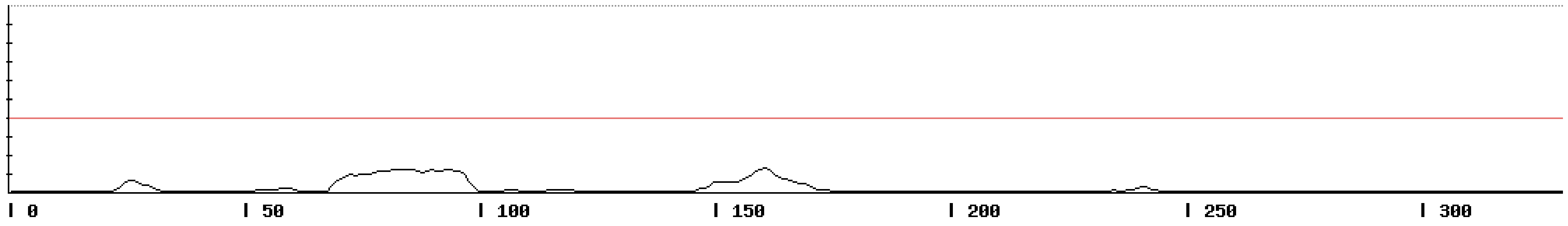

Predictions for Dianthuscaryophyllus_Dca52773.1

Viterbi Path	
Viterbi Path	
Posterior @ 0.4	
Posterior @ 0.4	
Posterior Graphic. Threshold Marked @ 0.4	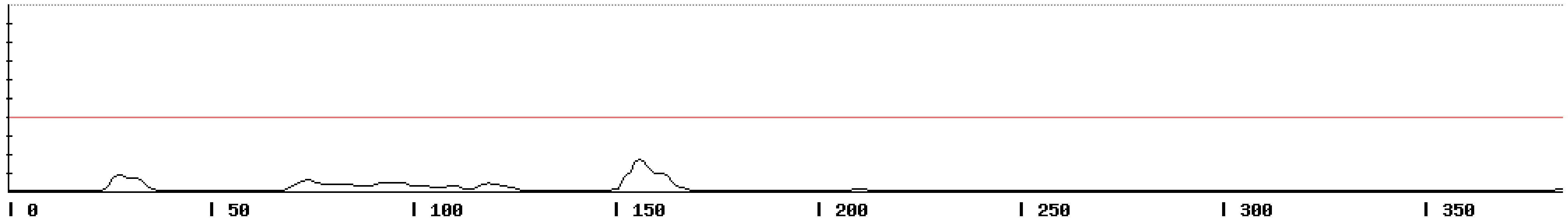

Predictions for Dianthuscaryophyllus_Dca53894.1

Viterbi Path	
Viterbi Path	
Posterior @ 0.4	
Posterior @ 0.4	

Posterior Graphic. Threshold Marked @ 0.4

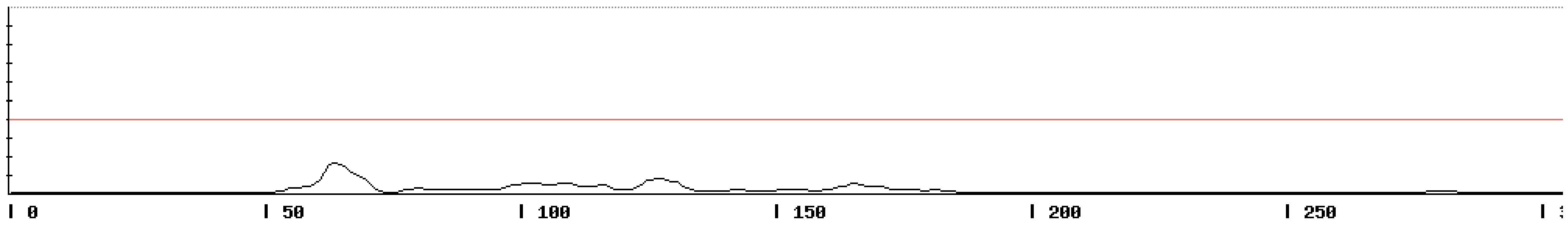

Predictions for Dianthuscaryophyllus_Dca53956.1

Viterbi Path

Viterbi Path

Posterior @ 0.4

Posterior @ 0.4

Posterior Graphic. Threshold Marked @ 0.4

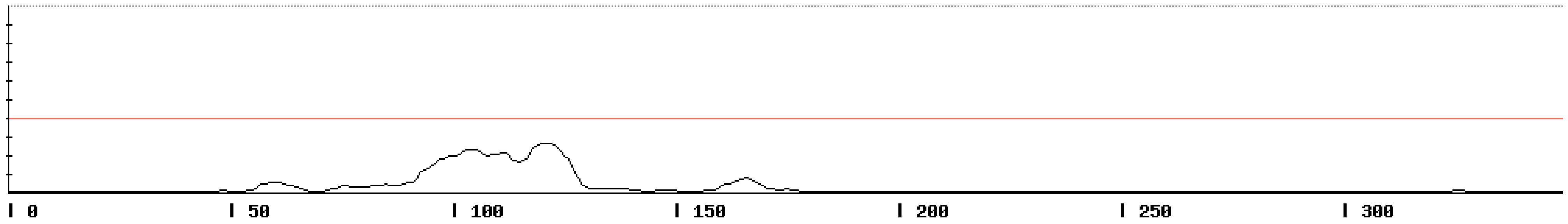

Predictions for Dianthuscaryophyllus_Dca54893.1

Viterbi Path

Viterbi Path

Posterior @ 0.4

Posterior @ 0.4

Posterior Graphic. Threshold Marked @ 0.4

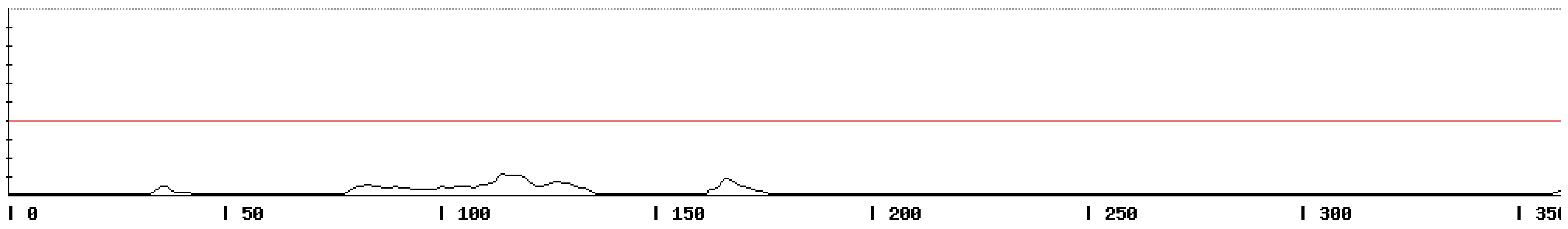

Predictions for Dianthuscaryophyllus_Dca5512.1

Viterbi Path

Viterbi Path

Posterior @ 0.4

Posterior @ 0.4

Posterior Graphic. Threshold Marked @ 0.4

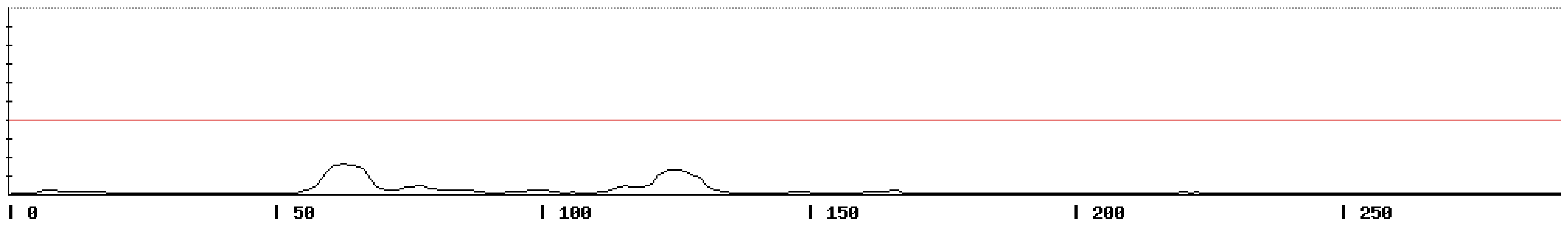

Predictions for Dianthuscaryophyllus_Dca56092.1

Viterbi Path

Viterbi Path

Posterior @ 0.4	
Posterior @ 0.4	
Posterior Graphic. Threshold Marked @ 0.4	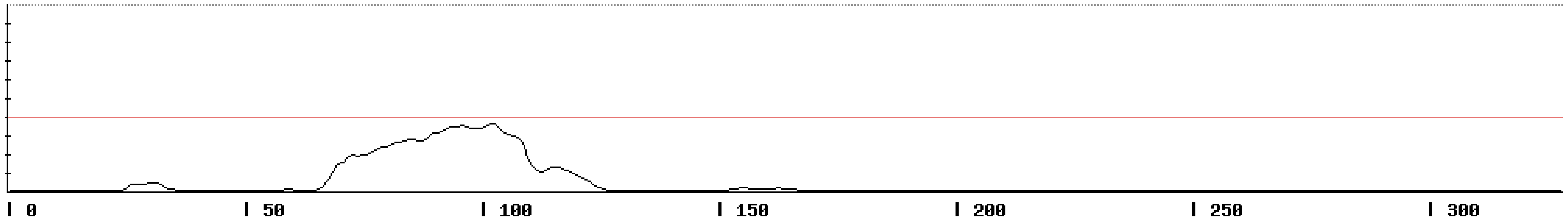

Predictions for Dianthuscaryophyllus_Dca5808.1	
Viterbi Path	
Viterbi Path	
Posterior @ 0.4	151 - GKRKRRRI - 158
Posterior @ 0.4	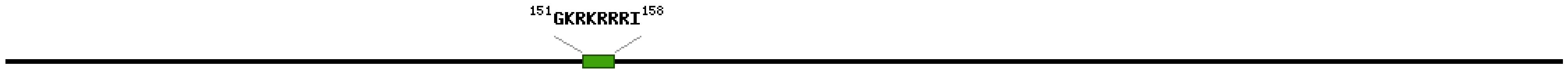
Posterior Graphic. Threshold Marked @ 0.4	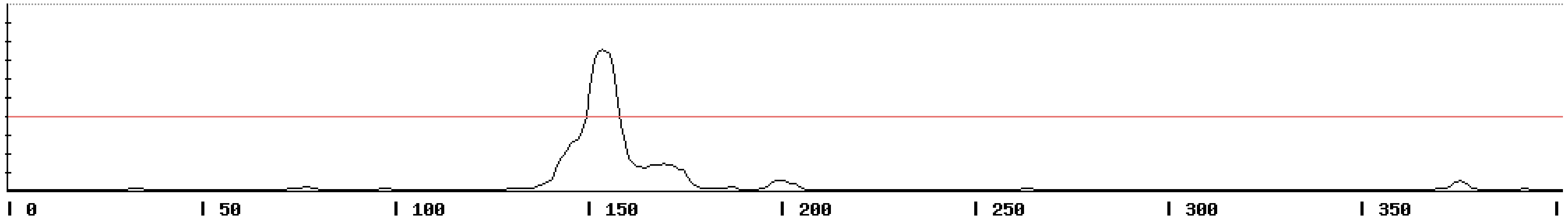

Predictions for Dianthuscaryophyllus_Dca58921.1	
Viterbi Path	
Viterbi Path	

Posterior @ 0.4

Posterior @ 0.4

Posterior Graphic. Threshold Marked @ 0.4

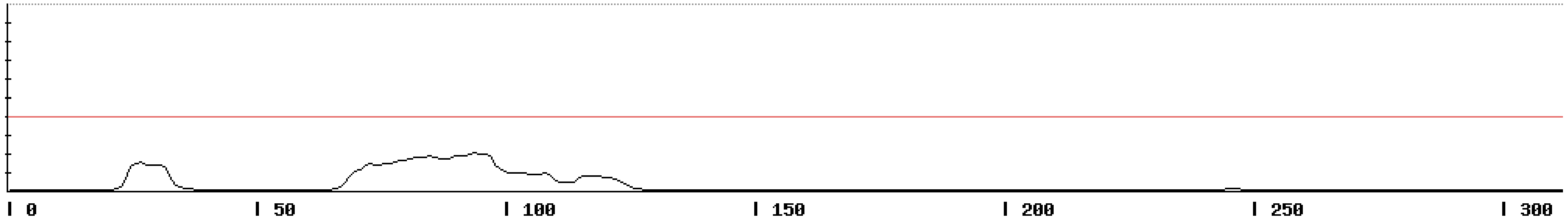

Predictions for Dianthuscaryophyllus_Dca59148.1

Viterbi Path

Viterbi Path

Posterior @ 0.4

Posterior @ 0.4

Posterior Graphic. Threshold Marked @ 0.4

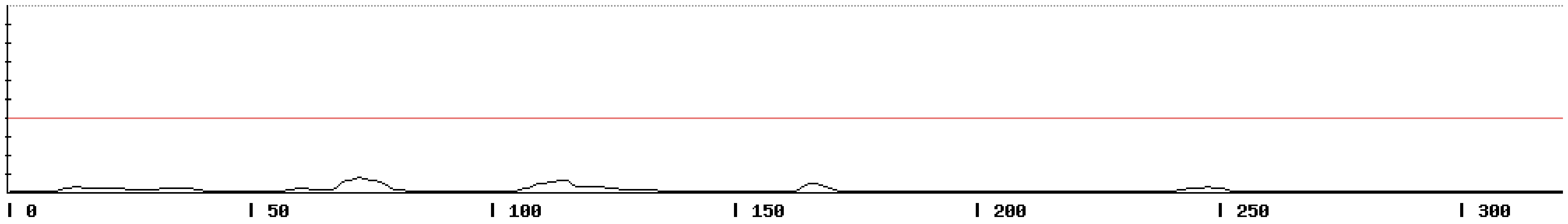

Predictions for Dianthuscaryophyllus_Dca5917.1

Viterbi Path

Viterbi Path

Posterior @ 0.4 — 79 - RKQK - 82

Posterior @ 0.4

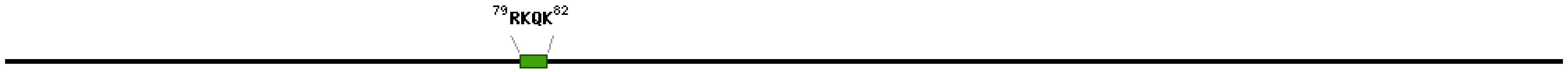

Posterior Graphic. Threshold Marked @ 0.4

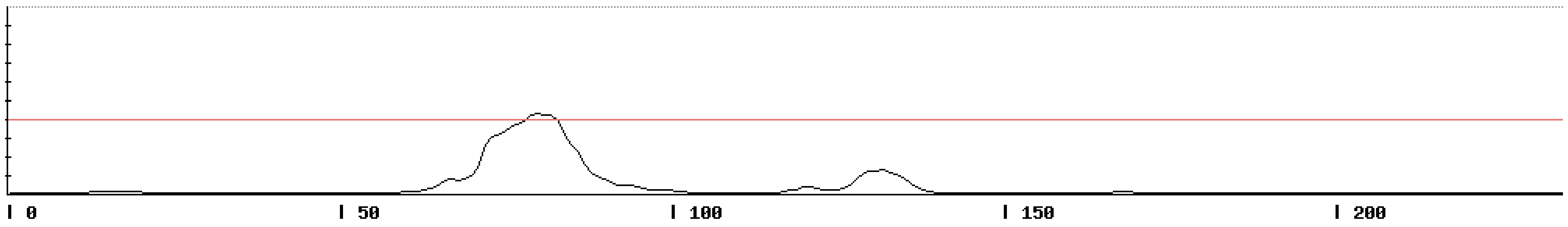

Predictions for Dianthuscaryophyllus_Dca59199.1

Viterbi Path

Viterbi Path

Posterior @ 0.4

Posterior @ 0.4

Posterior Graphic. Threshold Marked @ 0.4

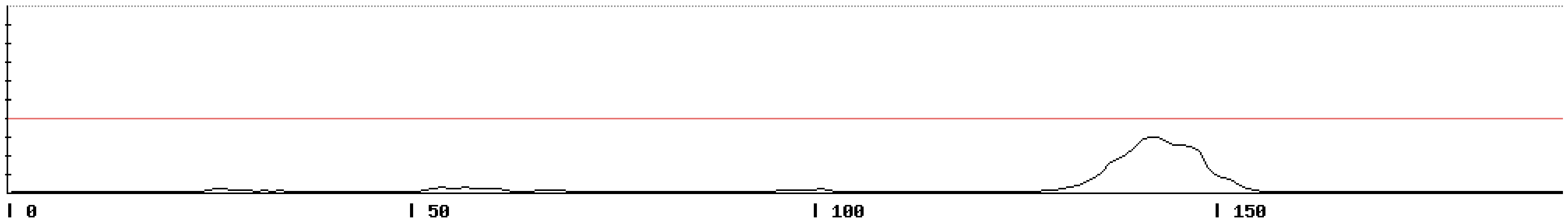

Predictions for Dianthuscaryophyllus_Dca59259.1

Viterbi Path	
Viterbi Path	
Posterior @ 0.4	
Posterior @ 0.4	
Posterior Graphic. Threshold Marked @ 0.4	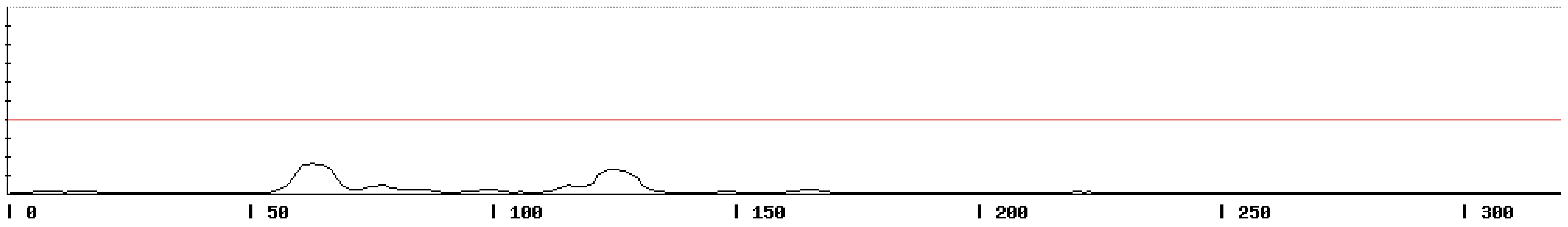

Predictions for Dianthuscaryophyllus_Dca59654.1

Viterbi Path	
Viterbi Path	
Posterior @ 0.4	257 - RRIRLRKLRR - 266
Posterior @ 0.4	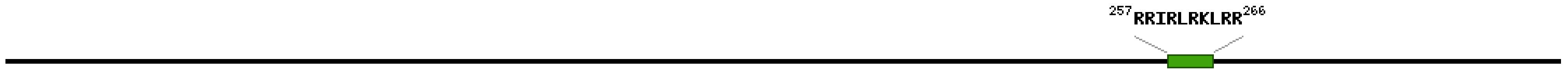
Posterior Graphic. Threshold Marked @ 0.4	

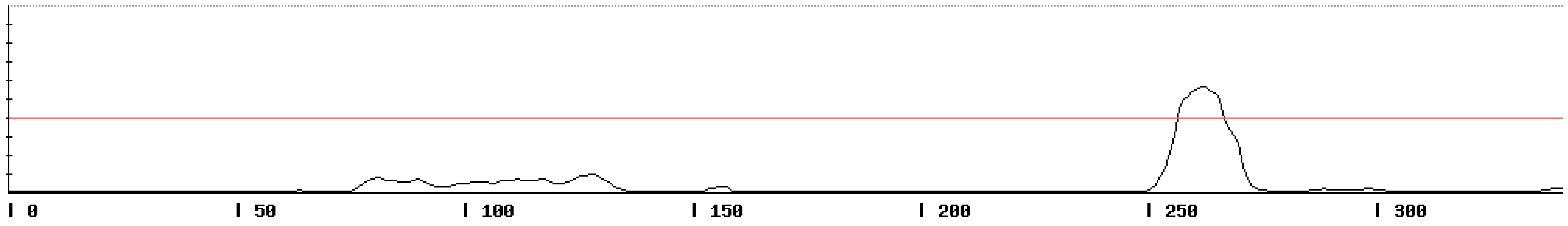

Predictions for Dianthuscaryophyllus_Dca6000.1

Viterbi Path

Viterbi Path

Posterior @ 0.4 — 97 - GKRKRRRI - 104

Posterior @ 0.4

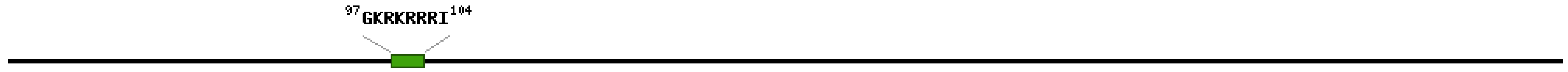

Posterior Graphic. Threshold Marked @ 0.4

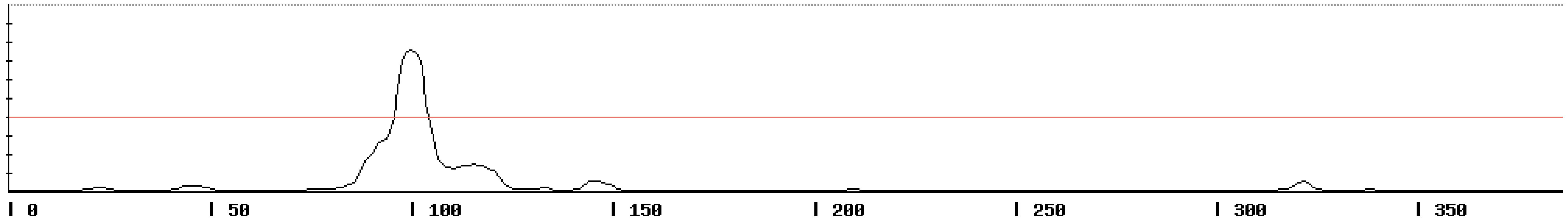

Predictions for Dianthuscaryophyllus_Dca60574.1

Viterbi Path

Viterbi Path

Posterior @ 0.4 — 327 - NRRIRLRKLRRVSPRKEE - 344

Posterior @ 0.4

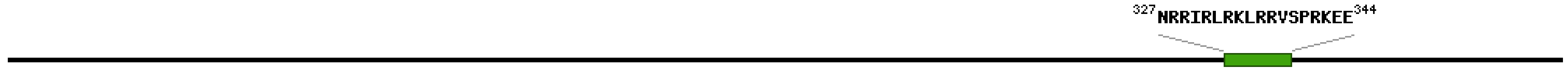

Posterior Graphic. Threshold Marked @ 0.4	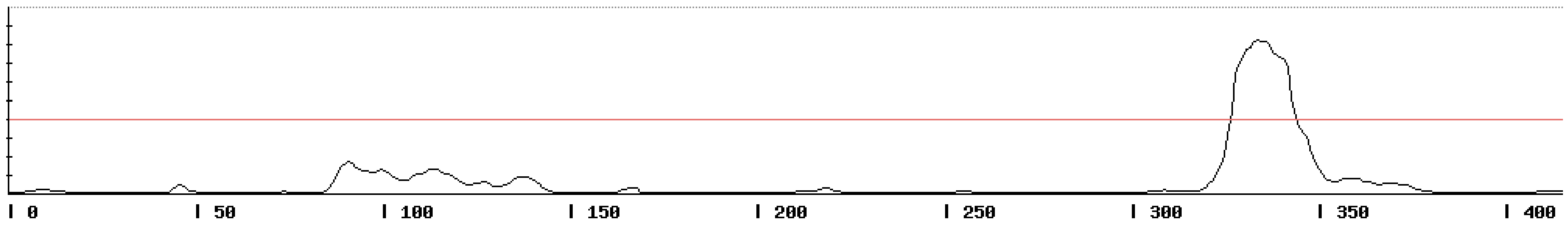

Predictions for Dianthuscaryophyllus_Dca62279.1

Viterbi Path	
Viterbi Path	
Posterior @ 0.4	
Posterior @ 0.4	
Posterior Graphic. Threshold Marked @ 0.4	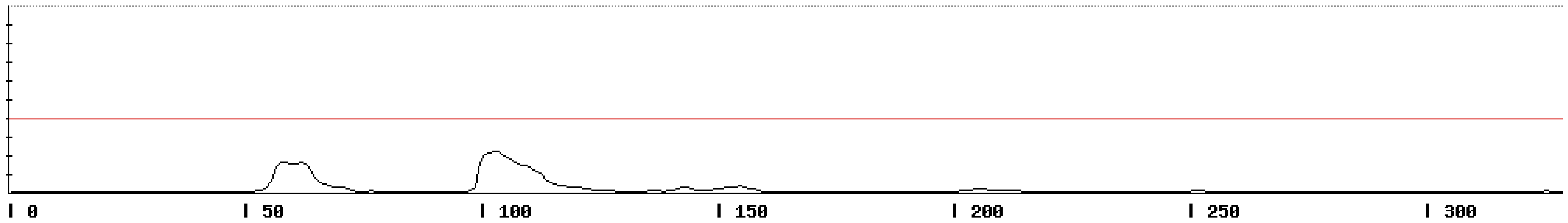

Predictions for Dianthuscaryophyllus_Dca6500.1

Viterbi Path	
Viterbi Path	
Posterior @ 0.4	
Posterior @ 0.4	

Posterior Graphic. Threshold Marked @ 0.4	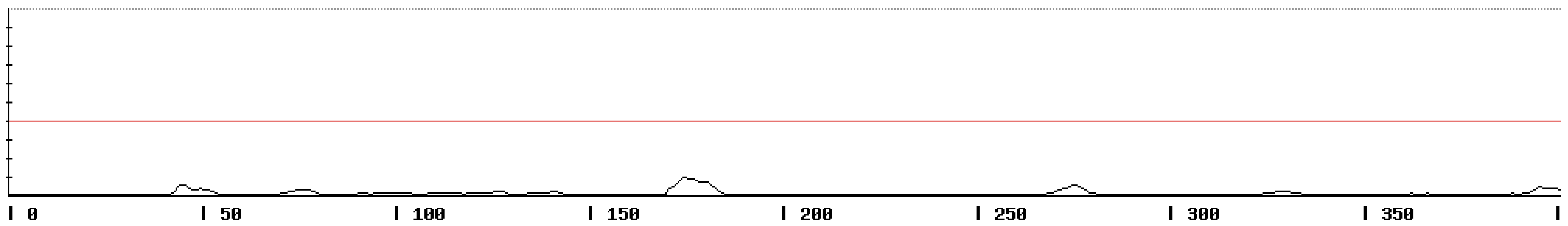

Predictions for Dianthuscaryophyllus_Dca6608.1

Revision r.9 - Last Updated on : February 24 2015 17:45:20.

alex.nguyenba@utoronto.ca

Intended to be used with Firefox

Viterbi Path

Posterior @ 0.4

Posterior @ 0.4

Posterior Graphic. Threshold Marked @ 0.4

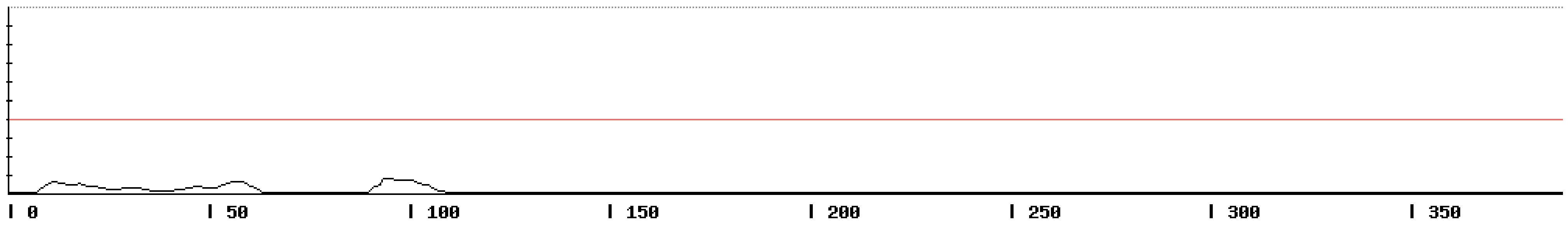

Predictions for Dichantheliumoligosanthes_Do000661.1

Viterbi Path

Viterbi Path

Posterior @ 0.4

Posterior @ 0.4

Posterior Graphic. Threshold Marked @ 0.4

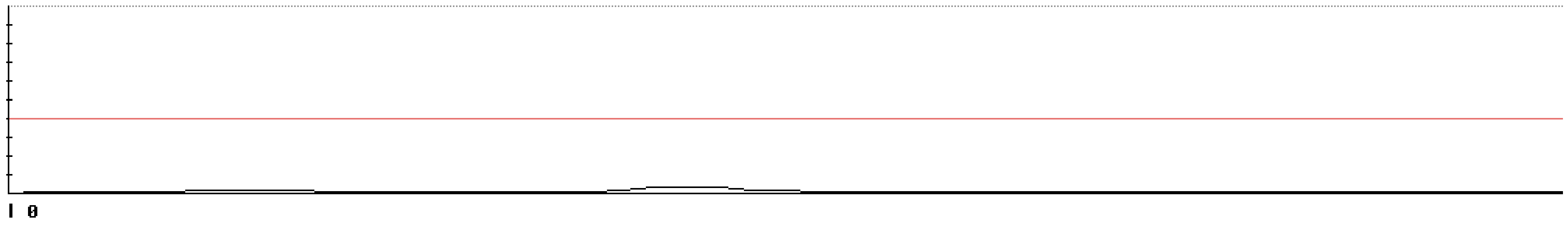

Predictions for Dichantheliumoligosanthes_Do000937.1

Viterbi Path

Viterbi Path

Posterior @ 0.4 297 - RRGKKARA - 304

Posterior @ 0.4

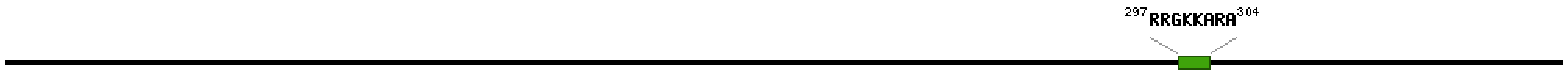

Posterior Graphic. Threshold Marked @ 0.4

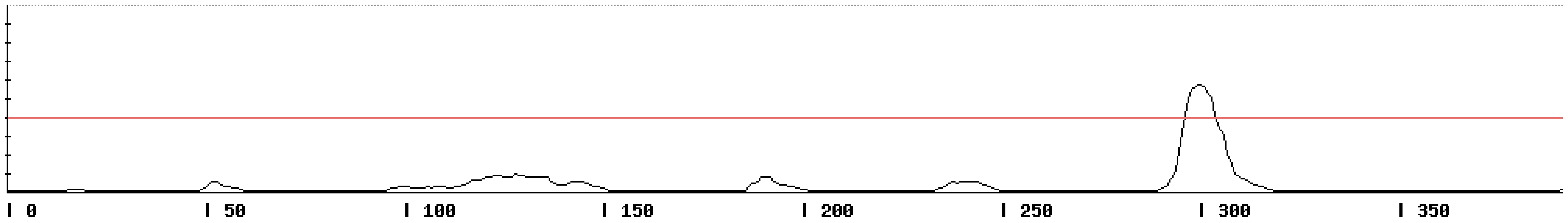

Predictions for Dichantheliumoligosanthes_Do001152.1

Viterbi Path

Viterbi Path

Posterior @ 0.4

Posterior @ 0.4

Posterior Graphic. Threshold Marked @ 0.4

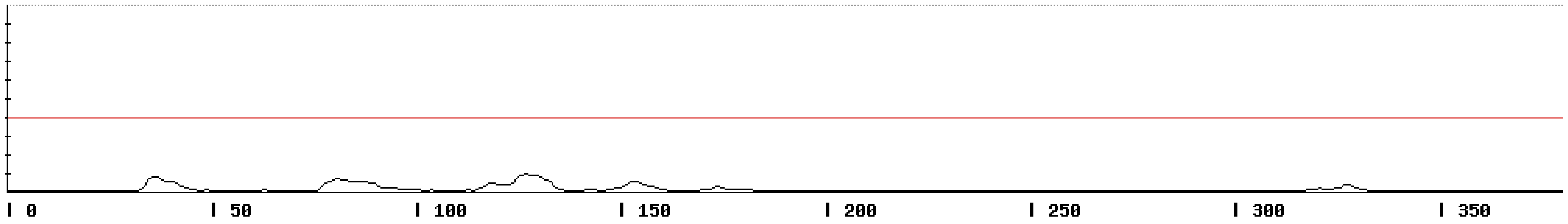

Predictions for Dichantheliumoligosanthes_Do001398.1

Viterbi Path

Viterbi Path

Posterior @ 0.4

Posterior @ 0.4

Posterior Graphic. Threshold Marked @ 0.4

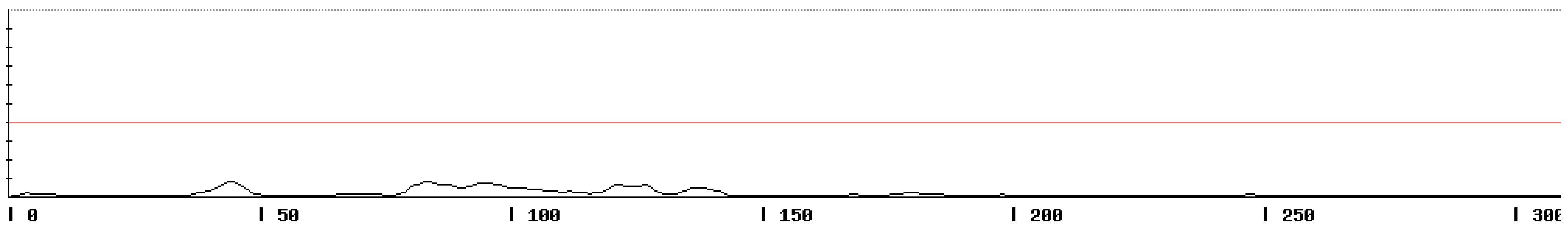

Predictions for Dichantheliumoligosanthes_Do001534.1

Viterbi Path

Viterbi Path

Posterior @ 0.4

Posterior @ 0.4

Posterior Graphic. Threshold Marked @ 0.4

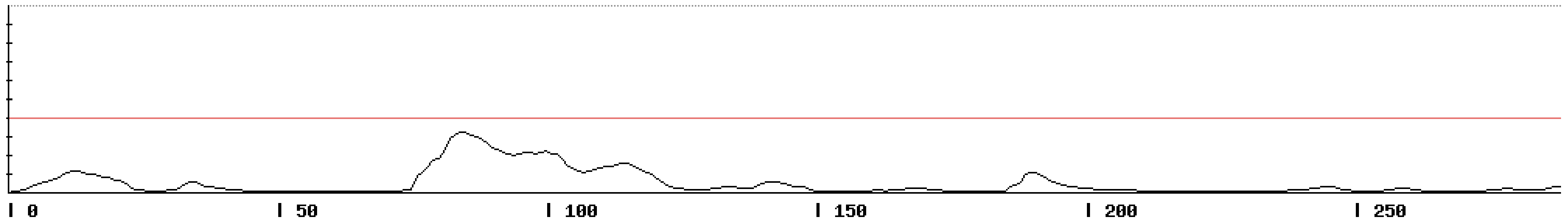

Predictions for Dichantheliumoligosanthes_Do001959.1

Viterbi Path	
Viterbi Path	
Posterior @ 0.4	
Posterior @ 0.4	
Posterior Graphic. Threshold Marked @ 0.4	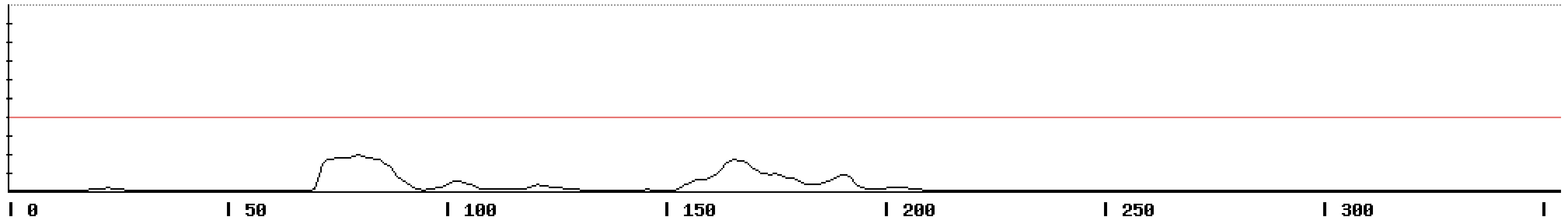

Predictions for Dichantheliumoligosanthes_Do002102.1

Viterbi Path	
Viterbi Path	
Posterior @ 0.4	
Posterior @ 0.4	

Posterior Graphic. Threshold Marked @ 0.4

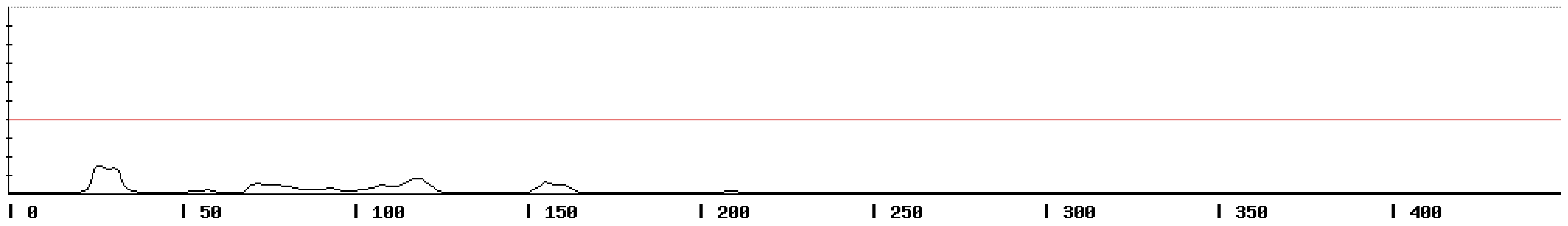

Predictions for Dichantheliumoligosanthes_Do002294.1

Viterbi Path

Viterbi Path

Posterior @ 0.4

104 - KLRIPGGGGKEAAWRKK - 120

333 - SGHGKKRKRG - 342

Posterior @ 0.4

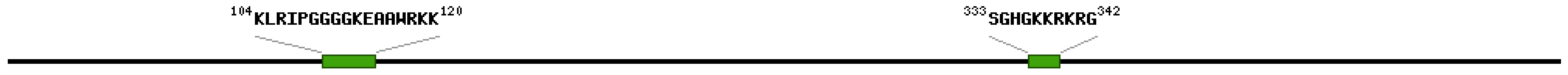

Posterior Graphic. Threshold Marked @ 0.4

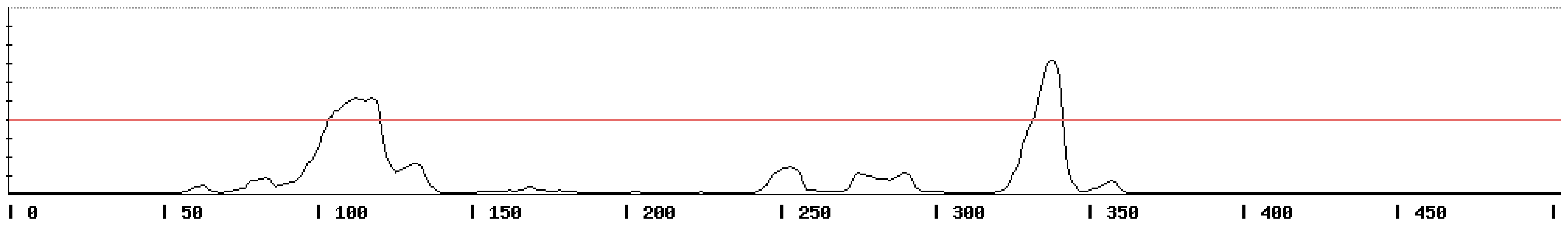

Predictions for Dichantheliumoligosanthes_Do002300.1

Viterbi Path

Viterbi Path

Posterior @ 0.4

330 - SGHGKKRKRG - 339

Posterior @ 0.4

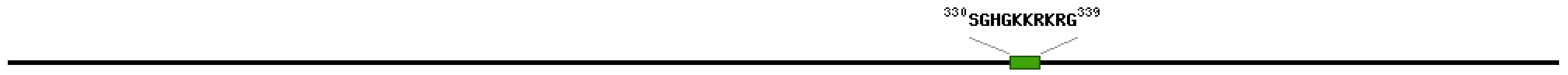

Posterior Graphic. Threshold Marked @ 0.4

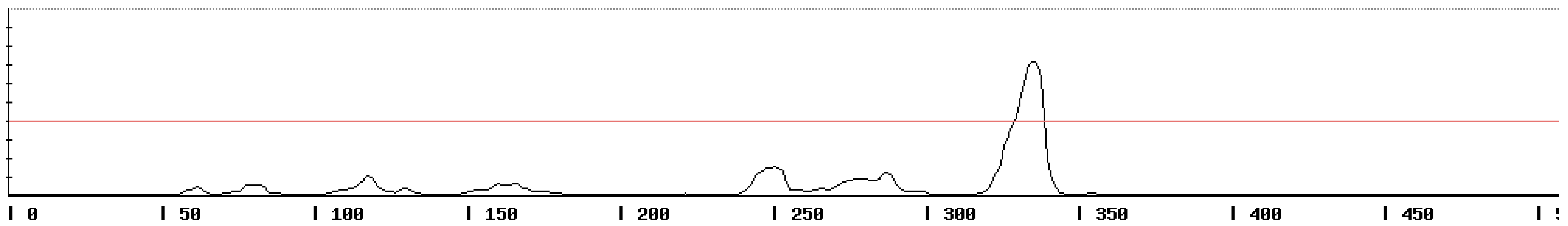

Predictions for Dichantheliumoligosanthes_Do002362.1

Viterbi Path

Viterbi Path

Posterior @ 0.4

Posterior @ 0.4

Posterior Graphic. Threshold Marked @ 0.4

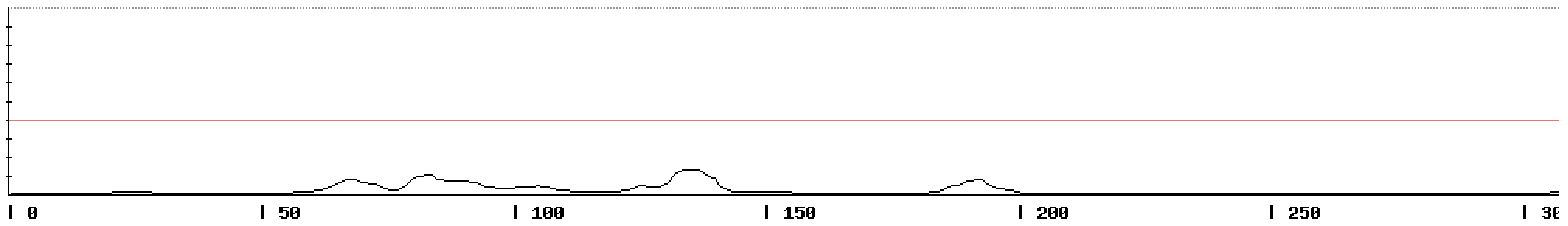

Predictions for Dichantheliumoligosanthes_Do002449.1

Viterbi Path

Viterbi Path

Posterior @ 0.4	
Posterior @ 0.4	
Posterior Graphic. Threshold Marked @ 0.4	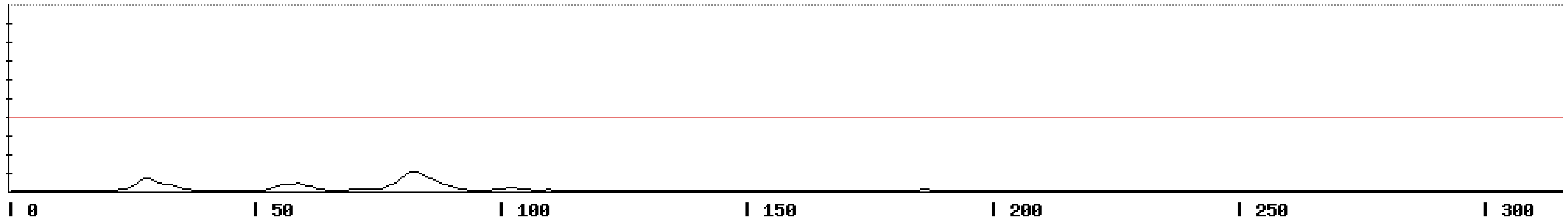

Predictions for Dichantheliumoligosanthes_Do002541.1	
Viterbi Path	
Viterbi Path	
Posterior @ 0.4	
Posterior @ 0.4	
Posterior Graphic. Threshold Marked @ 0.4	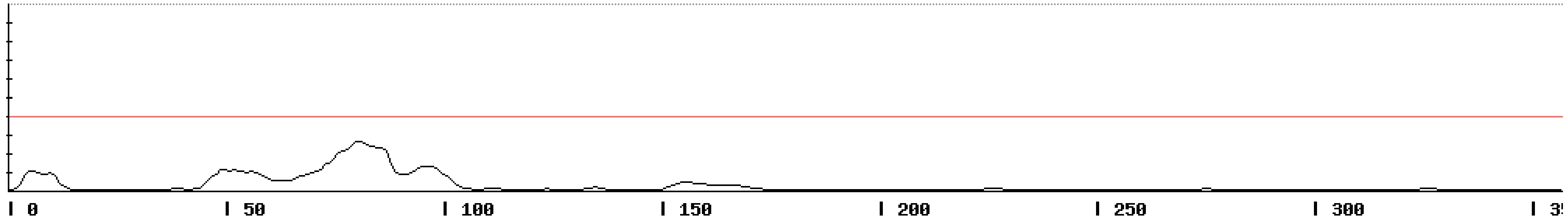

Predictions for Dichantheliumoligosanthes_Do002628.1	
Viterbi Path	
Viterbi Path	

Posterior @ 0.4

Posterior @ 0.4

Posterior Graphic. Threshold Marked @ 0.4

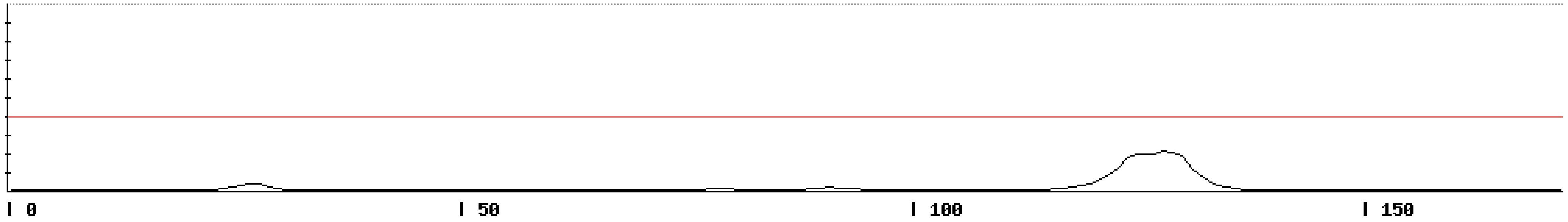

Predictions for Dichantheliumoligosanthes_Do002836.1

Viterbi Path	216 - KKSRRKRTKATNIDPGKTKKRRMNKK - 241
	536 - PRPARAEALRRCRVRRQRRKARRGSR - 561

Viterbi Path

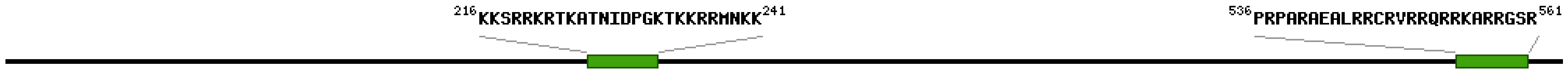

Posterior @ 0.4	214 - SSKKSRRKRTKATNIDPGKTKKRRMNKKAA - 243
	261 - KAPLKLKSRARKSATRRENK - 280
	537 - RPARAEALRRCRVRRQRRKARRGSR - 561

Posterior @ 0.4

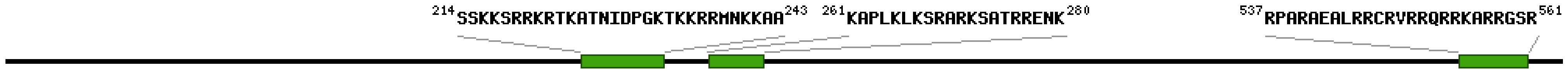

Posterior Graphic. Threshold Marked @ 0.4

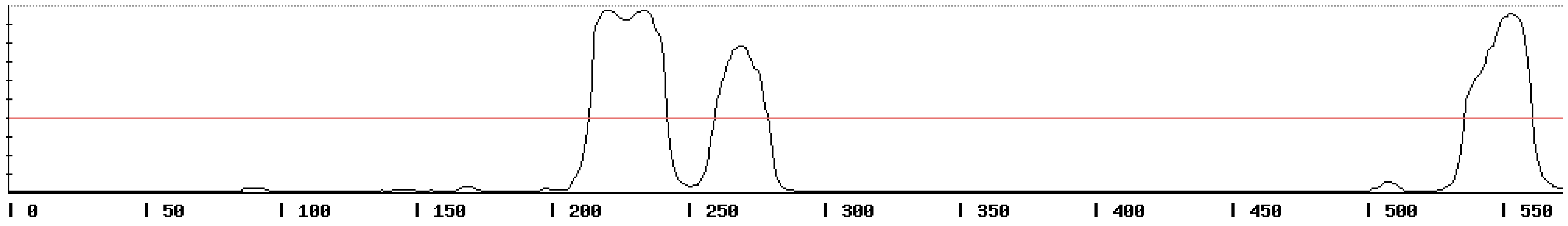

Predictions for Dichantheliumoligosanthes_Do003247.1

Viterbi Path	
Viterbi Path	
Posterior @ 0.4	174 - KKKHHHKESGGGSGGKHG - 191
Posterior @ 0.4	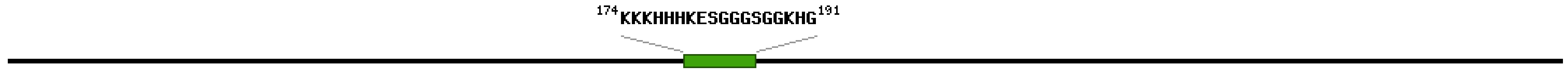
Posterior Graphic. Threshold Marked @ 0.4	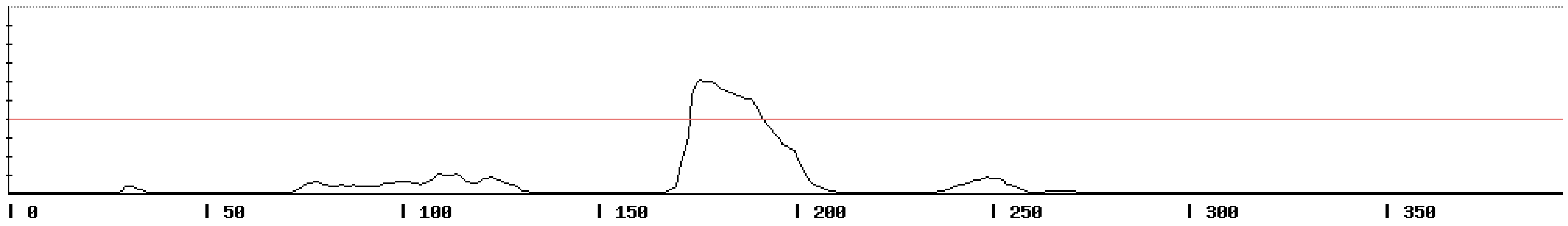

Predictions for Dichantheliumoligosanthes_Do003574.1

Viterbi Path	
Viterbi Path	
Posterior @ 0.4	
Posterior @ 0.4	

Posterior Graphic. Threshold Marked @ 0.4

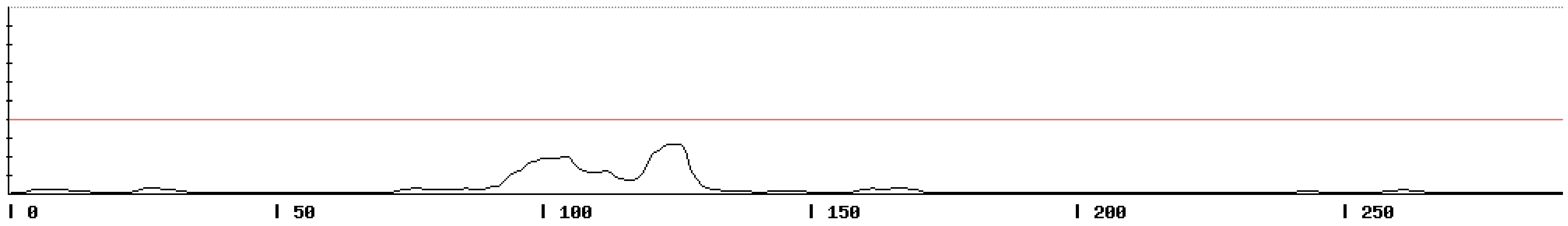

Predictions for Dichantheliumoligosanthes_Do003611.1

Viterbi Path

Viterbi Path

Posterior @ 0.4

Posterior @ 0.4

Posterior Graphic. Threshold Marked @ 0.4

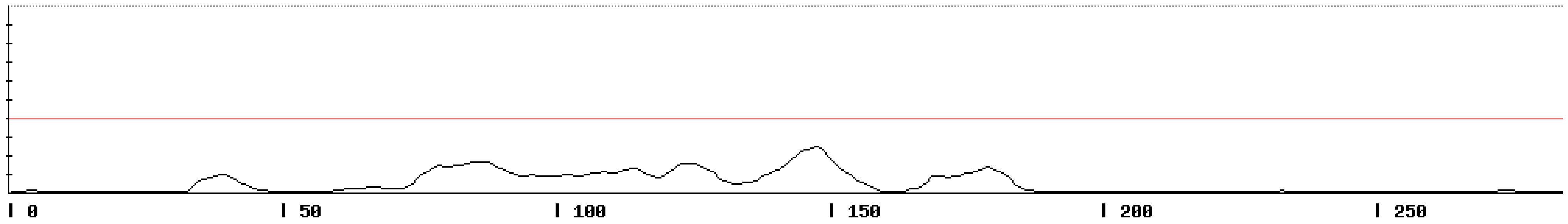

Predictions for Dichantheliumoligosanthes_Do003686.1

Viterbi Path

Viterbi Path

Posterior @ 0.4

Posterior @ 0.4

Posterior Graphic. Threshold Marked @ 0.4

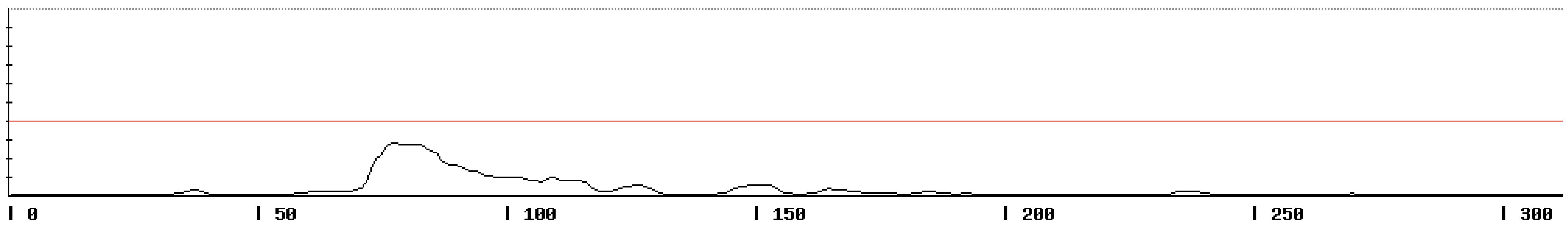

Predictions for Dichantheliumoligosanthes_Do003738.1

Viterbi Path

Viterbi Path

Posterior @ 0.4

85 - KYPNGSRPNRAAGRGYWKATGADKPIAPKGSGRAAGIKKA - 124

Posterior @ 0.4

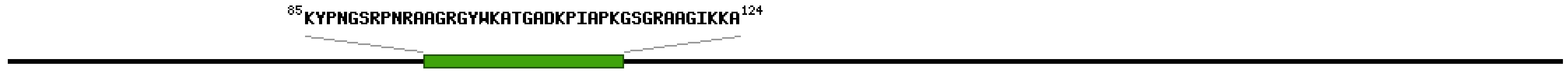

Posterior Graphic. Threshold Marked @ 0.4

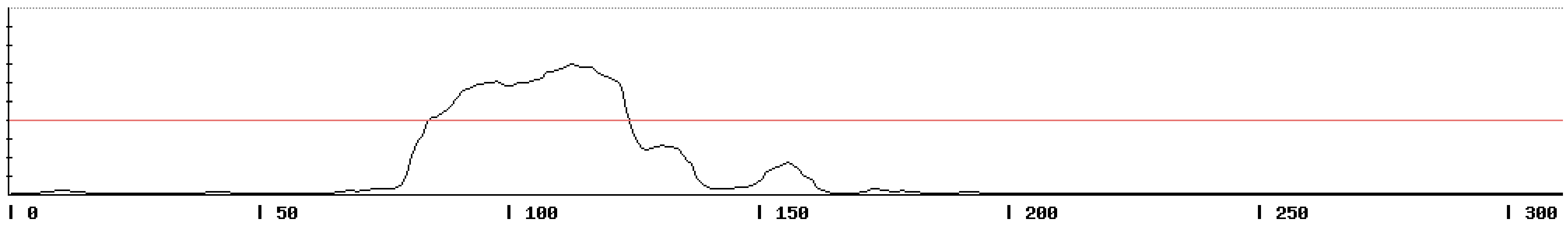

Predictions for Dichantheliumoligosanthes_Do003840.1

Viterbi Path

Viterbi Path

Posterior @ 0.4	
Posterior @ 0.4	
Posterior Graphic. Threshold Marked @ 0.4	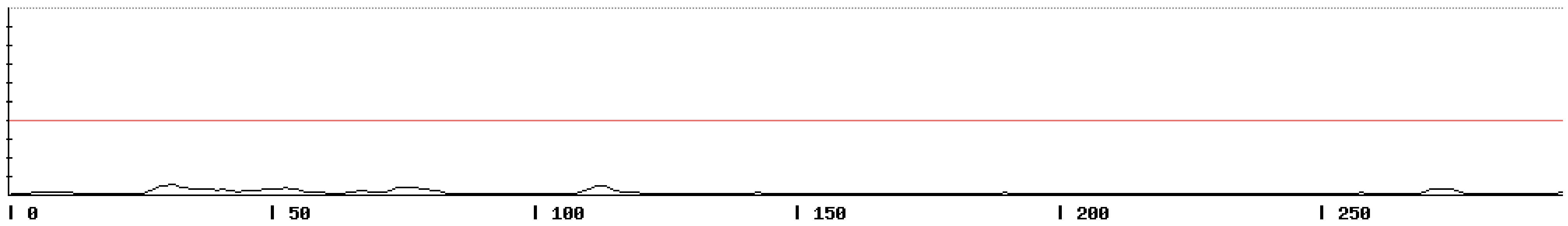

Predictions for Dichantheliumoligosanthes_Do003868.1

Viterbi Path	2382 - PRRKRKAIAKRSKAK - 2396
Viterbi Path	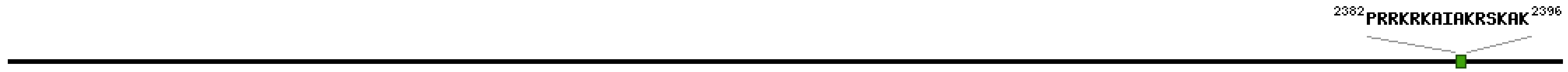
Posterior @ 0.4	101 - KHRRHGRERASHR - 113
	2382 - PRRKRKAIAKRSKAK - 2396
Posterior @ 0.4	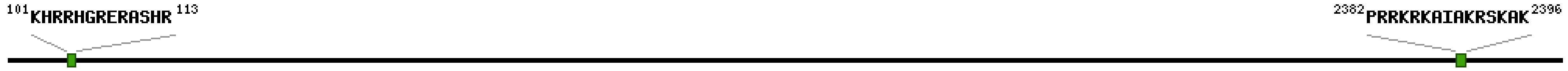
Posterior Graphic. Threshold Marked @ 0.4	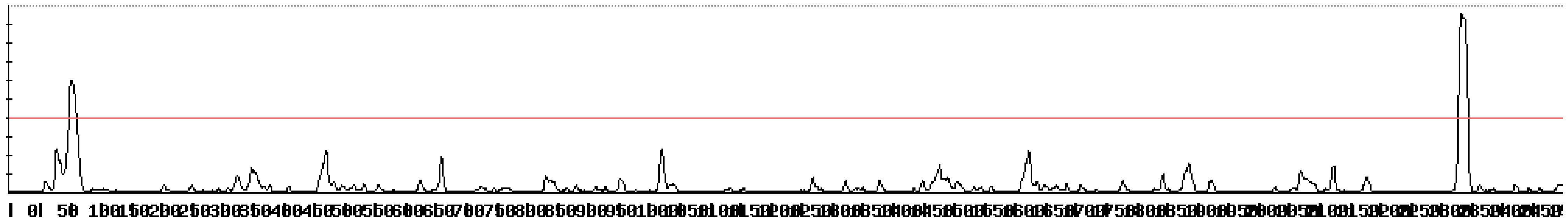

Predictions for Dichantheliumoligosanthes_Do003940.1

Viterbi Path	
Viterbi Path	

Posterior @ 0.4 105 - GSGKRIKVRR - 114

Posterior @ 0.4

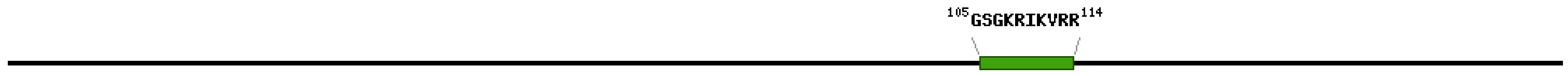

Posterior Graphic. Threshold Marked @ 0.4

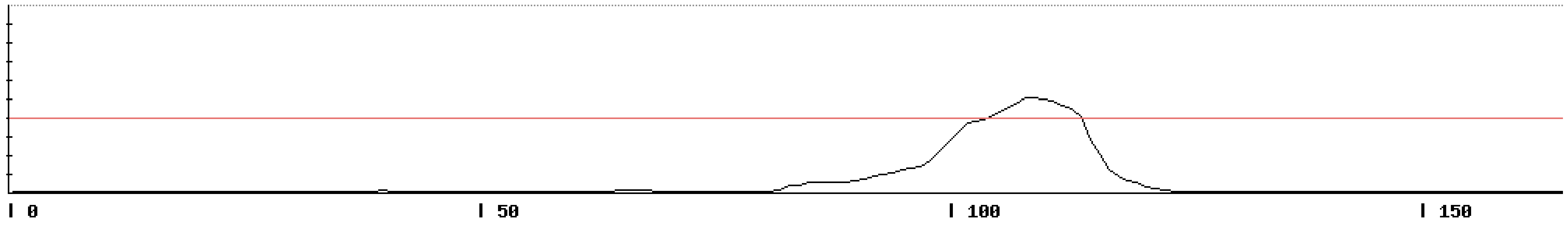

Predictions for Dichantheliumoligosanthes_Do003945.1

Viterbi Path

Viterbi Path

Posterior @ 0.4

Posterior @ 0.4

Posterior Graphic. Threshold Marked @ 0.4

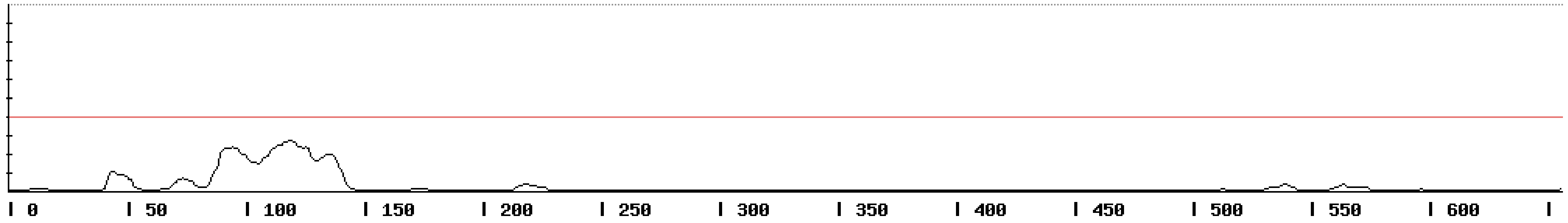

Predictions for Dichantheliumoligosanthes_Do004161.1

Viterbi Path

Viterbi Path

Posterior @ 0.4

Posterior @ 0.4

Posterior Graphic. Threshold Marked @ 0.4

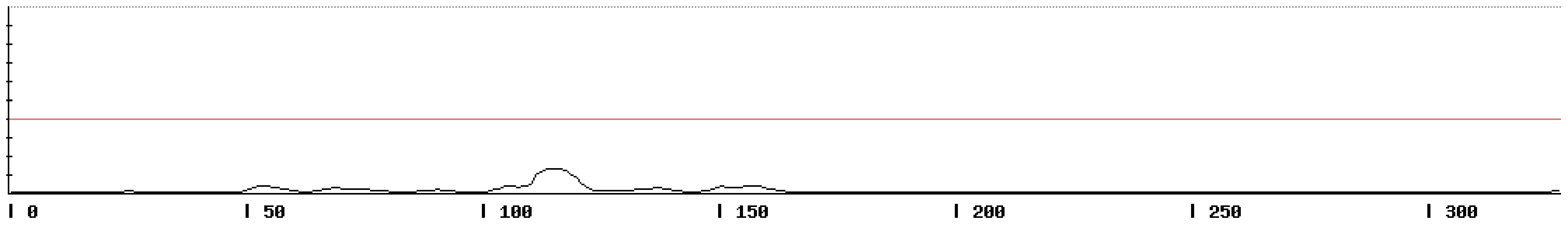

Predictions for Dichantheliumoligosanthes_Do004998.1

Viterbi Path

Viterbi Path

Posterior @ 0.4

Posterior @ 0.4

Posterior Graphic. Threshold Marked @ 0.4

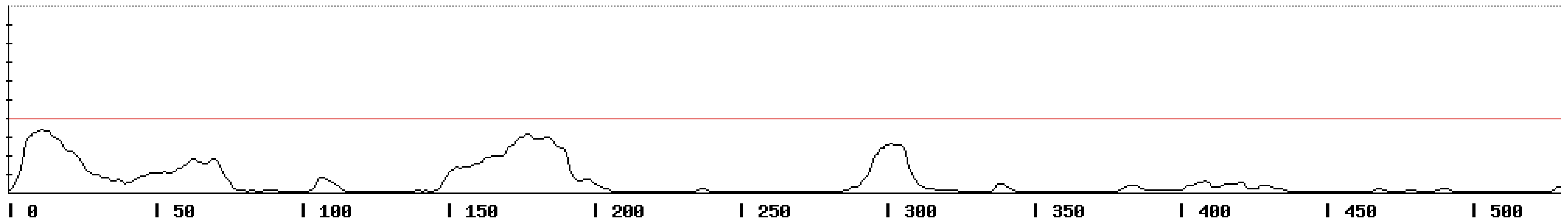

Predictions for Dichantheliumoligosanthes_Do005022.1	
Viterbi Path	
Viterbi Path	
Posterior @ 0.4	144 - RKRRK - 148
Posterior @ 0.4	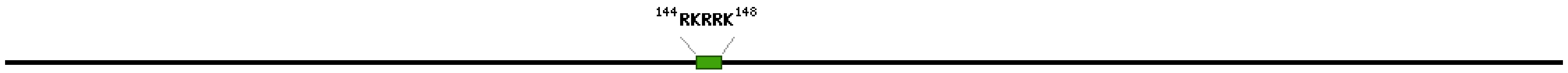
Posterior Graphic. Threshold Marked @ 0.4	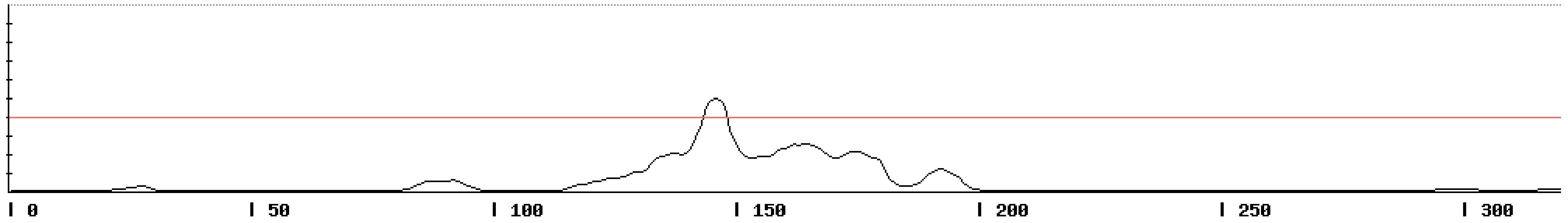

Predictions for Dichantheliumoligosanthes_Do005225.1	
Viterbi Path	
Viterbi Path	
Posterior @ 0.4	73 - APATGRGGRRRT - 84
Posterior @ 0.4	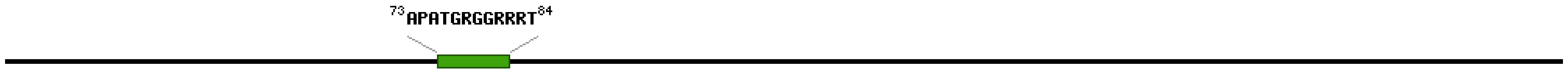
Posterior Graphic. Threshold Marked @ 0.4	

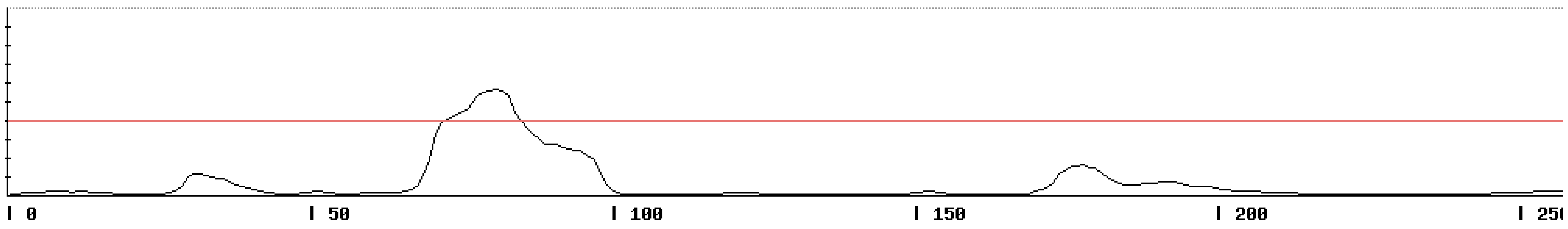

Predictions for Dichantheliumoligosanthes_Do005286.1

Viterbi Path

Viterbi Path

Posterior @ 0.4

483 - RRKR - 486

514 - RRRRKK - 519

Posterior @ 0.4

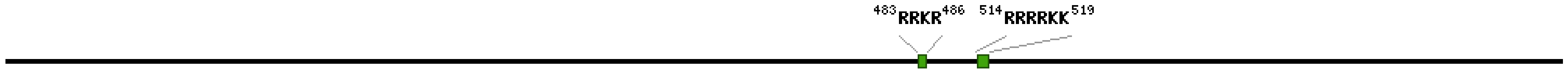

Posterior Graphic. Threshold Marked @ 0.4

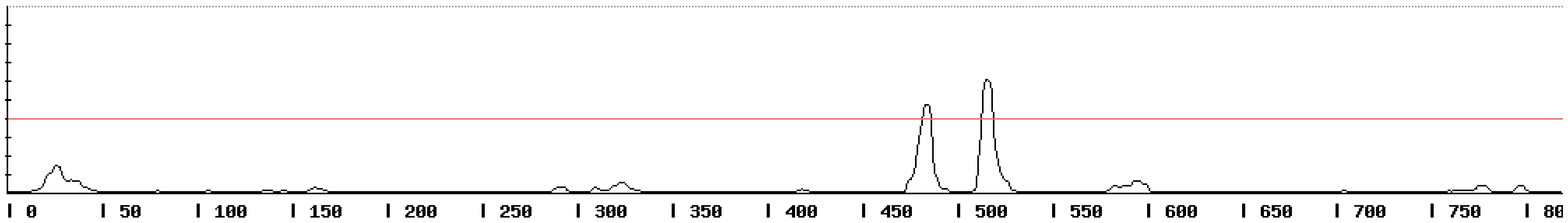

Predictions for Dichantheliumoligosanthes_Do005424.1

Viterbi Path

Viterbi Path

Posterior @ 0.4

Posterior @ 0.4

Posterior Graphic. Threshold Marked @ 0.4	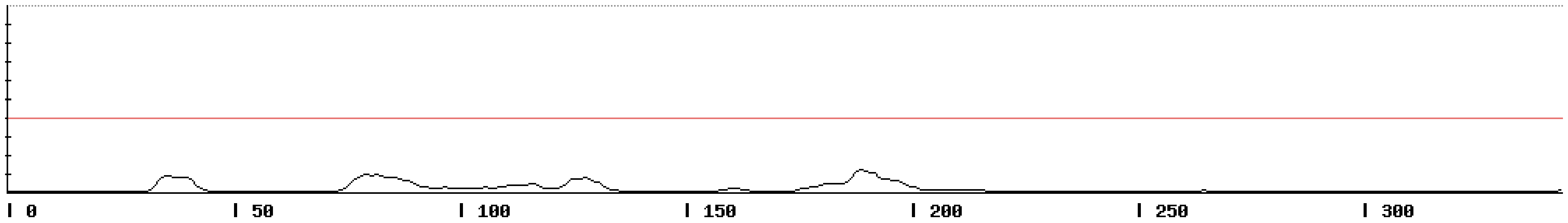

Predictions for Dichantheliumoligosanthes_Do006064.1	
Viterbi Path	
Viterbi Path	
Posterior @ 0.4	
Posterior @ 0.4	
Posterior Graphic. Threshold Marked @ 0.4	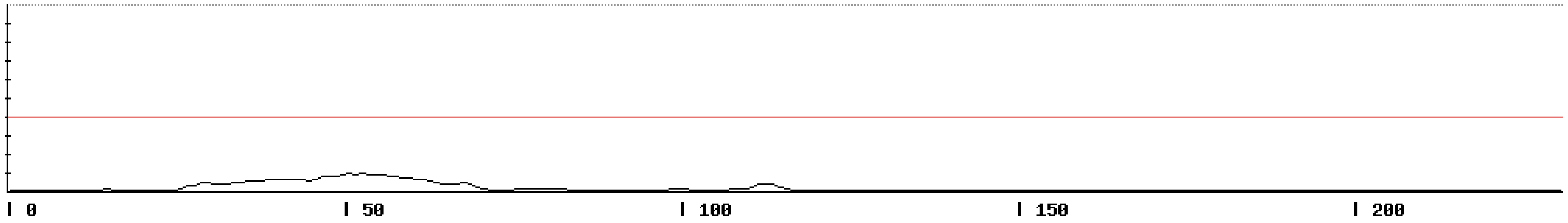

Predictions for Dichantheliumoligosanthes_Do006093.1	
Viterbi Path	
Viterbi Path	

Posterior @ 0.4

Posterior @ 0.4

Posterior Graphic. Threshold Marked @ 0.4

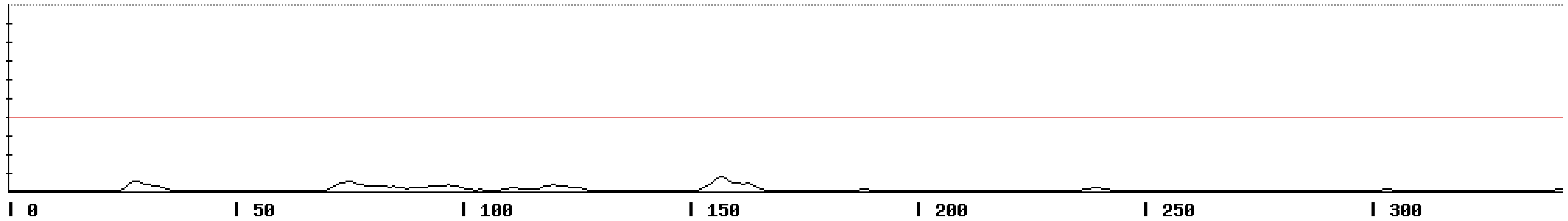

Predictions for Dichantheliumoligosanthes_Do006636.1

Viterbi Path

Viterbi Path

Posterior @ 0.4

Posterior @ 0.4

Posterior Graphic. Threshold Marked @ 0.4

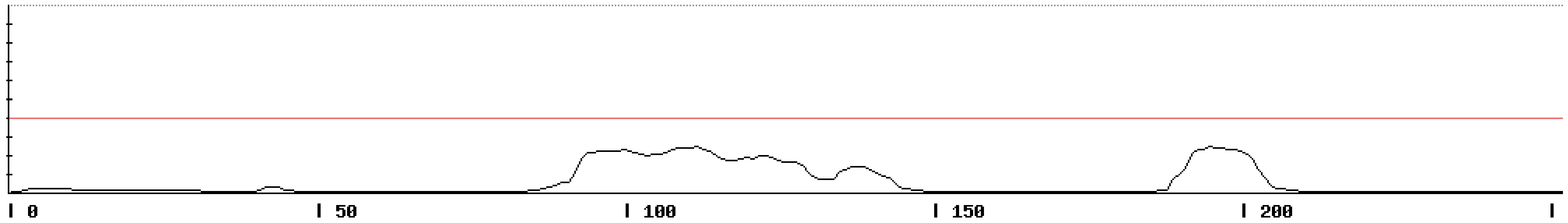

Predictions for Dichantheliumoligosanthes_Do006900.1

Viterbi Path

Viterbi Path

Posterior @ 0.4

Posterior @ 0.4

Posterior Graphic. Threshold Marked @ 0.4

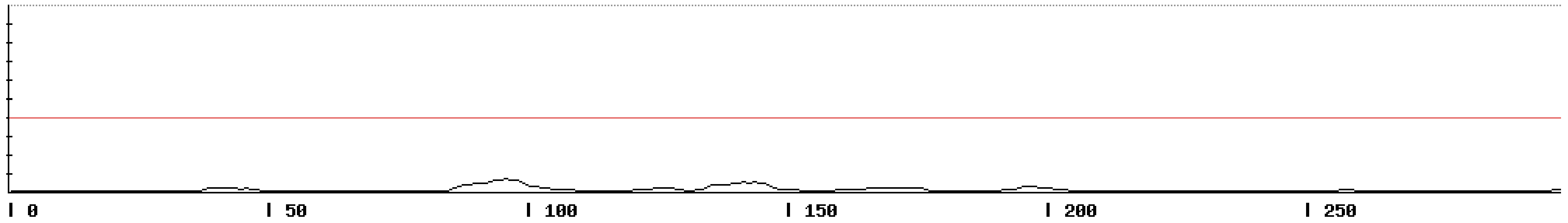

Predictions for Dichantheliumoligosanthes_Do007068.1

Viterbi Path

Viterbi Path

Posterior @ 0.4

90 - AGGRGGPGNRTNRA - 103

Posterior @ 0.4

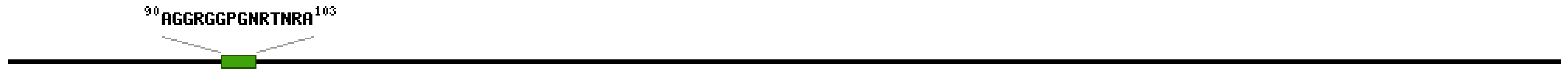

Posterior Graphic. Threshold Marked @ 0.4

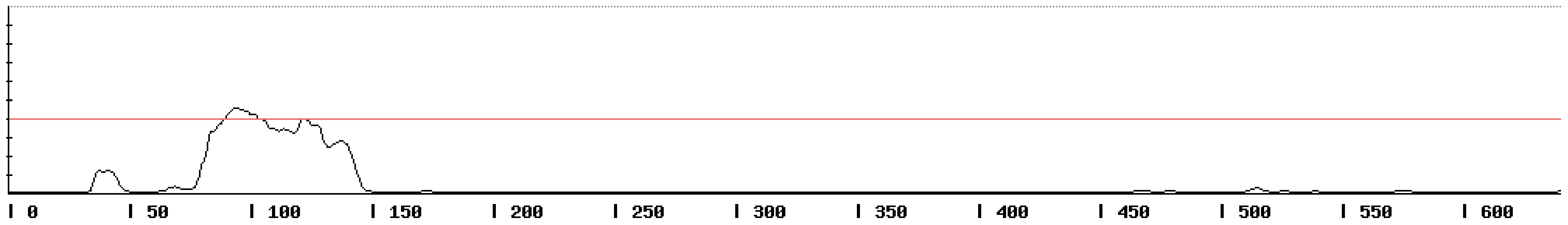

Predictions for Dichantheliumoligosanthes_Do007526.1

Viterbi Path

Viterbi Path

Posterior @ 0.4

Posterior @ 0.4

Posterior Graphic. Threshold Marked @ 0.4

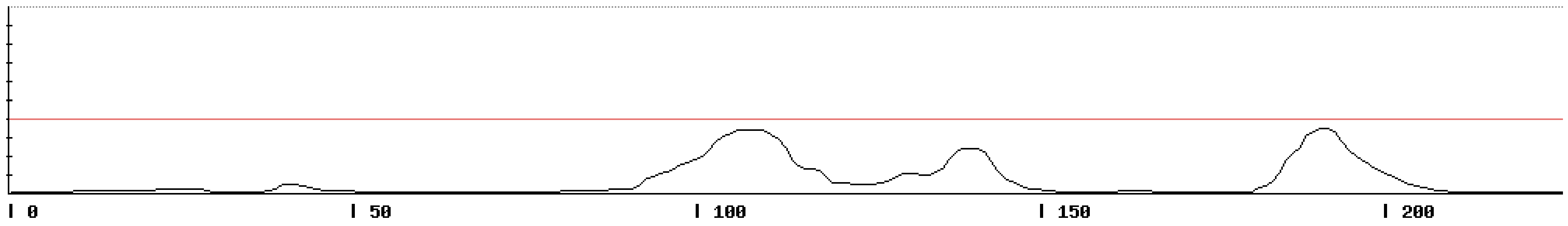

Predictions for Dichantheliumoligosanthes_Do007699.1

Viterbi Path

Viterbi Path

Posterior @ 0.4

Posterior @ 0.4

Posterior Graphic. Threshold Marked @ 0.4

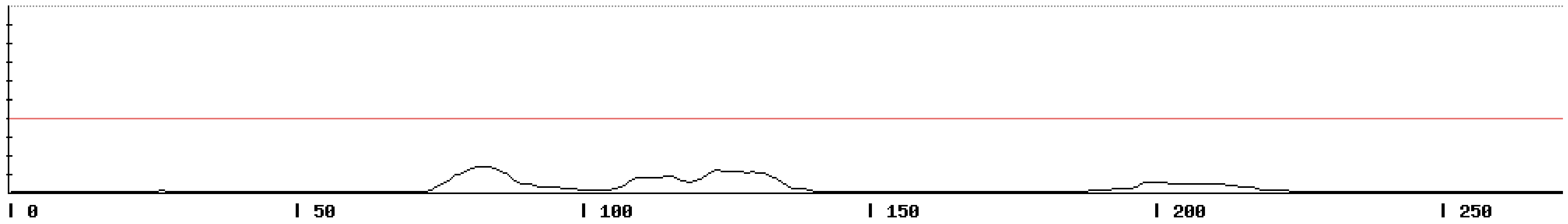

Predictions for Dichantheliumoligosanthes_Do007795.1	
Viterbi Path	
Viterbi Path	
Posterior @ 0.4	171 - REKKKQGKGK - 180
Posterior @ 0.4	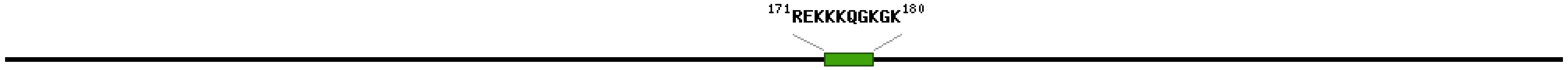
Posterior Graphic. Threshold Marked @ 0.4	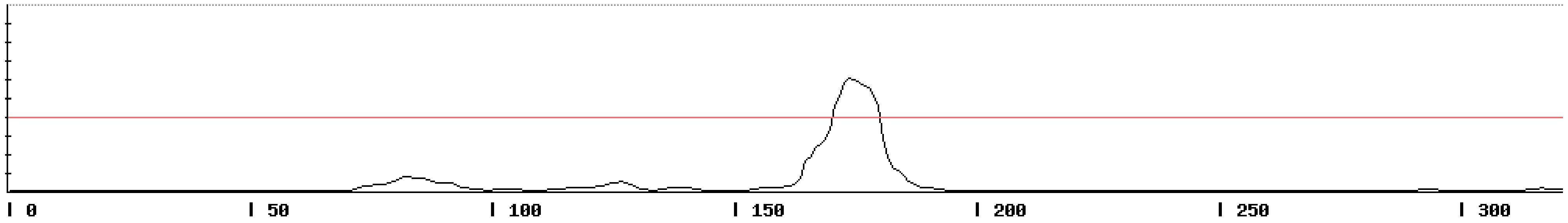

Predictions for Dichantheliumoligosanthes_Do007897.1	
Viterbi Path	
Viterbi Path	
Posterior @ 0.4	
Posterior @ 0.4	
Posterior Graphic. Threshold Marked @ 0.4	

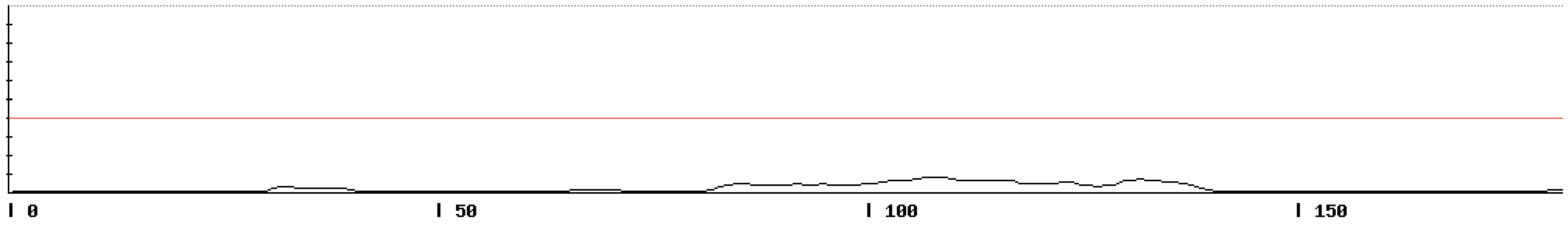

Predictions for Dichantheliumoligosanthes_Do008213.1

Viterbi Path	
Viterbi Path	
Posterior @ 0.4	191 - KSSKRPAVAPAAAKPASPPAKQRWRRLA - 218
Posterior @ 0.4	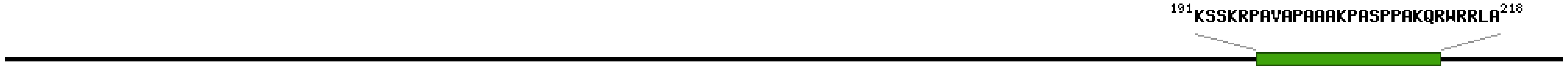
Posterior Graphic. Threshold Marked @ 0.4	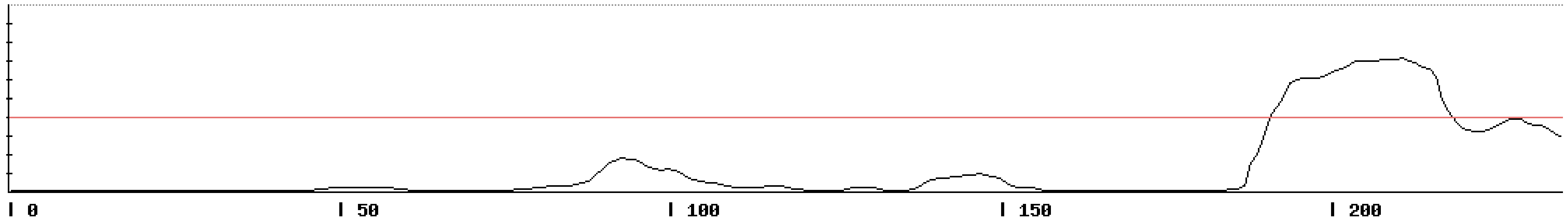

Predictions for Dichantheliumoligosanthes_Do008239.1

Viterbi Path	624 - PRSRRYPGGDRPSRRTADNRGRWKPSTGQSKPGKNAGAGNSKAKKGLEKDRR - 675
Viterbi Path	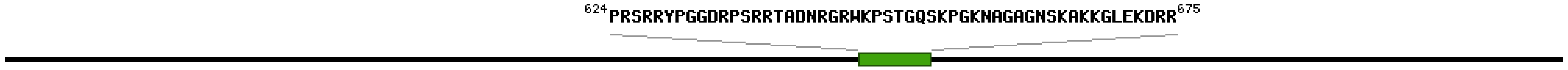
Posterior @ 0.4	59 - RLKEQIRRKAKAAAA - 73
	625 - RSRRYPGGDRPSRRTADNRGRWKPSTGQSKPGKNAGAGNSKAKKGLEKDRR - 675
Posterior @ 0.4	

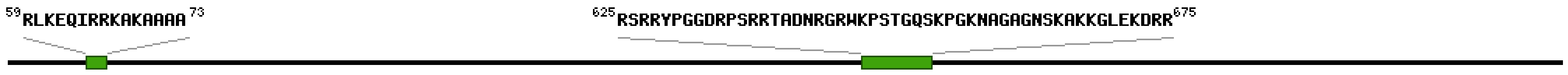

Posterior Graphic. Threshold Marked @ 0.4

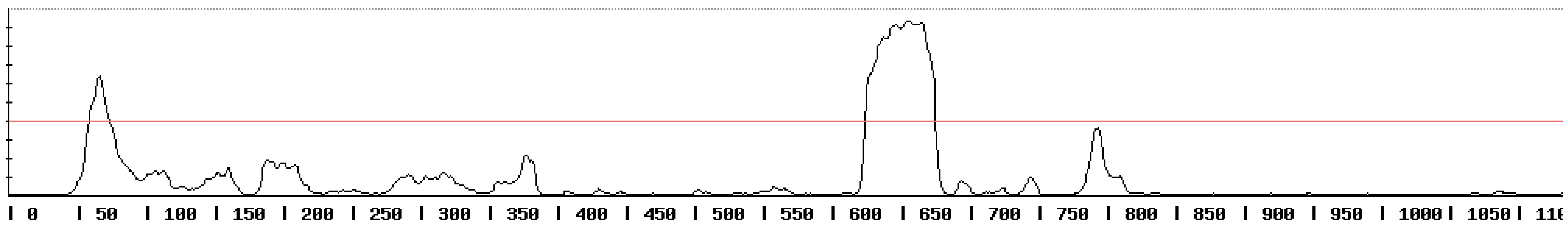

Predictions for Dichantheliumoligosanthes_Do008886.1

Viterbi Path	
Viterbi Path	
Posterior @ 0.4	
Posterior @ 0.4	

Posterior Graphic. Threshold Marked @ 0.4

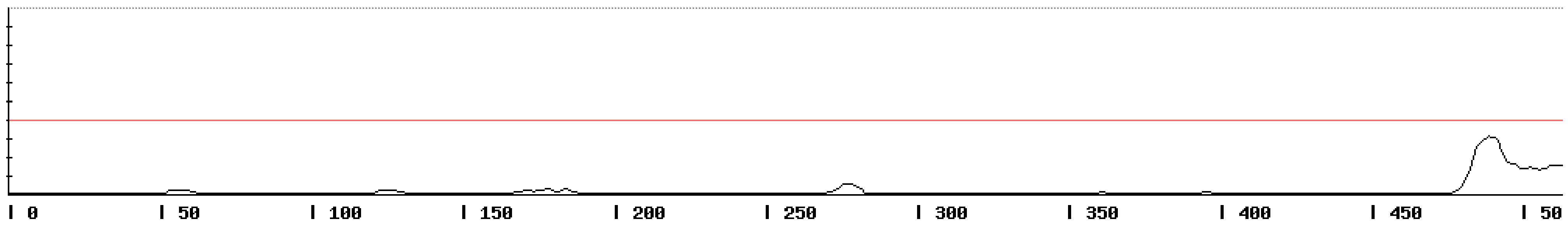

Predictions for Dichantheliumoligosanthes_Do009067.1

Viterbi Path	524 - PRGPRLRRRRREDPARRAR - 542

Viterbi Path

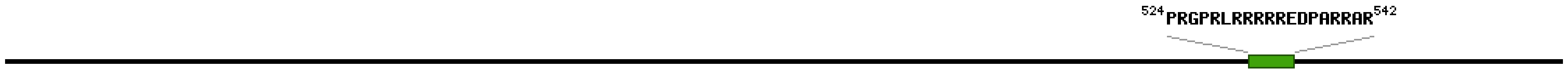

Posterior @ 0.4	517 - RSGATSSPRGPRLRRRRREDPARRARTTS - 545
Posterior @ 0.4	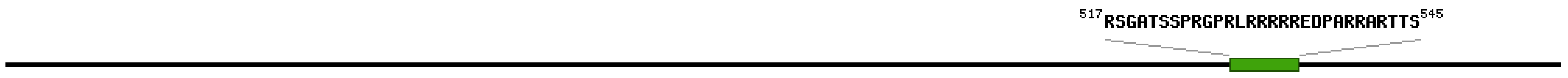
Posterior Graphic. Threshold Marked @ 0.4	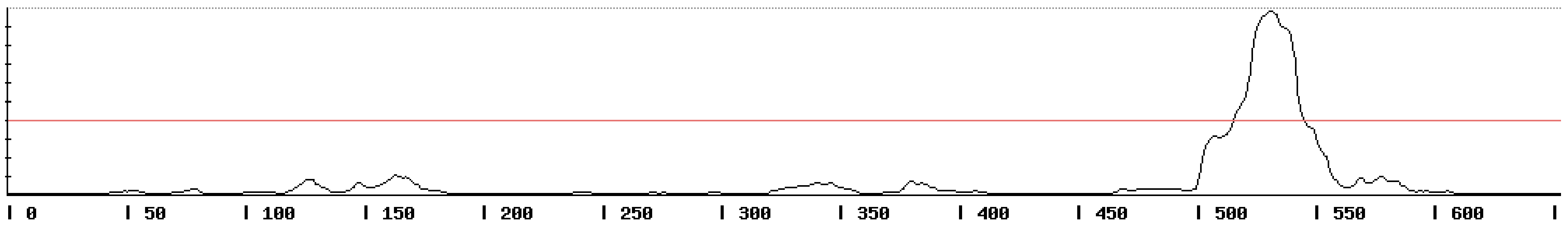

Predictions for Dichantheliumoligosanthes_Do009902.1

Viterbi Path	
Viterbi Path	
Posterior @ 0.4	146 - RKRRK - 150
Posterior @ 0.4	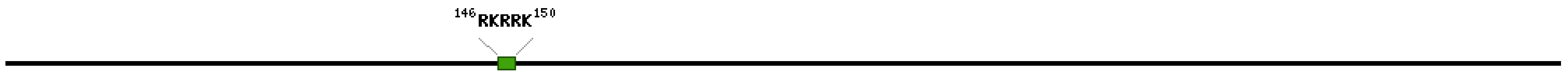
Posterior Graphic. Threshold Marked @ 0.4	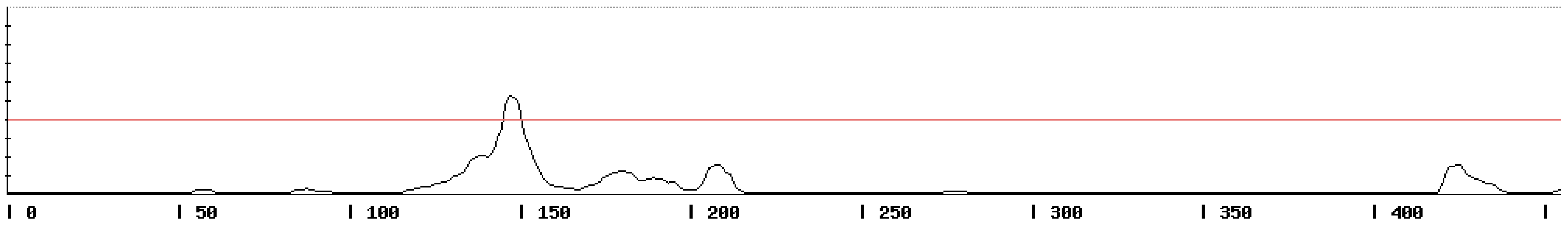

Predictions for Dichantheliumoligosanthes_Do009936.1

Viterbi Path	
Viterbi Path	

Posterior @ 0.4

Posterior @ 0.4

Posterior Graphic. Threshold Marked @ 0.4

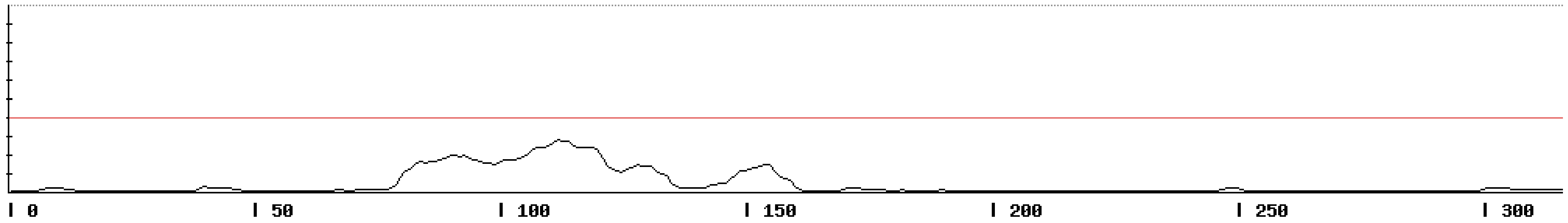

Predictions for Dichantheliumoligosanthes_Do009957.1

Viterbi Path

Viterbi Path

Posterior @ 0.4

Posterior @ 0.4

Posterior Graphic. Threshold Marked @ 0.4

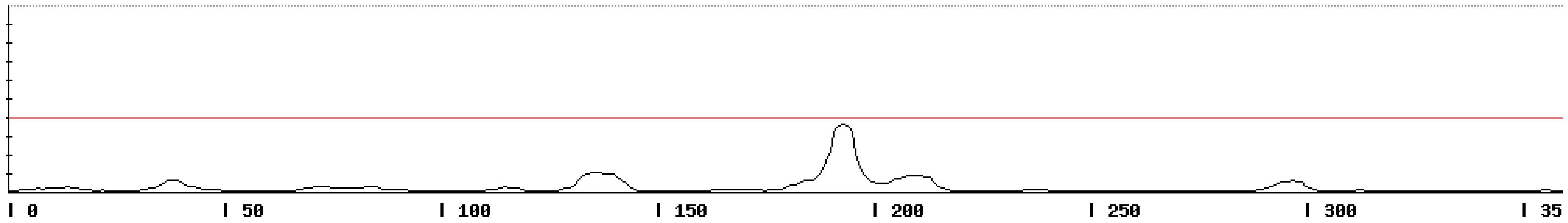

Predictions for Dichantheliumoligosanthes_Do010251.1

Viterbi Path

Viterbi Path

Posterior @ 0.4

Posterior @ 0.4

Posterior Graphic. Threshold Marked @ 0.4

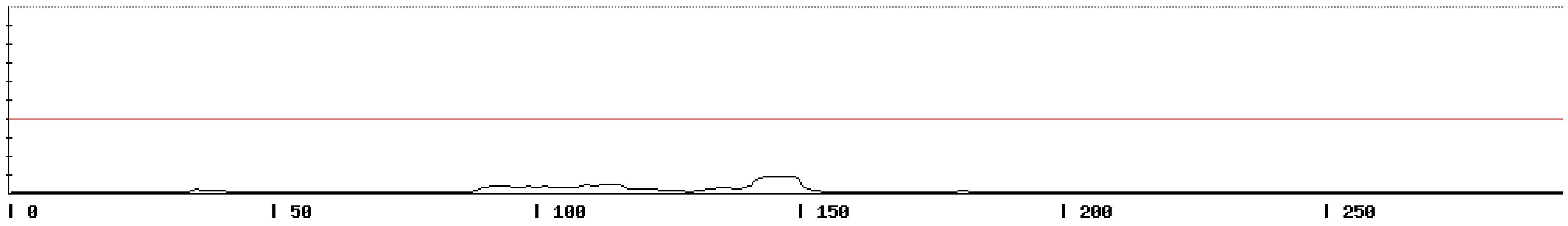

Predictions for Dichantheliumoligosanthes_Do010623.1

Viterbi Path

Viterbi Path

Posterior @ 0.4

Posterior @ 0.4

Posterior Graphic. Threshold Marked @ 0.4

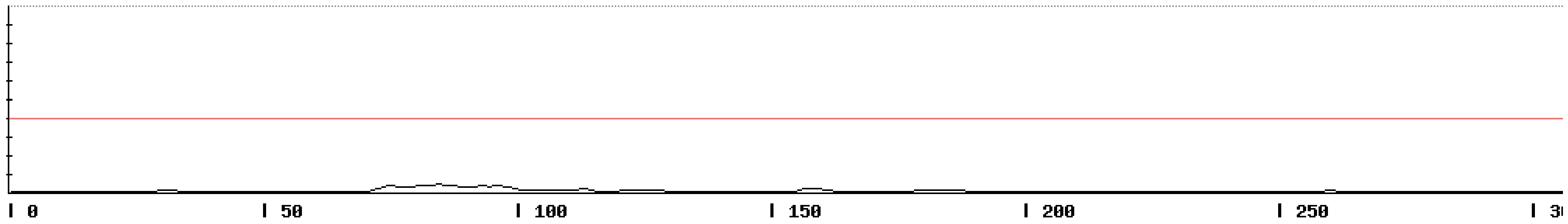

Predictions for Dichantheliumoligosanthes_Do010830.1

Viterbi Path	
Viterbi Path	
Posterior @ 0.4	330 - SGHGKKRKRG - 339<br>730 - GHAKKRRL - 737
Posterior @ 0.4	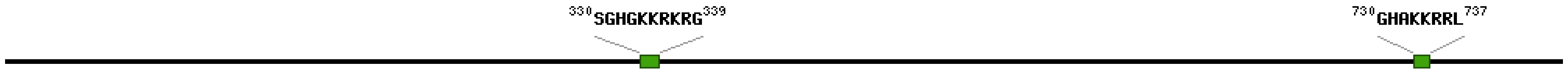
Posterior Graphic. Threshold Marked @ 0.4	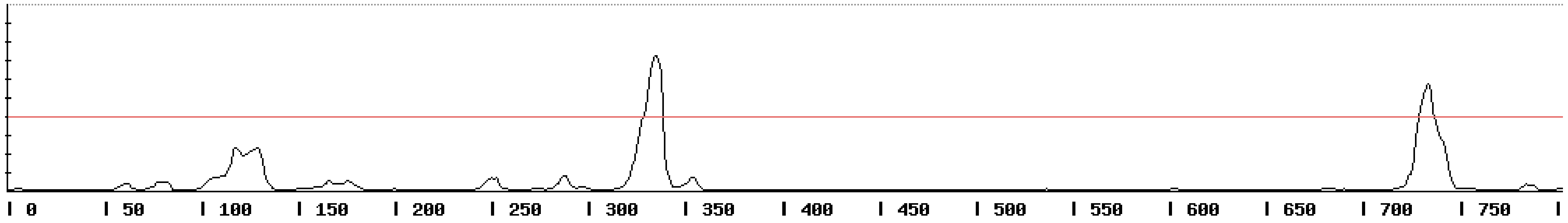

Predictions for Dichantheliumoligosanthes_Do010860.1

Viterbi Path	
Viterbi Path	
Posterior @ 0.4	
Posterior @ 0.4	
Posterior Graphic. Threshold Marked @ 0.4	

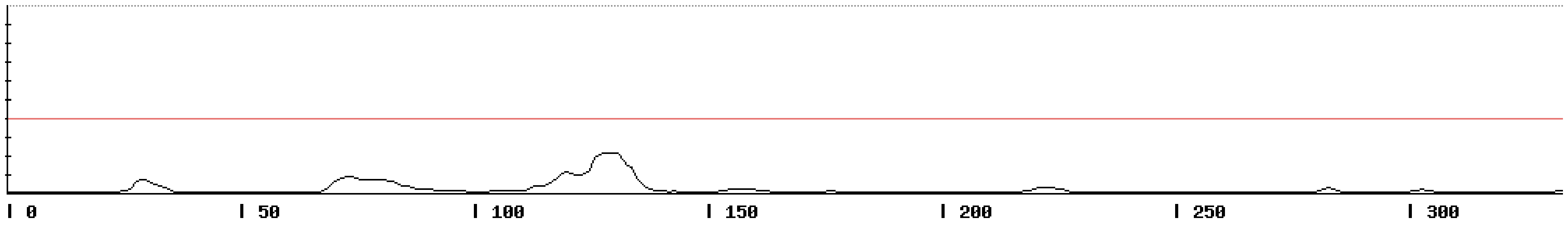

Predictions for Dichantheliumoligosanthes_Do010926.1

Viterbi Path	
Viterbi Path	
Posterior @ 0.4	139 - RKRRK - 143
Posterior @ 0.4	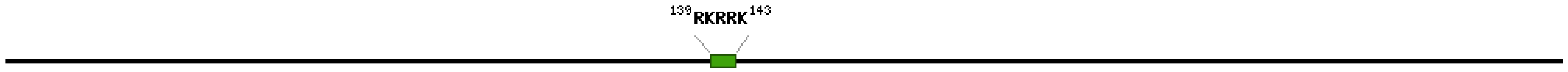
Posterior Graphic. Threshold Marked @ 0.4	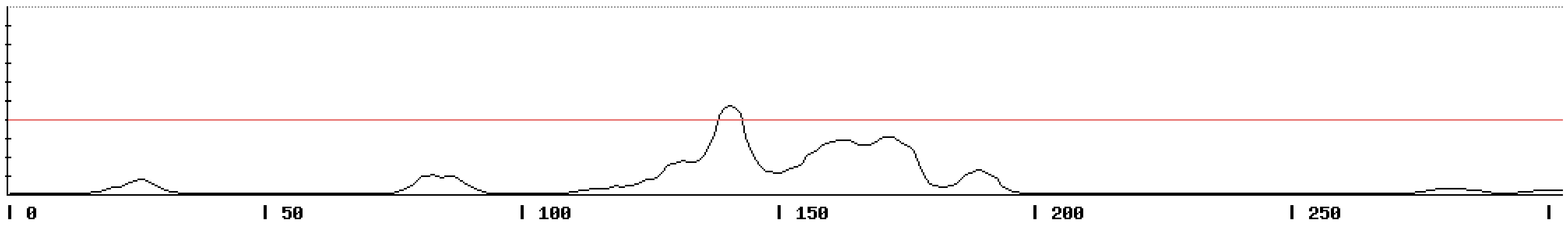

Predictions for Dichantheliumoligosanthes_Do011140.1

Viterbi Path	
Viterbi Path	
Posterior @ 0.4	
Posterior @ 0.4	

Posterior Graphic. Threshold Marked @ 0.4

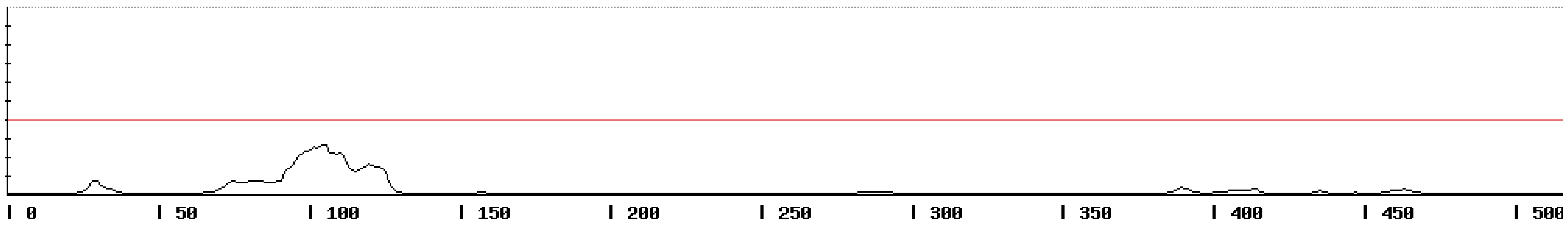

Predictions for Dichantheliumoligosanthes_Do011344.1

Viterbi Path	
Viterbi Path	
Posterior @ 0.4	85 - RGKVR - 89
Posterior @ 0.4	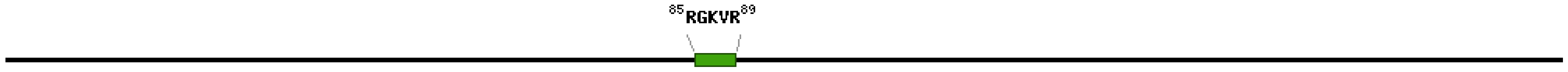
Posterior Graphic. Threshold Marked @ 0.4	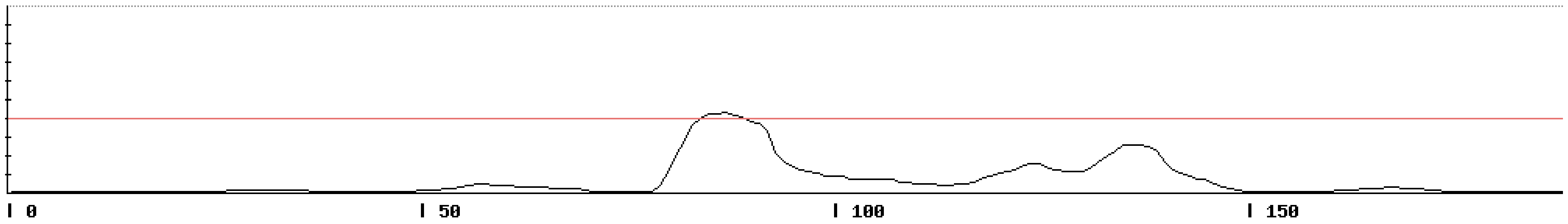

Predictions for Dichantheliumoligosanthes_Do011426.1

Viterbi Path	
Viterbi Path	
Posterior @ 0.4	187 - SRSARKRK - 194
Posterior @ 0.4	

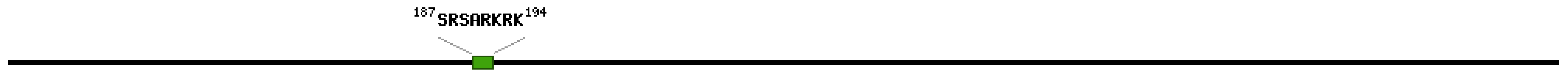

Posterior Graphic. Threshold Marked @ 0.4

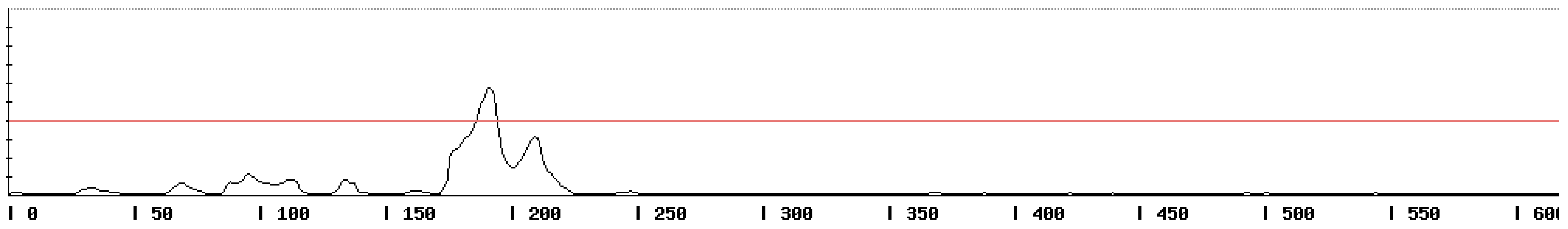

Predictions for Dichantheliumoligosanthes_Do011477.1

Viterbi Path	
Viterbi Path	
Posterior @ 0.4	80 - RHKSVRGKRKAR - 91
	283 - PARKRKAP - 290
	304 - PAPKRK - 309
	602 - PRLRGRRTTSTLVRRQRHRLDPRRRPSVRALVRPRLGNSAMTGGPLLMLRRKPRPQQQRRSPKISPN - 668
	685 - RKSRSPANPG - 694

Posterior @ 0.4

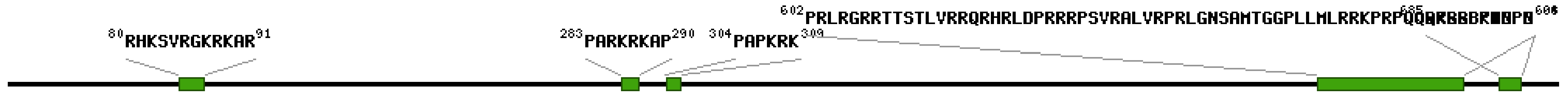

Posterior Graphic. Threshold Marked @ 0.4

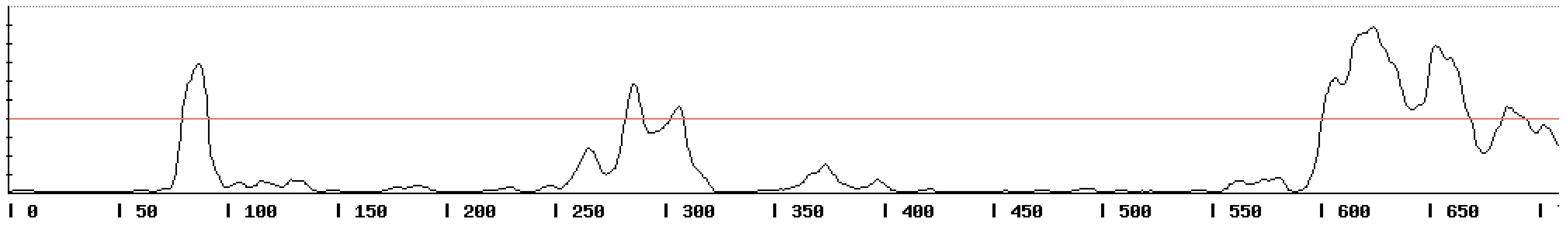

Predictions for Dichantheliumoligosanthes_Do011689.1

Viterbi Path

Viterbi Path

Posterior @ 0.4

Posterior @ 0.4

Posterior Graphic. Threshold Marked @ 0.4

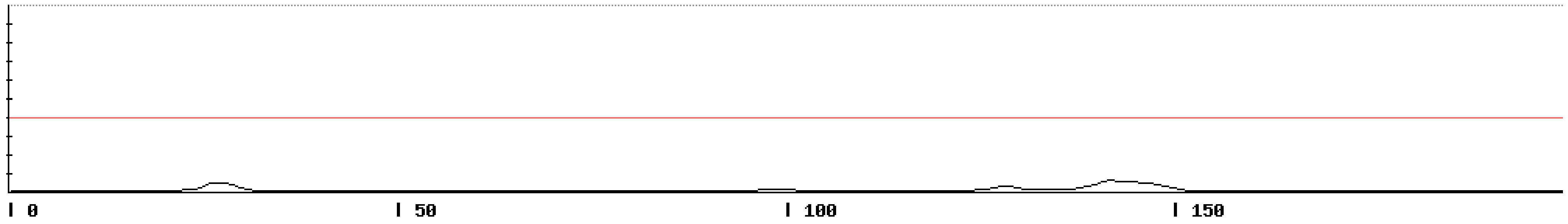

Predictions for Dichantheliumoligosanthes_Do011734.1

Viterbi Path

Viterbi Path

Posterior @ 0.4 | 70 - GKNAPRRRGRSHQRSWTVAGADGKKKGTWSPGKIKYKGRAG - 110

Posterior @ 0.4

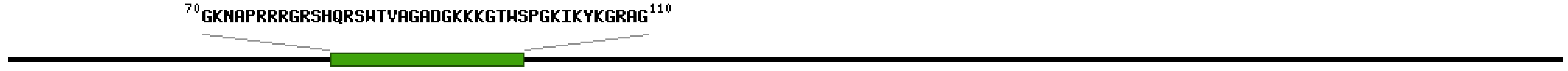

Posterior Graphic. Threshold Marked @ 0.4

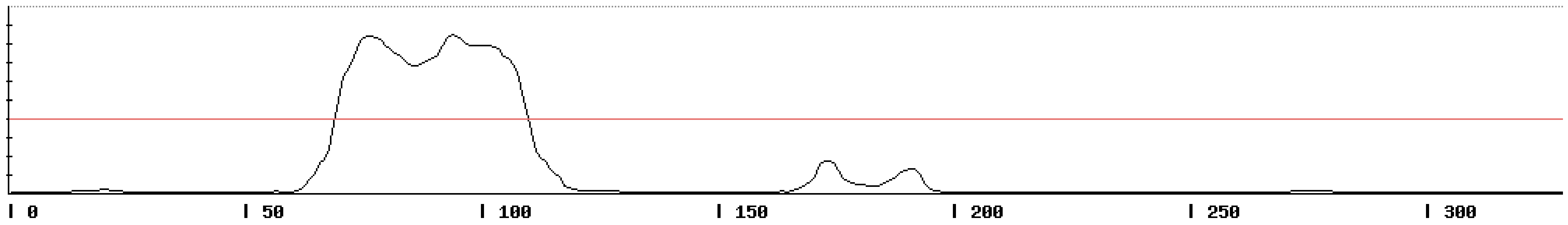

Predictions for Dichantheliumoligosanthes_Do011989.1	
Viterbi Path	
Viterbi Path	
Posterior @ 0.4	147 - KRR - 149
Posterior @ 0.4	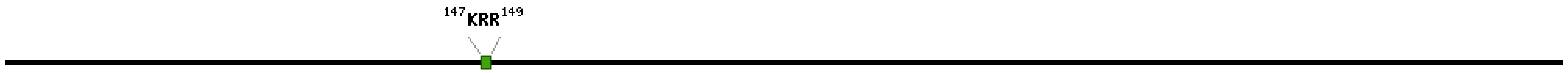
Posterior Graphic. Threshold Marked @ 0.4	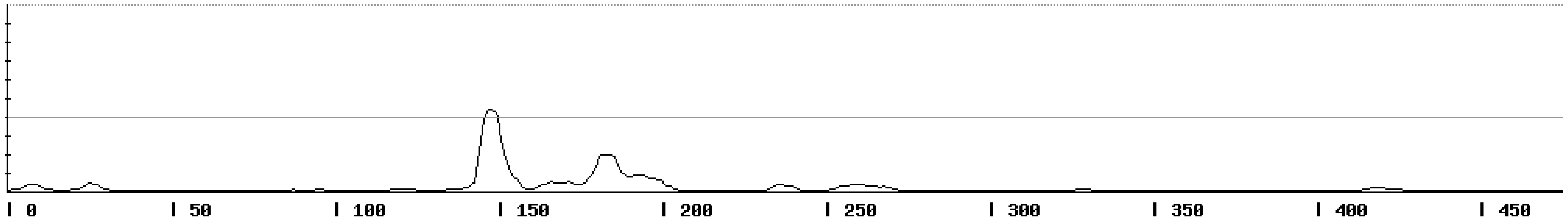

Predictions for Dichantheliumoligosanthes_Do012309.1	
Viterbi Path	
Viterbi Path	
Posterior @ 0.4	
Posterior @ 0.4	
Posterior Graphic. Threshold Marked @ 0.4	

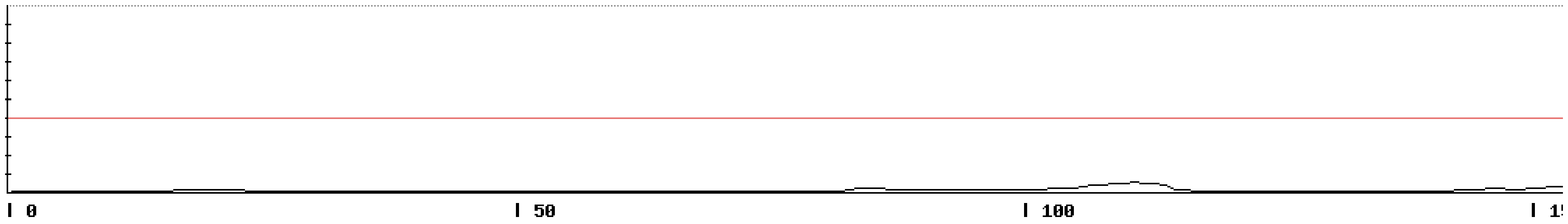

Predictions for Dichantheliumoligosanthes_Do013120.1

Viterbi Path	
Viterbi Path	
Posterior @ 0.4	
Posterior @ 0.4	
Posterior Graphic. Threshold Marked @ 0.4	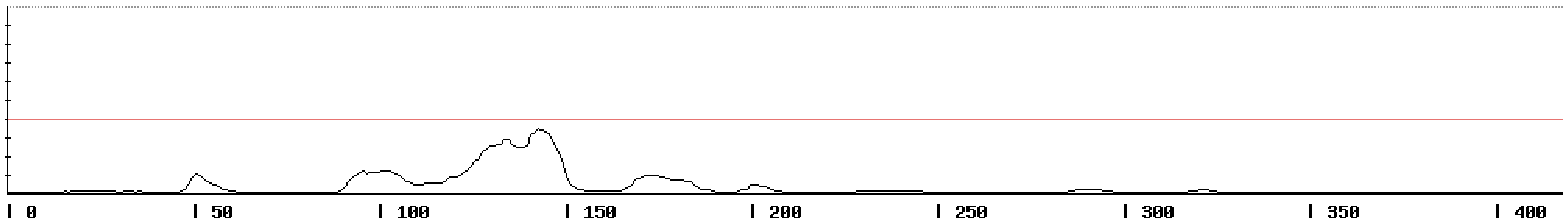

Predictions for Dichantheliumoligosanthes_Do013239.1

Viterbi Path	
Viterbi Path	
Posterior @ 0.4	
Posterior @ 0.4	

Posterior Graphic. Threshold Marked @ 0.4

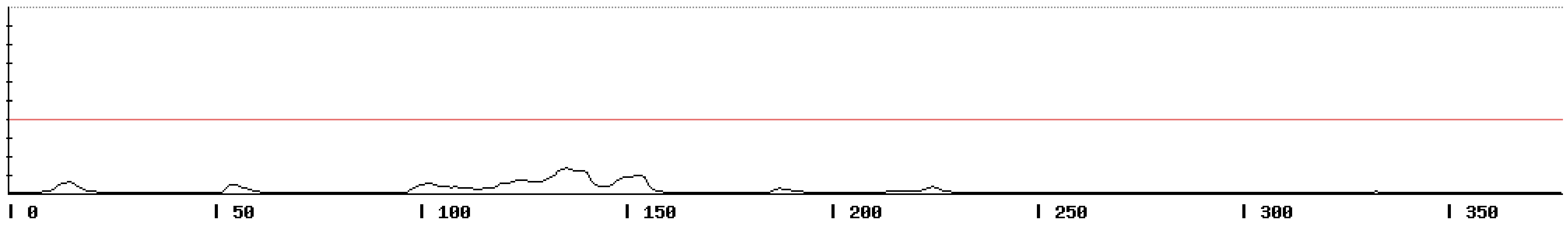

Predictions for Dichantheliumoligosanthes_Do013407.1

Viterbi Path

Viterbi Path

Posterior @ 0.4

Posterior @ 0.4

Posterior Graphic. Threshold Marked @ 0.4

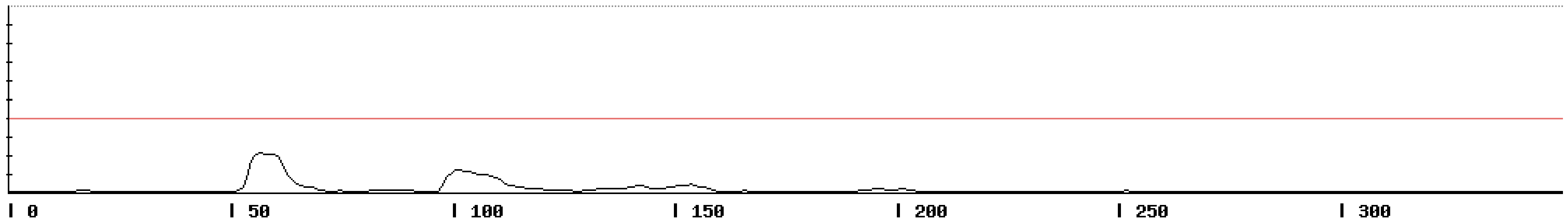

Predictions for Dichantheliumoligosanthes_Do013581.1

Viterbi Path

Viterbi Path

Posterior @ 0.4 125 - R - 125

Posterior @ 0.4

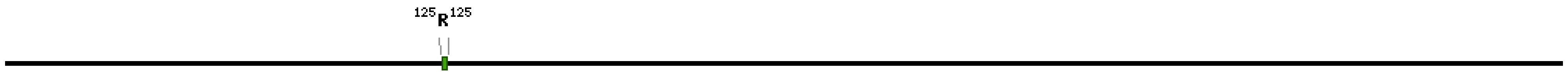

Posterior Graphic. Threshold Marked @ 0.4

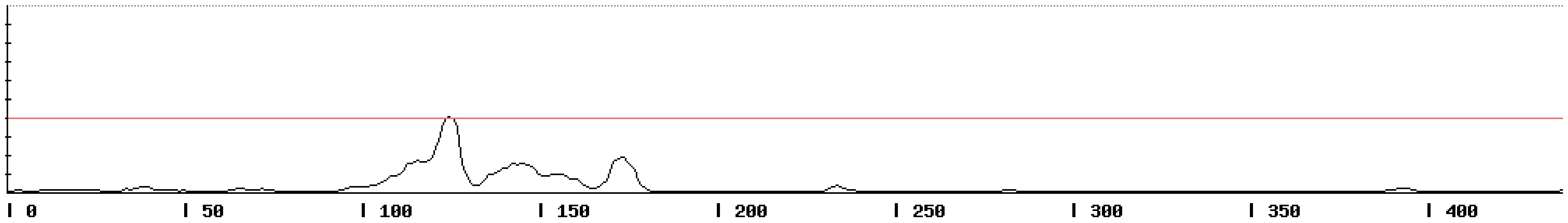

Predictions for Dichantheliumoligosanthes_Do013621.1

Viterbi Path

Viterbi Path

Posterior @ 0.4

Posterior @ 0.4

Posterior Graphic. Threshold Marked @ 0.4

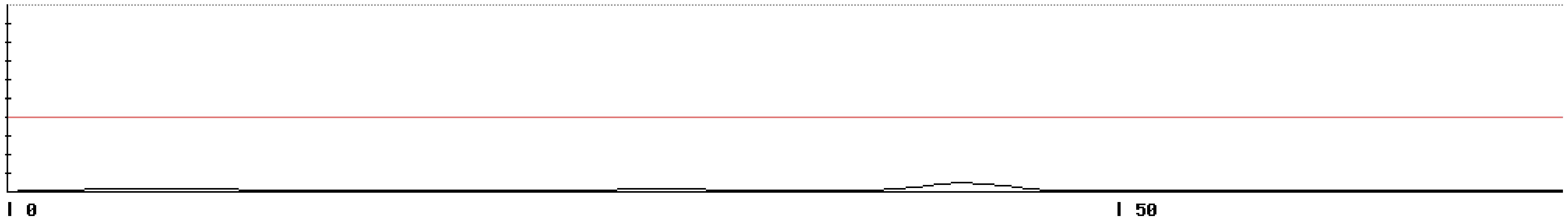

Predictions for Dichantheliumoligosanthes_Do013671.1

Viterbi Path

Viterbi Path

Posterior @ 0.4

Posterior @ 0.4

Posterior Graphic. Threshold Marked @ 0.4

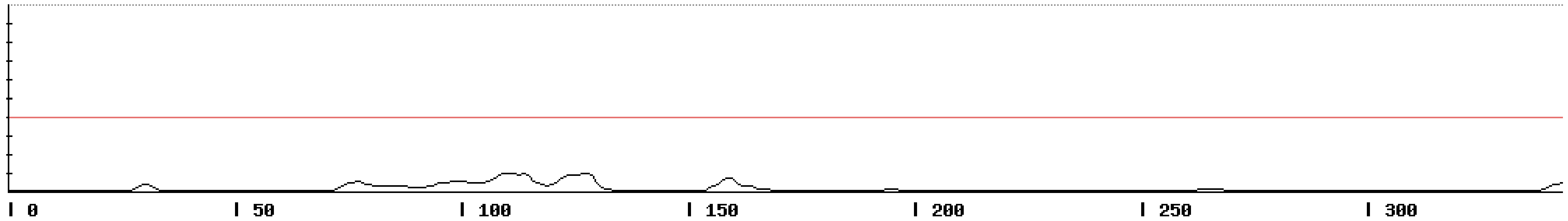

Predictions for Dichantheliumoligosanthes_Do013739.1

Viterbi Path

Viterbi Path

Posterior @ 0.4

Posterior @ 0.4

Posterior Graphic. Threshold Marked @ 0.4

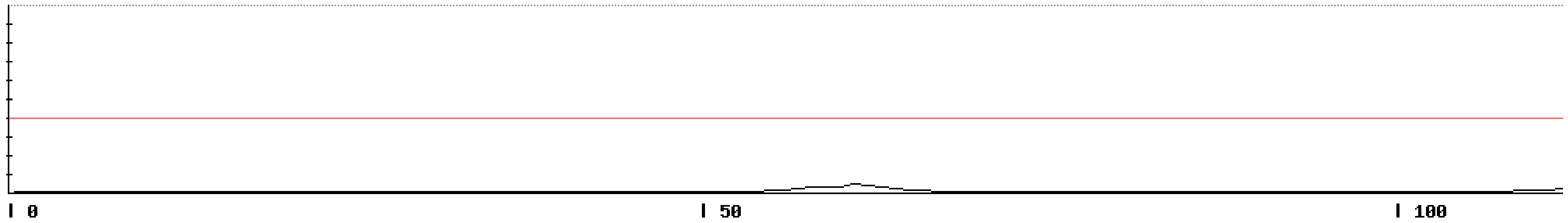

Predictions for Dichantheliumoligosanthes_Do013815.1

Viterbi Path

Viterbi Path

Posterior @ 0.4

Posterior @ 0.4

Posterior Graphic. Threshold Marked @ 0.4

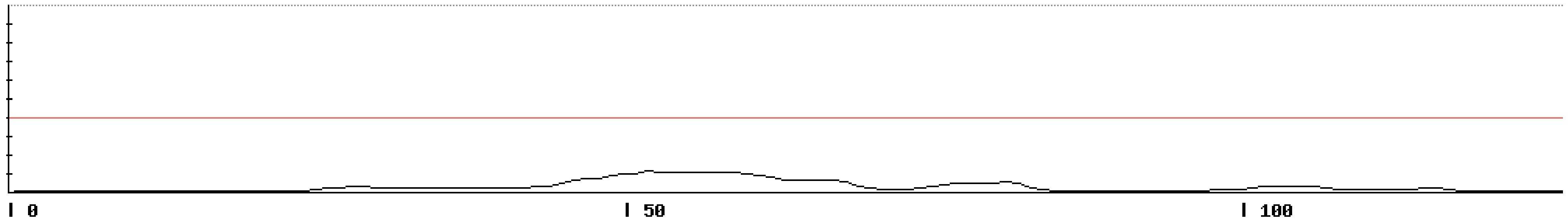

Predictions for Dichantheliumoligosanthes_Do014291.1

Viterbi Path

Viterbi Path

Posterior @ 0.4

Posterior @ 0.4

Posterior Graphic. Threshold Marked @ 0.4

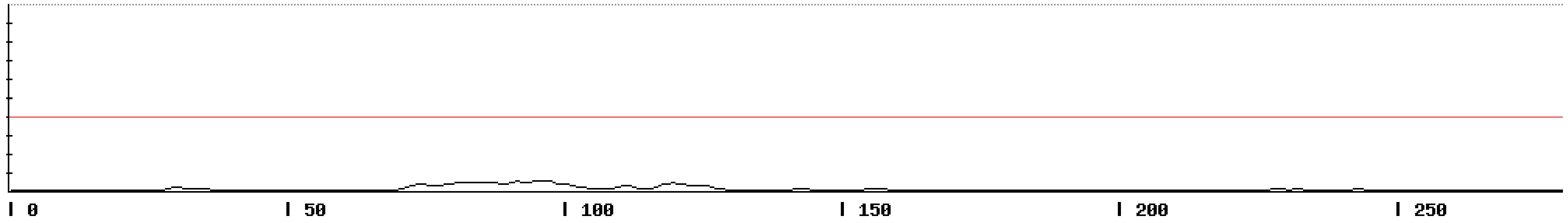

Predictions for Dichantheliumoligosanthes_Do015330.1

Viterbi Path

Viterbi Path

Posterior @ 0.4

Posterior @ 0.4

Posterior Graphic. Threshold Marked @ 0.4

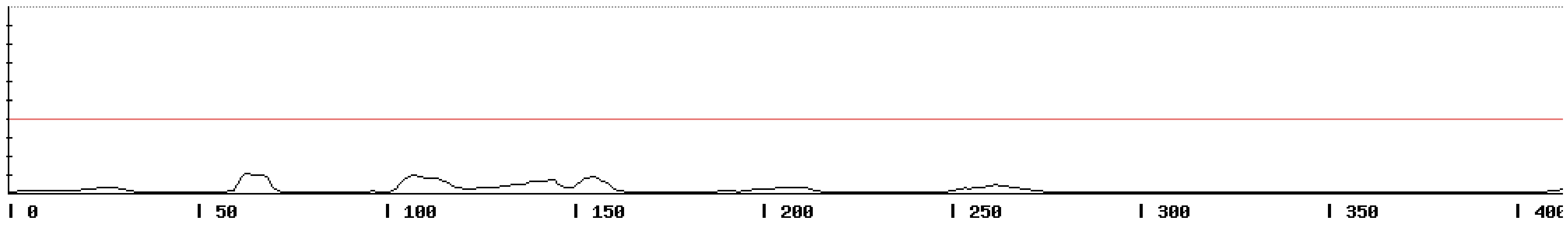

Predictions for Dichantheliumoligosanthes_Do015340.1

Viterbi Path

Viterbi Path

Posterior @ 0.4

Posterior @ 0.4

Posterior Graphic. Threshold Marked @ 0.4

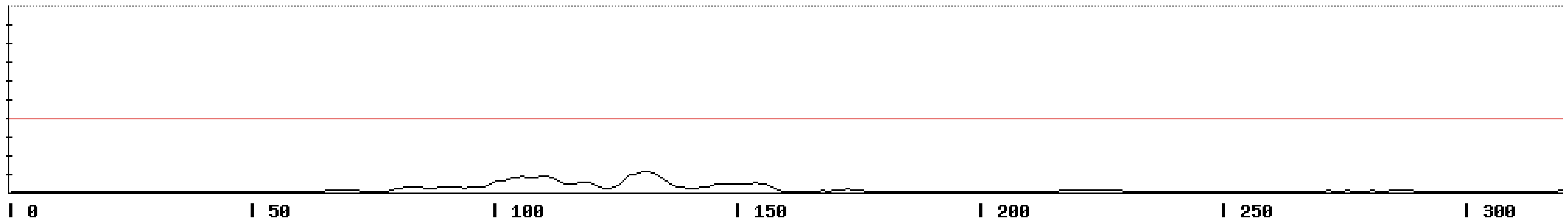

Predictions for Dichantheliumoligosanthes_Do015402.1

Viterbi Path

Viterbi Path

Posterior @ 0.4

177 - R - 177

558 - RG - 559

Posterior @ 0.4

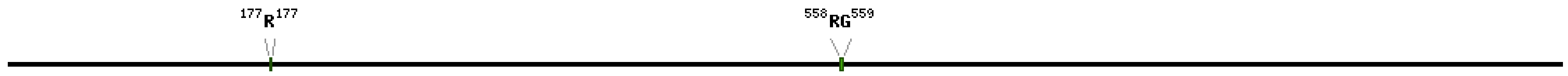

Posterior Graphic. Threshold Marked @ 0.4

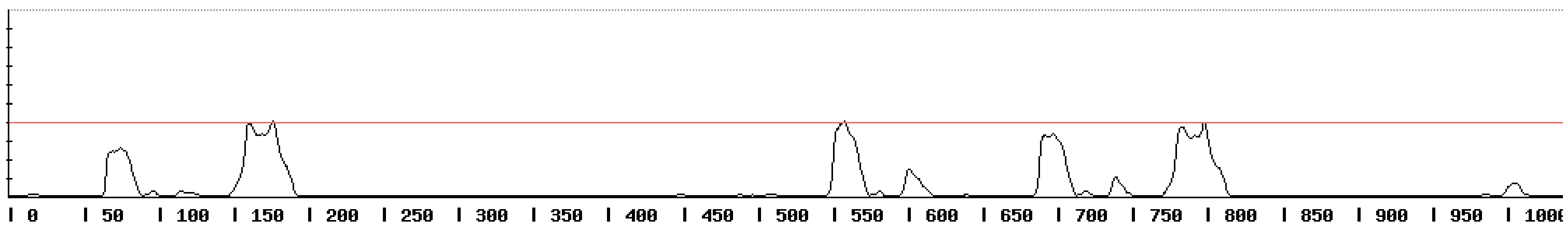

Predictions for Dichantheliumoligosanthes_Do015555.1

Viterbi Path

Viterbi Path

Posterior @ 0.4

Posterior @ 0.4

Posterior Graphic. Threshold Marked @ 0.4

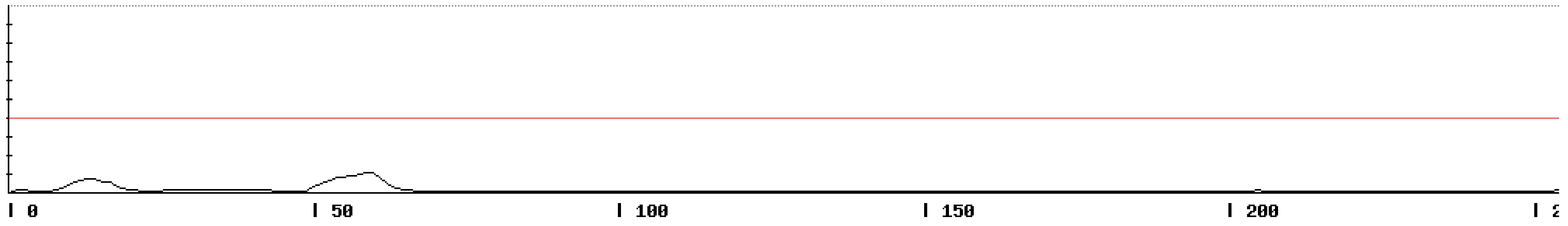

Predictions for Dichantheliumoligosanthes_Do015656.1

Viterbi Path

Viterbi Path

Posterior @ 0.4: 166 - RKRKR - 170

Posterior @ 0.4

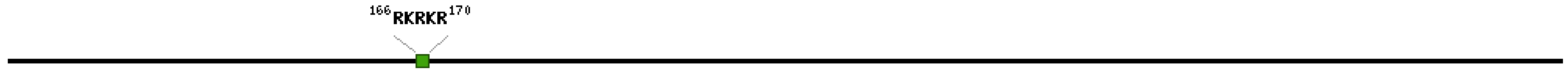

Posterior Graphic. Threshold Marked @ 0.4

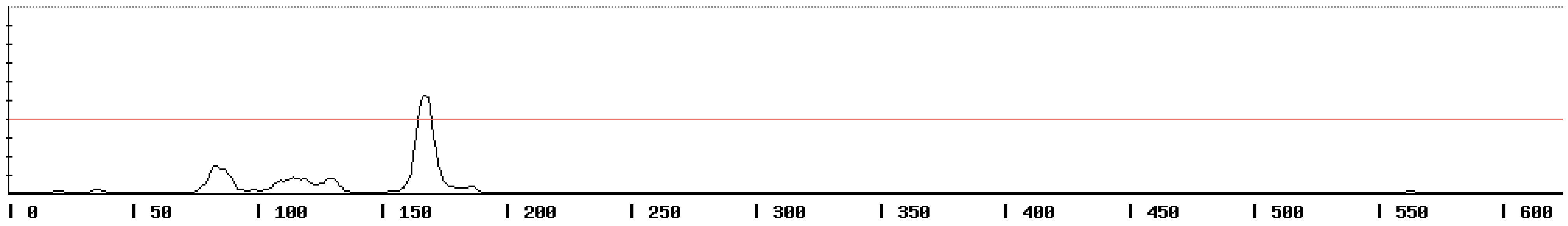

Predictions for Dichantheliumoligosanthes_Do015720.1

Viterbi Path

Viterbi Path

Posterior @ 0.4: 434 - PKSKGKGKAPKGVSRR - 449

Posterior @ 0.4

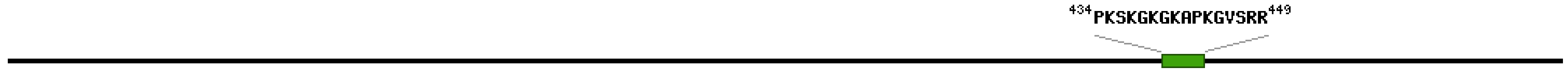

Posterior Graphic. Threshold Marked @ 0.4

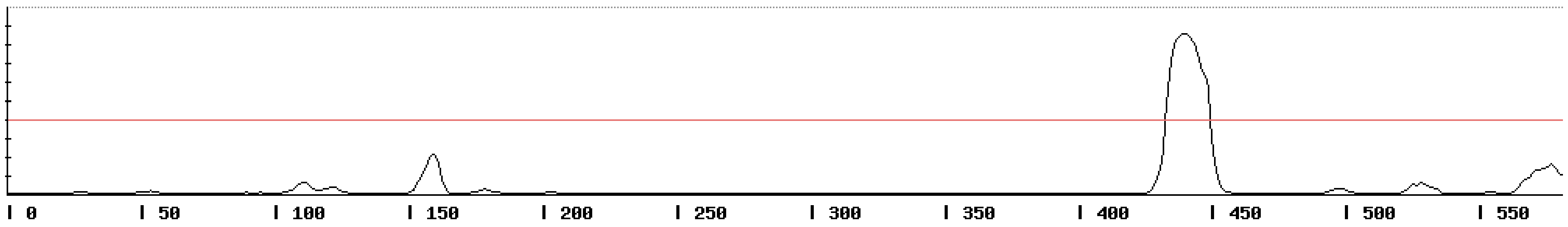

Predictions for Dichantheliumoligosanthes_Do015782.1

Viterbi Path	357 - RKKQGKGGLHPAKEPKKEQSNPTKKPSSSRRRR - 389
Viterbi Path	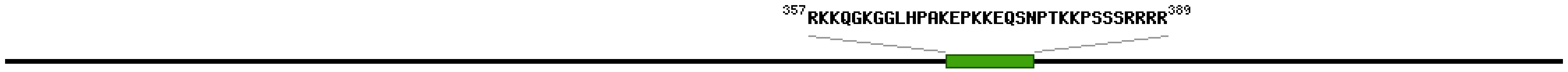
Posterior @ 0.4	163 - MLSRIRITGRAKKERKK - 179
	356 - VRKKQGKGGLHPAKEPKKEQSNPTKKPSSSRRRRV - 390
	563 - GRAKKER - 569
Posterior @ 0.4	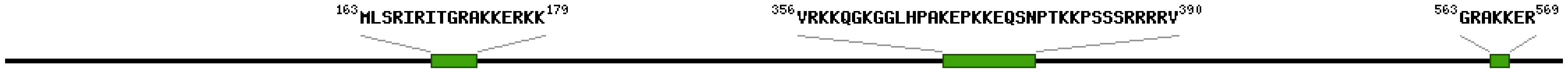
Posterior Graphic. Threshold Marked @ 0.4	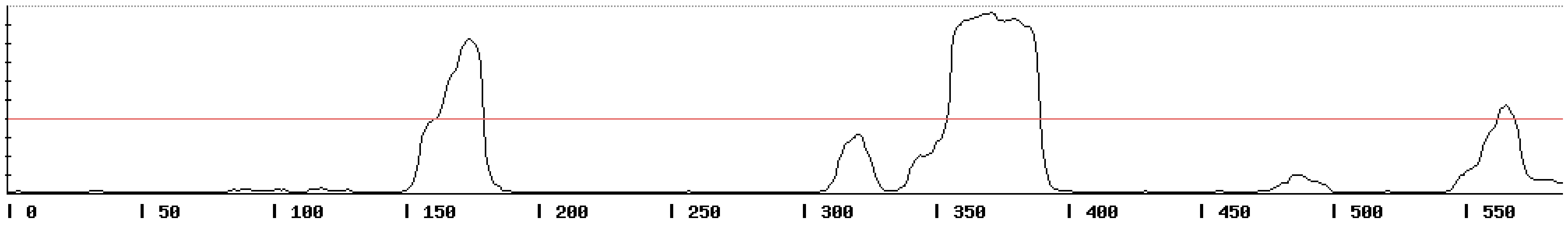

Predictions for Dichantheliumoligosanthes_Do015799.1

Viterbi Path	
Viterbi Path	
Posterior @ 0.4	90 - KATGKDRKVNSHRRA - 104

	624 - KKKAKKVMK - 632
Posterior @ 0.4	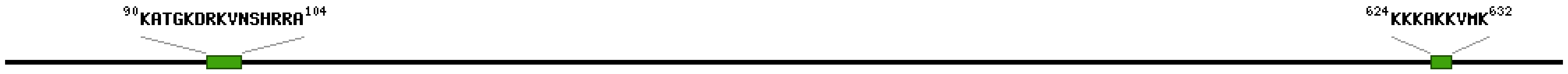
Posterior Graphic. Threshold Marked @ 0.4	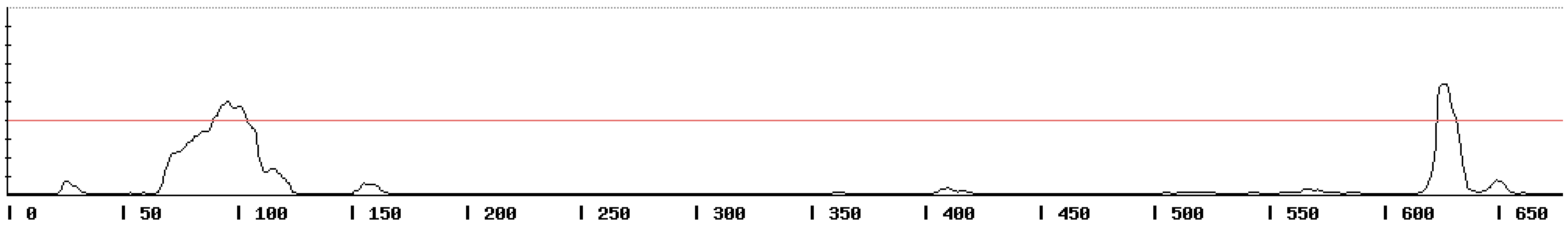

Predictions for Dichantheliumoligosanthes_Do015875.1

Viterbi Path	
Viterbi Path	
Posterior @ 0.4	
Posterior @ 0.4	
Posterior Graphic. Threshold Marked @ 0.4	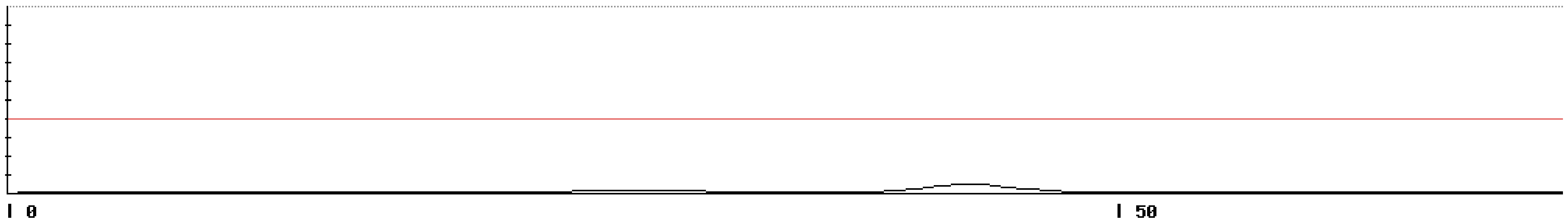

Predictions for Dichantheliumoligosanthes_Do015901.1

Viterbi Path	
Viterbi Path	

Posterior @ 0.4

Posterior @ 0.4

Posterior Graphic. Threshold Marked @ 0.4

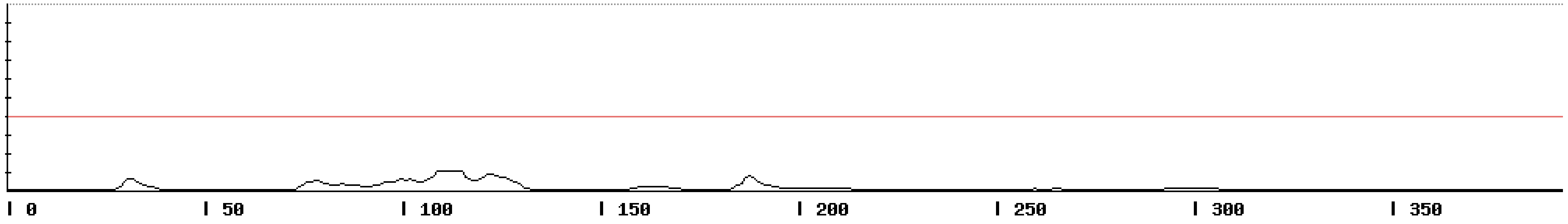

Predictions for Dichantheliumoligosanthes_Do016040.1

Viterbi Path

Viterbi Path

Posterior @ 0.4

Posterior @ 0.4

Posterior Graphic. Threshold Marked @ 0.4

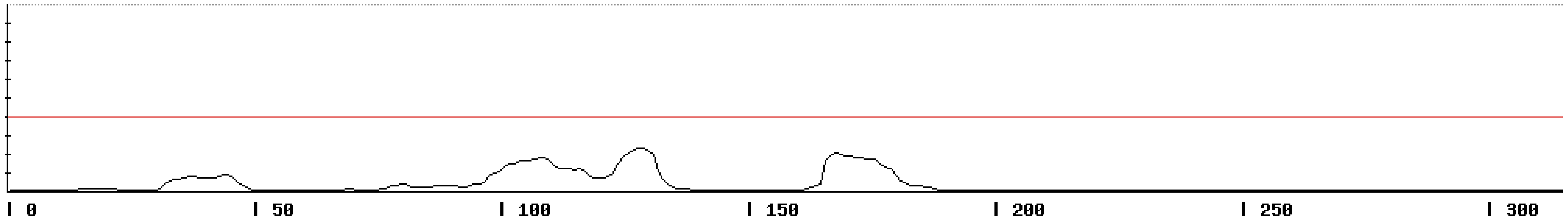

Predictions for Dichantheliumoligosanthes_Do016375.1

Viterbi Path

Viterbi Path

Posterior @ 0.4

Posterior @ 0.4

Posterior Graphic. Threshold Marked @ 0.4

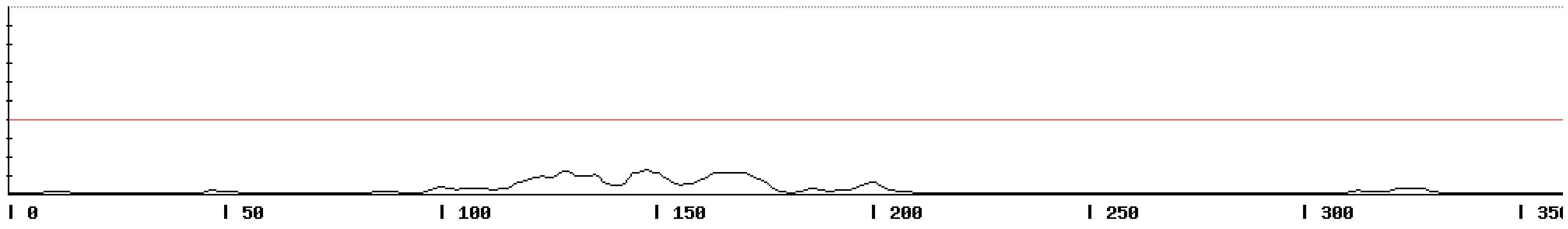

Predictions for Dichantheliumoligosanthes_Do016529.1

Viterbi Path

Viterbi Path

Posterior @ 0.4

Posterior @ 0.4

Posterior Graphic. Threshold Marked @ 0.4

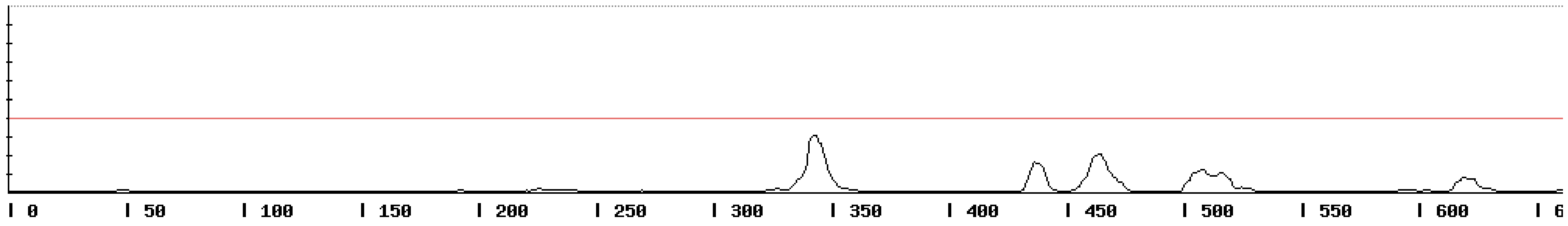

Predictions for Dichantheliumoligosanthes_Do016642.1	
Viterbi Path	
Viterbi Path	
Posterior @ 0.4	120 - RKRRK - 124
Posterior @ 0.4	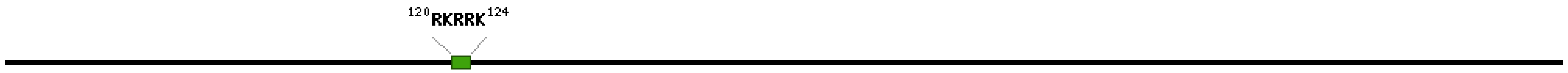
Posterior Graphic. Threshold Marked @ 0.4	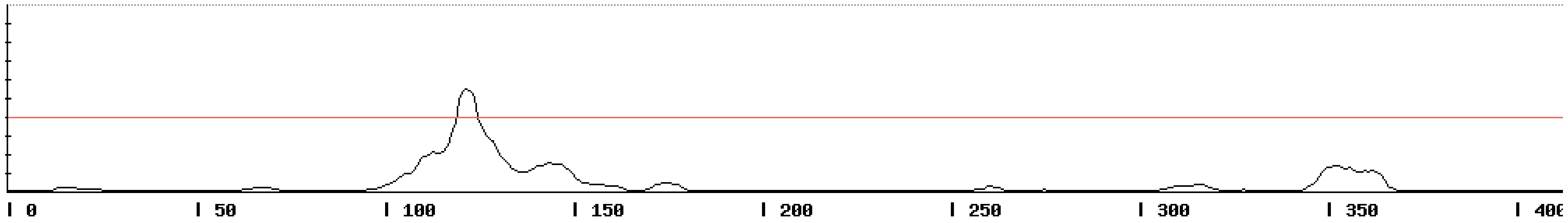

Predictions for Dichantheliumoligosanthes_Do017455.1	
Viterbi Path	
Viterbi Path	
Posterior @ 0.4	210 - KKKKRM - 215
Posterior @ 0.4	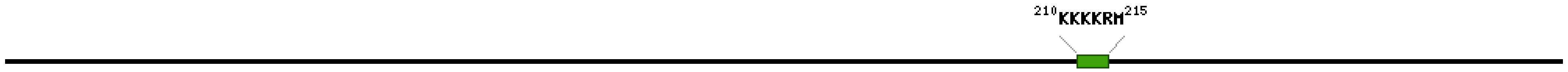
Posterior Graphic. Threshold Marked @ 0.4	

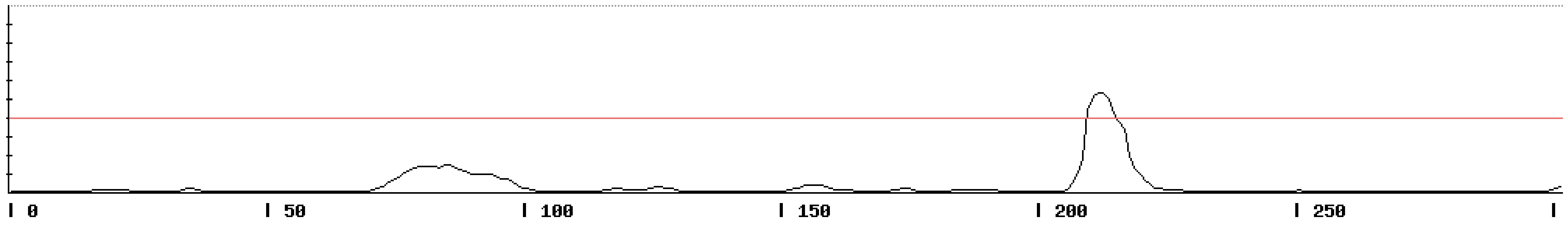

Predictions for Dichantheliumoligosanthes_Do017860.1

Viterbi Path

Viterbi Path

Posterior @ 0.4

Posterior @ 0.4

Posterior Graphic. Threshold Marked @ 0.4

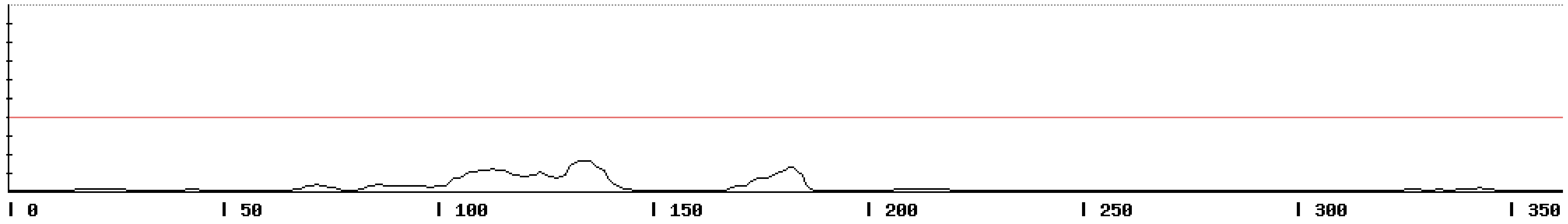

Predictions for Dichantheliumoligosanthes_Do017927.1

Viterbi Path

Viterbi Path

Posterior @ 0.4

83 - PSRKTRDGRGRWK - 95

210 - SEKQARKRPMAELPPRVRAAPARKQAR - 236

Posterior @ 0.4

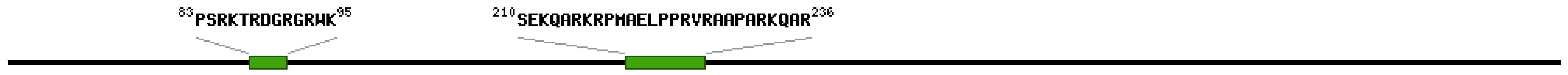

Posterior Graphic. Threshold Marked @ 0.4

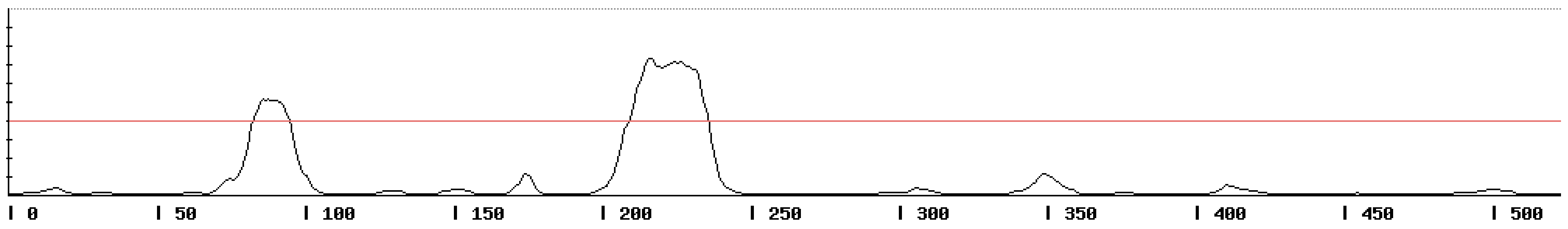

Predictions for Dichantheliumoligosanthes_Do018235.1

Viterbi Path

Viterbi Path

Posterior @ 0.4

Posterior @ 0.4

Posterior Graphic. Threshold Marked @ 0.4

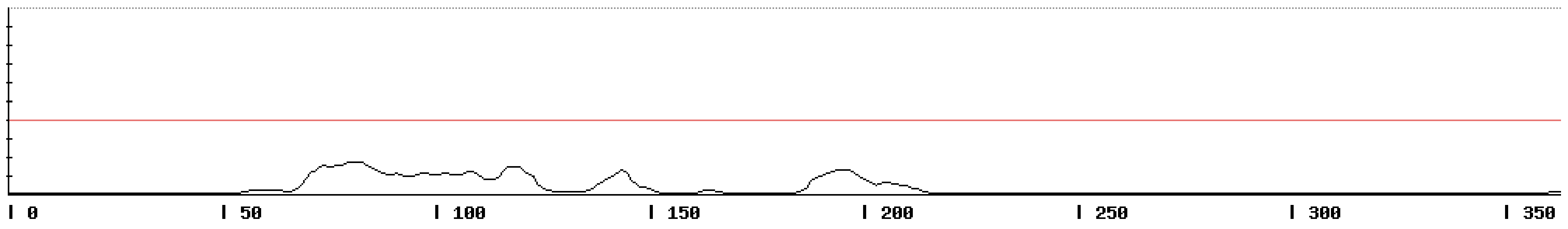

Predictions for Dichantheliumoligosanthes_Do018469.1

Viterbi Path

Viterbi Path

Posterior @ 0.4	85 - RTKSPRGRRKSRT - 97
Posterior @ 0.4	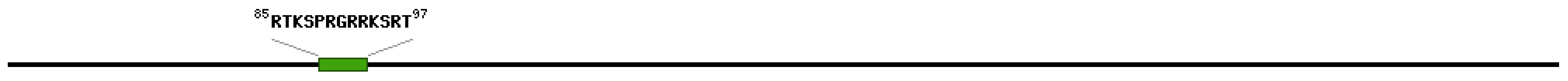
Posterior Graphic. Threshold Marked @ 0.4	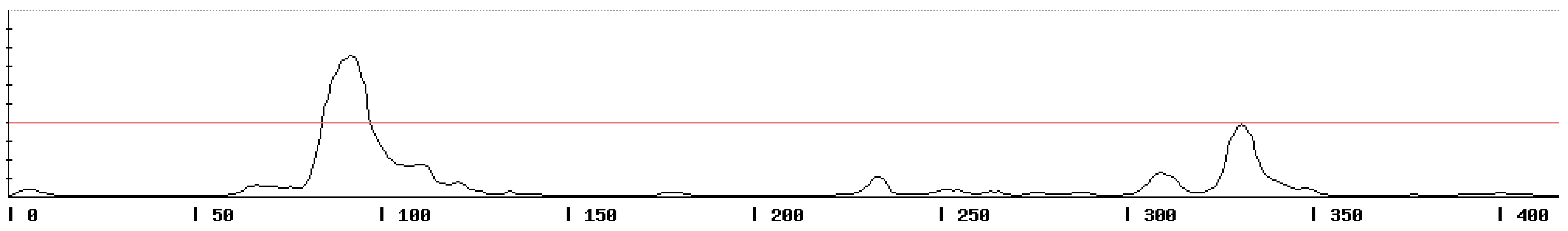

Predictions for Dichantheliumoligosanthes_Do018790.1

Viterbi Path	
Viterbi Path	
Posterior @ 0.4	
Posterior @ 0.4	
Posterior Graphic. Threshold Marked @ 0.4	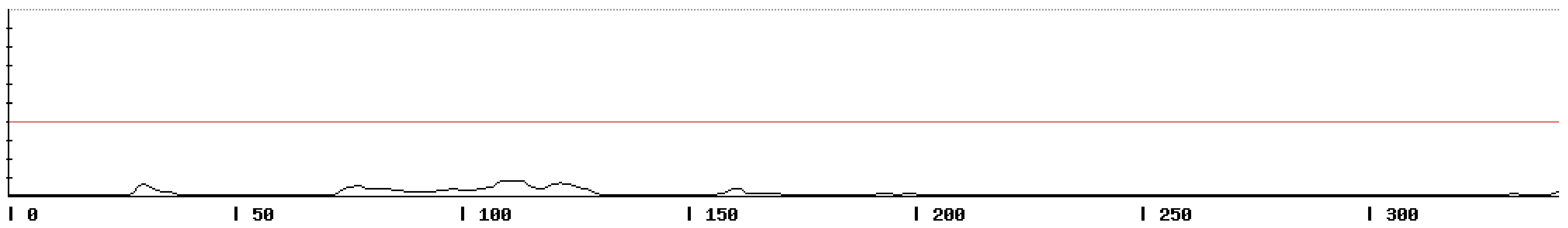

Predictions for Dichantheliumoligosanthes_Do019007.1

Viterbi Path	
Viterbi Path	

Posterior @ 0.4

Posterior @ 0.4

Posterior Graphic. Threshold Marked @ 0.4

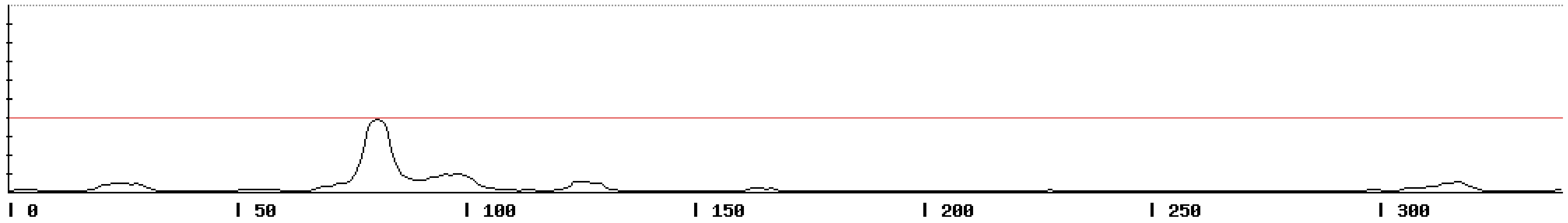

Predictions for Dichantheliumoligosanthes_Do019333.1

Viterbi Path

Viterbi Path

Posterior @ 0.4

Posterior @ 0.4

Posterior Graphic. Threshold Marked @ 0.4

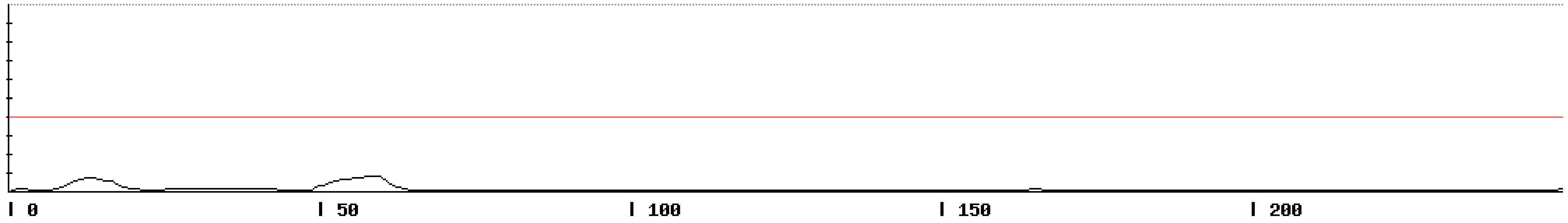

Predictions for Dichantheliumoligosanthes_Do019447.1

Viterbi Path

Viterbi Path

Posterior @ 0.4

Posterior @ 0.4

Posterior Graphic. Threshold Marked @ 0.4

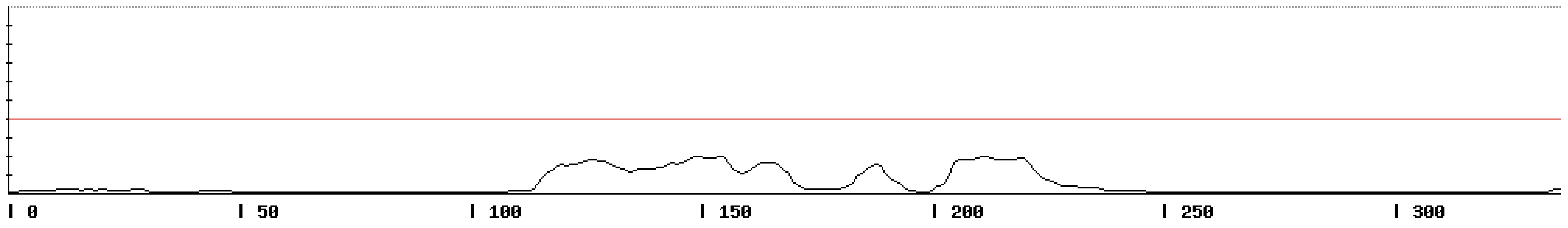

Predictions for Dichantheliumoligosanthes_Do020566.1

Viterbi Path

Viterbi Path

Posterior @ 0.4

Posterior @ 0.4

Posterior Graphic. Threshold Marked @ 0.4

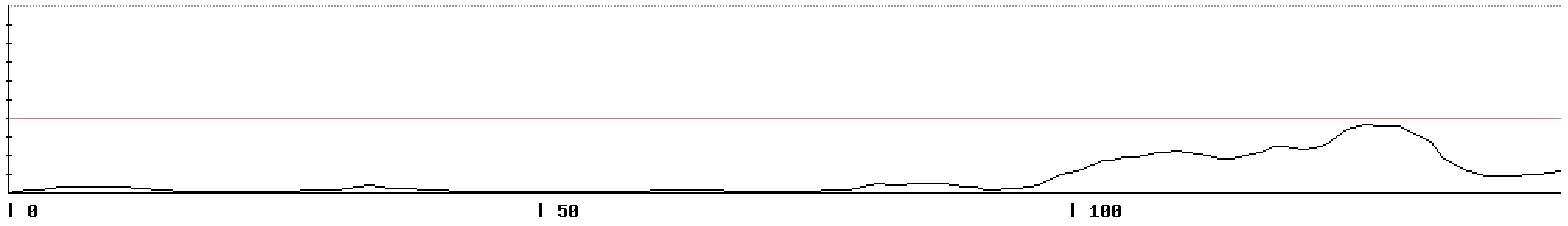

Predictions for Dichantheliumoligosanthes_Do020646.1	
Viterbi Path	
Viterbi Path	
Posterior @ 0.4	
Posterior @ 0.4	
Posterior Graphic. Threshold Marked @ 0.4	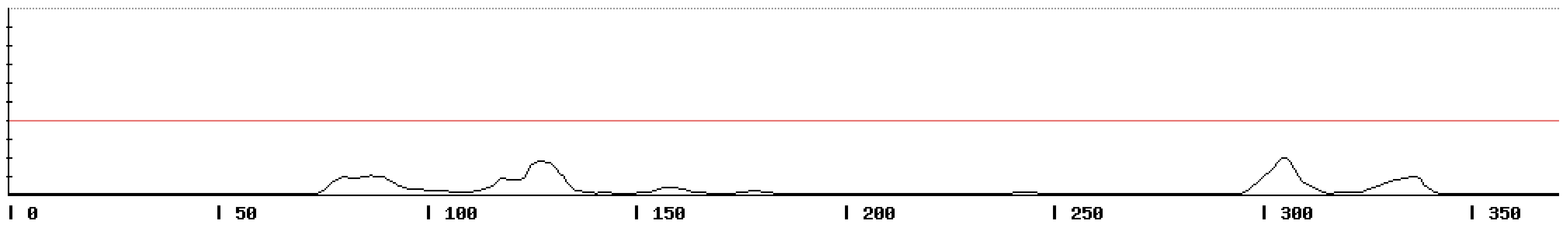

Predictions for Dichantheliumoligosanthes_Do020891.1	
Viterbi Path	
Viterbi Path	
Posterior @ 0.4	
Posterior @ 0.4	
Posterior Graphic. Threshold Marked @ 0.4	

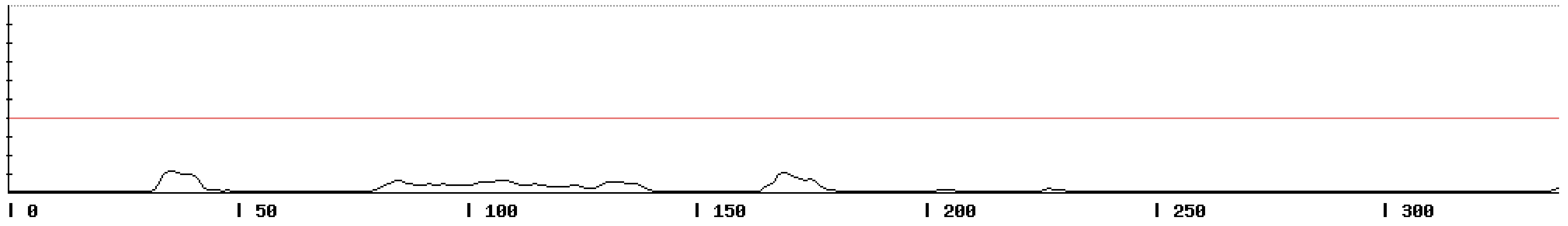

Predictions for Dichantheliumoligosanthes_Do020978.1

Viterbi Path

Viterbi Path

Posterior @ 0.4

Posterior @ 0.4

Posterior Graphic. Threshold Marked @ 0.4

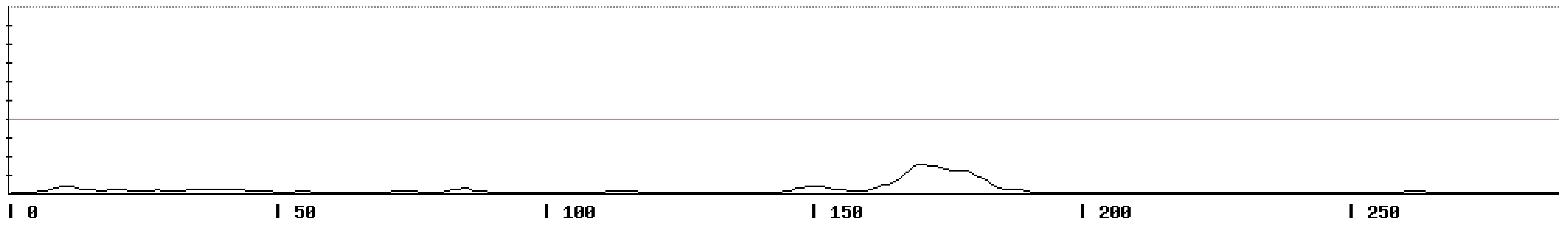

Predictions for Dichantheliumoligosanthes_Do021114.1

Viterbi Path

Viterbi Path

Posterior @ 0.4

Posterior @ 0.4

Posterior Graphic. Threshold Marked @ 0.4	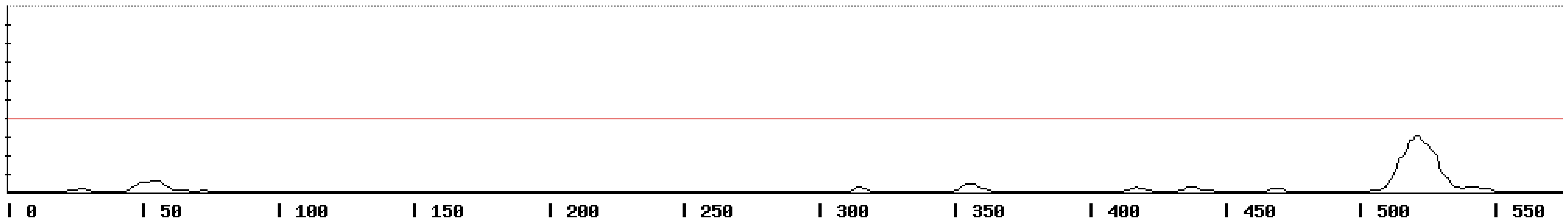

Predictions for Dichantheliumoligosanthes_Do021222.1

Viterbi Path	
Viterbi Path	
Posterior @ 0.4	
Posterior @ 0.4	
Posterior Graphic. Threshold Marked @ 0.4	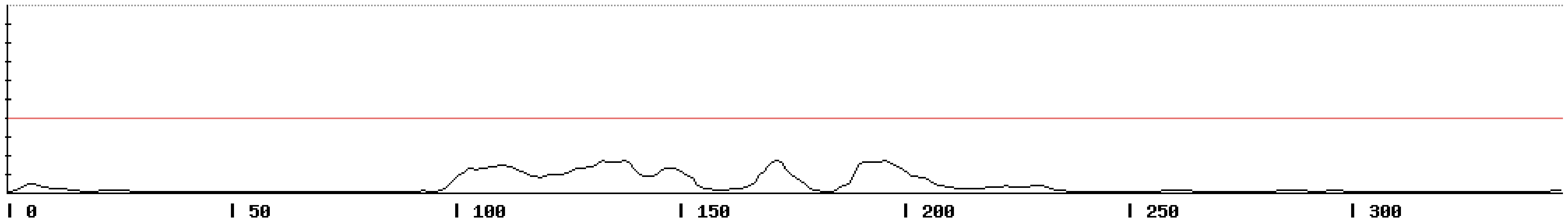

Predictions for Dichantheliumoligosanthes_Do021446.1

Viterbi Path	
Viterbi Path	
Posterior @ 0.4	
Posterior @ 0.4	

Posterior Graphic. Threshold Marked @ 0.4	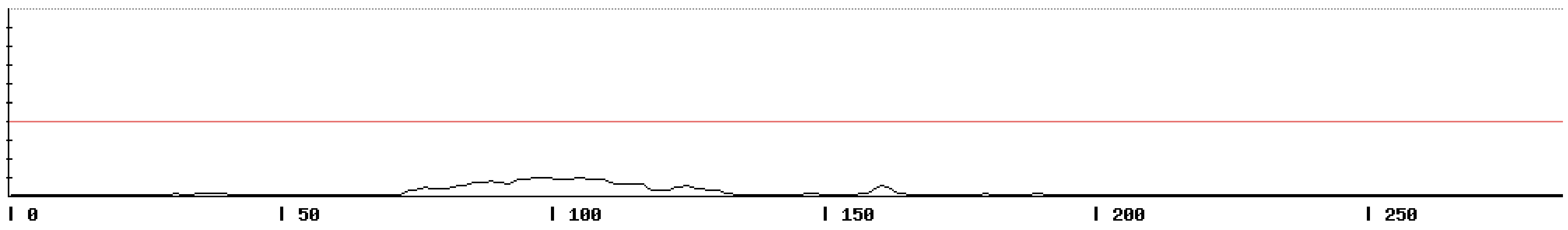

Predictions for Dichantheliumoligosanthes_Do021529.1

Viterbi Path

Viterbi Path

Posterior @ 0.4

Posterior @ 0.4

Posterior Graphic. Threshold Marked @ 0.4

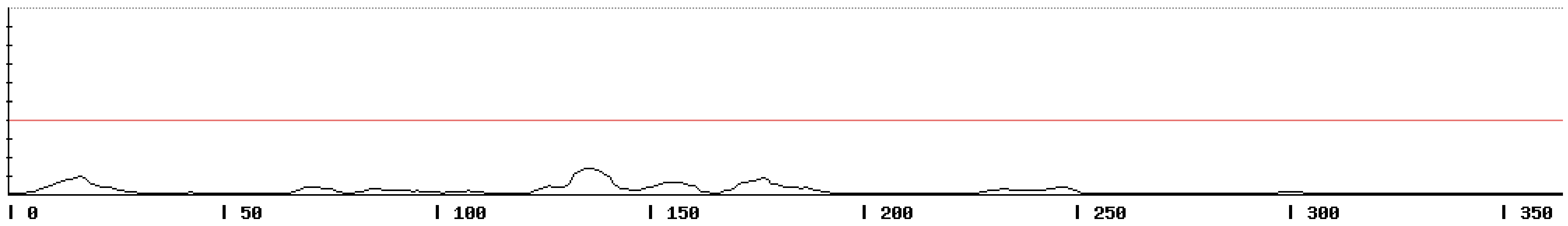

Predictions for Dichantheliumoligosanthes_Do021829.1

Viterbi Path

Viterbi Path

Posterior @ 0.4	101 - PRRKYKNGKRPAR - 113
Posterior @ 0.4	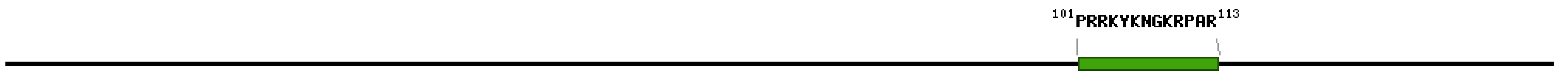
Posterior Graphic. Threshold Marked @ 0.4	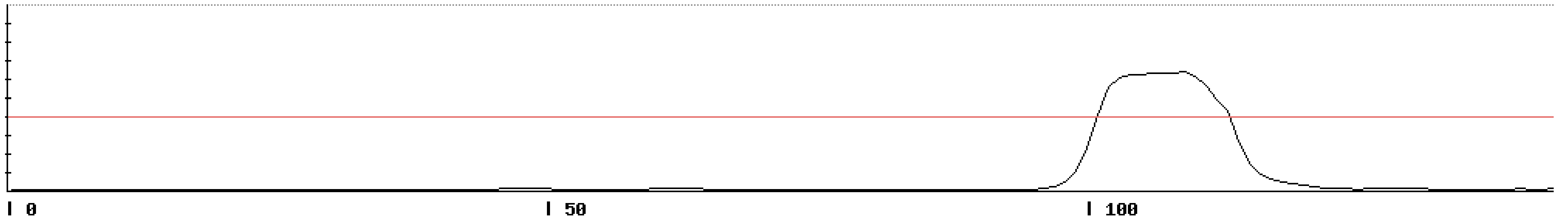

Predictions for Dichantheliumoligosanthes_Do021855.1

Viterbi Path	
Viterbi Path	
Posterior @ 0.4	
Posterior @ 0.4	
Posterior Graphic. Threshold Marked @ 0.4	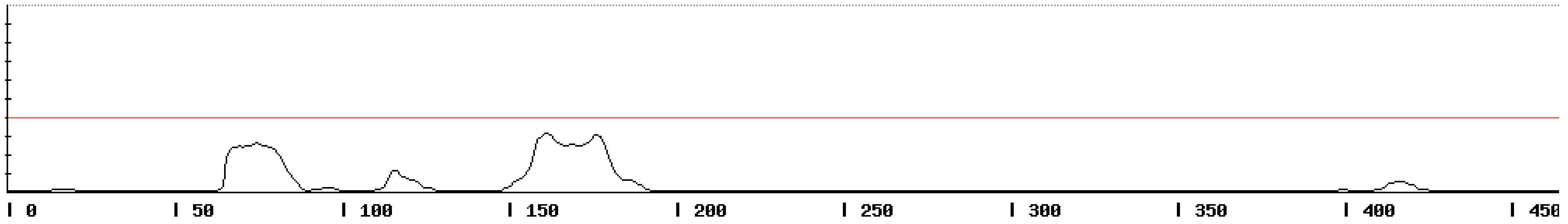

Predictions for Dichantheliumoligosanthes_Do021915.1

Viterbi Path	
Viterbi Path	

Posterior @ 0.4

Posterior @ 0.4

Posterior Graphic. Threshold Marked @ 0.4

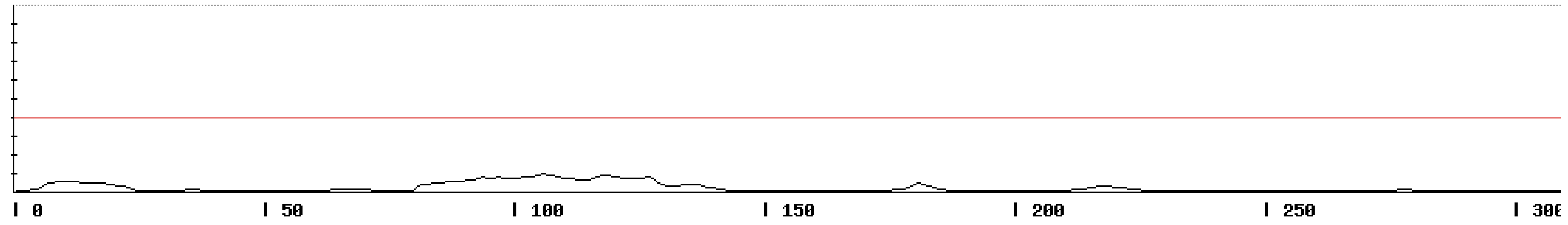

Predictions for Dichantheliumoligosanthes_Do022620.1

Viterbi Path

Viterbi Path

Posterior @ 0.4

Posterior @ 0.4

Posterior Graphic. Threshold Marked @ 0.4

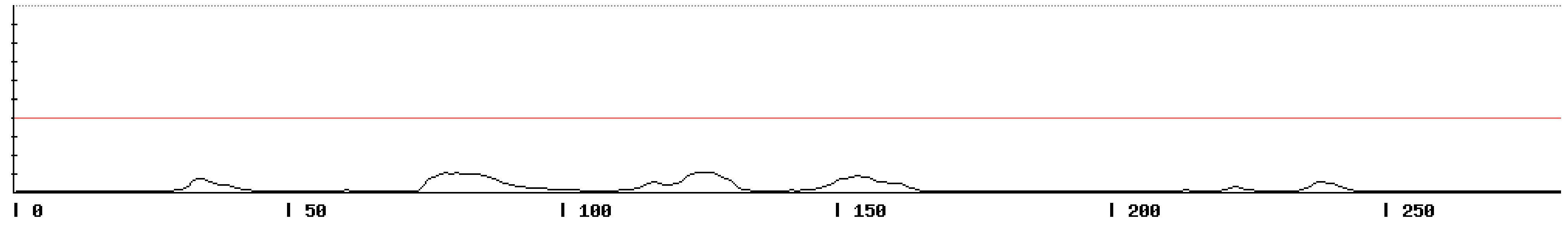

Predictions for Dichantheliumoligosanthes_Do022798.1

Viterbi Path

Viterbi Path

Posterior @ 0.4

606 - RSTRKRKP - 613

Posterior @ 0.4

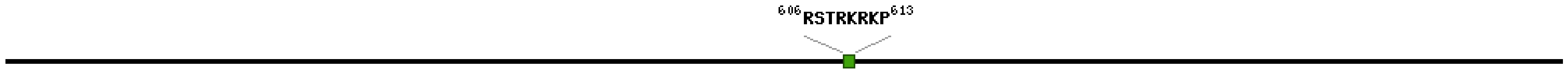

Posterior Graphic. Threshold Marked @ 0.4

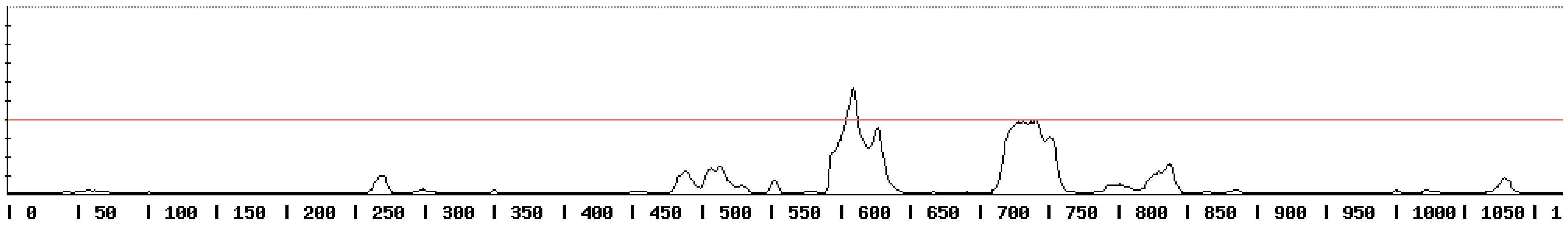

Predictions for Dichantheliumoligosanthes_Do023167.1

Viterbi Path

Viterbi Path

Posterior @ 0.4

Posterior @ 0.4

Posterior Graphic. Threshold Marked @ 0.4

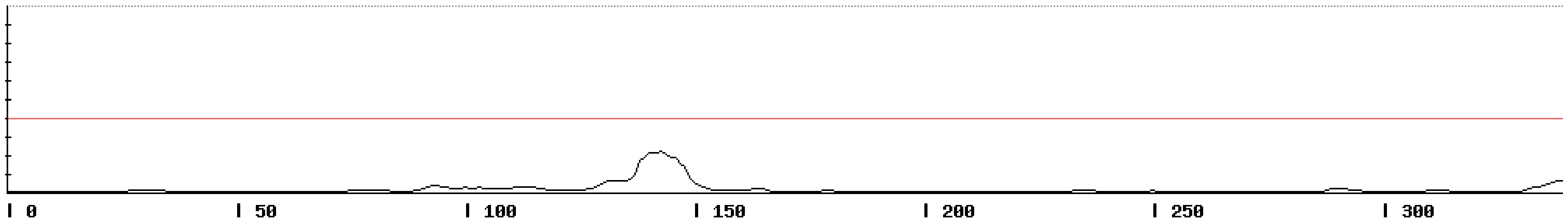

Predictions for Dichantheliumoligosanthes_Do023186.1	
Viterbi Path	
Viterbi Path	
Posterior @ 0.4	
Posterior @ 0.4	
Posterior Graphic. Threshold Marked @ 0.4	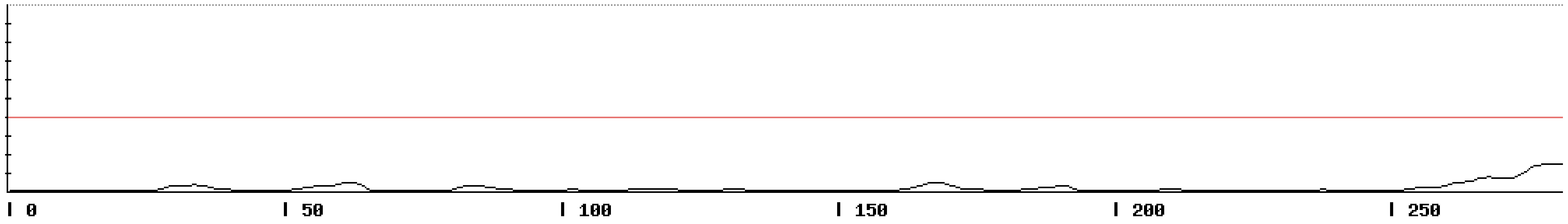

Predictions for Dichantheliumoligosanthes_Do023285.1	
Viterbi Path	
Viterbi Path	
Posterior @ 0.4	
Posterior @ 0.4	
Posterior Graphic. Threshold Marked @ 0.4	

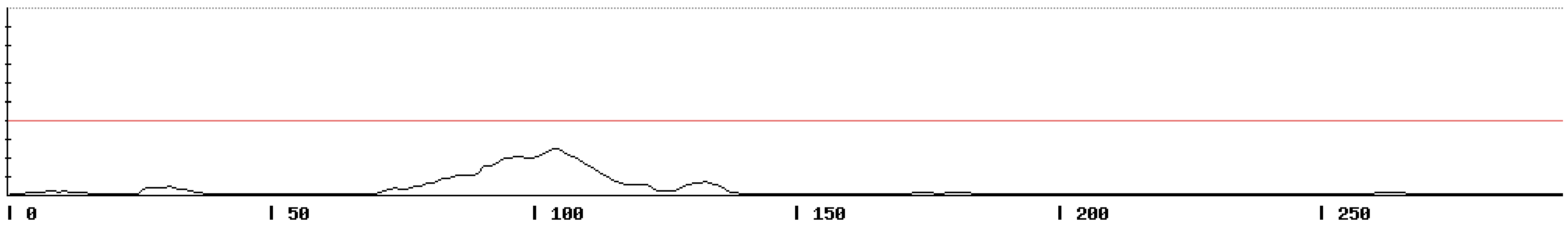

Predictions for Dichantheliumoligosanthes_Do023544.1

Viterbi Path

Viterbi Path

Posterior @ 0.4 — 77 - RRGRRGVKS - 85

Posterior @ 0.4

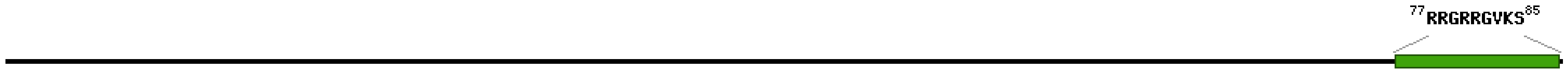

Posterior Graphic. Threshold Marked @ 0.4

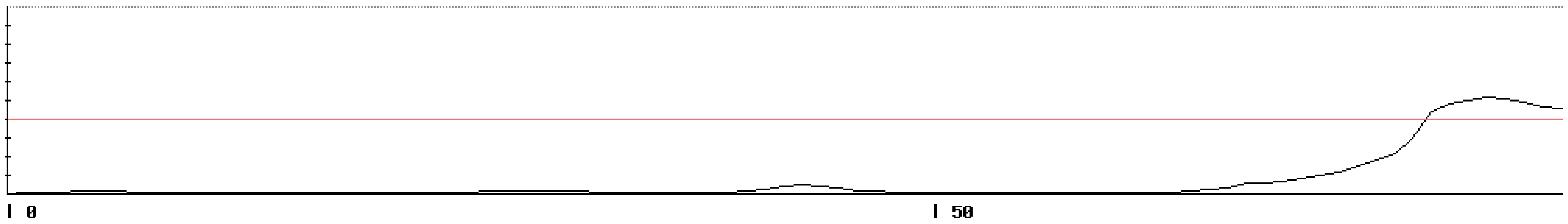

Predictions for Dichantheliumoligosanthes_Do023815.1

Viterbi Path

Viterbi Path

Posterior @ 0.4 — 190 - RNRKYKNGKRPAR - 202

Posterior @ 0.4

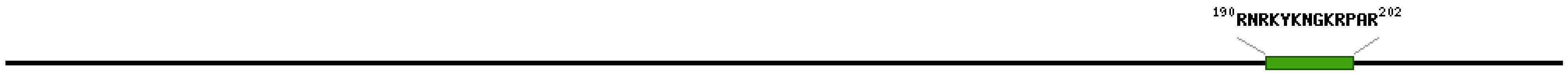

Posterior Graphic. Threshold Marked @ 0.4

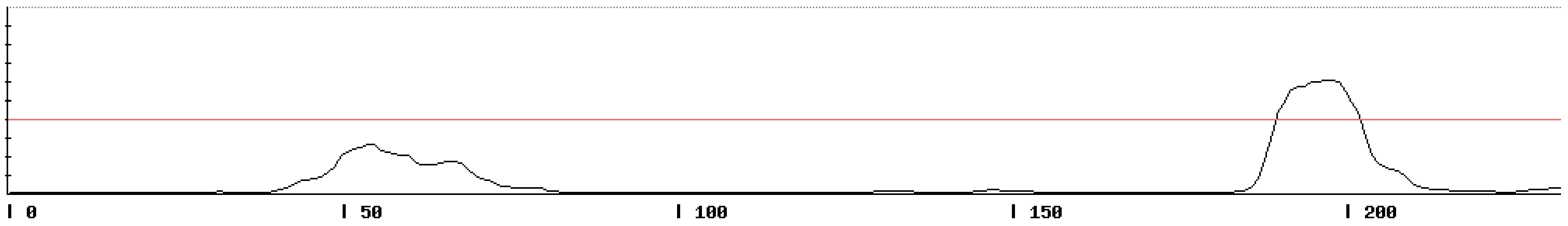

Predictions for Dichantheliumoligosanthes_Do023975.1

Viterbi Path

Viterbi Path

Posterior @ 0.4

186 - SSRSSRKRKPV - 196

210 - K - 210

Posterior @ 0.4

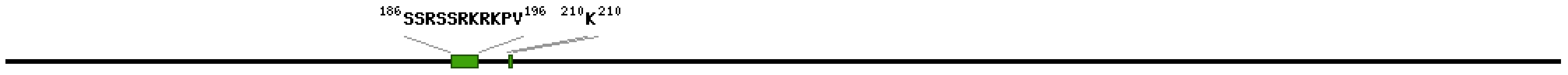

Posterior Graphic. Threshold Marked @ 0.4

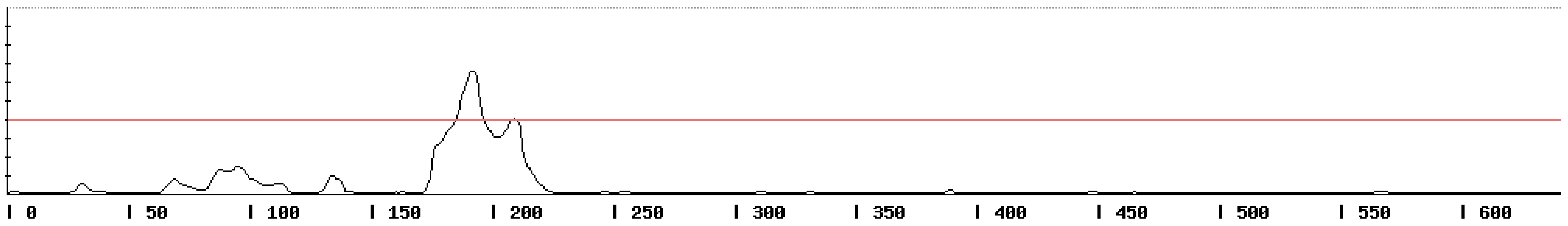

Predictions for Dichantheliumoligosanthes_Do024028.1

Viterbi Path

Viterbi Path

Posterior @ 0.4

499 - AYGRGQRKRRR - 509

Posterior @ 0.4

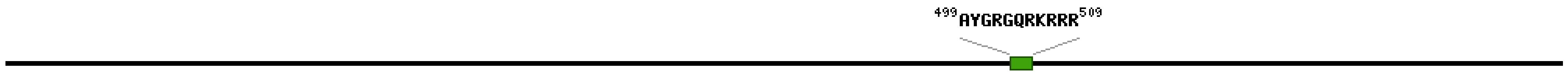

Posterior Graphic. Threshold Marked @ 0.4

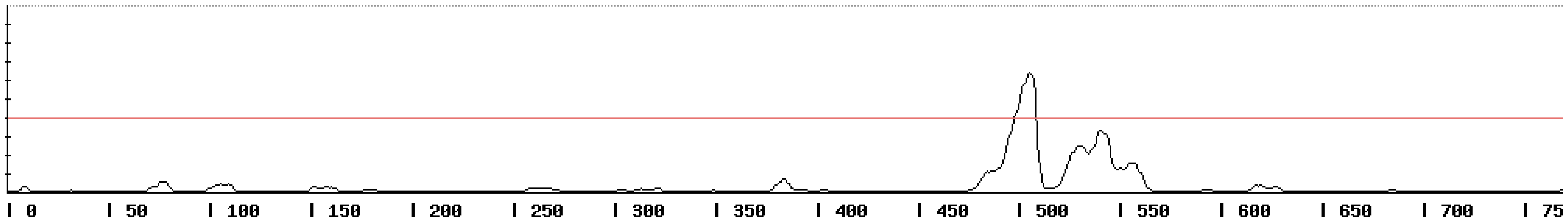

Predictions for Dichantheliumoligosanthes_Do024521.1

Viterbi Path

Viterbi Path

Posterior @ 0.4

Posterior @ 0.4

Posterior Graphic. Threshold Marked @ 0.4

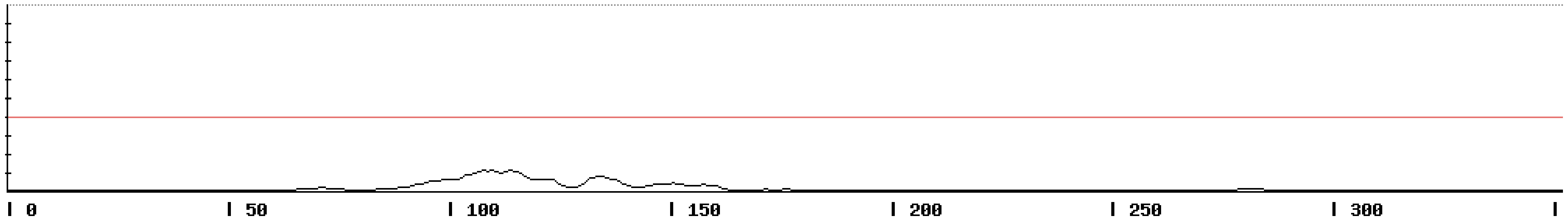

Predictions for Dichantheliumoligosanthes_Do025061.1

Viterbi Path

Viterbi Path

Posterior @ 0.4

Posterior @ 0.4

Posterior Graphic. Threshold Marked @ 0.4

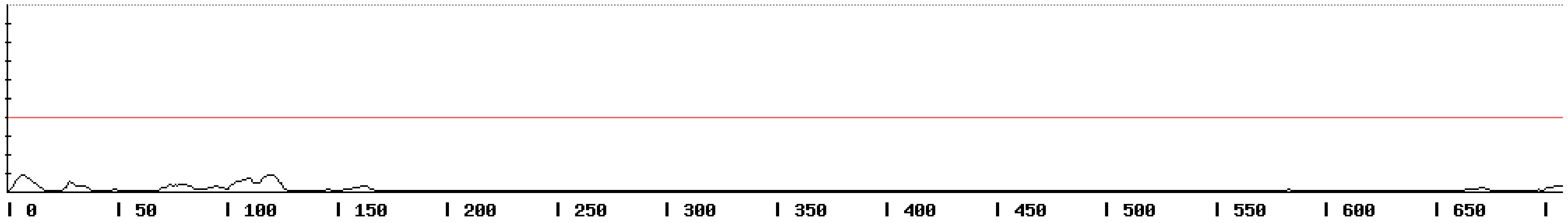

Predictions for Dichantheliumoligosanthes_Do025091.1

Viterbi Path: 222 - KKKQQKKEKQSEESKEGETKKKRTKRKAKEEGGKKKRKK - 260

Viterbi Path

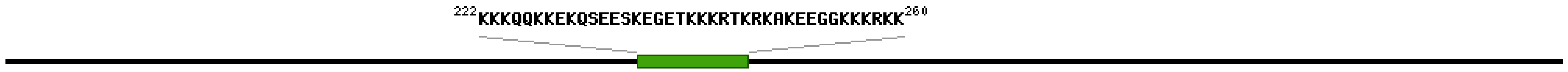

Posterior @ 0.4:

156 - RRKKK - 160

222 - KKKQQKKEKQSEESKEGETKKKRTKRKAKEEGGKKKRKKH - 261

Posterior @ 0.4

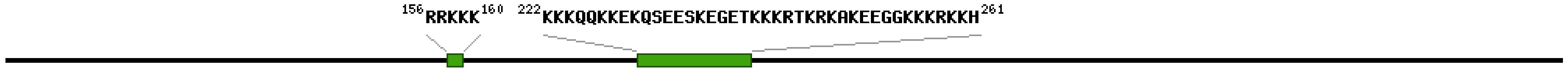

Posterior Graphic. Threshold Marked @ 0.4

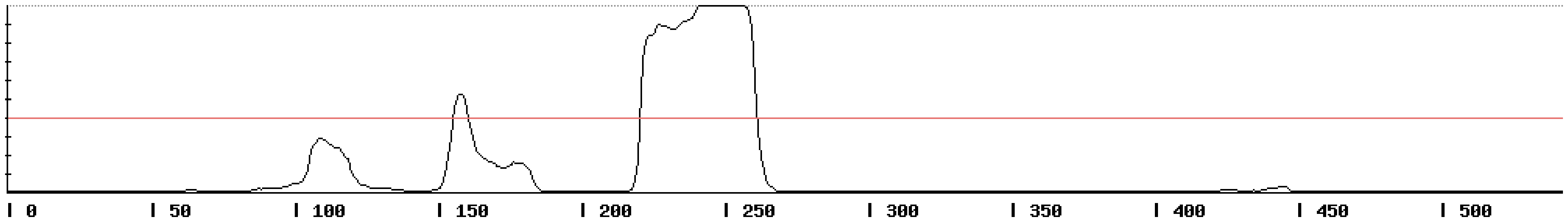

Predictions for Dichantheliumoligosanthes_Do025322.1

Viterbi Path

Viterbi Path

Posterior @ 0.4	305 - KRKR - 308
Posterior @ 0.4	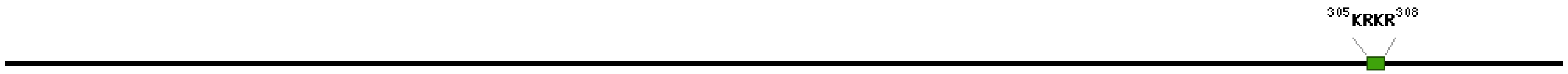
Posterior Graphic. Threshold Marked @ 0.4	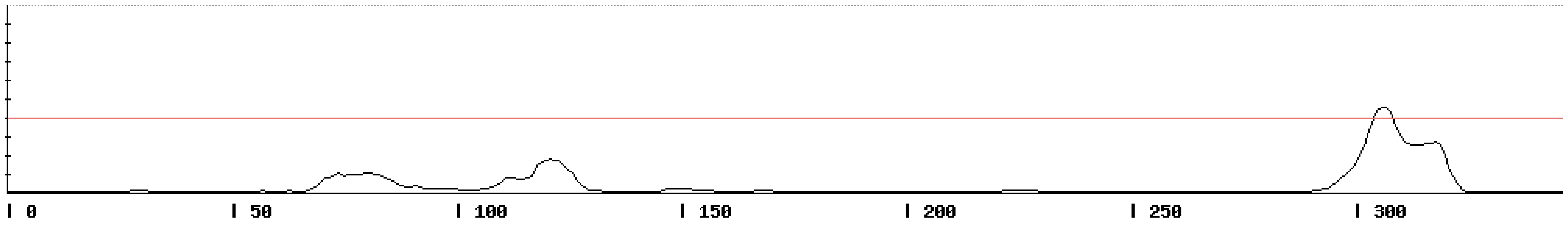

Predictions for Dichantheliumoligosanthes_Do025343.1

Viterbi Path	
Viterbi Path	
Posterior @ 0.4	
Posterior @ 0.4	
Posterior Graphic. Threshold Marked @ 0.4	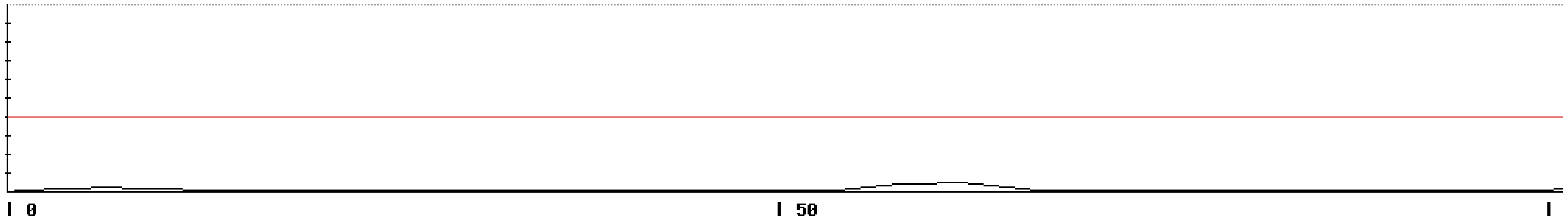

Predictions for Dichantheliumoligosanthes_Do025546.1

Viterbi Path

Viterbi Path

Posterior @ 0.4

Posterior @ 0.4

Posterior Graphic. Threshold Marked @ 0.4

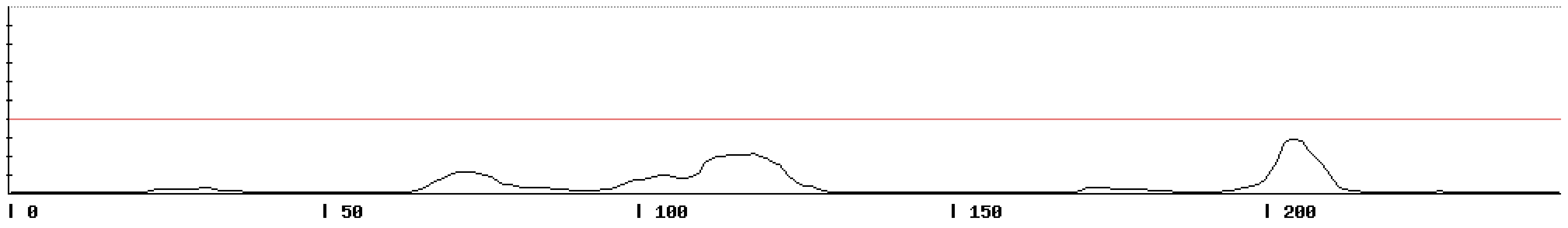

Predictions for Dichantheliumoligosanthes_Do025993.1

Viterbi Path

Viterbi Path

Posterior @ 0.4

Posterior @ 0.4

Posterior Graphic. Threshold Marked @ 0.4

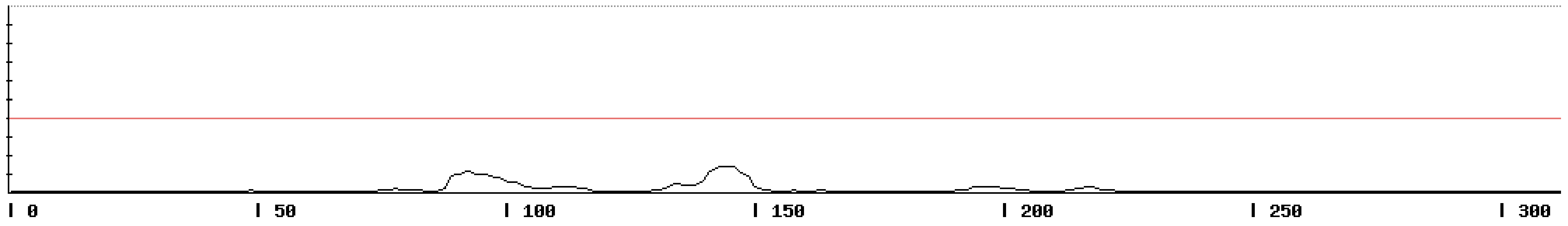

Predictions for Dichantheliumoligosanthes_Do026215.1

Viterbi Path	
Viterbi Path	
Posterior @ 0.4	57 - GRKSRKRG - 64
	78 - GSRQKR - 83
	91 - WEGQGKRKRAKGKGSEGE - 108
Posterior @ 0.4	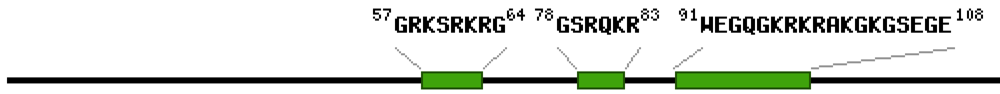
Posterior Graphic. Threshold Marked @ 0.4	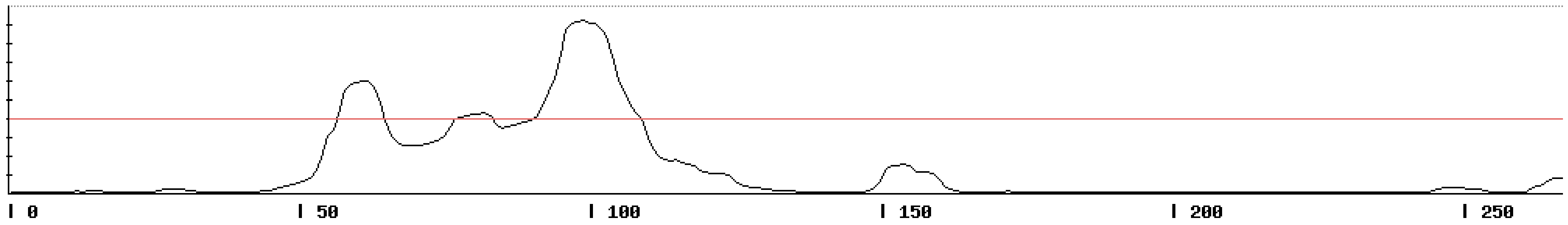

Predictions for Dichantheliumoligosanthes_Do026445.1

Viterbi Path	
Viterbi Path	
Posterior @ 0.4	
Posterior @ 0.4	
Posterior Graphic. Threshold Marked @ 0.4	

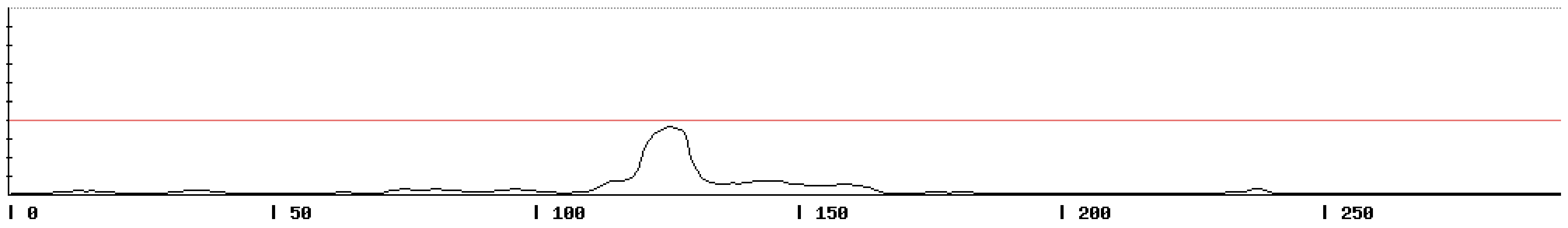

Predictions for Dichantheliumoligosanthes_Do027054.1

Viterbi Path

Viterbi Path

Posterior @ 0.4

Posterior @ 0.4

Posterior Graphic. Threshold Marked @ 0.4

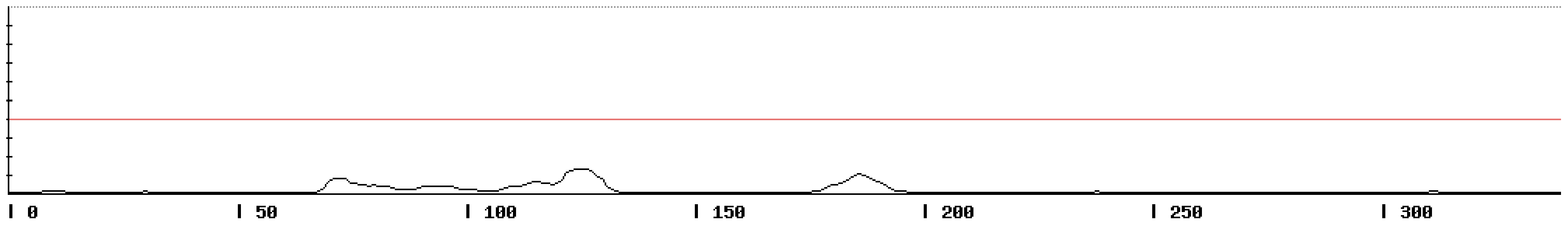

Predictions for Dichantheliumoligosanthes_Do027273.1

Viterbi Path

Viterbi Path

Posterior @ 0.4

Posterior @ 0.4

Posterior Graphic. Threshold Marked @ 0.4

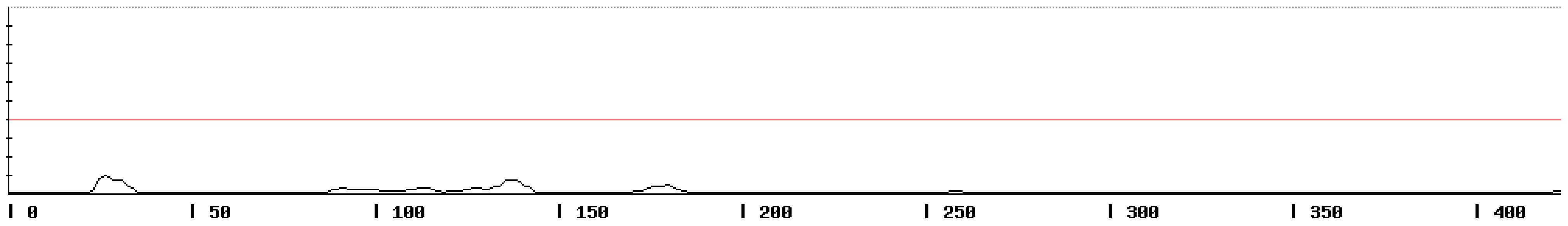

Predictions for Dichantheliumoligosanthes_Do027688.1

Viterbi Path

Viterbi Path

Posterior @ 0.4

80 - VRARRATRRGYWKSTGKDKAVH - 101

Posterior @ 0.4

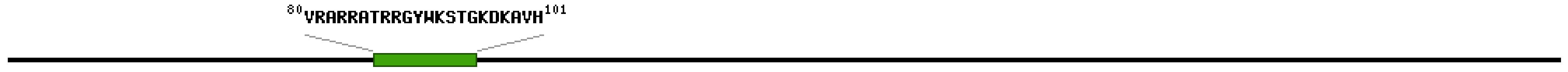

Posterior Graphic. Threshold Marked @ 0.4

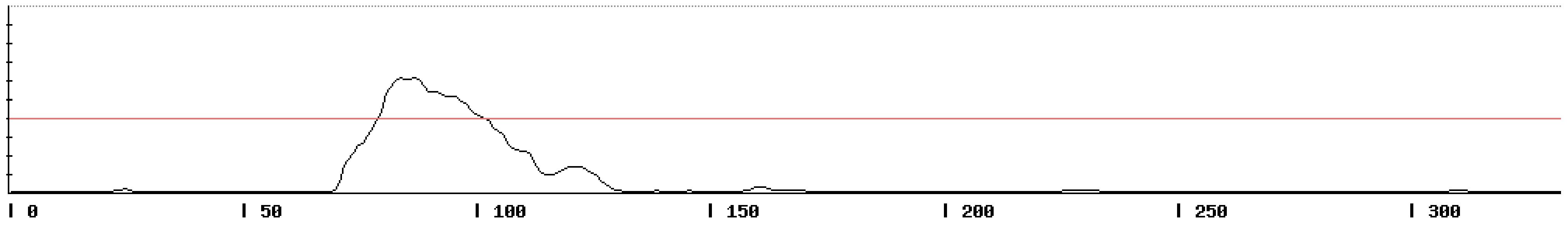

Predictions for Dichantheliumoligosanthes_Do027727.1

Viterbi Path

Viterbi Path

Posterior @ 0.4

Posterior @ 0.4

Posterior Graphic. Threshold Marked @ 0.4

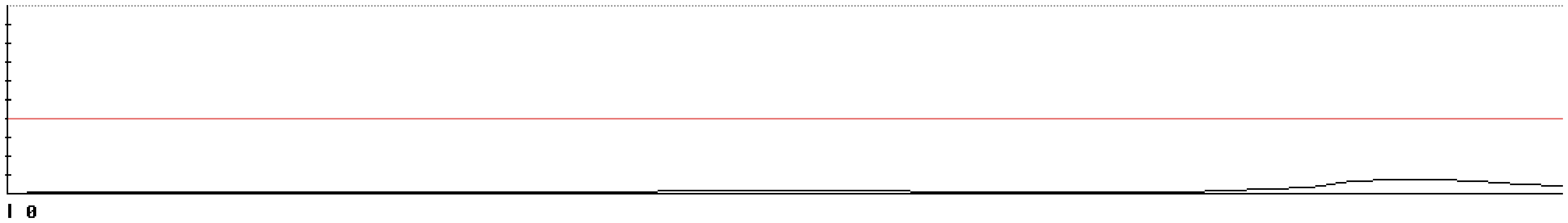

Predictions for Dichantheliumoligosanthes_Do027950.1

Viterbi Path

Viterbi Path

Posterior @ 0.4

Posterior @ 0.4

Posterior Graphic. Threshold Marked @ 0.4

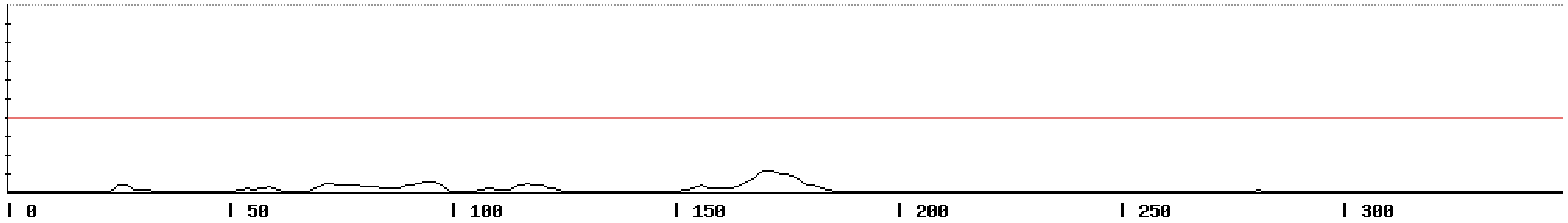

Predictions for Dichantheliumoligosanthes_Do028570.1

Viterbi Path

Viterbi Path

Posterior @ 0.4	164 - KLAPRKTAAAKPVFKPPKRAAARSNRAASP - 193
Posterior @ 0.4	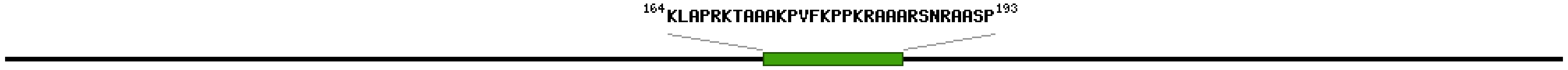
Posterior Graphic. Threshold Marked @ 0.4	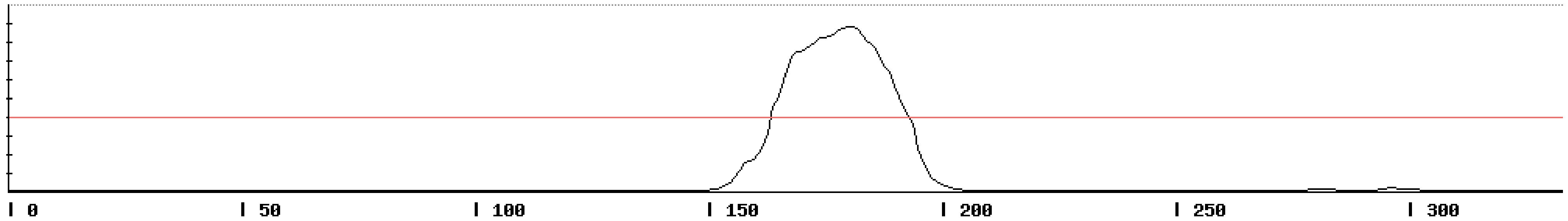

Predictions for Dichantheliumoligosanthes_Do028605.1

Viterbi Path	
Viterbi Path	
Posterior @ 0.4	
Posterior @ 0.4	
Posterior Graphic. Threshold Marked @ 0.4	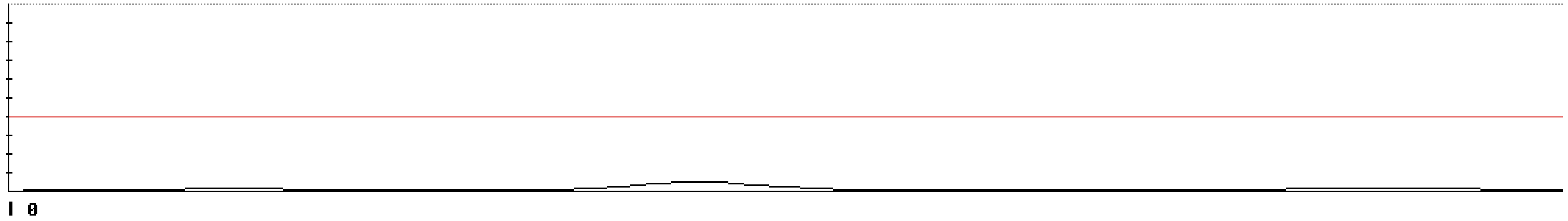

Predictions for Dichantheliumoligosanthes_Do028914.1

Viterbi Path	
Viterbi Path	

Posterior @ 0.4

Posterior @ 0.4

Posterior Graphic. Threshold Marked @ 0.4

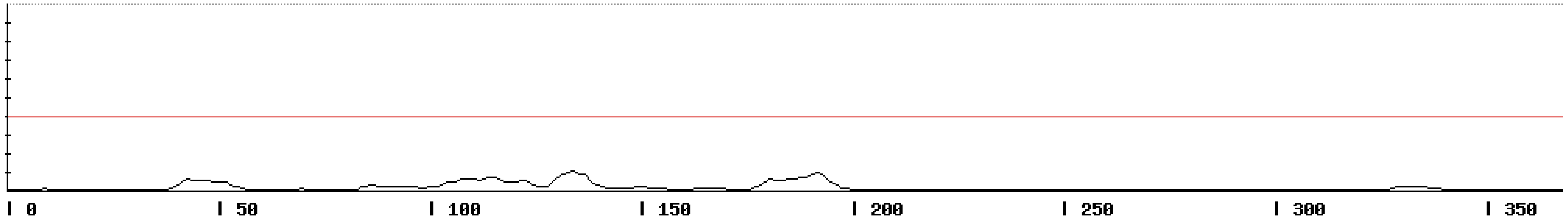

Predictions for Dichantheliumoligosanthes_Do028959.1

Viterbi Path

Viterbi Path

Posterior @ 0.4

Posterior @ 0.4

Posterior Graphic. Threshold Marked @ 0.4

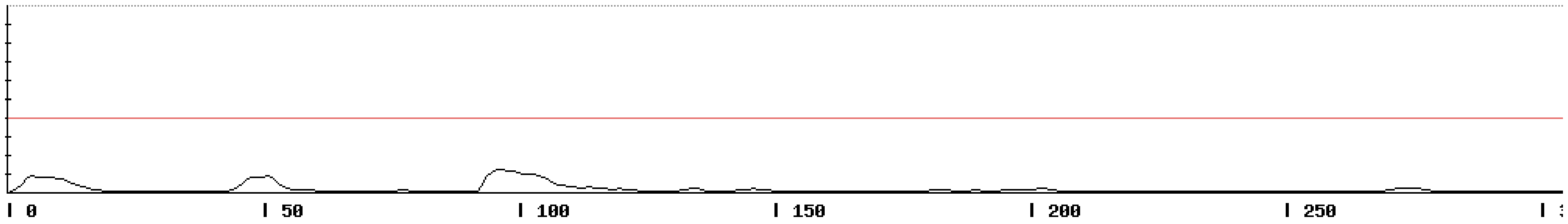

Predictions for Dichantheliumoligosanthes_Do029152.1

Viterbi Path	
Viterbi Path	
Posterior @ 0.4	
Posterior @ 0.4	
Posterior Graphic. Threshold Marked @ 0.4	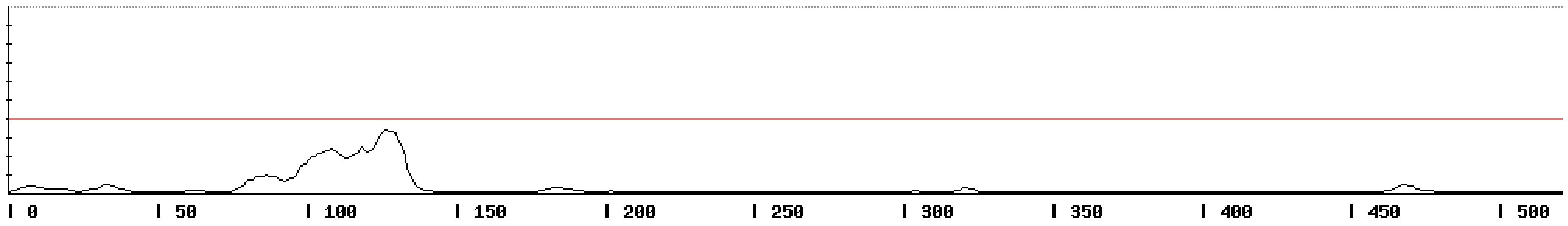

Predictions for Dichantheliumoligosanthes_Do029755.1	
Viterbi Path	

Revision r.9 - Last Updated on : February 24 2015 17:45:20.
alex.nguyenba@utoronto.ca

Intended to be used with Firefox

Viterbi Path

Posterior @ 0.4

Posterior @ 0.4

Posterior Graphic. Threshold Marked @ 0.4

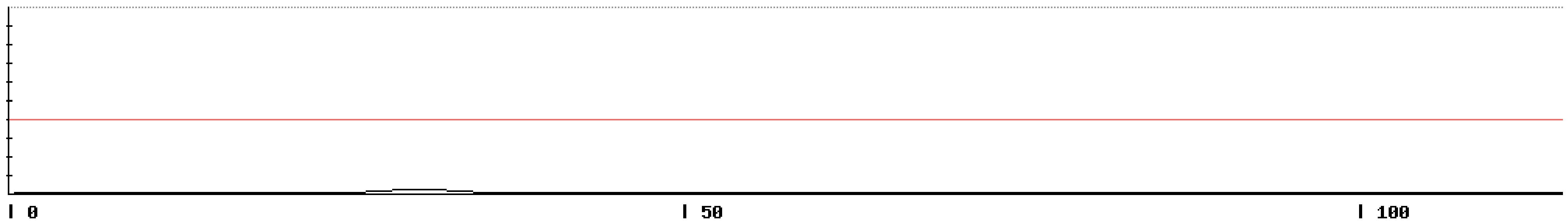

Predictions for Dorcocerashygrometricum_KZV13853.1

Viterbi Path

Viterbi Path

Posterior @ 0.4

Posterior @ 0.4

Posterior Graphic. Threshold Marked @ 0.4

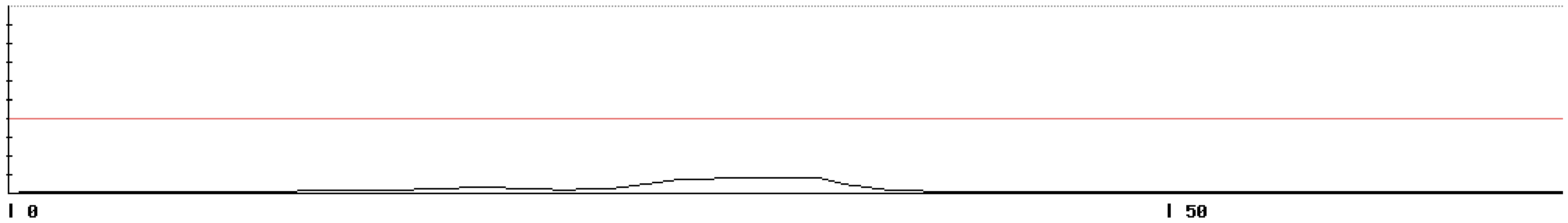

Predictions for Dorcocerashygrometricum_KZV14447.1

Viterbi Path	
Viterbi Path	
Posterior @ 0.4	122 - GSGRK - 126
Posterior @ 0.4	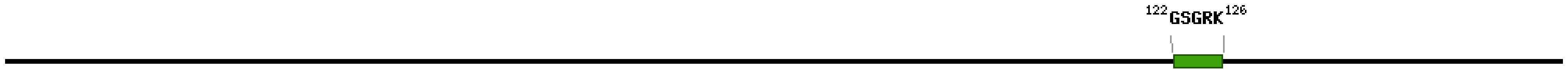
Posterior Graphic. Threshold Marked @ 0.4	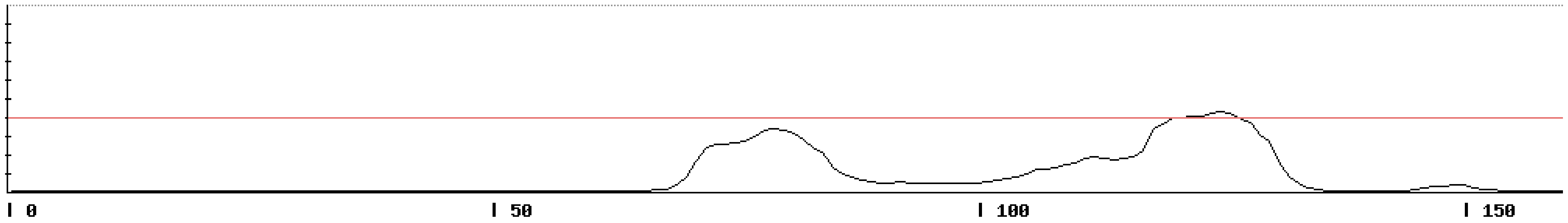

Predictions for Dorcocerashygrometricum_KZV14722.1

Viterbi Path	
Viterbi Path	
Posterior @ 0.4	
Posterior @ 0.4	
Posterior Graphic. Threshold Marked @ 0.4	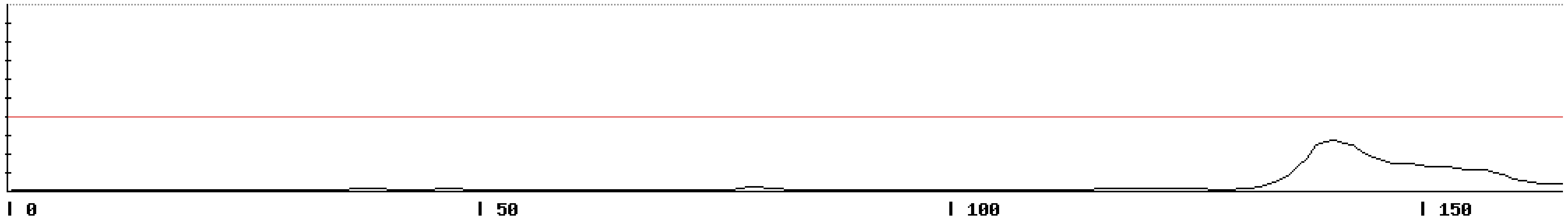

Predictions for Dorcocerashygrometricum_KZV15946.1

Viterbi Path

Viterbi Path

Posterior @ 0.4

Posterior @ 0.4

Posterior Graphic. Threshold Marked @ 0.4

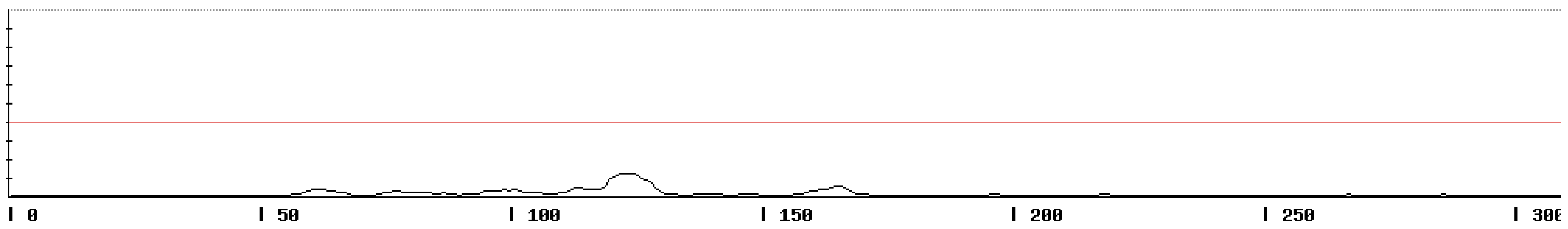

Predictions for Dorcocerashygrometricum_KZV15975.1

Viterbi Path

Viterbi Path

Posterior @ 0.4

Posterior @ 0.4

Posterior Graphic. Threshold Marked @ 0.4

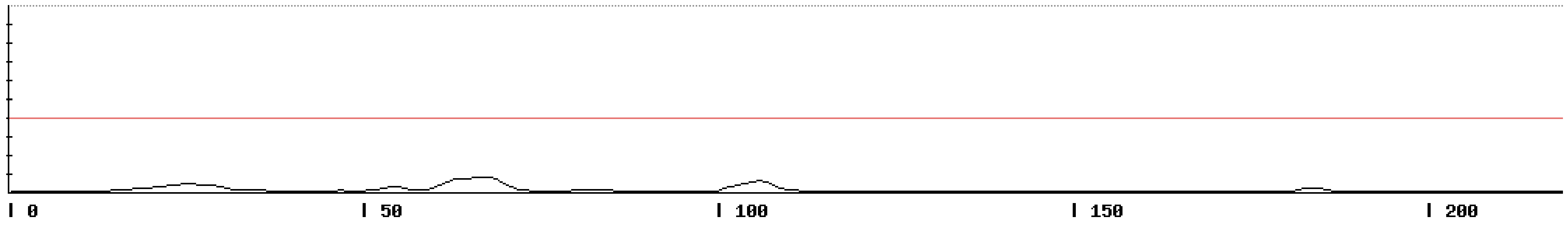

Predictions for Dorcocerashygrometricum_KZV15977.1

Viterbi Path

Viterbi Path

Posterior @ 0.4

Posterior @ 0.4

Posterior Graphic. Threshold Marked @ 0.4

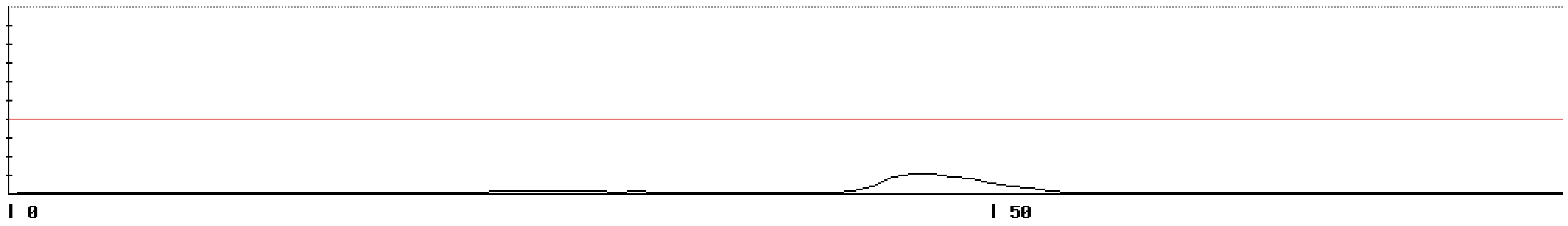

Predictions for Dorcocerashygrometricum_KZV15980.1

Viterbi Path

Viterbi Path

Posterior @ 0.4

Posterior @ 0.4

Posterior Graphic. Threshold Marked @ 0.4

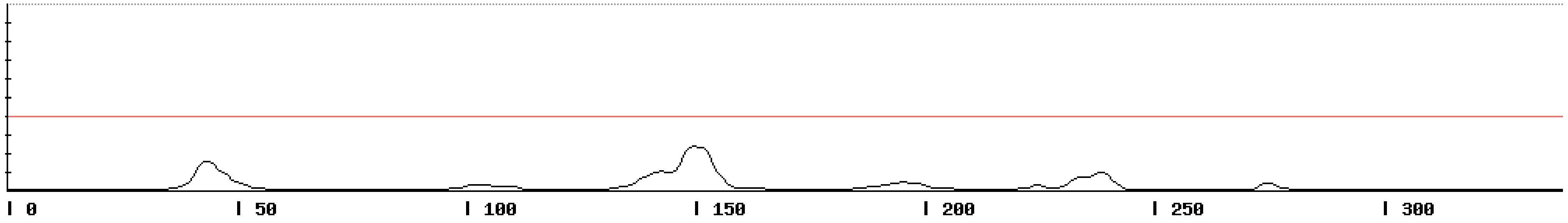

Predictions for Dorcocerashygrometricum_KZV15981.1

Viterbi Path

Viterbi Path

Posterior @ 0.4

Posterior @ 0.4

Posterior Graphic. Threshold Marked @ 0.4

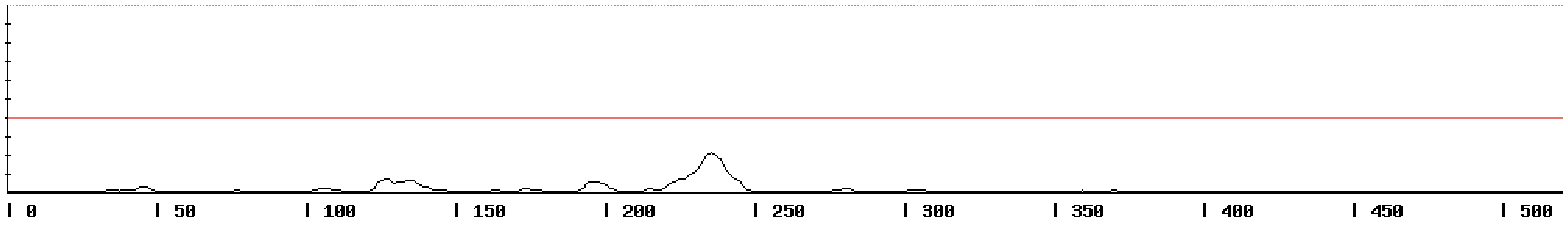

Predictions for Dorcocerashygrometricum_KZV16022.1

Viterbi Path

Viterbi Path

Posterior @ 0.4

Posterior @ 0.4

Posterior Graphic. Threshold Marked @ 0.4

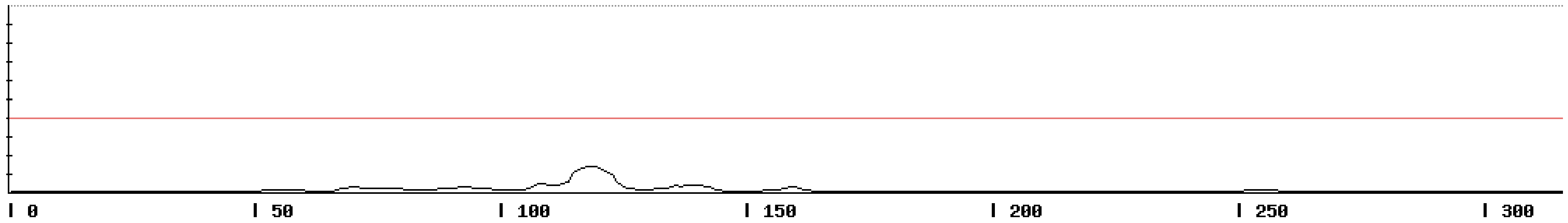

Predictions for Dorcocerashygrometricum_KZV17376.1

Viterbi Path

Viterbi Path

Posterior @ 0.4

Posterior @ 0.4

Posterior Graphic. Threshold Marked @ 0.4

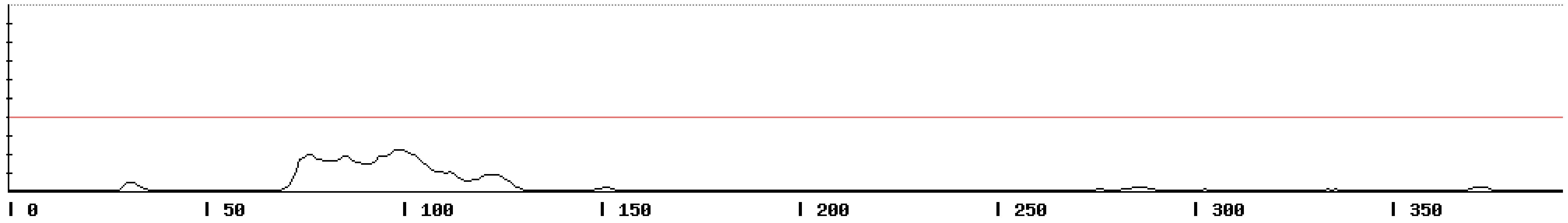

Predictions for Dorcocerashygrometricum_KZV17505.1

Viterbi Path

Viterbi Path

Posterior @ 0.4

Posterior @ 0.4

Posterior Graphic. Threshold Marked @ 0.4

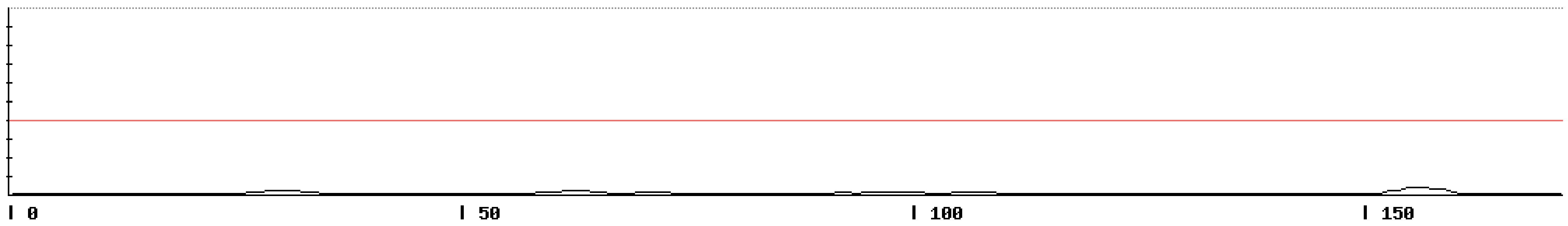

Predictions for Dorcocerashygrometricum_KZV17664.1

Viterbi Path

Viterbi Path

Posterior @ 0.4

Posterior @ 0.4

Posterior Graphic. Threshold Marked @ 0.4

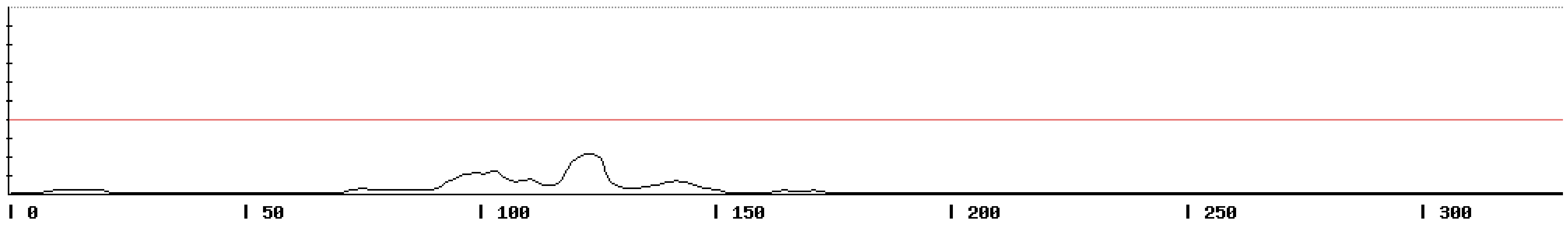

Predictions for Dorcocerashygrometricum_KZV17891.1

Viterbi Path

Viterbi Path

Posterior @ 0.4

Posterior @ 0.4

Posterior Graphic. Threshold Marked @ 0.4

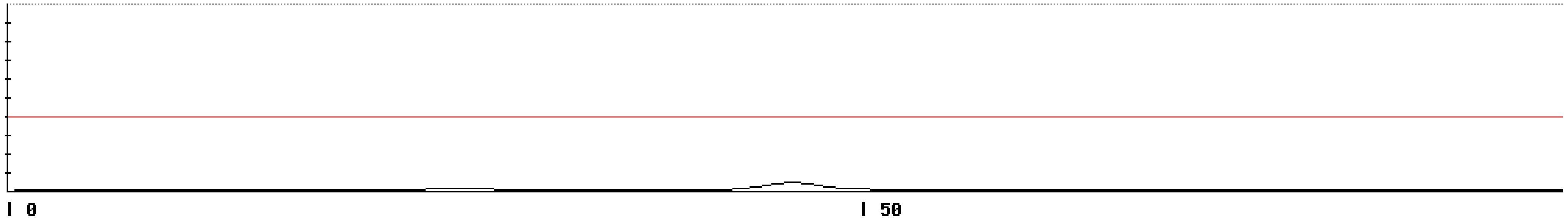

Predictions for Dorcocerashygrometricum_KZV20205.1

Viterbi Path

Viterbi Path

Posterior @ 0.4

Posterior @ 0.4

Posterior Graphic. Threshold Marked @ 0.4

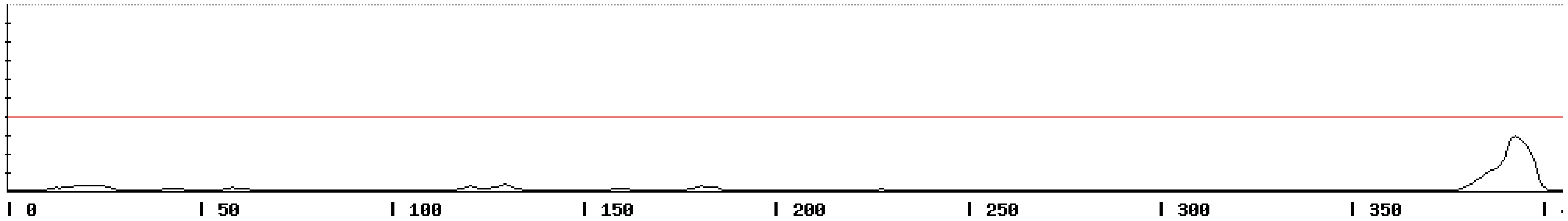

Predictions for Dorcocerashygrometricum_KZV22153.1

Viterbi Path

Viterbi Path

Posterior @ 0.4 | 122 - GSGRK - 126

Posterior @ 0.4

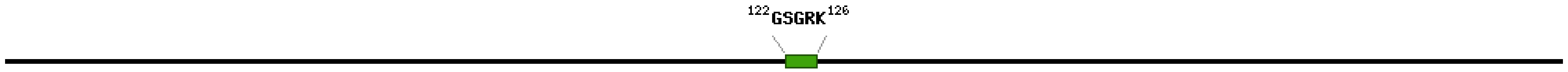

Posterior Graphic. Threshold Marked @ 0.4

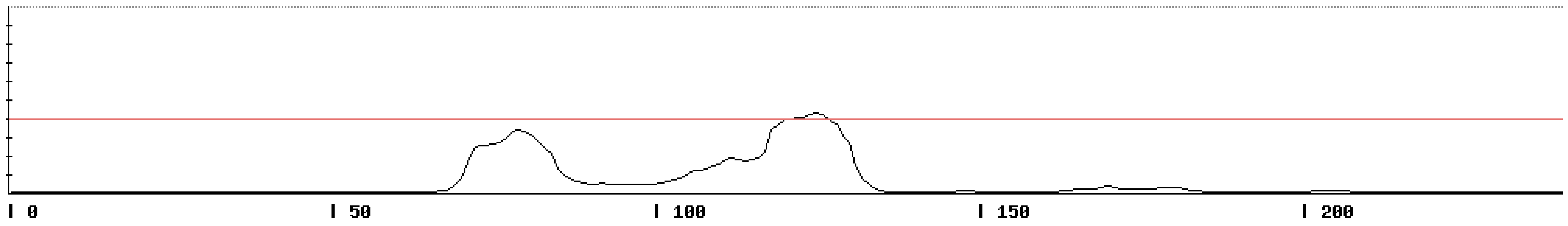

Predictions for Dorcocerashygrometricum_KZV22447.1

Viterbi Path

Viterbi Path

Posterior @ 0.4

Posterior @ 0.4

Posterior Graphic. Threshold Marked @ 0.4

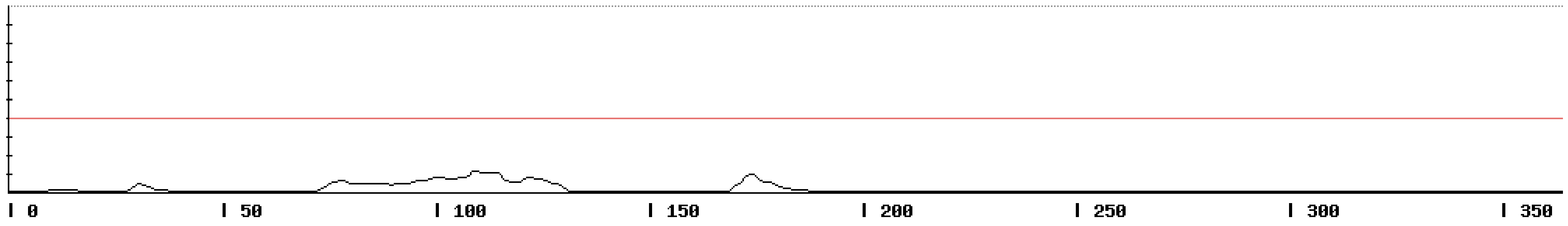

Predictions for Dorcocerashygrometricum_KZV22577.1	
Viterbi Path	
Viterbi Path	
Posterior @ 0.4	
Posterior @ 0.4	
Posterior Graphic. Threshold Marked @ 0.4	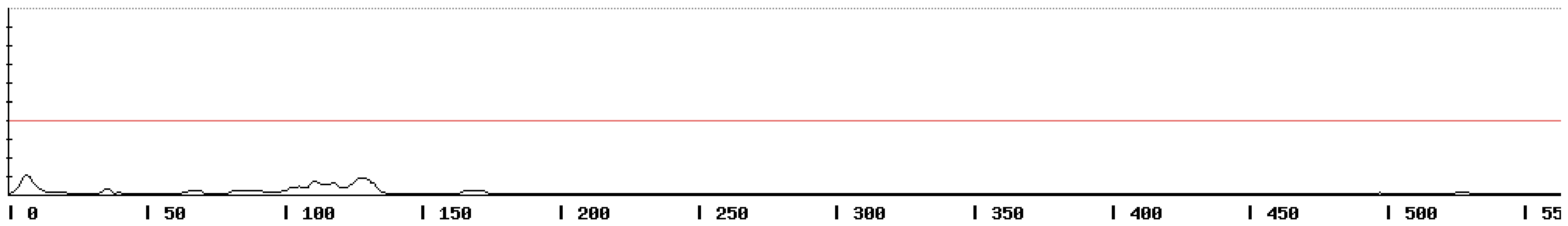

Predictions for Dorcocerashygrometricum_KZV23241.1	
Viterbi Path	
Viterbi Path	
Posterior @ 0.4	
Posterior @ 0.4	
Posterior Graphic. Threshold Marked @ 0.4	

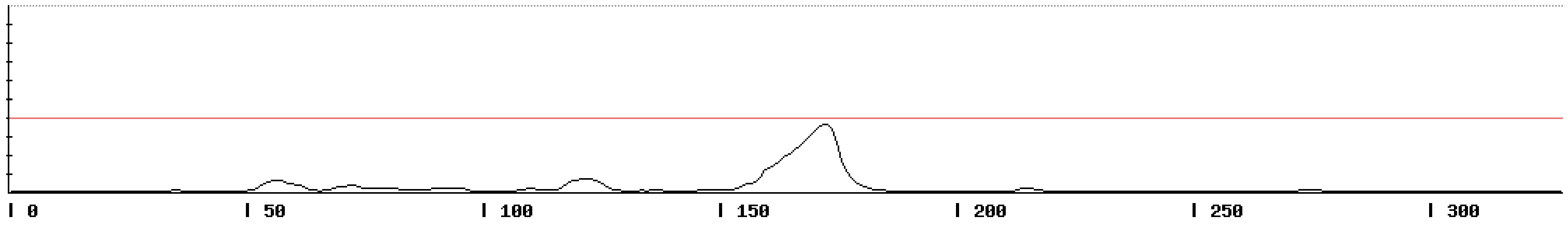

Predictions for Dorcocerashygrometricum_KZV23257.1

Viterbi Path

Viterbi Path

Posterior @ 0.4

Posterior @ 0.4

Posterior Graphic. Threshold Marked @ 0.4

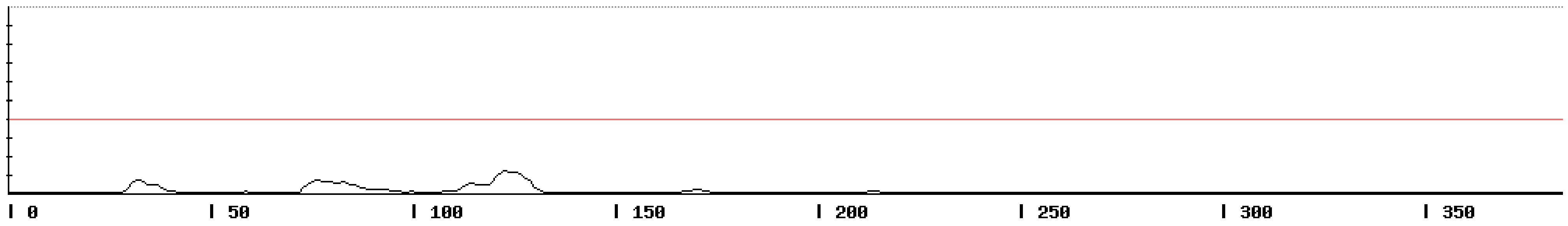

Predictions for Dorcocerashygrometricum_KZV27169.1

Viterbi Path

Viterbi Path

Posterior @ 0.4

Posterior @ 0.4

Posterior Graphic. Threshold Marked @ 0.4	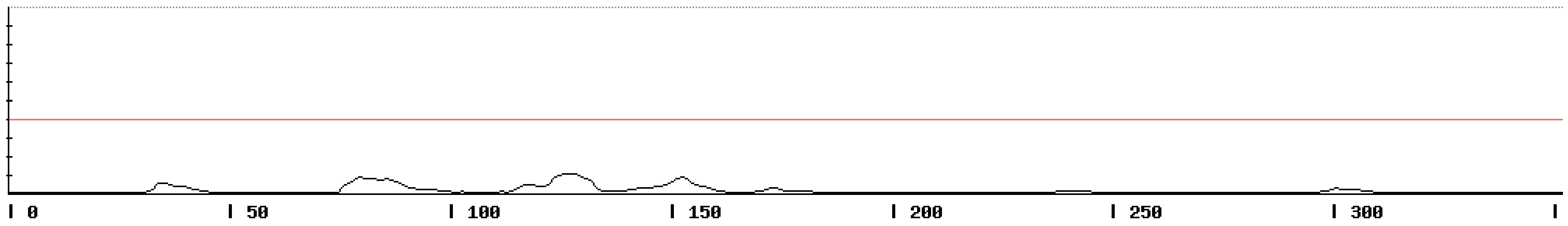

Predictions for Dorcocerashygrometricum_KZV27222.1	
Viterbi Path	
Viterbi Path	
Posterior @ 0.4	
Posterior @ 0.4	
Posterior Graphic. Threshold Marked @ 0.4	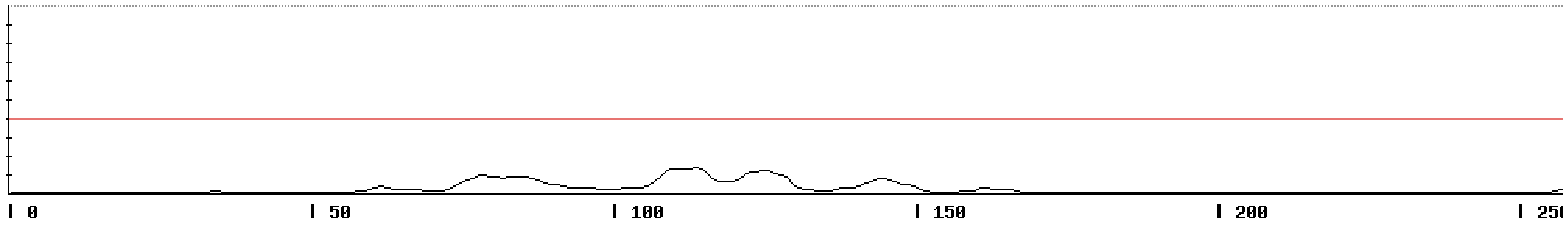

Predictions for Dorcocerashygrometricum_KZV27421.1	
Viterbi Path	
Viterbi Path	
Posterior @ 0.4	
Posterior @ 0.4	

Posterior Graphic. Threshold Marked @ 0.4

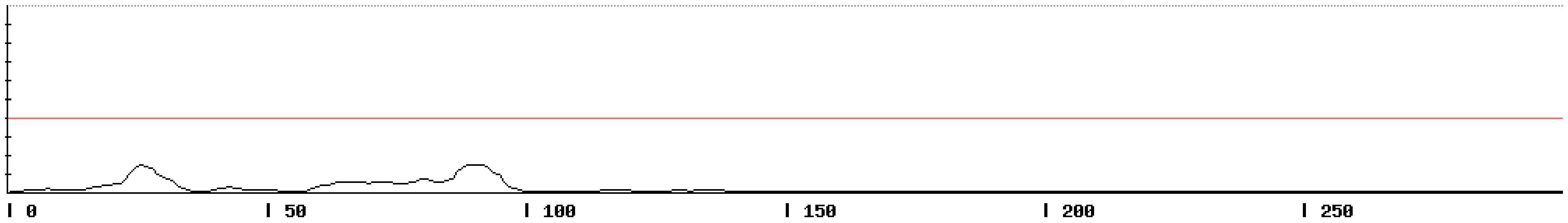

Predictions for Dorcocerashygrometricum_KZV27640.1

Viterbi Path

Viterbi Path

Posterior @ 0.4: 52 - QRKRRRISGRESTIKAHVRWHKTGKTK - 78

Posterior @ 0.4

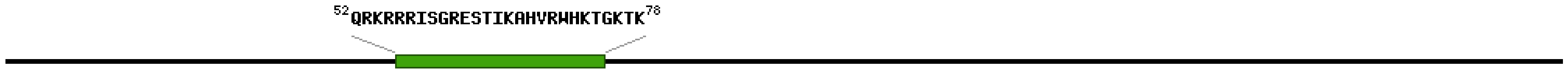

Posterior Graphic. Threshold Marked @ 0.4

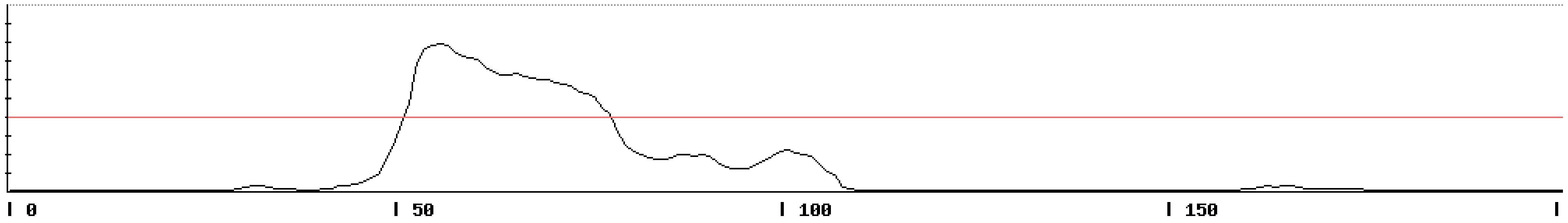

Predictions for Dorcocerashygrometricum_KZV28741.1

Viterbi Path

Viterbi Path

Posterior @ 0.4

Posterior @ 0.4

Posterior Graphic. Threshold Marked @ 0.4

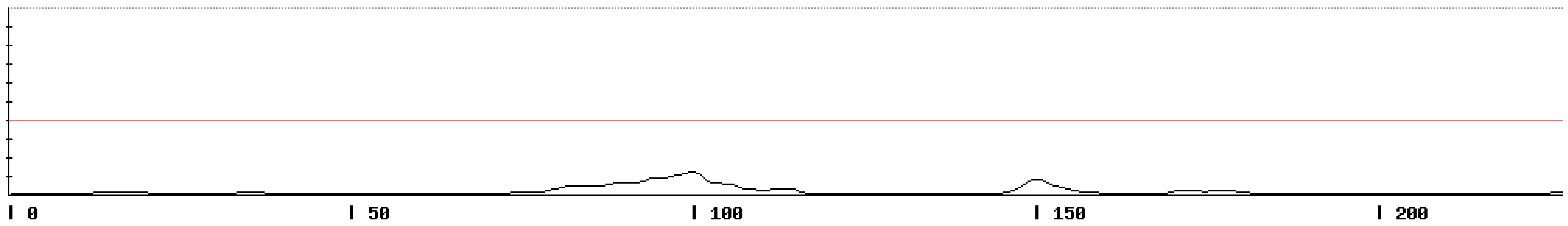

Predictions for Dorcocerashygrometricum_KZV28942.1

Viterbi Path

Viterbi Path

Posterior @ 0.4

Posterior @ 0.4

Posterior Graphic. Threshold Marked @ 0.4

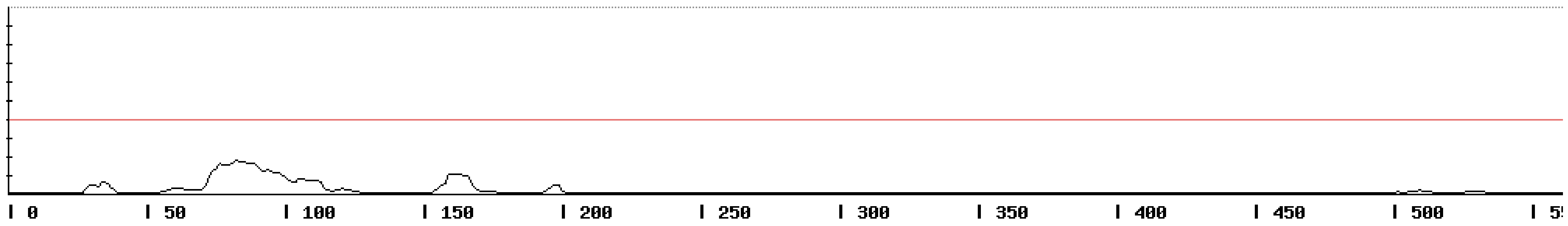

Predictions for Dorcocerashygrometricum_KZV29177.1

Viterbi Path

Viterbi Path

Posterior @ 0.4

Posterior @ 0.4

Posterior Graphic. Threshold Marked @ 0.4

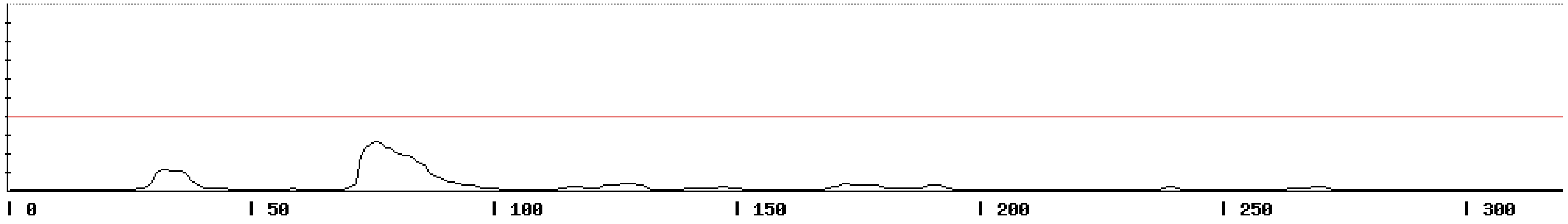

Predictions for Dorcocerashygrometricum_KZV30139.1

Viterbi Path

Viterbi Path

Posterior @ 0.4 | 159 - RKRRK - 163

Posterior @ 0.4

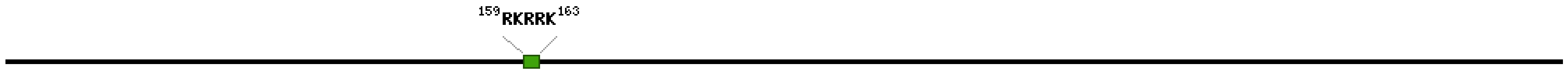

Posterior Graphic. Threshold Marked @ 0.4

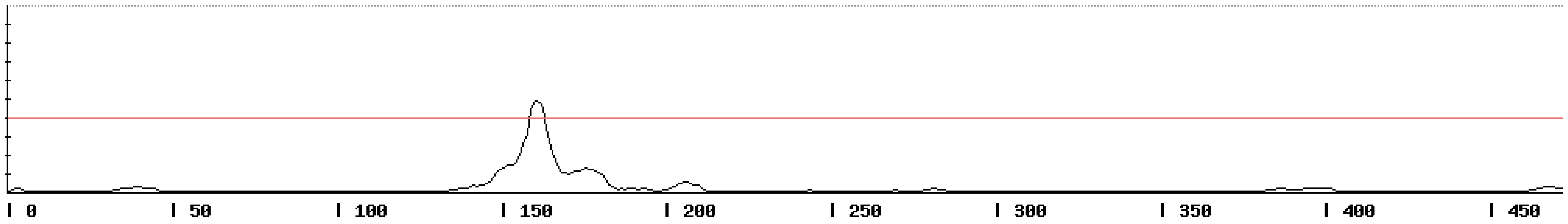

Predictions for Dorcocerashygrometricum_KZV32195.1

Viterbi Path	
Viterbi Path	
Posterior @ 0.4	776 - RKK - 778
Posterior @ 0.4	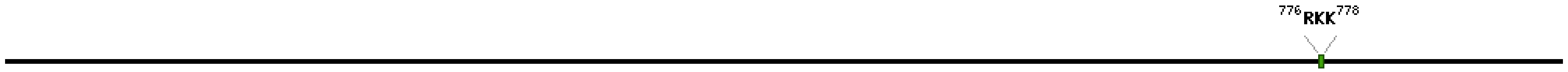
Posterior Graphic. Threshold Marked @ 0.4	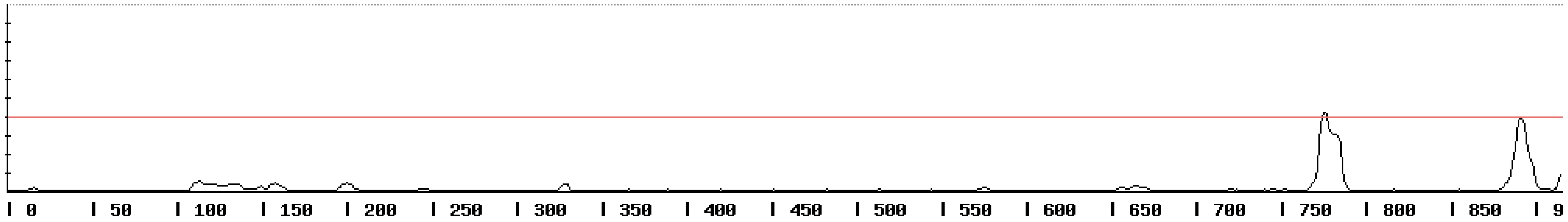

Predictions for Dorcocerashygrometricum_KZV32898.1

Viterbi Path	
Viterbi Path	
Posterior @ 0.4	
Posterior @ 0.4	
Posterior Graphic. Threshold Marked @ 0.4	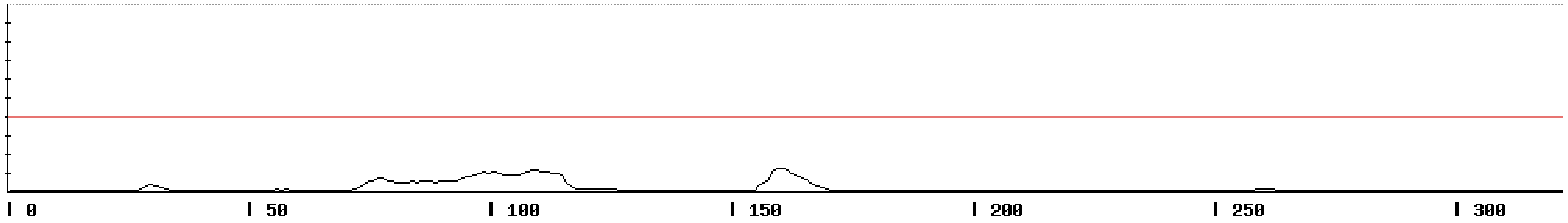

Predictions for Dorcocerashygrometricum_KZV32904.1	
Viterbi Path	
Viterbi Path	
Posterior @ 0.4	
Posterior @ 0.4	
Posterior Graphic. Threshold Marked @ 0.4	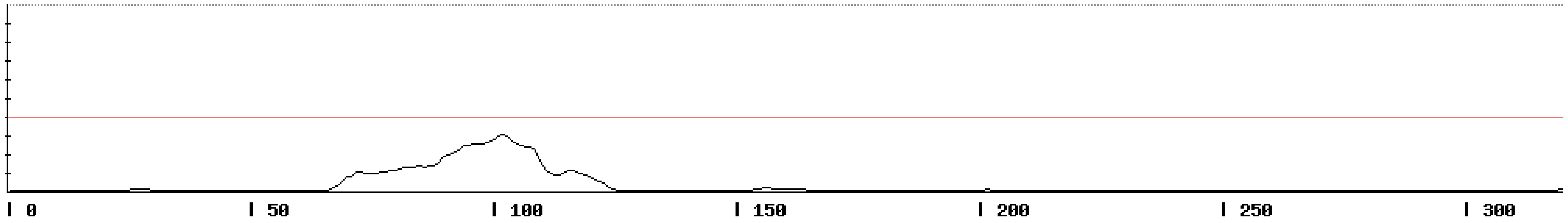

Predictions for Dorcocerashygrometricum_KZV32979.1	
Viterbi Path	
Viterbi Path	
Posterior @ 0.4	
Posterior @ 0.4	
Posterior Graphic. Threshold Marked @ 0.4	

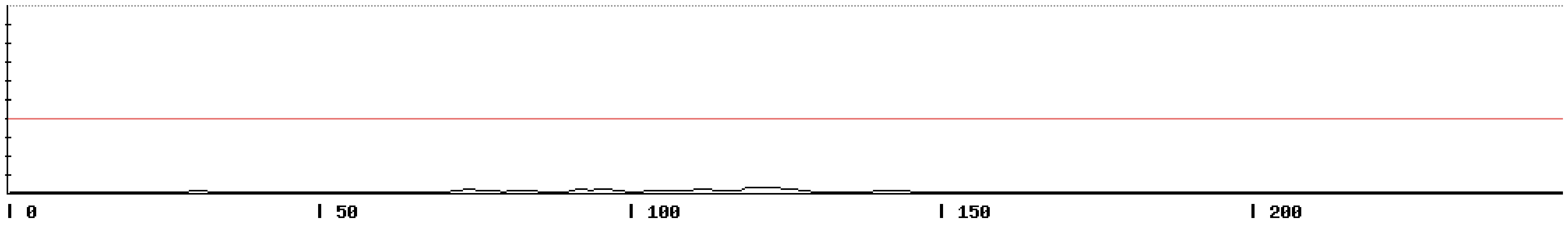

Predictions for Dorcocerashygrometricum_KZV33083.1

Viterbi Path	
Viterbi Path	
Posterior @ 0.4	
Posterior @ 0.4	
Posterior Graphic. Threshold Marked @ 0.4	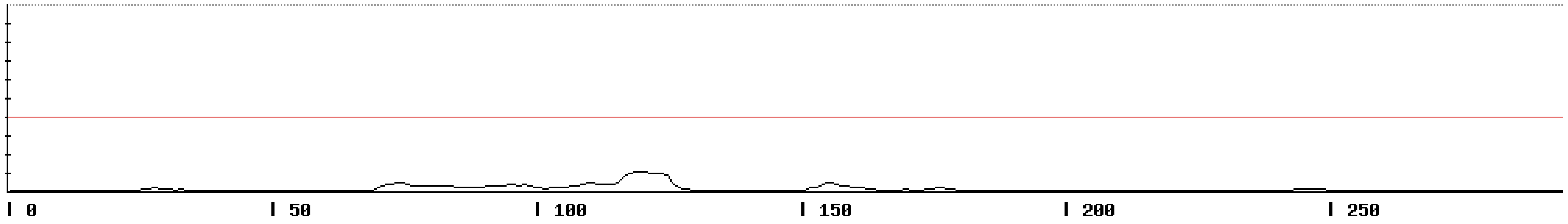

Predictions for Dorcocerashygrometricum_KZV34223.1

Viterbi Path	
Viterbi Path	
Posterior @ 0.4	361 - KK - 362
Posterior @ 0.4	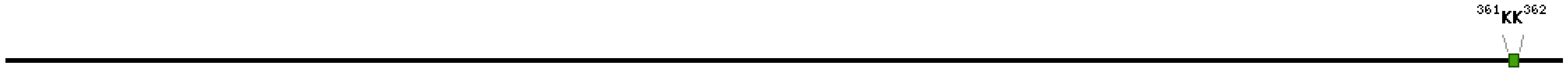

Posterior Graphic. Threshold Marked @ 0.4

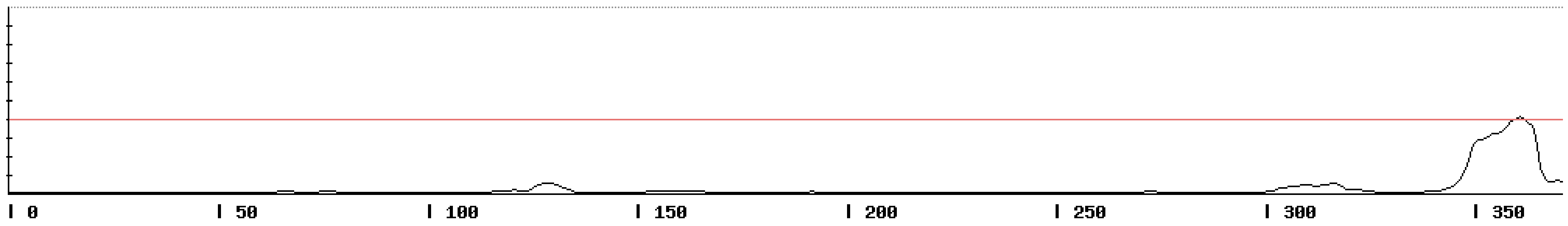

Predictions for Dorcocerashygrometricum_KZV34250.1

Viterbi Path

Viterbi Path

Posterior @ 0.4

Posterior @ 0.4

Posterior Graphic. Threshold Marked @ 0.4

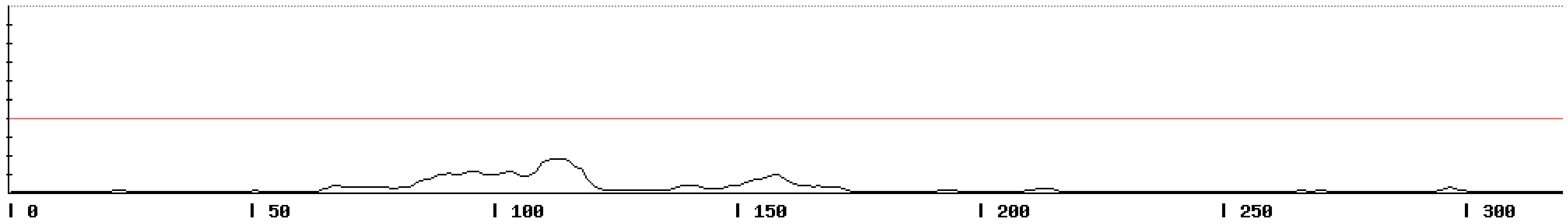

Predictions for Dorcocerashygrometricum_KZV36249.1

Viterbi Path

Viterbi Path

Posterior @ 0.4

Posterior @ 0.4

Posterior Graphic. Threshold Marked @ 0.4

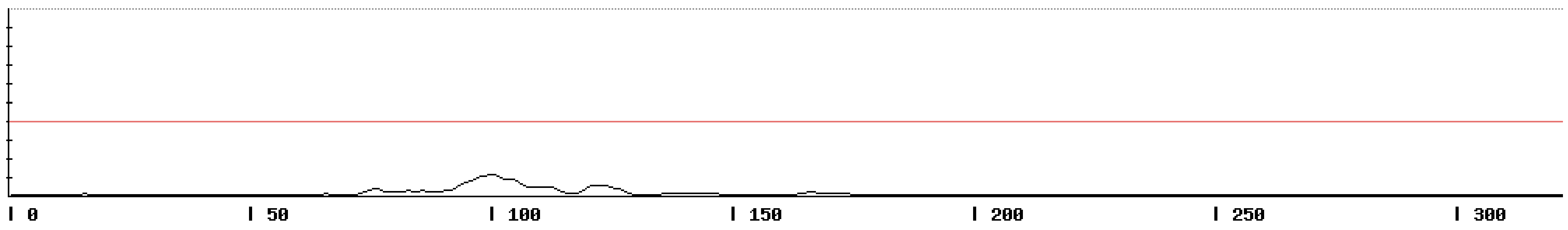

Predictions for Dorcocerashygrometricum_KZV36257.1

Viterbi Path

Viterbi Path

Posterior @ 0.4

Posterior @ 0.4

Posterior Graphic. Threshold Marked @ 0.4

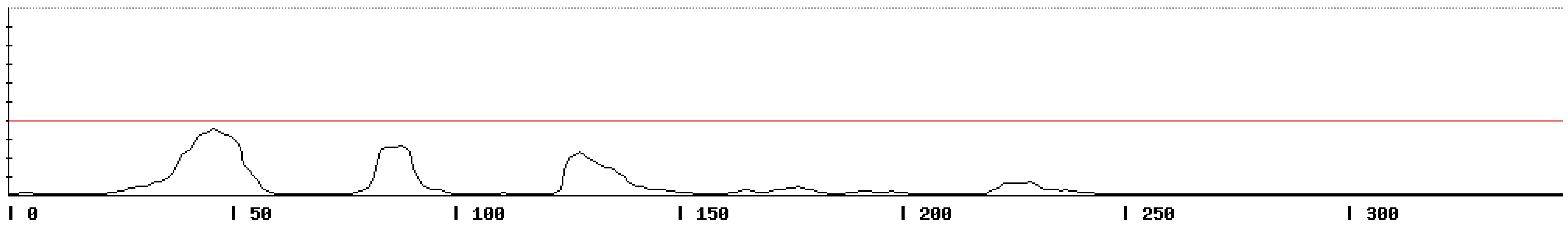

Predictions for Dorcocerashygrometricum_KZV36778.1

Viterbi Path

Viterbi Path

Posterior @ 0.4

Posterior @ 0.4

Posterior Graphic. Threshold Marked @ 0.4

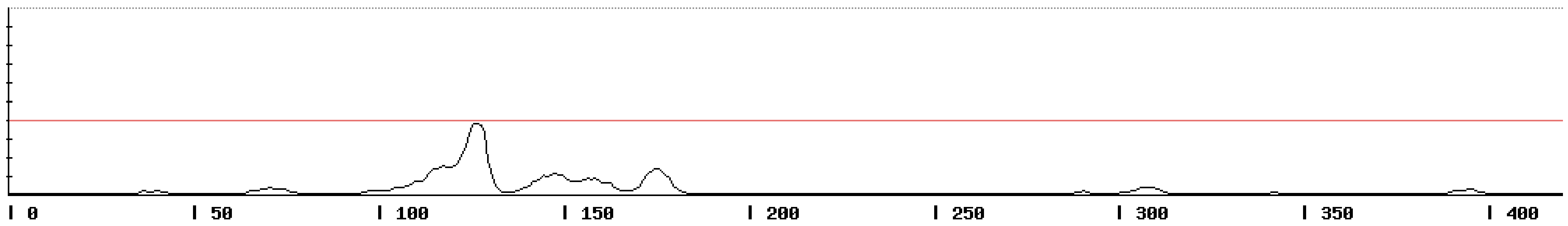

Predictions for Dorcocerashygrometricum_KZV37136.1

Viterbi Path

Viterbi Path

Posterior @ 0.4

Posterior @ 0.4

Posterior Graphic. Threshold Marked @ 0.4

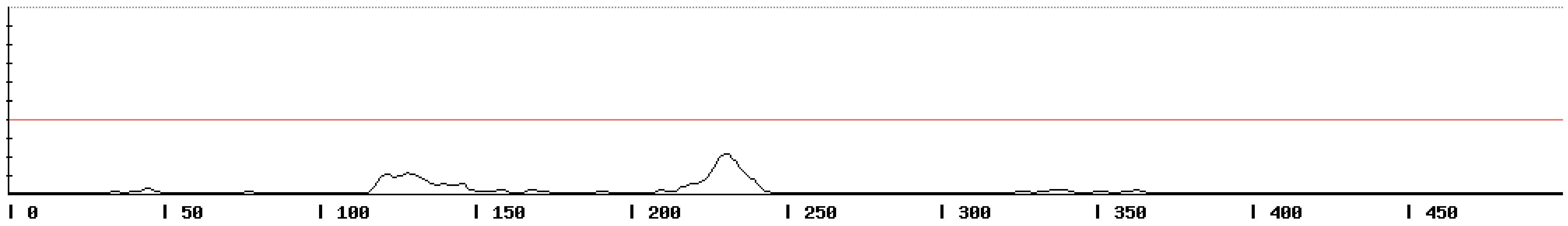

Predictions for Dorcocerashygrometricum_KZV37138.1

Viterbi Path

Viterbi Path

Posterior @ 0.4

Posterior @ 0.4

Posterior Graphic. Threshold Marked @ 0.4

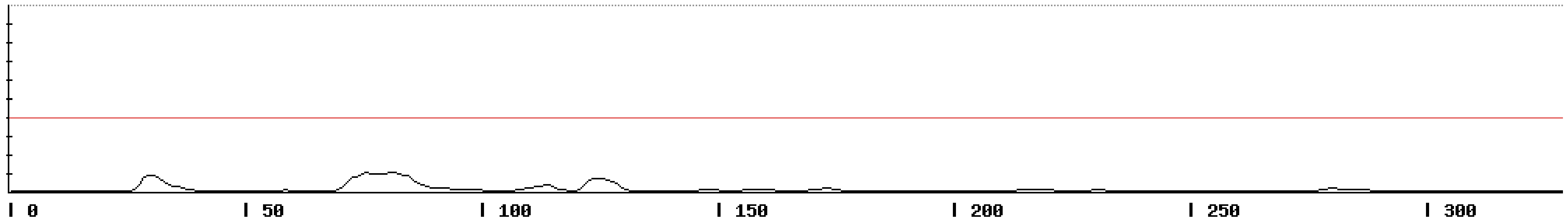

Predictions for Dorcocerashygrometricum_KZV37345.1

Viterbi Path

Viterbi Path

Posterior @ 0.4

Posterior @ 0.4

Posterior Graphic. Threshold Marked @ 0.4

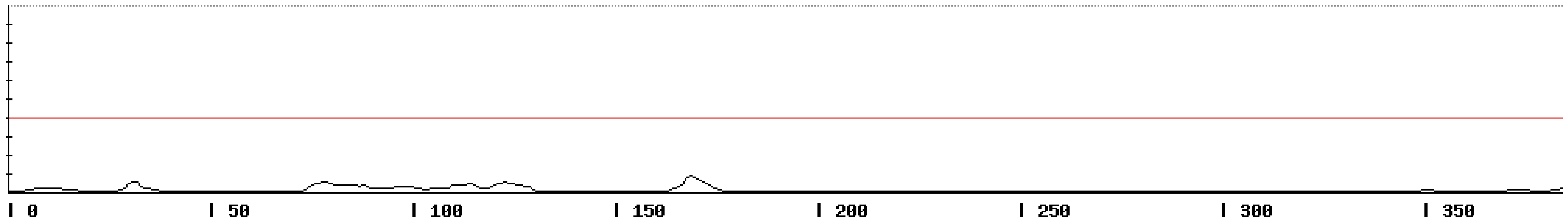

Predictions for Dorcocerashygrometricum_KZV37882.1

Viterbi Path

Viterbi Path

Posterior @ 0.4

Posterior @ 0.4

Posterior Graphic. Threshold Marked @ 0.4

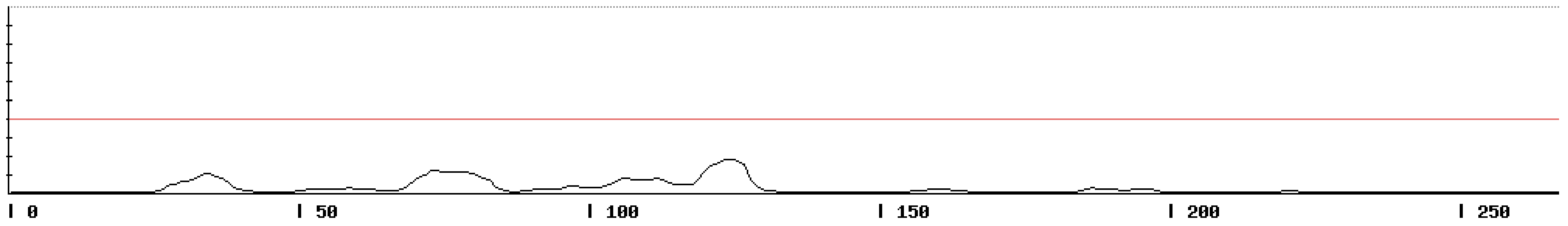

Predictions for Dorcocerashygrometricum_KZV38032.1

Viterbi Path

Viterbi Path

Posterior @ 0.4

Posterior @ 0.4

Posterior Graphic. Threshold Marked @ 0.4

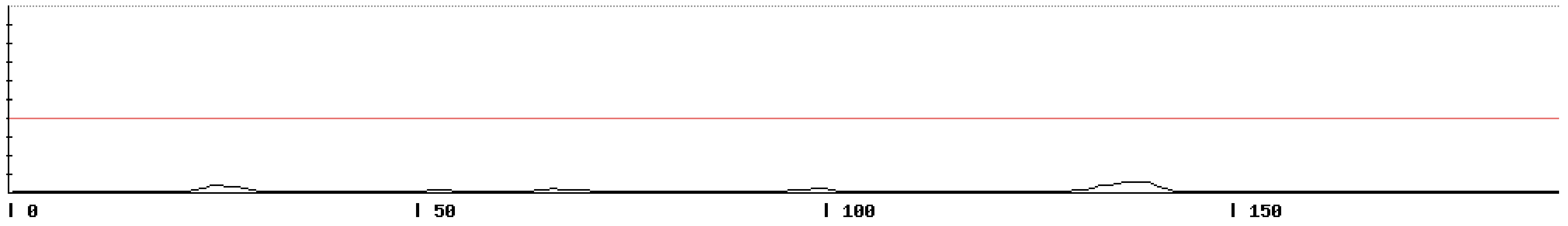

Predictions for Dorcocerashygrometricum_KZV39518.1

Viterbi Path

Viterbi Path

Posterior @ 0.4

Posterior @ 0.4

Posterior Graphic. Threshold Marked @ 0.4

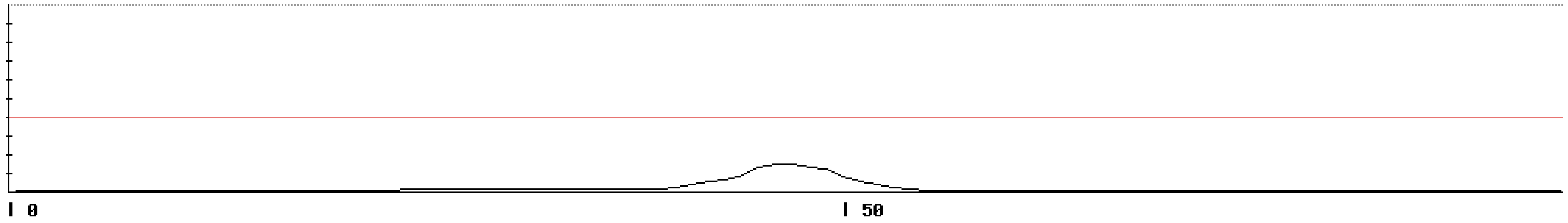

Predictions for Dorcocerashygrometricum_KZV39521.1

Viterbi Path

Viterbi Path

Posterior @ 0.4

Posterior @ 0.4

Posterior Graphic. Threshold Marked @ 0.4

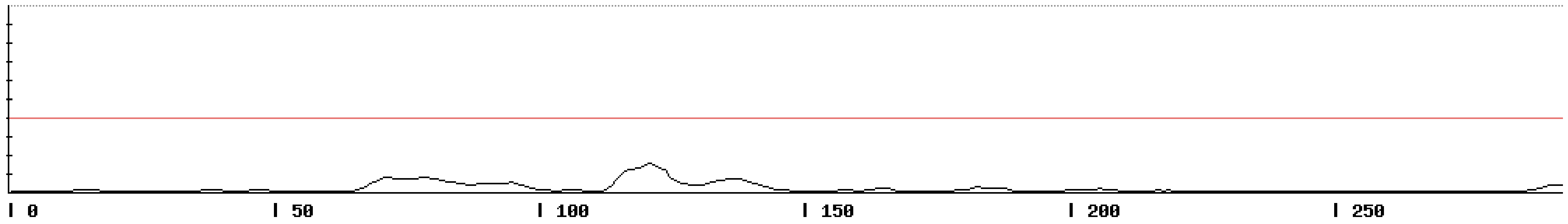

Predictions for Dorcocerashygrometricum_KZV39663.1

Viterbi Path

Viterbi Path

Posterior @ 0.4

Posterior @ 0.4

Posterior Graphic. Threshold Marked @ 0.4

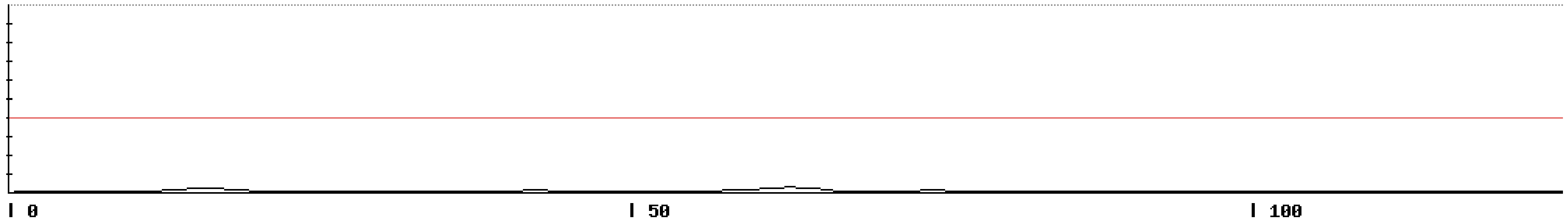

Predictions for Dorcocerashygrometricum_KZV39664.1

Viterbi Path

Viterbi Path

Posterior @ 0.4

Posterior @ 0.4

Posterior Graphic. Threshold Marked @ 0.4

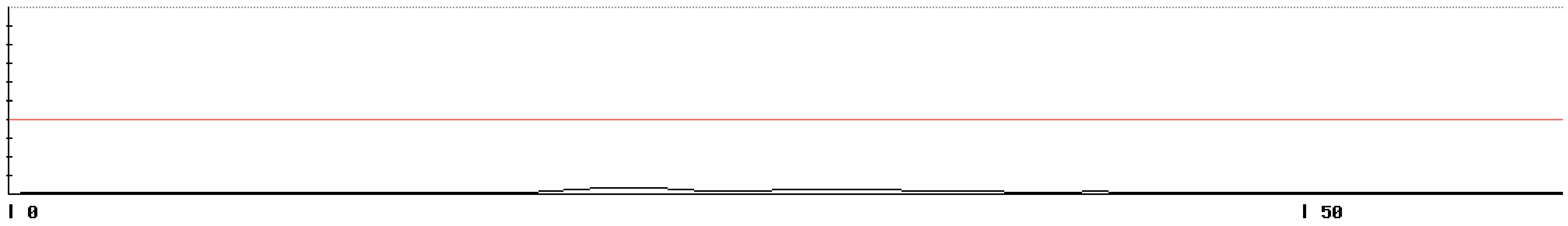

Predictions for Dorcocerashygrometricum_KZV39672.1

Viterbi Path

Viterbi Path

Posterior @ 0.4

Posterior @ 0.4

Posterior Graphic. Threshold Marked @ 0.4

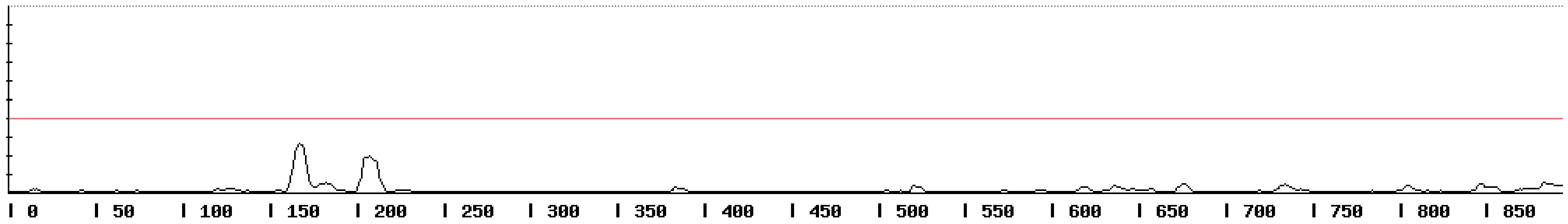

Predictions for Dorcocerashygrometricum_KZV40562.1

Viterbi Path

Viterbi Path

Posterior @ 0.4

Posterior @ 0.4

Posterior Graphic. Threshold Marked @ 0.4

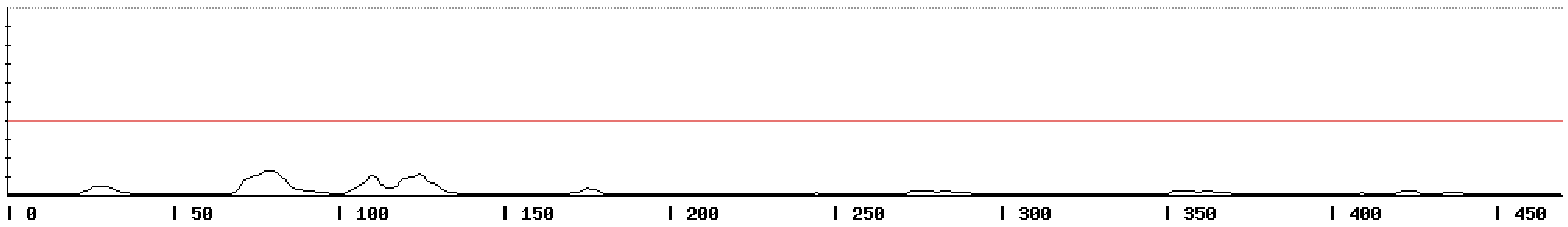

Predictions for Dorcocerashygrometricum_KZV40837.1

Viterbi Path

Viterbi Path

Posterior @ 0.4

Posterior @ 0.4

Posterior Graphic. Threshold Marked @ 0.4

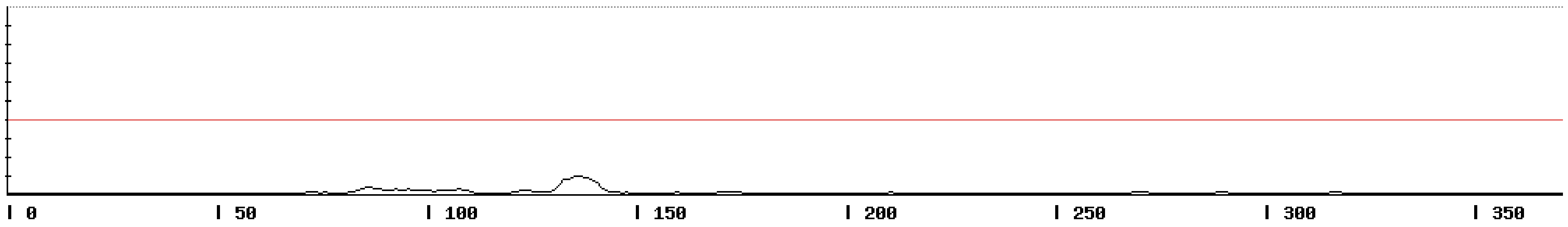

Predictions for Dorcocerashygrometricum_KZV40900.1

Viterbi Path

Viterbi Path

Posterior @ 0.4

Posterior @ 0.4

Posterior Graphic. Threshold Marked @ 0.4

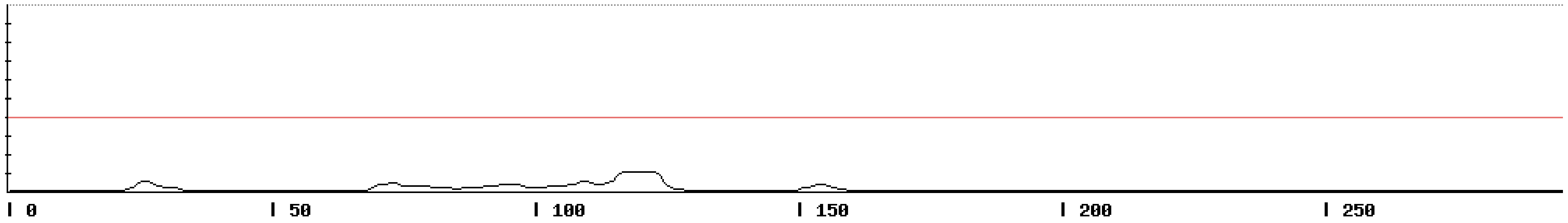

Predictions for Dorcocerashygrometricum_KZV40957.1

Viterbi Path

Viterbi Path

Posterior @ 0.4

Posterior @ 0.4

Posterior Graphic. Threshold Marked @ 0.4

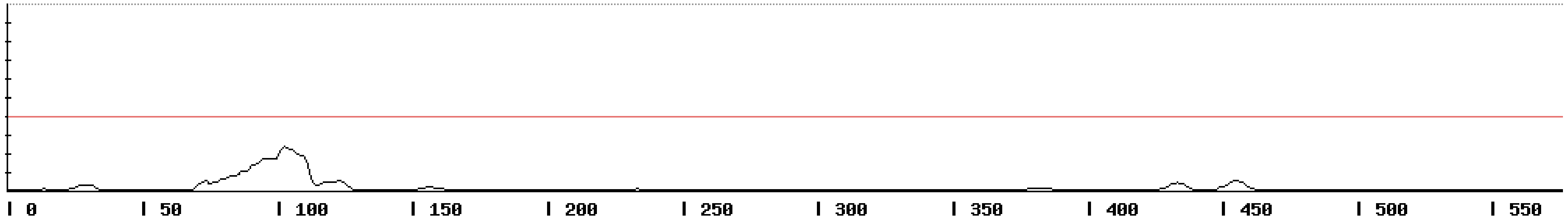

Predictions for Dorcocerashygrometricum_KZV40989.1

Viterbi Path

Viterbi Path

Posterior @ 0.4

Posterior @ 0.4

Posterior Graphic. Threshold Marked @ 0.4

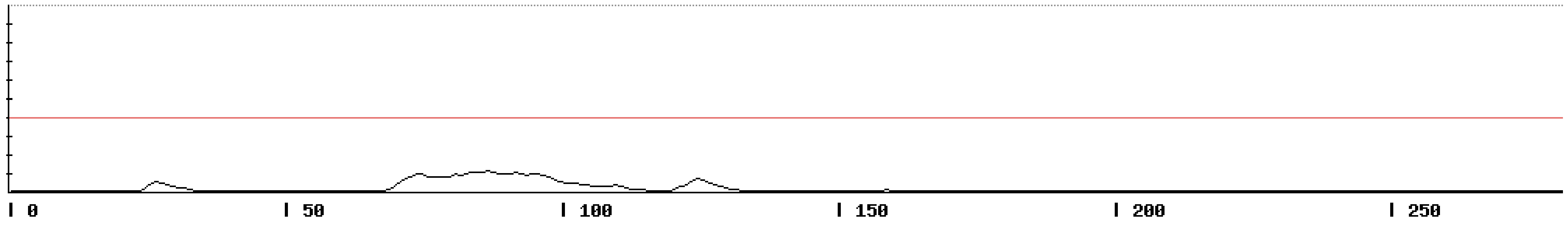

Predictions for Dorcocerashygrometricum_KZV41188.1

Viterbi Path

Viterbi Path

Posterior @ 0.4

Posterior @ 0.4

Posterior Graphic. Threshold Marked @ 0.4

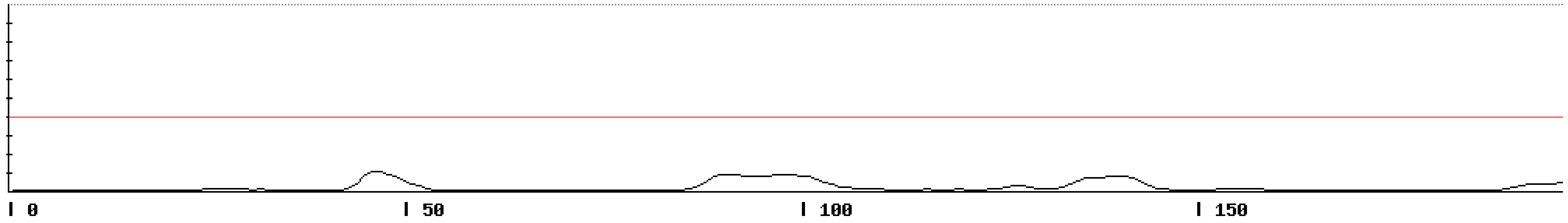

Predictions for Dorcocerashygrometricum_KZV41413.1

Viterbi Path

Viterbi Path

Posterior @ 0.4

Posterior @ 0.4

Posterior Graphic. Threshold Marked @ 0.4

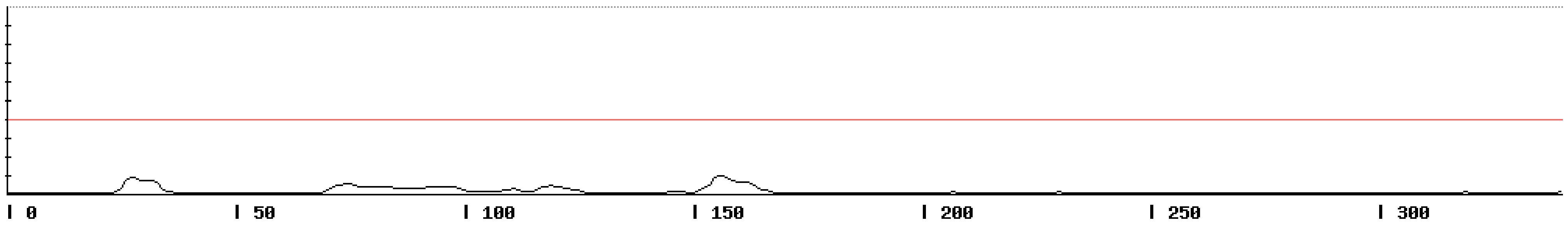

Predictions for Dorcocerashygrometricum_KZV41653.1

Viterbi Path

Viterbi Path

Posterior @ 0.4

Posterior @ 0.4

Posterior Graphic. Threshold Marked @ 0.4

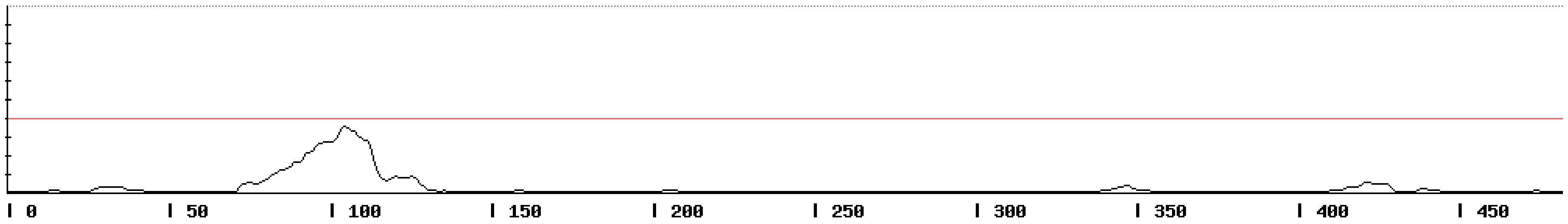

Predictions for Dorcocerashygrometricum_KZV43054.1	
Viterbi Path	
Viterbi Path	
Posterior @ 0.4	
Posterior @ 0.4	
Posterior Graphic. Threshold Marked @ 0.4	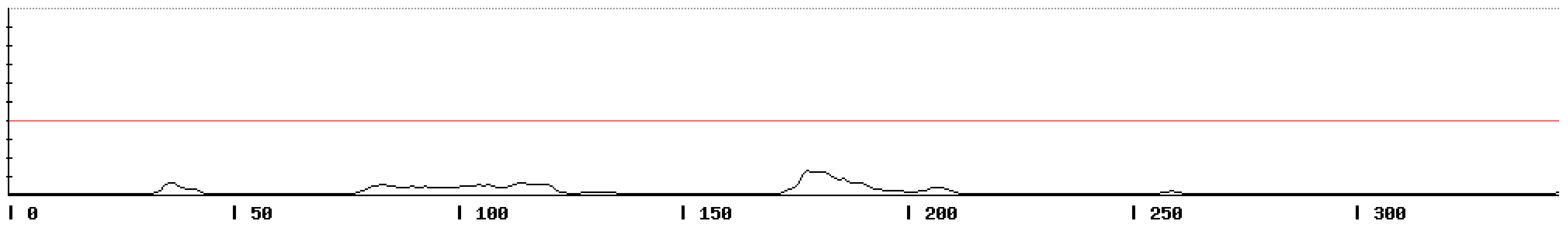

Predictions for Dorcocerashygrometricum_KZV43485.1	
Viterbi Path	
Viterbi Path	
Posterior @ 0.4	99 - RNIISKKGAKIGMK - 112
Posterior @ 0.4	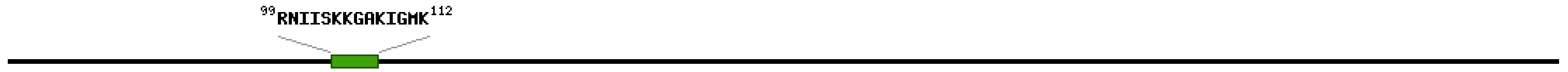
Posterior Graphic. Threshold Marked @ 0.4	

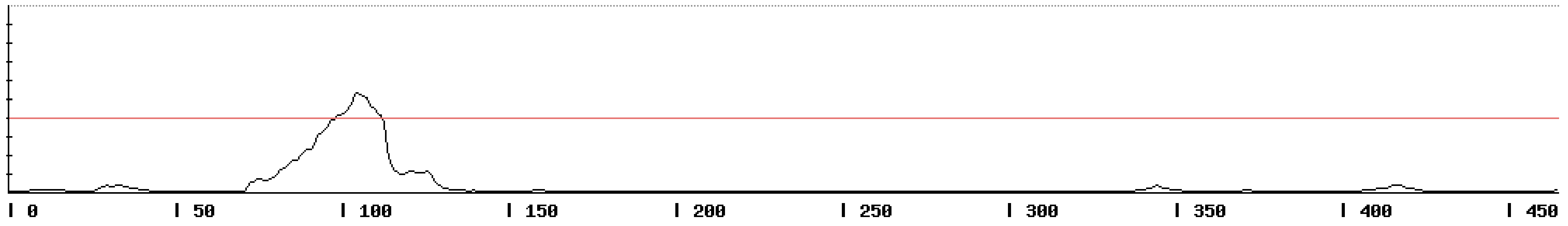

Predictions for Dorcocerashygrometricum_KZV43708.1

Viterbi Path	
Viterbi Path	
Posterior @ 0.4	194 - AKKNNRK - 200
Posterior @ 0.4	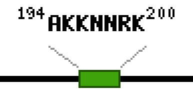
Posterior Graphic. Threshold Marked @ 0.4	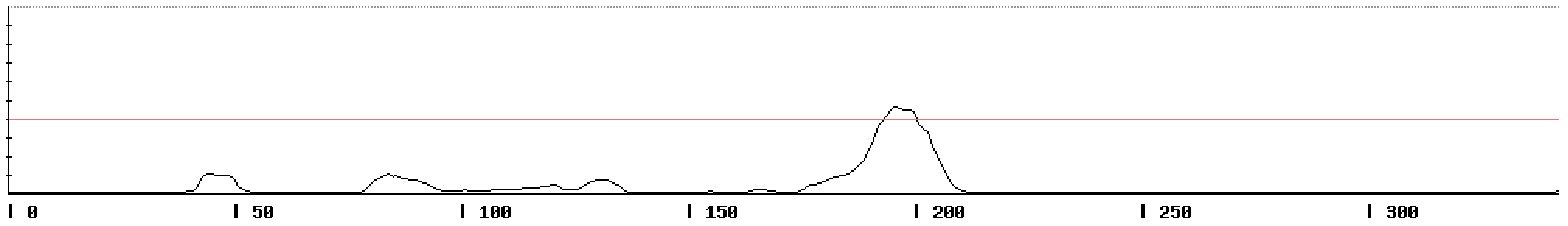

Predictions for Dorcocerashygrometricum_KZV45352.1

Viterbi Path	
Viterbi Path	
Posterior @ 0.4	
Posterior @ 0.4	

Posterior Graphic. Threshold Marked @ 0.4

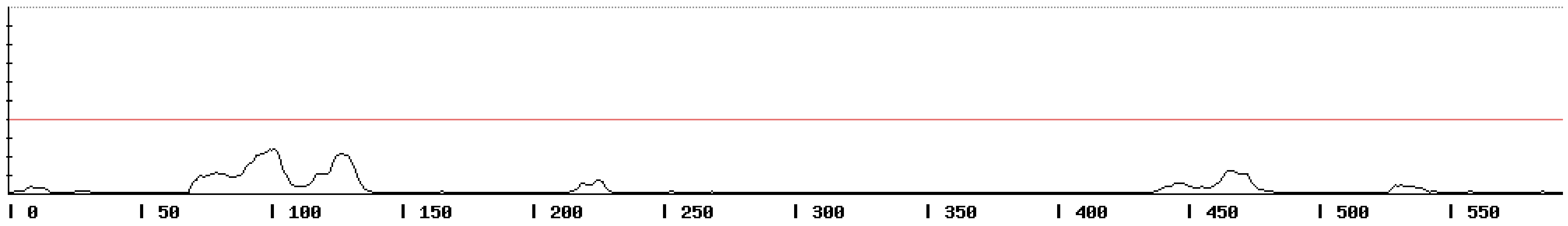

Predictions for Dorcocerashygrometricum_KZV45900.1

Viterbi Path

Viterbi Path

Posterior @ 0.4

Posterior @ 0.4

Posterior Graphic. Threshold Marked @ 0.4

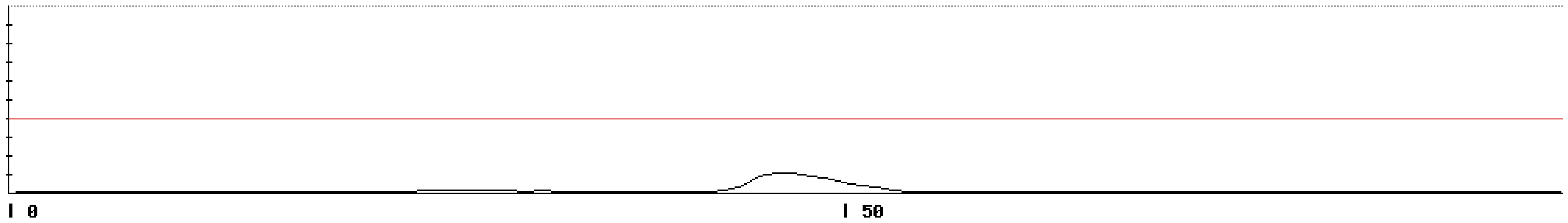

Predictions for Dorcocerashygrometricum_KZV45902.1

Viterbi Path

Viterbi Path

Posterior @ 0.4

Posterior @ 0.4

Posterior Graphic. Threshold Marked @ 0.4	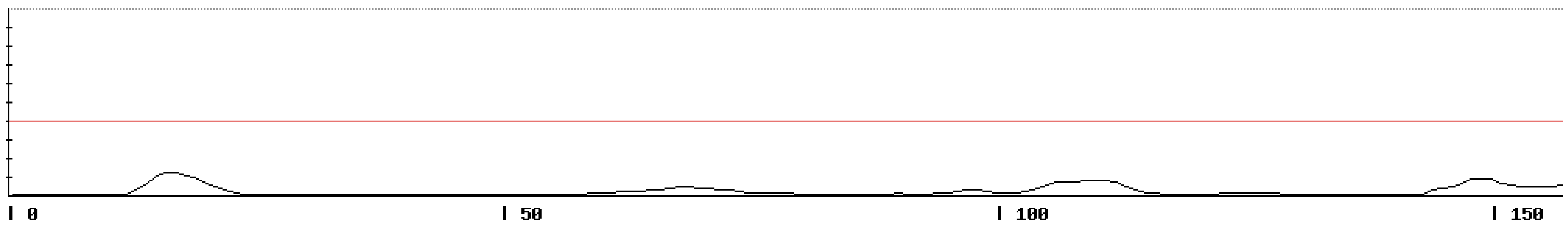

Predictions for Dorcocerashygrometricum_KZV46422.1	
Viterbi Path	
Viterbi Path	
Posterior @ 0.4	372 - QRKRRRISGRESTIKAHVRWHKTGKTK - 398
Posterior @ 0.4	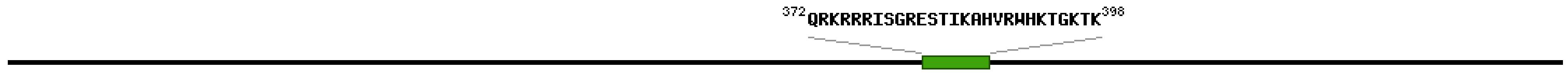
Posterior Graphic. Threshold Marked @ 0.4	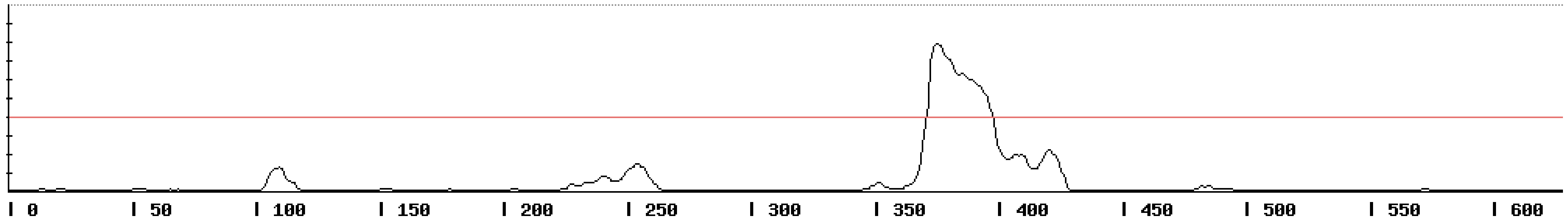

Predictions for Dorcocerashygrometricum_KZV47090.1	
Viterbi Path	
Viterbi Path	

Posterior @ 0.4

Posterior @ 0.4

Posterior Graphic. Threshold Marked @ 0.4

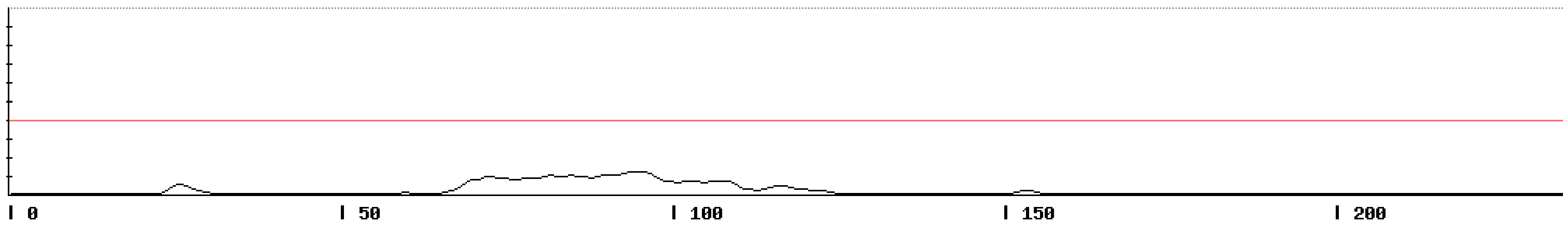

Predictions for Dorcocerashygrometricum_KZV47588.1

Viterbi Path

Viterbi Path

Posterior @ 0.4

Posterior @ 0.4

Posterior Graphic. Threshold Marked @ 0.4

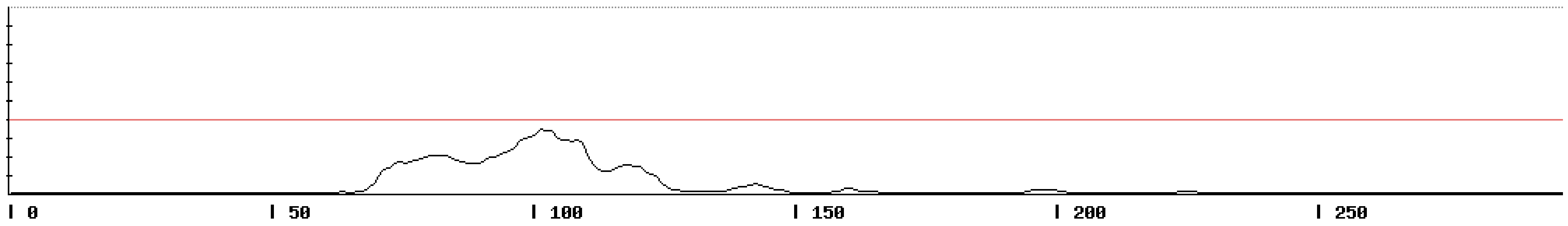

Predictions for Dorcocerashygrometricum_KZV48824.1

Viterbi Path

Viterbi Path

Posterior @ 0.4	
Posterior @ 0.4	
Posterior Graphic. Threshold Marked @ 0.4	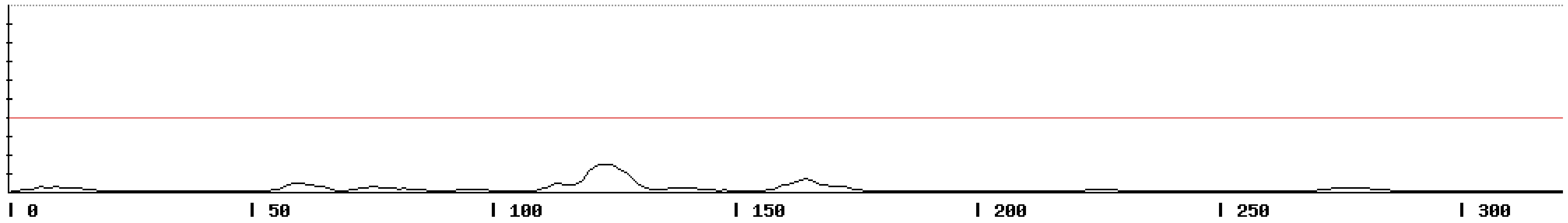

Predictions for Dorcocerashygrometricum_KZV51115.1

Viterbi Path	
Viterbi Path	
Posterior @ 0.4	136 - RKRRK - 140
Posterior @ 0.4	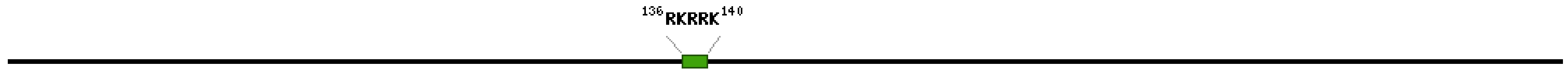
Posterior Graphic. Threshold Marked @ 0.4	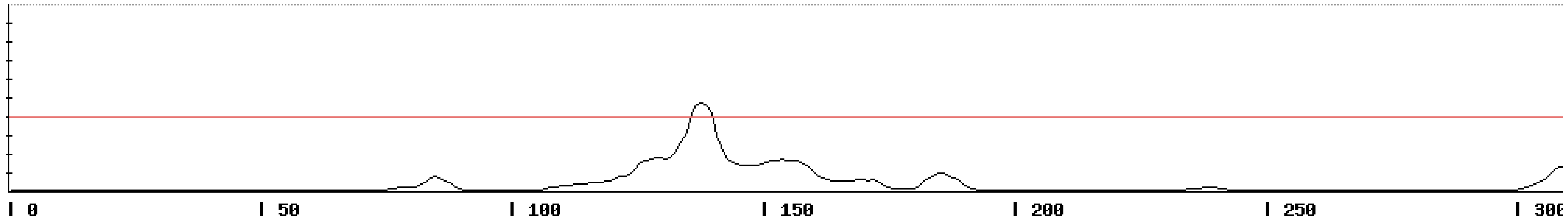

Predictions for Dorcocerashygrometricum_KZV51579.1

Viterbi Path	
Viterbi Path	
Posterior @ 0.4	
Posterior @ 0.4	
Posterior Graphic. Threshold Marked @ 0.4	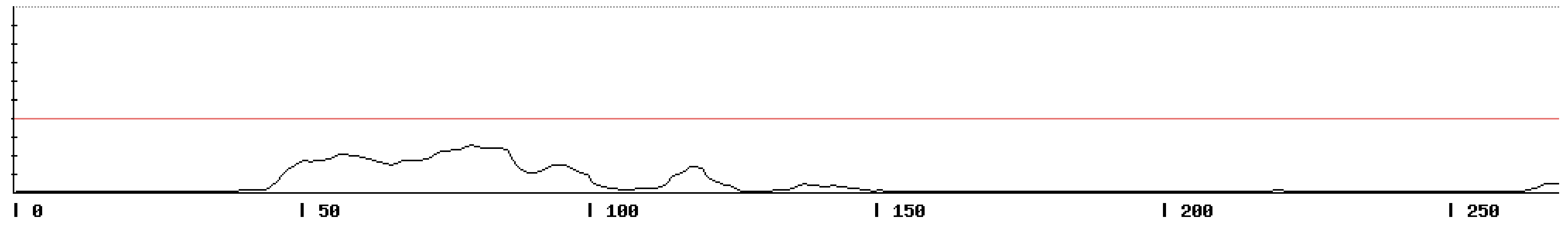

Predictions for Dorcocerashygrometricum_KZV52968.1

Viterbi Path	
Viterbi Path	
Posterior @ 0.4	116 - PK - 117
Posterior @ 0.4	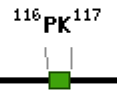
Posterior Graphic. Threshold Marked @ 0.4	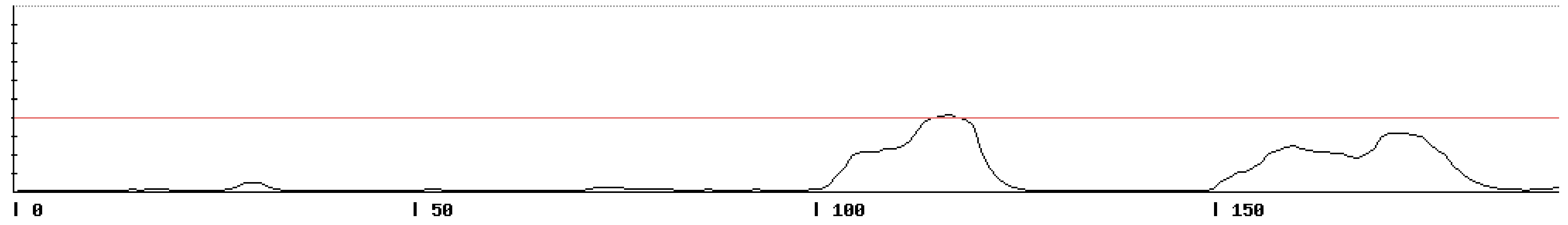

Predictions for Dorcocerashygrometricum_KZV52969.1

Viterbi Path	
Viterbi Path	
Posterior @ 0.4	
Posterior @ 0.4	
Posterior Graphic. Threshold Marked @ 0.4	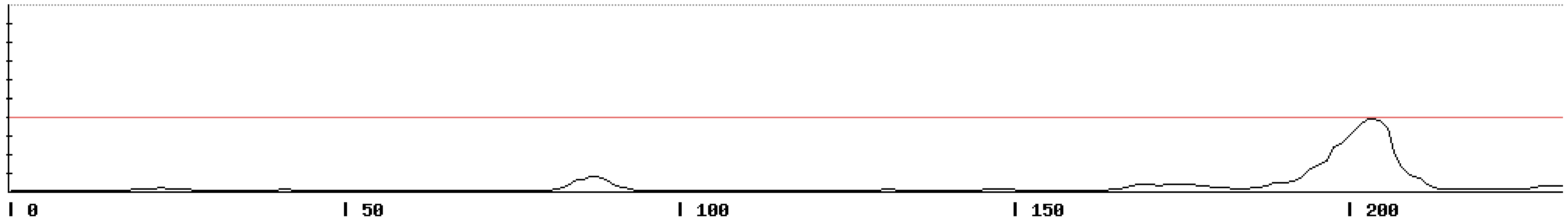

Predictions for Dorcocerashygrometricum_KZV53715.1

Viterbi Path	
Viterbi Path	
Posterior @ 0.4	
Posterior @ 0.4	
Posterior Graphic. Threshold Marked @ 0.4	

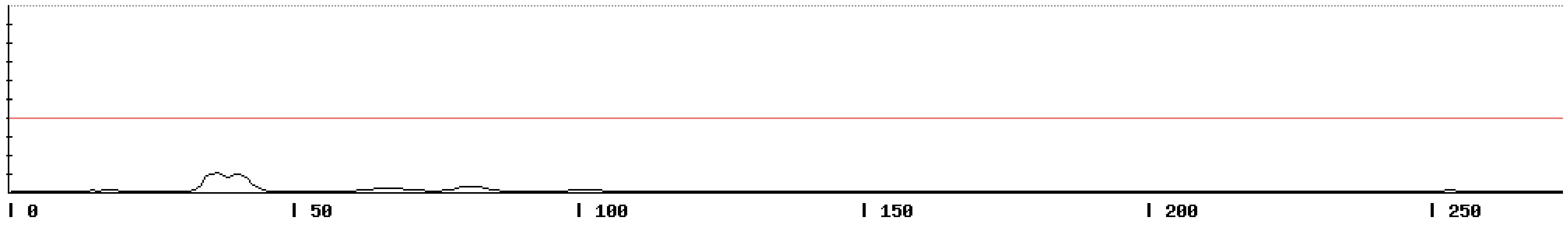

Predictions for Dorcocerashygrometricum_KZV53808.1	
Viterbi Path	
Viterbi Path	
Posterior @ 0.4	
Posterior @ 0.4	
Posterior Graphic. Threshold Marked @ 0.4	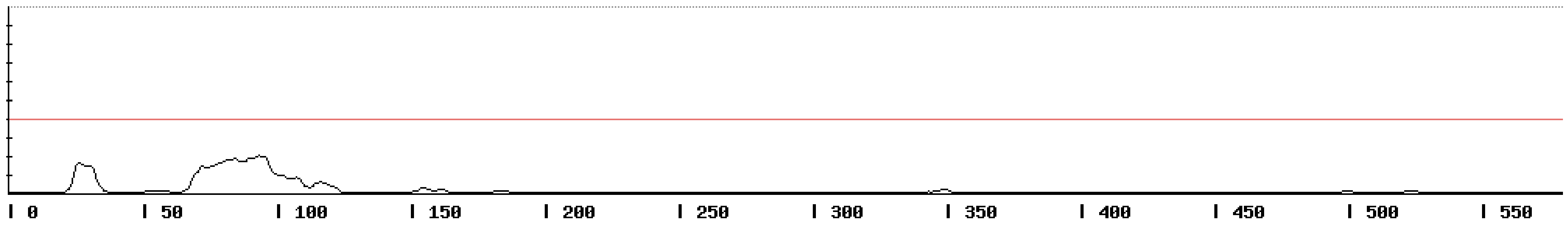

Predictions for Dorcocerashygrometricum_KZV53859.1	
Viterbi Path	
Viterbi Path	
Posterior @ 0.4	
Posterior @ 0.4	

Posterior Graphic. Threshold Marked @ 0.4

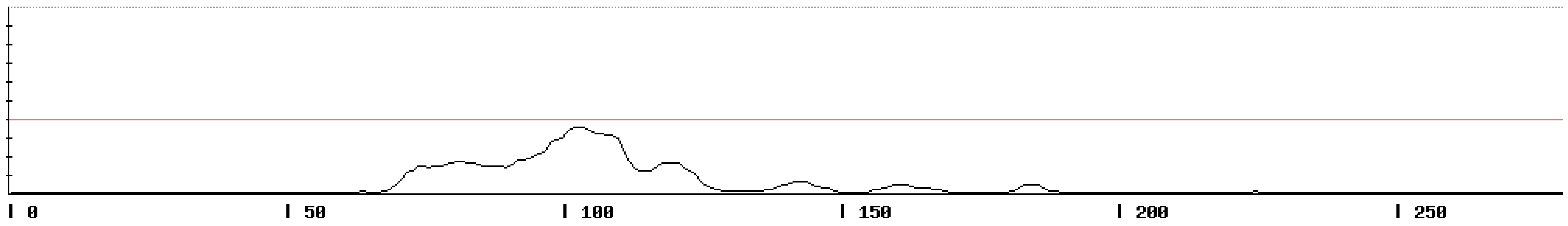

Predictions for Dorcocerashygrometricum_KZV55311.1

Viterbi Path

Viterbi Path

Posterior @ 0.4

Posterior @ 0.4

Posterior Graphic. Threshold Marked @ 0.4

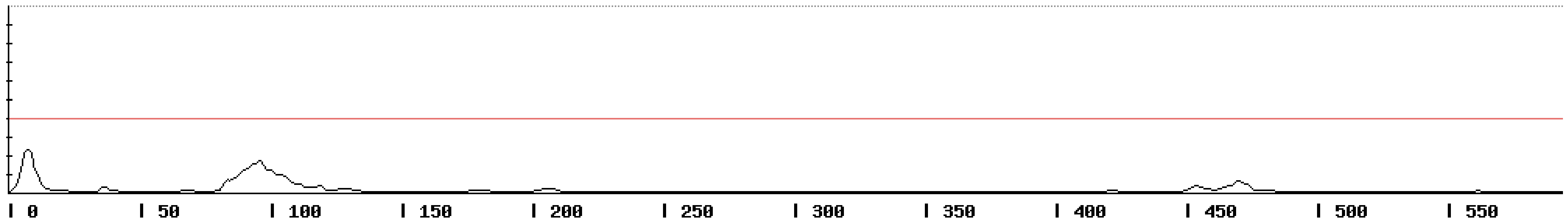

Predictions for Dorcocerashygrometricum_KZV55312.1

Viterbi Path

Viterbi Path

Posterior @ 0.4

Posterior @ 0.4

Posterior Graphic. Threshold Marked @ 0.4

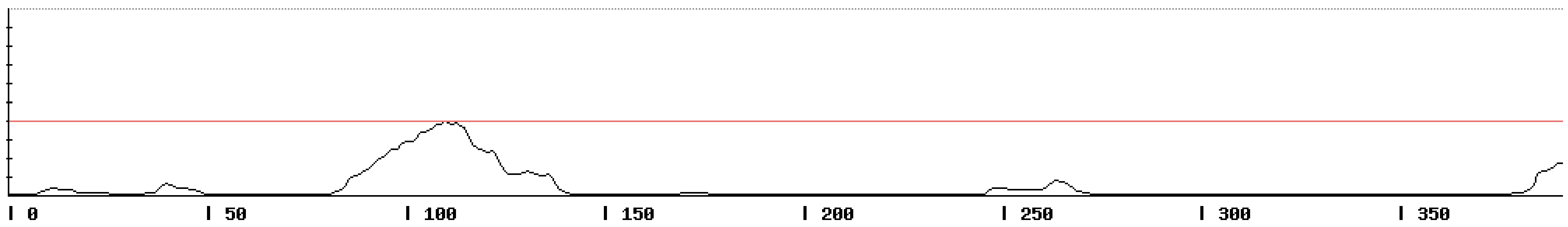

Predictions for Dorcocerashygrometricum_KZV56170.1

Viterbi Path

Viterbi Path

Posterior @ 0.4

Posterior @ 0.4

Posterior Graphic. Threshold Marked @ 0.4

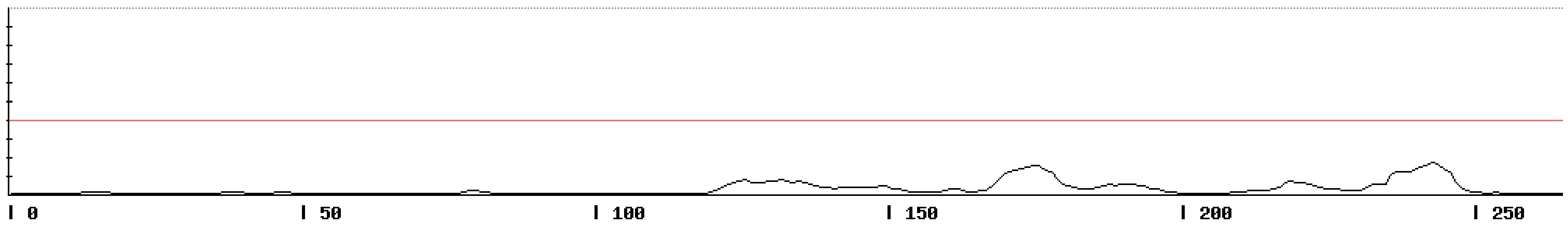

Predictions for Dorcocerashygrometricum_KZV56171.1

Viterbi Path

Viterbi Path

Posterior @ 0.4

Posterior @ 0.4

Posterior Graphic. Threshold Marked @ 0.4

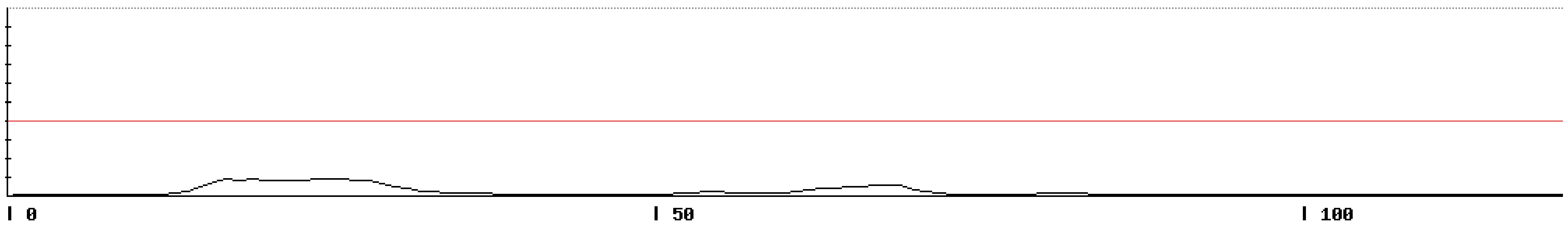

Predictions for Dorcocerashygrometricum_KZV56172.1

Viterbi Path

Viterbi Path

Posterior @ 0.4

Posterior @ 0.4

Posterior Graphic. Threshold Marked @ 0.4

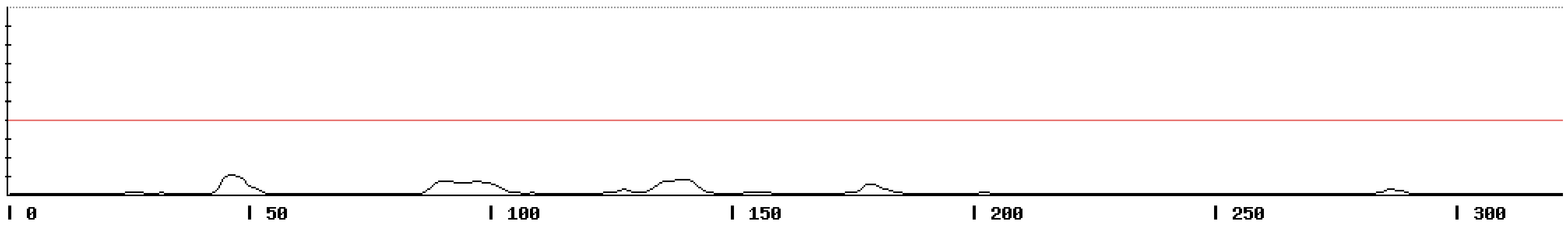

Predictions for Dorcocerashygrometricum_KZV56528.1

Viterbi Path

Viterbi Path

Posterior @ 0.4

Posterior @ 0.4

Posterior Graphic. Threshold Marked @ 0.4

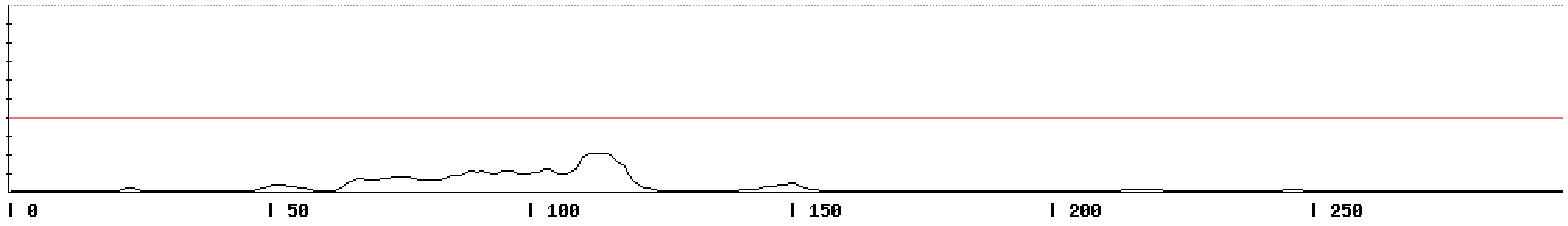

Predictions for Dorcocerashygrometricum_KZV56914.1

Viterbi Path

Viterbi Path

Posterior @ 0.4

Posterior @ 0.4

Posterior Graphic. Threshold Marked @ 0.4

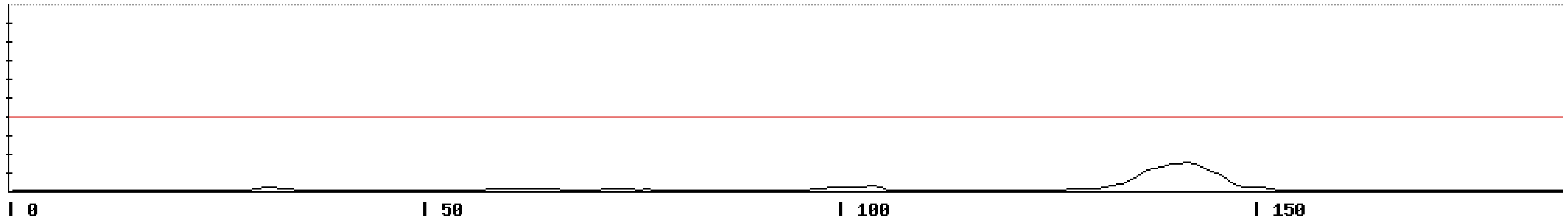

Predictions for Dorcocerashygrometricum_KZV56958.1

Viterbi Path	
Viterbi Path	
Posterior @ 0.4	
Posterior @ 0.4	
Posterior Graphic. Threshold Marked @ 0.4	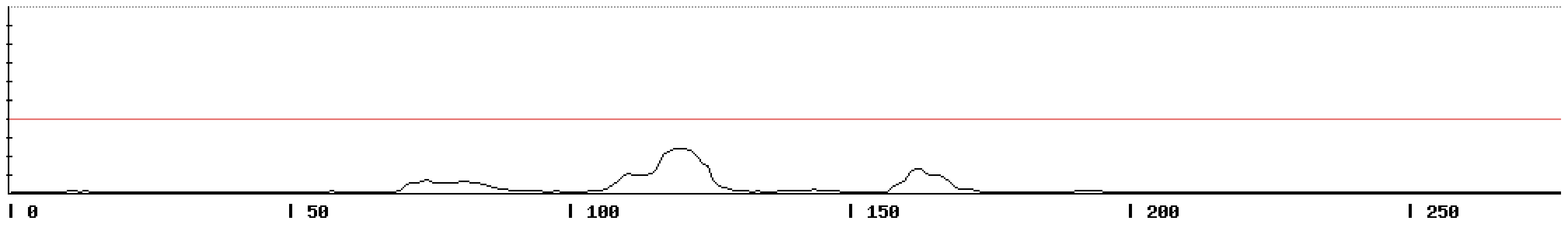

Predictions for Dorcocerashygrometricum_KZV57704.1

Revision r.9 - Last Updated on : February 24 2015 17:45:20.

alex.nguyenba@utoronto.ca

Intended to be used with Firefox

Viterbi Path	
Posterior @ 0.4	81 - RRAGLGR - 87
Posterior @ 0.4	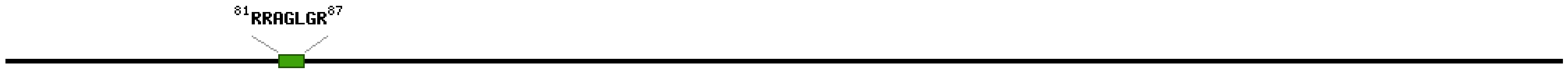
Posterior Graphic. Threshold Marked @ 0.4	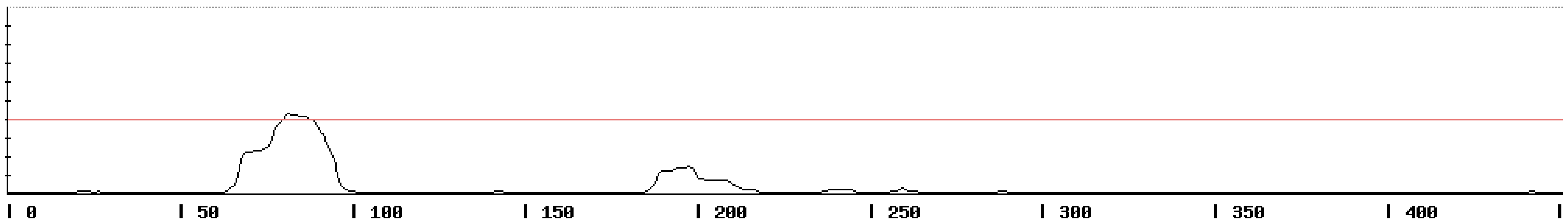

Predictions for Elaeisguineensis_XP_010904650.1

Viterbi Path	
Viterbi Path	
Posterior @ 0.4	
Posterior @ 0.4	
Posterior Graphic. Threshold Marked @ 0.4	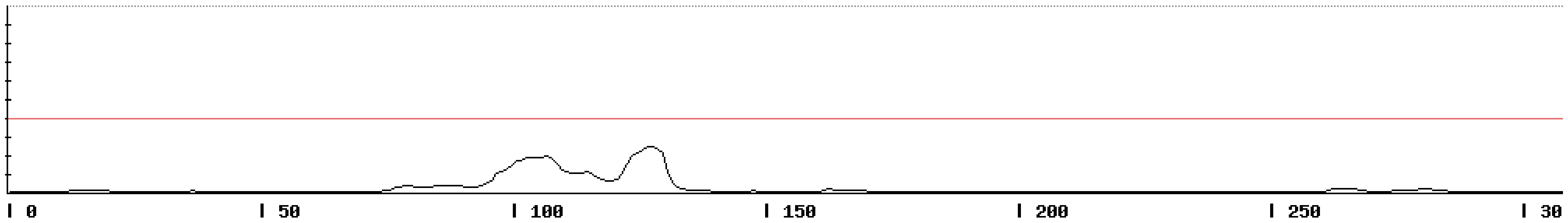

Predictions for Elaeisguineensis_XP_010904748.1

Viterbi Path	
Viterbi Path	
Posterior @ 0.4	
Posterior @ 0.4	
Posterior Graphic. Threshold Marked @ 0.4	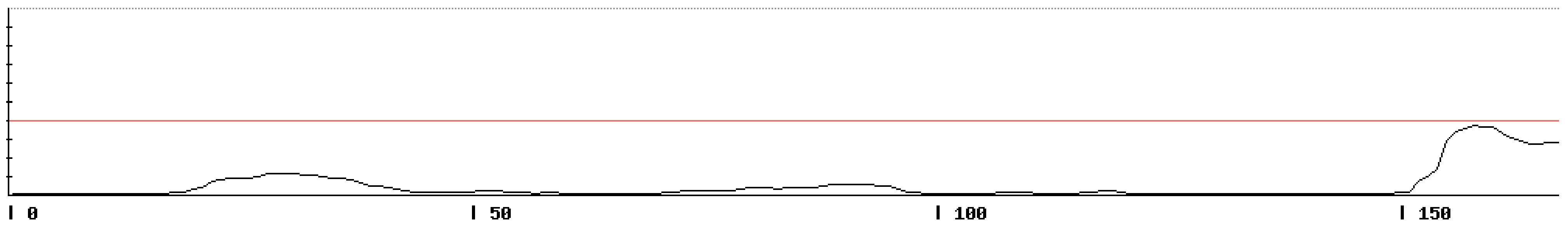

Predictions for Elaeisguineensis_XP_010904917.1

Viterbi Path	
Viterbi Path	
Posterior @ 0.4	
Posterior @ 0.4	
Posterior Graphic. Threshold Marked @ 0.4	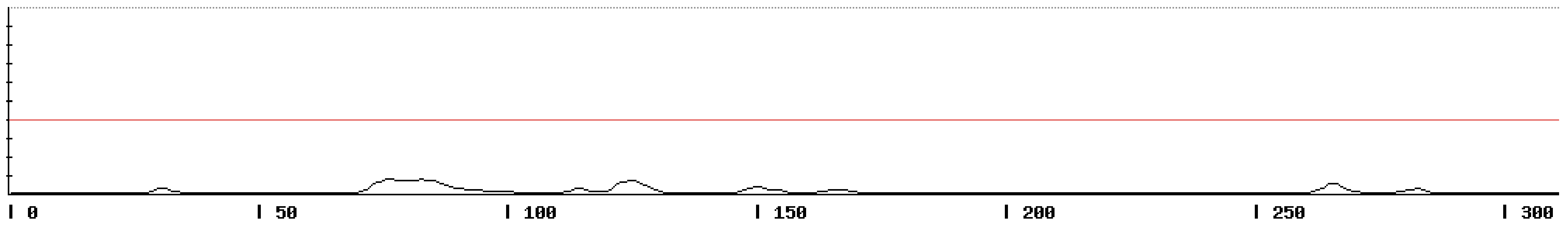

Predictions for Elaeisguineensis_XP_010905801.1	
Viterbi Path	
Viterbi Path	
Posterior @ 0.4	
Posterior @ 0.4	
Posterior Graphic. Threshold Marked @ 0.4	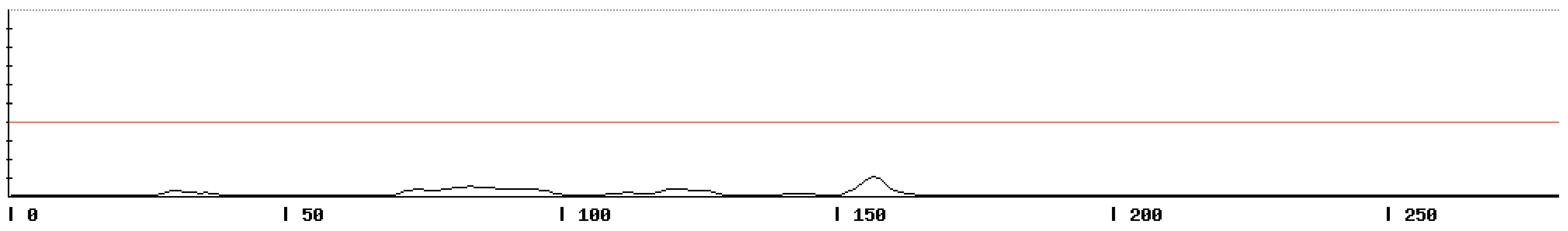

Predictions for Elaeisguineensis_XP_010906053.1	
Viterbi Path	
Viterbi Path	
Posterior @ 0.4	
Posterior @ 0.4	
Posterior Graphic. Threshold Marked @ 0.4	

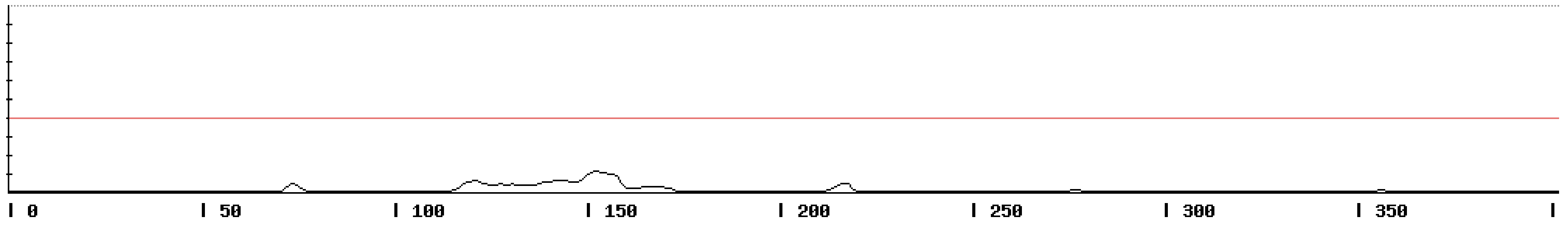

Predictions for Elaeisguineensis_XP_010906173.1

Viterbi Path	
Viterbi Path	
Posterior @ 0.4	49 - RRAGAGRWKARQR - 61<br>151 - KVAPEPAAKRRRMKE - 165
Posterior @ 0.4	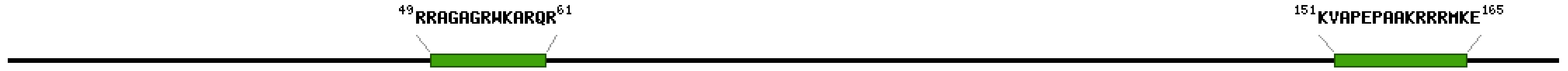
Posterior Graphic. Threshold Marked @ 0.4	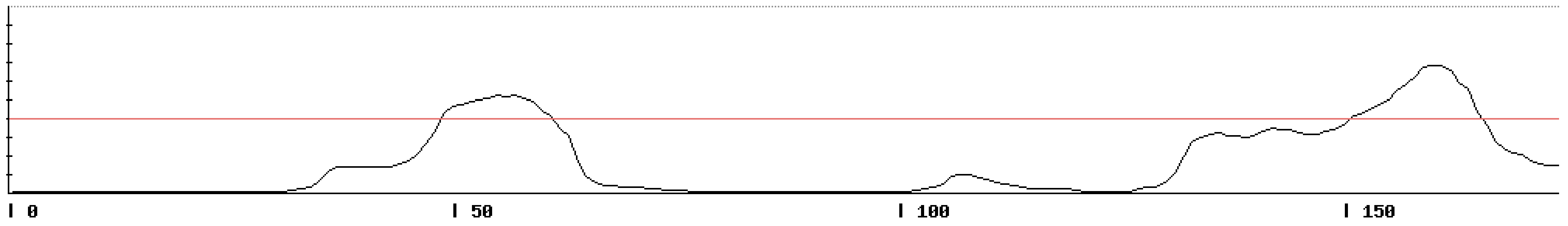

Predictions for Elaeisguineensis_XP_010906624.1

Viterbi Path	
Viterbi Path	
Posterior @ 0.4	
Posterior @ 0.4	

Posterior Graphic. Threshold Marked @ 0.4

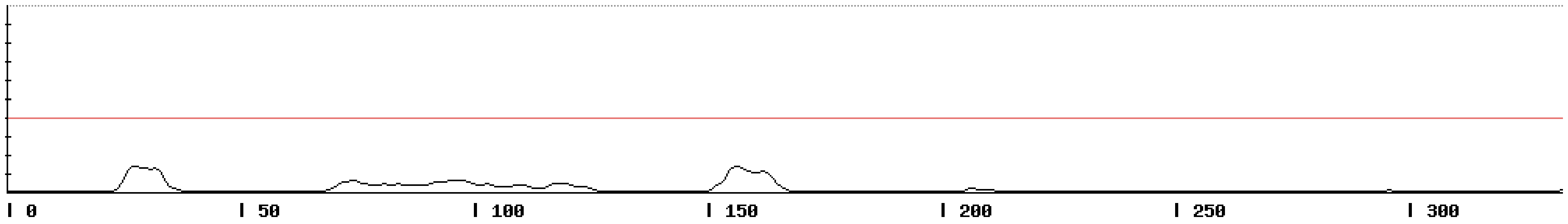

Predictions for Elaeisguineensis_XP_010906724.1

Viterbi Path

Viterbi Path

Posterior @ 0.4

Posterior @ 0.4

Posterior Graphic. Threshold Marked @ 0.4

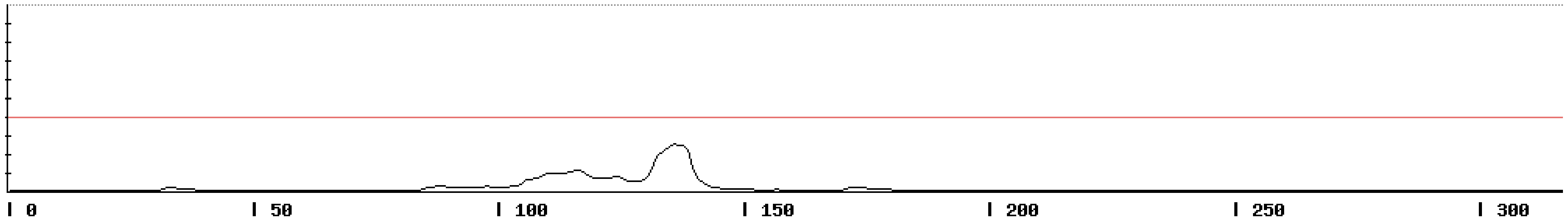

Predictions for Elaeisguineensis_XP_010906799.1

Viterbi Path

Viterbi Path

Posterior @ 0.4

Posterior @ 0.4

Posterior Graphic. Threshold Marked @ 0.4

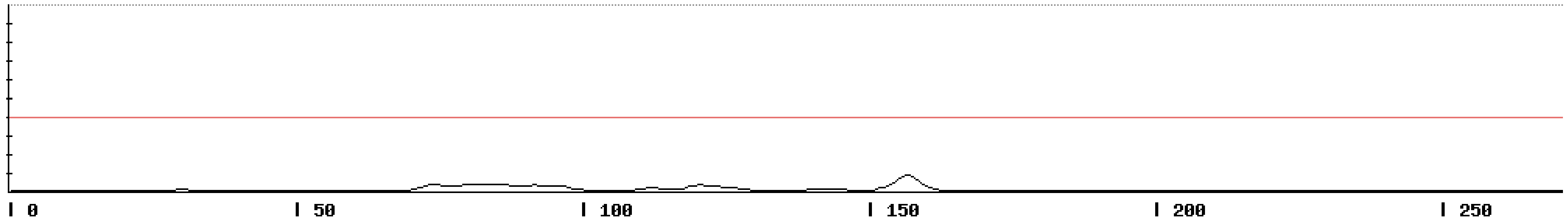

Predictions for Elaeisguineensis_XP_010907545.1

Viterbi Path

Viterbi Path

Posterior @ 0.4

Posterior @ 0.4

Posterior Graphic. Threshold Marked @ 0.4

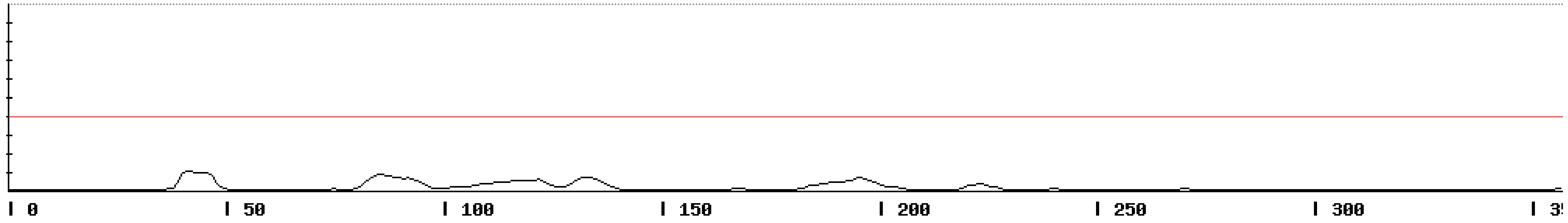

Predictions for Elaeisguineensis_XP_010907664.1

Viterbi Path

Viterbi Path

Posterior @ 0.4

Posterior @ 0.4

Posterior Graphic. Threshold Marked @ 0.4

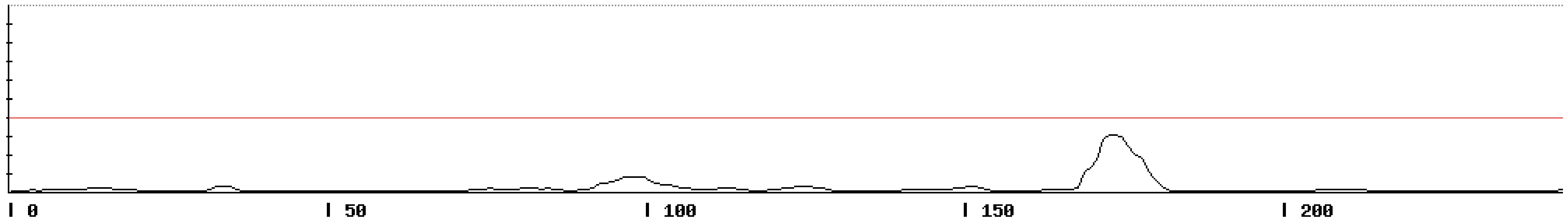

Predictions for Elaeisguineensis_XP_010907895.1

Viterbi Path

Viterbi Path

Posterior @ 0.4

Posterior @ 0.4

Posterior Graphic. Threshold Marked @ 0.4

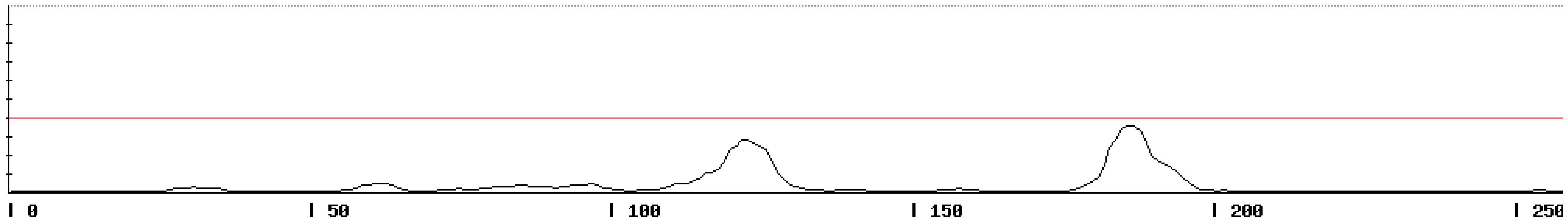

Predictions for Elaeisguineensis_XP_010908127.1

Viterbi Path

Viterbi Path

Posterior @ 0.4

Posterior @ 0.4

Posterior Graphic. Threshold Marked @ 0.4

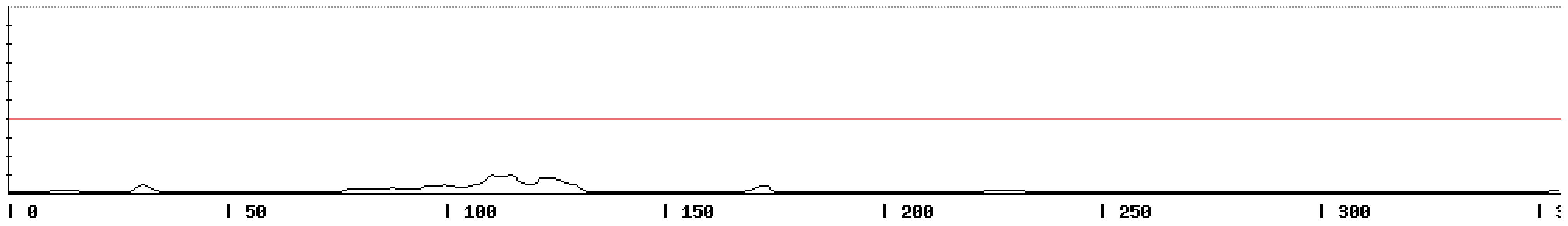

Predictions for Elaeisguineensis_XP_010908290.1

Viterbi Path

Viterbi Path

Posterior @ 0.4

Posterior @ 0.4

Posterior Graphic. Threshold Marked @ 0.4

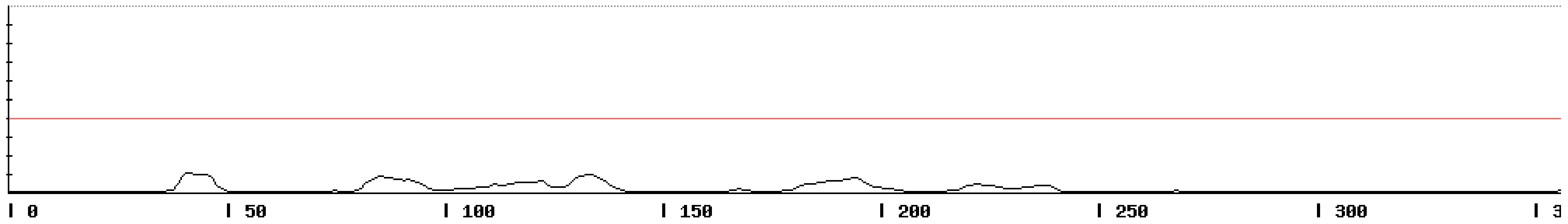

Predictions for Elaeisguineensis_XP_010908622.1	
Viterbi Path	
Viterbi Path	
Posterior @ 0.4	
Posterior @ 0.4	
Posterior Graphic. Threshold Marked @ 0.4	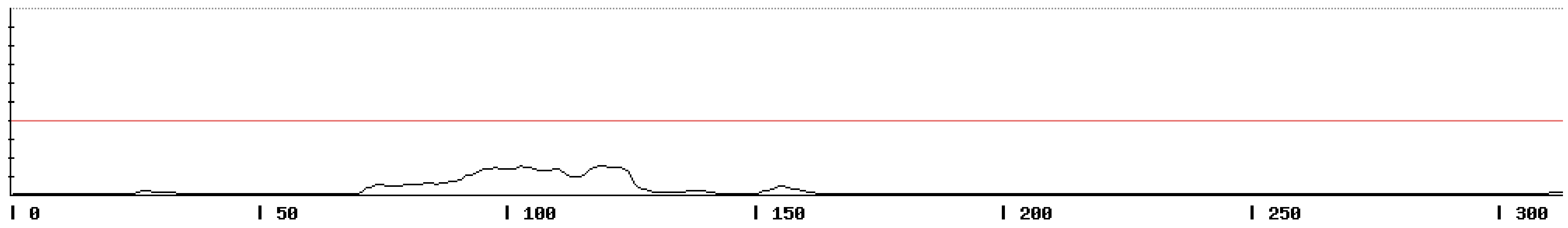

Predictions for Elaeisguineensis_XP_010908787.1	
Viterbi Path	
Viterbi Path	
Posterior @ 0.4	
Posterior @ 0.4	
Posterior Graphic. Threshold Marked @ 0.4	

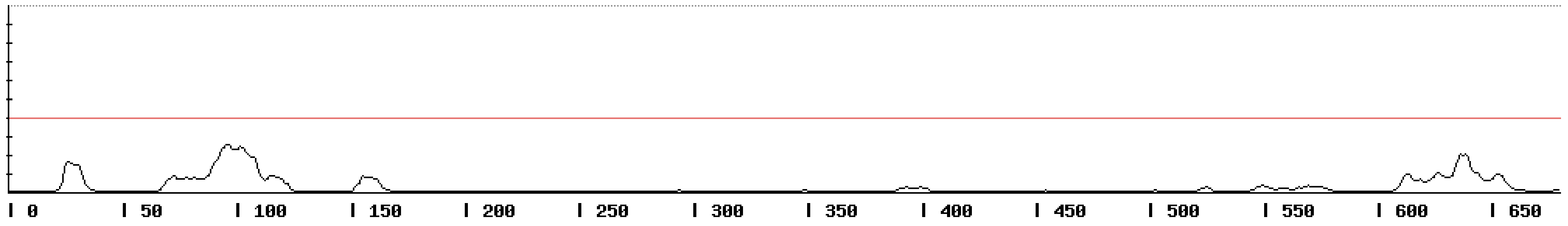

Predictions for Elaeisguineensis_XP_010908788.1

Viterbi Path

Viterbi Path

Posterior @ 0.4

Posterior @ 0.4

Posterior Graphic. Threshold Marked @ 0.4

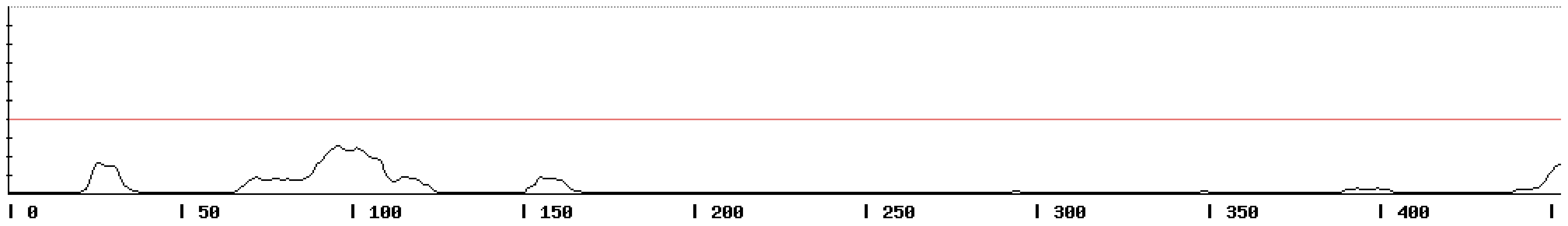

Predictions for Elaeisguineensis_XP_010909195.1

Viterbi Path

Viterbi Path

Posterior @ 0.4

Posterior @ 0.4

Posterior Graphic. Threshold Marked @ 0.4

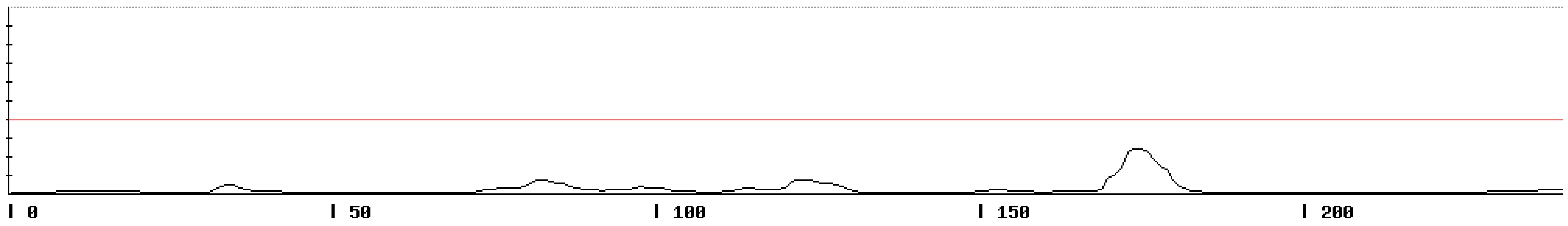

Predictions for Elaeisguineensis_XP_010909711.1

Viterbi Path

Viterbi Path

Posterior @ 0.4

Posterior @ 0.4

Posterior Graphic. Threshold Marked @ 0.4

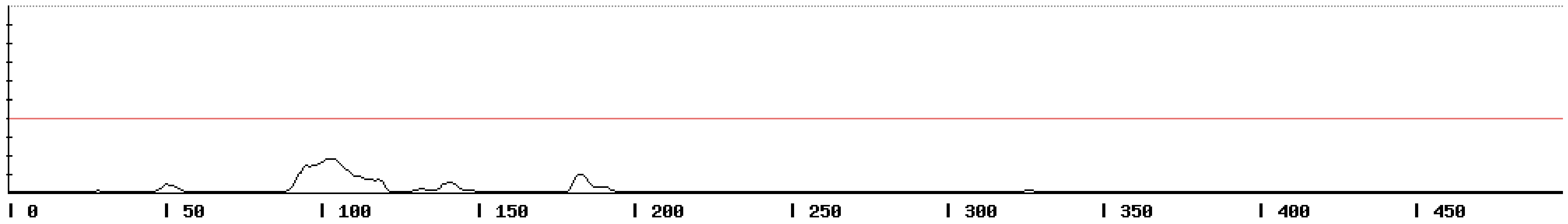

Predictions for Elaeisguineensis_XP_010909712.1

Viterbi Path

Viterbi Path

Posterior @ 0.4

Posterior @ 0.4

Posterior Graphic. Threshold Marked @ 0.4

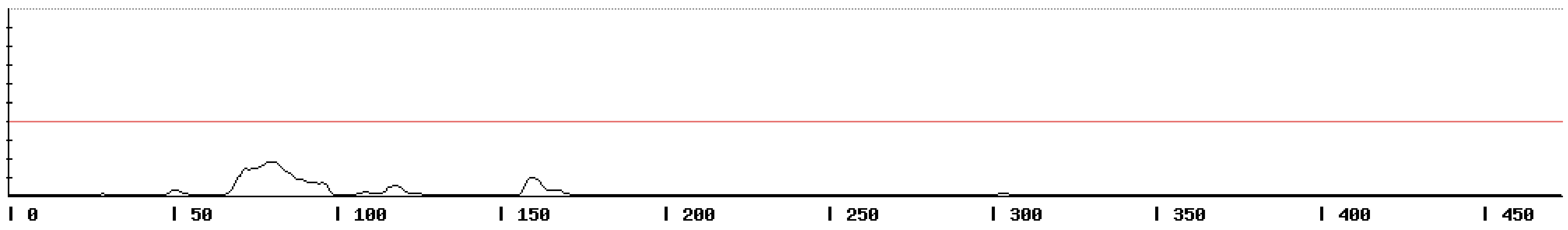

Predictions for Elaeisguineensis_XP_010909714.1

Viterbi Path

Viterbi Path

Posterior @ 0.4

Posterior @ 0.4

Posterior Graphic. Threshold Marked @ 0.4

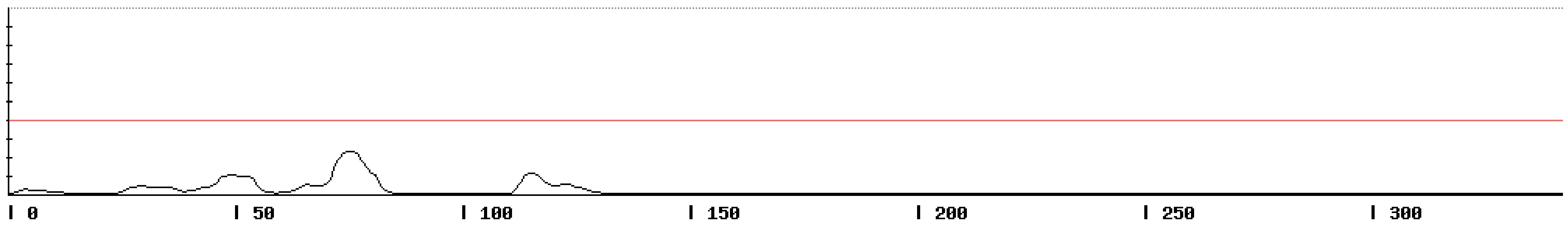

Predictions for Elaeisguineensis_XP_010909878.1

Viterbi Path

Viterbi Path

Posterior @ 0.4

Posterior @ 0.4

Posterior Graphic. Threshold Marked @ 0.4

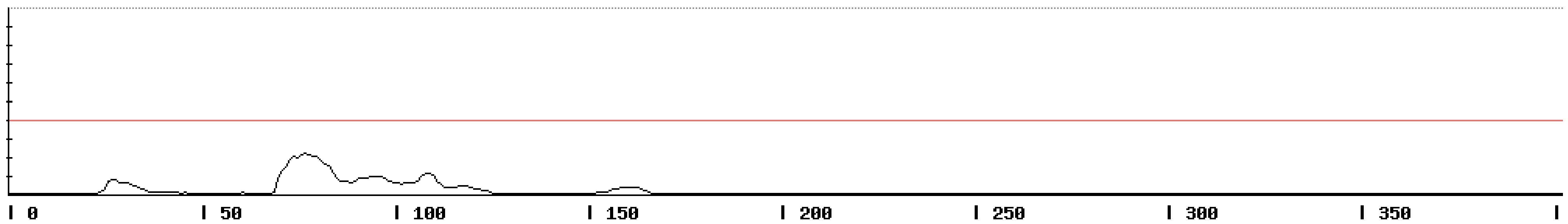

Predictions for Elaeisguineensis_XP_010909880.1

Viterbi Path

Viterbi Path

Posterior @ 0.4

Posterior @ 0.4

Posterior Graphic. Threshold Marked @ 0.4

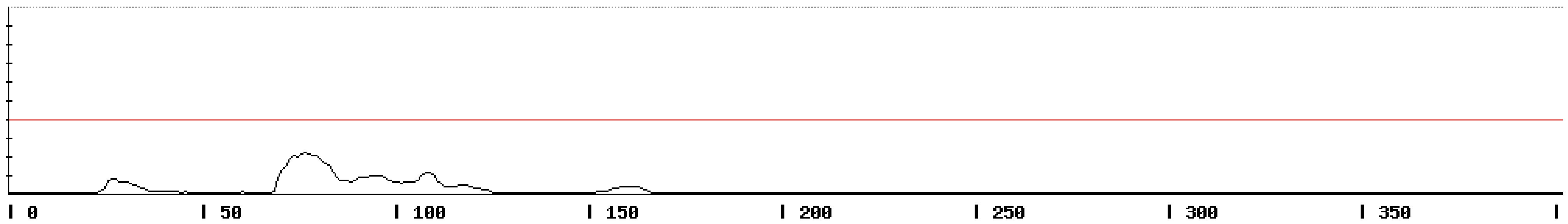

Predictions for Elaeisguineensis_XP_010909881.1

Viterbi Path

Viterbi Path

Posterior @ 0.4

Posterior @ 0.4

Posterior Graphic. Threshold Marked @ 0.4

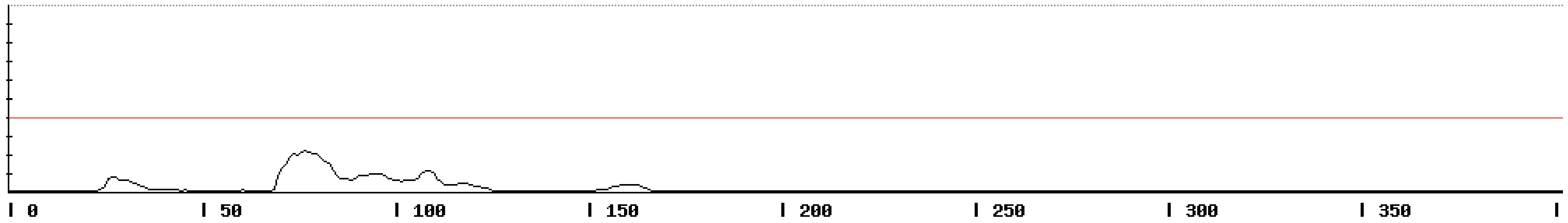

Predictions for Elaeisguineensis_XP_010910284.1

Viterbi Path

Viterbi Path

Posterior @ 0.4

111 - GKRKSGKKEPERTK - 124

Posterior @ 0.4

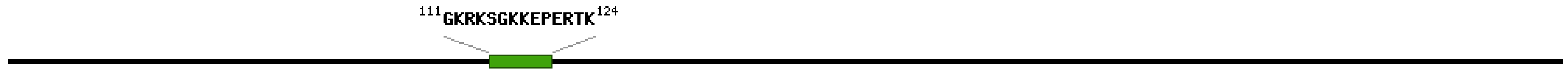

Posterior Graphic. Threshold Marked @ 0.4

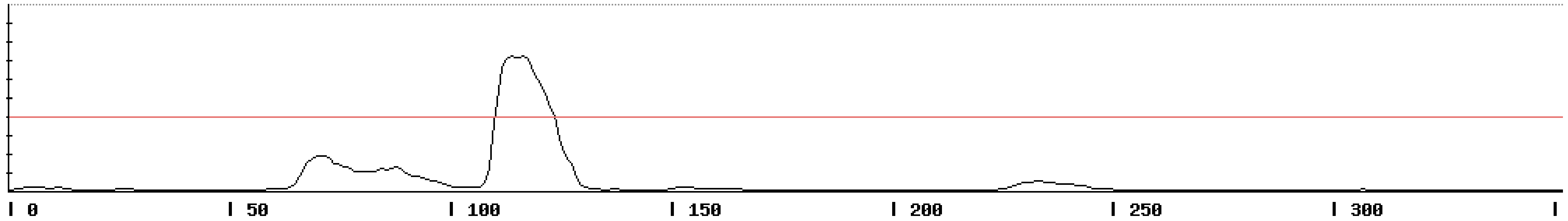

Predictions for Elaeisguineensis_XP_010910356.1

Viterbi Path

Viterbi Path

Posterior @ 0.4

Posterior @ 0.4

Posterior Graphic. Threshold Marked @ 0.4

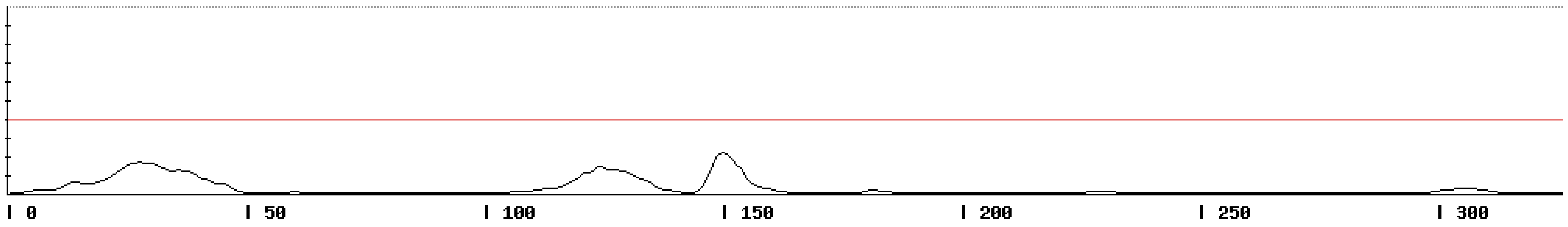

Predictions for Elaeisguineensis_XP_010910361.1

Viterbi Path

Viterbi Path

Posterior @ 0.4

Posterior @ 0.4

Posterior Graphic. Threshold Marked @ 0.4

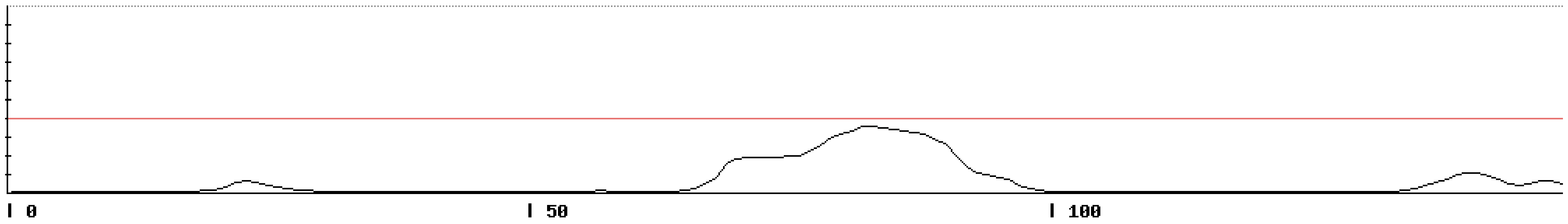

Predictions for Elaeisguineensis_XP_010910555.1

Viterbi Path	
Viterbi Path	
Posterior @ 0.4	
Posterior @ 0.4	
Posterior Graphic. Threshold Marked @ 0.4	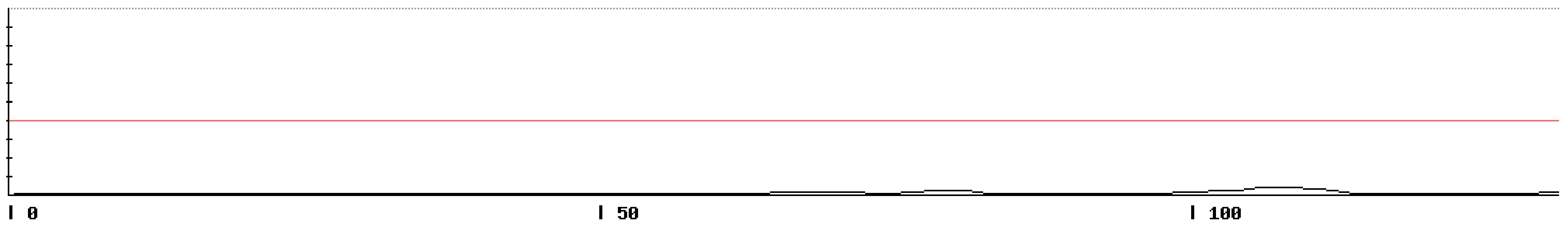

Predictions for Elaeisguineensis_XP_010910754.1

Viterbi Path	
Viterbi Path	
Posterior @ 0.4	
Posterior @ 0.4	
Posterior Graphic. Threshold Marked @ 0.4	

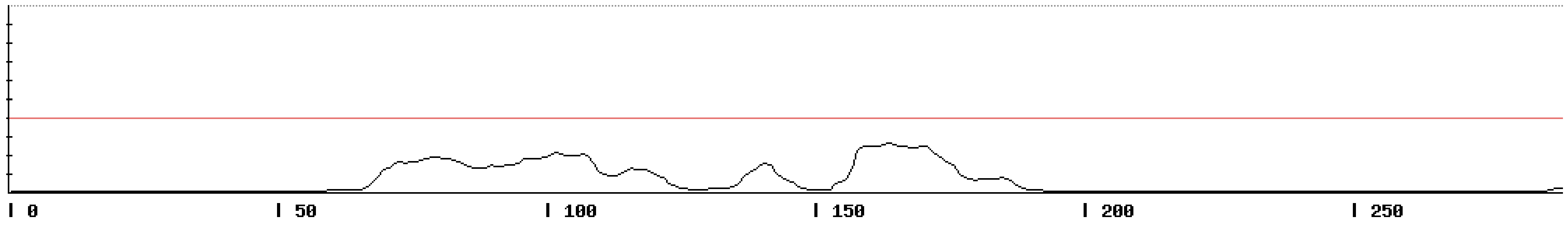

Predictions for Elaeisguineensis_XP_010911124.1	
Viterbi Path	
Viterbi Path	
Posterior @ 0.4	167 - GSDRKAAGWRMAGRPEP - 183
Posterior @ 0.4	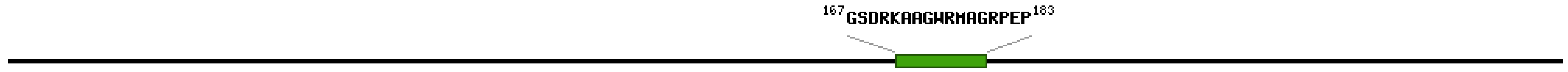
Posterior Graphic. Threshold Marked @ 0.4	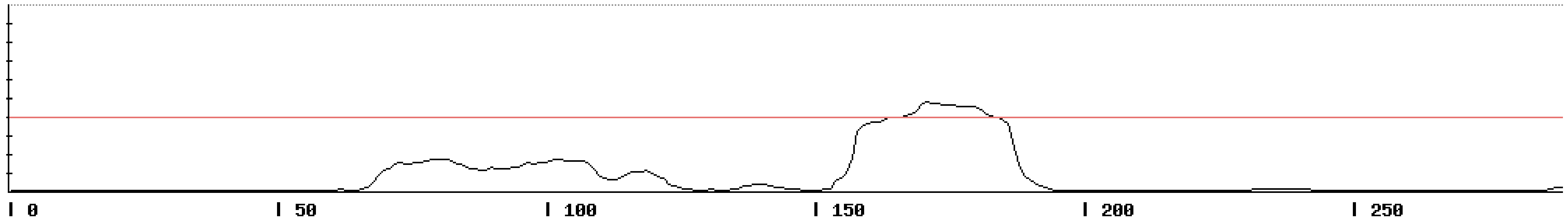

Predictions for Elaeisguineensis_XP_010911249.1	
Viterbi Path	
Viterbi Path	
Posterior @ 0.4	72 - ERFRNKKHRDRKVKNGGR - 89
Posterior @ 0.4	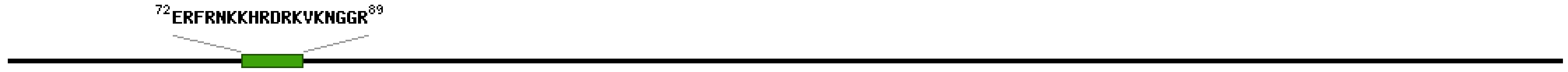

Posterior Graphic. Threshold Marked @ 0.4

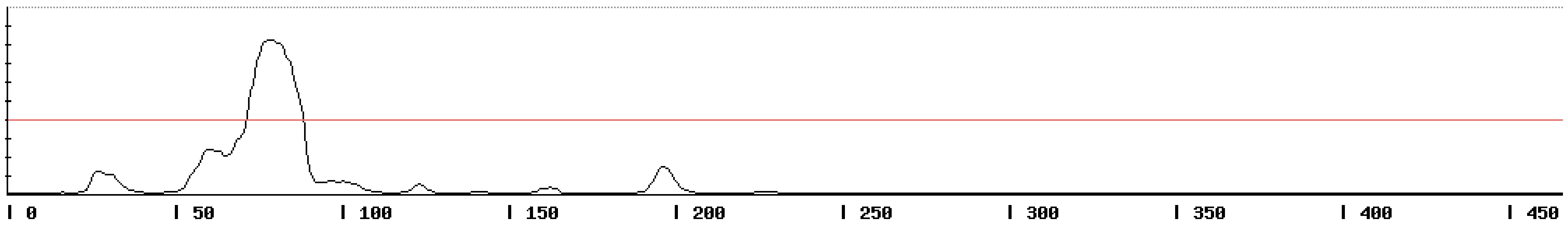

Predictions for Elaeisguineensis_XP_010911448.1

Viterbi Path

Viterbi Path

Posterior @ 0.4: 82 - RRAGAG - 87

Posterior @ 0.4

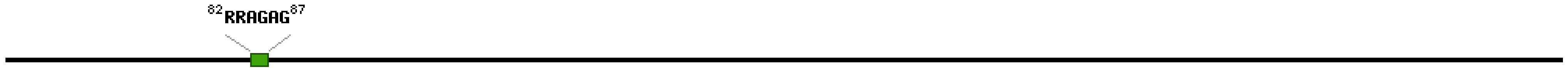

Posterior Graphic. Threshold Marked @ 0.4

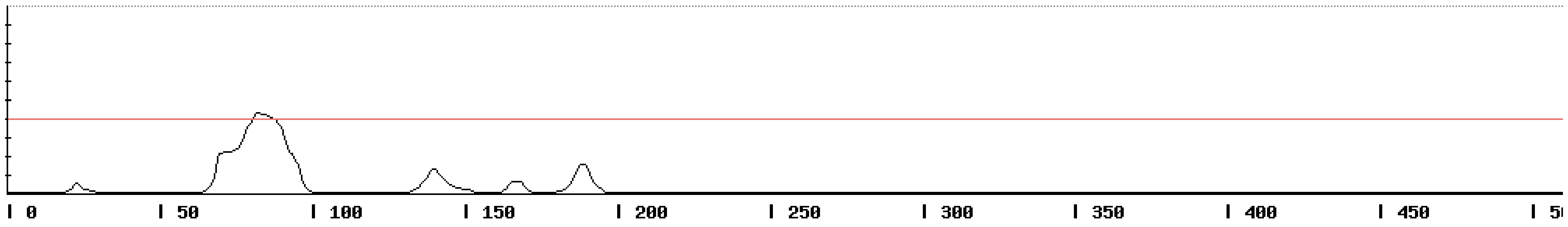

Predictions for Elaeisguineensis_XP_010911499.1

Viterbi Path

Viterbi Path

Posterior @ 0.4

Posterior @ 0.4

Posterior Graphic. Threshold Marked @ 0.4	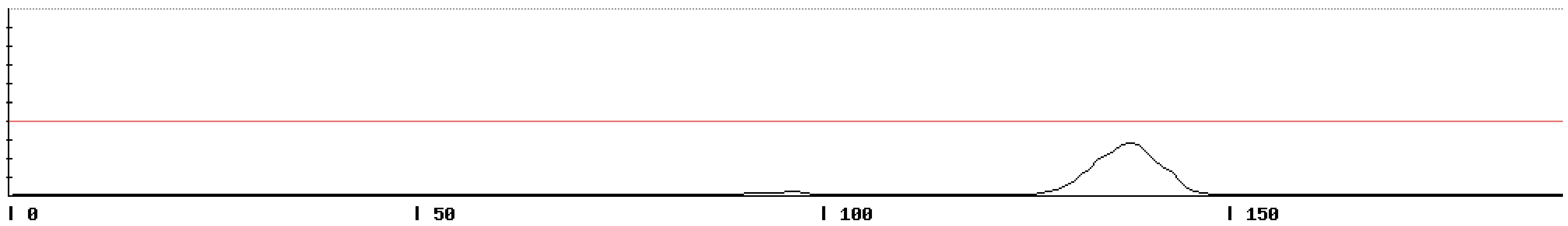

Predictions for Elaeisguineensis_XP_010911657.1

Viterbi Path	
Viterbi Path	
Posterior @ 0.4	
Posterior @ 0.4	
Posterior Graphic. Threshold Marked @ 0.4	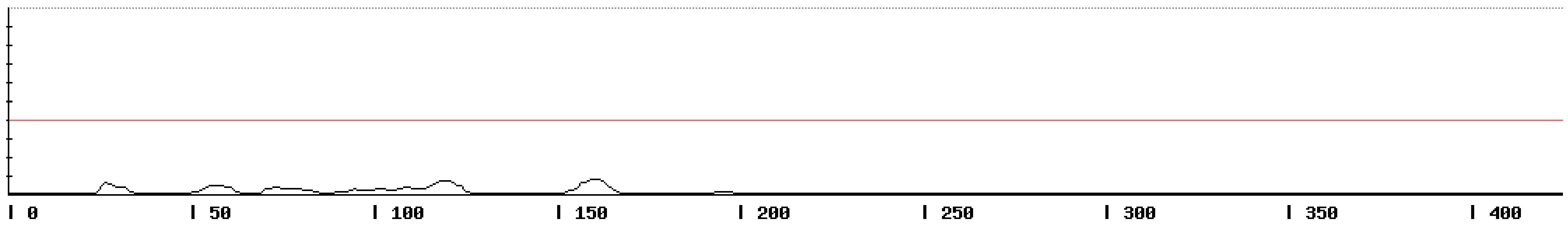

Predictions for Elaeisguineensis_XP_010911663.1

Viterbi Path	
Viterbi Path	

Posterior @ 0.4

Posterior @ 0.4

Posterior Graphic. Threshold Marked @ 0.4

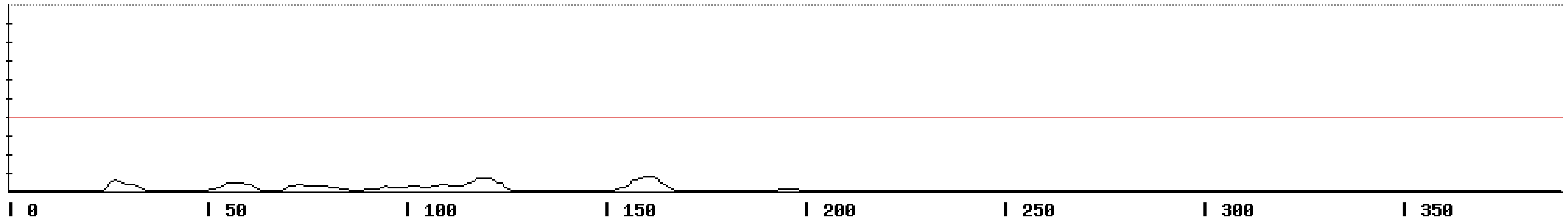

Predictions for Elaeisguineensis_XP_010911676.1

Viterbi Path

Viterbi Path

Posterior @ 0.4

Posterior @ 0.4

Posterior Graphic. Threshold Marked @ 0.4

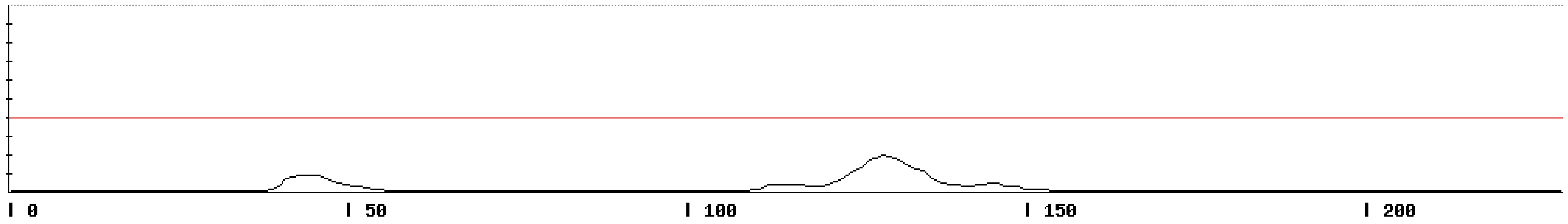

Predictions for Elaeisguineensis_XP_010911718.1

Viterbi Path

Viterbi Path

Posterior @ 0.4	80 - ASRRAGAGR - 88
Posterior @ 0.4	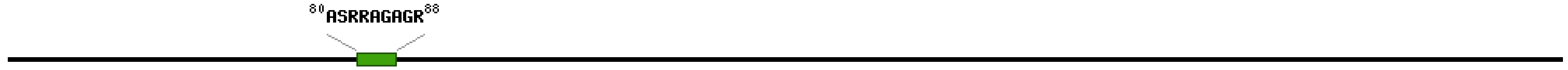
Posterior Graphic. Threshold Marked @ 0.4	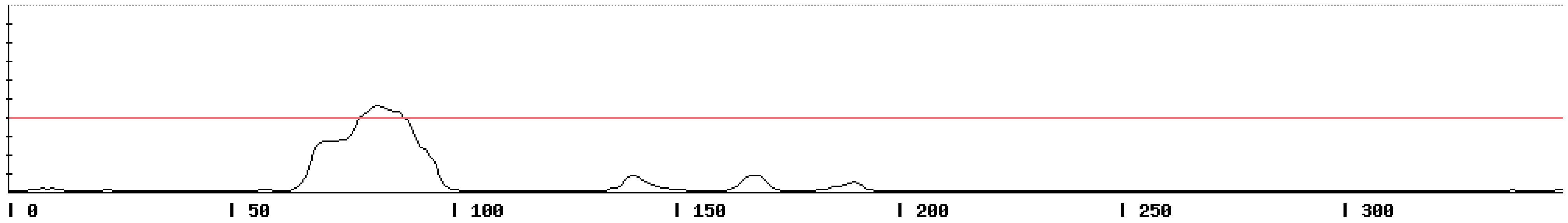

Predictions for Elaeisguineensis_XP_010911913.1

Viterbi Path	
Viterbi Path	
Posterior @ 0.4	
Posterior @ 0.4	
Posterior Graphic. Threshold Marked @ 0.4	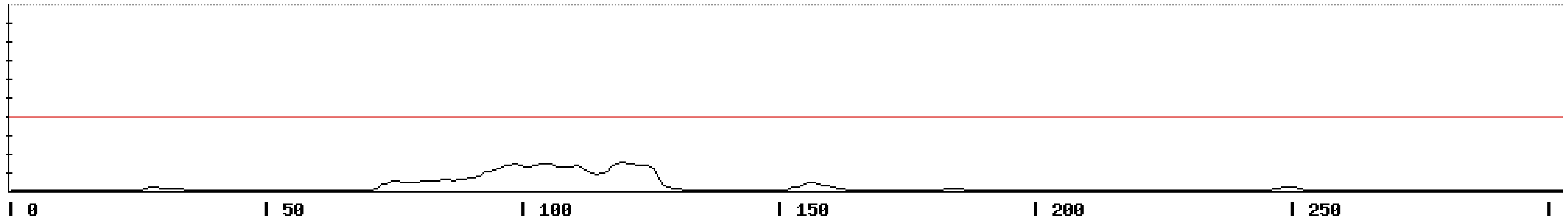

Predictions for Elaeisguineensis_XP_010912317.1

Viterbi Path

Viterbi Path

Posterior @ 0.4

Posterior @ 0.4

Posterior Graphic. Threshold Marked @ 0.4

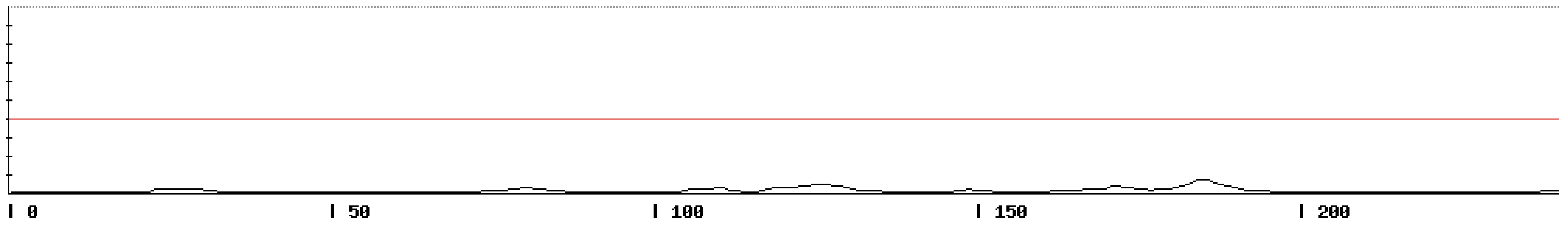

Predictions for Elaeisguineensis_XP_010912541.1

Viterbi Path

Viterbi Path

Posterior @ 0.4

Posterior @ 0.4

Posterior Graphic. Threshold Marked @ 0.4

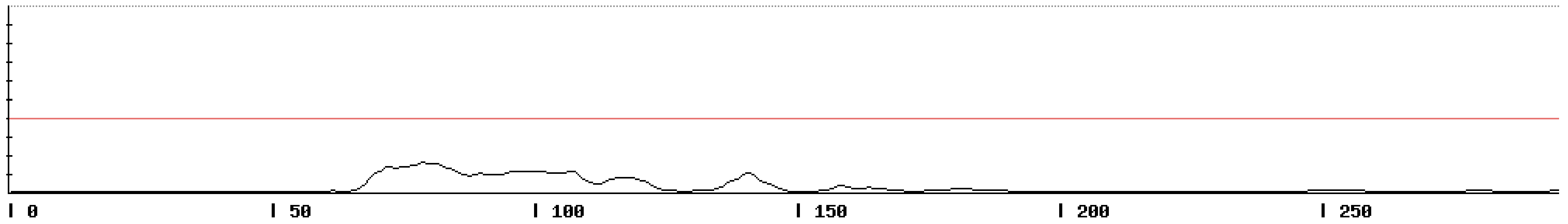

Predictions for Elaeisguineensis_XP_010912542.1	
Viterbi Path	
Viterbi Path	
Posterior @ 0.4	
Posterior @ 0.4	
Posterior Graphic. Threshold Marked @ 0.4	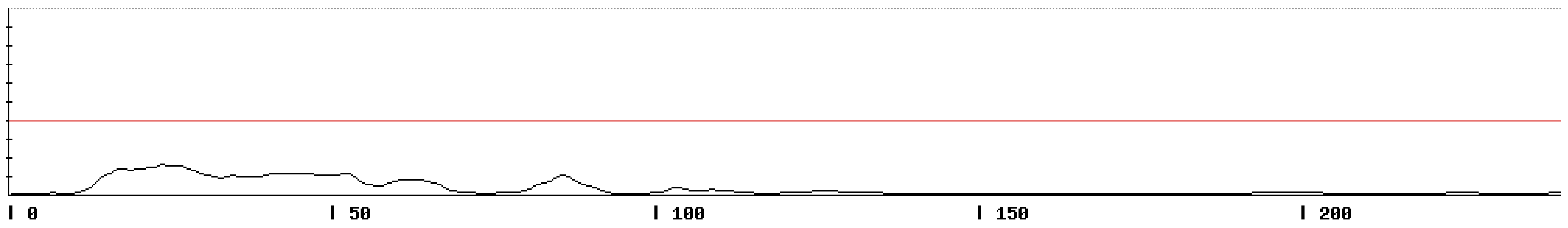

Predictions for Elaeisguineensis_XP_010912590.1	
Viterbi Path	
Viterbi Path	
Posterior @ 0.4	
Posterior @ 0.4	
Posterior Graphic. Threshold Marked @ 0.4	

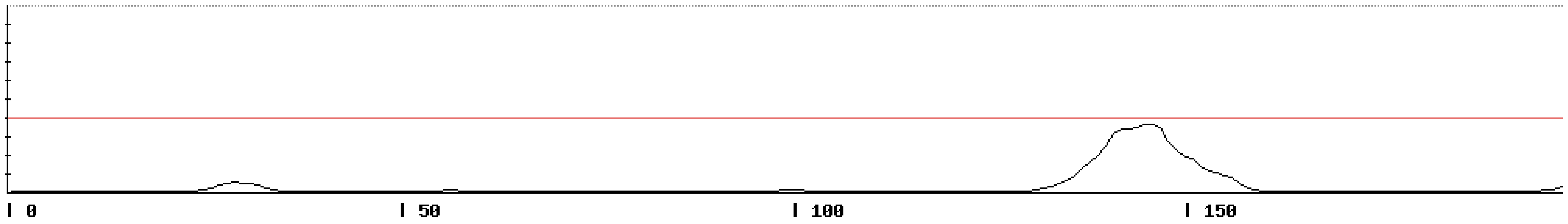

Predictions for Elaeisguineensis_XP_010912708.1

Viterbi Path

Viterbi Path

Posterior @ 0.4

Posterior @ 0.4

Posterior Graphic. Threshold Marked @ 0.4

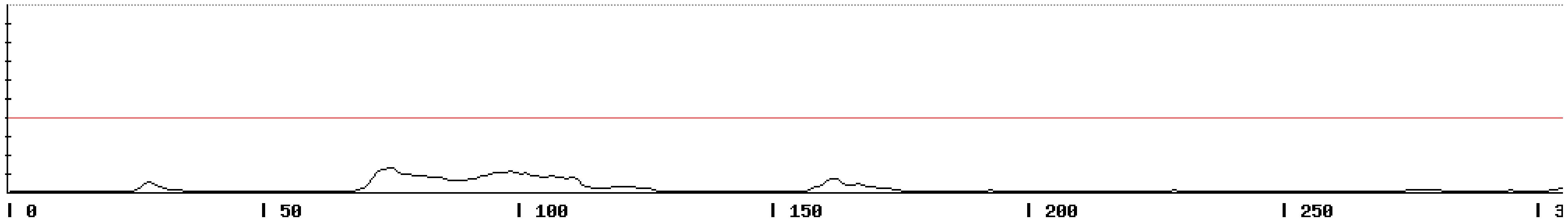

Predictions for Elaeisguineensis_XP_010912730.1

Viterbi Path

Viterbi Path

Posterior @ 0.4

Posterior @ 0.4

Posterior Graphic. Threshold Marked @ 0.4

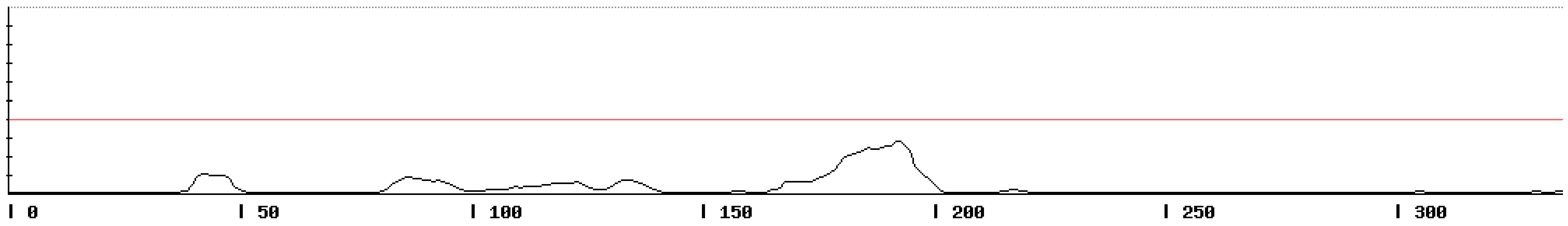

Predictions for Elaeisguineensis_XP_010912974.1

Viterbi Path

Viterbi Path

Posterior @ 0.4

134 - HRKRRKIH - 141

148 - EERVRWHKTGKTKPVRENGVHKGWKKIM - 175

181 - SKG - 183

Posterior @ 0.4

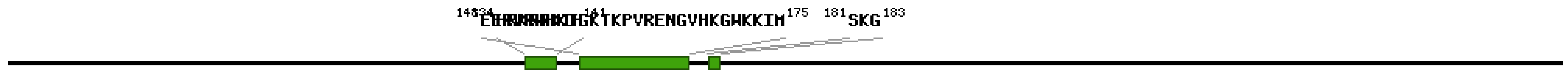

Posterior Graphic. Threshold Marked @ 0.4

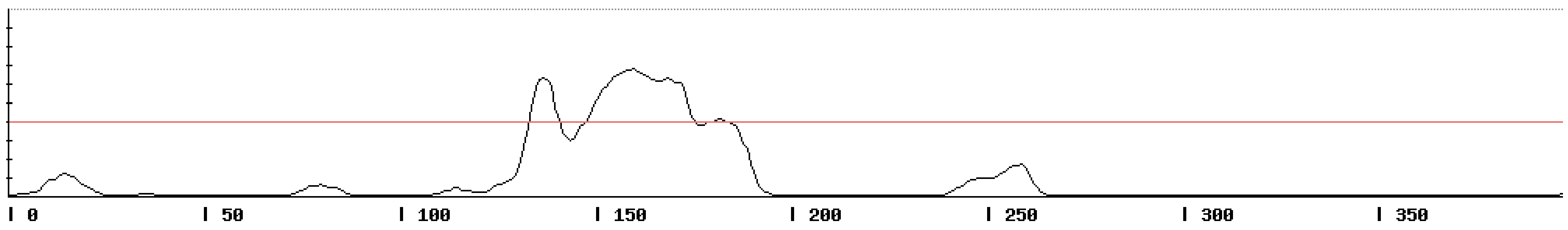

Predictions for Elaeisguineensis_XP_010913848.1

Viterbi Path

Viterbi Path

Posterior @ 0.4

Posterior @ 0.4

Posterior Graphic. Threshold Marked @ 0.4

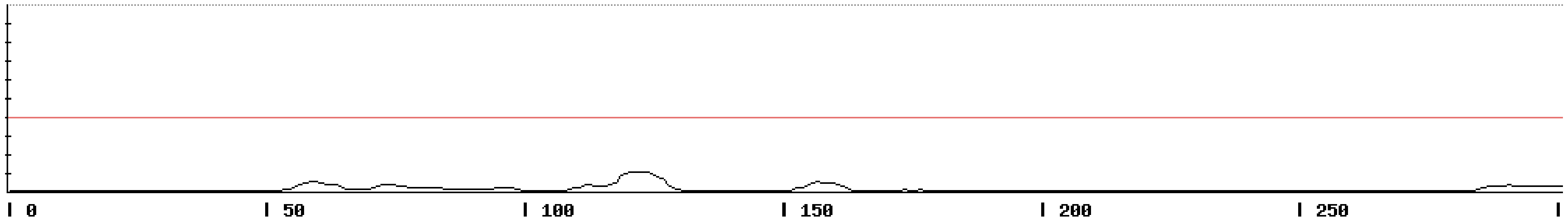

Predictions for Elaeisguineensis_XP_010915633.1

Viterbi Path

Viterbi Path

Posterior @ 0.4

Posterior @ 0.4

Posterior Graphic. Threshold Marked @ 0.4

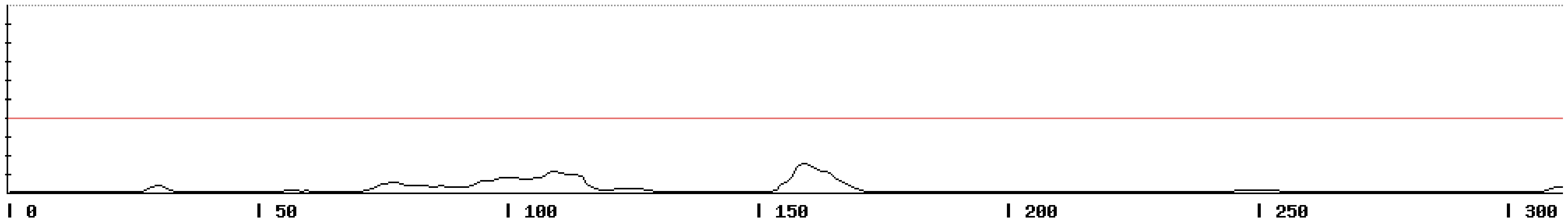

Predictions for Elaeisguineensis_XP_010916003.1

Viterbi Path

Viterbi Path

Posterior @ 0.4

Posterior @ 0.4

Posterior Graphic. Threshold Marked @ 0.4

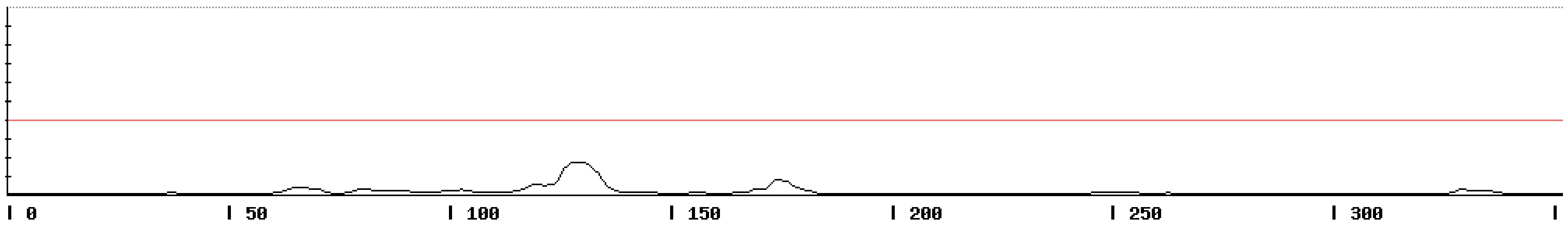

Predictions for Elaeisguineensis_XP_010916182.1

Viterbi Path

Viterbi Path

Posterior @ 0.4

197 - KKGKKP - 202

1005 - RRRRKK - 1010

Posterior @ 0.4

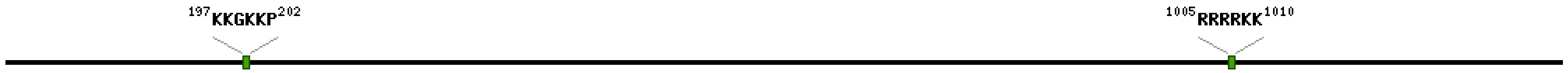

Posterior Graphic. Threshold Marked @ 0.4

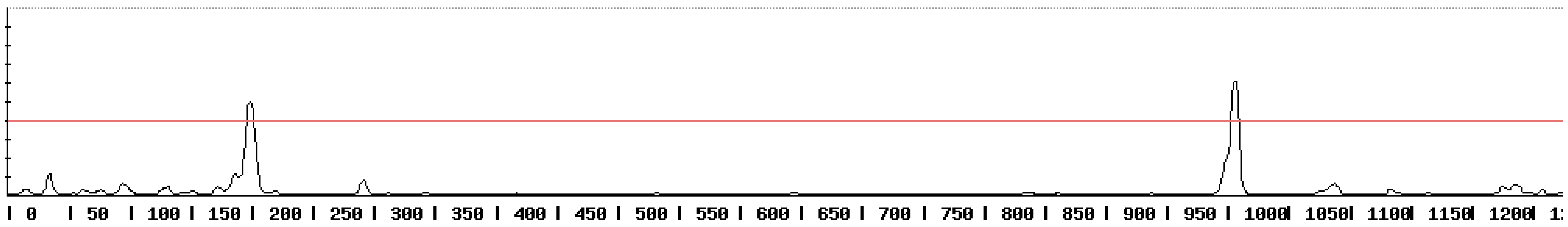

Predictions for Elaeisguineensis_XP_010916183.1

Viterbi Path

Viterbi Path

Posterior @ 0.4	196 - KKGKKP - 201
	1004 - RRRRKK - 1009
Posterior @ 0.4	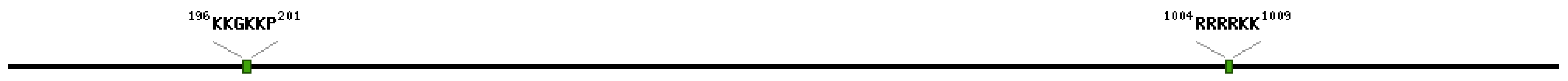
Posterior Graphic. Threshold Marked @ 0.4	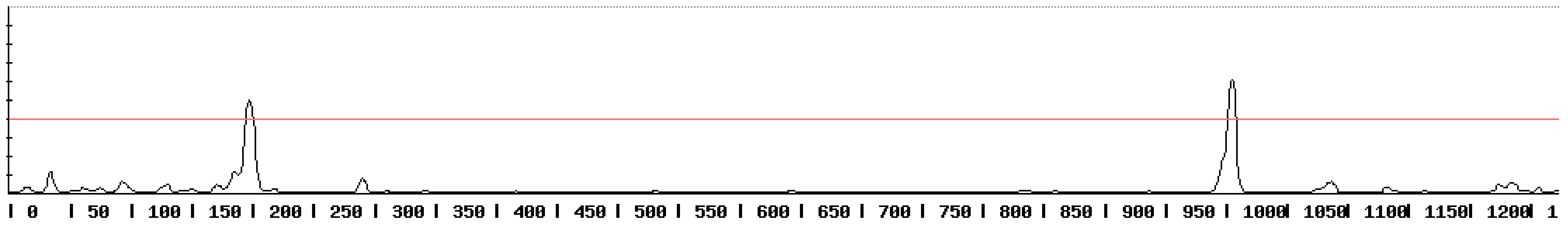

Predictions for Elaeisguineensis_XP_010916375.1

Viterbi Path	
Viterbi Path	
Posterior @ 0.4	
Posterior @ 0.4	
Posterior Graphic. Threshold Marked @ 0.4	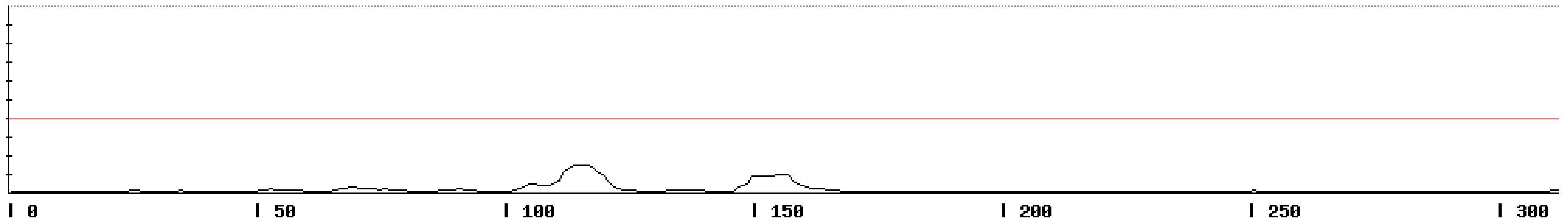

Predictions for Elaeisguineensis_XP_010917044.1	
Viterbi Path	
Viterbi Path	
Posterior @ 0.4	
Posterior @ 0.4	
Posterior Graphic. Threshold Marked @ 0.4	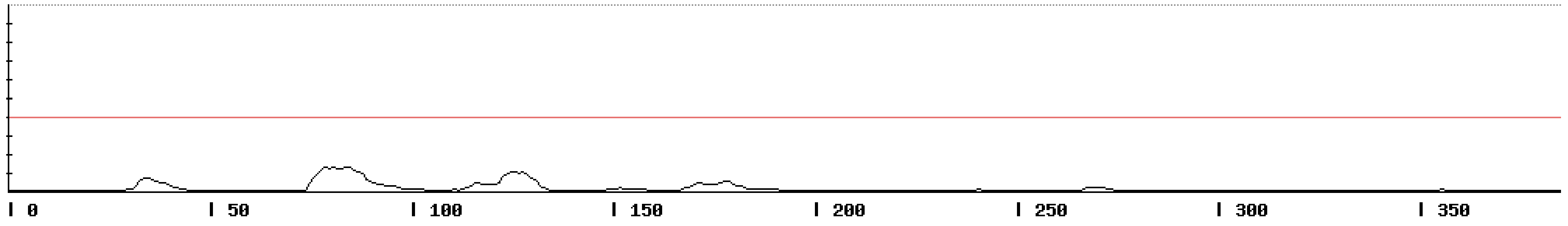

Predictions for Elaeisguineensis_XP_010917079.1	
Viterbi Path	
Viterbi Path	
Posterior @ 0.4	
Posterior @ 0.4	
Posterior Graphic. Threshold Marked @ 0.4	

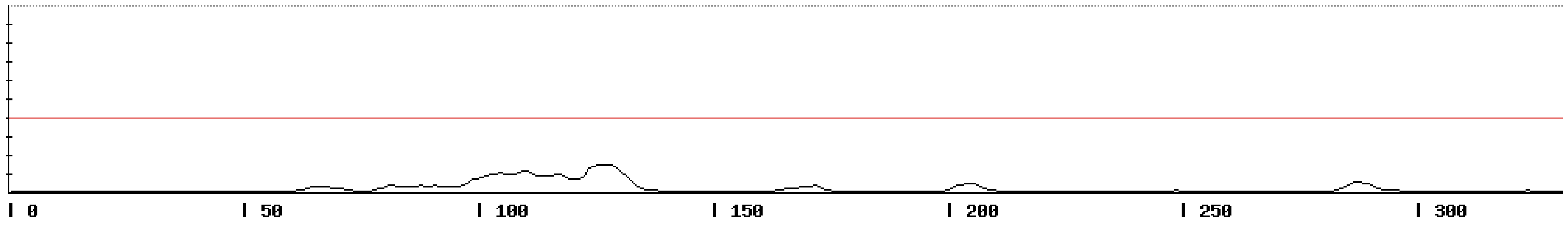

Predictions for Elaeisguineensis_XP_010917080.1

Viterbi Path

Viterbi Path

Posterior @ 0.4

Posterior @ 0.4

Posterior Graphic. Threshold Marked @ 0.4

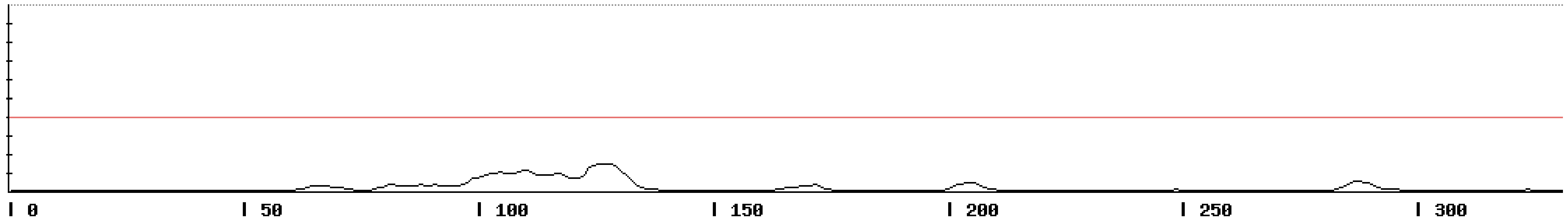

Predictions for Elaeisguineensis_XP_010917082.1

Viterbi Path

Viterbi Path

Posterior @ 0.4

Posterior @ 0.4

Posterior Graphic. Threshold Marked @ 0.4

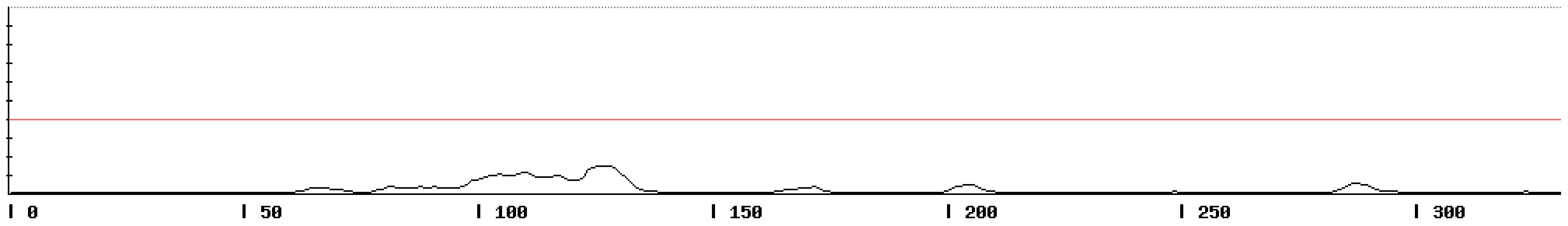

Predictions for Elaeisguineensis_XP_010917083.1

Viterbi Path

Viterbi Path

Posterior @ 0.4

Posterior @ 0.4

Posterior Graphic. Threshold Marked @ 0.4

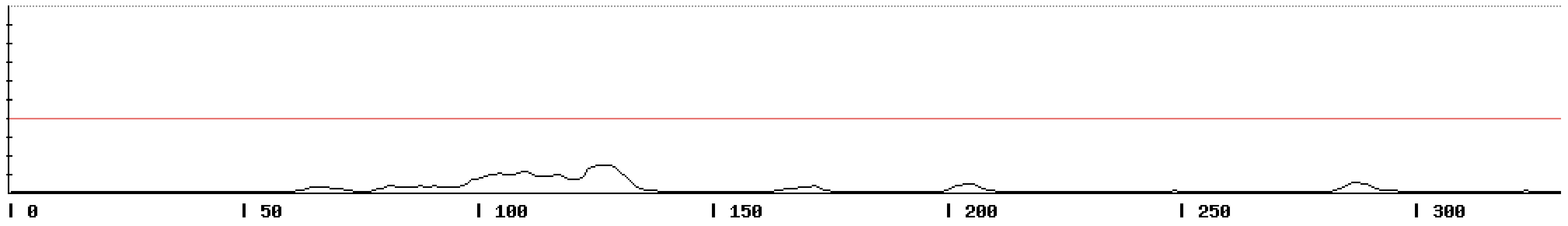

Predictions for Elaeisguineensis_XP_010917234.1

Viterbi Path

Viterbi Path

Posterior @ 0.4

Posterior @ 0.4

Posterior Graphic. Threshold Marked @ 0.4

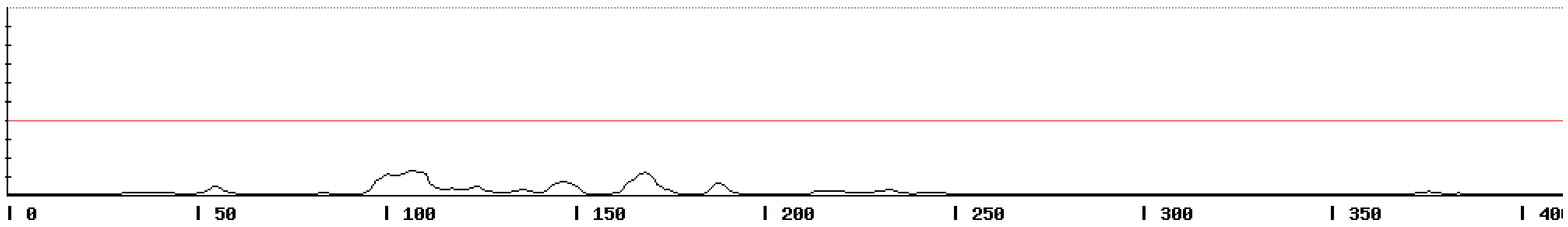

Predictions for Elaeisguineensis_XP_010918221.1

Viterbi Path

Viterbi Path

Posterior @ 0.4

Posterior @ 0.4

Posterior Graphic. Threshold Marked @ 0.4

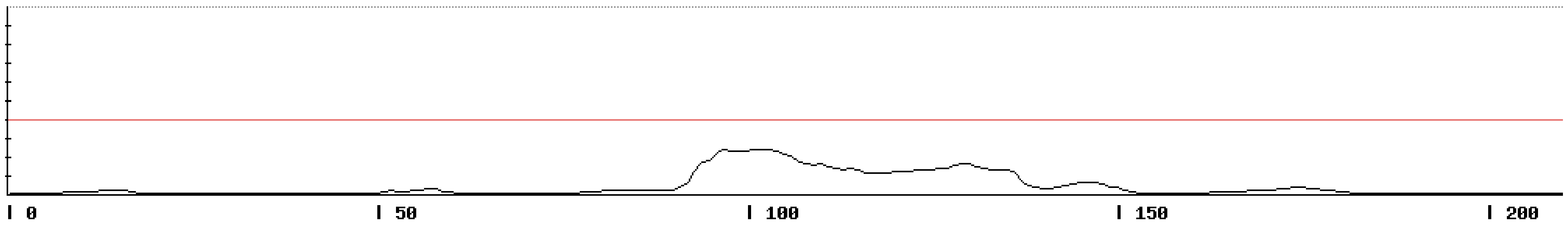

Predictions for Elaeisguineensis_XP_010919011.1

Viterbi Path

Viterbi Path

Posterior @ 0.4

Posterior @ 0.4

Posterior Graphic. Threshold Marked @ 0.4

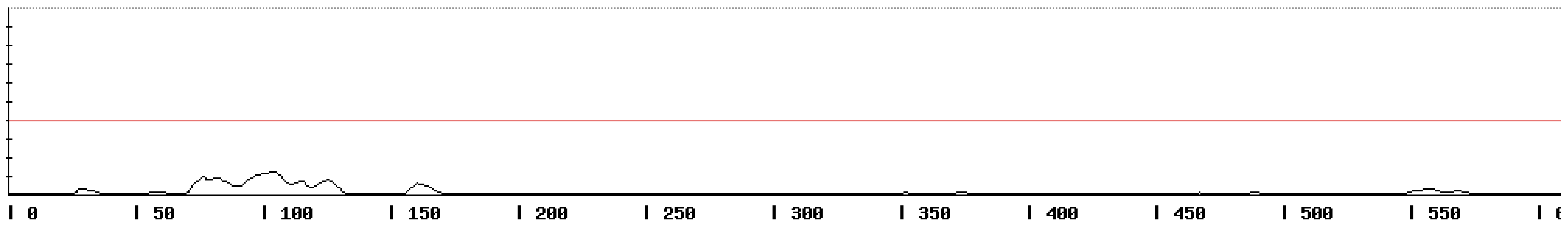

Predictions for Elaeisguineensis_XP_010919012.1

Viterbi Path

Viterbi Path

Posterior @ 0.4

Posterior @ 0.4

Posterior Graphic. Threshold Marked @ 0.4

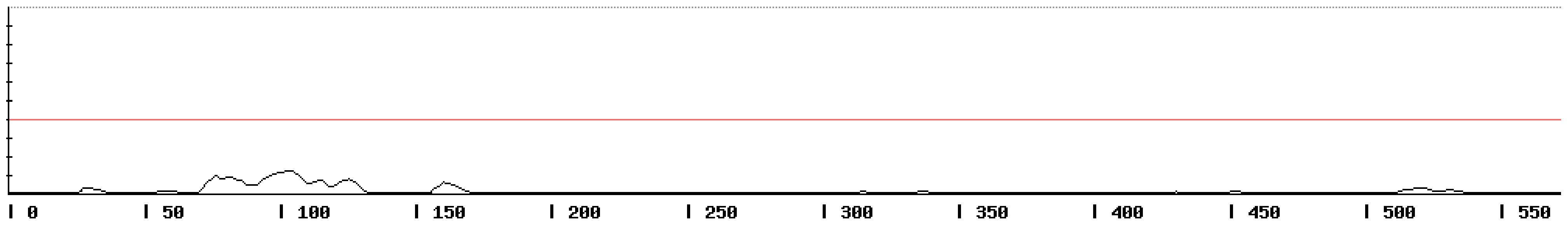

Predictions for Elaeisguineensis_XP_010919159.1

Viterbi Path

Viterbi Path

Posterior @ 0.4

Posterior @ 0.4

Posterior Graphic. Threshold Marked @ 0.4

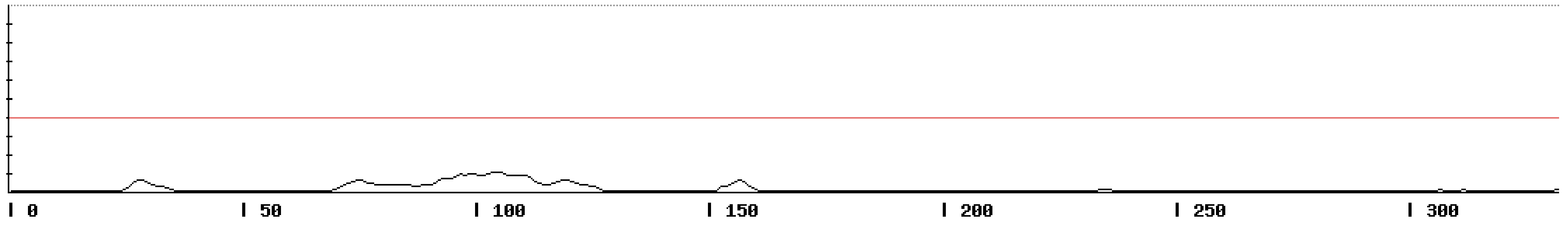

Predictions for Elaeisguineensis_XP_010919316.1

Viterbi Path

Viterbi Path

Posterior @ 0.4 145 - TRKRRKVHTDAHGGETRWHKTGKTRPVLAGGKLKGYK - 181

Posterior @ 0.4

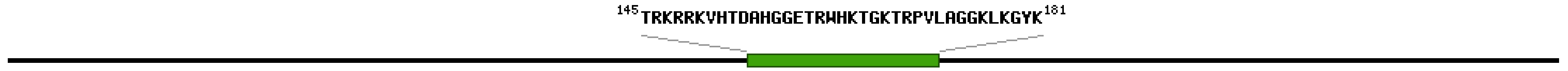

Posterior Graphic. Threshold Marked @ 0.4

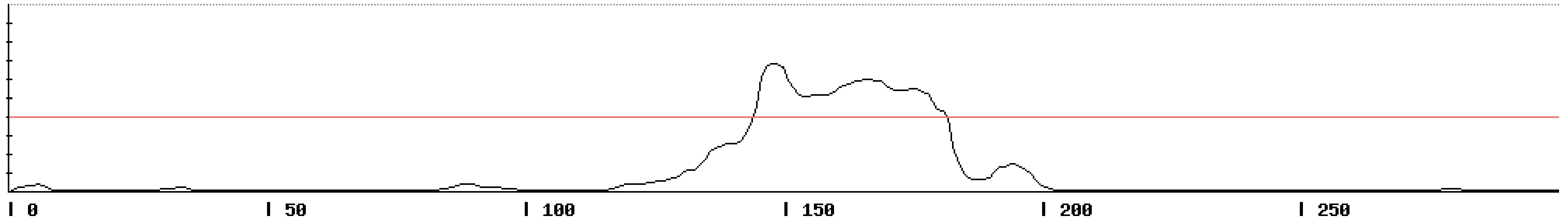

Predictions for Elaeisguineensis_XP_010919471.1

Viterbi Path

Viterbi Path

Posterior @ 0.4

Posterior @ 0.4

Posterior Graphic. Threshold Marked @ 0.4

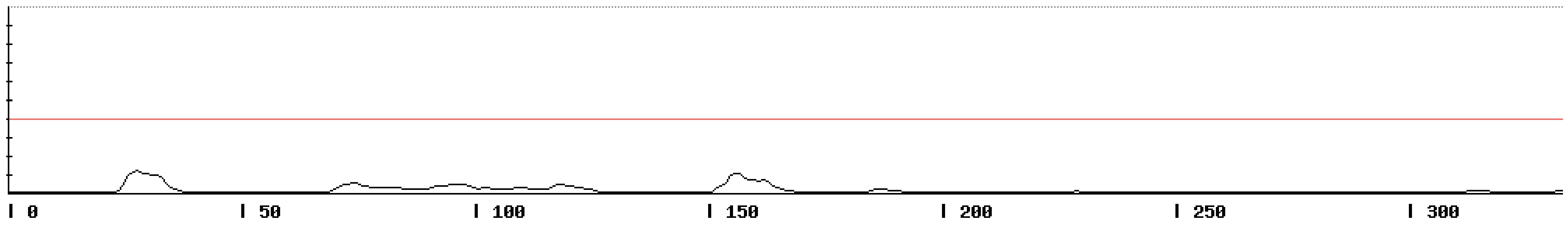

Predictions for Elaeisguineensis_XP_010919872.1

Viterbi Path

Viterbi Path

Posterior @ 0.4

Posterior @ 0.4

Posterior Graphic. Threshold Marked @ 0.4

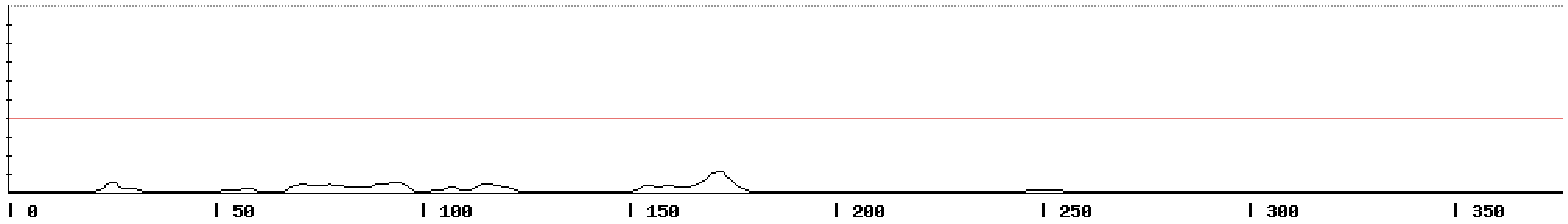

Predictions for Elaeisguineensis_XP_010919873.1

Viterbi Path

Viterbi Path

Posterior @ 0.4

Posterior @ 0.4

Posterior Graphic. Threshold Marked @ 0.4

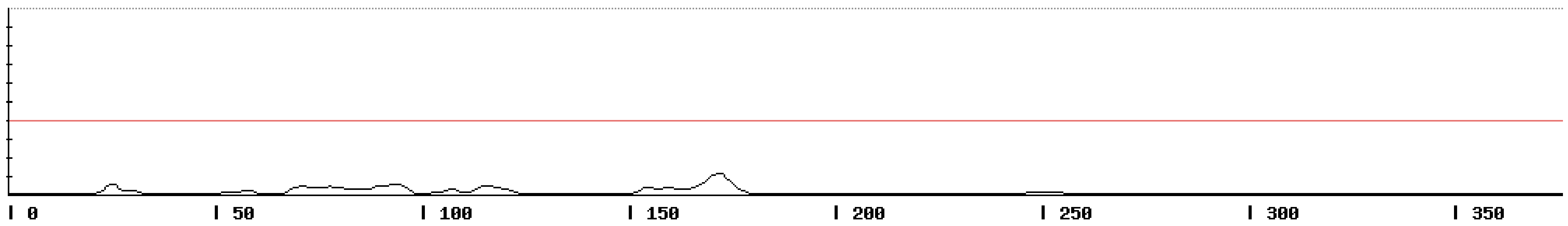

Predictions for Elaeisguineensis_XP_010919874.1

Viterbi Path

Viterbi Path

Posterior @ 0.4

Posterior @ 0.4

Posterior Graphic. Threshold Marked @ 0.4

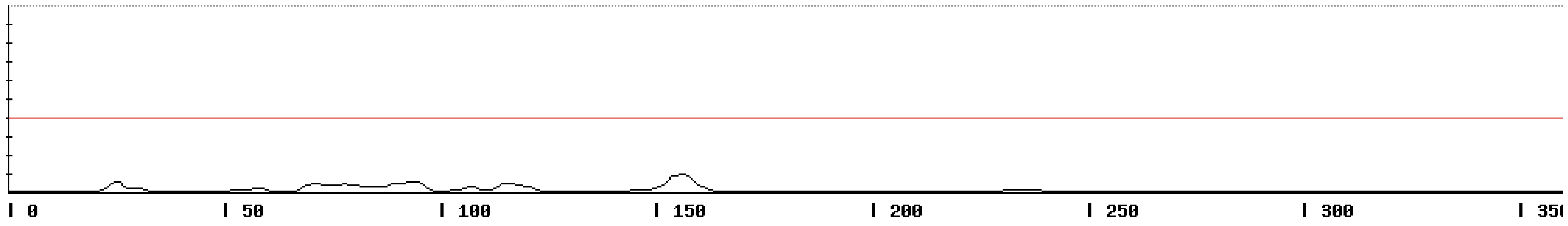

Predictions for Elaeisguineensis_XP_010919875.1

Viterbi Path

Viterbi Path

Posterior @ 0.4

Posterior @ 0.4

Posterior Graphic. Threshold Marked @ 0.4

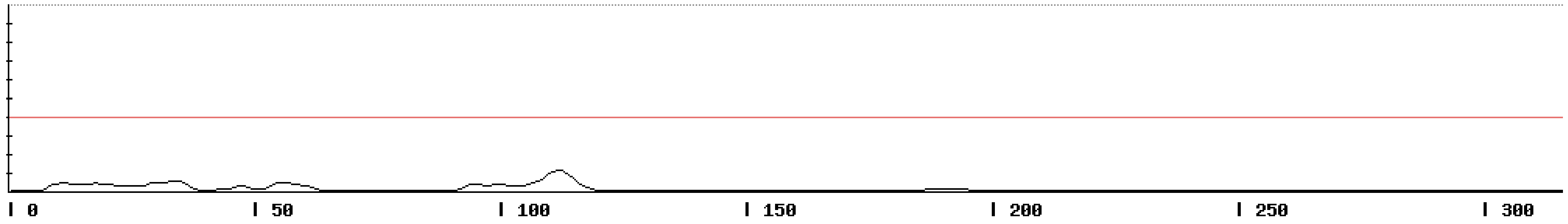

Predictions for Elaeisguineensis_XP_010921105.1

Viterbi Path

Viterbi Path

Posterior @ 0.4

Posterior @ 0.4

Posterior Graphic. Threshold Marked @ 0.4

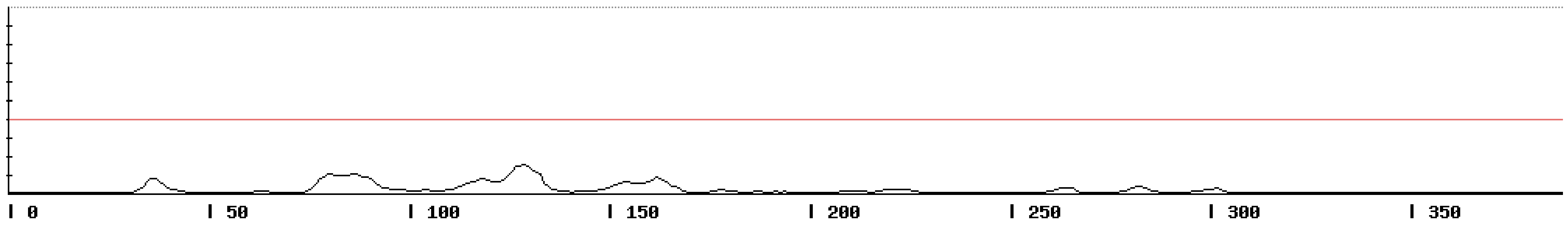

Predictions for Elaeisguineensis_XP_010921285.1

Viterbi Path

Viterbi Path

Posterior @ 0.4

Posterior @ 0.4

Posterior Graphic. Threshold Marked @ 0.4

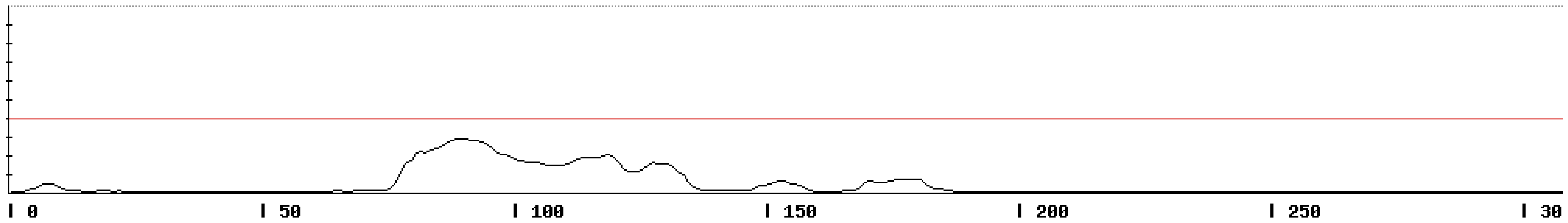

Predictions for Elaeisguineensis_XP_010921289.1

Viterbi Path

Viterbi Path

Posterior @ 0.4

Posterior @ 0.4

Posterior Graphic. Threshold Marked @ 0.4

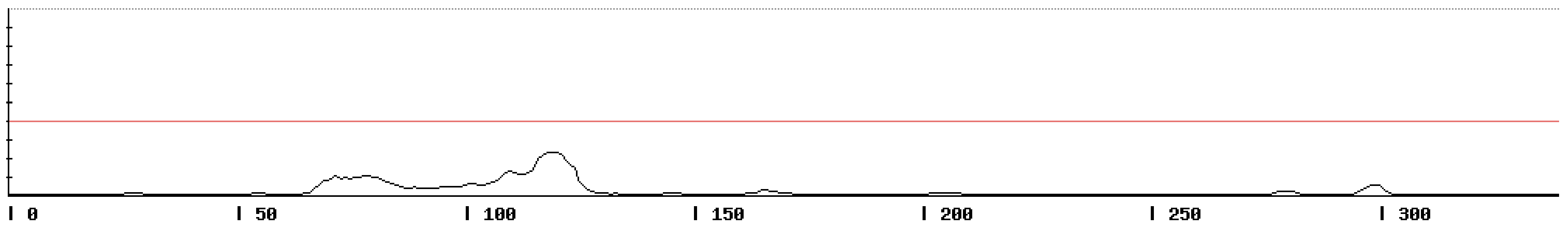

Predictions for Elaeisguineensis_XP_010921291.1

Viterbi Path

Viterbi Path

Posterior @ 0.4

Posterior @ 0.4

Posterior Graphic. Threshold Marked @ 0.4

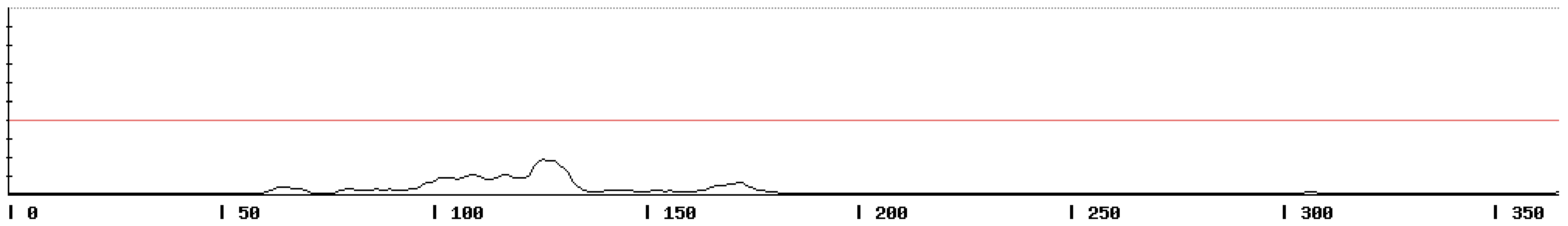

Predictions for Elaeisguineensis_XP_010921292.1

Viterbi Path

Viterbi Path

Posterior @ 0.4

Posterior @ 0.4

Posterior Graphic. Threshold Marked @ 0.4

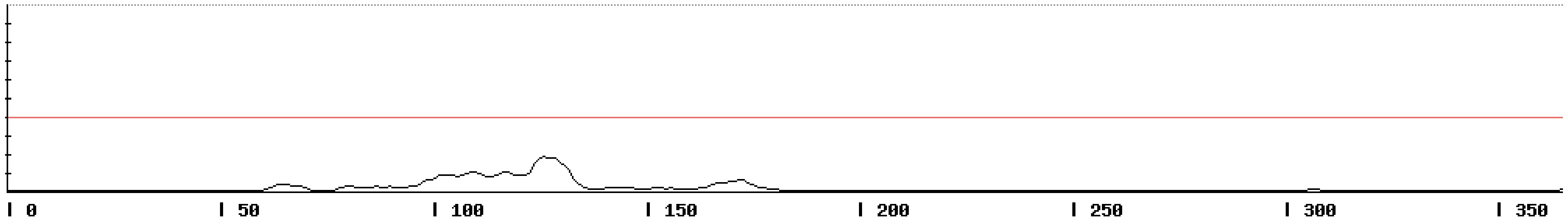

Predictions for Elaeisguineensis_XP_010921550.1

Viterbi Path

Viterbi Path

Posterior @ 0.4

Posterior @ 0.4

Posterior Graphic. Threshold Marked @ 0.4

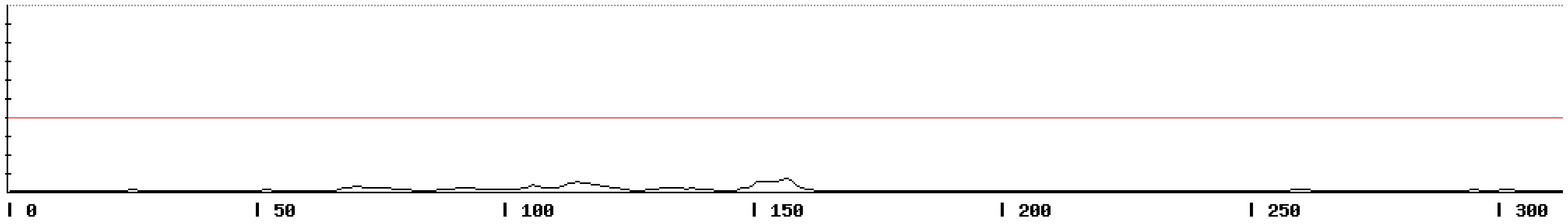

Predictions for Elaeisguineensis_XP_010922255.1

Viterbi Path

Viterbi Path

Posterior @ 0.4

Posterior @ 0.4

Posterior Graphic. Threshold Marked @ 0.4

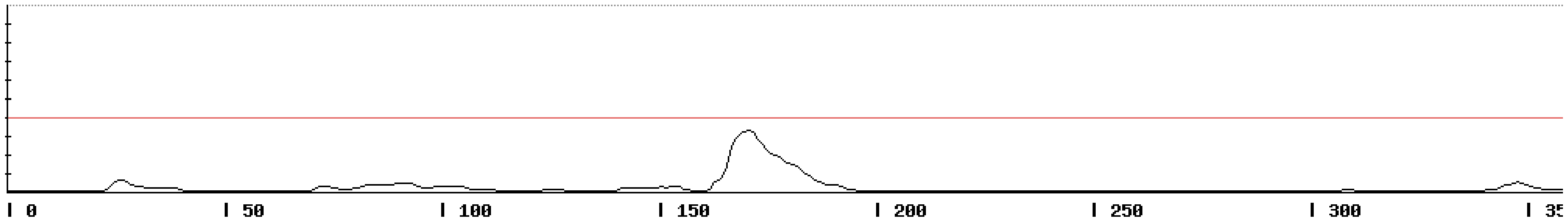

Predictions for Elaeisguineensis_XP_010922867.1

Viterbi Path

Viterbi Path

Posterior @ 0.4 87 - NRAAGR - 92

Posterior @ 0.4

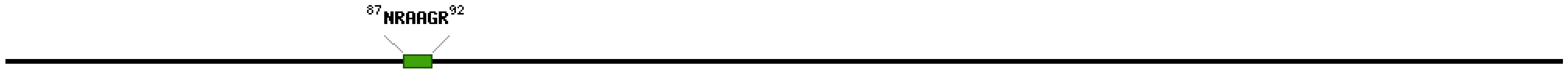

Posterior Graphic. Threshold Marked @ 0.4

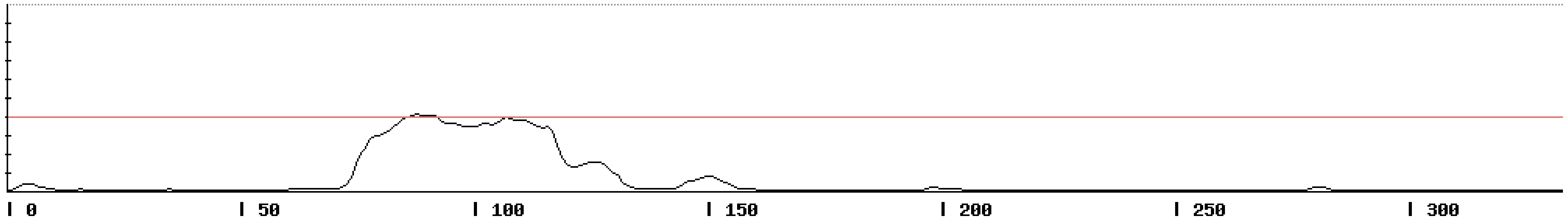

Predictions for Elaeisguineensis_XP_010922868.1

Viterbi Path

Viterbi Path

Posterior @ 0.4 — 87 - NRAAGR - 92

Posterior @ 0.4

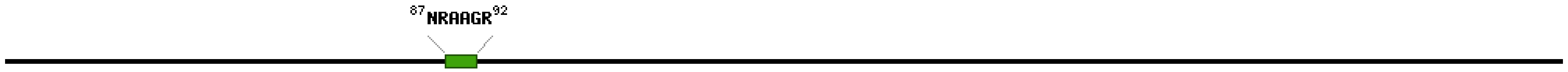

Posterior Graphic. Threshold Marked @ 0.4

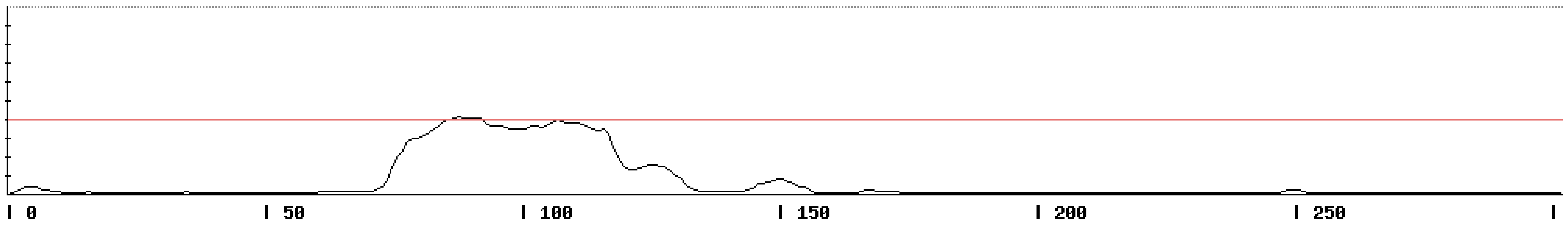

Predictions for Elaeisguineensis_XP_010922870.1

Viterbi Path

Viterbi Path

Posterior @ 0.4

Posterior @ 0.4

Posterior Graphic. Threshold Marked @ 0.4

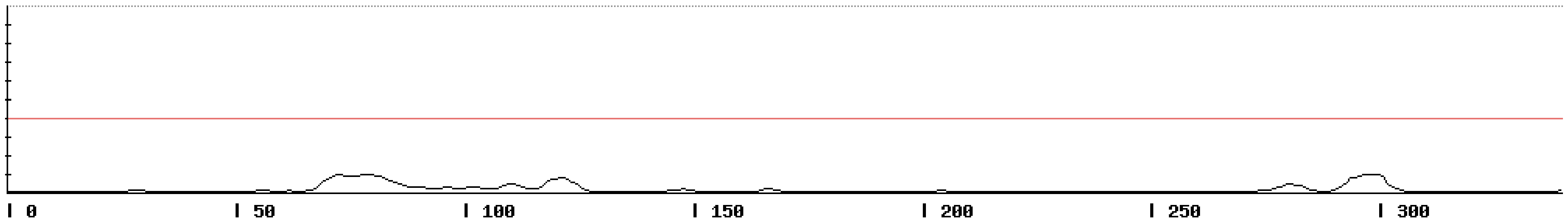

Predictions for Elaeisguineensis_XP_010922873.1	
Viterbi Path	
Viterbi Path	
Posterior @ 0.4	
Posterior @ 0.4	
Posterior Graphic. Threshold Marked @ 0.4	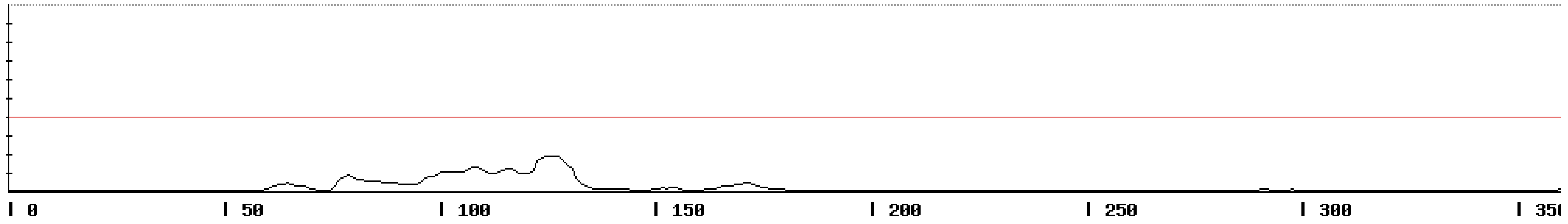

Predictions for Elaeisguineensis_XP_010923739.1	
Viterbi Path	
Viterbi Path	
Posterior @ 0.4	
Posterior @ 0.4	
Posterior Graphic. Threshold Marked @ 0.4	

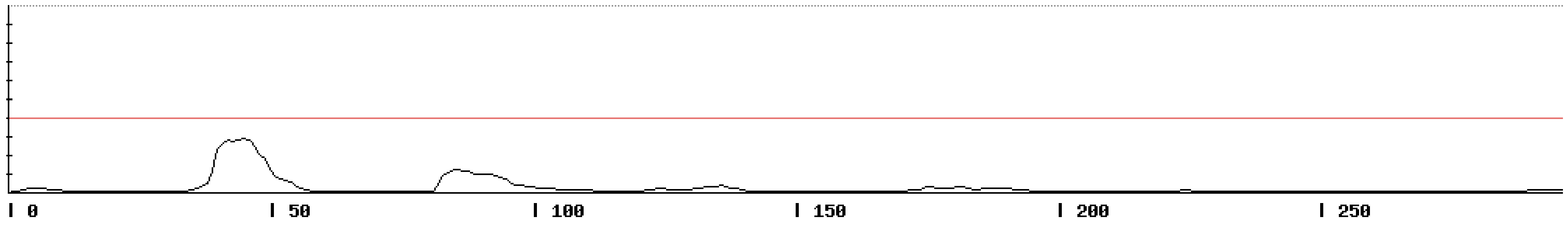

Predictions for Elaeisguineensis_XP_010924066.1

Viterbi Path

Viterbi Path

Posterior @ 0.4

Posterior @ 0.4

Posterior Graphic. Threshold Marked @ 0.4

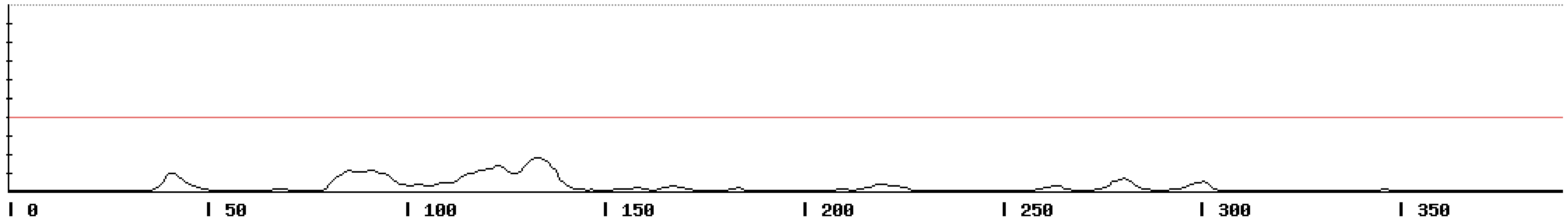

Predictions for Elaeisguineensis_XP_010924067.1

Viterbi Path

Viterbi Path

Posterior @ 0.4

Posterior @ 0.4

Posterior Graphic. Threshold Marked @ 0.4

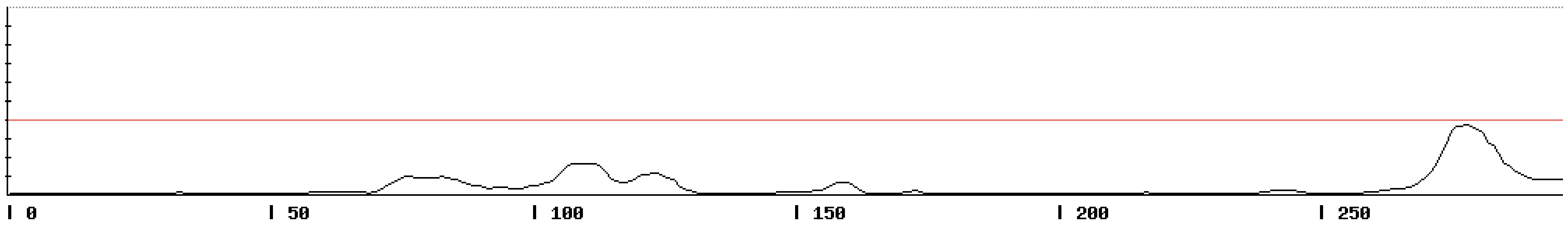

Predictions for Elaeisguineensis_XP_010924406.1

Viterbi Path

Viterbi Path

Posterior @ 0.4 — 139 - TRKRRKVSTDEHGRETRWHKTGKTRPVVAGGKLKGYK - 175

Posterior @ 0.4

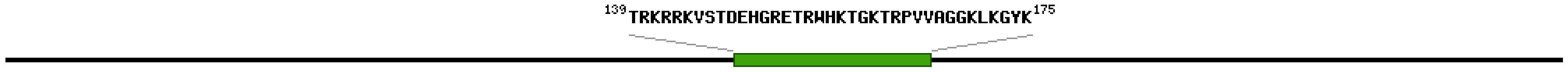

Posterior Graphic. Threshold Marked @ 0.4

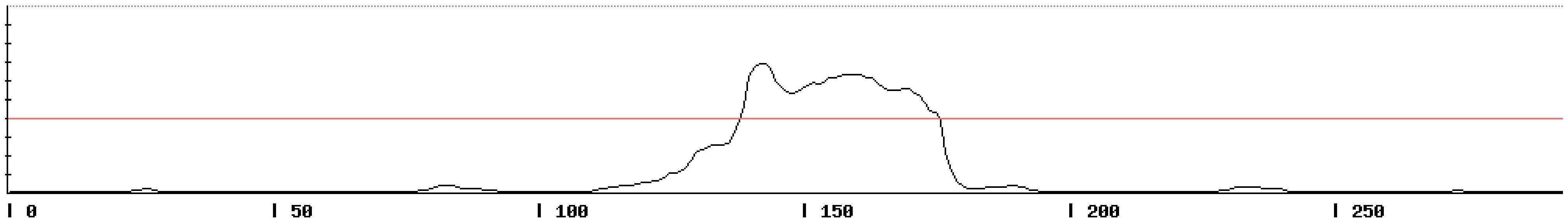

Predictions for Elaeisguineensis_XP_010925507.1

Viterbi Path

Viterbi Path

Posterior @ 0.4

Posterior @ 0.4

Posterior Graphic. Threshold Marked @ 0.4

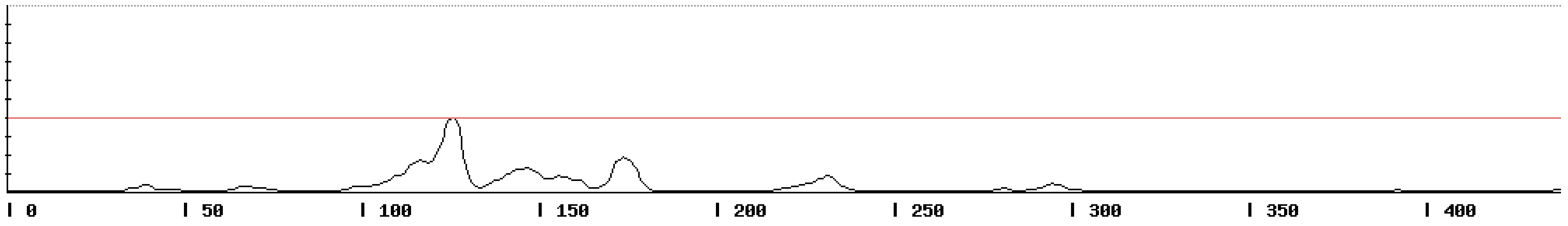

Predictions for Elaeisguineensis_XP_010925508.1

Viterbi Path

Viterbi Path

Posterior @ 0.4

Posterior @ 0.4

Posterior Graphic. Threshold Marked @ 0.4

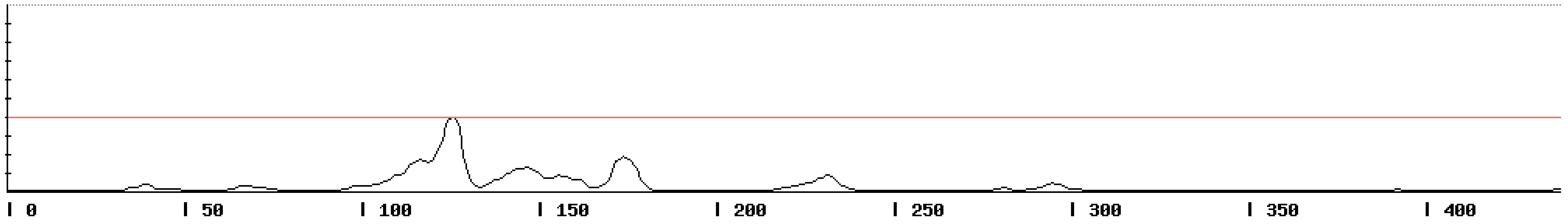

Predictions for Elaeisguineensis_XP_010925509.1

Viterbi Path

Viterbi Path

Posterior @ 0.4	
Posterior @ 0.4	
Posterior Graphic. Threshold Marked @ 0.4	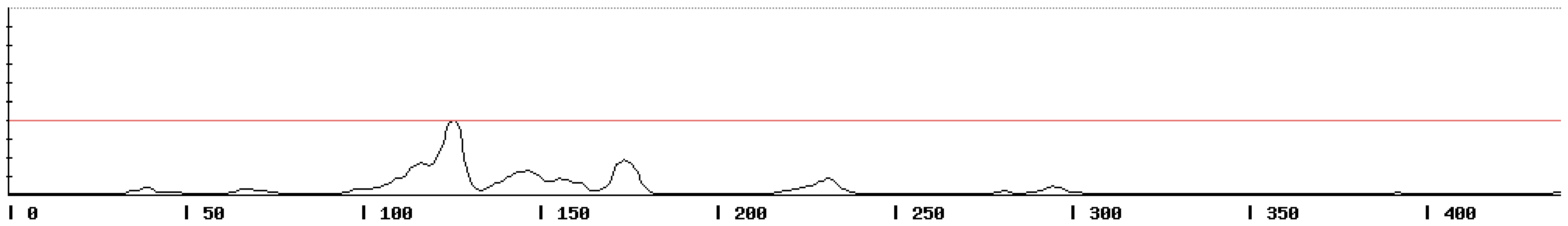

Predictions for Elaeisguineensis_XP_010925510.1

Viterbi Path	
Viterbi Path	
Posterior @ 0.4	
Posterior @ 0.4	
Posterior Graphic. Threshold Marked @ 0.4	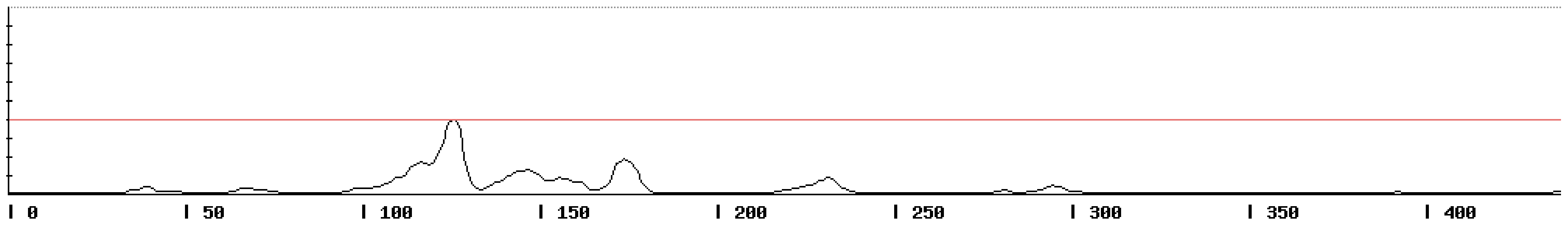

Predictions for Elaeisguineensis_XP_010925511.1

Viterbi Path	
Viterbi Path	

Posterior @ 0.4

Posterior @ 0.4

Posterior Graphic. Threshold Marked @ 0.4

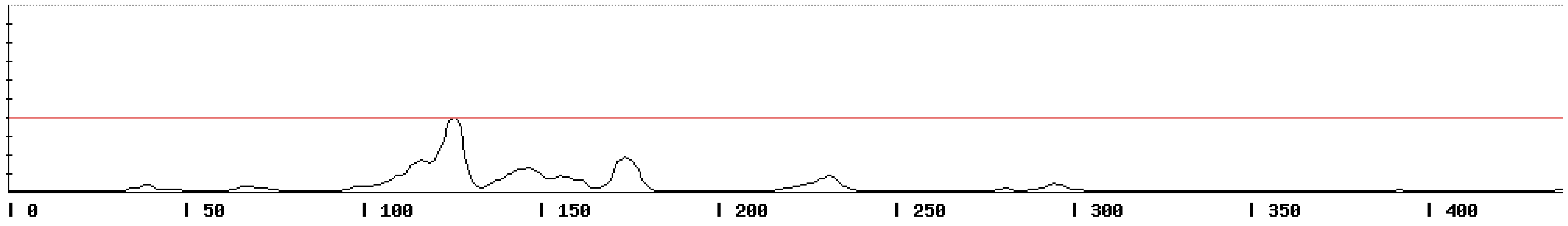

Predictions for Elaeisguineensis_XP_010925709.1

Viterbi Path

Viterbi Path

Posterior @ 0.4

Posterior @ 0.4

Posterior Graphic. Threshold Marked @ 0.4

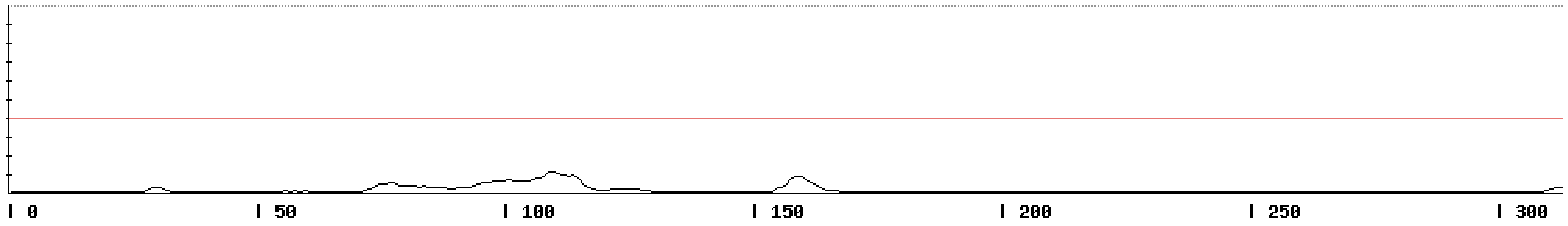

Predictions for Elaeisguineensis_XP_010925972.1

Viterbi Path

Viterbi Path

Posterior @ 0.4

Posterior @ 0.4

Posterior Graphic. Threshold Marked @ 0.4

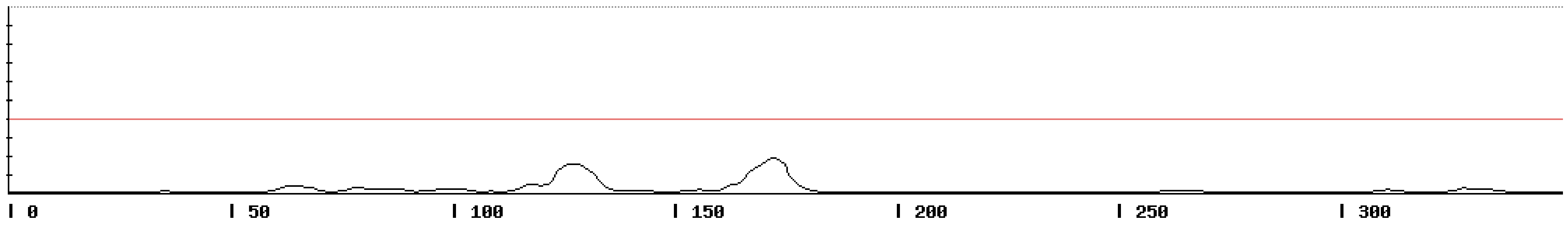

Predictions for Elaeisguineensis_XP_010926039.1

Viterbi Path

Viterbi Path

Posterior @ 0.4

Posterior @ 0.4

Posterior Graphic. Threshold Marked @ 0.4

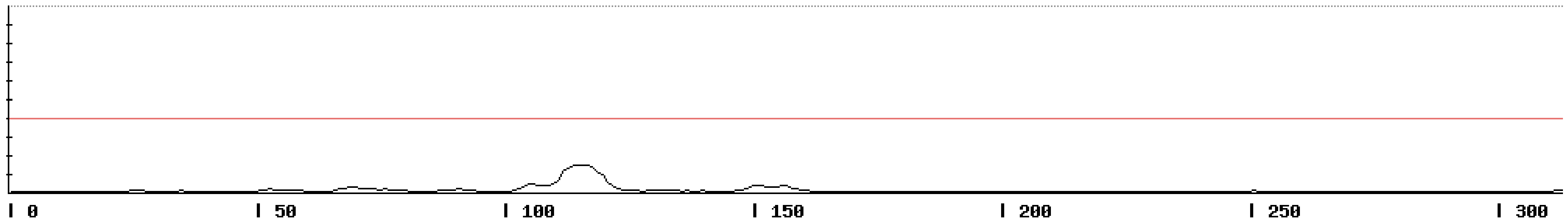

Predictions for Elaeisguineensis_XP_010926910.1	
Viterbi Path	
Viterbi Path	
Posterior @ 0.4	
Posterior @ 0.4	
Posterior Graphic. Threshold Marked @ 0.4	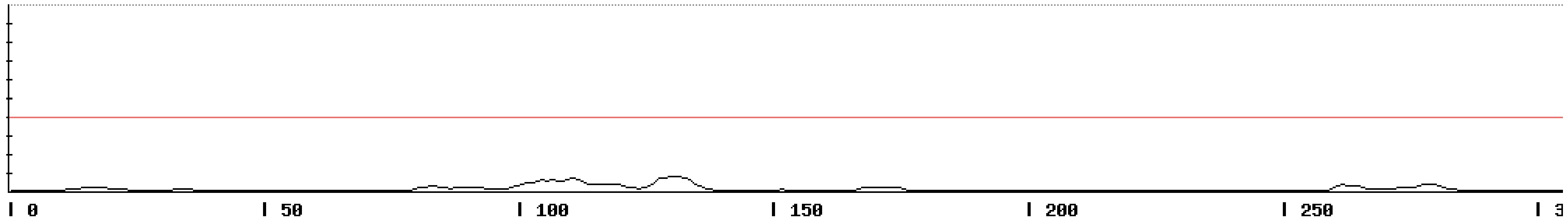

Predictions for Elaeisguineensis_XP_010927035.1	
Viterbi Path	
Viterbi Path	
Posterior @ 0.4	
Posterior @ 0.4	
Posterior Graphic. Threshold Marked @ 0.4	

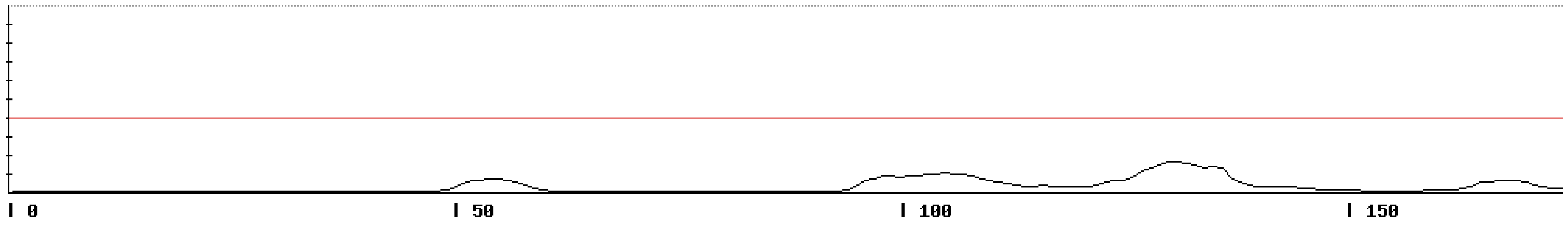

Predictions for Elaeisguineensis_XP_010927165.1

Viterbi Path

Viterbi Path

Posterior @ 0.4 — 246 - PRKKKYPKGTRPARETNGGYWKASGRDMIIKDKNKKEIGHKK - 287

Posterior @ 0.4

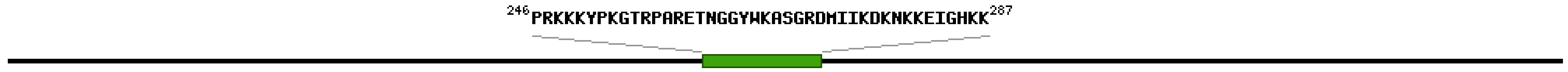

Posterior Graphic. Threshold Marked @ 0.4

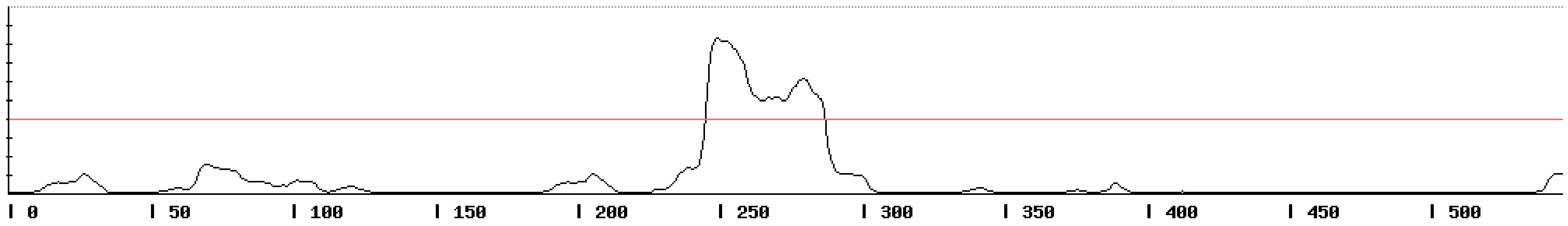

Predictions for Elaeisguineensis_XP_010927216.1

Viterbi Path

Viterbi Path

Posterior @ 0.4

Posterior @ 0.4

Posterior Graphic. Threshold Marked @ 0.4

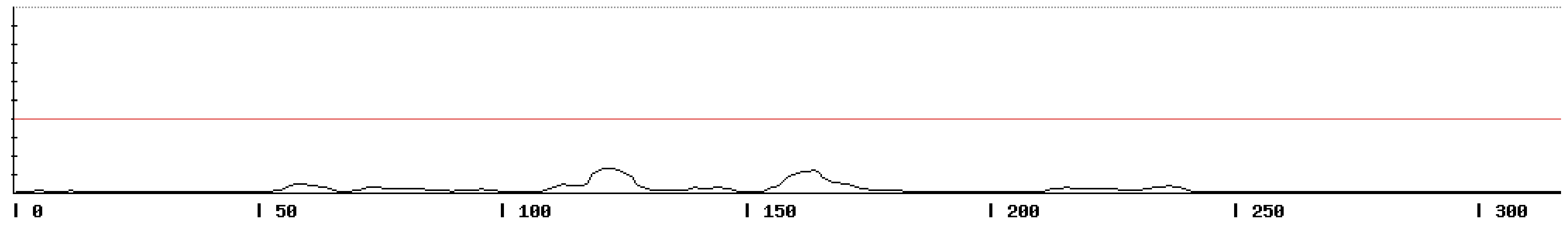

Predictions for Elaeisguineensis_XP_010927489.1

Viterbi Path

Viterbi Path

Posterior @ 0.4

Posterior @ 0.4

Posterior Graphic. Threshold Marked @ 0.4

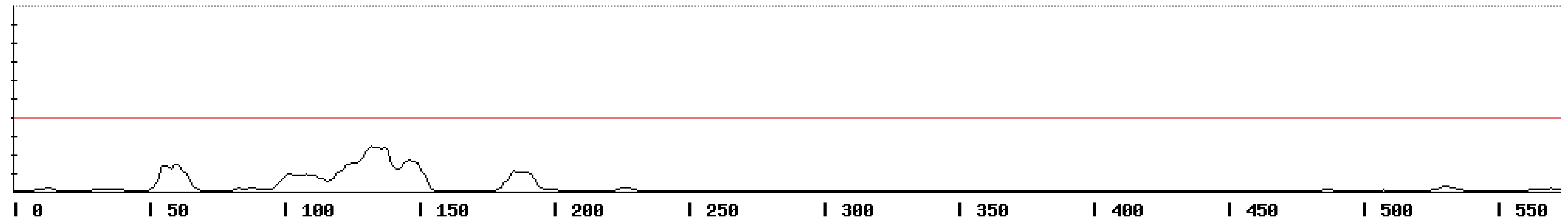

Predictions for Elaeisguineensis_XP_010928246.1

Viterbi Path

Viterbi Path

Posterior @ 0.4

Posterior @ 0.4

Posterior Graphic. Threshold Marked @ 0.4	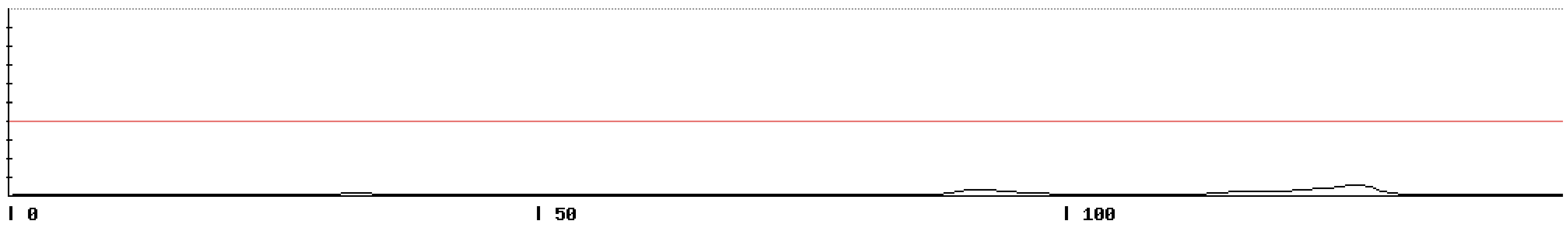

Predictions for Elaeisguineensis_XP_010928247.1	
Viterbi Path	
Viterbi Path	
Posterior @ 0.4	
Posterior @ 0.4	
Posterior Graphic. Threshold Marked @ 0.4	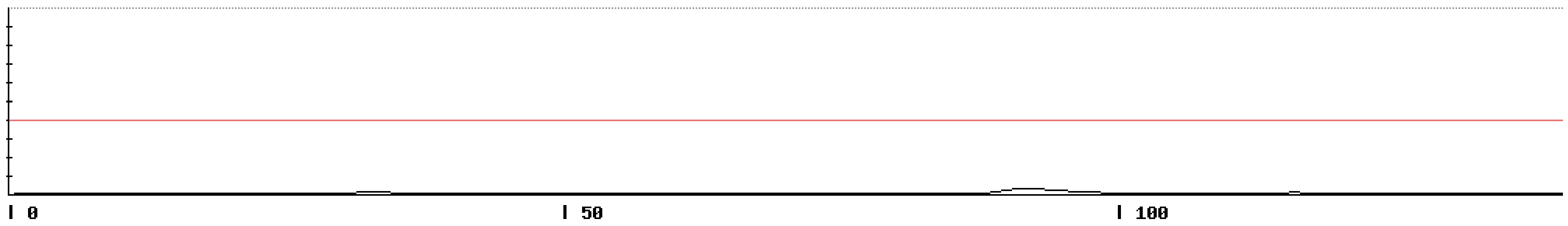

Predictions for Elaeisguineensis_XP_010929380.1	
Viterbi Path	
Viterbi Path	

Posterior @ 0.4

Posterior @ 0.4

Posterior Graphic. Threshold Marked @ 0.4

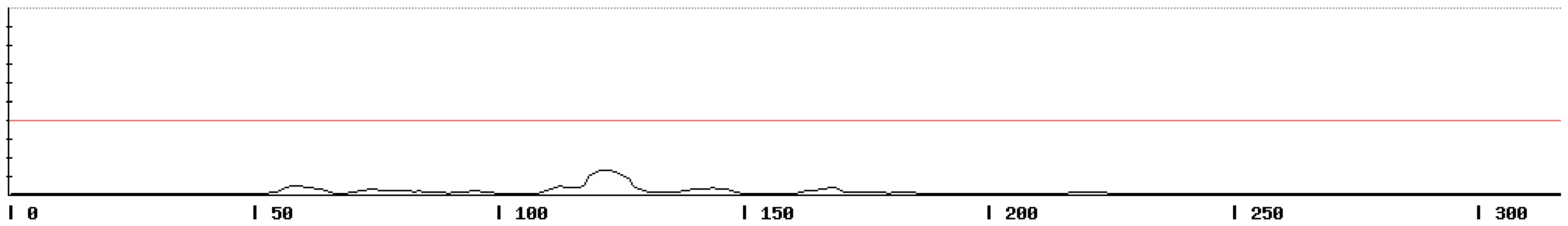

Predictions for Elaeisguineensis_XP_010929381.1

Viterbi Path

Viterbi Path

Posterior @ 0.4

Posterior @ 0.4

Posterior Graphic. Threshold Marked @ 0.4

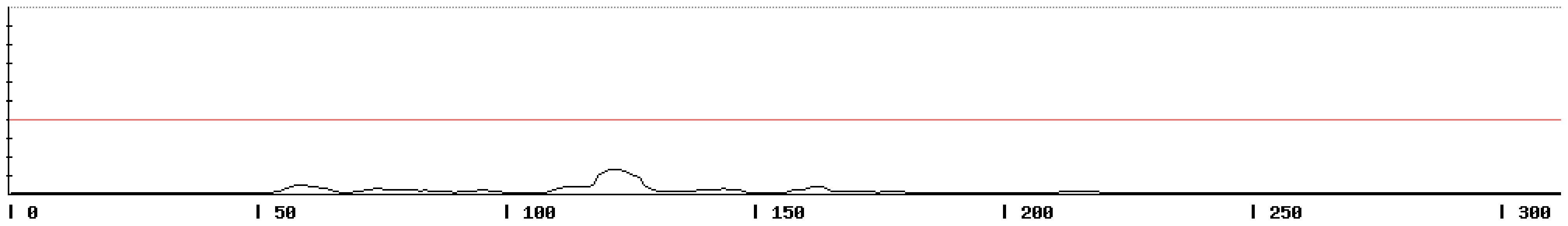

Predictions for Elaeisguineensis_XP_010929607.1

Viterbi Path

Viterbi Path

Posterior @ 0.4

Posterior @ 0.4

Posterior Graphic. Threshold Marked @ 0.4

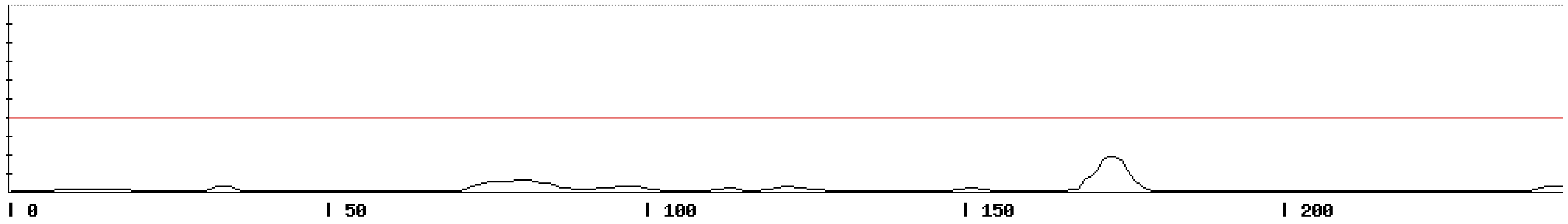

Predictions for Elaeisguineensis_XP_010930341.1

Viterbi Path

Viterbi Path

Posterior @ 0.4

Posterior @ 0.4

Posterior Graphic. Threshold Marked @ 0.4

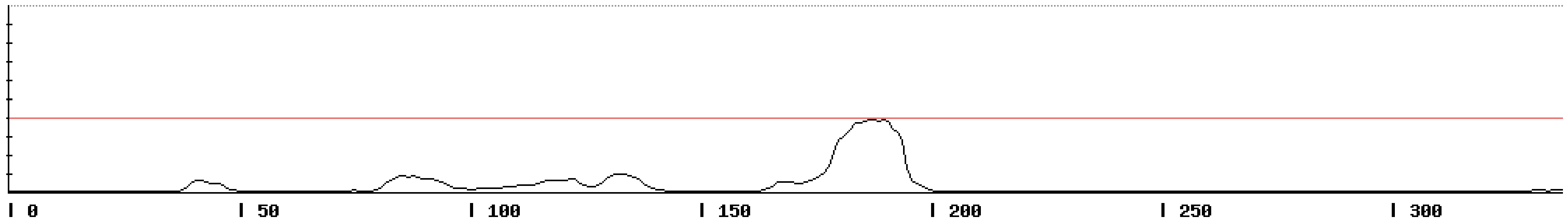

Predictions for Elaeisguineensis_XP_010930343.1

Viterbi Path

Viterbi Path

Posterior @ 0.4

Posterior @ 0.4

Posterior Graphic. Threshold Marked @ 0.4

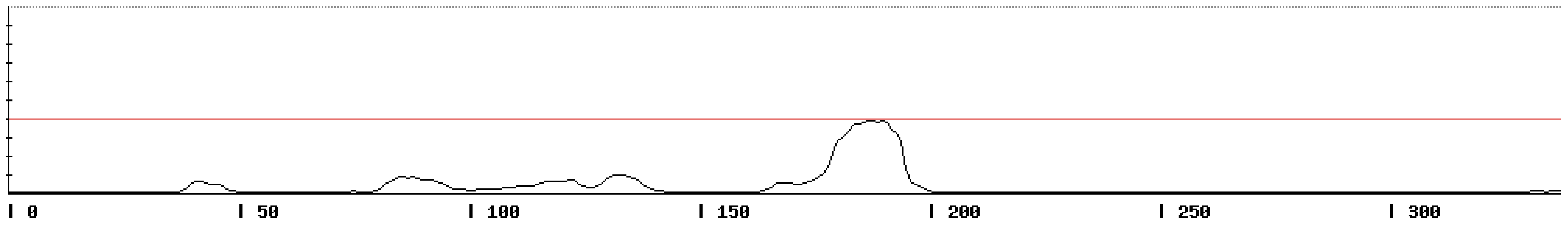

Predictions for Elaeisguineensis_XP_010930409.1

Viterbi Path

Viterbi Path

Posterior @ 0.4

Posterior @ 0.4

Posterior Graphic. Threshold Marked @ 0.4

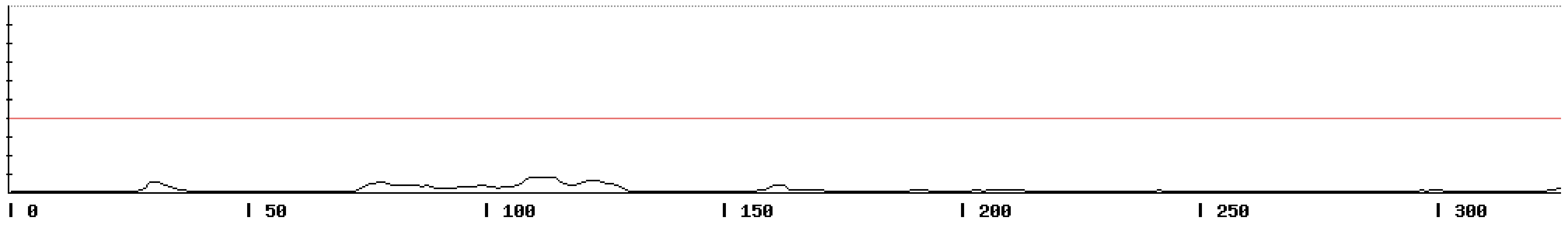

Predictions for Elaeisguineensis_XP_010930661.1	
Viterbi Path	
Viterbi Path	
Posterior @ 0.4	143 - RSLKKRRSPR - 152
Posterior @ 0.4	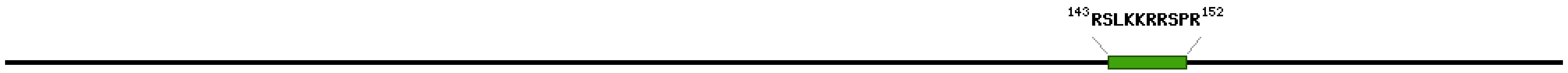
Posterior Graphic. Threshold Marked @ 0.4	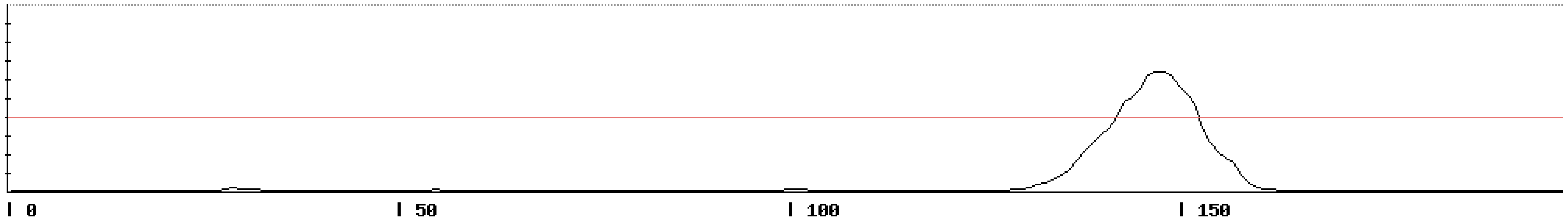

Predictions for Elaeisguineensis_XP_010930725.1	
Viterbi Path	
Viterbi Path	
Posterior @ 0.4	
Posterior @ 0.4	
Posterior Graphic. Threshold Marked @ 0.4	

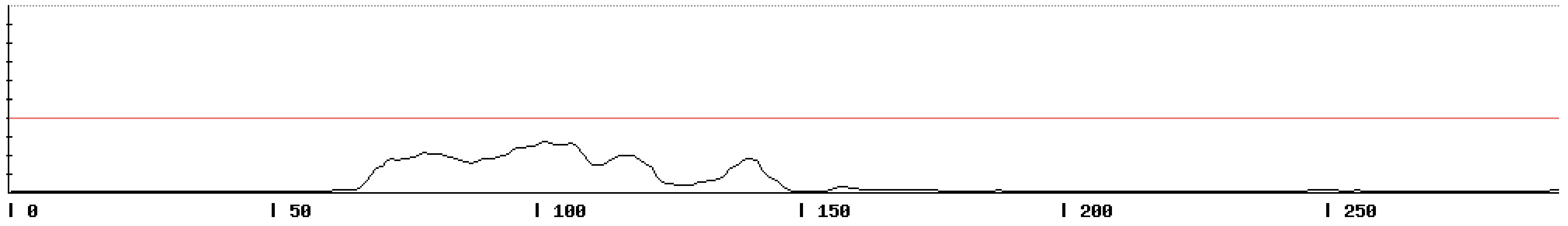

Predictions for Elaeisguineensis_XP_010930821.1

Viterbi Path

Viterbi Path

Posterior @ 0.4

Posterior @ 0.4

Posterior Graphic. Threshold Marked @ 0.4

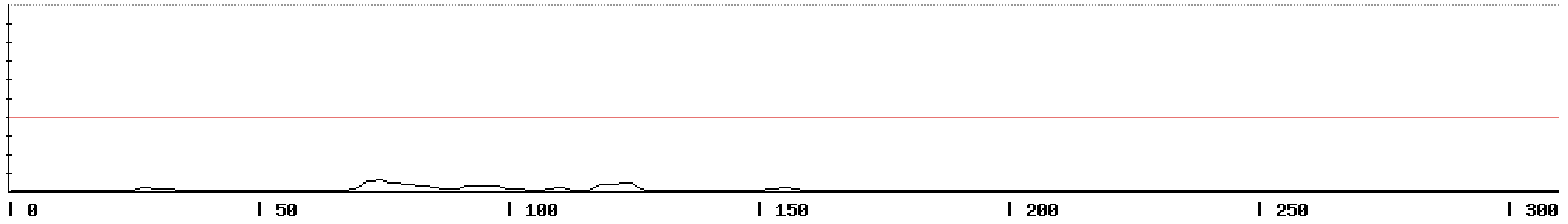

Predictions for Elaeisguineensis_XP_010931110.1

Viterbi Path

Viterbi Path

Posterior @ 0.4

Posterior @ 0.4

Posterior Graphic. Threshold Marked @ 0.4

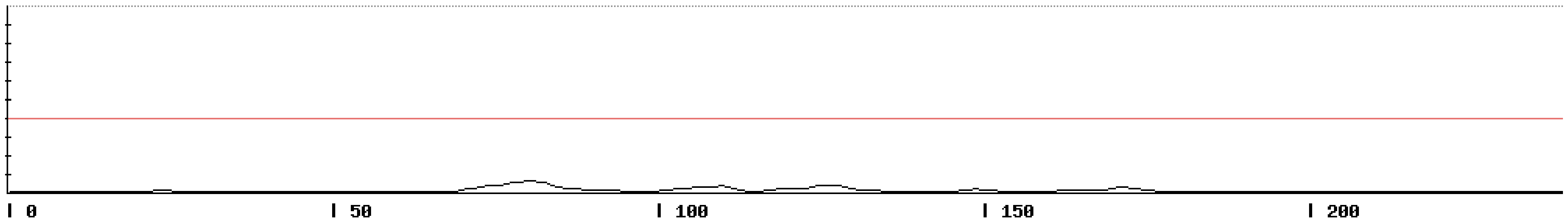

Predictions for Elaeisguineensis_XP_010931440.1

Viterbi Path

Viterbi Path

Posterior @ 0.4

Posterior @ 0.4

Posterior Graphic. Threshold Marked @ 0.4

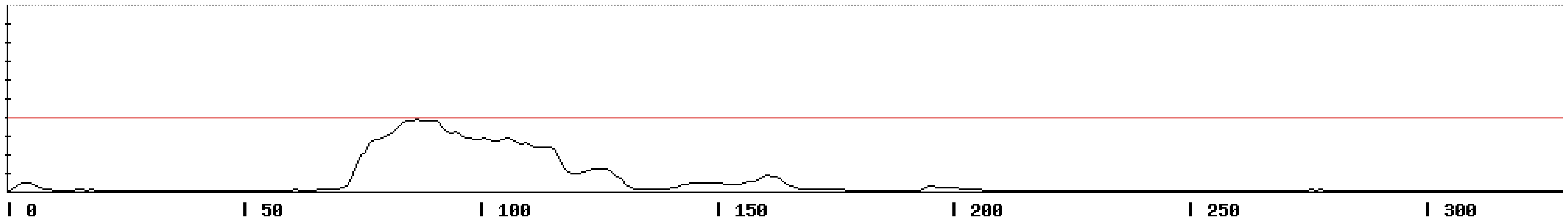

Predictions for Elaeisguineensis_XP_010931441.1

Viterbi Path

Viterbi Path

Posterior @ 0.4

Posterior @ 0.4

Posterior Graphic. Threshold Marked @ 0.4

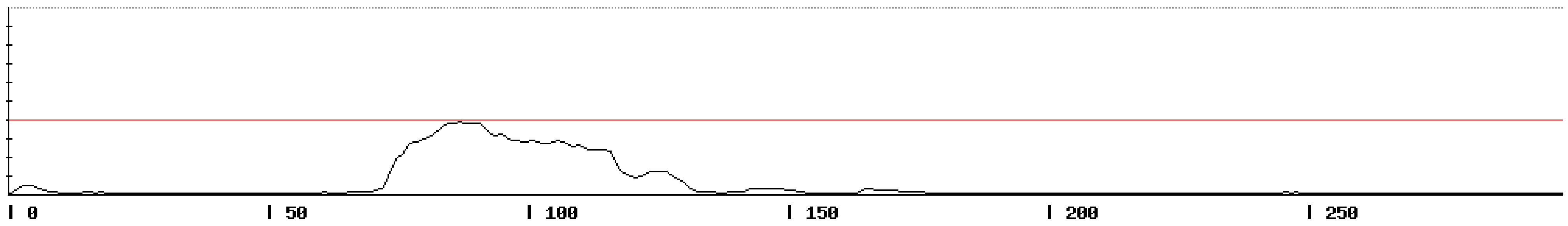

Predictions for Elaeisguineensis_XP_010931442.1

Viterbi Path

Viterbi Path

Posterior @ 0.4

Posterior @ 0.4

Posterior Graphic. Threshold Marked @ 0.4

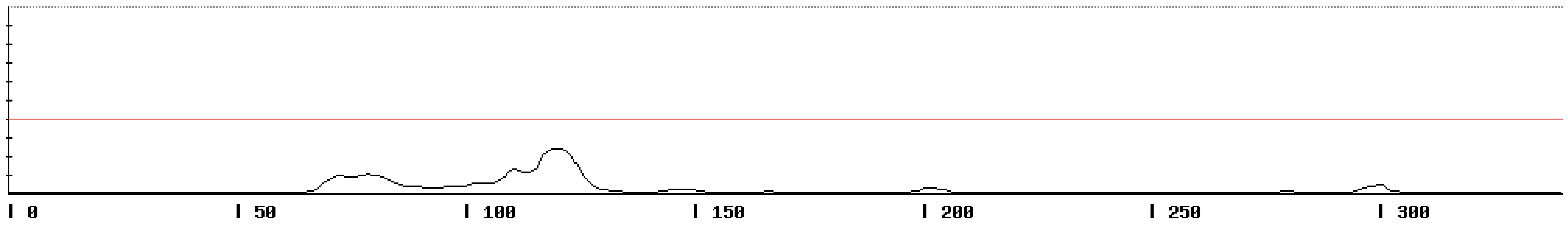

Predictions for Elaeisguineensis_XP_010931444.1

Viterbi Path

Viterbi Path

Posterior @ 0.4

Posterior @ 0.4

Posterior Graphic. Threshold Marked @ 0.4

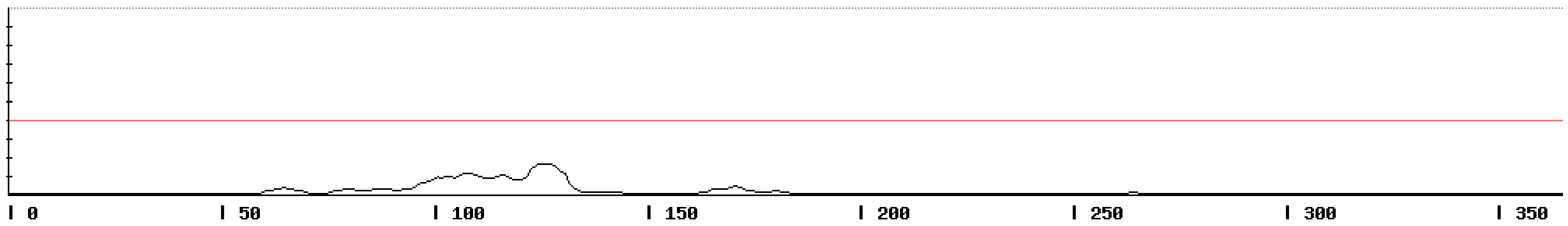

Predictions for Elaeisguineensis_XP_010932188.1

Viterbi Path

Viterbi Path

Posterior @ 0.4

Posterior @ 0.4

Posterior Graphic. Threshold Marked @ 0.4

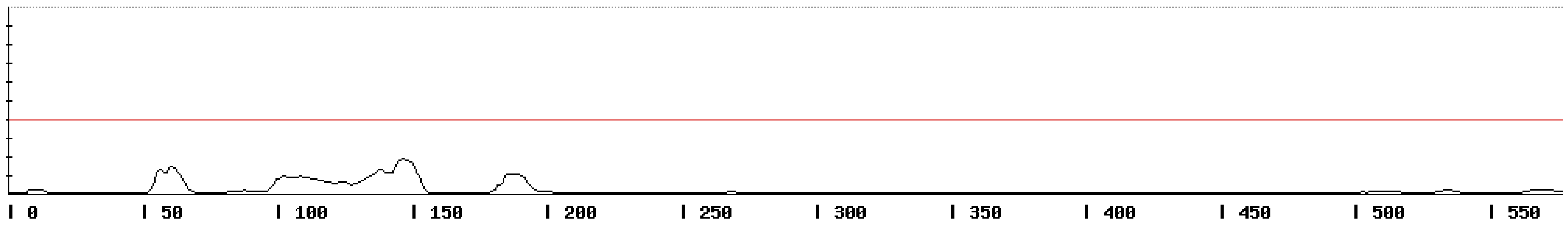

Predictions for Elaeisguineensis_XP_010932189.1

Viterbi Path

Viterbi Path

Posterior @ 0.4	
Posterior @ 0.4	
Posterior Graphic. Threshold Marked @ 0.4	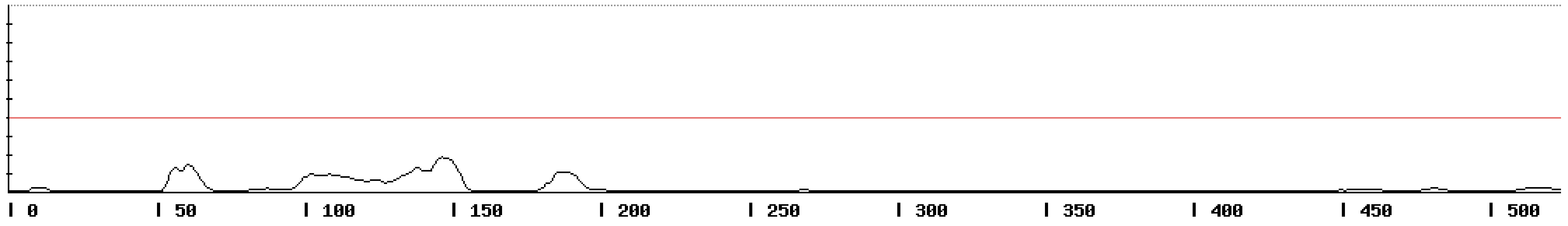

Predictions for Elaeisguineensis_XP_010932619.1

Viterbi Path	
Viterbi Path	
Posterior @ 0.4	
Posterior @ 0.4	
Posterior Graphic. Threshold Marked @ 0.4	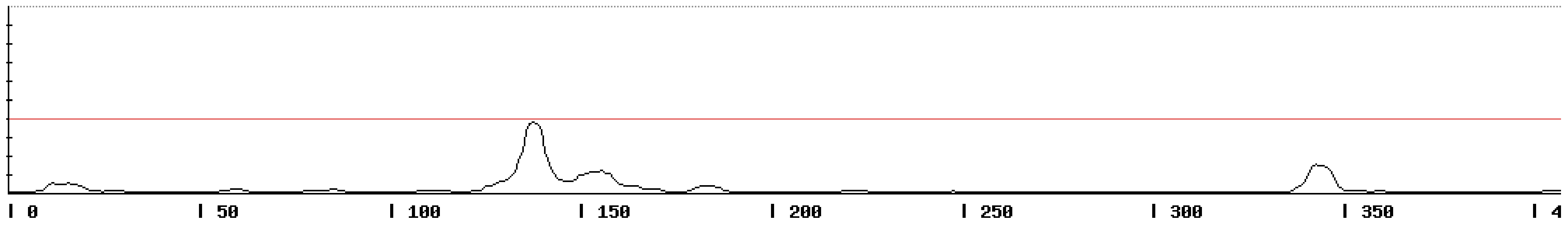

Predictions for Elaeisguineensis_XP_010932620.1

Viterbi Path

Viterbi Path

Posterior @ 0.4

Posterior @ 0.4

Posterior Graphic. Threshold Marked @ 0.4

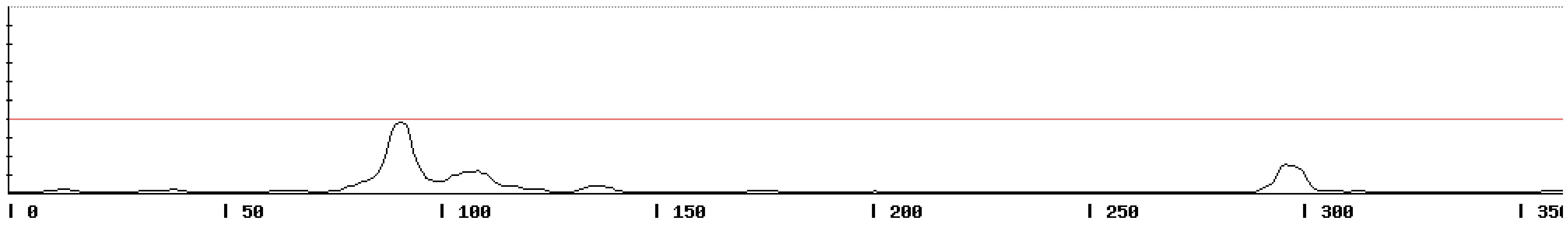

Predictions for Elaeisguineensis_XP_010933504.1

Viterbi Path

Viterbi Path

Posterior @ 0.4

Posterior @ 0.4

Posterior Graphic. Threshold Marked @ 0.4

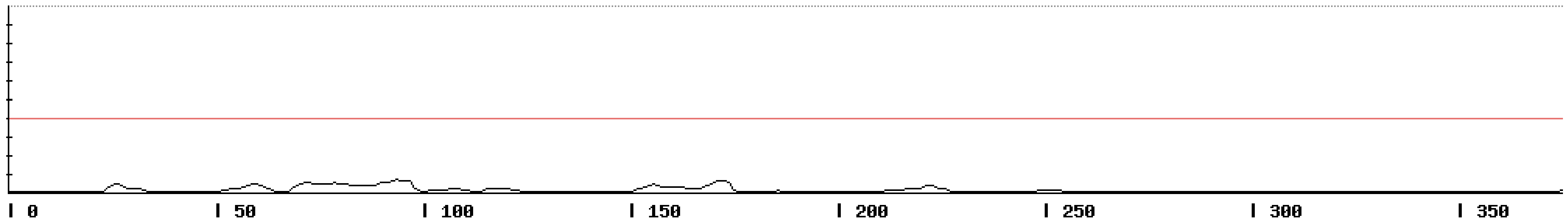

Predictions for Elaeisguineensis_XP_010933505.1

Viterbi Path

Viterbi Path

Posterior @ 0.4

Posterior @ 0.4

Posterior Graphic. Threshold Marked @ 0.4

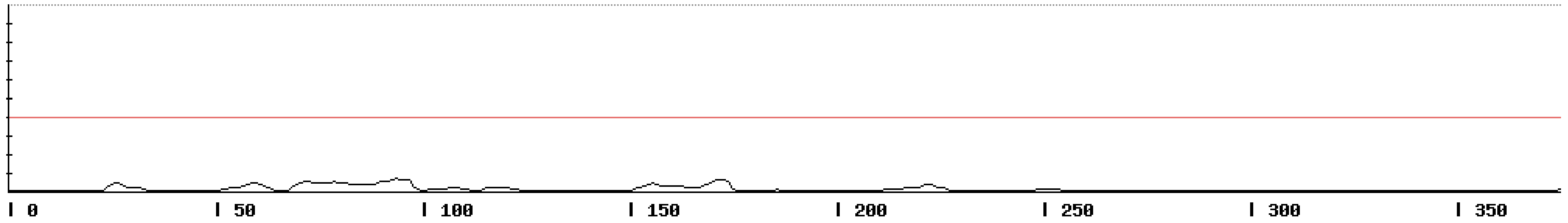

Predictions for Elaeisguineensis_XP_010933718.1

Viterbi Path

Viterbi Path

Posterior @ 0.4

Posterior @ 0.4

Posterior Graphic. Threshold Marked @ 0.4

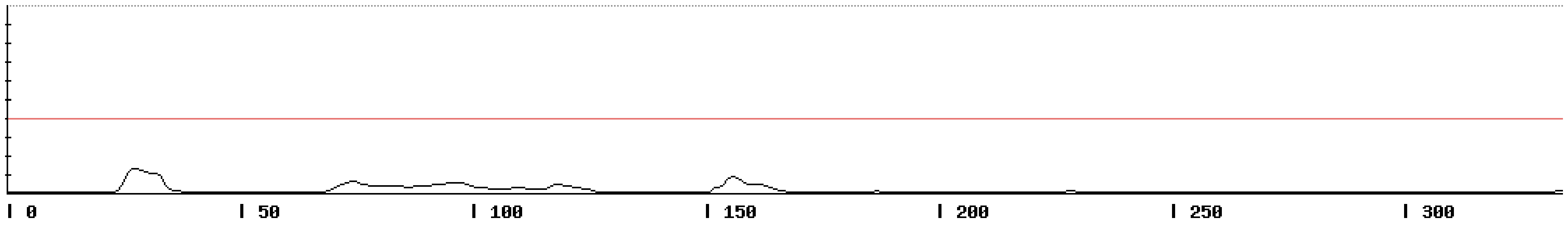

Predictions for Elaeisguineensis_XP_010933936.1

Viterbi Path	
Viterbi Path	
Posterior @ 0.4	
Posterior @ 0.4	
Posterior Graphic. Threshold Marked @ 0.4	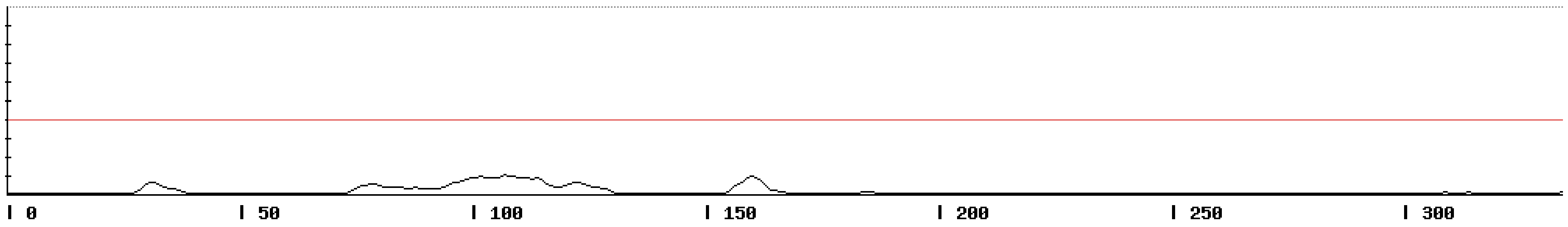

Predictions for Elaeisguineensis_XP_010934040.1

Viterbi Path	
Viterbi Path	
Posterior @ 0.4	
Posterior @ 0.4	

Posterior Graphic. Threshold Marked @ 0.4

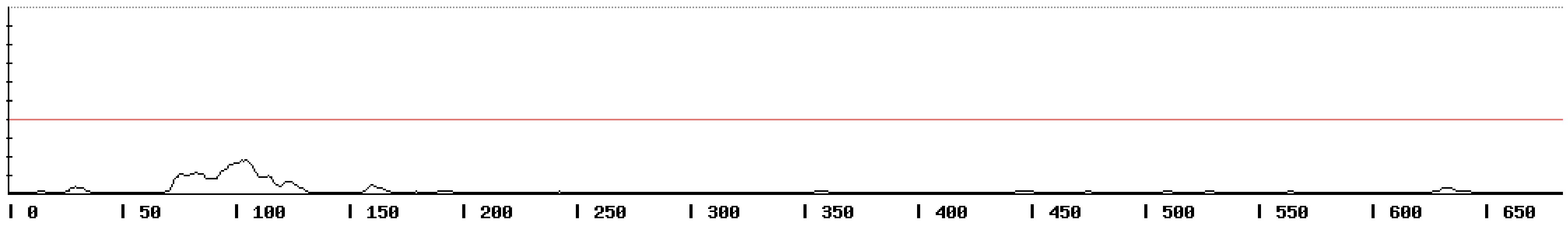

Predictions for Elaeisguineensis_XP_010934041.1

Viterbi Path

Viterbi Path

Posterior @ 0.4

Posterior @ 0.4

Posterior Graphic. Threshold Marked @ 0.4

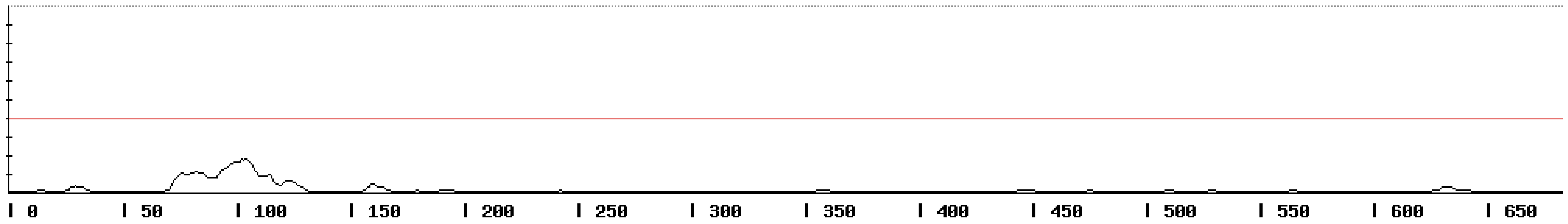

Predictions for Elaeisguineensis_XP_010934316.1

Viterbi Path

Viterbi Path

Posterior @ 0.4

Posterior @ 0.4

Posterior Graphic. Threshold Marked @ 0.4

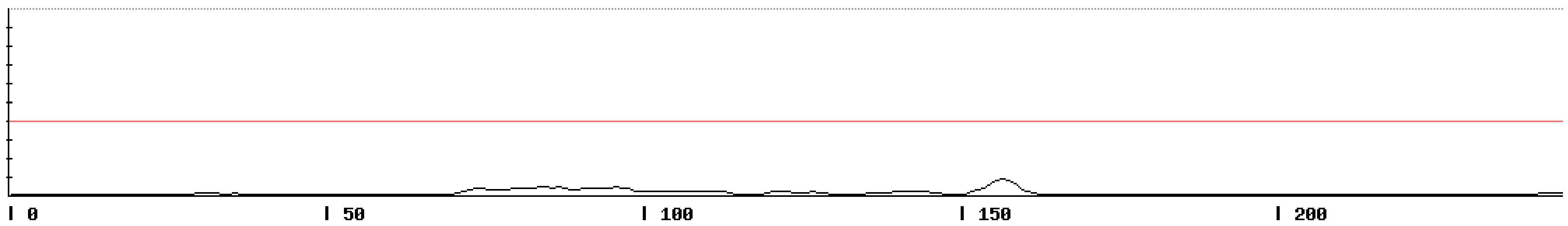

Predictions for Elaeisguineensis_XP_010934904.1

Viterbi Path

Viterbi Path

Posterior @ 0.4

105 - K - 105

123 - HRGRAPKG - 130

563 - IRTRKAHPPSLNKLSSKRGR - 582

Posterior @ 0.4

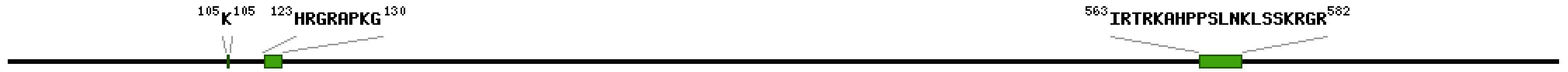

Posterior Graphic. Threshold Marked @ 0.4

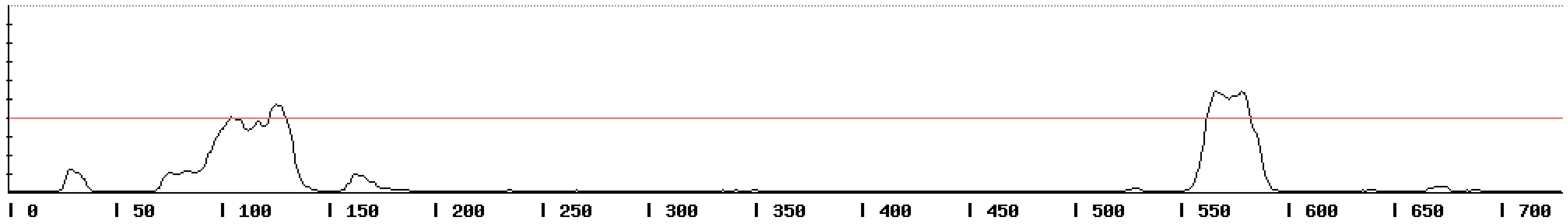

Predictions for Elaeisguineensis_XP_010934905.1

Viterbi Path

Viterbi Path

Posterior @ 0.4	105 - K - 105
	123 - HRGRAPKG - 130
	563 - IRTRKAHPPSLNKLSSKRGR - 582
Posterior @ 0.4	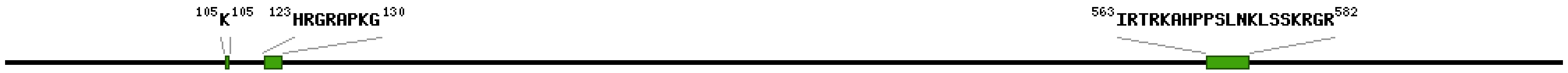
Posterior Graphic. Threshold Marked @ 0.4	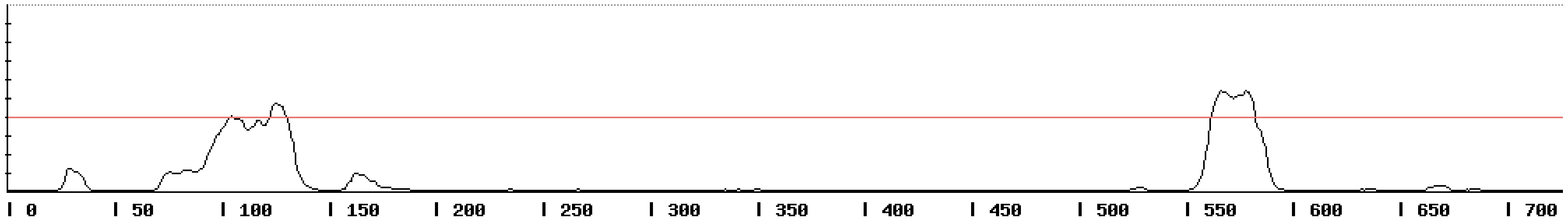

Predictions for Elaeisguineensis_XP_010934906.1	
Viterbi Path	
Viterbi Path	
Posterior @ 0.4	105 - K - 105
	123 - HRGRAPKG - 130
	532 - IRTRKAHPPSLNKLSSKRGR - 551
Posterior @ 0.4	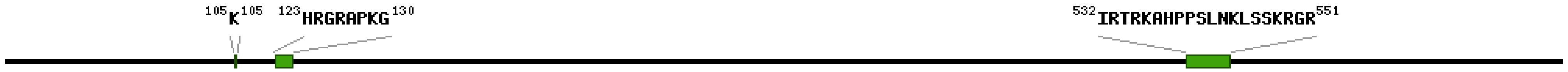
Posterior Graphic. Threshold Marked @ 0.4	

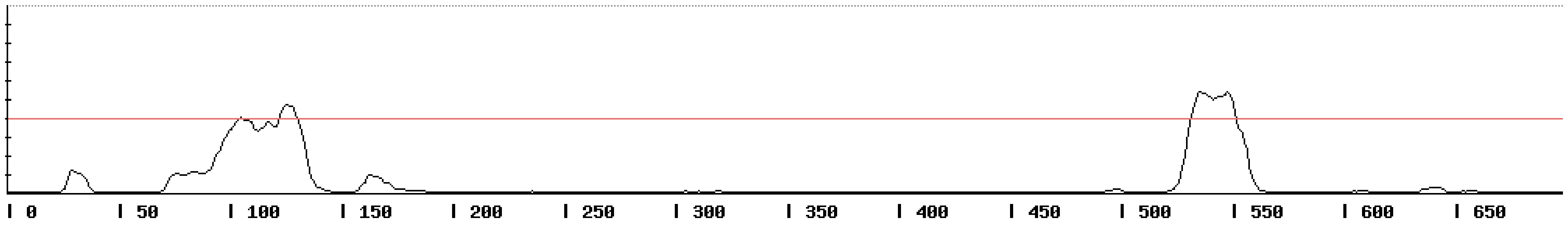

Predictions for Elaeisguineensis_XP_010935230.1	
Viterbi Path	
Viterbi Path	
Posterior @ 0.4	
Posterior @ 0.4	
Posterior Graphic. Threshold Marked @ 0.4	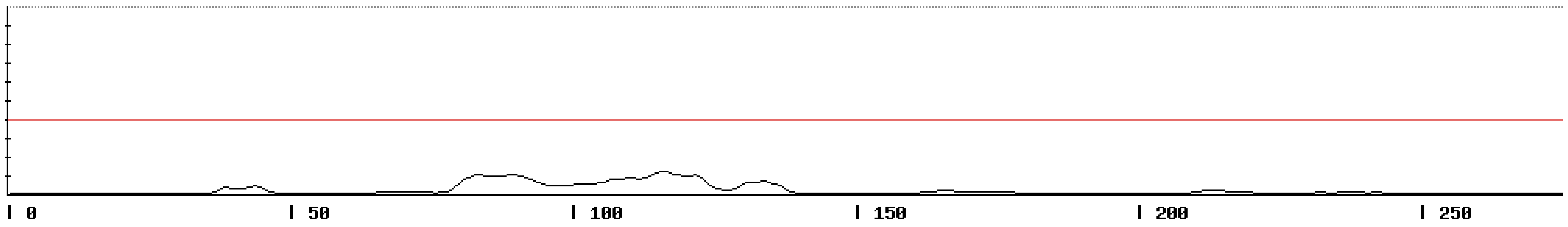

Predictions for Elaeisguineensis_XP_010935330.1	
Viterbi Path	
Viterbi Path	
Posterior @ 0.4	
Posterior @ 0.4	

Posterior Graphic. Threshold Marked @ 0.4	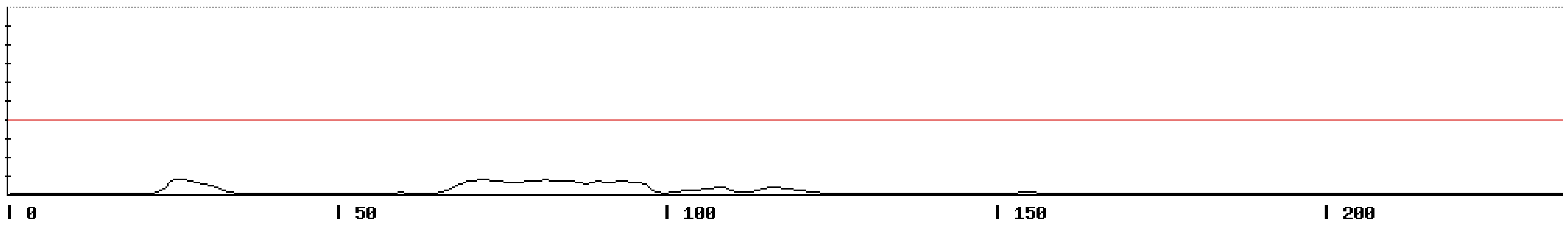

Predictions for Elaeisguineensis_XP_010935812.1

Viterbi Path	
Viterbi Path	
Posterior @ 0.4	
Posterior @ 0.4	
Posterior Graphic. Threshold Marked @ 0.4	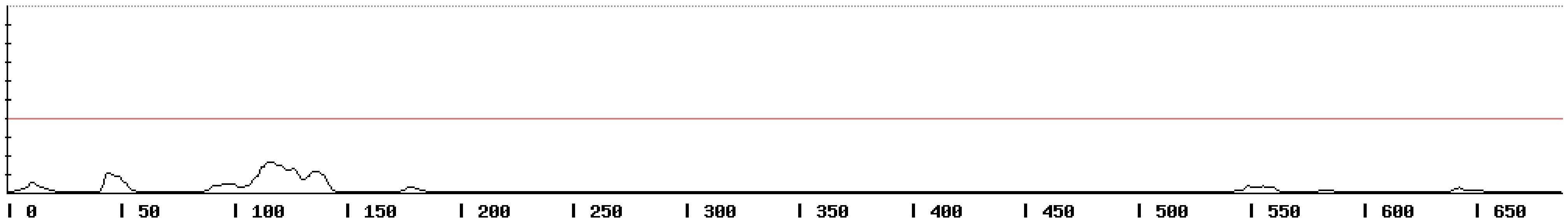

Predictions for Elaeisguineensis_XP_010935813.1

Viterbi Path	
Viterbi Path	
Posterior @ 0.4	
Posterior @ 0.4	

Posterior Graphic. Threshold Marked @ 0.4

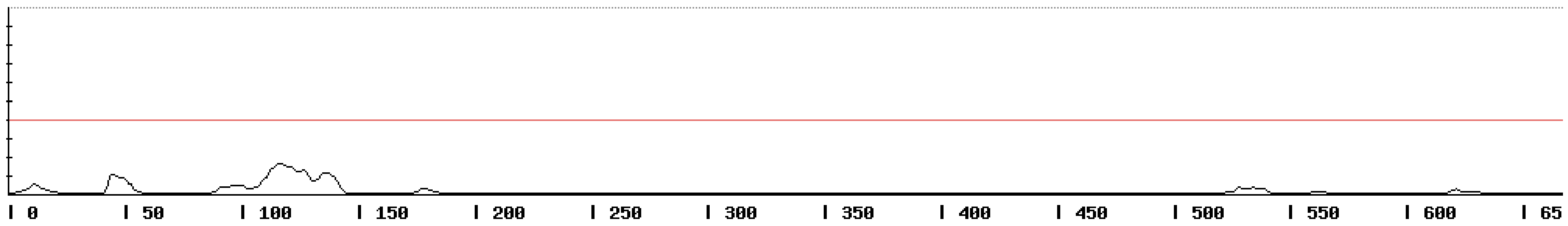

Predictions for Elaeisguineensis_XP_010935925.1

Viterbi Path

Viterbi Path

Posterior @ 0.4

Posterior @ 0.4

Posterior Graphic. Threshold Marked @ 0.4

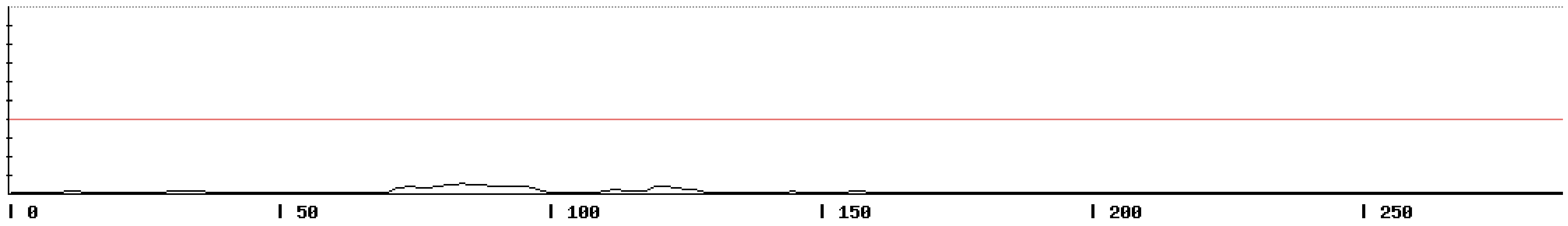

Predictions for Elaeisguineensis_XP_010936653.1

Viterbi Path

Viterbi Path

Posterior @ 0.4

Posterior @ 0.4

Posterior Graphic. Threshold Marked @ 0.4

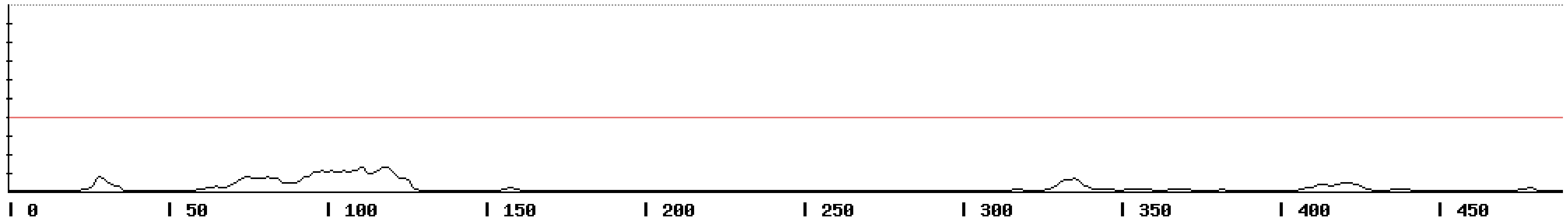

Predictions for Elaeisguineensis_XP_010936654.1

Viterbi Path

Viterbi Path

Posterior @ 0.4

Posterior @ 0.4

Posterior Graphic. Threshold Marked @ 0.4

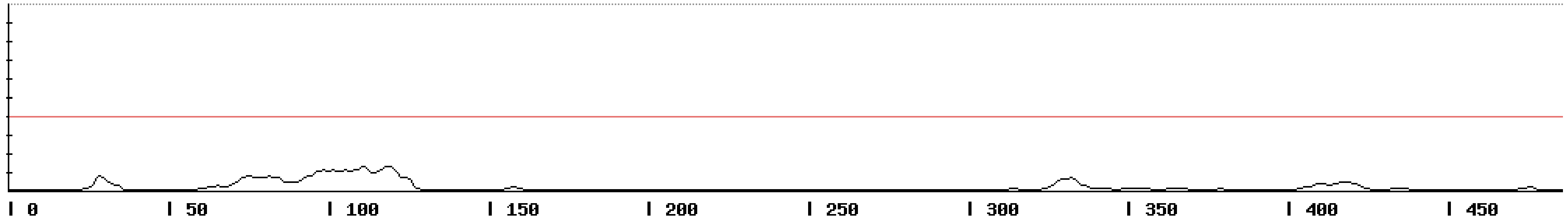

Predictions for Elaeisguineensis_XP_010937197.1

Viterbi Path

Viterbi Path

Posterior @ 0.4

Posterior @ 0.4

Posterior Graphic. Threshold Marked @ 0.4

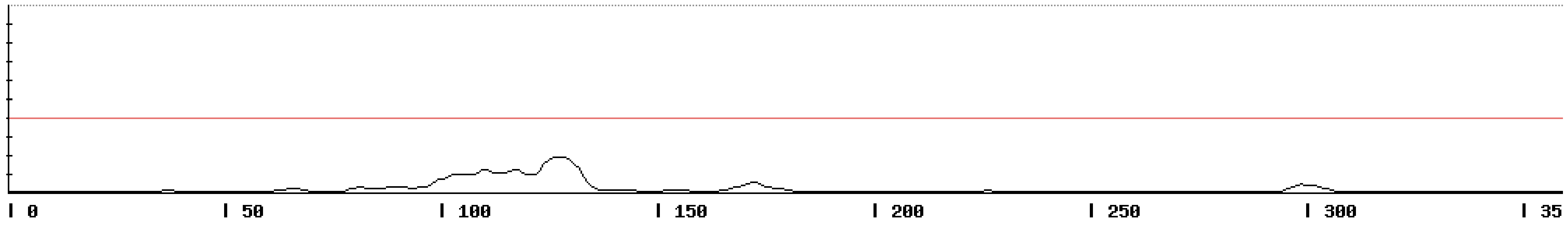

Predictions for Elaeisguineensis_XP_010937198.1

Viterbi Path

Viterbi Path

Posterior @ 0.4

Posterior @ 0.4

Posterior Graphic. Threshold Marked @ 0.4

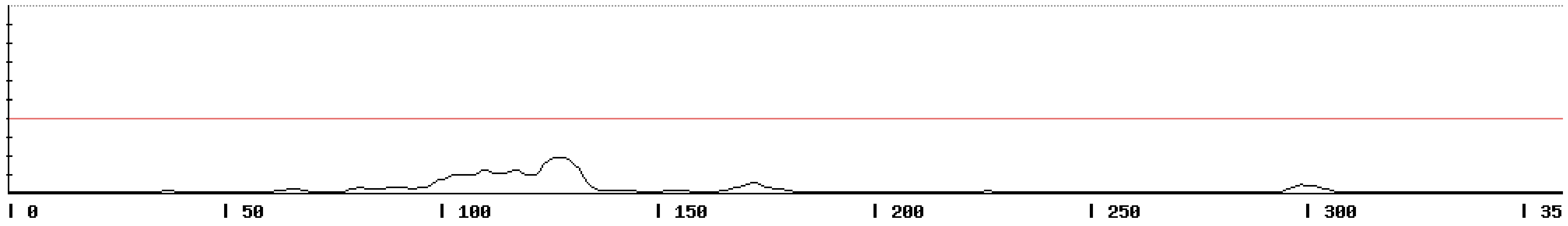

Predictions for Elaeisguineensis_XP_010937199.1

Viterbi Path

Viterbi Path

Posterior @ 0.4

Posterior @ 0.4

Posterior Graphic. Threshold Marked @ 0.4

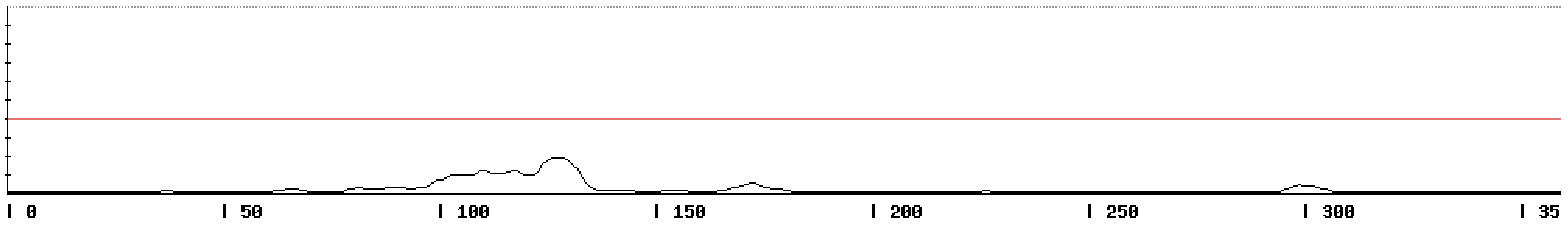

Predictions for Elaeisguineensis_XP_010937200.1

Viterbi Path

Viterbi Path

Posterior @ 0.4

Posterior @ 0.4

Posterior Graphic. Threshold Marked @ 0.4

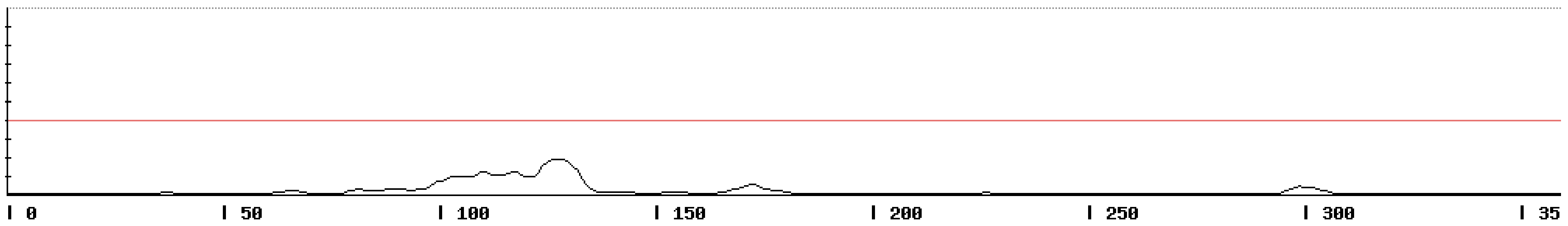

Predictions for Elaeisguineensis_XP_010937201.1	
Viterbi Path	
Viterbi Path	
Posterior @ 0.4	
Posterior @ 0.4	
Posterior Graphic. Threshold Marked @ 0.4	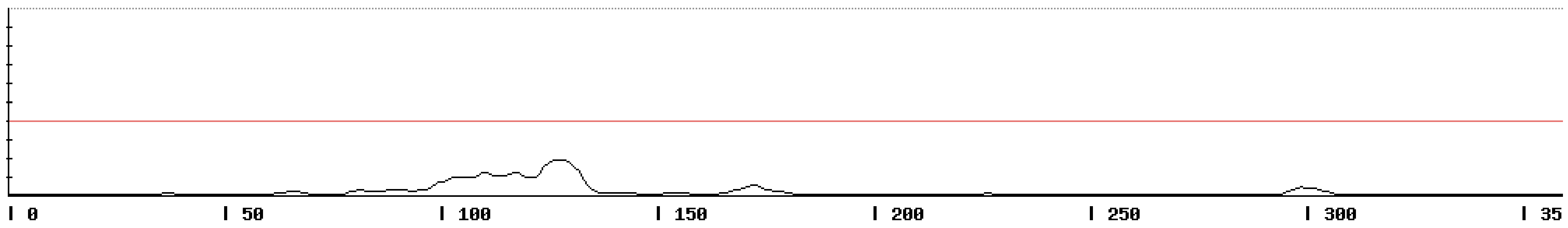

Predictions for Elaeisguineensis_XP_010937202.1	
Viterbi Path	
Viterbi Path	
Posterior @ 0.4	
Posterior @ 0.4	
Posterior Graphic. Threshold Marked @ 0.4	

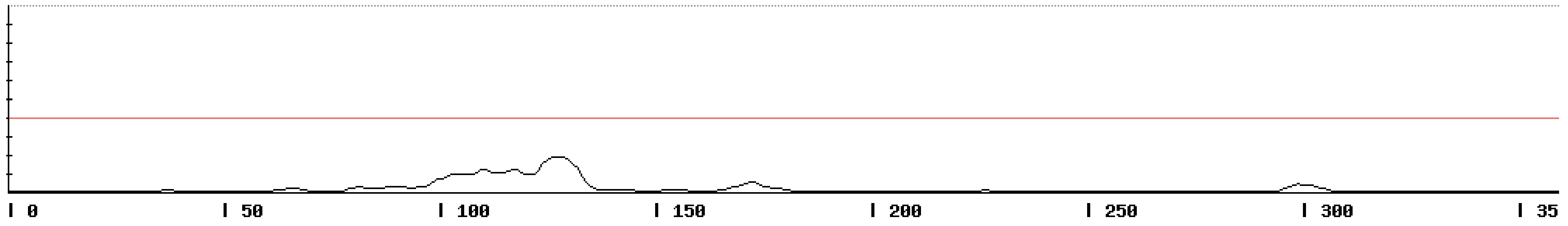

Predictions for Elaeisguineensis_XP_010937354.1

Viterbi Path	
Viterbi Path	
Posterior @ 0.4	
Posterior @ 0.4	
Posterior Graphic. Threshold Marked @ 0.4	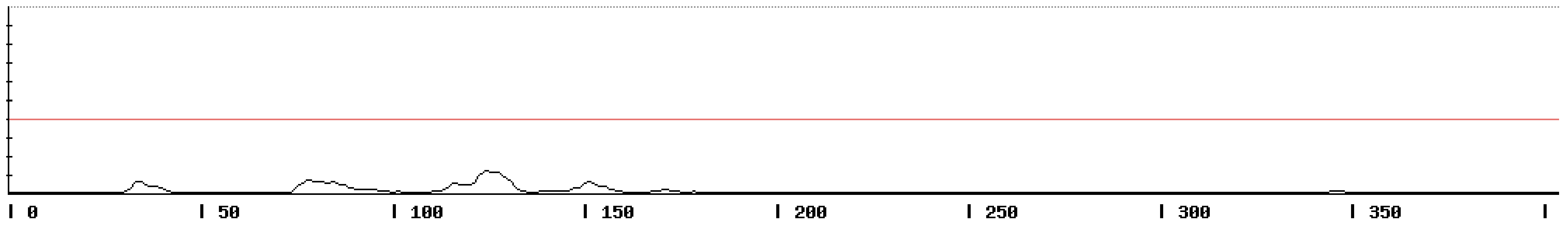

Predictions for Elaeisguineensis_XP_010938113.1

Viterbi Path	
Viterbi Path	
Posterior @ 0.4	
Posterior @ 0.4	

Posterior Graphic. Threshold Marked @ 0.4

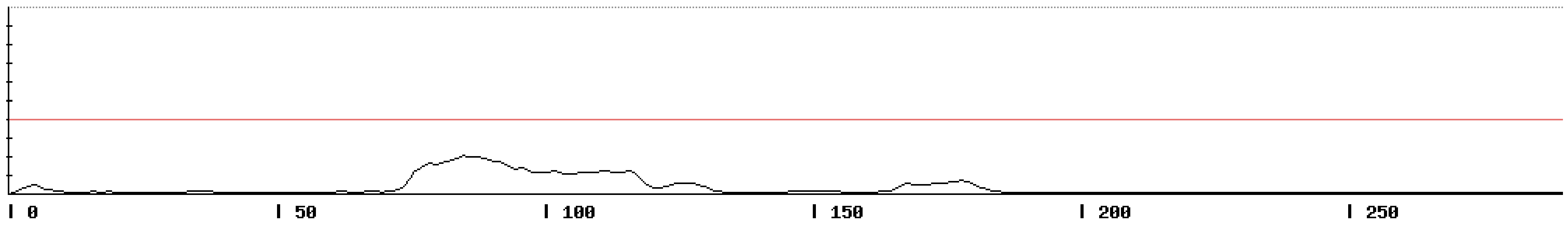

Predictions for Elaeisguineensis_XP_010938117.1

Viterbi Path

Viterbi Path

Posterior @ 0.4

Posterior @ 0.4

Posterior Graphic. Threshold Marked @ 0.4

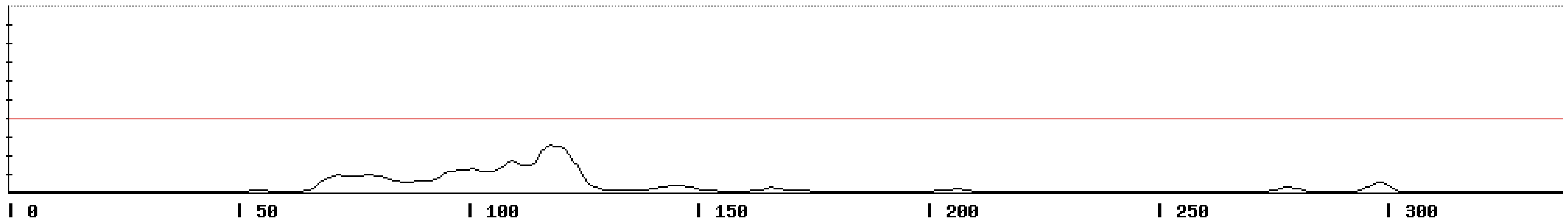

Predictions for Elaeisguineensis_XP_010938118.1

Viterbi Path

Viterbi Path

Posterior @ 0.4

Posterior @ 0.4

Posterior Graphic. Threshold Marked @ 0.4

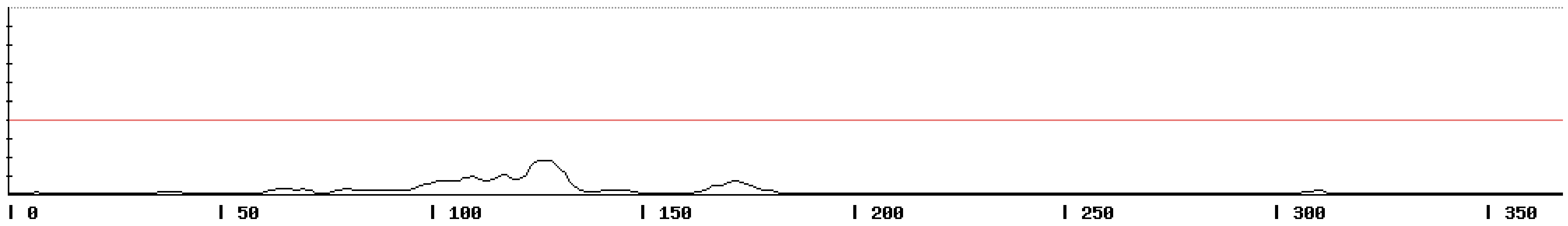

Predictions for Elaeisguineensis_XP_010939027.1

Viterbi Path

Viterbi Path

Posterior @ 0.4

Posterior @ 0.4

Posterior Graphic. Threshold Marked @ 0.4

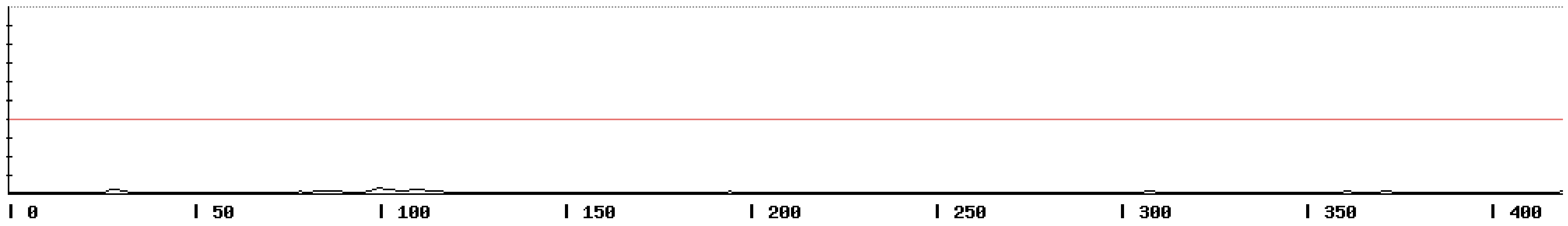

Predictions for Elaeisguineensis_XP_010939028.1

Viterbi Path

Viterbi Path

Posterior @ 0.4	
Posterior @ 0.4	
Posterior Graphic. Threshold Marked @ 0.4	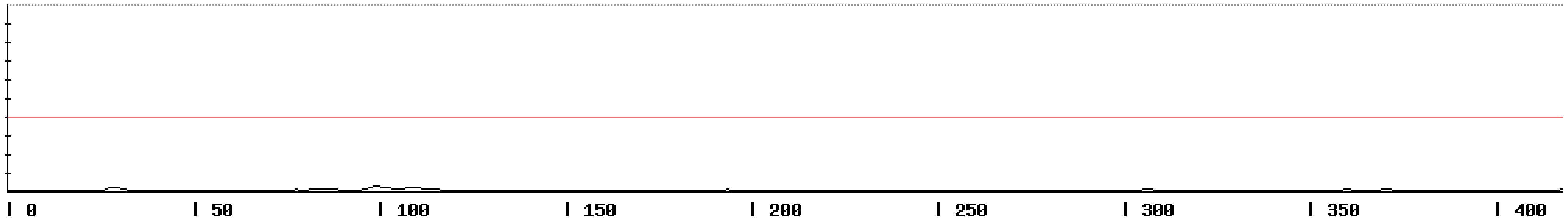

Predictions for Elaeisguineensis_XP_010939029.1	
Viterbi Path	
Viterbi Path	
Posterior @ 0.4	
Posterior @ 0.4	
Posterior Graphic. Threshold Marked @ 0.4	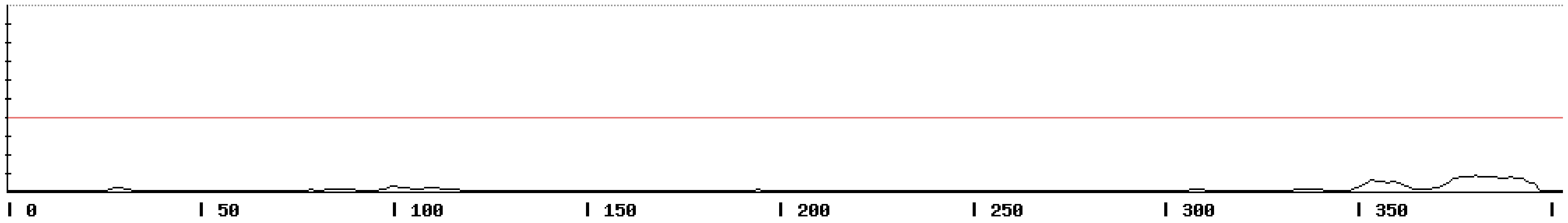

Predictions for Elaeisguineensis_XP_010939032.1	
Viterbi Path	
Viterbi Path	

Posterior @ 0.4

Posterior @ 0.4

Posterior Graphic. Threshold Marked @ 0.4

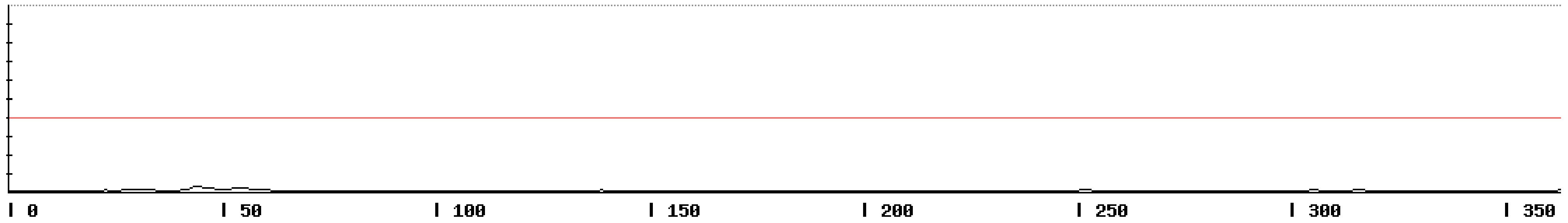

Predictions for Elaeisguineensis_XP_010939033.1

Viterbi Path

Viterbi Path

Posterior @ 0.4

Posterior @ 0.4

Posterior Graphic. Threshold Marked @ 0.4

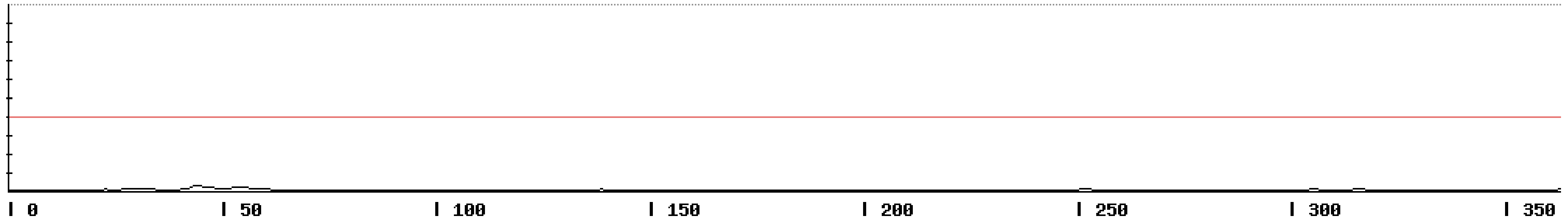

Predictions for Elaeisguineensis_XP_010939825.1

Viterbi Path

Viterbi Path

Posterior @ 0.4

Posterior @ 0.4

Posterior Graphic. Threshold Marked @ 0.4

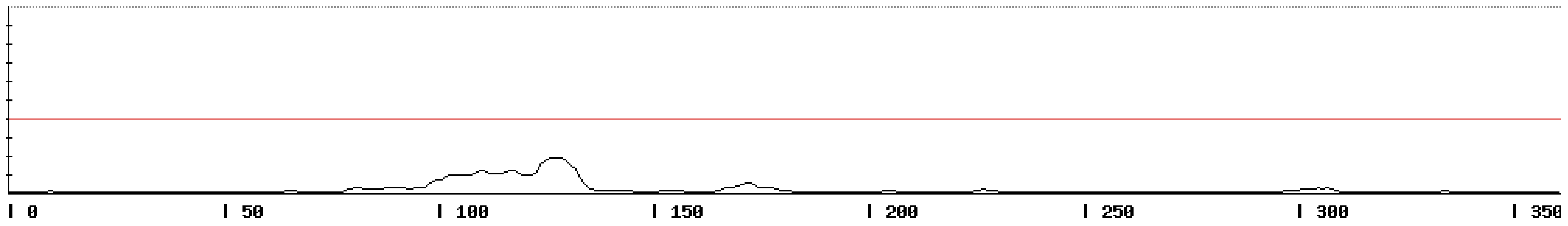

Predictions for Elaeisguineensis_XP_010939826.1

Viterbi Path

Viterbi Path

Posterior @ 0.4

Posterior @ 0.4

Posterior Graphic. Threshold Marked @ 0.4

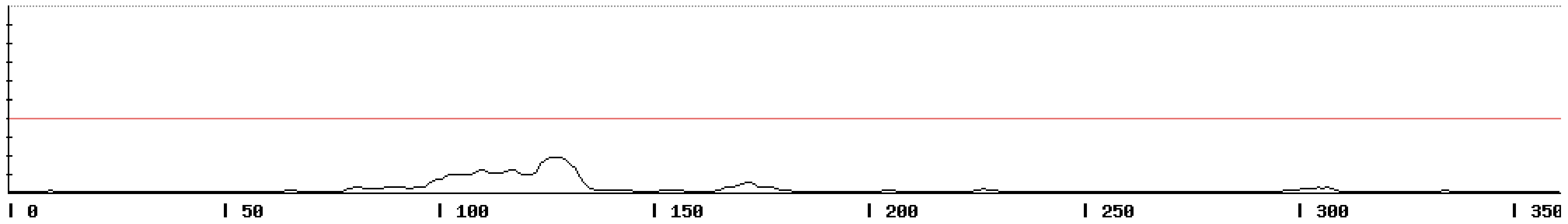

Predictions for Elaeisguineensis_XP_010939827.1

Viterbi Path

Viterbi Path

Posterior @ 0.4

Posterior @ 0.4

Posterior Graphic. Threshold Marked @ 0.4

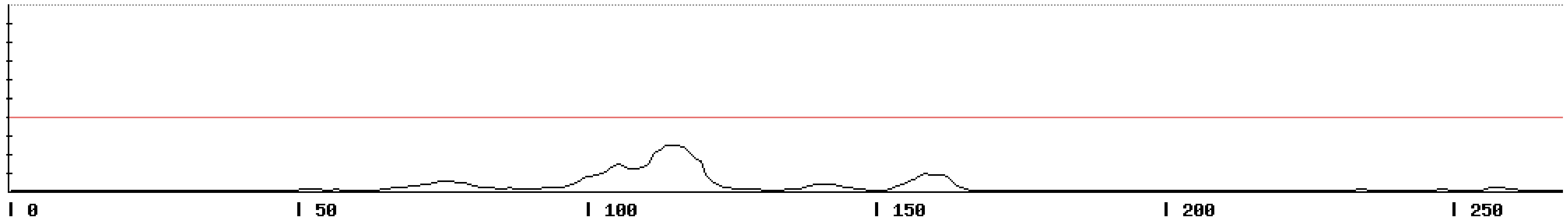

Predictions for Elaeisguineensis_XP_010939828.1

Viterbi Path

Viterbi Path

Posterior @ 0.4

Posterior @ 0.4

Posterior Graphic. Threshold Marked @ 0.4

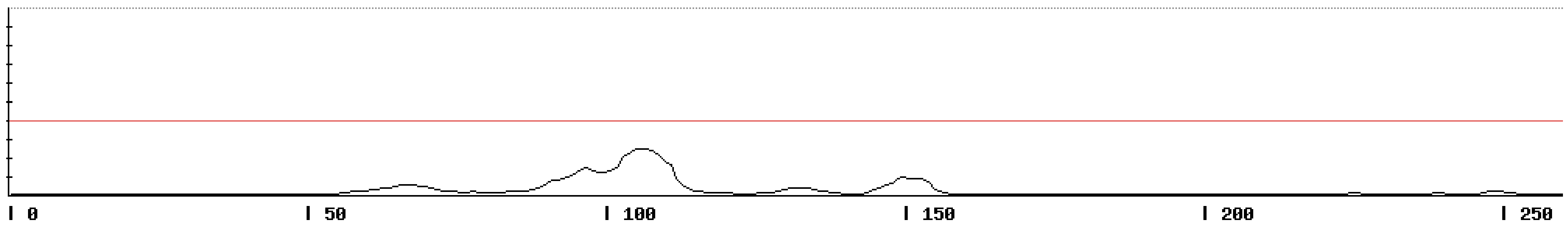

Predictions for Elaeisguineensis_XP_010940228.1

Viterbi Path

Viterbi Path

Posterior @ 0.4

Posterior @ 0.4

Posterior Graphic. Threshold Marked @ 0.4

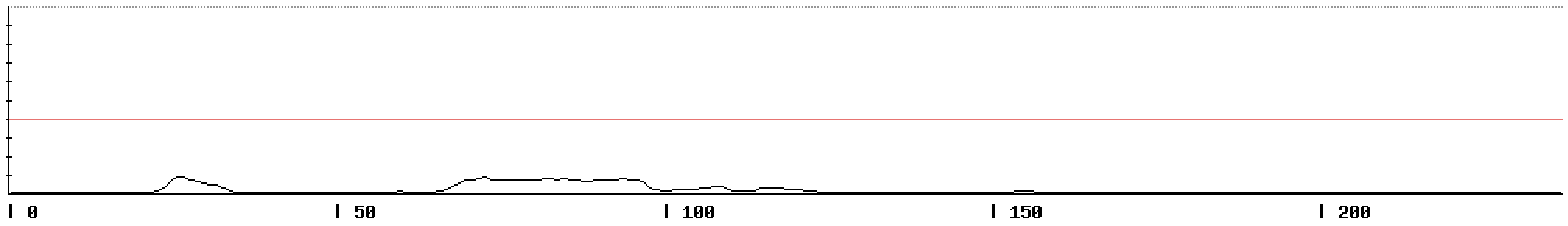

Predictions for Elaeisguineensis_XP_010940229.1

Viterbi Path

Viterbi Path

Posterior @ 0.4

Posterior @ 0.4

Posterior Graphic. Threshold Marked @ 0.4

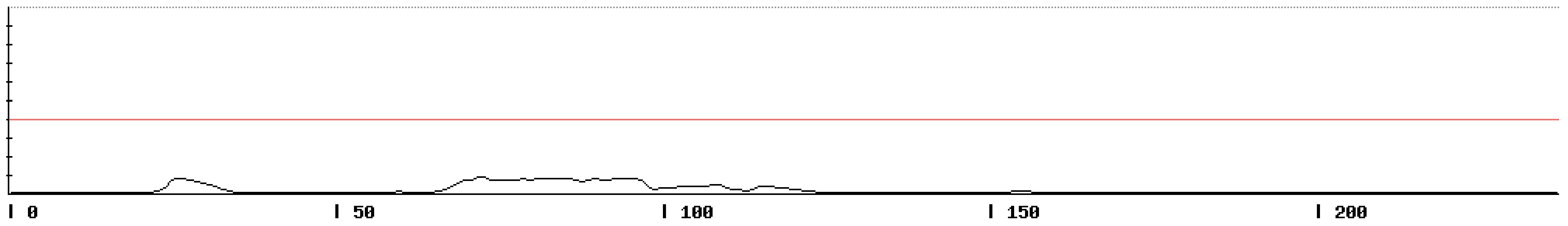

Predictions for Elaeisguineensis_XP_010940544.1

Viterbi Path

Viterbi Path

Posterior @ 0.4

Posterior @ 0.4

Posterior Graphic. Threshold Marked @ 0.4

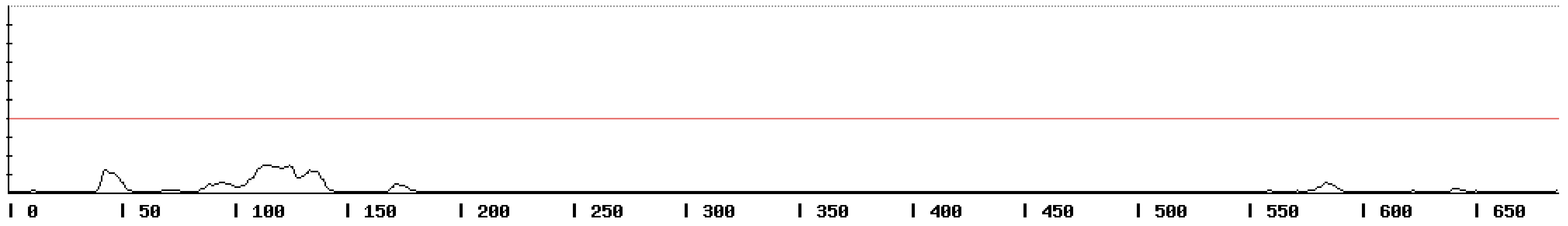

Predictions for Elaeisguineensis_XP_010940742.1

Viterbi Path

Viterbi Path

Posterior @ 0.4

Posterior @ 0.4

Posterior Graphic. Threshold Marked @ 0.4	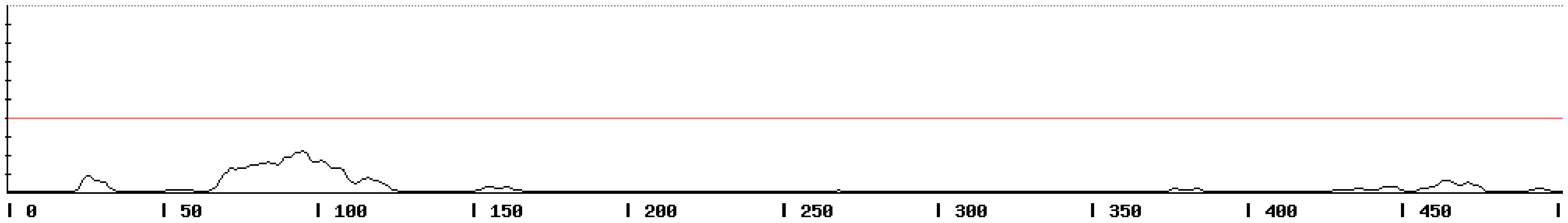

Predictions for Elaeisguineensis_XP_010940850.1

Viterbi Path	
Viterbi Path	
Posterior @ 0.4	
Posterior @ 0.4	
Posterior Graphic. Threshold Marked @ 0.4	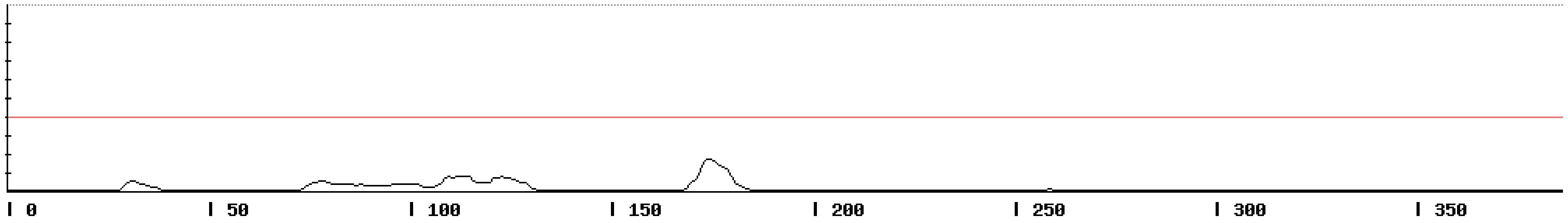

Predictions for Elaeisguineensis_XP_010941123.1

Viterbi Path	
Viterbi Path	

Posterior @ 0.4

Posterior @ 0.4

Posterior Graphic. Threshold Marked @ 0.4

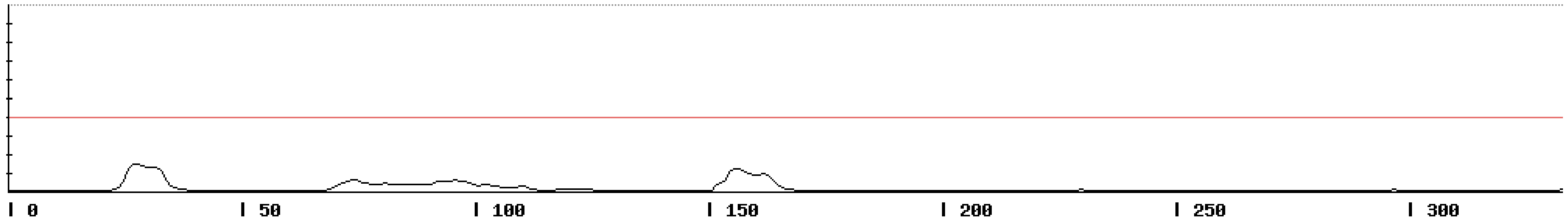

Predictions for Elaeisguineensis_XP_010941180.1

Viterbi Path

Viterbi Path

Posterior @ 0.4

Posterior @ 0.4

Posterior Graphic. Threshold Marked @ 0.4

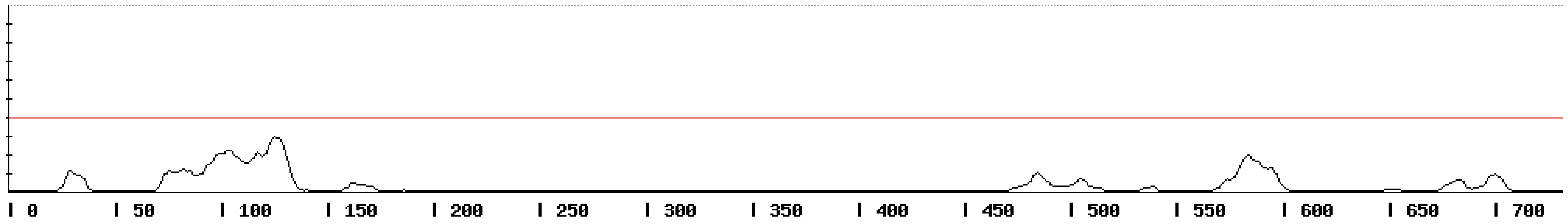

Predictions for Elaeisguineensis_XP_010941181.1

Viterbi Path

Viterbi Path

Posterior @ 0.4

Posterior @ 0.4

Posterior Graphic. Threshold Marked @ 0.4

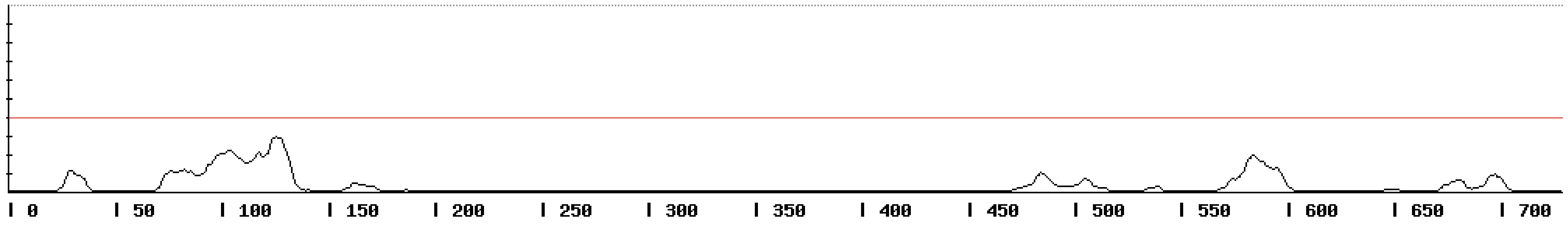

Predictions for Elaeisguineensis_XP_010941405.1

Viterbi Path

Viterbi Path

Posterior @ 0.4

Posterior @ 0.4

Posterior Graphic. Threshold Marked @ 0.4

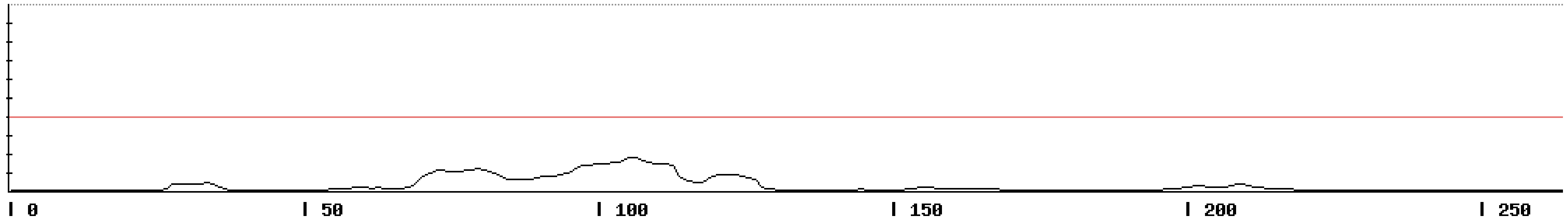

Predictions for Elaeisguineensis_XP_010941568.1

Viterbi Path

Viterbi Path

Posterior @ 0.4

Posterior @ 0.4

Posterior Graphic. Threshold Marked @ 0.4

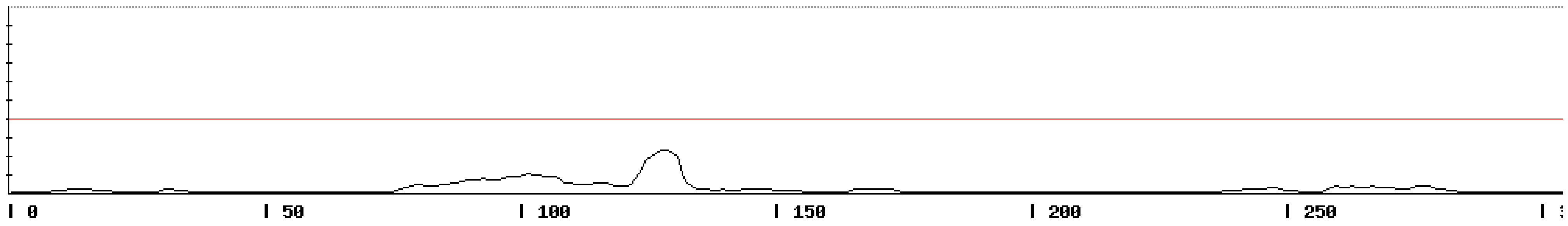

Predictions for Elaeisguineensis_XP_010942594.1

Viterbi Path

Viterbi Path

Posterior @ 0.4

Posterior @ 0.4

Posterior Graphic. Threshold Marked @ 0.4

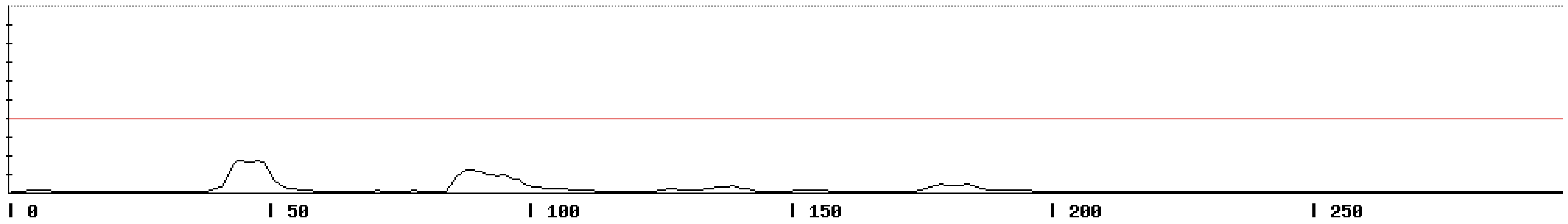

Predictions for Elaeisguineensis_XP_010943148.1	
Viterbi Path	
Viterbi Path	
Posterior @ 0.4	
Posterior @ 0.4	
Posterior Graphic. Threshold Marked @ 0.4	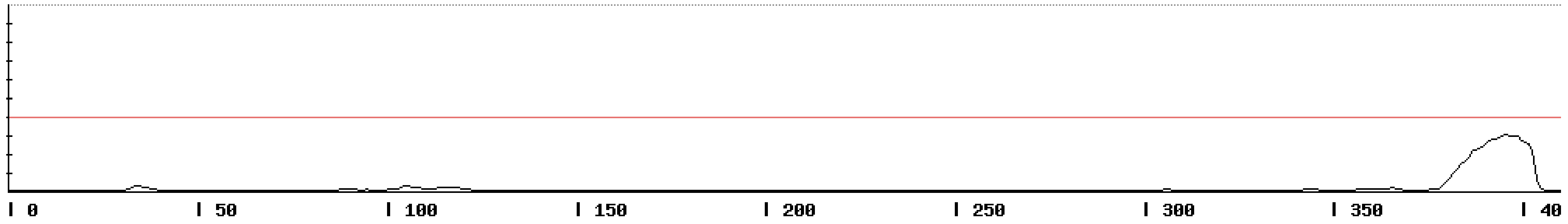

Predictions for Elaeisguineensis_XP_010943149.1	
Viterbi Path	
Viterbi Path	
Posterior @ 0.4	
Posterior @ 0.4	
Posterior Graphic. Threshold Marked @ 0.4	

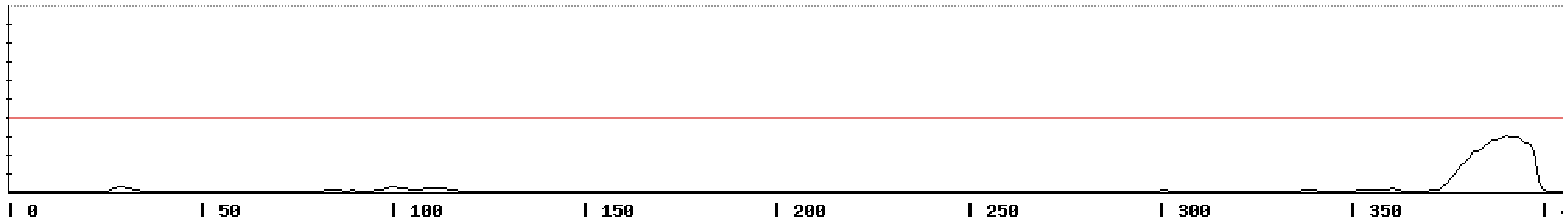

Predictions for Elaeisguineensis_XP_010943150.1

Viterbi Path

Viterbi Path

Posterior @ 0.4

Posterior @ 0.4

Posterior Graphic. Threshold Marked @ 0.4

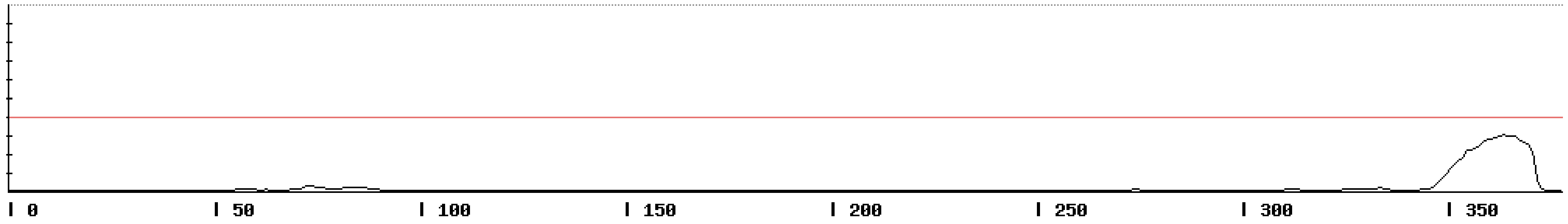

Predictions for Elaeisguineensis_XP_010943151.1

Viterbi Path

Viterbi Path

Posterior @ 0.4

Posterior @ 0.4

Posterior Graphic. Threshold Marked @ 0.4

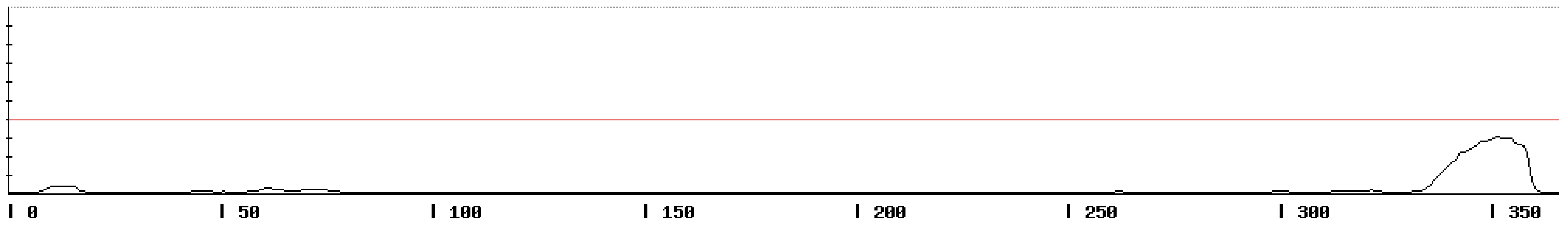

Predictions for Elaeisguineensis_XP_010943745.1

Viterbi Path

Viterbi Path

Posterior @ 0.4

Posterior @ 0.4

Posterior Graphic. Threshold Marked @ 0.4

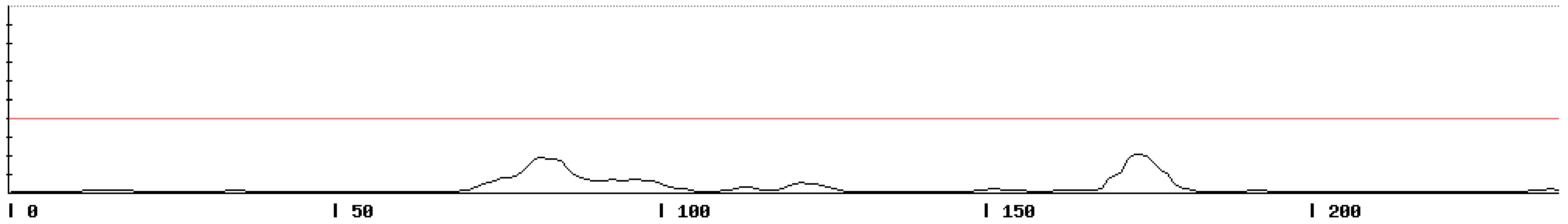

Predictions for Elaeisguineensis_XP_010943816.1

Viterbi Path

Viterbi Path

Posterior @ 0.4

Posterior @ 0.4

Posterior Graphic. Threshold Marked @ 0.4

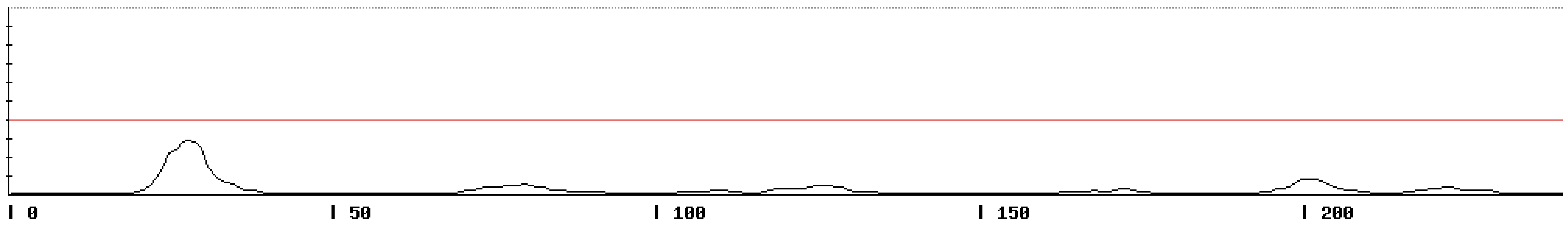

Predictions for Elaeisguineensis_XP_010943933.1

Viterbi Path	
Viterbi Path	
Posterior @ 0.4	
Posterior @ 0.4	
Posterior Graphic. Threshold Marked @ 0.4	

Revision r.9 - Last Updated on : February 24 2015 17:45:20.

alex.nguyenba@utoronto.ca

Intended to be used with Firefox

Viterbi Path

Posterior @ 0.4

Posterior @ 0.4

Posterior Graphic. Threshold Marked @ 0.4

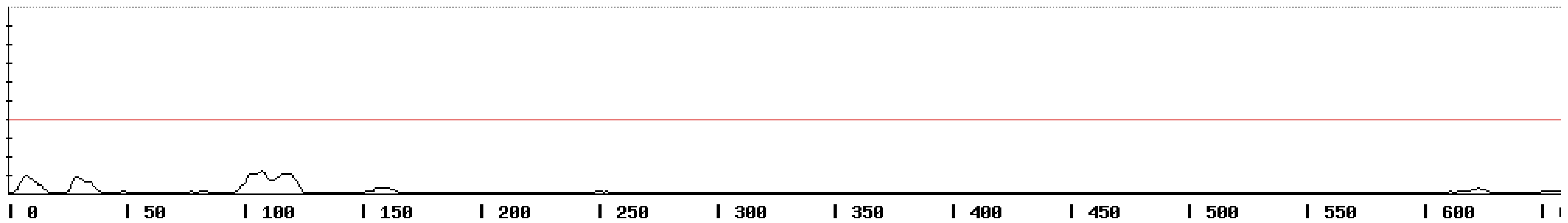

Predictions for Eragrostistef_462845257

Viterbi Path

Viterbi Path

Posterior @ 0.4

Posterior @ 0.4

Posterior Graphic. Threshold Marked @ 0.4

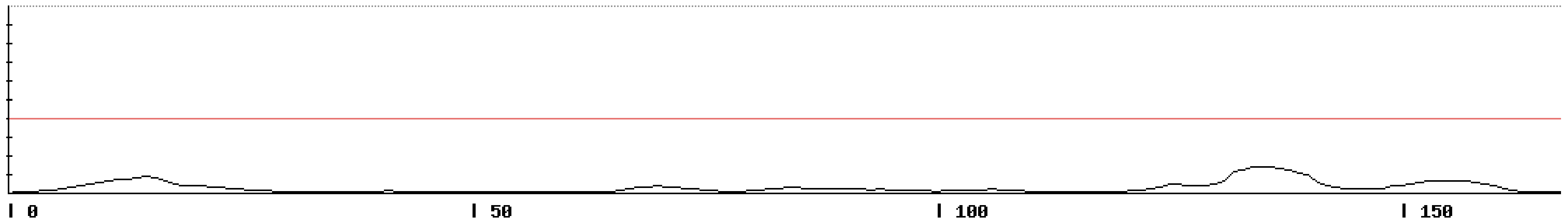

Predictions for Eragrostistef_462845378

Viterbi Path	
Viterbi Path	
Posterior @ 0.4	
Posterior @ 0.4	
Posterior Graphic. Threshold Marked @ 0.4	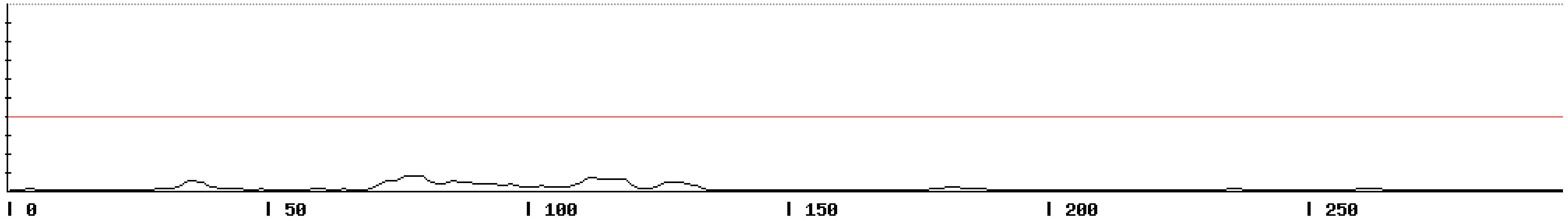

Predictions for Eragrostistef_462846211

Viterbi Path	
Viterbi Path	
Posterior @ 0.4	
Posterior @ 0.4	
Posterior Graphic. Threshold Marked @ 0.4	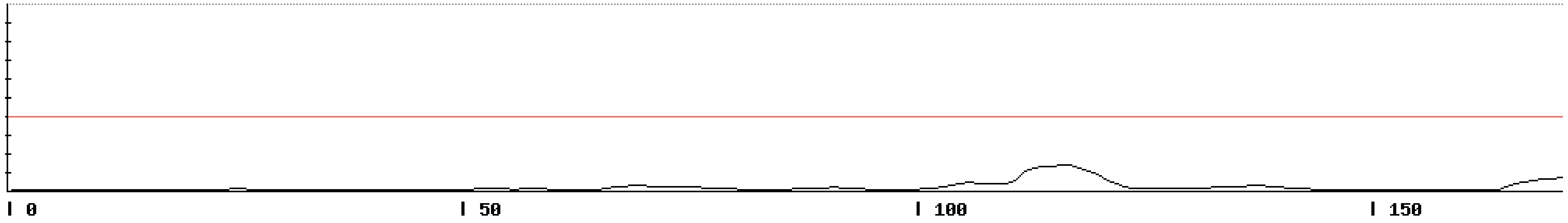

Predictions for Eragrostistef_462847284

Viterbi Path	
Viterbi Path	
Posterior @ 0.4	
Posterior @ 0.4	
Posterior Graphic. Threshold Marked @ 0.4	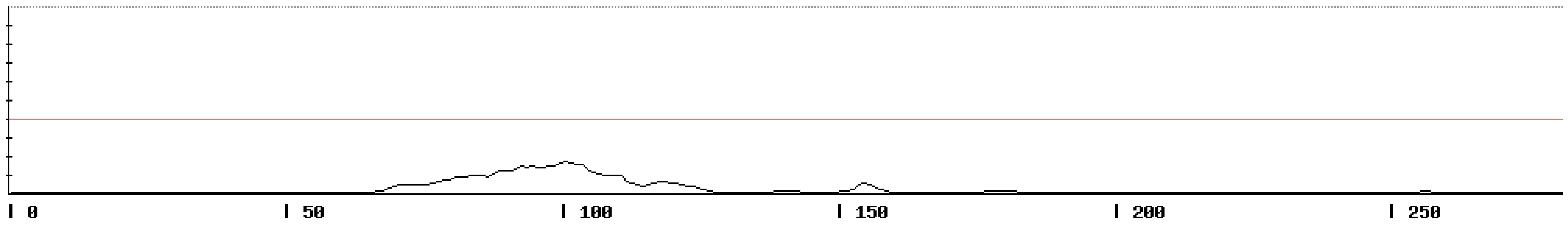

Predictions for Eragrostistef_462848454

Viterbi Path	
Viterbi Path	
Posterior @ 0.4	
Posterior @ 0.4	
Posterior Graphic. Threshold Marked @ 0.4	

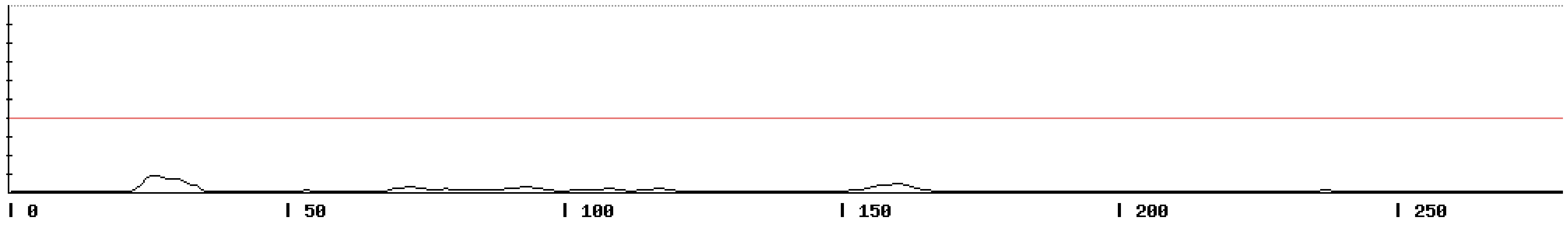

Predictions for Eragrostistef_462849304

Viterbi Path

Viterbi Path

Posterior @ 0.4

Posterior @ 0.4

Posterior Graphic. Threshold Marked @ 0.4

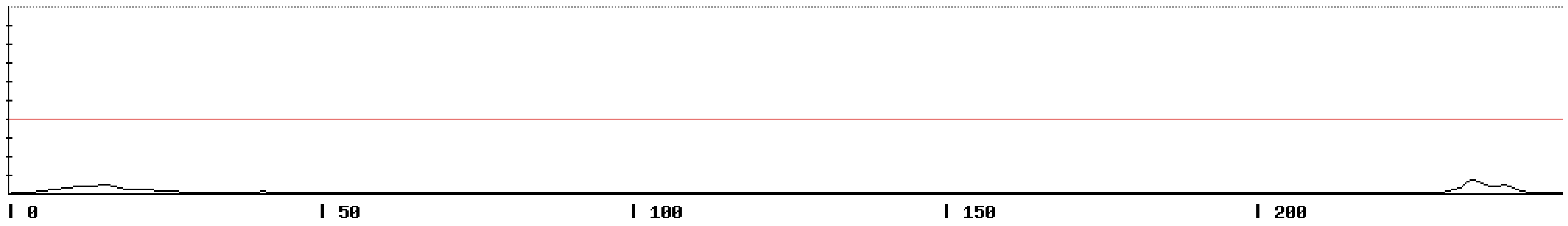

Predictions for Eragrostistef_462849307

Viterbi Path

Viterbi Path

Posterior @ 0.4

Posterior @ 0.4

Posterior Graphic. Threshold Marked @ 0.4

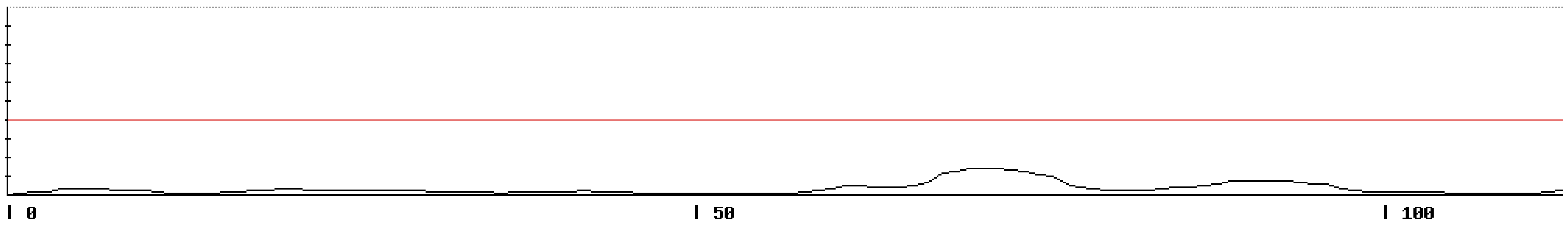

Predictions for Eragrostistef_462849400

Viterbi Path

Viterbi Path

Posterior @ 0.4: 137 - RKRRKI - 142

Posterior @ 0.4

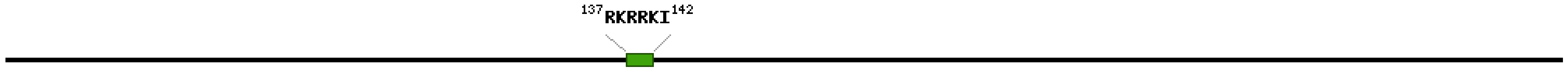

Posterior Graphic. Threshold Marked @ 0.4

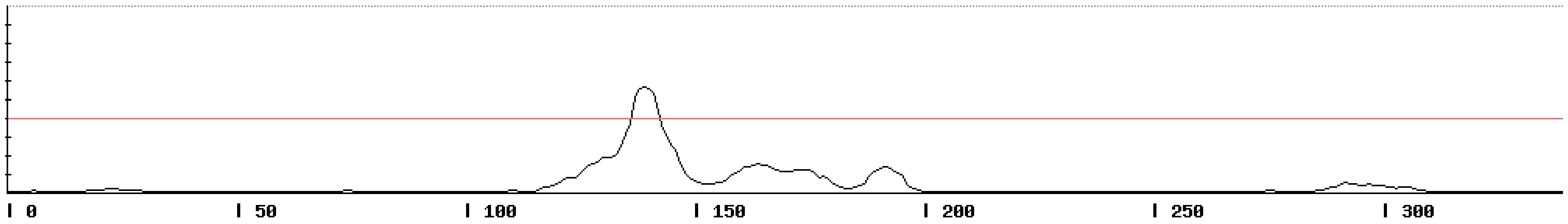

Predictions for Eragrostistef_462850766

Viterbi Path

Viterbi Path

Posterior @ 0.4

Posterior @ 0.4

Posterior Graphic. Threshold Marked @ 0.4

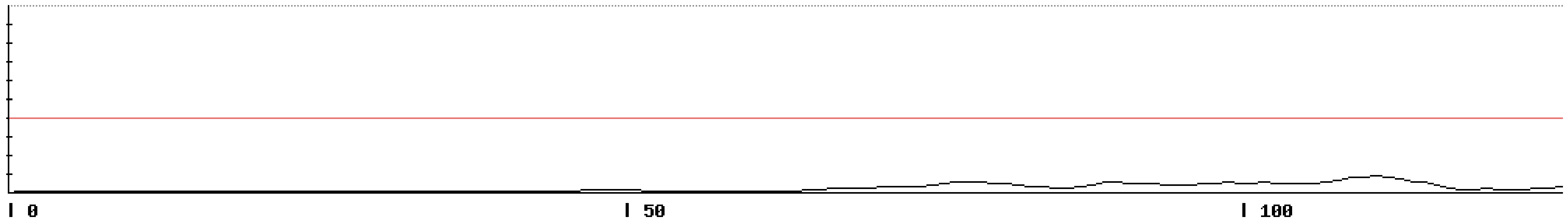

Predictions for Eragrostistef_462851085

Viterbi Path

Viterbi Path

Posterior @ 0.4

Posterior @ 0.4

Posterior Graphic. Threshold Marked @ 0.4

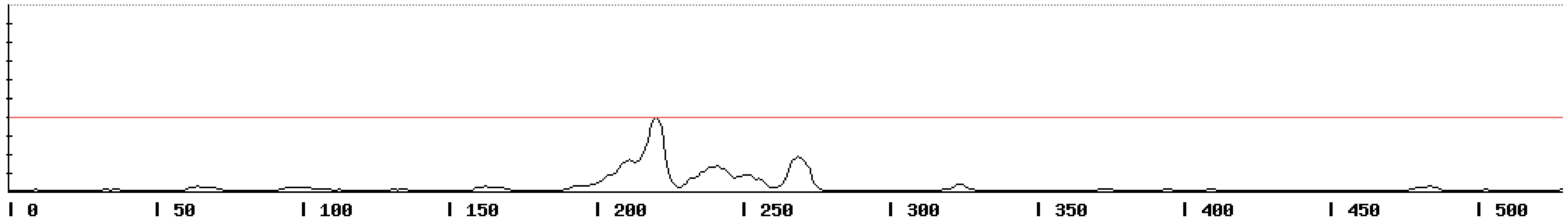

Predictions for Eragrostistef_462851776

Viterbi Path

Viterbi Path

Posterior @ 0.4

Posterior @ 0.4

Posterior Graphic. Threshold Marked @ 0.4

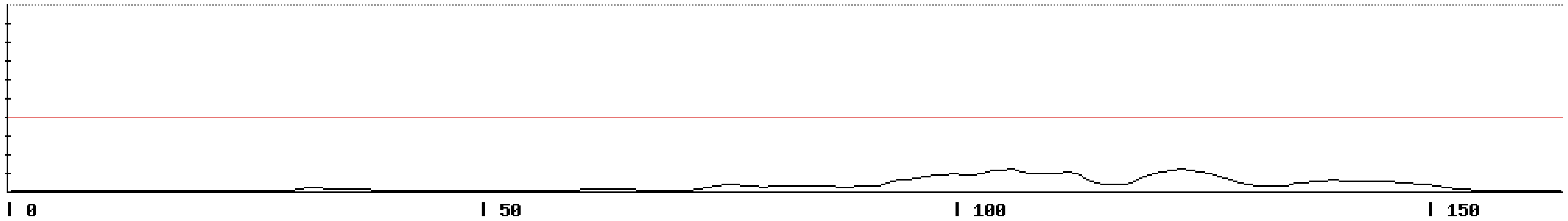

Predictions for Eragrostistef_462852590

Viterbi Path

Viterbi Path

Posterior @ 0.4

Posterior @ 0.4

Posterior Graphic. Threshold Marked @ 0.4

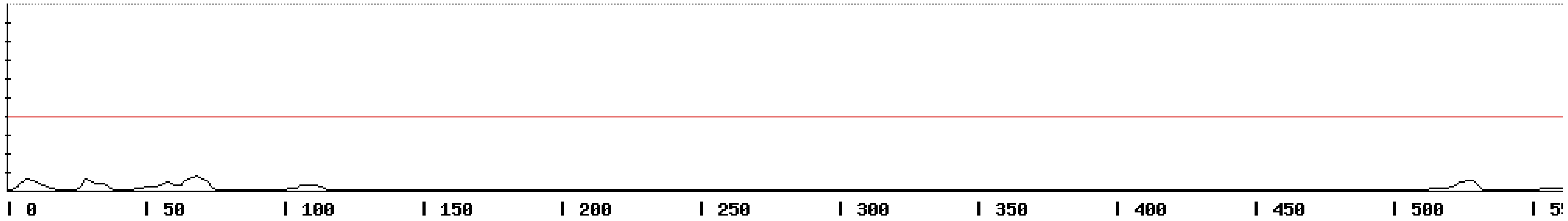

Predictions for Eragrostistef_462853180

Viterbi Path

Viterbi Path

Posterior @ 0.4

Posterior @ 0.4

Posterior Graphic. Threshold Marked @ 0.4

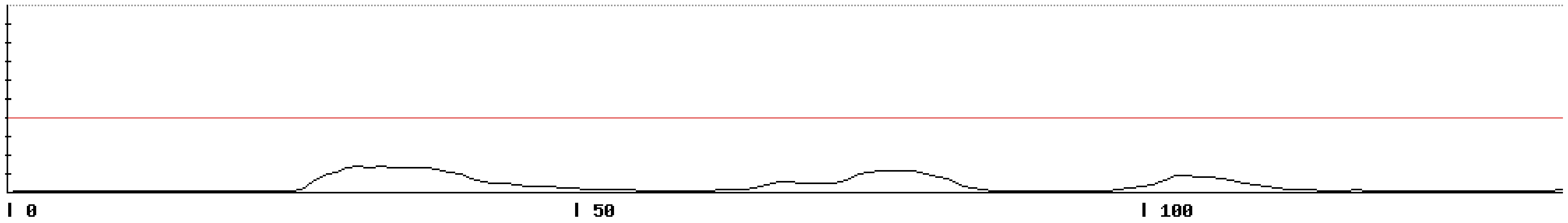

Predictions for Eragrostistef_462853715

Viterbi Path

Viterbi Path

Posterior @ 0.4 254 - RRPSSRR - 260

Posterior @ 0.4

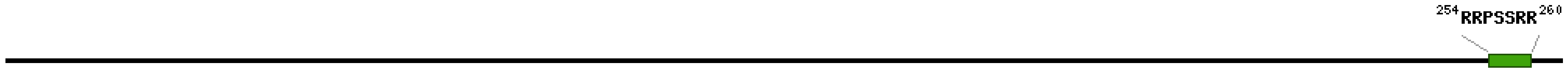

Posterior Graphic. Threshold Marked @ 0.4

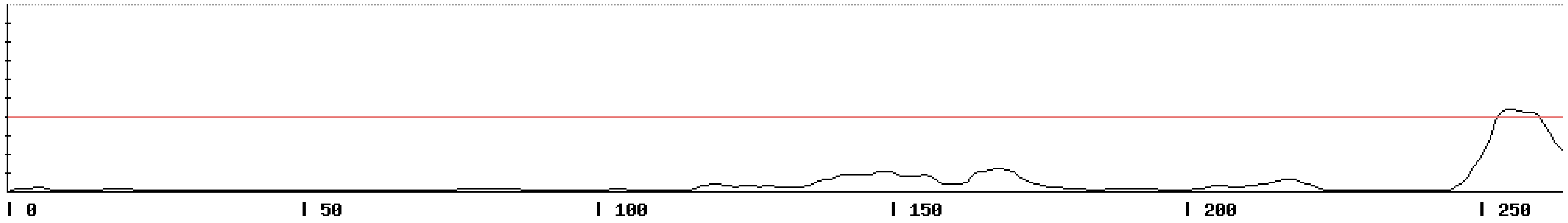

Predictions for Eragrostistef_462854596

Viterbi Path

Viterbi Path

Posterior @ 0.4

Posterior @ 0.4

Posterior Graphic. Threshold Marked @ 0.4

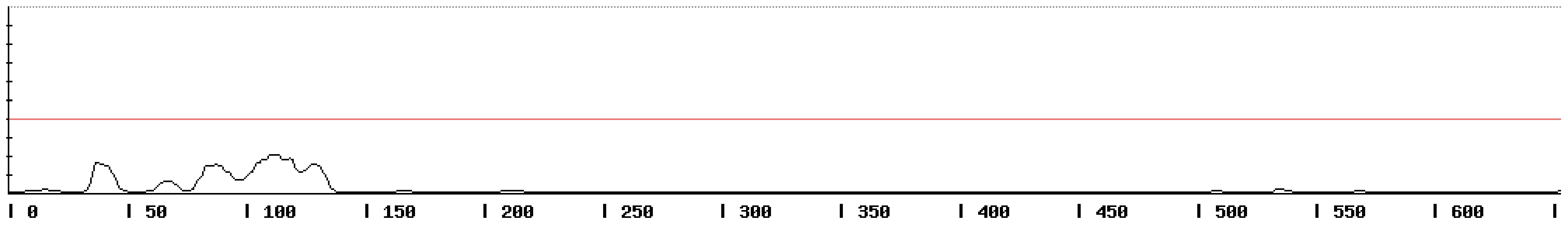

Predictions for Eragrostistef_462855362

Viterbi Path

Viterbi Path

Posterior @ 0.4

Posterior @ 0.4

Posterior Graphic. Threshold Marked @ 0.4

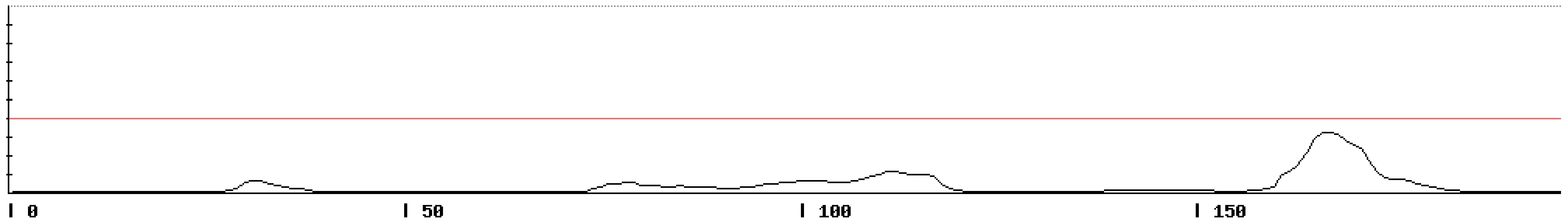

Predictions for Eragrostistef_462857692

Viterbi Path	
Viterbi Path	
Posterior @ 0.4	
Posterior @ 0.4	
Posterior Graphic. Threshold Marked @ 0.4	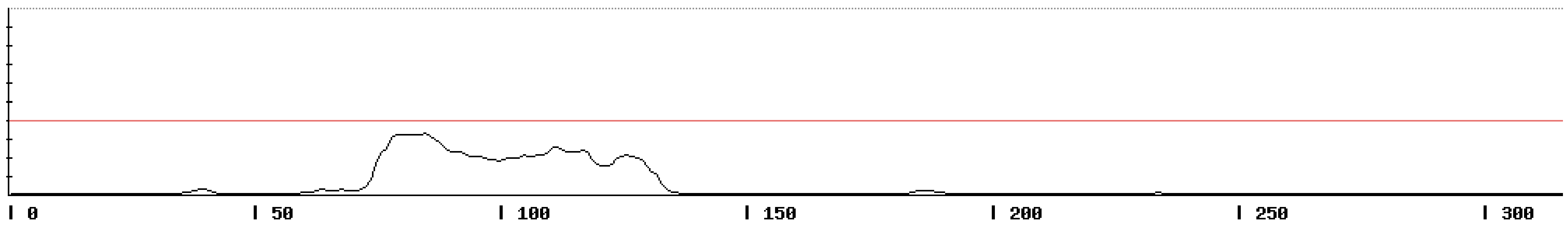

Predictions for Eragrostistef_462859342

Viterbi Path	
Viterbi Path	
Posterior @ 0.4	143 - KKATERAGAKSPKKVKVA - 160
Posterior @ 0.4	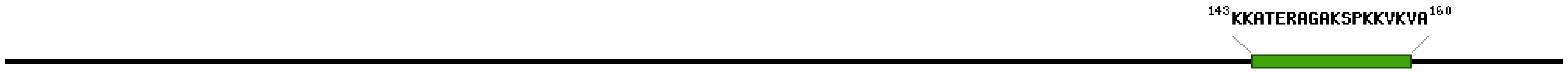
Posterior Graphic. Threshold Marked @ 0.4	

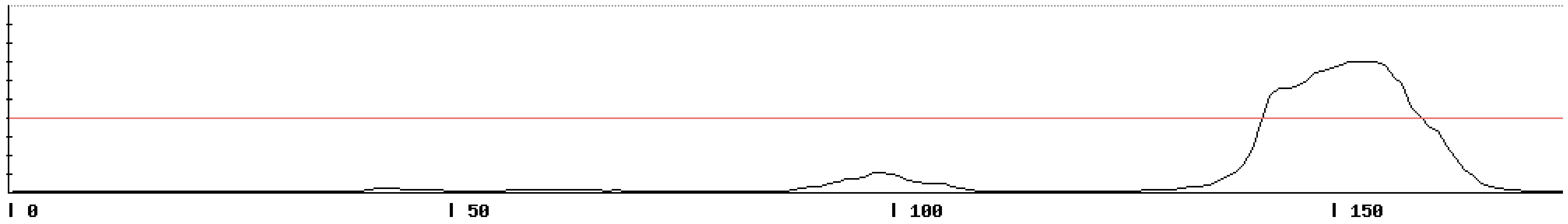

Predictions for Eragrostistef_462859354

Viterbi Path	
Viterbi Path	
Posterior @ 0.4	
Posterior @ 0.4	
Posterior Graphic. Threshold Marked @ 0.4	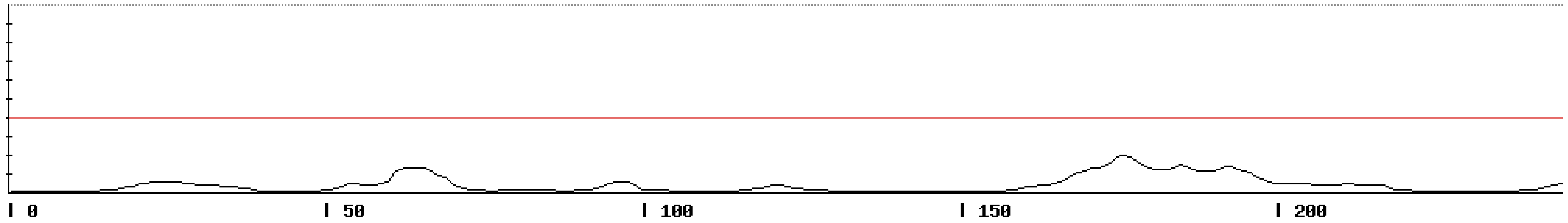

Predictions for Eragrostistef_462859360

Viterbi Path	
Viterbi Path	
Posterior @ 0.4	
Posterior @ 0.4	

Posterior Graphic. Threshold Marked @ 0.4

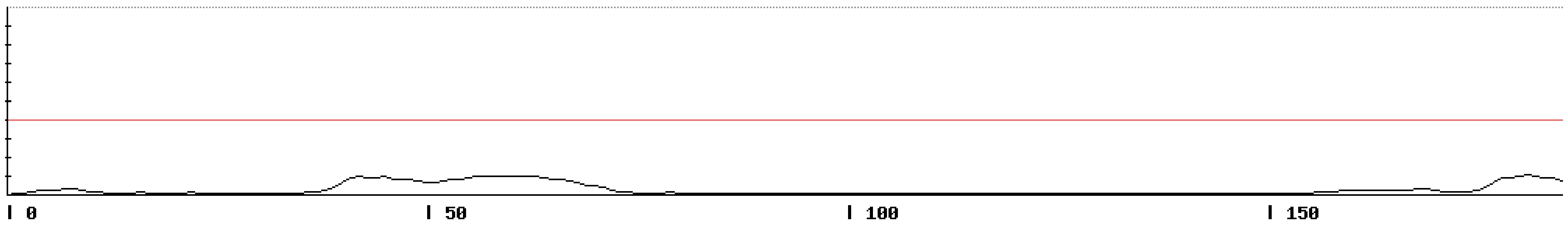

Predictions for Eragrostistef_462859722

Viterbi Path

Viterbi Path

Posterior @ 0.4

121 - RKRRK - 125

Posterior @ 0.4

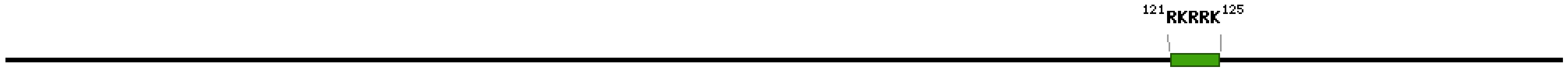

Posterior Graphic. Threshold Marked @ 0.4

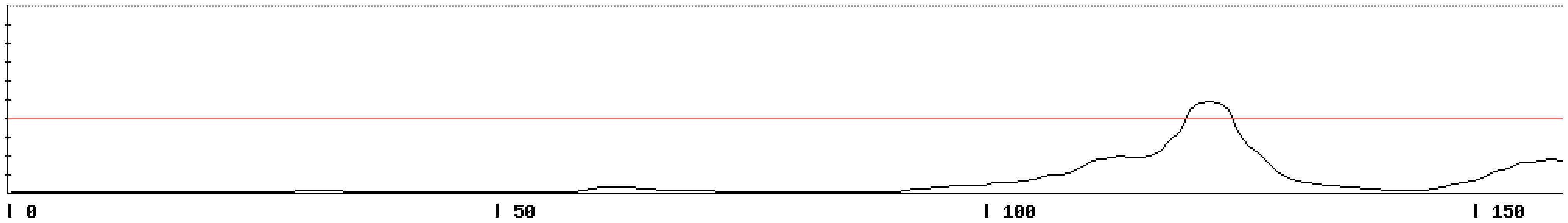

Predictions for Eragrostistef_462859892

Viterbi Path

Viterbi Path

Posterior @ 0.4

Posterior @ 0.4

Posterior Graphic. Threshold Marked @ 0.4

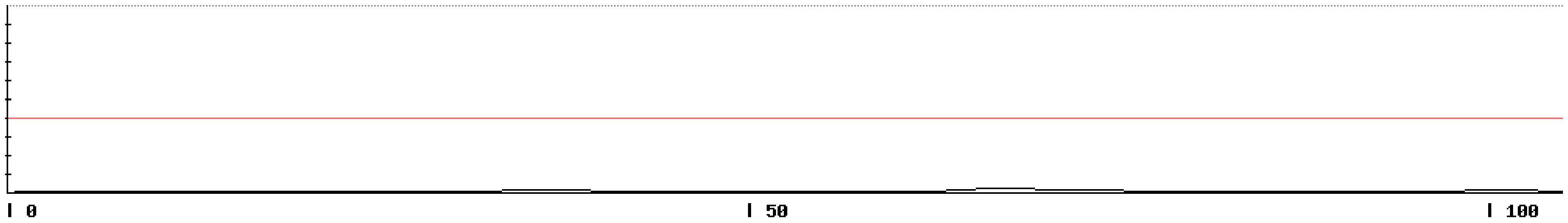

Predictions for Eragrostistef_462859904

Viterbi Path

Viterbi Path

Posterior @ 0.4

121 - RKRRK - 125

166 - GRQRGRKP - 173

Posterior @ 0.4

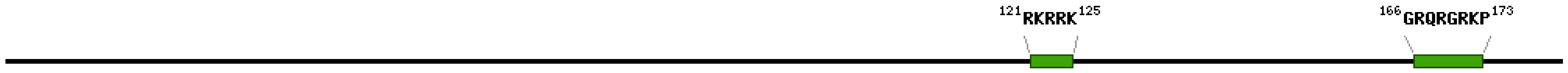

Posterior Graphic. Threshold Marked @ 0.4

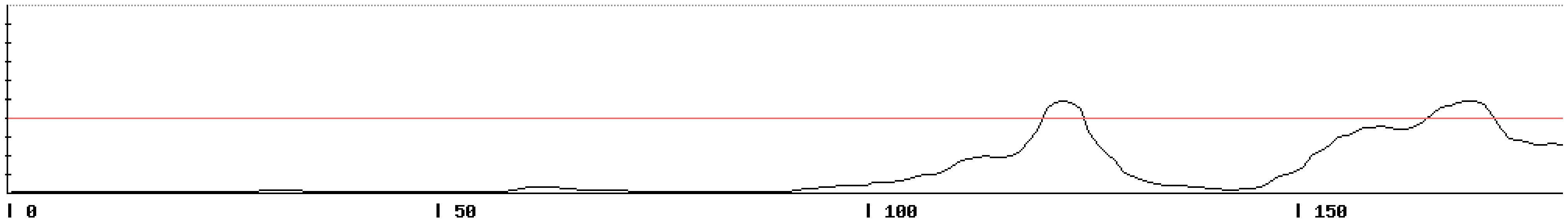

Predictions for Eragrostistef_462860543

Viterbi Path

Viterbi Path

Posterior @ 0.4	
Posterior @ 0.4	
Posterior Graphic. Threshold Marked @ 0.4	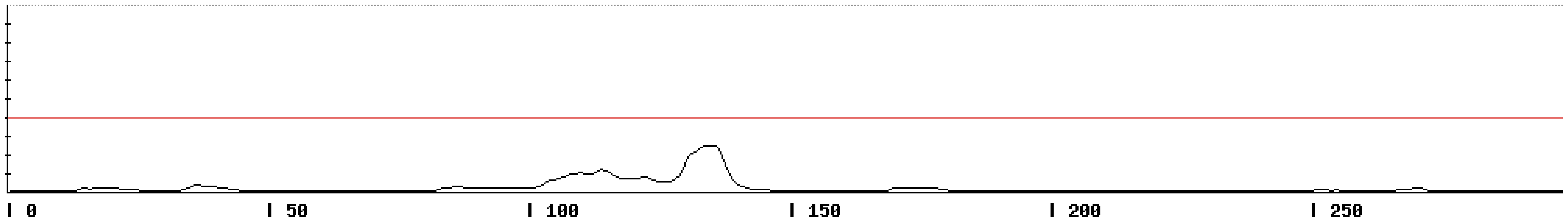

Predictions for Eragrostistef_462860887

Viterbi Path	
Viterbi Path	
Posterior @ 0.4	133 - PRRGEKKSSRA - 143
Posterior @ 0.4	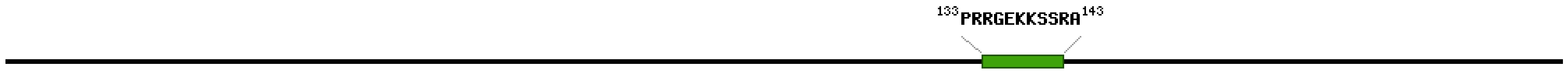
Posterior Graphic. Threshold Marked @ 0.4	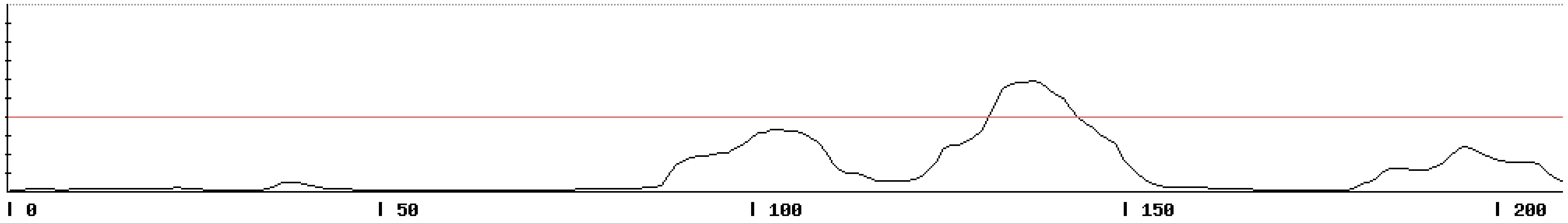

Predictions for Eragrostistef_462862046

Viterbi Path

Viterbi Path

Posterior @ 0.4

Posterior @ 0.4

Posterior Graphic. Threshold Marked @ 0.4

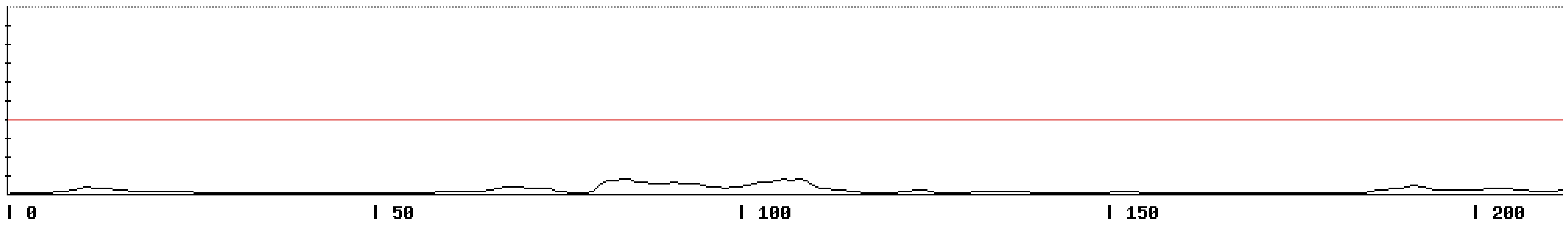

Predictions for Eragrostistef_462862308

Viterbi Path

Viterbi Path

Posterior @ 0.4

Posterior @ 0.4

Posterior Graphic. Threshold Marked @ 0.4

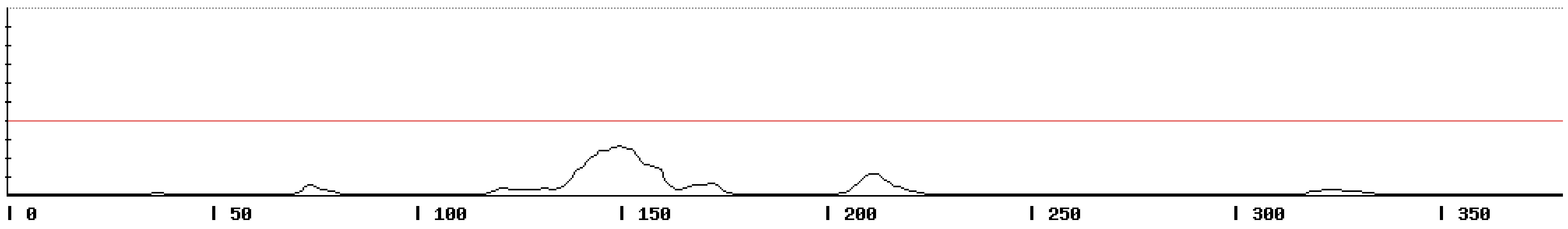

Predictions for Eragrostistef_462862329

Viterbi Path

Viterbi Path

Posterior @ 0.4

Posterior @ 0.4

Posterior Graphic. Threshold Marked @ 0.4

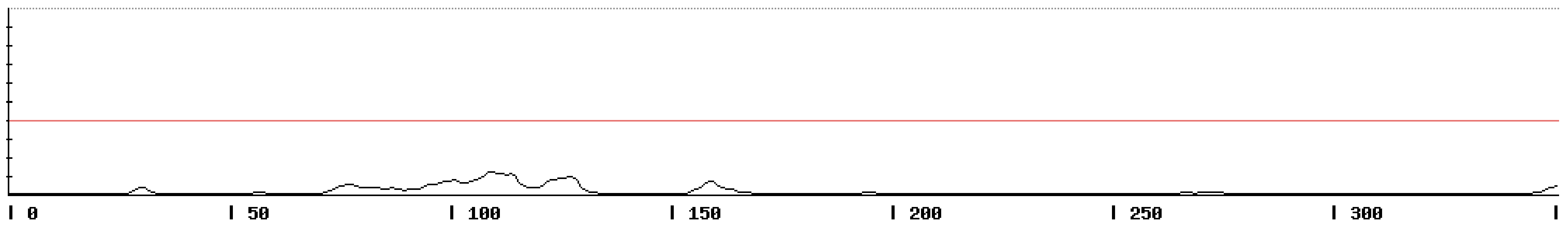

Predictions for Eragrostistef_462863766

Viterbi Path

Viterbi Path

Posterior @ 0.4

Posterior @ 0.4

Posterior Graphic. Threshold Marked @ 0.4

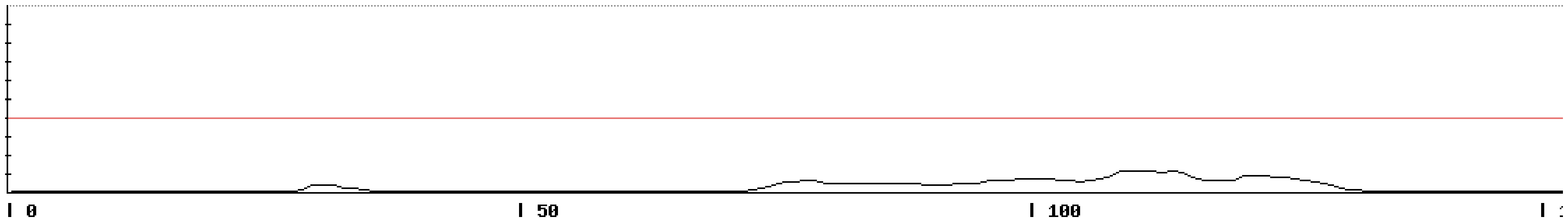

Predictions for Eragrostistef_462866365

Viterbi Path

Viterbi Path

Posterior @ 0.4

Posterior @ 0.4

Posterior Graphic. Threshold Marked @ 0.4

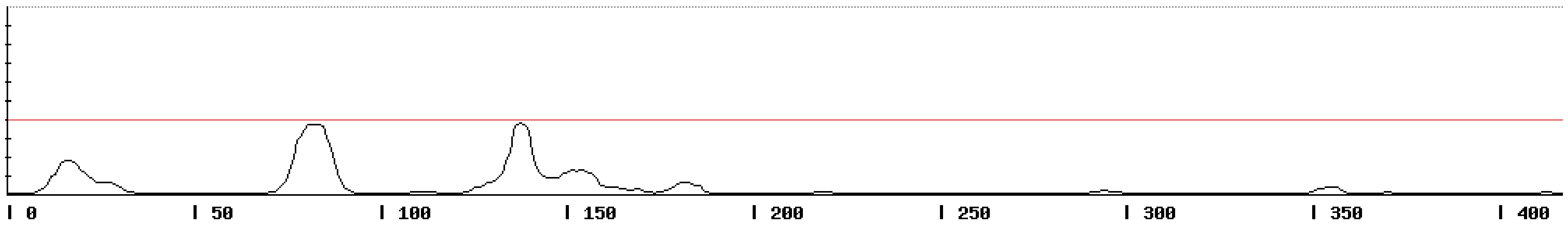

Predictions for Eragrostistef_462868670

Viterbi Path

Viterbi Path

Posterior @ 0.4

Posterior @ 0.4

Posterior Graphic. Threshold Marked @ 0.4

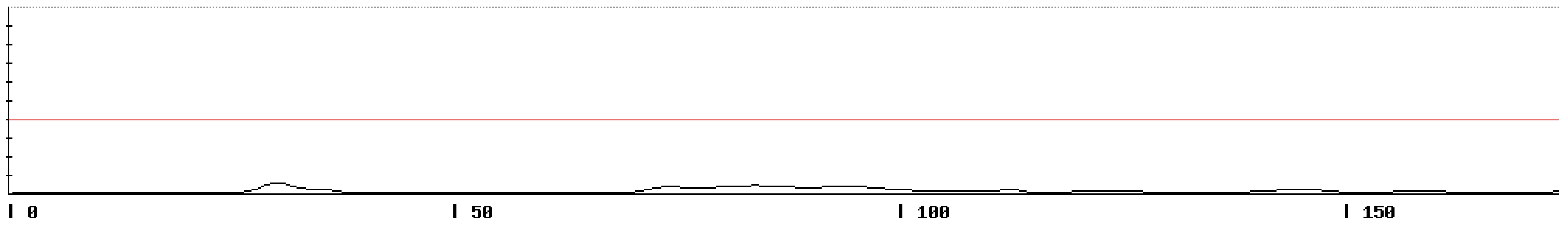

Predictions for Eragrostistef_462873660

Viterbi Path

Viterbi Path

Posterior @ 0.4

Posterior @ 0.4

Posterior Graphic. Threshold Marked @ 0.4

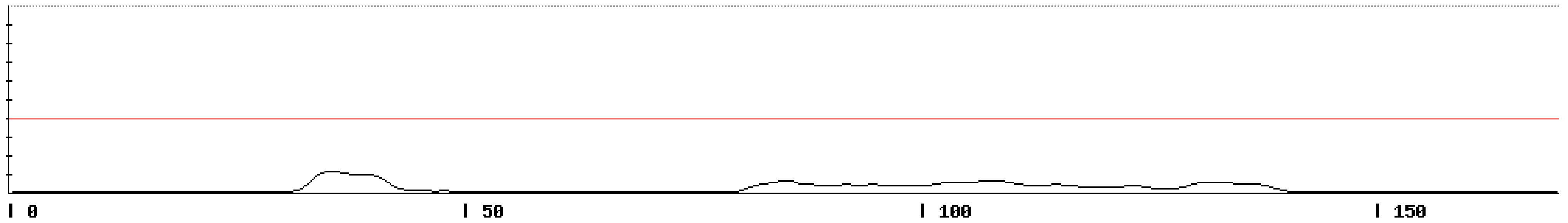

Predictions for Eragrostistef_462874350

Viterbi Path

Viterbi Path

Posterior @ 0.4

Posterior @ 0.4

Posterior Graphic. Threshold Marked @ 0.4	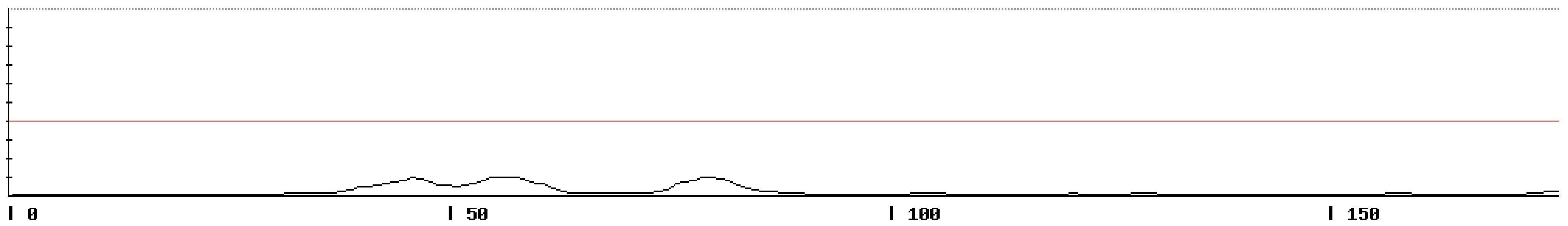

Predictions for Eragrostistef_462874904

Viterbi Path	
Viterbi Path	
Posterior @ 0.4	
Posterior @ 0.4	
Posterior Graphic. Threshold Marked @ 0.4	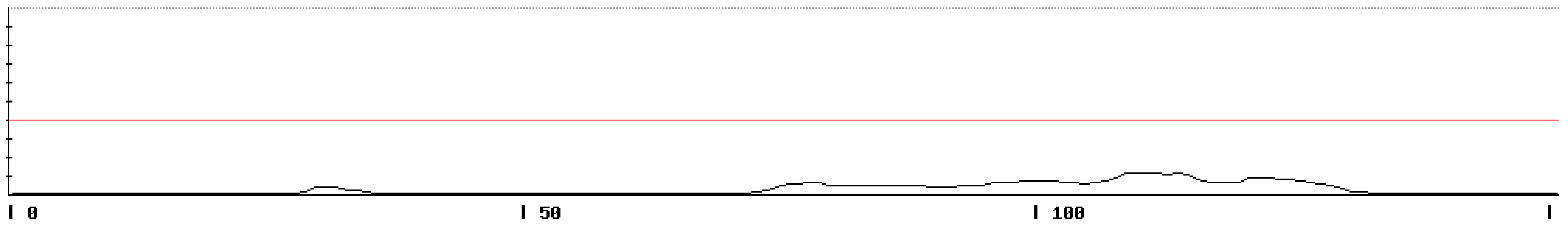

Predictions for Eragrostistef_462875034

Viterbi Path	
Viterbi Path	

Posterior @ 0.4

Posterior @ 0.4

Posterior Graphic. Threshold Marked @ 0.4

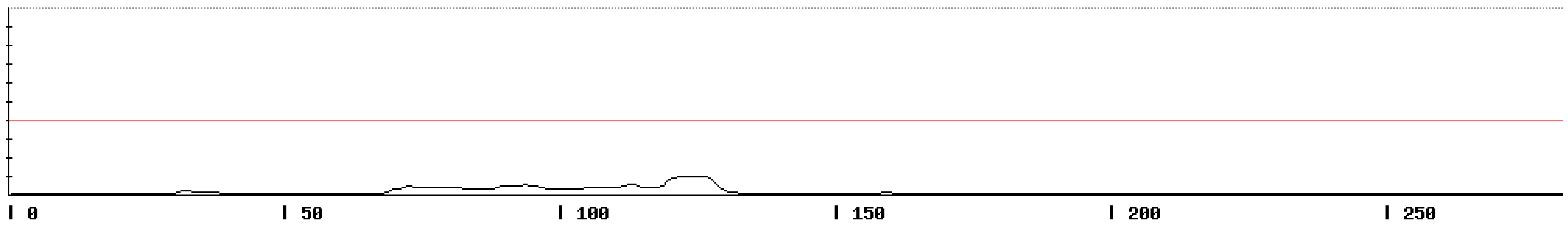

Predictions for Eragrostistef_462875202

Viterbi Path

Viterbi Path

Posterior @ 0.4

Posterior @ 0.4

Posterior Graphic. Threshold Marked @ 0.4

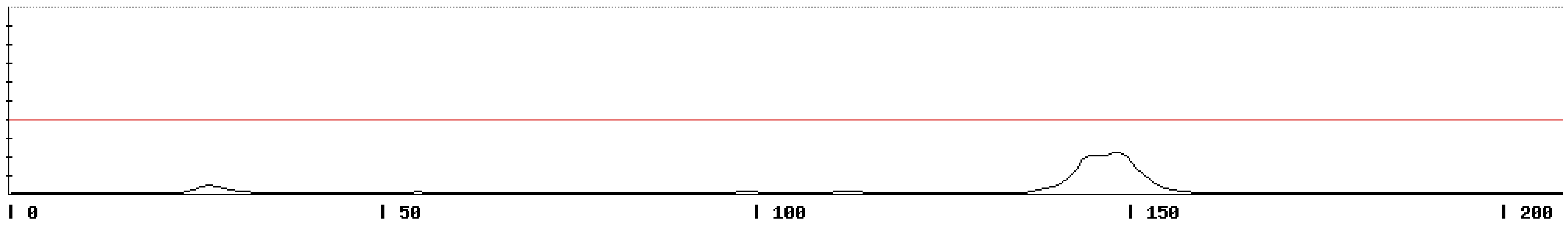

Predictions for Eragrostistef_462876216

Viterbi Path

Viterbi Path

Posterior @ 0.4	
Posterior @ 0.4	
Posterior Graphic. Threshold Marked @ 0.4	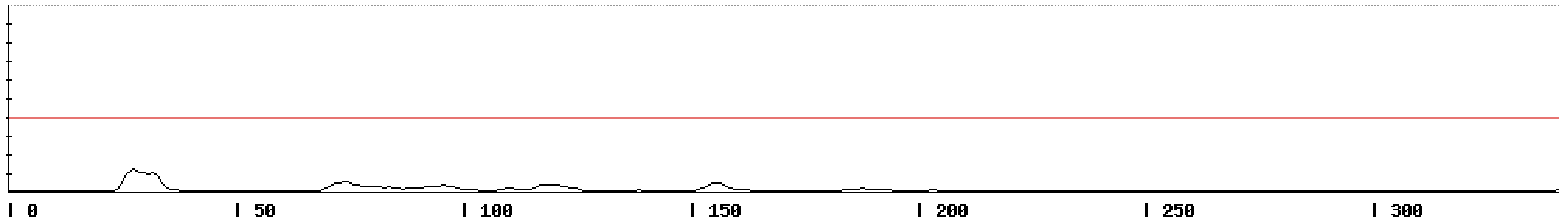

**Predictions for Eragrostistef_462876895**	
Viterbi Path	
Viterbi Path	
Posterior @ 0.4	99 - GAPKKPVGSPKPVAIKK - 115
Posterior @ 0.4	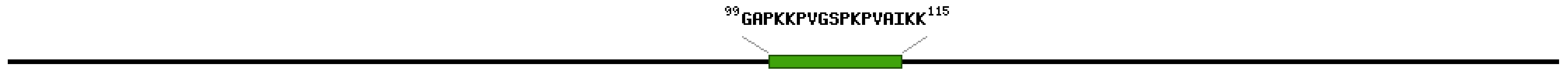
Posterior Graphic. Threshold Marked @ 0.4	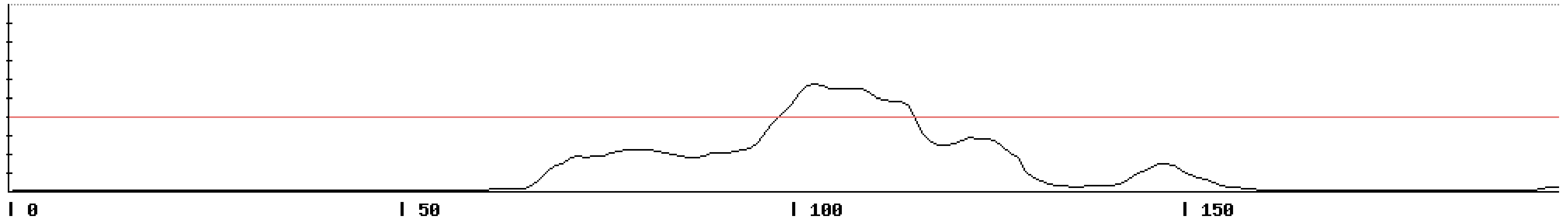

Predictions for Eragrostistef_462877018

Viterbi Path

Viterbi Path

Posterior @ 0.4

Posterior @ 0.4

Posterior Graphic. Threshold Marked @ 0.4

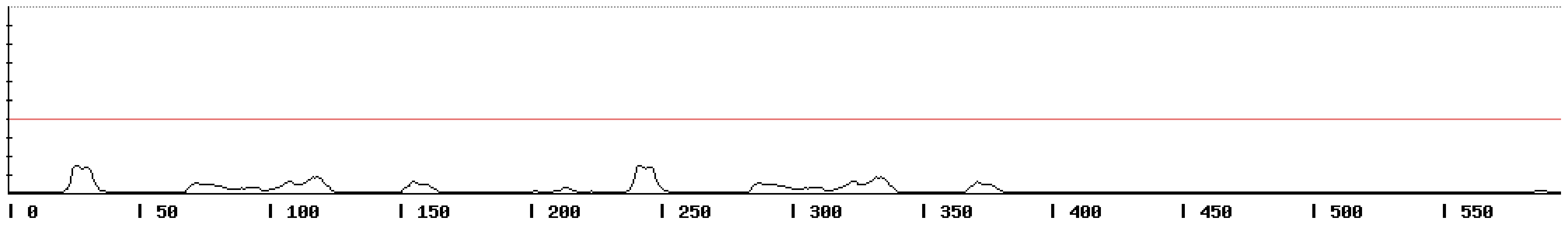

Predictions for Eragrostistef_462877364

Viterbi Path

Viterbi Path

Posterior @ 0.4

Posterior @ 0.4

Posterior Graphic. Threshold Marked @ 0.4

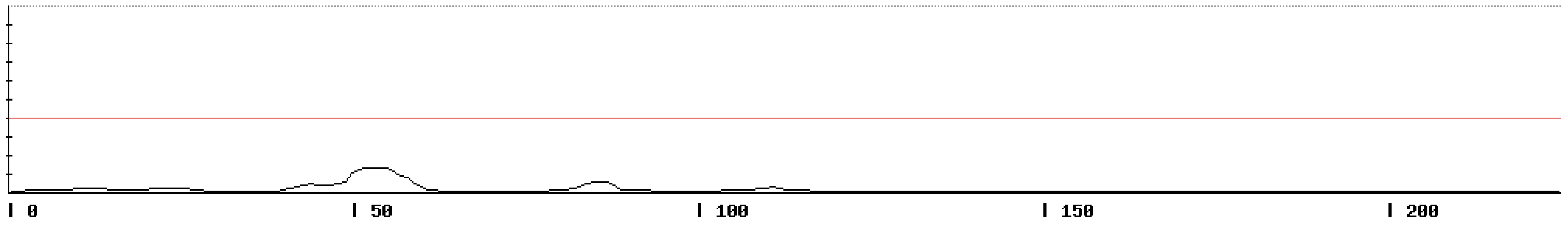

Predictions for Eragrostistef_462877666

Viterbi Path

Viterbi Path

Posterior @ 0.4

Posterior @ 0.4

Posterior Graphic. Threshold Marked @ 0.4

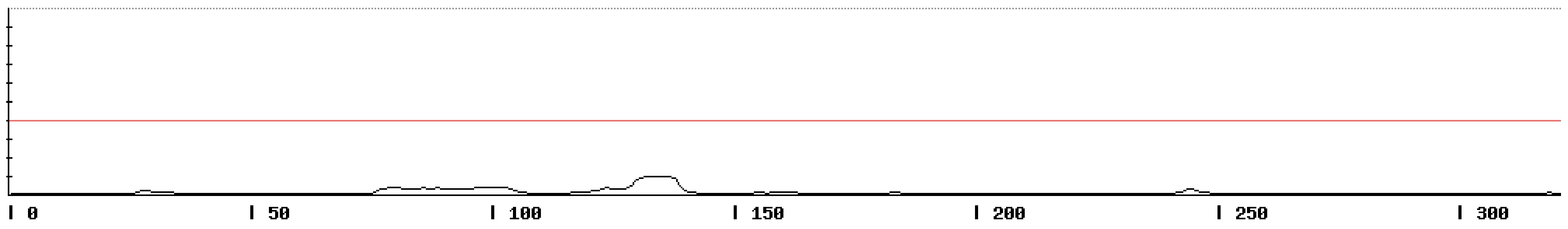

Predictions for Eragrostistef_462878241

Viterbi Path

Viterbi Path

Posterior @ 0.4

Posterior @ 0.4

Posterior Graphic. Threshold Marked @ 0.4

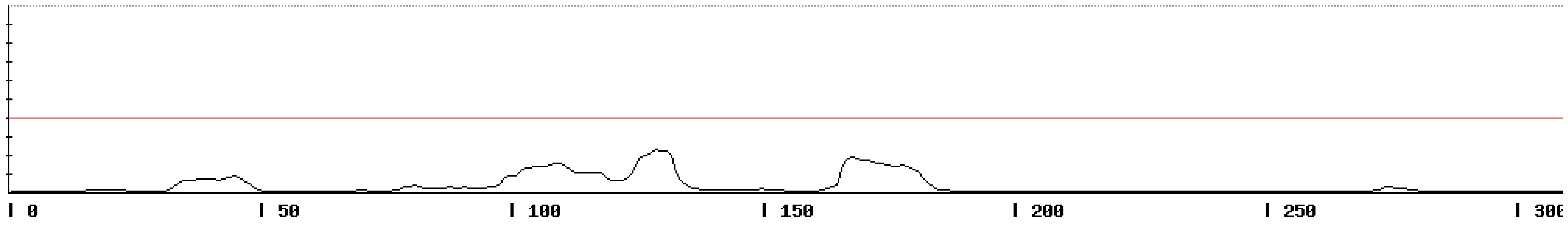

Predictions for Eragrostistef_462878398

Viterbi Path	
Viterbi Path	
Posterior @ 0.4	
Posterior @ 0.4	
Posterior Graphic. Threshold Marked @ 0.4	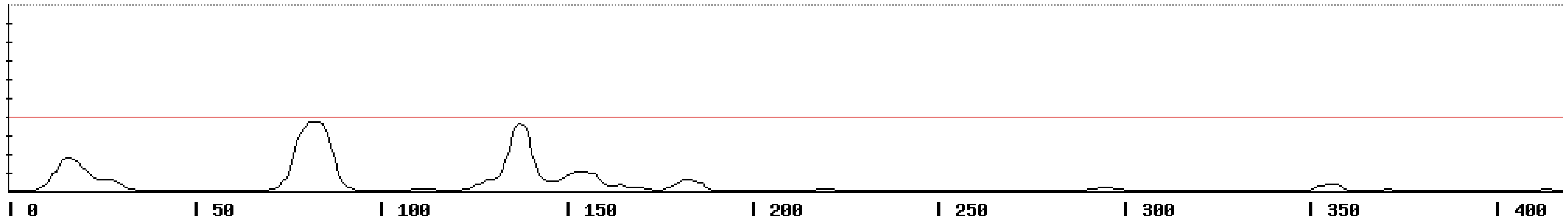

Predictions for Eragrostistef_462882337

Viterbi Path	
Viterbi Path	
Posterior @ 0.4	
Posterior @ 0.4	

Posterior Graphic. Threshold Marked @ 0.4

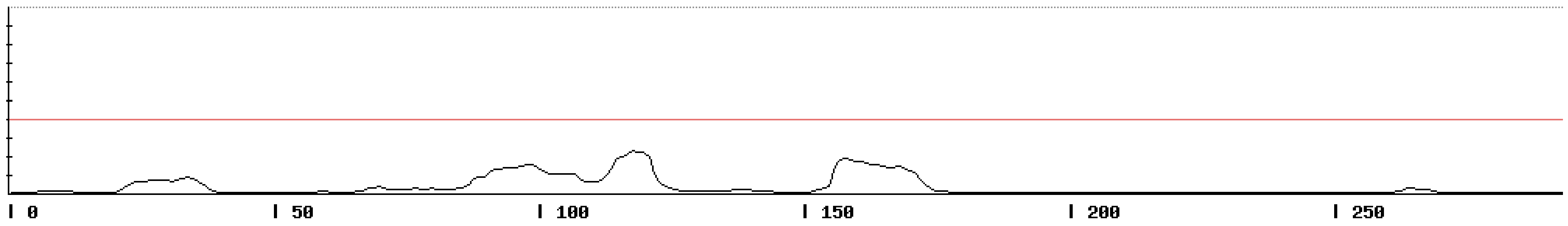

Predictions for Eragrostistef_462883251

Viterbi Path

Viterbi Path

Posterior @ 0.4

Posterior @ 0.4

Posterior Graphic. Threshold Marked @ 0.4

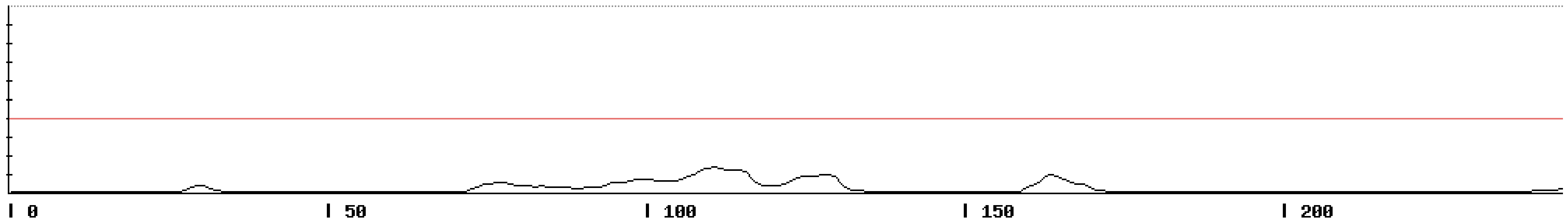

Predictions for Eragrostistef_462884464

Viterbi Path

Viterbi Path

Posterior @ 0.4

Posterior @ 0.4

Posterior Graphic. Threshold Marked @ 0.4

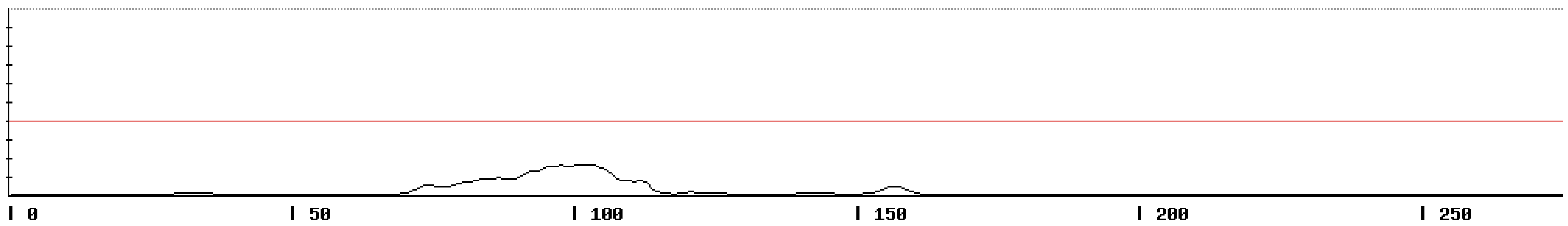

Predictions for Eragrostistef_462884854

Viterbi Path

Viterbi Path

Posterior @ 0.4

Posterior @ 0.4

Posterior Graphic. Threshold Marked @ 0.4

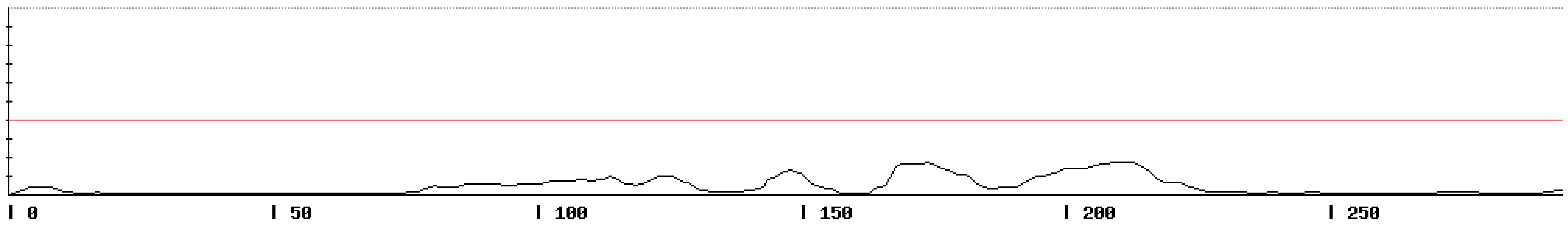

Predictions for Eragrostistef_462884889

Viterbi Path

Viterbi Path

Posterior @ 0.4

Posterior @ 0.4

Posterior Graphic. Threshold Marked @ 0.4

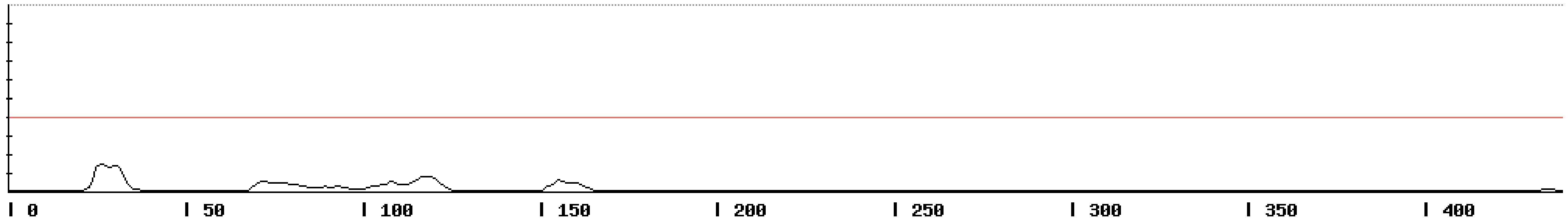

Predictions for Eragrostistef_462886054

Viterbi Path

Viterbi Path

Posterior @ 0.4

Posterior @ 0.4

Posterior Graphic. Threshold Marked @ 0.4

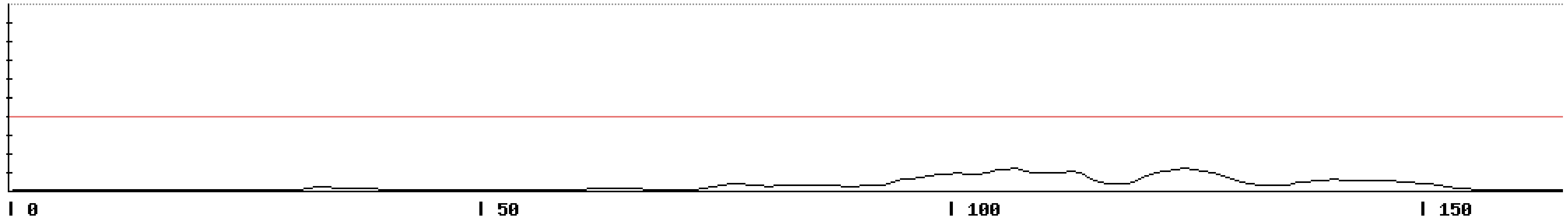

Predictions for Eragrostistef_462886115

Viterbi Path

Viterbi Path

Posterior @ 0.4

Posterior @ 0.4

Posterior Graphic. Threshold Marked @ 0.4

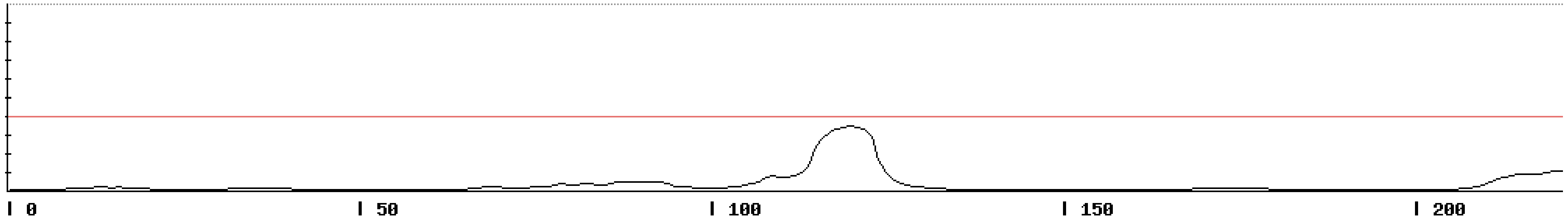

Predictions for Eragrostistef_462886118

Viterbi Path

Viterbi Path

Posterior @ 0.4

Posterior @ 0.4

Posterior Graphic. Threshold Marked @ 0.4

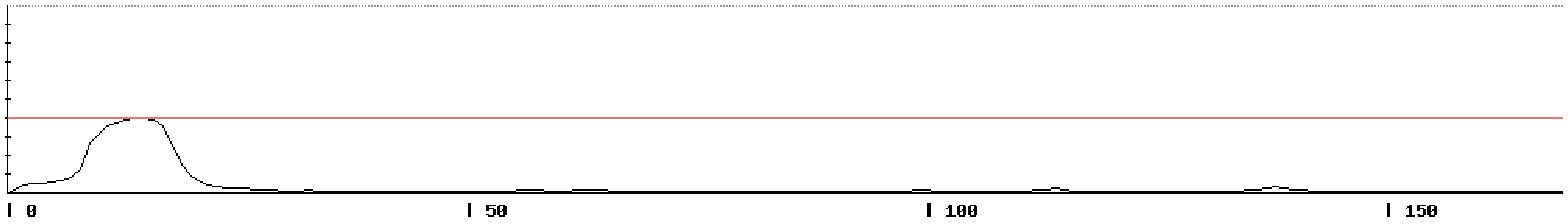

Predictions for Eragrostistef_462886127

Viterbi Path	
Viterbi Path	
Posterior @ 0.4	6 - RPRRQGRQTQRAAGGAGFVRHKLKRWRPPAAAPAVASRRR - 45
Posterior @ 0.4	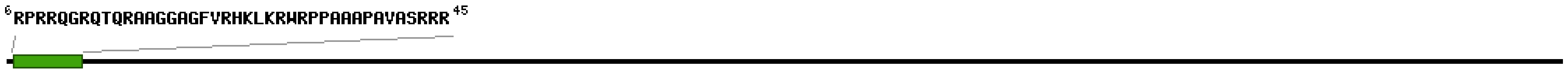
Posterior Graphic. Threshold Marked @ 0.4	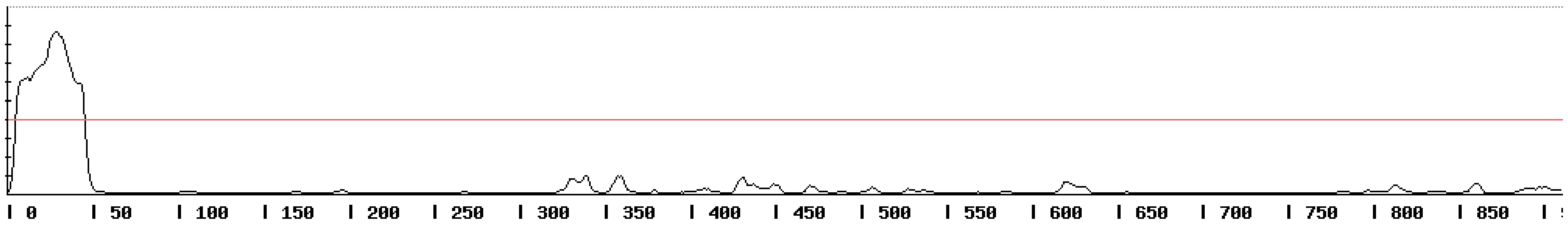

Predictions for Eragrostistef_462887616

Viterbi Path	
Viterbi Path	
Posterior @ 0.4	
Posterior @ 0.4	
Posterior Graphic. Threshold Marked @ 0.4	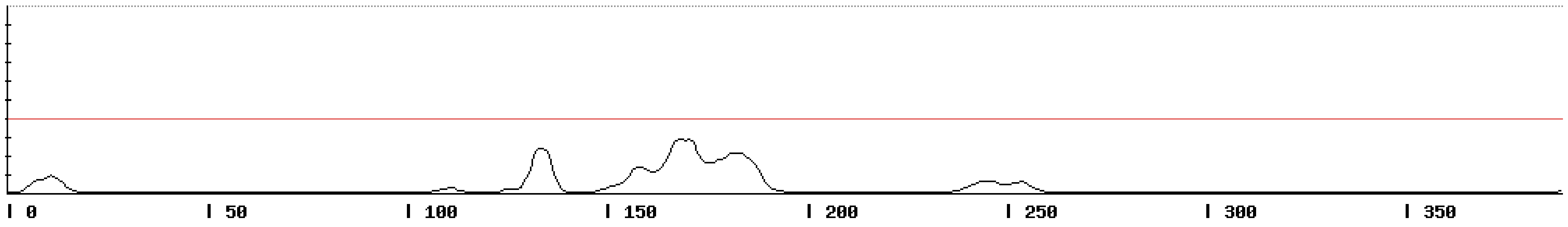

Predictions for Eragrostistef_462888232	
Viterbi Path	
Viterbi Path	
Posterior @ 0.4	
Posterior @ 0.4	
Posterior Graphic. Threshold Marked @ 0.4	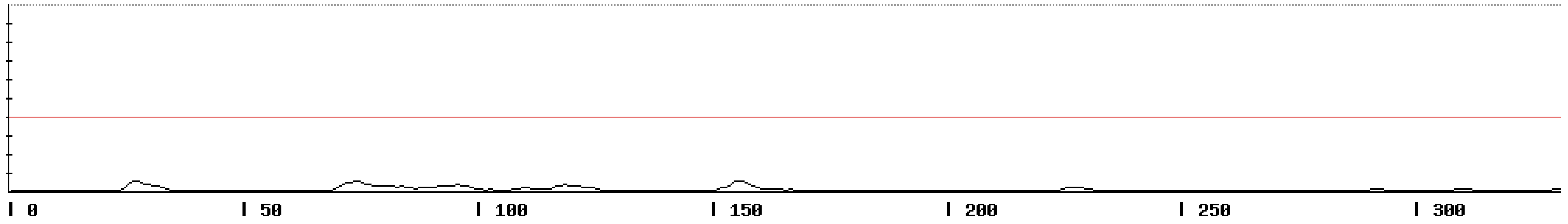

Predictions for Eragrostistef_462888815	
Viterbi Path	
Viterbi Path	
Posterior @ 0.4	
Posterior @ 0.4	
Posterior Graphic. Threshold Marked @ 0.4	

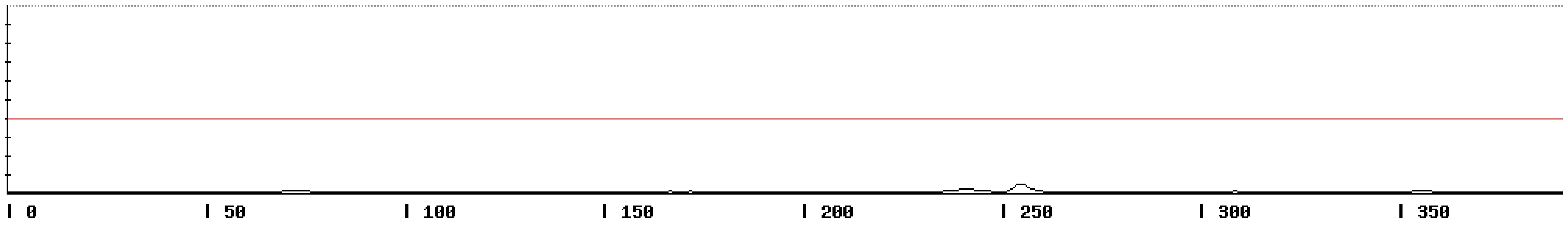

Predictions for Eragrostistef_462889166

Viterbi Path	
Viterbi Path	
Posterior @ 0.4	138 - AFAPRRGKKKNSNR - 151
Posterior @ 0.4	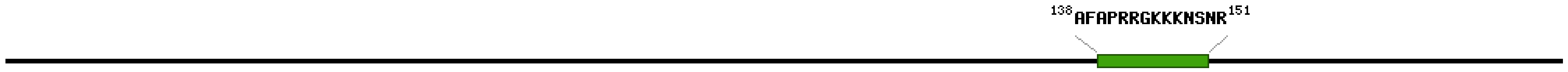
Posterior Graphic. Threshold Marked @ 0.4	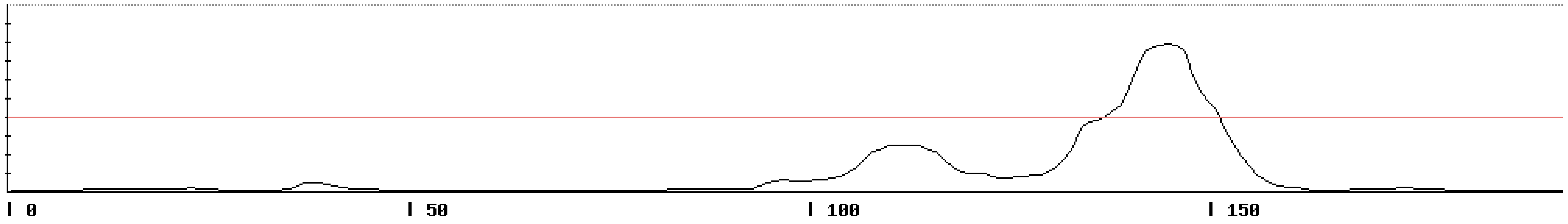

Predictions for Eragrostistef_462889309

Viterbi Path	
Viterbi Path	
Posterior @ 0.4	
Posterior @ 0.4	

Posterior Graphic. Threshold Marked @ 0.4

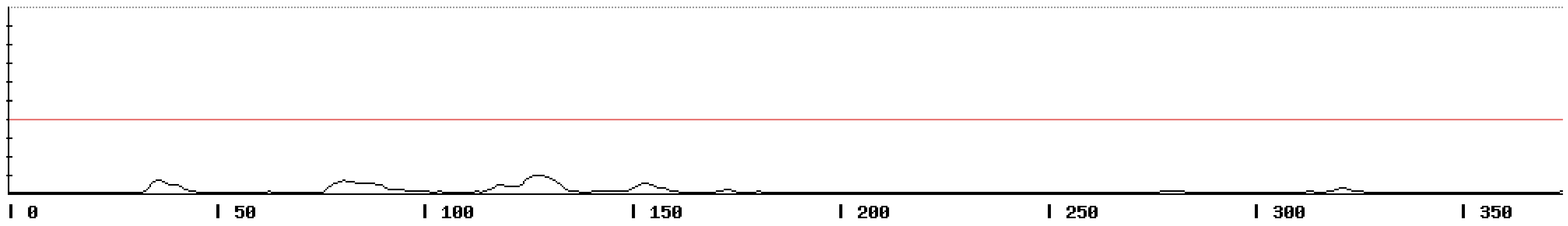

Predictions for Eragrostistef_462889453

Viterbi Path

Viterbi Path

Posterior @ 0.4

Posterior @ 0.4

Posterior Graphic. Threshold Marked @ 0.4

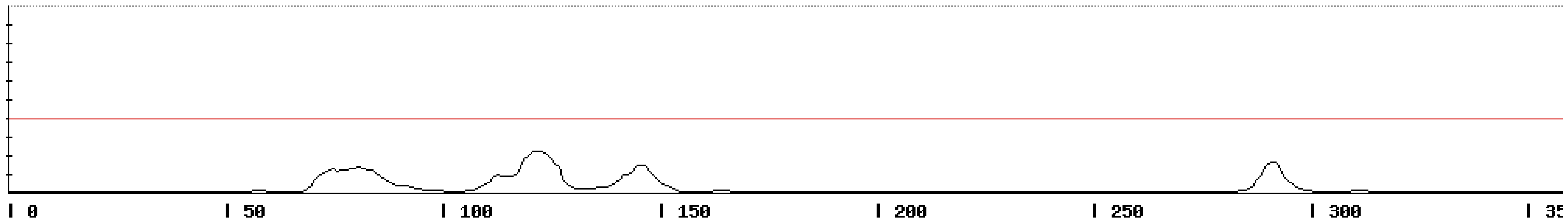

Predictions for Eragrostistef_462889474

Viterbi Path

Viterbi Path

Posterior @ 0.4

Posterior @ 0.4

Posterior Graphic. Threshold Marked @ 0.4

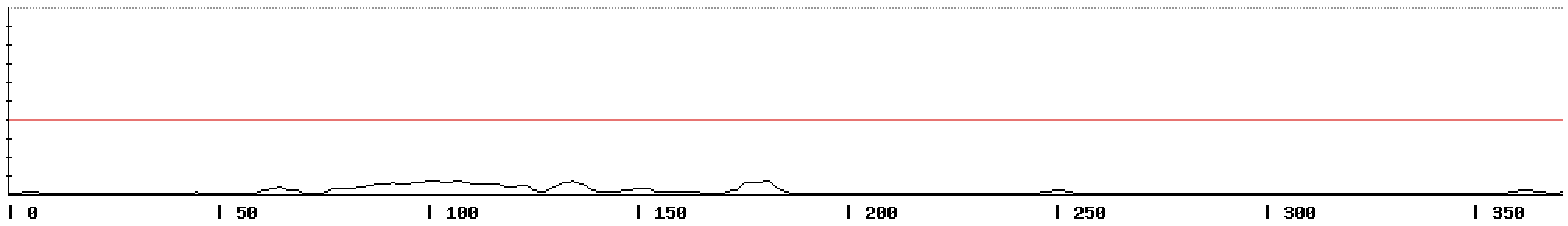

Predictions for Eragrostistef_462889477

Viterbi Path

Viterbi Path

Posterior @ 0.4

Posterior @ 0.4

Posterior Graphic. Threshold Marked @ 0.4

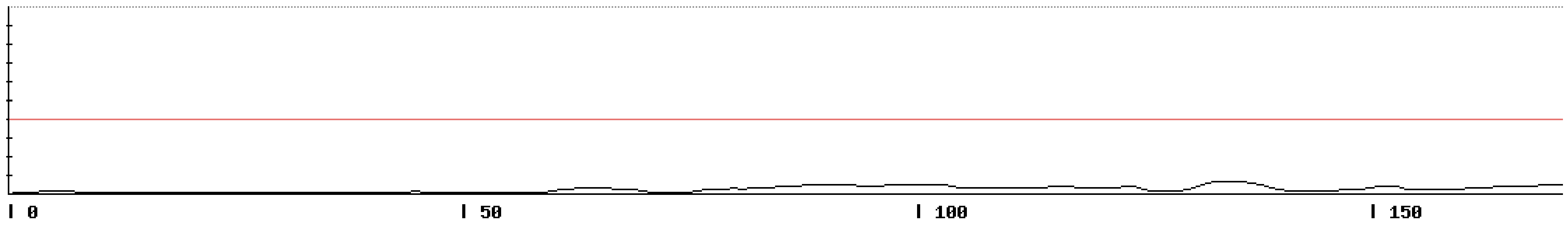

Predictions for Eragrostistef_462890006

Viterbi Path

Viterbi Path

Posterior @ 0.4

Posterior @ 0.4

Posterior Graphic. Threshold Marked @ 0.4

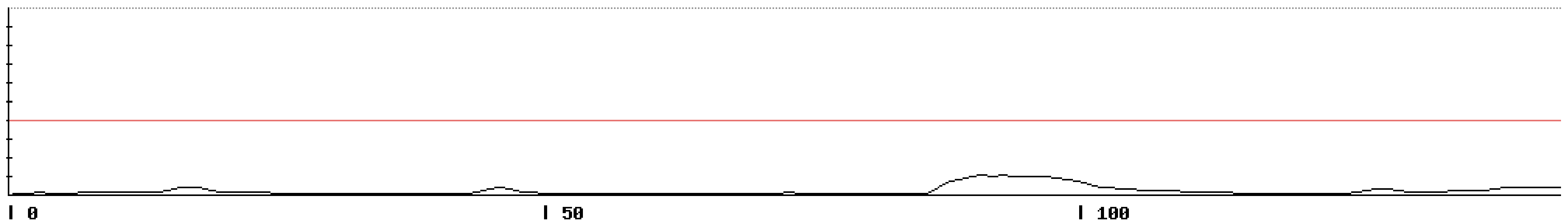

Predictions for Eragrostistef_462890505

Viterbi Path

Viterbi Path

Posterior @ 0.4

Posterior @ 0.4

Posterior Graphic. Threshold Marked @ 0.4

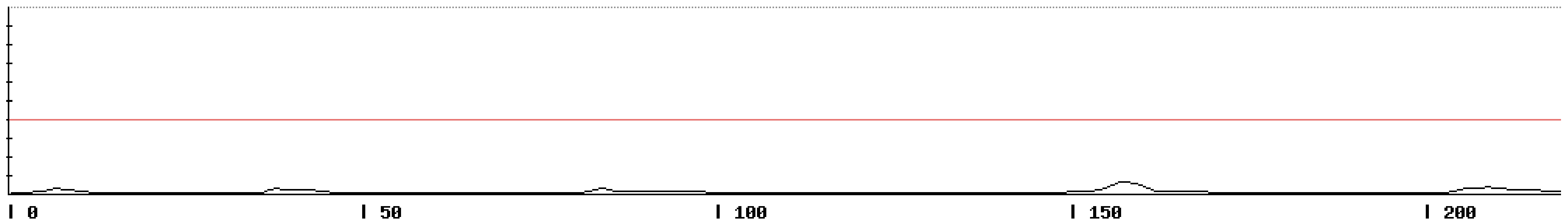

Predictions for Eragrostistef_462890696

Viterbi Path

Viterbi Path

Posterior @ 0.4

Posterior @ 0.4

Posterior Graphic. Threshold Marked @ 0.4

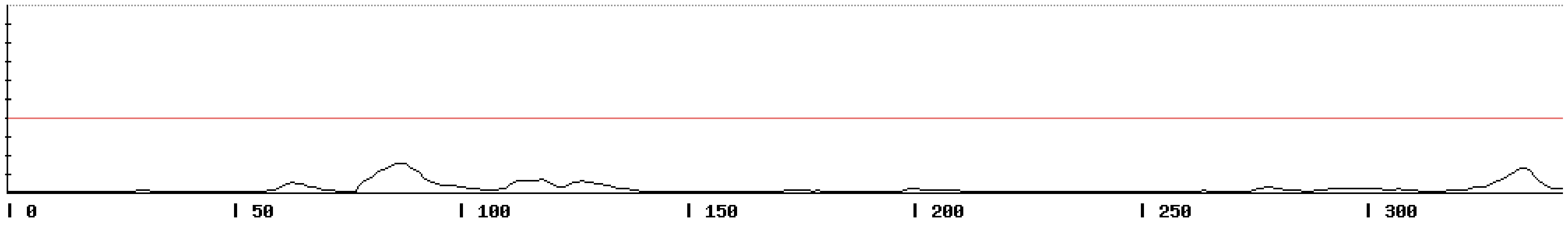

Predictions for Eragrostistef_462891236

Viterbi Path

Viterbi Path

Posterior @ 0.4

Posterior @ 0.4

Posterior Graphic. Threshold Marked @ 0.4

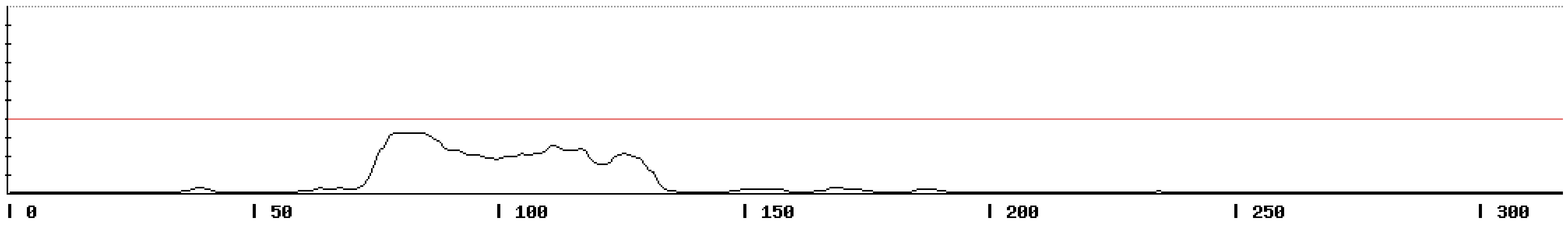

Predictions for Eragrostistef_462893056

Viterbi Path	
Viterbi Path	
Posterior @ 0.4	
Posterior @ 0.4	
Posterior Graphic. Threshold Marked @ 0.4	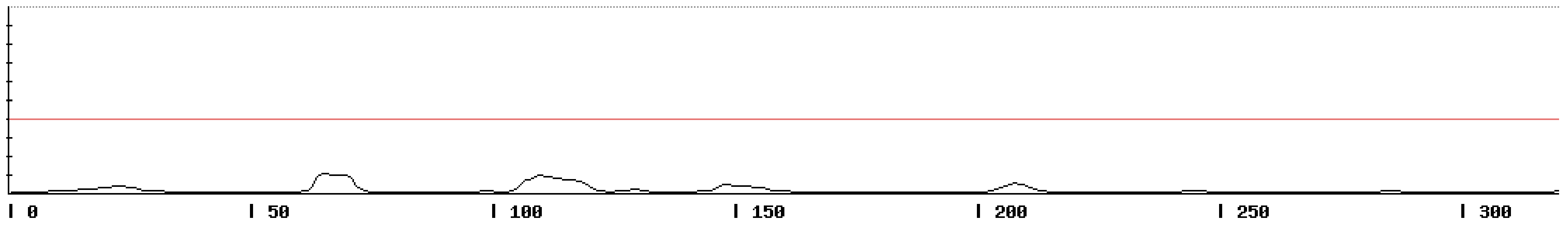

Predictions for Eragrostistef_462893665	
Viterbi Path	
Viterbi Path	
Posterior @ 0.4	
Posterior @ 0.4	
Posterior Graphic. Threshold Marked @ 0.4	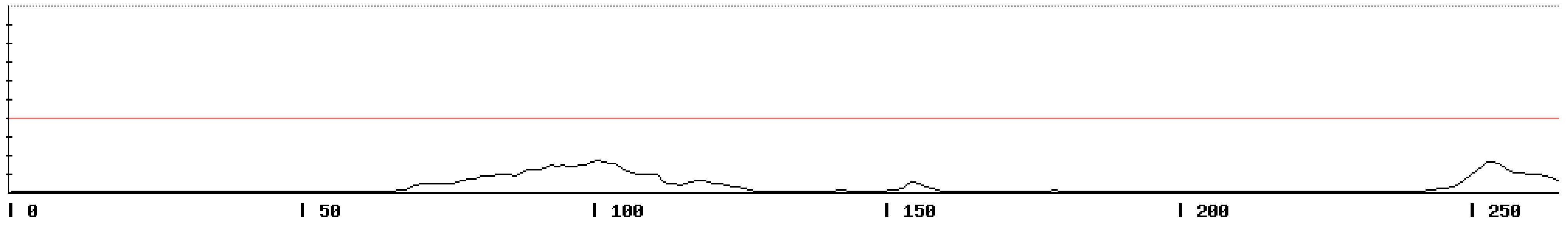

Predictions for Eragrostistef_462894879	
Viterbi Path	
Viterbi Path	
Posterior @ 0.4	
Posterior @ 0.4	
Posterior Graphic. Threshold Marked @ 0.4	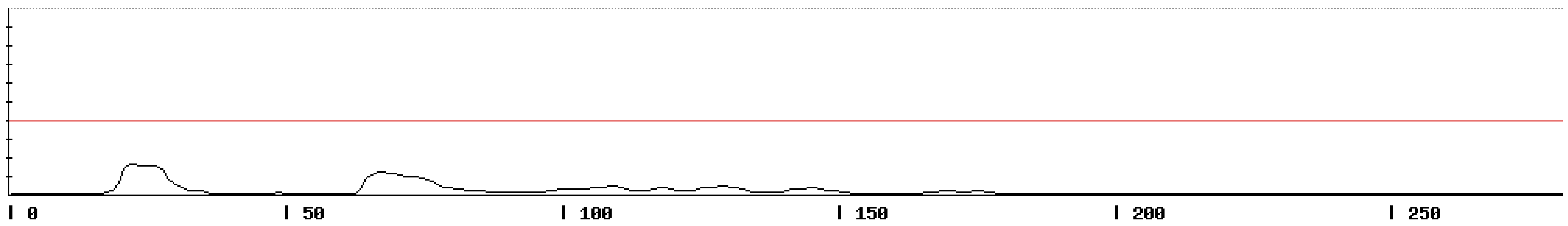

Predictions for Eragrostistef_462895220	
Viterbi Path	
Viterbi Path	
Posterior @ 0.4	128 - RKRRKI - 133
Posterior @ 0.4	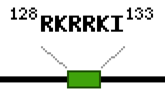
Posterior Graphic. Threshold Marked @ 0.4	

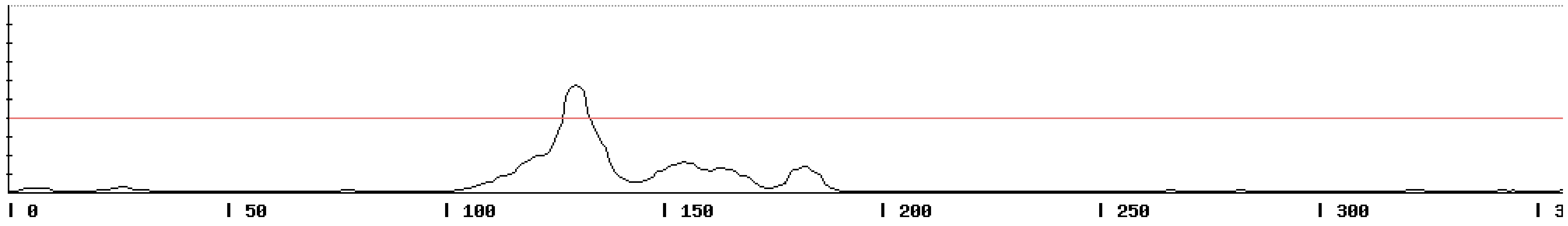

Predictions for Eragrostistef_462896090

Viterbi Path

Viterbi Path

Posterior @ 0.4

283 - R - 283

Posterior @ 0.4

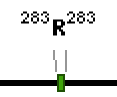

Posterior Graphic. Threshold Marked @ 0.4

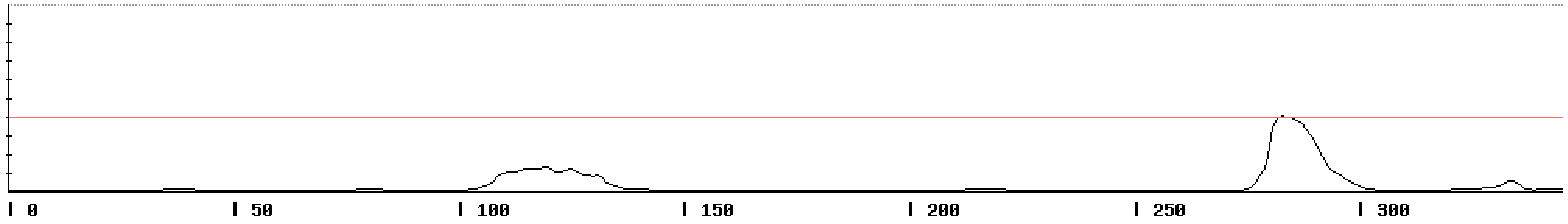

Predictions for Eragrostistef_462896237

Viterbi Path

Viterbi Path

Posterior @ 0.4

Posterior @ 0.4

Posterior Graphic. Threshold Marked @ 0.4

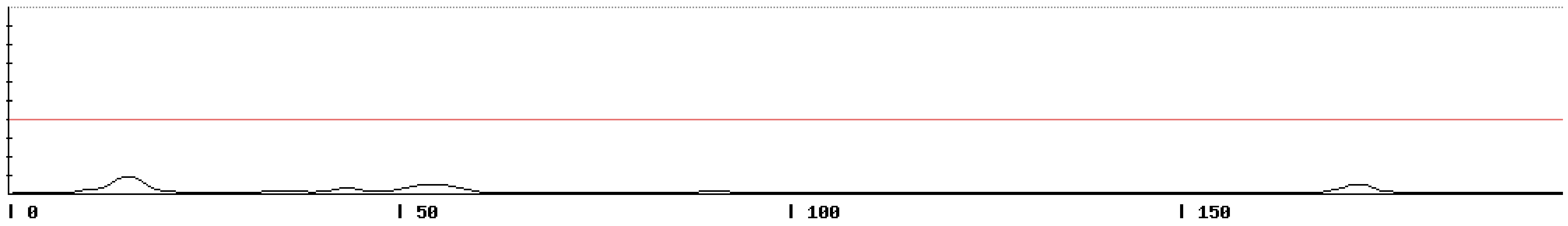

Predictions for Eragrostistef_462896630

Viterbi Path

Viterbi Path

Posterior @ 0.4

Posterior @ 0.4

Posterior Graphic. Threshold Marked @ 0.4

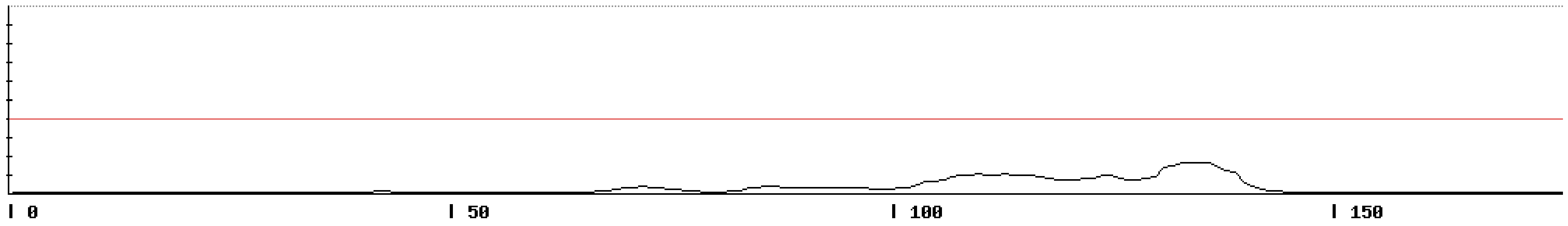

Predictions for Eragrostistef_462896806

Viterbi Path

Viterbi Path

Posterior @ 0.4

Posterior @ 0.4

Posterior Graphic. Threshold Marked @ 0.4

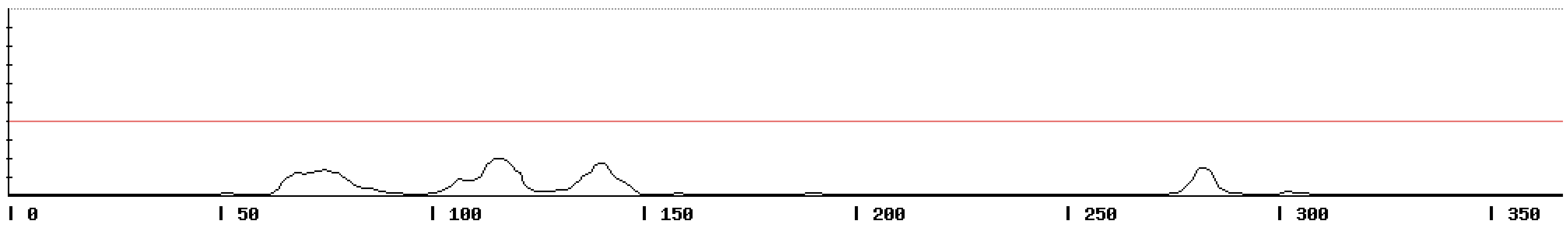

Predictions for Eragrostistef_462896821

Viterbi Path

Viterbi Path

Posterior @ 0.4

Posterior @ 0.4

Posterior Graphic. Threshold Marked @ 0.4

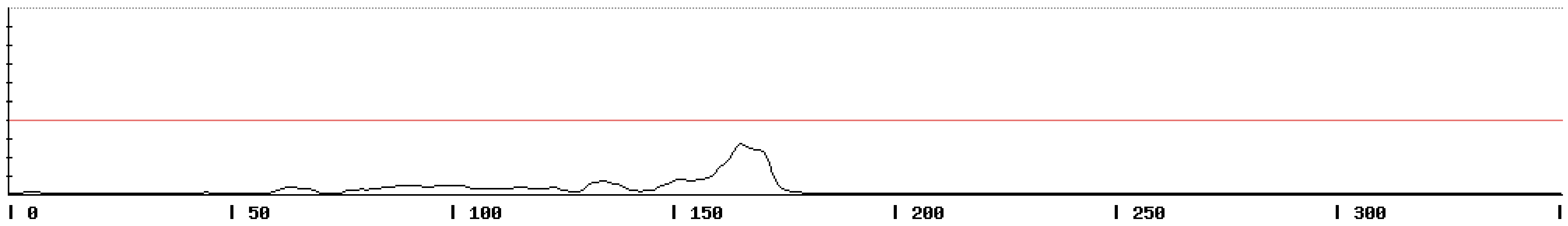

Predictions for Eragrostistef_462896955

Viterbi Path

Viterbi Path

Posterior @ 0.4

Posterior @ 0.4

Posterior Graphic. Threshold Marked @ 0.4

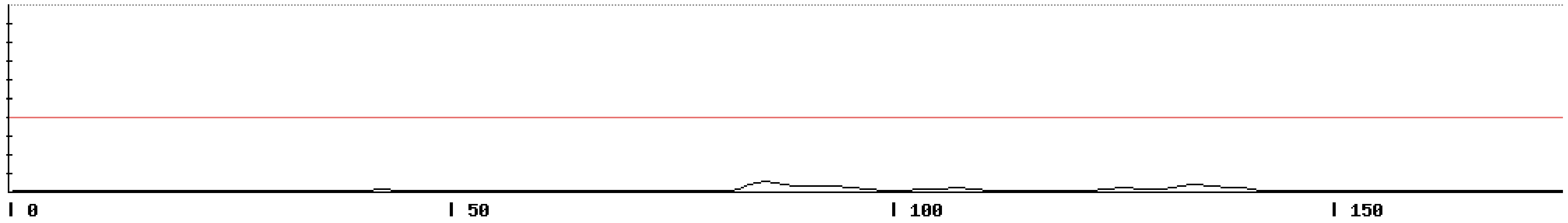

Predictions for Eragrostistef_462897429

Viterbi Path

Viterbi Path

Posterior @ 0.4

Posterior @ 0.4

Posterior Graphic. Threshold Marked @ 0.4

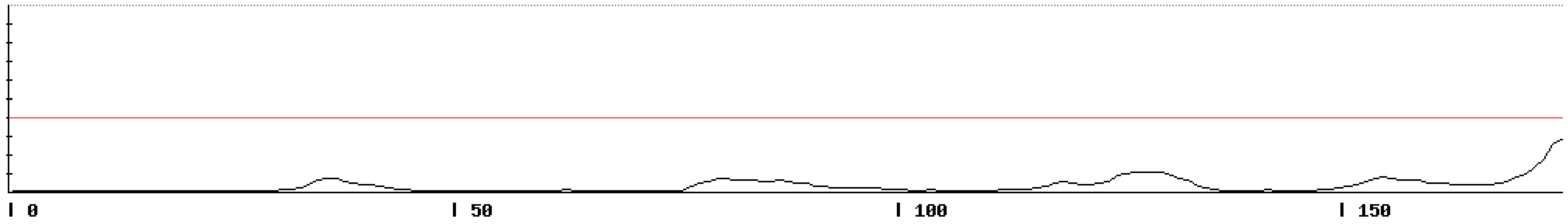

Predictions for Eragrostistef_462897554

Viterbi Path

Viterbi Path

Posterior @ 0.4

87 - RTRKYPGGTRPSRRTADDRGRWKPSTGQSAPKKGKSE - 123

268 - ATAPKRGRRPALPRGPPAPAHGR - 290

Posterior @ 0.4

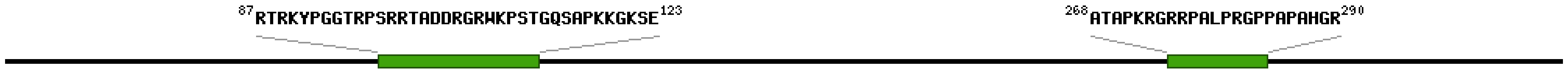

Posterior Graphic. Threshold Marked @ 0.4

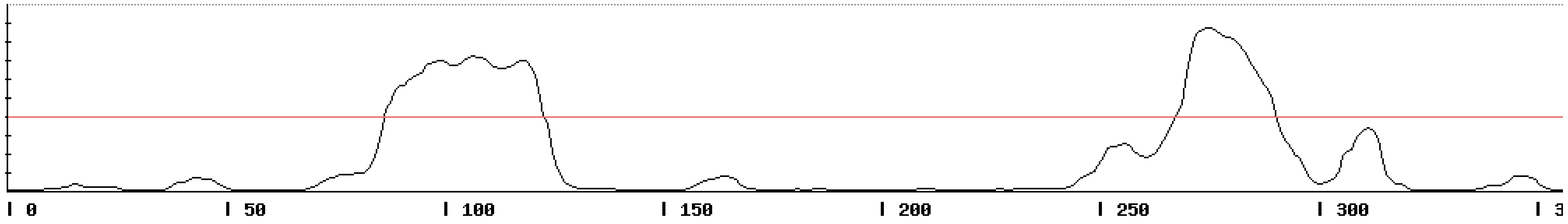

Predictions for Eragrostistef_462897883

Viterbi Path

Viterbi Path

Posterior @ 0.4

Posterior @ 0.4

Posterior Graphic. Threshold Marked @ 0.4

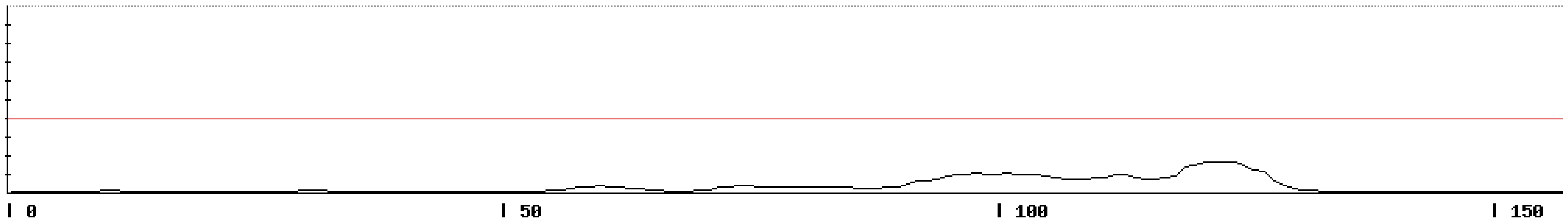

Predictions for Eragrostistef_462898851	
Viterbi Path	
Viterbi Path	
Posterior @ 0.4	
Posterior @ 0.4	
Posterior Graphic. Threshold Marked @ 0.4	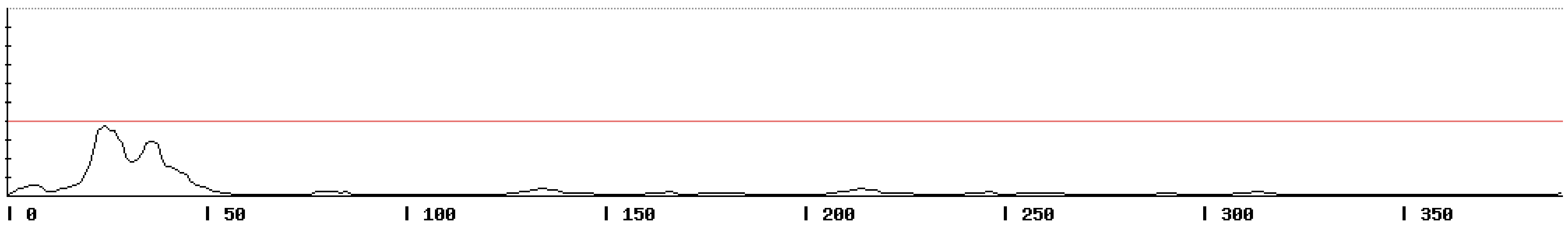

Predictions for Eragrostistef_462901323	
Viterbi Path	
Viterbi Path	
Posterior @ 0.4	213 - SRRRRRRK - 220
Posterior @ 0.4	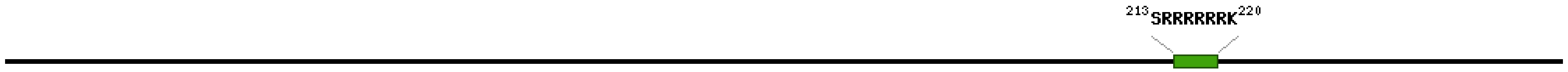
Posterior Graphic. Threshold Marked @ 0.4	

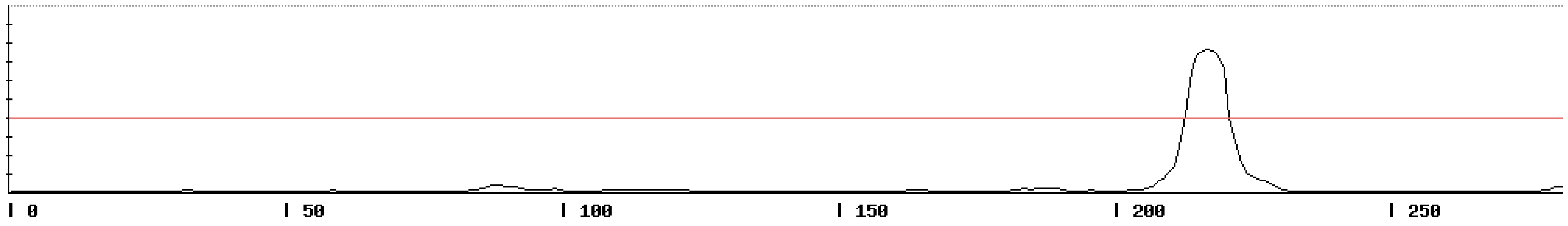

Predictions for Eragrostistef_462901884

Viterbi Path

Viterbi Path

Posterior @ 0.4

Posterior @ 0.4

Posterior Graphic. Threshold Marked @ 0.4

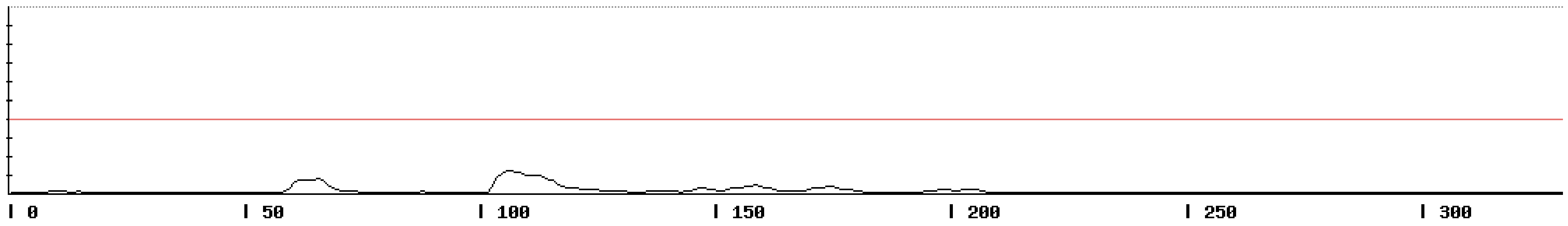

Predictions for Eragrostistef_462903459

Viterbi Path

Viterbi Path

Posterior @ 0.4: 56 - RKRGRKWLK - 64

Posterior @ 0.4

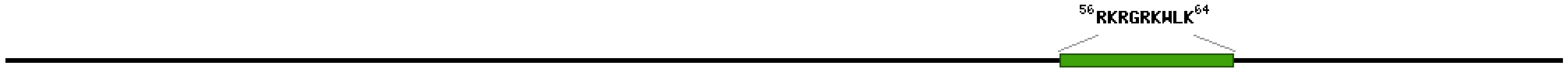

Posterior Graphic. Threshold Marked @ 0.4

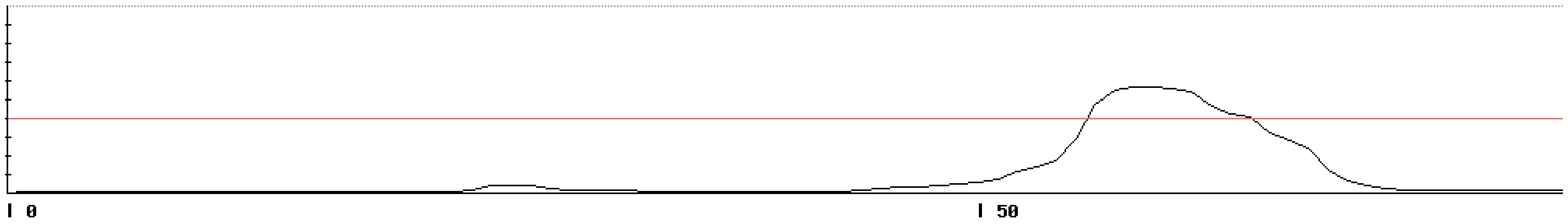

Predictions for Eragrostistef_462903983

Viterbi Path	
Viterbi Path	
Posterior @ 0.4	92 - RKRRK - 96
Posterior @ 0.4	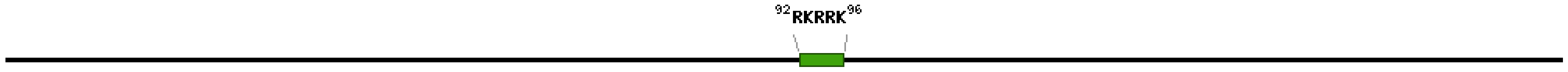
Posterior Graphic. Threshold Marked @ 0.4	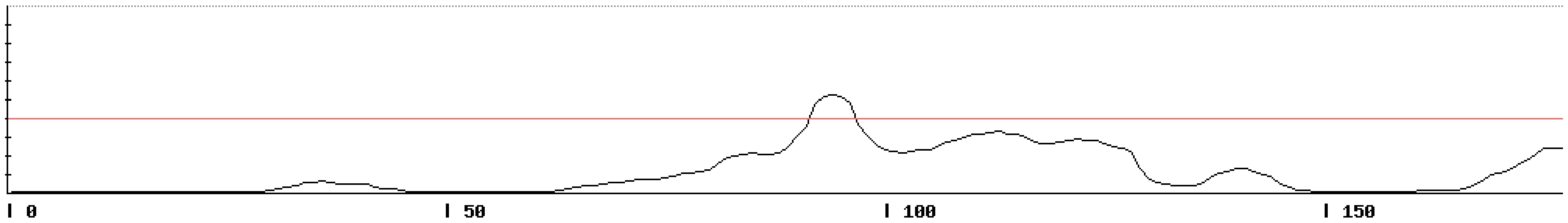

Predictions for Eragrostistef_462904384

Viterbi Path	
Viterbi Path	
Posterior @ 0.4	
Posterior @ 0.4	

Posterior Graphic. Threshold Marked @ 0.4

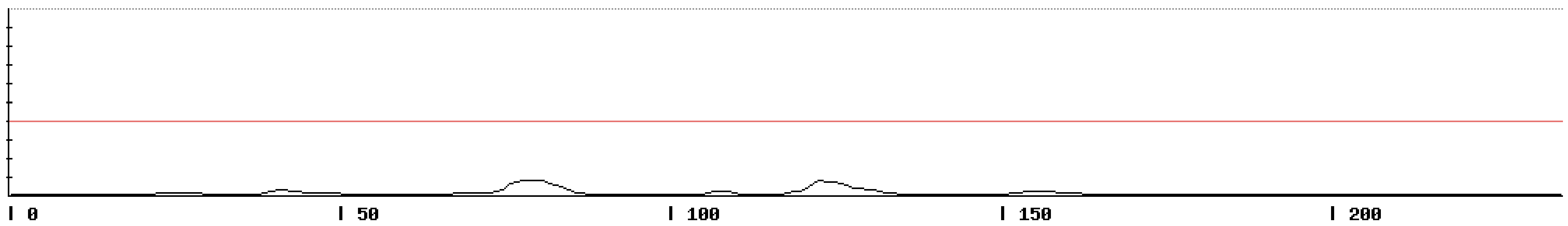

Predictions for Eragrostistef_462904624

Viterbi Path

Viterbi Path

Posterior @ 0.4

Posterior @ 0.4

Posterior Graphic. Threshold Marked @ 0.4

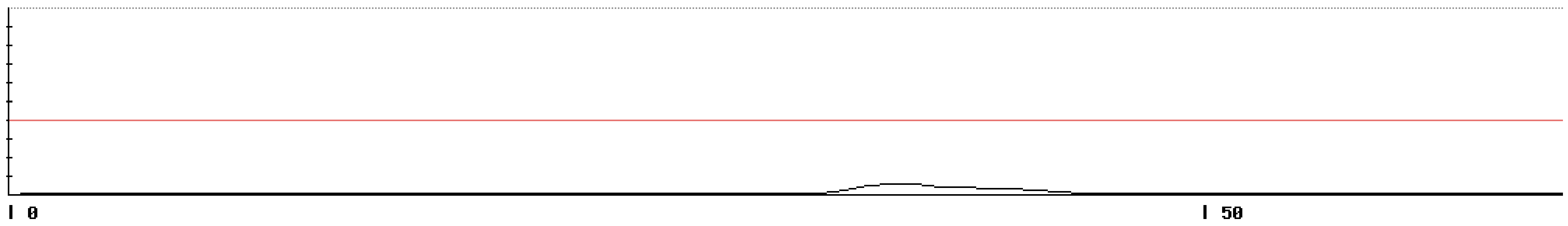

Predictions for Eragrostistef_462904909

Viterbi Path

Viterbi Path

Posterior @ 0.4

Posterior @ 0.4

Posterior Graphic. Threshold Marked @ 0.4

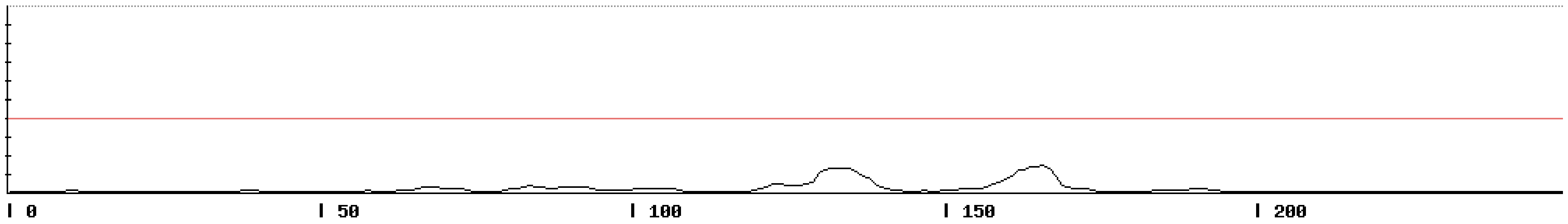

Predictions for Eragrostistef_462907634

Viterbi Path

Viterbi Path

Posterior @ 0.4

Posterior @ 0.4

Posterior Graphic. Threshold Marked @ 0.4

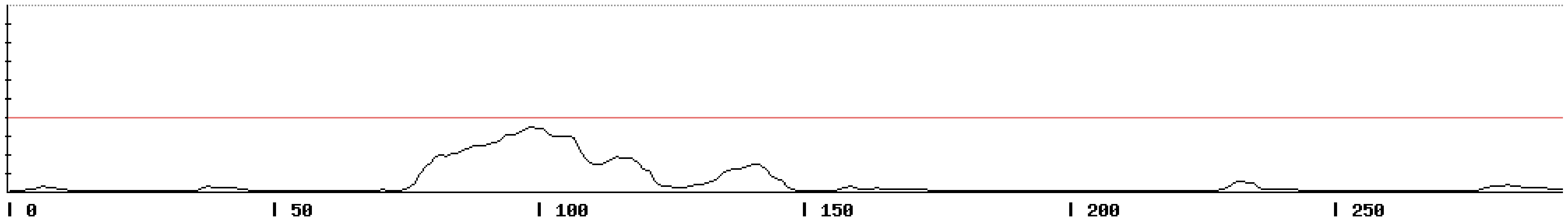

Predictions for Eragrostistef_462908578

Viterbi Path

Viterbi Path

Posterior @ 0.4

Posterior @ 0.4

Posterior Graphic. Threshold Marked @ 0.4

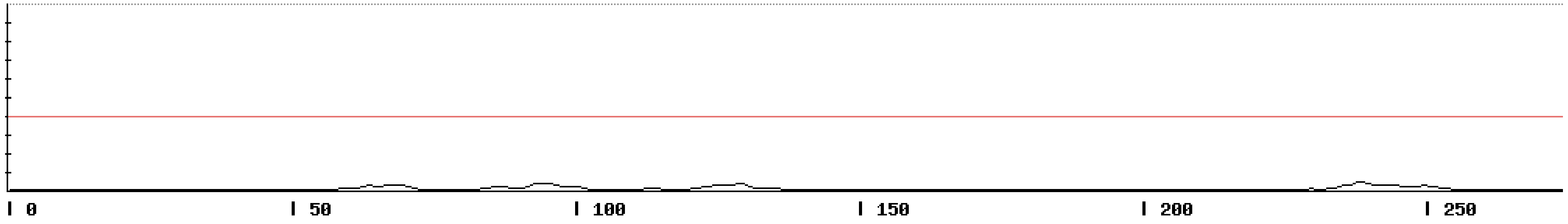

Predictions for Eragrostistef_462909266

Viterbi Path

Viterbi Path

Posterior @ 0.4

Posterior @ 0.4

Posterior Graphic. Threshold Marked @ 0.4

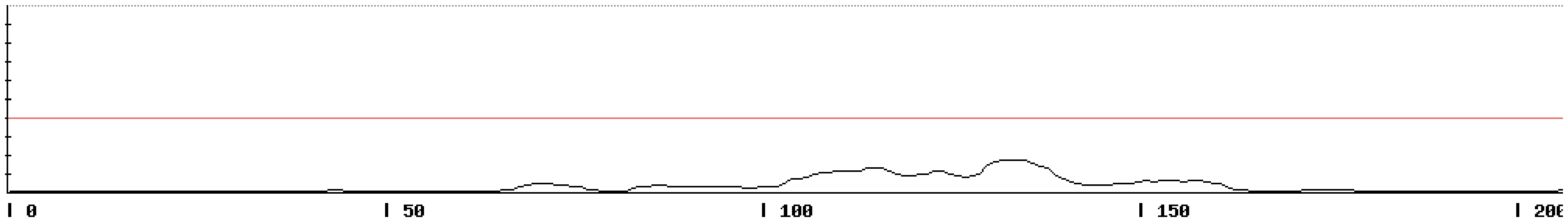

Predictions for Eragrostistef_462909305

Viterbi Path

Viterbi Path

Posterior @ 0.4

Posterior @ 0.4

Posterior Graphic. Threshold Marked @ 0.4

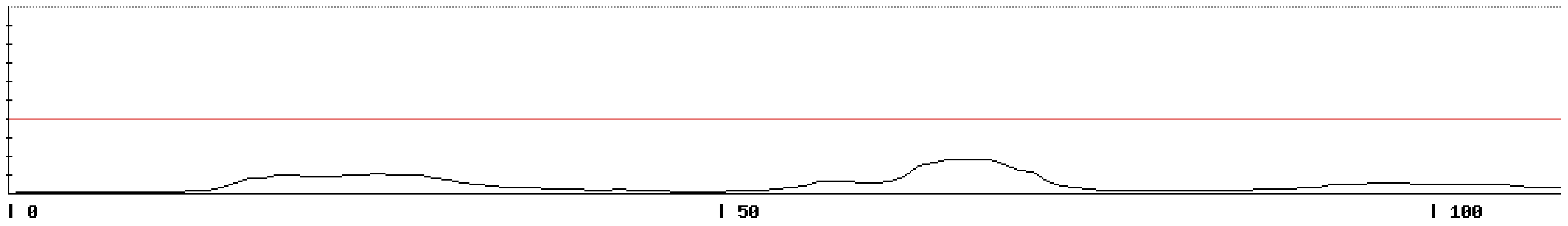

Predictions for Eragrostistef_462911983

Viterbi Path

Viterbi Path

Posterior @ 0.4

Posterior @ 0.4

Posterior Graphic. Threshold Marked @ 0.4

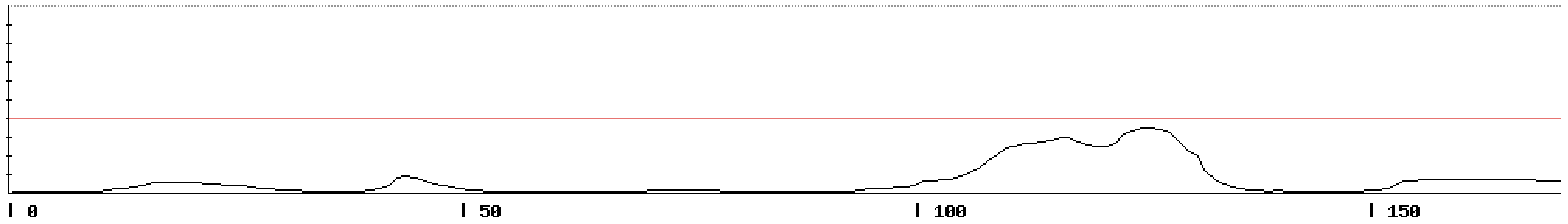

Predictions for Eragrostistef_462913088	
Viterbi Path	
Viterbi Path	
Posterior @ 0.4	
Posterior @ 0.4	
Posterior Graphic. Threshold Marked @ 0.4	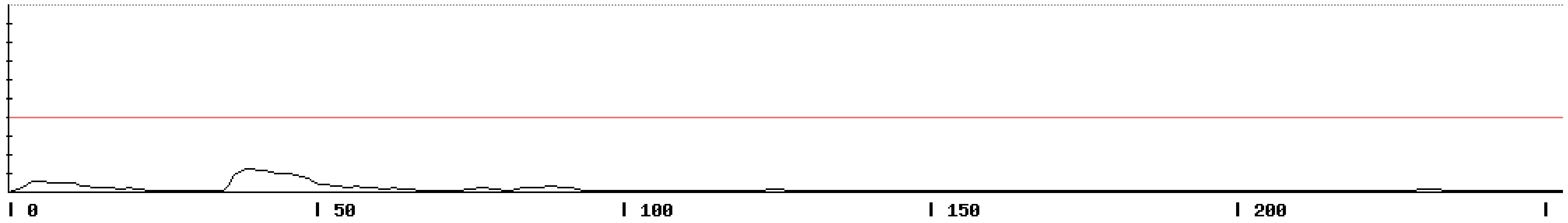

Predictions for Eragrostistef_462913356	
Viterbi Path	
Viterbi Path	
Posterior @ 0.4	168 - RTKPSGRGGR - 177
Posterior @ 0.4	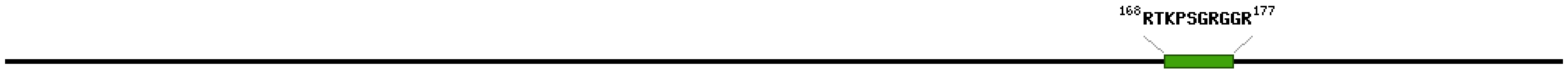
Posterior Graphic. Threshold Marked @ 0.4	

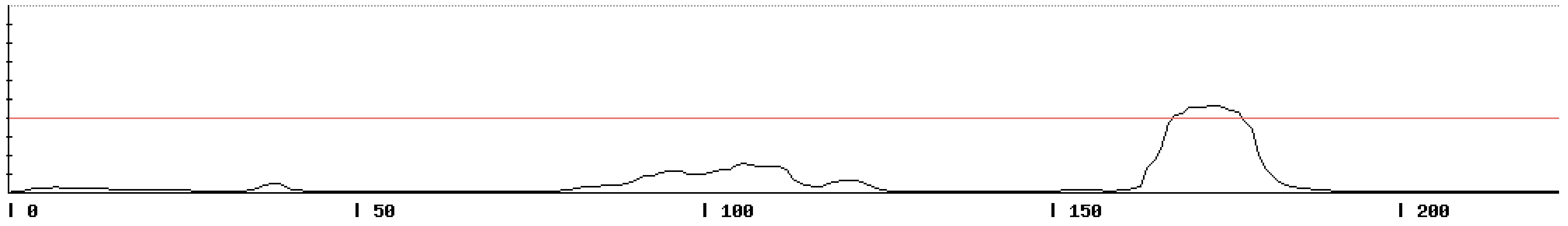

Predictions for Eragrostistef_462913543

Viterbi Path

Viterbi Path

Posterior @ 0.4: 536 - SRRRRRRKT - 544

Posterior @ 0.4

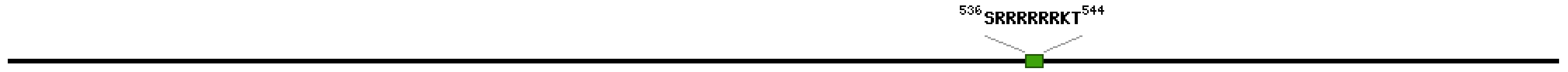

Posterior Graphic. Threshold Marked @ 0.4

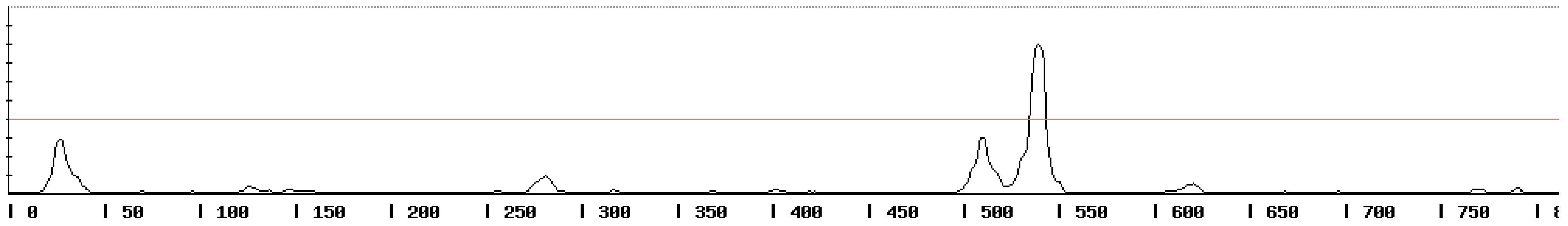

Predictions for Eragrostistef_462913881

Viterbi Path

Viterbi Path

Posterior @ 0.4

Posterior @ 0.4

Posterior Graphic. Threshold Marked @ 0.4

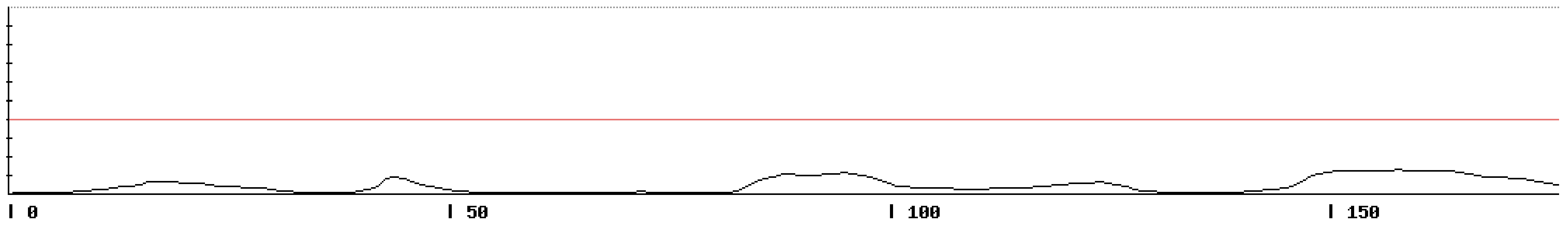

Predictions for Eragrostistef_462914386

Viterbi Path

Viterbi Path

Posterior @ 0.4

Posterior @ 0.4

Posterior Graphic. Threshold Marked @ 0.4

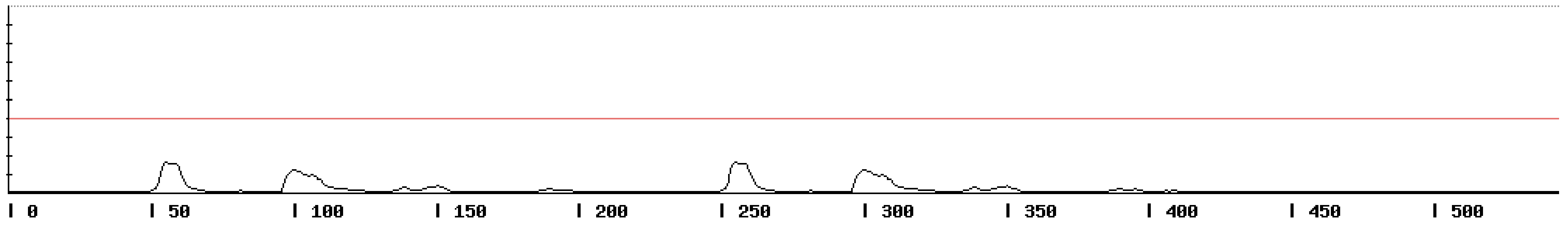

Predictions for Eragrostistef_462914484

Viterbi Path

Viterbi Path

Posterior @ 0.4

Posterior @ 0.4

Posterior Graphic. Threshold Marked @ 0.4

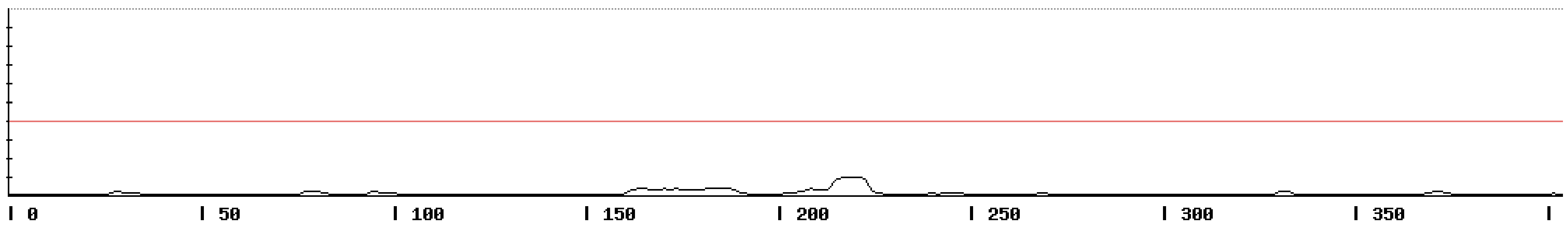

Predictions for Eragrostistef_462915047

Viterbi Path

Viterbi Path

Posterior @ 0.4

104 - SRPNRAAGRGYWKATGADKPIAPKGTARP - 132

152 - RRGPR - 156

Posterior @ 0.4

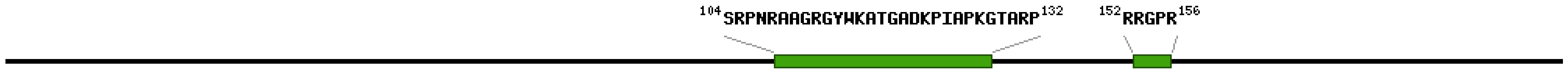

Posterior Graphic. Threshold Marked @ 0.4

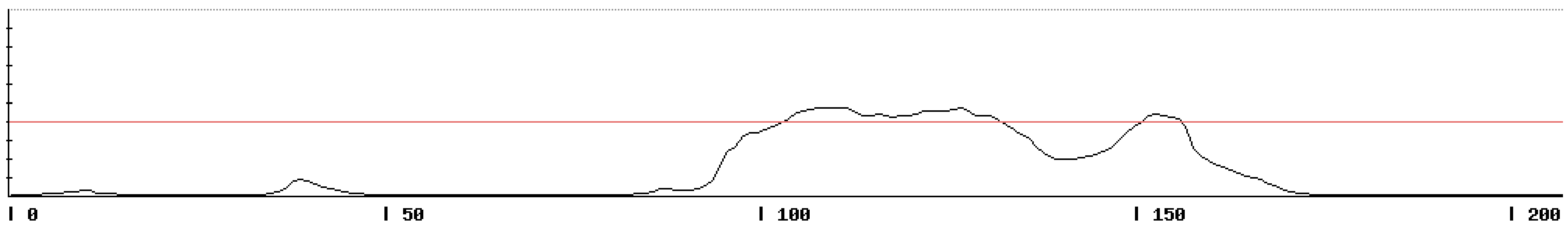

Predictions for Eragrostistef_462915132

Viterbi Path

Viterbi Path

Posterior @ 0.4

Posterior @ 0.4

Posterior Graphic. Threshold Marked @ 0.4

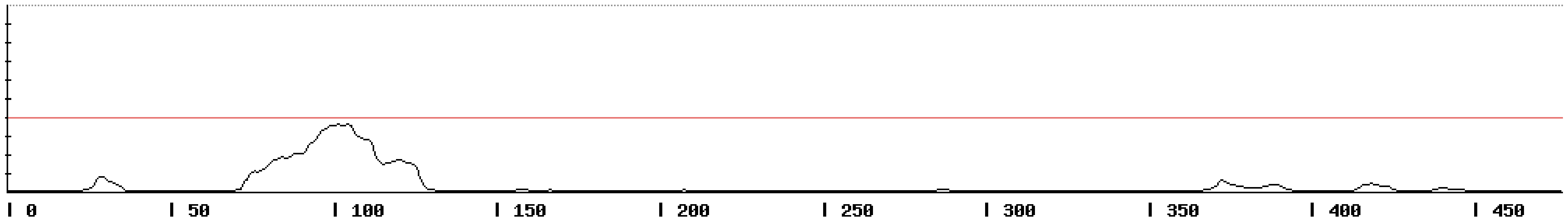

Predictions for Eragrostistef_462915318

Viterbi Path

Viterbi Path

Posterior @ 0.4 — 90 - KATGKDRKVNSHKRAVG - 106

Posterior @ 0.4

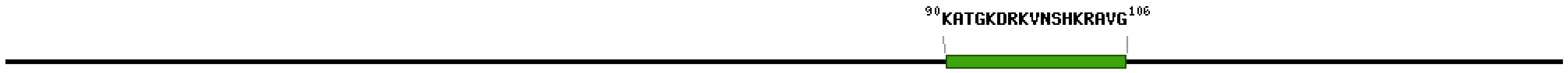

Posterior Graphic. Threshold Marked @ 0.4

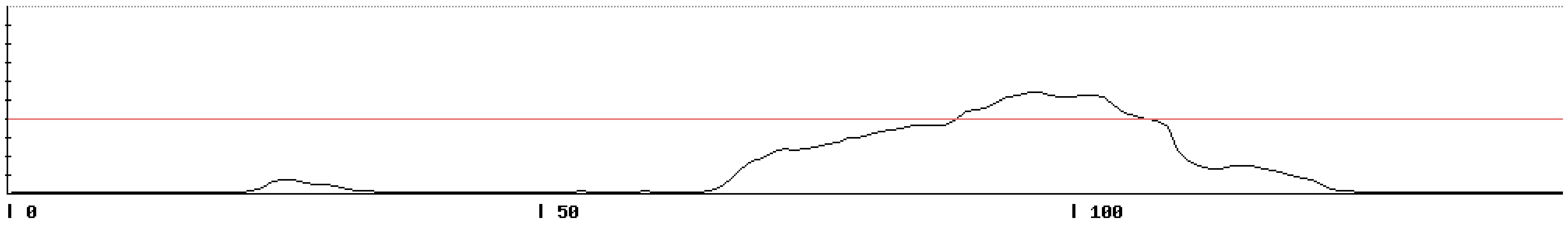

Predictions for Eragrostistef_462915481

Viterbi Path	
Viterbi Path	
Posterior @ 0.4	
Posterior @ 0.4	
Posterior Graphic. Threshold Marked @ 0.4	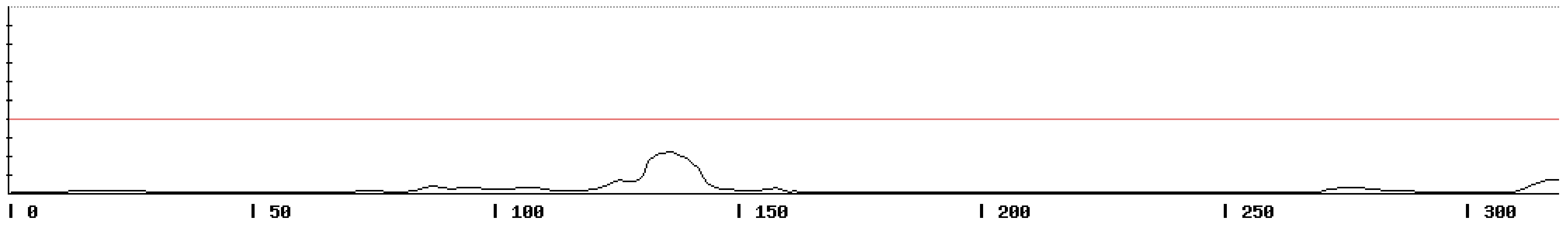

Predictions for Eragrostistef_462916120	
Viterbi Path	
Viterbi Path	
Posterior @ 0.4	300 - ARRPRRRLAQRHVRVPAEAVLKRP - 323
Posterior @ 0.4	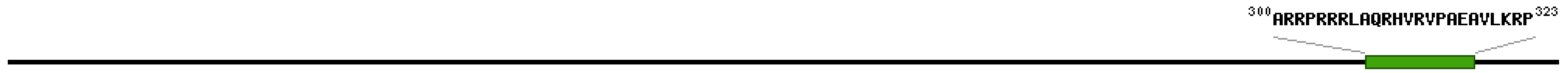
Posterior Graphic. Threshold Marked @ 0.4	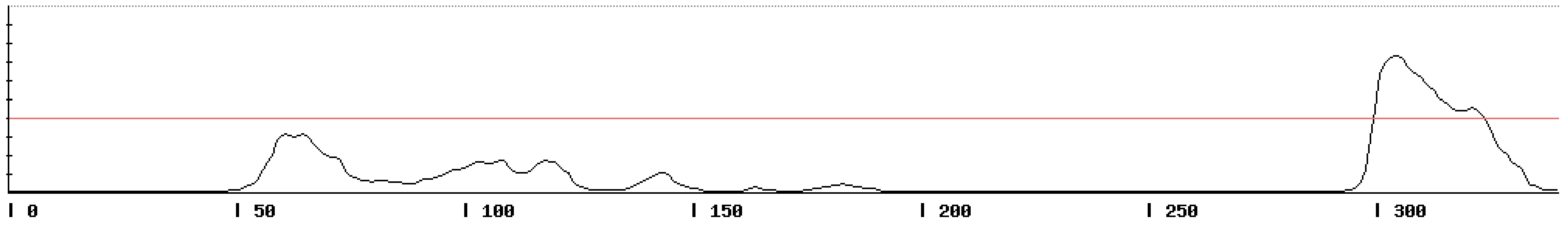

Predictions for Eragrostistef_462916219	
Viterbi Path	
Viterbi Path	
Posterior @ 0.4	
Posterior @ 0.4	
Posterior Graphic. Threshold Marked @ 0.4	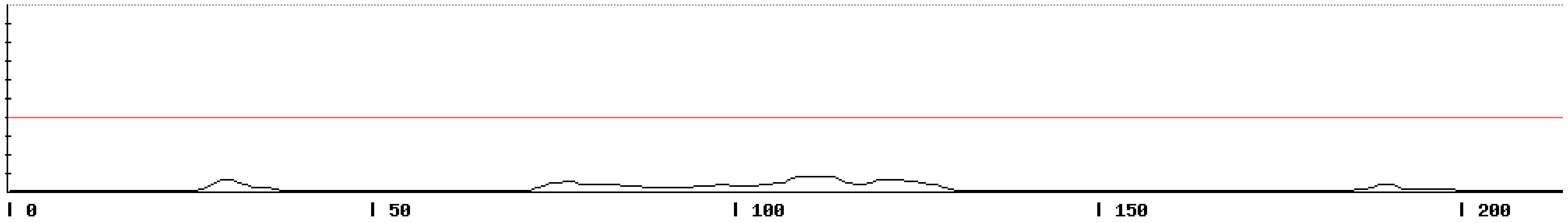

Predictions for Eragrostistef_462916573	
Viterbi Path	
Viterbi Path	
Posterior @ 0.4	38 - RRKGRLNFHRKAKVK - 52
Posterior @ 0.4	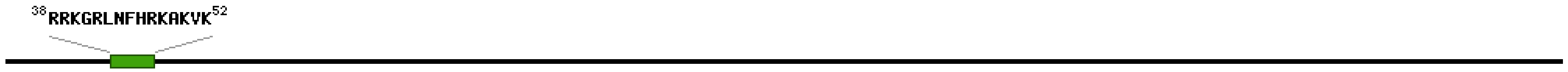
Posterior Graphic. Threshold Marked @ 0.4	

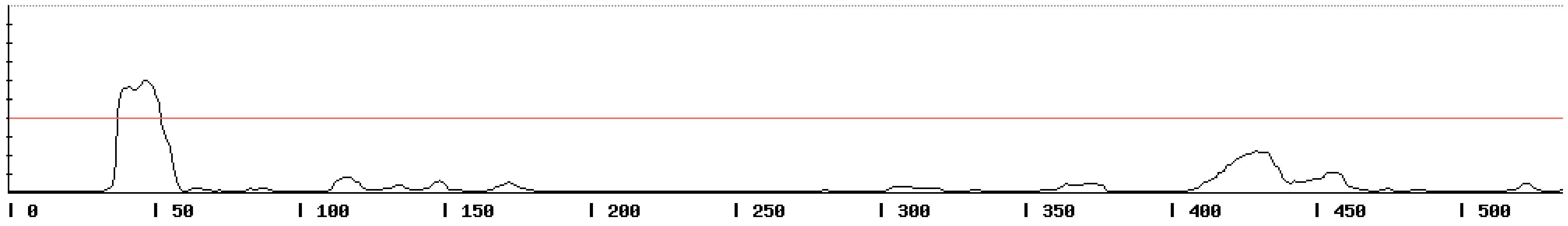

Predictions for Eragrostistef_462916576

Viterbi Path

Viterbi Path

Posterior @ 0.4

Posterior @ 0.4

Posterior Graphic. Threshold Marked @ 0.4

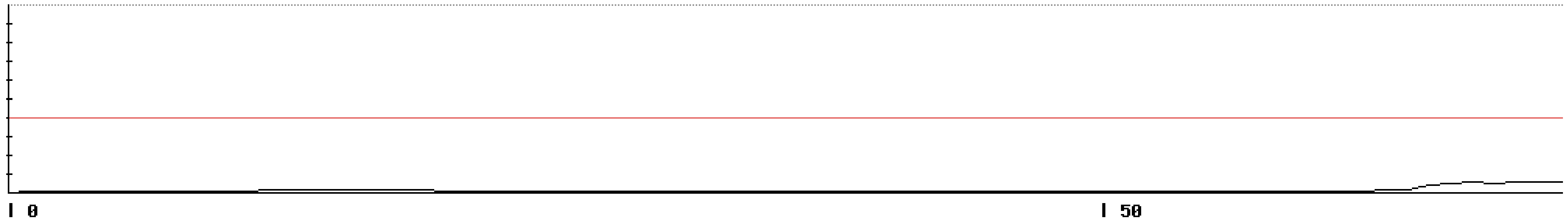

Predictions for Eragrostistef_462917501

Viterbi Path

Viterbi Path

Posterior @ 0.4 102 - RKRRK - 106

Posterior @ 0.4

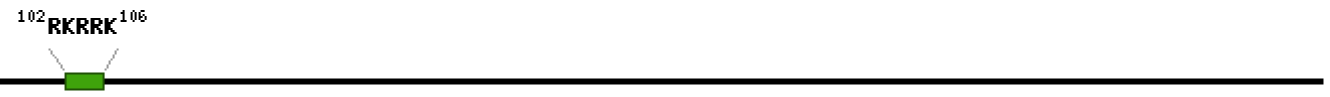

Posterior Graphic. Threshold Marked @ 0.4

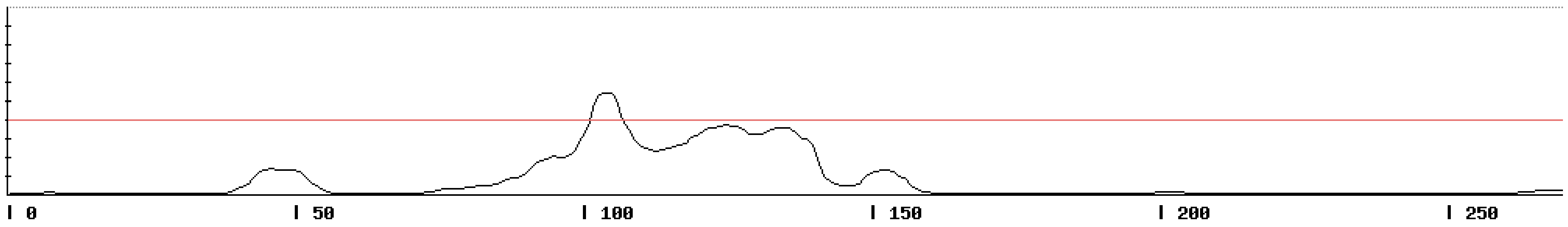

Predictions for Eragrostistef_462917732

Viterbi Path

Viterbi Path

Posterior @ 0.4

Posterior @ 0.4

Posterior Graphic. Threshold Marked @ 0.4

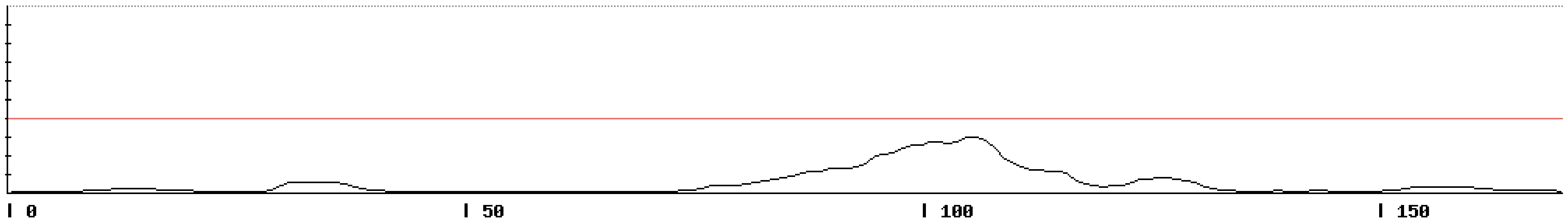

Predictions for Eragrostistef_462918821

Viterbi Path

Viterbi Path

Posterior @ 0.4

Posterior @ 0.4

Posterior Graphic. Threshold Marked @ 0.4

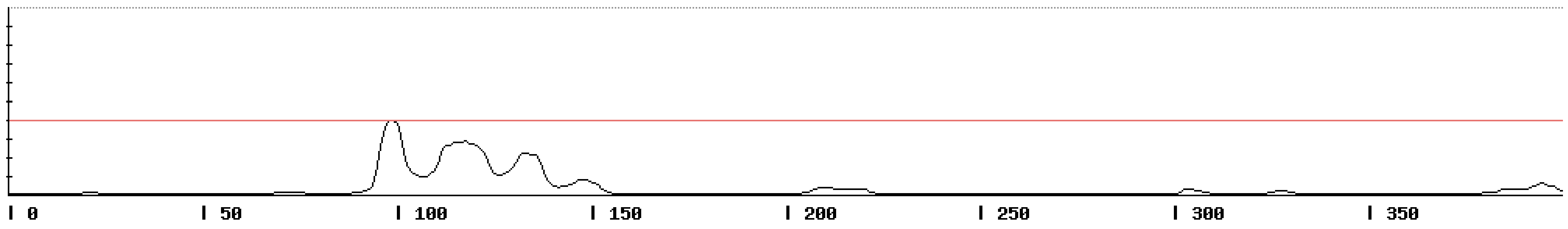

Predictions for Eragrostistef_462918993

Viterbi Path

Viterbi Path

Posterior @ 0.4: 113 - R - 113

Posterior @ 0.4

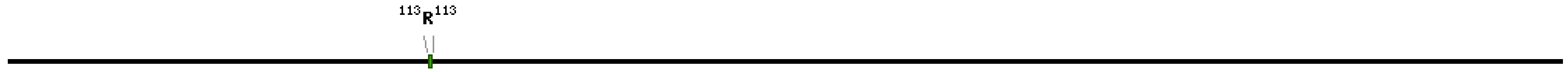

Posterior Graphic. Threshold Marked @ 0.4

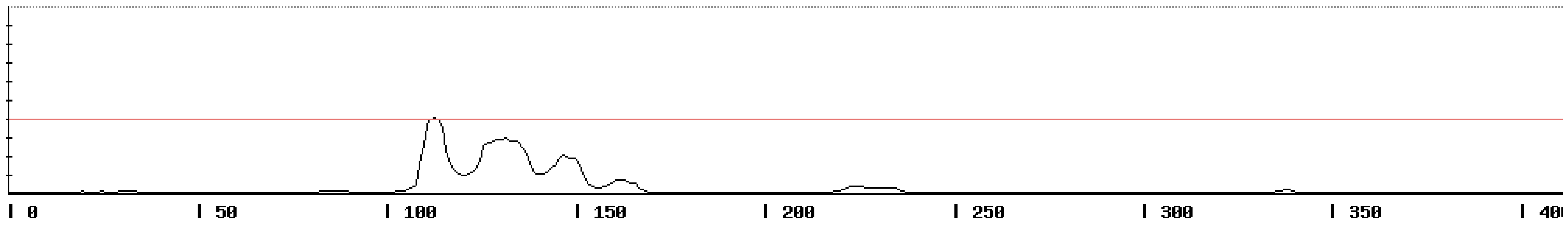

Predictions for Eragrostistef_462919686

Viterbi Path

Viterbi Path

Posterior @ 0.4

Posterior @ 0.4

Posterior Graphic. Threshold Marked @ 0.4

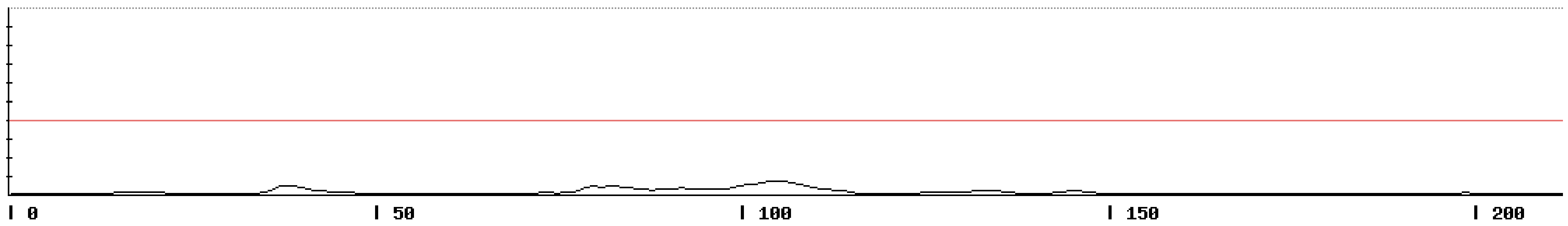

Predictions for Eragrostistef_462919689

Viterbi Path

Viterbi Path

Posterior @ 0.4

Posterior @ 0.4

Posterior Graphic. Threshold Marked @ 0.4

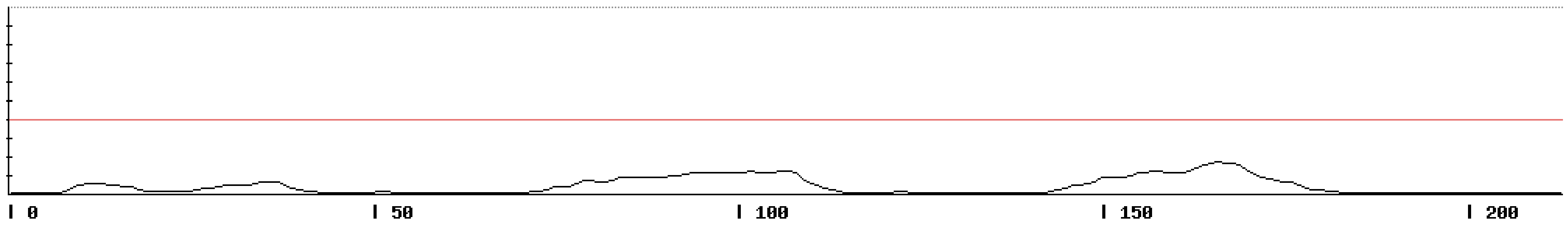

Predictions for Eragrostistef_462920247

Viterbi Path

Viterbi Path

Posterior @ 0.4

Posterior @ 0.4

Posterior Graphic. Threshold Marked @ 0.4

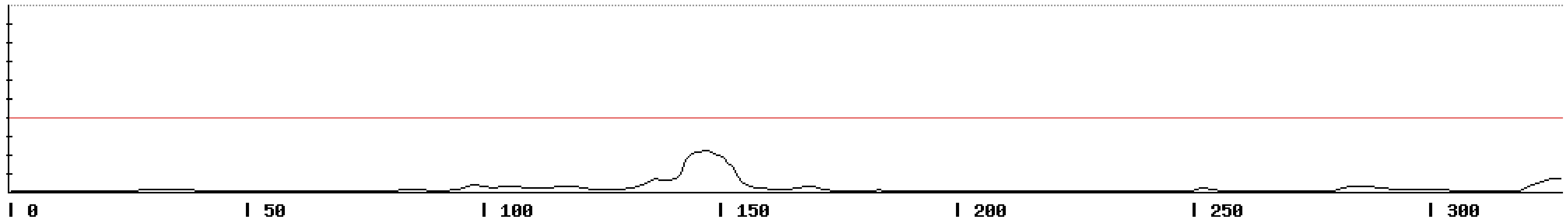

Predictions for Eragrostistef_462923579

Viterbi Path

Viterbi Path

Posterior @ 0.4

Posterior @ 0.4

Posterior Graphic. Threshold Marked @ 0.4

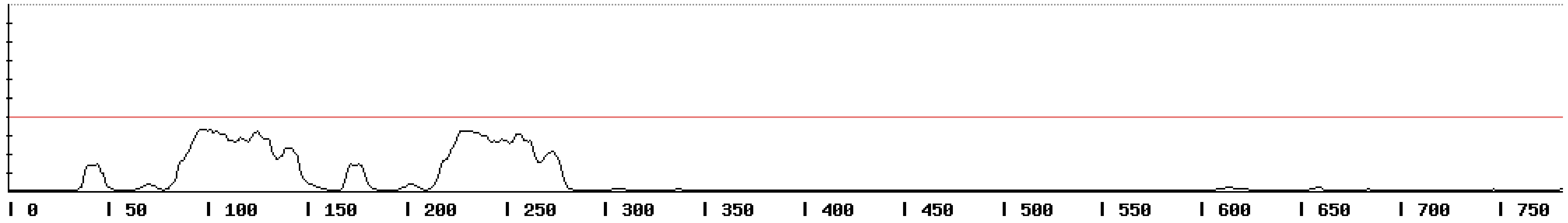

Predictions for Eragrostistef_462923865

Viterbi Path

Viterbi Path

Posterior @ 0.4

Posterior @ 0.4

Posterior Graphic. Threshold Marked @ 0.4

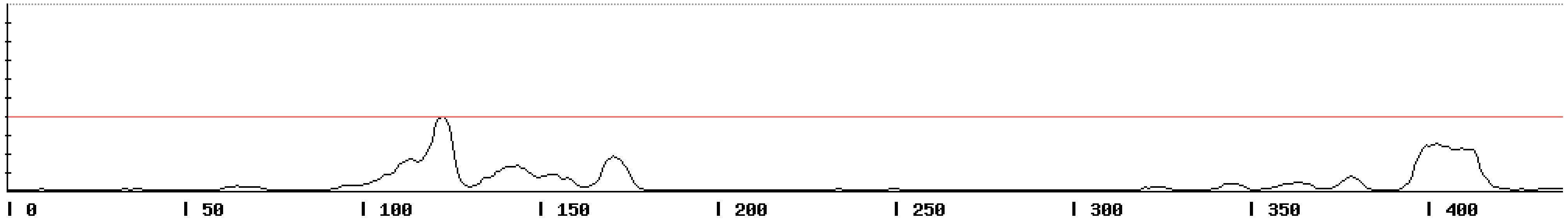

Predictions for Eragrostistef_462924105

Viterbi Path

Viterbi Path

Posterior @ 0.4

Posterior @ 0.4

Posterior Graphic. Threshold Marked @ 0.4

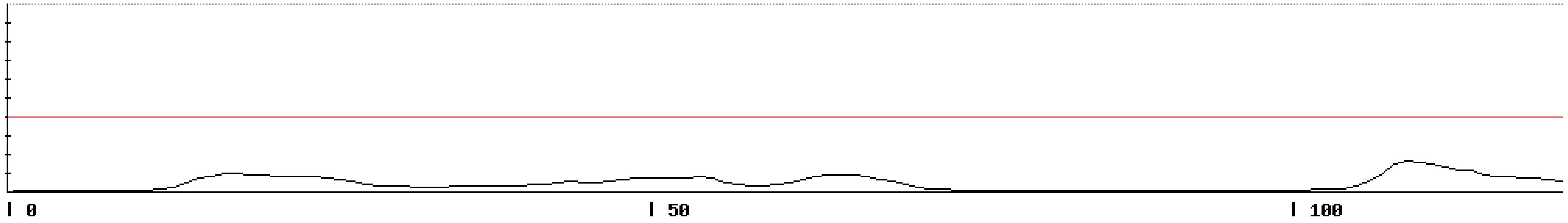

Predictions for Eragrostistef_462924531

Viterbi Path

Viterbi Path

Posterior @ 0.4

Posterior @ 0.4

Posterior Graphic. Threshold Marked @ 0.4

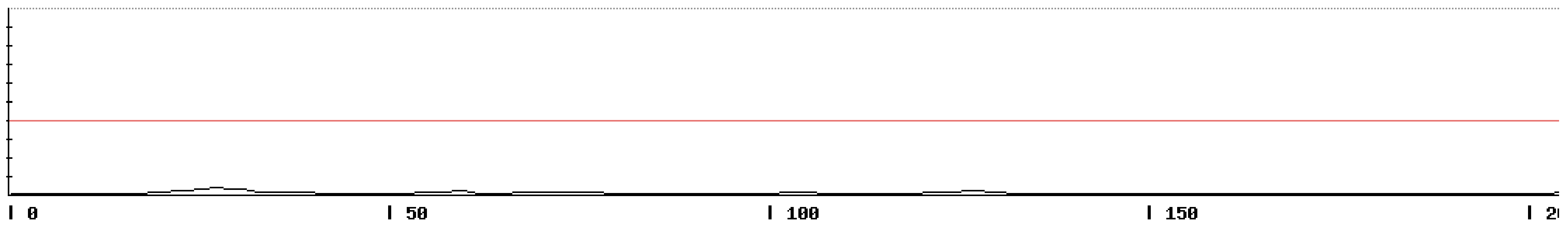

Predictions for Eragrostistef_462924962

Viterbi Path

Viterbi Path

Posterior @ 0.4

Posterior @ 0.4

Posterior Graphic. Threshold Marked @ 0.4

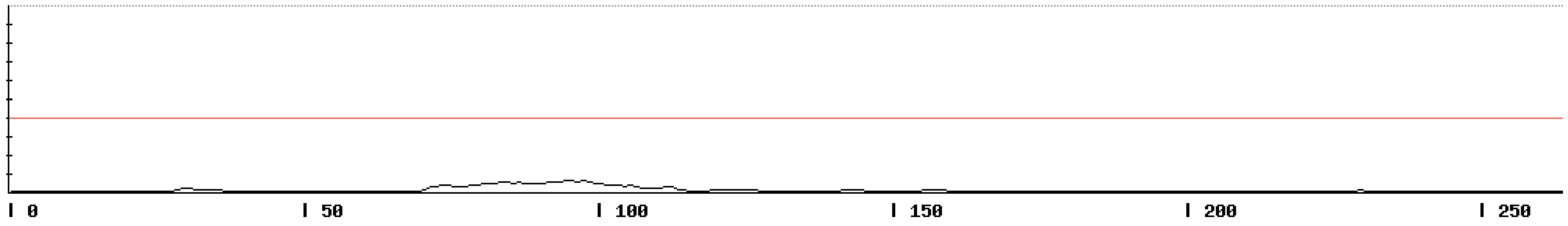

Predictions for Eragrostistef_462925586	
Viterbi Path	
Viterbi Path	
Posterior @ 0.4	
Posterior @ 0.4	
Posterior Graphic. Threshold Marked @ 0.4	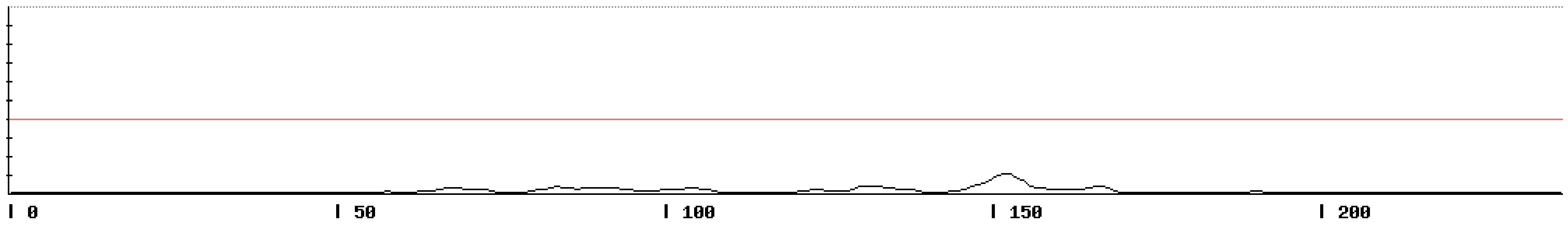

Predictions for Eragrostistef_462925604	
Viterbi Path	
Viterbi Path	
Posterior @ 0.4	
Posterior @ 0.4	

Posterior Graphic. Threshold Marked @ 0.4

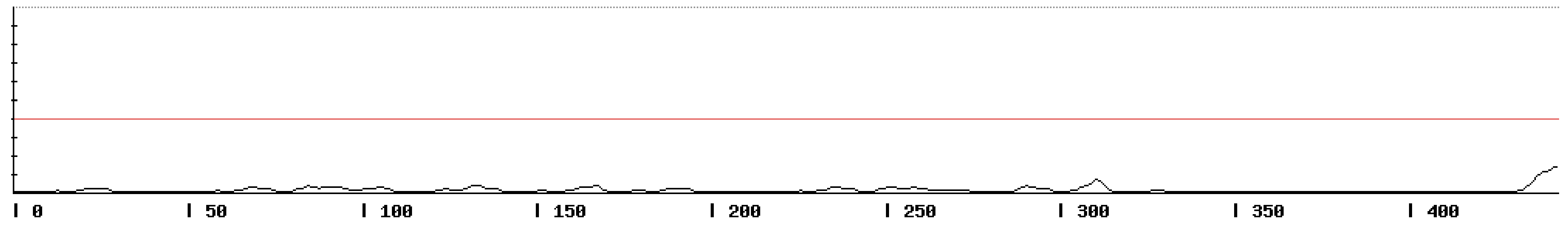

Predictions for Eragrostistef_462926622

Viterbi Path

Viterbi Path

Posterior @ 0.4 — 142 - RKRRK - 146

Posterior @ 0.4

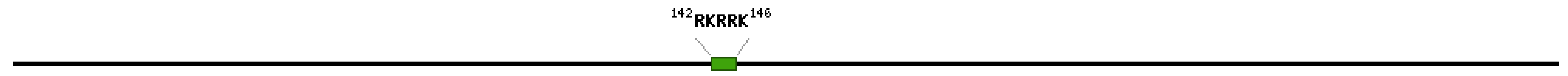

Posterior Graphic. Threshold Marked @ 0.4

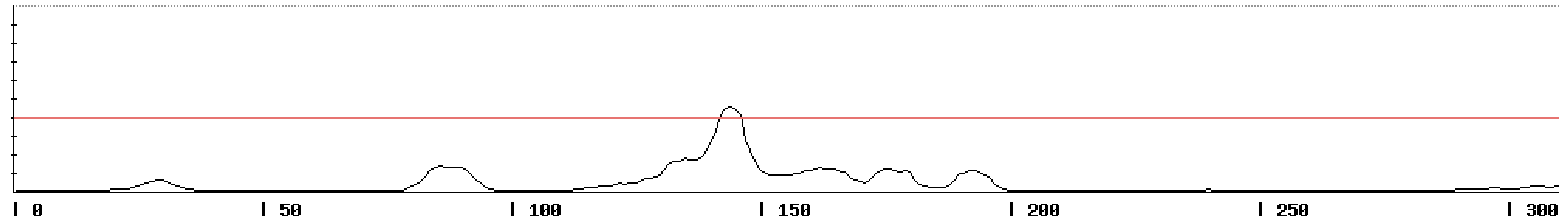

Predictions for Eragrostistef_462927251

Viterbi Path

Viterbi Path

Posterior @ 0.4

Posterior @ 0.4

Posterior Graphic. Threshold Marked @ 0.4

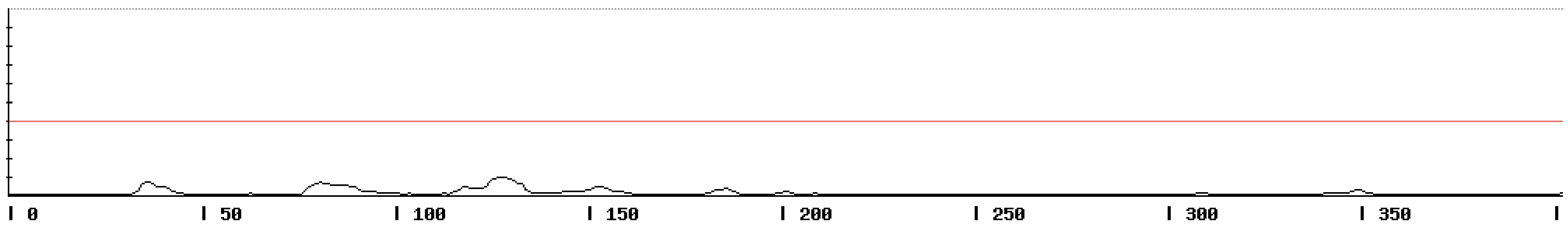

Predictions for Eragrostistef_462927431

Viterbi Path

Viterbi Path

Posterior @ 0.4

Posterior @ 0.4

Posterior Graphic. Threshold Marked @ 0.4

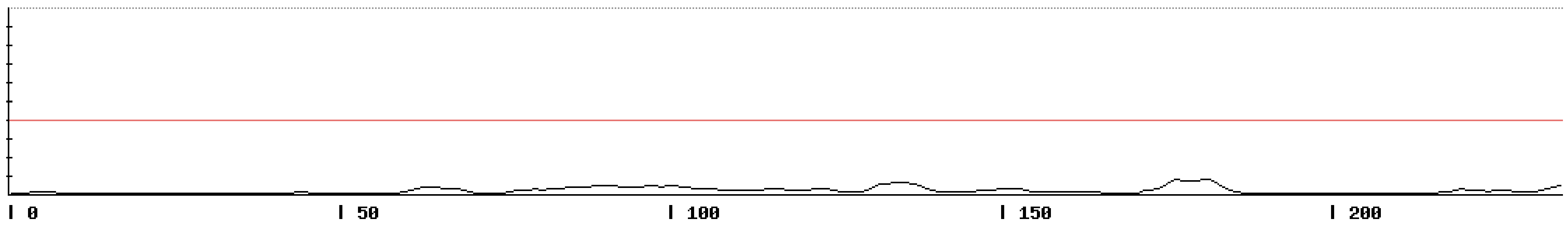

Predictions for Eragrostistef_462927434

Viterbi Path

Viterbi Path

Posterior @ 0.4

Posterior @ 0.4

Posterior Graphic. Threshold Marked @ 0.4

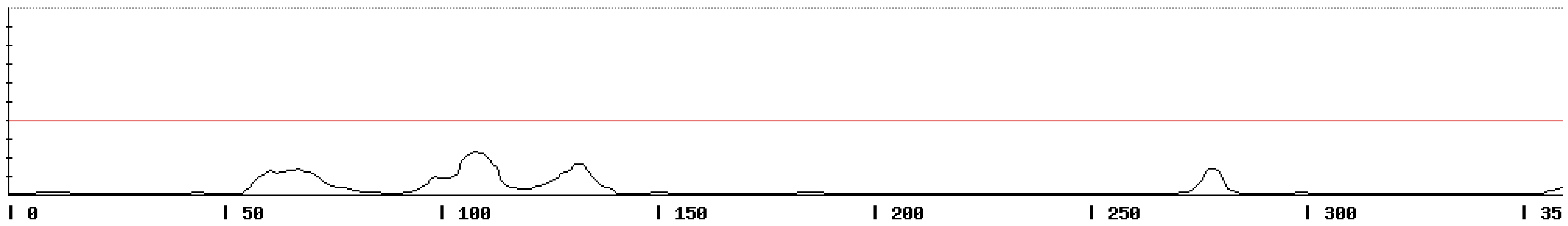

Predictions for Eragrostistef_462927625

Viterbi Path

Viterbi Path

Posterior @ 0.4

Posterior @ 0.4

Posterior Graphic. Threshold Marked @ 0.4

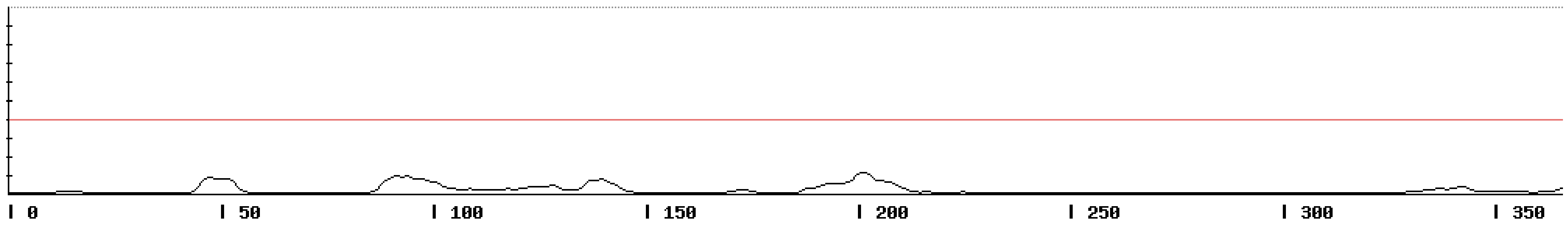

Predictions for Eragrostistef_462927994

Viterbi Path

Viterbi Path

Posterior @ 0.4

Posterior @ 0.4

Posterior Graphic. Threshold Marked @ 0.4

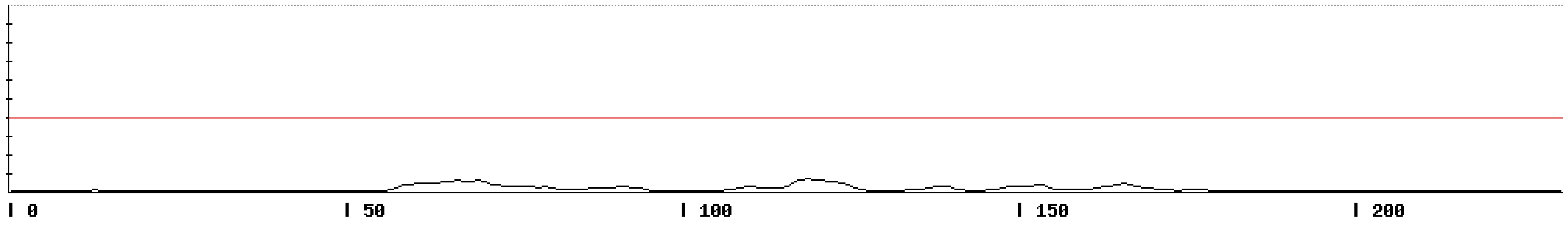

Predictions for Eragrostistef_462929130

Viterbi Path

Viterbi Path

Posterior @ 0.4

Posterior @ 0.4

Posterior Graphic. Threshold Marked @ 0.4

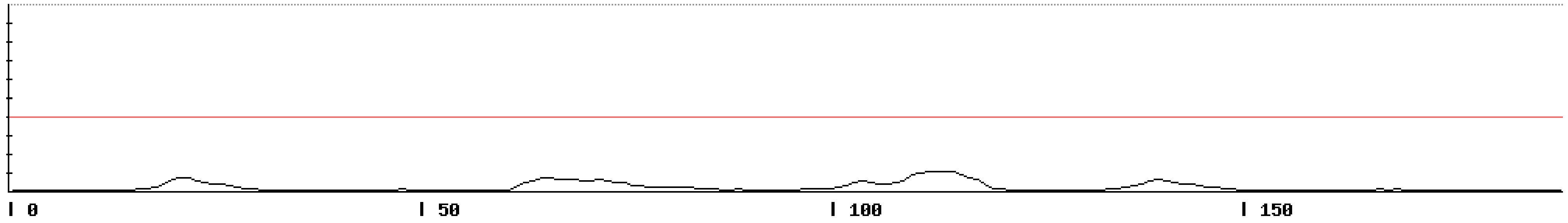

Predictions for Eragrostistef_462929476

Viterbi Path

Viterbi Path

Posterior @ 0.4

Posterior @ 0.4

Posterior Graphic. Threshold Marked @ 0.4

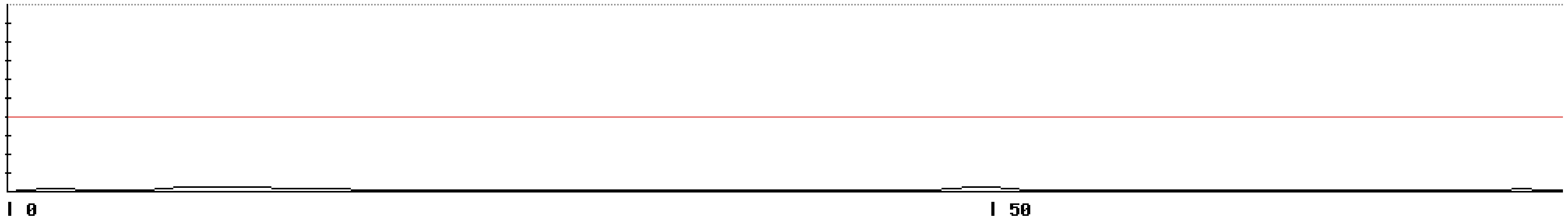

Predictions for Eragrostistef_462929491

Viterbi Path

Viterbi Path

Posterior @ 0.4

Posterior @ 0.4

Posterior Graphic. Threshold Marked @ 0.4

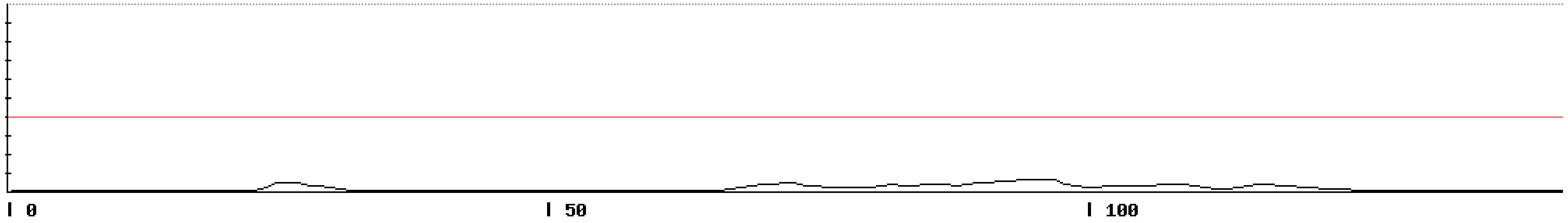

Predictions for Eragrostistef_462929533	
Viterbi Path	
Viterbi Path	
Posterior @ 0.4	
Posterior @ 0.4	
Posterior Graphic. Threshold Marked @ 0.4	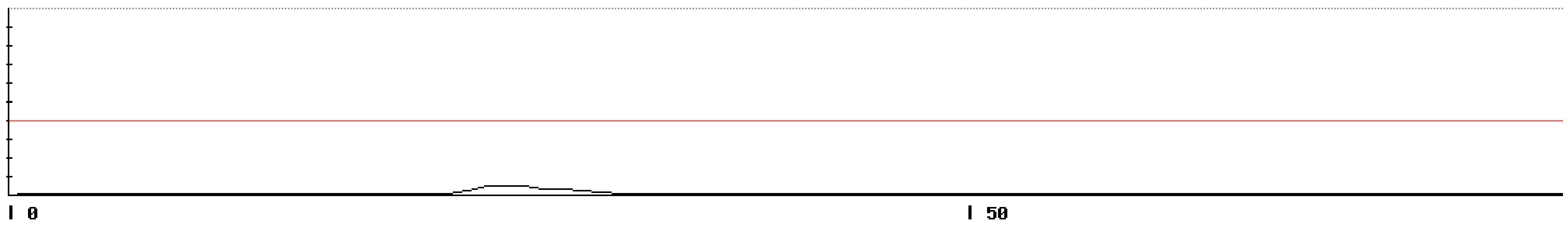

Predictions for Eragrostistef_462929575	
Viterbi Path	
Viterbi Path	
Posterior @ 0.4	
Posterior @ 0.4	
Posterior Graphic. Threshold Marked @ 0.4	

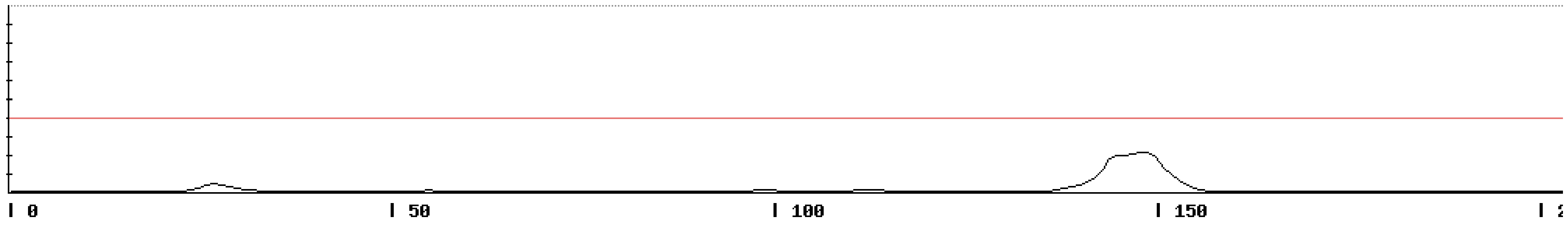

Predictions for Eragrostistef_462930844	
Viterbi Path	
Viterbi Path	
Posterior @ 0.4	
Posterior @ 0.4	
Posterior Graphic. Threshold Marked @ 0.4	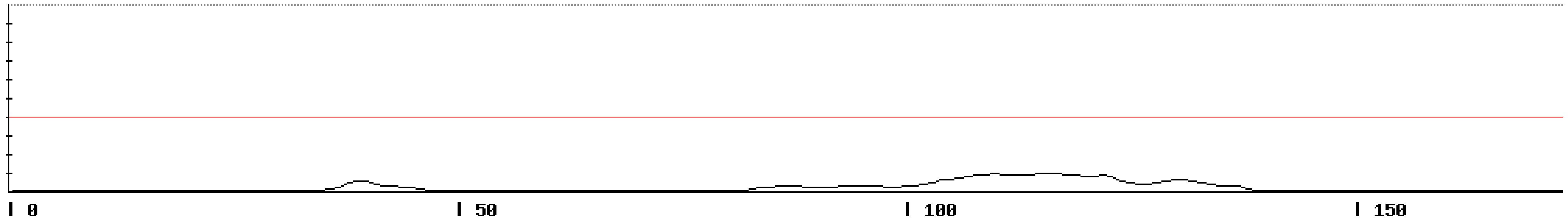

Predictions for Eragrostistef_462932187	
Viterbi Path	
Viterbi Path	
Posterior @ 0.4	
Posterior @ 0.4	

Posterior Graphic. Threshold Marked @ 0.4

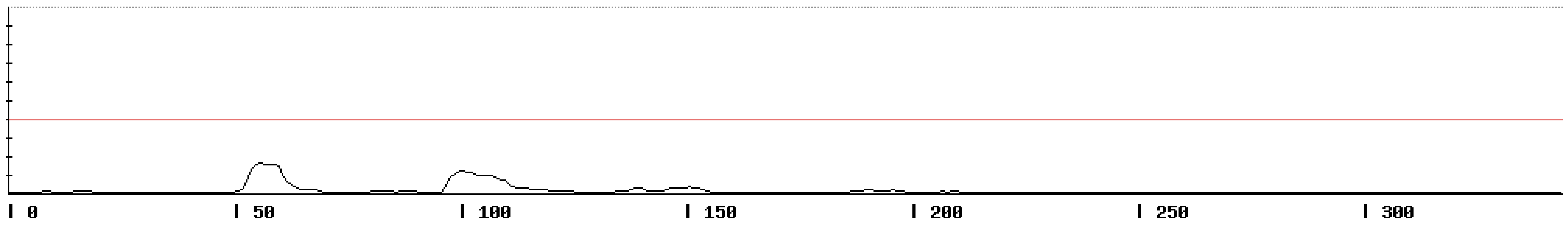

Predictions for Eragrostistef_462933116

Viterbi Path

Viterbi Path

Posterior @ 0.4

Posterior @ 0.4

Posterior Graphic. Threshold Marked @ 0.4

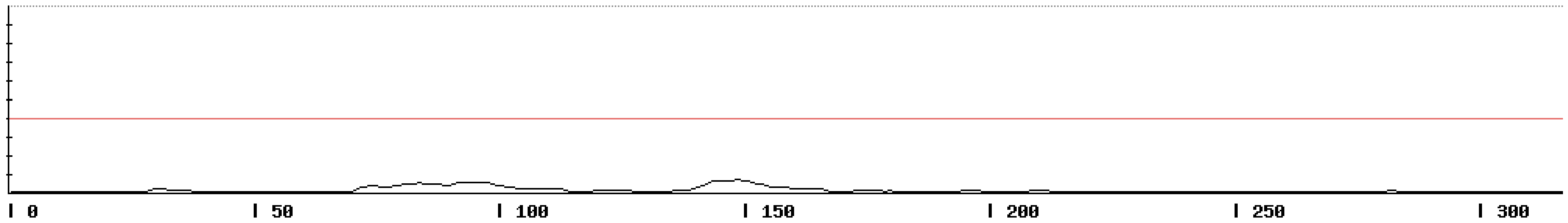

Predictions for Eragrostistef_462933929

Viterbi Path

Viterbi Path

Posterior @ 0.4

Posterior @ 0.4

Posterior Graphic. Threshold Marked @ 0.4

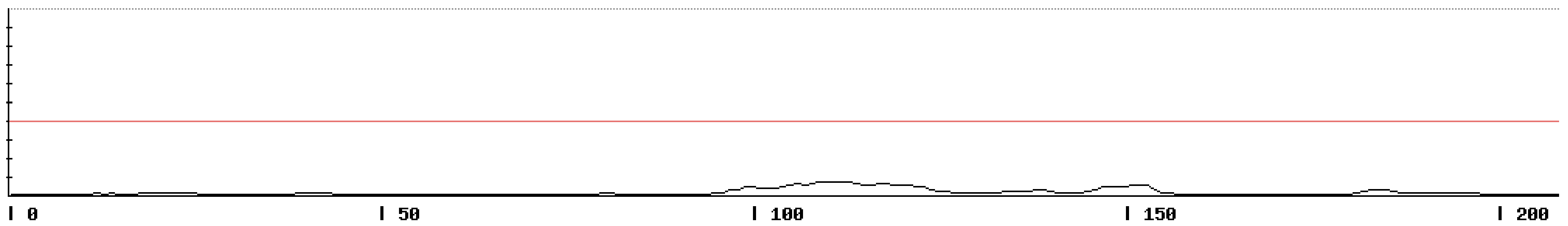

Predictions for Eragrostistef_462936145

Viterbi Path

Viterbi Path

Posterior @ 0.4 — 94 - KATGKDRVIKSKGDKRKQHIIGMKKTLVFHRGRAPKGE - 131

Posterior @ 0.4

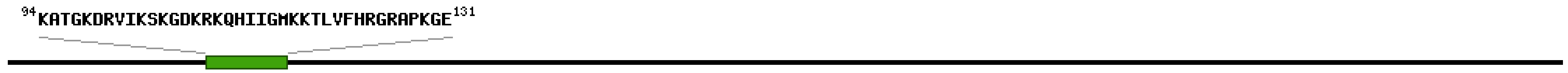

Posterior Graphic. Threshold Marked @ 0.4

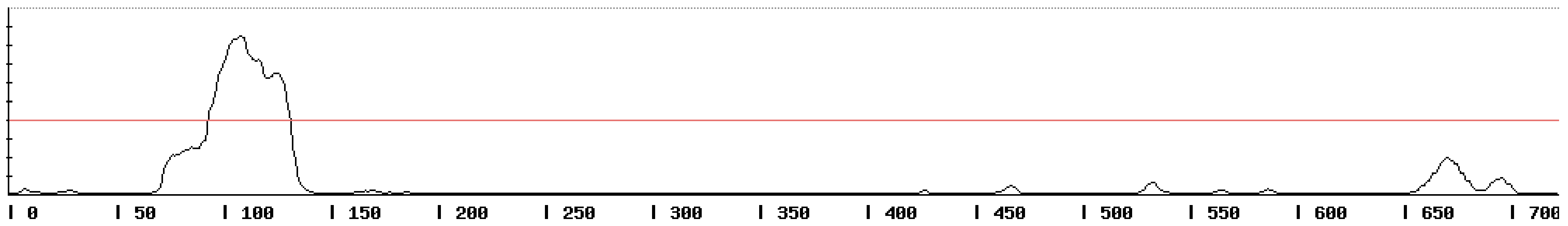

Predictions for Eragrostistef_462936202

Viterbi Path

Viterbi Path

Posterior @ 0.4	123 - R - 123
Posterior @ 0.4	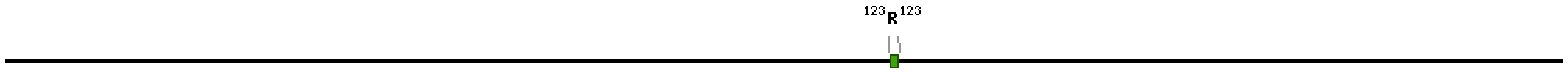
Posterior Graphic. Threshold Marked @ 0.4	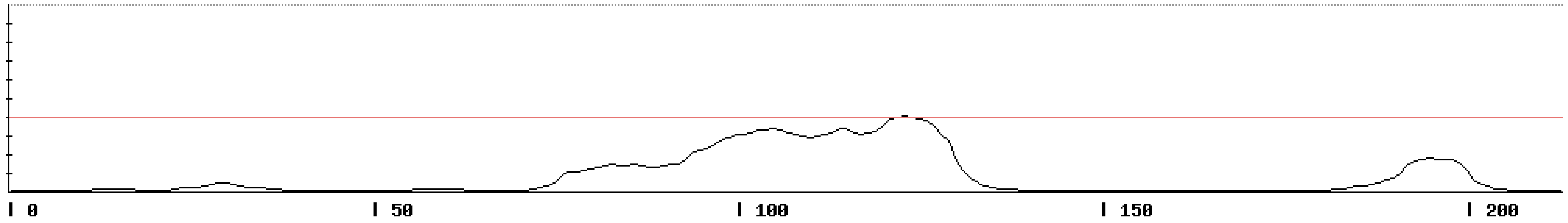

Predictions for Eragrostistef_462936314

Viterbi Path	
Viterbi Path	
Posterior @ 0.4	
Posterior @ 0.4	
Posterior Graphic. Threshold Marked @ 0.4	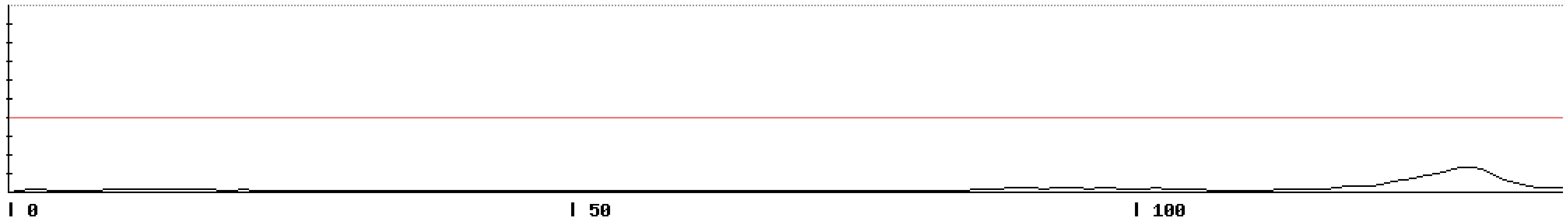

Predictions for Eragrostistef_462936936

Viterbi Path	
Viterbi Path	

Posterior @ 0.4

Posterior @ 0.4

Posterior Graphic. Threshold Marked @ 0.4

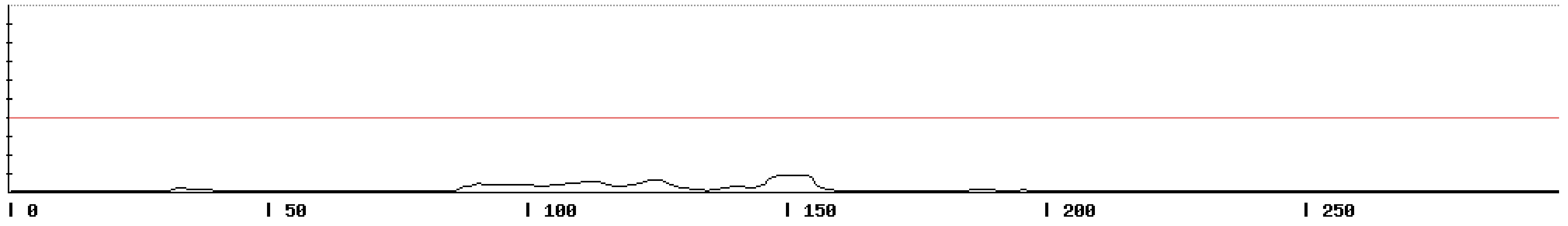

Predictions for Eragrostistef_462937525

Viterbi Path

Viterbi Path

Posterior @ 0.4

Posterior @ 0.4

Posterior Graphic. Threshold Marked @ 0.4

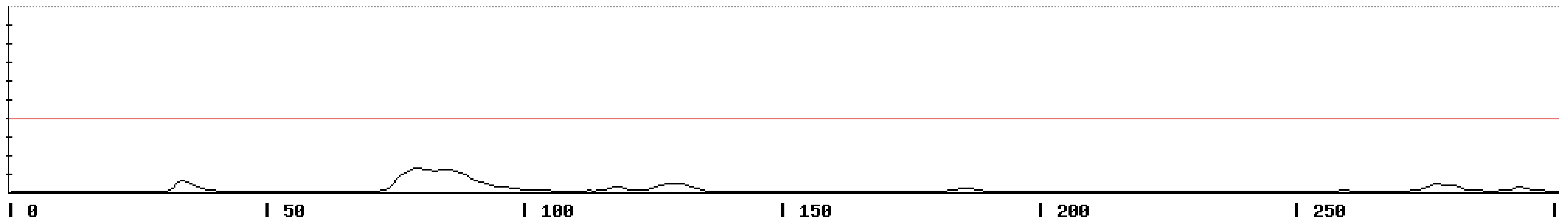

Predictions for Eragrostistef_462937657

Viterbi Path

Posterior @ 0.4

Posterior @ 0.4

Posterior Graphic. Threshold Marked @ 0.4

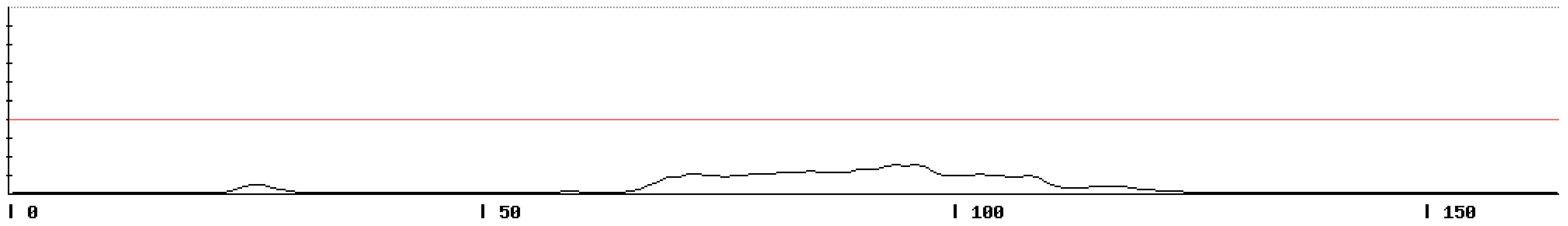

Predictions for Eucalyptuscamaldulensis_EcC000284.10

Viterbi Path

Viterbi Path

Posterior @ 0.4

Posterior @ 0.4

Posterior Graphic. Threshold Marked @ 0.4

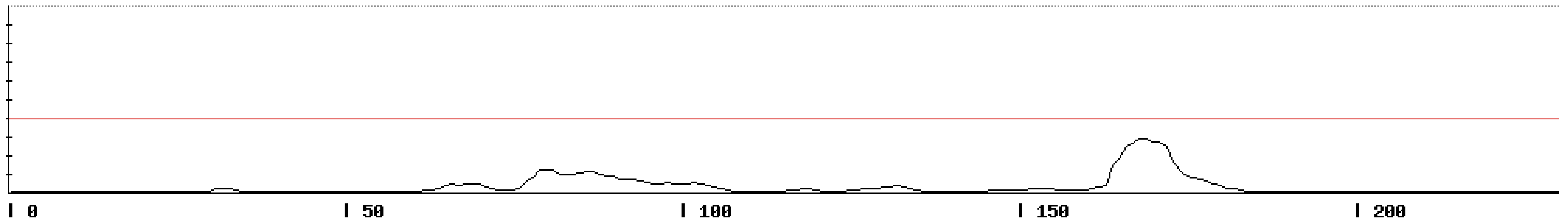

Predictions for Eucalyptuscamaldulensis_EcC000293.10

Viterbi Path

Viterbi Path

Posterior @ 0.4

Posterior @ 0.4

Posterior Graphic. Threshold Marked @ 0.4

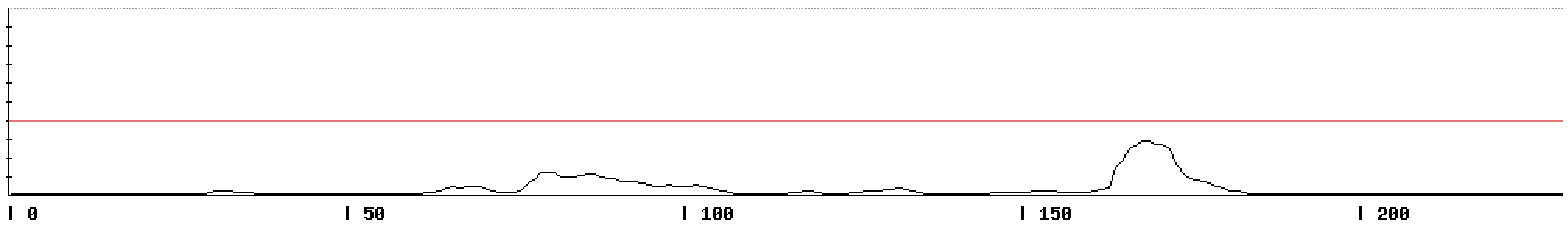

Predictions for Eucalyptuscamaldulensis_EcC000293.20

Viterbi Path

Viterbi Path

Posterior @ 0.4

Posterior @ 0.4

Posterior Graphic. Threshold Marked @ 0.4

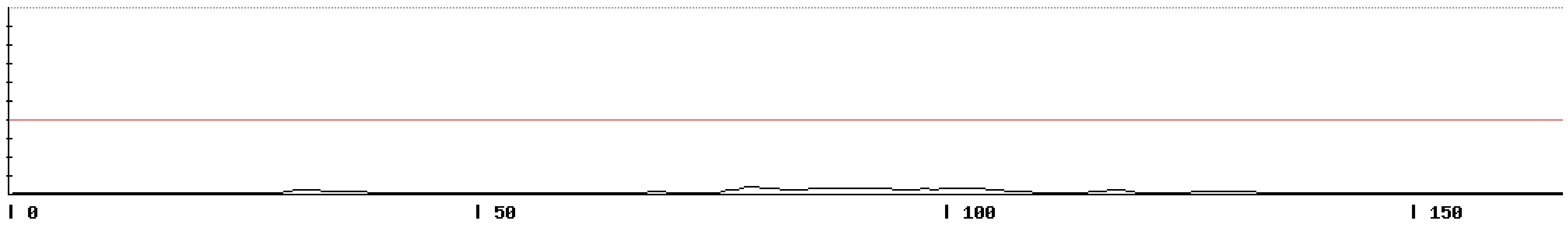

Predictions for Eucalyptuscamaldulensis_EcC000810.10

Viterbi Path	
Viterbi Path	
Posterior @ 0.4	
Posterior @ 0.4	
Posterior Graphic. Threshold Marked @ 0.4	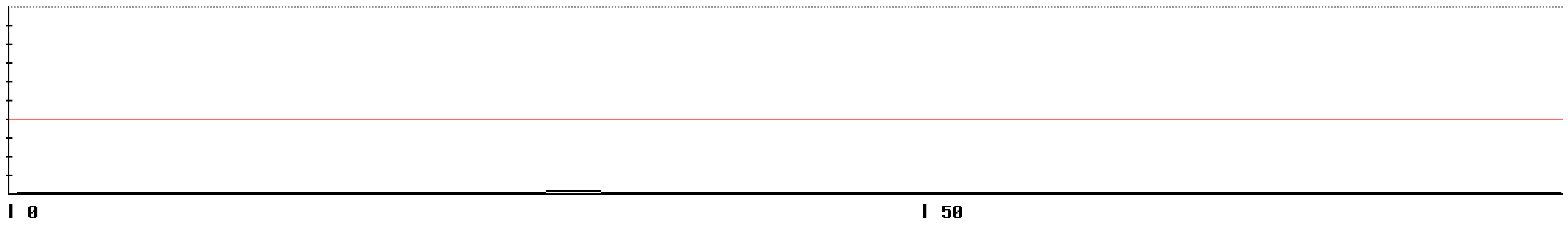

Predictions for Eucalyptuscamaldulensis_EcC002206.10

Viterbi Path	
Viterbi Path	
Posterior @ 0.4	
Posterior @ 0.4	
Posterior Graphic. Threshold Marked @ 0.4	

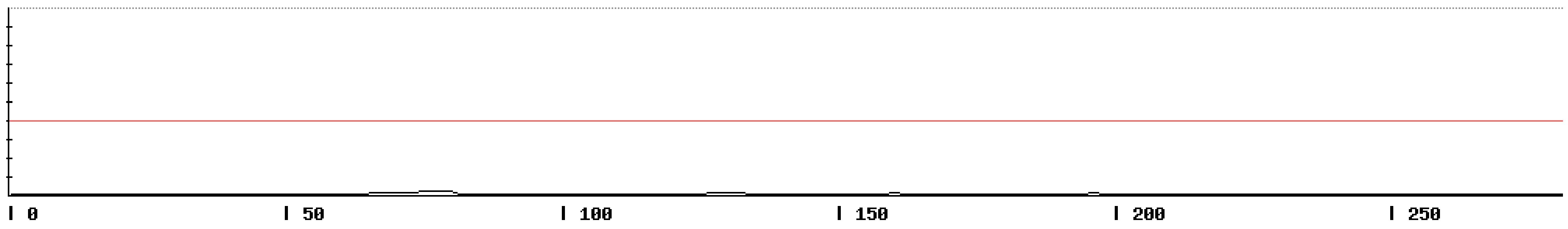

Predictions for Eucalyptuscamaldulensis_EcC002371.20

Viterbi Path	
Viterbi Path	
Posterior @ 0.4	
Posterior @ 0.4	
Posterior Graphic. Threshold Marked @ 0.4	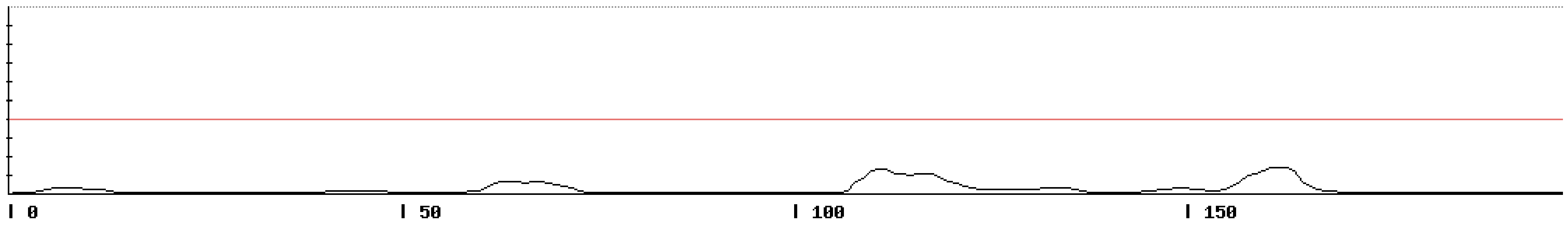

Predictions for Eucalyptuscamaldulensis_EcC002598.10

Viterbi Path	
Viterbi Path	
Posterior @ 0.4	98 - DRKIK - 102
Posterior @ 0.4	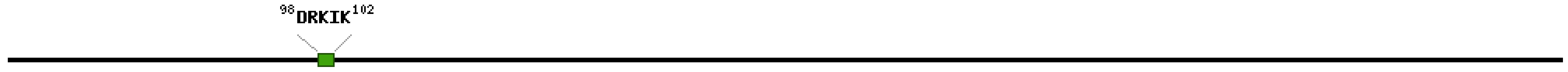

Posterior Graphic. Threshold Marked @ 0.4

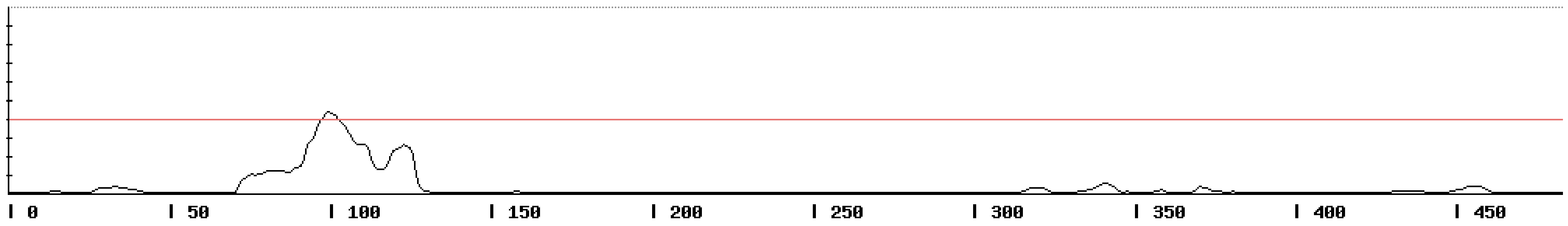

Predictions for Eucalyptuscamaldulensis_EcC003884.20

Viterbi Path

Viterbi Path

Posterior @ 0.4

Posterior @ 0.4

Posterior Graphic. Threshold Marked @ 0.4

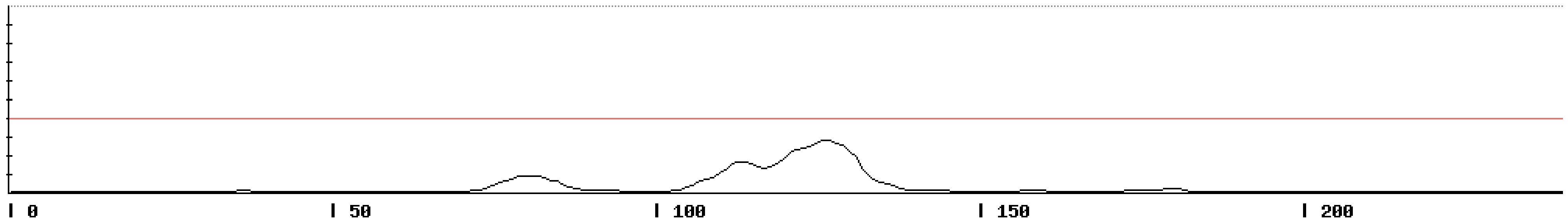

Predictions for Eucalyptuscamaldulensis_EcC003993.10

Viterbi Path

Viterbi Path

Posterior @ 0.4

Posterior @ 0.4

Posterior Graphic. Threshold Marked @ 0.4

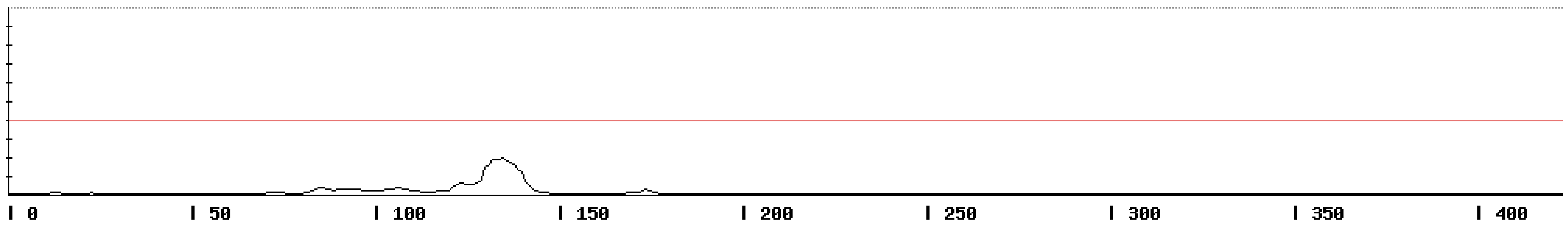

Predictions for Eucalyptuscamaldulensis_EcC004159.10

Viterbi Path

Viterbi Path

Posterior @ 0.4

Posterior @ 0.4

Posterior Graphic. Threshold Marked @ 0.4

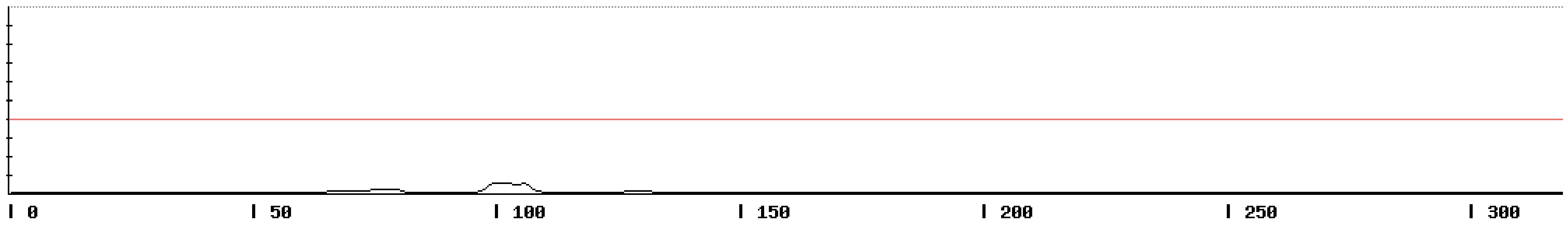

Predictions for Eucalyptuscamaldulensis_EcC004877.10

Viterbi Path

Viterbi Path

Posterior @ 0.4 — 95 - GKPHKIRTQDRKNTLGLKKILVFKKRQSPK - 124

Posterior @ 0.4

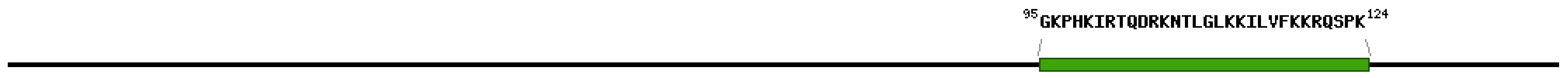

Posterior Graphic. Threshold Marked @ 0.4

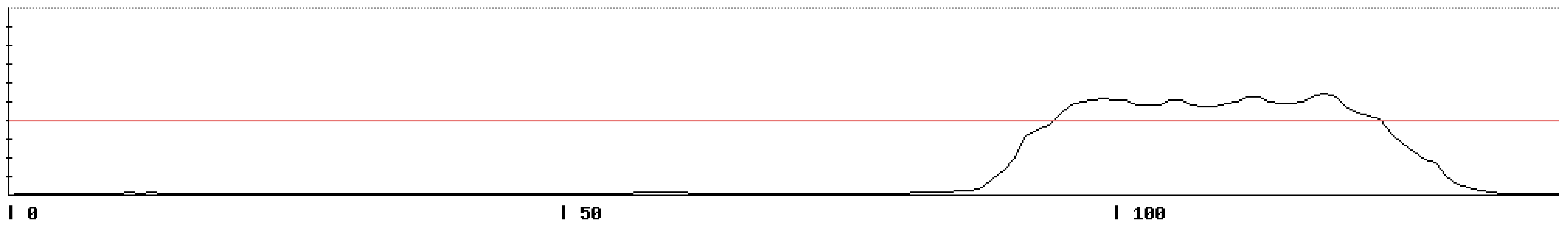

Predictions for Eucalyptuscamaldulensis_EcC004877.30

Viterbi Path

Viterbi Path

Posterior @ 0.4 — 66 - RKSTGKTHKIR - 76

Posterior @ 0.4

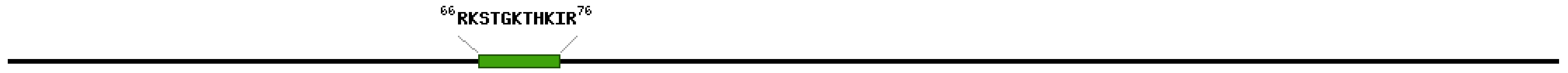

Posterior Graphic. Threshold Marked @ 0.4

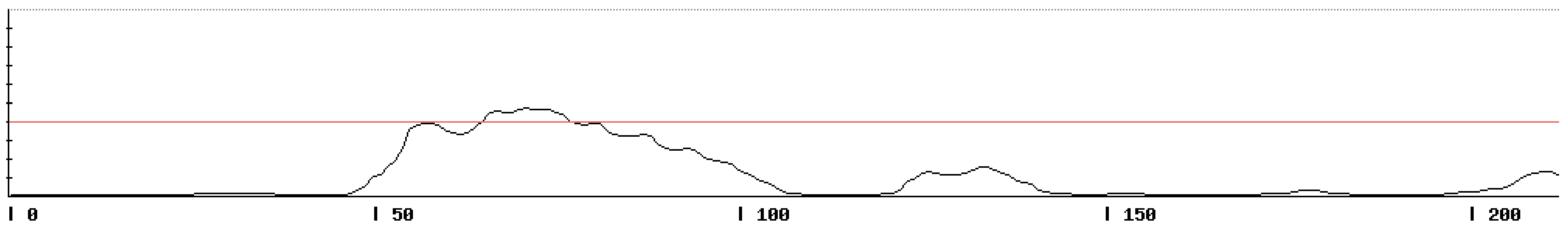

Predictions for Eucalyptuscamaldulensis_EcC004939.10

Viterbi Path

Viterbi Path

Posterior @ 0.4

Posterior @ 0.4

Posterior Graphic. Threshold Marked @ 0.4

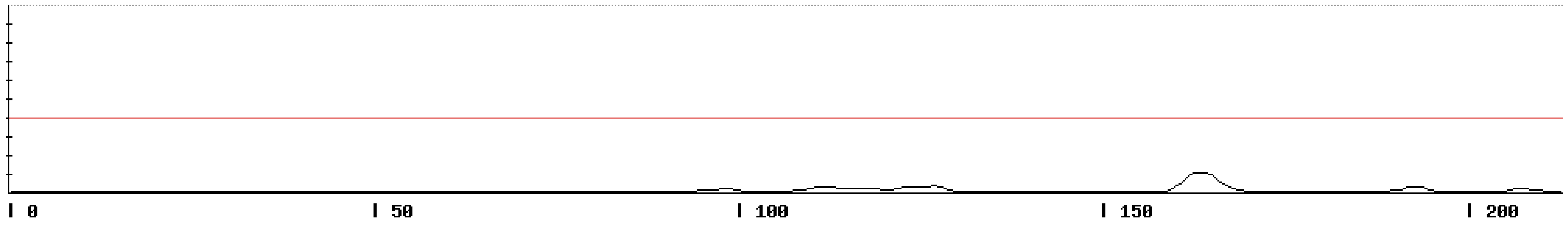

Predictions for Eucalyptuscamaldulensis_EcC006579.20

Viterbi Path

Viterbi Path

Posterior @ 0.4

Posterior @ 0.4

Posterior Graphic. Threshold Marked @ 0.4

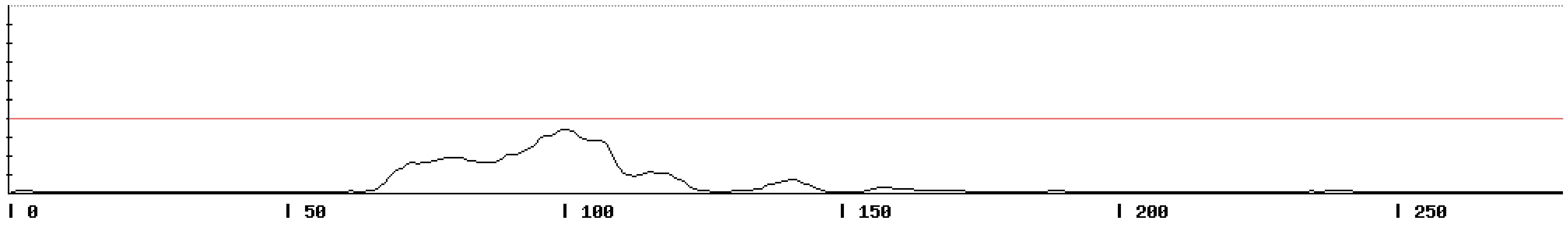

Predictions for Eucalyptuscamaldulensis_EcC007719.10

Viterbi Path	
Viterbi Path	
Posterior @ 0.4	
Posterior @ 0.4	
Posterior Graphic. Threshold Marked @ 0.4	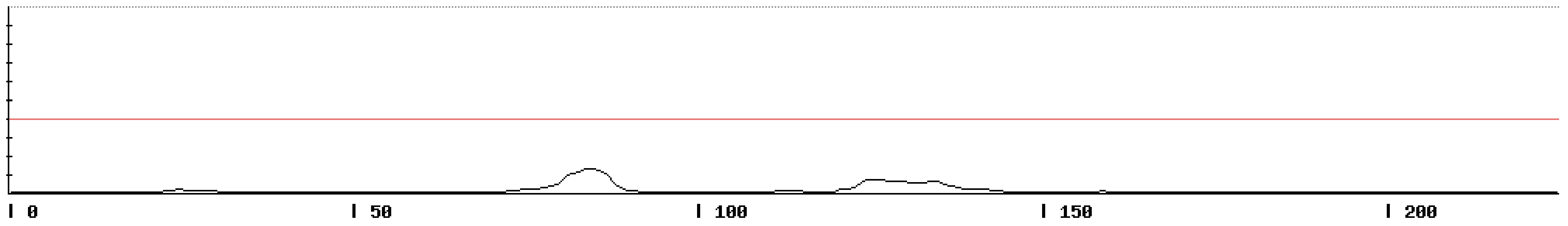

Predictions for Eucalyptuscamaldulensis_EcC008393.20

Viterbi Path	
Viterbi Path	
Posterior @ 0.4	168 - KKDKK - 172
Posterior @ 0.4	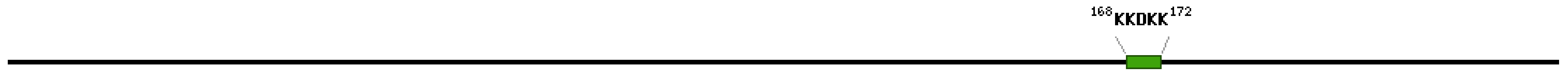
Posterior Graphic. Threshold Marked @ 0.4	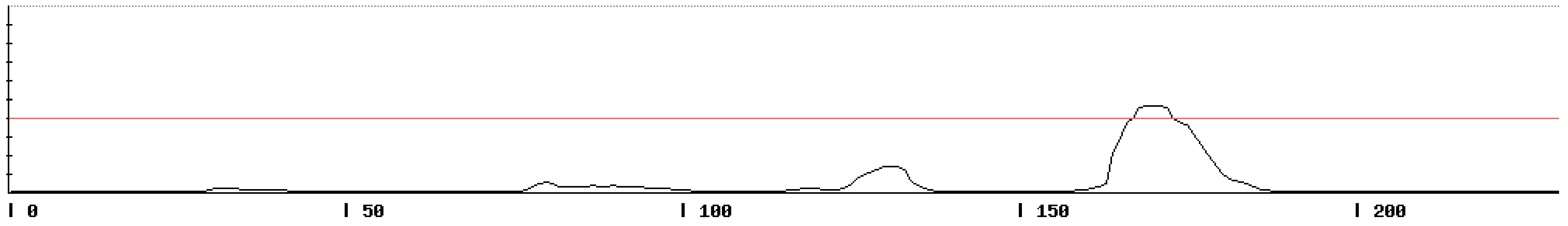

Predictions for Eucalyptuscamaldulensis_EcC008884.40

Viterbi Path	
Viterbi Path	
Posterior @ 0.4	82 - R - 82
Posterior @ 0.4	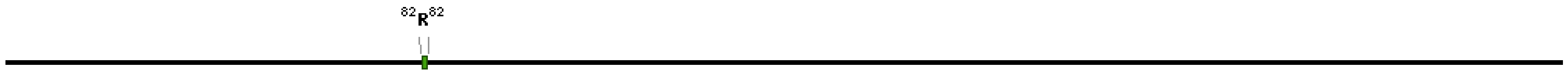
Posterior Graphic. Threshold Marked @ 0.4	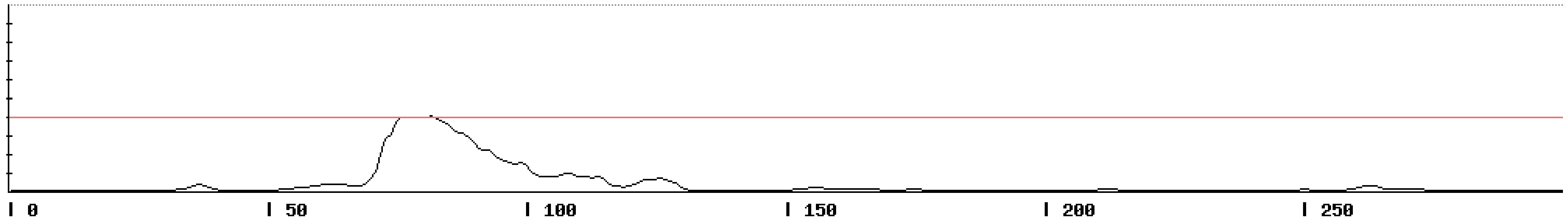

Predictions for Eucalyptuscamaldulensis_EcC009286.20

Viterbi Path	
Viterbi Path	
Posterior @ 0.4	
Posterior @ 0.4	
Posterior Graphic. Threshold Marked @ 0.4	

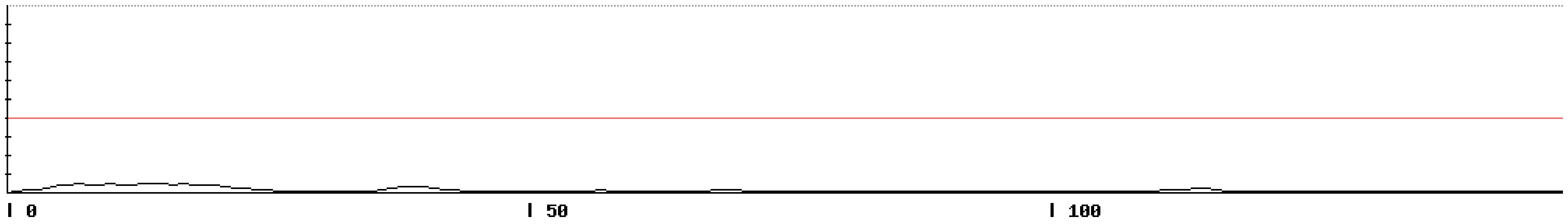

Predictions for Eucalyptuscamaldulensis_EcC010357.20

Viterbi Path	
Viterbi Path	
Posterior @ 0.4	
Posterior @ 0.4	
Posterior Graphic. Threshold Marked @ 0.4	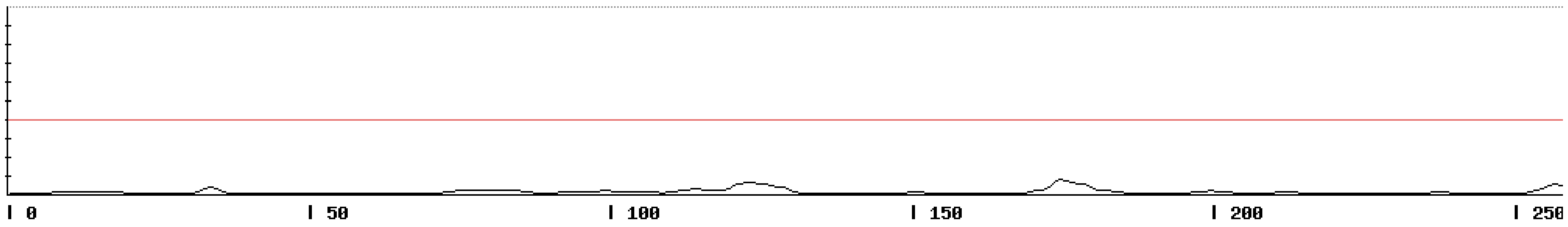

Predictions for Eucalyptuscamaldulensis_EcC010793.10

Viterbi Path	
Viterbi Path	
Posterior @ 0.4	76 - RGRKRKRK - 83
Posterior @ 0.4	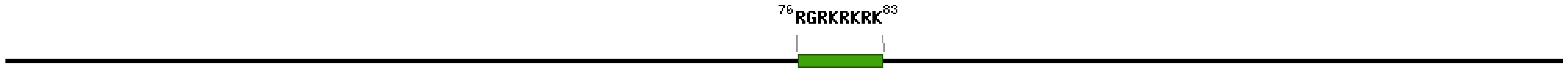

Posterior Graphic. Threshold Marked @ 0.4	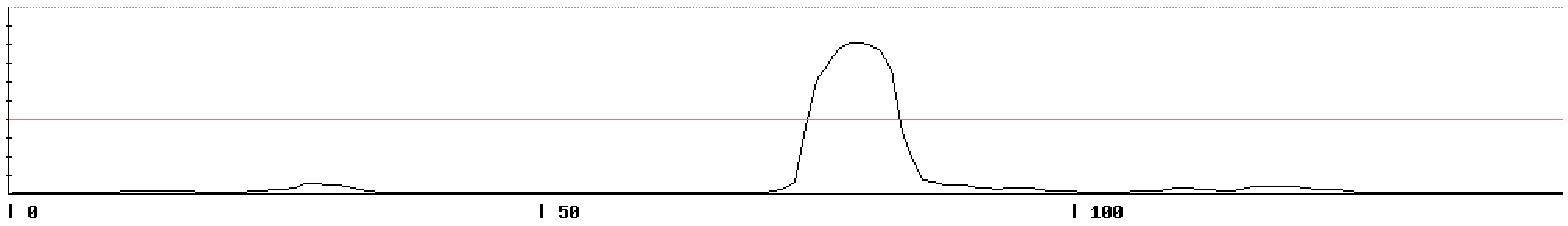

Predictions for Eucalyptuscamaldulensis_EcC010992.20

Viterbi Path	
Viterbi Path	
Posterior @ 0.4	
Posterior @ 0.4	
Posterior Graphic. Threshold Marked @ 0.4	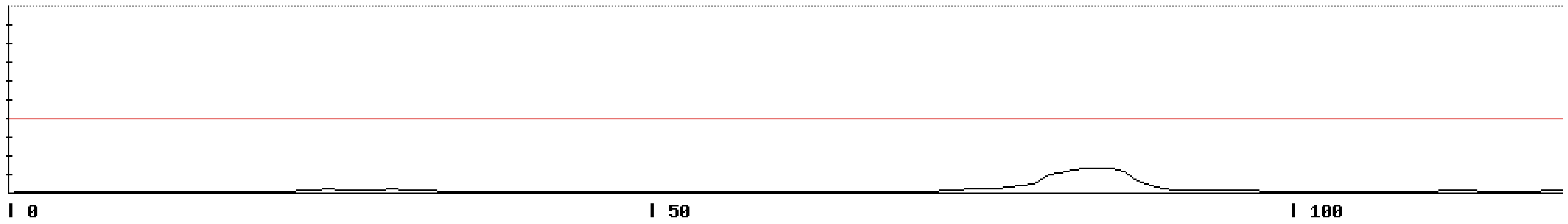

Predictions for Eucalyptuscamaldulensis_EcC011699.50

Viterbi Path	
Viterbi Path	
Posterior @ 0.4	80 - EGIKKKRKSK - 89
Posterior @ 0.4	

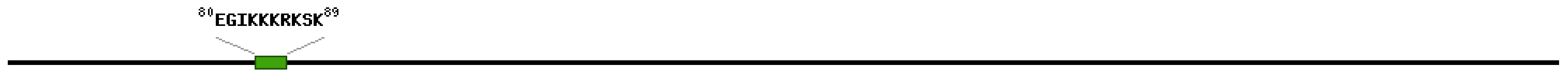

Posterior Graphic. Threshold Marked @ 0.4

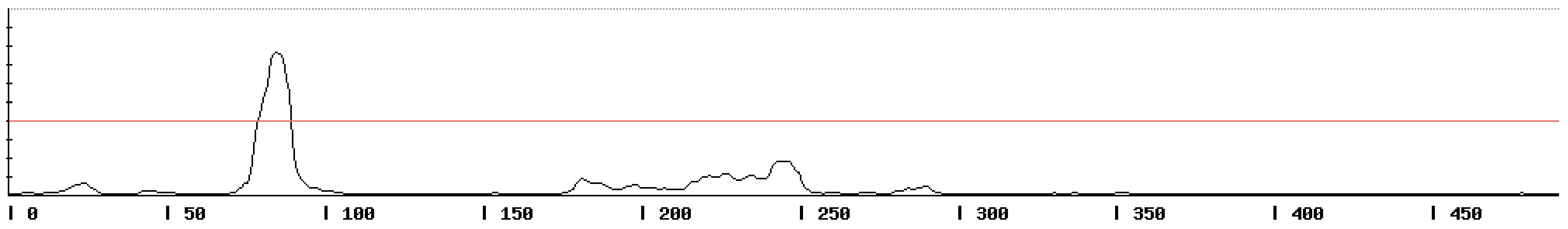

Predictions for Eucalyptuscamaldulensis_EcC011699.60

Viterbi Path

Viterbi Path

Posterior @ 0.4

Posterior @ 0.4

Posterior Graphic. Threshold Marked @ 0.4

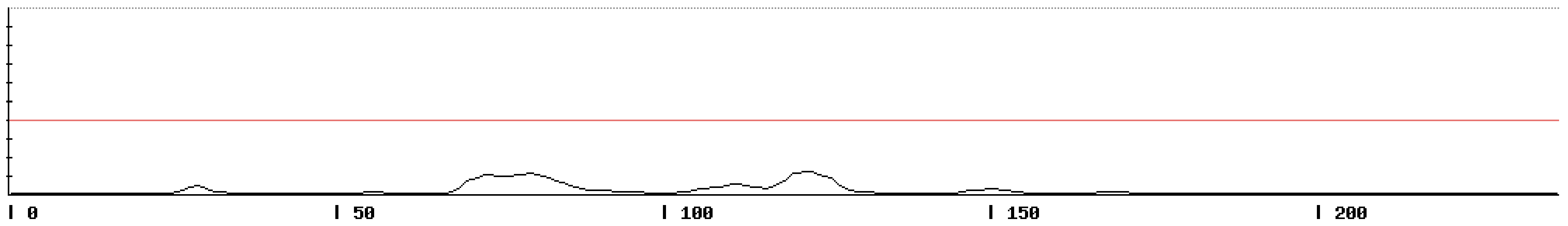

Predictions for Eucalyptuscamaldulensis_EcC011895.40

Viterbi Path

Viterbi Path

Posterior @ 0.4

Posterior @ 0.4

Posterior Graphic. Threshold Marked @ 0.4

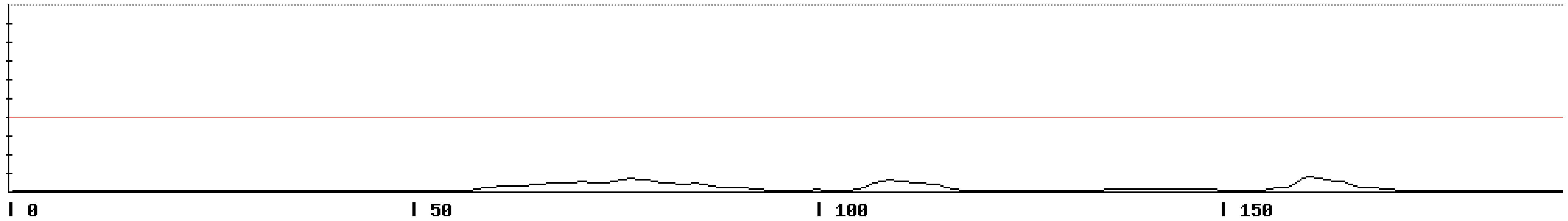

Predictions for Eucalyptuscamaldulensis_EcC013314.30

Viterbi Path

Viterbi Path

Posterior @ 0.4

Posterior @ 0.4

Posterior Graphic. Threshold Marked @ 0.4

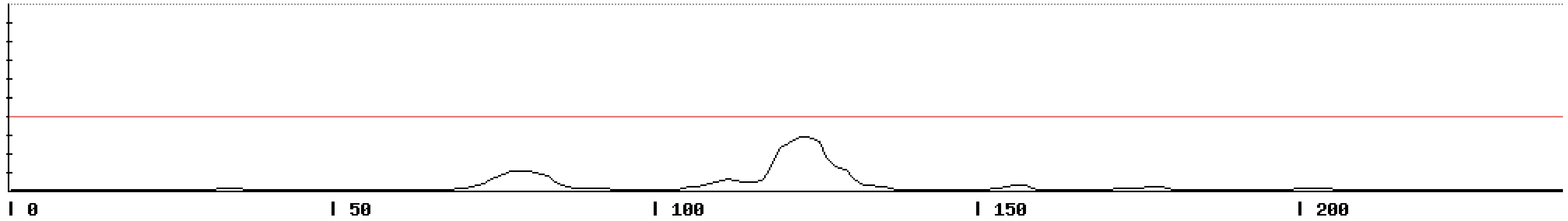

Predictions for Eucalyptuscamaldulensis_EcC013670.30

Viterbi Path

Viterbi Path

Posterior @ 0.4

Posterior @ 0.4

Posterior Graphic. Threshold Marked @ 0.4

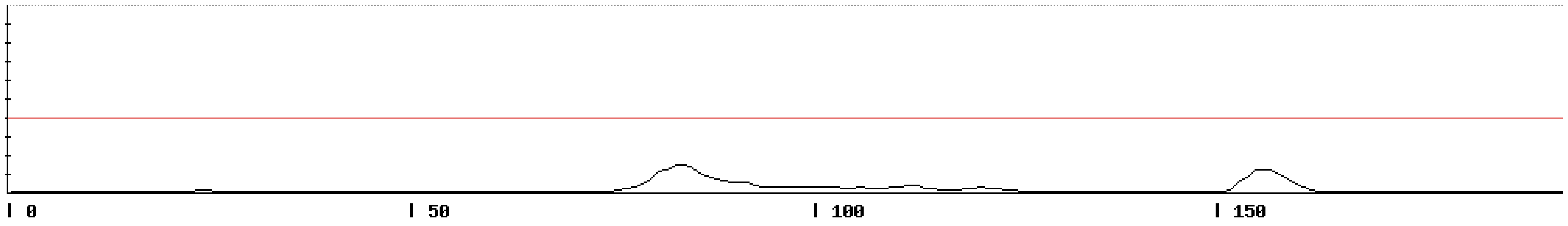

Predictions for Eucalyptuscamaldulensis_EcC014144.10

Viterbi Path

Viterbi Path

Posterior @ 0.4

Posterior @ 0.4

Posterior Graphic. Threshold Marked @ 0.4

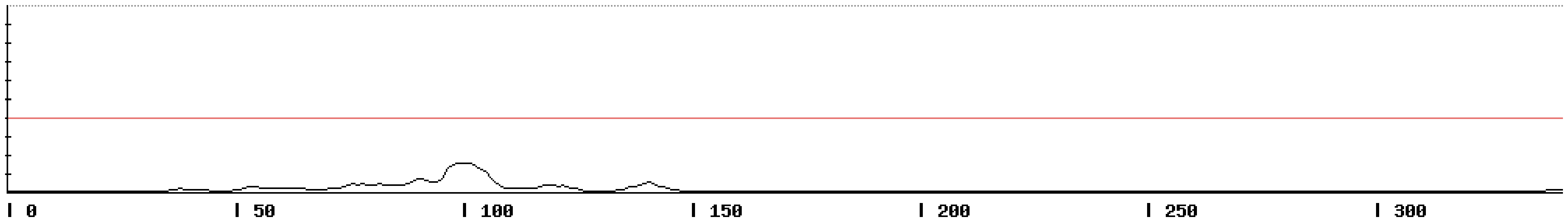

Predictions for Eucalyptuscamaldulensis_EcC014449.180

Viterbi Path	
Viterbi Path	
Posterior @ 0.4	
Posterior @ 0.4	
Posterior Graphic. Threshold Marked @ 0.4	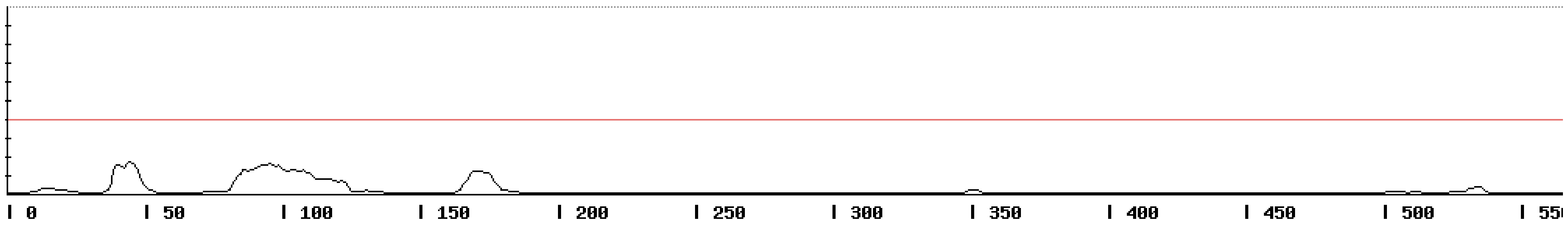

Predictions for Eucalyptuscamaldulensis_EcC014521.10

Viterbi Path	
Viterbi Path	
Posterior @ 0.4	166 - KKDKK - 170
Posterior @ 0.4	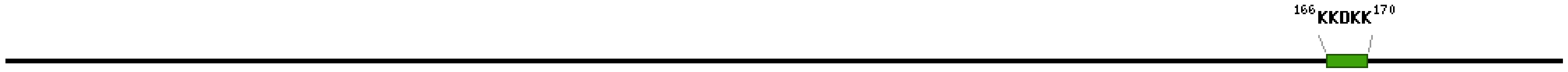
Posterior Graphic. Threshold Marked @ 0.4	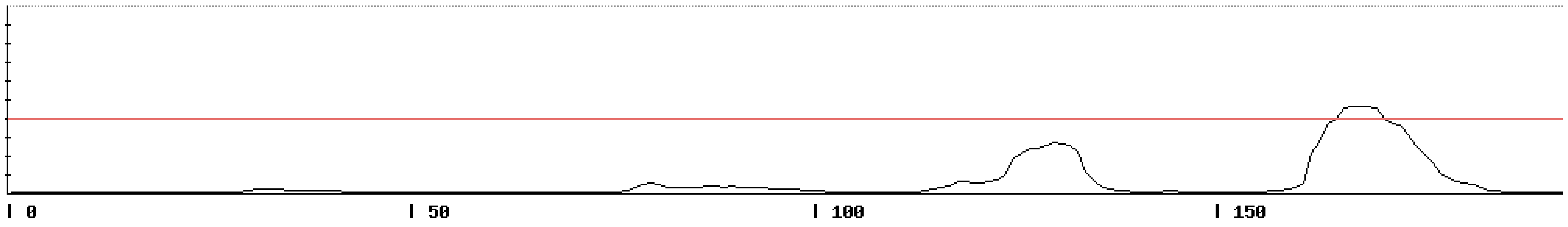

Predictions for Eucalyptuscamaldulensis_EcC014537.10	
Viterbi Path	
Viterbi Path	
Posterior @ 0.4	
Posterior @ 0.4	
Posterior Graphic. Threshold Marked @ 0.4	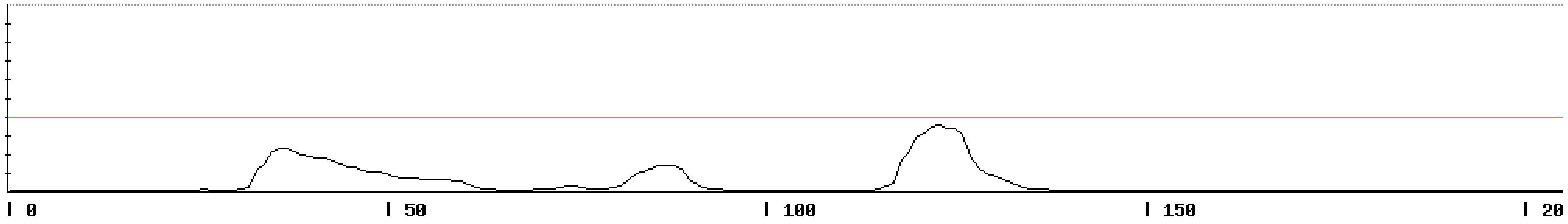

Predictions for Eucalyptuscamaldulensis_EcC014963.10	
Viterbi Path	
Viterbi Path	
Posterior @ 0.4	
Posterior @ 0.4	
Posterior Graphic. Threshold Marked @ 0.4	

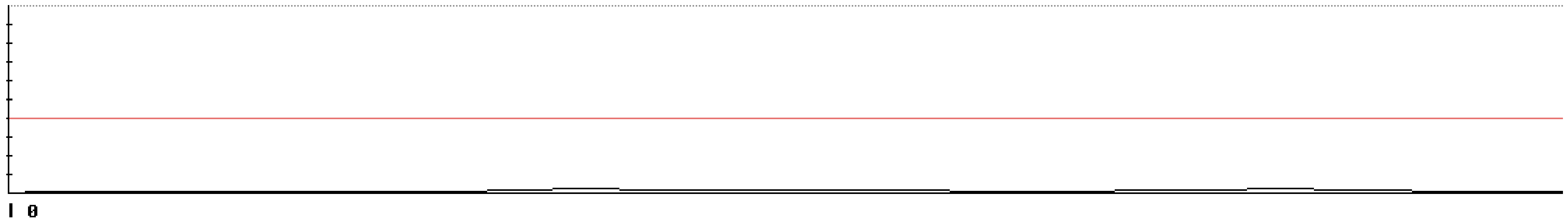

Predictions for Eucalyptuscamaldulensis_EcC015146.10	
Viterbi Path	
Viterbi Path	
Posterior @ 0.4	
Posterior @ 0.4	
Posterior Graphic. Threshold Marked @ 0.4	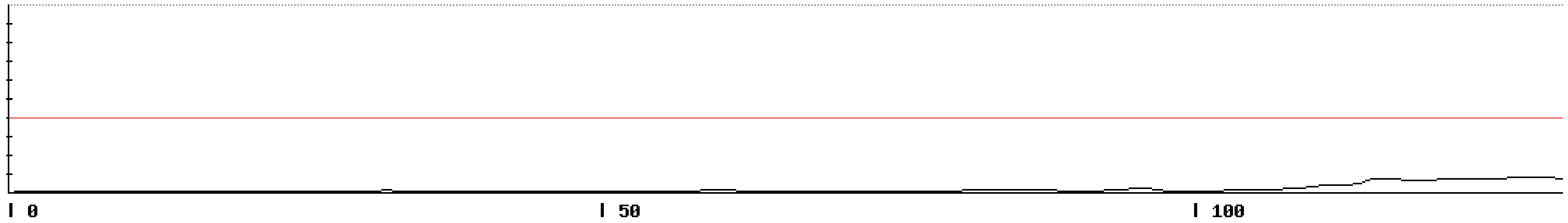

Predictions for Eucalyptuscamaldulensis_EcC015739.30	
Viterbi Path	
Viterbi Path	
Posterior @ 0.4	
Posterior @ 0.4	

Posterior Graphic. Threshold Marked @ 0.4

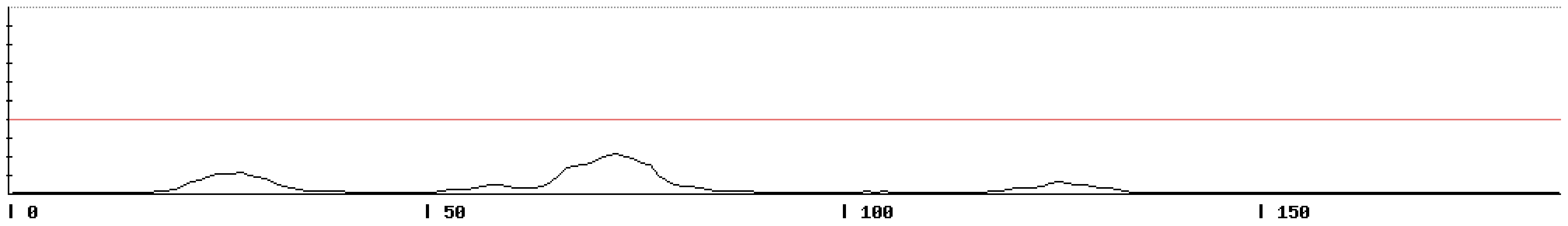

Predictions for Eucalyptuscamaldulensis_EcC016415.10

Viterbi Path

Viterbi Path

Posterior @ 0.4 — 71 - KK - 72

Posterior @ 0.4

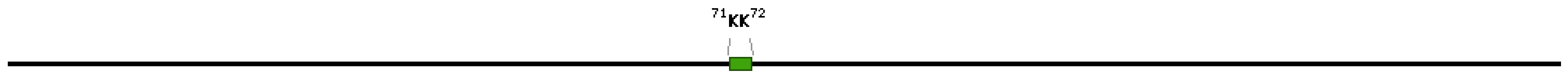

Posterior Graphic. Threshold Marked @ 0.4

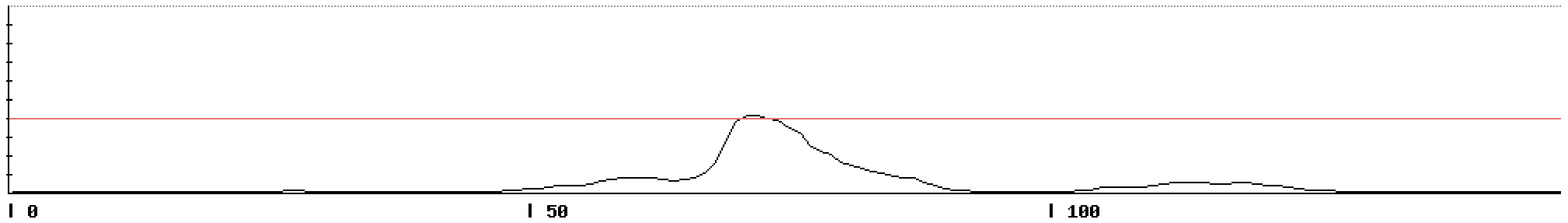

Predictions for Eucalyptuscamaldulensis_EcC019123.10

Viterbi Path

Viterbi Path

Posterior @ 0.4

Posterior @ 0.4

Posterior Graphic. Threshold Marked @ 0.4

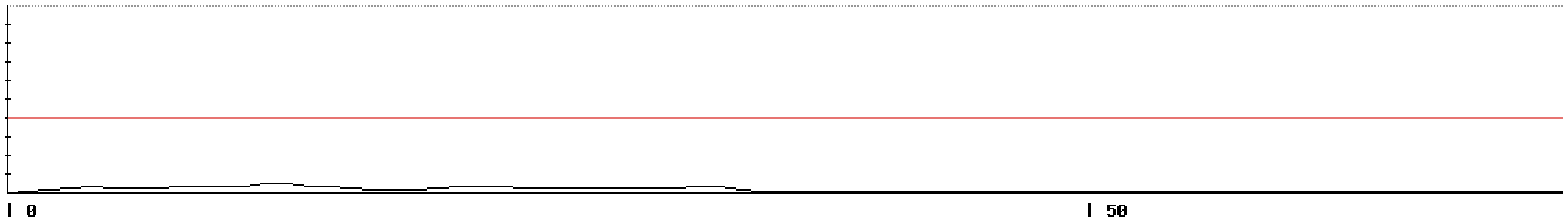

Predictions for Eucalyptuscamaldulensis_EcC019247.60

Viterbi Path

Viterbi Path

Posterior @ 0.4 — 236 - NRRIRLRRHKPERSSK - 251

Posterior @ 0.4

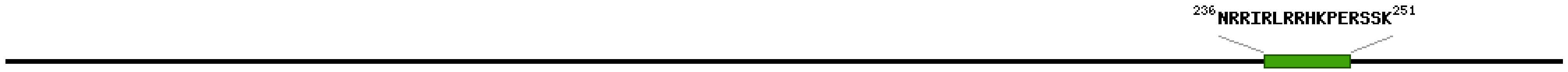

Posterior Graphic. Threshold Marked @ 0.4

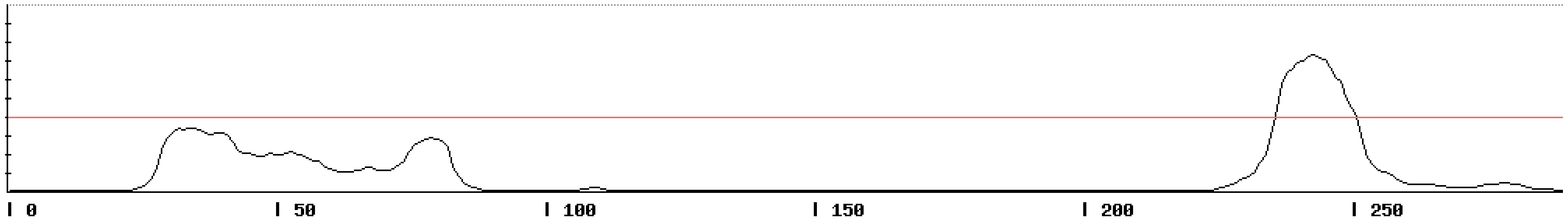

Predictions for Eucalyptuscamaldulensis_EcC019551.10

Viterbi Path

Viterbi Path

Posterior @ 0.4 | 85 - WKPKGG - 90

Posterior @ 0.4

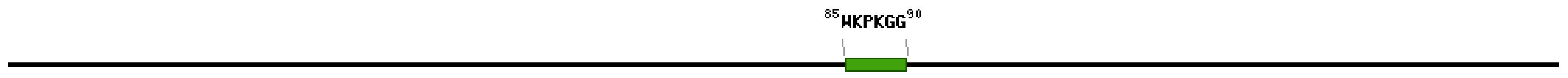

Posterior Graphic. Threshold Marked @ 0.4

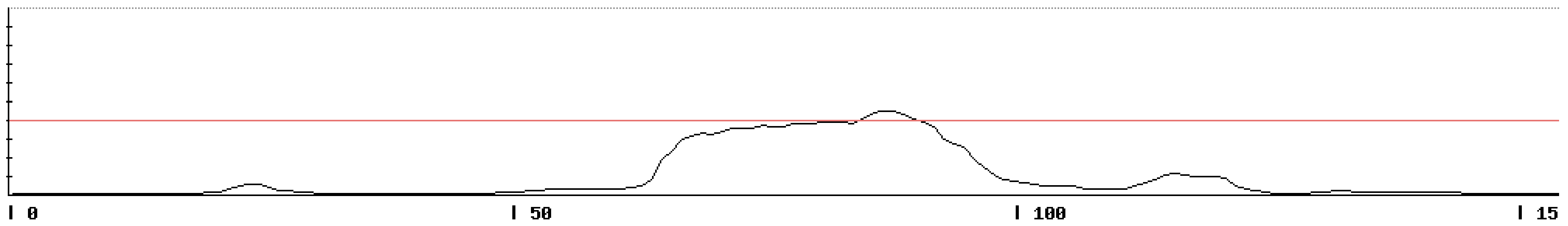

Predictions for Eucalyptuscamaldulensis_EcC021292.20

Viterbi Path

Viterbi Path

Posterior @ 0.4

Posterior @ 0.4

Posterior Graphic. Threshold Marked @ 0.4

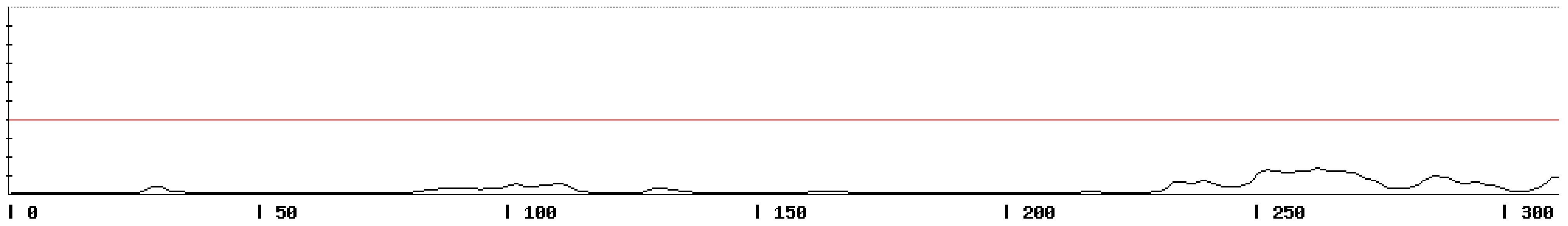

Predictions for Eucalyptuscamaldulensis_EcC021761.20

Viterbi Path

Viterbi Path

Posterior @ 0.4

Posterior @ 0.4

Posterior Graphic. Threshold Marked @ 0.4

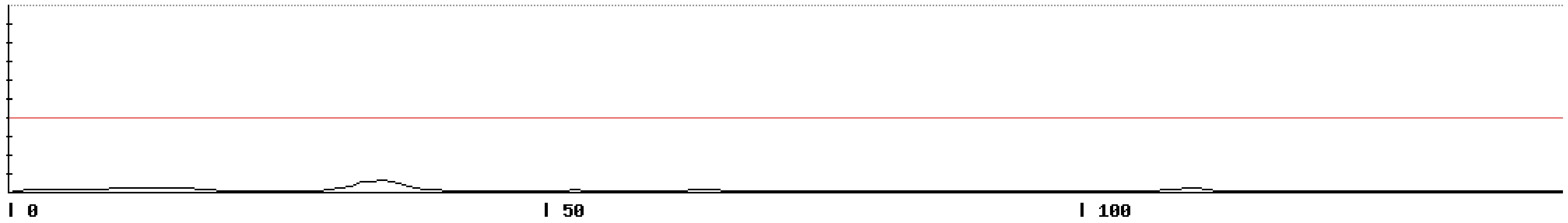

Predictions for Eucalyptuscamaldulensis_EcC021933.10

Viterbi Path

Viterbi Path

Posterior @ 0.4

Posterior @ 0.4

Posterior Graphic. Threshold Marked @ 0.4

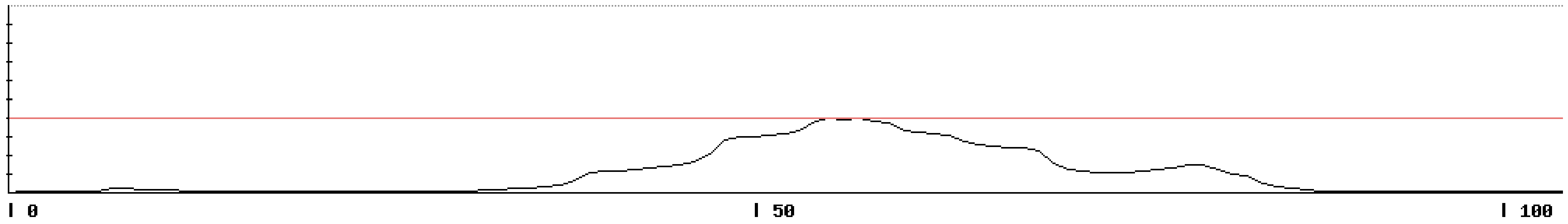

Predictions for Eucalyptuscamaldulensis_EcC022004.10

Viterbi Path

Viterbi Path

Posterior @ 0.4

121 - KDKEIRRMKKVV - 132

Posterior @ 0.4

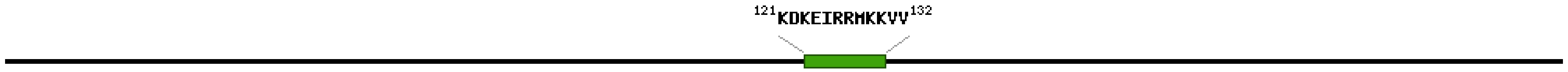

Posterior Graphic. Threshold Marked @ 0.4

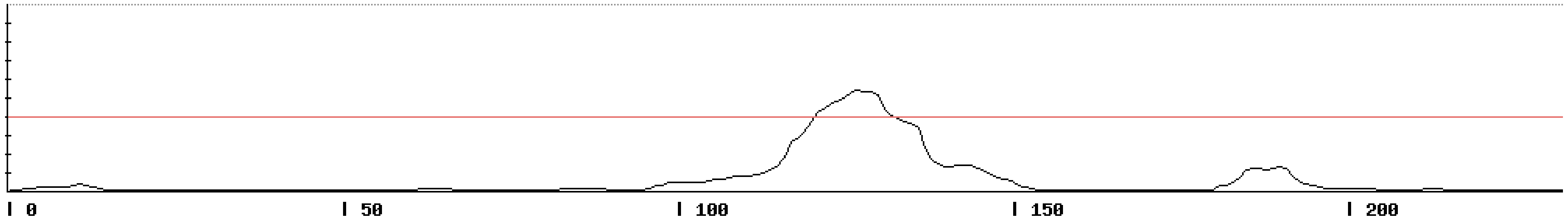

Predictions for Eucalyptuscamaldulensis_EcC025730.10

Viterbi Path

Viterbi Path

Posterior @ 0.4

Posterior @ 0.4

Posterior Graphic. Threshold Marked @ 0.4

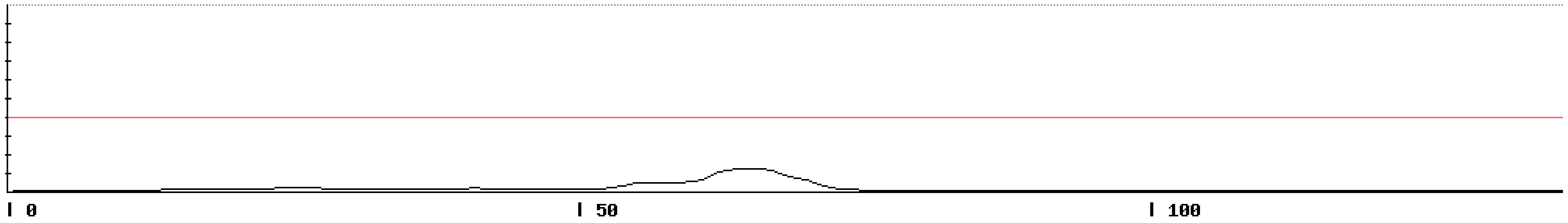

Predictions for Eucalyptuscamaldulensis_EcC025730.20	
Viterbi Path	
Viterbi Path	
Posterior @ 0.4	140 - RKRRK - 144
Posterior @ 0.4	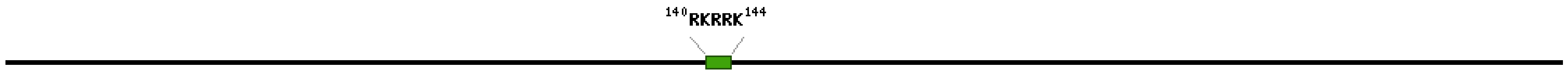
Posterior Graphic. Threshold Marked @ 0.4	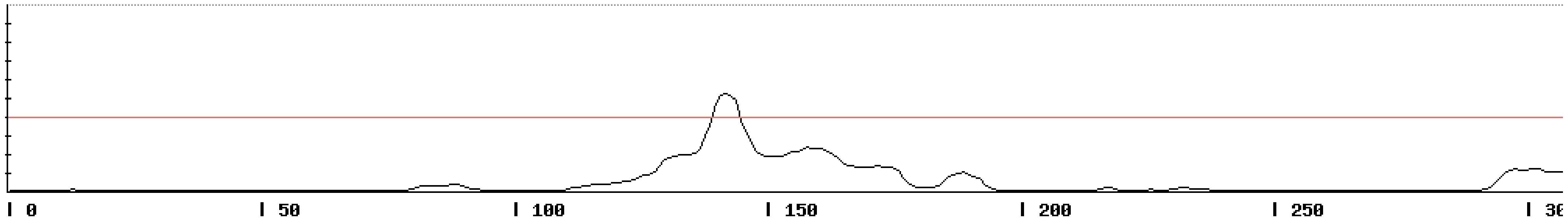

Predictions for Eucalyptuscamaldulensis_EcC026270.10	
Viterbi Path	
Viterbi Path	
Posterior @ 0.4	
Posterior @ 0.4	
Posterior Graphic. Threshold Marked @ 0.4	

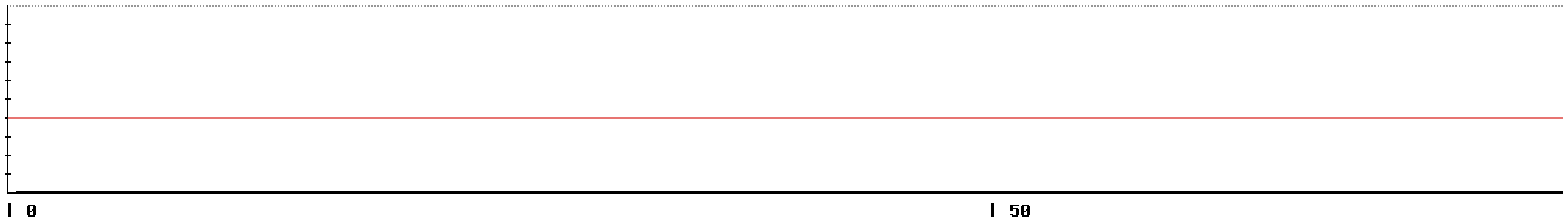

Predictions for Eucalyptuscamaldulensis_EcC026270.30

Viterbi Path

Viterbi Path

Posterior @ 0.4 65 - KKRTKRSPAKGKQIEIKK - 82

Posterior @ 0.4

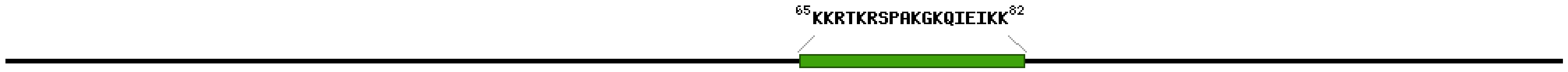

Posterior Graphic. Threshold Marked @ 0.4

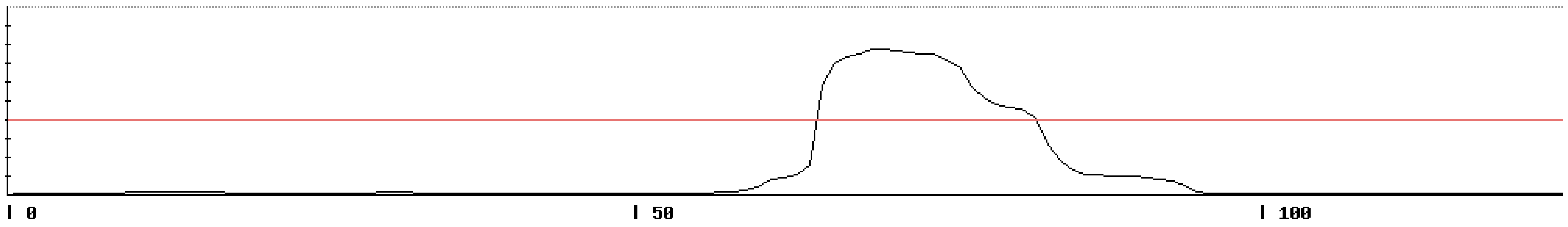

Predictions for Eucalyptuscamaldulensis_EcC026270.40

Viterbi Path

Viterbi Path

Posterior @ 0.4 161 - RKRKKVRSAK - 170

Posterior @ 0.4

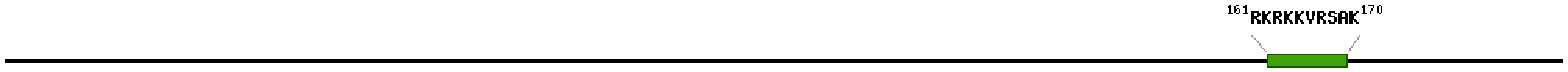

Posterior Graphic. Threshold Marked @ 0.4	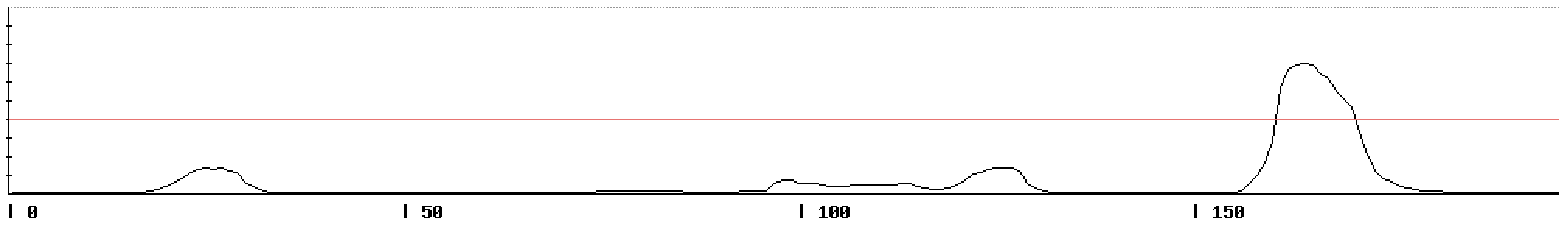

Predictions for Eucalyptuscamaldulensis_EcC026270.50	
Viterbi Path	
Viterbi Path	
Posterior @ 0.4	
Posterior @ 0.4	
Posterior Graphic. Threshold Marked @ 0.4	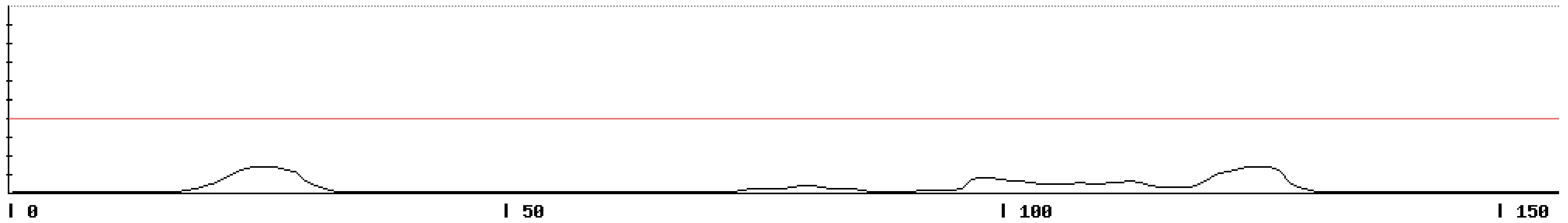

Predictions for Eucalyptuscamaldulensis_EcC026270.60	
Viterbi Path	
Viterbi Path	
Posterior @ 0.4	161 - RKRKKARSAK - 170
Posterior @ 0.4	

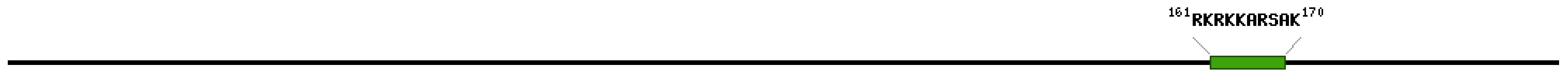

Posterior Graphic. Threshold Marked @ 0.4

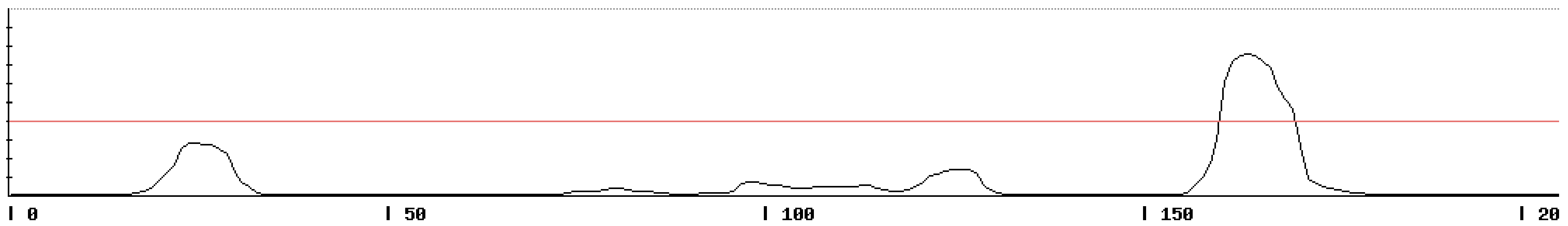

Predictions for Eucalyptuscamaldulensis_EcC026270.70

Viterbi Path

Viterbi Path

Posterior @ 0.4

Posterior @ 0.4

Posterior Graphic. Threshold Marked @ 0.4

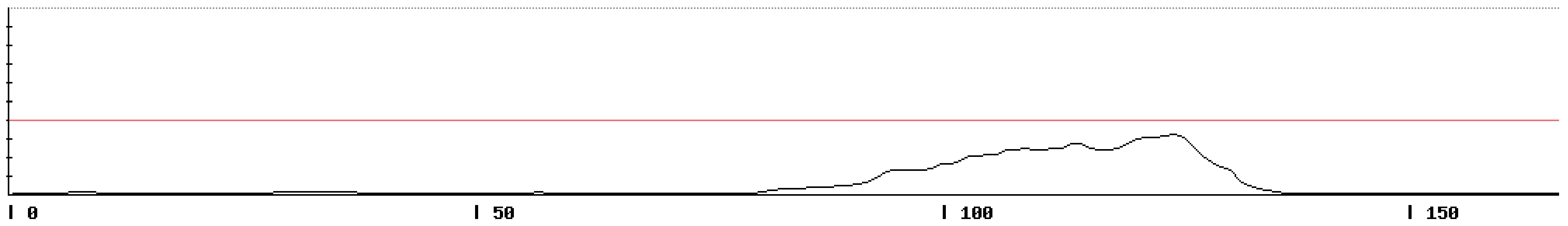

Predictions for Eucalyptuscamaldulensis_EcC026831.10

Viterbi Path

Viterbi Path

Posterior @ 0.4 | 121 - PPRGRK - 126

Posterior @ 0.4

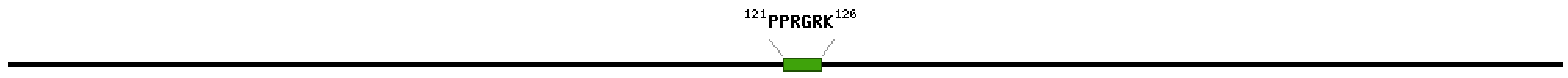

Posterior Graphic. Threshold Marked @ 0.4

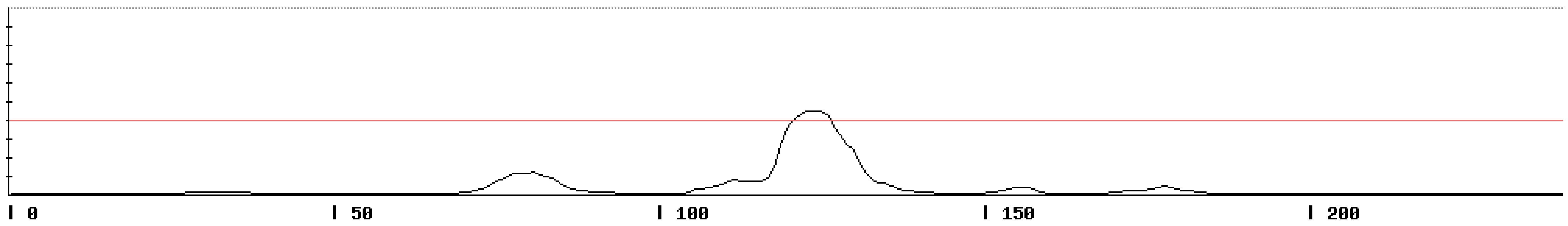

Predictions for Eucalyptuscamaldulensis_EcC026831.20

Viterbi Path

Viterbi Path

Posterior @ 0.4

Posterior @ 0.4

Posterior Graphic. Threshold Marked @ 0.4

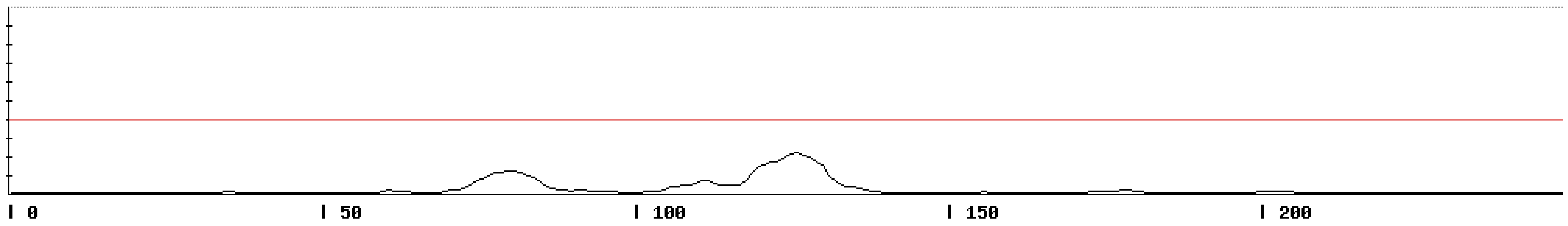

Predictions for Eucalyptuscamaldulensis_EcC028332.10

Viterbi Path

Viterbi Path

Posterior @ 0.4

Posterior @ 0.4

Posterior Graphic. Threshold Marked @ 0.4

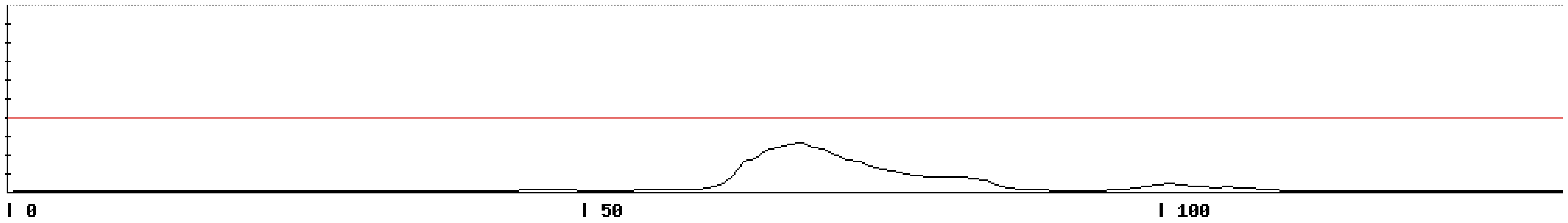

Predictions for Eucalyptuscamaldulensis_EcC028332.20

Viterbi Path

Viterbi Path

Posterior @ 0.4

Posterior @ 0.4

Posterior Graphic. Threshold Marked @ 0.4

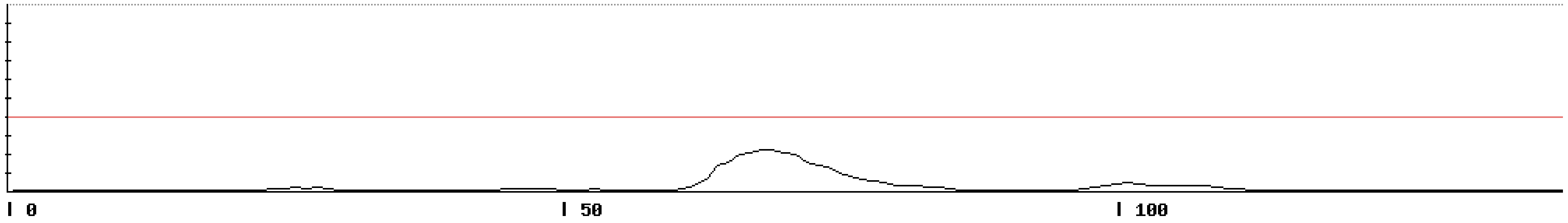

Predictions for Eucalyptuscamaldulensis_EcC028332.30

Viterbi Path

Viterbi Path

Posterior @ 0.4

Posterior @ 0.4

Posterior Graphic. Threshold Marked @ 0.4

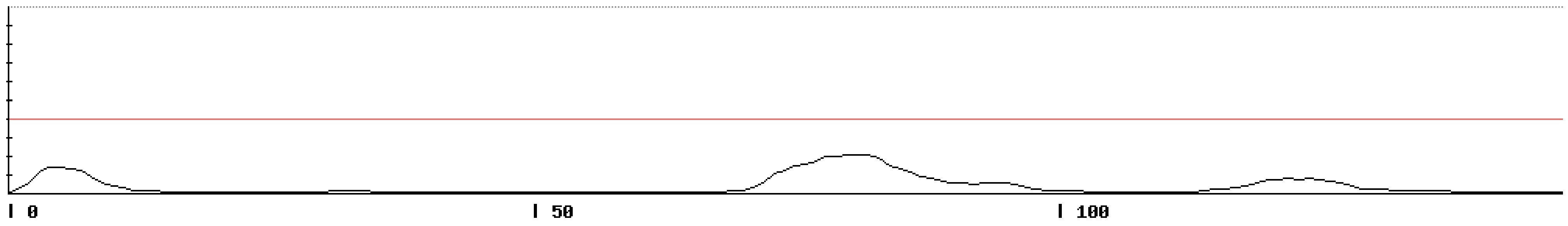

Predictions for Eucalyptuscamaldulensis_EcC028332.40

Viterbi Path

Viterbi Path

Posterior @ 0.4

Posterior @ 0.4

Posterior Graphic. Threshold Marked @ 0.4

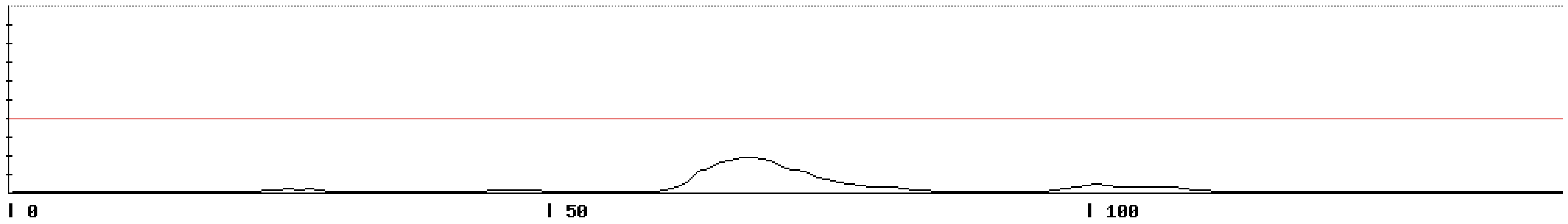

Predictions for Eucalyptuscamaldulensis_EcC029058.20

Viterbi Path

Viterbi Path

Posterior @ 0.4

Posterior @ 0.4

Posterior Graphic. Threshold Marked @ 0.4

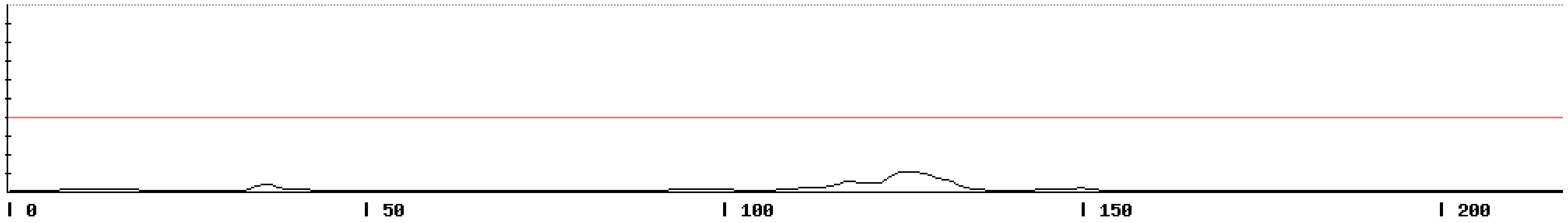

Predictions for Eucalyptuscamaldulensis_EcC029930.40

Viterbi Path

Viterbi Path

Posterior @ 0.4

Posterior @ 0.4

Posterior Graphic. Threshold Marked @ 0.4

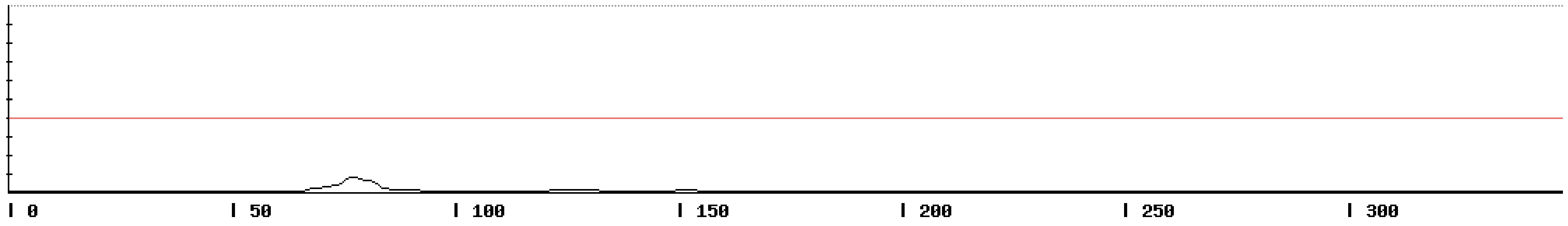

Predictions for Eucalyptuscamaldulensis_EcC031547.30

Viterbi Path

Viterbi Path

Posterior @ 0.4

Posterior @ 0.4

Posterior Graphic. Threshold Marked @ 0.4

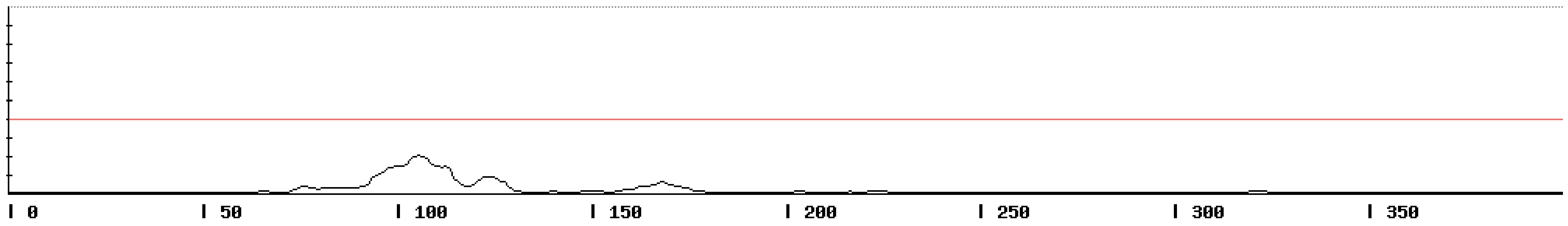

Predictions for Eucalyptuscamaldulensis_EcC032657.20

Viterbi Path

Viterbi Path

Posterior @ 0.4

Posterior @ 0.4

Posterior Graphic. Threshold Marked @ 0.4

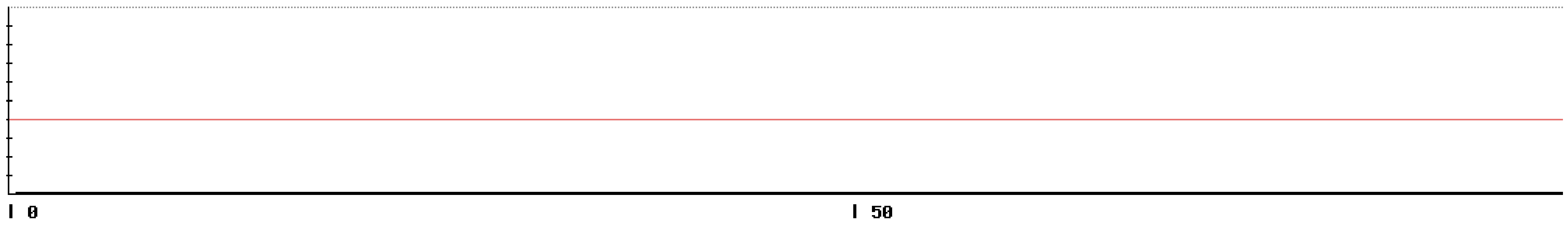

Predictions for Eucalyptuscamaldulensis_EcC032657.30

Viterbi Path	
Viterbi Path	
Posterior @ 0.4	11 - K - 11
Posterior @ 0.4	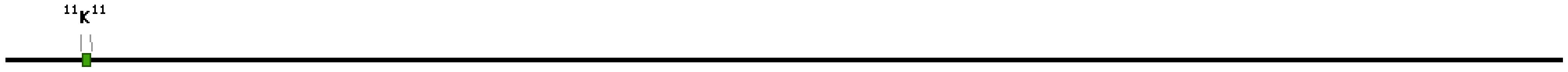
Posterior Graphic. Threshold Marked @ 0.4	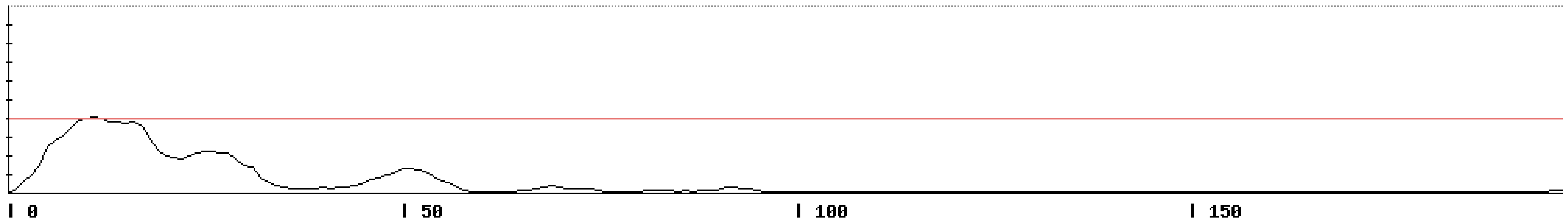

Predictions for Eucalyptuscamaldulensis_EcC033159.10

Viterbi Path	13 - KELPKKANRVEKRKREREKMKK - 34
Viterbi Path	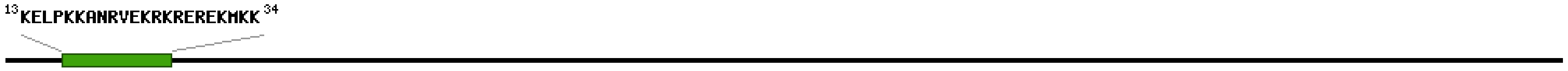
Posterior @ 0.4	13 - KELPKKANRVEKRKREREKMKKAQ - 36
Posterior @ 0.4	

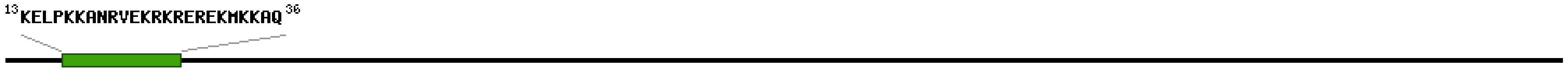

Posterior Graphic. Threshold Marked @ 0.4

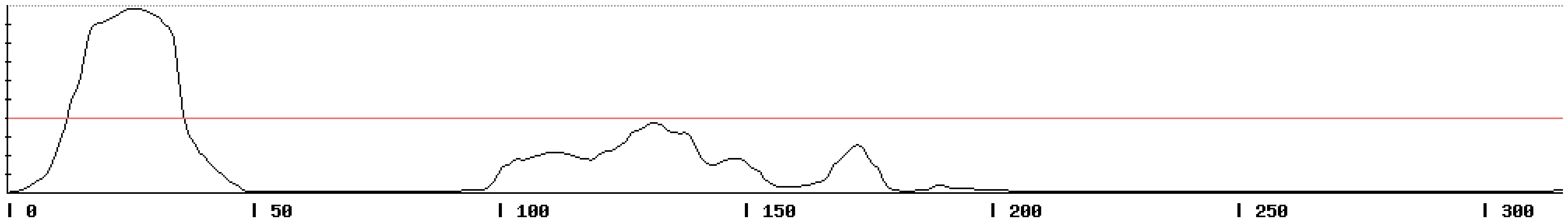

Predictions for Eucalyptuscamaldulensis_EcC034269.10

Viterbi Path	
Viterbi Path	
Posterior @ 0.4	140 - RKK - 142
Posterior @ 0.4	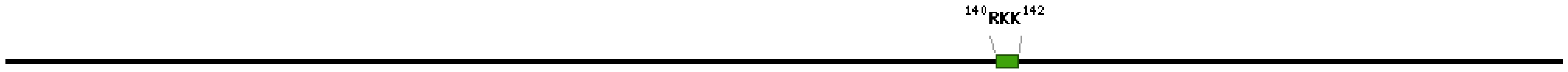
Posterior Graphic. Threshold Marked @ 0.4	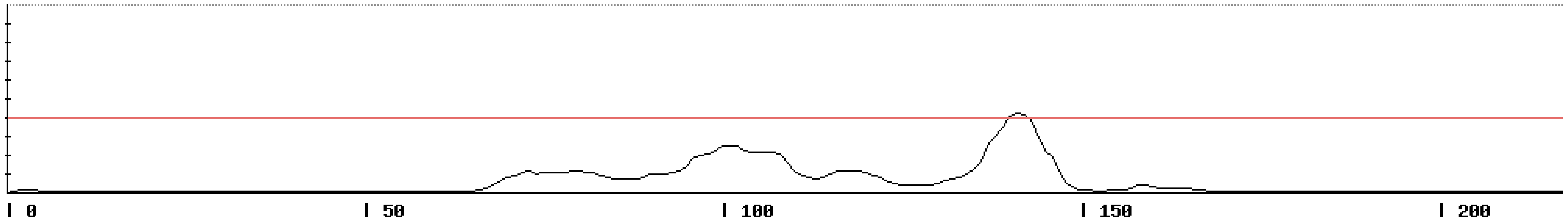

Predictions for Eucalyptuscamaldulensis_EcC034730.10

Viterbi Path	
Viterbi Path	

Posterior @ 0.4	
Posterior @ 0.4	
Posterior Graphic. Threshold Marked @ 0.4	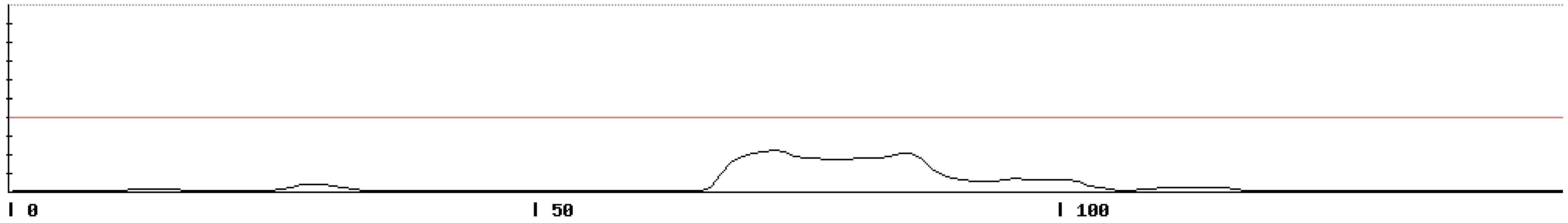

Predictions for Eucalyptuscamaldulensis_EcC035778.30	
Viterbi Path	
Viterbi Path	
Posterior @ 0.4	
Posterior @ 0.4	
Posterior Graphic. Threshold Marked @ 0.4	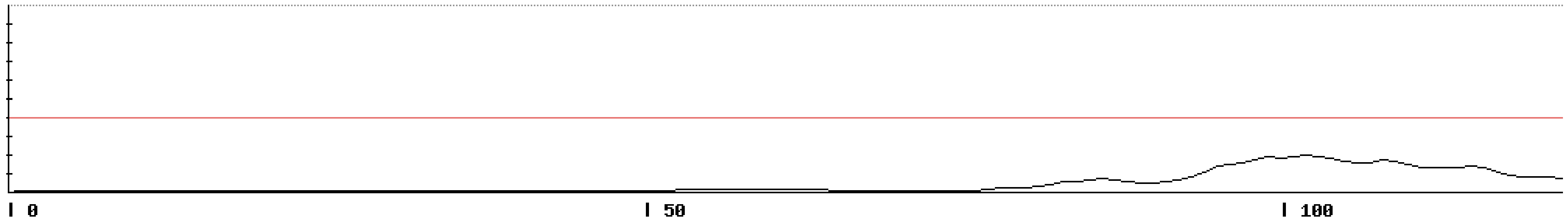

Predictions for Eucalyptuscamaldulensis_EcC035779.10	
Viterbi Path	
Viterbi Path	

Posterior @ 0.4

Posterior @ 0.4

Posterior Graphic. Threshold Marked @ 0.4

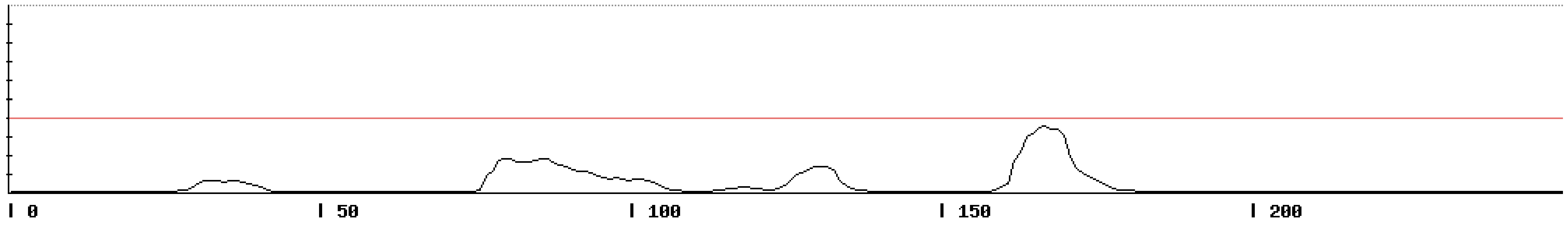

Predictions for Eucalyptuscamaldulensis_EcC035780.10

Viterbi Path

Viterbi Path

Posterior @ 0.4

Posterior @ 0.4

Posterior Graphic. Threshold Marked @ 0.4

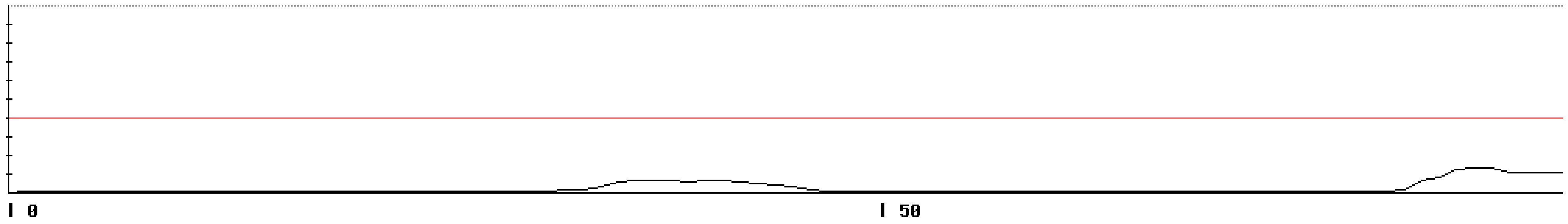

Predictions for Eucalyptuscamaldulensis_EcC037198.10

Viterbi Path

Viterbi Path

Posterior @ 0.4

Posterior @ 0.4

Posterior Graphic. Threshold Marked @ 0.4

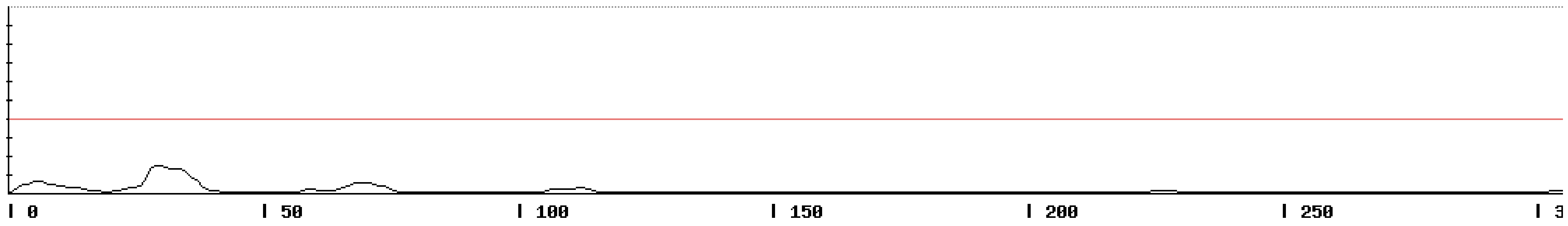

Predictions for Eucalyptuscamaldulensis_EcC037361.10

Viterbi Path

Viterbi Path

Posterior @ 0.4

Posterior @ 0.4

Posterior Graphic. Threshold Marked @ 0.4

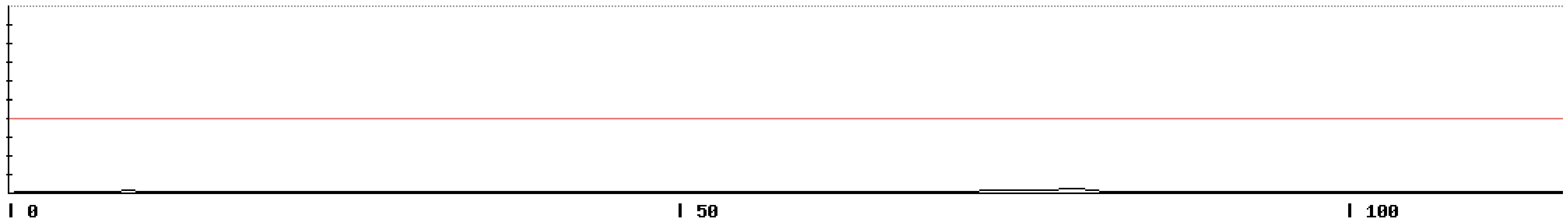

Predictions for Eucalyptuscamaldulensis_EcC037763.10

Viterbi Path

Viterbi Path

Posterior @ 0.4

Posterior @ 0.4

Posterior Graphic. Threshold Marked @ 0.4

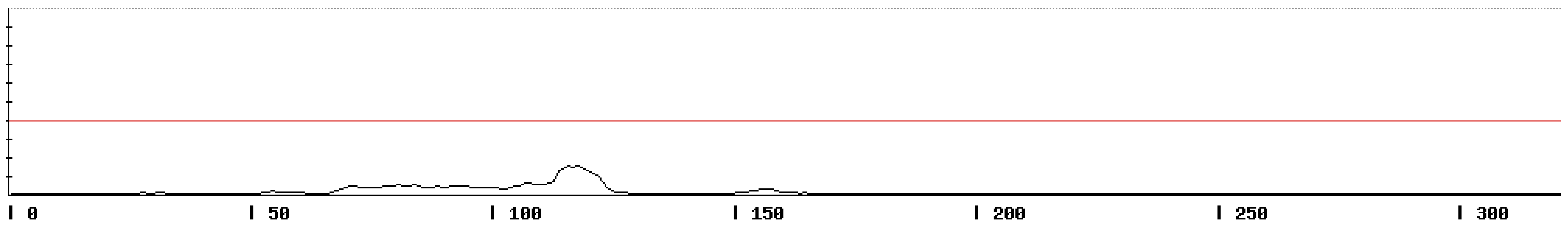

Predictions for Eucalyptuscamaldulensis_EcC038364.10

Viterbi Path

Viterbi Path

Posterior @ 0.4

Posterior @ 0.4

Posterior Graphic. Threshold Marked @ 0.4

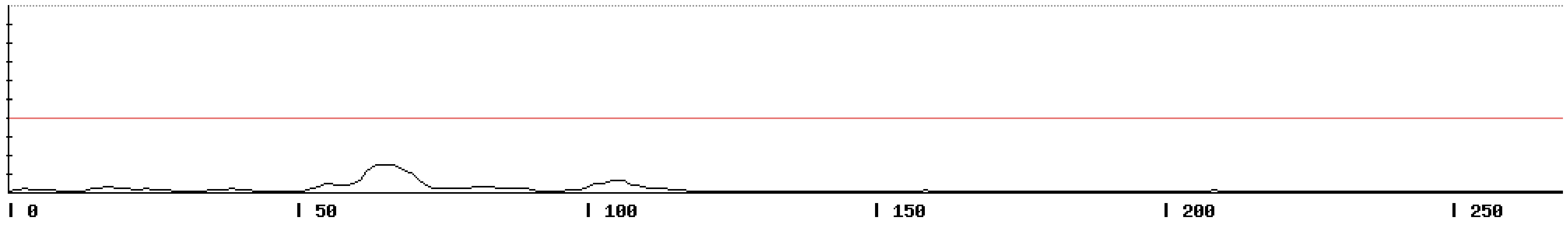

Predictions for Eucalyptuscamaldulensis_EcC038365.10

Viterbi Path	
Viterbi Path	
Posterior @ 0.4	
Posterior @ 0.4	
Posterior Graphic. Threshold Marked @ 0.4	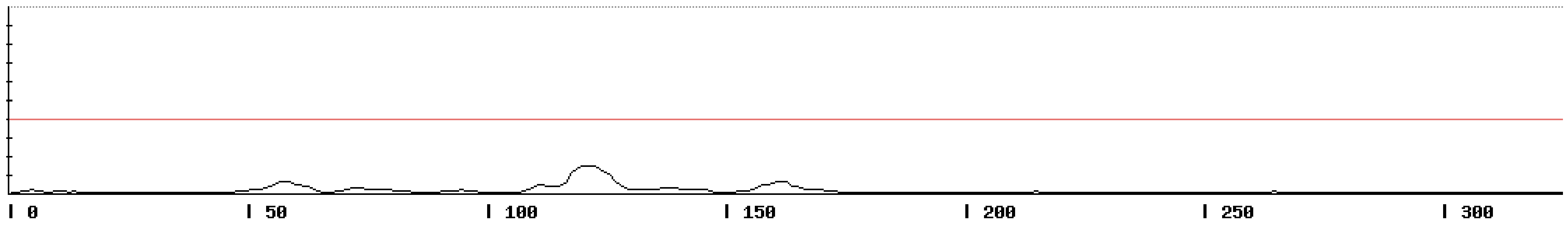

Predictions for Eucalyptuscamaldulensis_EcC038558.10

Viterbi Path	
Viterbi Path	
Posterior @ 0.4	104 - GKPHKIRTQDRKNTLGLKKILVFKKRQSPK - 133
Posterior @ 0.4	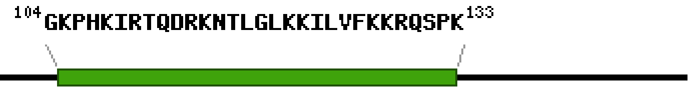

Posterior Graphic. Threshold Marked @ 0.4	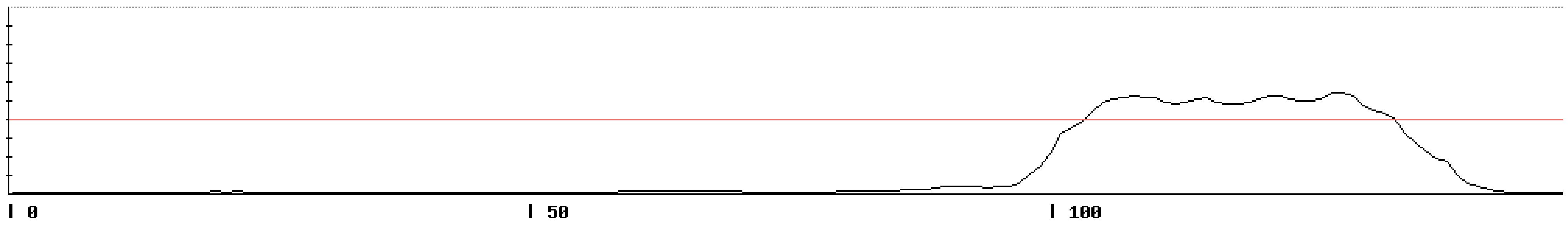

Predictions for Eucalyptuscamaldulensis_EcC038572.10

Viterbi Path	
Viterbi Path	
Posterior @ 0.4	
Posterior @ 0.4	
Posterior Graphic. Threshold Marked @ 0.4	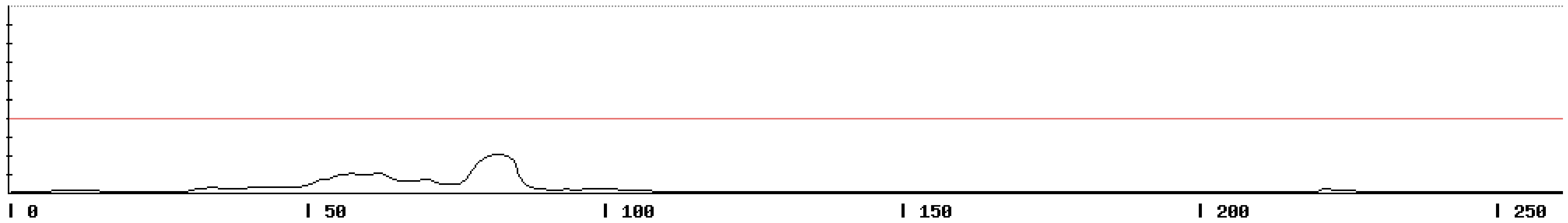

Predictions for Eucalyptuscamaldulensis_EcC039244.20

Viterbi Path	
Viterbi Path	
Posterior @ 0.4	
Posterior @ 0.4	

Posterior Graphic. Threshold Marked @ 0.4

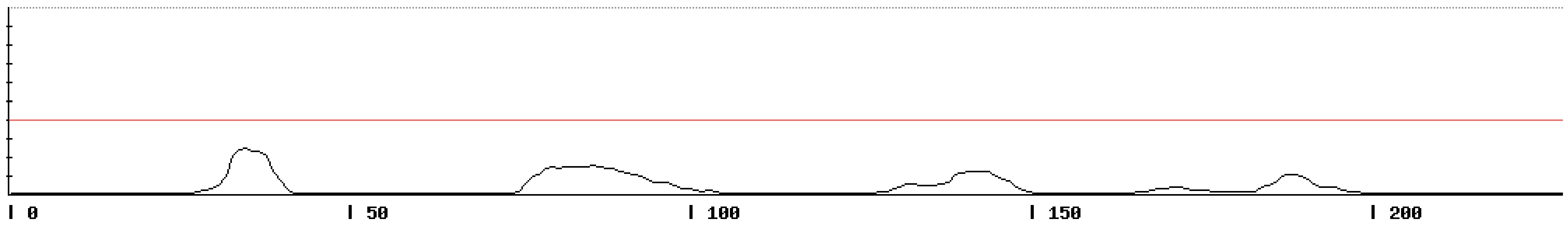

Predictions for Eucalyptuscamaldulensis_EcC039765.30

Viterbi Path

Viterbi Path

Posterior @ 0.4

Posterior @ 0.4

Posterior Graphic. Threshold Marked @ 0.4

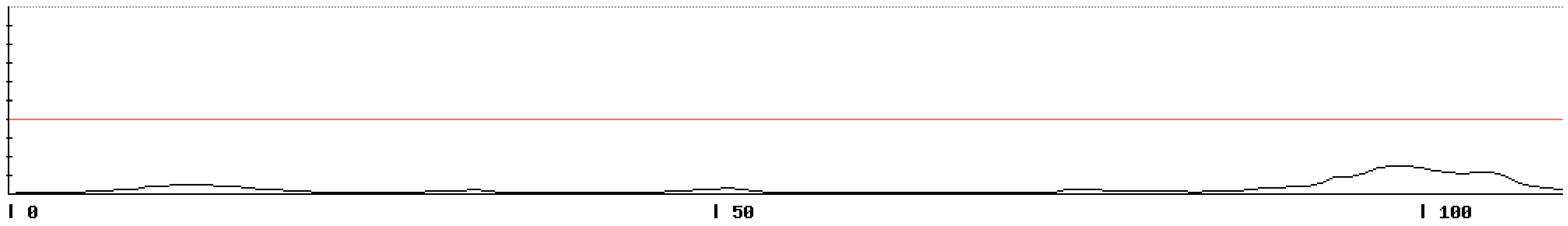

Predictions for Eucalyptuscamaldulensis_EcC040617.10

Viterbi Path

Viterbi Path

Posterior @ 0.4

Posterior @ 0.4

Posterior Graphic. Threshold Marked @ 0.4

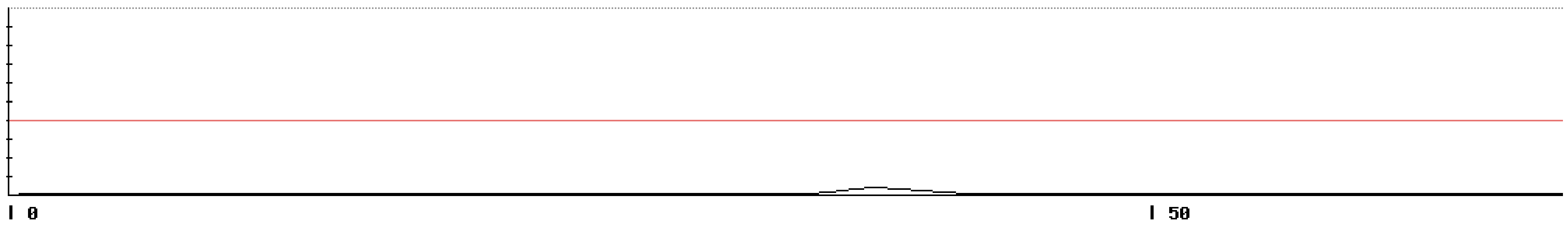

Predictions for Eucalyptuscamaldulensis_EcC042463.10

Viterbi Path

Viterbi Path

Posterior @ 0.4

Posterior @ 0.4

Posterior Graphic. Threshold Marked @ 0.4

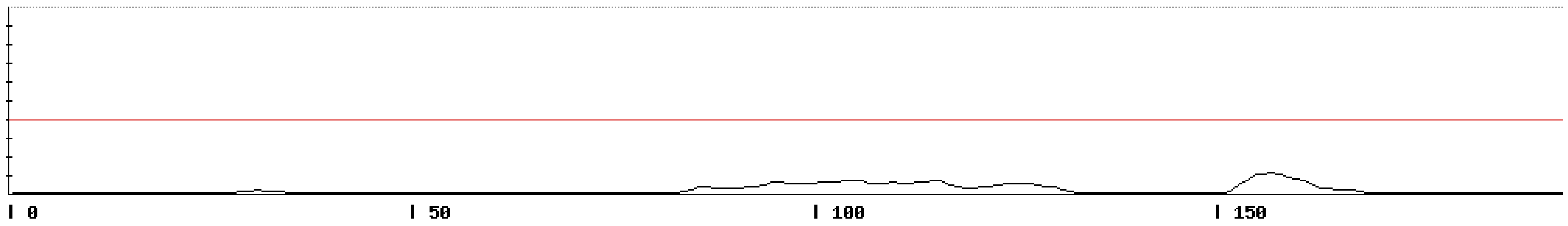

Predictions for Eucalyptuscamaldulensis_EcC043273.40

Viterbi Path

Viterbi Path

Posterior @ 0.4	128 - GKRKRRKI - 135
Posterior @ 0.4	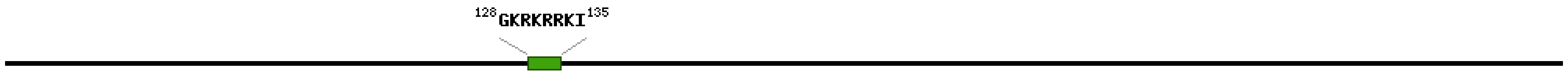
Posterior Graphic. Threshold Marked @ 0.4	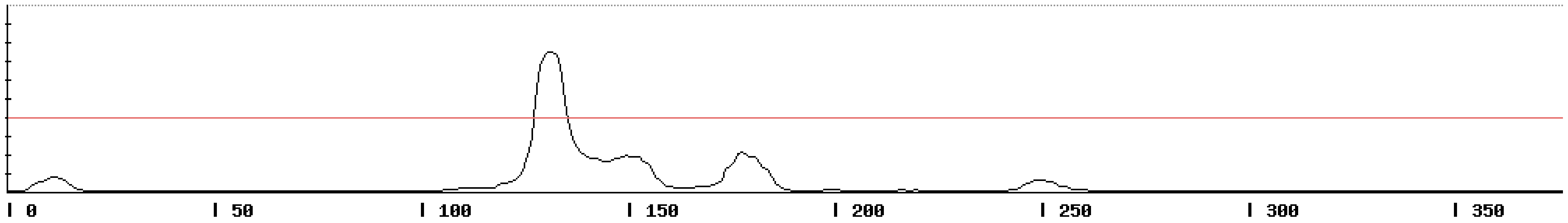

Predictions for Eucalyptuscamaldulensis_EcC044048.10

Viterbi Path	
Viterbi Path	
Posterior @ 0.4	
Posterior @ 0.4	
Posterior Graphic. Threshold Marked @ 0.4	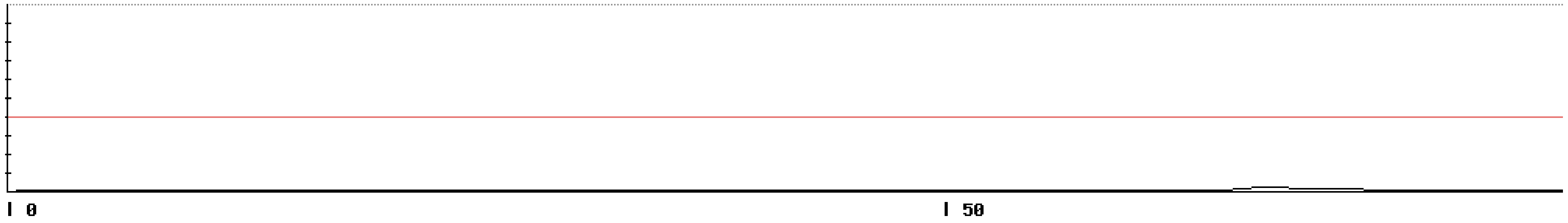

Predictions for Eucalyptuscamaldulensis_EcC044326.40

Viterbi Path

Viterbi Path

Posterior @ 0.4

Posterior @ 0.4

Posterior Graphic. Threshold Marked @ 0.4

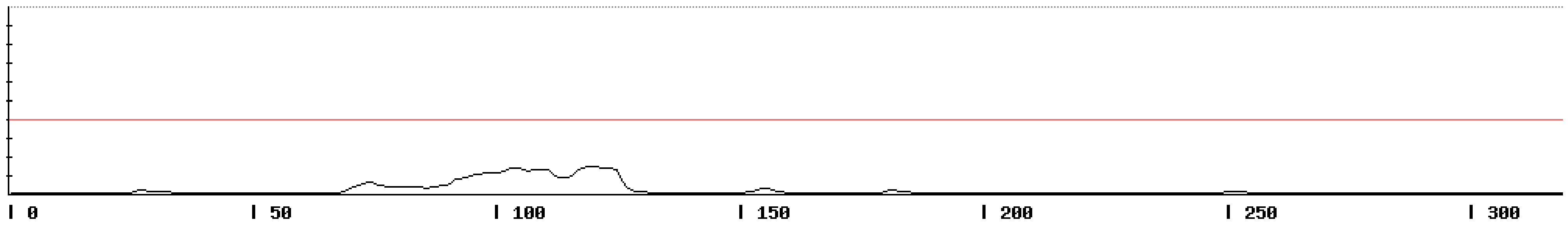

Predictions for Eucalyptuscamaldulensis_EcC045149.10

Viterbi Path

Viterbi Path

Posterior @ 0.4

Posterior @ 0.4

Posterior Graphic. Threshold Marked @ 0.4

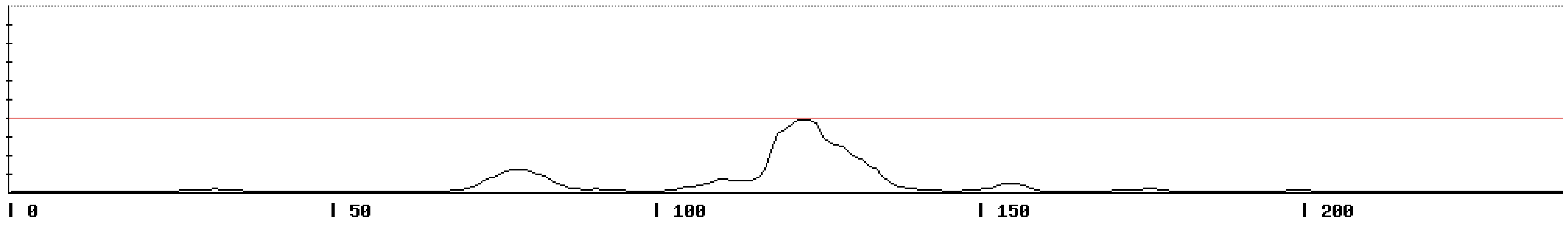

Predictions for Eucalyptuscamaldulensis_EcC045149.20	
Viterbi Path	
Viterbi Path	
Posterior @ 0.4	
Posterior @ 0.4	
Posterior Graphic. Threshold Marked @ 0.4	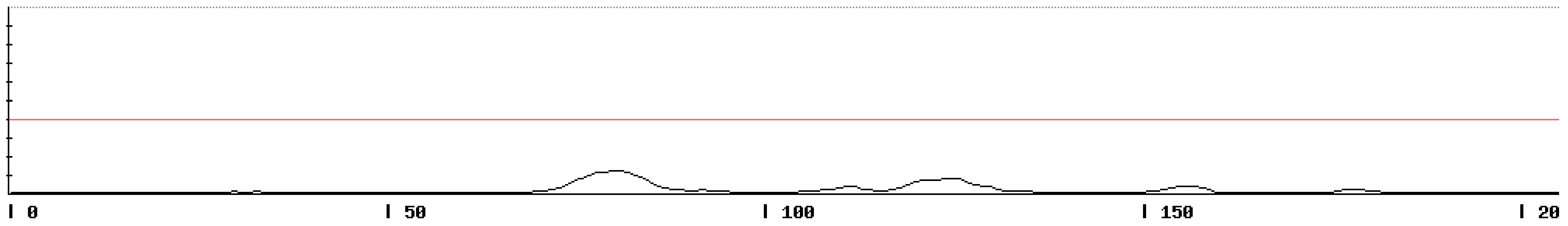

Predictions for Eucalyptuscamaldulensis_EcC045469.20	
Viterbi Path	
Viterbi Path	
Posterior @ 0.4	119 - KNGKR - 123
	213 - KRSKSELANRKEEGGETGQKKRKNMASTEGGRKAPHK - 249
Posterior @ 0.4	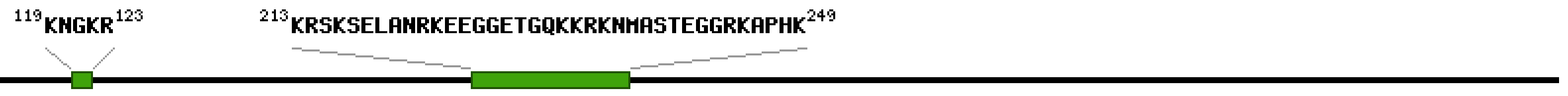
Posterior Graphic. Threshold Marked @ 0.4	

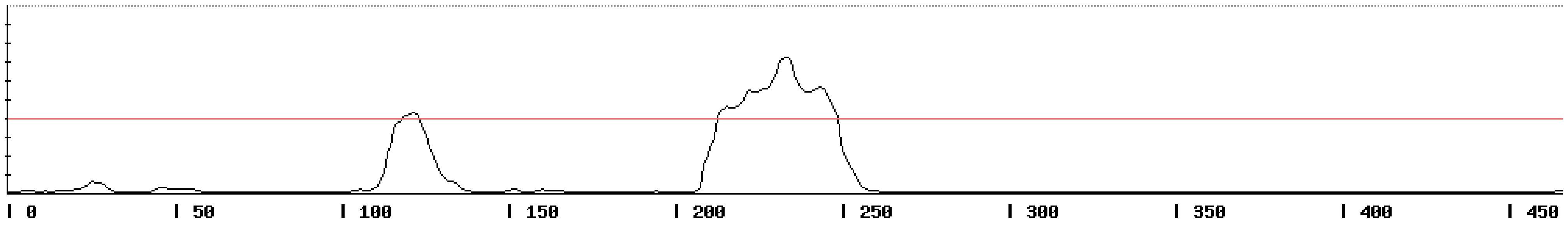

Predictions for Eucalyptuscamaldulensis_EcC045469.40	
Viterbi Path	
Viterbi Path	
Posterior @ 0.4	117 - KYKNGKRPNR - 126
Posterior @ 0.4	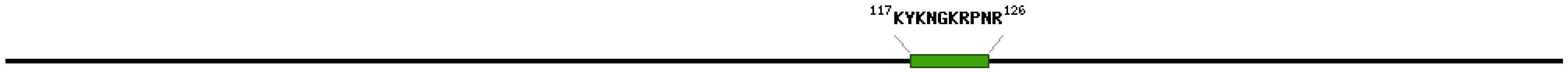
Posterior Graphic. Threshold Marked @ 0.4	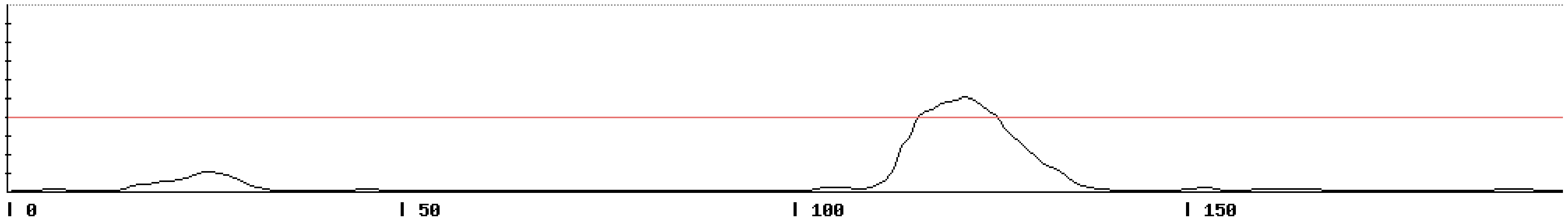

Predictions for Eucalyptuscamaldulensis_EcC045470.10	
Viterbi Path	
Viterbi Path	
Posterior @ 0.4	
Posterior @ 0.4	

Posterior Graphic. Threshold Marked @ 0.4

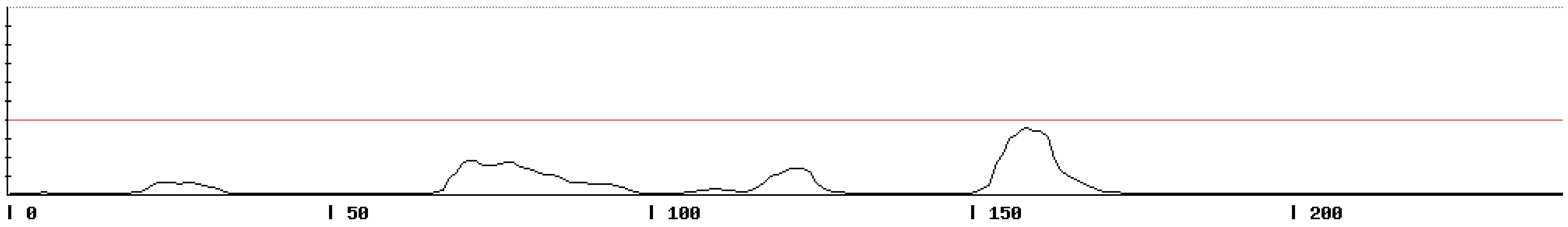

Predictions for Eucalyptuscamaldulensis_EcC045470.20

Viterbi Path

Viterbi Path

Posterior @ 0.4 — 99 - KPTGKTHKIRTQDKKNTLGLKKILVFKKRHSPKN - 132

Posterior @ 0.4

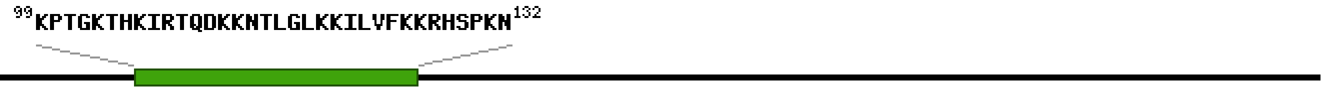

Posterior Graphic. Threshold Marked @ 0.4

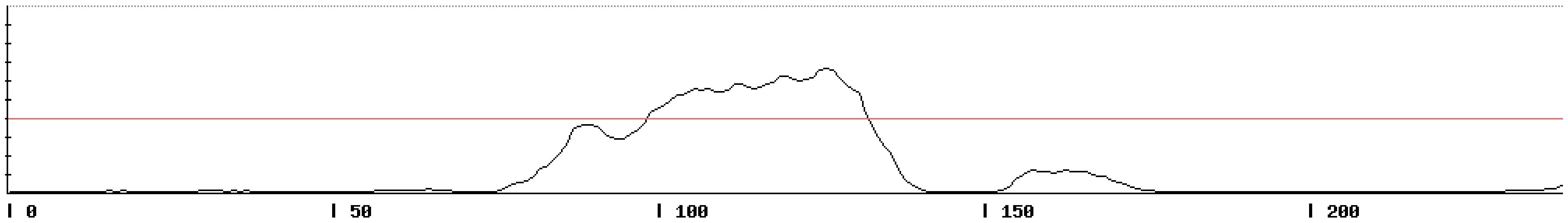

Predictions for Eucalyptuscamaldulensis_EcC045559.20

Viterbi Path

Viterbi Path

Posterior @ 0.4 — 195 - GGKKGVK - 201

Posterior @ 0.4

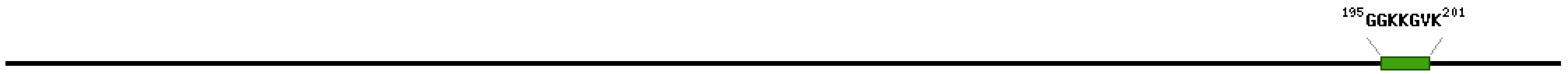

Posterior Graphic. Threshold Marked @ 0.4

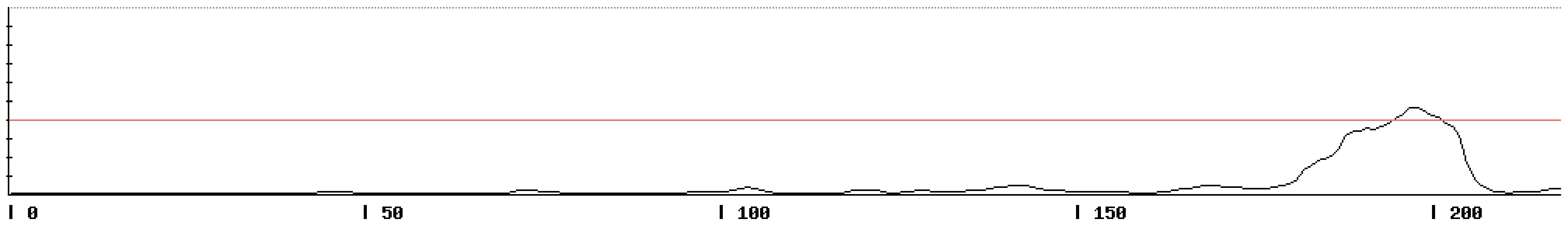

Predictions for Eucalyptuscamaldulensis_EcC045730.120

Viterbi Path

Viterbi Path

Posterior @ 0.4 115 - TRKRRRVQ - 122

Posterior @ 0.4

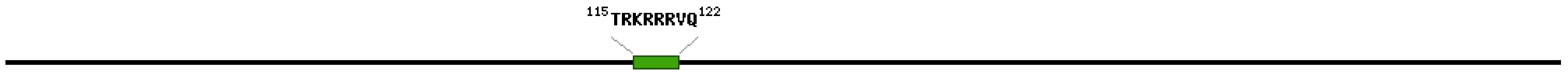

Posterior Graphic. Threshold Marked @ 0.4

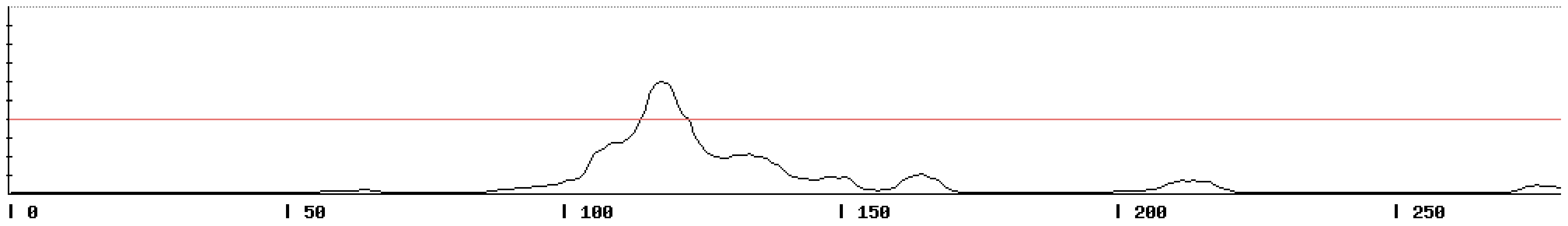

Predictions for Eucalyptuscamaldulensis_EcC045730.30

Viterbi Path

Viterbi Path

Posterior @ 0.4	134 - TRKRRRV - 140
Posterior @ 0.4	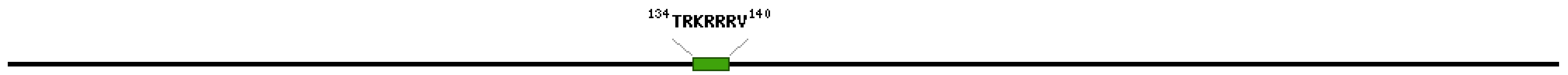
Posterior Graphic. Threshold Marked @ 0.4	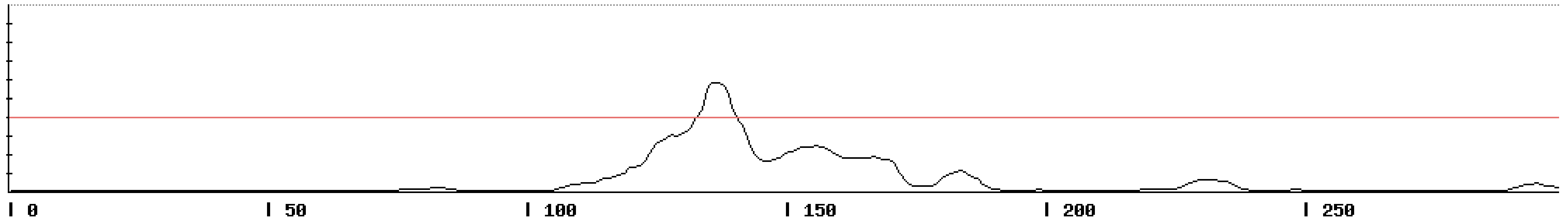

Predictions for Eucalyptuscamaldulensis_EcC045730.50

Viterbi Path	
Viterbi Path	
Posterior @ 0.4	
Posterior @ 0.4	
Posterior Graphic. Threshold Marked @ 0.4	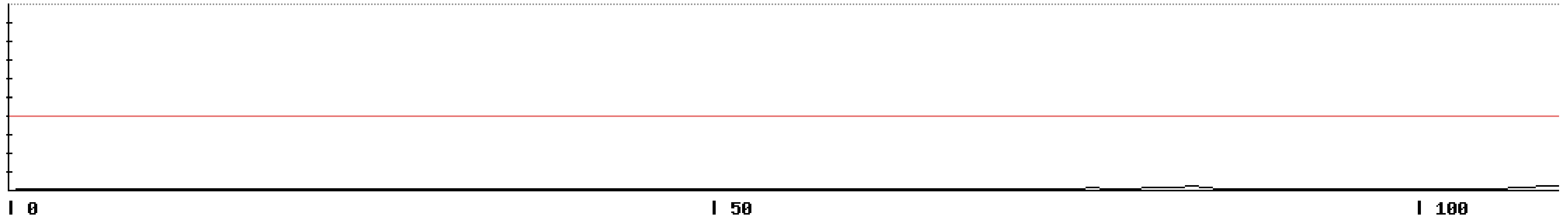

Predictions for Eucalyptuscamaldulensis_EcC045730.60

Viterbi Path	
Viterbi Path	

Posterior @ 0.4	142 - ATGTRKRRRVHKNADE - 157
	161 - RWHKTGK - 167
Posterior @ 0.4	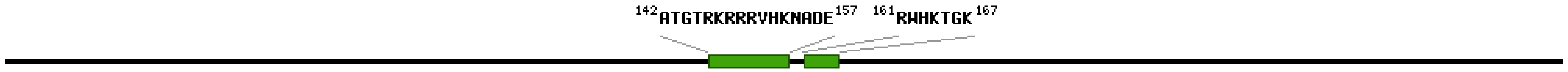
Posterior Graphic. Threshold Marked @ 0.4	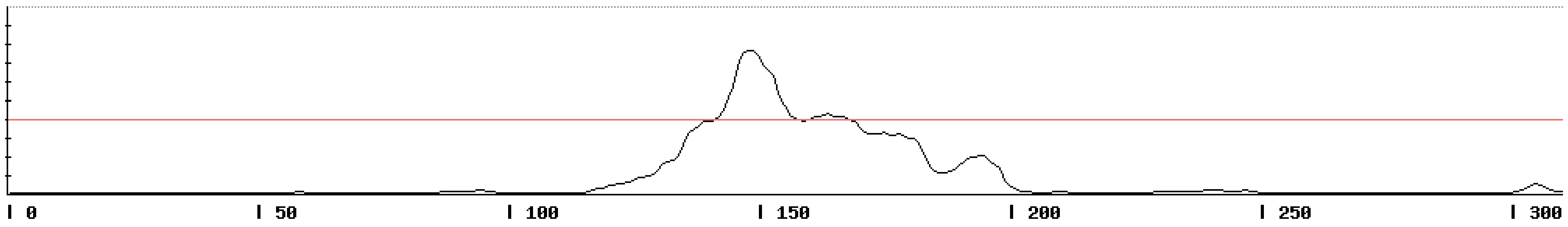

Predictions for Eucalyptuscamaldulensis_EcC046909.20

Viterbi Path	
Viterbi Path	
Posterior @ 0.4	
Posterior @ 0.4	
Posterior Graphic. Threshold Marked @ 0.4	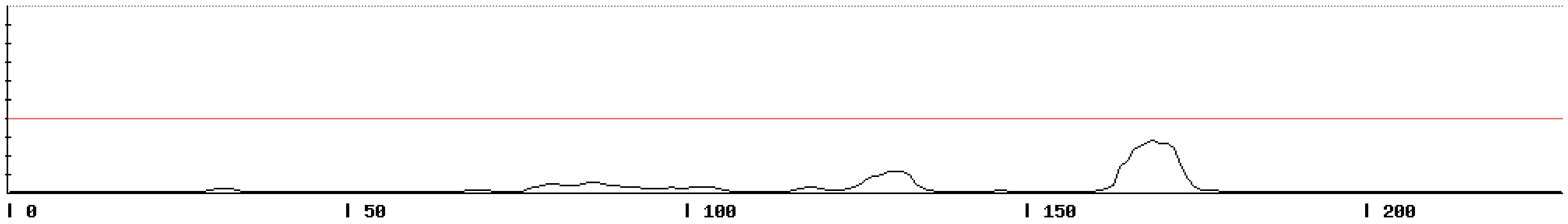

Predictions for Eucalyptuscamaldulensis_EcC046950.50	
Viterbi Path	
Viterbi Path	
Posterior @ 0.4	
Posterior @ 0.4	
Posterior Graphic. Threshold Marked @ 0.4	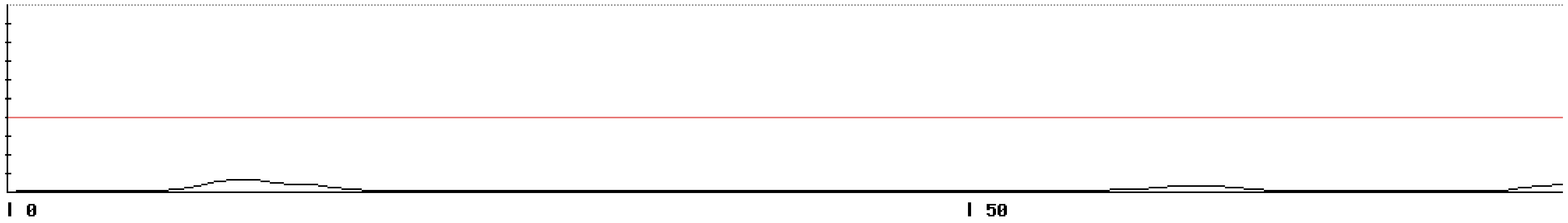

Predictions for Eucalyptuscamaldulensis_EcC046960.10	
Viterbi Path	
Viterbi Path	
Posterior @ 0.4	
Posterior @ 0.4	
Posterior Graphic. Threshold Marked @ 0.4	

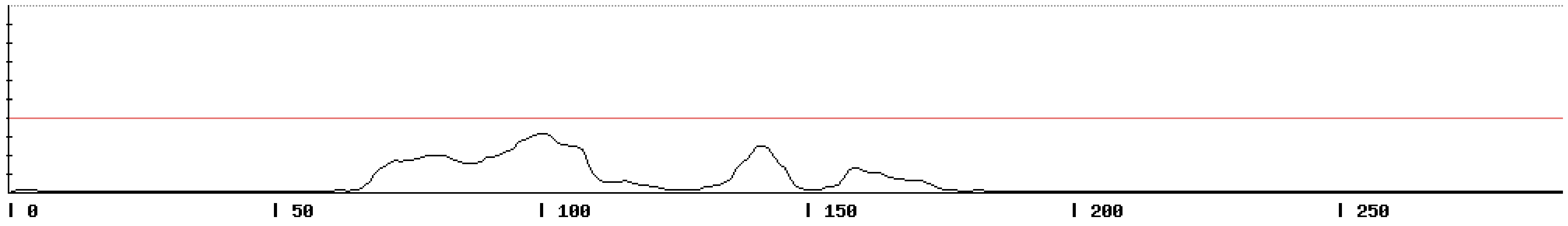

Predictions for Eucalyptuscamaldulensis_EcC047084.10

Viterbi Path

Viterbi Path

Posterior @ 0.4

Posterior @ 0.4

Posterior Graphic. Threshold Marked @ 0.4

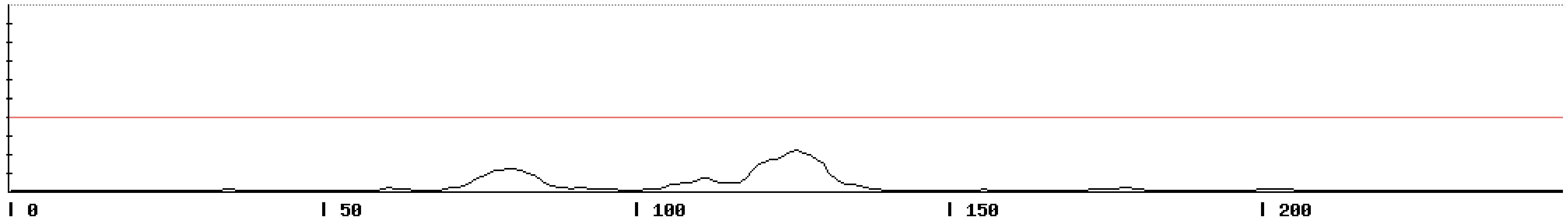

Predictions for Eucalyptuscamaldulensis_EcC047263.20

Viterbi Path

Viterbi Path

Posterior @ 0.4 | 140 - RKK - 142

Posterior @ 0.4

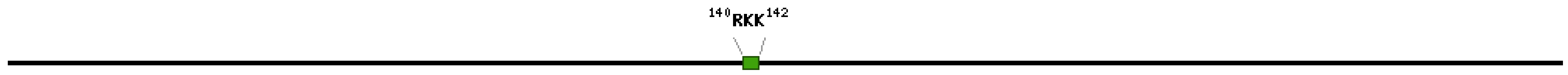

Posterior Graphic. Threshold Marked @ 0.4

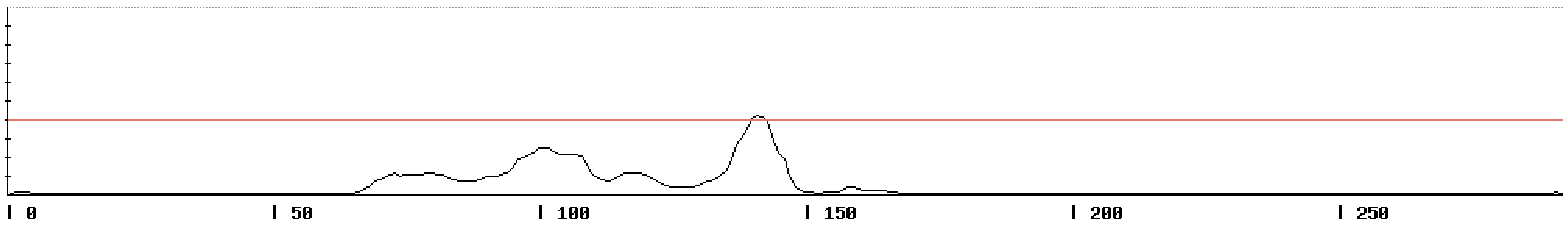

Predictions for Eucalyptuscamaldulensis_EcC047268.10

Viterbi Path

Viterbi Path

Posterior @ 0.4

Posterior @ 0.4

Posterior Graphic. Threshold Marked @ 0.4

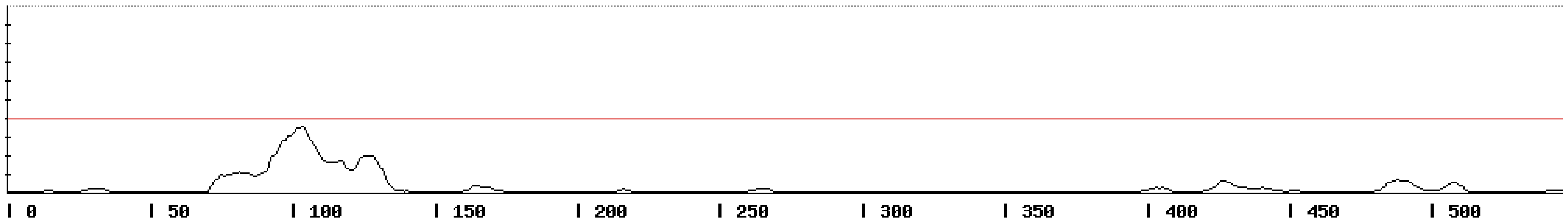

Predictions for Eucalyptuscamaldulensis_EcC047312.10

Viterbi Path

Viterbi Path

Posterior @ 0.4

Posterior @ 0.4

Posterior Graphic. Threshold Marked @ 0.4

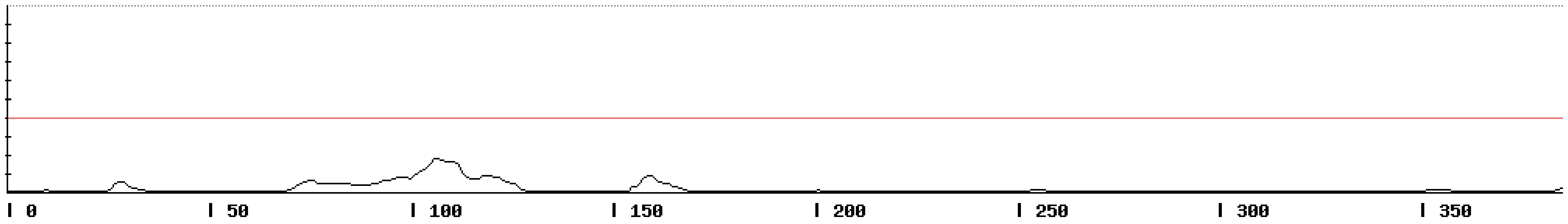

Predictions for Eucalyptuscamaldulensis_EcC047434.20

Viterbi Path

Viterbi Path

Posterior @ 0.4

Posterior @ 0.4

Posterior Graphic. Threshold Marked @ 0.4

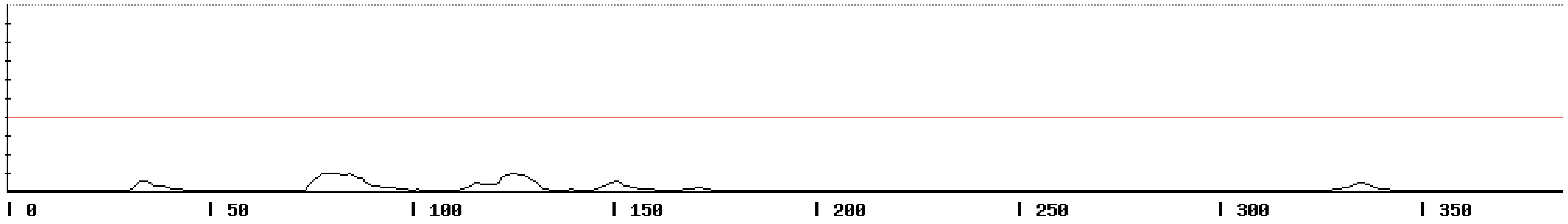

Predictions for Eucalyptuscamaldulensis_EcC047441.10

Viterbi Path

Viterbi Path

Posterior @ 0.4	
Posterior @ 0.4	
Posterior Graphic. Threshold Marked @ 0.4	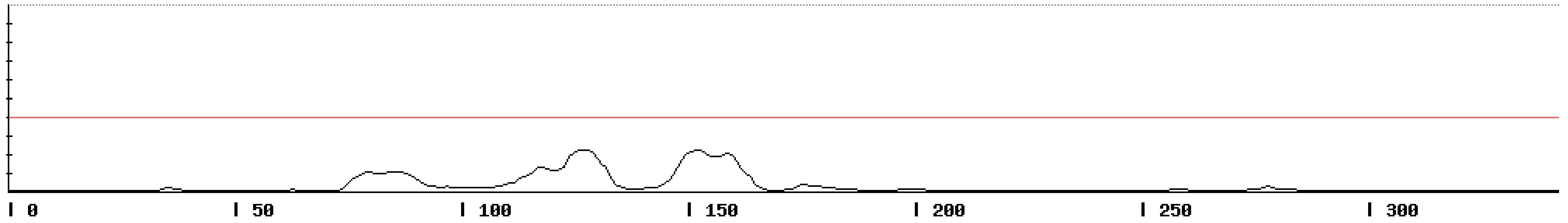

Predictions for Eucalyptuscamaldulensis_EcC047555.10

Viterbi Path	
Viterbi Path	
Posterior @ 0.4	
Posterior @ 0.4	
Posterior Graphic. Threshold Marked @ 0.4	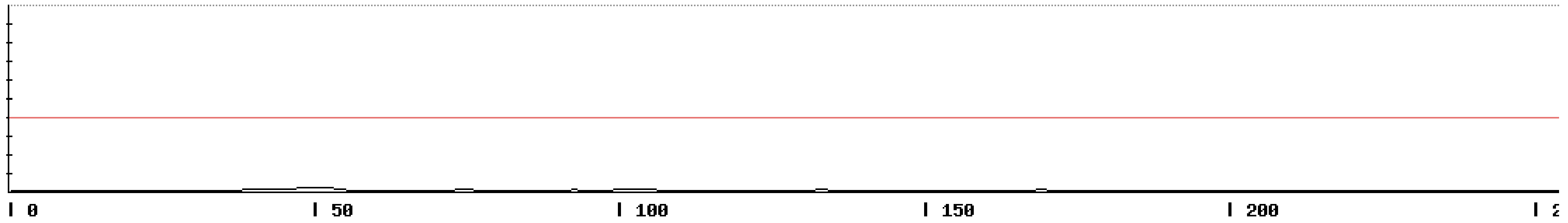

Predictions for Eucalyptuscamaldulensis_EcC047556.20

Viterbi Path	
Viterbi Path	

Posterior @ 0.4	
Posterior @ 0.4	
Posterior Graphic. Threshold Marked @ 0.4	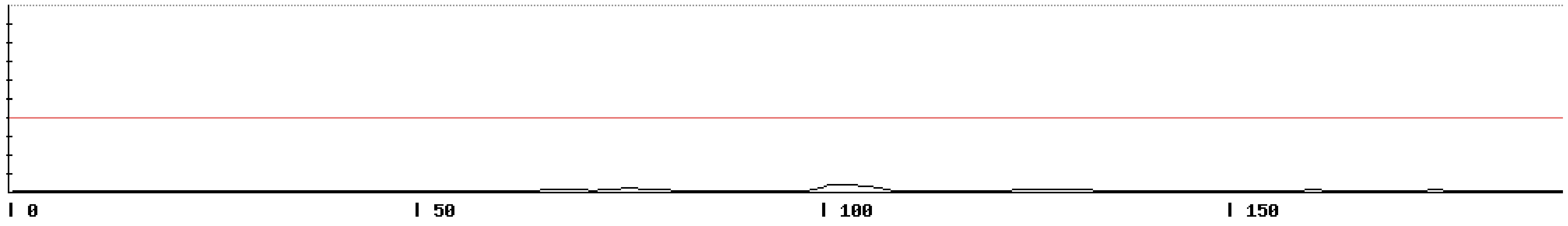

Predictions for Eucalyptuscamaldulensis_EcC048056.30

Viterbi Path	
Viterbi Path	
Posterior @ 0.4	
Posterior @ 0.4	
Posterior Graphic. Threshold Marked @ 0.4	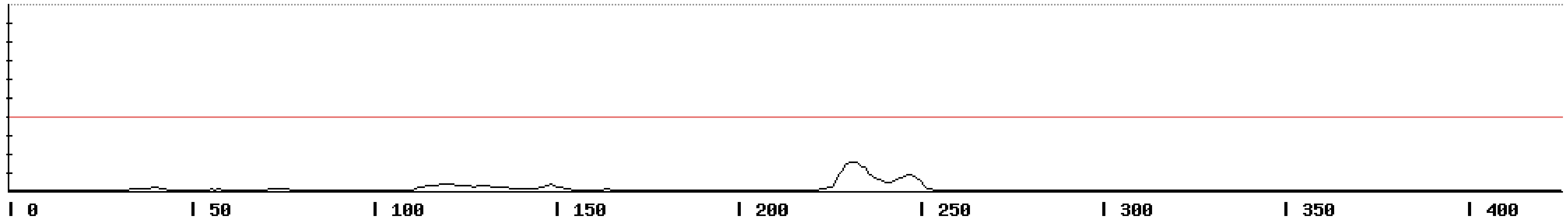

Predictions for Eucalyptuscamaldulensis_EcC048062.30

Viterbi Path	
Viterbi Path	
Posterior @ 0.4	
Posterior @ 0.4	
Posterior Graphic. Threshold Marked @ 0.4	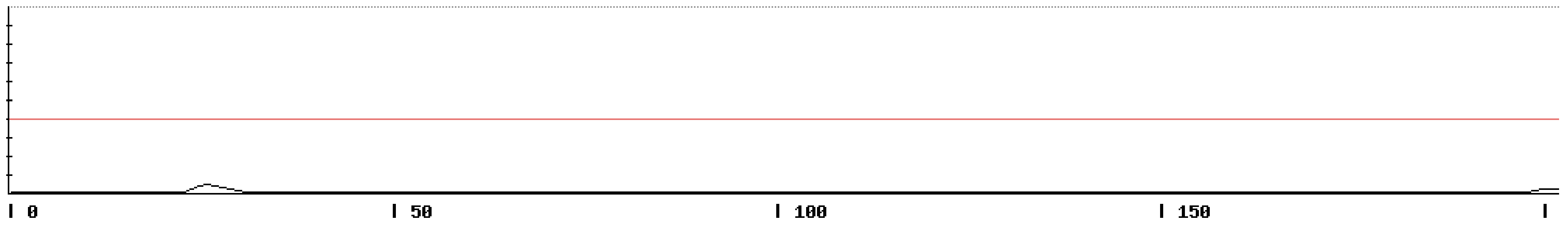

Predictions for Eucalyptuscamaldulensis_EcC048452.10

Viterbi Path	
Viterbi Path	
Posterior @ 0.4	
Posterior @ 0.4	
Posterior Graphic. Threshold Marked @ 0.4	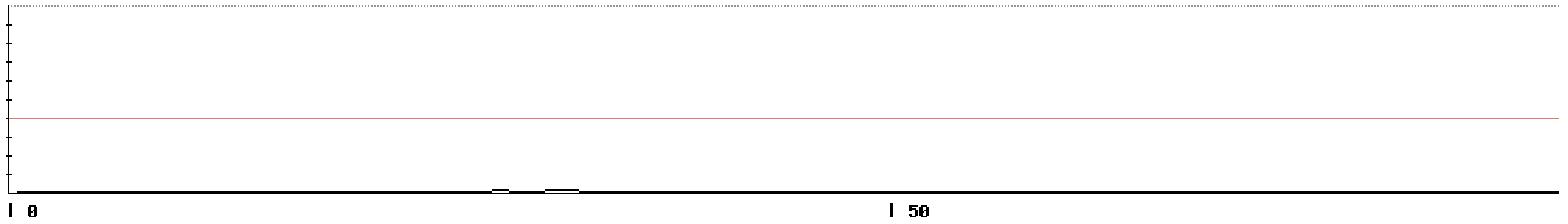

Predictions for Eucalyptuscamaldulensis_EcC048655.10	
Viterbi Path	
Viterbi Path	
Posterior @ 0.4	
Posterior @ 0.4	
Posterior Graphic. Threshold Marked @ 0.4	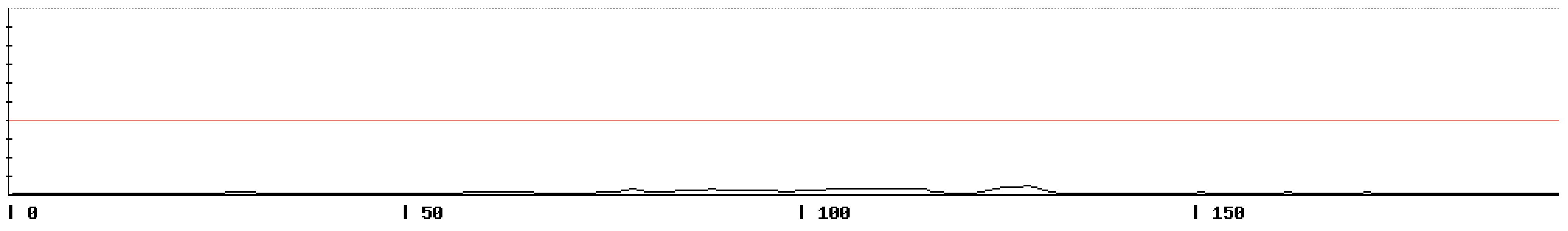

Predictions for Eucalyptuscamaldulensis_EcC048732.10	
Viterbi Path	
Viterbi Path	
Posterior @ 0.4	
Posterior @ 0.4	
Posterior Graphic. Threshold Marked @ 0.4	

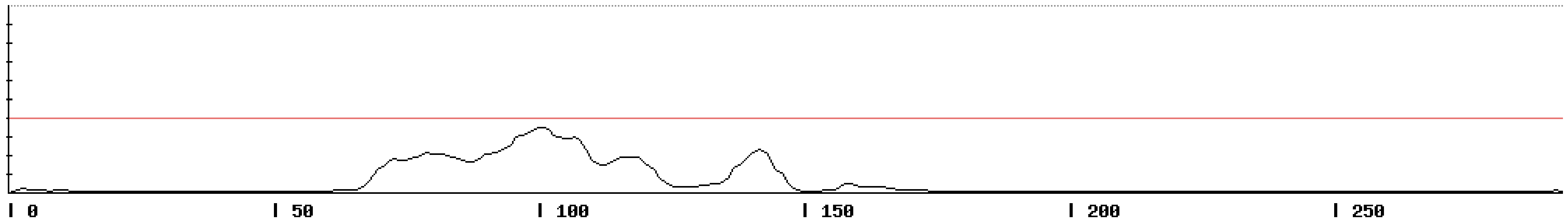

Predictions for Eucalyptuscamaldulensis_EcC049036.10

Viterbi Path

Viterbi Path

Posterior @ 0.4

Posterior @ 0.4

Posterior Graphic. Threshold Marked @ 0.4

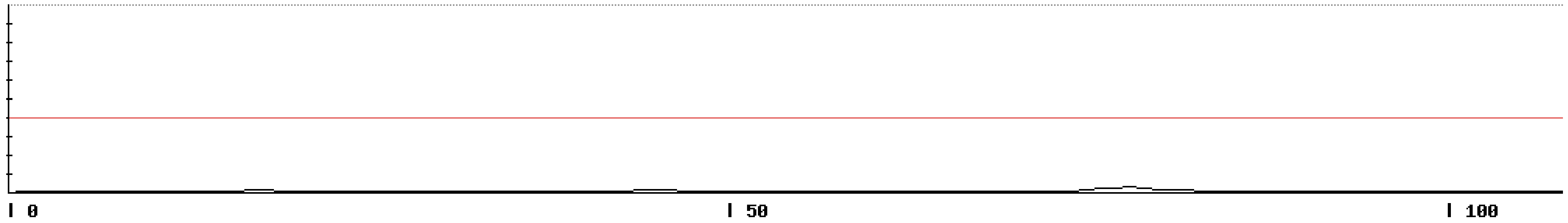

Predictions for Eucalyptuscamaldulensis_EcC049544.20

Viterbi Path

Viterbi Path

Posterior @ 0.4

Posterior @ 0.4

Posterior Graphic. Threshold Marked @ 0.4	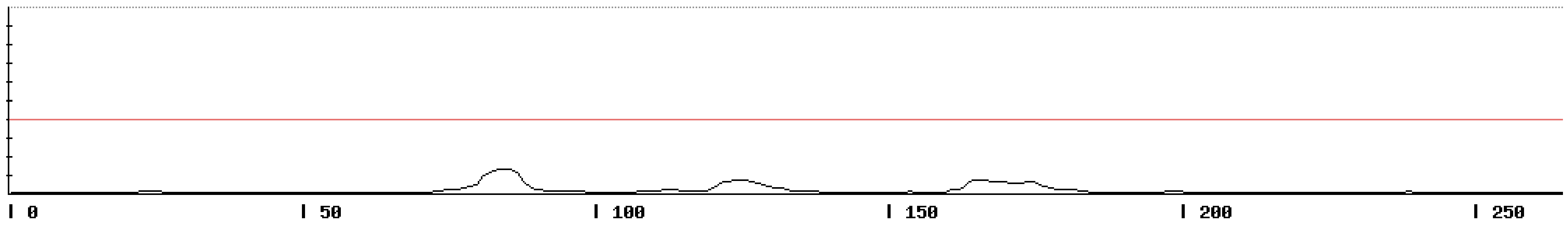

Predictions for Eucalyptuscamaldulensis_EcC049642.10	
Viterbi Path	
Viterbi Path	
Posterior @ 0.4	
Posterior @ 0.4	
Posterior Graphic. Threshold Marked @ 0.4	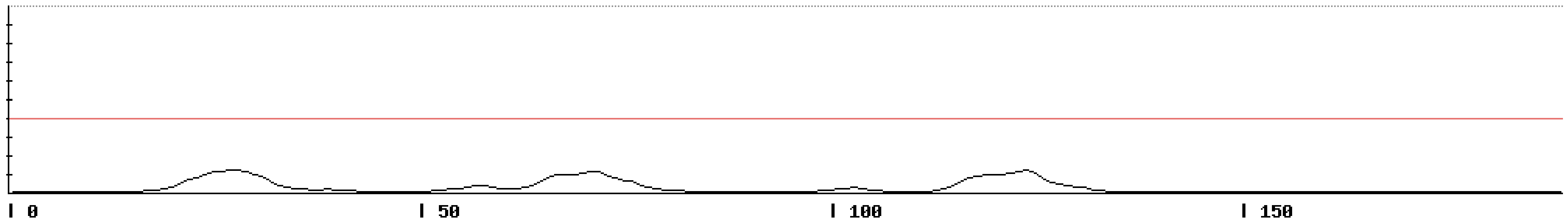

Predictions for Eucalyptuscamaldulensis_EcC049810.10	
Viterbi Path	
Viterbi Path	
Posterior @ 0.4	
Posterior @ 0.4	

Posterior Graphic. Threshold Marked @ 0.4

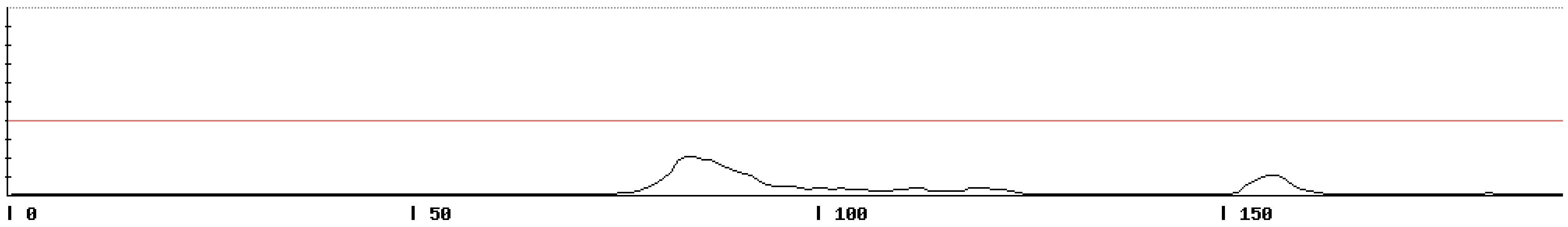

Predictions for Eucalyptuscamaldulensis_EcC049819.20

Viterbi Path

Viterbi Path

Posterior @ 0.4

Posterior @ 0.4

Posterior Graphic. Threshold Marked @ 0.4

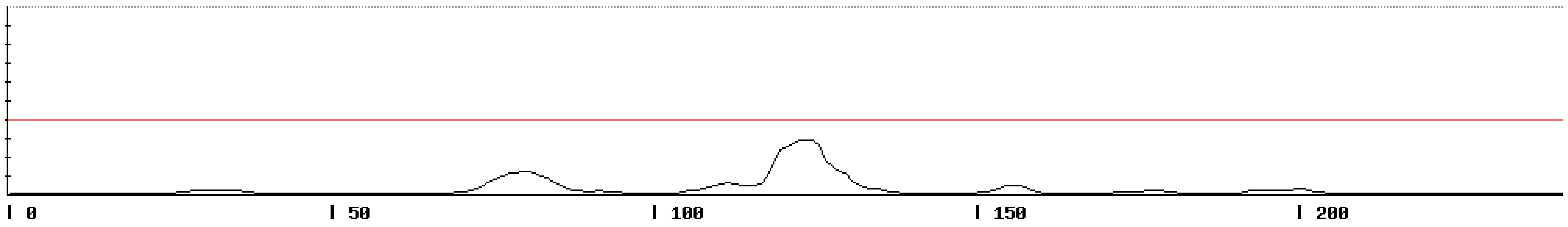

Predictions for Eucalyptuscamaldulensis_EcC050668.20

Viterbi Path

Viterbi Path

Posterior @ 0.4

Posterior @ 0.4

Posterior Graphic. Threshold Marked @ 0.4

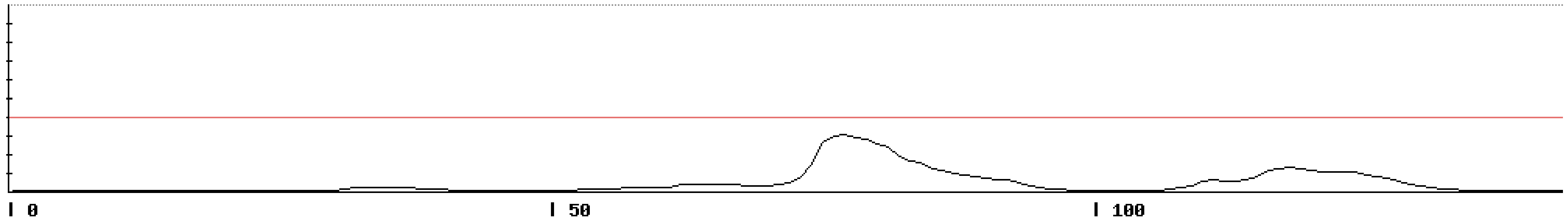

Predictions for Eucalyptuscamaldulensis_EcC051092.30

Viterbi Path

Viterbi Path

Posterior @ 0.4

Posterior @ 0.4

Posterior Graphic. Threshold Marked @ 0.4

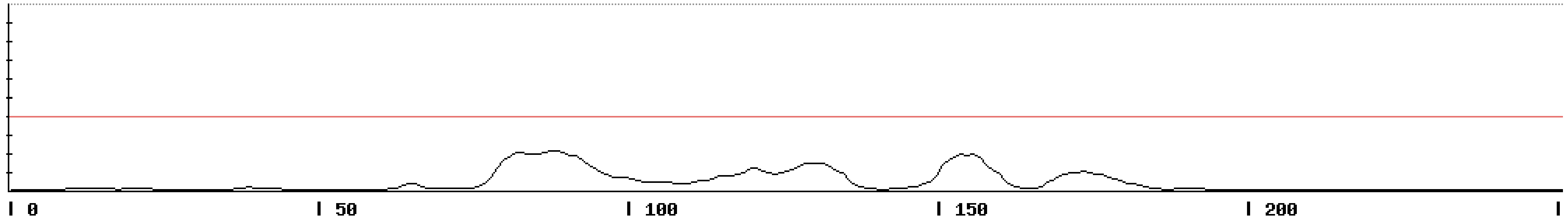

Predictions for Eucalyptuscamaldulensis_EcC051212.10

Viterbi Path

Viterbi Path

Posterior @ 0.4

Posterior @ 0.4

Posterior Graphic. Threshold Marked @ 0.4

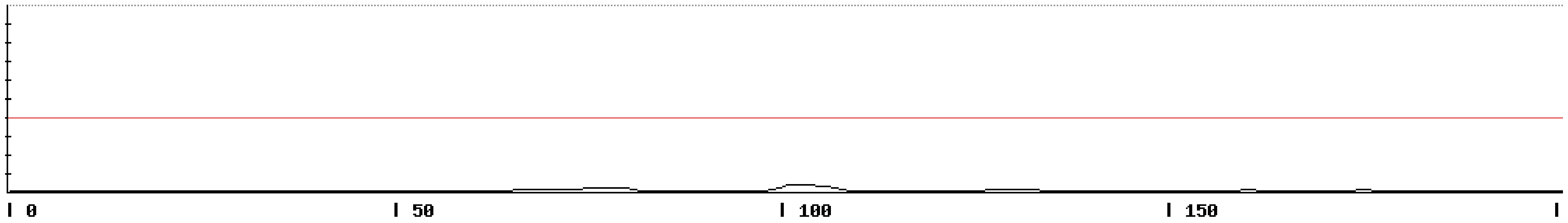

Predictions for Eucalyptuscamaldulensis_EcC051253.10

Viterbi Path

Viterbi Path

Posterior @ 0.4

Posterior @ 0.4

Posterior Graphic. Threshold Marked @ 0.4

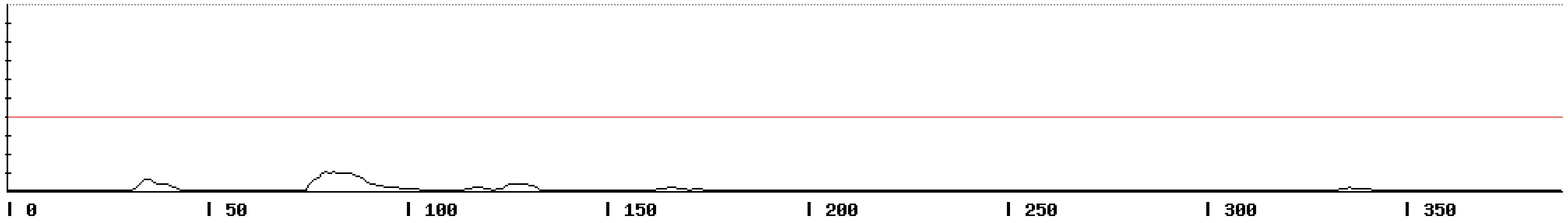

Predictions for Eucalyptuscamaldulensis_EcC052230.40

Viterbi Path

Viterbi Path

Posterior @ 0.4

Posterior @ 0.4

Posterior Graphic. Threshold Marked @ 0.4

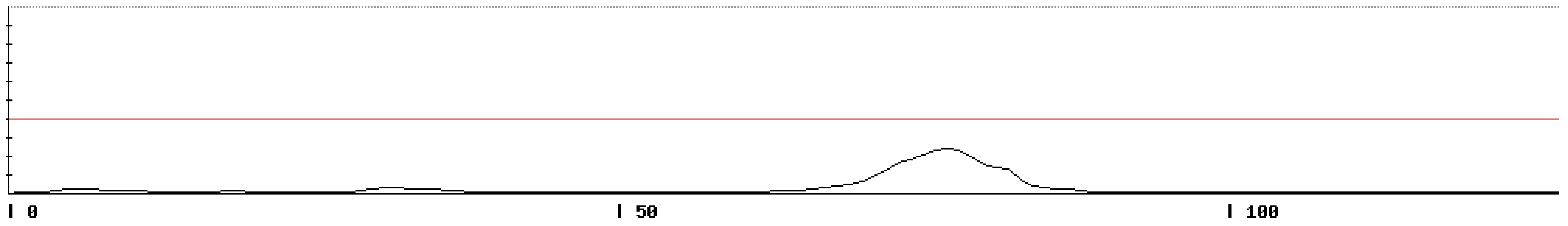

Predictions for Eucalyptuscamaldulensis_EcC052412.30

Viterbi Path

Viterbi Path

Posterior @ 0.4

Posterior @ 0.4

Posterior Graphic. Threshold Marked @ 0.4

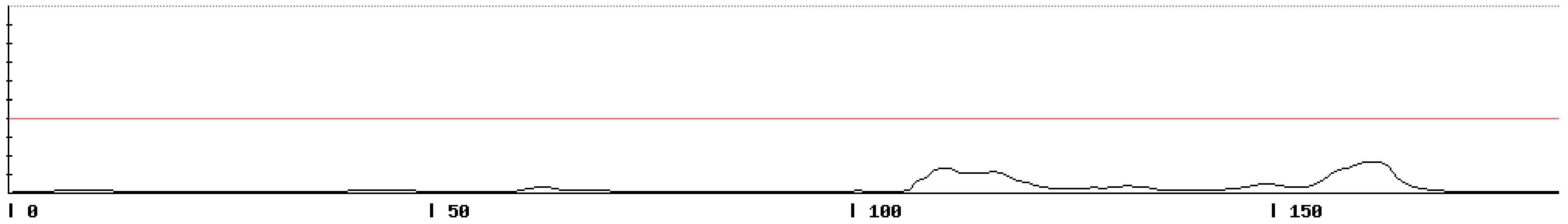

Predictions for Eucalyptuscamaldulensis_EcC052727.20

Viterbi Path

Viterbi Path

Posterior @ 0.4

Posterior @ 0.4

Posterior Graphic. Threshold Marked @ 0.4

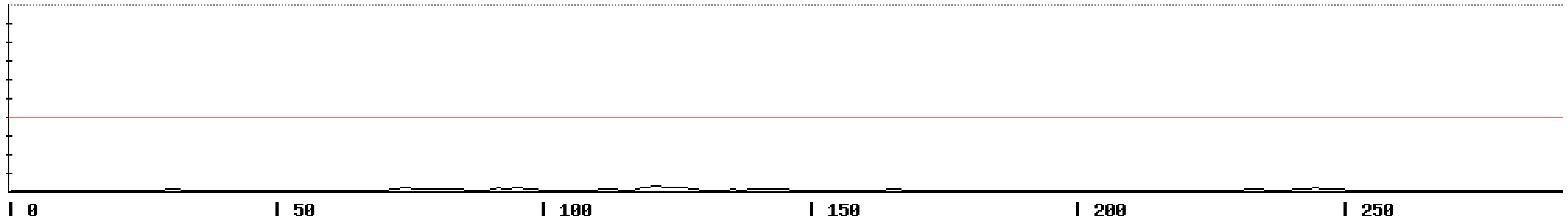

Predictions for Eucalyptuscamaldulensis_EcC052744.10

Viterbi Path

Viterbi Path

Posterior @ 0.4

Posterior @ 0.4

Posterior Graphic. Threshold Marked @ 0.4

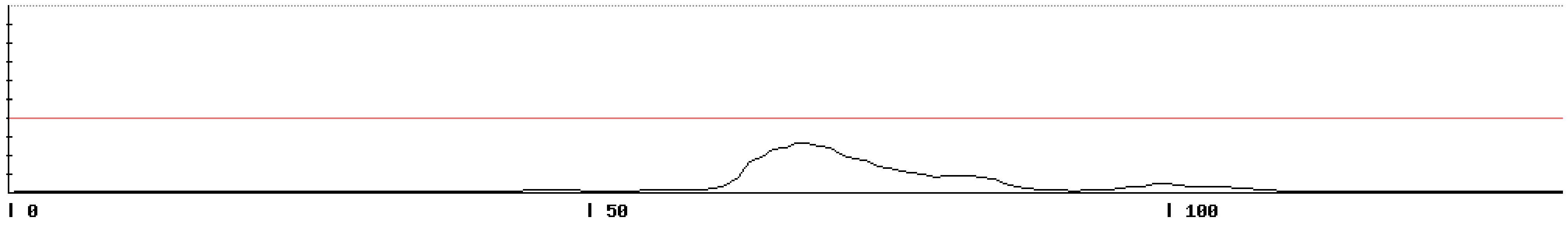

Predictions for Eucalyptuscamaldulensis_EcC052833.20

Viterbi Path	
Viterbi Path	
Posterior @ 0.4	
Posterior @ 0.4	
Posterior Graphic. Threshold Marked @ 0.4	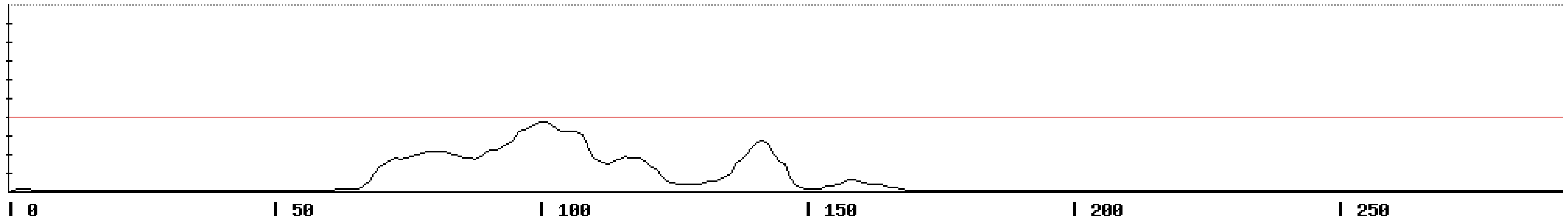

Predictions for Eucalyptuscamaldulensis_EcC052855.20

Viterbi Path	
Viterbi Path	
Posterior @ 0.4	
Posterior @ 0.4	

Posterior Graphic. Threshold Marked @ 0.4

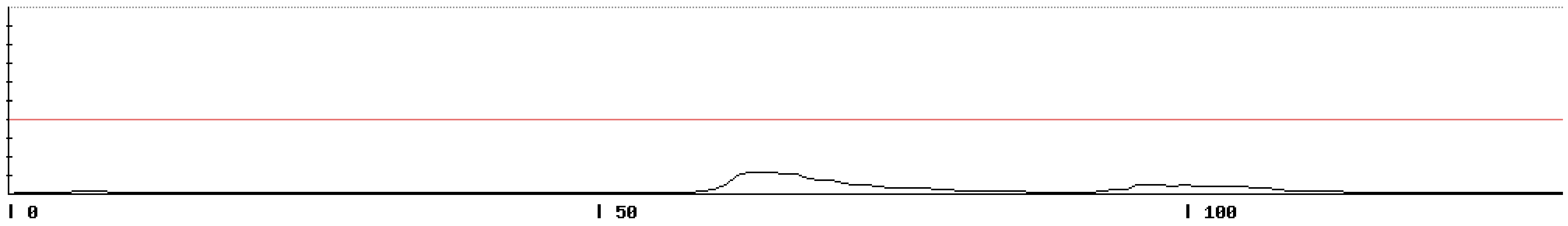

Predictions for Eucalyptuscamaldulensis_EcC052965.10

Viterbi Path

Viterbi Path

Posterior @ 0.4

Posterior @ 0.4

Posterior Graphic. Threshold Marked @ 0.4

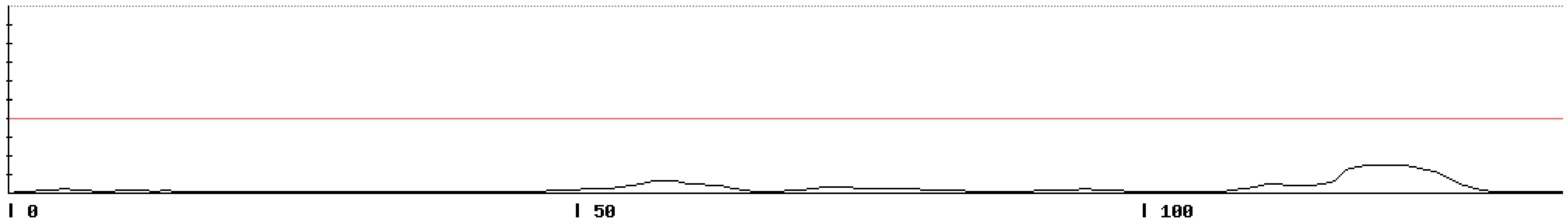

Predictions for Eucalyptuscamaldulensis_EcC053744.10

Viterbi Path

Viterbi Path

Posterior @ 0.4

Posterior @ 0.4

Posterior Graphic. Threshold Marked @ 0.4

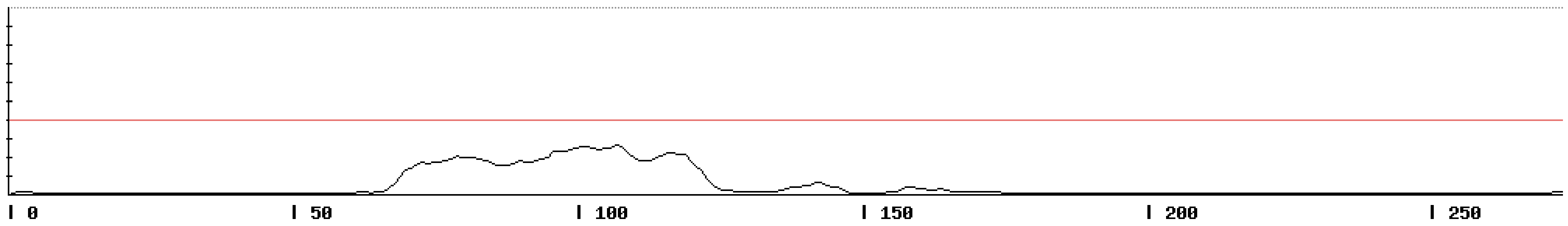

Predictions for Eucalyptuscamaldulensis_EcC053744.20

Viterbi Path

Viterbi Path

Posterior @ 0.4

Posterior @ 0.4

Posterior Graphic. Threshold Marked @ 0.4

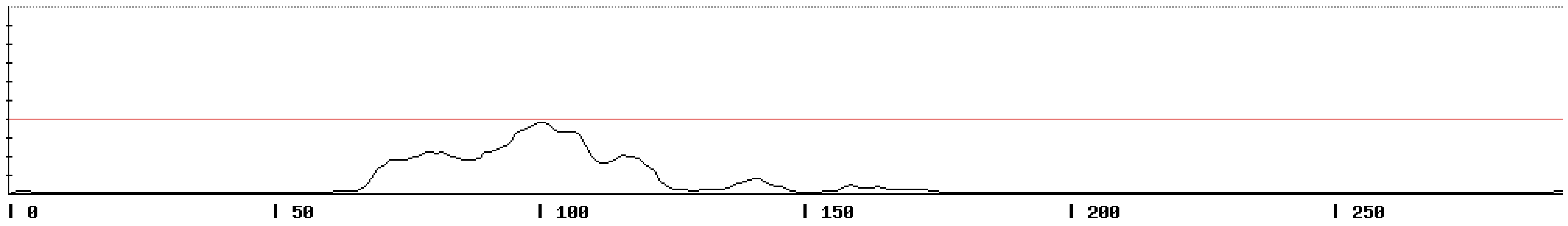

Predictions for Eucalyptuscamaldulensis_EcC053763.10

Viterbi Path

Viterbi Path

Posterior @ 0.4	
Posterior @ 0.4	
Posterior Graphic. Threshold Marked @ 0.4	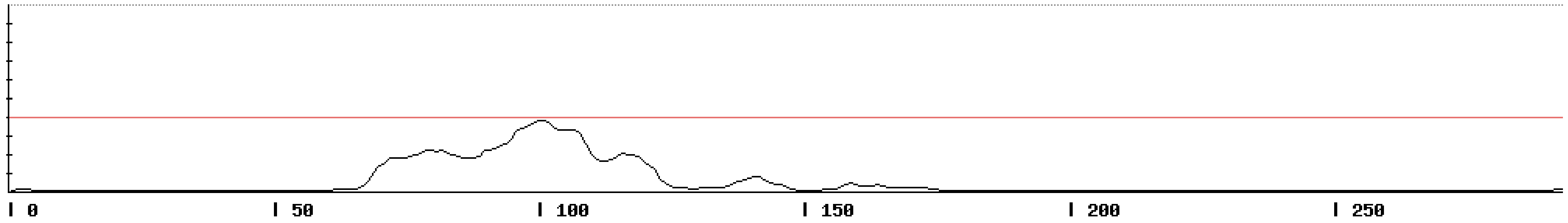

Predictions for Eucalyptuscamaldulensis_EcC053818.10

Viterbi Path	
Viterbi Path	
Posterior @ 0.4	68 - KK - 69
Posterior @ 0.4	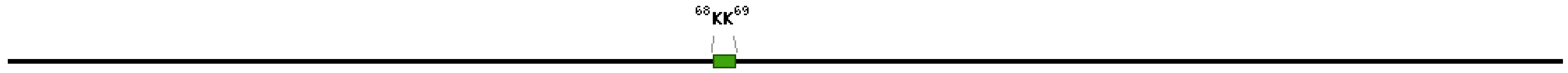
Posterior Graphic. Threshold Marked @ 0.4	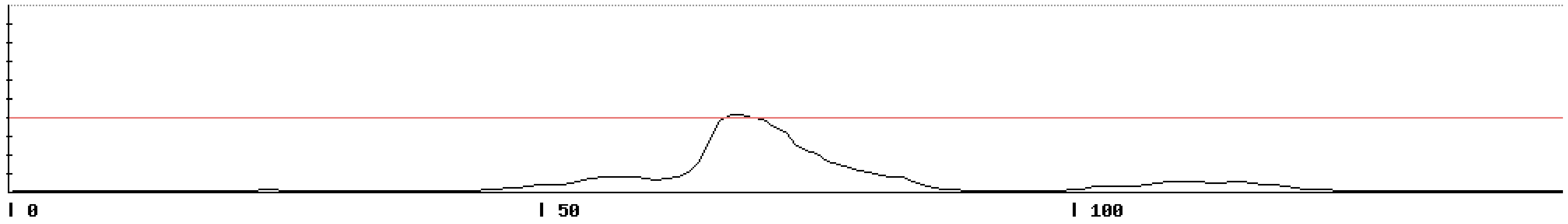

Predictions for Eucalyptuscamaldulensis_EcC053822.40

Viterbi Path	
Viterbi Path	

Posterior @ 0.4

Posterior @ 0.4

Posterior Graphic. Threshold Marked @ 0.4

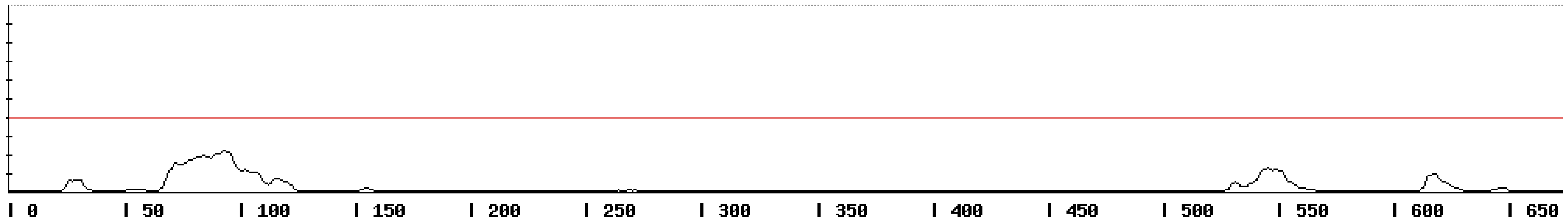

Predictions for Eucalyptuscamaldulensis_EcC053999.10

Viterbi Path

Viterbi Path

Posterior @ 0.4

Posterior @ 0.4

Posterior Graphic. Threshold Marked @ 0.4

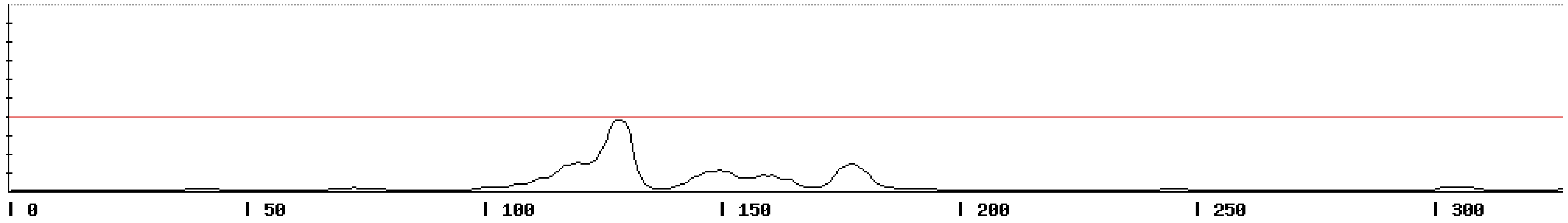

Predictions for Eucalyptuscamaldulensis_EcC054060.20

Viterbi Path

Viterbi Path

Posterior @ 0.4

100 - NRKRRK - 105

Posterior @ 0.4

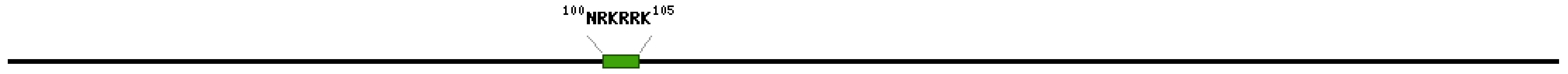

Posterior Graphic. Threshold Marked @ 0.4

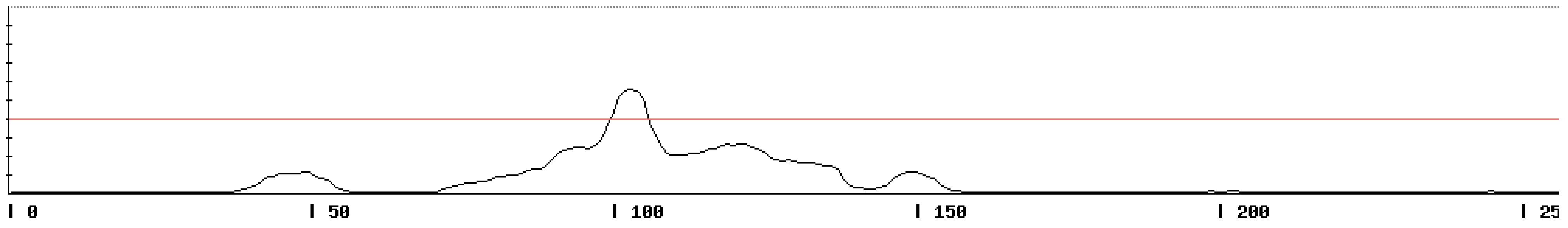

Predictions for Eucalyptuscamaldulensis_EcC054212.20

Viterbi Path

Viterbi Path

Posterior @ 0.4

Posterior @ 0.4

Posterior Graphic. Threshold Marked @ 0.4

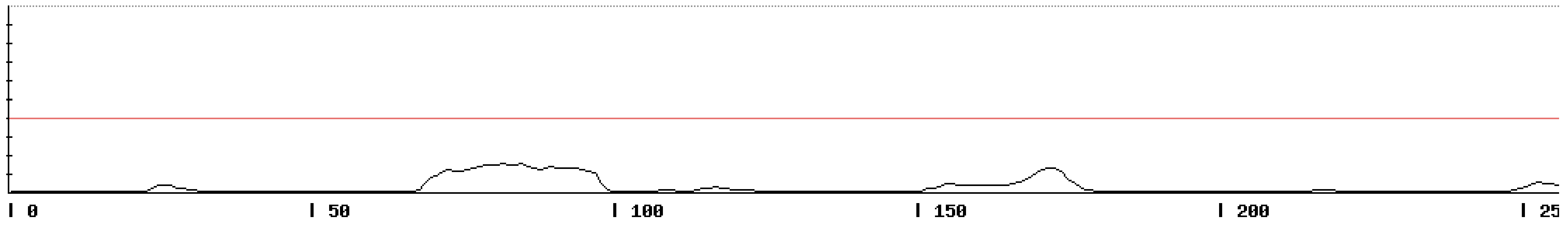

Predictions for Eucalyptuscamaldulensis_EcC054284.60

Viterbi Path

Viterbi Path

Posterior @ 0.4

Posterior @ 0.4

Posterior Graphic. Threshold Marked @ 0.4

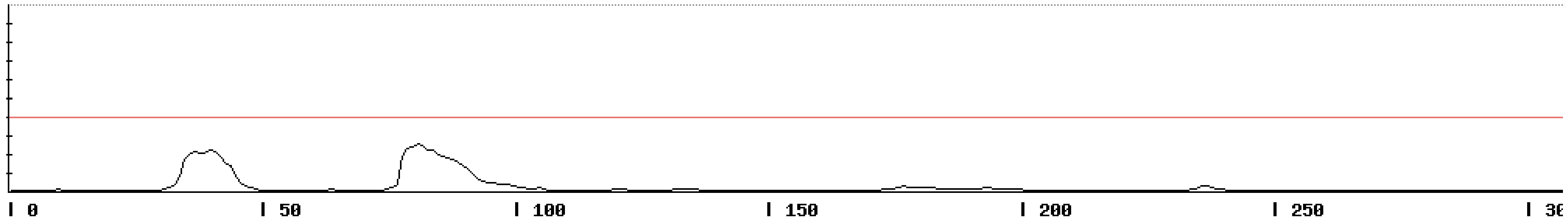

Predictions for Eucalyptuscamaldulensis_EcC054363.70

Viterbi Path

Viterbi Path

Posterior @ 0.4

Posterior @ 0.4

Posterior Graphic. Threshold Marked @ 0.4

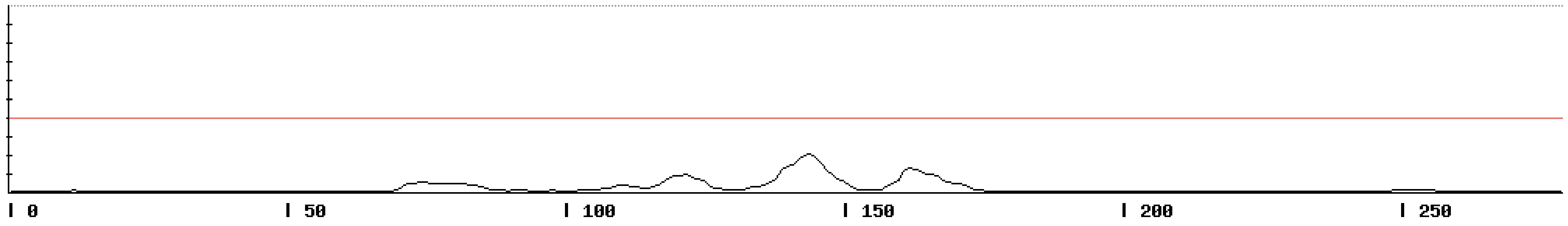

Predictions for Eucalyptuscamaldulensis_EcC054376.20

Viterbi Path

Viterbi Path

Posterior @ 0.4

Posterior @ 0.4

Posterior Graphic. Threshold Marked @ 0.4

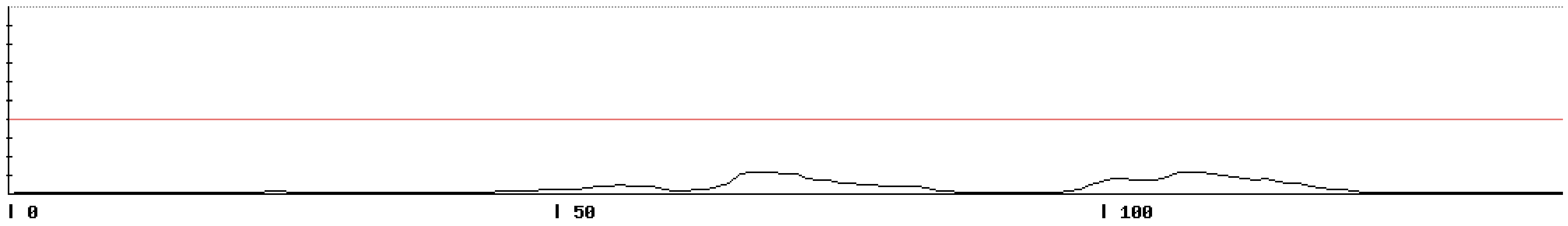

Predictions for Eucalyptuscamaldulensis_EcC054471.80

Viterbi Path

Viterbi Path

Posterior @ 0.4

Posterior @ 0.4

Posterior Graphic. Threshold Marked @ 0.4

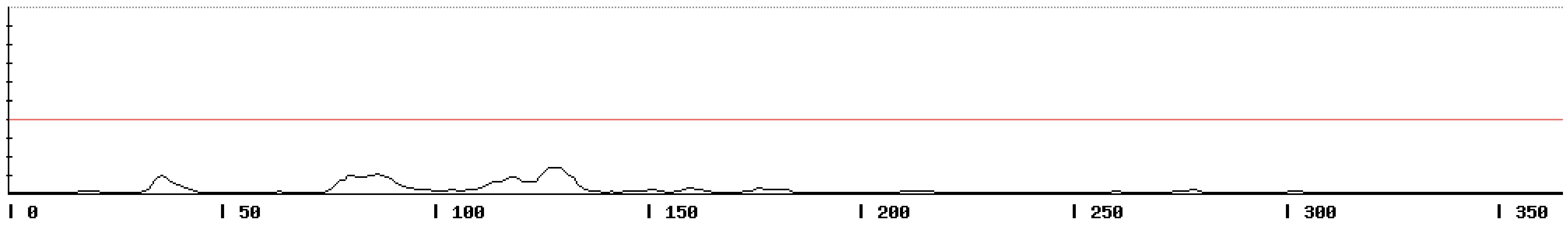

Predictions for Eucalyptuscamaldulensis_EcC054519.20

Viterbi Path

Viterbi Path

Posterior @ 0.4

Posterior @ 0.4

Posterior Graphic. Threshold Marked @ 0.4

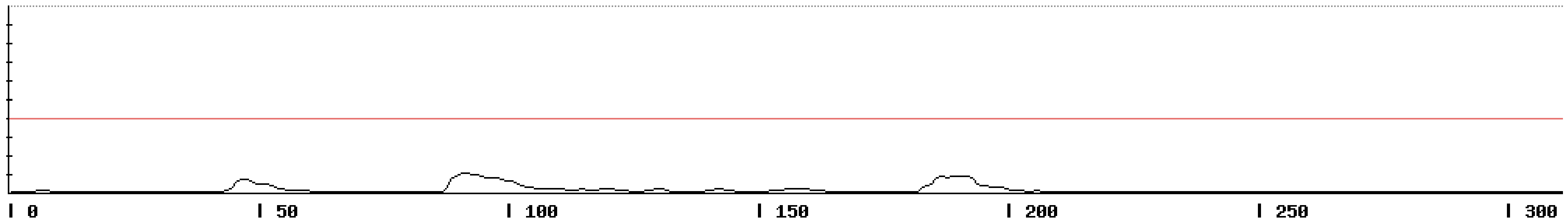

Predictions for Eucalyptuscamaldulensis_EcC054588.60

Viterbi Path

Viterbi Path

Posterior @ 0.4

Posterior @ 0.4

Posterior Graphic. Threshold Marked @ 0.4

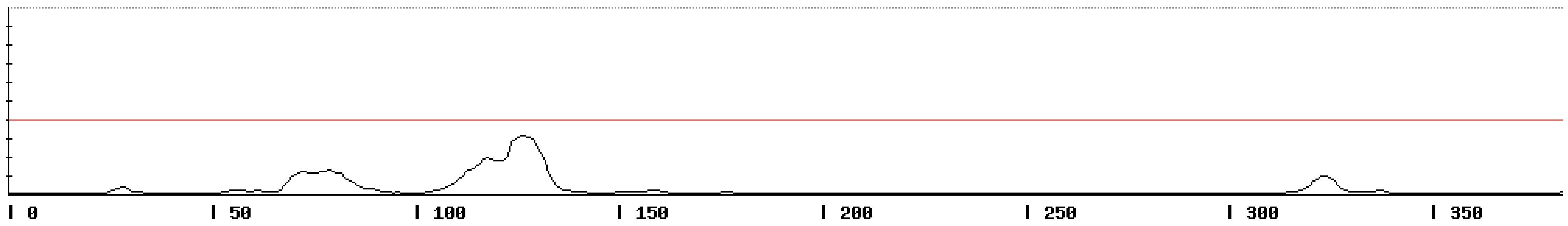

Predictions for Eucalyptuscamaldulensis_EcC054610.60

Viterbi Path

Viterbi Path

Posterior @ 0.4

Posterior @ 0.4

Posterior Graphic. Threshold Marked @ 0.4

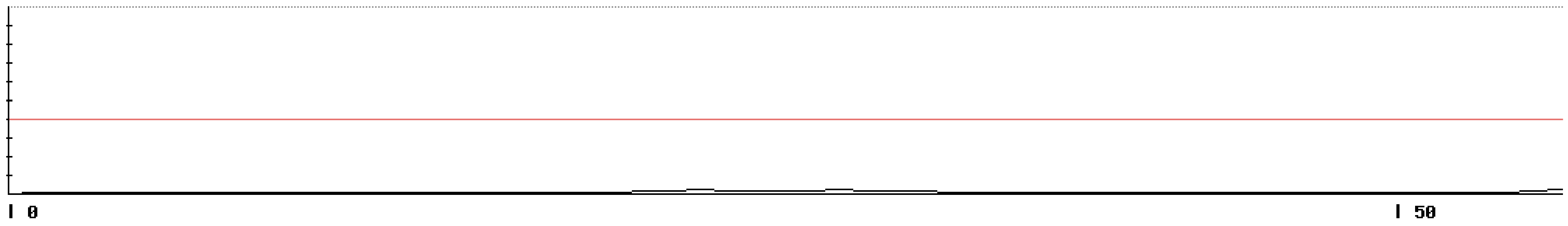

Predictions for Eucalyptuscamaldulensis_EcC054610.70

Viterbi Path

Viterbi Path

Posterior @ 0.4

Posterior @ 0.4

Posterior Graphic. Threshold Marked @ 0.4

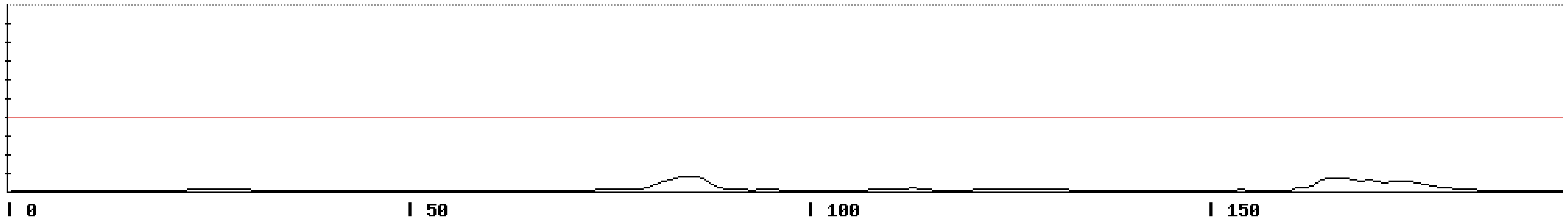

Predictions for Eucalyptuscamaldulensis_EcC054635.10

Viterbi Path

Viterbi Path

Posterior @ 0.4

Posterior @ 0.4

Posterior Graphic. Threshold Marked @ 0.4

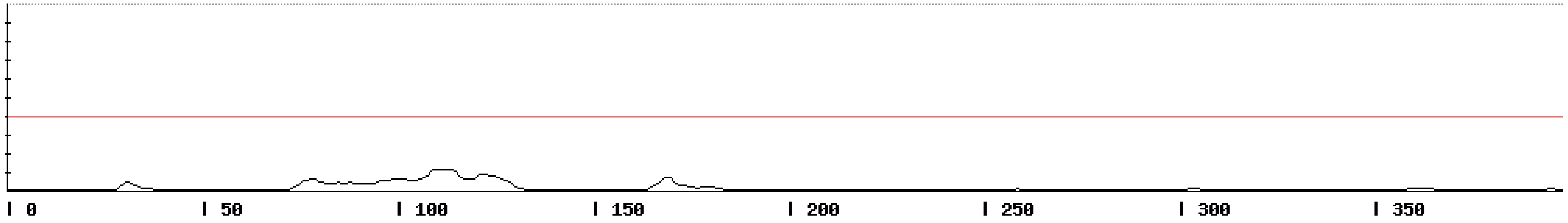

Predictions for Eucalyptuscamaldulensis_EcC054759.130

Viterbi Path

Viterbi Path

Posterior @ 0.4

Posterior @ 0.4

Posterior Graphic. Threshold Marked @ 0.4

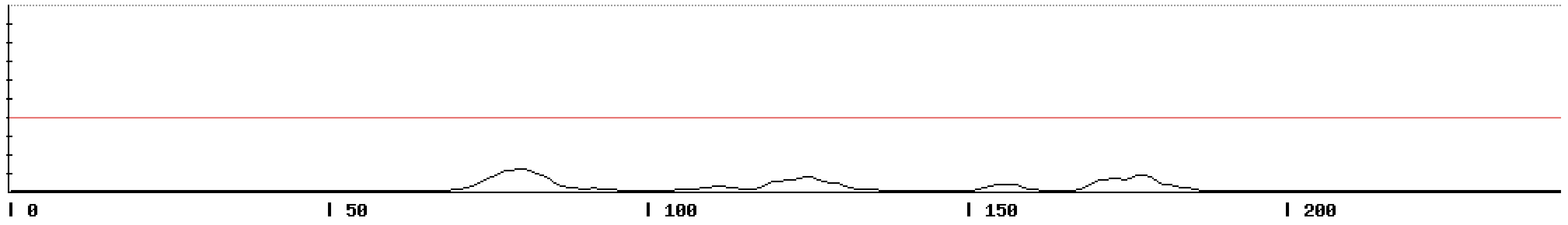

Predictions for Eucalyptuscamaldulensis_EcC054759.90

Viterbi Path

Viterbi Path

Posterior @ 0.4

Posterior @ 0.4

Posterior Graphic. Threshold Marked @ 0.4

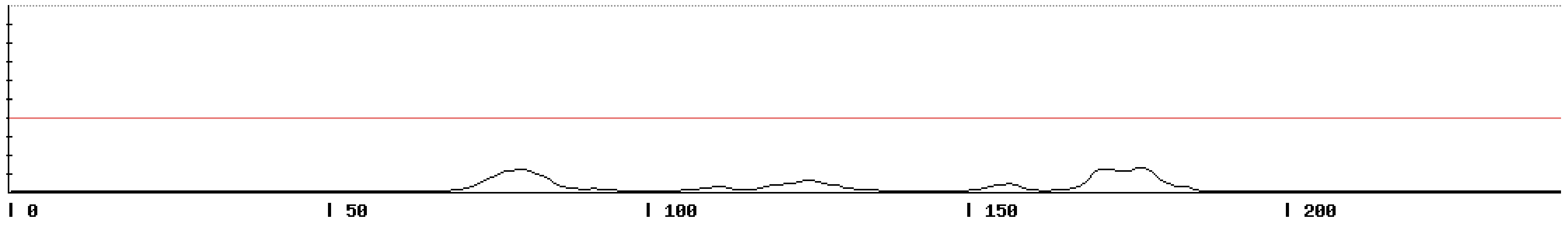

Predictions for Eucalyptuscamaldulensis_EcC054820.310

Viterbi Path

Viterbi Path

Posterior @ 0.4

Posterior @ 0.4

Posterior Graphic. Threshold Marked @ 0.4

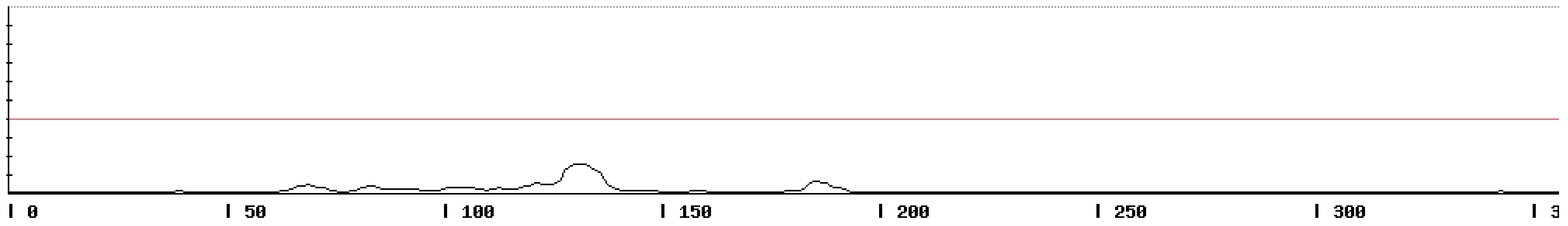

Predictions for Eucalyptuscamaldulensis_EcC054881.10

Viterbi Path

Viterbi Path

Posterior @ 0.4

Posterior @ 0.4

Posterior Graphic. Threshold Marked @ 0.4

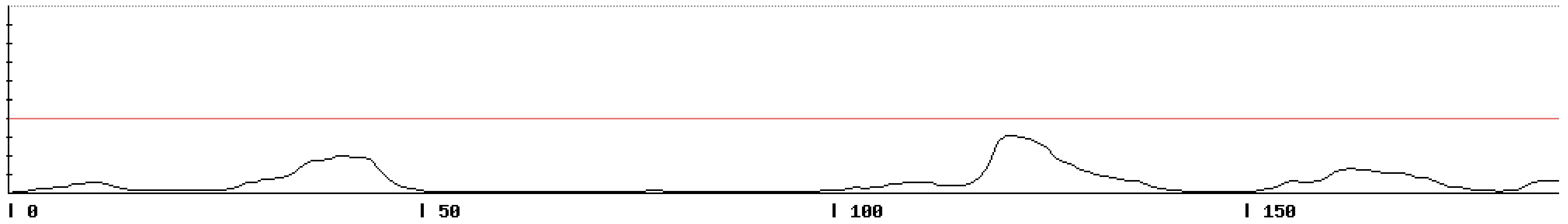

Predictions for Eucalyptuscamaldulensis_EcC054881.20

Viterbi Path	
Viterbi Path	
Posterior @ 0.4	
Posterior @ 0.4	
Posterior Graphic. Threshold Marked @ 0.4	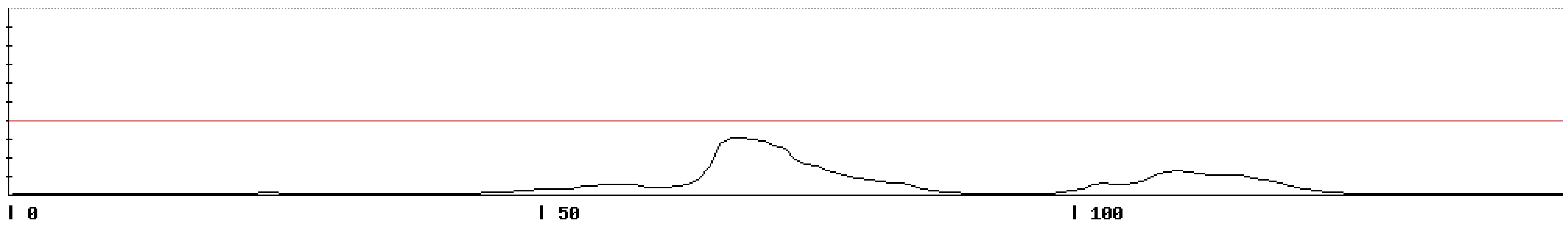

Predictions for Eucalyptuscamaldulensis_EcC054881.30

Viterbi Path	
Viterbi Path	
Posterior @ 0.4	
Posterior @ 0.4	
Posterior Graphic. Threshold Marked @ 0.4	

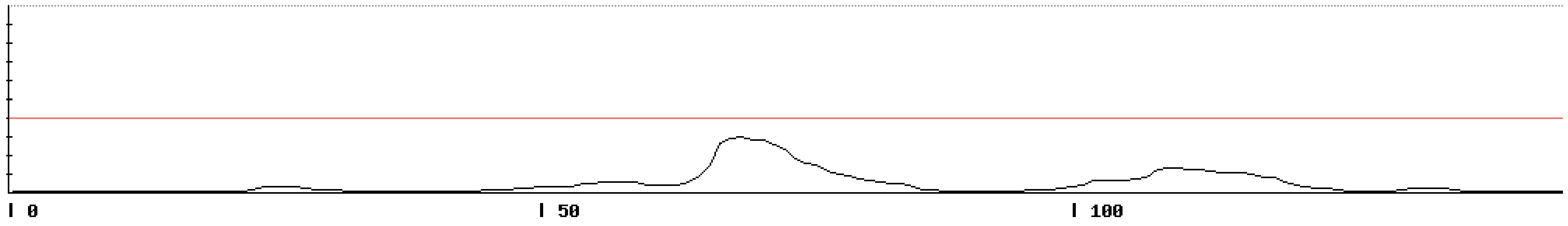

Predictions for Eucalyptuscamaldulensis_EcC054892.20

Viterbi Path

Viterbi Path

Posterior @ 0.4

Posterior @ 0.4

Posterior Graphic. Threshold Marked @ 0.4

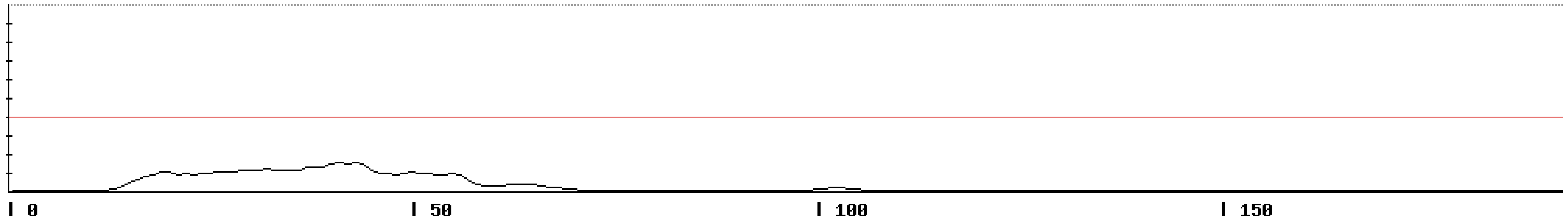

Predictions for Eucalyptuscamaldulensis_EcC054901.60

Viterbi Path

Viterbi Path

Posterior @ 0.4

Posterior @ 0.4

Posterior Graphic. Threshold Marked @ 0.4

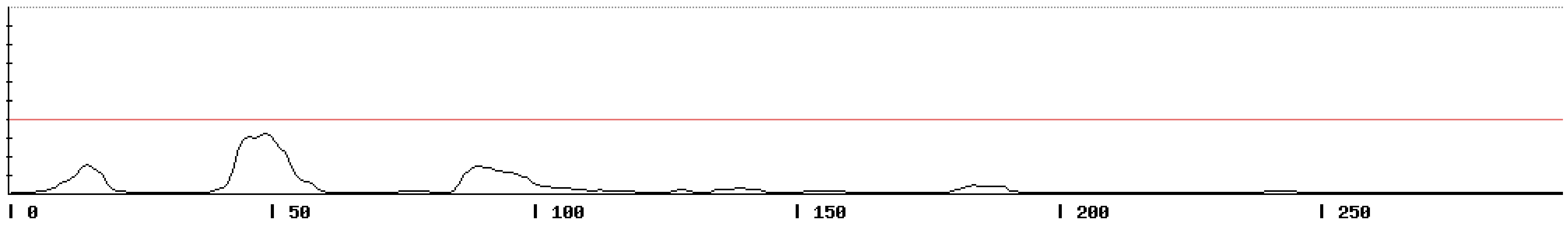

Predictions for Eucalyptuscamaldulensis_EcC054909.250

Viterbi Path

Viterbi Path

Posterior @ 0.4

208 - RHAKQSKNQRRKKAISH - 224

Posterior @ 0.4

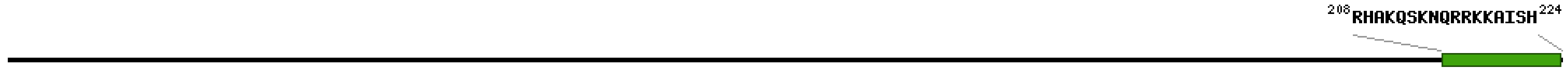

Posterior Graphic. Threshold Marked @ 0.4

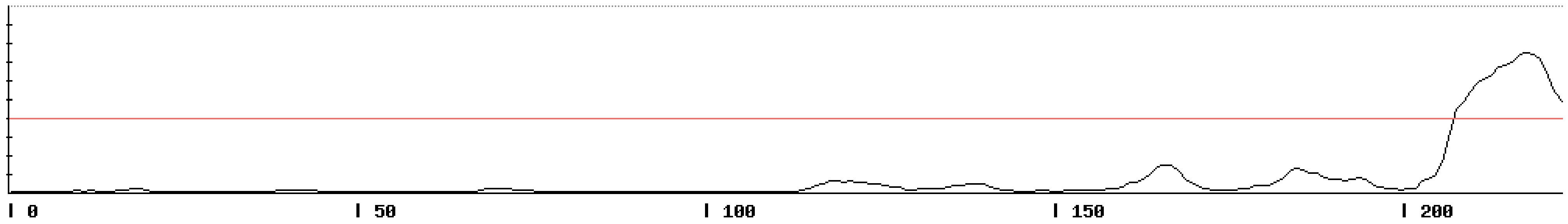

Predictions for Eucalyptuscamaldulensis_EcC054949.10

Viterbi Path

Viterbi Path

Posterior @ 0.4

Posterior @ 0.4

Posterior Graphic. Threshold Marked @ 0.4

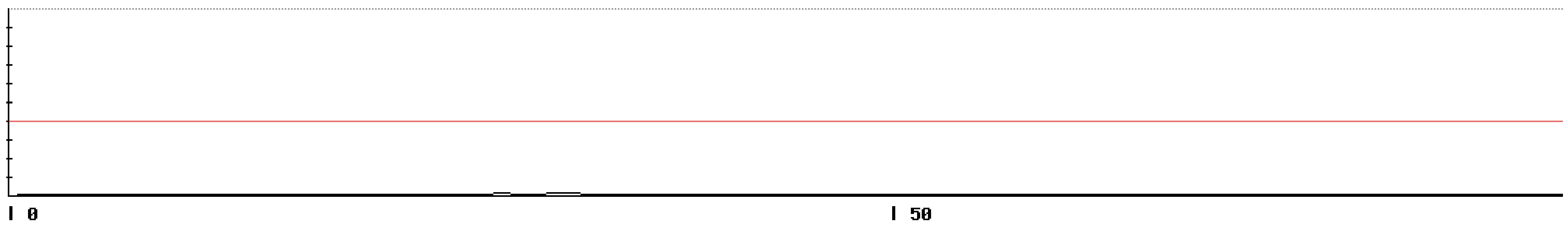

Predictions for Eucalyptuscamaldulensis_EcC055029.30

Viterbi Path

Viterbi Path

Posterior @ 0.4

Posterior @ 0.4

Posterior Graphic. Threshold Marked @ 0.4

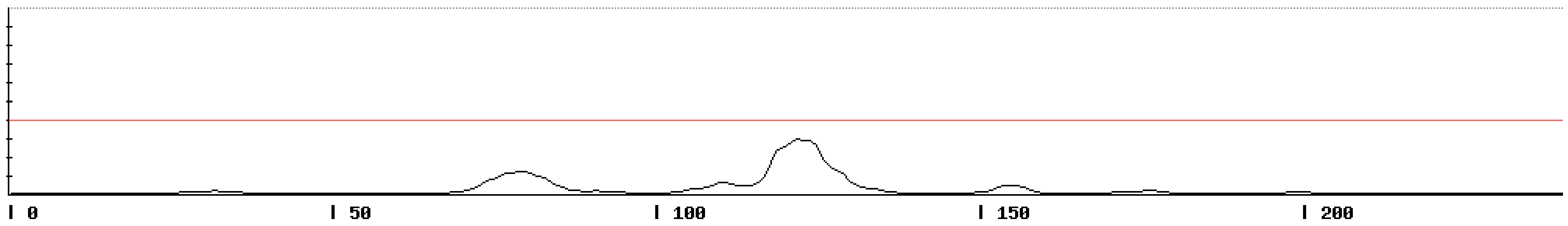

Predictions for Eucalyptuscamaldulensis_EcC055085.10

Viterbi Path

Viterbi Path

Posterior @ 0.4

Posterior @ 0.4

Posterior Graphic. Threshold Marked @ 0.4

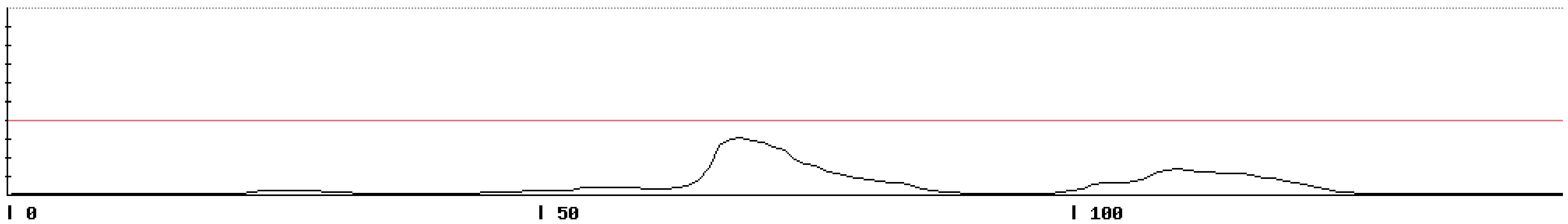

Predictions for Eucalyptuscamaldulensis_EcC055085.30

Viterbi Path

Viterbi Path

Posterior @ 0.4

Posterior @ 0.4

Posterior Graphic. Threshold Marked @ 0.4

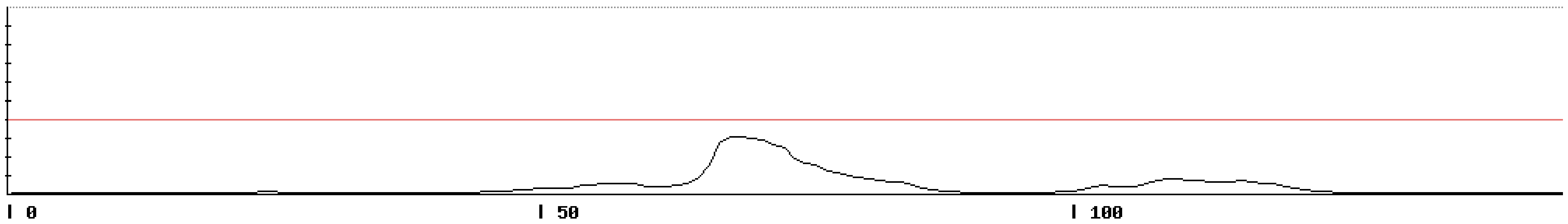

Predictions for Eucalyptuscamaldulensis_EcC055115.80

Viterbi Path

Viterbi Path

Posterior @ 0.4 | 137 - KRR - 139

Posterior @ 0.4

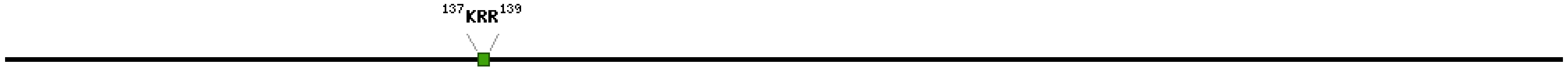

Posterior Graphic. Threshold Marked @ 0.4

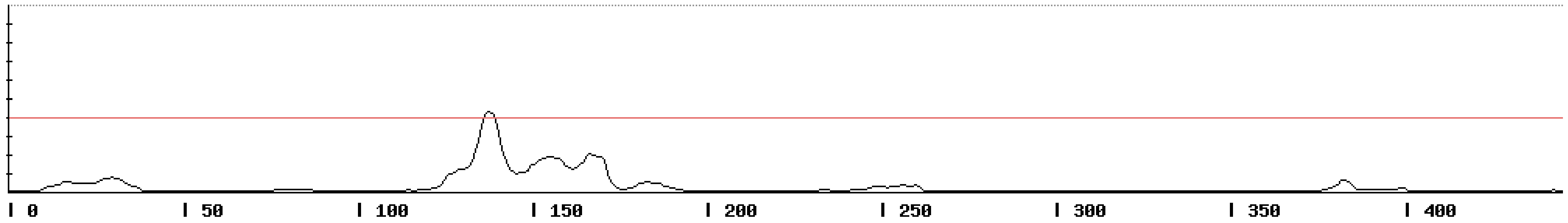

Predictions for Eucalyptuscamaldulensis_EcC055170.30

Viterbi Path

Viterbi Path

Posterior @ 0.4

Posterior @ 0.4

Posterior Graphic. Threshold Marked @ 0.4

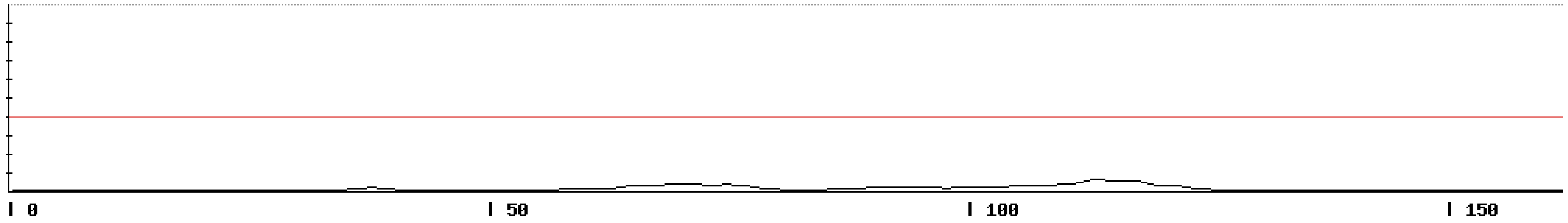

Predictions for Eucalyptuscamaldulensis_EcC055192.60

Viterbi Path

Viterbi Path

Posterior @ 0.4

Posterior @ 0.4

Posterior Graphic. Threshold Marked @ 0.4

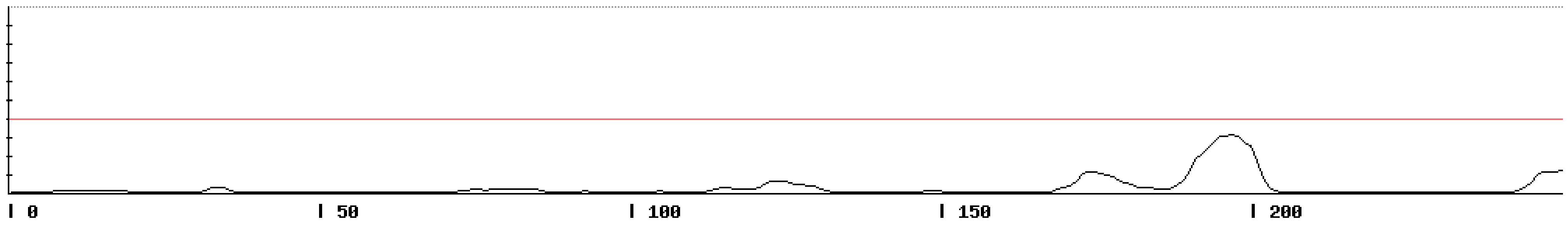

Predictions for Eucalyptuscamaldulensis_EcC055219.60

Viterbi Path

Viterbi Path

Posterior @ 0.4

Posterior @ 0.4

Posterior Graphic. Threshold Marked @ 0.4

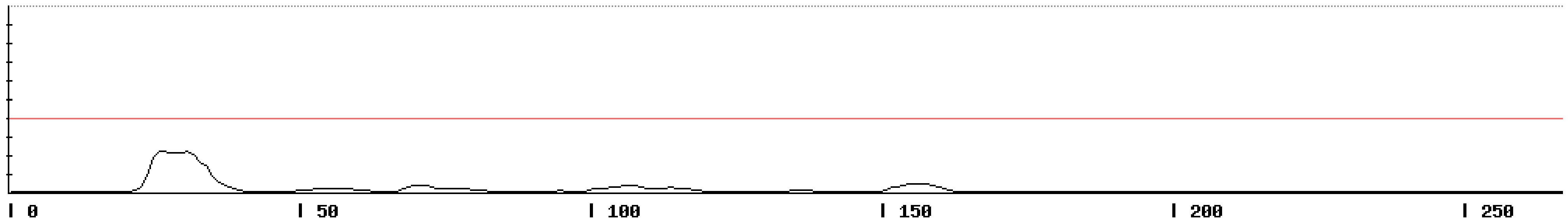

Predictions for Eucalyptuscamaldulensis_EcC064305.10	
Viterbi Path	
Viterbi Path	
Posterior @ 0.4	
Posterior @ 0.4	
Posterior Graphic. Threshold Marked @ 0.4	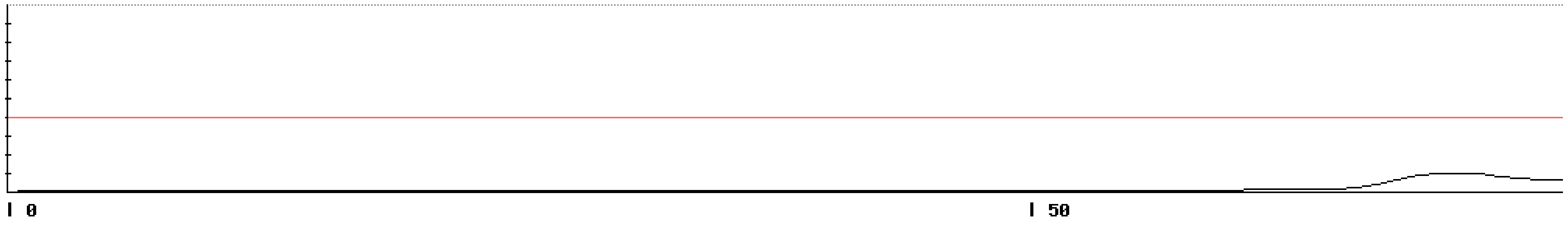

Predictions for Eucalyptuscamaldulensis_EcC065589.10	
Viterbi Path	
Viterbi Path	
Posterior @ 0.4	94 - KHAKHSKNGRKS - 105
Posterior @ 0.4	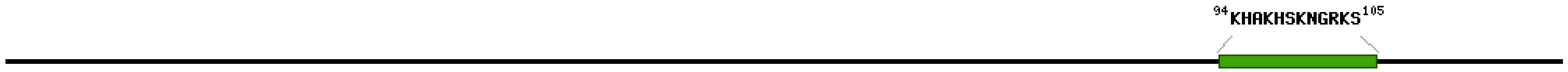
Posterior Graphic. Threshold Marked @ 0.4	

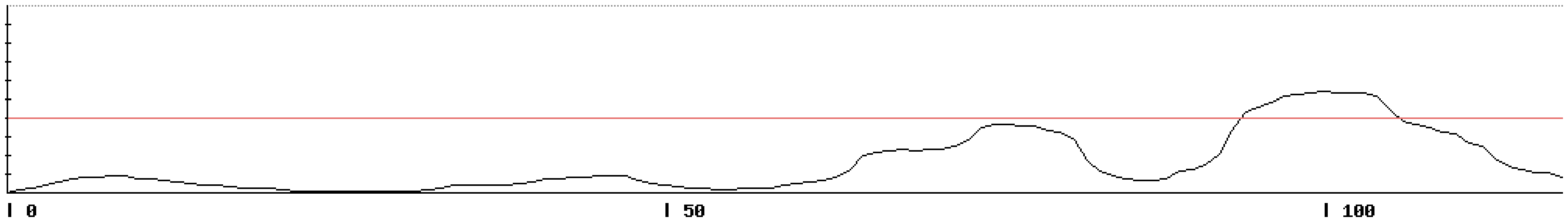

Predictions for Eucalyptuscamaldulensis_EcC073358.10	
Viterbi Path	
Viterbi Path	
Posterior @ 0.4	
Posterior @ 0.4	
Posterior Graphic. Threshold Marked @ 0.4	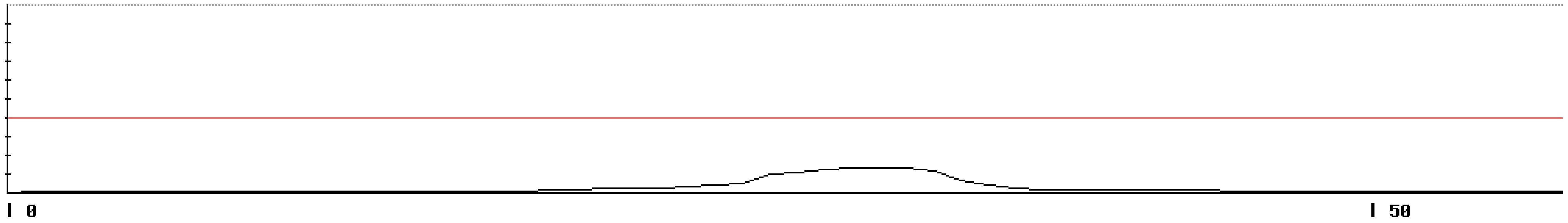

Predictions for Eucalyptuscamaldulensis_EcC074539.10	
Viterbi Path	
Viterbi Path	
Posterior @ 0.4	
Posterior @ 0.4	

Posterior Graphic. Threshold Marked @ 0.4	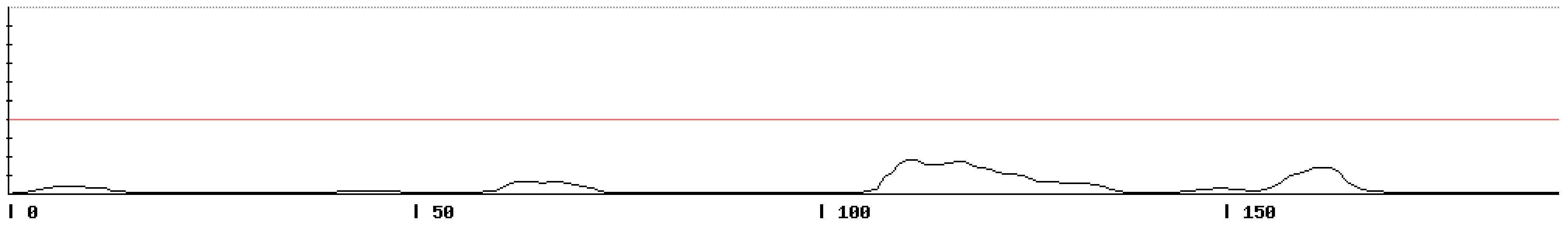

Predictions for Eucalyptuscamaldulensis_EcC074612.10	
Viterbi Path	
Viterbi Path	
Posterior @ 0.4	
Posterior @ 0.4	
Posterior Graphic. Threshold Marked @ 0.4	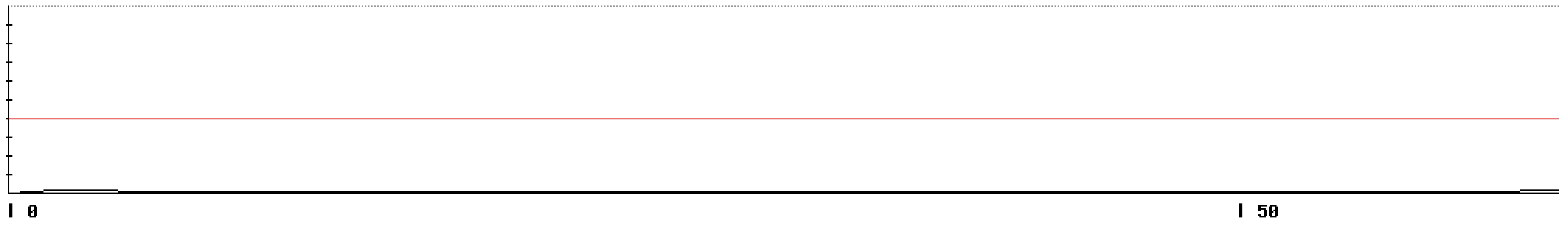

Predictions for Eucalyptuscamaldulensis_EcC080461.10	
Viterbi Path	
Viterbi Path	
Posterior @ 0.4	
Posterior @ 0.4	

Posterior Graphic. Threshold Marked @ 0.4

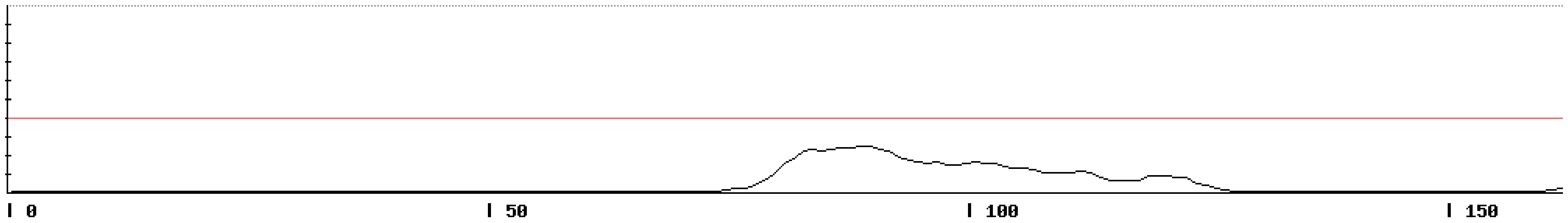

Predictions for Eucalyptuscamaldulensis_EcC081179.10

Viterbi Path

Viterbi Path

Posterior @ 0.4

Posterior @ 0.4

Posterior Graphic. Threshold Marked @ 0.4

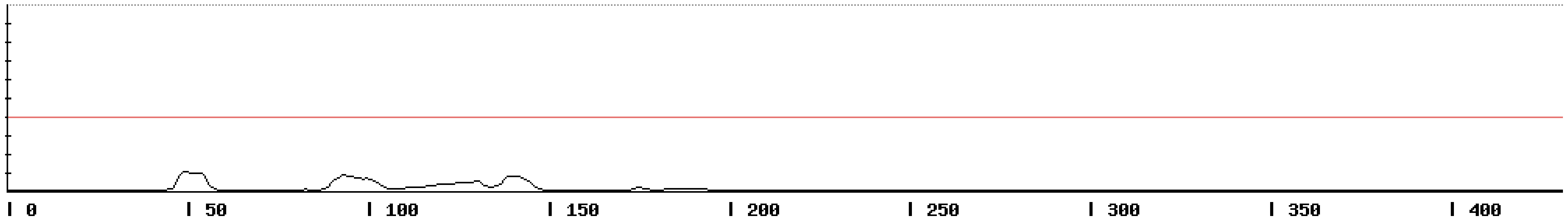

Predictions for Eucalyptuscamaldulensis_EcS504758.10

Viterbi Path

Viterbi Path

Posterior @ 0.4

Posterior @ 0.4

Posterior Graphic. Threshold Marked @ 0.4

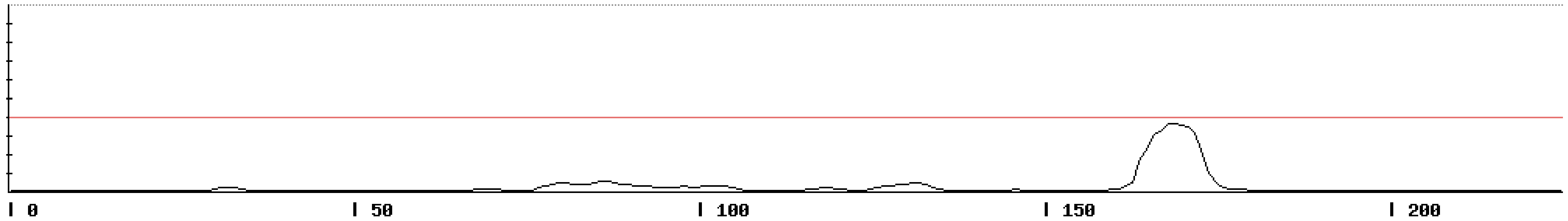

Predictions for Eucalyptuscamaldulensis_EcS504776.10

Viterbi Path

Viterbi Path

Posterior @ 0.4

Posterior @ 0.4

Posterior Graphic. Threshold Marked @ 0.4

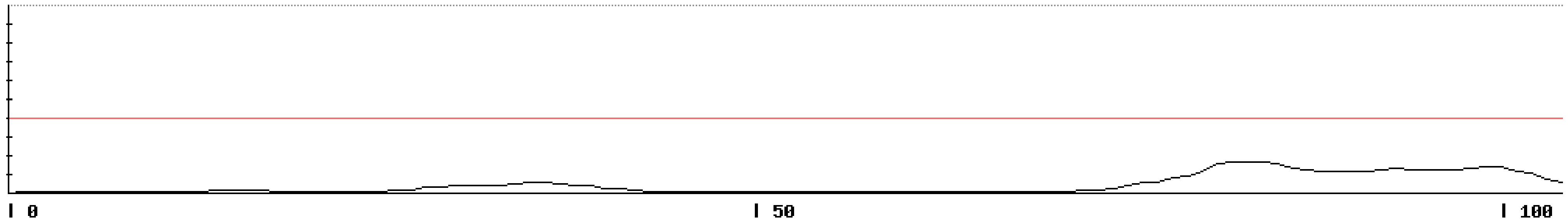

Predictions for Eucalyptuscamaldulensis_EcS505036.10

Viterbi Path

Viterbi Path

Posterior @ 0.4

Posterior @ 0.4

Posterior Graphic. Threshold Marked @ 0.4

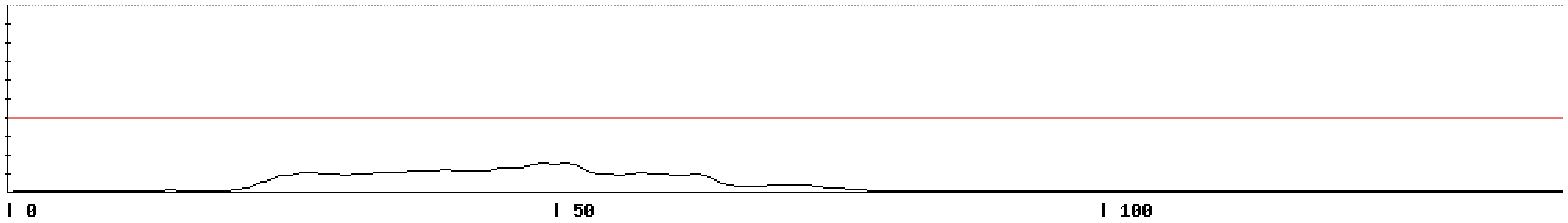

Predictions for Eucalyptuscamaldulensis_EcS506387.10

Viterbi Path

Viterbi Path

Posterior @ 0.4

Posterior @ 0.4

Posterior Graphic. Threshold Marked @ 0.4

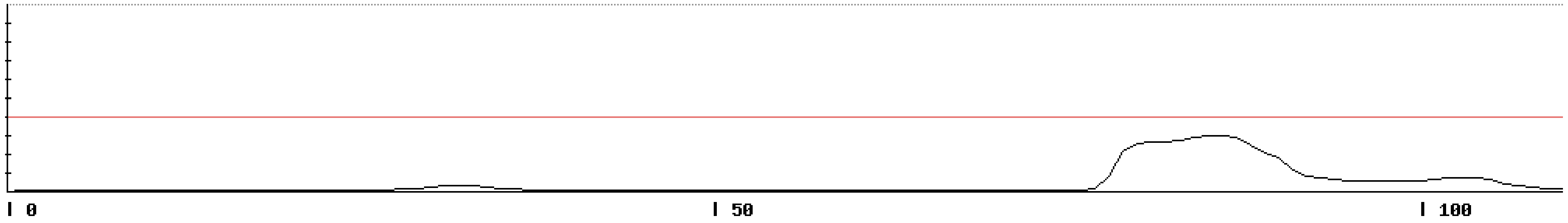

Predictions for Eucalyptuscamaldulensis_EcS516652.10

Viterbi Path

Viterbi Path

Posterior @ 0.4

Posterior @ 0.4

Posterior Graphic. Threshold Marked @ 0.4

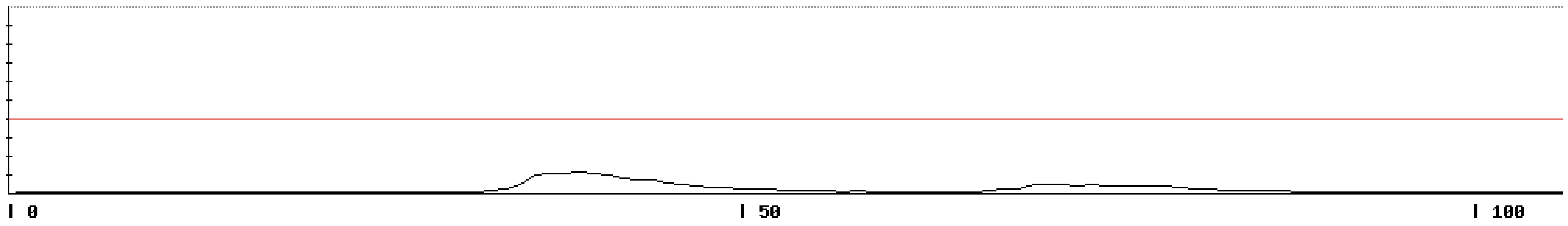

Predictions for Eucalyptuscamaldulensis_EcS531726.10

Viterbi Path

Viterbi Path

Posterior @ 0.4

Posterior @ 0.4

Posterior Graphic. Threshold Marked @ 0.4

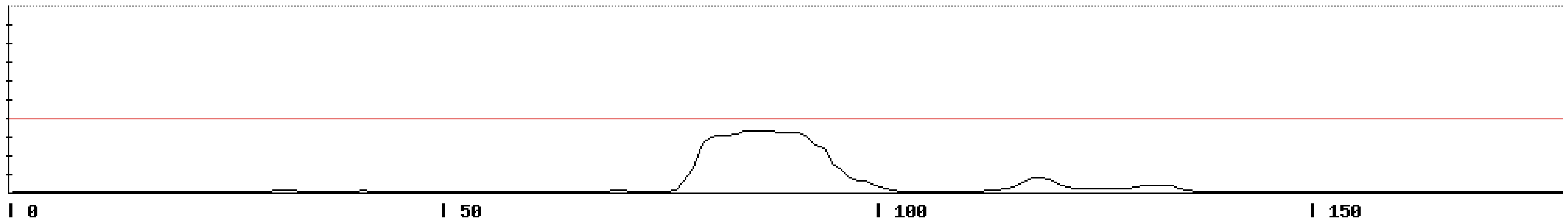

Predictions for Eucalyptuscamaldulensis_EcS534536.10

Viterbi Path

Viterbi Path

Posterior @ 0.4

Posterior @ 0.4

Posterior Graphic. Threshold Marked @ 0.4

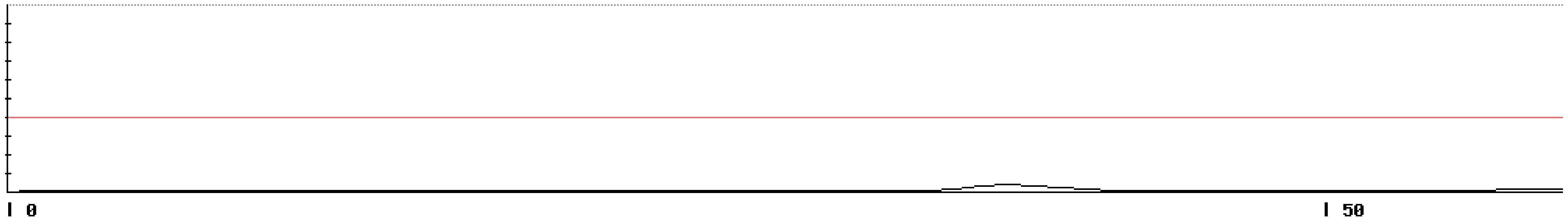

Predictions for Eucalyptuscamaldulensis_EcS542481.10

Viterbi Path

Viterbi Path

Posterior @ 0.4

Posterior @ 0.4

Posterior Graphic. Threshold Marked @ 0.4

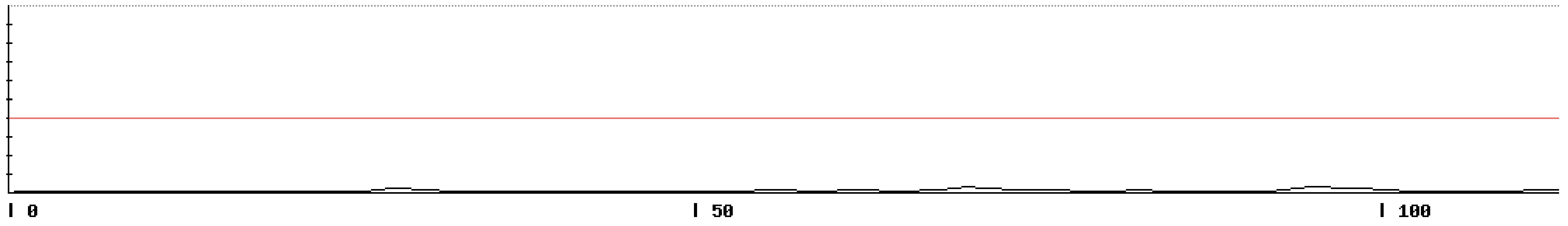

Predictions for Eucalyptuscamaldulensis_EcS561877.10

Viterbi Path	
Viterbi Path	
Posterior @ 0.4	
Posterior @ 0.4	
Posterior Graphic. Threshold Marked @ 0.4	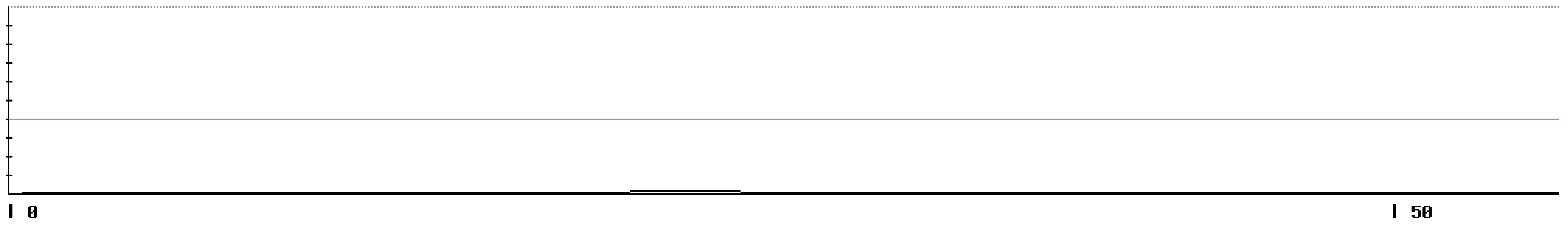

Predictions for Eucalyptuscamaldulensis_EcS577108.10

Viterbi Path	
Viterbi Path	
Posterior @ 0.4	36 - PAKAYATGTRKRRRVHKSADERETRWHKTGKTRSVLAGRVVK - 77
Posterior @ 0.4	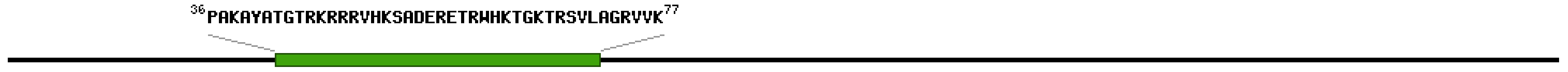

Posterior Graphic. Threshold Marked @ 0.4

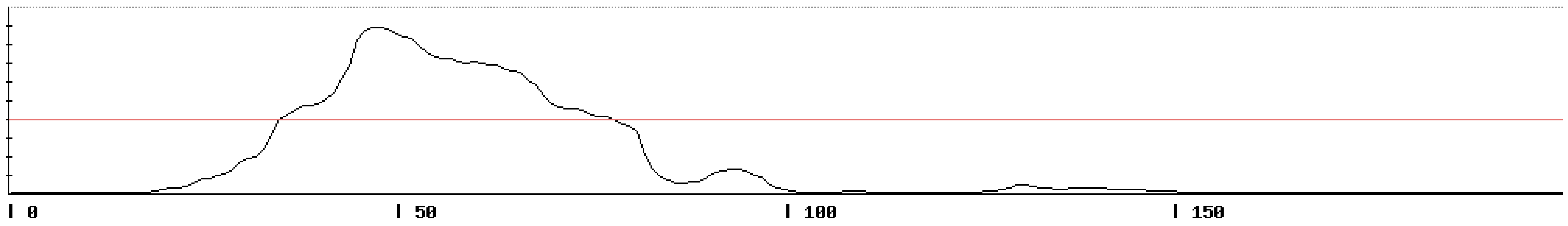

Predictions for Eucalyptuscamaldulensis_EcS581349.10

Viterbi Path

Viterbi Path

Posterior @ 0.4

Posterior @ 0.4

Posterior Graphic. Threshold Marked @ 0.4

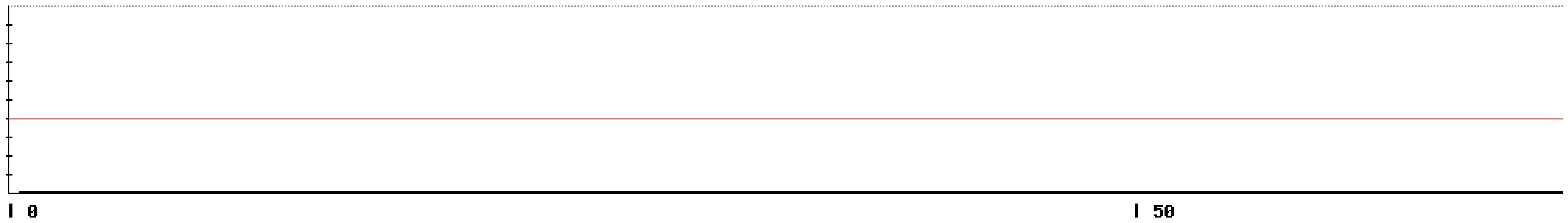

Predictions for Eucalyptuscamaldulensis_EcS582569.10

Viterbi Path

Viterbi Path

Posterior @ 0.4

Posterior @ 0.4

Posterior Graphic. Threshold Marked @ 0.4

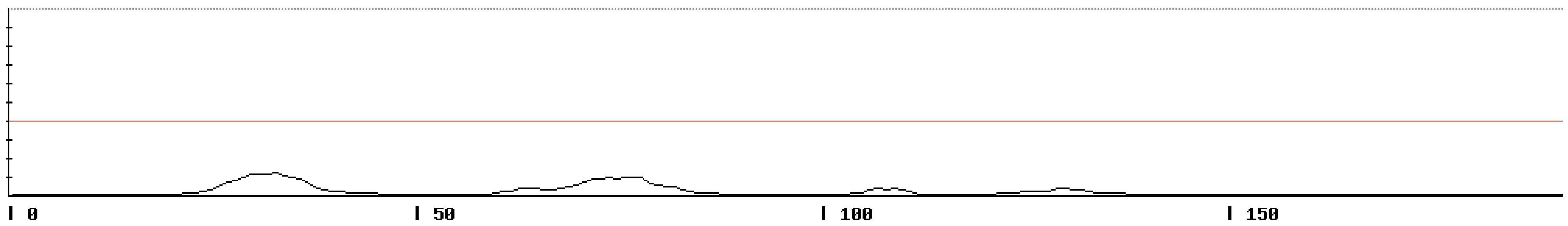

Predictions for Eucalyptuscamaldulensis_EcS628566.10

Viterbi Path

Viterbi Path

Posterior @ 0.4

Posterior @ 0.4

Posterior Graphic. Threshold Marked @ 0.4

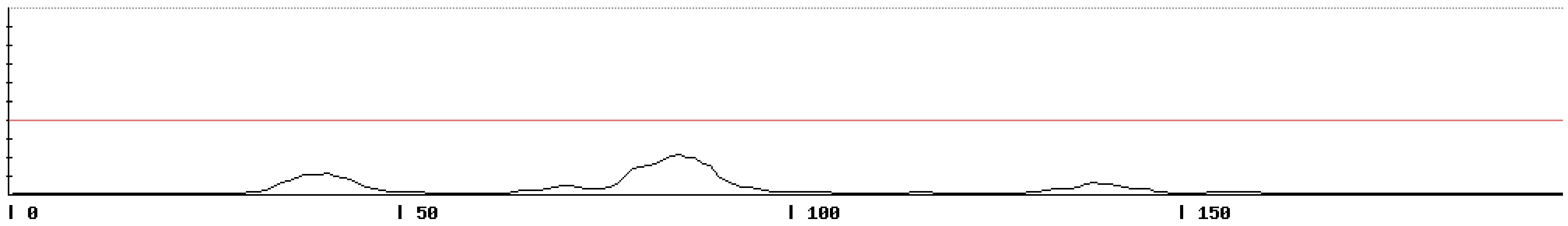

Predictions for Eucalyptuscamaldulensis_EcS632222.10

Viterbi Path

Viterbi Path

Posterior @ 0.4	
Posterior @ 0.4	
Posterior Graphic. Threshold Marked @ 0.4	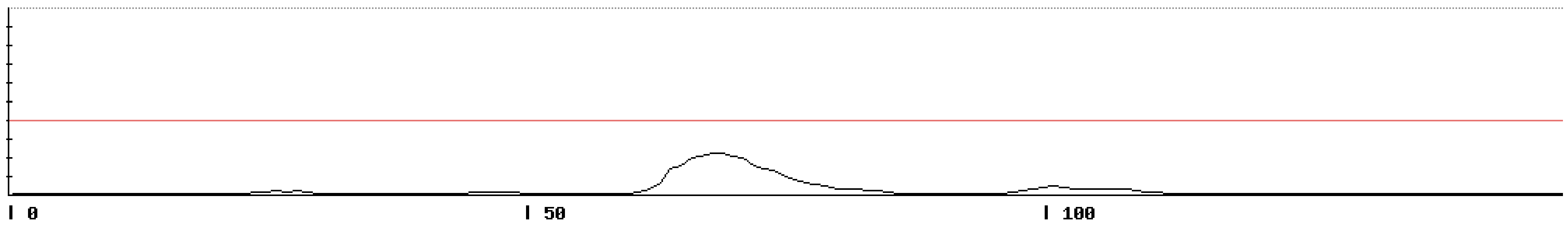

Predictions for Eucalyptuscamaldulensis_EcS658905.10	
Viterbi Path	
Viterbi Path	
Posterior @ 0.4	
Posterior @ 0.4	
Posterior Graphic. Threshold Marked @ 0.4	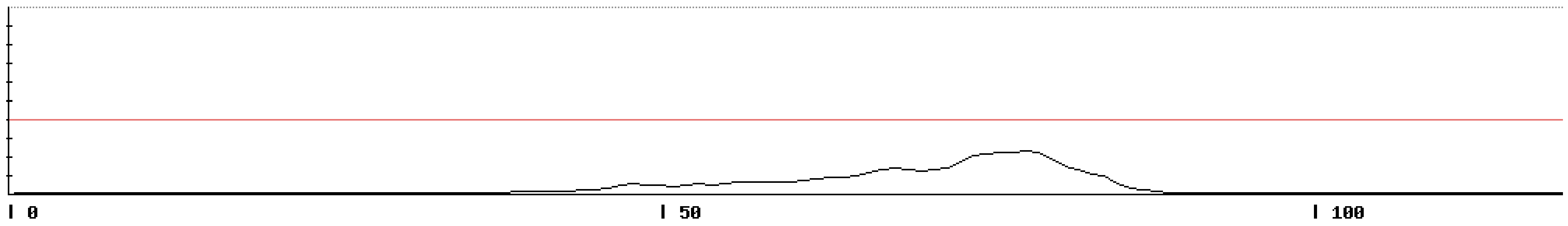

Predictions for Eucalyptuscamaldulensis_EcS701937.10	
Viterbi Path	
Viterbi Path	

Posterior @ 0.4	
Posterior @ 0.4	
Posterior Graphic. Threshold Marked @ 0.4	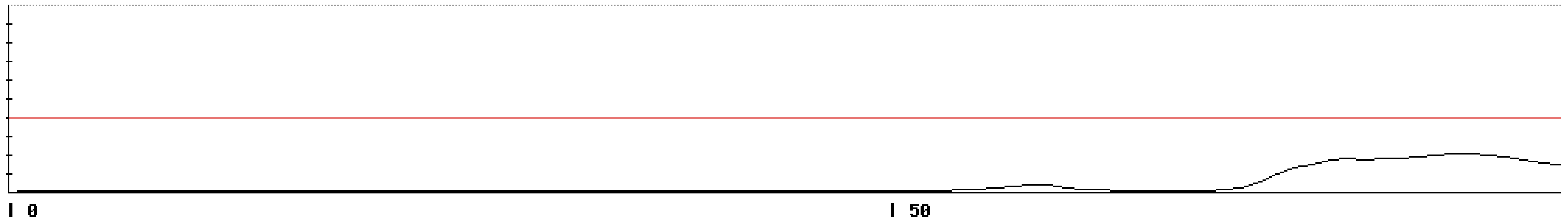

Predictions for Eucalyptuscamaldulensis_EcS702709.10

Viterbi Path	
Viterbi Path	
Posterior @ 0.4	
Posterior @ 0.4	
Posterior Graphic. Threshold Marked @ 0.4	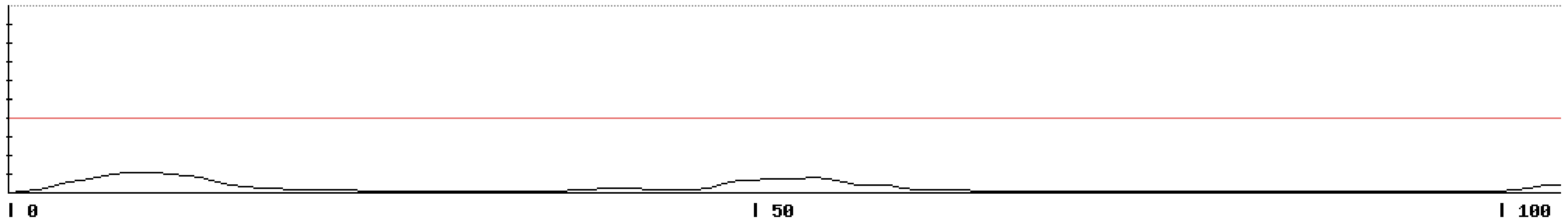

Predictions for Eucalyptuscamaldulensis_EcS720278.10

Viterbi Path

Viterbi Path

Posterior @ 0.4

Posterior @ 0.4

Posterior Graphic. Threshold Marked @ 0.4

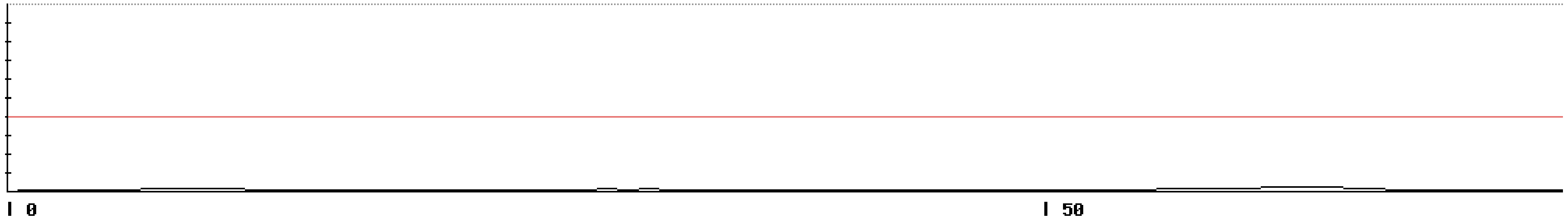

Predictions for Eucalyptuscamaldulensis_EcS722800.10

Viterbi Path

Viterbi Path

Posterior @ 0.4

90 - RR - 91

95 - HGSWKPTGENRKIRTQDKKKILGLKKILVFKKH - 127

Posterior @ 0.4

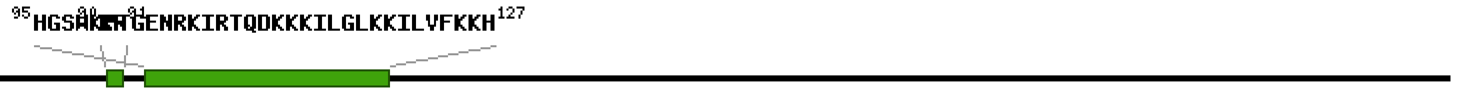

Posterior Graphic. Threshold Marked @ 0.4

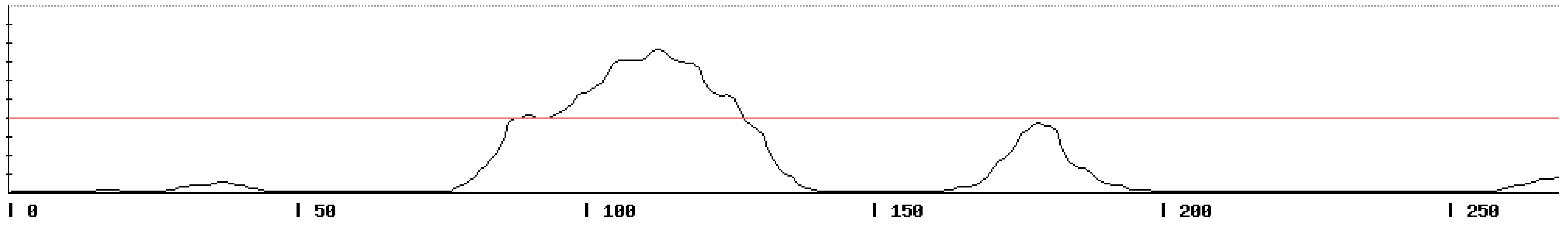

Predictions for Eucalyptuscamaldulensis_EcS722803.10

Viterbi Path

Viterbi Path

Posterior @ 0.4

Posterior @ 0.4

Posterior Graphic. Threshold Marked @ 0.4

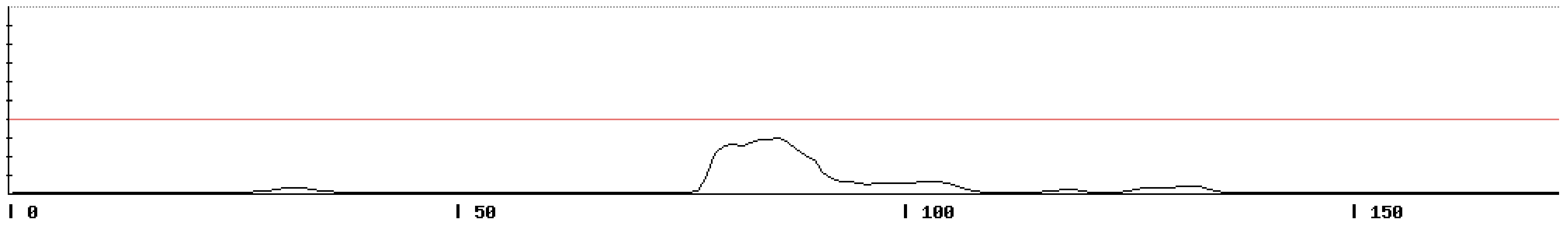

Predictions for Eucalyptuscamaldulensis_EcS724721.10

Viterbi Path

Viterbi Path

Posterior @ 0.4

17 - RGRKRKRK - 24

Posterior @ 0.4

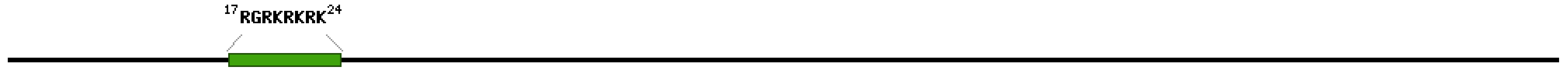

Posterior Graphic. Threshold Marked @ 0.4

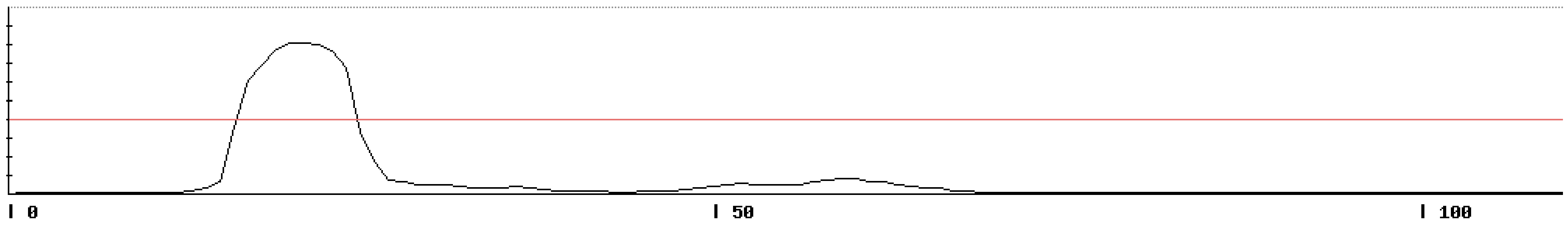

Predictions for Eucalyptuscamaldulensis_EcS724722.10

Viterbi Path

Viterbi Path

Posterior @ 0.4 76 - RGRKRKRK - 83

Posterior @ 0.4

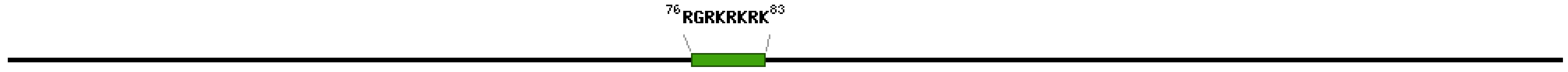

Posterior Graphic. Threshold Marked @ 0.4

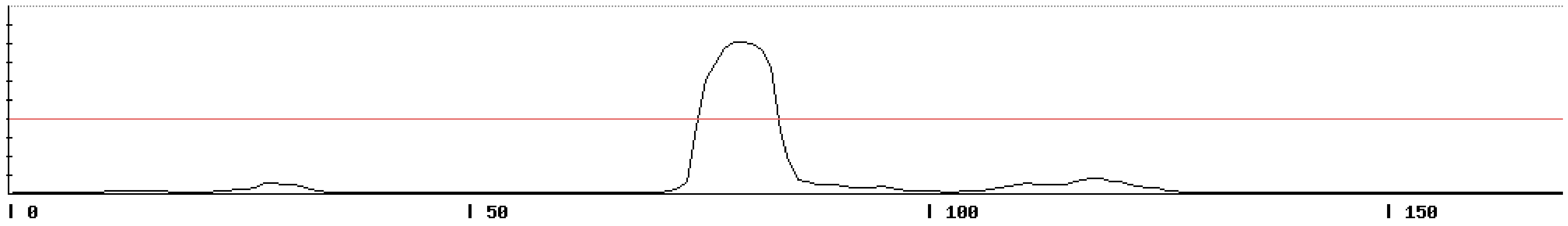

Predictions for Eucalyptuscamaldulensis_EcS725039.10

Viterbi Path

Viterbi Path

Posterior @ 0.4

Posterior @ 0.4

Posterior Graphic. Threshold Marked @ 0.4	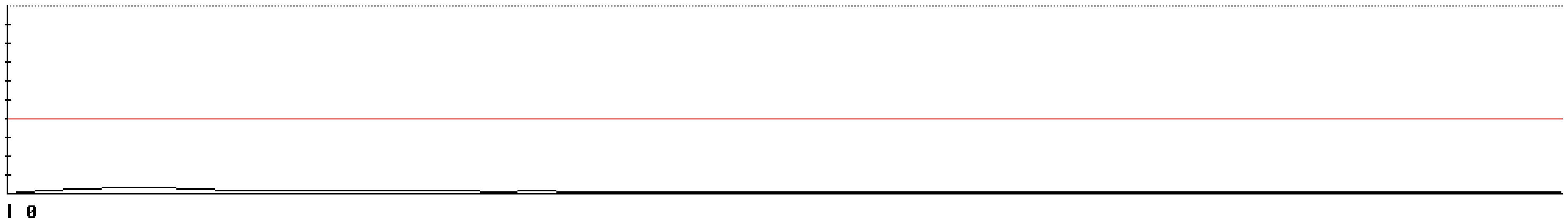

Predictions for Eucalyptuscamaldulensis_EcS732568.10

Viterbi Path	
Viterbi Path	
Posterior @ 0.4	
Posterior @ 0.4	
Posterior Graphic. Threshold Marked @ 0.4	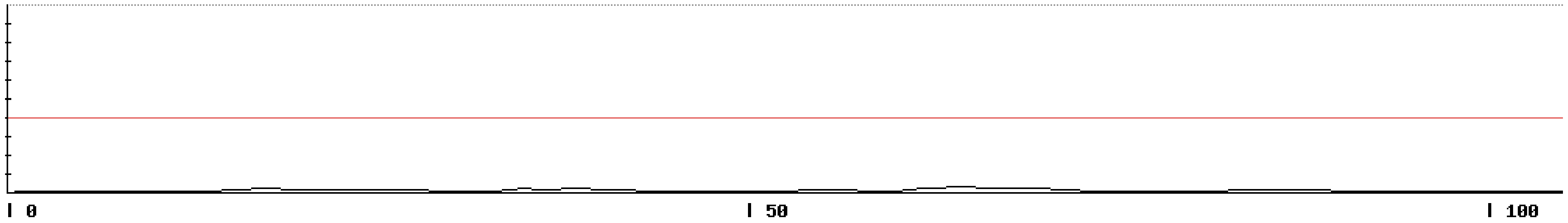

Predictions for Eucalyptuscamaldulensis_EcS732569.10

Viterbi Path	
Viterbi Path	

Posterior @ 0.4

Posterior @ 0.4

Posterior Graphic. Threshold Marked @ 0.4

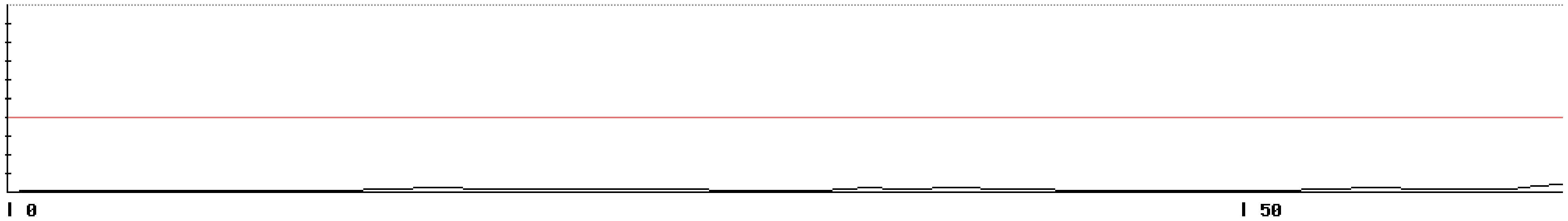

Predictions for Eucalyptuscamaldulensis_EcS734564.10

Viterbi Path

Viterbi Path

Posterior @ 0.4

Posterior @ 0.4

Posterior Graphic. Threshold Marked @ 0.4

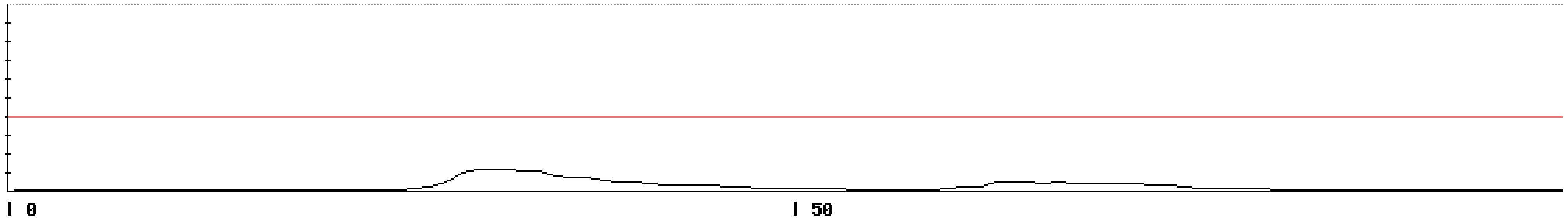

Predictions for Eucalyptuscamaldulensis_EcS738470.10

Viterbi Path

Viterbi Path

Posterior @ 0.4	
Posterior @ 0.4	
Posterior Graphic. Threshold Marked @ 0.4	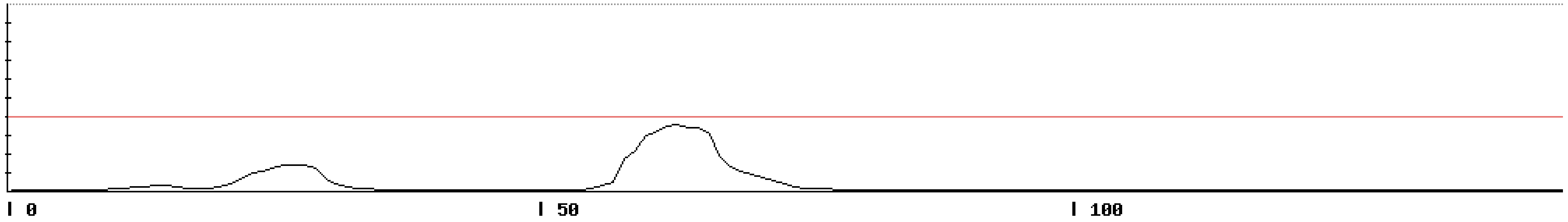

Predictions for Eucalyptuscamaldulensis_EcS744841.10	
Viterbi Path	
Viterbi Path	
Posterior @ 0.4	
Posterior @ 0.4	
Posterior Graphic. Threshold Marked @ 0.4	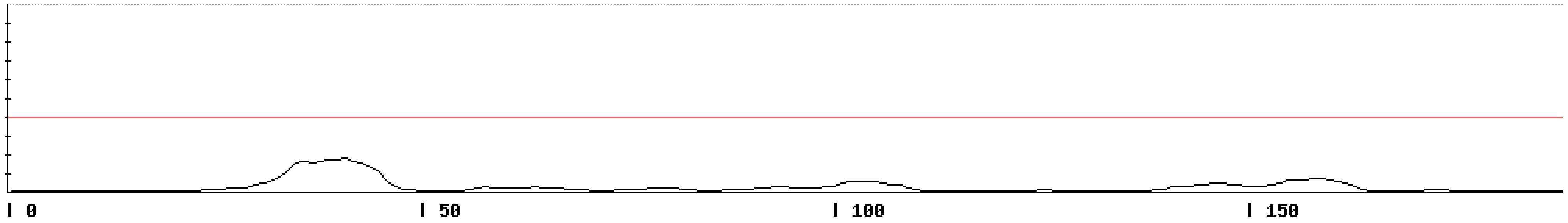

Predictions for Eucalyptuscamaldulensis_EcS751831.10

Viterbi Path

Viterbi Path

Posterior @ 0.4

Posterior @ 0.4

Posterior Graphic. Threshold Marked @ 0.4

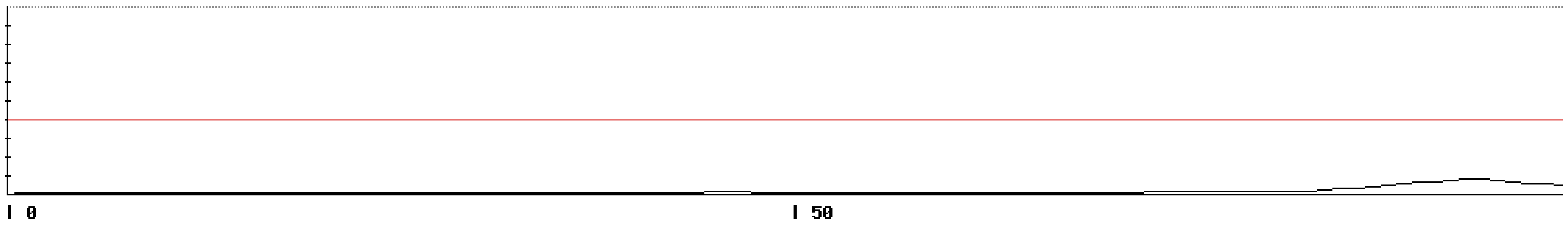

Predictions for Eucalyptuscamaldulensis_EcS754350.20

Viterbi Path

9 - KFKARKKRARTRESQKKTNRVEKRP - 33

Viterbi Path

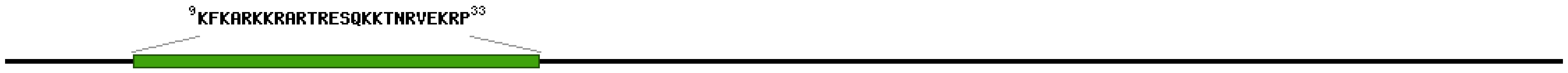

Posterior @ 0.4

6 - ELSKFKARKKRARTRESQKKTNRVEKRP - 33

Posterior @ 0.4

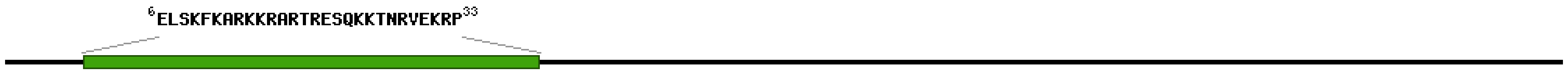

Posterior Graphic. Threshold Marked @ 0.4

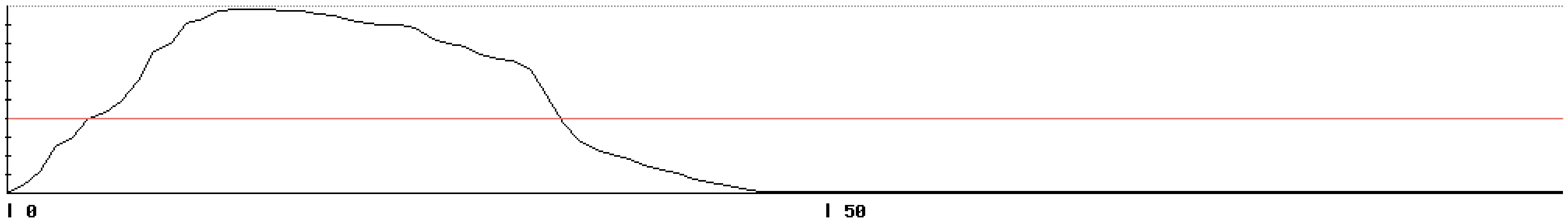

Predictions for Eucalyptuscamaldulensis_EcS756720.10	
Viterbi Path	
Viterbi Path	
Posterior @ 0.4	
Posterior @ 0.4	
Posterior Graphic. Threshold Marked @ 0.4	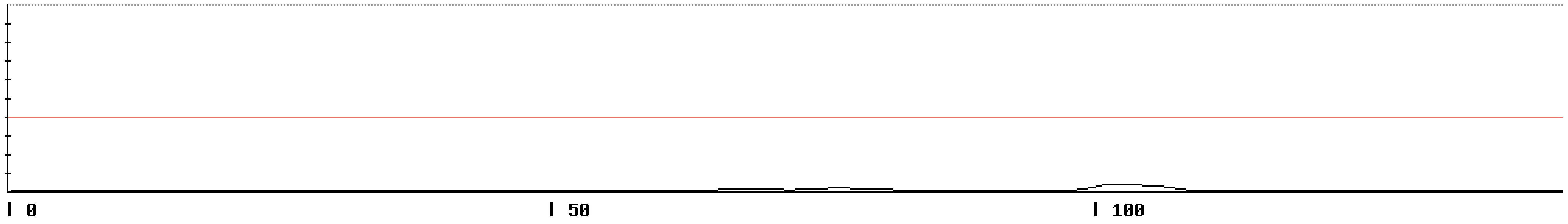

Predictions for Eucalyptuscamaldulensis_EcS756885.10	
Viterbi Path	
Viterbi Path	
Posterior @ 0.4	
Posterior @ 0.4	
Posterior Graphic. Threshold Marked @ 0.4	

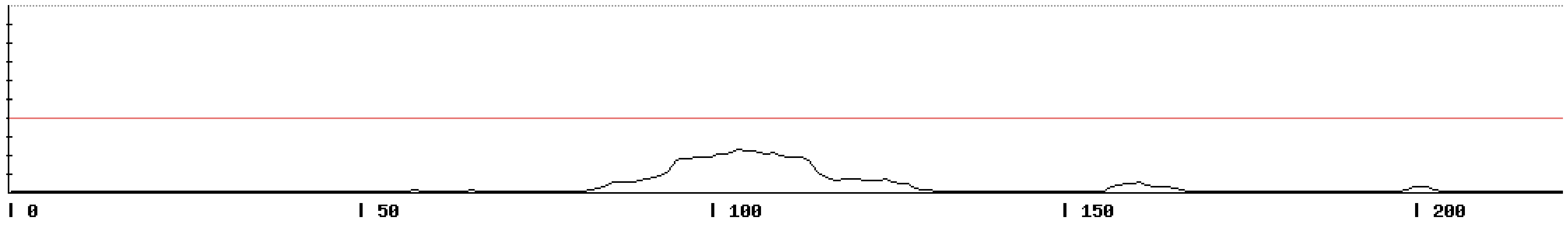

Predictions for Eucalyptuscamaldulensis_EcS756886.10

Viterbi Path

Viterbi Path

Posterior @ 0.4

Posterior @ 0.4

Posterior Graphic. Threshold Marked @ 0.4

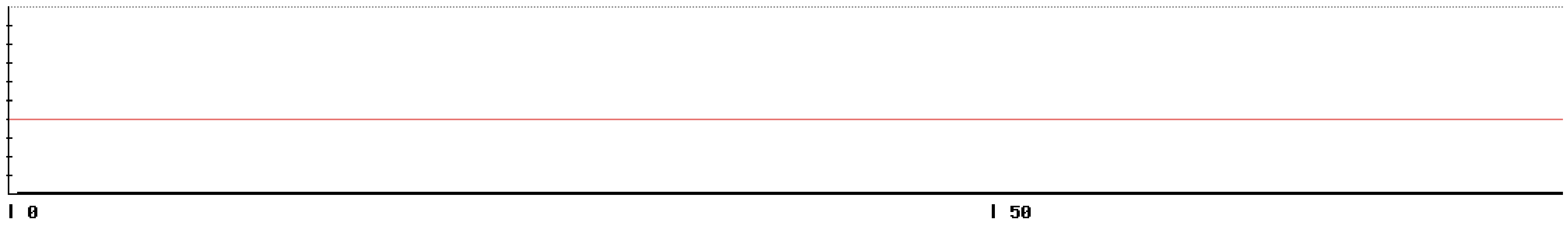

Predictions for Eucalyptuscamaldulensis_EcS760789.10

Viterbi Path

Viterbi Path

Revision r.9 - Last Updated on : February 24 2015 17:45:20.

alex.nguyenba@utoronto.ca

Intended to be used with Firefox

Viterbi Path	
Posterior @ 0.4	100 - PTGKTHKIRTQDKKNTLGLKKILVFKKRQSPK - 131
Posterior @ 0.4	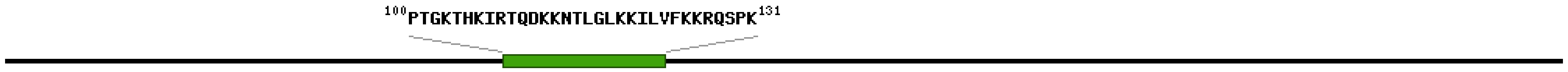
Posterior Graphic. Threshold Marked @ 0.4	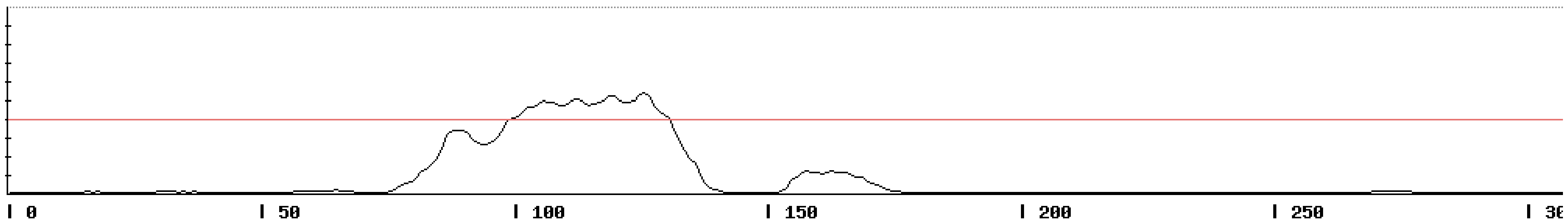

Predictions for Eucalyptusgrandis_Eucgr.A00359.1.p

Viterbi Path	
Viterbi Path	
Posterior @ 0.4	
Posterior @ 0.4	
Posterior Graphic. Threshold Marked @ 0.4	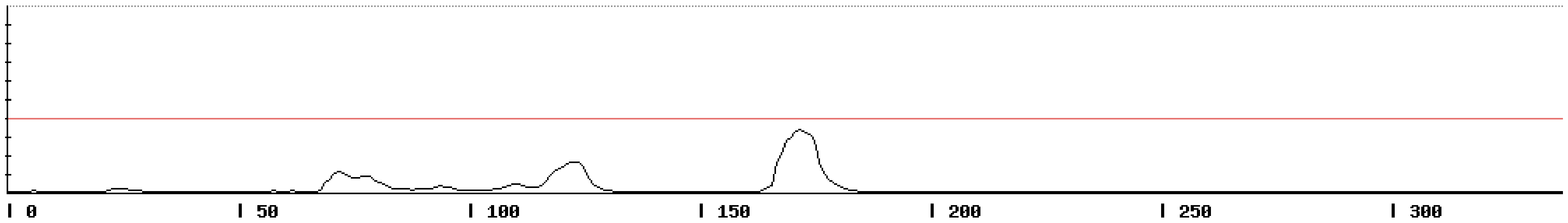

Predictions for Eucalyptusgrandis_Eucgr.A00362.1.p

Viterbi Path	
Viterbi Path	
Posterior @ 0.4	81 - PHK - 83
Posterior @ 0.4	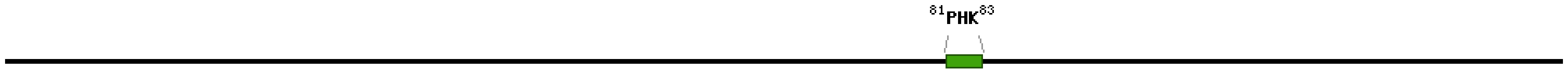
Posterior Graphic. Threshold Marked @ 0.4	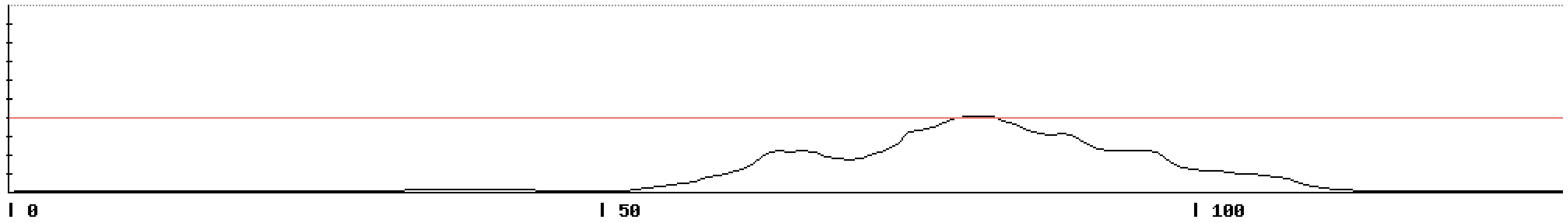

Predictions for Eucalyptusgrandis_Eucgr.A00365.1.p

Viterbi Path	
Viterbi Path	
Posterior @ 0.4	
Posterior @ 0.4	
Posterior Graphic. Threshold Marked @ 0.4	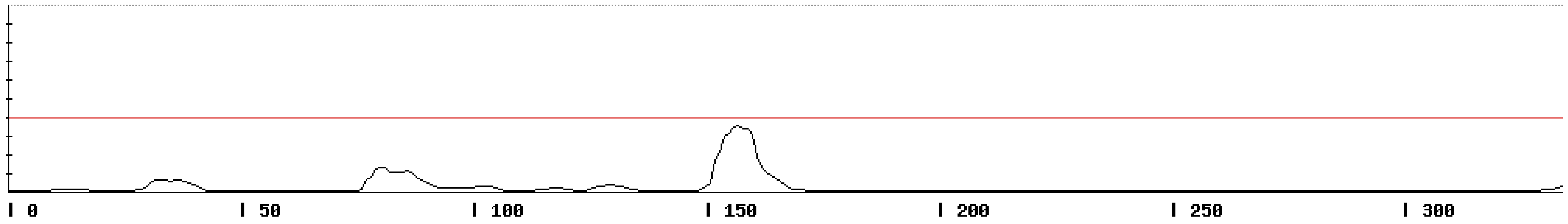

Predictions for Eucalyptusgrandis_Eucgr.A00368.1.p

Viterbi Path	
Viterbi Path	
Posterior @ 0.4	168 - KKDKK - 172
Posterior @ 0.4	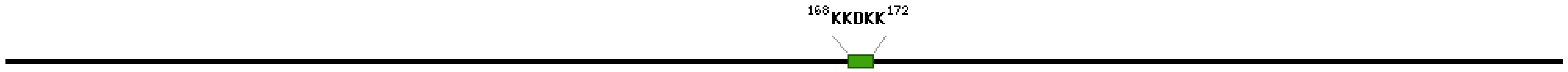
Posterior Graphic. Threshold Marked @ 0.4	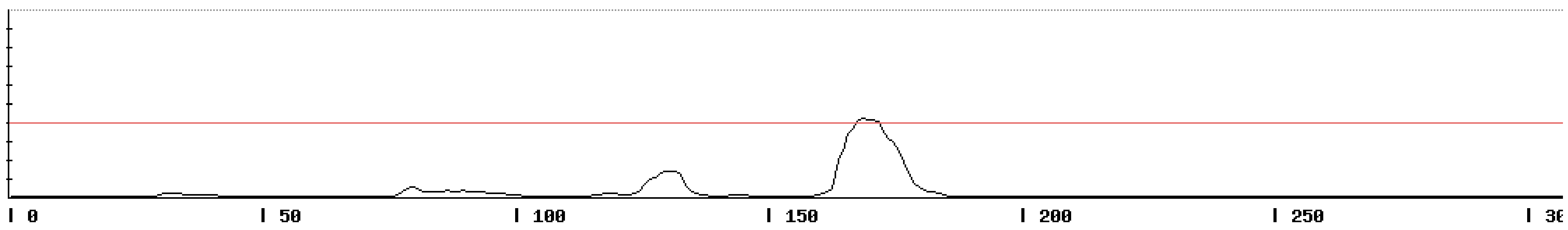

Predictions for Eucalyptusgrandis_Eucgr.A00369.1.p

Viterbi Path	
Viterbi Path	
Posterior @ 0.4	
Posterior @ 0.4	
Posterior Graphic. Threshold Marked @ 0.4	

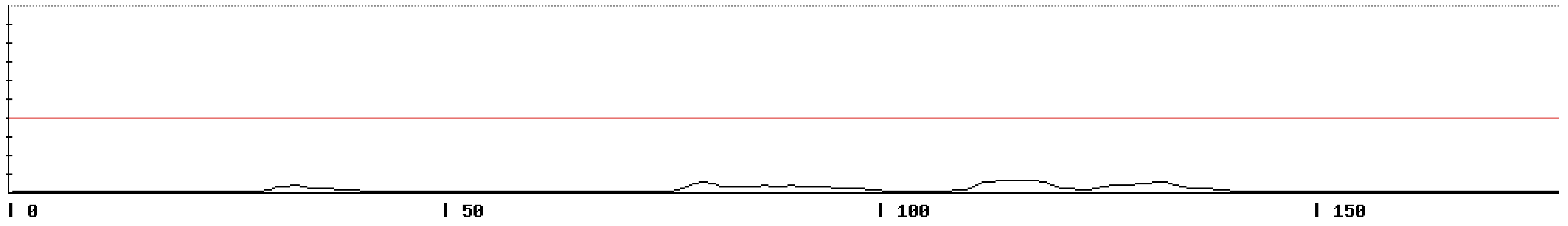

Predictions for Eucalyptusgrandis_Eucgr.A00370.1.p	
Viterbi Path	
Viterbi Path	
Posterior @ 0.4	166 - KHKKDKKLMK - 175
Posterior @ 0.4	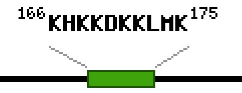
Posterior Graphic. Threshold Marked @ 0.4	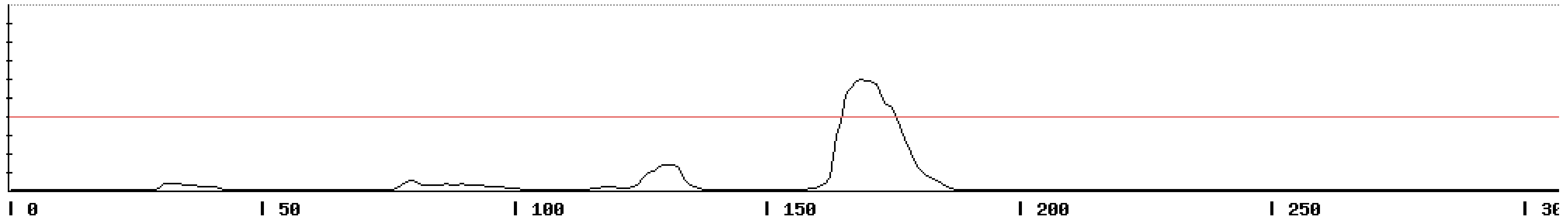

Predictions for Eucalyptusgrandis_Eucgr.A00371.1.p	
Viterbi Path	
Viterbi Path	
Posterior @ 0.4	168 - KKDKKLMKGR - 177
Posterior @ 0.4	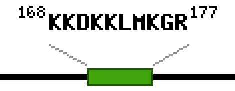

Posterior Graphic. Threshold Marked @ 0.4

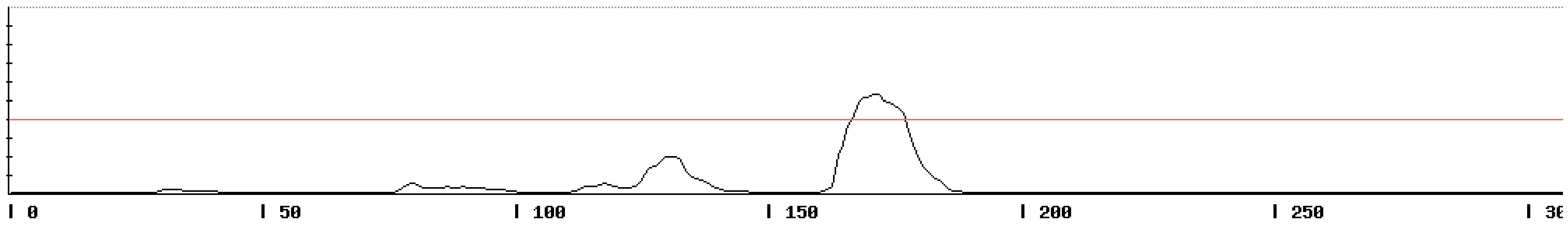

Predictions for Eucalyptusgrandis_Eucgr.A00435.1.p

Viterbi Path

Viterbi Path

Posterior @ 0.4

Posterior @ 0.4

Posterior Graphic. Threshold Marked @ 0.4

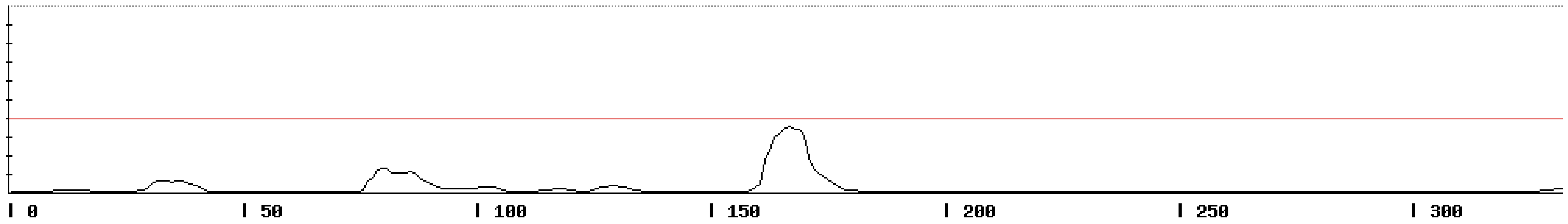

Predictions for Eucalyptusgrandis_Eucgr.A00437.1.p

Viterbi Path

Viterbi Path

Posterior @ 0.4

Posterior @ 0.4

Posterior Graphic. Threshold Marked @ 0.4

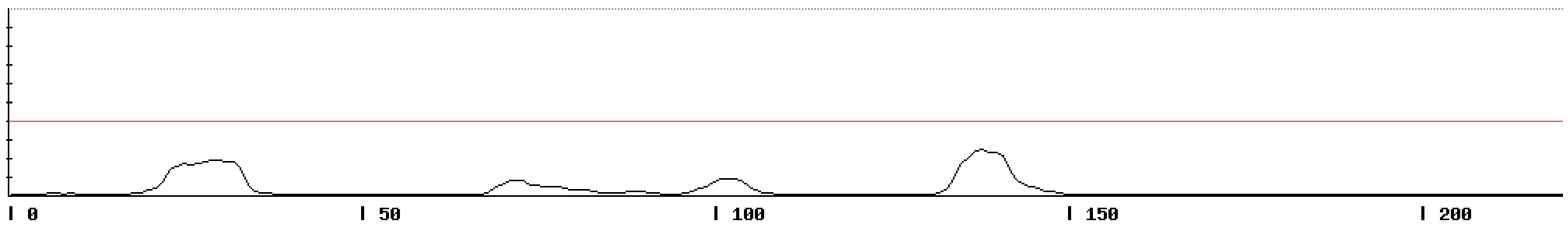

Predictions for Eucalyptusgrandis_Eucgr.A00494.1.p

Viterbi Path

Viterbi Path

Posterior @ 0.4 — 351 - RRRKSDKKNKMFKK - 364

Posterior @ 0.4

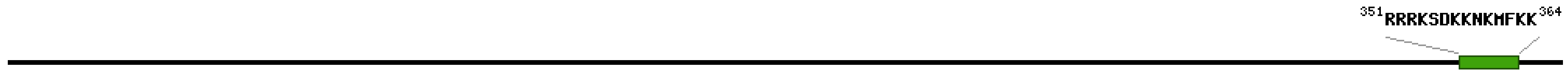

Posterior Graphic. Threshold Marked @ 0.4

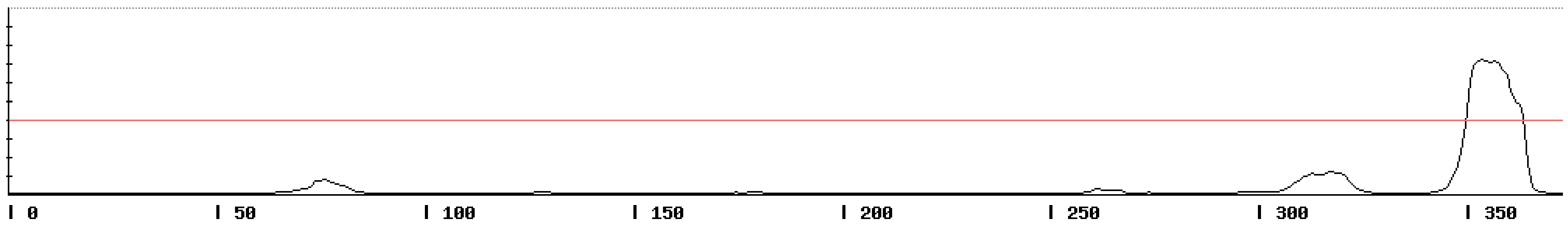

Predictions for Eucalyptusgrandis_Eucgr.A00969.1.p

Viterbi Path

Viterbi Path

Posterior @ 0.4

Posterior @ 0.4

Posterior Graphic. Threshold Marked @ 0.4

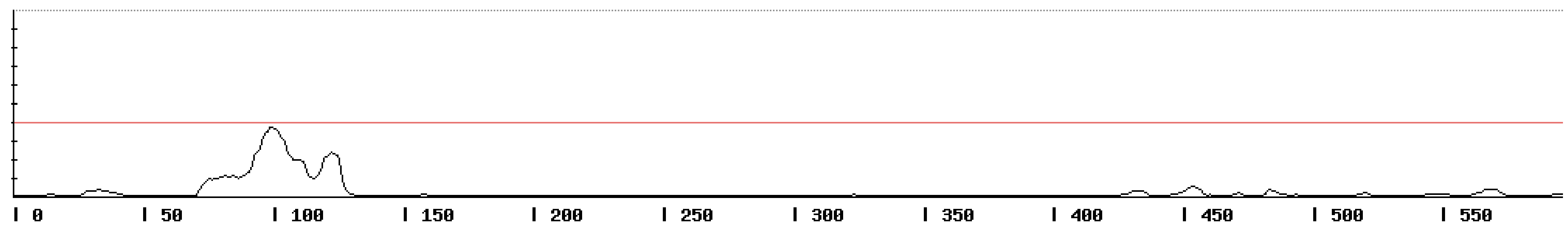

Predictions for Eucalyptusgrandis_Eucgr.A02028.1.p

Viterbi Path

Viterbi Path

Posterior @ 0.4

Posterior @ 0.4

Posterior Graphic. Threshold Marked @ 0.4

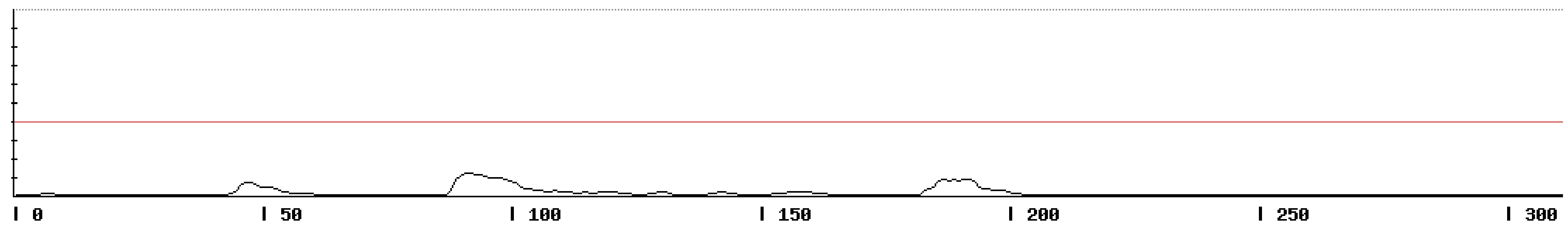

Predictions for Eucalyptusgrandis_Eucgr.A02070.1.p

Viterbi Path

Viterbi Path

Posterior @ 0.4

Posterior @ 0.4

Posterior Graphic. Threshold Marked @ 0.4

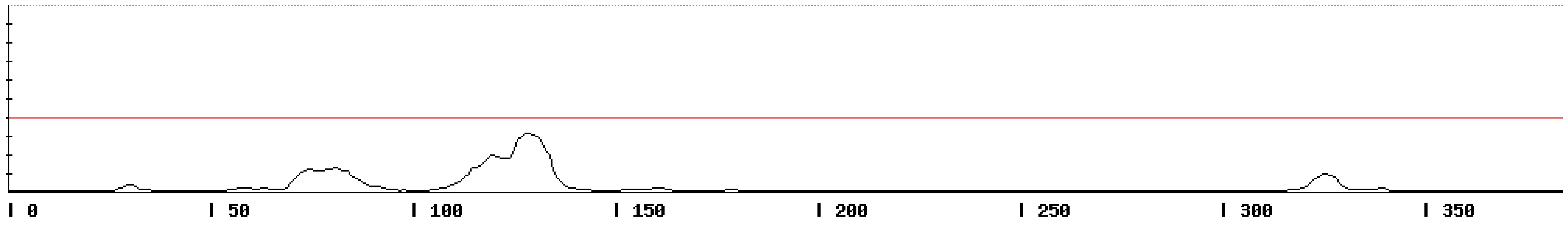

Predictions for Eucalyptusgrandis_Eucgr.A02074.1.p

Viterbi Path

Viterbi Path

Posterior @ 0.4

Posterior @ 0.4

Posterior Graphic. Threshold Marked @ 0.4

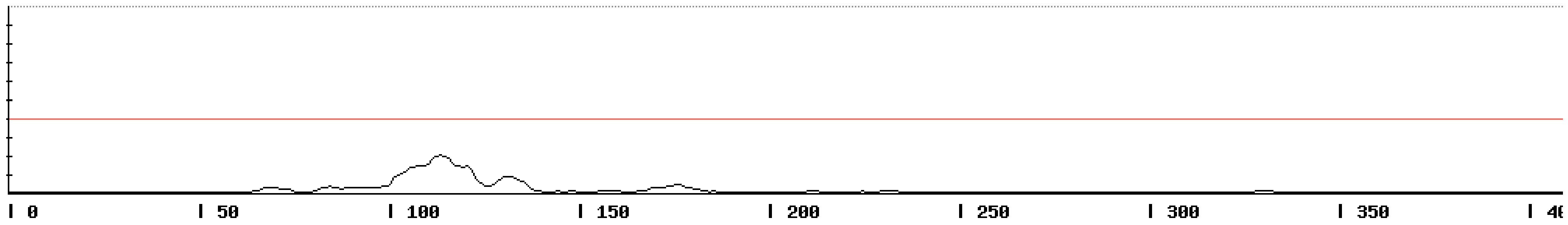

Predictions for Eucalyptusgrandis_Eucgr.A02635.1.p

Viterbi Path	
Viterbi Path	
Posterior @ 0.4	
Posterior @ 0.4	
Posterior Graphic. Threshold Marked @ 0.4	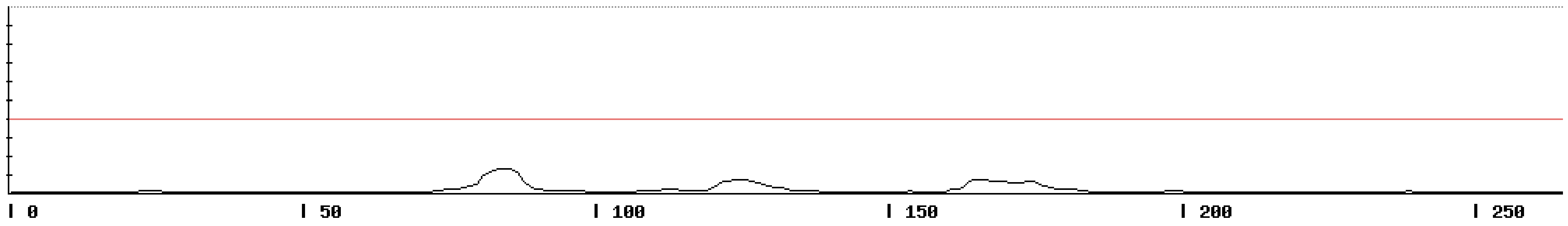

Predictions for Eucalyptusgrandis_Eucgr.A02636.1.p	
Viterbi Path	
Viterbi Path	
Posterior @ 0.4	
Posterior @ 0.4	
Posterior Graphic. Threshold Marked @ 0.4	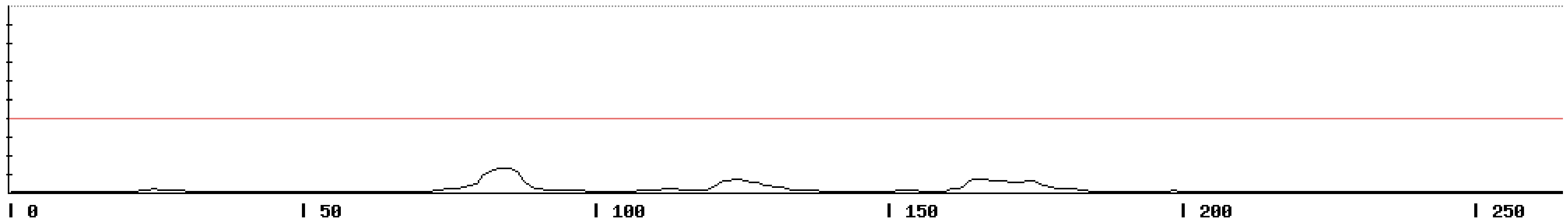

Predictions for Eucalyptusgrandis_Eucgr.A02637.1.p	
Viterbi Path	
Viterbi Path	
Posterior @ 0.4	
Posterior @ 0.4	
Posterior Graphic. Threshold Marked @ 0.4	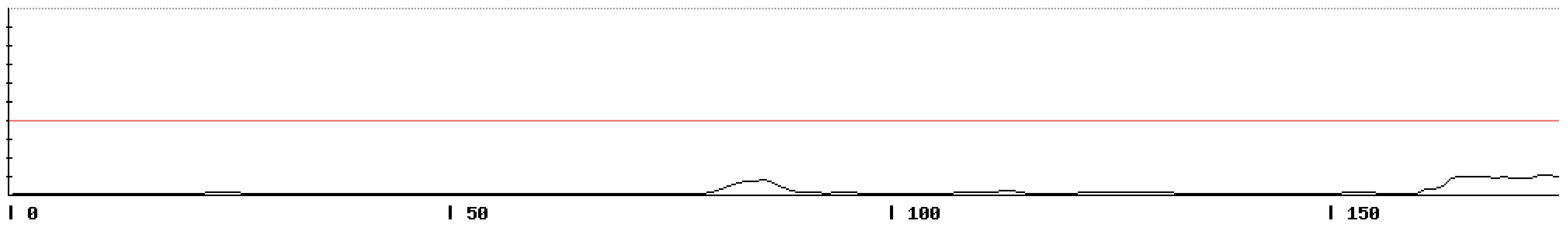

Predictions for Eucalyptusgrandis_Eucgr.A02638.1.p	
Viterbi Path	
Viterbi Path	
Posterior @ 0.4	
Posterior @ 0.4	
Posterior Graphic. Threshold Marked @ 0.4	

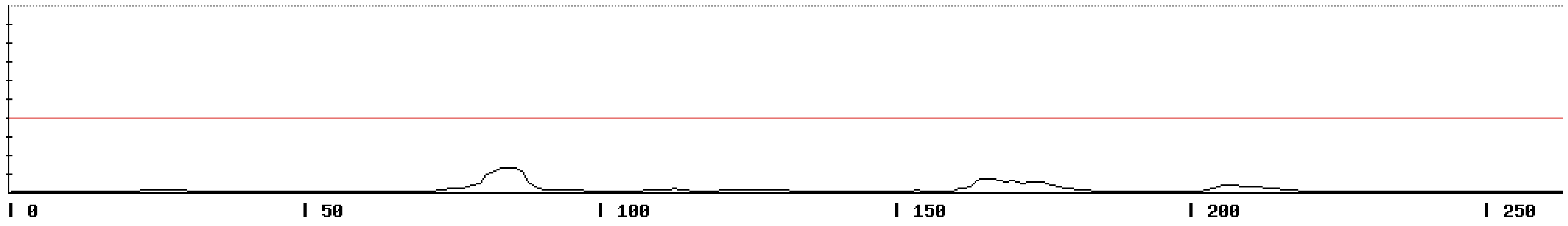

Predictions for Eucalyptusgrandis_Eucgr.A02639.1.p

Viterbi Path

Viterbi Path

Posterior @ 0.4

Posterior @ 0.4

Posterior Graphic. Threshold Marked @ 0.4

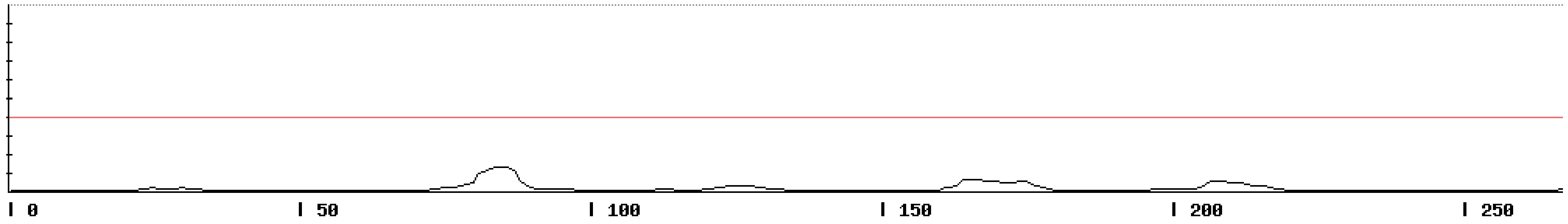

Predictions for Eucalyptusgrandis_Eucgr.A02887.1.p

Viterbi Path

Viterbi Path

Posterior @ 0.4

Posterior @ 0.4

Posterior Graphic. Threshold Marked @ 0.4

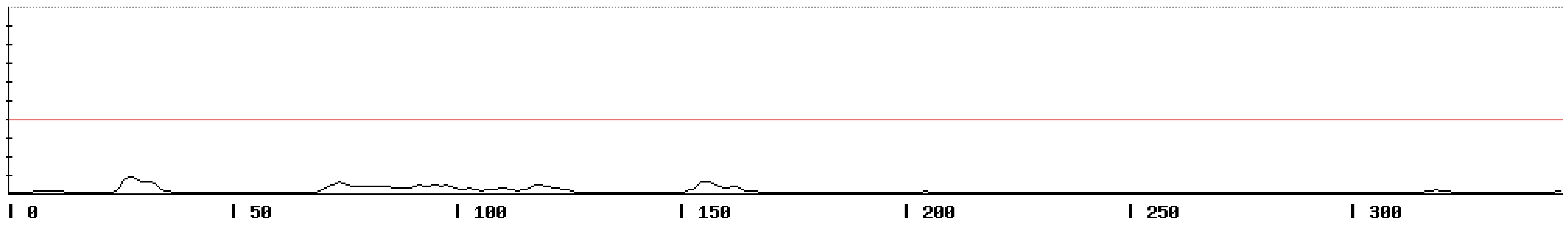

Predictions for Eucalyptusgrandis_Eucgr.B00529.1.p

Viterbi Path

Viterbi Path

Posterior @ 0.4

Posterior @ 0.4

Posterior Graphic. Threshold Marked @ 0.4

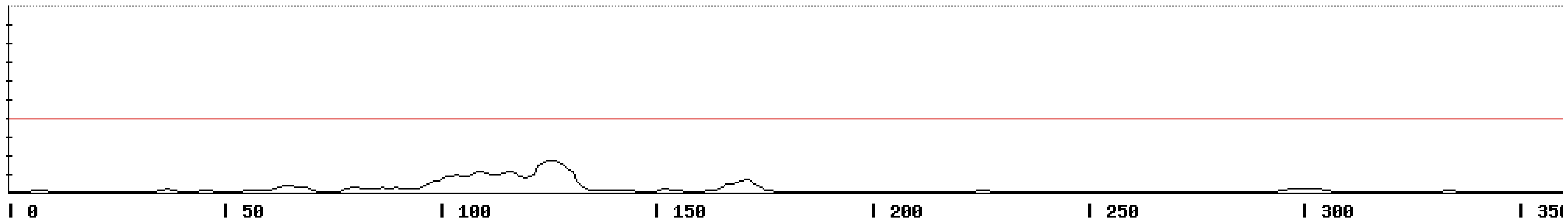

Predictions for Eucalyptusgrandis_Eucgr.B00724.1.p

Viterbi Path

Viterbi Path

Posterior @ 0.4

Posterior @ 0.4

Posterior Graphic. Threshold Marked @ 0.4

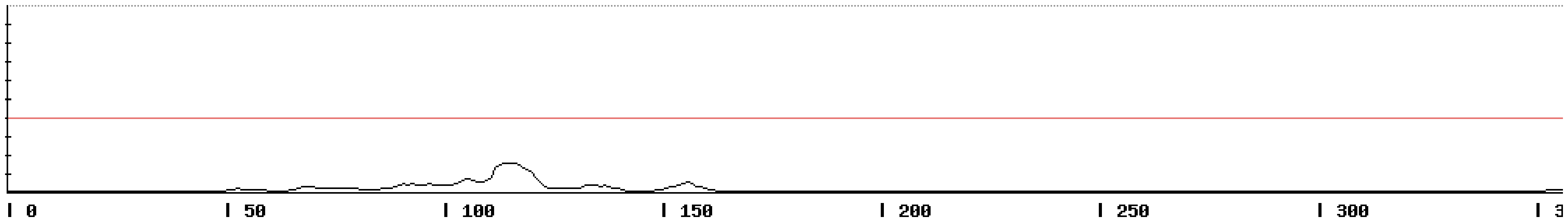

Predictions for Eucalyptusgrandis_Eucgr.B01624.1.p

Viterbi Path

Viterbi Path

Posterior @ 0.4

Posterior @ 0.4

Posterior Graphic. Threshold Marked @ 0.4

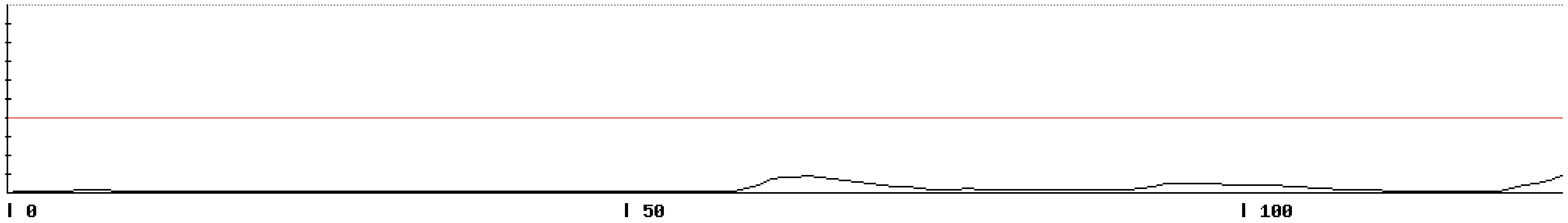

Predictions for Eucalyptusgrandis_Eucgr.B02485.1.p

Viterbi Path

Viterbi Path

Posterior @ 0.4

Posterior @ 0.4

Posterior Graphic. Threshold Marked @ 0.4

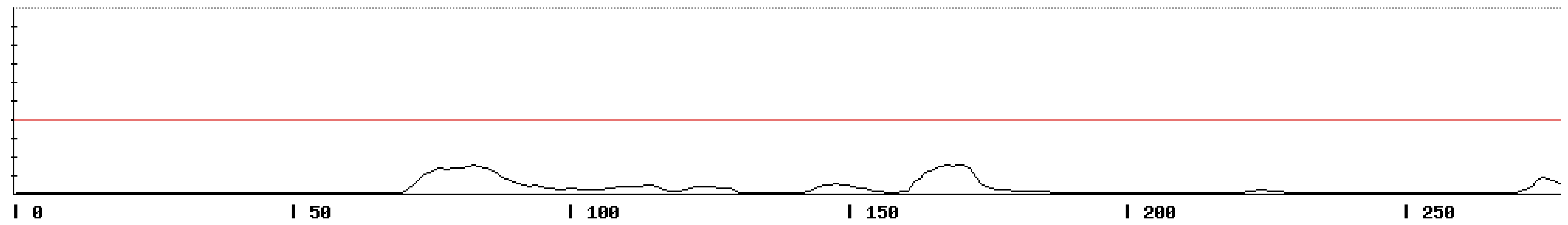

Predictions for Eucalyptusgrandis_Eucgr.B03208.1.p

Viterbi Path

Viterbi Path

Posterior @ 0.4

Posterior @ 0.4

Posterior Graphic. Threshold Marked @ 0.4

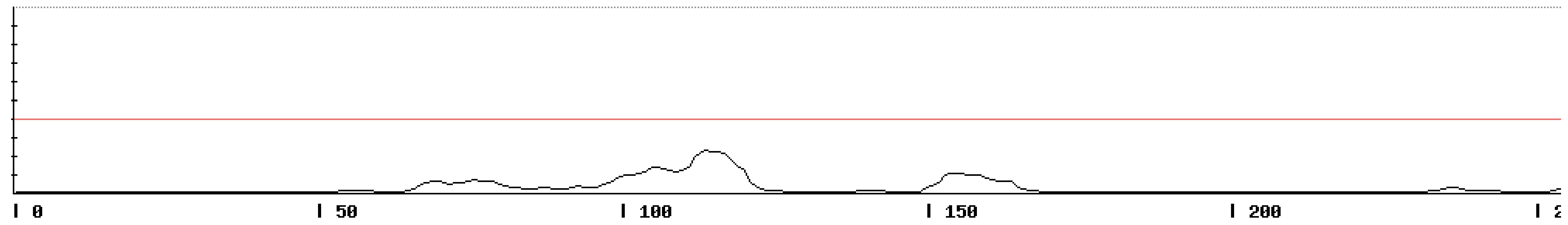

Predictions for Eucalyptusgrandis_Eucgr.B03537.1.p

Viterbi Path

Viterbi Path

Posterior @ 0.4

Posterior @ 0.4

Posterior Graphic. Threshold Marked @ 0.4

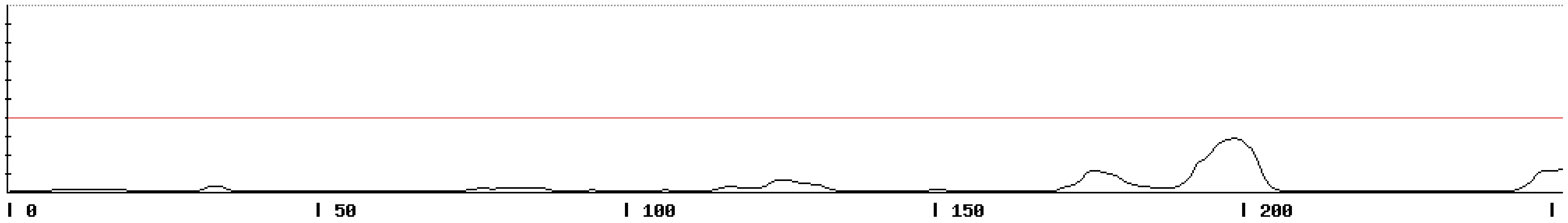

Predictions for Eucalyptusgrandis_Eucgr.B03693.1.p

Viterbi Path

Viterbi Path

Posterior @ 0.4 189 - KHAKHSKNGRK - 199

Posterior @ 0.4

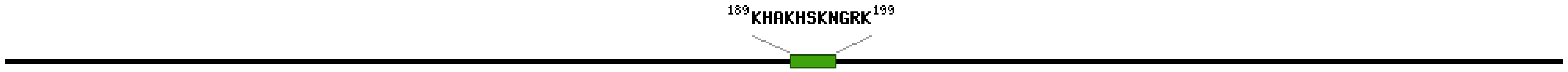

Posterior Graphic. Threshold Marked @ 0.4

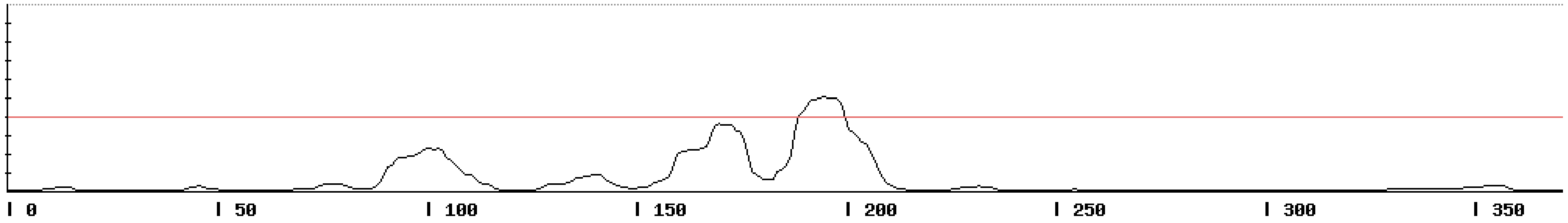

Predictions for Eucalyptusgrandis_Eucgr.B03703.1.p

Viterbi Path	
Viterbi Path	
Posterior @ 0.4	
Posterior @ 0.4	
Posterior Graphic. Threshold Marked @ 0.4	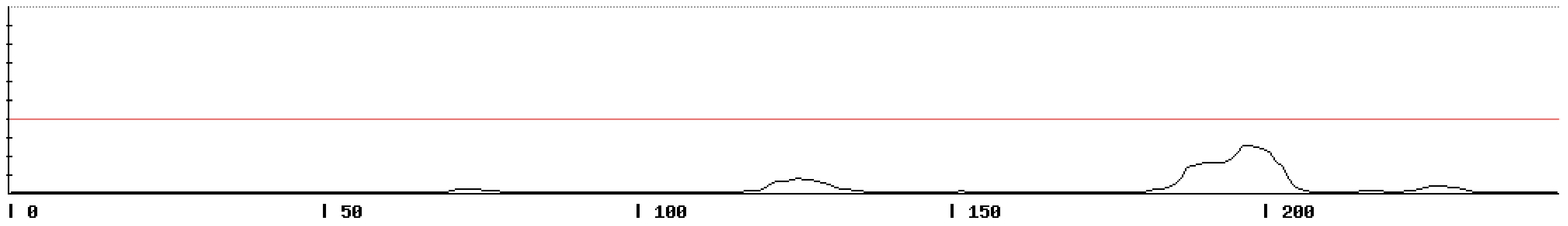

Predictions for Eucalyptusgrandis_Eucgr.B03823.1.p

Viterbi Path	
Viterbi Path	
Posterior @ 0.4	293 - NRRIRLRRHKPERSSK - 308
Posterior @ 0.4	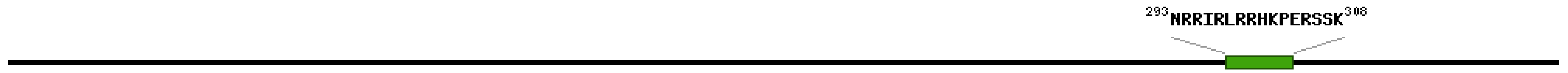
Posterior Graphic. Threshold Marked @ 0.4	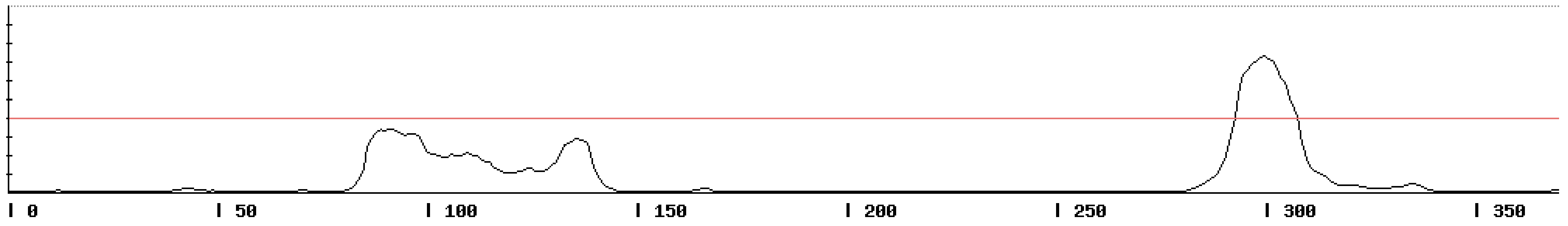

Predictions for Eucalyptusgrandis_Eucgr.C00958.1.p	
Viterbi Path	
Viterbi Path	
Posterior @ 0.4	
Posterior @ 0.4	
Posterior Graphic. Threshold Marked @ 0.4	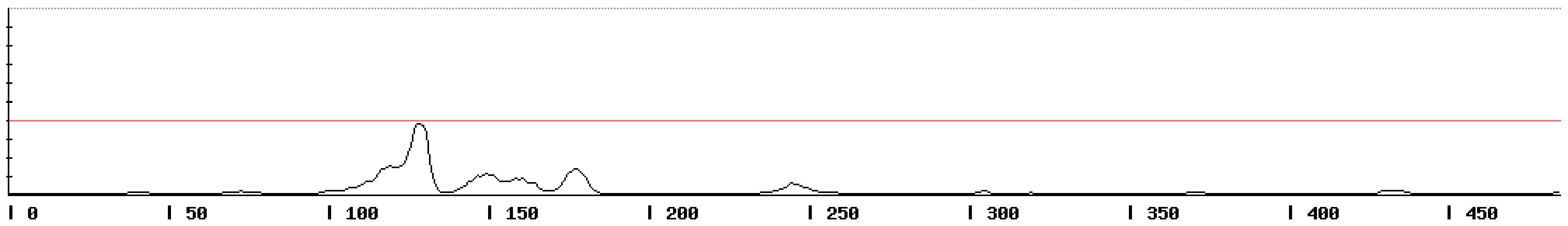

Predictions for Eucalyptusgrandis_Eucgr.C01264.1.p	
Viterbi Path	
Viterbi Path	
Posterior @ 0.4	
Posterior @ 0.4	
Posterior Graphic. Threshold Marked @ 0.4	

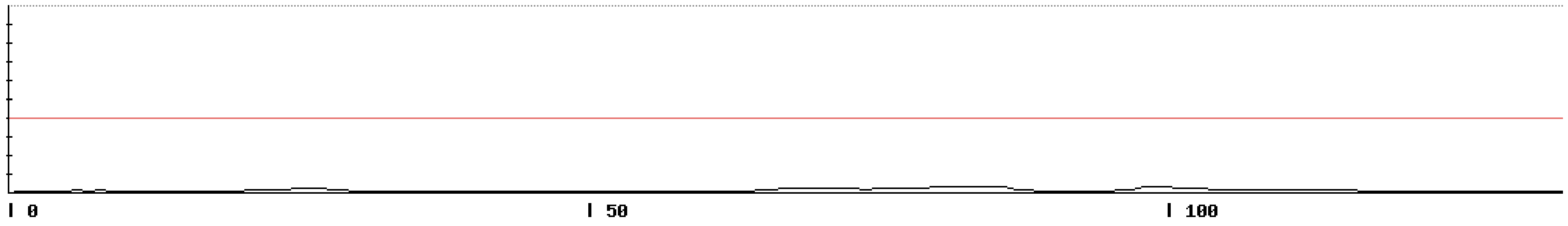

Predictions for Eucalyptusgrandis_Eucgr.C02105.1.p	
Viterbi Path	
Viterbi Path	
Posterior @ 0.4	
Posterior @ 0.4	
Posterior Graphic. Threshold Marked @ 0.4	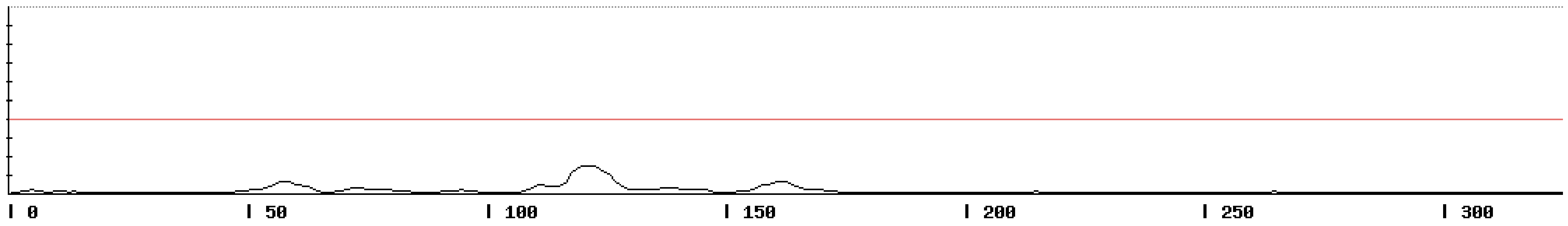

Predictions for Eucalyptusgrandis_Eucgr.C02446.1.p	
Viterbi Path	
Viterbi Path	
Posterior @ 0.4	
Posterior @ 0.4	

Posterior Graphic. Threshold Marked @ 0.4

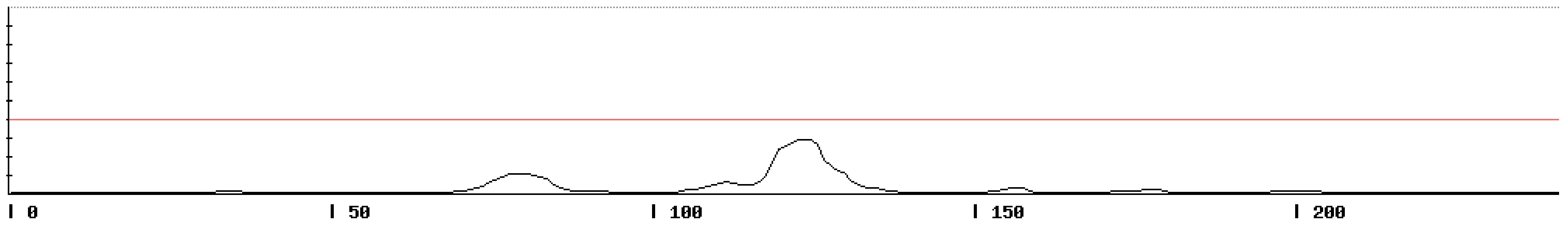

Predictions for Eucalyptusgrandis_Eucgr.D00591.1.p

Viterbi Path

Viterbi Path

Posterior @ 0.4 — 134 - TRKRRRV - 140

Posterior @ 0.4

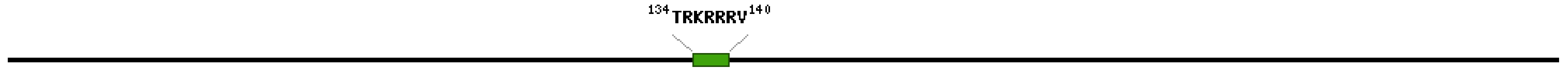

Posterior Graphic. Threshold Marked @ 0.4

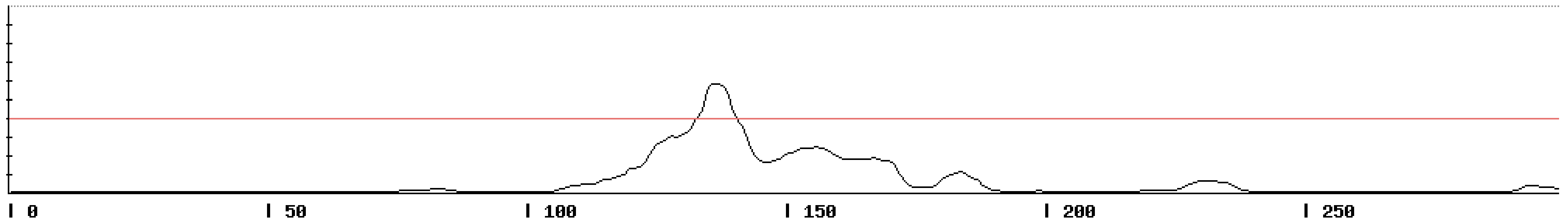

Predictions for Eucalyptusgrandis_Eucgr.D00592.1.p

Viterbi Path

Viterbi Path

Posterior @ 0.4

133 - GTRKRRRVHNNA - 144

147 - R - 147

149 - TRWHKTGK - 156

Posterior @ 0.4	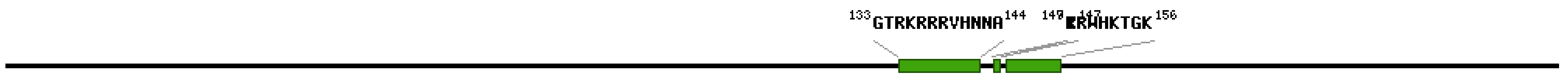
Posterior Graphic. Threshold Marked @ 0.4	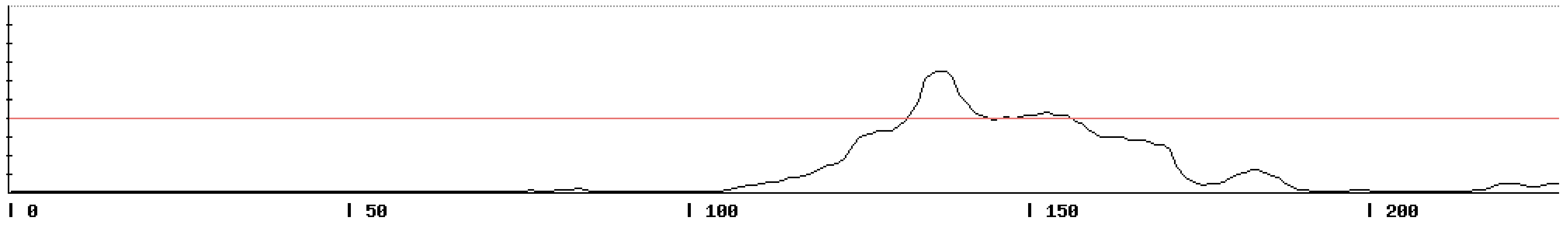

Predictions for Eucalyptusgrandis_Eucgr.D00593.1.p	
Viterbi Path	
Viterbi Path	
Posterior @ 0.4	
Posterior @ 0.4	
Posterior Graphic. Threshold Marked @ 0.4	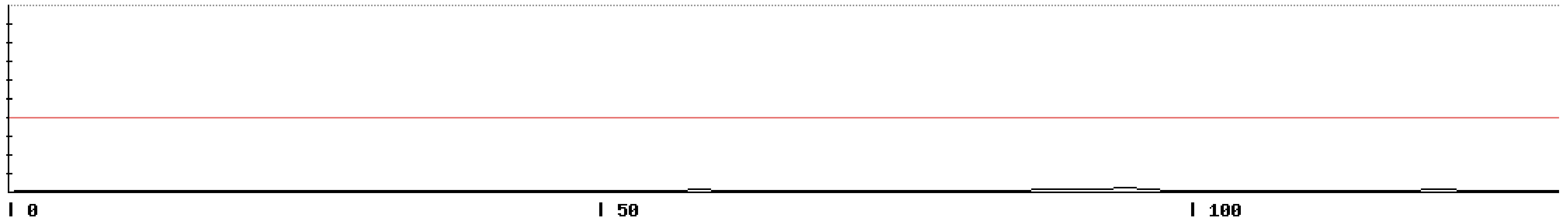

Predictions for Eucalyptusgrandis_Eucgr.D00594.1.p	
Viterbi Path	
Viterbi Path	

Posterior @ 0.4	128 - TGTRKRRRVHKN - 139
Posterior @ 0.4	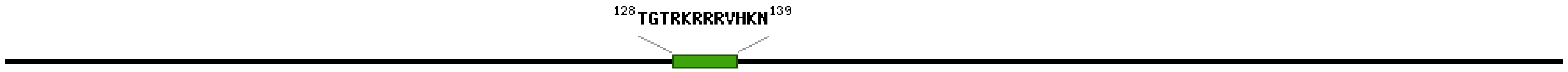
Posterior Graphic. Threshold Marked @ 0.4	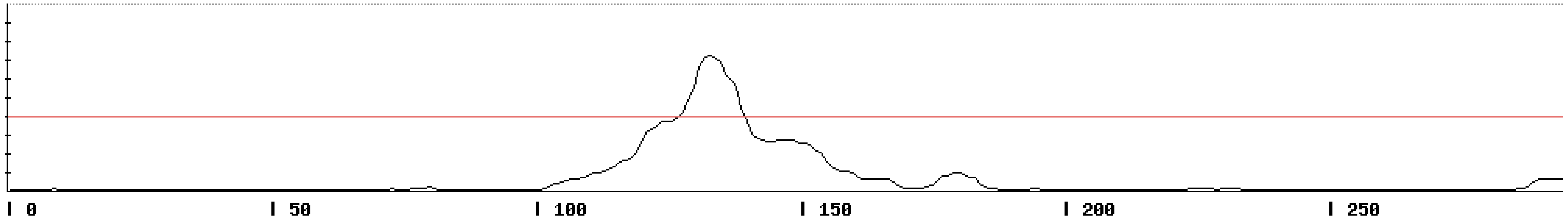

Predictions for Eucalyptusgrandis_Eucgr.D00595.1.p

Viterbi Path	
Viterbi Path	
Posterior @ 0.4	134 - TRKRRRVQ - 141
Posterior @ 0.4	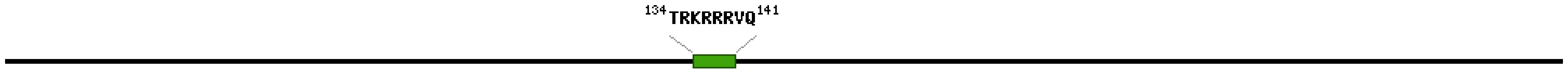
Posterior Graphic. Threshold Marked @ 0.4	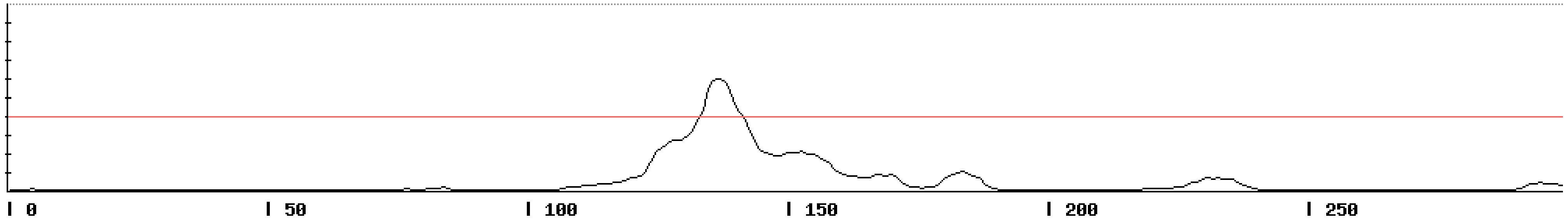

Predictions for Eucalyptusgrandis_Eucgr.D00665.1.p

Viterbi Path	
Viterbi Path	

Posterior @ 0.4

Posterior @ 0.4

Posterior Graphic. Threshold Marked @ 0.4

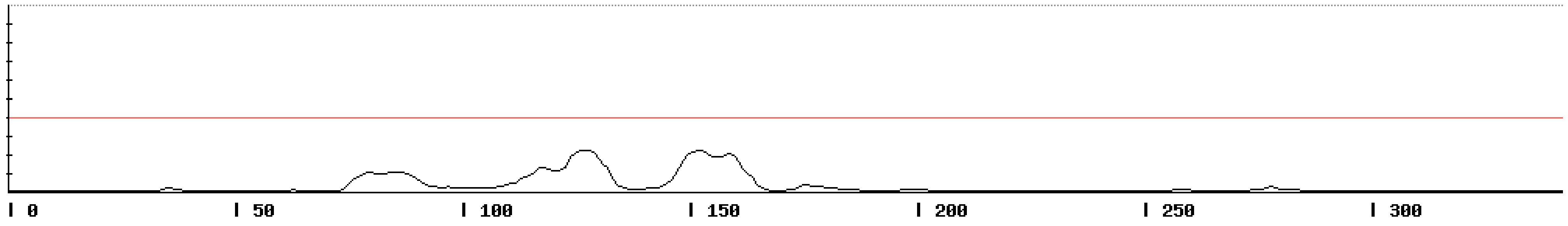

Predictions for Eucalyptusgrandis_Eucgr.D01671.1.p

Viterbi Path

Viterbi Path

Posterior @ 0.4

Posterior @ 0.4

Posterior Graphic. Threshold Marked @ 0.4

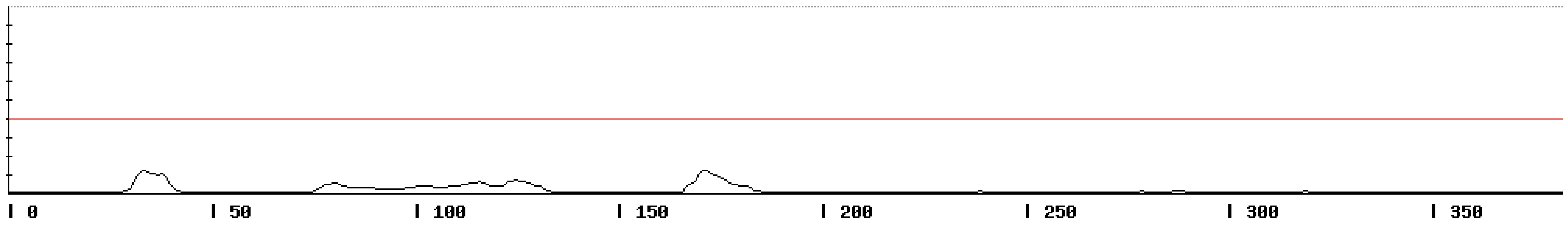

Predictions for Eucalyptusgrandis_Eucgr.D02027.1.p

Viterbi Path

Viterbi Path

Posterior @ 0.4

Posterior @ 0.4

Posterior Graphic. Threshold Marked @ 0.4

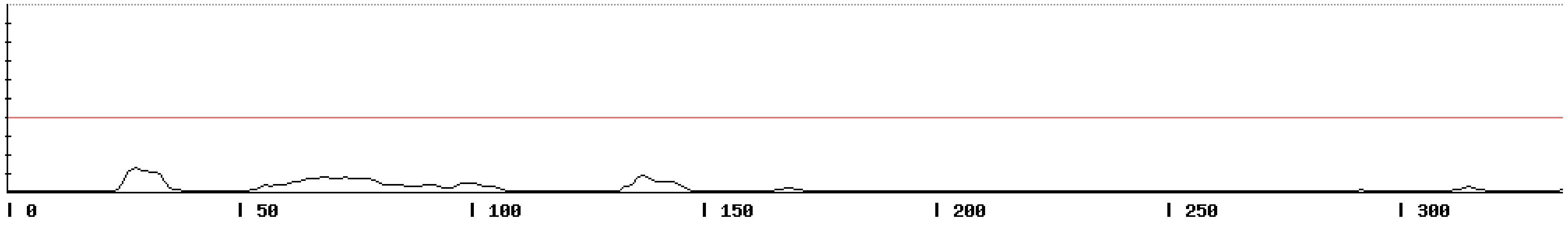

Predictions for Eucalyptusgrandis_Eucgr.D02182.1.p

Viterbi Path

Viterbi Path

Posterior @ 0.4

Posterior @ 0.4

Posterior Graphic. Threshold Marked @ 0.4

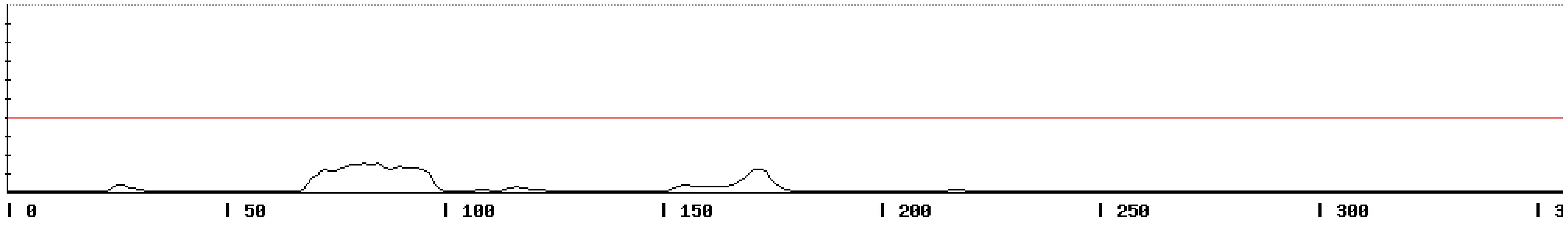

Predictions for Eucalyptusgrandis_Eucgr.E00298.1.p

Viterbi Path	
Viterbi Path	
Posterior @ 0.4	
Posterior @ 0.4	
Posterior Graphic. Threshold Marked @ 0.4	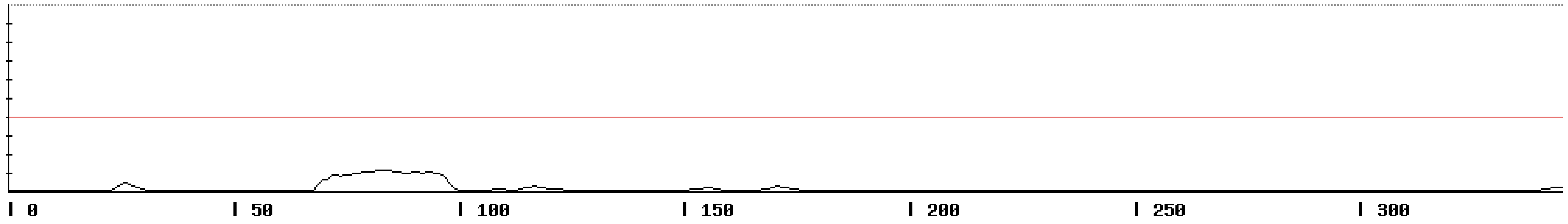

Predictions for Eucalyptusgrandis_Eucgr.E00541.1.p

Viterbi Path	
Viterbi Path	
Posterior @ 0.4	
Posterior @ 0.4	
Posterior Graphic. Threshold Marked @ 0.4	

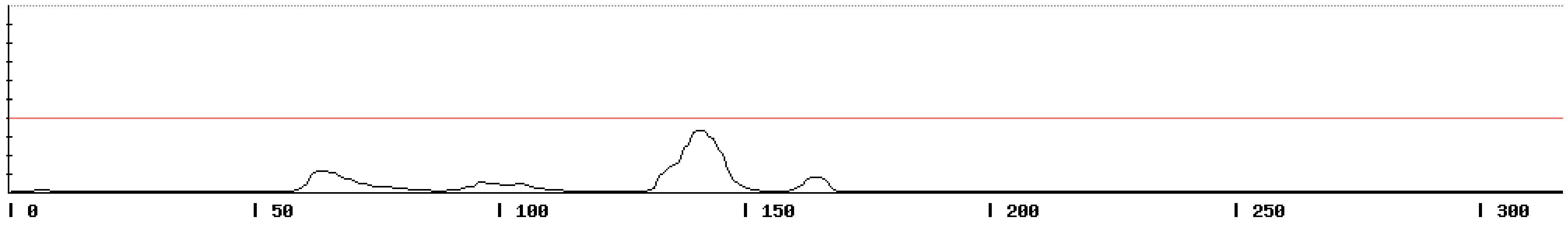

Predictions for Eucalyptusgrandis_Eucgr.E00542.1.p

Viterbi Path

Viterbi Path

Posterior @ 0.4

Posterior @ 0.4

Posterior Graphic. Threshold Marked @ 0.4

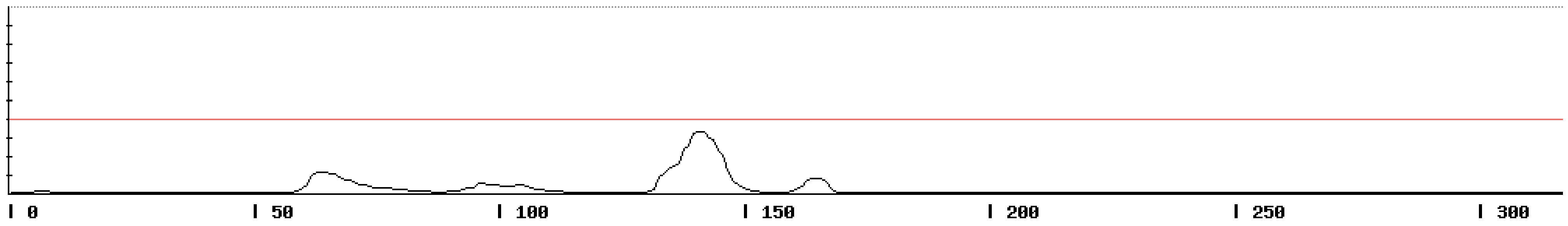

Predictions for Eucalyptusgrandis_Eucgr.E00545.1.p

Viterbi Path

Viterbi Path

Posterior @ 0.4

Posterior @ 0.4

Posterior Graphic. Threshold Marked @ 0.4

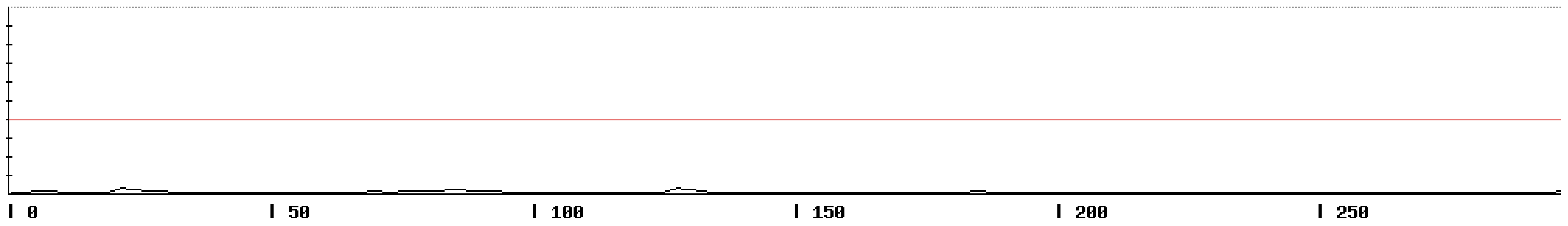

Predictions for Eucalyptusgrandis_Eucgr.E00551.1.p

Viterbi Path

Viterbi Path

Posterior @ 0.4

Posterior @ 0.4

Posterior Graphic. Threshold Marked @ 0.4

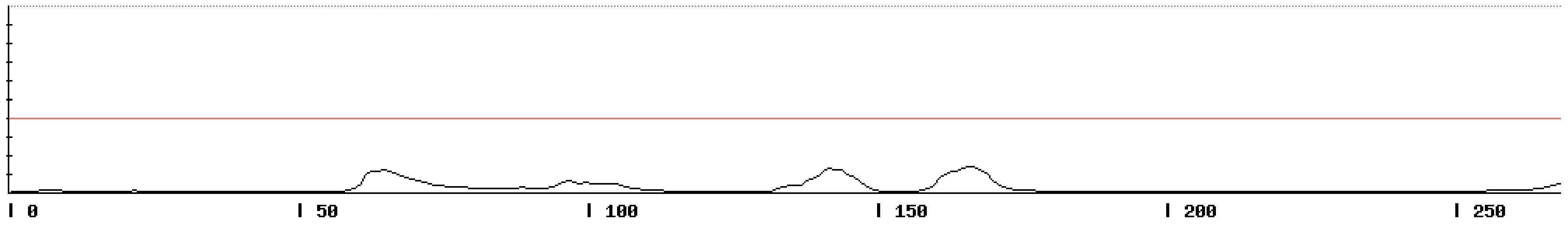

Predictions for Eucalyptusgrandis_Eucgr.E01053.1.p

Viterbi Path

Viterbi Path

Posterior @ 0.4

Posterior @ 0.4

Posterior Graphic. Threshold Marked @ 0.4

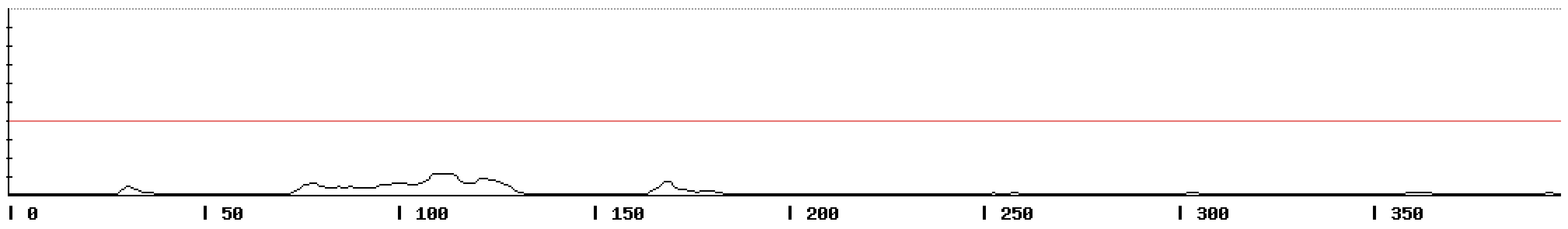

Predictions for Eucalyptusgrandis_Eucgr.E01095.1.p

Viterbi Path

Viterbi Path

Posterior @ 0.4 102 - KSRK - 105

Posterior @ 0.4

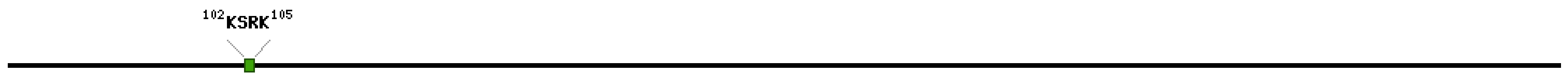

Posterior Graphic. Threshold Marked @ 0.4

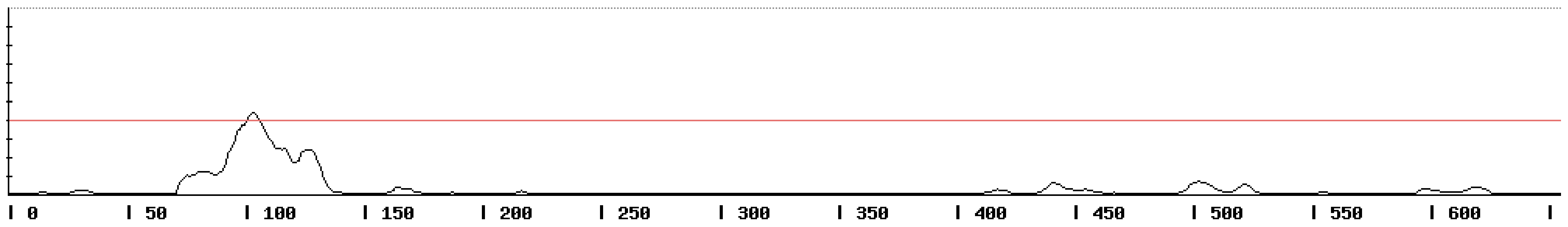

Predictions for Eucalyptusgrandis_Eucgr.E03226.1.p

Viterbi Path

Viterbi Path

Posterior @ 0.4	139 - RKRRK - 143
Posterior @ 0.4	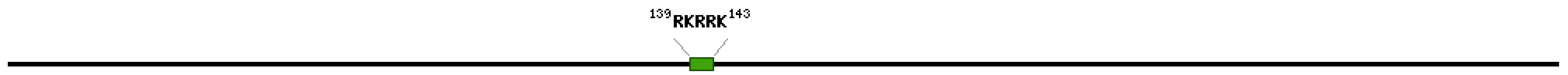
Posterior Graphic. Threshold Marked @ 0.4	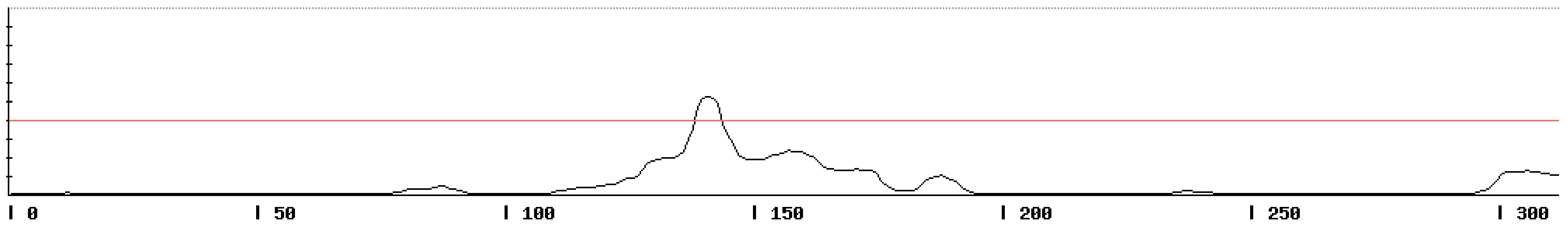

Predictions for Eucalyptusgrandis_Eucgr.F01091.1.p

Viterbi Path	
Viterbi Path	
Posterior @ 0.4	
Posterior @ 0.4	
Posterior Graphic. Threshold Marked @ 0.4	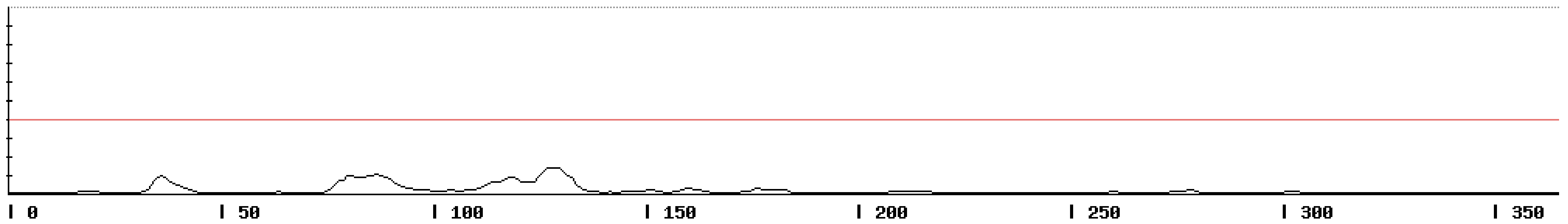

Predictions for Eucalyptusgrandis_Eucgr.F01093.1.p

Viterbi Path	
Viterbi Path	

Posterior @ 0.4

Posterior @ 0.4

Posterior Graphic. Threshold Marked @ 0.4

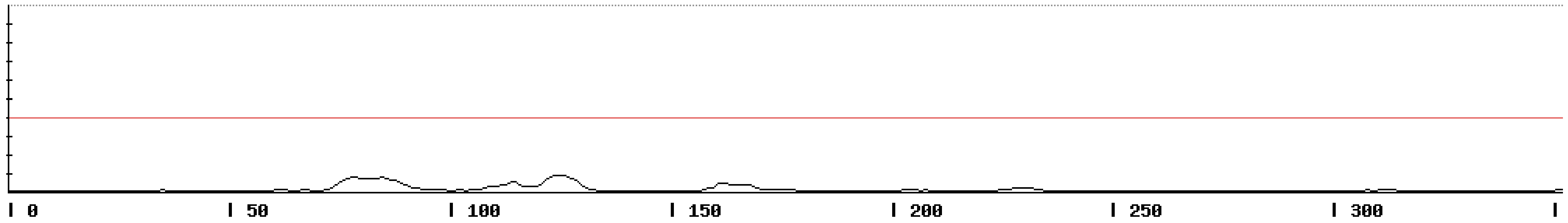

Predictions for Eucalyptusgrandis_Eucgr.F01170.1.p

Viterbi Path

Viterbi Path

Posterior @ 0.4

Posterior @ 0.4

Posterior Graphic. Threshold Marked @ 0.4

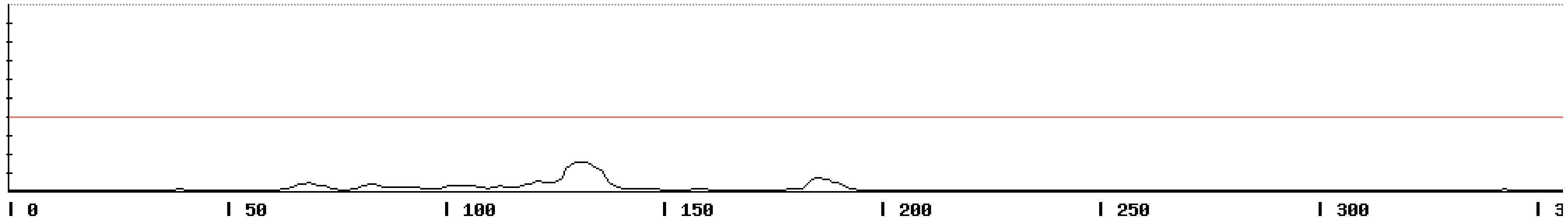

Predictions for Eucalyptusgrandis_Eucgr.F01449.1.p

Viterbi Path

Viterbi Path

Posterior @ 0.4

Posterior @ 0.4

Posterior Graphic. Threshold Marked @ 0.4

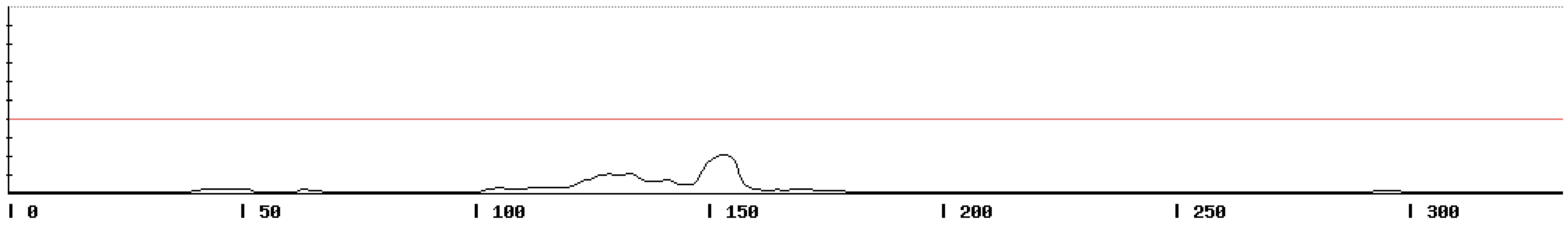

Predictions for Eucalyptusgrandis_Eucgr.F01463.1.p

Viterbi Path

Viterbi Path

Posterior @ 0.4

Posterior @ 0.4

Posterior Graphic. Threshold Marked @ 0.4

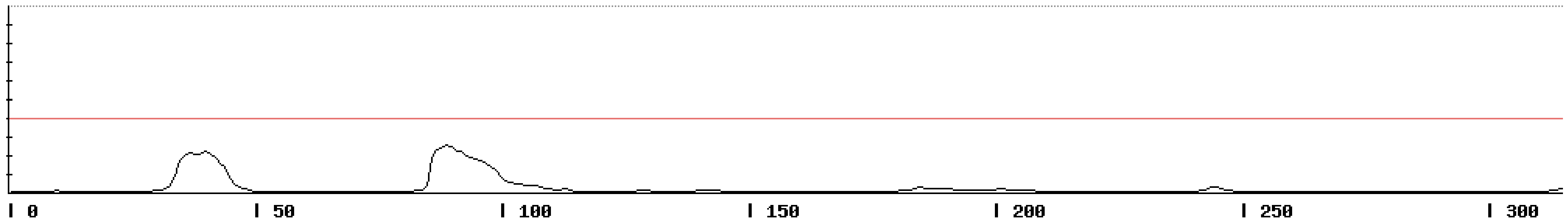

Predictions for Eucalyptusgrandis_Eucgr.F01535.1.p	
Viterbi Path	
Viterbi Path	
Posterior @ 0.4	
Posterior @ 0.4	
Posterior Graphic. Threshold Marked @ 0.4	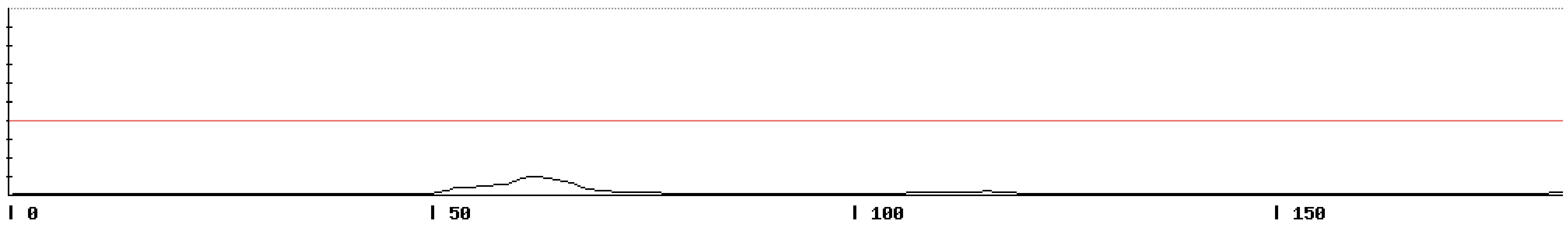

Predictions for Eucalyptusgrandis_Eucgr.F01536.1.p	
Viterbi Path	
Viterbi Path	
Posterior @ 0.4	
Posterior @ 0.4	
Posterior Graphic. Threshold Marked @ 0.4	

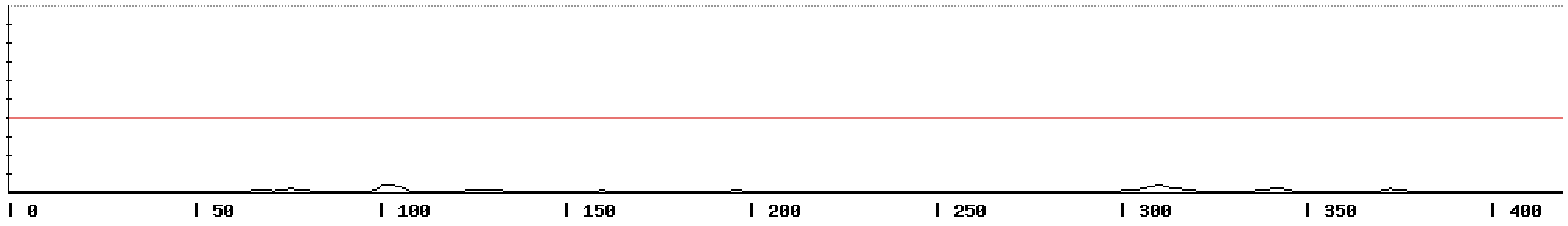

Predictions for Eucalyptusgrandis_Eucgr.F01537.1.p

Viterbi Path	
Viterbi Path	
Posterior @ 0.4	348 - RAPSPGPGGRRKTDGAFKKLRKK - 370
Posterior @ 0.4	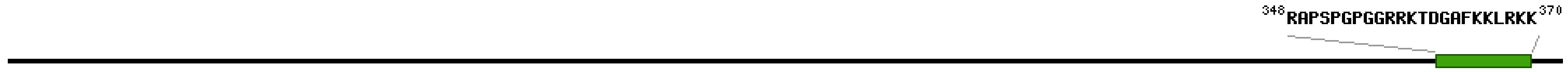
Posterior Graphic. Threshold Marked @ 0.4	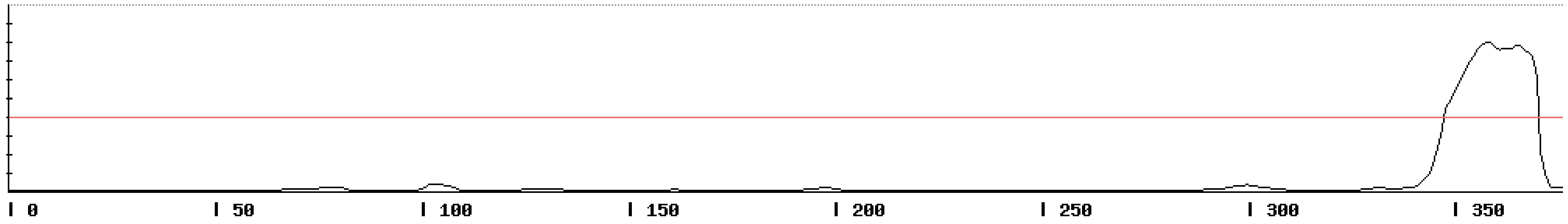

Predictions for Eucalyptusgrandis_Eucgr.F01538.1.p

Viterbi Path	
Viterbi Path	
Posterior @ 0.4	345 - TDGRLKKLKRK - 355
	372 - RAPSPGPGGRRKTDGALKKLQK - 393
Posterior @ 0.4	

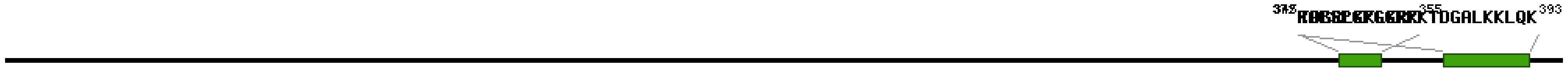

Posterior Graphic. Threshold Marked @ 0.4

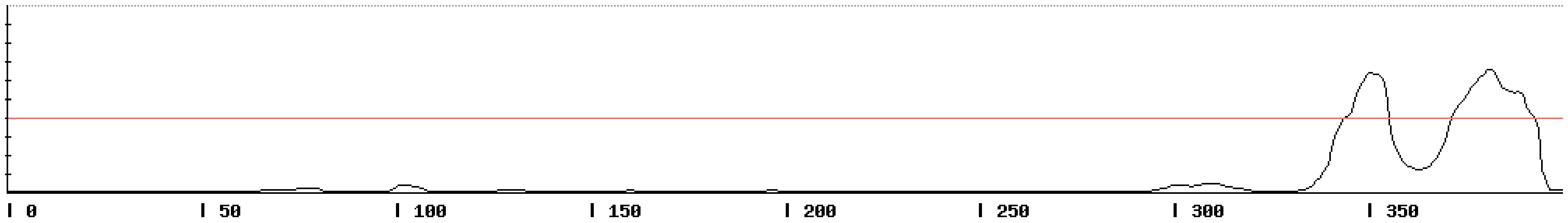

Predictions for Eucalyptusgrandis_Eucgr.F01539.1.p

Viterbi Path

Viterbi Path

Posterior @ 0.4

346 - TDGRLKKLKRK - 356

372 - GRAPSPGPGGRRKTDGAFKKLRKK - 395

Posterior @ 0.4

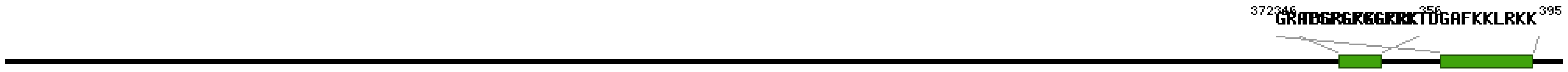

Posterior Graphic. Threshold Marked @ 0.4

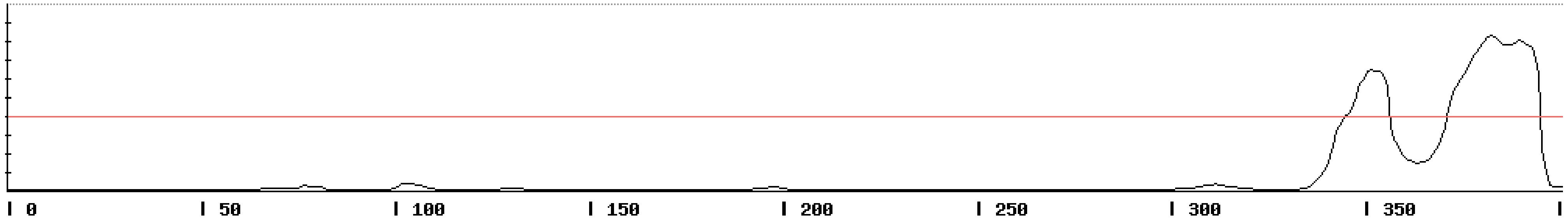

Predictions for Eucalyptusgrandis_Eucgr.F02615.1.p

Viterbi Path

Viterbi Path

Posterior @ 0.4

Posterior @ 0.4

Posterior Graphic. Threshold Marked @ 0.4

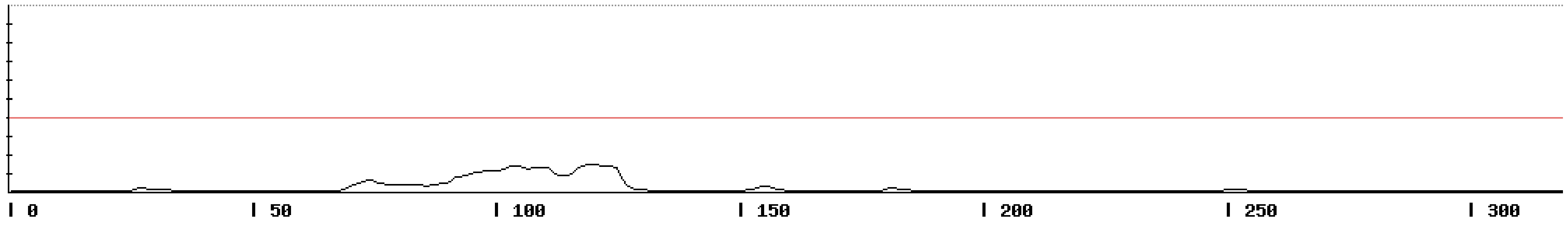

Predictions for Eucalyptusgrandis_Eucgr.F02771.1.p

Viterbi Path

Viterbi Path

Posterior @ 0.4

Posterior @ 0.4

Posterior Graphic. Threshold Marked @ 0.4

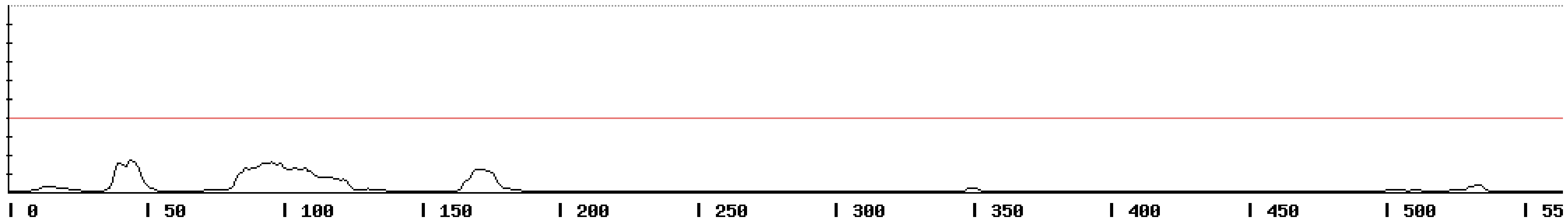

Predictions for Eucalyptusgrandis_Eucgr.F02910.1.p

Viterbi Path	
Viterbi Path	
Posterior @ 0.4	
Posterior @ 0.4	
Posterior Graphic. Threshold Marked @ 0.4	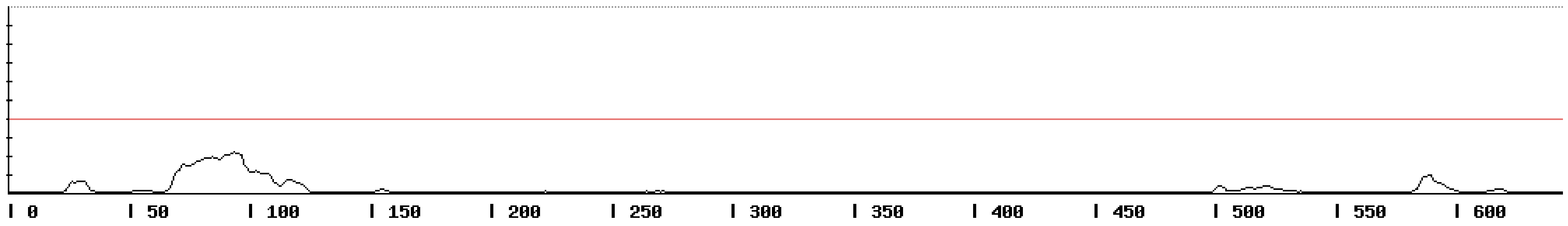

Predictions for Eucalyptusgrandis_Eucgr.F03588.1.p

Viterbi Path	
Viterbi Path	
Posterior @ 0.4	
Posterior @ 0.4	
Posterior Graphic. Threshold Marked @ 0.4	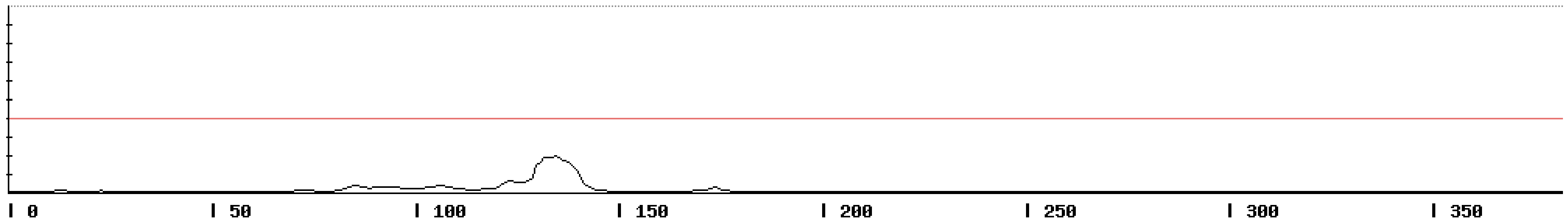

Predictions for Eucalyptusgrandis_Eucgr.F03962.1.p

Viterbi Path	
Viterbi Path	
Posterior @ 0.4	
Posterior @ 0.4	
Posterior Graphic. Threshold Marked @ 0.4	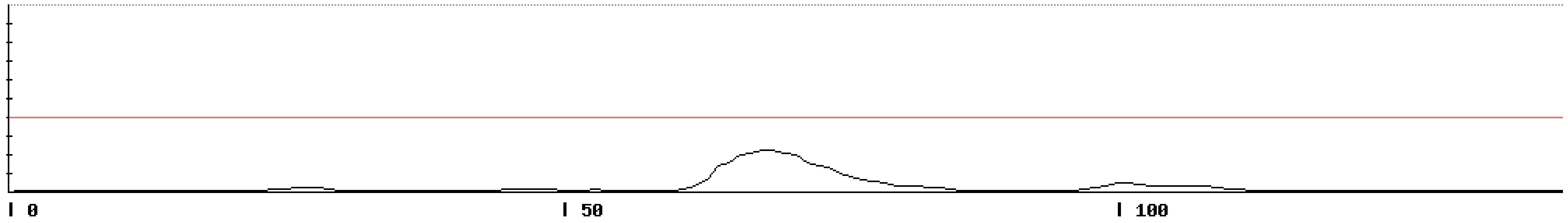

Predictions for Eucalyptusgrandis_Eucgr.F03963.1.p

Viterbi Path	
Viterbi Path	
Posterior @ 0.4	
Posterior @ 0.4	
Posterior Graphic. Threshold Marked @ 0.4	

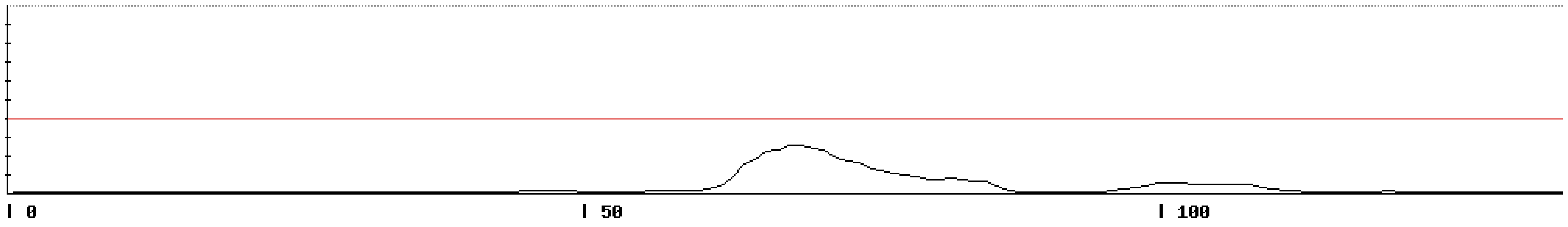

Predictions for Eucalyptusgrandis_Eucgr.F04097.1.p	
Viterbi Path	
Viterbi Path	
Posterior @ 0.4	
Posterior @ 0.4	
Posterior Graphic. Threshold Marked @ 0.4	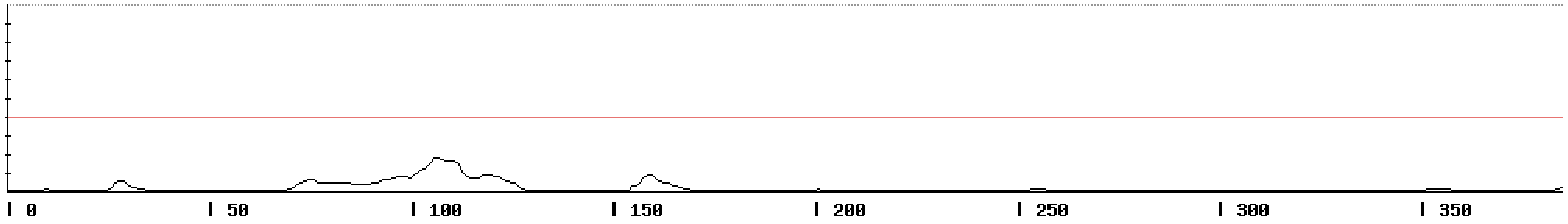

Predictions for Eucalyptusgrandis_Eucgr.F04341.1.p	
Viterbi Path	
Viterbi Path	
Posterior @ 0.4	
Posterior @ 0.4	

Posterior Graphic. Threshold Marked @ 0.4

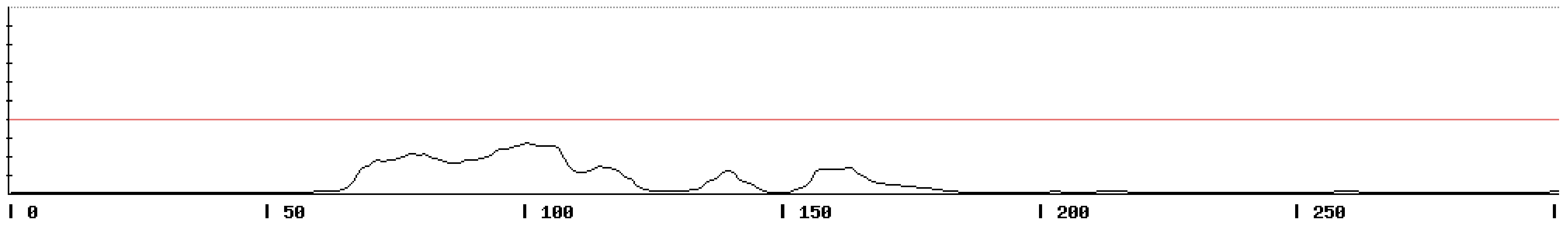

Predictions for Eucalyptusgrandis_Eucgr.G00054.1.p

Viterbi Path

Viterbi Path

Posterior @ 0.4

Posterior @ 0.4

Posterior Graphic. Threshold Marked @ 0.4

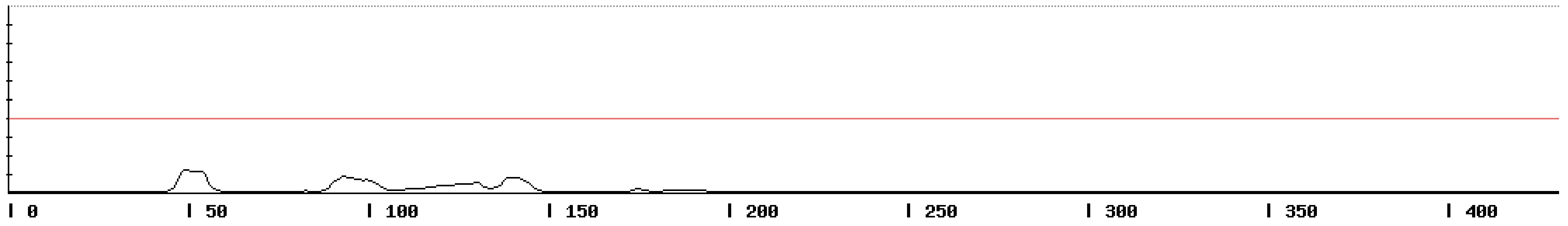

Predictions for Eucalyptusgrandis_Eucgr.G01047.1.p

Viterbi Path

Viterbi Path

Posterior @ 0.4

Posterior @ 0.4

Posterior Graphic. Threshold Marked @ 0.4

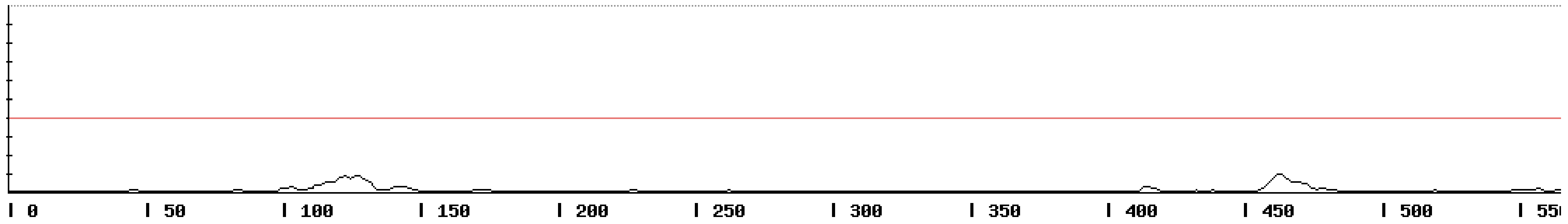

Predictions for Eucalyptusgrandis_Eucgr.G01049.1.p

Viterbi Path

Viterbi Path

Posterior @ 0.4

Posterior @ 0.4

Posterior Graphic. Threshold Marked @ 0.4

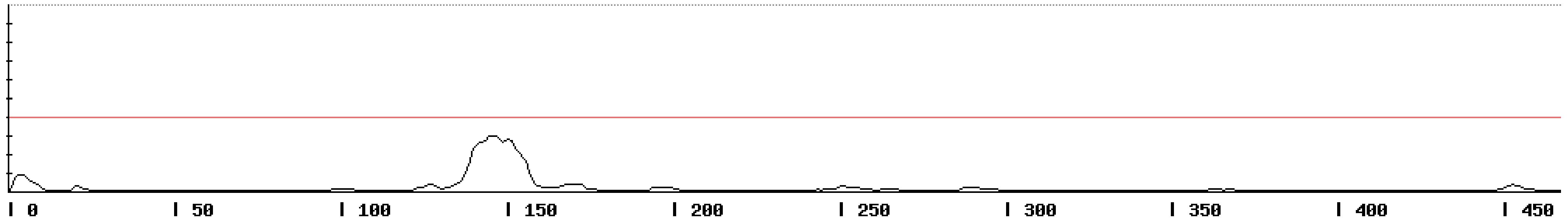

Predictions for Eucalyptusgrandis_Eucgr.G01052.1.p

Viterbi Path

Viterbi Path

Posterior @ 0.4

Posterior @ 0.4

Posterior Graphic. Threshold Marked @ 0.4

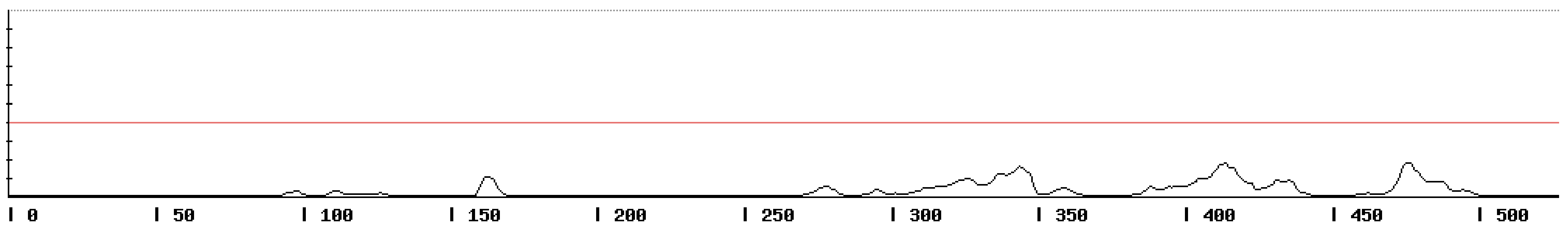

Predictions for Eucalyptusgrandis_Eucgr.G01053.1.p

Viterbi Path

Viterbi Path

Posterior @ 0.4

Posterior @ 0.4

Posterior Graphic. Threshold Marked @ 0.4

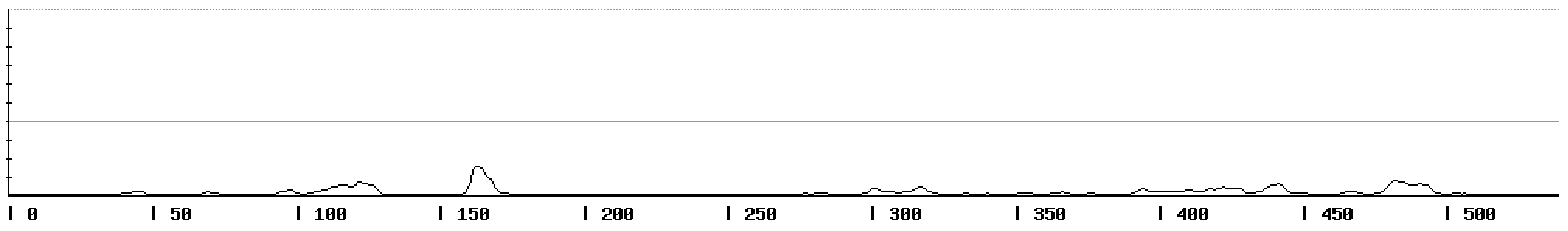

Predictions for Eucalyptusgrandis_Eucgr.G01060.1.p

Viterbi Path

Viterbi Path

Posterior @ 0.4	
Posterior @ 0.4	
Posterior Graphic. Threshold Marked @ 0.4	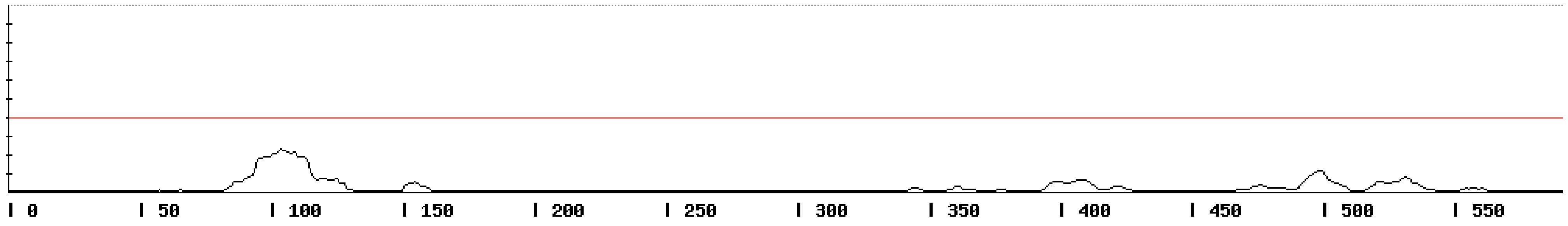

Predictions for Eucalyptusgrandis_Eucgr.G01061.1.p

Viterbi Path	
Viterbi Path	
Posterior @ 0.4	161 - RKRKKVRSAK - 170
Posterior @ 0.4	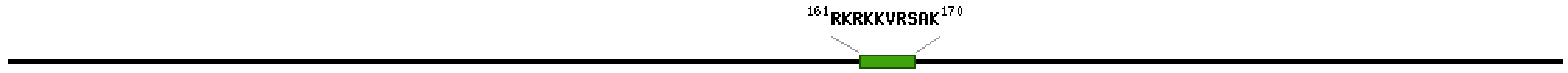
Posterior Graphic. Threshold Marked @ 0.4	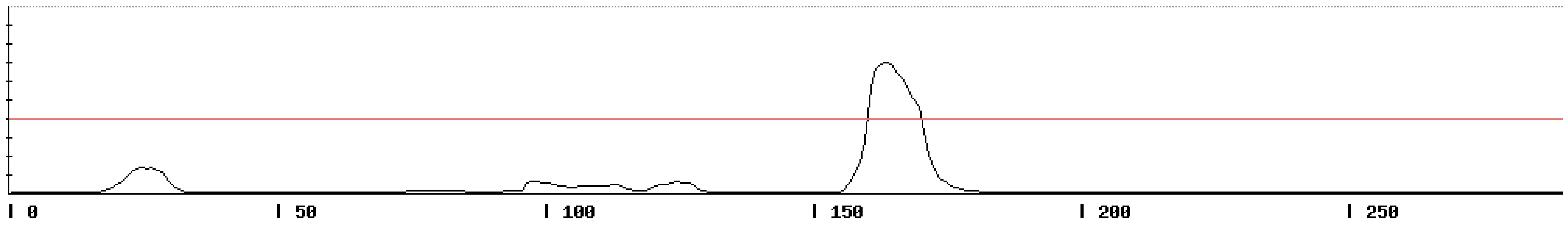

Predictions for Eucalyptusgrandis_Eucgr.G01063.1.p

Viterbi Path

Viterbi Path

Posterior @ 0.4 76 - RKRKKARSAK - 85

Posterior @ 0.4

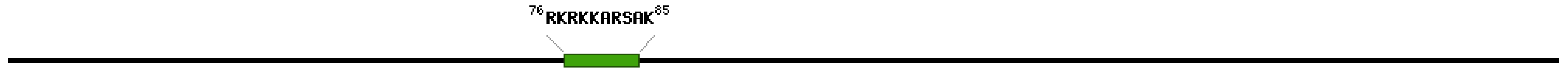

Posterior Graphic. Threshold Marked @ 0.4

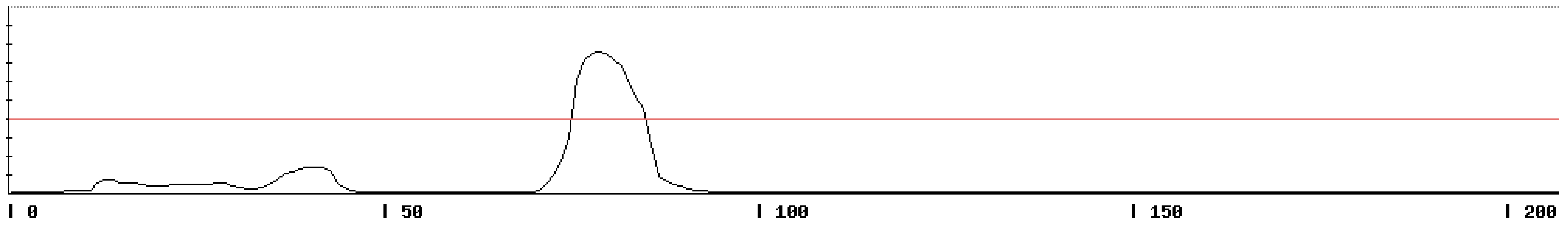

Predictions for Eucalyptusgrandis_Eucgr.G01066.1.p

Viterbi Path

Viterbi Path

Posterior @ 0.4

Posterior @ 0.4

Posterior Graphic. Threshold Marked @ 0.4

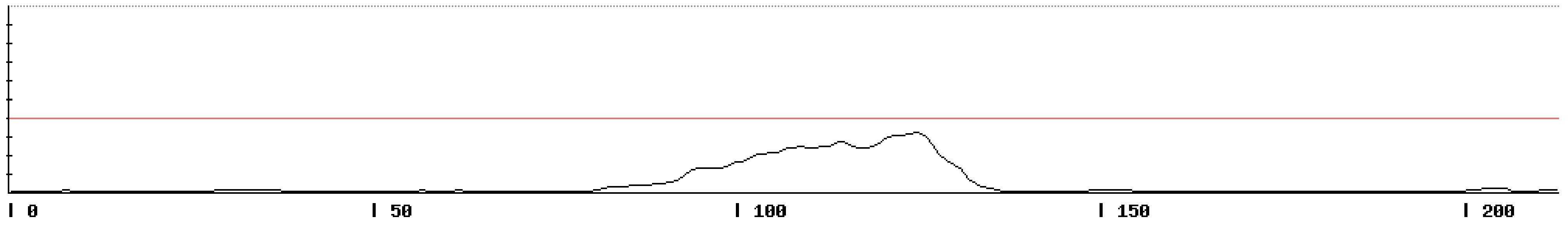

Predictions for Eucalyptusgrandis_Eucgr.G01067.1.p	
Viterbi Path	
Viterbi Path	
Posterior @ 0.4	
Posterior @ 0.4	
Posterior Graphic. Threshold Marked @ 0.4	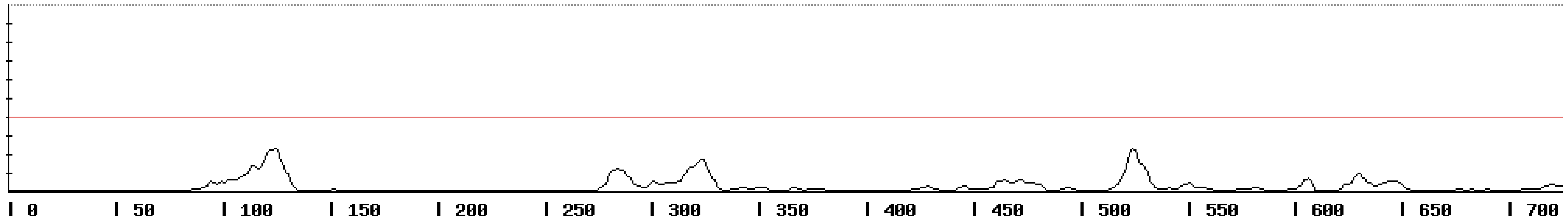

Predictions for Eucalyptusgrandis_Eucgr.G01073.1.p	
Viterbi Path	
Viterbi Path	
Posterior @ 0.4	
Posterior @ 0.4	
Posterior Graphic. Threshold Marked @ 0.4	

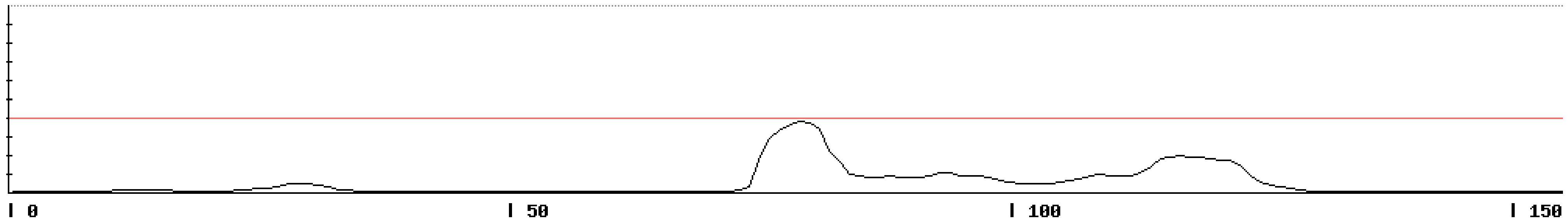

Predictions for Eucalyptusgrandis_Eucgr.G01074.1.p

Viterbi Path	
Viterbi Path	
Posterior @ 0.4	75 - RGHKRKRK - 82
	158 - KSKK - 161
Posterior @ 0.4	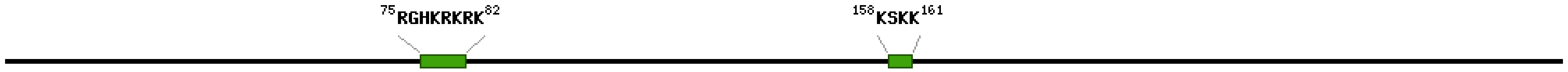
Posterior Graphic. Threshold Marked @ 0.4	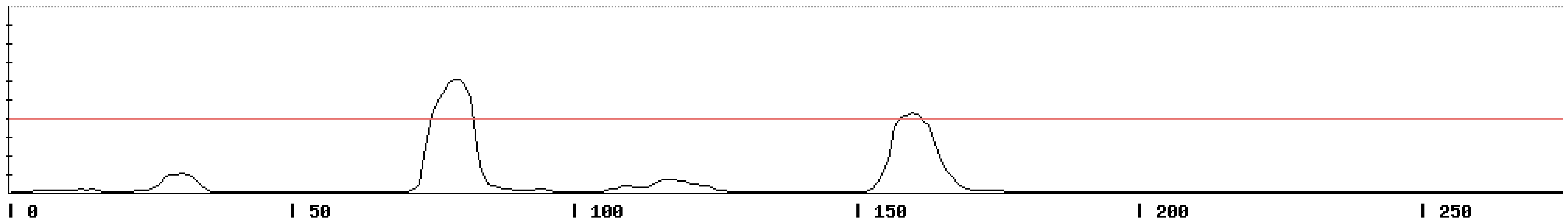

Predictions for Eucalyptusgrandis_Eucgr.G01075.1.p

Viterbi Path	
Viterbi Path	
Posterior @ 0.4	154 - KNRKKEKAK - 162
Posterior @ 0.4	

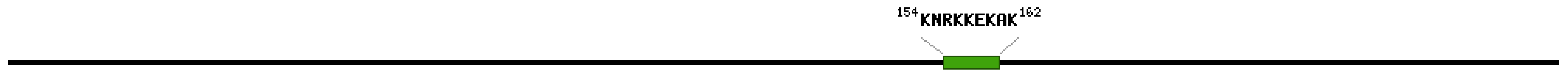

Posterior Graphic. Threshold Marked @ 0.4

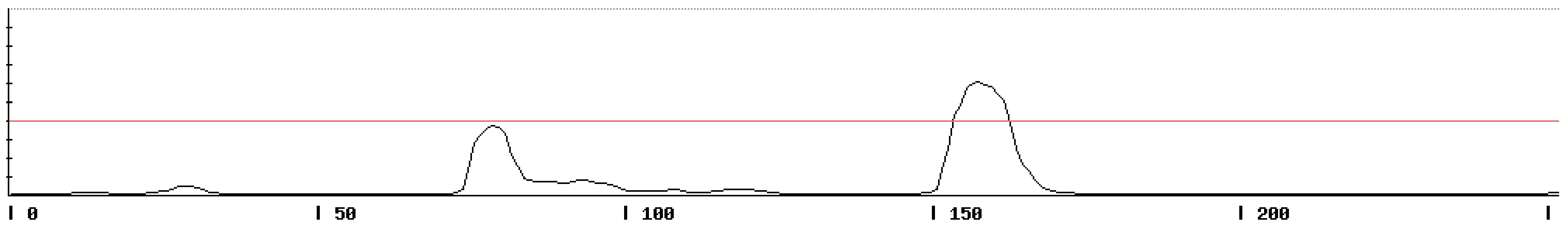

Predictions for Eucalyptusgrandis_Eucgr.G01077.1.p

Viterbi Path

Viterbi Path

Posterior @ 0.4

20 - RGHKRKRKIE - 29

103 - KSKK - 106

Posterior @ 0.4

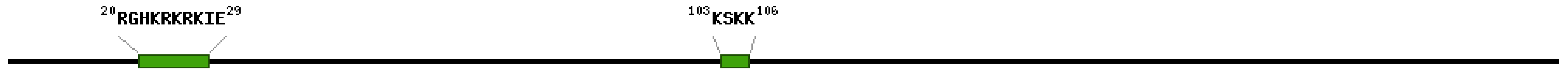

Posterior Graphic. Threshold Marked @ 0.4

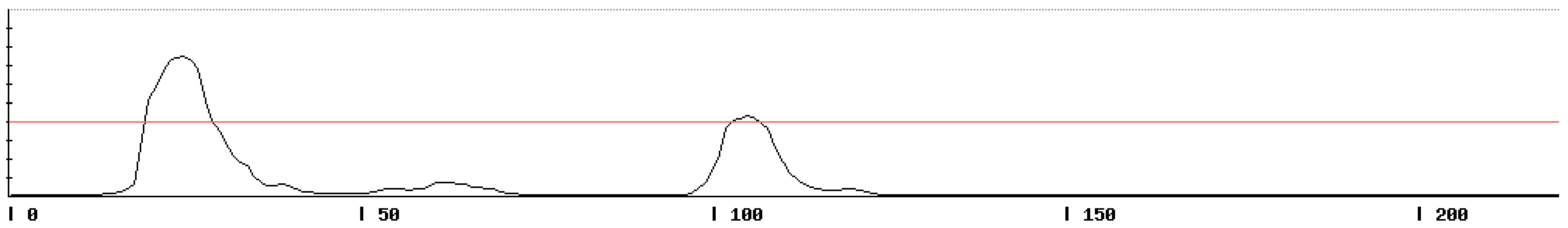

Predictions for Eucalyptusgrandis_Eucgr.G01078.1.p

Viterbi Path

Viterbi Path

Posterior @ 0.4 | 13 - RIRRPSLAKKAERRKG - 28

Posterior @ 0.4

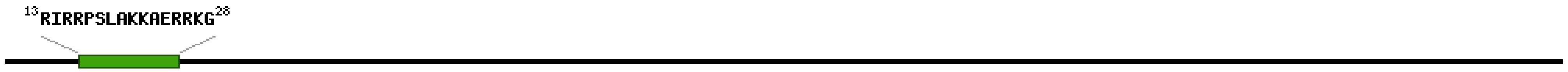

Posterior Graphic. Threshold Marked @ 0.4

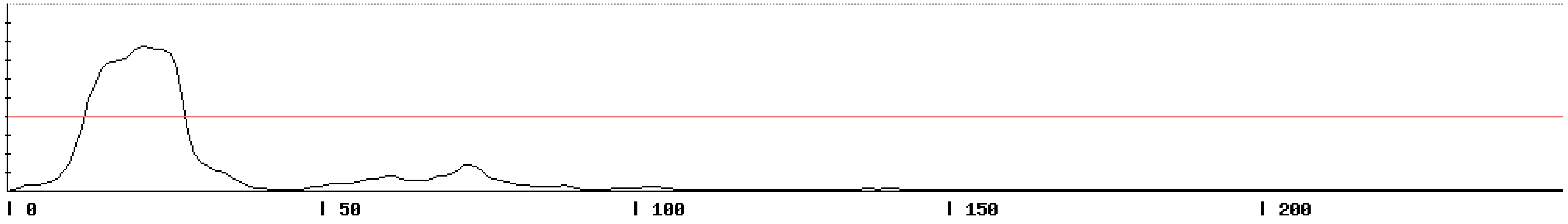

Predictions for Eucalyptusgrandis_Eucgr.G01081.1.p

Viterbi Path

Viterbi Path

Posterior @ 0.4

Posterior @ 0.4

Posterior Graphic. Threshold Marked @ 0.4

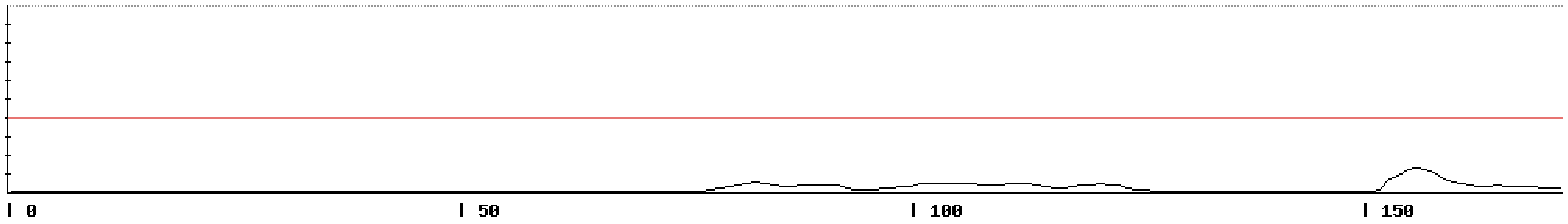

Predictions for Eucalyptusgrandis_Eucgr.G01082.1.p

Viterbi Path

Viterbi Path

Posterior @ 0.4

Posterior @ 0.4

Posterior Graphic. Threshold Marked @ 0.4

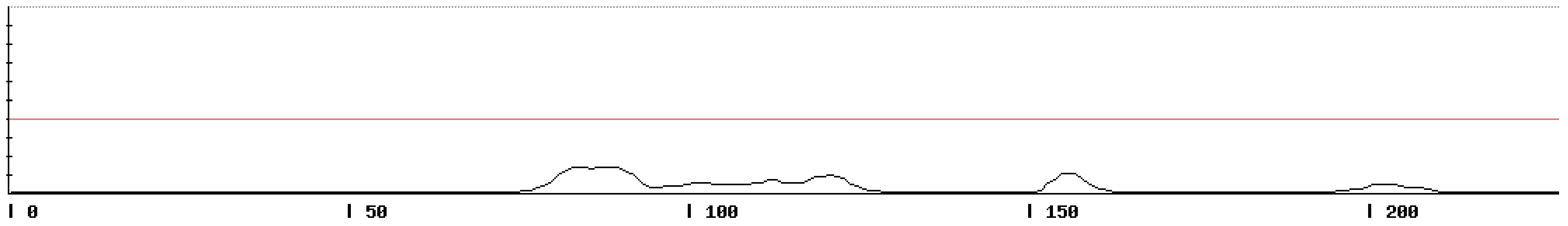

Predictions for Eucalyptusgrandis_Eucgr.G01083.1.p

Viterbi Path

Viterbi Path

Posterior @ 0.4

Posterior @ 0.4

Posterior Graphic. Threshold Marked @ 0.4

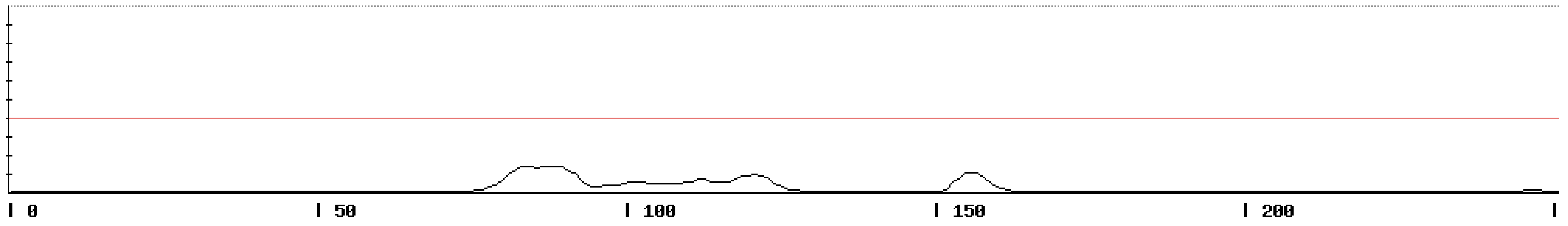

Predictions for Eucalyptusgrandis_Eucgr.G01507.1.p

Viterbi Path

Viterbi Path

Posterior @ 0.4 — 71 - RGRKRKTQLGFWKETSKKRVIRA - 93

Posterior @ 0.4

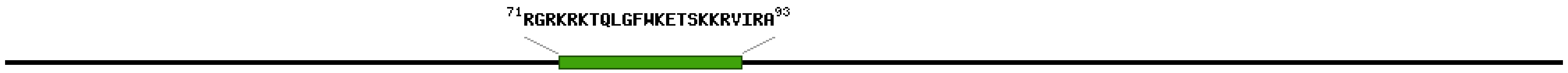

Posterior Graphic. Threshold Marked @ 0.4

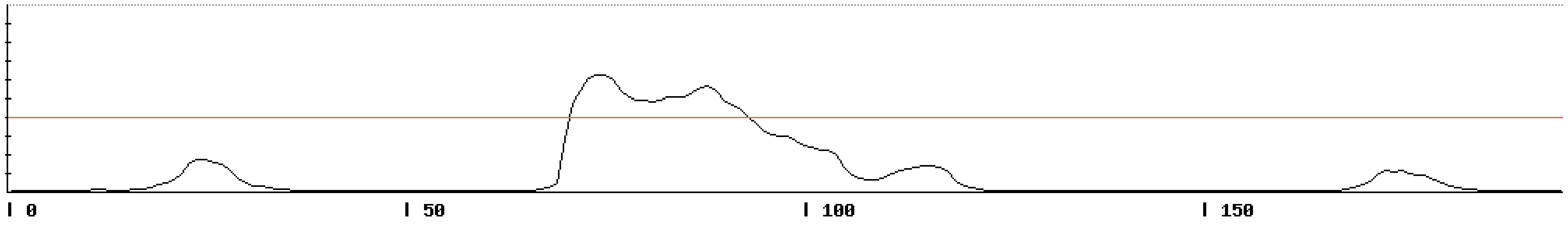

Predictions for Eucalyptusgrandis_Eucgr.G01548.1.p

Viterbi Path

Viterbi Path

Posterior @ 0.4

Posterior @ 0.4

Posterior Graphic. Threshold Marked @ 0.4

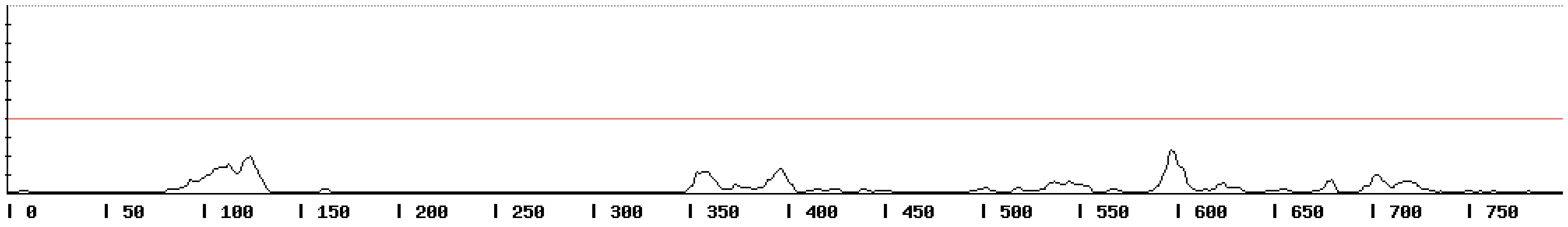

Predictions for Eucalyptusgrandis_Eucgr.G01550.1.p

Viterbi Path

Viterbi Path

Posterior @ 0.4

Posterior @ 0.4

Posterior Graphic. Threshold Marked @ 0.4

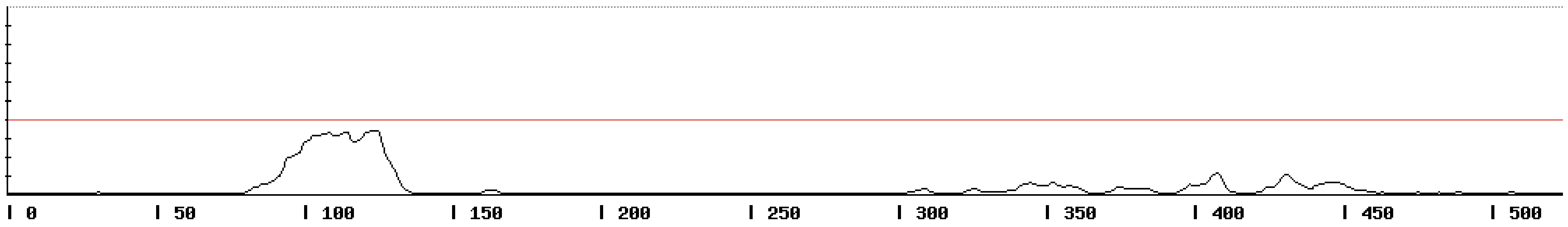

Predictions for Eucalyptusgrandis_Eucgr.G01551.1.p

Viterbi Path

Viterbi Path

Posterior @ 0.4

Posterior @ 0.4

Posterior Graphic. Threshold Marked @ 0.4

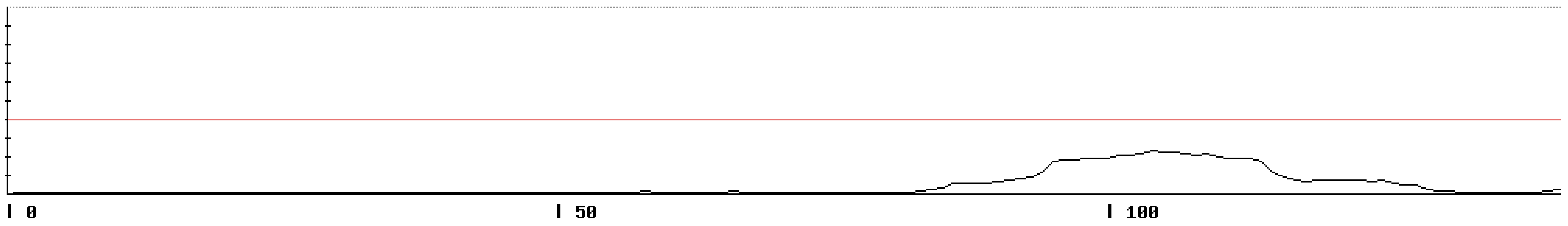

Predictions for Eucalyptusgrandis_Eucgr.G01758.1.p

Viterbi Path

Viterbi Path

Posterior @ 0.4 414 - RAKVRKYKERSPDRN - 428

Posterior @ 0.4

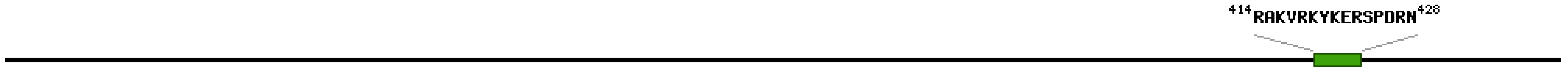

Posterior Graphic. Threshold Marked @ 0.4

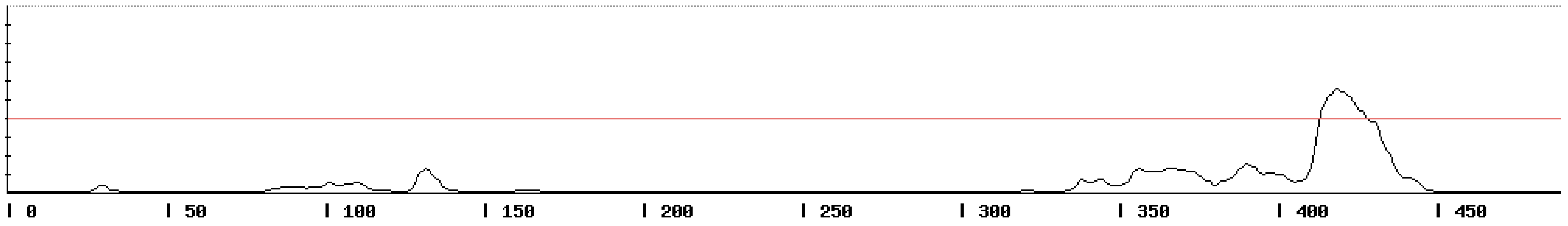

Predictions for Eucalyptusgrandis_Eucgr.G01984.1.p

Viterbi Path

Viterbi Path

Posterior @ 0.4

Posterior @ 0.4

Posterior Graphic. Threshold Marked @ 0.4

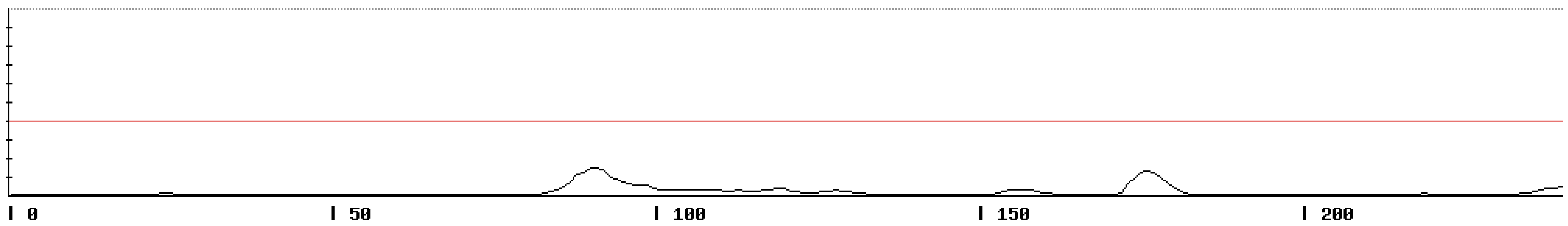

Predictions for Eucalyptusgrandis_Eucgr.G02349.1.p

Viterbi Path

Viterbi Path

Posterior @ 0.4 — 137 - KRR - 139

Posterior @ 0.4

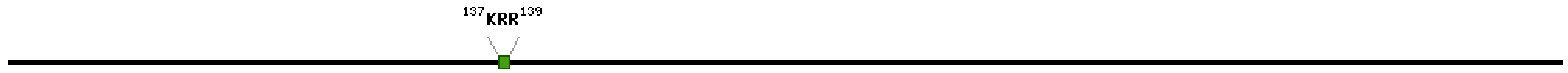

Posterior Graphic. Threshold Marked @ 0.4

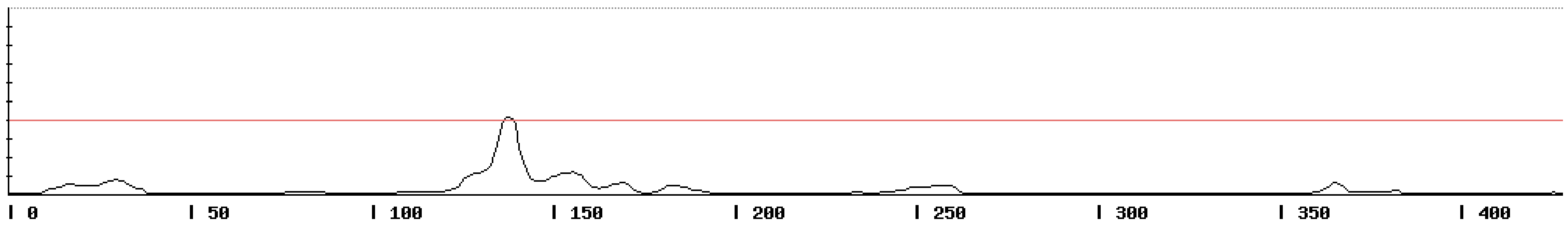

Predictions for Eucalyptusgrandis_Eucgr.G02486.1.p

Viterbi Path

Viterbi Path

Posterior @ 0.4

Posterior @ 0.4

Posterior Graphic. Threshold Marked @ 0.4

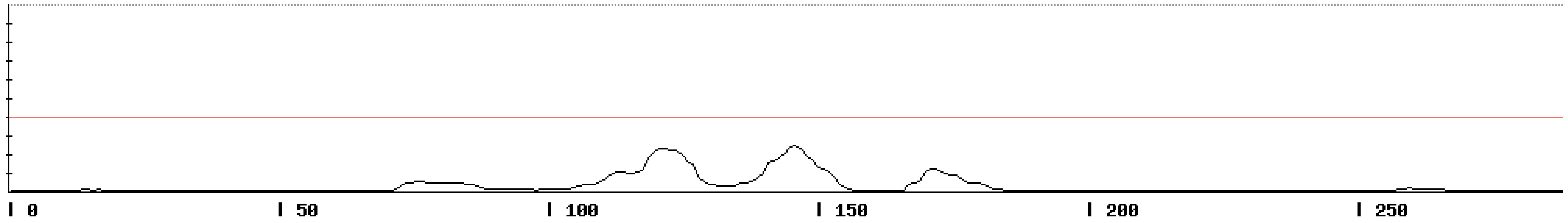

Predictions for Eucalyptusgrandis_Eucgr.G02506.1.p

Viterbi Path

Viterbi Path

Posterior @ 0.4

Posterior @ 0.4

Posterior Graphic. Threshold Marked @ 0.4

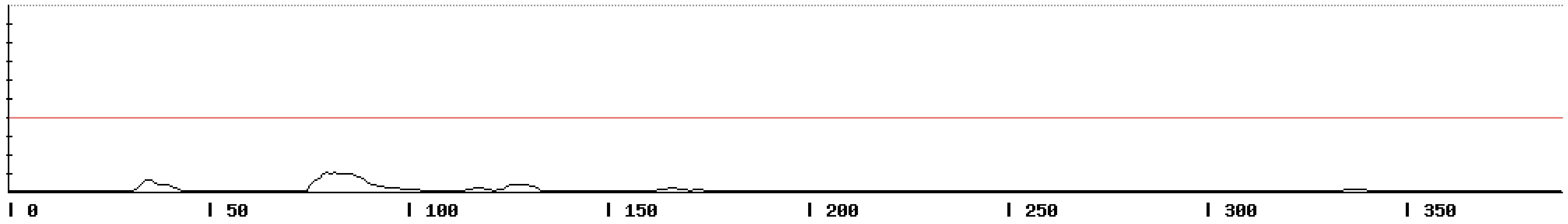

Predictions for Eucalyptusgrandis_Eucgr.G02740.1.p

Viterbi Path

Viterbi Path

Posterior @ 0.4

Posterior @ 0.4

Posterior Graphic. Threshold Marked @ 0.4

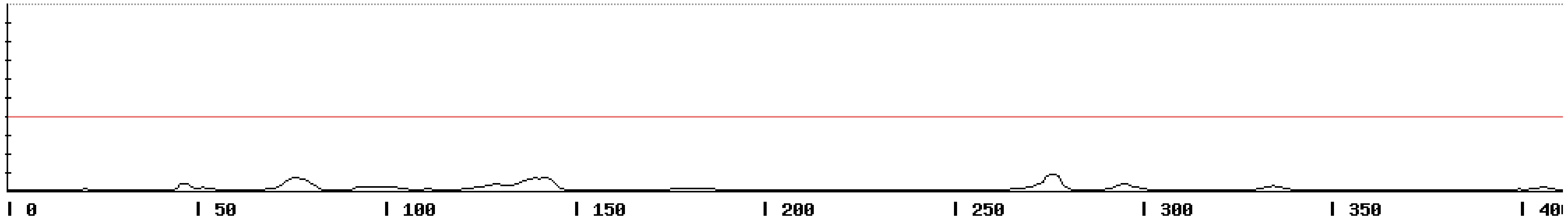

Predictions for Eucalyptusgrandis_Eucgr.H00614.1.p

Viterbi Path

Viterbi Path

Posterior @ 0.4

Posterior @ 0.4

Posterior Graphic. Threshold Marked @ 0.4

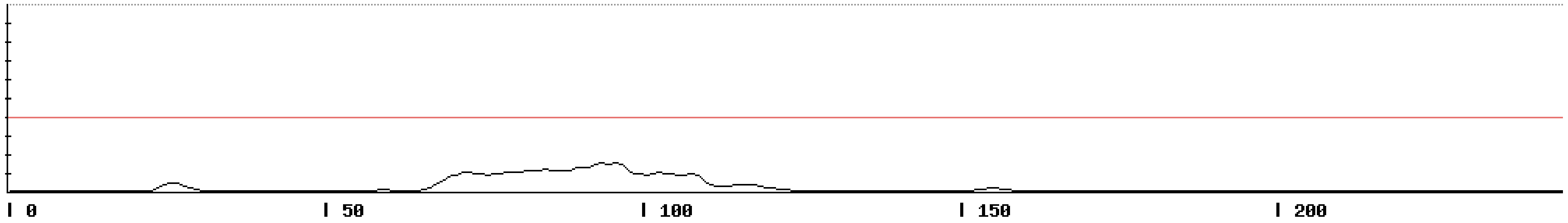

Predictions for Eucalyptusgrandis_Eucgr.H00826.1.p

Viterbi Path

Viterbi Path

Posterior @ 0.4

Posterior @ 0.4

Posterior Graphic. Threshold Marked @ 0.4

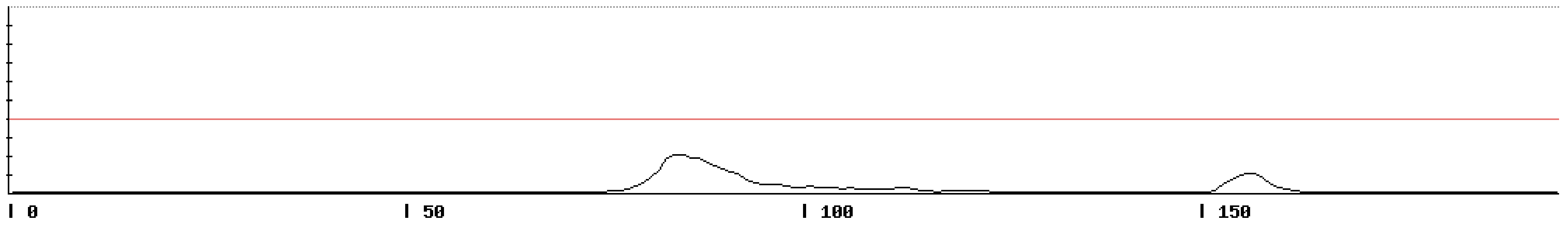

Predictions for Eucalyptusgrandis_Eucgr.H03362.1.p

Viterbi Path

Viterbi Path

Posterior @ 0.4

171 - KKMRPKRSDNGNGDFSHRKRKKS - 193

Posterior @ 0.4

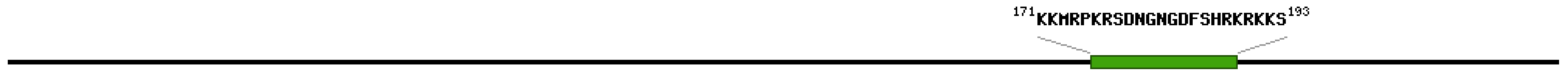

Posterior Graphic. Threshold Marked @ 0.4

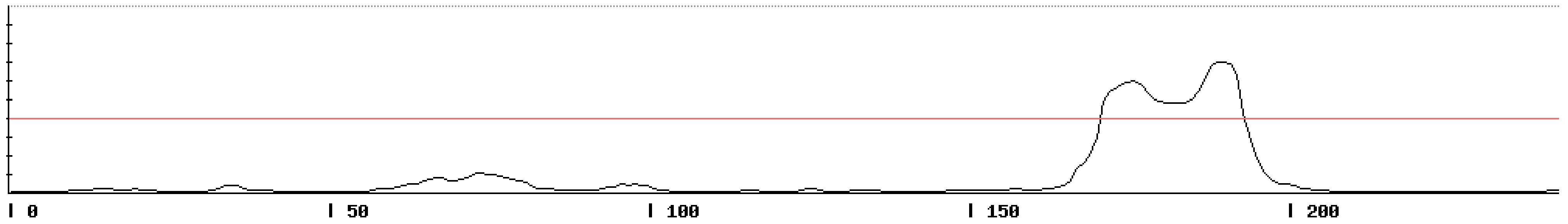

Predictions for Eucalyptusgrandis_Eucgr.H03387.1.p	
Viterbi Path	
Viterbi Path	
Posterior @ 0.4	
Posterior @ 0.4	
Posterior Graphic. Threshold Marked @ 0.4	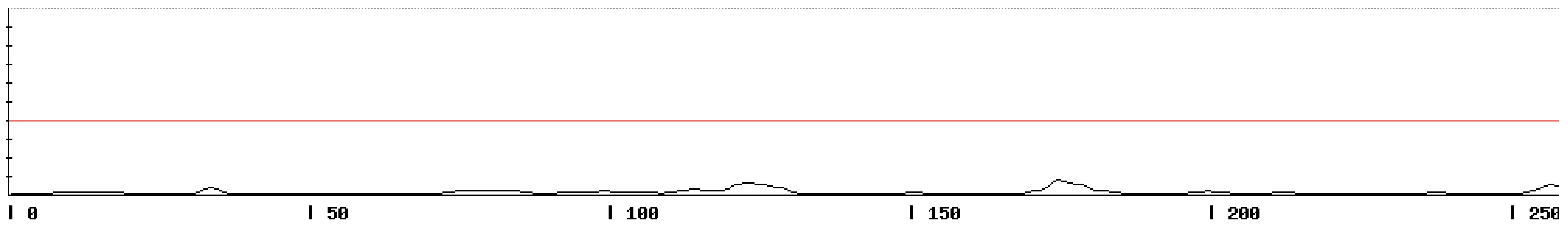

Predictions for Eucalyptusgrandis_Eucgr.H05089.1.p	
Viterbi Path	
Viterbi Path	
Posterior @ 0.4	94 - DKPIGRPKPLGIKKA - 108
Posterior @ 0.4	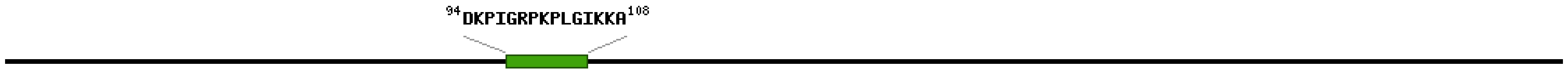
Posterior Graphic. Threshold Marked @ 0.4	

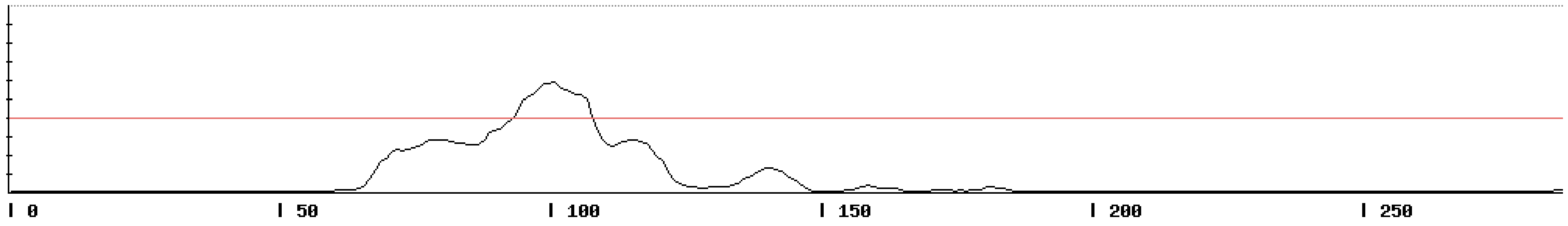

Predictions for Eucalyptusgrandis_Eucgr.I00056.1.p

Viterbi Path	
Viterbi Path	
Posterior @ 0.4	
Posterior @ 0.4	
Posterior Graphic. Threshold Marked @ 0.4	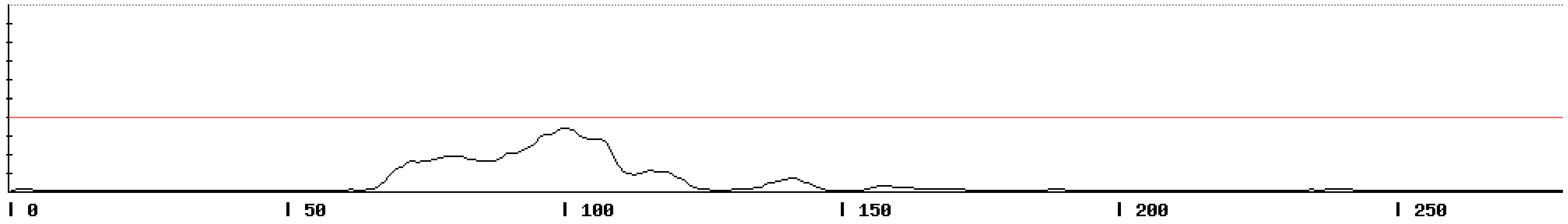

Predictions for Eucalyptusgrandis_Eucgr.I00057.1.p

Viterbi Path	
Viterbi Path	
Posterior @ 0.4	
Posterior @ 0.4	

Posterior Graphic. Threshold Marked @ 0.4

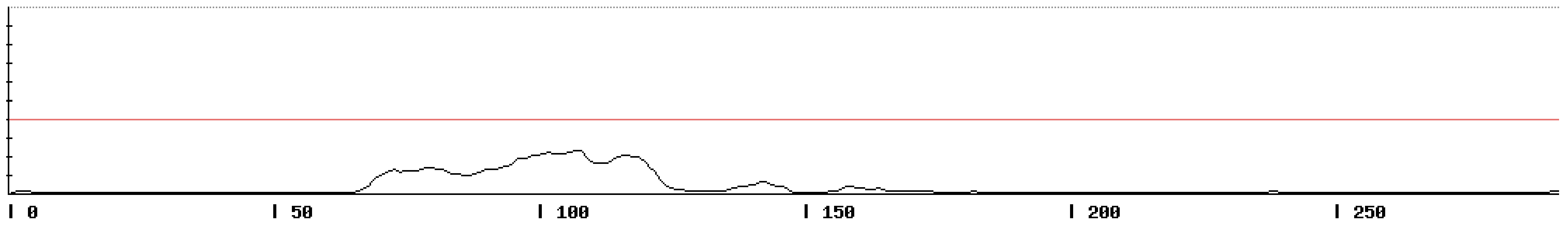

Predictions for Eucalyptusgrandis_Eucgr.I00058.1.p

Viterbi Path

Viterbi Path

Posterior @ 0.4

Posterior @ 0.4

Posterior Graphic. Threshold Marked @ 0.4

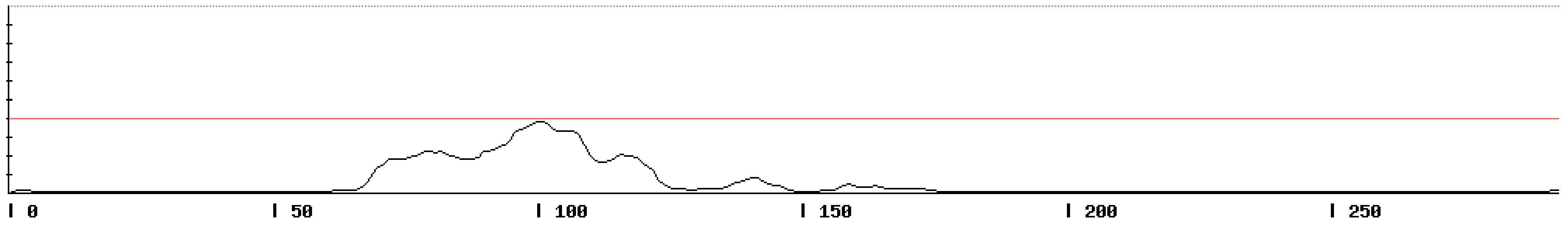

Predictions for Eucalyptusgrandis_Eucgr.I00059.1.p

Viterbi Path

Viterbi Path

Posterior @ 0.4

Posterior @ 0.4

Posterior Graphic. Threshold Marked @ 0.4

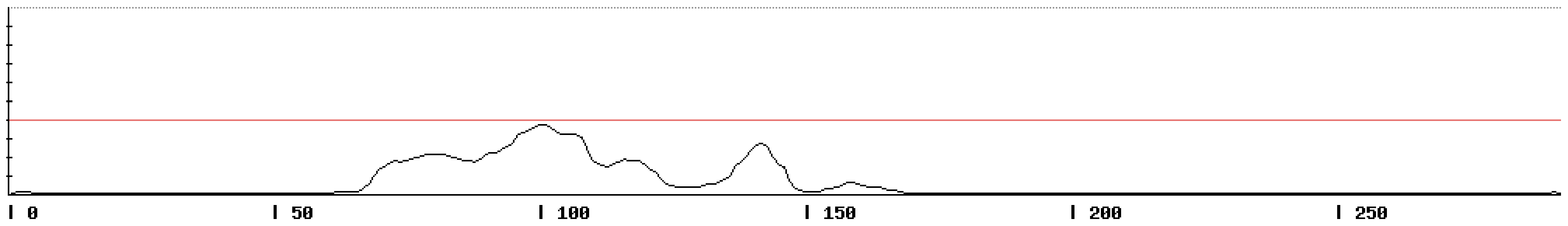

Predictions for Eucalyptusgrandis_Eucgr.I00060.1.p

Viterbi Path

Viterbi Path

Posterior @ 0.4

Posterior @ 0.4

Posterior Graphic. Threshold Marked @ 0.4

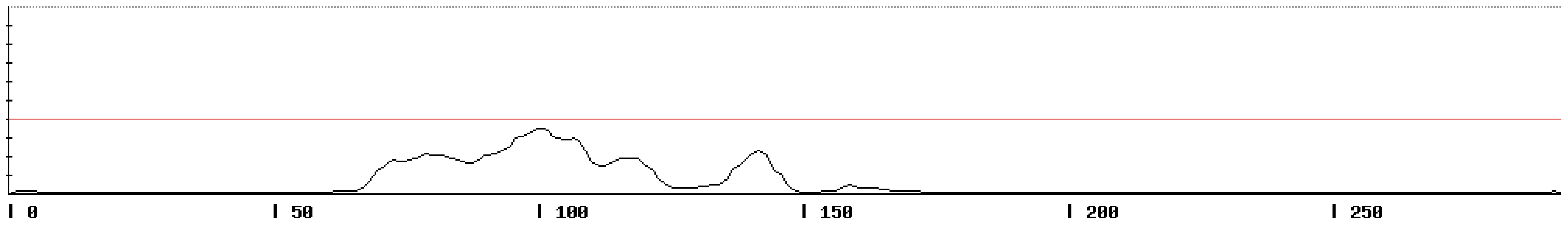

Predictions for Eucalyptusgrandis_Eucgr.I00095.1.p

Viterbi Path

Viterbi Path

Posterior @ 0.4

Posterior @ 0.4

Posterior Graphic. Threshold Marked @ 0.4

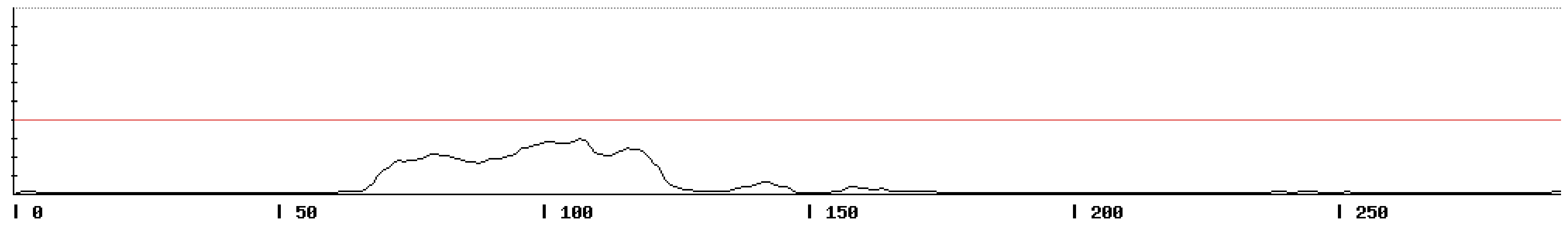

Predictions for Eucalyptusgrandis_Eucgr.I00097.1.p

Viterbi Path

Viterbi Path

Posterior @ 0.4

Posterior @ 0.4

Posterior Graphic. Threshold Marked @ 0.4

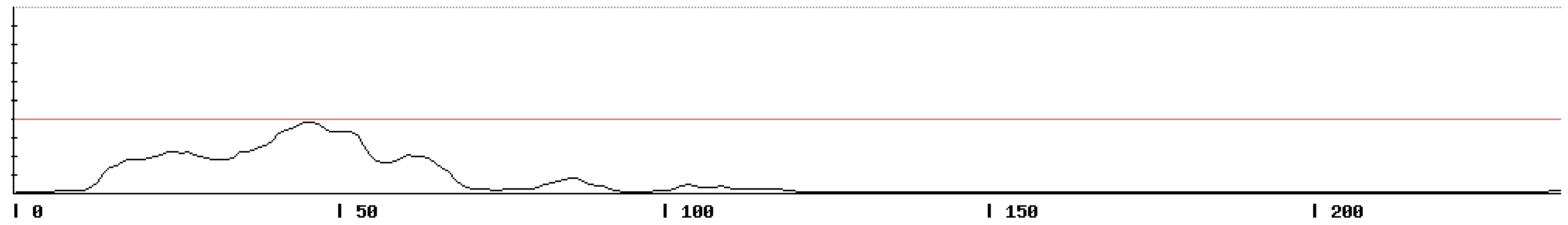

Predictions for Eucalyptusgrandis_Eucgr.I00098.1.p

Viterbi Path

Viterbi Path

Posterior @ 0.4 — 17 - RVSRRRRTESREERERERKMKKA - 40

Posterior @ 0.4

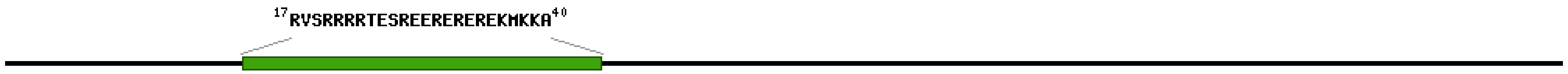

Posterior Graphic. Threshold Marked @ 0.4

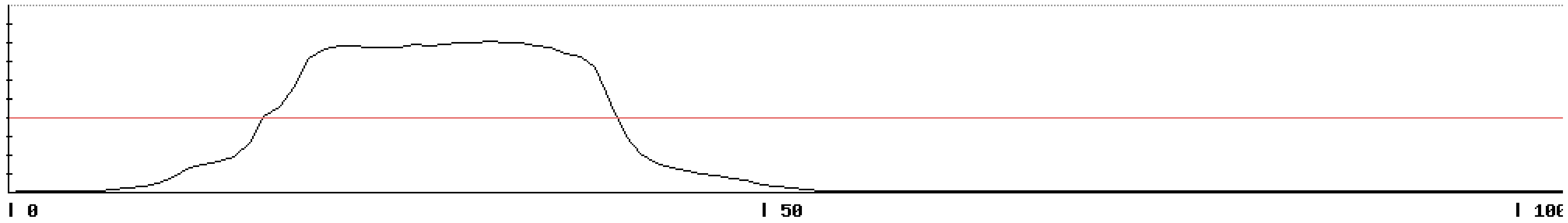

Predictions for Eucalyptusgrandis_Eucgr.I00099.1.p

Viterbi Path

Viterbi Path

Posterior @ 0.4 — 140 - RKK - 142

Posterior @ 0.4

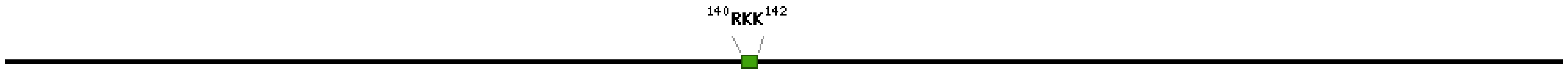

Posterior Graphic. Threshold Marked @ 0.4

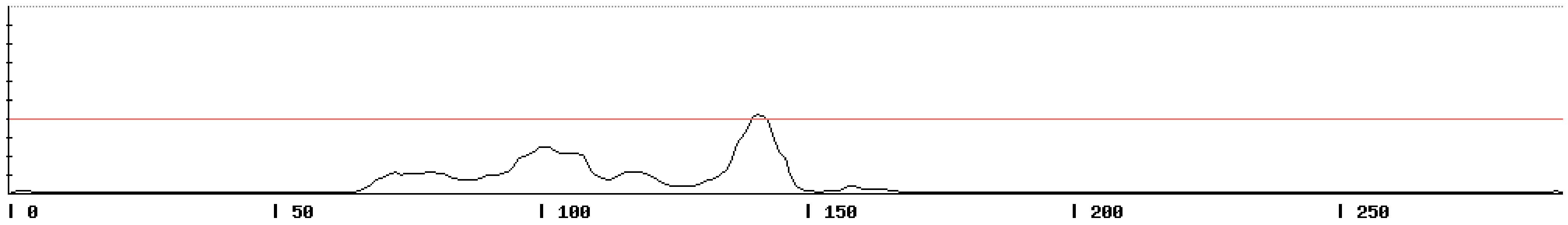

Predictions for Eucalyptusgrandis_Eucgr.I00100.1.p

Viterbi Path	
Viterbi Path	
Posterior @ 0.4	
Posterior @ 0.4	
Posterior Graphic. Threshold Marked @ 0.4	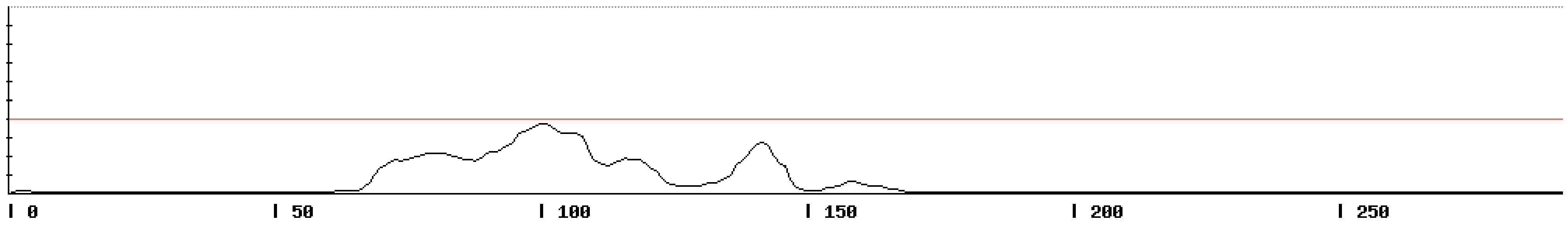

Predictions for Eucalyptusgrandis_Eucgr.I00101.1.p

Viterbi Path	
Viterbi Path	
Posterior @ 0.4	
Posterior @ 0.4	
Posterior Graphic. Threshold Marked @ 0.4	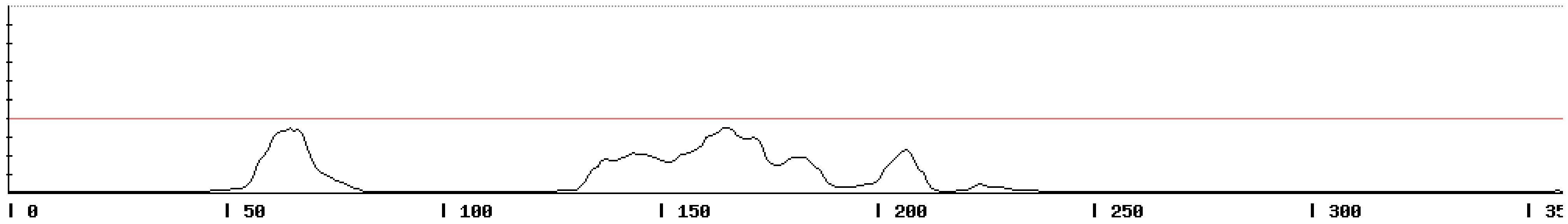

Predictions for Eucalyptusgrandis_Eucgr.I00102.1.p

Viterbi Path

Viterbi Path

Posterior @ 0.4

Posterior @ 0.4

Posterior Graphic. Threshold Marked @ 0.4

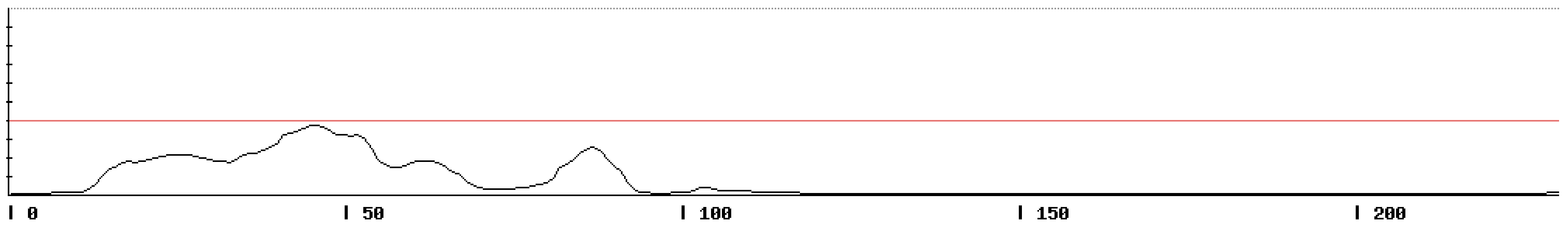

Predictions for Eucalyptusgrandis_Eucgr.I00191.1.p

Viterbi Path

Viterbi Path

Posterior @ 0.4

Posterior @ 0.4

Posterior Graphic. Threshold Marked @ 0.4

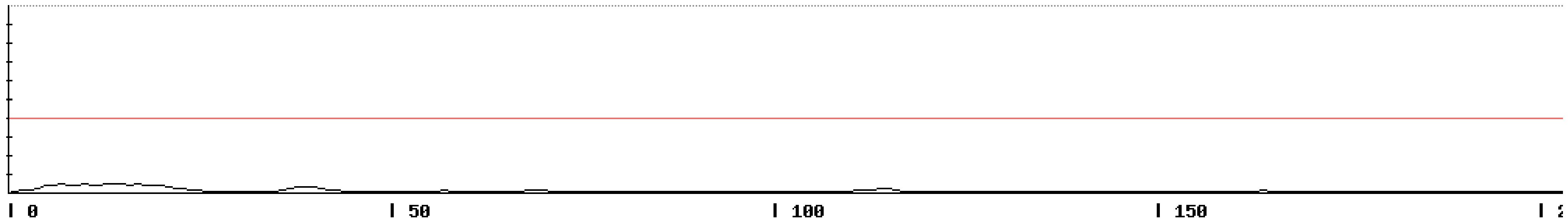

Predictions for Eucalyptusgrandis_Eucgr.I00192.1.p

Viterbi Path

Viterbi Path

Posterior @ 0.4

Posterior @ 0.4

Posterior Graphic. Threshold Marked @ 0.4

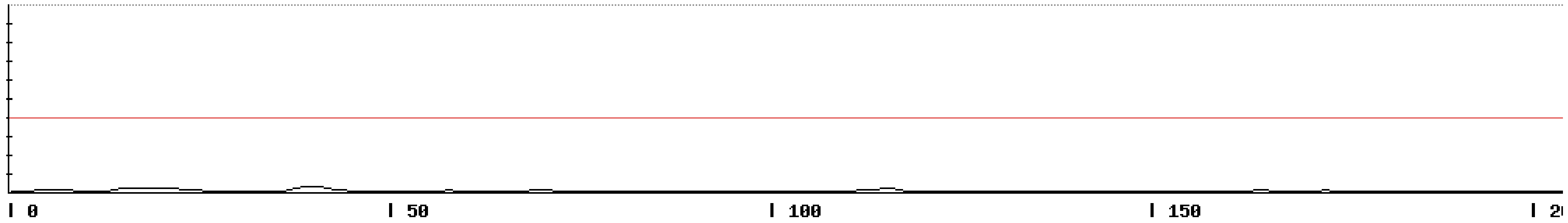

Predictions for Eucalyptusgrandis_Eucgr.I00193.1.p

Viterbi Path

Viterbi Path

Posterior @ 0.4

Posterior @ 0.4

Posterior Graphic. Threshold Marked @ 0.4	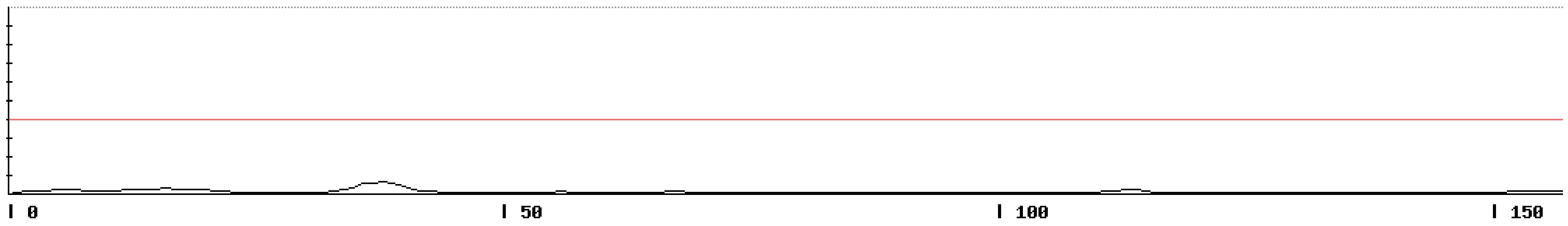

Predictions for Eucalyptusgrandis_Eucgr.I00213.1.p

Viterbi Path	
Viterbi Path	
Posterior @ 0.4	
Posterior @ 0.4	
Posterior Graphic. Threshold Marked @ 0.4	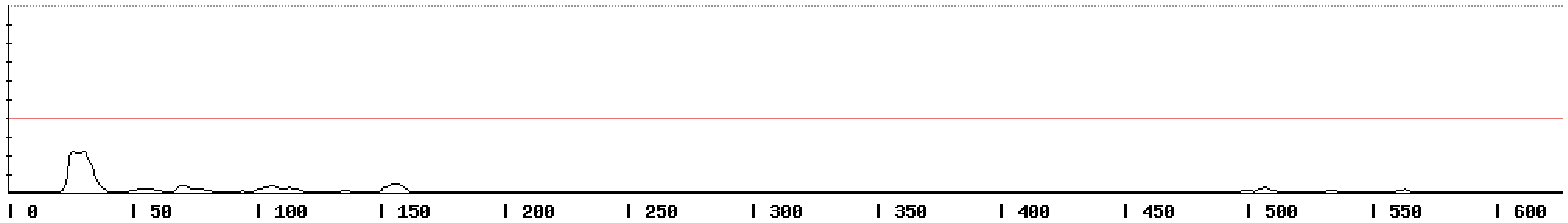

Predictions for Eucalyptusgrandis_Eucgr.I00583.1.p

Viterbi Path	
Viterbi Path	
Posterior @ 0.4	
Posterior @ 0.4	

Posterior Graphic. Threshold Marked @ 0.4

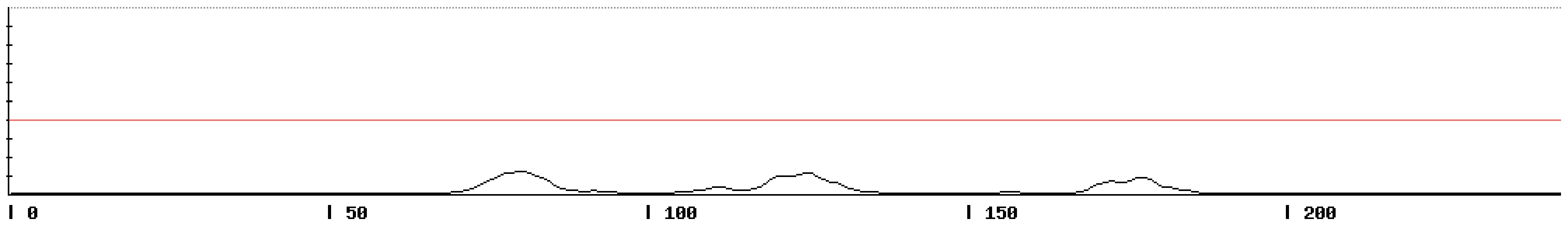

Predictions for Eucalyptusgrandis_Eucgr.I00587.1.p

Viterbi Path

Viterbi Path

Posterior @ 0.4

Posterior @ 0.4

Posterior Graphic. Threshold Marked @ 0.4

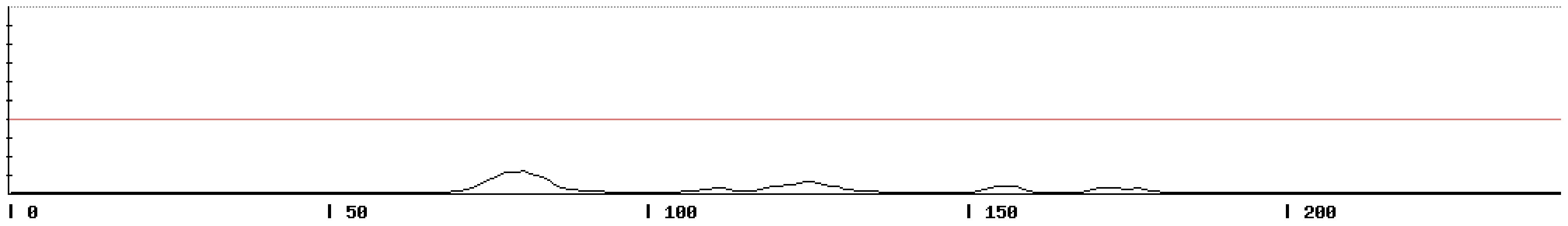

Predictions for Eucalyptusgrandis_Eucgr.I01494.1.p

Viterbi Path

Viterbi Path

Posterior @ 0.4 — 82 - R - 82

Posterior @ 0.4

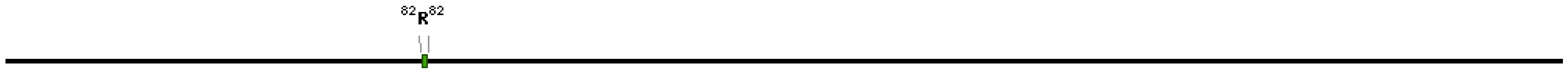

Posterior Graphic. Threshold Marked @ 0.4

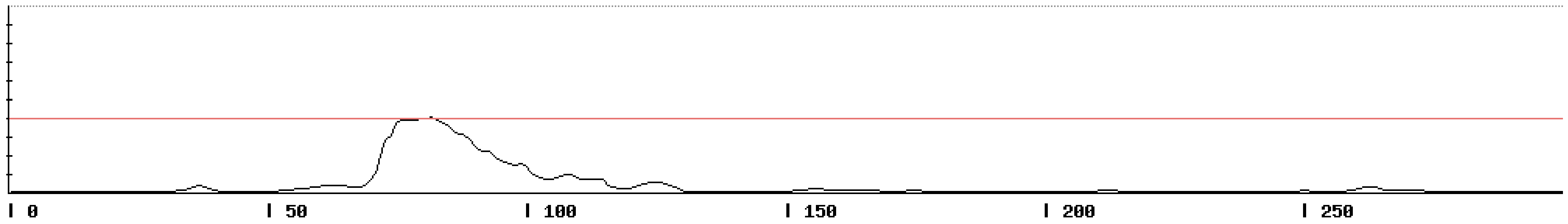

Predictions for Eucalyptusgrandis_Eucgr.I01940.1.p

Viterbi Path

Viterbi Path

Posterior @ 0.4

Posterior @ 0.4

Posterior Graphic. Threshold Marked @ 0.4

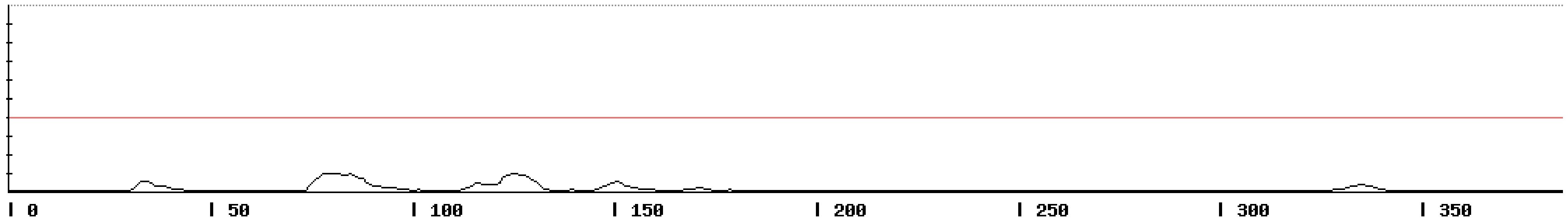

Predictions for Eucalyptusgrandis_Eucgr.I01958.1.p

Viterbi Path

Viterbi Path

Posterior @ 0.4	102 - KDKEIRRMKKVV - 113
Posterior @ 0.4	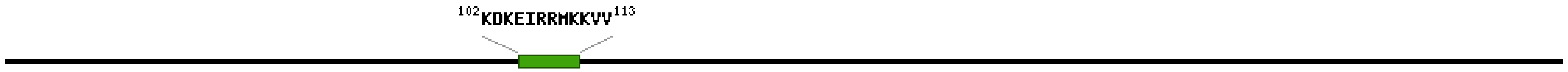
Posterior Graphic. Threshold Marked @ 0.4	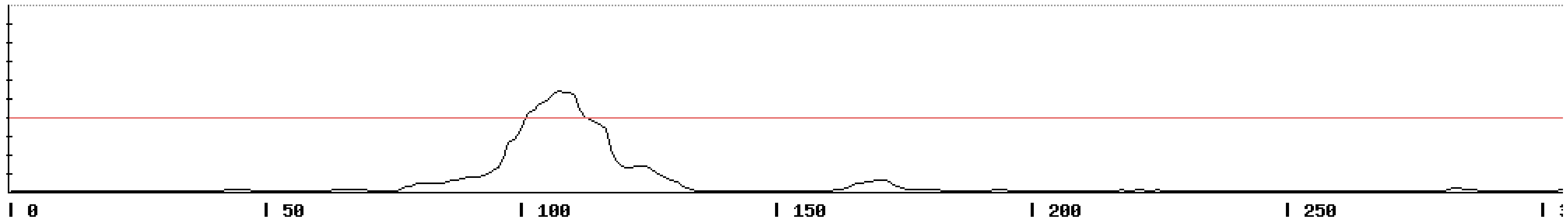

Predictions for Eucalyptusgrandis_Eucgr.I02366.1.p

Viterbi Path	
Viterbi Path	
Posterior @ 0.4	
Posterior @ 0.4	
Posterior Graphic. Threshold Marked @ 0.4	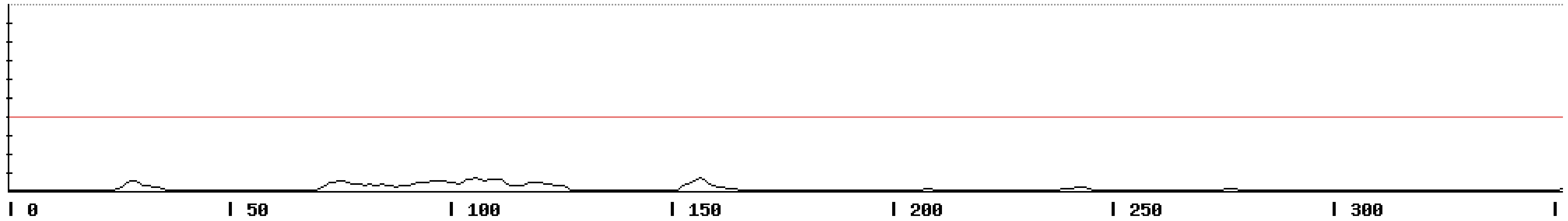

Predictions for Eucalyptusgrandis_Eucgr.I02571.1.p

Viterbi Path

Viterbi Path

Posterior @ 0.4

Posterior @ 0.4

Posterior Graphic. Threshold Marked @ 0.4

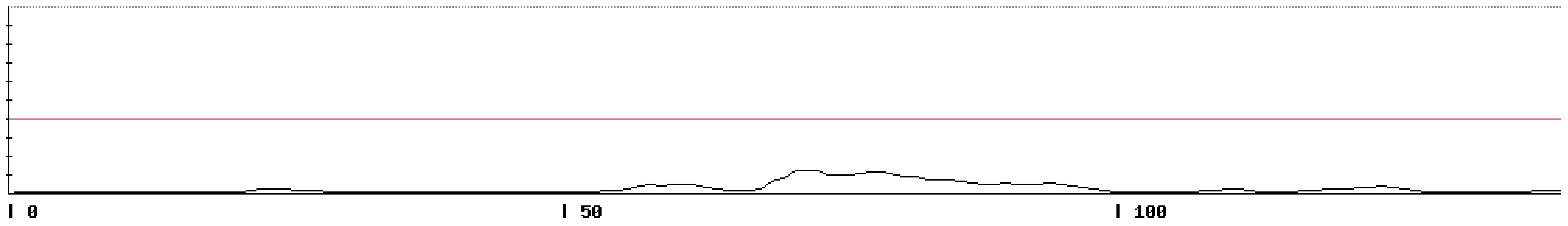

Predictions for Eucalyptusgrandis_Eucgr.I02578.1.p

Viterbi Path

Viterbi Path

Posterior @ 0.4

Posterior @ 0.4

Posterior Graphic. Threshold Marked @ 0.4

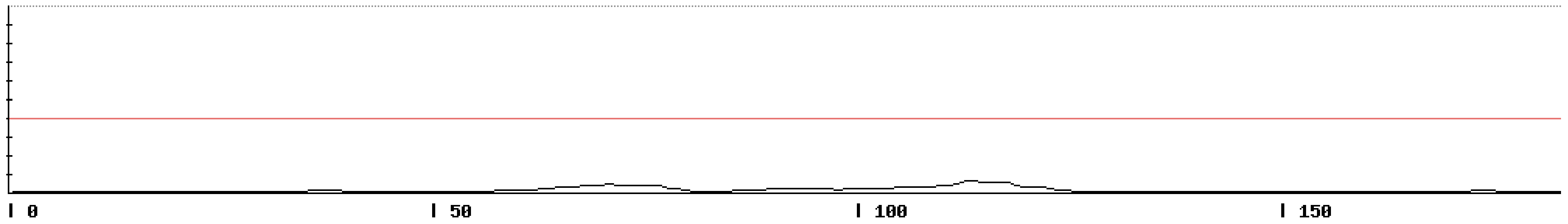

Predictions for Eucalyptusgrandis_Eucgr.I02695.1.p

Viterbi Path	
Viterbi Path	
Posterior @ 0.4	
Posterior @ 0.4	
Posterior Graphic. Threshold Marked @ 0.4	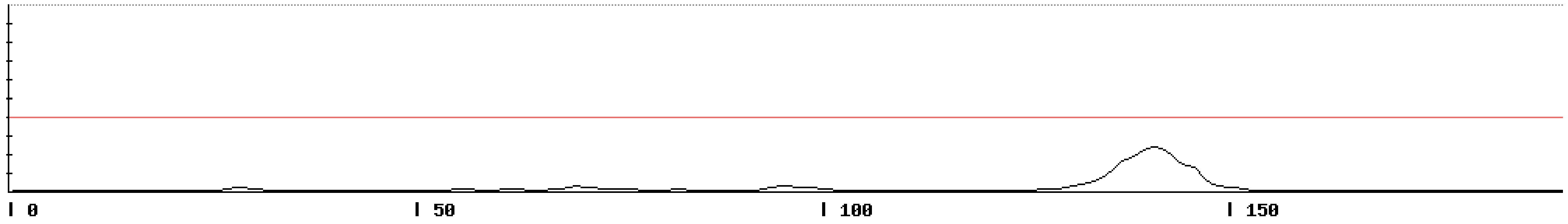

Predictions for Eucalyptusgrandis_Eucgr.J00505.1.p

Viterbi Path	
Viterbi Path	
Posterior @ 0.4	
Posterior @ 0.4	
Posterior Graphic. Threshold Marked @ 0.4	

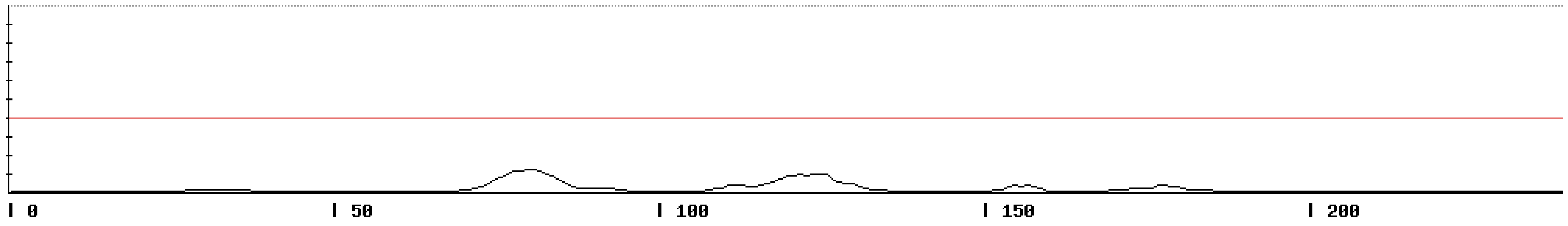

Predictions for Eucalyptusgrandis_Eucgr.J00508.1.p

Viterbi Path	
Viterbi Path	
Posterior @ 0.4	
Posterior @ 0.4	
Posterior Graphic. Threshold Marked @ 0.4	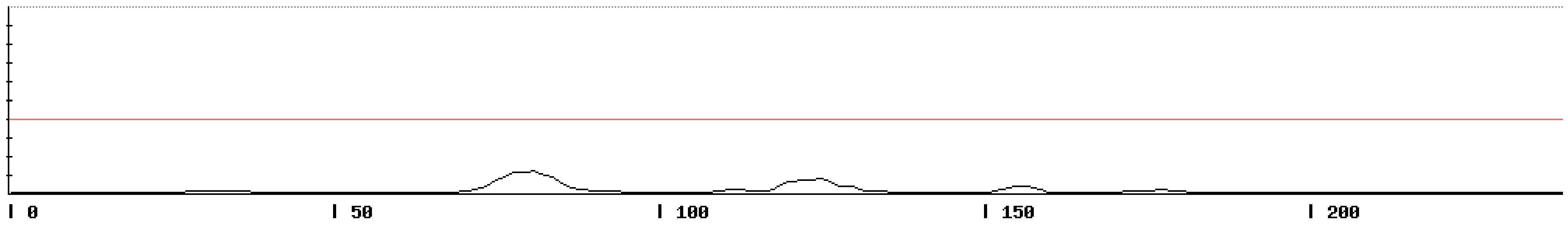

Predictions for Eucalyptusgrandis_Eucgr.J00509.1.p

Viterbi Path	
Viterbi Path	
Posterior @ 0.4	
Posterior @ 0.4	

Posterior Graphic. Threshold Marked @ 0.4

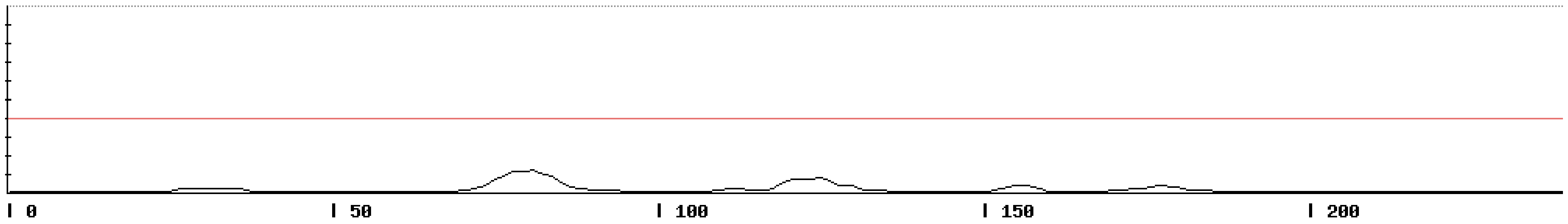

Predictions for Eucalyptusgrandis_Eucgr.J00511.1.p

Viterbi Path

Viterbi Path

Posterior @ 0.4

Posterior @ 0.4

Posterior Graphic. Threshold Marked @ 0.4

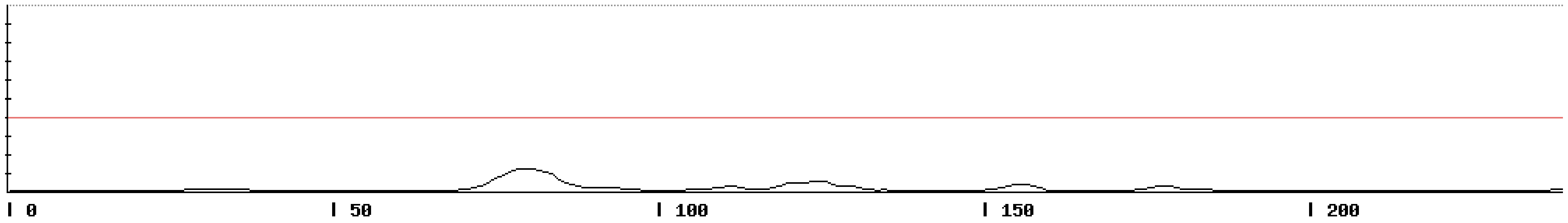

Predictions for Eucalyptusgrandis_Eucgr.J00512.1.p

Viterbi Path

Viterbi Path

Posterior @ 0.4

Posterior @ 0.4

Posterior Graphic. Threshold Marked @ 0.4

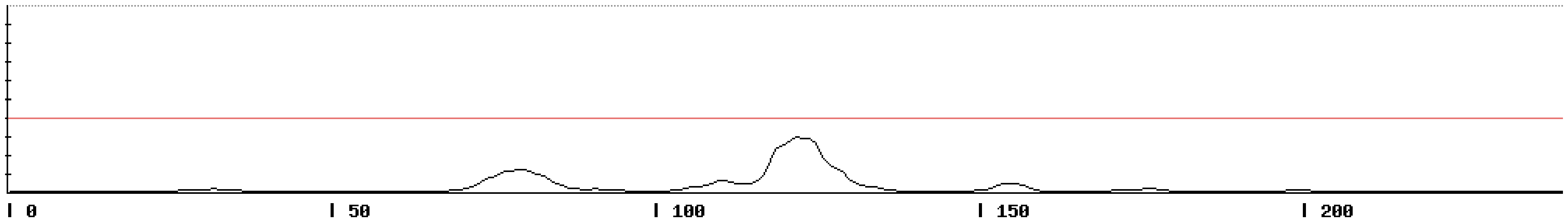

Predictions for Eucalyptusgrandis_Eucgr.J00513.1.p

Viterbi Path

Viterbi Path

Posterior @ 0.4

Posterior @ 0.4

Posterior Graphic. Threshold Marked @ 0.4

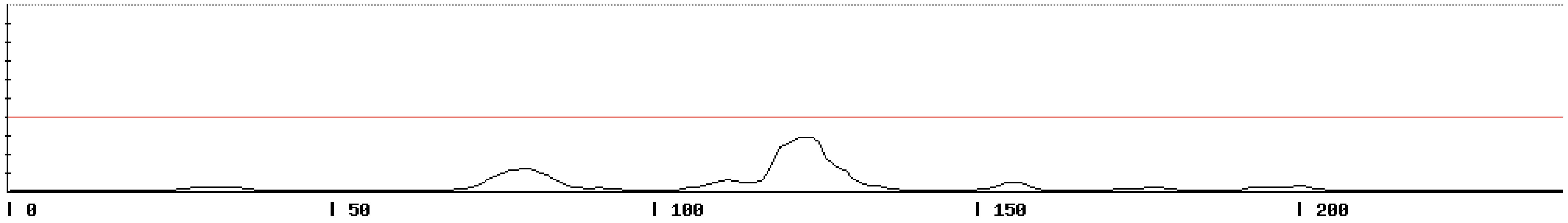

Predictions for Eucalyptusgrandis_Eucgr.J00514.1.p

Viterbi Path

Viterbi Path

Posterior @ 0.4

Posterior @ 0.4

Posterior Graphic. Threshold Marked @ 0.4

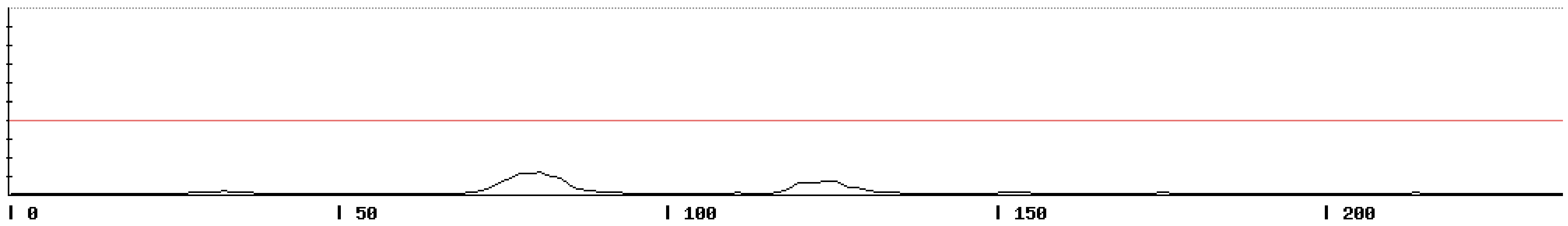

Predictions for Eucalyptusgrandis_Eucgr.J00516.1.p

Viterbi Path

Viterbi Path

Posterior @ 0.4 — 122 - PRGRK - 126

Posterior @ 0.4

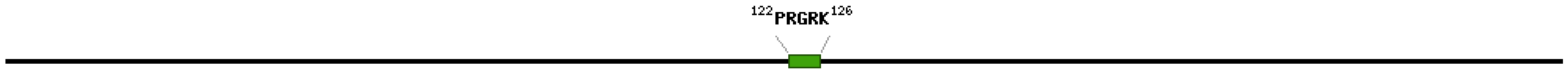

Posterior Graphic. Threshold Marked @ 0.4

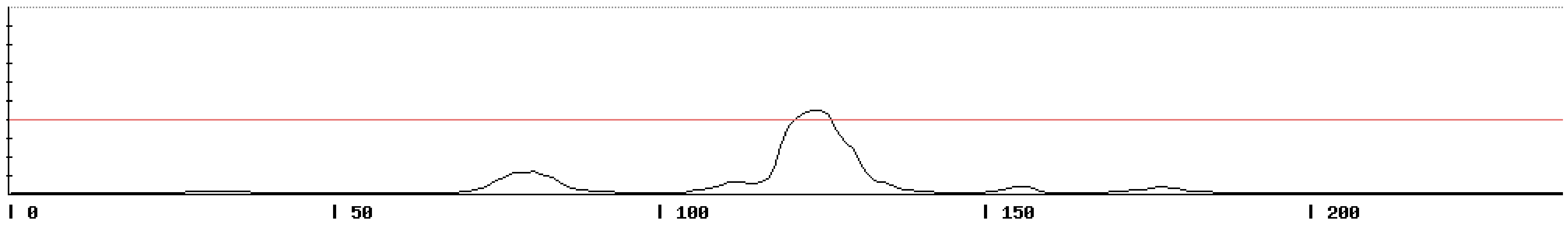

Predictions for Eucalyptusgrandis_Eucgr.J00517.1.p

Viterbi Path

Viterbi Path

Posterior @ 0.4	
Posterior @ 0.4	
Posterior Graphic. Threshold Marked @ 0.4	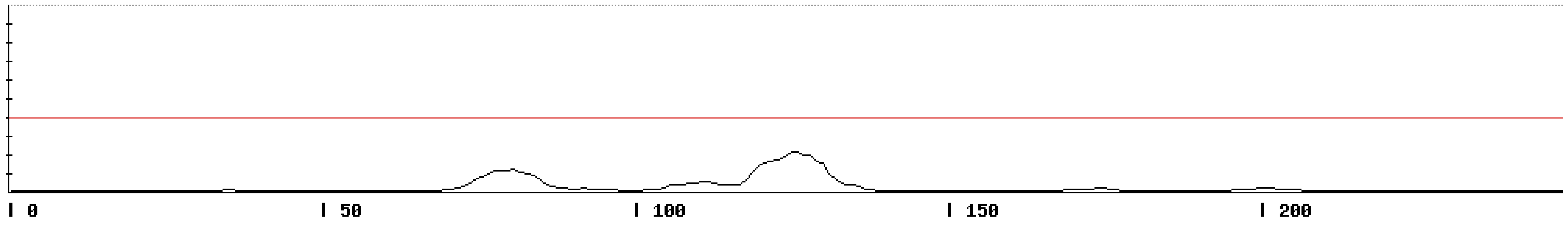

Predictions for Eucalyptusgrandis_Eucgr.J00518.1.p	
Viterbi Path	
Viterbi Path	
Posterior @ 0.4	
Posterior @ 0.4	
Posterior Graphic. Threshold Marked @ 0.4	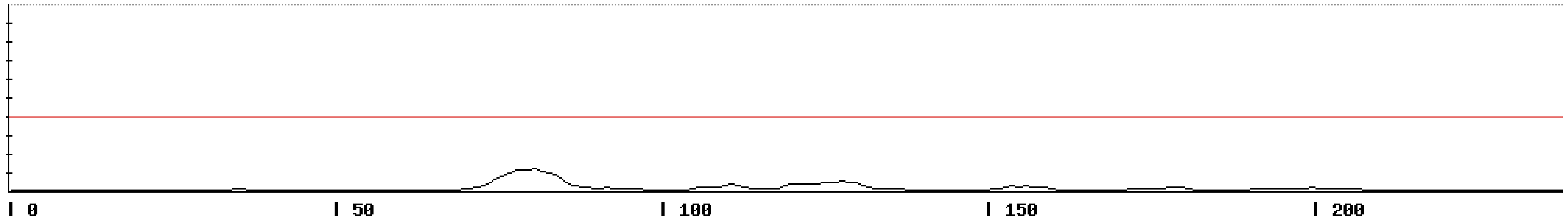

Predictions for Eucalyptusgrandis_Eucgr.J00519.1.p

Viterbi Path

Viterbi Path

Posterior @ 0.4

Posterior @ 0.4

Posterior Graphic. Threshold Marked @ 0.4

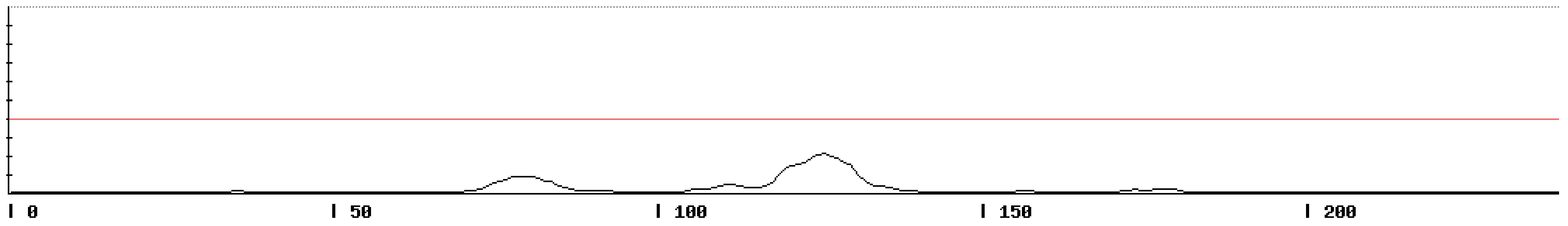

Predictions for Eucalyptusgrandis_Eucgr.J00520.1.p

Viterbi Path

Viterbi Path

Posterior @ 0.4

Posterior @ 0.4

Posterior Graphic. Threshold Marked @ 0.4

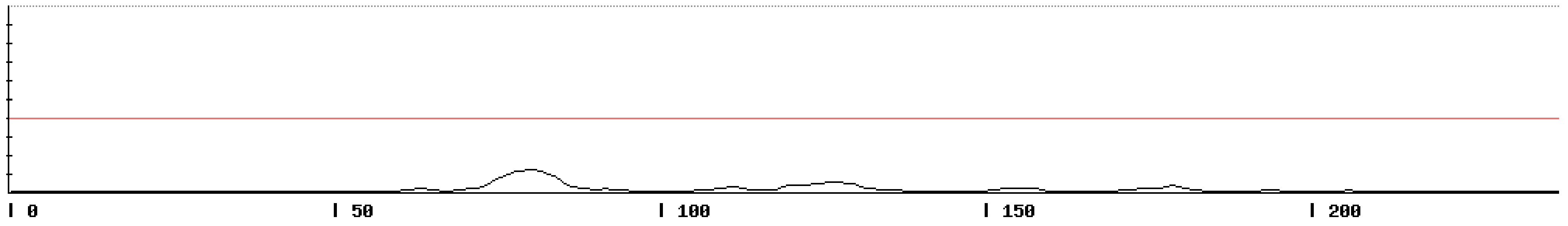

Predictions for Eucalyptusgrandis_Eucgr.J00521.1.p

Viterbi Path

Viterbi Path

Posterior @ 0.4

Posterior @ 0.4

Posterior Graphic. Threshold Marked @ 0.4

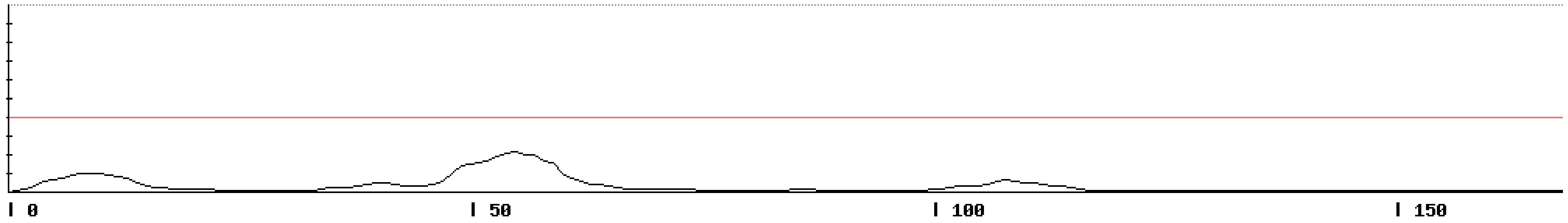

Predictions for Eucalyptusgrandis_Eucgr.J00531.1.p

Viterbi Path

Viterbi Path

Posterior @ 0.4

Posterior @ 0.4

Posterior Graphic. Threshold Marked @ 0.4

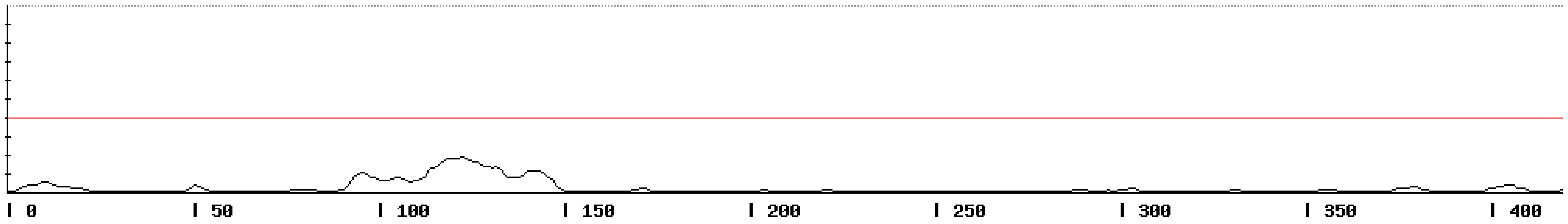

Predictions for Eucalyptusgrandis_Eucgr.J00940.1.p	
Viterbi Path	
Viterbi Path	
Posterior @ 0.4	
Posterior @ 0.4	
Posterior Graphic. Threshold Marked @ 0.4	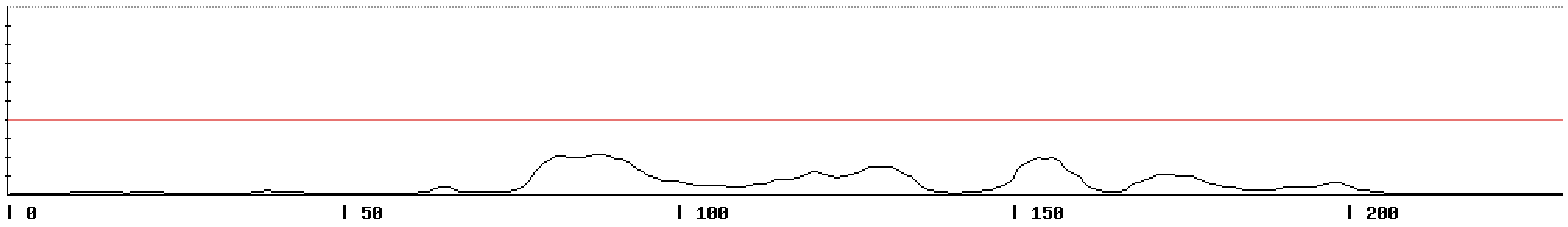

Predictions for Eucalyptusgrandis_Eucgr.J01038.1.p	
Viterbi Path	
Viterbi Path	
Posterior @ 0.4	
Posterior @ 0.4	

Posterior Graphic. Threshold Marked @ 0.4

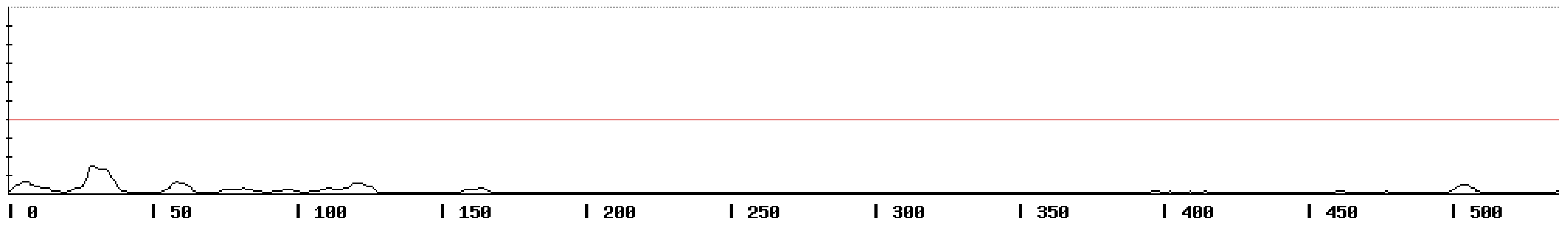

Predictions for Eucalyptusgrandis_Eucgr.J02254.1.p

Viterbi Path

Viterbi Path

Posterior @ 0.4 167 - KRR - 169

Posterior @ 0.4

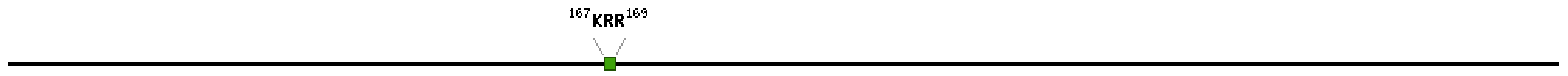

Posterior Graphic. Threshold Marked @ 0.4

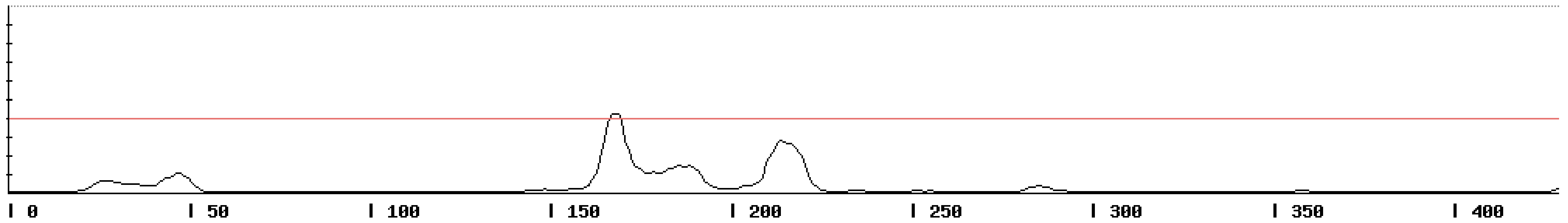

Predictions for Eucalyptusgrandis_Eucgr.K01061.1.p

Viterbi Path

Viterbi Path

Posterior @ 0.4 139 - NRKRRK - 144

Posterior @ 0.4

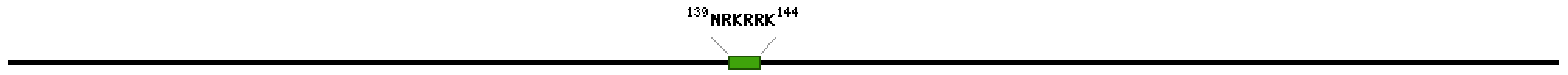

Posterior Graphic. Threshold Marked @ 0.4	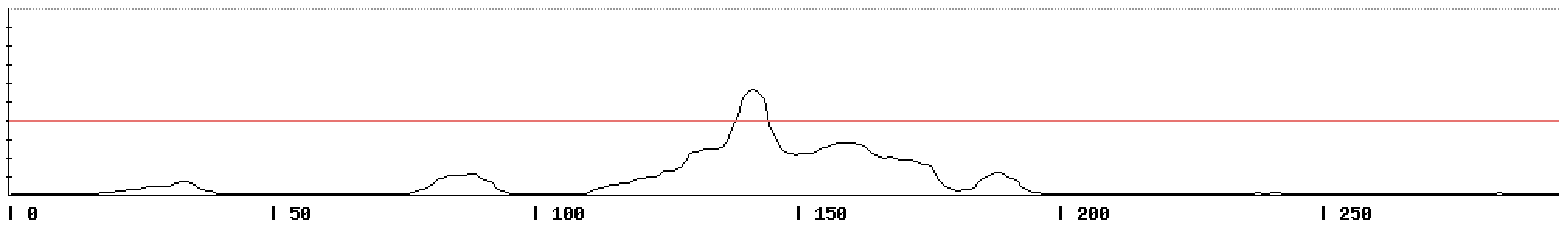

Predictions for Eucalyptusgrandis_Eucgr.K01228.1.p	
Viterbi Path	
Viterbi Path	
Posterior @ 0.4	
Posterior @ 0.4	
Posterior Graphic. Threshold Marked @ 0.4	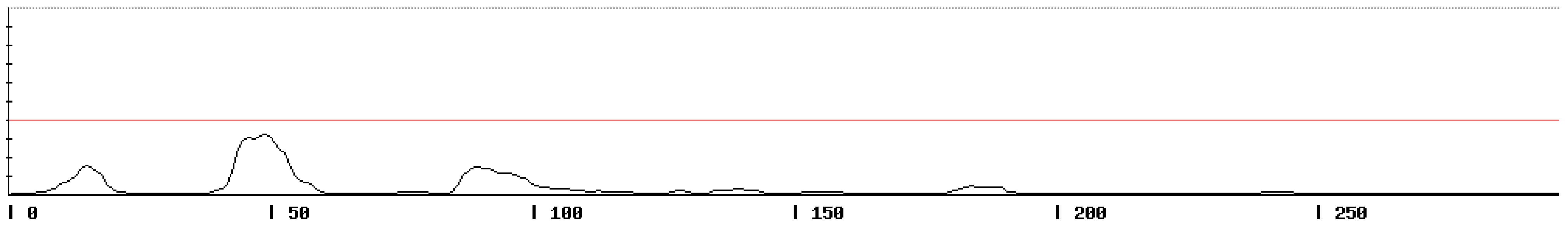

Predictions for Eucalyptusgrandis_Eucgr.K01471.1.p	
Viterbi Path	
Viterbi Path	

Posterior @ 0.4

Posterior @ 0.4

Posterior Graphic. Threshold Marked @ 0.4

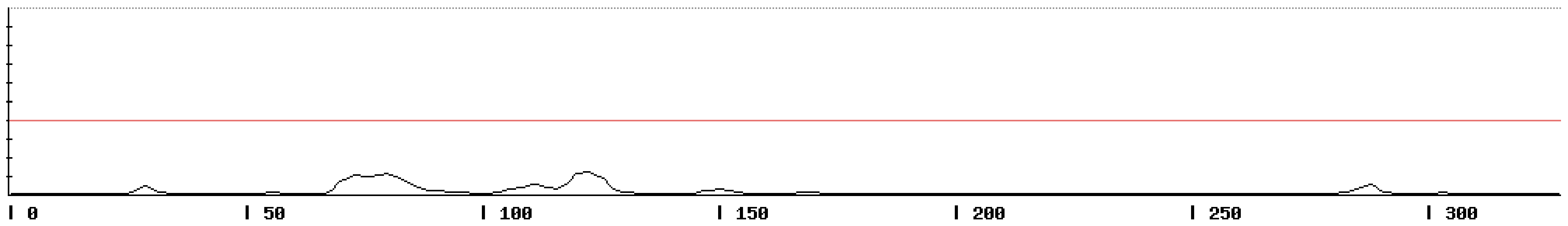

Predictions for Eucalyptusgrandis_Eucgr.K01472.1.p

Viterbi Path

Viterbi Path

Posterior @ 0.4

Posterior @ 0.4

Posterior Graphic. Threshold Marked @ 0.4

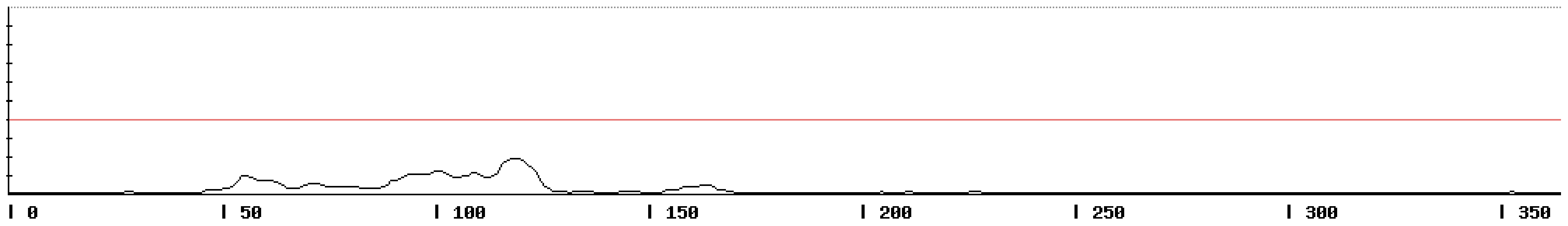

Predictions for Eucalyptusgrandis_Eucgr.K02205.1.p

Viterbi Path

Viterbi Path

Posterior @ 0.4

Posterior @ 0.4

Posterior Graphic. Threshold Marked @ 0.4

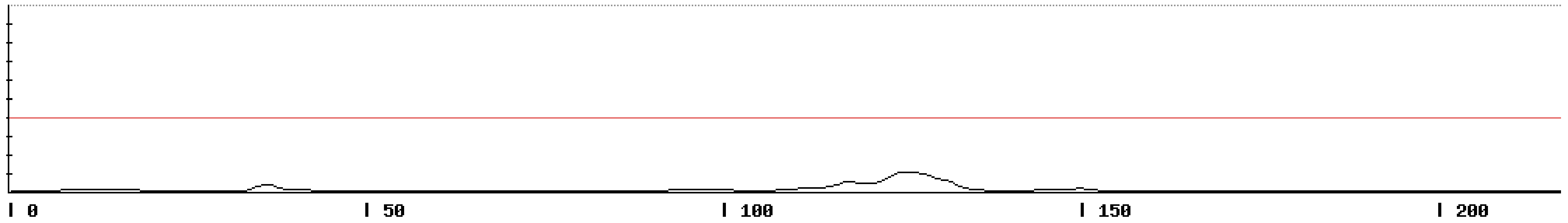

Predictions for Eucalyptusgrandis_Eucgr.K02225.1.p

Viterbi Path

Viterbi Path

Posterior @ 0.4

Posterior @ 0.4

Posterior Graphic. Threshold Marked @ 0.4

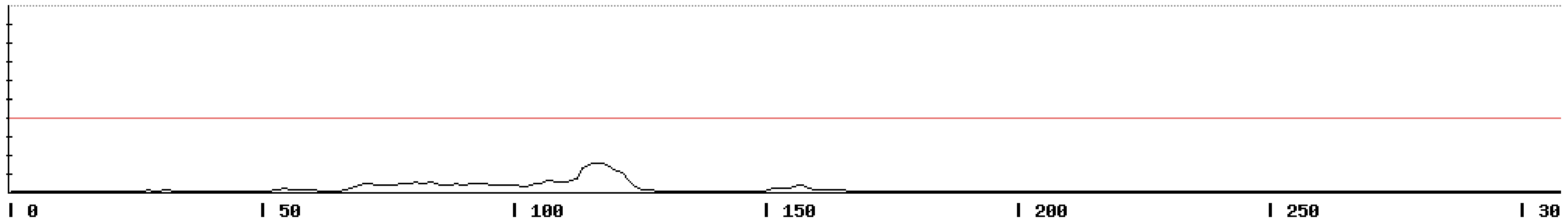

Predictions for Eucalyptusgrandis_Eucgr.K02303.1.p

Viterbi Path

Viterbi Path

Posterior @ 0.4

Posterior @ 0.4

Posterior Graphic. Threshold Marked @ 0.4

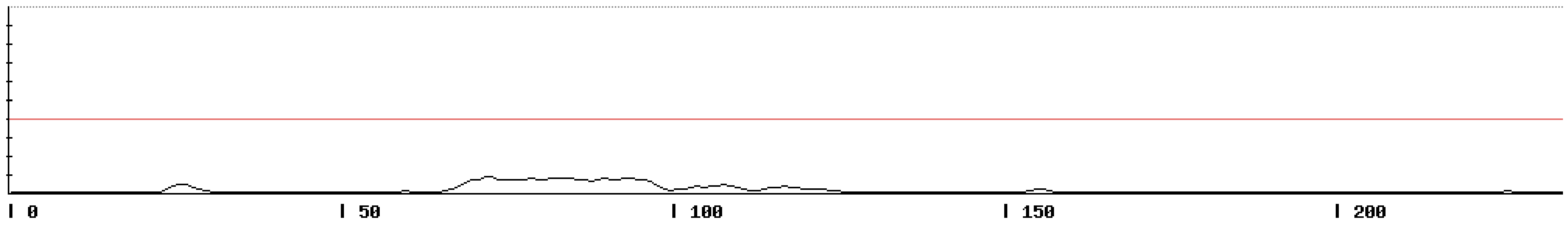

Predictions for Eucalyptusgrandis_Eucgr.K03256.1.p

Viterbi Path

Viterbi Path

Posterior @ 0.4

Posterior @ 0.4

Posterior Graphic. Threshold Marked @ 0.4

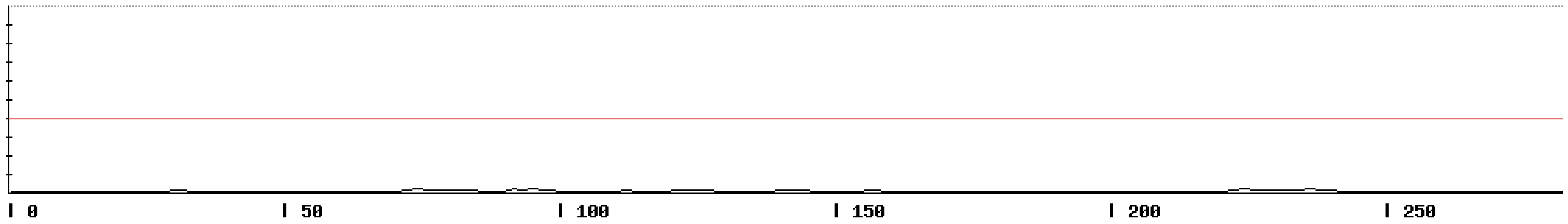

Predictions for Eucalyptusgrandis_Eucgr.L01867.1.p	
Viterbi Path	
Viterbi Path	
Posterior @ 0.4	
Posterior @ 0.4	
Posterior Graphic. Threshold Marked @ 0.4	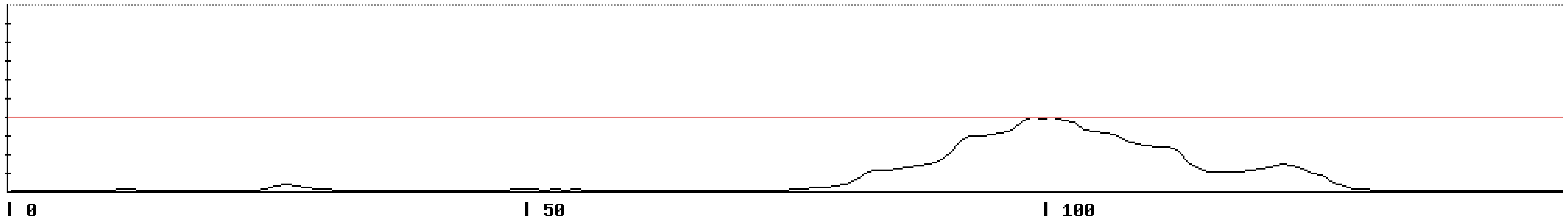

Predictions for Eucalyptusgrandis_Eucgr.L01924.1.p	
Viterbi Path	
Viterbi Path	
Posterior @ 0.4	161 - RKRKKVRSAK - 170
Posterior @ 0.4	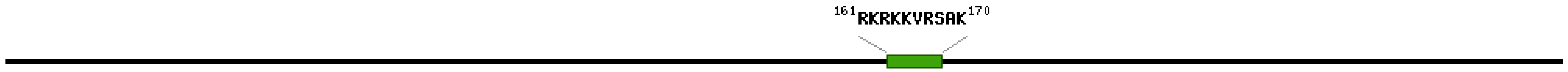
Posterior Graphic. Threshold Marked @ 0.4	

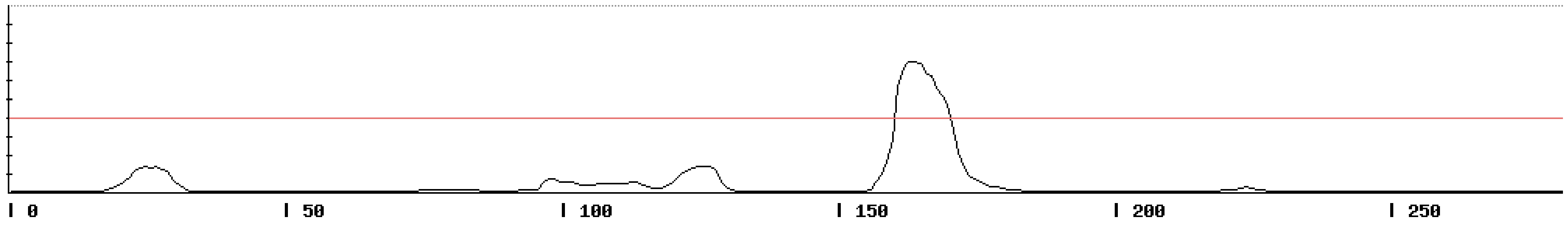

Predictions for Eucalyptusgrandis_Eucgr.L01925.1.p

Viterbi Path

Viterbi Path

Posterior @ 0.4

Posterior @ 0.4

Posterior Graphic. Threshold Marked @ 0.4

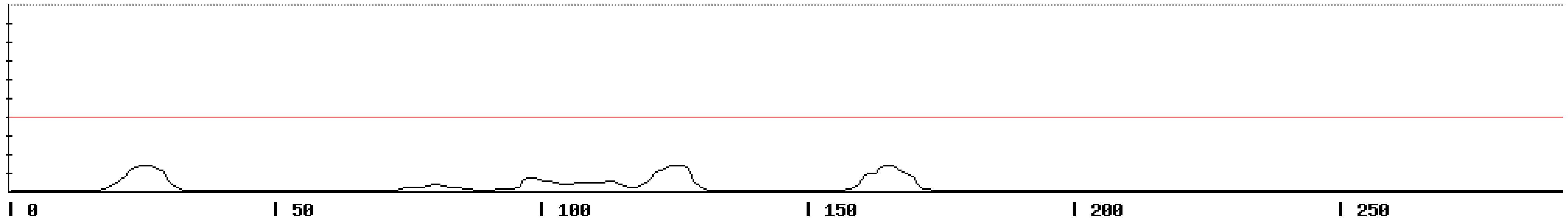

Predictions for Eucalyptusgrandis_Eucgr.L02201.1.p

Viterbi Path

Viterbi Path

Posterior @ 0.4

154 - KNRKKEKAK - 162

Posterior @ 0.4

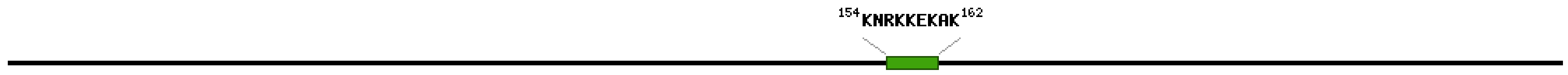

Posterior Graphic. Threshold Marked @ 0.4

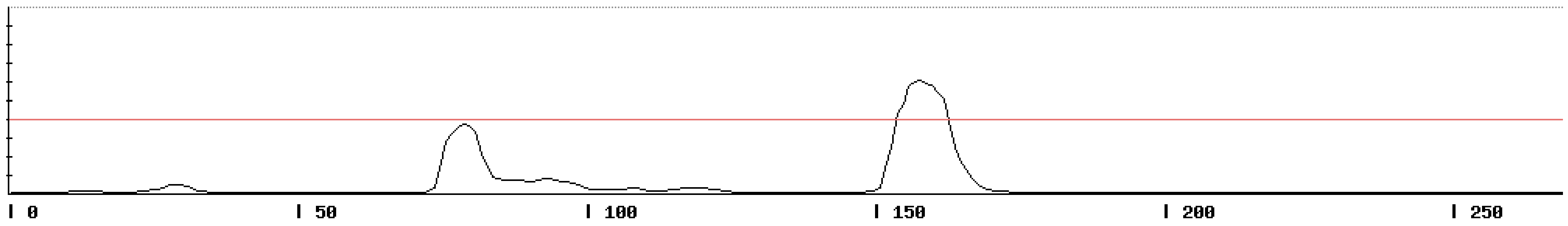

Predictions for Eucalyptusgrandis_Eucgr.L02202.1.p

Viterbi Path

Viterbi Path

Posterior @ 0.4

Posterior @ 0.4

Posterior Graphic. Threshold Marked @ 0.4

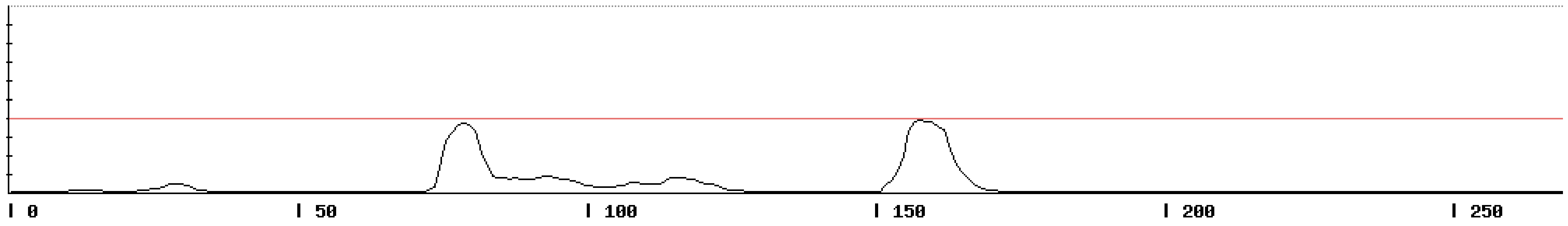

Predictions for Eucalyptusgrandis_Eucgr.L02499.1.p

Viterbi Path

Viterbi Path

Posterior @ 0.4

Posterior @ 0.4

Posterior Graphic. Threshold Marked @ 0.4

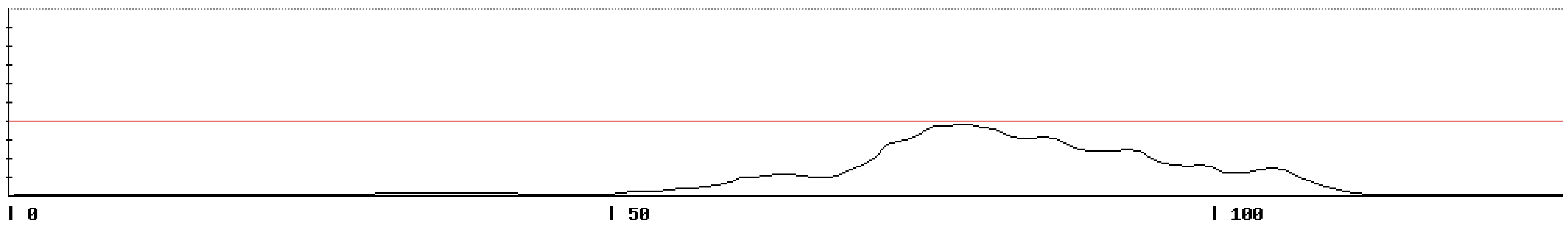

Predictions for Eucalyptusgrandis_Eucgr.L02501.1.p

Viterbi Path

Viterbi Path

Posterior @ 0.4

Posterior @ 0.4

Posterior Graphic. Threshold Marked @ 0.4

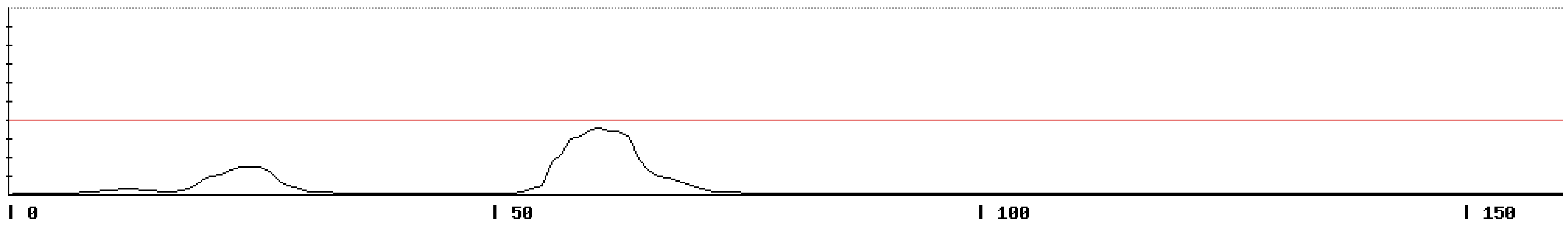

Predictions for Eucalyptusgrandis_Eucgr.L02674.1.p

Viterbi Path

Viterbi Path

Posterior @ 0.4	207 - AKHPKNGQKSDVPPKK - 222
	272 - KKSVLPTKKRSKQK - 285
Posterior @ 0.4	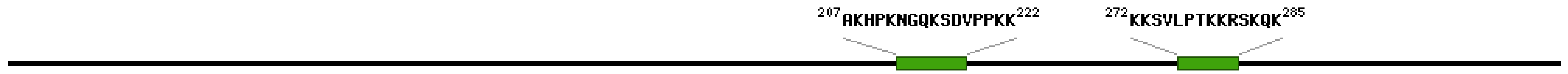
Posterior Graphic. Threshold Marked @ 0.4	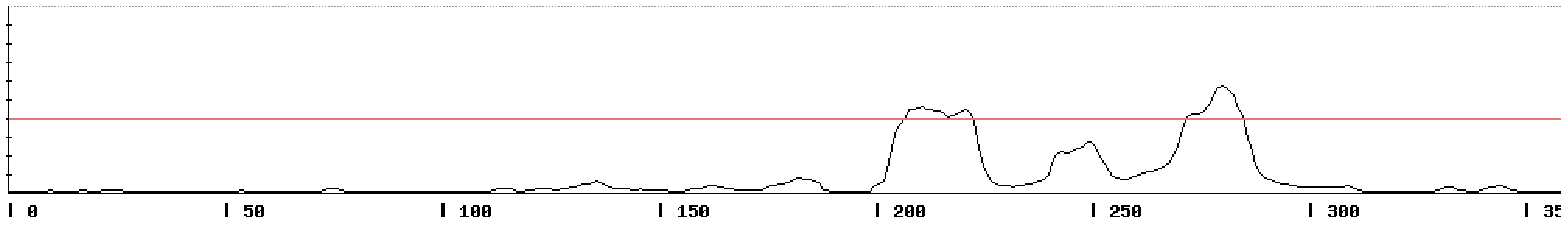

Predictions for Eucalyptusgrandis_Eucgr.L02683.1.p

Viterbi Path	
Viterbi Path	
Posterior @ 0.4	
Posterior @ 0.4	
Posterior Graphic. Threshold Marked @ 0.4	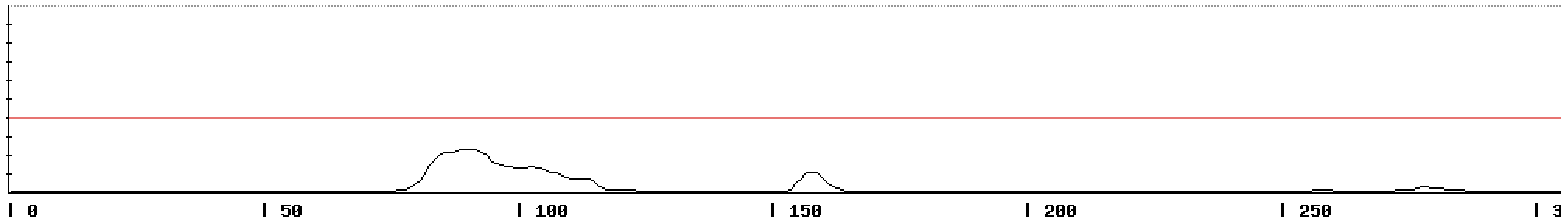

Predictions for Eucalyptusgrandis_Eucgr.L02695.1.p

Viterbi Path

Revision r.9 - Last Updated on : February 24 2015 17:45:20.
alex.nguyenba@utoronto.ca
Intended to be used with Firefox

Viterbi Path

Posterior @ 0.4

Posterior @ 0.4

Posterior Graphic. Threshold Marked @ 0.4

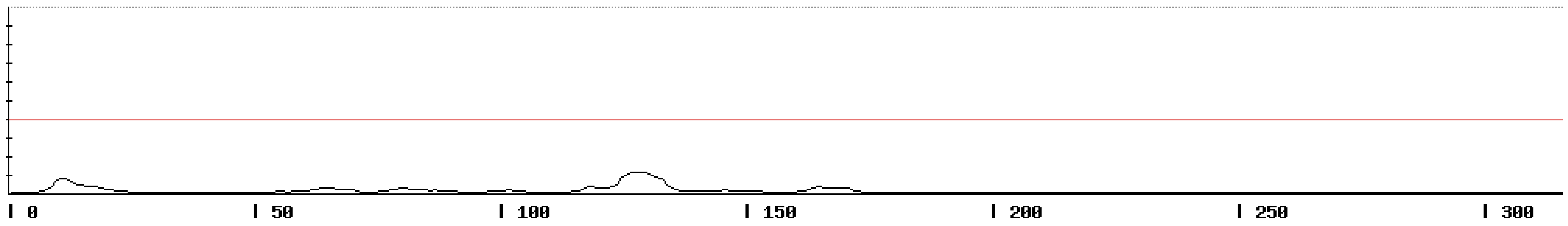

Predictions for Eutremasalsugineum_Thhalv10001200m

Viterbi Path

Viterbi Path

Posterior @ 0.4

Posterior @ 0.4

Posterior Graphic. Threshold Marked @ 0.4

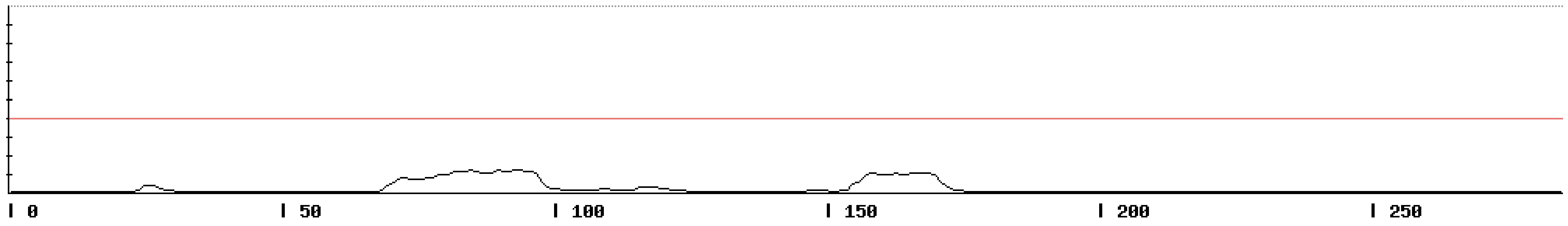

Predictions for Eutremasalsugineum_Thhalv10001506m

Viterbi Path

Viterbi Path

Posterior @ 0.4

Posterior @ 0.4

Posterior Graphic. Threshold Marked @ 0.4

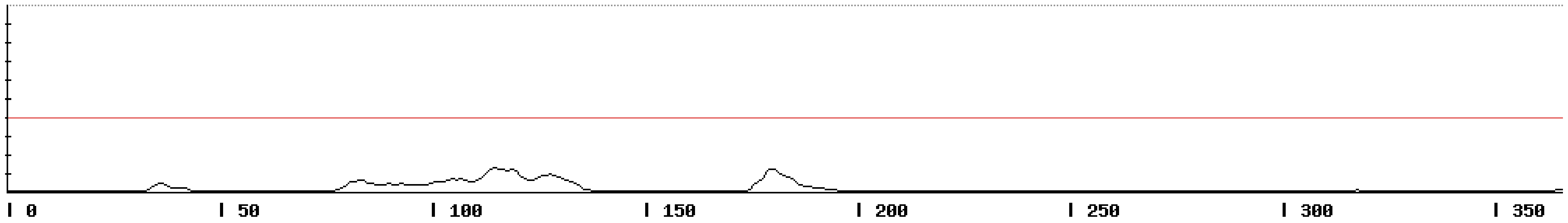

Predictions for Eutremasalsugineum_Thhalv10002027m

Viterbi Path

Viterbi Path

Posterior @ 0.4

Posterior @ 0.4

Posterior Graphic. Threshold Marked @ 0.4

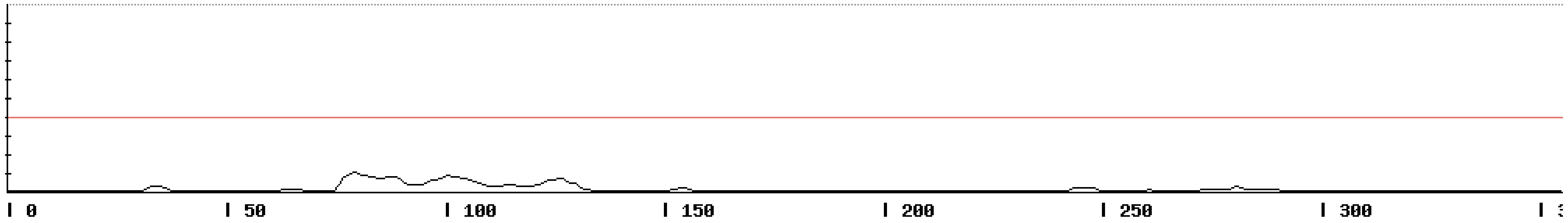

Predictions for Eutremasalsugineum_Thhalv10002040m	
Viterbi Path	
Viterbi Path	
Posterior @ 0.4	
Posterior @ 0.4	
Posterior Graphic. Threshold Marked @ 0.4	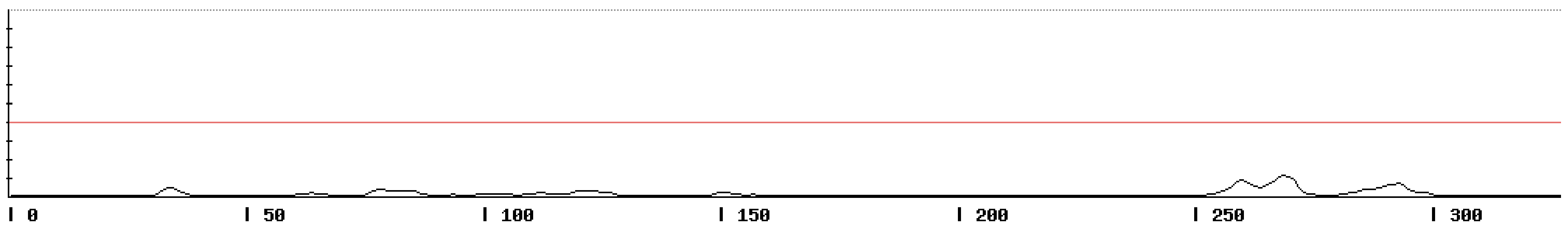

Predictions for Eutremasalsugineum_Thhalv10002300m	
Viterbi Path	261 - KKRSKRGSRTLPKPPPRAKRMR - 282
Viterbi Path	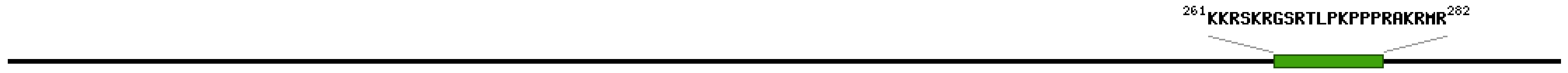
Posterior @ 0.4	259 - SSKKRSKRGSRTLPKPPPRAKRMRN - 283
Posterior @ 0.4	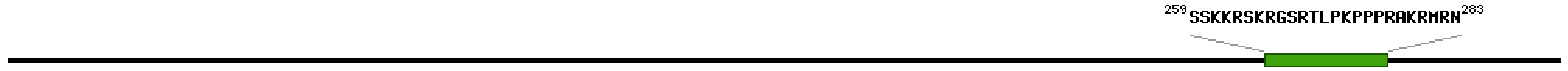
Posterior Graphic. Threshold Marked @ 0.4	

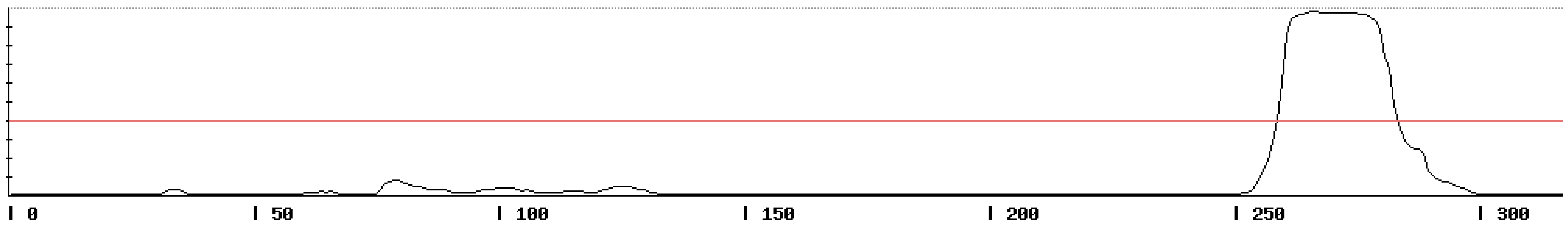

Predictions for Eutremasalsugineum_Thhalv10004105m

Viterbi Path	
Viterbi Path	
Posterior @ 0.4	
Posterior @ 0.4	
Posterior Graphic. Threshold Marked @ 0.4	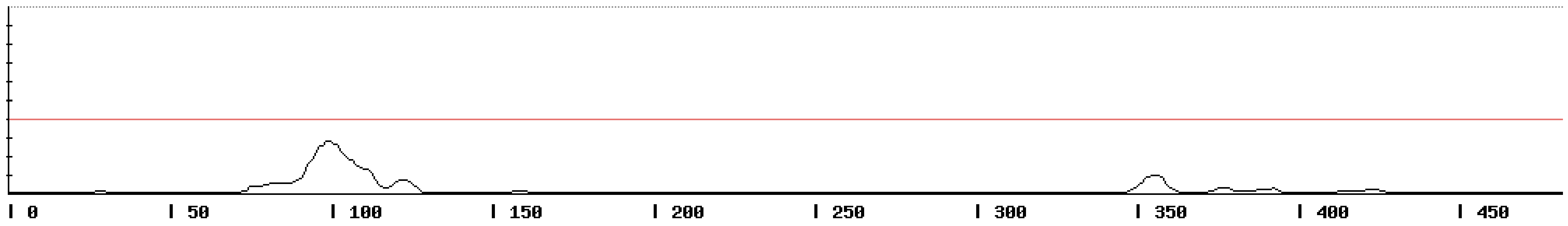

Predictions for Eutremasalsugineum_Thhalv10004177m

Viterbi Path	
Viterbi Path	
Posterior @ 0.4	
Posterior @ 0.4	

Posterior Graphic. Threshold Marked @ 0.4

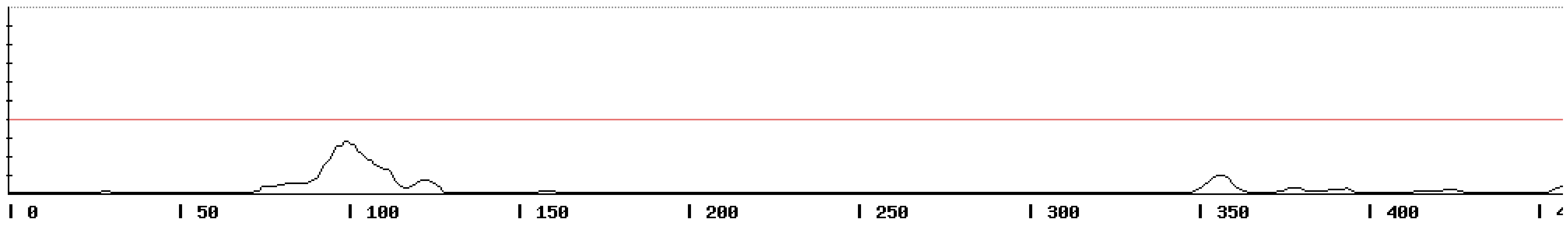

Predictions for Eutremasalsugineum_Thhalv10004330m

Viterbi Path

Viterbi Path

Posterior @ 0.4

Posterior @ 0.4

Posterior Graphic. Threshold Marked @ 0.4

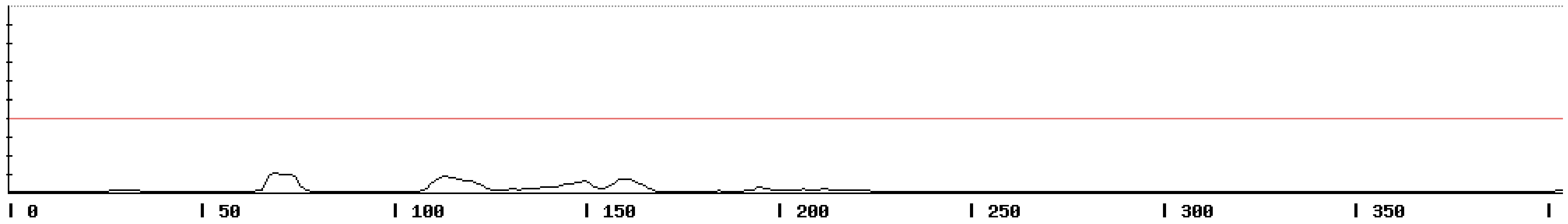

Predictions for Eutremasalsugineum_Thhalv10004488m

Viterbi Path

Viterbi Path

Posterior @ 0.4

Posterior @ 0.4

Posterior Graphic. Threshold Marked @ 0.4

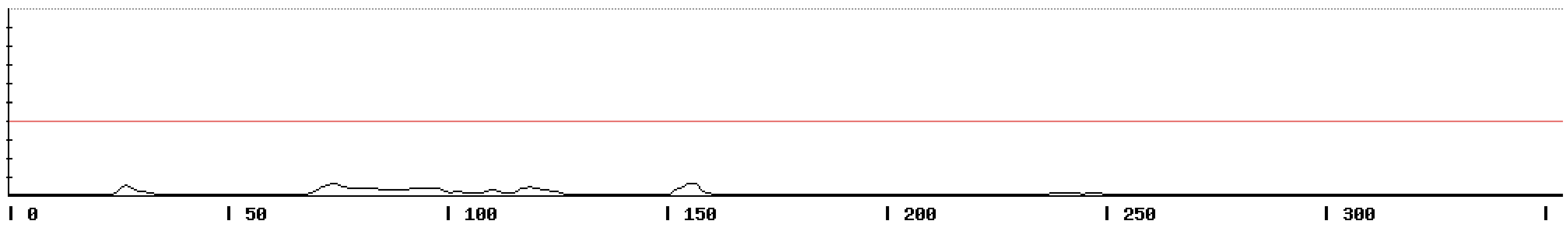

Predictions for Eutremasalsugineum_Thhalv10004501m

Viterbi Path

Viterbi Path

Posterior @ 0.4 71 - EKKYPKSAKAKR - 82

Posterior @ 0.4

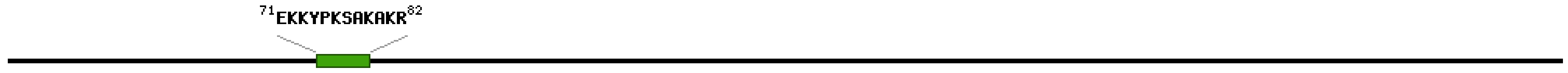

Posterior Graphic. Threshold Marked @ 0.4

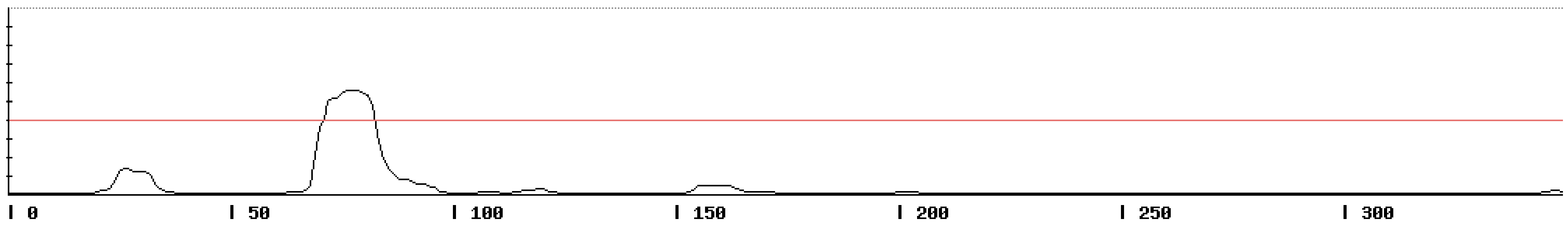

Predictions for Eutremasalsugineum_Thhalv10004550m

Viterbi Path

Viterbi Path

Posterior @ 0.4	
Posterior @ 0.4	
Posterior Graphic. Threshold Marked @ 0.4	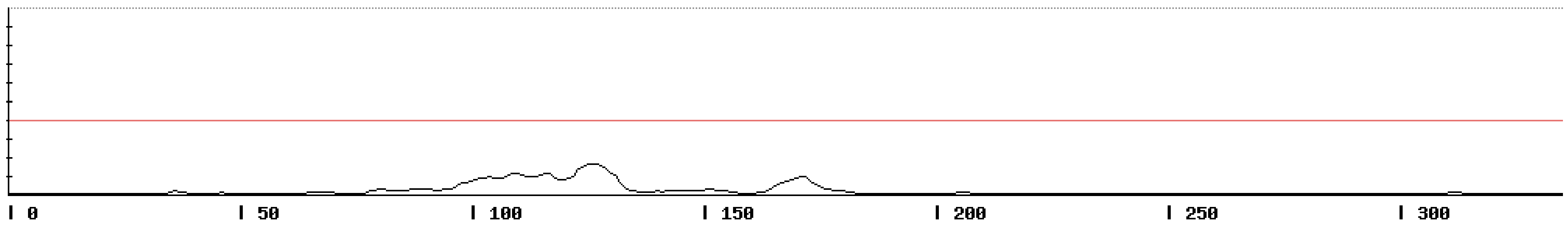

Predictions for Eutremasalsugineum_Thhalv10004583m

Viterbi Path	
Viterbi Path	
Posterior @ 0.4	
Posterior @ 0.4	
Posterior Graphic. Threshold Marked @ 0.4	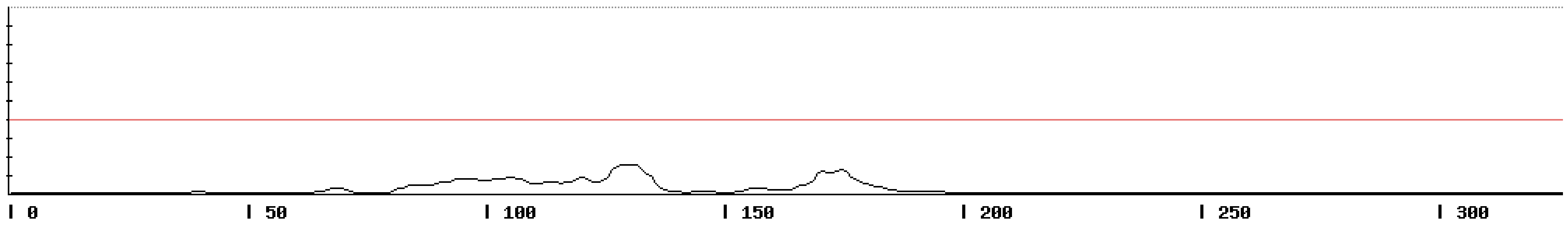

Predictions for Eutremasalsugineum_Thhalv10004786m

Viterbi Path	
Viterbi Path	

Posterior @ 0.4 | 98 - GKPK - 101

Posterior @ 0.4

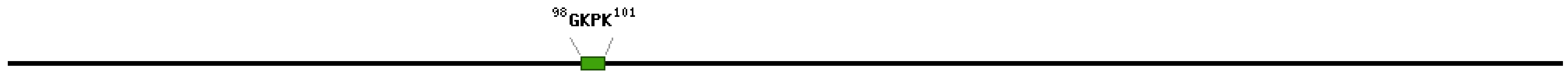

Posterior Graphic. Threshold Marked @ 0.4

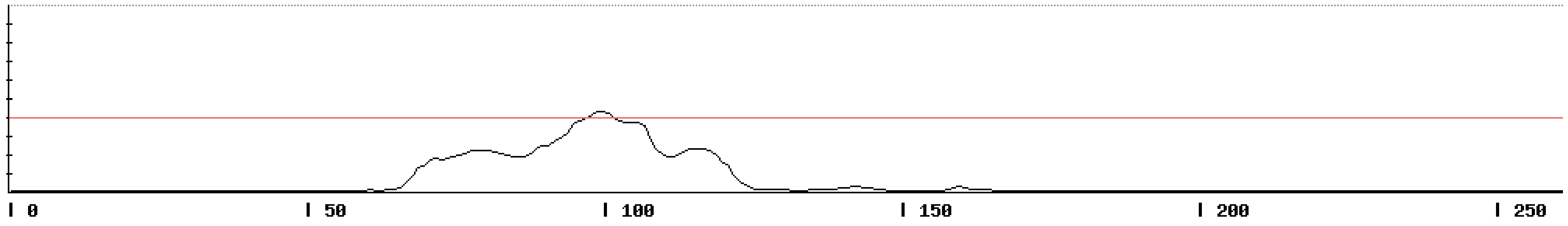

Predictions for Eutremasalsugineum_Thhalv10004805m

Viterbi Path

Viterbi Path

Posterior @ 0.4

Posterior @ 0.4

Posterior Graphic. Threshold Marked @ 0.4

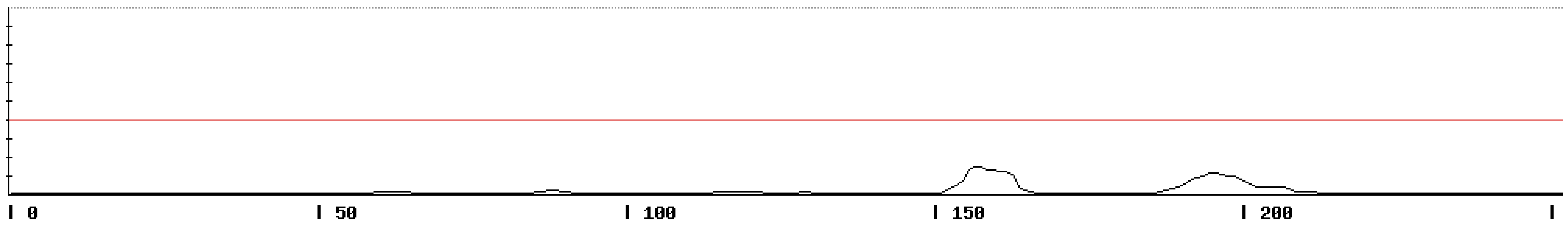

Predictions for Eutremasalsugineum_Thhalv10005380m

Viterbi Path

Viterbi Path

Posterior @ 0.4

Posterior @ 0.4

Posterior Graphic. Threshold Marked @ 0.4

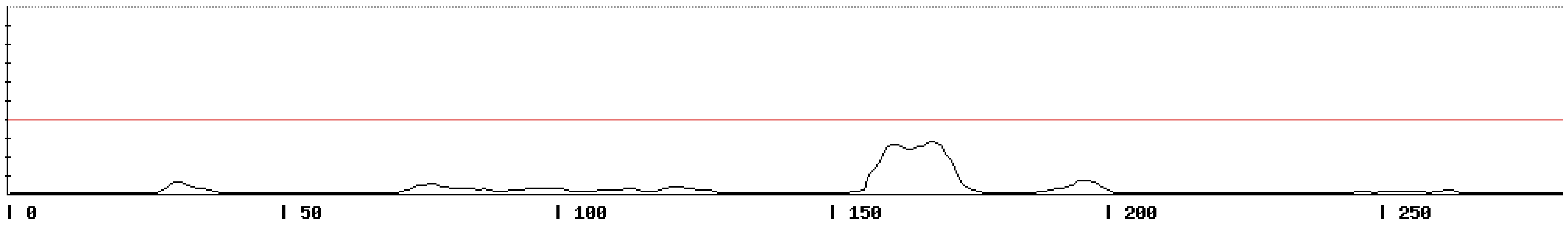

Predictions for Eutremasalsugineum_Thhalv10005552m

Viterbi Path

Viterbi Path

Posterior @ 0.4

Posterior @ 0.4

Posterior Graphic. Threshold Marked @ 0.4

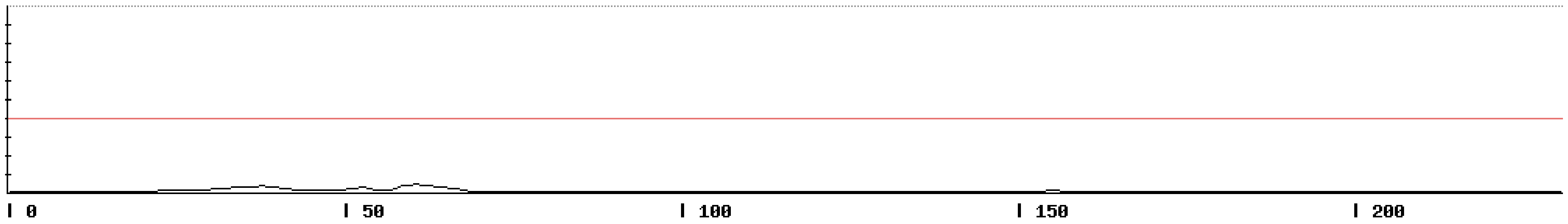

Predictions for Eutremasalsugineum_Thhalv10006408m

Viterbi Path	
Viterbi Path	
Posterior @ 0.4	198 - KKKKK - 202
Posterior @ 0.4	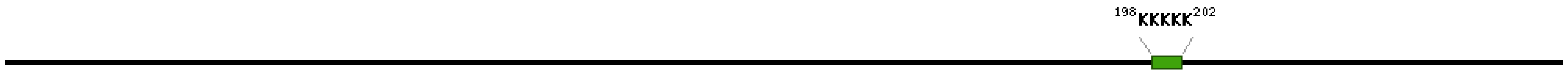
Posterior Graphic. Threshold Marked @ 0.4	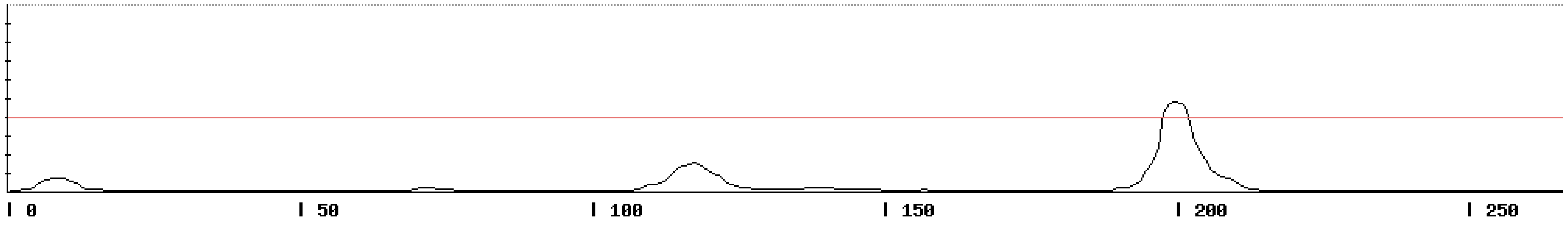

Predictions for Eutremasalsugineum_Thhalv10006451m

Viterbi Path	
Viterbi Path	
Posterior @ 0.4	
Posterior @ 0.4	
Posterior Graphic. Threshold Marked @ 0.4	

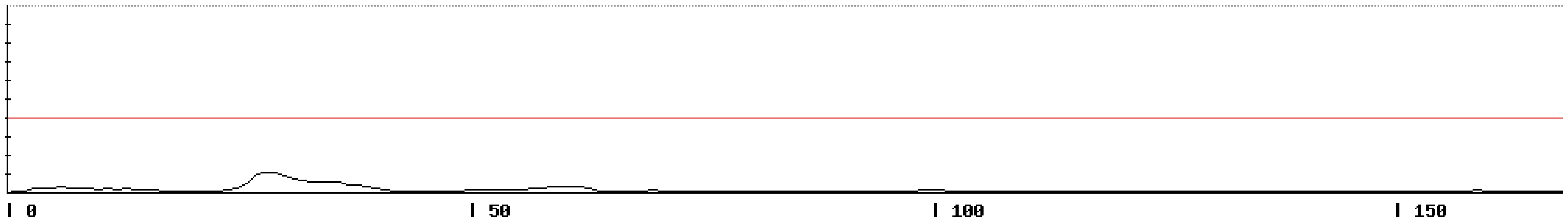

Predictions for Eutremasalsugineum_Thhalv10007021m

Viterbi Path	
Viterbi Path	
Posterior @ 0.4	
Posterior @ 0.4	
Posterior Graphic. Threshold Marked @ 0.4	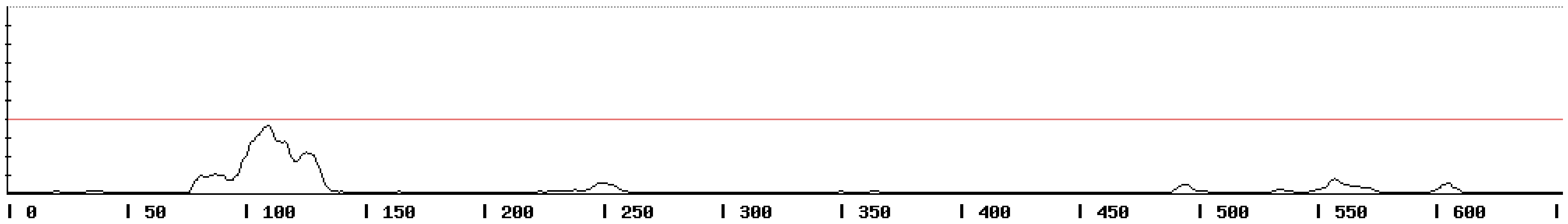

Predictions for Eutremasalsugineum_Thhalv10007196m

Viterbi Path	
Viterbi Path	
Posterior @ 0.4	
Posterior @ 0.4	

Posterior Graphic. Threshold Marked @ 0.4

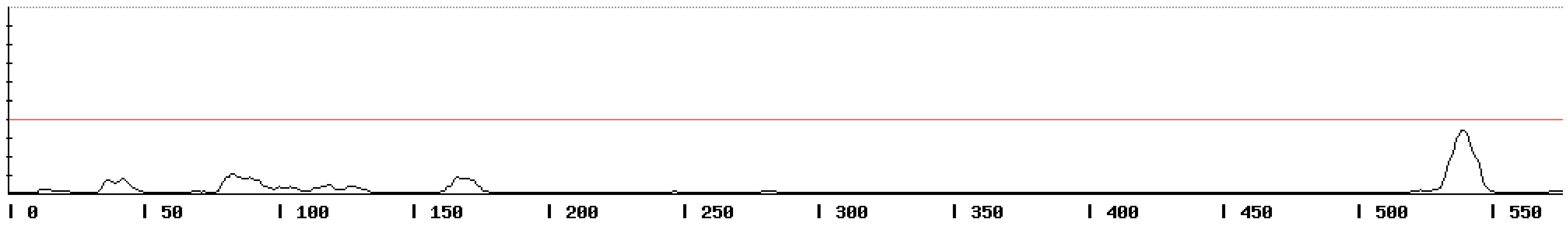

Predictions for Eutremasalsugineum_Thhalv10007221m

Viterbi Path

Viterbi Path

Posterior @ 0.4

Posterior @ 0.4

Posterior Graphic. Threshold Marked @ 0.4

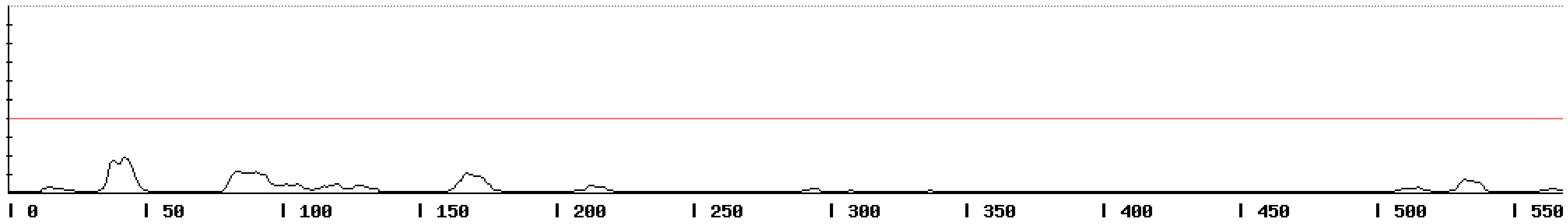

Predictions for Eutremasalsugineum_Thhalv10007369m

Viterbi Path

Viterbi Path

Posterior @ 0.4

36 - KRKIRRKKMR - 45

84 - KHGGRSSR - 91

Posterior @ 0.4

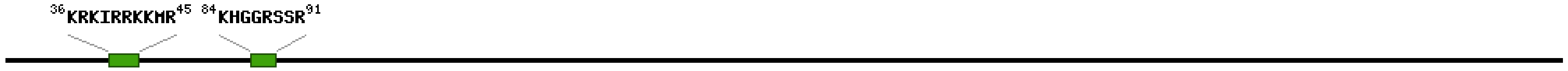

Posterior Graphic. Threshold Marked @ 0.4

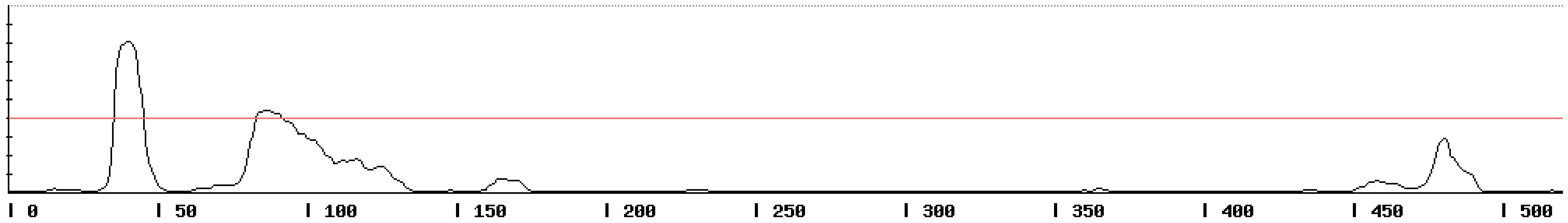

Predictions for Eutremasalsugineum_Thhalv10007551m

Viterbi Path	
Viterbi Path	
Posterior @ 0.4	137 - KR - 138
Posterior @ 0.4	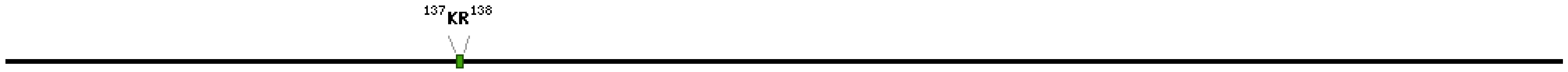
Posterior Graphic. Threshold Marked @ 0.4	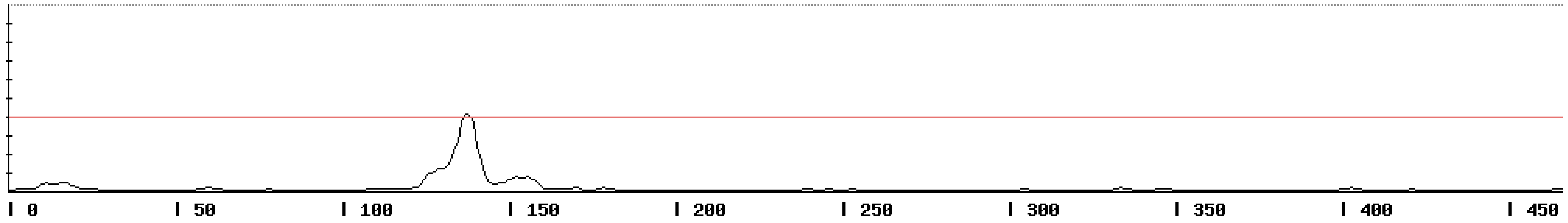

Predictions for Eutremasalsugineum_Thhalv10007665m

Viterbi Path	
Viterbi Path	

Posterior @ 0.4

Posterior @ 0.4

Posterior Graphic. Threshold Marked @ 0.4

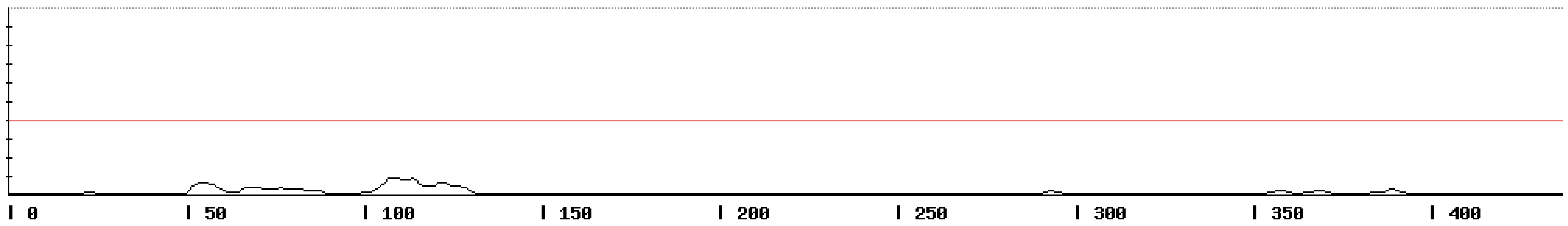

Predictions for Eutremasalsugineum_Thhalv10007709m

Viterbi Path

Viterbi Path

Posterior @ 0.4

Posterior @ 0.4

Posterior Graphic. Threshold Marked @ 0.4

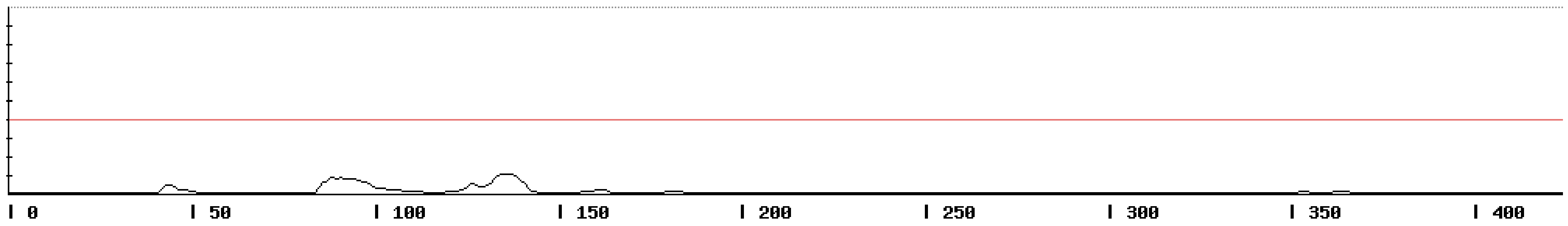

Predictions for Eutremasalsugineum_Thhalv10007823m

Viterbi Path

Viterbi Path

Posterior @ 0.4

Posterior @ 0.4

Posterior Graphic. Threshold Marked @ 0.4

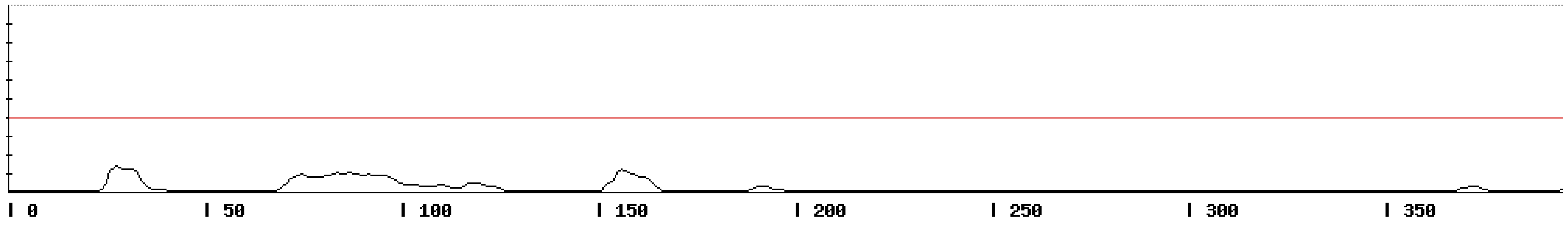

Predictions for Eutremasalsugineum_Thhalv10008226m

Viterbi Path

Viterbi Path

Posterior @ 0.4

Posterior @ 0.4

Posterior Graphic. Threshold Marked @ 0.4

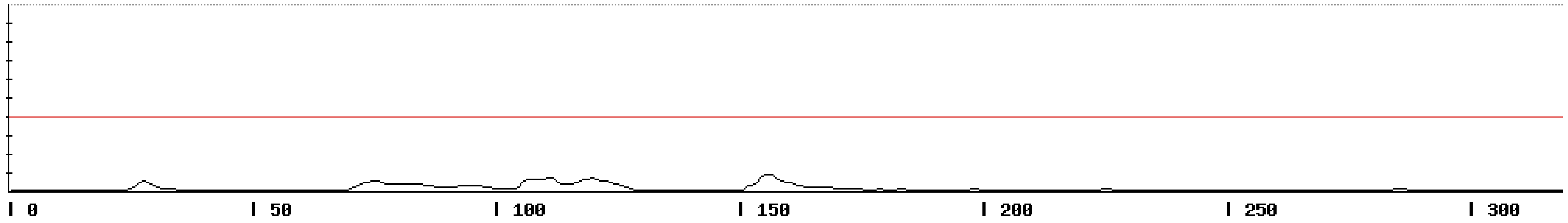

Predictions for Eutremasalsugineum_Thhalv10008249m

Viterbi Path	
Viterbi Path	
Posterior @ 0.4	156 - KRR - 158
Posterior @ 0.4	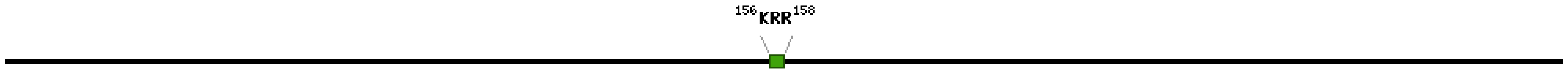
Posterior Graphic. Threshold Marked @ 0.4	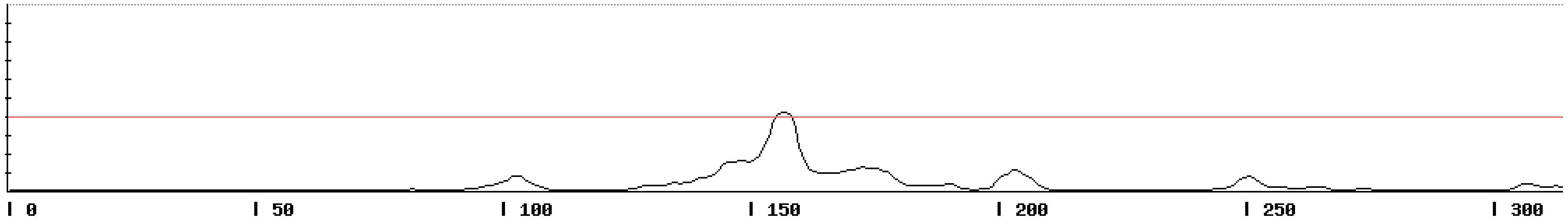

Predictions for Eutremasalsugineum_Thhalv10008371m

Viterbi Path	
Viterbi Path	
Posterior @ 0.4	
Posterior @ 0.4	
Posterior Graphic. Threshold Marked @ 0.4	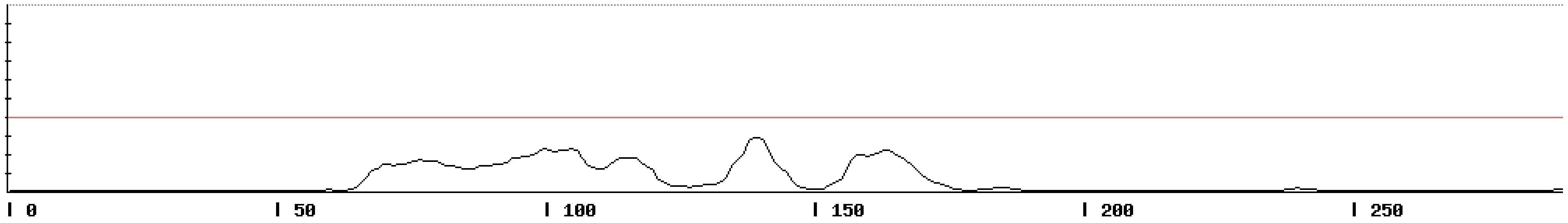

Predictions for Eutremasalsugineum_Thhalv10009336m	
Viterbi Path	
Viterbi Path	
Posterior @ 0.4	
Posterior @ 0.4	
Posterior Graphic. Threshold Marked @ 0.4	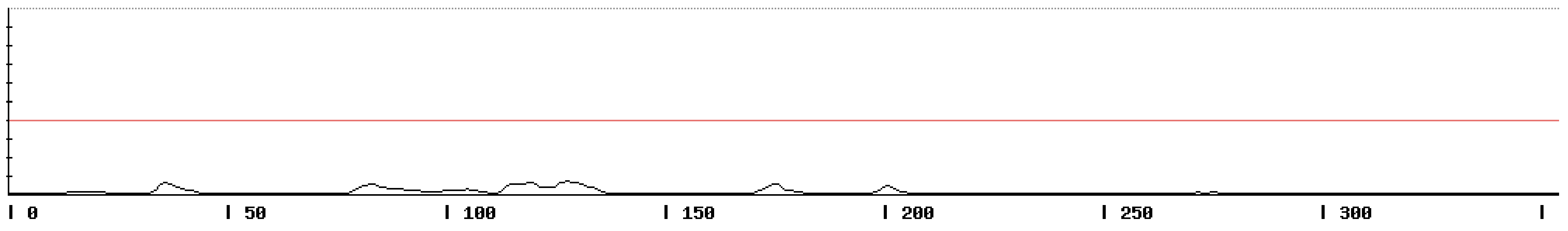

Predictions for Eutremasalsugineum_Thhalv10009374m	
Viterbi Path	
Viterbi Path	
Posterior @ 0.4	
Posterior @ 0.4	
Posterior Graphic. Threshold Marked @ 0.4	

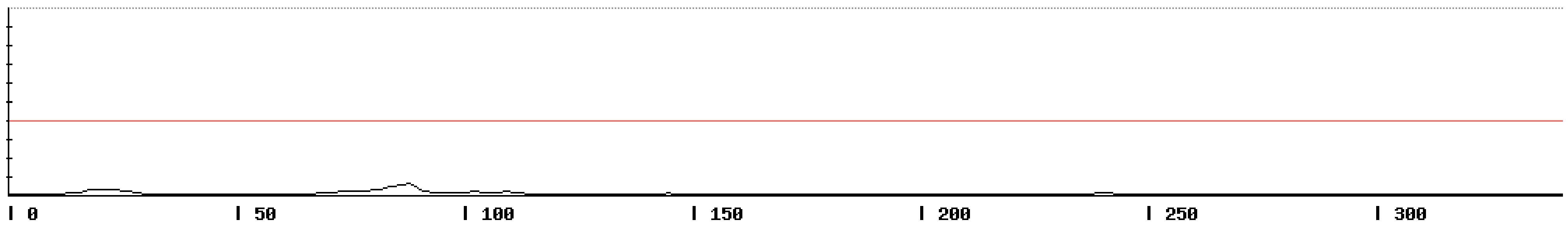

Predictions for Eutremasalsugineum_Thhalv10009466m

Viterbi Path

Viterbi Path

Posterior @ 0.4

Posterior @ 0.4

Posterior Graphic. Threshold Marked @ 0.4

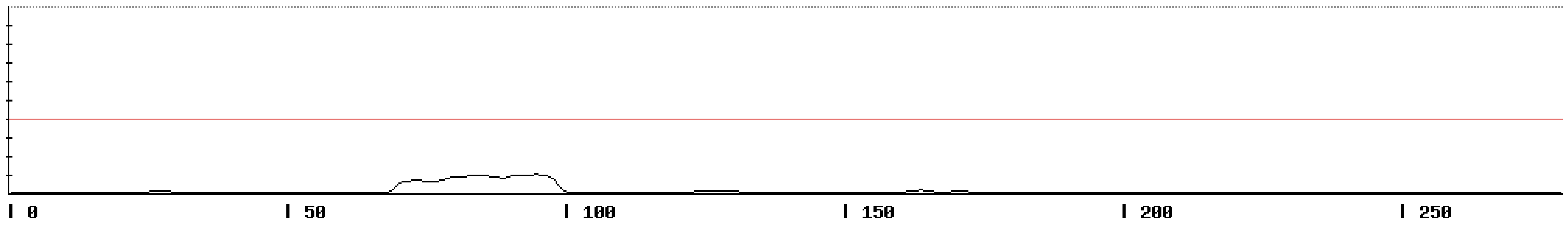

Predictions for Eutremasalsugineum_Thhalv10010340m

Viterbi Path

Viterbi Path

Posterior @ 0.4 415 - KKGKPMRSK - 423

Posterior @ 0.4

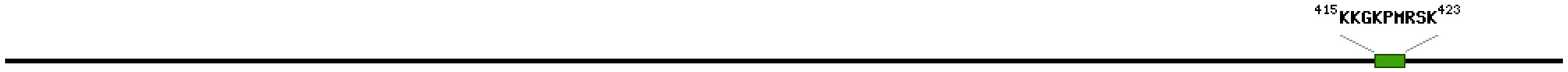

Posterior Graphic. Threshold Marked @ 0.4

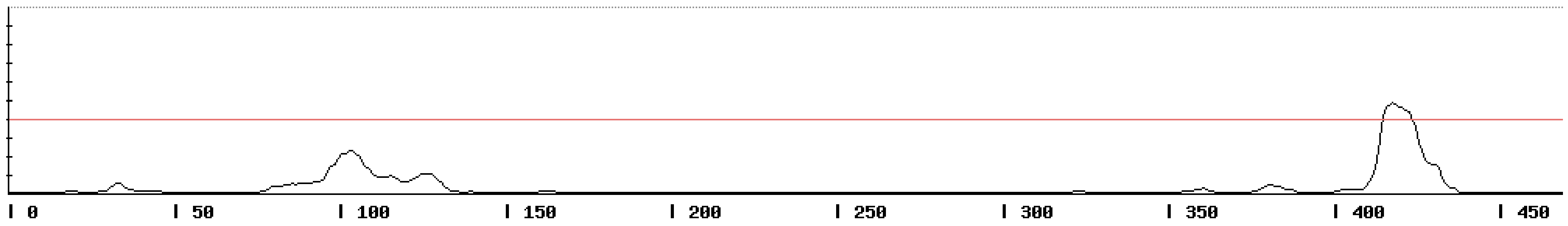

Predictions for Eutremasalsugineum_Thhalv10010503m

Viterbi Path

Viterbi Path

Posterior @ 0.4

Posterior @ 0.4

Posterior Graphic. Threshold Marked @ 0.4

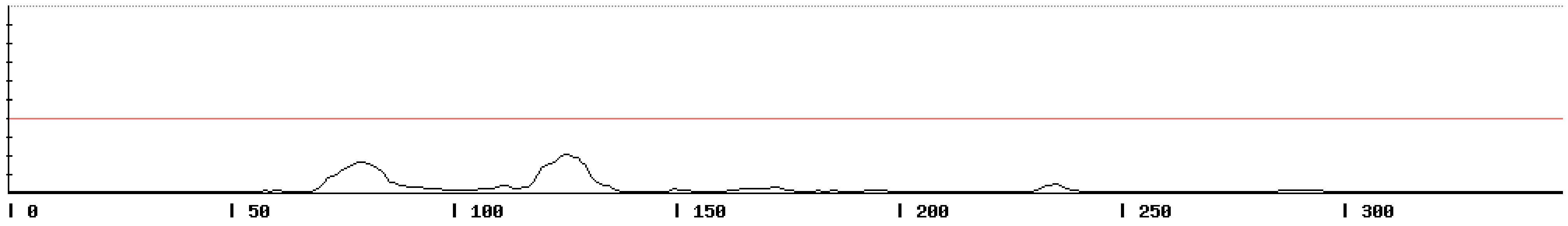

Predictions for Eutremasalsugineum_Thhalv10010675m

Viterbi Path

Viterbi Path

Posterior @ 0.4

Posterior @ 0.4

Posterior Graphic. Threshold Marked @ 0.4	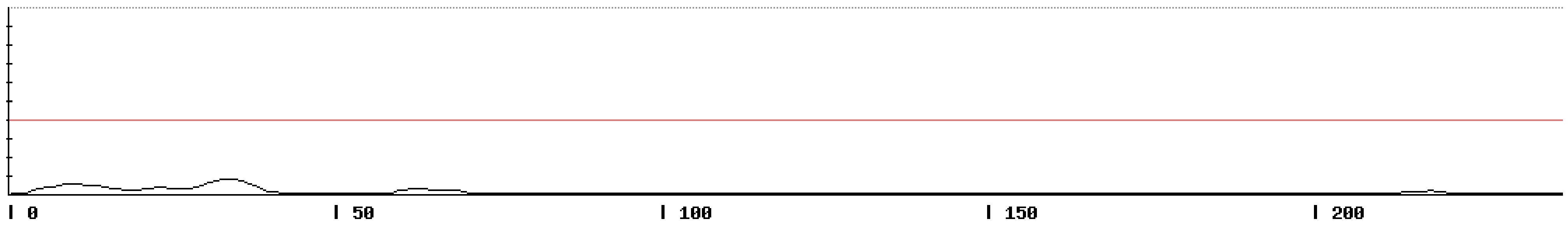

Predictions for Eutremasalsugineum_Thhalv10011123m

Viterbi Path	
Viterbi Path	
Posterior @ 0.4	
Posterior @ 0.4	
Posterior Graphic. Threshold Marked @ 0.4	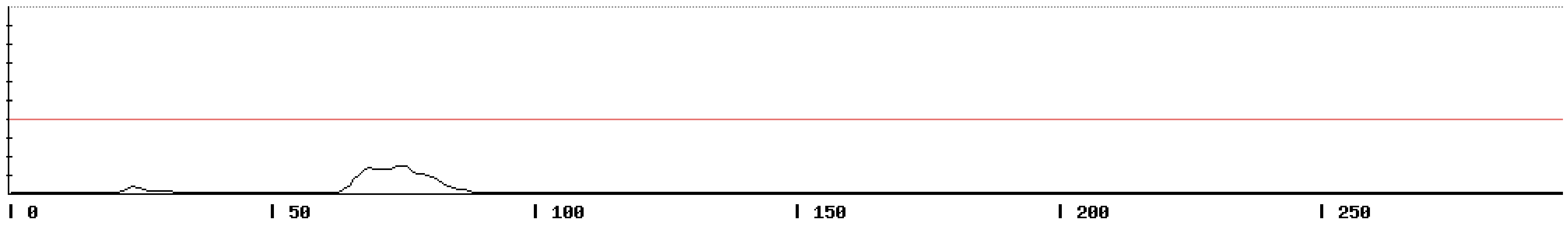

Predictions for Eutremasalsugineum_Thhalv10011659m

Viterbi Path	
Viterbi Path	

Posterior @ 0.4

Posterior @ 0.4

Posterior Graphic. Threshold Marked @ 0.4

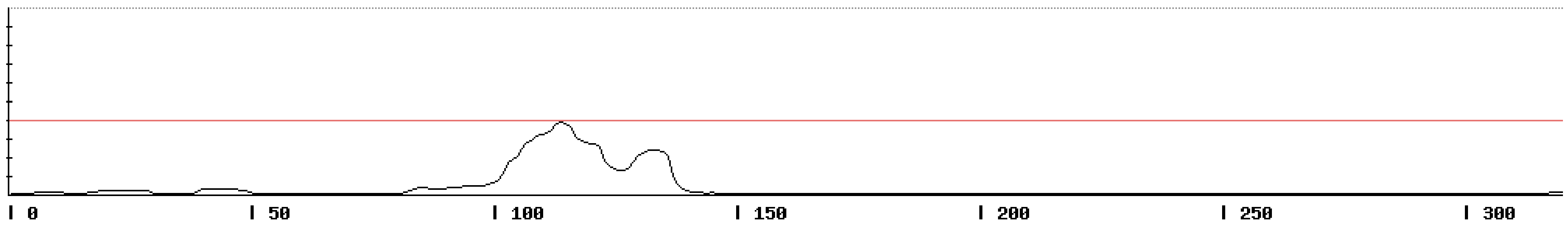

Predictions for Eutremasalsugineum_Thhalv10011676m

Viterbi Path

Viterbi Path

Posterior @ 0.4

Posterior @ 0.4

Posterior Graphic. Threshold Marked @ 0.4

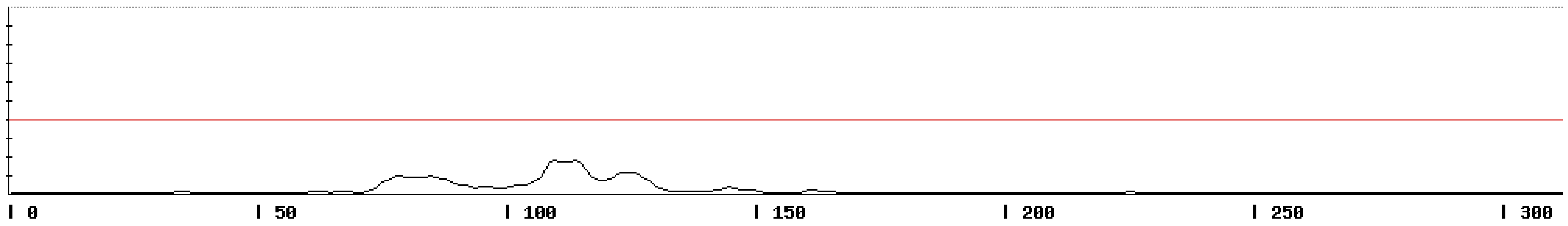

Predictions for Eutremasalsugineum_Thhalv10011708m

Viterbi Path

Viterbi Path

Posterior @ 0.4

Posterior @ 0.4

Posterior Graphic. Threshold Marked @ 0.4

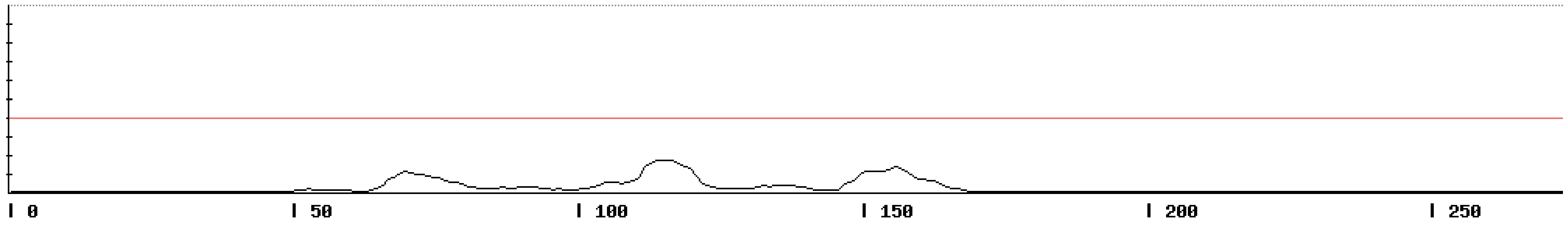

Predictions for Eutremasalsugineum_Thhalv10012158m

Viterbi Path

Viterbi Path

Posterior @ 0.4

Posterior @ 0.4

Posterior Graphic. Threshold Marked @ 0.4

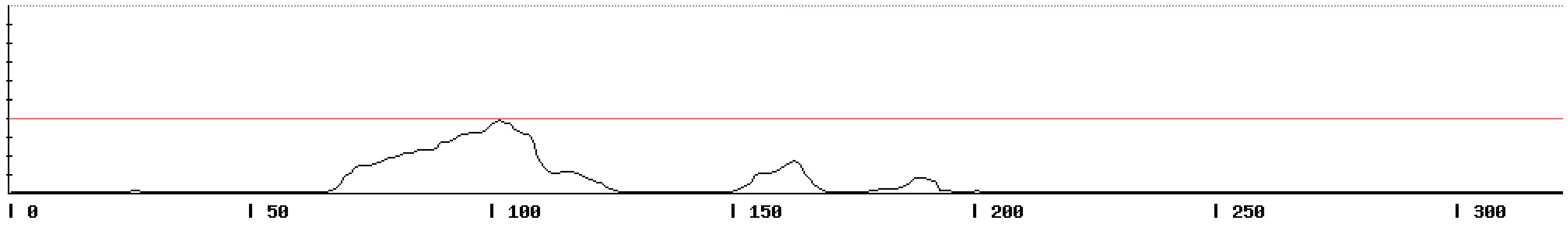

Predictions for Eutremasalsugineum_Thhalv10012308m

Viterbi Path

Viterbi Path

Posterior @ 0.4

Posterior @ 0.4

Posterior Graphic. Threshold Marked @ 0.4

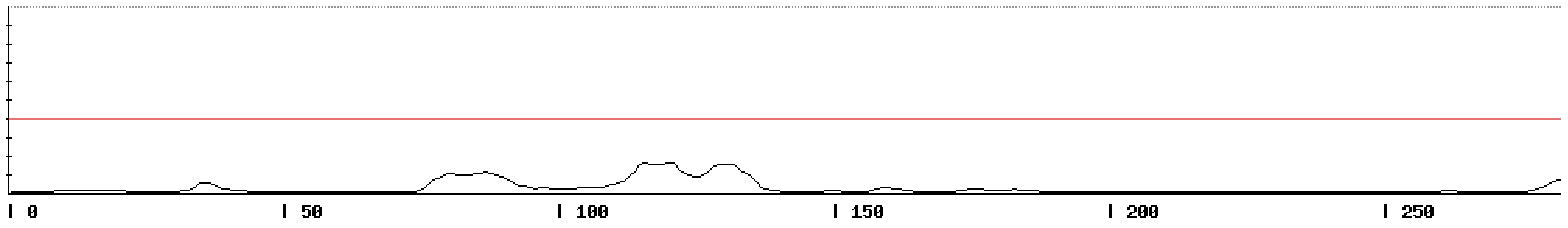

Predictions for Eutremasalsugineum_Thhalv10013110m

Viterbi Path

Viterbi Path

Posterior @ 0.4

Posterior @ 0.4

Posterior Graphic. Threshold Marked @ 0.4

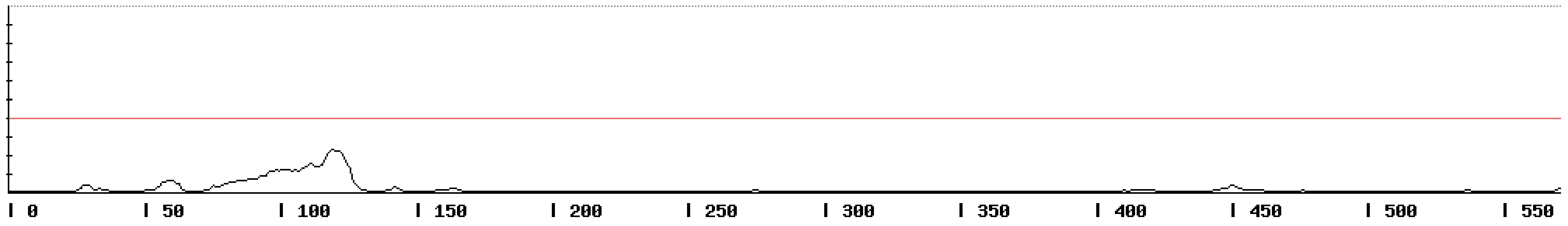

Predictions for Eutremasalsugineum_Thhalv10013344m

Viterbi Path	
Viterbi Path	
Posterior @ 0.4	
Posterior @ 0.4	
Posterior Graphic. Threshold Marked @ 0.4	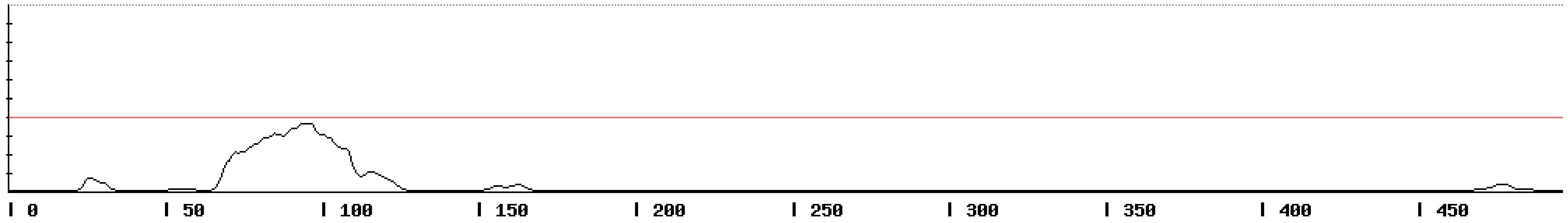

Predictions for Eutremasalsugineum_Thhalv10013778m

Viterbi Path	
Viterbi Path	
Posterior @ 0.4	
Posterior @ 0.4	
Posterior Graphic. Threshold Marked @ 0.4	

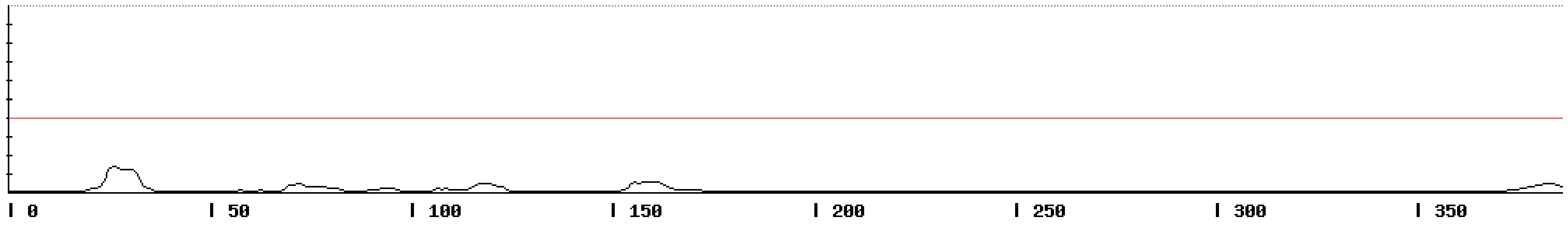

Predictions for Eutremasalsugineum_Thhalv10013784m

Viterbi Path

Viterbi Path

Posterior @ 0.4

Posterior @ 0.4

Posterior Graphic. Threshold Marked @ 0.4

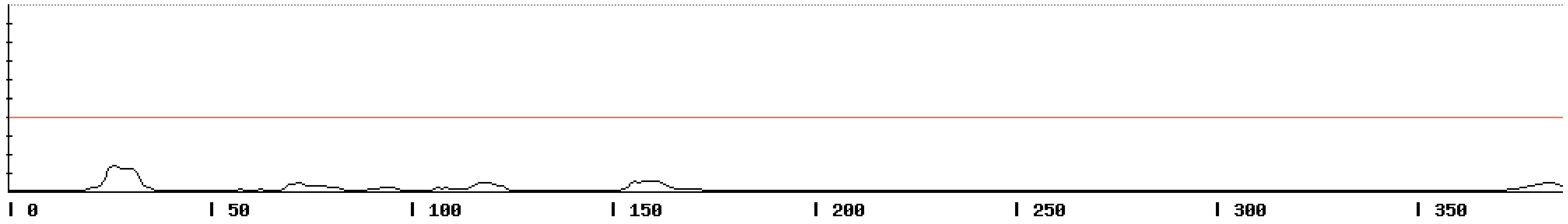

Predictions for Eutremasalsugineum_Thhalv10013785m

Viterbi Path

Viterbi Path

Posterior @ 0.4

Posterior @ 0.4

Posterior Graphic. Threshold Marked @ 0.4

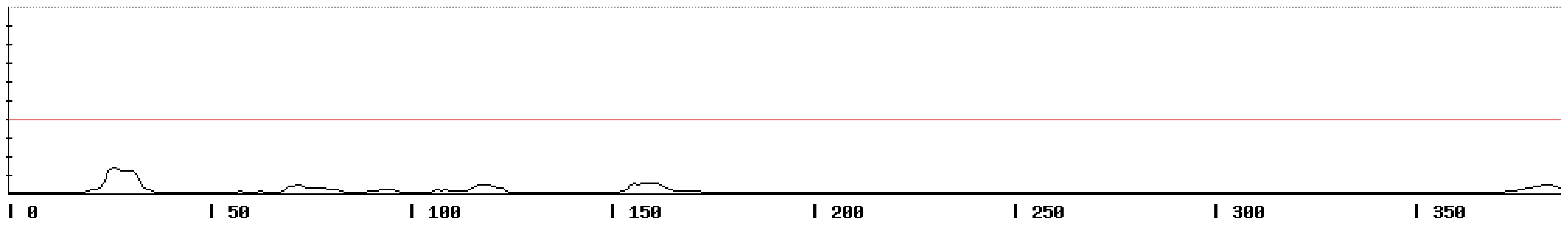

Predictions for Eutremasalsugineum_Thhalv10013863m

Viterbi Path

Viterbi Path

Posterior @ 0.4

Posterior @ 0.4

Posterior Graphic. Threshold Marked @ 0.4

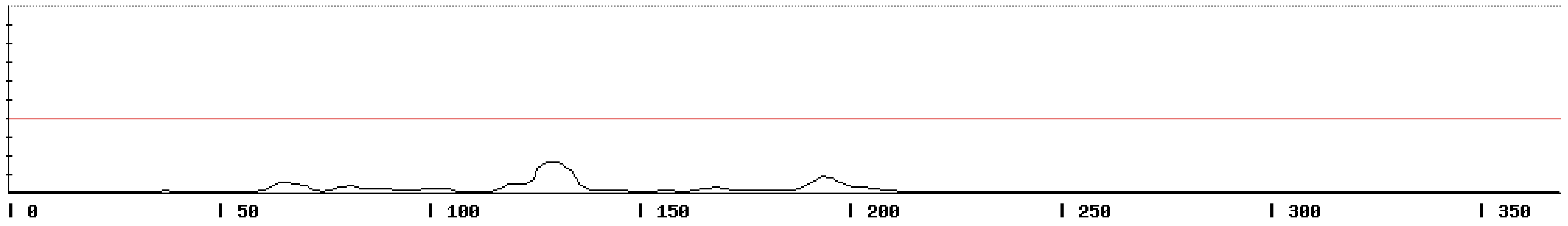

Predictions for Eutremasalsugineum_Thhalv10013903m

Viterbi Path

Viterbi Path

Posterior @ 0.4

Posterior @ 0.4

Posterior Graphic. Threshold Marked @ 0.4	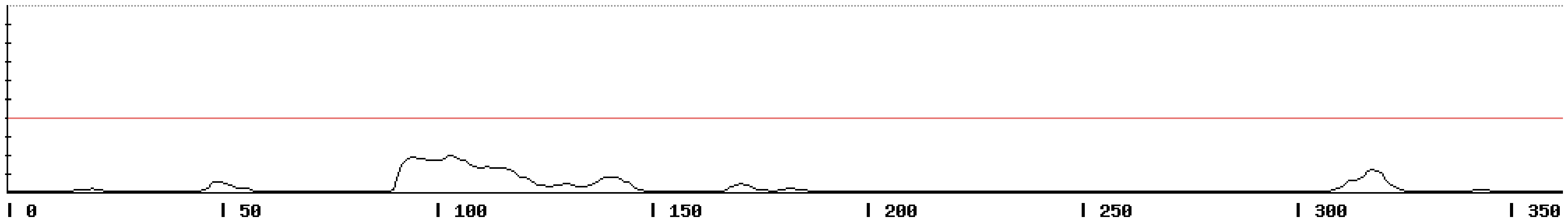

Predictions for Eutremasalsugineum_Thhalv10014060m

Viterbi Path	
Viterbi Path	
Posterior @ 0.4	
Posterior @ 0.4	
Posterior Graphic. Threshold Marked @ 0.4	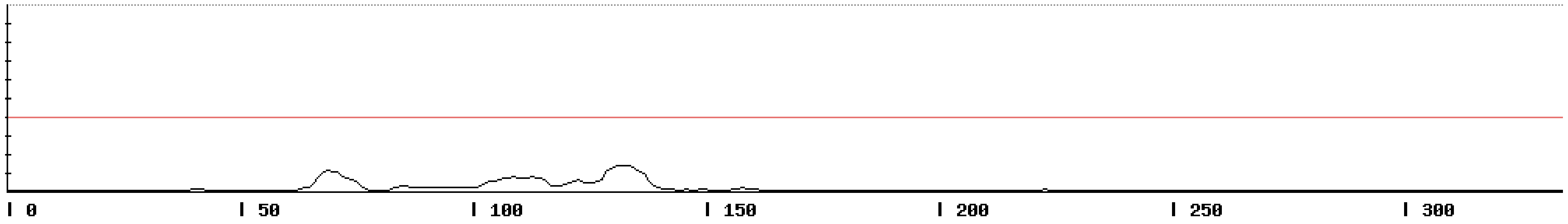

Predictions for Eutremasalsugineum_Thhalv10014081m

Viterbi Path	
Viterbi Path	

Posterior @ 0.4

Posterior @ 0.4

Posterior Graphic. Threshold Marked @ 0.4

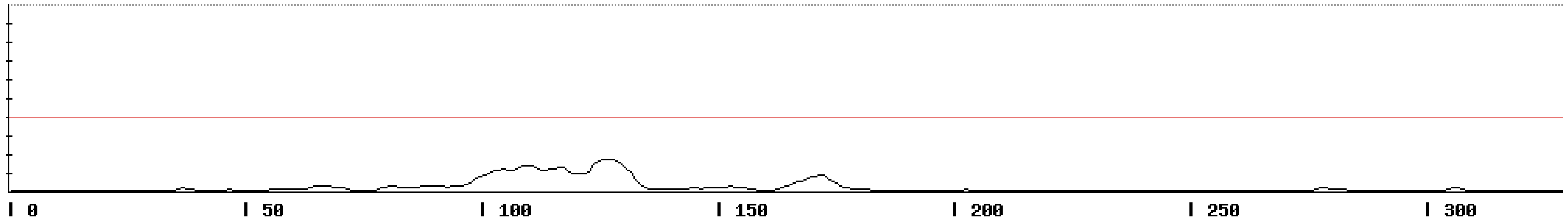

Predictions for Eutremasalsugineum_Thhalv10014174m

Viterbi Path

Viterbi Path

Posterior @ 0.4

Posterior @ 0.4

Posterior Graphic. Threshold Marked @ 0.4

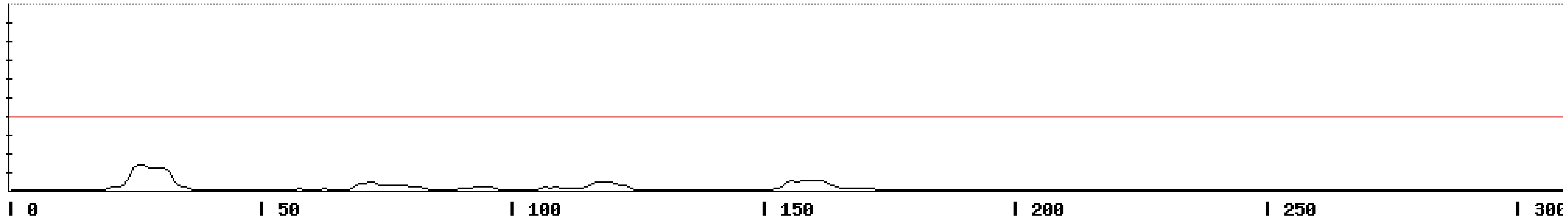

Predictions for Eutremasalsugineum_Thhalv10014325m

Viterbi Path

Viterbi Path

Posterior @ 0.4	98 - GKPK - 101
Posterior @ 0.4	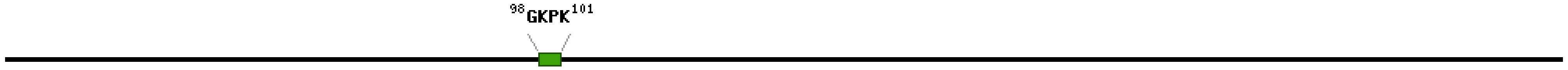
Posterior Graphic. Threshold Marked @ 0.4	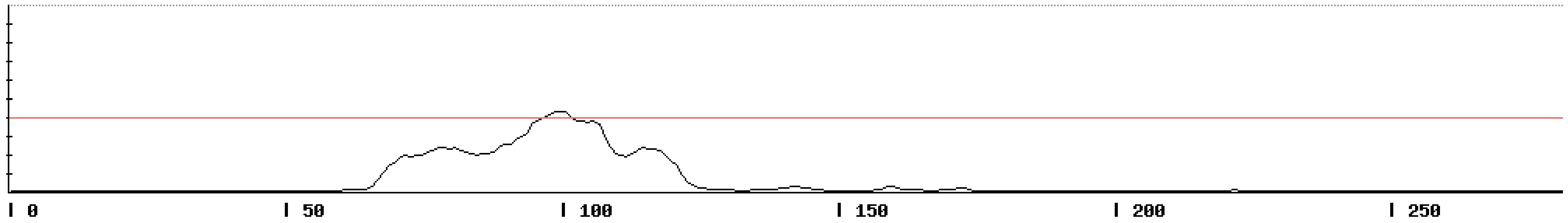

Predictions for Eutremasalsugineum_Thhalv10014495m

Viterbi Path	
Viterbi Path	
Posterior @ 0.4	
Posterior @ 0.4	
Posterior Graphic. Threshold Marked @ 0.4	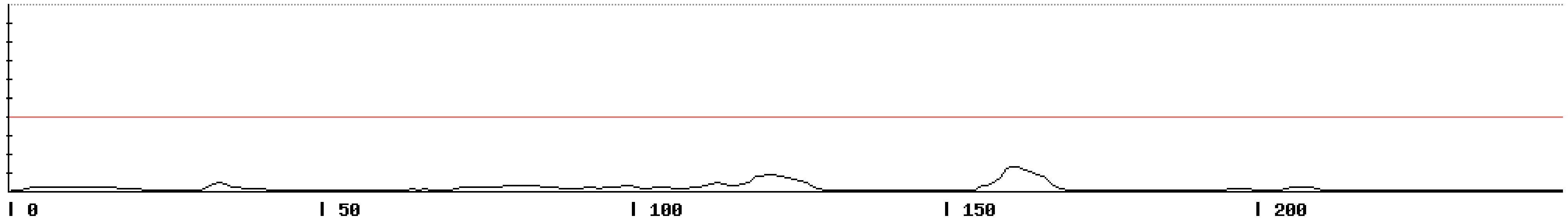

Predictions for Eutremasalsugineum_Thhalv10014563m

Viterbi Path

Viterbi Path

Posterior @ 0.4

Posterior @ 0.4

Posterior Graphic. Threshold Marked @ 0.4

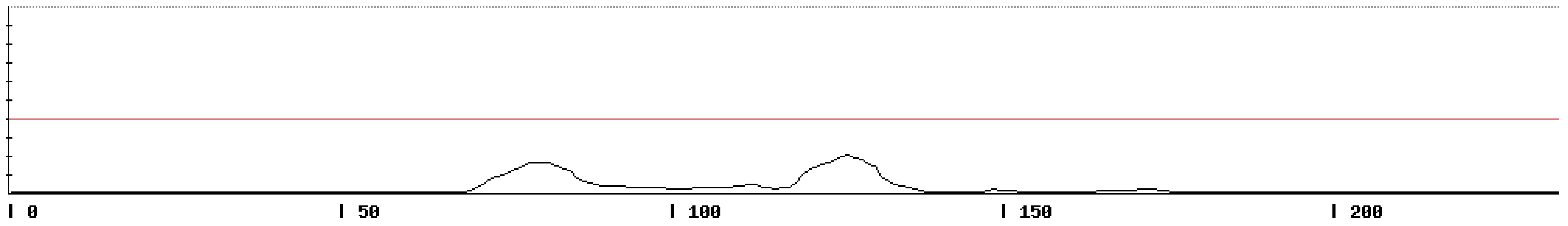

Predictions for Eutremasalsugineum_Thhalv10014659m

Viterbi Path

Viterbi Path

Posterior @ 0.4 | 163 - KVSKGRMQRKAKG - 175

Posterior @ 0.4

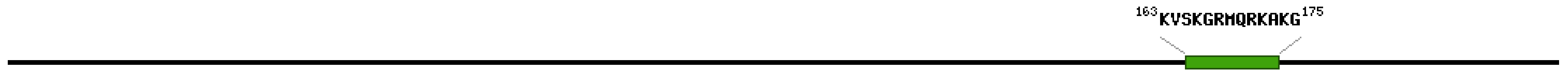

Posterior Graphic. Threshold Marked @ 0.4

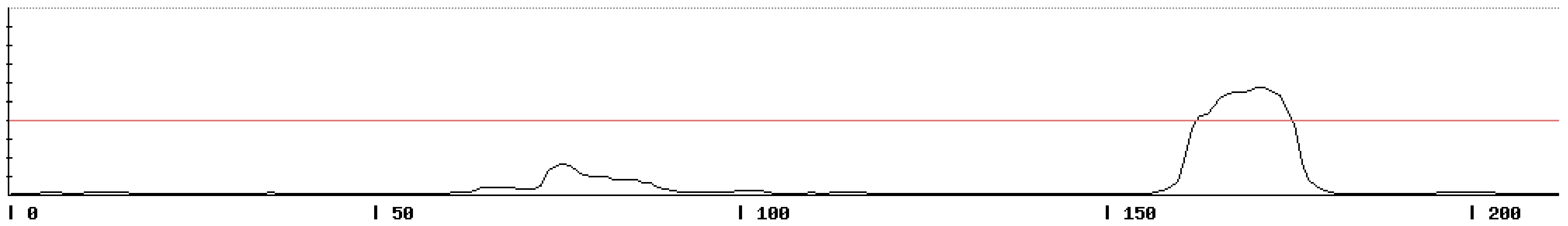

Predictions for Eutremasalsugineum_Thhalv10014770m	
Viterbi Path	
Viterbi Path	
Posterior @ 0.4	
Posterior @ 0.4	
Posterior Graphic. Threshold Marked @ 0.4	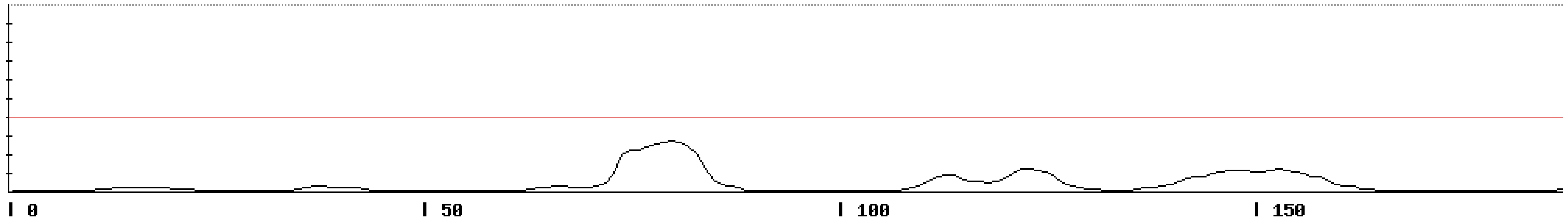

Predictions for Eutremasalsugineum_Thhalv10014889m	
Viterbi Path	
Viterbi Path	
Posterior @ 0.4	
Posterior @ 0.4	
Posterior Graphic. Threshold Marked @ 0.4	

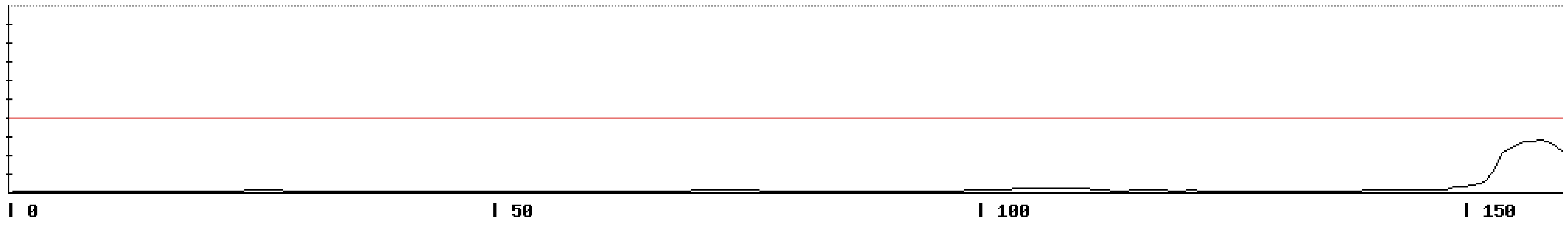

Predictions for Eutremasalsugineum_Thhalv10015280m

Viterbi Path	
Viterbi Path	
Posterior @ 0.4	
Posterior @ 0.4	
Posterior Graphic. Threshold Marked @ 0.4	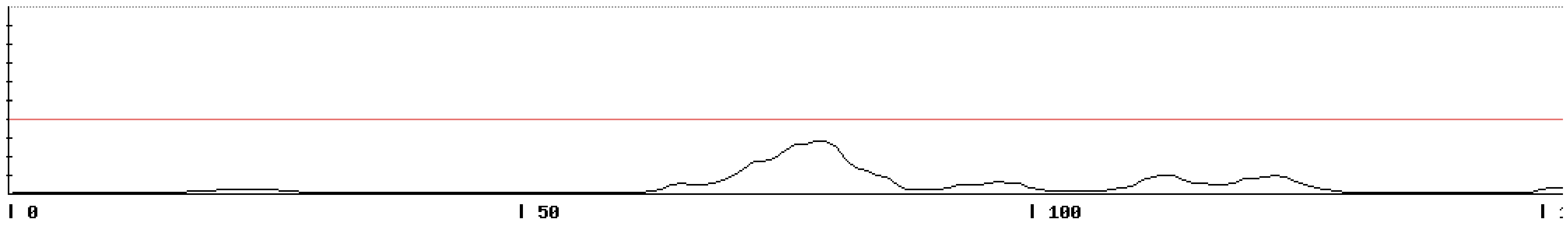

Predictions for Eutremasalsugineum_Thhalv10015349m

Viterbi Path	
Viterbi Path	
Posterior @ 0.4	78 - KKLGRGKNSKRR - 89
	164 - DKSKKKK - 170
Posterior @ 0.4	

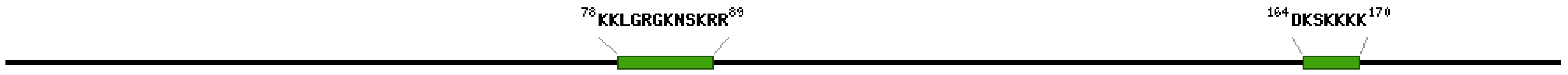

Posterior Graphic. Threshold Marked @ 0.4

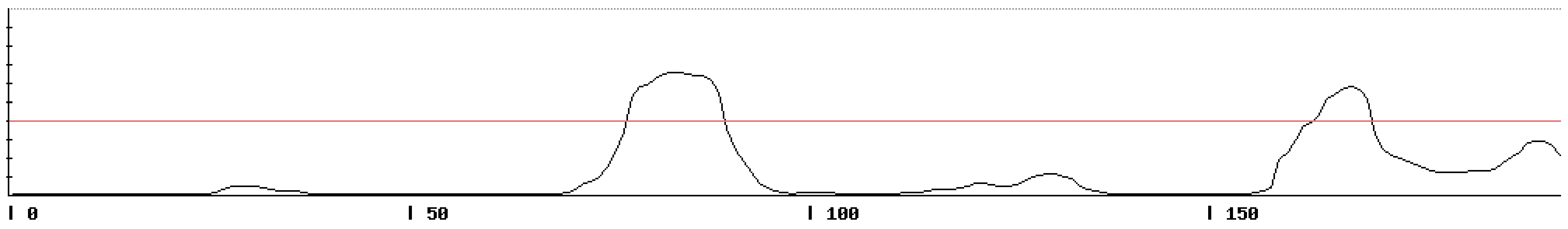

Predictions for Eutremasalsugineum_Thhalv10015488m

Viterbi Path

Viterbi Path

Posterior @ 0.4 — 114 - WKKTGRDRKIKSG - 126

Posterior @ 0.4

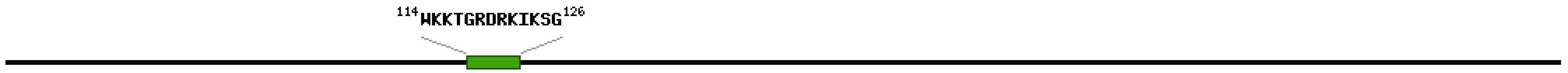

Posterior Graphic. Threshold Marked @ 0.4

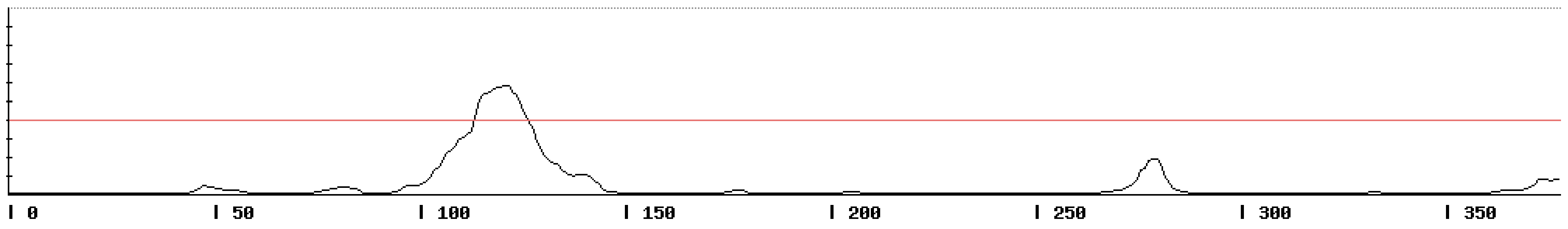

Predictions for Eutremasalsugineum_Thhalv10015599m

Viterbi Path

Viterbi Path

Posterior @ 0.4

22 - HKIPKINRSKKKDDRHRPRE - 41

62 - KRKLKGKSK - 70

156 - TKKTLTFTKSKNKKKKQK - 173

Posterior @ 0.4

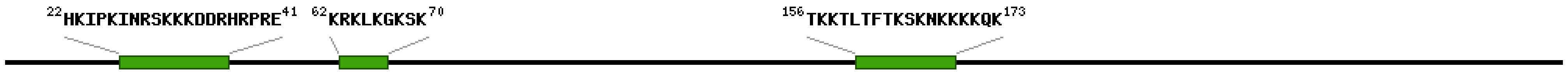

Posterior Graphic. Threshold Marked @ 0.4

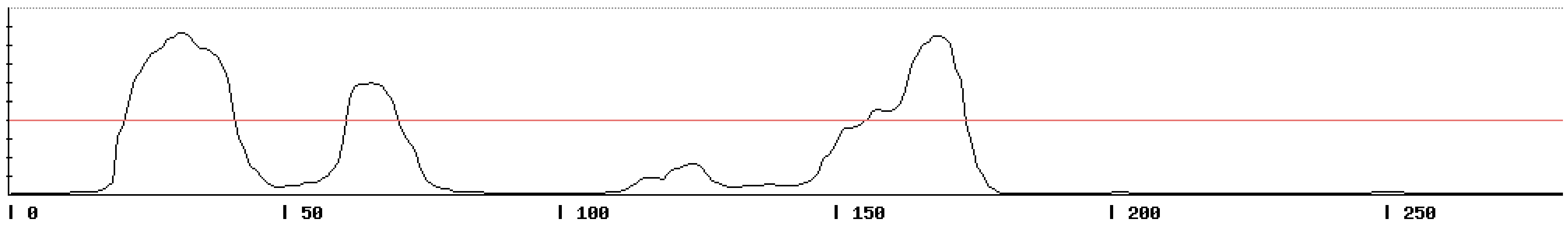

Predictions for Eutremasalsugineum_Thhalv10015728m

Viterbi Path

Viterbi Path

Posterior @ 0.4

130 - PKGQRKRRRITPSGQKDETVRWRKTGKTKPVKLNGVQRGSKKFMVLYKSAKKG - 182

Posterior @ 0.4

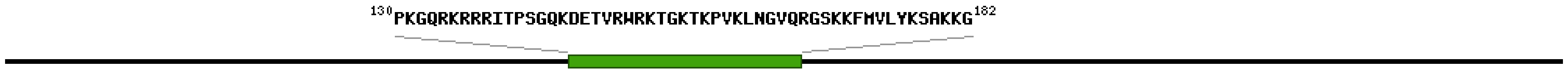

Posterior Graphic. Threshold Marked @ 0.4

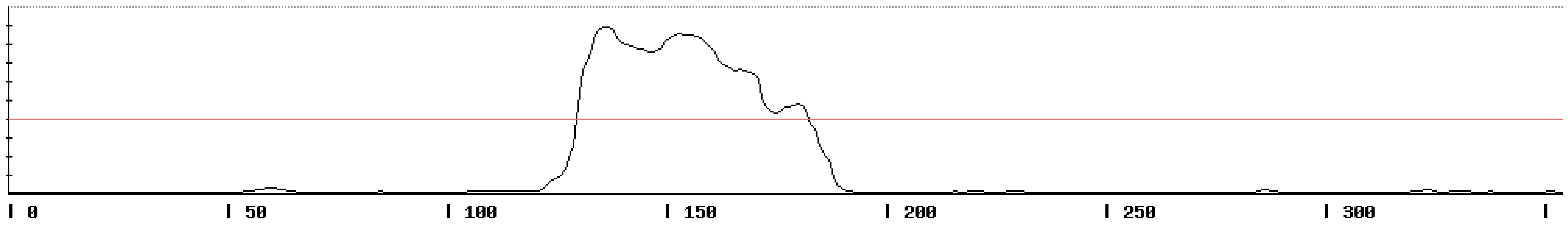

Predictions for Eutremasalsugineum_Thhalv10015750m

Viterbi Path

Viterbi Path

Posterior @ 0.4

77 - KKIGRGKNSKRR - 88

127 - KSKKTKK - 133

Posterior @ 0.4

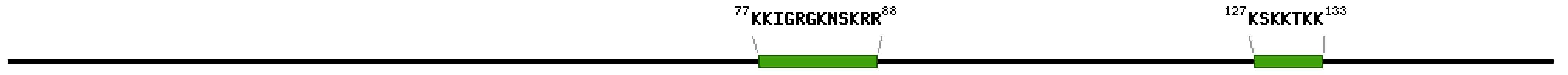

Posterior Graphic. Threshold Marked @ 0.4

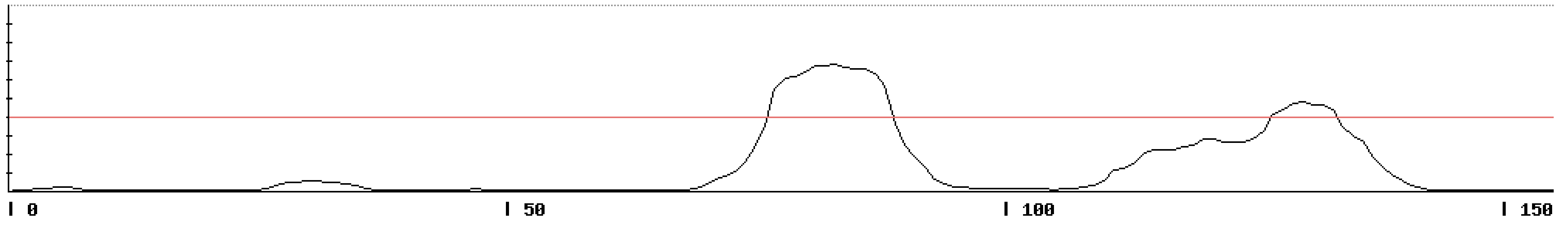

Predictions for Eutremasalsugineum_Thhalv10015892m

Viterbi Path

Viterbi Path

Posterior @ 0.4

127 - KR - 128

Posterior @ 0.4

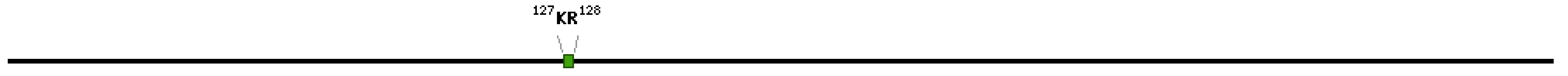

Posterior Graphic. Threshold Marked @ 0.4

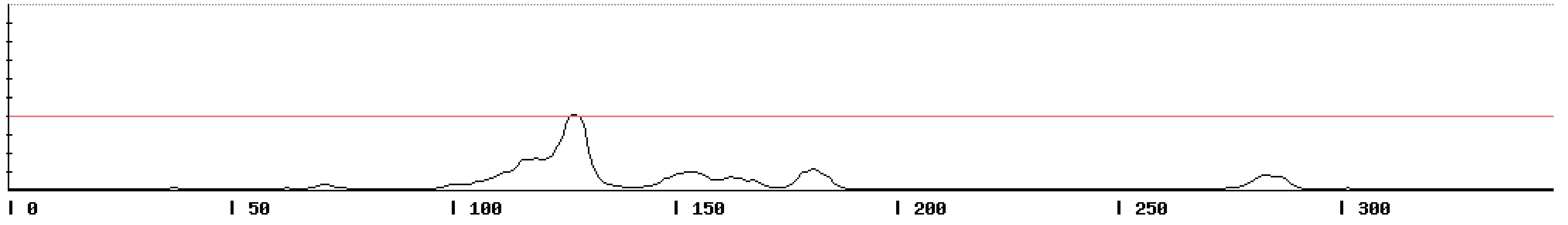

Predictions for Eutremasalsugineum_Thhalv10015954m

Viterbi Path	
Viterbi Path	
Posterior @ 0.4	77 - KKIGRGKNSKRR - 88<br>127 - KSKKTKKQQK - 136
Posterior @ 0.4	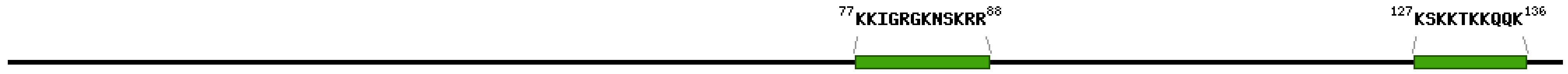
Posterior Graphic. Threshold Marked @ 0.4	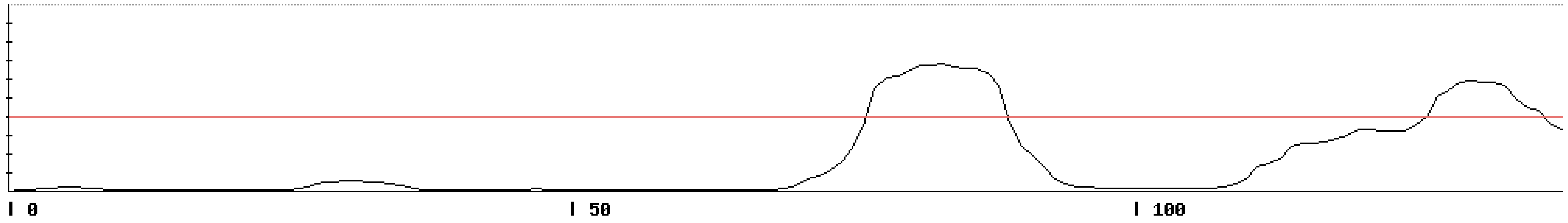

Predictions for Eutremasalsugineum_Thhalv10017054m

Viterbi Path	
Viterbi Path	
Posterior @ 0.4	
Posterior @ 0.4	
Posterior Graphic. Threshold Marked @ 0.4	

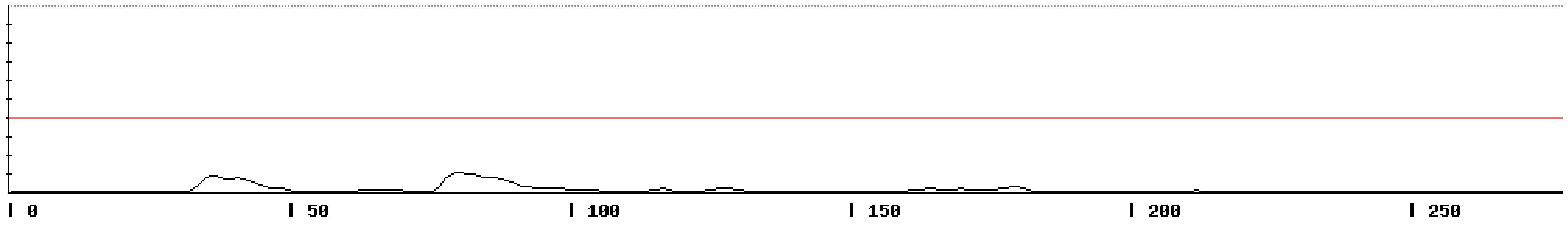

Predictions for Eutremasalsugineum_Thhalv10017056m

Viterbi Path

Viterbi Path

Posterior @ 0.4

74 - GGKKPKRTVPGR - 85

Posterior @ 0.4

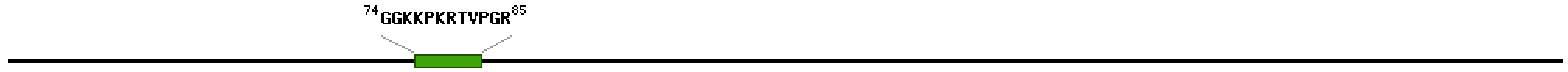

Posterior Graphic. Threshold Marked @ 0.4

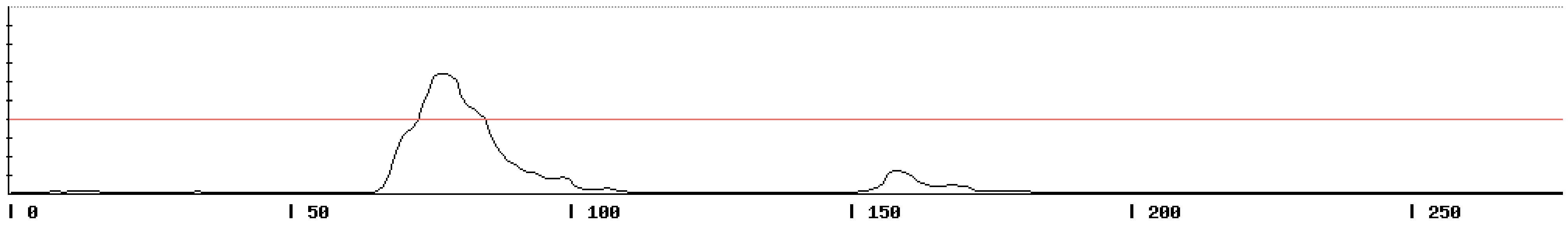

Predictions for Eutremasalsugineum_Thhalv10017152m

Viterbi Path

Viterbi Path

Posterior @ 0.4

Posterior @ 0.4

Posterior Graphic. Threshold Marked @ 0.4

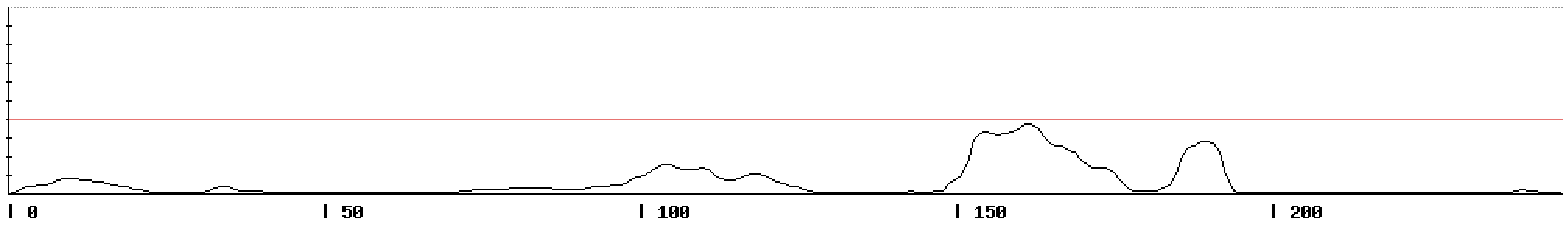

Predictions for Eutremasalsugineum_Thhalv10017577m

Viterbi Path	
Viterbi Path	
Posterior @ 0.4	38 - PRPKIGGKKPKRTVPGRG - 55
Posterior @ 0.4	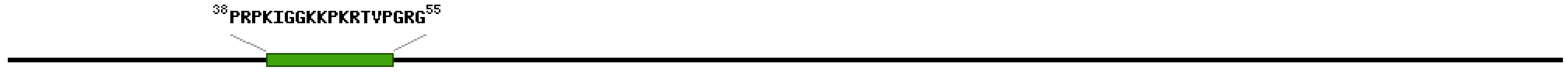
Posterior Graphic. Threshold Marked @ 0.4	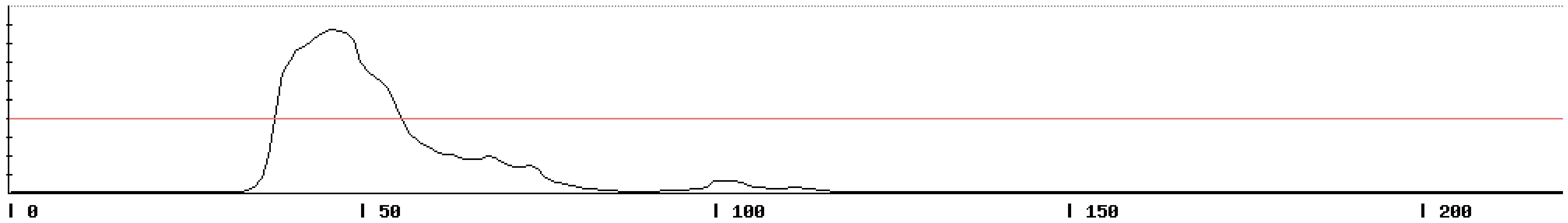

Predictions for Eutremasalsugineum_Thhalv10017700m

Viterbi Path	
Viterbi Path	
Posterior @ 0.4	74 - GGKKPKRTVPGR - 85
Posterior @ 0.4	

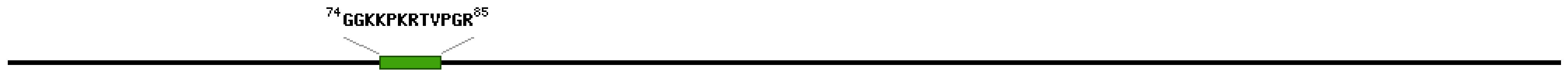

Posterior Graphic. Threshold Marked @ 0.4

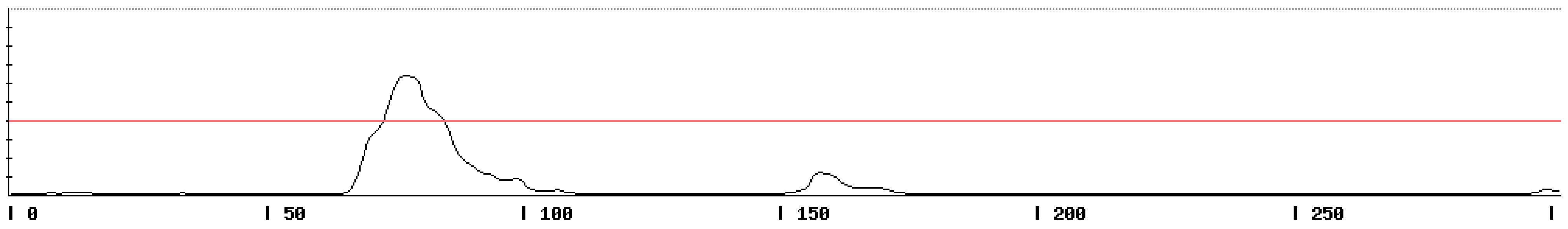

Predictions for Eutremasalsugineum_Thhalv10017733m

Viterbi Path

Viterbi Path

Posterior @ 0.4 — 74 - GGKKPKRTVPGR - 85

Posterior @ 0.4

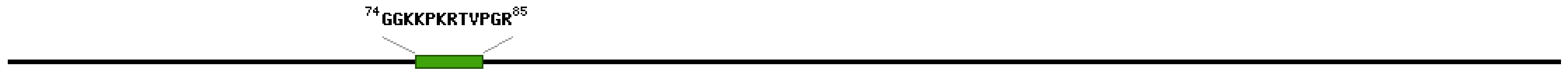

Posterior Graphic. Threshold Marked @ 0.4

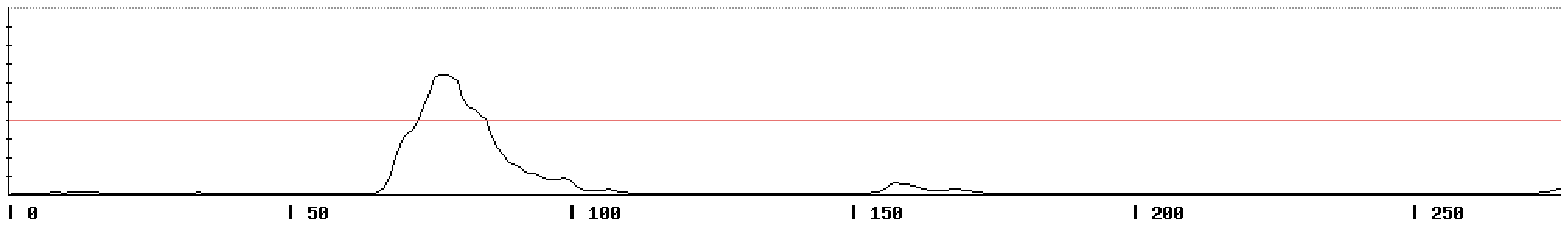

Predictions for Eutremasalsugineum_Thhalv10018275m

Viterbi Path

Viterbi Path

Posterior @ 0.4	
Posterior @ 0.4	
Posterior Graphic. Threshold Marked @ 0.4	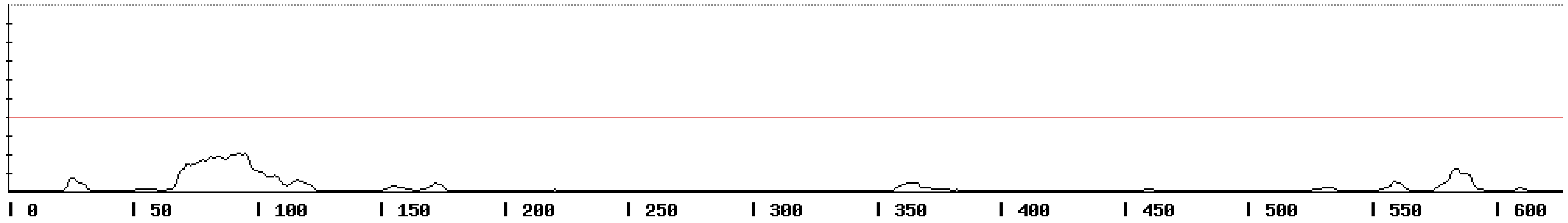

Predictions for Eutremasalsugineum_Thhalv10018853m

Viterbi Path	
Viterbi Path	
Posterior @ 0.4	
Posterior @ 0.4	
Posterior Graphic. Threshold Marked @ 0.4	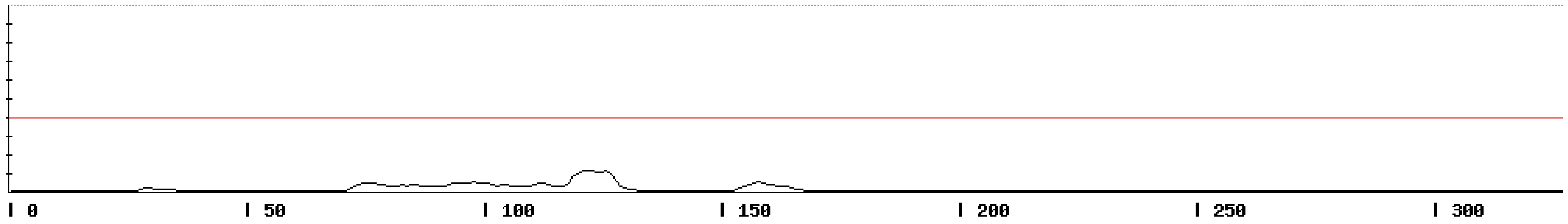

Predictions for Eutremasalsugineum_Thhalv10018997m

Viterbi Path	
Viterbi Path	

Posterior @ 0.4

Posterior @ 0.4

Posterior Graphic. Threshold Marked @ 0.4

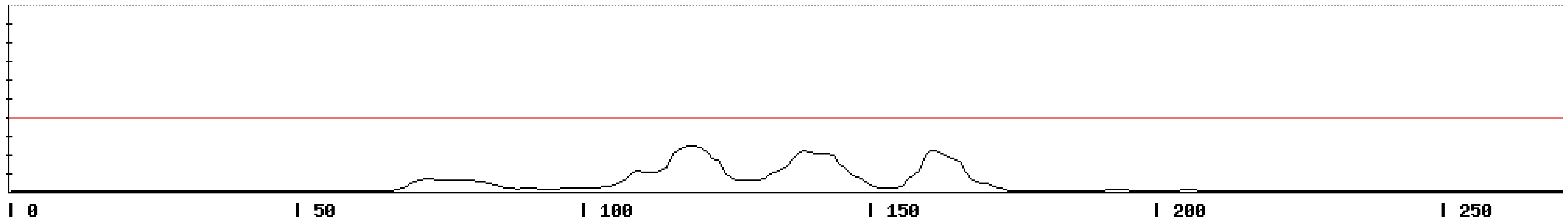

Predictions for Eutremasalsugineum_Thhalv10018998m

Viterbi Path

Viterbi Path

Posterior @ 0.4

98 - KPIGRPKPVGIKK - 110

Posterior @ 0.4

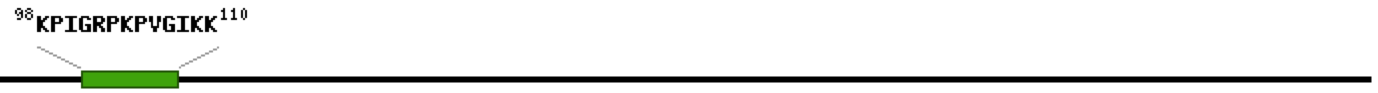

Posterior Graphic. Threshold Marked @ 0.4

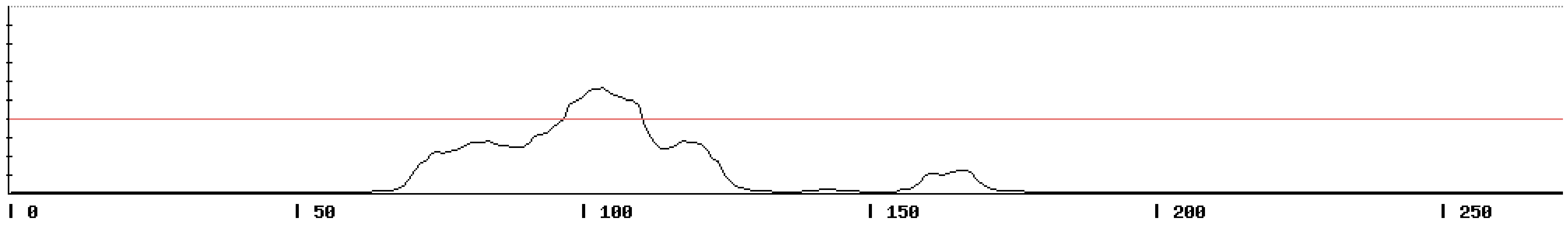

Predictions for Eutremasalsugineum_Thhalv10019493m

Viterbi Path

Viterbi Path

Posterior @ 0.4

Posterior @ 0.4

Posterior Graphic. Threshold Marked @ 0.4

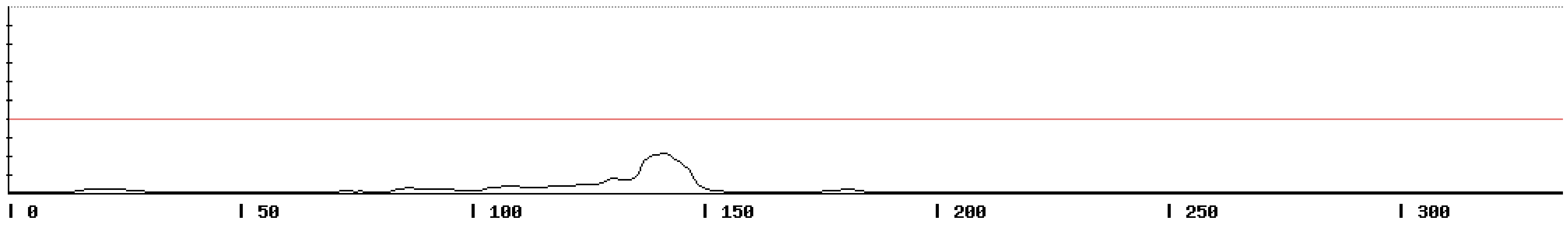

Predictions for Eutremasalsugineum_Thhalv10019534m

Viterbi Path

Viterbi Path

Posterior @ 0.4

Posterior @ 0.4

Posterior Graphic. Threshold Marked @ 0.4

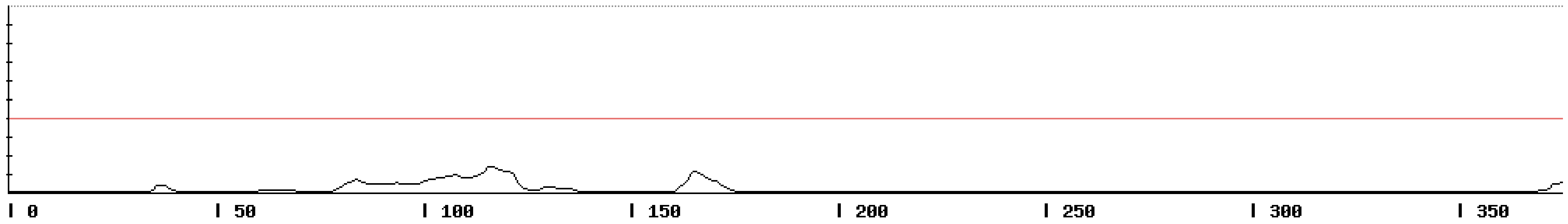

Predictions for Eutremasalsugineum_Thhalv10019631m

Viterbi Path	
Viterbi Path	
Posterior @ 0.4	
Posterior @ 0.4	
Posterior Graphic. Threshold Marked @ 0.4	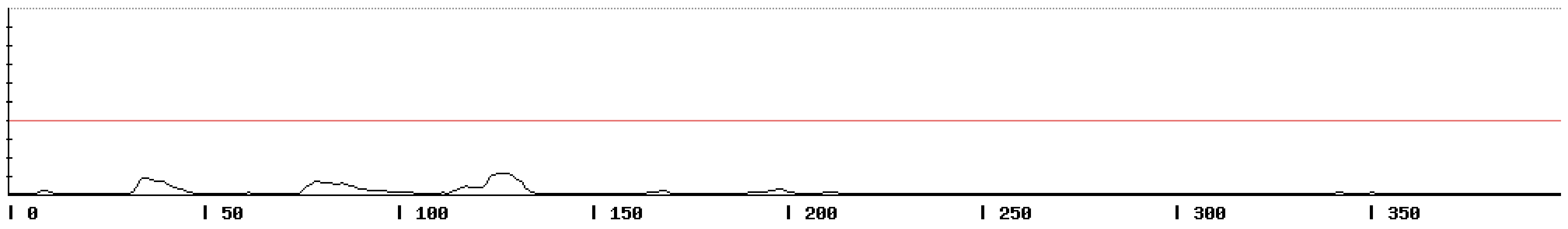

Predictions for Eutremasalsugineum_Thhalv10020438m

Viterbi Path	
Viterbi Path	
Posterior @ 0.4	
Posterior @ 0.4	
Posterior Graphic. Threshold Marked @ 0.4	

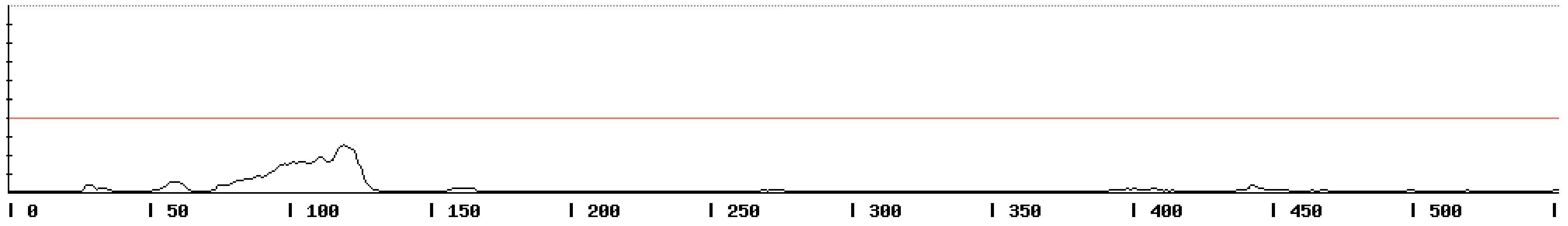

Predictions for Eutremasalsugineum_Thhalv10020717m

Viterbi Path

Viterbi Path

Posterior @ 0.4

Posterior @ 0.4

Posterior Graphic. Threshold Marked @ 0.4

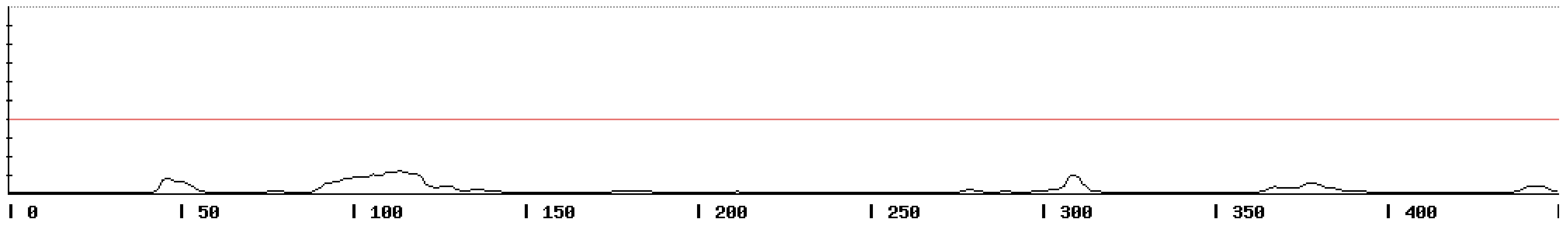

Predictions for Eutremasalsugineum_Thhalv10020745m

Viterbi Path

Viterbi Path

Posterior @ 0.4

Posterior @ 0.4

Posterior Graphic. Threshold Marked @ 0.4

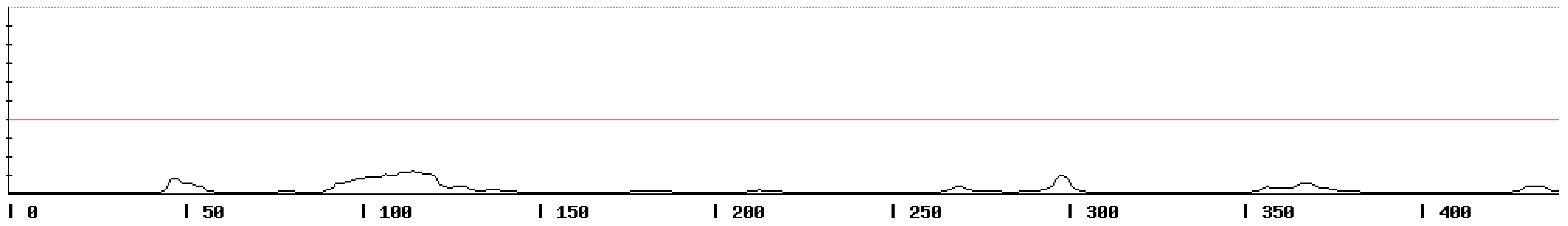

Predictions for Eutremasalsugineum_Thhalv10020756m

Viterbi Path

Viterbi Path

Posterior @ 0.4

Posterior @ 0.4

Posterior Graphic. Threshold Marked @ 0.4

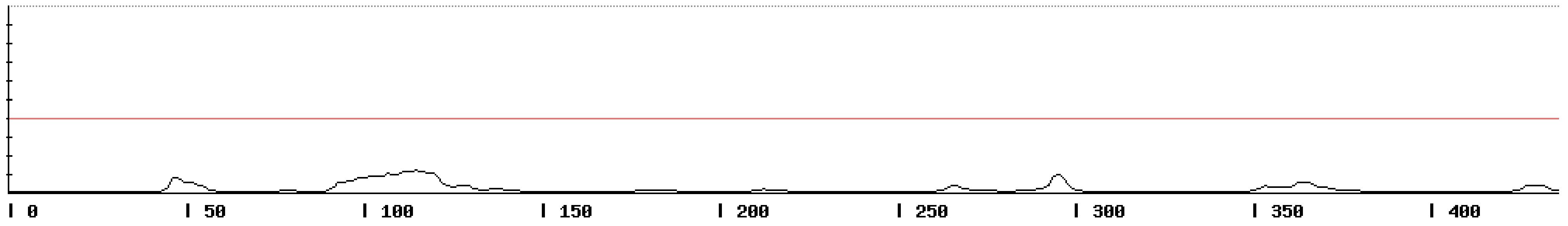

Predictions for Eutremasalsugineum_Thhalv10021011m

Viterbi Path

Viterbi Path

Posterior @ 0.4

Posterior @ 0.4

Posterior Graphic. Threshold Marked @ 0.4	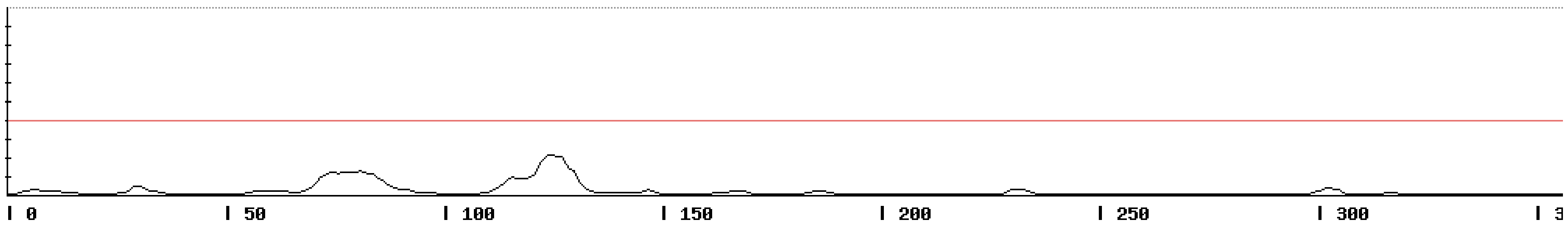

Predictions for Eutremasalsugineum_Thhalv10021125m	
Viterbi Path	
Viterbi Path	
Posterior @ 0.4	
Posterior @ 0.4	
Posterior Graphic. Threshold Marked @ 0.4	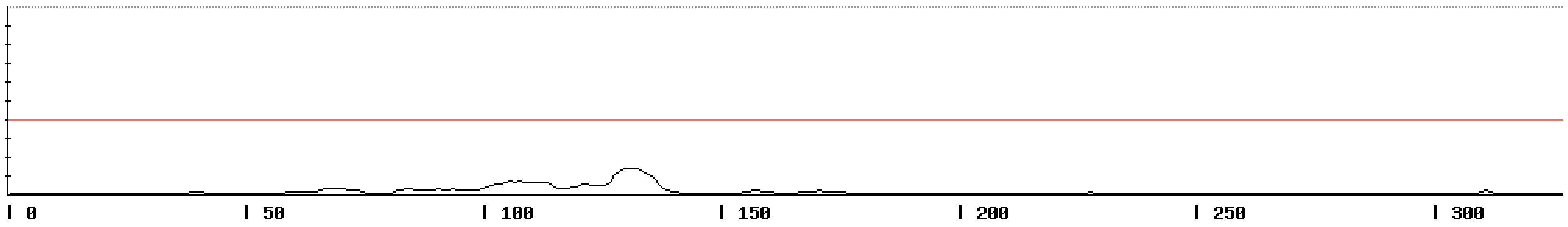

Predictions for Eutremasalsugineum_Thhalv10021174m	
Viterbi Path	
Viterbi Path	

Posterior @ 0.4

Posterior @ 0.4

Posterior Graphic. Threshold Marked @ 0.4

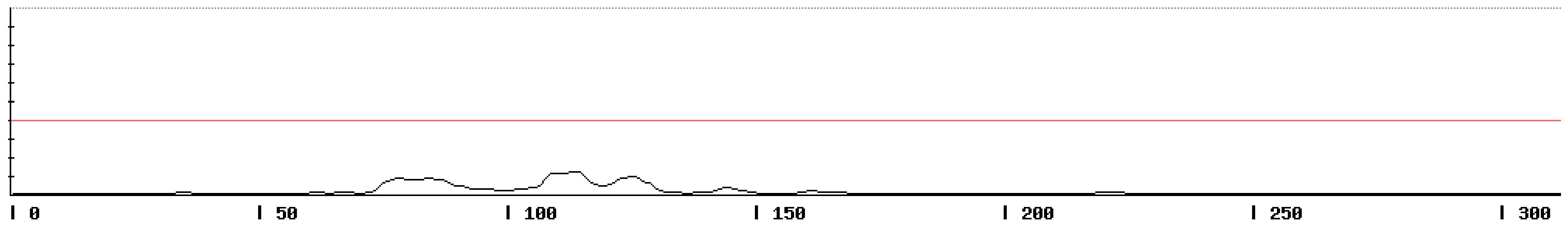

Predictions for Eutremasalsugineum_Thhalv10021186m

Viterbi Path

Viterbi Path

Posterior @ 0.4

Posterior @ 0.4

Posterior Graphic. Threshold Marked @ 0.4

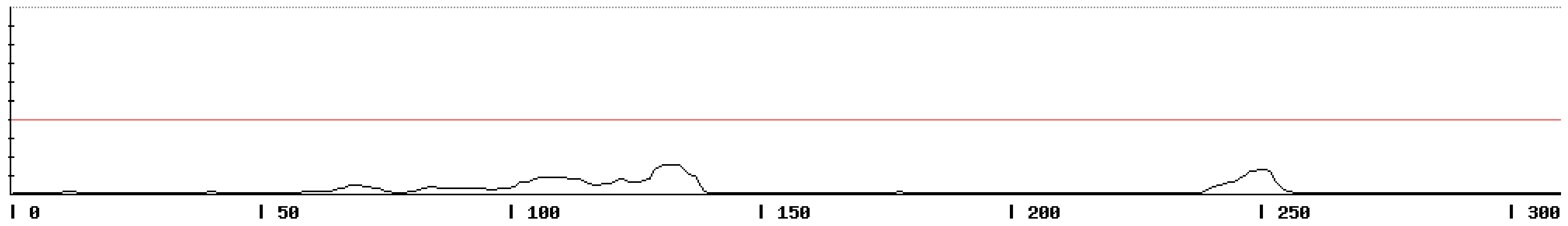

Predictions for Eutremasalsugineum_Thhalv10021200m

Viterbi Path

Viterbi Path

Posterior @ 0.4

Posterior @ 0.4

Posterior Graphic. Threshold Marked @ 0.4

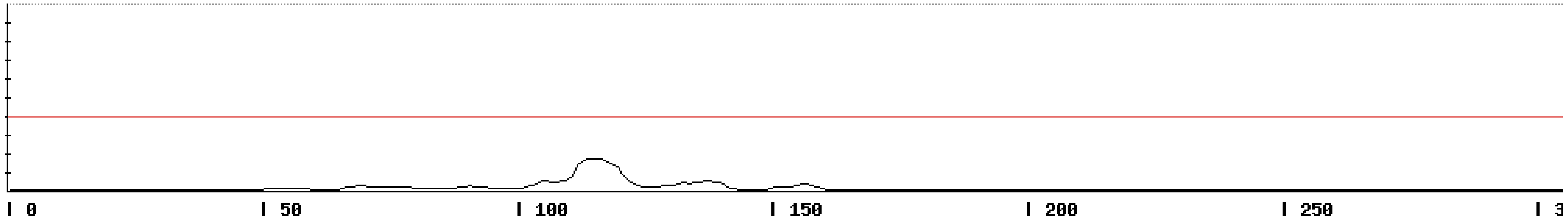

Predictions for Eutremasalsugineum_Thhalv10021225m

Viterbi Path

Viterbi Path

Posterior @ 0.4

Posterior @ 0.4

Posterior Graphic. Threshold Marked @ 0.4

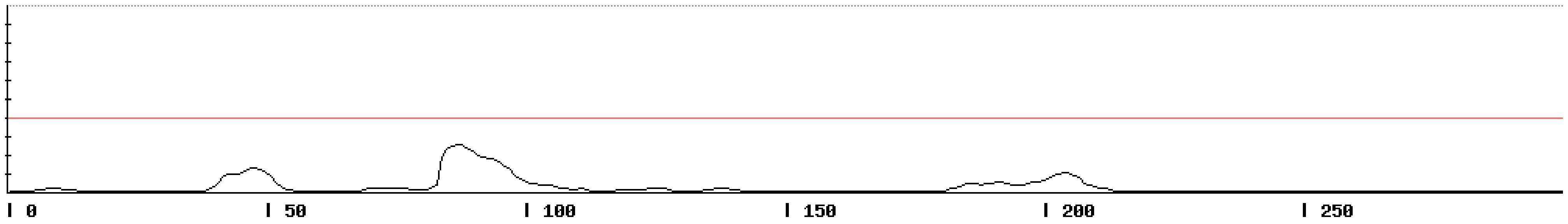

Predictions for Eutremasalsugineum_Thhalv10021297m

Viterbi Path	
Viterbi Path	
Posterior @ 0.4	
Posterior @ 0.4	
Posterior Graphic. Threshold Marked @ 0.4	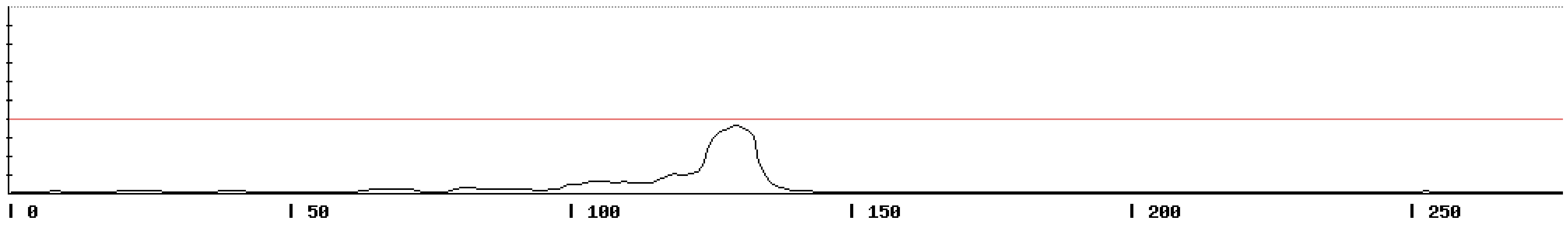

Predictions for Eutremasalsugineum_Thhalv10021408m

Viterbi Path	
Viterbi Path	
Posterior @ 0.4	
Posterior @ 0.4	
Posterior Graphic. Threshold Marked @ 0.4	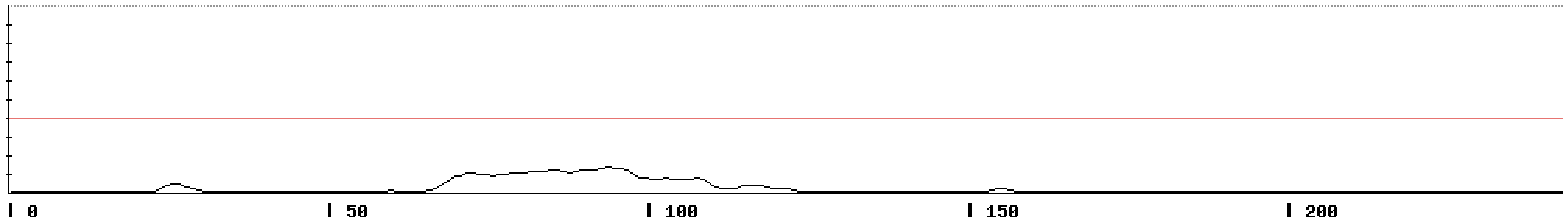

Predictions for Eutremasalsugineum_Thhalv10022055m	
Viterbi Path	
Viterbi Path	
Posterior @ 0.4	
Posterior @ 0.4	
Posterior Graphic. Threshold Marked @ 0.4	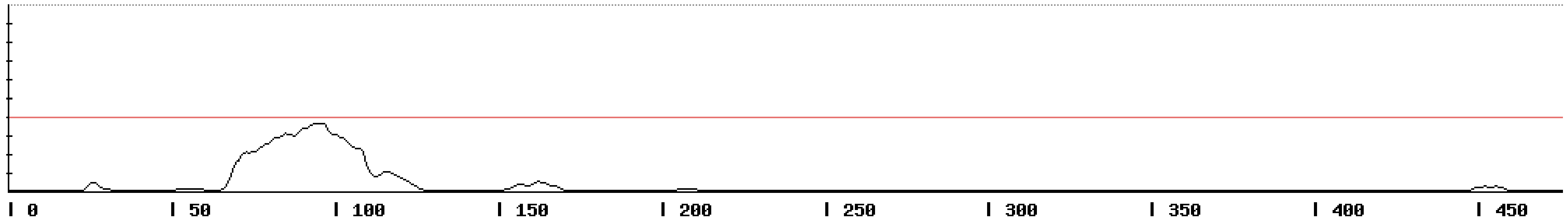

Predictions for Eutremasalsugineum_Thhalv10022099m	
Viterbi Path	
Viterbi Path	
Posterior @ 0.4	
Posterior @ 0.4	
Posterior Graphic. Threshold Marked @ 0.4	

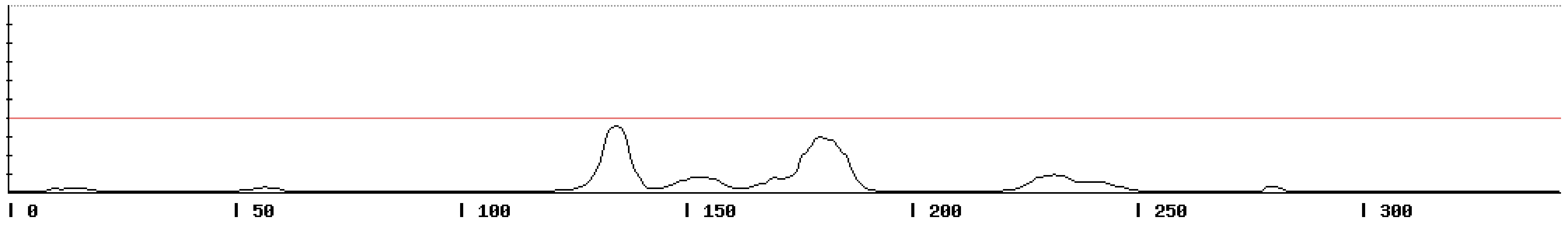

Predictions for Eutremasalsugineum_Thhalv10022353m

Viterbi Path

Viterbi Path

Posterior @ 0.4

Posterior @ 0.4

Posterior Graphic. Threshold Marked @ 0.4

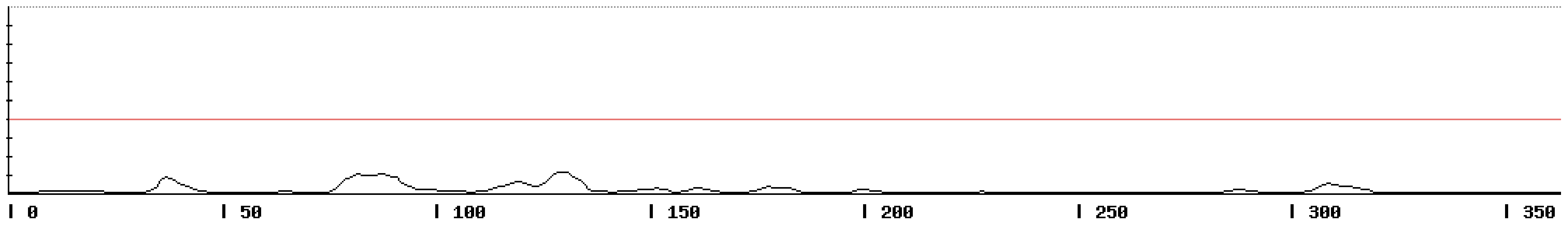

Predictions for Eutremasalsugineum_Thhalv10022756m

Viterbi Path

Viterbi Path

Posterior @ 0.4 213 - KENKRKKKNK - 222

Posterior @ 0.4

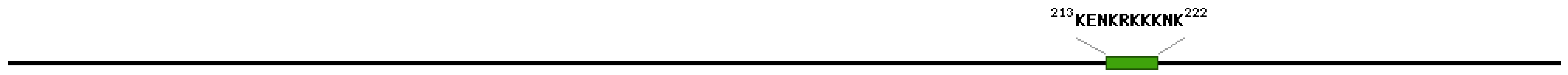

Posterior Graphic. Threshold Marked @ 0.4	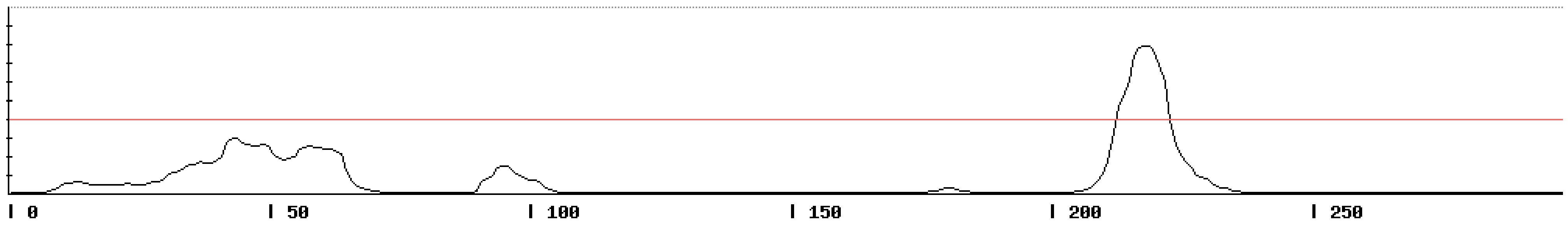

Predictions for Eutremasalsugineum_Thhalv10022800m	
Viterbi Path	
Viterbi Path	
Posterior @ 0.4	
Posterior @ 0.4	
Posterior Graphic. Threshold Marked @ 0.4	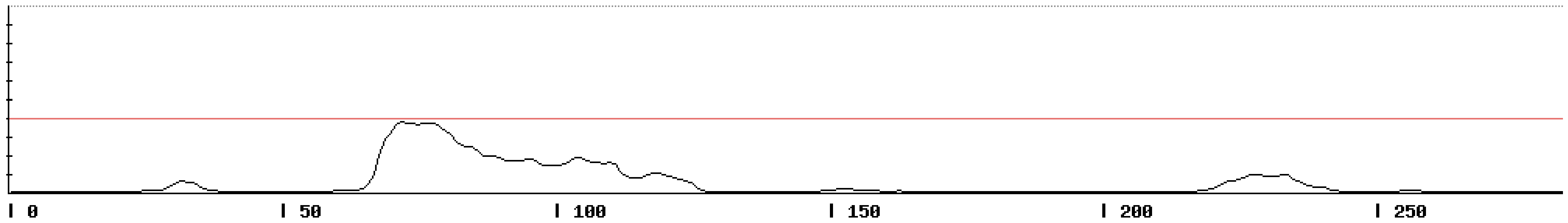

Predictions for Eutremasalsugineum_Thhalv10023058m	
Viterbi Path	
Viterbi Path	
Posterior @ 0.4	
Posterior @ 0.4	

Posterior Graphic. Threshold Marked @ 0.4

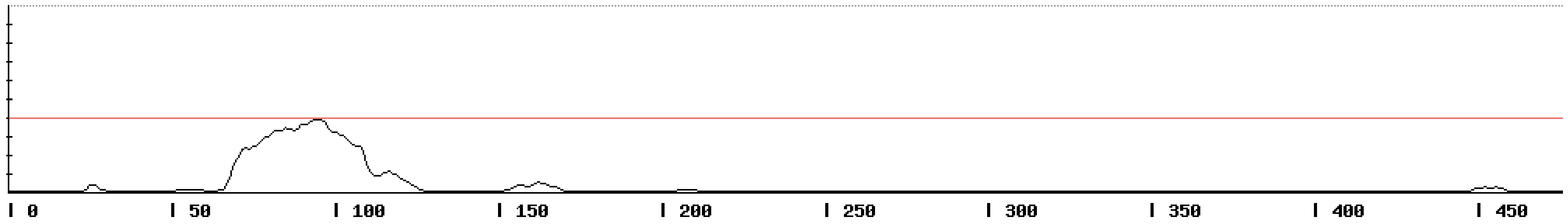

Predictions for Eutremasalsugineum_Thhalv10023502m

Viterbi Path

Viterbi Path

Posterior @ 0.4

Posterior @ 0.4

Posterior Graphic. Threshold Marked @ 0.4

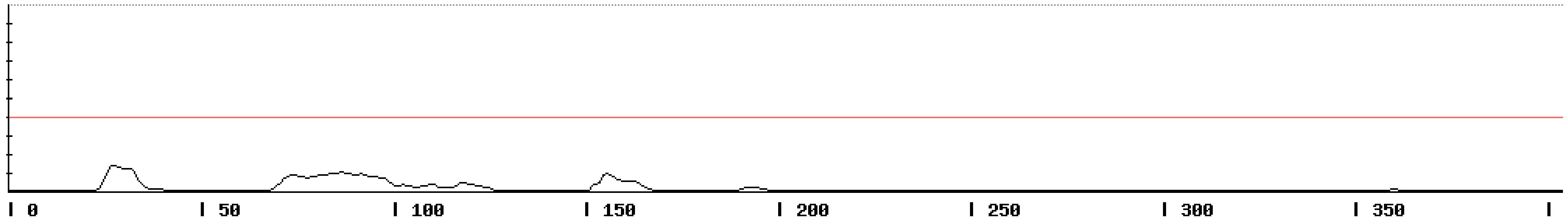

Predictions for Eutremasalsugineum_Thhalv10023591m

Viterbi Path

Viterbi Path

Posterior @ 0.4

Posterior @ 0.4

Posterior Graphic. Threshold Marked @ 0.4

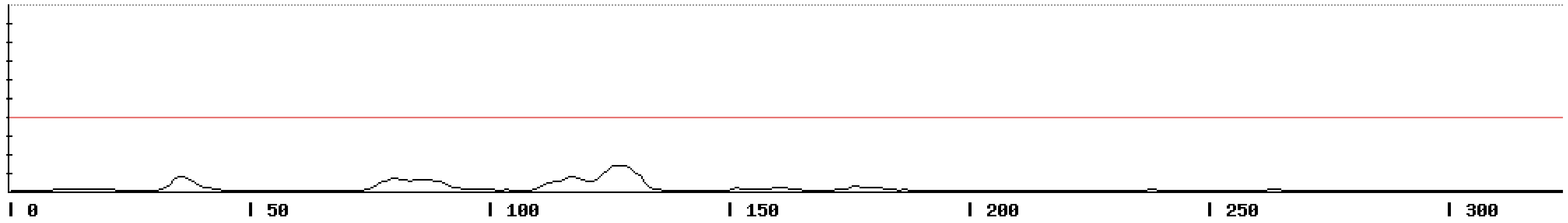

Predictions for Eutremasalsugineum_Thhalv10023723m

Viterbi Path

Viterbi Path

Posterior @ 0.4 121 - KRRV - 124

Posterior @ 0.4

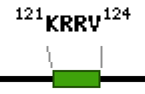

Posterior Graphic. Threshold Marked @ 0.4

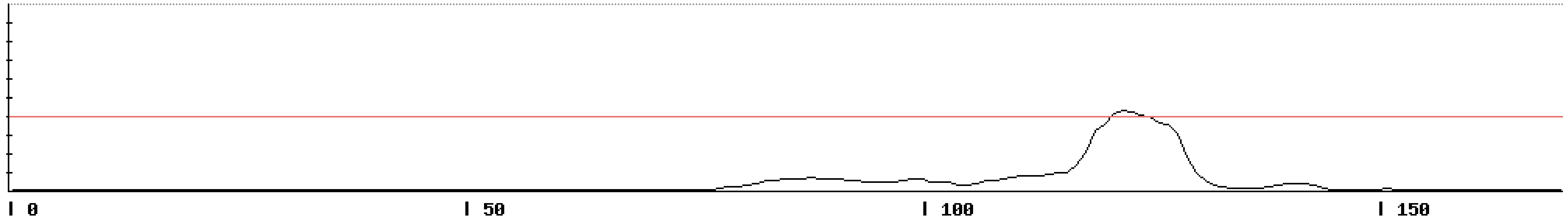

Predictions for Eutremasalsugineum_Thhalv10023876m

Viterbi Path

Viterbi Path

Posterior @ 0.4

Posterior @ 0.4

Posterior Graphic. Threshold Marked @ 0.4

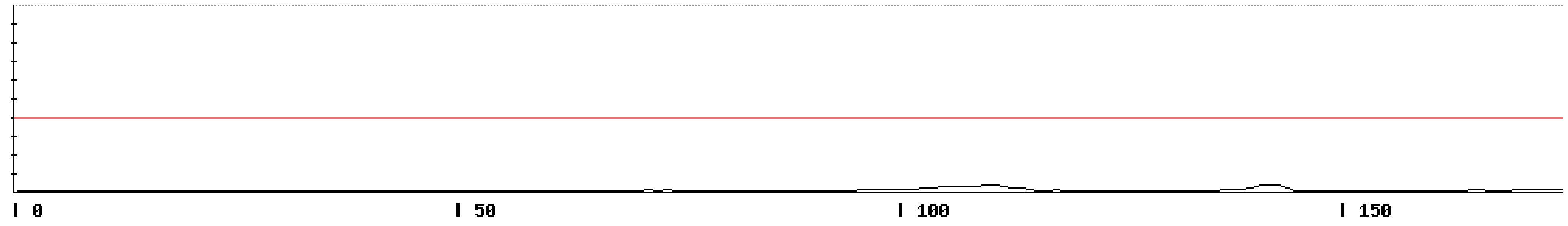

Predictions for Eutremasalsugineum_Thhalv10023895m

Viterbi Path

Viterbi Path

Posterior @ 0.4

Posterior @ 0.4

Posterior Graphic. Threshold Marked @ 0.4

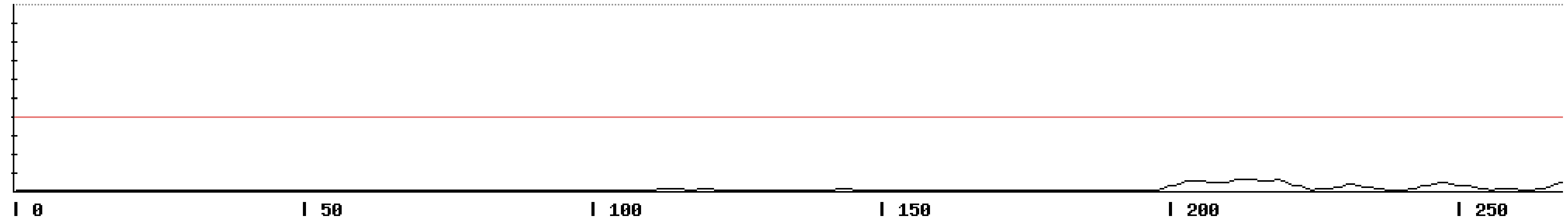

Predictions for Eutremasalsugineum_Thhalv10024006m

Viterbi Path	
Viterbi Path	
Posterior @ 0.4	
Posterior @ 0.4	
Posterior Graphic. Threshold Marked @ 0.4	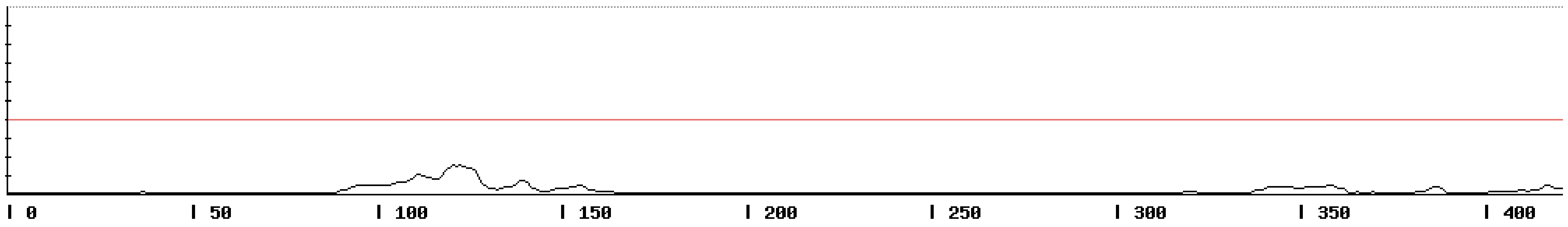

Predictions for Eutremasalsugineum_Thhalv10024128m

Viterbi Path	
Viterbi Path	
Posterior @ 0.4	
Posterior @ 0.4	
Posterior Graphic. Threshold Marked @ 0.4	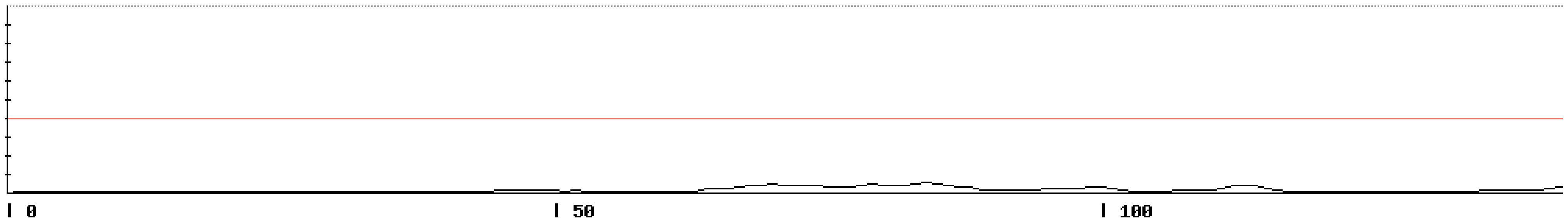

Predictions for Eutremasalsugineum_Thhalv10024148m

Viterbi Path	
Viterbi Path	
Posterior @ 0.4	
Posterior @ 0.4	
Posterior Graphic. Threshold Marked @ 0.4	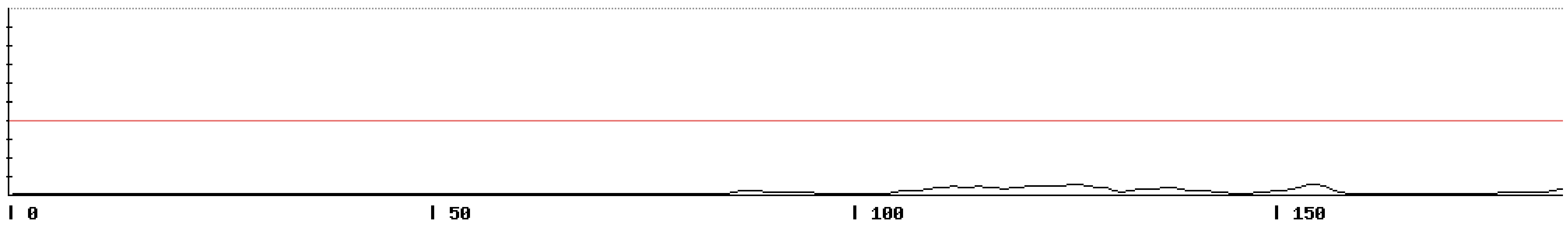

Predictions for Eutremasalsugineum_Thhalv10024149m

Viterbi Path	
Viterbi Path	
Posterior @ 0.4	
Posterior @ 0.4	
Posterior Graphic. Threshold Marked @ 0.4	

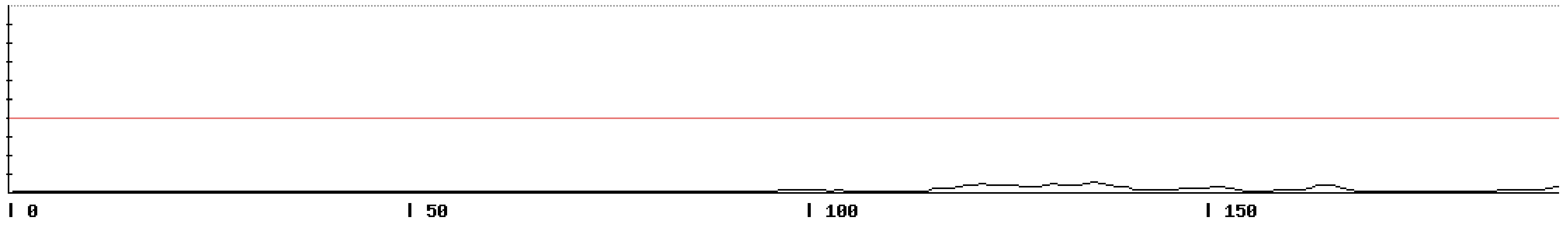

Predictions for Eutremasalsugineum_Thhalv10024980m

Viterbi Path

Viterbi Path

Posterior @ 0.4

Posterior @ 0.4

Posterior Graphic. Threshold Marked @ 0.4

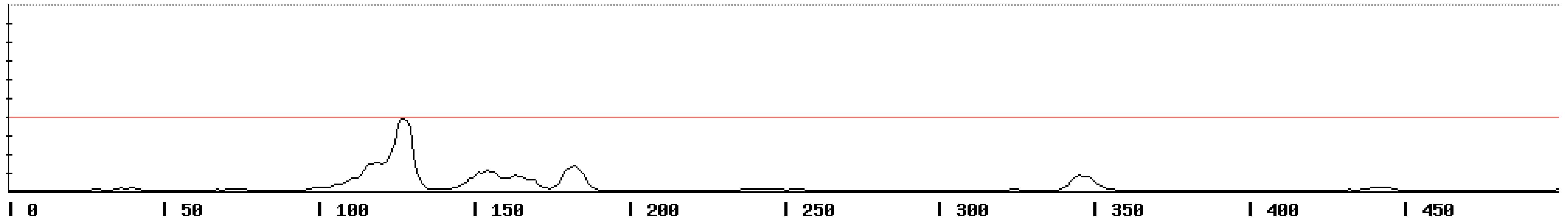

Predictions for Eutremasalsugineum_Thhalv10025510m

Viterbi Path

Viterbi Path

Posterior @ 0.4

281 - KDKKKK - 286

Posterior @ 0.4

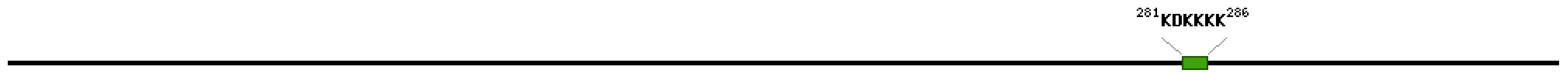

Posterior Graphic. Threshold Marked @ 0.4

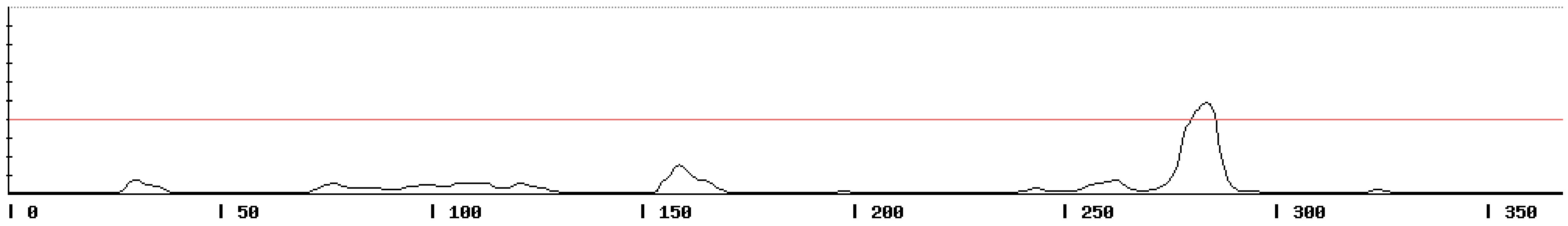

Predictions for Eutremasalsugineum_Thhalv10025606m

Viterbi Path

Viterbi Path

Posterior @ 0.4

Posterior @ 0.4

Posterior Graphic. Threshold Marked @ 0.4

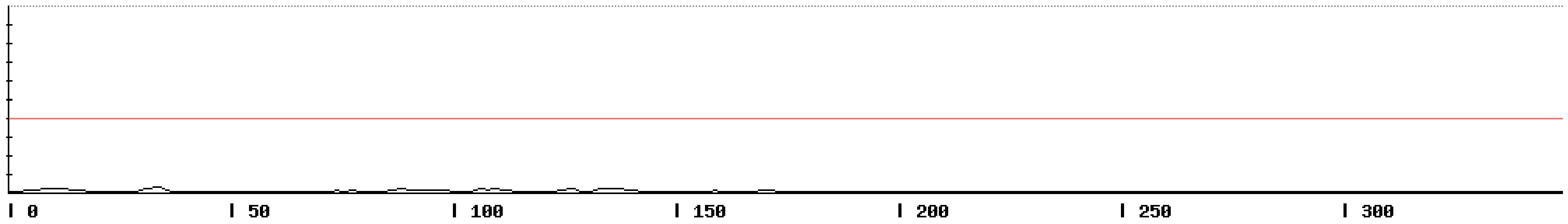

Predictions for Eutremasalsugineum_Thhalv10025818m

Viterbi Path

Viterbi Path

Posterior @ 0.4

Posterior @ 0.4

Posterior Graphic. Threshold Marked @ 0.4

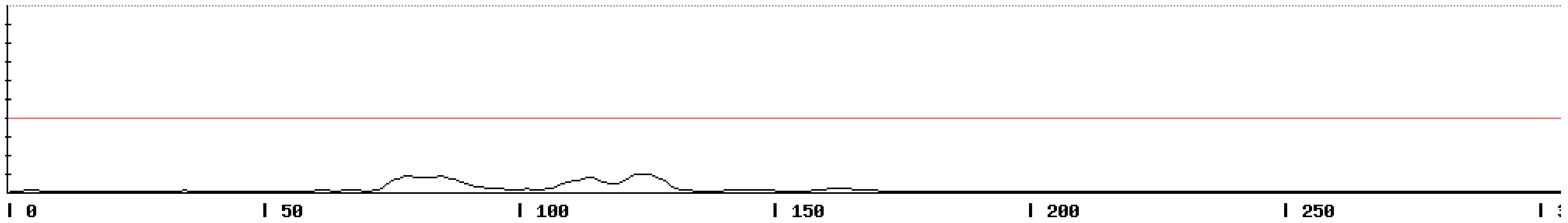

Predictions for Eutremasalsugineum_Thhalv10025819m

Viterbi Path

Viterbi Path

Posterior @ 0.4 137 - RKRRK - 141

Posterior @ 0.4

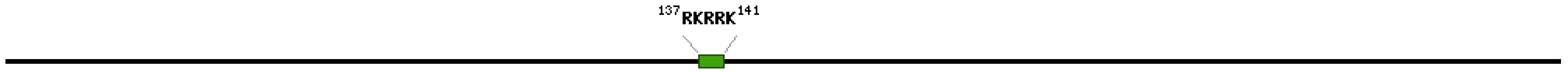

Posterior Graphic. Threshold Marked @ 0.4

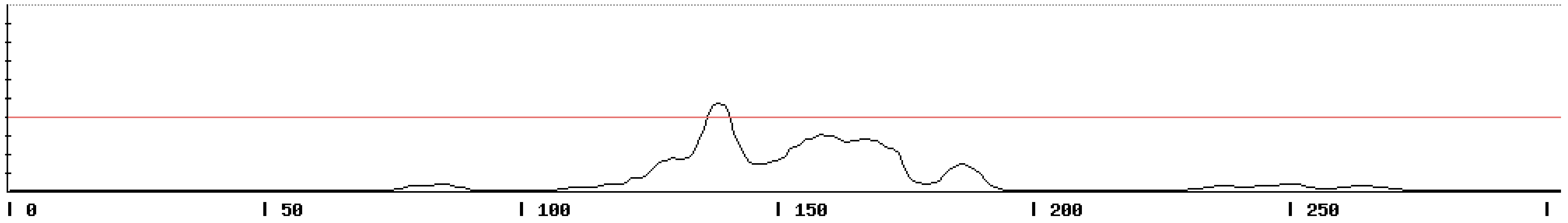

Predictions for Eutremasalsugineum_Thhalv10027064m

Viterbi Path

Viterbi Path

Posterior @ 0.4 158 - KKNERKAKALKN - 169

Posterior @ 0.4

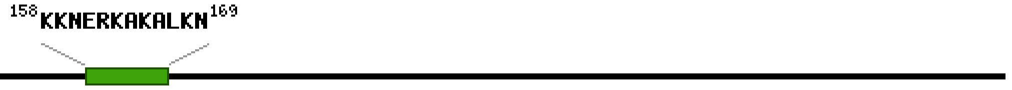

Posterior Graphic. Threshold Marked @ 0.4

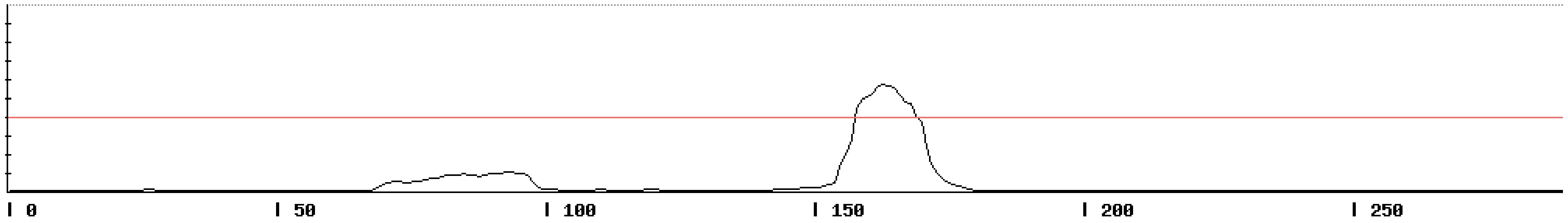

Predictions for Eutremasalsugineum_Thhalv10027479m

Viterbi Path

Viterbi Path

Posterior @ 0.4

Posterior @ 0.4

Posterior Graphic. Threshold Marked @ 0.4

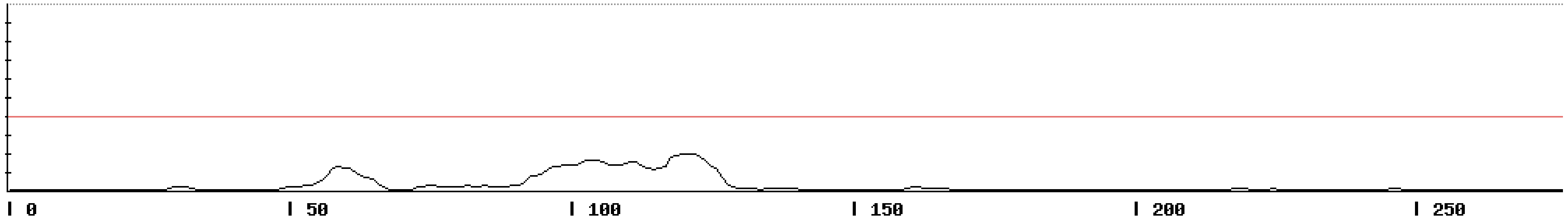

Predictions for Eutremasalsugineum_Thhalv10027860m

Viterbi Path

Viterbi Path

Posterior @ 0.4

Posterior @ 0.4

Posterior Graphic. Threshold Marked @ 0.4

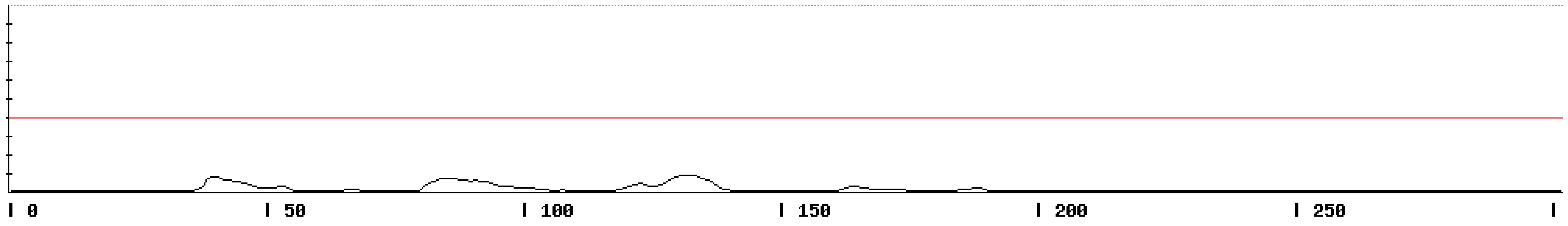

Predictions for Eutremasalsugineum_Thhalv10027874m

Viterbi Path

Viterbi Path

Posterior @ 0.4

Posterior @ 0.4

Posterior Graphic. Threshold Marked @ 0.4

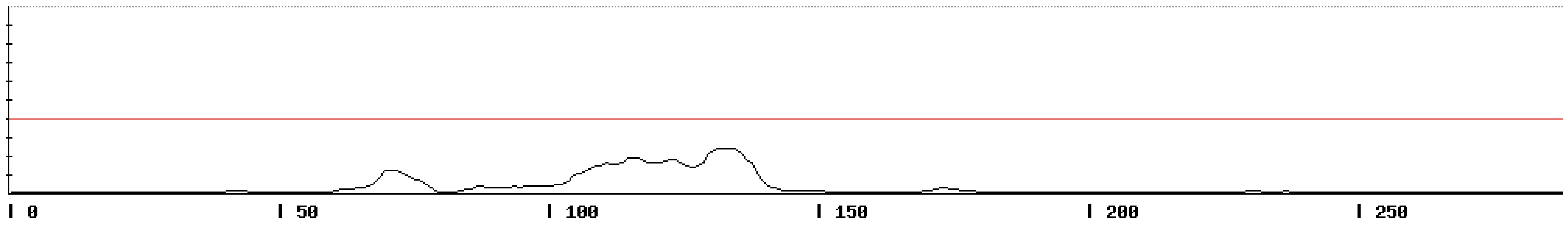

Predictions for Eutremasalsugineum_Thhalv10027901m

Viterbi Path

Viterbi Path

Posterior @ 0.4

Posterior @ 0.4

Posterior Graphic. Threshold Marked @ 0.4

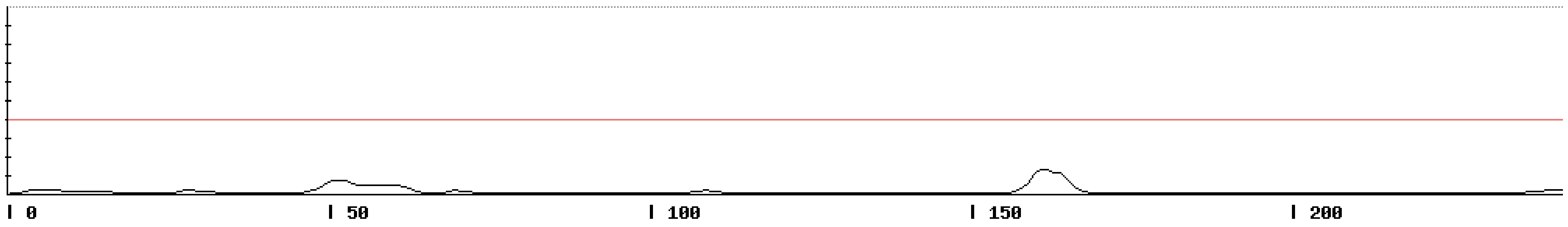

Predictions for Eutremasalsugineum_Thhalv10028104m

Viterbi Path

85 - GKSKKKKKKKQK - 96

Viterbi Path

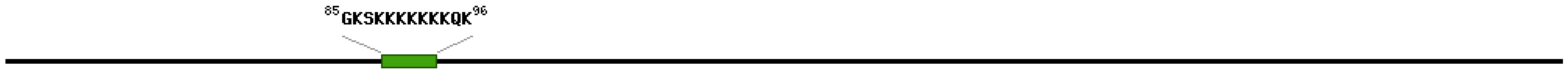

Posterior @ 0.4

79 - KRTLTFGKSKKKKKKKQK - 96

Posterior @ 0.4

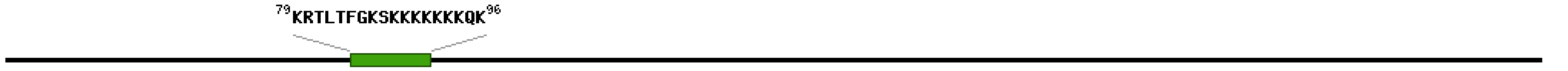

Posterior Graphic. Threshold Marked @ 0.4

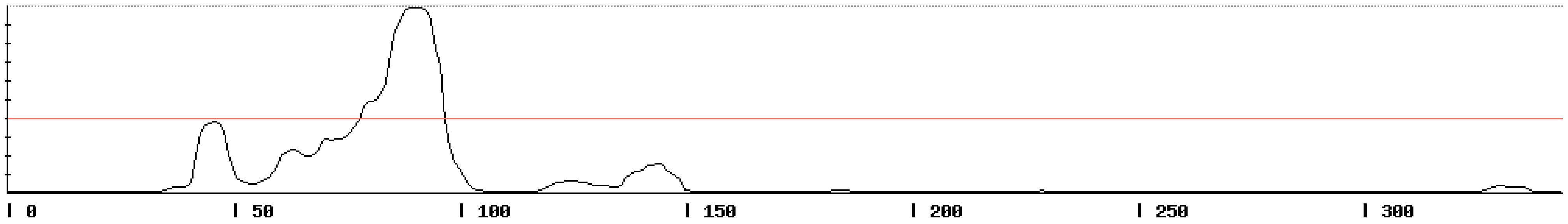

Predictions for Eutremasalsugineum_Thhalv10028345m

Viterbi Path: 42 - RKKAKRKSKK - 51

Viterbi Path

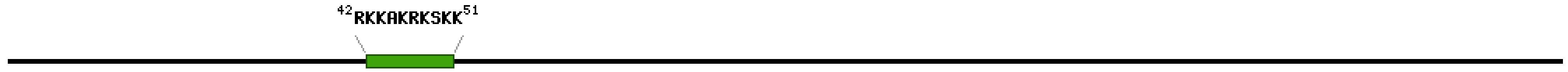

Posterior @ 0.4: 42 - RKKAKRKSKKEESTDRKTG - 60

Posterior @ 0.4

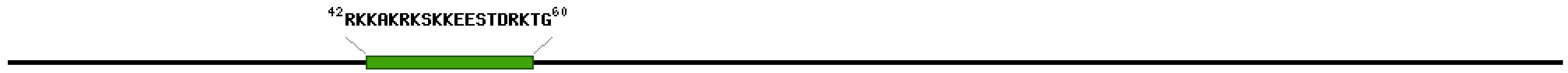

Posterior Graphic. Threshold Marked @ 0.4

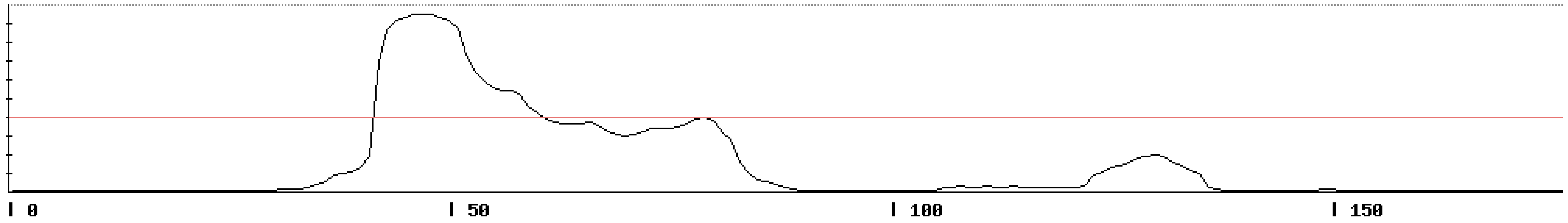

Predictions for Eutremasalsugineum_Thhalv10028684m

Viterbi Path

Viterbi Path

Posterior @ 0.4: 75 - KKNATKRTTRFGFWKSTGKDRKIKDKRGEIIGN - 107

Posterior @ 0.4

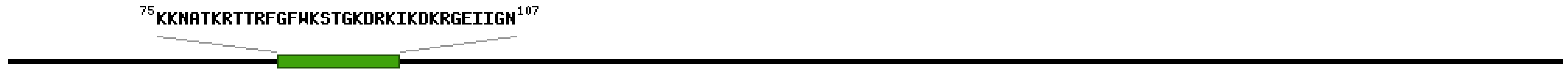

Posterior Graphic. Threshold Marked @ 0.4

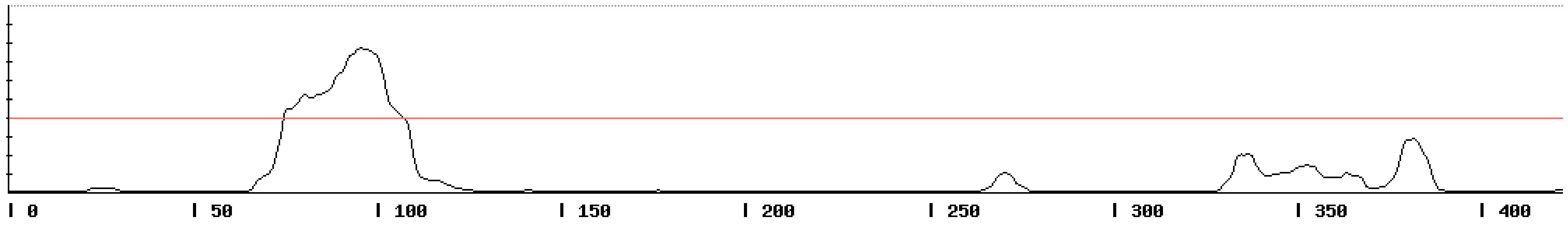

Predictions for Eutremasalsugineum_Thhalv10029162m

Revision r.9 - Last Updated on : February 24 2015 17:45:20.

alex.nguyenba@utoronto.ca

Intended to be used with Firefox

Viterbi Path	
Posterior @ 0.4	77 - KKKKTARSSRASRT - 90<br>175 - KKRKR - 179
Posterior @ 0.4	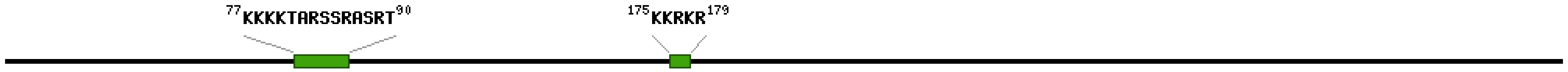
Posterior Graphic. Threshold Marked @ 0.4	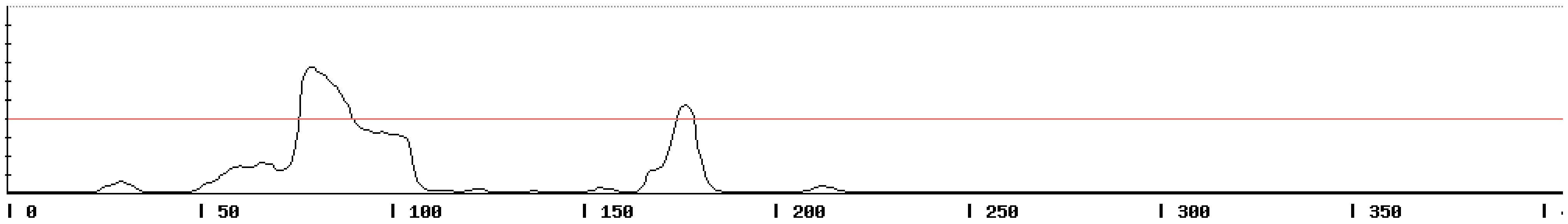

Predictions for Fragariavesca_mrna00252.1-v1.0-hybrid

Viterbi Path	
Viterbi Path	
Posterior @ 0.4	
Posterior @ 0.4	
Posterior Graphic. Threshold Marked @ 0.4	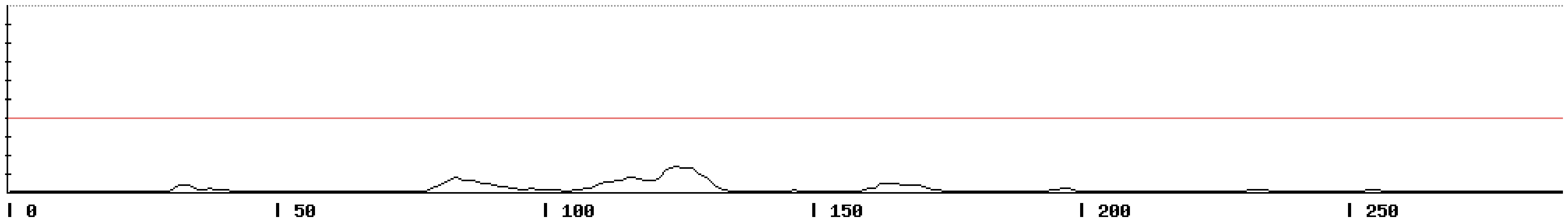

Predictions for Fragariavesca_mrna00951.1-v1.0-hybrid

Viterbi Path	
Viterbi Path	
Posterior @ 0.4	85 - RKRKYQNGRRPNRK - 98<br>161 - RK - 162
Posterior @ 0.4	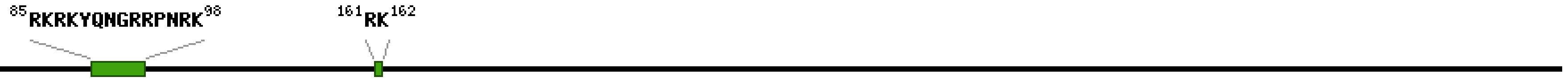
Posterior Graphic. Threshold Marked @ 0.4	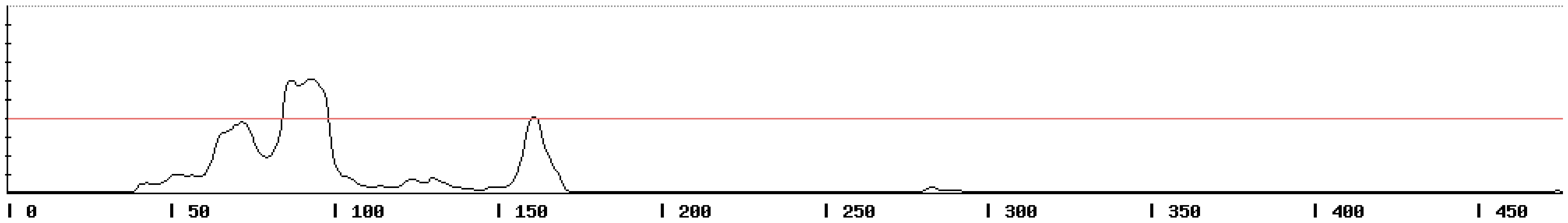

Predictions for Fragariavesca_mrna00971.1-v1.0-hybrid

Viterbi Path	
Viterbi Path	
Posterior @ 0.4	
Posterior @ 0.4	
Posterior Graphic. Threshold Marked @ 0.4	

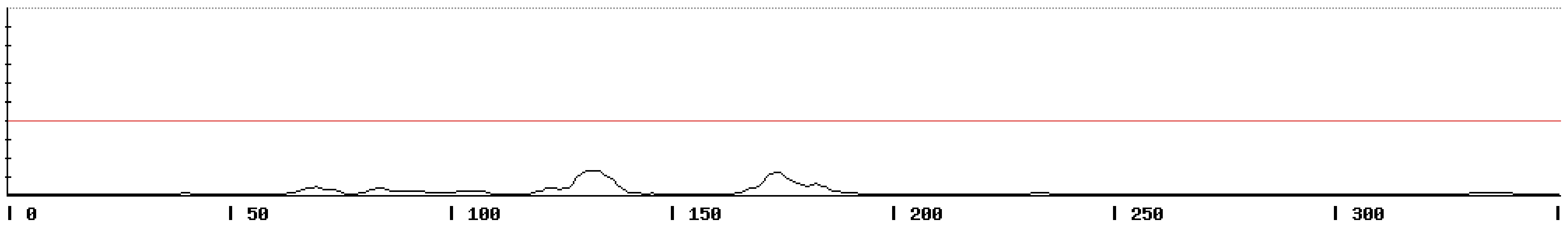

Predictions for Fragariavesca_mrna01023.1-v1.0-hybrid

Viterbi Path	
Viterbi Path	
Posterior @ 0.4	
Posterior @ 0.4	
Posterior Graphic. Threshold Marked @ 0.4	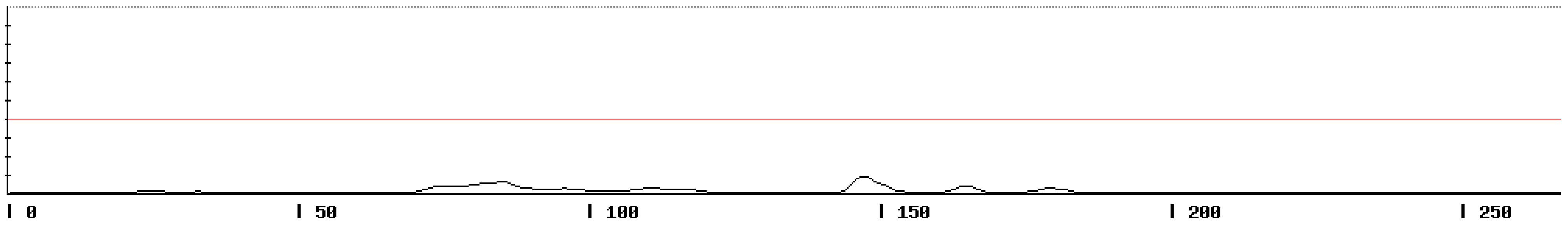

Predictions for Fragariavesca_mrna01364.1-v1.0-hybrid

Viterbi Path	
Viterbi Path	
Posterior @ 0.4	193 - KRRKLTSKKRGR - 204
Posterior @ 0.4	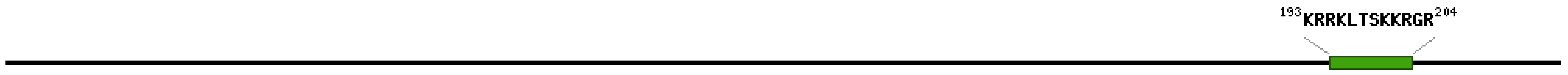

Posterior Graphic. Threshold Marked @ 0.4

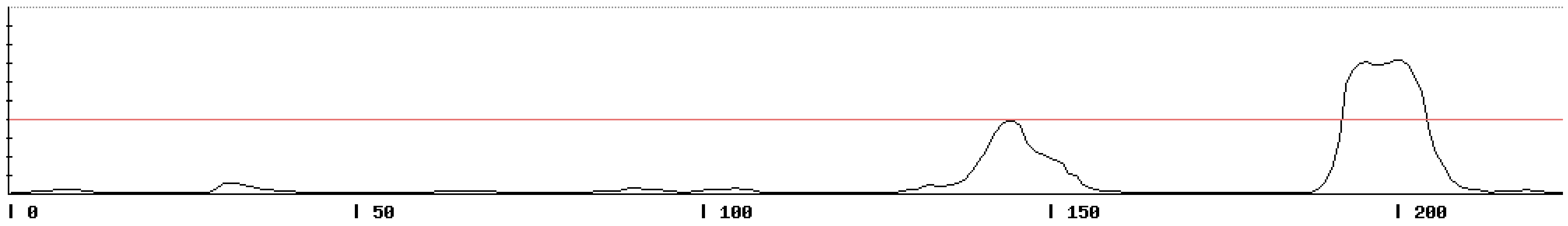

Predictions for Fragariavesca_mrna01851.1-v1.0-hybrid

Viterbi Path

Viterbi Path

Posterior @ 0.4

Posterior @ 0.4

Posterior Graphic. Threshold Marked @ 0.4

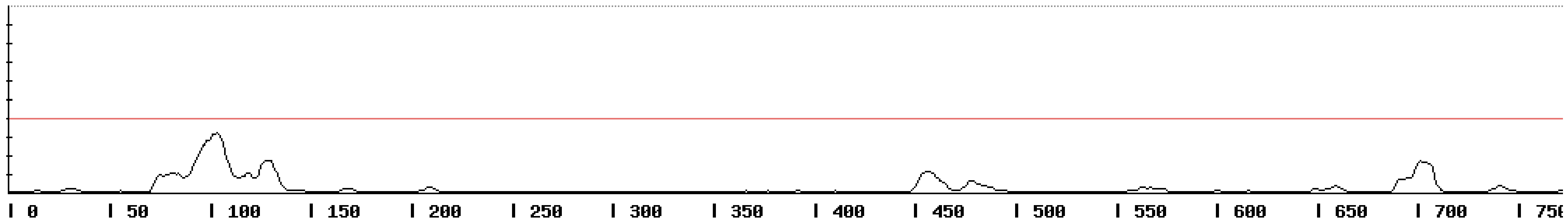

Predictions for Fragariavesca_mrna01881.1-v1.0-hybrid

Viterbi Path

Viterbi Path

Posterior @ 0.4

Posterior @ 0.4

Posterior Graphic. Threshold Marked @ 0.4

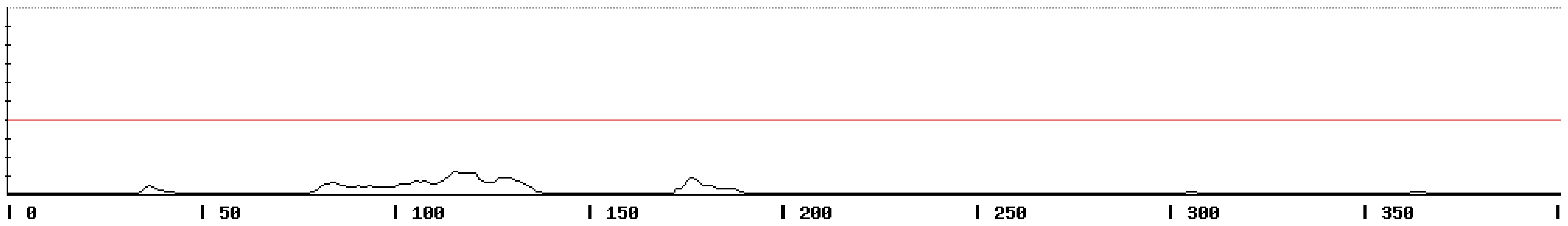

Predictions for Fragariavesca_mrna02016.1-v1.0-hybrid

Viterbi Path

Viterbi Path

Posterior @ 0.4 — 109 - KTKLSKR - 115

Posterior @ 0.4

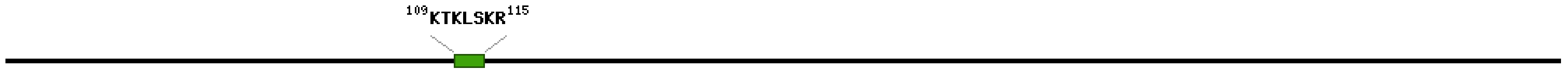

Posterior Graphic. Threshold Marked @ 0.4

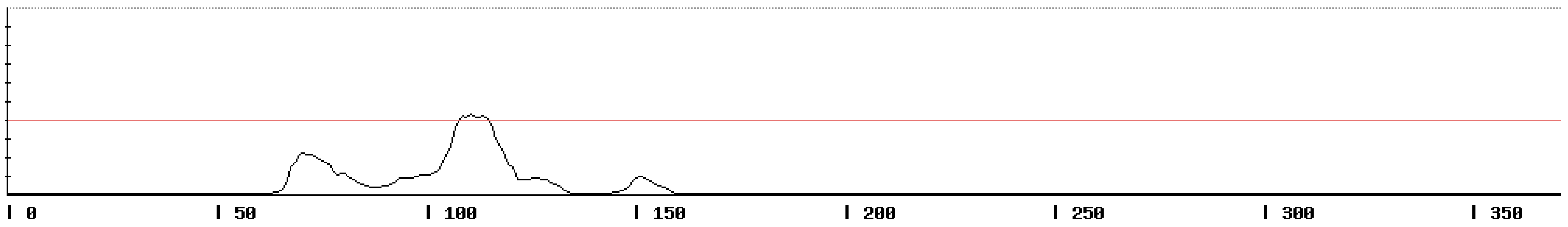

Predictions for Fragariavesca_mrna02444.1-v1.0-hybrid

Viterbi Path

Viterbi Path

Posterior @ 0.4

Posterior @ 0.4

Posterior Graphic. Threshold Marked @ 0.4

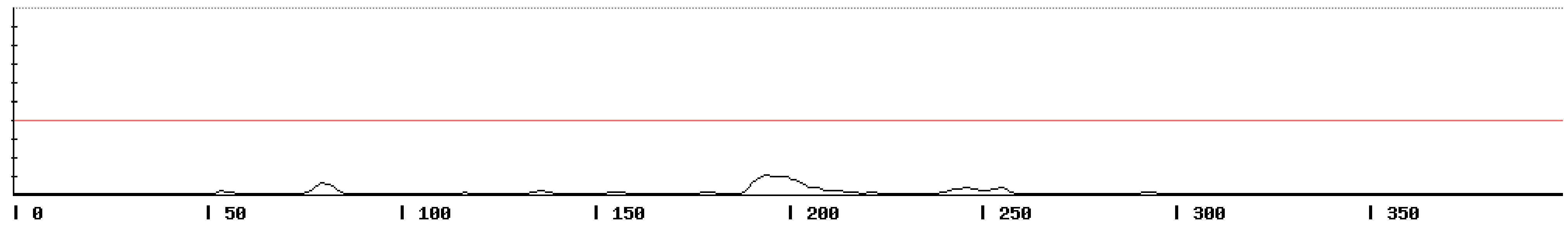

Predictions for Fragariavesca_mrna03833.1-v1.0-hybrid

Viterbi Path

Viterbi Path

Posterior @ 0.4

Posterior @ 0.4

Posterior Graphic. Threshold Marked @ 0.4

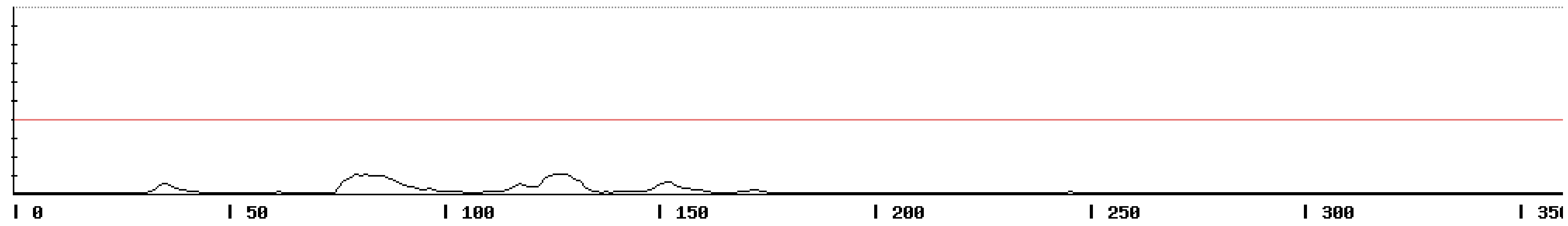

Predictions for Fragariavesca_mrna03875.1-v1.0-hybrid

Viterbi Path

Viterbi Path

Posterior @ 0.4

Posterior @ 0.4

Posterior Graphic. Threshold Marked @ 0.4

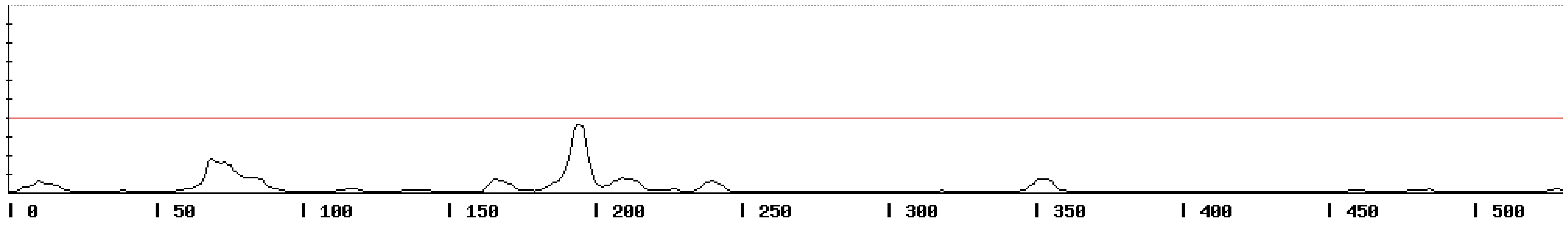

Predictions for Fragariavesca_mrna03910.1-v1.0-hybrid

Viterbi Path

Viterbi Path

Posterior @ 0.4

65 - KRKN - 68

71 - R - 71

83 - KKKT - 86

166 - KKK - 168

Posterior @ 0.4

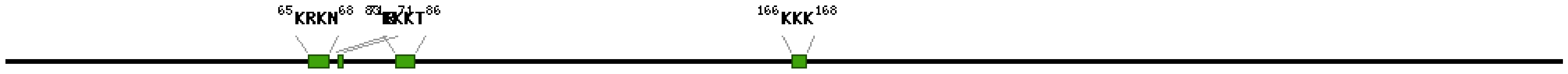

Posterior Graphic. Threshold Marked @ 0.4

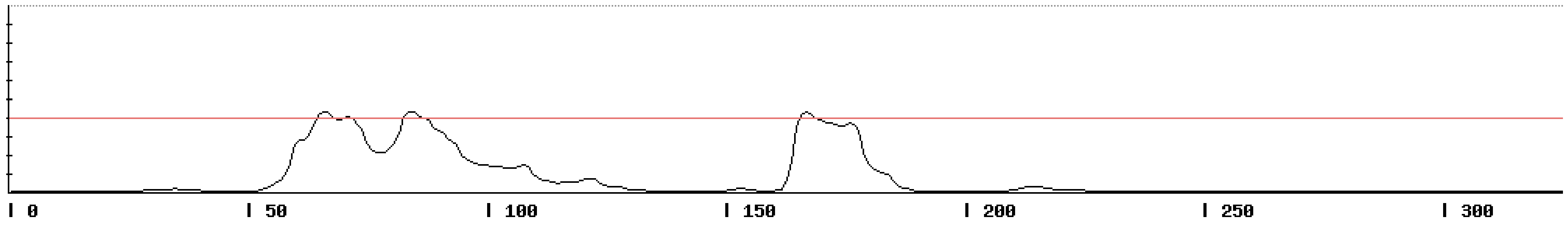

Predictions for Fragariavesca_mrna04249.1-v1.0-hybrid

Viterbi Path

Viterbi Path

Posterior @ 0.4

Posterior @ 0.4

Posterior Graphic. Threshold Marked @ 0.4

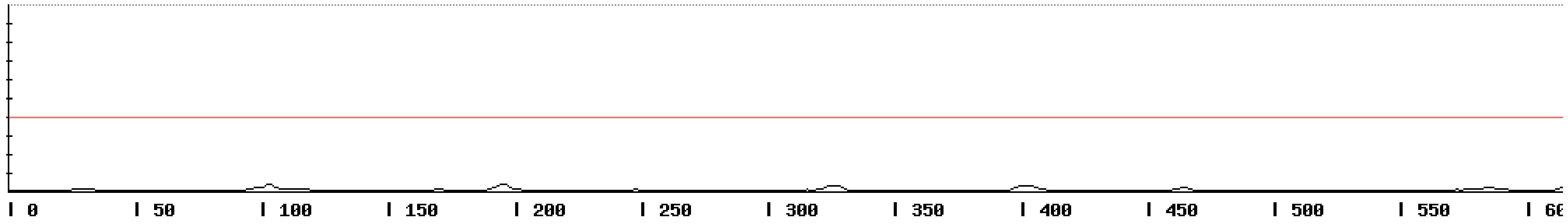

Predictions for Fragariavesca_mrna04303.1-v1.0-hybrid

Viterbi Path 1941 - KRKRPAPRKGRKSGVR - 1956

Viterbi Path

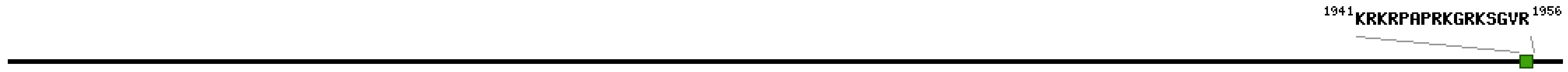

Posterior @ 0.4

414 - SRKPKAKRKA - 423

1940 - VKRKRPAPRKGRKSG - 1954

Posterior @ 0.4

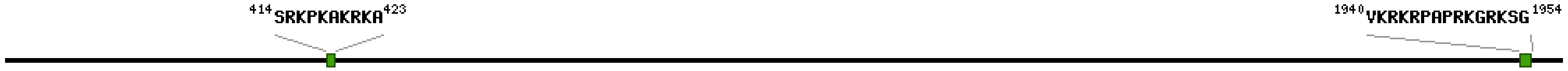

Posterior Graphic. Threshold Marked @ 0.4

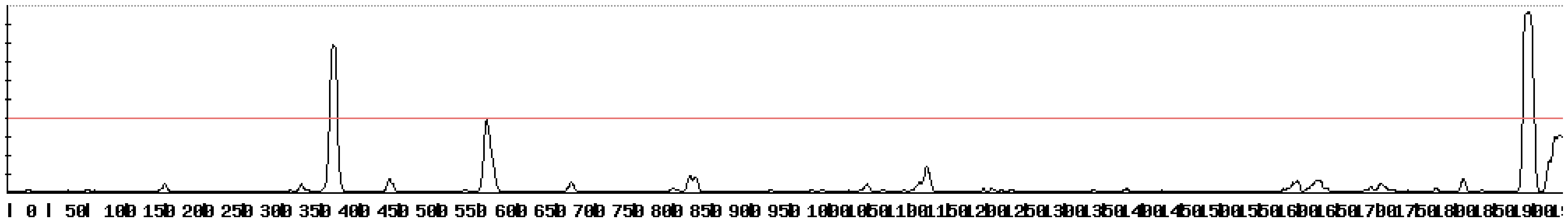

Predictions for Fragariavesca_mrna04424.1-v1.0-hybrid

Viterbi Path

Viterbi Path

Posterior @ 0.4

Posterior @ 0.4

Posterior Graphic. Threshold Marked @ 0.4

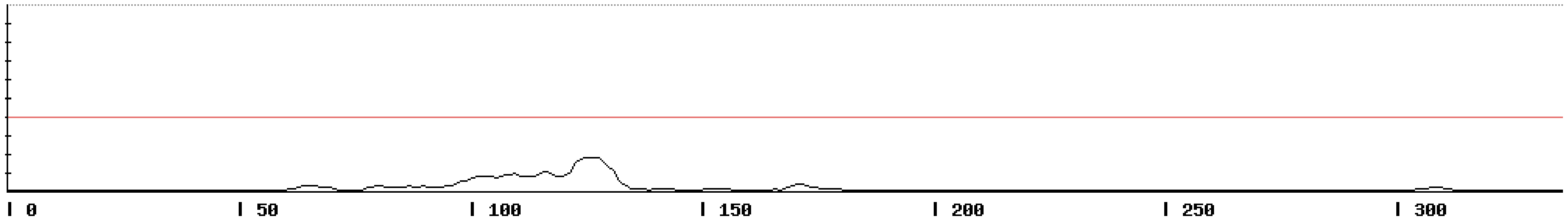

Predictions for Fragariavesca_mrna04766.1-v1.0-hybrid

Viterbi Path

Viterbi Path

Posterior @ 0.4

Posterior @ 0.4

Posterior Graphic. Threshold Marked @ 0.4

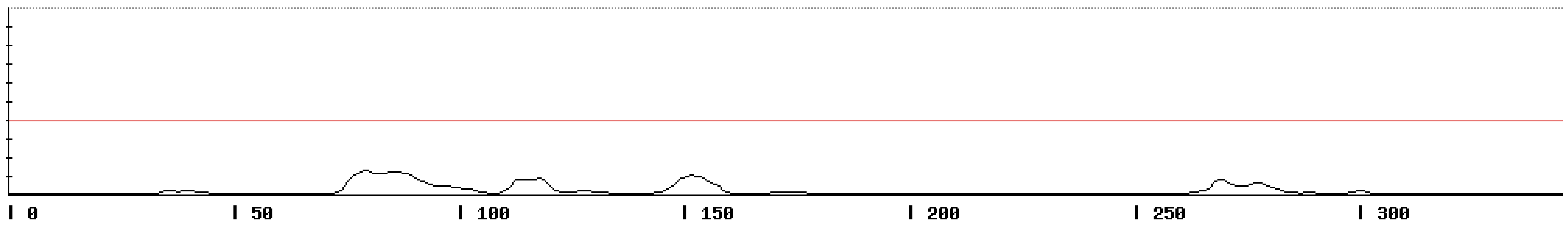

Predictions for Fragariavesca_mrna06460.1-v1.0-hybrid

Viterbi Path

Viterbi Path

Posterior @ 0.4

Posterior @ 0.4

Posterior Graphic. Threshold Marked @ 0.4

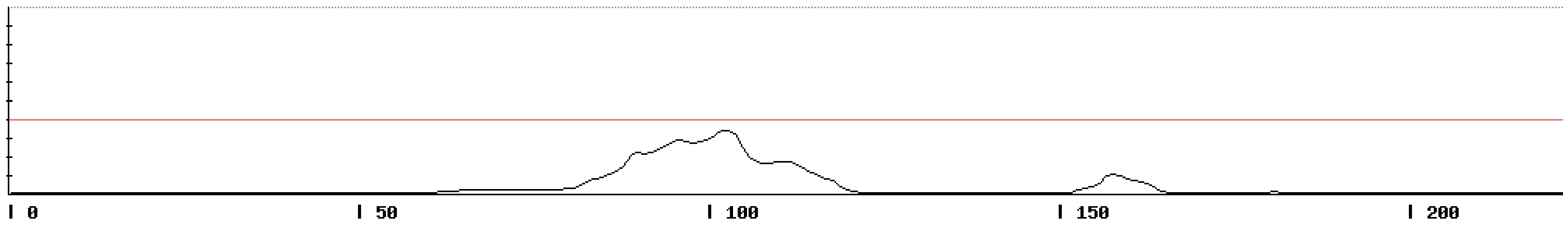

Predictions for Fragariavesca_mrna07815.1-v1.0-hybrid

Viterbi Path

Viterbi Path

Posterior @ 0.4	93 - RLKKLSPKGKGKRSSR - 108
Posterior @ 0.4	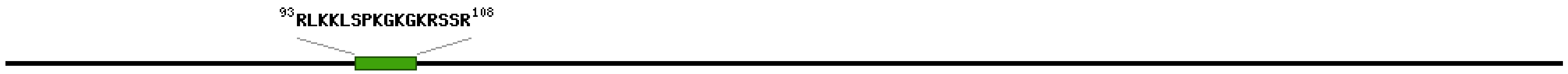
Posterior Graphic. Threshold Marked @ 0.4	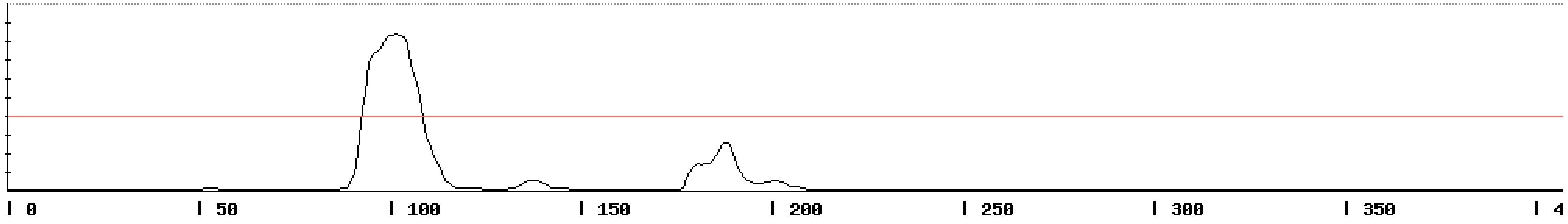

Predictions for Fragariavesca_mrna07822.1-v1.0-hybrid

Viterbi Path	
Viterbi Path	
Posterior @ 0.4	77 - KKHKNRINR - 85<br>152 - NPSKRPSPPSSKPSDKKRKCQHDPEPSKKPK - 182
Posterior @ 0.4	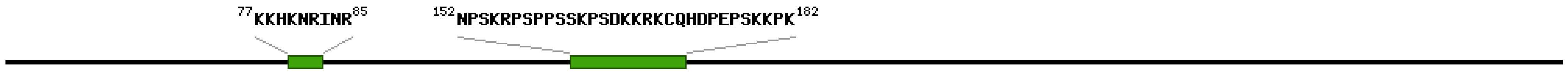
Posterior Graphic. Threshold Marked @ 0.4	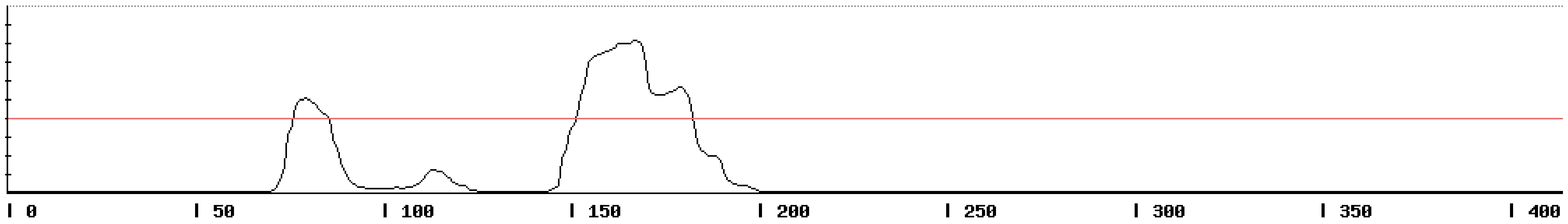

Predictions for Fragariavesca_mrna08112.1-v1.0-hybrid	
Viterbi Path	
Viterbi Path	
Posterior @ 0.4	
Posterior @ 0.4	
Posterior Graphic. Threshold Marked @ 0.4	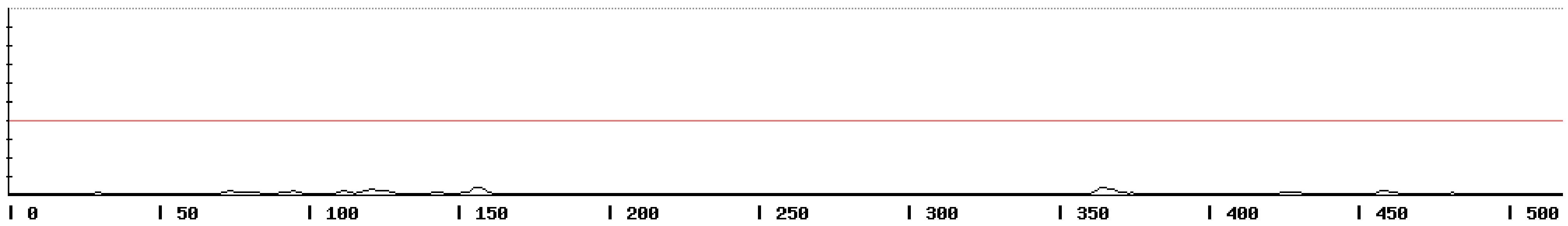

Predictions for Fragariavesca_mrna08427.1-v1.0-hybrid	
Viterbi Path	
Viterbi Path	
Posterior @ 0.4	
Posterior @ 0.4	
Posterior Graphic. Threshold Marked @ 0.4	

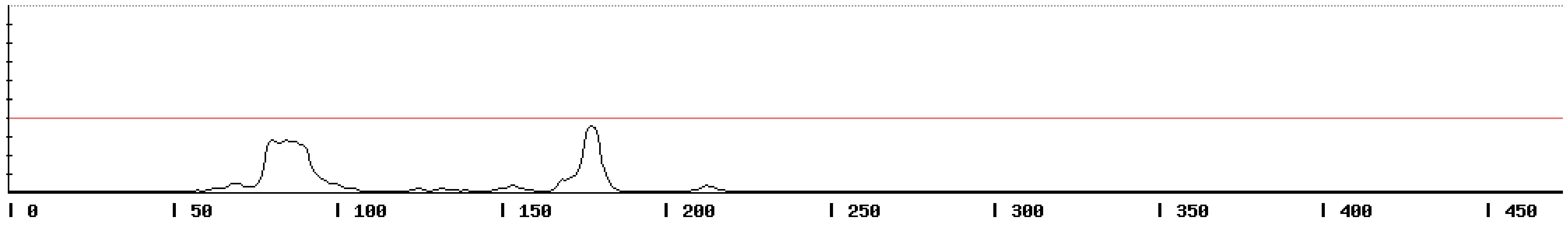

Predictions for Fragariavesca_mrna08448.1-v1.0-hybrid

Viterbi Path

Viterbi Path

Posterior @ 0.4

Posterior @ 0.4

Posterior Graphic. Threshold Marked @ 0.4

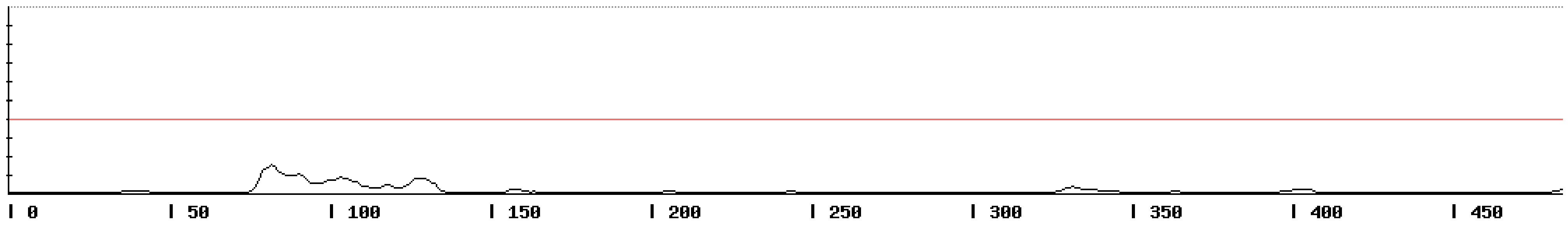

Predictions for Fragariavesca_mrna08765.1-v1.0-hybrid

Viterbi Path

Viterbi Path

Posterior @ 0.4

Posterior @ 0.4

Posterior Graphic. Threshold Marked @ 0.4	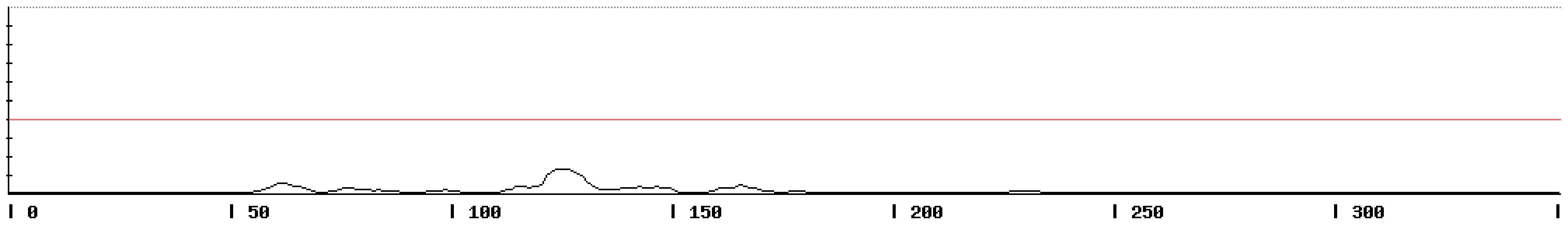

Predictions for Fragariavesca_mrna09127.1-v1.0-hybrid

Viterbi Path	
Viterbi Path	
Posterior @ 0.4	
Posterior @ 0.4	
Posterior Graphic. Threshold Marked @ 0.4	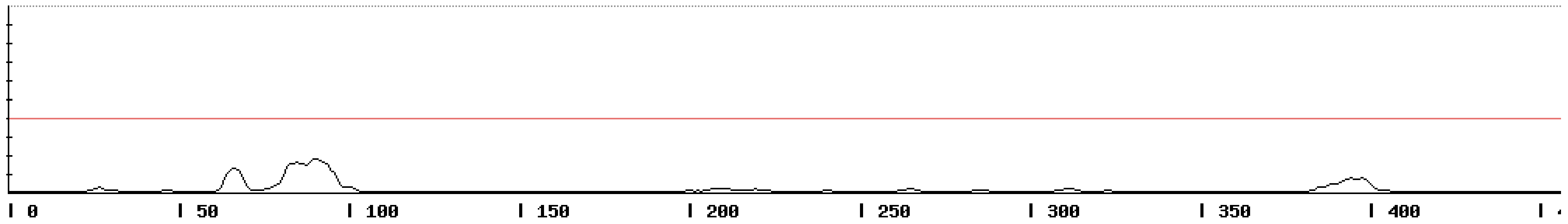

Predictions for Fragariavesca_mrna09293.1-v1.0-hybrid

Viterbi Path	
Viterbi Path	
Posterior @ 0.4	
Posterior @ 0.4	

Posterior Graphic. Threshold Marked @ 0.4

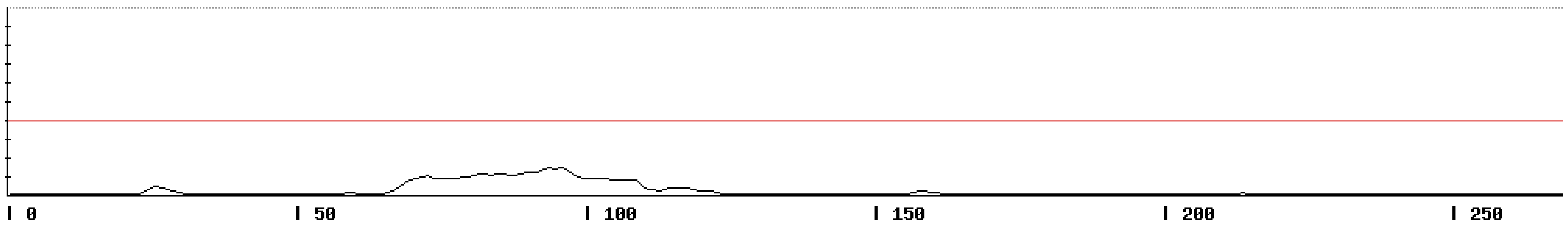

Predictions for Fragariavesca_mrna09702.1-v1.0-hybrid

Viterbi Path

Viterbi Path

Posterior @ 0.4

Posterior @ 0.4

Posterior Graphic. Threshold Marked @ 0.4

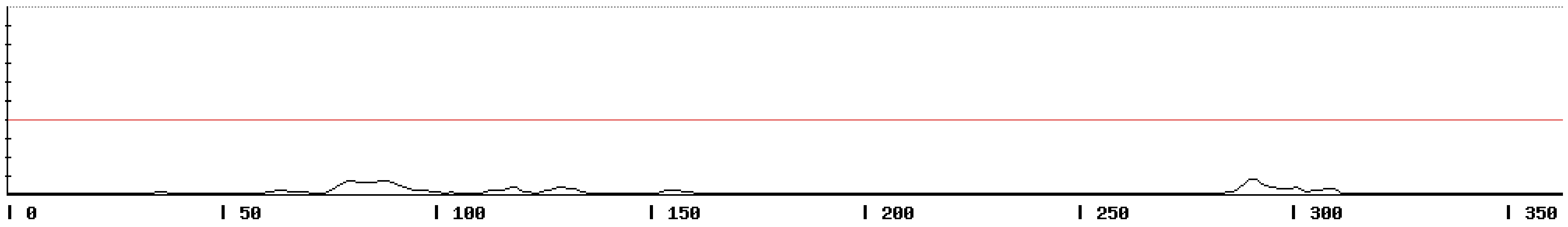

Predictions for Fragariavesca_mrna10469.1-v1.0-hybrid

Viterbi Path

Viterbi Path

Posterior @ 0.4

Posterior @ 0.4

Posterior Graphic. Threshold Marked @ 0.4

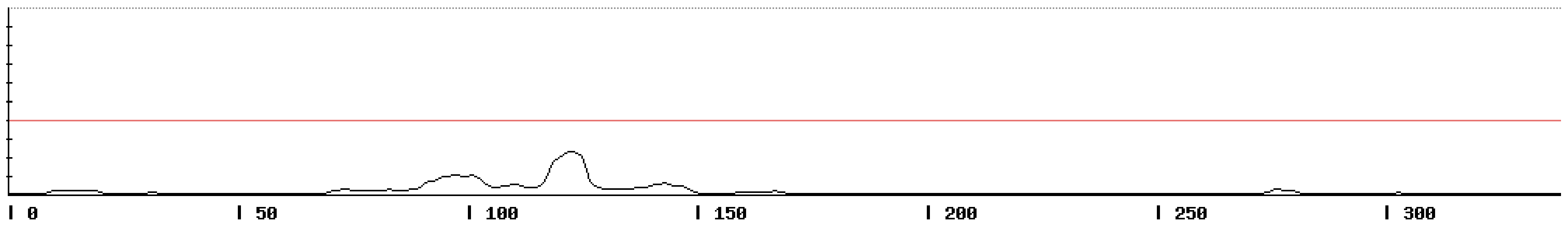

Predictions for Fragariavesca_mrna10722.1-v1.0-hybrid

Viterbi Path

Viterbi Path

Posterior @ 0.4

Posterior @ 0.4

Posterior Graphic. Threshold Marked @ 0.4

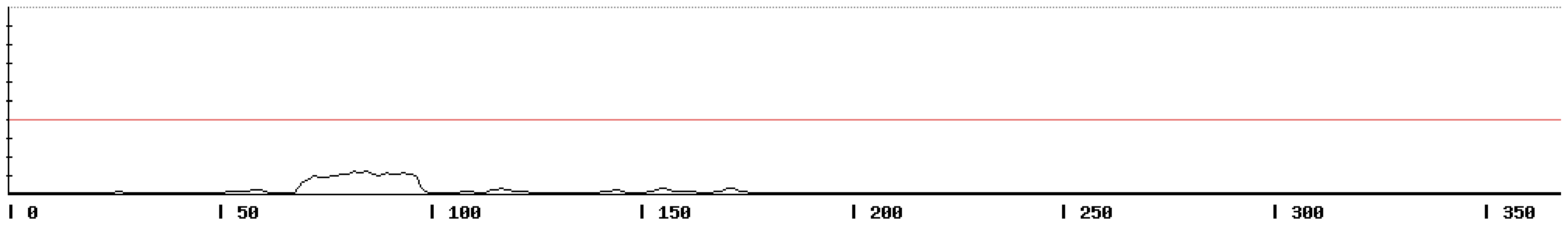

Predictions for Fragariavesca_mrna10901.1-v1.0-hybrid

Viterbi Path

Viterbi Path

Posterior @ 0.4	165 - RDRKYRNGKRPNRKA - 179
Posterior @ 0.4	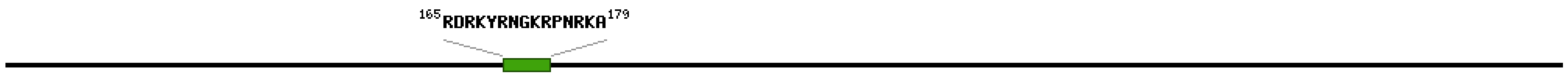
Posterior Graphic. Threshold Marked @ 0.4	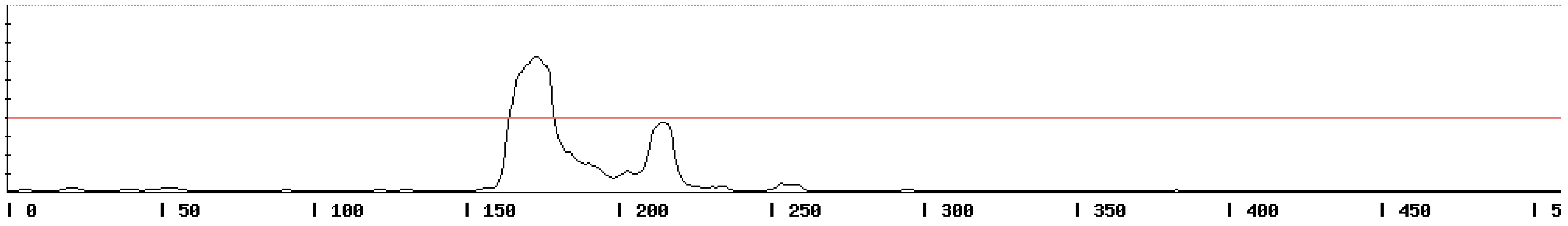

Predictions for Fragariavesca_mrna10904.1-v1.0-hybrid

Viterbi Path	
Viterbi Path	
Posterior @ 0.4	
Posterior @ 0.4	
Posterior Graphic. Threshold Marked @ 0.4	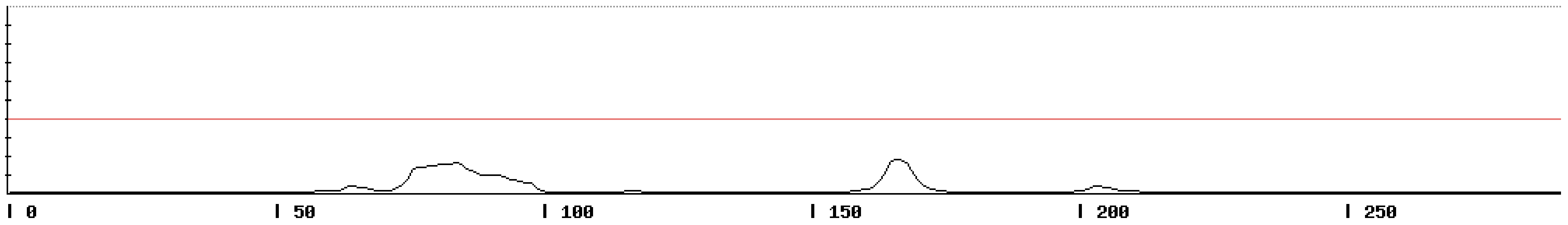

Predictions for Fragariavesca_mrna11226.1-v1.0-hybrid

Viterbi Path

Viterbi Path

Posterior @ 0.4

Posterior @ 0.4

Posterior Graphic. Threshold Marked @ 0.4

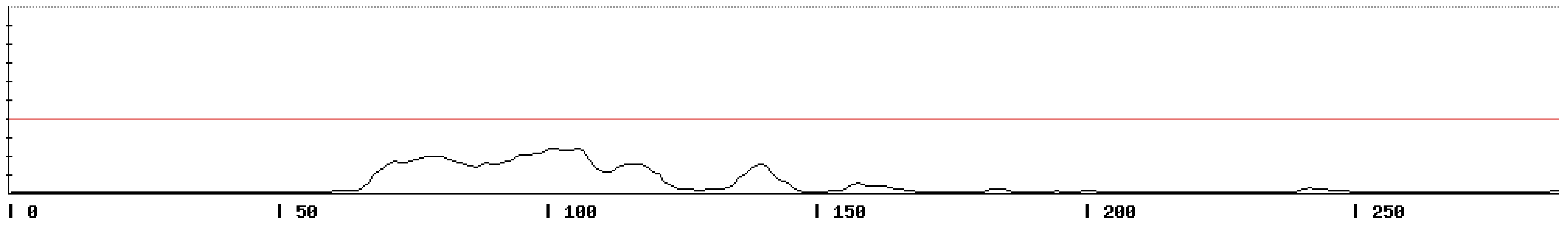

Predictions for Fragariavesca_mrna11344.1-v1.0-hybrid

Viterbi Path

Viterbi Path

Posterior @ 0.4

Posterior @ 0.4

Posterior Graphic. Threshold Marked @ 0.4

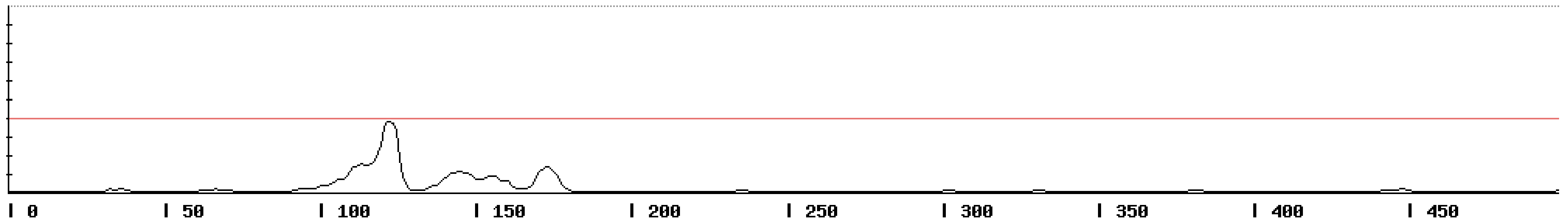

Predictions for Fragariavesca_mrna11780.1-v1.0-hybrid

Viterbi Path

Viterbi Path

Posterior @ 0.4

Posterior @ 0.4

Posterior Graphic. Threshold Marked @ 0.4

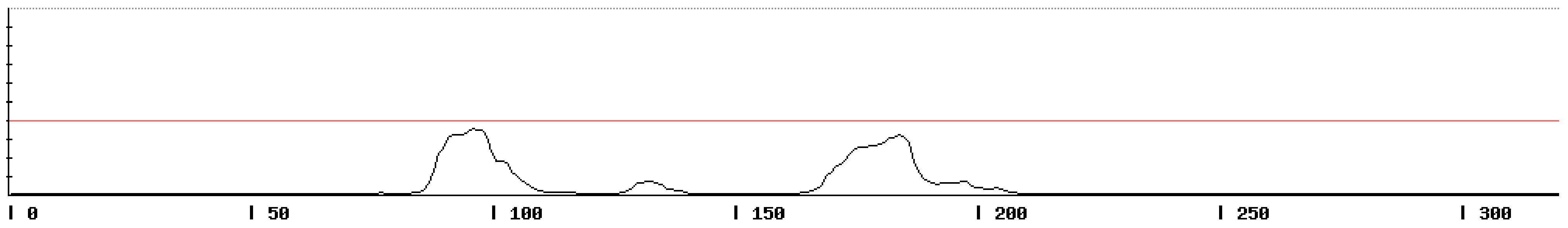

Predictions for Fragariavesca_mrna12133.1-v1.0-hybrid

Viterbi Path

Viterbi Path

Posterior @ 0.4

147 - NPGKRPSPPPSSKPSDNKRKCQAESRPSKKTK - 178

Posterior @ 0.4

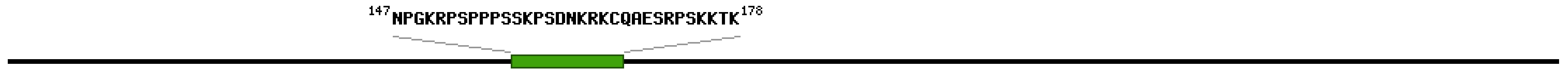

Posterior Graphic. Threshold Marked @ 0.4

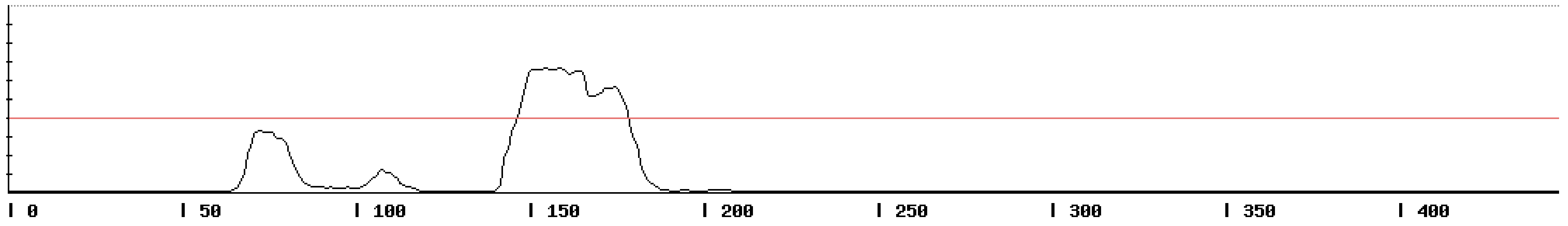

Predictions for Fragariavesca_mrna12140.1-v1.0-hybrid

Viterbi Path	
Viterbi Path	
Posterior @ 0.4	98 - KRLSAKGKGKR - 108
	181 - KRNGKSNNKKRKA - 193
Posterior @ 0.4	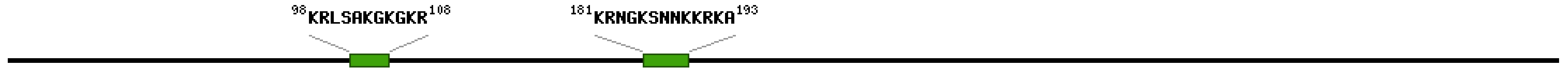
Posterior Graphic. Threshold Marked @ 0.4	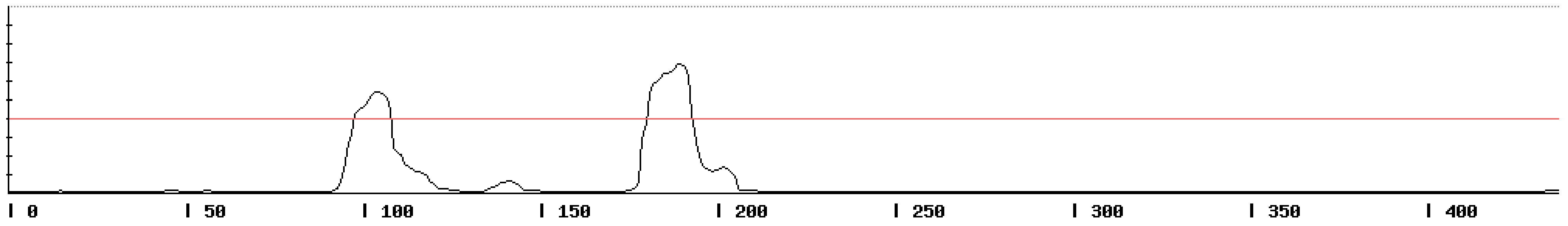

Predictions for Fragariavesca_mrna12168.1-v1.0-hybrid

Viterbi Path	
Viterbi Path	
Posterior @ 0.4	148 - PDKRPSPPPLSKPSDKKRKCQADPKPSKKTKS - 179
Posterior @ 0.4	

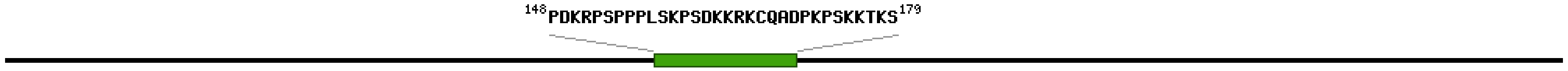

Posterior Graphic. Threshold Marked @ 0.4

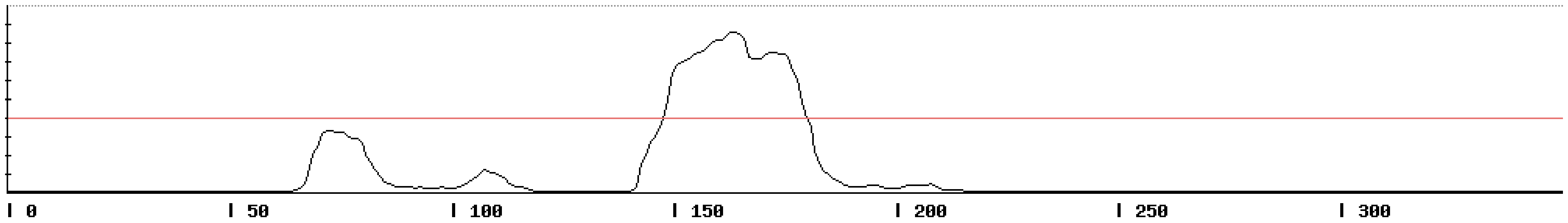

Predictions for Fragariavesca_mrna12442.1-v1.0-hybrid

Viterbi Path

Viterbi Path

Posterior @ 0.4 — 101 - KDRRIKSGANLVGMKKTLVFHKGRAPKGKR - 130

Posterior @ 0.4

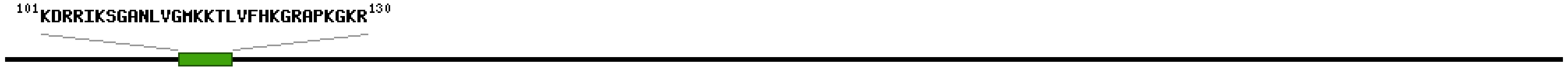

Posterior Graphic. Threshold Marked @ 0.4

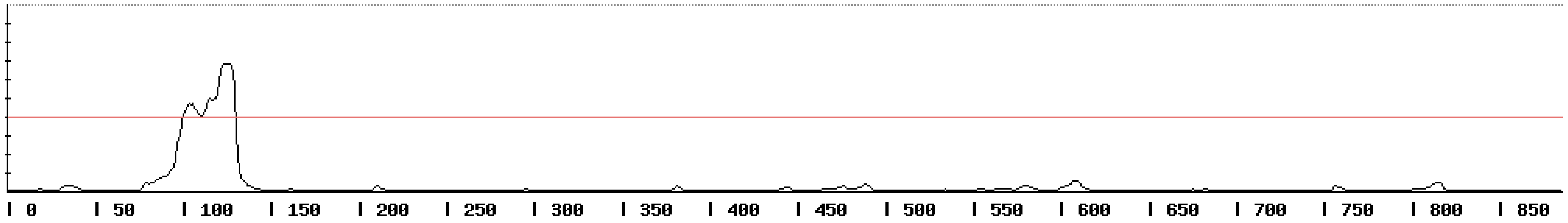

Predictions for Fragariavesca_mrna12934.1-v1.0-hybrid

Viterbi Path

Viterbi Path

Posterior @ 0.4

Posterior @ 0.4

Posterior Graphic. Threshold Marked @ 0.4

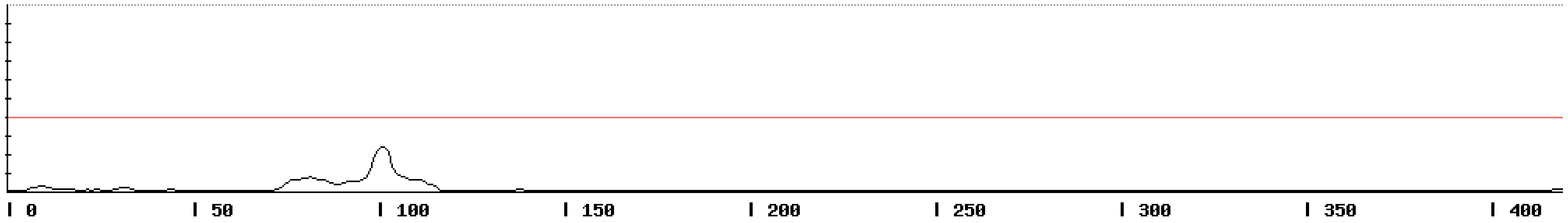

Predictions for Fragariavesca_mrna14066.1-v1.0-hybrid

Viterbi Path

Viterbi Path

Posterior @ 0.4

Posterior @ 0.4

Posterior Graphic. Threshold Marked @ 0.4

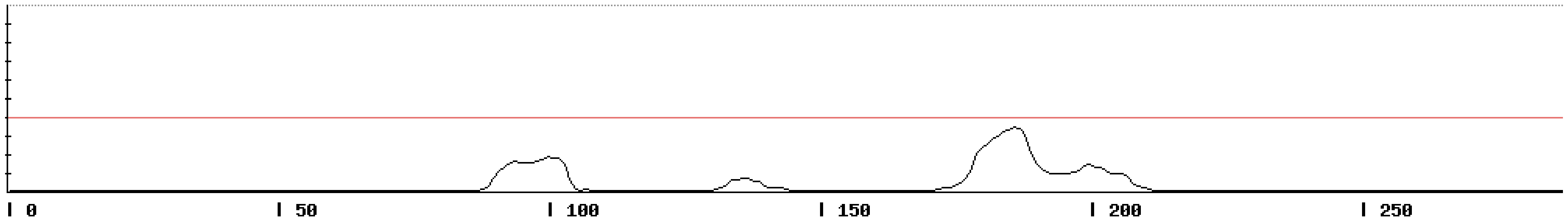

Predictions for Fragariavesca_mrna14711.1-v1.0-hybrid

Viterbi Path

Viterbi Path

Posterior @ 0.4 | 204 - RGKPPKGKK - 212

Posterior @ 0.4

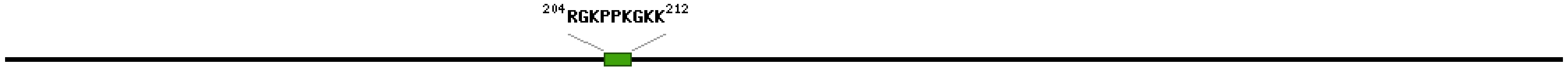

Posterior Graphic. Threshold Marked @ 0.4

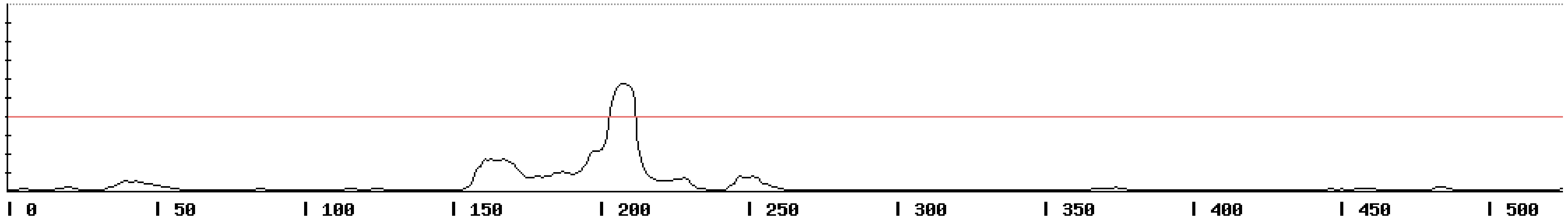

Predictions for Fragariavesca_mrna14869.1-v1.0-hybrid

Viterbi Path

Viterbi Path

Posterior @ 0.4

Posterior @ 0.4

Posterior Graphic. Threshold Marked @ 0.4

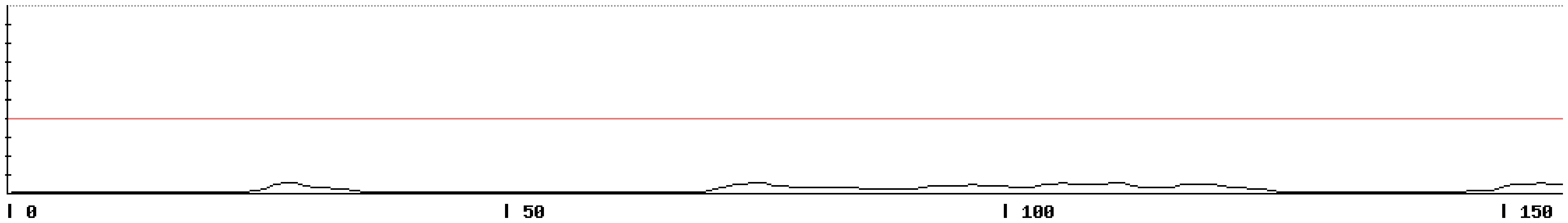

Predictions for Fragariavesca_mrna15095.1-v1.0-hybrid

Viterbi Path	
Viterbi Path	
Posterior @ 0.4	345 - GKKATVVKTKKLKKR - 359
Posterior @ 0.4	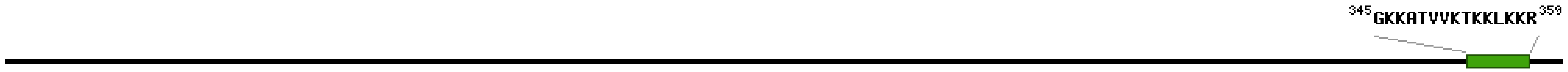
Posterior Graphic. Threshold Marked @ 0.4	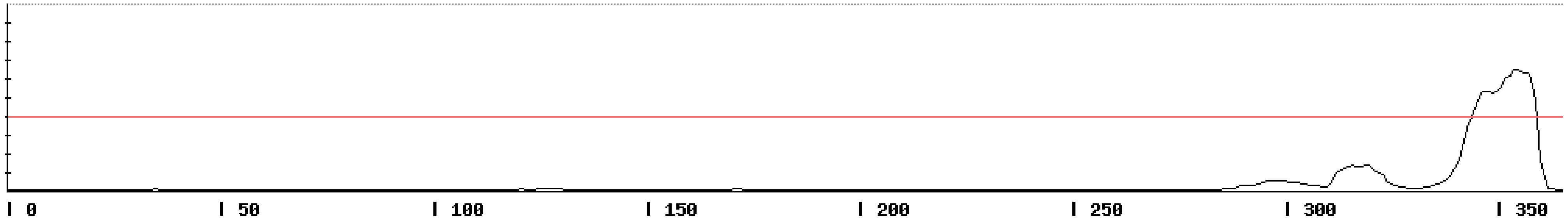

Predictions for Fragariavesca_mrna15644.1-v1.0-hybrid

Viterbi Path	
Viterbi Path	
Posterior @ 0.4	
Posterior @ 0.4	
Posterior Graphic. Threshold Marked @ 0.4	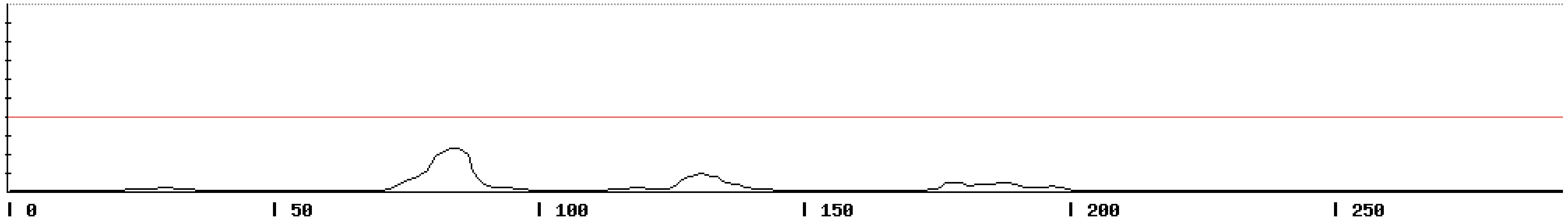

Predictions for Fragariavesca_mrna17203.1-v1.0-hybrid	
Viterbi Path	
Viterbi Path	
Posterior @ 0.4	
Posterior @ 0.4	
Posterior Graphic. Threshold Marked @ 0.4	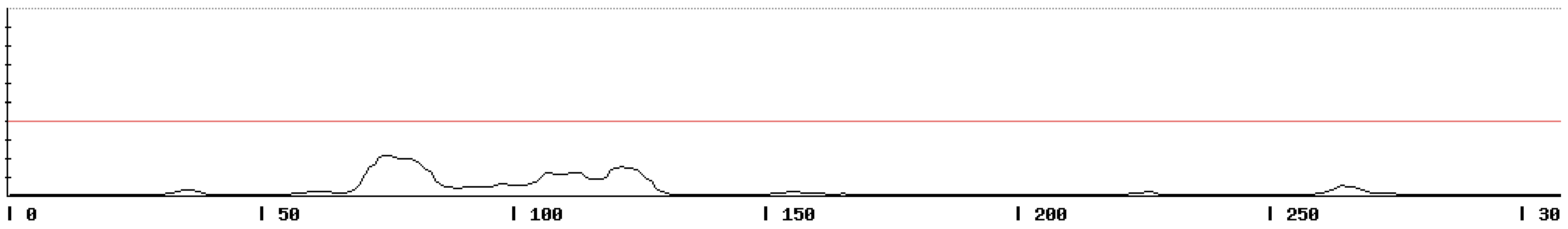

Predictions for Fragariavesca_mrna17589.1-v1.0-hybrid	
Viterbi Path	
Viterbi Path	
Posterior @ 0.4	363 - RKDRKRK - 369
Posterior @ 0.4	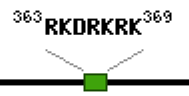
Posterior Graphic. Threshold Marked @ 0.4	

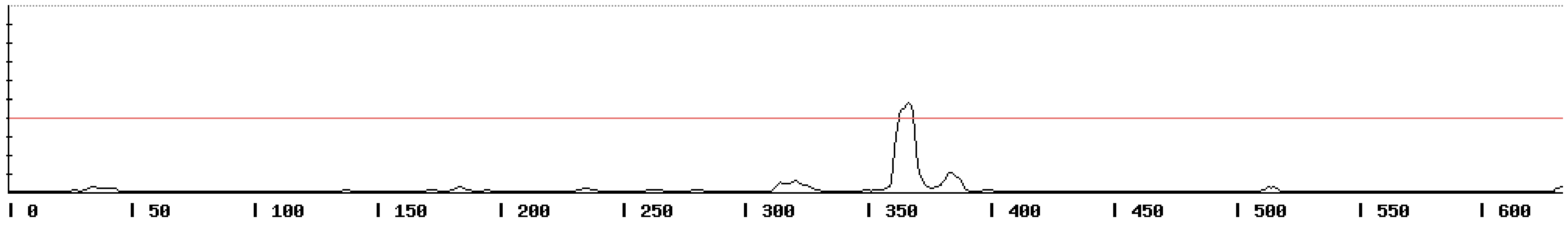

Predictions for Fragariavesca_mrna17610.1-v1.0-hybrid

Viterbi Path	
Viterbi Path	
Posterior @ 0.4	158 - RIRKDRKRK - 166
	480 - LKKSK - 484
Posterior @ 0.4	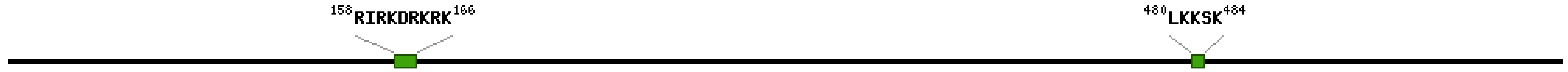
Posterior Graphic. Threshold Marked @ 0.4	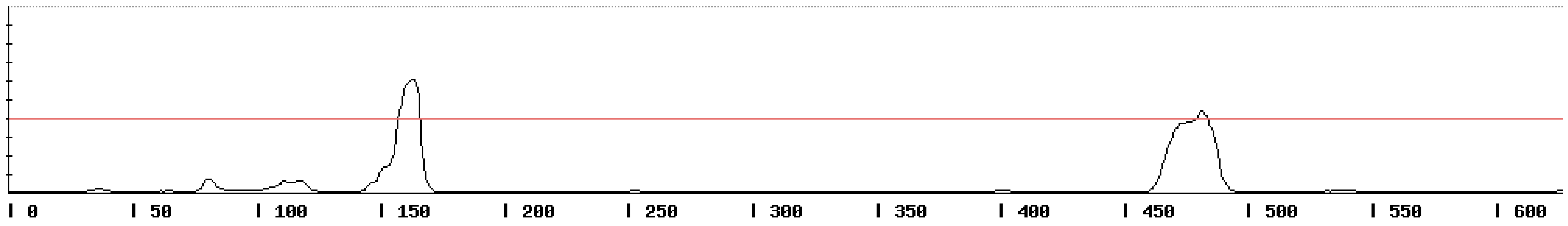

Predictions for Fragariavesca_mrna17720.1-v1.0-hybrid

Viterbi Path	
Viterbi Path	
Posterior @ 0.4	106 - GEKKNKTPIGRKRKFR - 121
	285 - KKKDKGKEKK - 294
	407 - GRKRKL - 412

Posterior @ 0.4

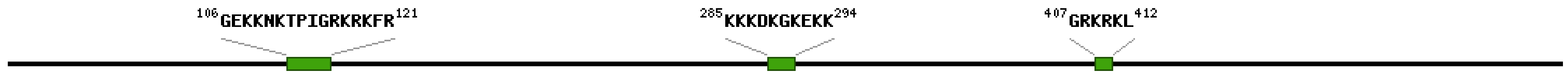

Posterior Graphic. Threshold Marked @ 0.4

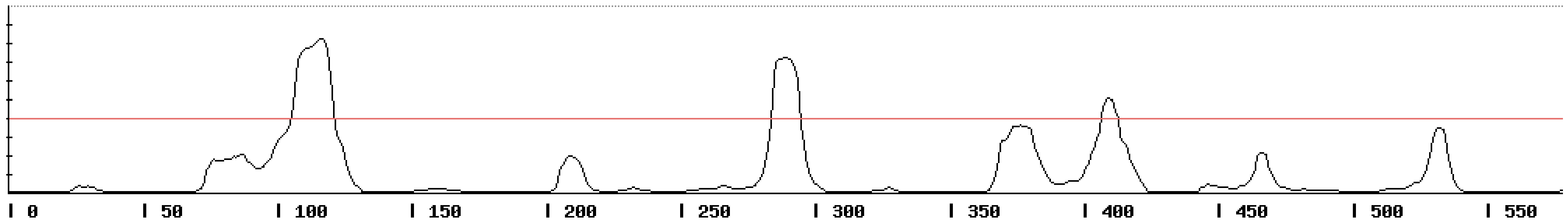

Predictions for Fragariavesca_mrna17721.1-v1.0-hybrid

Viterbi Path

Viterbi Path

Posterior @ 0.4

122 - KRKFR - 126

161 - NIRKGRKRKE - 170

283 - EAKQKHIGRRRKLR - 296

Posterior @ 0.4

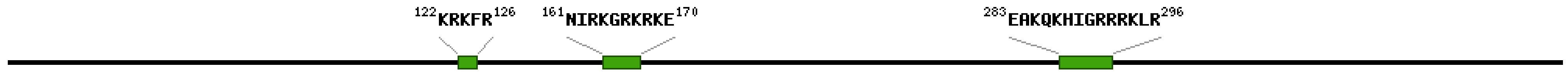

Posterior Graphic. Threshold Marked @ 0.4

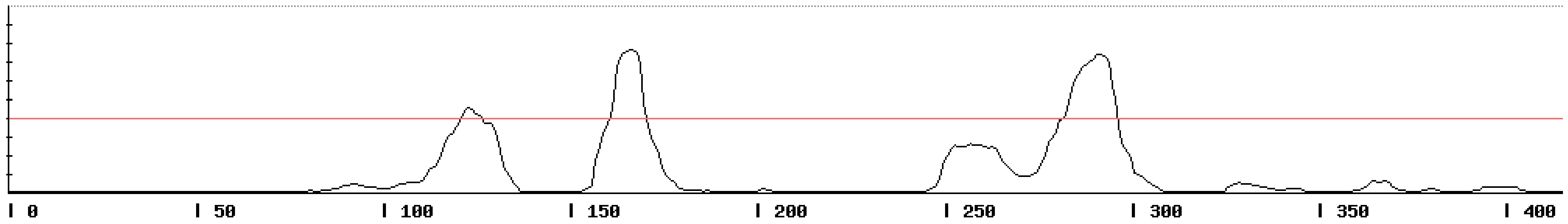

Predictions for Fragariavesca_mrna17724.1-v1.0-hybrid

Viterbi Path

Viterbi Path	
Posterior @ 0.4	
Posterior @ 0.4	
Posterior Graphic. Threshold Marked @ 0.4	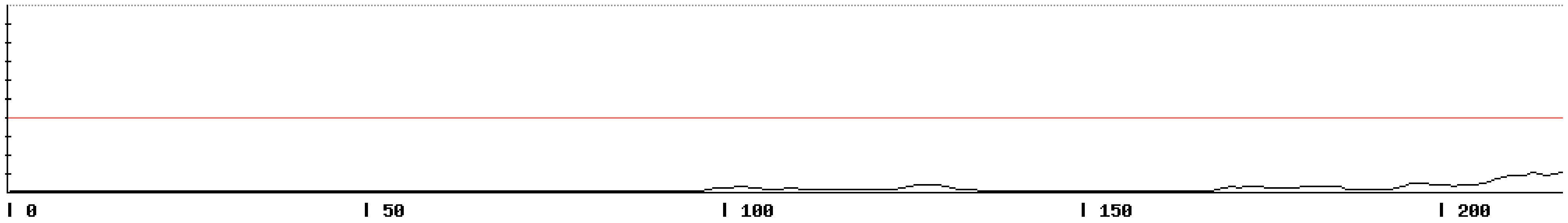

Predictions for Fragariavesca_mrna17762.1-v1.0-hybrid

Viterbi Path	
Viterbi Path	
Posterior @ 0.4	130 - SRKRRKV - 136
Posterior @ 0.4	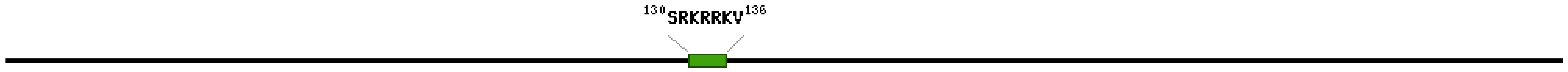
Posterior Graphic. Threshold Marked @ 0.4	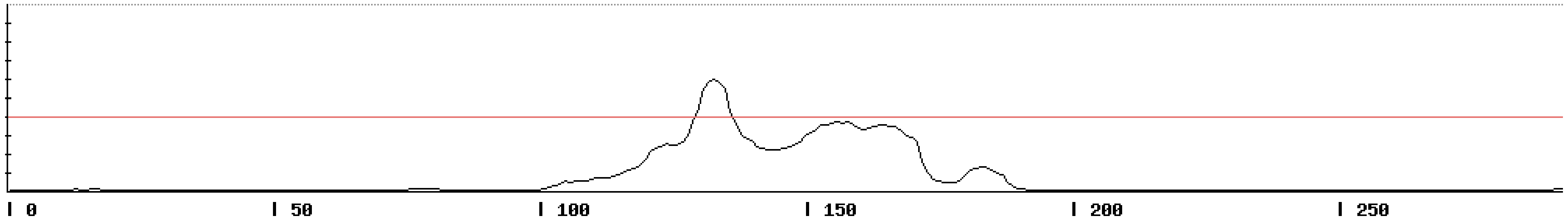

Predictions for Fragariavesca_mrna18589.1-v1.0-hybrid

Viterbi Path	
Viterbi Path	
Posterior @ 0.4	
Posterior @ 0.4	
Posterior Graphic. Threshold Marked @ 0.4	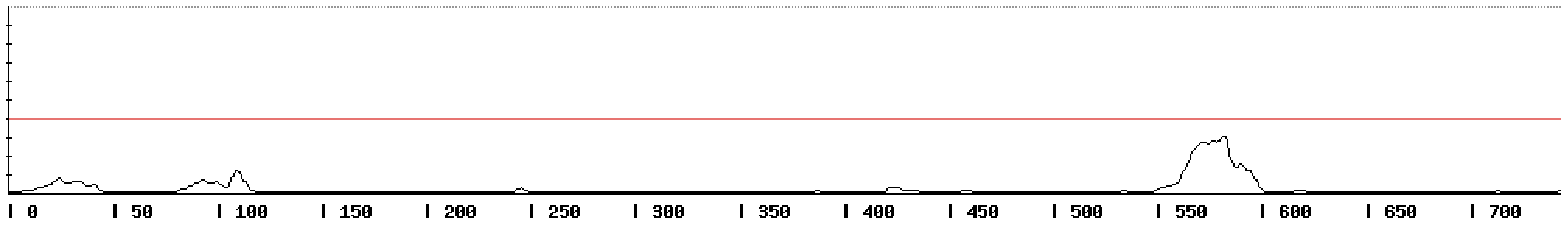

Predictions for Fragariavesca_mrna18590.1-v1.0-hybrid

Viterbi Path	
Viterbi Path	
Posterior @ 0.4	
Posterior @ 0.4	
Posterior Graphic. Threshold Marked @ 0.4	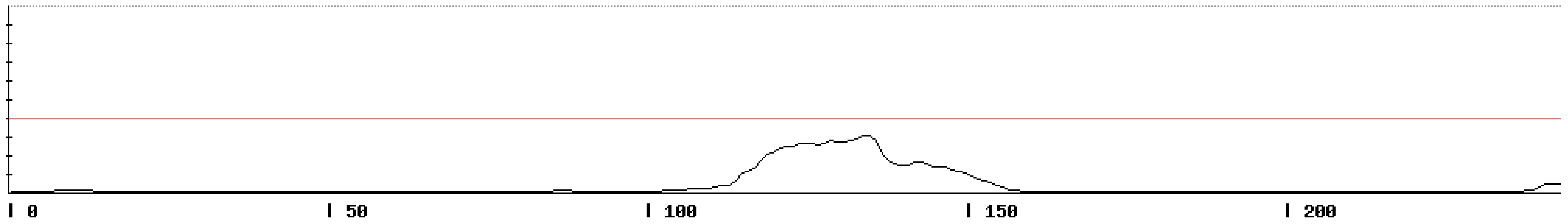

Predictions for Fragariavesca_mrna18635.1-v1.0-hybrid

Viterbi Path

Viterbi Path

Posterior @ 0.4

Posterior @ 0.4

Posterior Graphic. Threshold Marked @ 0.4

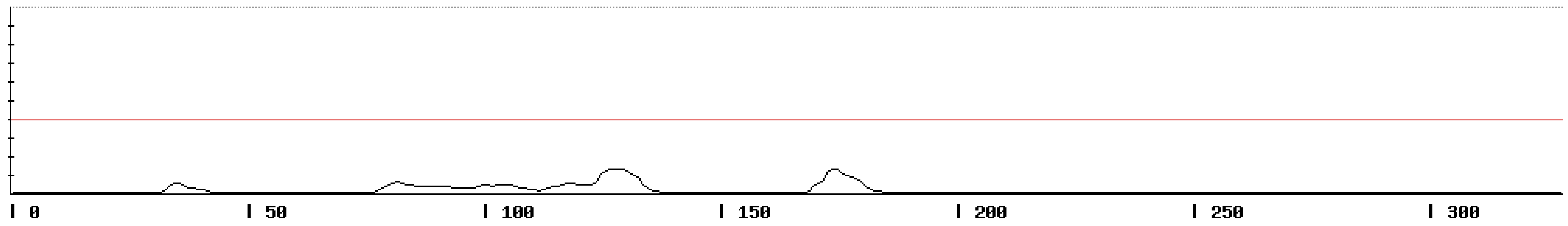

Predictions for Fragariavesca_mrna18636.1-v1.0-hybrid

Viterbi Path

Viterbi Path

Posterior @ 0.4

Posterior @ 0.4

Posterior Graphic. Threshold Marked @ 0.4

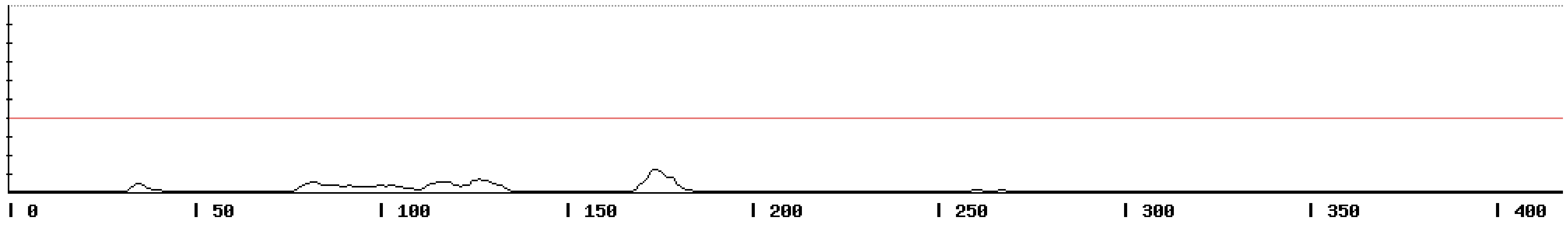

Predictions for Fragariavesca_mrna19382.1-v1.0-hybrid

Viterbi Path

298 - RKPKRIGKKRKLR - 310

357 - PKEKKRKRQQIDQLEDKKPKRKK - 379

Viterbi Path

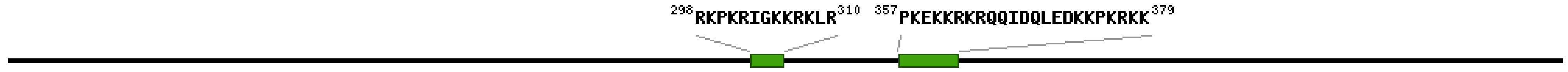

Posterior @ 0.4

297 - ERKPKRIGKKRKLR - 310

357 - PKEKKRKRQQIDQLEDKKPKRKKVV - 381

Posterior @ 0.4

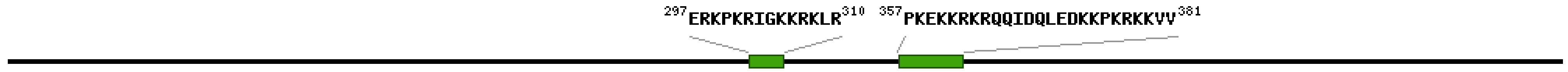

Posterior Graphic. Threshold Marked @ 0.4

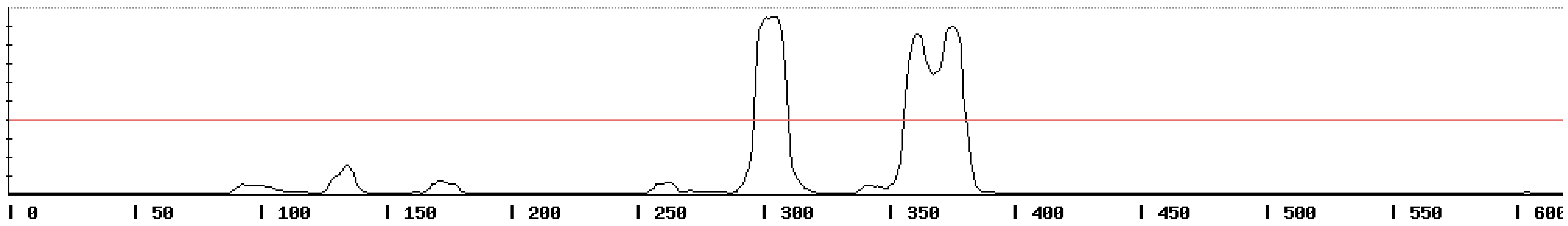

Predictions for Fragariavesca_mrna19716.1-v1.0-hybrid

Viterbi Path

Viterbi Path

Posterior @ 0.4

Posterior @ 0.4

Posterior Graphic. Threshold Marked @ 0.4

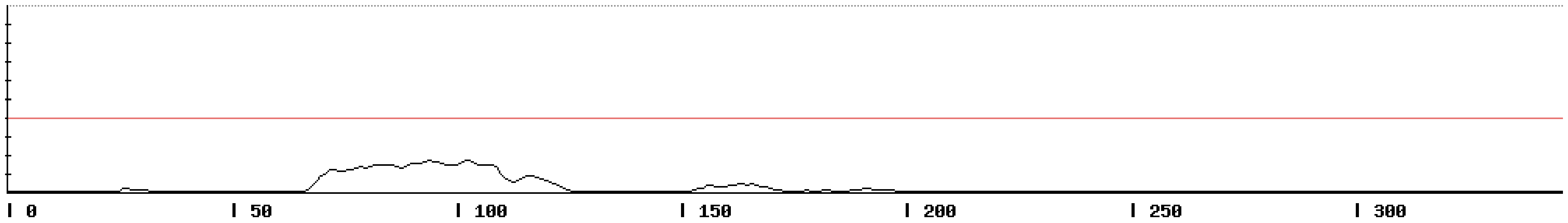

Predictions for Fragariavesca_mrna19898.1-v1.0-hybrid

Viterbi Path

Viterbi Path

Posterior @ 0.4

Posterior @ 0.4

Posterior Graphic. Threshold Marked @ 0.4

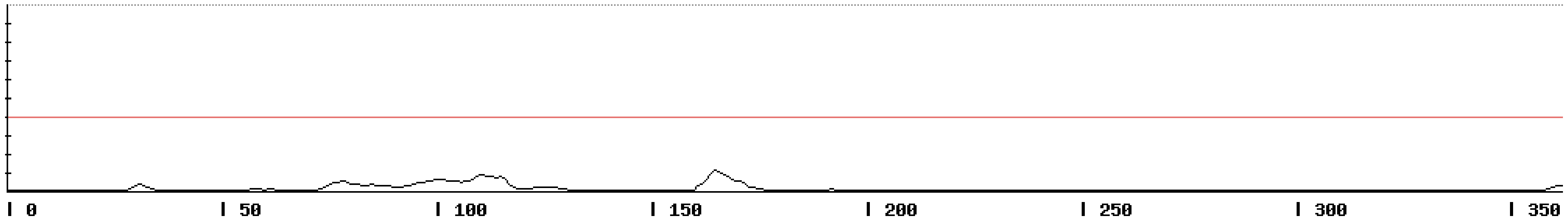

Predictions for Fragariavesca_mrna20078.1-v1.0-hybrid

Viterbi Path

Viterbi Path

Posterior @ 0.4	
Posterior @ 0.4	
Posterior Graphic. Threshold Marked @ 0.4	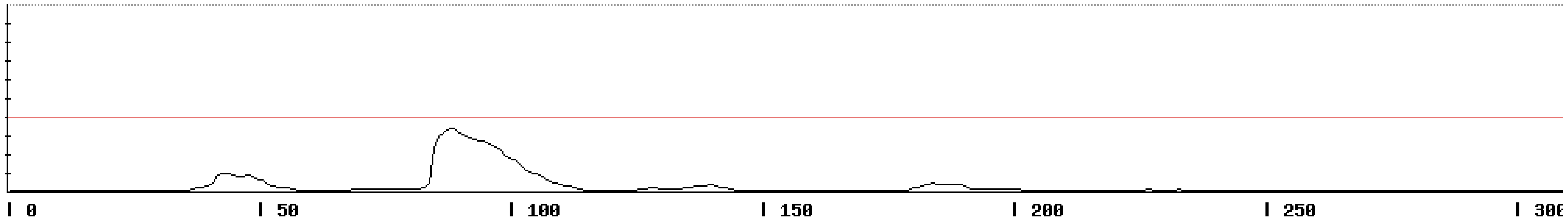

Predictions for Fragariavesca_mrna20482.1-v1.0-hybrid

Viterbi Path	
Viterbi Path	
Posterior @ 0.4	150 - KKRKL - 154<br>163 - RKNGEPKTKIAKKRKQR - 179
Posterior @ 0.4	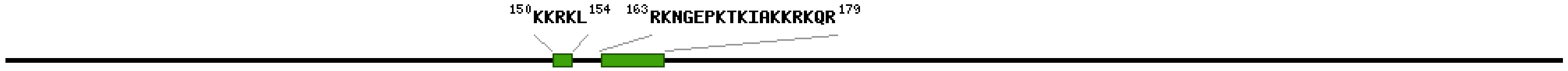
Posterior Graphic. Threshold Marked @ 0.4	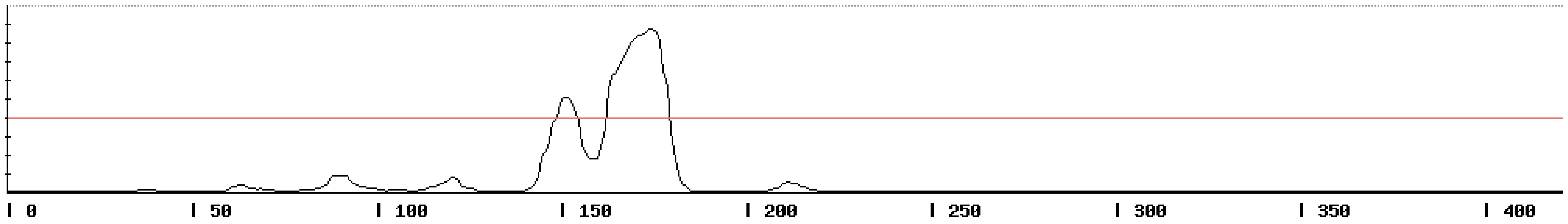

Predictions for Fragariavesca_mrna20500.1-v1.0-hybrid

Viterbi Path	
Viterbi Path	

Posterior @ 0.4 | 163 - RKNGEPKTKIAKKRKQ - 178

Posterior @ 0.4

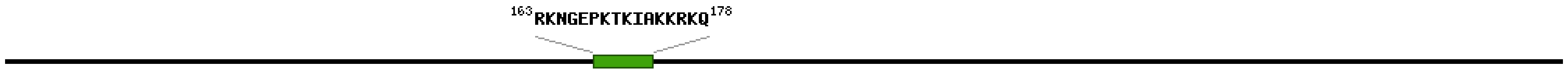

Posterior Graphic. Threshold Marked @ 0.4

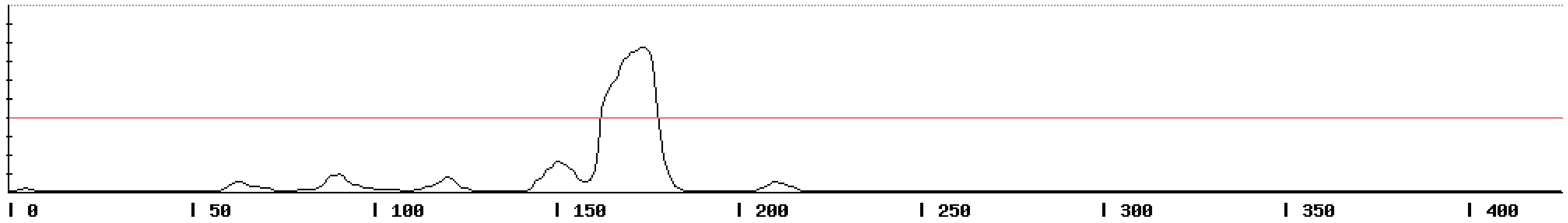

Predictions for Fragariavesca_mrna20686.1-v1.0-hybrid

Viterbi Path

Viterbi Path

Posterior @ 0.4

Posterior @ 0.4

Posterior Graphic. Threshold Marked @ 0.4

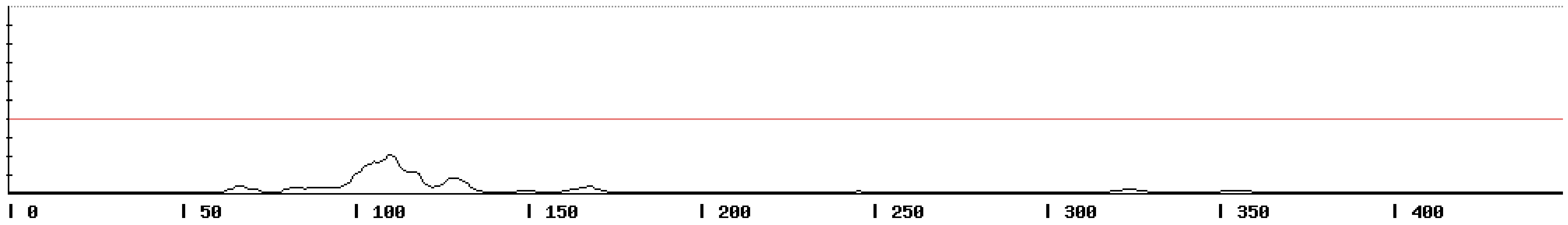

Predictions for Fragariavesca_mrna21248.1-v1.0-hybrid

Viterbi Path

Viterbi Path

Posterior @ 0.4

Posterior @ 0.4

Posterior Graphic. Threshold Marked @ 0.4

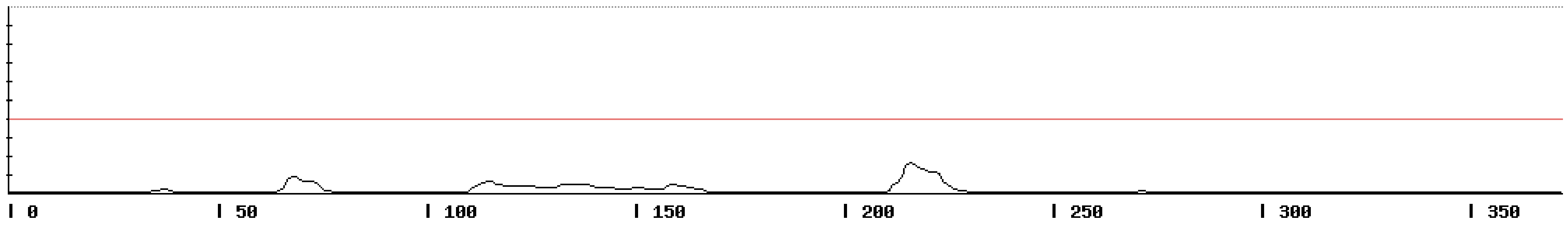

Predictions for Fragariavesca_mrna21734.1-v1.0-hybrid

Viterbi Path

Viterbi Path

Posterior @ 0.4

Posterior @ 0.4

Posterior Graphic. Threshold Marked @ 0.4

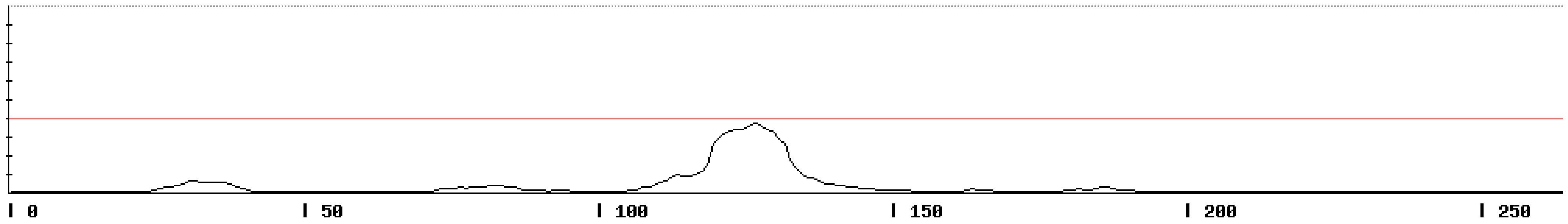

Predictions for Fragariavesca_mrna21755.1-v1.0-hybrid	
Viterbi Path	222 - KKRKTIVDKPKAKRHKKK - 239
Viterbi Path	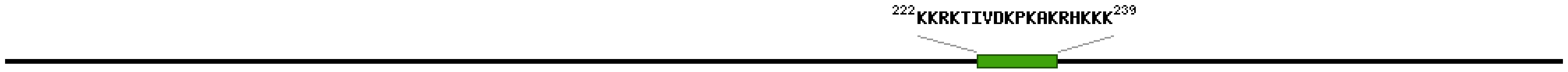
Posterior @ 0.4	218 - RVDVKKRKTIVDKPKAKRHKKKE - 240
Posterior @ 0.4	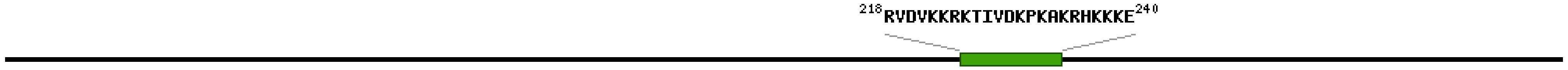
Posterior Graphic. Threshold Marked @ 0.4	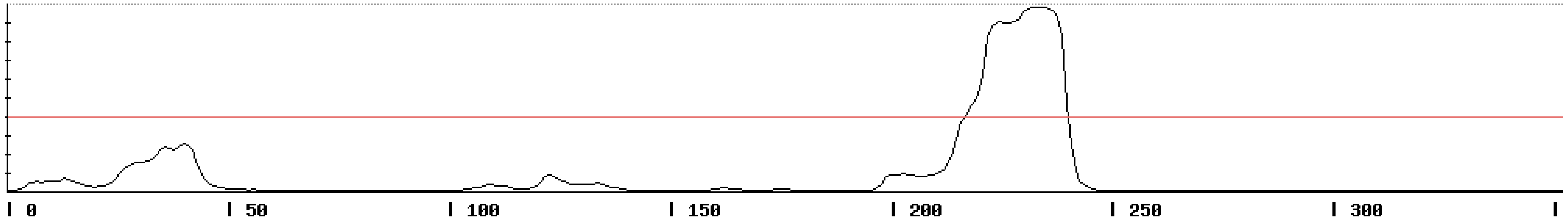

Predictions for Fragariavesca_mrna21872.1-v1.0-hybrid	
Viterbi Path	
Viterbi Path	
Posterior @ 0.4	
Posterior @ 0.4	
Posterior Graphic. Threshold Marked @ 0.4	

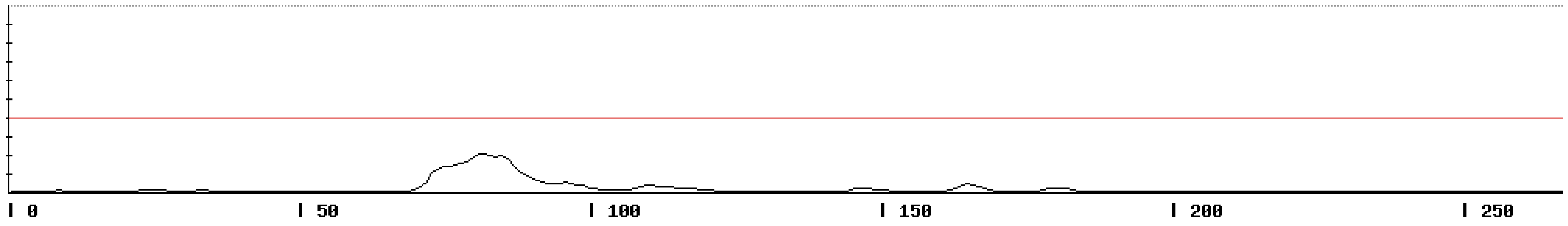

Predictions for Fragariavesca_mrna22092.1-v1.0-hybrid

Viterbi Path	
Viterbi Path	
Posterior @ 0.4	206 - KFRYEKKKNKK - 216
Posterior @ 0.4	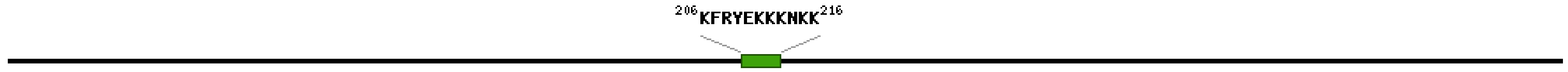
Posterior Graphic. Threshold Marked @ 0.4	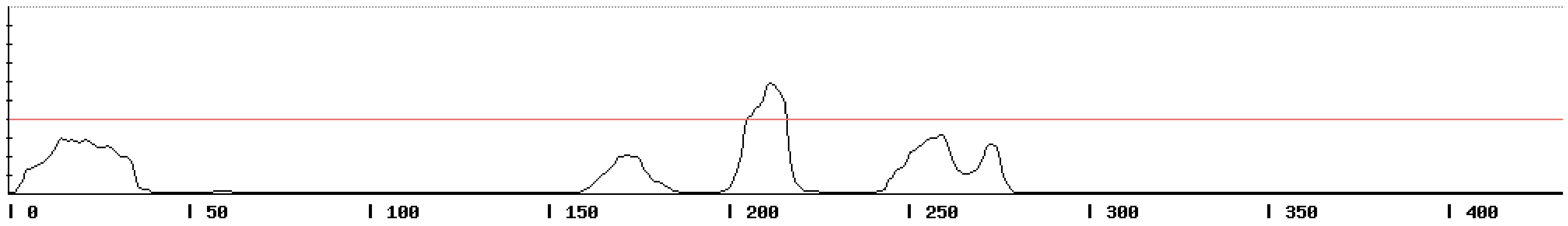

Predictions for Fragariavesca_mrna22095.1-v1.0-hybrid

Viterbi Path	
Viterbi Path	
Posterior @ 0.4	185 - QKKESKNEKDKK - 196
	231 - KGGDRKRKS - 239
	253 - RSKQSLKKRKLEPRR - 267
Posterior @ 0.4	

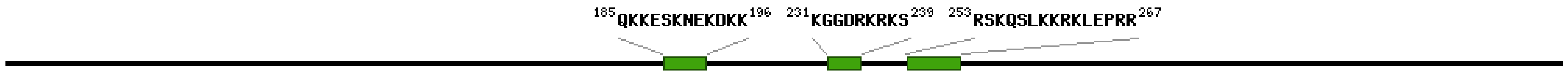

Posterior Graphic. Threshold Marked @ 0.4	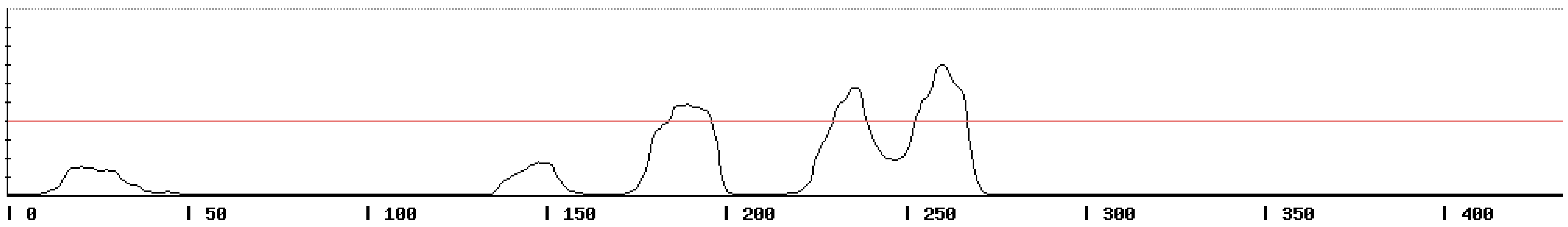

Predictions for Fragariavesca_mrna22097.1-v1.0-hybrid

Viterbi Path	
Viterbi Path	
Posterior @ 0.4	179 - DRKRK - 183
	198 - RSKKRKLEPRR - 208
Posterior @ 0.4	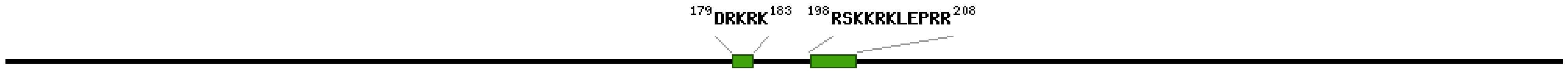
Posterior Graphic. Threshold Marked @ 0.4	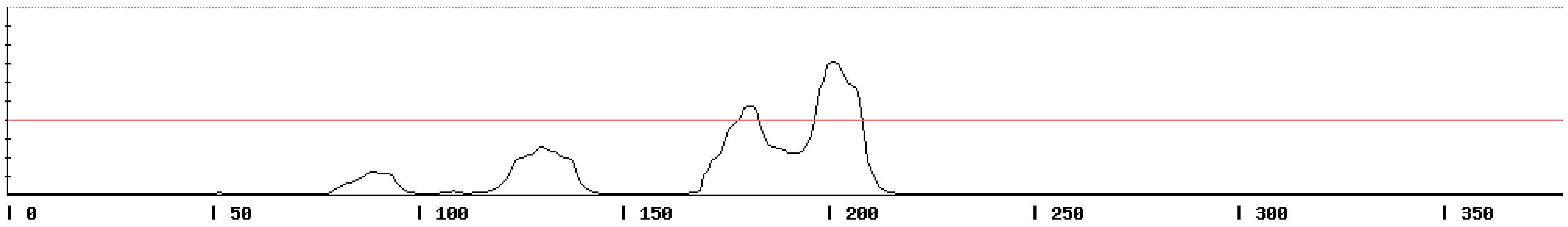

Predictions for Fragariavesca_mrna22263.1-v1.0-hybrid

Viterbi Path	
Viterbi Path	

Posterior @ 0.4	81 - TPKGKHIKR - 89<br>115 - KRR - 117
Posterior @ 0.4	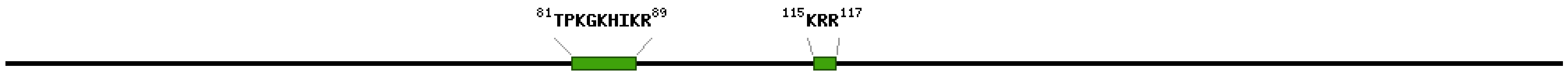
Posterior Graphic. Threshold Marked @ 0.4	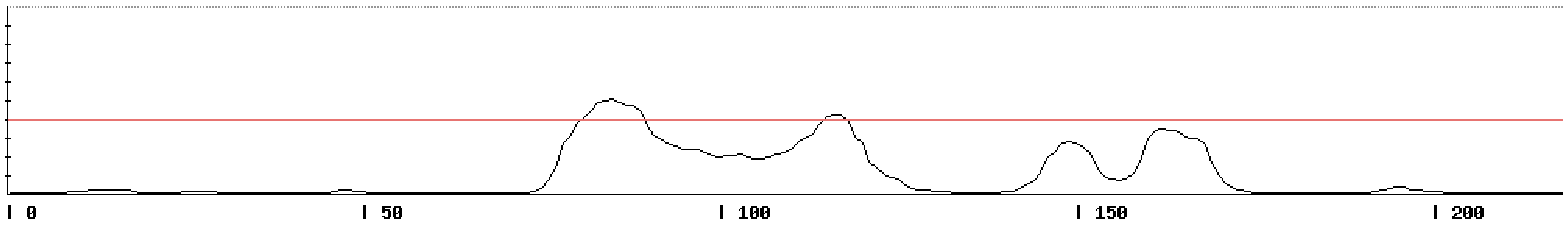

Predictions for Fragariavesca_mrna22522.1-v1.0-hybrid

Viterbi Path	
Viterbi Path	
Posterior @ 0.4	
Posterior @ 0.4	
Posterior Graphic. Threshold Marked @ 0.4	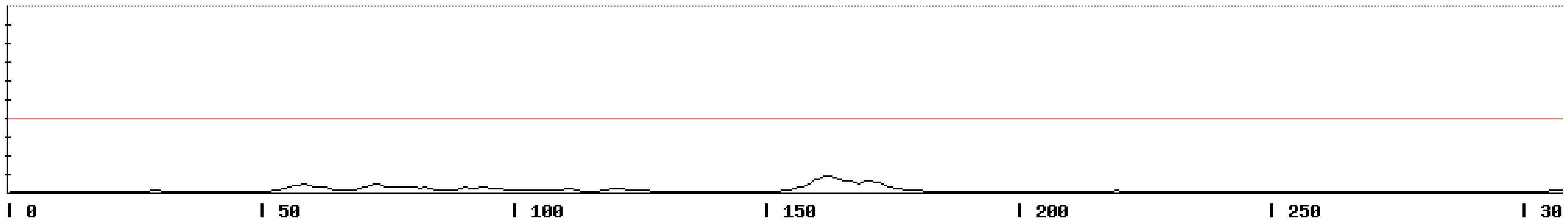

Predictions for Fragariavesca_mrna22558.1-v1.0-hybrid

Viterbi Path

Viterbi Path

Posterior @ 0.4

71 - KRKKLNPKGSRF - 82

150 - KPPPKTNETKRK - 161

Posterior @ 0.4

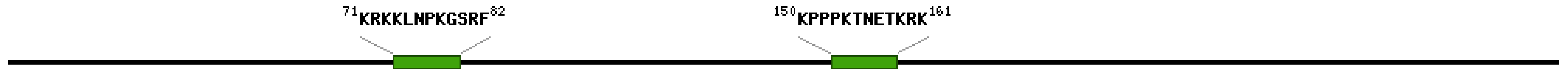

Posterior Graphic. Threshold Marked @ 0.4

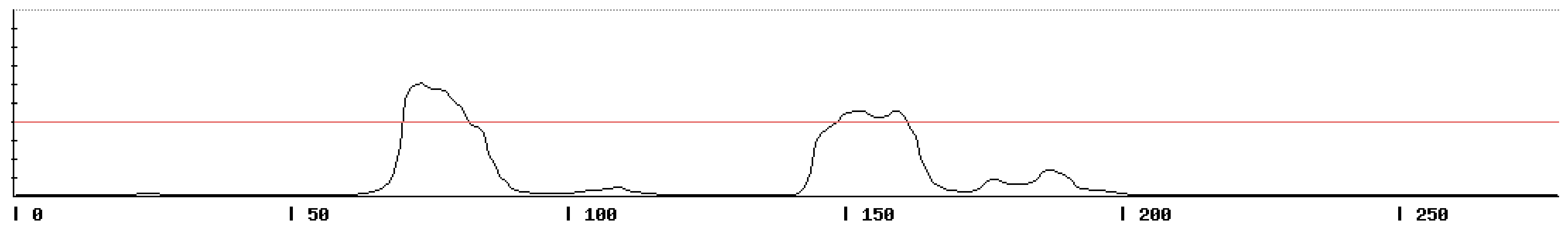

Predictions for Fragariavesca_mrna22559.1-v1.0-hybrid

Viterbi Path

177 - KKNSVKKRKSASFVDHQSRKRKKKLK - 202

Viterbi Path

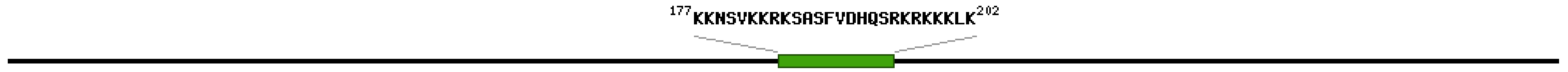

Posterior @ 0.4

177 - KKNSVKKRKSASFVDHQSRKRKKKLK - 202

Posterior @ 0.4

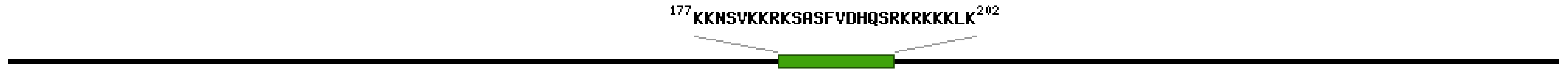

Posterior Graphic. Threshold Marked @ 0.4

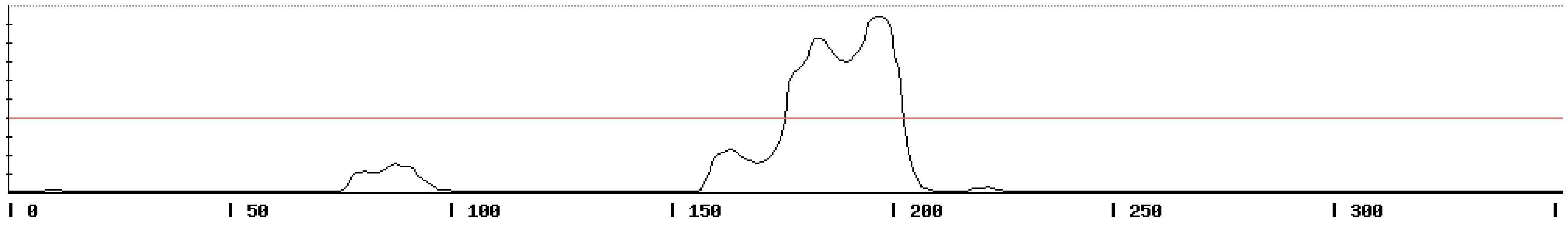

Predictions for Fragariavesca_mrna22722.1-v1.0-hybrid

Viterbi Path	
Viterbi Path	
Posterior @ 0.4	165 - DSKTKIEKKRKQR - 177
Posterior @ 0.4	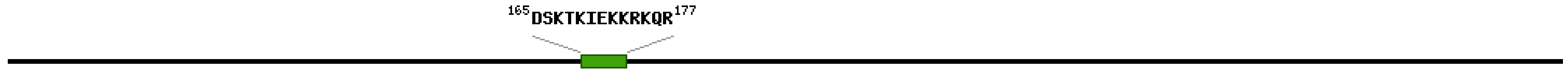
Posterior Graphic. Threshold Marked @ 0.4	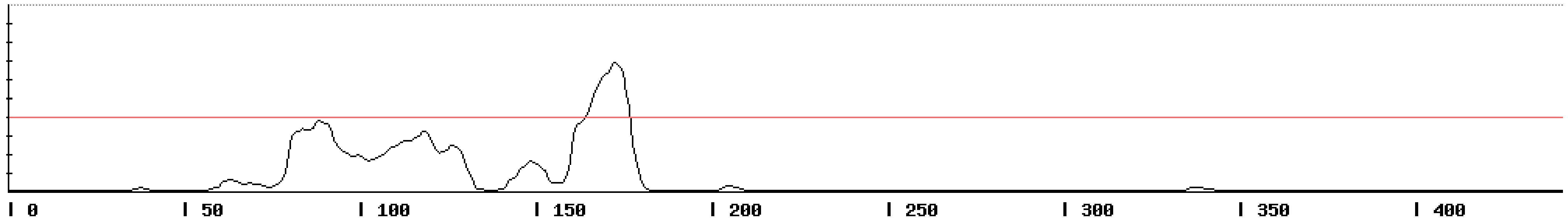

Predictions for Fragariavesca_mrna22723.1-v1.0-hybrid

Viterbi Path	
Viterbi Path	
Posterior @ 0.4	165 - DSKTKIEKKRKQR - 177
Posterior @ 0.4	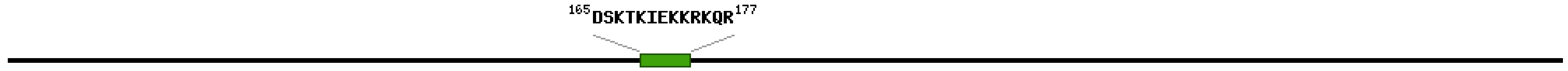

Posterior Graphic. Threshold Marked @ 0.4	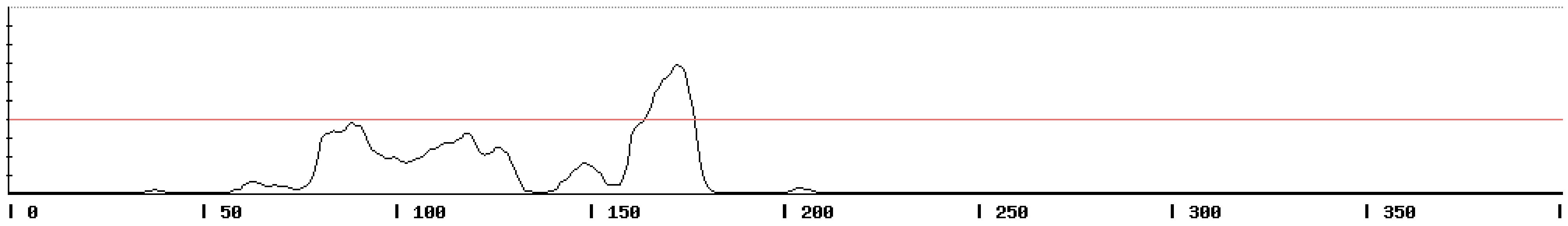

Predictions for Fragariavesca_mrna23099.1-v1.0-hybrid

Viterbi Path	
Viterbi Path	
Posterior @ 0.4	86 - KKHTKGTRKKR - 96
Posterior @ 0.4	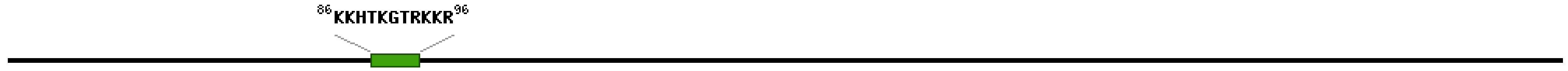
Posterior Graphic. Threshold Marked @ 0.4	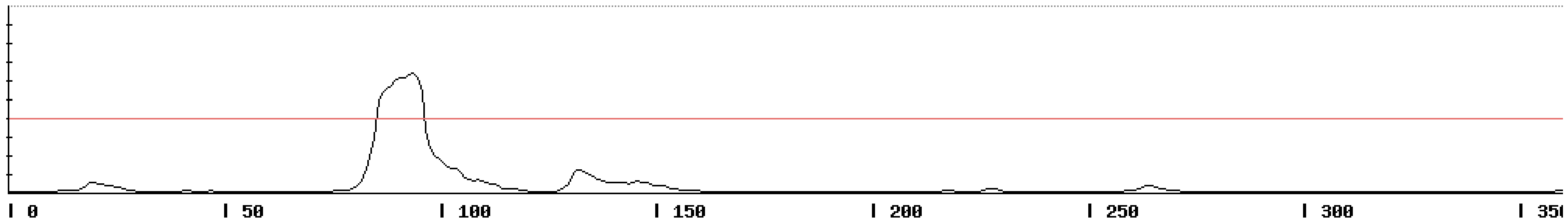

Predictions for Fragariavesca_mrna23394.1-v1.0-hybrid

Viterbi Path	
Viterbi Path	
Posterior @ 0.4	600 - VEREIKARRSKALIGKKR - 617
Posterior @ 0.4	

	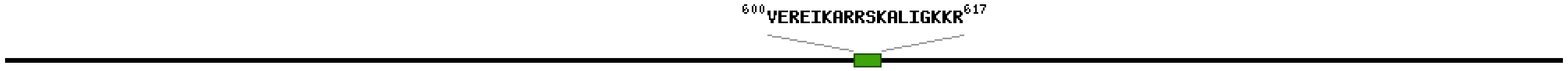
Posterior Graphic. Threshold Marked @ 0.4	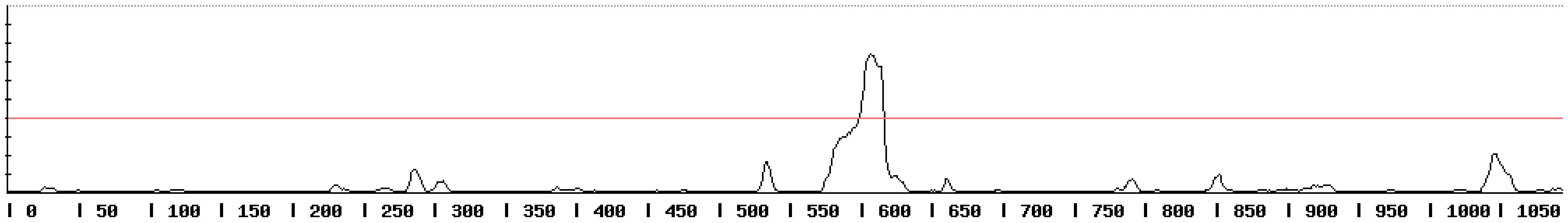

Predictions for Fragariavesca_mrna23552.1-v1.0-hybrid	
Viterbi Path	
Viterbi Path	
Posterior @ 0.4	75 - RKKLNPKAKRFHRKV - 89
Posterior @ 0.4	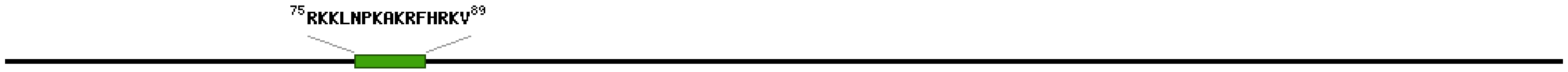
Posterior Graphic. Threshold Marked @ 0.4	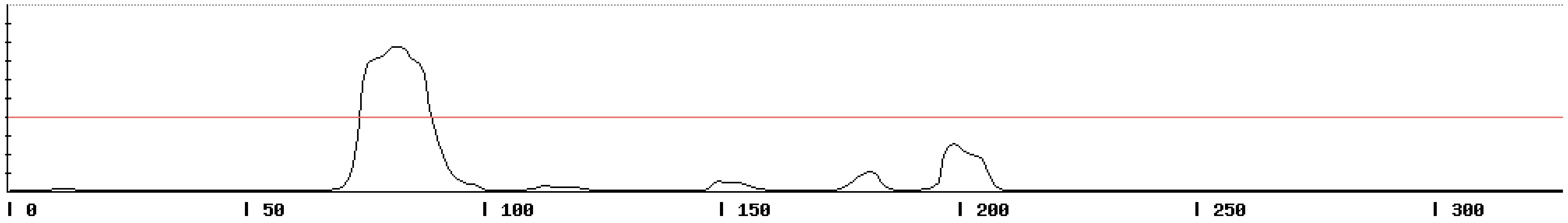

Predictions for Fragariavesca_mrna23560.1-v1.0-hybrid	
Viterbi Path	
Viterbi Path	

Posterior @ 0.4

Posterior @ 0.4

Posterior Graphic. Threshold Marked @ 0.4

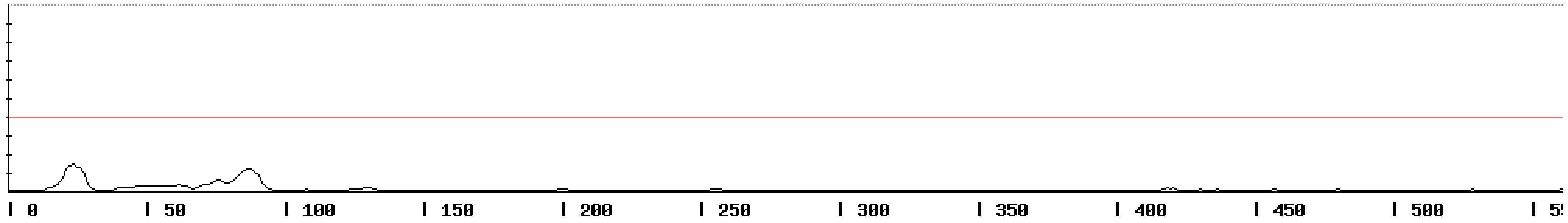

Predictions for Fragariavesca_mrna23561.1-v1.0-hybrid

Viterbi Path

Viterbi Path

Posterior @ 0.4

Posterior @ 0.4

Posterior Graphic. Threshold Marked @ 0.4

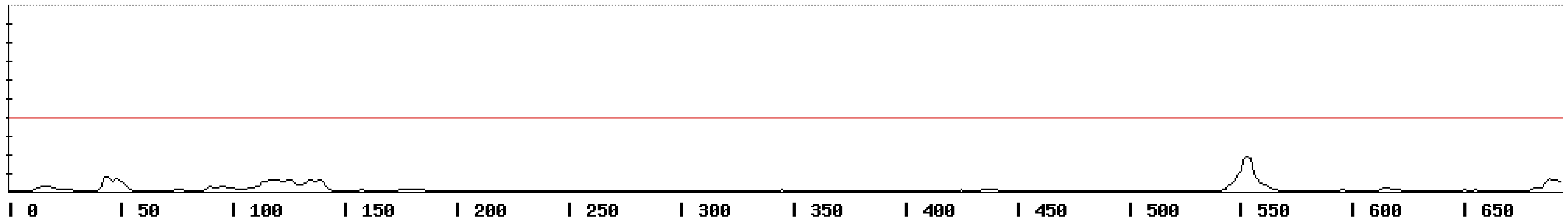

Predictions for Fragariavesca_mrna24179.1-v1.0-hybrid

Viterbi Path

Viterbi Path

Posterior @ 0.4	95 - KHIGKPKALGIKK - 107
	656 - GRKPFKNIREVKK - 668
	720 - SKKKKRKVR - 728
Posterior @ 0.4	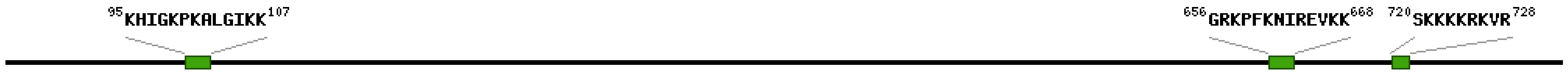
Posterior Graphic. Threshold Marked @ 0.4	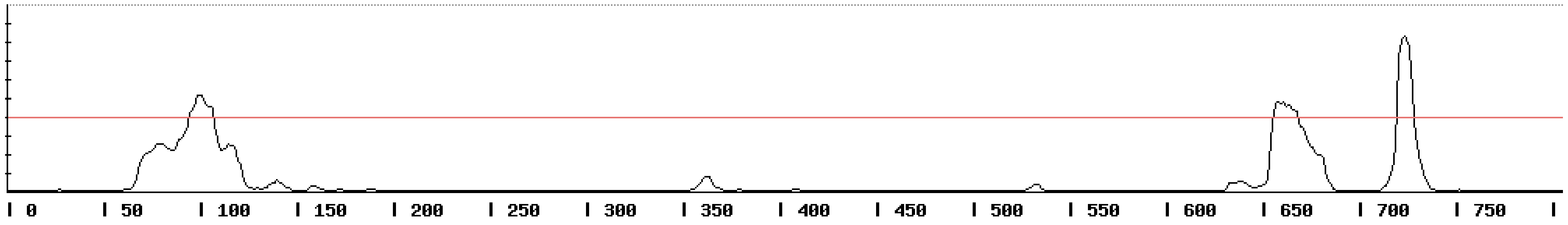

Predictions for Fragariavesca_mrna24287.1-v1.0-hybrid

Viterbi Path	
Viterbi Path	
Posterior @ 0.4	236 - LRSKKRKL - 243
Posterior @ 0.4	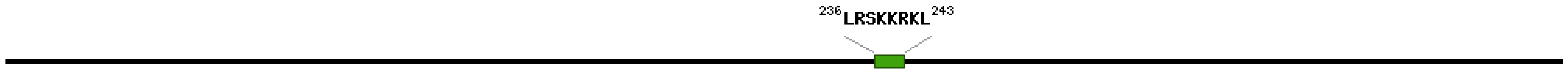
Posterior Graphic. Threshold Marked @ 0.4	

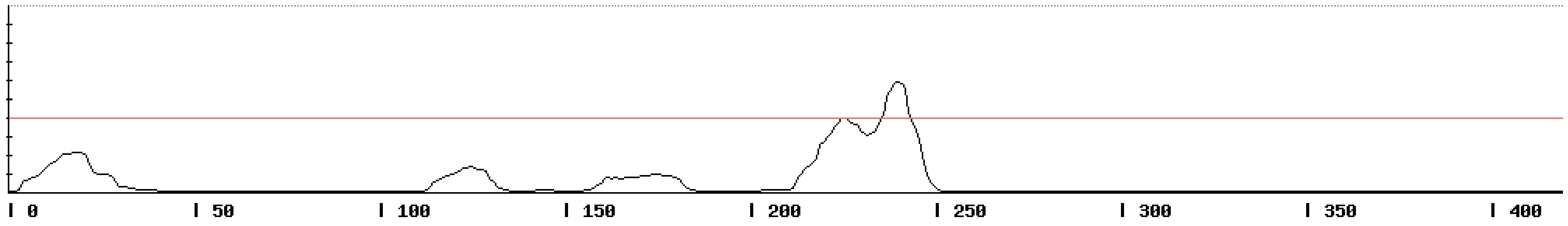

Predictions for Fragariavesca_mrna24342.1-v1.0-hybrid

Viterbi Path

Viterbi Path

Posterior @ 0.4

81 - KKMKSTDKRIRRTVGSGTWKGNDSGKPVKSSGRKIGLKKRF - 121

166 - SKSKIEKKRKQR - 177

Posterior @ 0.4

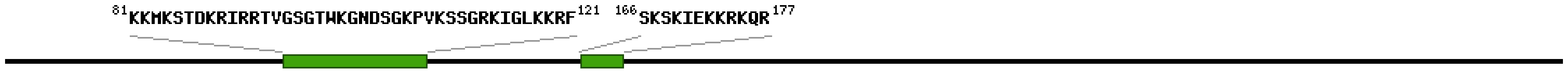

Posterior Graphic. Threshold Marked @ 0.4

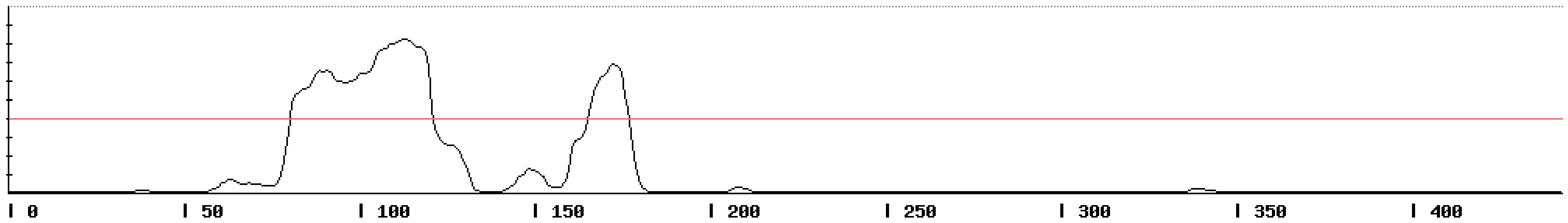

Predictions for Fragariavesca_mrna24344.1-v1.0-hybrid

Viterbi Path

Viterbi Path

Posterior @ 0.4

163 - RKNGEPKTKIEKKRKQR - 179

Posterior @ 0.4

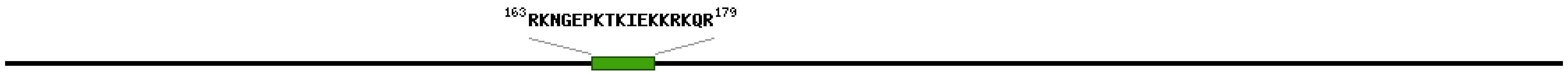

Posterior Graphic. Threshold Marked @ 0.4

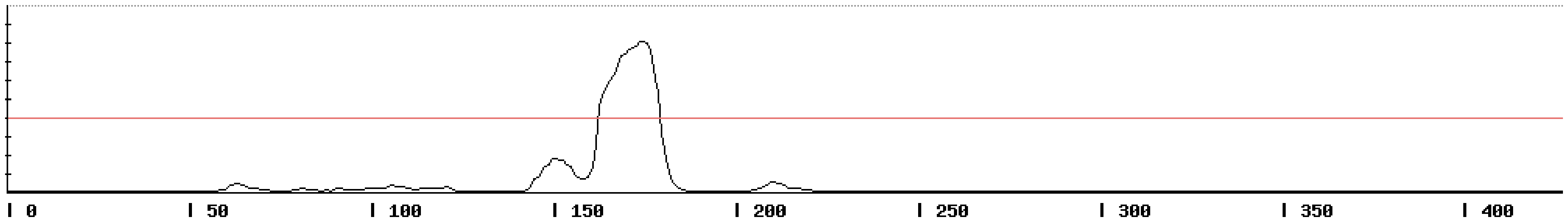

Predictions for Fragariavesca_mrna25163.1-v1.0-hybrid

Viterbi Path

Viterbi Path

Posterior @ 0.4

Posterior @ 0.4

Posterior Graphic. Threshold Marked @ 0.4

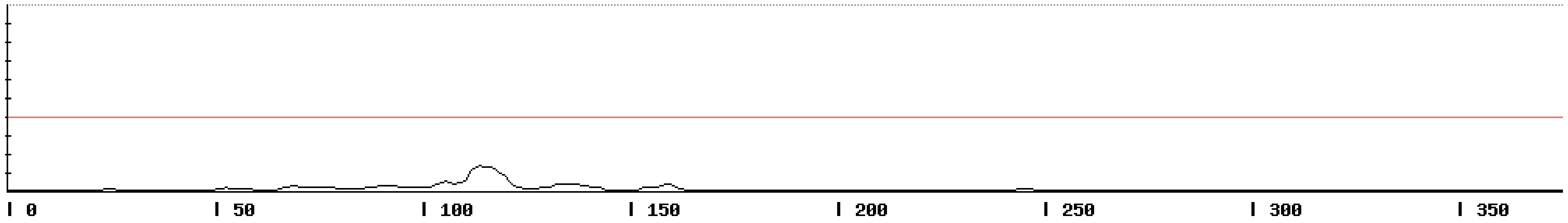

Predictions for Fragariavesca_mrna25225.1-v1.0-hybrid

Viterbi Path

Viterbi Path

Posterior @ 0.4

Posterior @ 0.4

Posterior Graphic. Threshold Marked @ 0.4

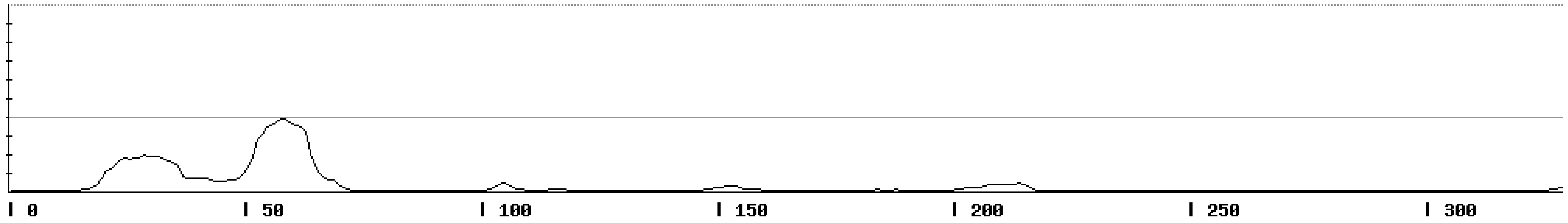

Predictions for Fragariavesca_mrna25284.1-v1.0-hybrid

Viterbi Path

Viterbi Path

Posterior @ 0.4

Posterior @ 0.4

Posterior Graphic. Threshold Marked @ 0.4

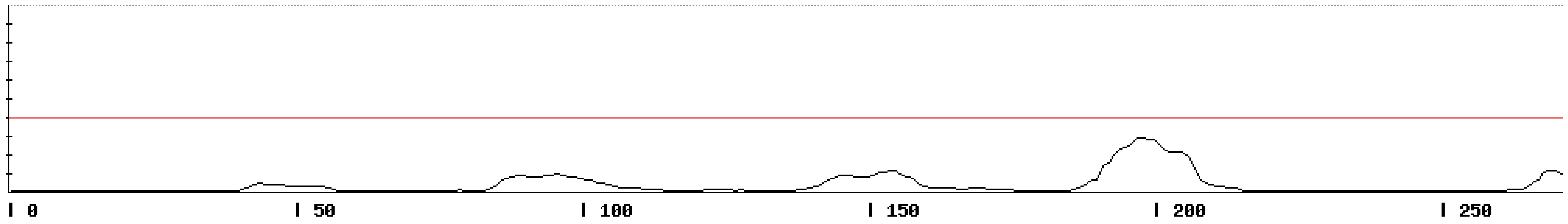

Predictions for Fragariavesca_mrna25783.1-v1.0-hybrid

Viterbi Path

Viterbi Path

Posterior @ 0.4

Posterior @ 0.4

Posterior Graphic. Threshold Marked @ 0.4

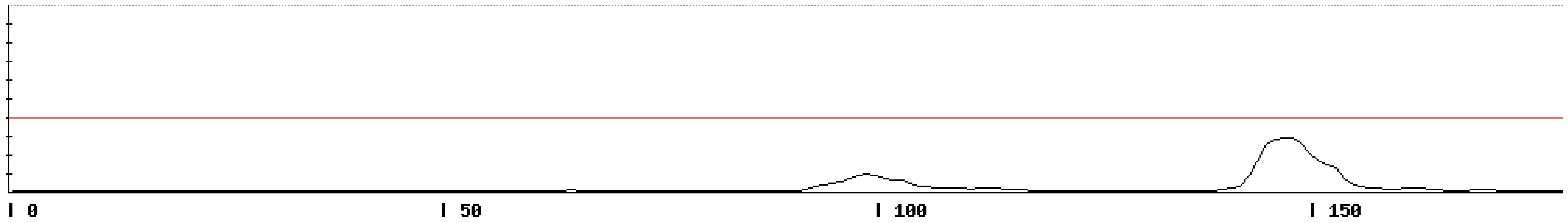

Predictions for Fragariavesca_mrna25794.1-v1.0-hybrid

Viterbi Path

Viterbi Path

Posterior @ 0.4

Posterior @ 0.4

Posterior Graphic. Threshold Marked @ 0.4

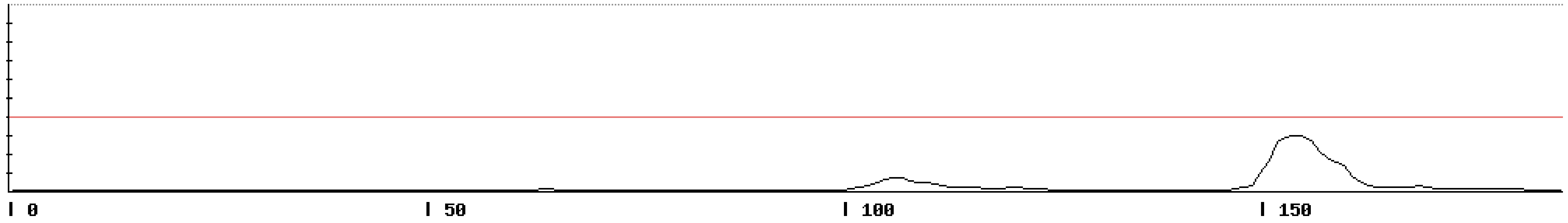

Predictions for Fragariavesca_mrna26043.1-v1.0-hybrid

Viterbi Path

Viterbi Path

Posterior @ 0.4

Posterior @ 0.4

Posterior Graphic. Threshold Marked @ 0.4

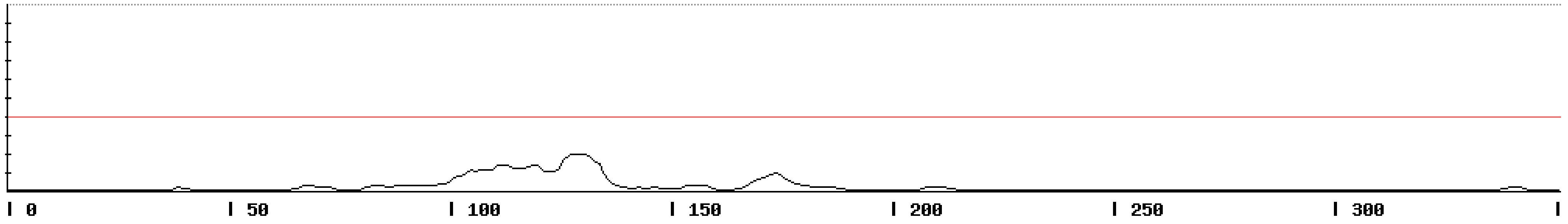

Predictions for Fragariavesca_mrna26542.1-v1.0-hybrid

Viterbi Path 251 - RKSKTAVKKPRELKKMKKKMAR - 272

Viterbi Path

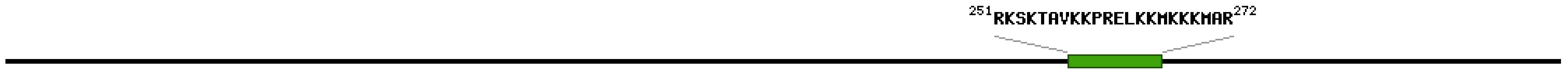

Posterior @ 0.4 248 - RLVRKSKTAVKKPRELKKMKKKMAR - 272

Posterior @ 0.4

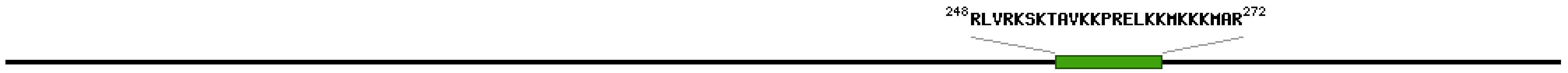

Posterior Graphic. Threshold Marked @ 0.4

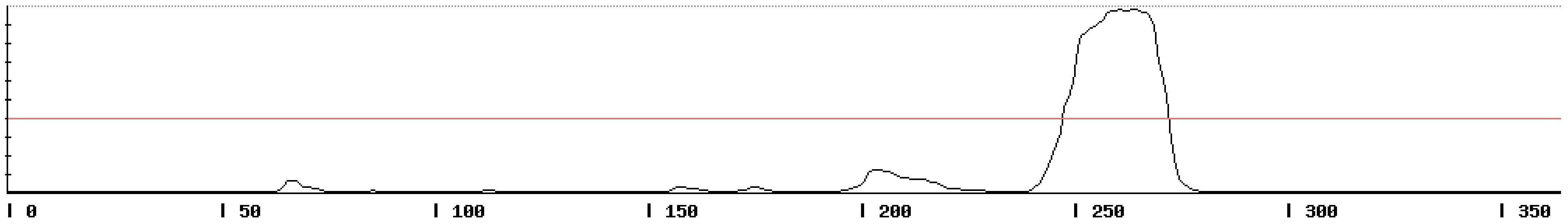

Predictions for Fragariavesca_mrna27184.1-v1.0-hybrid

Viterbi Path	
Viterbi Path	
Posterior @ 0.4	77 - KKKKTAR - 83<br>174 - KKRK - 177
Posterior @ 0.4	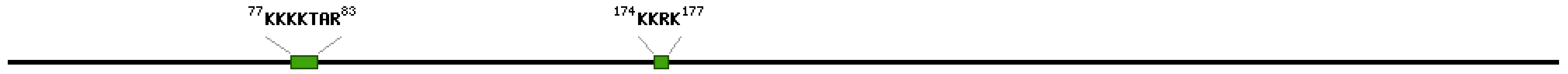
Posterior Graphic. Threshold Marked @ 0.4	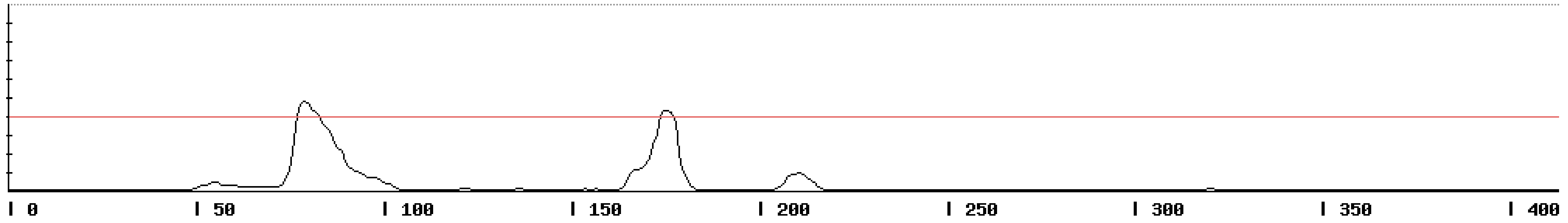

Predictions for Fragariavesca_mrna27240.1-v1.0-hybrid

Viterbi Path	
Viterbi Path	
Posterior @ 0.4	83 - KKVSAKGSRICRKVGKGTWKGQDKA - 107<br>180 - KKRK - 183
Posterior @ 0.4	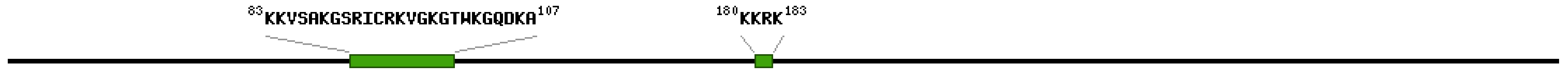
Posterior Graphic. Threshold Marked @ 0.4	

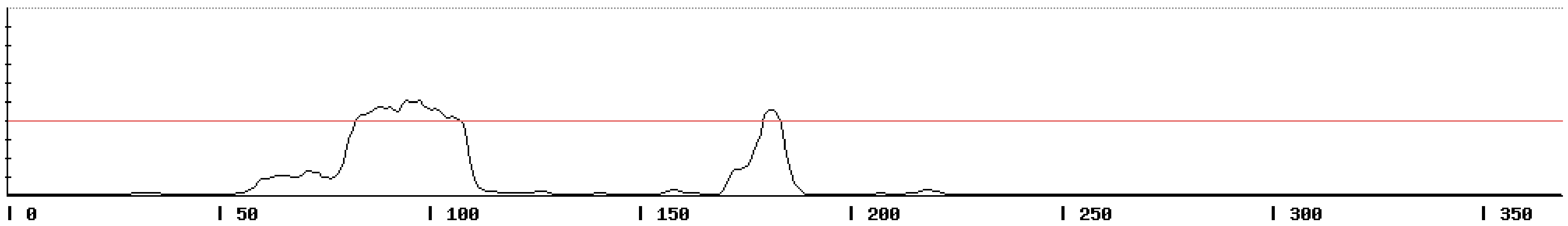

Predictions for Fragariavesca_mrna27279.1-v1.0-hybrid

Viterbi Path

Viterbi Path

Posterior @ 0.4 — 158 - LRKNKGSKEKGKR - 170

Posterior @ 0.4

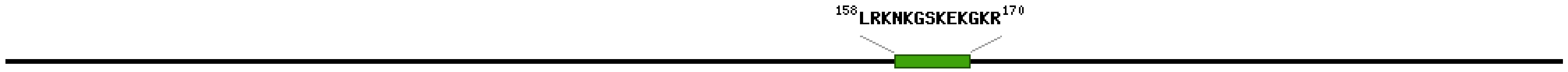

Posterior Graphic. Threshold Marked @ 0.4

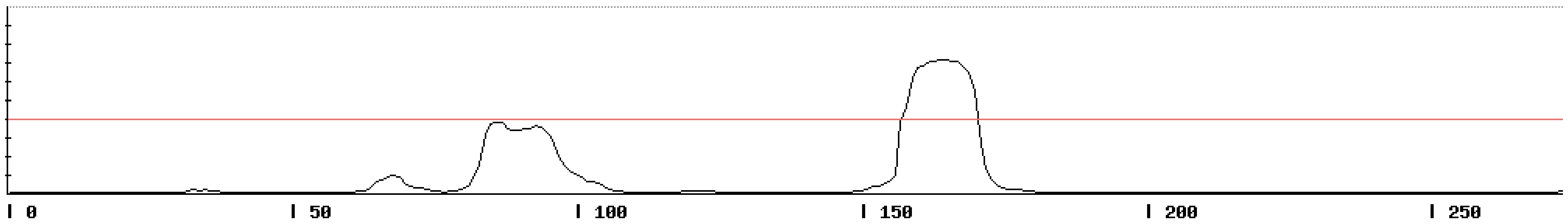

Predictions for Fragariavesca_mrna27539.1-v1.0-hybrid

Viterbi Path

Viterbi Path

Posterior @ 0.4 — 38 - KVGRHRR - 44

Posterior @ 0.4

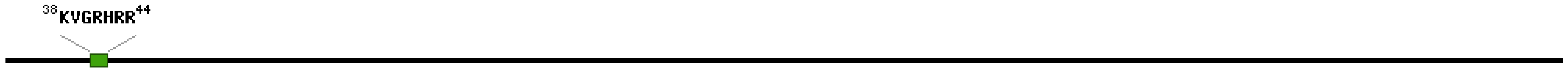

Posterior Graphic. Threshold Marked @ 0.4

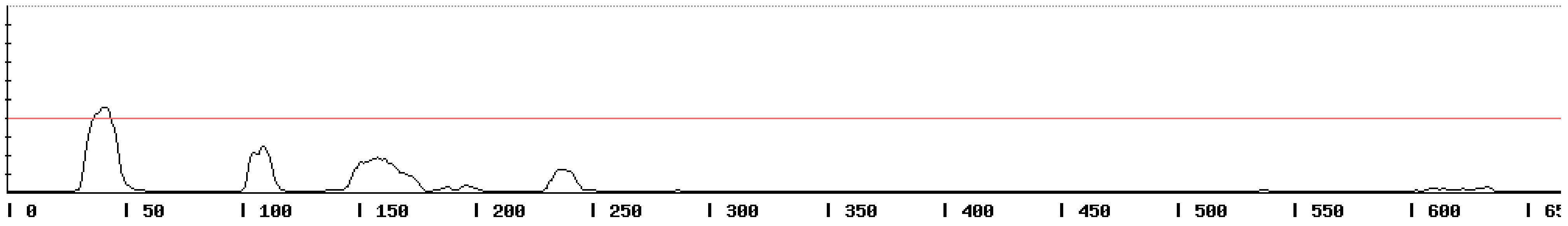

Predictions for Fragariavesca_mrna27614.1-v1.0-hybrid

Viterbi Path

Viterbi Path

Posterior @ 0.4

Posterior @ 0.4

Posterior Graphic. Threshold Marked @ 0.4

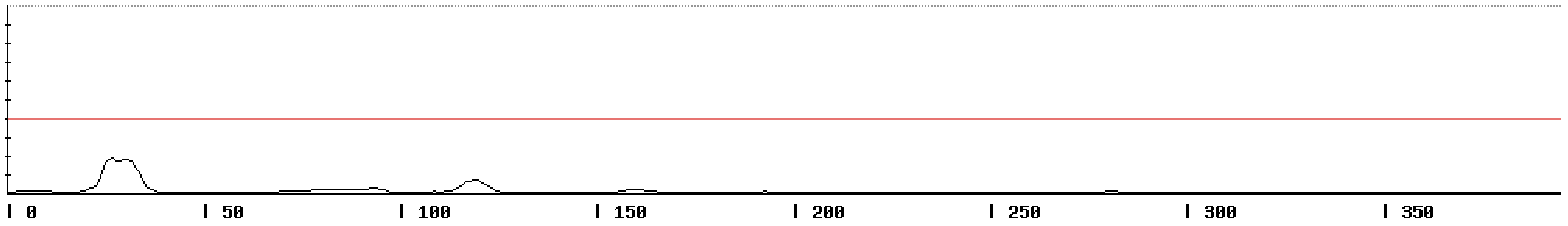

Predictions for Fragariavesca_mrna27615.1-v1.0-hybrid

Viterbi Path

Viterbi Path

Posterior @ 0.4

Posterior @ 0.4

Posterior Graphic. Threshold Marked @ 0.4	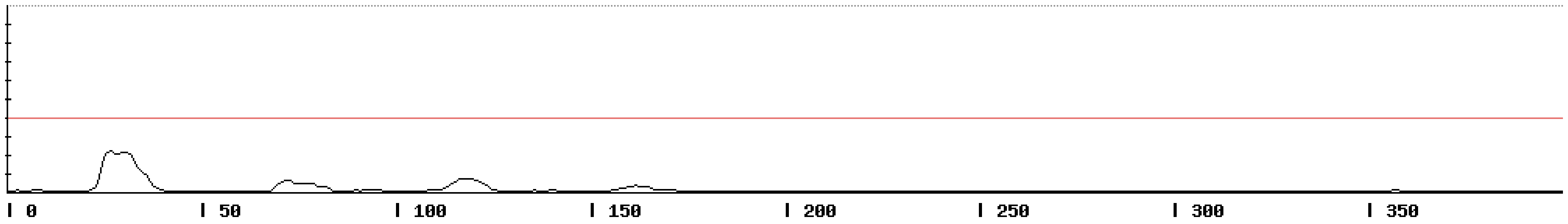

Predictions for Fragariavesca_mrna27793.1-v1.0-hybrid

Viterbi Path	2 - GRRGPSGATRKEKERERKRRVVRGGR - 27
Viterbi Path	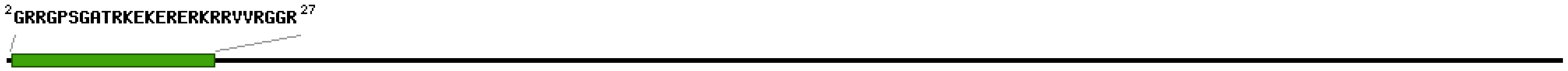
Posterior @ 0.4	3 - RRGPSGATRKEKERERKRRVVRGGR - 27
Posterior @ 0.4	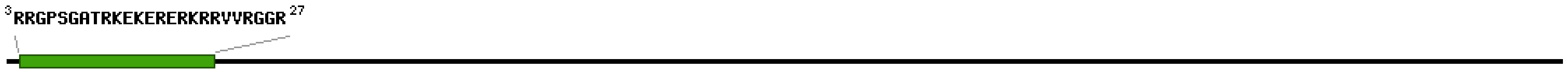
Posterior Graphic. Threshold Marked @ 0.4	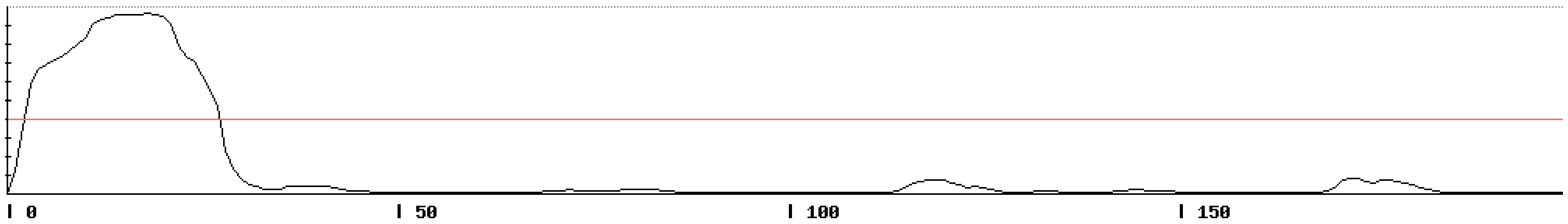

Predictions for Fragariavesca_mrna27940.1-v1.0-hybrid

Viterbi Path	
Viterbi Path	

Posterior @ 0.4 | 578 - RKRRKIDKE - 586

595 - RWHKTGKTKAIKGGVHPGWKK - 615

Posterior @ 0.4

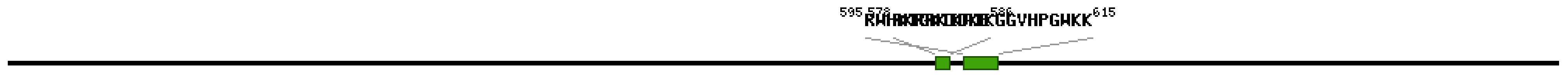

Posterior Graphic. Threshold Marked @ 0.4

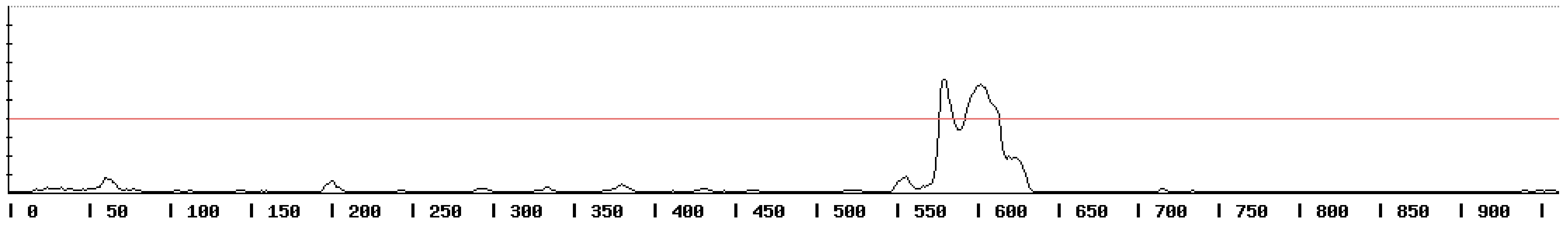

Predictions for Fragariavesca_mrna28487.1-v1.0-hybrid

Viterbi Path

Viterbi Path

Posterior @ 0.4 | 74 - KLNPKGKRID - 83

Posterior @ 0.4

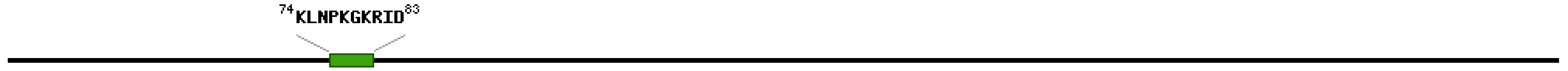

Posterior Graphic. Threshold Marked @ 0.4

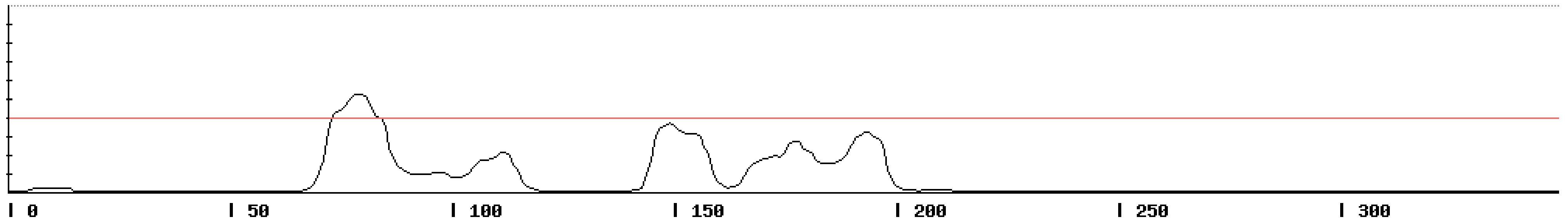

Predictions for Fragariavesca_mrna28762.1-v1.0-hybrid

Viterbi Path

Viterbi Path

Posterior @ 0.4

77 - GR - 78

Posterior @ 0.4

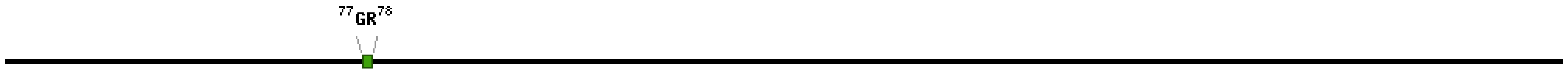

Posterior Graphic. Threshold Marked @ 0.4

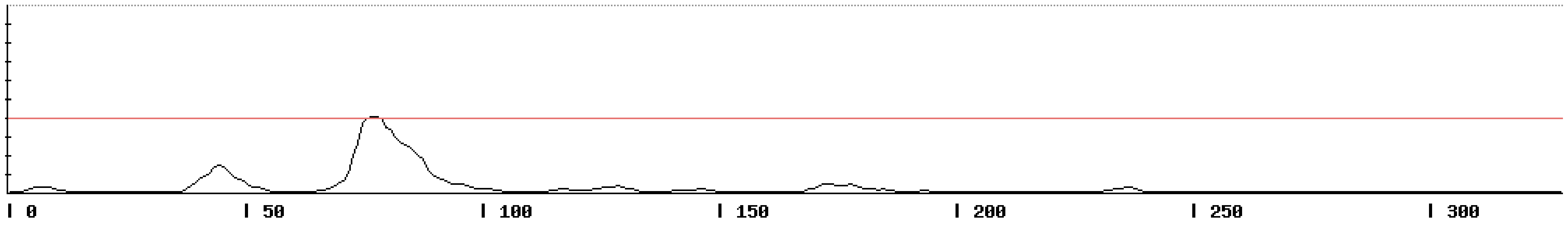

Predictions for Fragariavesca_mrna29085.1-v1.0-hybrid

Viterbi Path

Viterbi Path

Posterior @ 0.4

Posterior @ 0.4

Posterior Graphic. Threshold Marked @ 0.4

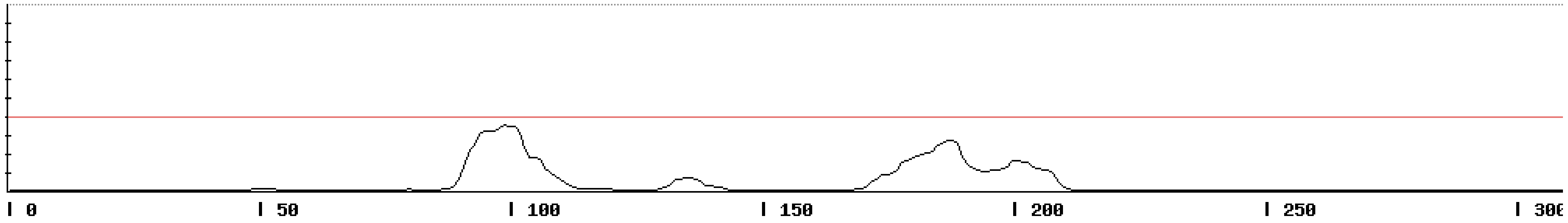

Predictions for Fragariavesca_mrna29106.1-v1.0-hybrid

Viterbi Path

Viterbi Path

Posterior @ 0.4

Posterior @ 0.4

Posterior Graphic. Threshold Marked @ 0.4

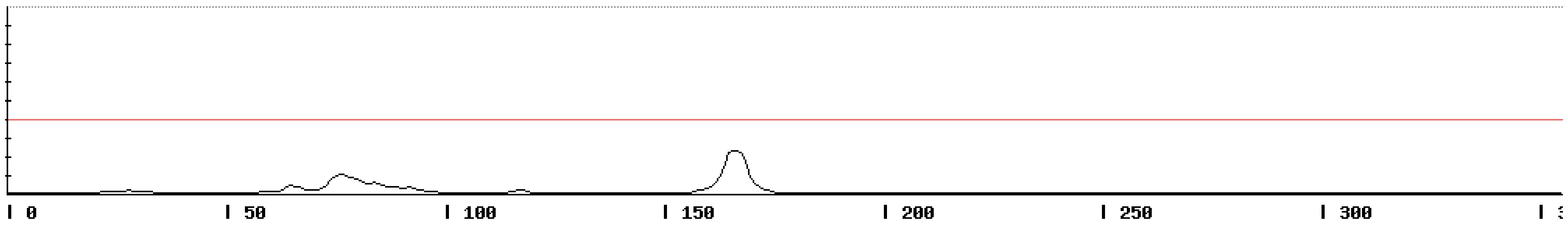

Predictions for Fragariavesca_mrna29122.1-v1.0-hybrid

Viterbi Path

Viterbi Path

Posterior @ 0.4

Posterior @ 0.4

Posterior Graphic. Threshold Marked @ 0.4

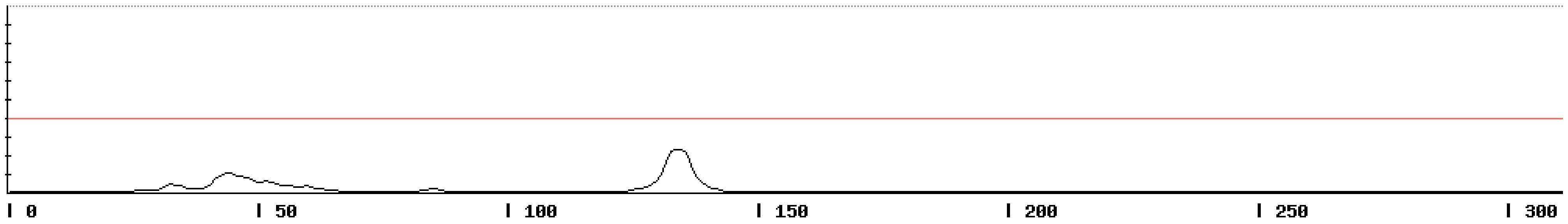

Predictions for Fragariavesca_mrna29260.1-v1.0-hybrid

Viterbi Path

Viterbi Path

Posterior @ 0.4

Posterior @ 0.4

Posterior Graphic. Threshold Marked @ 0.4

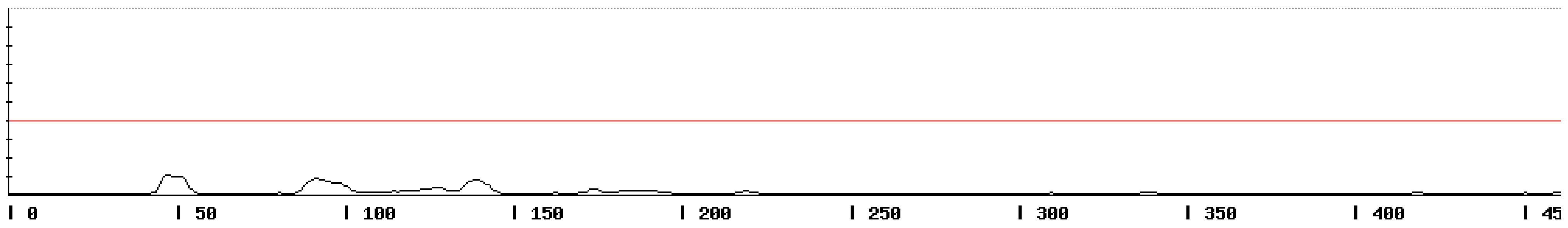

Predictions for Fragariavesca_mrna29354.1-v1.0-hybrid

Viterbi Path

Viterbi Path

Posterior @ 0.4

72 - SRRKKRTRRGGK - 83

Posterior @ 0.4

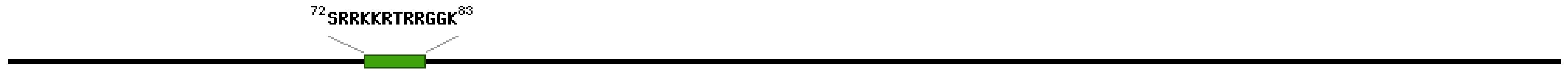

Posterior Graphic. Threshold Marked @ 0.4

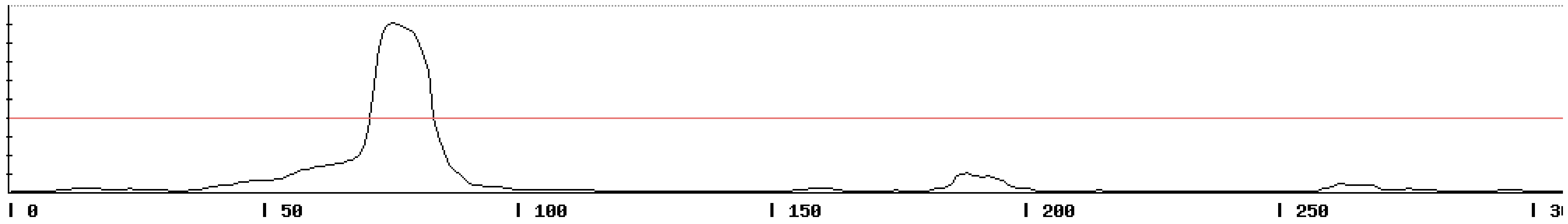

Predictions for Fragariavesca_mrna29766.1-v1.0-hybrid

Viterbi Path	
Viterbi Path	
Posterior @ 0.4	291 - RKRRK - 295
Posterior @ 0.4	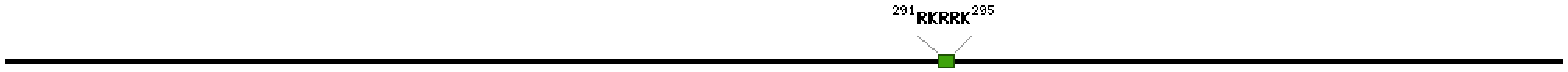
Posterior Graphic. Threshold Marked @ 0.4	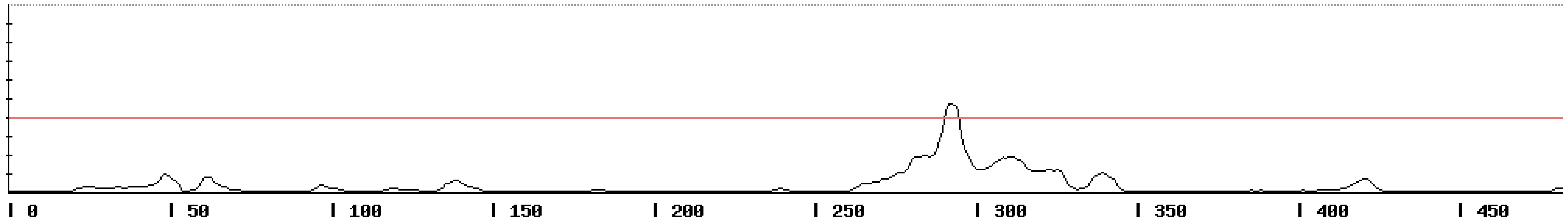

Predictions for Fragariavesca_mrna30018.1-v1.0-hybrid

Viterbi Path	
Viterbi Path	
Posterior @ 0.4	169 - NAKGGDRKRKSSAAEKNVEDENLTRRQSLKKRKREPRRS - 207
Posterior @ 0.4	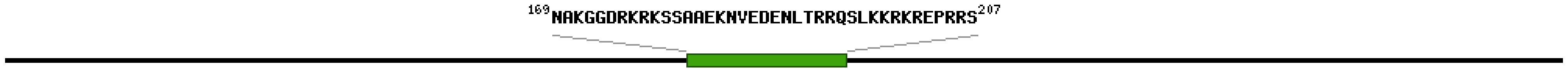

Posterior Graphic. Threshold Marked @ 0.4

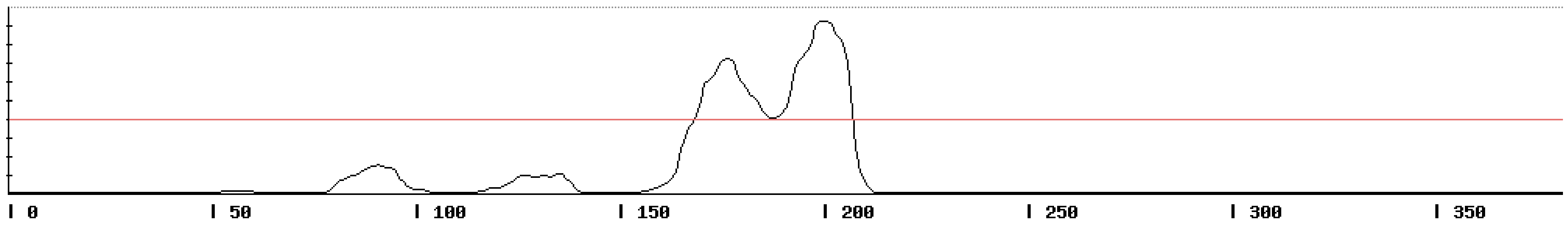

Predictions for Fragariavesca_mrna30051.1-v1.0-hybrid

Viterbi Path

Viterbi Path

Posterior @ 0.4

Posterior @ 0.4

Posterior Graphic. Threshold Marked @ 0.4

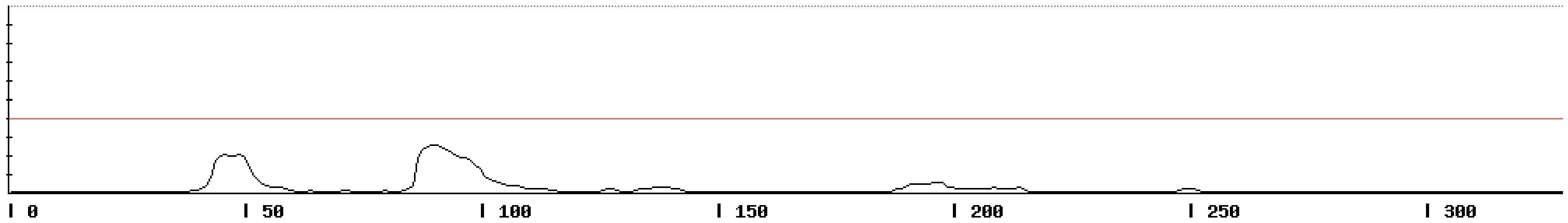

Predictions for Fragariavesca_mrna30439.1-v1.0-hybrid

Viterbi Path

Viterbi Path

Posterior @ 0.4

Posterior @ 0.4

Posterior Graphic. Threshold Marked @ 0.4

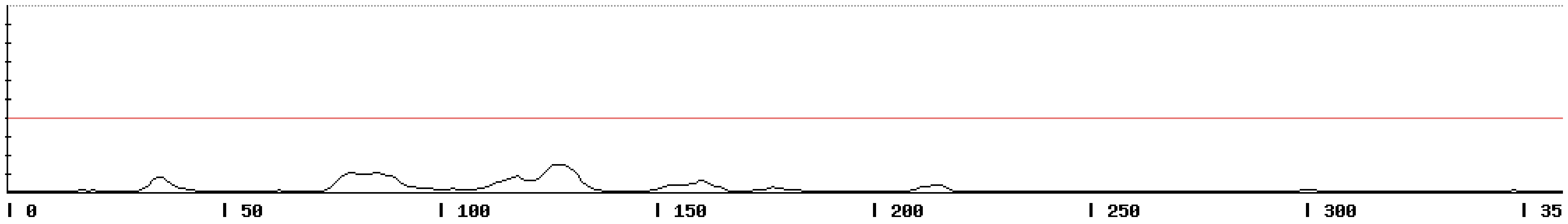

Predictions for Fragariavesca_mrna30746.1-v1.0-hybrid

Viterbi Path

Viterbi Path

Posterior @ 0.4

Posterior @ 0.4

Posterior Graphic. Threshold Marked @ 0.4

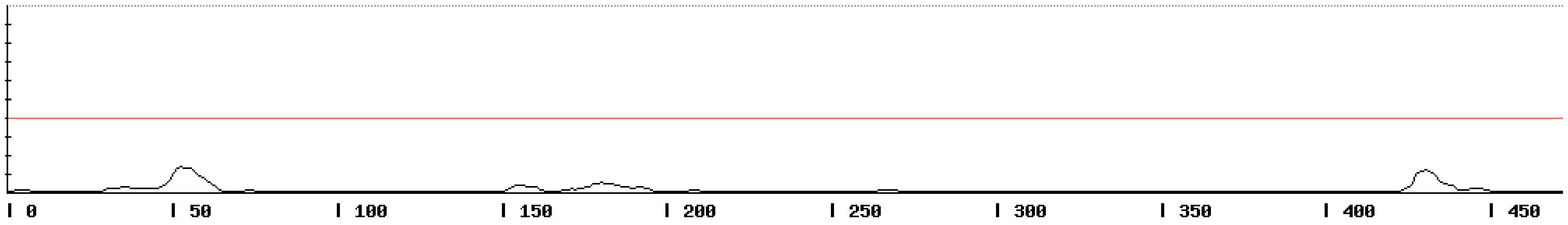

Predictions for Fragariavesca_mrna30749.1-v1.0-hybrid

Viterbi Path

Viterbi Path

Posterior @ 0.4

Posterior @ 0.4

Posterior Graphic. Threshold Marked @ 0.4

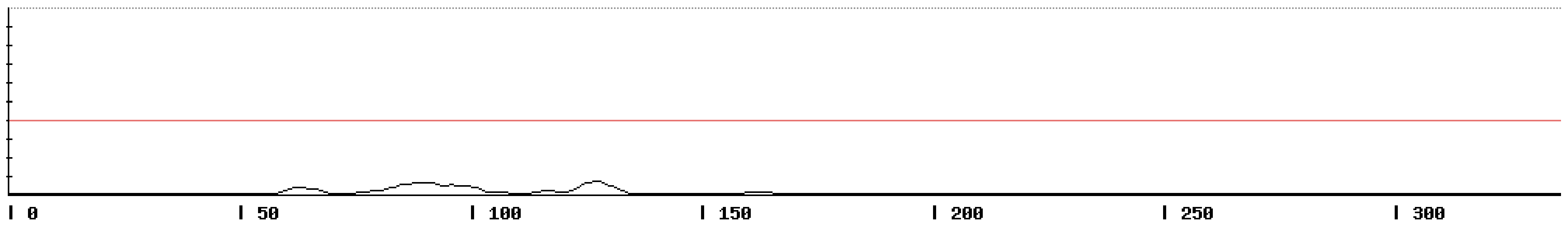

Predictions for Fragariavesca_mrna31149.1-v1.0-hybrid

Viterbi Path

Viterbi Path

Posterior @ 0.4

Posterior @ 0.4

Posterior Graphic. Threshold Marked @ 0.4

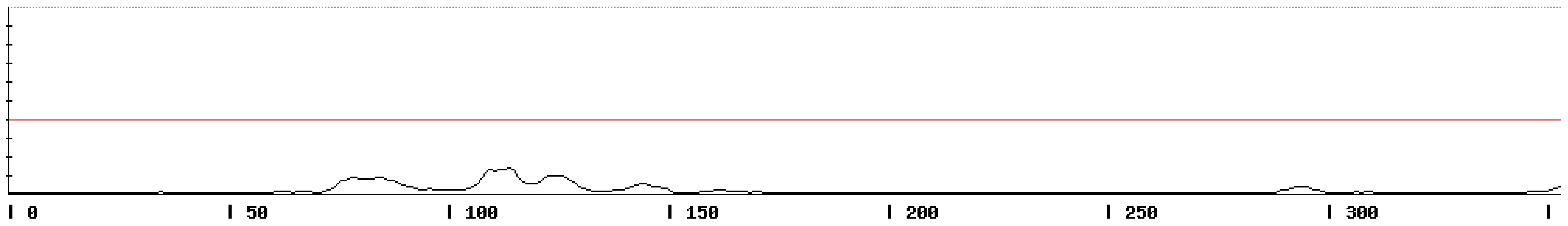

Predictions for Fragariavesca_mrna31150.1-v1.0-hybrid

Viterbi Path

Viterbi Path

Posterior @ 0.4

Posterior @ 0.4

Posterior Graphic. Threshold Marked @ 0.4

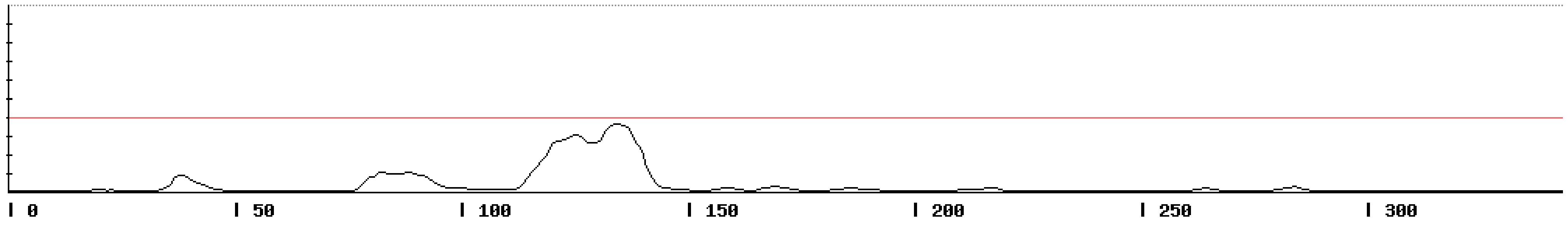

Predictions for Fragariavesca_mrna31175.1-v1.0-hybrid

Viterbi Path

Viterbi Path

Posterior @ 0.4

Posterior @ 0.4

Posterior Graphic. Threshold Marked @ 0.4

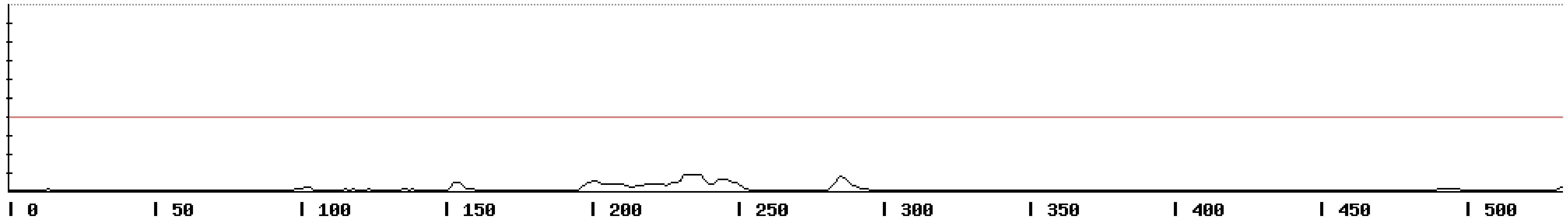

Predictions for Fragariavesca_mrna31367.1-v1.0-hybrid

Viterbi Path

Viterbi Path

Posterior @ 0.4 — 80 - KTPRARGKRI - 89

Posterior @ 0.4

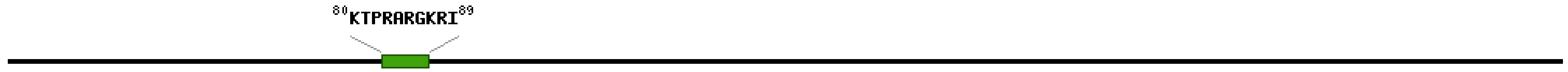

Posterior Graphic. Threshold Marked @ 0.4

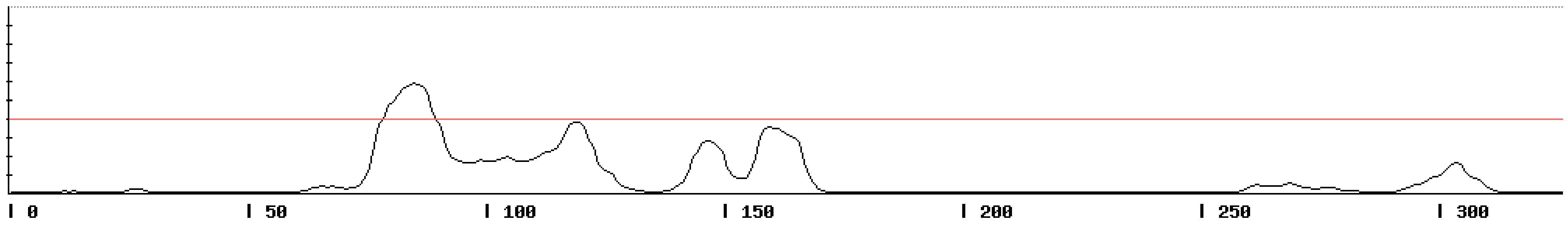

Predictions for Fragariavesca_mrna31474.1-v1.0-hybrid

Viterbi Path

Viterbi Path

Posterior @ 0.4

Posterior @ 0.4

Posterior Graphic. Threshold Marked @ 0.4

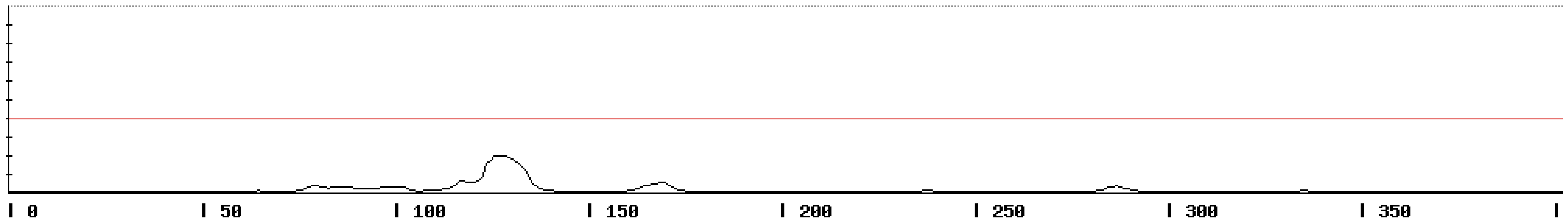

Predictions for Fragariavesca_mrna31658.1-v1.0-hybrid	
Viterbi Path	
Viterbi Path	
Posterior @ 0.4	
Posterior @ 0.4	
Posterior Graphic. Threshold Marked @ 0.4	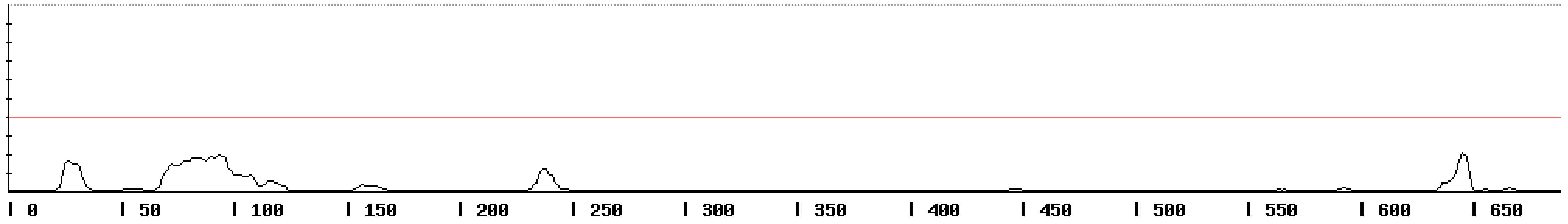

Predictions for Fragariavesca_mrna31798.1-v1.0-hybrid	
Viterbi Path	
Viterbi Path	
Posterior @ 0.4	
Posterior @ 0.4	
Posterior Graphic. Threshold Marked @ 0.4	

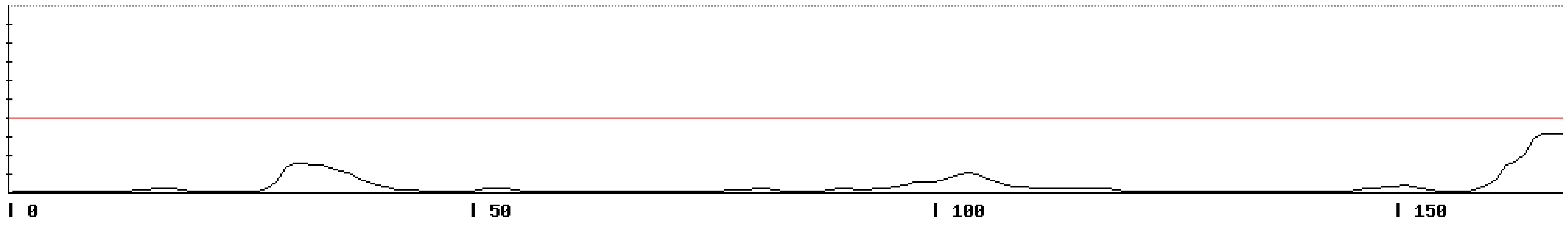

Predictions for Fragariavesca_mrna32254.1-v1.0-hybrid	
Viterbi Path	
Viterbi Path	
Posterior @ 0.4	
Posterior @ 0.4	
Posterior Graphic. Threshold Marked @ 0.4	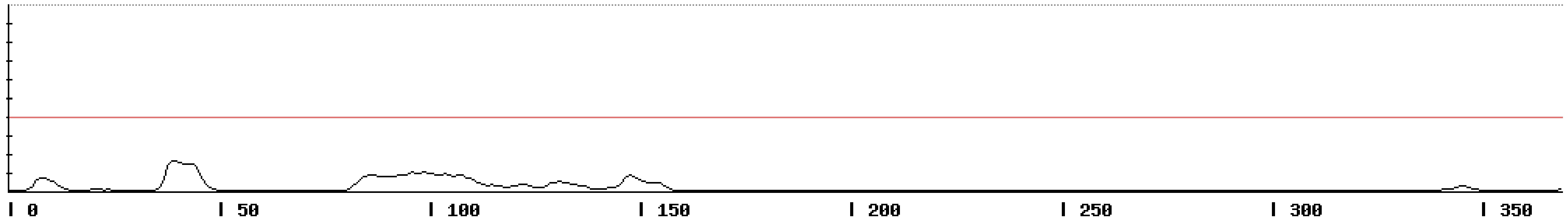

Predictions for Fragariavesca_mrna34004.1-v1.0-hybrid	
Viterbi Path	
Viterbi Path	
Posterior @ 0.4	
Posterior @ 0.4	

Posterior Graphic. Threshold Marked @ 0.4

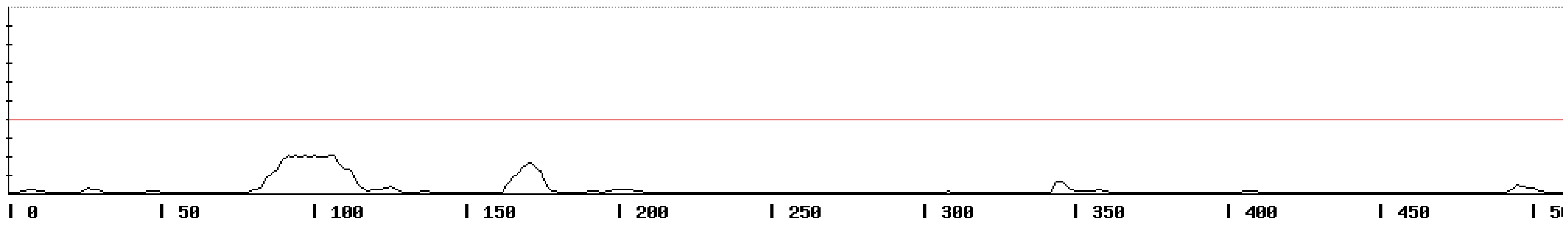

Predictions for Fragariavesca_mrna34005.1-v1.0-hybrid

Viterbi Path

638 - RSNRSTKKGFWKITGKERGIKARRSKAVIGKKRTLTFYQGRGEAKKKK - 685

Viterbi Path

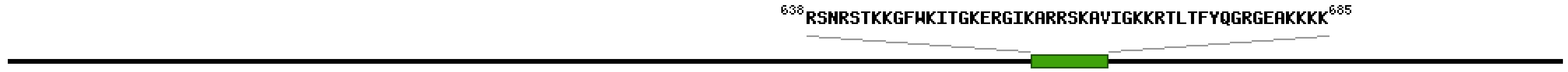

Posterior @ 0.4

148 - HRRRRRRGGRIDRGWSKGG - 165

640 - NRSTKKGFWKITGKERGIKARRSKAVIGKKRTLTFYQGRGEAKKKK - 685

Posterior @ 0.4

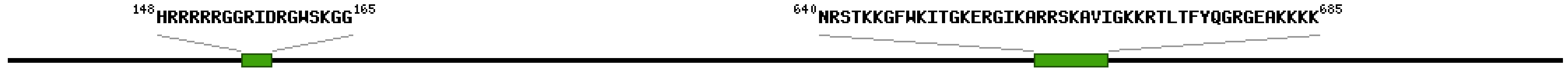

Posterior Graphic. Threshold Marked @ 0.4

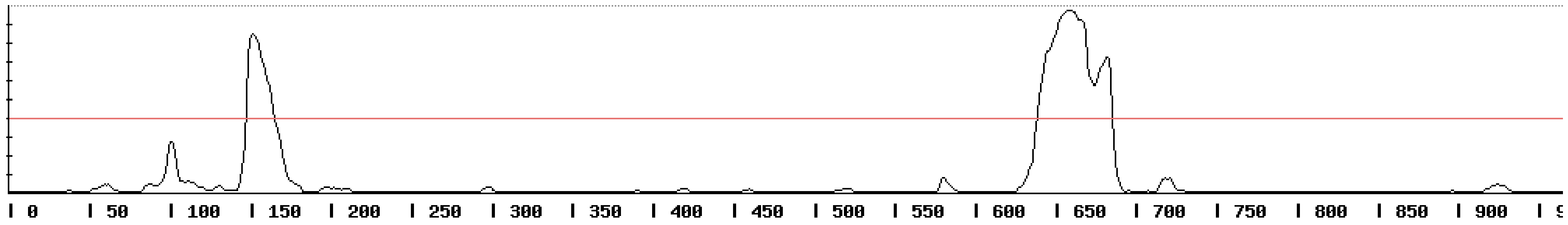

Predictions for Fragariavesca_mrna34006.1-v1.0-hybrid

Viterbi Path

Viterbi Path

Posterior @ 0.4

173 - RLKKKSGK - 180

Posterior @ 0.4

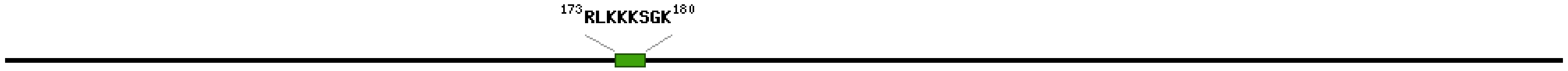

Posterior Graphic. Threshold Marked @ 0.4

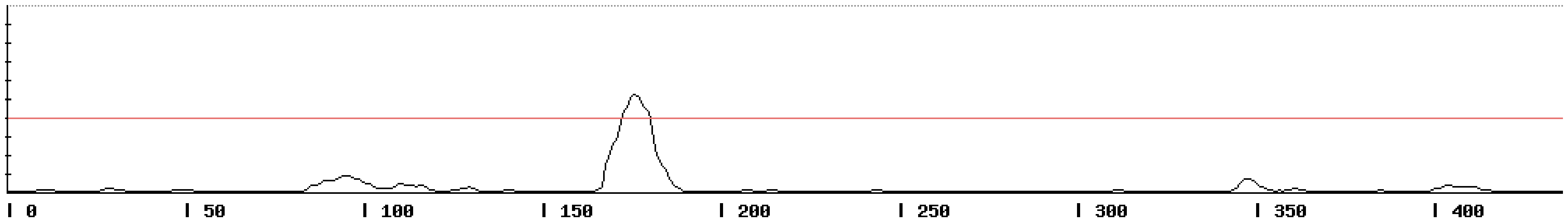

Predictions for Fragariavesca_mrna35011.1-v1.0-hybrid

Viterbi Path: 79 - KSRRSNRSTKKGFWKITGKERGIKARRSKAVIGKKRTLTFYQGRGKAKKK - 128

Viterbi Path

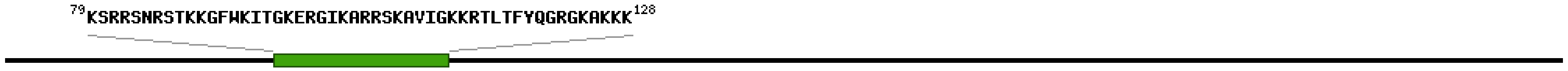

Posterior @ 0.4: 78 - NKSRRSNRSTKKGFWKITGKERGIKARRSKAVIGKKRTLTFYQGRGKAKKK - 128

Posterior @ 0.4

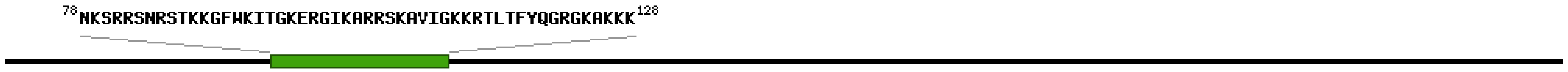

Posterior Graphic. Threshold Marked @ 0.4

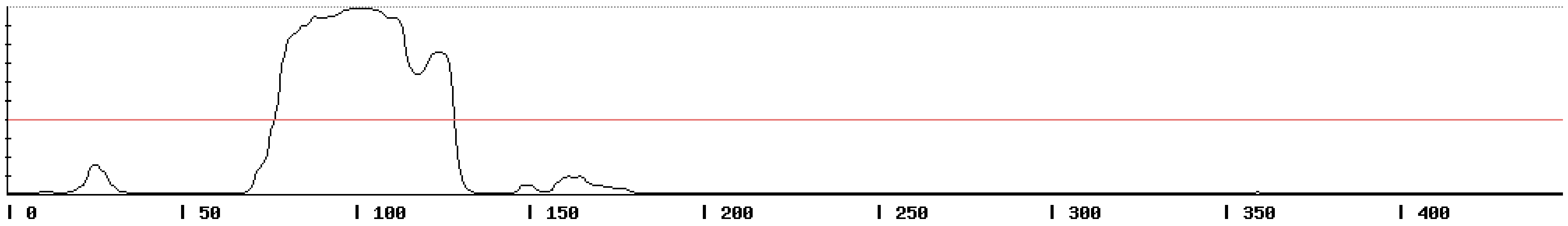

Predictions for Fragariavesca_mrna35012.1-v1.0-hybrid

Viterbi Path

Viterbi Path

Posterior @ 0.4

Posterior @ 0.4

Posterior Graphic. Threshold Marked @ 0.4

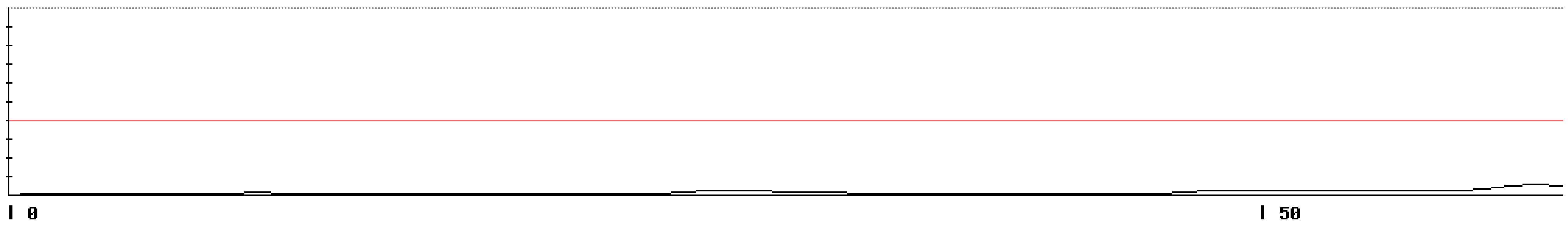

Predictions for Fragariavesca_mrna35013.1-v1.0-hybrid

Viterbi Path

Viterbi Path

Posterior @ 0.4

Posterior @ 0.4

Posterior Graphic. Threshold Marked @ 0.4

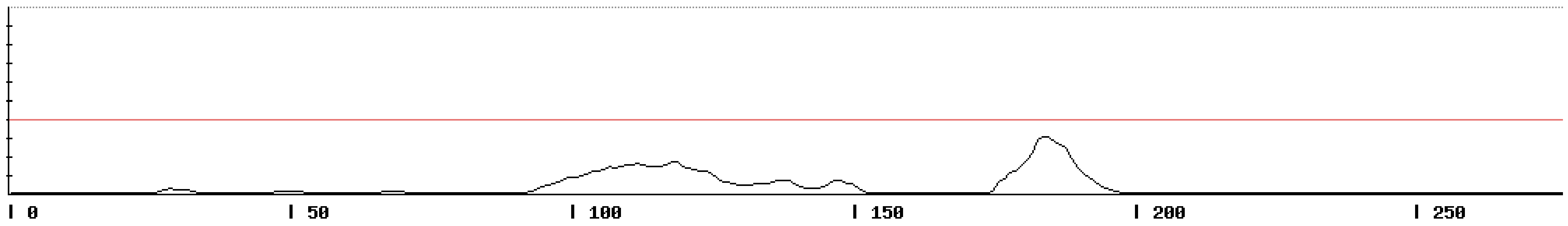

Predictions for Fragariavesca_mrna35014.1-v1.0-hybrid

Viterbi Path

Viterbi Path

Posterior @ 0.4	174 - KKK - 176
Posterior @ 0.4	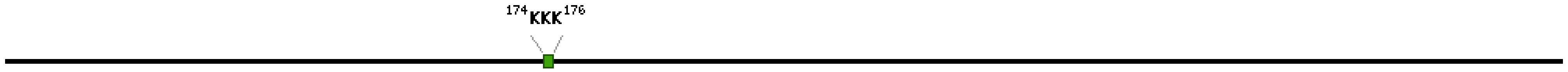
Posterior Graphic. Threshold Marked @ 0.4	

Revision r.9 - Last Updated on : February 24 2015 17:45:20.

alex.nguyenba@utoronto.ca

Intended to be used with Firefox

Viterbi Path	
Posterior @ 0.4	
Posterior @ 0.4	
Posterior Graphic. Threshold Marked @ 0.4	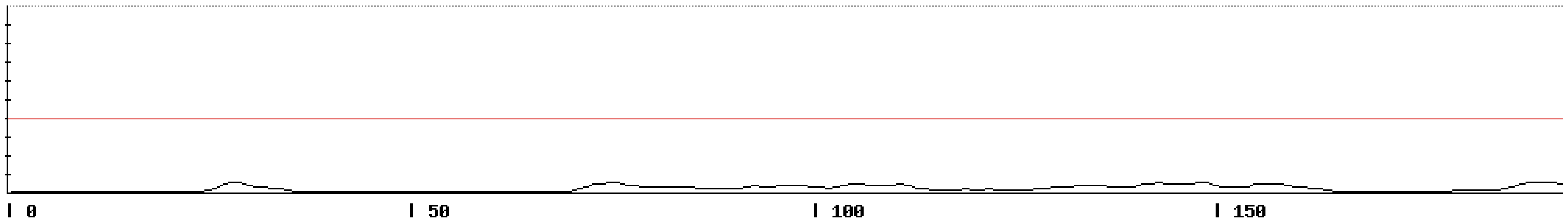

Predictions for Fragariaxananassa_FANhyb_icon00000825_a.1.g00001.1

Viterbi Path	
Viterbi Path	
Posterior @ 0.4	79 - GRRPN - 83<br>146 - PKRKKGKGN - 154
Posterior @ 0.4	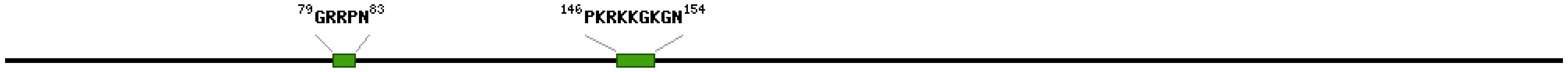
Posterior Graphic. Threshold Marked @ 0.4	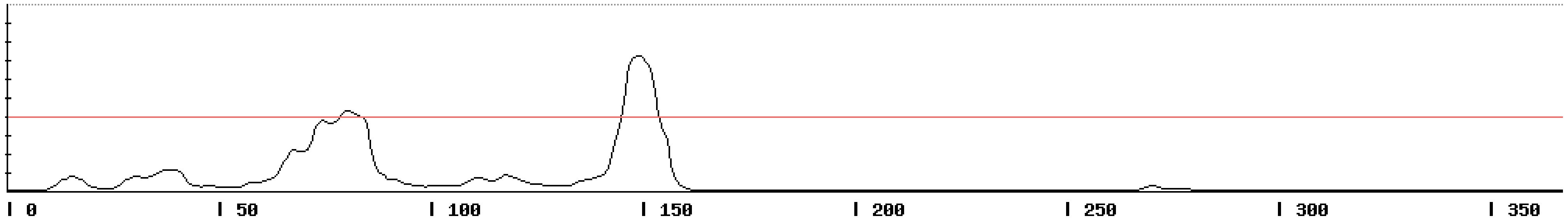

Predictions for Fragariaxananassa_FANhyb_icon00001173_a.1.g00001.1

Viterbi Path

Viterbi Path

Posterior @ 0.4

Posterior @ 0.4

Posterior Graphic. Threshold Marked @ 0.4

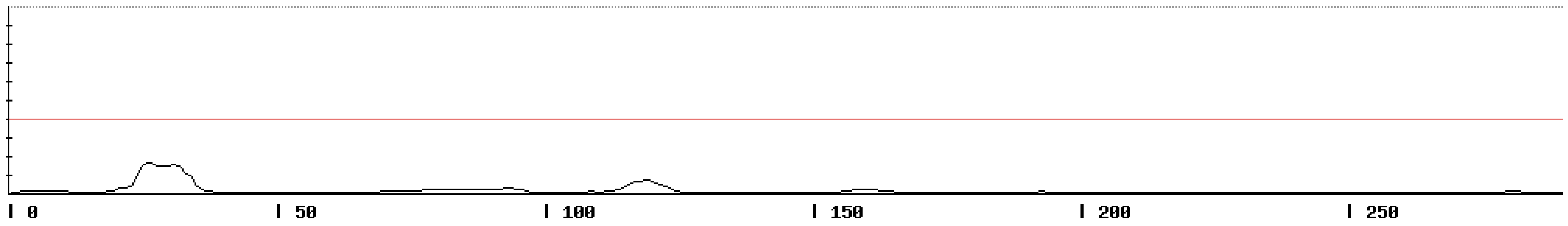

Predictions for Fragariaxananassa_FANhyb_icon00001436_a.1.g00001.1

Viterbi Path

Viterbi Path

Posterior @ 0.4

Posterior @ 0.4

Posterior Graphic. Threshold Marked @ 0.4

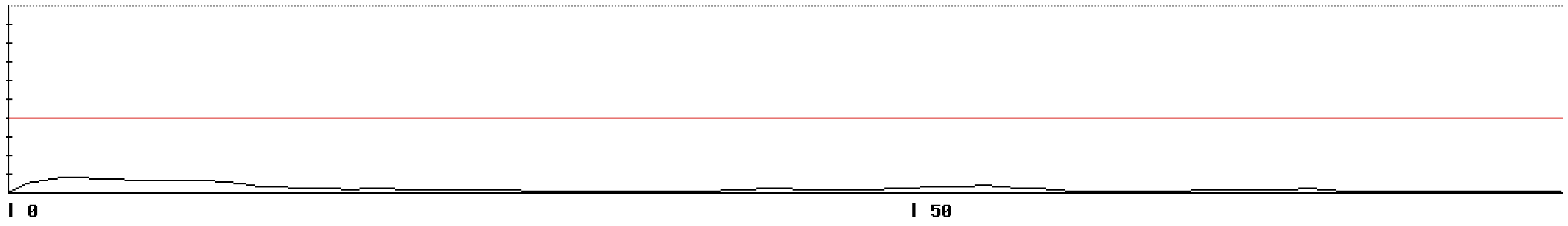

Predictions for Fragariaxananassa_FANhyb_icon00002021_a.1.g00001.1

Viterbi Path	298 - RKPKRIGKKRKLR - 310<br>357 - PKEKKRKRQQIDQLEDKKPKRKK - 379
Viterbi Path	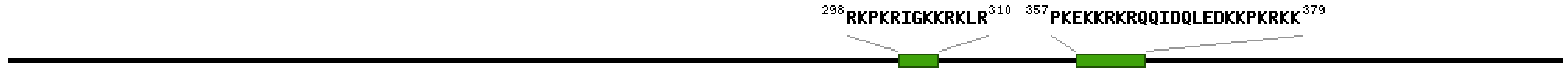
Posterior @ 0.4	297 - ERKPKRIGKKRKLR - 310<br>357 - PKEKKRKRQQIDQLEDKKPKRKKVV - 381
Posterior @ 0.4	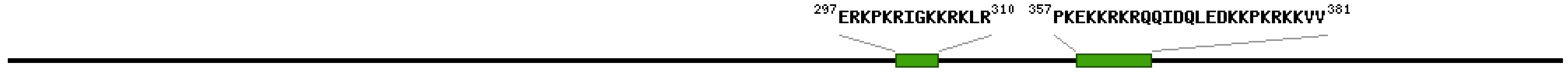
Posterior Graphic. Threshold Marked @ 0.4	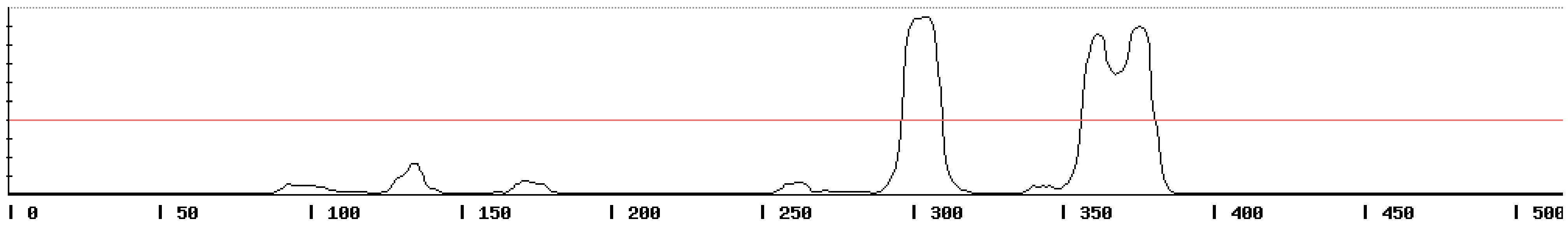

Predictions for Fragariaxananassa_FANhyb_icon00002805_a.1.g00001.1

Viterbi Path	
Viterbi Path	
Posterior @ 0.4	108 - PGKRPSPPPLSKPSDNKRKCQADPKPSKKTK - 138
Posterior @ 0.4	

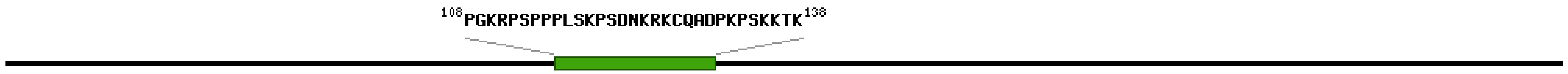

Posterior Graphic. Threshold Marked @ 0.4

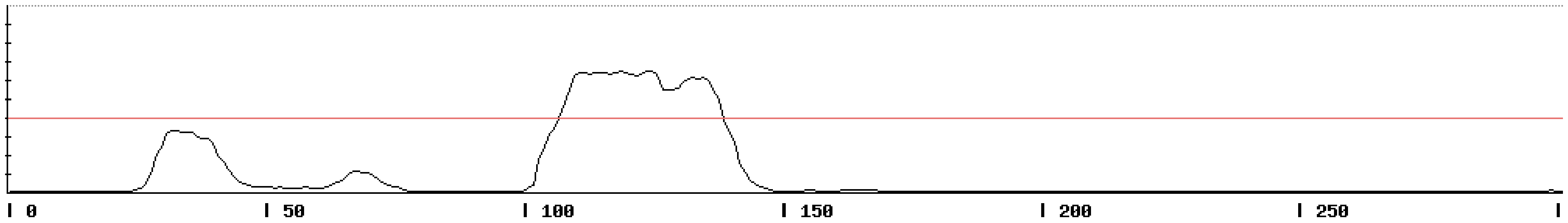

Predictions for Fragariaxananassa_FANhyb_icon00003337_a.1.g00001.1

Viterbi Path

Viterbi Path

Posterior @ 0.4

Posterior @ 0.4

Posterior Graphic. Threshold Marked @ 0.4

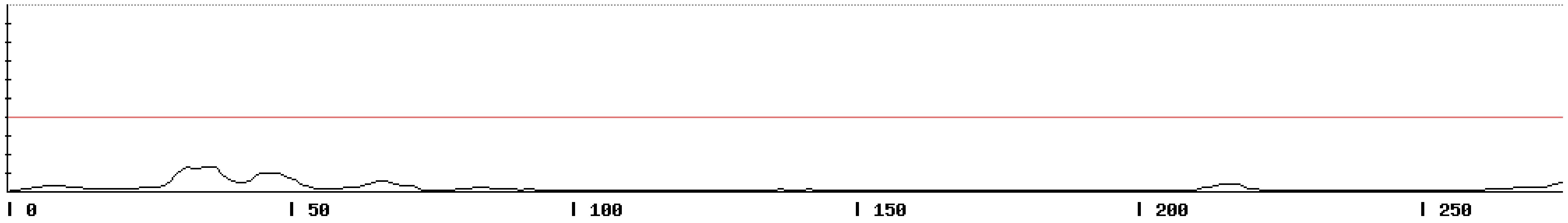

Predictions for Fragariaxananassa_FANhyb_icon00003954_a.1.g00001.1

Viterbi Path

Viterbi Path

Posterior @ 0.4

Posterior @ 0.4

Posterior Graphic. Threshold Marked @ 0.4

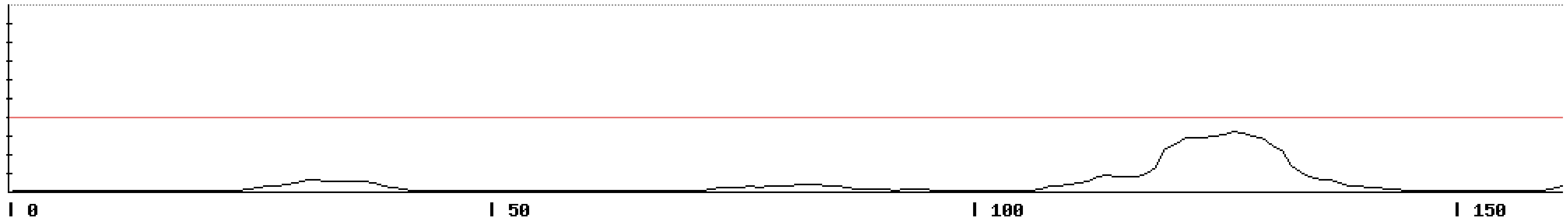

Predictions for Fragariaxananassa_FANhyb_icon00004712_a.1.g00001.1

Viterbi Path: 176 - KKNSVKKRKSASFVDHQSRKRKKKLK - 201

Viterbi Path

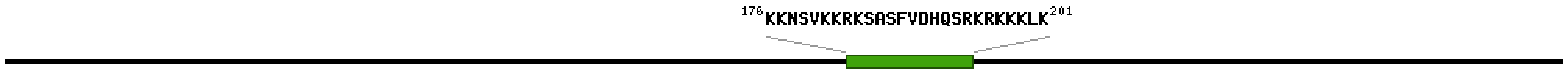

Posterior @ 0.4: 176 - KKNSVKKRKSASFVDHQSRKRKKKLK - 201

Posterior @ 0.4

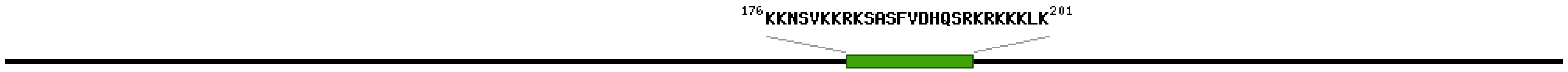

Posterior Graphic. Threshold Marked @ 0.4

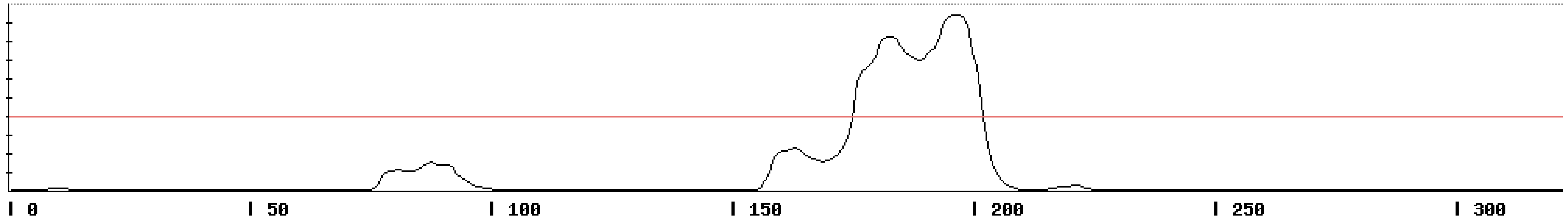

Predictions for Fragariaxananassa_FANhyb_icon00005788_a.1.g00001.1

Viterbi Path

Viterbi Path

Posterior @ 0.4	14 - NIRKGRKRKE - 23
	136 - EAKQKHIGRRRKLR - 149

Posterior @ 0.4

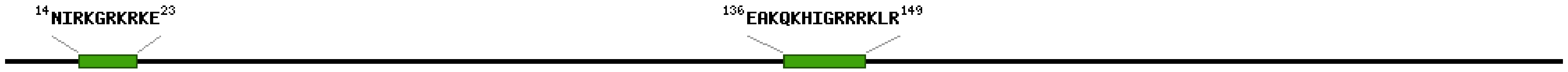

Posterior Graphic. Threshold Marked @ 0.4

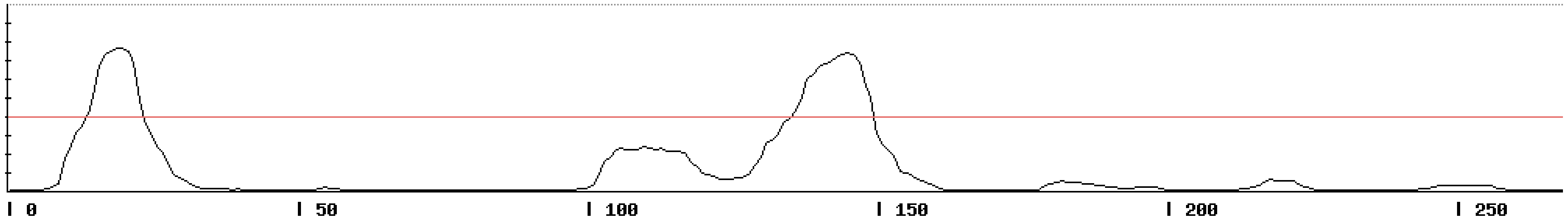

Predictions for Fragariaxananassa_FANhyb_icon00006293_a.1.g00001.1

Viterbi Path

Viterbi Path

Posterior @ 0.4 — 75 - RKKLNPKAKRFHRKV - 89

Posterior @ 0.4

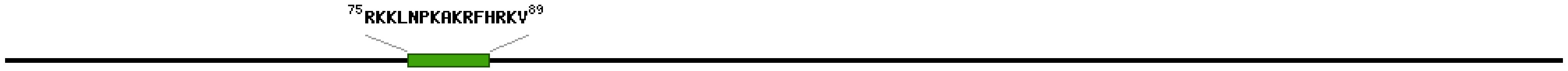

Posterior Graphic. Threshold Marked @ 0.4

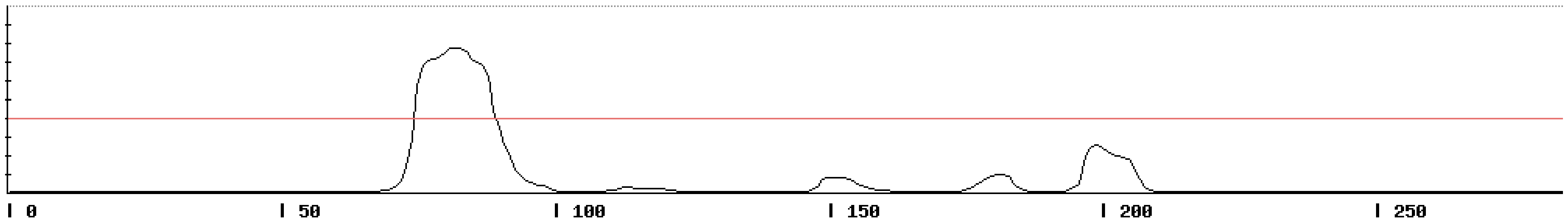

Predictions for Fragariaxananassa_FANhyb_icon00006922_a.1.g00001.1

Viterbi Path

Viterbi Path

Posterior @ 0.4: 136 - RKNGEPKTKIAKKRKQR - 152

Posterior @ 0.4

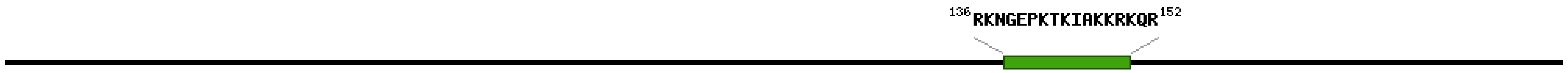

Posterior Graphic. Threshold Marked @ 0.4

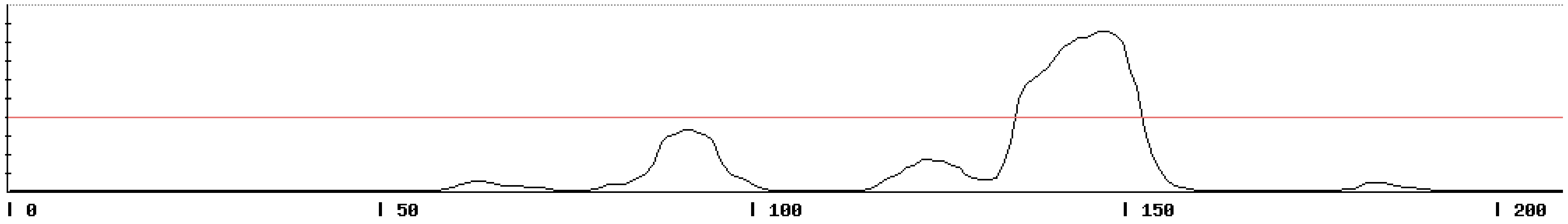

Predictions for Fragariaxananassa_FANhyb_icon00007937_a.1.g00001.1

Viterbi Path

Viterbi Path

Posterior @ 0.4

Posterior @ 0.4

Posterior Graphic. Threshold Marked @ 0.4

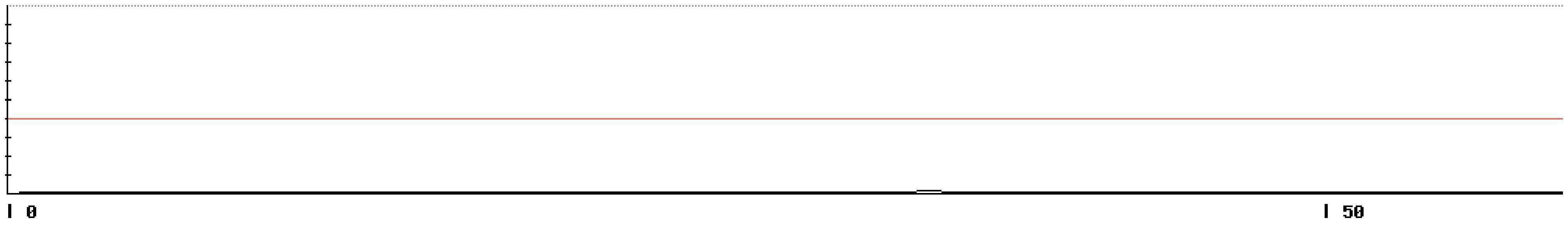

Predictions for Fragariaxananassa_FANhyb_icon00009395_a.1.g00001.1

Viterbi Path

Viterbi Path

Posterior @ 0.4

Posterior @ 0.4

Posterior Graphic. Threshold Marked @ 0.4

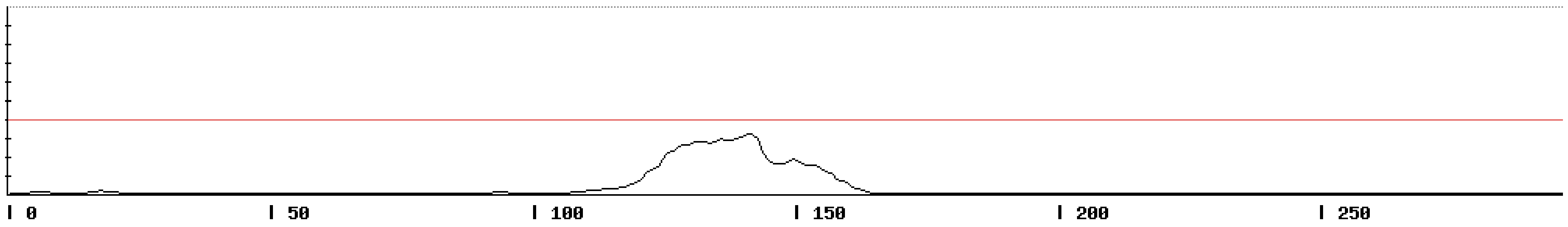

Predictions for Fragariaxananassa_FANhyb_icon00009606_a.1.g00001.1

Viterbi Path

Viterbi Path

Posterior @ 0.4

Posterior @ 0.4

Posterior Graphic. Threshold Marked @ 0.4

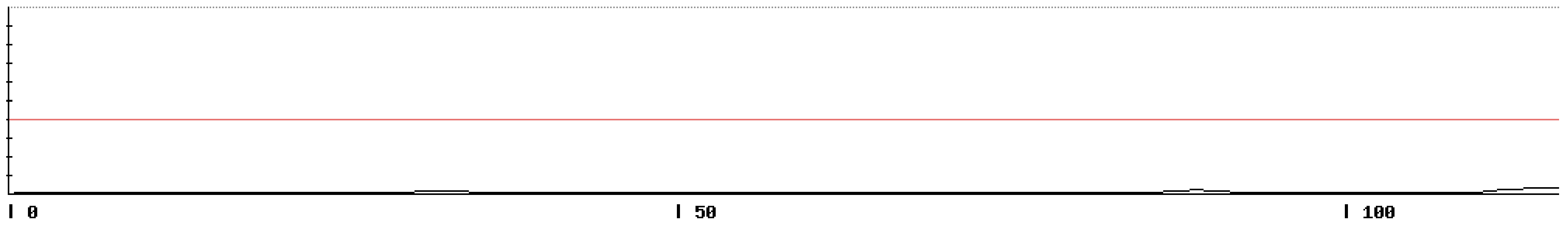

Predictions for Fragariaxananassa_FANhyb_icon00010638_a.1.g00001.1

Viterbi Path

Viterbi Path

Posterior @ 0.4

Posterior @ 0.4

Posterior Graphic. Threshold Marked @ 0.4

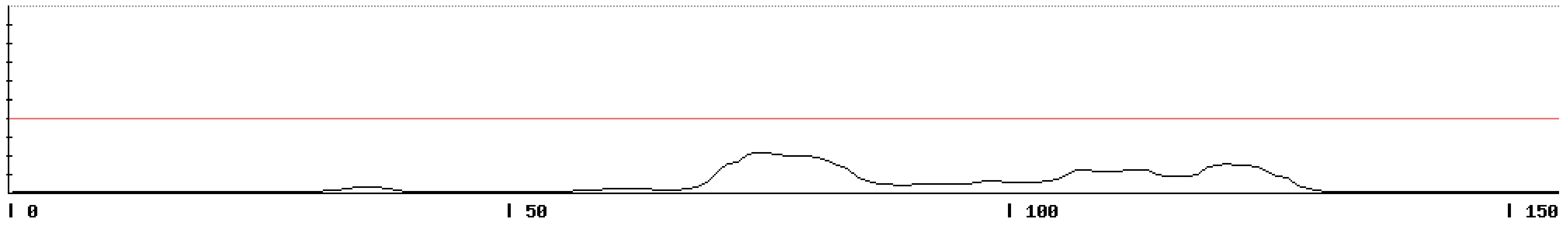

Predictions for Fragariaxananassa_FANhyb_icon00013874_a.1.g00001.1

Viterbi Path

Viterbi Path

Posterior @ 0.4

Posterior @ 0.4

Posterior Graphic. Threshold Marked @ 0.4

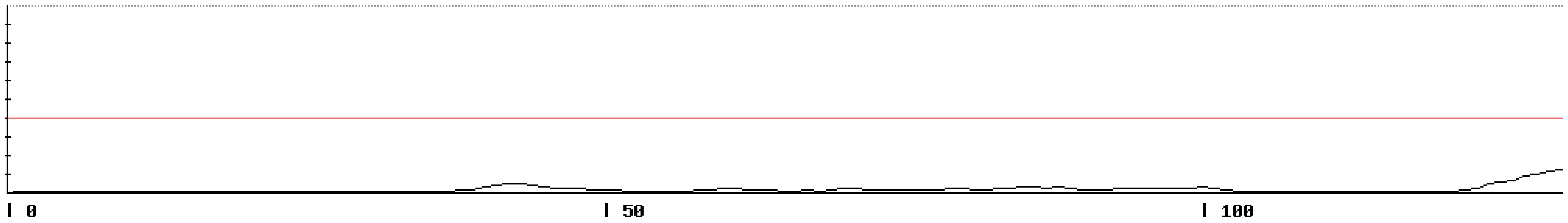

Predictions for Fragariaxananassa_FANhyb_icon00013945_a.1.g00001.1

Viterbi Path

Viterbi Path

Posterior @ 0.4

Posterior @ 0.4

Posterior Graphic. Threshold Marked @ 0.4

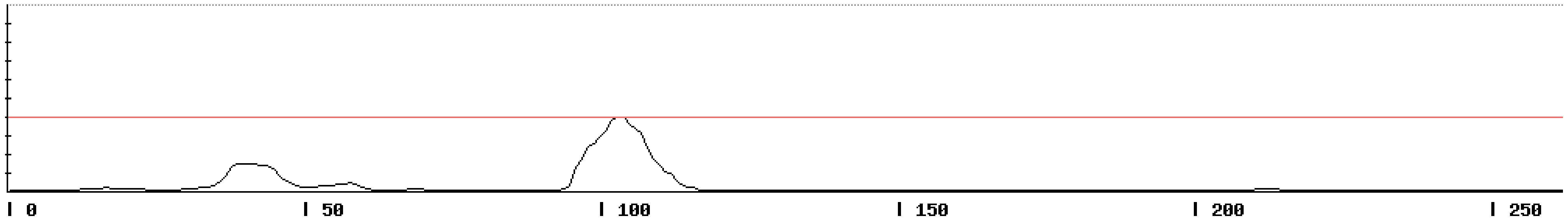

Predictions for Fragariaxananassa_FANhyb_icon00016851_a.1.g00001.1

Viterbi Path

Viterbi Path

Posterior @ 0.4	
Posterior @ 0.4	
Posterior Graphic. Threshold Marked @ 0.4	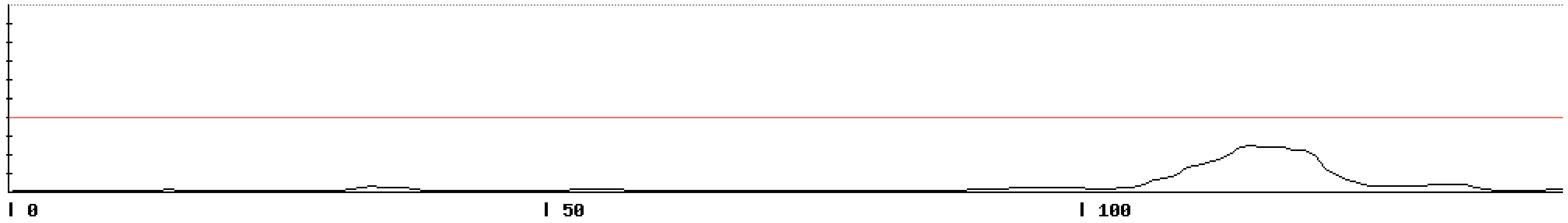

Predictions for Fragariaxananassa_FANhyb_icon00018154_a.1.g00001.1

Viterbi Path	
Viterbi Path	
Posterior @ 0.4	
Posterior @ 0.4	
Posterior Graphic. Threshold Marked @ 0.4	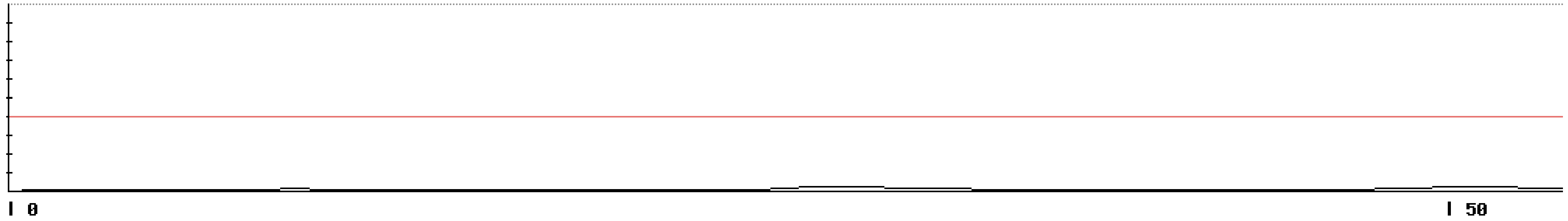

Predictions for Fragariaxananassa_FANhyb_icon00020054_a.1.g00001.1

Viterbi Path	79 - KSRRSNRSTKKGFWKITGKERGIKARRSKAVIGKKRTLTFYQGRGEAKKKKP - 130
Viterbi Path	

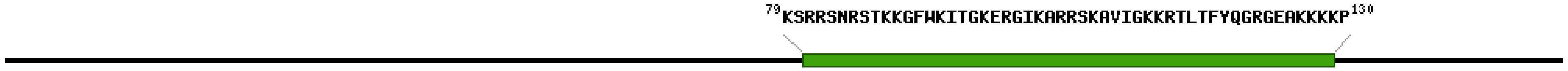

Posterior @ 0.4	77 - YNKSRRSNRSTKKGFWKITGKERGIKARRSKAVIGKKRTLTFYQGRGEAKKKKPA - 131

Posterior @ 0.4

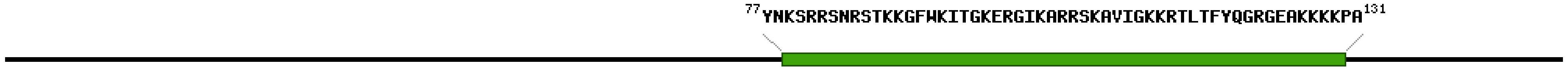

Posterior Graphic. Threshold Marked @ 0.4

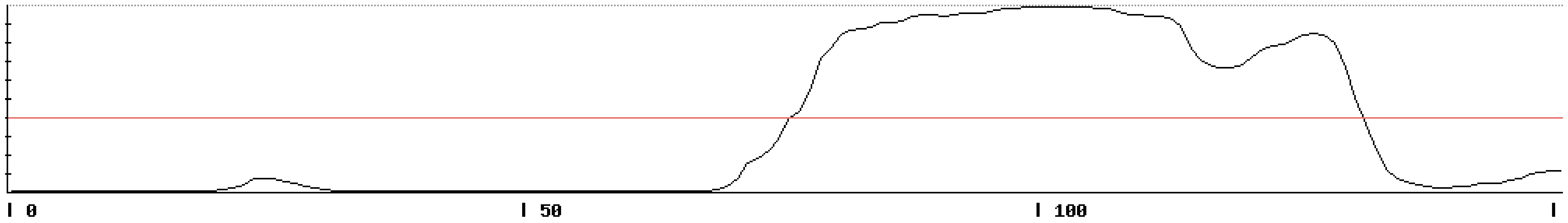

Predictions for Fragariaxananassa_FANhyb_icon00021436_a.1.g00001.1

Viterbi Path

Viterbi Path

Posterior @ 0.4

Posterior @ 0.4

Posterior Graphic. Threshold Marked @ 0.4

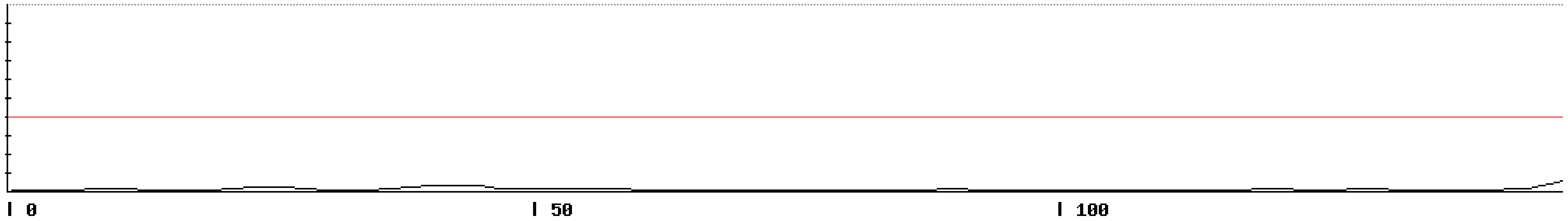

Predictions for Fragariaxananassa_FANhyb_icon00024708_a.1.g00001.1

Viterbi Path

Viterbi Path

Posterior @ 0.4

Posterior @ 0.4

Posterior Graphic. Threshold Marked @ 0.4

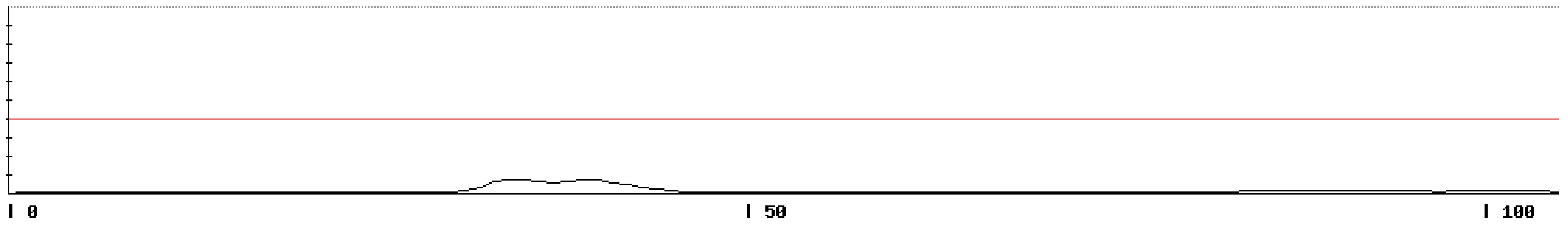

Predictions for Fragariaxananassa_FANhyb_icon00025050_a.1.g00001.1

Viterbi Path

Viterbi Path

Posterior @ 0.4

88 - KRLSAKGKGKR - 98

171 - KRNGKSNNKKRKA - 183

Posterior @ 0.4

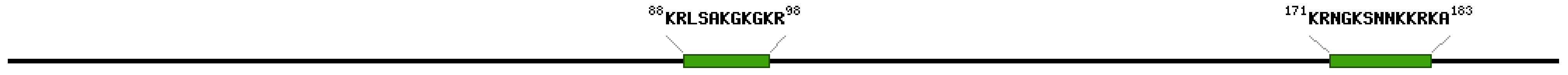

Posterior Graphic. Threshold Marked @ 0.4

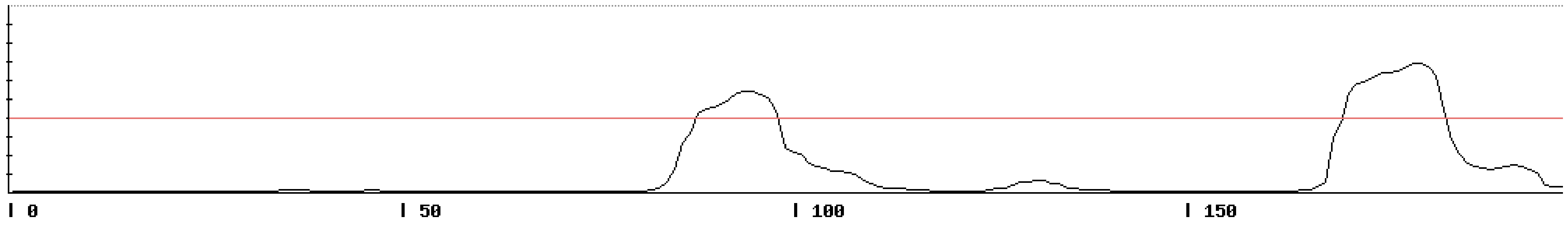

Predictions for Fragariaxananassa_FANhyb_icon00027555_a.1.g00001.1

Viterbi Path

Viterbi Path

Posterior @ 0.4

Posterior @ 0.4

Posterior Graphic. Threshold Marked @ 0.4

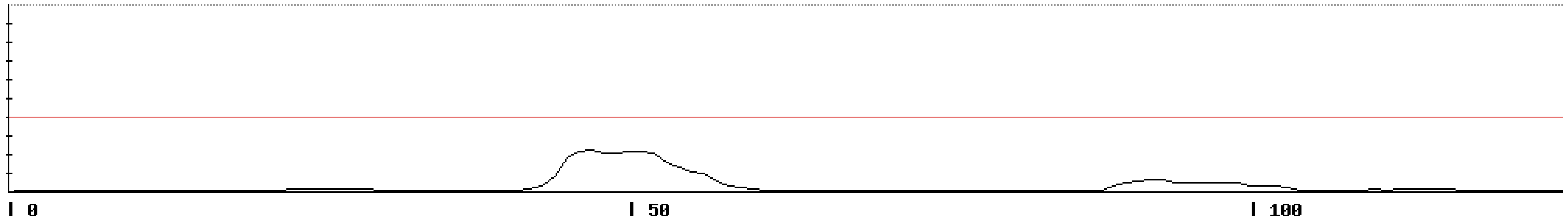

Predictions for Fragariaxananassa_FANhyb_icon00029204_a.1.g00001.1

Viterbi Path

Viterbi Path

Posterior @ 0.4 109 - RLKKKSGK - 116

Posterior @ 0.4

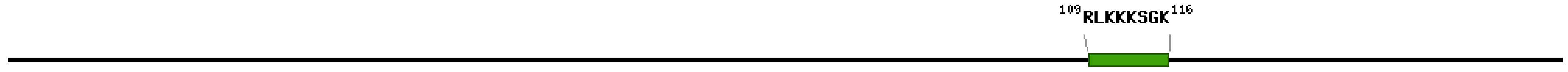

Posterior Graphic. Threshold Marked @ 0.4	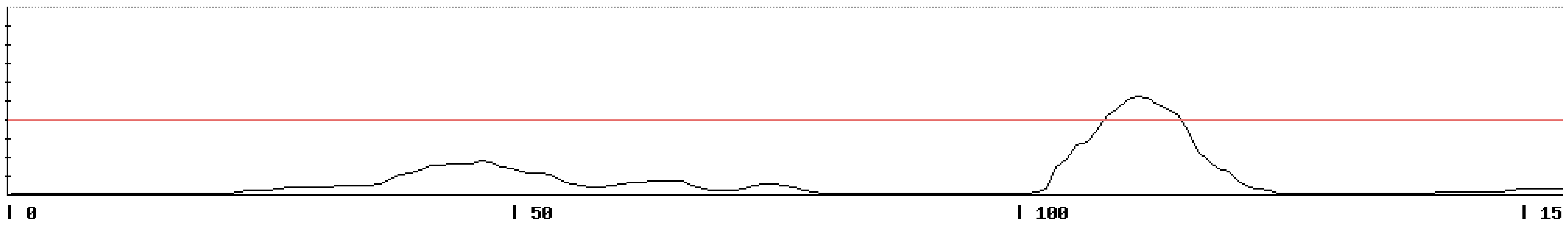

Predictions for Fragariaxananassa_FANhyb_icon00034378_a.1.g00001.1

Viterbi Path	
Viterbi Path	
Posterior @ 0.4	
Posterior @ 0.4	
Posterior Graphic. Threshold Marked @ 0.4	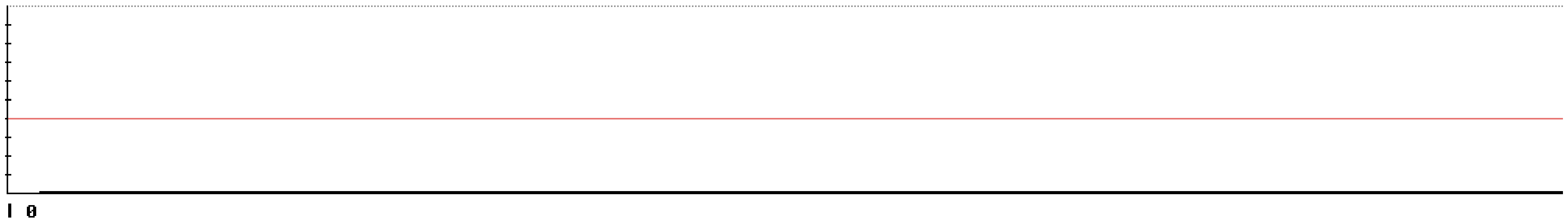

Predictions for Fragariaxananassa_FANhyb_icon00039646_a.1.g00001.1

Viterbi Path	
Viterbi Path	
Posterior @ 0.4	
Posterior @ 0.4	

Posterior Graphic. Threshold Marked @ 0.4

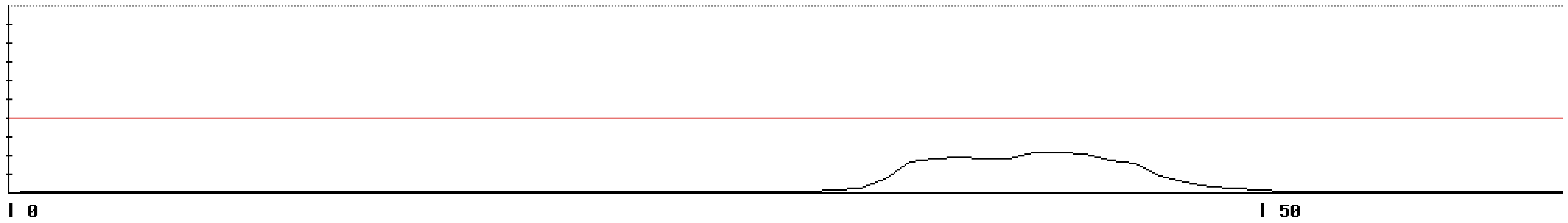

Predictions for Fragariaxananassa_FANhyb_icon00043296_a.1.g00001.1

Viterbi Path

Viterbi Path

Posterior @ 0.4

Posterior @ 0.4

Posterior Graphic. Threshold Marked @ 0.4

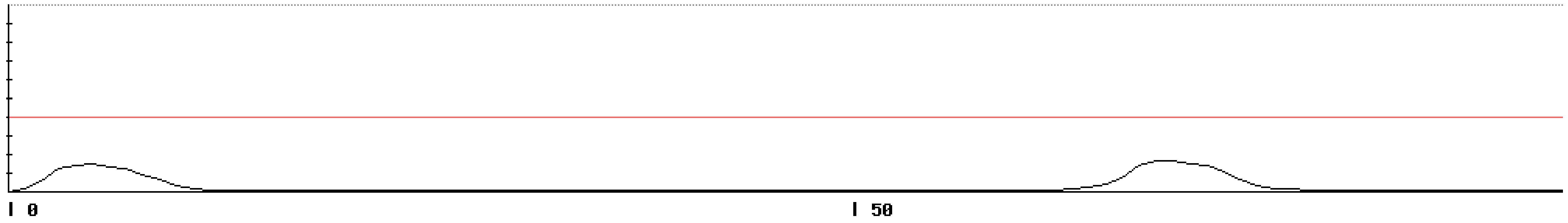

Predictions for Fragariaxananassa_FANhyb_icon00048378_a.1.g00001.1

Viterbi Path

Viterbi Path

Posterior @ 0.4	
Posterior @ 0.4	
Posterior Graphic. Threshold Marked @ 0.4	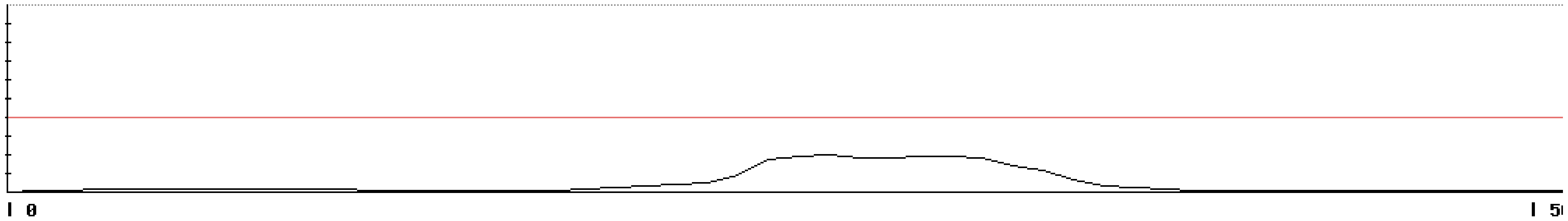

Predictions for Fragariaxananassa_FANhyb_icon16706045_s.1.g00001.1

Viterbi Path	
Viterbi Path	
Posterior @ 0.4	21 - NSKRKSKSKSTKI - 33
Posterior @ 0.4	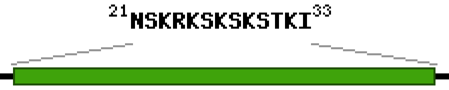
Posterior Graphic. Threshold Marked @ 0.4	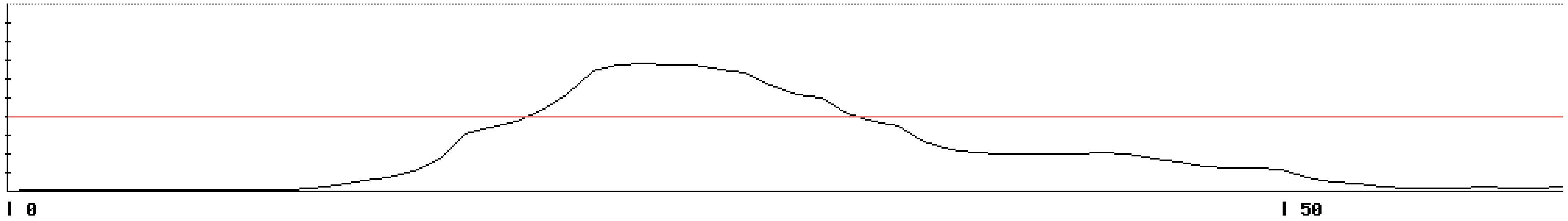

Predictions for Fragariaxananassa_FANhyb_icon18303609_s.1.g00001.1

Viterbi Path	
Viterbi Path	

Posterior @ 0.4

Posterior @ 0.4

Posterior Graphic. Threshold Marked @ 0.4

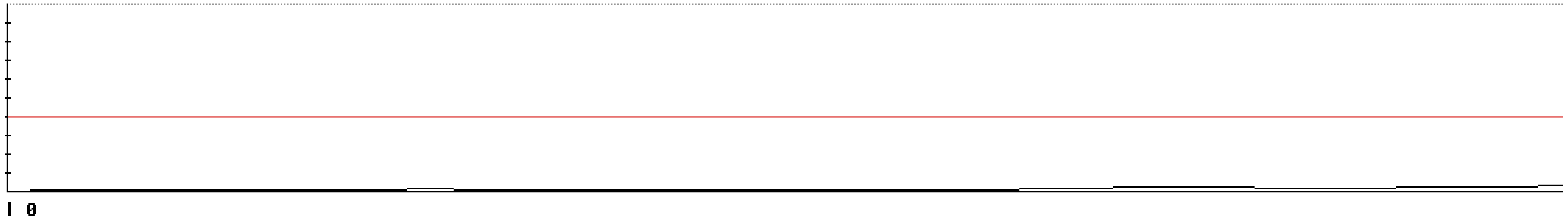

Predictions for Fragariaxananassa_FANhyb_icon19146025_s.1.g00001.1

Viterbi Path

Viterbi Path

Posterior @ 0.4

Posterior @ 0.4

Posterior Graphic. Threshold Marked @ 0.4

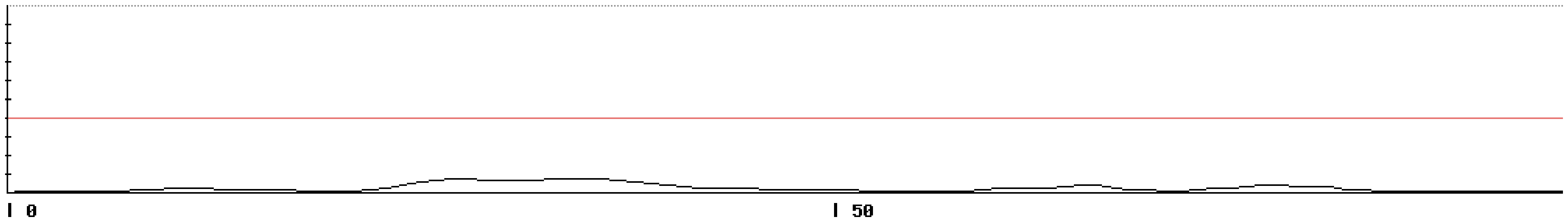

Predictions for Fragariaxananassa_FANhyb_icon19146413_o.1.g00001.1

Viterbi Path

Viterbi Path

Posterior @ 0.4

Posterior @ 0.4

Posterior Graphic. Threshold Marked @ 0.4

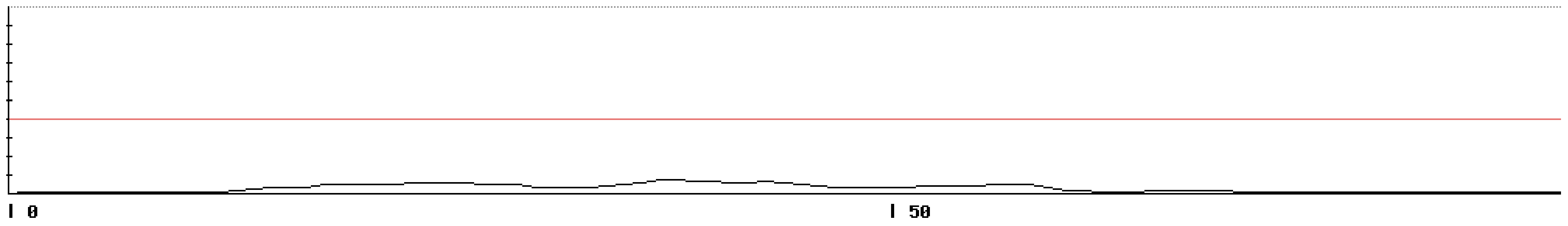

Predictions for Fragariaxananassa_FANhyb_icon19559383_s.1.g00001.1

Viterbi Path

Viterbi Path

Posterior @ 0.4

Posterior @ 0.4

Posterior Graphic. Threshold Marked @ 0.4

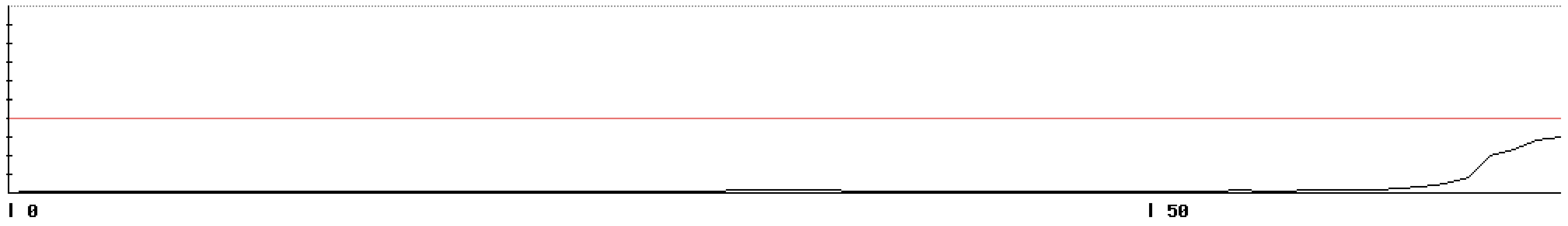

Predictions for Fragariaxananassa_FANhyb_icon19828183_s.1.g00001.1

Viterbi Path

Viterbi Path

Posterior @ 0.4

Posterior @ 0.4

Posterior Graphic. Threshold Marked @ 0.4

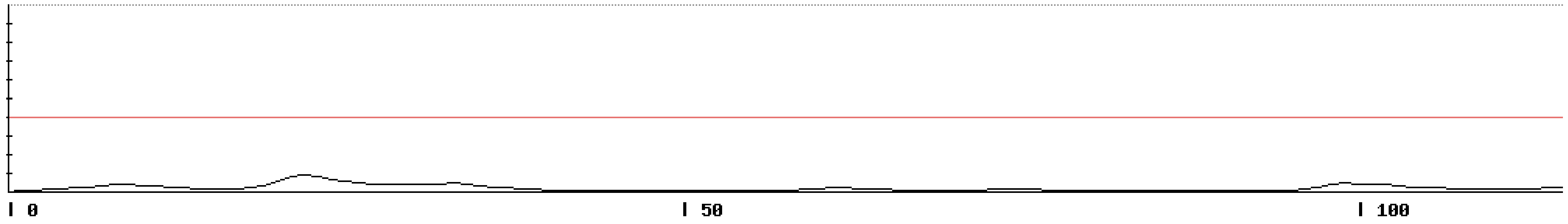

Predictions for Fragariaxananassa_FANhyb_rscf00000010.1.g00037.1

Viterbi Path

Viterbi Path

Posterior @ 0.4

Posterior @ 0.4

Posterior Graphic. Threshold Marked @ 0.4

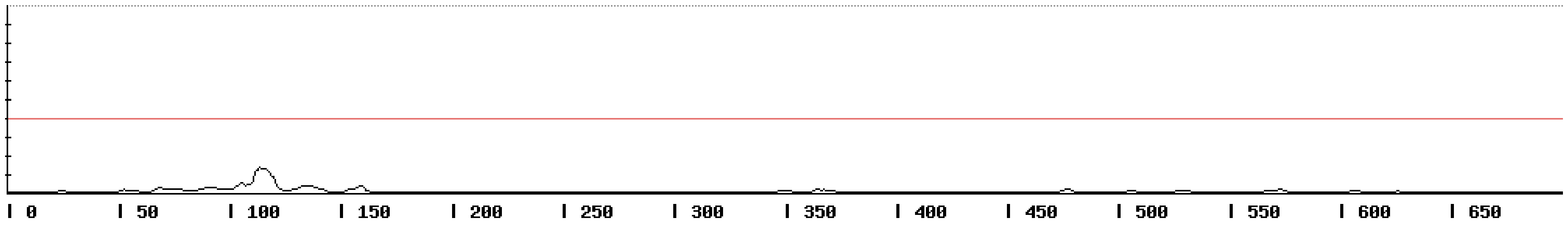

Predictions for Fragariaxananassa_FANhyb_rscf00000018.1.g00013.1

Viterbi Path	
Viterbi Path	
Posterior @ 0.4	
Posterior @ 0.4	
Posterior Graphic. Threshold Marked @ 0.4	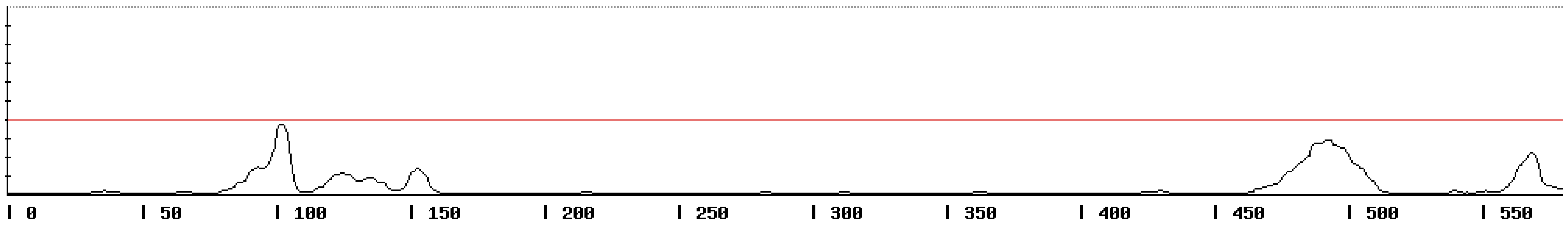

Predictions for Fragariaxananassa_FANhyb_rscf00000034.1.g00014.1

Viterbi Path	
Viterbi Path	
Posterior @ 0.4	8 - FKKKKA - 13
Posterior @ 0.4	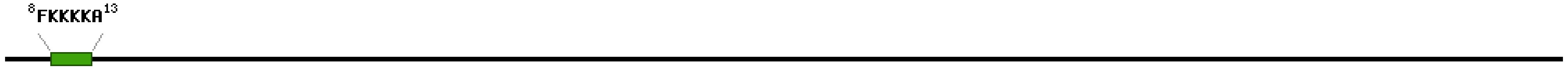

Posterior Graphic. Threshold Marked @ 0.4

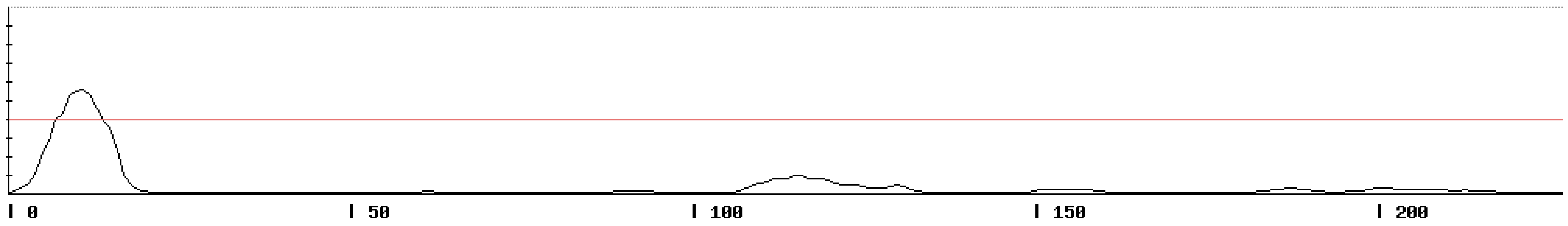

Predictions for Fragariaxananassa_FANhyb_rscf00000056.1.g00008.1

Viterbi Path

Viterbi Path

Posterior @ 0.4

Posterior @ 0.4

Posterior Graphic. Threshold Marked @ 0.4

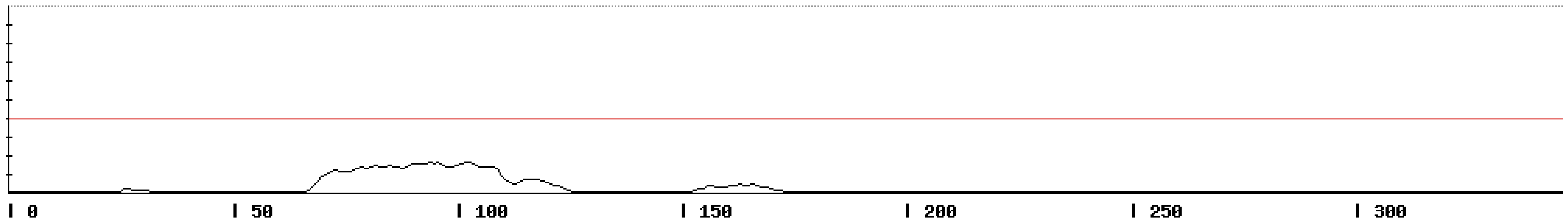

Predictions for Fragariaxananassa_FANhyb_rscf00000062.1.g00018.1

Viterbi Path

Viterbi Path

Posterior @ 0.4

Posterior @ 0.4

Posterior Graphic. Threshold Marked @ 0.4

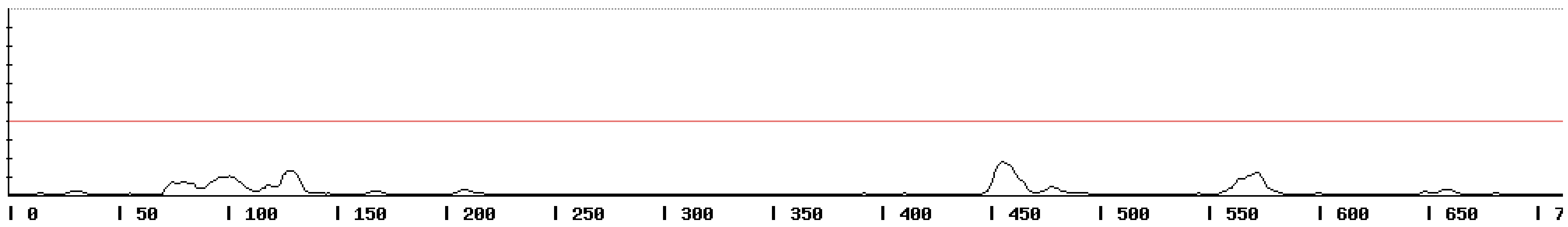

Predictions for Fragariaxananassa_FANhyb_rscf00000078.1.g00011.1

Viterbi Path

Viterbi Path

Posterior @ 0.4

Posterior @ 0.4

Posterior Graphic. Threshold Marked @ 0.4

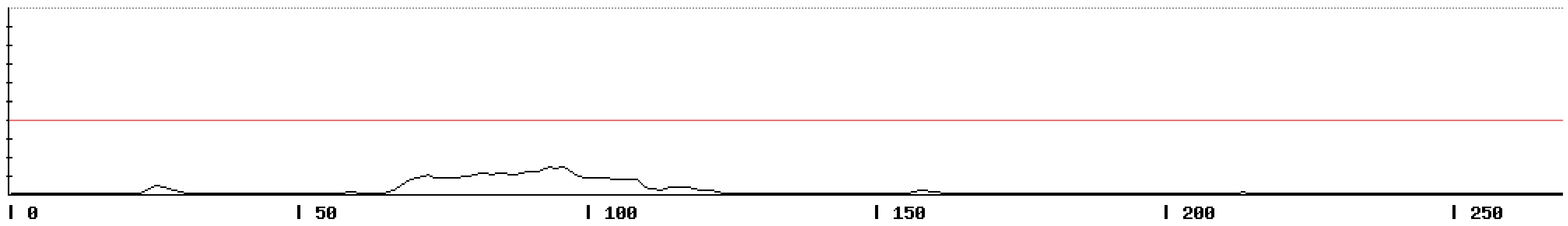

Predictions for Fragariaxananassa_FANhyb_rscf00000096.1.g00005.1

Viterbi Path

Viterbi Path

Posterior @ 0.4	72 - GRRRRKRSRDIGK - 84
	120 - RR - 121
	257 - RSGYWRRKGRVR - 268
Posterior @ 0.4	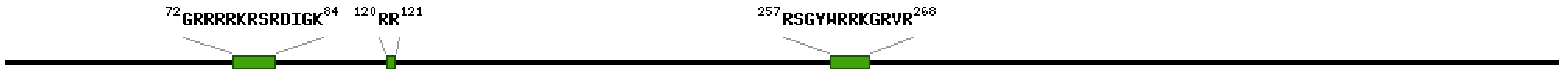
Posterior Graphic. Threshold Marked @ 0.4	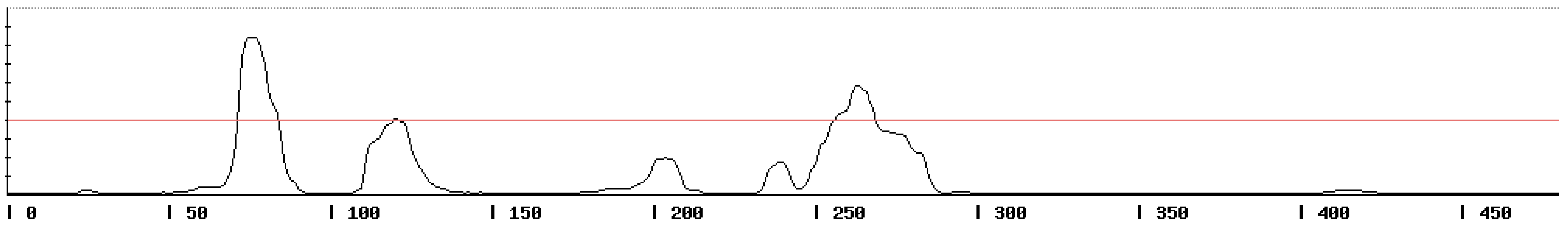

Predictions for Fragariaxananassa_FANhyb_rscf00000097.1.g00004.1

Viterbi Path	
Viterbi Path	
Posterior @ 0.4	
Posterior @ 0.4	
Posterior Graphic. Threshold Marked @ 0.4	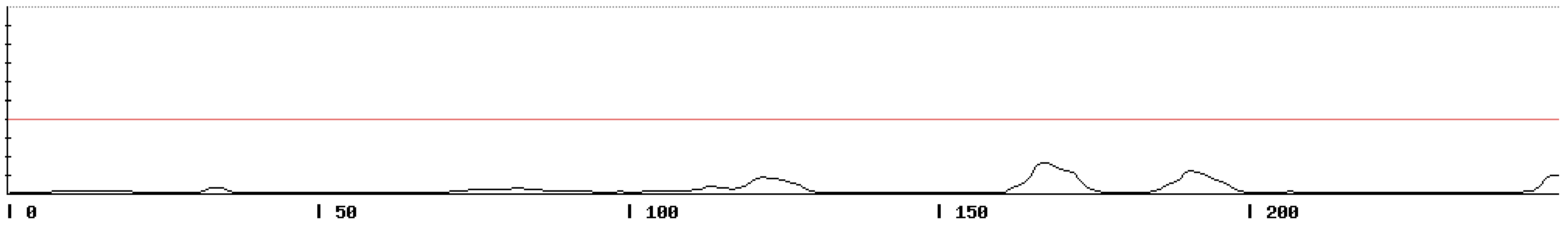

Predictions for Fragariaxananassa_FANhyb_rscf00000099.1.g00003.1

Viterbi Path	

Viterbi Path

Posterior @ 0.4

Posterior @ 0.4

Posterior Graphic. Threshold Marked @ 0.4

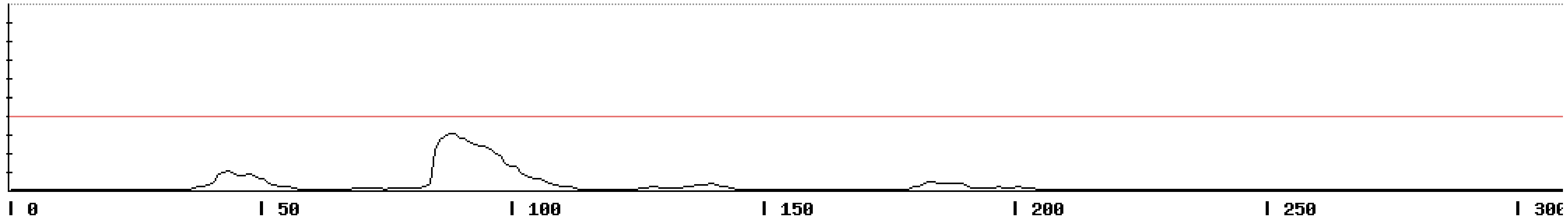

Predictions for Fragariaxananassa_FANhyb_rscf00000100.1.g00014.1

Viterbi Path

Viterbi Path

Posterior @ 0.4

Posterior @ 0.4

Posterior Graphic. Threshold Marked @ 0.4

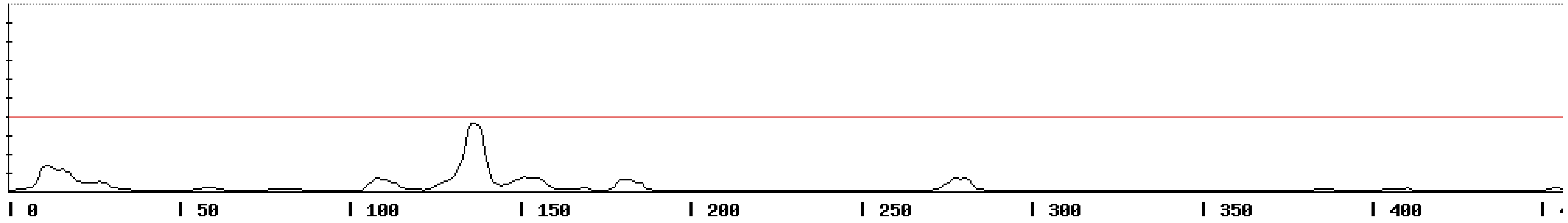

Predictions for Fragariaxananassa_FANhyb_rscf00000118.1.g00010.1

Viterbi Path

Viterbi Path

Posterior @ 0.4 — 87 - PKKLNPKGKRIDRRT - 101

Posterior @ 0.4

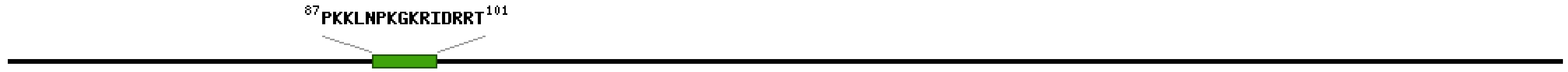

Posterior Graphic. Threshold Marked @ 0.4

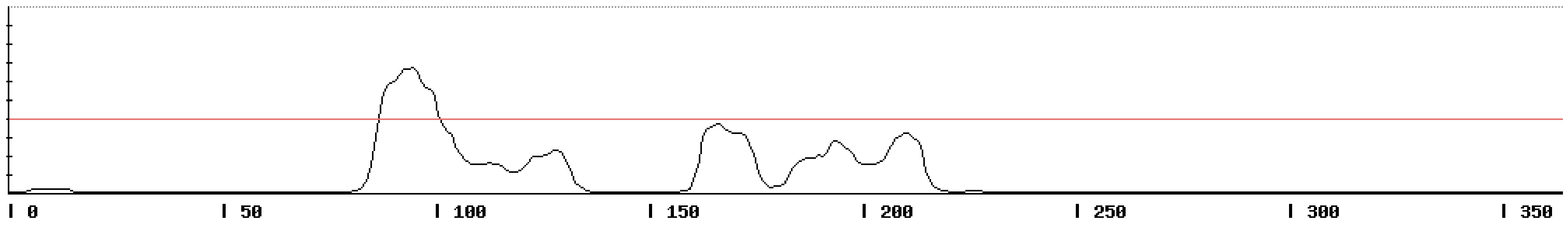

Predictions for Fragariaxananassa_FANhyb_rscf00000139.1.g00001.1

Viterbi Path

Viterbi Path

Posterior @ 0.4

Posterior @ 0.4

Posterior Graphic. Threshold Marked @ 0.4

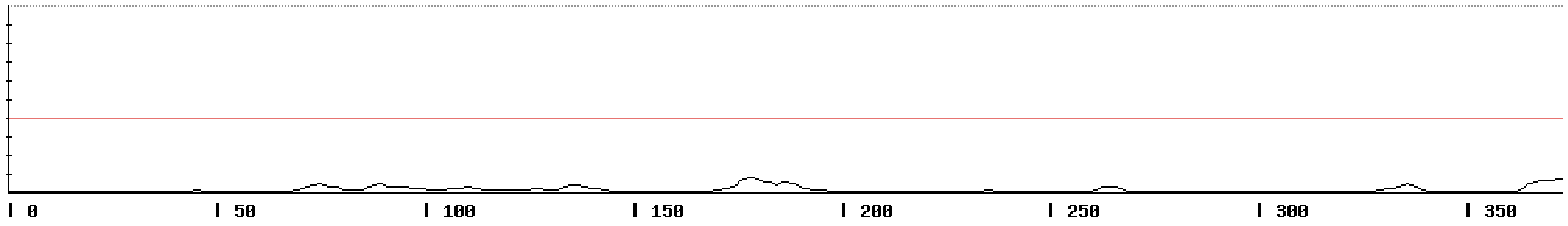

Predictions for Fragariaxananassa_FANhyb_rscf00000142.1.g00004.1

Viterbi Path

Viterbi Path

Posterior @ 0.4

Posterior @ 0.4

Posterior Graphic. Threshold Marked @ 0.4

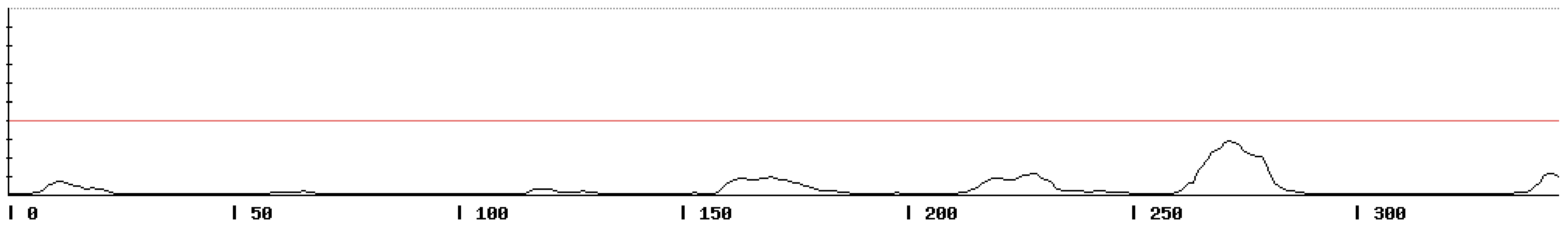

Predictions for Fragariaxananassa_FANhyb_rscf00000154.1.g00016.1

Viterbi Path

Viterbi Path

Posterior @ 0.4

71 - KRKKLNPKGSRF - 82

150 - KPPPK - 154

159 - KR - 160

Posterior @ 0.4

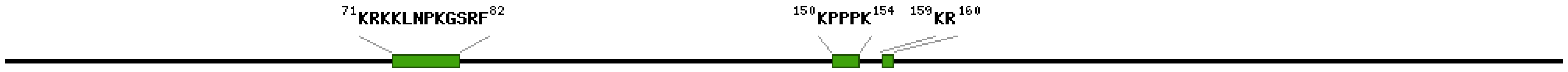

Posterior Graphic. Threshold Marked @ 0.4

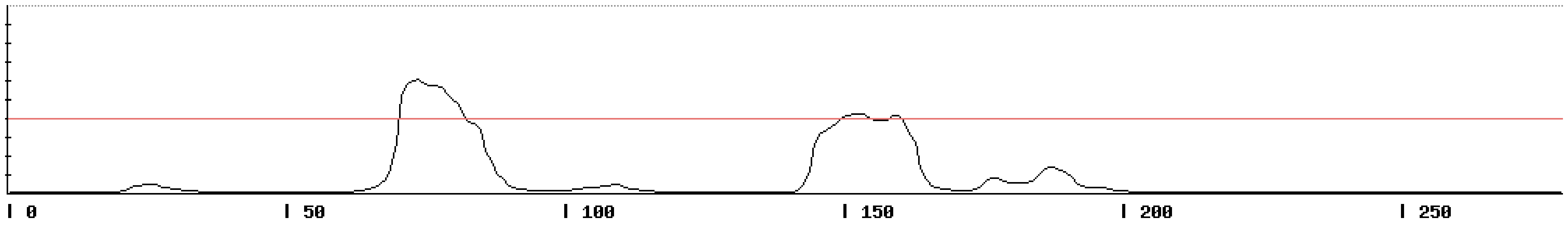

Predictions for Fragariaxananassa_FANhyb_rscf00000163.1.g00004.1	
Viterbi Path	
Viterbi Path	
Posterior @ 0.4	
Posterior @ 0.4	
Posterior Graphic. Threshold Marked @ 0.4	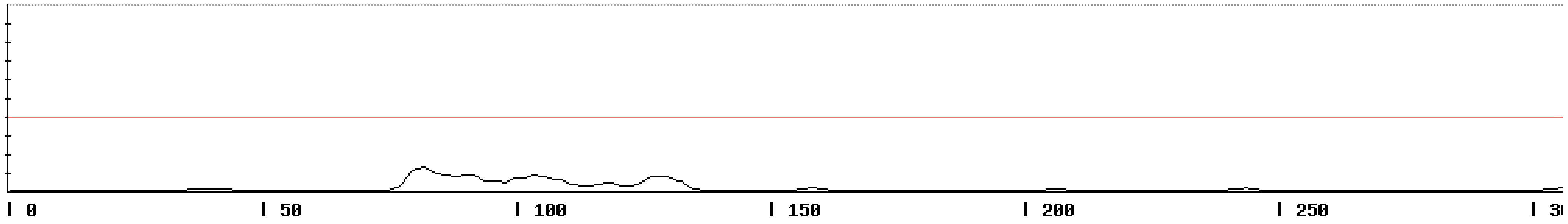

Predictions for Fragariaxananassa_FANhyb_rscf00000163.1.g00013.1	
Viterbi Path	
Viterbi Path	
Posterior @ 0.4	176 - KKRK - 179
Posterior @ 0.4	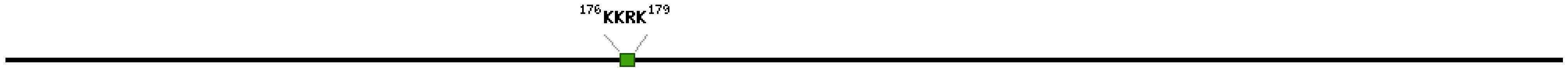

Posterior Graphic. Threshold Marked @ 0.4	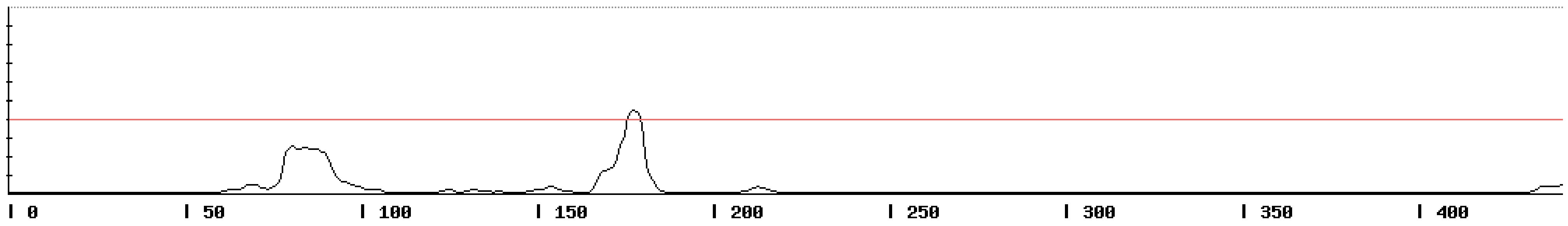

Predictions for Fragariaxananassa_FANhyb_rscf00000189.1.g00004.1

Viterbi Path	
Viterbi Path	
Posterior @ 0.4	
Posterior @ 0.4	
Posterior Graphic. Threshold Marked @ 0.4	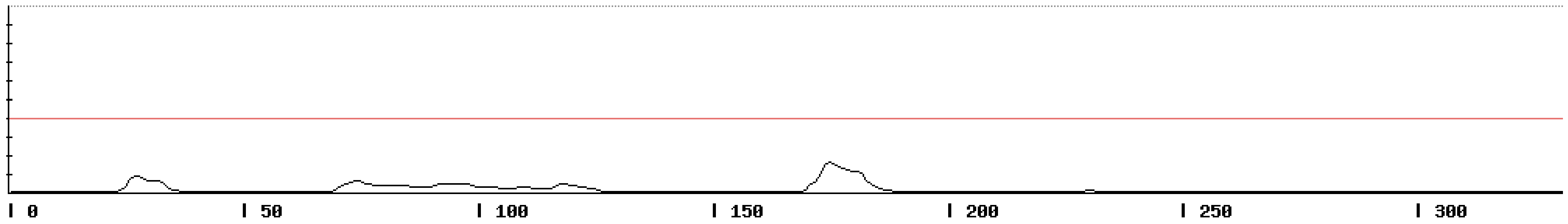

Predictions for Fragariaxananassa_FANhyb_rscf00000190.1.g00002.1

Viterbi Path	
Viterbi Path	
Posterior @ 0.4	
Posterior @ 0.4	

Posterior Graphic. Threshold Marked @ 0.4

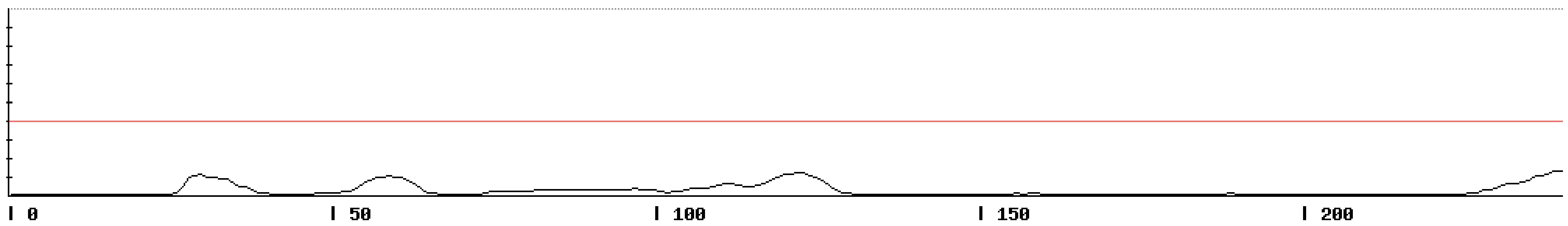

Predictions for Fragariaxananassa_FANhyb_rscf00000190.1.g00003.1

Viterbi Path

Viterbi Path

Posterior @ 0.4

Posterior @ 0.4

Posterior Graphic. Threshold Marked @ 0.4

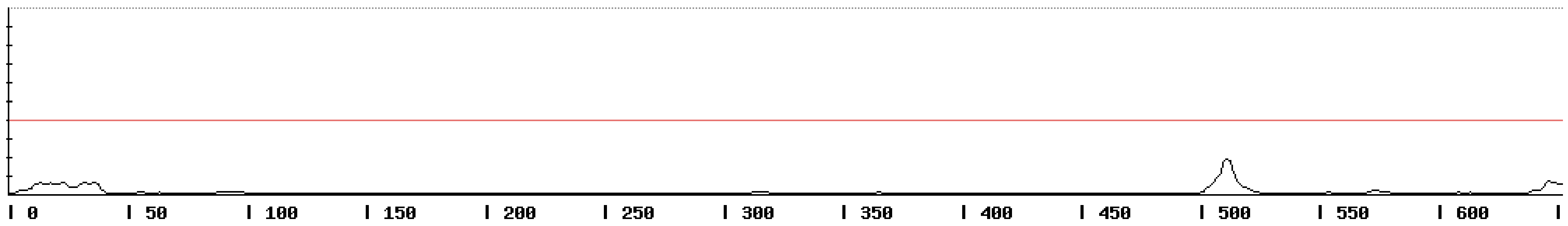

Predictions for Fragariaxananassa_FANhyb_rscf00000192.1.g00017.1

Viterbi Path

Viterbi Path

Posterior @ 0.4	164 - RKDRKRK - 170
Posterior @ 0.4	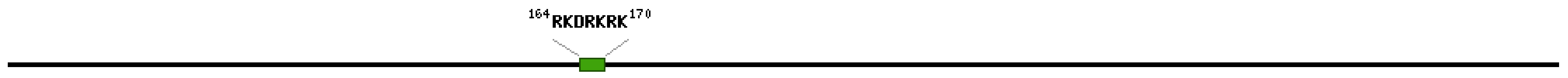
Posterior Graphic. Threshold Marked @ 0.4	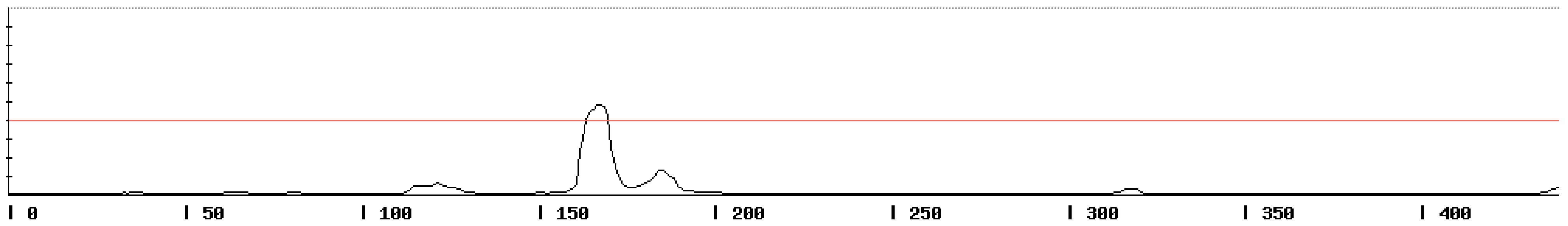

Predictions for Fragariaxananassa_FANhyb_rscf00000207.1.g00009.1

Viterbi Path	
Viterbi Path	
Posterior @ 0.4	
Posterior @ 0.4	
Posterior Graphic. Threshold Marked @ 0.4	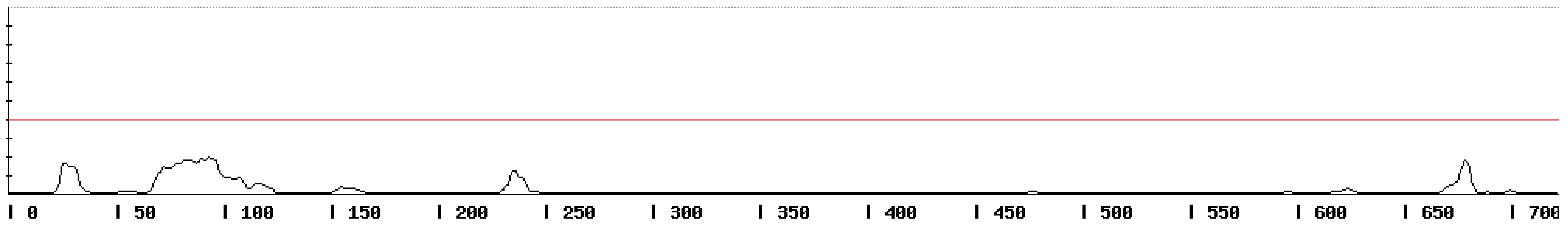

Predictions for Fragariaxananassa_FANhyb_rscf00000255.1.g00001.1

Viterbi Path	447 - RRKKKHCDSLSRLGRRRKRERKRER - 471
Viterbi Path	

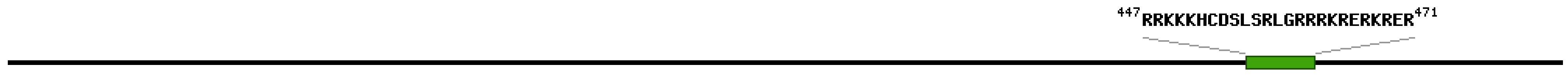

Posterior @ 0.4 — 447 - RRKKKHCDSLSRLGRRRKRERKRER - 471

Posterior @ 0.4

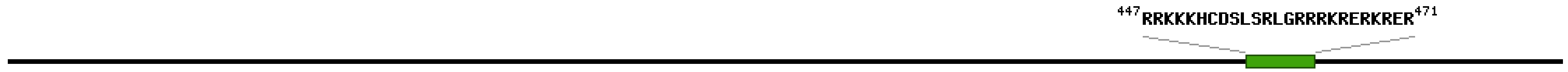

Posterior Graphic. Threshold Marked @ 0.4

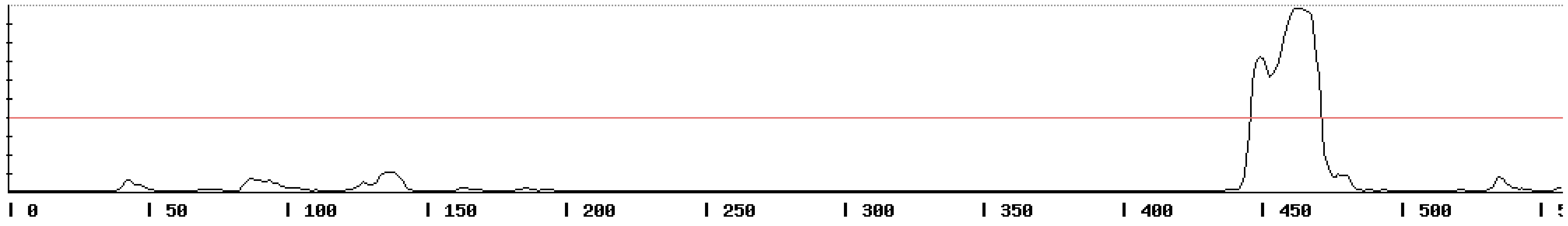

Predictions for Fragariaxananassa_FANhyb_rscf00000334.1.g00003.1

Viterbi Path

Viterbi Path

Posterior @ 0.4

Posterior @ 0.4

Posterior Graphic. Threshold Marked @ 0.4

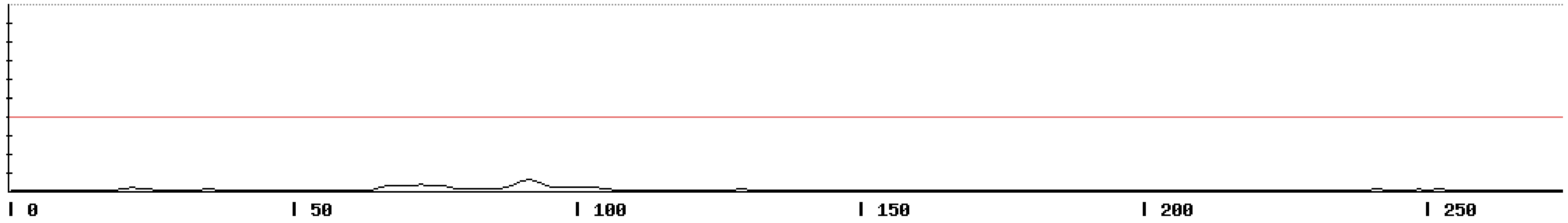

Predictions for Fragariaxananassa_FANhyb_rscf00000355.1.g00002.1

Viterbi Path

Viterbi Path

Posterior @ 0.4

Posterior @ 0.4

Posterior Graphic. Threshold Marked @ 0.4

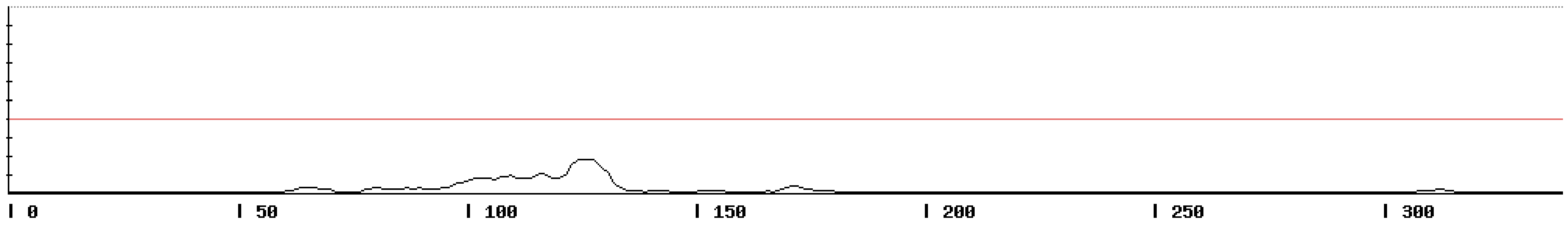

Predictions for Fragariaxananassa_FANhyb_rscf00000363.1.g00004.1

Viterbi Path

Viterbi Path

Posterior @ 0.4

Posterior @ 0.4

Posterior Graphic. Threshold Marked @ 0.4

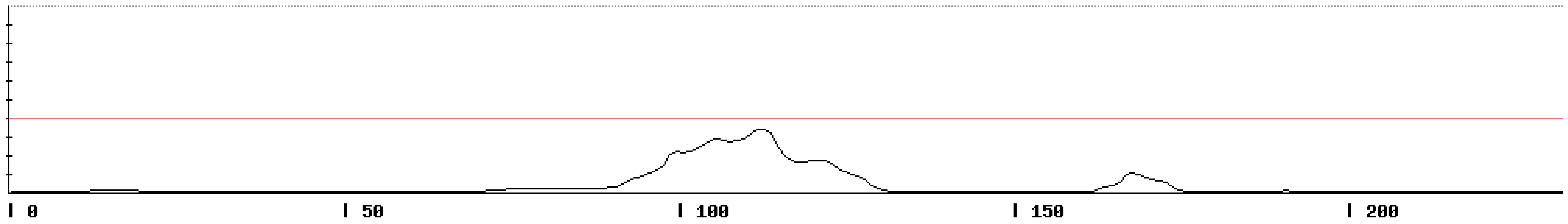

Predictions for Fragariaxananassa_FANhyb_rscf00000370.1.g00001.1

Viterbi Path

Viterbi Path

Posterior @ 0.4

Posterior @ 0.4

Posterior Graphic. Threshold Marked @ 0.4

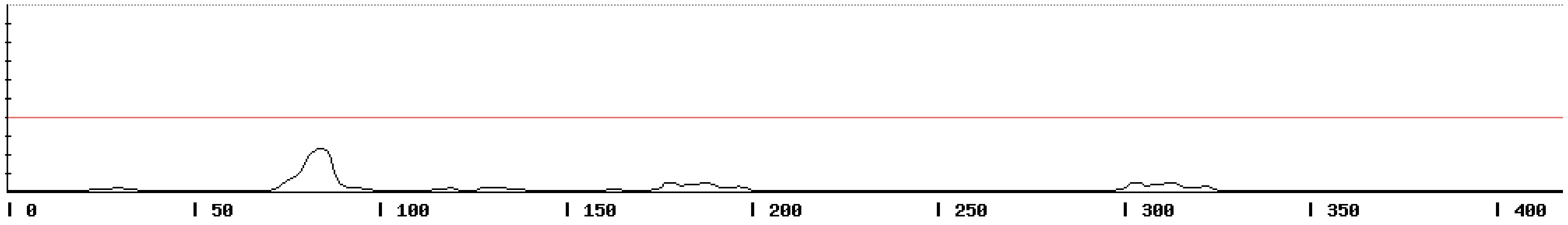

Predictions for Fragariaxananassa_FANhyb_rscf00000399.1.g00005.1

Viterbi Path

Viterbi Path

Posterior @ 0.4

Posterior @ 0.4

Posterior Graphic. Threshold Marked @ 0.4

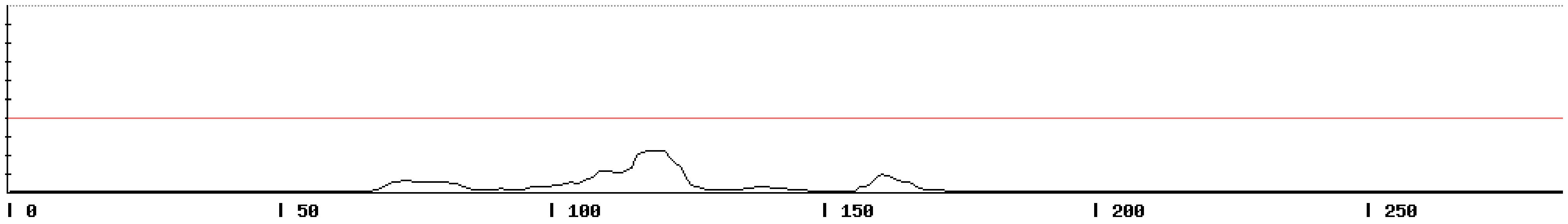

Predictions for Fragariaxananassa_FANhyb_rscf00000426.1.g00005.1

Viterbi Path	
Viterbi Path	
Posterior @ 0.4	
Posterior @ 0.4	
Posterior Graphic. Threshold Marked @ 0.4	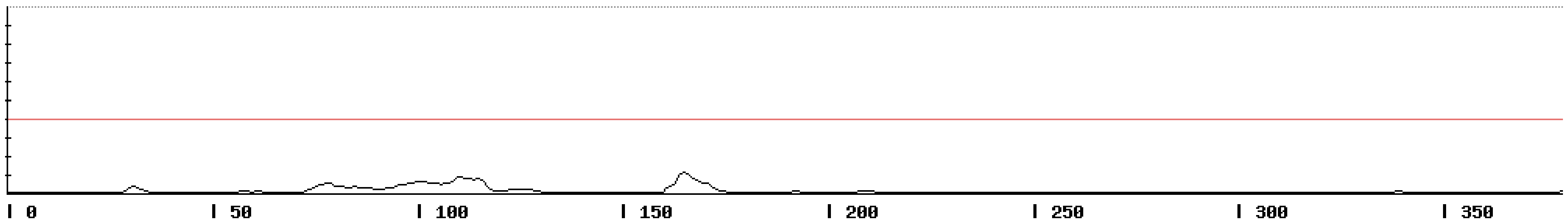

Predictions for Fragariaxananassa_FANhyb_rscf00000529.1.g00004.1

Viterbi Path	
Viterbi Path	
Posterior @ 0.4	122 - RIRKDRKRK - 130
Posterior @ 0.4	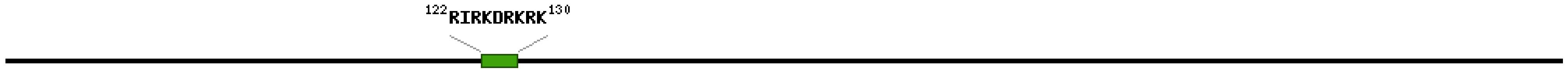

Posterior Graphic. Threshold Marked @ 0.4	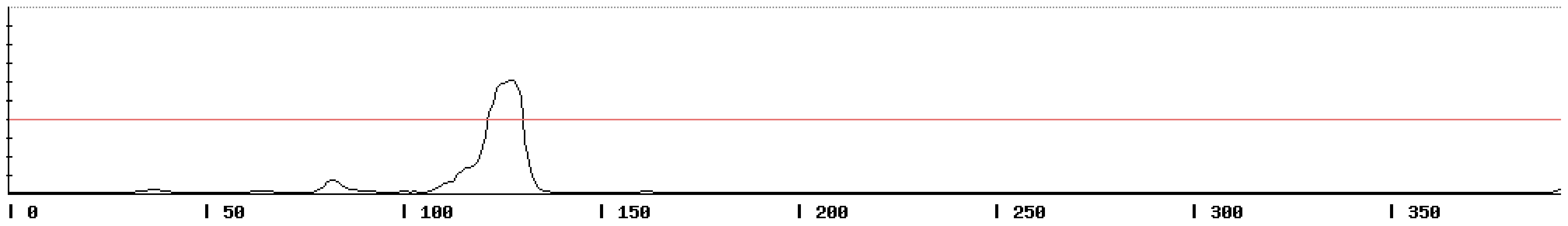

Predictions for Fragariaxananassa_FANhyb_rscf00000534.1.g00007.1	
Viterbi Path	
Viterbi Path	
Posterior @ 0.4	
Posterior @ 0.4	
Posterior Graphic. Threshold Marked @ 0.4	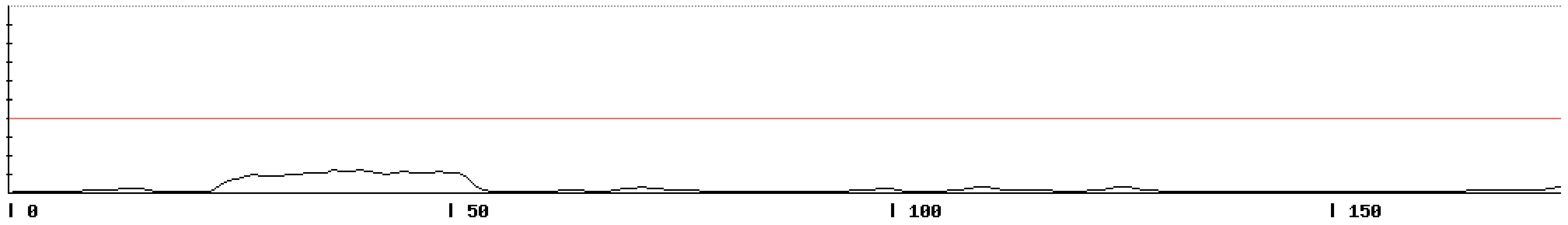

Predictions for Fragariaxananassa_FANhyb_rscf00000590.1.g00003.1	
Viterbi Path	
Viterbi Path	
Posterior @ 0.4	
Posterior @ 0.4	

Posterior Graphic. Threshold Marked @ 0.4

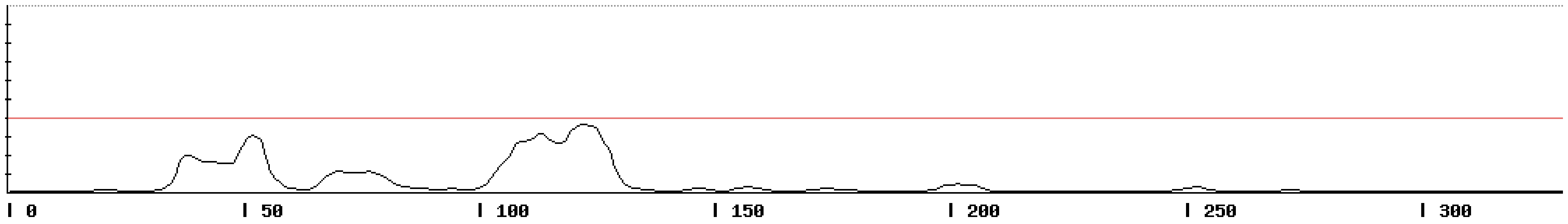

Predictions for Fragariaxananassa_FANhyb_rscf00000597.1.g00005.1

Viterbi Path

Viterbi Path

Posterior @ 0.4

Posterior @ 0.4

Posterior Graphic. Threshold Marked @ 0.4

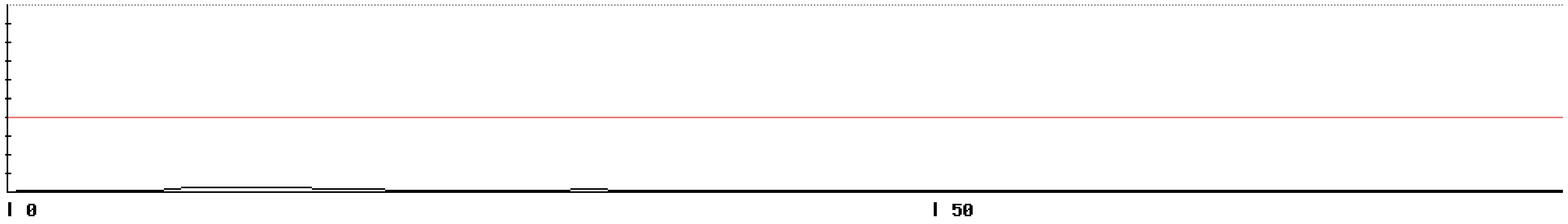

Predictions for Fragariaxananassa_FANhyb_rscf00000642.1.g00006.1

Viterbi Path

Viterbi Path

Posterior @ 0.4

Posterior @ 0.4

Posterior Graphic. Threshold Marked @ 0.4

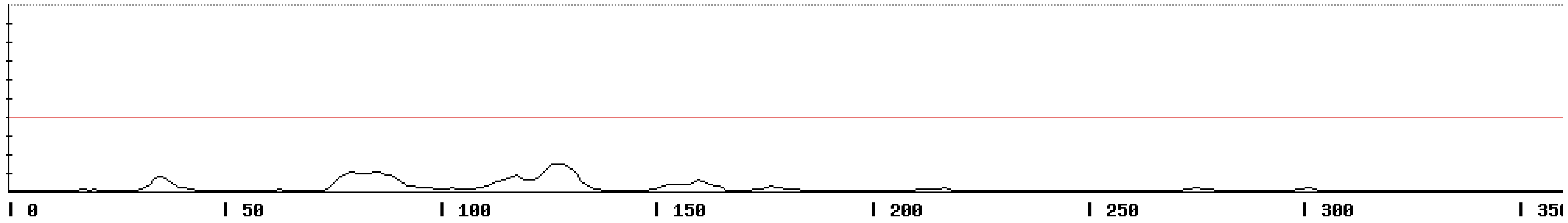

Predictions for Fragariaxananassa_FANhyb_rscf00000663.1.g00006.1

Viterbi Path

Viterbi Path

Posterior @ 0.4 — 219 - KRRGHRK - 225

Posterior @ 0.4

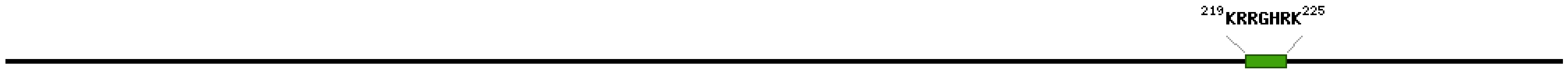

Posterior Graphic. Threshold Marked @ 0.4

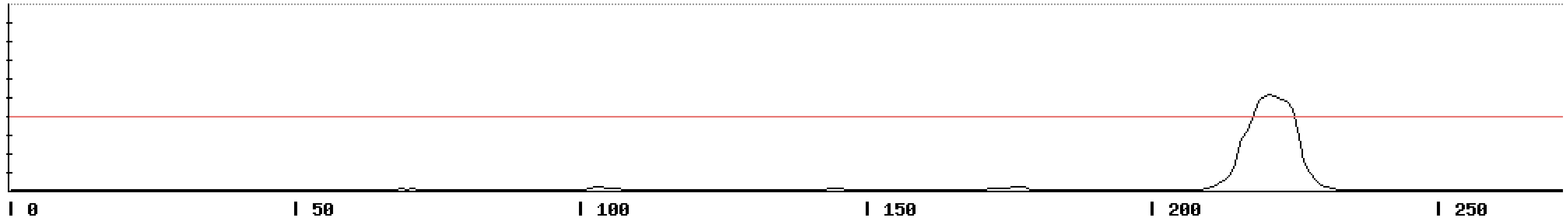

Predictions for Fragariaxananassa_FANhyb_rscf00000665.1.g00005.1

Viterbi Path

Viterbi Path

Posterior @ 0.4

121 - RIRYGGRYRSRAAKAKRVNKEGQIRHFFHRPSKAYTTGTRKRRKV - 165

Posterior @ 0.4

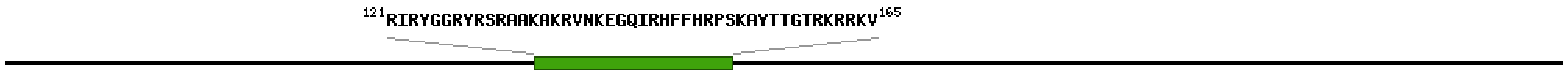

Posterior Graphic. Threshold Marked @ 0.4

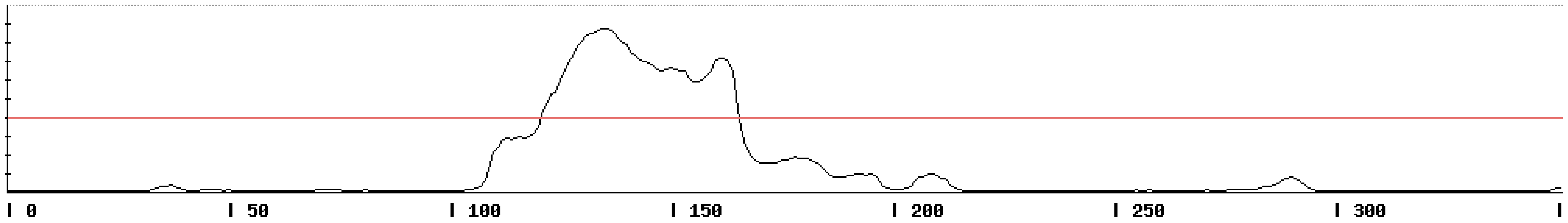

Predictions for Fragariaxananassa_FANhyb_rscf00000673.1.g00001.1

Viterbi Path

Viterbi Path

Posterior @ 0.4

333 - SRKRRKVH - 340

356 - KTGKTR - 361

Posterior @ 0.4

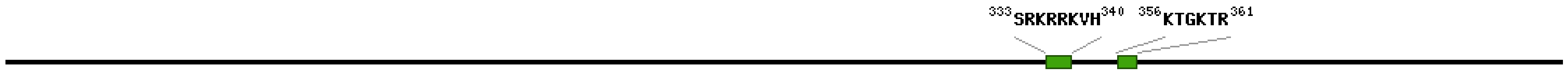

Posterior Graphic. Threshold Marked @ 0.4

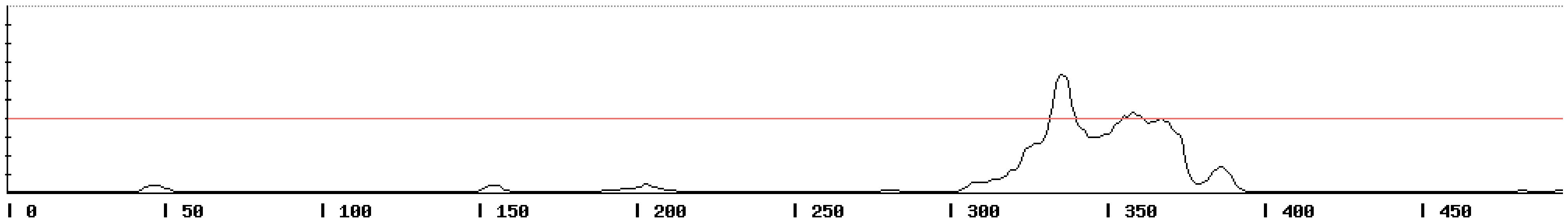

Predictions for Fragariaxananassa_FANhyb_rscf00000760.1.g00005.1

Viterbi Path	
Viterbi Path	
Posterior @ 0.4	
Posterior @ 0.4	
Posterior Graphic. Threshold Marked @ 0.4	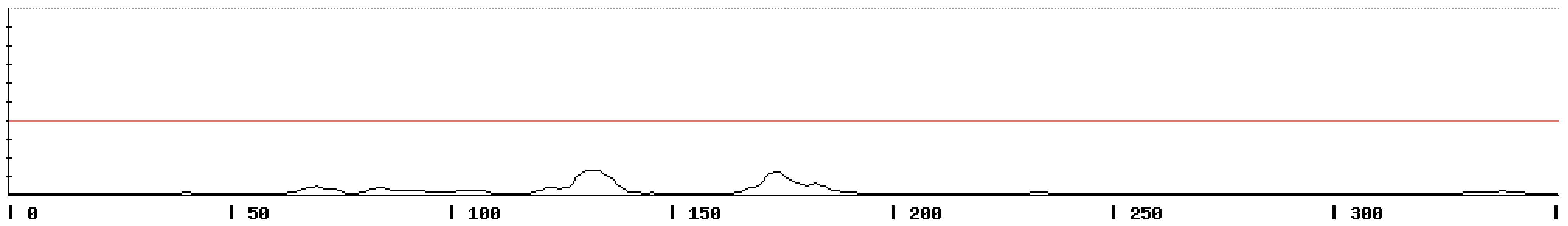

Predictions for Fragariaxananassa_FANhyb_rscf00000878.1.g00001.1

Viterbi Path	283 - KKKKEKGKEKK - 293
Viterbi Path	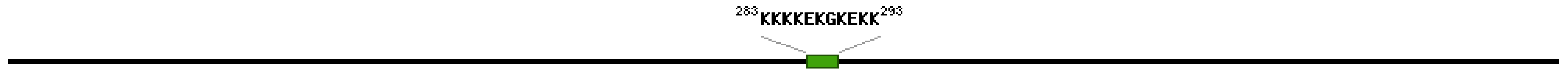
Posterior @ 0.4	106 - GEKKNKTPIGRKRKFR - 121
	282 - EKKKKEKGKEKKT - 294
	406 - GRKRKL - 411
	495 - ASSKAKRRR - 503
Posterior @ 0.4	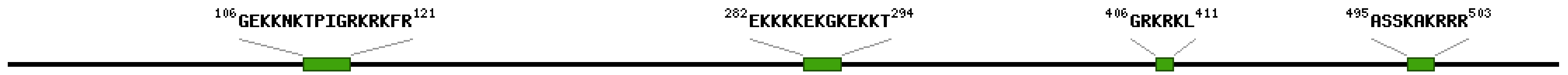
Posterior Graphic. Threshold Marked @ 0.4	

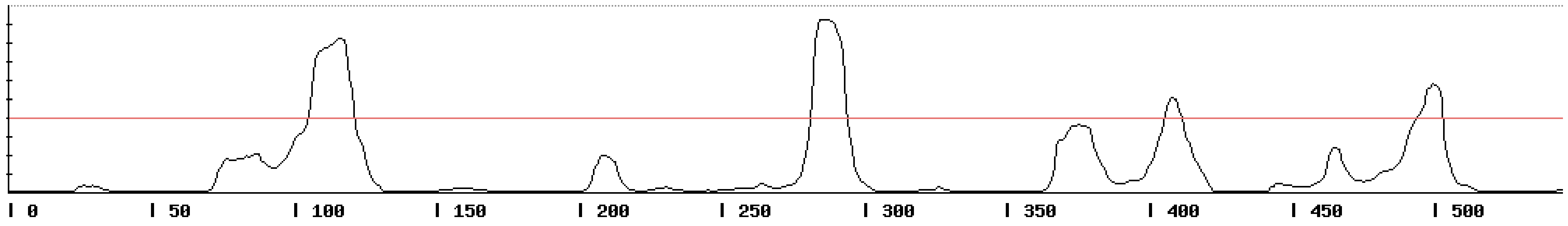

Predictions for Fragariaxananassa_FANhyb_rscf00001076.1.g00002.1

Viterbi Path

Viterbi Path

Posterior @ 0.4

Posterior @ 0.4

Posterior Graphic. Threshold Marked @ 0.4

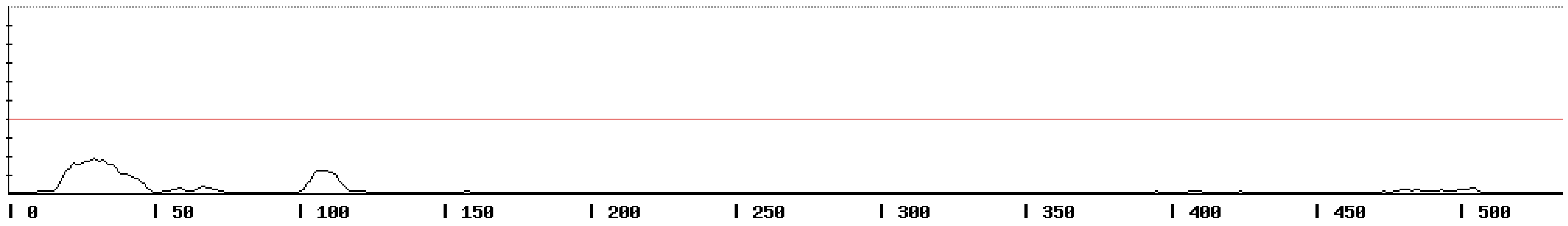

Predictions for Fragariaxananassa_FANhyb_rscf00001108.1.g00002.1

Viterbi Path

Viterbi Path

Posterior @ 0.4

Posterior @ 0.4

Posterior Graphic. Threshold Marked @ 0.4

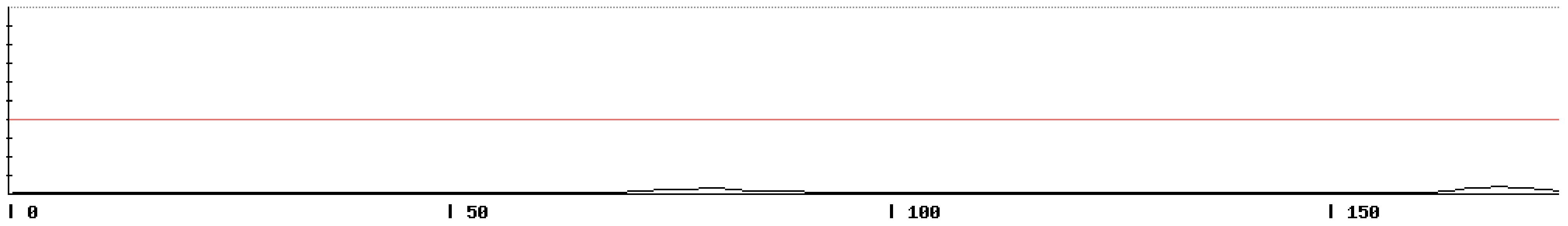

Predictions for Fragariaxananassa_FANhyb_rscf00001150.1.g00006.1

Viterbi Path: 486 - KHGLRKMKRKKKIR - 499

Viterbi Path

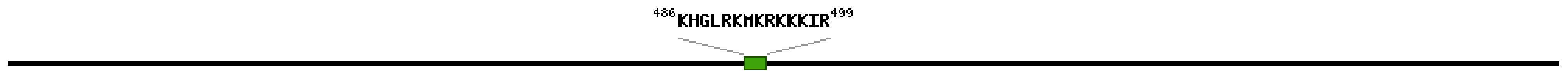

Posterior @ 0.4

486 - KHGLRKMKRKKKIR - 499

529 - WKITGVEREIKARRSKALIGKKRP - 552

Posterior @ 0.4

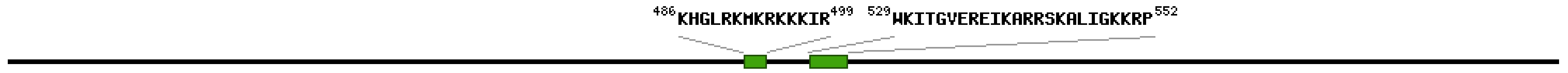

Posterior Graphic. Threshold Marked @ 0.4

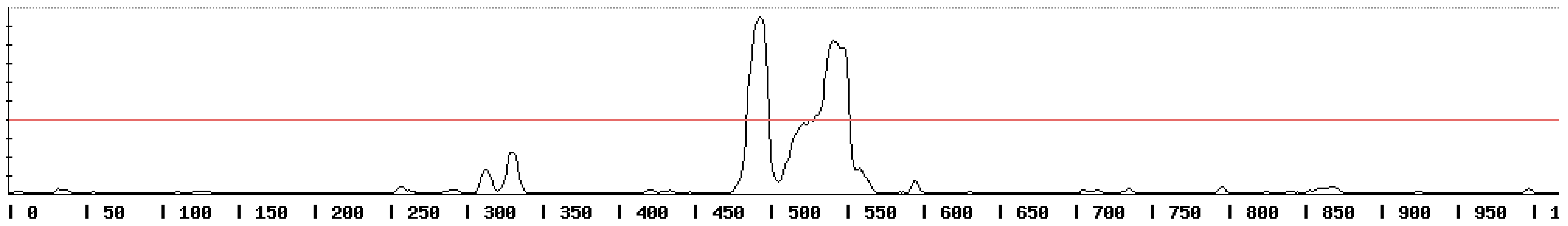

Predictions for Fragariaxananassa_FANhyb_rscf00001175.1.g00004.1

Viterbi Path

Viterbi Path

Posterior @ 0.4

65 - KRKN - 68

71 - R - 71

	83 - KKKT - 86
	166 - KKK - 168

Posterior @ 0.4

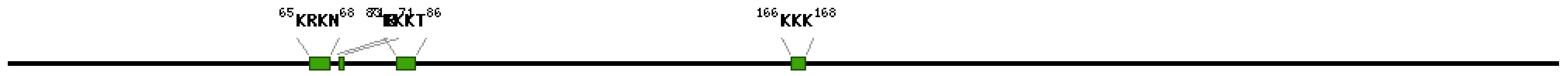

Posterior Graphic. Threshold Marked @ 0.4

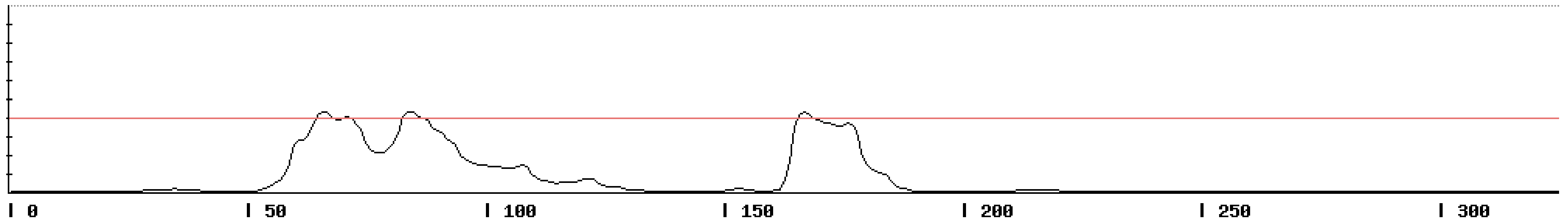

Predictions for Fragariaxananassa_FANhyb_rscf00001352.1.g00001.1

Viterbi Path

Viterbi Path

Posterior @ 0.4

Posterior @ 0.4

Posterior Graphic. Threshold Marked @ 0.4

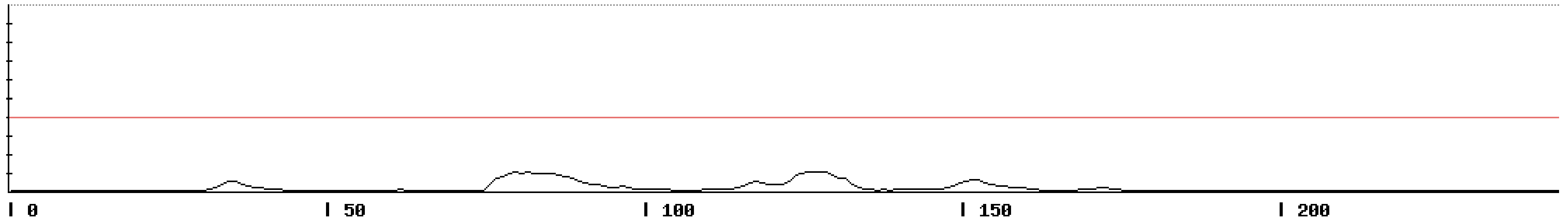

Predictions for Fragariaxananassa_FANhyb_rscf00001689.1.g00002.1

Viterbi Path

Viterbi Path

Posterior @ 0.4	41 - KHIGKPKALGIKK - 53
Posterior @ 0.4	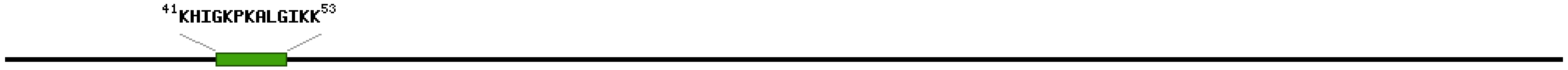
Posterior Graphic. Threshold Marked @ 0.4	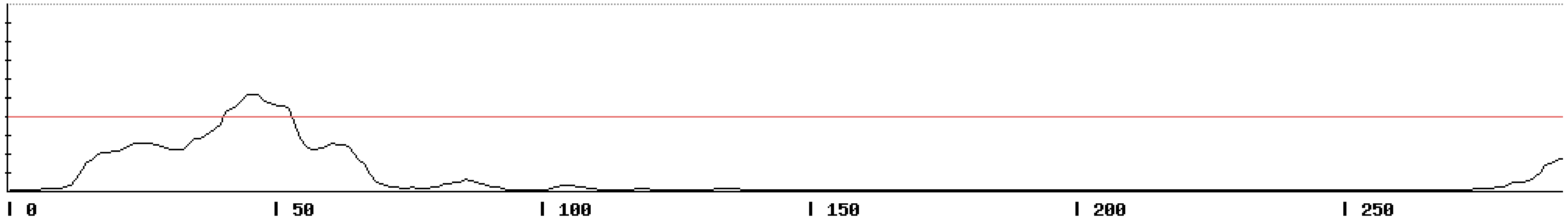

Predictions for Fragariaxananassa_FANhyb_rscf00001754.1.g00001.1

Viterbi Path	
Viterbi Path	
Posterior @ 0.4	
Posterior @ 0.4	
Posterior Graphic. Threshold Marked @ 0.4	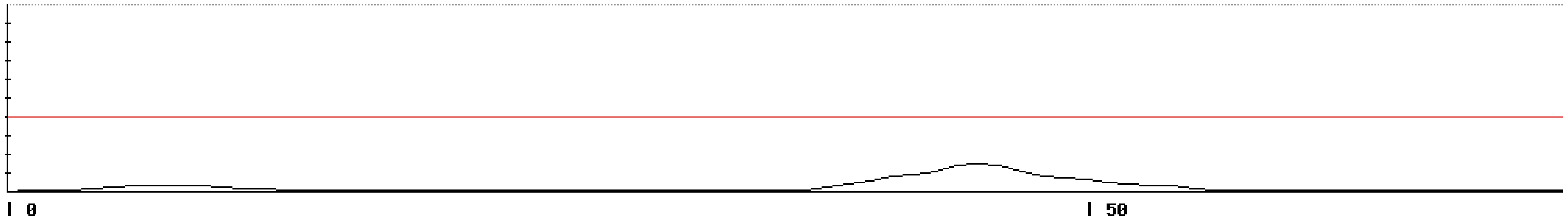

Predictions for Fragariaxananassa_FANhyb_rscf00002063.1.g00001.1

Viterbi Path	
Viterbi Path	
Posterior @ 0.4	
Posterior @ 0.4	
Posterior Graphic. Threshold Marked @ 0.4	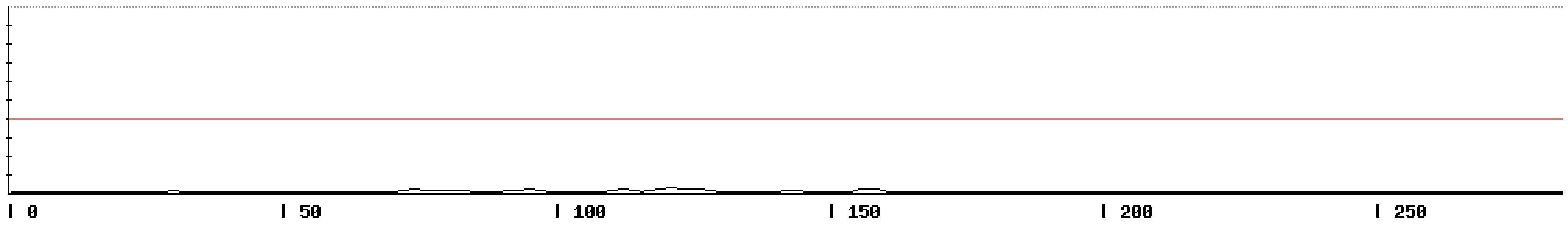

Predictions for Fragariaxananassa_FANhyb_rscf00002170.1.g00001.1

Viterbi Path	
Viterbi Path	
Posterior @ 0.4	150 - RSKGSMKKW - 158
Posterior @ 0.4	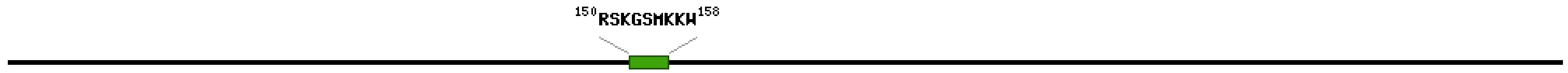
Posterior Graphic. Threshold Marked @ 0.4	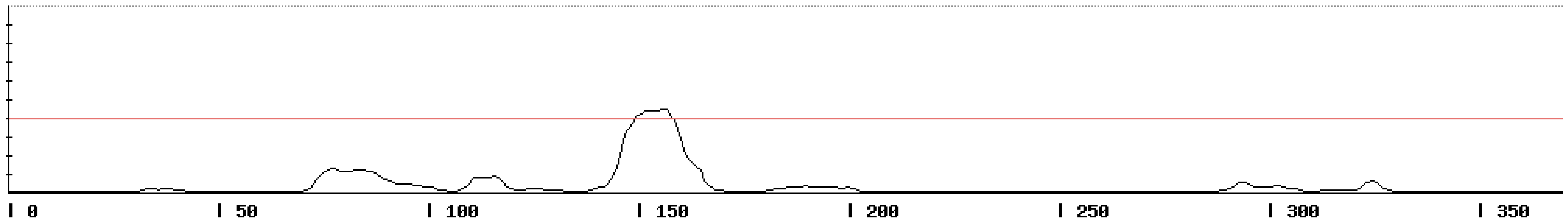

Predictions for Fragariaxananassa_FANhyb_rscf00002690.1.g00001.1

Viterbi Path	
Viterbi Path	
Posterior @ 0.4	
Posterior @ 0.4	
Posterior Graphic. Threshold Marked @ 0.4	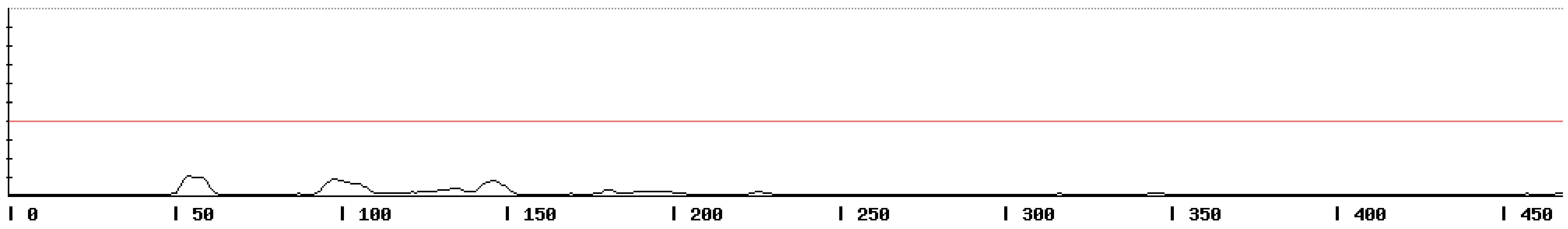

Predictions for Fragariaxananassa_FANhyb_rscf00002764.1.g00003.1

Viterbi Path	
Viterbi Path	
Posterior @ 0.4	77 - KKKKTAR - 83
	174 - KKRK - 177
Posterior @ 0.4	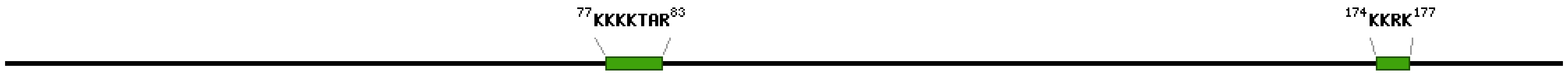
Posterior Graphic. Threshold Marked @ 0.4	

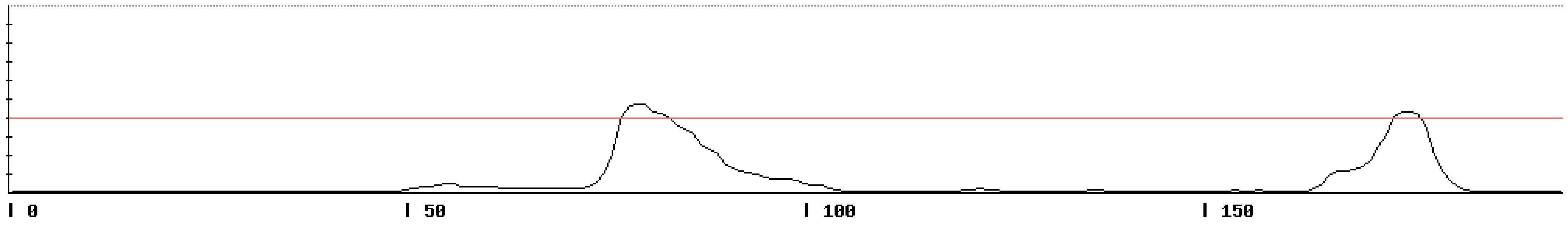

Predictions for Fragariaxananassa_FANhyb_rscf00002828.1.g00001.1

Viterbi Path	
Viterbi Path	
Posterior @ 0.4	
Posterior @ 0.4	
Posterior Graphic. Threshold Marked @ 0.4	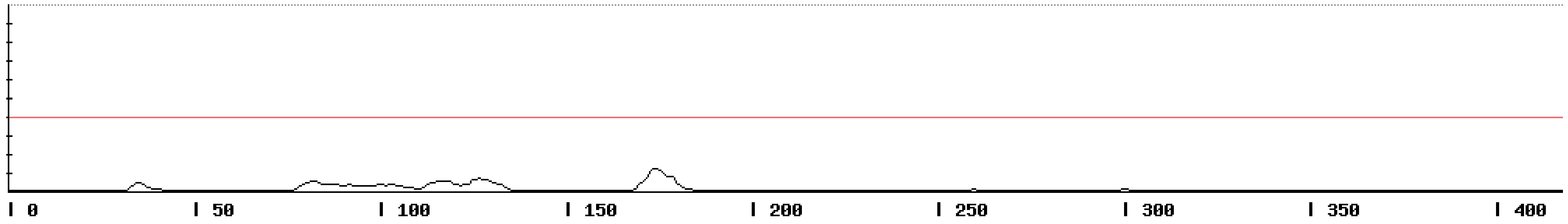

Predictions for Fragariaxananassa_FANhyb_rscf00003745.1.g00001.1

Viterbi Path	
Viterbi Path	
Posterior @ 0.4	
Posterior @ 0.4	

Posterior Graphic. Threshold Marked @ 0.4	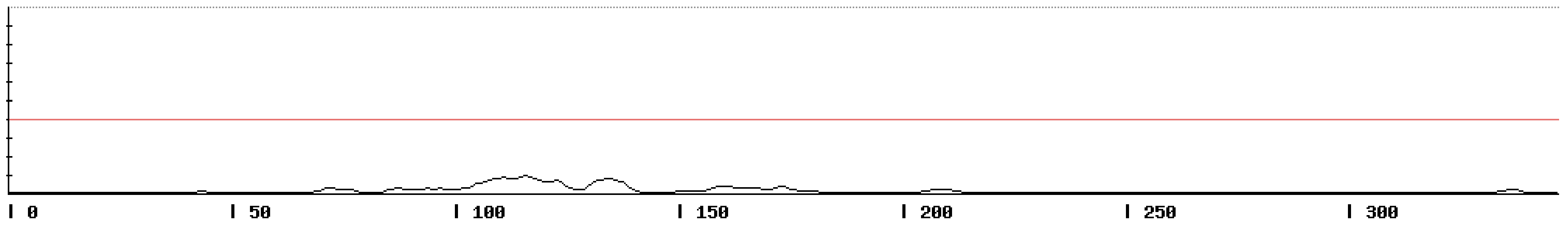

Predictions for Fragariaxananassa_FANhyb_rscf00003916.1.g00001.1

Viterbi Path	
Viterbi Path	
Posterior @ 0.4	
Posterior @ 0.4	
Posterior Graphic. Threshold Marked @ 0.4	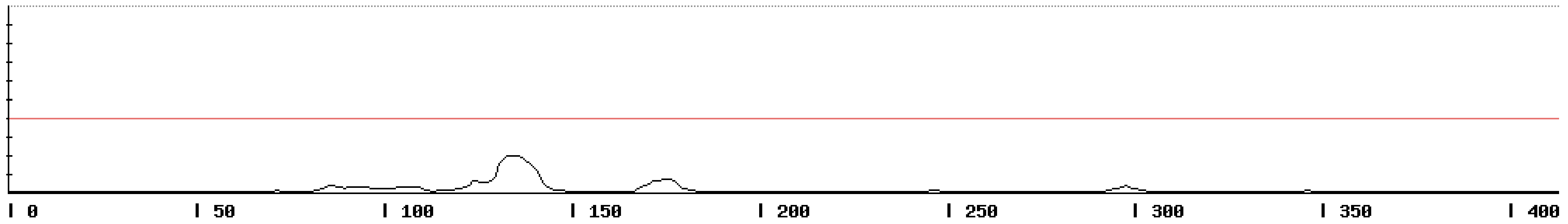

Predictions for Fragariaxananassa_FANhyb_rscf00004051.1.g00002.1

Viterbi Path	
Viterbi Path	
Posterior @ 0.4	
Posterior @ 0.4	

Posterior Graphic. Threshold Marked @ 0.4

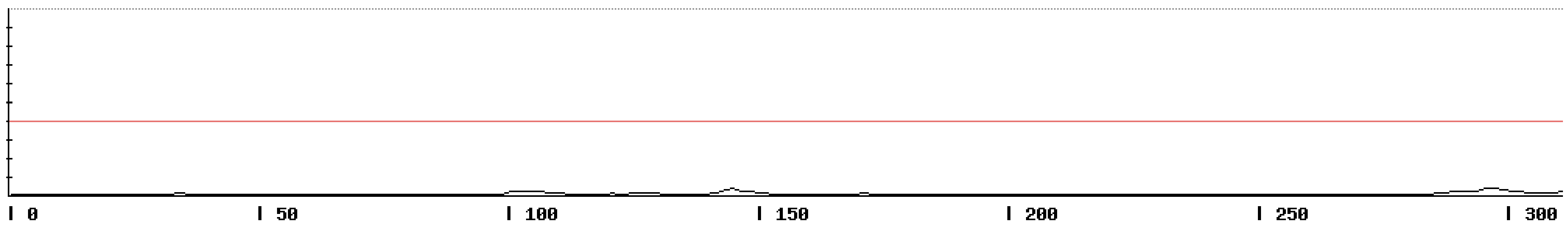

Predictions for Fragariaxananassa_FANhyb_rscf00004108.1.g00001.1

Viterbi Path

Viterbi Path

Posterior @ 0.4

Posterior @ 0.4

Posterior Graphic. Threshold Marked @ 0.4

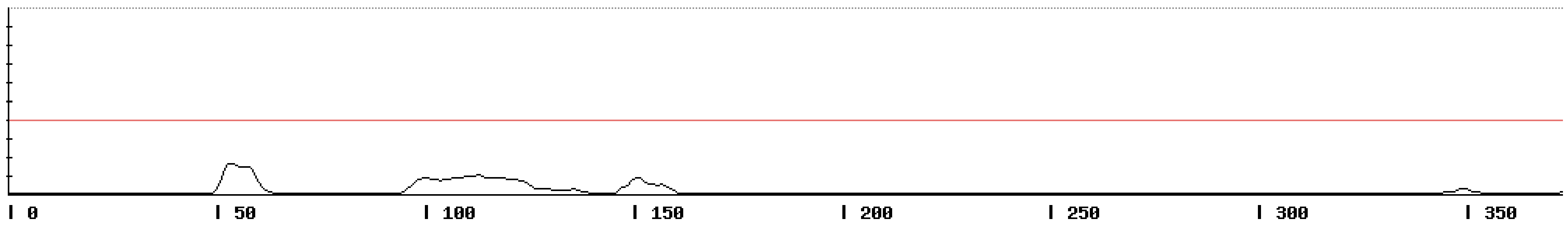

Predictions for Fragariaxananassa_FANhyb_rscf00004258.1.g00001.1

Viterbi Path

Viterbi Path

Posterior @ 0.4

Posterior @ 0.4

Posterior Graphic. Threshold Marked @ 0.4

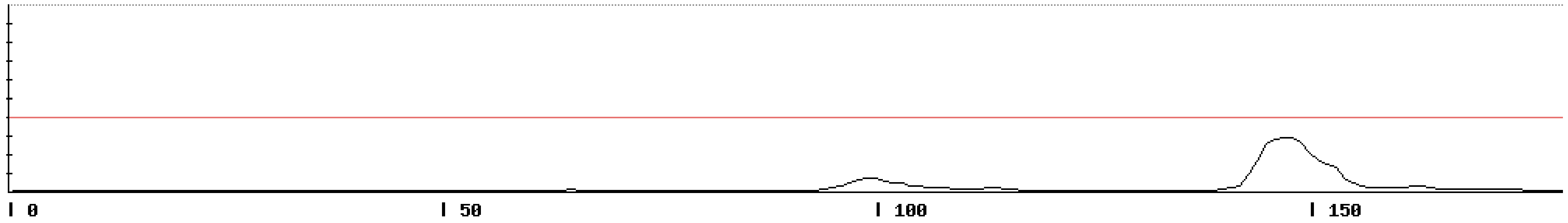

Predictions for Fragariaxananassa_FANhyb_rscf00004597.1.g00001.1

Viterbi Path

Viterbi Path

Posterior @ 0.4

Posterior @ 0.4

Posterior Graphic. Threshold Marked @ 0.4

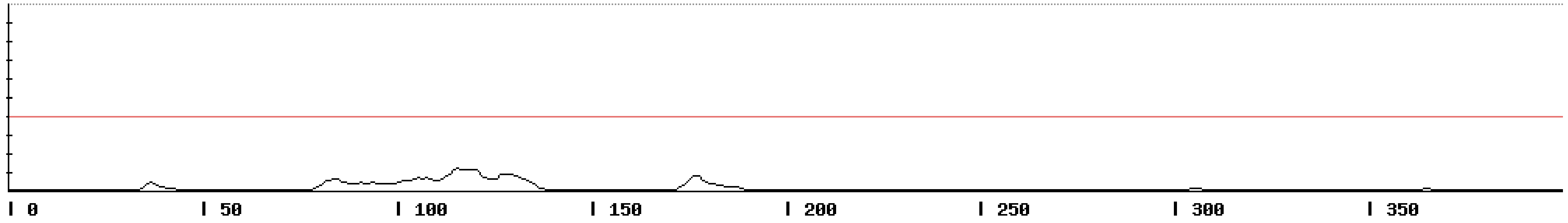

Predictions for Fragariaxananassa_FANhyb_rscf00004677.1.g00001.1

Viterbi Path

Viterbi Path

Posterior @ 0.4

Posterior @ 0.4

Posterior Graphic. Threshold Marked @ 0.4

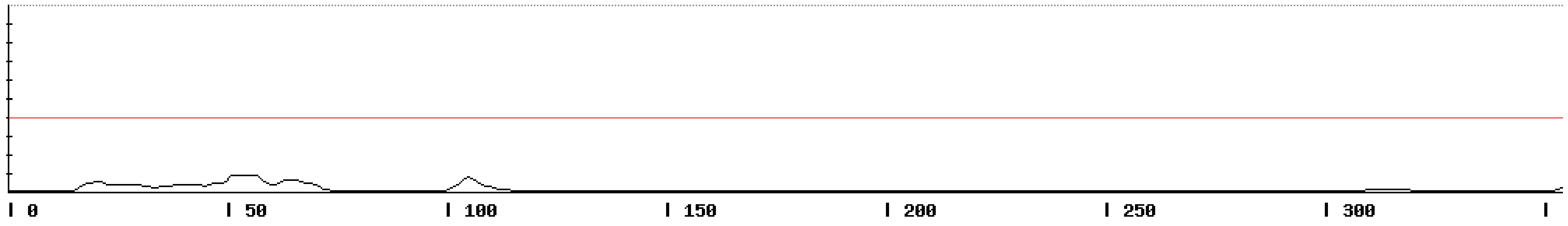

Predictions for Fragariaxananassa_FANhyb_rscf00004853.1.g00001.1

Viterbi Path

Viterbi Path

Posterior @ 0.4

166 - RKRRKIDKE - 174

183 - RWHKTGKTKAIKGGVHPGWKK - 203

Posterior @ 0.4

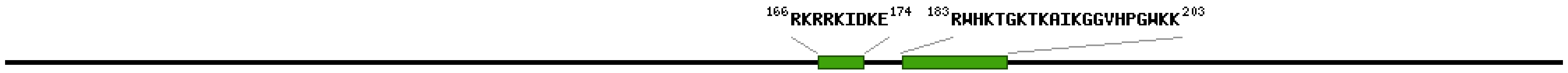

Posterior Graphic. Threshold Marked @ 0.4

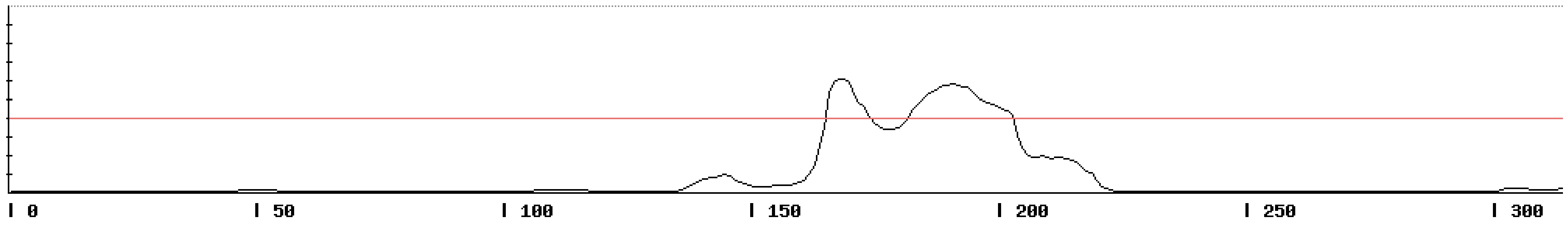

Predictions for Fragariaxananassa_FANhyb_rscf00004923.1.g00001.1	
Viterbi Path	
Viterbi Path	
Posterior @ 0.4	
Posterior @ 0.4	

Revision r.9 - Last Updated on : February 24 2015 17:45:20.

alex.nguyenba@utoronto.ca

Intended to be used with Firefox

Viterbi Path

Posterior @ 0.6

Posterior @ 0.6

Posterior Graphic. Threshold Marked @ 0.6

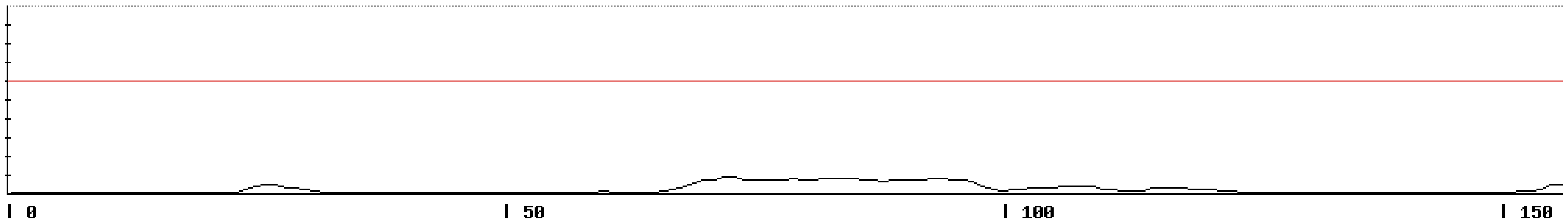

Predictions for Genliseaaurea_EPS57769.1

Viterbi Path

Viterbi Path

Posterior @ 0.6

Posterior @ 0.6

Posterior Graphic. Threshold Marked @ 0.6

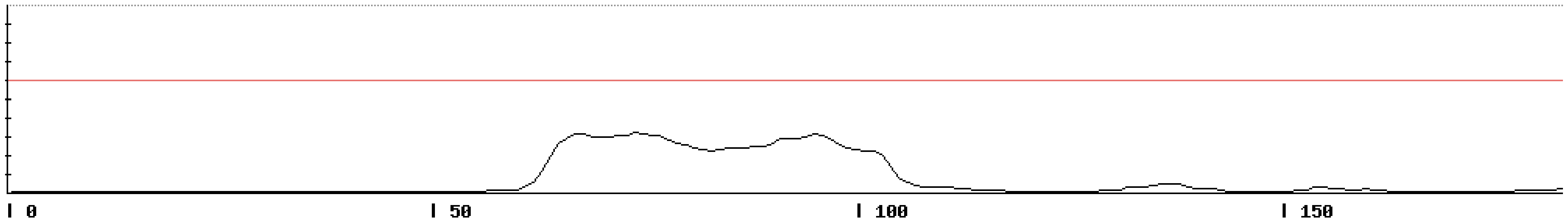

Predictions for Genliseaaurea_EPS58964.1

Viterbi Path	
Viterbi Path	
Posterior @ 0.6	
Posterior @ 0.6	
Posterior Graphic. Threshold Marked @ 0.6	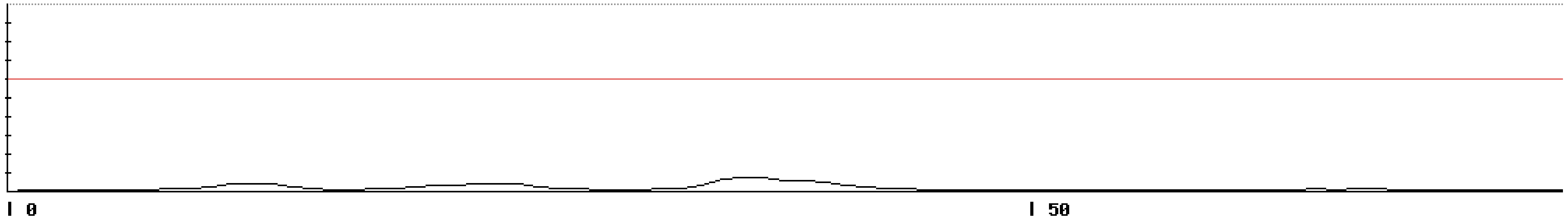

Predictions for Genliseaaurea_EPS59021.1

Viterbi Path	
Viterbi Path	
Posterior @ 0.6	
Posterior @ 0.6	
Posterior Graphic. Threshold Marked @ 0.6	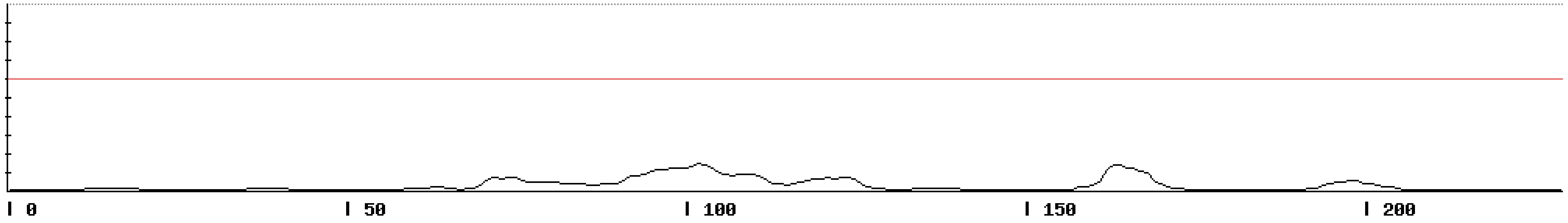

Predictions for Genliseaaurea_EPS59231.1	
Viterbi Path	
Viterbi Path	
Posterior @ 0.6	
Posterior @ 0.6	
Posterior Graphic. Threshold Marked @ 0.6	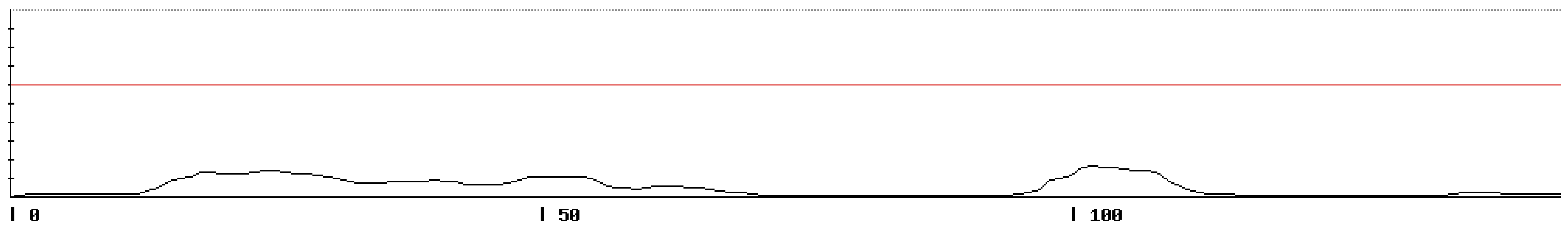

Predictions for Genliseaaurea_EPS59407.1	
Viterbi Path	
Viterbi Path	
Posterior @ 0.6	
Posterior @ 0.6	
Posterior Graphic. Threshold Marked @ 0.6	

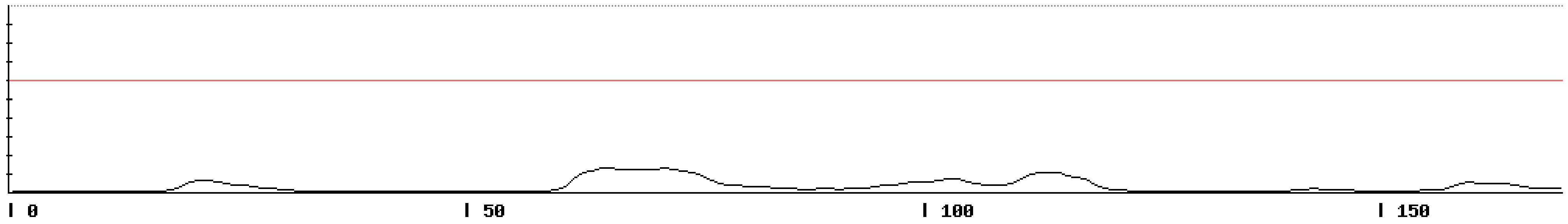

Predictions for Genliseaaurea_EPS60726.1

Viterbi Path	
Viterbi Path	
Posterior @ 0.6	
Posterior @ 0.6	
Posterior Graphic. Threshold Marked @ 0.6	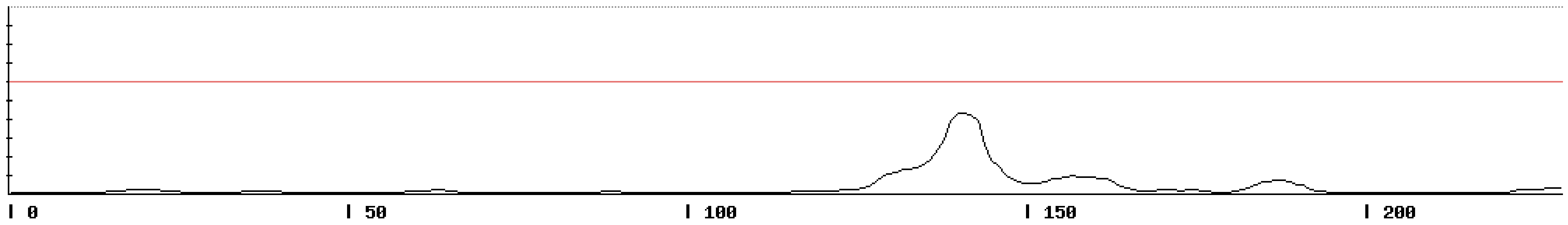

Predictions for Genliseaaurea_EPS61345.1

Viterbi Path	
Viterbi Path	
Posterior @ 0.6	
Posterior @ 0.6	

Posterior Graphic. Threshold Marked @ 0.6

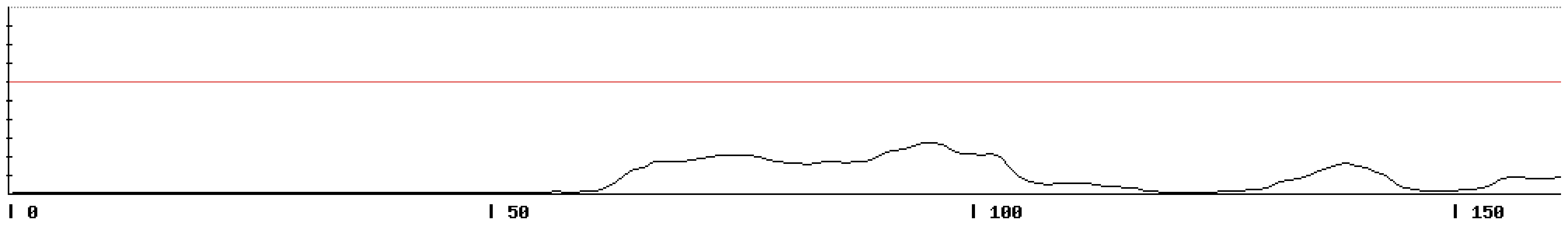

Predictions for Genliseaaurea_EPS62083.1

Viterbi Path

Viterbi Path

Posterior @ 0.6

Posterior @ 0.6

Posterior Graphic. Threshold Marked @ 0.6

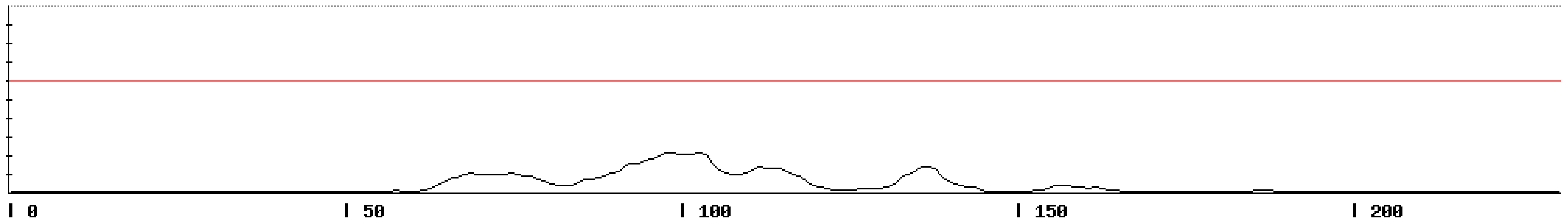

Predictions for Genliseaaurea_EPS62404.1

Viterbi Path

Viterbi Path

Posterior @ 0.6

Posterior @ 0.6

Posterior Graphic. Threshold Marked @ 0.6

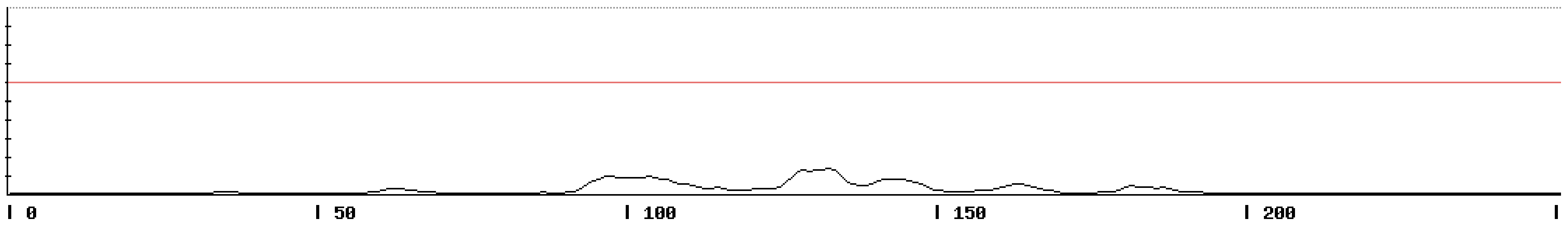

Predictions for Genliseaaurea_EPS62577.1

Viterbi Path

Viterbi Path

Posterior @ 0.6

Posterior @ 0.6

Posterior Graphic. Threshold Marked @ 0.6

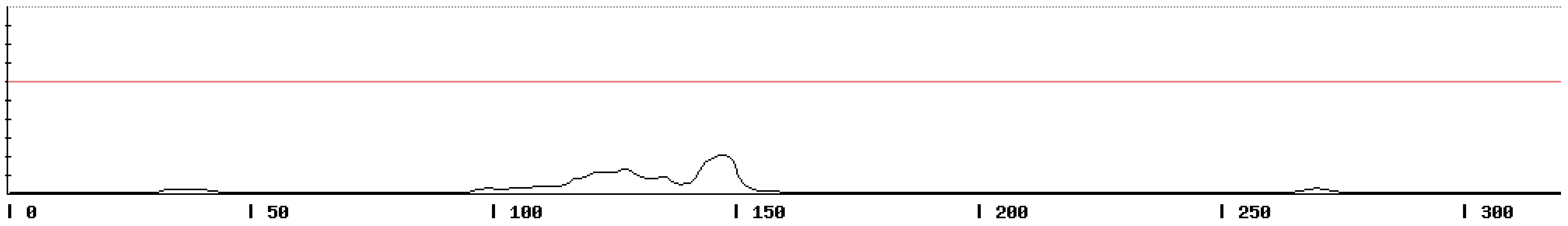

Predictions for Genliseaaurea_EPS62860.1

Viterbi Path

Viterbi Path

Posterior @ 0.6

Posterior @ 0.6

Posterior Graphic. Threshold Marked @ 0.6

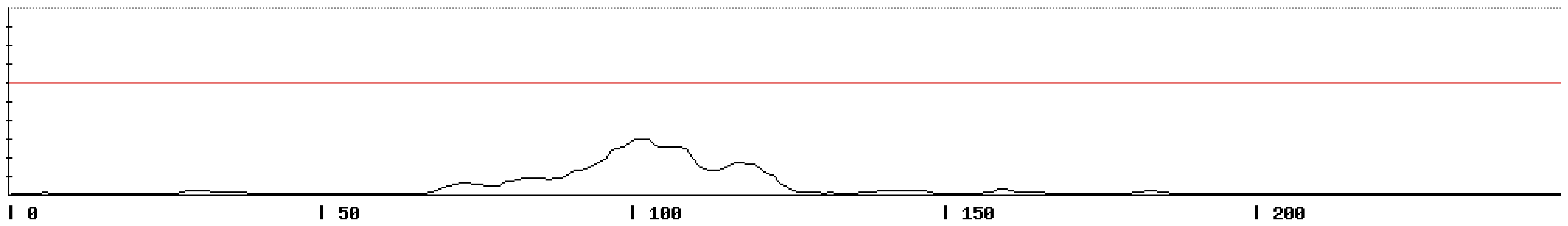

Predictions for Genliseaaurea_EPS62927.1

Viterbi Path

Viterbi Path

Posterior @ 0.6

Posterior @ 0.6

Posterior Graphic. Threshold Marked @ 0.6

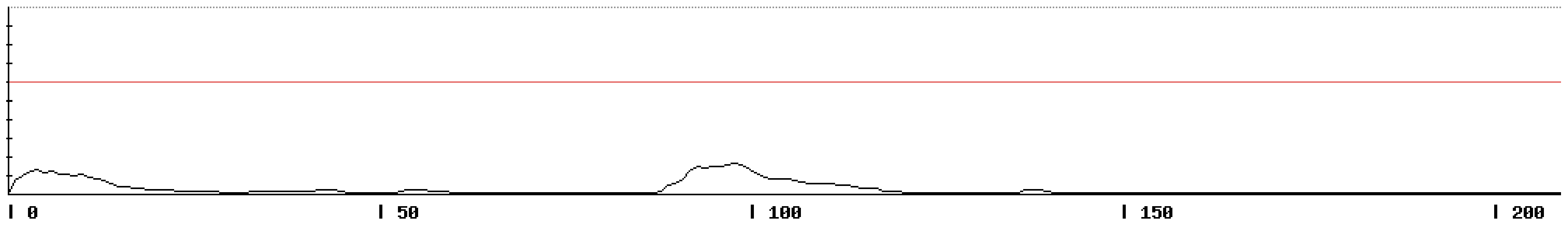

Predictions for Genliseaaurea_EPS63500.1

Viterbi Path

Viterbi Path

Posterior @ 0.6

Posterior @ 0.6

Posterior Graphic. Threshold Marked @ 0.6

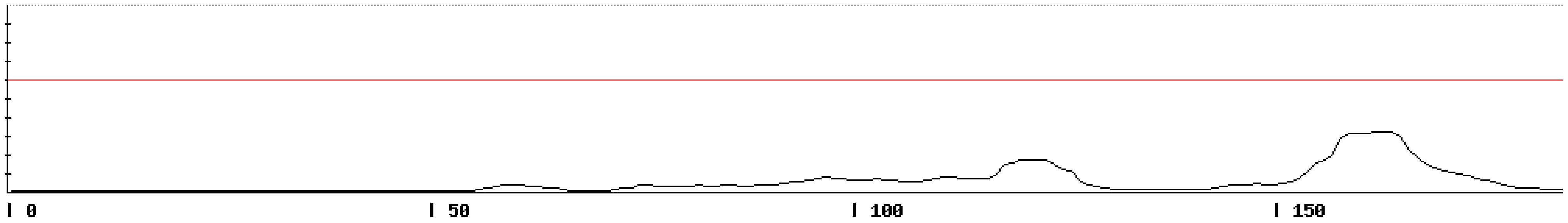

Predictions for Genliseaaurea_EPS63592.1

Viterbi Path

Viterbi Path

Posterior @ 0.6

Posterior @ 0.6

Posterior Graphic. Threshold Marked @ 0.6

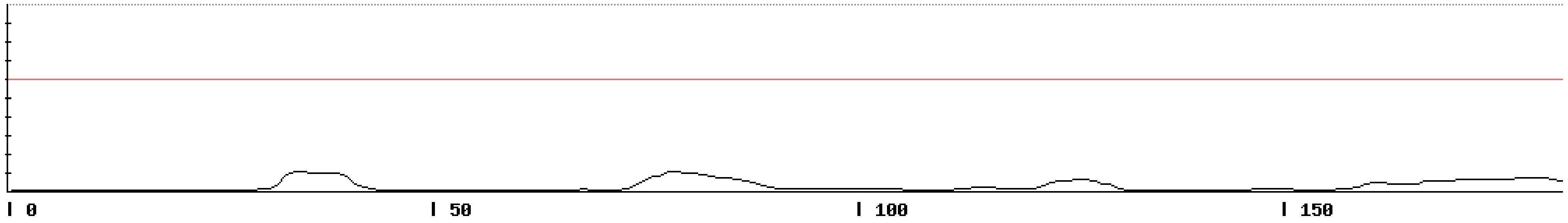

Predictions for Genliseaaurea_EPS63665.1

Viterbi Path	
Viterbi Path	
Posterior @ 0.6	
Posterior @ 0.6	
Posterior Graphic. Threshold Marked @ 0.6	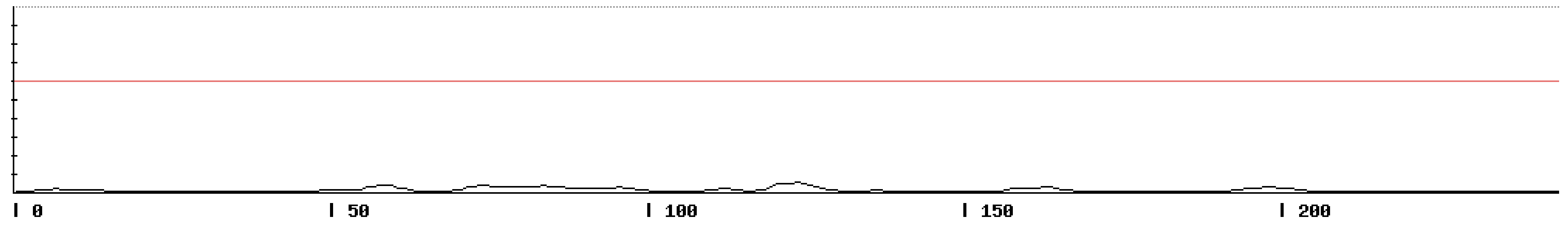

Predictions for Genliseaaurea_EPS63677.1

Viterbi Path	
Viterbi Path	
Posterior @ 0.6	
Posterior @ 0.6	
Posterior Graphic. Threshold Marked @ 0.6	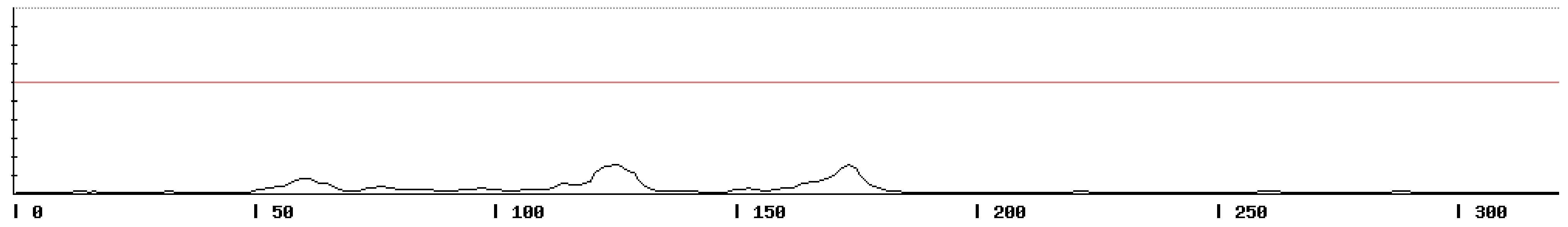

Predictions for Genliseaaurea_EPS63856.1	
Viterbi Path	
Viterbi Path	
Posterior @ 0.6	
Posterior @ 0.6	
Posterior Graphic. Threshold Marked @ 0.6	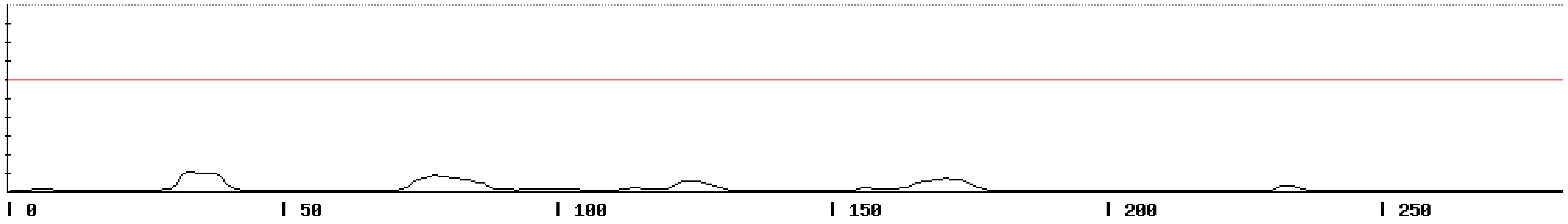

Predictions for Genliseaaurea_EPS63951.1	
Viterbi Path	
Viterbi Path	
Posterior @ 0.6	
Posterior @ 0.6	
Posterior Graphic. Threshold Marked @ 0.6	

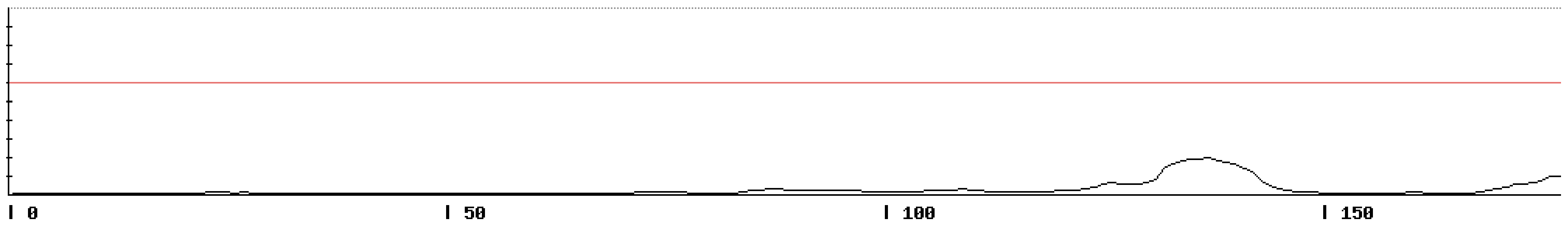

Predictions for Genliseaaurea_EPS64303.1

Viterbi Path	
Viterbi Path	
Posterior @ 0.6	
Posterior @ 0.6	
Posterior Graphic. Threshold Marked @ 0.6	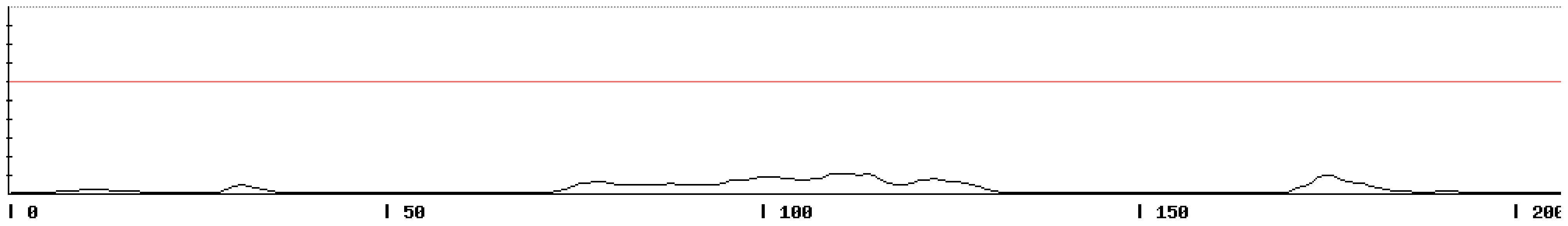

Predictions for Genliseaaurea_EPS64902.1

Viterbi Path	
Viterbi Path	
Posterior @ 0.6	
Posterior @ 0.6	

Posterior Graphic. Threshold Marked @ 0.6

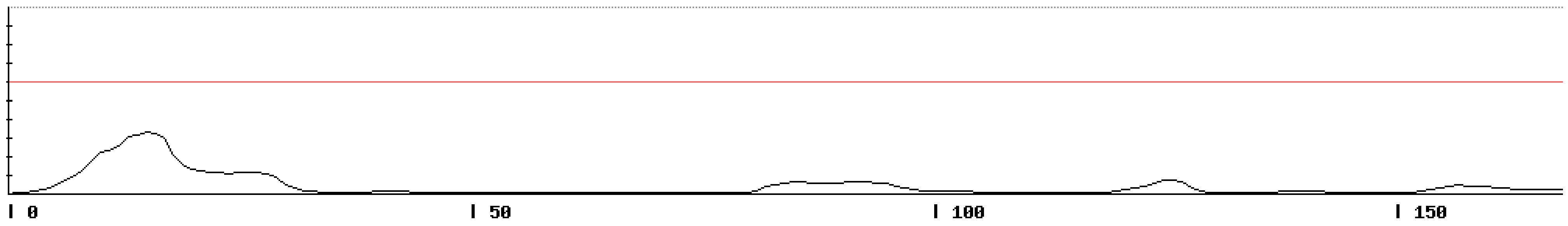

Predictions for Genliseaaurea_EPS65413.1

Viterbi Path

Viterbi Path

Posterior @ 0.6

Posterior @ 0.6

Posterior Graphic. Threshold Marked @ 0.6

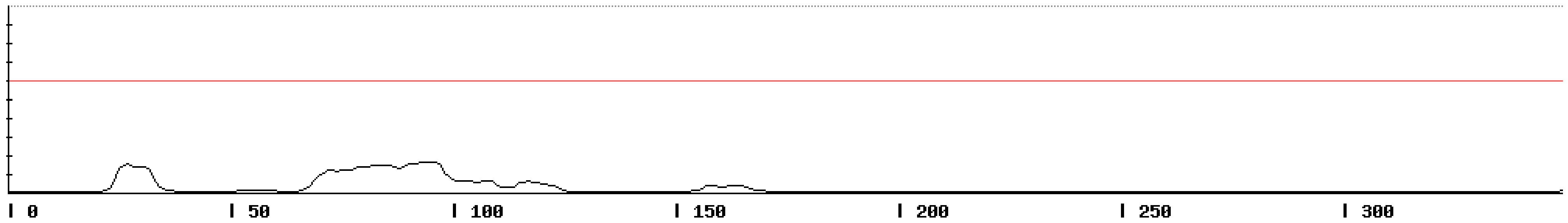

Predictions for Genliseaaurea_EPS65686.1

Viterbi Path

Viterbi Path

Posterior @ 0.6

Posterior @ 0.6

Posterior Graphic. Threshold Marked @ 0.6

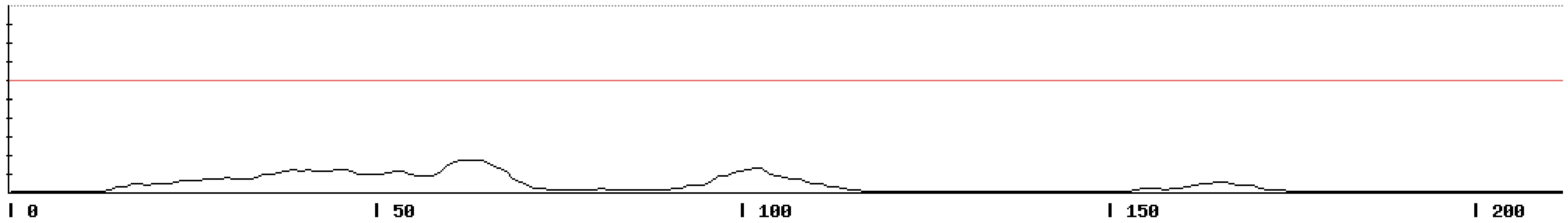

Predictions for Genliseaaurea_EPS65703.1

Viterbi Path

Viterbi Path

Posterior @ 0.6

Posterior @ 0.6

Posterior Graphic. Threshold Marked @ 0.6

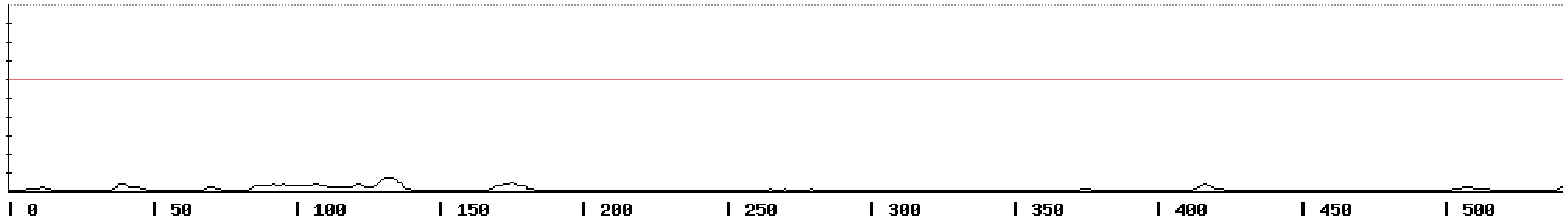

Predictions for Genliseaaurea_EPS65704.1

Viterbi Path

Viterbi Path

Posterior @ 0.6	
Posterior @ 0.6	
Posterior Graphic. Threshold Marked @ 0.6	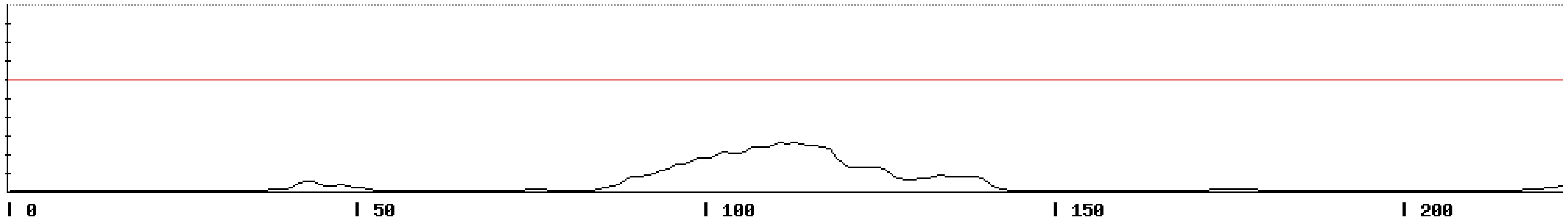

Predictions for Genliseaaurea_EPS65849.1

Viterbi Path	
Viterbi Path	
Posterior @ 0.6	
Posterior @ 0.6	
Posterior Graphic. Threshold Marked @ 0.6	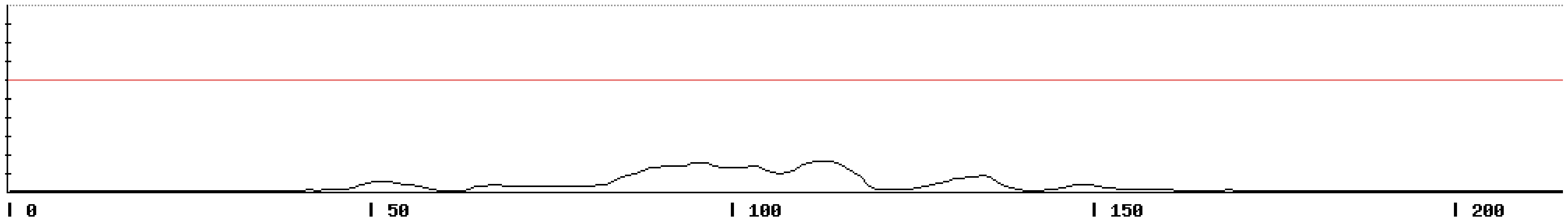

Predictions for Genliseaaurea_EPS66983.1

Viterbi Path	
Viterbi Path	

Posterior @ 0.6

Posterior @ 0.6

Posterior Graphic. Threshold Marked @ 0.6

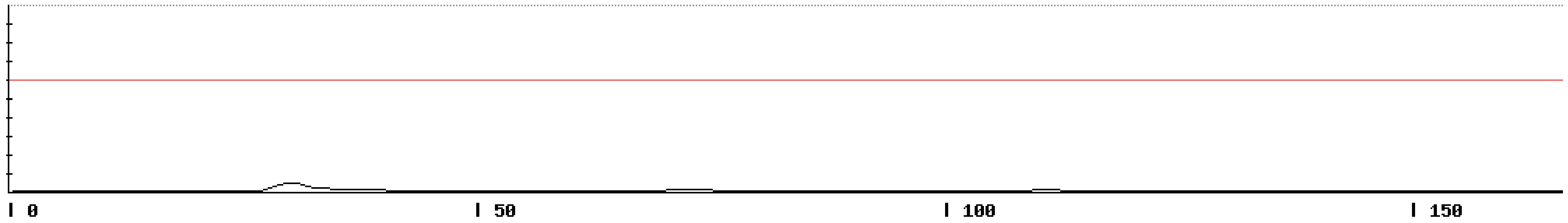

Predictions for Genliseaaurea_EPS67020.1

Viterbi Path

Viterbi Path

Posterior @ 0.6

Posterior @ 0.6

Posterior Graphic. Threshold Marked @ 0.6

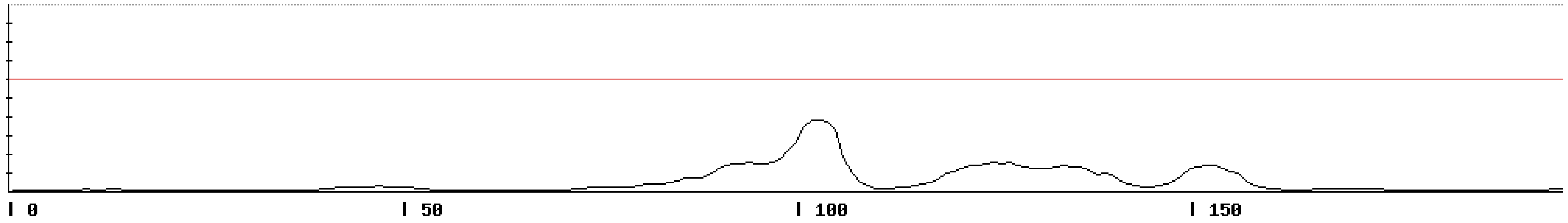

Predictions for Genliseaaurea_EPS67213.1

Viterbi Path

Viterbi Path

Posterior @ 0.6

Posterior @ 0.6

Posterior Graphic. Threshold Marked @ 0.6

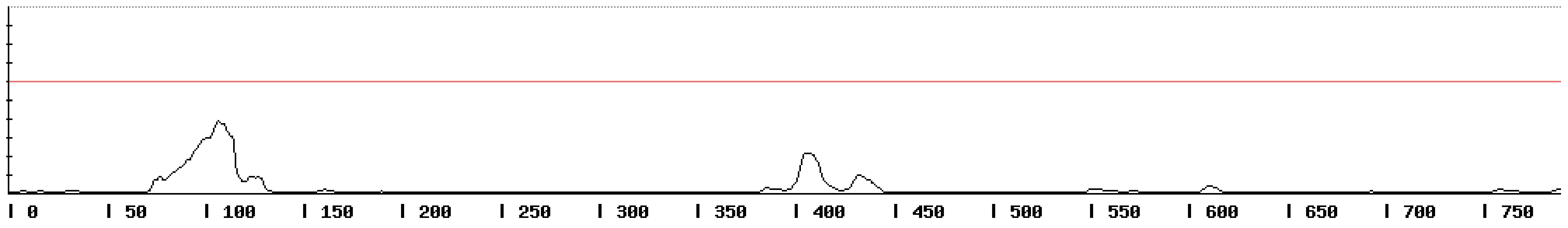

Predictions for Genliseaaurea_EPS68202.1

Viterbi Path

Viterbi Path

Posterior @ 0.6

Posterior @ 0.6

Posterior Graphic. Threshold Marked @ 0.6

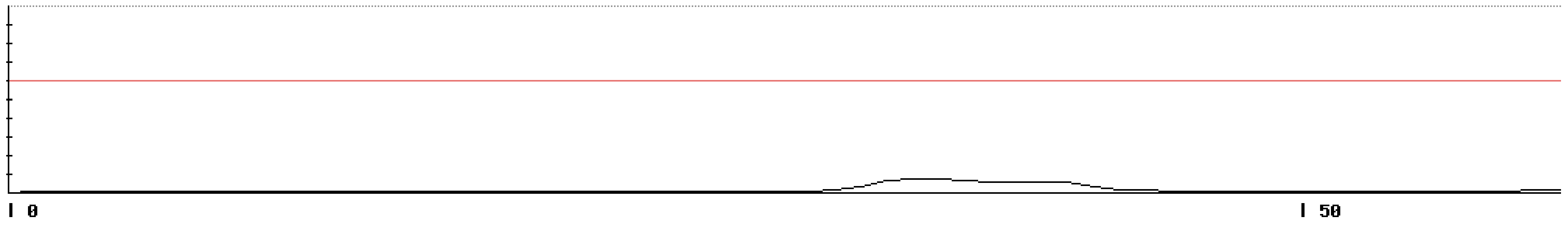

Predictions for Genliseaaurea_EPS68229.1	
Viterbi Path	
Viterbi Path	
Posterior @ 0.6	
Posterior @ 0.6	
Posterior Graphic. Threshold Marked @ 0.6	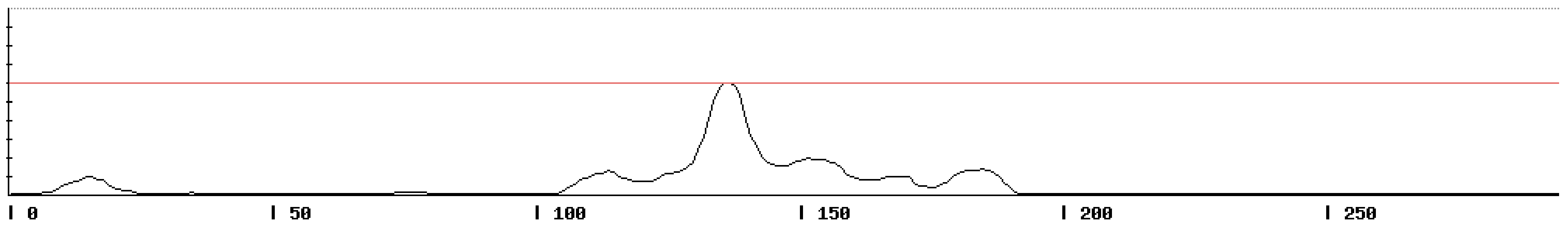

Predictions for Genliseaaurea_EPS68490.1	
Viterbi Path	
Viterbi Path	
Posterior @ 0.6	
Posterior @ 0.6	
Posterior Graphic. Threshold Marked @ 0.6	

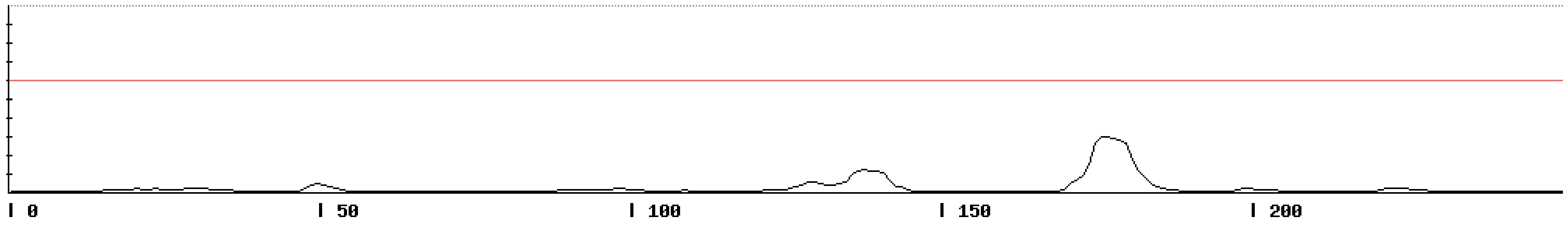

Predictions for Genliseaaurea_EPS69041.1

Viterbi Path	
Viterbi Path	
Posterior @ 0.6	
Posterior @ 0.6	
Posterior Graphic. Threshold Marked @ 0.6	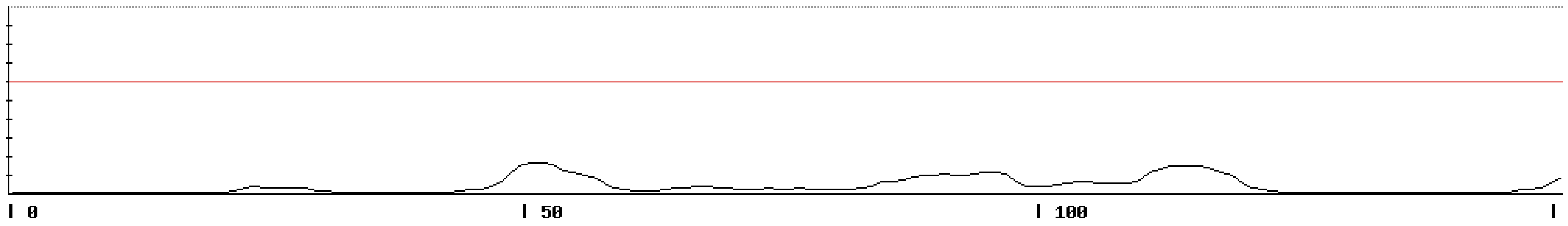

Predictions for Genliseaaurea_EPS69130.1

Viterbi Path	
Viterbi Path	
Posterior @ 0.6	
Posterior @ 0.6	

Posterior Graphic. Threshold Marked @ 0.6

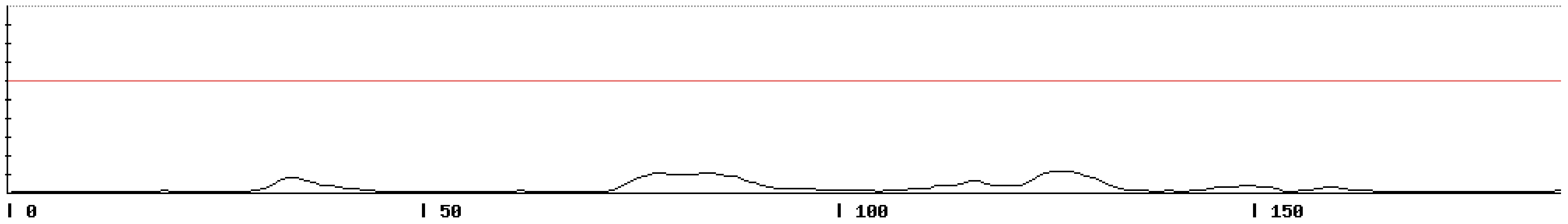

Predictions for Genliseaaurea_EPS69330.1

Viterbi Path

Viterbi Path

Posterior @ 0.6

Posterior @ 0.6

Posterior Graphic. Threshold Marked @ 0.6

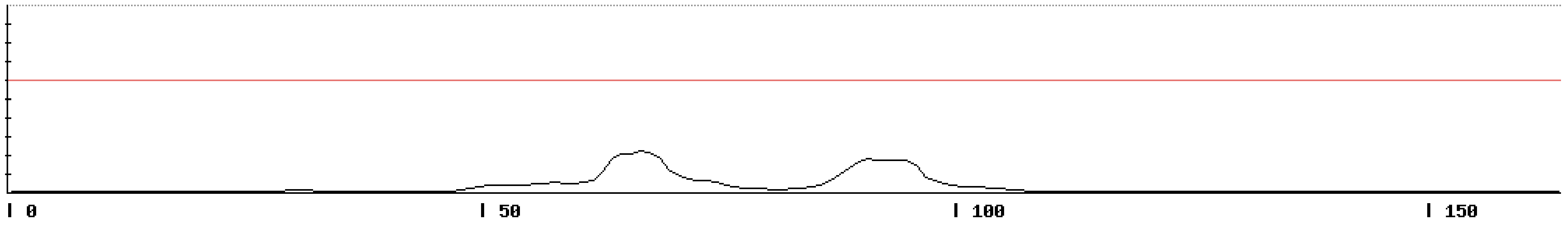

Predictions for Genliseaaurea_EPS70002.1

Viterbi Path

Viterbi Path

Posterior @ 0.6

Posterior @ 0.6

Posterior Graphic. Threshold Marked @ 0.6

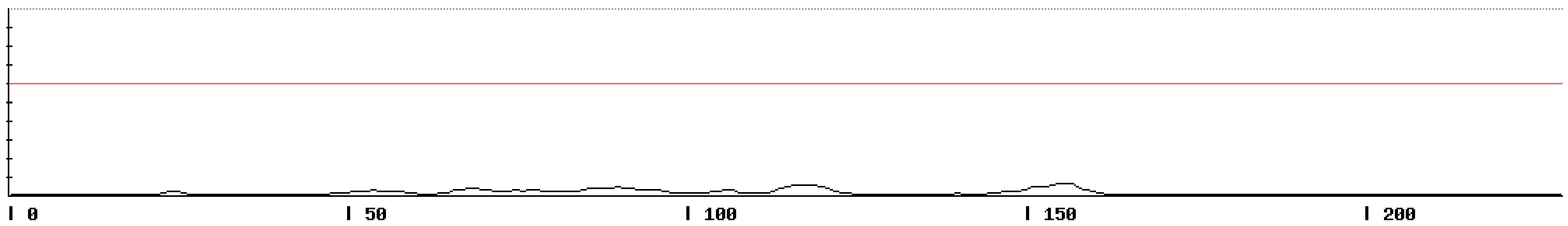

Predictions for Genliseaaurea_EPS70298.1

Viterbi Path

Viterbi Path

Posterior @ 0.6

Posterior @ 0.6

Posterior Graphic. Threshold Marked @ 0.6

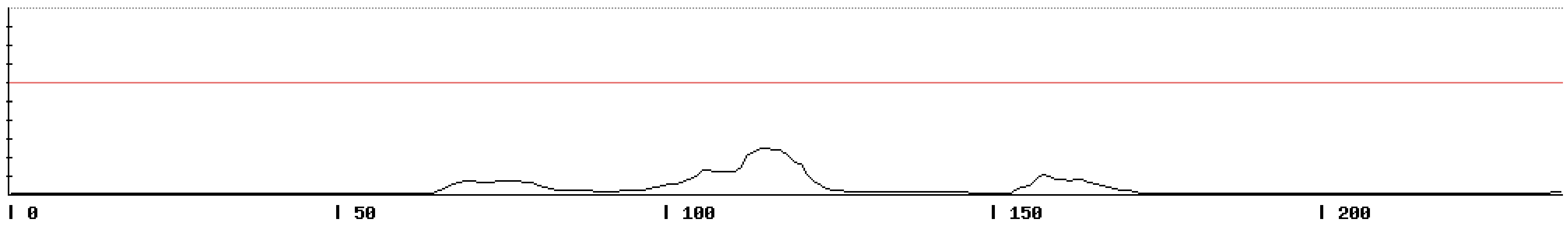

Predictions for Genliseaaurea_EPS70444.1

Viterbi Path

Viterbi Path

Posterior @ 0.6

Posterior @ 0.6

Posterior Graphic. Threshold Marked @ 0.6

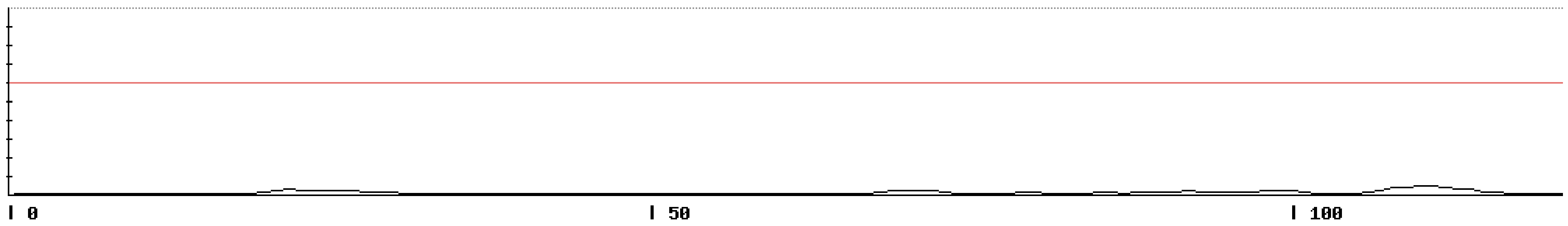

Predictions for Genliseaaurea_EPS70863.1

Viterbi Path

Viterbi Path

Posterior @ 0.6

Posterior @ 0.6

Posterior Graphic. Threshold Marked @ 0.6

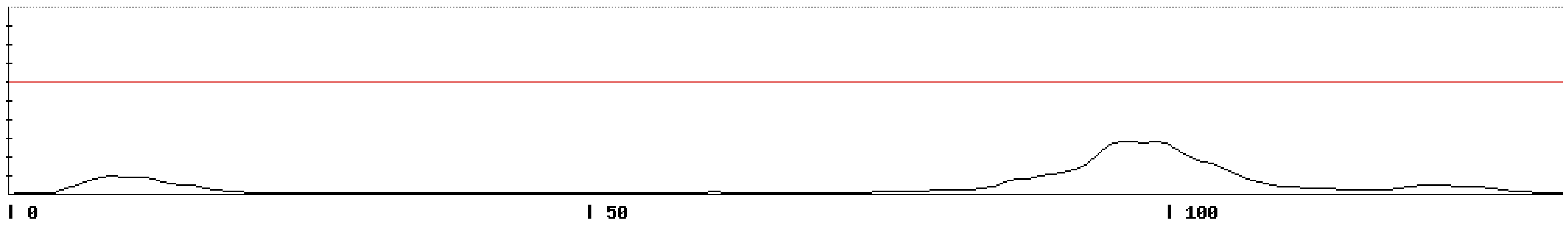

Predictions for Genliseaaurea_EPS70912.1

Viterbi Path

Viterbi Path

Posterior @ 0.6

Posterior @ 0.6

Posterior Graphic. Threshold Marked @ 0.6

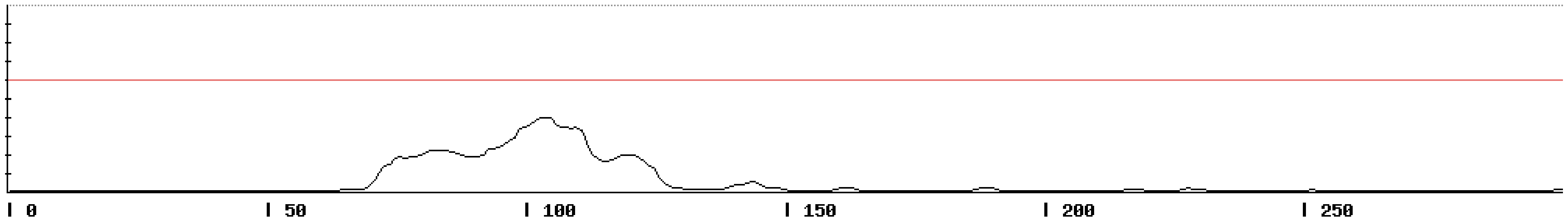

Predictions for Genliseaaurea_EPS71066.1

Viterbi Path

Viterbi Path

Posterior @ 0.6

Posterior @ 0.6

Posterior Graphic. Threshold Marked @ 0.6

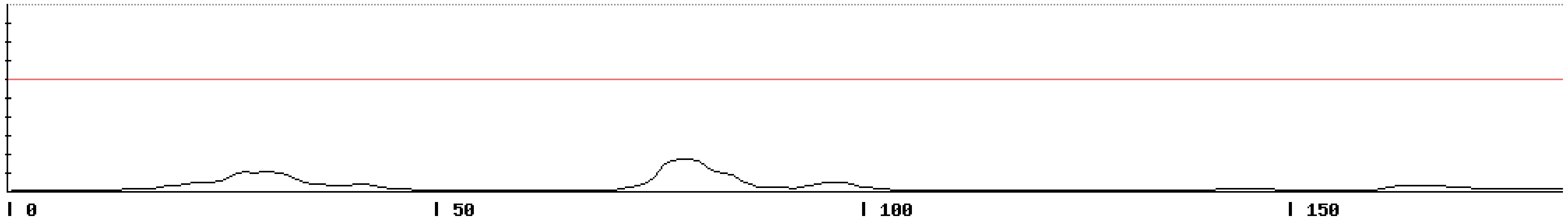

Predictions for Genliseaaurea_EPS72463.1

Viterbi Path

Viterbi Path

Posterior @ 0.6

Posterior @ 0.6

Posterior Graphic. Threshold Marked @ 0.6

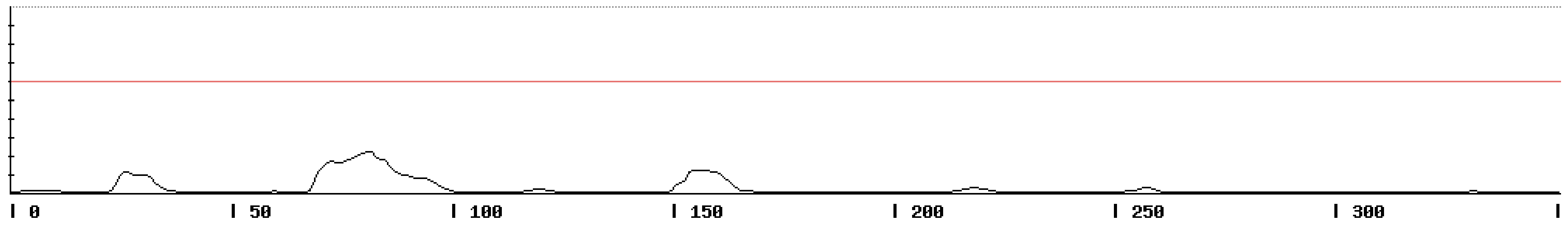

Predictions for Genliseaaurea_EPS72690.1

Viterbi Path

Viterbi Path

Posterior @ 0.6

Posterior @ 0.6

Posterior Graphic. Threshold Marked @ 0.6

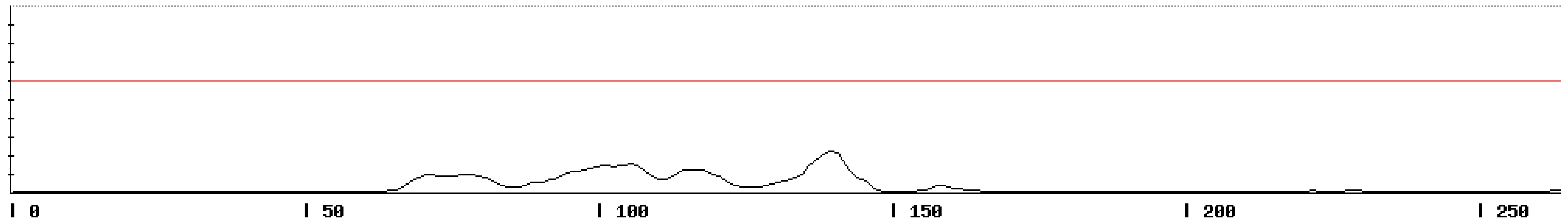

Predictions for Genliseaaurea_EPS72933.1

Viterbi Path	
Viterbi Path	
Posterior @ 0.6	
Posterior @ 0.6	
Posterior Graphic. Threshold Marked @ 0.6	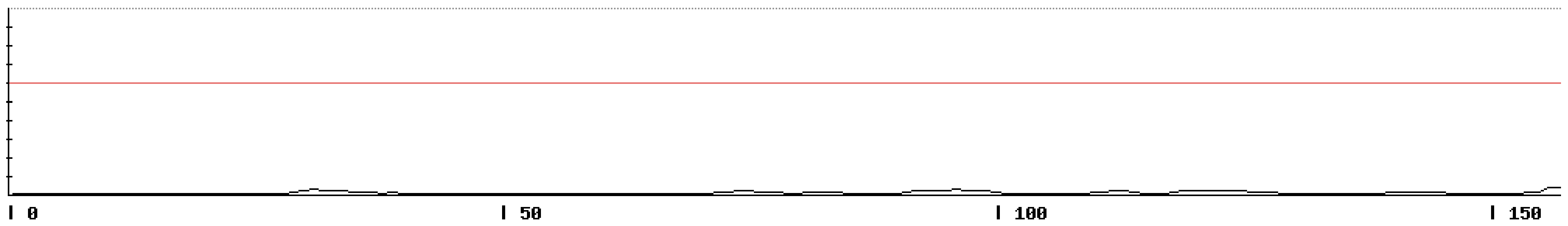

Predictions for Genliseaaurea_EPS73268.1

Viterbi Path	
Viterbi Path	
Posterior @ 0.6	95 - RKRRK - 99
	111 - WHKTGKTRPISSARKVKG - 128
Posterior @ 0.6	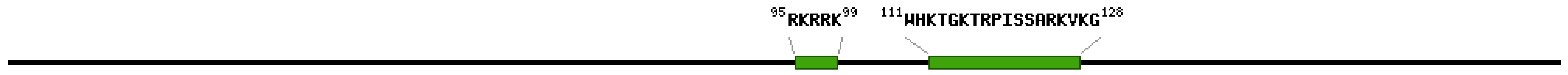

Revision r.9 - Last Updated on : February 24 2015 17:45:20. alex.nguyenba@utoronto.ca

Intended to be used with Firefox

Viterbi Path	
Posterior @ 0.4	
Posterior @ 0.4	
Posterior Graphic. Threshold Marked @ 0.4	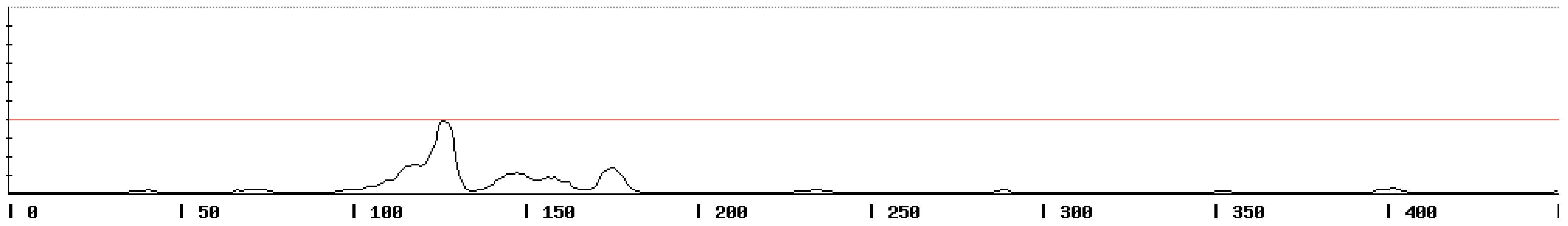

Predictions for Glycinemax_Glyma.01G046800.1.p

Viterbi Path	
Viterbi Path	
Posterior @ 0.4	
Posterior @ 0.4	
Posterior Graphic. Threshold Marked @ 0.4	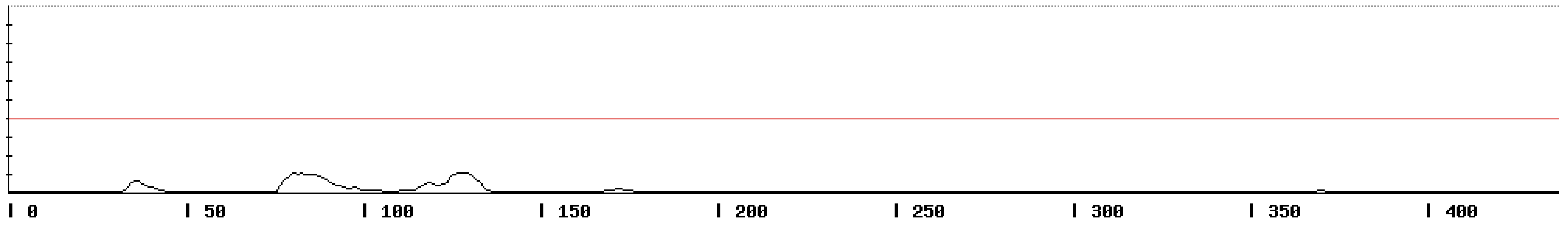

Predictions for Glycinemax_Glyma.01G051300.1.p

Viterbi Path

Viterbi Path

Posterior @ 0.4

Posterior @ 0.4

Posterior Graphic. Threshold Marked @ 0.4

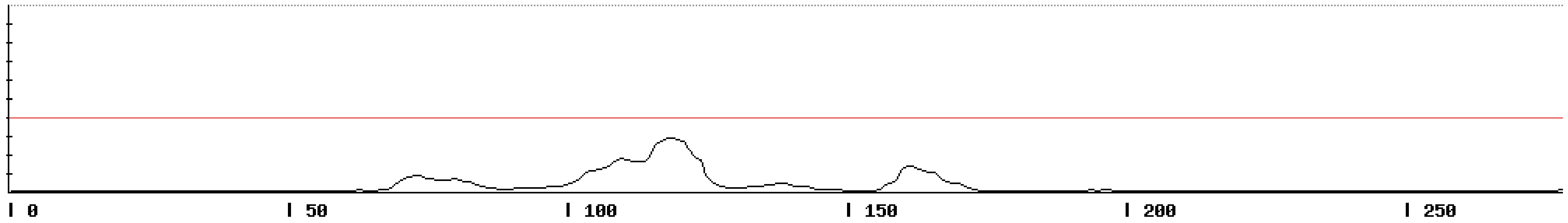

Predictions for Glycinemax_Glyma.01G088200.1.p

Viterbi Path

Viterbi Path

Posterior @ 0.4

Posterior @ 0.4

Posterior Graphic. Threshold Marked @ 0.4

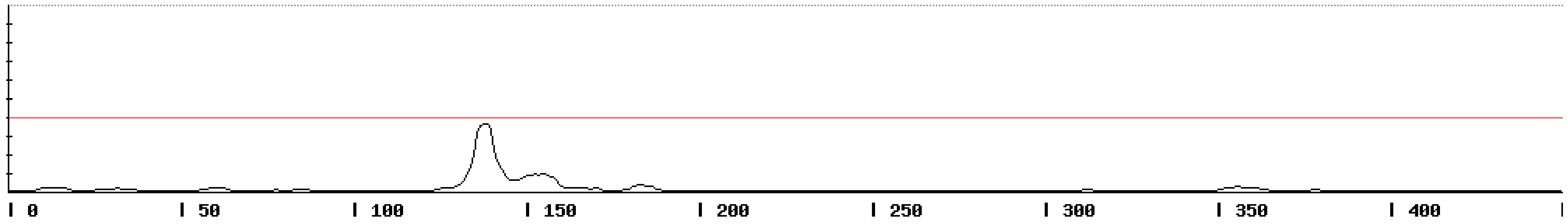

Predictions for Glycinemax_Glyma.01G167900.1.p

Viterbi Path

Viterbi Path

Posterior @ 0.4

Posterior @ 0.4

Posterior Graphic. Threshold Marked @ 0.4

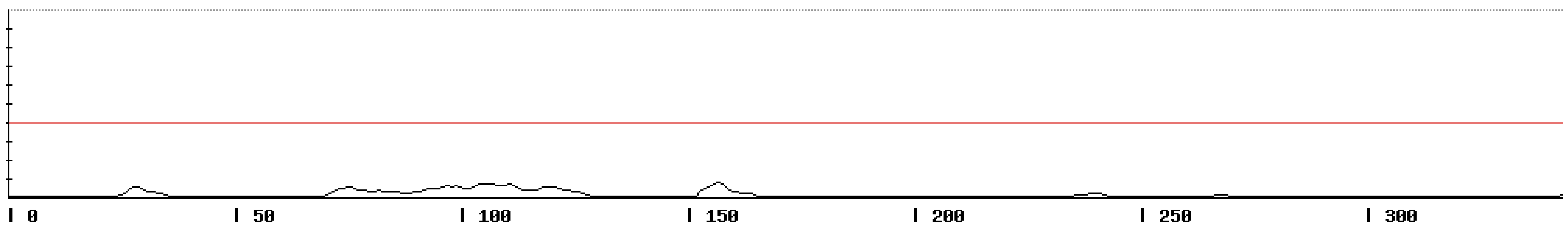

Predictions for Glycinemax_Glyma.02G050100.1.p

Viterbi Path

Viterbi Path

Posterior @ 0.4

Posterior @ 0.4

Posterior Graphic. Threshold Marked @ 0.4

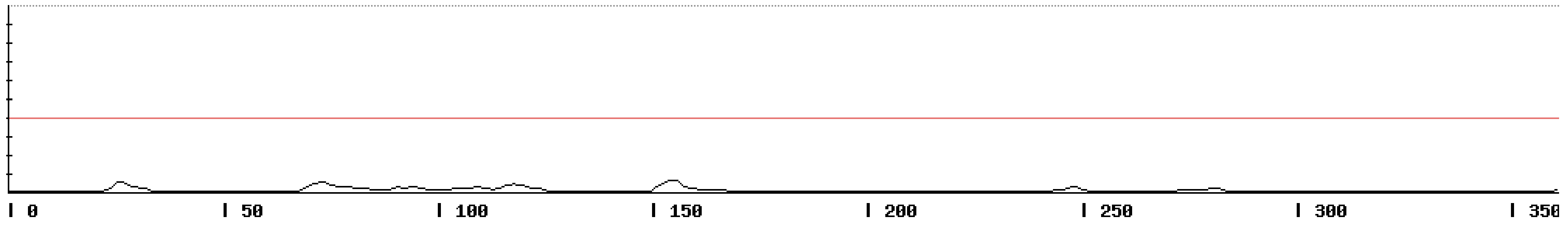

Predictions for Glycinemax_Glyma.02G070000.1.p

Viterbi Path

Viterbi Path

Posterior @ 0.4

Posterior @ 0.4

Posterior Graphic. Threshold Marked @ 0.4

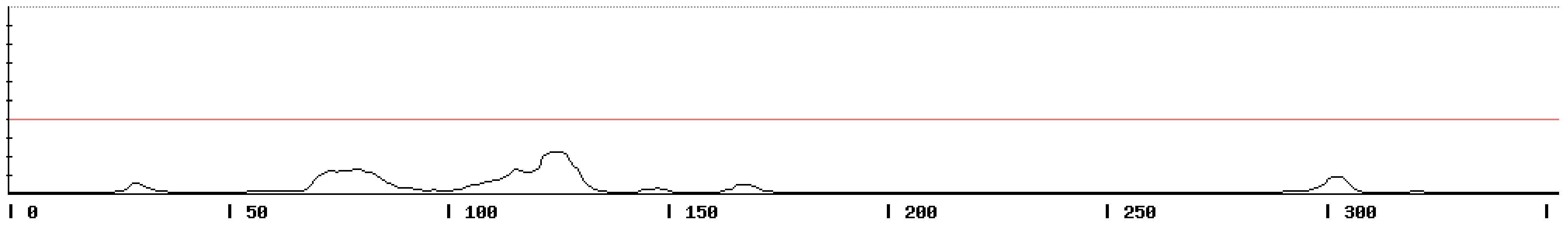

Predictions for Glycinemax_Glyma.02G070600.1.p

Viterbi Path

Viterbi Path

Posterior @ 0.4

Posterior @ 0.4

Posterior Graphic. Threshold Marked @ 0.4	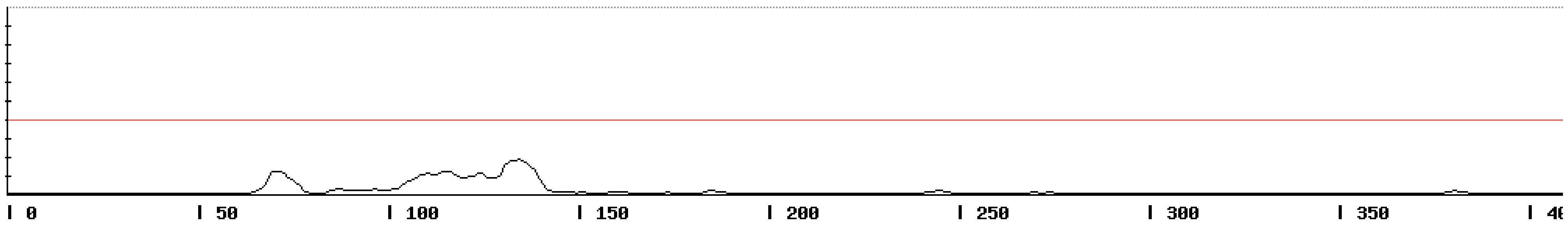

Predictions for Glycinemax_Glyma.02G100200.1.p	
Viterbi Path	
Viterbi Path	
Posterior @ 0.4	137 - KRR - 139
Posterior @ 0.4	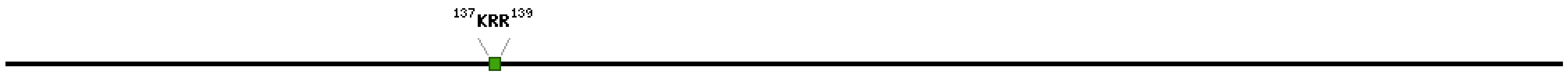
Posterior Graphic. Threshold Marked @ 0.4	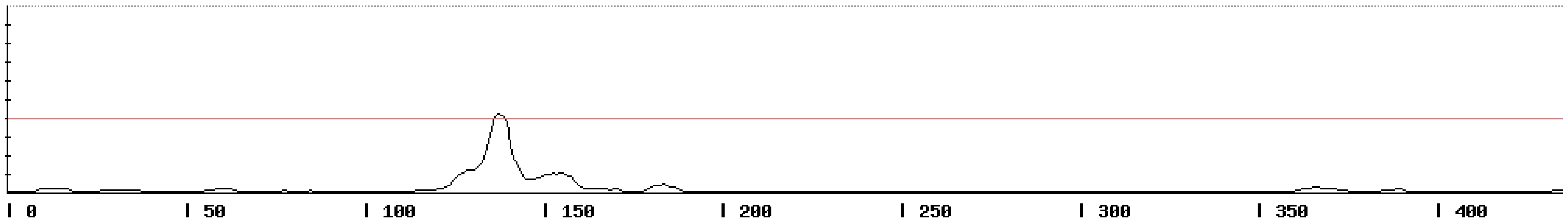

Predictions for Glycinemax_Glyma.02G107000.1.p	
Viterbi Path	
Viterbi Path	
Posterior @ 0.4	
Posterior @ 0.4	

Posterior Graphic. Threshold Marked @ 0.4

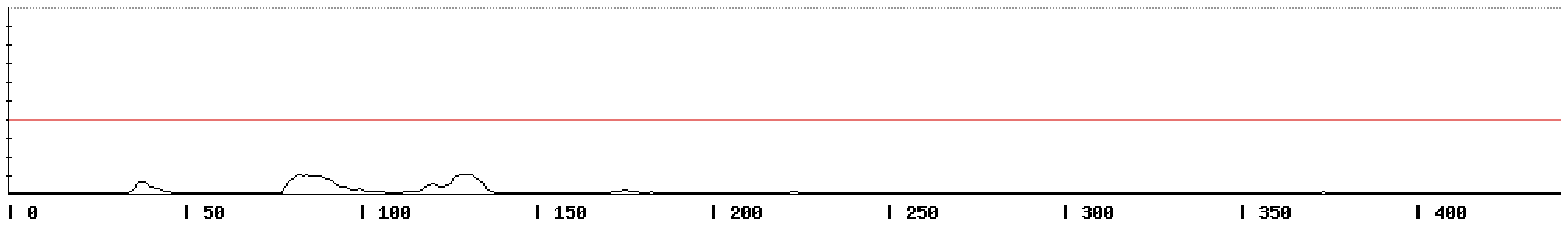

Predictions for Glycinemax_Glyma.02G109800.1.p

Viterbi Path

Viterbi Path

Posterior @ 0.4

Posterior @ 0.4

Posterior Graphic. Threshold Marked @ 0.4

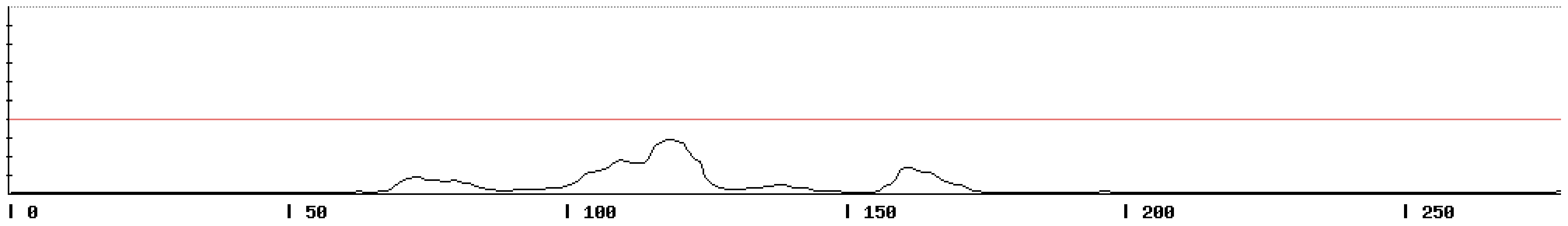

Predictions for Glycinemax_Glyma.02G222300.1.p

Viterbi Path

Viterbi Path

Posterior @ 0.4

Posterior @ 0.4

Posterior Graphic. Threshold Marked @ 0.4

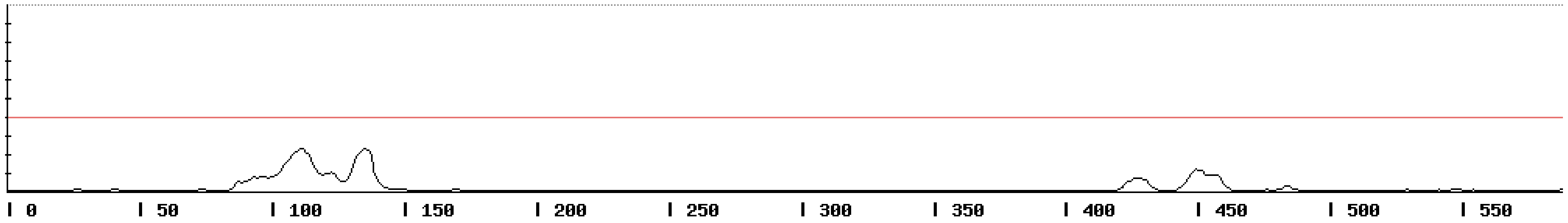

Predictions for Glycinemax_Glyma.02G240500.1.p

Viterbi Path

Viterbi Path

Posterior @ 0.4

Posterior @ 0.4

Posterior Graphic. Threshold Marked @ 0.4

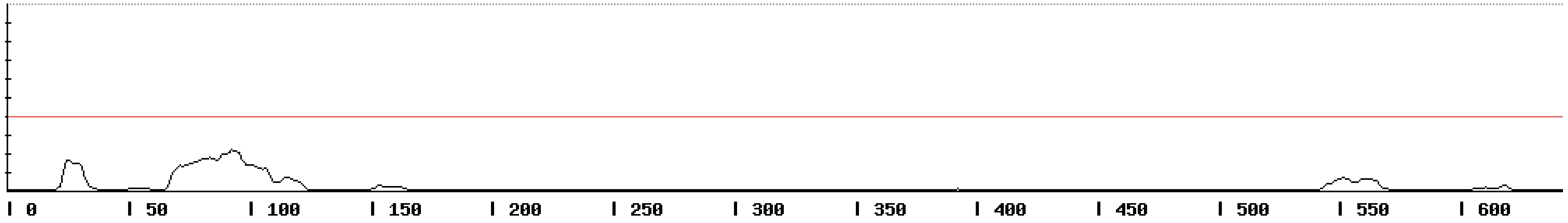

Predictions for Glycinemax_Glyma.02G284300.1.p

Viterbi Path

Viterbi Path

Posterior @ 0.4	
Posterior @ 0.4	
Posterior Graphic. Threshold Marked @ 0.4	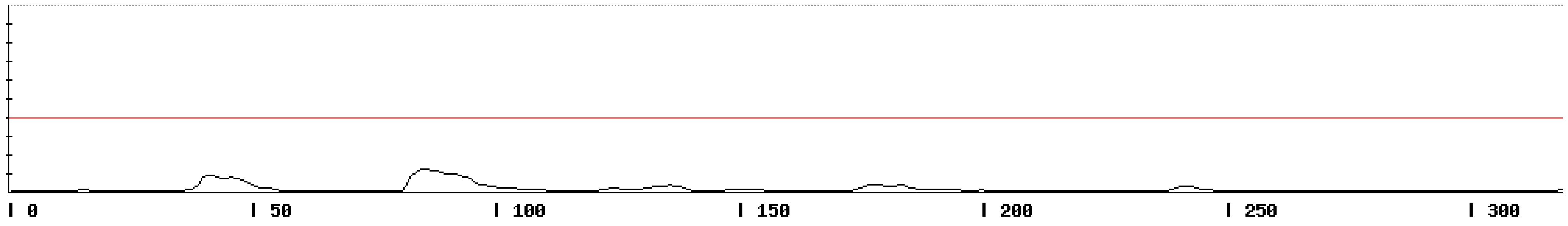

Predictions for Glycinemax_Glyma.03G164200.1.p

Viterbi Path	
Viterbi Path	
Posterior @ 0.4	
Posterior @ 0.4	
Posterior Graphic. Threshold Marked @ 0.4	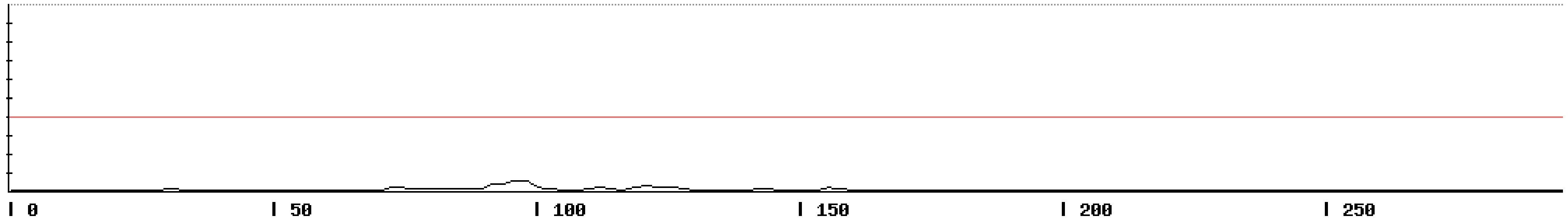

Predictions for Glycinemax_Glyma.03G179600.1.p

Viterbi Path

Viterbi Path

Posterior @ 0.4

Posterior @ 0.4

Posterior Graphic. Threshold Marked @ 0.4

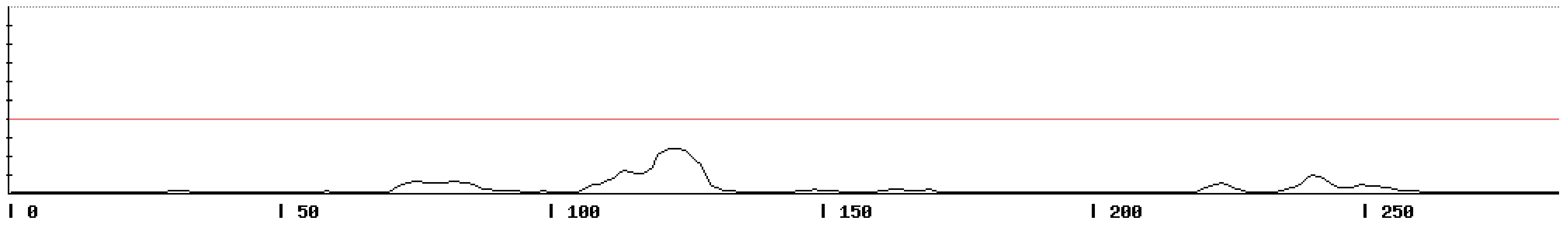

Predictions for Glycinemax_Glyma.03G197900.1.p

Viterbi Path

Viterbi Path

Posterior @ 0.4

Posterior @ 0.4

Posterior Graphic. Threshold Marked @ 0.4

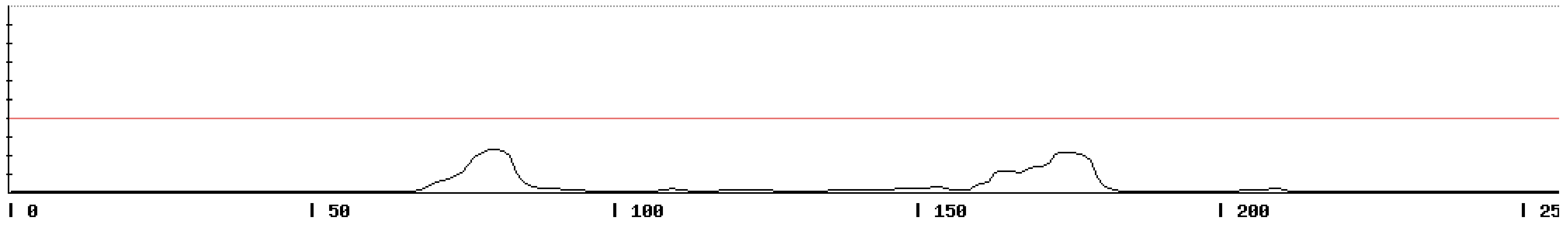

Predictions for Glycinemax_Glyma.04G014900.1.p

Viterbi Path	
Viterbi Path	
Posterior @ 0.4	
Posterior @ 0.4	
Posterior Graphic. Threshold Marked @ 0.4	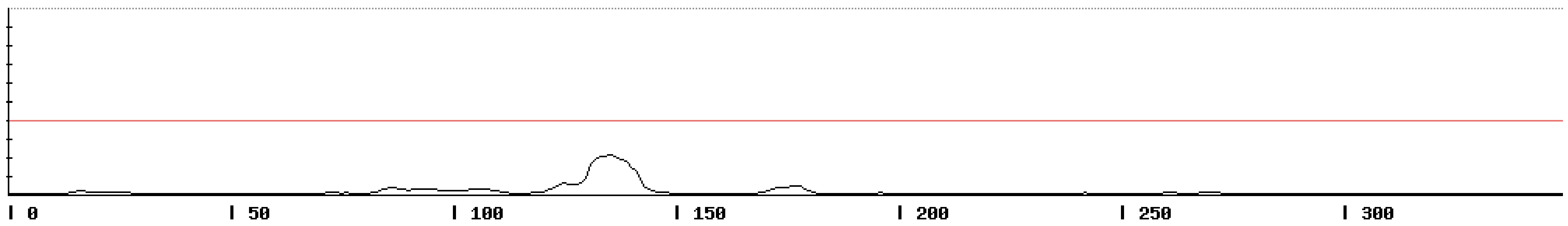

Predictions for Glycinemax_Glyma.04G078600.1.p

Viterbi Path	
Viterbi Path	
Posterior @ 0.4	
Posterior @ 0.4	
Posterior Graphic. Threshold Marked @ 0.4	

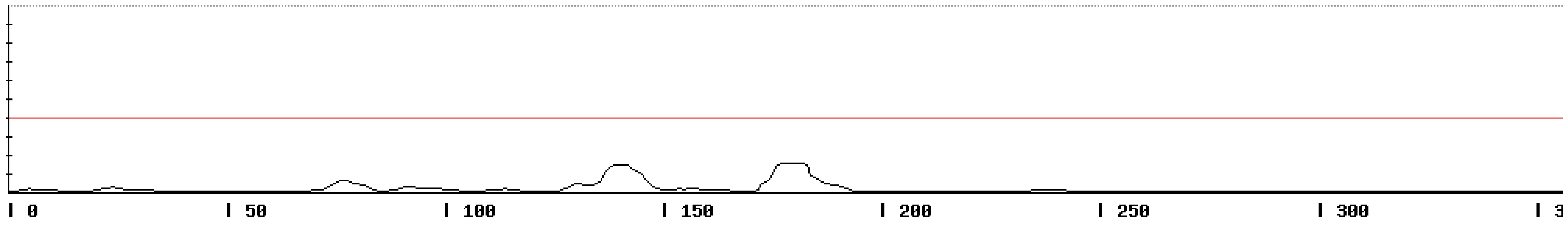

Predictions for Glycinemax_Glyma.04G119500.1.p

Viterbi Path

Viterbi Path

Posterior @ 0.4

Posterior @ 0.4

Posterior Graphic. Threshold Marked @ 0.4

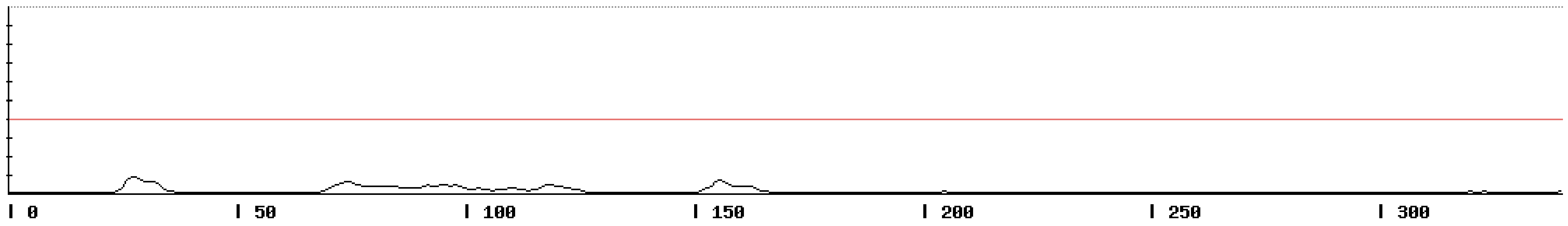

Predictions for Glycinemax_Glyma.04G167200.1.p

Viterbi Path

Viterbi Path

Posterior @ 0.4

Posterior @ 0.4

Posterior Graphic. Threshold Marked @ 0.4

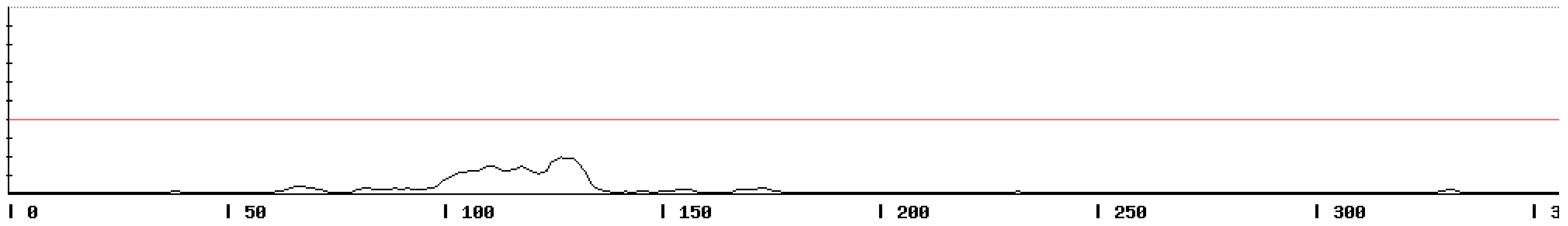

Predictions for Glycinemax_Glyma.04G175800.1.p

Viterbi Path

Viterbi Path

Posterior @ 0.4

Posterior @ 0.4

Posterior Graphic. Threshold Marked @ 0.4

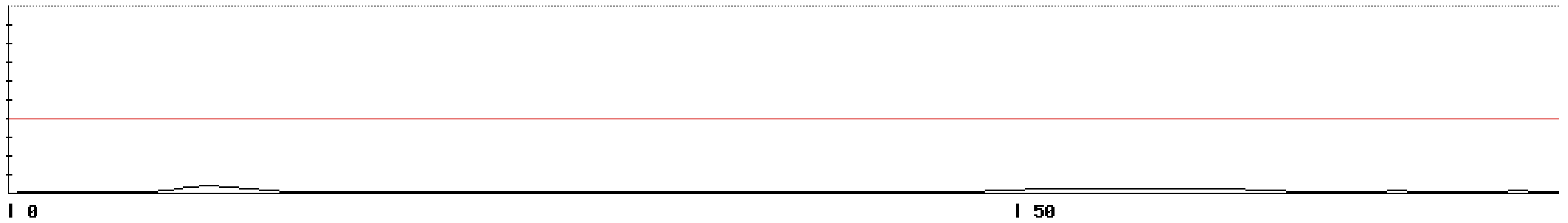

Predictions for Glycinemax_Glyma.04G199000.1.p

Viterbi Path

Viterbi Path

Posterior @ 0.4

Posterior @ 0.4

Posterior Graphic. Threshold Marked @ 0.4

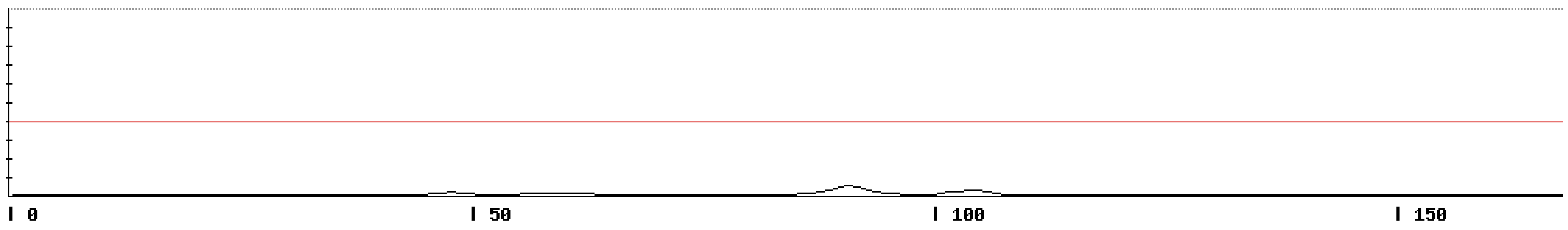

Predictions for Glycinemax_Glyma.04G208300.1.p

Viterbi Path

Viterbi Path

Posterior @ 0.4 — 92 - GADKPIGKPKALGIKKA - 108

Posterior @ 0.4

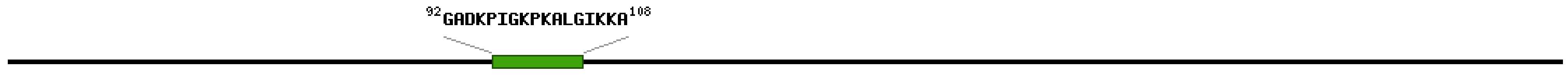

Posterior Graphic. Threshold Marked @ 0.4

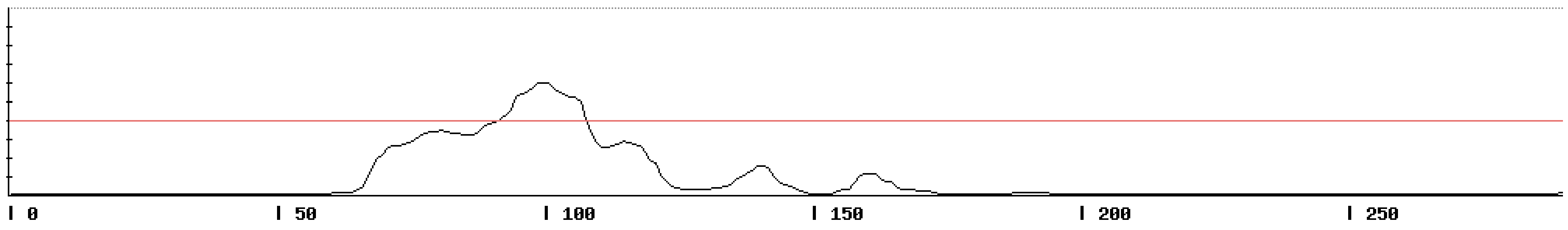

Predictions for Glycinemax_Glyma.04G212000.1.p

Viterbi Path

Viterbi Path

Posterior @ 0.4	140 - RRRRK - 144
Posterior @ 0.4	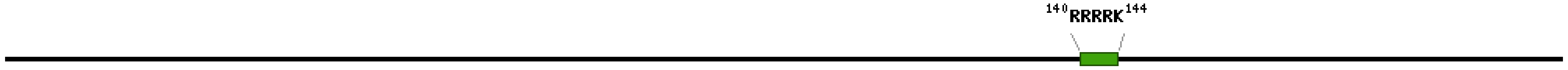
Posterior Graphic. Threshold Marked @ 0.4	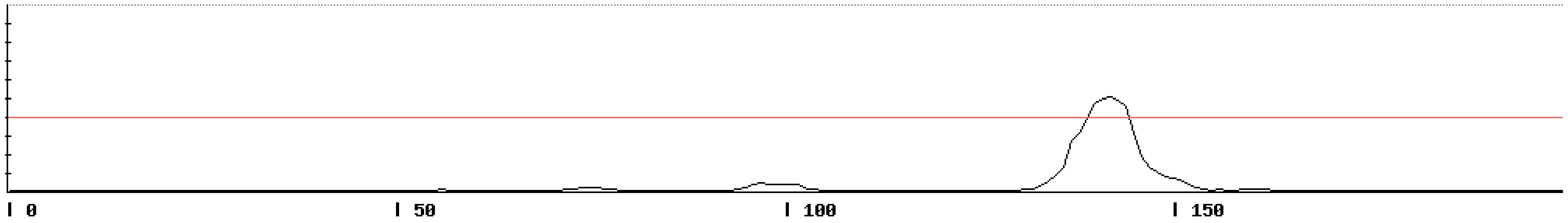

Predictions for Glycinemax_Glyma.04G213300.1.p

Viterbi Path	
Viterbi Path	
Posterior @ 0.4	25 - RKVRGKK - 31
Posterior @ 0.4	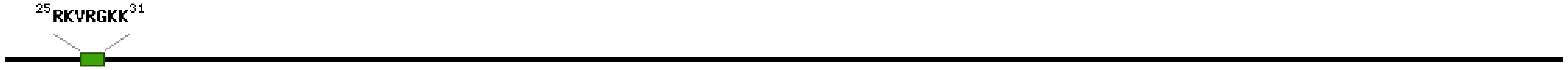
Posterior Graphic. Threshold Marked @ 0.4	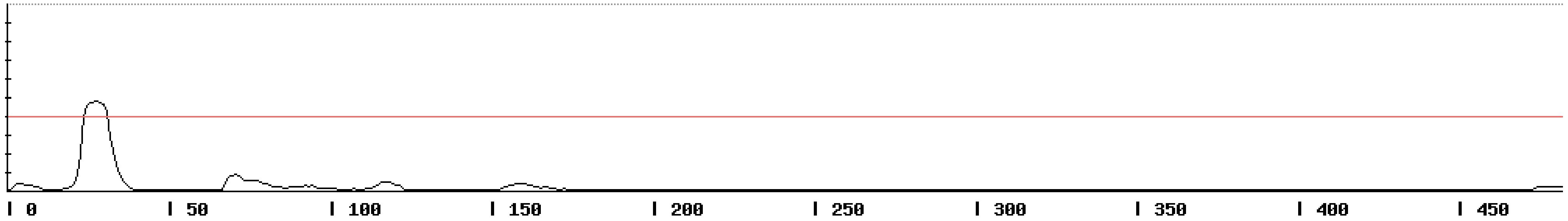

Predictions for Glycinemax_Glyma.04G226700.1.p

Viterbi Path	
Viterbi Path	

Posterior @ 0.4	
Posterior @ 0.4	
Posterior Graphic. Threshold Marked @ 0.4	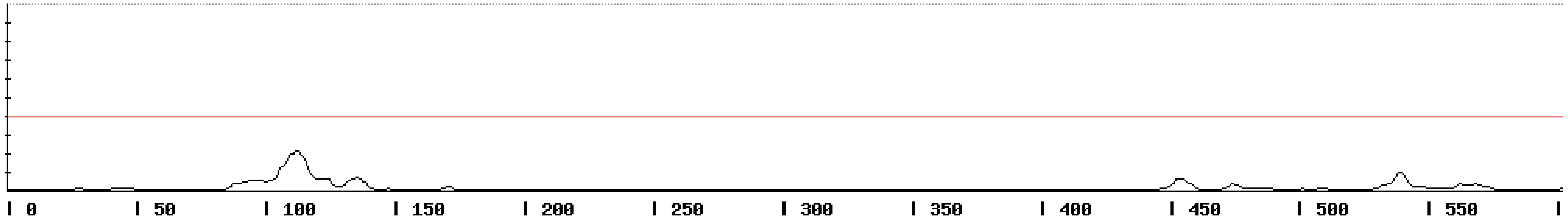

Predictions for Glycinemax_Glyma.04G249000.1.p

Viterbi Path	
Viterbi Path	
Posterior @ 0.4	
Posterior @ 0.4	
Posterior Graphic. Threshold Marked @ 0.4	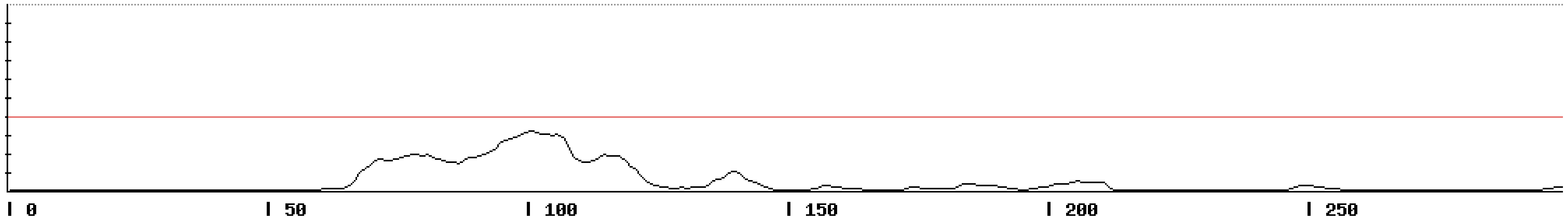

Predictions for Glycinemax_Glyma.05G002700.1.p

Viterbi Path

Viterbi Path

Posterior @ 0.4 — 132 - PRKRRK - 137

Posterior @ 0.4

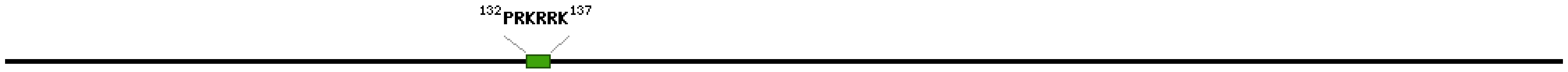

Posterior Graphic. Threshold Marked @ 0.4

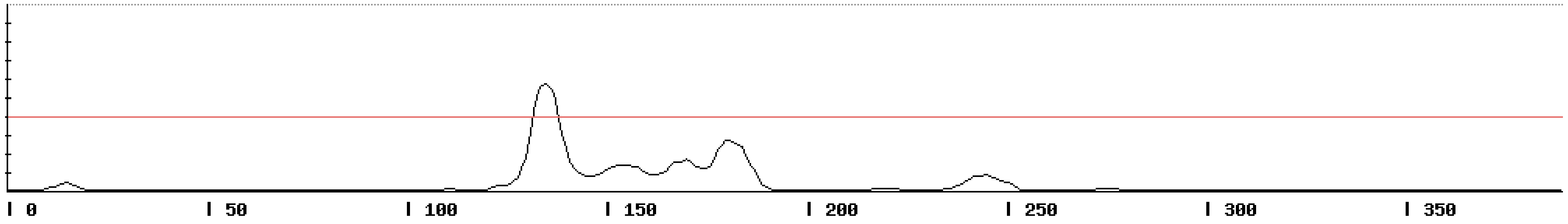

Predictions for Glycinemax_Glyma.05G025500.1.p

Viterbi Path

Viterbi Path

Posterior @ 0.4

Posterior @ 0.4

Posterior Graphic. Threshold Marked @ 0.4

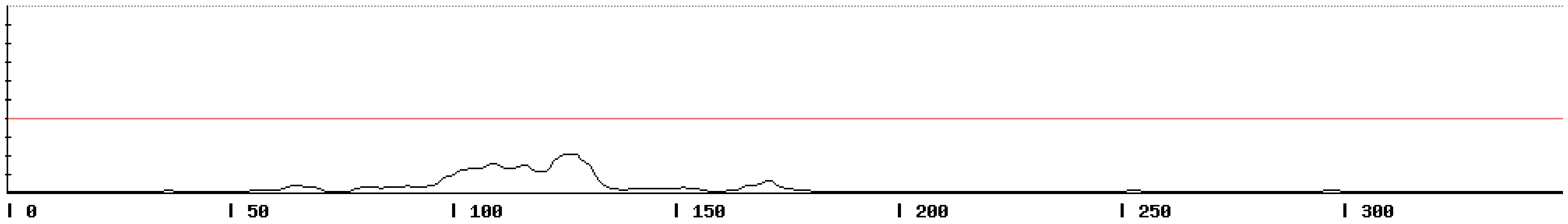

Predictions for Glycinemax_Glyma.05G055900.1.p

Viterbi Path

Viterbi Path

Posterior @ 0.4

Posterior @ 0.4

Posterior Graphic. Threshold Marked @ 0.4

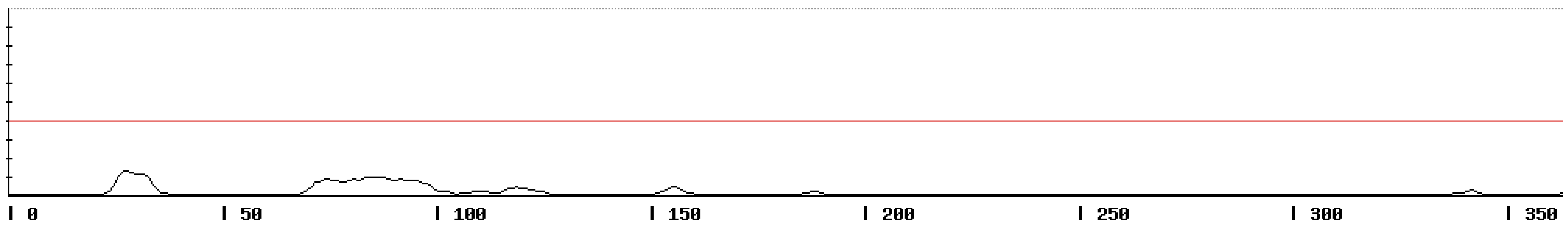

Predictions for Glycinemax_Glyma.05G086000.1.p

Viterbi Path

Viterbi Path

Posterior @ 0.4

Posterior @ 0.4

Posterior Graphic. Threshold Marked @ 0.4

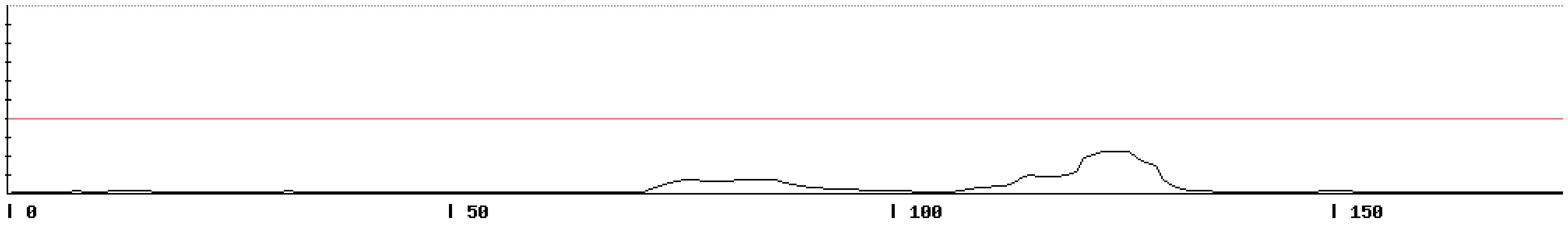

Predictions for Glycinemax_Glyma.05G108700.1.p

Viterbi Path

Viterbi Path

Posterior @ 0.4

Posterior @ 0.4

Posterior Graphic. Threshold Marked @ 0.4

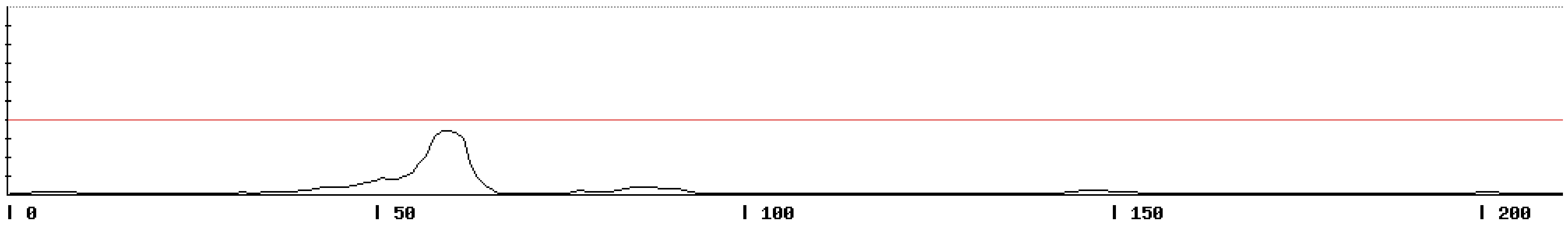

Predictions for Glycinemax_Glyma.05G113000.1.p

Viterbi Path

Viterbi Path

Posterior @ 0.4

Posterior @ 0.4

Posterior Graphic. Threshold Marked @ 0.4

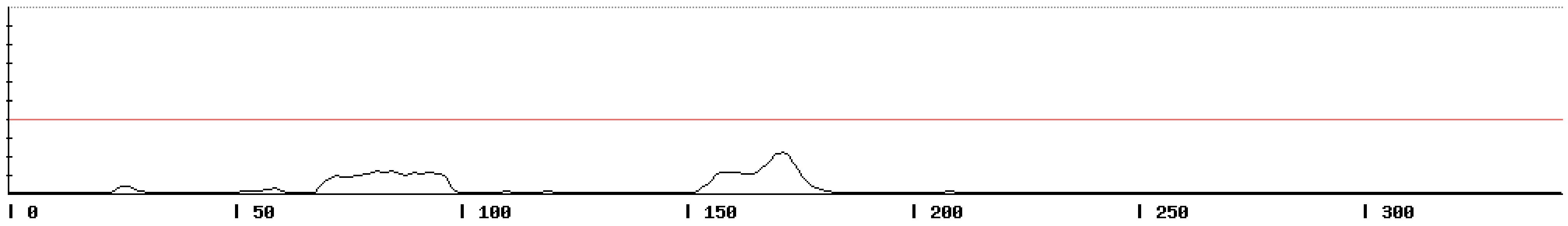

Predictions for Glycinemax_Glyma.05G120500.1.p

Viterbi Path

Viterbi Path

Posterior @ 0.4

Posterior @ 0.4

Posterior Graphic. Threshold Marked @ 0.4

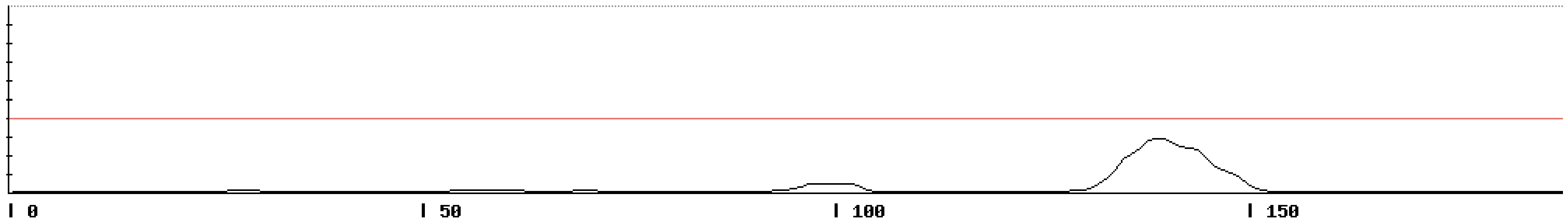

Predictions for Glycinemax_Glyma.05G191300.1.p

Viterbi Path

Viterbi Path

Posterior @ 0.4

Posterior @ 0.4

Posterior Graphic. Threshold Marked @ 0.4

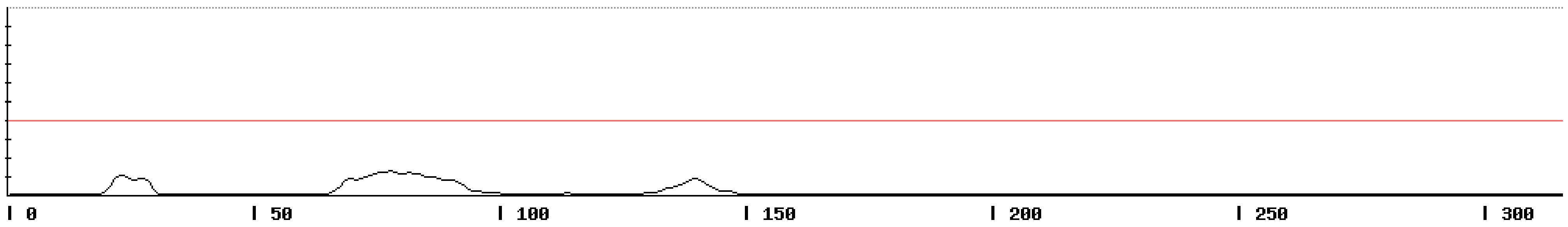

Predictions for Glycinemax_Glyma.05G192500.1.p

Viterbi Path

Viterbi Path

Posterior @ 0.4

Posterior @ 0.4

Posterior Graphic. Threshold Marked @ 0.4

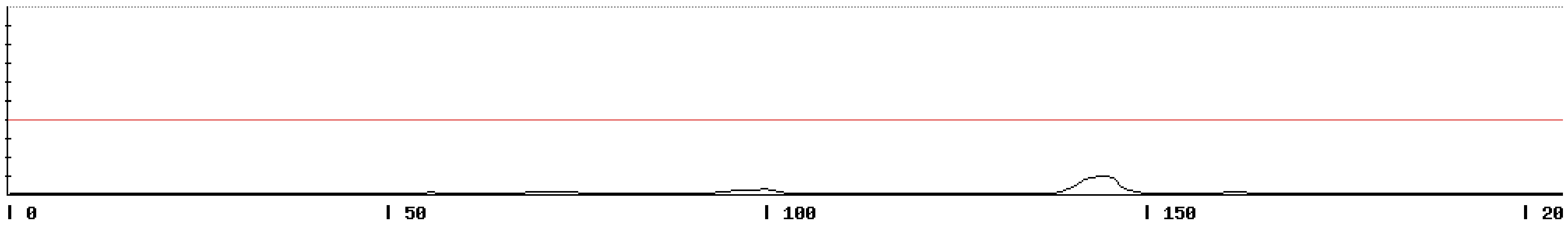

Predictions for Glycinemax_Glyma.05G195000.1.p

Viterbi Path

Viterbi Path

Posterior @ 0.4 92 - GADKPIGKPKALGIKK - 107

Posterior @ 0.4

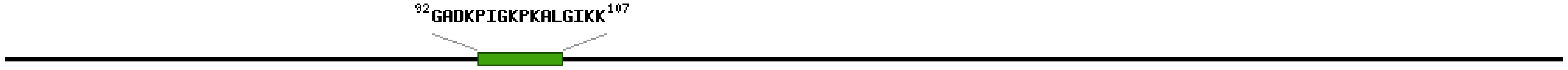

Posterior Graphic. Threshold Marked @ 0.4

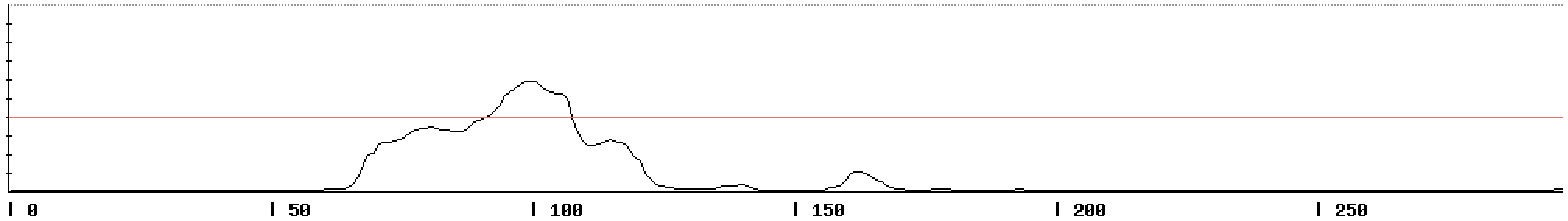

Predictions for Glycinemax_Glyma.05G202300.1.p

Viterbi Path

Viterbi Path

Posterior @ 0.4

Posterior @ 0.4

Posterior Graphic. Threshold Marked @ 0.4

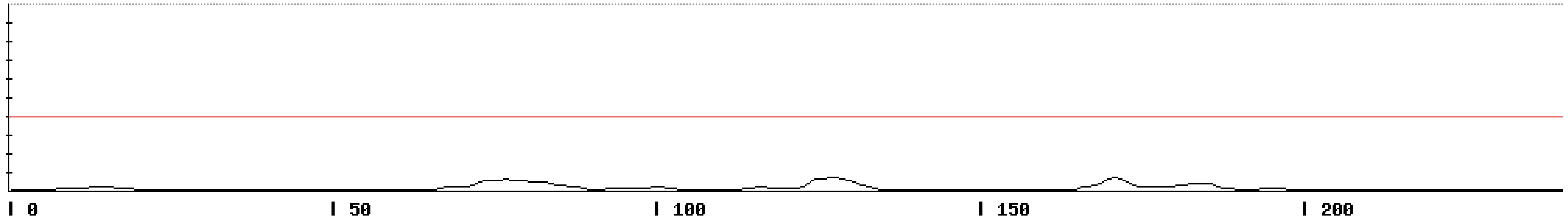

Predictions for Glycinemax_Glyma.05G225100.1.p

Viterbi Path

Viterbi Path

Posterior @ 0.4

Posterior @ 0.4

Posterior Graphic. Threshold Marked @ 0.4

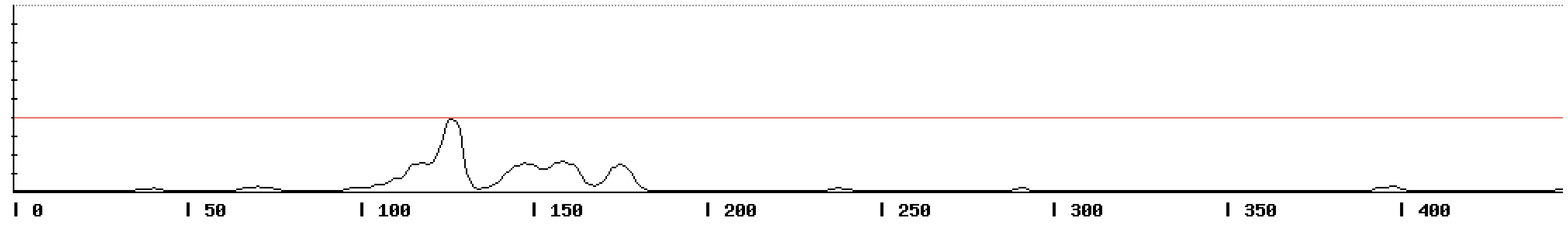

Predictions for Glycinemax_Glyma.05G234200.1.p

Viterbi Path

Viterbi Path

Posterior @ 0.4

Posterior @ 0.4

Posterior Graphic. Threshold Marked @ 0.4

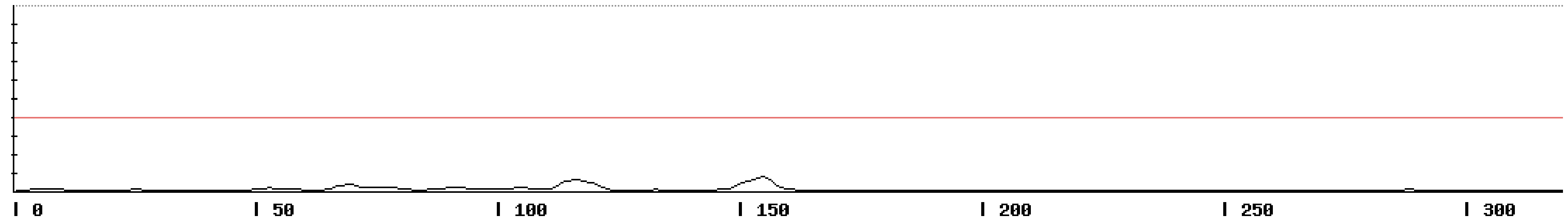

Predictions for Glycinemax_Glyma.06G014900.1.p

Viterbi Path

Viterbi Path

Posterior @ 0.4

Posterior @ 0.4

Posterior Graphic. Threshold Marked @ 0.4

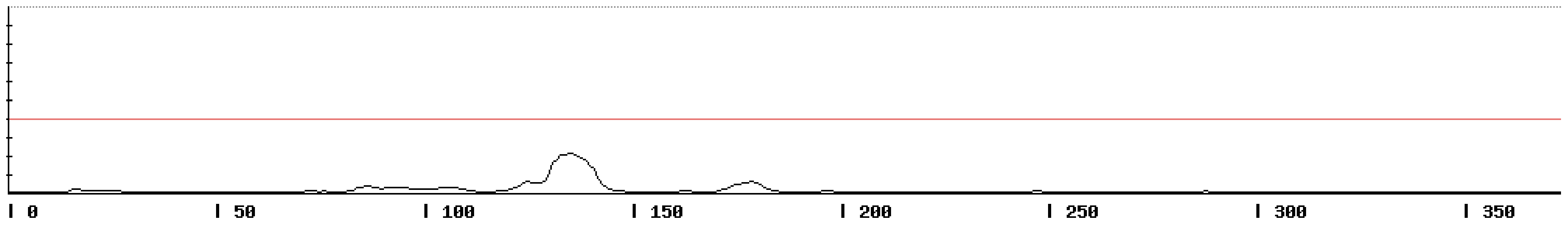

Predictions for Glycinemax_Glyma.06G080200.1.p

Viterbi Path

Viterbi Path

Posterior @ 0.4

Posterior @ 0.4

Posterior Graphic. Threshold Marked @ 0.4

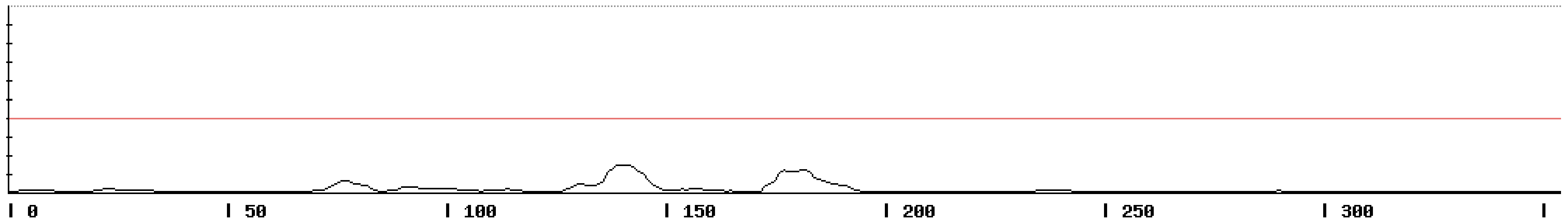

Predictions for Glycinemax_Glyma.06G114000.1.p	
Viterbi Path	
Viterbi Path	
Posterior @ 0.4	
Posterior @ 0.4	
Posterior Graphic. Threshold Marked @ 0.4	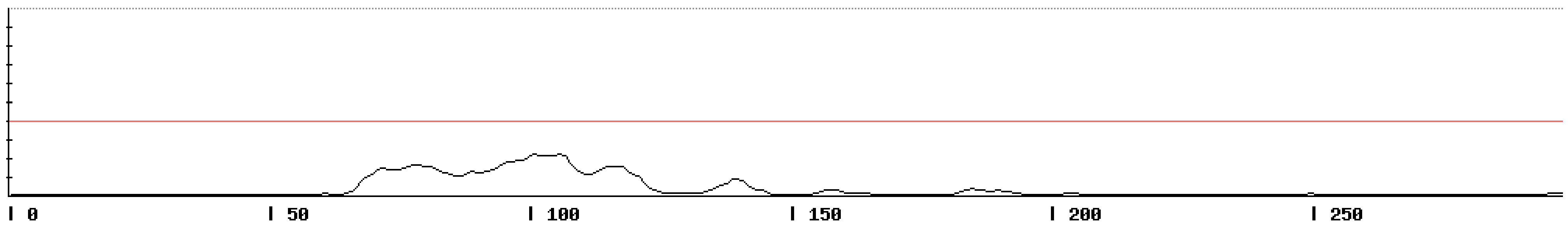

Predictions for Glycinemax_Glyma.06G138100.1.p	
Viterbi Path	
Viterbi Path	
Posterior @ 0.4	532 - SGKRKI - 537
Posterior @ 0.4	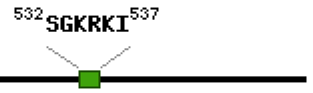
Posterior Graphic. Threshold Marked @ 0.4	

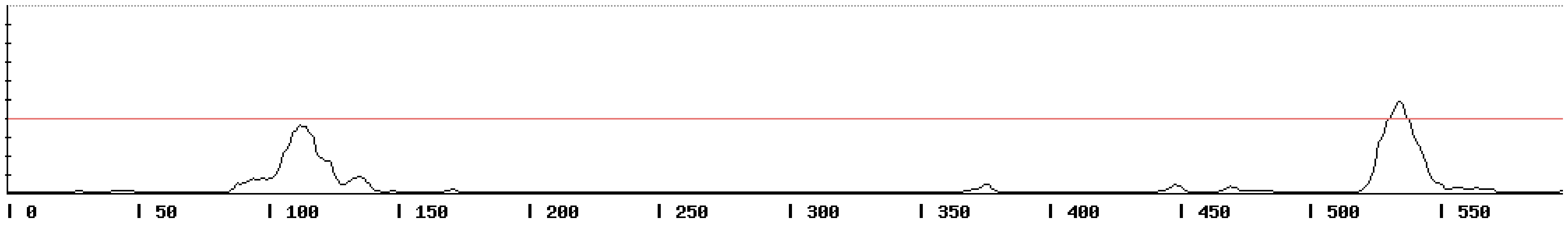

Predictions for Glycinemax_Glyma.06G152900.1.p

Viterbi Path	
Viterbi Path	
Posterior @ 0.4	25 - RKVRGKK - 31
Posterior @ 0.4	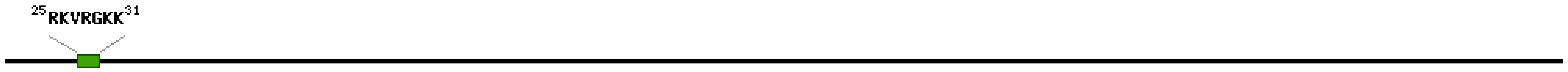
Posterior Graphic. Threshold Marked @ 0.4	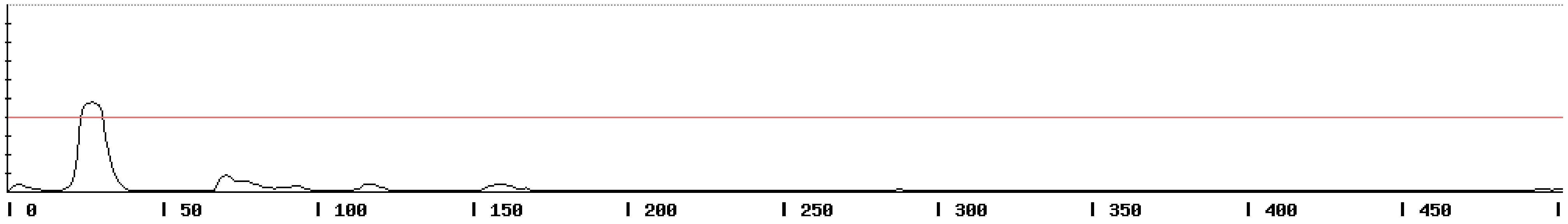

Predictions for Glycinemax_Glyma.06G154400.1.p

Viterbi Path	
Viterbi Path	
Posterior @ 0.4	
Posterior @ 0.4	

Posterior Graphic. Threshold Marked @ 0.4

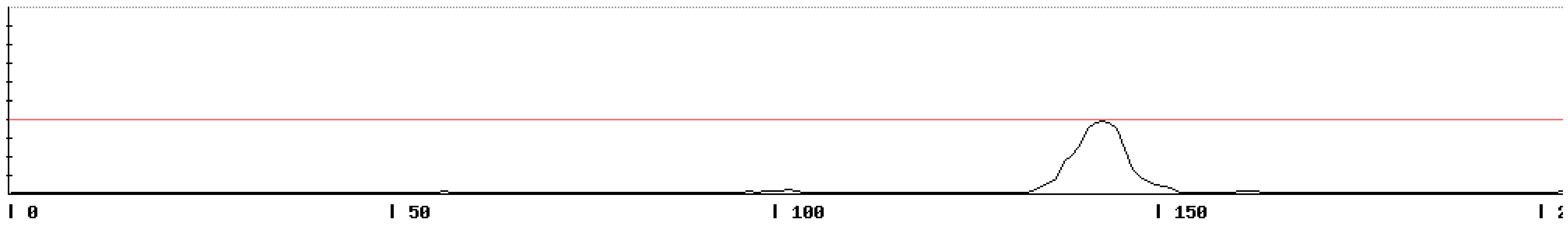

Predictions for Glycinemax_Glyma.06G157400.1.p

Viterbi Path

Viterbi Path

Posterior @ 0.4 95 - KAIGKPKALGIKK - 107

Posterior @ 0.4

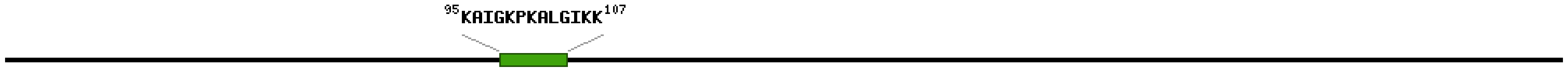

Posterior Graphic. Threshold Marked @ 0.4

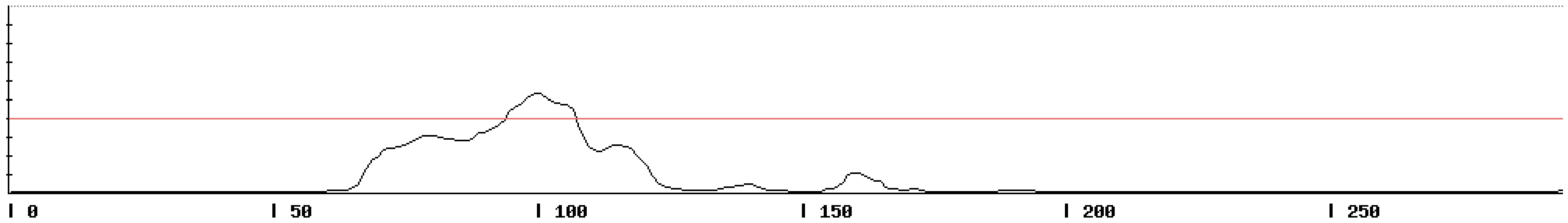

Predictions for Glycinemax_Glyma.06G166500.1.p

Viterbi Path

Viterbi Path

Posterior @ 0.4

Posterior @ 0.4

Posterior Graphic. Threshold Marked @ 0.4

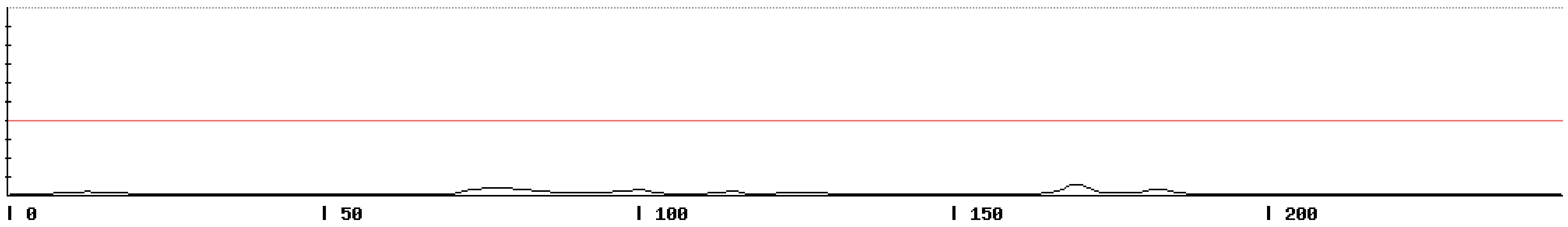

Predictions for Glycinemax_Glyma.06G195500.1.p

Viterbi Path

Viterbi Path

Posterior @ 0.4

Posterior @ 0.4

Posterior Graphic. Threshold Marked @ 0.4

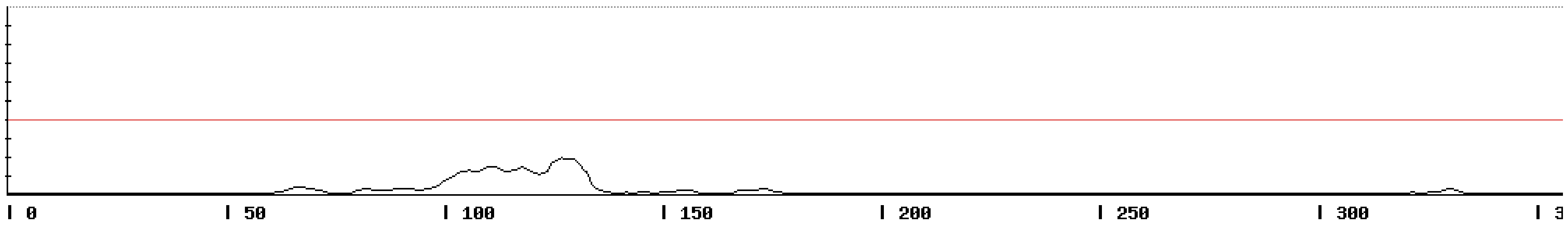

Predictions for Glycinemax_Glyma.06G236000.1.p

Viterbi Path

Viterbi Path

Posterior @ 0.4

Posterior @ 0.4

Posterior Graphic. Threshold Marked @ 0.4

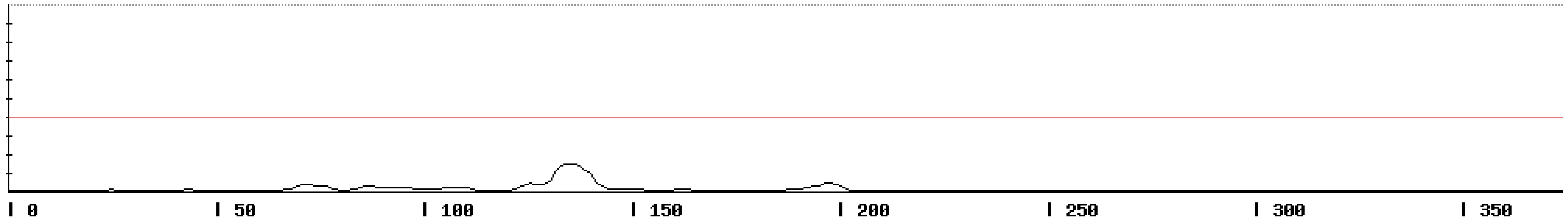

Predictions for Glycinemax_Glyma.06G248900.1.p

Viterbi Path

Viterbi Path

Posterior @ 0.4

Posterior @ 0.4

Posterior Graphic. Threshold Marked @ 0.4

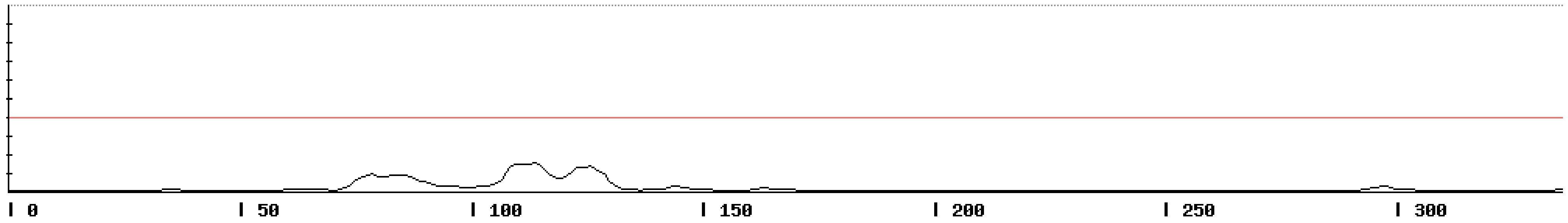

Predictions for Glycinemax_Glyma.06G249100.1.p

Viterbi Path

Viterbi Path

Posterior @ 0.4	
Posterior @ 0.4	
Posterior Graphic. Threshold Marked @ 0.4	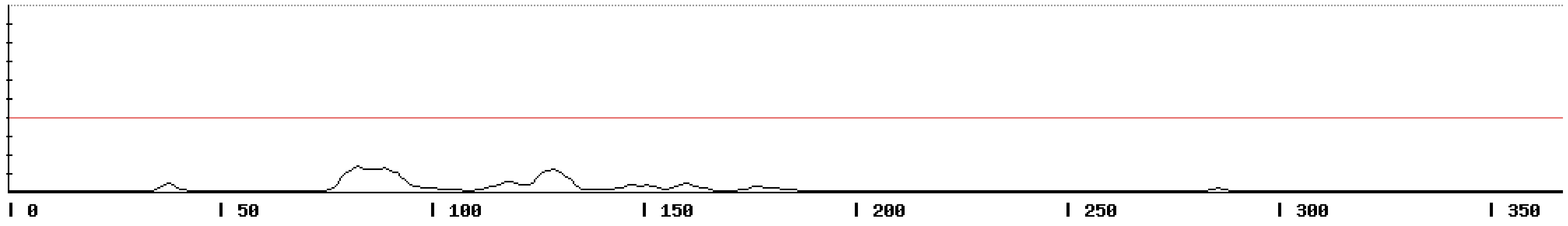

Predictions for Glycinemax_Glyma.06G288500.1.p

Viterbi Path	
Viterbi Path	
Posterior @ 0.4	127 - RKRRK - 131
Posterior @ 0.4	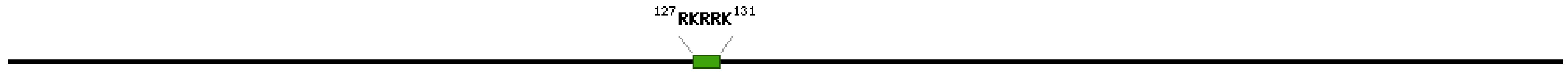
Posterior Graphic. Threshold Marked @ 0.4	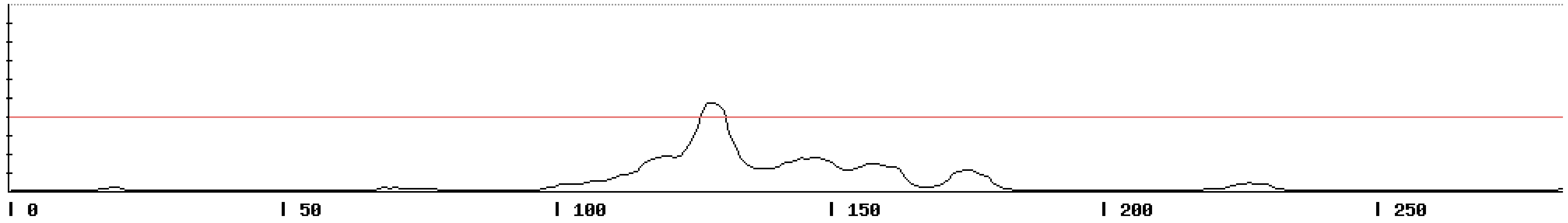

Predictions for Glycinemax_Glyma.06G318900.1.p

Viterbi Path

Viterbi Path

Posterior @ 0.4

Posterior @ 0.4

Posterior Graphic. Threshold Marked @ 0.4

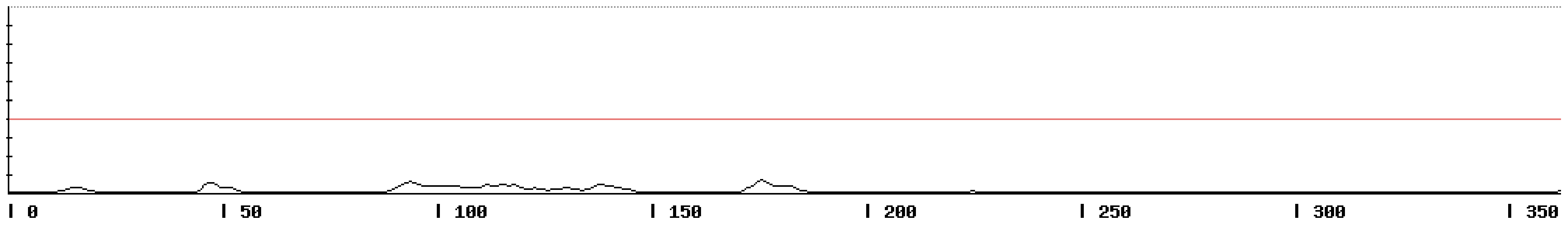

Predictions for Glycinemax_Glyma.07G047900.1.p

Viterbi Path

Viterbi Path

Posterior @ 0.4

Posterior @ 0.4

Posterior Graphic. Threshold Marked @ 0.4

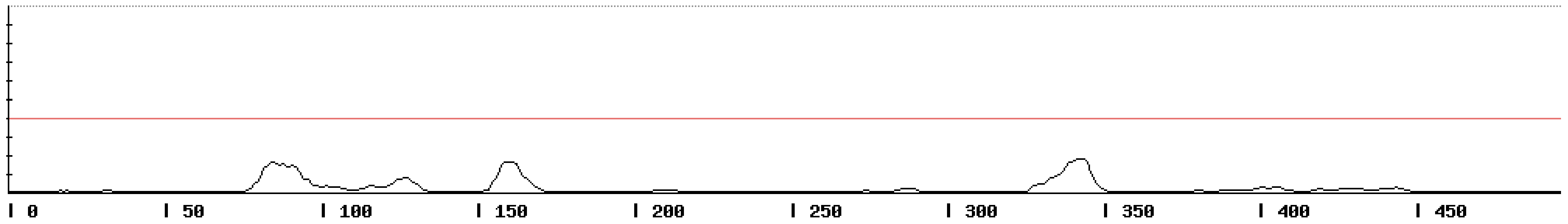

Predictions for Glycinemax_Glyma.07G048000.1.p

Viterbi Path

Viterbi Path

Posterior @ 0.4

Posterior @ 0.4

Posterior Graphic. Threshold Marked @ 0.4

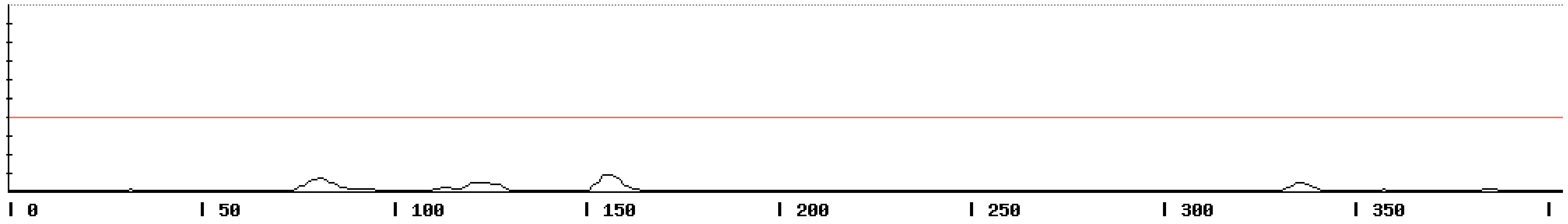

Predictions for Glycinemax_Glyma.07G048100.1.p

Viterbi Path

Viterbi Path

Posterior @ 0.4

Posterior @ 0.4

Posterior Graphic. Threshold Marked @ 0.4

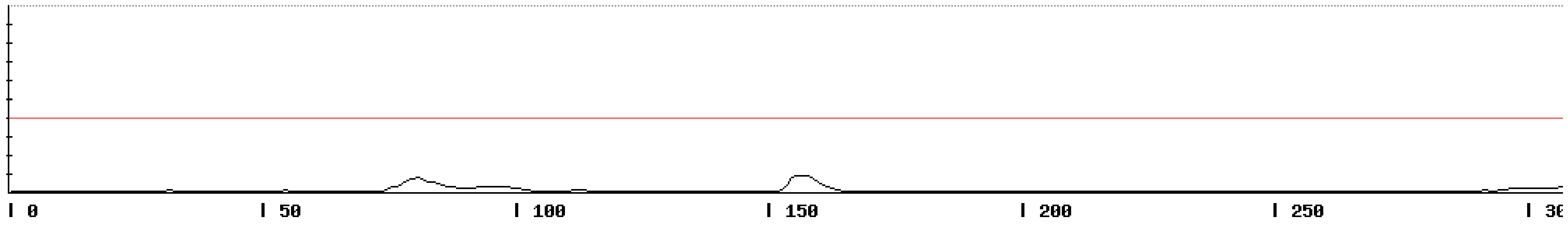

Predictions for Glycinemax_Glyma.07G050600.1.p

Viterbi Path

Viterbi Path

Posterior @ 0.4

Posterior @ 0.4

Posterior Graphic. Threshold Marked @ 0.4

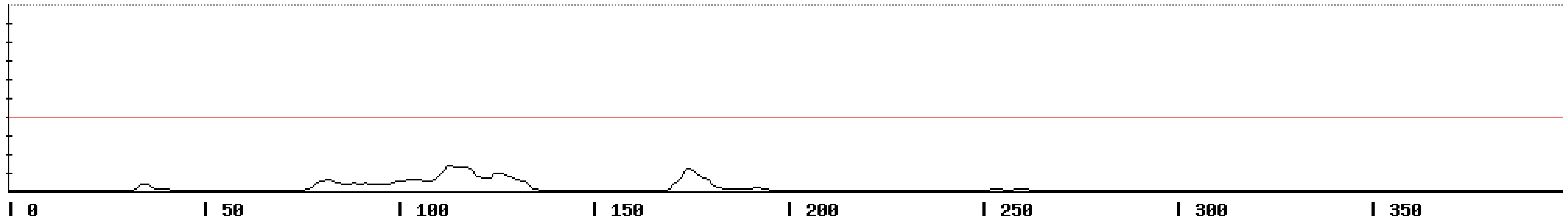

Predictions for Glycinemax_Glyma.07G092000.1.p

Viterbi Path

Viterbi Path

Posterior @ 0.4

Posterior @ 0.4

Posterior Graphic. Threshold Marked @ 0.4

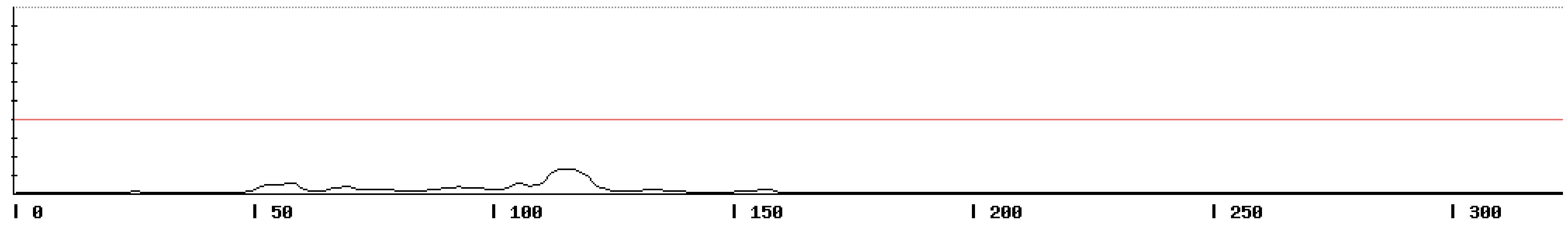

Predictions for Glycinemax_Glyma.07G126500.1.p

Viterbi Path

Viterbi Path

Posterior @ 0.4

Posterior @ 0.4

Posterior Graphic. Threshold Marked @ 0.4

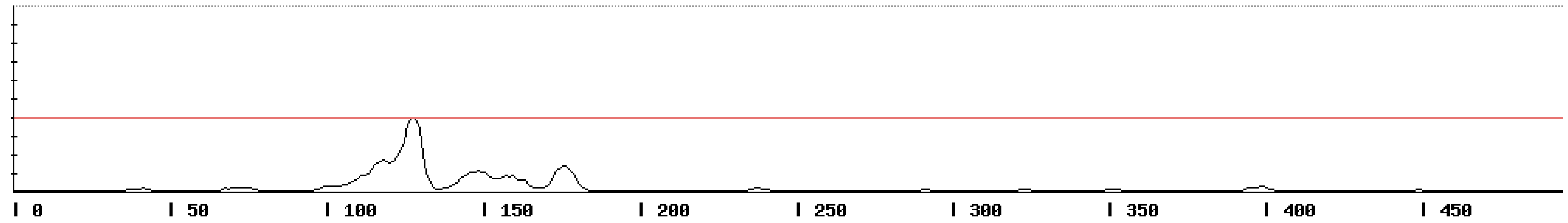

Predictions for Glycinemax_Glyma.07G192900.1.p

Viterbi Path

Viterbi Path

Posterior @ 0.4

Posterior @ 0.4

Posterior Graphic. Threshold Marked @ 0.4

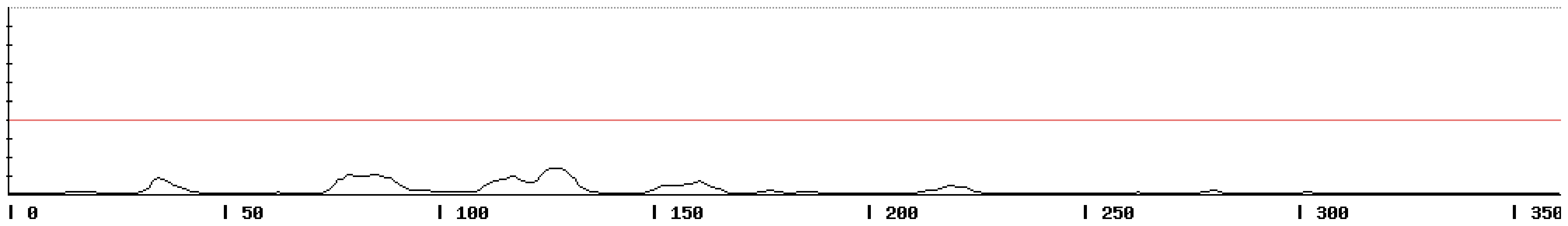

Predictions for Glycinemax_Glyma.07G201800.1.p

Viterbi Path	
Viterbi Path	
Posterior @ 0.4	146 - RKRRK - 150

Posterior @ 0.4

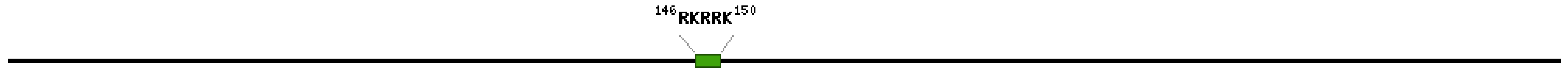

Posterior Graphic. Threshold Marked @ 0.4

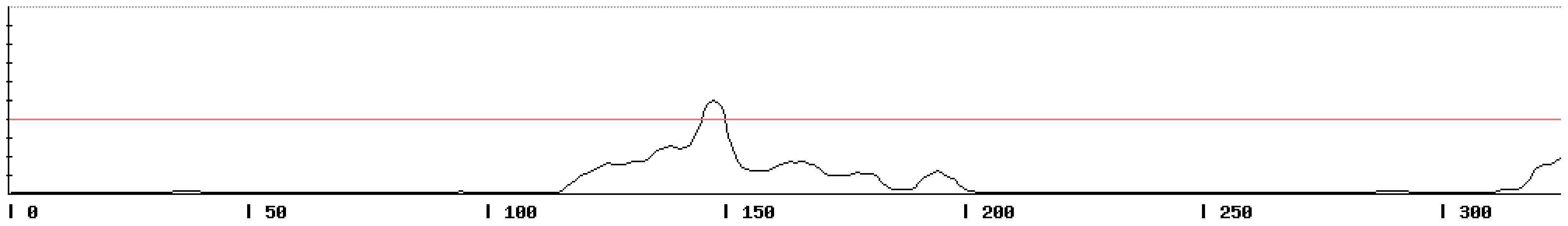

Predictions for Glycinemax_Glyma.07G229100.1.p

Viterbi Path	
Viterbi Path	

Posterior @ 0.4

Posterior @ 0.4

Posterior Graphic. Threshold Marked @ 0.4

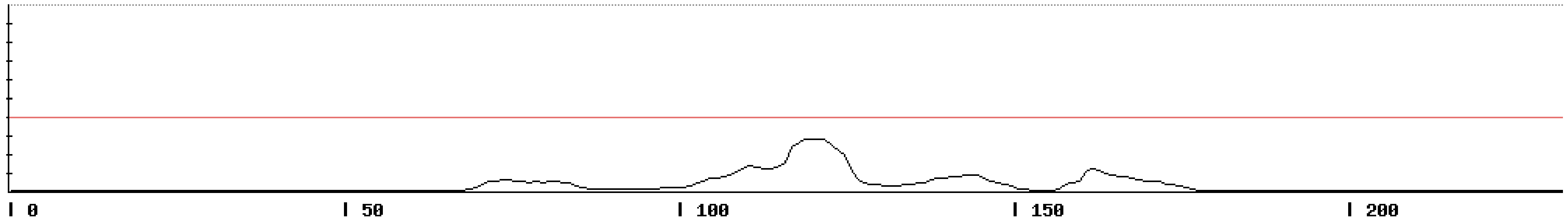

Predictions for Glycinemax_Glyma.07G271100.1.p

Viterbi Path

Viterbi Path

Posterior @ 0.4

Posterior @ 0.4

Posterior Graphic. Threshold Marked @ 0.4

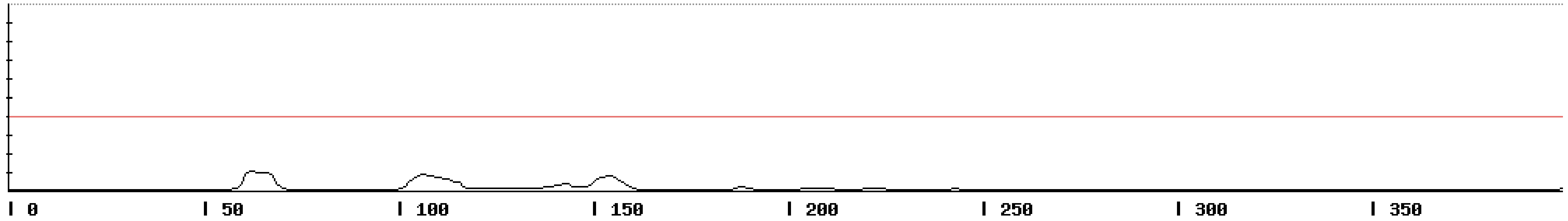

Predictions for Glycinemax_Glyma.08G009700.1.p

Viterbi Path

Viterbi Path

Posterior @ 0.4

Posterior @ 0.4

Posterior Graphic. Threshold Marked @ 0.4

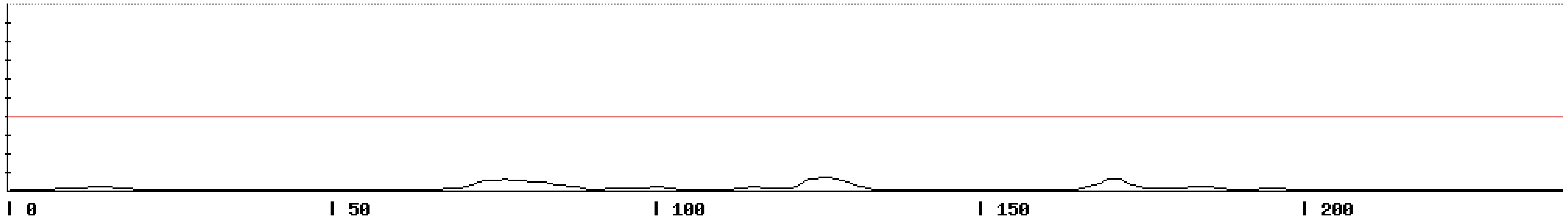

Predictions for Glycinemax_Glyma.08G031900.1.p

Viterbi Path

Viterbi Path

Posterior @ 0.4

Posterior @ 0.4

Posterior Graphic. Threshold Marked @ 0.4

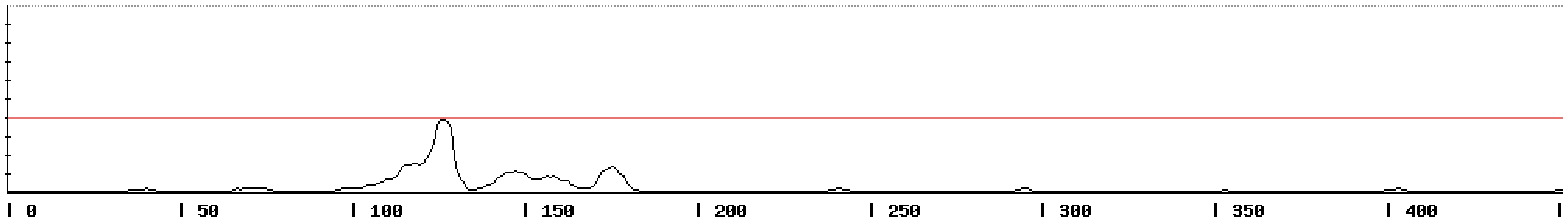

Predictions for Glycinemax_Glyma.08G041500.1.p

Viterbi Path

Viterbi Path

Posterior @ 0.4

Posterior @ 0.4

Posterior Graphic. Threshold Marked @ 0.4

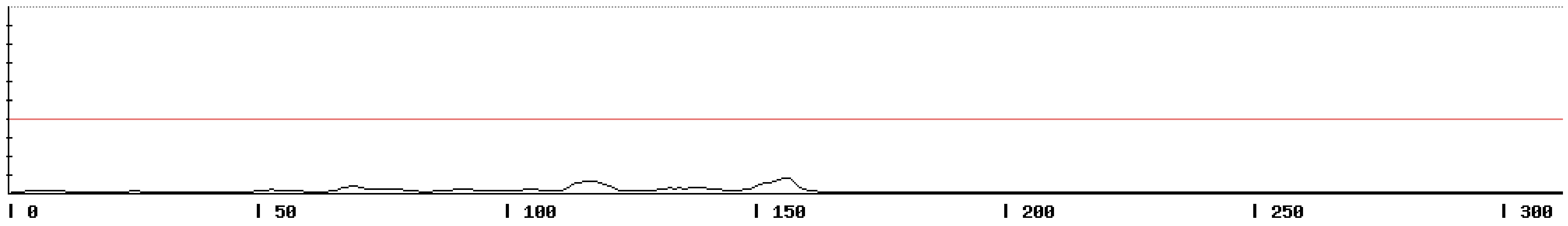

Predictions for Glycinemax_Glyma.08G075300.1.p

Viterbi Path

Viterbi Path

Posterior @ 0.4

Posterior @ 0.4

Posterior Graphic. Threshold Marked @ 0.4

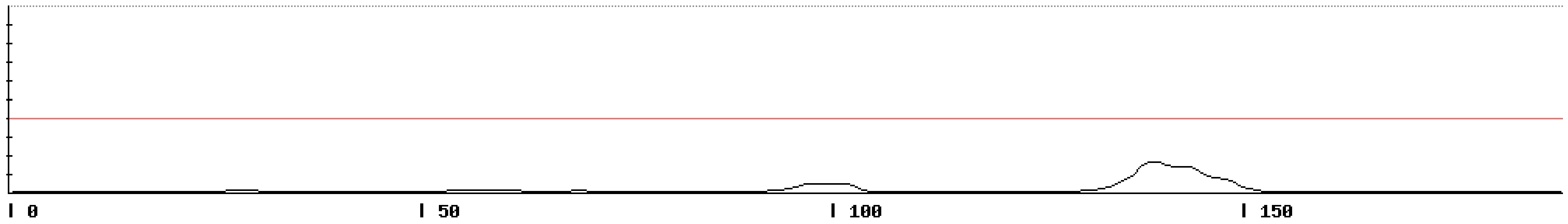

Predictions for Glycinemax_Glyma.08G156500.1.p	
Viterbi Path	
Viterbi Path	
Posterior @ 0.4	
Posterior @ 0.4	
Posterior Graphic. Threshold Marked @ 0.4	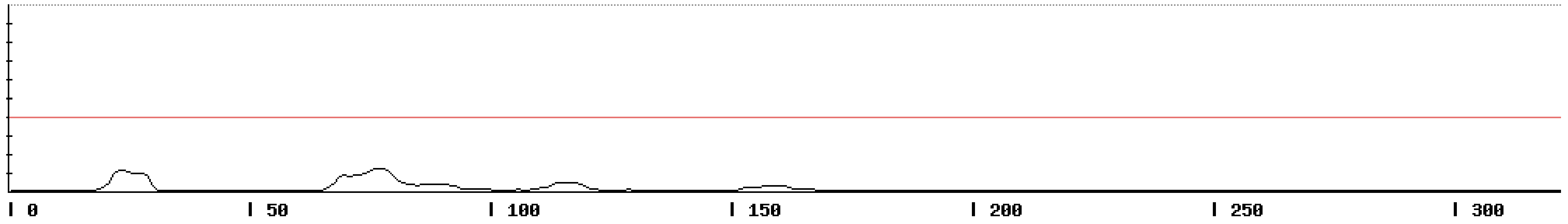

Predictions for Glycinemax_Glyma.08G161300.1.p	
Viterbi Path	
Viterbi Path	
Posterior @ 0.4	
Posterior @ 0.4	
Posterior Graphic. Threshold Marked @ 0.4	

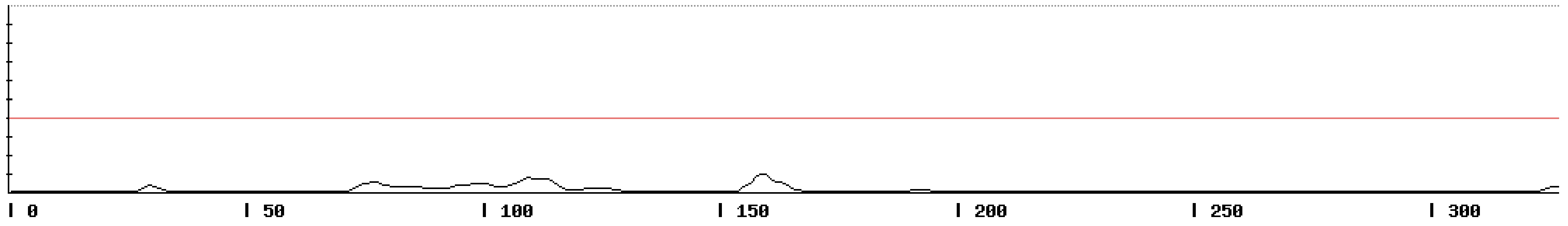

Predictions for Glycinemax_Glyma.08G163100.1.p

Viterbi Path

Viterbi Path

Posterior @ 0.4

Posterior @ 0.4

Posterior Graphic. Threshold Marked @ 0.4

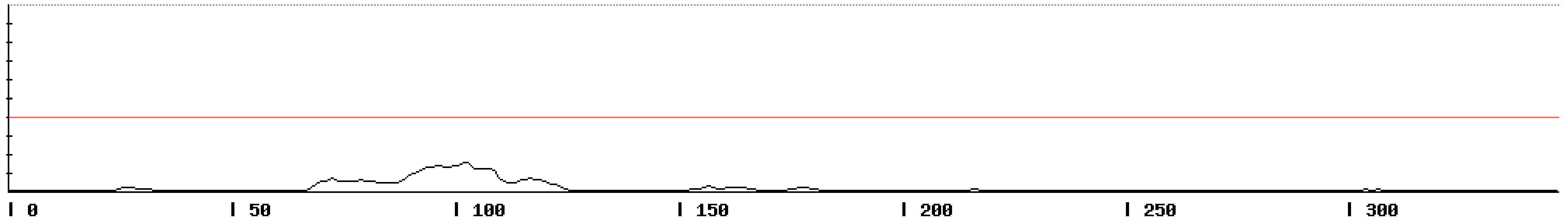

Predictions for Glycinemax_Glyma.08G169400.1.p

Viterbi Path

Viterbi Path

Posterior @ 0.4

Posterior @ 0.4

Posterior Graphic. Threshold Marked @ 0.4

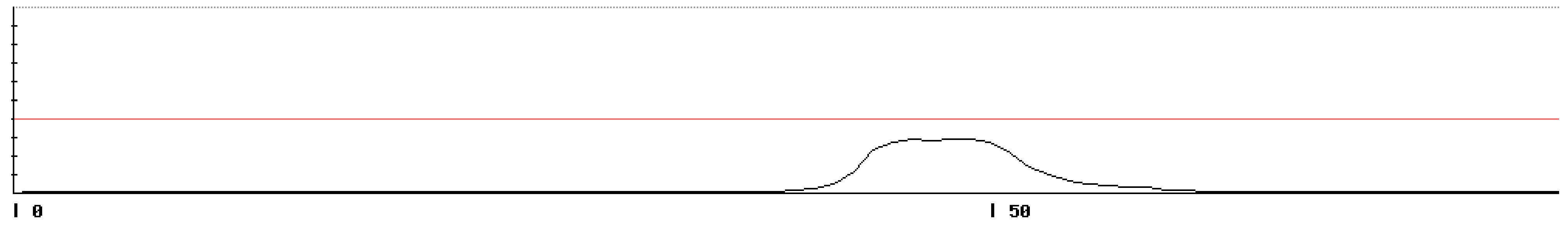

Predictions for Glycinemax_Glyma.08G173400.1.p

Viterbi Path

Viterbi Path

Posterior @ 0.4

Posterior @ 0.4

Posterior Graphic. Threshold Marked @ 0.4

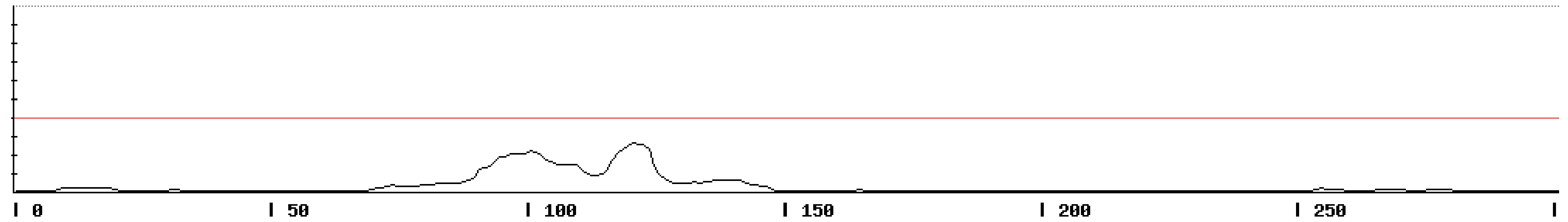

Predictions for Glycinemax_Glyma.08G181100.1.p

Viterbi Path

Viterbi Path

Posterior @ 0.4

Posterior @ 0.4

Posterior Graphic. Threshold Marked @ 0.4

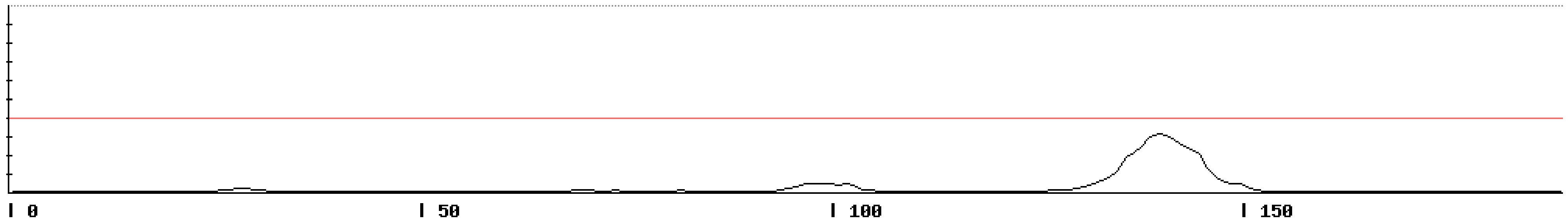

Predictions for Glycinemax_Glyma.08G301100.1.p

Viterbi Path

Viterbi Path

Posterior @ 0.4

Posterior @ 0.4

Posterior Graphic. Threshold Marked @ 0.4

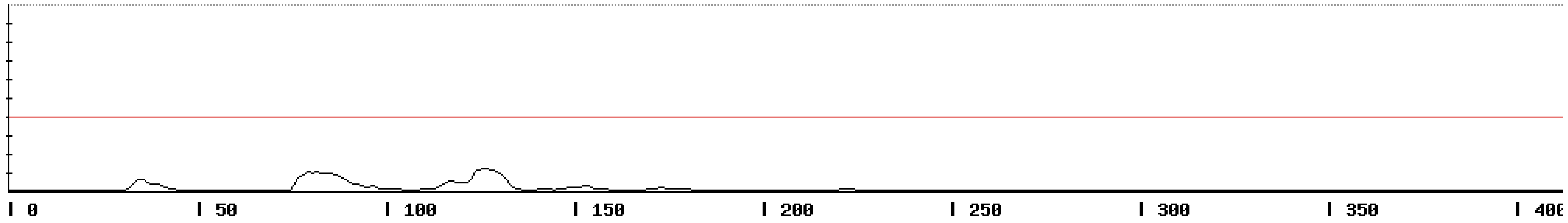

Predictions for Glycinemax_Glyma.08G307100.1.p

Viterbi Path

Viterbi Path

Posterior @ 0.4	
Posterior @ 0.4	
Posterior Graphic. Threshold Marked @ 0.4	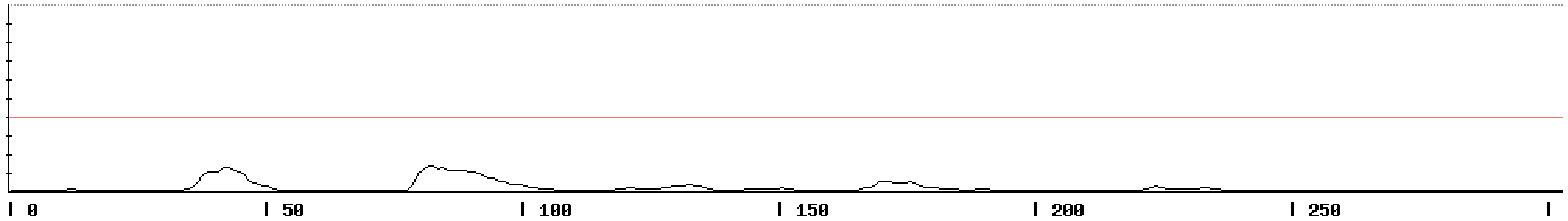

Predictions for Glycinemax_Glyma.08G360200.1.p	
Viterbi Path	
Viterbi Path	
Posterior @ 0.4	
Posterior @ 0.4	
Posterior Graphic. Threshold Marked @ 0.4	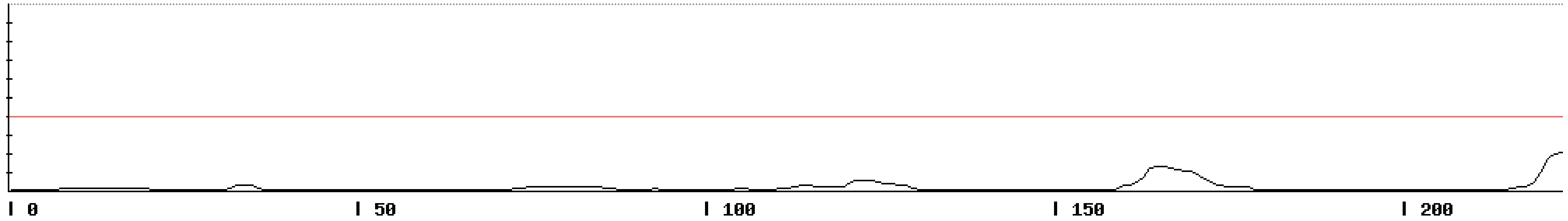

Predictions for Glycinemax_Glyma.09G167400.1.p	
Viterbi Path	17 - KKRKKGRREEKK - 28
Viterbi Path	

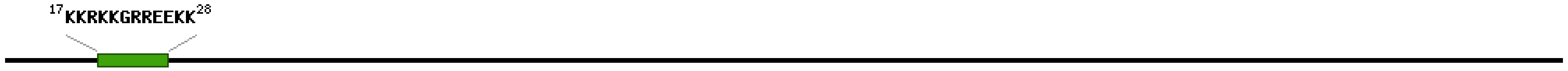

Posterior @ 0.4	16 - NKKRKKGRREEKKMSP - 31

Posterior @ 0.4

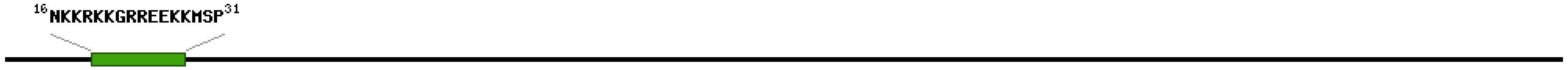

Posterior Graphic. Threshold Marked @ 0.4

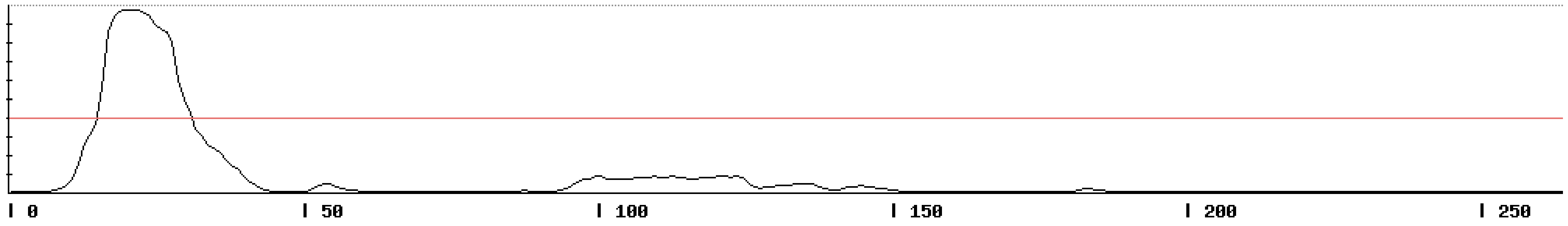

Predictions for Glycinemax_Glyma.09G184100.1.p

Viterbi Path

Viterbi Path

Posterior @ 0.4

Posterior @ 0.4

Posterior Graphic. Threshold Marked @ 0.4

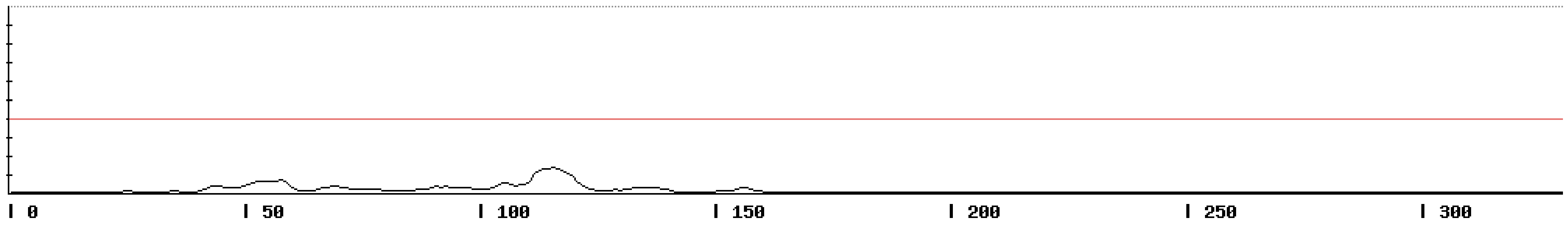

Predictions for Glycinemax_Glyma.09G231700.1.p

Viterbi Path

Viterbi Path

Posterior @ 0.4

Posterior @ 0.4

Posterior Graphic. Threshold Marked @ 0.4

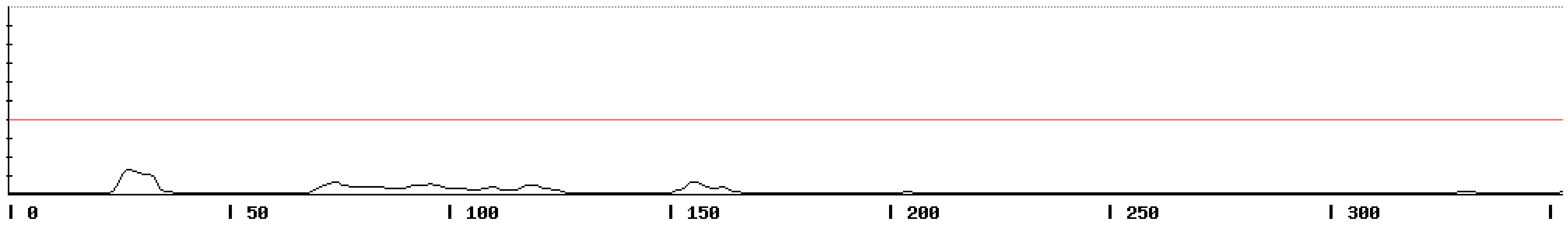

Predictions for Glycinemax_Glyma.09G233600.1.p

Viterbi Path

Viterbi Path

Posterior @ 0.4

Posterior @ 0.4

Posterior Graphic. Threshold Marked @ 0.4

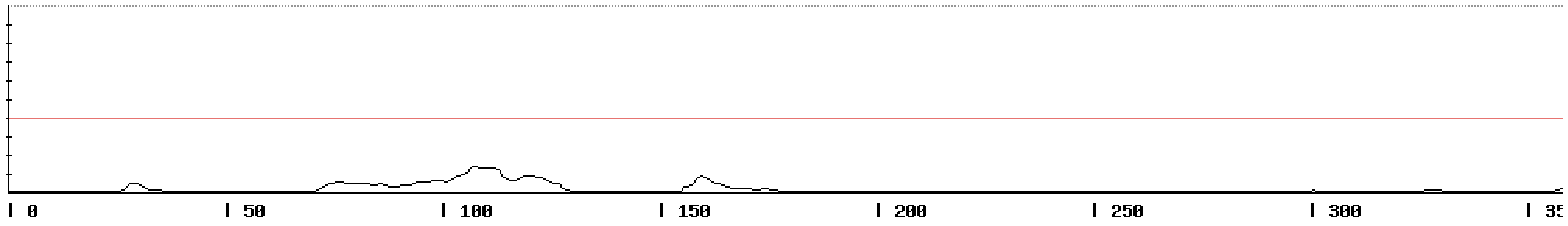

Predictions for Glycinemax_Glyma.09G235700.1.p	
Viterbi Path	
Viterbi Path	
Posterior @ 0.4	
Posterior @ 0.4	
Posterior Graphic. Threshold Marked @ 0.4	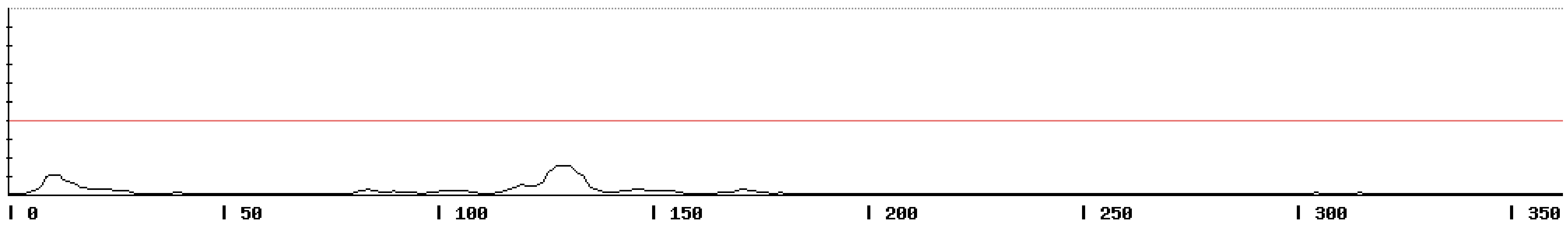

Predictions for Glycinemax_Glyma.10G037700.1.p	
Viterbi Path	
Viterbi Path	
Posterior @ 0.4	
Posterior @ 0.4	
Posterior Graphic. Threshold Marked @ 0.4	

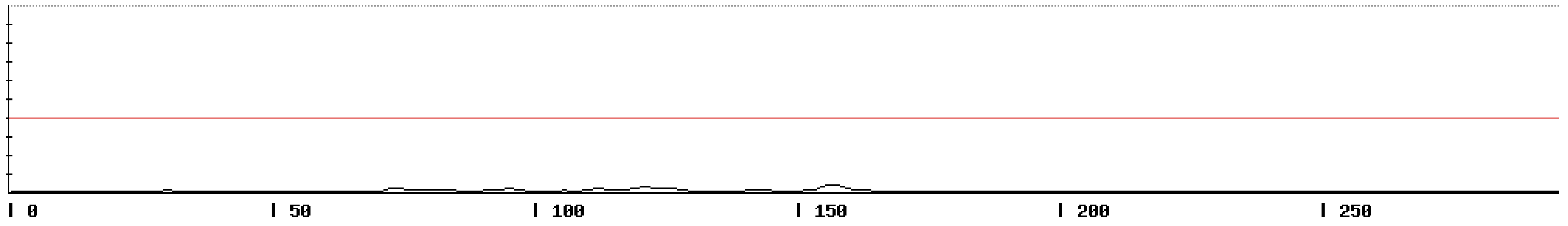

Predictions for Glycinemax_Glyma.10G077000.1.p	
Viterbi Path	
Viterbi Path	
Posterior @ 0.4	
Posterior @ 0.4	
Posterior Graphic. Threshold Marked @ 0.4	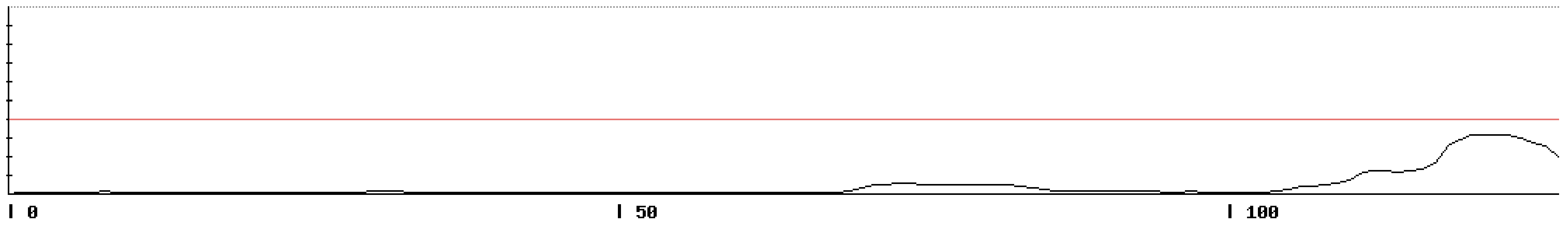

Predictions for Glycinemax_Glyma.10G077400.1.p	
Viterbi Path	
Viterbi Path	
Posterior @ 0.4	
Posterior @ 0.4	

Posterior Graphic. Threshold Marked @ 0.4

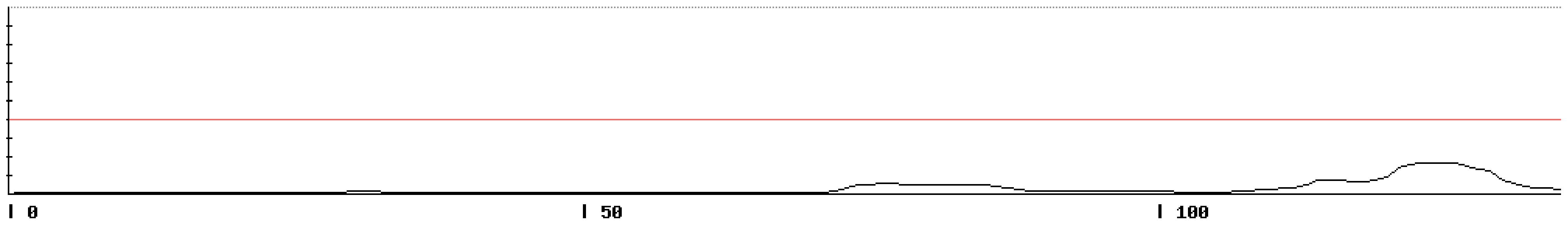

Predictions for Glycinemax_Glyma.10G197500.1.p

Viterbi Path

Viterbi Path

Posterior @ 0.4

Posterior @ 0.4

Posterior Graphic. Threshold Marked @ 0.4

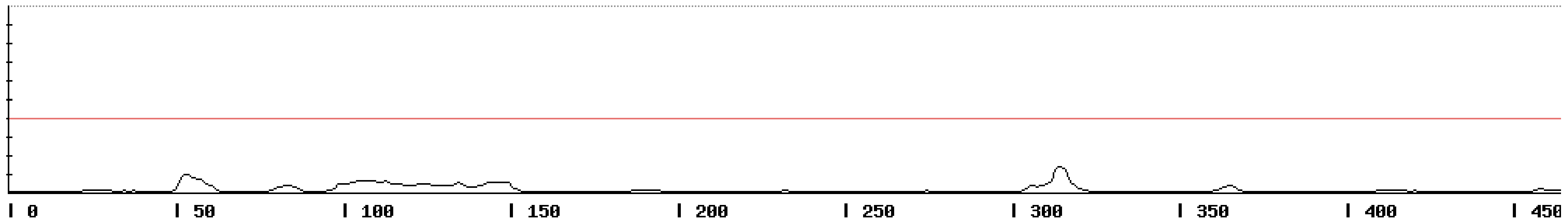

Predictions for Glycinemax_Glyma.10G197600.1.p

Viterbi Path

Viterbi Path

Posterior @ 0.4

Posterior @ 0.4

Posterior Graphic. Threshold Marked @ 0.4

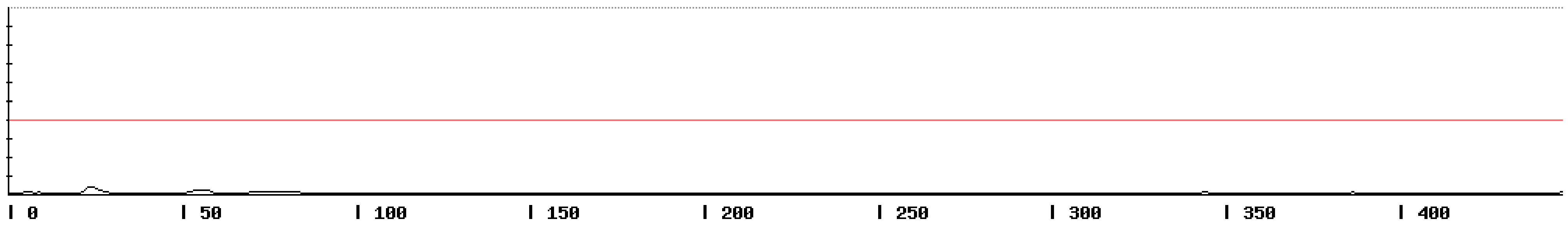

Predictions for Glycinemax_Glyma.10G204700.1.p

Viterbi Path

Viterbi Path

Posterior @ 0.4

Posterior @ 0.4

Posterior Graphic. Threshold Marked @ 0.4

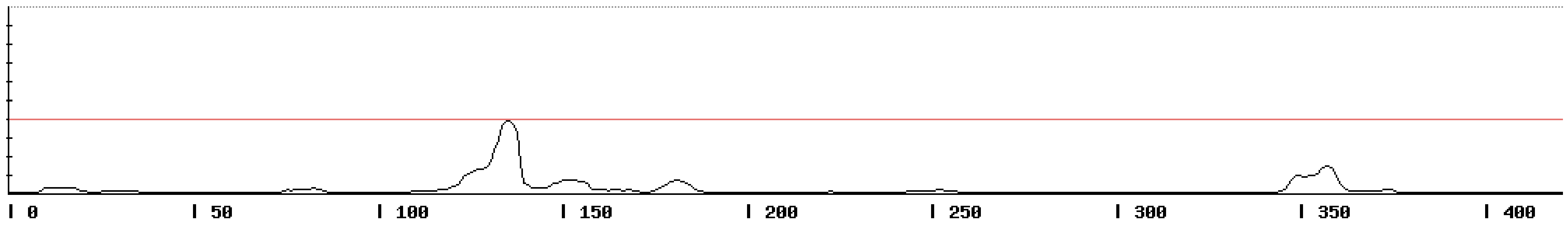

Predictions for Glycinemax_Glyma.10G216400.1.p

Viterbi Path

Viterbi Path

Posterior @ 0.4	
Posterior @ 0.4	
Posterior Graphic. Threshold Marked @ 0.4	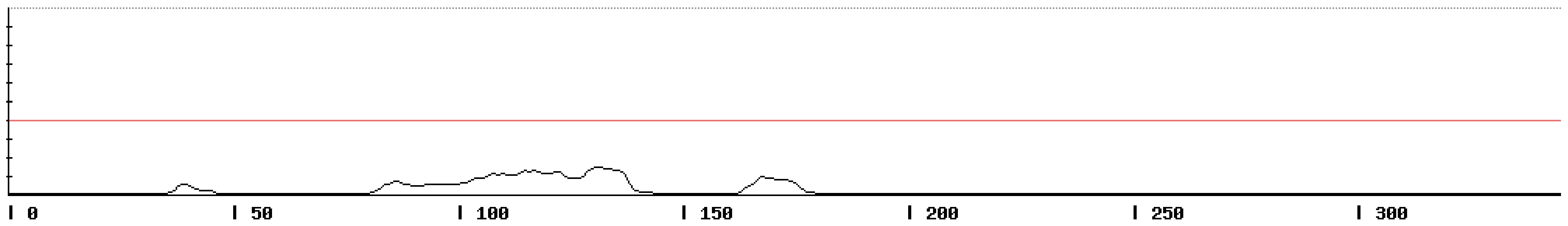

Predictions for Glycinemax_Glyma.10G219600.1.p	
Viterbi Path	
Viterbi Path	
Posterior @ 0.4	
Posterior @ 0.4	
Posterior Graphic. Threshold Marked @ 0.4	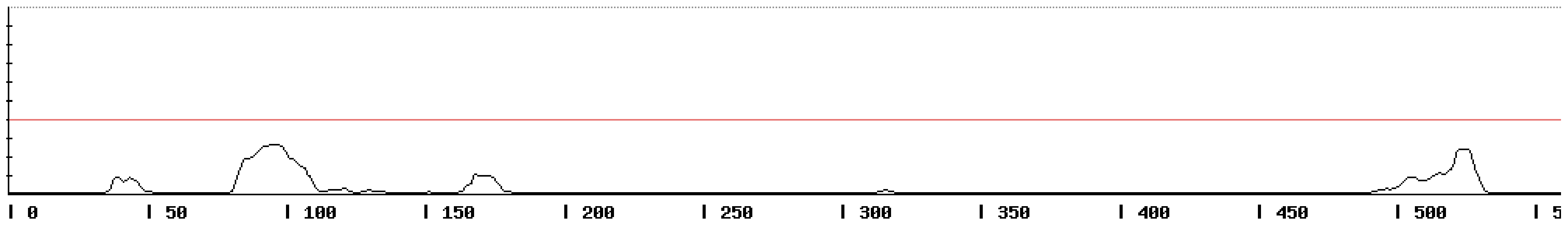

Predictions for Glycinemax_Glyma.11G030600.1.p	
Viterbi Path	
Viterbi Path	

Posterior @ 0.4

Posterior @ 0.4

Posterior Graphic. Threshold Marked @ 0.4

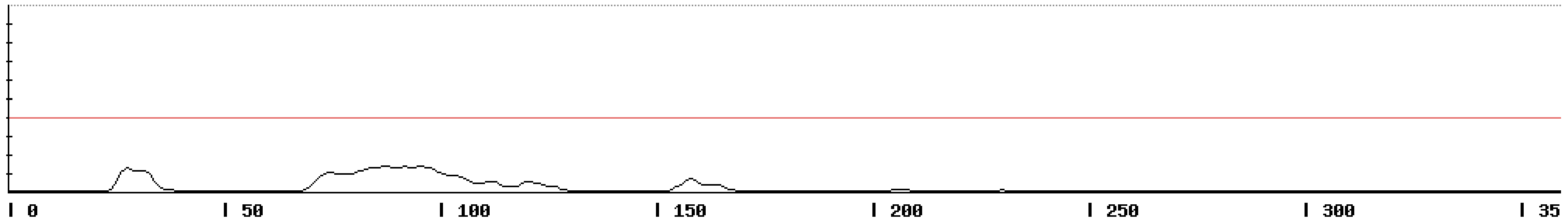

Predictions for Glycinemax_Glyma.11G075400.1.p

Viterbi Path

Viterbi Path

Posterior @ 0.4

Posterior @ 0.4

Posterior Graphic. Threshold Marked @ 0.4

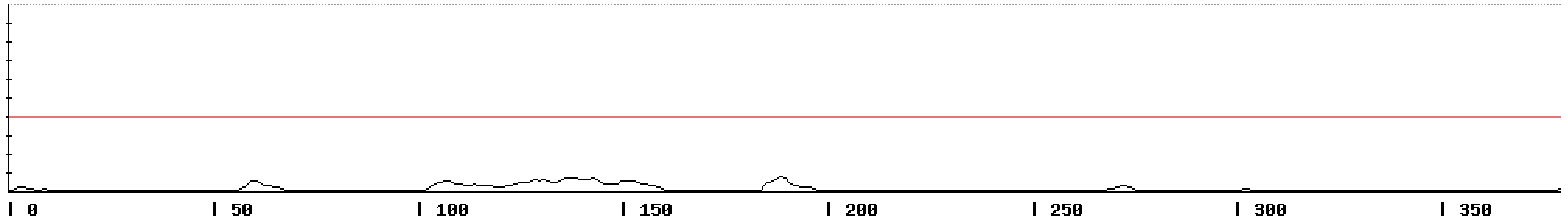

Predictions for Glycinemax_Glyma.11G096600.1.p

Viterbi Path	
Viterbi Path	
Posterior @ 0.4	
Posterior @ 0.4	
Posterior Graphic. Threshold Marked @ 0.4	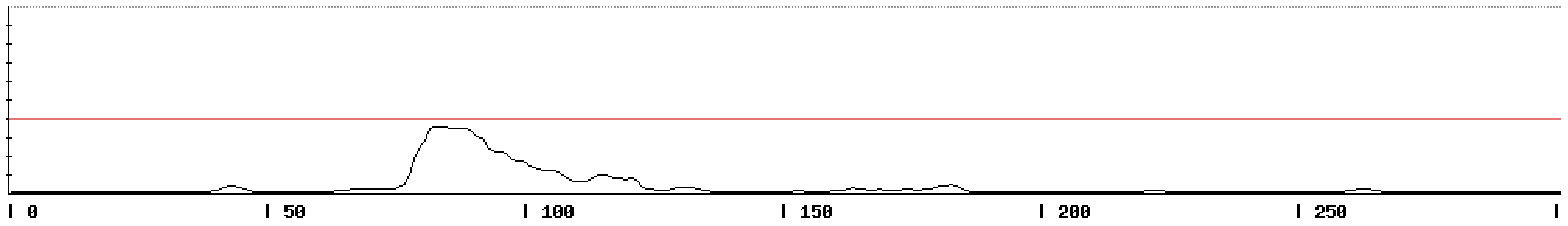

Predictions for Glycinemax_Glyma.11G182000.1.p

Viterbi Path	
Viterbi Path	
Posterior @ 0.4	
Posterior @ 0.4	
Posterior Graphic. Threshold Marked @ 0.4	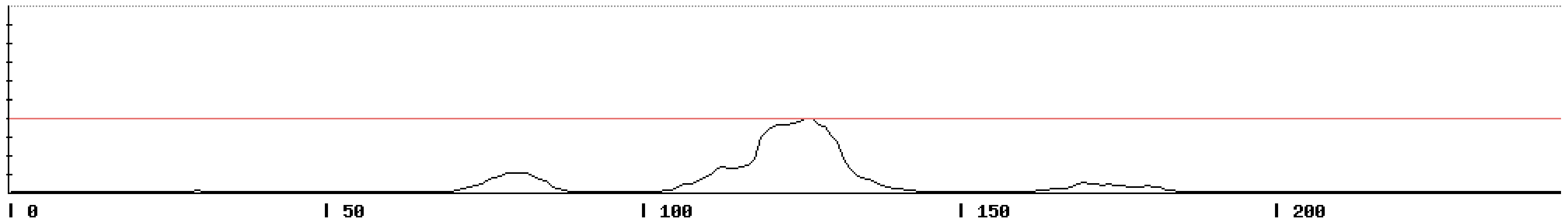

Predictions for Glycinemax_Glyma.11G212400.1.p

Viterbi Path

Viterbi Path

Posterior @ 0.4

Posterior @ 0.4

Posterior Graphic. Threshold Marked @ 0.4

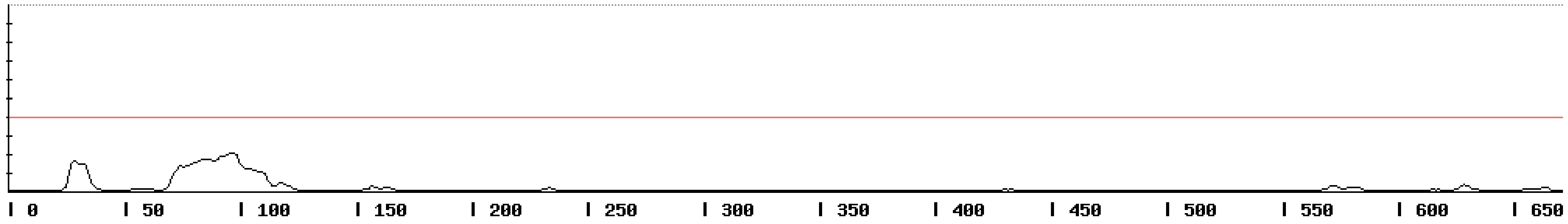

Predictions for Glycinemax_Glyma.12G003200.1.p

Viterbi Path

Viterbi Path

Posterior @ 0.4

Posterior @ 0.4

Posterior Graphic. Threshold Marked @ 0.4

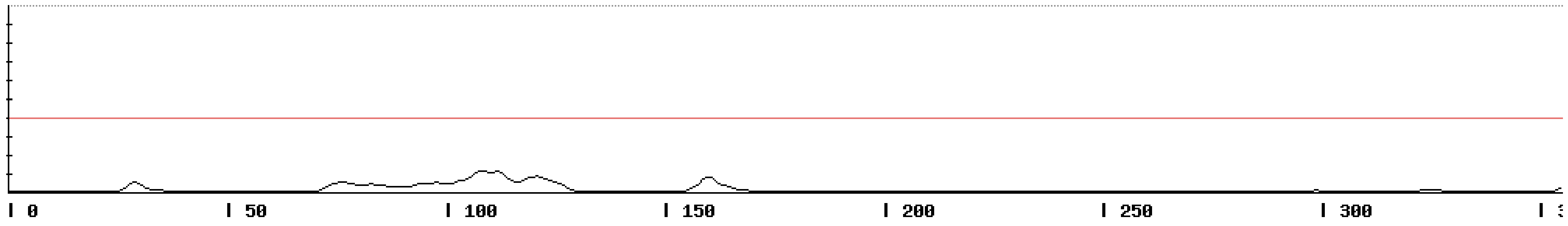

Predictions for Glycinemax_Glyma.12G004900.1.p	
Viterbi Path	
Viterbi Path	
Posterior @ 0.4	
Posterior @ 0.4	
Posterior Graphic. Threshold Marked @ 0.4	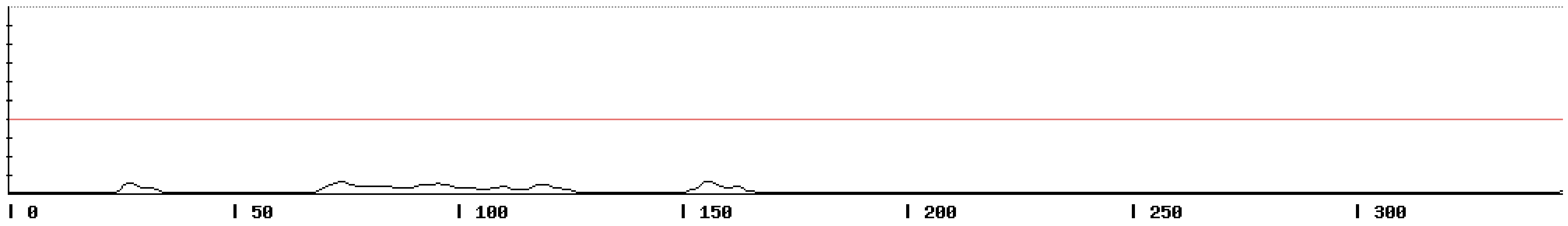

Predictions for Glycinemax_Glyma.12G022700.1.p	
Viterbi Path	
Viterbi Path	
Posterior @ 0.4	
Posterior @ 0.4	

Posterior Graphic. Threshold Marked @ 0.4

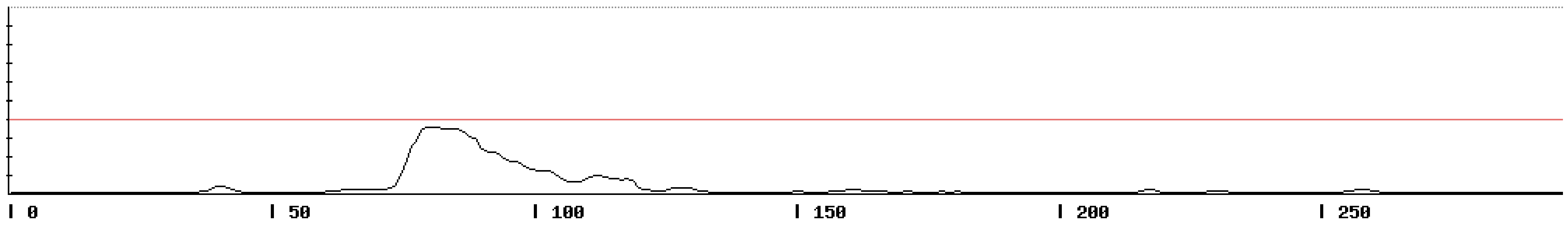

Predictions for Glycinemax_Glyma.12G091200.1.p

Viterbi Path	
Viterbi Path	
Posterior @ 0.4	
Posterior @ 0.4	
Posterior Graphic. Threshold Marked @ 0.4	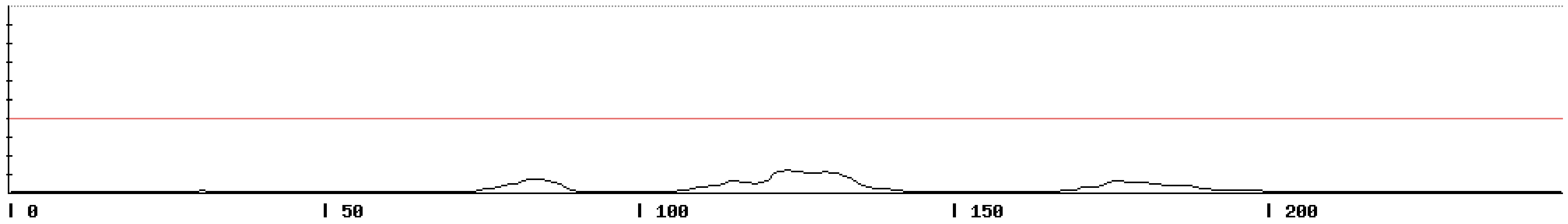

Predictions for Glycinemax_Glyma.12G118700.1.p

Viterbi Path	
Viterbi Path	
Posterior @ 0.4	127 - RKRRK - 131
Posterior @ 0.4	

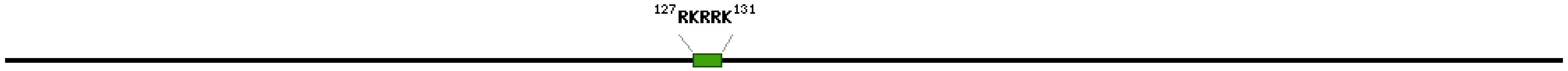

Posterior Graphic. Threshold Marked @ 0.4

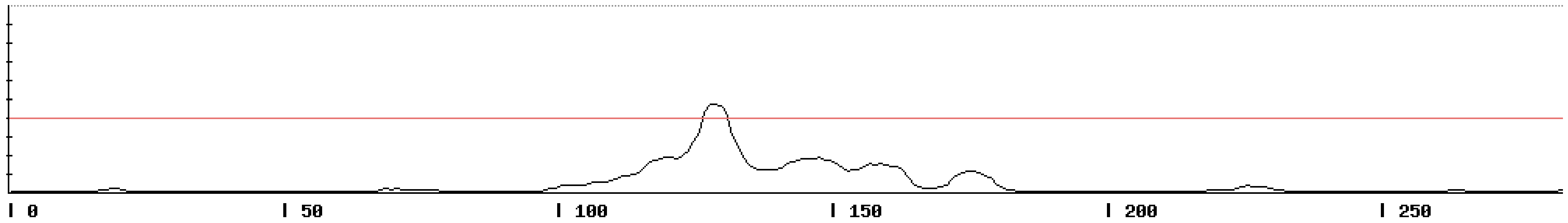

Predictions for Glycinemax_Glyma.12G145100.1.p

Viterbi Path

Viterbi Path

Posterior @ 0.4

Posterior @ 0.4

Posterior Graphic. Threshold Marked @ 0.4

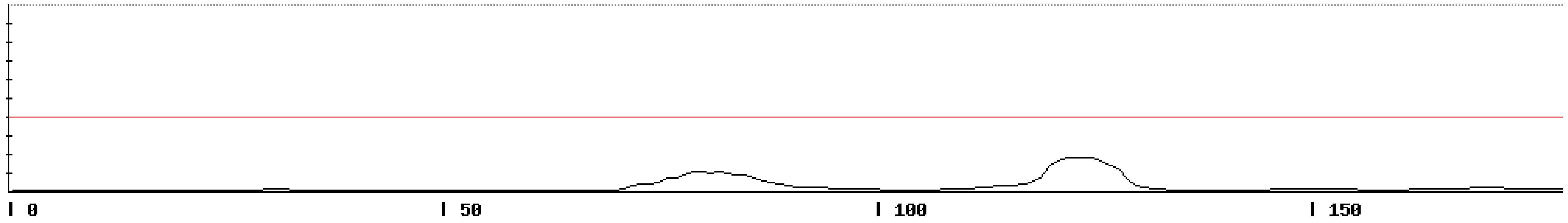

Predictions for Glycinemax_Glyma.12G148900.1.p

Viterbi Path

Viterbi Path

Posterior @ 0.4	
Posterior @ 0.4	
Posterior Graphic. Threshold Marked @ 0.4	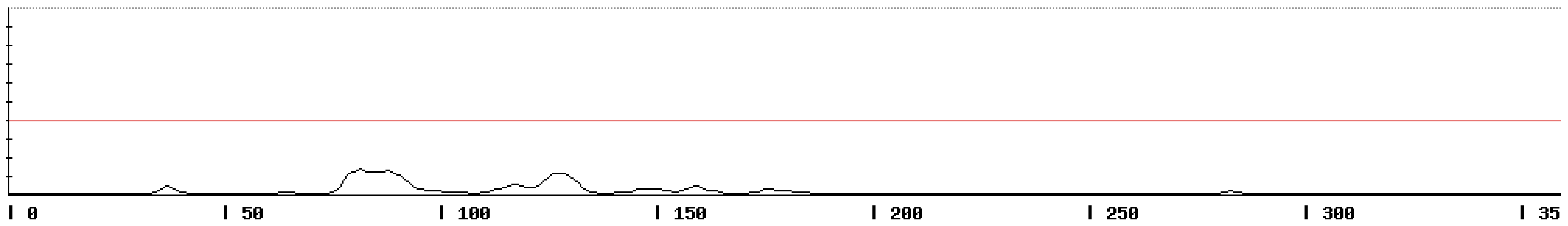

Predictions for Glycinemax_Glyma.12G149100.1.p

Viterbi Path	
Viterbi Path	
Posterior @ 0.4	
Posterior @ 0.4	
Posterior Graphic. Threshold Marked @ 0.4	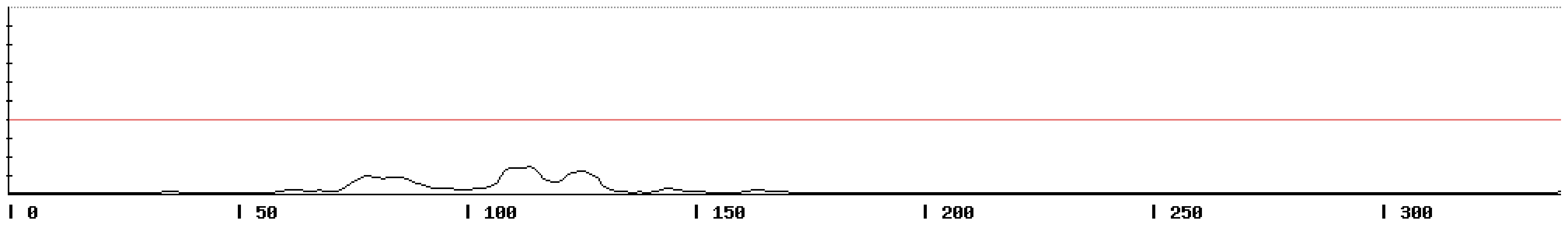

Predictions for Glycinemax_Glyma.12G160100.1.p

Viterbi Path	
Viterbi Path	

Posterior @ 0.4

Posterior @ 0.4

Posterior Graphic. Threshold Marked @ 0.4

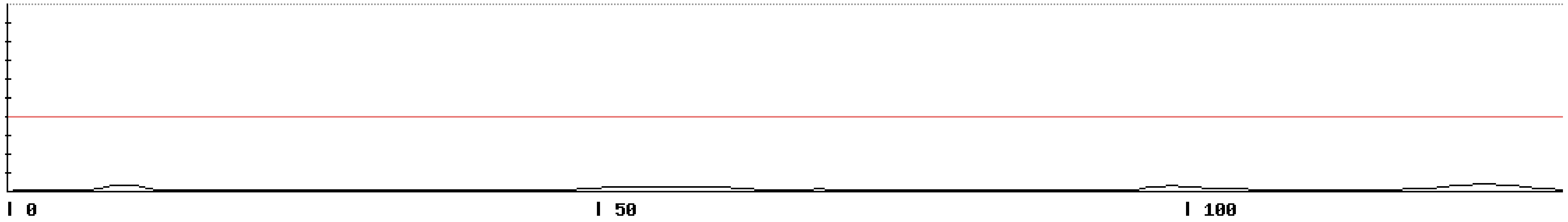

Predictions for Glycinemax_Glyma.12G161700.1.p

Viterbi Path

Viterbi Path

Posterior @ 0.4

Posterior @ 0.4

Posterior Graphic. Threshold Marked @ 0.4

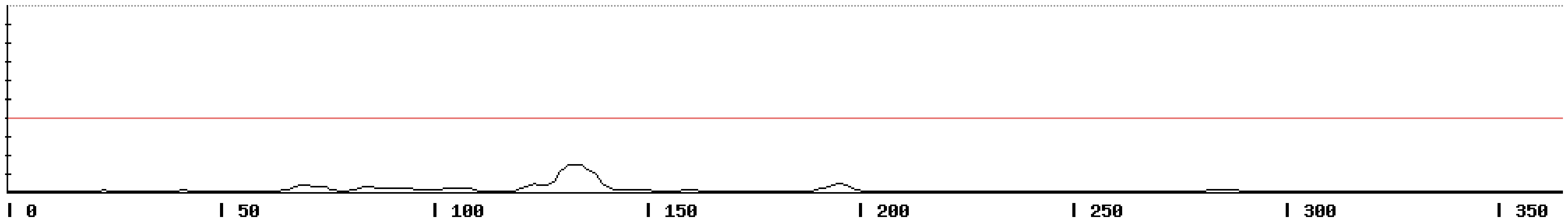

Predictions for Glycinemax_Glyma.12G171600.1.p

Viterbi Path	
Viterbi Path	
Posterior @ 0.4	
Posterior @ 0.4	
Posterior Graphic. Threshold Marked @ 0.4	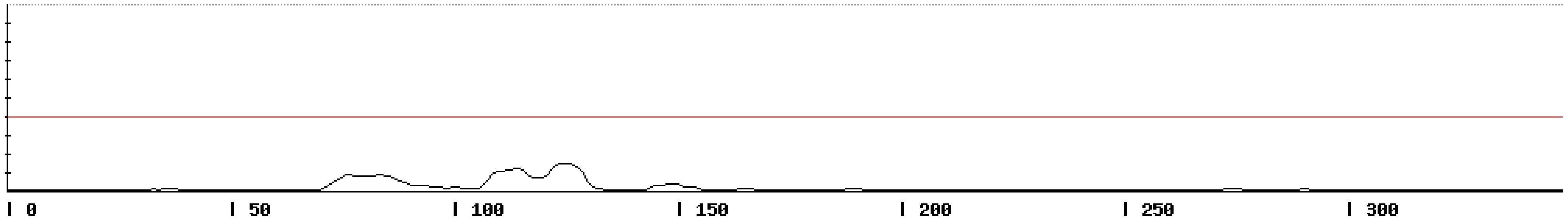

Predictions for Glycinemax_Glyma.12G186200.1.p

Viterbi Path	
Viterbi Path	
Posterior @ 0.4	124 - RK - 125
Posterior @ 0.4	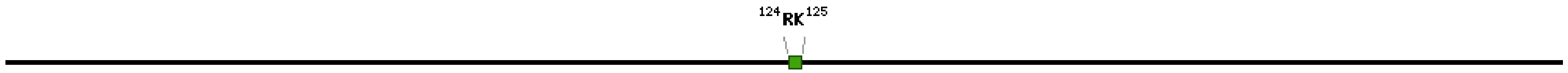
Posterior Graphic. Threshold Marked @ 0.4	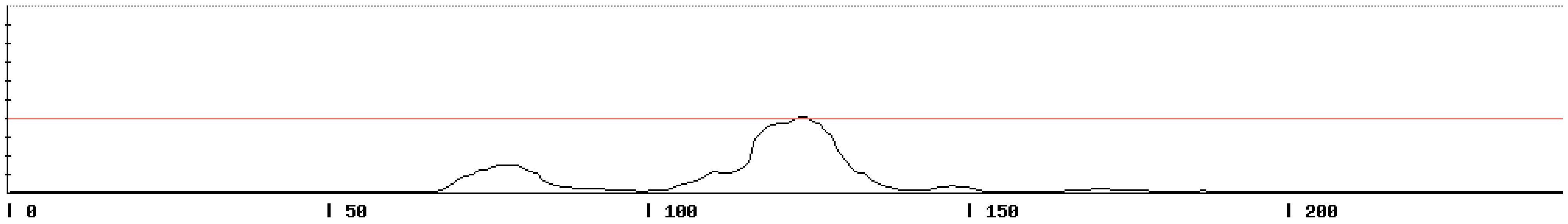

Predictions for Glycinemax_Glyma.12G186900.1.p	
Viterbi Path	
Viterbi Path	
Posterior @ 0.4	
Posterior @ 0.4	
Posterior Graphic. Threshold Marked @ 0.4	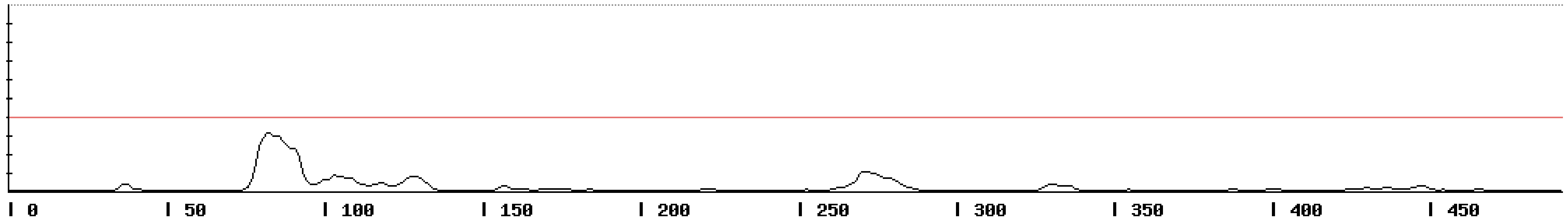

Predictions for Glycinemax_Glyma.12G206900.1.p	
Viterbi Path	
Viterbi Path	
Posterior @ 0.4	126 - RKRRK - 130
Posterior @ 0.4	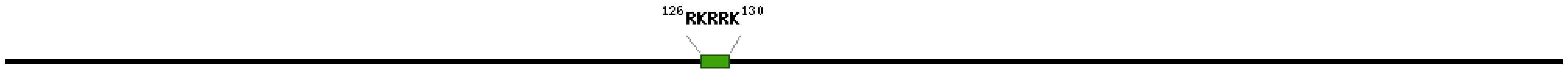
Posterior Graphic. Threshold Marked @ 0.4	

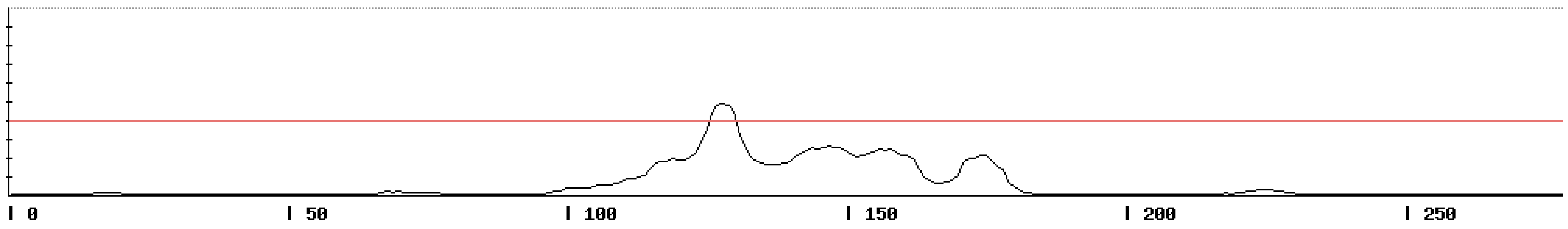

Predictions for Glycinemax_Glyma.12G221400.1.p

Viterbi Path

Viterbi Path

Posterior @ 0.4

Posterior @ 0.4

Posterior Graphic. Threshold Marked @ 0.4

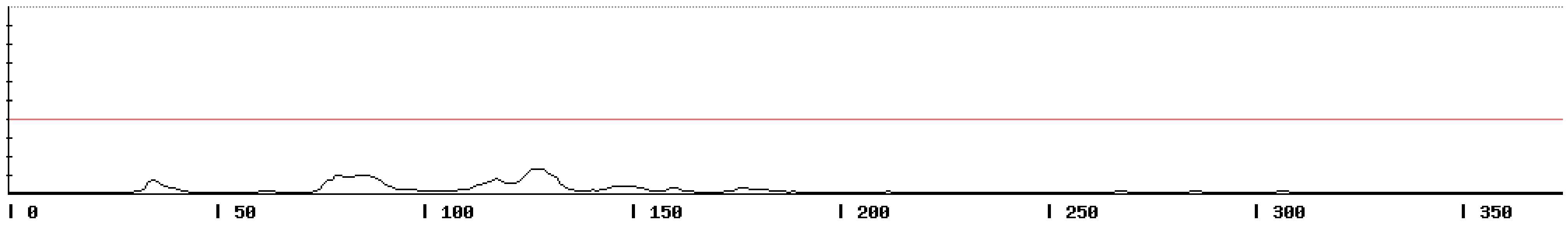

Predictions for Glycinemax_Glyma.12G221500.1.p

Viterbi Path

Viterbi Path

Posterior @ 0.4

Posterior @ 0.4

Posterior Graphic. Threshold Marked @ 0.4	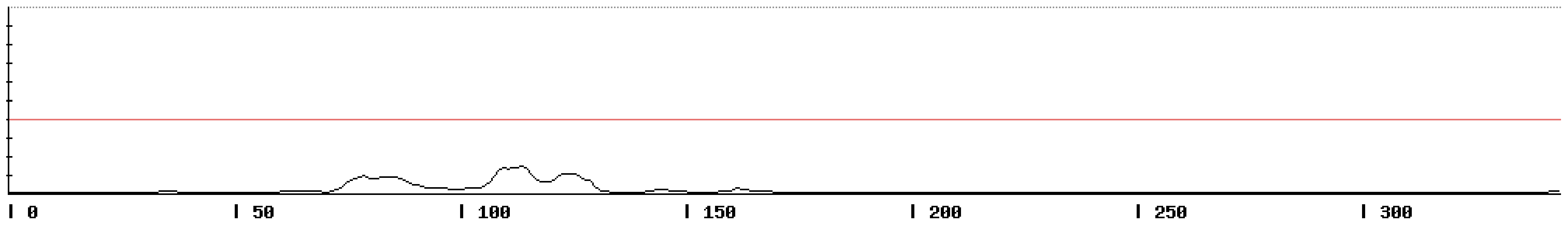

Predictions for Glycinemax_Glyma.12G226500.1.p

Viterbi Path	
Viterbi Path	
Posterior @ 0.4	
Posterior @ 0.4	
Posterior Graphic. Threshold Marked @ 0.4	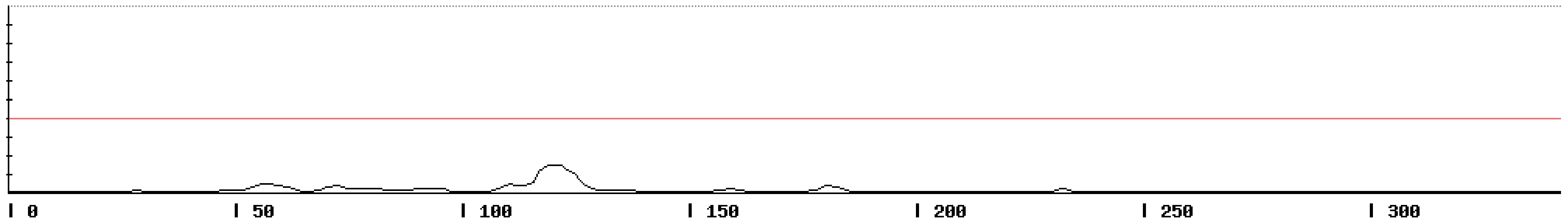

Predictions for Glycinemax_Glyma.13G030900.1.p

Viterbi Path	
Viterbi Path	
Posterior @ 0.4	97 - KPVGKPKPVGIKK - 109
Posterior @ 0.4	

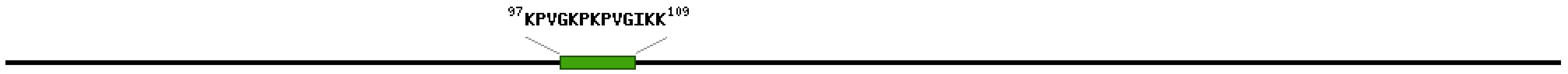

Posterior Graphic. Threshold Marked @ 0.4

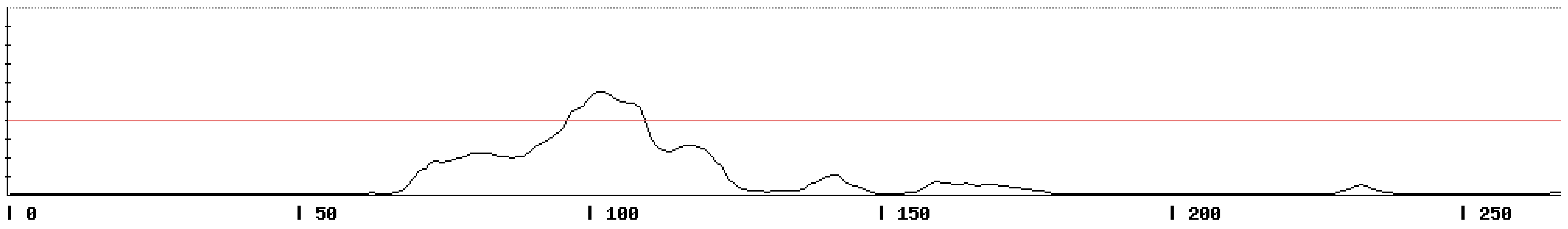

Predictions for Glycinemax_Glyma.13G062000.1.p

Viterbi Path

Viterbi Path

Posterior @ 0.4

Posterior @ 0.4

Posterior Graphic. Threshold Marked @ 0.4

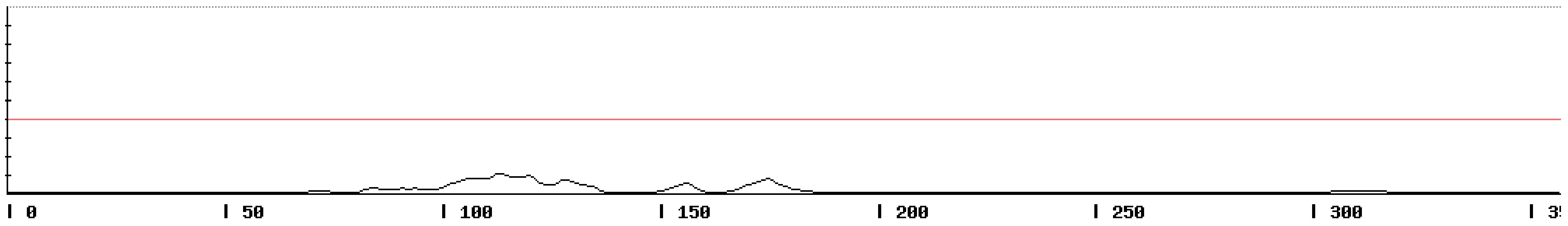

Predictions for Glycinemax_Glyma.13G063300.1.p

Viterbi Path

Viterbi Path

Posterior @ 0.4

Posterior @ 0.4

Posterior Graphic. Threshold Marked @ 0.4

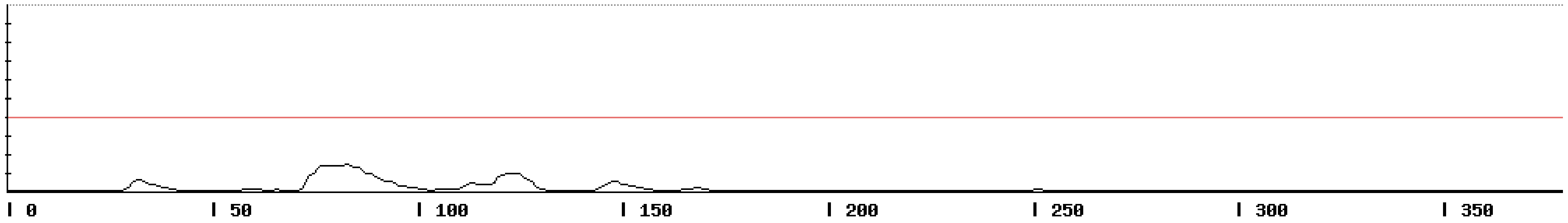

Predictions for Glycinemax_Glyma.13G174700.1.p

Viterbi Path

Viterbi Path

Posterior @ 0.4 — 146 - TRKRRKVHTDKE - 157

Posterior @ 0.4

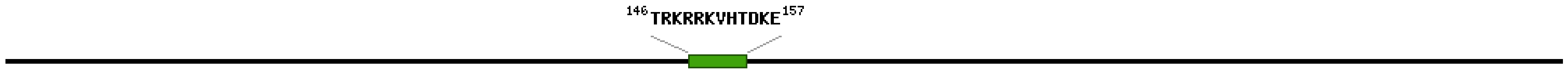

Posterior Graphic. Threshold Marked @ 0.4

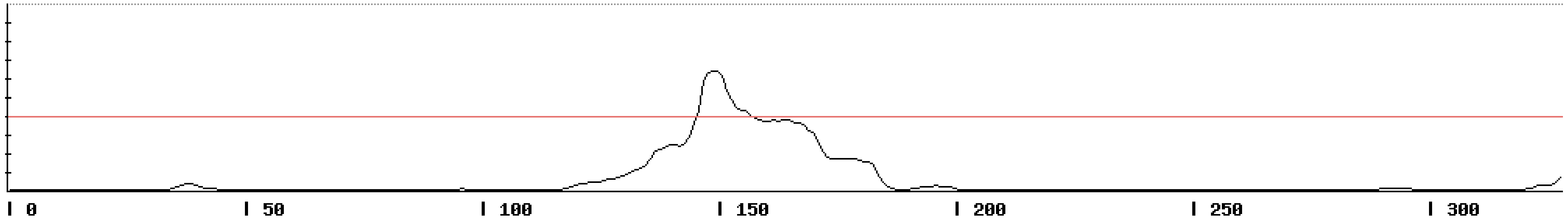

Predictions for Glycinemax_Glyma.13G234700.1.p

Viterbi Path

Viterbi Path

Posterior @ 0.4 | 152 - KRR - 154

Posterior @ 0.4

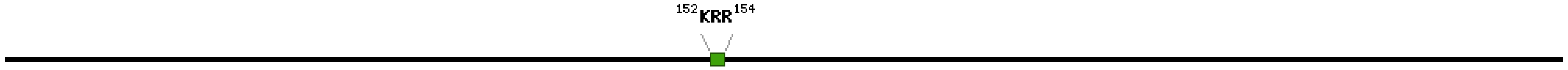

Posterior Graphic. Threshold Marked @ 0.4

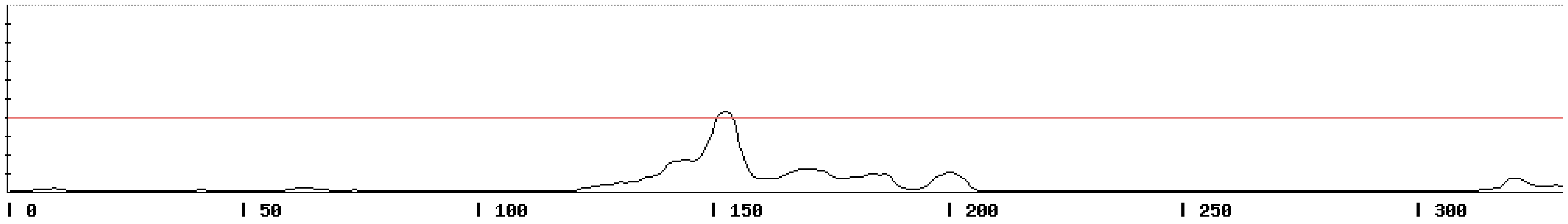

Predictions for Glycinemax_Glyma.13G243200.1.p

Viterbi Path

Viterbi Path

Posterior @ 0.4

Posterior @ 0.4

Posterior Graphic. Threshold Marked @ 0.4

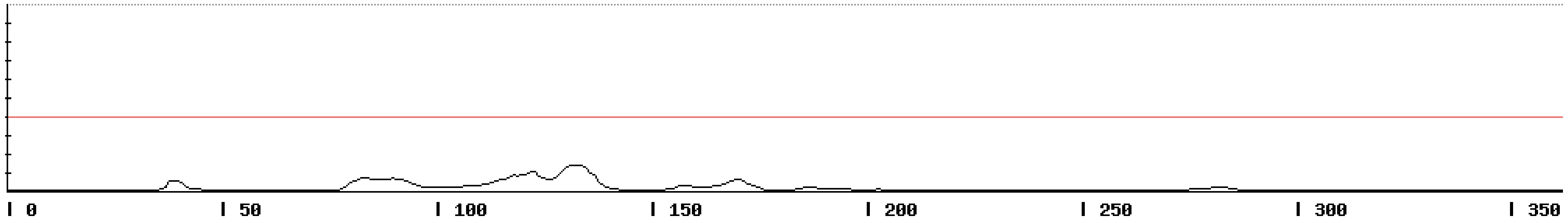

Predictions for Glycinemax_Glyma.13G274300.1.p

Viterbi Path

Viterbi Path

Posterior @ 0.4

Posterior @ 0.4

Posterior Graphic. Threshold Marked @ 0.4

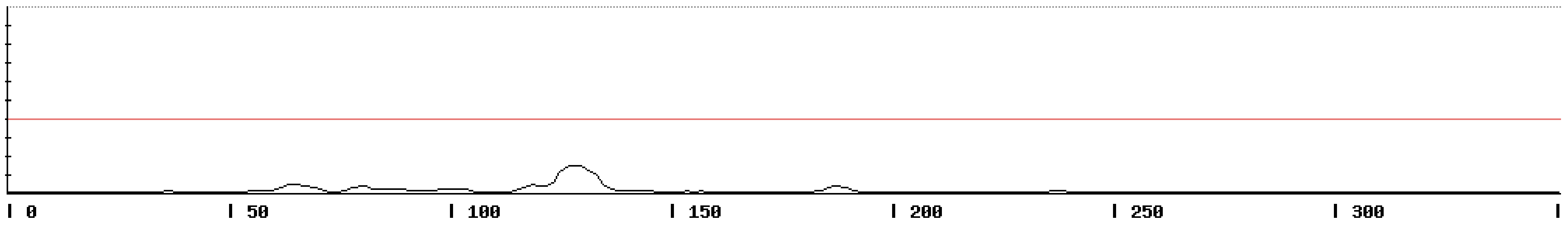

Predictions for Glycinemax_Glyma.13G279900.1.p

Viterbi Path

Viterbi Path

Posterior @ 0.4

Posterior @ 0.4

Posterior Graphic. Threshold Marked @ 0.4

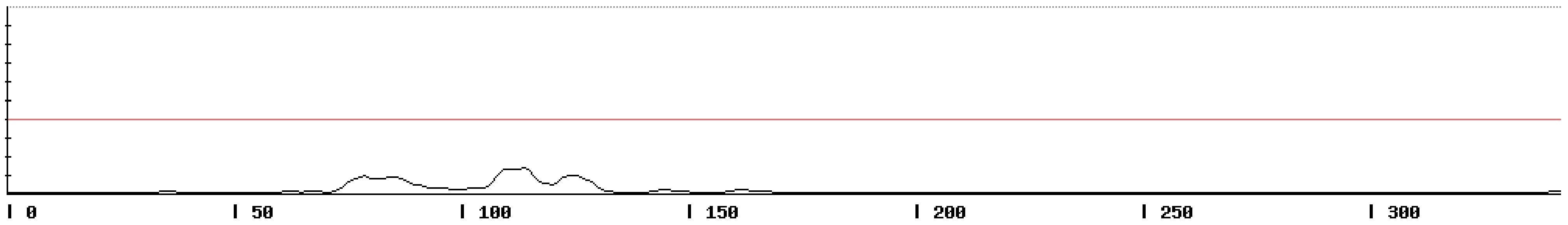

Predictions for Glycinemax_Glyma.13G280000.1.p	
Viterbi Path	
Viterbi Path	
Posterior @ 0.4	
Posterior @ 0.4	
Posterior Graphic. Threshold Marked @ 0.4	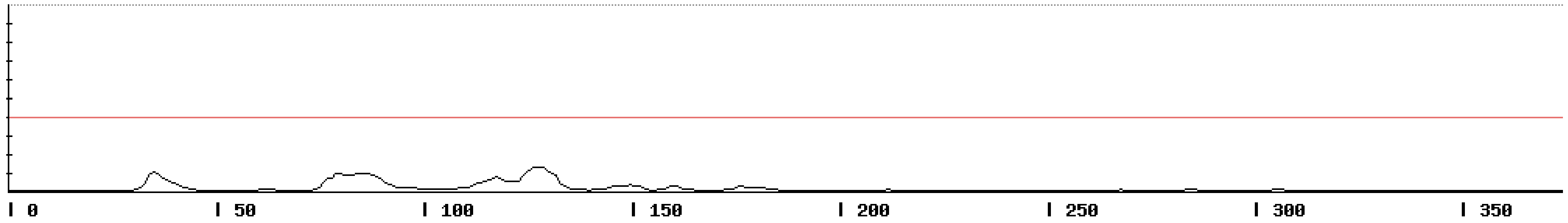

Predictions for Glycinemax_Glyma.13G294000.1.p	
Viterbi Path	
Viterbi Path	
Posterior @ 0.4	126 - RKRRK - 130
Posterior @ 0.4	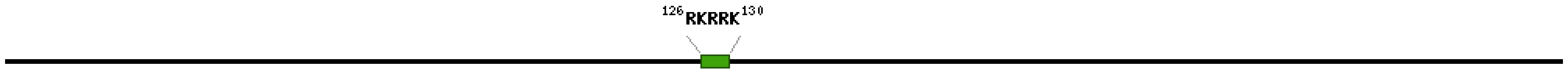
Posterior Graphic. Threshold Marked @ 0.4	

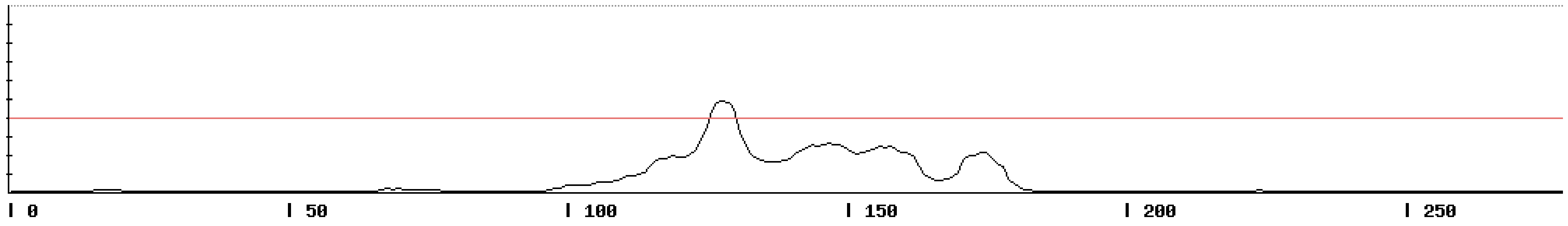

Predictions for Glycinemax_Glyma.13G314600.1.p

Viterbi Path	
Viterbi Path	
Posterior @ 0.4	
Posterior @ 0.4	
Posterior Graphic. Threshold Marked @ 0.4	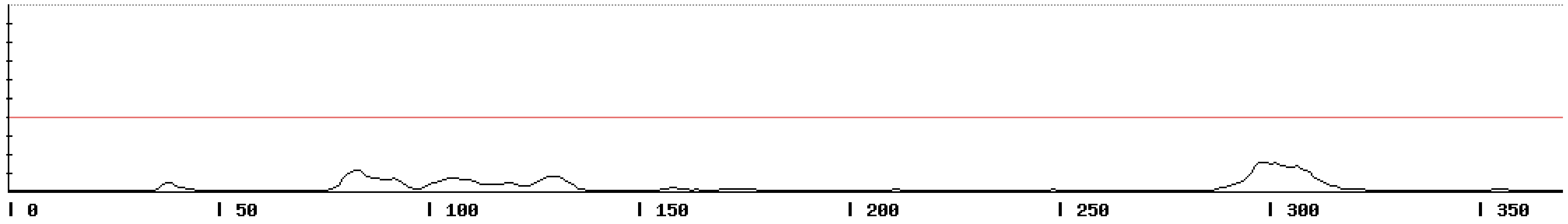

Predictions for Glycinemax_Glyma.13G315300.1.p

Viterbi Path	
Viterbi Path	
Posterior @ 0.4	124 - RK - 125
Posterior @ 0.4	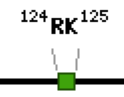

Posterior Graphic. Threshold Marked @ 0.4	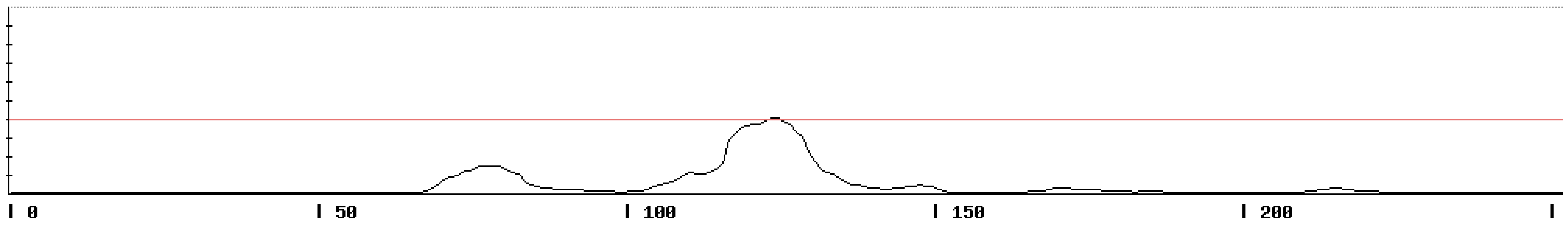

**Predictions for Glycinemax_Glyma.13G327600.1.p**	
Viterbi Path	
Viterbi Path	
Posterior @ 0.4	
Posterior @ 0.4	
Posterior Graphic. Threshold Marked @ 0.4	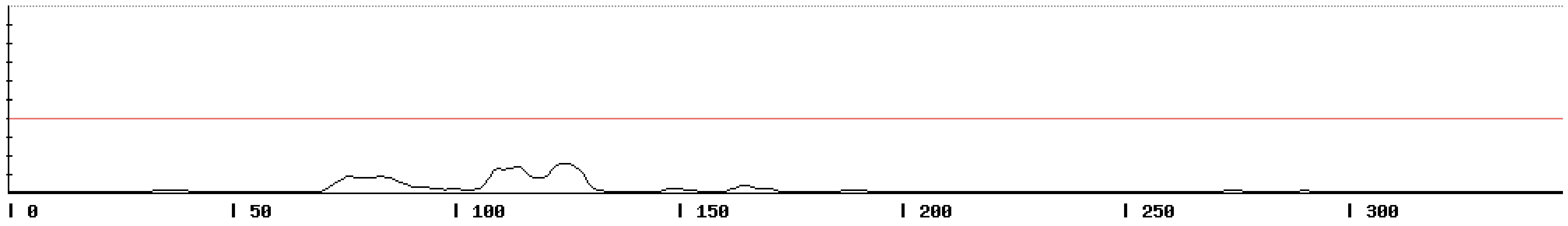

**Predictions for Glycinemax_Glyma.14G030700.1.p**	
Viterbi Path	
Viterbi Path	
Posterior @ 0.4	
Posterior @ 0.4	

Posterior Graphic. Threshold Marked @ 0.4

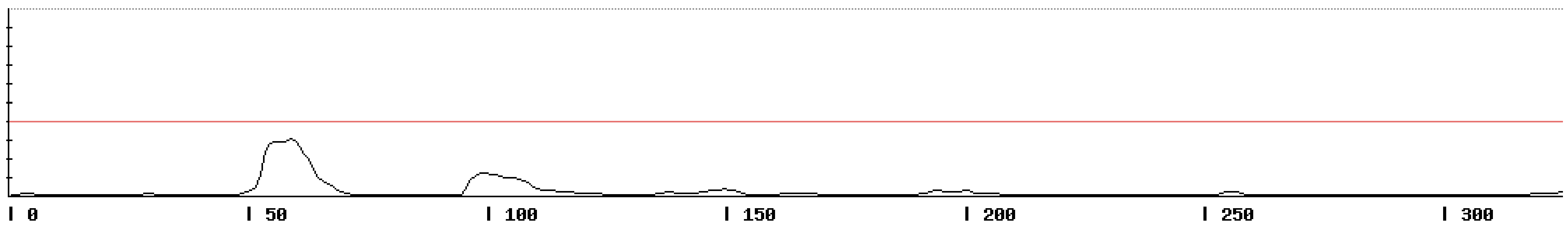

Predictions for Glycinemax_Glyma.14G084300.1.p

Viterbi Path: 82 - KRRNRKRKVKSGFWLRKGRVR - 102

Viterbi Path

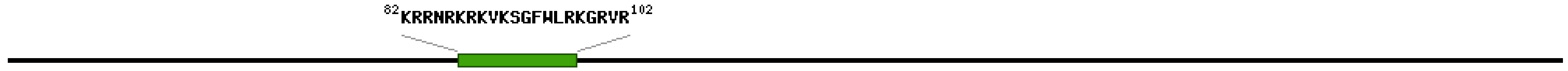

Posterior @ 0.4: 71 - KK - 72

78 - AKETKRRNRKRKVKSGFWLRKGRV - 101

Posterior @ 0.4

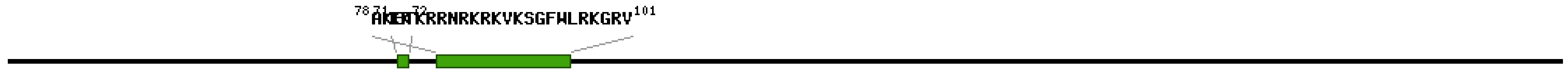

Posterior Graphic. Threshold Marked @ 0.4

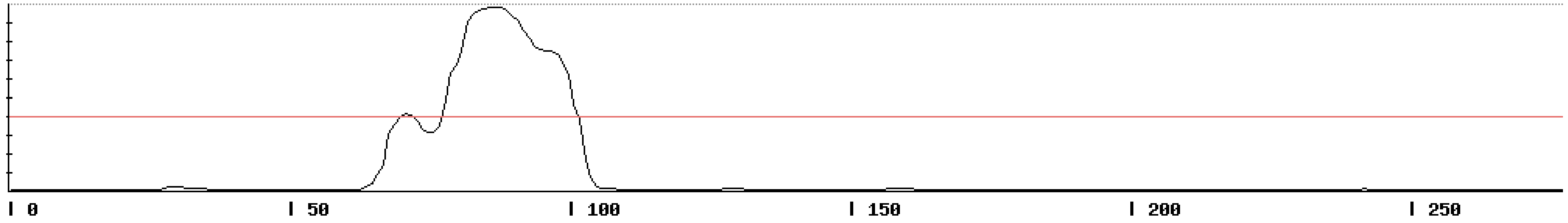

Predictions for Glycinemax_Glyma.14G140100.1.p

Viterbi Path

Viterbi Path

Posterior @ 0.4

Posterior @ 0.4

Posterior Graphic. Threshold Marked @ 0.4

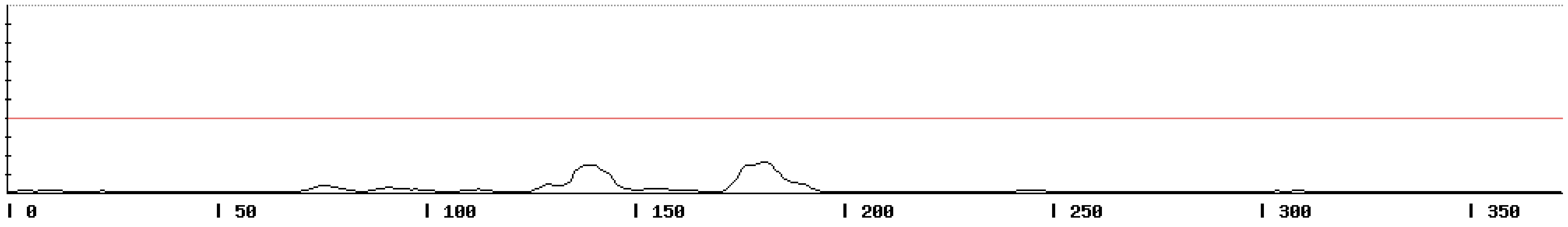

Predictions for Glycinemax_Glyma.14G152700.1.p

Viterbi Path

Viterbi Path

Posterior @ 0.4

97 - KPVGKPKPVGIKK - 109

Posterior @ 0.4

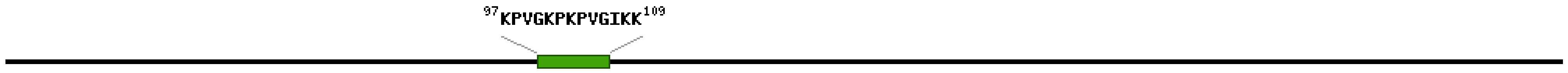

Posterior Graphic. Threshold Marked @ 0.4

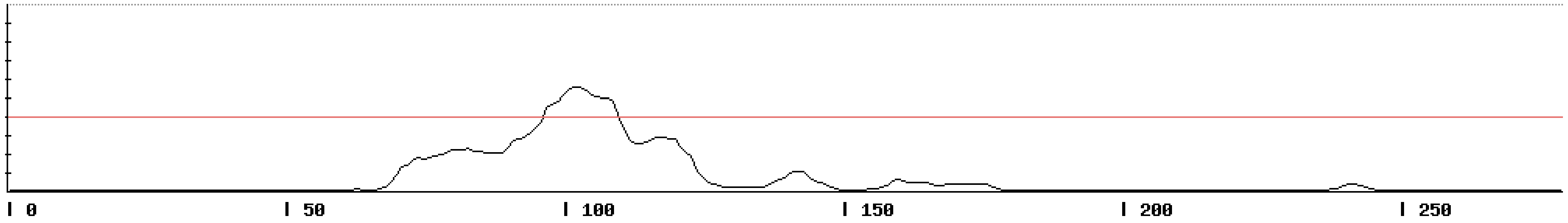

Predictions for Glycinemax_Glyma.14G189300.1.p

Viterbi Path

Viterbi Path

Posterior @ 0.4

Posterior @ 0.4

Posterior Graphic. Threshold Marked @ 0.4

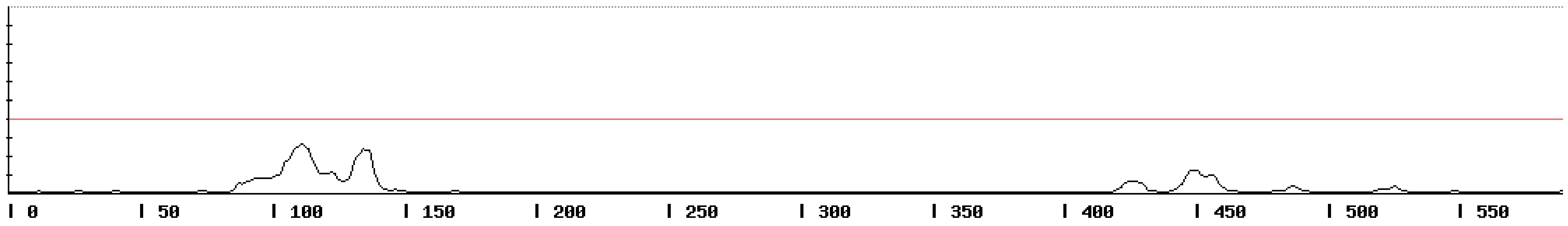

Predictions for Glycinemax_Glyma.14G210000.1.p

Viterbi Path

Viterbi Path

Posterior @ 0.4

Posterior @ 0.4

Posterior Graphic. Threshold Marked @ 0.4

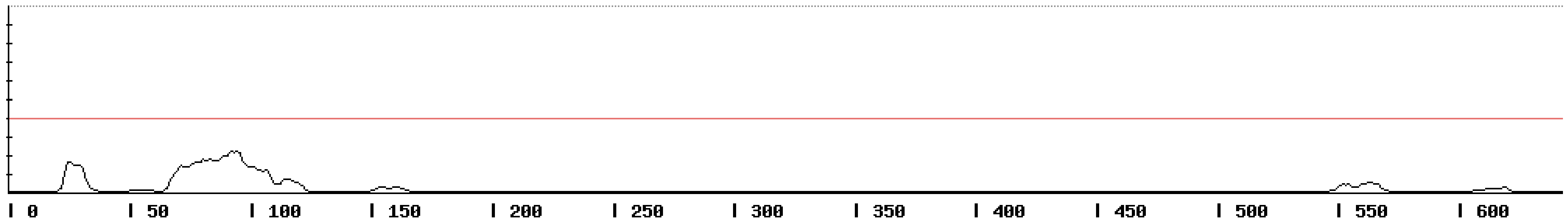

Predictions for Glycinemax_Glyma.15G051200.1.p	
Viterbi Path	
Viterbi Path	
Posterior @ 0.4	
Posterior @ 0.4	
Posterior Graphic. Threshold Marked @ 0.4	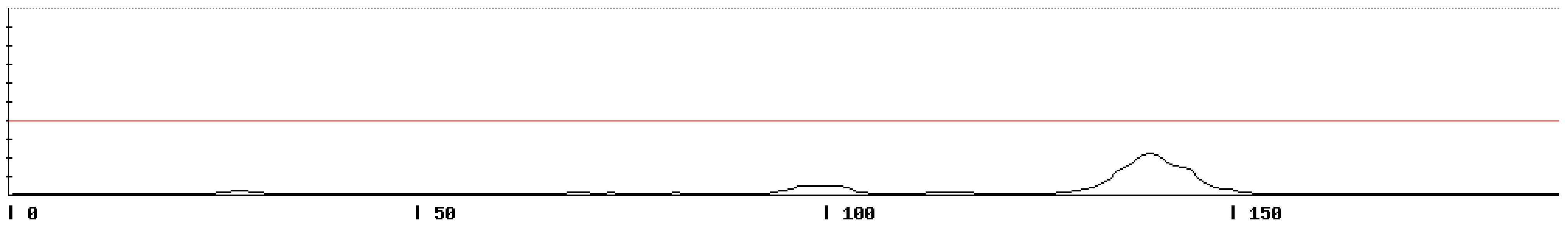

Predictions for Glycinemax_Glyma.15G070300.1.p	
Viterbi Path	
Viterbi Path	
Posterior @ 0.4	
Posterior @ 0.4	
Posterior Graphic. Threshold Marked @ 0.4	

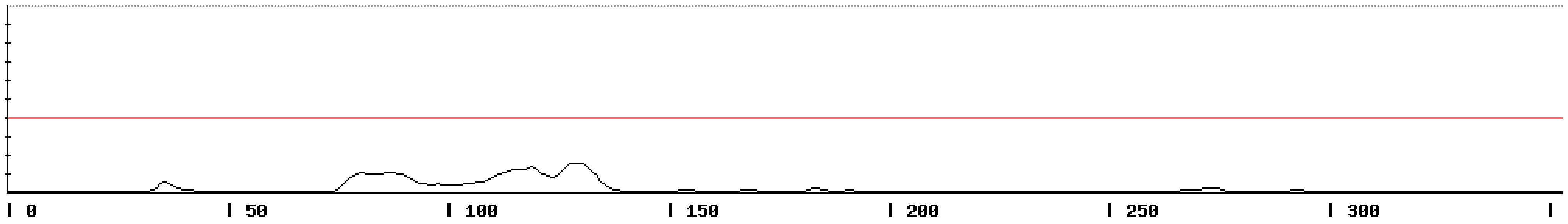

Predictions for Glycinemax_Glyma.15G078300.1.p

Viterbi Path	
Viterbi Path	
Posterior @ 0.4	140 - KRR - 142
Posterior @ 0.4	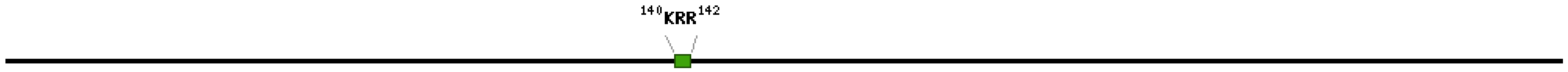
Posterior Graphic. Threshold Marked @ 0.4	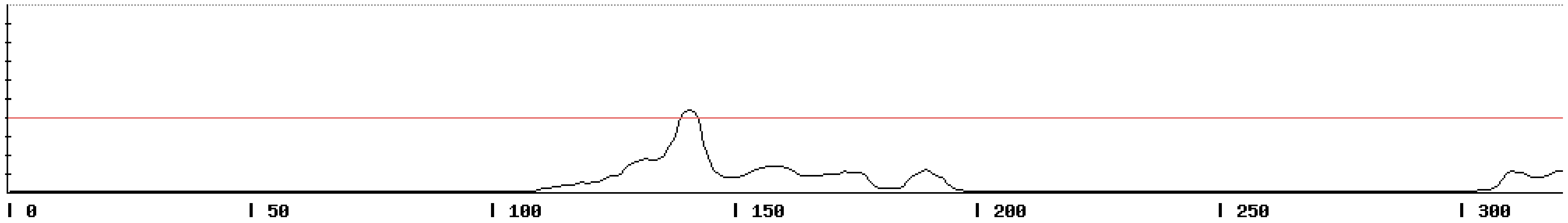

Predictions for Glycinemax_Glyma.15G254000.1.p

Viterbi Path	
Viterbi Path	
Posterior @ 0.4	
Posterior @ 0.4	

Posterior Graphic. Threshold Marked @ 0.4

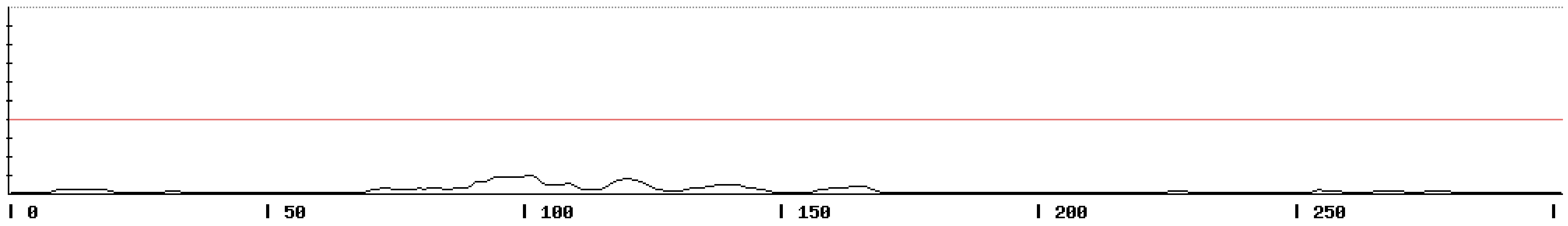

Predictions for Glycinemax_Glyma.15G257700.1.p

Viterbi Path

Viterbi Path

Posterior @ 0.4

Posterior @ 0.4

Posterior Graphic. Threshold Marked @ 0.4

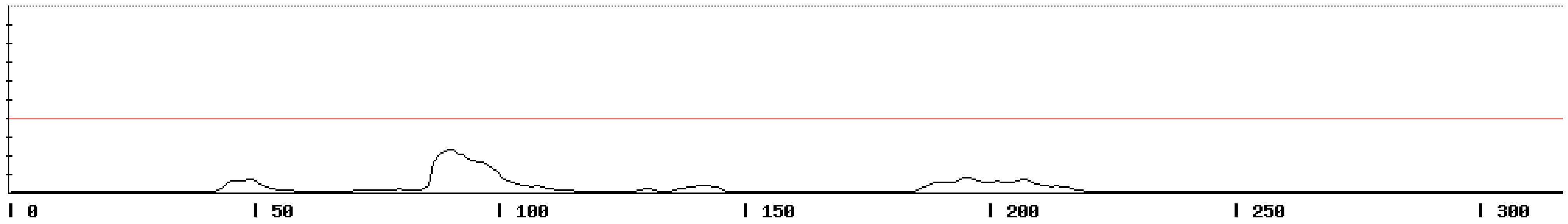

Predictions for Glycinemax_Glyma.15G264100.1.p

Viterbi Path

Viterbi Path

Posterior @ 0.4

Posterior @ 0.4

Posterior Graphic. Threshold Marked @ 0.4

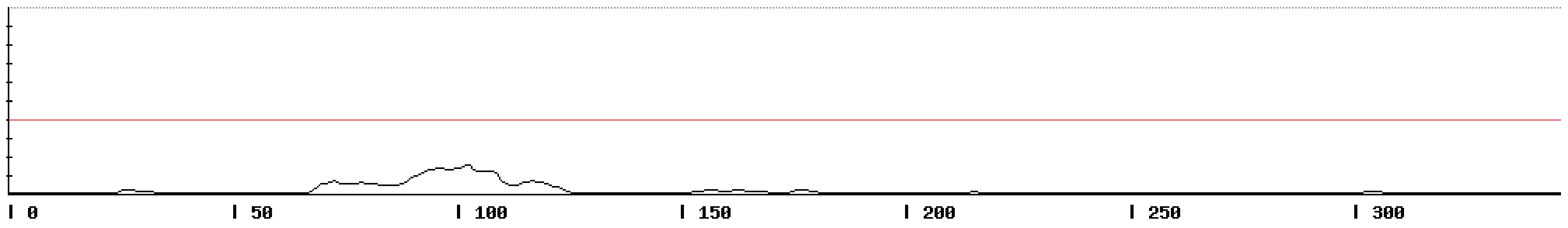

Predictions for Glycinemax_Glyma.15G266500.1.p

Viterbi Path

Viterbi Path

Posterior @ 0.4

Posterior @ 0.4

Posterior Graphic. Threshold Marked @ 0.4

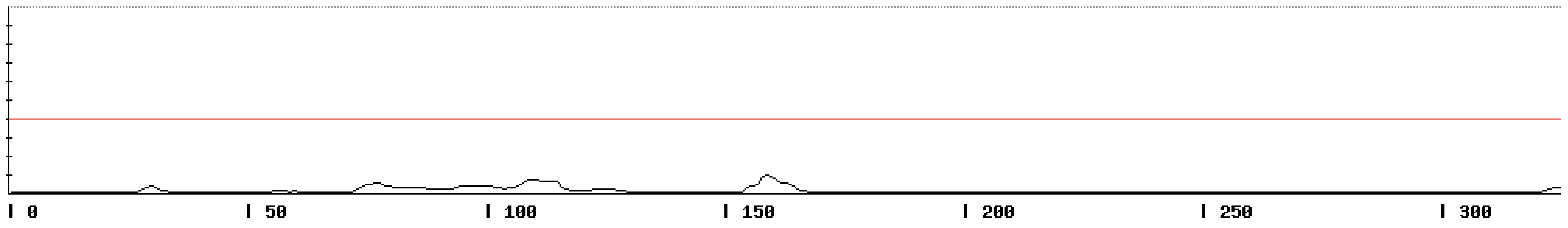

Predictions for Glycinemax_Glyma.16G016400.1.p

Viterbi Path

Viterbi Path

Posterior @ 0.4

Posterior @ 0.4

Posterior Graphic. Threshold Marked @ 0.4

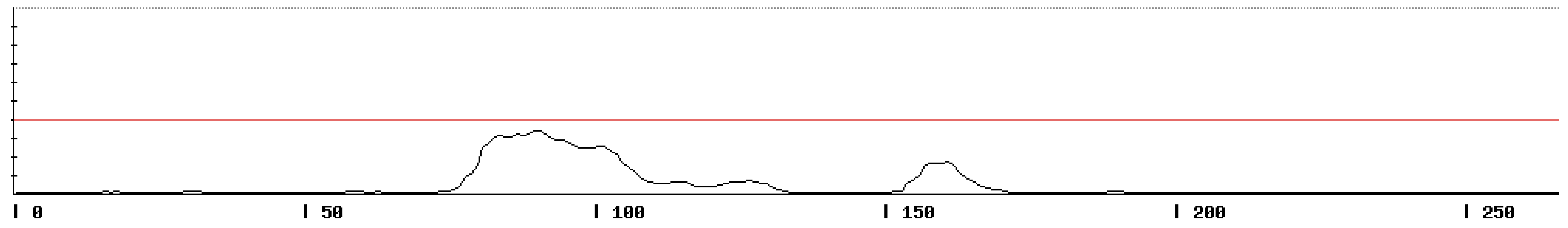

Predictions for Glycinemax_Glyma.16G016600.1.p

Viterbi Path

Viterbi Path

Posterior @ 0.4

Posterior @ 0.4

Posterior Graphic. Threshold Marked @ 0.4

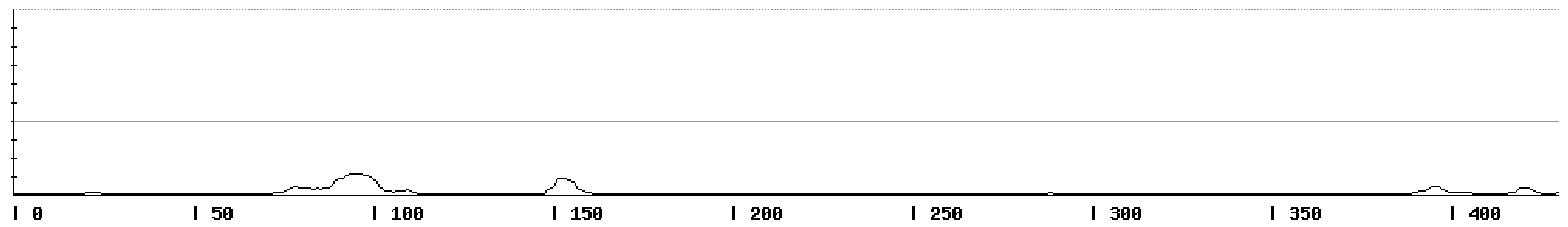

Predictions for Glycinemax_Glyma.16G016700.1.p

Viterbi Path

Viterbi Path

Posterior @ 0.4

Posterior @ 0.4

Posterior Graphic. Threshold Marked @ 0.4

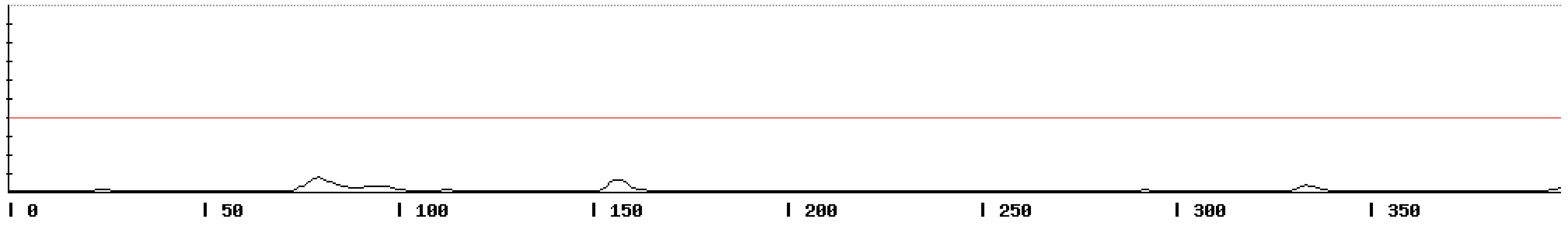

Predictions for Glycinemax_Glyma.16G019400.1.p

Viterbi Path

Viterbi Path

Posterior @ 0.4

Posterior @ 0.4

Posterior Graphic. Threshold Marked @ 0.4

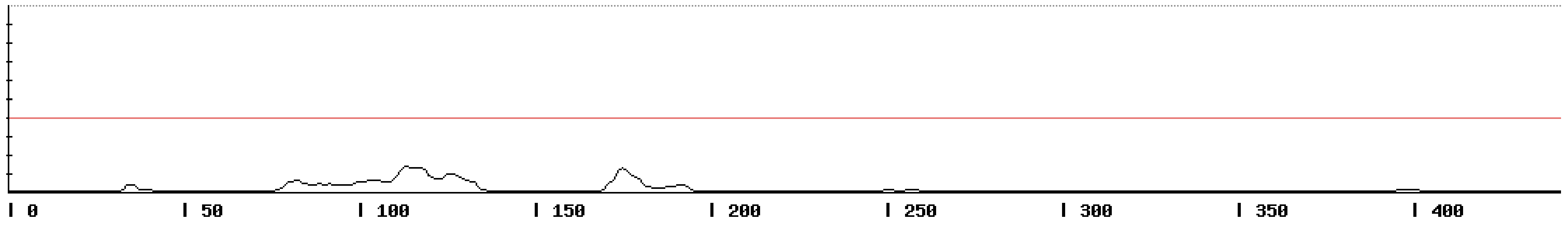

Predictions for Glycinemax_Glyma.16G042900.1.p

Viterbi Path

Viterbi Path

Posterior @ 0.4

Posterior @ 0.4

Posterior Graphic. Threshold Marked @ 0.4

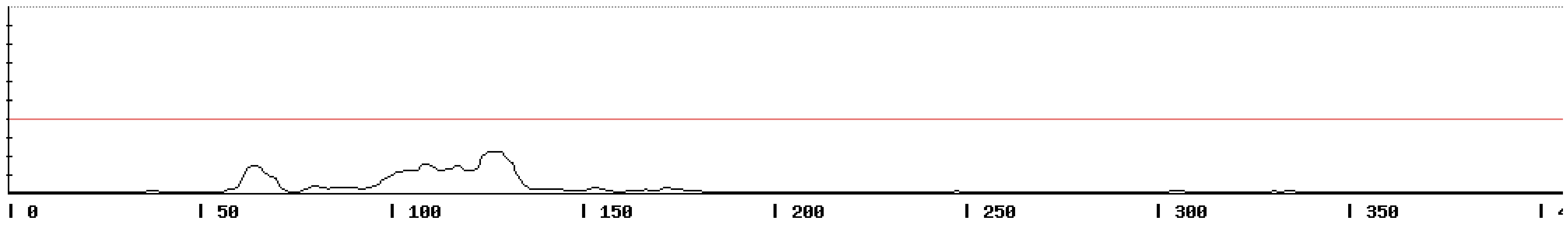

Predictions for Glycinemax_Glyma.16G043200.1.p

Viterbi Path

Viterbi Path

Posterior @ 0.4

Posterior @ 0.4

Posterior Graphic. Threshold Marked @ 0.4

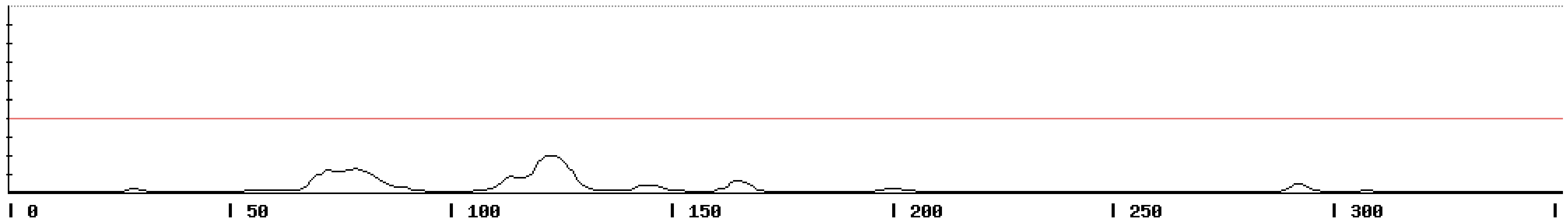

Predictions for Glycinemax_Glyma.16G051800.1.p	
Viterbi Path	159 - KTKKEGRKGKKAKR - 172
Viterbi Path	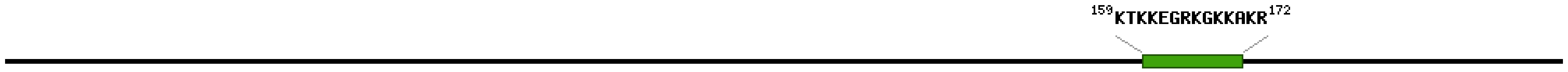
Posterior @ 0.4	158 - FKTKKEGRKGKKAKRS - 173
Posterior @ 0.4	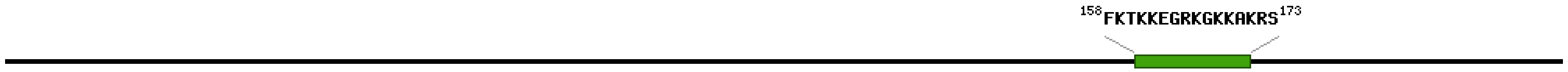
Posterior Graphic. Threshold Marked @ 0.4	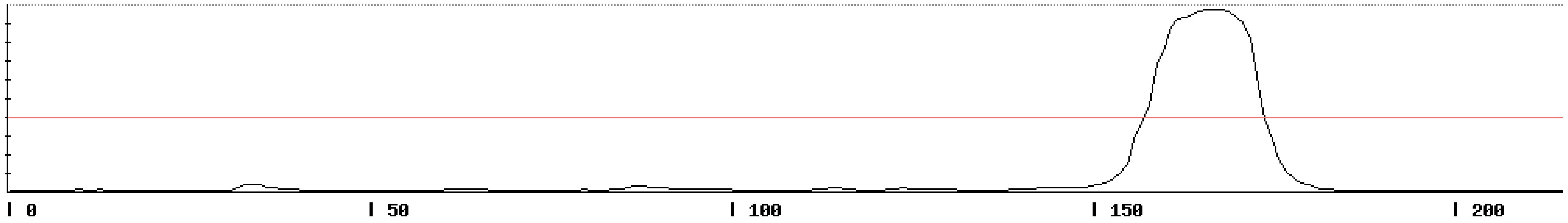

Predictions for Glycinemax_Glyma.16G069300.1.p	
Viterbi Path	
Viterbi Path	
Posterior @ 0.4	134 - KRRK - 137
Posterior @ 0.4	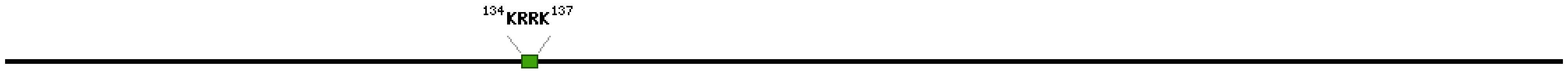
Posterior Graphic. Threshold Marked @ 0.4	

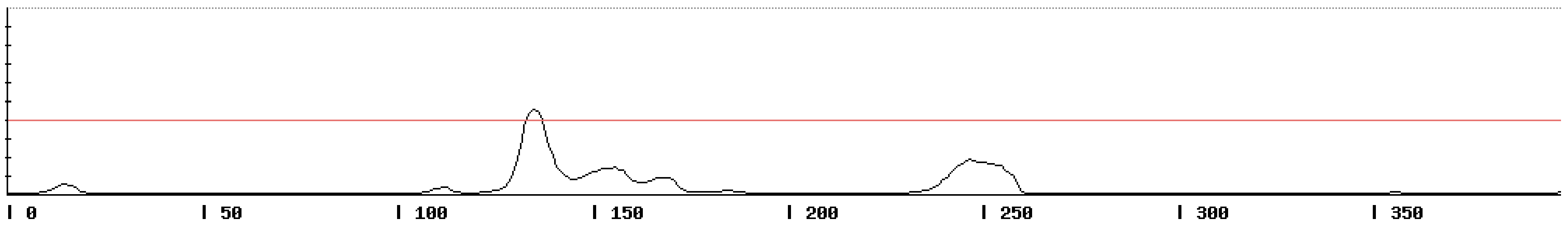

Predictions for Glycinemax_Glyma.16G130200.1.p

Viterbi Path

Viterbi Path

Posterior @ 0.4

Posterior @ 0.4

Posterior Graphic. Threshold Marked @ 0.4

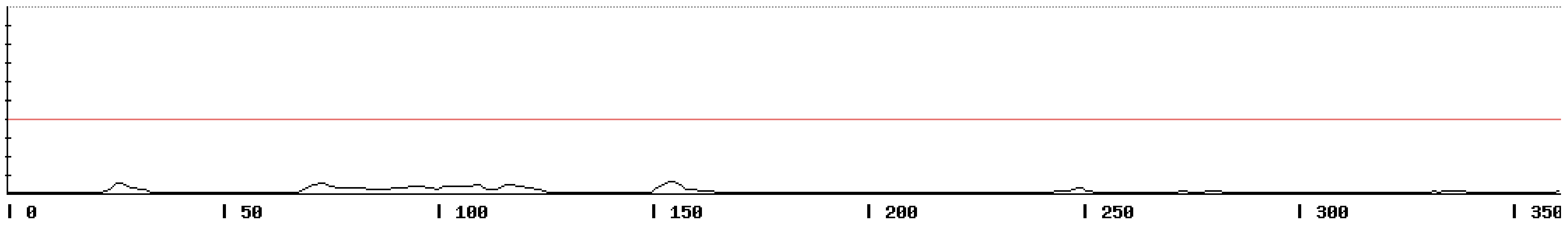

Predictions for Glycinemax_Glyma.16G151500.1.p

Viterbi Path

Viterbi Path

Posterior @ 0.4

Posterior @ 0.4

Posterior Graphic. Threshold Marked @ 0.4

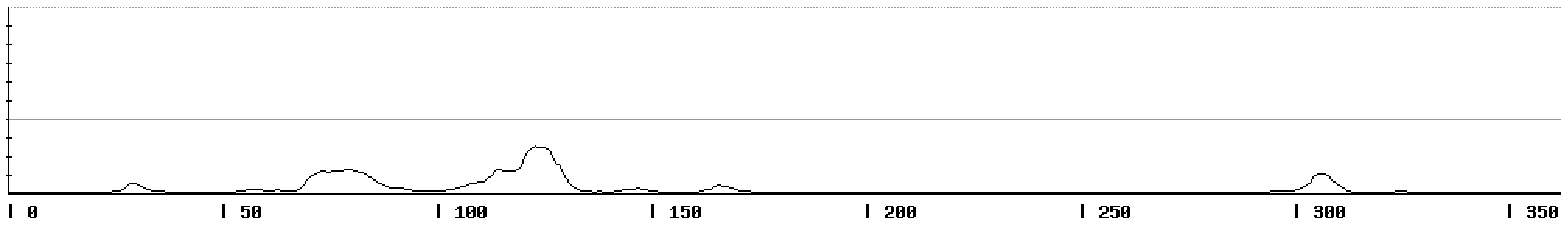

Predictions for Glycinemax_Glyma.16G152100.1.p

Viterbi Path

Viterbi Path

Posterior @ 0.4

Posterior @ 0.4

Posterior Graphic. Threshold Marked @ 0.4

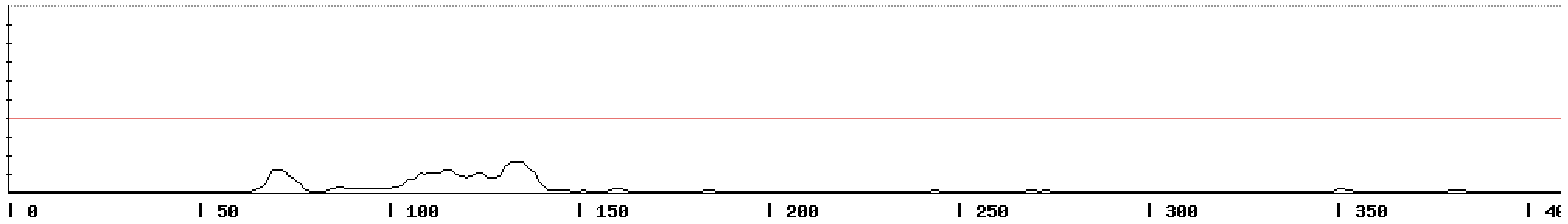

Predictions for Glycinemax_Glyma.16G217400.1.p

Viterbi Path: 17 - KKRKKGRREKK - 27

Viterbi Path

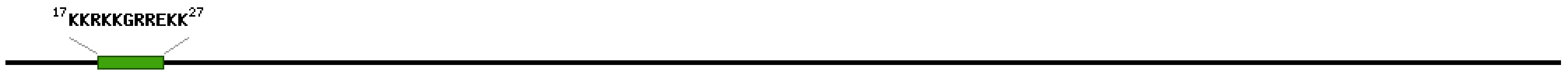

Posterior @ 0.4: 16 - NKKRKKGRREKKMSP - 30

Posterior @ 0.4

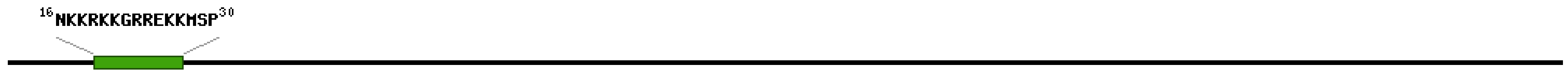

Posterior Graphic. Threshold Marked @ 0.4

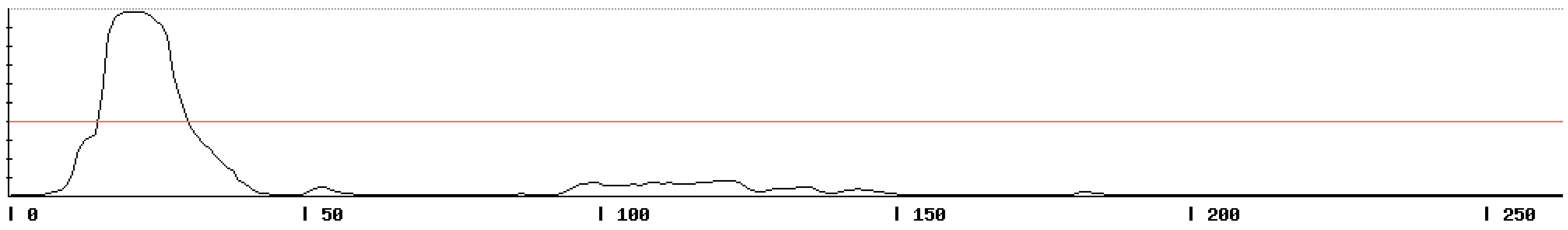

Predictions for Glycinemax_Glyma.17G002800.1.p

Viterbi Path

Viterbi Path

Posterior @ 0.4

Posterior @ 0.4

Posterior Graphic. Threshold Marked @ 0.4

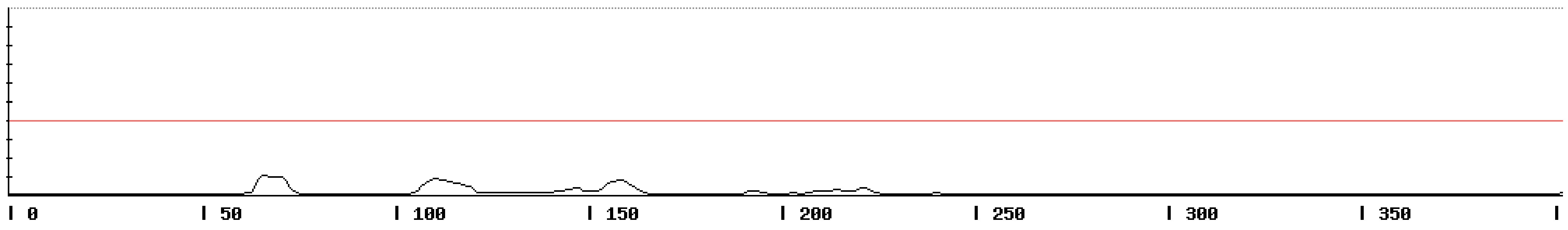

Predictions for Glycinemax_Glyma.17G101500.1.p

Viterbi Path

Viterbi Path

Posterior @ 0.4

Posterior @ 0.4

Posterior Graphic. Threshold Marked @ 0.4

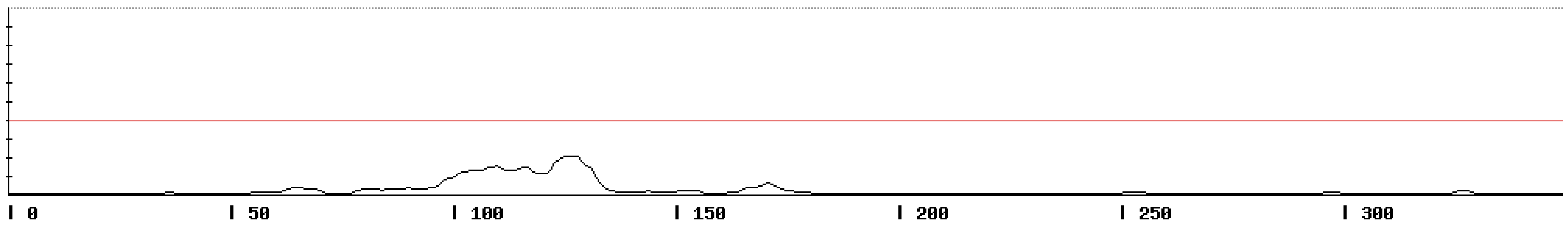

Predictions for Glycinemax_Glyma.17G138100.1.p

Viterbi Path

Viterbi Path

Posterior @ 0.4

Posterior @ 0.4

Posterior Graphic. Threshold Marked @ 0.4

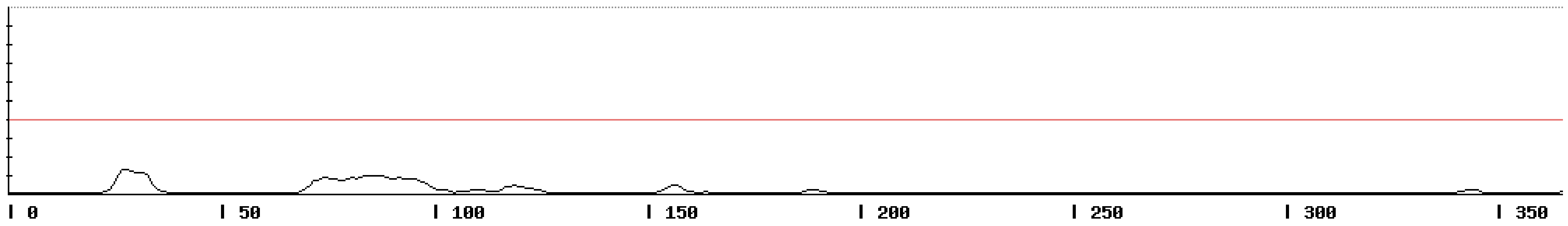

Predictions for Glycinemax_Glyma.17G154100.1.p

Viterbi Path

Viterbi Path

Posterior @ 0.4	
Posterior @ 0.4	
Posterior Graphic. Threshold Marked @ 0.4	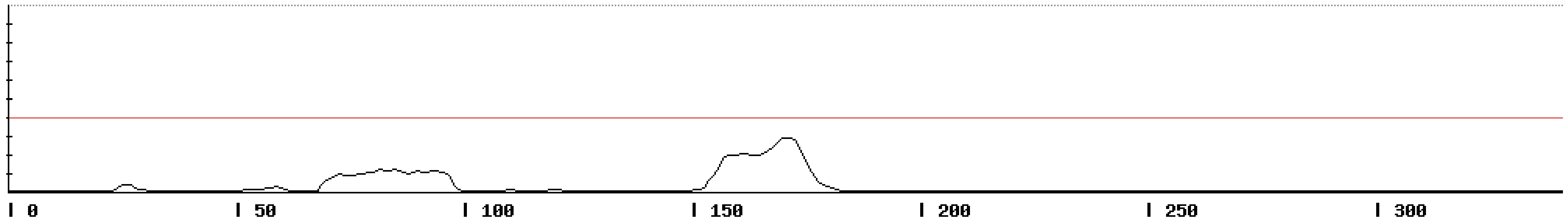

Predictions for Glycinemax_Glyma.17G185000.1.p	
Viterbi Path	
Viterbi Path	
Posterior @ 0.4	153 - KNKMKTRRSHGKKPSS - 168
Posterior @ 0.4	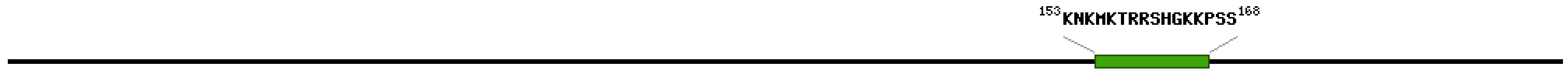
Posterior Graphic. Threshold Marked @ 0.4	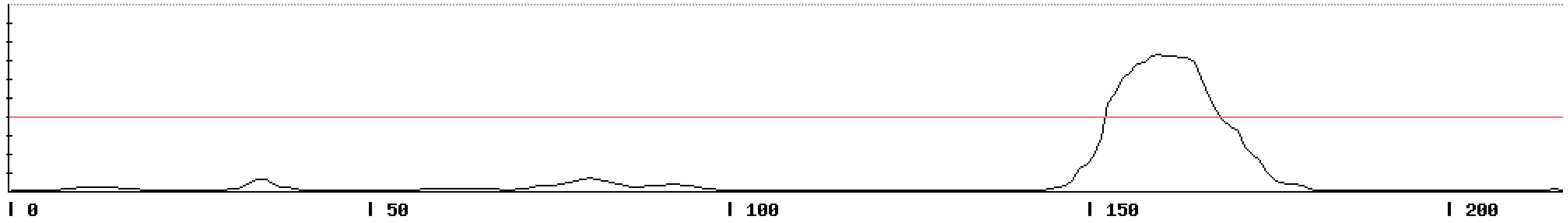

Predictions for Glycinemax_Glyma.17G240700.1.p

Viterbi Path	
Viterbi Path	
Posterior @ 0.4	72 - K - 72
	79 - AKETKRRNRRKVKSGFWLRKGKVR - 102
Posterior @ 0.4	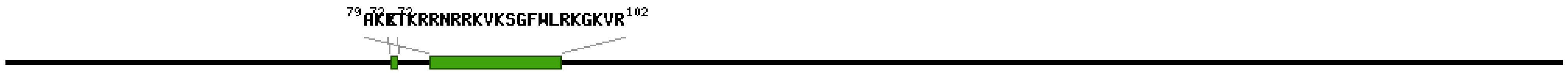
Posterior Graphic. Threshold Marked @ 0.4	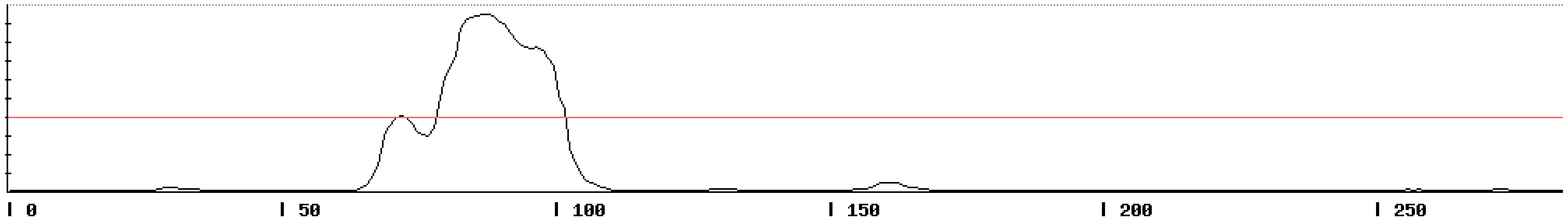

Predictions for Glycinemax_Glyma.18G043900.1.p

Viterbi Path	
Viterbi Path	
Posterior @ 0.4	
Posterior @ 0.4	
Posterior Graphic. Threshold Marked @ 0.4	

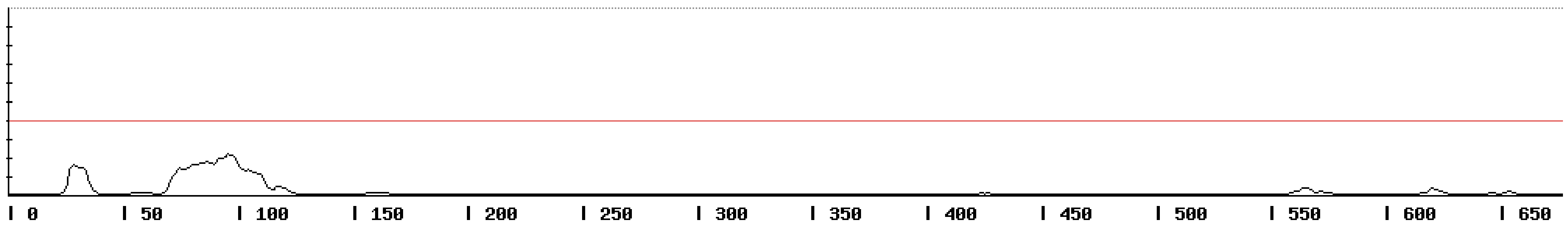

Predictions for Glycinemax_Glyma.18G110700.1.p

Viterbi Path

Viterbi Path

Posterior @ 0.4

Posterior @ 0.4

Posterior Graphic. Threshold Marked @ 0.4

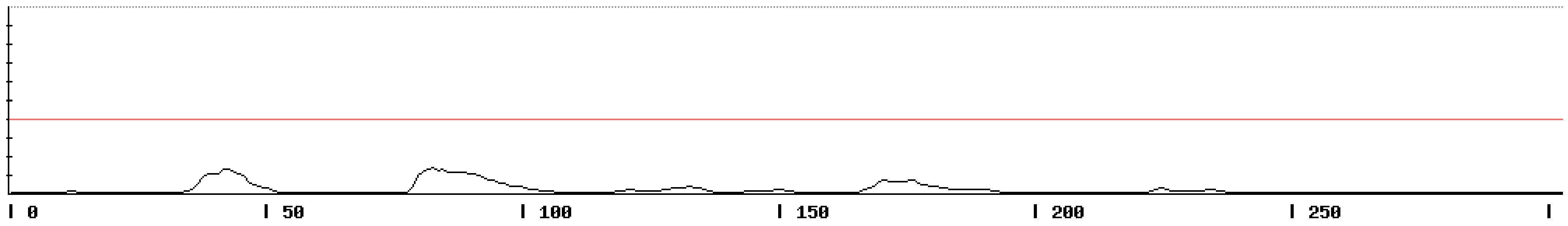

Predictions for Glycinemax_Glyma.18G119300.1.p

Viterbi Path

Viterbi Path

Posterior @ 0.4

Posterior @ 0.4

Posterior Graphic. Threshold Marked @ 0.4

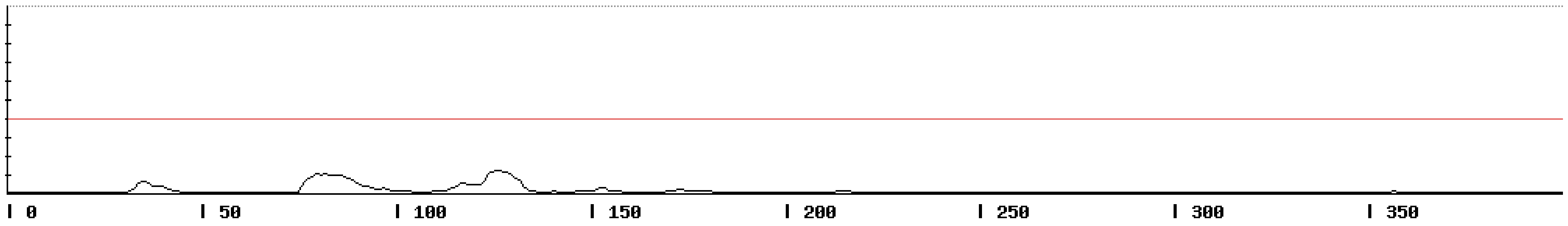

Predictions for Glycinemax_Glyma.18G261300.1.p

Viterbi Path

Viterbi Path

Posterior @ 0.4

Posterior @ 0.4

Posterior Graphic. Threshold Marked @ 0.4

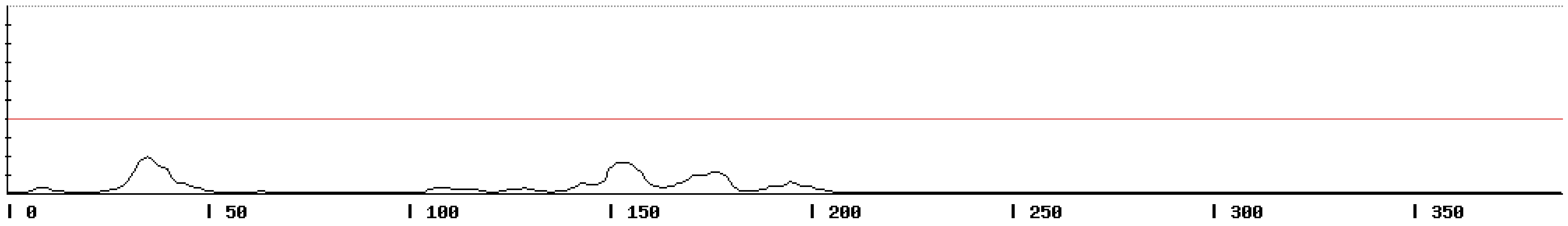

Predictions for Glycinemax_Glyma.18G301500.1.p

Viterbi Path

Viterbi Path

Posterior @ 0.4

Posterior @ 0.4

Posterior Graphic. Threshold Marked @ 0.4

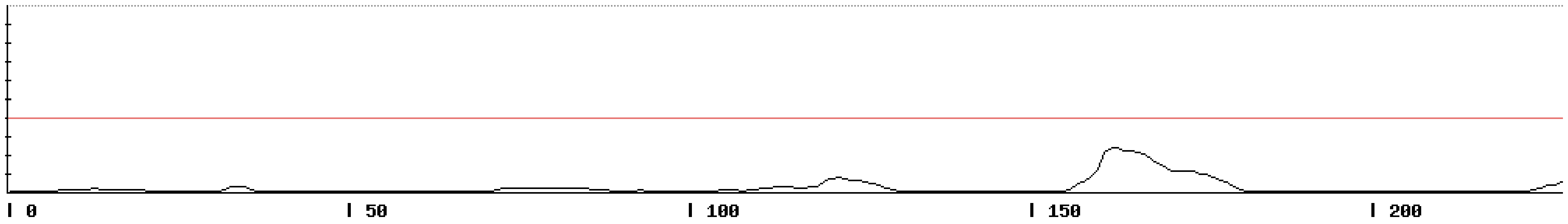

Predictions for Glycinemax_Glyma.19G002900.1.p

Viterbi Path

Viterbi Path

Posterior @ 0.4

131 - GRRKRRKI - 138

Posterior @ 0.4

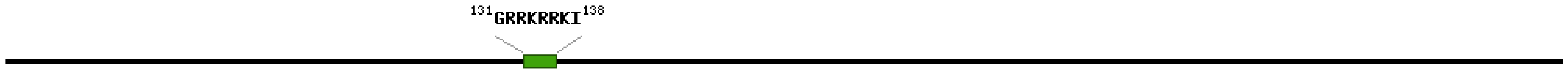

Posterior Graphic. Threshold Marked @ 0.4

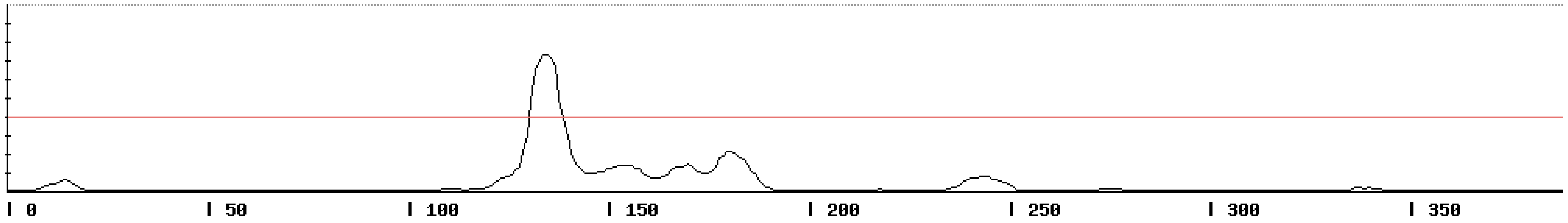

Predictions for Glycinemax_Glyma.19G021900.1.p

Viterbi Path

Viterbi Path

Posterior @ 0.4

Posterior @ 0.4

Posterior Graphic. Threshold Marked @ 0.4

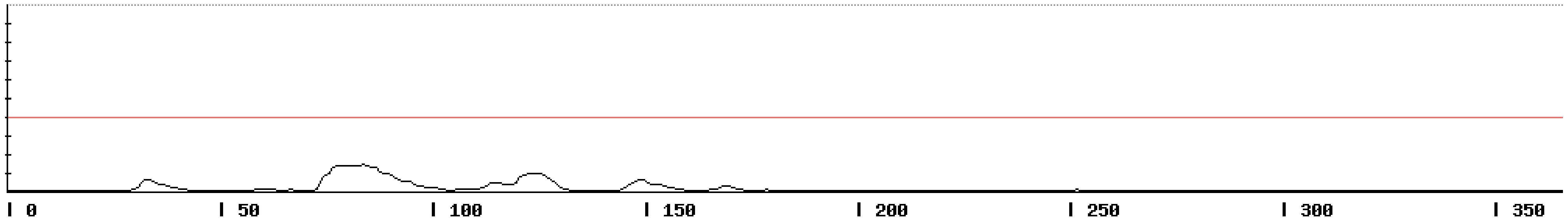

Predictions for Glycinemax_Glyma.19G024500.1.p

Viterbi Path

Viterbi Path

Posterior @ 0.4

Posterior @ 0.4

Posterior Graphic. Threshold Marked @ 0.4

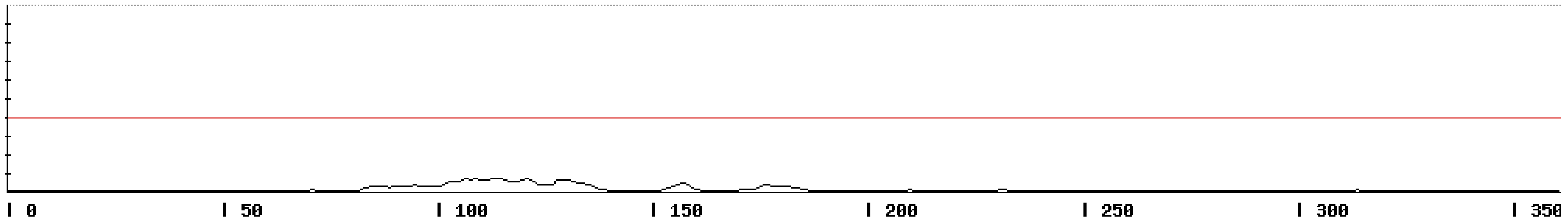

Predictions for Glycinemax_Glyma.19G056400.1.p

Viterbi Path

Viterbi Path

Posterior @ 0.4 | 134 - KRRK - 137

Posterior @ 0.4

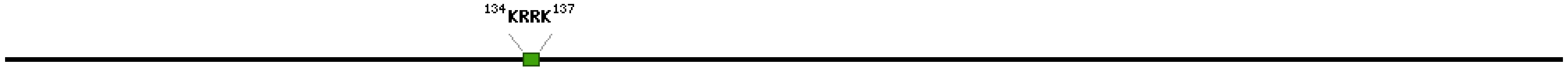

Posterior Graphic. Threshold Marked @ 0.4

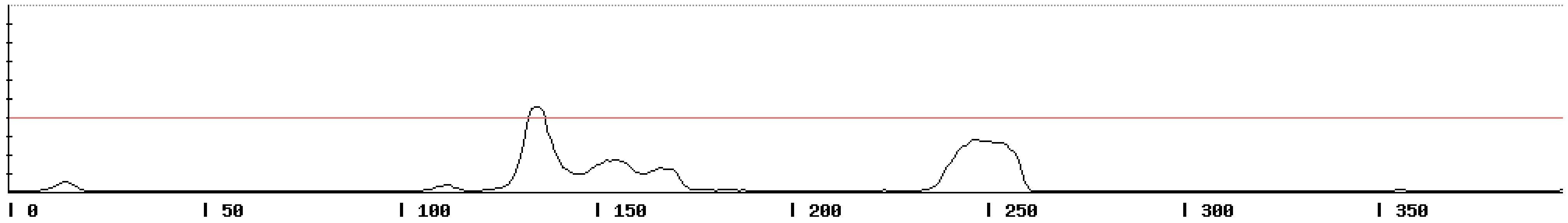

Predictions for Glycinemax_Glyma.19G097700.1.p

Viterbi Path

Viterbi Path

Posterior @ 0.4 | 159 - EKKEGRKGKKAKGS - 172

Posterior @ 0.4

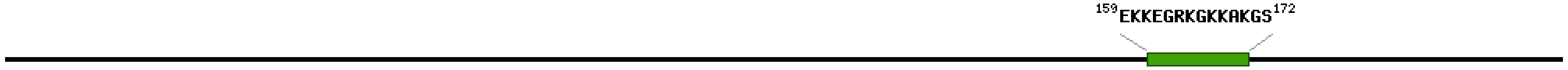

Posterior Graphic. Threshold Marked @ 0.4

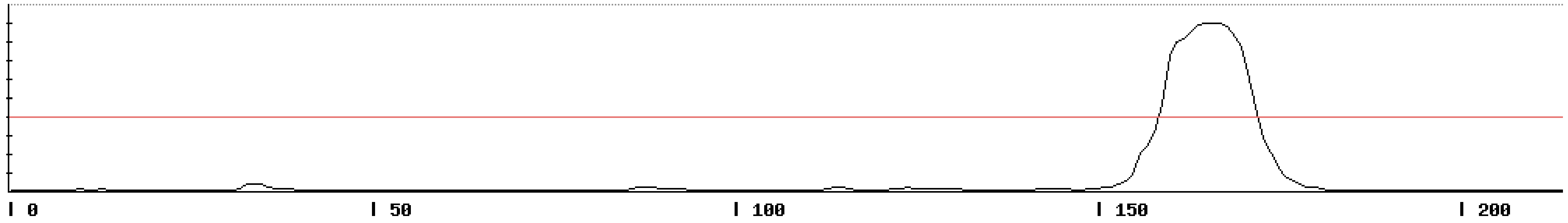

Predictions for Glycinemax_Glyma.19G108800.1.p

Viterbi Path

Viterbi Path

Posterior @ 0.4

Posterior @ 0.4

Posterior Graphic. Threshold Marked @ 0.4

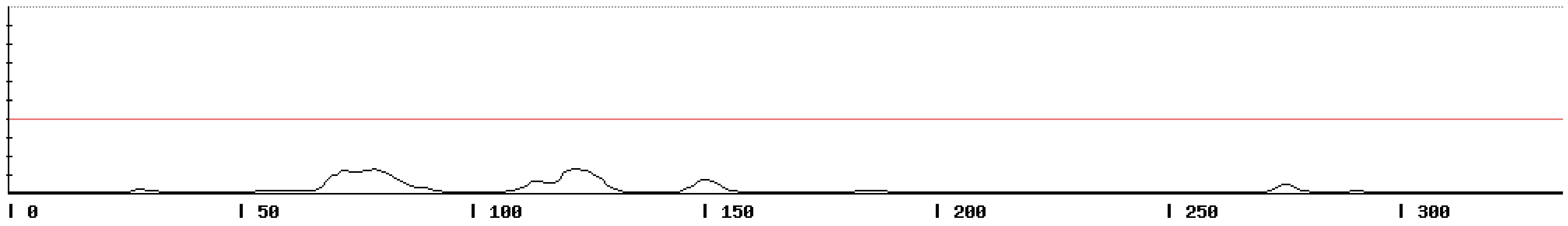

Predictions for Glycinemax_Glyma.19G109100.1.p

Viterbi Path

Viterbi Path

Posterior @ 0.4

Posterior @ 0.4

Posterior Graphic. Threshold Marked @ 0.4

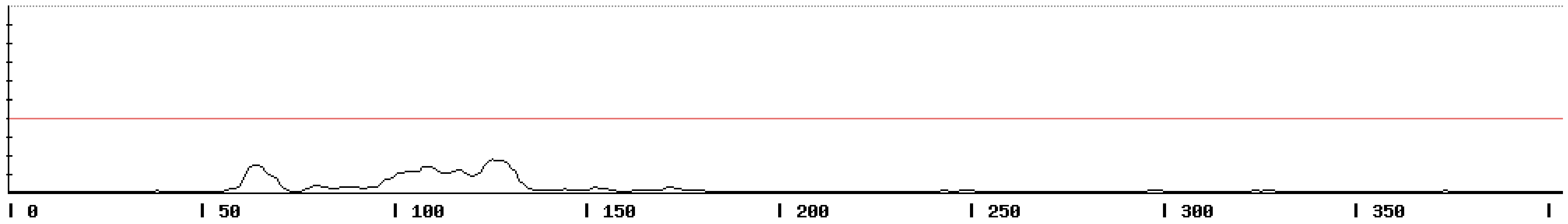

Predictions for Glycinemax_Glyma.19G165600.1.p

Viterbi Path

Viterbi Path

Posterior @ 0.4

Posterior @ 0.4

Posterior Graphic. Threshold Marked @ 0.4

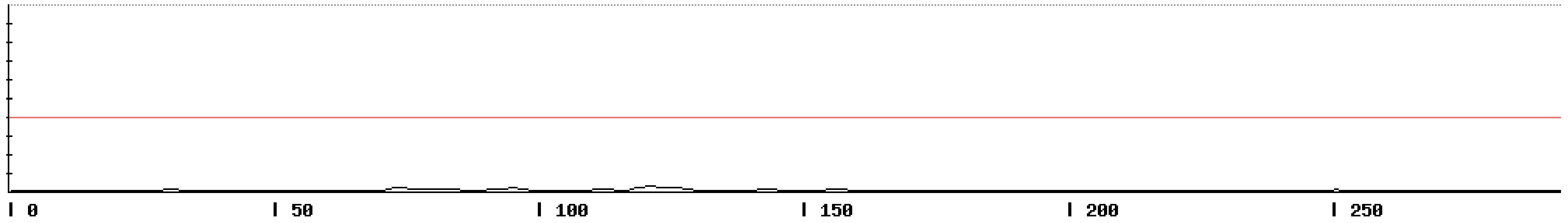

Predictions for Glycinemax_Glyma.19G180300.1.p

Viterbi Path

Viterbi Path

Posterior @ 0.4

Posterior @ 0.4

Posterior Graphic. Threshold Marked @ 0.4

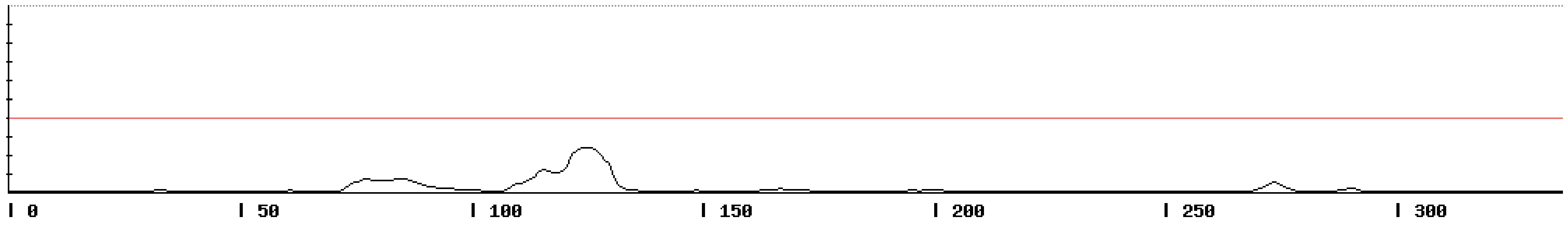

Predictions for Glycinemax_Glyma.19G195800.1.p

Viterbi Path

Viterbi Path

Posterior @ 0.4

Posterior @ 0.4

Posterior Graphic. Threshold Marked @ 0.4

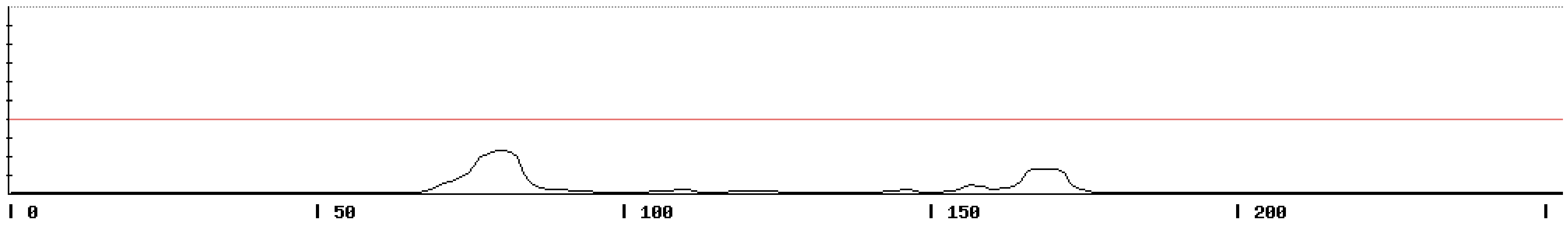

Predictions for Glycinemax_Glyma.19G259500.1.p

Viterbi Path

Viterbi Path

Posterior @ 0.4

Posterior @ 0.4

Posterior Graphic. Threshold Marked @ 0.4

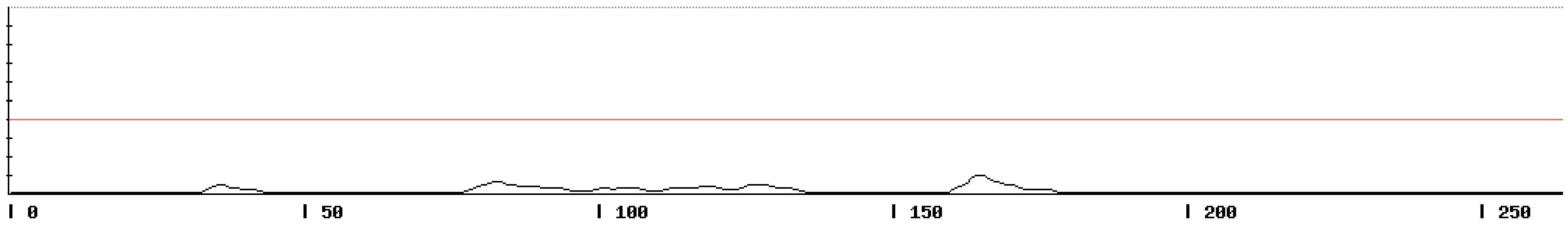

Predictions for Glycinemax_Glyma.19G259700.1.p

Viterbi Path

Viterbi Path

Posterior @ 0.4

Posterior @ 0.4

Posterior Graphic. Threshold Marked @ 0.4

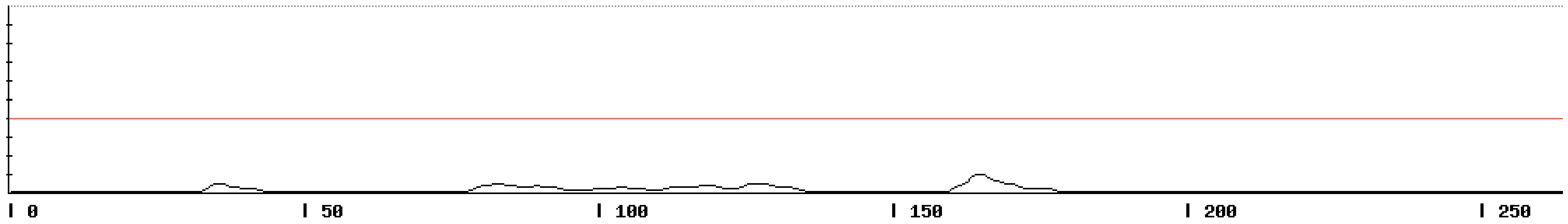

Predictions for Glycinemax_Glyma.20G033300.1.p

Viterbi Path

Viterbi Path

Posterior @ 0.4

Posterior @ 0.4

Posterior Graphic. Threshold Marked @ 0.4

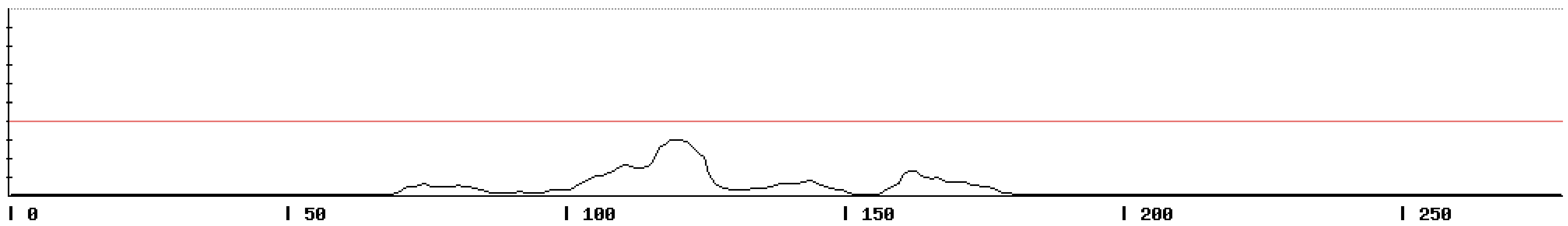

Predictions for Glycinemax_Glyma.20G172100.1.p

Viterbi Path

Viterbi Path

Posterior @ 0.4

Posterior @ 0.4

Posterior Graphic. Threshold Marked @ 0.4

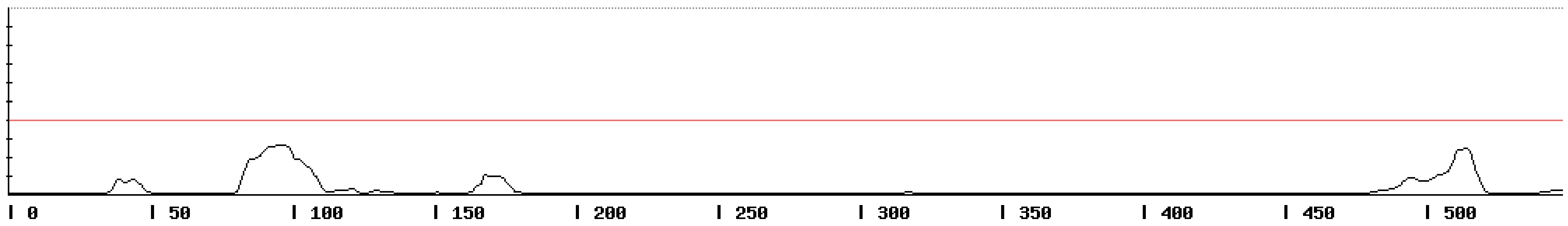

Predictions for Glycinemax_Glyma.20G175500.1.p

Viterbi Path

Viterbi Path

Posterior @ 0.4	
Posterior @ 0.4	
Posterior Graphic. Threshold Marked @ 0.4	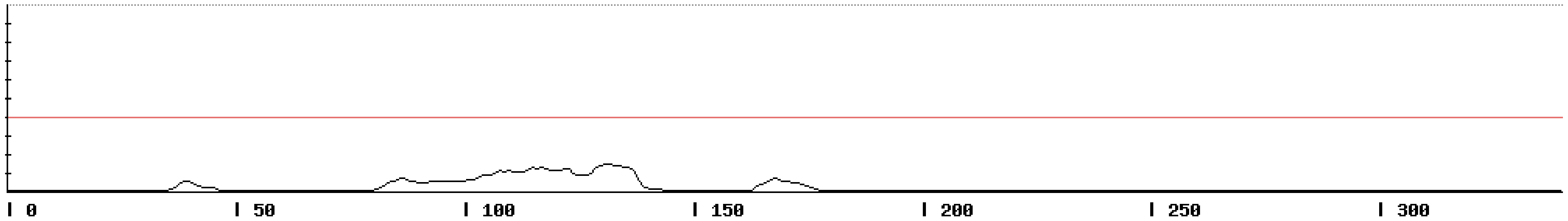

Predictions for Glycinemax_Glyma.20G185800.1.p	
Viterbi Path	
Viterbi Path	
Posterior @ 0.4	
Posterior @ 0.4	
Posterior Graphic. Threshold Marked @ 0.4	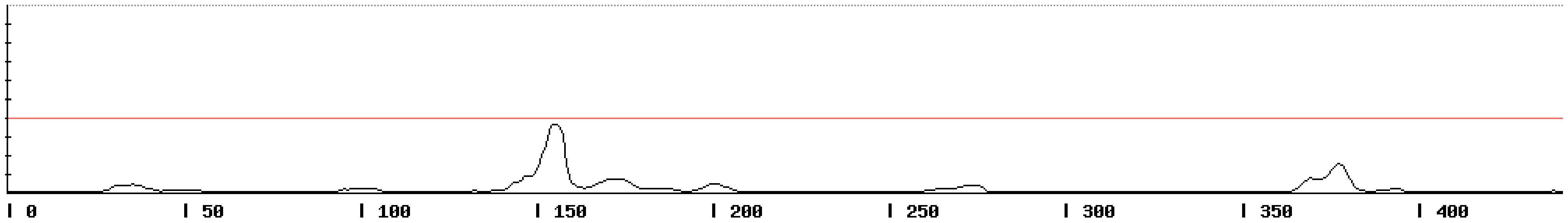

Predictions for Glycinemax_Glyma.20G192300.1.p	
Viterbi Path	
Viterbi Path	

Posterior @ 0.4

Posterior @ 0.4

Posterior Graphic. Threshold Marked @ 0.4

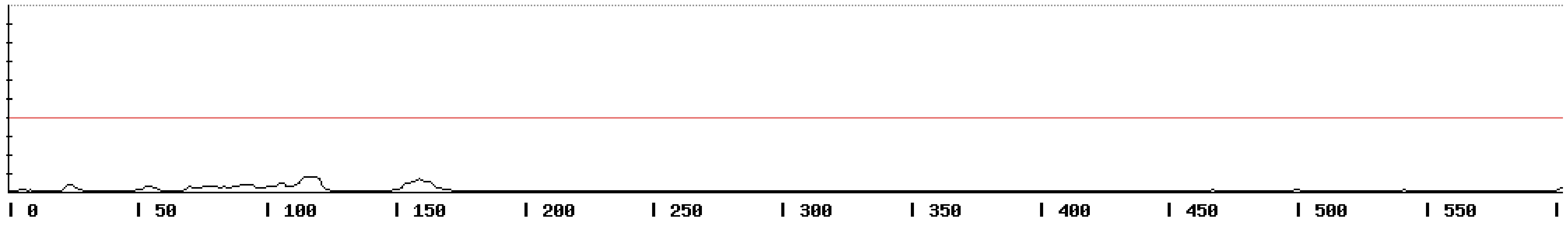

Predictions for Glycinemax_Glyma.20G192500.1.p

Viterbi Path

Viterbi Path

Posterior @ 0.4

Posterior @ 0.4

Posterior Graphic. Threshold Marked @ 0.4

Revision r.9 - Last Updated on : February 24 2015 17:45:20.

alex.nguyenba@utoronto.ca

Intended to be used with Firefox

Viterbi Path	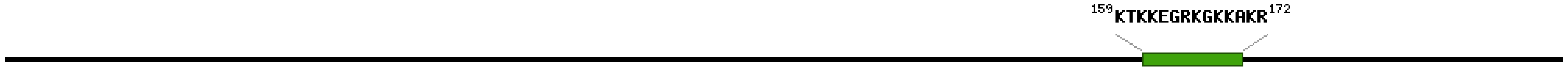
Posterior @ 0.4	158 - FKTKKEGRKGKKAKRS - 173
Posterior @ 0.4	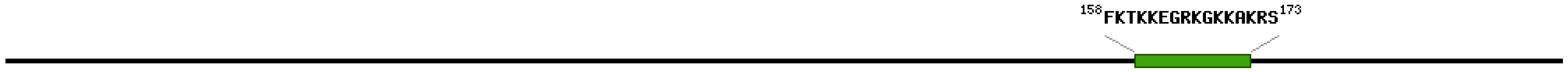
Posterior Graphic. Threshold Marked @ 0.4	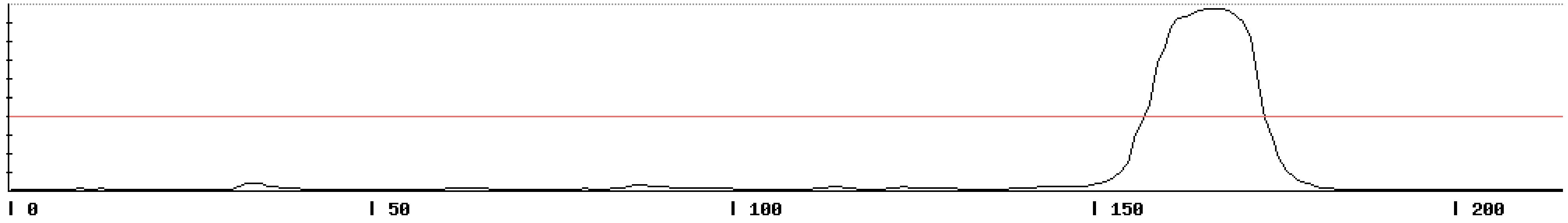

Predictions for Glycinesoja_KHM99647.1

Viterbi Path	
Viterbi Path	
Posterior @ 0.4	
Posterior @ 0.4	
Posterior Graphic. Threshold Marked @ 0.4	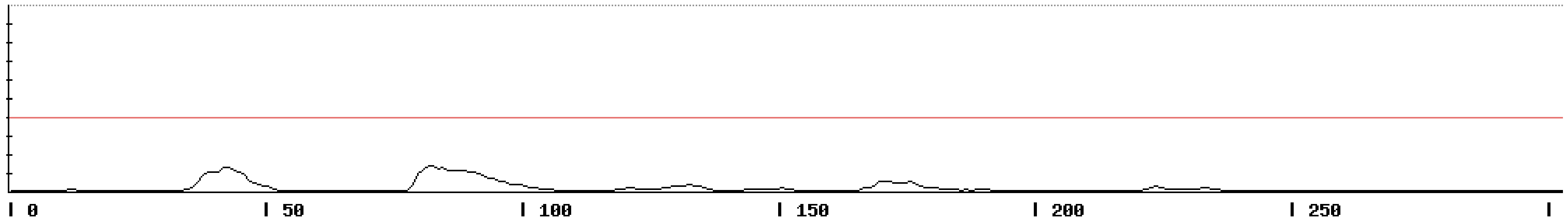

Predictions for Glycinesoja_KHM99823.1

Viterbi Path

Viterbi Path

Posterior @ 0.4

Posterior @ 0.4

Posterior Graphic. Threshold Marked @ 0.4

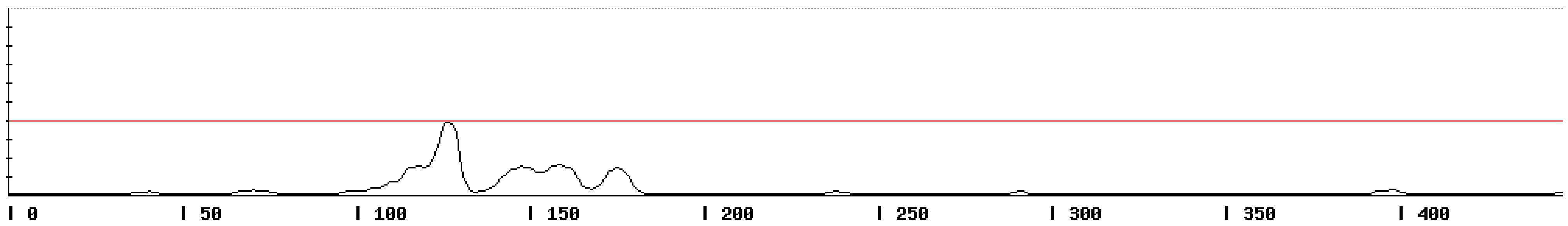

Predictions for Glycinesoja_KHN00492.1

Viterbi Path

Viterbi Path

Posterior @ 0.4

Posterior @ 0.4

Posterior Graphic. Threshold Marked @ 0.4

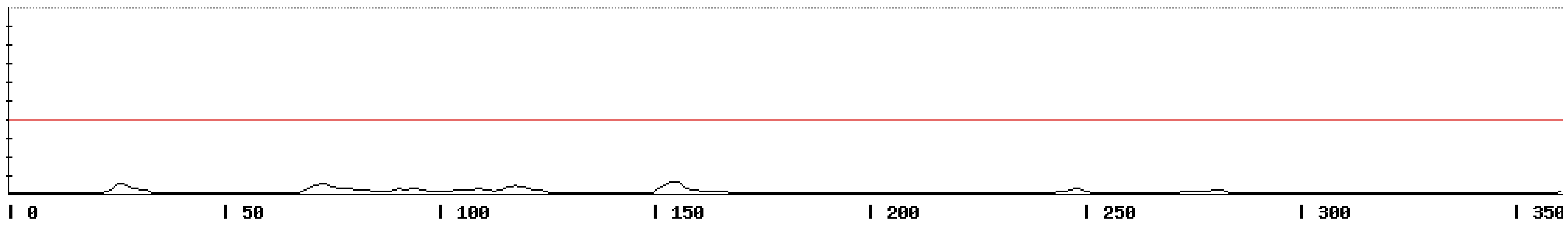

Predictions for Glycinesoja_KHN00659.1	
Viterbi Path	
Viterbi Path	
Posterior @ 0.4	
Posterior @ 0.4	
Posterior Graphic. Threshold Marked @ 0.4	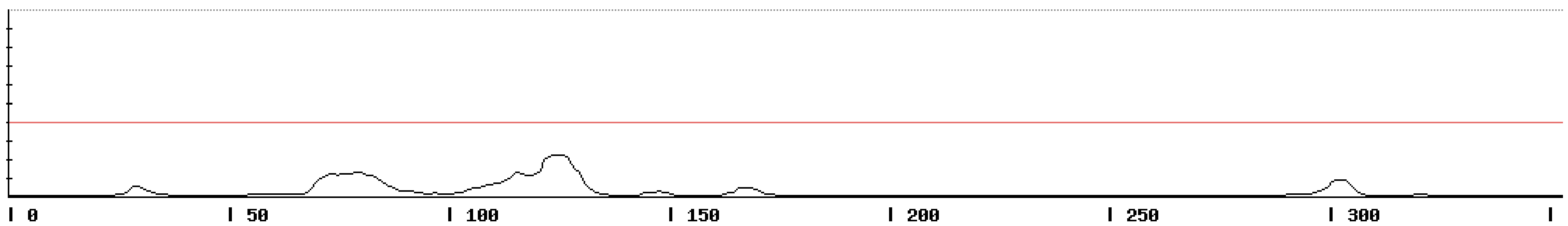

Predictions for Glycinesoja_KHN00664.1	
Viterbi Path	
Viterbi Path	
Posterior @ 0.4	
Posterior @ 0.4	
Posterior Graphic. Threshold Marked @ 0.4	

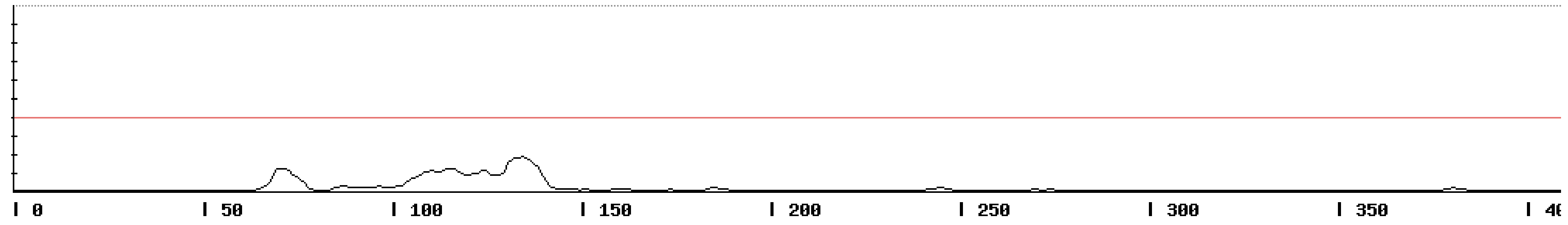

Predictions for Glycinesoja_KHN00769.1

Viterbi Path

Viterbi Path

Posterior @ 0.4

Posterior @ 0.4

Posterior Graphic. Threshold Marked @ 0.4

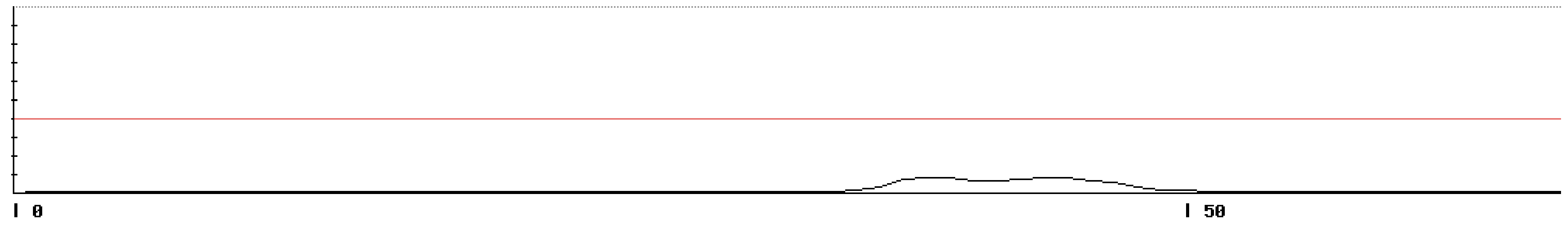

Predictions for Glycinesoja_KHN00770.1

Viterbi Path

Viterbi Path

Posterior @ 0.4

Posterior @ 0.4

Posterior Graphic. Threshold Marked @ 0.4

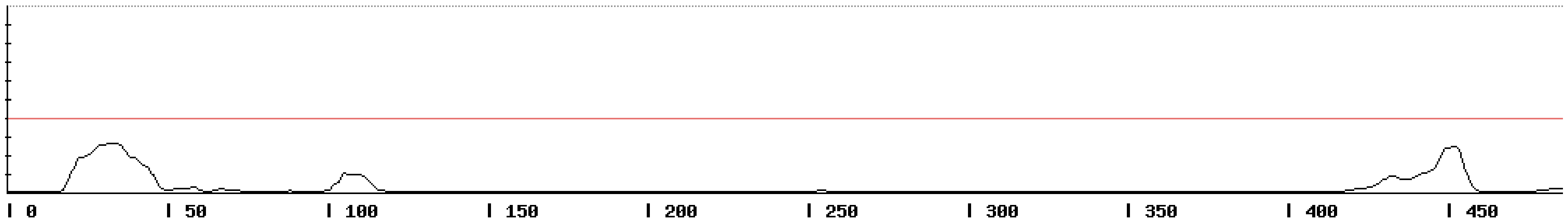

Predictions for Glycinesoja_KHN00798.1

Viterbi Path

Viterbi Path

Posterior @ 0.4

Posterior @ 0.4

Posterior Graphic. Threshold Marked @ 0.4

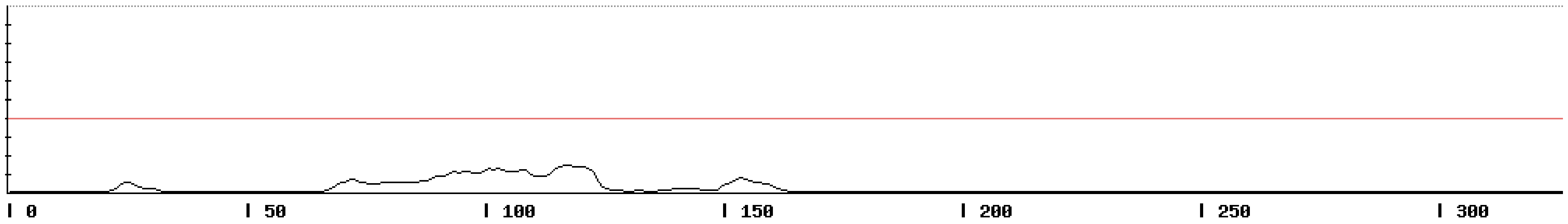

Predictions for Glycinesoja_KHN00898.1

Viterbi Path

Viterbi Path

Posterior @ 0.4

Posterior @ 0.4

Posterior Graphic. Threshold Marked @ 0.4

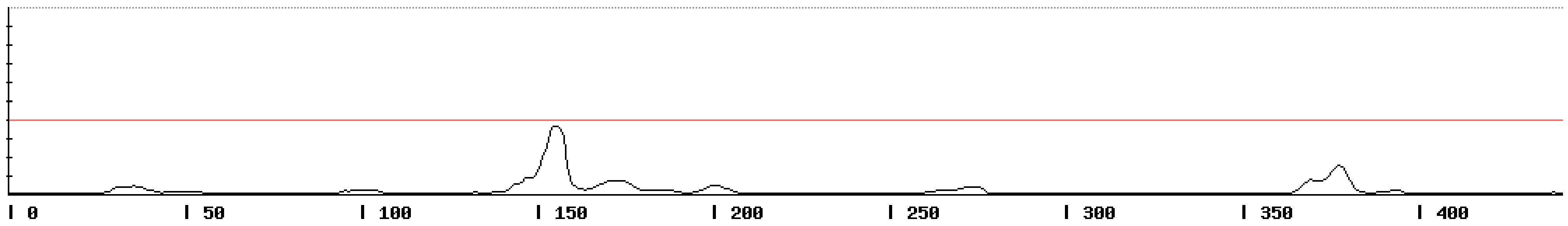

Predictions for Glycinesoja_KHN00952.1

Viterbi Path

Viterbi Path

Posterior @ 0.4

Posterior @ 0.4

Posterior Graphic. Threshold Marked @ 0.4

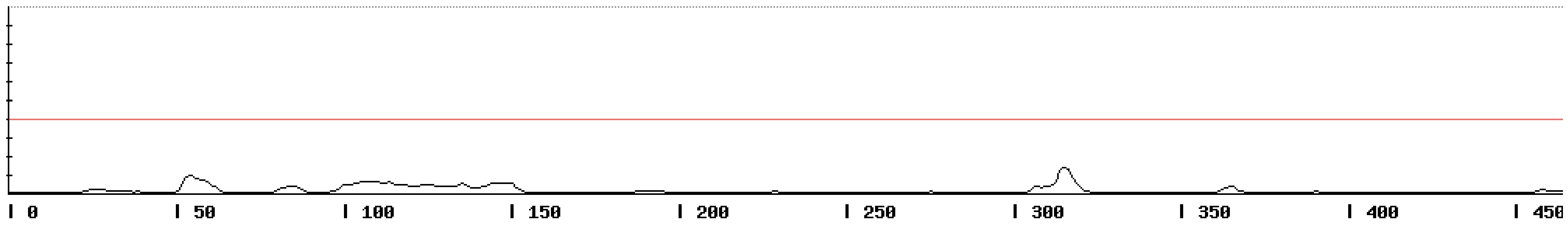

Predictions for Glycinesoja_KHN01093.1

Viterbi Path

Viterbi Path

Posterior @ 0.4 | 42 - RKRRK - 46

Posterior @ 0.4

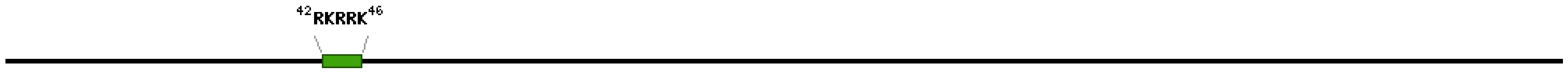

Posterior Graphic. Threshold Marked @ 0.4

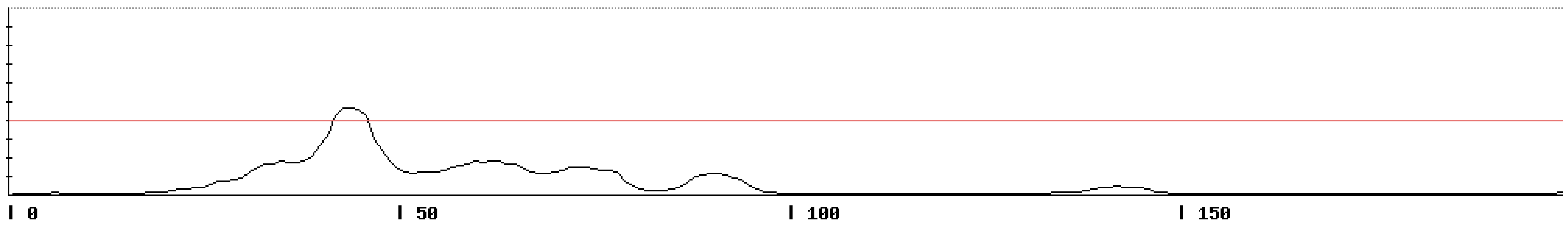

Predictions for Glycinesoja_KHN01217.1

Viterbi Path

Viterbi Path

Posterior @ 0.4

Posterior @ 0.4

Posterior Graphic. Threshold Marked @ 0.4

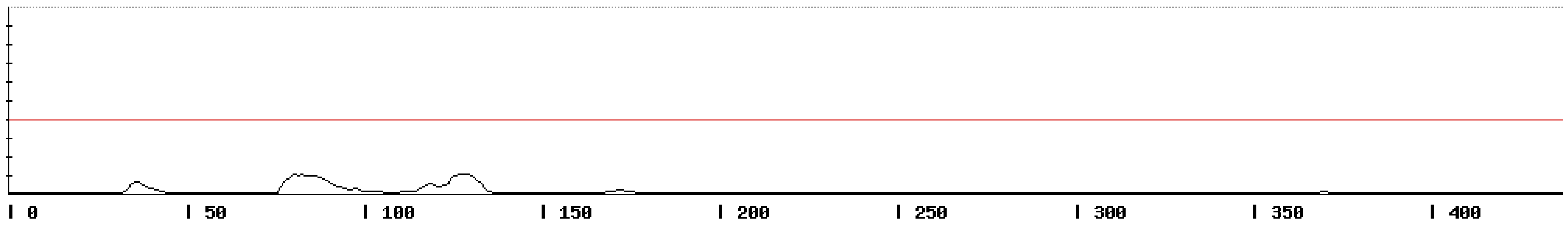

Predictions for Glycinesoja_KHN01256.1

Viterbi Path

Viterbi Path

Posterior @ 0.4

Posterior @ 0.4

Posterior Graphic. Threshold Marked @ 0.4

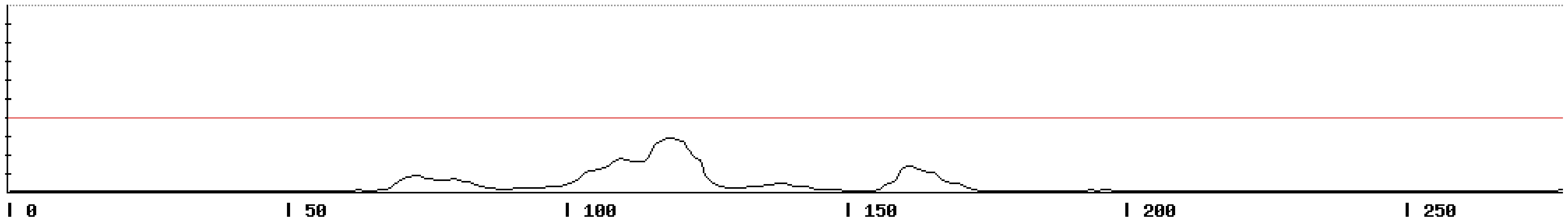

Predictions for Glycinesoja_KHN01494.1

Viterbi Path

Viterbi Path

Posterior @ 0.4

Posterior @ 0.4

Posterior Graphic. Threshold Marked @ 0.4

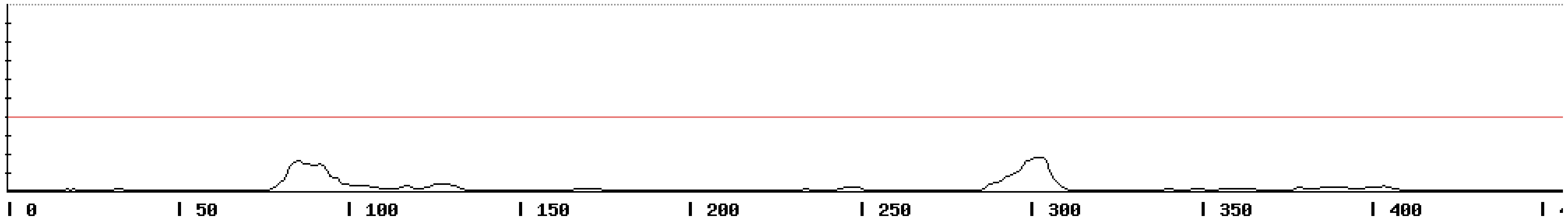

Predictions for Glycinesoja_KHN01680.1

Viterbi Path

Viterbi Path

Posterior @ 0.4

Posterior @ 0.4

Posterior Graphic. Threshold Marked @ 0.4

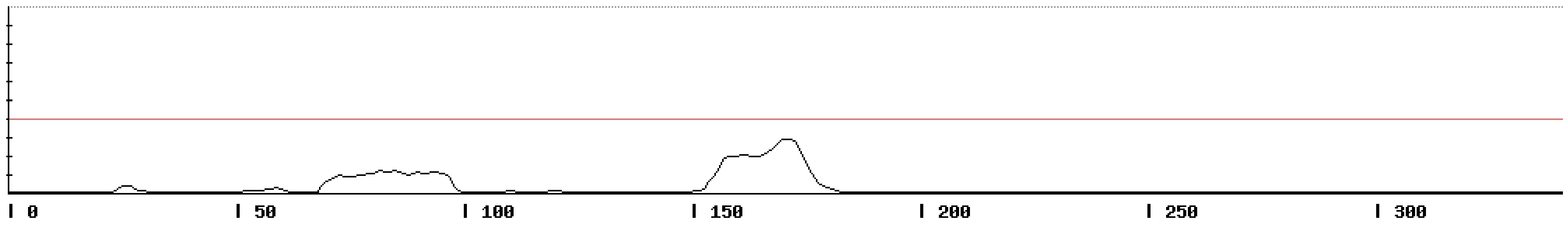

Predictions for Glycinesoja_KHN02203.1

Viterbi Path

Viterbi Path

Posterior @ 0.4

Posterior @ 0.4

Posterior Graphic. Threshold Marked @ 0.4

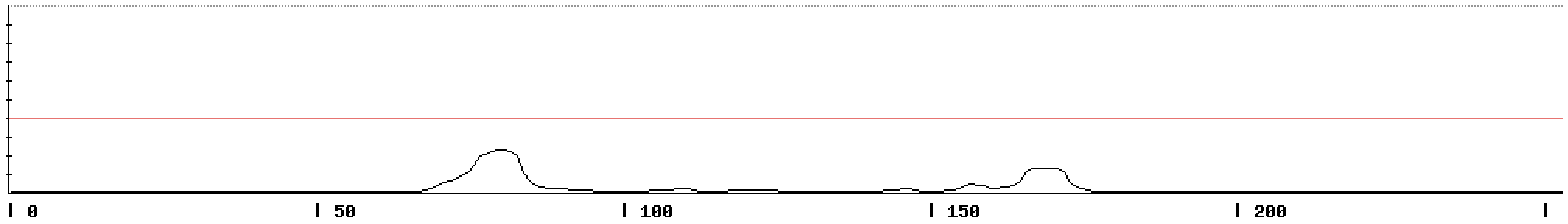

Predictions for Glycinesoja_KHN02351.1

Viterbi Path

Viterbi Path

Posterior @ 0.4

Posterior @ 0.4

Posterior Graphic. Threshold Marked @ 0.4

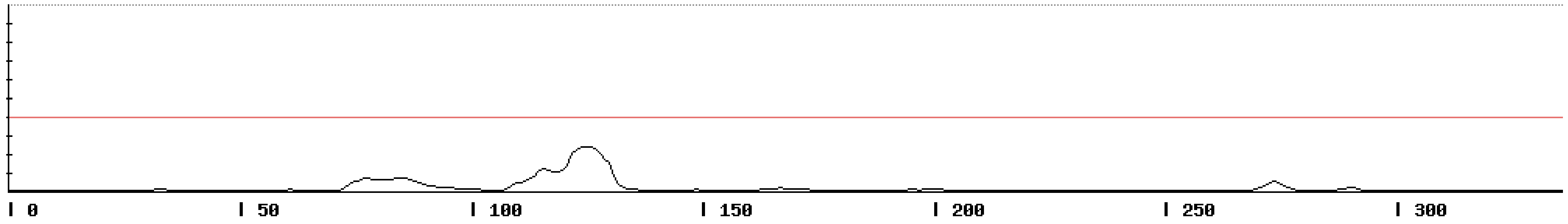

Predictions for Glycinesoja_KHN02495.1

Viterbi Path

Viterbi Path

Posterior @ 0.4

Posterior @ 0.4

Posterior Graphic. Threshold Marked @ 0.4

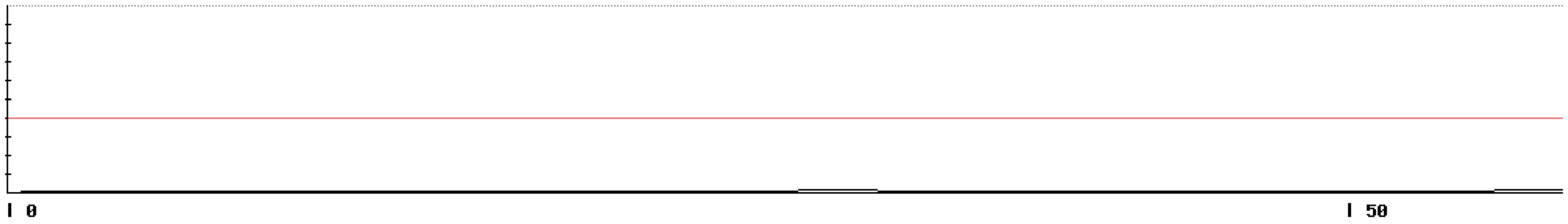

Predictions for Glycinesoja_KHN02496.1

Viterbi Path

Viterbi Path

Posterior @ 0.4

Posterior @ 0.4

Posterior Graphic. Threshold Marked @ 0.4

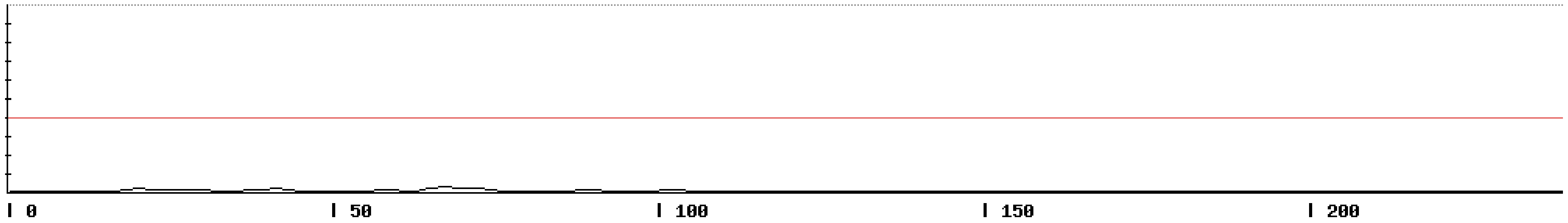

Predictions for Glycinesoja_KHN03193.1

Viterbi Path

Viterbi Path

Posterior @ 0.4

Posterior @ 0.4

Posterior Graphic. Threshold Marked @ 0.4	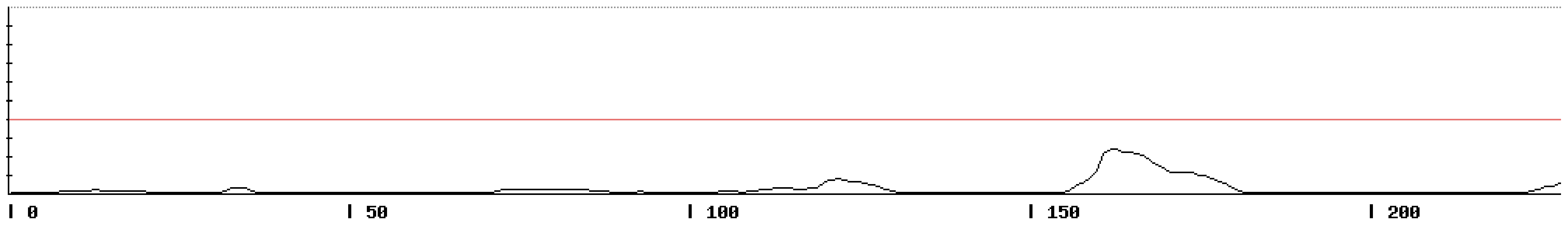

Predictions for Glycinesoja_KHN03216.1	
Viterbi Path	
Viterbi Path	
Posterior @ 0.4	
Posterior @ 0.4	
Posterior Graphic. Threshold Marked @ 0.4	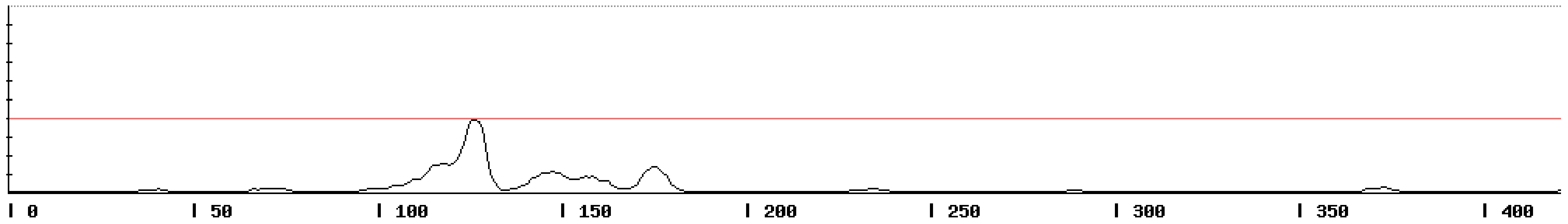

Predictions for Glycinesoja_KHN03571.1	
Viterbi Path	
Viterbi Path	
Posterior @ 0.4	
Posterior @ 0.4	

Posterior Graphic. Threshold Marked @ 0.4

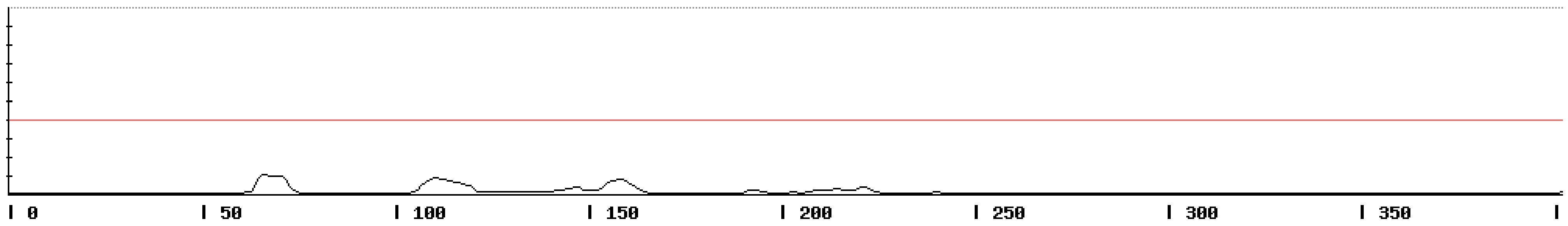

Predictions for Glycinesoja_KHN04148.1

Viterbi Path

Viterbi Path

Posterior @ 0.4

Posterior @ 0.4

Posterior Graphic. Threshold Marked @ 0.4

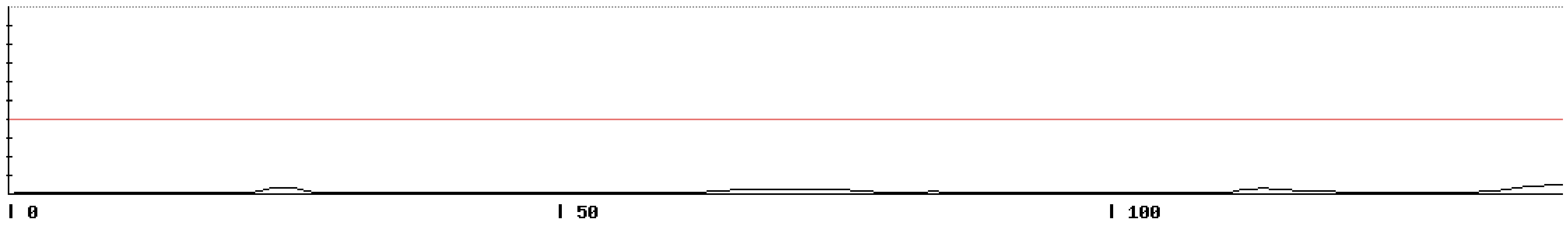

Predictions for Glycinesoja_KHN04334.1

Viterbi Path

Viterbi Path

Posterior @ 0.4

Posterior @ 0.4

Posterior Graphic. Threshold Marked @ 0.4

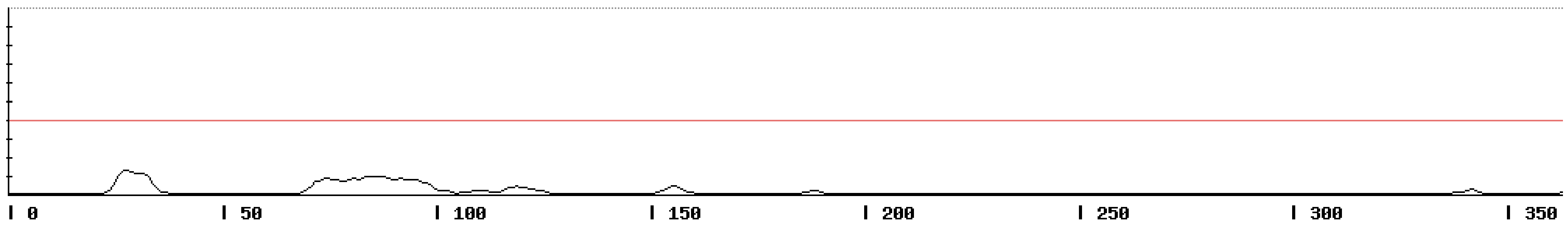

Predictions for Glycinesoja_KHN05484.1

Viterbi Path

Viterbi Path

Posterior @ 0.4

Posterior @ 0.4

Posterior Graphic. Threshold Marked @ 0.4

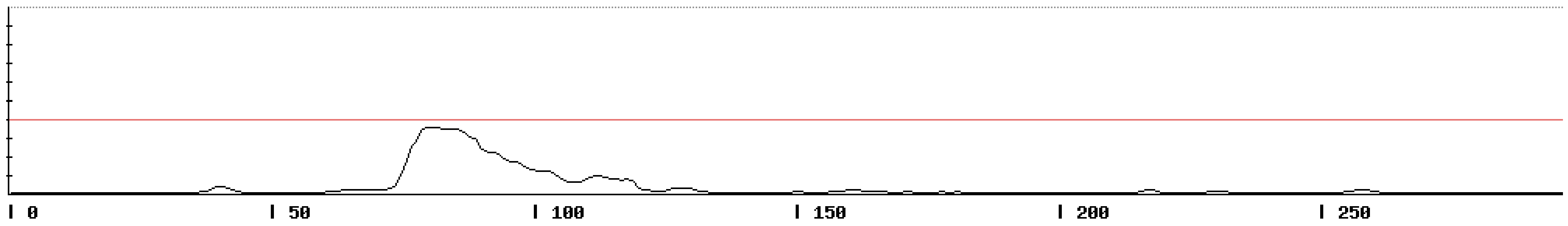

Predictions for Glycinesoja_KHN05857.1

Viterbi Path

Viterbi Path

Posterior @ 0.4	137 - KRR - 139
Posterior @ 0.4	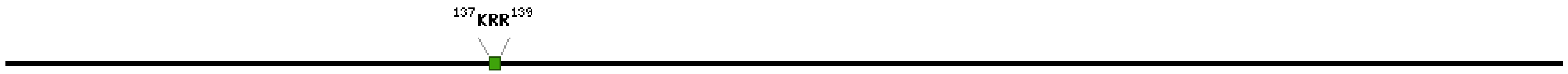
Posterior Graphic. Threshold Marked @ 0.4	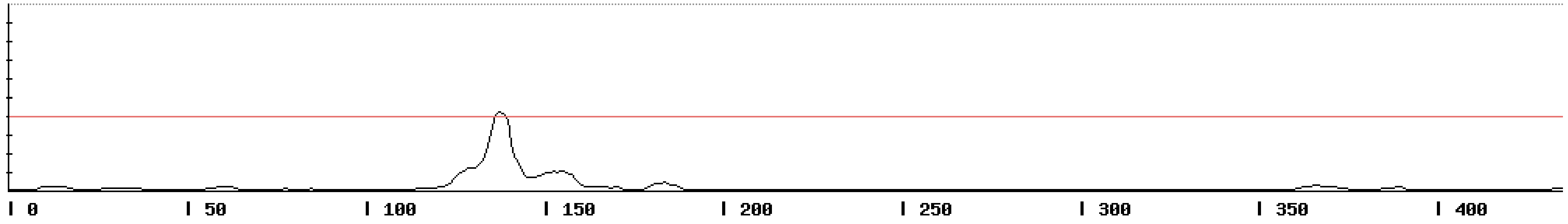

Predictions for Glycinesoja_KHN05861.1	
Viterbi Path	
Viterbi Path	
Posterior @ 0.4	
Posterior @ 0.4	
Posterior Graphic. Threshold Marked @ 0.4	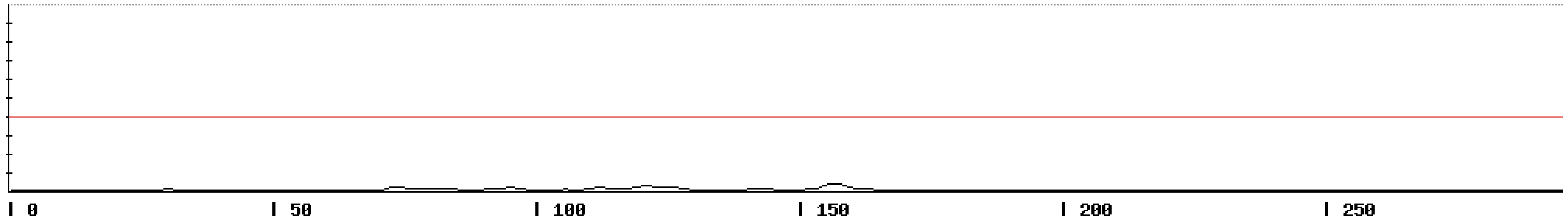

Predictions for Glycinesoja_KHN06358.1

Viterbi Path

Viterbi Path

Posterior @ 0.4

Posterior @ 0.4

Posterior Graphic. Threshold Marked @ 0.4

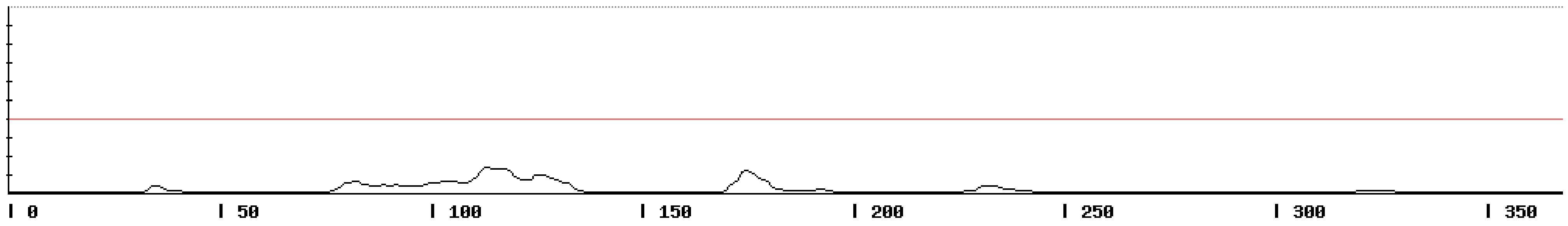

Predictions for Glycinesoja_KHN06383.1

Viterbi Path

Viterbi Path

Posterior @ 0.4

Posterior @ 0.4

Posterior Graphic. Threshold Marked @ 0.4

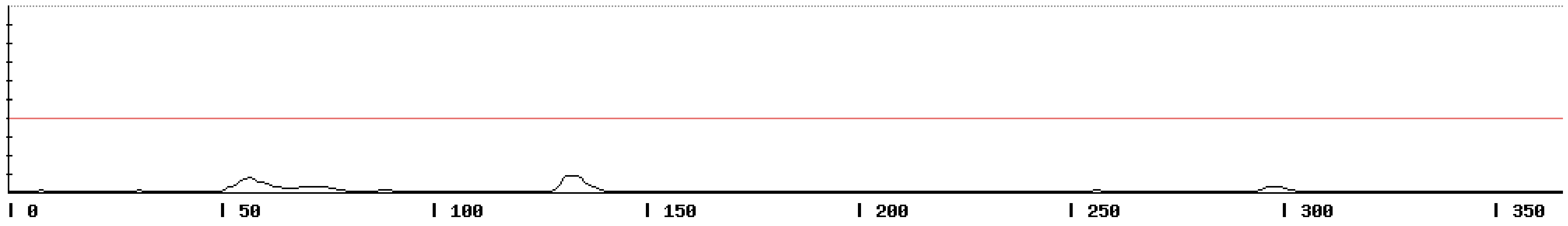

Predictions for Glycinesoja_KHN06858.1	
Viterbi Path	
Viterbi Path	
Posterior @ 0.4	
Posterior @ 0.4	
Posterior Graphic. Threshold Marked @ 0.4	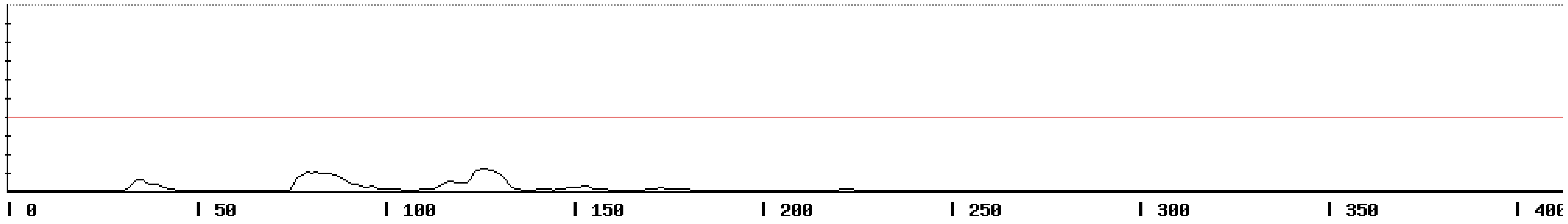

Predictions for Glycinesoja_KHN07294.1	
Viterbi Path	
Viterbi Path	
Posterior @ 0.4	
Posterior @ 0.4	
Posterior Graphic. Threshold Marked @ 0.4	

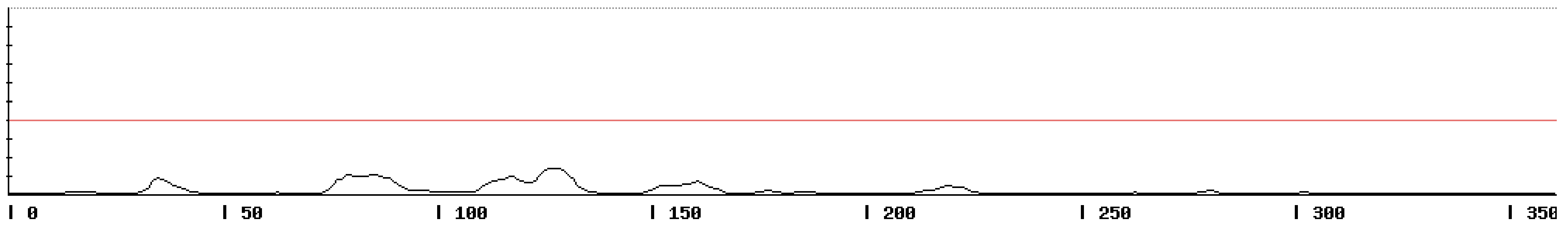

Predictions for Glycinesoja_KHN08165.1

Viterbi Path

Viterbi Path

Posterior @ 0.4

Posterior @ 0.4

Posterior Graphic. Threshold Marked @ 0.4

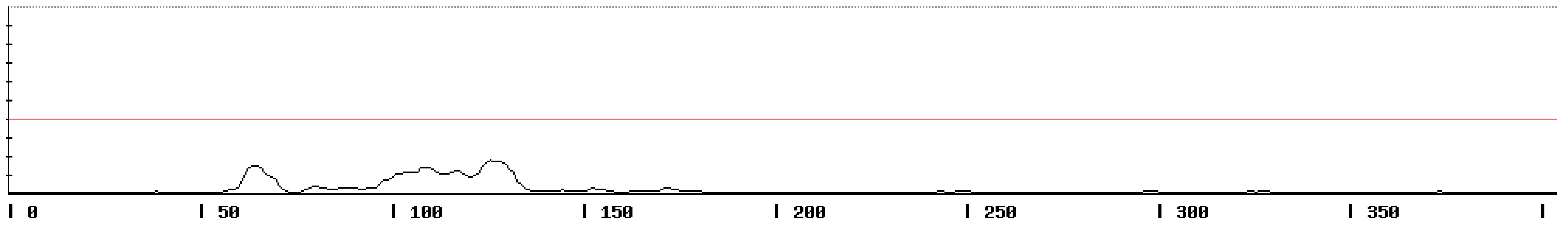

Predictions for Glycinesoja_KHN08169.1

Viterbi Path

Viterbi Path

Posterior @ 0.4

Posterior @ 0.4

Posterior Graphic. Threshold Marked @ 0.4

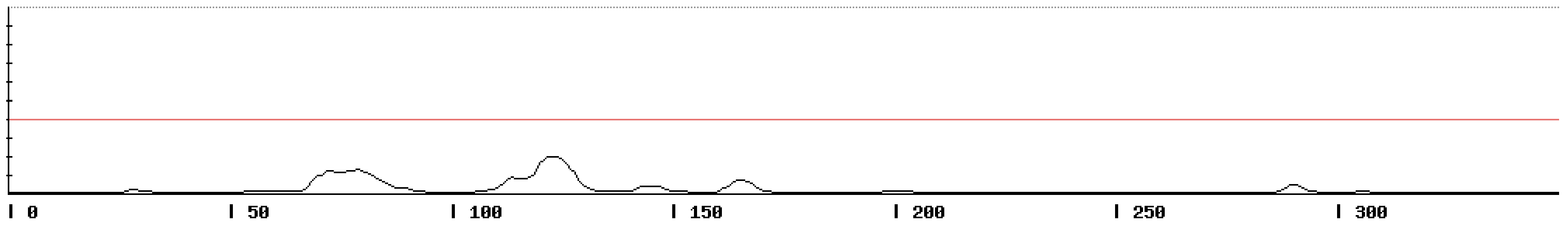

Predictions for Glycinesoja_KHN08484.1

Viterbi Path

Viterbi Path

Posterior @ 0.4 — 140 - KRR - 142

Posterior @ 0.4

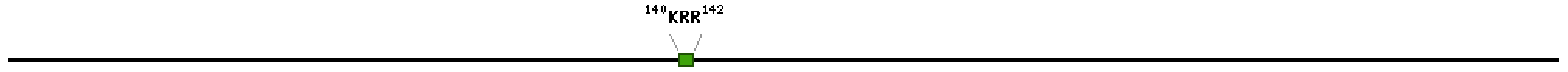

Posterior Graphic. Threshold Marked @ 0.4

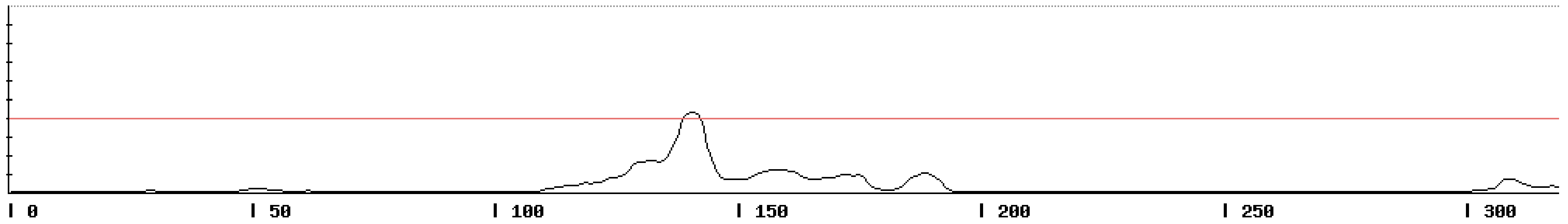

Predictions for Glycinesoja_KHN08560.1

Viterbi Path

Viterbi Path

Posterior @ 0.4

Posterior @ 0.4

Posterior Graphic. Threshold Marked @ 0.4

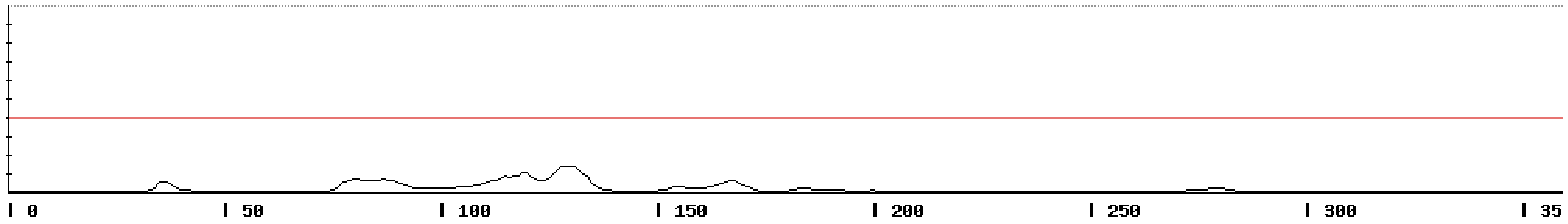

Predictions for Glycinesoja_KHN08773.1

Viterbi Path

Viterbi Path

Posterior @ 0.4

Posterior @ 0.4

Posterior Graphic. Threshold Marked @ 0.4

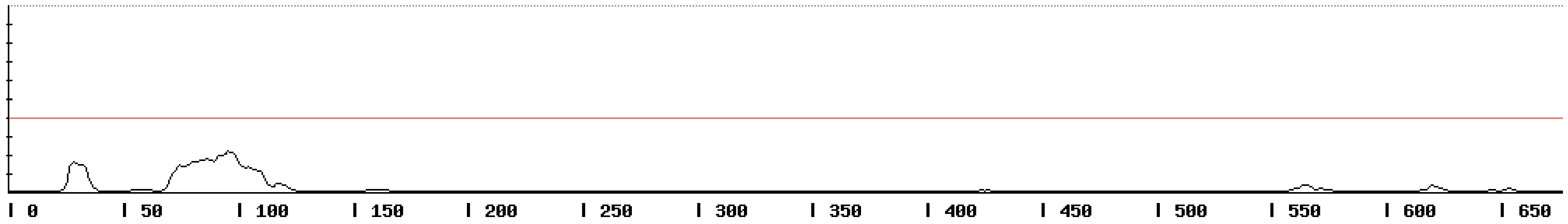

Predictions for Glycinesoja_KHN08926.1

Viterbi Path

Viterbi Path

Posterior @ 0.4

Posterior @ 0.4

Posterior Graphic. Threshold Marked @ 0.4

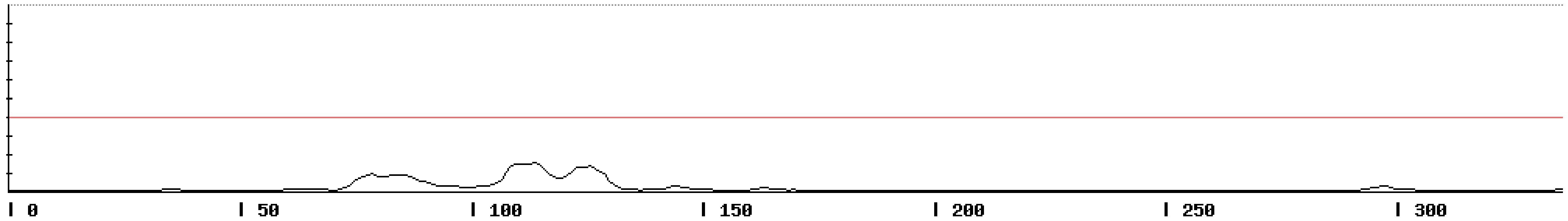

Predictions for Glycinesoja_KHN09257.1

Viterbi Path

Viterbi Path

Posterior @ 0.4

Posterior @ 0.4

Posterior Graphic. Threshold Marked @ 0.4

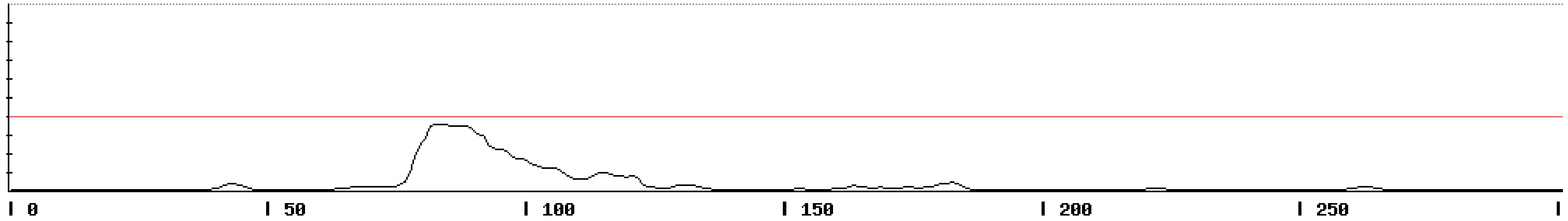

Predictions for Glycinesoja_KHN09490.1

Viterbi Path

Viterbi Path

Posterior @ 0.4

Posterior @ 0.4

Posterior Graphic. Threshold Marked @ 0.4

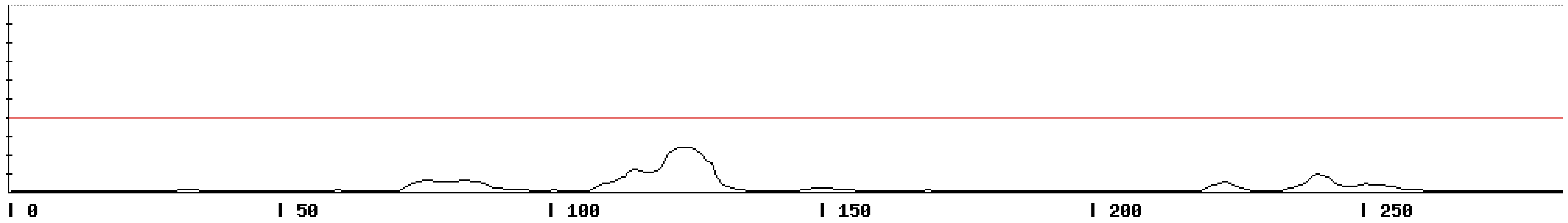

Predictions for Glycinesoja_KHN09607.1

Viterbi Path

Viterbi Path

Posterior @ 0.4 25 - RKVRGKK - 31

Posterior @ 0.4

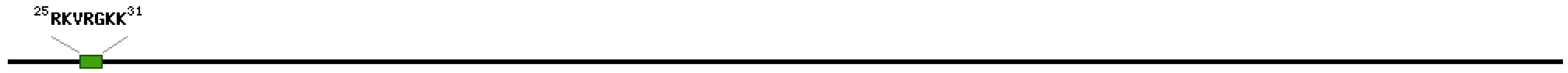

Posterior Graphic. Threshold Marked @ 0.4

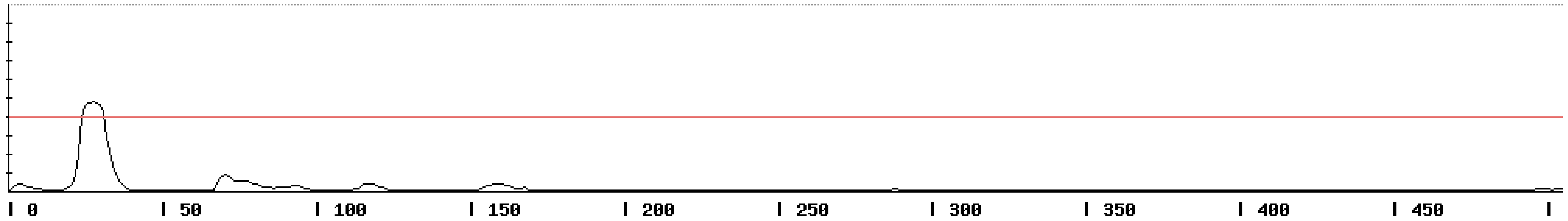

Predictions for Glycinesoja_KHN09619.1

Viterbi Path

Viterbi Path

Posterior @ 0.4

Posterior @ 0.4

Posterior Graphic. Threshold Marked @ 0.4

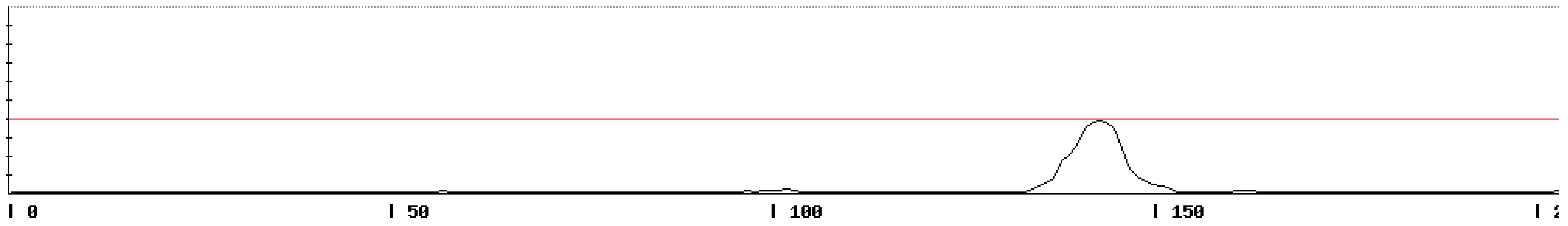

Predictions for Glycinesoja_KHN09647.1

Viterbi Path

Viterbi Path

Posterior @ 0.4 95 - KAIGKPKALGIKK - 107

Posterior @ 0.4

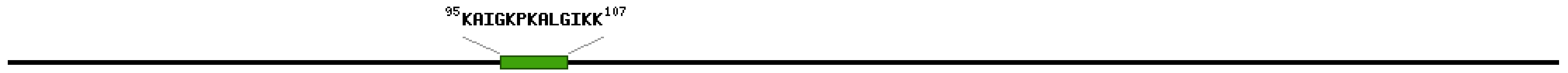

Posterior Graphic. Threshold Marked @ 0.4

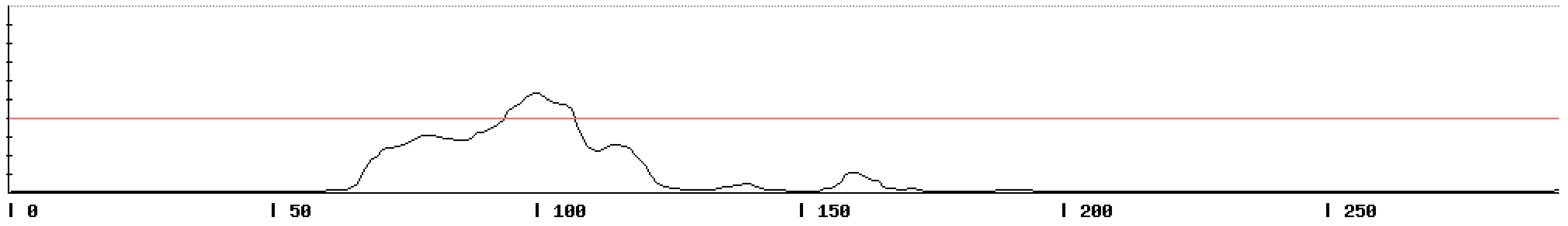

Predictions for Glycinesoja_KHN09787.1	
Viterbi Path	
Viterbi Path	
Posterior @ 0.4	
Posterior @ 0.4	
Posterior Graphic. Threshold Marked @ 0.4	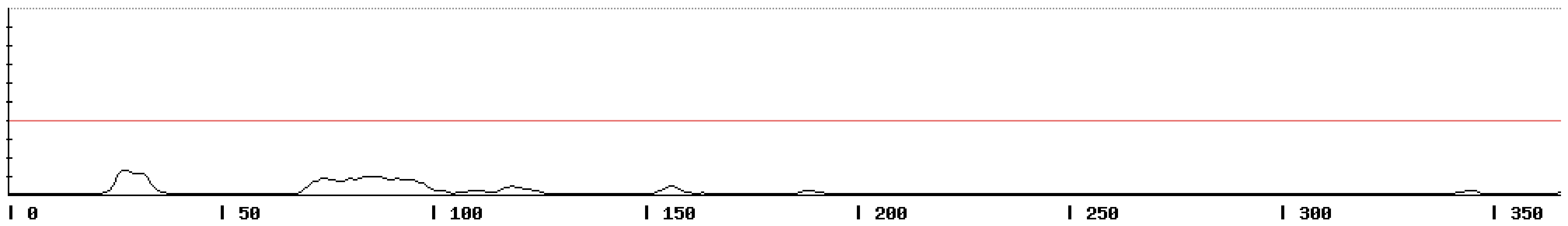

Predictions for Glycinesoja_KHN09961.1	
Viterbi Path	
Viterbi Path	
Posterior @ 0.4	145 - TRKRRKVHTDKE - 156
Posterior @ 0.4	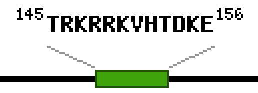
Posterior Graphic. Threshold Marked @ 0.4	

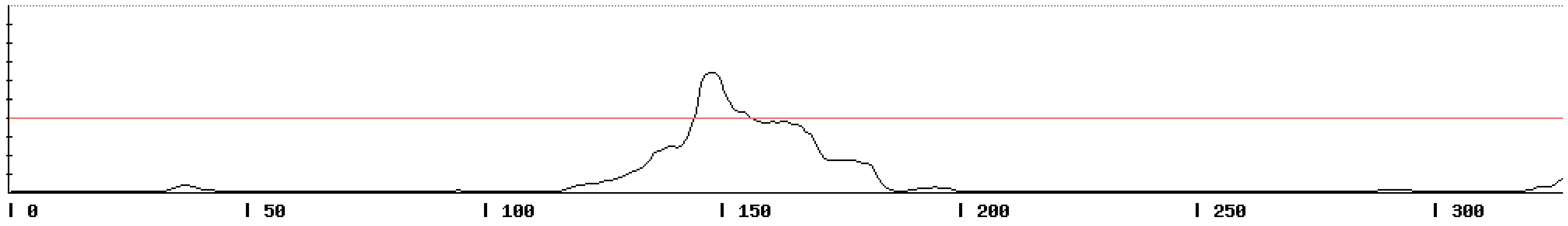

Predictions for Glycinesoja_KHN10245.1

Viterbi Path	
Viterbi Path	
Posterior @ 0.4	
Posterior @ 0.4	
Posterior Graphic. Threshold Marked @ 0.4	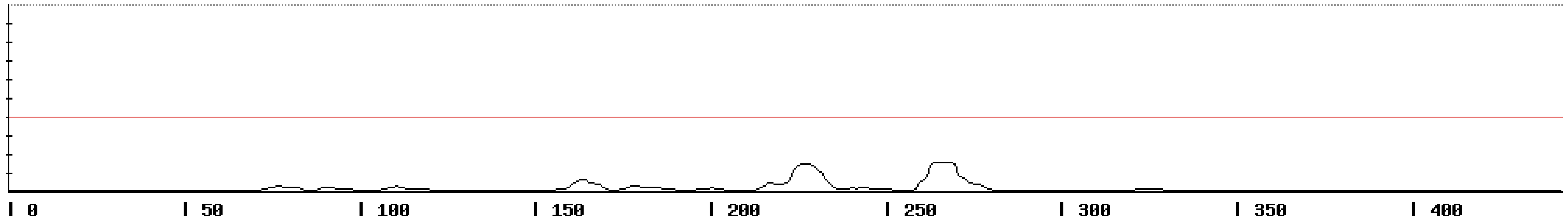

Predictions for Glycinesoja_KHN10750.1

Viterbi Path	
Viterbi Path	
Posterior @ 0.4	
Posterior @ 0.4	

Posterior Graphic. Threshold Marked @ 0.4

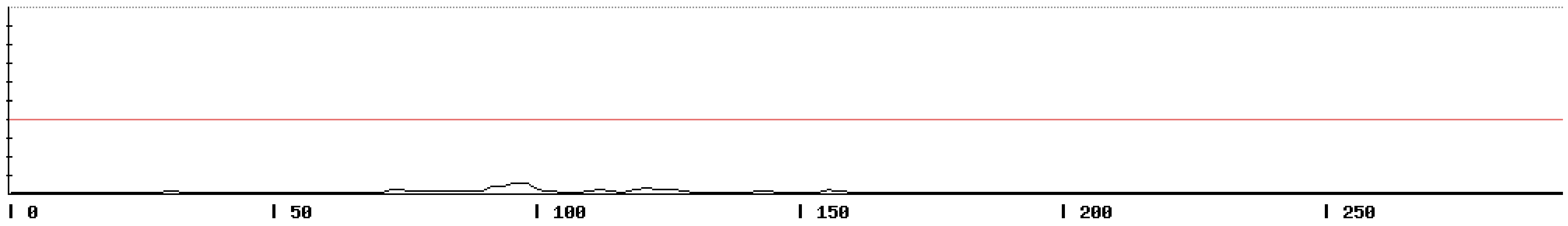

Predictions for Glycinesoja_KHN10787.1

Viterbi Path

Viterbi Path

Posterior @ 0.4

Posterior @ 0.4

Posterior Graphic. Threshold Marked @ 0.4

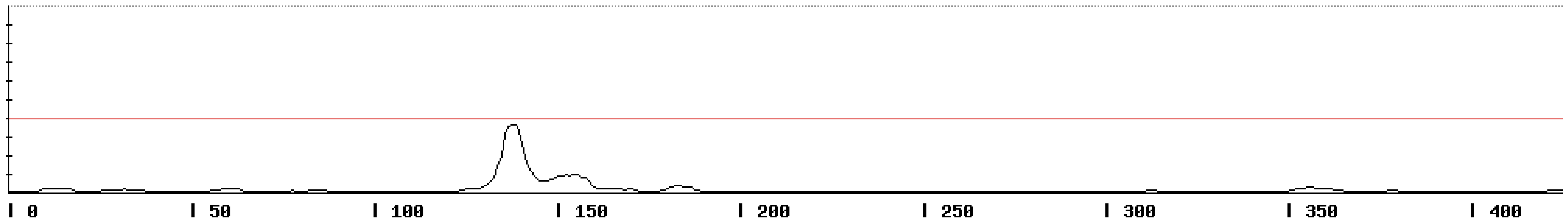

Predictions for Glycinesoja_KHN11377.1

Viterbi Path

Viterbi Path

Posterior @ 0.4

Posterior @ 0.4

Posterior Graphic. Threshold Marked @ 0.4

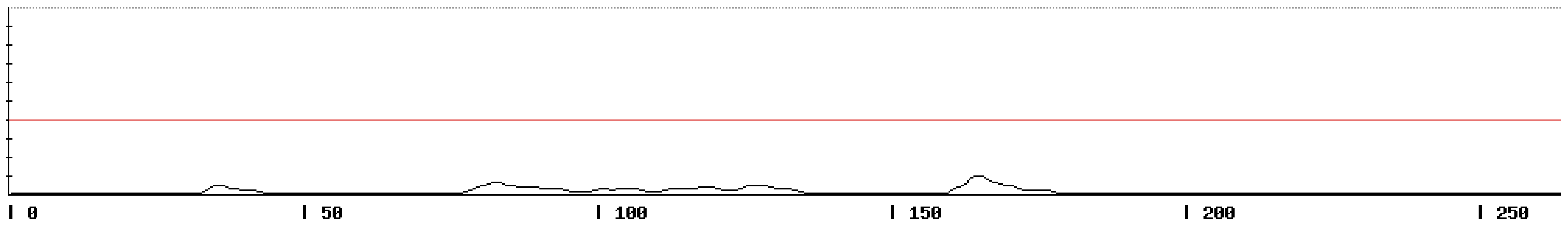

Predictions for Glycinesoja_KHN11379.1

Viterbi Path

Viterbi Path

Posterior @ 0.4

Posterior @ 0.4

Posterior Graphic. Threshold Marked @ 0.4

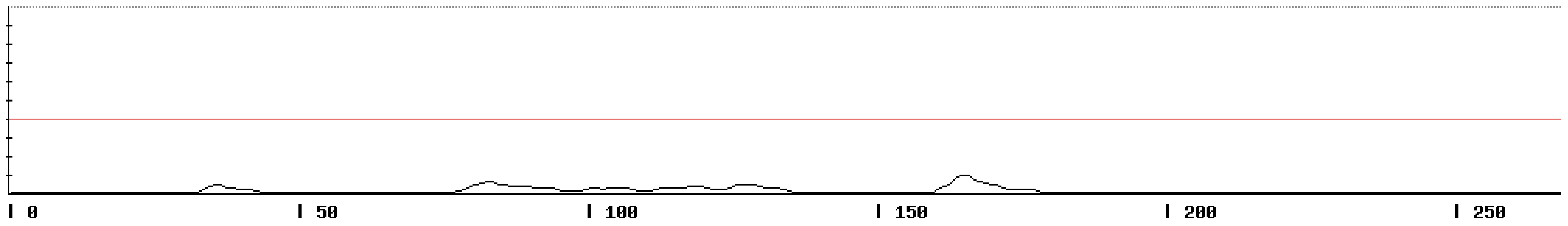

Predictions for Glycinesoja_KHN11625.1

Viterbi Path

Viterbi Path

Posterior @ 0.4

Posterior @ 0.4

Posterior Graphic. Threshold Marked @ 0.4

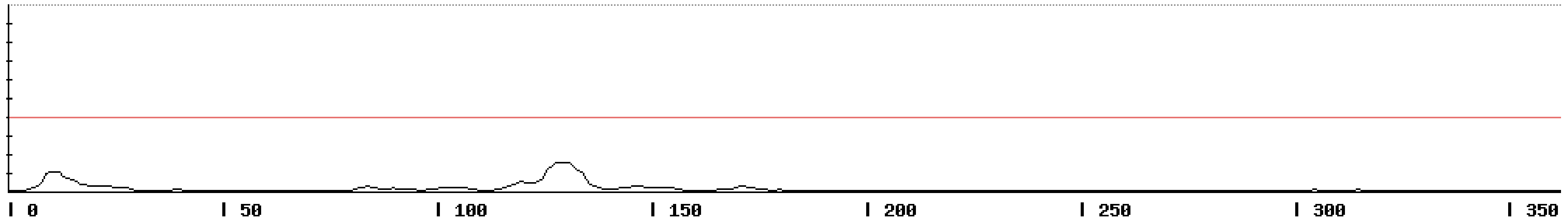

Predictions for Glycinesoja_KHN11644.1

Viterbi Path

Viterbi Path

Posterior @ 0.4

Posterior @ 0.4

Posterior Graphic. Threshold Marked @ 0.4

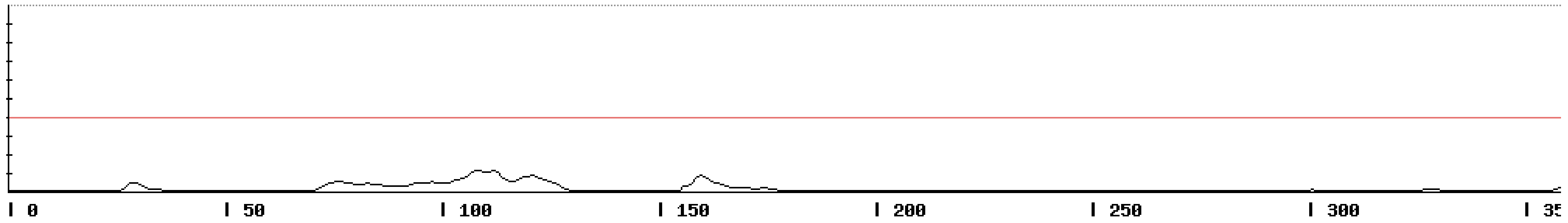

Predictions for Glycinesoja_KHN11660.1

Viterbi Path

Viterbi Path

Posterior @ 0.4	
Posterior @ 0.4	
Posterior Graphic. Threshold Marked @ 0.4	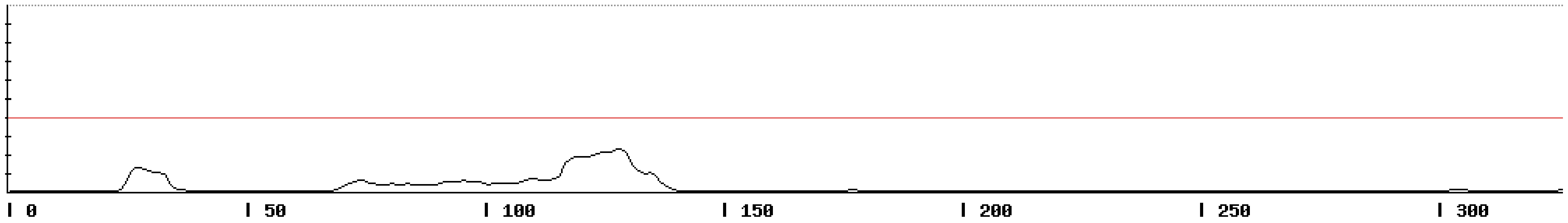

Predictions for Glycinesoja_KHN11983.1

Viterbi Path	
Viterbi Path	
Posterior @ 0.4	
Posterior @ 0.4	
Posterior Graphic. Threshold Marked @ 0.4	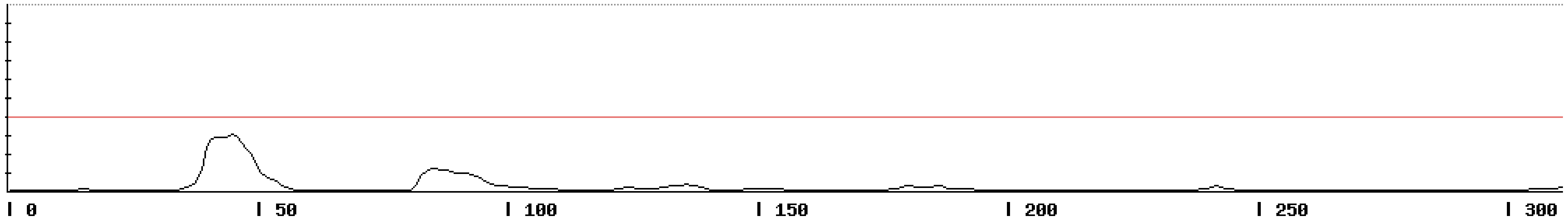

Predictions for Glycinesoja_KHN12212.1

Viterbi Path

Viterbi Path

Posterior @ 0.4

Posterior @ 0.4

Posterior Graphic. Threshold Marked @ 0.4

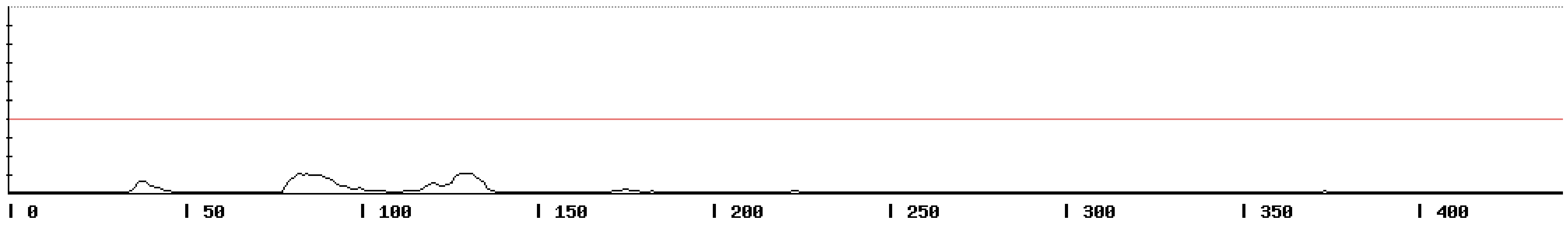

Predictions for Glycinesoja_KHN12796.1

Viterbi Path

Viterbi Path

Posterior @ 0.4

72 - K - 72

79 - AKETKRRNRRKVKSGFWLRKGKVR - 102

Posterior @ 0.4

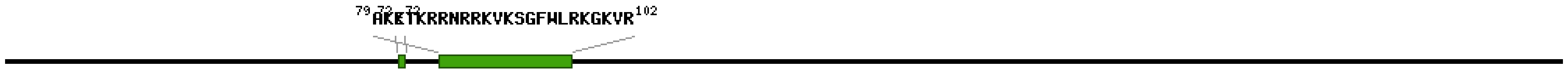

Posterior Graphic. Threshold Marked @ 0.4

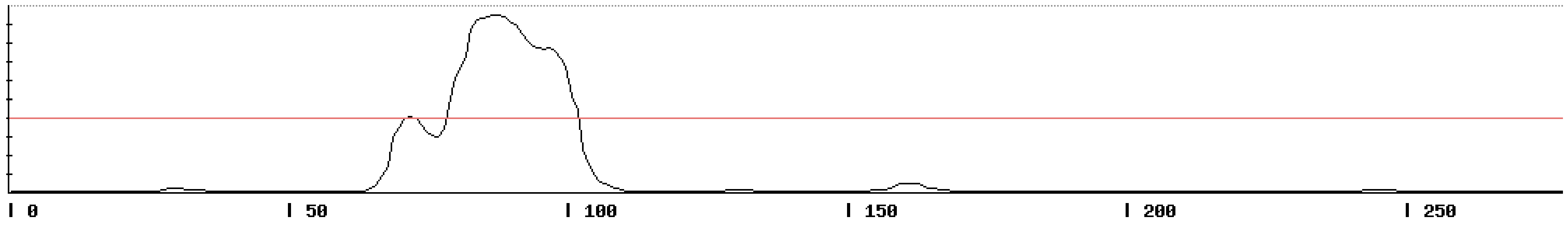

Predictions for Glycinesoja_KHN13213.1	
Viterbi Path	
Viterbi Path	
Posterior @ 0.4	
Posterior @ 0.4	
Posterior Graphic. Threshold Marked @ 0.4	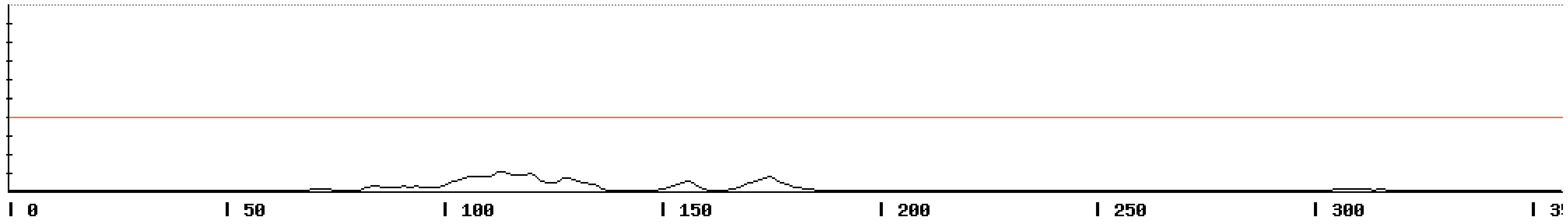

Predictions for Glycinesoja_KHN13226.1	
Viterbi Path	
Viterbi Path	
Posterior @ 0.4	
Posterior @ 0.4	

Posterior Graphic. Threshold Marked @ 0.4

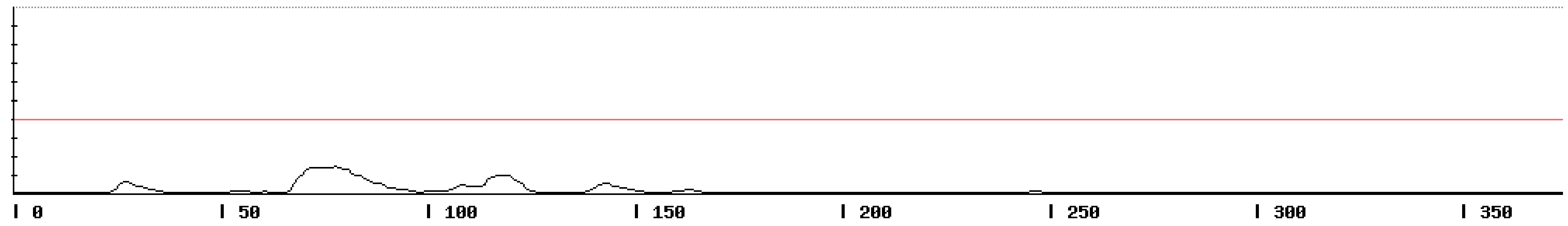

Predictions for Glycinesoja_KHN13600.1

Viterbi Path

Viterbi Path

Posterior @ 0.4

Posterior @ 0.4

Posterior Graphic. Threshold Marked @ 0.4

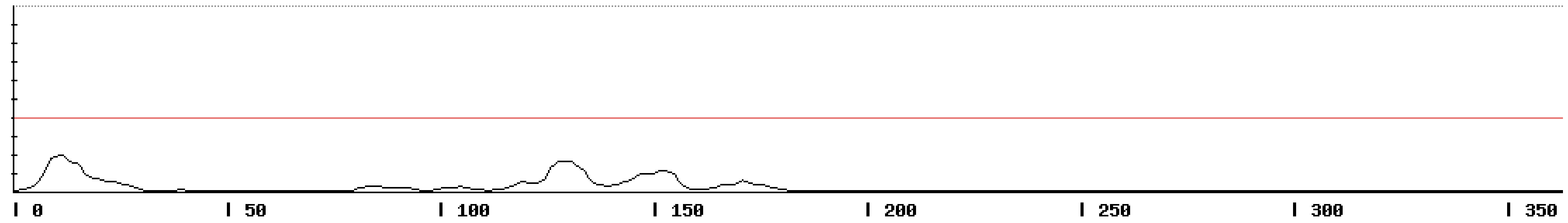

Predictions for Glycinesoja_KHN14494.1

Viterbi Path

Viterbi Path

Posterior @ 0.4

Posterior @ 0.4

Posterior Graphic. Threshold Marked @ 0.4

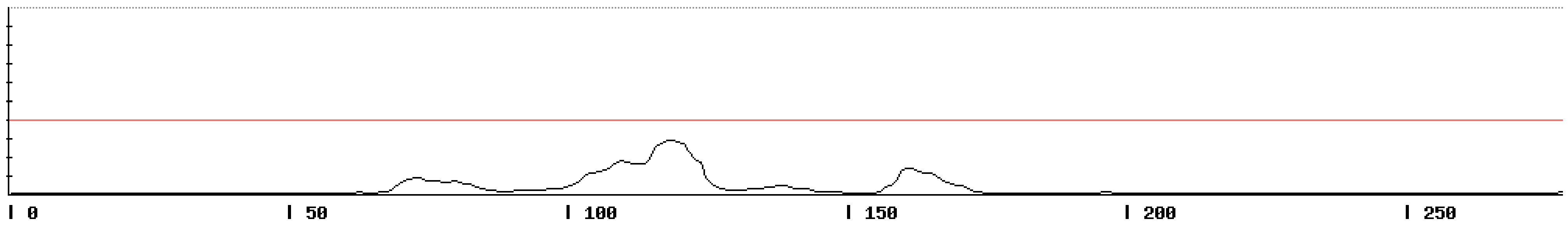

Predictions for Glycinesoja_KHN15010.1

Viterbi Path

Viterbi Path

Posterior @ 0.4

Posterior @ 0.4

Posterior Graphic. Threshold Marked @ 0.4

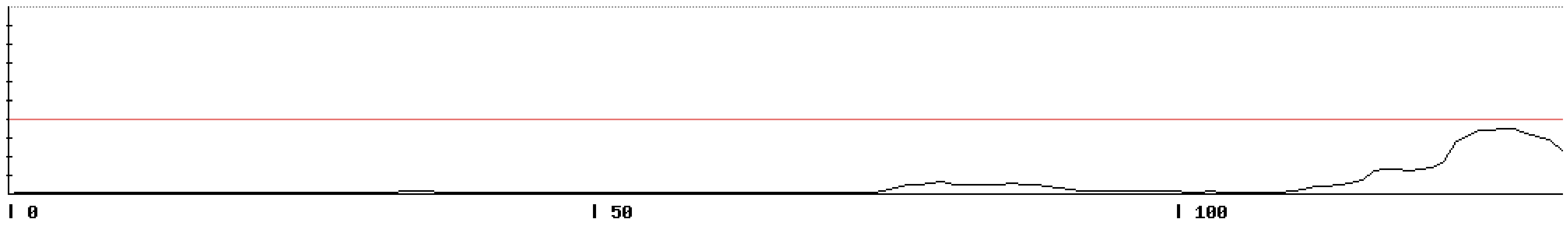

Predictions for Glycinesoja_KHN15359.1

Viterbi Path

Viterbi Path

Posterior @ 0.4	
Posterior @ 0.4	
Posterior Graphic. Threshold Marked @ 0.4	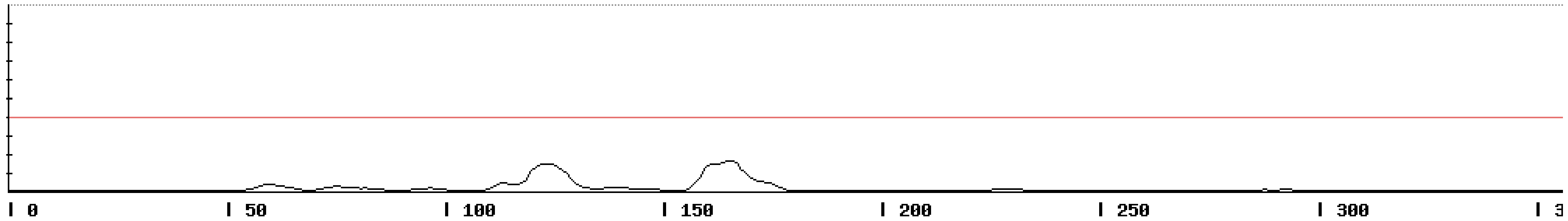

Predictions for Glycinesoja_KHN15497.1	
Viterbi Path	
Viterbi Path	
Posterior @ 0.4	146 - RKRRK - 150
Posterior @ 0.4	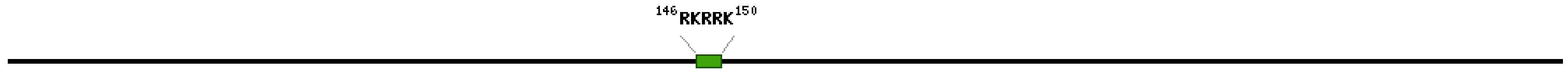
Posterior Graphic. Threshold Marked @ 0.4	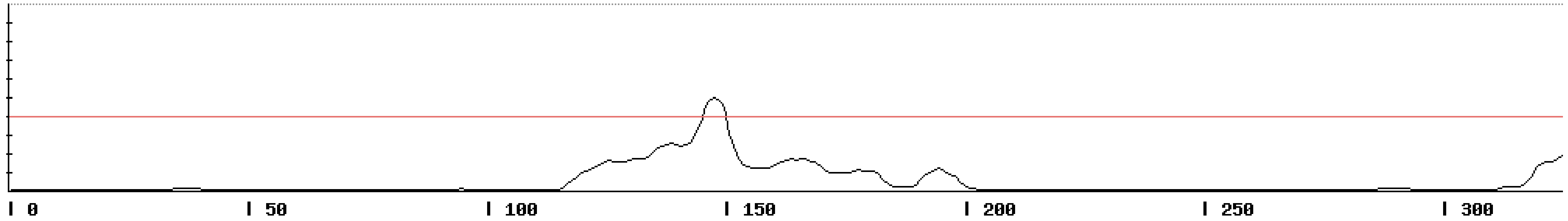

Predictions for Glycinesoja_KHN15864.1	
Viterbi Path	
Viterbi Path	

Posterior @ 0.4

Posterior @ 0.4

Posterior Graphic. Threshold Marked @ 0.4

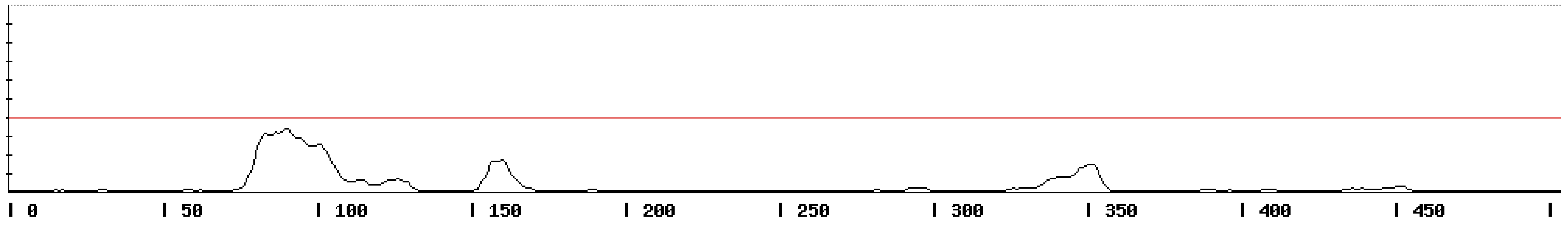

Predictions for Glycinesoja_KHN15865.1

Viterbi Path

Viterbi Path

Posterior @ 0.4

Posterior @ 0.4

Posterior Graphic. Threshold Marked @ 0.4

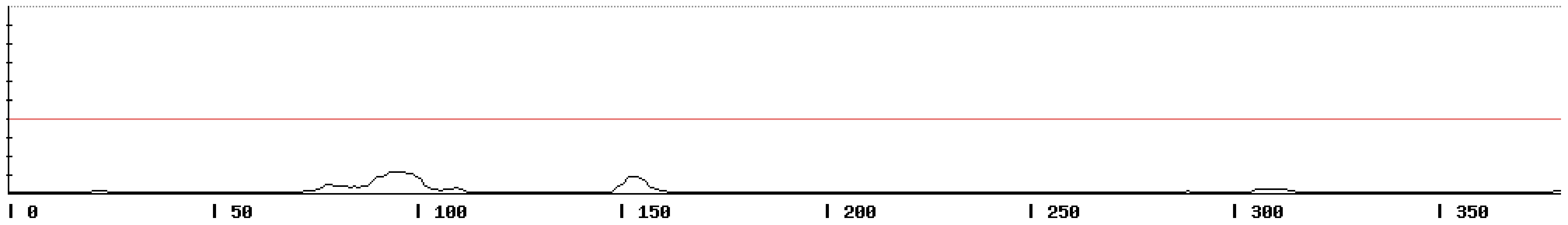

Predictions for Glycinesoja_KHN15866.1

Viterbi Path	
Viterbi Path	
Posterior @ 0.4	
Posterior @ 0.4	
Posterior Graphic. Threshold Marked @ 0.4	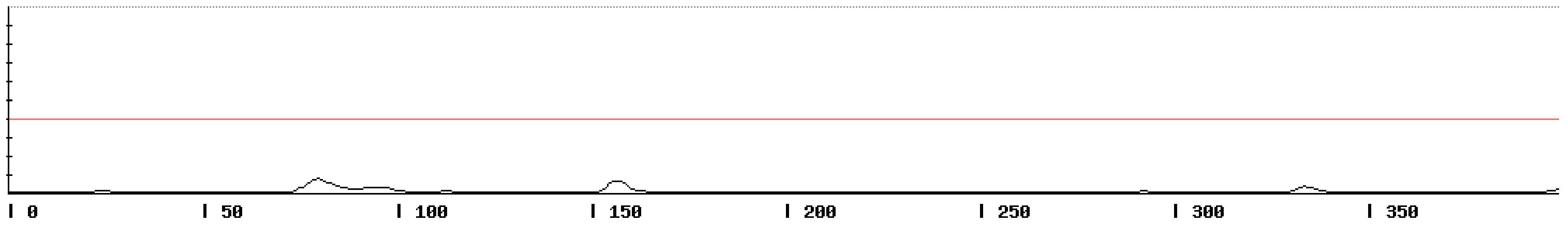

Predictions for Glycinesoja_KHN15891.1	
Viterbi Path	
Viterbi Path	
Posterior @ 0.4	
Posterior @ 0.4	
Posterior Graphic. Threshold Marked @ 0.4	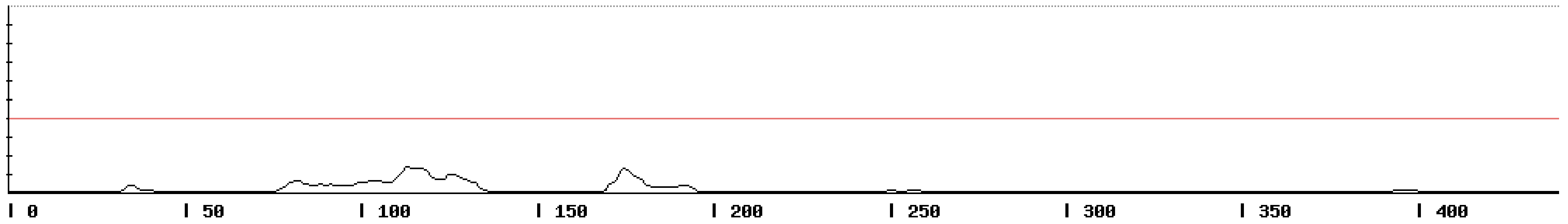

Predictions for Glycinesoja_KHN16438.1	
Viterbi Path	
Viterbi Path	
Posterior @ 0.4	159 - EKKEGRKGKKAKGS - 172
Posterior @ 0.4	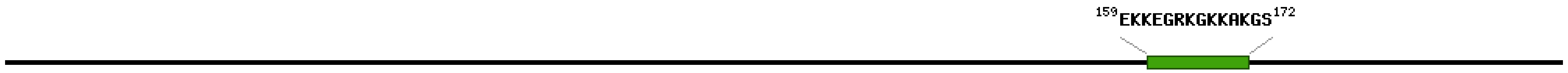
Posterior Graphic. Threshold Marked @ 0.4	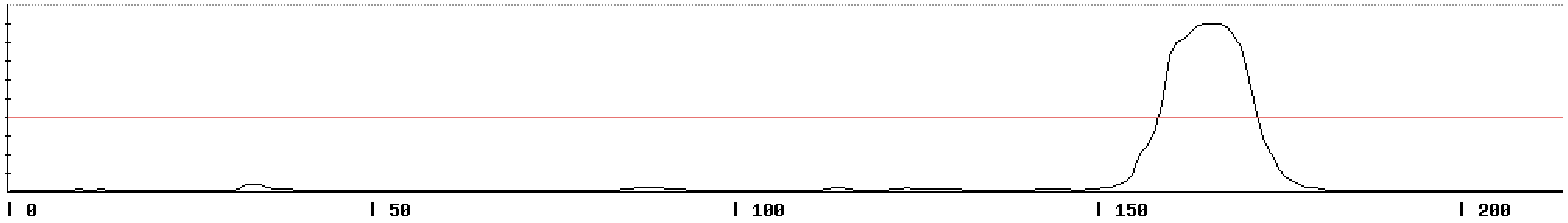

Predictions for Glycinesoja_KHN16575.1	
Viterbi Path	
Viterbi Path	
Posterior @ 0.4	
Posterior @ 0.4	
Posterior Graphic. Threshold Marked @ 0.4	

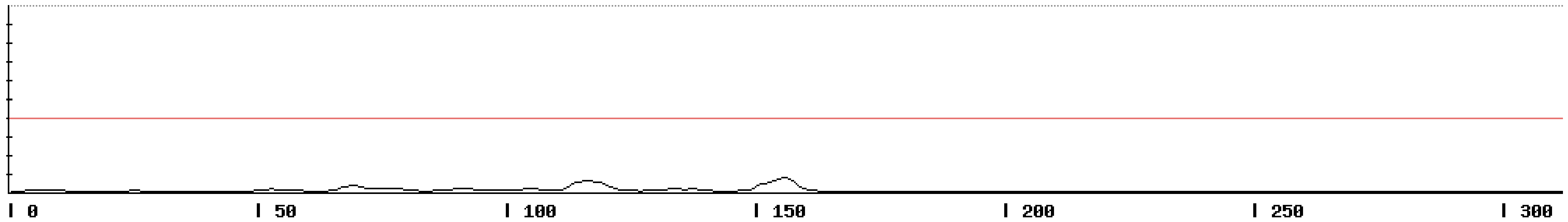

Predictions for Glycinesoja_KHN16660.1

Viterbi Path	
Viterbi Path	
Posterior @ 0.4	
Posterior @ 0.4	
Posterior Graphic. Threshold Marked @ 0.4	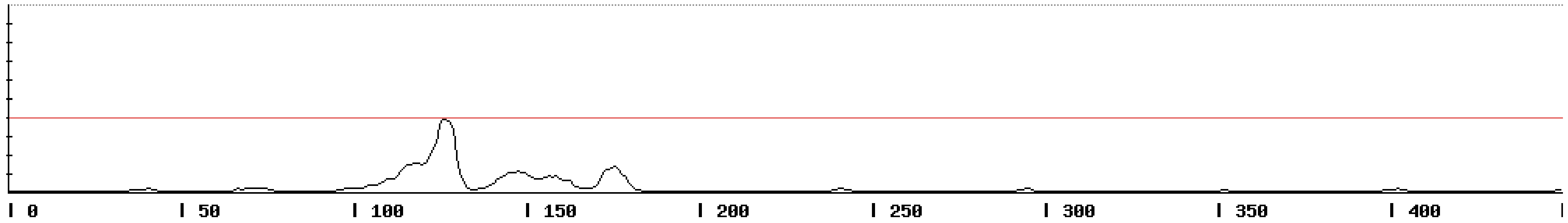

Predictions for Glycinesoja_KHN16868.1

Viterbi Path	
Viterbi Path	
Posterior @ 0.4	
Posterior @ 0.4	

Posterior Graphic. Threshold Marked @ 0.4

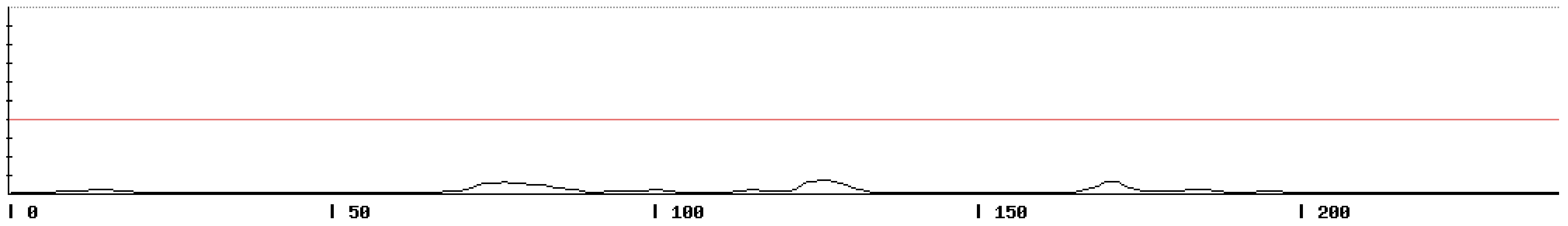

Predictions for Glycinesoja_KHN17058.1

Viterbi Path

Viterbi Path

Posterior @ 0.4

Posterior @ 0.4

Posterior Graphic. Threshold Marked @ 0.4

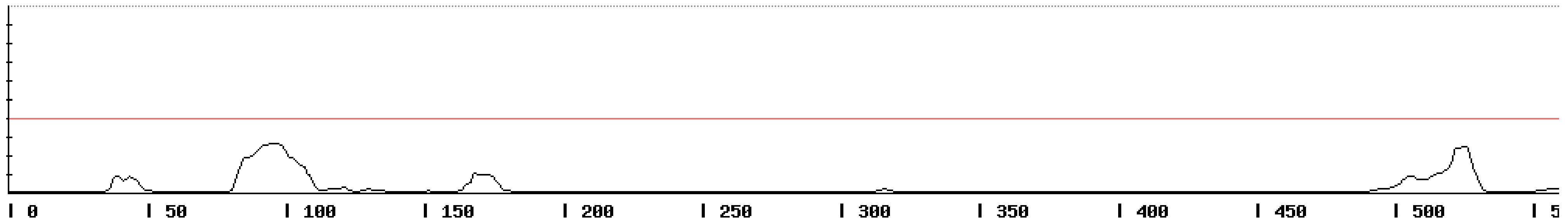

Predictions for Glycinesoja_KHN17084.1

Viterbi Path

Viterbi Path

Posterior @ 0.4

Posterior @ 0.4

Posterior Graphic. Threshold Marked @ 0.4

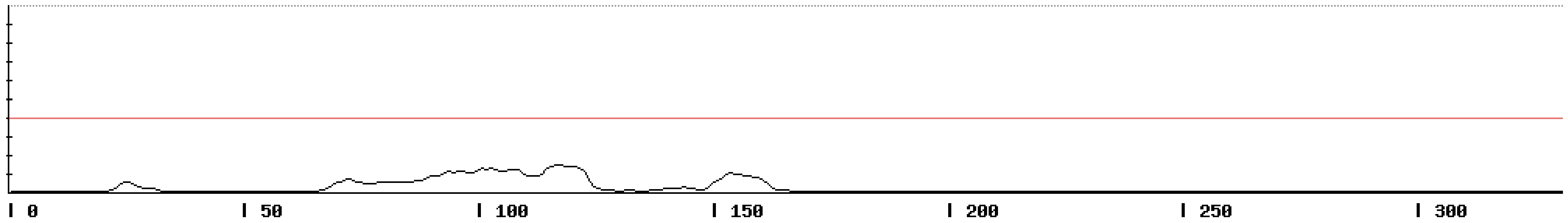

Predictions for Glycinesoja_KHN17368.1

Viterbi Path

Viterbi Path

Posterior @ 0.4

Posterior @ 0.4

Posterior Graphic. Threshold Marked @ 0.4

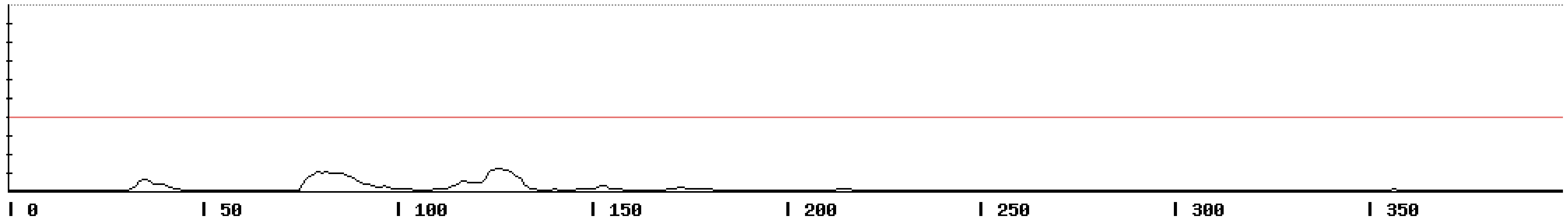

Predictions for Glycinesoja_KHN17402.1

Viterbi Path

Viterbi Path

Posterior @ 0.4	92 - GADKPIGKPKALGIKK - 107
Posterior @ 0.4	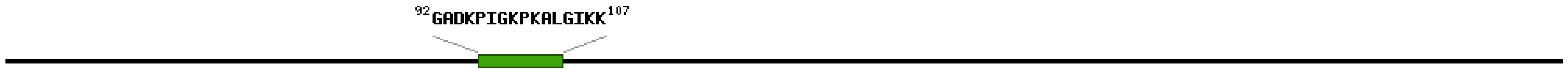
Posterior Graphic. Threshold Marked @ 0.4	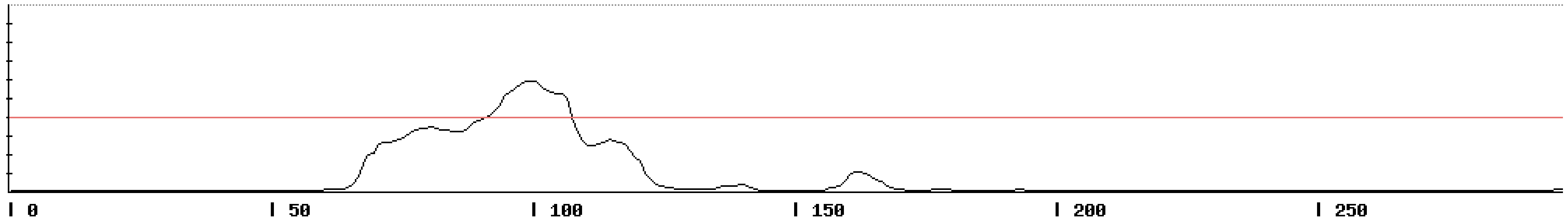

Predictions for Glycinesoja_KHN17478.1

Viterbi Path	
Viterbi Path	
Posterior @ 0.4	
Posterior @ 0.4	
Posterior Graphic. Threshold Marked @ 0.4	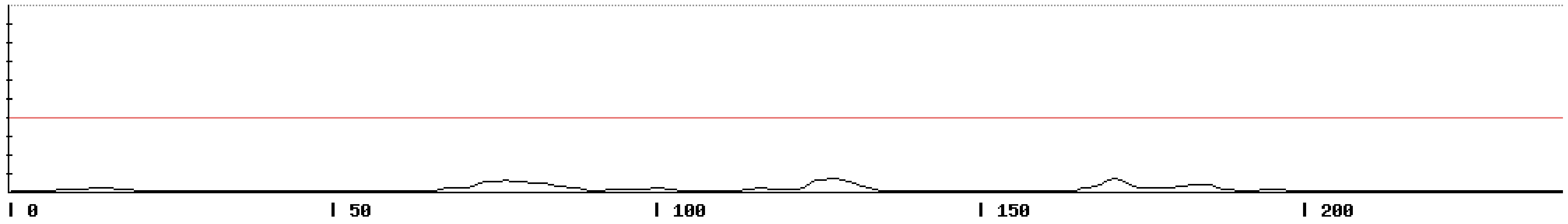

Predictions for Glycinesoja_KHN18216.1

Viterbi Path	
Viterbi Path	

Posterior @ 0.4	
Posterior @ 0.4	
Posterior Graphic. Threshold Marked @ 0.4	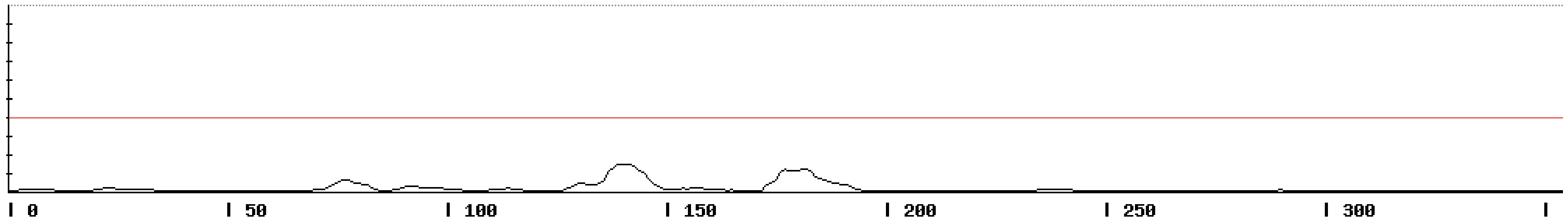

Predictions for Glycinesoja_KHN18563.1	
Viterbi Path	
Viterbi Path	
Posterior @ 0.4	
Posterior @ 0.4	
Posterior Graphic. Threshold Marked @ 0.4	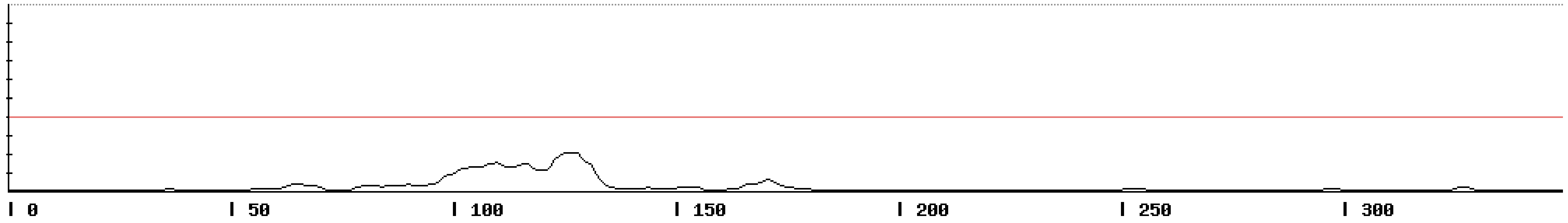

Predictions for Glycinesoja_KHN18791.1

Viterbi Path

Viterbi Path

Posterior @ 0.4

Posterior @ 0.4

Posterior Graphic. Threshold Marked @ 0.4

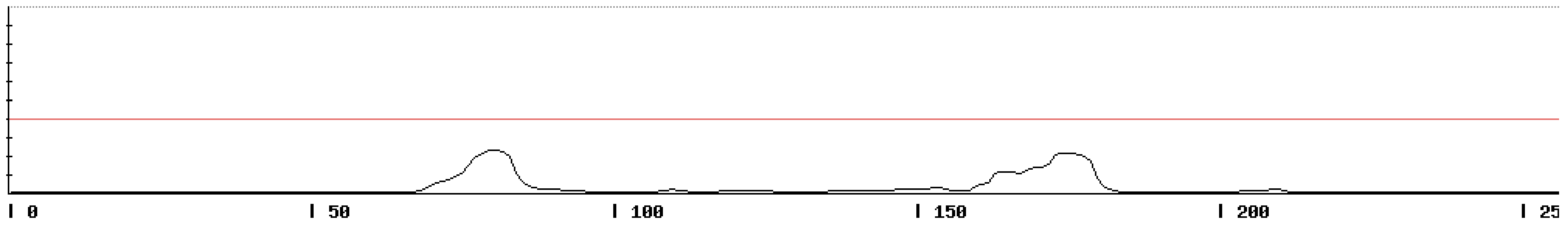

Predictions for Glycinesoja_KHN18942.1

Viterbi Path

Viterbi Path

Posterior @ 0.4

140 - KRR - 142

Posterior @ 0.4

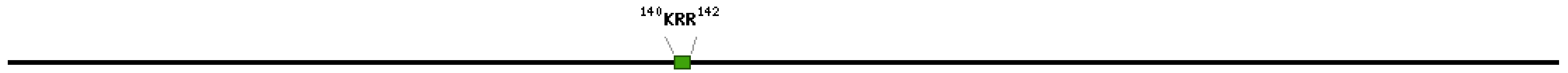

Posterior Graphic. Threshold Marked @ 0.4

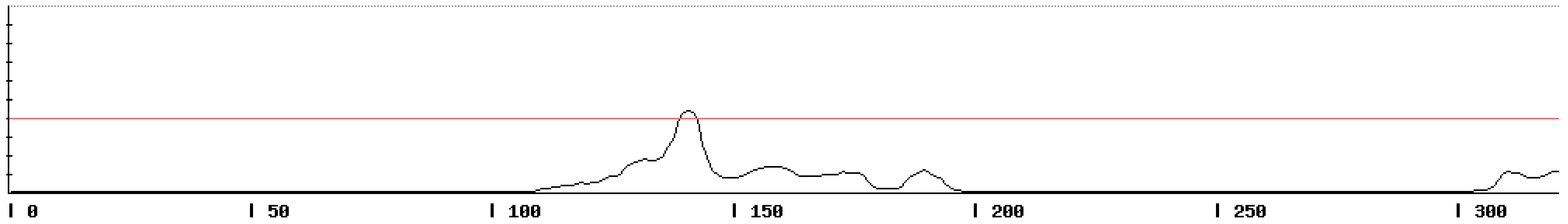

Predictions for Glycinesoja_KHN19577.1	
Viterbi Path	
Viterbi Path	
Posterior @ 0.4	532 - SGKRKI - 537
Posterior @ 0.4	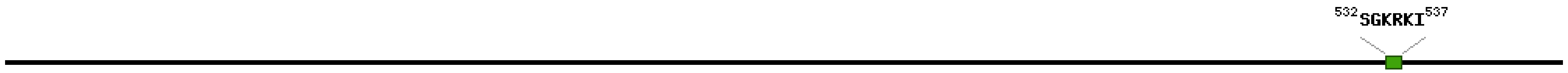
Posterior Graphic. Threshold Marked @ 0.4	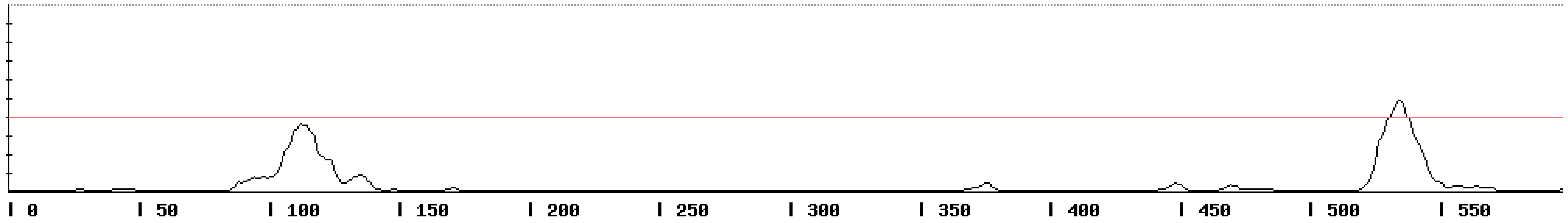

Predictions for Glycinesoja_KHN19686.1	
Viterbi Path	
Viterbi Path	
Posterior @ 0.4	131 - GRRKRRKI - 138
Posterior @ 0.4	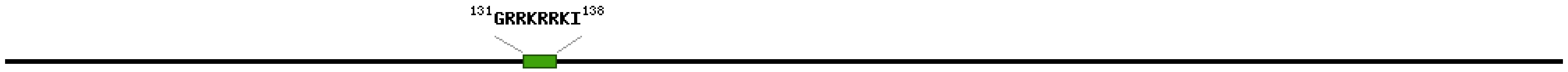
Posterior Graphic. Threshold Marked @ 0.4	

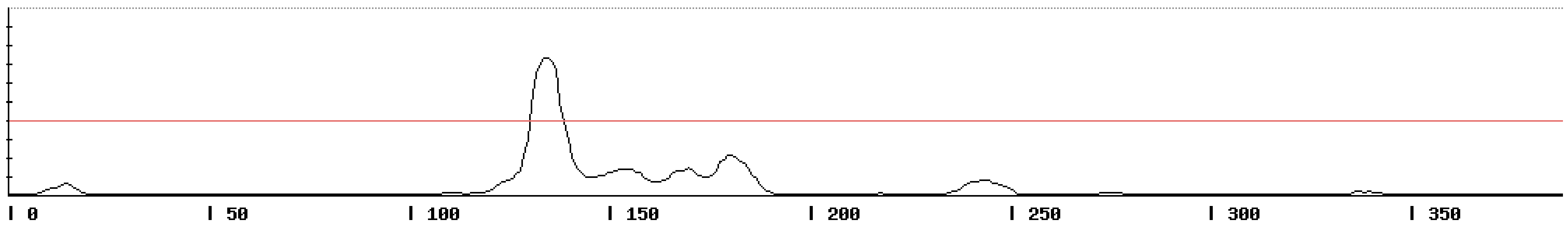

Predictions for Glycinesoja_KHN20086.1

Viterbi Path	
Viterbi Path	
Posterior @ 0.4	
Posterior @ 0.4	
Posterior Graphic. Threshold Marked @ 0.4	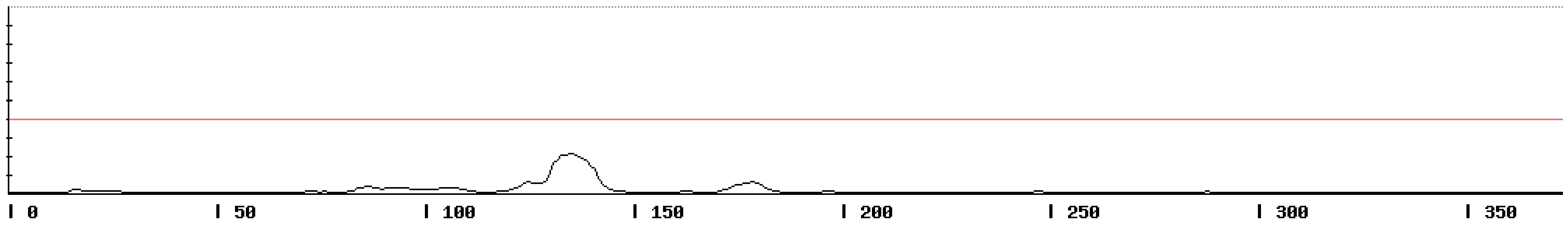

Predictions for Glycinesoja_KHN20606.1

Viterbi Path	
Viterbi Path	
Posterior @ 0.4	97 - KPVGKPKPVGIKK - 109
Posterior @ 0.4	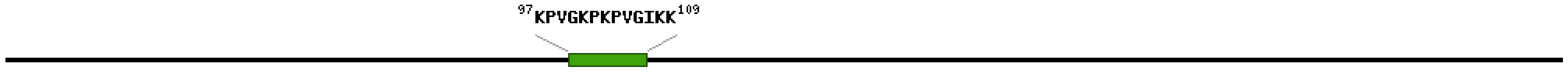

Posterior Graphic. Threshold Marked @ 0.4

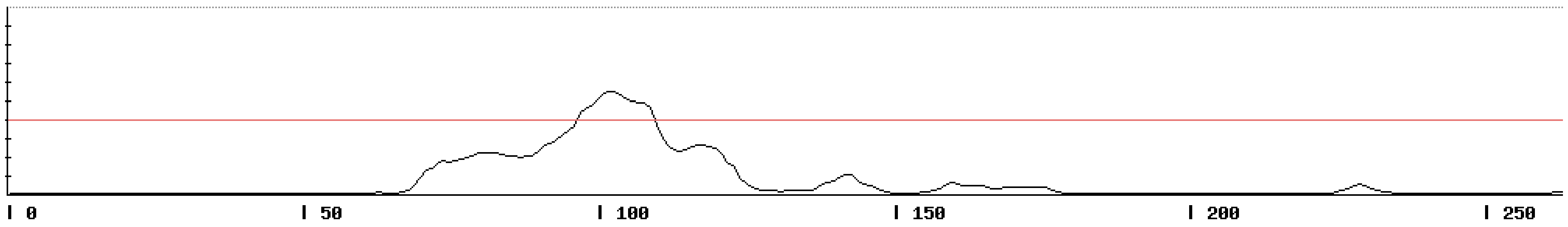

Predictions for Glycinesoja_KHN20732.1

Viterbi Path

Viterbi Path

Posterior @ 0.4

Posterior @ 0.4

Posterior Graphic. Threshold Marked @ 0.4

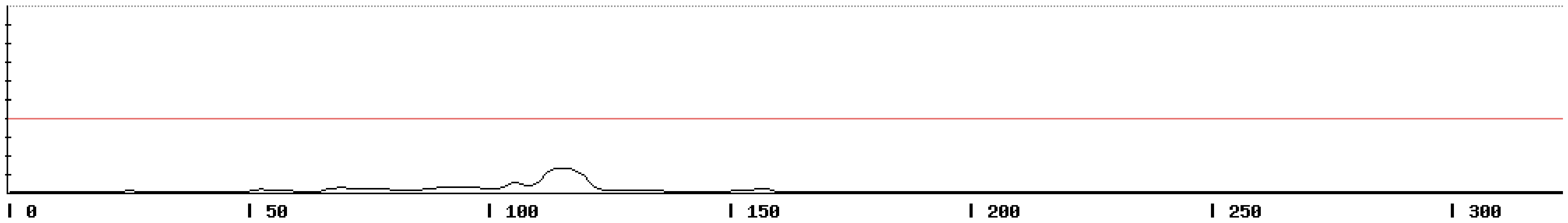

Predictions for Glycinesoja_KHN21163.1

Viterbi Path

Viterbi Path

Posterior @ 0.4

Posterior @ 0.4

Posterior Graphic. Threshold Marked @ 0.4

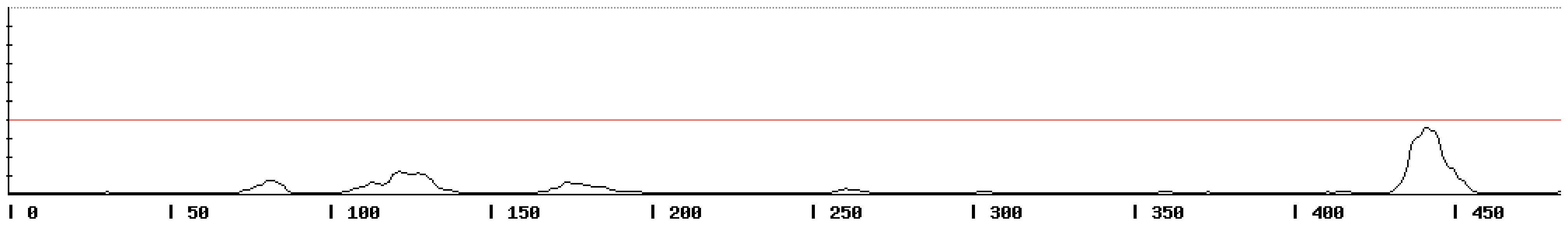

Predictions for Glycinesoja_KHN21353.1

Viterbi Path

Viterbi Path

Posterior @ 0.4

Posterior @ 0.4

Posterior Graphic. Threshold Marked @ 0.4

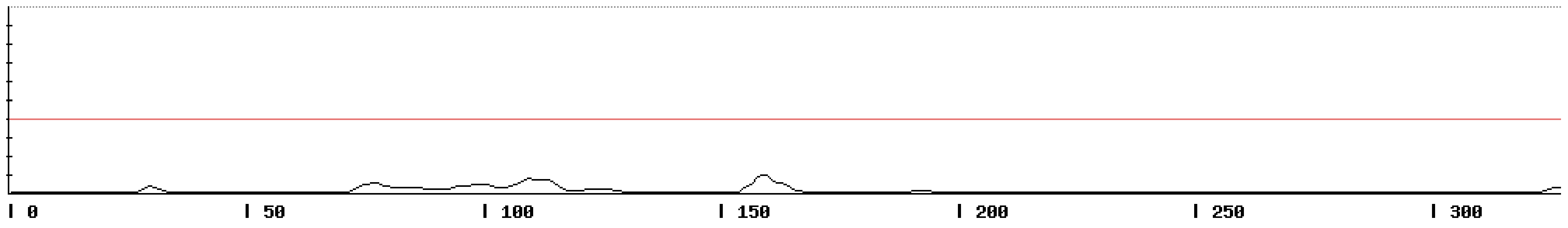

Predictions for Glycinesoja_KHN21369.1

Viterbi Path

Viterbi Path

Posterior @ 0.4

Posterior @ 0.4

Posterior Graphic. Threshold Marked @ 0.4

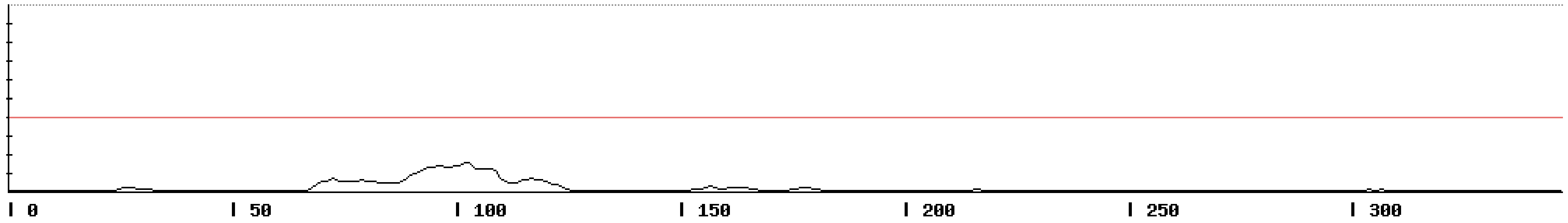

Predictions for Glycinesoja_KHN21530.1

Viterbi Path

Viterbi Path

Posterior @ 0.4

Posterior @ 0.4

Posterior Graphic. Threshold Marked @ 0.4

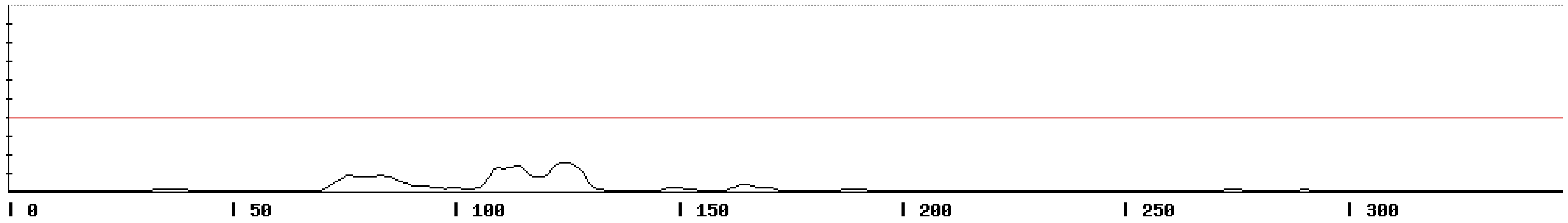

Predictions for Glycinesoja_KHN21567.1

Viterbi Path

Viterbi Path

Posterior @ 0.4

Posterior @ 0.4

Posterior Graphic. Threshold Marked @ 0.4

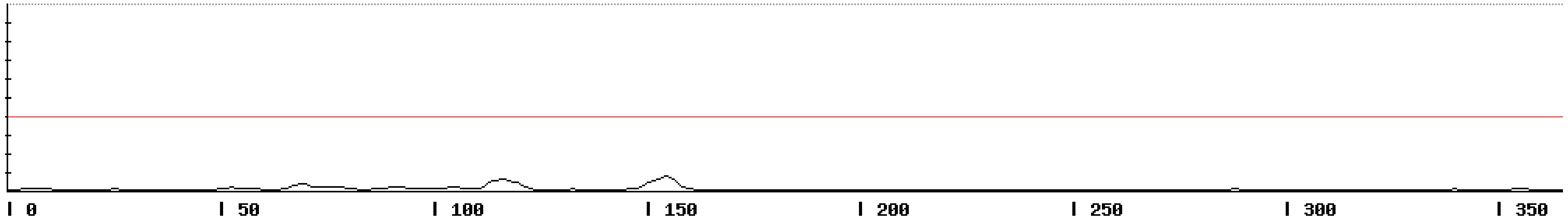

Predictions for Glycinesoja_KHN21713.1

Viterbi Path

Viterbi Path

Posterior @ 0.4

Posterior @ 0.4

Posterior Graphic. Threshold Marked @ 0.4

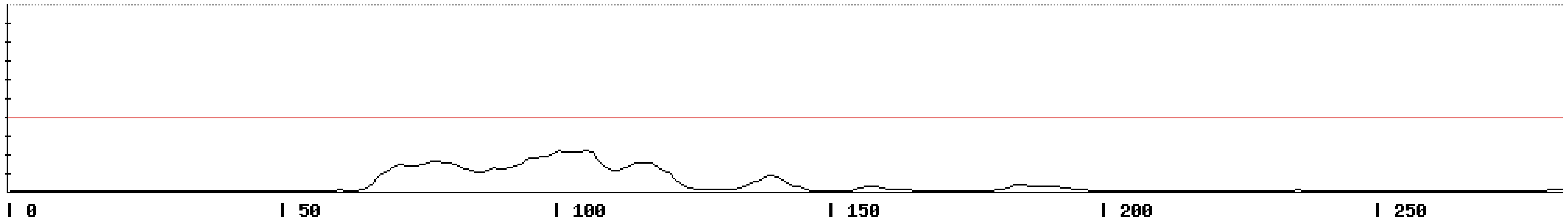

Predictions for Glycinesoja_KHN23594.1

Viterbi Path

Viterbi Path

Posterior @ 0.4

Posterior @ 0.4

Posterior Graphic. Threshold Marked @ 0.4

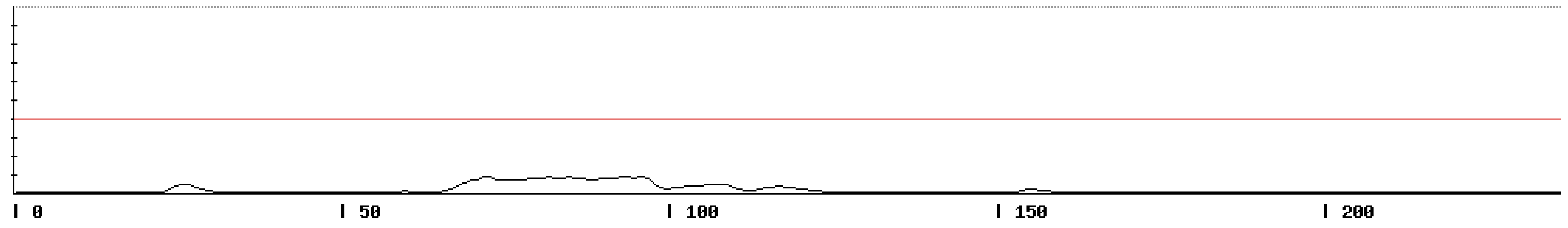

Predictions for Glycinesoja_KHN23958.1

Viterbi Path

Viterbi Path

Posterior @ 0.4

Posterior @ 0.4

Posterior Graphic. Threshold Marked @ 0.4

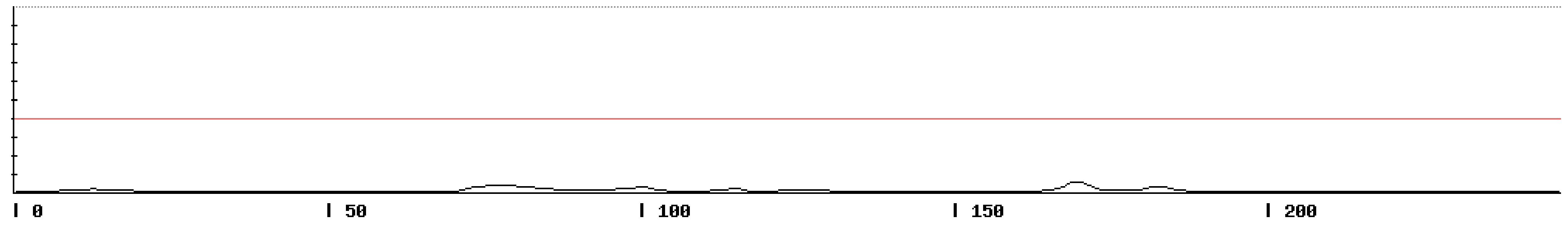

Predictions for Glycinesoja_KHN24171.1	
Viterbi Path	
Viterbi Path	
Posterior @ 0.4	
Posterior @ 0.4	
Posterior Graphic. Threshold Marked @ 0.4	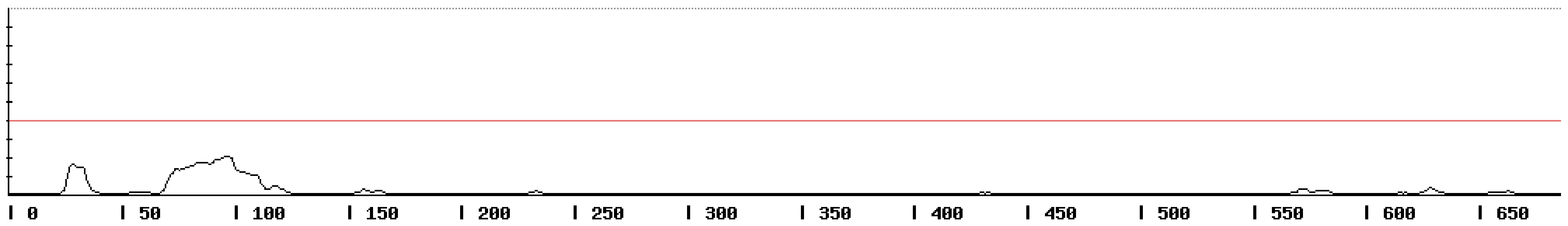

Predictions for Glycinesoja_KHN24309.1	
Viterbi Path	
Viterbi Path	
Posterior @ 0.4	
Posterior @ 0.4	
Posterior Graphic. Threshold Marked @ 0.4	

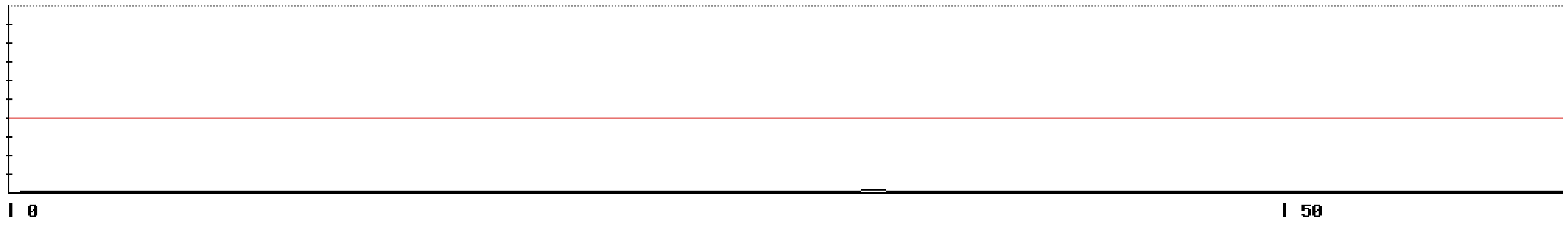

Predictions for Glycinesoja_KHN24532.1

Viterbi Path	
Viterbi Path	
Posterior @ 0.4	
Posterior @ 0.4	
Posterior Graphic. Threshold Marked @ 0.4	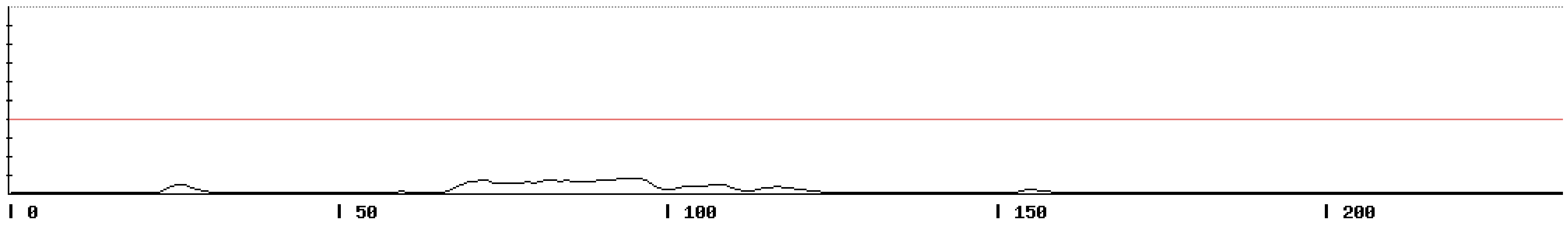

Predictions for Glycinesoja_KHN24582.1

Viterbi Path	
Viterbi Path	
Posterior @ 0.4	
Posterior @ 0.4	

Posterior Graphic. Threshold Marked @ 0.4

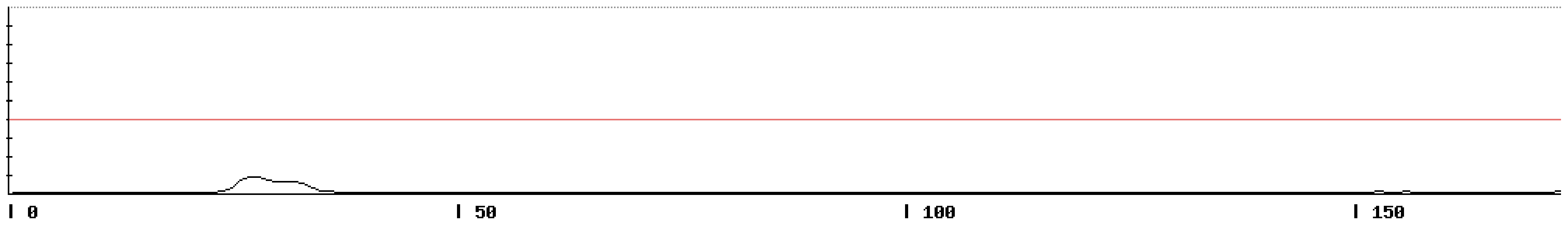

Predictions for Glycinesoja_KHN24977.1

Viterbi Path

Viterbi Path

Posterior @ 0.4

Posterior @ 0.4

Posterior Graphic. Threshold Marked @ 0.4

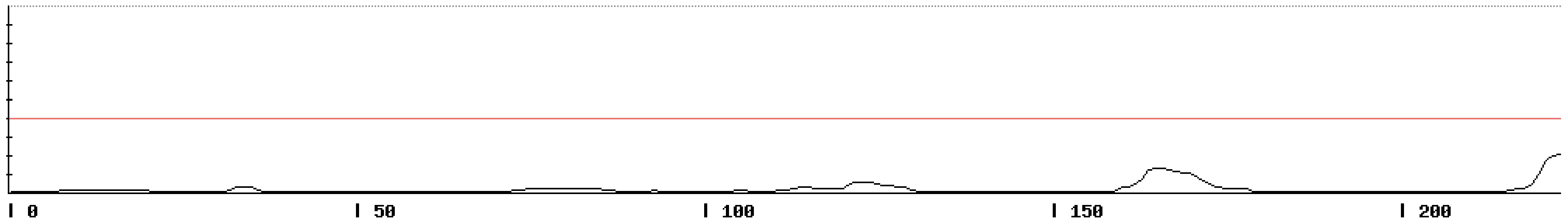

Predictions for Glycinesoja_KHN25034.1

Viterbi Path

Viterbi Path

Posterior @ 0.4

Posterior @ 0.4

Posterior Graphic. Threshold Marked @ 0.4

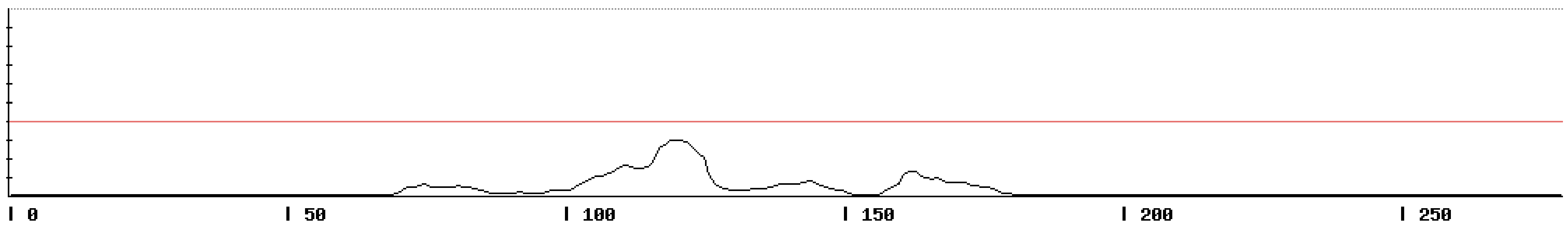

Predictions for Glycinesoja_KHN25045.1

Viterbi Path

Viterbi Path

Posterior @ 0.4

Posterior @ 0.4

Posterior Graphic. Threshold Marked @ 0.4

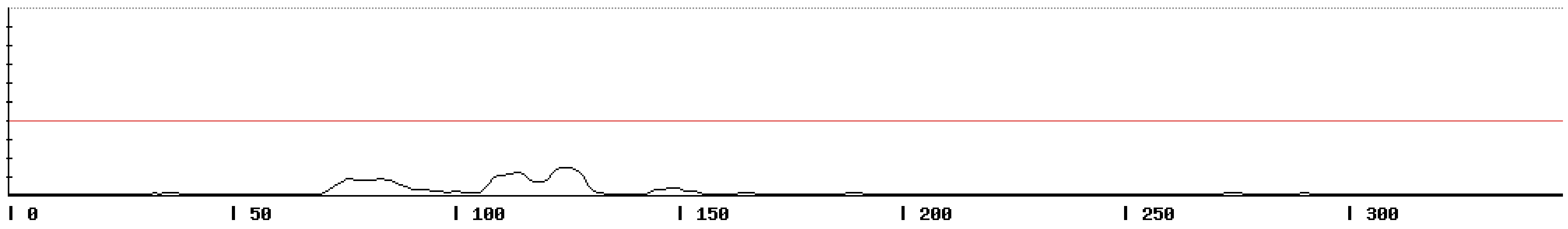

Predictions for Glycinesoja_KHN25522.1

Viterbi Path

Viterbi Path

Posterior @ 0.4

Posterior @ 0.4

Posterior Graphic. Threshold Marked @ 0.4

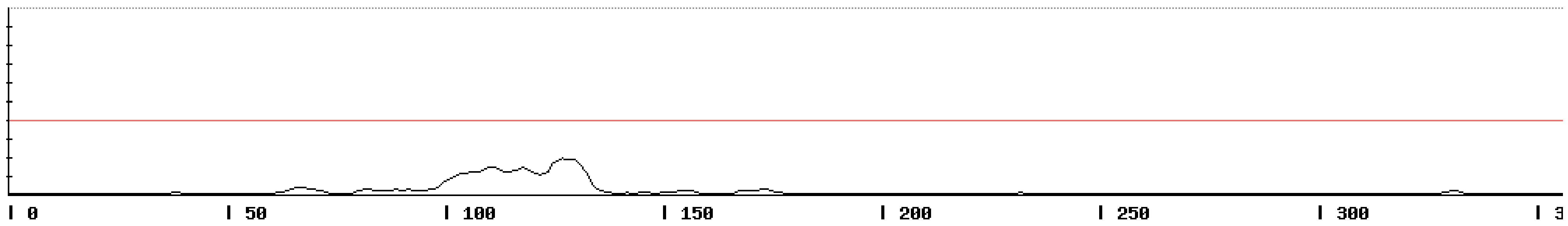

Predictions for Glycinesoja_KHN26260.1

Viterbi Path

Viterbi Path

Posterior @ 0.4

Posterior @ 0.4

Posterior Graphic. Threshold Marked @ 0.4

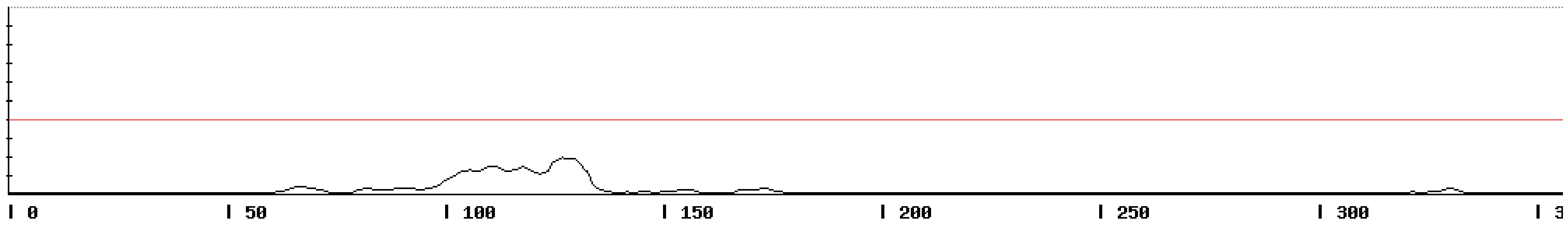

Predictions for Glycinesoja_KHN26726.1

Viterbi Path

Viterbi Path

Posterior @ 0.4

Posterior @ 0.4

Posterior Graphic. Threshold Marked @ 0.4

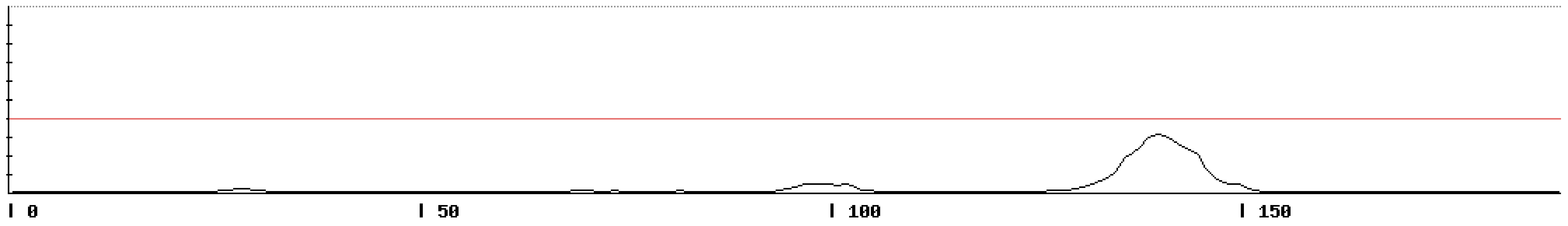

Predictions for Glycinesoja_KHN26800.1

Viterbi Path

Viterbi Path

Posterior @ 0.4

Posterior @ 0.4

Posterior Graphic. Threshold Marked @ 0.4

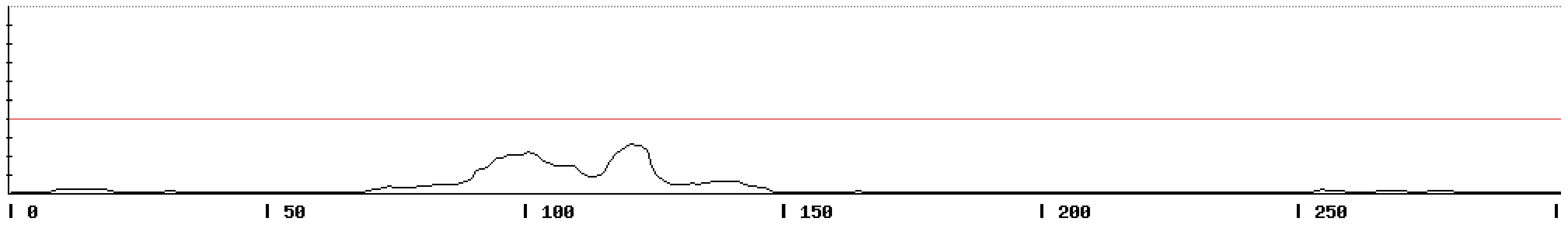

Predictions for Glycinesoja_KHN26839.1

Viterbi Path	
Viterbi Path	
Posterior @ 0.4	
Posterior @ 0.4	
Posterior Graphic. Threshold Marked @ 0.4	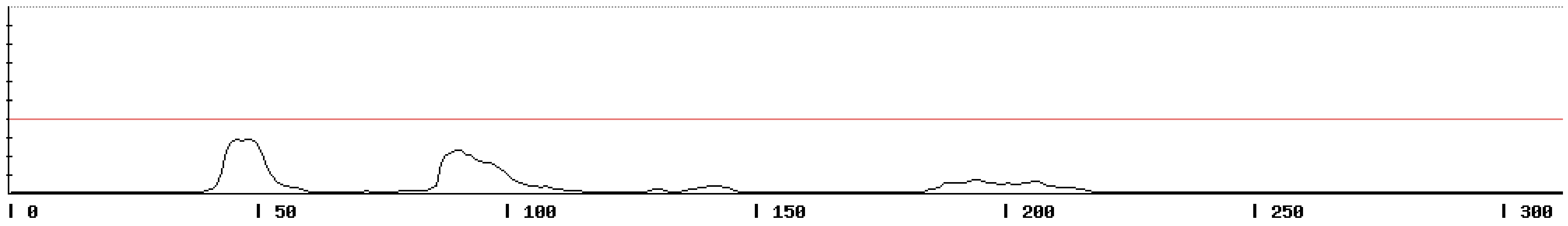

Predictions for Glycinesoja_KHN27473.1

Viterbi Path	
Viterbi Path	
Posterior @ 0.4	48 - PRKRRK - 53
Posterior @ 0.4	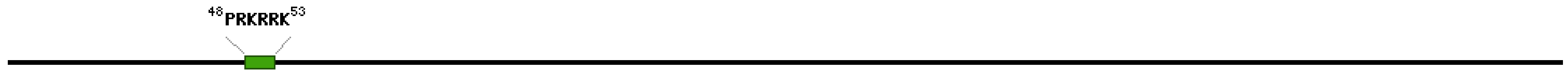
Posterior Graphic. Threshold Marked @ 0.4	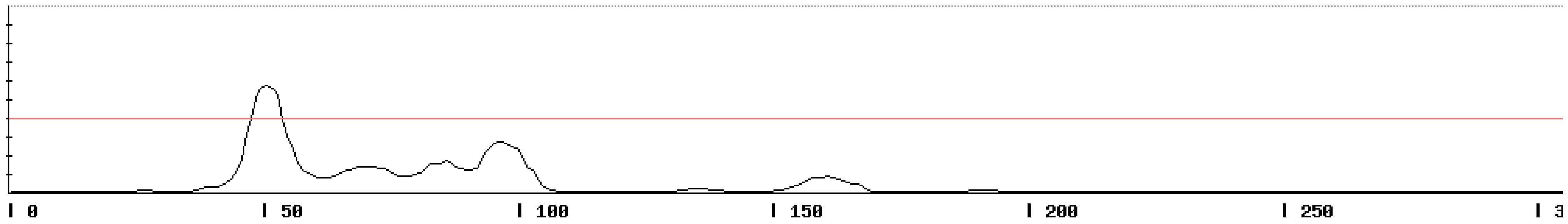

Predictions for Glycinesoja_KHN27560.1	
Viterbi Path	
Viterbi Path	
Posterior @ 0.4	151 - KRRK - 154
Posterior @ 0.4	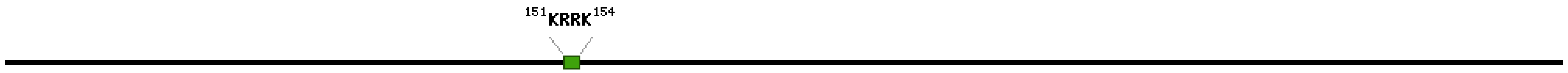
Posterior Graphic. Threshold Marked @ 0.4	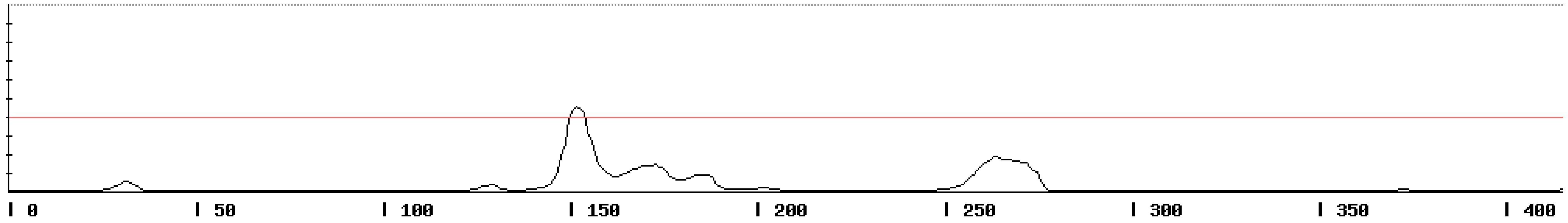

Predictions for Glycinesoja_KHN27636.1	
Viterbi Path	
Viterbi Path	
Posterior @ 0.4	124 - RK - 125
Posterior @ 0.4	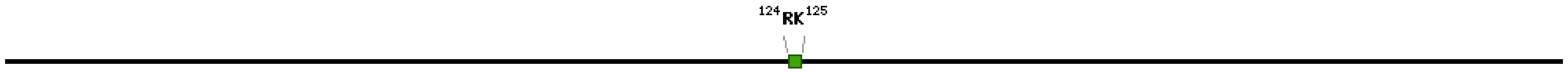
Posterior Graphic. Threshold Marked @ 0.4	

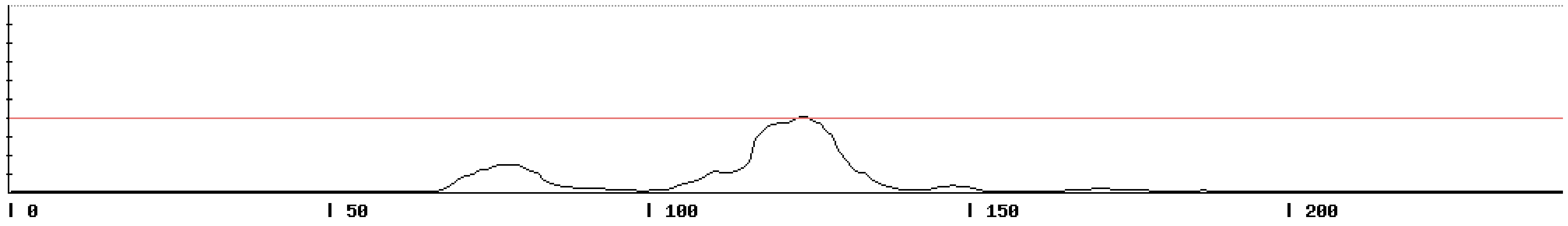

Predictions for Glycinesoja_KHN27713.1

Viterbi Path

Viterbi Path

Posterior @ 0.4

Posterior @ 0.4

Posterior Graphic. Threshold Marked @ 0.4

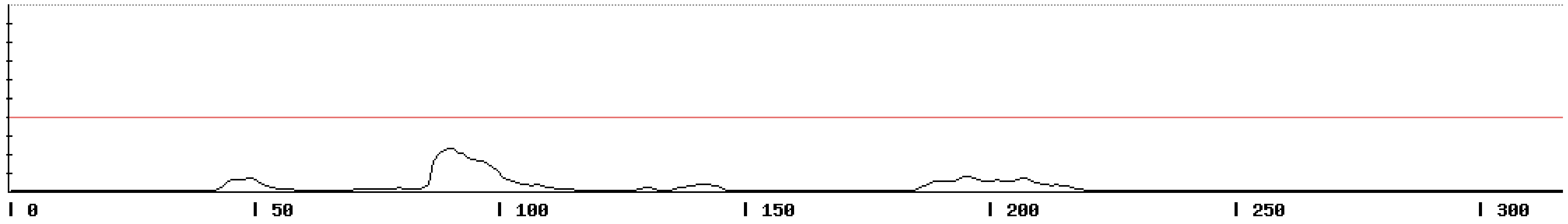

Predictions for Glycinesoja_KHN27750.1

Viterbi Path

Viterbi Path

Posterior @ 0.4

Posterior @ 0.4

Posterior Graphic. Threshold Marked @ 0.4	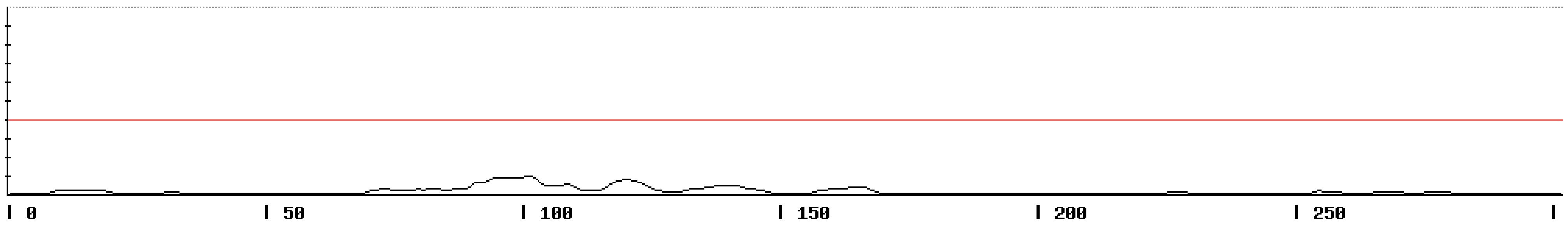

Predictions for Glycinesoja_KHN27913.1

Viterbi Path	
Viterbi Path	
Posterior @ 0.4	126 - RKRRK - 130
Posterior @ 0.4	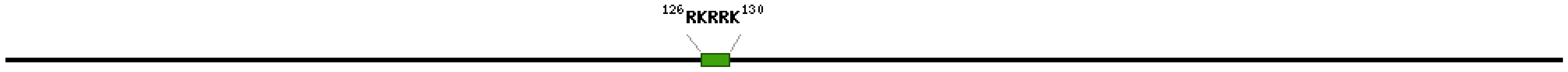
Posterior Graphic. Threshold Marked @ 0.4	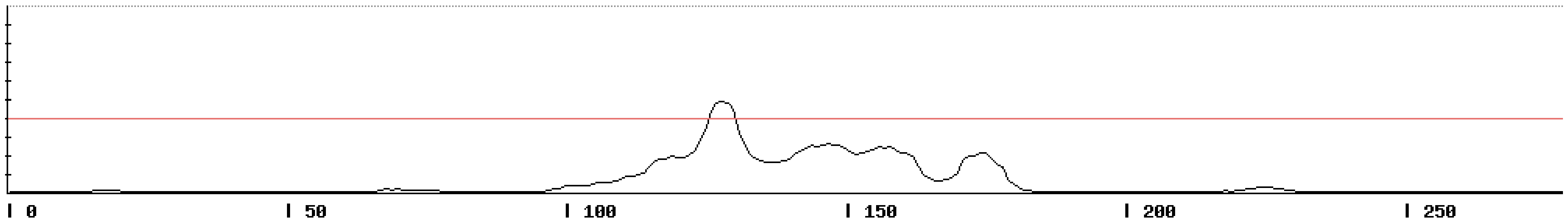

Predictions for Glycinesoja_KHN28035.1

Viterbi Path	
Viterbi Path	
Posterior @ 0.4	
Posterior @ 0.4	

Posterior Graphic. Threshold Marked @ 0.4	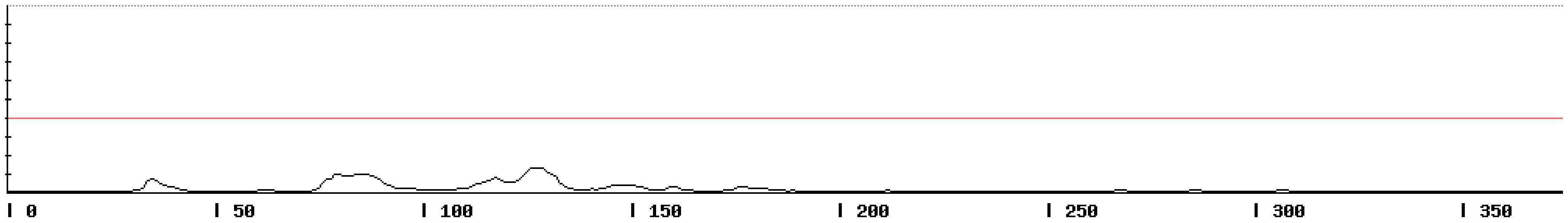

Predictions for Glycinesoja_KHN28036.1

Viterbi Path	
Viterbi Path	
Posterior @ 0.4	
Posterior @ 0.4	
Posterior Graphic. Threshold Marked @ 0.4	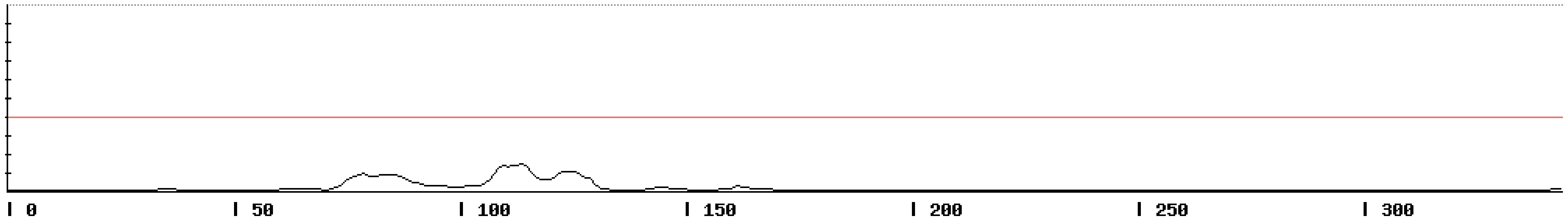

Predictions for Glycinesoja_KHN28079.1

Viterbi Path	
Viterbi Path	

Posterior @ 0.4

Posterior @ 0.4

Posterior Graphic. Threshold Marked @ 0.4

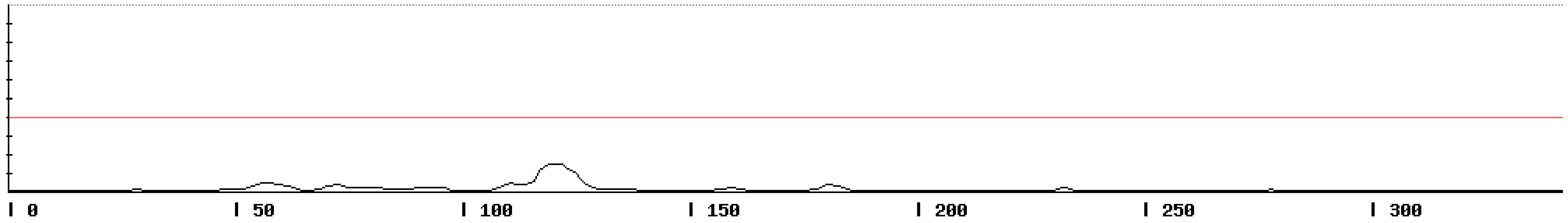

Predictions for Glycinesoja_KHN28123.1

Viterbi Path

Viterbi Path

Posterior @ 0.4 153 - KNKMKTRRSHGKKPSS - 168

Posterior @ 0.4

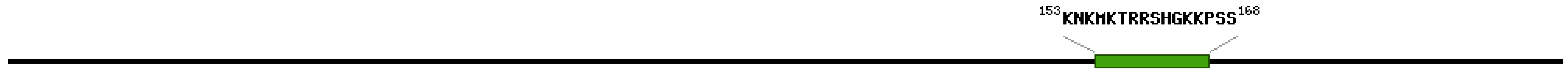

Posterior Graphic. Threshold Marked @ 0.4

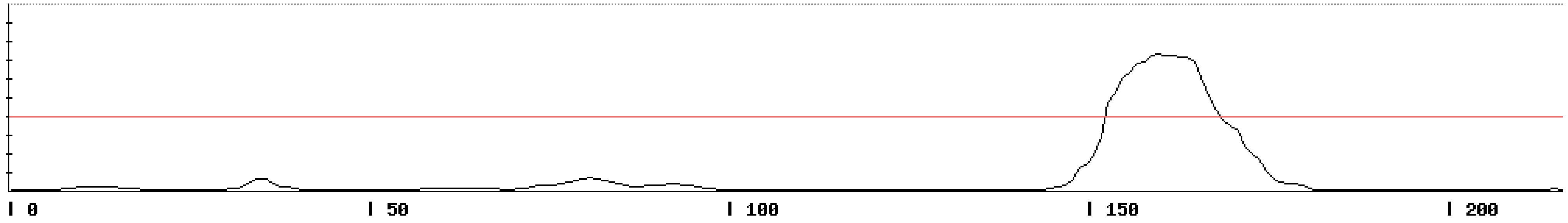

Predictions for Glycinesoja_KHN28140.1

Viterbi Path

Viterbi Path

Posterior @ 0.4

Posterior @ 0.4

Posterior Graphic. Threshold Marked @ 0.4

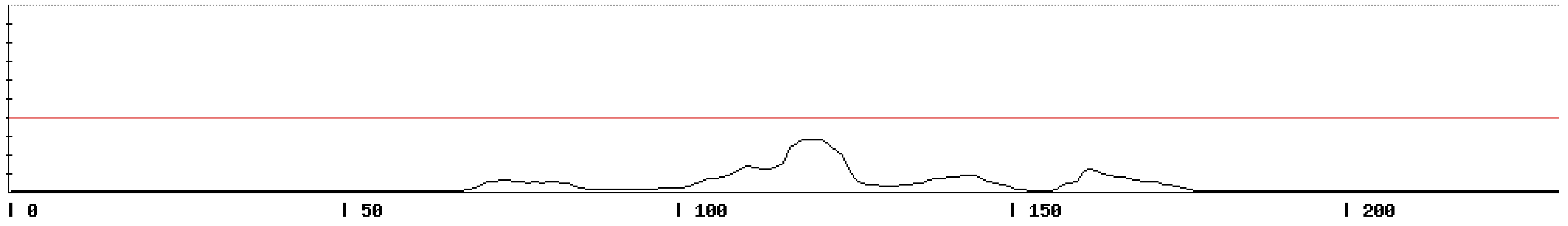

Predictions for Glycinesoja_KHN28504.1

Viterbi Path

Viterbi Path

Posterior @ 0.4

Posterior @ 0.4

Posterior Graphic. Threshold Marked @ 0.4

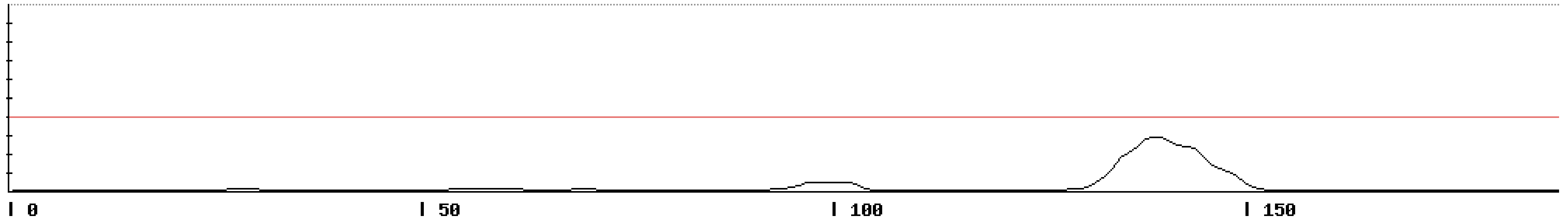

Predictions for Glycinesoja_KHN29242.1

Viterbi Path	
Viterbi Path	
Posterior @ 0.4	
Posterior @ 0.4	
Posterior Graphic. Threshold Marked @ 0.4	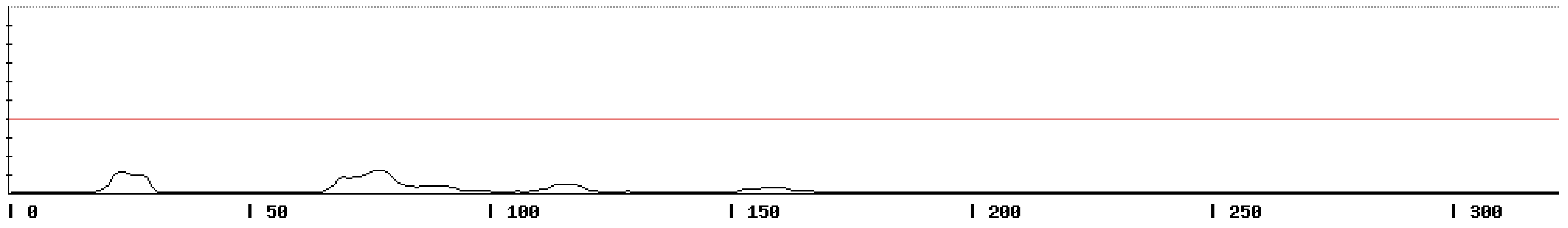

Predictions for Glycinesoja_KHN31143.1	
Viterbi Path	
Viterbi Path	
Posterior @ 0.4	25 - RKVRGKK - 31
Posterior @ 0.4	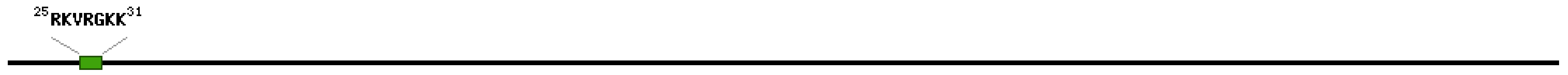
Posterior Graphic. Threshold Marked @ 0.4	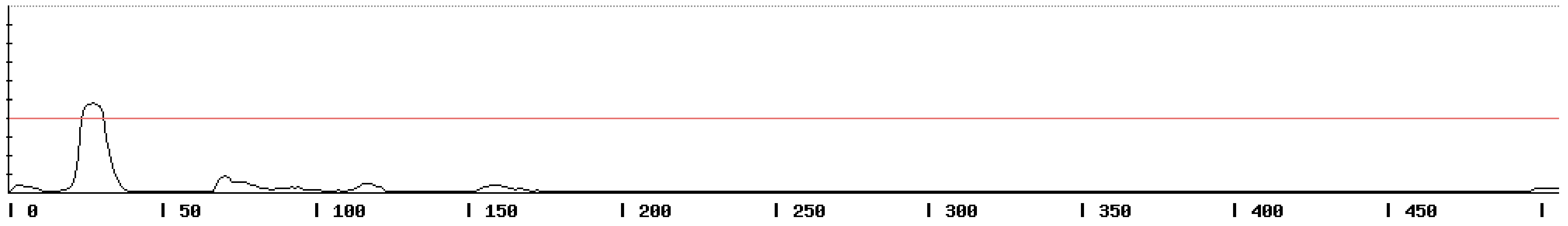

Predictions for Glycinesoja_KHN31158.1	
Viterbi Path	
Viterbi Path	
Posterior @ 0.4	140 - RRRRK - 144
Posterior @ 0.4	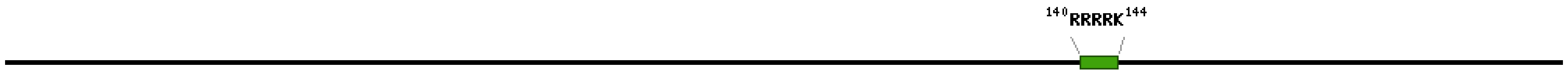
Posterior Graphic. Threshold Marked @ 0.4	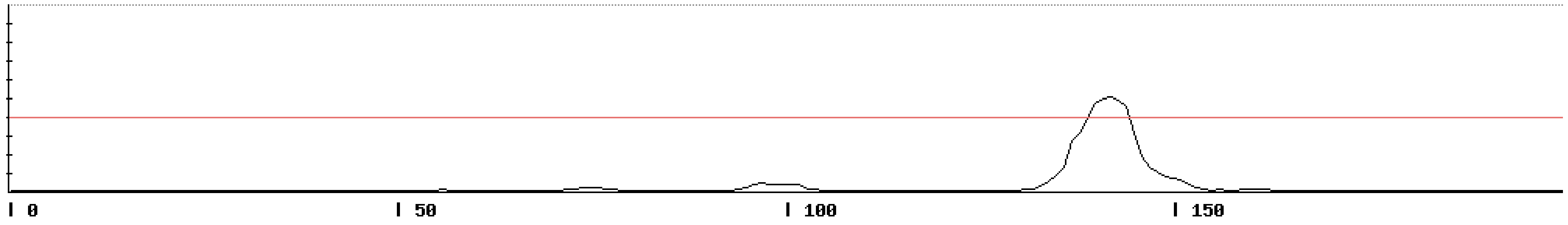

Predictions for Glycinesoja_KHN31193.1	
Viterbi Path	
Viterbi Path	
Posterior @ 0.4	92 - GADKPIGKPKALGIKKA - 108
Posterior @ 0.4	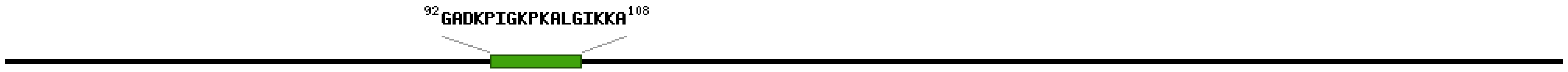
Posterior Graphic. Threshold Marked @ 0.4	

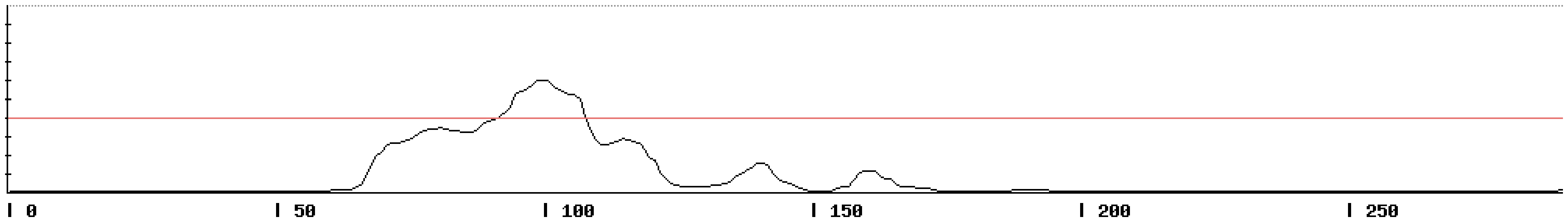

Predictions for Glycinesoja_KHN32045.1	
Viterbi Path	
Viterbi Path	
Posterior @ 0.4	
Posterior @ 0.4	
Posterior Graphic. Threshold Marked @ 0.4	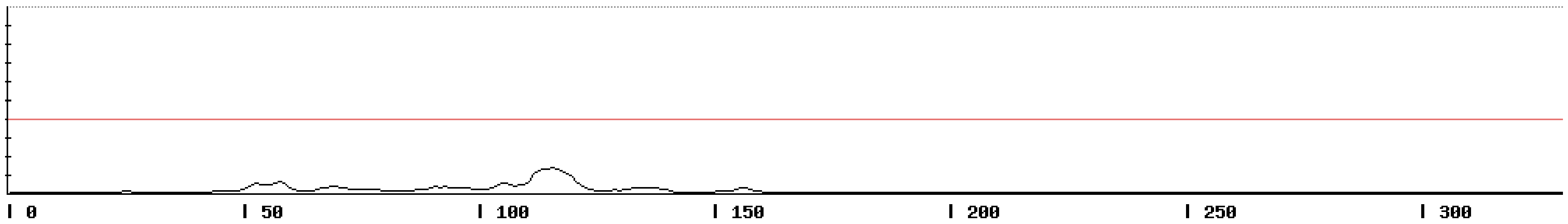

Predictions for Glycinesoja_KHN32454.1	
Viterbi Path	
Viterbi Path	
Posterior @ 0.4	
Posterior @ 0.4	

Posterior Graphic. Threshold Marked @ 0.4	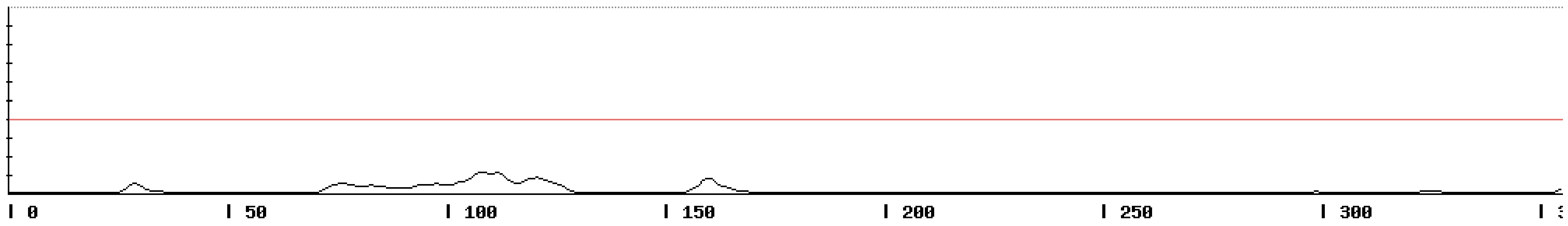

Predictions for Glycinesoja_KHN32743.1	
Viterbi Path	
Viterbi Path	
Posterior @ 0.4	
Posterior @ 0.4	
Posterior Graphic. Threshold Marked @ 0.4	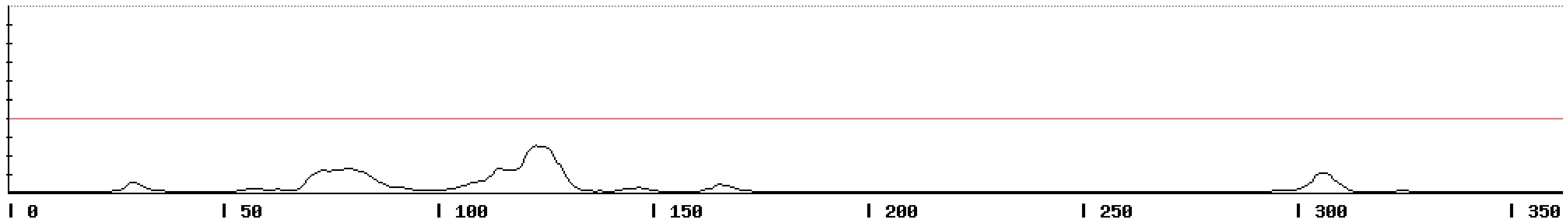

Predictions for Glycinesoja_KHN32748.1	
Viterbi Path	
Viterbi Path	
Posterior @ 0.4	
Posterior @ 0.4	

Posterior Graphic. Threshold Marked @ 0.4

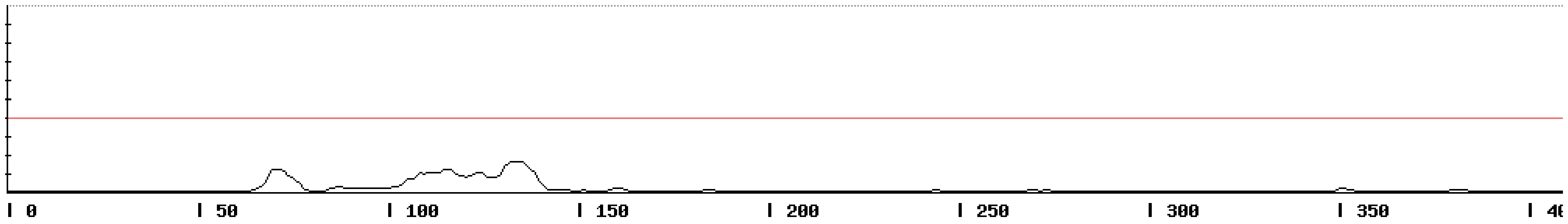

Predictions for Glycinesoja_KHN32858.1

Viterbi Path

Viterbi Path

Posterior @ 0.4

Posterior @ 0.4

Posterior Graphic. Threshold Marked @ 0.4

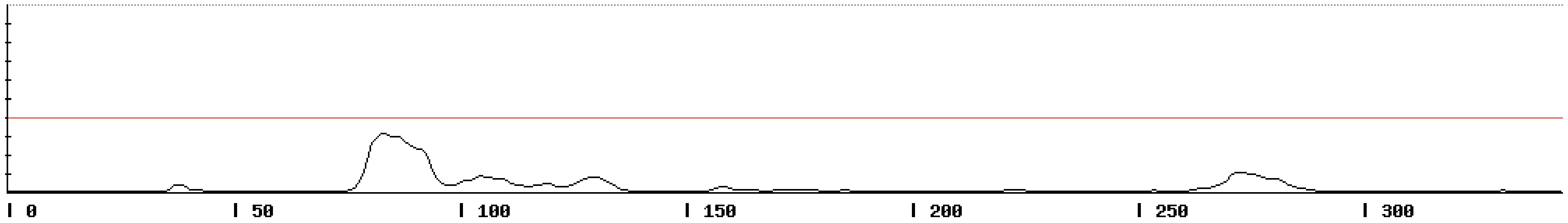

Predictions for Glycinesoja_KHN32865.1

Viterbi Path

Viterbi Path

Posterior @ 0.4	124 - RK - 125
Posterior @ 0.4	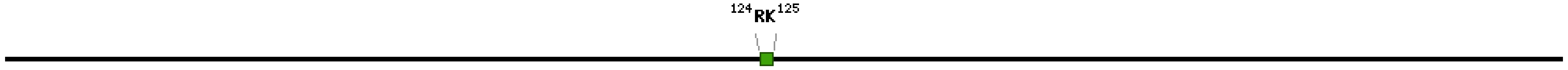
Posterior Graphic. Threshold Marked @ 0.4	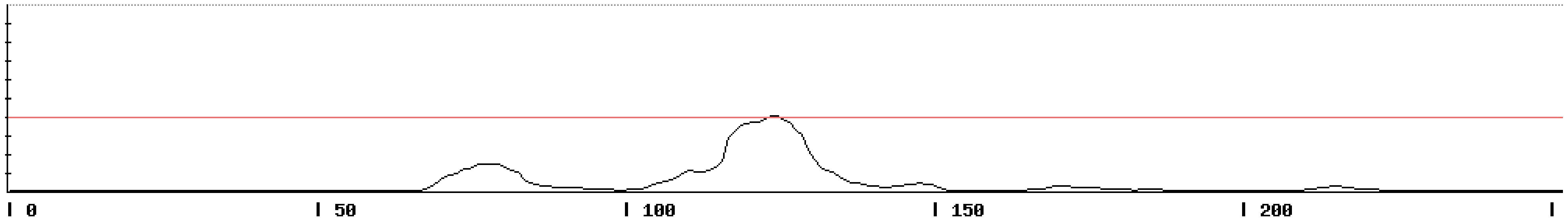

Predictions for Glycinesoja_KHN33501.1

Viterbi Path	
Viterbi Path	
Posterior @ 0.4	
Posterior @ 0.4	
Posterior Graphic. Threshold Marked @ 0.4	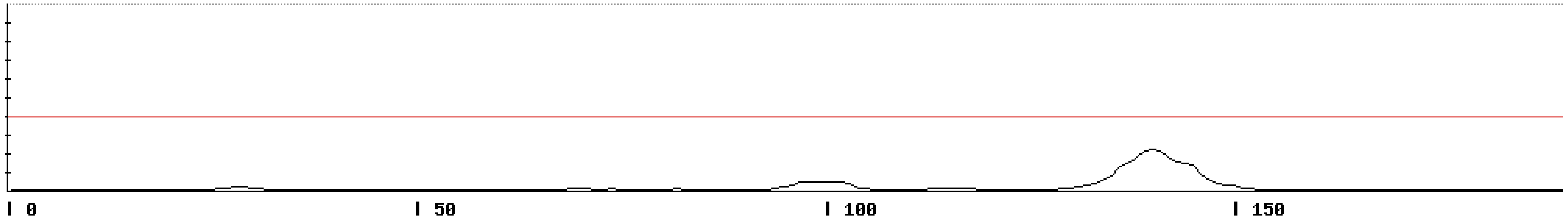

Predictions for Glycinesoja_KHN33774.1

Viterbi Path	
Viterbi Path	

Posterior @ 0.4

Posterior @ 0.4

Posterior Graphic. Threshold Marked @ 0.4

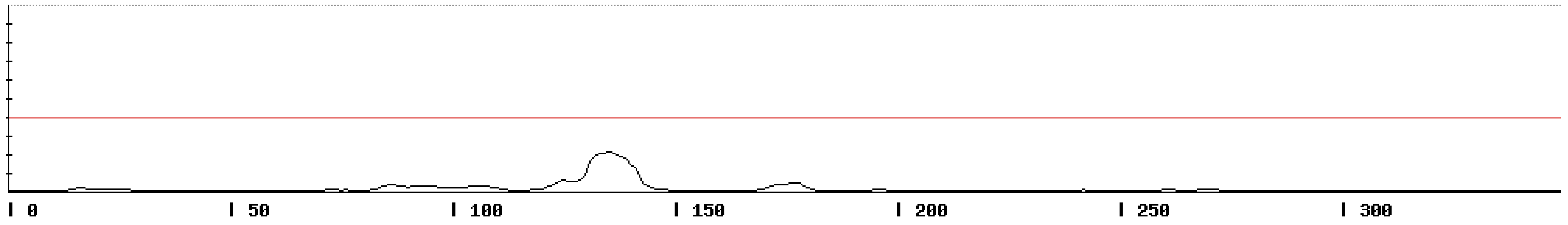

Predictions for Glycinesoja_KHN34075.1

Viterbi Path

Viterbi Path

Posterior @ 0.4

Posterior @ 0.4

Posterior Graphic. Threshold Marked @ 0.4

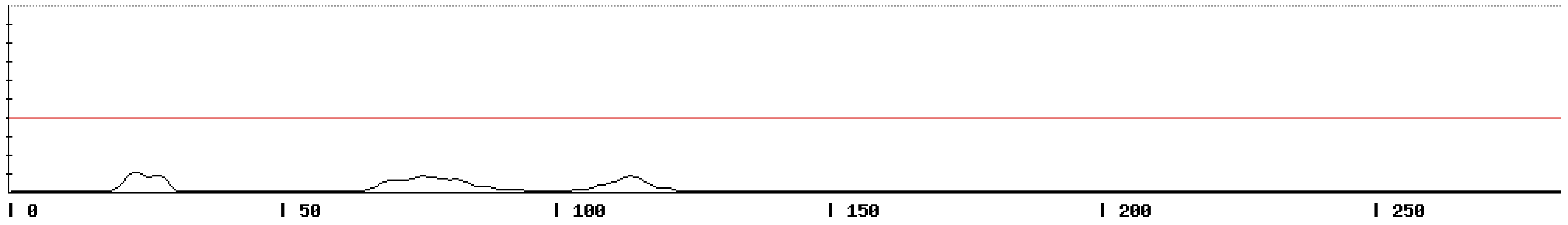

Predictions for Glycinesoja_KHN34085.1

Viterbi Path

Viterbi Path

Posterior @ 0.4

Posterior @ 0.4

Posterior Graphic. Threshold Marked @ 0.4

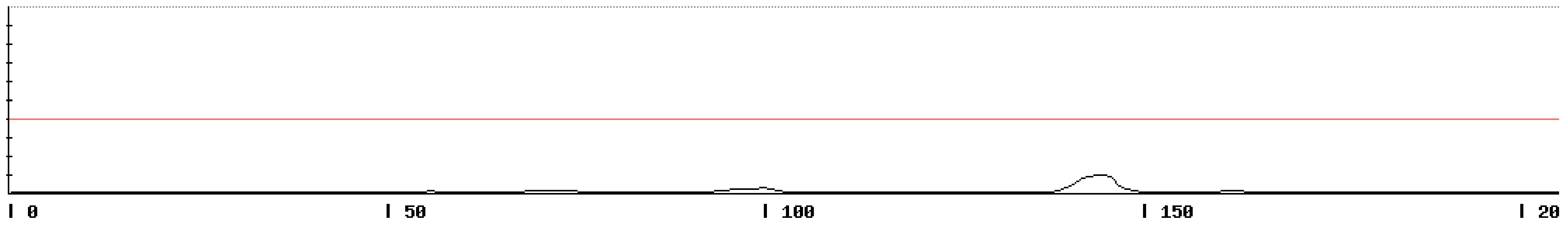

Predictions for Glycinesoja_KHN34302.1

Viterbi Path

Viterbi Path

Posterior @ 0.4

Posterior @ 0.4

Posterior Graphic. Threshold Marked @ 0.4

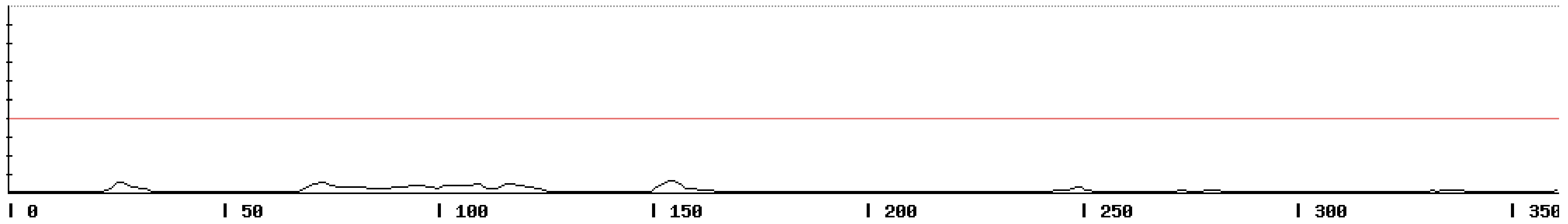

Predictions for Glycinesoja_KHN34454.1	
Viterbi Path	
Viterbi Path	
Posterior @ 0.4	105 - KRRK - 108
Posterior @ 0.4	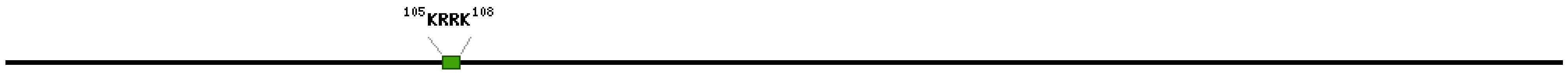
Posterior Graphic. Threshold Marked @ 0.4	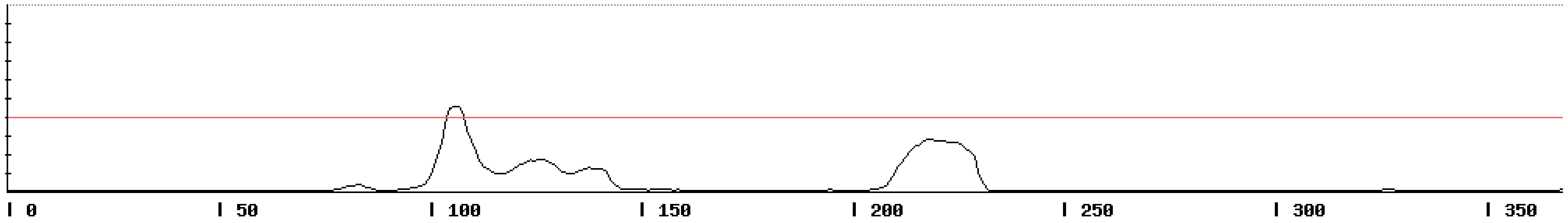

Predictions for Glycinesoja_KHN34958.1	
Viterbi Path	
Viterbi Path	
Posterior @ 0.4	
Posterior @ 0.4	
Posterior Graphic. Threshold Marked @ 0.4	

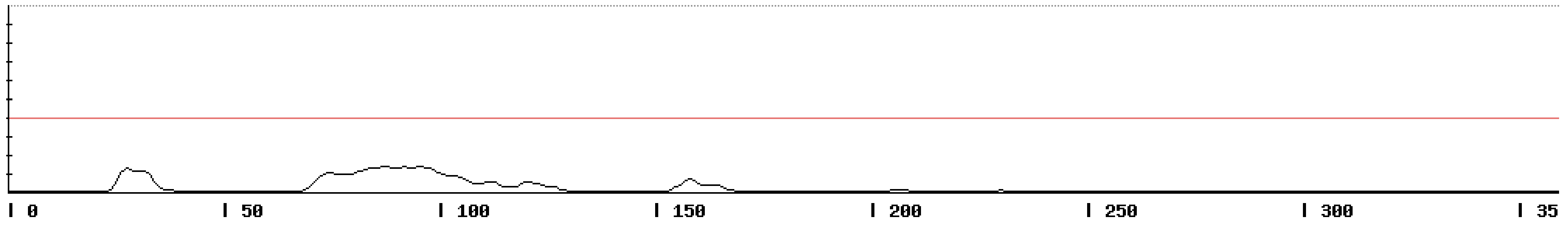

Predictions for Glycinesoja_KHN36047.1

Viterbi Path

Viterbi Path

Posterior @ 0.4

Posterior @ 0.4

Posterior Graphic. Threshold Marked @ 0.4

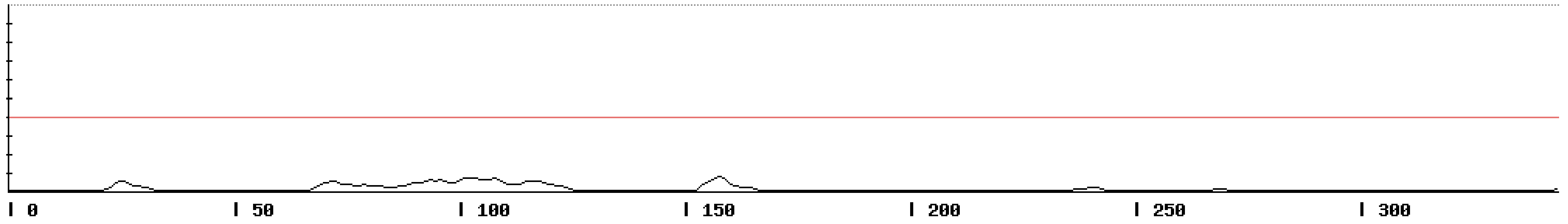

Predictions for Glycinesoja_KHN36348.1

Viterbi Path

Viterbi Path

Posterior @ 0.4

Posterior @ 0.4

Posterior Graphic. Threshold Marked @ 0.4

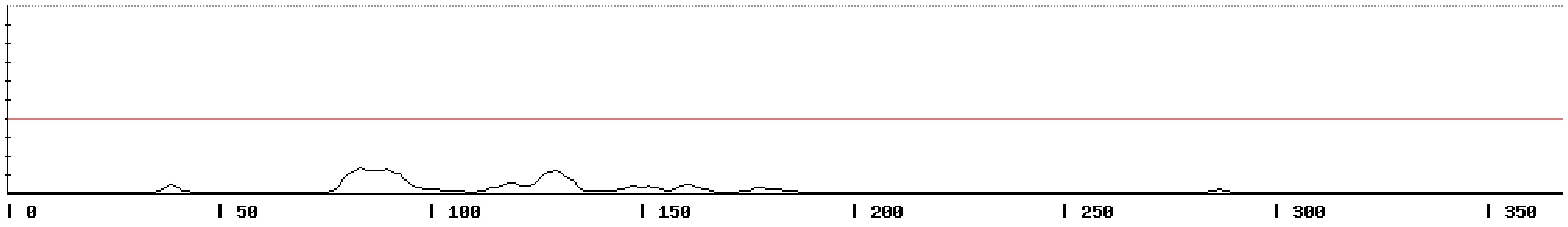

Predictions for Glycinesoja_KHN36474.1

Viterbi Path

Viterbi Path

Posterior @ 0.4

Posterior @ 0.4

Posterior Graphic. Threshold Marked @ 0.4

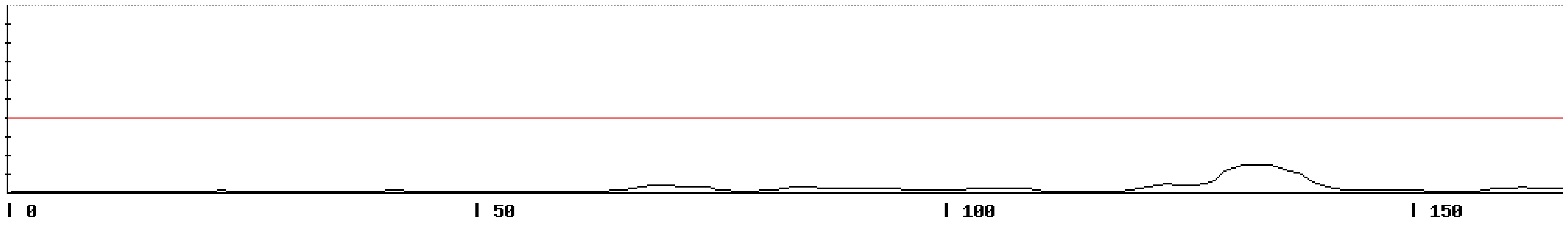

Predictions for Glycinesoja_KHN36489.1

Viterbi Path

Viterbi Path

Posterior @ 0.4

Posterior @ 0.4

Posterior Graphic. Threshold Marked @ 0.4

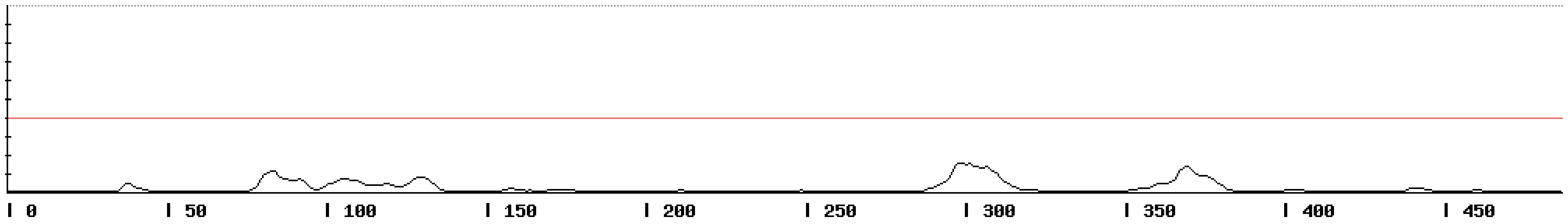

Predictions for Glycinesoja_KHN36667.1

Viterbi Path

Viterbi Path

Posterior @ 0.4 | 126 - RKRRK - 130

Posterior @ 0.4

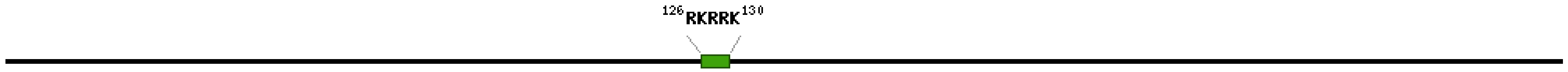

Posterior Graphic. Threshold Marked @ 0.4

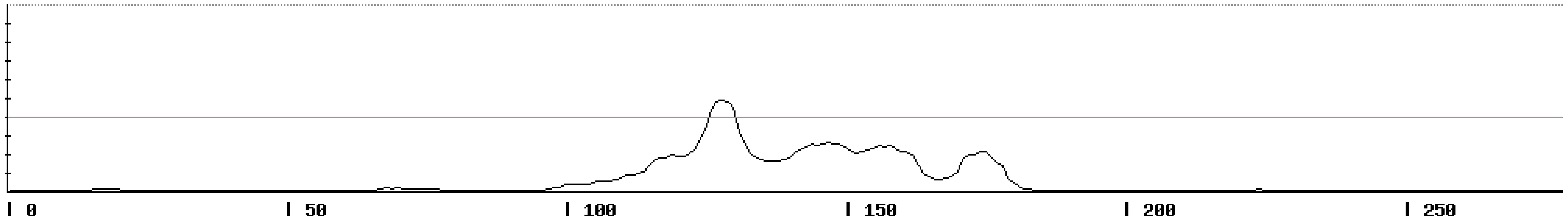

Predictions for Glycinesoja_KHN36793.1

Viterbi Path

Viterbi Path

Posterior @ 0.4	
Posterior @ 0.4	
Posterior Graphic. Threshold Marked @ 0.4	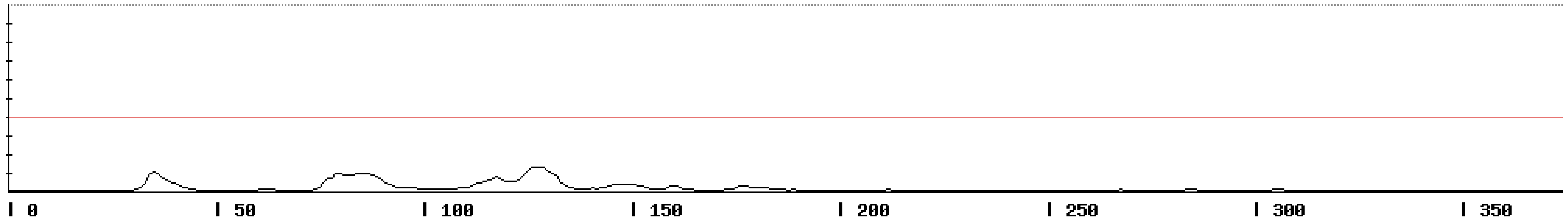

Predictions for Glycinesoja_KHN36794.1

Viterbi Path	
Viterbi Path	
Posterior @ 0.4	
Posterior @ 0.4	
Posterior Graphic. Threshold Marked @ 0.4	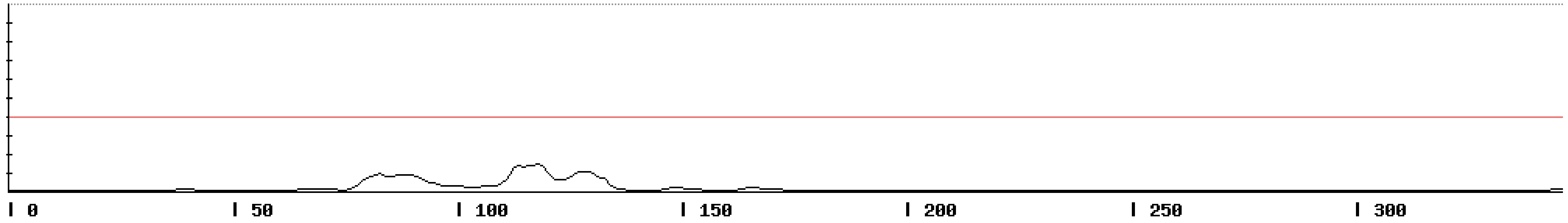

Predictions for Glycinesoja_KHN36848.1

Viterbi Path	
Viterbi Path	

Posterior @ 0.4

Posterior @ 0.4

Posterior Graphic. Threshold Marked @ 0.4

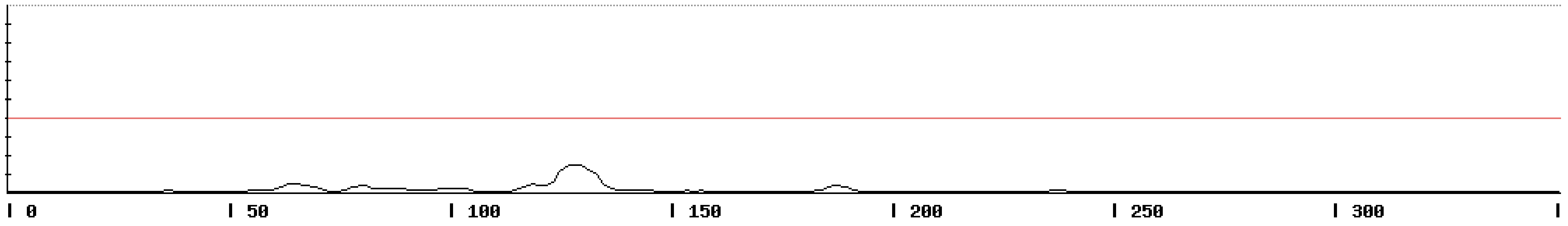

Predictions for Glycinesoja_KHN37822.1

Viterbi Path

Viterbi Path

Posterior @ 0.4

Posterior @ 0.4

Posterior Graphic. Threshold Marked @ 0.4

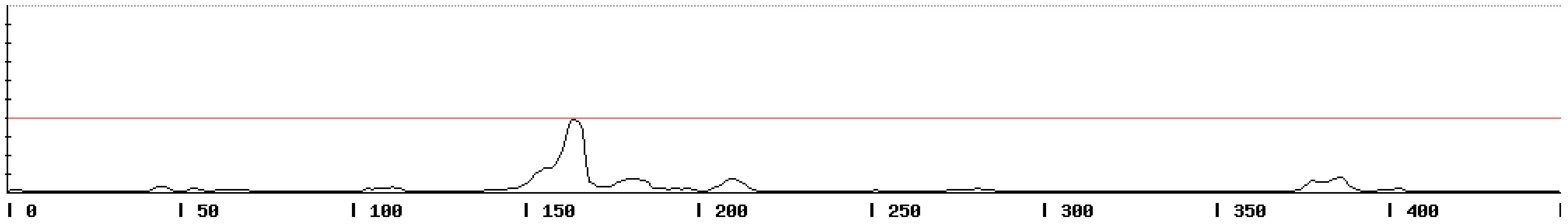

Predictions for Glycinesoja_KHN37882.1

Viterbi Path

Viterbi Path

Posterior @ 0.4

Posterior @ 0.4

Posterior Graphic. Threshold Marked @ 0.4

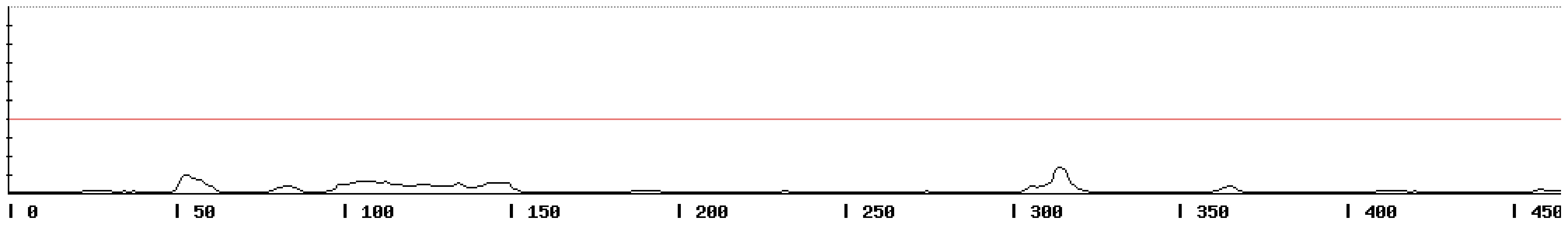

Predictions for Glycinesoja_KHN38340.1

Viterbi Path

Viterbi Path

Posterior @ 0.4

Posterior @ 0.4

Posterior Graphic. Threshold Marked @ 0.4

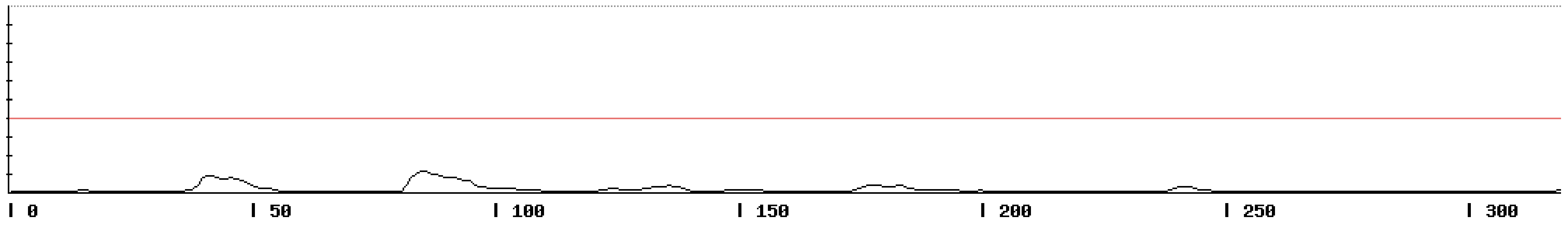

Predictions for Glycinesoja_KHN38619.1	
Viterbi Path	
Viterbi Path	
Posterior @ 0.4	
Posterior @ 0.4	
Posterior Graphic. Threshold Marked @ 0.4	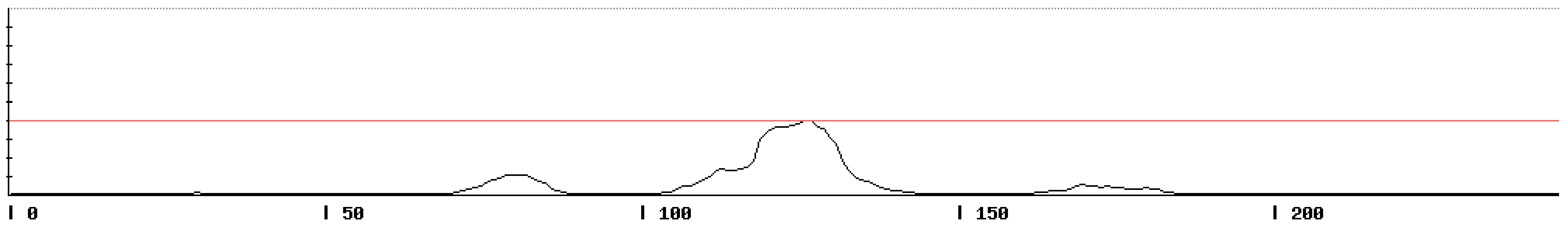

Predictions for Glycinesoja_KHN39199.1	
Viterbi Path	
Viterbi Path	
Posterior @ 0.4	
Posterior @ 0.4	
Posterior Graphic. Threshold Marked @ 0.4	

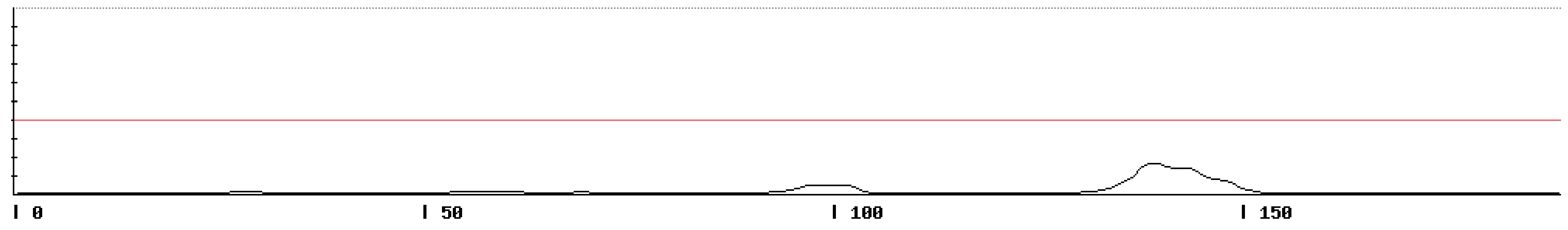

Predictions for Glycinesoja_KHN39501.1

Viterbi Path	
Viterbi Path	
Posterior @ 0.4	
Posterior @ 0.4	
Posterior Graphic. Threshold Marked @ 0.4	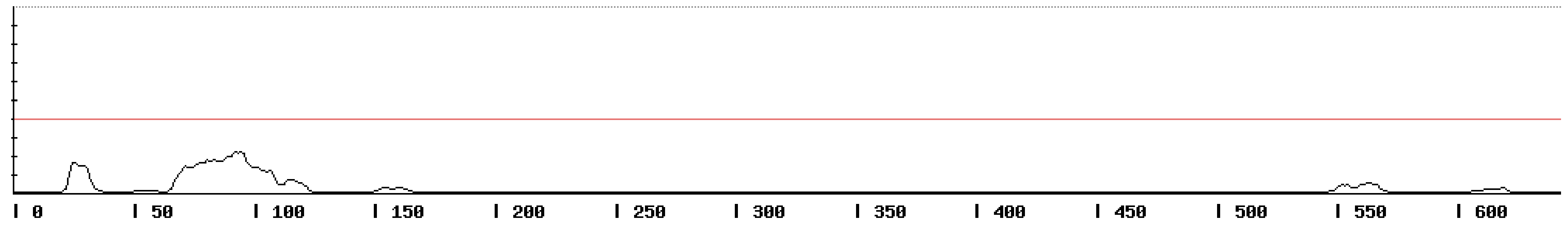

Predictions for Glycinesoja_KHN40025.1

Viterbi Path	
Viterbi Path	
Posterior @ 0.4	
Posterior @ 0.4	

Posterior Graphic. Threshold Marked @ 0.4

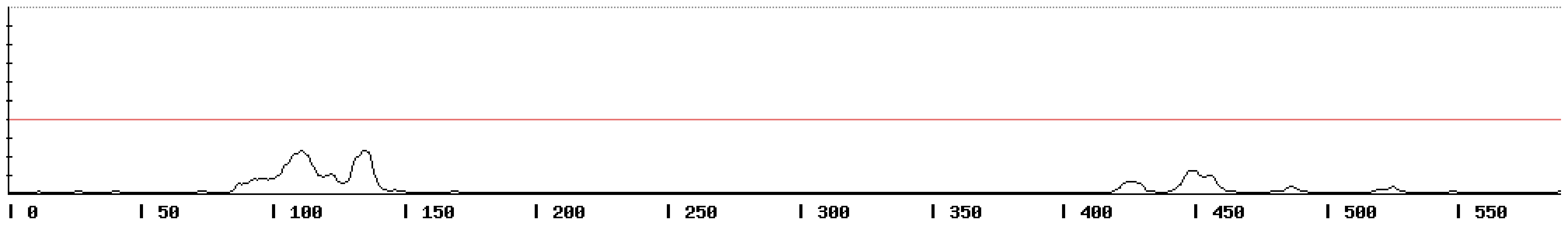

Predictions for Glycinesoja_KHN40175.1

Viterbi Path

Viterbi Path

Posterior @ 0.4

Posterior @ 0.4

Posterior Graphic. Threshold Marked @ 0.4

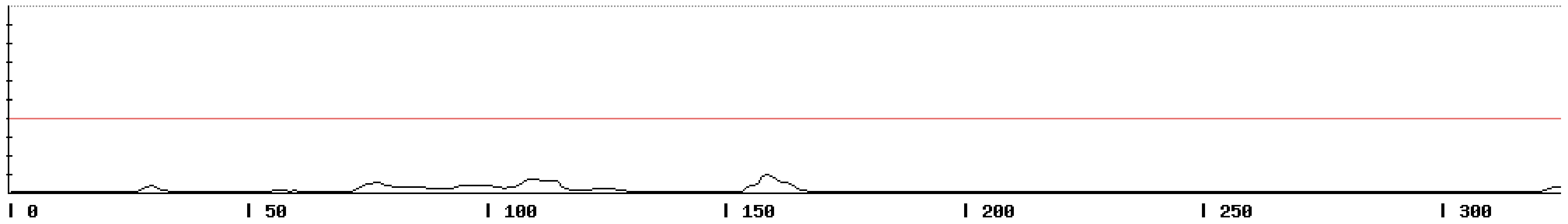

Predictions for Glycinesoja_KHN40210.1

Viterbi Path

Viterbi Path

Posterior @ 0.4

Posterior @ 0.4

Posterior Graphic. Threshold Marked @ 0.4

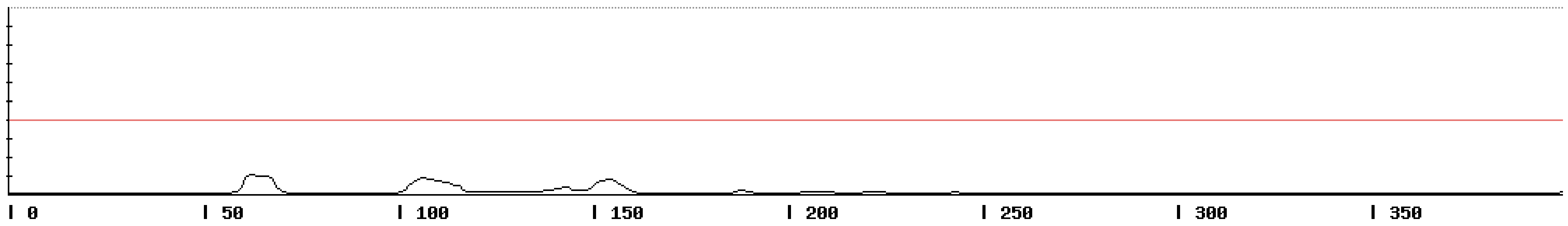

Predictions for Glycinesoja_KHN40359.1

Viterbi Path

Viterbi Path

Posterior @ 0.4

Posterior @ 0.4

Posterior Graphic. Threshold Marked @ 0.4

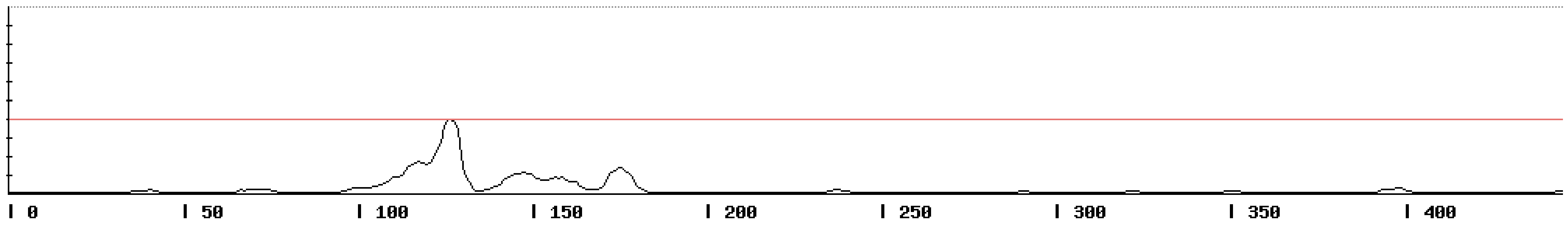

Predictions for Glycinesoja_KHN41028.1

Viterbi Path

Viterbi Path

Posterior @ 0.4

Posterior @ 0.4

Posterior Graphic. Threshold Marked @ 0.4

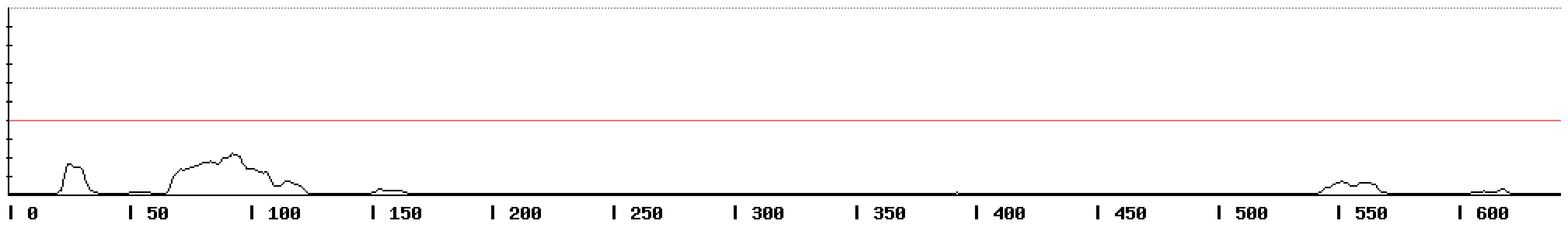

Predictions for Glycinesoja_KHN41910.1

Viterbi Path

Viterbi Path

Posterior @ 0.4

Posterior @ 0.4

Posterior Graphic. Threshold Marked @ 0.4

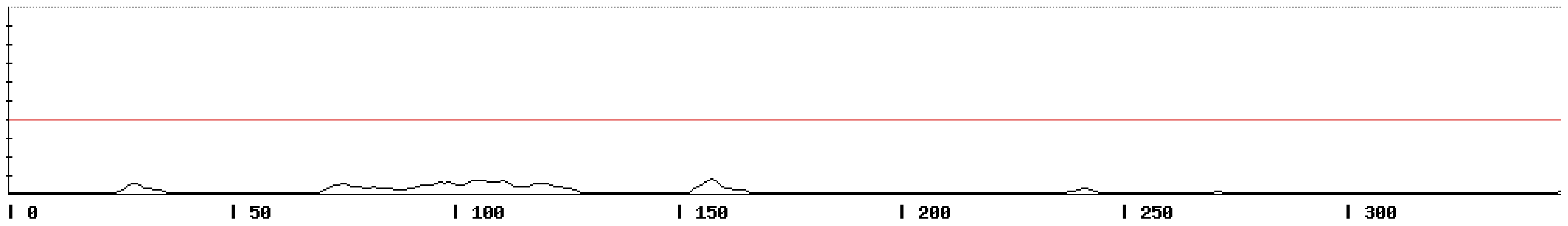

Predictions for Glycinesoja_KHN42768.1

Viterbi Path

Viterbi Path

Posterior @ 0.4

Posterior @ 0.4

Posterior Graphic. Threshold Marked @ 0.4

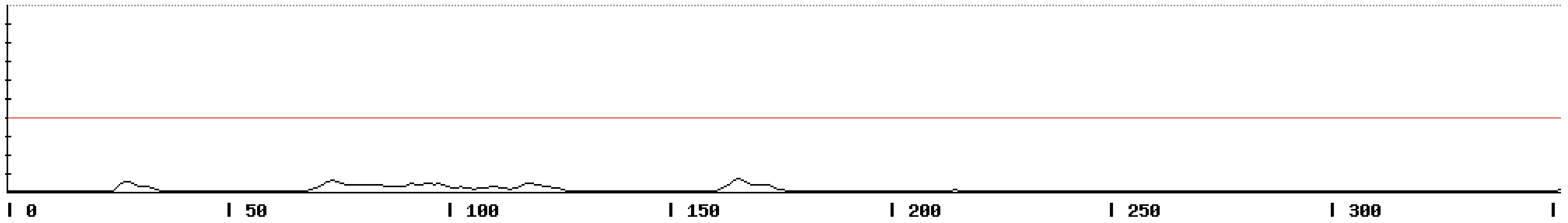

Predictions for Glycinesoja_KHN44912.1

Viterbi Path

Viterbi Path

Posterior @ 0.4

41 - KPVGKPKPVGIKK - 53

Posterior @ 0.4

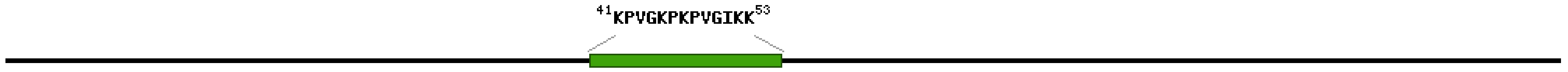

Posterior Graphic. Threshold Marked @ 0.4

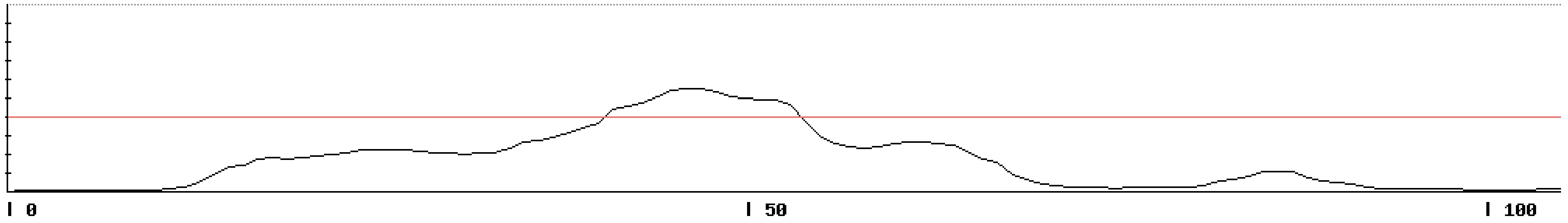

Predictions for Glycinesoja_KHN45185.1

Viterbi Path

Viterbi Path

Posterior @ 0.4

Posterior @ 0.4

Posterior Graphic. Threshold Marked @ 0.4

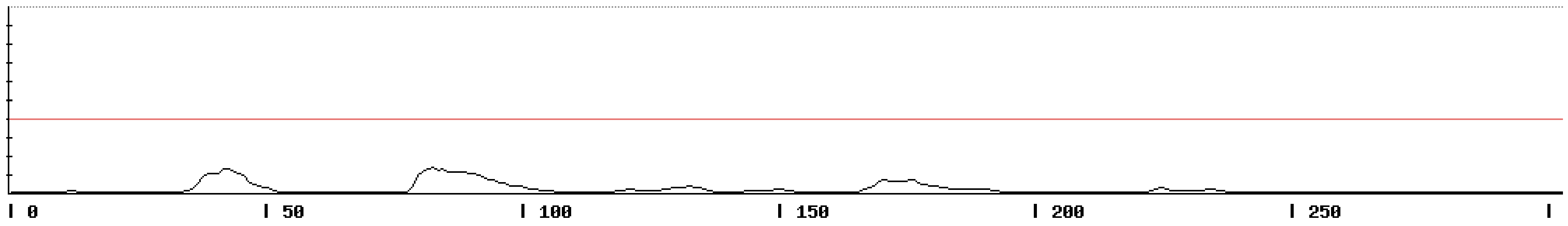

Predictions for Glycinesoja_KHN45496.1

Viterbi Path

Viterbi Path

Posterior @ 0.4

Posterior @ 0.4

Posterior Graphic. Threshold Marked @ 0.4

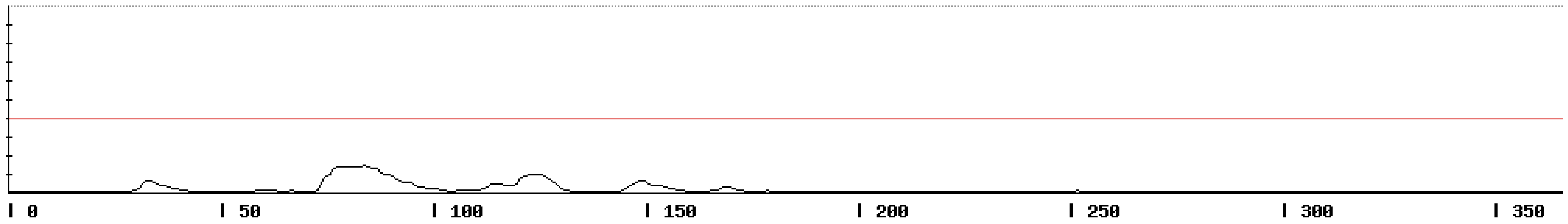

Predictions for Glycinesoja_KHN45516.1

Viterbi Path	
Viterbi Path	
Posterior @ 0.4	
Posterior @ 0.4	
Posterior Graphic. Threshold Marked @ 0.4	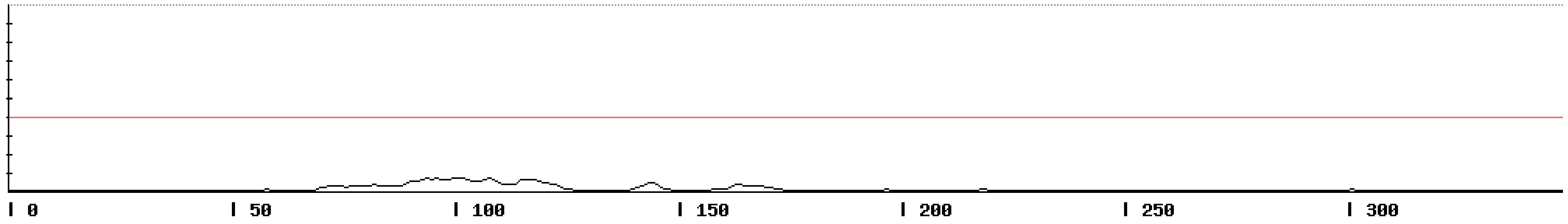

Predictions for Glycinesoja_KHN45836.1

Viterbi Path	
Viterbi Path	
Posterior @ 0.4	
Posterior @ 0.4	
Posterior Graphic. Threshold Marked @ 0.4	

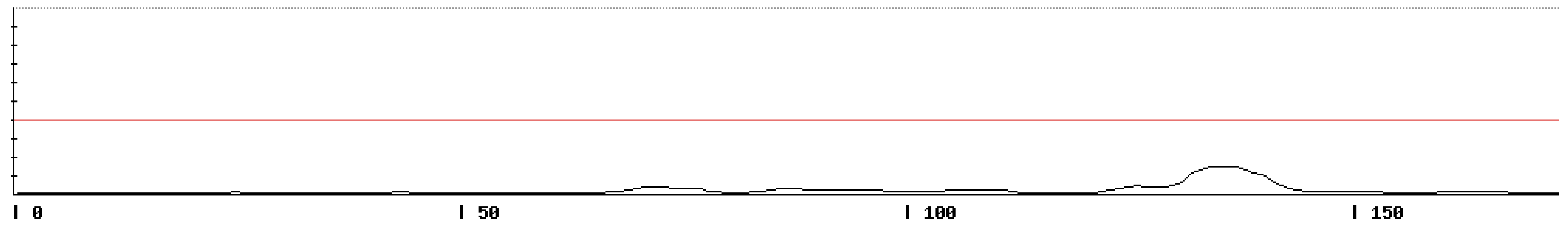

Predictions for Glycinesoja_KHN45976.1

Viterbi Path

Viterbi Path

Posterior @ 0.4

Posterior @ 0.4

Posterior Graphic. Threshold Marked @ 0.4

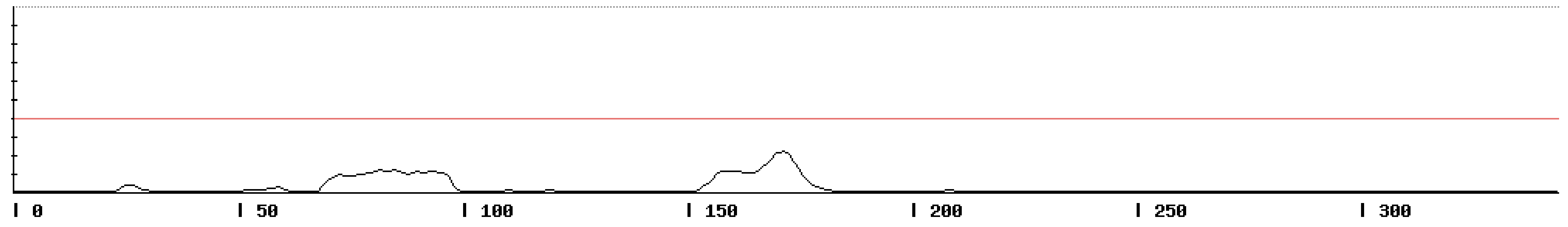

Predictions for Glycinesoja_KHN46203.1

Viterbi Path

Viterbi Path

Posterior @ 0.4

Posterior @ 0.4

Posterior Graphic. Threshold Marked @ 0.4

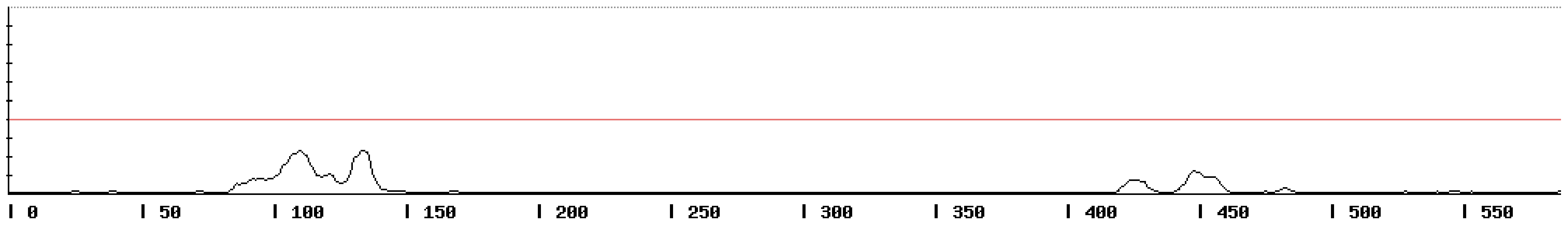

Predictions for Glycinesoja_KHN46796.1

Viterbi Path

Viterbi Path

Posterior @ 0.4

Posterior @ 0.4

Posterior Graphic. Threshold Marked @ 0.4

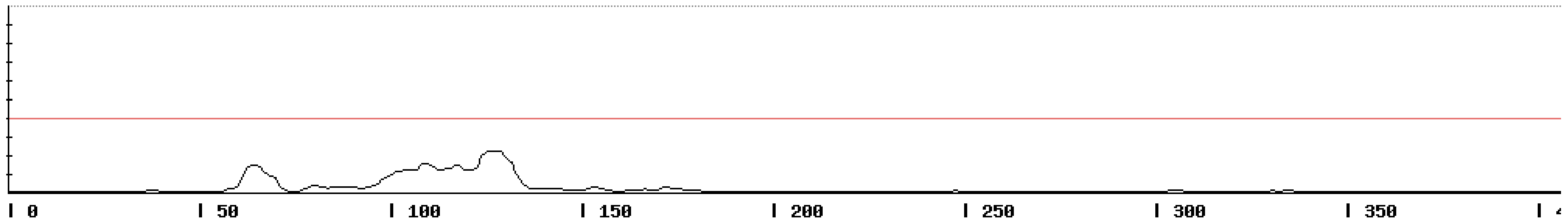

Predictions for Glycinesoja_KHN46799.1

Viterbi Path

Viterbi Path

Posterior @ 0.4

Posterior @ 0.4

Posterior Graphic. Threshold Marked @ 0.4

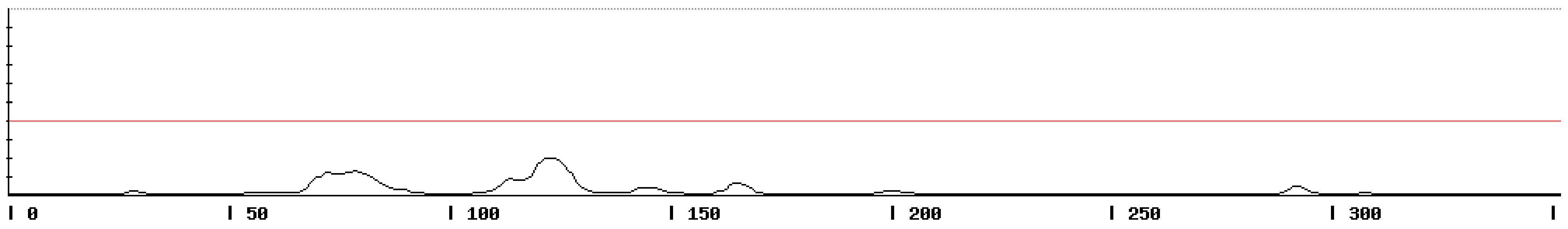

Predictions for Glycinesoja_KHN47015.1

Viterbi Path

Viterbi Path

Posterior @ 0.4

Posterior @ 0.4

Posterior Graphic. Threshold Marked @ 0.4

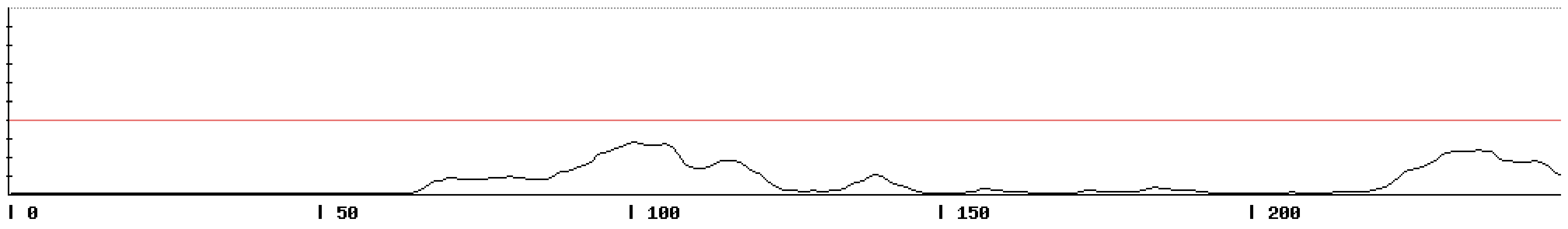

Predictions for Glycinesoja_KHN47410.1

Viterbi Path

Viterbi Path

Posterior @ 0.4

Posterior @ 0.4

Posterior Graphic. Threshold Marked @ 0.4

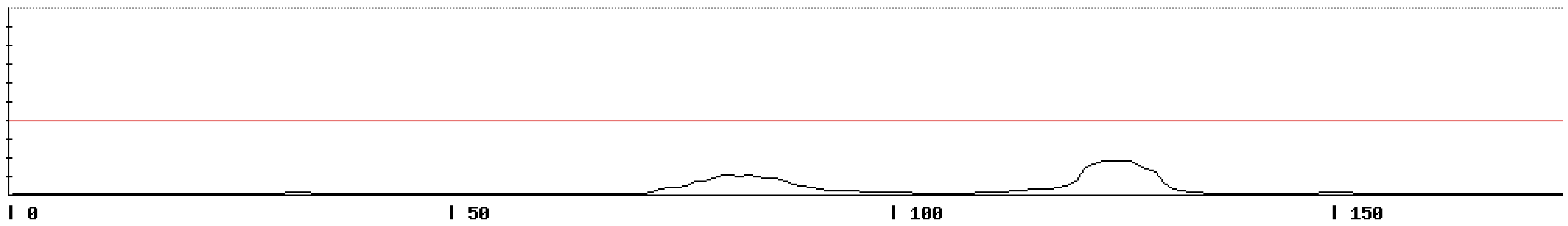

Predictions for Glycinesoja_KHN47549.1

Viterbi Path

Viterbi Path

Posterior @ 0.4 — 127 - RKRRK - 131

Posterior @ 0.4

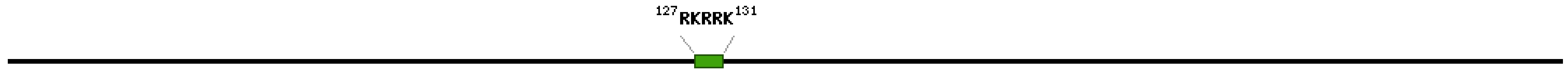

Posterior Graphic. Threshold Marked @ 0.4

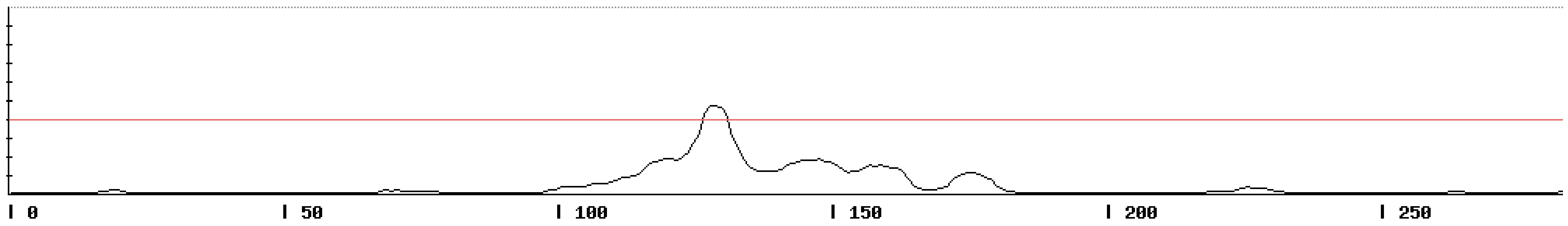

Predictions for Glycinesoja_KHN48695.1

Viterbi Path

Viterbi Path

Posterior @ 0.4

Posterior @ 0.4

Posterior Graphic. Threshold Marked @ 0.4

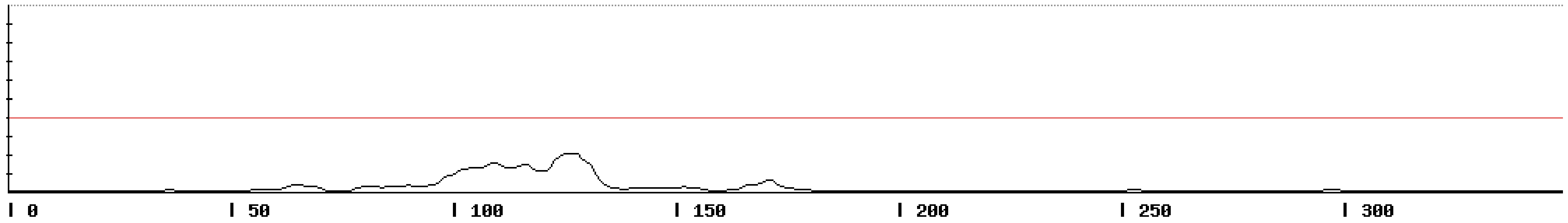

Predictions for Glycinesoja_KHN48759.1

Viterbi Path

Viterbi Path

Posterior @ 0.4

Posterior @ 0.4

Posterior Graphic. Threshold Marked @ 0.4

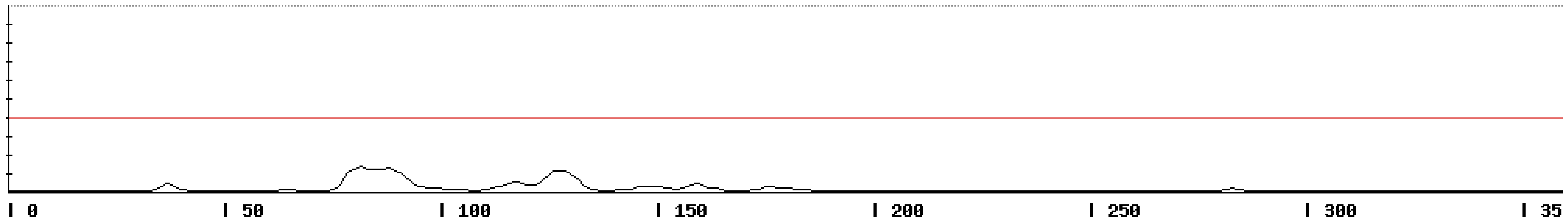

Predictions for Glycinesoja_KHN48762.1

Viterbi Path

Viterbi Path

Posterior @ 0.4

Posterior @ 0.4

Posterior Graphic. Threshold Marked @ 0.4

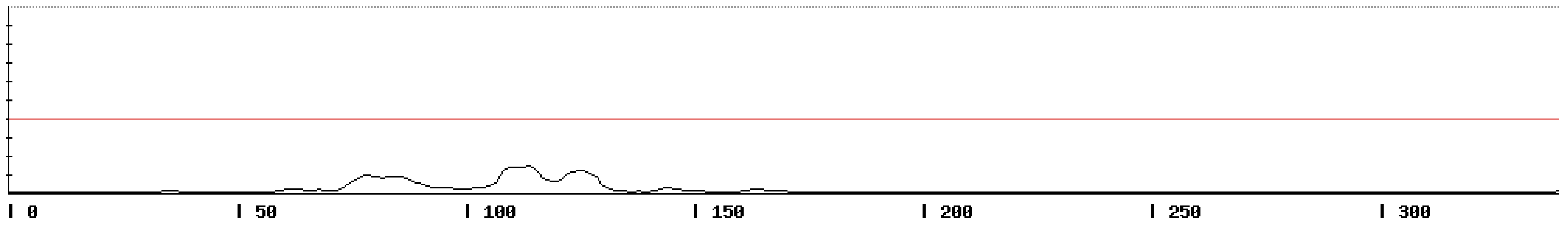

Predictions for Glycinesoja_KHN48855.1

Viterbi Path

Viterbi Path

Posterior @ 0.4

Posterior @ 0.4

Revision r.9 - Last Updated on : February 24 2015 17:45:20.

alex.nguyenba@utoronto.ca

Intended to be used with Firefox

Viterbi Path

Posterior @ 0.4 — 543 - RGRGEAQRGSRRGGNRGKIIMLPRG - 567

Posterior @ 0.4

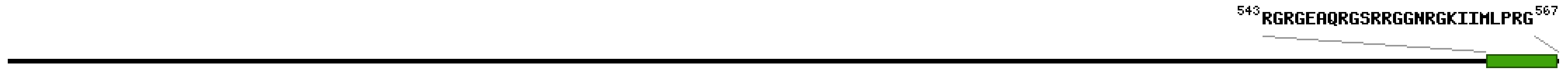

Posterior Graphic. Threshold Marked @ 0.4

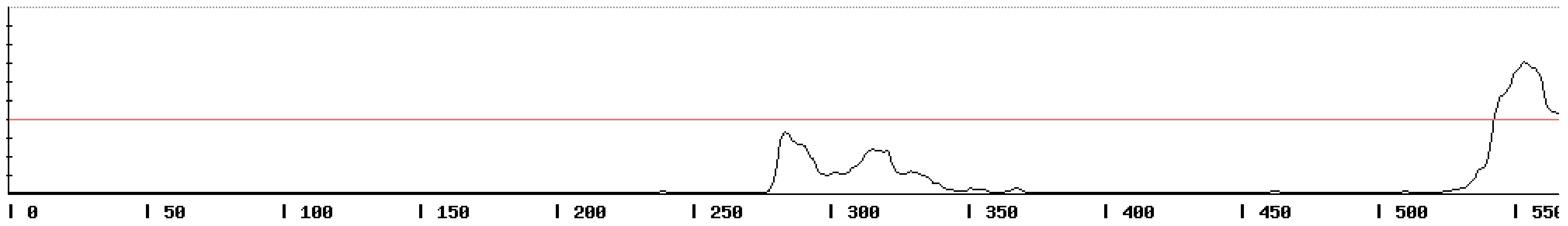

Predictions for Gossypiumarboreum_Cotton_A_00256_BGI-A2_v1.0

Viterbi Path

Viterbi Path

Posterior @ 0.4 — 74 - RKHGKGGRPNR - 84

221 - KKQK - 224

Posterior @ 0.4

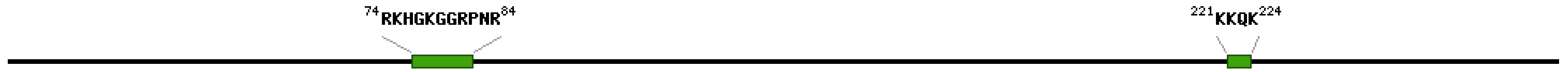

Posterior Graphic. Threshold Marked @ 0.4

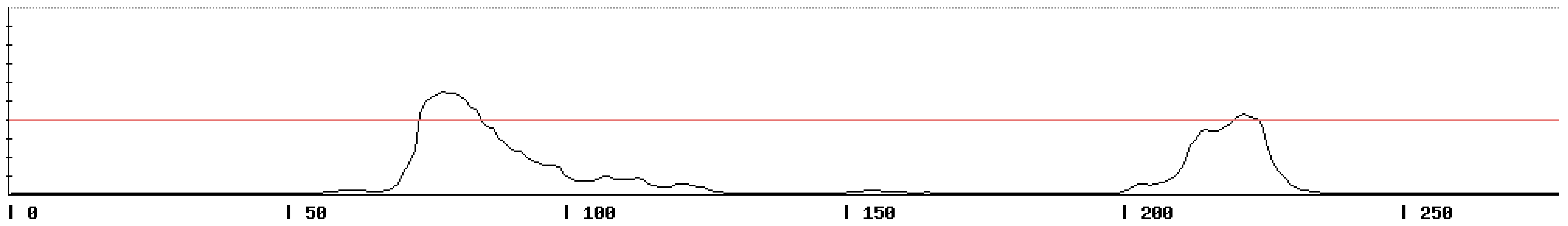

Predictions for Gossypiumarboreum_Cotton_A_00602_BGI-A2_v1.0

Viterbi Path

Viterbi Path

Posterior @ 0.4

Posterior @ 0.4

Posterior Graphic. Threshold Marked @ 0.4

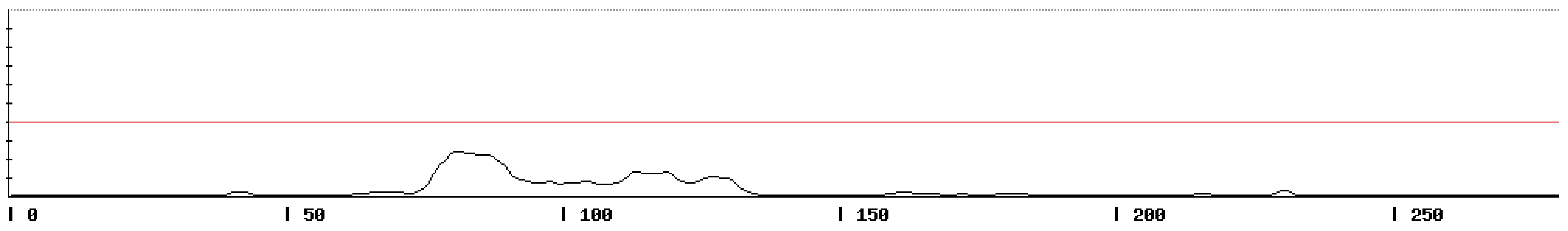

Predictions for Gossypiumarboreum_Cotton_A_00831_BGI-A2_v1.0

Viterbi Path

Viterbi Path

Posterior @ 0.4

Posterior @ 0.4

Posterior Graphic. Threshold Marked @ 0.4

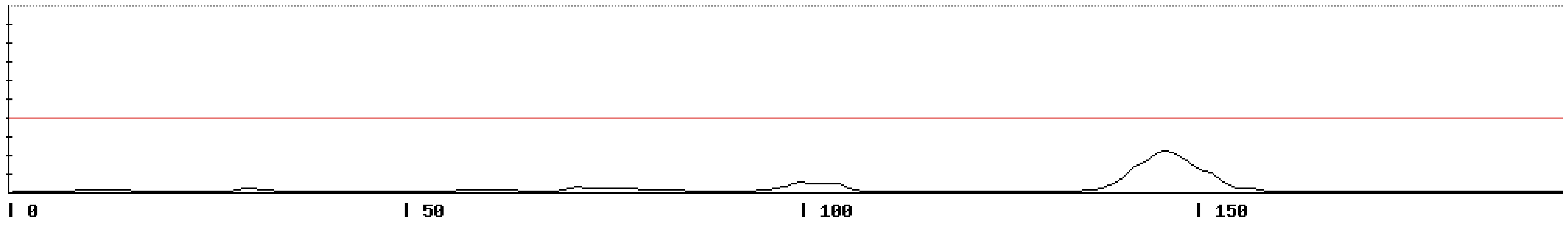

Predictions for Gossypiumarboreum_Cotton_A_00841_BGI-A2_v1.0

Viterbi Path	
Viterbi Path	
Posterior @ 0.4	21 - LKKKSKSR - 28<br>115 - GEIKKNKKRK - 124
Posterior @ 0.4	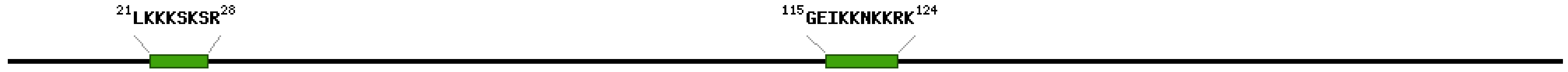
Posterior Graphic. Threshold Marked @ 0.4	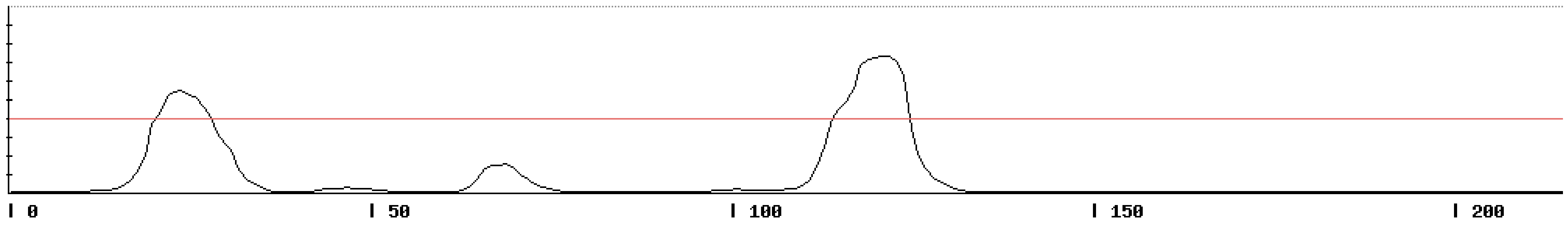

Predictions for Gossypiumarboreum_Cotton_A_01505_BGI-A2_v1.0

Viterbi Path	
Viterbi Path	
Posterior @ 0.4	
Posterior @ 0.4	

Posterior Graphic. Threshold Marked @ 0.4	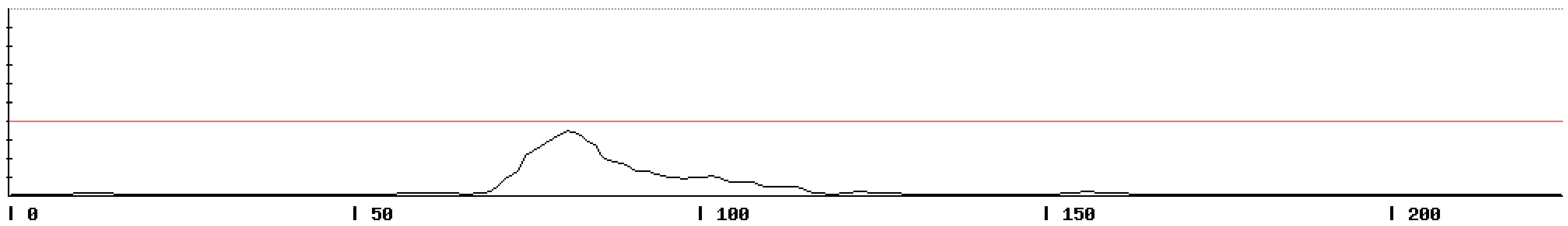

Predictions for Gossypiumarboreum_Cotton_A_01569_BGI-A2_v1.0

Viterbi Path	
Viterbi Path	
Posterior @ 0.4	
Posterior @ 0.4	
Posterior Graphic. Threshold Marked @ 0.4	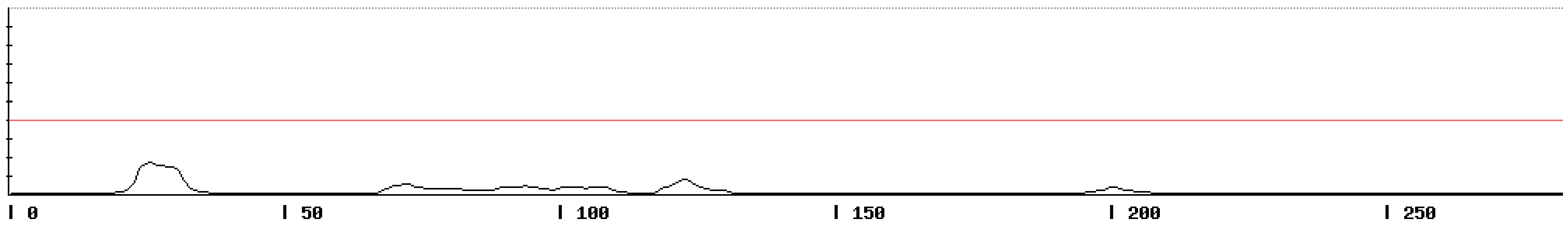

Predictions for Gossypiumarboreum_Cotton_A_01813_BGI-A2_v1.0

Viterbi Path	
Viterbi Path	

Posterior @ 0.4

Posterior @ 0.4

Posterior Graphic. Threshold Marked @ 0.4

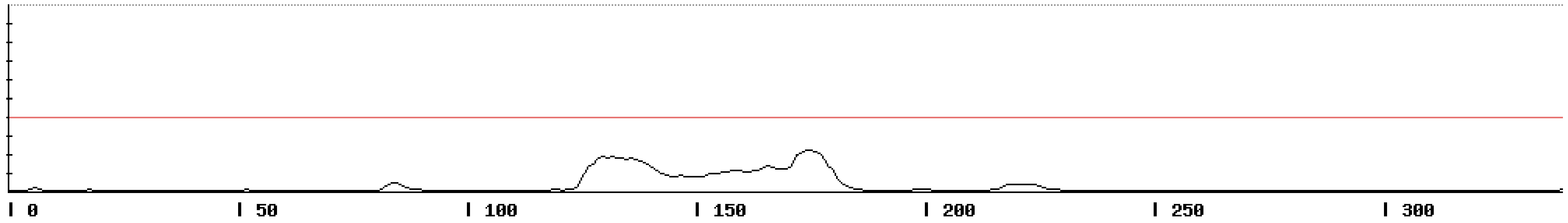

Predictions for Gossypiumarboreum_Cotton_A_01943_BGI-A2_v1.0

Viterbi Path

Viterbi Path

Posterior @ 0.4

Posterior @ 0.4

Posterior Graphic. Threshold Marked @ 0.4

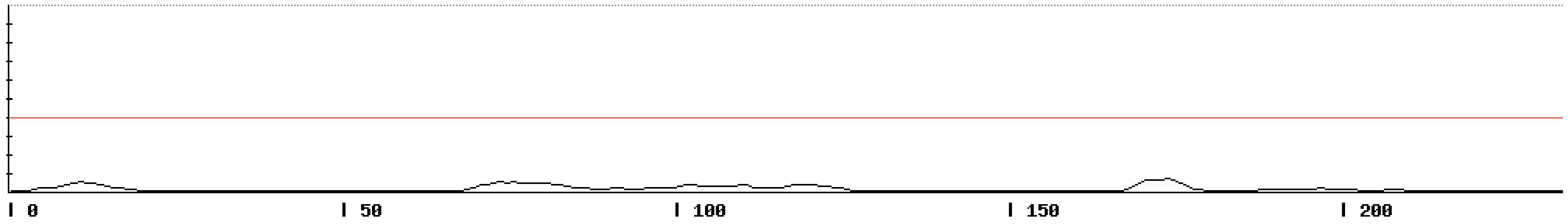

Predictions for Gossypiumarboreum_Cotton_A_01989_BGI-A2_v1.0

Viterbi Path

Viterbi Path

Posterior @ 0.4	
Posterior @ 0.4	
Posterior Graphic. Threshold Marked @ 0.4	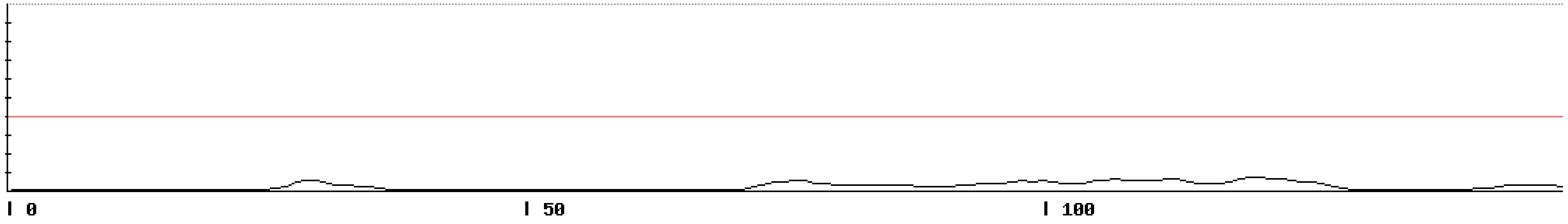

Predictions for Gossypiumarboreum_Cotton_A_02191_BGI-A2_v1.0

Viterbi Path	
Viterbi Path	
Posterior @ 0.4	
Posterior @ 0.4	
Posterior Graphic. Threshold Marked @ 0.4	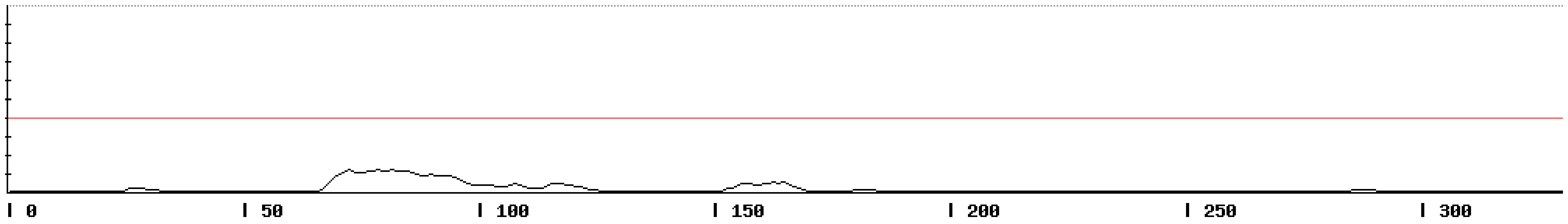

Predictions for Gossypiumarboreum_Cotton_A_02365_BGI-A2_v1.0

Viterbi Path

Viterbi Path

Posterior @ 0.4

Posterior @ 0.4

Posterior Graphic. Threshold Marked @ 0.4

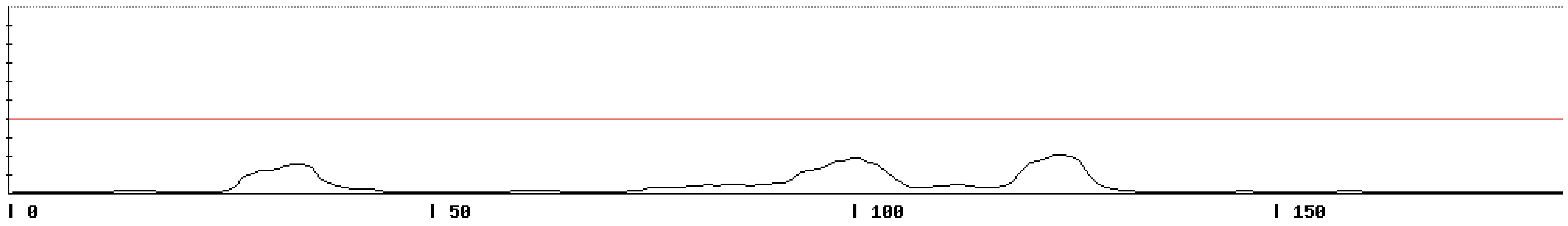

Predictions for Gossypiumarboreum_Cotton_A_02366_BGI-A2_v1.0

Viterbi Path

Viterbi Path

Posterior @ 0.4

Posterior @ 0.4

Posterior Graphic. Threshold Marked @ 0.4

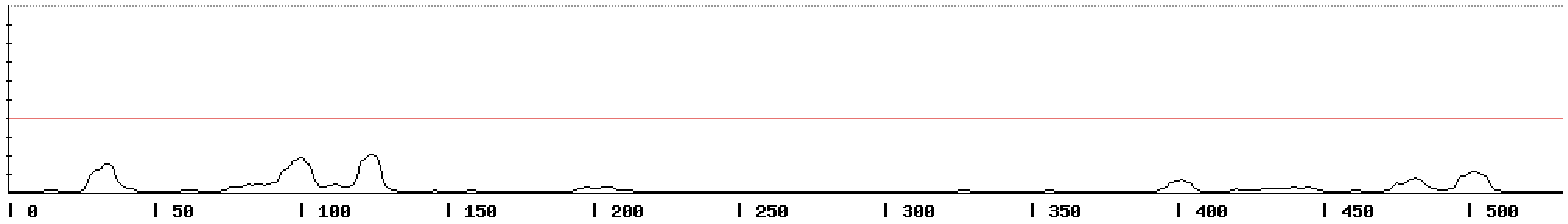

Predictions for Gossypiumarboreum_Cotton_A_02718_BGI-A2_v1.0

Viterbi Path

Viterbi Path

Posterior @ 0.4

Posterior @ 0.4

Posterior Graphic. Threshold Marked @ 0.4

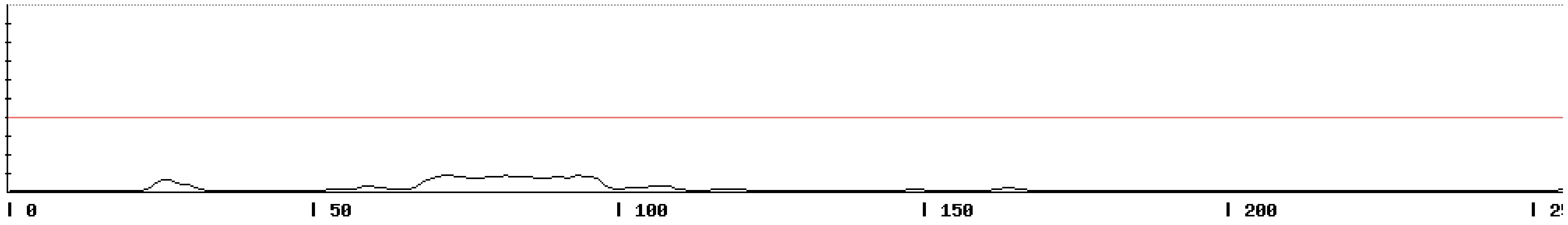

Predictions for Gossypiumarboreum_Cotton_A_03013_BGI-A2_v1.0

Viterbi Path

Viterbi Path

Posterior @ 0.4

Posterior @ 0.4

Posterior Graphic. Threshold Marked @ 0.4

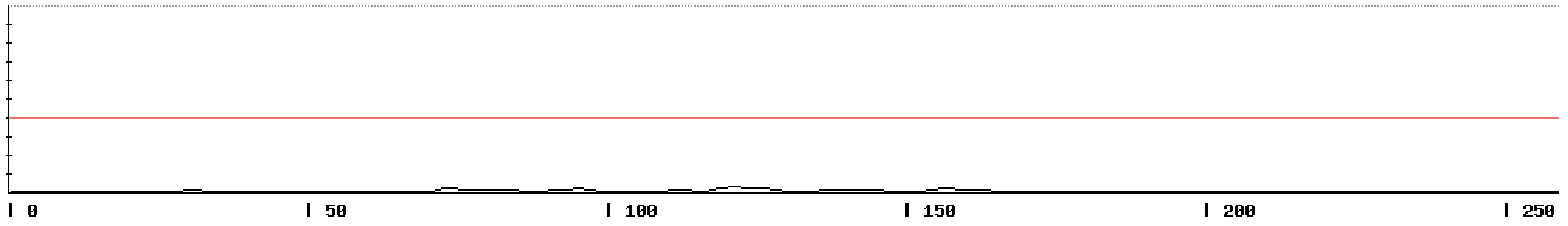

Predictions for Gossypiumarboreum_Cotton_A_03573_BGI-A2_v1.0

Viterbi Path

Viterbi Path

Posterior @ 0.4

Posterior @ 0.4

Posterior Graphic. Threshold Marked @ 0.4

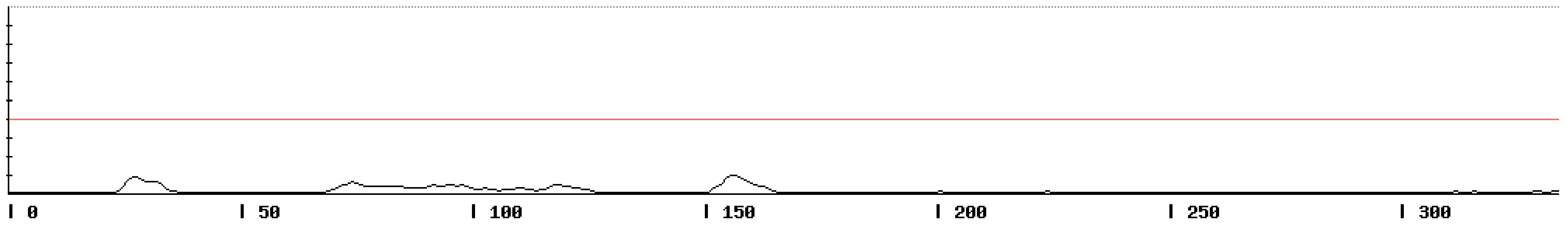

Predictions for Gossypiumarboreum_Cotton_A_03930_BGI-A2_v1.0

Viterbi Path

Viterbi Path

Posterior @ 0.4

Posterior @ 0.4

Posterior Graphic. Threshold Marked @ 0.4

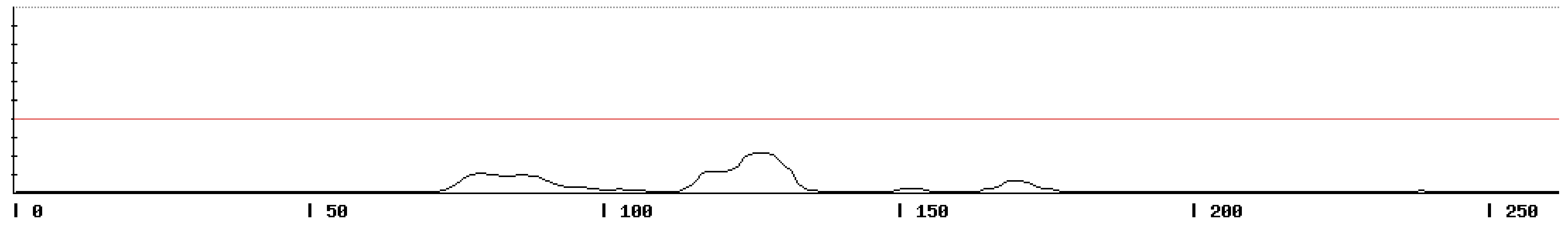

Predictions for Gossypiumarboreum_Cotton_A_04401_BGI-A2_v1.0

Viterbi Path

Viterbi Path

Posterior @ 0.4

Posterior @ 0.4

Posterior Graphic. Threshold Marked @ 0.4

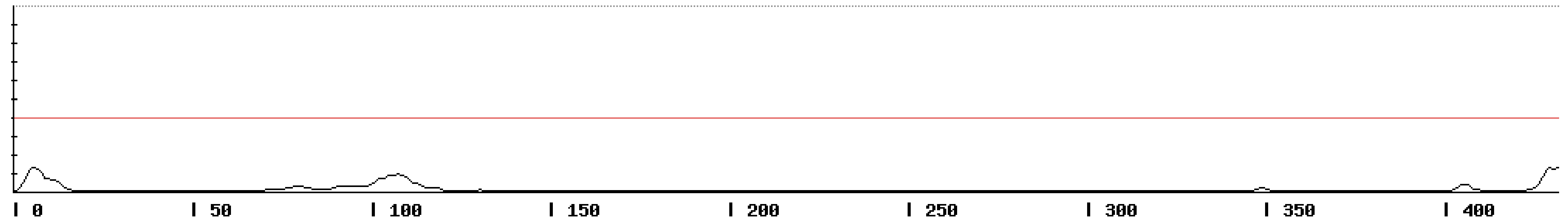

Predictions for Gossypiumarboreum_Cotton_A_04422_BGI-A2_v1.0

Viterbi Path

Viterbi Path

Posterior @ 0.4

70 - KKKSK - 74

163 - KR - 164

Posterior @ 0.4

	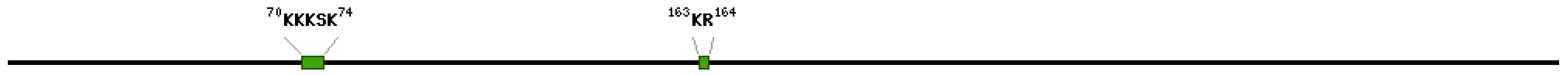
Posterior Graphic. Threshold Marked @ 0.4	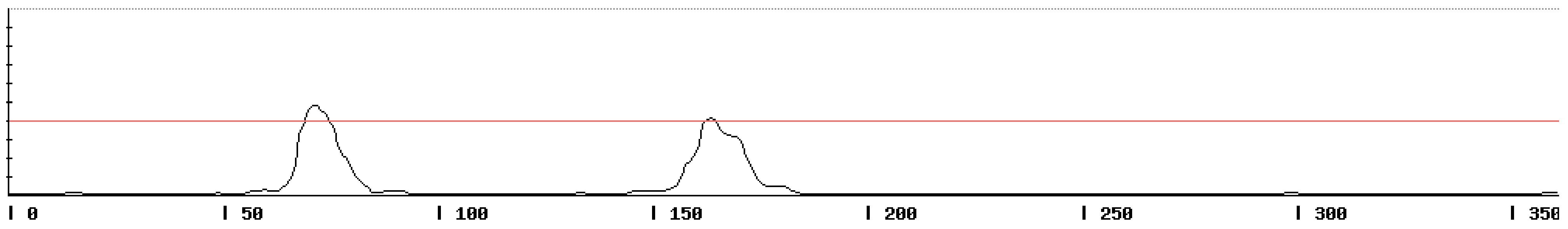

Predictions for Gossypiumarboreum_Cotton_A_04481_BGI-A2_v1.0

Viterbi Path	
Viterbi Path	
Posterior @ 0.4	137 - RKRRK - 141
Posterior @ 0.4	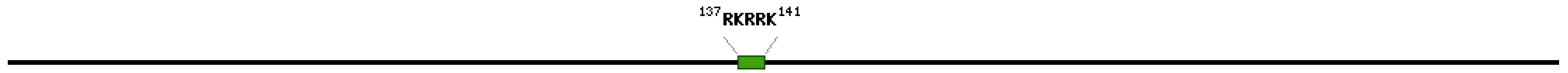
Posterior Graphic. Threshold Marked @ 0.4	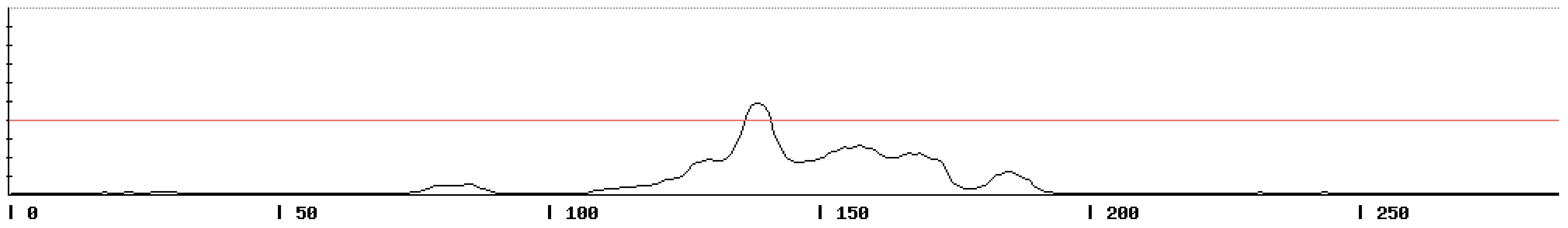

Predictions for Gossypiumarboreum_Cotton_A_04509_BGI-A2_v1.0

Viterbi Path	
Viterbi Path	

Posterior @ 0.4

Posterior @ 0.4

Posterior Graphic. Threshold Marked @ 0.4

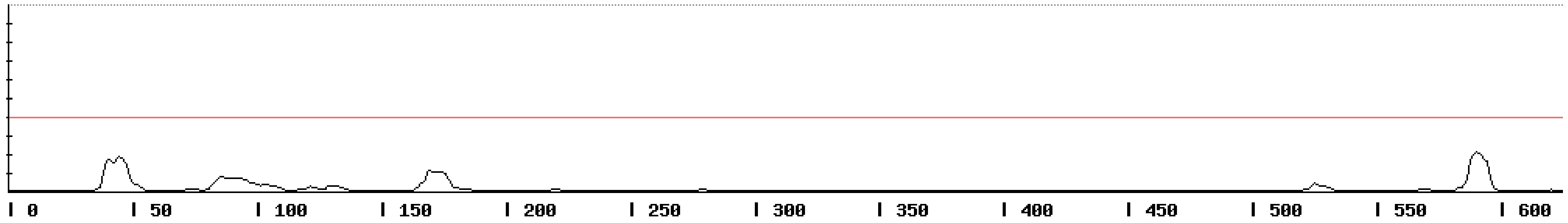

Predictions for Gossypiumarboreum_Cotton_A_04612_BGI-A2_v1.0

Viterbi Path

Viterbi Path

Posterior @ 0.4

Posterior @ 0.4

Posterior Graphic. Threshold Marked @ 0.4

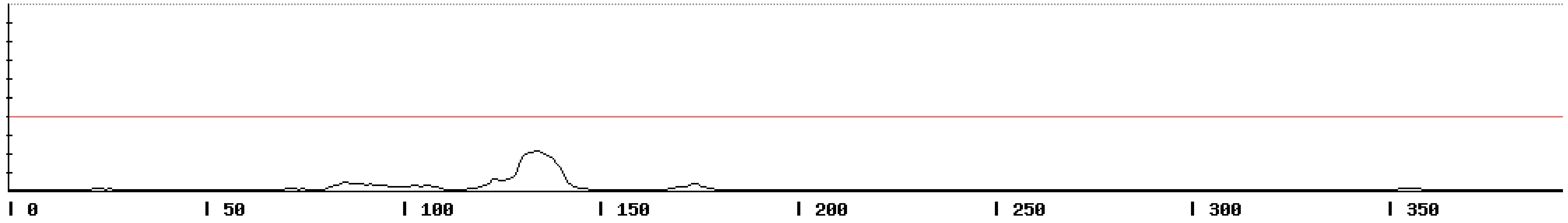

Predictions for Gossypiumarboreum_Cotton_A_04939_BGI-A2_v1.0

Viterbi Path

Viterbi Path

Posterior @ 0.4	77 - KLKKKGKGKN - 86
Posterior @ 0.4	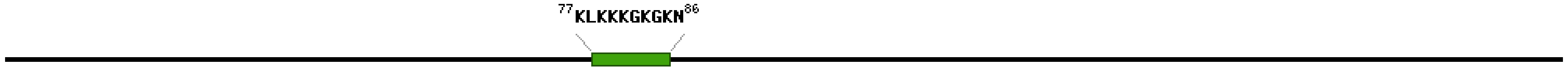
Posterior Graphic. Threshold Marked @ 0.4	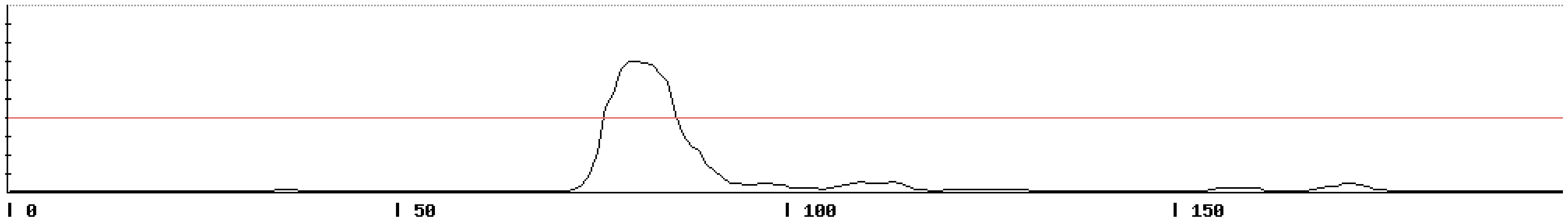

Predictions for Gossypiumarboreum_Cotton_A_05099_BGI-A2_v1.0

Viterbi Path	
Viterbi Path	
Posterior @ 0.4	
Posterior @ 0.4	
Posterior Graphic. Threshold Marked @ 0.4	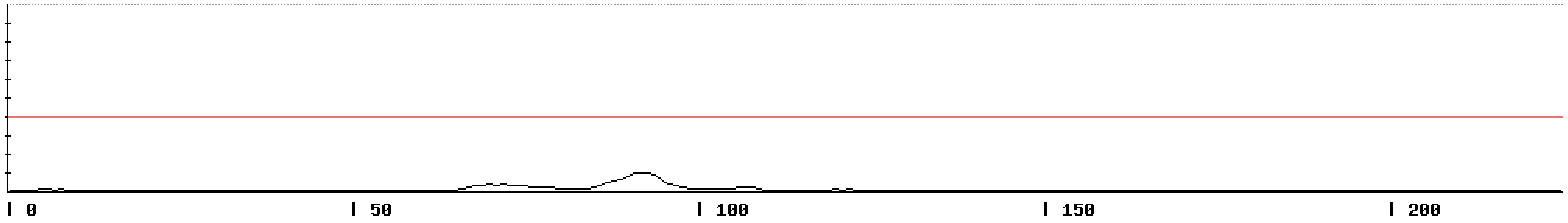

Predictions for Gossypiumarboreum_Cotton_A_05272_BGI-A2_v1.0

Viterbi Path	
Viterbi Path	
Posterior @ 0.4	
Posterior @ 0.4	
Posterior Graphic. Threshold Marked @ 0.4	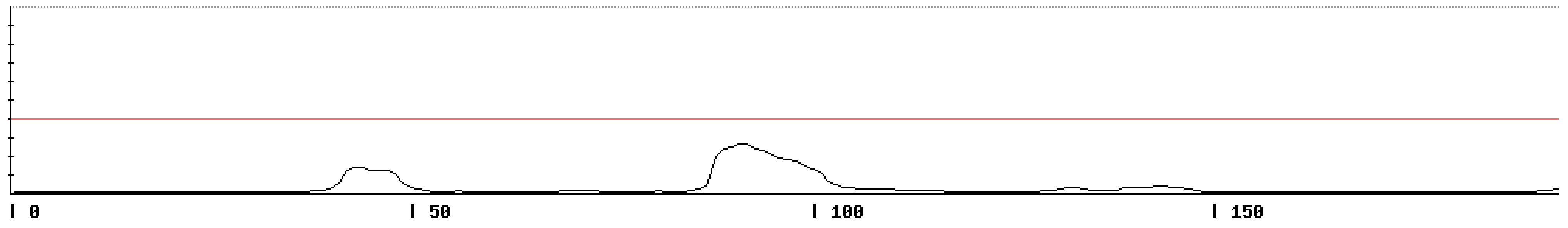

Predictions for Gossypiumarboreum_Cotton_A_05740_BGI-A2_v1.0

Viterbi Path	
Viterbi Path	
Posterior @ 0.4	
Posterior @ 0.4	
Posterior Graphic. Threshold Marked @ 0.4	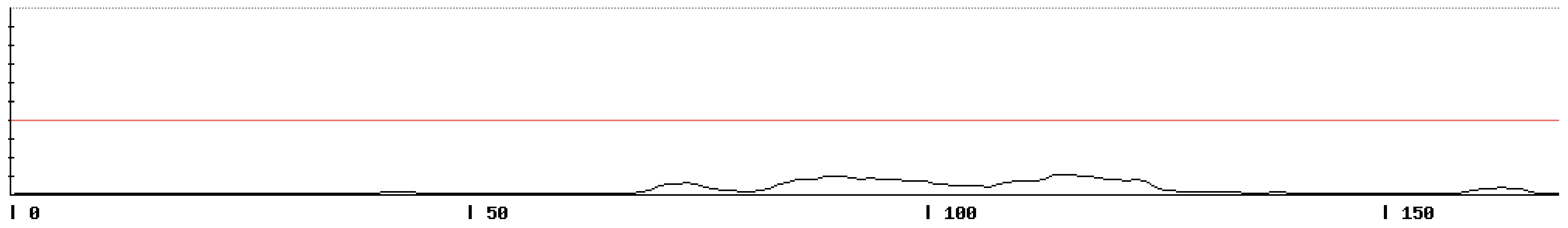

Predictions for Gossypiumarboreum_Cotton_A_05832_BGI-A2_v1.0	
Viterbi Path	
Viterbi Path	
Posterior @ 0.4	
Posterior @ 0.4	
Posterior Graphic. Threshold Marked @ 0.4	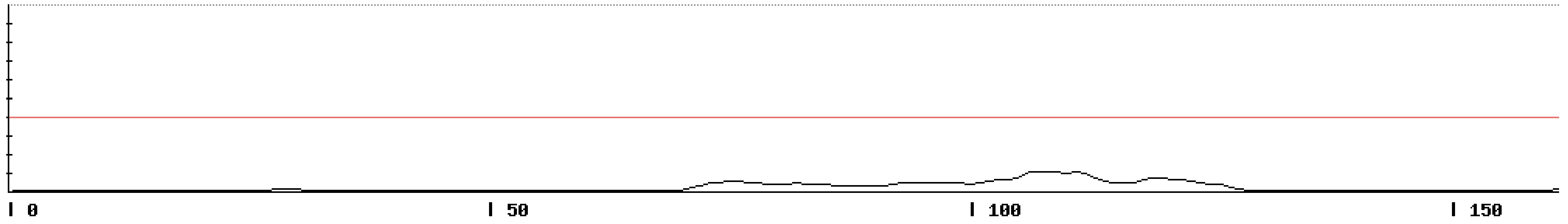

Predictions for Gossypiumarboreum_Cotton_A_05844_BGI-A2_v1.0	
Viterbi Path	
Viterbi Path	
Posterior @ 0.4	
Posterior @ 0.4	
Posterior Graphic. Threshold Marked @ 0.4	

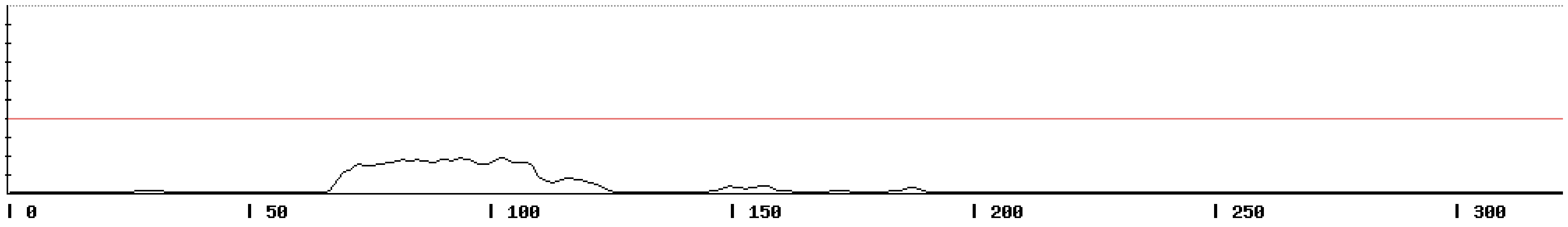

Predictions for Gossypiumarboreum_Cotton_A_05845_BGI-A2_v1.0

Viterbi Path	
Viterbi Path	
Posterior @ 0.4	
Posterior @ 0.4	
Posterior Graphic. Threshold Marked @ 0.4	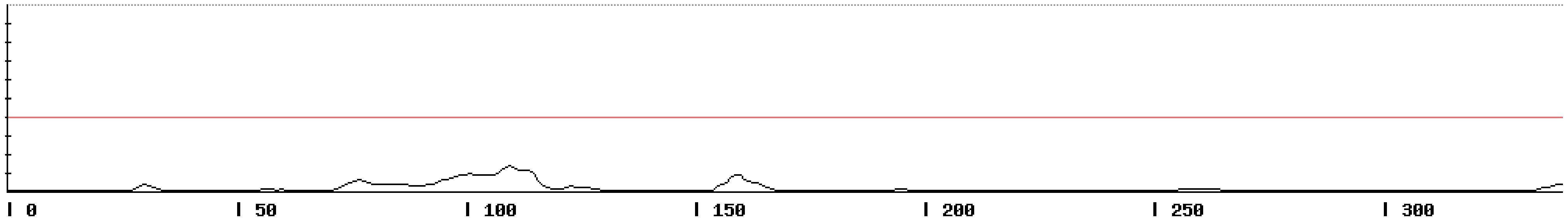

Predictions for Gossypiumarboreum_Cotton_A_06086_BGI-A2_v1.0

Viterbi Path	
Viterbi Path	
Posterior @ 0.4	351 - KKKKP - 355
Posterior @ 0.4	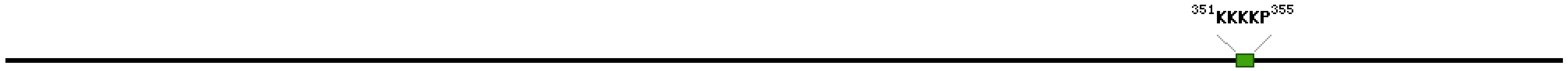

Posterior Graphic. Threshold Marked @ 0.4

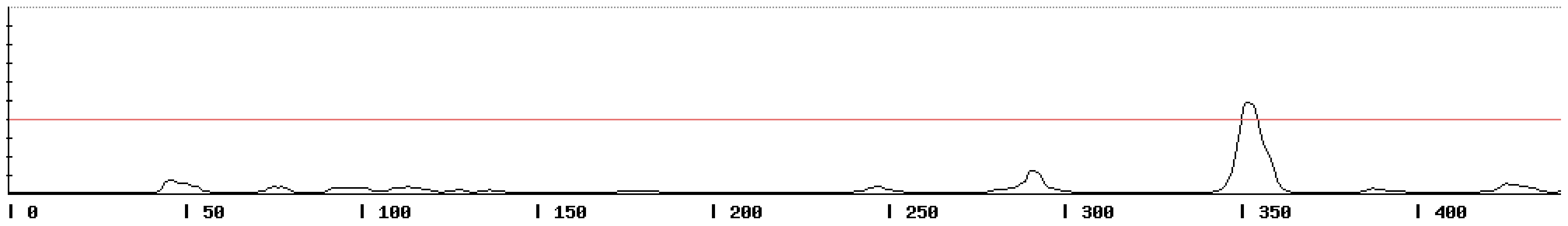

Predictions for Gossypiumarboreum_Cotton_A_06161_BGI-A2_v1.0

Viterbi Path

Viterbi Path

Posterior @ 0.4

Posterior @ 0.4

Posterior Graphic. Threshold Marked @ 0.4

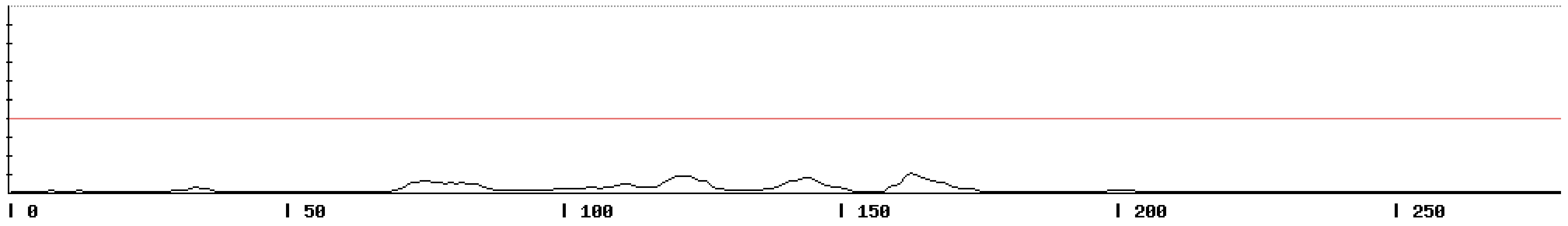

Predictions for Gossypiumarboreum_Cotton_A_06256_BGI-A2_v1.0

Viterbi Path

Viterbi Path

Posterior @ 0.4

Posterior @ 0.4

Posterior Graphic. Threshold Marked @ 0.4

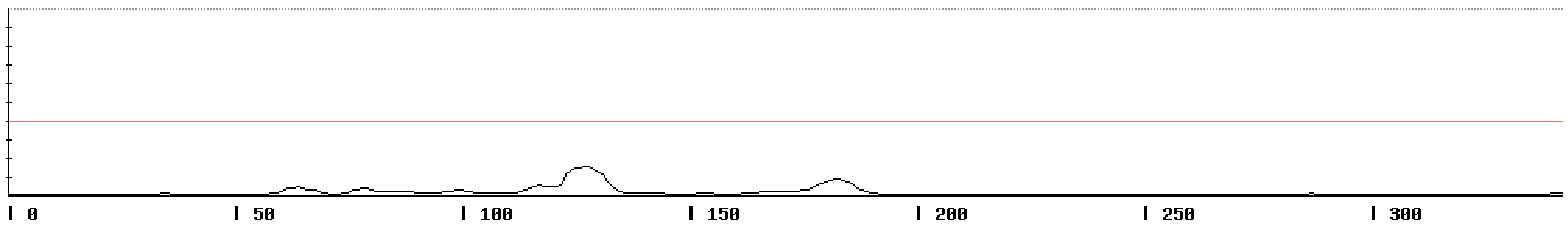

Predictions for Gossypiumarboreum_Cotton_A_06324_BGI-A2_v1.0

Viterbi Path

Viterbi Path

Posterior @ 0.4

Posterior @ 0.4

Posterior Graphic. Threshold Marked @ 0.4

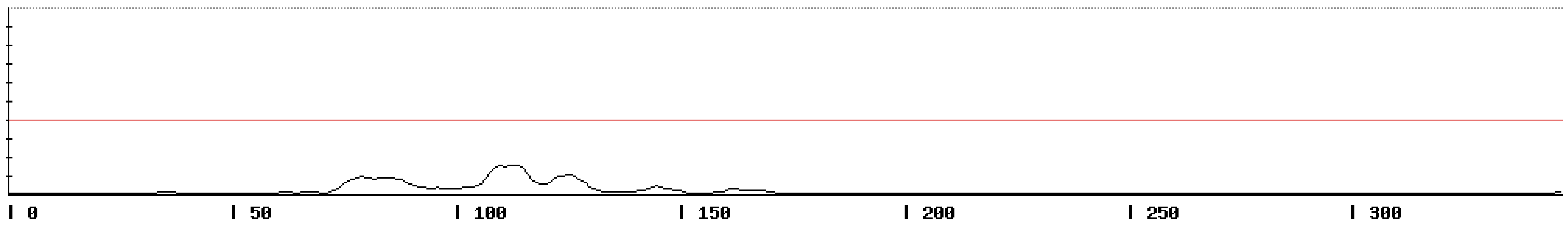

Predictions for Gossypiumarboreum_Cotton_A_06325_BGI-A2_v1.0

Viterbi Path

Viterbi Path

Posterior @ 0.4

Posterior @ 0.4

Posterior Graphic. Threshold Marked @ 0.4

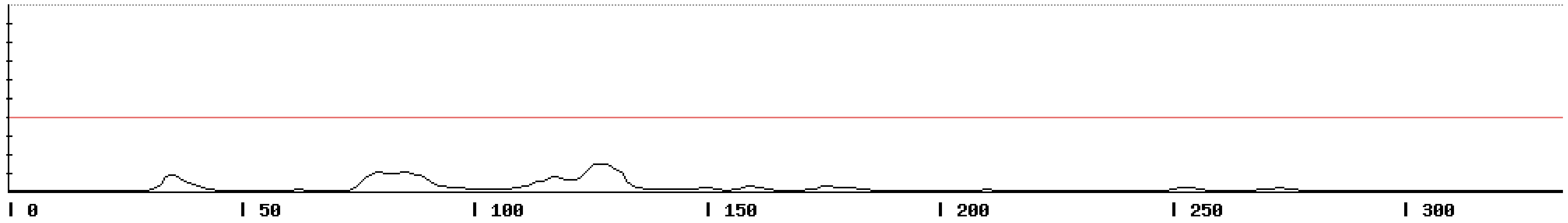

Predictions for Gossypiumarboreum_Cotton_A_06877_BGI-A2_v1.0

Viterbi Path

Viterbi Path

Posterior @ 0.4

Posterior @ 0.4

Posterior Graphic. Threshold Marked @ 0.4

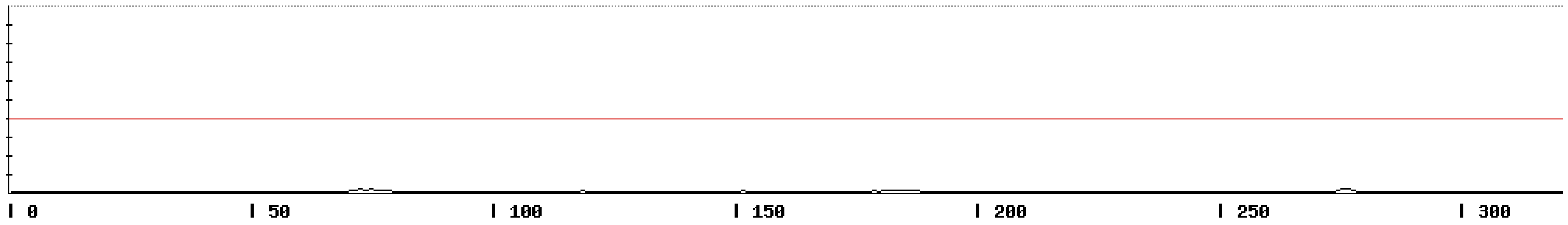

Predictions for Gossypiumarboreum_Cotton_A_07124_BGI-A2_v1.0

Viterbi Path

Viterbi Path

Posterior @ 0.4

Posterior @ 0.4

Posterior Graphic. Threshold Marked @ 0.4

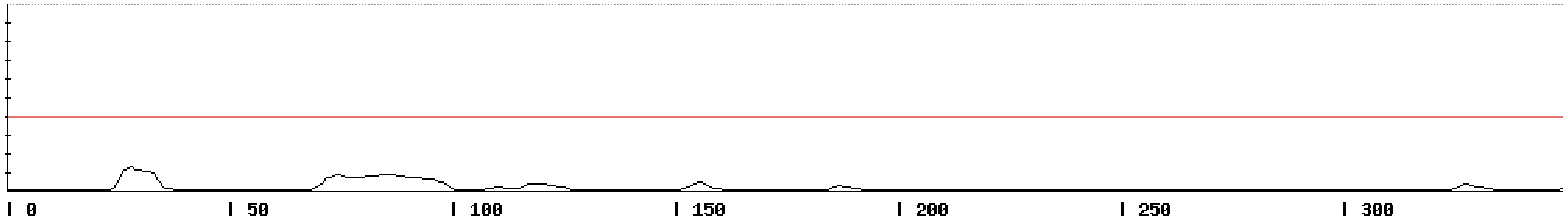

Predictions for Gossypiumarboreum_Cotton_A_07511_BGI-A2_v1.0

Viterbi Path

Viterbi Path

Posterior @ 0.4 166 - QRKRSRRGKKH - 176

Posterior @ 0.4

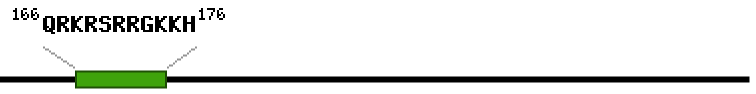

Posterior Graphic. Threshold Marked @ 0.4

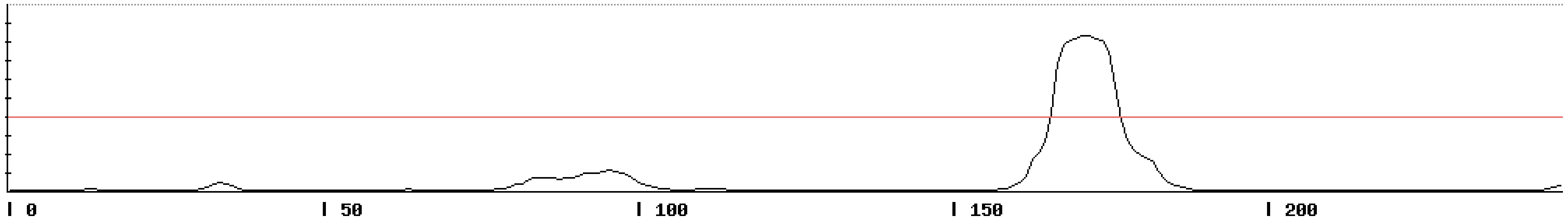

Predictions for Gossypiumarboreum_Cotton_A_07824_BGI-A2_v1.0

Viterbi Path	
Viterbi Path	
Posterior @ 0.4	
Posterior @ 0.4	
Posterior Graphic. Threshold Marked @ 0.4	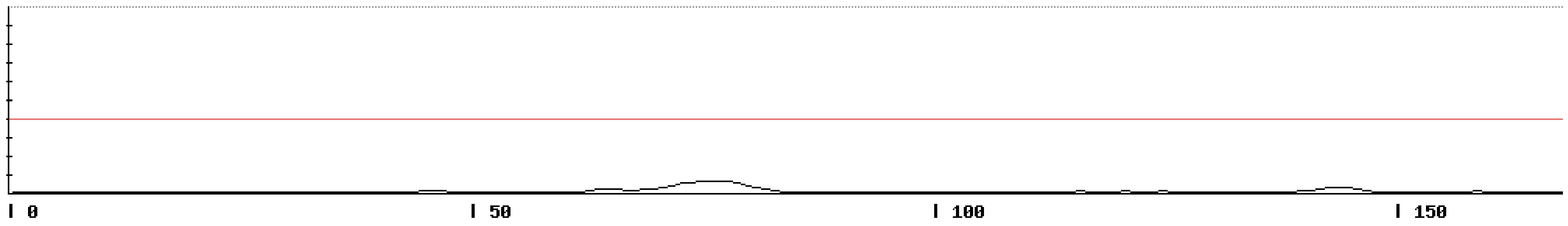

Predictions for Gossypiumarboreum_Cotton_A_07894_BGI-A2_v1.0	
Viterbi Path	
Viterbi Path	
Posterior @ 0.4	
Posterior @ 0.4	
Posterior Graphic. Threshold Marked @ 0.4	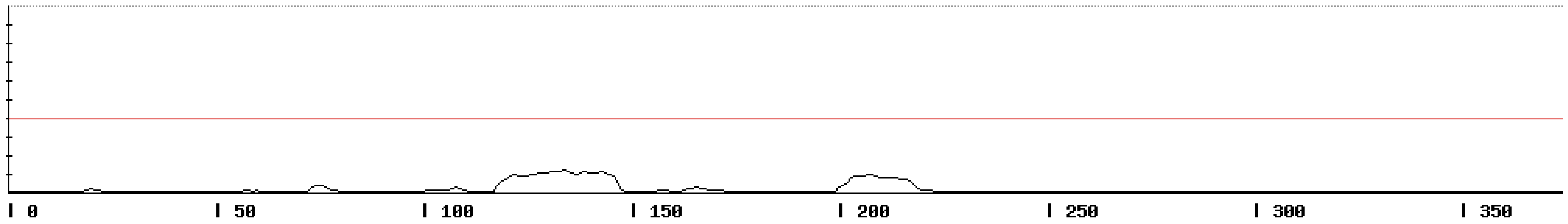

Predictions for Gossypiumarboreum_Cotton_A_08115_BGI-A2_v1.0

Viterbi Path

Viterbi Path

Posterior @ 0.4

Posterior @ 0.4

Posterior Graphic. Threshold Marked @ 0.4

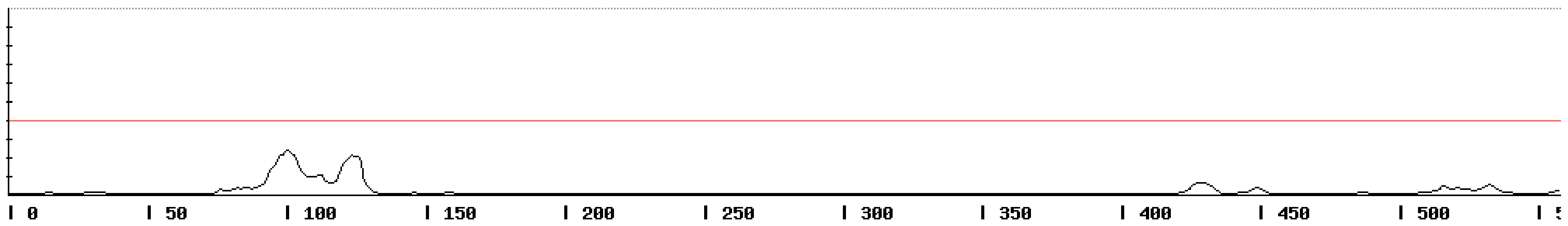

Predictions for Gossypiumarboreum_Cotton_A_08128_BGI-A2_v1.0

Viterbi Path

Viterbi Path

Posterior @ 0.4

Posterior @ 0.4

Posterior Graphic. Threshold Marked @ 0.4

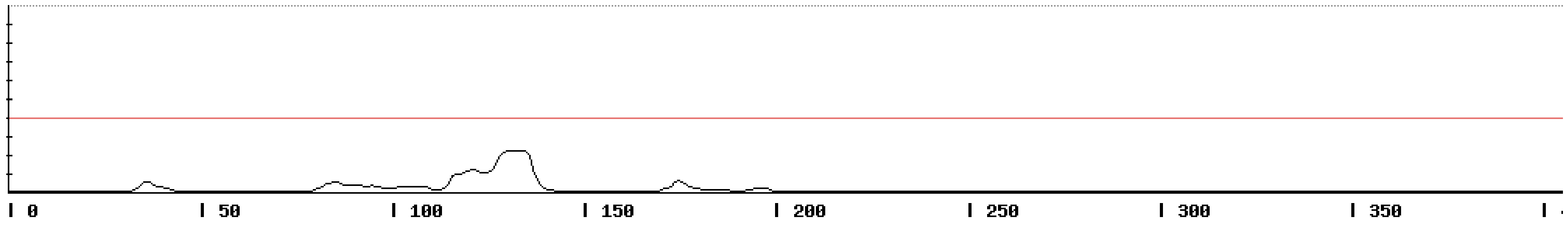

Predictions for Gossypiumarboreum_Cotton_A_08671_BGI-A2_v1.0

Viterbi Path	100 - KSKGGRGRRRAKGGFWRKIGKVR - 122
Viterbi Path	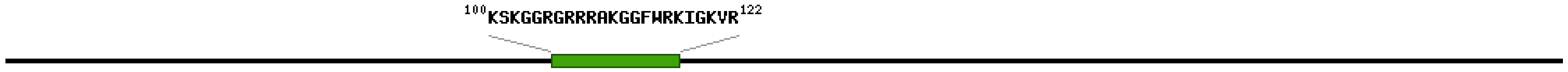
Posterior @ 0.4	99 - TKSKGGRGRRRAKGGFWRKIGKVR - 122
Posterior @ 0.4	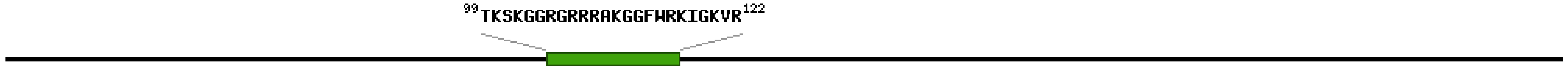
Posterior Graphic. Threshold Marked @ 0.4	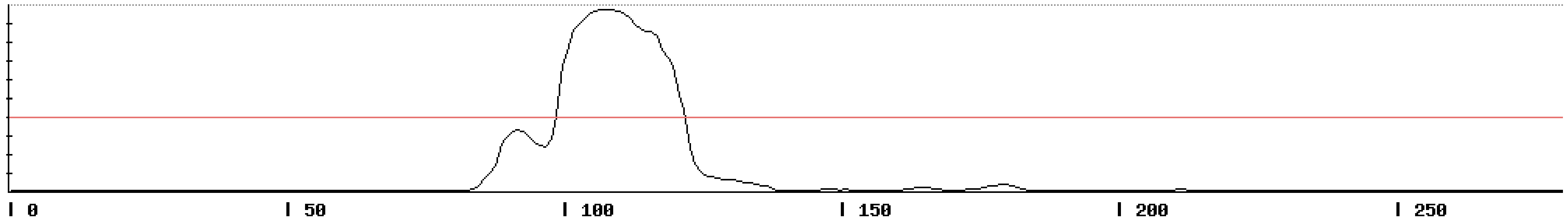

Predictions for Gossypiumarboreum_Cotton_A_08816_BGI-A2_v1.0

Viterbi Path	
Viterbi Path	
Posterior @ 0.4	
Posterior @ 0.4	

Posterior Graphic. Threshold Marked @ 0.4	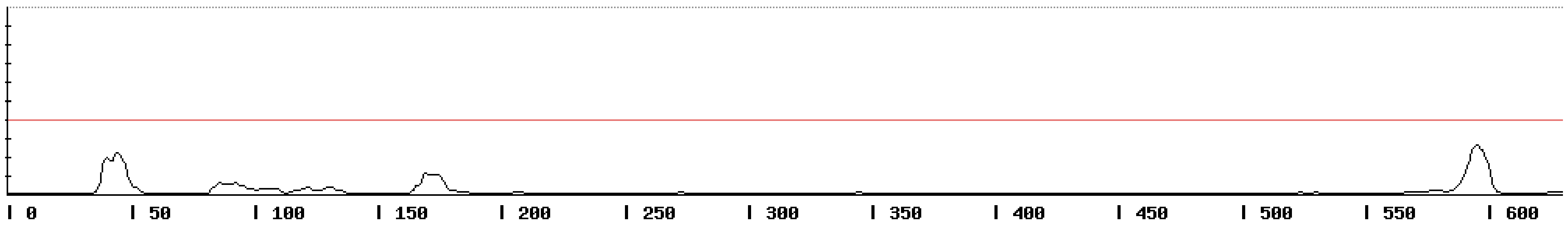

Predictions for Gossypiumarboreum_Cotton_A_09163_BGI-A2_v1.0

Viterbi Path	
Viterbi Path	
Posterior @ 0.4	
Posterior @ 0.4	
Posterior Graphic. Threshold Marked @ 0.4	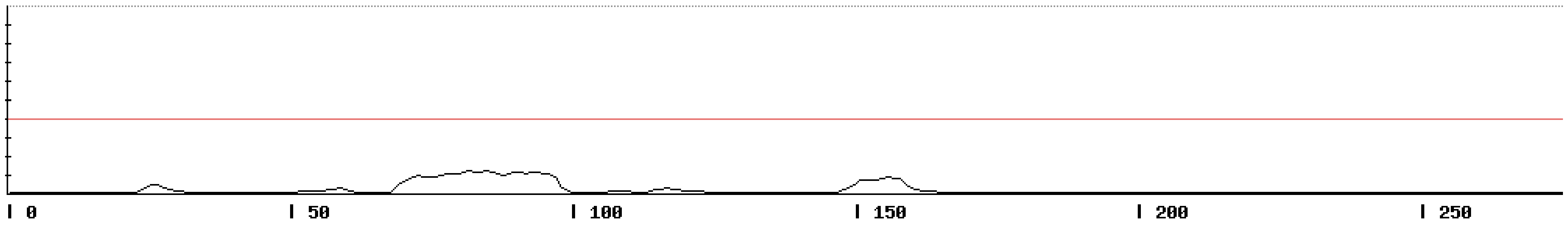

Predictions for Gossypiumarboreum_Cotton_A_09393_BGI-A2_v1.0

Viterbi Path	
Viterbi Path	
Posterior @ 0.4	
Posterior @ 0.4	

Posterior Graphic. Threshold Marked @ 0.4

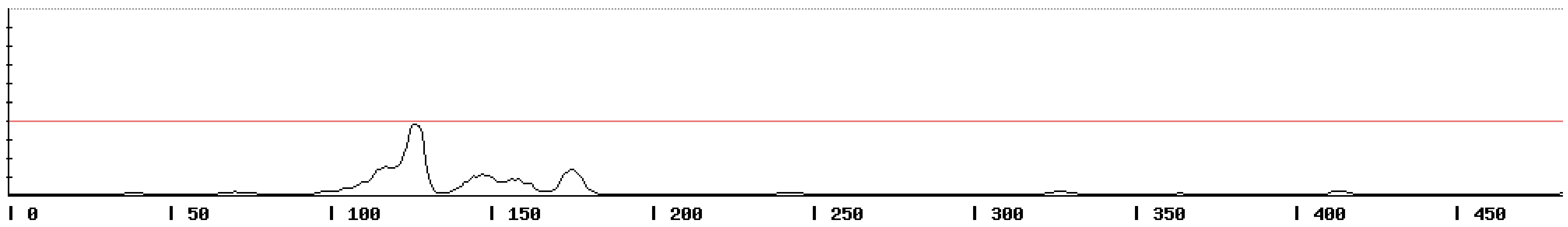

Predictions for Gossypiumarboreum_Cotton_A_09515_BGI-A2_v1.0

Viterbi Path

Viterbi Path

Posterior @ 0.4

Posterior @ 0.4

Posterior Graphic. Threshold Marked @ 0.4

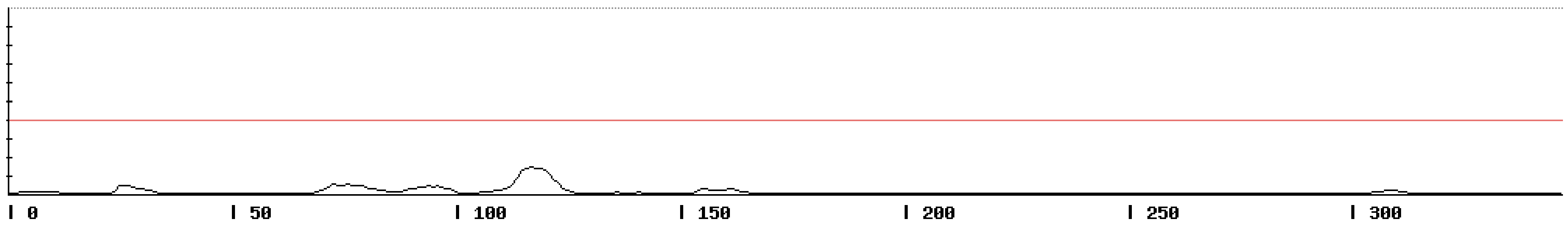

Predictions for Gossypiumarboreum_Cotton_A_09599_BGI-A2_v1.0

Viterbi Path

Viterbi Path

Posterior @ 0.4	118 - KAPSGKKTK - 126
Posterior @ 0.4	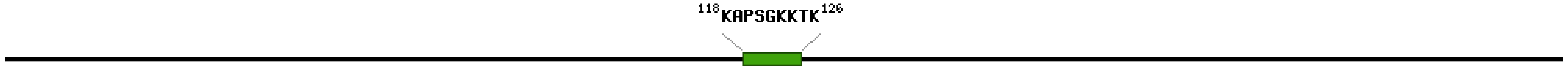
Posterior Graphic. Threshold Marked @ 0.4	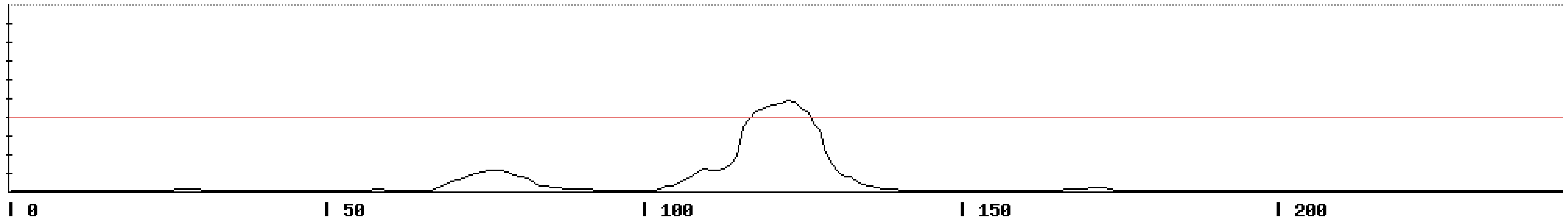

Predictions for Gossypiumarboreum_Cotton_A_09649_BGI-A2_v1.0

Viterbi Path	
Viterbi Path	
Posterior @ 0.4	
Posterior @ 0.4	
Posterior Graphic. Threshold Marked @ 0.4	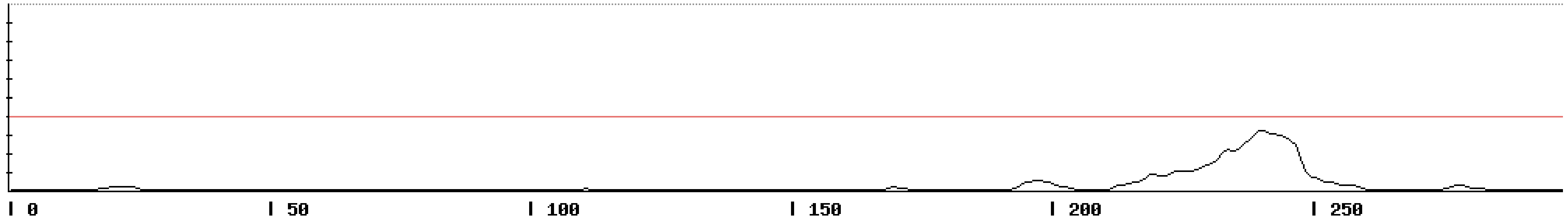

Predictions for Gossypiumarboreum_Cotton_A_10020_BGI-A2_v1.0

Viterbi Path	
Viterbi Path	

Posterior @ 0.4

Posterior @ 0.4

Posterior Graphic. Threshold Marked @ 0.4

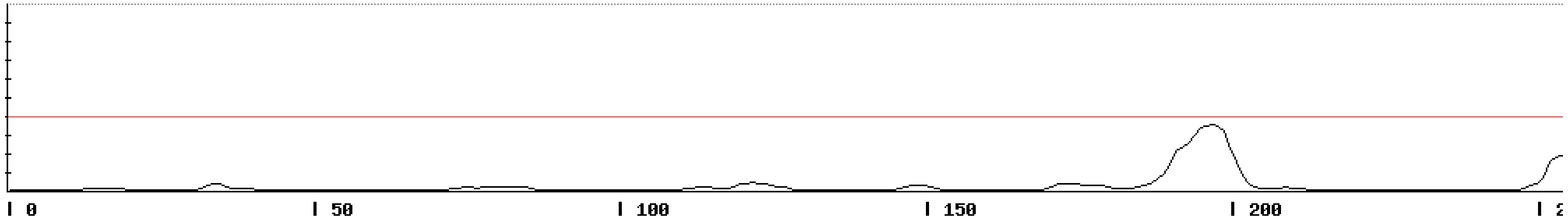

Predictions for Gossypiumarboreum_Cotton_A_10022_BGI-A2_v1.0

Viterbi Path

Viterbi Path

Posterior @ 0.4

Posterior @ 0.4

Posterior Graphic. Threshold Marked @ 0.4

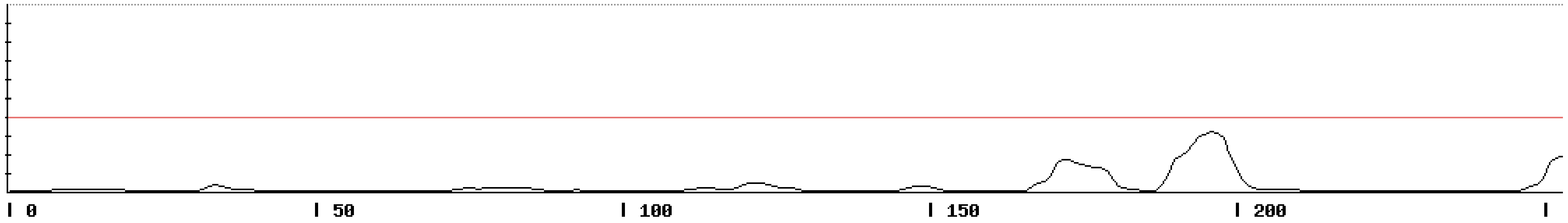

Predictions for Gossypiumarboreum_Cotton_A_10170_BGI-A2_v1.0

Viterbi Path	
Viterbi Path	
Posterior @ 0.4	
Posterior @ 0.4	
Posterior Graphic. Threshold Marked @ 0.4	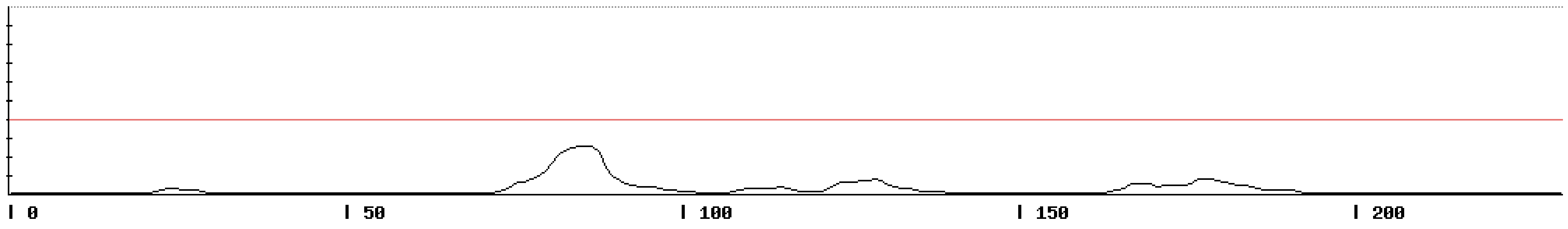

Predictions for Gossypiumarboreum_Cotton_A_11313_BGI-A2_v1.0

Viterbi Path	
Viterbi Path	
Posterior @ 0.4	146 - RKRRK - 150
Posterior @ 0.4	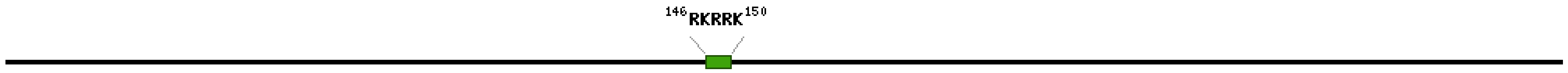
Posterior Graphic. Threshold Marked @ 0.4	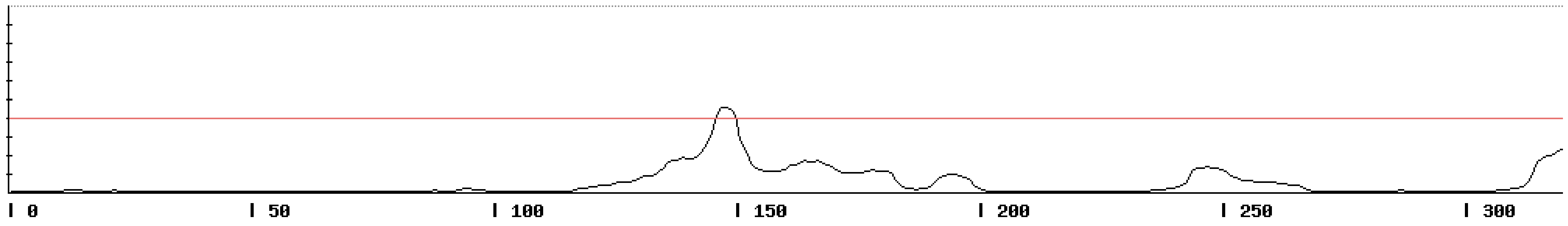

Predictions for Gossypiumarboreum_Cotton_A_11365_BGI-A2_v1.0

Viterbi Path

Viterbi Path

Posterior @ 0.4

Posterior @ 0.4

Posterior Graphic. Threshold Marked @ 0.4

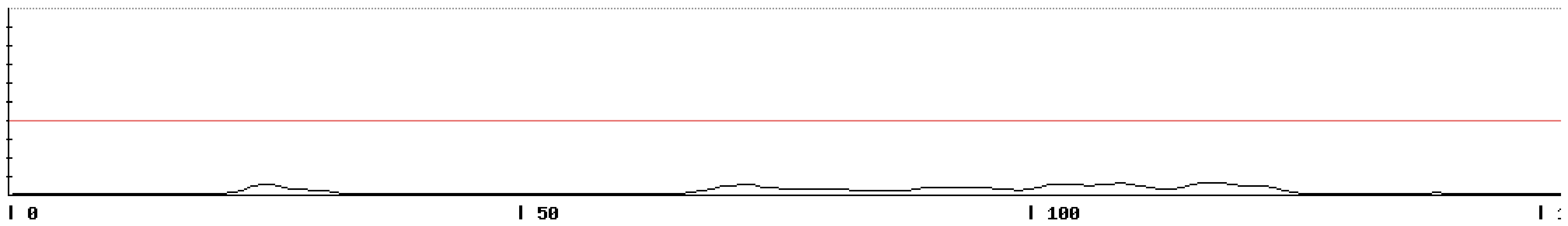

Predictions for Gossypiumarboreum_Cotton_A_12607_BGI-A2_v1.0

Viterbi Path

Viterbi Path

Posterior @ 0.4

Posterior @ 0.4

Posterior Graphic. Threshold Marked @ 0.4

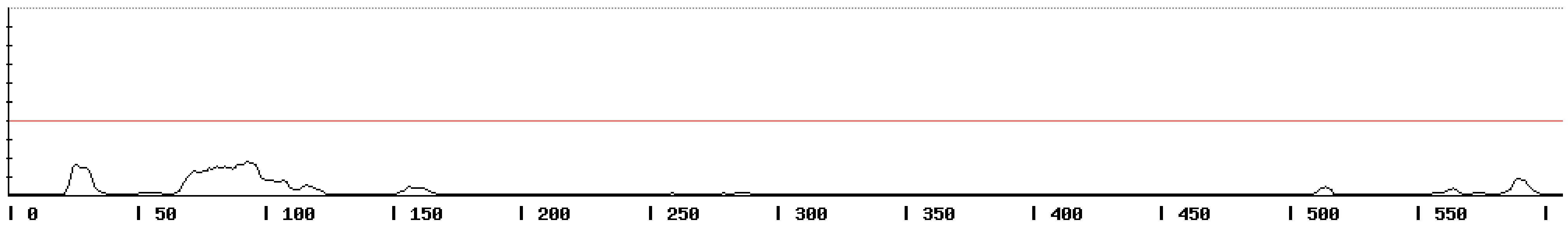

Predictions for Gossypiumarboreum_Cotton_A_12683_BGI-A2_v1.0

Viterbi Path

Viterbi Path

Posterior @ 0.4

Posterior @ 0.4

Posterior Graphic. Threshold Marked @ 0.4

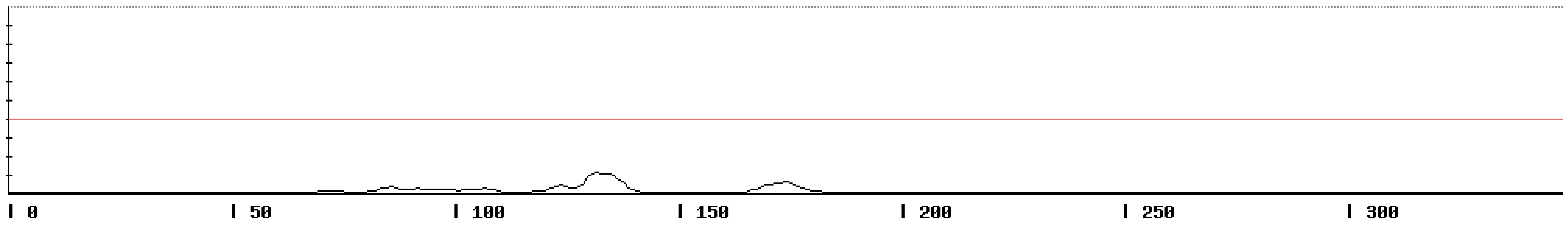

Predictions for Gossypiumarboreum_Cotton_A_13074_BGI-A2_v1.0

Viterbi Path

Viterbi Path

Posterior @ 0.4

Posterior @ 0.4

Posterior Graphic. Threshold Marked @ 0.4

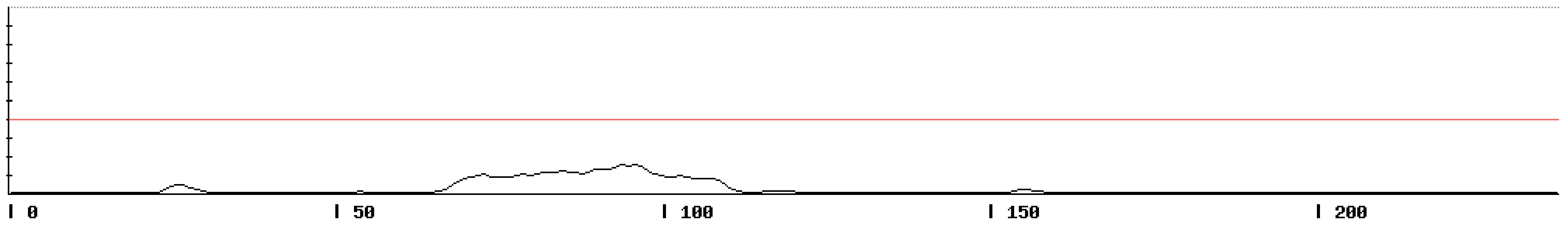

Predictions for Gossypiumarboreum_Cotton_A_13127_BGI-A2_v1.0

Viterbi Path

Viterbi Path

Posterior @ 0.4

Posterior @ 0.4

Posterior Graphic. Threshold Marked @ 0.4

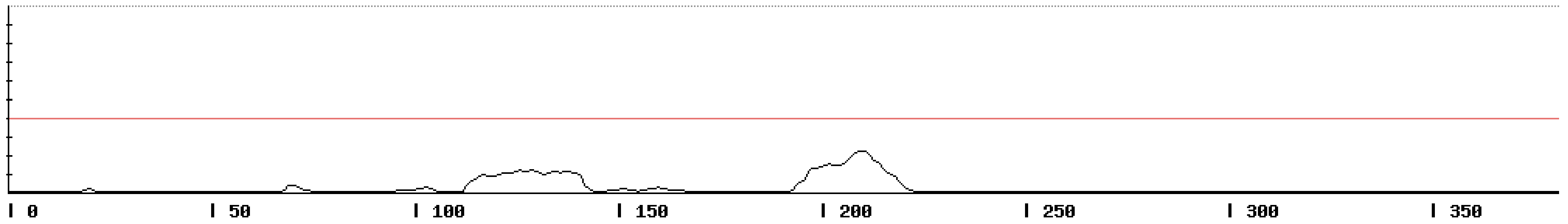

Predictions for Gossypiumarboreum_Cotton_A_13198_BGI-A2_v1.0

Viterbi Path

Viterbi Path

Posterior @ 0.4

Posterior @ 0.4

Posterior Graphic. Threshold Marked @ 0.4

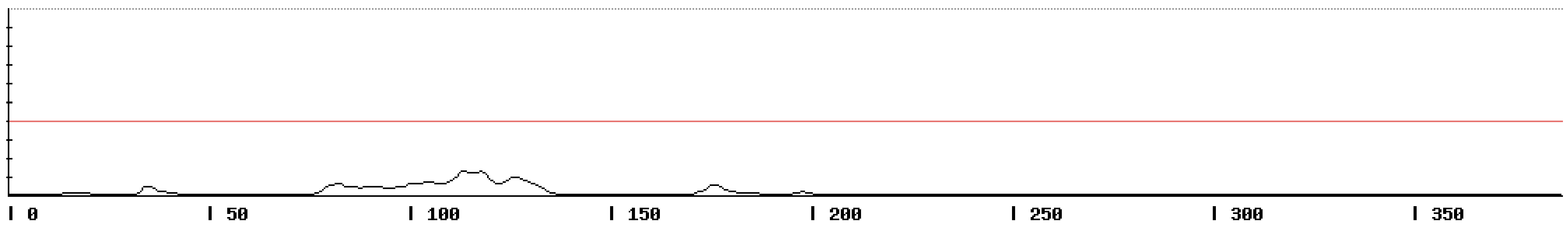

Predictions for Gossypiumarboreum_Cotton_A_13395_BGI-A2_v1.0

Viterbi Path

Viterbi Path

Posterior @ 0.4

Posterior @ 0.4

Posterior Graphic. Threshold Marked @ 0.4

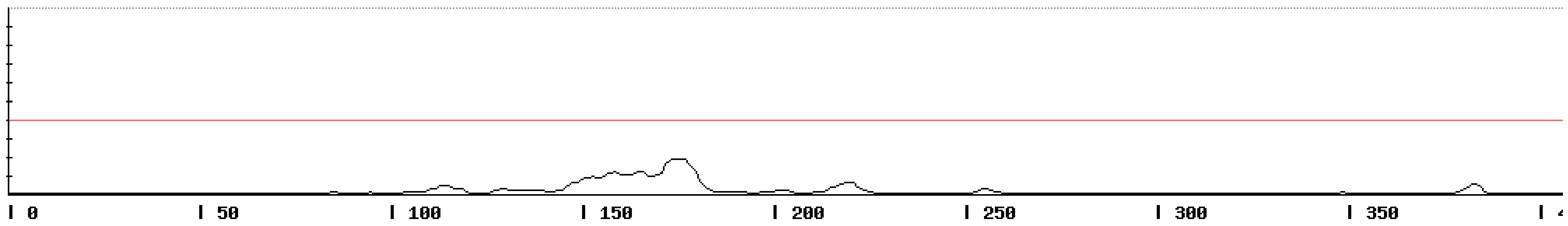

Predictions for Gossypiumarboreum_Cotton_A_13487_BGI-A2_v1.0

Viterbi Path

Viterbi Path

Posterior @ 0.4

Posterior @ 0.4

Posterior Graphic. Threshold Marked @ 0.4

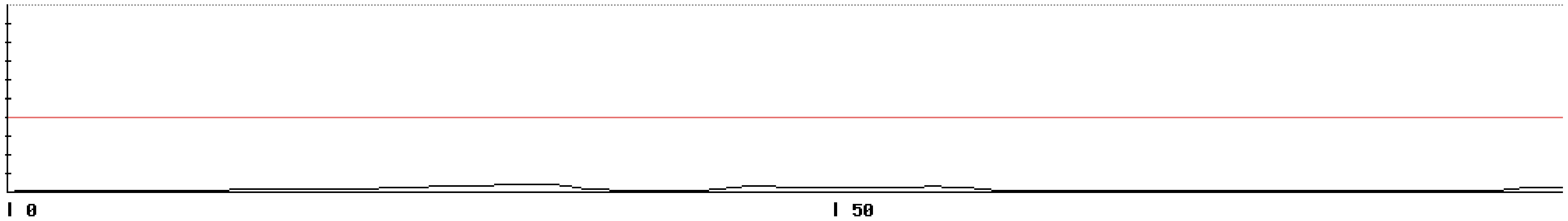

Predictions for Gossypiumarboreum_Cotton_A_13494_BGI-A2_v1.0

Viterbi Path

Viterbi Path

Posterior @ 0.4 114 - RKGKKIGLKK - 123

Posterior @ 0.4

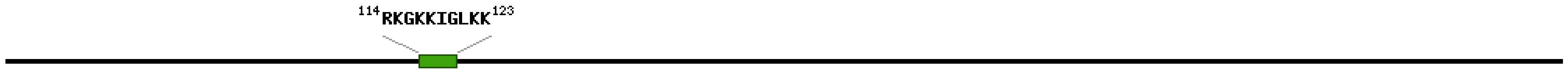

Posterior Graphic. Threshold Marked @ 0.4

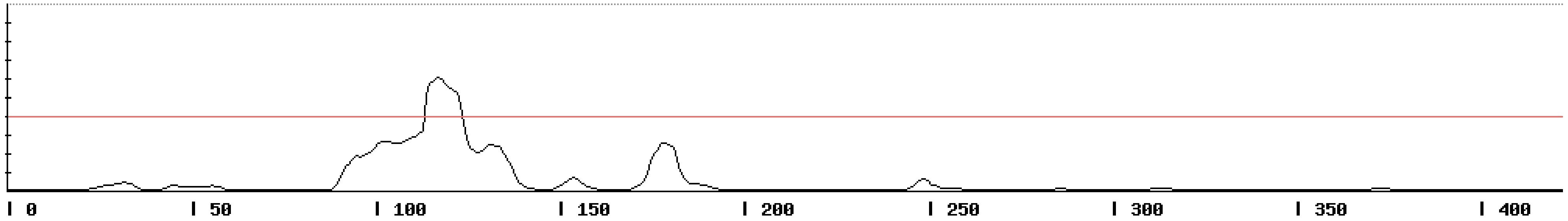

Predictions for Gossypiumarboreum_Cotton_A_13667_BGI-A2_v1.0

Viterbi Path

Viterbi Path

Posterior @ 0.4

Posterior @ 0.4

Posterior Graphic. Threshold Marked @ 0.4

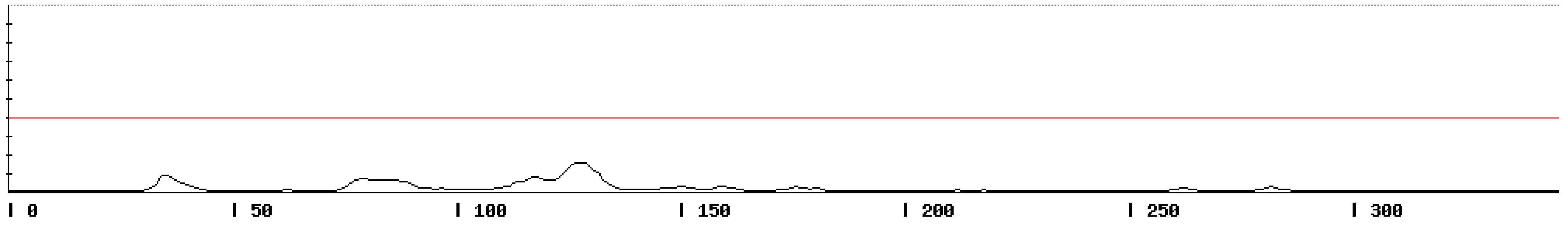

Predictions for Gossypiumarboreum_Cotton_A_13669_BGI-A2_v1.0

Viterbi Path

Viterbi Path

Posterior @ 0.4

Posterior @ 0.4

Posterior Graphic. Threshold Marked @ 0.4

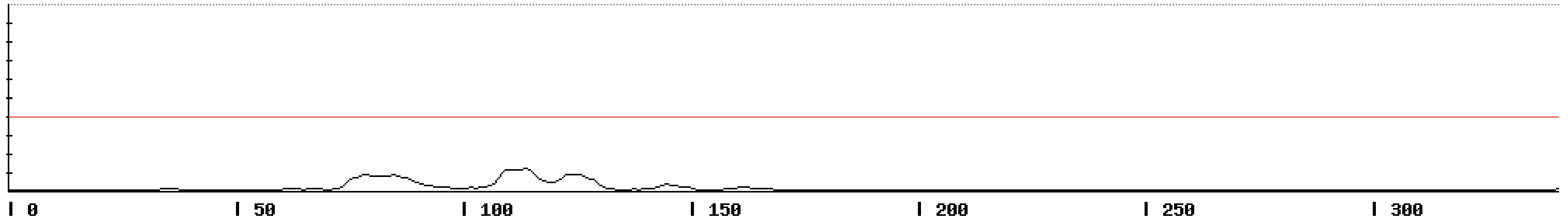

Predictions for Gossypiumarboreum_Cotton_A_13728_BGI-A2_v1.0

Viterbi Path

Viterbi Path

Posterior @ 0.4 — 311 - KRAKVEEGPSSSRKPSESHGGRRKGGSLGGMLRK - 344

Posterior @ 0.4

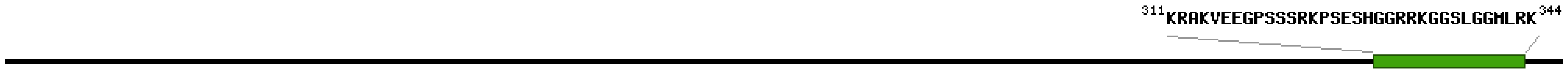

Posterior Graphic. Threshold Marked @ 0.4

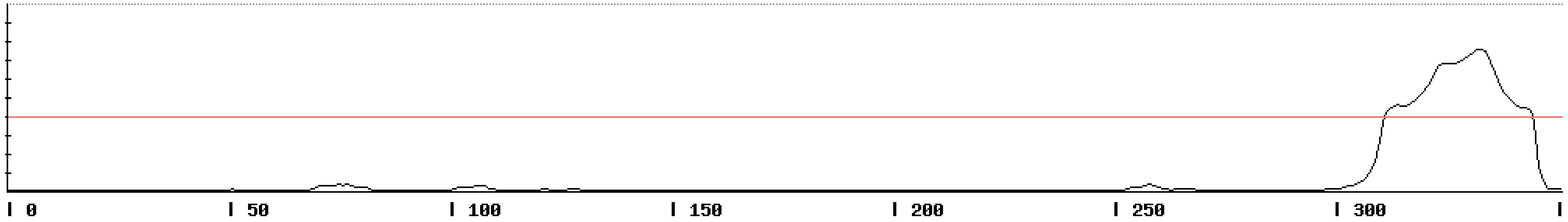

Predictions for Gossypiumarboreum_Cotton_A_13859_BGI-A2_v1.0

Viterbi Path

Viterbi Path

Posterior @ 0.4 — 301 - NRRIKLRKPKA - 311

Posterior @ 0.4

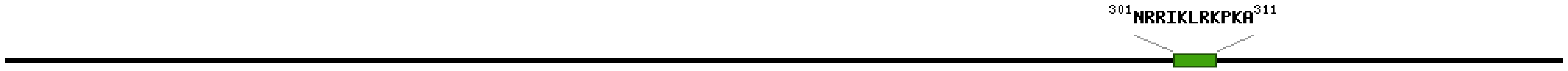

Posterior Graphic. Threshold Marked @ 0.4

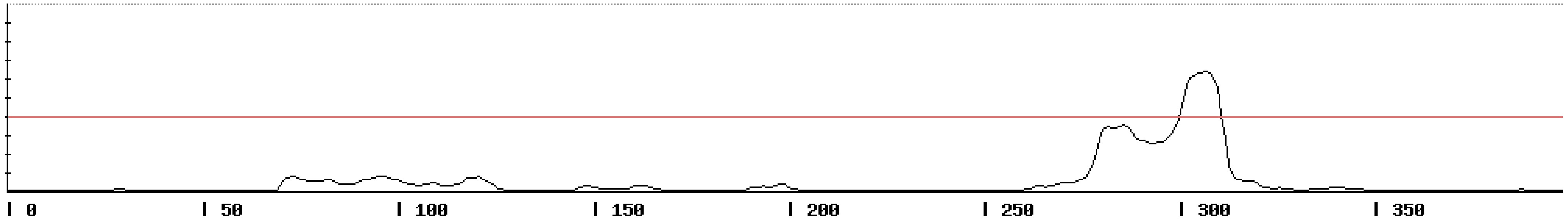

Predictions for Gossypiumarboreum_Cotton_A_14093_BGI-A2_v1.0

Viterbi Path

Viterbi Path

Posterior @ 0.4

Posterior @ 0.4

Posterior Graphic. Threshold Marked @ 0.4

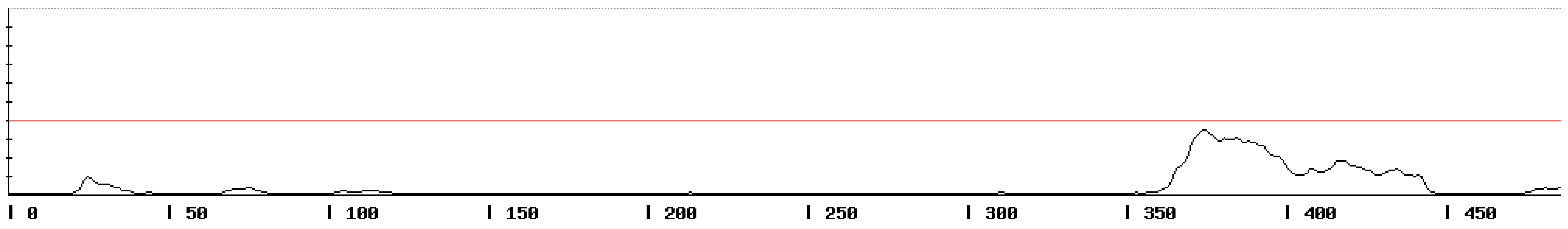

Predictions for Gossypiumarboreum_Cotton_A_15184_BGI-A2_v1.0

Viterbi Path

Viterbi Path

Posterior @ 0.4

Posterior @ 0.4

Posterior Graphic. Threshold Marked @ 0.4

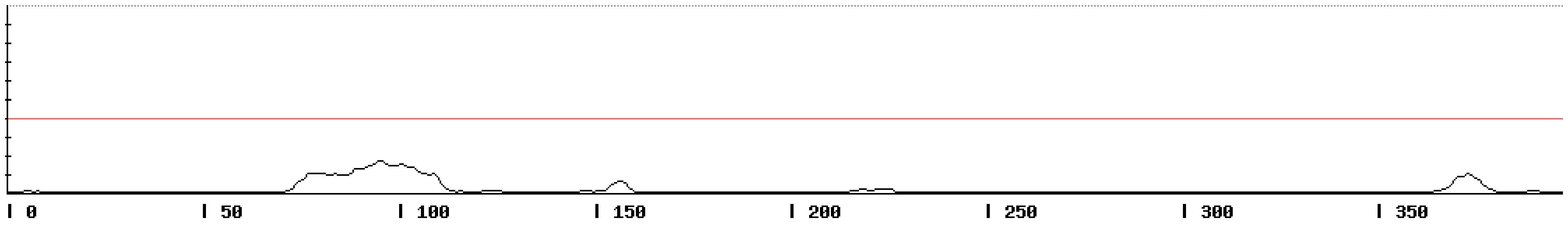

Predictions for Gossypiumarboreum_Cotton_A_15474_BGI-A2_v1.0

Viterbi Path

Viterbi Path

Posterior @ 0.4

Posterior @ 0.4

Posterior Graphic. Threshold Marked @ 0.4

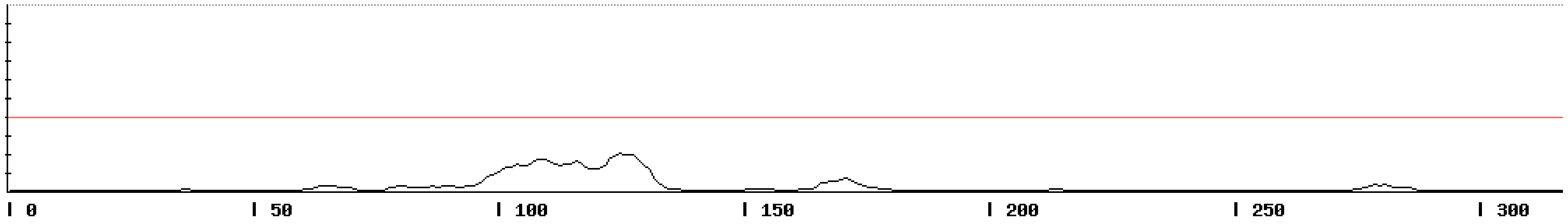

Predictions for Gossypiumarboreum_Cotton_A_15646_BGI-A2_v1.0

Viterbi Path

Viterbi Path

Posterior @ 0.4

Posterior @ 0.4

Posterior Graphic. Threshold Marked @ 0.4

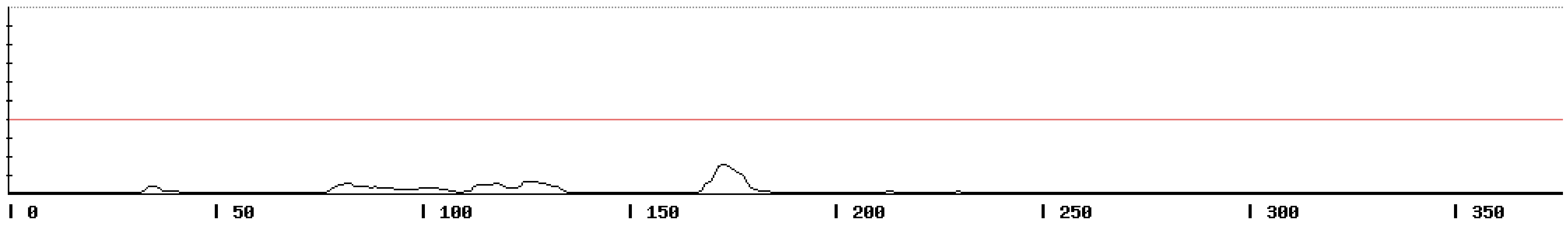

Predictions for Gossypiumarboreum_Cotton_A_15664_BGI-A2_v1.0

Viterbi Path	
Viterbi Path	
Posterior @ 0.4	80 - RNSKRKNRVTKK - 91
Posterior @ 0.4	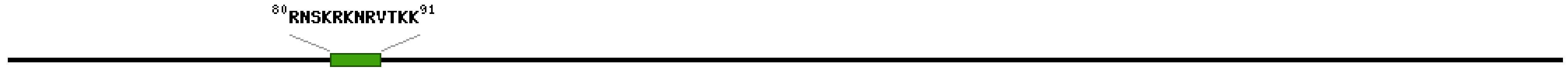
Posterior Graphic. Threshold Marked @ 0.4	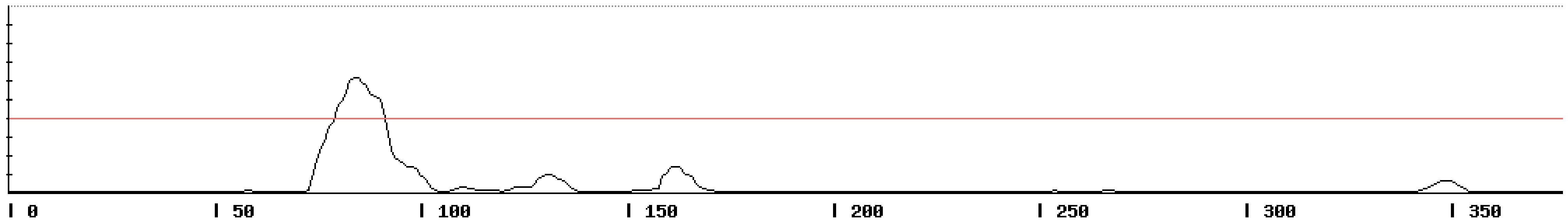

Predictions for Gossypiumarboreum_Cotton_A_15665_BGI-A2_v1.0

Viterbi Path	
Viterbi Path	
Posterior @ 0.4	
Posterior @ 0.4	

Posterior Graphic. Threshold Marked @ 0.4

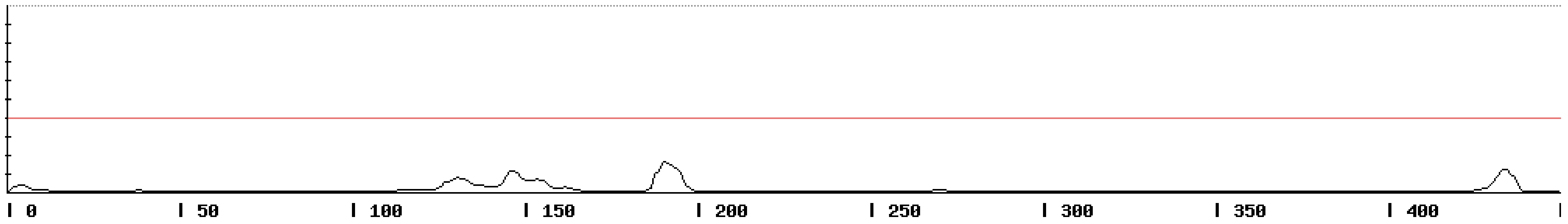

Predictions for Gossypiumarboreum_Cotton_A_15785_BGI-A2_v1.0

Viterbi Path

Viterbi Path

Posterior @ 0.4

Posterior @ 0.4

Posterior Graphic. Threshold Marked @ 0.4

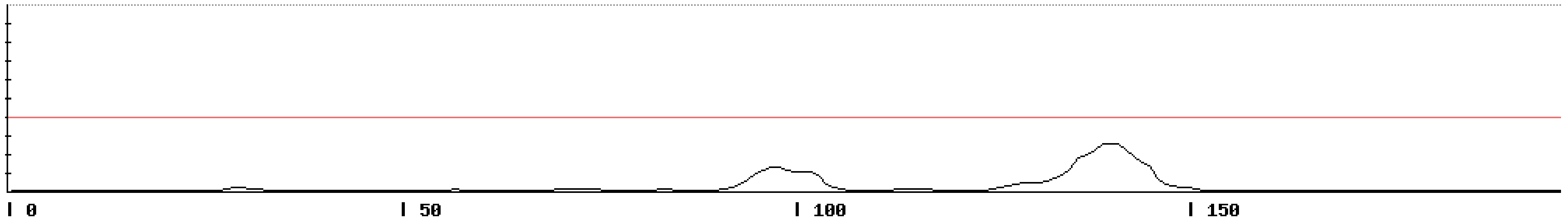

Predictions for Gossypiumarboreum_Cotton_A_15893_BGI-A2_v1.0

Viterbi Path

Viterbi Path

Posterior @ 0.4

Posterior @ 0.4

Posterior Graphic. Threshold Marked @ 0.4

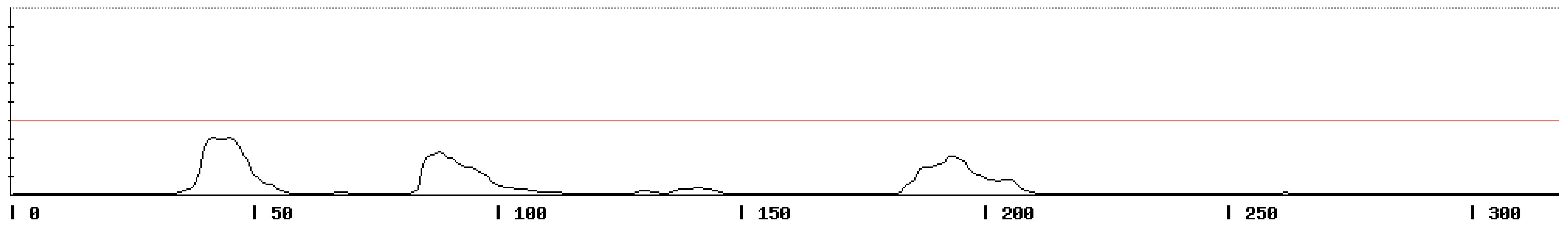

Predictions for Gossypiumarboreum_Cotton_A_16296_BGI-A2_v1.0

Viterbi Path

Viterbi Path

Posterior @ 0.4

Posterior @ 0.4

Posterior Graphic. Threshold Marked @ 0.4

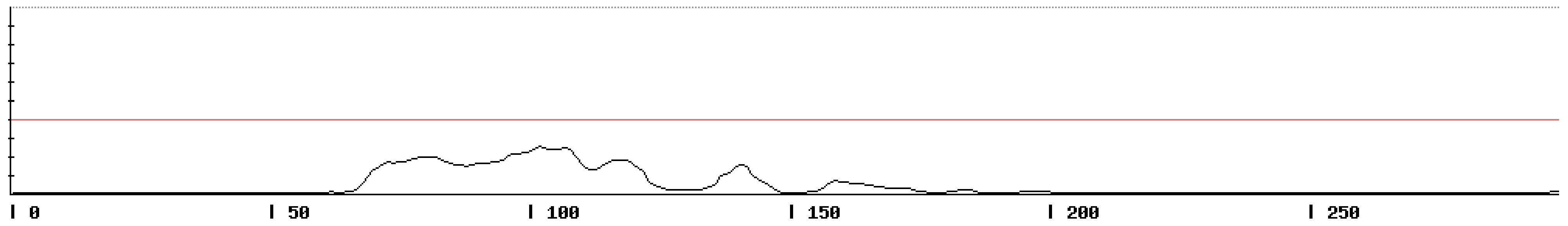

Predictions for Gossypiumarboreum_Cotton_A_16773_BGI-A2_v1.0

Viterbi Path

Viterbi Path

Posterior @ 0.4

Posterior @ 0.4

Posterior Graphic. Threshold Marked @ 0.4

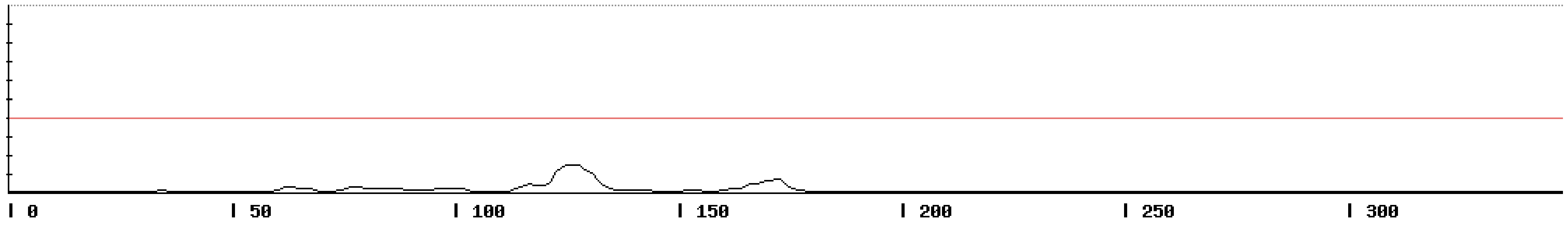

Predictions for Gossypiumarboreum_Cotton_A_16813_BGI-A2_v1.0

Viterbi Path

Viterbi Path

Posterior @ 0.4

Posterior @ 0.4

Posterior Graphic. Threshold Marked @ 0.4

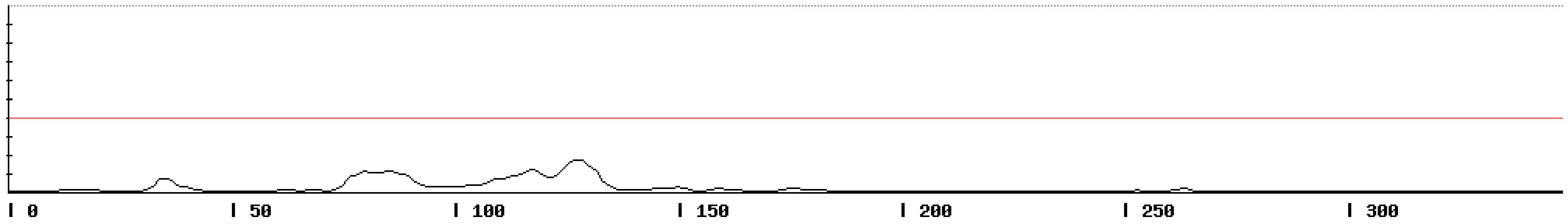

Predictions for Gossypiumarboreum_Cotton_A_17125_BGI-A2_v1.0

Viterbi Path

Viterbi Path

Posterior @ 0.4

Posterior @ 0.4

Posterior Graphic. Threshold Marked @ 0.4

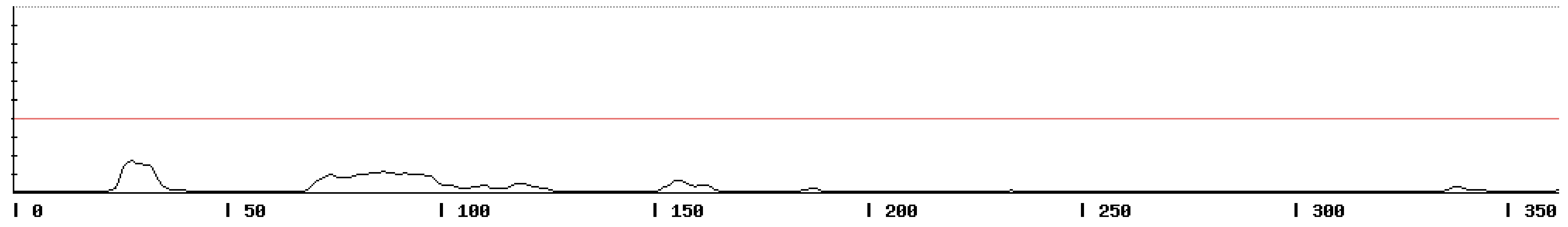

Predictions for Gossypiumarboreum_Cotton_A_17275_BGI-A2_v1.0

Viterbi Path

Viterbi Path

Posterior @ 0.4

Posterior @ 0.4

Posterior Graphic. Threshold Marked @ 0.4

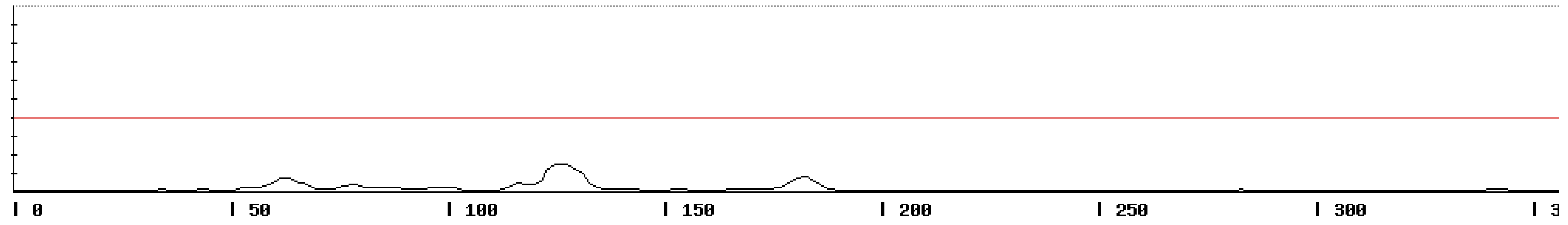

Predictions for Gossypiumarboreum_Cotton_A_17362_BGI-A2_v1.0

Viterbi Path

Viterbi Path

Posterior @ 0.4 — 93 - KATGKDRTIKSKKSLIGMKK - 112

Posterior @ 0.4

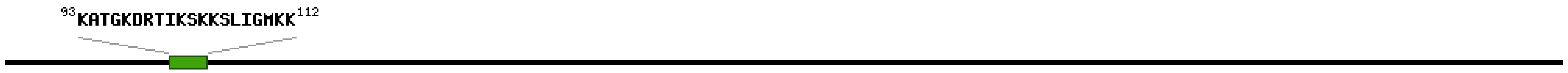

Posterior Graphic. Threshold Marked @ 0.4

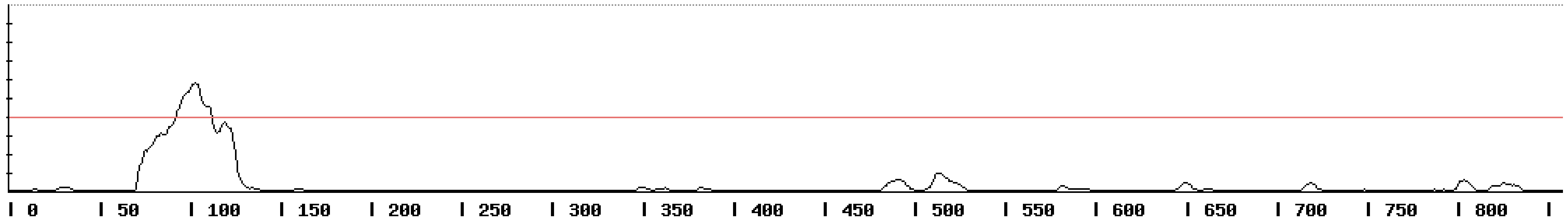

Predictions for Gossypiumarboreum_Cotton_A_18089_BGI-A2_v1.0

Viterbi Path

Viterbi Path

Posterior @ 0.4

Posterior @ 0.4

Posterior Graphic. Threshold Marked @ 0.4

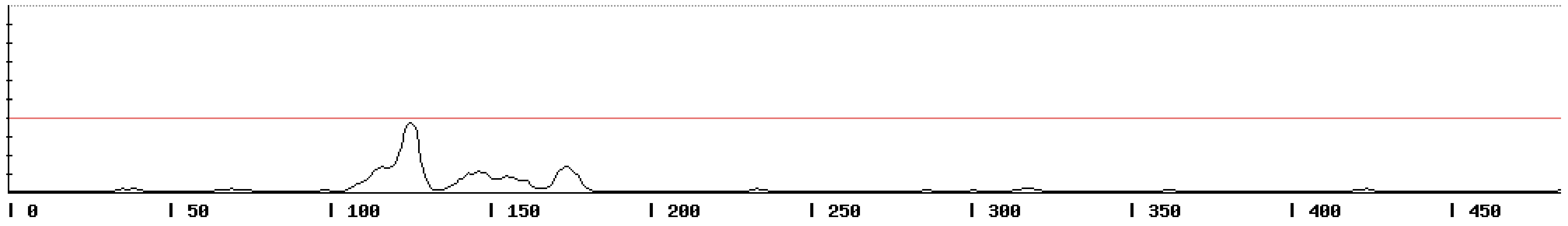

Predictions for Gossypiumarboreum_Cotton_A_18123_BGI-A2_v1.0

Viterbi Path

Viterbi Path

Posterior @ 0.4

Posterior @ 0.4

Posterior Graphic. Threshold Marked @ 0.4

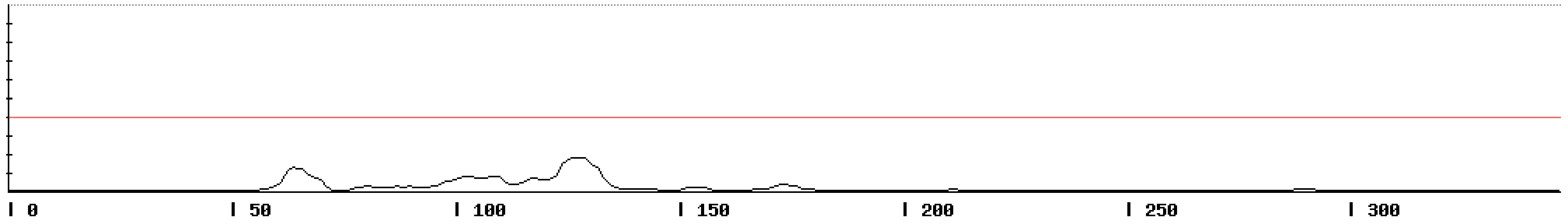

Predictions for Gossypiumarboreum_Cotton_A_18125_BGI-A2_v1.0

Viterbi Path

Viterbi Path

Posterior @ 0.4

Posterior @ 0.4

Posterior Graphic. Threshold Marked @ 0.4

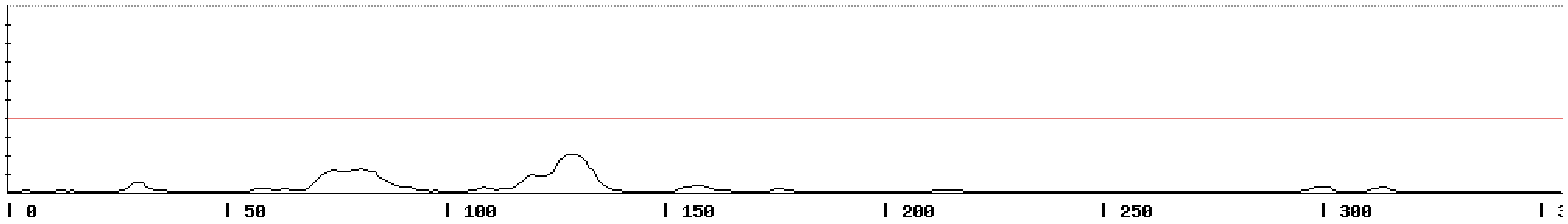

Predictions for Gossypiumarboreum_Cotton_A_18426_BGI-A2_v1.0

Viterbi Path

Viterbi Path

Posterior @ 0.4

Posterior @ 0.4

Posterior Graphic. Threshold Marked @ 0.4

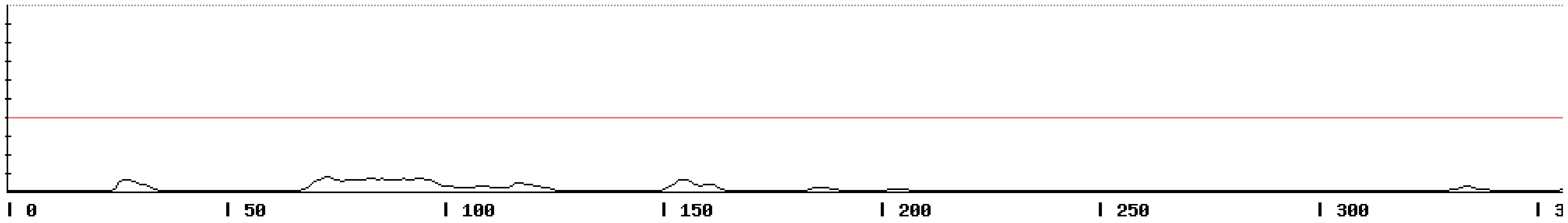

Predictions for Gossypiumarboreum_Cotton_A_19336_BGI-A2_v1.0

Viterbi Path

Viterbi Path

Posterior @ 0.4

Posterior @ 0.4

Posterior Graphic. Threshold Marked @ 0.4

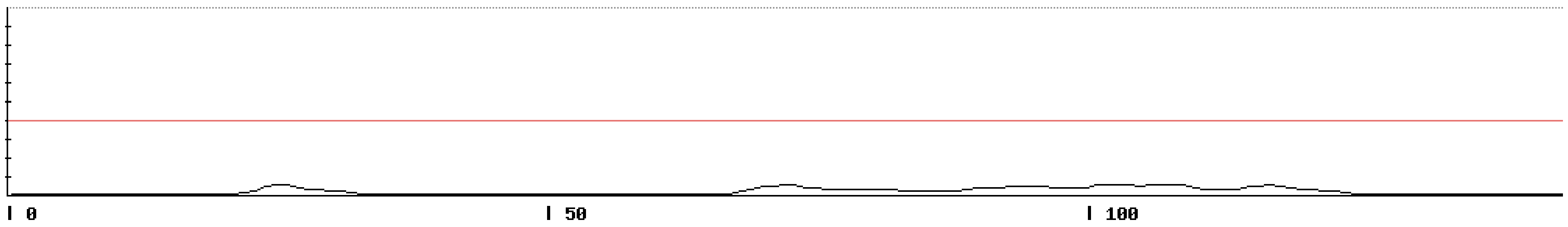

Predictions for Gossypiumarboreum_Cotton_A_19848_BGI-A2_v1.0

Viterbi Path

Viterbi Path

Posterior @ 0.4

Posterior @ 0.4

Posterior Graphic. Threshold Marked @ 0.4

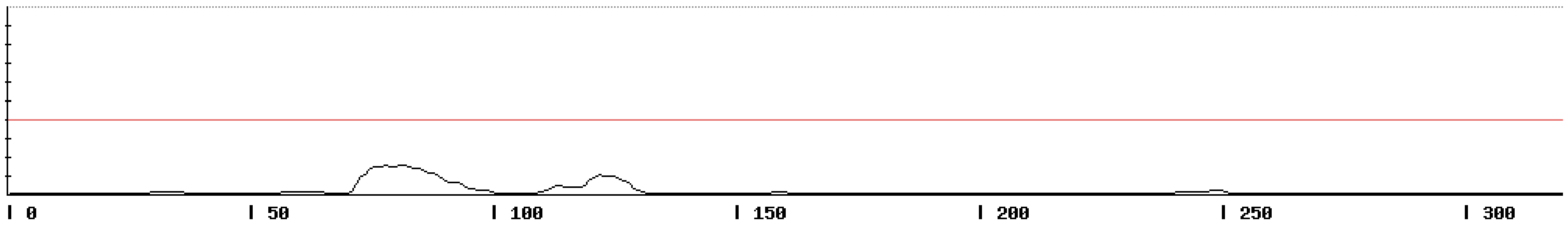

Predictions for Gossypiumarboreum_Cotton_A_20638_BGI-A2_v1.0

Viterbi Path

Viterbi Path

Posterior @ 0.4	160 - RKRRRIQNRH - 169
	176 - VRWHKTGKTKPVIENGVRKGWKKIMVLYKRSNR - 208
Posterior @ 0.4	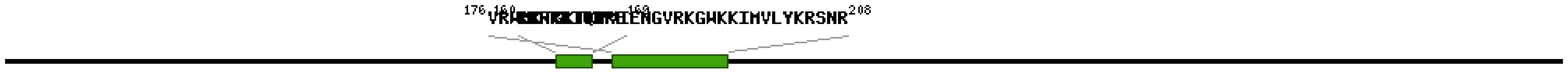
Posterior Graphic. Threshold Marked @ 0.4	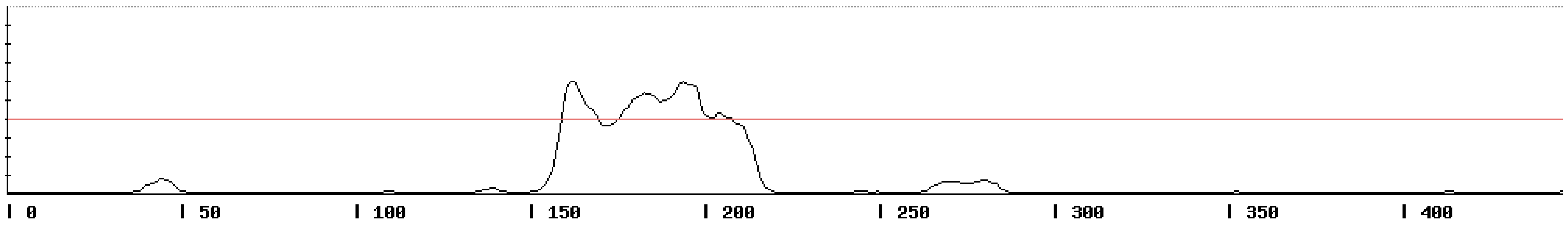

Predictions for Gossypiumarboreum_Cotton_A_20658_BGI-A2_v1.0

Viterbi Path	
Viterbi Path	
Posterior @ 0.4	
Posterior @ 0.4	
Posterior Graphic. Threshold Marked @ 0.4	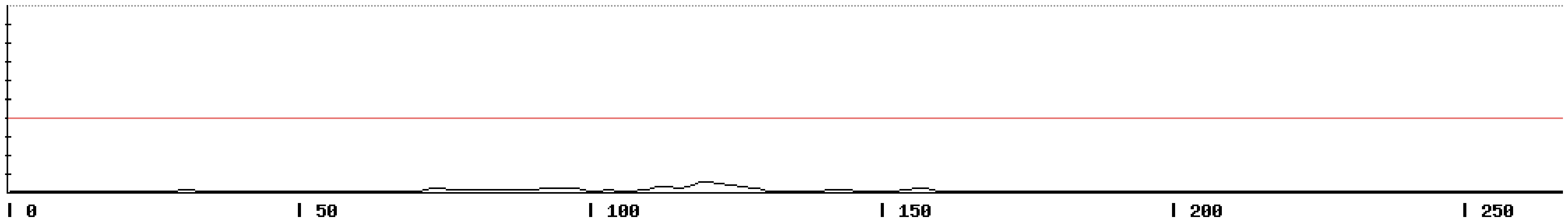

Predictions for Gossypiumarboreum_Cotton_A_21002_BGI-A2_v1.0

Viterbi Path	
Viterbi Path	

Posterior @ 0.4

Posterior @ 0.4

Posterior Graphic. Threshold Marked @ 0.4

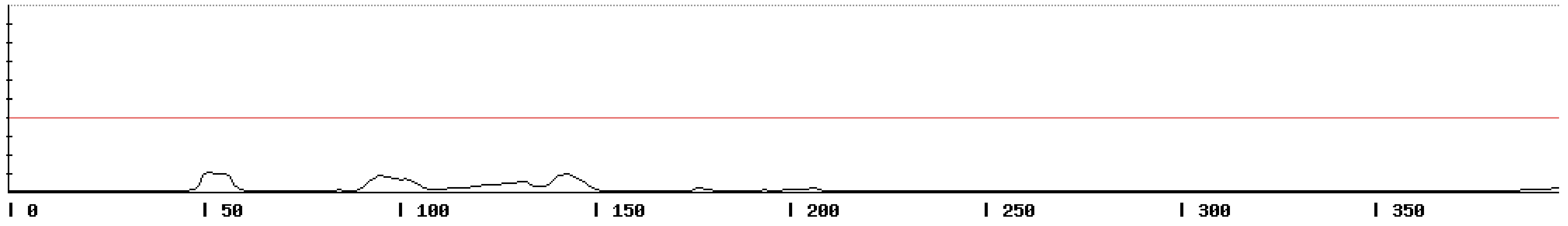

Predictions for Gossypiumarboreum_Cotton_A_22700_BGI-A2_v1.0

Viterbi Path

Viterbi Path

Posterior @ 0.4 | 81 - KKRKR - 85

Posterior @ 0.4

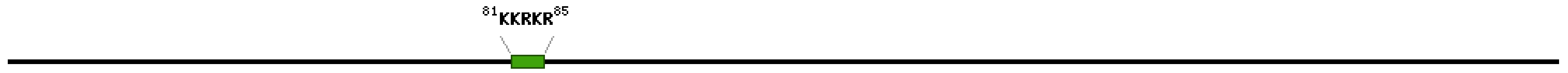

Posterior Graphic. Threshold Marked @ 0.4

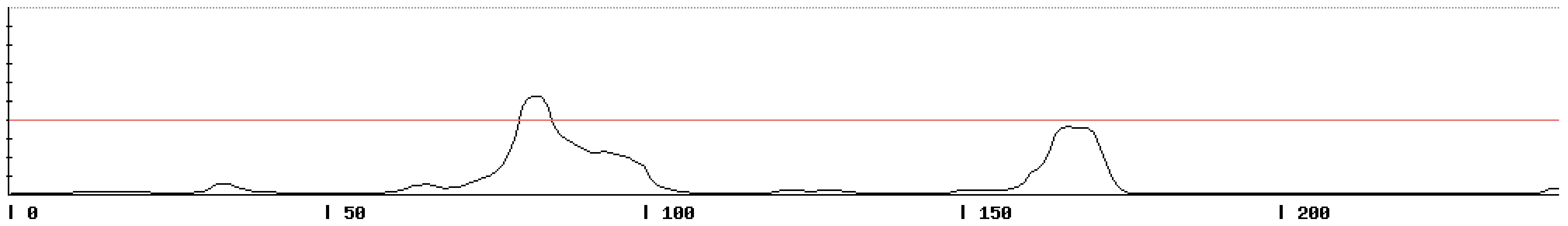

Predictions for Gossypiumarboreum_Cotton_A_23128_BGI-A2_v1.0

Viterbi Path

Viterbi Path

Posterior @ 0.4

Posterior @ 0.4

Posterior Graphic. Threshold Marked @ 0.4

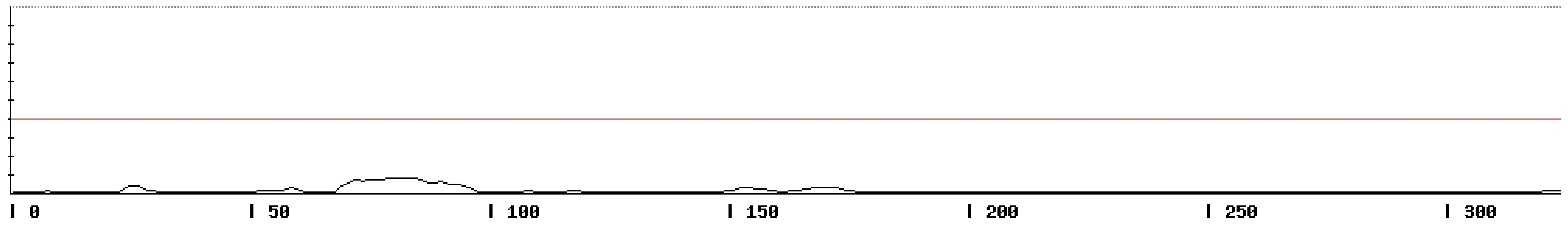

Predictions for Gossypiumarboreum_Cotton_A_23892_BGI-A2_v1.0

Viterbi Path

Viterbi Path

Posterior @ 0.4

Posterior @ 0.4

Posterior Graphic. Threshold Marked @ 0.4

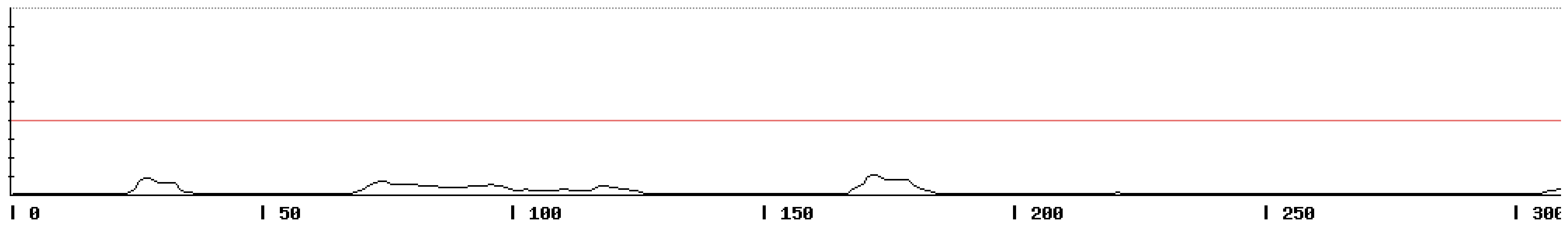

Predictions for Gossypiumarboreum_Cotton_A_23920_BGI-A2_v1.0	
Viterbi Path	
Viterbi Path	
Posterior @ 0.4	
Posterior @ 0.4	
Posterior Graphic. Threshold Marked @ 0.4	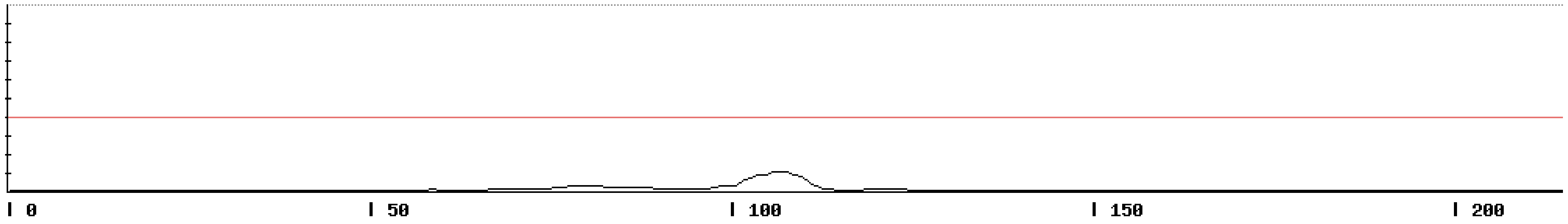

Predictions for Gossypiumarboreum_Cotton_A_24225_BGI-A2_v1.0	
Viterbi Path	
Viterbi Path	
Posterior @ 0.4	
Posterior @ 0.4	
Posterior Graphic. Threshold Marked @ 0.4	

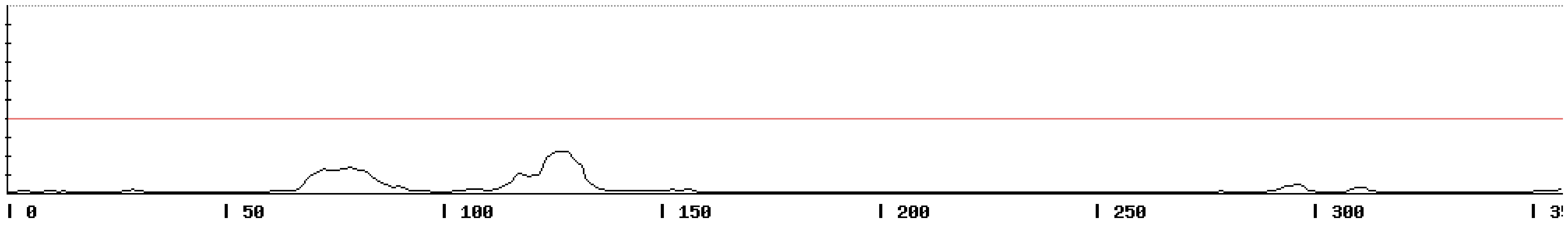

Predictions for Gossypiumarboreum_Cotton_A_24792_BGI-A2_v1.0

Viterbi Path

Viterbi Path

Posterior @ 0.4 163 - KRNKKIKQDSRQGAAKR - 179

Posterior @ 0.4

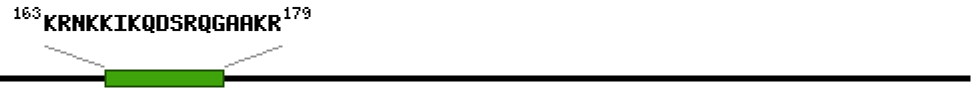

Posterior Graphic. Threshold Marked @ 0.4

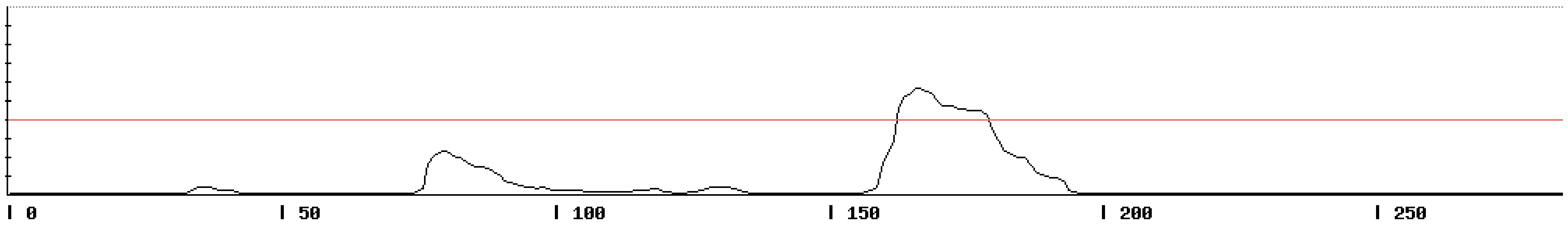

Predictions for Gossypiumarboreum_Cotton_A_24793_BGI-A2_v1.0

Viterbi Path

Viterbi Path

Posterior @ 0.4 166 - RK - 167

Posterior @ 0.4

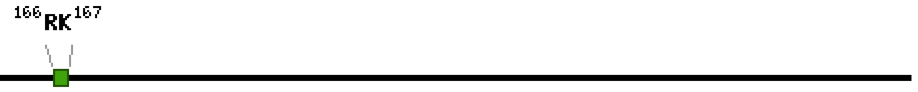

Posterior Graphic. Threshold Marked @ 0.4

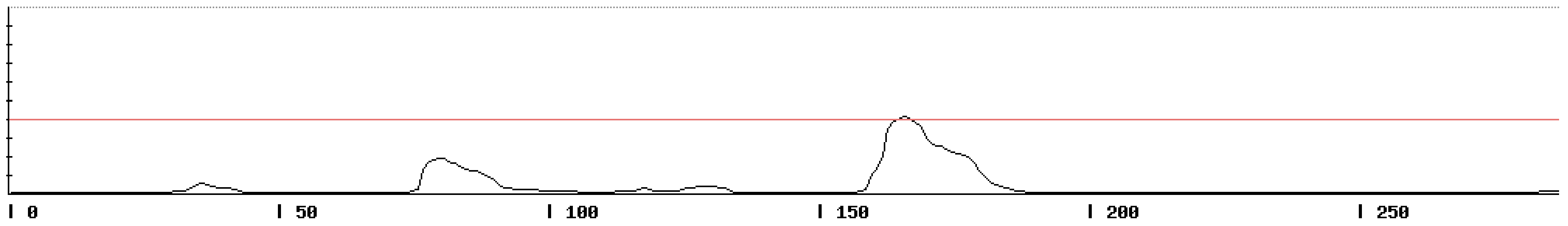

Predictions for Gossypiumarboreum_Cotton_A_24794_BGI-A2_v1.0

Viterbi Path

Viterbi Path

Posterior @ 0.4 163 - KRHKKNKQE - 171

Posterior @ 0.4

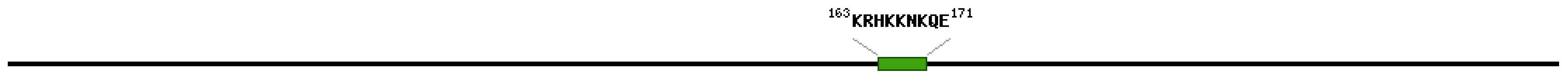

Posterior Graphic. Threshold Marked @ 0.4

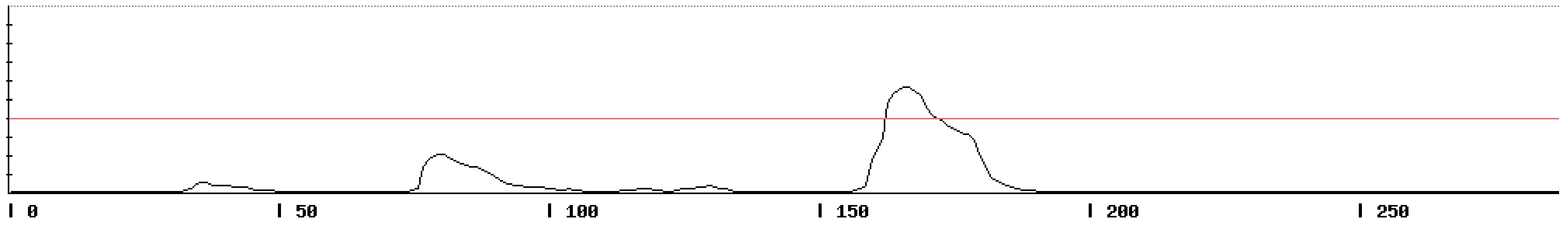

Predictions for Gossypiumarboreum_Cotton_A_24945_BGI-A2_v1.0

Viterbi Path

Viterbi Path

Posterior @ 0.4

Posterior @ 0.4

Posterior Graphic. Threshold Marked @ 0.4

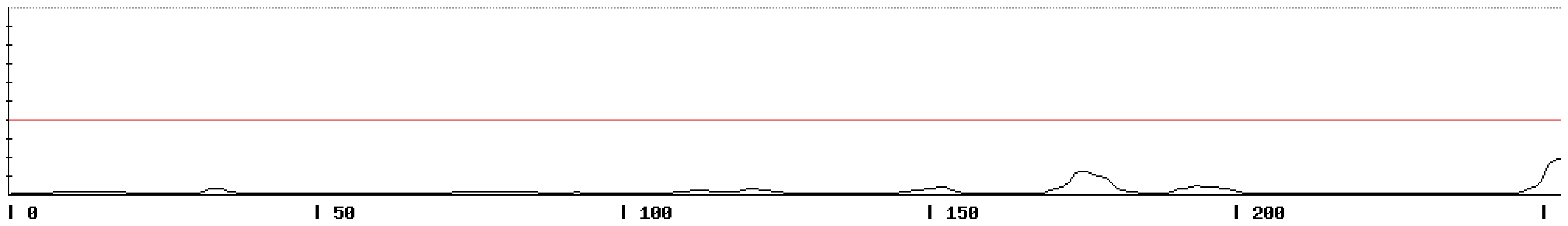

Predictions for Gossypiumarboreum_Cotton_A_25050_BGI-A2_v1.0

Viterbi Path

Viterbi Path

Posterior @ 0.4

Posterior @ 0.4

Posterior Graphic. Threshold Marked @ 0.4

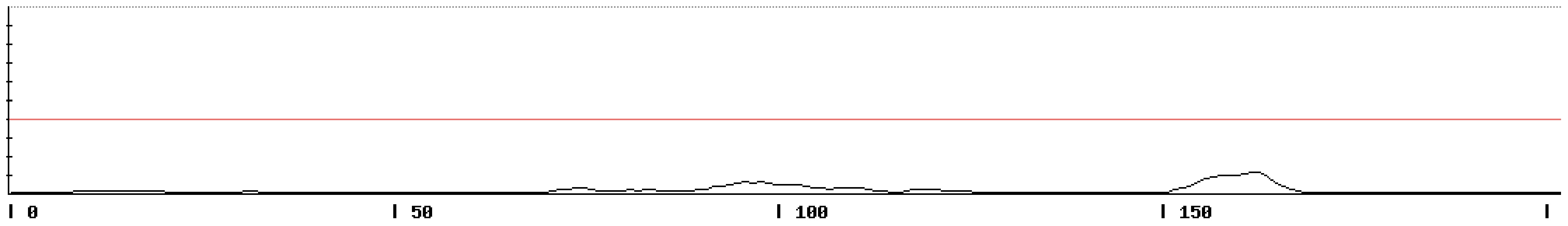

Predictions for Gossypiumarboreum_Cotton_A_25101_BGI-A2_v1.0

Viterbi Path

Viterbi Path

Posterior @ 0.4	
Posterior @ 0.4	
Posterior Graphic. Threshold Marked @ 0.4	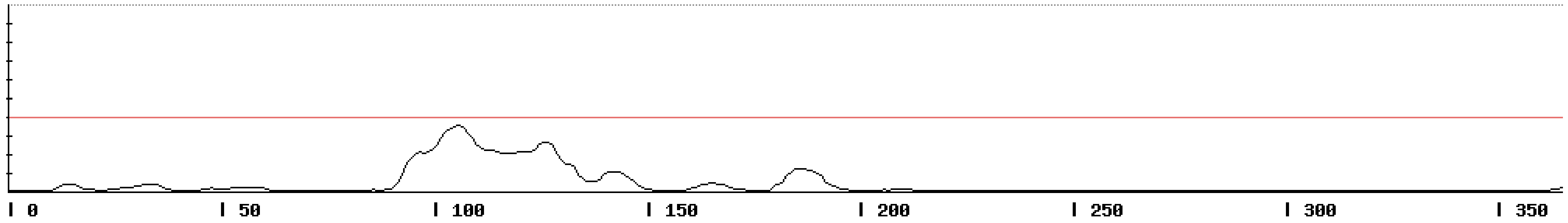

Predictions for Gossypiumarboreum_Cotton_A_25115_BGI-A2_v1.0

Viterbi Path	
Viterbi Path	
Posterior @ 0.4	
Posterior @ 0.4	
Posterior Graphic. Threshold Marked @ 0.4	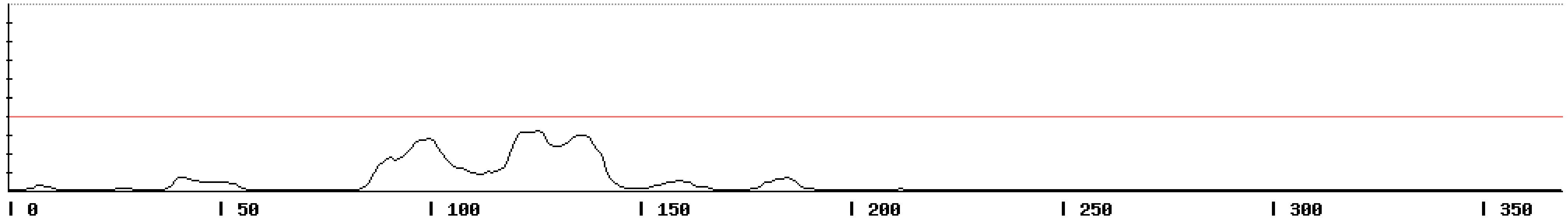

Predictions for Gossypiumarboreum_Cotton_A_25243_BGI-A2_v1.0

Viterbi Path	
Viterbi Path	

Posterior @ 0.4 — 101 - RKHPQGKRPNR - 111

Posterior @ 0.4

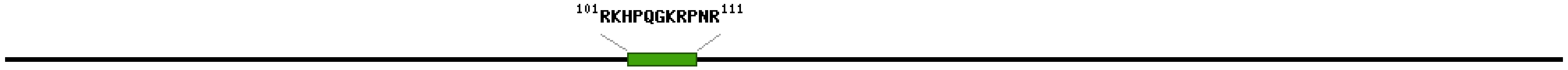

Posterior Graphic. Threshold Marked @ 0.4

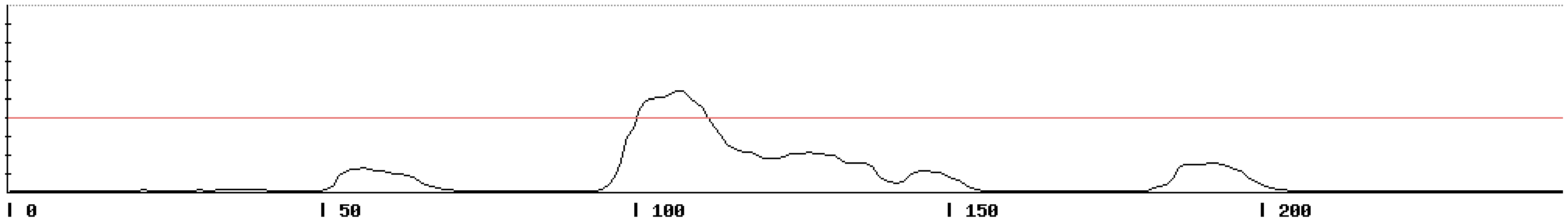

Predictions for Gossypiumarboreum_Cotton_A_25450_BGI-A2_v1.0

Viterbi Path

Viterbi Path

Posterior @ 0.4 — 97 - IGKPKALGIK - 106

Posterior @ 0.4

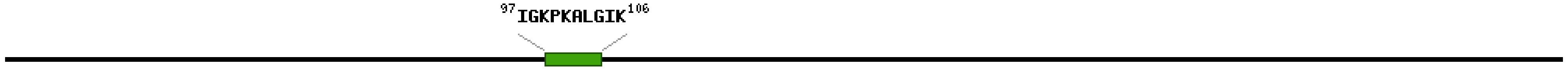

Posterior Graphic. Threshold Marked @ 0.4

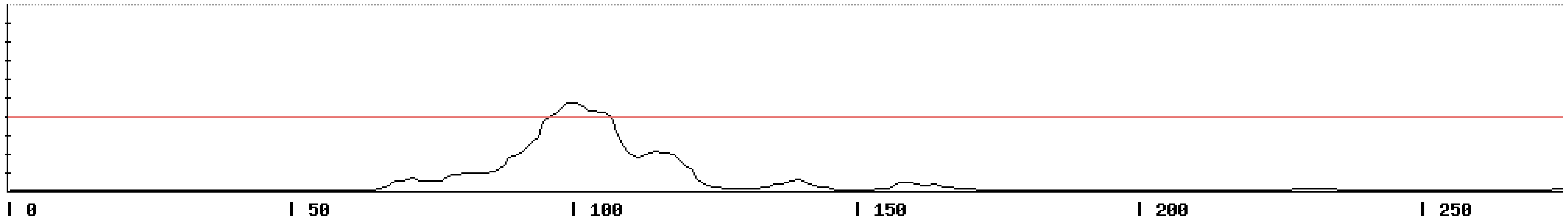

Predictions for Gossypiumarboreum_Cotton_A_26263_BGI-A2_v1.0

Viterbi Path	
Viterbi Path	
Posterior @ 0.4	
Posterior @ 0.4	
Posterior Graphic. Threshold Marked @ 0.4	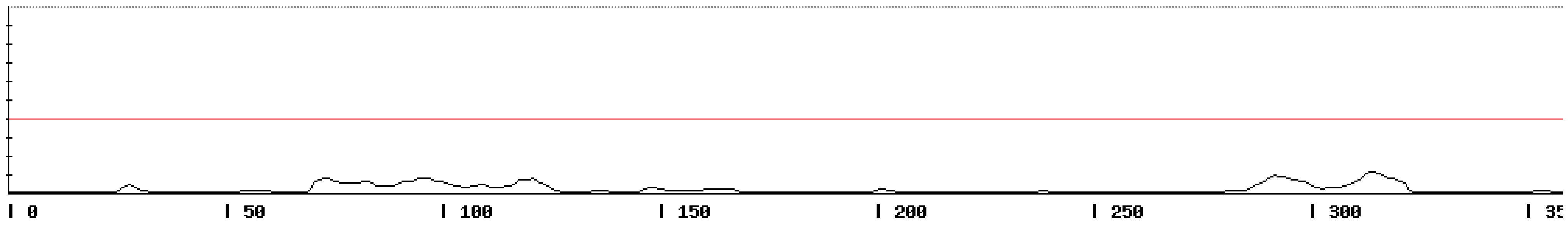

Predictions for Gossypiumarboreum_Cotton_A_26270_BGI-A2_v1.0

Viterbi Path	
Viterbi Path	
Posterior @ 0.4	123 - GRK - 125
Posterior @ 0.4	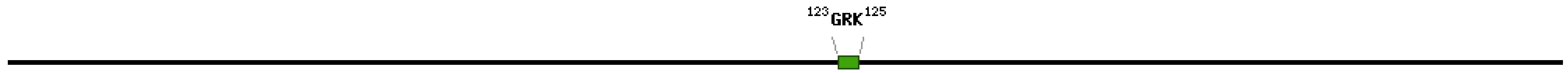
Posterior Graphic. Threshold Marked @ 0.4	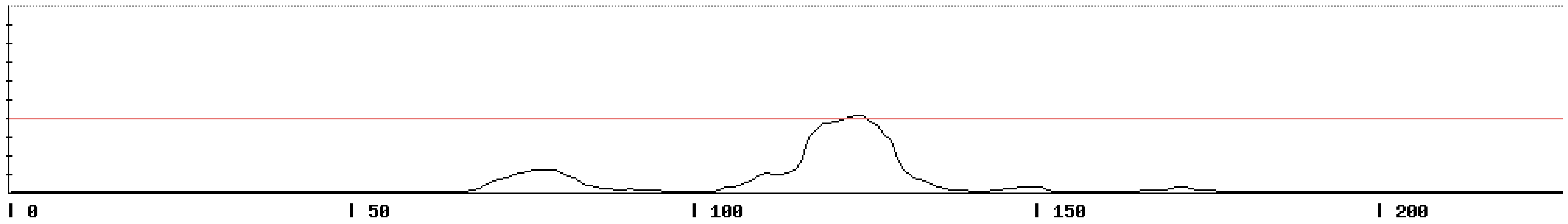

Predictions for Gossypiumarboreum_Cotton_A_26426_BGI-A2_v1.0

Viterbi Path

Viterbi Path

Posterior @ 0.4

Posterior @ 0.4

Posterior Graphic. Threshold Marked @ 0.4

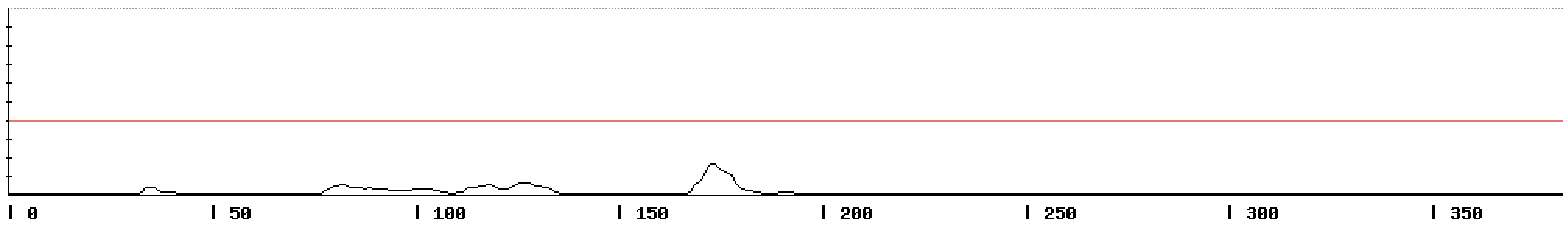

Predictions for Gossypiumarboreum_Cotton_A_27343_BGI-A2_v1.0

Viterbi Path

Viterbi Path

Posterior @ 0.4

Posterior @ 0.4

Posterior Graphic. Threshold Marked @ 0.4

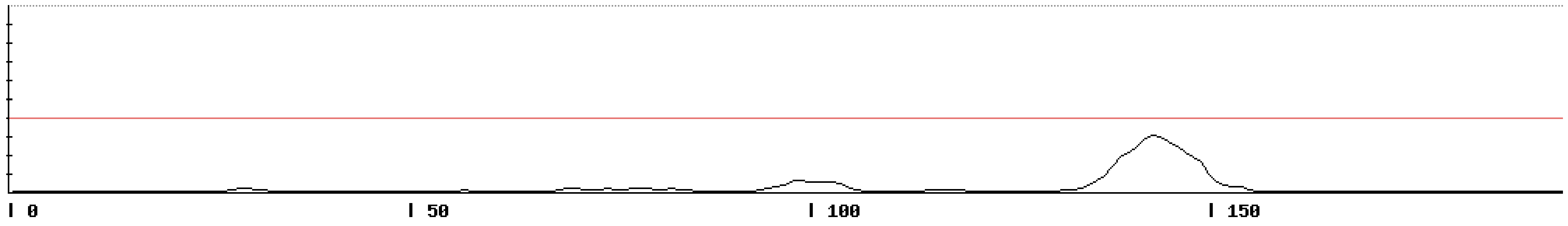

Predictions for Gossypiumarboreum_Cotton_A_27805_BGI-A2_v1.0

Viterbi Path

Viterbi Path

Posterior @ 0.4: 72 - LKKKSKSR - 79

Posterior @ 0.4

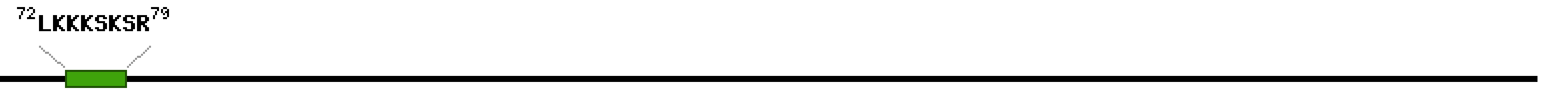

Posterior Graphic. Threshold Marked @ 0.4

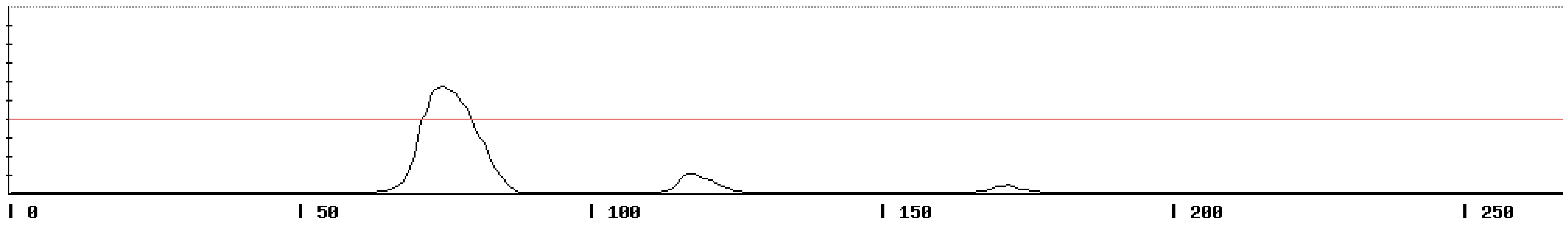

Predictions for Gossypiumarboreum_Cotton_A_27806_BGI-A2_v1.0

Viterbi Path

Viterbi Path

Posterior @ 0.4: 72 - LKKKSKSR - 79

Posterior @ 0.4

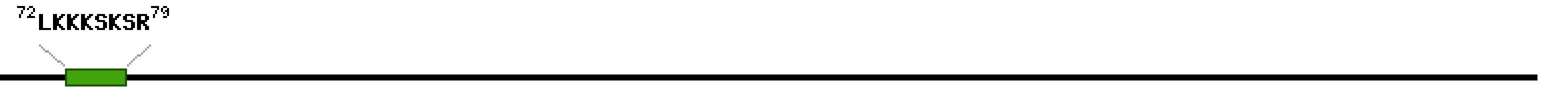

Posterior Graphic. Threshold Marked @ 0.4

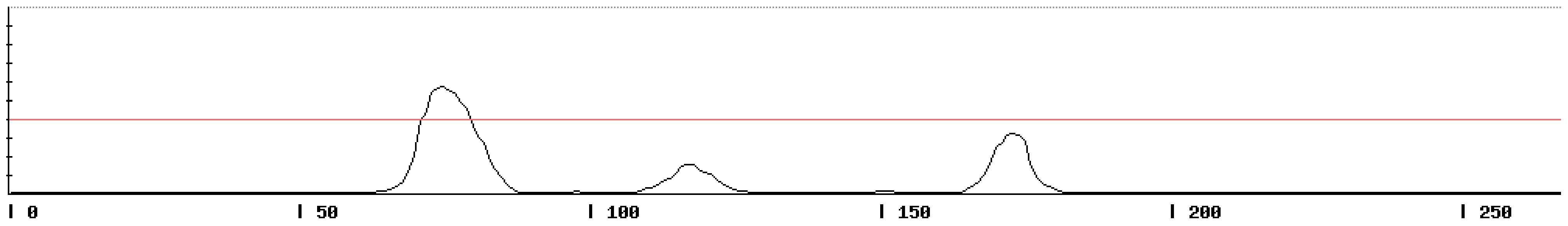

Predictions for Gossypiumarboreum_Cotton_A_28228_BGI-A2_v1.0

Viterbi Path

Viterbi Path

Posterior @ 0.4

68 - LKKKSKSR - 75

113 - KKKKK - 117

165 - KKNKKRK - 171

Posterior @ 0.4

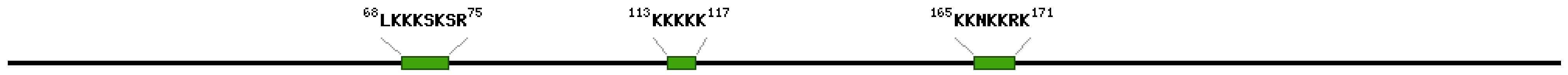

Posterior Graphic. Threshold Marked @ 0.4

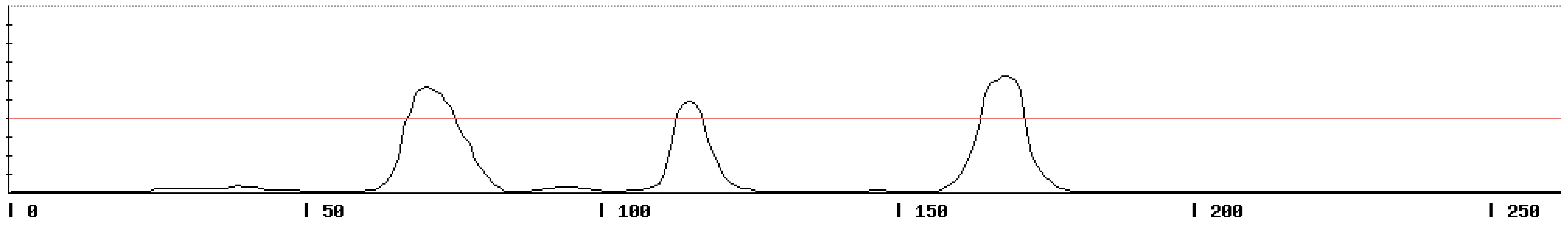

Predictions for Gossypiumarboreum_Cotton_A_28334_BGI-A2_v1.0

Viterbi Path

Viterbi Path

Posterior @ 0.4

Posterior @ 0.4

Posterior Graphic. Threshold Marked @ 0.4

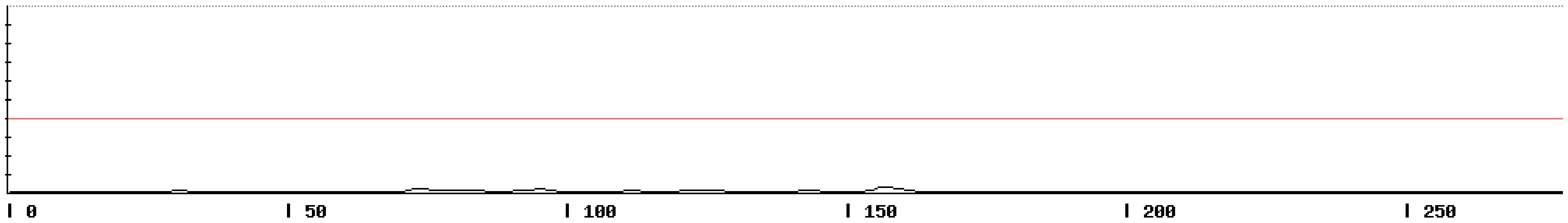

Predictions for Gossypiumarboreum_Cotton_A_28452_BGI-A2_v1.0

Viterbi Path

Viterbi Path

Posterior @ 0.4

Posterior @ 0.4

Posterior Graphic. Threshold Marked @ 0.4

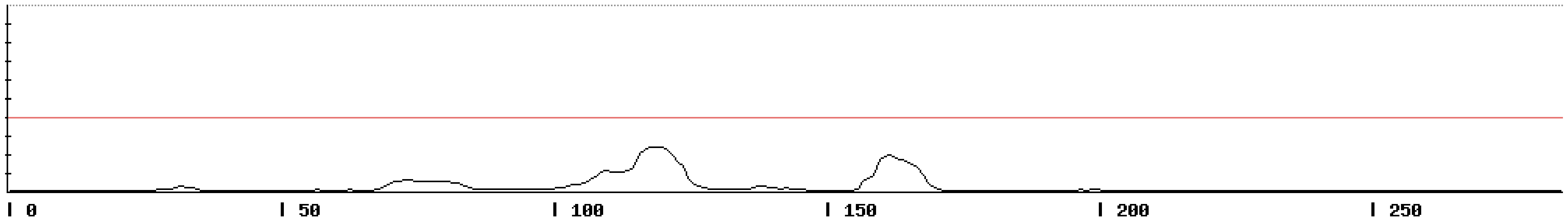

Predictions for Gossypiumarboreum_Cotton_A_28834_BGI-A2_v1.0

Viterbi Path

Viterbi Path

Posterior @ 0.4	
Posterior @ 0.4	
Posterior Graphic. Threshold Marked @ 0.4	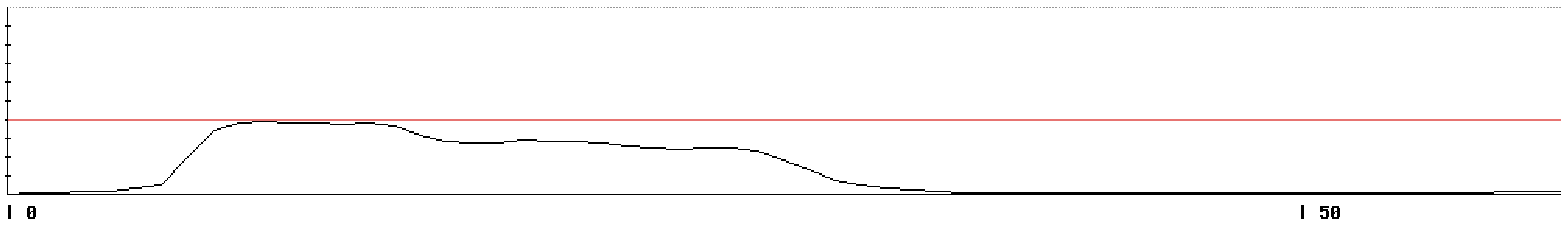

Predictions for Gossypiumarboreum_Cotton_A_29050_BGI-A2_v1.0	
Viterbi Path	
Viterbi Path	
Posterior @ 0.4	141 - QRKRKARKHAAK - 152
Posterior @ 0.4	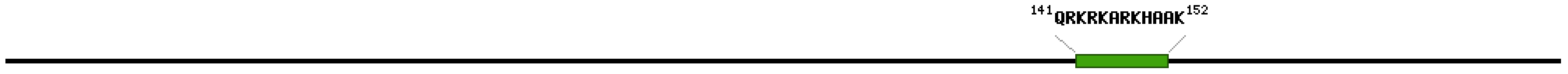
Posterior Graphic. Threshold Marked @ 0.4	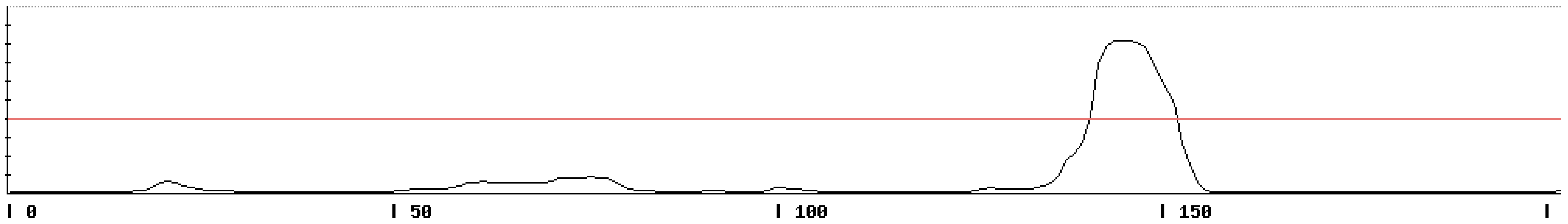

Predictions for Gossypiumarboreum_Cotton_A_29108_BGI-A2_v1.0	
Viterbi Path	
Viterbi Path	

Posterior @ 0.4

Posterior @ 0.4

Posterior Graphic. Threshold Marked @ 0.4

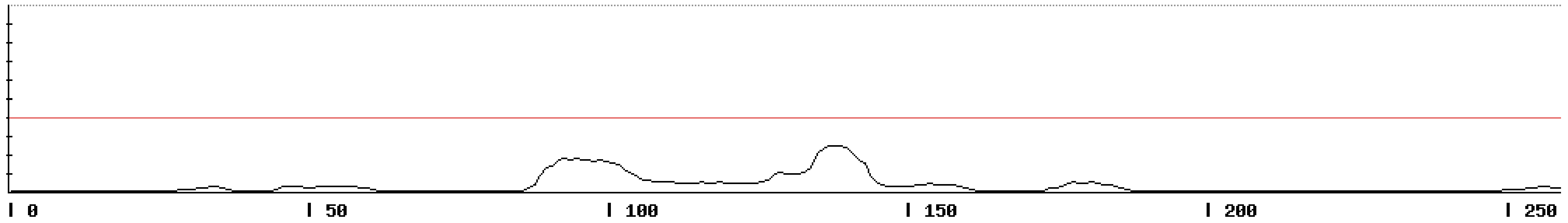

Predictions for Gossypiumarboreum_Cotton_A_29173_BGI-A2_v1.0

Viterbi Path

Viterbi Path

Posterior @ 0.4

Posterior @ 0.4

Posterior Graphic. Threshold Marked @ 0.4

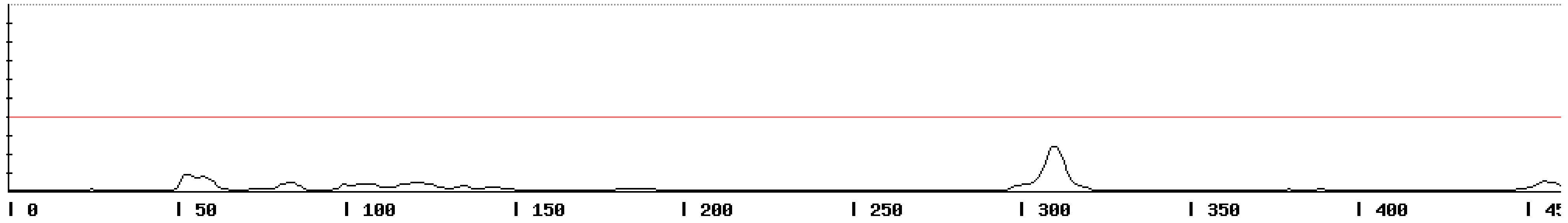

Predictions for Gossypiumarboreum_Cotton_A_29176_BGI-A2_v1.0

Viterbi Path	
Viterbi Path	
Posterior @ 0.4	
Posterior @ 0.4	
Posterior Graphic. Threshold Marked @ 0.4	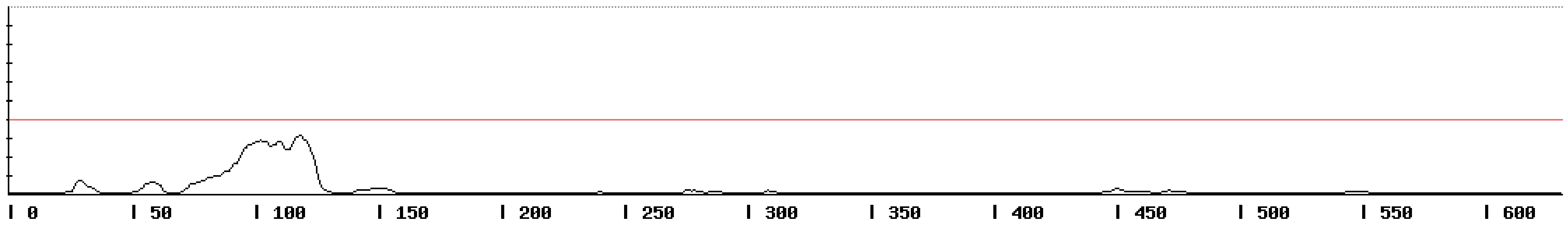

Predictions for Gossypiumarboreum_Cotton_A_29202_BGI-A2_v1.0

Viterbi Path	
Viterbi Path	
Posterior @ 0.4	
Posterior @ 0.4	
Posterior Graphic. Threshold Marked @ 0.4	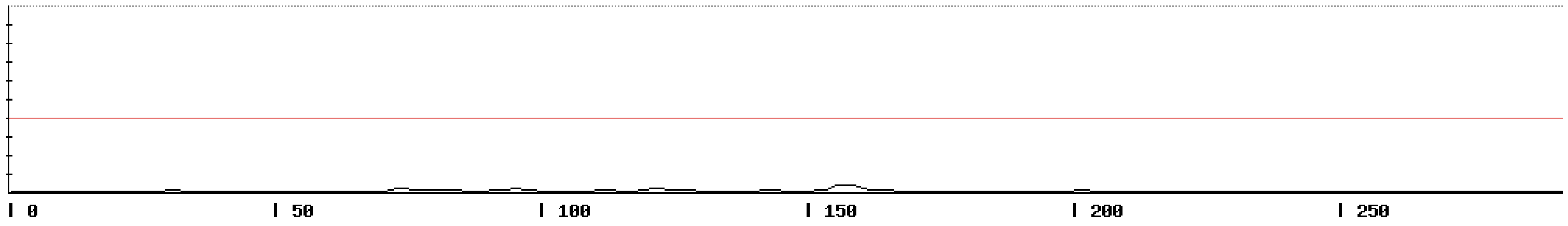

Predictions for Gossypiumarboreum_Cotton_A_29356_BGI-A2_v1.0	
Viterbi Path	
Viterbi Path	
Posterior @ 0.4	129 - KKKRKM - 134
Posterior @ 0.4	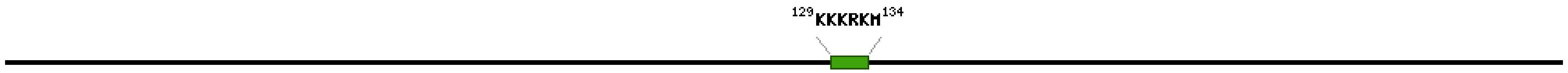
Posterior Graphic. Threshold Marked @ 0.4	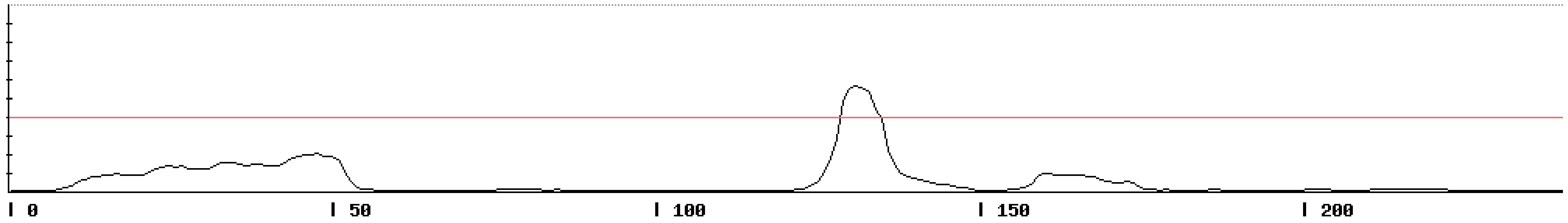

Predictions for Gossypiumarboreum_Cotton_A_29358_BGI-A2_v1.0	
Viterbi Path	
Viterbi Path	
Posterior @ 0.4	
Posterior @ 0.4	
Posterior Graphic. Threshold Marked @ 0.4	

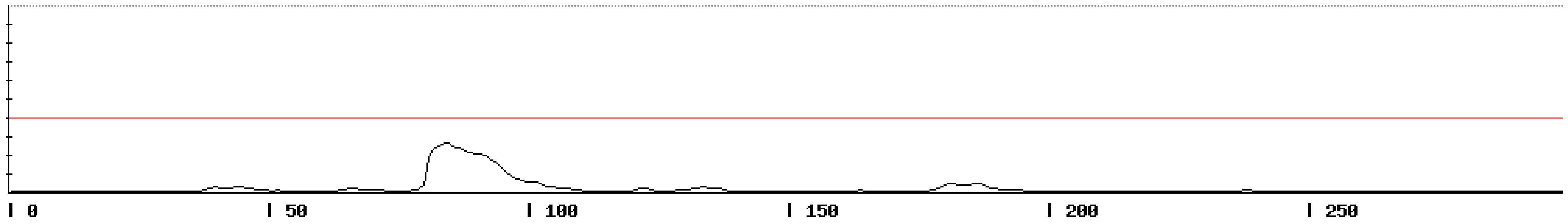

Predictions for Gossypiumarboreum_Cotton_A_30404_BGI-A2_v1.0

Viterbi Path

Viterbi Path

Posterior @ 0.4

Posterior @ 0.4

Posterior Graphic. Threshold Marked @ 0.4

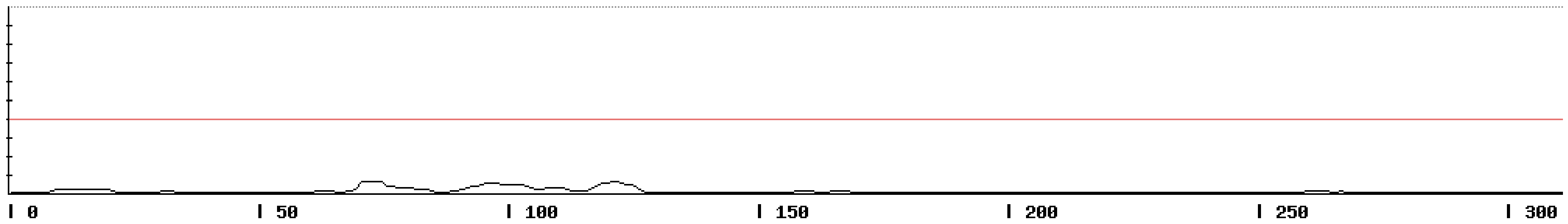

Predictions for Gossypiumarboreum_Cotton_A_30603_BGI-A2_v1.0

Viterbi Path

Viterbi Path

Posterior @ 0.4

Posterior @ 0.4

Posterior Graphic. Threshold Marked @ 0.4	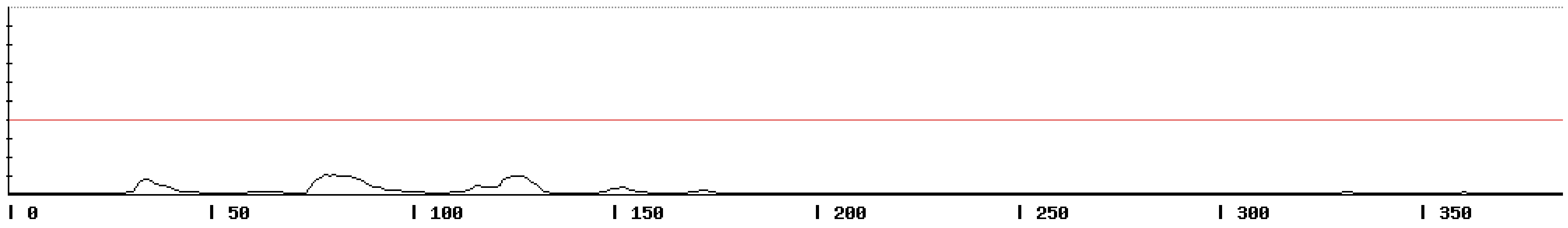

Predictions for Gossypiumarboreum_Cotton_A_31522_BGI-A2_v1.0

Viterbi Path	
Viterbi Path	
Posterior @ 0.4	
Posterior @ 0.4	
Posterior Graphic. Threshold Marked @ 0.4	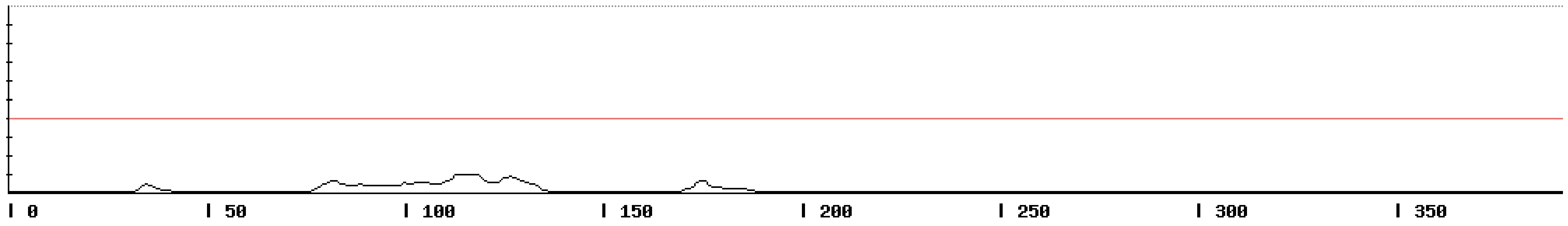

Predictions for Gossypiumarboreum_Cotton_A_32448_BGI-A2_v1.0

Viterbi Path	
Viterbi Path	
Posterior @ 0.4	
Posterior @ 0.4	

Posterior Graphic. Threshold Marked @ 0.4

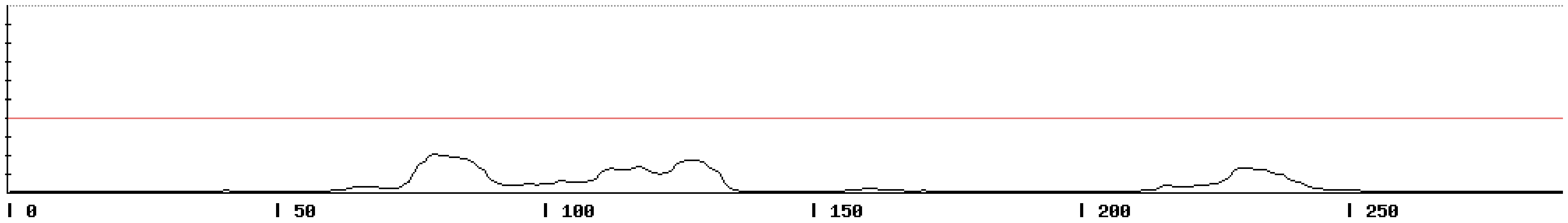

Predictions for Gossypiumarboreum_Cotton_A_32478_BGI-A2_v1.0

Viterbi Path

Viterbi Path

Posterior @ 0.4

Posterior @ 0.4

Posterior Graphic. Threshold Marked @ 0.4

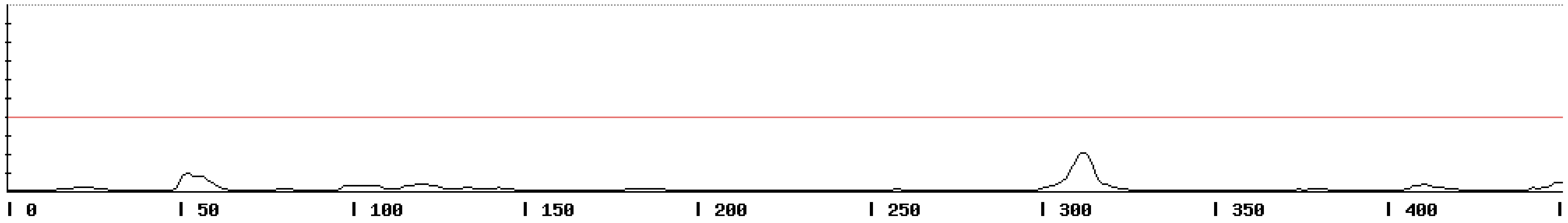

Predictions for Gossypiumarboreum_Cotton_A_32870_BGI-A2_v1.0

Viterbi Path

Viterbi Path

Posterior @ 0.4

Posterior @ 0.4

Posterior Graphic. Threshold Marked @ 0.4

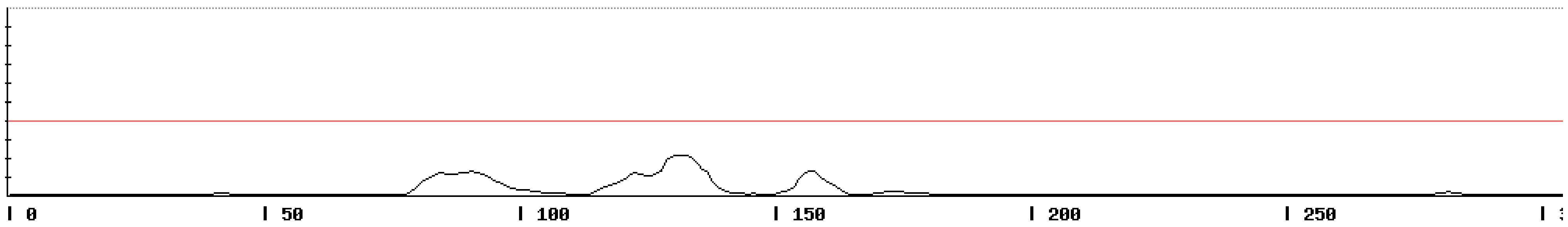

Predictions for Gossypiumarboreum_Cotton_A_33158_BGI-A2_v1.0

Viterbi Path

Viterbi Path

Posterior @ 0.4

Posterior @ 0.4

Posterior Graphic. Threshold Marked @ 0.4

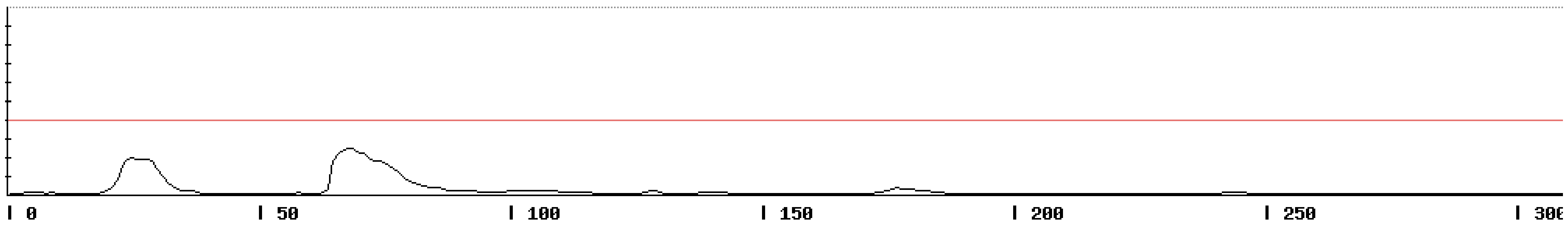

Predictions for Gossypiumarboreum_Cotton_A_33477_BGI-A2_v1.0

Viterbi Path

Viterbi Path

Posterior @ 0.4

Posterior @ 0.4

Posterior Graphic. Threshold Marked @ 0.4

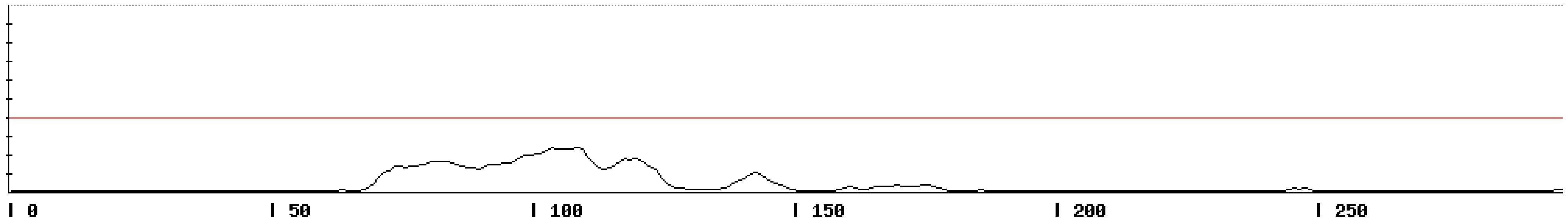

Predictions for Gossypiumarboreum_Cotton_A_33555_BGI-A2_v1.0

Viterbi Path

Viterbi Path

Posterior @ 0.4 98 - KDRKI - 102

Posterior @ 0.4

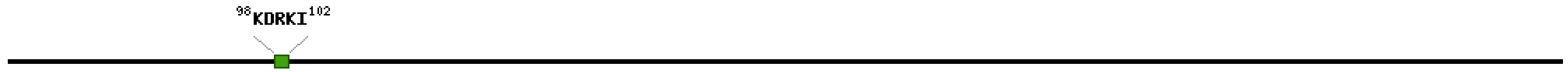

Posterior Graphic. Threshold Marked @ 0.4

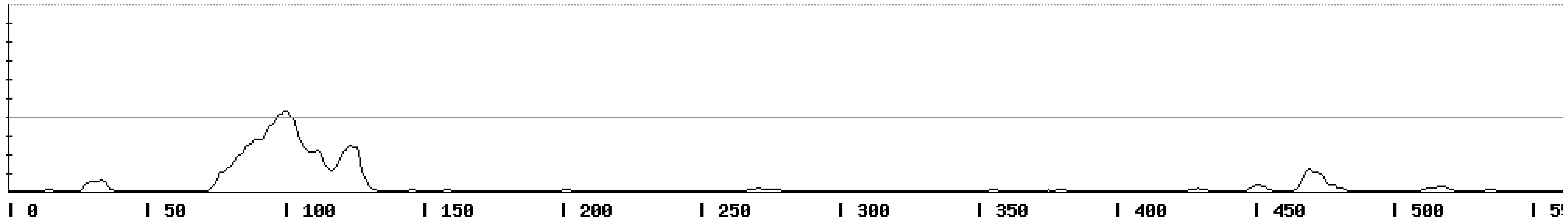

Predictions for Gossypiumarboreum_Cotton_A_34222_BGI-A2_v1.0

Viterbi Path	
Viterbi Path	
Posterior @ 0.4	
Posterior @ 0.4	
Posterior Graphic. Threshold Marked @ 0.4	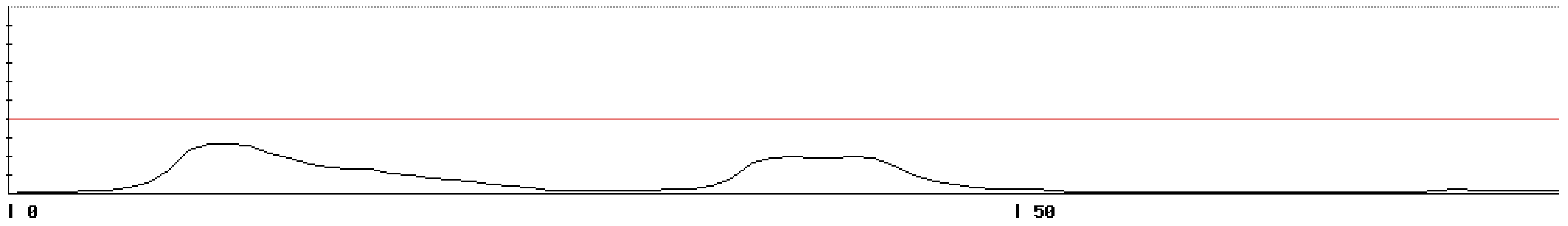

Predictions for Gossypiumarboreum_Cotton_A_34465_BGI-A2_v1.0

Viterbi Path	
Viterbi Path	
Posterior @ 0.4	129 - RKRRK - 133
Posterior @ 0.4	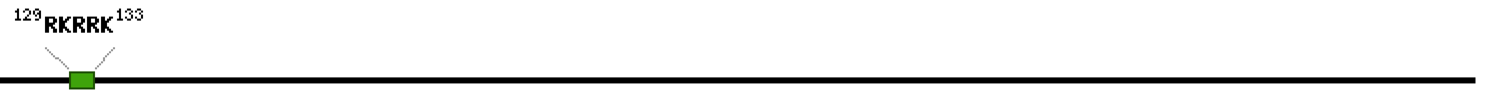
Posterior Graphic. Threshold Marked @ 0.4	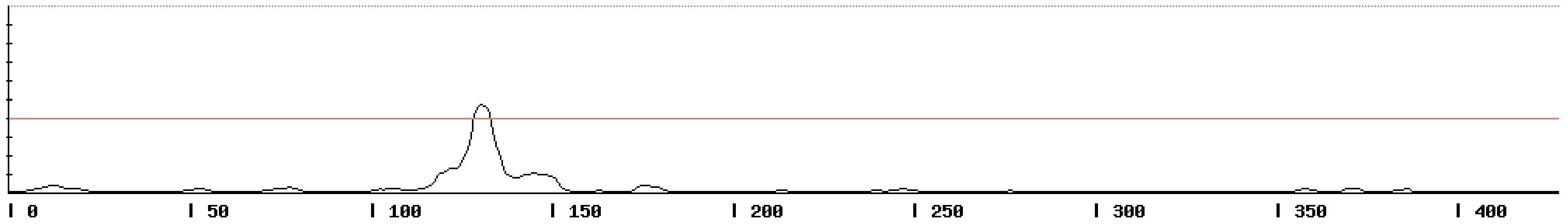

Predictions for Gossypiumarboreum_Cotton_A_34560_BGI-A2_v1.0	
Viterbi Path	
Viterbi Path	
Posterior @ 0.4	72 - LKKKSKSR - 79
Posterior @ 0.4	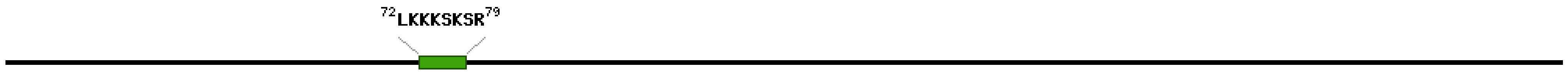
Posterior Graphic. Threshold Marked @ 0.4	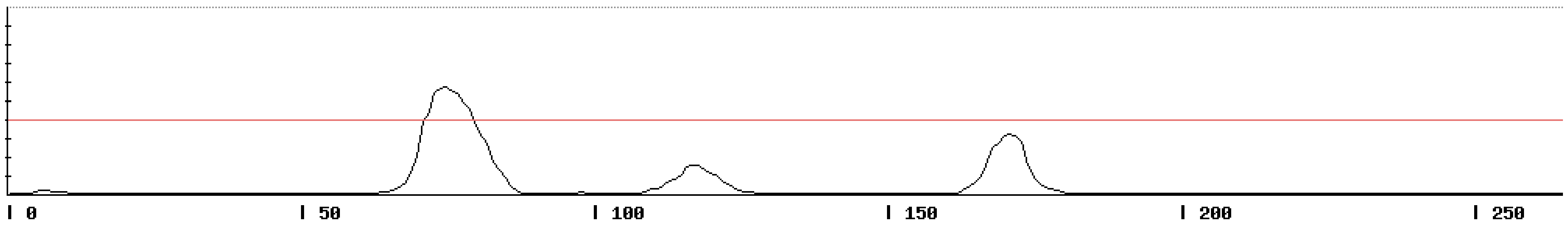

Predictions for Gossypiumarboreum_Cotton_A_35053_BGI-A2_v1.0	
Viterbi Path	
Viterbi Path	
Posterior @ 0.4	
Posterior @ 0.4	
Posterior Graphic. Threshold Marked @ 0.4	

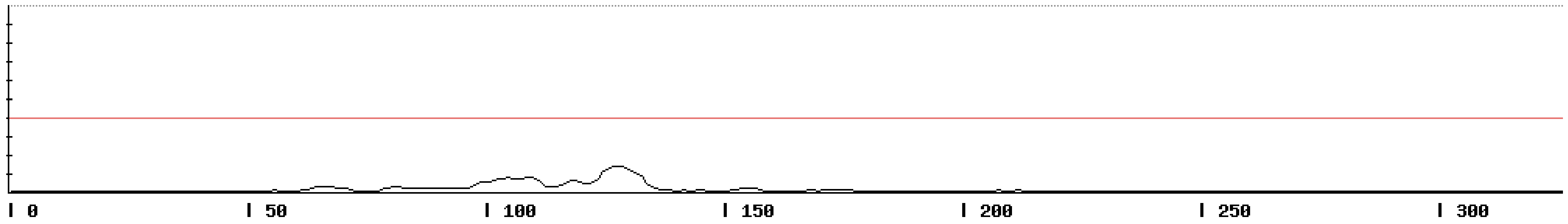

Predictions for Gossypiumarboreum_Cotton_A_35198_BGI-A2_v1.0

Viterbi Path

Viterbi Path

Posterior @ 0.4

Posterior @ 0.4

Posterior Graphic. Threshold Marked @ 0.4

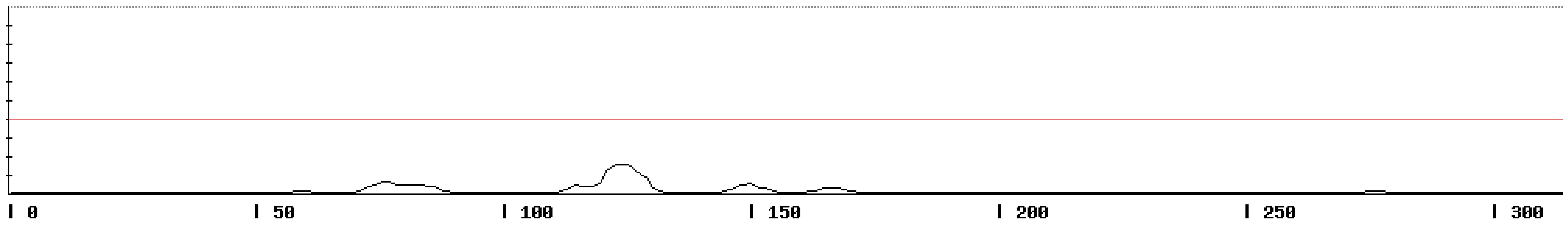

Predictions for Gossypiumarboreum_Cotton_A_35235_BGI-A2_v1.0

Viterbi Path

Viterbi Path

Posterior @ 0.4 97 - KDRTIKSKKSLIG - 109

Posterior @ 0.4

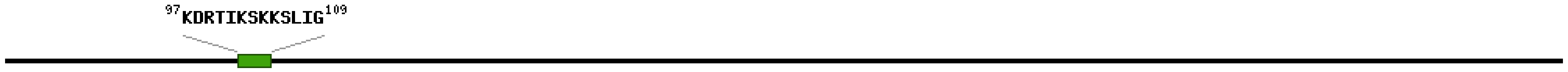

Posterior Graphic. Threshold Marked @ 0.4

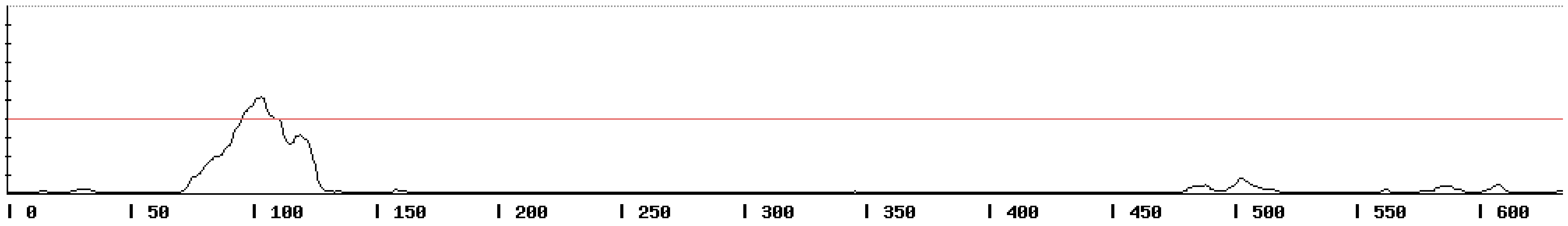

Predictions for Gossypiumarboreum_Cotton_A_35583_BGI-A2_v1.0

Viterbi Path

Viterbi Path

Posterior @ 0.4

Posterior @ 0.4

Posterior Graphic. Threshold Marked @ 0.4

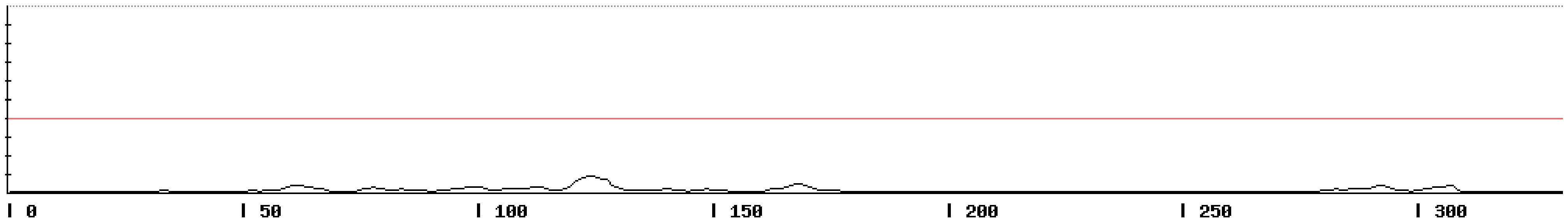

Predictions for Gossypiumarboreum_Cotton_A_35637_BGI-A2_v1.0

Viterbi Path

Viterbi Path

Posterior @ 0.4

Posterior @ 0.4

Posterior Graphic. Threshold Marked @ 0.4

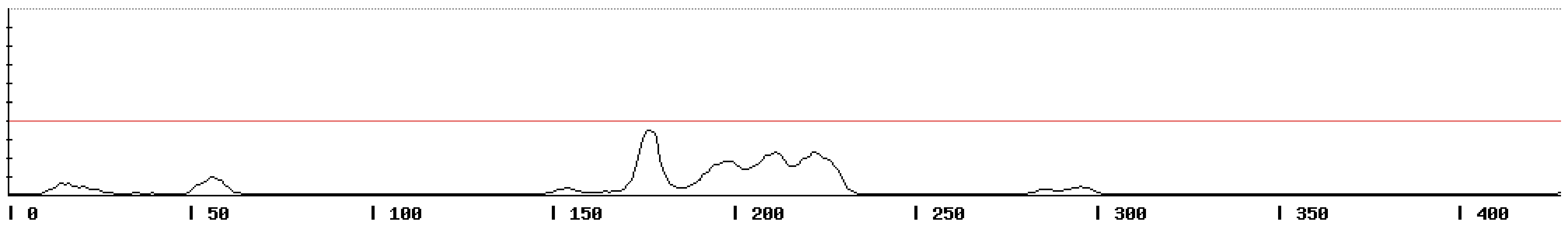

Predictions for Gossypiumarboreum_Cotton_A_36063_BGI-A2_v1.0

Viterbi Path

Viterbi Path

Posterior @ 0.4

Posterior @ 0.4

Posterior Graphic. Threshold Marked @ 0.4

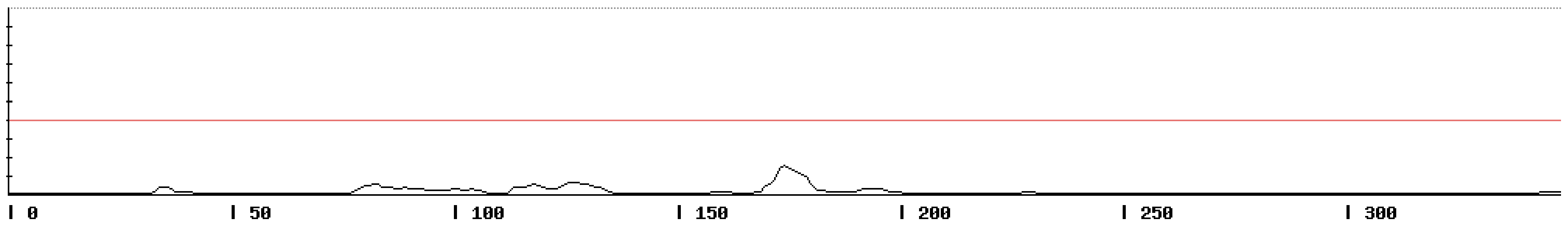

Predictions for Gossypiumarboreum_Cotton_A_36235_BGI-A2_v1.0

Viterbi Path

Viterbi Path

Posterior @ 0.4

Posterior @ 0.4

Posterior Graphic. Threshold Marked @ 0.4

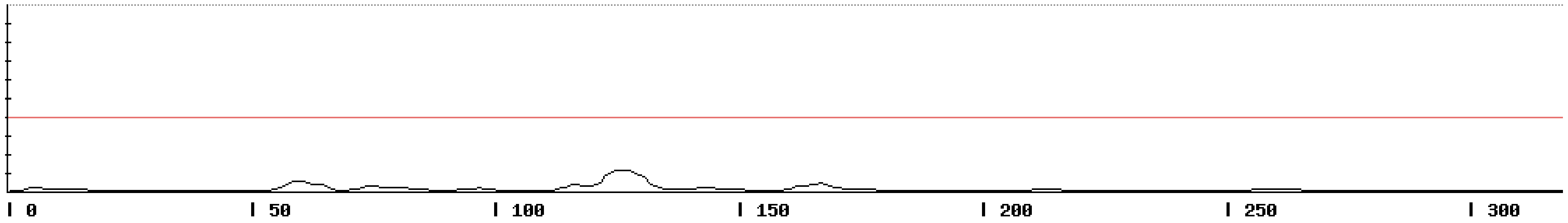

Predictions for Gossypiumarboreum_Cotton_A_36402_BGI-A2_v1.0

Viterbi Path

Viterbi Path

Posterior @ 0.4

Posterior @ 0.4

Posterior Graphic. Threshold Marked @ 0.4

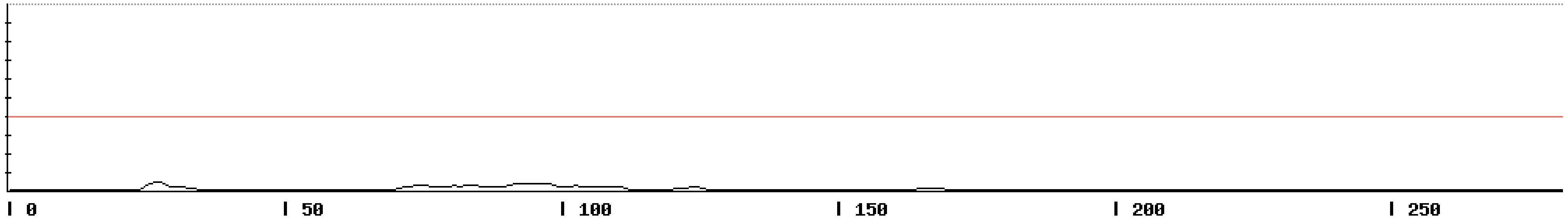

Predictions for Gossypiumarboreum_Cotton_A_36420_BGI-A2_v1.0

Viterbi Path

Viterbi Path

Posterior @ 0.4	78 - RGGKPKRR - 85
Posterior @ 0.4	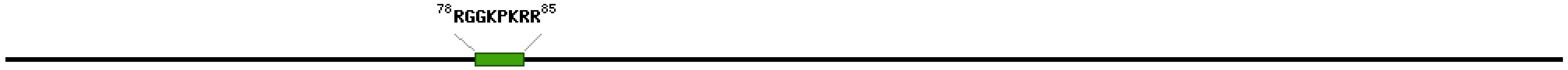
Posterior Graphic. Threshold Marked @ 0.4	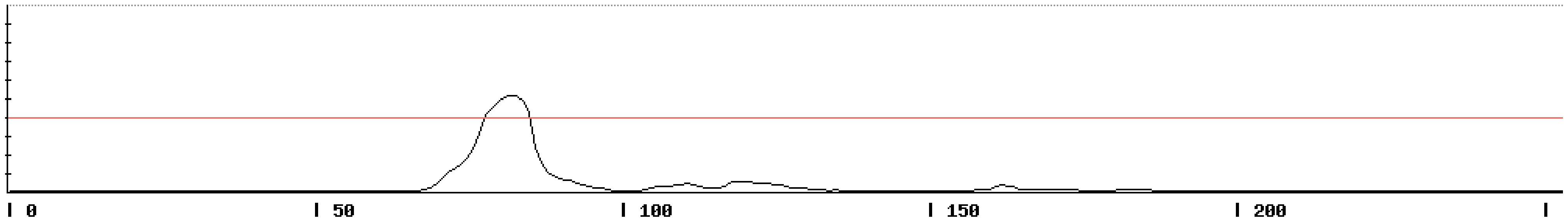

Predictions for Gossypiumarboreum_Cotton_A_36695_BGI-A2_v1.0

Viterbi Path	
Viterbi Path	
Posterior @ 0.4	
Posterior @ 0.4	
Posterior Graphic. Threshold Marked @ 0.4	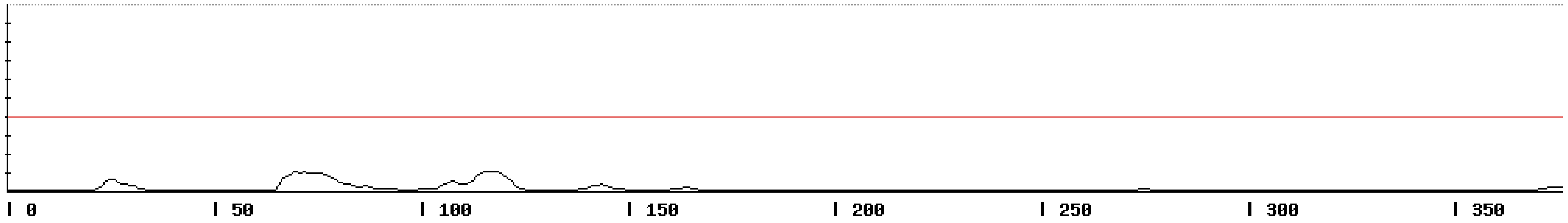

Predictions for Gossypiumarboreum_Cotton_A_36752_BGI-A2_v1.0

Viterbi Path

Viterbi Path

Posterior @ 0.4

Posterior @ 0.4

Posterior Graphic. Threshold Marked @ 0.4

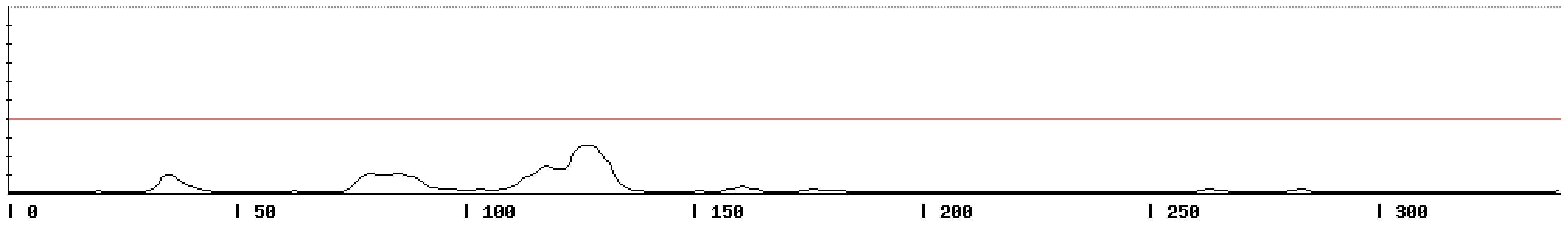

Predictions for Gossypiumarboreum_Cotton_A_37610_BGI-A2_v1.0

Viterbi Path

Viterbi Path

Posterior @ 0.4

Posterior @ 0.4

Posterior Graphic. Threshold Marked @ 0.4

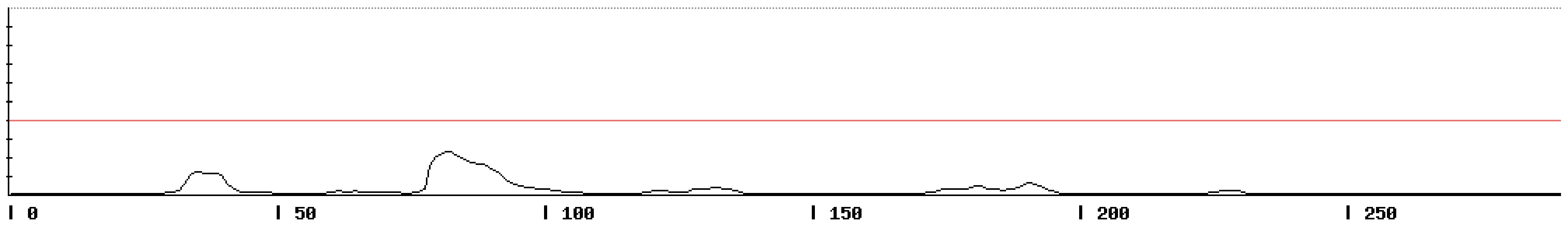

Predictions for Gossypiumarboreum_Cotton_A_37659_BGI-A2_v1.0

Viterbi Path	
Viterbi Path	
Posterior @ 0.4	135 - RKRRK - 139
Posterior @ 0.4	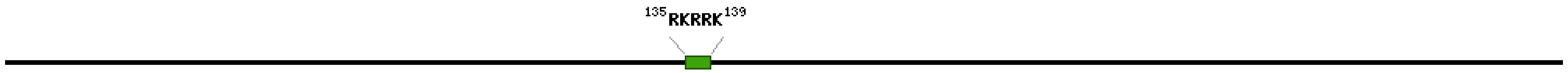
Posterior Graphic. Threshold Marked @ 0.4	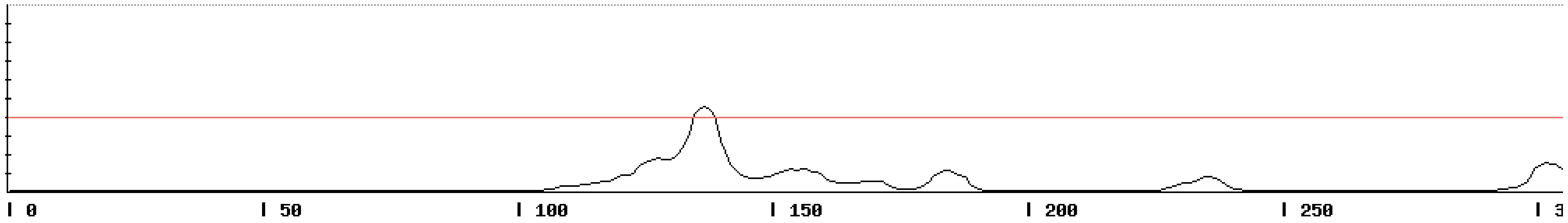

Predictions for Gossypiumarboreum_Cotton_A_38266_BGI-A2_v1.0

Viterbi Path	73 - RRGDNKRVKRTTDKGFWKVTGKPRKVKGKKGCAAKK - 108
Viterbi Path	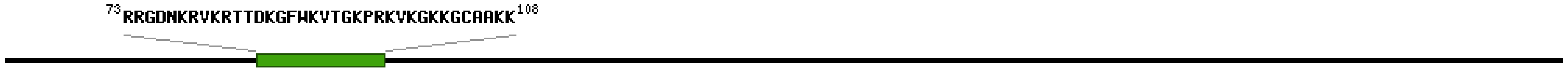
Posterior @ 0.4	73 - RRGDNKRVKRTTDKGFWKVTGKPRKVKGKKGCAAKK - 108
Posterior @ 0.4	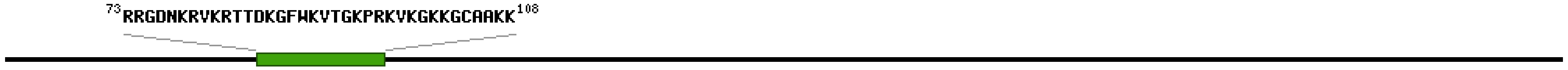
Posterior Graphic. Threshold Marked @ 0.4	

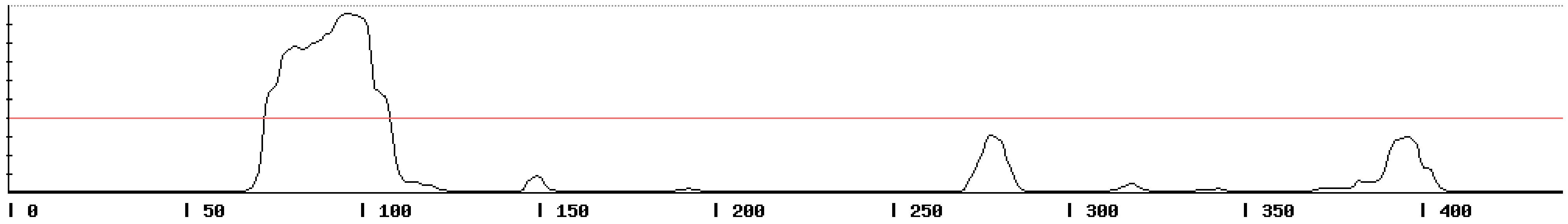

Predictions for Gossypiumarboreum_Cotton_A_38770_BGI-A2_v1.0

Viterbi Path

Viterbi Path

Posterior @ 0.4

Posterior @ 0.4

Posterior Graphic. Threshold Marked @ 0.4

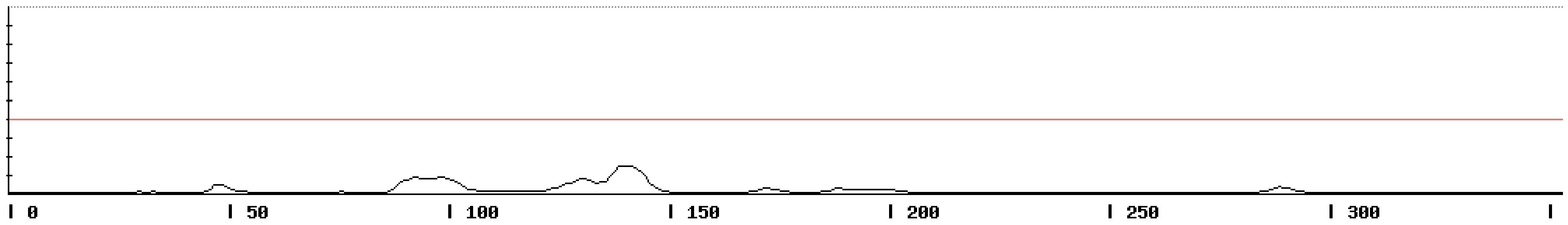

Predictions for Gossypiumarboreum_Cotton_A_39298_BGI-A2_v1.0

Viterbi Path

Viterbi Path

Posterior @ 0.4

Posterior @ 0.4

Posterior Graphic. Threshold Marked @ 0.4	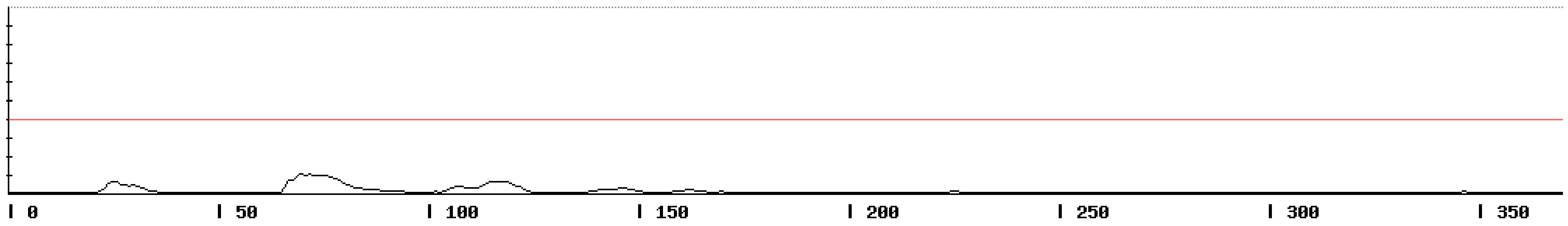

Predictions for Gossypiumarboreum_Cotton_A_39966_BGI-A2_v1.0

Viterbi Path	
Viterbi Path	
Posterior @ 0.4	
Posterior @ 0.4	
Posterior Graphic. Threshold Marked @ 0.4	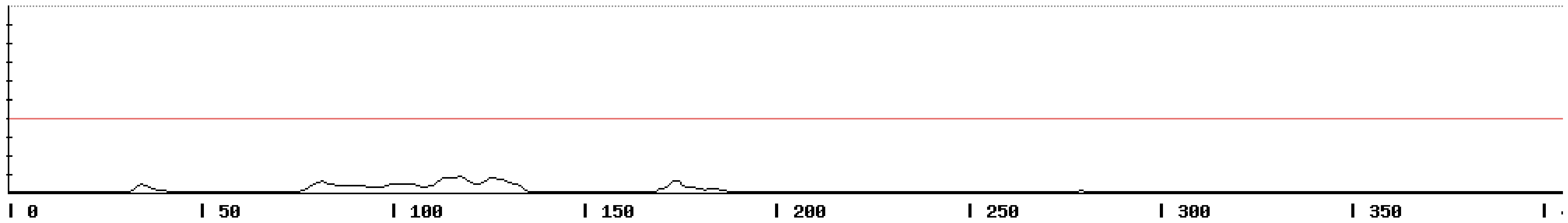

Predictions for Gossypiumarboreum_Cotton_A_40150_BGI-A2_v1.0

Viterbi Path	
Viterbi Path	
Posterior @ 0.4	
Posterior @ 0.4	

Posterior Graphic. Threshold Marked @ 0.4

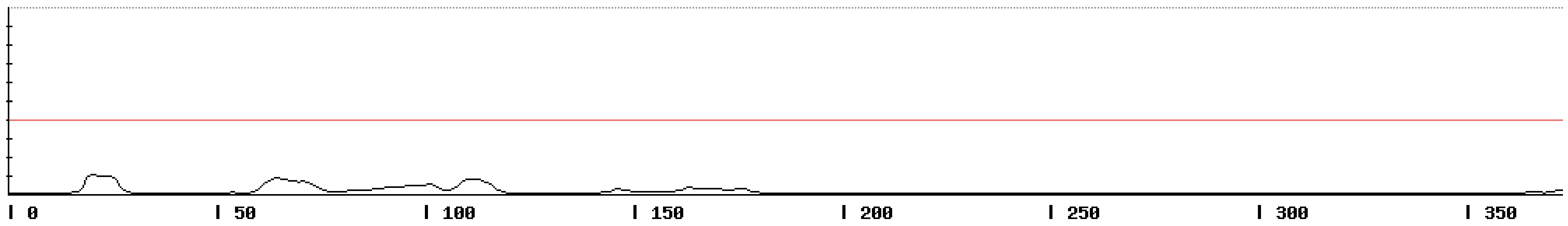

Predictions for Gossypiumarboreum_Cotton_A_40947_BGI-A2_v1.0	
Viterbi Path	
Viterbi Path	
Posterior @ 0.4	
Posterior @ 0.4	

Revision r.9 - Last Updated on : February 24 2015 17:45:20.

alex.nguyenba@utoronto.ca

Intended to be used with Firefox

Viterbi Path	
Posterior @ 0.4	74 - RKHGKGGRPNR - 84
Posterior @ 0.4	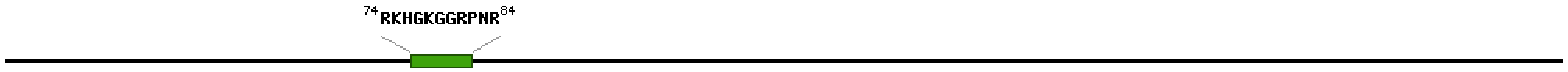
Posterior Graphic. Threshold Marked @ 0.4	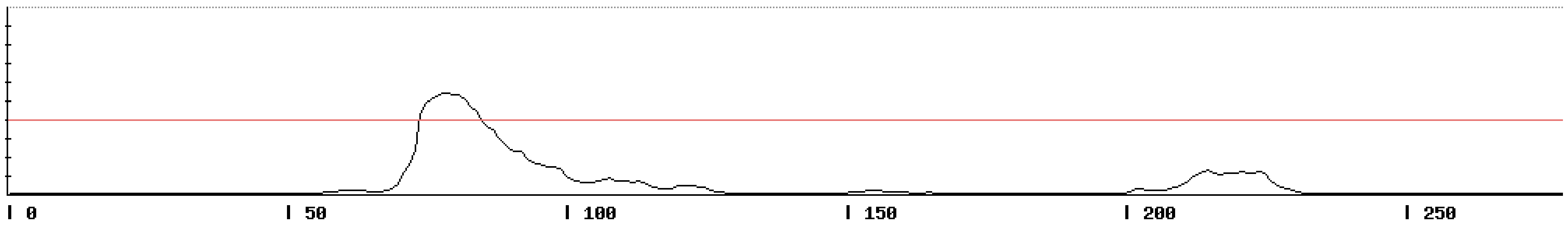

Predictions for Gossypiumhirsutum_Gh_A01G0131

Viterbi Path	
Viterbi Path	
Posterior @ 0.4	69 - LKKRSKSR - 76
Posterior @ 0.4	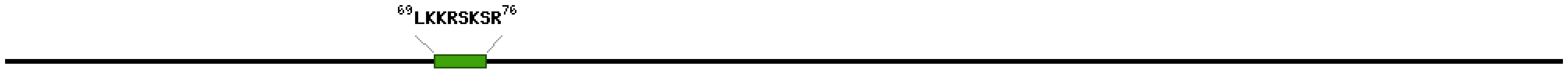
Posterior Graphic. Threshold Marked @ 0.4	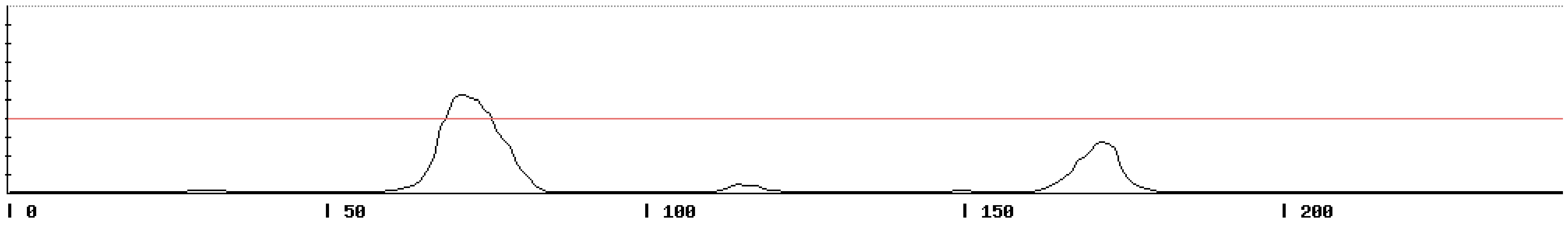

Predictions for Gossypiumhirsutum_Gh_A01G0250

Viterbi Path

Viterbi Path

Posterior @ 0.4

Posterior @ 0.4

Posterior Graphic. Threshold Marked @ 0.4

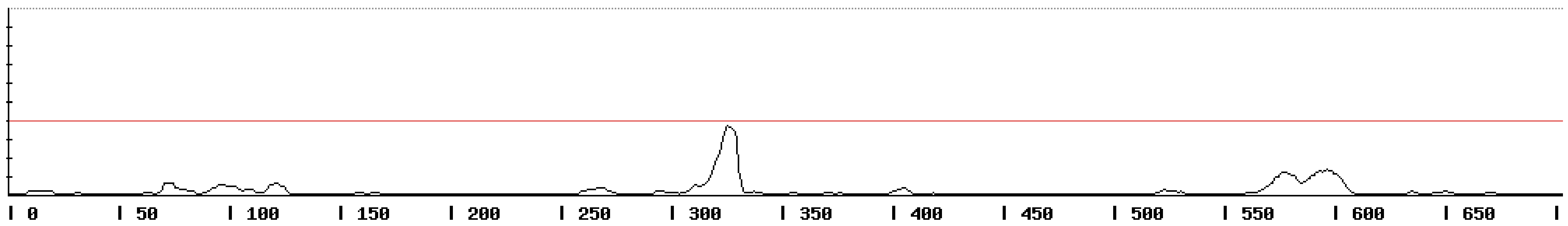

Predictions for Gossypiumhirsutum_Gh_A01G0267

Viterbi Path

Viterbi Path

Posterior @ 0.4

Posterior @ 0.4

Posterior Graphic. Threshold Marked @ 0.4

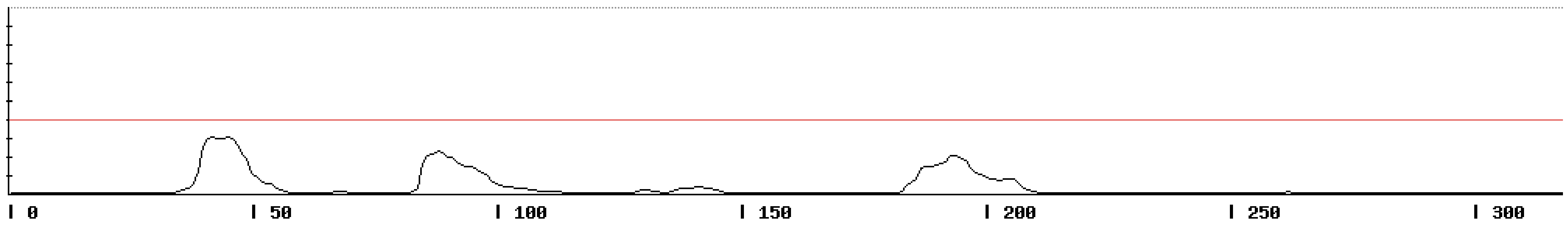

Predictions for Gossypiumhirsutum_Gh_A01G0298	
Viterbi Path	
Viterbi Path	
Posterior @ 0.4	
Posterior @ 0.4	
Posterior Graphic. Threshold Marked @ 0.4	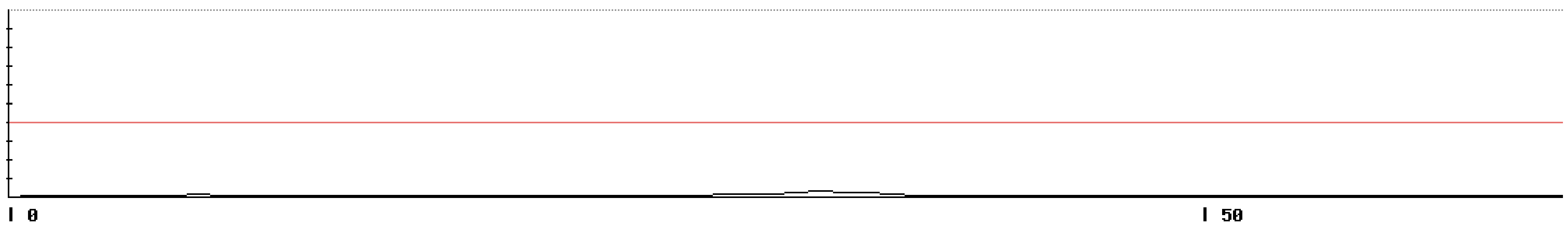

Predictions for Gossypiumhirsutum_Gh_A01G0440	
Viterbi Path	
Viterbi Path	
Posterior @ 0.4	
Posterior @ 0.4	
Posterior Graphic. Threshold Marked @ 0.4	

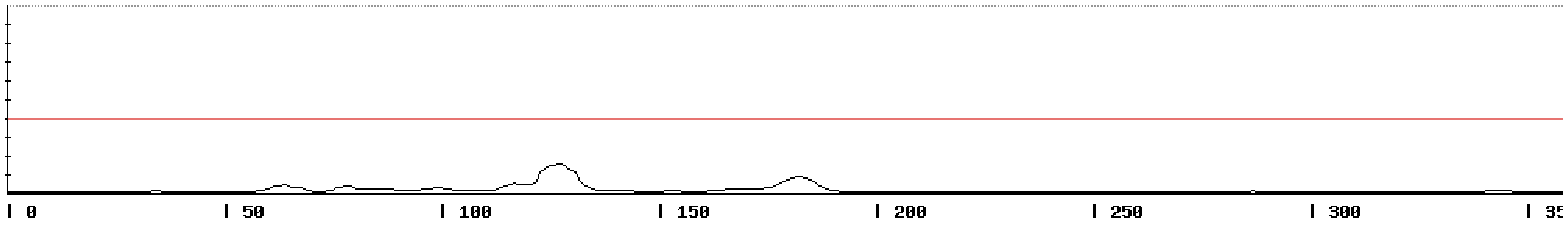

Predictions for Gossypiumhirsutum_Gh_A01G0497	
Viterbi Path	
Viterbi Path	
Posterior @ 0.4	
Posterior @ 0.4	
Posterior Graphic. Threshold Marked @ 0.4	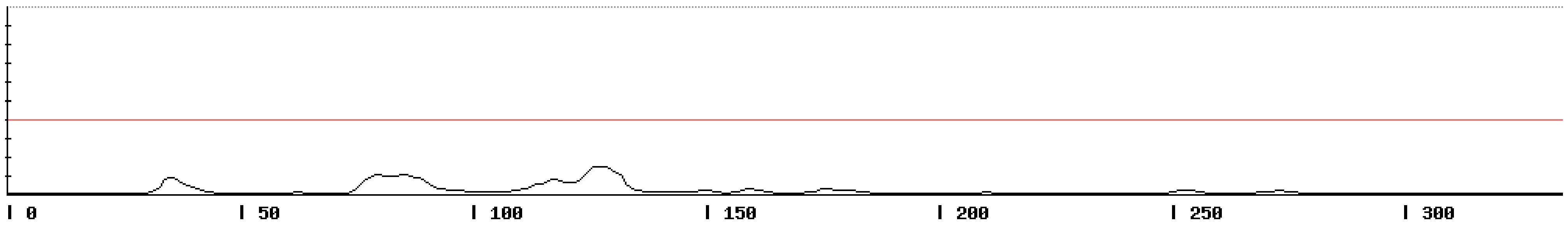

Predictions for Gossypiumhirsutum_Gh_A01G0806	
Viterbi Path	
Viterbi Path	
Posterior @ 0.4	
Posterior @ 0.4	

Posterior Graphic. Threshold Marked @ 0.4

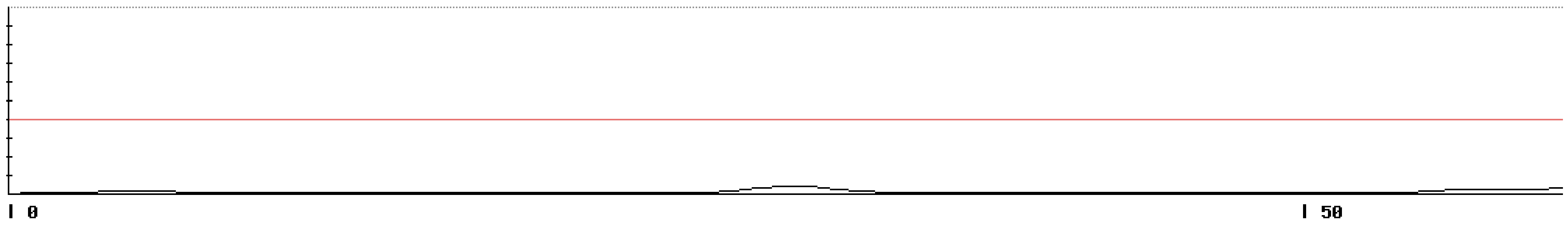

Predictions for Gossypiumhirsutum_Gh_A01G0819

Viterbi Path

Viterbi Path

Posterior @ 0.4

Posterior @ 0.4

Posterior Graphic. Threshold Marked @ 0.4

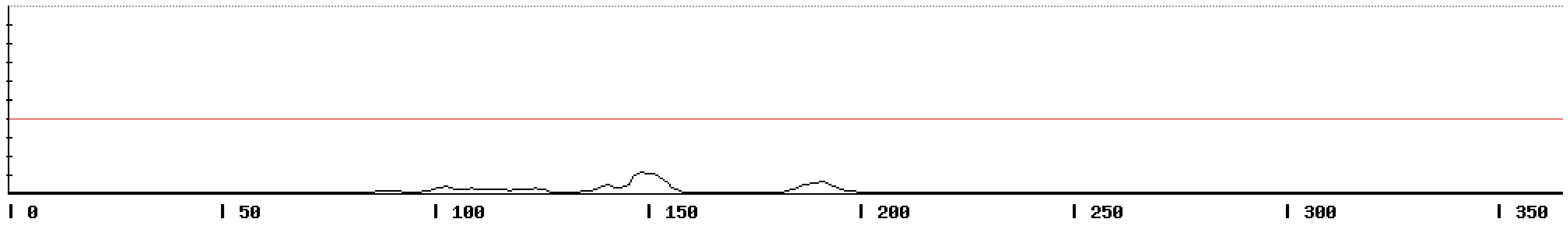

Predictions for Gossypiumhirsutum_Gh_A01G1139

Viterbi Path

Viterbi Path

Posterior @ 0.4

Posterior @ 0.4

Posterior Graphic. Threshold Marked @ 0.4

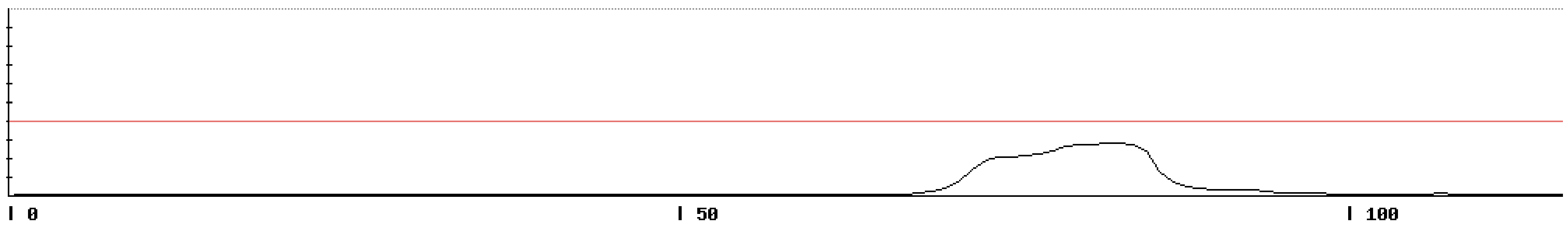

Predictions for Gossypiumhirsutum_Gh_A01G2064

Viterbi Path

Viterbi Path

Posterior @ 0.4

Posterior @ 0.4

Posterior Graphic. Threshold Marked @ 0.4

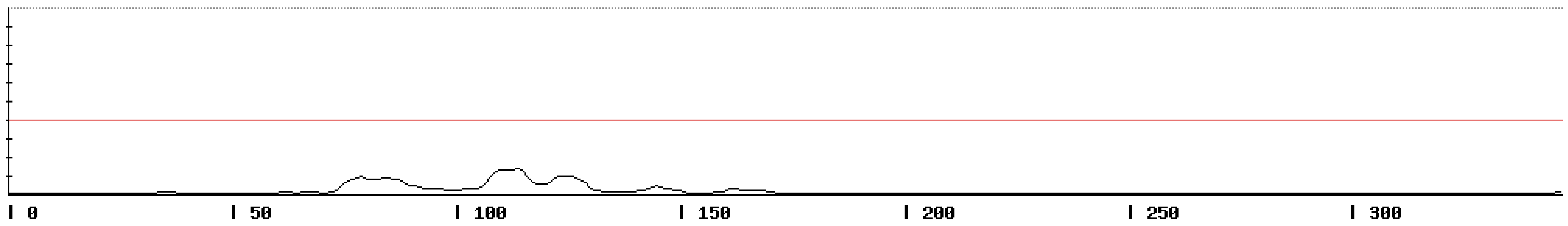

Predictions for Gossypiumhirsutum_Gh_A01G2142

Viterbi Path

Viterbi Path

Posterior @ 0.4

Posterior @ 0.4

Posterior Graphic. Threshold Marked @ 0.4

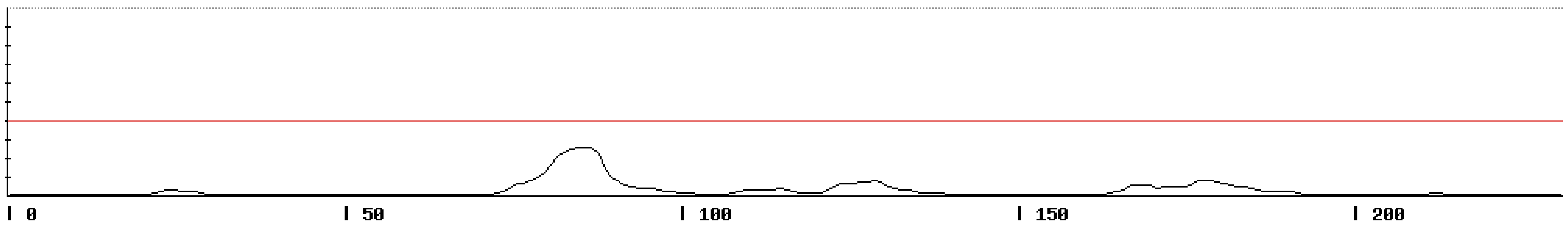

Predictions for Gossypiumhirsutum_Gh_A02G0101

Viterbi Path

Viterbi Path

Posterior @ 0.4

712 - KTRERGQKRKRLVQKVGKTARR - 733

Posterior @ 0.4

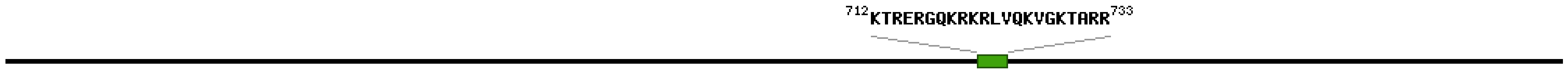

Posterior Graphic. Threshold Marked @ 0.4

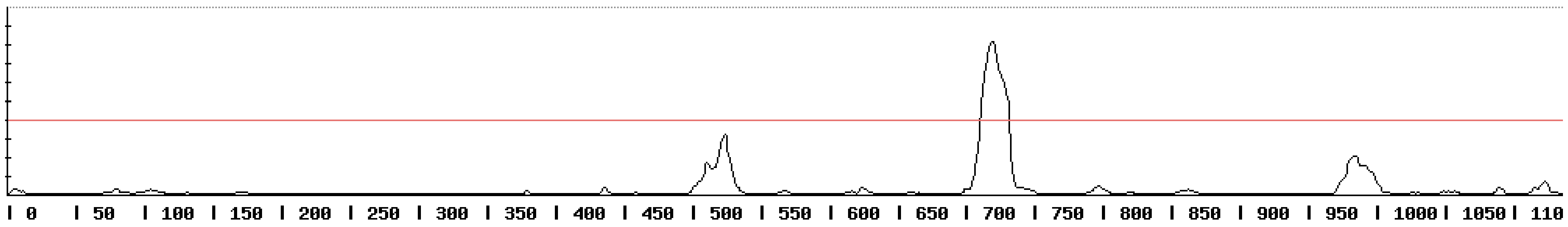

Predictions for Gossypiumhirsutum_Gh_A02G0114

Viterbi Path

Viterbi Path

Posterior @ 0.4

70 - KKKSK - 74

163 - KR - 164

Posterior @ 0.4

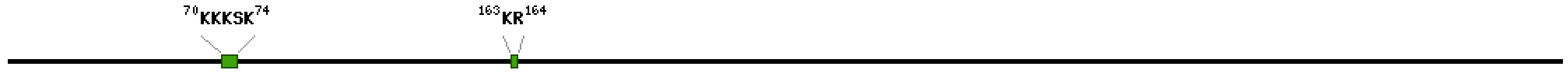

Posterior Graphic. Threshold Marked @ 0.4

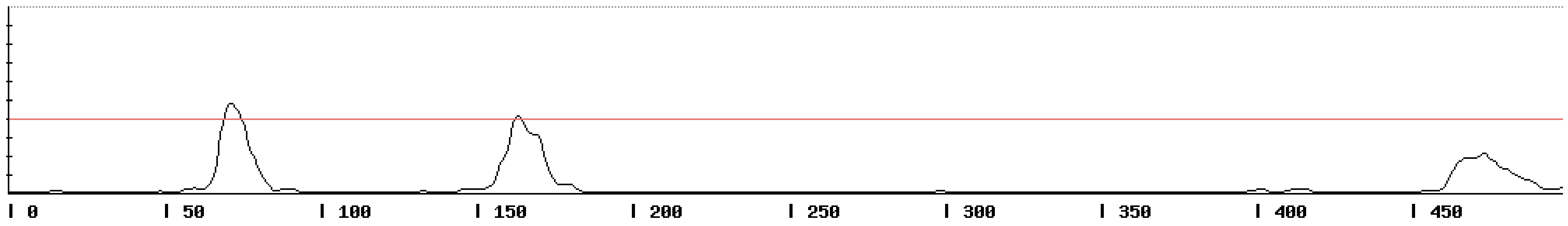

Predictions for Gossypiumhirsutum_Gh_A02G0150

Viterbi Path

Viterbi Path

Posterior @ 0.4

Posterior @ 0.4

Posterior Graphic. Threshold Marked @ 0.4

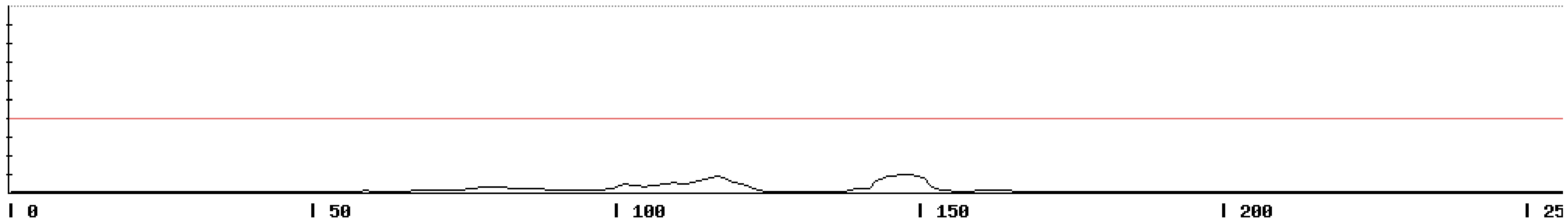

Predictions for Gossypiumhirsutum_Gh_A02G0634	
Viterbi Path	
Viterbi Path	
Posterior @ 0.4	
Posterior @ 0.4	
Posterior Graphic. Threshold Marked @ 0.4	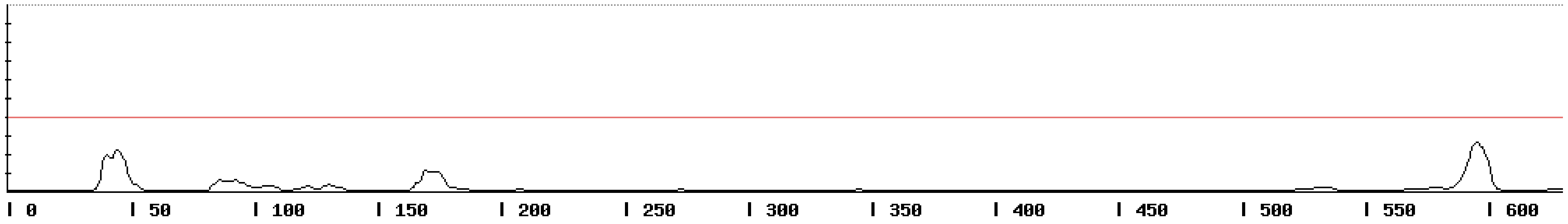

Predictions for Gossypiumhirsutum_Gh_A02G0749	
Viterbi Path	
Viterbi Path	
Posterior @ 0.4	
Posterior @ 0.4	
Posterior Graphic. Threshold Marked @ 0.4	

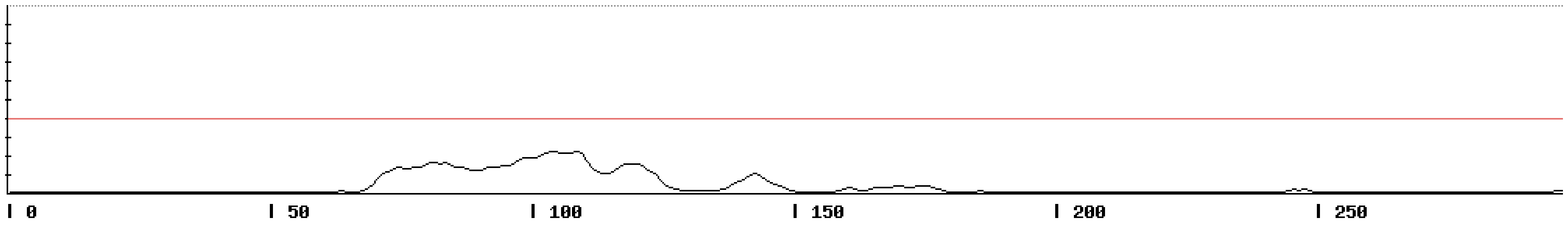

Predictions for Gossypiumhirsutum_Gh_A02G0941

Viterbi Path	
Viterbi Path	
Posterior @ 0.4	97 - KDRTIKSKKSLIG - 109
Posterior @ 0.4	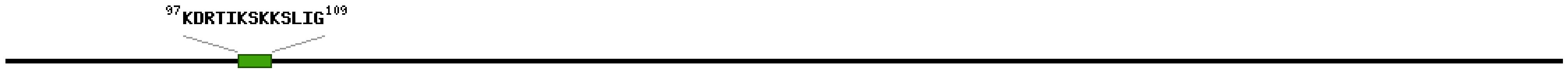
Posterior Graphic. Threshold Marked @ 0.4	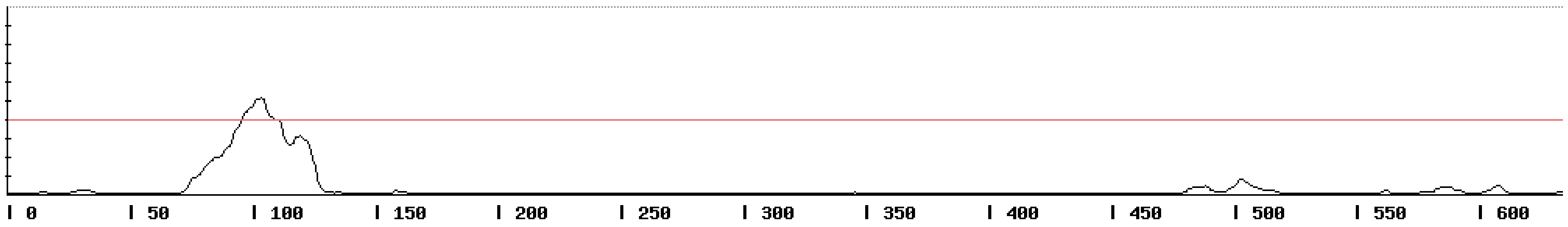

Predictions for Gossypiumhirsutum_Gh_A02G0977

Viterbi Path	
Viterbi Path	
Posterior @ 0.4	
Posterior @ 0.4	

Posterior Graphic. Threshold Marked @ 0.4

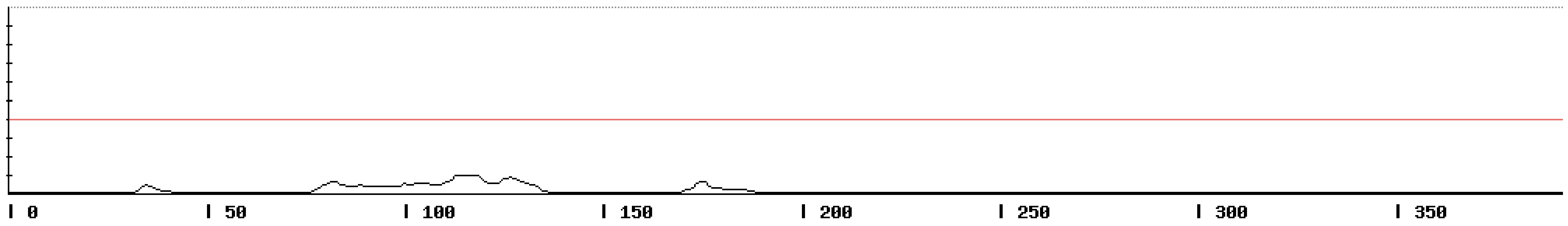

Predictions for Gossypiumhirsutum_Gh_A02G1625

Viterbi Path

Viterbi Path

Posterior @ 0.4

Posterior @ 0.4

Posterior Graphic. Threshold Marked @ 0.4

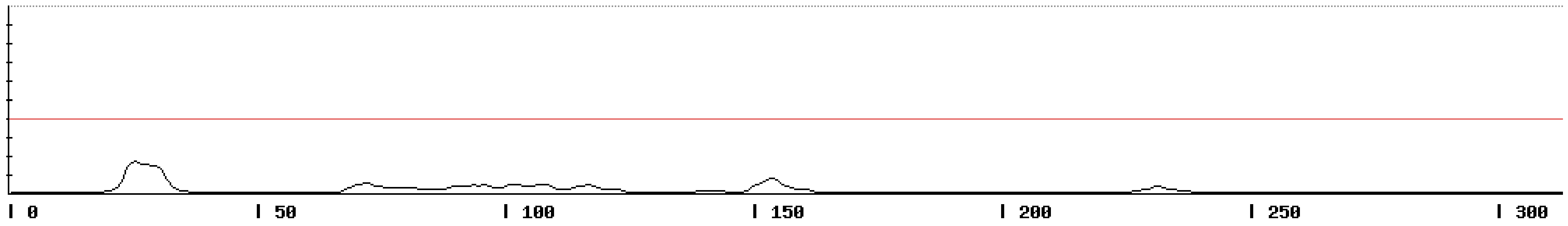

Predictions for Gossypiumhirsutum_Gh_A02G1687

Viterbi Path

Viterbi Path

Posterior @ 0.4

Posterior @ 0.4

Posterior Graphic. Threshold Marked @ 0.4

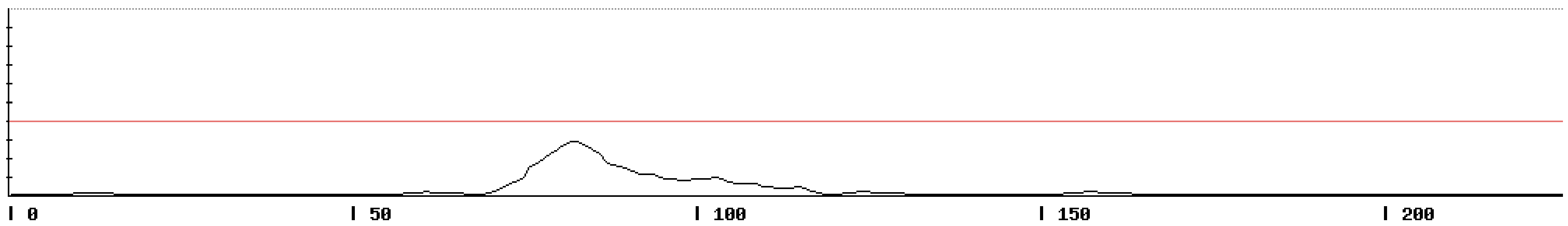

Predictions for Gossypiumhirsutum_Gh_A03G0024

Viterbi Path

Viterbi Path

Posterior @ 0.4

Posterior @ 0.4

Posterior Graphic. Threshold Marked @ 0.4

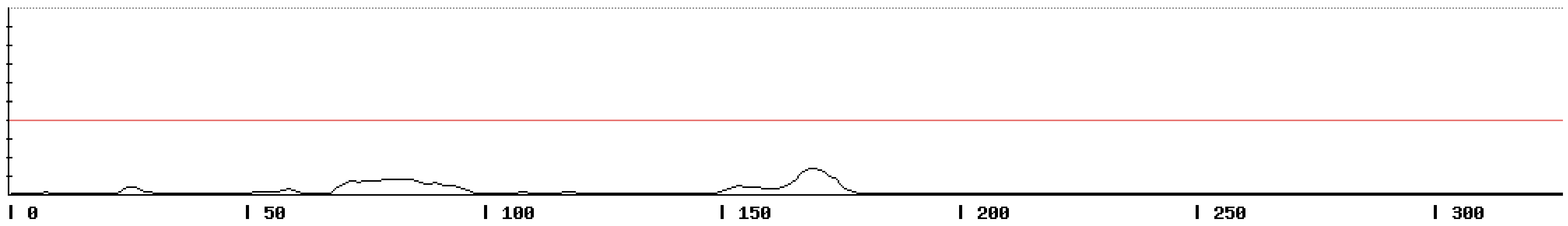

Predictions for Gossypiumhirsutum_Gh_A03G0506

Viterbi Path

Viterbi Path

Posterior @ 0.4

Posterior @ 0.4

Posterior Graphic. Threshold Marked @ 0.4

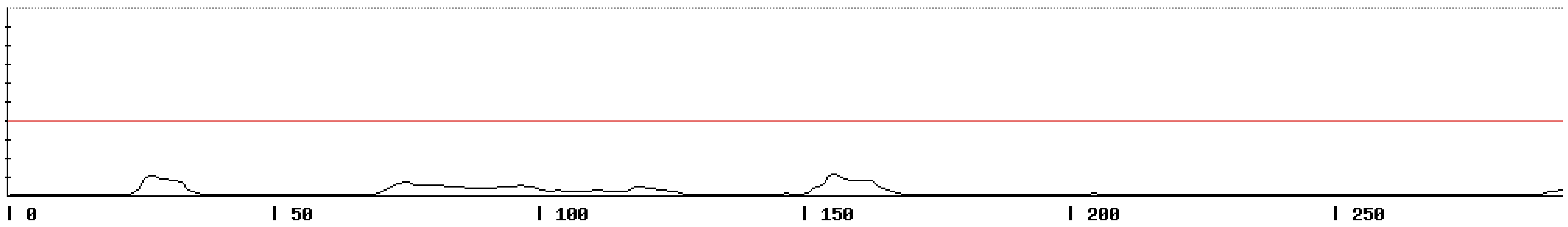

Predictions for Gossypiumhirsutum_Gh_A03G0874

Viterbi Path

Viterbi Path

Posterior @ 0.4

Posterior @ 0.4

Posterior Graphic. Threshold Marked @ 0.4

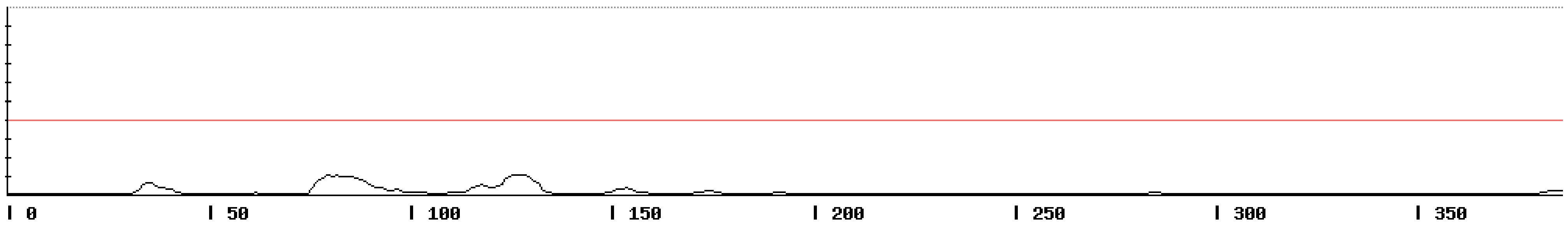

Predictions for Gossypiumhirsutum_Gh_A03G0887

Viterbi Path

Viterbi Path

Posterior @ 0.4

Posterior @ 0.4

Posterior Graphic. Threshold Marked @ 0.4

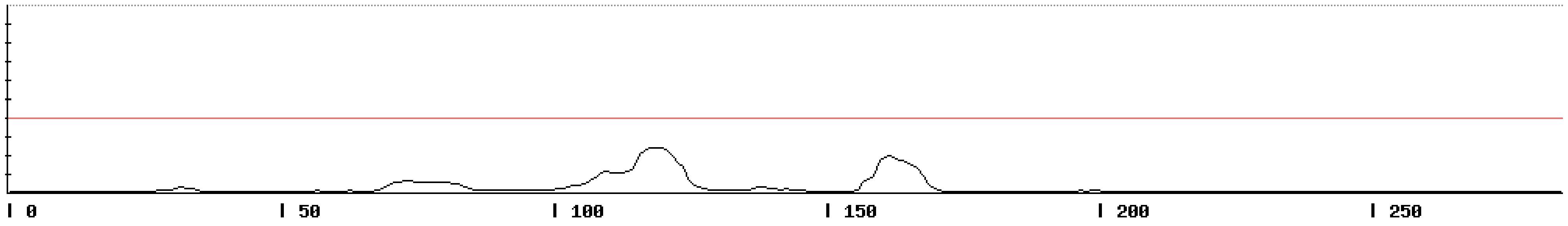

Predictions for Gossypiumhirsutum_Gh_A03G1332

Viterbi Path

Viterbi Path

Posterior @ 0.4

Posterior @ 0.4

Posterior Graphic. Threshold Marked @ 0.4

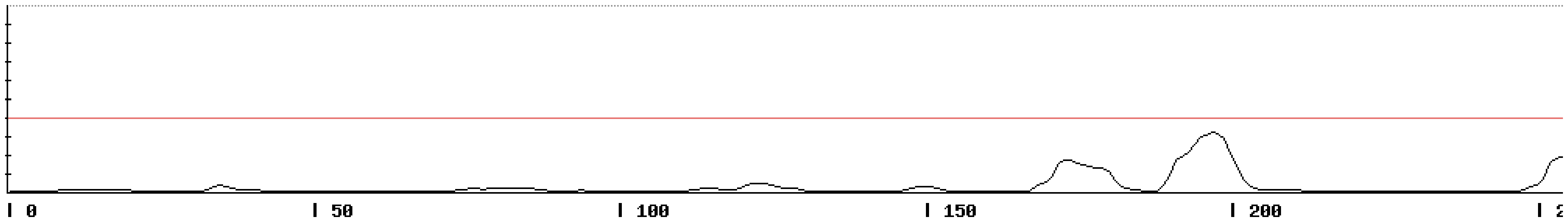

Predictions for Gossypiumhirsutum_Gh_A03G1333

Viterbi Path

Viterbi Path

Posterior @ 0.4

Posterior @ 0.4

Posterior Graphic. Threshold Marked @ 0.4

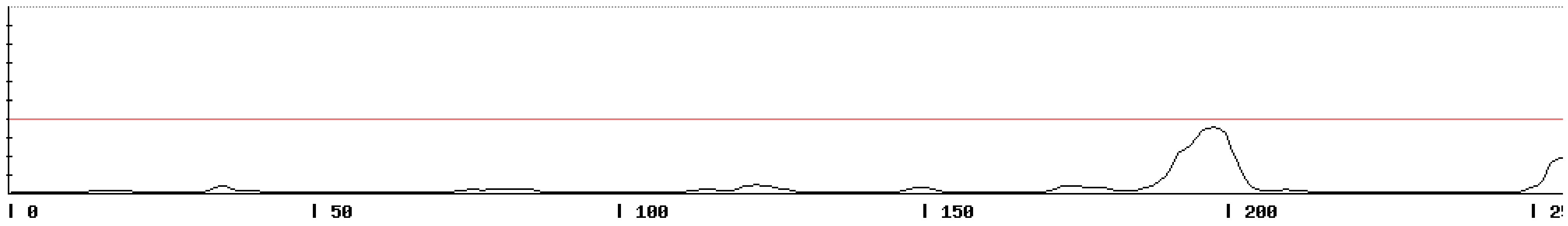

Predictions for Gossypiumhirsutum_Gh_A03G1512

Viterbi Path

Viterbi Path

Posterior @ 0.4

Posterior @ 0.4

Posterior Graphic. Threshold Marked @ 0.4

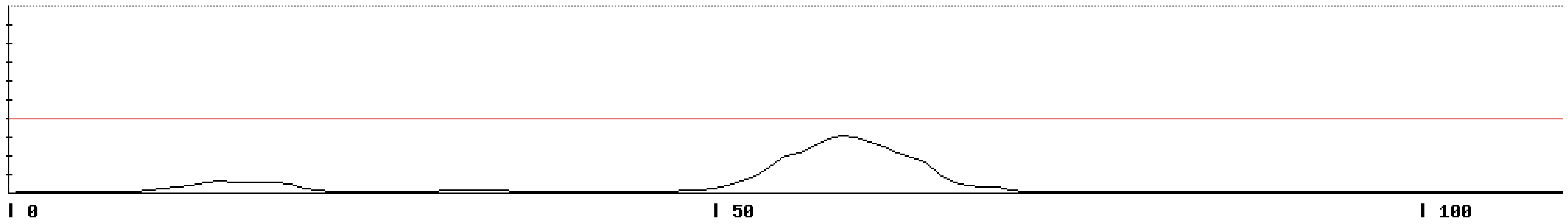

Predictions for Gossypiumhirsutum_Gh_A03G1843	
Viterbi Path	
Viterbi Path	
Posterior @ 0.4	
Posterior @ 0.4	
Posterior Graphic. Threshold Marked @ 0.4	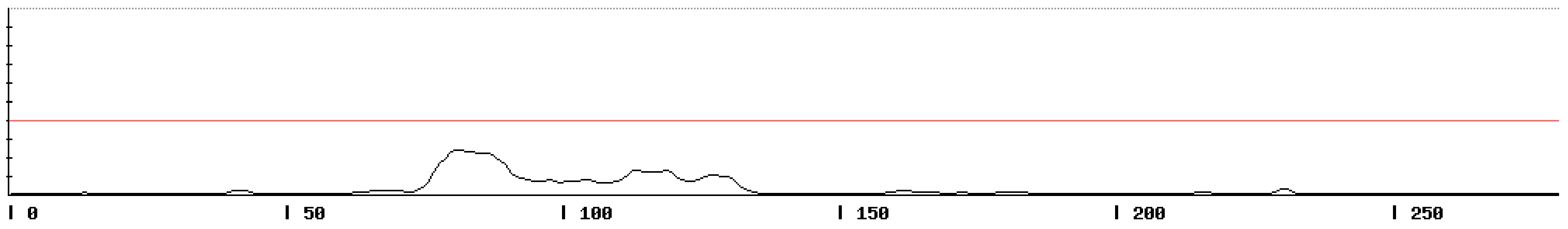

Predictions for Gossypiumhirsutum_Gh_A03G1986	
Viterbi Path	
Viterbi Path	
Posterior @ 0.4	
Posterior @ 0.4	
Posterior Graphic. Threshold Marked @ 0.4	

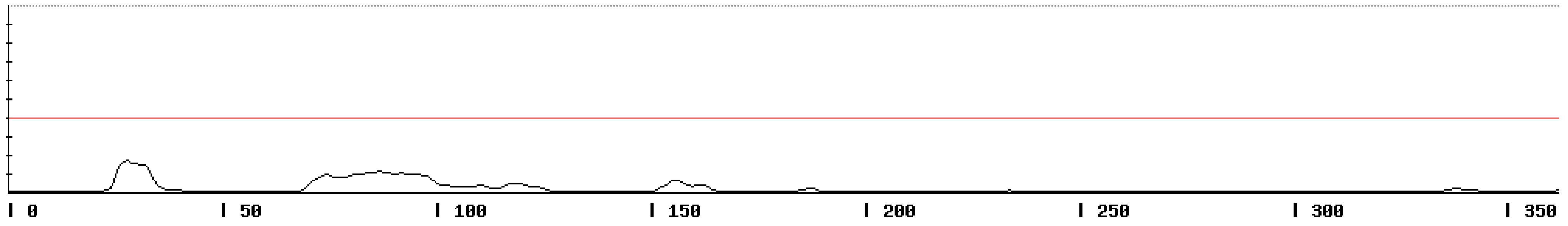

Predictions for Gossypiumhirsutum_Gh_A04G0414

Viterbi Path

Viterbi Path

Posterior @ 0.4

Posterior @ 0.4

Posterior Graphic. Threshold Marked @ 0.4

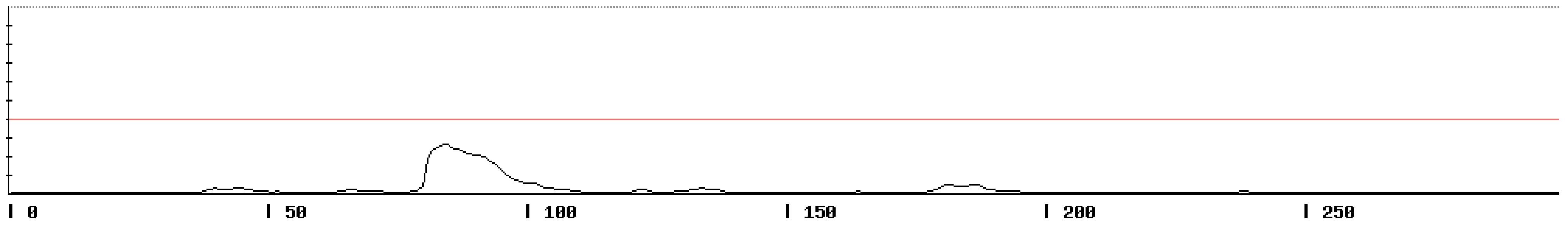

Predictions for Gossypiumhirsutum_Gh_A04G0422

Viterbi Path

Viterbi Path

Posterior @ 0.4

Posterior @ 0.4

Posterior Graphic. Threshold Marked @ 0.4

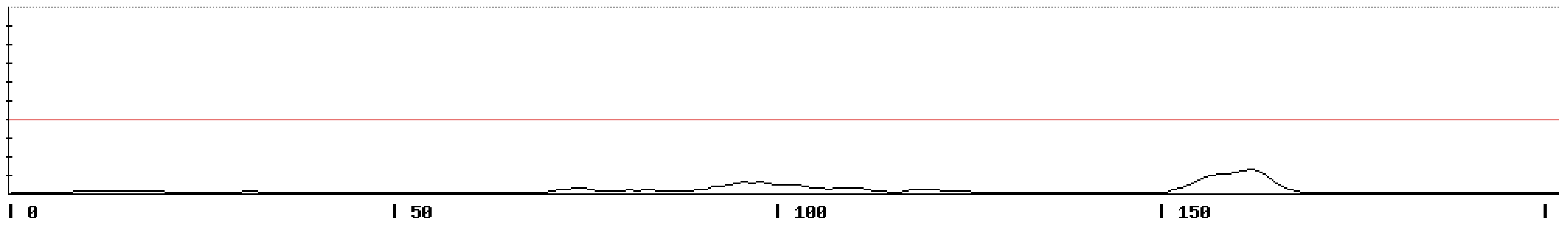

Predictions for Gossypiumhirsutum_Gh_A04G0481

Viterbi Path

Viterbi Path

Posterior @ 0.4

81 - RGGKPKRR - 88

Posterior @ 0.4

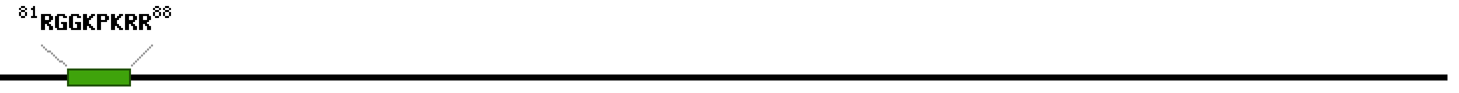

Posterior Graphic. Threshold Marked @ 0.4

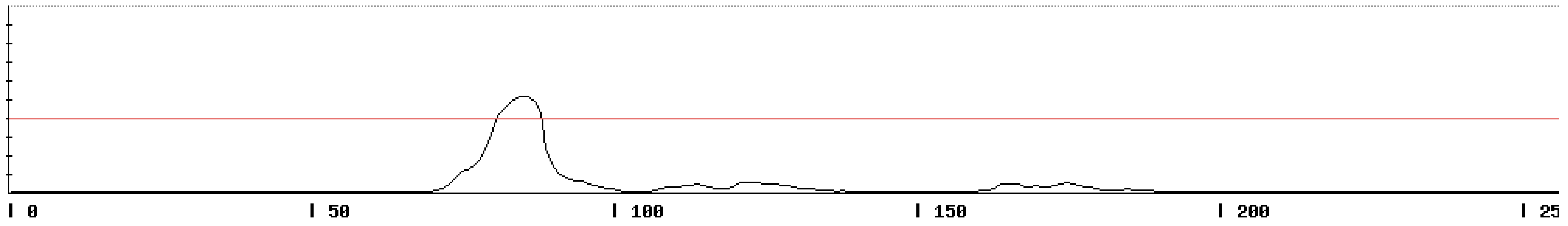

Predictions for Gossypiumhirsutum_Gh_A04G0634

Viterbi Path

Viterbi Path

Posterior @ 0.4

Posterior @ 0.4

Posterior Graphic. Threshold Marked @ 0.4

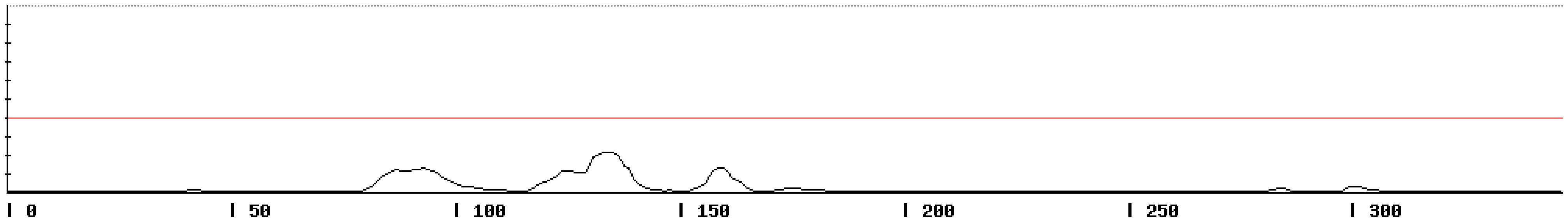

Predictions for Gossypiumhirsutum_Gh_A04G0868

Viterbi Path

Viterbi Path

Posterior @ 0.4

Posterior @ 0.4

Posterior Graphic. Threshold Marked @ 0.4

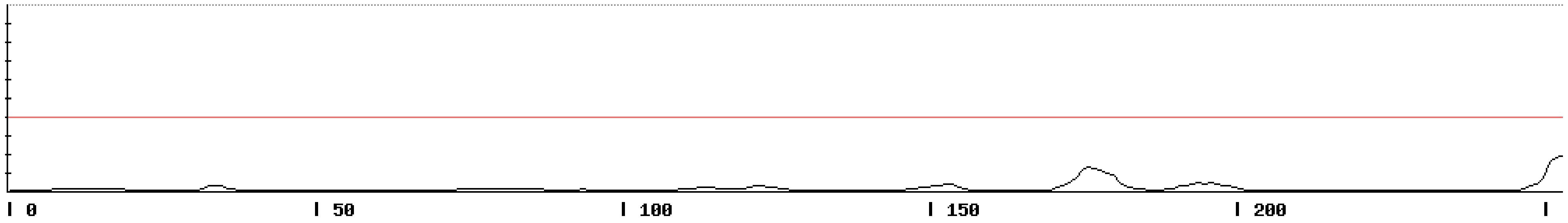

Predictions for Gossypiumhirsutum_Gh_A04G1143

Viterbi Path

Viterbi Path

Posterior @ 0.4

Posterior @ 0.4

Posterior Graphic. Threshold Marked @ 0.4

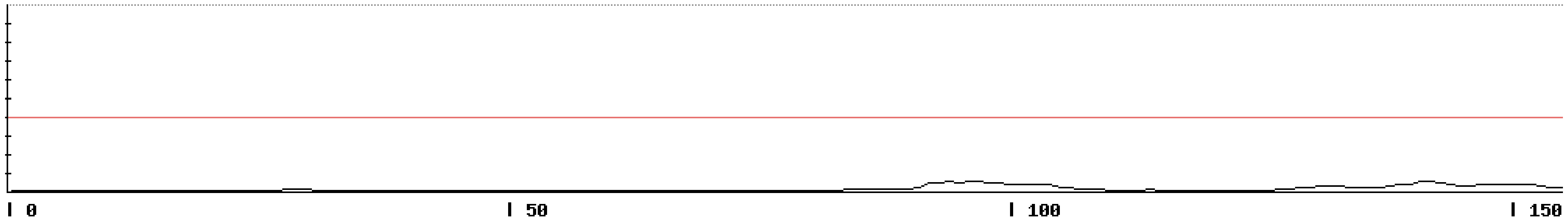

Predictions for Gossypiumhirsutum_Gh_A04G1299

Viterbi Path

Viterbi Path

Posterior @ 0.4

Posterior @ 0.4

Posterior Graphic. Threshold Marked @ 0.4

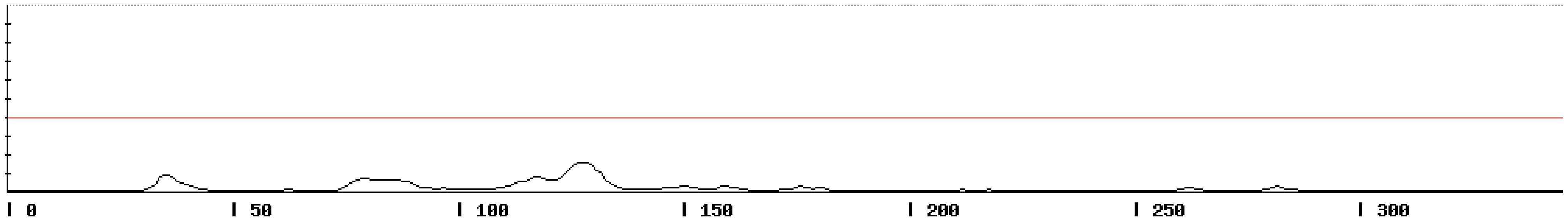

Predictions for Gossypiumhirsutum_Gh_A04G1303

Viterbi Path

Viterbi Path

Posterior @ 0.4

Posterior @ 0.4

Posterior Graphic. Threshold Marked @ 0.4

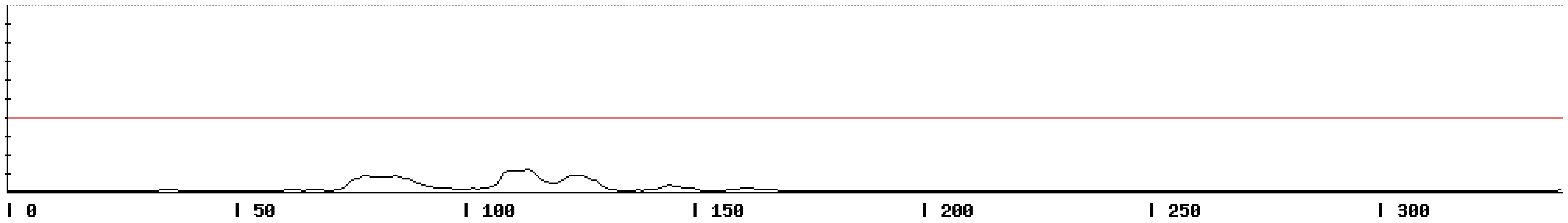

Predictions for Gossypiumhirsutum_Gh_A05G0197

Viterbi Path

Viterbi Path

Posterior @ 0.4

Posterior @ 0.4

Posterior Graphic. Threshold Marked @ 0.4

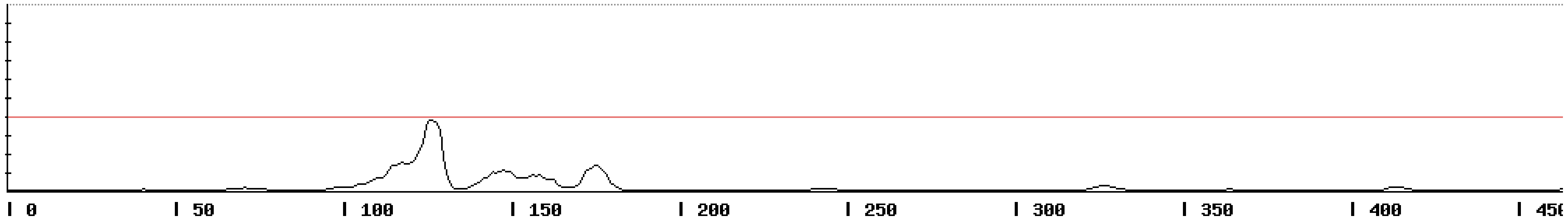

Predictions for Gossypiumhirsutum_Gh_A05G0652

Viterbi Path

Viterbi Path

Posterior @ 0.4 — 312 - NRRIKLRKPK - 321

Posterior @ 0.4

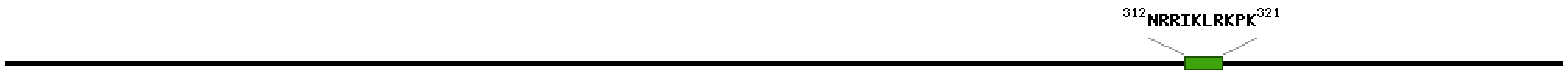

Posterior Graphic. Threshold Marked @ 0.4

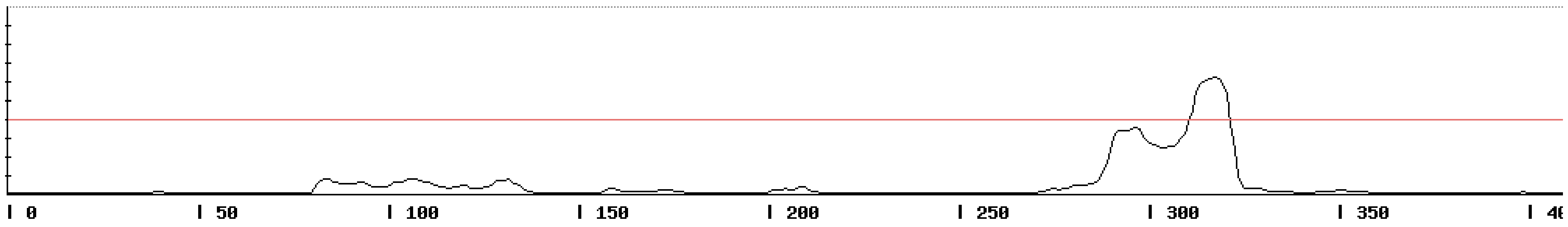

Predictions for Gossypiumhirsutum_Gh_A05G1339

Viterbi Path

Viterbi Path

Posterior @ 0.4 — 146 - RKRRK - 150

Posterior @ 0.4

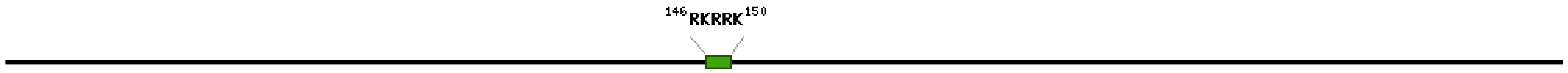

Posterior Graphic. Threshold Marked @ 0.4

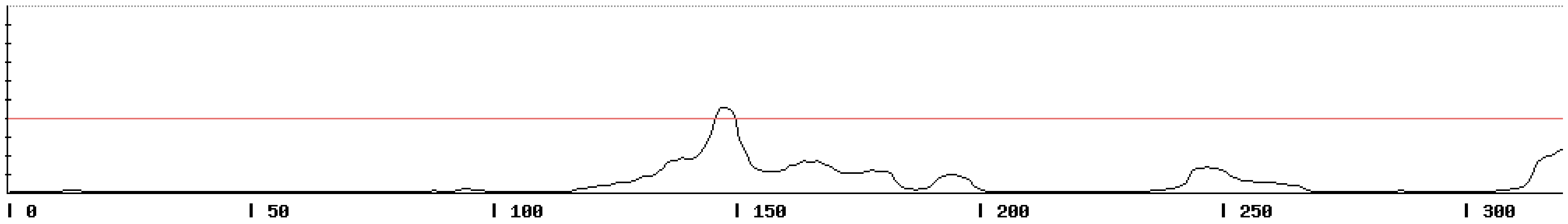

Predictions for Gossypiumhirsutum_Gh_A05G1381

Viterbi Path	
Viterbi Path	
Posterior @ 0.4	
Posterior @ 0.4	
Posterior Graphic. Threshold Marked @ 0.4	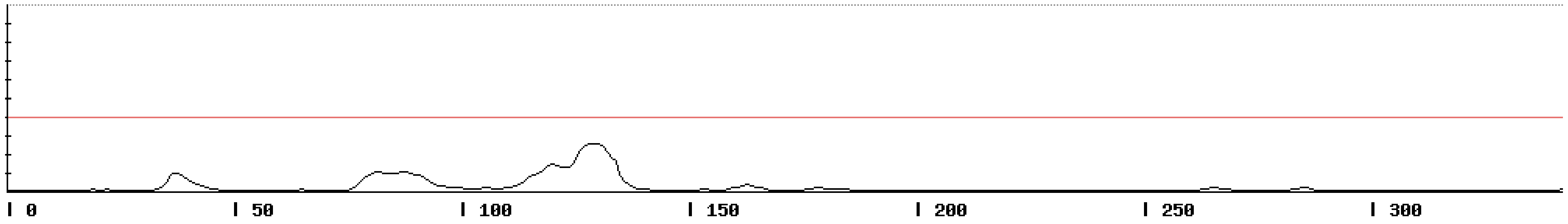

Predictions for Gossypiumhirsutum_Gh_A05G1526

Viterbi Path	
Viterbi Path	
Posterior @ 0.4	
Posterior @ 0.4	
Posterior Graphic. Threshold Marked @ 0.4	

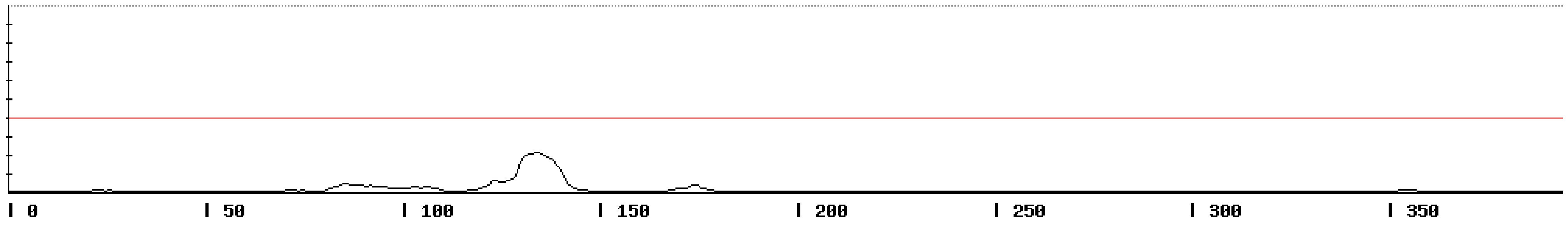

Predictions for Gossypiumhirsutum_Gh_A05G1684

Viterbi Path	
Viterbi Path	
Posterior @ 0.4	322 - SSRKPSESHGGRRKGGSLGGMLR - 344
Posterior @ 0.4	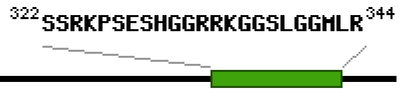
Posterior Graphic. Threshold Marked @ 0.4	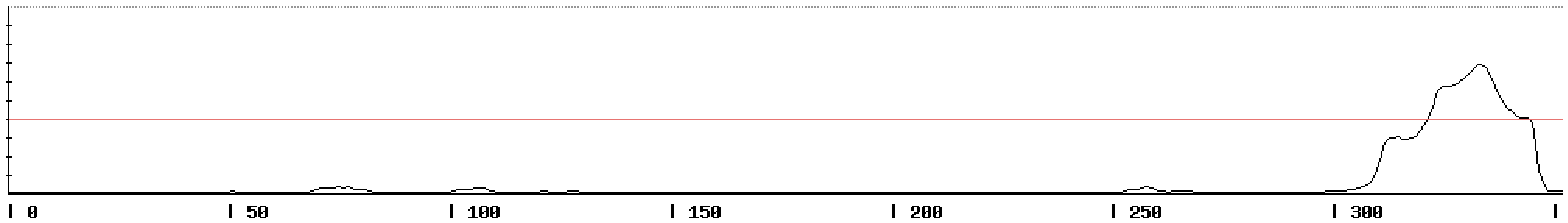

Predictions for Gossypiumhirsutum_Gh_A05G1766

Viterbi Path	100 - KSKGGRGRRRAKGGVWRKIGKVR - 122
Viterbi Path	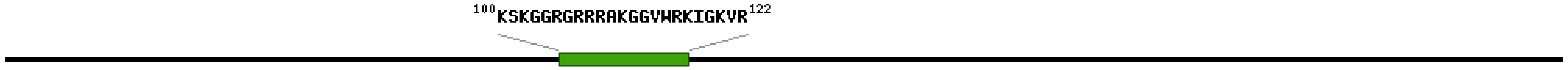
Posterior @ 0.4	99 - TKSKGGRGRRRAKGGVWRKIGKVR - 122
Posterior @ 0.4	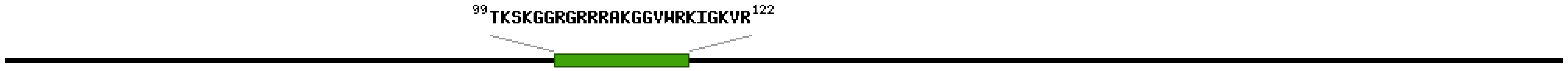

Posterior Graphic. Threshold Marked @ 0.4

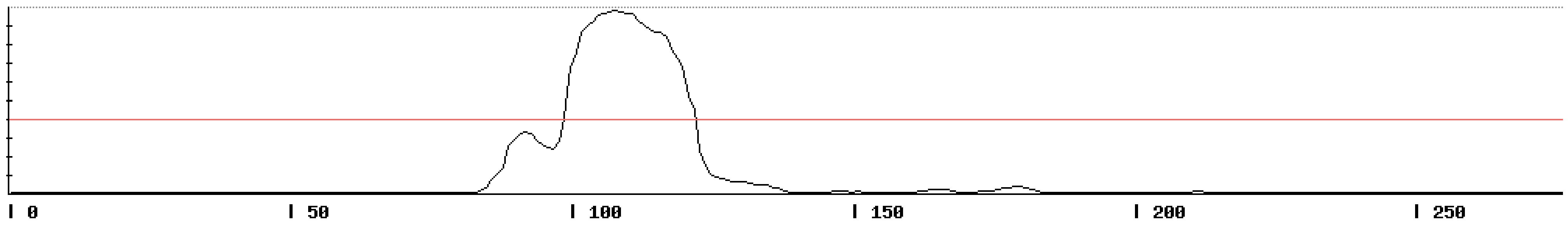

Predictions for Gossypiumhirsutum_Gh_A05G2095

Viterbi Path

Viterbi Path

Posterior @ 0.4

Posterior @ 0.4

Posterior Graphic. Threshold Marked @ 0.4

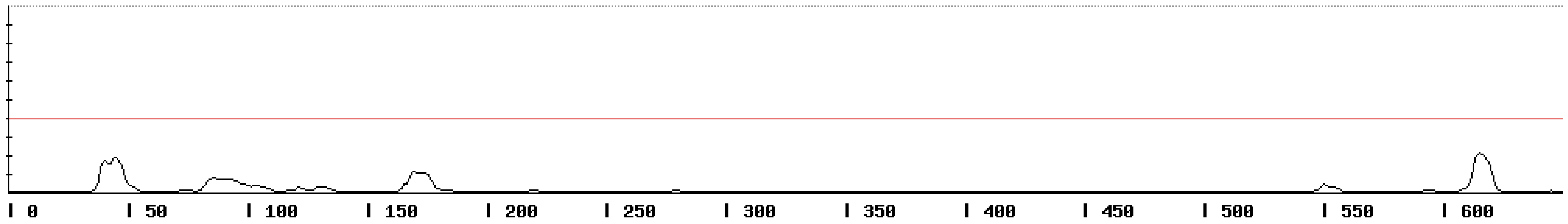

Predictions for Gossypiumhirsutum_Gh_A05G2506

Viterbi Path

Viterbi Path

Posterior @ 0.4

Posterior @ 0.4

Posterior Graphic. Threshold Marked @ 0.4	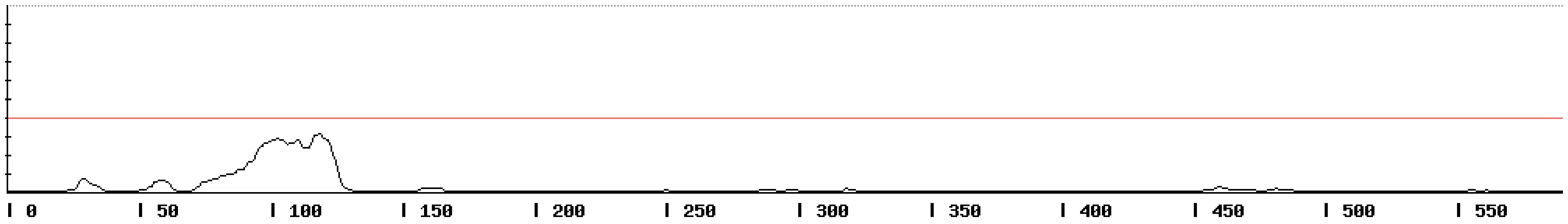

Predictions for Gossypiumhirsutum_Gh_A05G2509	
Viterbi Path	
Viterbi Path	
Posterior @ 0.4	
Posterior @ 0.4	
Posterior Graphic. Threshold Marked @ 0.4	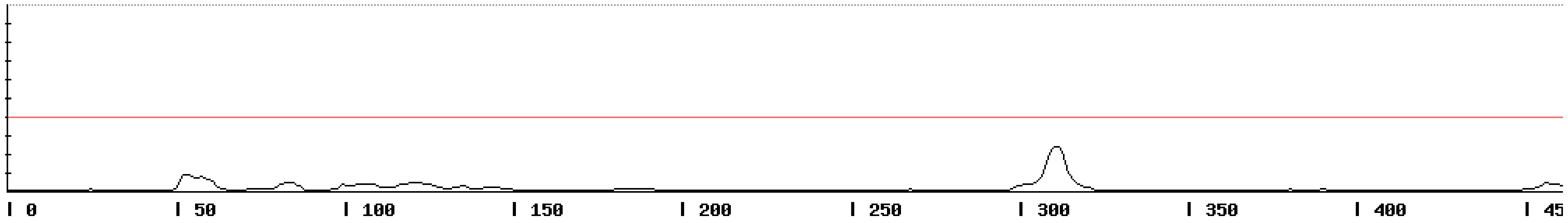

Predictions for Gossypiumhirsutum_Gh_A05G2928	
Viterbi Path	
Viterbi Path	

Posterior @ 0.4	143 - QRKRKARKHAAK - 154
Posterior @ 0.4	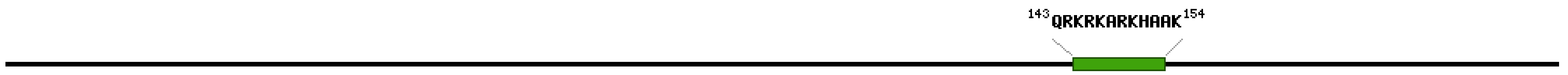
Posterior Graphic. Threshold Marked @ 0.4	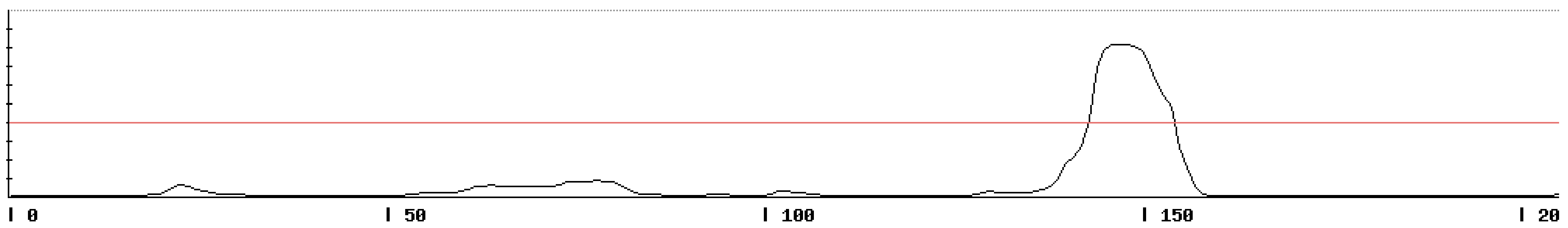

Predictions for Gossypiumhirsutum_Gh_A05G3023

Viterbi Path	
Viterbi Path	
Posterior @ 0.4	93 - RKHPQGKRPNR - 103
Posterior @ 0.4	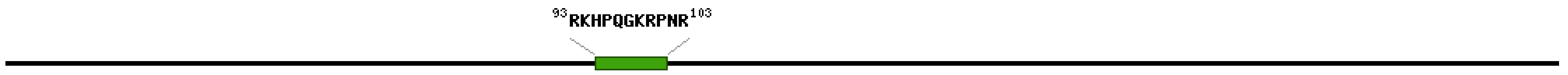
Posterior Graphic. Threshold Marked @ 0.4	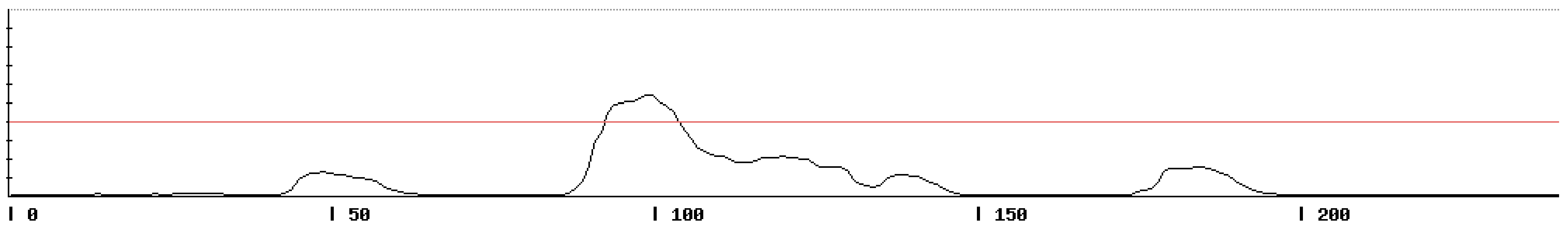

Predictions for Gossypiumhirsutum_Gh_A05G3310

Viterbi Path	
Viterbi Path	

Posterior @ 0.4	97 - IGKPKALGIK - 106
Posterior @ 0.4	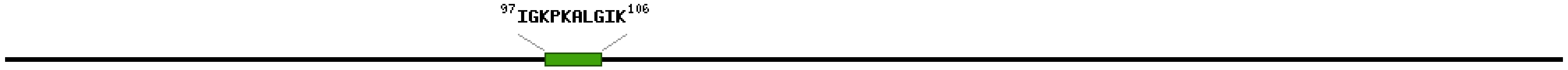
Posterior Graphic. Threshold Marked @ 0.4	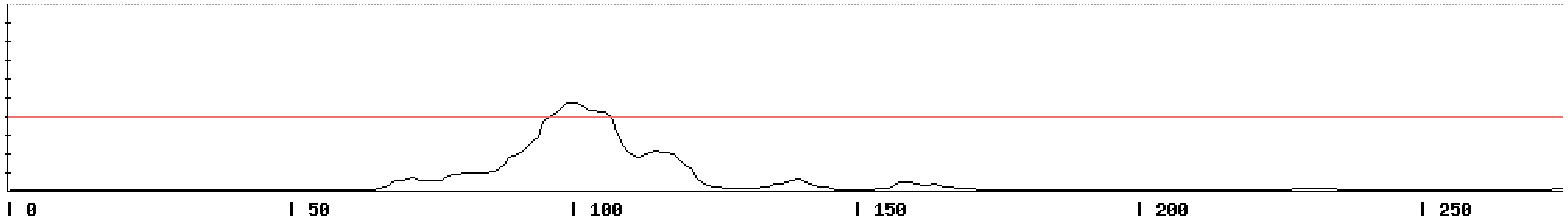

Predictions for Gossypiumhirsutum_Gh_A05G3322

Viterbi Path	
Viterbi Path	
Posterior @ 0.4	
Posterior @ 0.4	
Posterior Graphic. Threshold Marked @ 0.4	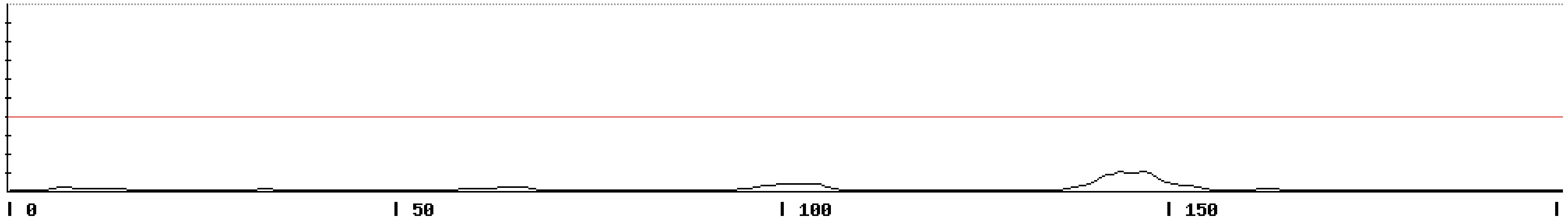

Predictions for Gossypiumhirsutum_Gh_A05G3555

Viterbi Path	
Viterbi Path	
Posterior @ 0.4	351 - KKKKP - 355
Posterior @ 0.4	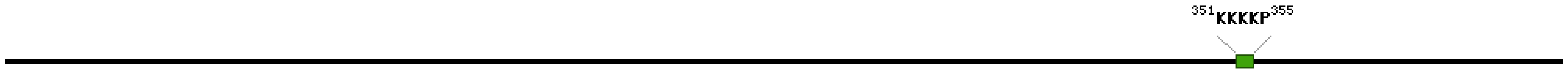
Posterior Graphic. Threshold Marked @ 0.4	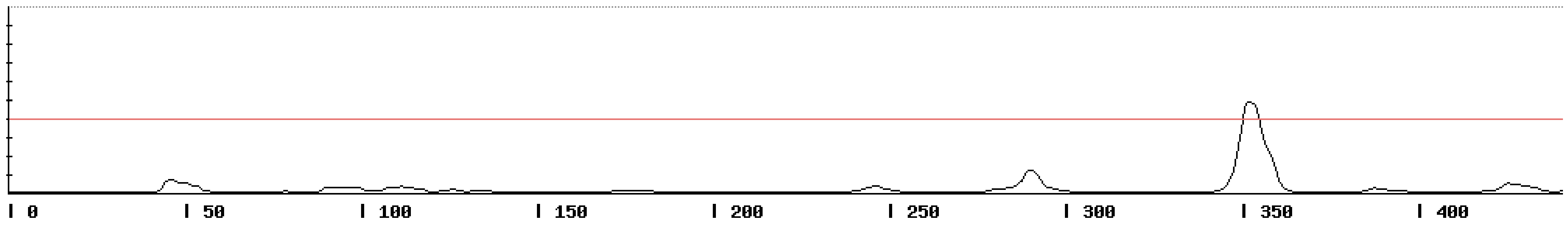

Predictions for Gossypiumhirsutum_Gh_A06G0097

Viterbi Path	367 - KPKSSRRKRDALGRMKELRKK - 387
Viterbi Path	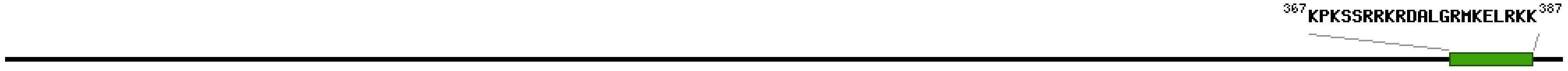
Posterior @ 0.4	366 - VKPKSSRRKRDALGRMKELRKK - 387
Posterior @ 0.4	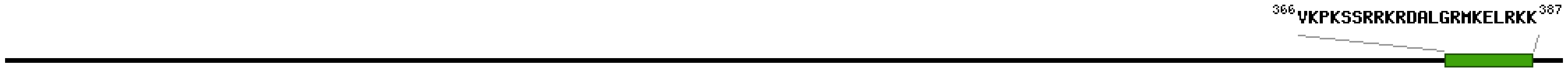
Posterior Graphic. Threshold Marked @ 0.4	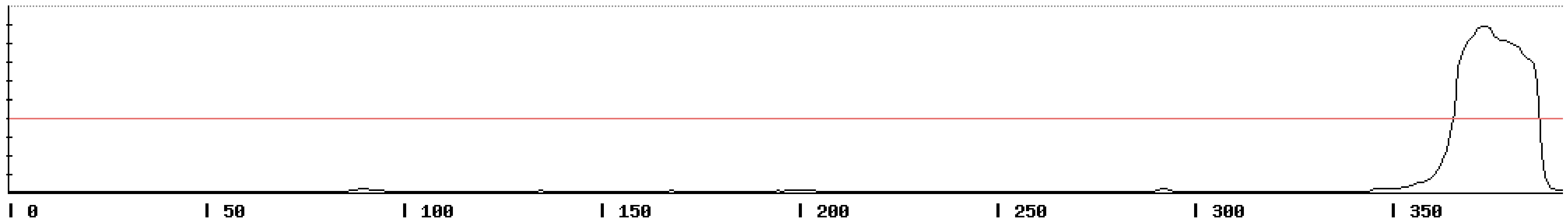

Predictions for Gossypiumhirsutum_Gh_A06G0601	
Viterbi Path	
Viterbi Path	
Posterior @ 0.4	
Posterior @ 0.4	
Posterior Graphic. Threshold Marked @ 0.4	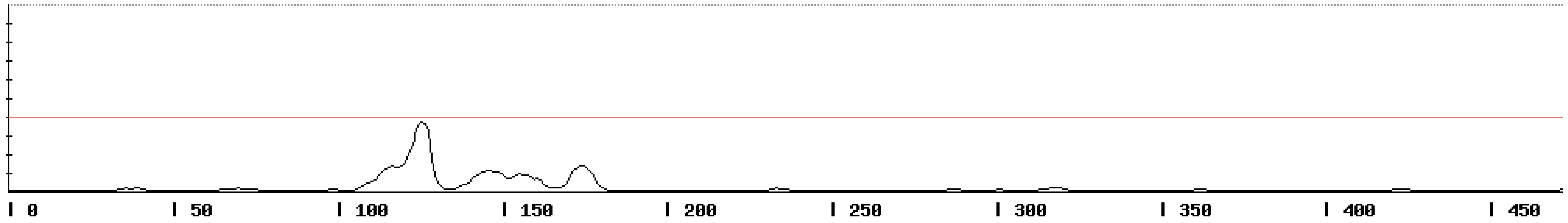

Predictions for Gossypiumhirsutum_Gh_A06G0970	
Viterbi Path	
Viterbi Path	
Posterior @ 0.4	
Posterior @ 0.4	
Posterior Graphic. Threshold Marked @ 0.4	

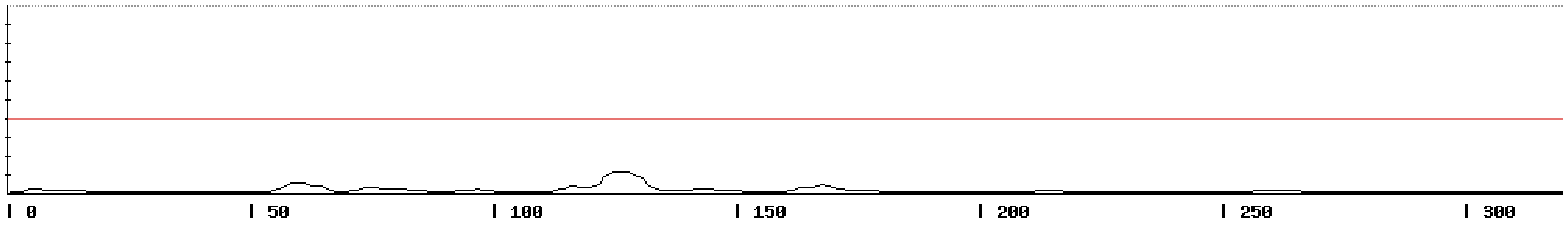

Predictions for Gossypiumhirsutum_Gh_A06G1226

Viterbi Path	
Viterbi Path	
Posterior @ 0.4	
Posterior @ 0.4	
Posterior Graphic. Threshold Marked @ 0.4	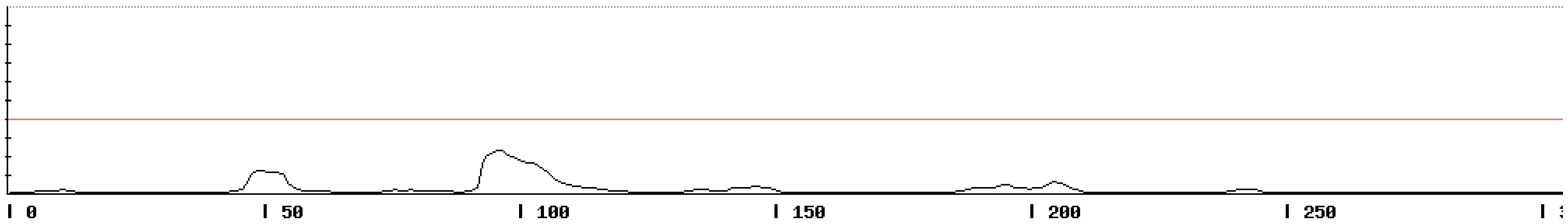

Predictions for Gossypiumhirsutum_Gh_A06G1758

Viterbi Path	
Viterbi Path	
Posterior @ 0.4	66 - KLKKKGKGKN - 75
Posterior @ 0.4	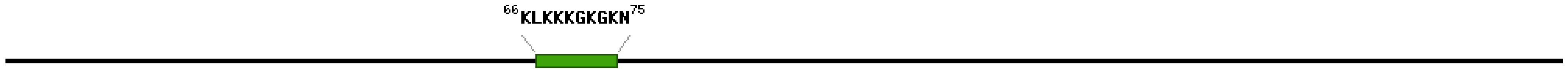

Posterior Graphic. Threshold Marked @ 0.4

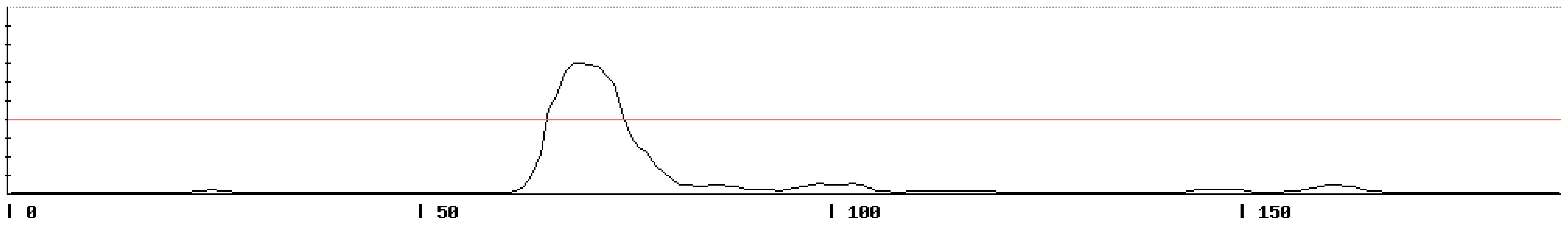

Predictions for Gossypiumhirsutum_Gh_A06G1759

Viterbi Path

Viterbi Path

Posterior @ 0.4 — 66 - KLKKKGKGKN - 75

Posterior @ 0.4

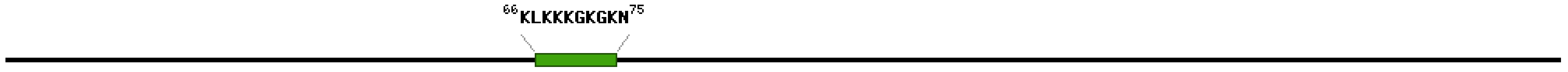

Posterior Graphic. Threshold Marked @ 0.4

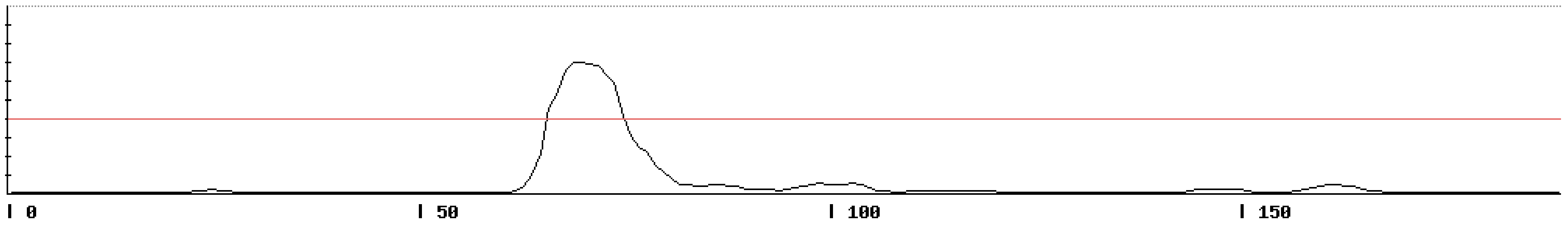

Predictions for Gossypiumhirsutum_Gh_A06G1910

Viterbi Path

Viterbi Path

Posterior @ 0.4

Posterior @ 0.4

Posterior Graphic. Threshold Marked @ 0.4

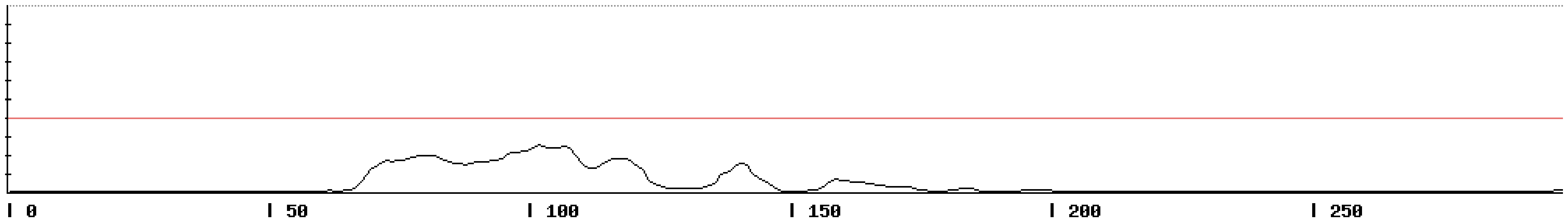

Predictions for Gossypiumhirsutum_Gh_A06G1947

Viterbi Path

Viterbi Path

Posterior @ 0.4

Posterior @ 0.4

Posterior Graphic. Threshold Marked @ 0.4

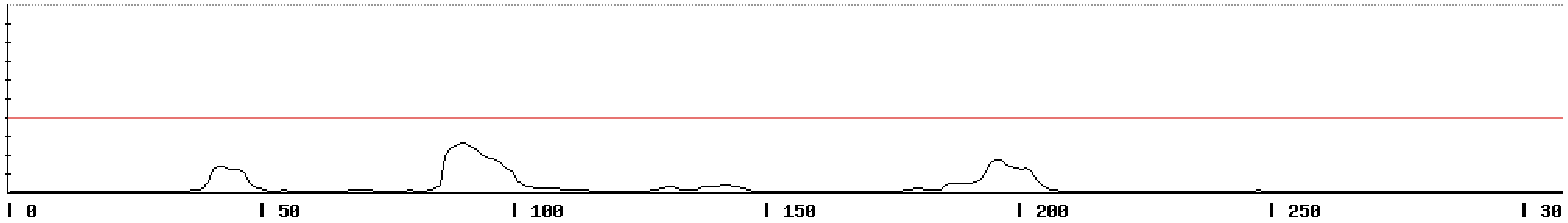

Predictions for Gossypiumhirsutum_Gh_A06G2028

Viterbi Path

Viterbi Path

Posterior @ 0.4	135 - RKRRK - 139
Posterior @ 0.4	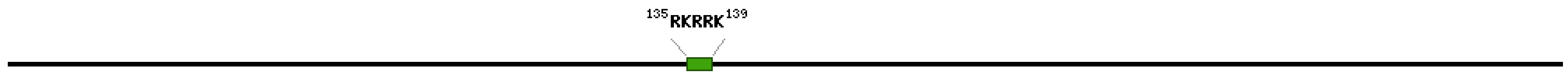
Posterior Graphic. Threshold Marked @ 0.4	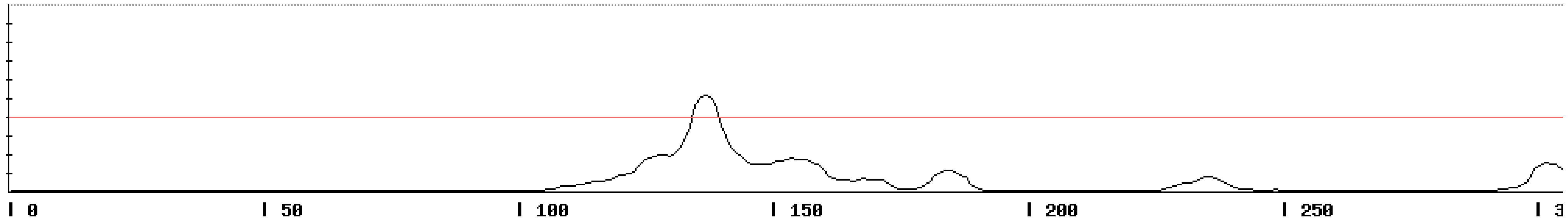

Predictions for Gossypiumhirsutum_Gh_A06G2114

Viterbi Path	
Viterbi Path	
Posterior @ 0.4	
Posterior @ 0.4	
Posterior Graphic. Threshold Marked @ 0.4	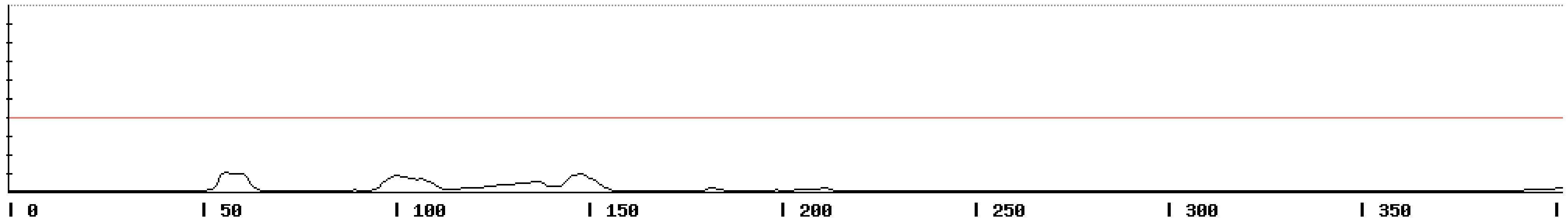

Predictions for Gossypiumhirsutum_Gh_A07G0349

Viterbi Path	
Viterbi Path	

Posterior @ 0.4

Posterior @ 0.4

Posterior Graphic. Threshold Marked @ 0.4

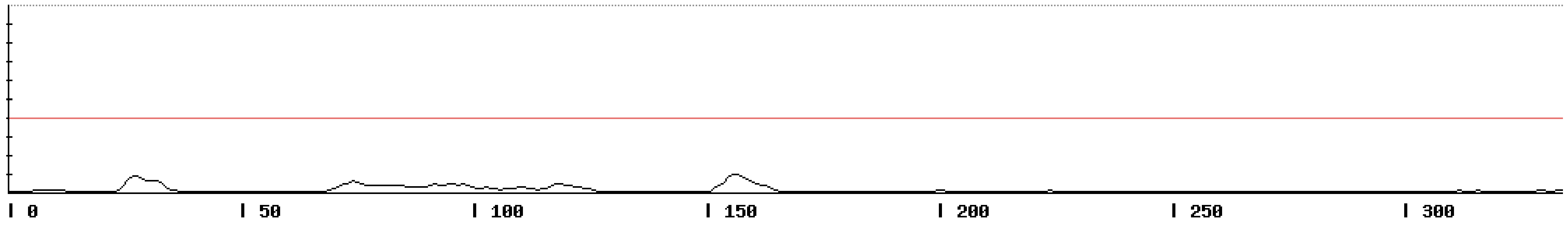

Predictions for Gossypiumhirsutum_Gh_A07G0558

Viterbi Path

Viterbi Path

Posterior @ 0.4

Posterior @ 0.4

Posterior Graphic. Threshold Marked @ 0.4

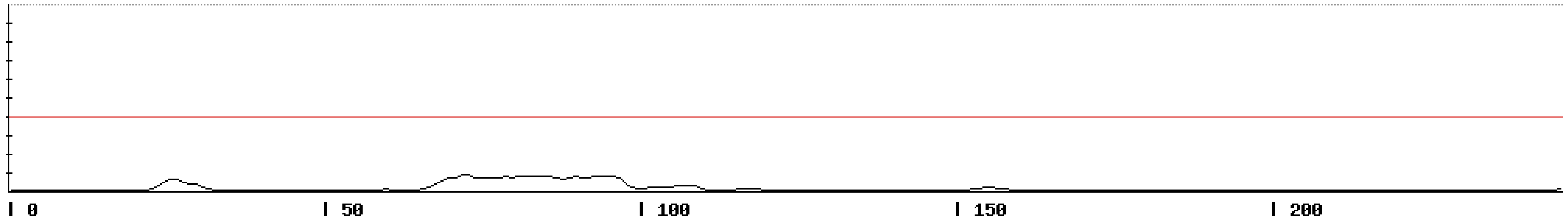

Predictions for Gossypiumhirsutum_Gh_A07G0739

Viterbi Path

Viterbi Path

Posterior @ 0.4

Posterior @ 0.4

Posterior Graphic. Threshold Marked @ 0.4

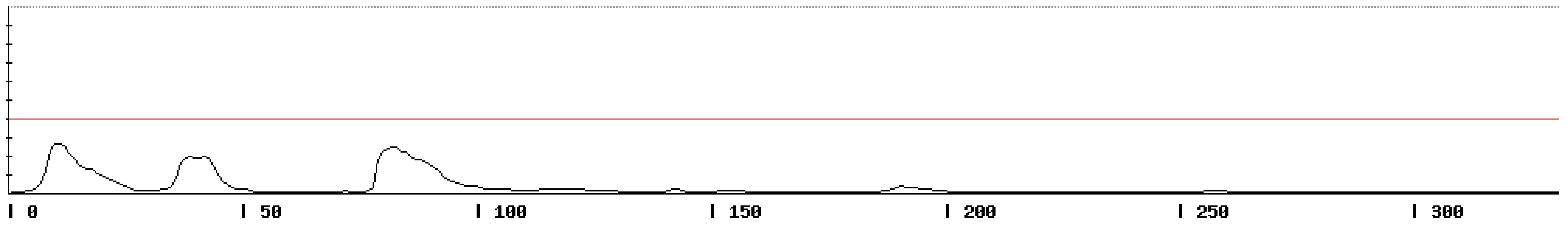

Predictions for Gossypiumhirsutum_Gh_A07G1009

Viterbi Path

Viterbi Path

Posterior @ 0.4

118 - KAPSGKKTK - 126

Posterior @ 0.4

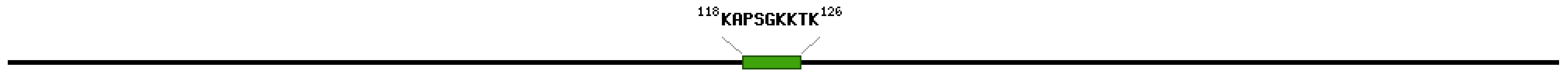

Posterior Graphic. Threshold Marked @ 0.4

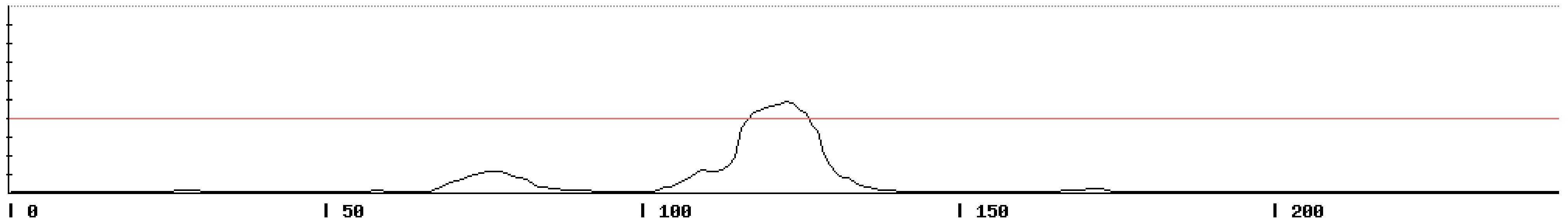

Predictions for Gossypiumhirsutum_Gh_A07G1218	
Viterbi Path	
Viterbi Path	
Posterior @ 0.4	
Posterior @ 0.4	
Posterior Graphic. Threshold Marked @ 0.4	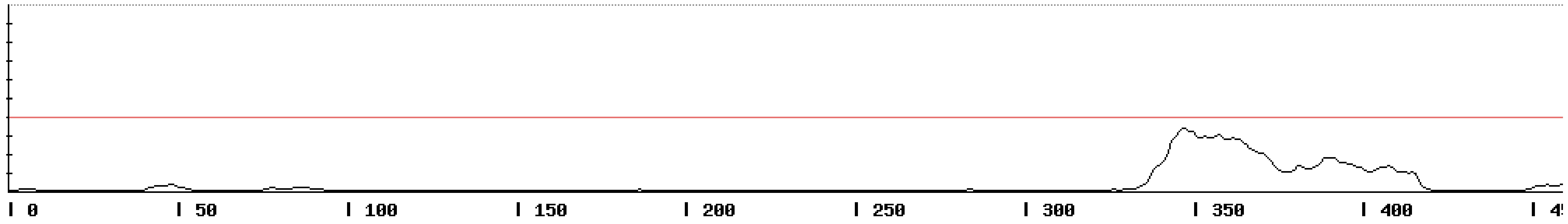

Predictions for Gossypiumhirsutum_Gh_A07G1226	
Viterbi Path	
Viterbi Path	
Posterior @ 0.4	
Posterior @ 0.4	
Posterior Graphic. Threshold Marked @ 0.4	

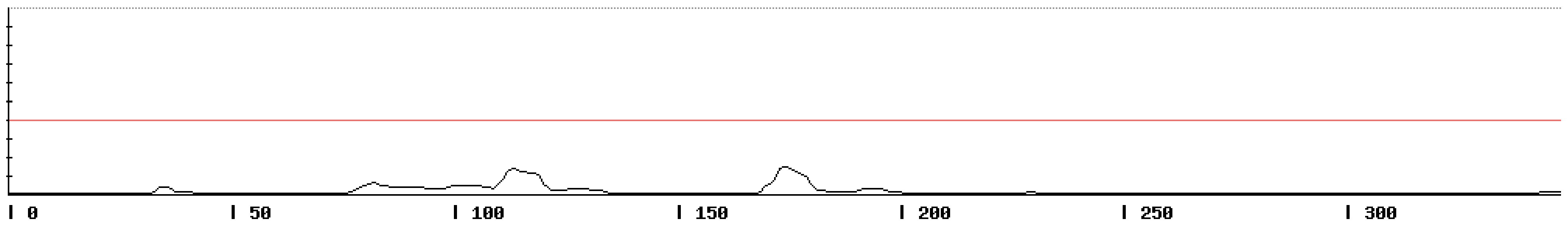

Predictions for Gossypiumhirsutum_Gh_A07G1402

Viterbi Path

Viterbi Path

Posterior @ 0.4

Posterior @ 0.4

Posterior Graphic. Threshold Marked @ 0.4

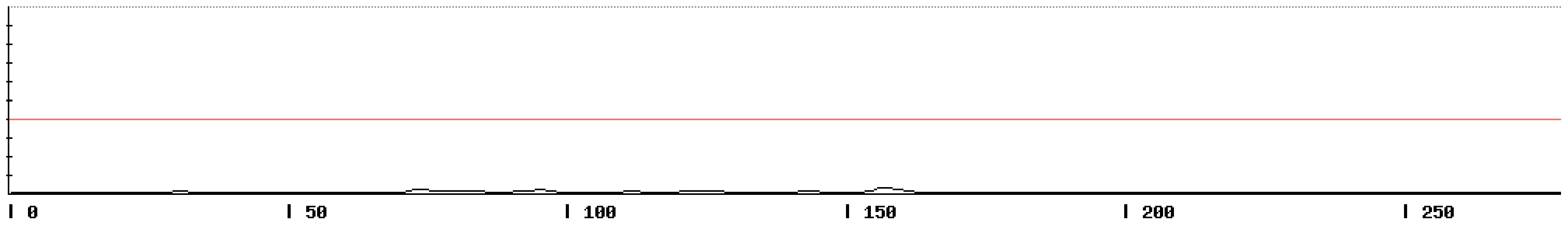

Predictions for Gossypiumhirsutum_Gh_A07G1811

Viterbi Path

Viterbi Path

Posterior @ 0.4

Posterior @ 0.4

Posterior Graphic. Threshold Marked @ 0.4

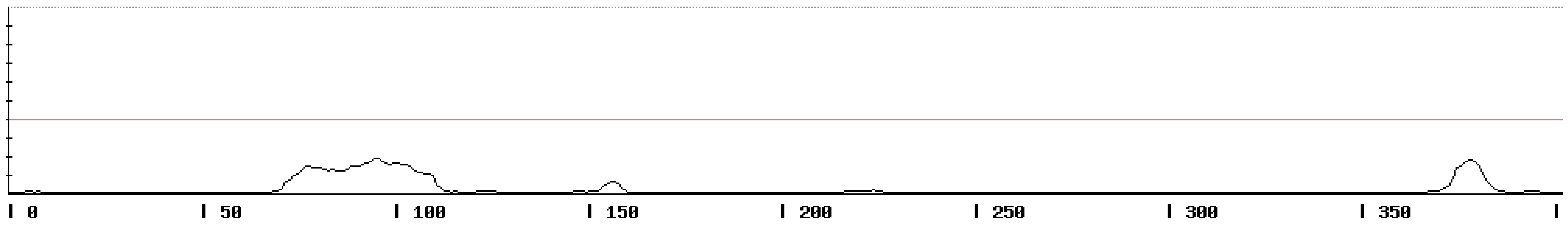

Predictions for Gossypiumhirsutum_Gh_A07G1948

Viterbi Path	
Viterbi Path	
Posterior @ 0.4	
Posterior @ 0.4	
Posterior Graphic. Threshold Marked @ 0.4	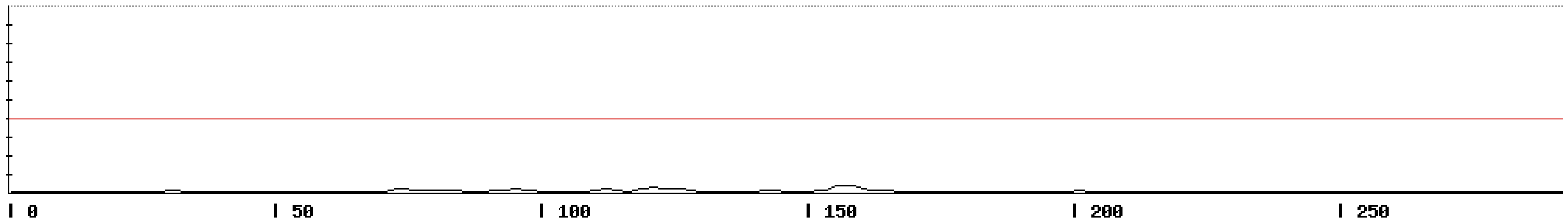

Predictions for Gossypiumhirsutum_Gh_A07G2132

Viterbi Path	
Viterbi Path	
Posterior @ 0.4	81 - KKRKR - 85
Posterior @ 0.4	

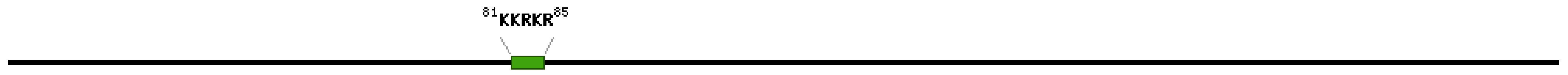

Posterior Graphic. Threshold Marked @ 0.4

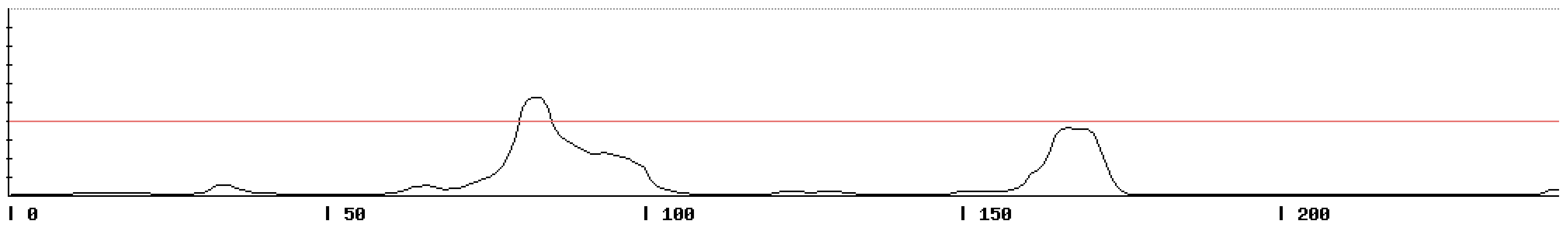

Predictions for Gossypiumhirsutum_Gh_A08G0336

Viterbi Path

Viterbi Path

Posterior @ 0.4

Posterior @ 0.4

Posterior Graphic. Threshold Marked @ 0.4

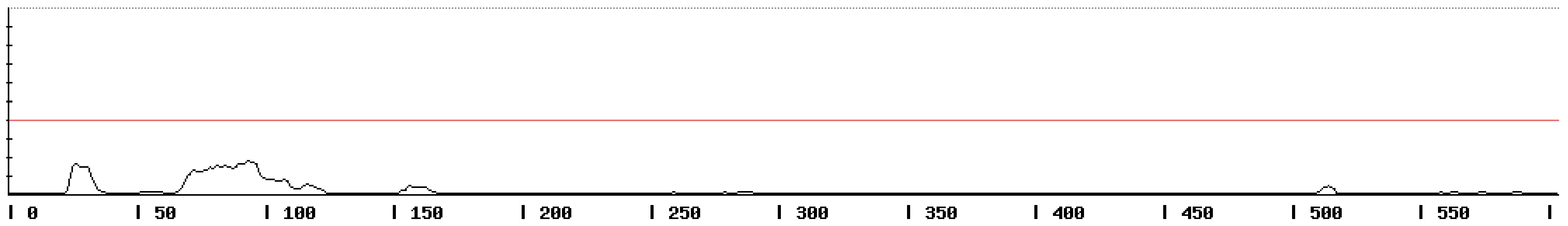

Predictions for Gossypiumhirsutum_Gh_A08G0961

Viterbi Path

Viterbi Path

Posterior @ 0.4

Posterior @ 0.4

Posterior Graphic. Threshold Marked @ 0.4

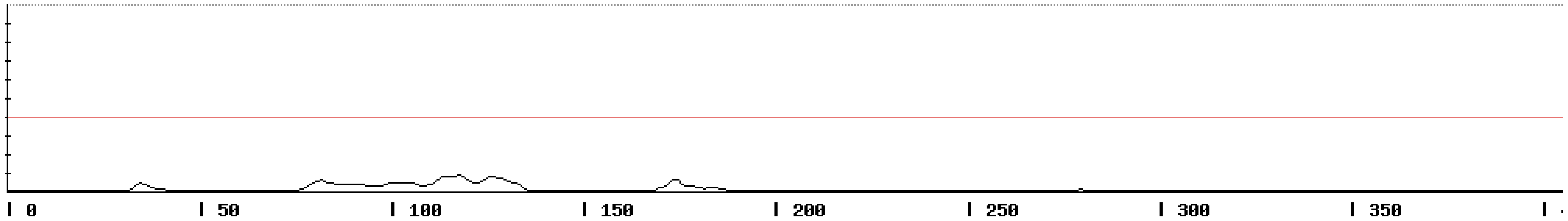

Predictions for Gossypiumhirsutum_Gh_A08G1086

Viterbi Path: 73 - RRGDNKRVKRTTDKGFWKVTGKPRKVKGKKGCAAKK - 108

Viterbi Path

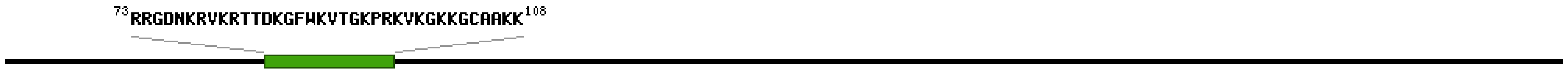

Posterior @ 0.4: 73 - RRGDNKRVKRTTDKGFWKVTGKPRKVKGKKGCAAKK - 108

Posterior @ 0.4

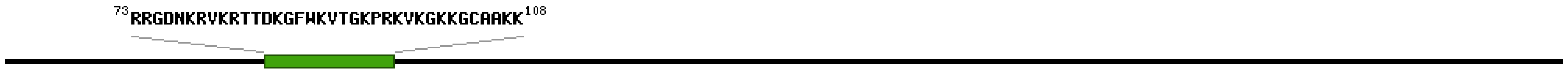

Posterior Graphic. Threshold Marked @ 0.4

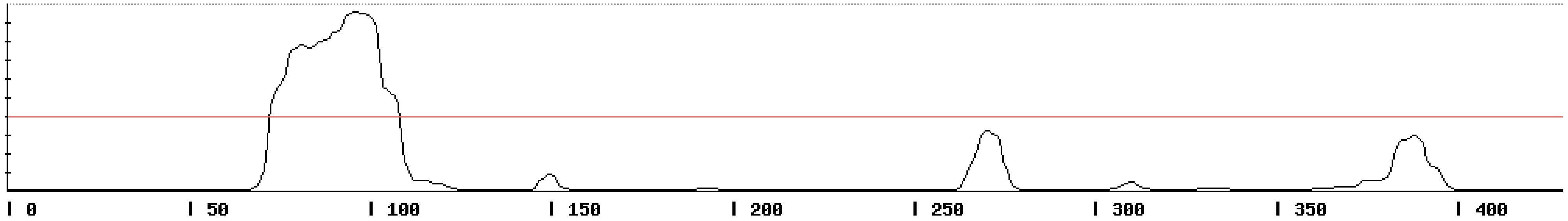

Predictions for Gossypiumhirsutum_Gh_A08G1428

Viterbi Path

Viterbi Path

Posterior @ 0.4

Posterior @ 0.4

Posterior Graphic. Threshold Marked @ 0.4

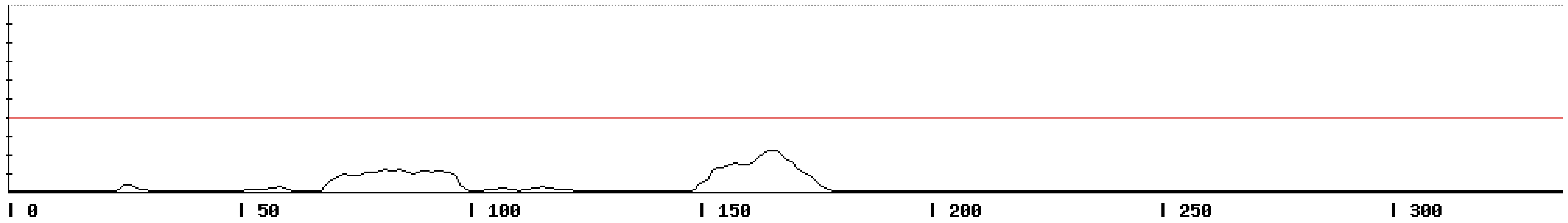

Predictions for Gossypiumhirsutum_Gh_A08G1552

Viterbi Path

Viterbi Path

Posterior @ 0.4

Posterior @ 0.4

Posterior Graphic. Threshold Marked @ 0.4

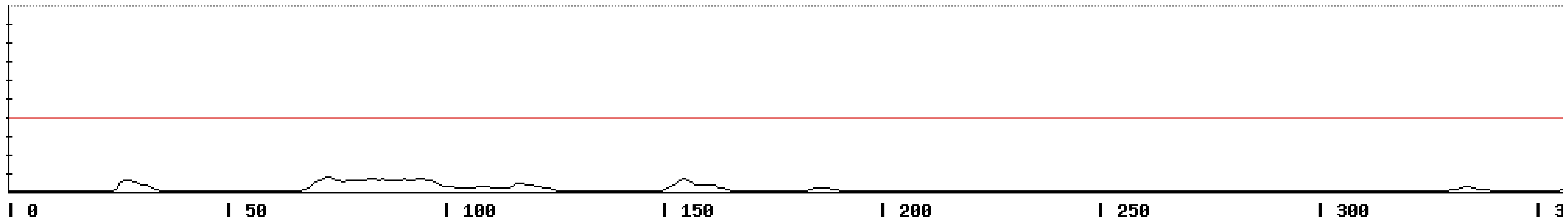

Predictions for Gossypiumhirsutum_Gh_A08G1691

Viterbi Path	
Viterbi Path	
Posterior @ 0.4	
Posterior @ 0.4	
Posterior Graphic. Threshold Marked @ 0.4	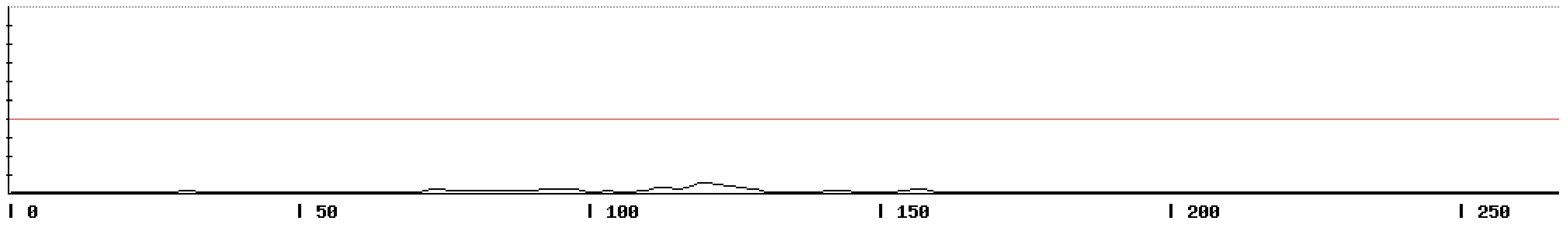

Predictions for Gossypiumhirsutum_Gh_A08G1743

Viterbi Path	
Viterbi Path	
Posterior @ 0.4	475 - SKRPRRM - 481
Posterior @ 0.4	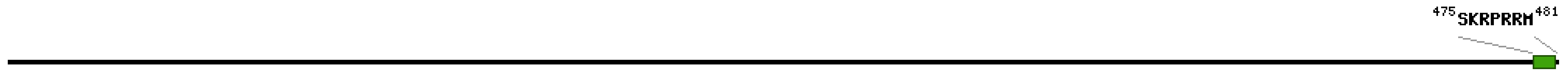
Posterior Graphic. Threshold Marked @ 0.4	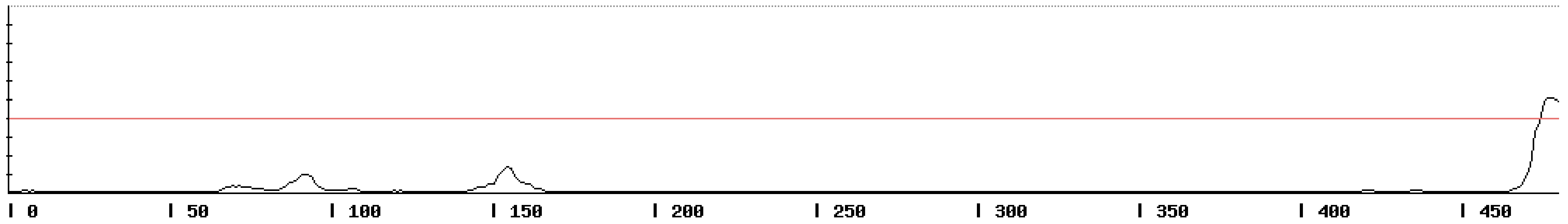

Predictions for Gossypiumhirsutum_Gh_A08G1953

Viterbi Path

Viterbi Path

Posterior @ 0.4

Posterior @ 0.4

Posterior Graphic. Threshold Marked @ 0.4

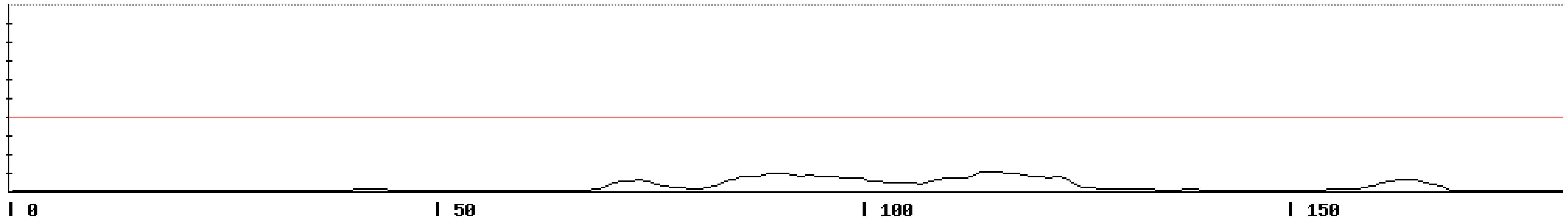

Predictions for Gossypiumhirsutum_Gh_A08G2042

Viterbi Path

Viterbi Path

Posterior @ 0.4

Posterior @ 0.4

Posterior Graphic. Threshold Marked @ 0.4

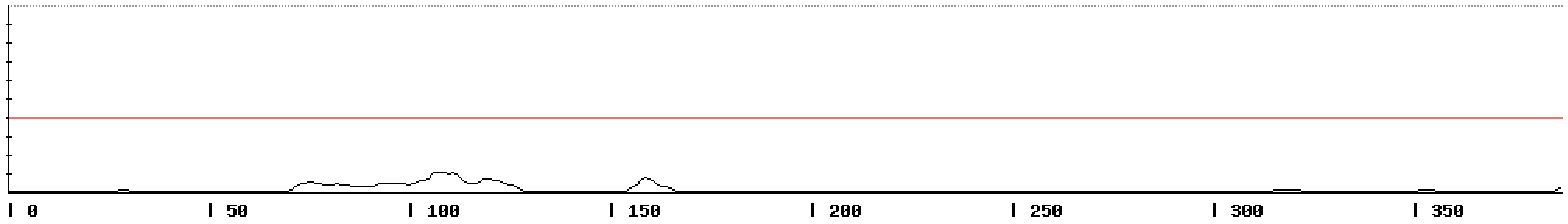

Predictions for Gossypiumhirsutum_Gh_A08G2055

Viterbi Path

Viterbi Path

Posterior @ 0.4: 163 - KRNKKIKQDSRQGAAKR - 179

Posterior @ 0.4

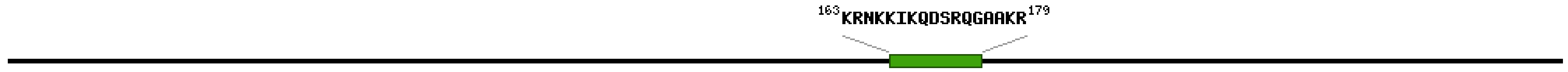

Posterior Graphic. Threshold Marked @ 0.4

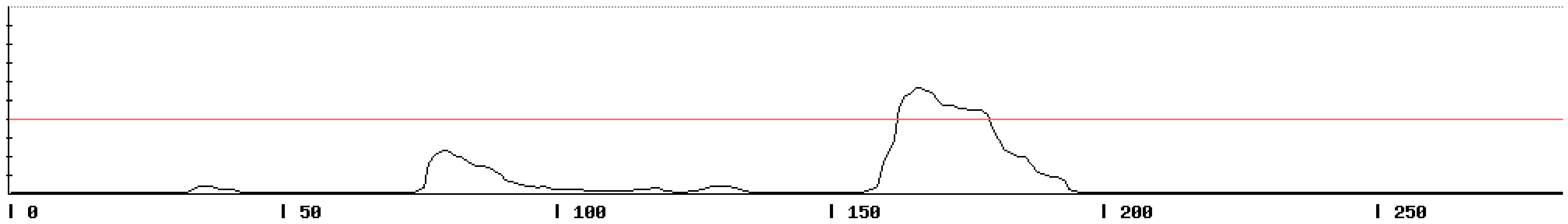

Predictions for Gossypiumhirsutum_Gh_A08G2056

Viterbi Path

Viterbi Path

Posterior @ 0.4: 166 - RK - 167

Posterior @ 0.4

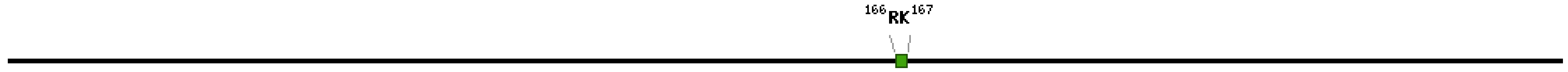

Posterior Graphic. Threshold Marked @ 0.4

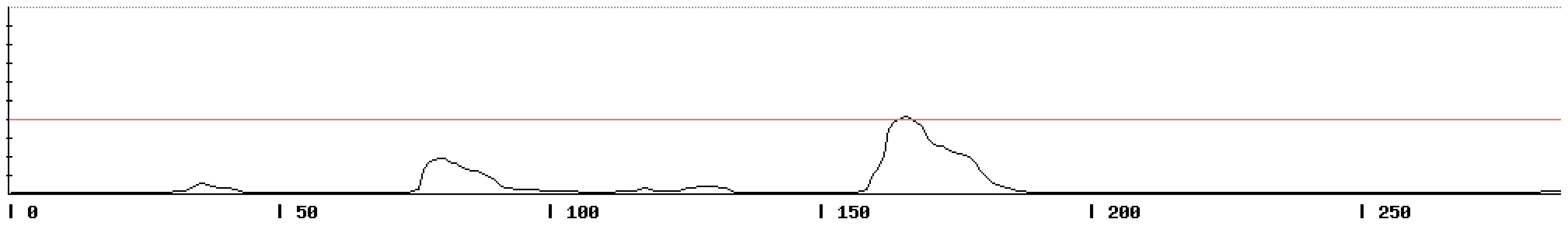

Predictions for Gossypiumhirsutum_Gh_A08G2057

Viterbi Path

Viterbi Path

Posterior @ 0.4 163 - KRHKKNKQE - 171

Posterior @ 0.4

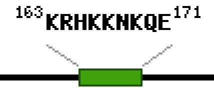

Posterior Graphic. Threshold Marked @ 0.4

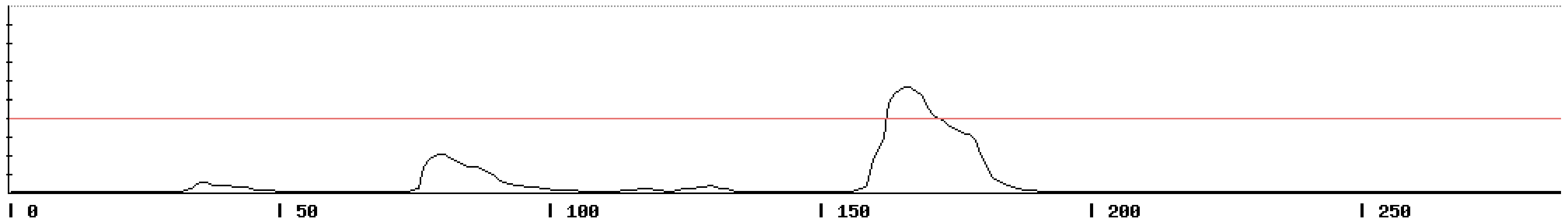

Predictions for Gossypiumhirsutum_Gh_A08G2288

Viterbi Path

Viterbi Path

Posterior @ 0.4

Posterior @ 0.4

Posterior Graphic. Threshold Marked @ 0.4

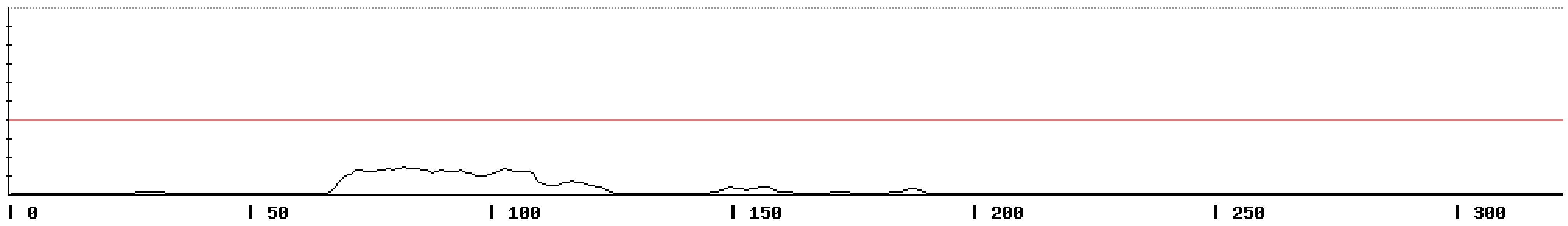

Predictions for Gossypiumhirsutum_Gh_A08G2289

Viterbi Path

Viterbi Path

Posterior @ 0.4

Posterior @ 0.4

Posterior Graphic. Threshold Marked @ 0.4

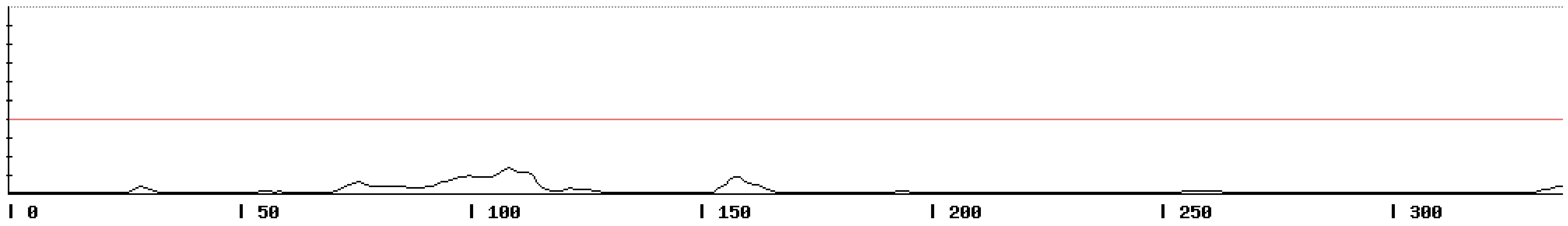

Predictions for Gossypiumhirsutum_Gh_A09G0040

Viterbi Path

Viterbi Path

Posterior @ 0.4

Posterior @ 0.4

Posterior Graphic. Threshold Marked @ 0.4

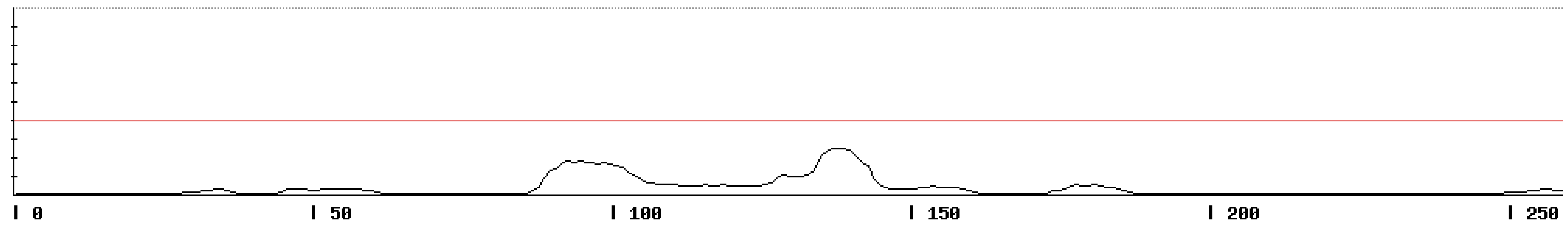

Predictions for Gossypiumhirsutum_Gh_A09G0159

Viterbi Path

Viterbi Path

Posterior @ 0.4

Posterior @ 0.4

Posterior Graphic. Threshold Marked @ 0.4

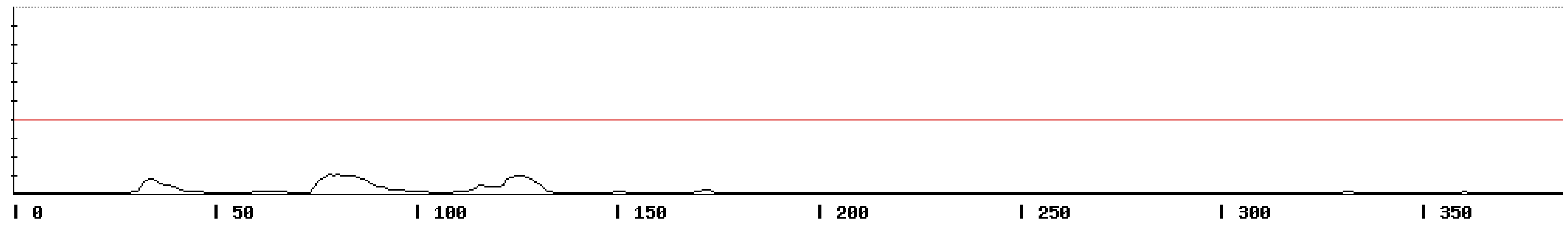

Predictions for Gossypiumhirsutum_Gh_A09G0294

Viterbi Path

Viterbi Path

Posterior @ 0.4

Posterior @ 0.4

Posterior Graphic. Threshold Marked @ 0.4

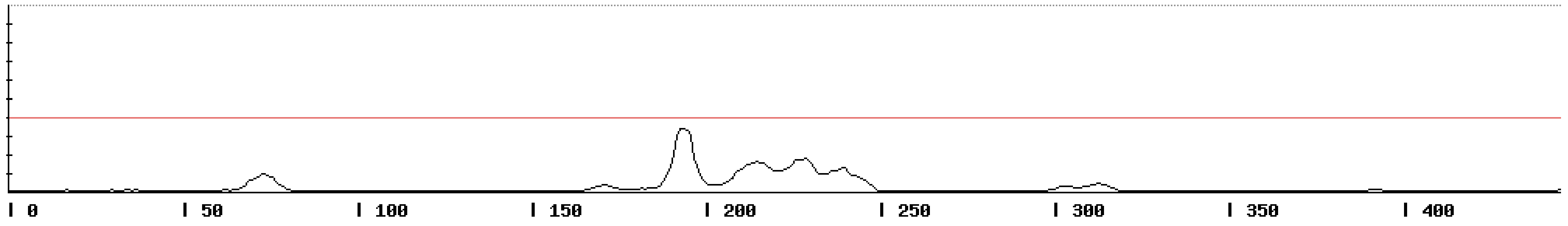

Predictions for Gossypiumhirsutum_Gh_A09G0474

Viterbi Path

Viterbi Path

Posterior @ 0.4

Posterior @ 0.4

Posterior Graphic. Threshold Marked @ 0.4

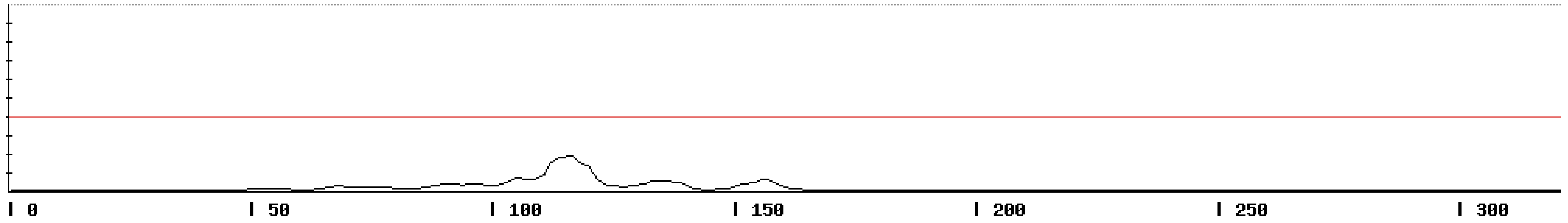

Predictions for Gossypiumhirsutum_Gh_A09G0703

Viterbi Path	
Viterbi Path	
Posterior @ 0.4	
Posterior @ 0.4	
Posterior Graphic. Threshold Marked @ 0.4	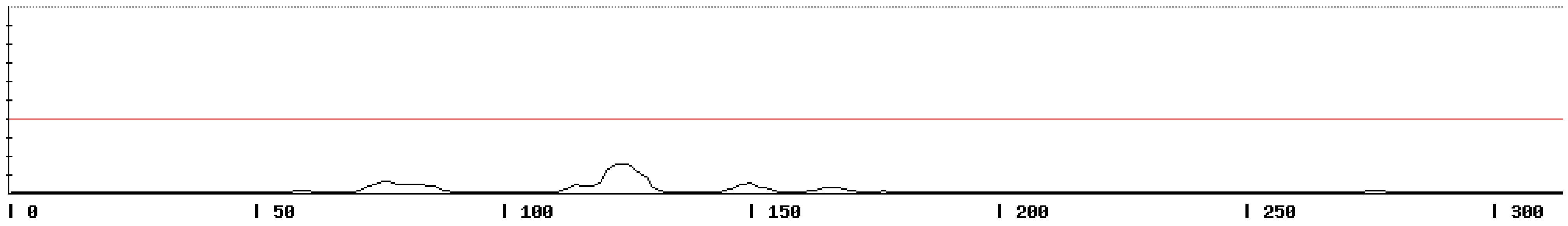

Predictions for Gossypiumhirsutum_Gh_A09G0913

Viterbi Path	
Viterbi Path	
Posterior @ 0.4	123 - GRK - 125
Posterior @ 0.4	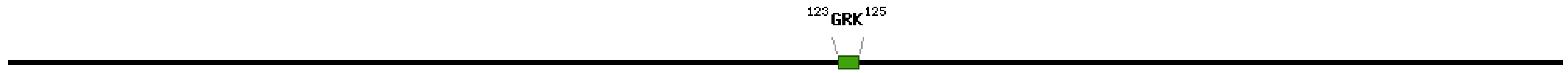
Posterior Graphic. Threshold Marked @ 0.4	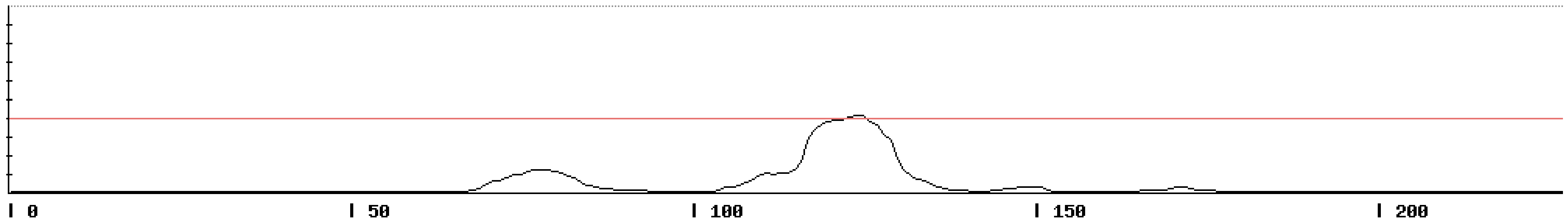

Predictions for Gossypiumhirsutum_Gh_A09G0920

Viterbi Path

Viterbi Path

Posterior @ 0.4

Posterior @ 0.4

Posterior Graphic. Threshold Marked @ 0.4

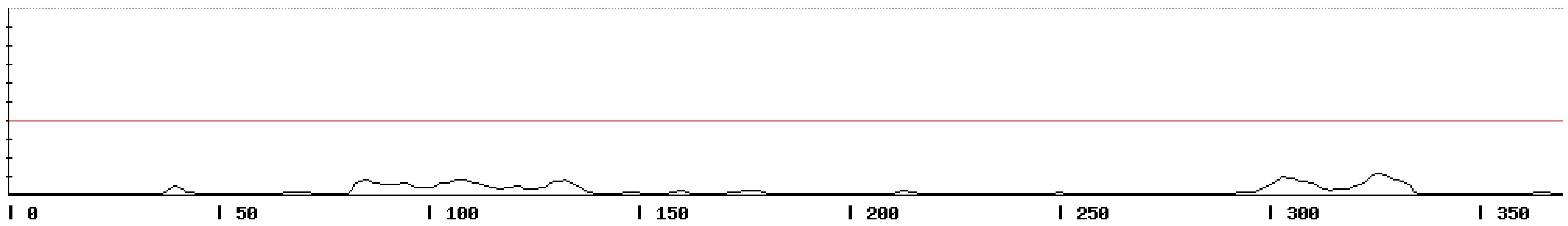

Predictions for Gossypiumhirsutum_Gh_A09G1062

Viterbi Path

Viterbi Path

Posterior @ 0.4

Posterior @ 0.4

Posterior Graphic. Threshold Marked @ 0.4

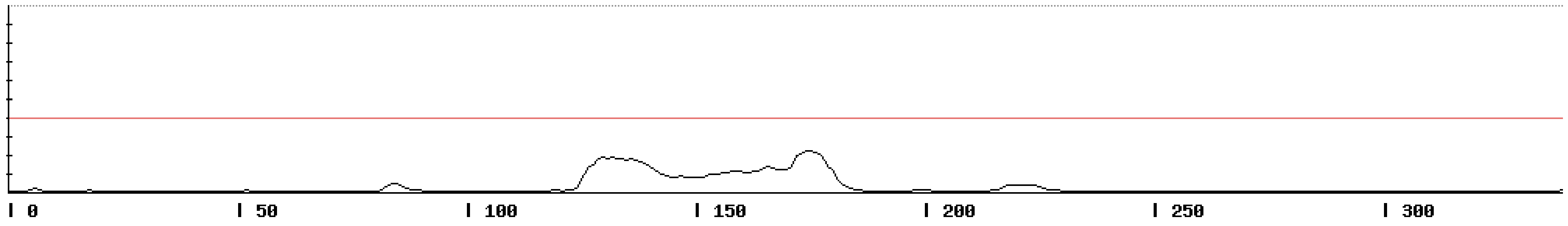

Predictions for Gossypiumhirsutum_Gh_A09G1149

Viterbi Path	
Viterbi Path	
Posterior @ 0.4	
Posterior @ 0.4	
Posterior Graphic. Threshold Marked @ 0.4	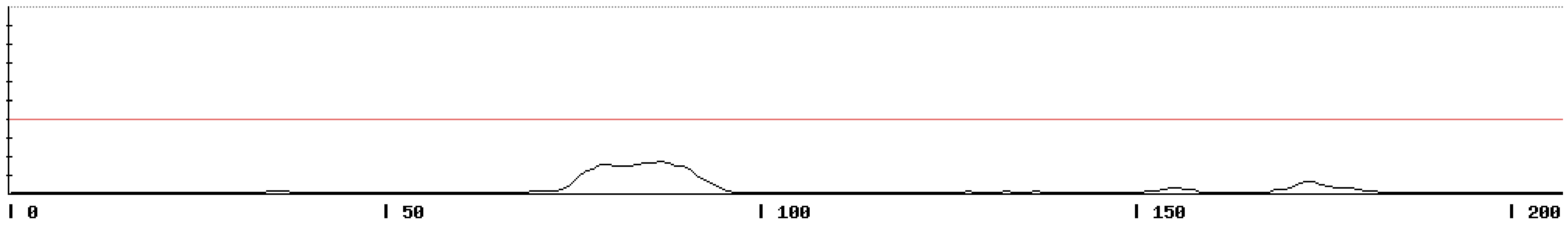

Predictions for Gossypiumhirsutum_Gh_A09G1559

Viterbi Path	
Viterbi Path	
Posterior @ 0.4	
Posterior @ 0.4	

Posterior Graphic. Threshold Marked @ 0.4	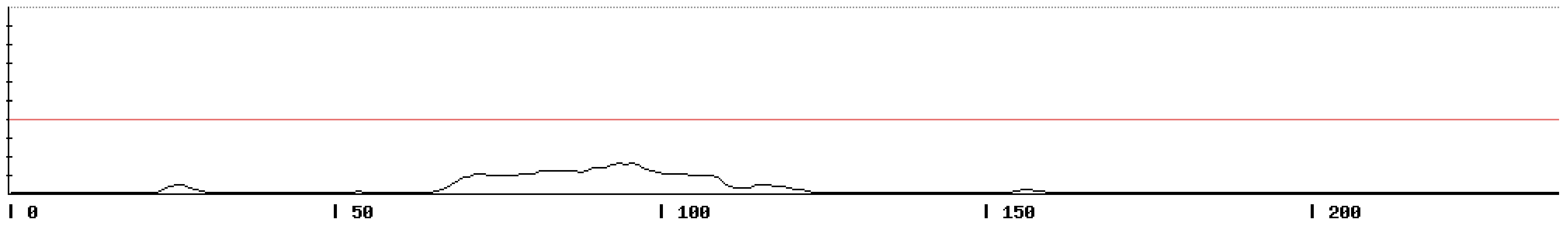

Predictions for Gossypiumhirsutum_Gh_A09G1677

Viterbi Path	
Viterbi Path	
Posterior @ 0.4	
Posterior @ 0.4	
Posterior Graphic. Threshold Marked @ 0.4	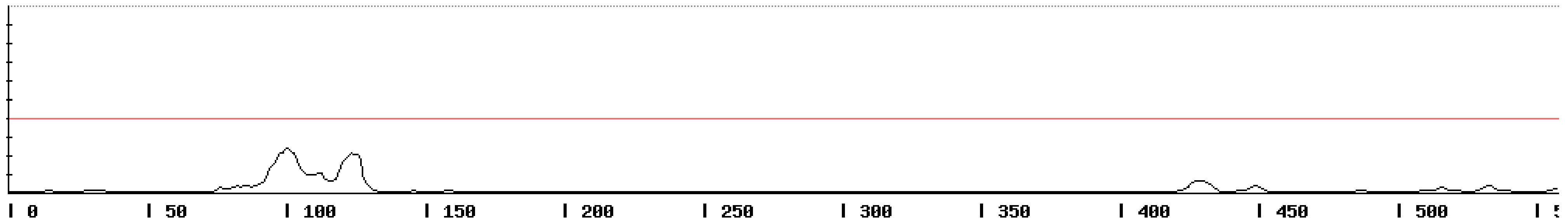

Predictions for Gossypiumhirsutum_Gh_A09G2152

Viterbi Path	
Viterbi Path	
Posterior @ 0.4	
Posterior @ 0.4	

Posterior Graphic. Threshold Marked @ 0.4

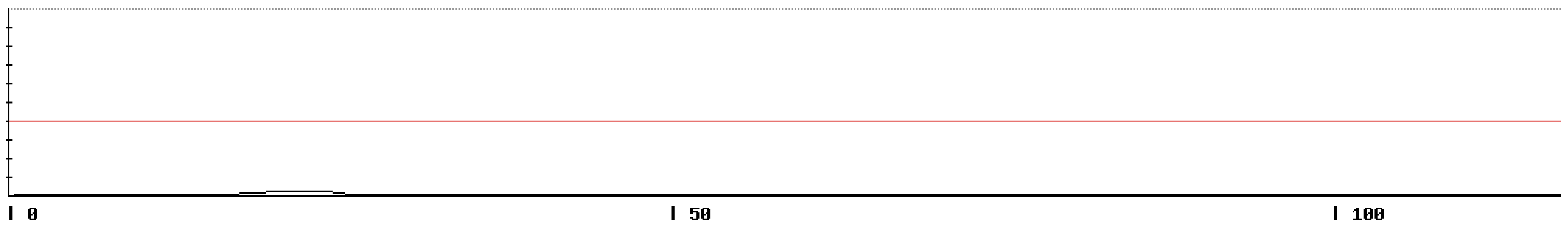

Predictions for Gossypiumhirsutum_Gh_A09G2371

Viterbi Path

Viterbi Path

Posterior @ 0.4

Posterior @ 0.4

Posterior Graphic. Threshold Marked @ 0.4

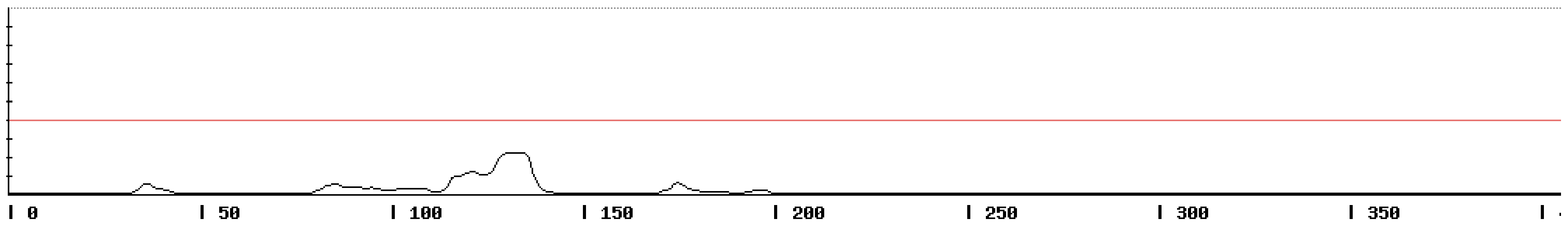

Predictions for Gossypiumhirsutum_Gh_A10G0263

Viterbi Path

Viterbi Path

Posterior @ 0.4

Posterior @ 0.4

Posterior Graphic. Threshold Marked @ 0.4

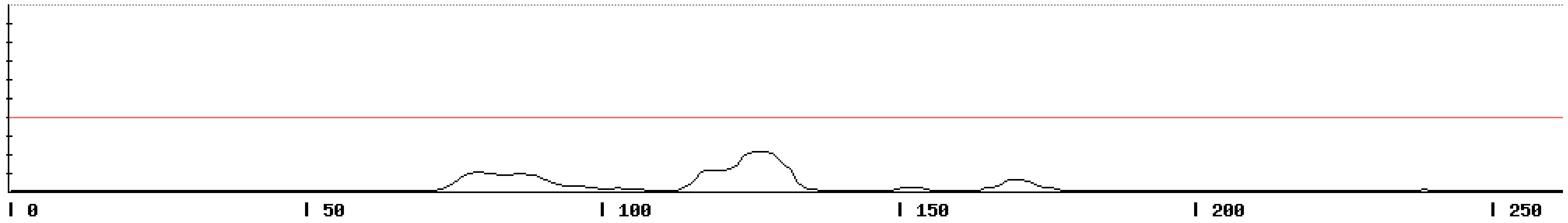

Predictions for Gossypiumhirsutum_Gh_A10G0607

Viterbi Path

Viterbi Path

Posterior @ 0.4

Posterior @ 0.4

Posterior Graphic. Threshold Marked @ 0.4

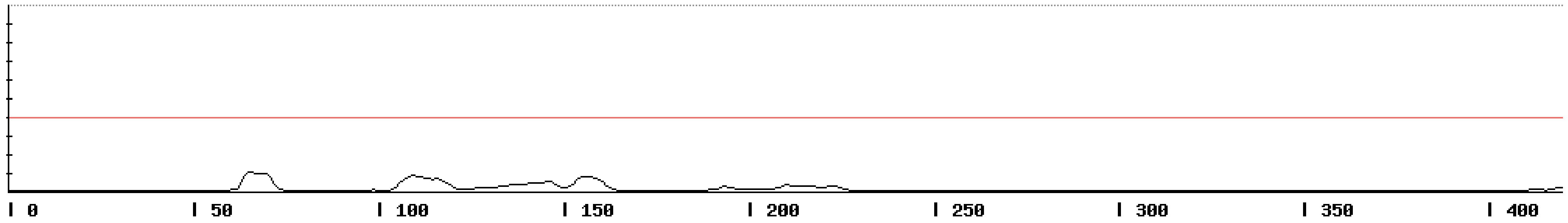

Predictions for Gossypiumhirsutum_Gh_A10G0627

Viterbi Path

Viterbi Path

Posterior @ 0.4	166 - QRKRSRRGKKH - 176
Posterior @ 0.4	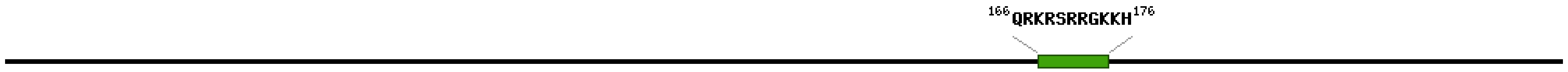
Posterior Graphic. Threshold Marked @ 0.4	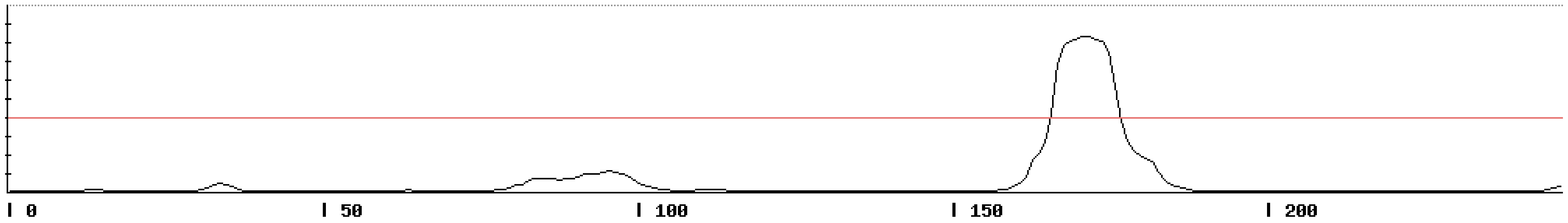

Predictions for Gossypiumhirsutum_Gh_A10G1796

Viterbi Path	
Viterbi Path	
Posterior @ 0.4	
Posterior @ 0.4	
Posterior Graphic. Threshold Marked @ 0.4	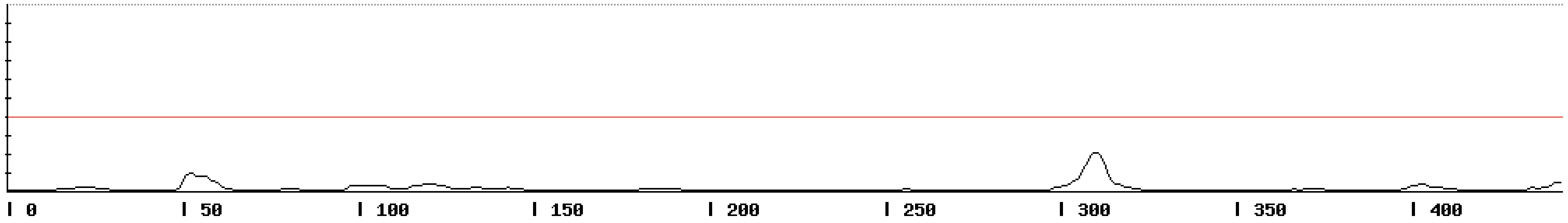

Predictions for Gossypiumhirsutum_Gh_A10G1944

Viterbi Path	
Viterbi Path	
Posterior @ 0.4	282 - LKKKSKSR - 289
Posterior @ 0.4	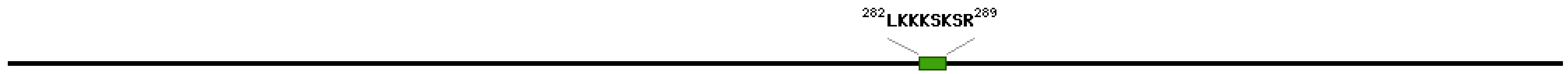
Posterior Graphic. Threshold Marked @ 0.4	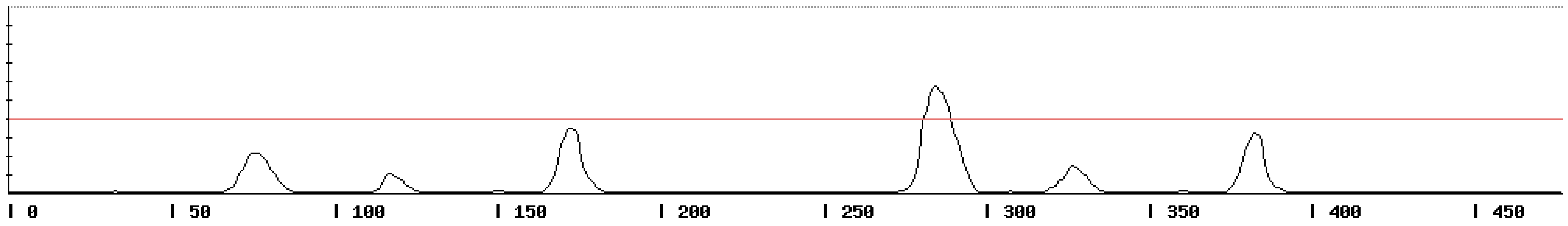

Predictions for Gossypiumhirsutum_Gh_A10G2008

Viterbi Path	
Viterbi Path	
Posterior @ 0.4	
Posterior @ 0.4	
Posterior Graphic. Threshold Marked @ 0.4	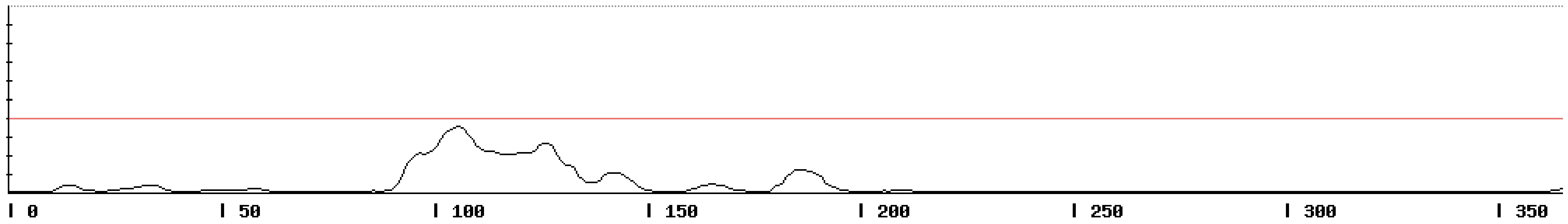

Predictions for Gossypiumhirsutum_Gh_A10G2021

Viterbi Path	
Viterbi Path	
Posterior @ 0.4	
Posterior @ 0.4	
Posterior Graphic. Threshold Marked @ 0.4	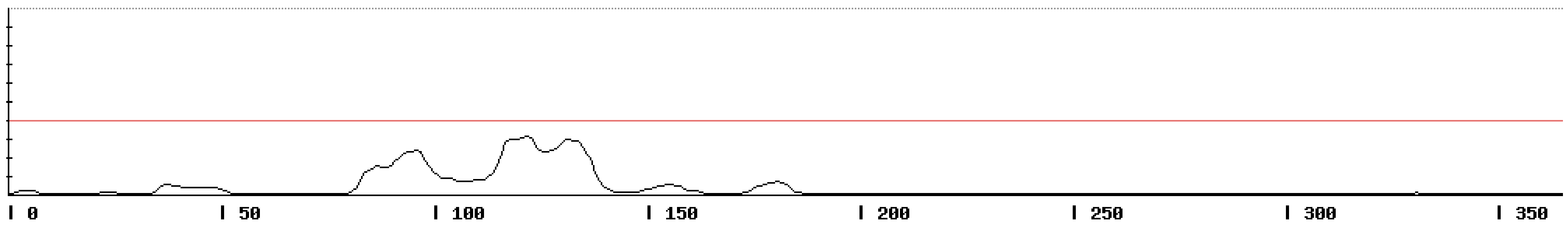

Predictions for Gossypiumhirsutum_Gh_A10G2022

Viterbi Path	
Viterbi Path	
Posterior @ 0.4	115 - RKGKKIGLKK - 124
Posterior @ 0.4	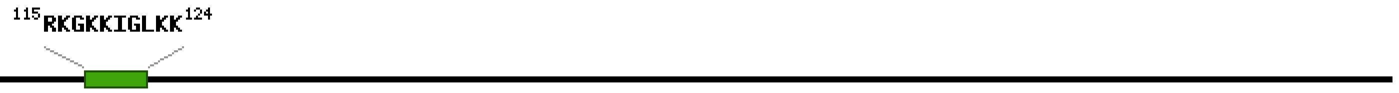
Posterior Graphic. Threshold Marked @ 0.4	

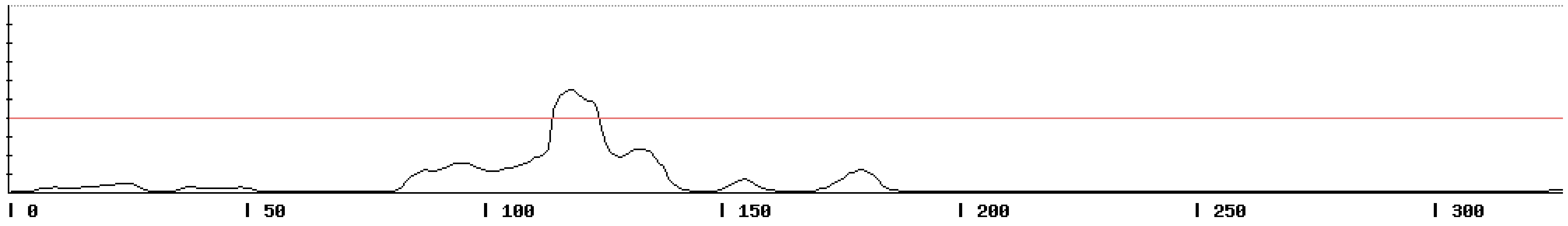

Predictions for Gossypiumhirsutum_Gh_A10G2023

Viterbi Path	
Viterbi Path	
Posterior @ 0.4	115 - RKGKKIGLKK - 124
Posterior @ 0.4	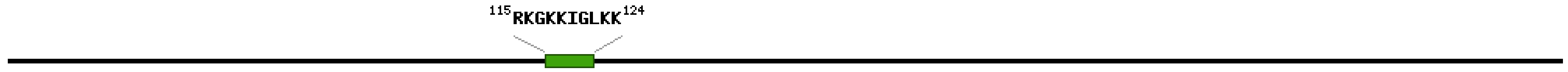
Posterior Graphic. Threshold Marked @ 0.4	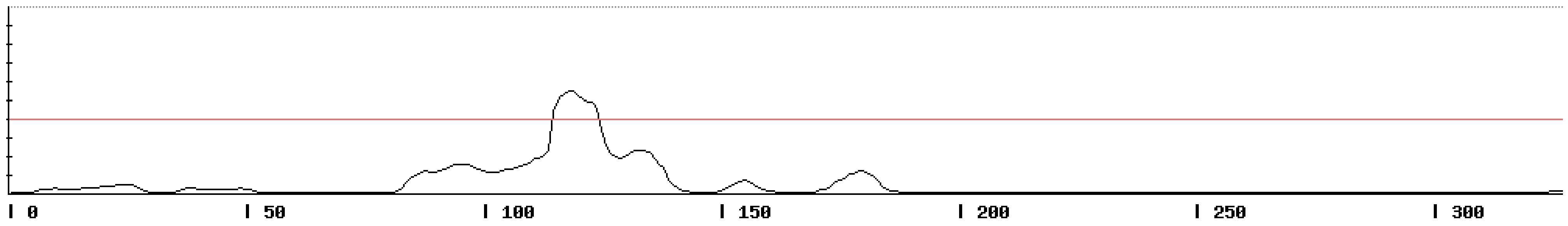

Predictions for Gossypiumhirsutum_Gh_A11G0147

Viterbi Path	
Viterbi Path	
Posterior @ 0.4	
Posterior @ 0.4	

Posterior Graphic. Threshold Marked @ 0.4

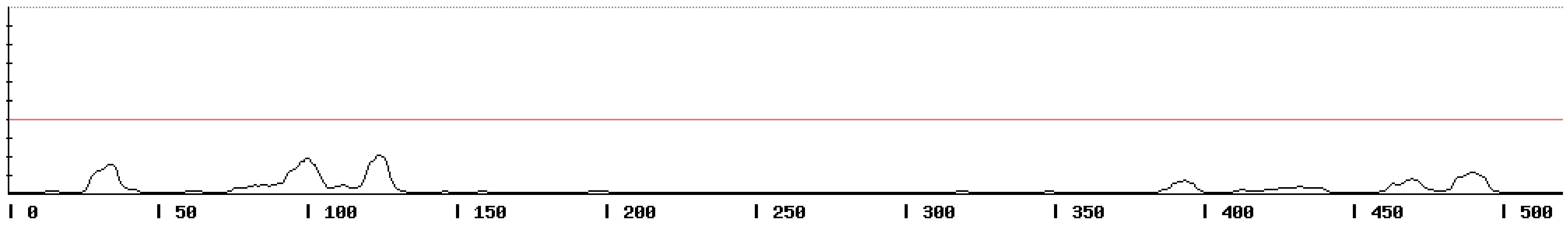

Predictions for Gossypiumhirsutum_Gh_A11G0266

Viterbi Path

Viterbi Path

Posterior @ 0.4

Posterior @ 0.4

Posterior Graphic. Threshold Marked @ 0.4

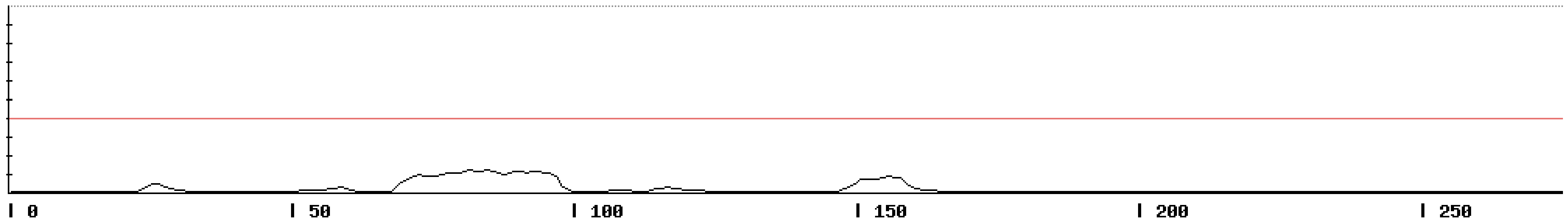

Predictions for Gossypiumhirsutum_Gh_A11G0290

Viterbi Path

Viterbi Path

Posterior @ 0.4

Posterior @ 0.4

Posterior Graphic. Threshold Marked @ 0.4

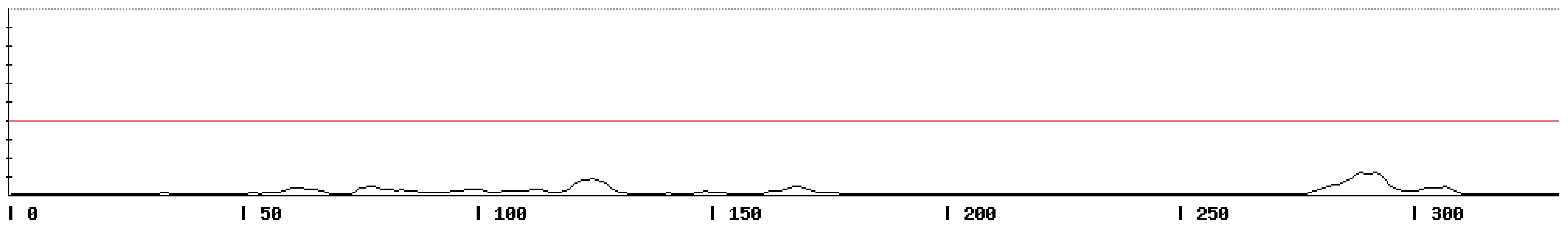

Predictions for Gossypiumhirsutum_Gh_A11G0348

Viterbi Path

Viterbi Path

Posterior @ 0.4

175 - KKDKKKKFLQKA - 186

Posterior @ 0.4

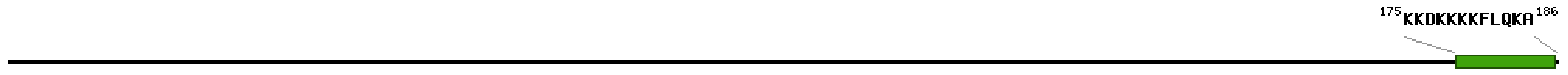

Posterior Graphic. Threshold Marked @ 0.4

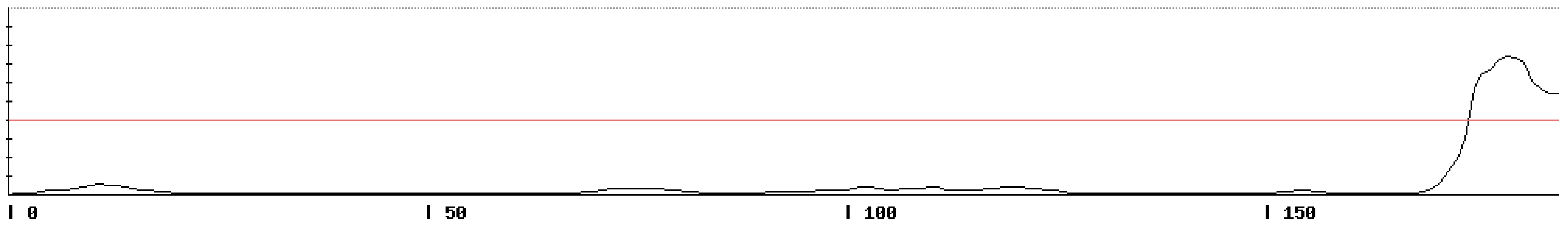

Predictions for Gossypiumhirsutum_Gh_A11G0386

Viterbi Path

Viterbi Path

Posterior @ 0.4

Posterior @ 0.4

Posterior Graphic. Threshold Marked @ 0.4

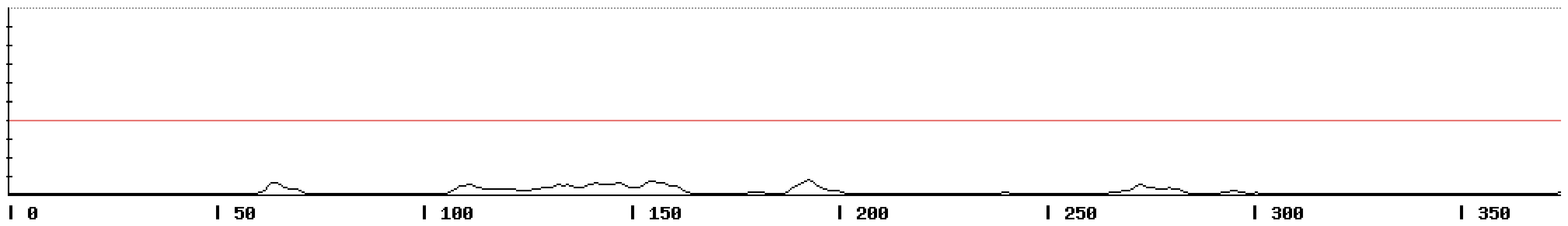

Predictions for Gossypiumhirsutum_Gh_A11G0524

Viterbi Path

Viterbi Path

Posterior @ 0.4

Posterior @ 0.4

Posterior Graphic. Threshold Marked @ 0.4

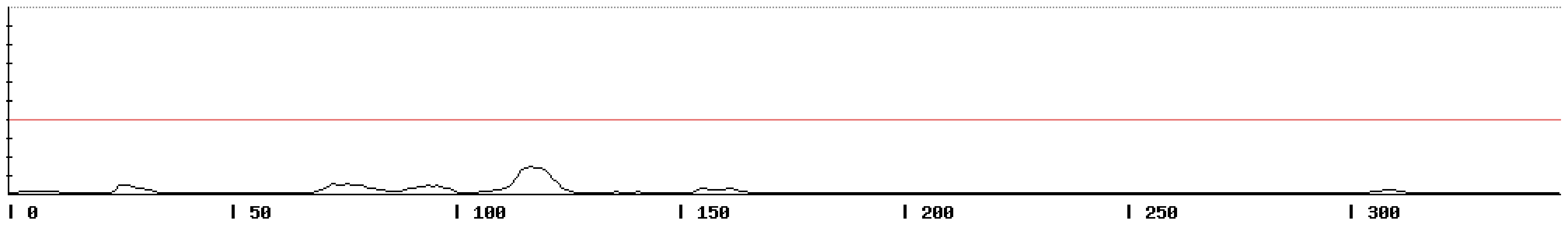

Predictions for Gossypiumhirsutum_Gh_A11G0613

Viterbi Path

Viterbi Path

Posterior @ 0.4	131 - RKRRRIQNRH - 140<br>147 - VRWHKTGKTKPVIENGVRKGWKKIMVLYKRSNR - 179
Posterior @ 0.4	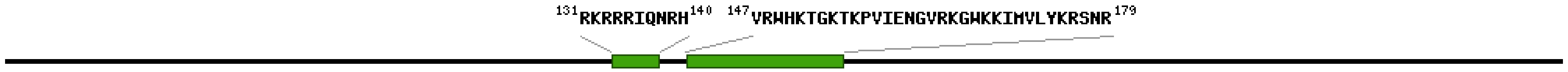
Posterior Graphic. Threshold Marked @ 0.4	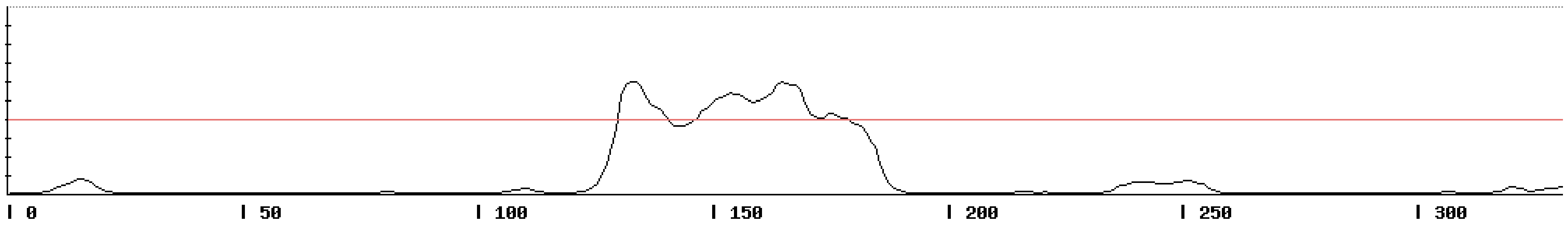

Predictions for Gossypiumhirsutum_Gh_A11G0633

Viterbi Path	
Viterbi Path	
Posterior @ 0.4	
Posterior @ 0.4	
Posterior Graphic. Threshold Marked @ 0.4	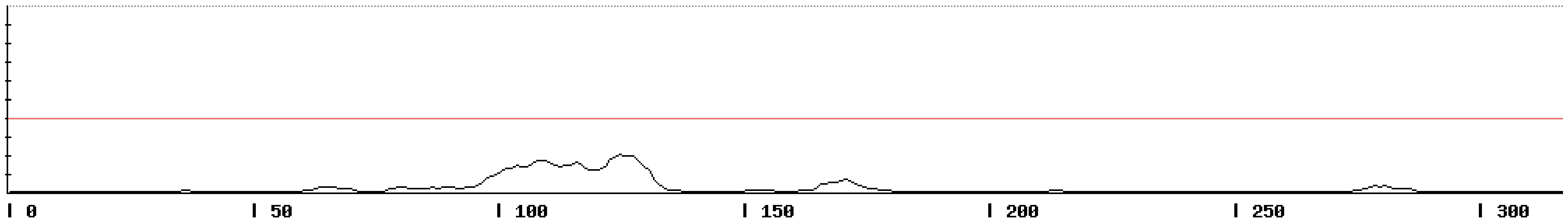

Predictions for Gossypiumhirsutum_Gh_A11G0915	
Viterbi Path	
Viterbi Path	
Posterior @ 0.4	
Posterior @ 0.4	
Posterior Graphic. Threshold Marked @ 0.4	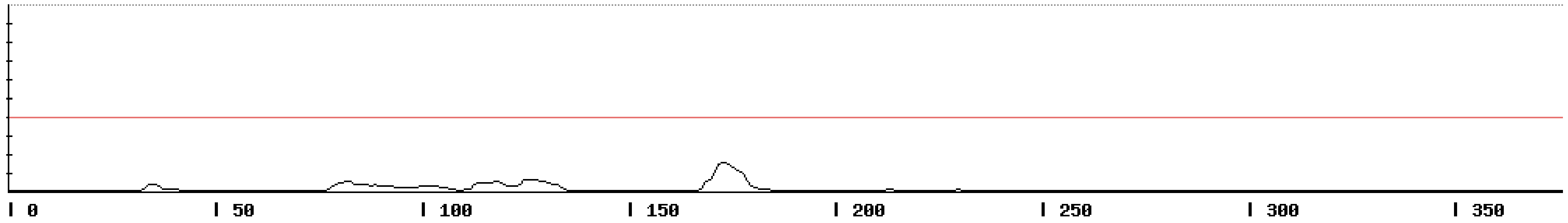

Predictions for Gossypiumhirsutum_Gh_A11G0931	
Viterbi Path	
Viterbi Path	
Posterior @ 0.4	80 - RNSKRKNRVTKK - 91
Posterior @ 0.4	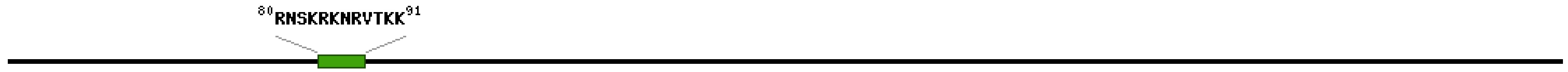
Posterior Graphic. Threshold Marked @ 0.4	

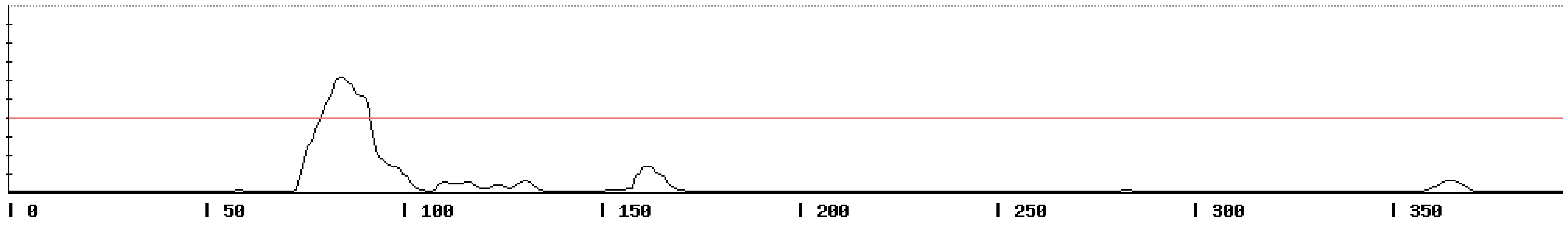

Predictions for Gossypiumhirsutum_Gh_A11G0932

Viterbi Path	
Viterbi Path	
Posterior @ 0.4	
Posterior @ 0.4	
Posterior Graphic. Threshold Marked @ 0.4	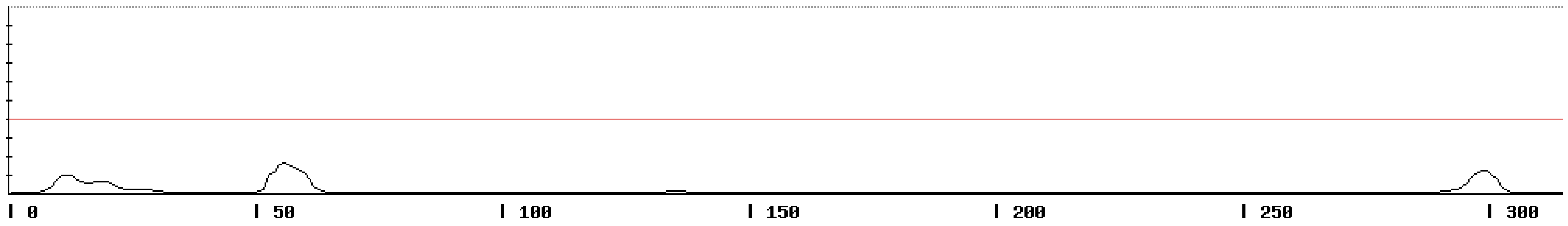

Predictions for Gossypiumhirsutum_Gh_A11G1568

Viterbi Path	
Viterbi Path	
Posterior @ 0.4	
Posterior @ 0.4	

Posterior Graphic. Threshold Marked @ 0.4

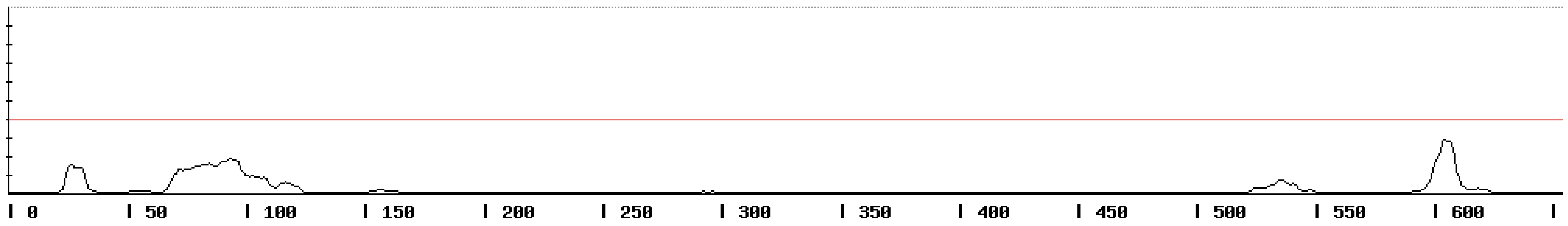

Predictions for Gossypiumhirsutum_Gh_A11G1635

Viterbi Path

Viterbi Path

Posterior @ 0.4

Posterior @ 0.4

Posterior Graphic. Threshold Marked @ 0.4

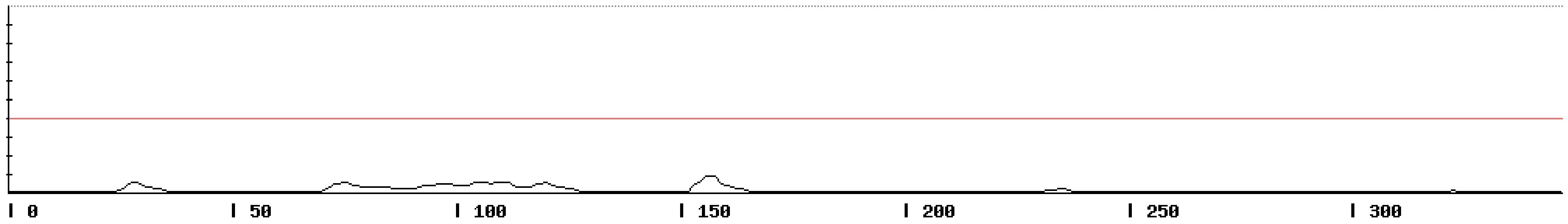

Predictions for Gossypiumhirsutum_Gh_A11G2165

Viterbi Path

Viterbi Path

Posterior @ 0.4

Posterior @ 0.4

Posterior Graphic. Threshold Marked @ 0.4

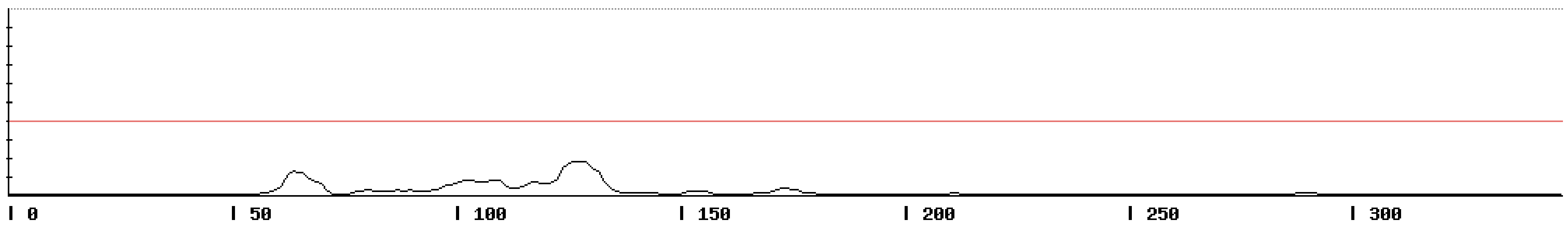

Predictions for Gossypiumhirsutum_Gh_A11G2168

Viterbi Path

Viterbi Path

Posterior @ 0.4

Posterior @ 0.4

Posterior Graphic. Threshold Marked @ 0.4

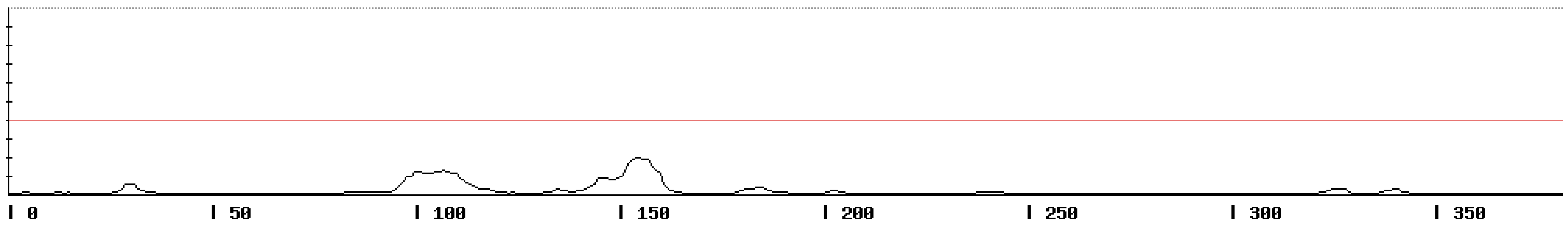

Predictions for Gossypiumhirsutum_Gh_A11G2580

Viterbi Path

Viterbi Path

Posterior @ 0.4	
Posterior @ 0.4	
Posterior Graphic. Threshold Marked @ 0.4	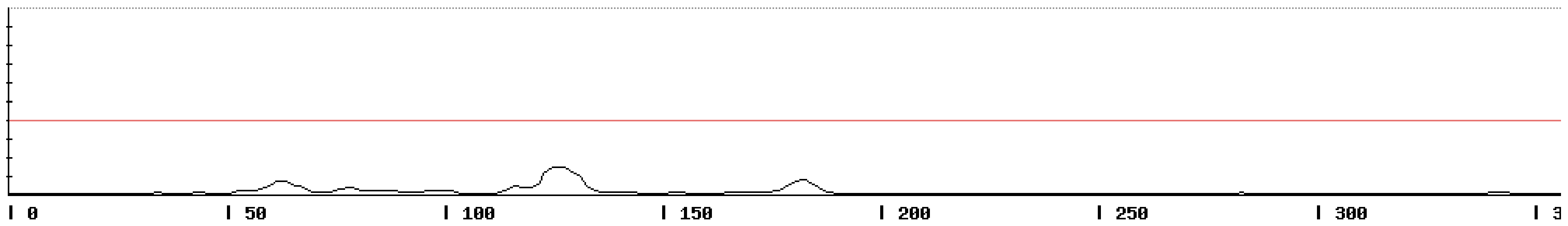

Predictions for Gossypiumhirsutum_Gh_A11G3079	
Viterbi Path	
Viterbi Path	
Posterior @ 0.4	
Posterior @ 0.4	
Posterior Graphic. Threshold Marked @ 0.4	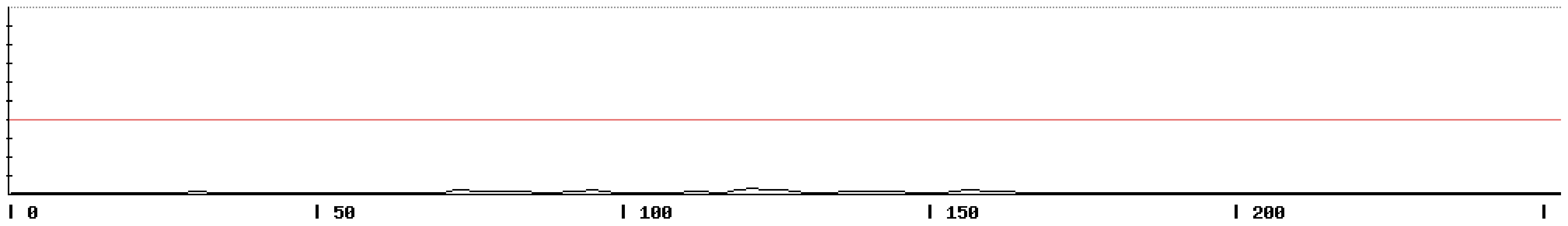

Predictions for Gossypiumhirsutum_Gh_A12G0118	
Viterbi Path	
Viterbi Path	

Posterior @ 0.4

Posterior @ 0.4

Posterior Graphic. Threshold Marked @ 0.4

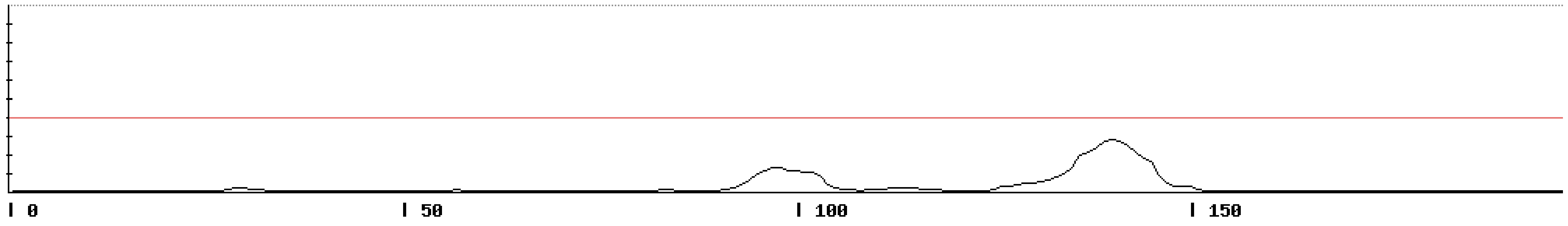

Predictions for Gossypiumhirsutum_Gh_A12G0319

Viterbi Path

Viterbi Path

Posterior @ 0.4

Posterior @ 0.4

Posterior Graphic. Threshold Marked @ 0.4

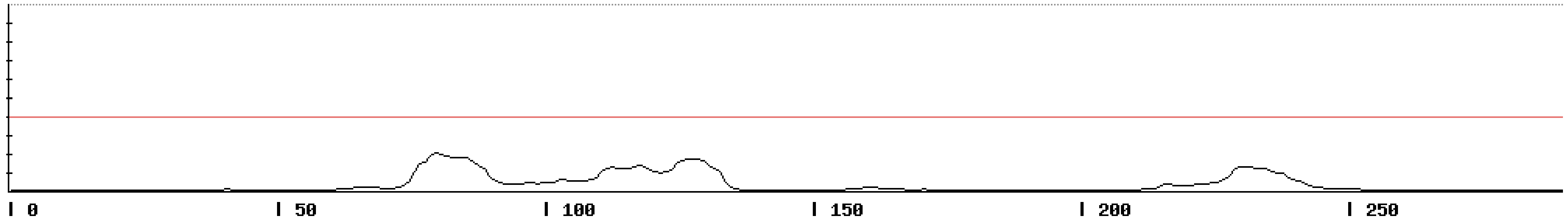

Predictions for Gossypiumhirsutum_Gh_A12G0557

Viterbi Path

Viterbi Path

Posterior @ 0.4

Posterior @ 0.4

Posterior Graphic. Threshold Marked @ 0.4

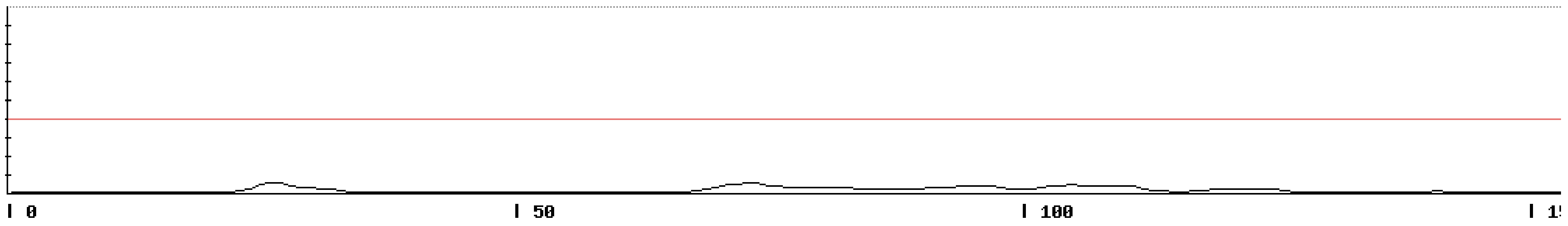

Predictions for Gossypiumhirsutum_Gh_A12G1049

Viterbi Path

Viterbi Path

Posterior @ 0.4

Posterior @ 0.4

Posterior Graphic. Threshold Marked @ 0.4

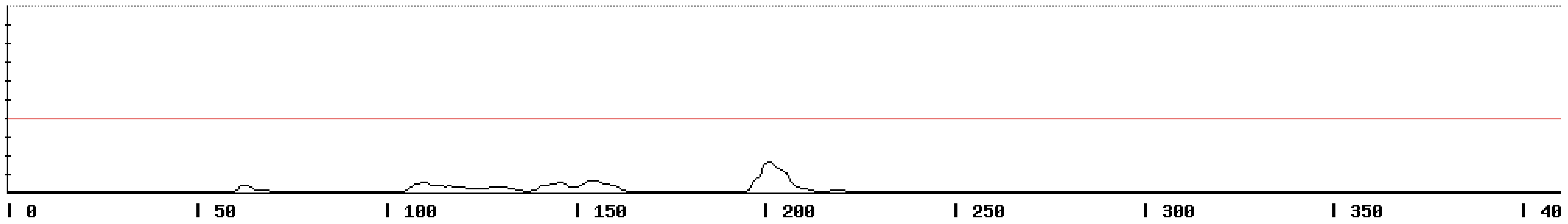

Predictions for Gossypiumhirsutum_Gh_A12G1285	
Viterbi Path	
Viterbi Path	
Posterior @ 0.4	137 - RKRRK - 141
Posterior @ 0.4	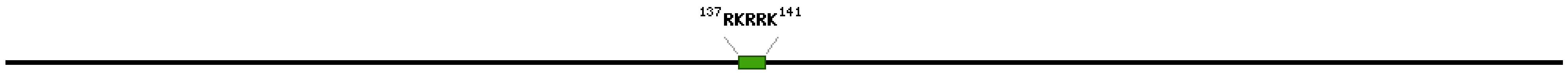
Posterior Graphic. Threshold Marked @ 0.4	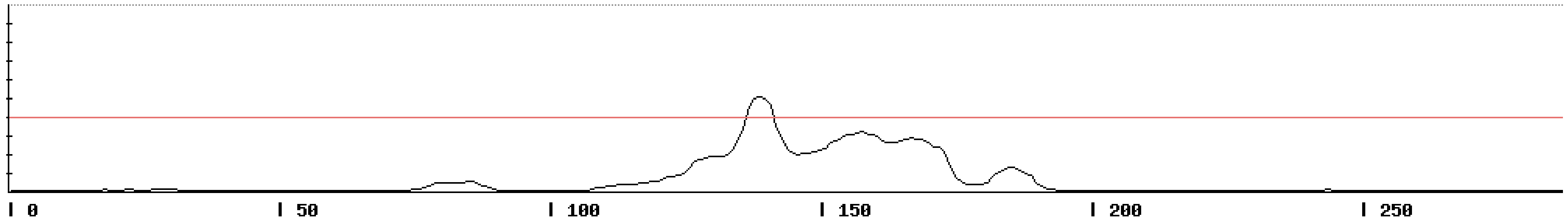

Predictions for Gossypiumhirsutum_Gh_A12G1505	
Viterbi Path	
Viterbi Path	
Posterior @ 0.4	
Posterior @ 0.4	
Posterior Graphic. Threshold Marked @ 0.4	

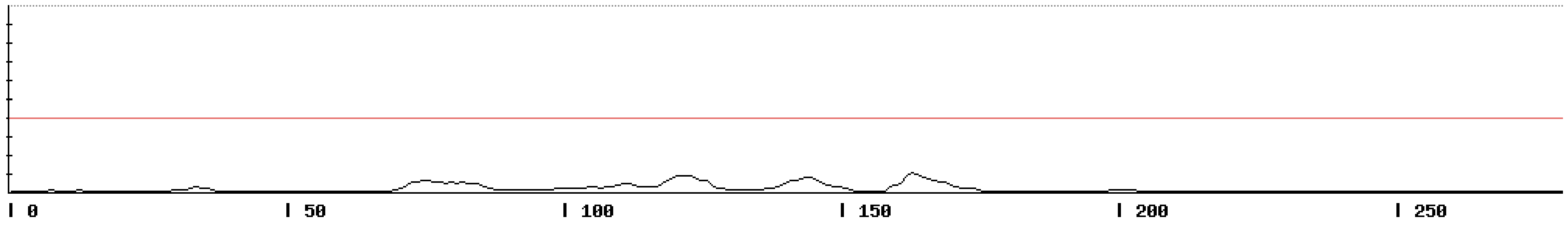

Predictions for Gossypiumhirsutum_Gh_A12G1557

Viterbi Path	
Viterbi Path	
Posterior @ 0.4	132 - RKRRK - 136
Posterior @ 0.4	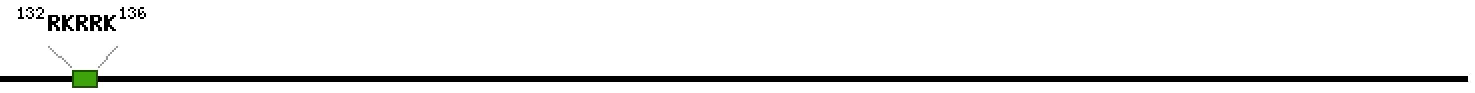
Posterior Graphic. Threshold Marked @ 0.4	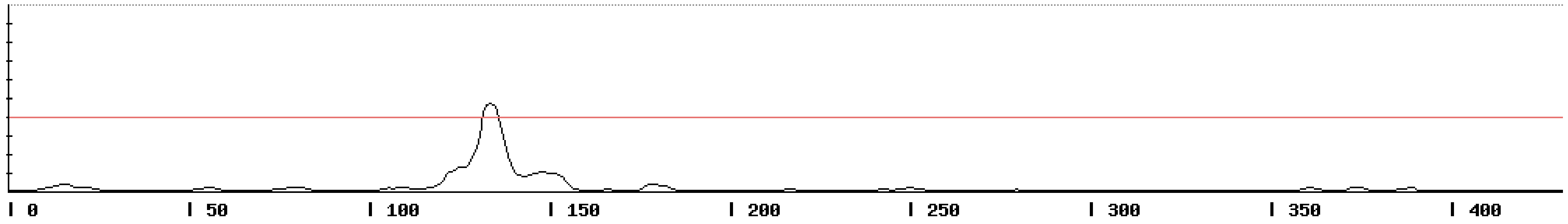

Predictions for Gossypiumhirsutum_Gh_A12G1620

Viterbi Path	
Viterbi Path	
Posterior @ 0.4	
Posterior @ 0.4	

Posterior Graphic. Threshold Marked @ 0.4

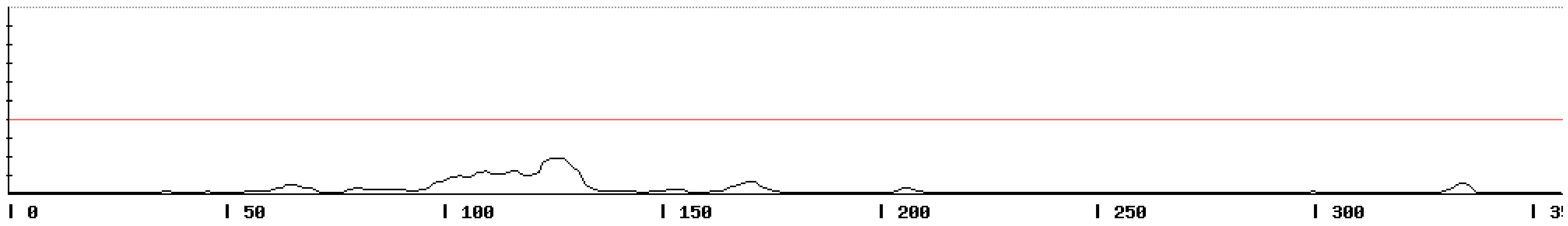

Predictions for Gossypiumhirsutum_Gh_A12G1649

Viterbi Path

Viterbi Path

Posterior @ 0.4

Posterior @ 0.4

Posterior Graphic. Threshold Marked @ 0.4

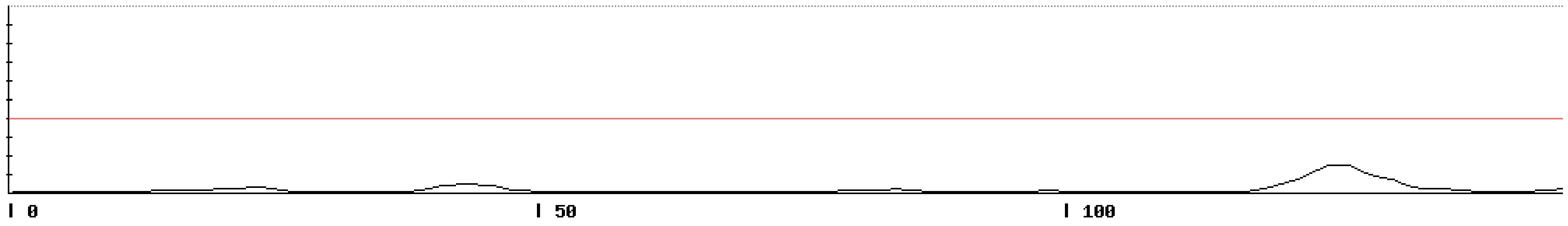

Predictions for Gossypiumhirsutum_Gh_A12G1908

Viterbi Path

Viterbi Path

Posterior @ 0.4

Posterior @ 0.4

Posterior Graphic. Threshold Marked @ 0.4

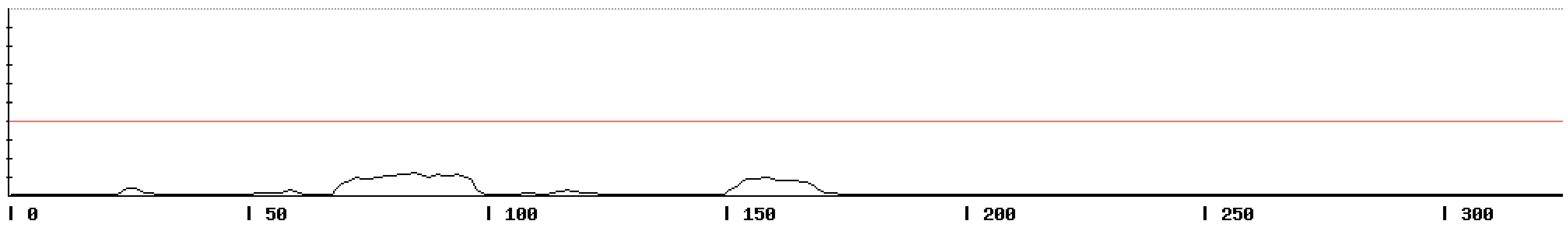

Predictions for Gossypiumhirsutum_Gh_A12G1990

Viterbi Path

Viterbi Path

Posterior @ 0.4

Posterior @ 0.4

Posterior Graphic. Threshold Marked @ 0.4

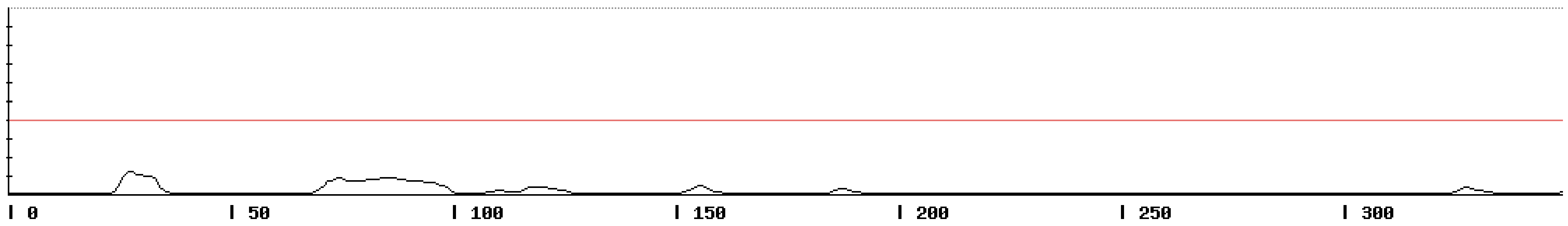

Predictions for Gossypiumhirsutum_Gh_A12G2179

Viterbi Path

Viterbi Path

Posterior @ 0.4	
Posterior @ 0.4	
Posterior Graphic. Threshold Marked @ 0.4	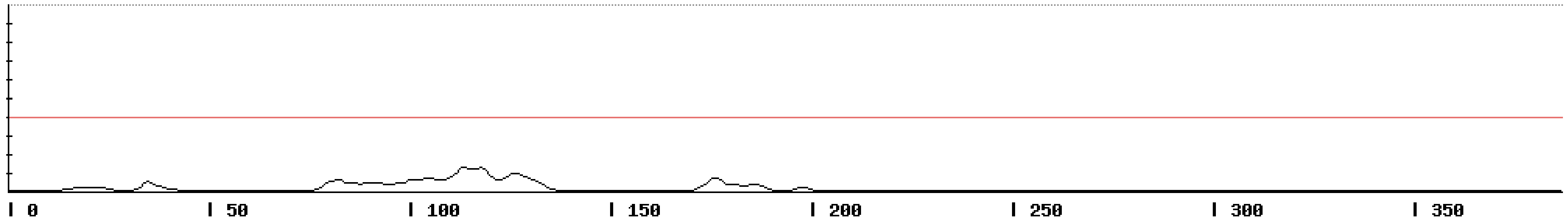

Predictions for Gossypiumhirsutum_Gh_A12G2195	
Viterbi Path	
Viterbi Path	
Posterior @ 0.4	93 - KATGKDRTIKSKKSLIGMKK - 112
Posterior @ 0.4	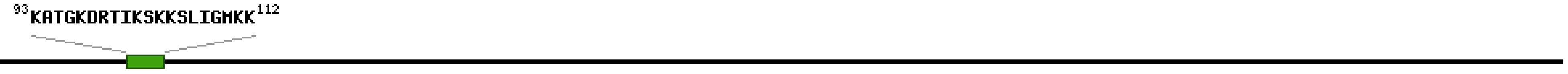
Posterior Graphic. Threshold Marked @ 0.4	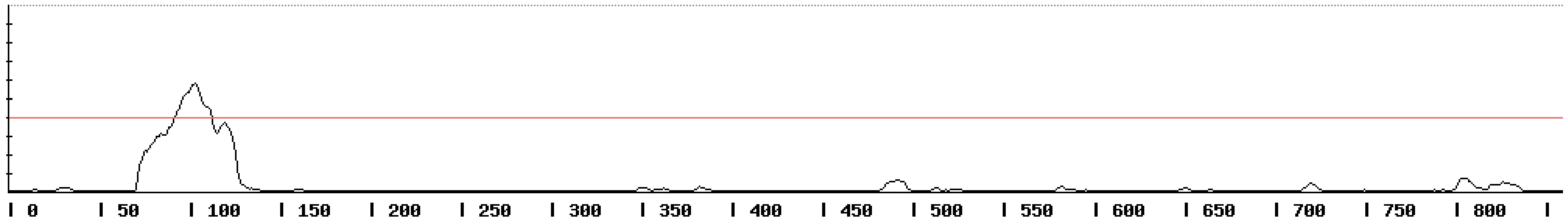

Predictions for Gossypiumhirsutum_Gh_A13G0551	
Viterbi Path	
Viterbi Path	

Posterior @ 0.4	68 - LKKKSKSR - 75
	113 - KKKKK - 117
	165 - KKNKKRK - 171
Posterior @ 0.4	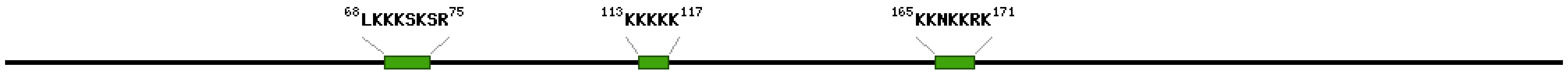
Posterior Graphic. Threshold Marked @ 0.4	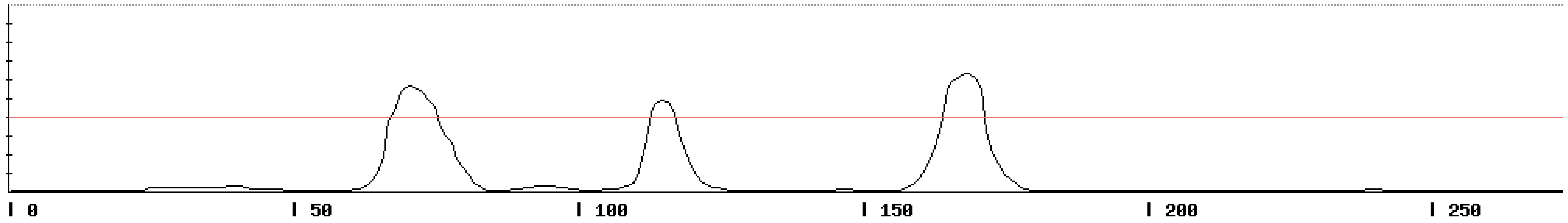

Predictions for Gossypiumhirsutum_Gh_A13G0565

Viterbi Path	
Viterbi Path	
Posterior @ 0.4	123 - LKKKSKSR - 130
	217 - GEIKKNKKRK - 226
Posterior @ 0.4	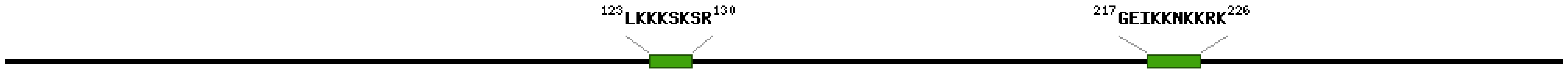
Posterior Graphic. Threshold Marked @ 0.4	

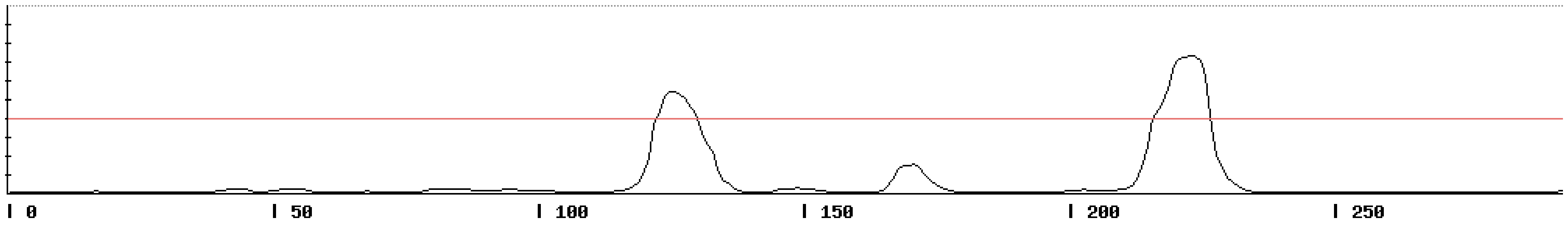

Predictions for Gossypiumhirsutum_Gh_A13G0575

Viterbi Path	
Viterbi Path	
Posterior @ 0.4	
Posterior @ 0.4	
Posterior Graphic. Threshold Marked @ 0.4	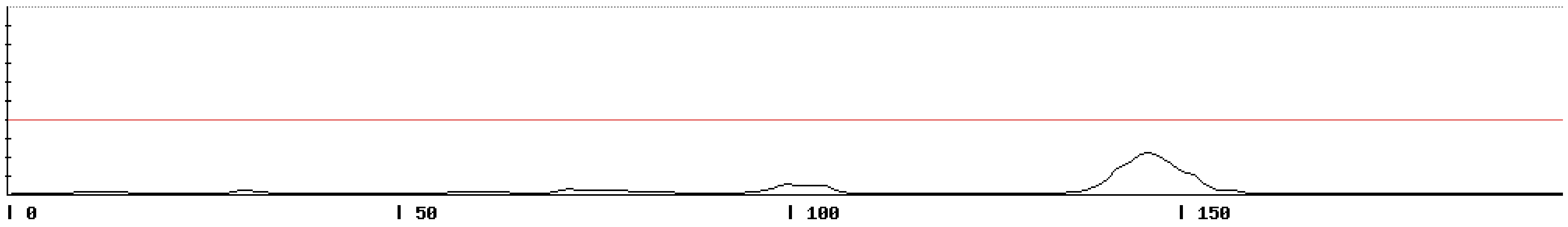

Predictions for Gossypiumhirsutum_Gh_A13G0655

Viterbi Path	
Viterbi Path	
Posterior @ 0.4	
Posterior @ 0.4	

Posterior Graphic. Threshold Marked @ 0.4

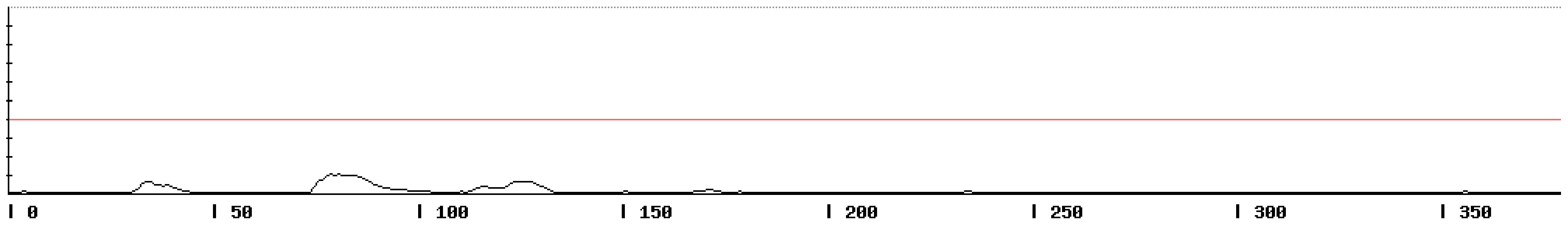

Predictions for Gossypiumhirsutum_Gh_A13G0828

Viterbi Path

Viterbi Path

Posterior @ 0.4

Posterior @ 0.4

Posterior Graphic. Threshold Marked @ 0.4

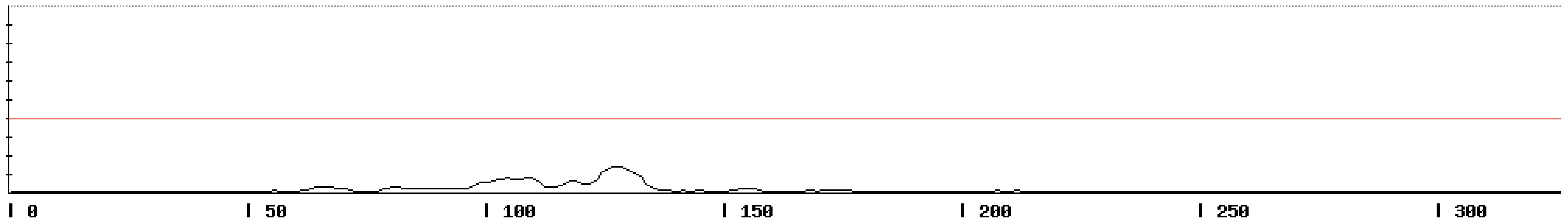

Predictions for Gossypiumhirsutum_Gh_A13G1020

Viterbi Path

Viterbi Path

Posterior @ 0.4

Posterior @ 0.4

Posterior Graphic. Threshold Marked @ 0.4

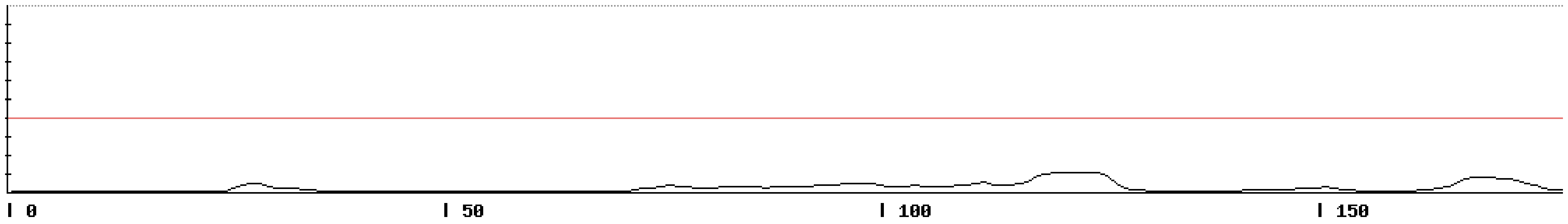

Predictions for Gossypiumhirsutum_Gh_A13G1061

Viterbi Path

Viterbi Path

Posterior @ 0.4

Posterior @ 0.4

Posterior Graphic. Threshold Marked @ 0.4

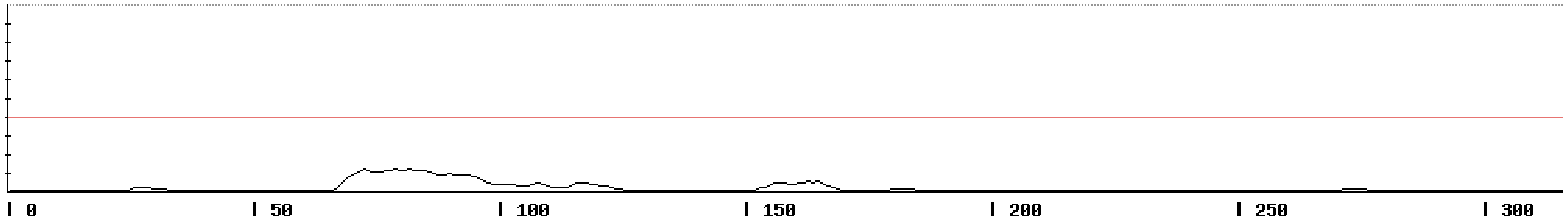

Predictions for Gossypiumhirsutum_Gh_A13G1229

Viterbi Path

Viterbi Path

Posterior @ 0.4

Posterior @ 0.4

Posterior Graphic. Threshold Marked @ 0.4

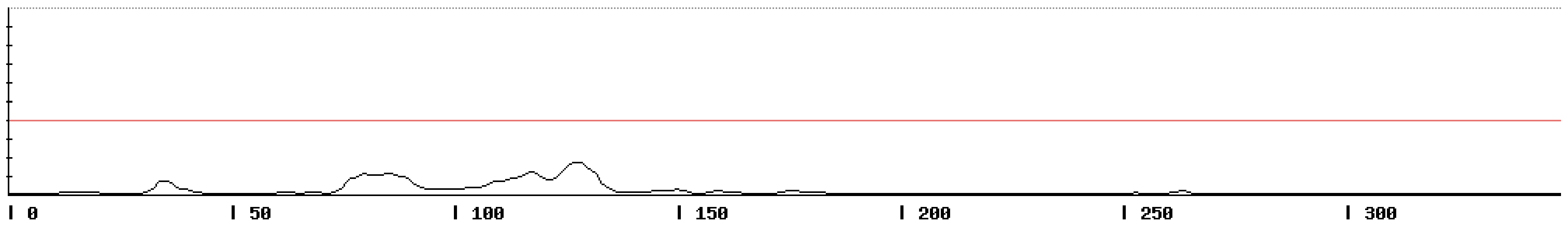

Predictions for Gossypiumhirsutum_Gh_A13G1230

Viterbi Path

Viterbi Path

Posterior @ 0.4

Posterior @ 0.4

Posterior Graphic. Threshold Marked @ 0.4

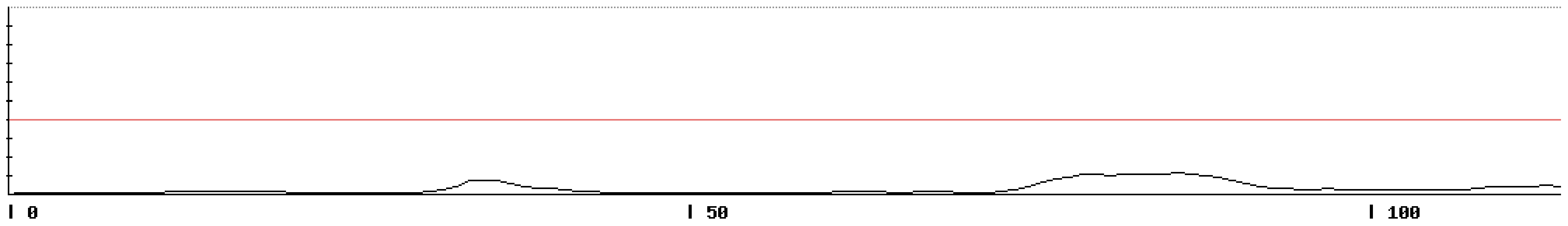

Predictions for Gossypiumhirsutum_Gh_A13G1259

Viterbi Path

Viterbi Path

Posterior @ 0.4	
Posterior @ 0.4	
Posterior Graphic. Threshold Marked @ 0.4	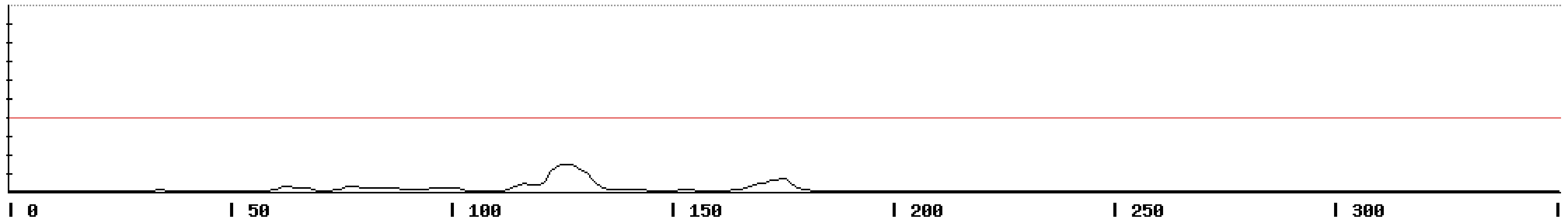

Predictions for Gossypiumhirsutum_Gh_A13G1439

Viterbi Path	
Viterbi Path	
Posterior @ 0.4	
Posterior @ 0.4	
Posterior Graphic. Threshold Marked @ 0.4	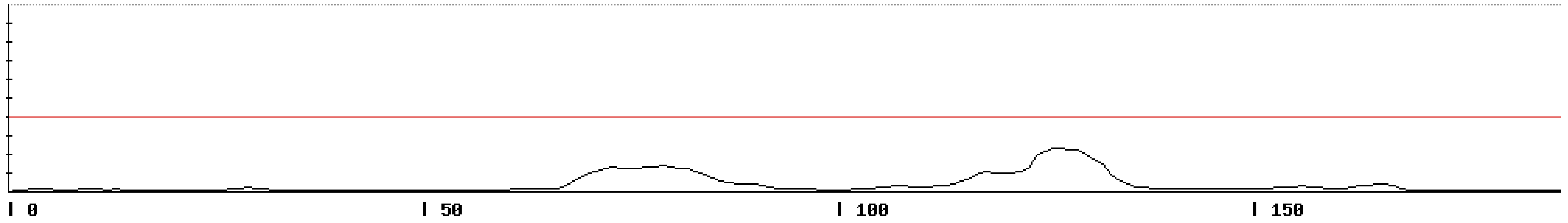

Predictions for Gossypiumhirsutum_Gh_D01G0012

Viterbi Path	
Viterbi Path	
Posterior @ 0.4	74 - RKHGKGGRPNR - 84
Posterior @ 0.4	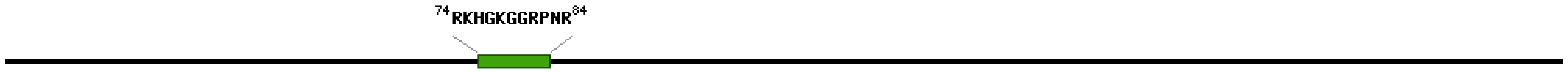
Posterior Graphic. Threshold Marked @ 0.4	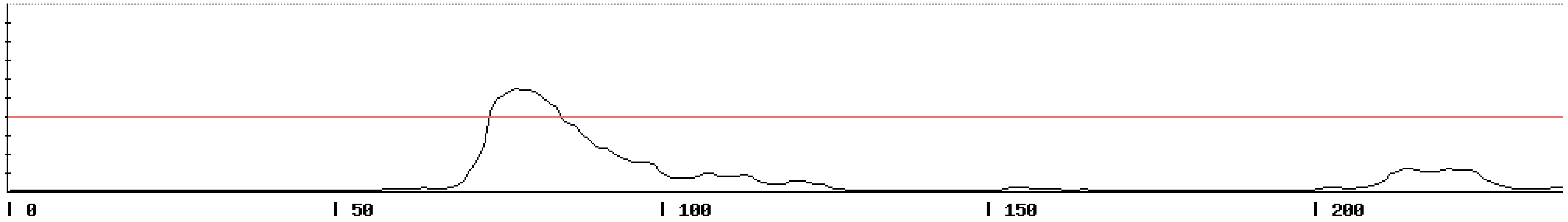

Predictions for Gossypiumhirsutum_Gh_D01G0073

Viterbi Path	
Viterbi Path	
Posterior @ 0.4	526 - RGRGEAQRGSRRGGNRGKIIMLPRG - 550
Posterior @ 0.4	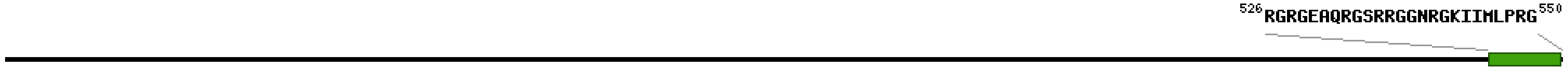
Posterior Graphic. Threshold Marked @ 0.4	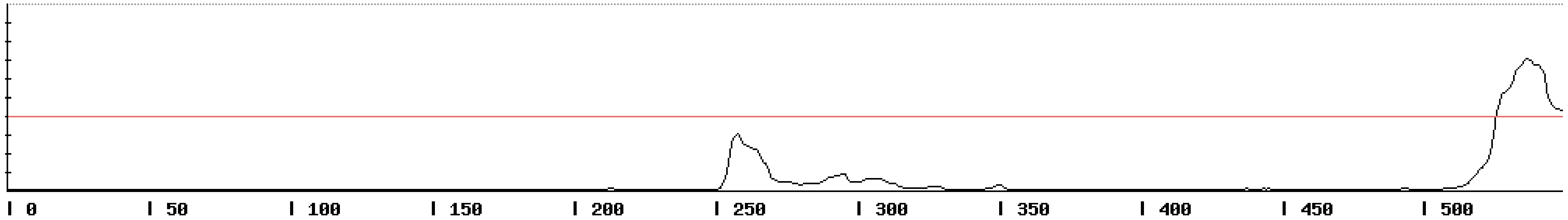

Predictions for Gossypiumhirsutum_Gh_D01G0249

Viterbi Path	

Viterbi Path	
Posterior @ 0.4	
Posterior @ 0.4	
Posterior Graphic. Threshold Marked @ 0.4	

**Predictions for Gossypiumhirsutum_Gh_D01G0278**	
Viterbi Path	
Viterbi Path	
Posterior @ 0.4	
Posterior @ 0.4	
Posterior Graphic. Threshold Marked @ 0.4	

**Predictions for Gossypiumhirsutum_Gh_D01G0448**	
Viterbi Path	
Viterbi Path	
Posterior @ 0.4	
Posterior @ 0.4	
Posterior Graphic. Threshold Marked @ 0.4	

**Predictions for Gossypiumhirsutum_Gh_D01G0514**	
Viterbi Path	
Viterbi Path	
Posterior @ 0.4	
Posterior @ 0.4	
Posterior Graphic. Threshold Marked @ 0.4	

Predictions for Gossypiumhirsutum_Gh_D01G0835	
Viterbi Path	
Viterbi Path	
Posterior @ 0.4	
Posterior @ 0.4	
Posterior Graphic. Threshold Marked @ 0.4	

Predictions for Gossypiumhirsutum_Gh_D01G0847	
Viterbi Path	
Viterbi Path	
Posterior @ 0.4	
Posterior @ 0.4	
Posterior Graphic. Threshold Marked @ 0.4	

Predictions for Gossypiumhirsutum_Gh_D01G2346	
Viterbi Path	
Viterbi Path	
Posterior @ 0.4	
Posterior @ 0.4	
Posterior Graphic. Threshold Marked @ 0.4	

Predictions for Gossypiumhirsutum_Gh_D02G0122	
Viterbi Path	
Viterbi Path	
Posterior @ 0.4	
Posterior @ 0.4	
Posterior Graphic. Threshold Marked @ 0.4	

Predictions for Gossypiumhirsutum_Gh_D02G0123

Viterbi Path	
Viterbi Path	
Posterior @ 0.4	
Posterior @ 0.4	
Posterior Graphic. Threshold Marked @ 0.4	

Predictions for Gossypiumhirsutum_Gh_D02G0124	
Viterbi Path	
Viterbi Path	
Posterior @ 0.4	
Posterior @ 0.4	
Posterior Graphic. Threshold Marked @ 0.4	

Predictions for Gossypiumhirsutum_Gh_D02G0149	
Viterbi Path	
Viterbi Path	
Posterior @ 0.4	70 - KKKS - 73
Posterior @ 0.4	
Posterior Graphic. Threshold Marked @ 0.4	

Predictions for Gossypiumhirsutum_Gh_D02G0187	
Viterbi Path	
Viterbi Path	
Posterior @ 0.4	
Posterior @ 0.4	
Posterior Graphic. Threshold Marked @ 0.4	

Predictions for Gossypiumhirsutum_Gh_D02G0682	
Viterbi Path	

Viterbi Path	
Posterior @ 0.4	
Posterior @ 0.4	
Posterior Graphic. Threshold Marked @ 0.4	

**Predictions for Gossypiumhirsutum_Gh_D02G0790**	
Viterbi Path	
Viterbi Path	
Posterior @ 0.4	
Posterior @ 0.4	
Posterior Graphic. Threshold Marked @ 0.4	

**Predictions for Gossypiumhirsutum_Gh_D02G1257**	
Viterbi Path	
Viterbi Path	
Posterior @ 0.4	
Posterior @ 0.4	
Posterior Graphic. Threshold Marked @ 0.4	

**Predictions for Gossypiumhirsutum_Gh_D02G1270**	
Viterbi Path	
Viterbi Path	
Posterior @ 0.4	
Posterior @ 0.4	
Posterior Graphic. Threshold Marked @ 0.4	

**Predictions for Gossypiumhirsutum_Gh_D02G1769**	
Viterbi Path	
Viterbi Path	

Posterior @ 0.4	
Posterior @ 0.4	
Posterior Graphic. Threshold Marked @ 0.4	

**Predictions for Gossypiumhirsutum_Gh_D02G1981**	
Viterbi Path	
Viterbi Path	
Posterior @ 0.4	
Posterior @ 0.4	
Posterior Graphic. Threshold Marked @ 0.4	

**Predictions for Gossypiumhirsutum_Gh_D02G2283**	
Viterbi Path	
Viterbi Path	
Posterior @ 0.4	
Posterior @ 0.4	
Posterior Graphic. Threshold Marked @ 0.4	

**Predictions for Gossypiumhirsutum_Gh_D03G0032**	
Viterbi Path	
Viterbi Path	
Posterior @ 0.4	
Posterior @ 0.4	
Posterior Graphic. Threshold Marked @ 0.4	

**Predictions for Gossypiumhirsutum_Gh_D03G0096**	
Viterbi Path	
Viterbi Path	
Posterior @ 0.4	

Posterior @ 0.4	
Posterior Graphic. Threshold Marked @ 0.4	

Predictions for Gossypiumhirsutum_Gh_D03G0775	
Viterbi Path	
Viterbi Path	
Posterior @ 0.4	
Posterior @ 0.4	
Posterior Graphic. Threshold Marked @ 0.4	

Predictions for Gossypiumhirsutum_Gh_D03G0824	
Viterbi Path	
Viterbi Path	
Posterior @ 0.4	97 - KDRTIKSKKSLIG - 109
Posterior @ 0.4	
Posterior Graphic. Threshold Marked @ 0.4	

Predictions for Gossypiumhirsutum_Gh_D03G1021	
Viterbi Path	
Viterbi Path	
Posterior @ 0.4	
Posterior @ 0.4	
Posterior Graphic. Threshold Marked @ 0.4	

Predictions for Gossypiumhirsutum_Gh_D03G1542	
Viterbi Path	
Viterbi Path	
Posterior @ 0.4	
Posterior @ 0.4	

Posterior Graphic. Threshold Marked @ 0.4	

Predictions for Gossypiumhirsutum_Gh_D03G1627	
Viterbi Path	
Viterbi Path	
Posterior @ 0.4	
Posterior @ 0.4	
Posterior Graphic. Threshold Marked @ 0.4	

Predictions for Gossypiumhirsutum_Gh_D04G0050	
Viterbi Path	
Viterbi Path	
Posterior @ 0.4	352 - KKKKP - 356
Posterior @ 0.4	
Posterior Graphic. Threshold Marked @ 0.4	

Predictions for Gossypiumhirsutum_Gh_D04G0278	
Viterbi Path	
Viterbi Path	
Posterior @ 0.4	
Posterior @ 0.4	
Posterior Graphic. Threshold Marked @ 0.4	

Predictions for Gossypiumhirsutum_Gh_D04G0293	
Viterbi Path	
Viterbi Path	
Posterior @ 0.4	
Posterior @ 0.4	
Posterior Graphic. Threshold Marked @ 0.4	

Predictions for Gossypiumhirsutum_Gh_D04G0450	
Viterbi Path	
Viterbi Path	
Posterior @ 0.4	
Posterior @ 0.4	
Posterior Graphic. Threshold Marked @ 0.4	

Predictions for Gossypiumhirsutum_Gh_D04G0624	
Viterbi Path	
Viterbi Path	
Posterior @ 0.4	101 - RKHPQGKRPNR - 111
Posterior @ 0.4	
Posterior Graphic. Threshold Marked @ 0.4	

Predictions for Gossypiumhirsutum_Gh_D04G0712	
Viterbi Path	
Viterbi Path	
Posterior @ 0.4	144 - RKRKARKHAAK - 154
Posterior @ 0.4	
Posterior Graphic. Threshold Marked @ 0.4	

Predictions for Gossypiumhirsutum_Gh_D04G1097	
Viterbi Path	
Viterbi Path	
Posterior @ 0.4	
Posterior @ 0.4	
Posterior Graphic. Threshold Marked @ 0.4	

Predictions for Gossypiumhirsutum_Gh_D04G1368	

Viterbi Path	
Viterbi Path	
Posterior @ 0.4	
Posterior @ 0.4	
Posterior Graphic. Threshold Marked @ 0.4	

Predictions for Gossypiumhirsutum_Gh_D04G1749	
Viterbi Path	
Viterbi Path	
Posterior @ 0.4	
Posterior @ 0.4	
Posterior Graphic. Threshold Marked @ 0.4	

Predictions for Gossypiumhirsutum_Gh_D04G1757	
Viterbi Path	
Viterbi Path	
Posterior @ 0.4	
Posterior @ 0.4	
Posterior Graphic. Threshold Marked @ 0.4	

Predictions for Gossypiumhirsutum_Gh_D04G1974	
Viterbi Path	
Viterbi Path	
Posterior @ 0.4	
Posterior @ 0.4	
Posterior Graphic. Threshold Marked @ 0.4	

Predictions for Gossypiumhirsutum_Gh_D05G0270	
Viterbi Path	

Viterbi Path	
Posterior @ 0.4	
Posterior @ 0.4	
Posterior Graphic. Threshold Marked @ 0.4	

**Predictions for Gossypiumhirsutum_Gh_D05G0797**	
Viterbi Path	
Viterbi Path	
Posterior @ 0.4	312 - NRRIKLRKPK - 321
Posterior @ 0.4	
Posterior Graphic. Threshold Marked @ 0.4	

**Predictions for Gossypiumhirsutum_Gh_D05G1508**	
Viterbi Path	
Viterbi Path	
Posterior @ 0.4	146 - RKRRK - 150
Posterior @ 0.4	
Posterior Graphic. Threshold Marked @ 0.4	

**Predictions for Gossypiumhirsutum_Gh_D05G1549**	
Viterbi Path	
Viterbi Path	
Posterior @ 0.4	
Posterior @ 0.4	
Posterior Graphic. Threshold Marked @ 0.4	

**Predictions for Gossypiumhirsutum_Gh_D05G1697**	
Viterbi Path	
Viterbi Path	

Posterior @ 0.4	
Posterior @ 0.4	
Posterior Graphic. Threshold Marked @ 0.4	

**Predictions for Gossypiumhirsutum_Gh_D05G1875**	
Viterbi Path	
Viterbi Path	
Posterior @ 0.4	380 - RAKVEEGPSSSRKPSESHGGRRKGGSLGGVLRK - 412
Posterior @ 0.4	
Posterior Graphic. Threshold Marked @ 0.4	

**Predictions for Gossypiumhirsutum_Gh_D05G1960**	
Viterbi Path	100 - KSKGGRGRRRAKGGFWRKIGKVR - 122
Viterbi Path	
Posterior @ 0.4	99 - TKSKGGRGRRRAKGGFWRKIGKVR - 122
Posterior @ 0.4	
Posterior Graphic. Threshold Marked @ 0.4	

**Predictions for Gossypiumhirsutum_Gh_D05G2348**	
Viterbi Path	
Viterbi Path	
Posterior @ 0.4	
Posterior @ 0.4	
Posterior Graphic. Threshold Marked @ 0.4	

**Predictions for Gossypiumhirsutum_Gh_D05G2784**	
Viterbi Path	
Viterbi Path	
Posterior @ 0.4	

Posterior @ 0.4	
Posterior Graphic. Threshold Marked @ 0.4	

Predictions for Gossypiumhirsutum_Gh_D05G2787	
Viterbi Path	
Viterbi Path	
Posterior @ 0.4	
Posterior @ 0.4	
Posterior Graphic. Threshold Marked @ 0.4	

Predictions for Gossypiumhirsutum_Gh_D05G3197	
Viterbi Path	
Viterbi Path	
Posterior @ 0.4	
Posterior @ 0.4	
Posterior Graphic. Threshold Marked @ 0.4	

Predictions for Gossypiumhirsutum_Gh_D05G3221	
Viterbi Path	
Viterbi Path	
Posterior @ 0.4	
Posterior @ 0.4	
Posterior Graphic. Threshold Marked @ 0.4	

Predictions for Gossypiumhirsutum_Gh_D05G3474	
Viterbi Path	
Viterbi Path	
Posterior @ 0.4	
Posterior @ 0.4	

Posterior Graphic. Threshold Marked @ 0.4	

Predictions for Gossypiumhirsutum_Gh_D05G3475	
Viterbi Path	
Viterbi Path	
Posterior @ 0.4	
Posterior @ 0.4	
Posterior Graphic. Threshold Marked @ 0.4	

Predictions for Gossypiumhirsutum_Gh_D06G0070	
Viterbi Path	364 - KPKSSRRKRDALGRVKELRKK - 384
Viterbi Path	
Posterior @ 0.4	362 - SAKPKSSRRKRDALGRVKELRKK - 384
Posterior @ 0.4	
Posterior Graphic. Threshold Marked @ 0.4	

Predictions for Gossypiumhirsutum_Gh_D06G0417	
Viterbi Path	
Viterbi Path	
Posterior @ 0.4	
Posterior @ 0.4	
Posterior Graphic. Threshold Marked @ 0.4	

Predictions for Gossypiumhirsutum_Gh_D06G0681	
Viterbi Path	
Viterbi Path	
Posterior @ 0.4	
Posterior @ 0.4	
Posterior Graphic. Threshold Marked @ 0.4	

Predictions for Gossypiumhirsutum_Gh_D06G1146	
Viterbi Path	
Viterbi Path	
Posterior @ 0.4	
Posterior @ 0.4	
Posterior Graphic. Threshold Marked @ 0.4	

Predictions for Gossypiumhirsutum_Gh_D06G1277	
Viterbi Path	
Viterbi Path	
Posterior @ 0.4	
Posterior @ 0.4	
Posterior Graphic. Threshold Marked @ 0.4	

Predictions for Gossypiumhirsutum_Gh_D06G1417	
Viterbi Path	
Viterbi Path	
Posterior @ 0.4	135 - RKRRK - 139
Posterior @ 0.4	
Posterior Graphic. Threshold Marked @ 0.4	

Predictions for Gossypiumhirsutum_Gh_D06G1546	
Viterbi Path	
Viterbi Path	
Posterior @ 0.4	
Posterior @ 0.4	
Posterior Graphic. Threshold Marked @ 0.4	

Predictions for Gossypiumhirsutum_Gh_D06G2096	

Viterbi Path	
Viterbi Path	
Posterior @ 0.4	
Posterior @ 0.4	
Posterior Graphic. Threshold Marked @ 0.4	

Predictions for Gossypiumhirsutum_Gh_D06G2219	
Viterbi Path	
Viterbi Path	
Posterior @ 0.4	77 - KLKKKSKGK - 85
Posterior @ 0.4	
Posterior Graphic. Threshold Marked @ 0.4	

Predictions for Gossypiumhirsutum_Gh_D07G0411	
Viterbi Path	
Viterbi Path	
Posterior @ 0.4	
Posterior @ 0.4	
Posterior Graphic. Threshold Marked @ 0.4	

Predictions for Gossypiumhirsutum_Gh_D07G0624	
Viterbi Path	
Viterbi Path	
Posterior @ 0.4	
Posterior @ 0.4	
Posterior Graphic. Threshold Marked @ 0.4	

Predictions for Gossypiumhirsutum_Gh_D07G0802	
Viterbi Path	

Viterbi Path	
Posterior @ 0.4	
Posterior @ 0.4	
Posterior Graphic. Threshold Marked @ 0.4	

**Predictions for Gossypiumhirsutum_Gh_D07G1086**	
Viterbi Path	
Viterbi Path	
Posterior @ 0.4	118 - KAPSGKKTK - 126
Posterior @ 0.4	
Posterior Graphic. Threshold Marked @ 0.4	

**Predictions for Gossypiumhirsutum_Gh_D07G1320**	
Viterbi Path	
Viterbi Path	
Posterior @ 0.4	
Posterior @ 0.4	
Posterior Graphic. Threshold Marked @ 0.4	

**Predictions for Gossypiumhirsutum_Gh_D07G1330**	
Viterbi Path	
Viterbi Path	
Posterior @ 0.4	
Posterior @ 0.4	
Posterior Graphic. Threshold Marked @ 0.4	

**Predictions for Gossypiumhirsutum_Gh_D07G1502**	
Viterbi Path	
Viterbi Path	

Posterior @ 0.4	
Posterior @ 0.4	
Posterior Graphic. Threshold Marked @ 0.4	

Predictions for Gossypiumhirsutum_Gh_D07G2014	
Viterbi Path	
Viterbi Path	
Posterior @ 0.4	
Posterior @ 0.4	
Posterior Graphic. Threshold Marked @ 0.4	

Predictions for Gossypiumhirsutum_Gh_D07G2165	
Viterbi Path	
Viterbi Path	
Posterior @ 0.4	
Posterior @ 0.4	
Posterior Graphic. Threshold Marked @ 0.4	

Predictions for Gossypiumhirsutum_Gh_D07G2341	
Viterbi Path	
Viterbi Path	
Posterior @ 0.4	81 - KKRKR - 85
Posterior @ 0.4	
Posterior Graphic. Threshold Marked @ 0.4	

Predictions for Gossypiumhirsutum_Gh_D08G0433	
Viterbi Path	
Viterbi Path	
Posterior @ 0.4	

Posterior @ 0.4	
Posterior Graphic. Threshold Marked @ 0.4	

Predictions for Gossypiumhirsutum_Gh_D08G1138	
Viterbi Path	
Viterbi Path	
Posterior @ 0.4	
Posterior @ 0.4	
Posterior Graphic. Threshold Marked @ 0.4	

Predictions for Gossypiumhirsutum_Gh_D08G1172	
Viterbi Path	
Viterbi Path	
Posterior @ 0.4	
Posterior @ 0.4	
Posterior Graphic. Threshold Marked @ 0.4	

Predictions for Gossypiumhirsutum_Gh_D08G1370	
Viterbi Path	73 - RRGDNKRVKRTTDKGFWKVTGKPRKVKGKKGFAAKK - 108
Viterbi Path	
Posterior @ 0.4	73 - RRGDNKRVKRTTDKGFWKVTGKPRKVKGKKGFAAKKS - 109
Posterior @ 0.4	
Posterior Graphic. Threshold Marked @ 0.4	

Predictions for Gossypiumhirsutum_Gh_D08G1721	
Viterbi Path	
Viterbi Path	
Posterior @ 0.4	
Posterior @ 0.4	

Posterior Graphic. Threshold Marked @ 0.4	

Predictions for Gossypiumhirsutum_Gh_D08G1858	
Viterbi Path	
Viterbi Path	
Posterior @ 0.4	
Posterior @ 0.4	
Posterior Graphic. Threshold Marked @ 0.4	

Predictions for Gossypiumhirsutum_Gh_D08G2049	
Viterbi Path	
Viterbi Path	
Posterior @ 0.4	
Posterior @ 0.4	
Posterior Graphic. Threshold Marked @ 0.4	

Predictions for Gossypiumhirsutum_Gh_D08G2092	
Viterbi Path	
Viterbi Path	
Posterior @ 0.4	
Posterior @ 0.4	
Posterior Graphic. Threshold Marked @ 0.4	

Predictions for Gossypiumhirsutum_Gh_D08G2434	
Viterbi Path	
Viterbi Path	
Posterior @ 0.4	
Posterior @ 0.4	
Posterior Graphic. Threshold Marked @ 0.4	

Predictions for Gossypiumhirsutum_Gh_D08G2446	
Viterbi Path	
Viterbi Path	
Posterior @ 0.4	
Posterior @ 0.4	
Posterior Graphic. Threshold Marked @ 0.4	

Predictions for Gossypiumhirsutum_Gh_D08G2447	
Viterbi Path	
Viterbi Path	
Posterior @ 0.4	
Posterior @ 0.4	
Posterior Graphic. Threshold Marked @ 0.4	

Predictions for Gossypiumhirsutum_Gh_D08G2669	
Viterbi Path	
Viterbi Path	
Posterior @ 0.4	178 - KRNKKIKQ - 185
Posterior @ 0.4	
Posterior Graphic. Threshold Marked @ 0.4	

Predictions for Gossypiumhirsutum_Gh_D08G2670	
Viterbi Path	
Viterbi Path	
Posterior @ 0.4	
Posterior @ 0.4	
Posterior Graphic. Threshold Marked @ 0.4	

Predictions for Gossypiumhirsutum_Gh_D08G2671	

Viterbi Path	
Viterbi Path	
Posterior @ 0.4	163 - KRHKKNKQEWRQGAAKR - 179
Posterior @ 0.4	
Posterior Graphic. Threshold Marked @ 0.4	

**Predictions for Gossypiumhirsutum_Gh_D08G2683**	
Viterbi Path	
Viterbi Path	
Posterior @ 0.4	
Posterior @ 0.4	
Posterior Graphic. Threshold Marked @ 0.4	

**Predictions for Gossypiumhirsutum_Gh_D09G0038**	
Viterbi Path	
Viterbi Path	
Posterior @ 0.4	
Posterior @ 0.4	
Posterior Graphic. Threshold Marked @ 0.4	

**Predictions for Gossypiumhirsutum_Gh_D09G0151**	
Viterbi Path	
Viterbi Path	
Posterior @ 0.4	
Posterior @ 0.4	
Posterior Graphic. Threshold Marked @ 0.4	

**Predictions for Gossypiumhirsutum_Gh_D09G0298**	
Viterbi Path	

Viterbi Path	
Posterior @ 0.4	
Posterior @ 0.4	
Posterior Graphic. Threshold Marked @ 0.4	

**Predictions for Gossypiumhirsutum_Gh_D09G0480**	
Viterbi Path	
Viterbi Path	
Posterior @ 0.4	
Posterior @ 0.4	
Posterior Graphic. Threshold Marked @ 0.4	

**Predictions for Gossypiumhirsutum_Gh_D09G0716**	
Viterbi Path	
Viterbi Path	
Posterior @ 0.4	
Posterior @ 0.4	
Posterior Graphic. Threshold Marked @ 0.4	

**Predictions for Gossypiumhirsutum_Gh_D09G0943**	
Viterbi Path	
Viterbi Path	
Posterior @ 0.4	123 - GRK - 125
Posterior @ 0.4	
Posterior Graphic. Threshold Marked @ 0.4	

**Predictions for Gossypiumhirsutum_Gh_D09G0950**	
Viterbi Path	
Viterbi Path	

Posterior @ 0.4	
Posterior @ 0.4	
Posterior Graphic. Threshold Marked @ 0.4	

**Predictions for Gossypiumhirsutum_Gh_D09G1069**	
Viterbi Path	
Viterbi Path	
Posterior @ 0.4	
Posterior @ 0.4	
Posterior Graphic. Threshold Marked @ 0.4	

**Predictions for Gossypiumhirsutum_Gh_D09G1631**	
Viterbi Path	
Viterbi Path	
Posterior @ 0.4	
Posterior @ 0.4	
Posterior Graphic. Threshold Marked @ 0.4	

**Predictions for Gossypiumhirsutum_Gh_D09G1773**	
Viterbi Path	
Viterbi Path	
Posterior @ 0.4	
Posterior @ 0.4	
Posterior Graphic. Threshold Marked @ 0.4	

**Predictions for Gossypiumhirsutum_Gh_D09G1785**	
Viterbi Path	
Viterbi Path	
Posterior @ 0.4	

Posterior @ 0.4	
Posterior Graphic. Threshold Marked @ 0.4	

Predictions for Gossypiumhirsutum_Gh_D09G2356	
Viterbi Path	
Viterbi Path	
Posterior @ 0.4	
Posterior @ 0.4	
Posterior Graphic. Threshold Marked @ 0.4	

Predictions for Gossypiumhirsutum_Gh_D09G2357	
Viterbi Path	
Viterbi Path	
Posterior @ 0.4	176 - RRKK - 179
Posterior @ 0.4	
Posterior Graphic. Threshold Marked @ 0.4	

Predictions for Gossypiumhirsutum_Gh_D10G0263	
Viterbi Path	
Viterbi Path	
Posterior @ 0.4	
Posterior @ 0.4	
Posterior Graphic. Threshold Marked @ 0.4	

Predictions for Gossypiumhirsutum_Gh_D10G0772	
Viterbi Path	
Viterbi Path	
Posterior @ 0.4	166 - QRKRSRRGKKH - 176
Posterior @ 0.4	

Posterior Graphic. Threshold Marked @ 0.4	

Predictions for Gossypiumhirsutum_Gh_D10G0792	
Viterbi Path	
Viterbi Path	
Posterior @ 0.4	
Posterior @ 0.4	
Posterior Graphic. Threshold Marked @ 0.4	

Predictions for Gossypiumhirsutum_Gh_D10G2317	
Viterbi Path	
Viterbi Path	
Posterior @ 0.4	90 - NGSRPARKAGKGYWKATGA - 108
	110 - K - 110
	115 - KGKK - 118
Posterior @ 0.4	
Posterior Graphic. Threshold Marked @ 0.4	

Predictions for Gossypiumhirsutum_Gh_D10G2319	
Viterbi Path	
Viterbi Path	
Posterior @ 0.4	
Posterior @ 0.4	
Posterior Graphic. Threshold Marked @ 0.4	

Predictions for Gossypiumhirsutum_Gh_D10G2512	
Viterbi Path	
Viterbi Path	
Posterior @ 0.4	109 - RKGKKIGLKK - 118

Posterior @ 0.4	
Posterior Graphic. Threshold Marked @ 0.4	

Predictions for Gossypiumhirsutum_Gh_D10G2514	
Viterbi Path	
Viterbi Path	
Posterior @ 0.4	115 - RKGKKIGLKK - 124
Posterior @ 0.4	
Posterior Graphic. Threshold Marked @ 0.4	

Predictions for Gossypiumhirsutum_Gh_D10G2538	
Viterbi Path	
Viterbi Path	
Posterior @ 0.4	
Posterior @ 0.4	
Posterior Graphic. Threshold Marked @ 0.4	

Predictions for Gossypiumhirsutum_Gh_D11G0160	
Viterbi Path	
Viterbi Path	
Posterior @ 0.4	
Posterior @ 0.4	
Posterior Graphic. Threshold Marked @ 0.4	

Predictions for Gossypiumhirsutum_Gh_D11G0285	
Viterbi Path	
Viterbi Path	
Posterior @ 0.4	
Posterior @ 0.4	

Posterior Graphic. Threshold Marked @ 0.4	

Predictions for Gossypiumhirsutum_Gh_D11G0347	
Viterbi Path	
Viterbi Path	
Posterior @ 0.4	
Posterior @ 0.4	
Posterior Graphic. Threshold Marked @ 0.4	

Predictions for Gossypiumhirsutum_Gh_D11G0405	
Viterbi Path	
Viterbi Path	
Posterior @ 0.4	
Posterior @ 0.4	
Posterior Graphic. Threshold Marked @ 0.4	

Predictions for Gossypiumhirsutum_Gh_D11G0448	
Viterbi Path	
Viterbi Path	
Posterior @ 0.4	
Posterior @ 0.4	
Posterior Graphic. Threshold Marked @ 0.4	

Predictions for Gossypiumhirsutum_Gh_D11G0608	
Viterbi Path	
Viterbi Path	
Posterior @ 0.4	
Posterior @ 0.4	
Posterior Graphic. Threshold Marked @ 0.4	

Predictions for Gossypiumhirsutum_Gh_D11G0701	
Viterbi Path	
Viterbi Path	
Posterior @ 0.4	579 - PSKSKKEKKSKIK - 591
	965 - RKRRRIQNRHS - 975
	980 - HVRWHKTGKTKPVIENGVRKGWKKIMVLYKRSNRAHKP - 1017
Posterior @ 0.4	
Posterior Graphic. Threshold Marked @ 0.4	

Predictions for Gossypiumhirsutum_Gh_D11G0743	
Viterbi Path	
Viterbi Path	
Posterior @ 0.4	
Posterior @ 0.4	
Posterior Graphic. Threshold Marked @ 0.4	

Predictions for Gossypiumhirsutum_Gh_D11G1062	
Viterbi Path	
Viterbi Path	
Posterior @ 0.4	
Posterior @ 0.4	
Posterior Graphic. Threshold Marked @ 0.4	

Predictions for Gossypiumhirsutum_Gh_D11G1073	
Viterbi Path	
Viterbi Path	
Posterior @ 0.4	
Posterior @ 0.4	

Posterior Graphic. Threshold Marked @ 0.4	

Predictions for Gossypiumhirsutum_Gh_D11G1074	
Viterbi Path	
Viterbi Path	
Posterior @ 0.4	
Posterior @ 0.4	
Posterior Graphic. Threshold Marked @ 0.4	

Predictions for Gossypiumhirsutum_Gh_D11G1725	
Viterbi Path	
Viterbi Path	
Posterior @ 0.4	
Posterior @ 0.4	
Posterior Graphic. Threshold Marked @ 0.4	

Predictions for Gossypiumhirsutum_Gh_D11G1793	
Viterbi Path	
Viterbi Path	
Posterior @ 0.4	
Posterior @ 0.4	
Posterior Graphic. Threshold Marked @ 0.4	

Predictions for Gossypiumhirsutum_Gh_D11G2467	
Viterbi Path	
Viterbi Path	
Posterior @ 0.4	
Posterior @ 0.4	
Posterior Graphic. Threshold Marked @ 0.4	

Predictions for Gossypiumhirsutum_Gh_D11G2469	
Viterbi Path	
Viterbi Path	
Posterior @ 0.4	
Posterior @ 0.4	
Posterior Graphic. Threshold Marked @ 0.4	

Predictions for Gossypiumhirsutum_Gh_D11G2947	
Viterbi Path	
Viterbi Path	
Posterior @ 0.4	
Posterior @ 0.4	
Posterior Graphic. Threshold Marked @ 0.4	

Predictions for Gossypiumhirsutum_Gh_D11G3512	
Viterbi Path	
Viterbi Path	
Posterior @ 0.4	
Posterior @ 0.4	
Posterior Graphic. Threshold Marked @ 0.4	

Predictions for Gossypiumhirsutum_Gh_D12G0132	
Viterbi Path	
Viterbi Path	
Posterior @ 0.4	
Posterior @ 0.4	
Posterior Graphic. Threshold Marked @ 0.4	

Predictions for Gossypiumhirsutum_Gh_D12G0347

Viterbi Path	
Viterbi Path	
Posterior @ 0.4	
Posterior @ 0.4	
Posterior Graphic. Threshold Marked @ 0.4	

Predictions for Gossypiumhirsutum_Gh_D12G0574	
Viterbi Path	
Viterbi Path	
Posterior @ 0.4	
Posterior @ 0.4	
Posterior Graphic. Threshold Marked @ 0.4	

Predictions for Gossypiumhirsutum_Gh_D12G1169	
Viterbi Path	
Viterbi Path	
Posterior @ 0.4	
Posterior @ 0.4	
Posterior Graphic. Threshold Marked @ 0.4	

Predictions for Gossypiumhirsutum_Gh_D12G1407	
Viterbi Path	
Viterbi Path	
Posterior @ 0.4	137 - RKRRK - 141
Posterior @ 0.4	
Posterior Graphic. Threshold Marked @ 0.4	

Predictions for Gossypiumhirsutum_Gh_D12G1684	
Viterbi Path	

Viterbi Path	
Posterior @ 0.4	132 - RKRRK - 136
Posterior @ 0.4	
Posterior Graphic. Threshold Marked @ 0.4	

Predictions for Gossypiumhirsutum_Gh_D12G1761	
Viterbi Path	
Viterbi Path	
Posterior @ 0.4	
Posterior @ 0.4	
Posterior Graphic. Threshold Marked @ 0.4	

Predictions for Gossypiumhirsutum_Gh_D12G2088	
Viterbi Path	
Viterbi Path	
Posterior @ 0.4	
Posterior @ 0.4	
Posterior Graphic. Threshold Marked @ 0.4	

Predictions for Gossypiumhirsutum_Gh_D12G2169	
Viterbi Path	
Viterbi Path	
Posterior @ 0.4	
Posterior @ 0.4	
Posterior Graphic. Threshold Marked @ 0.4	

Predictions for Gossypiumhirsutum_Gh_D12G2359	
Viterbi Path	
Viterbi Path	

Posterior @ 0.4	
Posterior @ 0.4	
Posterior Graphic. Threshold Marked @ 0.4	

Predictions for Gossypiumhirsutum_Gh_D12G2375	
Viterbi Path	
Viterbi Path	
Posterior @ 0.4	93 - KATGKDRTIKSKKSLIGMKK - 112
Posterior @ 0.4	
Posterior Graphic. Threshold Marked @ 0.4	

Predictions for Gossypiumhirsutum_Gh_D12G2767	
Viterbi Path	
Viterbi Path	
Posterior @ 0.4	
Posterior @ 0.4	
Posterior Graphic. Threshold Marked @ 0.4	

Predictions for Gossypiumhirsutum_Gh_D13G0769	
Viterbi Path	
Viterbi Path	
Posterior @ 0.4	
Posterior @ 0.4	
Posterior Graphic. Threshold Marked @ 0.4	

Predictions for Gossypiumhirsutum_Gh_D13G1072	
Viterbi Path	
Viterbi Path	
Posterior @ 0.4	

Posterior @ 0.4	
Posterior Graphic. Threshold Marked @ 0.4	

Predictions for Gossypiumhirsutum_Gh_D13G1271	
Viterbi Path	
Viterbi Path	
Posterior @ 0.4	
Posterior @ 0.4	
Posterior Graphic. Threshold Marked @ 0.4	

Predictions for Gossypiumhirsutum_Gh_D13G1327	
Viterbi Path	
Viterbi Path	
Posterior @ 0.4	
Posterior @ 0.4	
Posterior Graphic. Threshold Marked @ 0.4	

Predictions for Gossypiumhirsutum_Gh_D13G1526	
Viterbi Path	
Viterbi Path	
Posterior @ 0.4	
Posterior @ 0.4	
Posterior Graphic. Threshold Marked @ 0.4	

Predictions for Gossypiumhirsutum_Gh_D13G1561	
Viterbi Path	
Viterbi Path	
Posterior @ 0.4	
Posterior @ 0.4	

Viterbi Path

Posterior @ 0.4

Posterior @ 0.4

Posterior Graphic. Threshold Marked @ 0.4

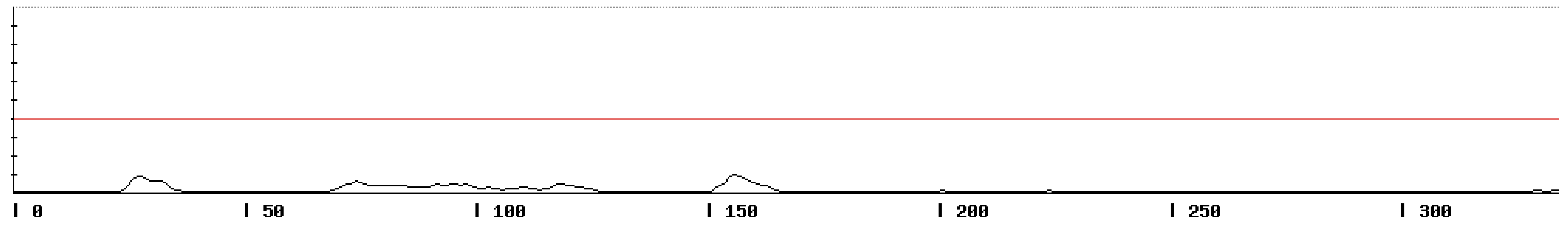

Predictions for Gossypiumraimondii_Gorai.001G070700.1

Viterbi Path

Viterbi Path

Posterior @ 0.4

Posterior @ 0.4

Posterior Graphic. Threshold Marked @ 0.4

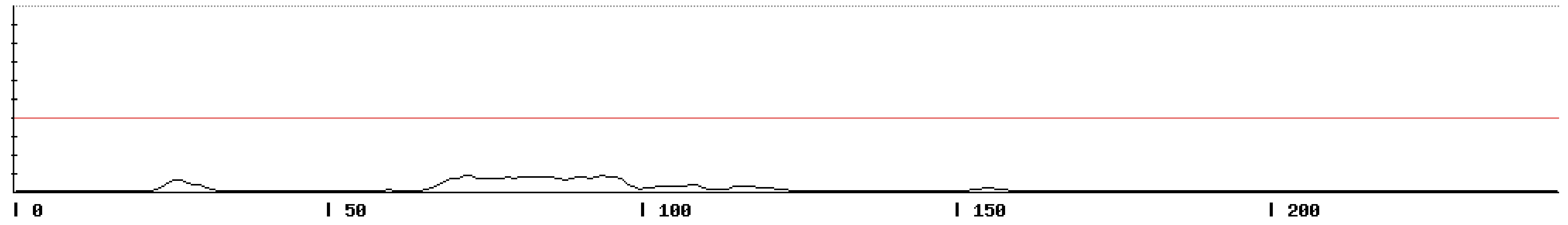

Predictions for Gossypiumraimondii_Gorai.001G091900.1

Viterbi Path

Viterbi Path

Posterior @ 0.4

Posterior @ 0.4

Posterior Graphic. Threshold Marked @ 0.4

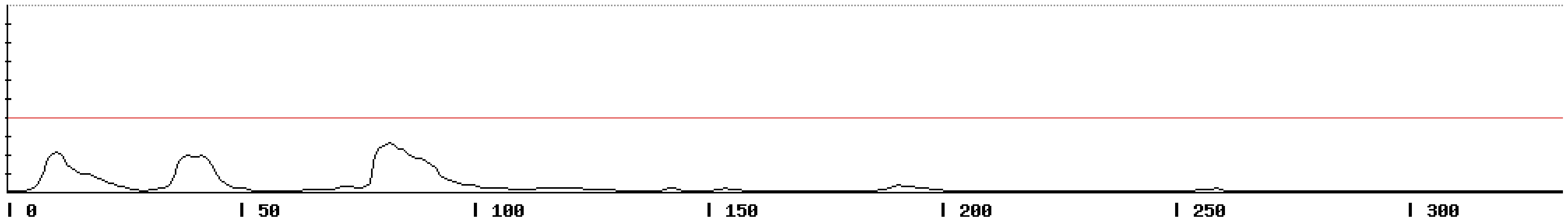

Predictions for Gossypiumraimondii_Gorai.001G122800.1

Viterbi Path

Viterbi Path

Posterior @ 0.4

118 - KAPSGKKTK - 126

Posterior @ 0.4

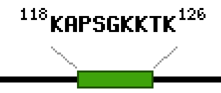

Posterior Graphic. Threshold Marked @ 0.4

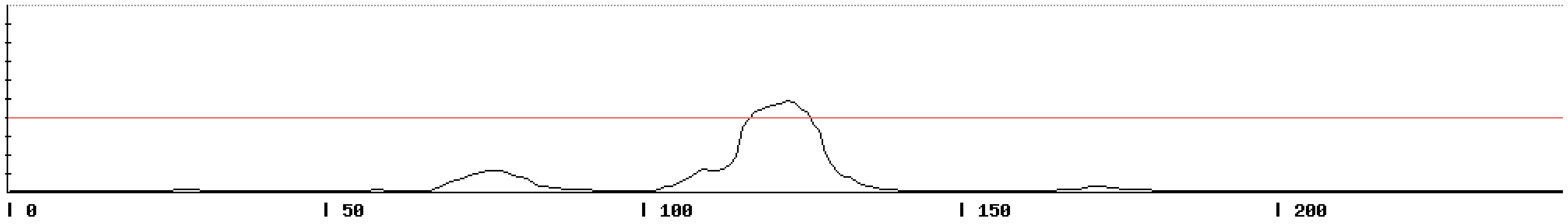

Predictions for Gossypiumraimondii_Gorai.001G150000.1

Viterbi Path

Viterbi Path

Posterior @ 0.4

Posterior @ 0.4

Posterior Graphic. Threshold Marked @ 0.4

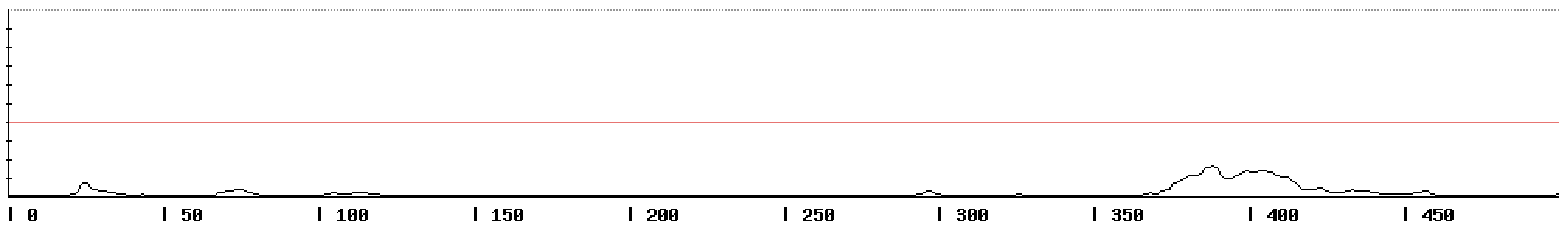

Predictions for Gossypiumraimondii_Gorai.001G150800.1

Viterbi Path

Viterbi Path

Posterior @ 0.4

Posterior @ 0.4

Posterior Graphic. Threshold Marked @ 0.4

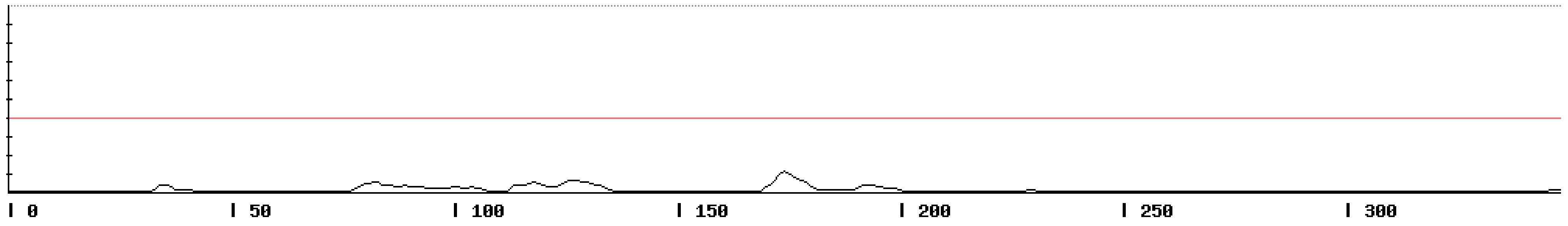

Predictions for Gossypiumraimondii_Gorai.001G178800.1

Viterbi Path

Viterbi Path

Posterior @ 0.4

Posterior @ 0.4

Posterior Graphic. Threshold Marked @ 0.4

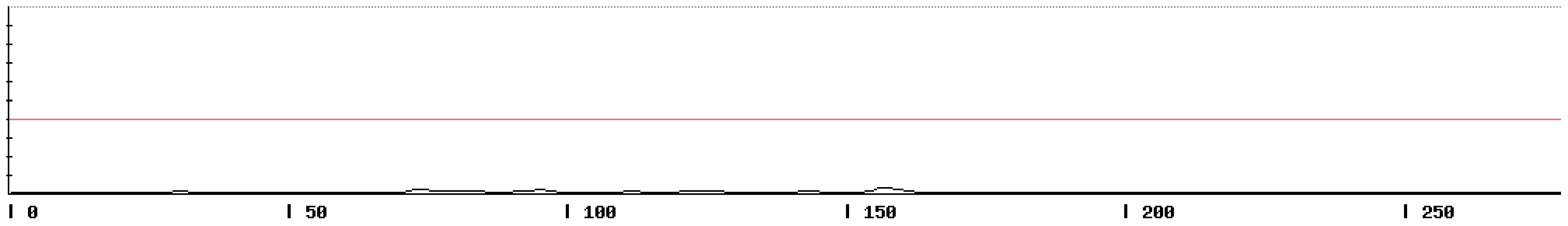

Predictions for Gossypiumraimondii_Gorai.001G231400.1

Viterbi Path

Viterbi Path

Posterior @ 0.4

Posterior @ 0.4

Posterior Graphic. Threshold Marked @ 0.4

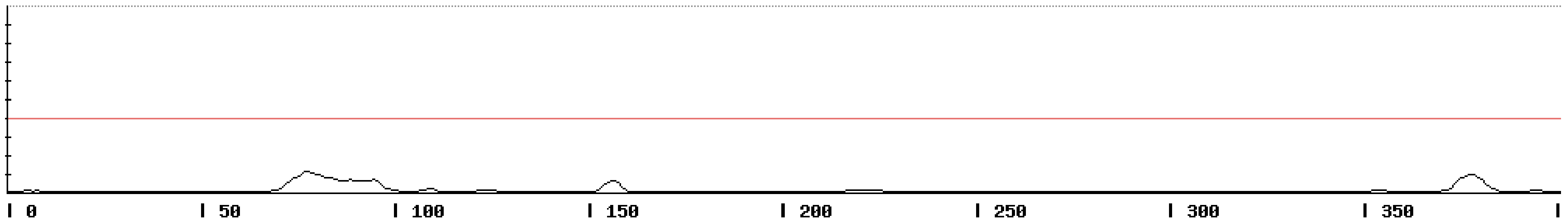

Predictions for Gossypiumraimondii_Gorai.001G254300.1

Viterbi Path	
Viterbi Path	
Posterior @ 0.4	
Posterior @ 0.4	
Posterior Graphic. Threshold Marked @ 0.4	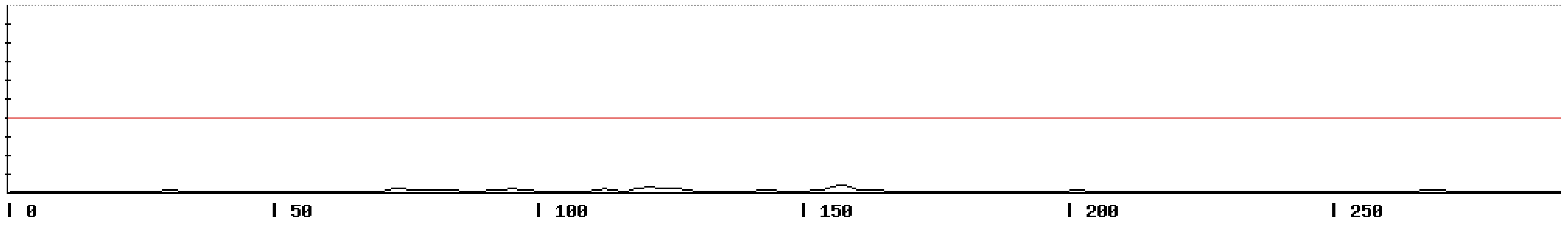

Predictions for Gossypiumraimondii_Gorai.001G274500.1

Viterbi Path	
Viterbi Path	
Posterior @ 0.4	81 - KKRKRV - 86
Posterior @ 0.4	

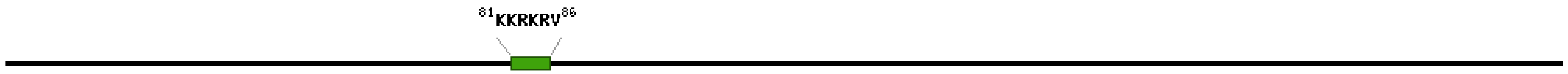

Posterior Graphic. Threshold Marked @ 0.4

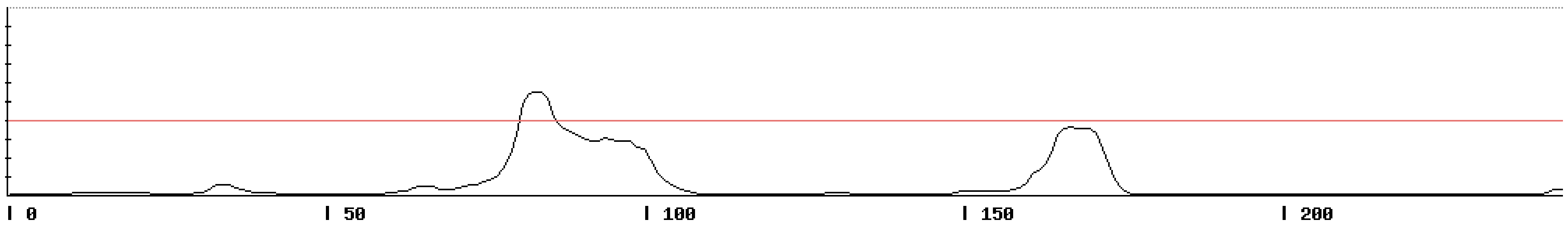

Predictions for Gossypiumraimondii_Gorai.002G001900.1

Viterbi Path

Viterbi Path

Posterior @ 0.4 — 74 - RKHGKGGRPNR - 84

Posterior @ 0.4

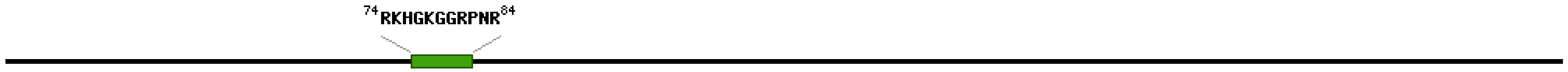

Posterior Graphic. Threshold Marked @ 0.4

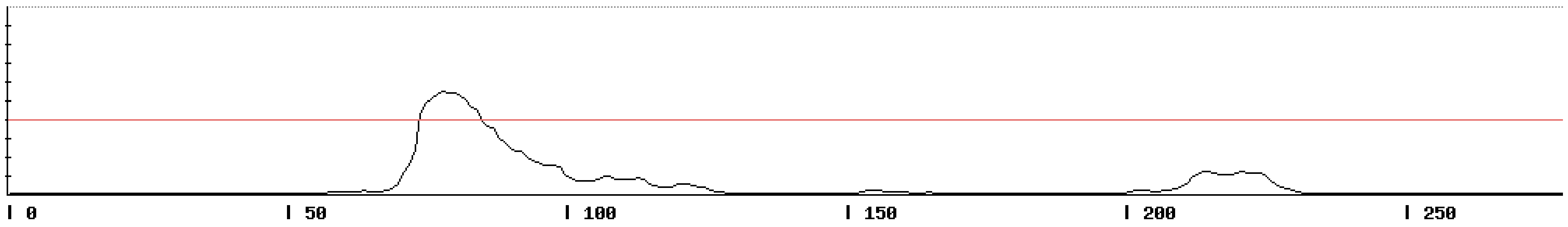

Predictions for Gossypiumraimondii_Gorai.002G008500.1

Viterbi Path

Viterbi Path

Posterior @ 0.4	526 - RGRGEAQRGSRRGGNRGKIIMLPRG - 550
Posterior @ 0.4	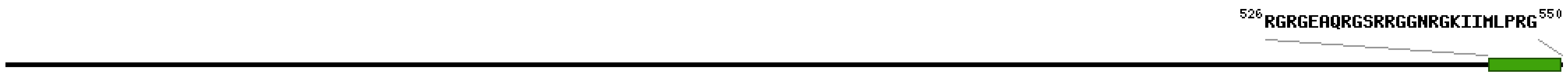
Posterior Graphic. Threshold Marked @ 0.4	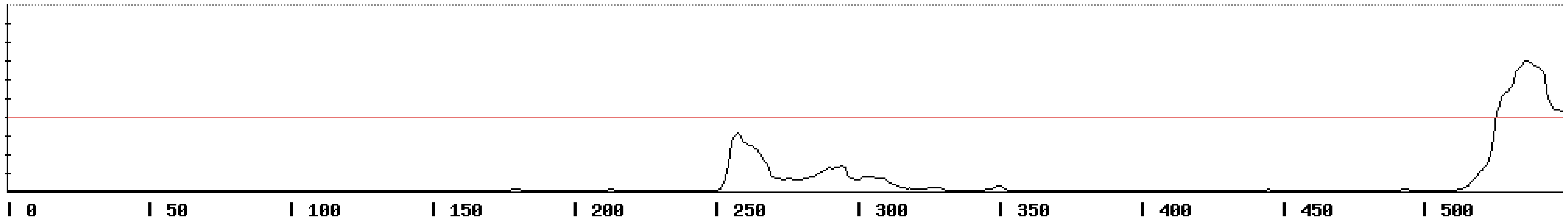

Predictions for Gossypiumraimondii_Gorai.002G019700.1

Viterbi Path	
Viterbi Path	
Posterior @ 0.4	70 - KKRSKSR - 76<br>170 - KKNKKRK - 176
Posterior @ 0.4	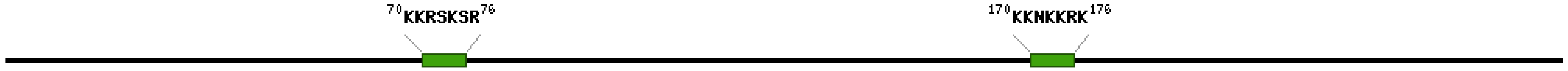
Posterior Graphic. Threshold Marked @ 0.4	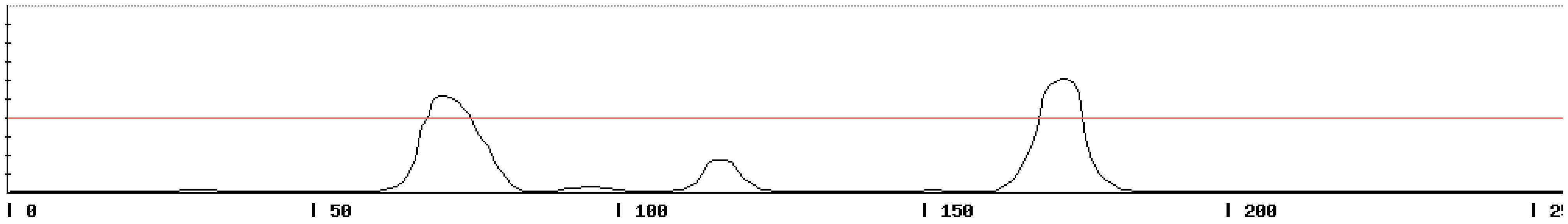

Predictions for Gossypiumraimondii_Gorai.002G033100.1

Viterbi Path	
Viterbi Path	

Posterior @ 0.4	
Posterior @ 0.4	
Posterior Graphic. Threshold Marked @ 0.4	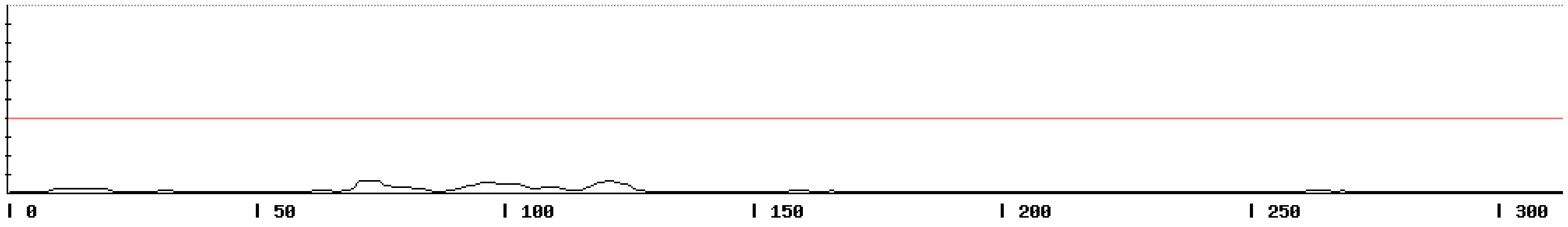

Predictions for Gossypiumraimondii_Gorai.002G036700.1	
Viterbi Path	
Viterbi Path	
Posterior @ 0.4	
Posterior @ 0.4	
Posterior Graphic. Threshold Marked @ 0.4	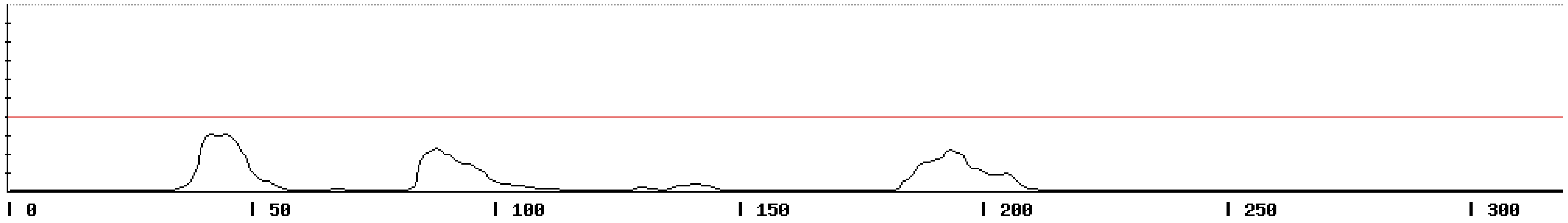

Predictions for Gossypiumraimondii_Gorai.002G067300.1

Viterbi Path

Viterbi Path

Posterior @ 0.4

Posterior @ 0.4

Posterior Graphic. Threshold Marked @ 0.4

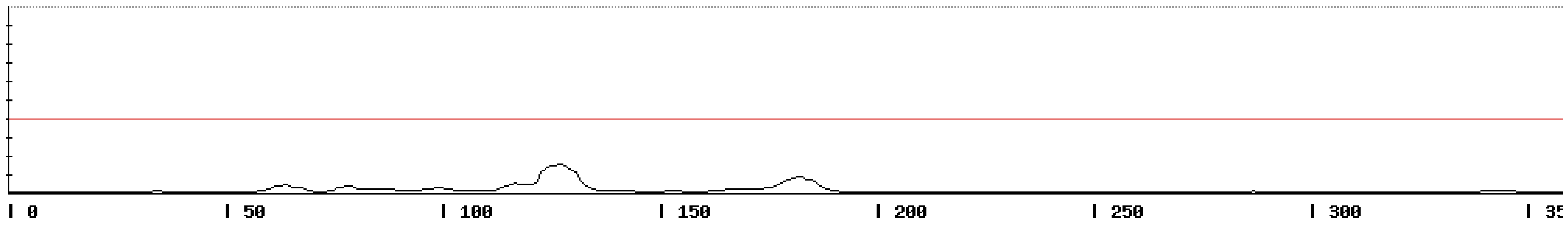

Predictions for Gossypiumraimondii_Gorai.002G073700.1

Viterbi Path

Viterbi Path

Posterior @ 0.4

Posterior @ 0.4

Posterior Graphic. Threshold Marked @ 0.4

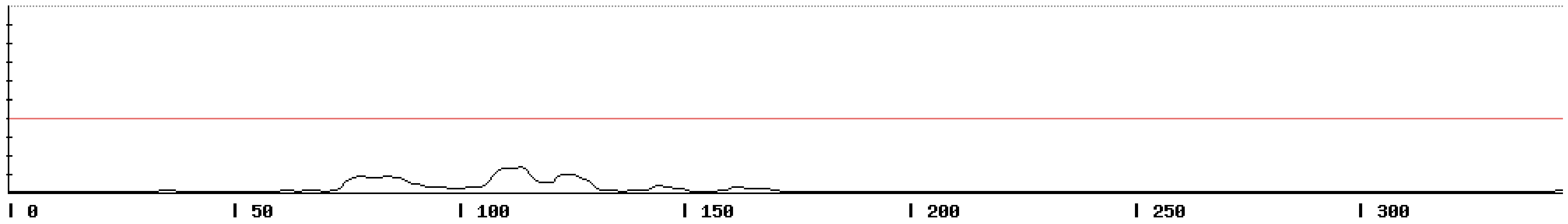

Predictions for Gossypiumraimondii_Gorai.002G073800.1

Viterbi Path

Viterbi Path

Posterior @ 0.4

Posterior @ 0.4

Posterior Graphic. Threshold Marked @ 0.4

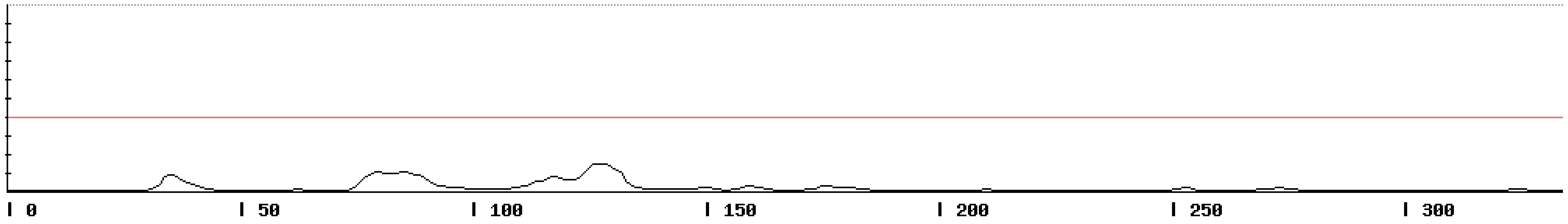

Predictions for Gossypiumraimondii_Gorai.002G111200.1

Viterbi Path

Viterbi Path

Posterior @ 0.4

Posterior @ 0.4

Posterior Graphic. Threshold Marked @ 0.4

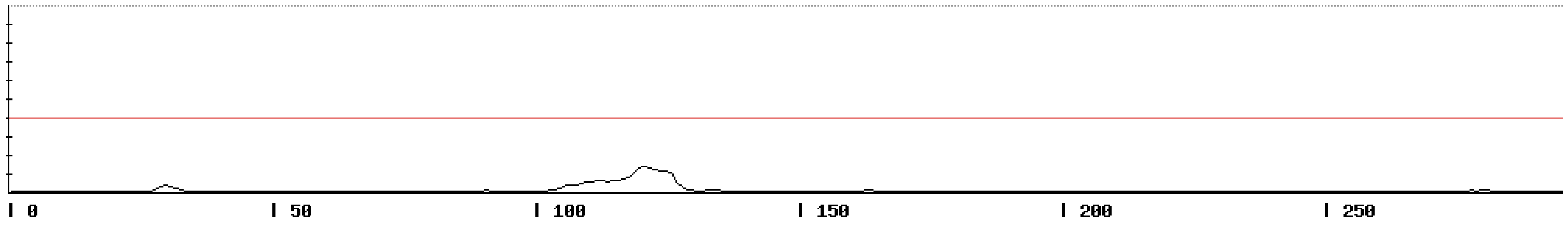

Predictions for Gossypiumraimondii_Gorai.002G113300.1

Viterbi Path	
Viterbi Path	
Posterior @ 0.4	
Posterior @ 0.4	
Posterior Graphic. Threshold Marked @ 0.4	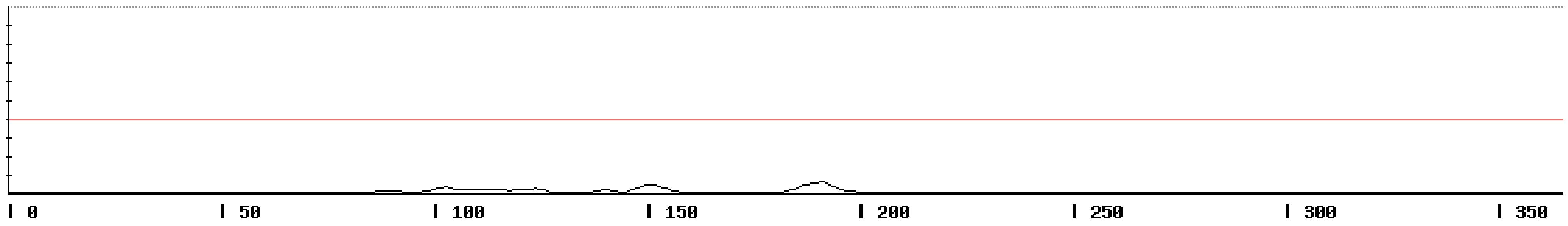

Predictions for Gossypiumraimondii_Gorai.002G238300.1

Viterbi Path	
Viterbi Path	
Posterior @ 0.4	
Posterior @ 0.4	

Posterior Graphic. Threshold Marked @ 0.4	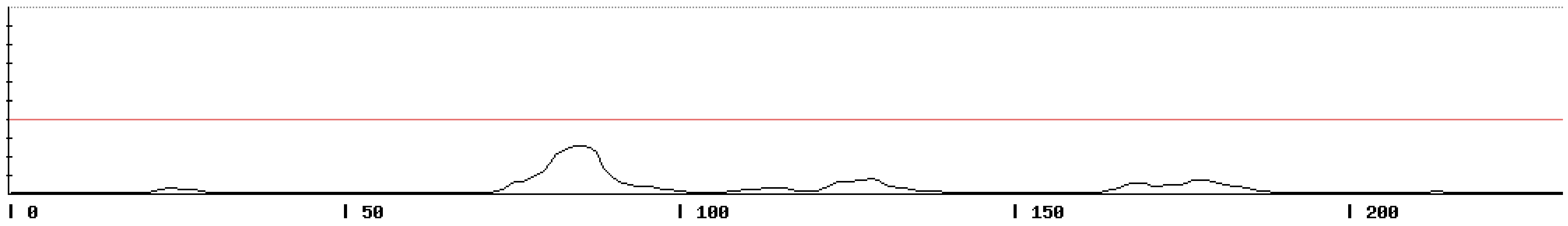

Predictions for Gossypiumraimondii_Gorai.003G003900.1	
Viterbi Path	
Viterbi Path	
Posterior @ 0.4	
Posterior @ 0.4	
Posterior Graphic. Threshold Marked @ 0.4	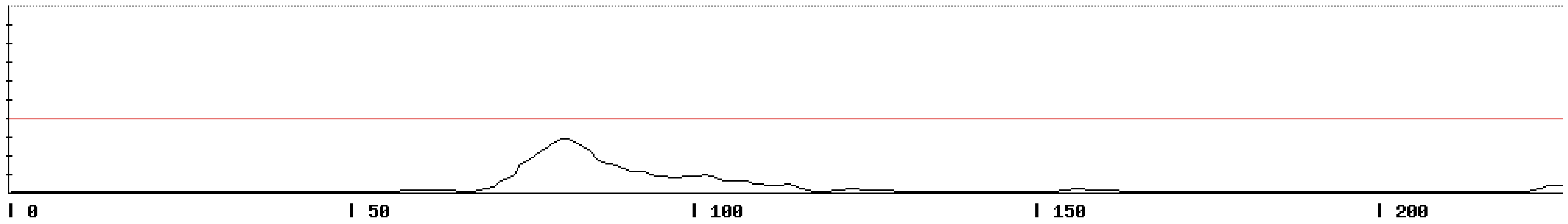

Predictions for Gossypiumraimondii_Gorai.003G010700.1	
Viterbi Path	
Viterbi Path	
Posterior @ 0.4	
Posterior @ 0.4	

Posterior Graphic. Threshold Marked @ 0.4

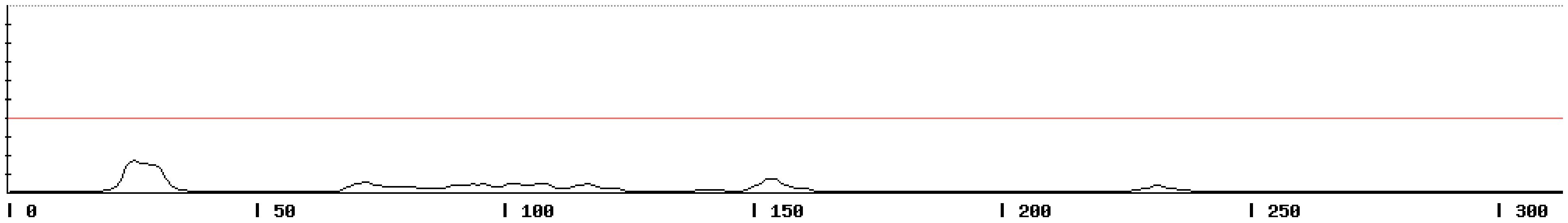

Predictions for Gossypiumraimondii_Gorai.003G073300.1

Viterbi Path

Viterbi Path

Posterior @ 0.4 — 97 - KDRTIKSKKSLIG - 109

Posterior @ 0.4

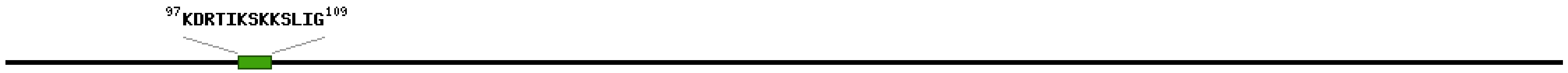

Posterior Graphic. Threshold Marked @ 0.4

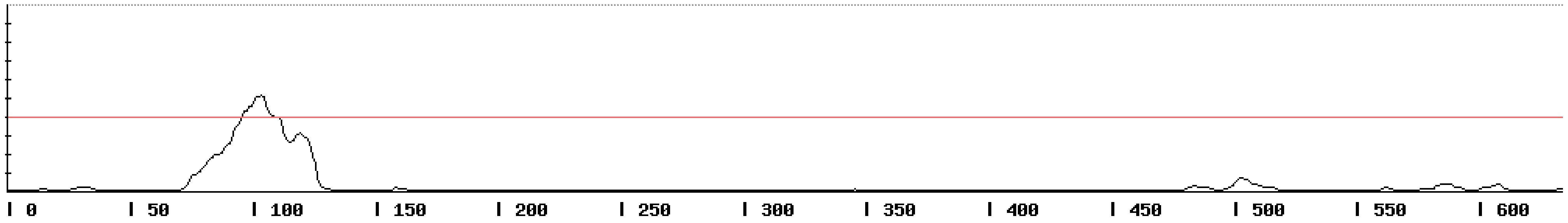

Predictions for Gossypiumraimondii_Gorai.003G077700.1

Viterbi Path

Viterbi Path

Posterior @ 0.4

Posterior @ 0.4

Posterior Graphic. Threshold Marked @ 0.4

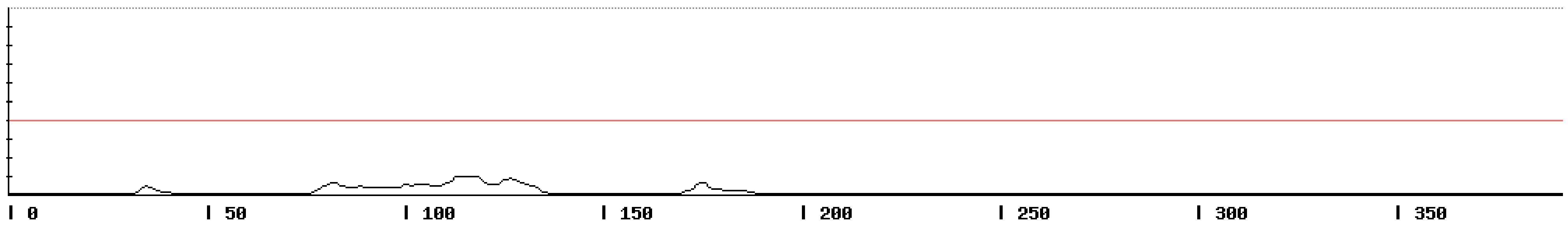

Predictions for Gossypiumraimondii_Gorai.003G114100.1

Viterbi Path

Viterbi Path

Posterior @ 0.4

Posterior @ 0.4

Posterior Graphic. Threshold Marked @ 0.4

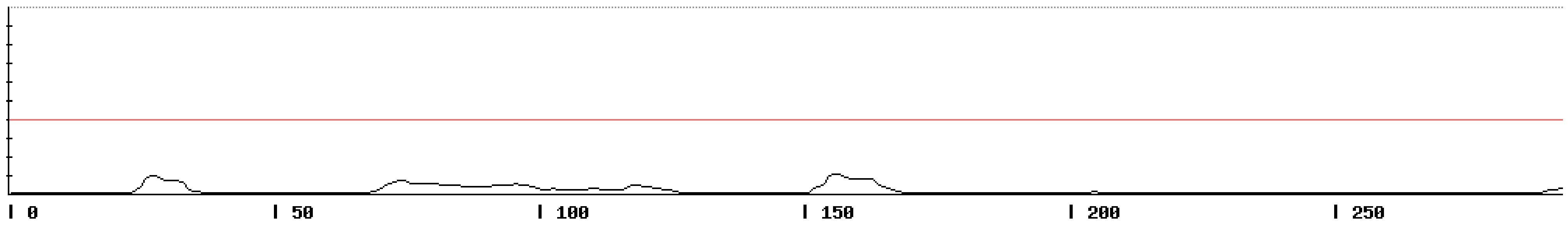

Predictions for Gossypiumraimondii_Gorai.003G169000.1

Viterbi Path

Viterbi Path

Posterior @ 0.4

Posterior @ 0.4

Posterior Graphic. Threshold Marked @ 0.4

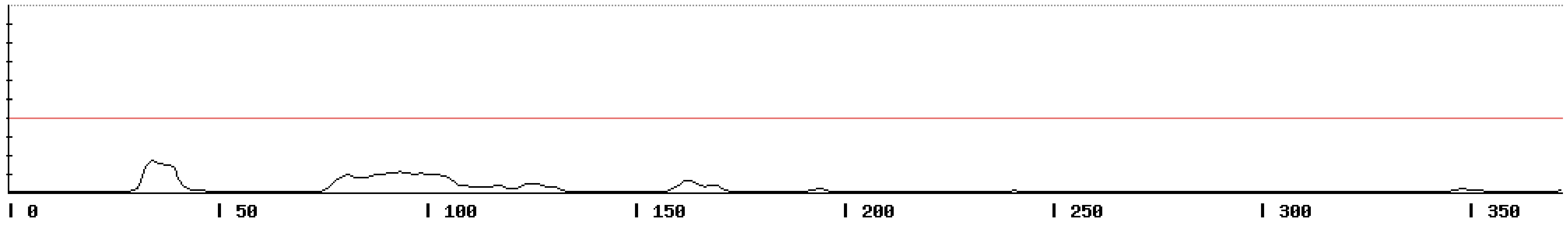

Predictions for Gossypiumraimondii_Gorai.003G180600.1

Viterbi Path

Viterbi Path

Posterior @ 0.4

Posterior @ 0.4

Posterior Graphic. Threshold Marked @ 0.4

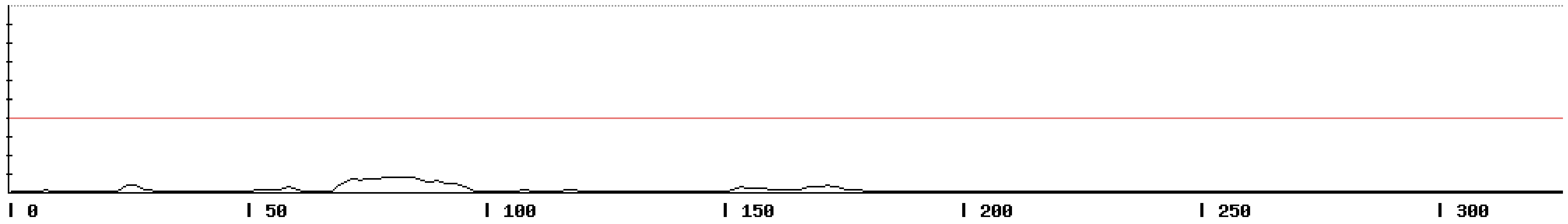

Predictions for Gorai.004G048200.1

Viterbi Path

Viterbi Path

Posterior @ 0.4

Posterior @ 0.4

Posterior Graphic. Threshold Marked @ 0.4

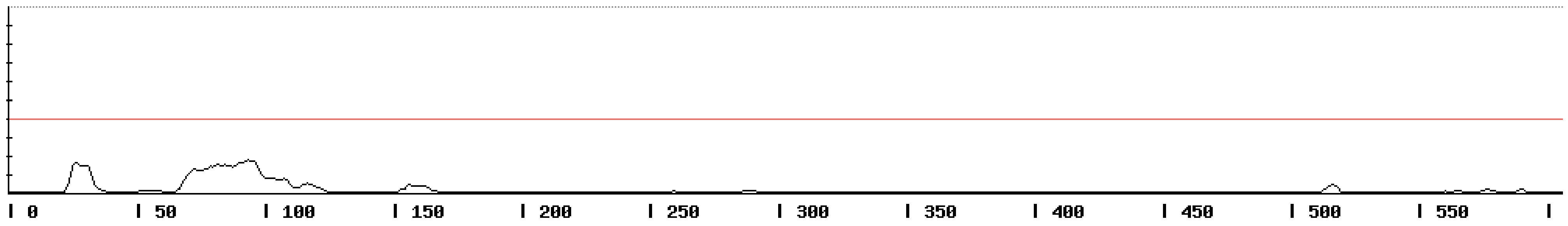

Predictions for Gossypiumraimondii_Gorai.004G125900.1

Viterbi Path

Viterbi Path

Posterior @ 0.4

Posterior @ 0.4

Posterior Graphic. Threshold Marked @ 0.4

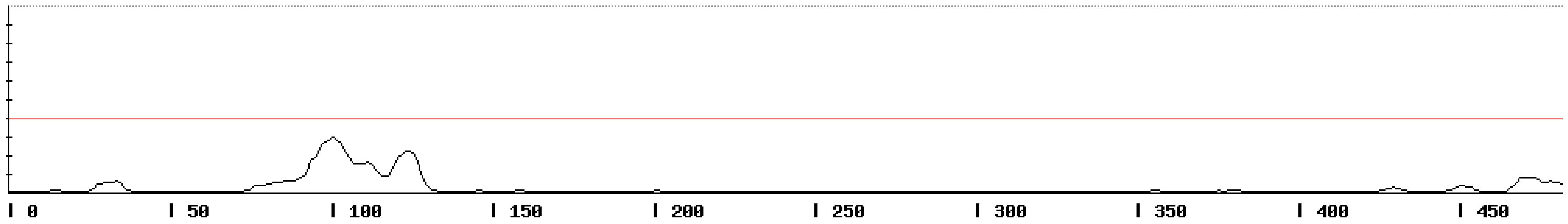

Predictions for Gossypiumraimondii_Gorai.004G129200.1	
Viterbi Path	
Viterbi Path	
Posterior @ 0.4	
Posterior @ 0.4	
Posterior Graphic. Threshold Marked @ 0.4	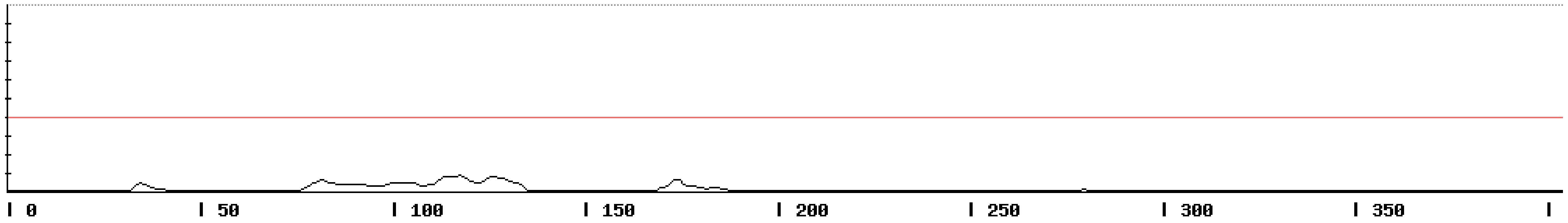

Predictions for Gossypiumraimondii_Gorai.004G149000.1	
Viterbi Path	73 - RRGDNKRVKRTTDKGFWKVTGKPRKVKGKKGCAAKK - 108
Viterbi Path	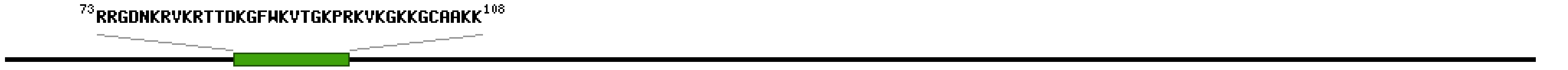
Posterior @ 0.4	73 - RRGDNKRVKRTTDKGFWKVTGKPRKVKGKKGCAAKK - 108
Posterior @ 0.4	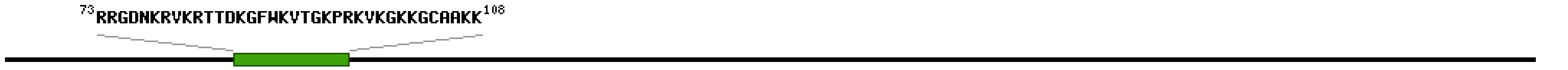
Posterior Graphic. Threshold Marked @ 0.4	

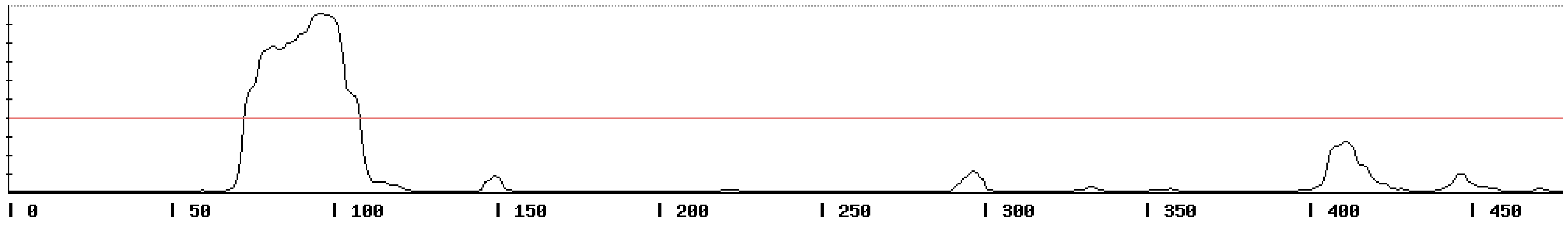

Predictions for Gossypiumraimondii_Gorai.004G186700.1

Viterbi Path

Viterbi Path

Posterior @ 0.4

Posterior @ 0.4

Posterior Graphic. Threshold Marked @ 0.4

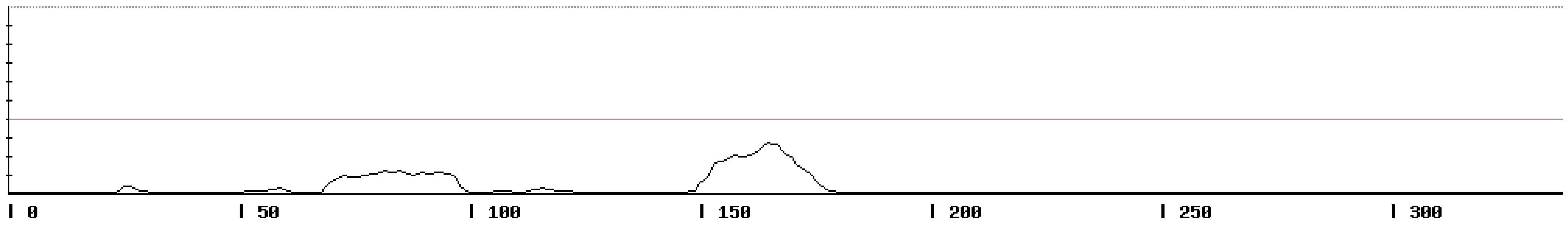

Predictions for Gossypiumraimondii_Gorai.004G201100.1

Viterbi Path

Viterbi Path

Posterior @ 0.4

Posterior @ 0.4

Posterior Graphic. Threshold Marked @ 0.4

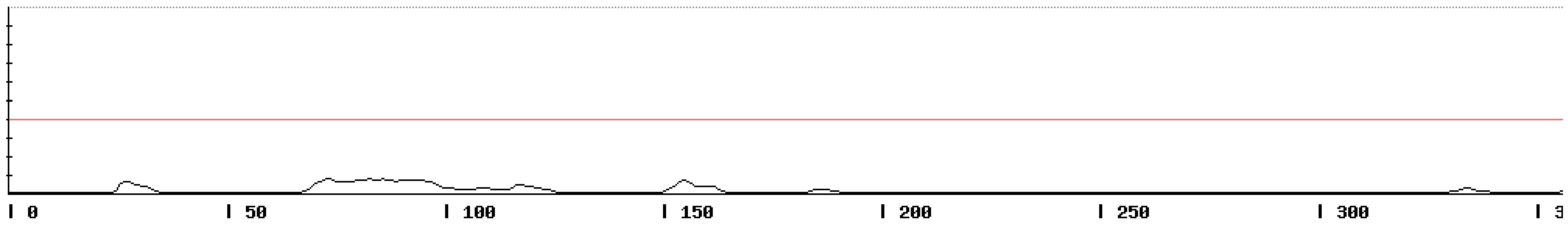

Predictions for Gossypiumraimondii_Gorai.004G221300.1

Viterbi Path

Viterbi Path

Posterior @ 0.4

Posterior @ 0.4

Posterior Graphic. Threshold Marked @ 0.4

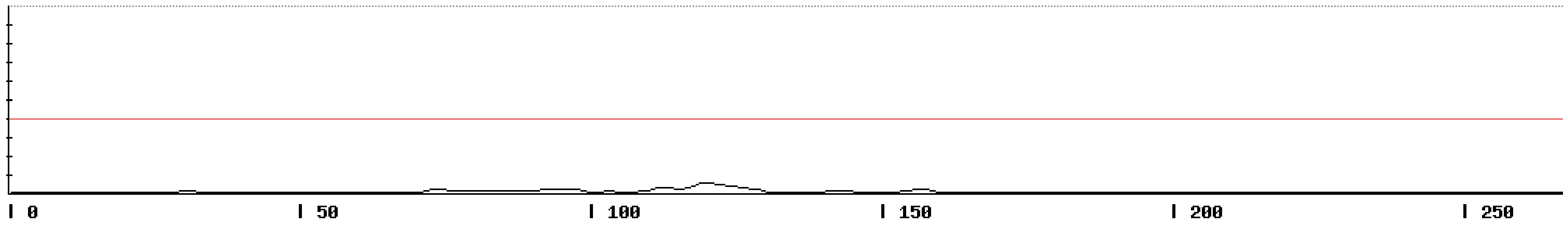

Predictions for Gossypiumraimondii_Gorai.004G227000.1

Viterbi Path

Viterbi Path

Posterior @ 0.4

475 - SKRPRRM - 481

Posterior @ 0.4

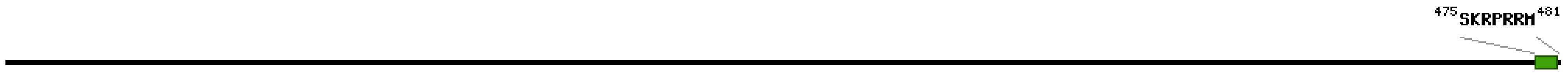

Posterior Graphic. Threshold Marked @ 0.4

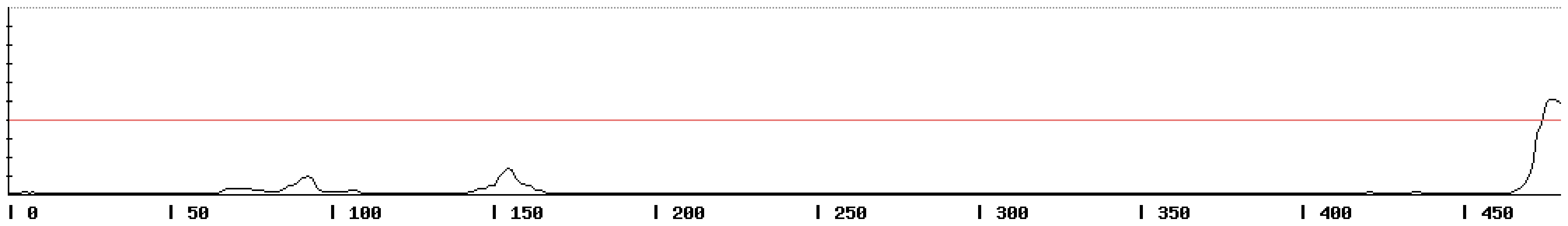

Predictions for Gossypiumraimondii_Gorai.004G258200.1

Viterbi Path

Viterbi Path

Posterior @ 0.4

Posterior @ 0.4

Posterior Graphic. Threshold Marked @ 0.4

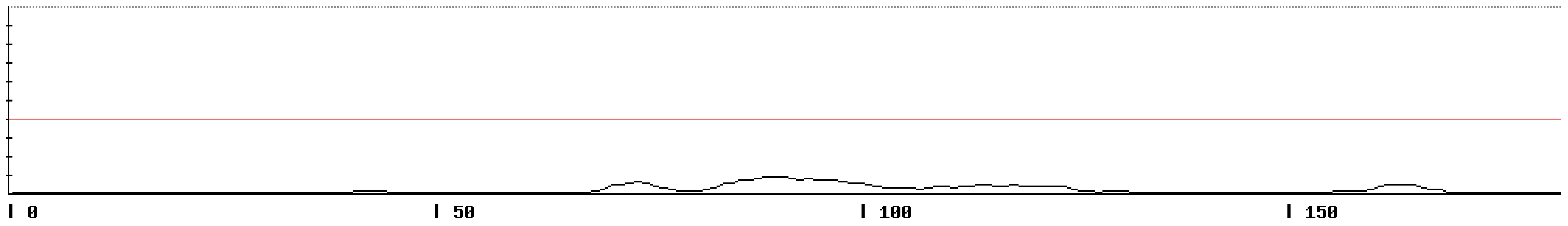

Predictions for Gossypiumraimondii_Gorai.004G267300.1

Viterbi Path

Viterbi Path

Posterior @ 0.4

Posterior @ 0.4

Posterior Graphic. Threshold Marked @ 0.4

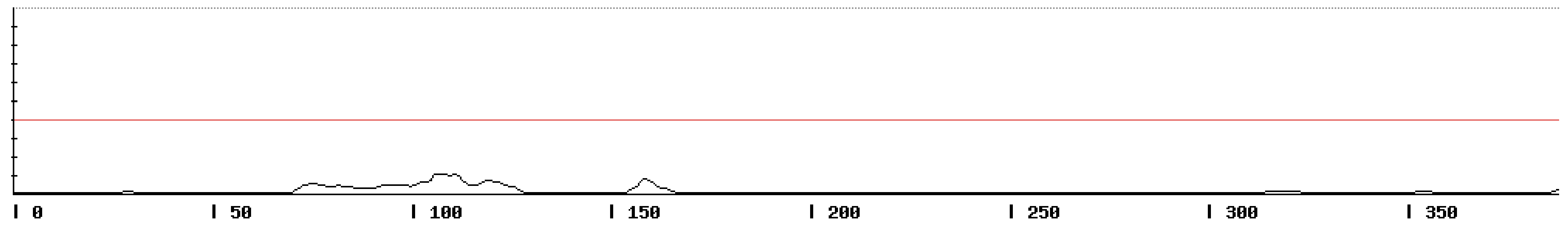

Predictions for Gossypiumraimondii_Gorai.004G268400.1

Viterbi Path

Viterbi Path

Posterior @ 0.4

Posterior @ 0.4

Posterior Graphic. Threshold Marked @ 0.4

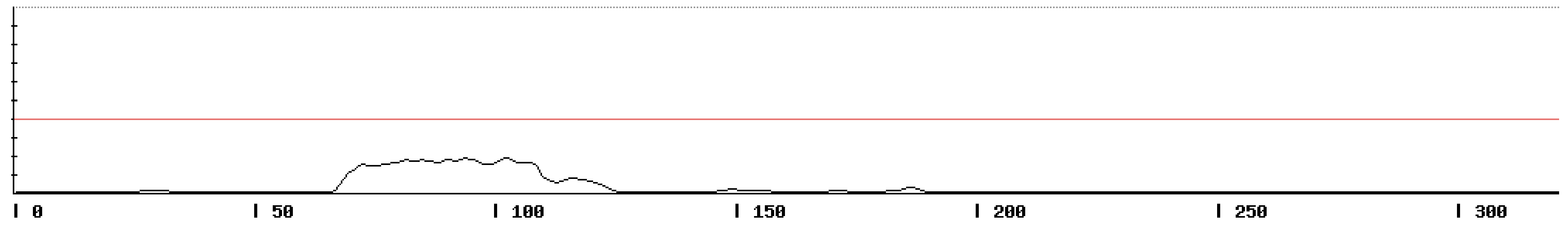

Predictions for Gossypiumraimondii_Gorai.004G268500.1

Viterbi Path

Viterbi Path

Posterior @ 0.4

Posterior @ 0.4

Posterior Graphic. Threshold Marked @ 0.4

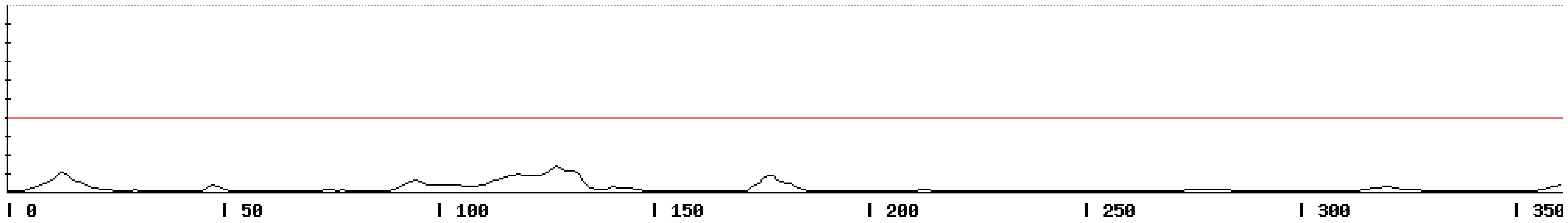

Predictions for Gossypiumraimondii_Gorai.004G270200.1

Viterbi Path

Viterbi Path

Posterior @ 0.4 — 178 - KRNKKIKQ - 185

Posterior @ 0.4

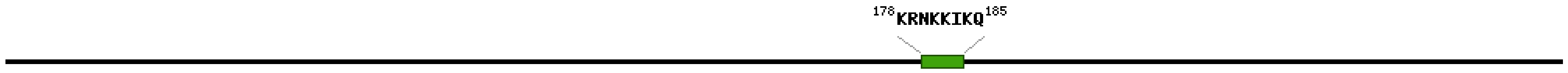

Posterior Graphic. Threshold Marked @ 0.4

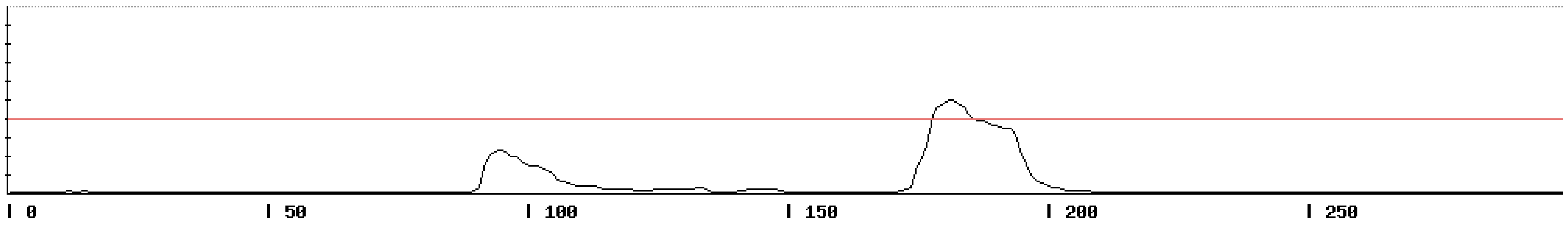

Predictions for Gossypiumraimondii_Gorai.004G270300.1

Viterbi Path	
Viterbi Path	
Posterior @ 0.4	166 - RK - 167
Posterior @ 0.4	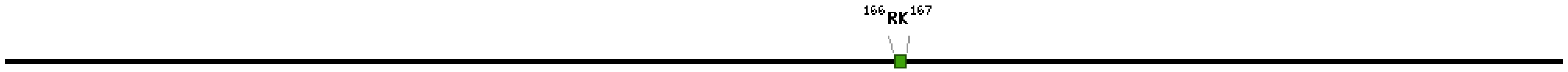
Posterior Graphic. Threshold Marked @ 0.4	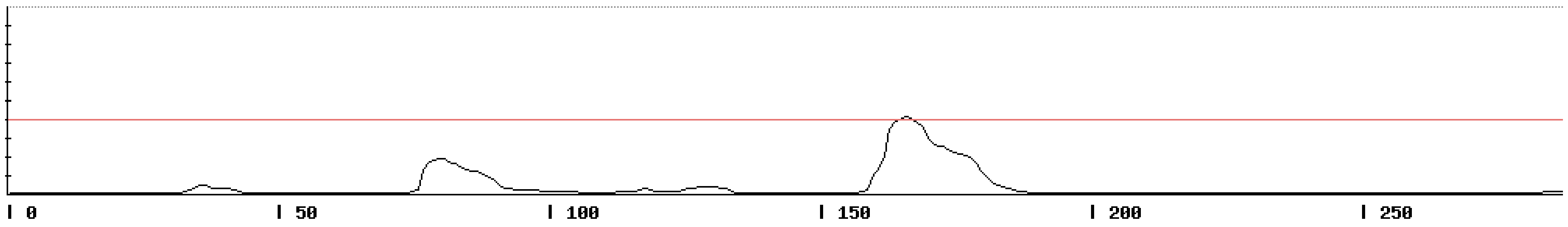

Predictions for Gossypiumraimondii_Gorai.004G270400.1

Viterbi Path	
Viterbi Path	
Posterior @ 0.4	163 - KRHKKNKQEWRQGAAKR - 179
Posterior @ 0.4	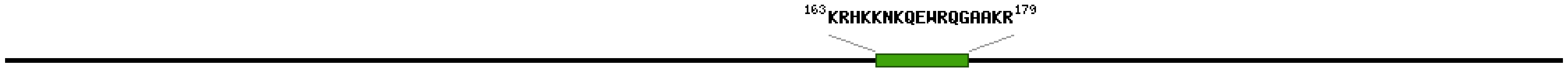
Posterior Graphic. Threshold Marked @ 0.4	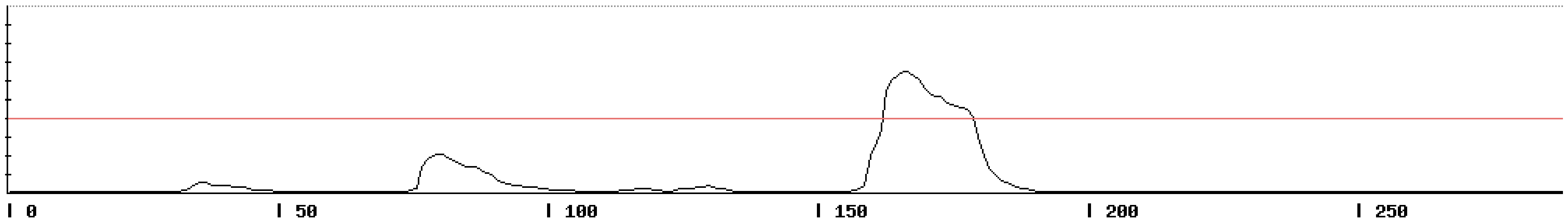

Predictions for Gossypiumraimondii_Gorai.005G013000.1	
Viterbi Path	
Viterbi Path	
Posterior @ 0.4	
Posterior @ 0.4	
Posterior Graphic. Threshold Marked @ 0.4	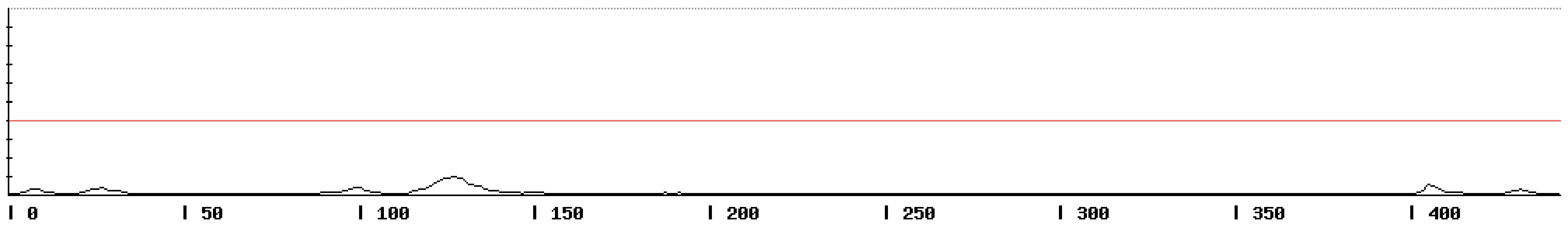

Predictions for Gossypiumraimondii_Gorai.005G013100.1	
Viterbi Path	
Viterbi Path	
Posterior @ 0.4	
Posterior @ 0.4	
Posterior Graphic. Threshold Marked @ 0.4	

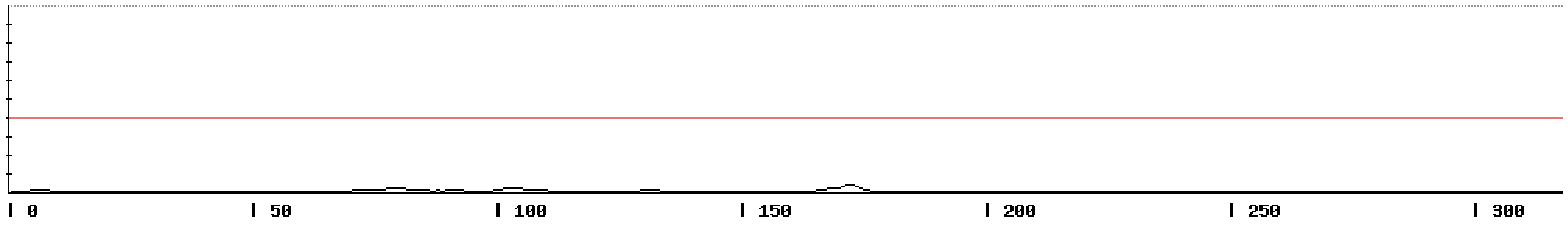

Predictions for Gossypiumraimondii_Gorai.005G013300.1

Viterbi Path	
Viterbi Path	
Posterior @ 0.4	
Posterior @ 0.4	
Posterior Graphic. Threshold Marked @ 0.4	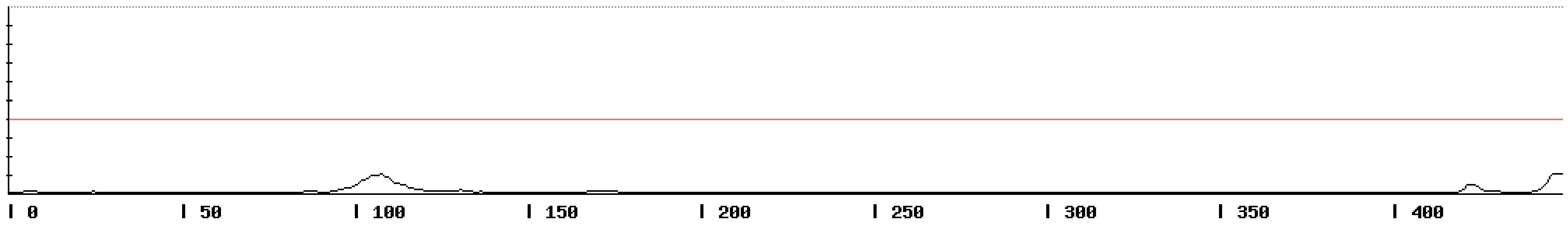

Predictions for Gossypiumraimondii_Gorai.005G016000.1

Viterbi Path	485 - KKKPKK - 490
Viterbi Path	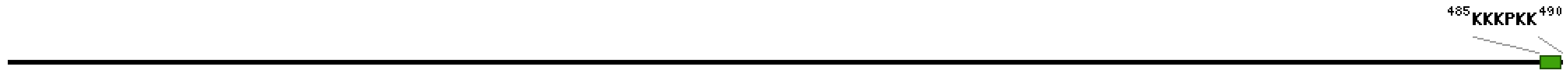
Posterior @ 0.4	70 - KKKSR - 74
	353 - KMRKWRERERNRRKSQRMG - 371
	478 - RFKSSVVKKKPKK - 490
Posterior @ 0.4	

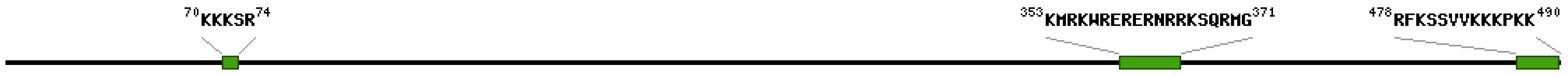

Posterior Graphic. Threshold Marked @ 0.4

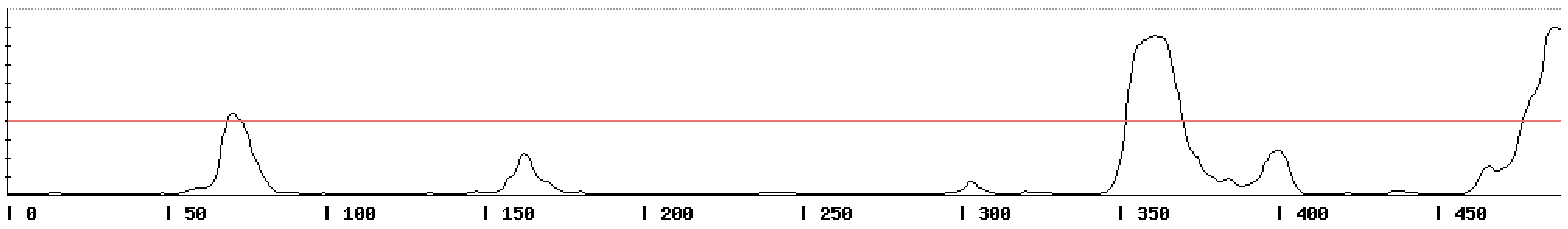

Predictions for Gossypiumraimondii_Gorai.005G020300.1

Viterbi Path

Viterbi Path

Posterior @ 0.4

Posterior @ 0.4

Posterior Graphic. Threshold Marked @ 0.4

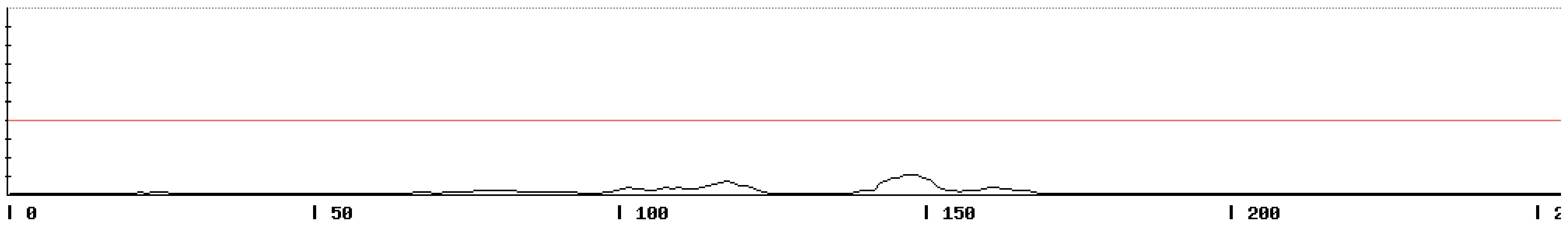

Predictions for Gossypiumraimondii_Gorai.005G020600.1

Viterbi Path

Viterbi Path

Posterior @ 0.4

Posterior @ 0.4

Posterior Graphic. Threshold Marked @ 0.4

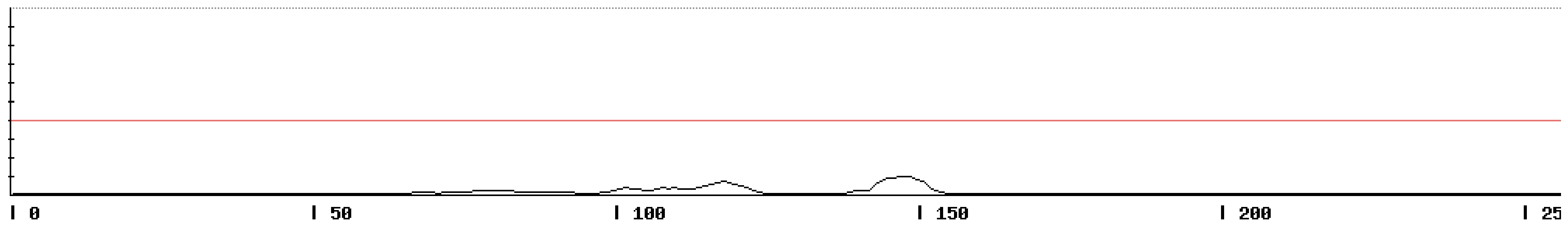

Predictions for Gossypiumraimondii_Gorai.005G076200.1

Viterbi Path

Viterbi Path

Posterior @ 0.4

Posterior @ 0.4

Posterior Graphic. Threshold Marked @ 0.4

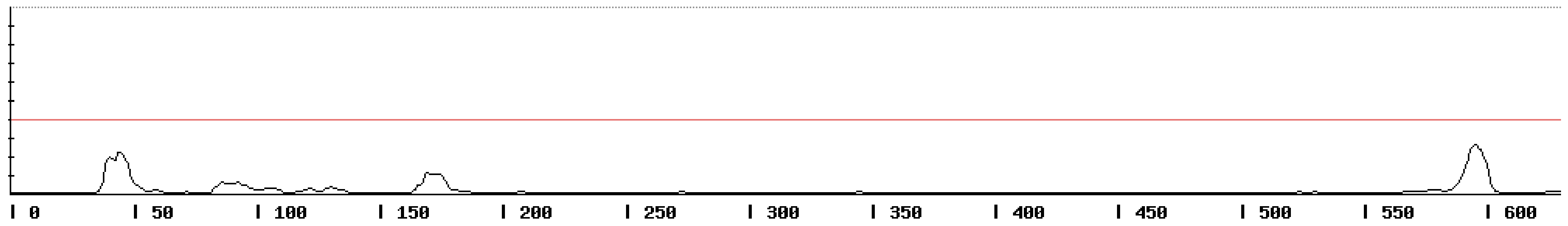

Predictions for Gossypiumraimondii_Gorai.005G088800.1

Viterbi Path

Viterbi Path

Posterior @ 0.4

Posterior @ 0.4

Posterior Graphic. Threshold Marked @ 0.4

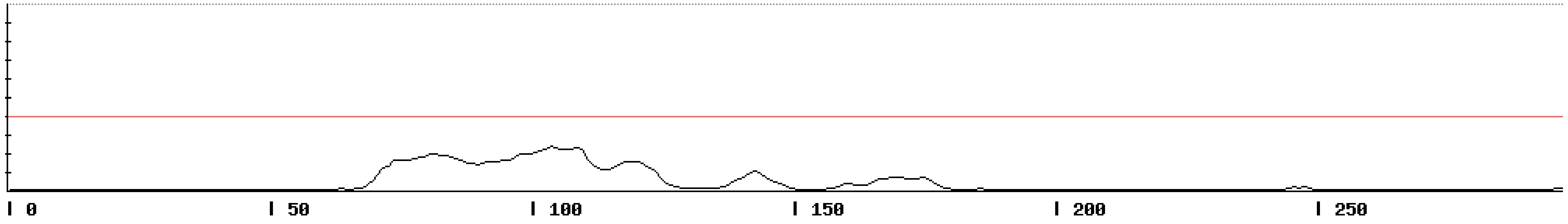

Predictions for Gossypiumraimondii_Gorai.005G141200.1

Viterbi Path

Viterbi Path

Posterior @ 0.4

Posterior @ 0.4

Posterior Graphic. Threshold Marked @ 0.4

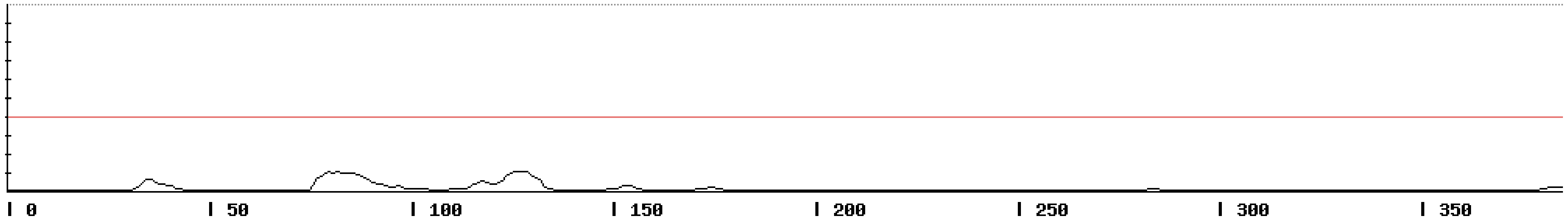

Predictions for Gossypiumraimondii_Gorai.005G142300.1

Viterbi Path

Viterbi Path

Posterior @ 0.4

Posterior @ 0.4

Posterior Graphic. Threshold Marked @ 0.4

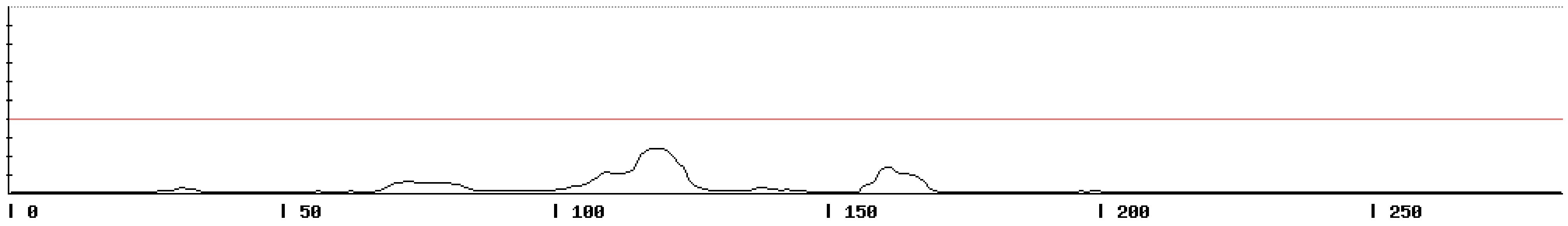

Predictions for Gossypiumraimondii_Gorai.005G195300.1

Viterbi Path

Viterbi Path

Posterior @ 0.4

191 - KGVGKARRK - 199

Posterior @ 0.4

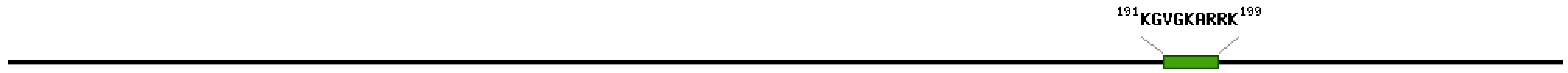

Posterior Graphic. Threshold Marked @ 0.4

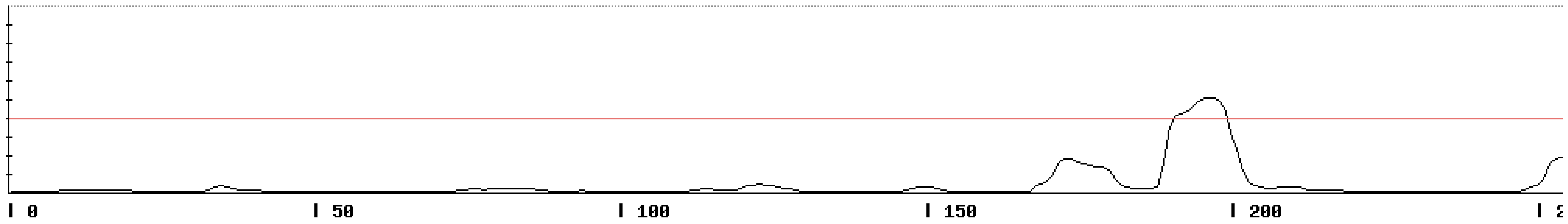

Predictions for Gossypiumraimondii_Gorai.005G195400.1	
Viterbi Path	
Viterbi Path	
Posterior @ 0.4	
Posterior @ 0.4	
Posterior Graphic. Threshold Marked @ 0.4	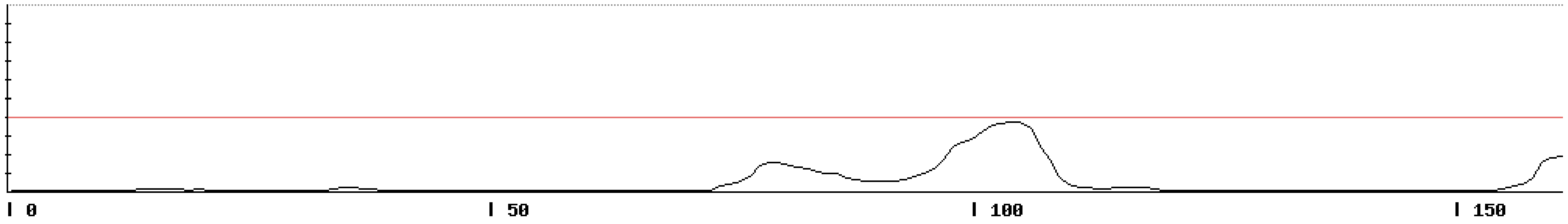

Predictions for Gossypiumraimondii_Gorai.005G217400.1	
Viterbi Path	
Viterbi Path	
Posterior @ 0.4	
Posterior @ 0.4	
Posterior Graphic. Threshold Marked @ 0.4	

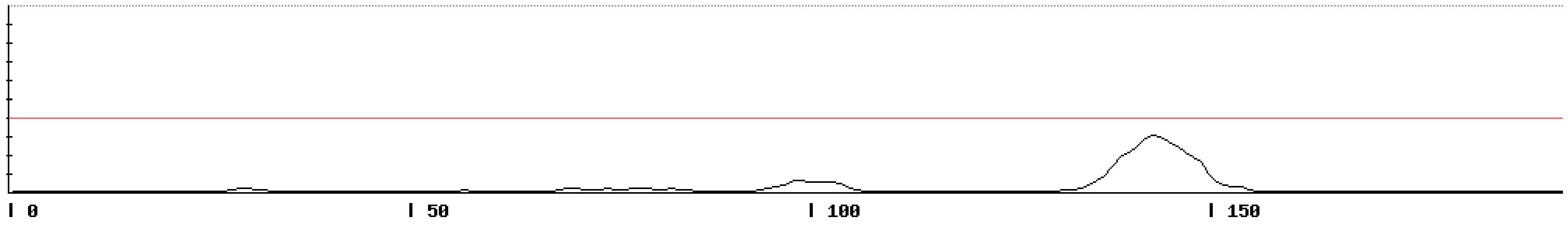

Predictions for Gossypiumraimondii_Gorai.005G257800.1

Viterbi Path

Viterbi Path

Posterior @ 0.4

Posterior @ 0.4

Posterior Graphic. Threshold Marked @ 0.4

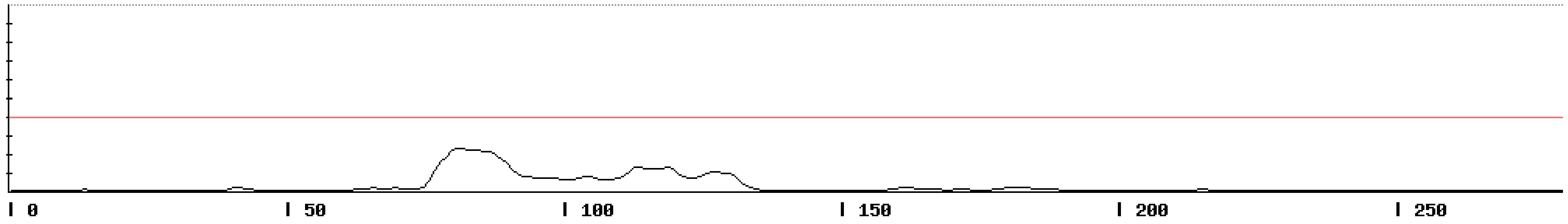

Predictions for Gossypiumraimondii_Gorai.006G005100.1

Viterbi Path

Viterbi Path

Posterior @ 0.4

Posterior @ 0.4

Posterior Graphic. Threshold Marked @ 0.4

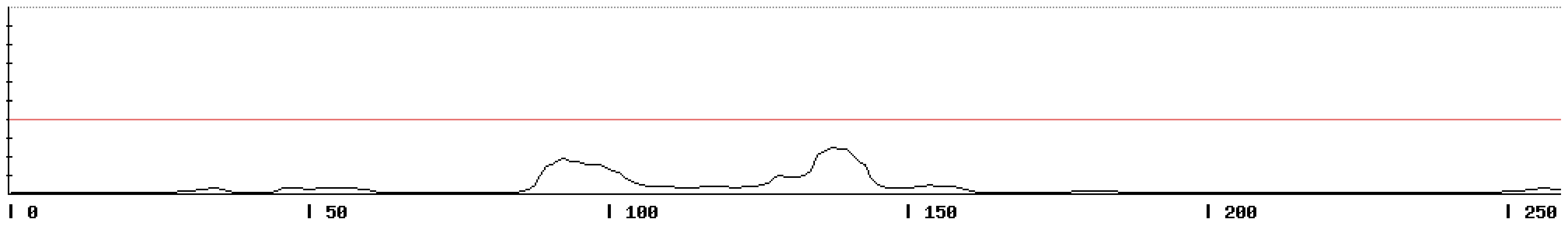

Predictions for Gossypiumraimondii_Gorai.006G017700.1

Viterbi Path

Viterbi Path

Posterior @ 0.4

Posterior @ 0.4

Posterior Graphic. Threshold Marked @ 0.4

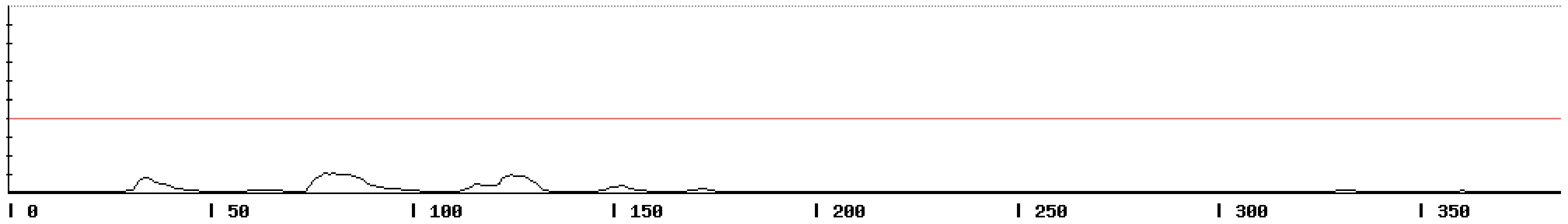

Predictions for Gossypiumraimondii_Gorai.006G034600.1

Viterbi Path

Viterbi Path

Posterior @ 0.4

Posterior @ 0.4

Posterior Graphic. Threshold Marked @ 0.4

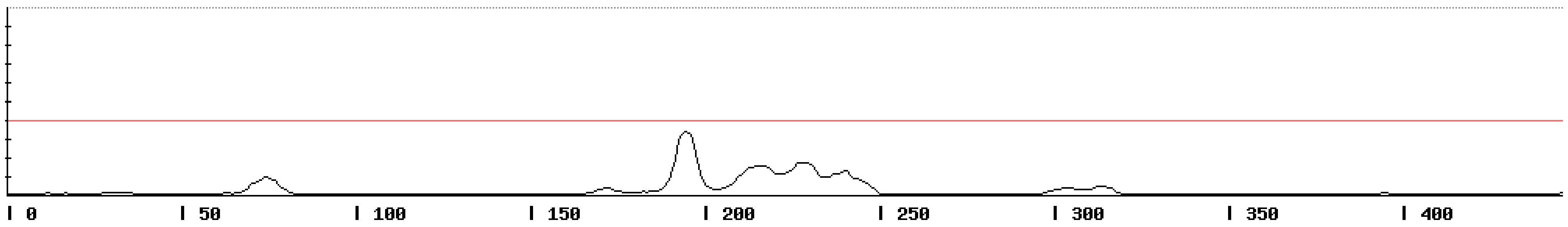

Predictions for Gossypiumraimondii_Gorai.006G060900.1

Viterbi Path

Viterbi Path

Posterior @ 0.4

Posterior @ 0.4

Posterior Graphic. Threshold Marked @ 0.4

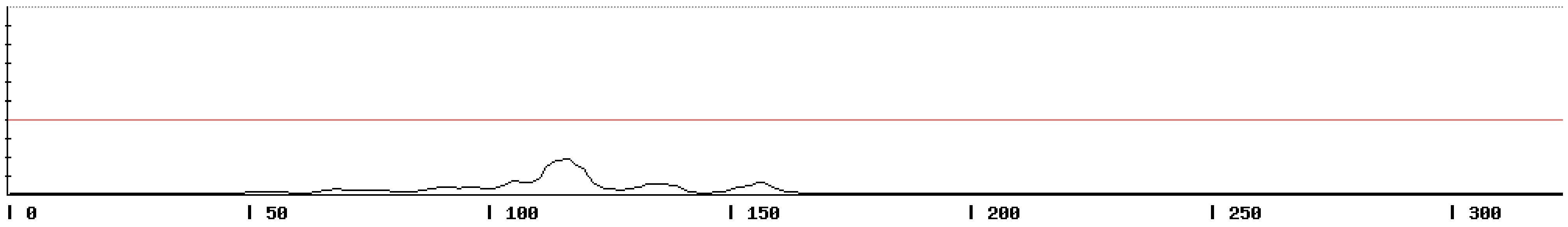

Predictions for Gossypiumraimondii_Gorai.006G088400.1

Viterbi Path

Viterbi Path

Posterior @ 0.4

Posterior @ 0.4

Posterior Graphic. Threshold Marked @ 0.4

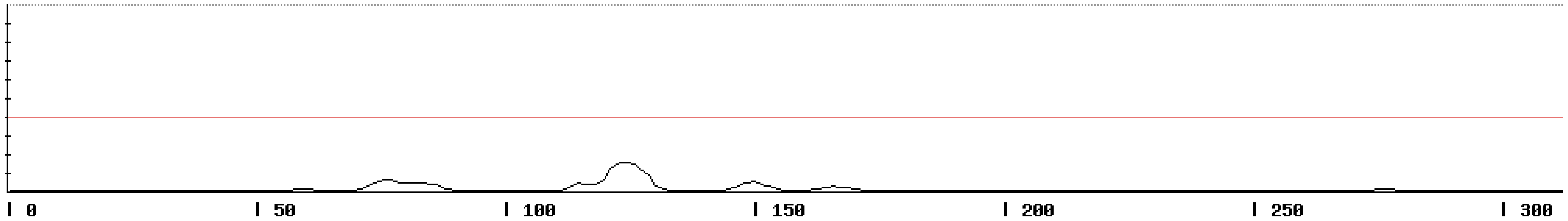

Predictions for Gossypiumraimondii_Gorai.006G113000.1

Viterbi Path

Viterbi Path

Posterior @ 0.4 123 - GRK - 125

Posterior @ 0.4

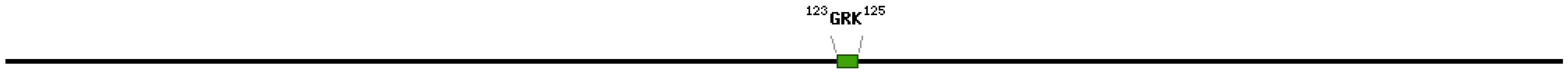

Posterior Graphic. Threshold Marked @ 0.4

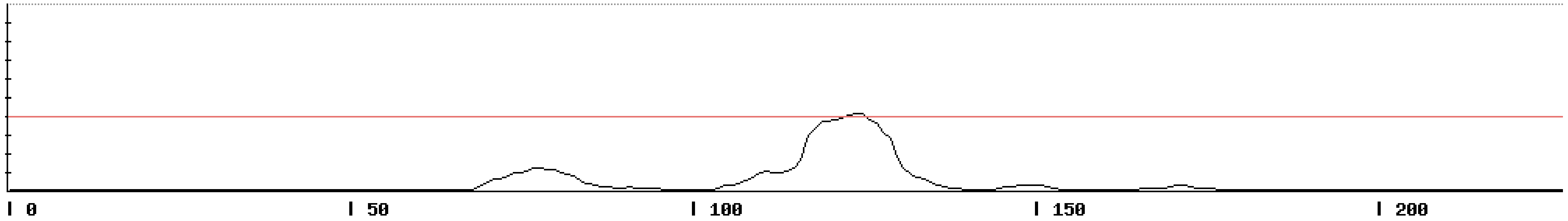

Predictions for Gossypiumraimondii_Gorai.006G113700.1

Viterbi Path

Viterbi Path

Posterior @ 0.4

Posterior @ 0.4

Posterior Graphic. Threshold Marked @ 0.4

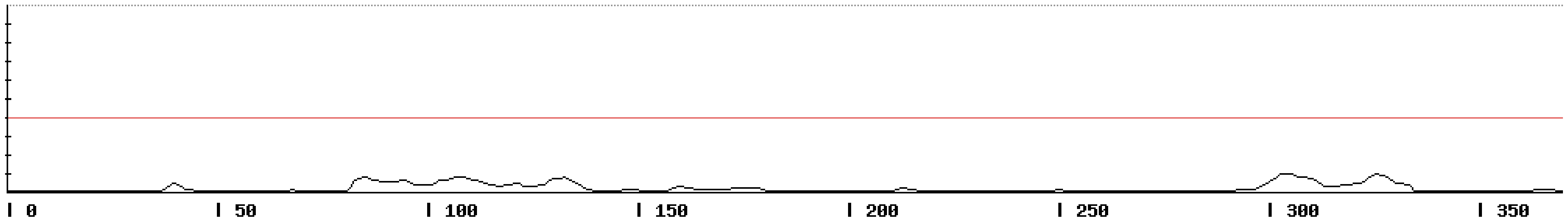

Predictions for Gossypiumraimondii_Gorai.006G127700.1

Viterbi Path

Viterbi Path

Posterior @ 0.4

Posterior @ 0.4

Posterior Graphic. Threshold Marked @ 0.4

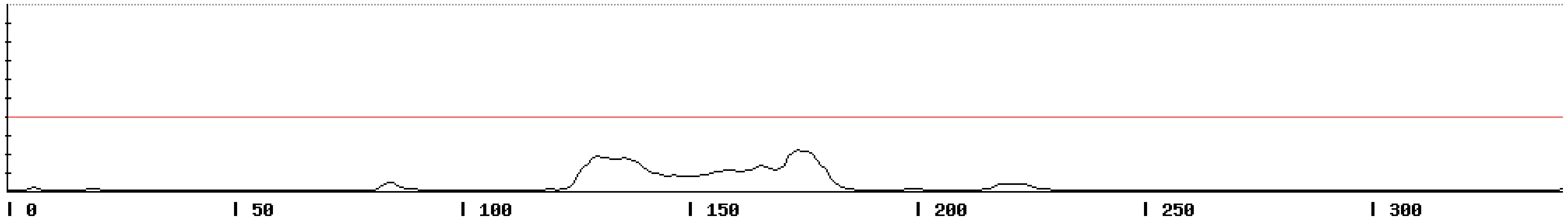

Predictions for Gossypiumraimondii_Gorai.006G189600.1

Viterbi Path

Viterbi Path

Posterior @ 0.4

Posterior @ 0.4

Posterior Graphic. Threshold Marked @ 0.4

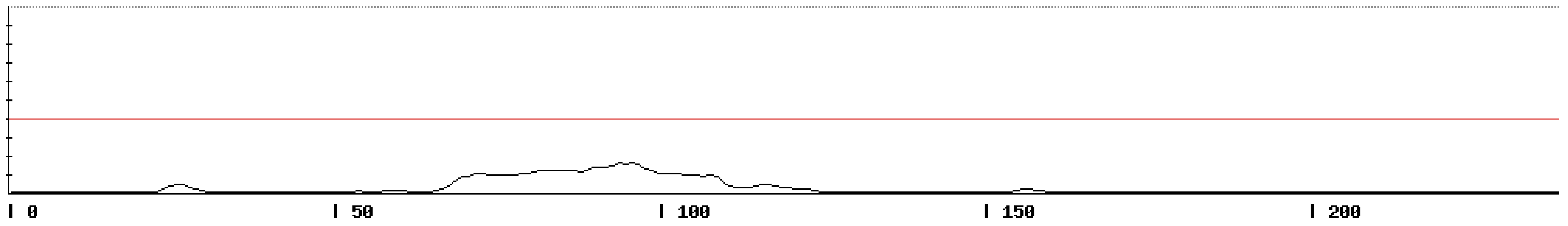

Predictions for Gossypiumraimondii_Gorai.006G203800.1

Viterbi Path

Viterbi Path

Posterior @ 0.4

Posterior @ 0.4

Posterior Graphic. Threshold Marked @ 0.4

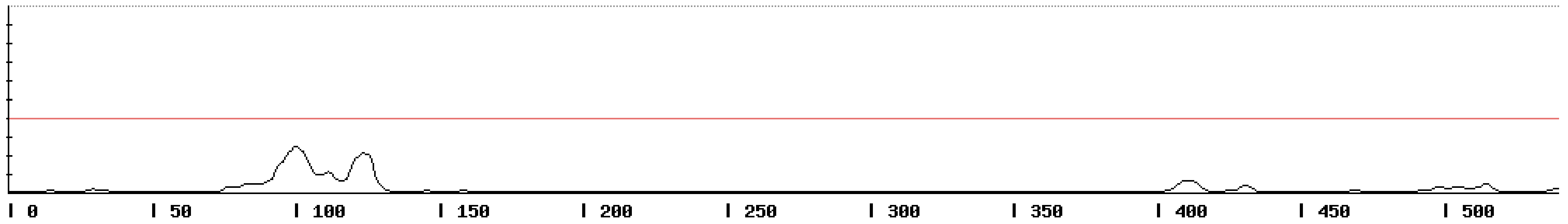

Predictions for Gossypiumraimondii_Gorai.006G205300.1	
Viterbi Path	
Viterbi Path	
Posterior @ 0.4	
Posterior @ 0.4	
Posterior Graphic. Threshold Marked @ 0.4	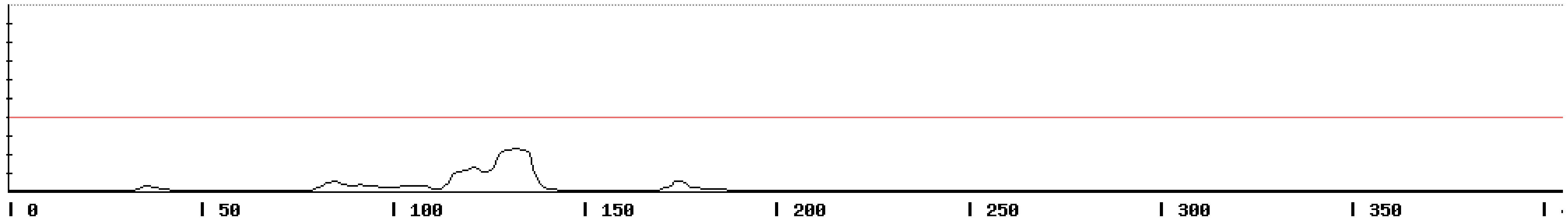

Predictions for Gossypiumraimondii_Gorai.006G267800.1	
Viterbi Path	
Viterbi Path	
Posterior @ 0.4	
Posterior @ 0.4	
Posterior Graphic. Threshold Marked @ 0.4	

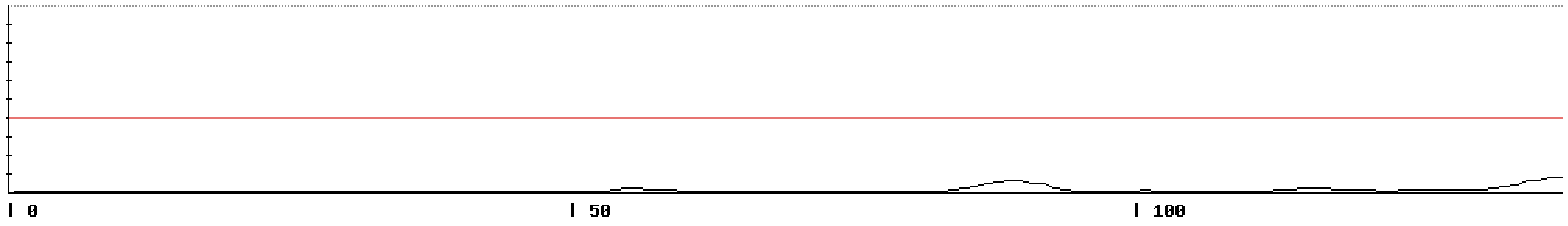

Predictions for Gossypiumraimondii_Gorai.006G267900.1

Viterbi Path

Viterbi Path

Posterior @ 0.4 — 100 - RRKK - 103

Posterior @ 0.4

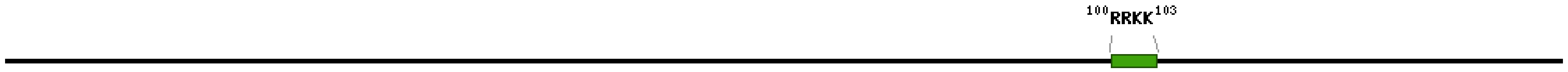

Posterior Graphic. Threshold Marked @ 0.4

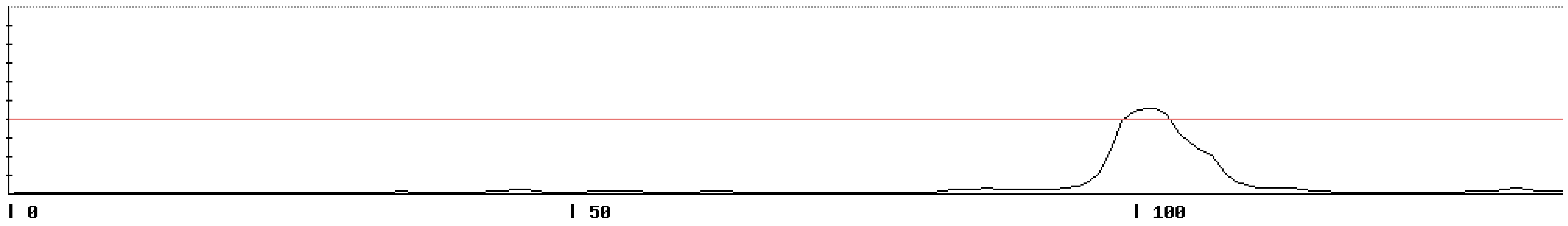

Predictions for Gossypiumraimondii_Gorai.007G017500.1

Viterbi Path

Viterbi Path

Posterior @ 0.4

Posterior @ 0.4

Posterior Graphic. Threshold Marked @ 0.4

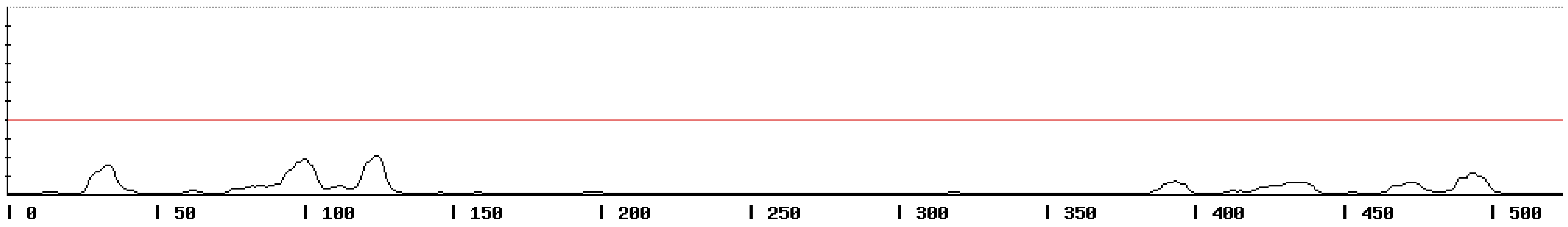

Predictions for Gossypiumraimondii_Gorai.007G031300.1

Viterbi Path	
Viterbi Path	
Posterior @ 0.4	
Posterior @ 0.4	
Posterior Graphic. Threshold Marked @ 0.4	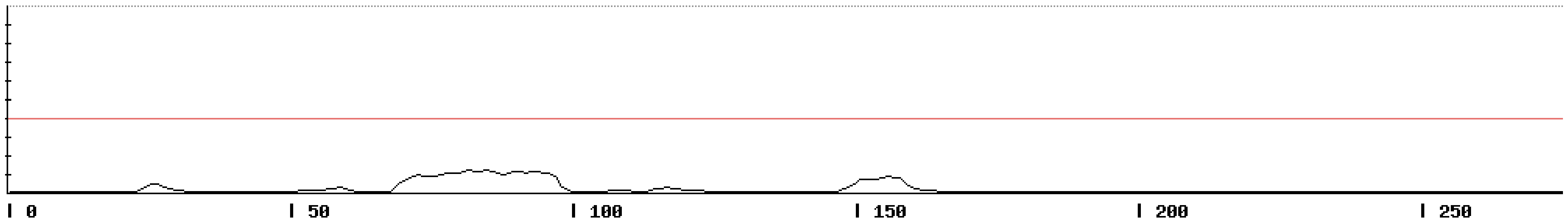

Predictions for Gossypiumraimondii_Gorai.007G038100.1

Viterbi Path	
Viterbi Path	
Posterior @ 0.4	
Posterior @ 0.4	

Posterior Graphic. Threshold Marked @ 0.4

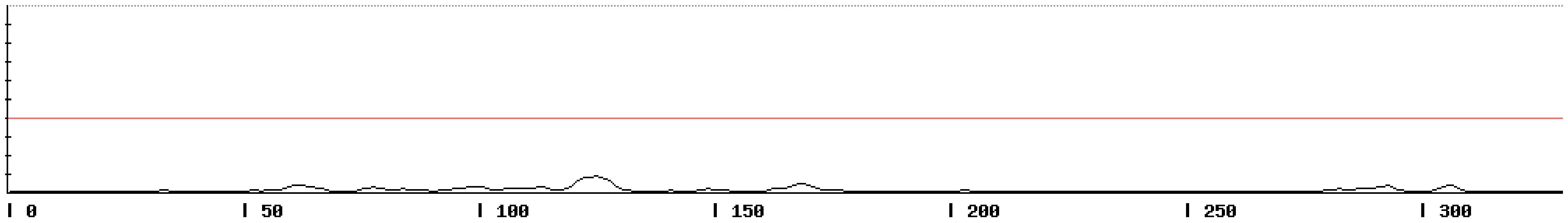

Predictions for Gossypiumraimondii_Gorai.007G043900.1

Viterbi Path

Viterbi Path

Posterior @ 0.4

Posterior @ 0.4

Posterior Graphic. Threshold Marked @ 0.4

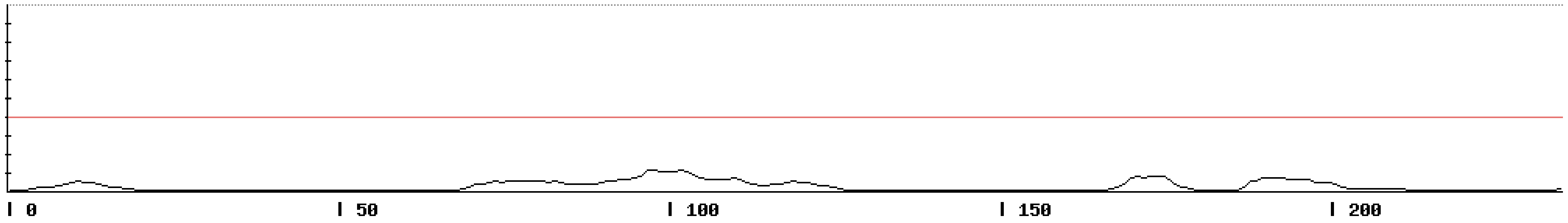

Predictions for Gossypiumraimondii_Gorai.007G048000.1

Viterbi Path

Viterbi Path

Posterior @ 0.4

Posterior @ 0.4

Posterior Graphic. Threshold Marked @ 0.4

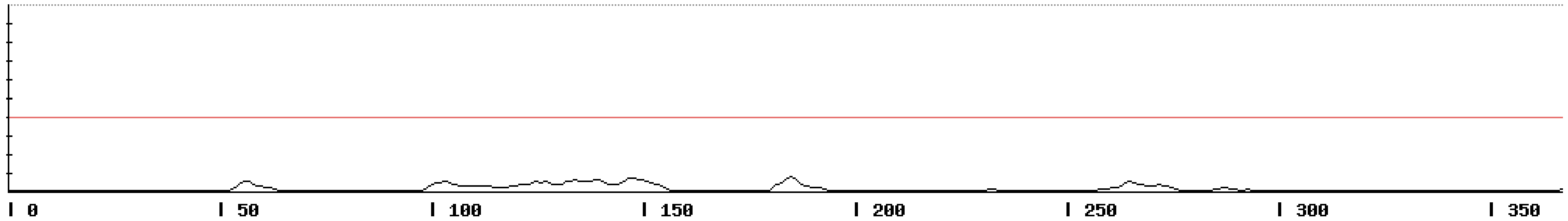

Predictions for Gossypiumraimondii_Gorai.007G065300.1

Viterbi Path

Viterbi Path

Posterior @ 0.4

Posterior @ 0.4

Posterior Graphic. Threshold Marked @ 0.4

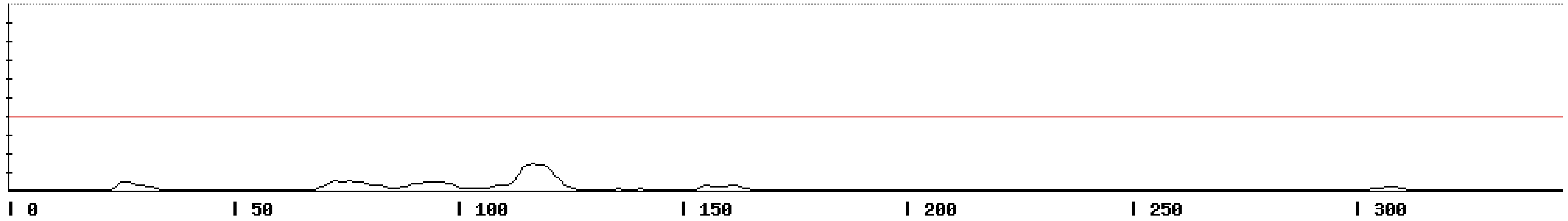

Predictions for Gossypiumraimondii_Gorai.007G075300.1

Viterbi Path

Viterbi Path

Posterior @ 0.4	129 - RKRRRIQNRHS - 139
	144 - HVRWHKTGKTKPVIENGVRKGWKKIMVLYKRSNRAHKP - 181

Posterior @ 0.4

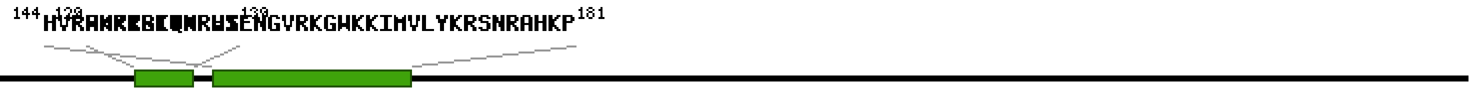

Posterior Graphic. Threshold Marked @ 0.4

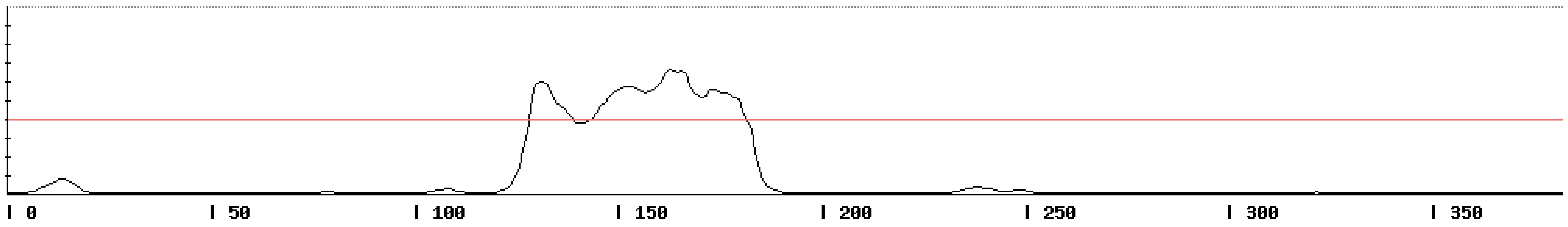

Predictions for Gossypiumraimondii_Gorai.007G079900.1

Viterbi Path

Viterbi Path

Posterior @ 0.4

Posterior @ 0.4

Posterior Graphic. Threshold Marked @ 0.4

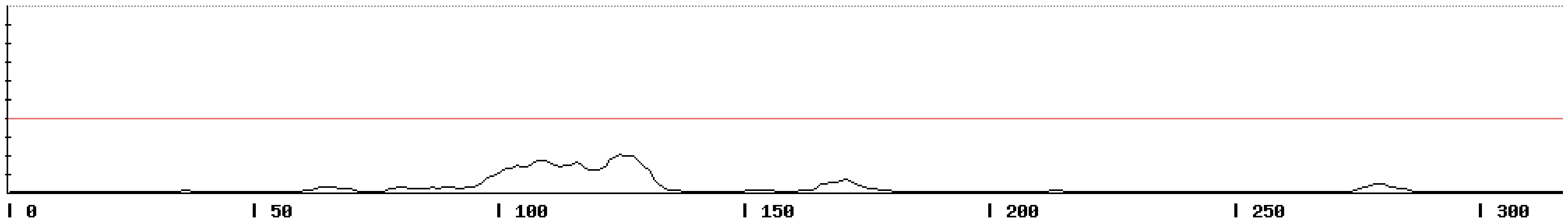

Predictions for Gossypiumraimondii_Gorai.007G112500.1

Viterbi Path	
Viterbi Path	
Posterior @ 0.4	
Posterior @ 0.4	
Posterior Graphic. Threshold Marked @ 0.4	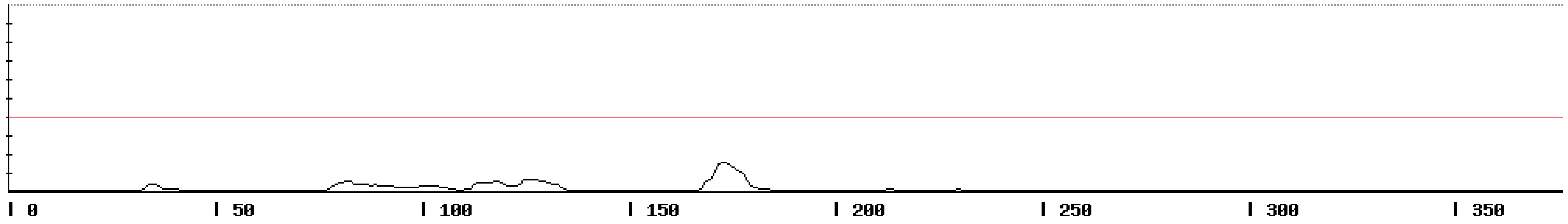

Predictions for Gossypiumraimondii_Gorai.007G114400.1

Viterbi Path	
Viterbi Path	
Posterior @ 0.4	
Posterior @ 0.4	
Posterior Graphic. Threshold Marked @ 0.4	

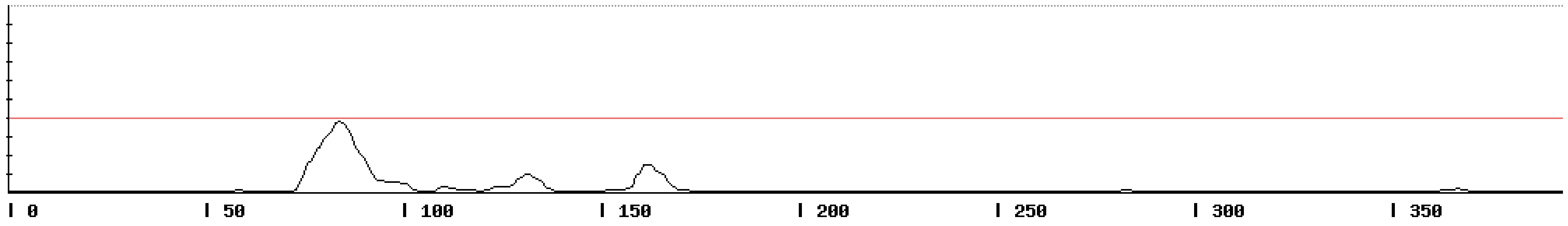

Predictions for Gossypiumraimondii_Gorai.007G114500.1

Viterbi Path	
Viterbi Path	
Posterior @ 0.4	
Posterior @ 0.4	
Posterior Graphic. Threshold Marked @ 0.4	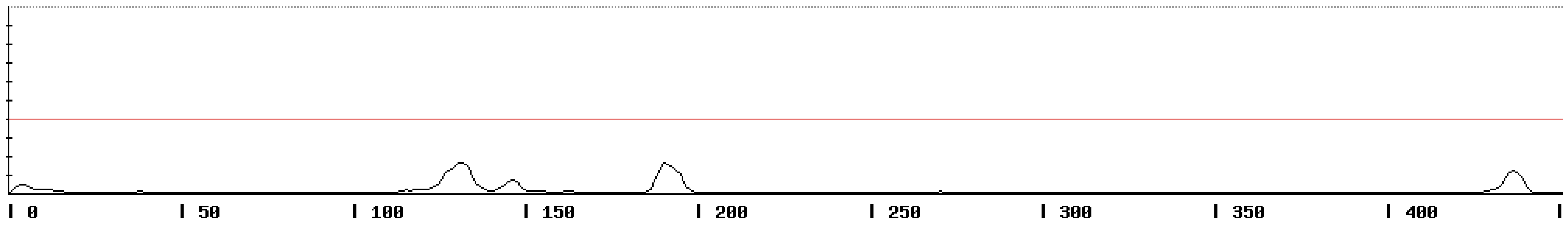

Predictions for Gossypiumraimondii_Gorai.007G147300.1

Viterbi Path	
Viterbi Path	
Posterior @ 0.4	
Posterior @ 0.4	

Posterior Graphic. Threshold Marked @ 0.4

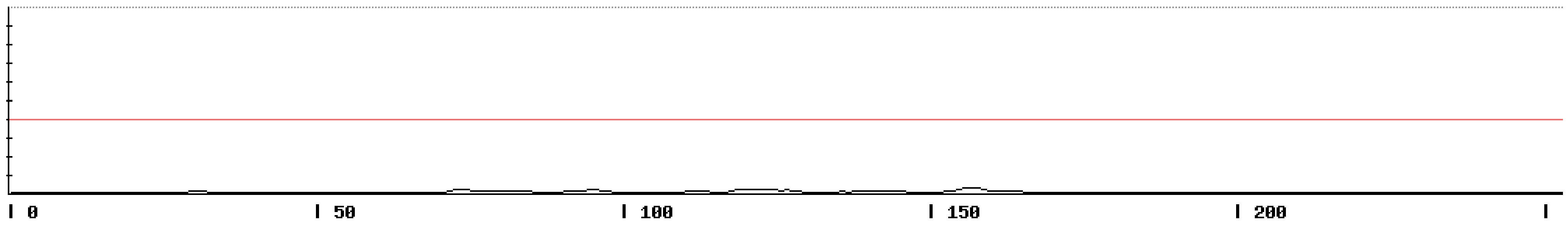

Predictions for Gossypiumraimondii_Gorai.007G188800.1

Viterbi Path

Viterbi Path

Posterior @ 0.4

Posterior @ 0.4

Posterior Graphic. Threshold Marked @ 0.4

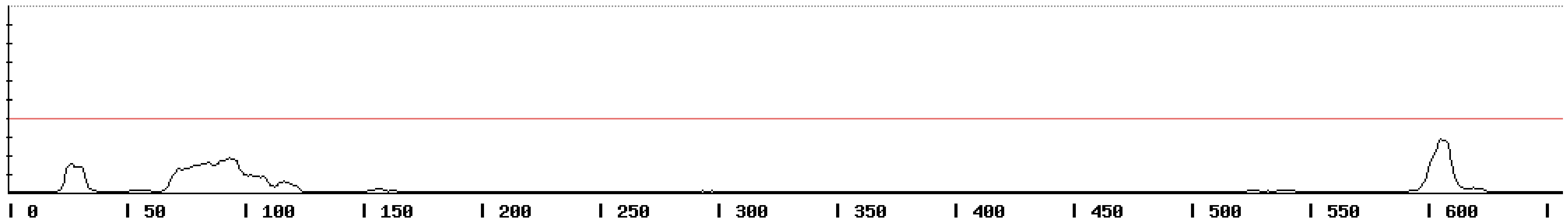

Predictions for Gossypiumraimondii_Gorai.007G196400.1

Viterbi Path

Viterbi Path

Posterior @ 0.4

Posterior @ 0.4

Posterior Graphic. Threshold Marked @ 0.4

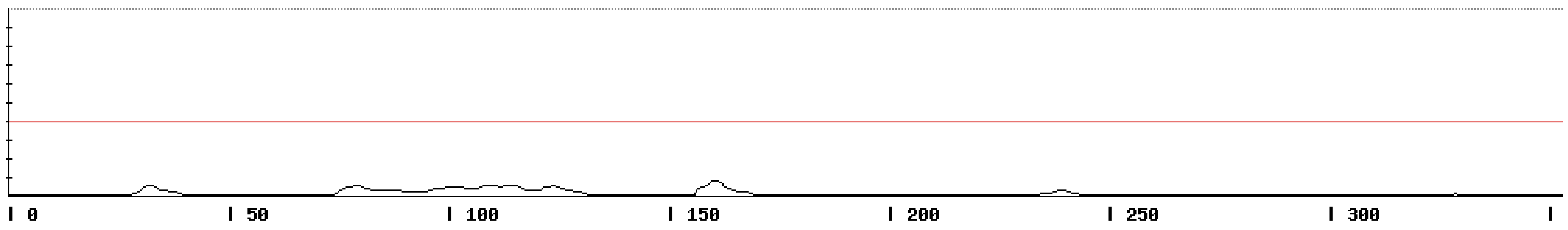

Predictions for Gossypiumraimondii_Gorai.007G267700.1

Viterbi Path

Viterbi Path

Posterior @ 0.4

Posterior @ 0.4

Posterior Graphic. Threshold Marked @ 0.4

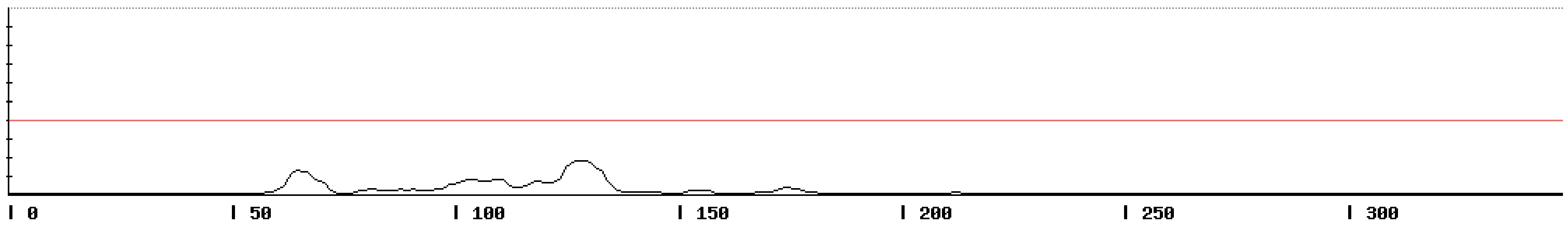

Predictions for Gossypiumraimondii_Gorai.007G267900.1

Viterbi Path

Viterbi Path

Posterior @ 0.4

Posterior @ 0.4

Posterior Graphic. Threshold Marked @ 0.4

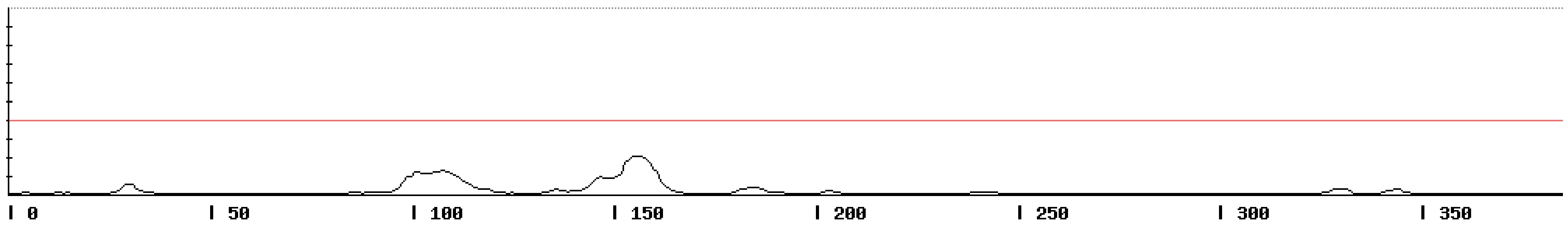

Predictions for Gossypiumraimondii_Gorai.007G323900.1

Viterbi Path

Viterbi Path

Posterior @ 0.4

Posterior @ 0.4

Posterior Graphic. Threshold Marked @ 0.4

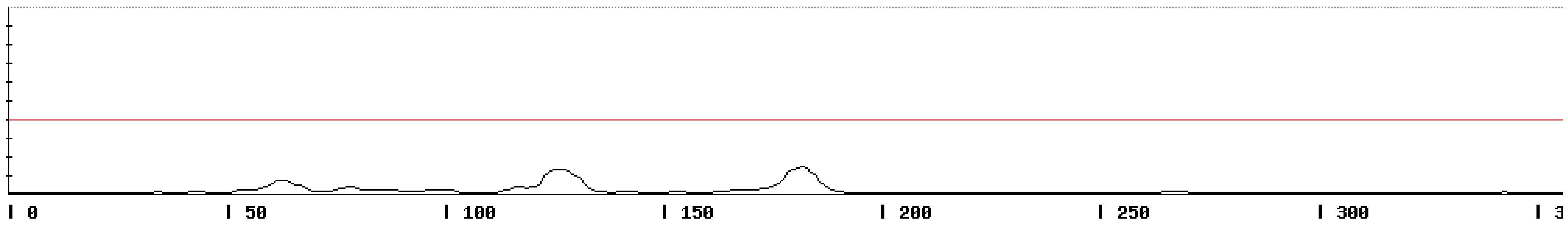

Predictions for Gossypiumraimondii_Gorai.008G014700.1

Viterbi Path

Viterbi Path

Posterior @ 0.4	
Posterior @ 0.4	
Posterior Graphic. Threshold Marked @ 0.4	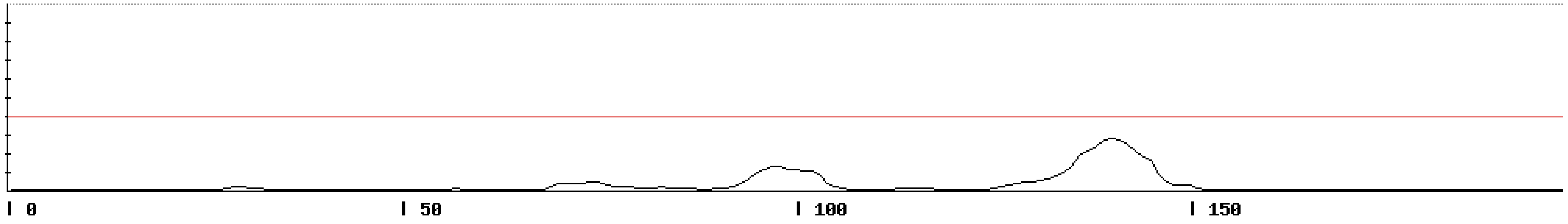

Predictions for Gossypiumraimondii_Gorai.008G038800.1	
Viterbi Path	
Viterbi Path	
Posterior @ 0.4	
Posterior @ 0.4	
Posterior Graphic. Threshold Marked @ 0.4	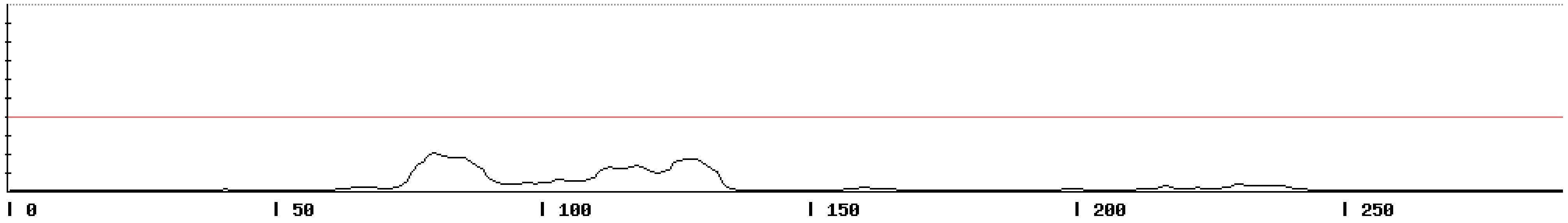

Predictions for Gossypiumraimondii_Gorai.008G063600.1

Viterbi Path

Viterbi Path

Posterior @ 0.4

Posterior @ 0.4

Posterior Graphic. Threshold Marked @ 0.4

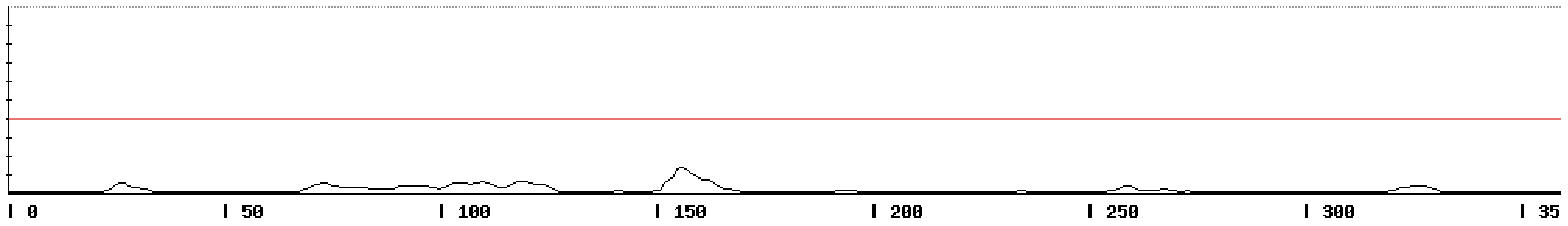

Predictions for Gossypiumraimondii_Gorai.008G130300.1

Viterbi Path

Viterbi Path

Posterior @ 0.4

Posterior @ 0.4

Posterior Graphic. Threshold Marked @ 0.4

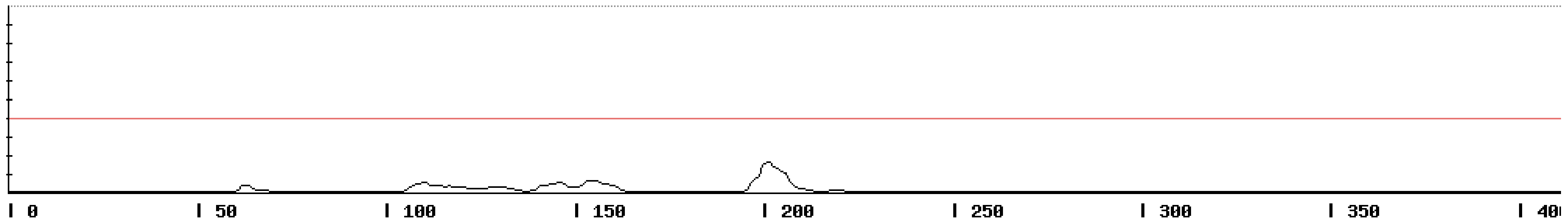

Predictions for Gossypiumraimondii_Gorai.008G155200.1

Viterbi Path	
Viterbi Path	
Posterior @ 0.4	137 - RKRRK - 141
Posterior @ 0.4	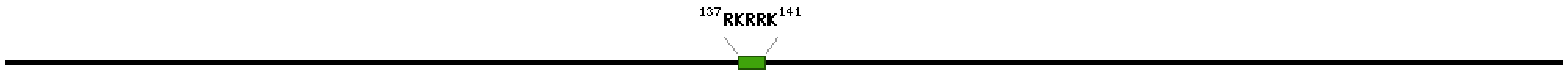
Posterior Graphic. Threshold Marked @ 0.4	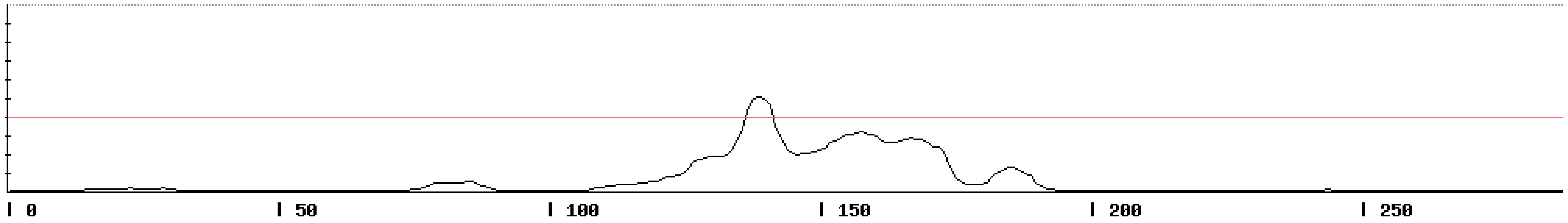

Predictions for Gossypiumraimondii_Gorai.008G179900.1

Viterbi Path	
Viterbi Path	
Posterior @ 0.4	
Posterior @ 0.4	
Posterior Graphic. Threshold Marked @ 0.4	

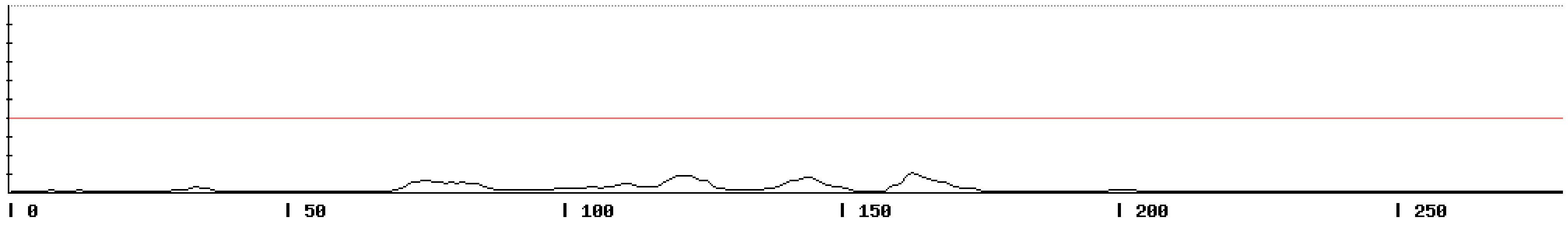

Predictions for Gossypiumraimondii_Gorai.008G186300.1

Viterbi Path	
Viterbi Path	
Posterior @ 0.4	132 - RKRRK - 136
Posterior @ 0.4	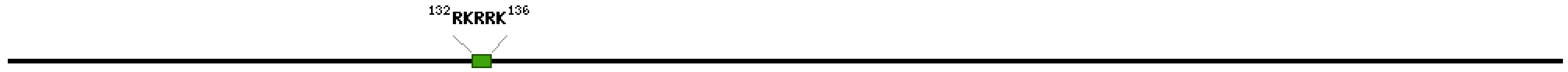
Posterior Graphic. Threshold Marked @ 0.4	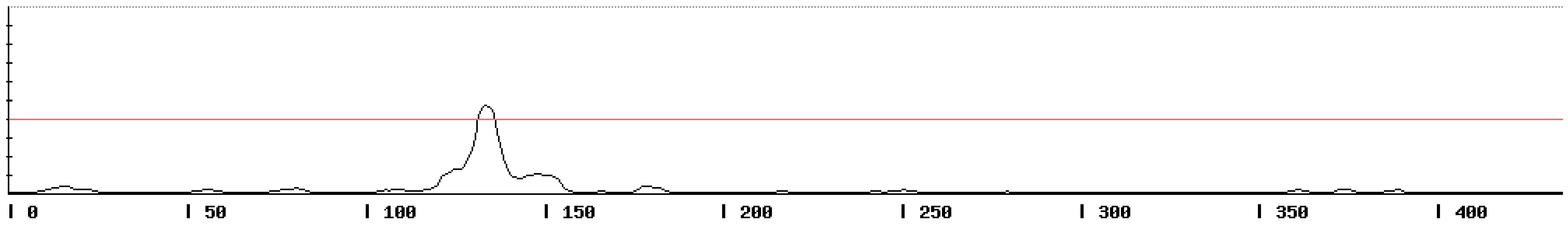

Predictions for Gossypiumraimondii_Gorai.008G194400.1

Viterbi Path	
Viterbi Path	
Posterior @ 0.4	
Posterior @ 0.4	

Posterior Graphic. Threshold Marked @ 0.4

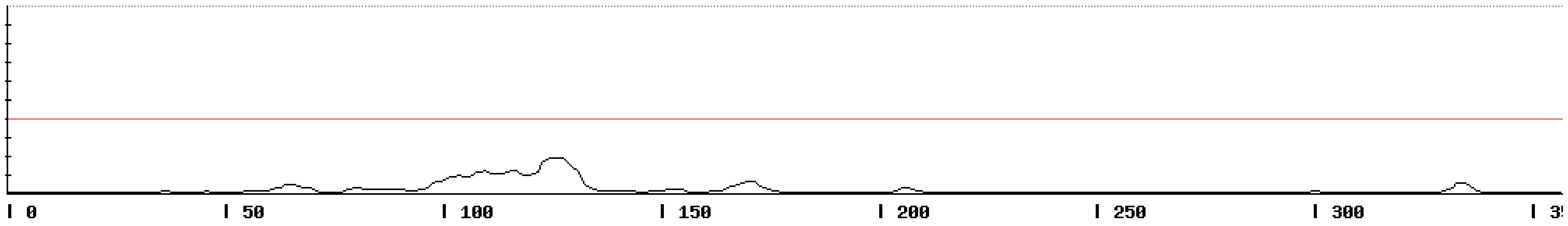

Predictions for Gossypiumraimondii_Gorai.008G197500.1	
Viterbi Path	
Viterbi Path	
Posterior @ 0.4	
Posterior @ 0.4	
Posterior Graphic. Threshold Marked @ 0.4	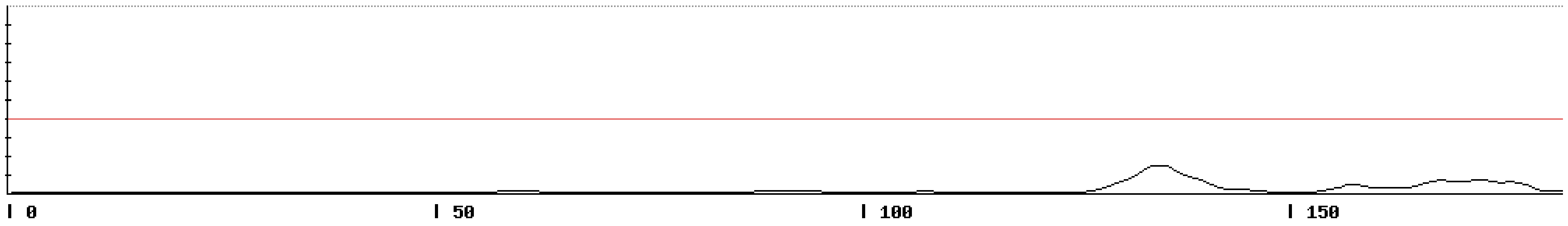

Predictions for Gossypiumraimondii_Gorai.008G227800.1	
Viterbi Path	
Viterbi Path	
Posterior @ 0.4	
Posterior @ 0.4	

Posterior Graphic. Threshold Marked @ 0.4	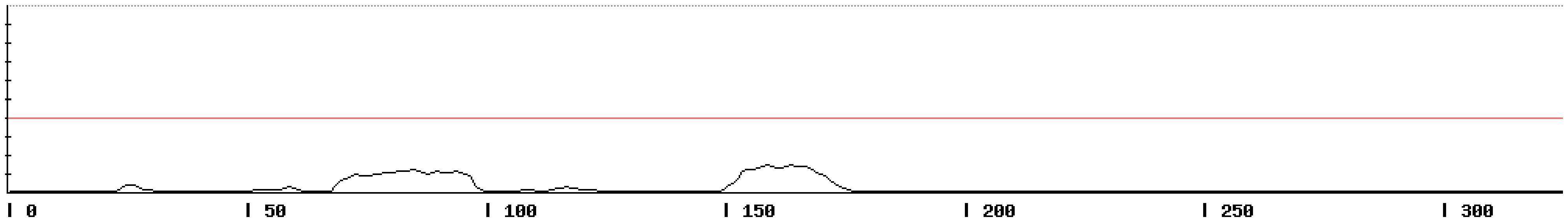

Predictions for Gossypiumraimondii_Gorai.008G236400.1

Viterbi Path	
Viterbi Path	
Posterior @ 0.4	
Posterior @ 0.4	
Posterior Graphic. Threshold Marked @ 0.4	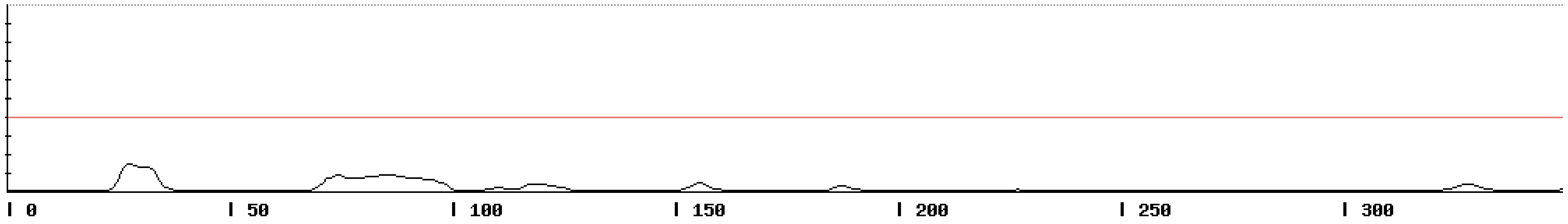

Predictions for Gossypiumraimondii_Gorai.008G259700.1

Viterbi Path	
Viterbi Path	

Posterior @ 0.4

Posterior @ 0.4

Posterior Graphic. Threshold Marked @ 0.4

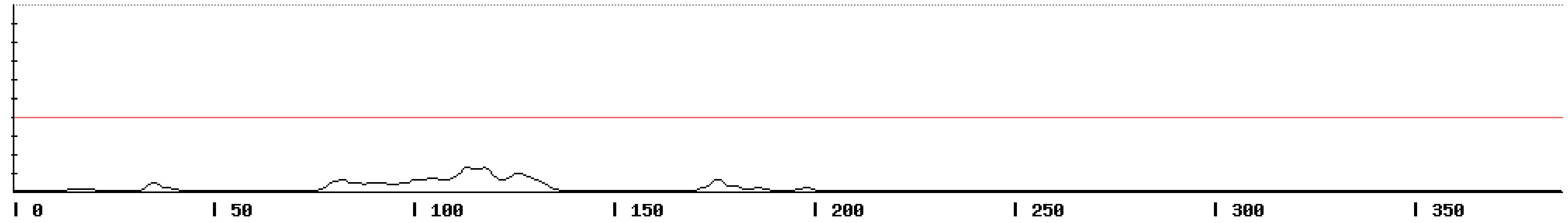

Predictions for Gossypiumraimondii_Gorai.008G261400.1

Viterbi Path

Viterbi Path

Posterior @ 0.4 — 93 - KATGKDRTIKSKKSLIGMKK - 112

Posterior @ 0.4

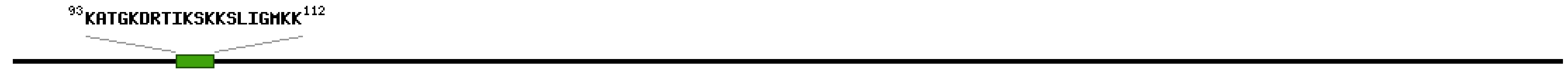

Posterior Graphic. Threshold Marked @ 0.4

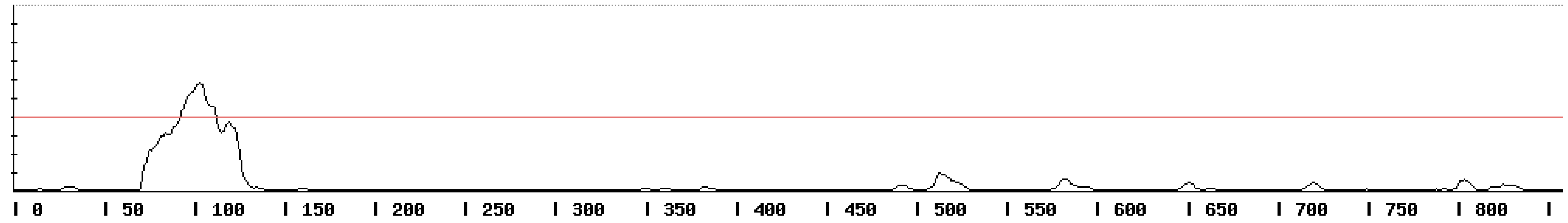

Predictions for Gossypiumraimondii_Gorai.009G028900.1

Viterbi Path

Viterbi Path

Posterior @ 0.4	
Posterior @ 0.4	
Posterior Graphic. Threshold Marked @ 0.4	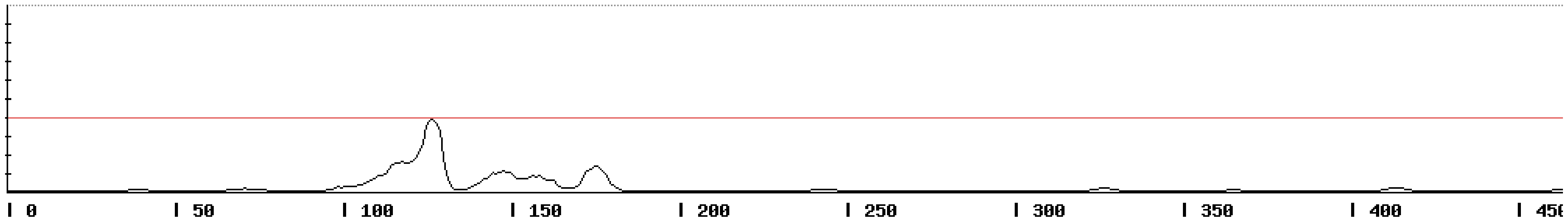

Predictions for Gossypiumraimondii_Gorai.009G083500.1

Viterbi Path	
Viterbi Path	
Posterior @ 0.4	312 - NRRIKLRKPK - 321
Posterior @ 0.4	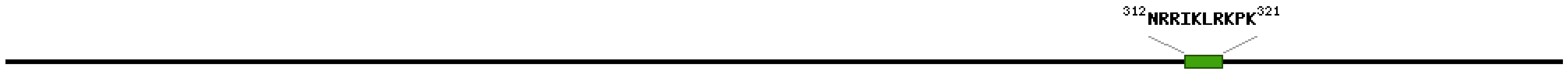
Posterior Graphic. Threshold Marked @ 0.4	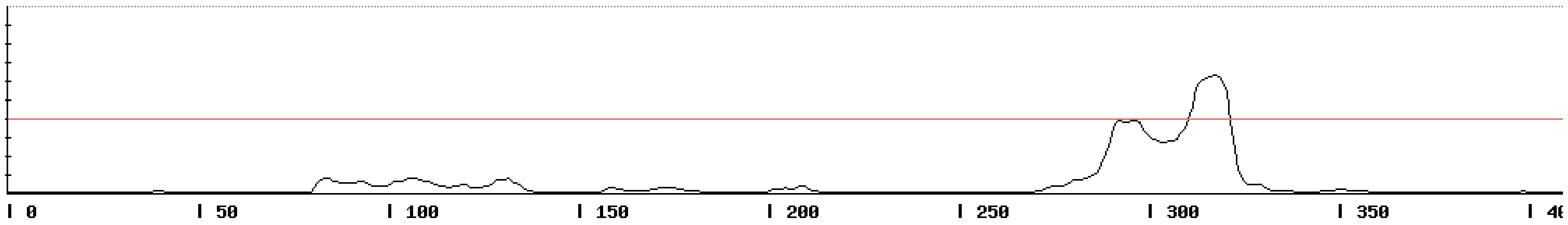

Predictions for Gossypiumraimondii_Gorai.009G166300.1

Viterbi Path	
Viterbi Path	
Posterior @ 0.4	146 - RKRRK - 150
Posterior @ 0.4	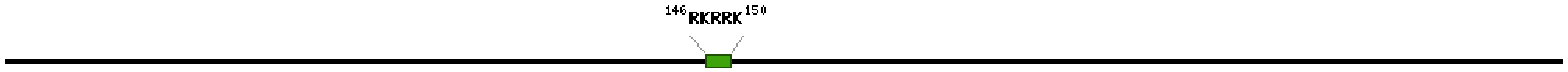
Posterior Graphic. Threshold Marked @ 0.4	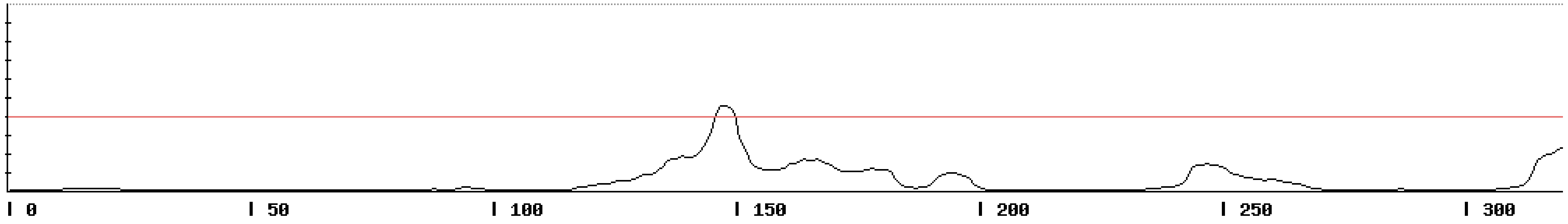

Predictions for Gossypiumraimondii_Gorai.009G170700.1

Viterbi Path	
Viterbi Path	
Posterior @ 0.4	
Posterior @ 0.4	
Posterior Graphic. Threshold Marked @ 0.4	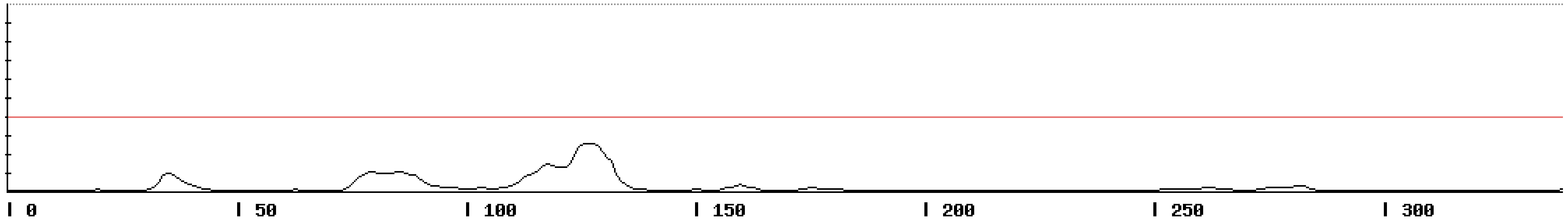

Predictions for Gossypiumraimondii_Gorai.009G186000.1	
Viterbi Path	
Viterbi Path	
Posterior @ 0.4	
Posterior @ 0.4	
Posterior Graphic. Threshold Marked @ 0.4	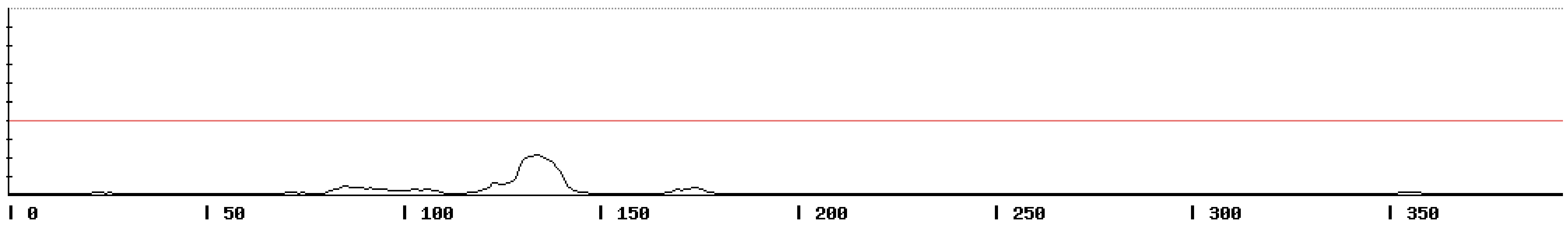

Predictions for Gossypiumraimondii_Gorai.009G204700.1	
Viterbi Path	
Viterbi Path	
Posterior @ 0.4	312 - KRAKVEEGPSSSRKPSESHGGRRKGGSLGGMLRK - 345
Posterior @ 0.4	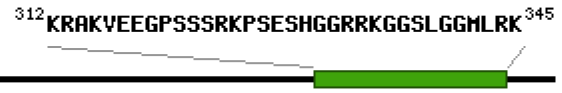
Posterior Graphic. Threshold Marked @ 0.4	

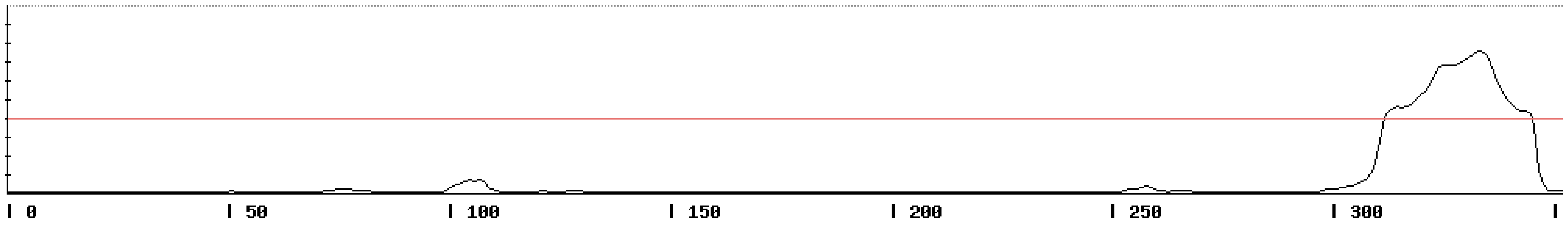

**Predictions for Gossypiumraimondii_Gorai.009G214000.1**	
Viterbi Path	100 - KSKGGRGRRRAKGGFWRKIGKVK - 122
Viterbi Path	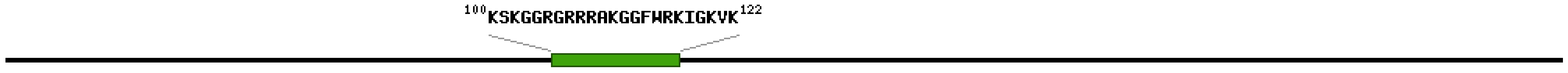
Posterior @ 0.4	99 - TKSKGGRGRRRAKGGFWRKIGKVK - 122
Posterior @ 0.4	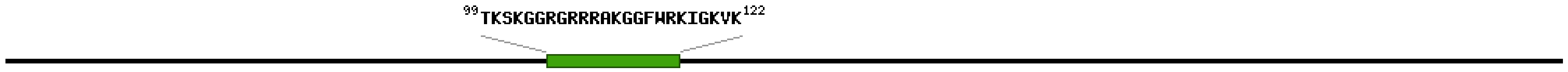
Posterior Graphic. Threshold Marked @ 0.4	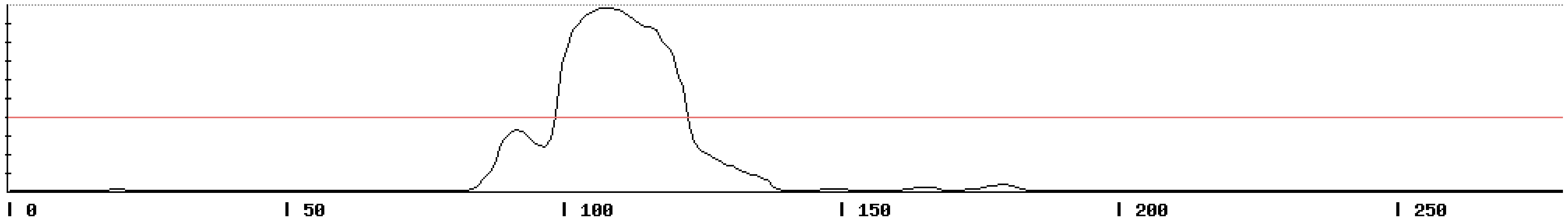

**Predictions for Gossypiumraimondii_Gorai.009G260000.1**	
Viterbi Path	
Viterbi Path	
Posterior @ 0.4	
Posterior @ 0.4	

Posterior Graphic. Threshold Marked @ 0.4	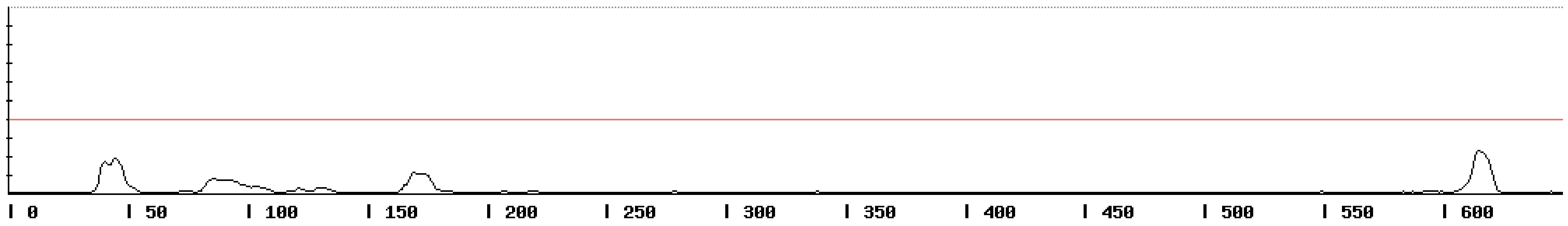

Predictions for Gossypiumraimondii_Gorai.009G309000.1	
Viterbi Path	
Viterbi Path	
Posterior @ 0.4	
Posterior @ 0.4	
Posterior Graphic. Threshold Marked @ 0.4	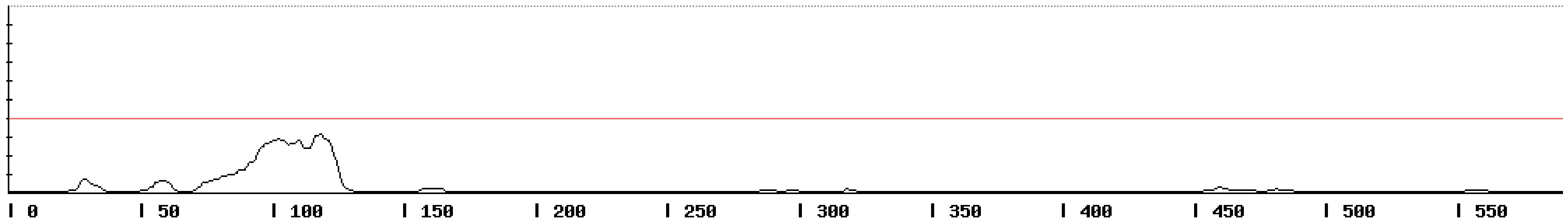

Predictions for Gossypiumraimondii_Gorai.009G309300.1	
Viterbi Path	
Viterbi Path	
Posterior @ 0.4	
Posterior @ 0.4	

Posterior Graphic. Threshold Marked @ 0.4

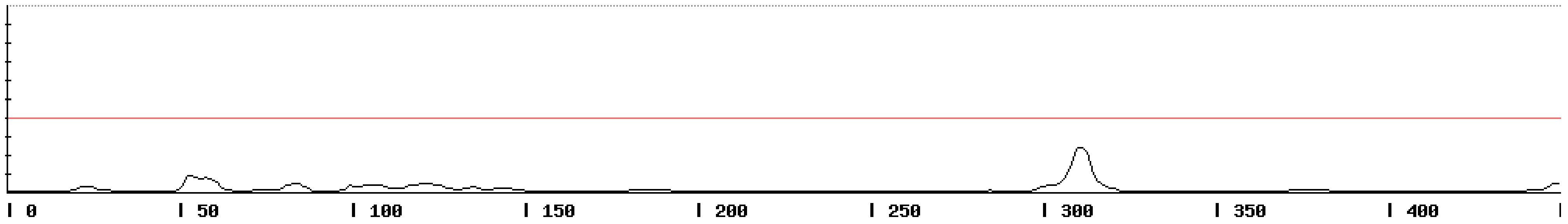

Predictions for Gossypiumraimondii_Gorai.009G354900.1

Viterbi Path

Viterbi Path

Posterior @ 0.4

Posterior @ 0.4

Posterior Graphic. Threshold Marked @ 0.4

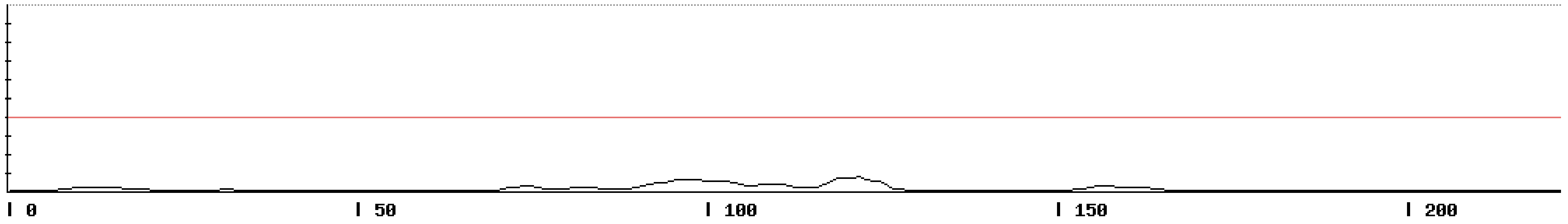

Predictions for Gossypiumraimondii_Gorai.009G357700.1

Viterbi Path

Viterbi Path

Posterior @ 0.4

Posterior @ 0.4

Posterior Graphic. Threshold Marked @ 0.4

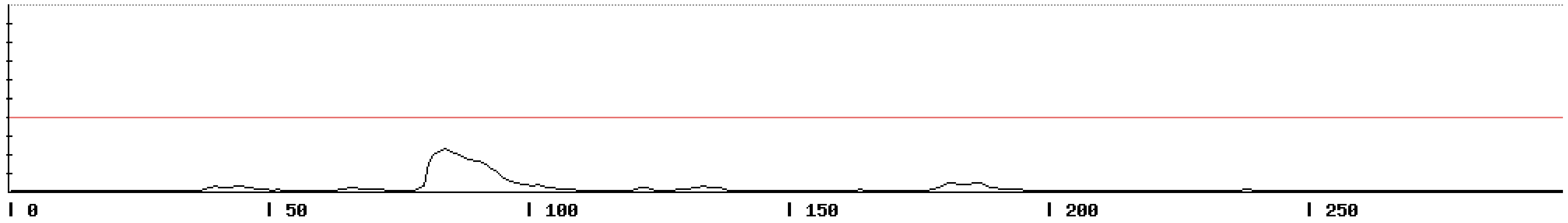

Predictions for Gossypiumraimondii_Gorai.009G399900.1

Viterbi Path

Viterbi Path

Posterior @ 0.4

Posterior @ 0.4

Posterior Graphic. Threshold Marked @ 0.4

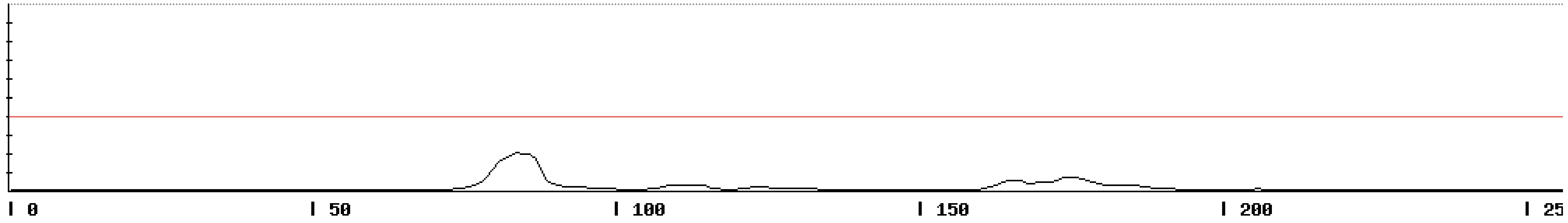

Predictions for Gossypiumraimondii_Gorai.009G418700.1

Viterbi Path

Viterbi Path

Posterior @ 0.4

Posterior @ 0.4

Posterior Graphic. Threshold Marked @ 0.4

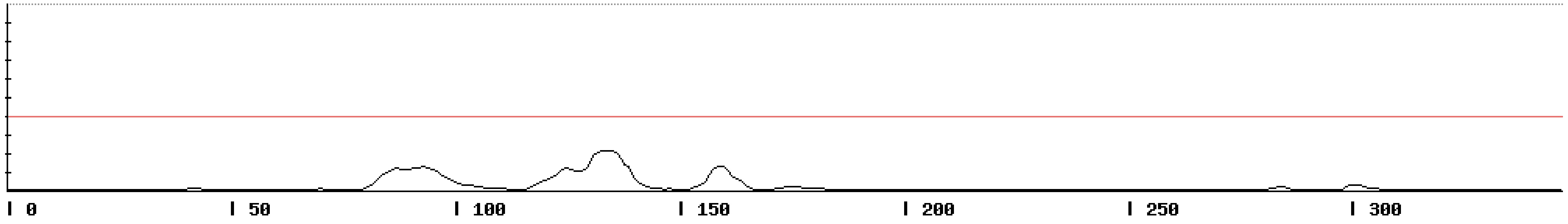

Predictions for Gossypiumraimondii_Gorai.009G433100.1

Viterbi Path

Viterbi Path

Posterior @ 0.4

Posterior @ 0.4

Posterior Graphic. Threshold Marked @ 0.4

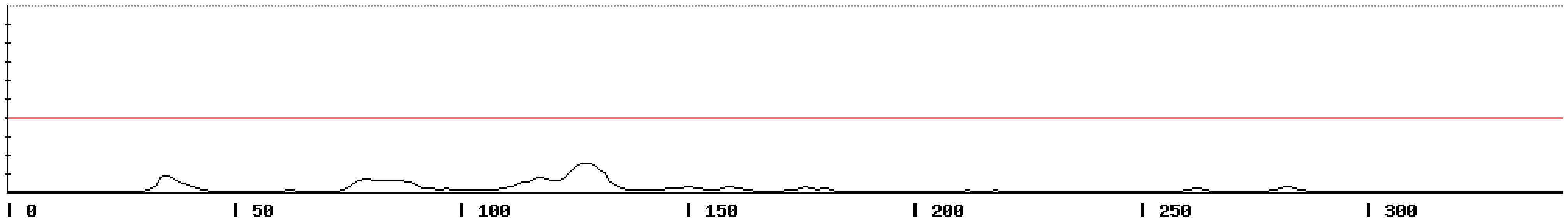

Predictions for Gossypiumraimondii_Gorai.009G433200.1

Viterbi Path

Viterbi Path

Posterior @ 0.4

Posterior @ 0.4

Posterior Graphic. Threshold Marked @ 0.4

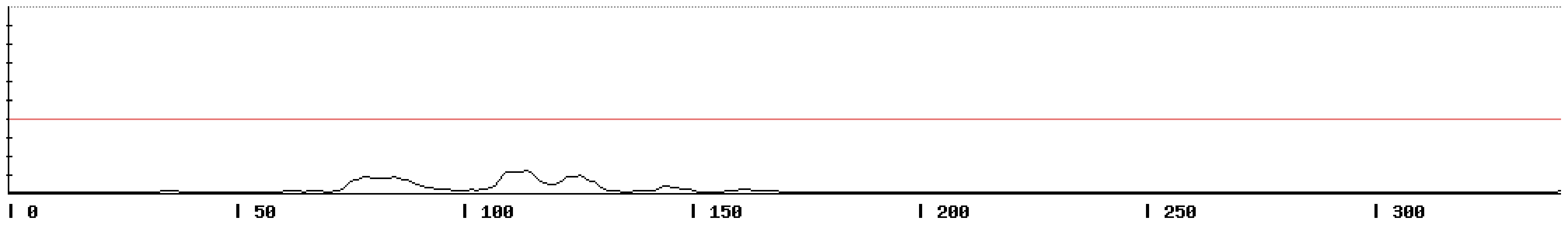

Predictions for Gossypiumraimondii_Gorai.010G012000.1

Viterbi Path

Viterbi Path

Posterior @ 0.4

Posterior @ 0.4

Posterior Graphic. Threshold Marked @ 0.4

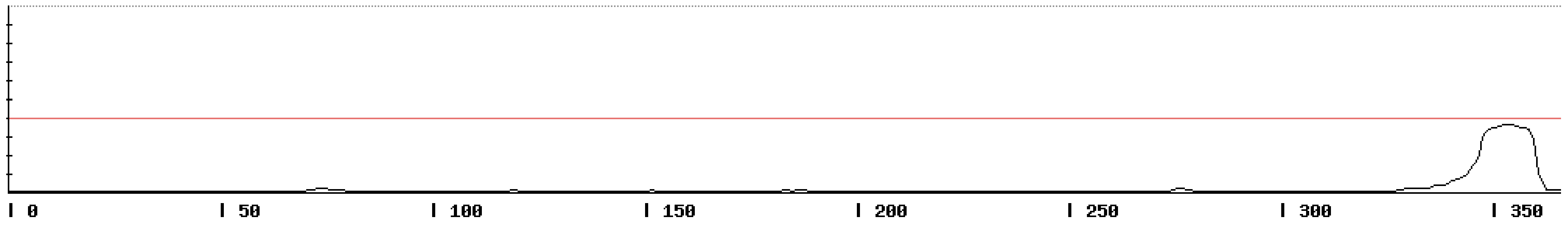

Predictions for Gossypiumraimondii_Gorai.010G051900.1	
Viterbi Path	
Viterbi Path	
Posterior @ 0.4	
Posterior @ 0.4	
Posterior Graphic. Threshold Marked @ 0.4	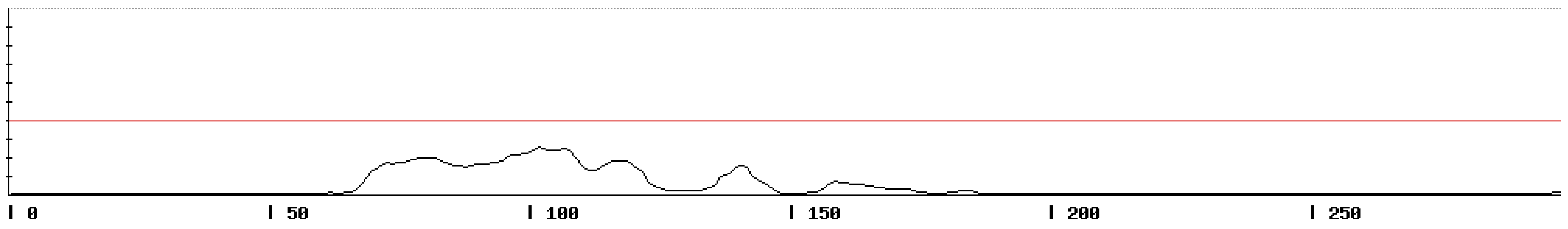

Predictions for Gossypiumraimondii_Gorai.010G078900.1	
Viterbi Path	
Viterbi Path	
Posterior @ 0.4	
Posterior @ 0.4	
Posterior Graphic. Threshold Marked @ 0.4	

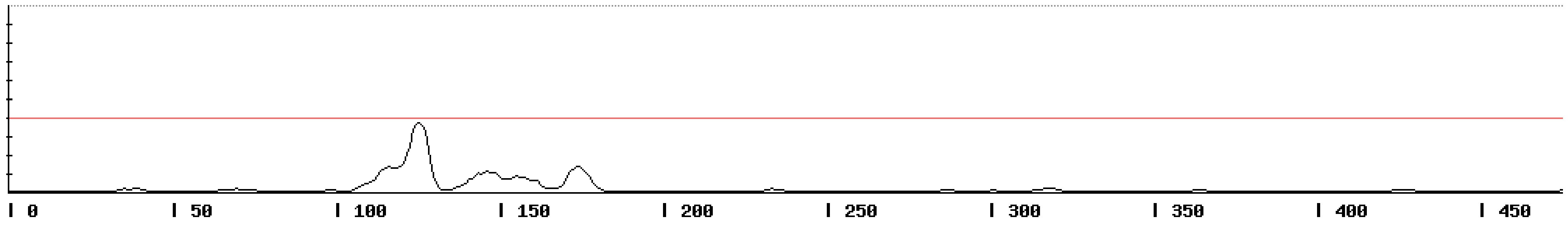

Predictions for Gossypiumraimondii_Gorai.010G124100.1

Viterbi Path	
Viterbi Path	
Posterior @ 0.4	
Posterior @ 0.4	
Posterior Graphic. Threshold Marked @ 0.4	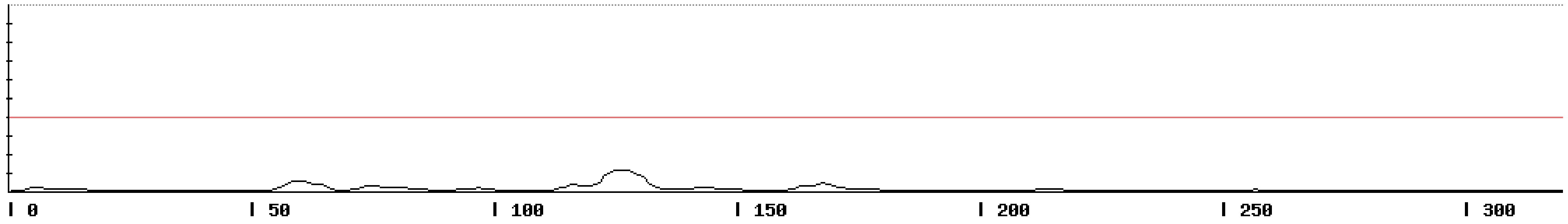

Predictions for Gossypiumraimondii_Gorai.010G142400.1

Viterbi Path	
Viterbi Path	
Posterior @ 0.4	
Posterior @ 0.4	

Posterior Graphic. Threshold Marked @ 0.4

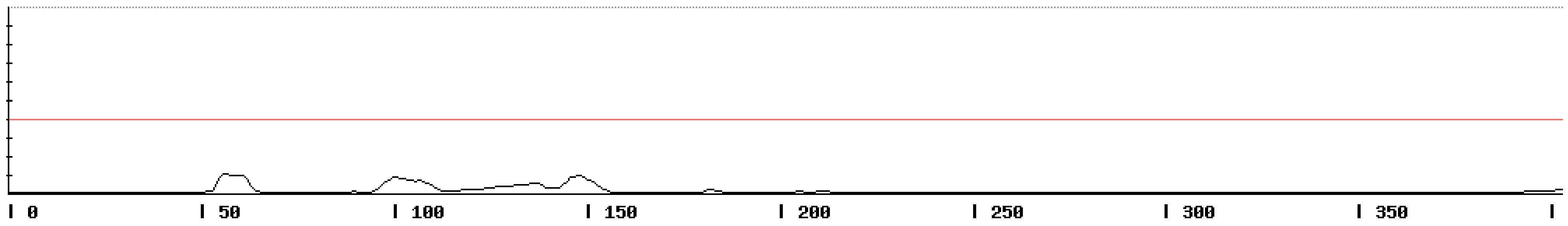

Predictions for Gossypiumraimondii_Gorai.010G151400.1

Viterbi Path

Viterbi Path

Posterior @ 0.4

Posterior @ 0.4

Posterior Graphic. Threshold Marked @ 0.4

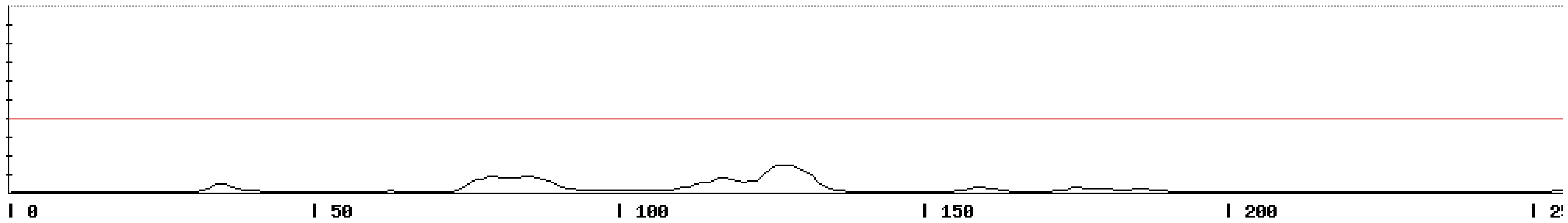

Predictions for Gossypiumraimondii_Gorai.010G157600.1

Viterbi Path

Viterbi Path

Posterior @ 0.4 — 135 - RKRRK - 139

Posterior @ 0.4

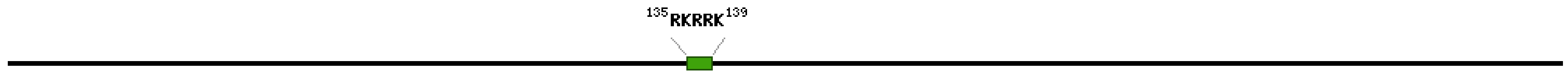

Posterior Graphic. Threshold Marked @ 0.4

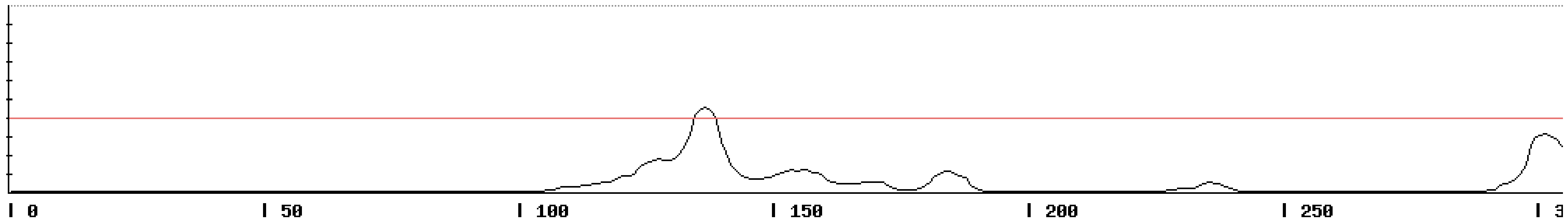

Predictions for Gossypiumraimondii_Gorai.010G171100.1

Viterbi Path

Viterbi Path

Posterior @ 0.4

Posterior @ 0.4

Posterior Graphic. Threshold Marked @ 0.4

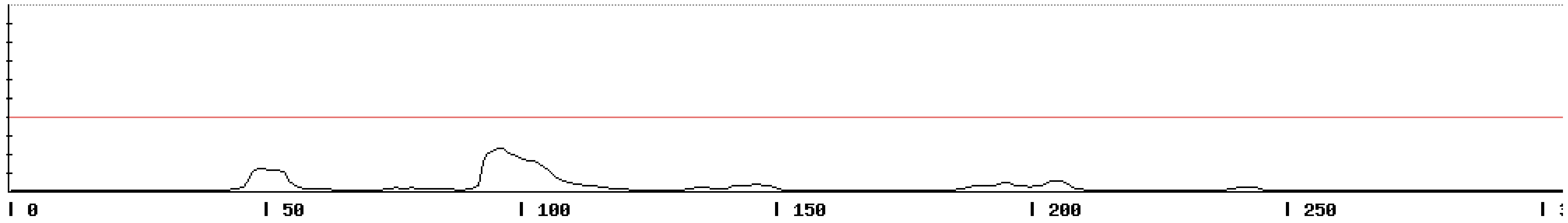

Predictions for Gossypiumraimondii_Gorai.010G236700.1

Viterbi Path

Viterbi Path

Posterior @ 0.4

Posterior @ 0.4

Posterior Graphic. Threshold Marked @ 0.4

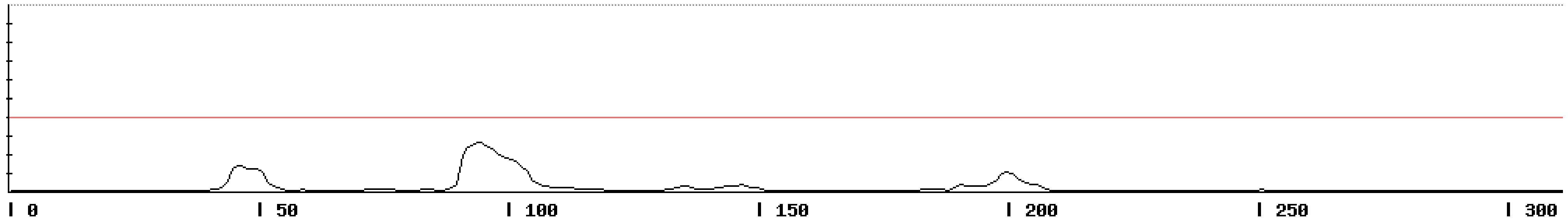

Predictions for Gossypiumraimondii_Gorai.010G251100.1

Viterbi Path

Viterbi Path

Posterior @ 0.4 77 - KLKKKSKGK - 85

Posterior @ 0.4

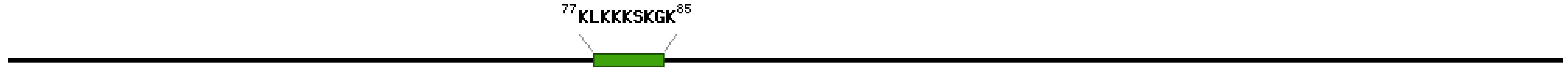

Posterior Graphic. Threshold Marked @ 0.4

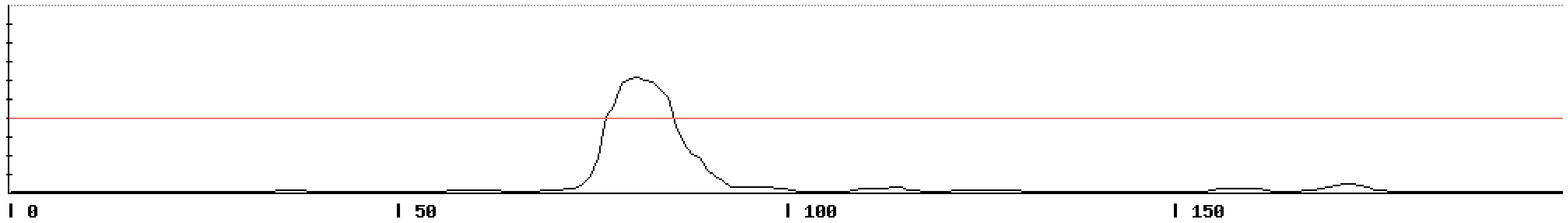

Predictions for Gossypiumraimondii_Gorai.011G030600.1

Viterbi Path

Viterbi Path

Posterior @ 0.4

Posterior @ 0.4

Posterior Graphic. Threshold Marked @ 0.4

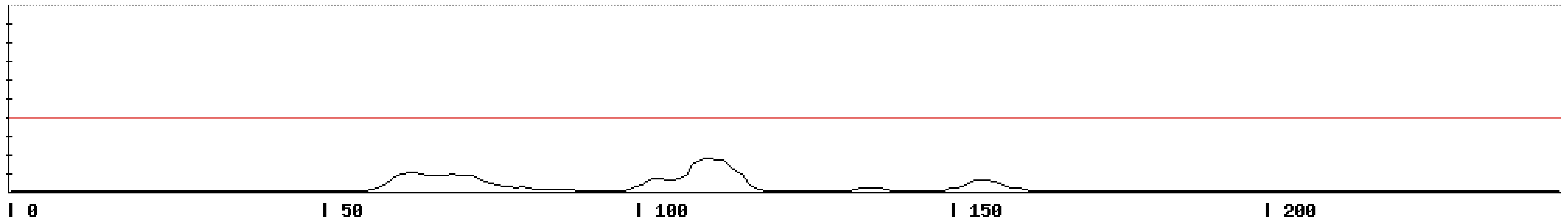

Predictions for Gossypiumraimondii_Gorai.011G087700.1

Viterbi Path

Viterbi Path

Posterior @ 0.4

166 - QRKRSRRGKKH - 176

Posterior @ 0.4

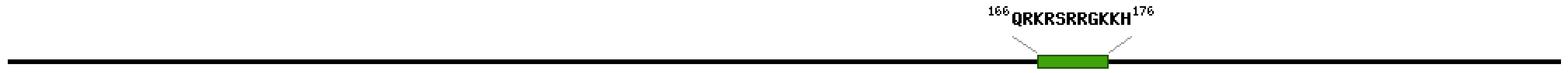

Posterior Graphic. Threshold Marked @ 0.4

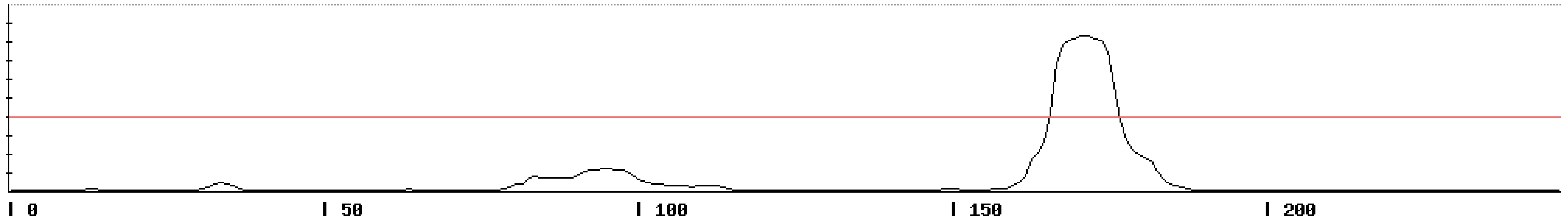

Predictions for Gossypiumraimondii_Gorai.011G090000.1

Viterbi Path

Viterbi Path

Posterior @ 0.4

Posterior @ 0.4

Posterior Graphic. Threshold Marked @ 0.4

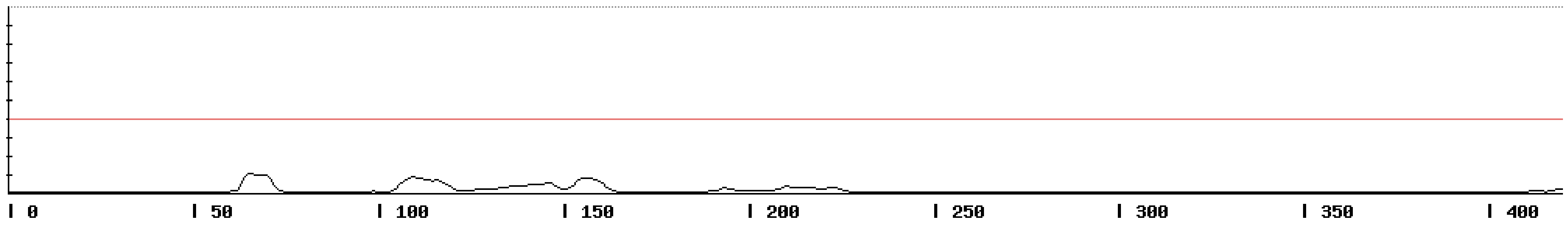

Predictions for Gossypiumraimondii_Gorai.011G234200.1

Viterbi Path

Viterbi Path

Posterior @ 0.4

Posterior @ 0.4

Posterior Graphic. Threshold Marked @ 0.4

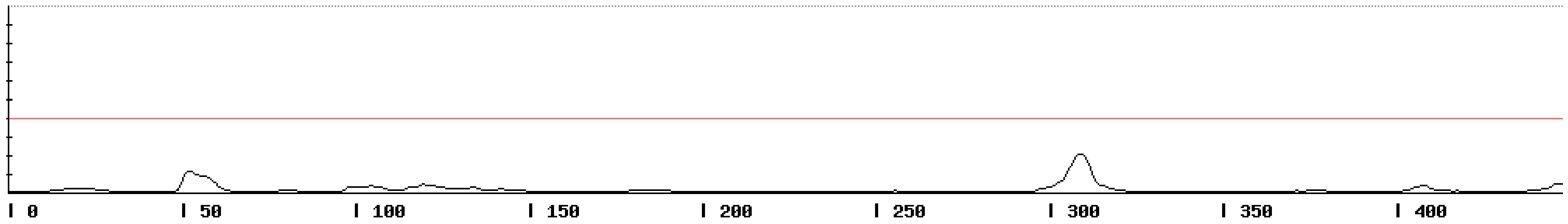

Predictions for Gossypiumraimondii_Gorai.011G239800.1	
Viterbi Path	
Viterbi Path	
Posterior @ 0.4	
Posterior @ 0.4	
Posterior Graphic. Threshold Marked @ 0.4	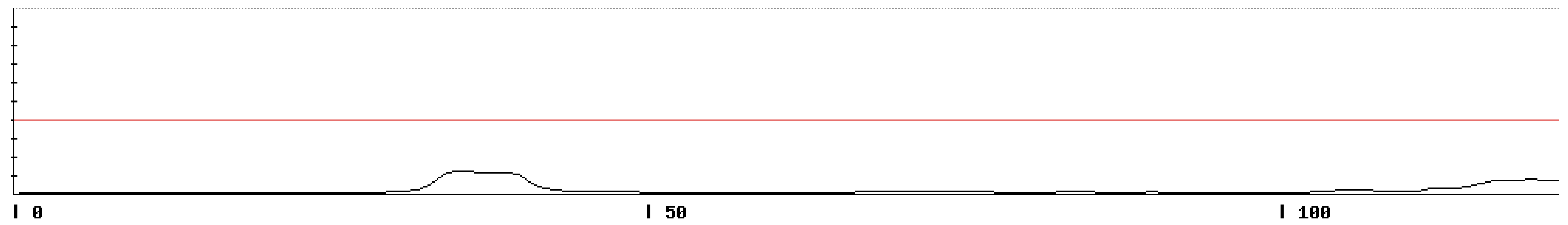

Predictions for Gossypiumraimondii_Gorai.011G257000.1	
Viterbi Path	
Viterbi Path	
Posterior @ 0.4	95 - LKKKSKS - 101<br>193 - KKKKRK - 198
Posterior @ 0.4	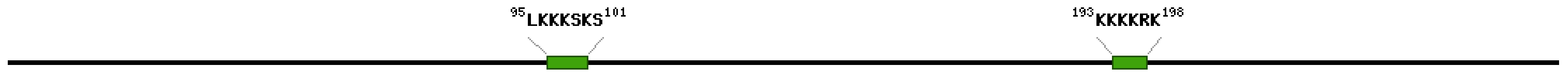
Posterior Graphic. Threshold Marked @ 0.4	

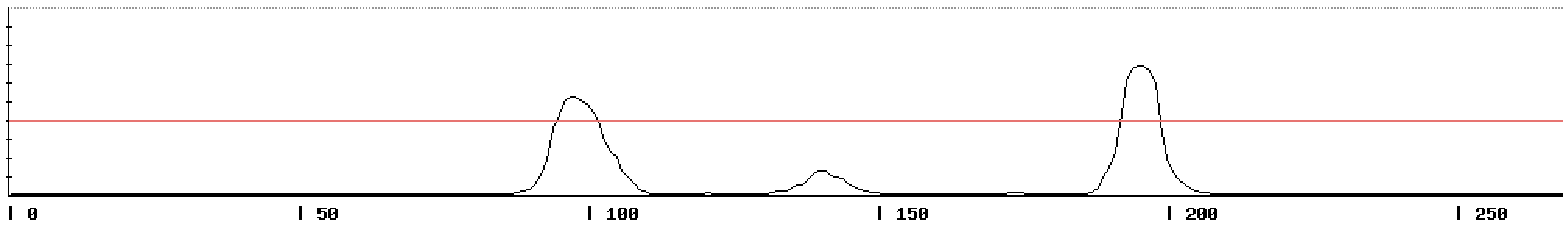

Predictions for Gossypiumraimondii_Gorai.011G269400.1

Viterbi Path

Viterbi Path

Posterior @ 0.4

Posterior @ 0.4

Posterior Graphic. Threshold Marked @ 0.4

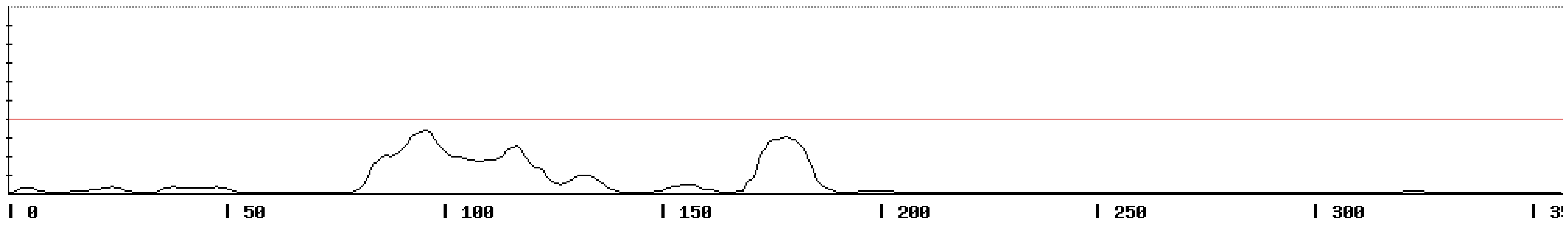

Predictions for Gossypiumraimondii_Gorai.011G269500.1

Viterbi Path

Viterbi Path

Posterior @ 0.4

Posterior @ 0.4

Posterior Graphic. Threshold Marked @ 0.4

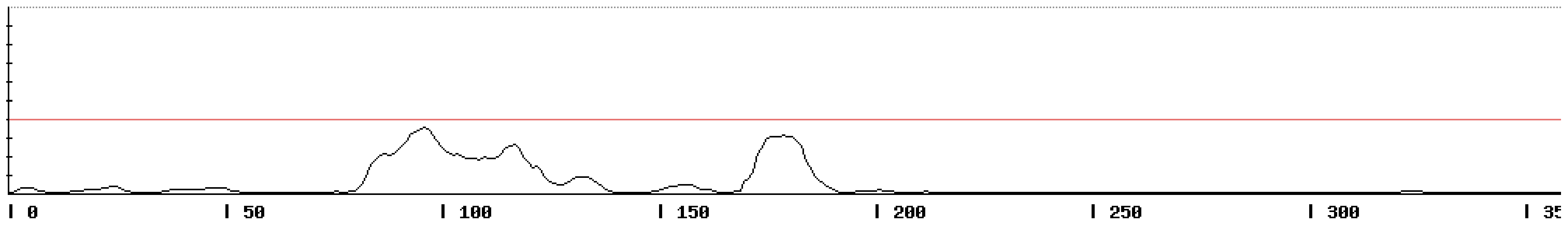

Predictions for Gossypiumraimondii_Gorai.011G271300.1

Viterbi Path

Viterbi Path

Posterior @ 0.4 — 109 - RKGKKIGLKK - 118

Posterior @ 0.4

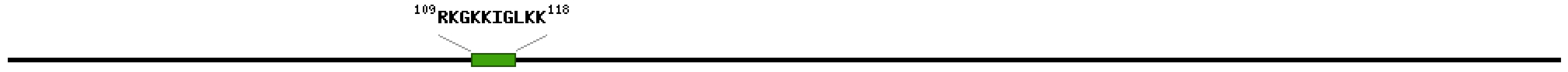

Posterior Graphic. Threshold Marked @ 0.4

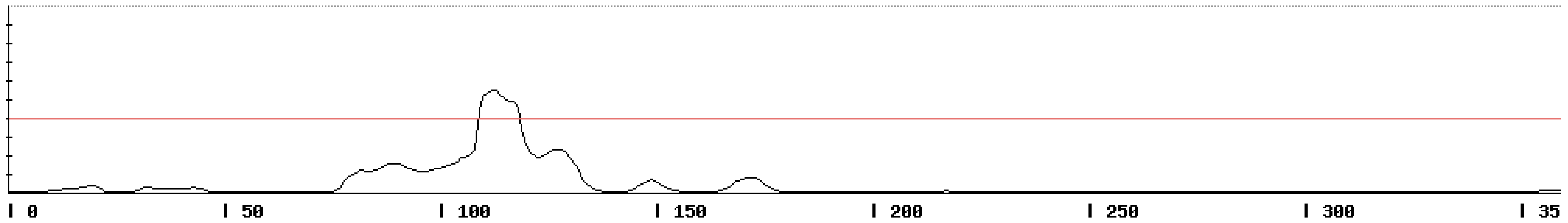

Predictions for Gossypiumraimondii_Gorai.011G271500.1

Viterbi Path

Viterbi Path

Posterior @ 0.4 — 109 - RKGKKIGLKK - 118

Posterior @ 0.4

	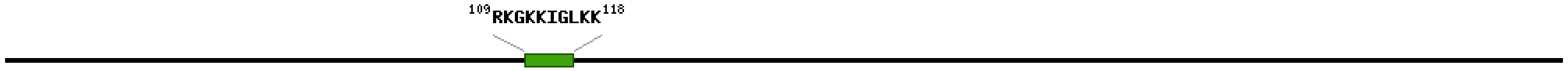
Posterior Graphic. Threshold Marked @ 0.4	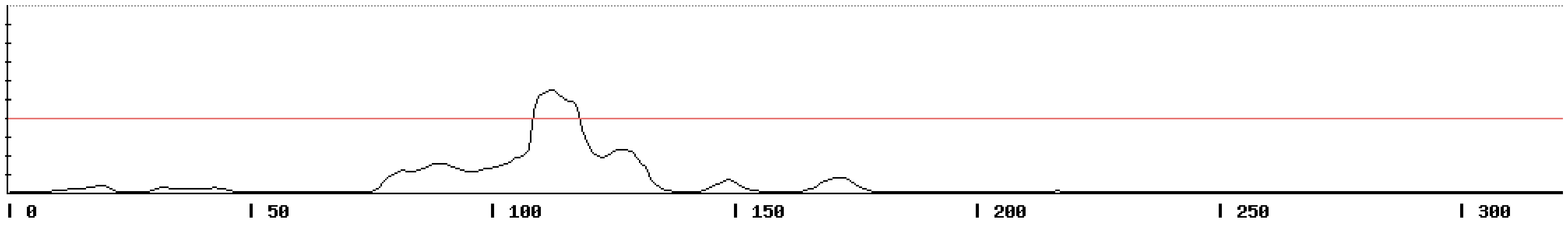

Predictions for Gossypiumraimondii_Gorai.012G007000.1	
Viterbi Path	
Viterbi Path	
Posterior @ 0.4	352 - KKKKP - 356
Posterior @ 0.4	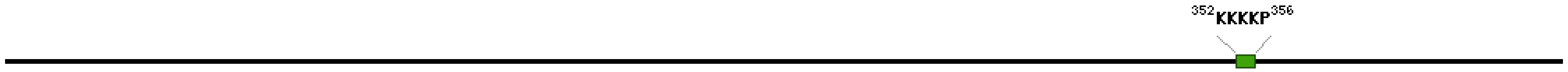
Posterior Graphic. Threshold Marked @ 0.4	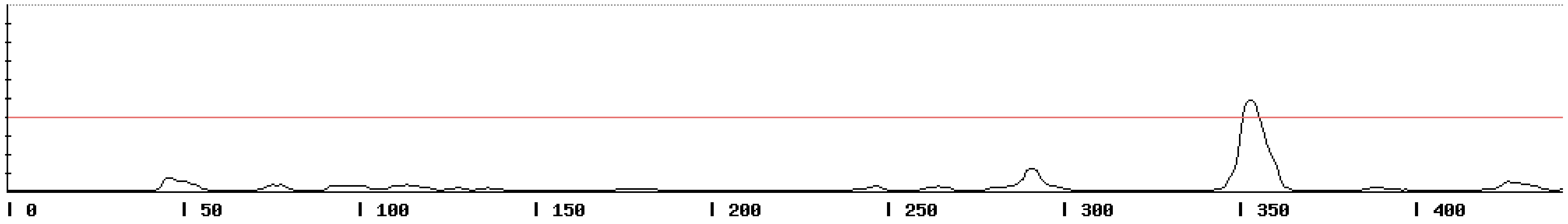

Predictions for Gossypiumraimondii_Gorai.012G036100.1	
Viterbi Path	
Viterbi Path	

Posterior @ 0.4

Posterior @ 0.4

Posterior Graphic. Threshold Marked @ 0.4

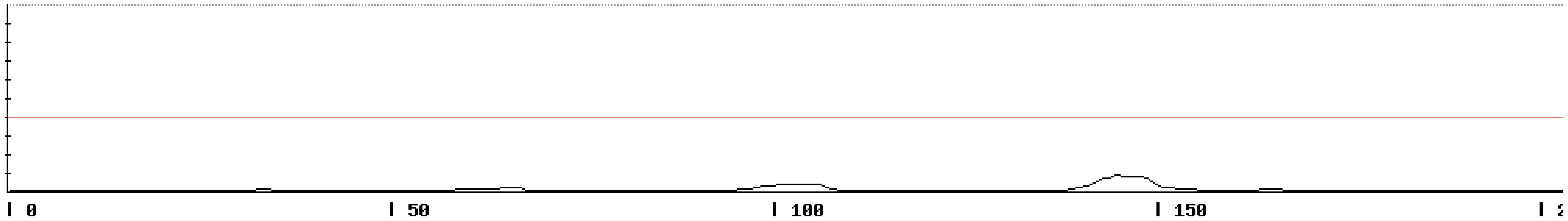

Predictions for Gossypiumraimondii_Gorai.012G037600.1

Viterbi Path

Viterbi Path

Posterior @ 0.4

Posterior @ 0.4

Posterior Graphic. Threshold Marked @ 0.4

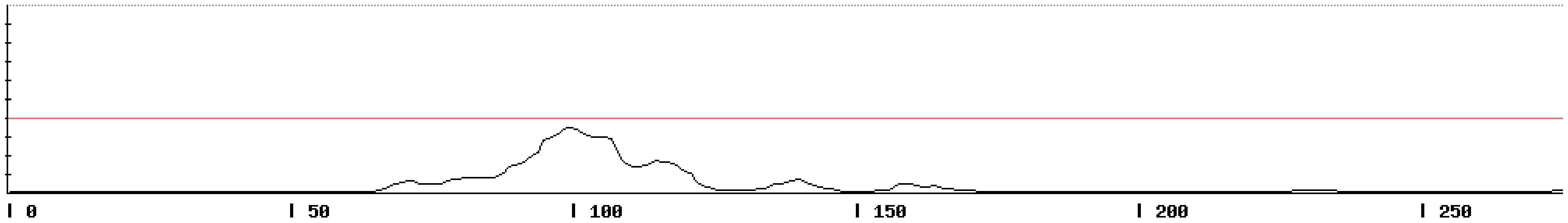

Predictions for Gossypiumraimondii_Gorai.012G054800.1

Viterbi Path

Viterbi Path

Posterior @ 0.4

Posterior @ 0.4

Posterior Graphic. Threshold Marked @ 0.4

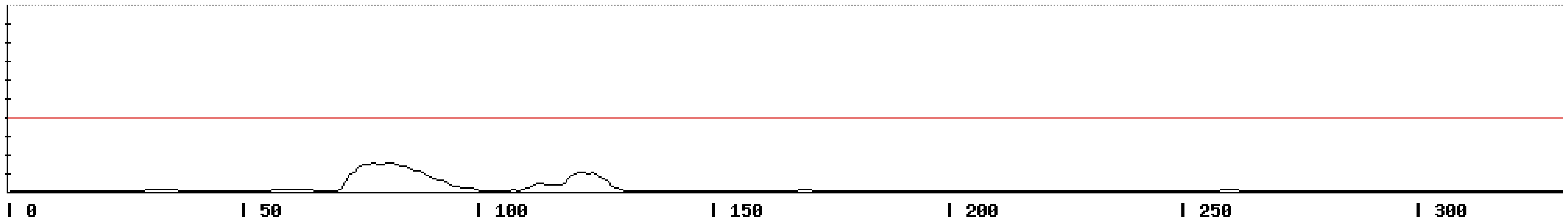

Predictions for Gossypiumraimondii_Gorai.012G073400.1

Viterbi Path

Viterbi Path

Posterior @ 0.4 — 101 - RKHPQGKRPNR - 111

Posterior @ 0.4

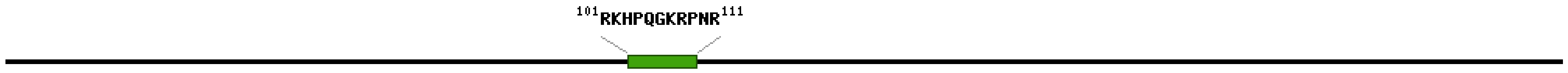

Posterior Graphic. Threshold Marked @ 0.4

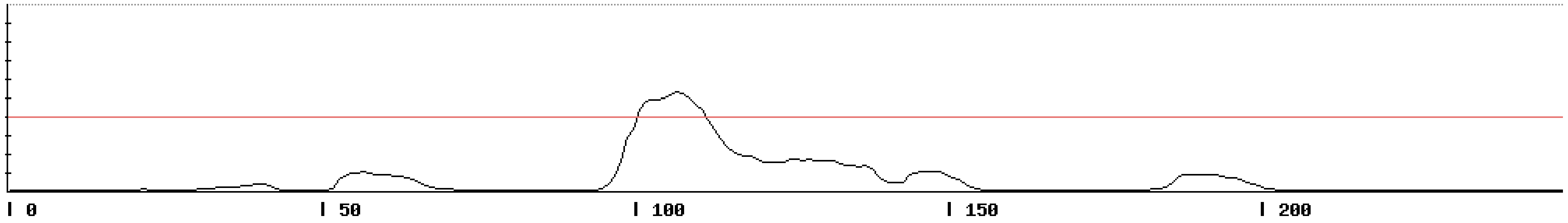

Predictions for Gossypiumraimondii_Gorai.012G083700.1

Viterbi Path

Viterbi Path

Posterior @ 0.4 | 144 - RKRKARKHAAK - 154

Posterior @ 0.4

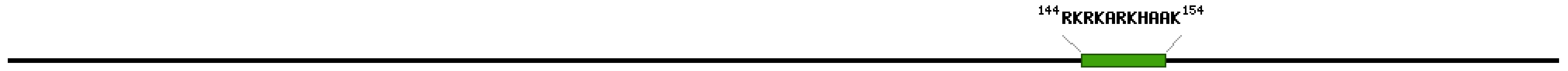

Posterior Graphic. Threshold Marked @ 0.4

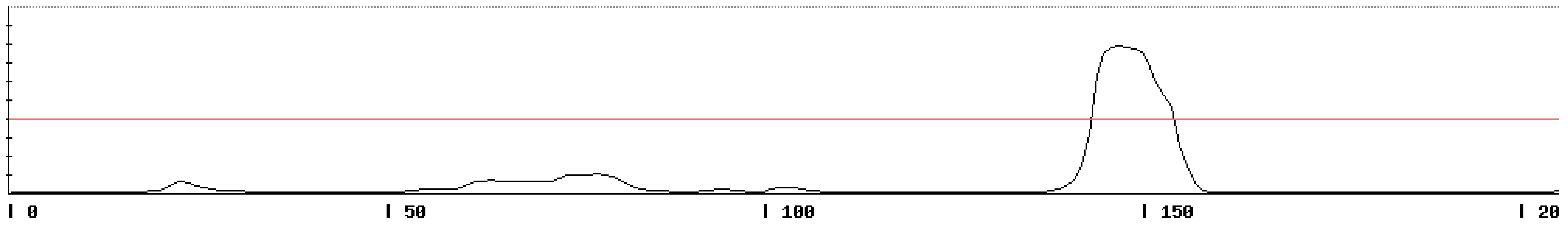

Predictions for Gossypiumraimondii_Gorai.012G125500.1

Viterbi Path

Viterbi Path

Posterior @ 0.4

Posterior @ 0.4

Posterior Graphic. Threshold Marked @ 0.4

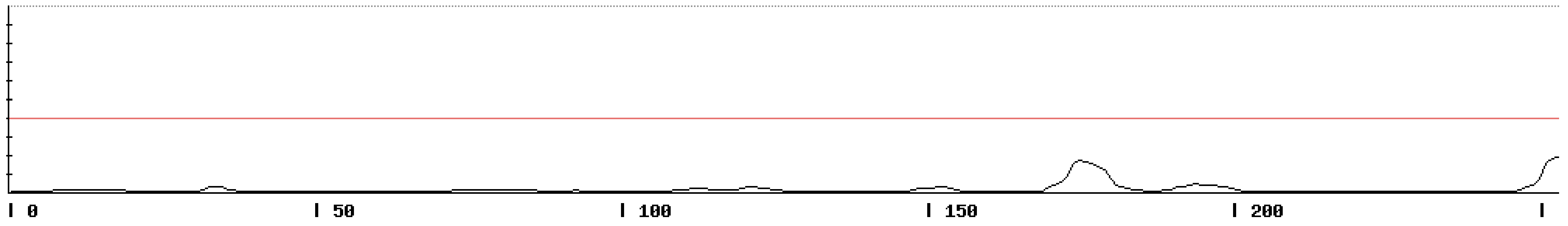

Predictions for Gossypiumraimondii_Gorai.012G169300.1	
Viterbi Path	
Viterbi Path	
Posterior @ 0.4	
Posterior @ 0.4	
Posterior Graphic. Threshold Marked @ 0.4	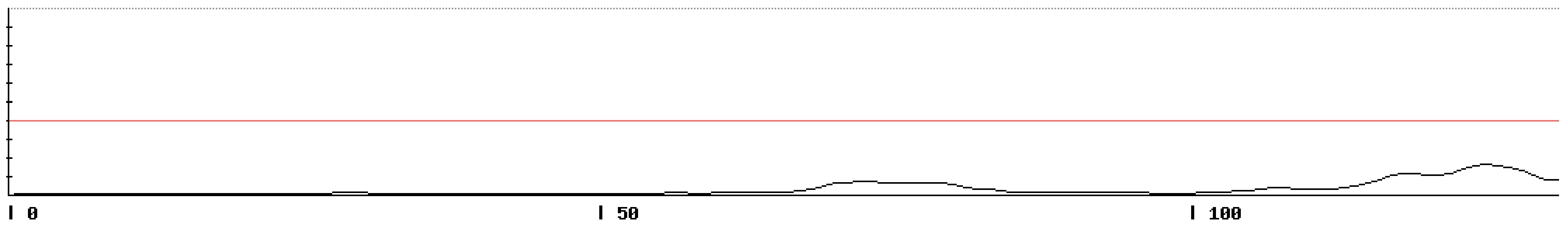

Predictions for Gossypiumraimondii_Gorai.013G063500.1	
Viterbi Path	
Viterbi Path	
Posterior @ 0.4	
Posterior @ 0.4	
Posterior Graphic. Threshold Marked @ 0.4	

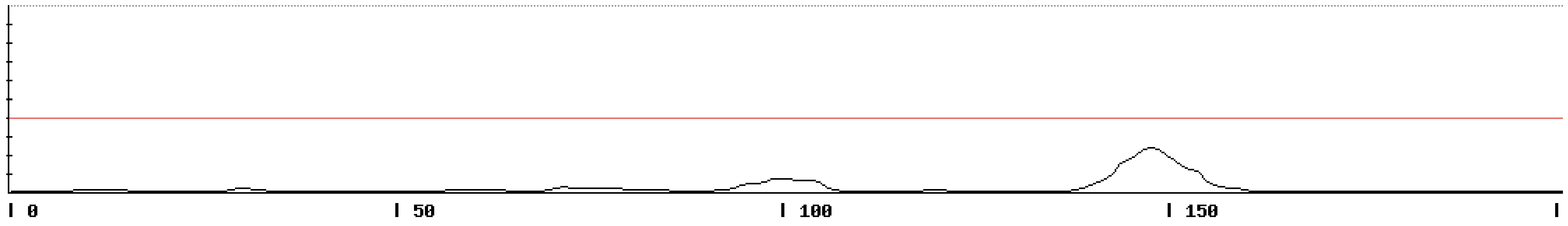

Predictions for Gossypiumraimondii_Gorai.013G066200.1

Viterbi Path	
Viterbi Path	
Posterior @ 0.4	167 - LKKKSKSR - 174
	264 - KKNKKRK - 270
Posterior @ 0.4	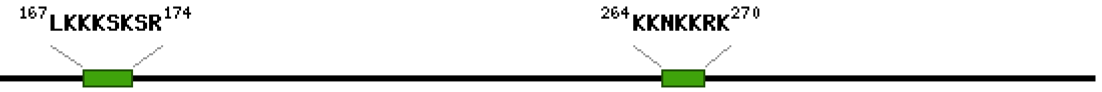
Posterior Graphic. Threshold Marked @ 0.4	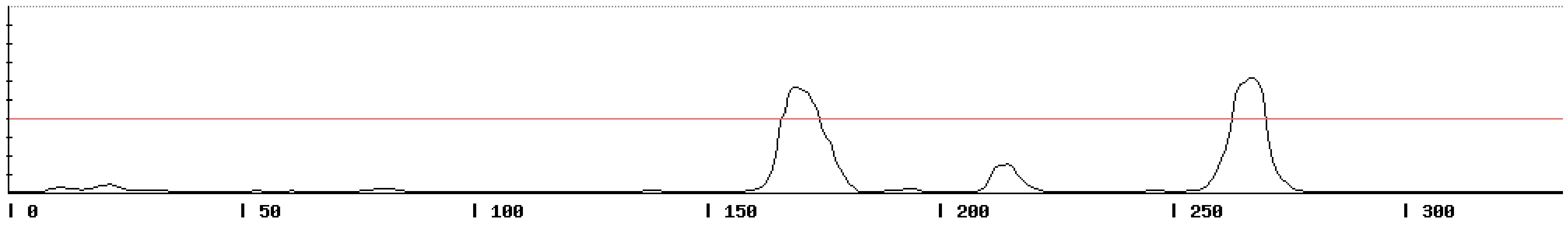

Predictions for Gossypiumraimondii_Gorai.013G085000.1

Viterbi Path	
Viterbi Path	
Posterior @ 0.4	
Posterior @ 0.4	

Posterior Graphic. Threshold Marked @ 0.4

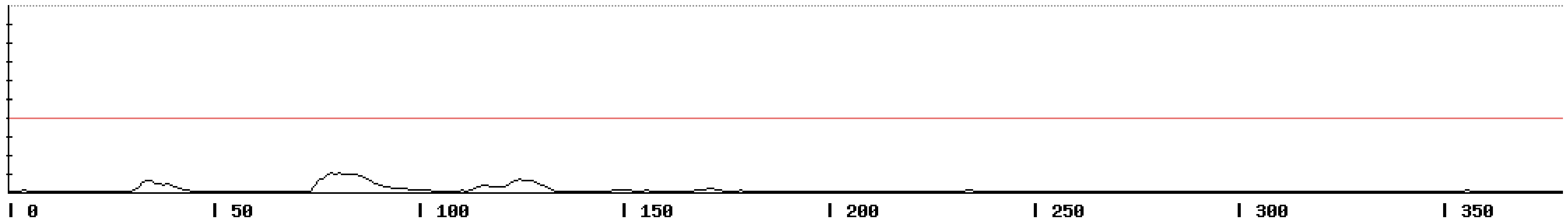

Predictions for Gossypiumraimondii_Gorai.013G118000.1

Viterbi Path

Viterbi Path

Posterior @ 0.4

Posterior @ 0.4

Posterior Graphic. Threshold Marked @ 0.4

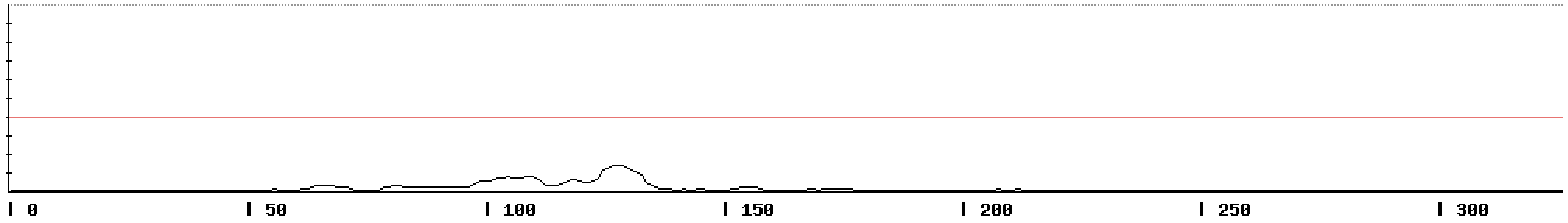

Predictions for Gossypiumraimondii_Gorai.013G140700.1

Viterbi Path

Viterbi Path

Posterior @ 0.4

Posterior @ 0.4

Posterior Graphic. Threshold Marked @ 0.4

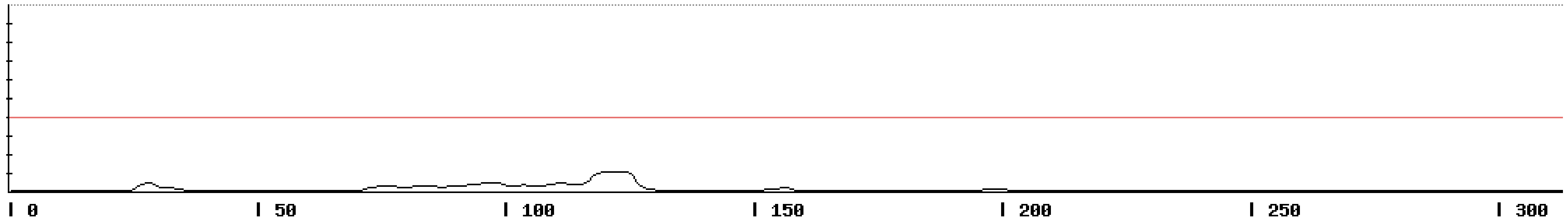

Predictions for Gossypiumraimondii_Gorai.013G146300.1

Viterbi Path

Viterbi Path

Posterior @ 0.4

Posterior @ 0.4

Posterior Graphic. Threshold Marked @ 0.4

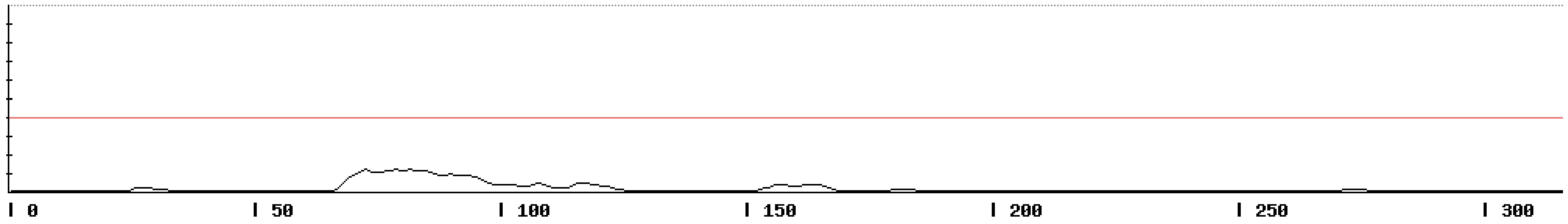

Predictions for Gossypiumraimondii_Gorai.013G167100.1

Revision r.9 - Last Updated on : February 24 2015 17:45:20.

alex.nguyenba@utoronto.ca

Intended to be used with Firefox

Viterbi Path

Posterior @ 0.4

Posterior @ 0.4

Posterior Graphic. Threshold Marked @ 0.4

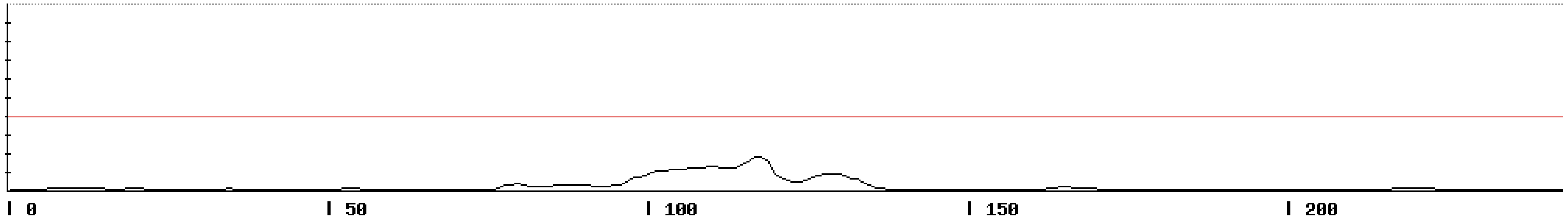

Predictions for Helianthusannuus_Han000596

Viterbi Path

Viterbi Path

Posterior @ 0.4

Posterior @ 0.4

Posterior Graphic. Threshold Marked @ 0.4

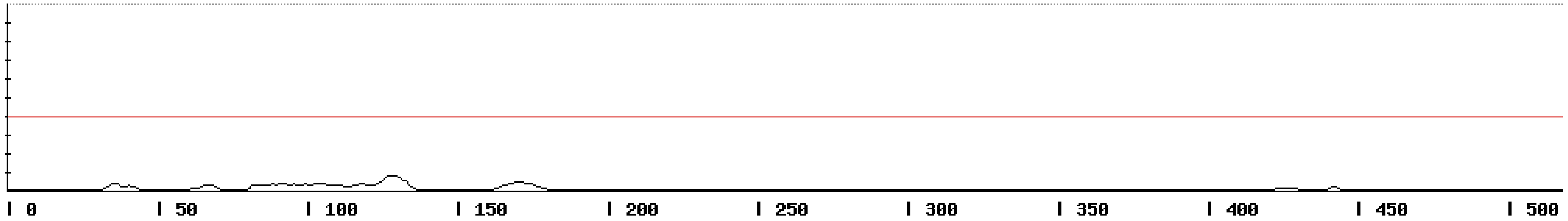

Predictions for Helianthusannuus_Han000682

Viterbi Path

Viterbi Path

Posterior @ 0.4

Posterior @ 0.4

Posterior Graphic. Threshold Marked @ 0.4

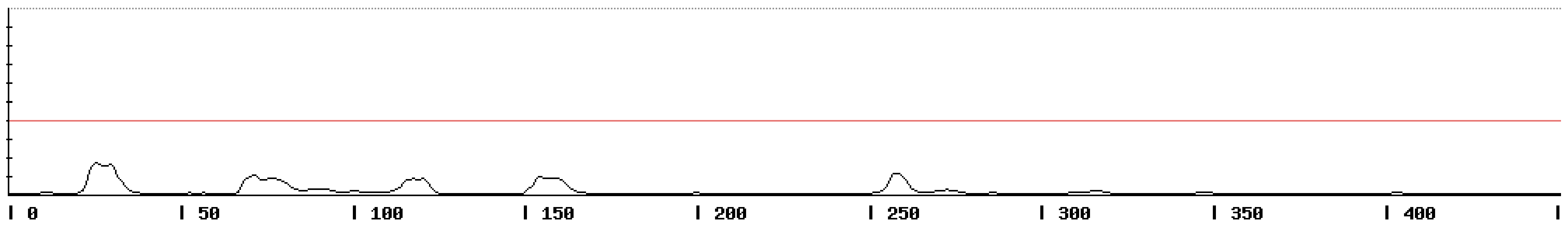

Predictions for Helianthusannuus_Han001383

Viterbi Path

Viterbi Path

Posterior @ 0.4

Posterior @ 0.4

Posterior Graphic. Threshold Marked @ 0.4

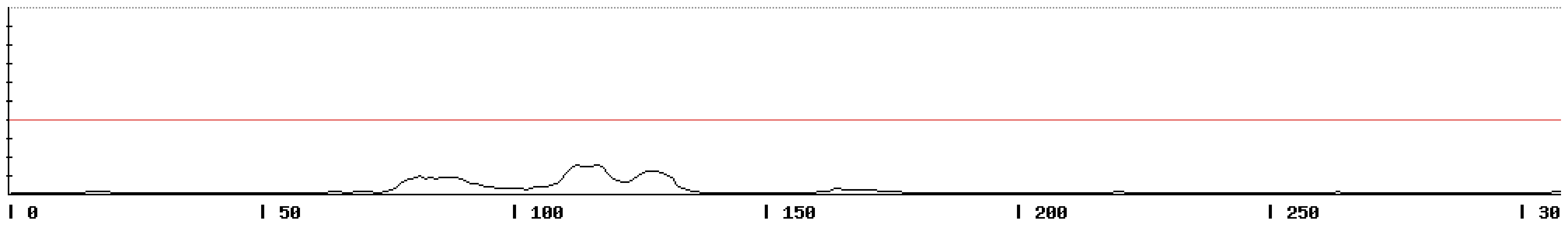

Predictions for Helianthusannuus_Han002027

Viterbi Path	
Viterbi Path	
Posterior @ 0.4	
Posterior @ 0.4	
Posterior Graphic. Threshold Marked @ 0.4	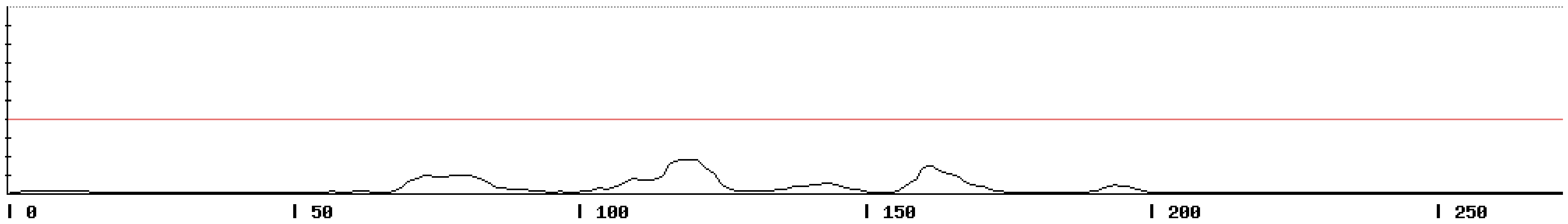

Predictions for Helianthusannuus_Han002034

Viterbi Path	
Viterbi Path	
Posterior @ 0.4	95 - DKPIGKPKPVGIKK - 108
Posterior @ 0.4	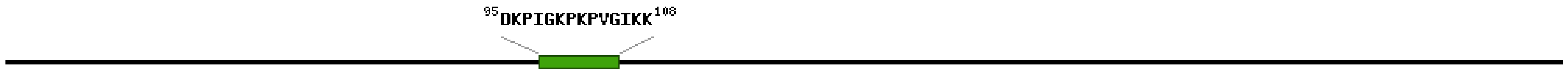
Posterior Graphic. Threshold Marked @ 0.4	

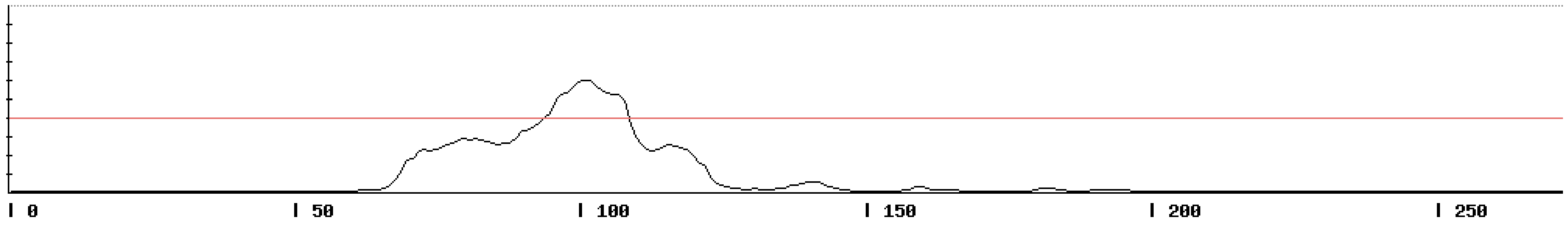

Predictions for Helianthusannuus_Han002216

Viterbi Path	
Viterbi Path	
Posterior @ 0.4	
Posterior @ 0.4	
Posterior Graphic. Threshold Marked @ 0.4	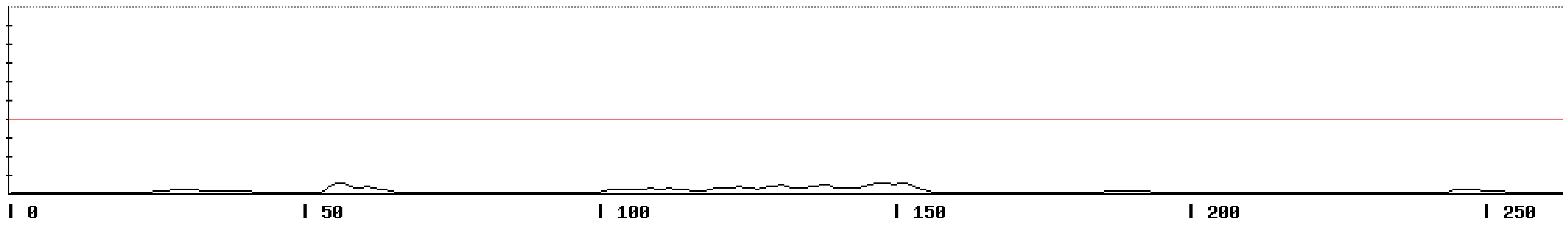

Predictions for Helianthusannuus_Han002537

Viterbi Path	
Viterbi Path	
Posterior @ 0.4	
Posterior @ 0.4	

Posterior Graphic. Threshold Marked @ 0.4

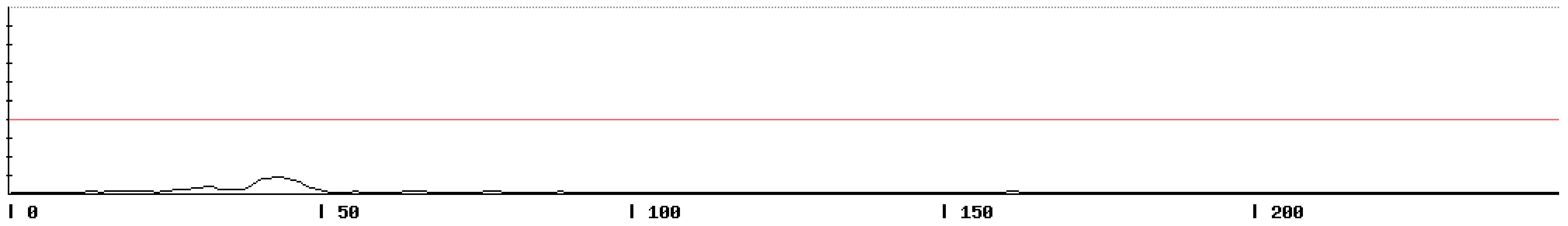

Predictions for Helianthusannuus_Han002724

Viterbi Path

Viterbi Path

Posterior @ 0.4

Posterior @ 0.4

Posterior Graphic. Threshold Marked @ 0.4

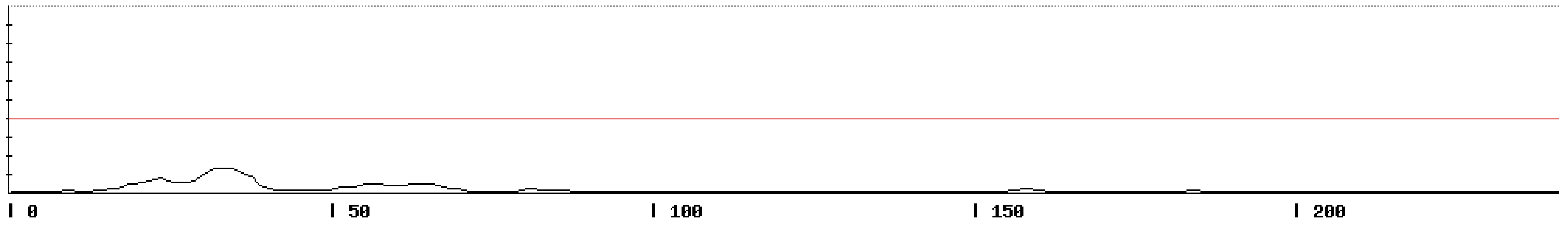

Predictions for Helianthusannuus_Han002848

Viterbi Path

Viterbi Path

Posterior @ 0.4

Posterior @ 0.4

Posterior Graphic. Threshold Marked @ 0.4

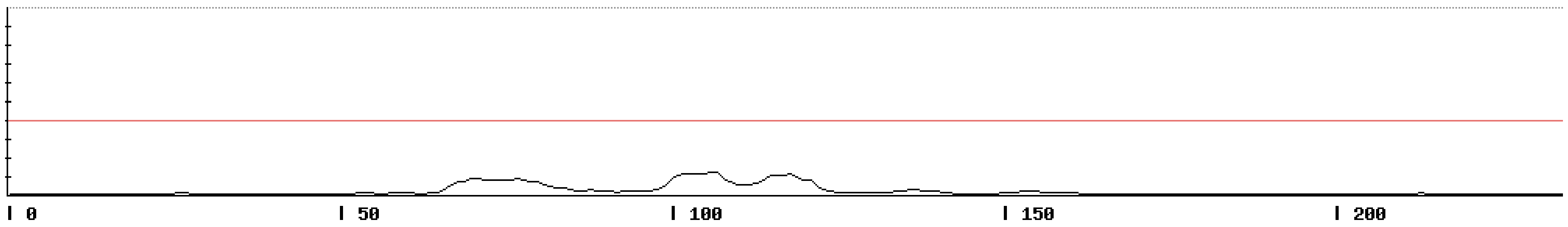

Predictions for Helianthusannuus_Han002889

Viterbi Path

Viterbi Path

Posterior @ 0.4

Posterior @ 0.4

Posterior Graphic. Threshold Marked @ 0.4

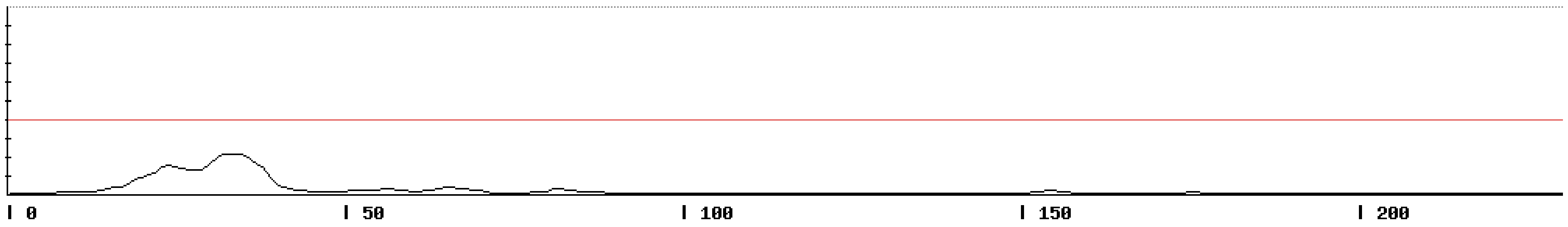

Predictions for Helianthusannuus_Han003293

Viterbi Path

Viterbi Path

Posterior @ 0.4

Posterior @ 0.4

Posterior Graphic. Threshold Marked @ 0.4

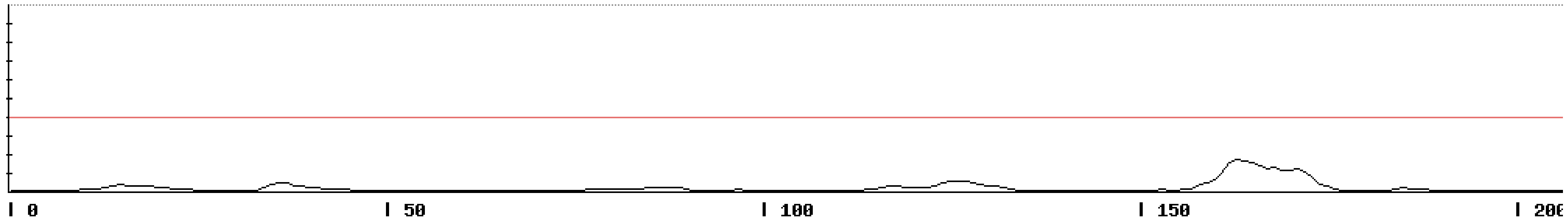

Predictions for Helianthusannuus_Han004179

Viterbi Path

Viterbi Path

Posterior @ 0.4 — 374 - RLEKPKPSRSSRR - 386

Posterior @ 0.4

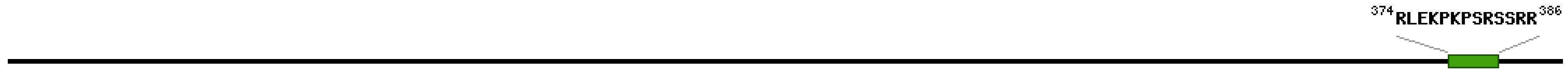

Posterior Graphic. Threshold Marked @ 0.4

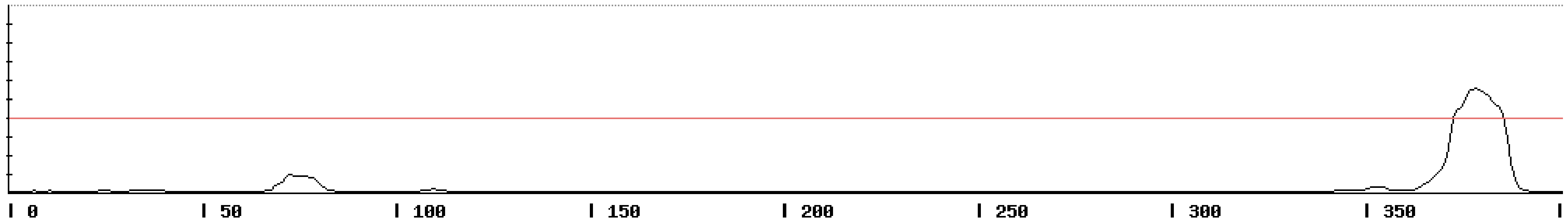

Predictions for Helianthusannuus_Han005492

Viterbi Path

Viterbi Path

Posterior @ 0.4 | 86 - KPIGKPKAVGIKK - 98

Posterior @ 0.4

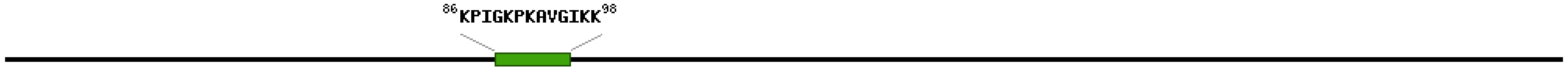

Posterior Graphic. Threshold Marked @ 0.4

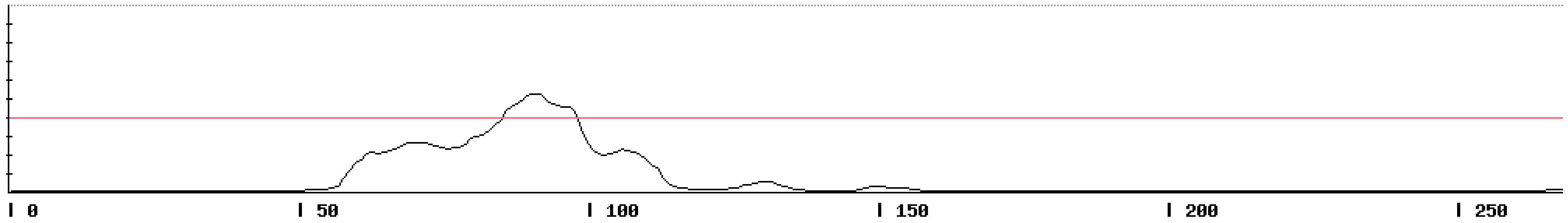

Predictions for Helianthusannuus_Han005788

Viterbi Path

Viterbi Path

Posterior @ 0.4

Posterior @ 0.4

Posterior Graphic. Threshold Marked @ 0.4

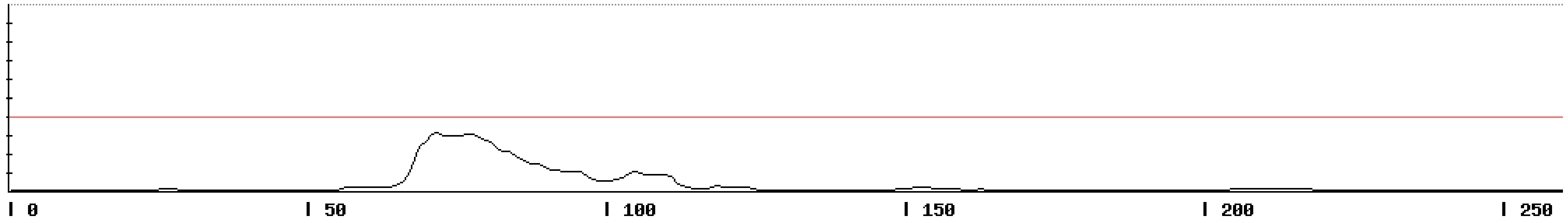

Predictions for Helianthusannuus_Han005958

Viterbi Path

Viterbi Path

Posterior @ 0.4

Posterior @ 0.4

Posterior Graphic. Threshold Marked @ 0.4

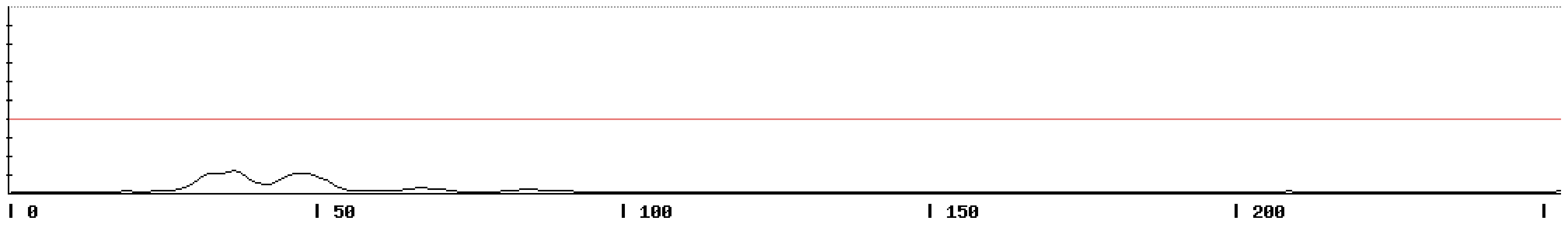

Predictions for Helianthusannuus_Han006107

Viterbi Path

Viterbi Path

Posterior @ 0.4

Posterior @ 0.4

Posterior Graphic. Threshold Marked @ 0.4

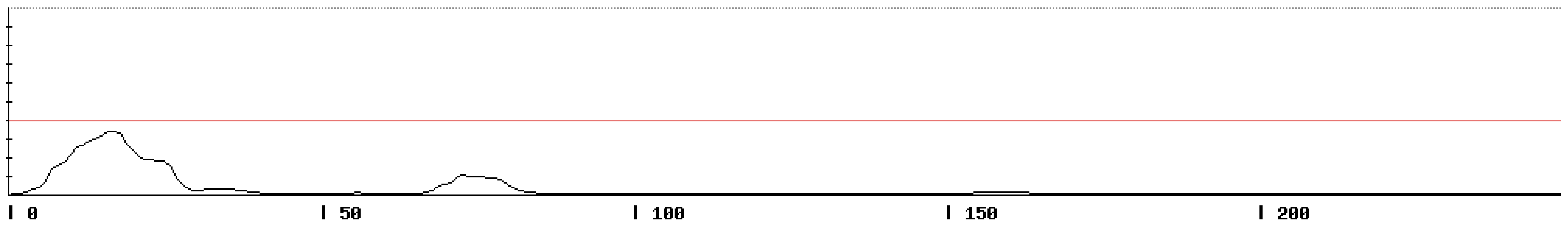

Predictions for Helianthusannuus_Han006117

Viterbi Path	
Viterbi Path	
Posterior @ 0.4	
Posterior @ 0.4	
Posterior Graphic. Threshold Marked @ 0.4	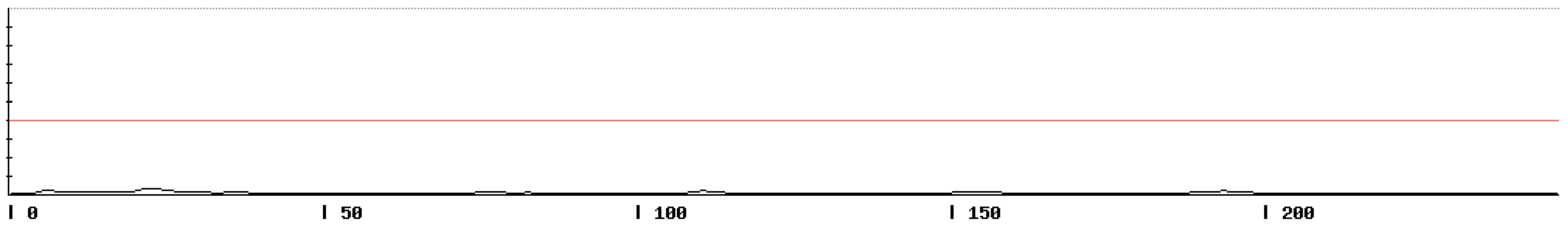

Predictions for Helianthusannuus_Han006317

Viterbi Path	
Viterbi Path	
Posterior @ 0.4	
Posterior @ 0.4	
Posterior Graphic. Threshold Marked @ 0.4	

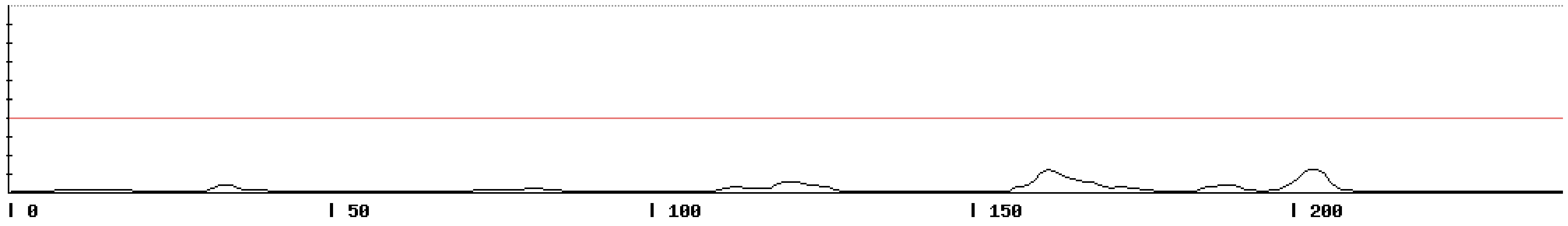

Predictions for Helianthusannuus_Han006448

</

Viterbi Path

Viterbi Path

Revision r.9 - Last Updated on : February 24 2015 17:45:20.

alex.nguyenba@utoronto.ca

Intended to be used with Firefox

Viterbi Path	
Posterior @ 0.4	190 - KKRR - 193
Posterior @ 0.4	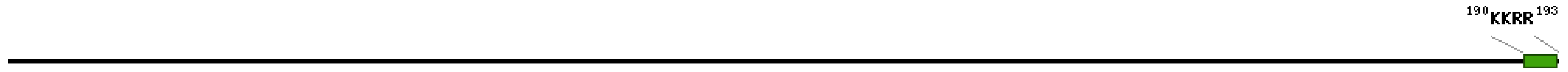
Posterior Graphic. Threshold Marked @ 0.4	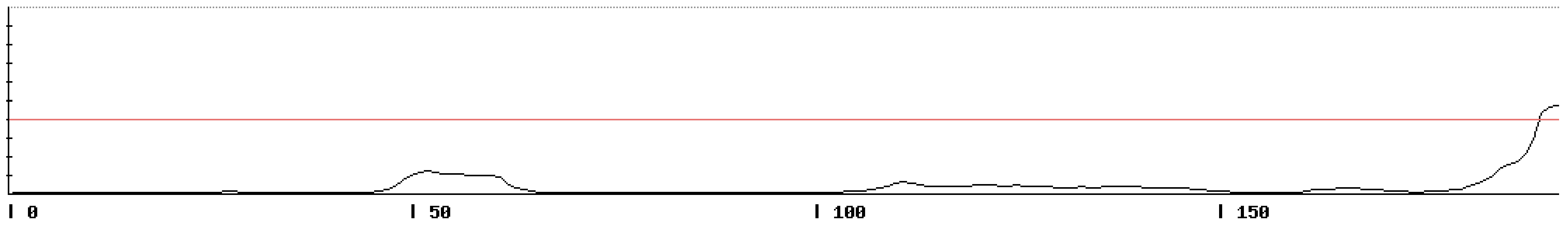

Predictions for Hordeumvulgare_MLOC_11732.1

Viterbi Path	
Viterbi Path	
Posterior @ 0.4	259 - KKGGGKTKK - 267
Posterior @ 0.4	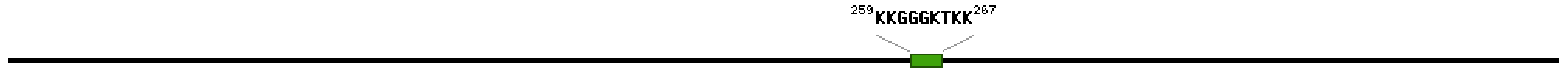
Posterior Graphic. Threshold Marked @ 0.4	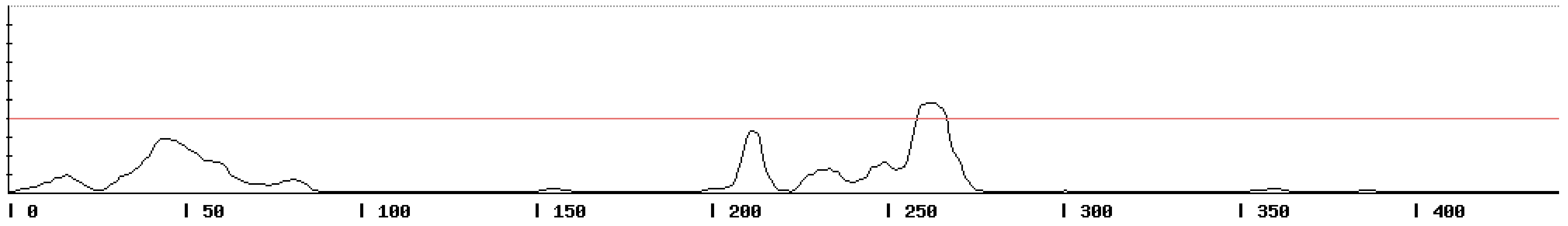

Predictions for Hordeumvulgare_MLOC_12466.1

Viterbi Path

Viterbi Path

Posterior @ 0.4

Posterior @ 0.4

Posterior Graphic. Threshold Marked @ 0.4

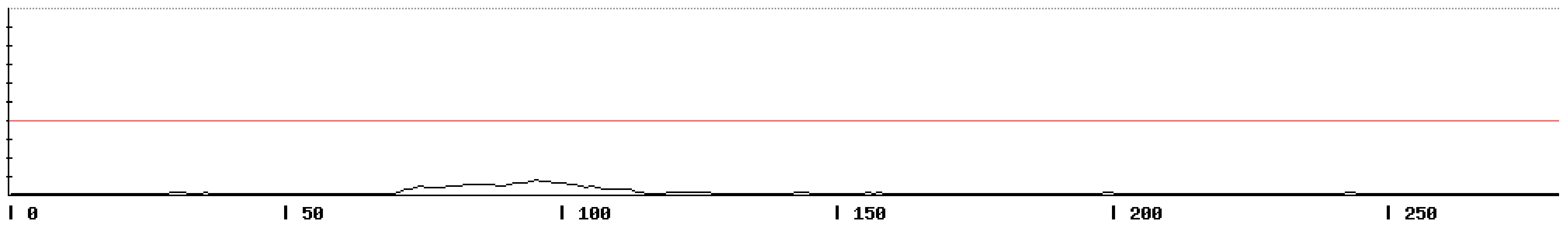

Predictions for Hordeumvulgare_MLOC_13932.1

Viterbi Path

Viterbi Path

Posterior @ 0.4

Posterior @ 0.4

Posterior Graphic. Threshold Marked @ 0.4

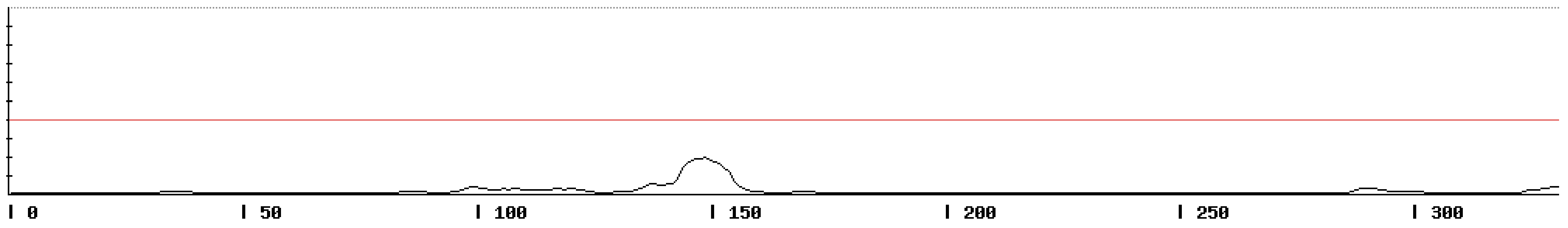

Predictions for Hordeumvulgare_MLOC_15184.1

Viterbi Path	
Viterbi Path	
Posterior @ 0.4	
Posterior @ 0.4	
Posterior Graphic. Threshold Marked @ 0.4	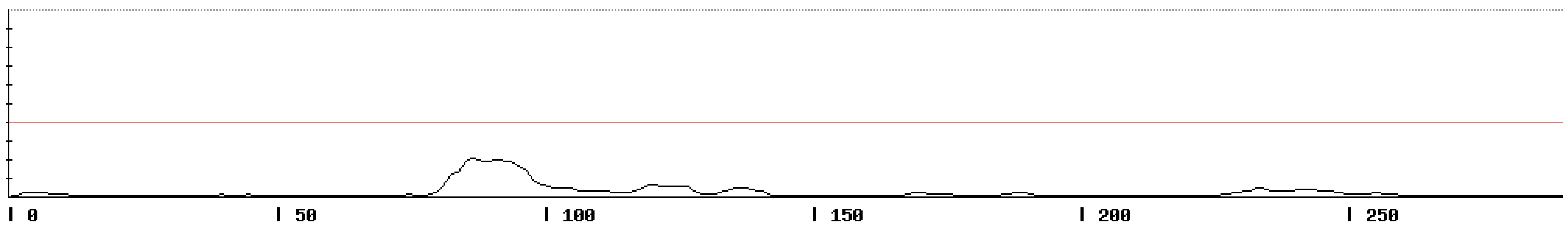

Predictions for Hordeumvulgare_MLOC_15681.2

Viterbi Path	
Viterbi Path	
Posterior @ 0.4	137 - SRKRSHKKKGHP - 148
Posterior @ 0.4	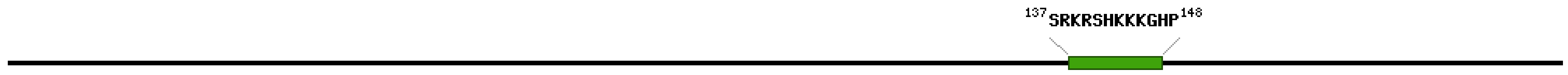
Posterior Graphic. Threshold Marked @ 0.4	

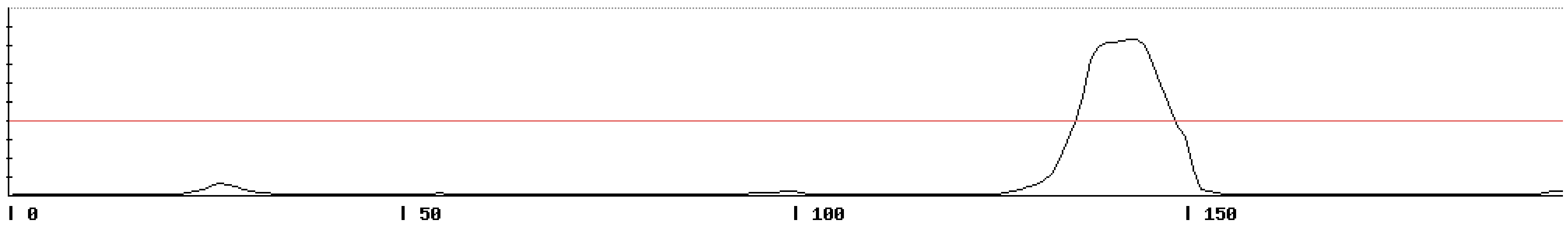

Predictions for Hordeumvulgare_MLOC_15746.1

Viterbi Path

Viterbi Path

Posterior @ 0.4

Posterior @ 0.4

Posterior Graphic. Threshold Marked @ 0.4

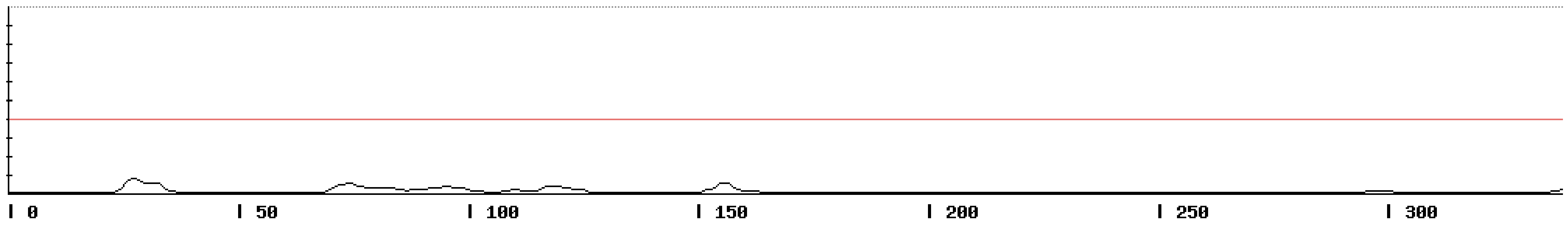

Predictions for Hordeumvulgare_MLOC_17128.1

Viterbi Path

Viterbi Path

Posterior @ 0.4

Posterior @ 0.4

Posterior Graphic. Threshold Marked @ 0.4

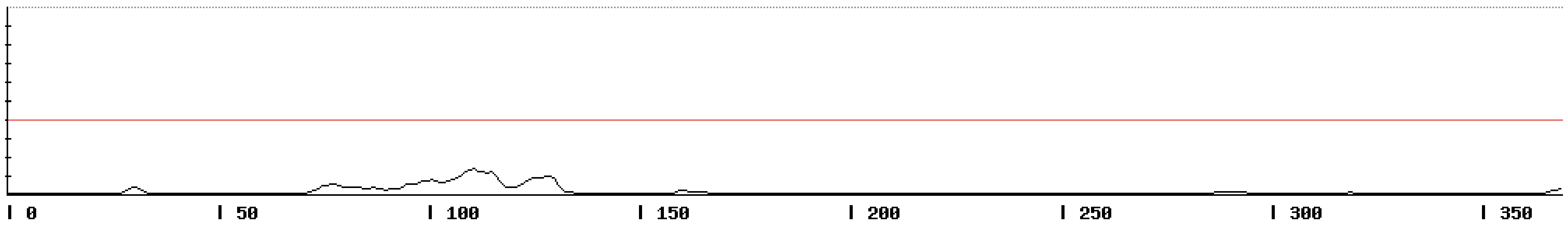

Predictions for Hordeumvulgare_MLOC_19175.1

Viterbi Path

Viterbi Path

Posterior @ 0.4

Posterior @ 0.4

Posterior Graphic. Threshold Marked @ 0.4

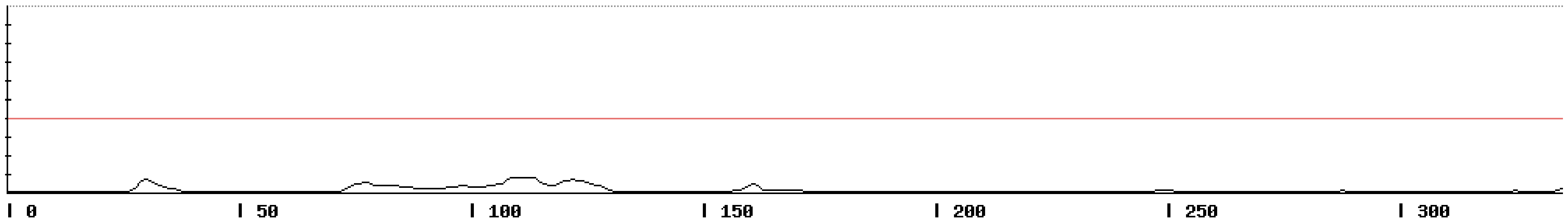

Predictions for Hordeumvulgare_MLOC_19933.1

Viterbi Path

Viterbi Path

Posterior @ 0.4

Posterior @ 0.4

Posterior Graphic. Threshold Marked @ 0.4

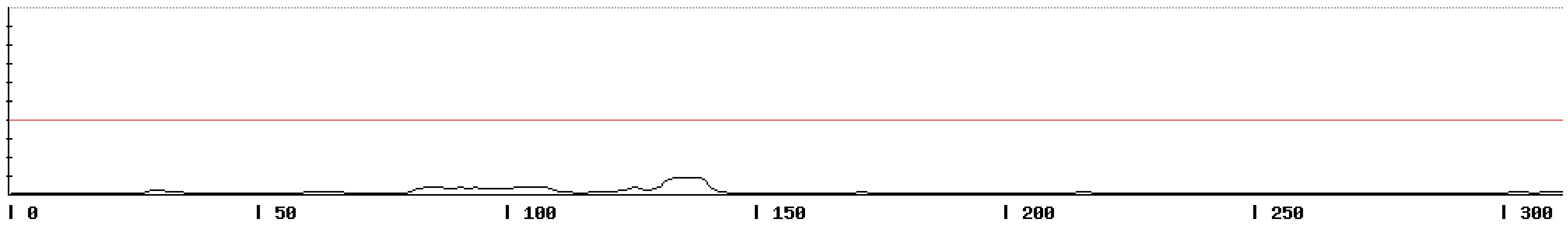

Predictions for Hordeumvulgare_MLOC_20905.1

Viterbi Path

Viterbi Path

Posterior @ 0.4

Posterior @ 0.4

Posterior Graphic. Threshold Marked @ 0.4

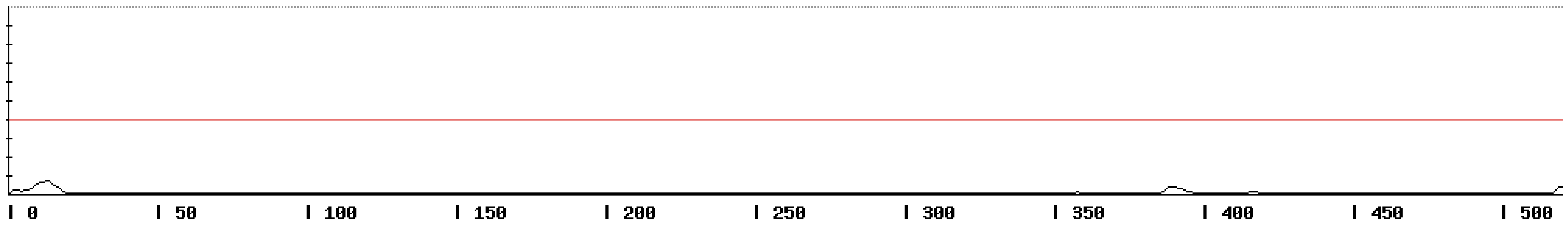

Predictions for Hordeumvulgare_MLOC_21966.1

Viterbi Path

Viterbi Path

Posterior @ 0.4	89 - GKRKRRKI - 96
Posterior @ 0.4	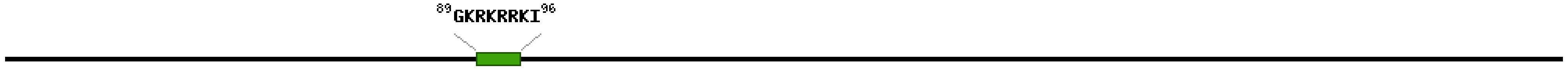
Posterior Graphic. Threshold Marked @ 0.4	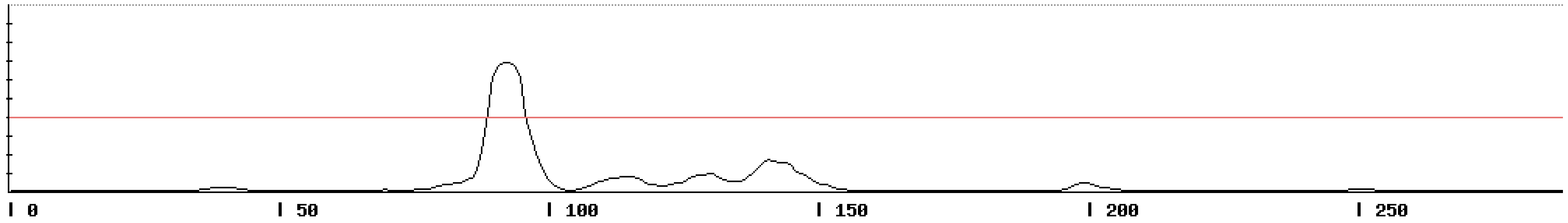

Predictions for Hordeumvulgare_MLOC_25708.1

Viterbi Path	
Viterbi Path	
Posterior @ 0.4	
Posterior @ 0.4	
Posterior Graphic. Threshold Marked @ 0.4	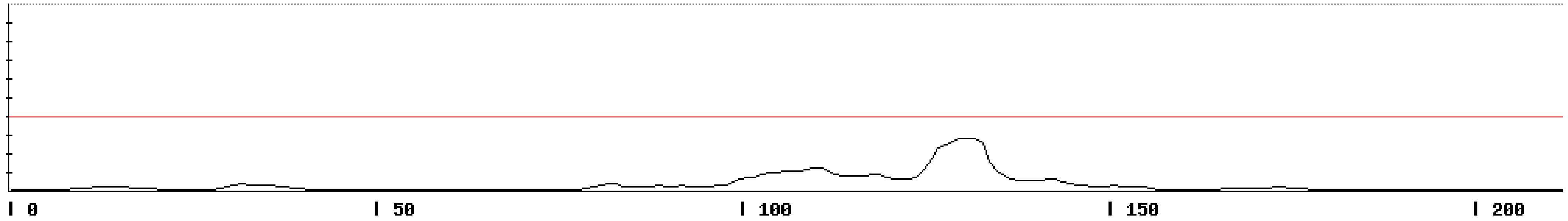

Predictions for Hordeumvulgare_MLOC_36501.1

Viterbi Path	
Viterbi Path	

Posterior @ 0.4

Posterior @ 0.4

Posterior Graphic. Threshold Marked @ 0.4

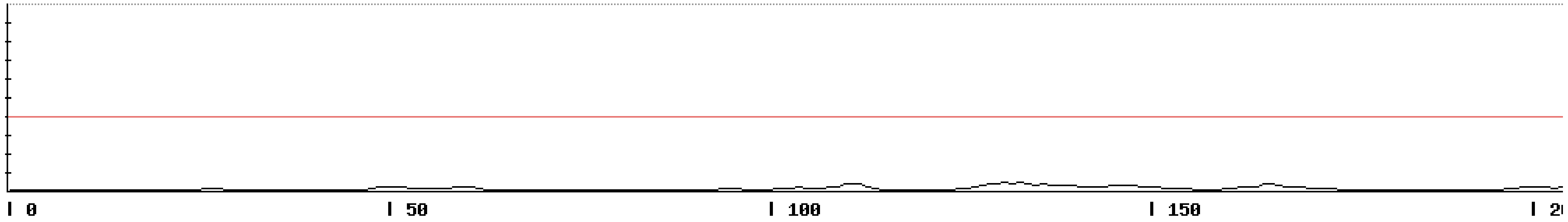

Predictions for Hordeumvulgare_MLOC_36942.1

Viterbi Path

Viterbi Path

Posterior @ 0.4

Posterior @ 0.4

Posterior Graphic. Threshold Marked @ 0.4

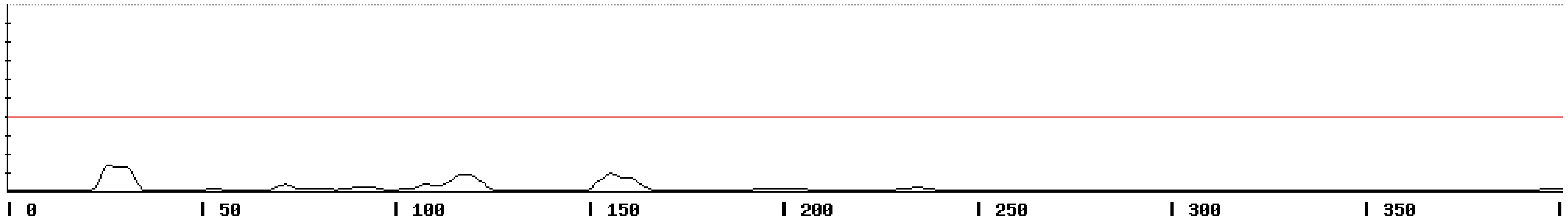

Predictions for Hordeumvulgare_MLOC_37104.1

Viterbi Path

Viterbi Path

Posterior @ 0.4

Posterior @ 0.4

Posterior Graphic. Threshold Marked @ 0.4

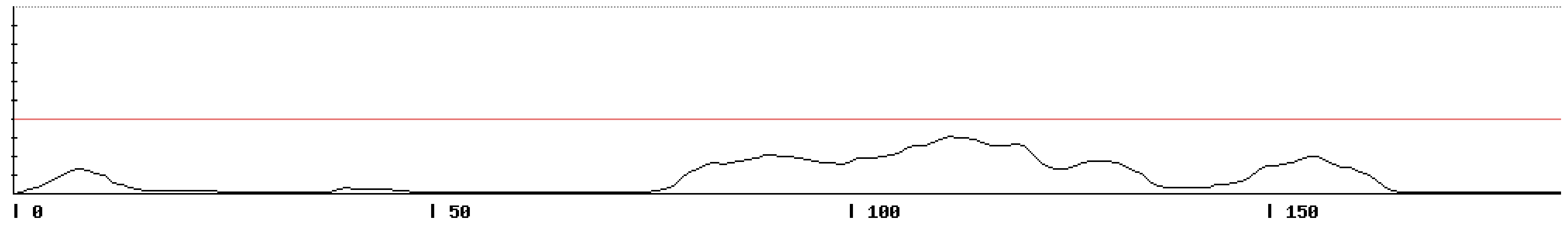

Predictions for Hordeumvulgare_MLOC_37364.1

Viterbi Path

Viterbi Path

Posterior @ 0.4

Posterior @ 0.4

Posterior Graphic. Threshold Marked @ 0.4

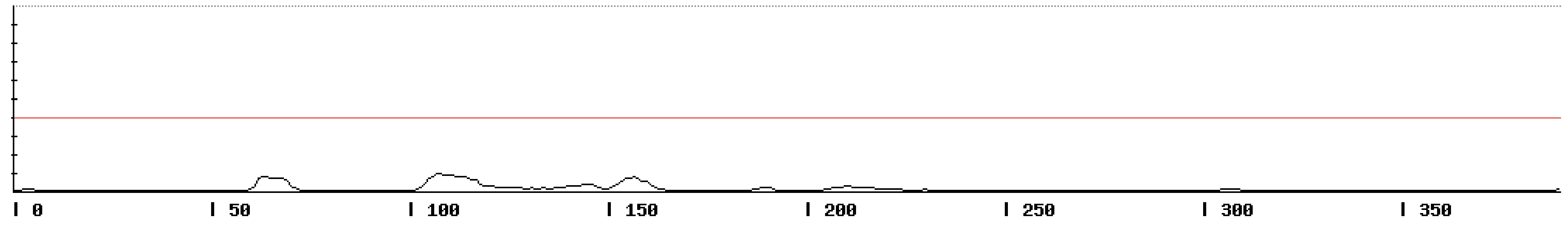

Predictions for Hordeumvulgare_MLOC_37843.1	
Viterbi Path	
Viterbi Path	
Posterior @ 0.4	
Posterior @ 0.4	
Posterior Graphic. Threshold Marked @ 0.4	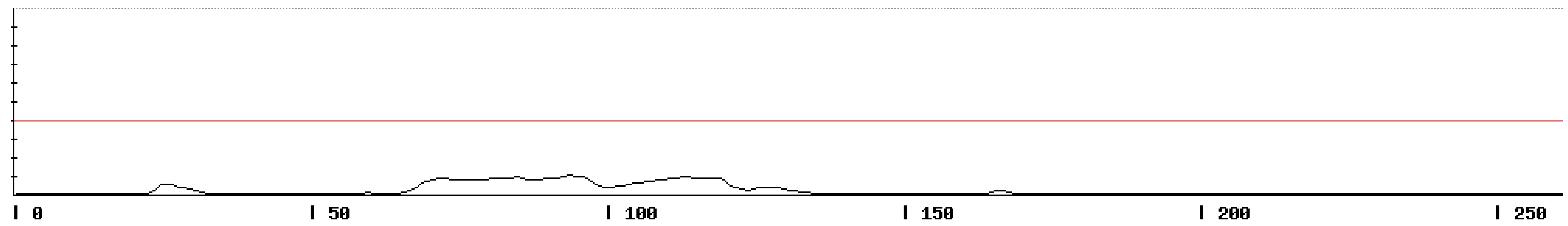

Predictions for Hordeumvulgare_MLOC_37844.1	
Viterbi Path	
Viterbi Path	
Posterior @ 0.4	
Posterior @ 0.4	
Posterior Graphic. Threshold Marked @ 0.4	

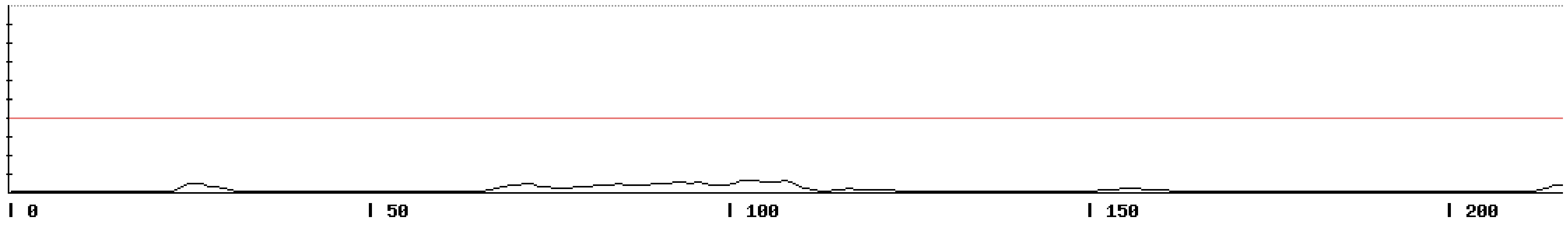

Predictions for Hordeumvulgare_MLOC_40602.1

Viterbi Path	
Viterbi Path	
Posterior @ 0.4	
Posterior @ 0.4	
Posterior Graphic. Threshold Marked @ 0.4	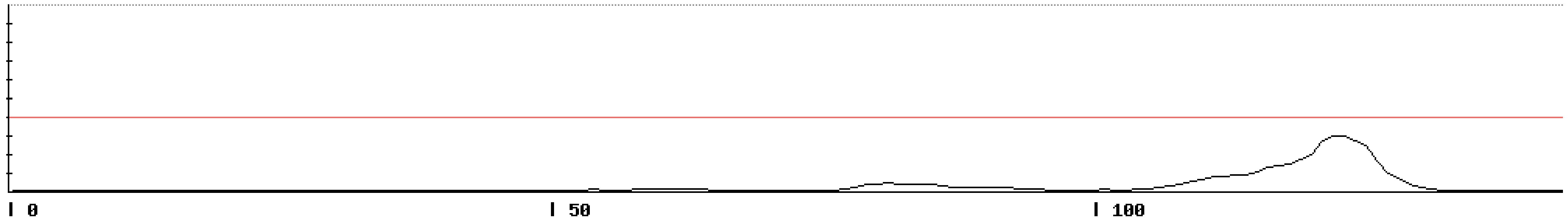

Predictions for Hordeumvulgare_MLOC_43934.1

Viterbi Path	
Viterbi Path	
Posterior @ 0.4	136 - GKRKRRK - 142
Posterior @ 0.4	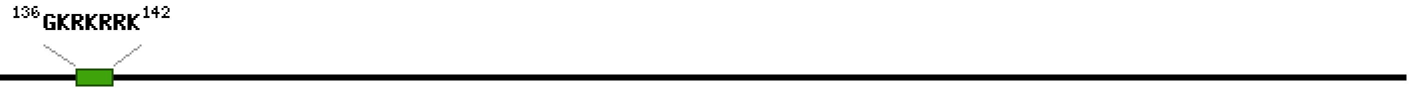

Posterior Graphic. Threshold Marked @ 0.4

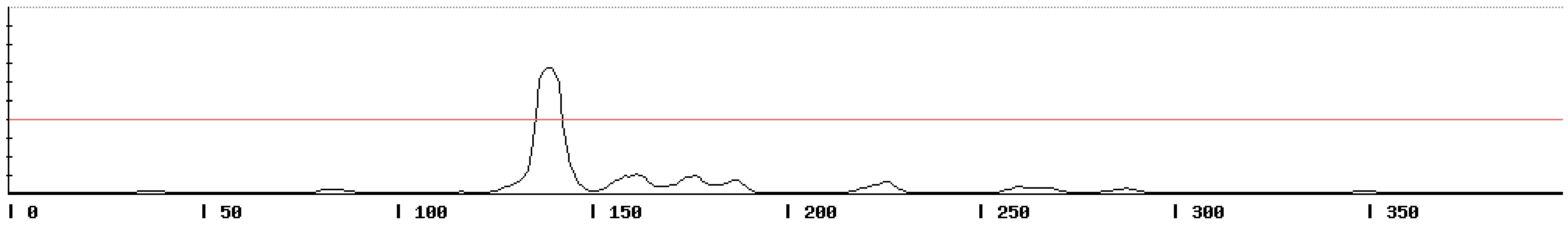

Predictions for Hordeumvulgare_MLOC_4747.1

Viterbi Path

Viterbi Path

Posterior @ 0.4

Posterior @ 0.4

Posterior Graphic. Threshold Marked @ 0.4

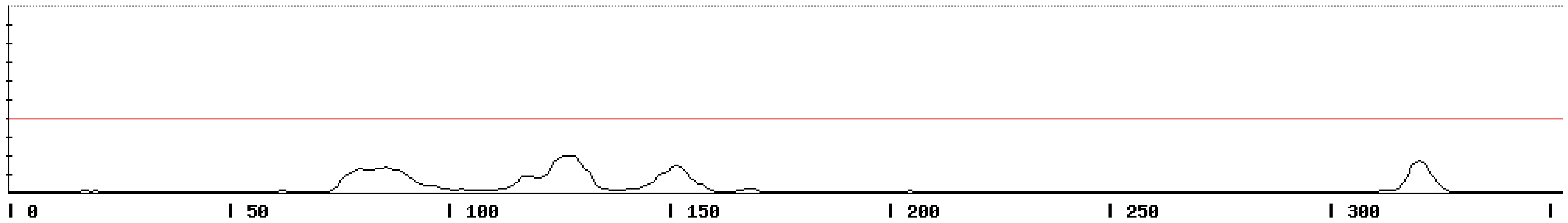

Predictions for Hordeumvulgare_MLOC_4952.1

Viterbi Path

Viterbi Path

Posterior @ 0.4

Posterior @ 0.4

Posterior Graphic. Threshold Marked @ 0.4

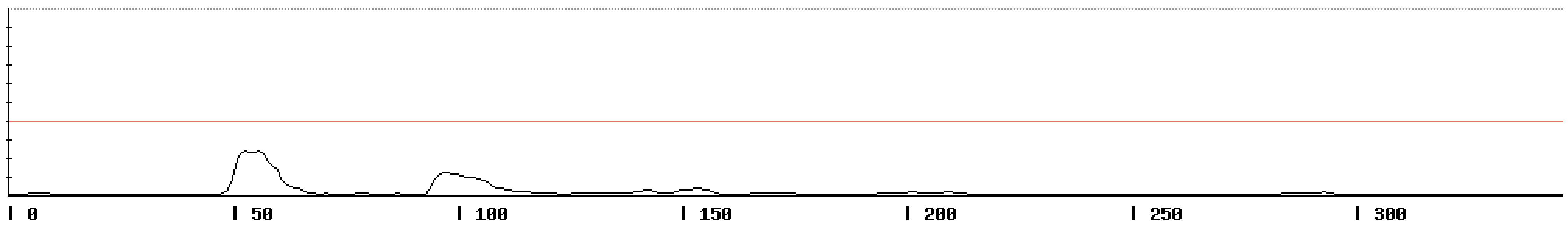

Predictions for Hordeumvulgare_MLOC_50406.1

Viterbi Path

Viterbi Path

Posterior @ 0.4

94 - KATGKDRVIKSKGEKKKQHMIGMKKTLVFHRGRAPKGE - 131

Posterior @ 0.4

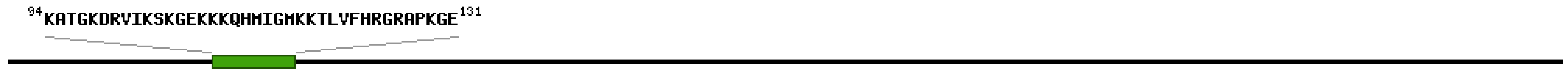

Posterior Graphic. Threshold Marked @ 0.4

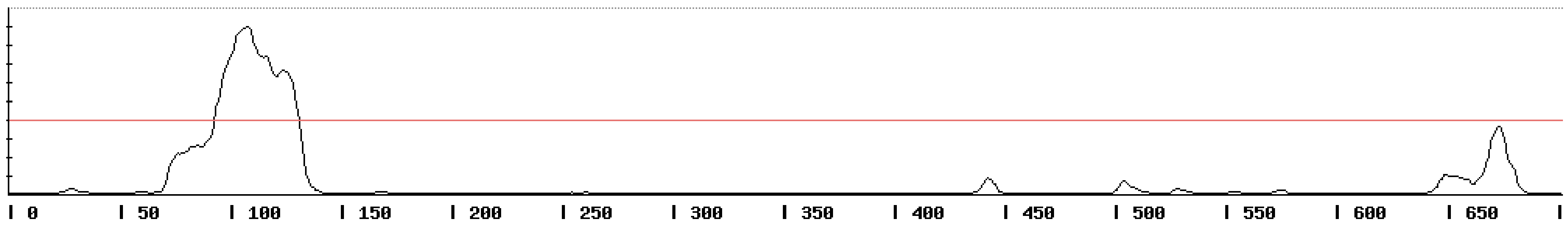

Predictions for Hordeumvulgare_MLOC_50895.1

Viterbi Path

Viterbi Path

Posterior @ 0.4 | 27 - PRRGRPLRPSP - 37

563 - PKKKRK - 568

594 - RQKRKKK - 600

Posterior @ 0.4

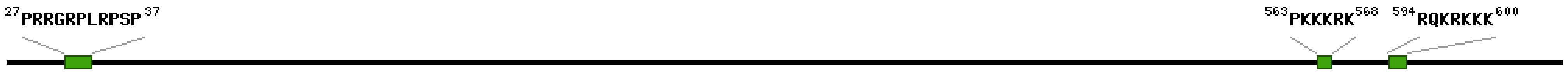

Posterior Graphic. Threshold Marked @ 0.4

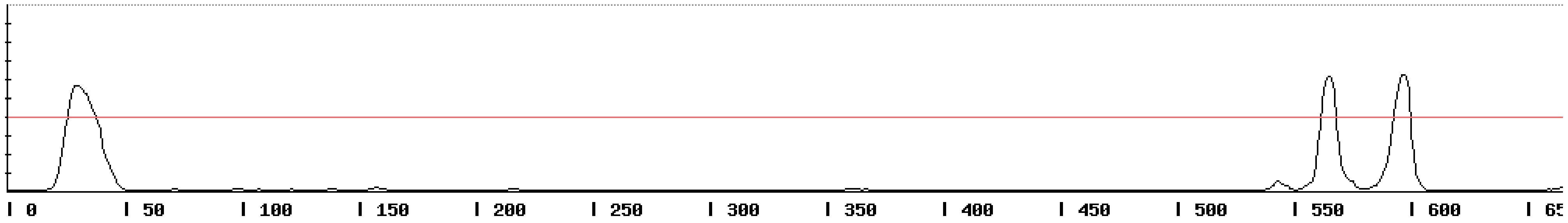

Predictions for Hordeumvulgare_MLOC_5165.1

Viterbi Path

Viterbi Path

Posterior @ 0.4

Posterior @ 0.4

Posterior Graphic. Threshold Marked @ 0.4

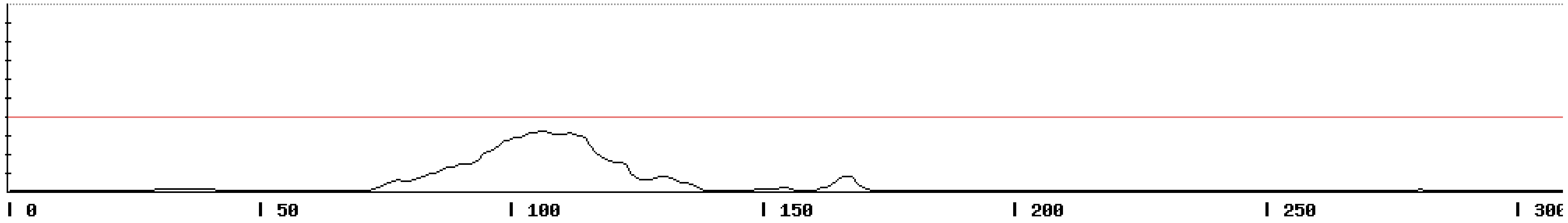

Predictions for Hordeumvulgare_MLOC_53086.1

Viterbi Path

Viterbi Path

Posterior @ 0.4

Posterior @ 0.4

Posterior Graphic. Threshold Marked @ 0.4

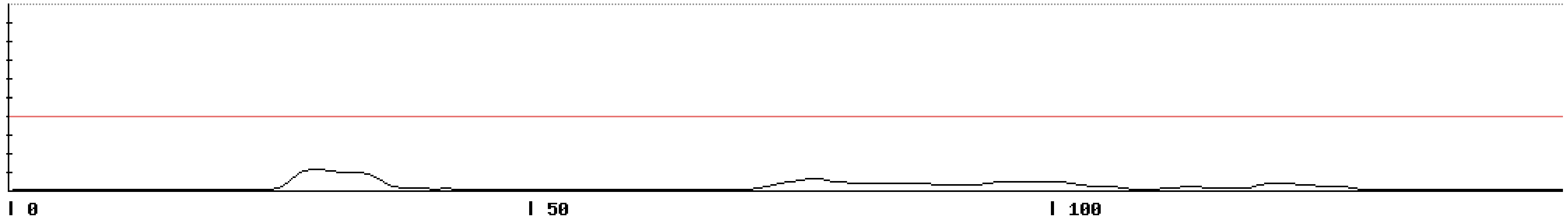

Predictions for Hordeumvulgare_MLOC_5374.1

Viterbi Path

Viterbi Path

Posterior @ 0.4

Posterior @ 0.4

Posterior Graphic. Threshold Marked @ 0.4

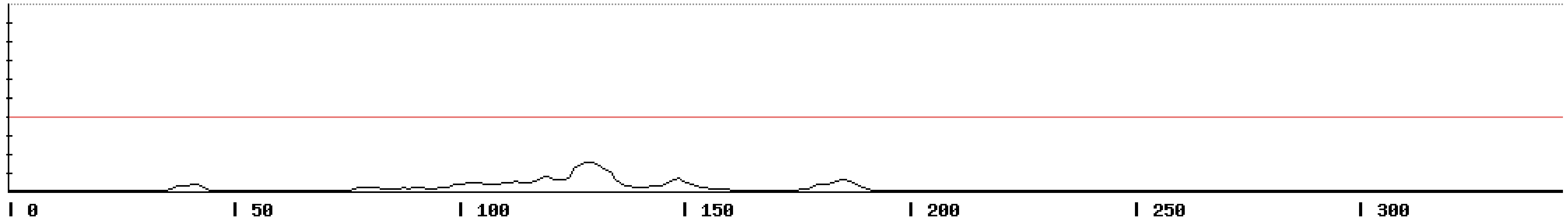

Predictions for Hordeumvulgare_MLOC_53744.1

Viterbi Path	
Viterbi Path	
Posterior @ 0.4	
Posterior @ 0.4	
Posterior Graphic. Threshold Marked @ 0.4	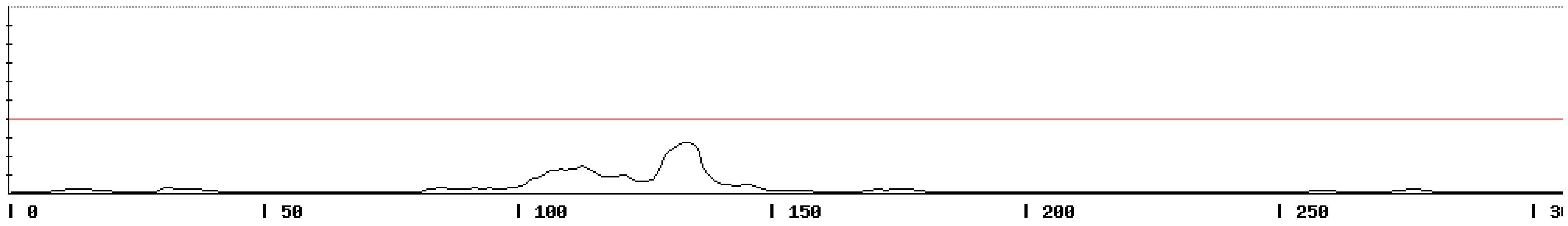

Predictions for Hordeumvulgare_MLOC_55957.1

Viterbi Path	
Viterbi Path	
Posterior @ 0.4	38 - GRGGRRRRAASGYWKA - 53
Posterior @ 0.4	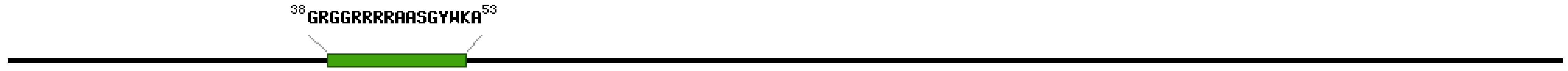
Posterior Graphic. Threshold Marked @ 0.4	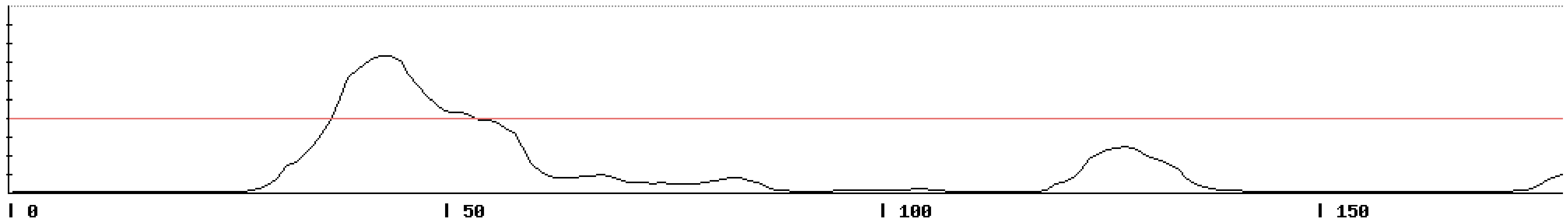

Predictions for Hordeumvulgare_MLOC_56103.1	
Viterbi Path	
Viterbi Path	
Posterior @ 0.4	
Posterior @ 0.4	
Posterior Graphic. Threshold Marked @ 0.4	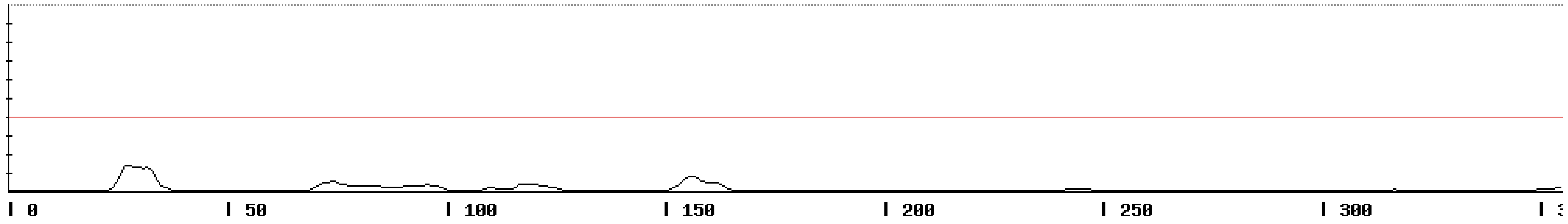

Predictions for Hordeumvulgare_MLOC_56769.1	
Viterbi Path	
Viterbi Path	
Posterior @ 0.4	242 - LPRKKARAPEEEDAASGASRRRPRK - 266
Posterior @ 0.4	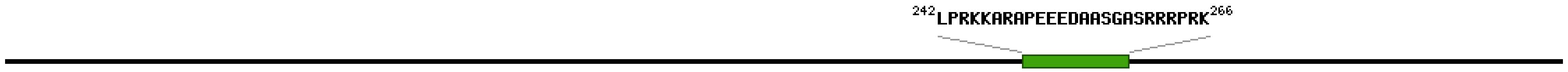
Posterior Graphic. Threshold Marked @ 0.4	

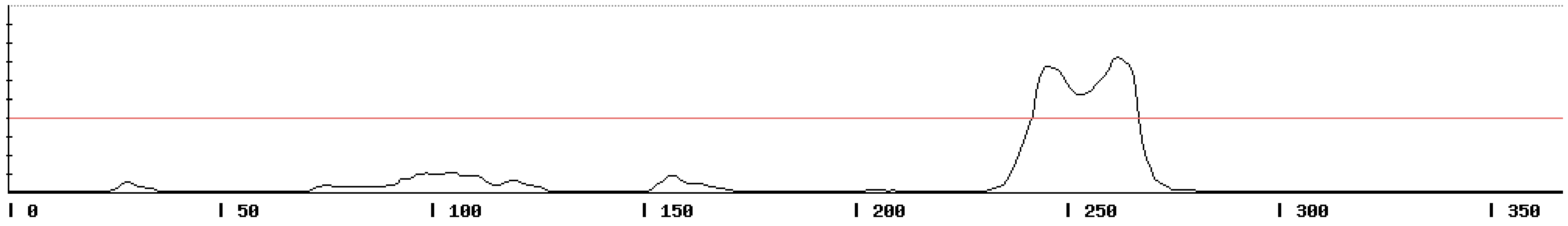

Predictions for Hordeumvulgare_MLOC_56943.1

Viterbi Path

Viterbi Path

Posterior @ 0.4

Posterior @ 0.4

Posterior Graphic. Threshold Marked @ 0.4

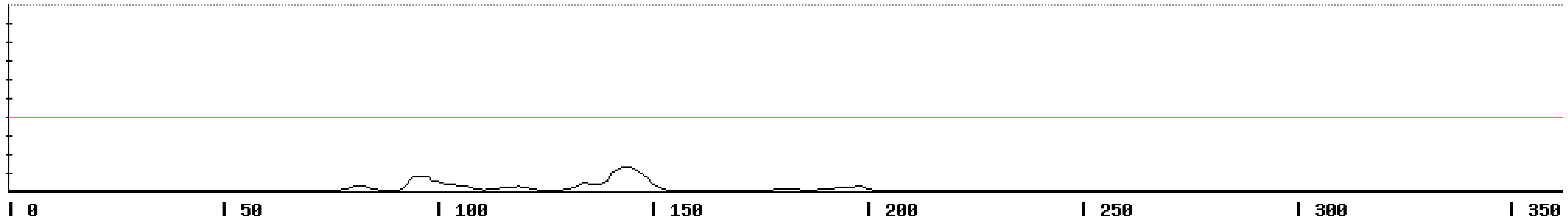

Predictions for Hordeumvulgare_MLOC_57215.1

Viterbi Path

Viterbi Path

Posterior @ 0.4

Posterior @ 0.4

Posterior Graphic. Threshold Marked @ 0.4

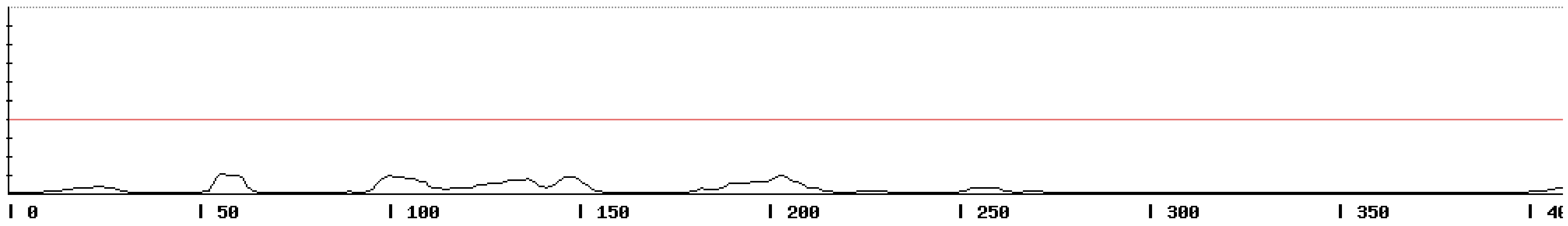

Predictions for Hordeumvulgare_MLOC_57427.1

Viterbi Path

Viterbi Path

Posterior @ 0.4

Posterior @ 0.4

Posterior Graphic. Threshold Marked @ 0.4

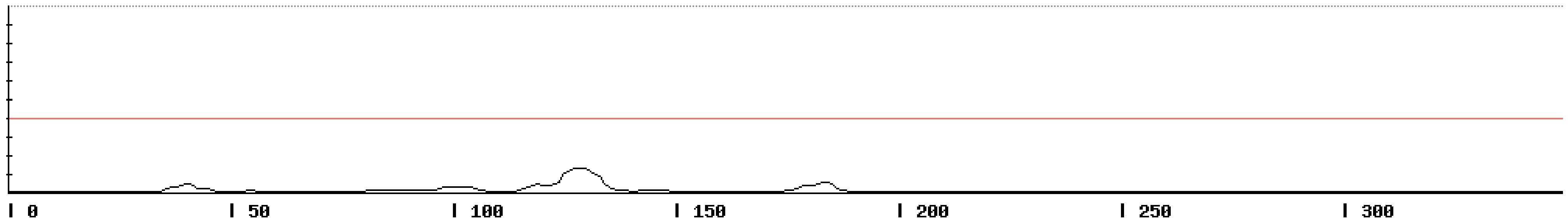

Predictions for Hordeumvulgare_MLOC_58272.1

Viterbi Path

Viterbi Path

Posterior @ 0.4

Posterior @ 0.4

Posterior Graphic. Threshold Marked @ 0.4

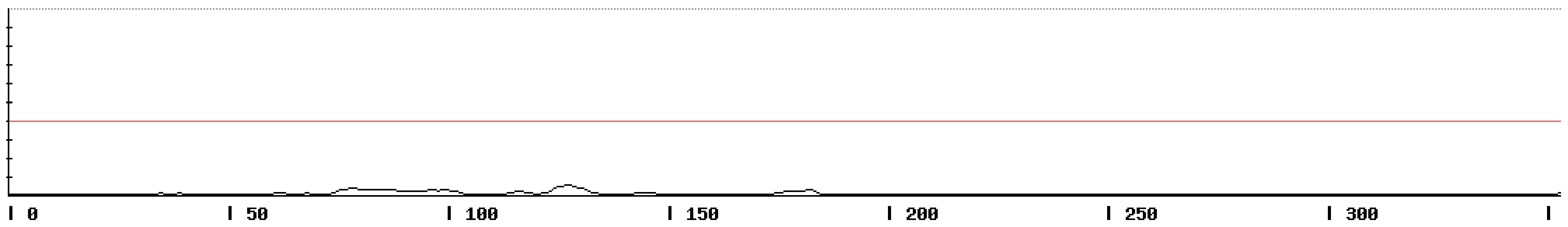

Predictions for Hordeumvulgare_MLOC_5832.1

Viterbi Path

Viterbi Path

Posterior @ 0.4

Posterior @ 0.4

Posterior Graphic. Threshold Marked @ 0.4

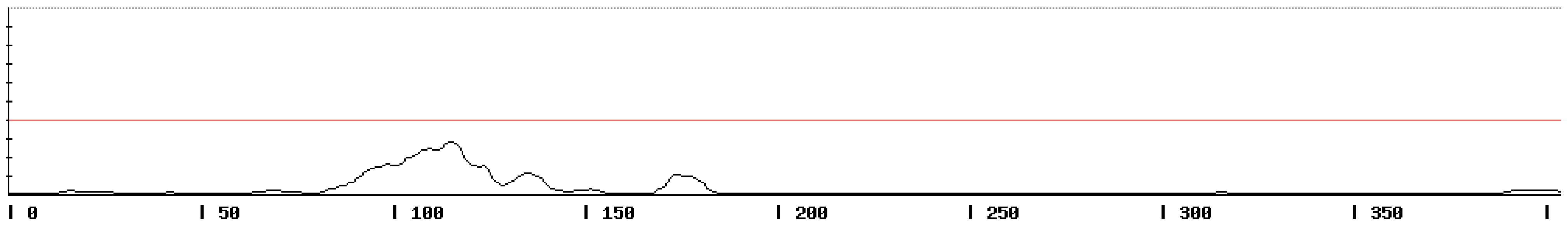

Predictions for Hordeumvulgare_MLOC_58950.1

Viterbi Path

Viterbi Path

Posterior @ 0.4

Posterior @ 0.4

Posterior Graphic. Threshold Marked @ 0.4

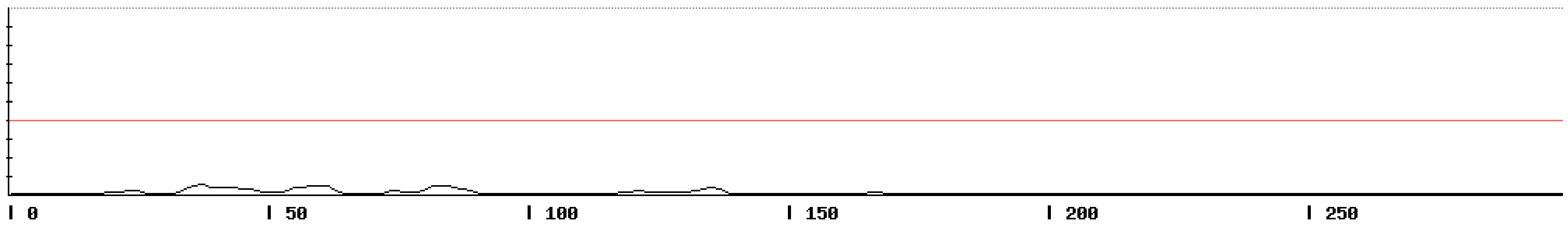

Predictions for Hordeumvulgare_MLOC_59162.1

Viterbi Path

Viterbi Path

Posterior @ 0.4

Posterior @ 0.4

Posterior Graphic. Threshold Marked @ 0.4

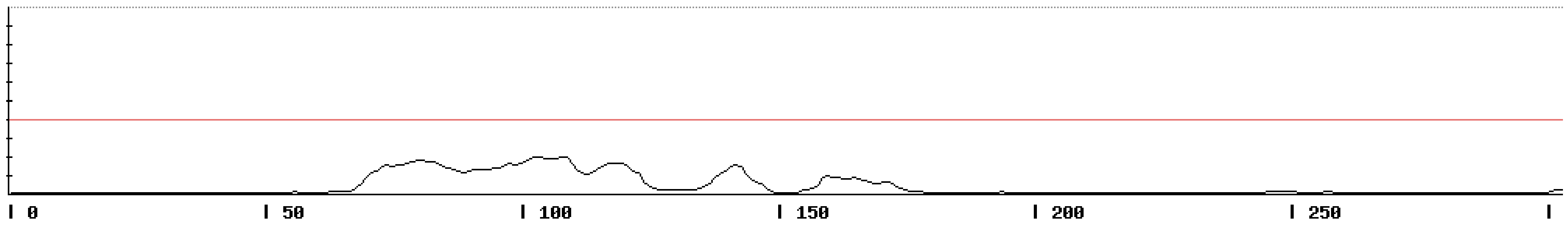

Predictions for Hordeumvulgare_MLOC_59591.1

Viterbi Path

Viterbi Path

Posterior @ 0.4	197 - PRPNKR - 202
Posterior @ 0.4	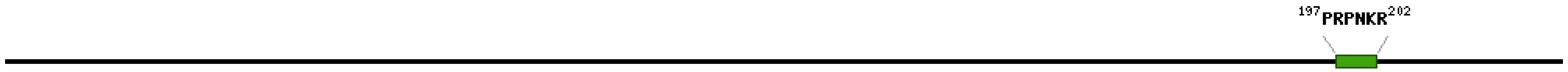
Posterior Graphic. Threshold Marked @ 0.4	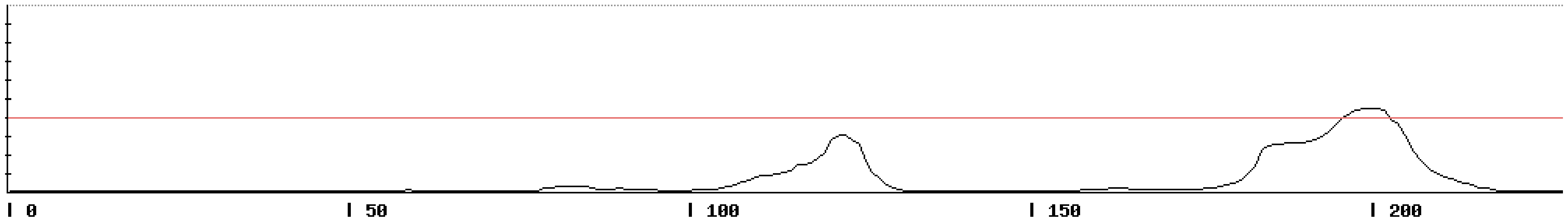

Predictions for Hordeumvulgare_MLOC_60079.1

Viterbi Path	
Viterbi Path	
Posterior @ 0.4	
Posterior @ 0.4	
Posterior Graphic. Threshold Marked @ 0.4	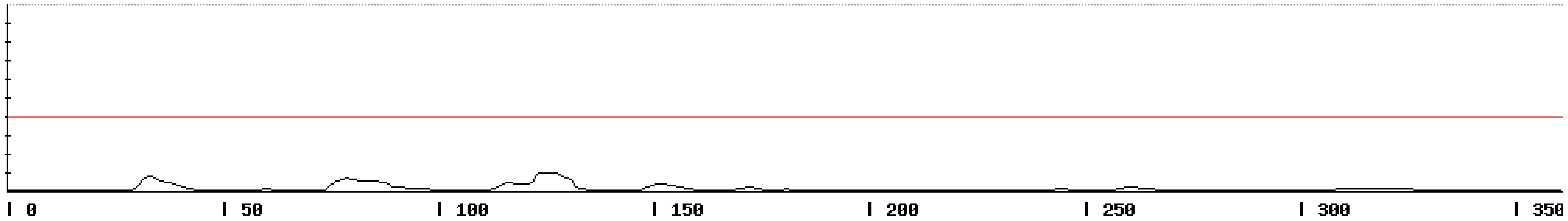

Predictions for Hordeumvulgare_MLOC_60153.1

Viterbi Path

Viterbi Path

Posterior @ 0.4

Posterior @ 0.4

Posterior Graphic. Threshold Marked @ 0.4

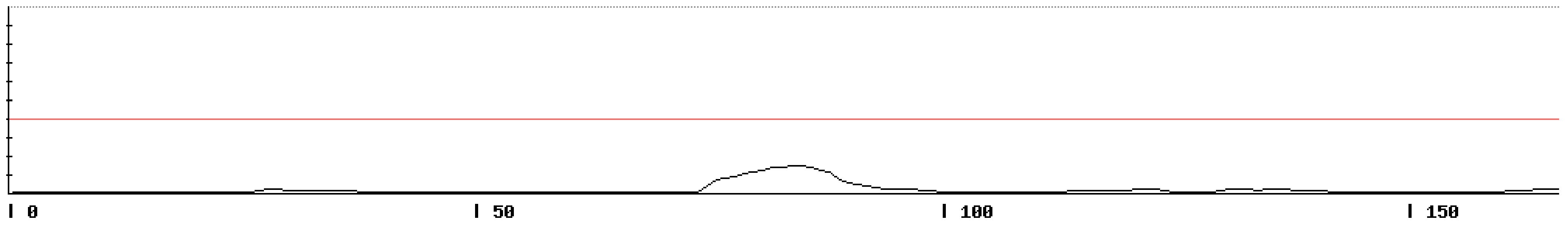

Predictions for Hordeumvulgare_MLOC_6066.1

Viterbi Path

Viterbi Path

Posterior @ 0.4

Posterior @ 0.4

Posterior Graphic. Threshold Marked @ 0.4

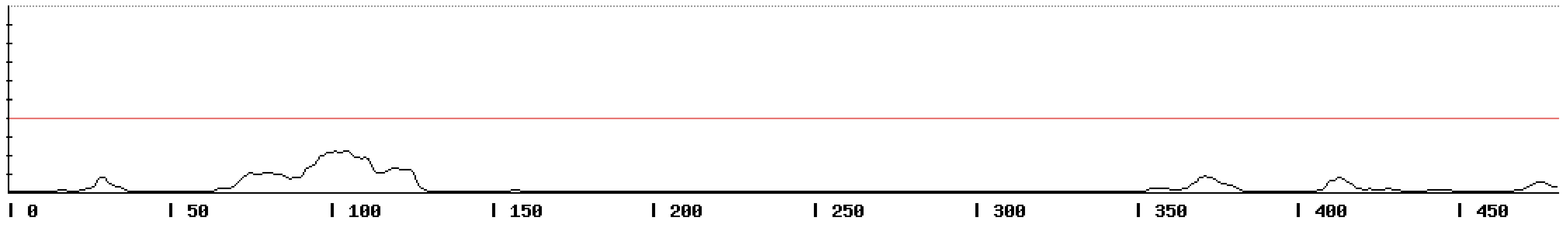

Predictions for Hordeumvulgare_MLOC_61270.1

Viterbi Path	
Viterbi Path	
Posterior @ 0.4	
Posterior @ 0.4	
Posterior Graphic. Threshold Marked @ 0.4	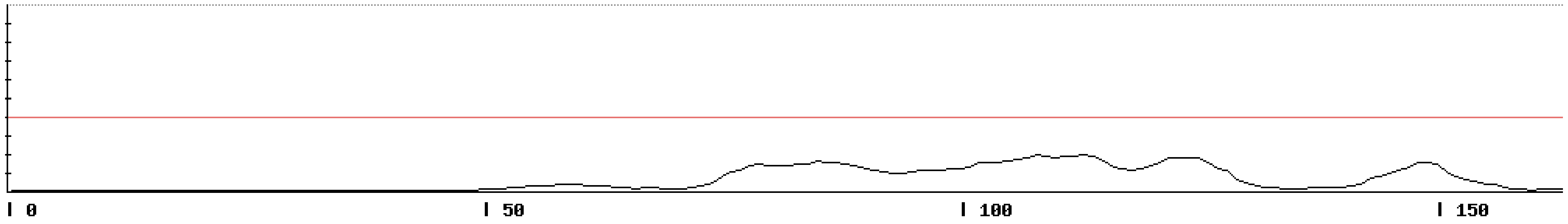

Predictions for Hordeumvulgare_MLOC_63121.1

Viterbi Path	
Viterbi Path	
Posterior @ 0.4	
Posterior @ 0.4	
Posterior Graphic. Threshold Marked @ 0.4	

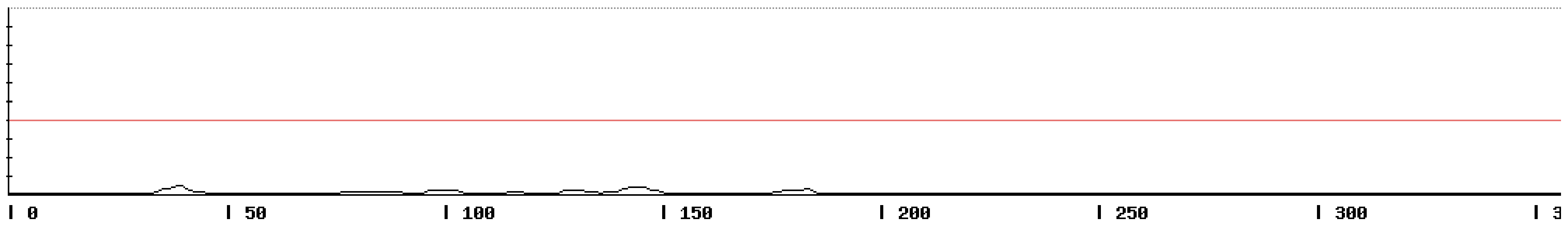

Predictions for Hordeumvulgare_MLOC_63743.1

Viterbi Path

Viterbi Path

Posterior @ 0.4

Posterior @ 0.4

Posterior Graphic. Threshold Marked @ 0.4

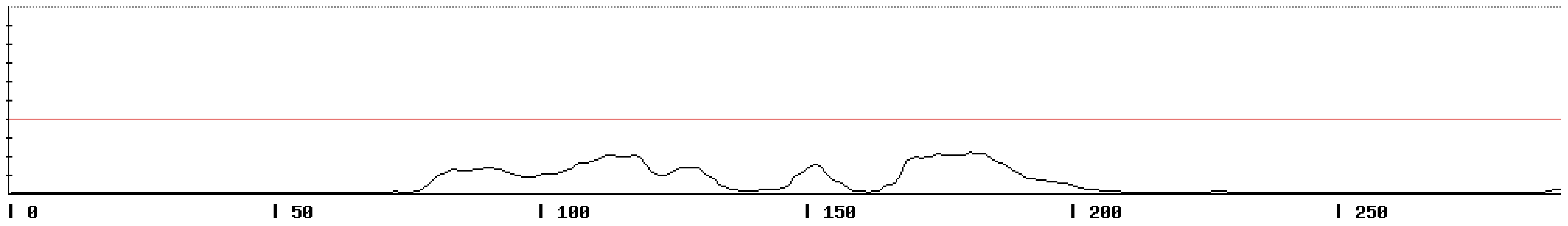

Predictions for Hordeumvulgare_MLOC_64057.1

Viterbi Path

Viterbi Path

Posterior @ 0.4

Posterior @ 0.4

Posterior Graphic. Threshold Marked @ 0.4

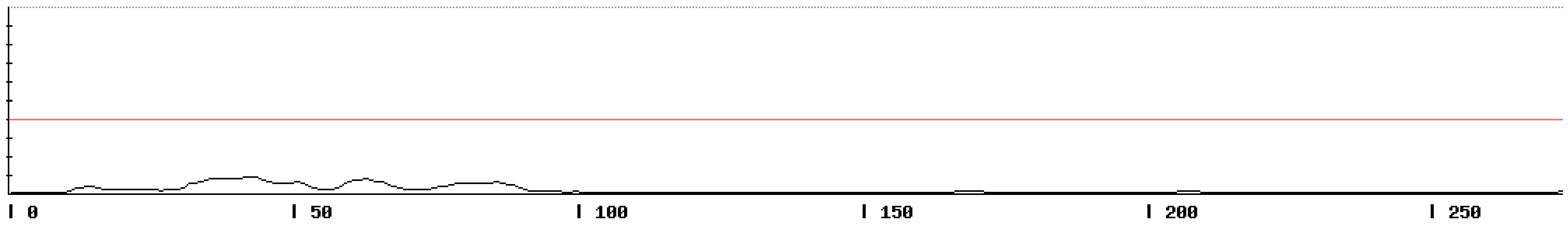

Predictions for Hordeumvulgare_MLOC_64240.1

Viterbi Path

Viterbi Path

Posterior @ 0.4

Posterior @ 0.4

Posterior Graphic. Threshold Marked @ 0.4

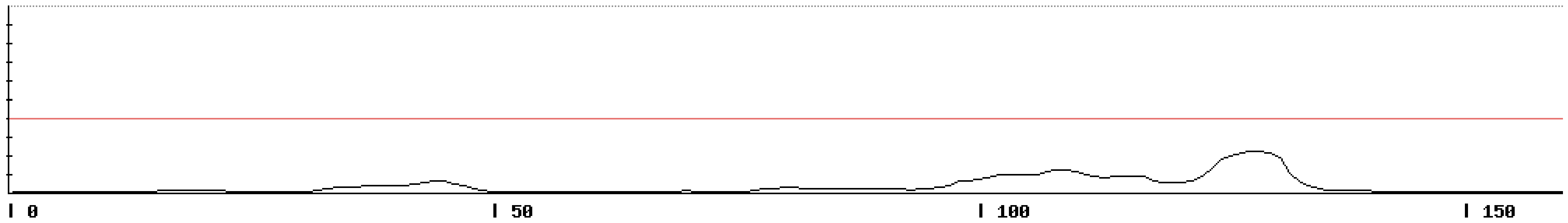

Predictions for Hordeumvulgare_MLOC_64644.1

Viterbi Path

Viterbi Path

Posterior @ 0.4

Posterior @ 0.4

Posterior Graphic. Threshold Marked @ 0.4

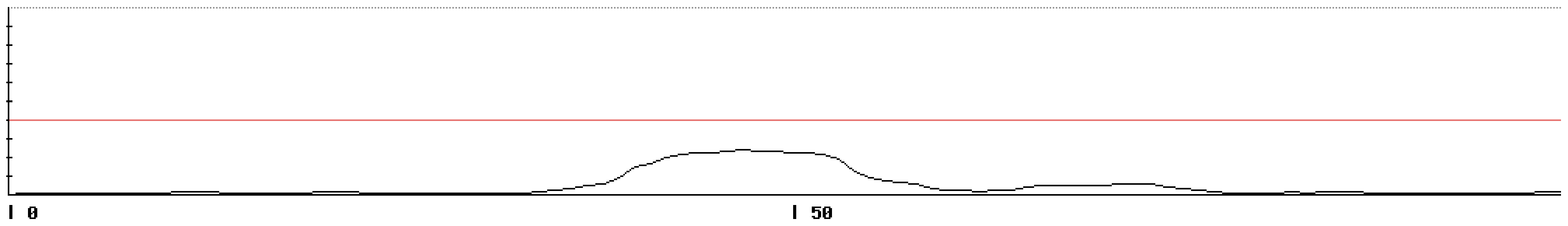

Predictions for Hordeumvulgare_MLOC_65101.1

Viterbi Path

Viterbi Path

Posterior @ 0.4

Posterior @ 0.4

Posterior Graphic. Threshold Marked @ 0.4

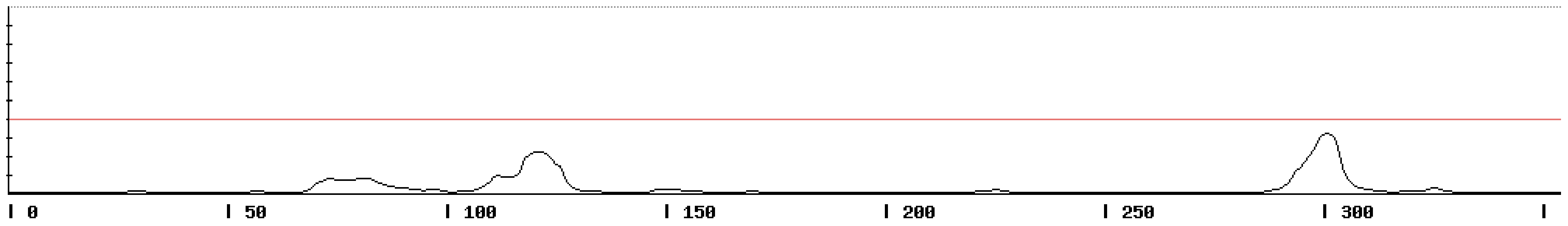

Predictions for Hordeumvulgare_MLOC_65286.1

Viterbi Path

Viterbi Path

Posterior @ 0.4

Posterior @ 0.4

Posterior Graphic. Threshold Marked @ 0.4

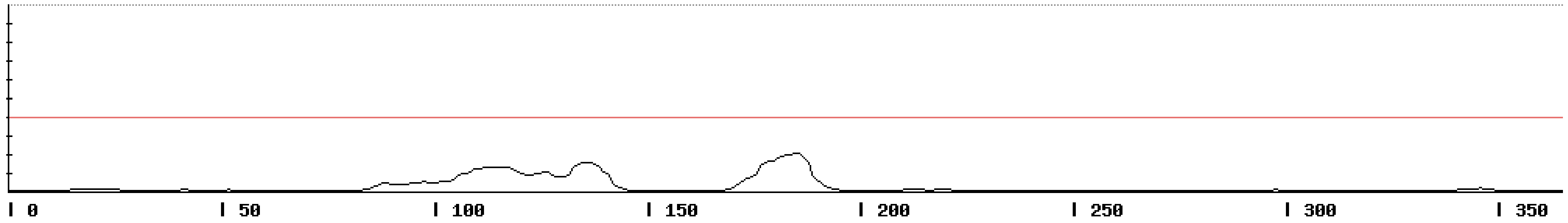

Predictions for Hordeumvulgare_MLOC_65400.1

Viterbi Path

Viterbi Path

Posterior @ 0.4

Posterior @ 0.4

Posterior Graphic. Threshold Marked @ 0.4

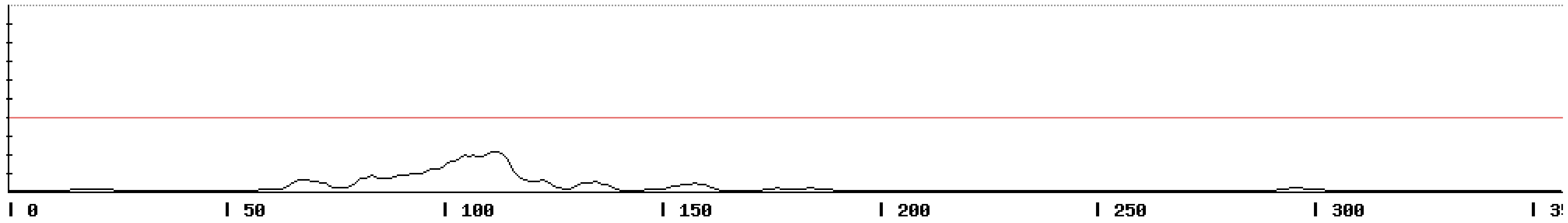

Predictions for Hordeumvulgare_MLOC_65456.1

Viterbi Path

Viterbi Path

Posterior @ 0.4

Posterior @ 0.4

Posterior Graphic. Threshold Marked @ 0.4

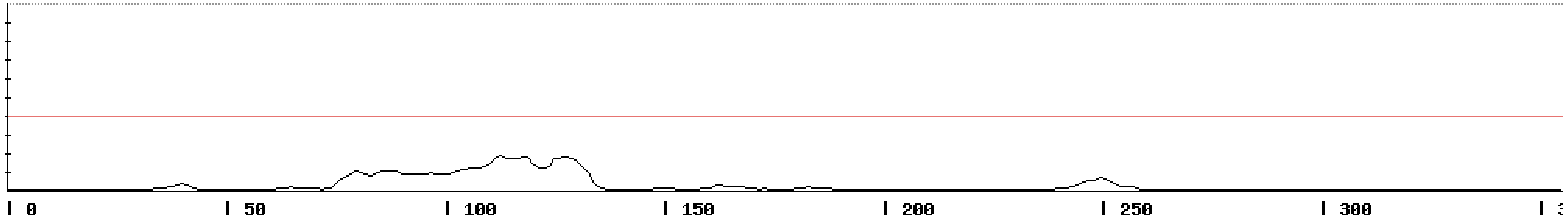

Predictions for Hordeumvulgare_MLOC_65480.1

Viterbi Path

Viterbi Path

Posterior @ 0.4

Posterior @ 0.4

Posterior Graphic. Threshold Marked @ 0.4

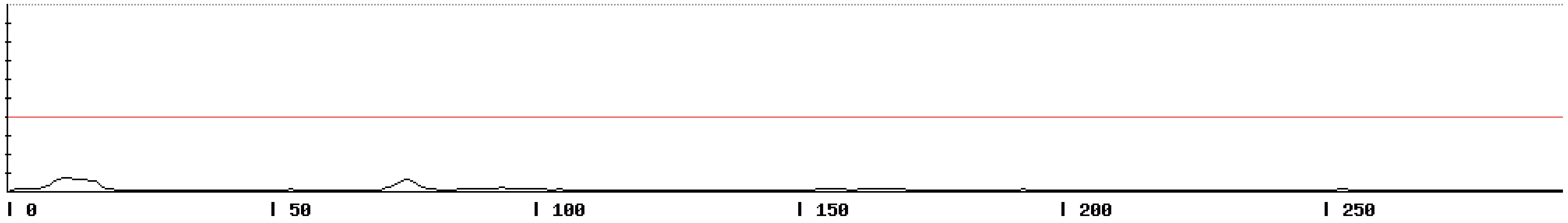

Predictions for Hordeumvulgare_MLOC_65714.1

Viterbi Path

Viterbi Path

Posterior @ 0.4

Posterior @ 0.4

Posterior Graphic. Threshold Marked @ 0.4

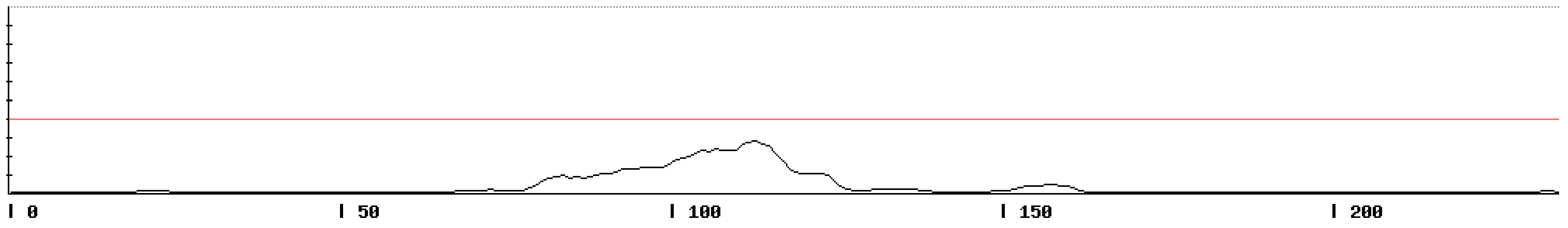

Predictions for Hordeumvulgare_MLOC_66083.1

Viterbi Path

Viterbi Path

Posterior @ 0.4

Posterior @ 0.4

Posterior Graphic. Threshold Marked @ 0.4

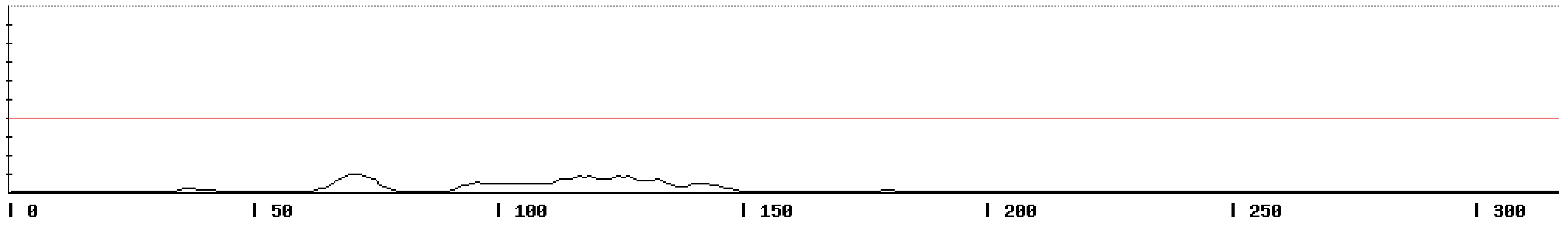

Predictions for Hordeumvulgare_MLOC_66348.1	
Viterbi Path	
Viterbi Path	
Posterior @ 0.4	
Posterior @ 0.4	
Posterior Graphic. Threshold Marked @ 0.4	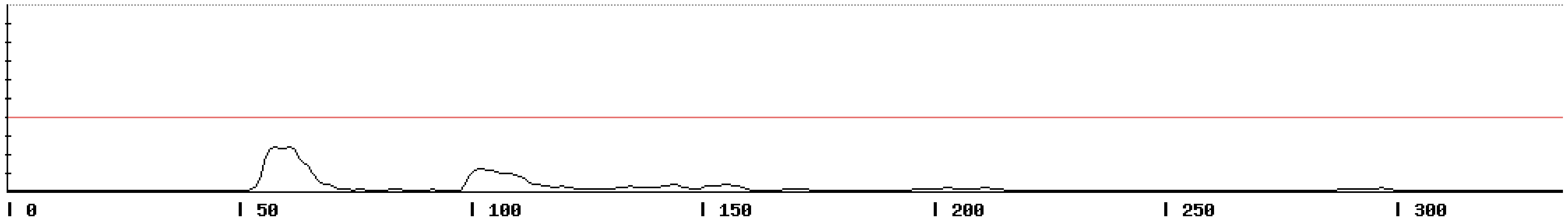

Predictions for Hordeumvulgare_MLOC_66537.1	
Viterbi Path	
Viterbi Path	
Posterior @ 0.4	119 - RKRRKI - 124
Posterior @ 0.4	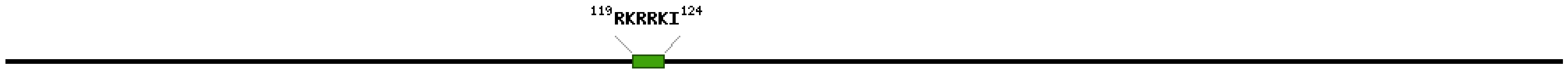
Posterior Graphic. Threshold Marked @ 0.4	

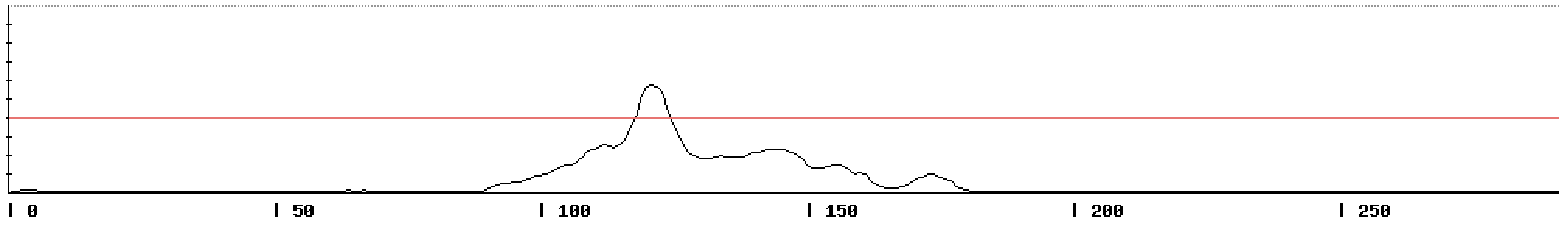

Predictions for Hordeumvulgare_MLOC_66924.1	
Viterbi Path	
Viterbi Path	
Posterior @ 0.4	
Posterior @ 0.4	
Posterior Graphic. Threshold Marked @ 0.4	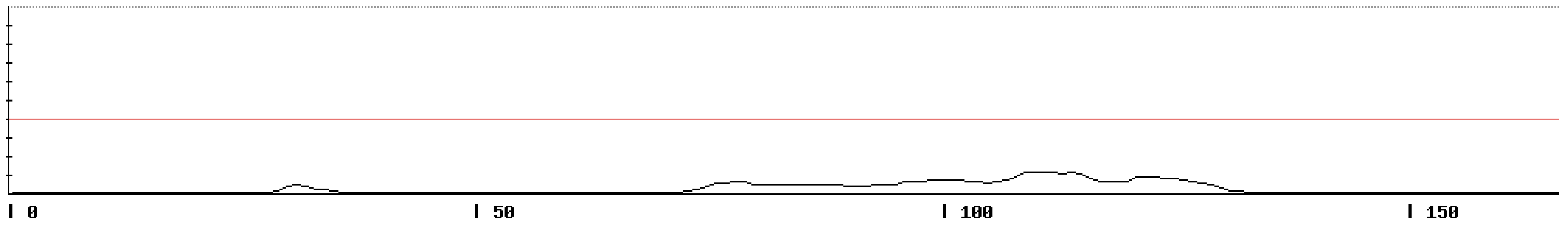

Predictions for Hordeumvulgare_MLOC_67250.1	
Viterbi Path	
Viterbi Path	
Posterior @ 0.4	
Posterior @ 0.4	

Posterior Graphic. Threshold Marked @ 0.4	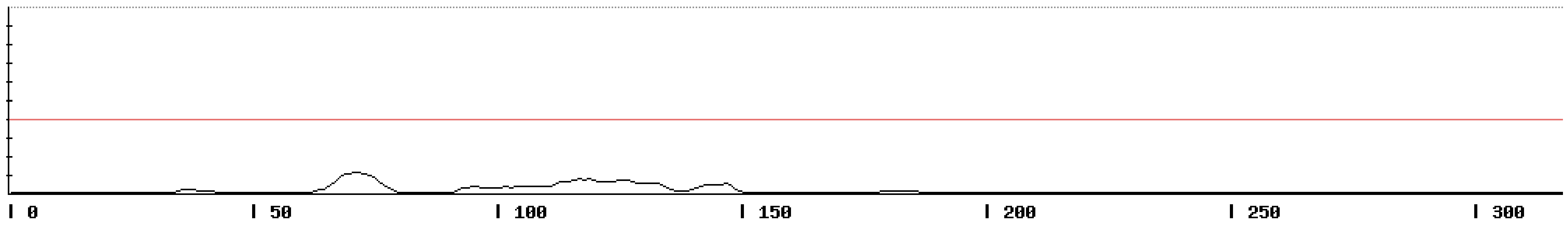

Predictions for Hordeumvulgare_MLOC_67296.1

Viterbi Path	
Viterbi Path	
Posterior @ 0.4	127 - VPRRAKGNKGSKA - 139
Posterior @ 0.4	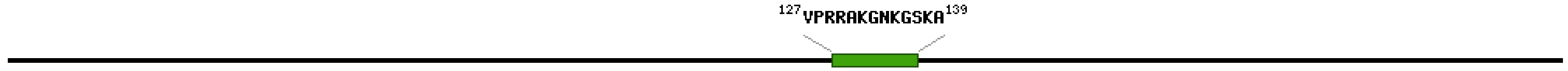
Posterior Graphic. Threshold Marked @ 0.4	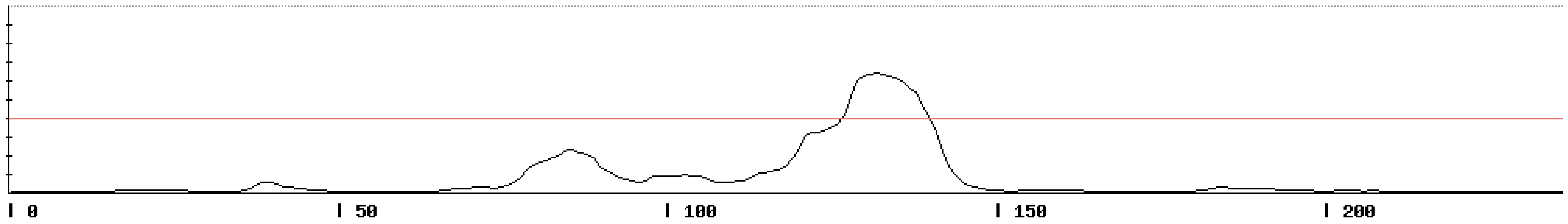

Predictions for Hordeumvulgare_MLOC_68185.1

Viterbi Path	
Viterbi Path	
Posterior @ 0.4	12 - SKKKKSSK - 19
Posterior @ 0.4	

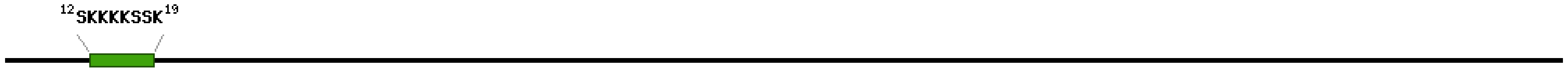

Posterior Graphic. Threshold Marked @ 0.4

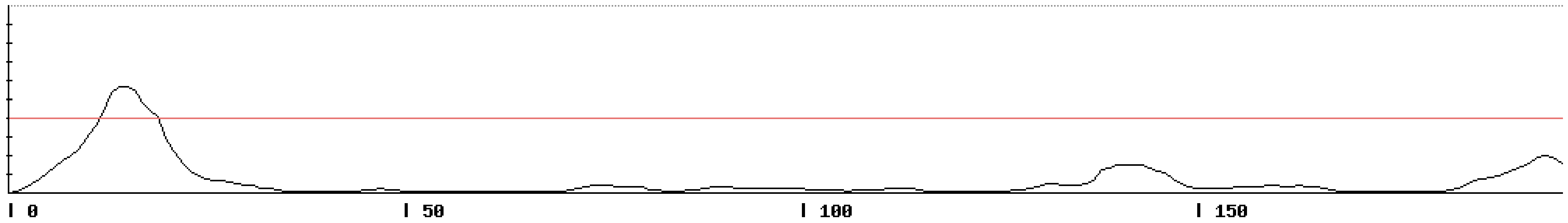

Predictions for Hordeumvulgare_MLOC_68284.1

Viterbi Path

Viterbi Path

Posterior @ 0.4

Posterior @ 0.4

Posterior Graphic. Threshold Marked @ 0.4

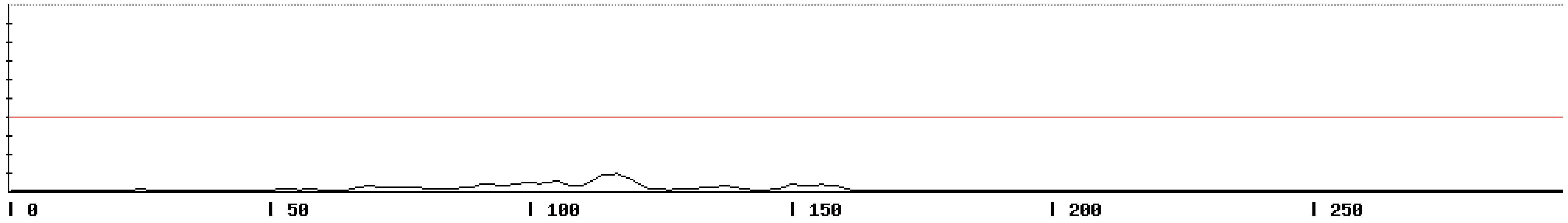

Predictions for Hordeumvulgare_MLOC_6901.1

Viterbi Path

Viterbi Path

Posterior @ 0.4

Posterior @ 0.4

Posterior Graphic. Threshold Marked @ 0.4

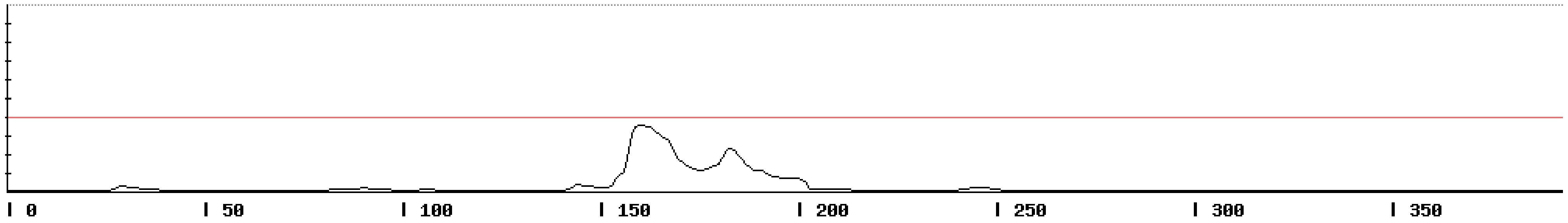

Predictions for Hordeumvulgare_MLOC_69621.1

Viterbi Path

Viterbi Path

Posterior @ 0.4

Posterior @ 0.4

Posterior Graphic. Threshold Marked @ 0.4

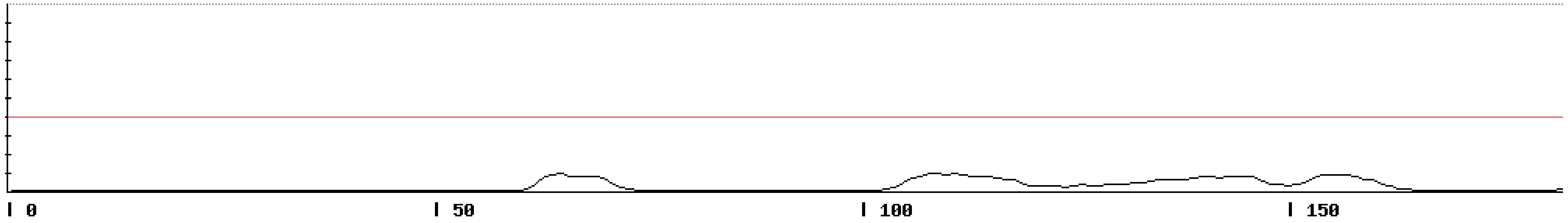

Predictions for Hordeumvulgare_MLOC_70929.1

Viterbi Path

Viterbi Path

Posterior @ 0.4	
Posterior @ 0.4	
Posterior Graphic. Threshold Marked @ 0.4	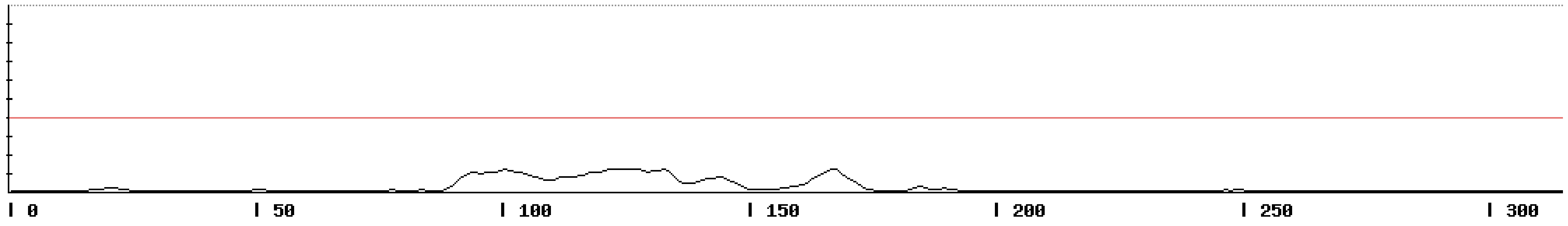

Predictions for Hordeumvulgare_MLOC_71128.1

Viterbi Path	
Viterbi Path	
Posterior @ 0.4	93 - GKDRKVN - 99<br>102 - K - 102
Posterior @ 0.4	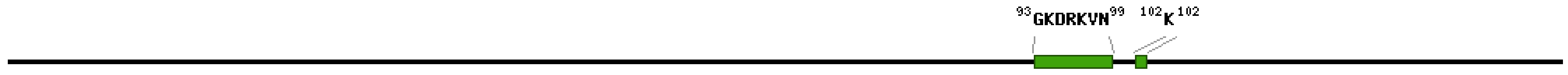
Posterior Graphic. Threshold Marked @ 0.4	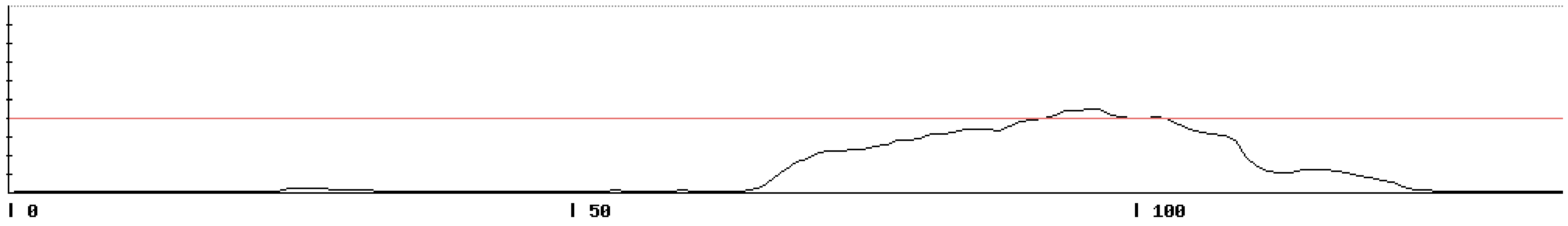

Predictions for Hordeumvulgare_MLOC_72553.1	
Viterbi Path	
Viterbi Path	
Posterior @ 0.4	137 - RKRRK - 141
Posterior @ 0.4	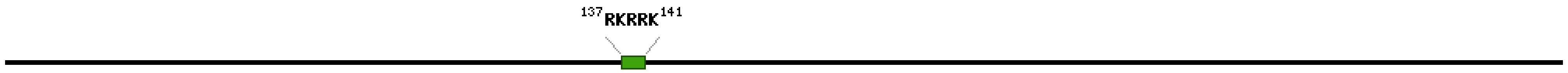
Posterior Graphic. Threshold Marked @ 0.4	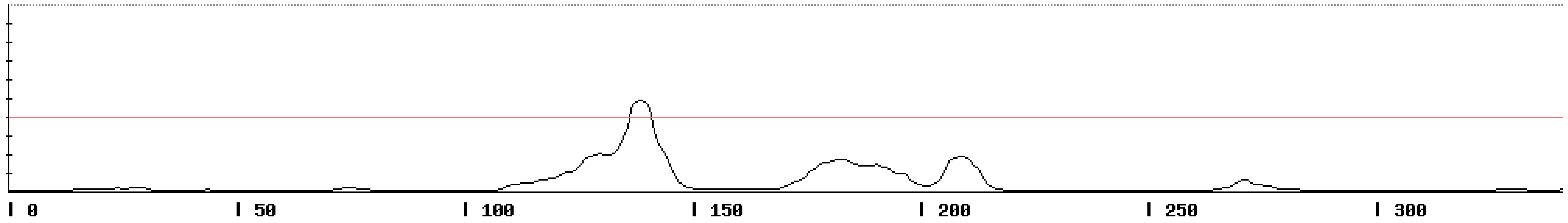

Predictions for Hordeumvulgare_MLOC_72737.1	
Viterbi Path	
Viterbi Path	
Posterior @ 0.4	
Posterior @ 0.4	
Posterior Graphic. Threshold Marked @ 0.4	

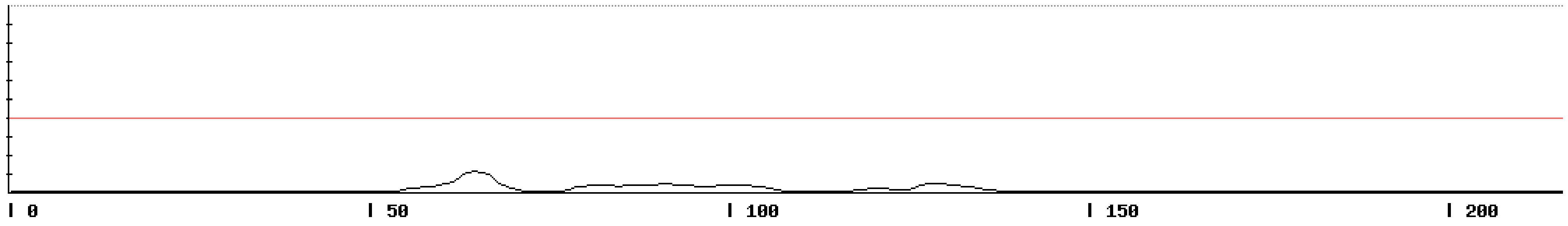

Predictions for Hordeumvulgare_MLOC_74840.1

Viterbi Path	254 - GRKKRKTDSRNPGPVLRPARGVLKLTRPATPGRKKK - 289
Viterbi Path	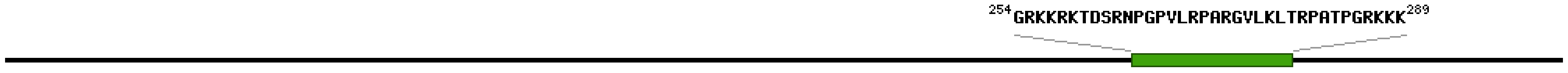
Posterior @ 0.4	202 - VEPKAAAGRKRNSKRR - 217<br>253 - SGRKKRKTDSRNPGPVLRPARGVLKLTRPATPGRKKK - 289
Posterior @ 0.4	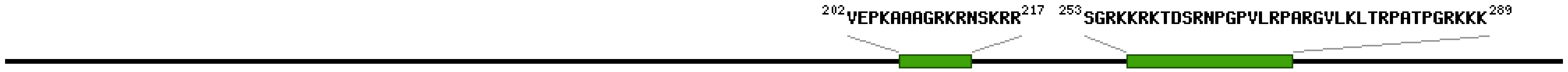
Posterior Graphic. Threshold Marked @ 0.4	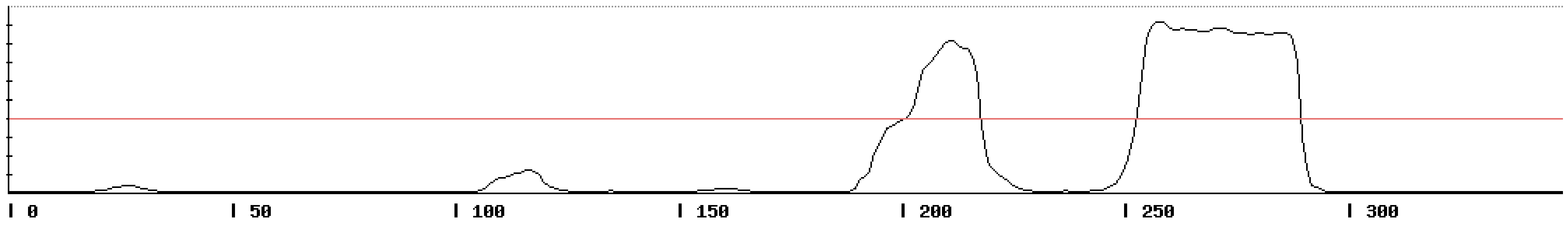

Predictions for Hordeumvulgare_MLOC_75297.2

Viterbi Path	
Viterbi Path	
Posterior @ 0.4	144 - RGRAPR - 149
Posterior @ 0.4	

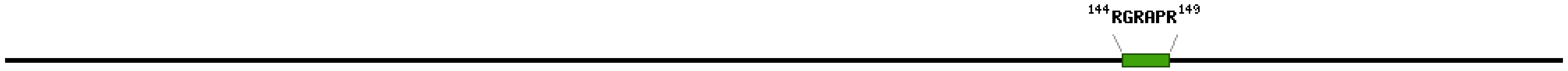

Posterior Graphic. Threshold Marked @ 0.4

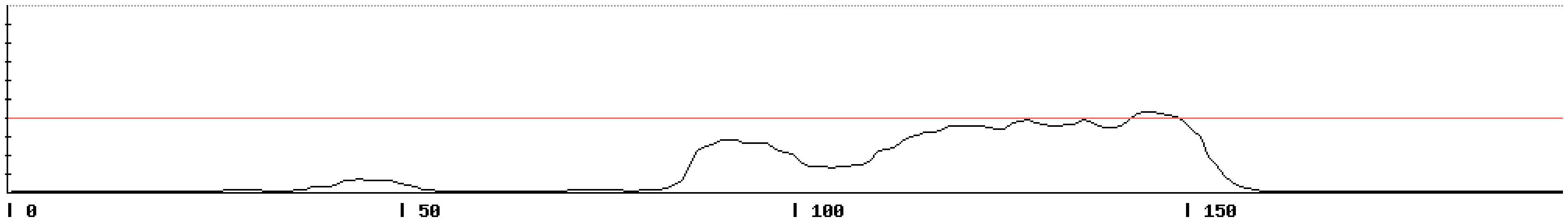

Predictions for Hordeumvulgare_MLOC_75524.1

Viterbi Path

Viterbi Path

Posterior @ 0.4

Posterior @ 0.4

Posterior Graphic. Threshold Marked @ 0.4

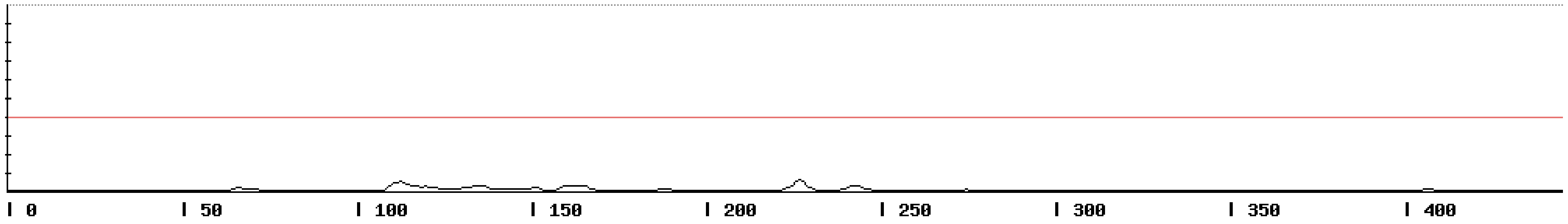

Predictions for Hordeumvulgare_MLOC_75795.1

Viterbi Path

Viterbi Path

Posterior @ 0.4

Posterior @ 0.4

Posterior Graphic. Threshold Marked @ 0.4

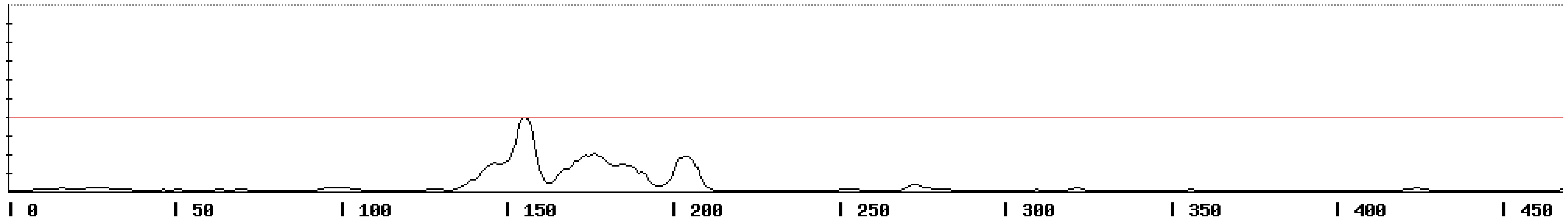

Predictions for Hordeumvulgare_MLOC_7757.1

Viterbi Path

Viterbi Path

Posterior @ 0.4

213 - KRKR - 216

Posterior @ 0.4

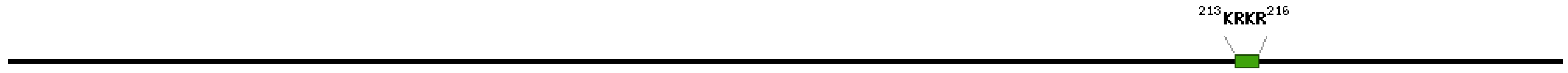

Posterior Graphic. Threshold Marked @ 0.4

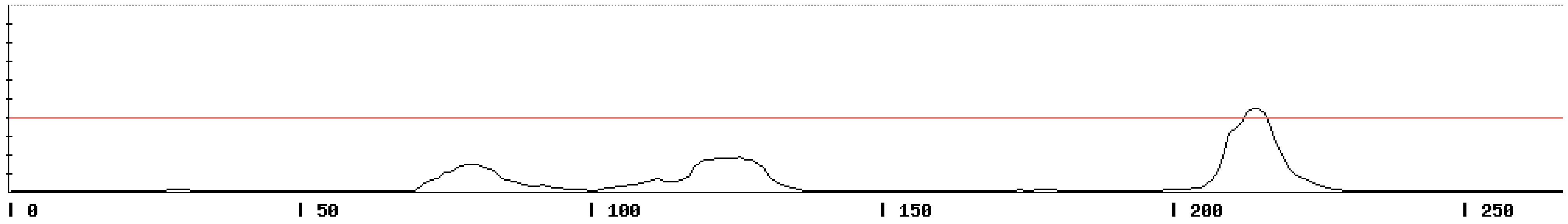

Predictions for Hordeumvulgare_MLOC_79696.1

Viterbi Path

Viterbi Path

Posterior @ 0.4	80 - GRRRKRTVK - 88
Posterior @ 0.4	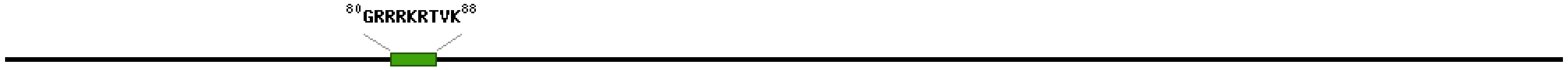
Posterior Graphic. Threshold Marked @ 0.4	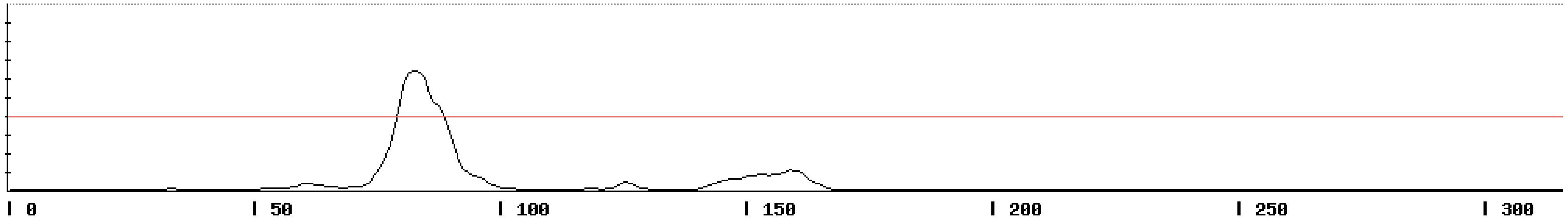

Revision r.9 - Last Updated on : February 24 2015 17:45:20. alex.nguyenba@utoronto.ca

Intended to be used with Firefox

Viterbi Path

Posterior @ 0.4

Posterior @ 0.4

Posterior Graphic. Threshold Marked @ 0.4

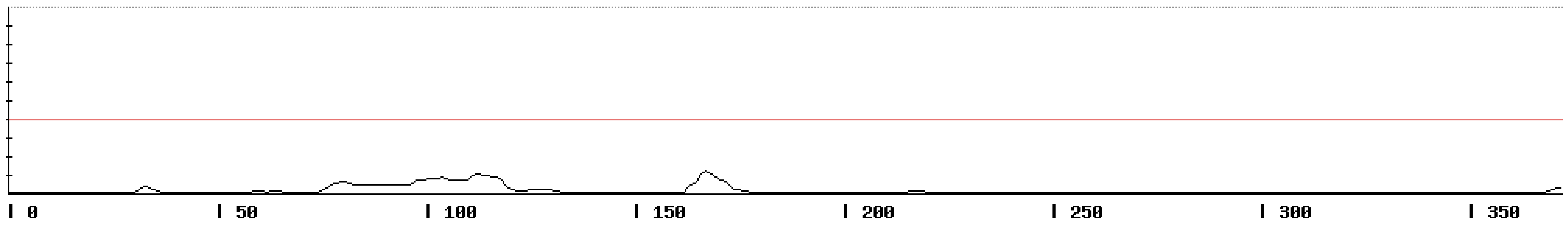

Predictions for Humuluslupulus_HL.SW.v1.0.G000619.1

Viterbi Path

Viterbi Path

Posterior @ 0.4

Posterior @ 0.4

Posterior Graphic. Threshold Marked @ 0.4

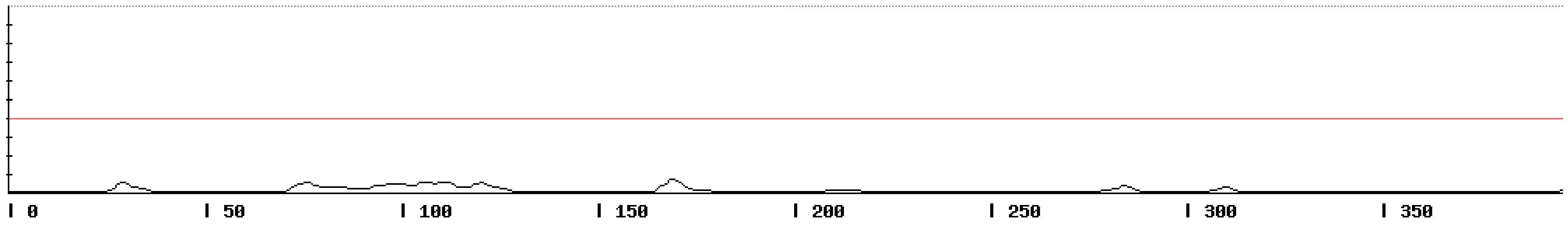

Predictions for Humuluslupulus_HL.SW.v1.0.G000878.1

Viterbi Path

Viterbi Path

Posterior @ 0.4

Posterior @ 0.4

Posterior Graphic. Threshold Marked @ 0.4

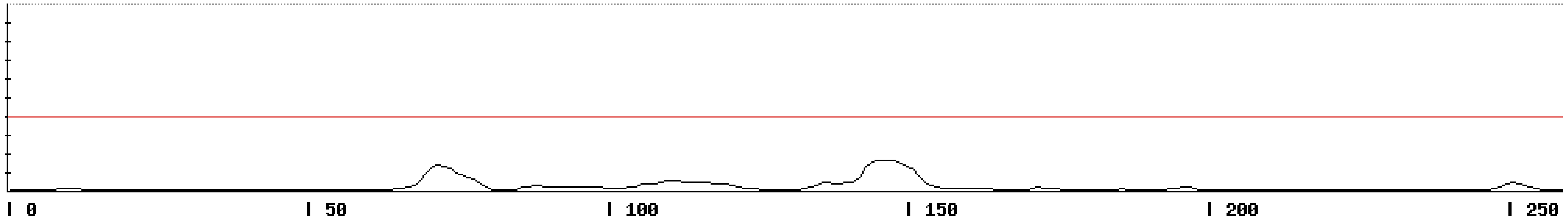

Predictions for Humuluslupulus_HL.SW.v1.0.G000888.1

Viterbi Path

Viterbi Path

Posterior @ 0.4

Posterior @ 0.4

Posterior Graphic. Threshold Marked @ 0.4

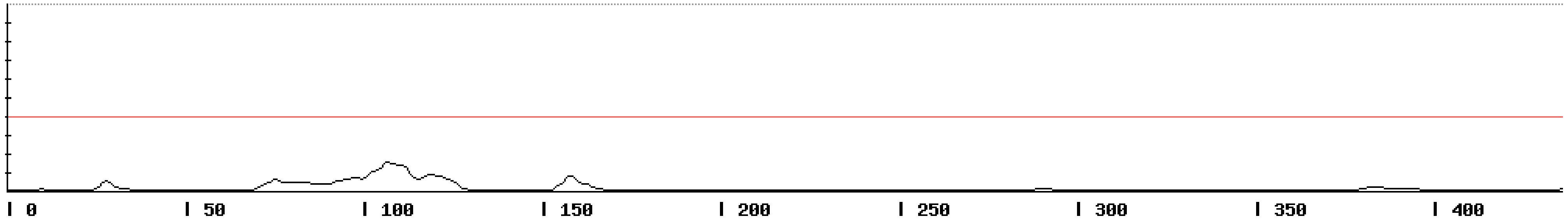

Predictions for Humuluslupulus_HL.SW.v1.0.G001728.1

Viterbi Path	
Viterbi Path	
Posterior @ 0.4	107 - KGK - 109
Posterior @ 0.4	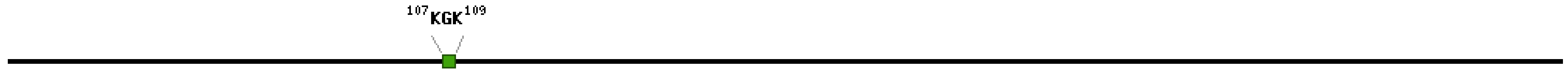
Posterior Graphic. Threshold Marked @ 0.4	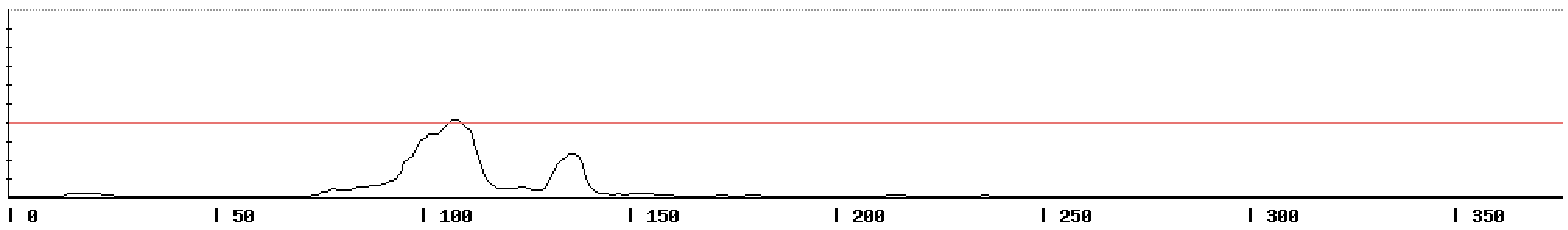

Predictions for Humuluslupulus_HL.SW.v1.0.G002087.1

Viterbi Path	
Viterbi Path	
Posterior @ 0.4	
Posterior @ 0.4	
Posterior Graphic. Threshold Marked @ 0.4	

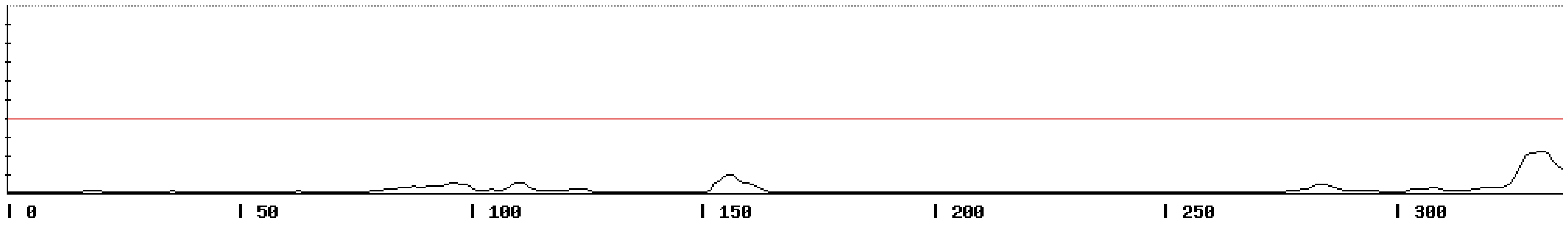

Predictions for Humuluslupulus_HL.SW.v1.0.G004549.1

Viterbi Path

Viterbi Path

Posterior @ 0.4

Posterior @ 0.4

Posterior Graphic. Threshold Marked @ 0.4

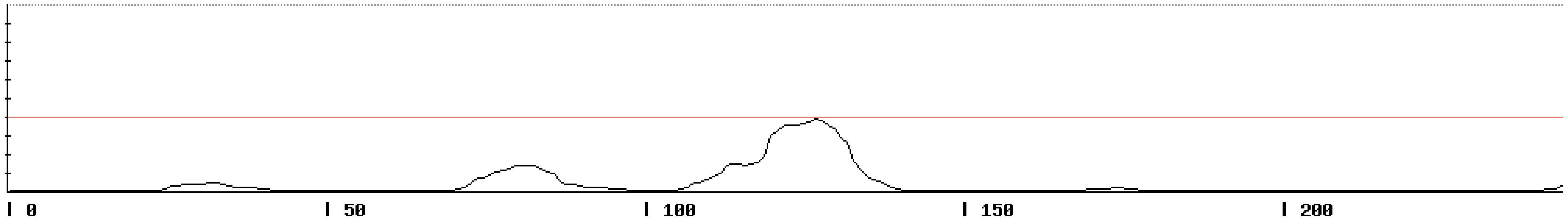

Predictions for Humuluslupulus_HL.SW.v1.0.G005163.1

Viterbi Path

Viterbi Path

Posterior @ 0.4

Posterior @ 0.4

Posterior Graphic. Threshold Marked @ 0.4

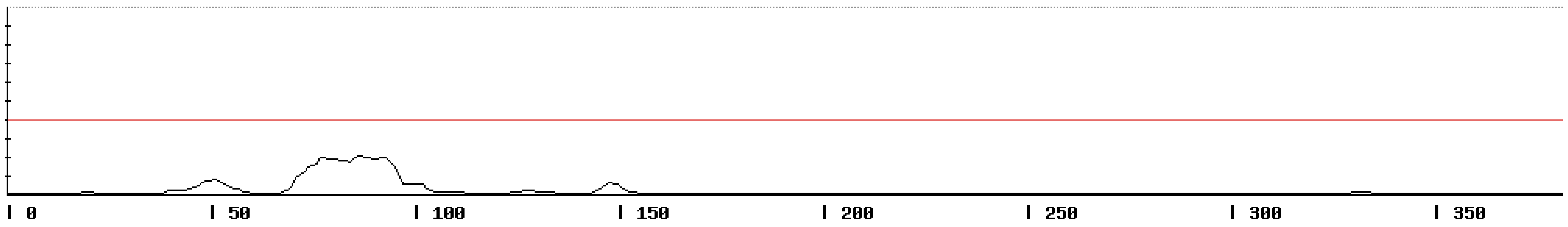

Predictions for Humuluslupulus_HL.SW.v1.0.G005365.1

Viterbi Path

Viterbi Path

Posterior @ 0.4

95 - ADKPIGKPKPLGIKKA - 110

Posterior @ 0.4

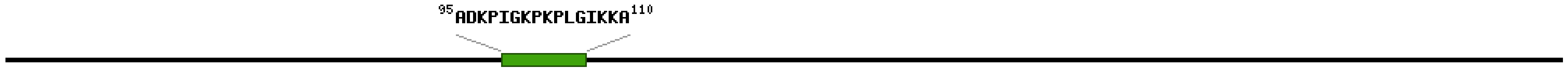

Posterior Graphic. Threshold Marked @ 0.4

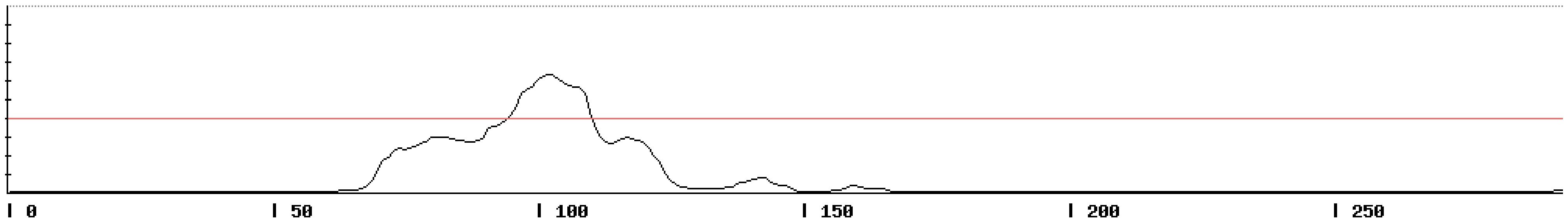

Predictions for Humuluslupulus_HL.SW.v1.0.G005390.1

Viterbi Path

Viterbi Path

Posterior @ 0.4

Posterior @ 0.4

Posterior Graphic. Threshold Marked @ 0.4

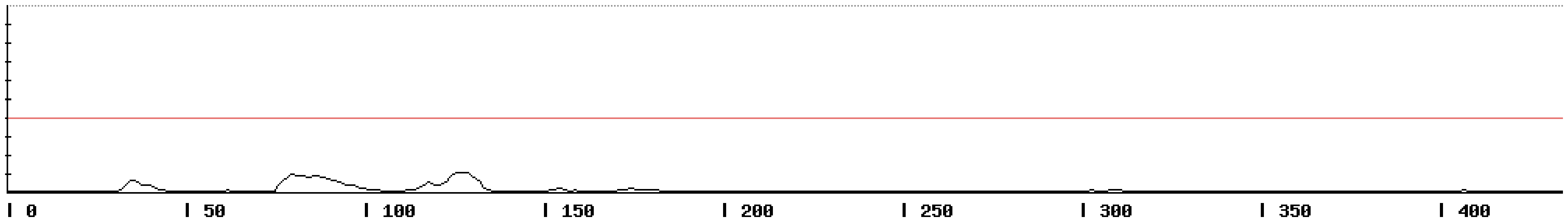

Predictions for Humuluslupulus_HL.SW.v1.0.G005640.1

Viterbi Path

Viterbi Path

Posterior @ 0.4

Posterior @ 0.4

Posterior Graphic. Threshold Marked @ 0.4

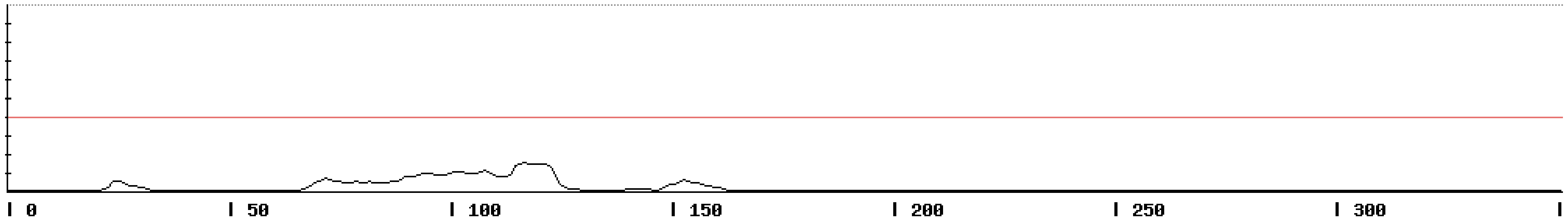

Predictions for Humuluslupulus_HL.SW.v1.0.G006494.1

Viterbi Path

Viterbi Path

Posterior @ 0.4

Posterior @ 0.4

Posterior Graphic. Threshold Marked @ 0.4

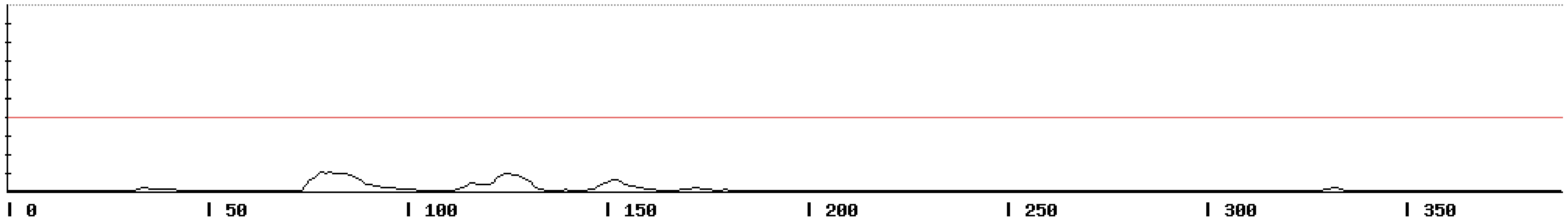

Predictions for Humuluslupulus_HL.SW.v1.0.G008530.1

Viterbi Path

Viterbi Path

Posterior @ 0.4

Posterior @ 0.4

Posterior Graphic. Threshold Marked @ 0.4

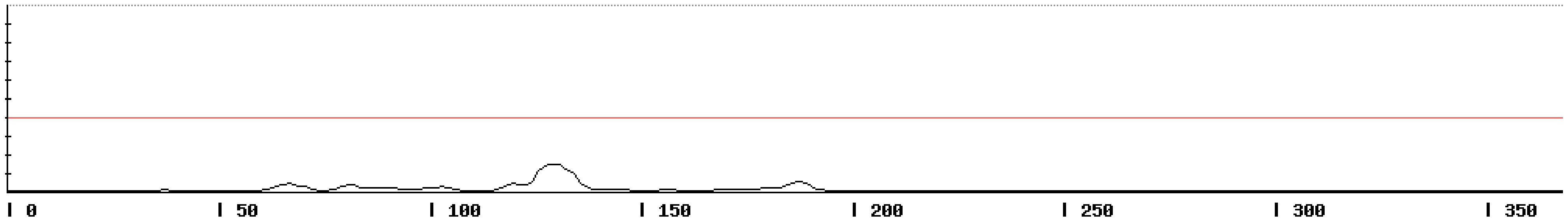

Predictions for Humuluslupulus_HL.SW.v1.0.G008629.1

Viterbi Path

Viterbi Path

Posterior @ 0.4	
Posterior @ 0.4	
Posterior Graphic. Threshold Marked @ 0.4	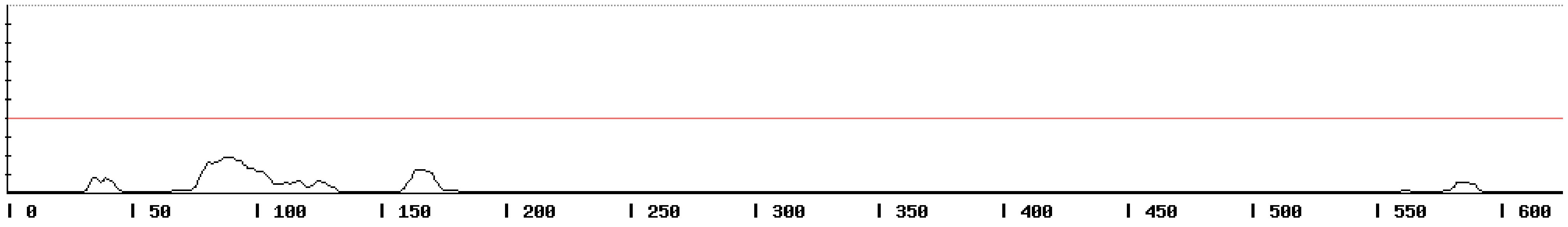

Predictions for Humuluslupulus_HL.SW.v1.0.G009272.1

Viterbi Path	
Viterbi Path	
Posterior @ 0.4	145 - RKRRK - 149
Posterior @ 0.4	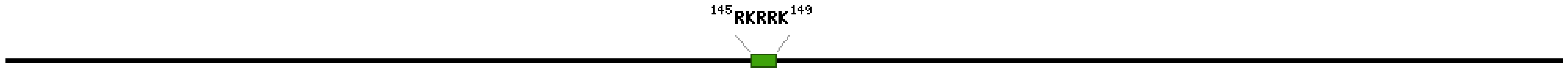
Posterior Graphic. Threshold Marked @ 0.4	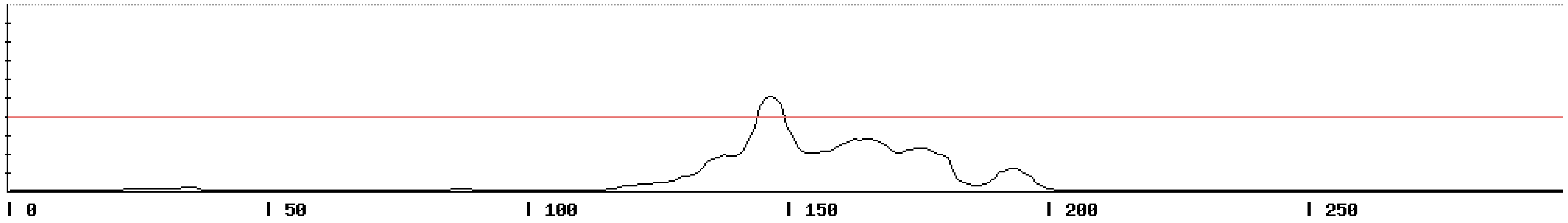

Predictions for Humuluslupulus_HL.SW.v1.0.G009399.1

Viterbi Path

Viterbi Path

Posterior @ 0.4

Posterior @ 0.4

Posterior Graphic. Threshold Marked @ 0.4

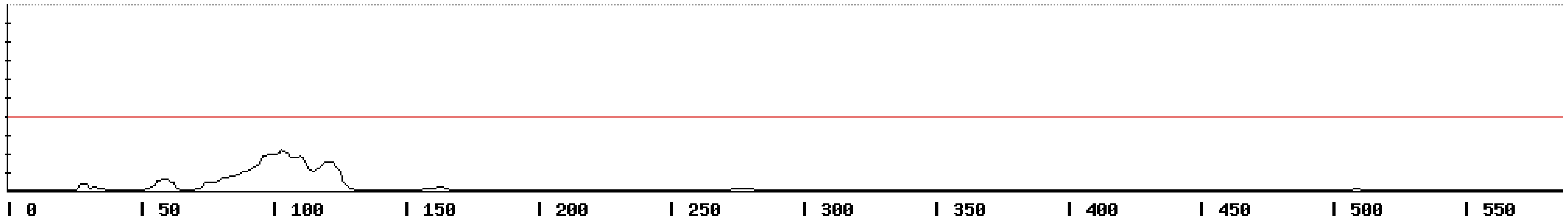

Predictions for Humuluslupulus_HL.SW.v1.0.G009400.1

Viterbi Path

Viterbi Path

Posterior @ 0.4

Posterior @ 0.4

Posterior Graphic. Threshold Marked @ 0.4

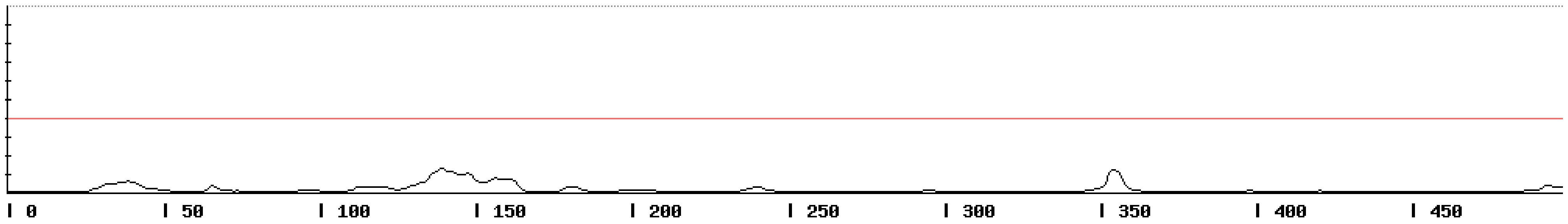

Predictions for Humuluslupulus_HL.SW.v1.0.G009649.1	
Viterbi Path	
Viterbi Path	
Posterior @ 0.4	
Posterior @ 0.4	
Posterior Graphic. Threshold Marked @ 0.4	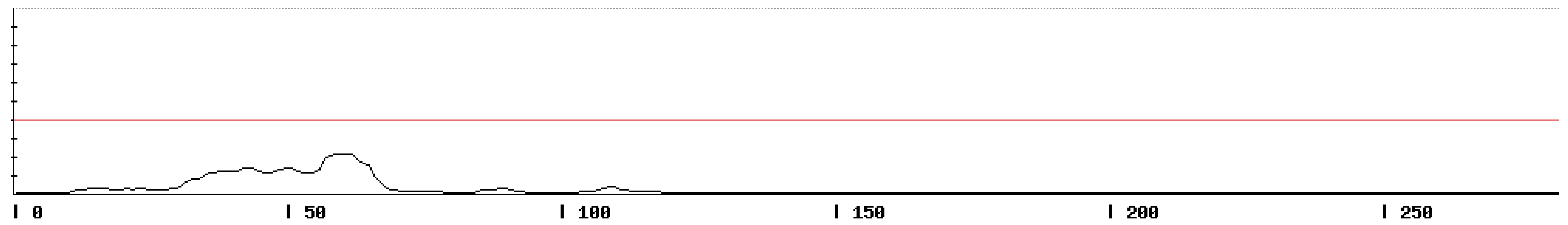

Predictions for Humuluslupulus_HL.SW.v1.0.G009784.1	
Viterbi Path	
Viterbi Path	
Posterior @ 0.4	
Posterior @ 0.4	
Posterior Graphic. Threshold Marked @ 0.4	

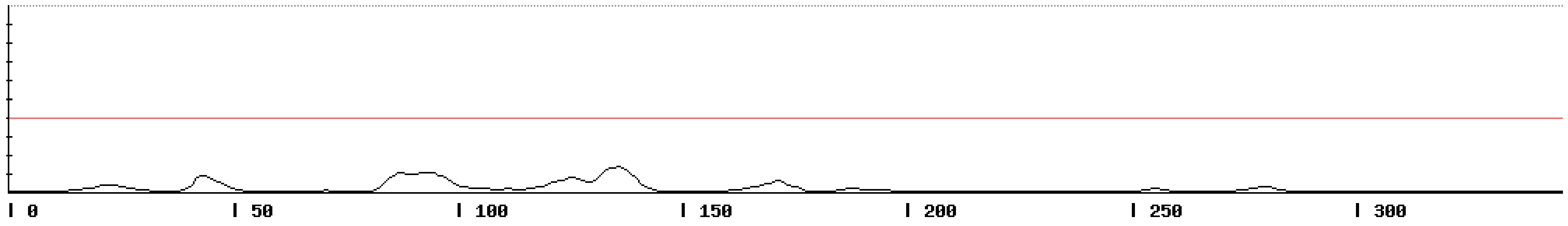

Predictions for Humuluslupulus_HL.SW.v1.0.G009856.1

Viterbi Path

Viterbi Path

Posterior @ 0.4

Posterior @ 0.4

Posterior Graphic. Threshold Marked @ 0.4

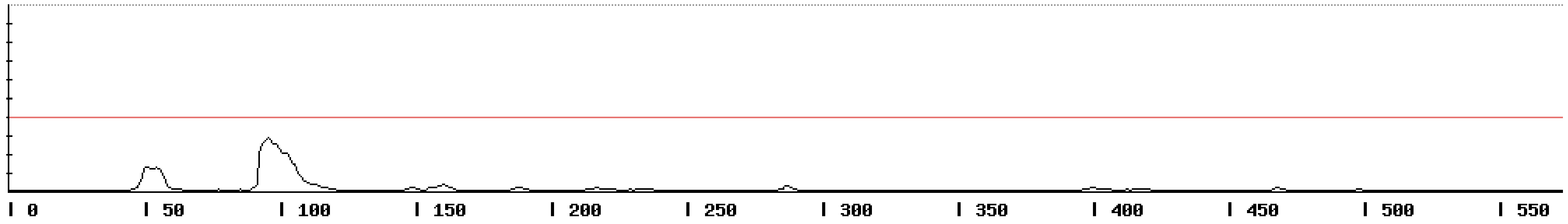

Predictions for Humuluslupulus_HL.SW.v1.0.G010657.1

Viterbi Path

Viterbi Path

Posterior @ 0.4

Posterior @ 0.4

Posterior Graphic. Threshold Marked @ 0.4	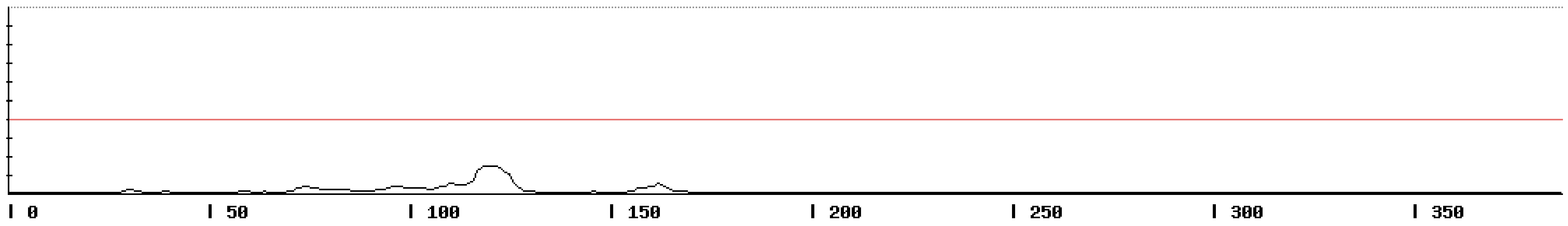

Predictions for Humuluslupulus_HL.SW.v1.0.G011042.1

Viterbi Path	
Viterbi Path	
Posterior @ 0.4	
Posterior @ 0.4	
Posterior Graphic. Threshold Marked @ 0.4	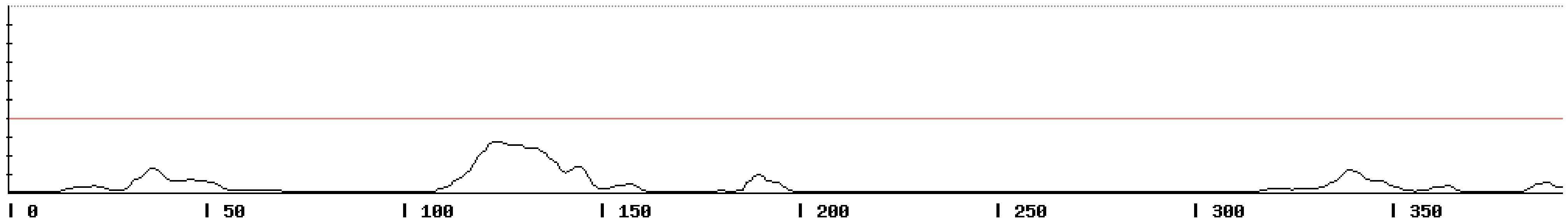

Predictions for Humuluslupulus_HL.SW.v1.0.G011145.1

Viterbi Path	
Viterbi Path	
Posterior @ 0.4	
Posterior @ 0.4	

Posterior Graphic. Threshold Marked @ 0.4

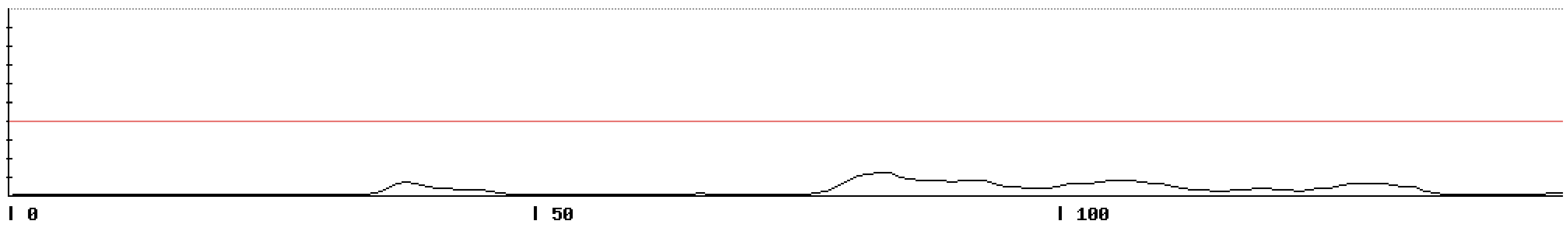

Predictions for Humuluslupulus_HL.SW.v1.0.G011193.1

Viterbi Path

Viterbi Path

Posterior @ 0.4

Posterior @ 0.4

Posterior Graphic. Threshold Marked @ 0.4

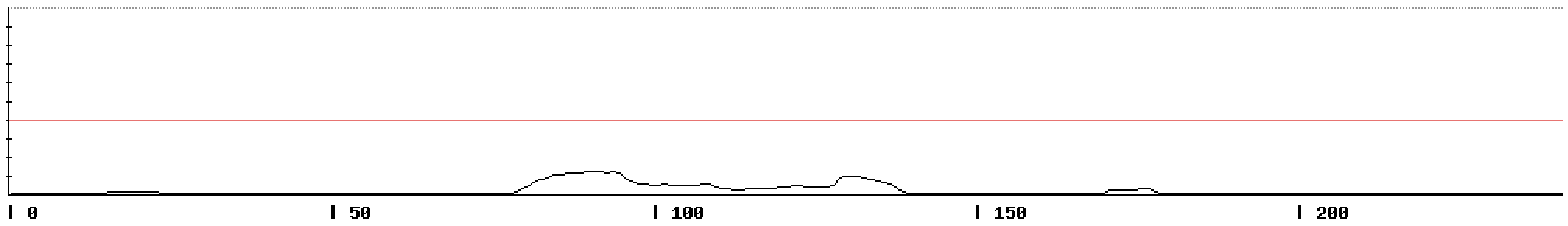

Predictions for Humuluslupulus_HL.SW.v1.0.G012590.1

Viterbi Path

Viterbi Path

Posterior @ 0.4

Posterior @ 0.4

Posterior Graphic. Threshold Marked @ 0.4

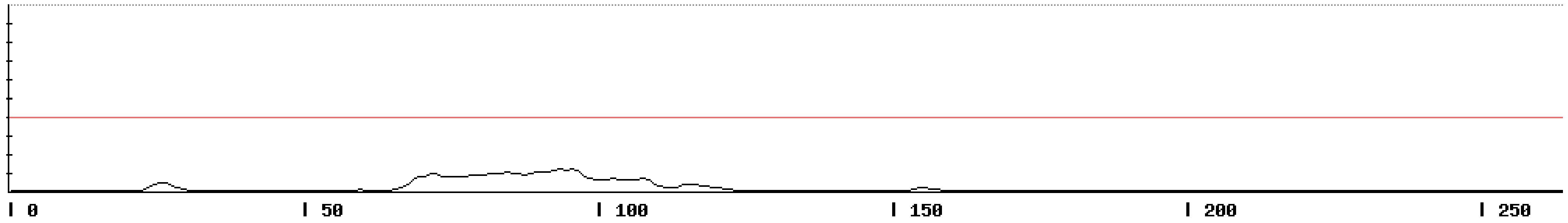

Predictions for Humuluslupulus_HL.SW.v1.0.G013166.1

Viterbi Path

Viterbi Path

Posterior @ 0.4

Posterior @ 0.4

Posterior Graphic. Threshold Marked @ 0.4

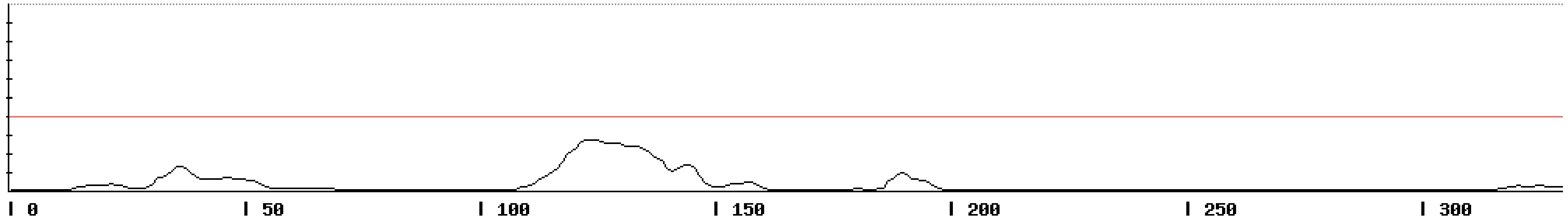

Predictions for Humuluslupulus_HL.SW.v1.0.G013425.1

Viterbi Path

Viterbi Path

Posterior @ 0.4	
Posterior @ 0.4	
Posterior Graphic. Threshold Marked @ 0.4	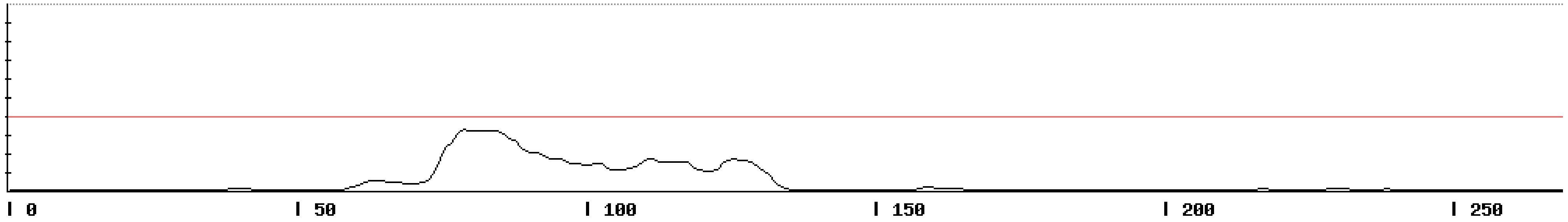

Predictions for Humuluslupulus_HL.SW.v1.0.G014470.1

Viterbi Path	
Viterbi Path	
Posterior @ 0.4	
Posterior @ 0.4	
Posterior Graphic. Threshold Marked @ 0.4	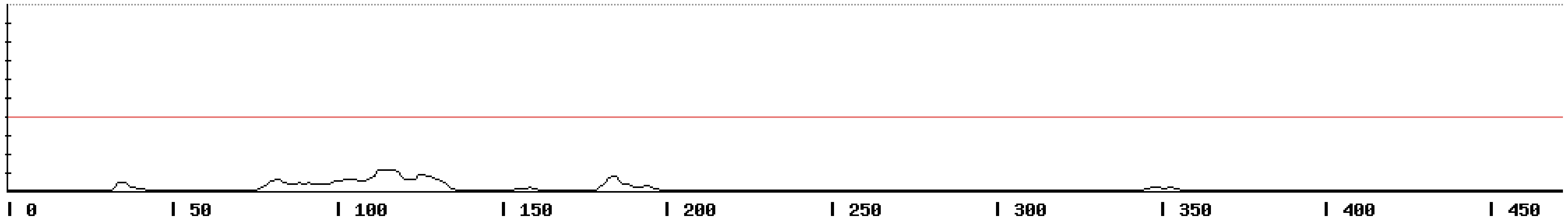

Predictions for Humuluslupulus_HL.SW.v1.0.G016378.1

Viterbi Path	
Viterbi Path	
Posterior @ 0.4	
Posterior @ 0.4	
Posterior Graphic. Threshold Marked @ 0.4	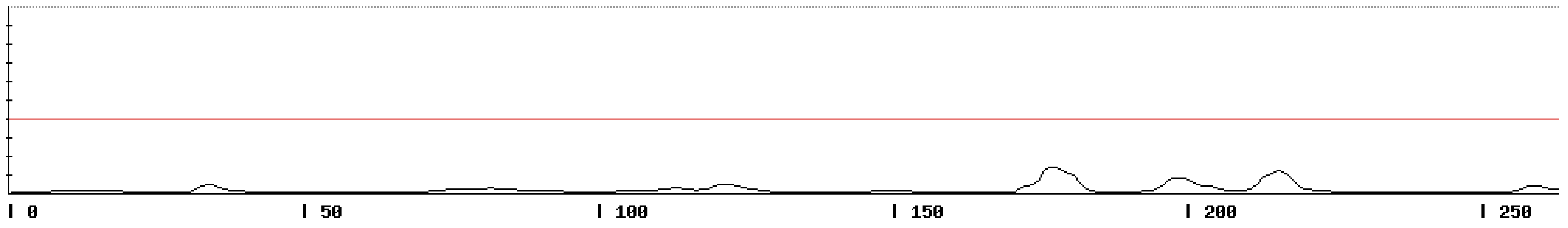

Predictions for Humuluslupulus_HL.SW.v1.0.G016542.1

Viterbi Path	
Viterbi Path	
Posterior @ 0.4	171 - KKRKLNNSKQRKR - 183
Posterior @ 0.4	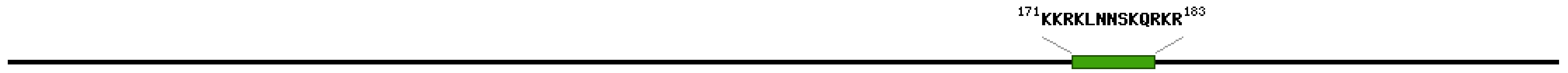
Posterior Graphic. Threshold Marked @ 0.4	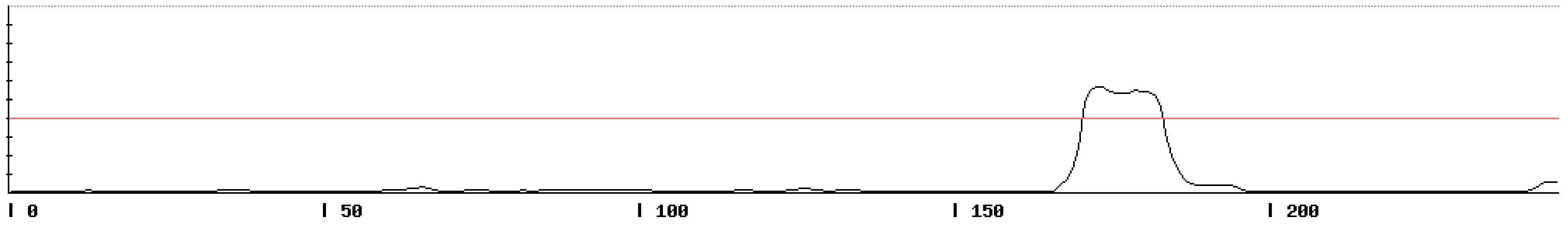

Predictions for Humuluslupulus_HL.SW.v1.0.G017885.1

Viterbi Path	
Viterbi Path	
Posterior @ 0.4	
Posterior @ 0.4	
Posterior Graphic. Threshold Marked @ 0.4	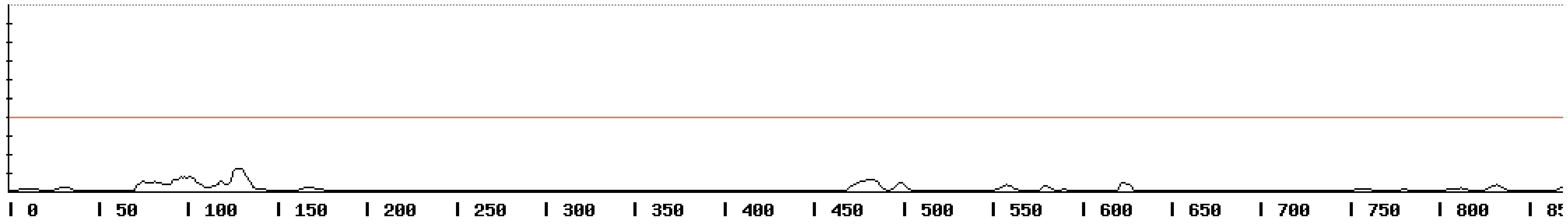

Predictions for Humuluslupulus_HL.SW.v1.0.G018182.1

Viterbi Path	120 - KGRKANKQKSARVISHSKKKGRK - 142
Viterbi Path	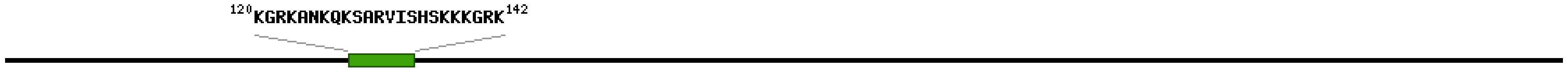
Posterior @ 0.4	50 - RKGRKTETGSWKITGKRKKIG - 70<br>118 - DNKGRKANKQKSARVISHSKKKGRKA - 143
Posterior @ 0.4	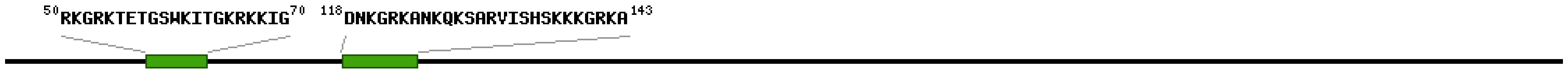
Posterior Graphic. Threshold Marked @ 0.4	

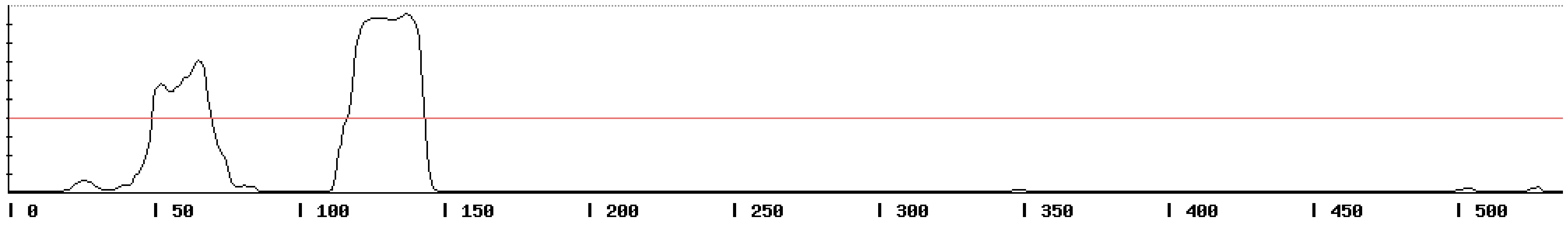

Predictions for Humuluslupulus_HL.SW.v1.0.G018728.1

Viterbi Path

Viterbi Path

Posterior @ 0.4

Posterior @ 0.4

Posterior Graphic. Threshold Marked @ 0.4

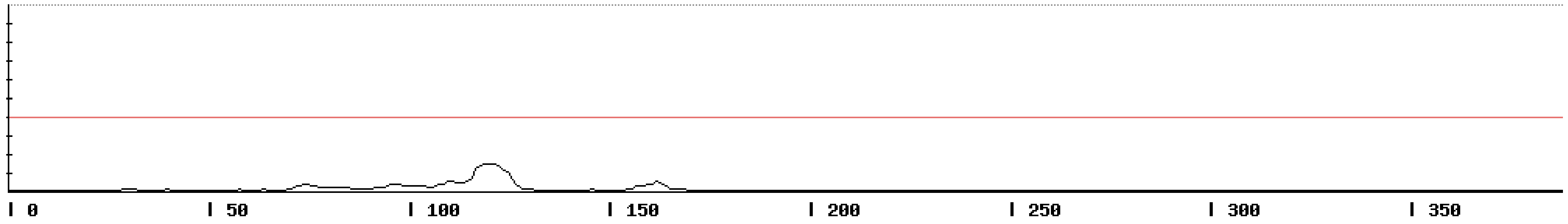

Predictions for Humuluslupulus_HL.SW.v1.0.G019335.1

Viterbi Path

Viterbi Path

Posterior @ 0.4

Posterior @ 0.4

Posterior Graphic. Threshold Marked @ 0.4

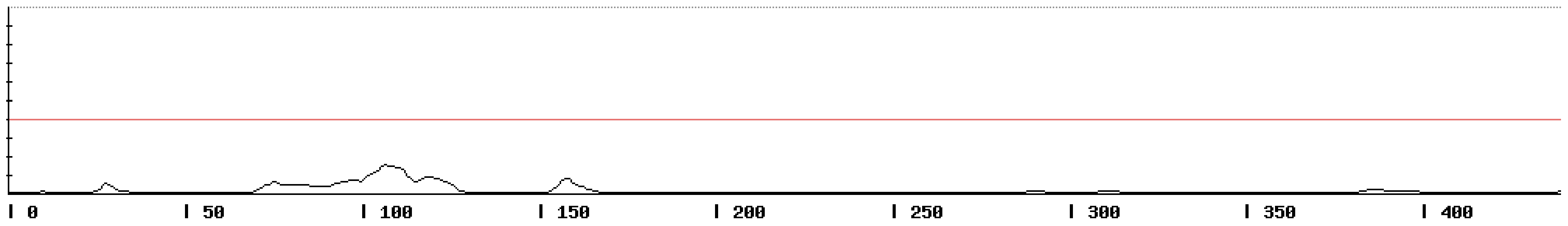

Predictions for Humuluslupulus_HL.SW.v1.0.G019549.1

Viterbi Path

Viterbi Path

Posterior @ 0.4

Posterior @ 0.4

Posterior Graphic. Threshold Marked @ 0.4

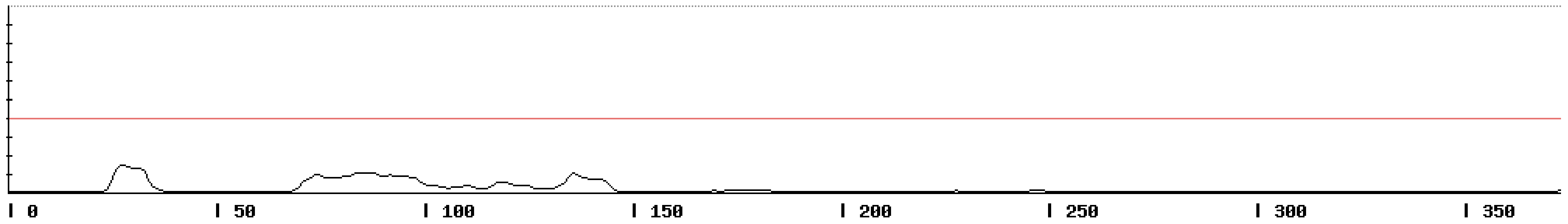

Predictions for Humuluslupulus_HL.SW.v1.0.G020386.1

Viterbi Path

Viterbi Path

Posterior @ 0.4

Posterior @ 0.4

Posterior Graphic. Threshold Marked @ 0.4

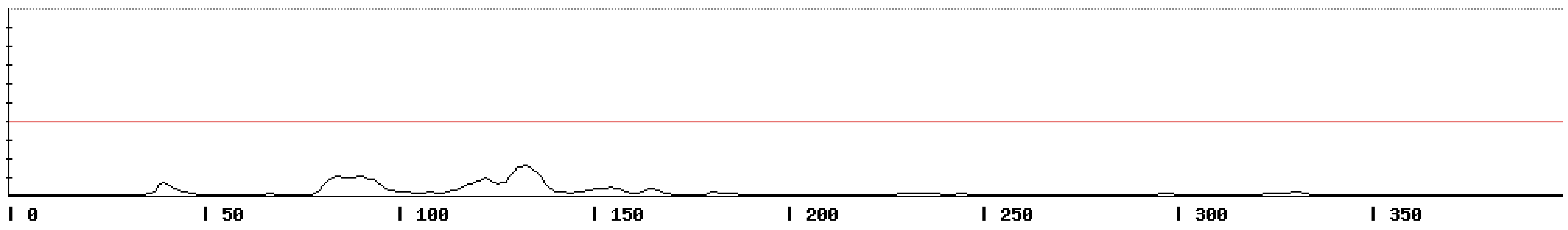

Predictions for Humuluslupulus_HL.SW.v1.0.G020409.1

Viterbi Path

Viterbi Path

Posterior @ 0.4

Posterior @ 0.4

Posterior Graphic. Threshold Marked @ 0.4

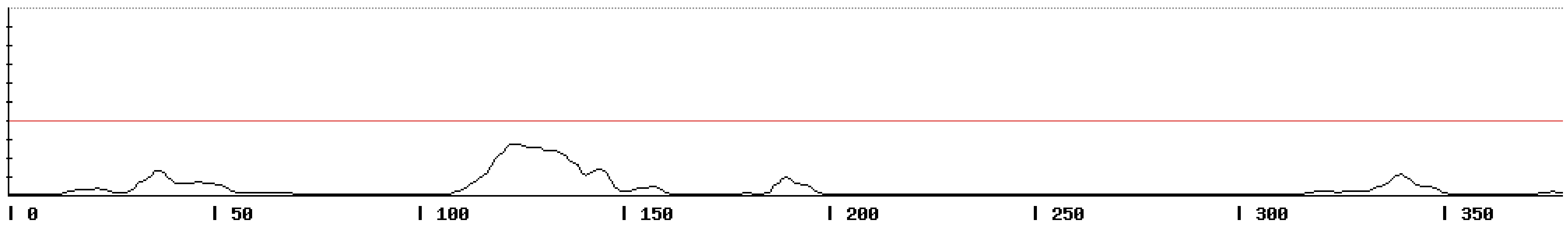

Predictions for Humuluslupulus_HL.SW.v1.0.G020466.1

Viterbi Path

Viterbi Path

Posterior @ 0.4

Posterior @ 0.4

Posterior Graphic. Threshold Marked @ 0.4

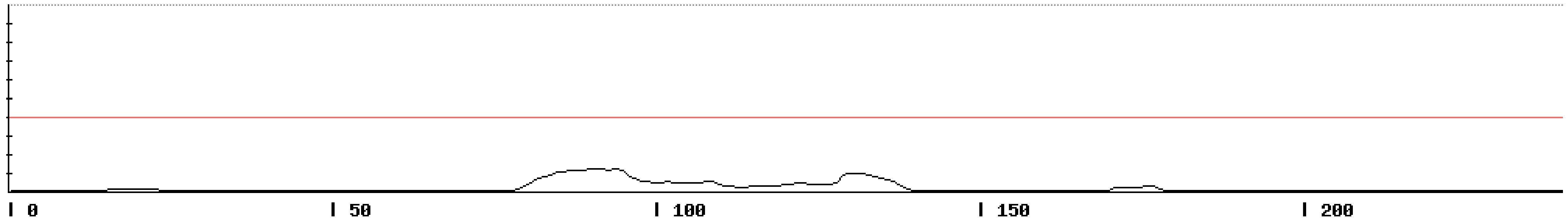

Predictions for Humuluslupulus_HL.SW.v1.0.G020849.1

Viterbi Path | 360 - KREAHGRKKKLRKK - 373

Viterbi Path

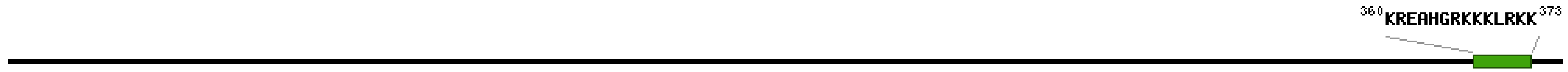

Posterior @ 0.4 | 360 - KREAHGRKKKLRKK - 373

Posterior @ 0.4

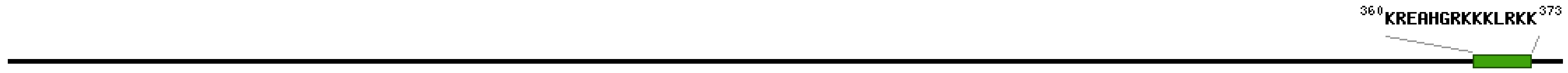

Posterior Graphic. Threshold Marked @ 0.4

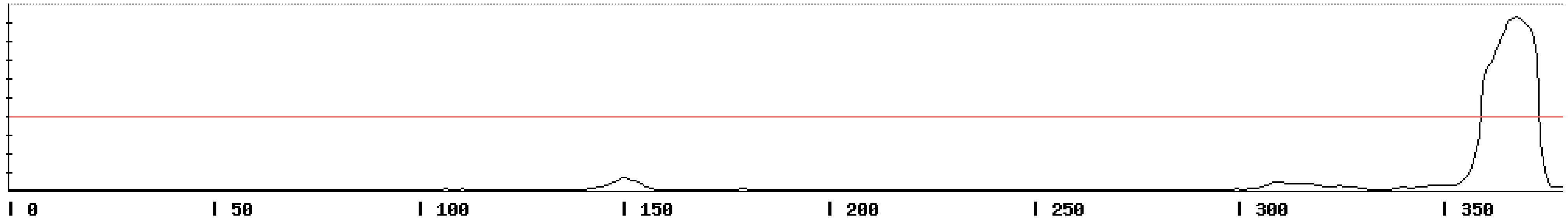

Predictions for Humuluslupulus_HL.SW.v1.0.G020941.1

Viterbi Path

Viterbi Path

Posterior @ 0.4

Posterior @ 0.4

Posterior Graphic. Threshold Marked @ 0.4

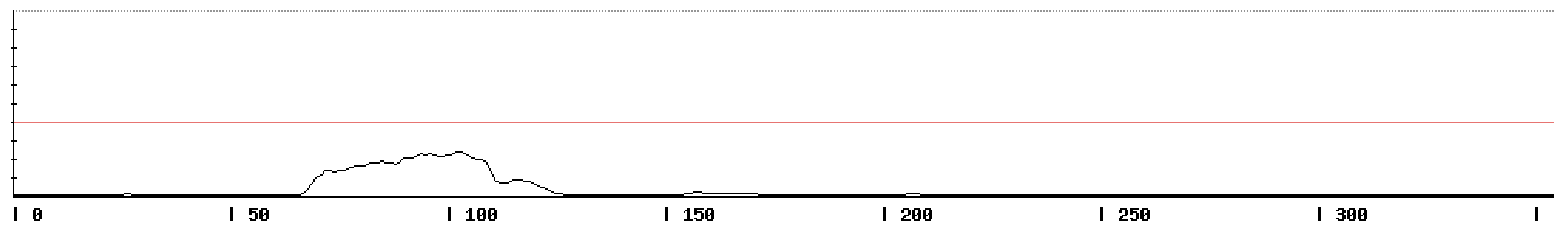

Predictions for Humuluslupulus_HL.SW.v1.0.G021104.1

Viterbi Path

Viterbi Path

Posterior @ 0.4

Posterior @ 0.4

Posterior Graphic. Threshold Marked @ 0.4

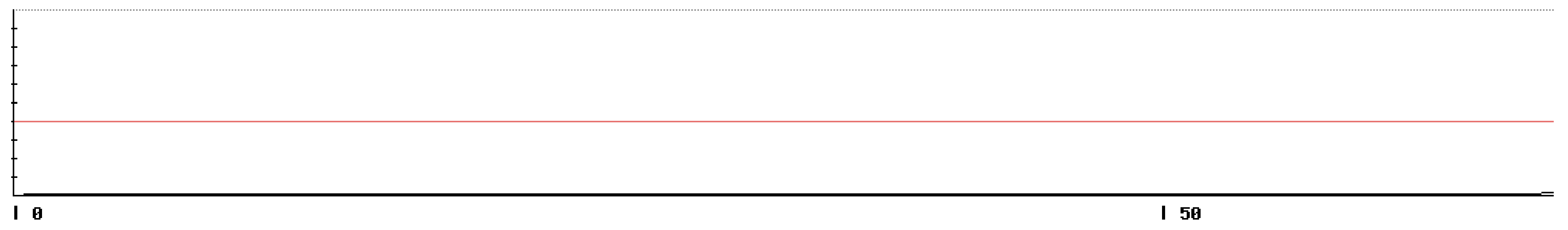

Predictions for Humuluslupulus_HL.SW.v1.0.G021105.1

Viterbi Path

Viterbi Path

Posterior @ 0.4

Posterior @ 0.4

Posterior Graphic. Threshold Marked @ 0.4

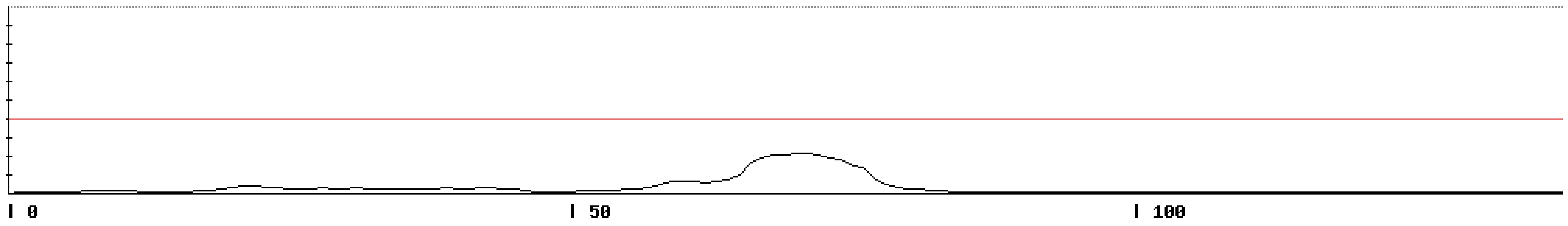

Predictions for Humuluslupulus_HL.SW.v1.0.G021106.1

Viterbi Path

Viterbi Path

Posterior @ 0.4

Posterior @ 0.4

Posterior Graphic. Threshold Marked @ 0.4

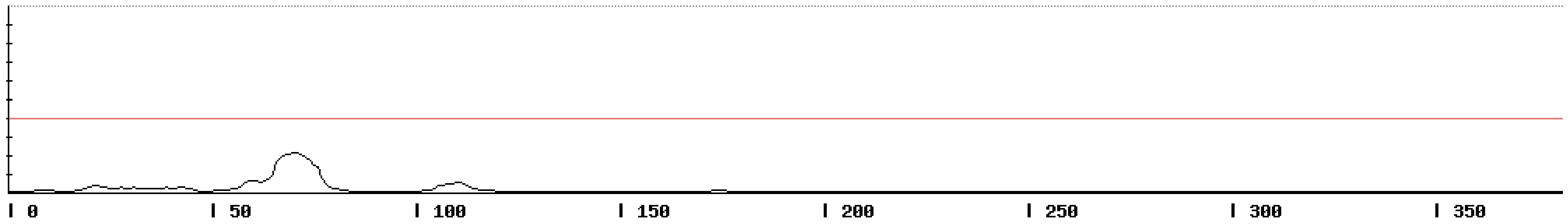

Predictions for Humuluslupulus_HL.SW.v1.0.G021766.1

Viterbi Path

Viterbi Path

Posterior @ 0.4

Posterior @ 0.4

Posterior Graphic. Threshold Marked @ 0.4

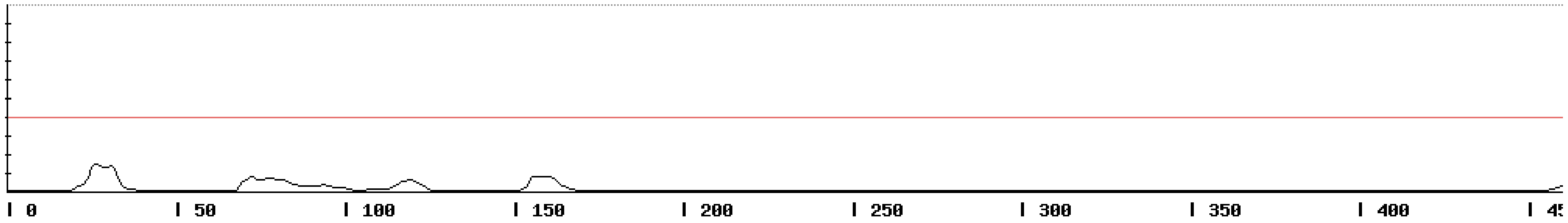

Predictions for Humuluslupulus_HL.SW.v1.0.G025856.1

Viterbi Path

Viterbi Path

Posterior @ 0.4 132 - KKSSGGK - 138

Posterior @ 0.4

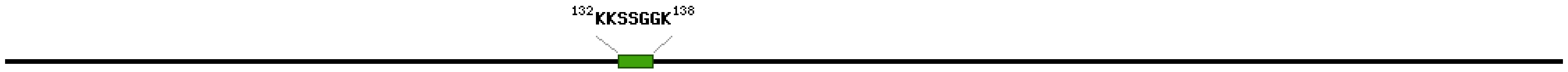

Posterior Graphic. Threshold Marked @ 0.4

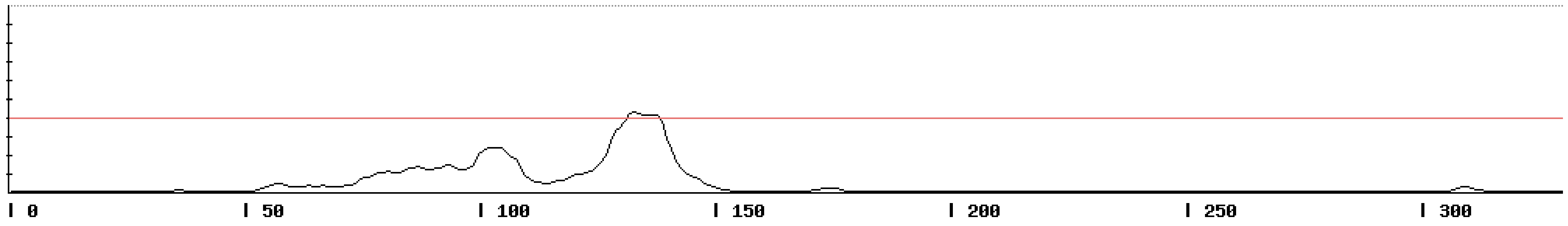

Predictions for Humuluslupulus_HL.SW.v1.0.G025992.1

Viterbi Path	
Viterbi Path	
Posterior @ 0.4	
Posterior @ 0.4	
Posterior Graphic. Threshold Marked @ 0.4	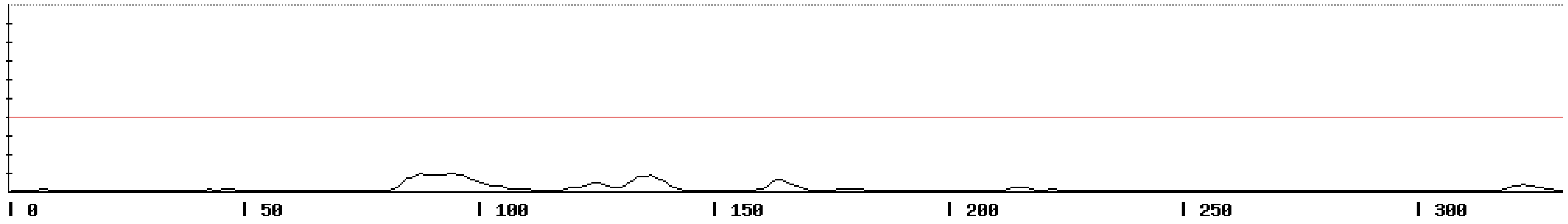

Predictions for Humuluslupulus_HL.SW.v1.0.G026083.1

Viterbi Path	
Viterbi Path	
Posterior @ 0.4	
Posterior @ 0.4	

Posterior Graphic. Threshold Marked @ 0.4	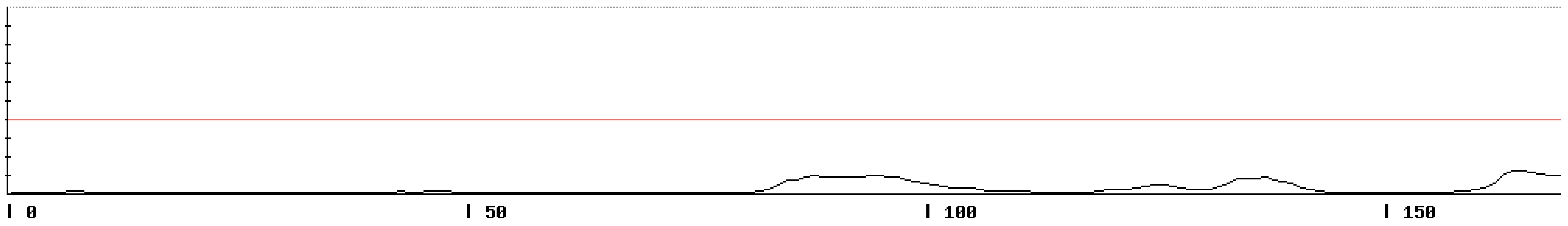

Predictions for Humuluslupulus_HL.SW.v1.0.G026417.1

Viterbi Path	
Viterbi Path	
Posterior @ 0.4	147 - R - 147
Posterior @ 0.4	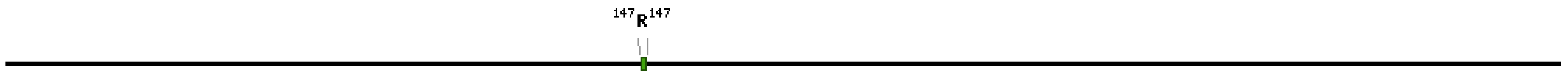
Posterior Graphic. Threshold Marked @ 0.4	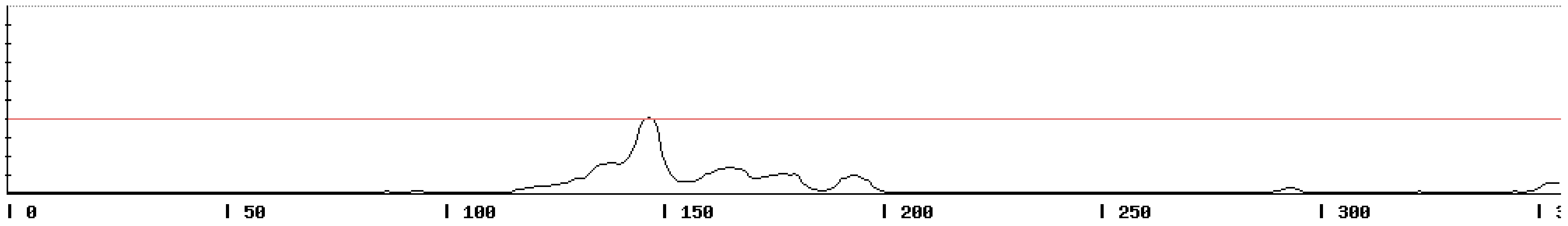

Predictions for Humuluslupulus_HL.SW.v1.0.G027235.1

Viterbi Path	
Viterbi Path	
Posterior @ 0.4	
Posterior @ 0.4	

Posterior Graphic. Threshold Marked @ 0.4

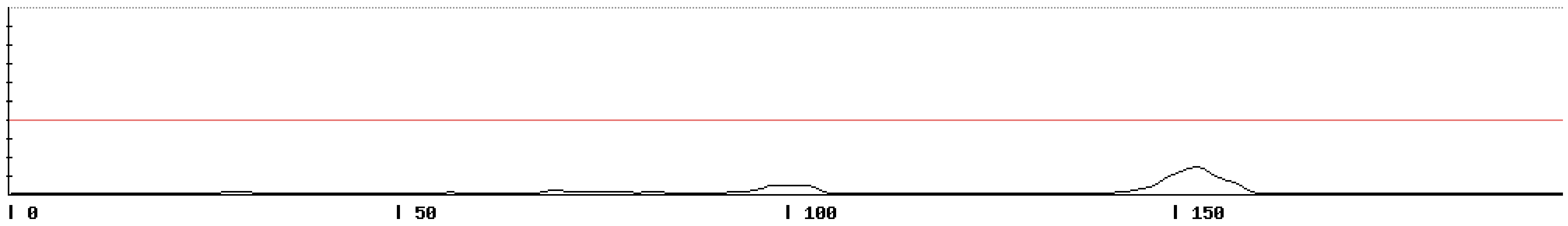

Predictions for Humuluslupulus_HL.SW.v1.0.G028514.1

Viterbi Path

Viterbi Path

Posterior @ 0.4

Posterior @ 0.4

Posterior Graphic. Threshold Marked @ 0.4

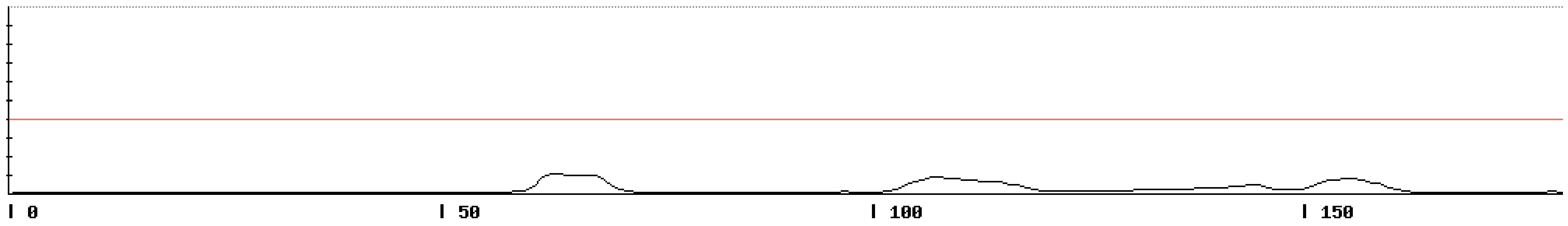

Predictions for Humuluslupulus_HL.SW.v1.0.G031438.1

Viterbi Path

Viterbi Path

Posterior @ 0.4

Posterior @ 0.4

Posterior Graphic. Threshold Marked @ 0.4

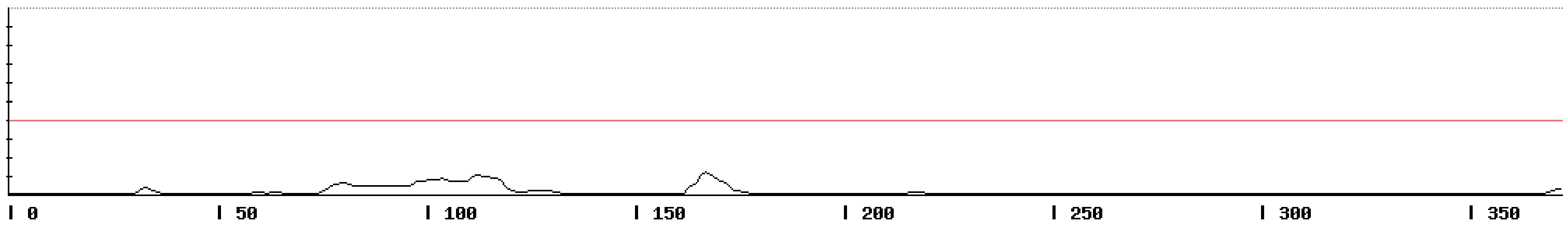

Predictions for Humuluslupulus_HL.SW.v1.0.G031875.1

Viterbi Path

Viterbi Path

Posterior @ 0.4

Posterior @ 0.4

Posterior Graphic. Threshold Marked @ 0.4

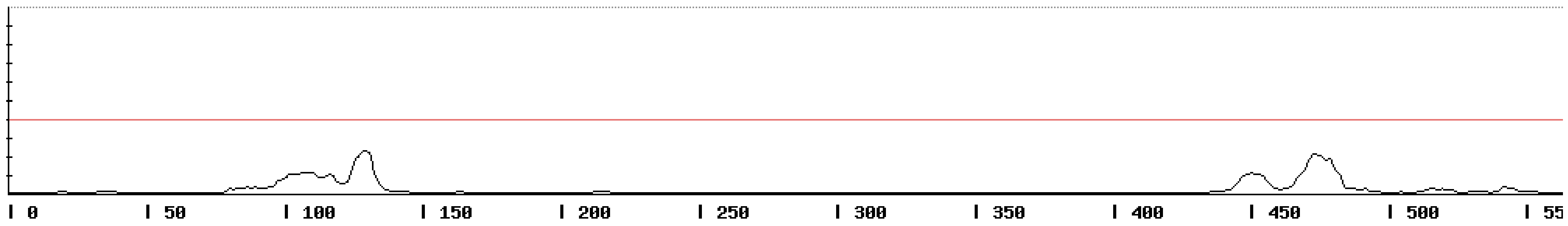

Predictions for Humuluslupulus_HL.SW.v1.0.G032767.1

Viterbi Path

Viterbi Path

Posterior @ 0.4	
Posterior @ 0.4	
Posterior Graphic. Threshold Marked @ 0.4	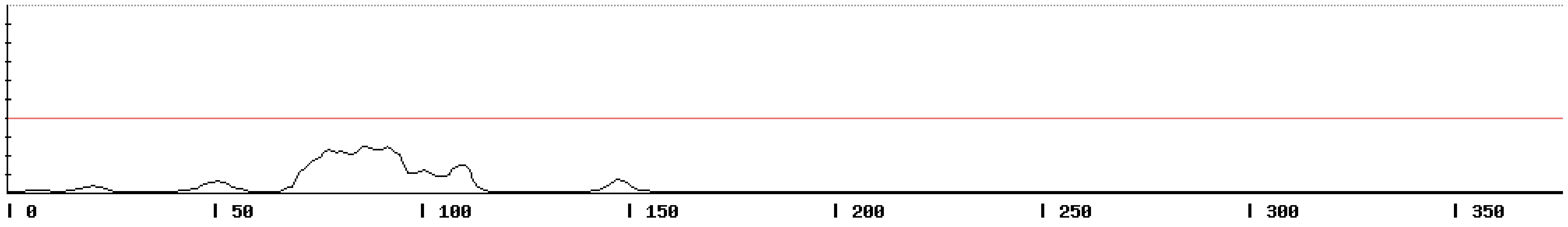

Predictions for Humuluslupulus_HL.SW.v1.0.G034009.1	
Viterbi Path	152 - KGRKANKQKSARVISHSKKKGRK - 174
Viterbi Path	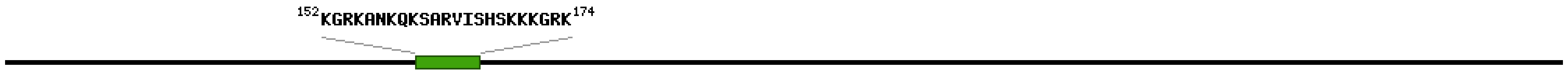
Posterior @ 0.4	82 - RKGRKTETGSWKITGKRKKIG - 102<br>150 - DNKGRKANKQKSARVISHSKKKGRKA - 175
Posterior @ 0.4	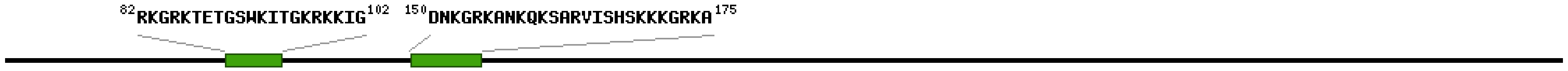
Posterior Graphic. Threshold Marked @ 0.4	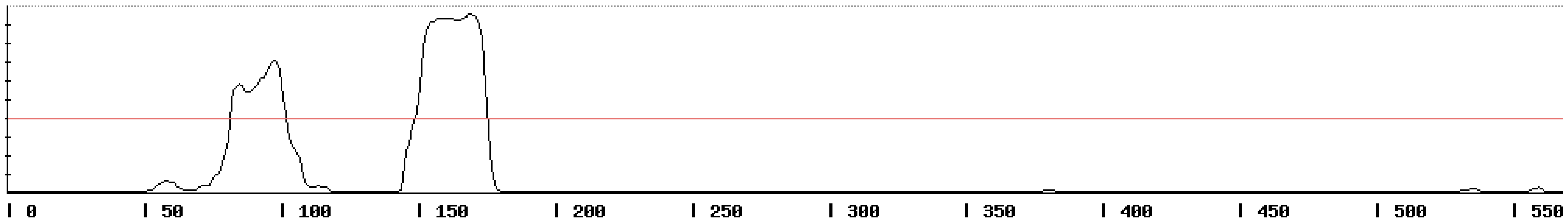

Predictions for Humuluslupulus_HL.SW.v1.0.G034029.1

Viterbi Path

Viterbi Path

Posterior @ 0.4

Posterior @ 0.4

Posterior Graphic. Threshold Marked @ 0.4

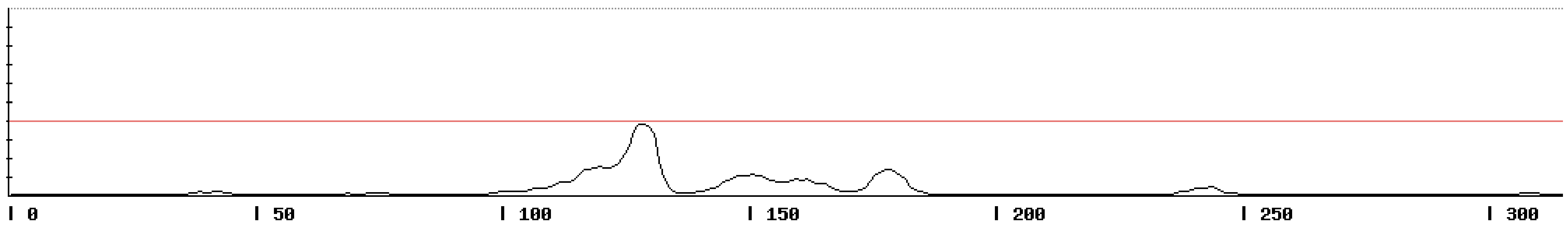

Predictions for Humuluslupulus_HL.SW.v1.0.G034044.1

Viterbi Path

Viterbi Path

Posterior @ 0.4

Posterior @ 0.4

Posterior Graphic. Threshold Marked @ 0.4

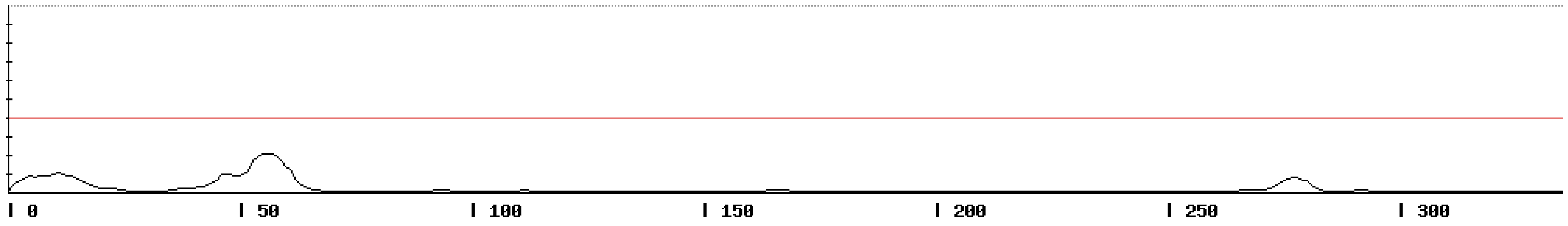

Predictions for Humuluslupulus_HL.SW.v1.0.G034382.1

Viterbi Path

Viterbi Path

Posterior @ 0.4

Posterior @ 0.4

Posterior Graphic. Threshold Marked @ 0.4

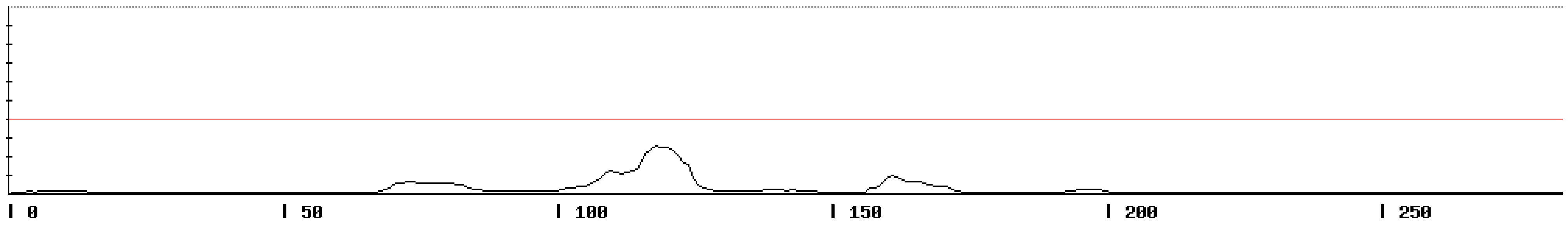

Predictions for Humuluslupulus_HL.SW.v1.0.G034529.1

Viterbi Path

Viterbi Path

Posterior @ 0.4

Posterior @ 0.4

Posterior Graphic. Threshold Marked @ 0.4

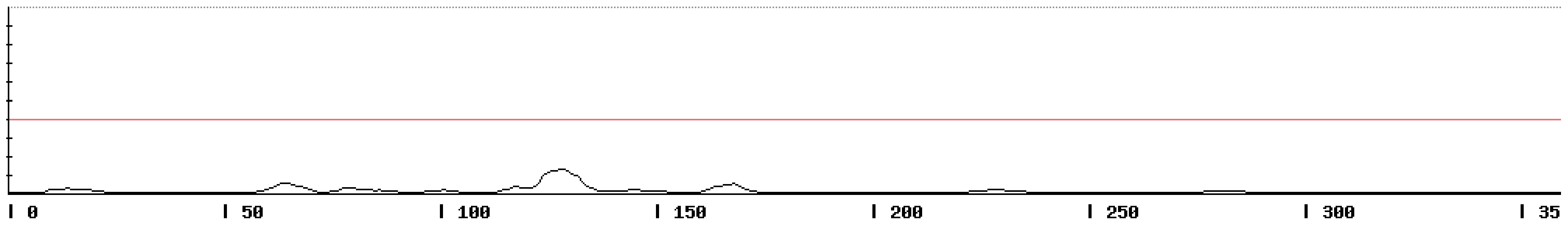

Predictions for Humuluslupulus_HL.SW.v1.0.G035058.1

Viterbi Path

Viterbi Path

Posterior @ 0.4 — 149 - RRRK - 152

Posterior @ 0.4

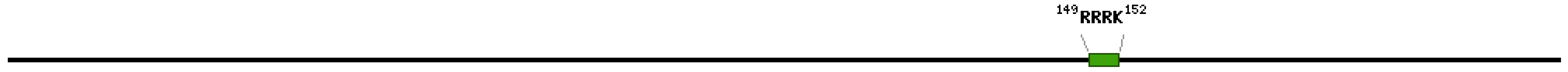

Posterior Graphic. Threshold Marked @ 0.4

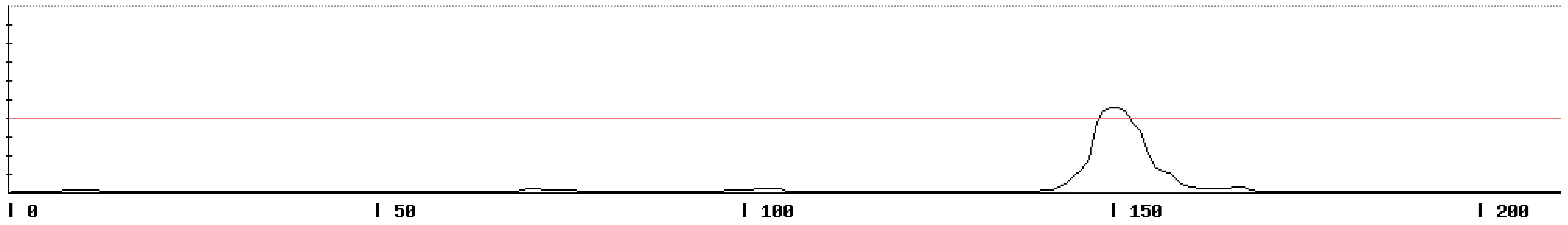

Predictions for Humuluslupulus_HL.SW.v1.0.G035065.1

Viterbi Path

Viterbi Path

Posterior @ 0.4

Posterior @ 0.4

Posterior Graphic. Threshold Marked @ 0.4

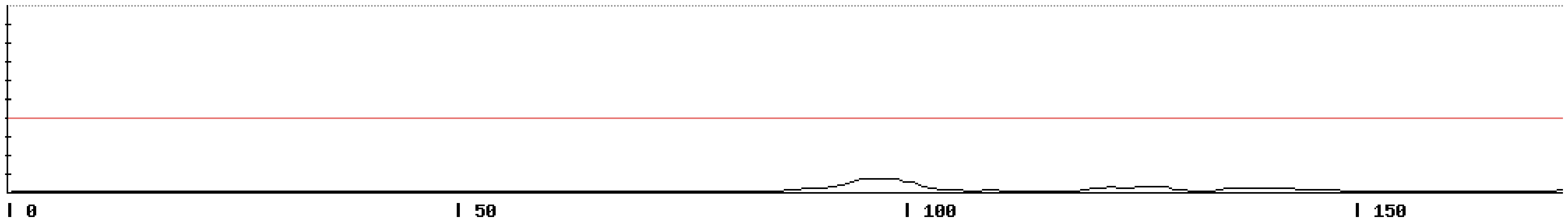

Predictions for Humuluslupulus_HL.SW.v1.0.G035408.1

Viterbi Path

Viterbi Path

Posterior @ 0.4

Posterior @ 0.4

Posterior Graphic. Threshold Marked @ 0.4

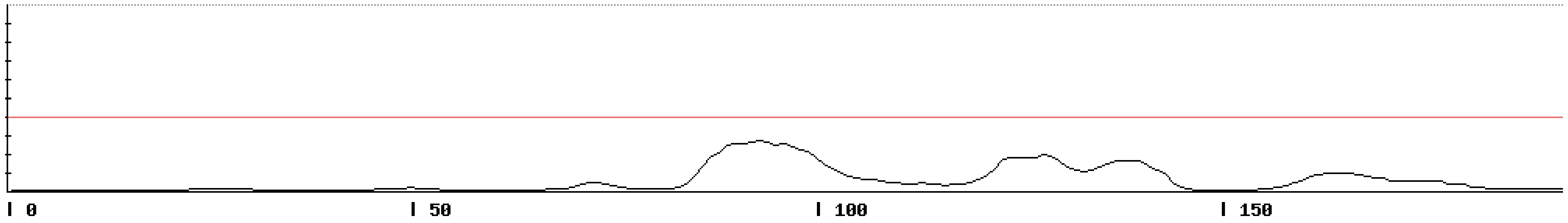

Predictions for Humuluslupulus_HL.SW.v1.0.G035931.1

Viterbi Path

Viterbi Path

Posterior @ 0.4

Posterior @ 0.4

Posterior Graphic. Threshold Marked @ 0.4

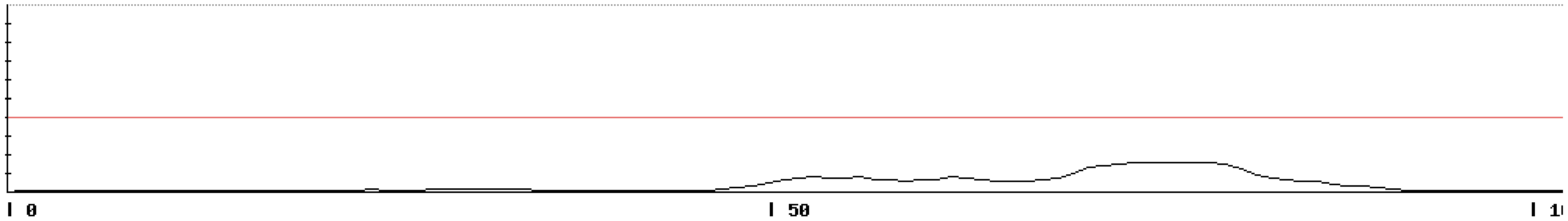

Predictions for Humuluslupulus_HL.SW.v1.0.G036073.1

Viterbi Path

Viterbi Path

Posterior @ 0.4

Posterior @ 0.4

Posterior Graphic. Threshold Marked @ 0.4

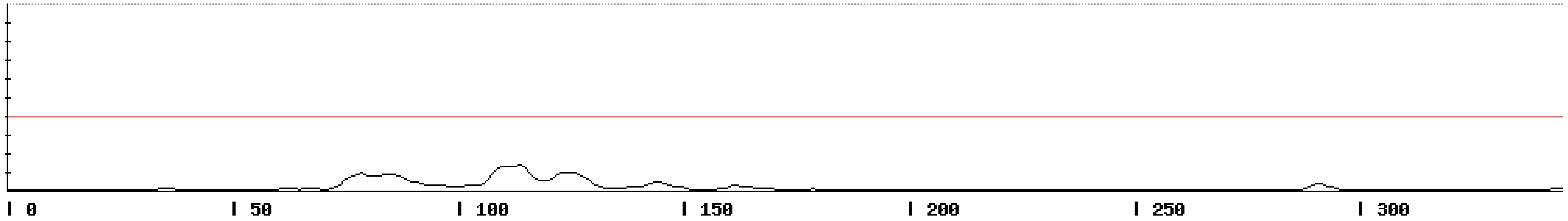

Predictions for Humuluslupulus_HL.SW.v1.0.G036702.1

Viterbi Path

Viterbi Path

Posterior @ 0.4 | 86 - RSNRKTKTGYWKITGKGQKIKS - 107

Posterior @ 0.4

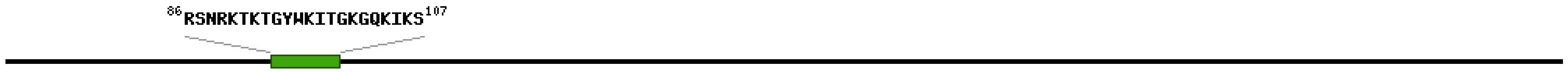

Posterior Graphic. Threshold Marked @ 0.4

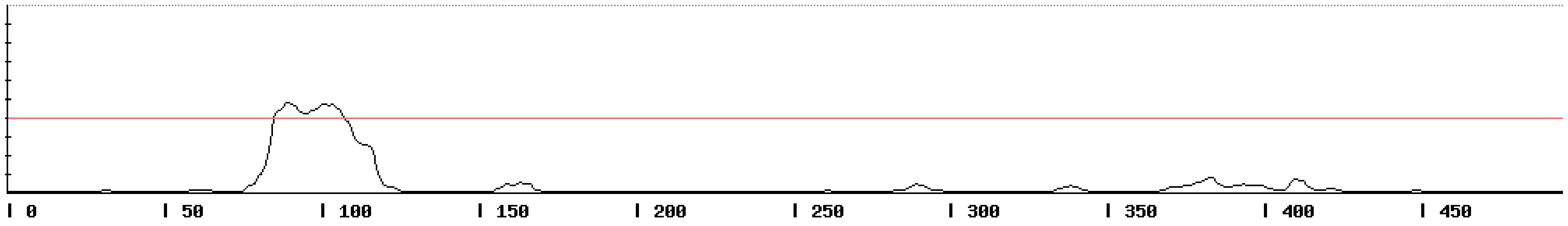

Predictions for Humuluslupulus_HL.SW.v1.0.G036821.1

Viterbi Path

Viterbi Path

Posterior @ 0.4

Posterior @ 0.4

Posterior Graphic. Threshold Marked @ 0.4

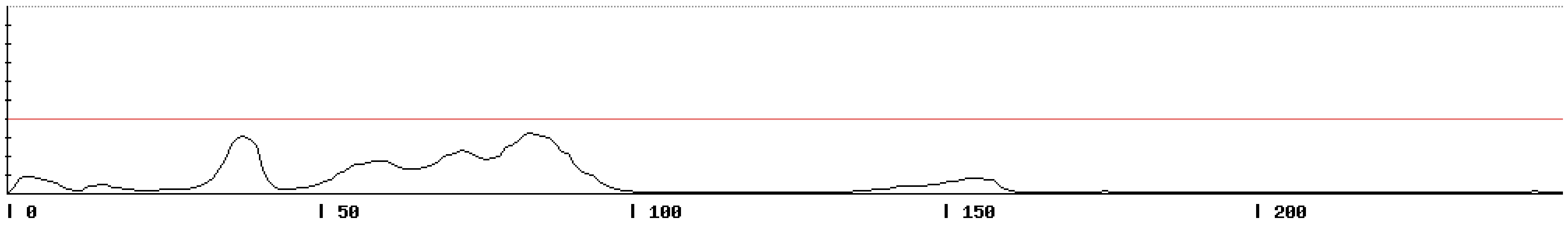

Predictions for Humuluslupulus_HL.SW.v1.0.G038119.1

Viterbi Path

Viterbi Path

Posterior @ 0.4

Posterior @ 0.4

Posterior Graphic. Threshold Marked @ 0.4

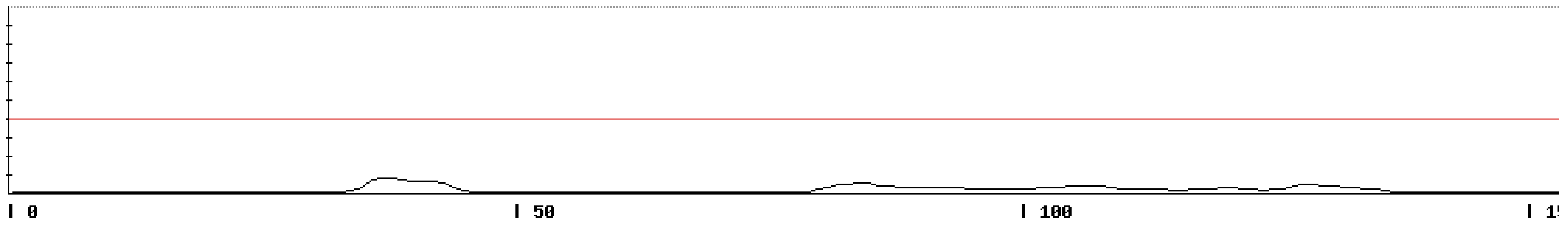

Predictions for Humuluslupulus_HL.SW.v1.0.G040977.1

Viterbi Path

Viterbi Path

Posterior @ 0.4

Posterior @ 0.4

Posterior Graphic. Threshold Marked @ 0.4

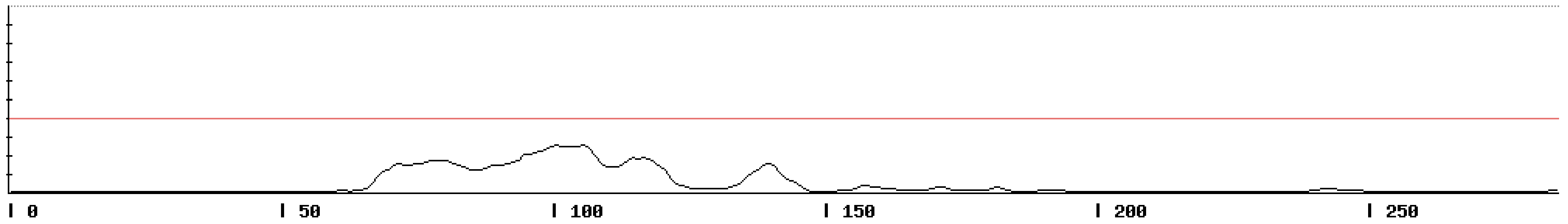

Predictions for Humuluslupulus_HL.SW.v1.0.G041306.1

Viterbi Path

Viterbi Path

Posterior @ 0.4

Posterior @ 0.4

Posterior Graphic. Threshold Marked @ 0.4

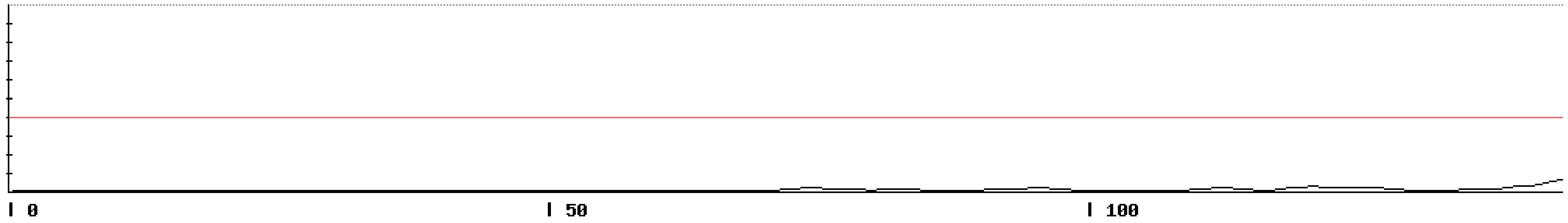

Predictions for Humuluslupulus_HL.SW.v1.0.G044172.1

Viterbi Path

Viterbi Path

Posterior @ 0.4

Posterior @ 0.4

Posterior Graphic. Threshold Marked @ 0.4

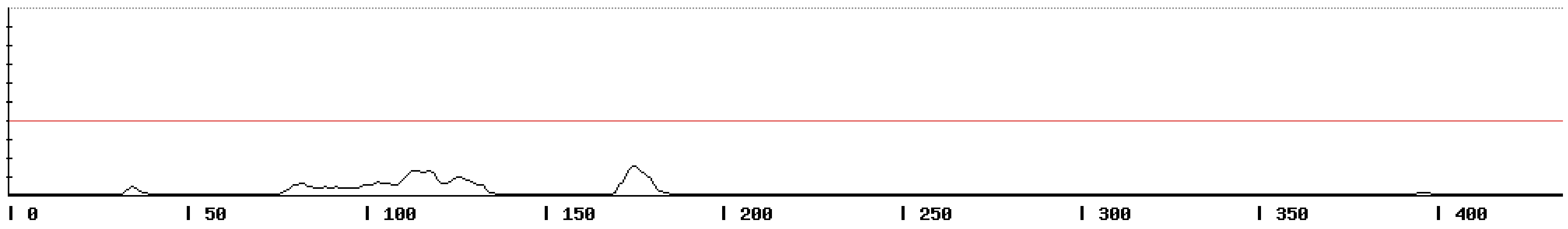

Predictions for Humuluslupulus_HL.SW.v1.0.G044249.1

Viterbi Path	
Viterbi Path	
Posterior @ 0.4	
Posterior @ 0.4	
Posterior Graphic. Threshold Marked @ 0.4	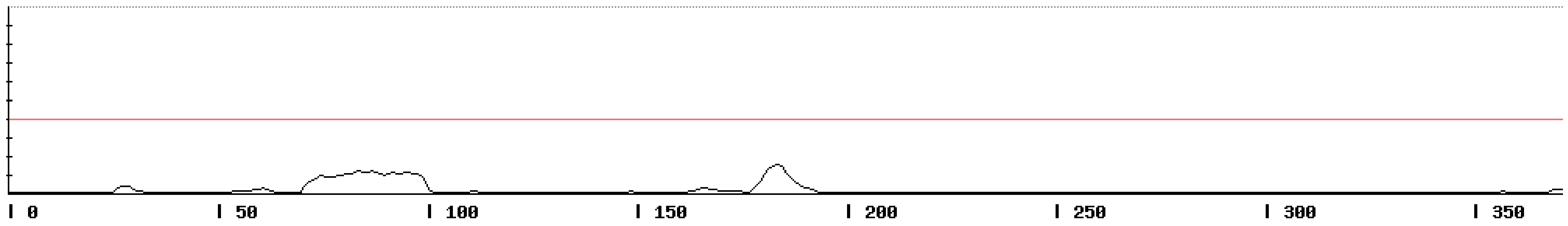

Predictions for Humuluslupulus_HL.SW.v1.0.G044515.1

Viterbi Path	
Viterbi Path	
Posterior @ 0.4	149 - RRRK - 152
Posterior @ 0.4	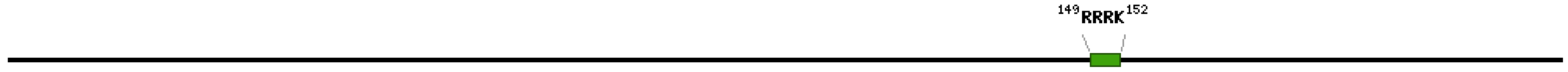

Posterior Graphic. Threshold Marked @ 0.4

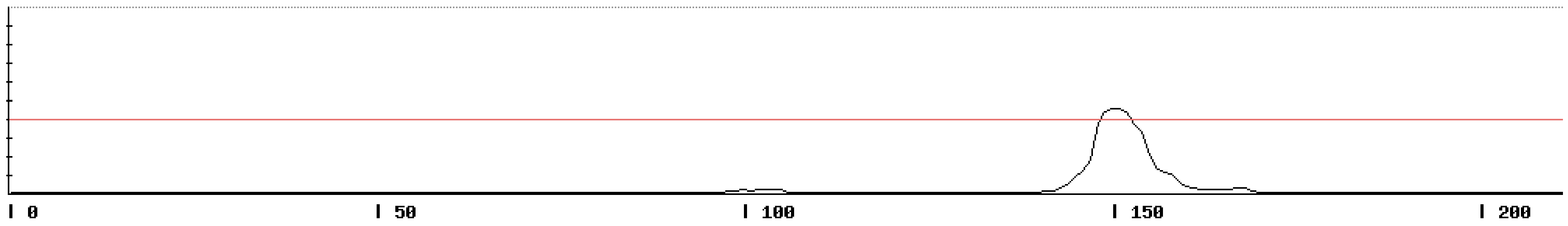

Predictions for Humuluslupulus_HL.SW.v1.0.G044753.1

Viterbi Path

Viterbi Path

Posterior @ 0.4

Posterior @ 0.4

Posterior Graphic. Threshold Marked @ 0.4

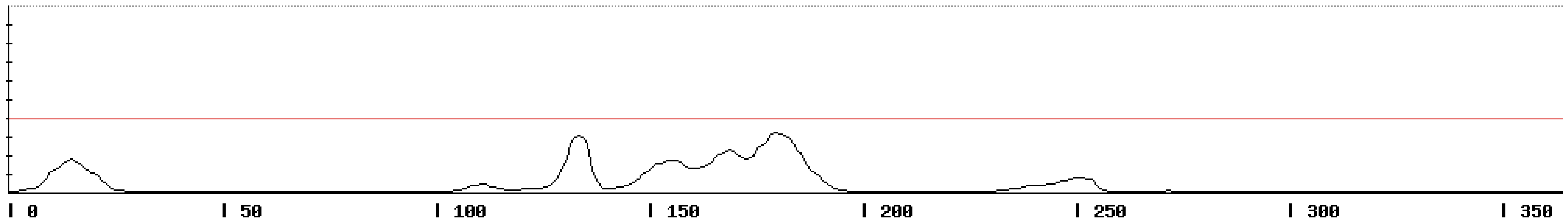

Predictions for Humuluslupulus_HL.SW.v1.0.G045589.1

Viterbi Path

Viterbi Path

Posterior @ 0.4

Posterior @ 0.4

Posterior Graphic. Threshold Marked @ 0.4

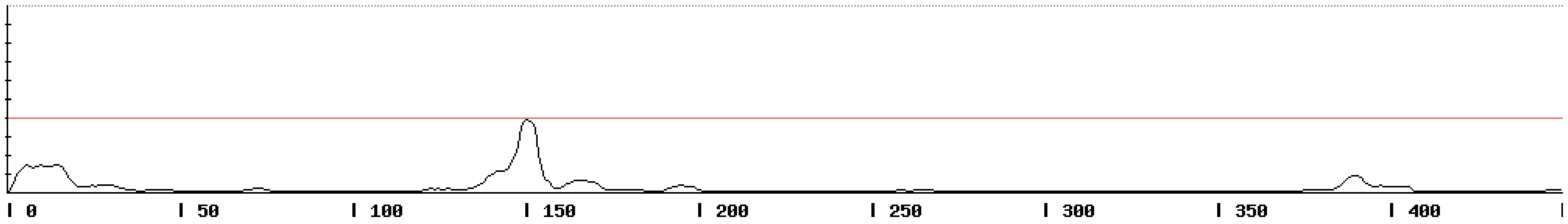

Predictions for Humuluslupulus_HL.SW.v1.0.G046733.1

Viterbi Path	
Viterbi Path	
Posterior @ 0.4	
Posterior @ 0.4	
Posterior Graphic. Threshold Marked @ 0.4	

Revision r.9 - Last Updated on : February 24 2015 17:45:20. alex.nguyenba@utoronto.ca

Intended to be used with Firefox

Viterbi Path

Posterior @ 0.4

Posterior @ 0.4

Posterior Graphic. Threshold Marked @ 0.4

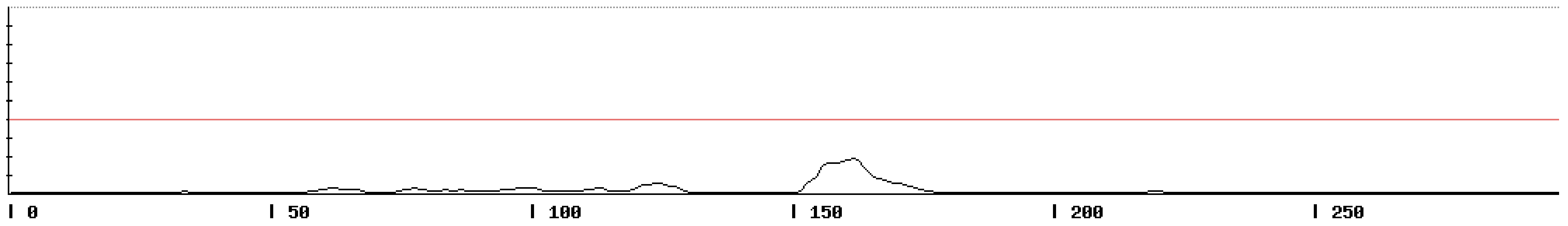

Predictions for Ipomoeatrifida_Itr_sc000018.1_g00020.1

Viterbi Path

Viterbi Path

Posterior @ 0.4

Posterior @ 0.4

Posterior Graphic. Threshold Marked @ 0.4

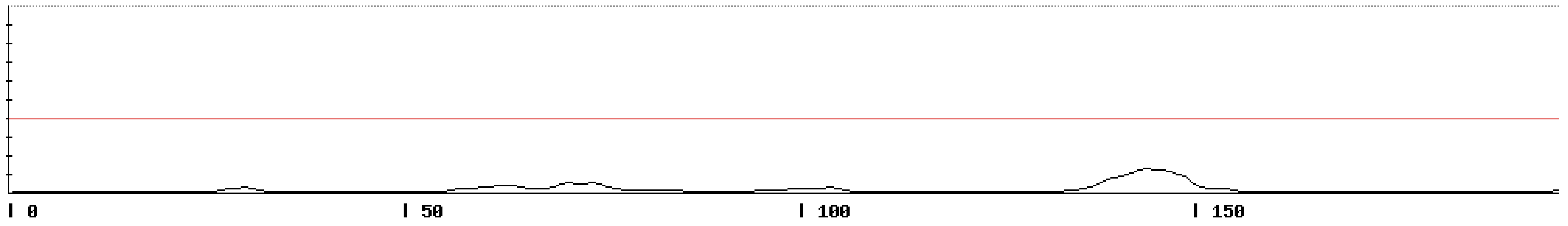

Predictions for Ipomoeatrifida_Itr_sc000021.1_g00002.1

Viterbi Path	
Viterbi Path	
Posterior @ 0.4	
Posterior @ 0.4	
Posterior Graphic. Threshold Marked @ 0.4	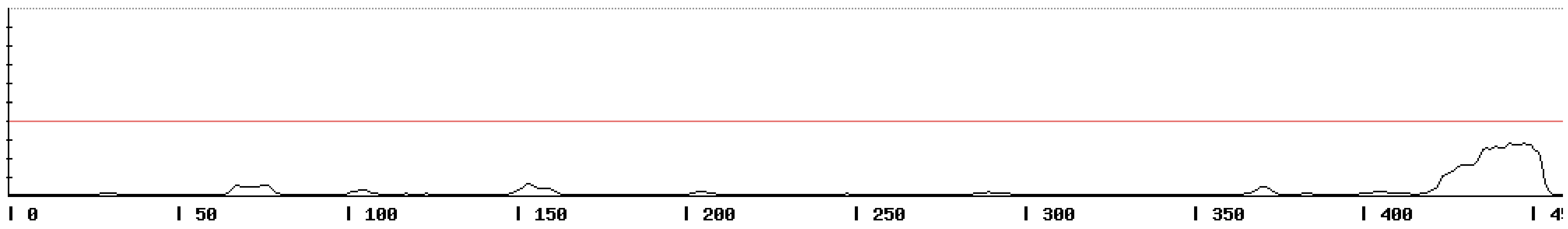

Predictions for Ipomoeatrifida_Itr_sc000023.1_g00005.1

Viterbi Path	
Viterbi Path	
Posterior @ 0.4	140 - KKRVKI - 145
Posterior @ 0.4	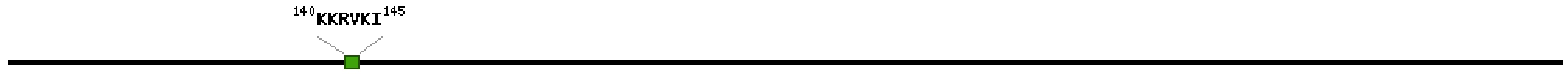
Posterior Graphic. Threshold Marked @ 0.4	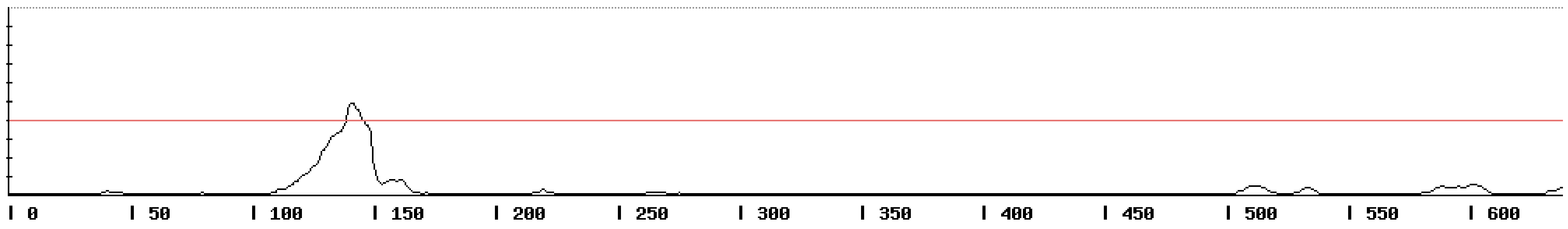

Predictions for Ipomoeatrifida_Itr_sc000033.1_g00037.1	
Viterbi Path	
Viterbi Path	
Posterior @ 0.4	
Posterior @ 0.4	
Posterior Graphic. Threshold Marked @ 0.4	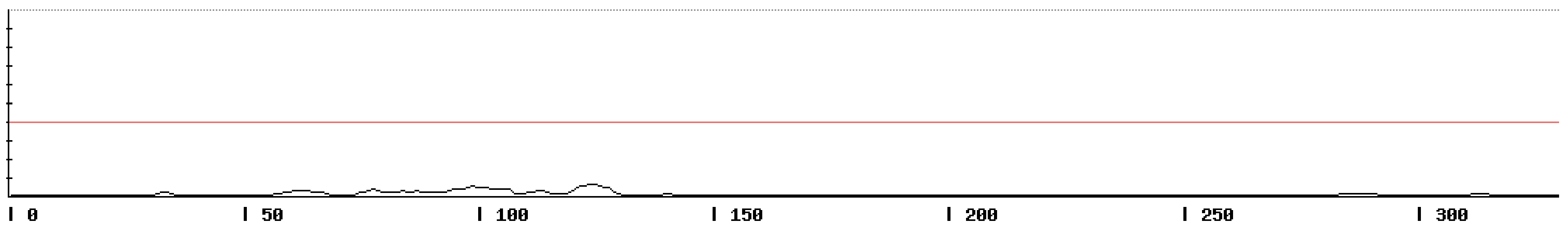

Predictions for Ipomoeatrifida_Itr_sc000040.1_g00038.1	
Viterbi Path	
Viterbi Path	
Posterior @ 0.4	
Posterior @ 0.4	
Posterior Graphic. Threshold Marked @ 0.4	

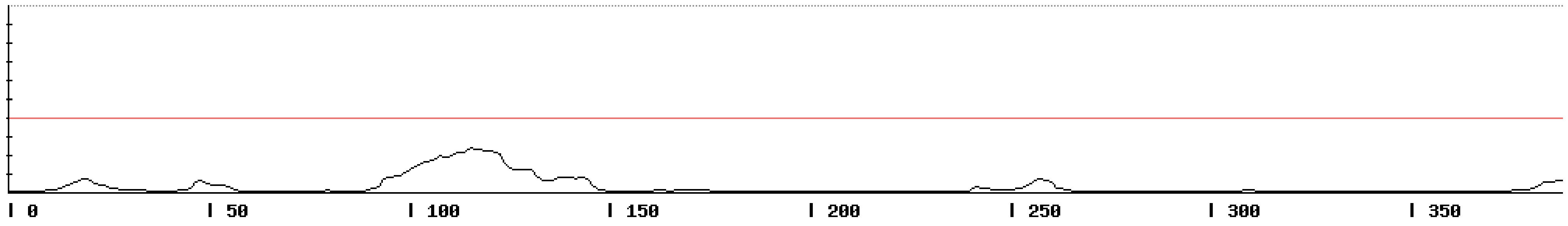

Predictions for Ipomoeatrifida_Itr_sc000044.1_g00049.1

Viterbi Path

Viterbi Path

Posterior @ 0.4

Posterior @ 0.4

Posterior Graphic. Threshold Marked @ 0.4

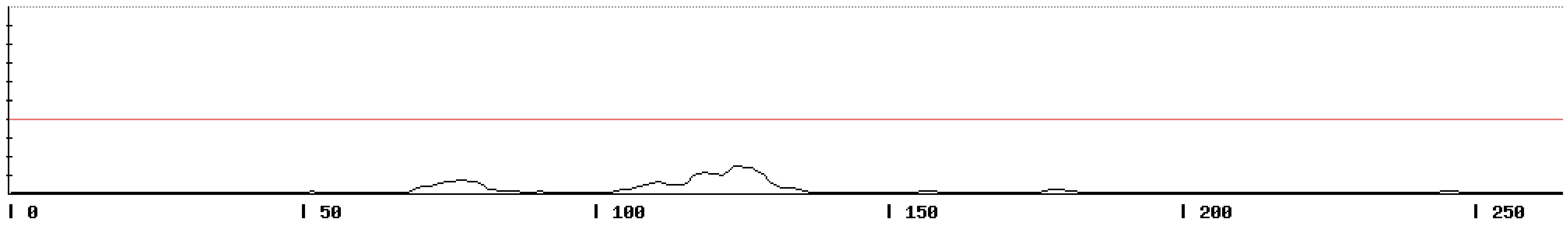

Predictions for Ipomoeatrifida_Itr_sc000044.1_g00054.1

Viterbi Path

Viterbi Path

Posterior @ 0.4

Posterior @ 0.4

Posterior Graphic. Threshold Marked @ 0.4

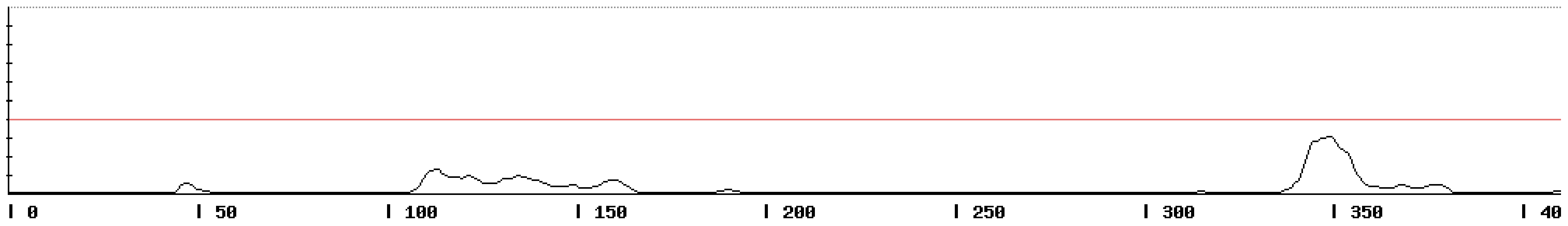

Predictions for Ipomoeatrifida_Itr_sc000045.1_g00004.1

Viterbi Path

Viterbi Path

Posterior @ 0.4

Posterior @ 0.4

Posterior Graphic. Threshold Marked @ 0.4

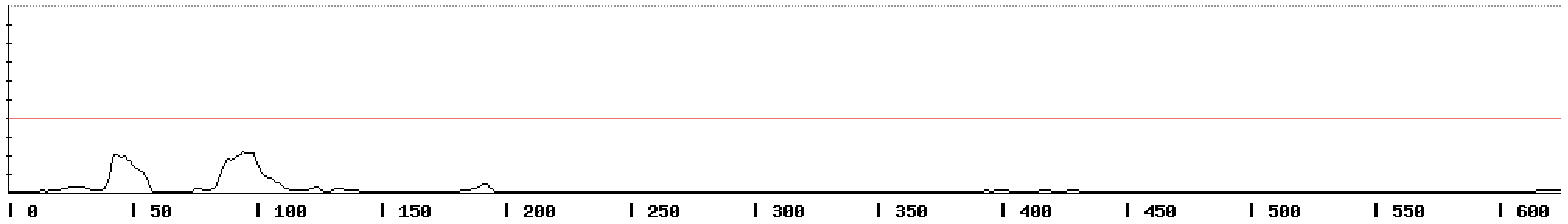

Predictions for Ipomoeatrifida_Itr_sc000047.1_g00055.1

Viterbi Path

Viterbi Path

Posterior @ 0.4

Posterior @ 0.4

Posterior Graphic. Threshold Marked @ 0.4

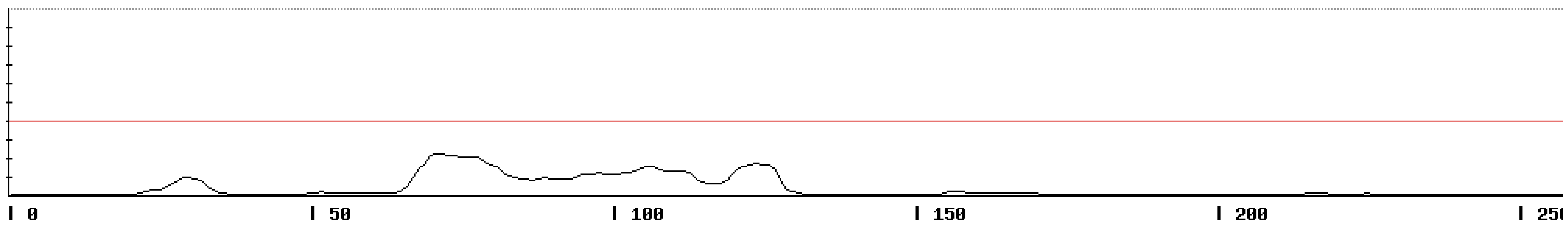

Predictions for Ipomoeatrifida_Itr_sc000049.1_g00003.1

Viterbi Path

Viterbi Path

Posterior @ 0.4

Posterior @ 0.4

Posterior Graphic. Threshold Marked @ 0.4

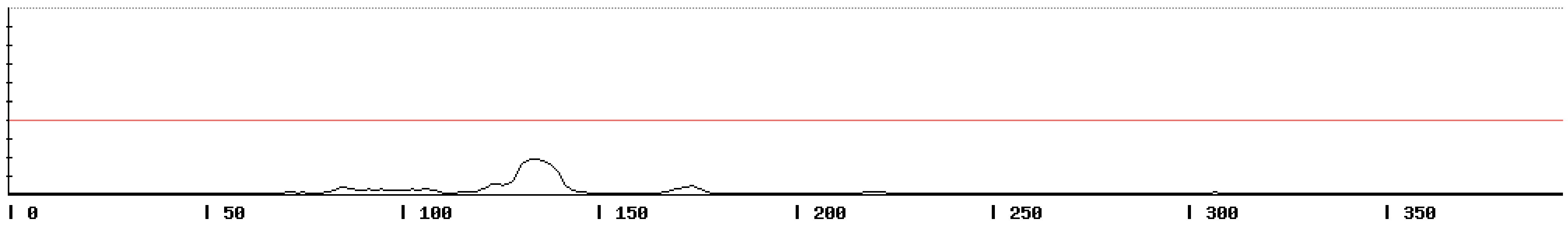

Predictions for Ipomoeatrifida_Itr_sc000051.1_g00029.1

Viterbi Path

Viterbi Path

Posterior @ 0.4	
Posterior @ 0.4	
Posterior Graphic. Threshold Marked @ 0.4	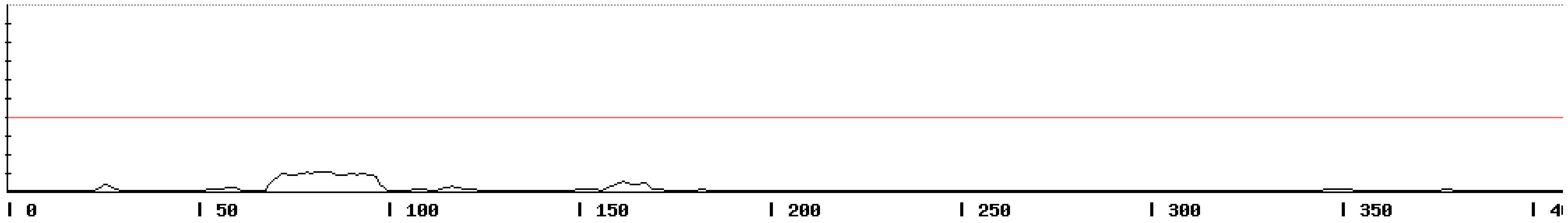

Predictions for Ipomoeatrifida_Itr_sc000054.1_g00015.1

Viterbi Path	
Viterbi Path	
Posterior @ 0.4	
Posterior @ 0.4	
Posterior Graphic. Threshold Marked @ 0.4	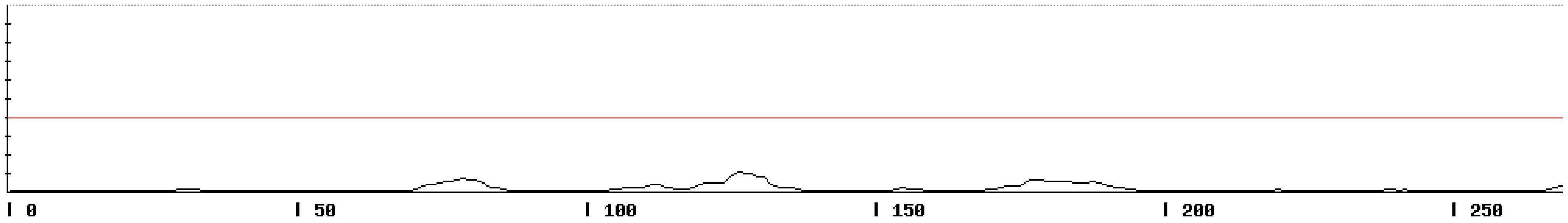

Predictions for Ipomoeatrifida_Itr_sc000055.1_g00009.1

Viterbi Path	
Viterbi Path	

Posterior @ 0.4

Posterior @ 0.4

Posterior Graphic. Threshold Marked @ 0.4

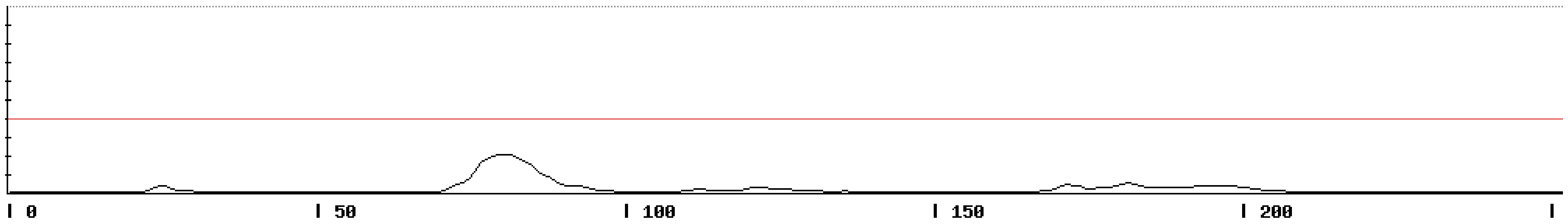

Predictions for Ipomoeatrifida_Itr_sc000061.1_g00003.1

Viterbi Path

Viterbi Path

Posterior @ 0.4

Posterior @ 0.4

Posterior Graphic. Threshold Marked @ 0.4

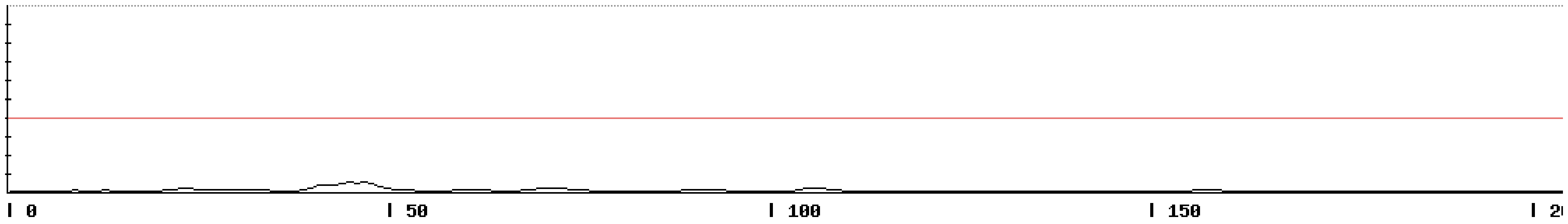

Predictions for Ipomoeatrifida_Itr_sc000065.1_g00033.1

Viterbi Path	
Viterbi Path	
Posterior @ 0.4	134 - RKRRK - 138
Posterior @ 0.4	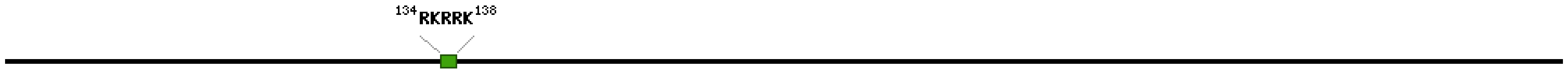
Posterior Graphic. Threshold Marked @ 0.4	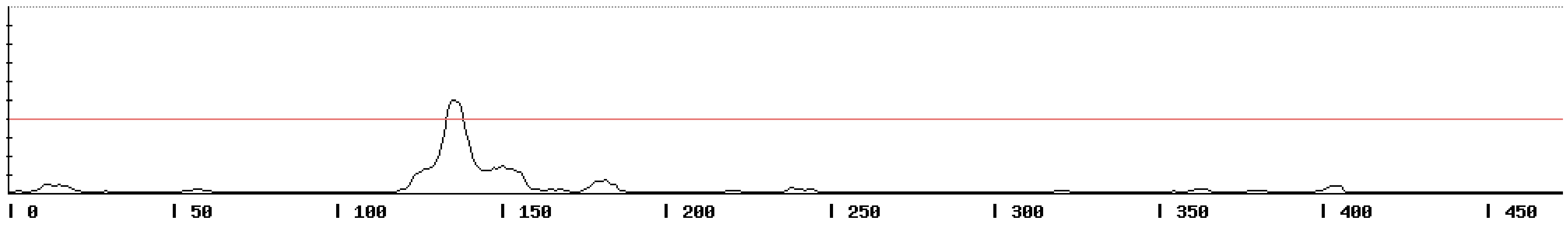

Predictions for Ipomoeatrifida_Itr_sc000066.1_g00032.1

Viterbi Path	
Viterbi Path	
Posterior @ 0.4	136 - GTRKRRKISN - 145
Posterior @ 0.4	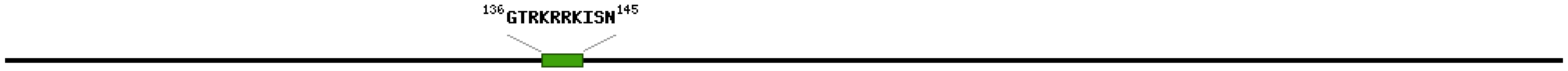
Posterior Graphic. Threshold Marked @ 0.4	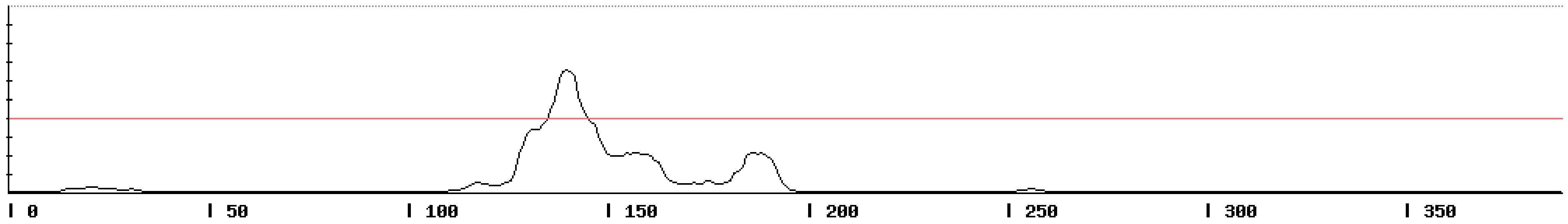

Predictions for Ipomoeatrifida_Itr_sc000070.1_g00008.1	
Viterbi Path	
Viterbi Path	
Posterior @ 0.4	
Posterior @ 0.4	
Posterior Graphic. Threshold Marked @ 0.4	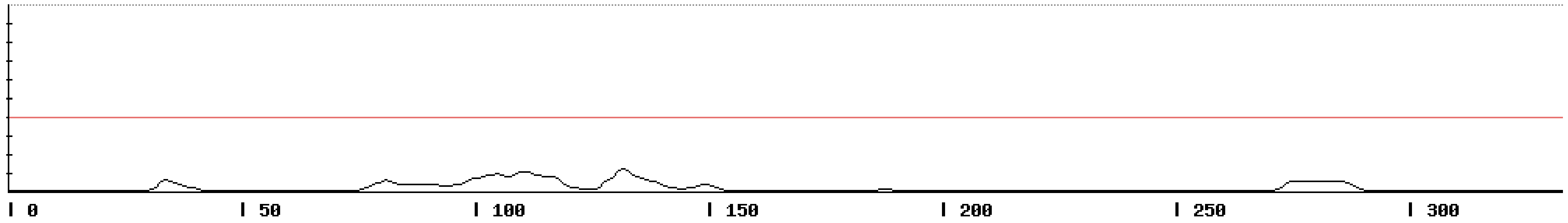

Predictions for Ipomoeatrifida_Itr_sc000081.1_g00015.1	
Viterbi Path	
Viterbi Path	
Posterior @ 0.4	
Posterior @ 0.4	
Posterior Graphic. Threshold Marked @ 0.4	

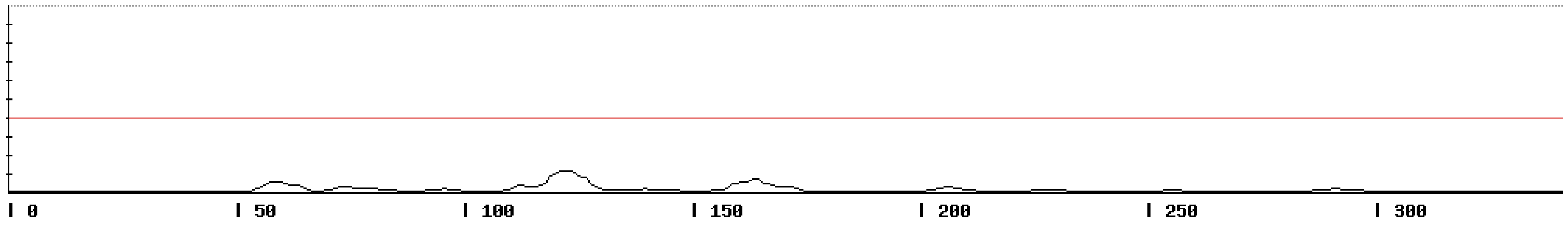

Predictions for Ipomoeatrifida_Itr_sc000087.1_g00007.1

Viterbi Path

Viterbi Path

Posterior @ 0.4

Posterior @ 0.4

Posterior Graphic. Threshold Marked @ 0.4

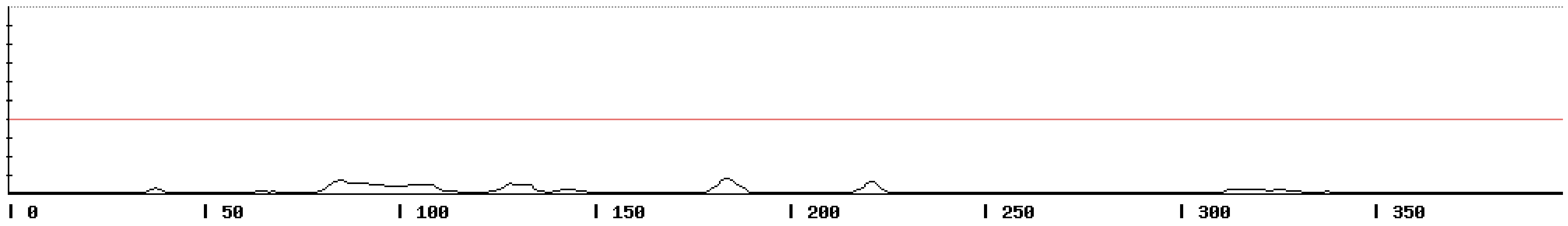

Predictions for Ipomoeatrifida_Itr_sc000088.1_g00031.1

Viterbi Path

Viterbi Path

Posterior @ 0.4

Posterior @ 0.4

Posterior Graphic. Threshold Marked @ 0.4

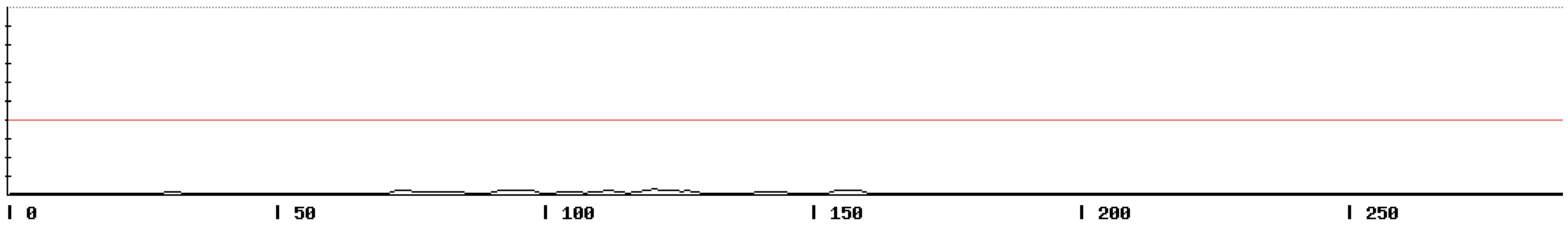

Predictions for Ipomoeatrifida_Itr_sc000102.1_g00017.1

Viterbi Path

Viterbi Path

Posterior @ 0.4 | 28 - RKVRR - 32

Posterior @ 0.4

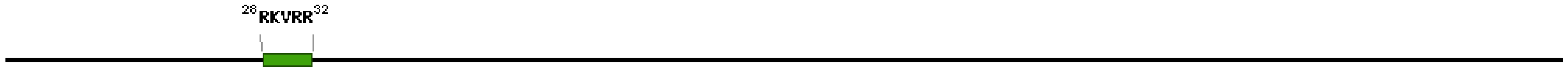

Posterior Graphic. Threshold Marked @ 0.4

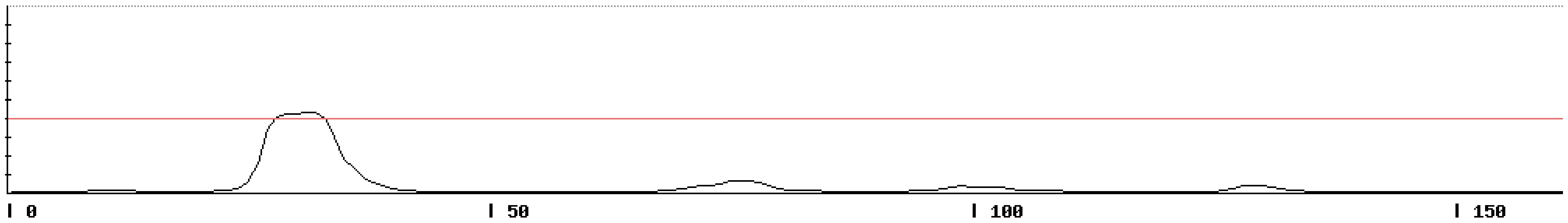

Predictions for Ipomoeatrifida_Itr_sc000102.1_g00018.1

Viterbi Path

Viterbi Path

Posterior @ 0.4

Posterior @ 0.4

Posterior Graphic. Threshold Marked @ 0.4

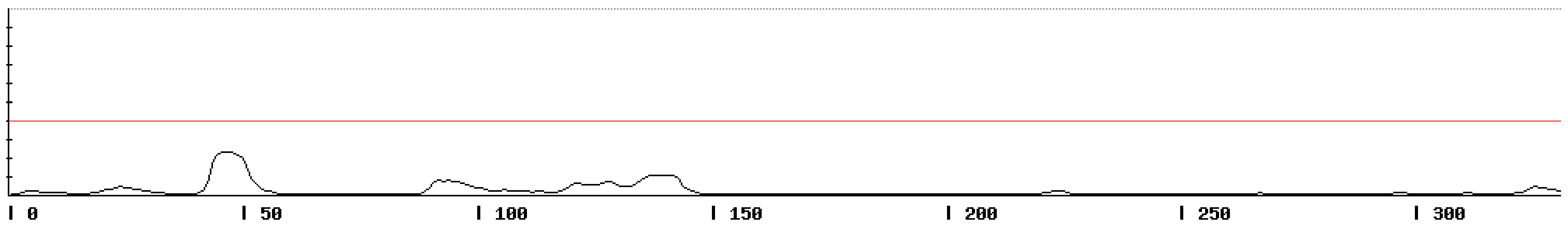

Predictions for Ipomoeatrifida_Itr_sc000102.1_g00019.1

Viterbi Path

Viterbi Path

Posterior @ 0.4

Posterior @ 0.4

Posterior Graphic. Threshold Marked @ 0.4

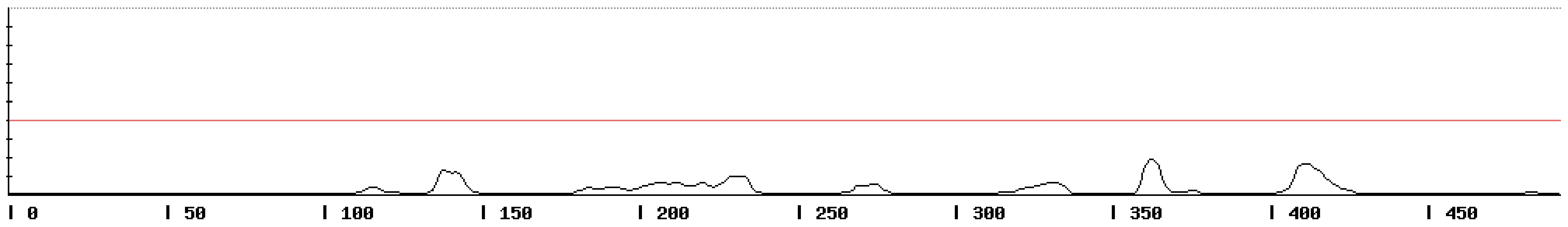

Predictions for Ipomoeatrifida_Itr_sc000114.1_g00031.1

Viterbi Path

Viterbi Path

Posterior @ 0.4

Posterior @ 0.4

Posterior Graphic. Threshold Marked @ 0.4

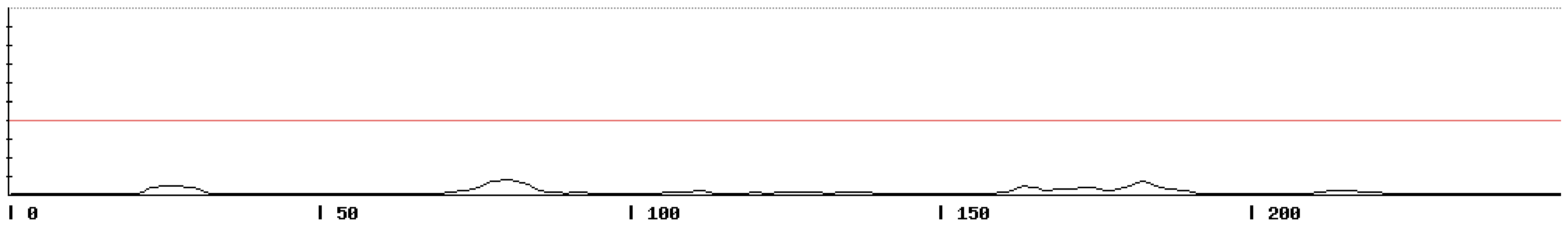

Predictions for Ipomoeatrifida_Itr_sc000114.1_g00032.1

Viterbi Path

Viterbi Path

Posterior @ 0.4

Posterior @ 0.4

Posterior Graphic. Threshold Marked @ 0.4

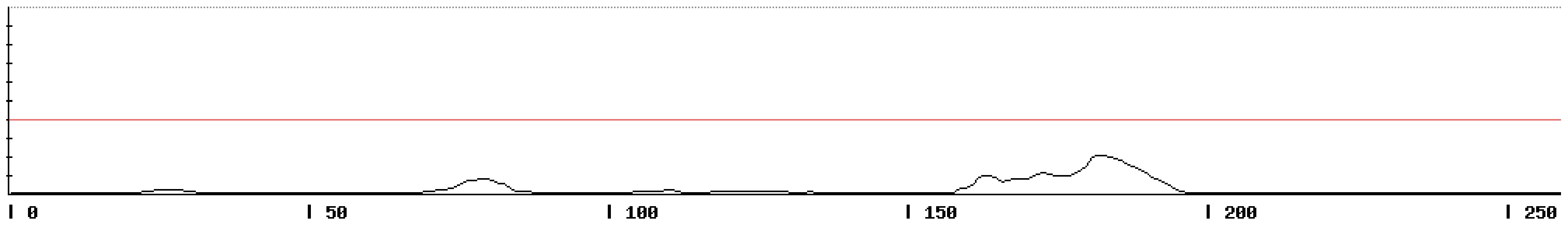

Predictions for Ipomoeatrifida_Itr_sc000134.1_g00027.1

Viterbi Path

Viterbi Path

Posterior @ 0.4

Posterior @ 0.4

Posterior Graphic. Threshold Marked @ 0.4

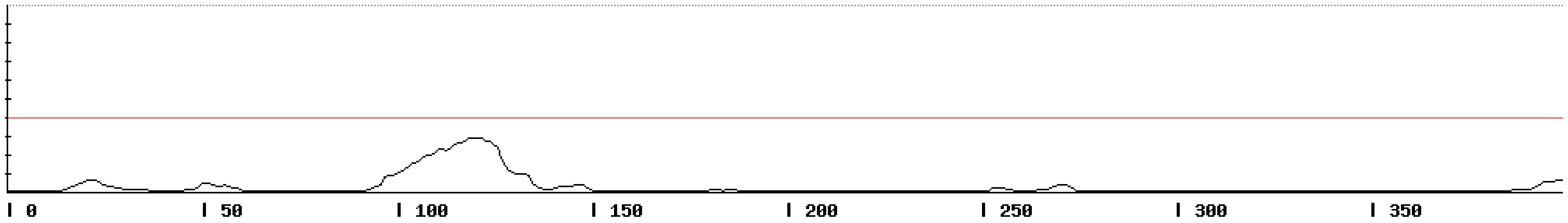

Predictions for Ipomoeatrifida_Itr_sc000134.1_g00028.1

Viterbi Path

Viterbi Path

Posterior @ 0.4

Posterior @ 0.4

Posterior Graphic. Threshold Marked @ 0.4

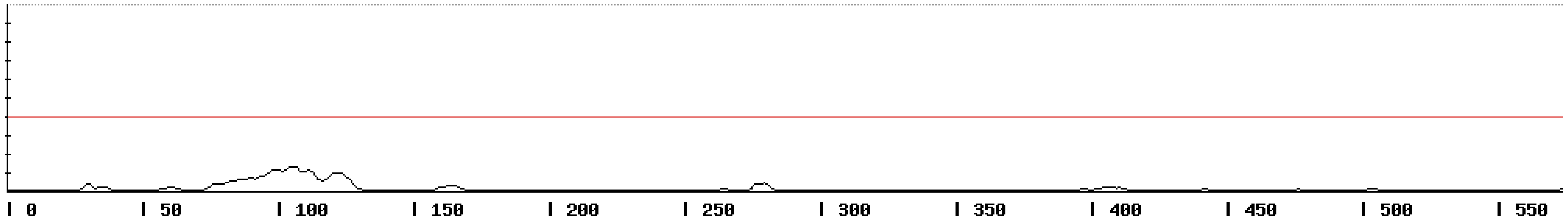

Predictions for Ipomoeatrifida_Itr_sc000140.1_g00008.1

Viterbi Path	
Viterbi Path	
Posterior @ 0.4	
Posterior @ 0.4	
Posterior Graphic. Threshold Marked @ 0.4	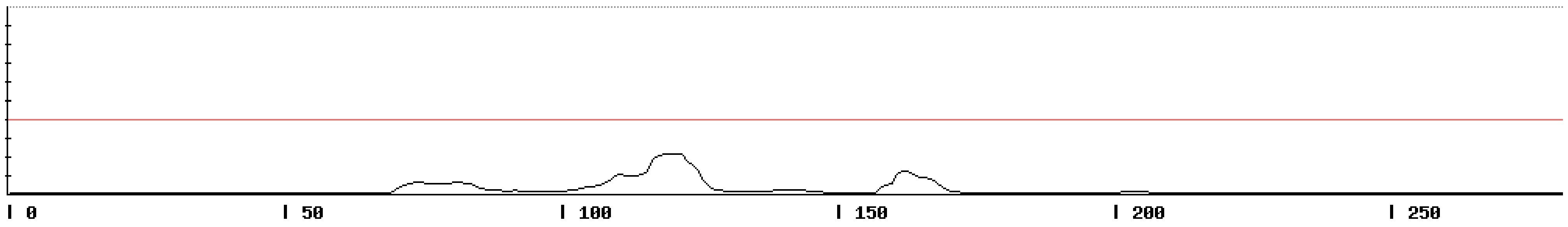

Predictions for Ipomoeatrifida_Itr_sc000157.1_g00002.1

Viterbi Path	
Viterbi Path	
Posterior @ 0.4	135 - RKRRK - 139
Posterior @ 0.4	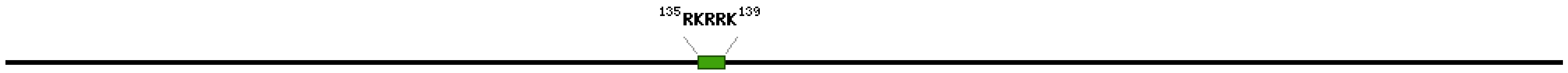
Posterior Graphic. Threshold Marked @ 0.4	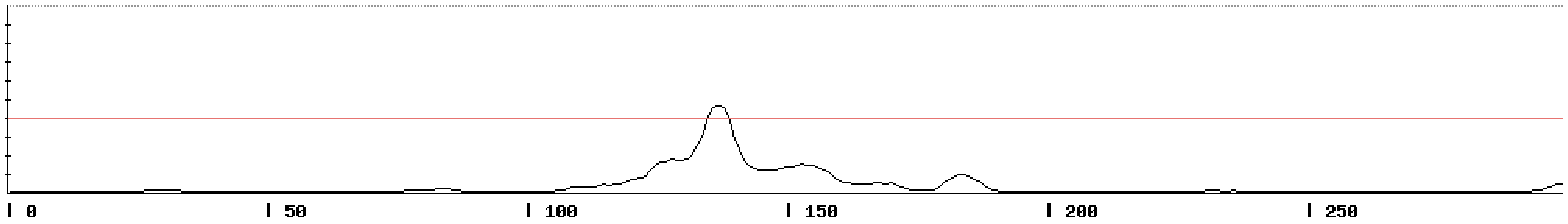

Predictions for Ipomoeatrifida_Itr_sc000157.1_g00004.1

Viterbi Path

Viterbi Path

Posterior @ 0.4

Posterior @ 0.4

Posterior Graphic. Threshold Marked @ 0.4

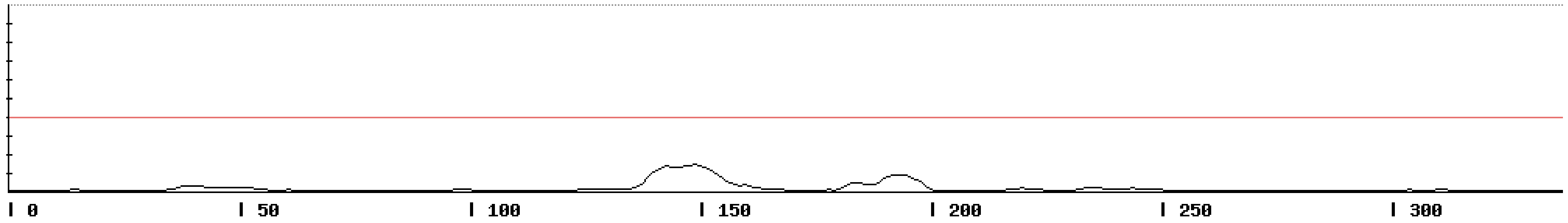

Predictions for Ipomoeatrifida_Itr_sc000185.1_g00013.1

Viterbi Path

Viterbi Path

Posterior @ 0.4

Posterior @ 0.4

Posterior Graphic. Threshold Marked @ 0.4

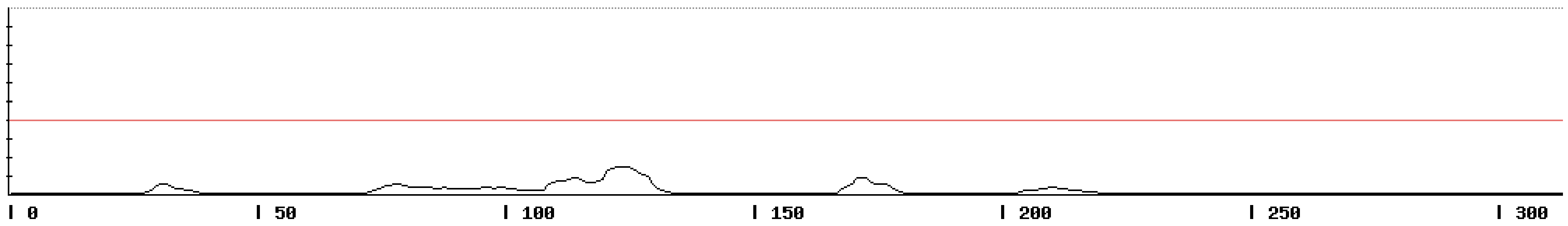

Predictions for Ipomoeatrifida_Itr_sc000185.1_g00020.1

Viterbi Path

Viterbi Path

Posterior @ 0.4 — 66 - KLKKKNPRNSRYRRSIGDGKGTWKAQDKGKAISRSGKIIGYKR - 108

Posterior @ 0.4

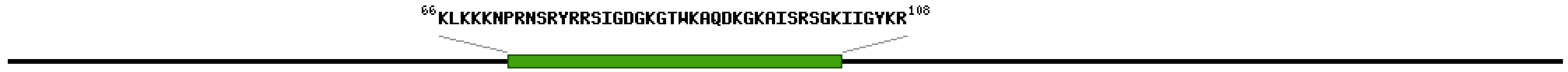

Posterior Graphic. Threshold Marked @ 0.4

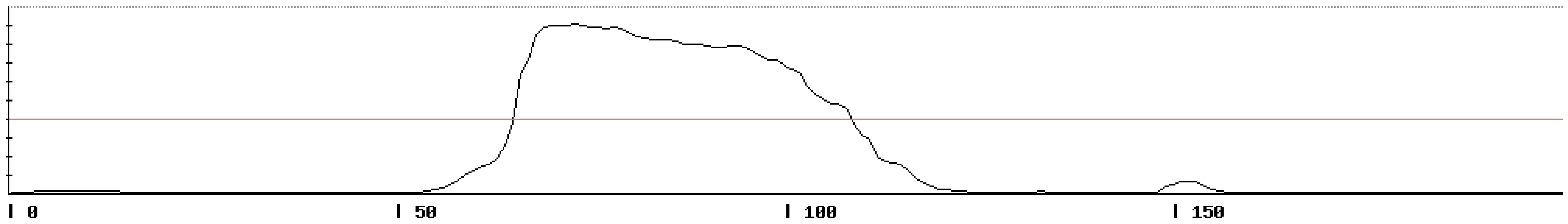

Predictions for Ipomoeatrifida_Itr_sc000189.1_g00014.1

Viterbi Path

Viterbi Path

Posterior @ 0.4 — 163 - KRPGK - 167

Posterior @ 0.4

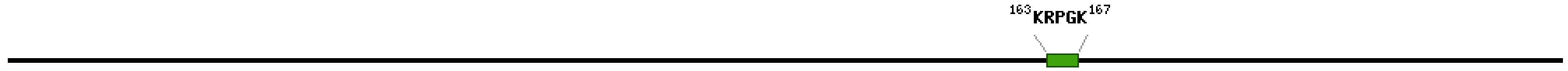

Posterior Graphic. Threshold Marked @ 0.4

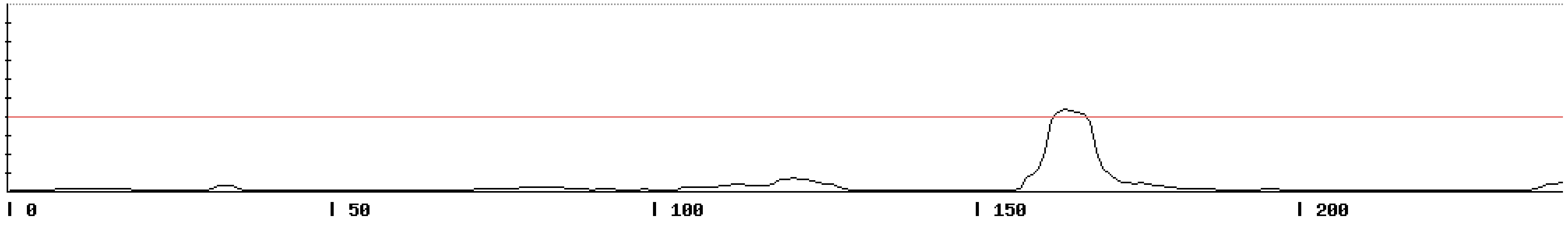

Predictions for Ipomoeatrifida_Itr_sc000194.1_g00003.1

Viterbi Path

Viterbi Path

Posterior @ 0.4

Posterior @ 0.4

Posterior Graphic. Threshold Marked @ 0.4

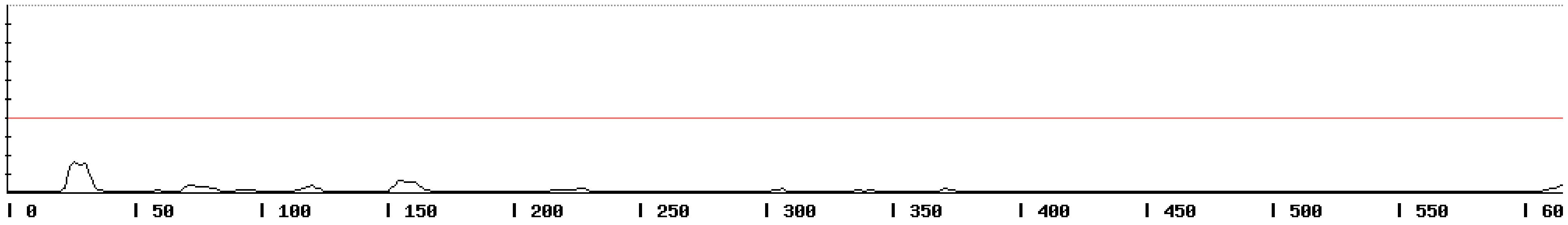

Predictions for Ipomoeatrifida_Itr_sc000194.1_g00020.1

Viterbi Path

Viterbi Path

Posterior @ 0.4

Posterior @ 0.4

Posterior Graphic. Threshold Marked @ 0.4

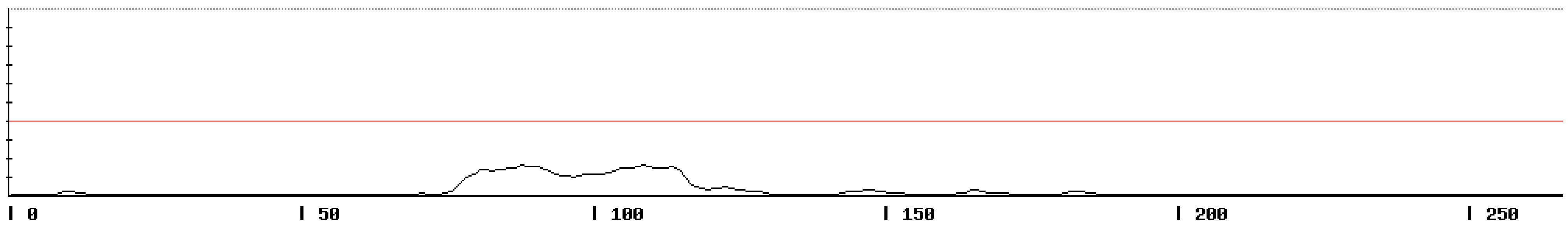

Predictions for Ipomoeatrifida_Itr_sc000244.1_g00012.1

Viterbi Path

Viterbi Path

Posterior @ 0.4

Posterior @ 0.4

Posterior Graphic. Threshold Marked @ 0.4

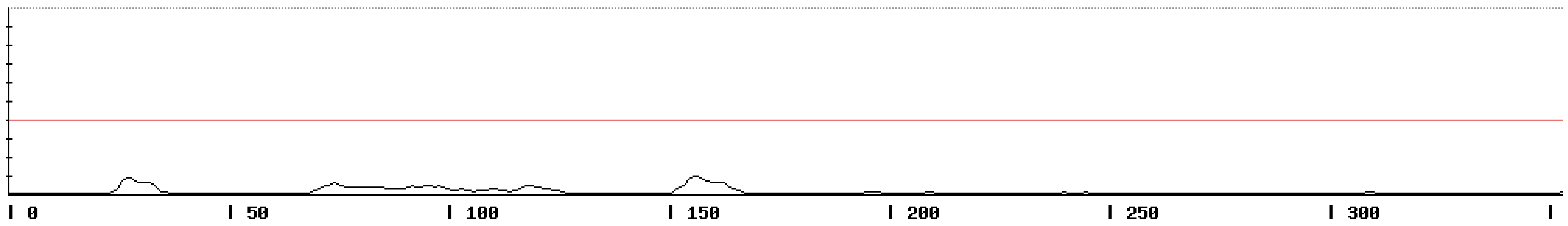

Predictions for Ipomoeatrifida_Itr_sc000257.1_g00022.1

Viterbi Path 68 - KLKKKGKLKTGCRVERTVGKKVKGKKPG - 95

Viterbi Path

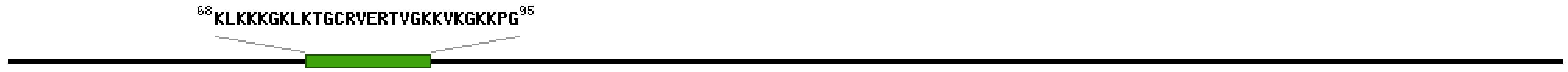

Posterior @ 0.4 | 68 - KLKKKGKLKTGCRVERTVGKKVKGKKPGN - 96

Posterior @ 0.4

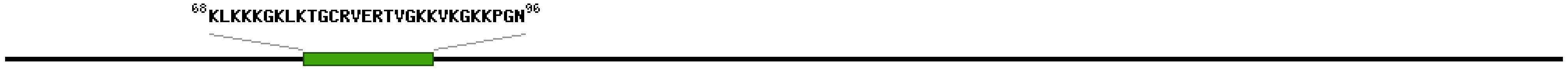

Posterior Graphic. Threshold Marked @ 0.4

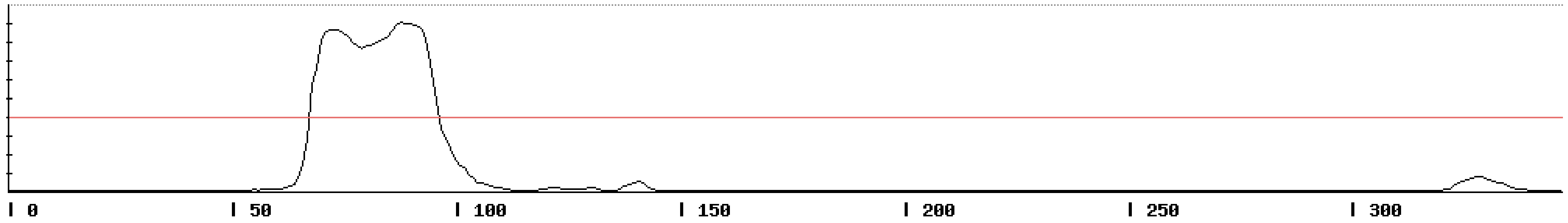

Predictions for Ipomoeatrifida_Itr_sc000259.1_g00017.1

Viterbi Path

Viterbi Path

Posterior @ 0.4

Posterior @ 0.4

Posterior Graphic. Threshold Marked @ 0.4

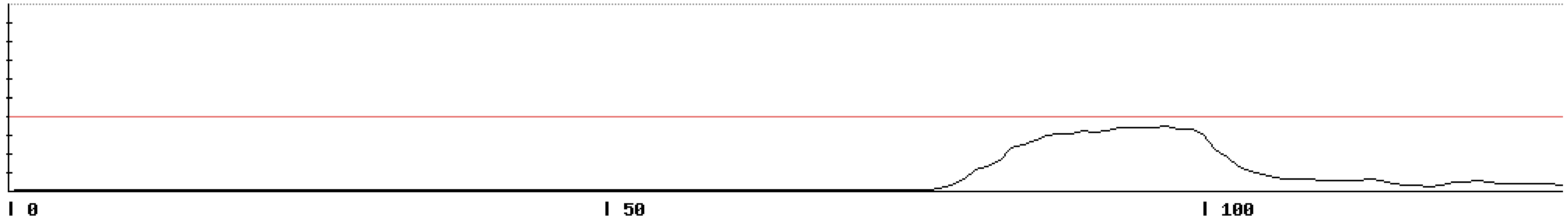

Predictions for Ipomoeatrifida_Itr_sc000264.1_g00005.1

Viterbi Path

Viterbi Path

Posterior @ 0.4

Posterior @ 0.4

Posterior Graphic. Threshold Marked @ 0.4

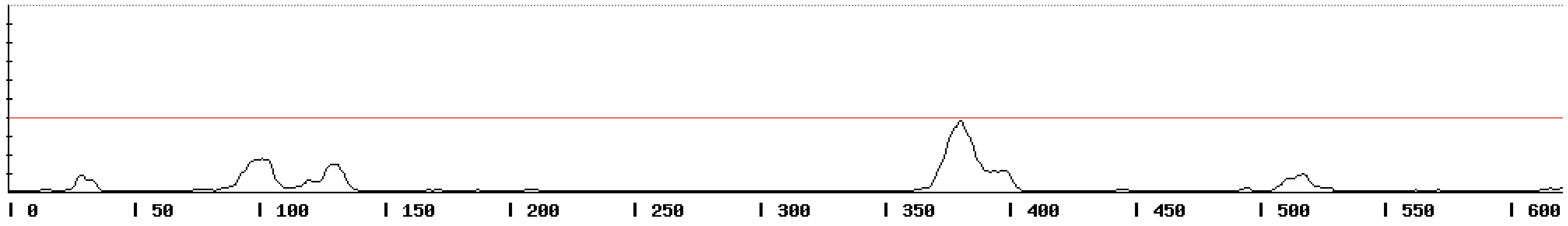

Predictions for Ipomoeatrifida_Itr_sc000264.1_g00019.1

Viterbi Path

Viterbi Path

Posterior @ 0.4

Posterior @ 0.4

Posterior Graphic. Threshold Marked @ 0.4

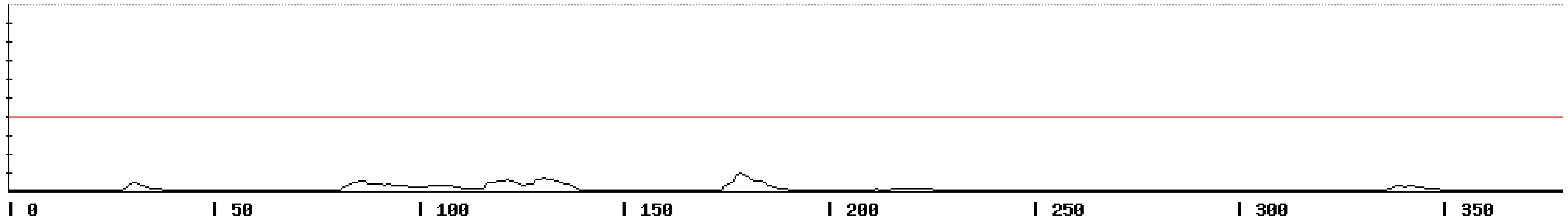

Predictions for Ipomoeatrifida_Itr_sc000277.1_g00004.1

Viterbi Path

Viterbi Path

Posterior @ 0.4

Posterior @ 0.4

Posterior Graphic. Threshold Marked @ 0.4

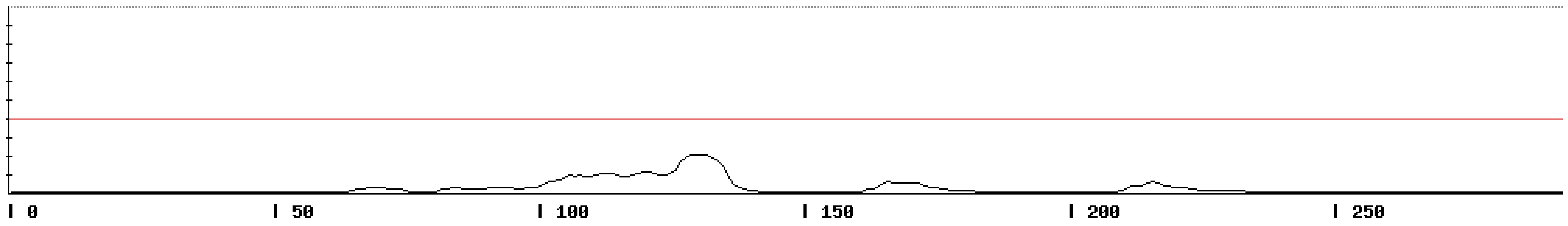

Predictions for Ipomoeatrifida_Itr_sc000283.1_g00018.1

Viterbi Path

Viterbi Path

Posterior @ 0.4

Posterior @ 0.4

Posterior Graphic. Threshold Marked @ 0.4

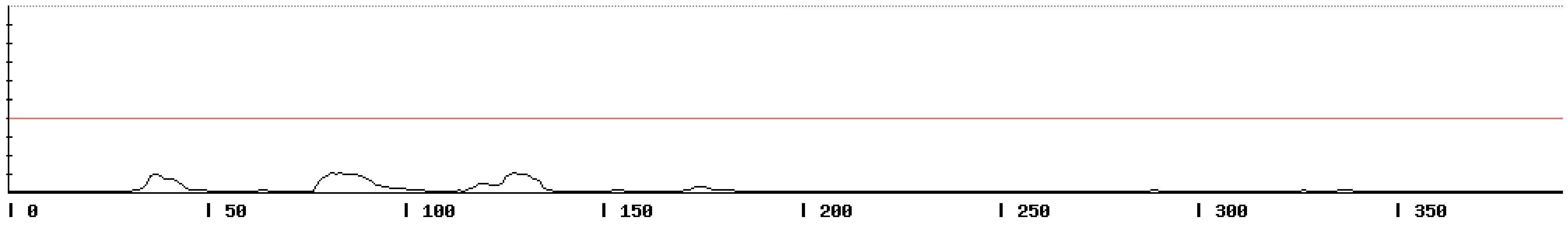

Predictions for Ipomoeatrifida_Itr_sc000322.1_g00018.1	
Viterbi Path	
Viterbi Path	
Posterior @ 0.4	221 - KKLGK - 225
Posterior @ 0.4	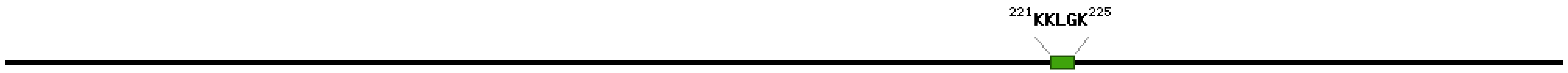
Posterior Graphic. Threshold Marked @ 0.4	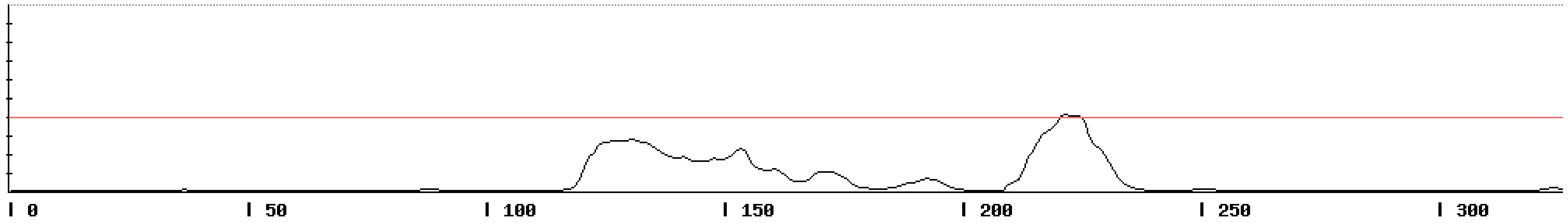

Predictions for Ipomoeatrifida_Itr_sc000325.1_g00018.1	
Viterbi Path	
Viterbi Path	
Posterior @ 0.4	221 - RSRKYRNGKRPNRA - 234
Posterior @ 0.4	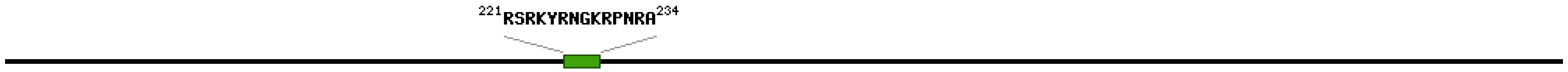
Posterior Graphic. Threshold Marked @ 0.4	

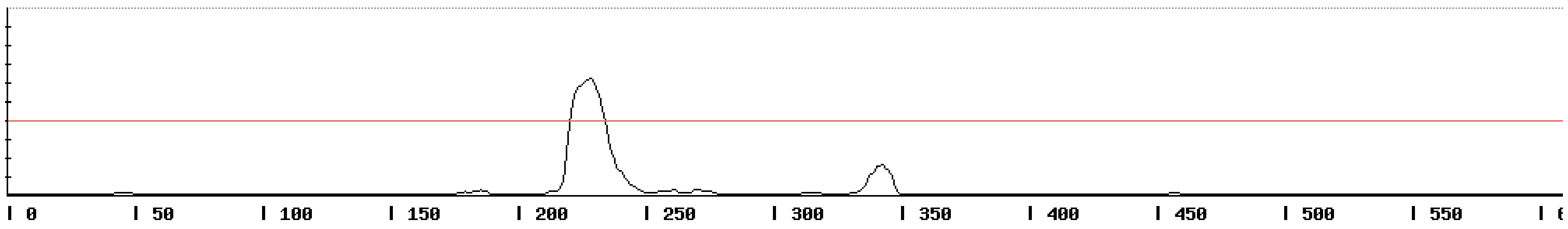

Predictions for Ipomoeatrifida_Itr_sc000335.1_g00025.1

Viterbi Path	
Viterbi Path	
Posterior @ 0.4	
Posterior @ 0.4	
Posterior Graphic. Threshold Marked @ 0.4	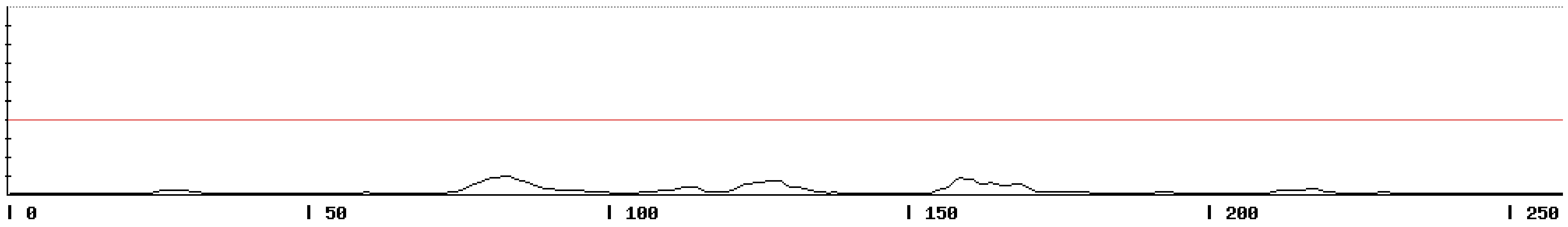

Predictions for Ipomoeatrifida_Itr_sc000358.1_g00015.1

Viterbi Path	
Viterbi Path	
Posterior @ 0.4	74 - RNGTRPKR - 81
Posterior @ 0.4	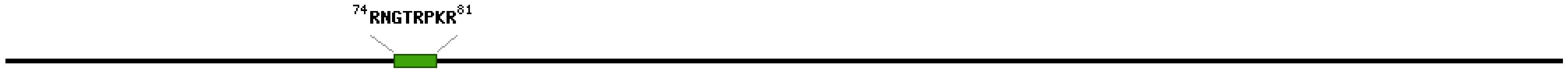

Posterior Graphic. Threshold Marked @ 0.4

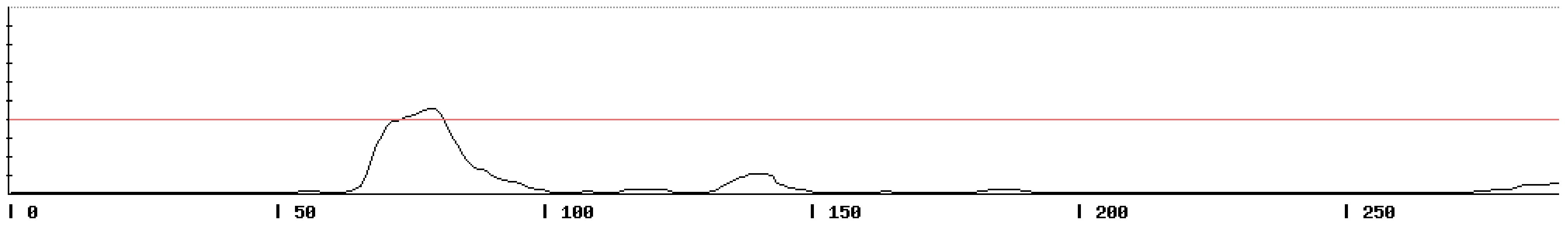

Predictions for Ipomoeatrifida_Itr_sc000358.1_g00016.1

Viterbi Path

Viterbi Path

Posterior @ 0.4

Posterior @ 0.4

Posterior Graphic. Threshold Marked @ 0.4

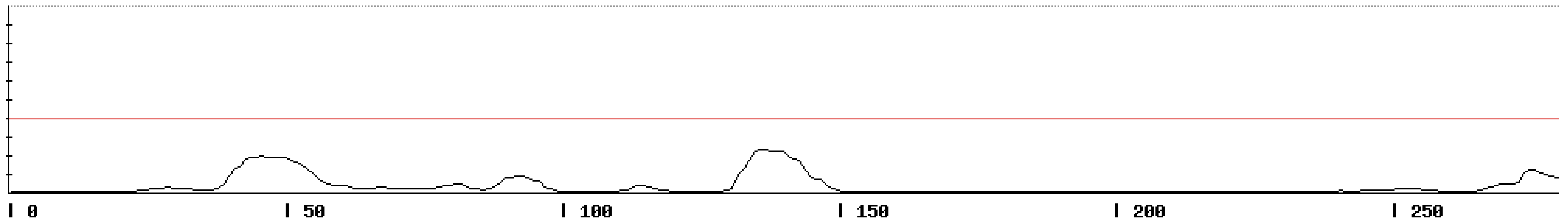

Predictions for Ipomoeatrifida_Itr_sc000361.1_g00027.1

Viterbi Path

Viterbi Path

Posterior @ 0.4

Posterior @ 0.4

Posterior Graphic. Threshold Marked @ 0.4

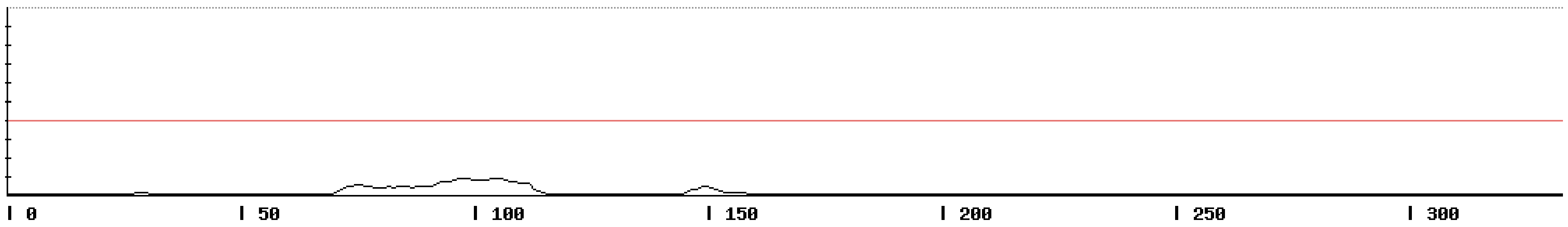

Predictions for Ipomoeatrifida_Itr_sc000397.1_g00016.1

Viterbi Path

Viterbi Path

Posterior @ 0.4

Posterior @ 0.4

Posterior Graphic. Threshold Marked @ 0.4

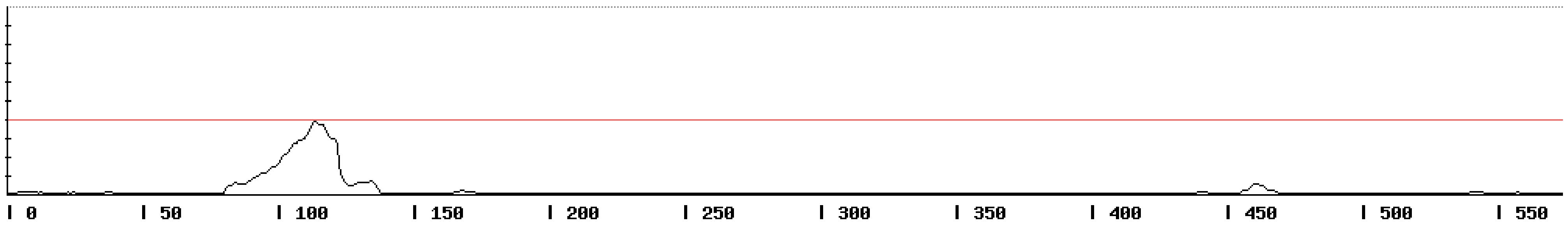

Predictions for Ipomoeatrifida_Itr_sc000403.1_g00012.1

Viterbi Path

Viterbi Path

Posterior @ 0.4

Posterior @ 0.4

Posterior Graphic. Threshold Marked @ 0.4

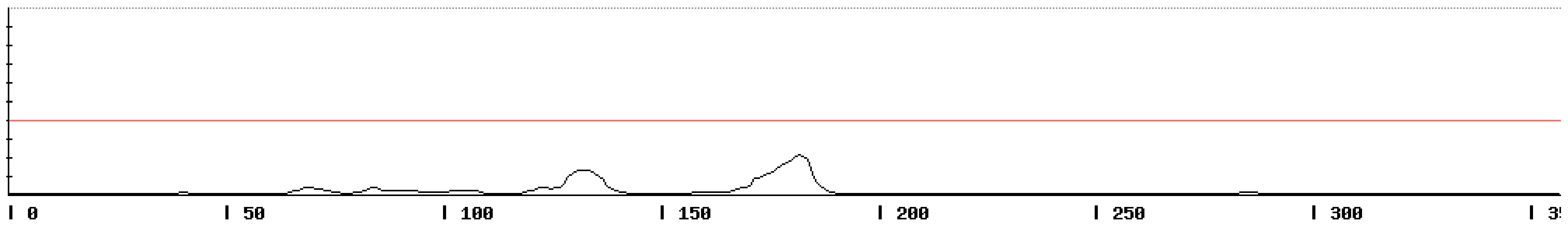

Predictions for Ipomoeatrifida_Itr_sc000419.1_g00006.1

Viterbi Path

Viterbi Path

Posterior @ 0.4

Posterior @ 0.4

Posterior Graphic. Threshold Marked @ 0.4

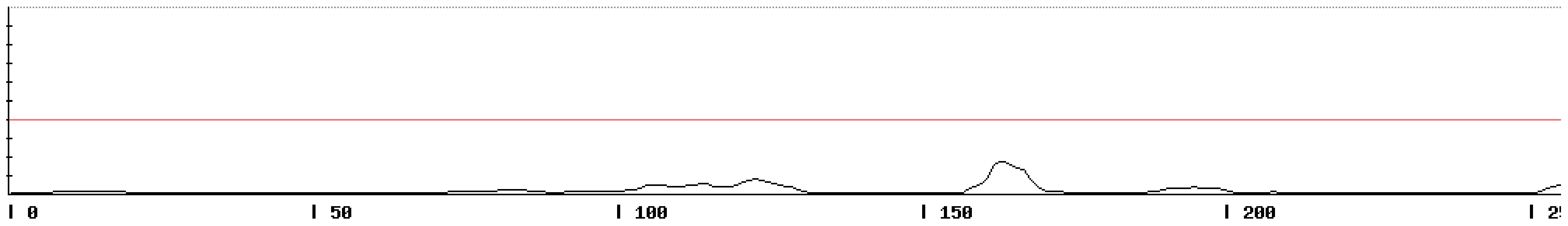

Predictions for Ipomoeatrifida_Itr_sc000436.1_g00007.1

Viterbi Path

Viterbi Path

Posterior @ 0.4	
Posterior @ 0.4	
Posterior Graphic. Threshold Marked @ 0.4	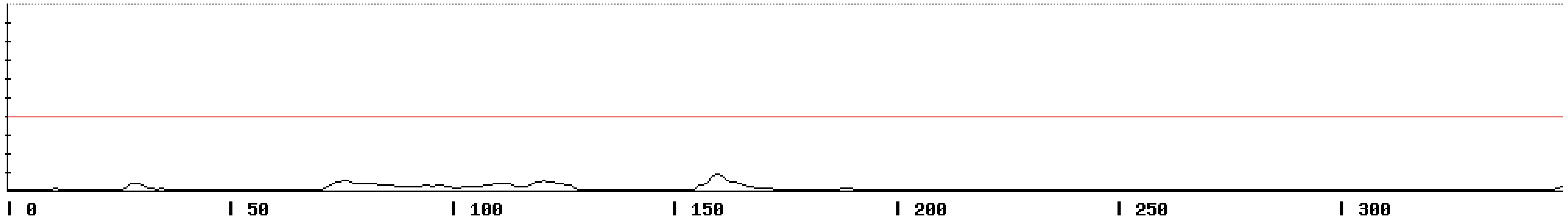

Predictions for Ipomoeatrifida_Itr_sc000440.1_g00020.1

Viterbi Path	
Viterbi Path	
Posterior @ 0.4	
Posterior @ 0.4	
Posterior Graphic. Threshold Marked @ 0.4	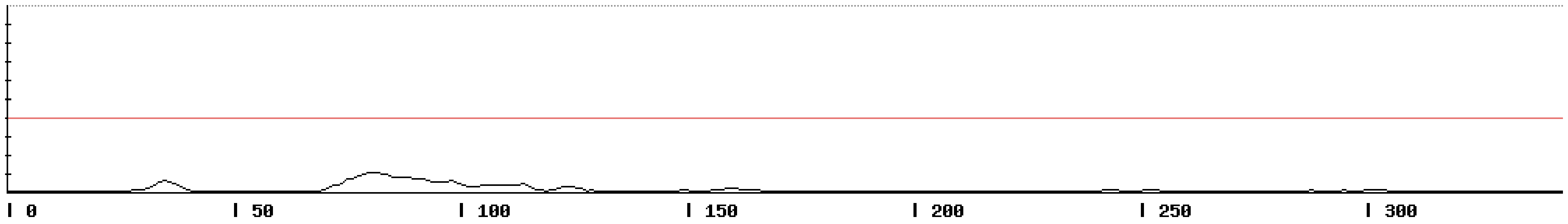

Predictions for Ipomoeatrifida_Itr_sc000448.1_g00012.1

Viterbi Path

Viterbi Path

Posterior @ 0.4

Posterior @ 0.4

Posterior Graphic. Threshold Marked @ 0.4

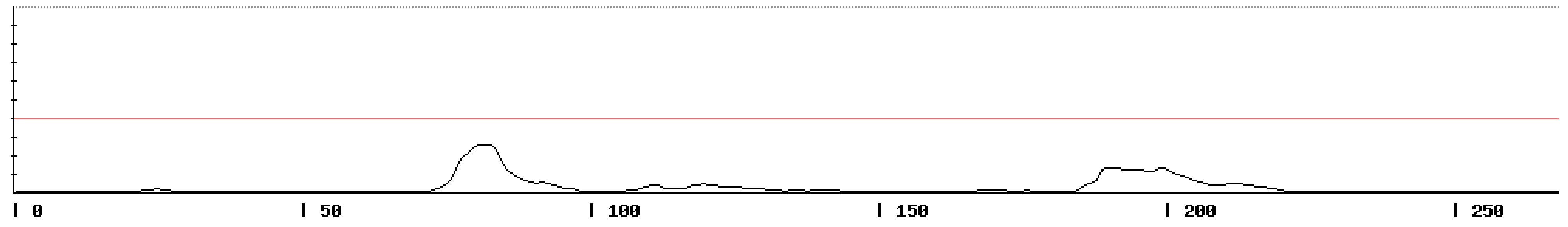

Predictions for Ipomoeatrifida_Itr_sc000462.1_g00010.1

Viterbi Path

Viterbi Path

Posterior @ 0.4

Posterior @ 0.4

Posterior Graphic. Threshold Marked @ 0.4

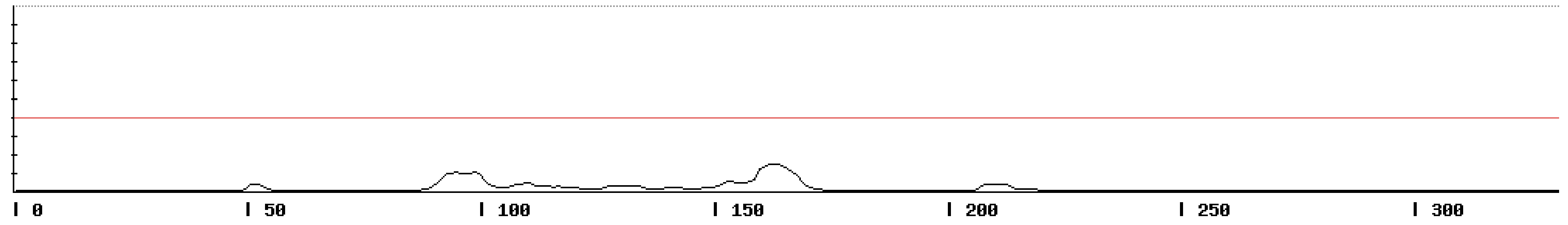

Predictions for Ipomoeatrifida_Itr_sc000509.1_g00002.1

Viterbi Path

Viterbi Path

Posterior @ 0.4

Posterior @ 0.4

Posterior Graphic. Threshold Marked @ 0.4

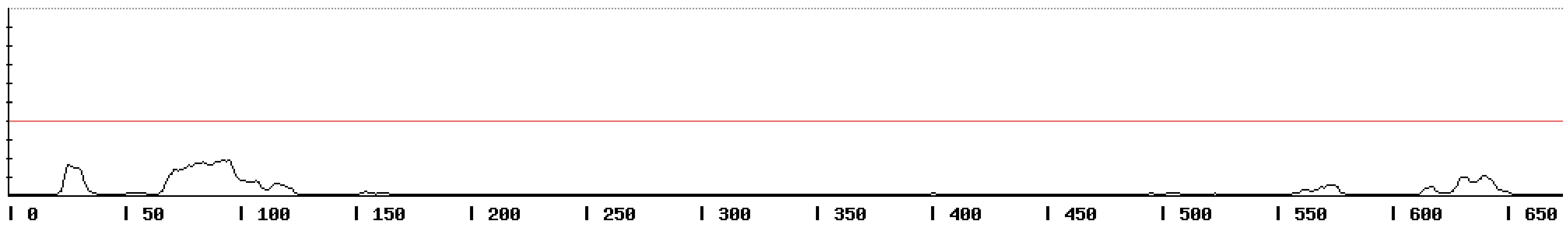

Predictions for Ipomoeatrifida_Itr_sc000521.1_g00006.1

Viterbi Path

Viterbi Path

Posterior @ 0.4

Posterior @ 0.4

Posterior Graphic. Threshold Marked @ 0.4

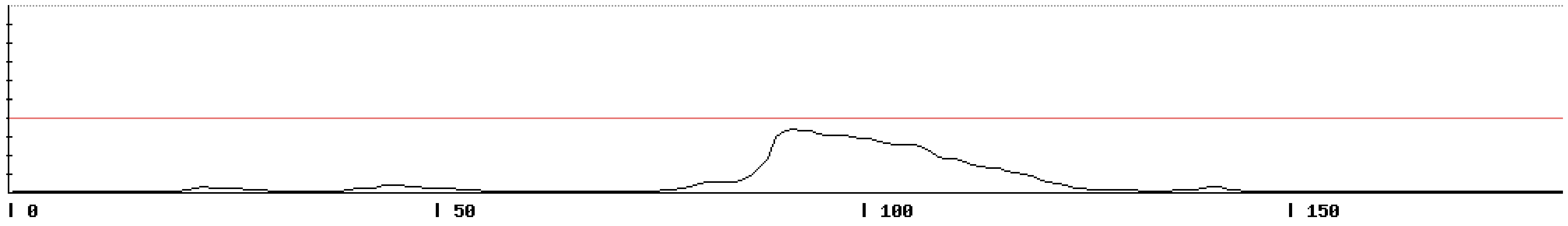

Predictions for Ipomoeatrifida_Itr_sc000535.1_g00006.1

Viterbi Path

Viterbi Path

Posterior @ 0.4

Posterior @ 0.4

Posterior Graphic. Threshold Marked @ 0.4

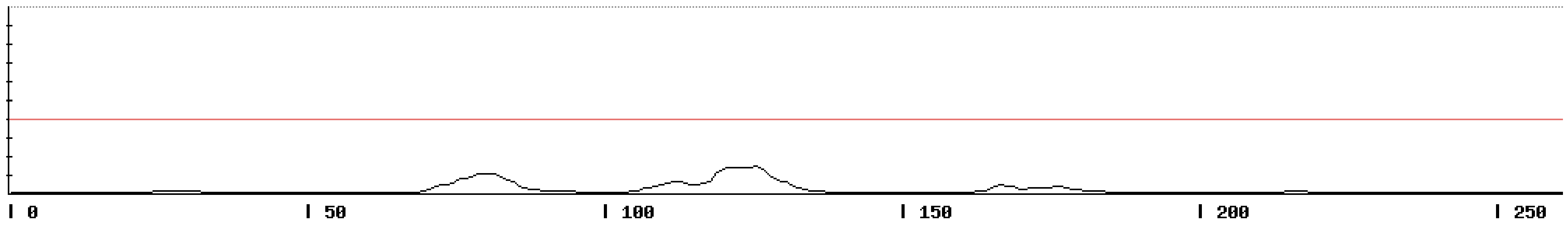

Predictions for Ipomoeatrifida_Itr_sc000539.1_g00012.1

Viterbi Path

Viterbi Path

Posterior @ 0.4

65 - KRKSKSMKGSRIN - 77

158 - LKRKKRAG - 165

Posterior @ 0.4

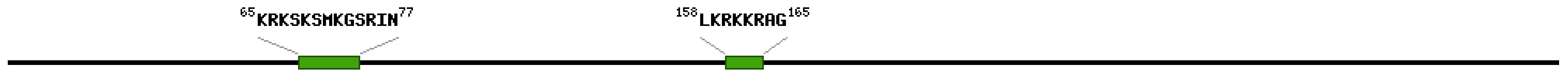

Posterior Graphic. Threshold Marked @ 0.4

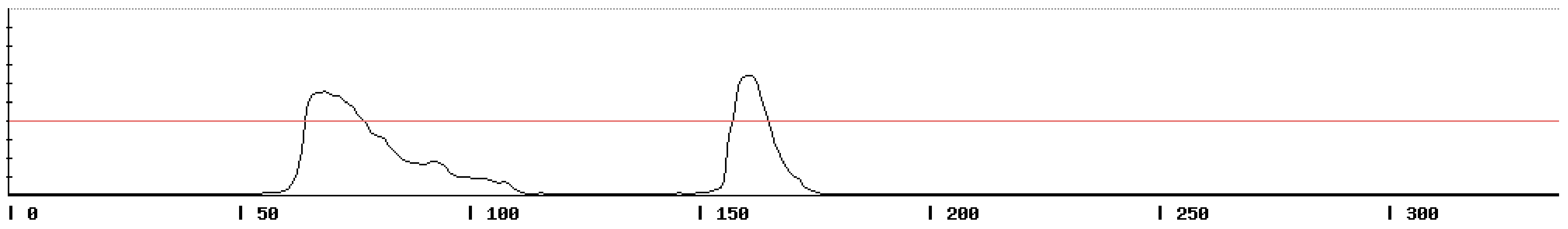

Predictions for Ipomoeatrifida_Itr_sc000539.1_g00013.1

Viterbi Path

Viterbi Path

Posterior @ 0.4

63 - KLKRKSKTMKGSRIDRTVGKRPGTWHGQDKAKP - 95

155 - RRKKR - 159

Posterior @ 0.4

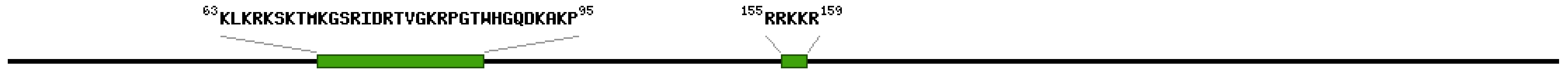

Posterior Graphic. Threshold Marked @ 0.4

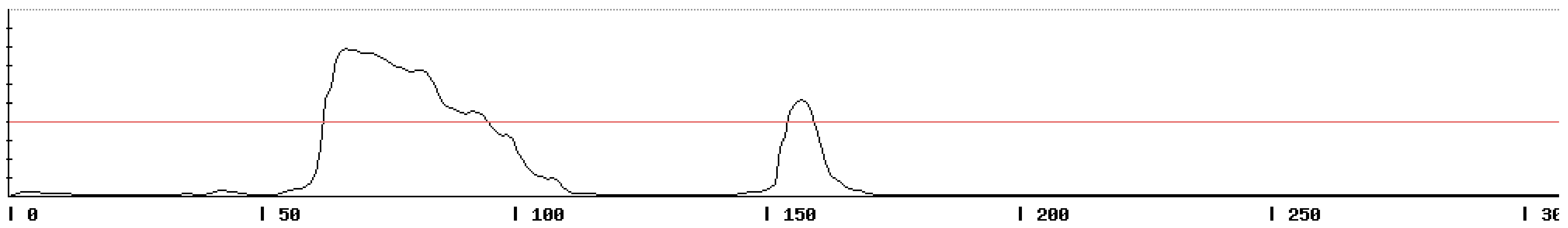

Predictions for Ipomoeatrifida_Itr_sc000543.1_g00010.1

Viterbi Path

Viterbi Path

Posterior @ 0.4	
Posterior @ 0.4	
Posterior Graphic. Threshold Marked @ 0.4	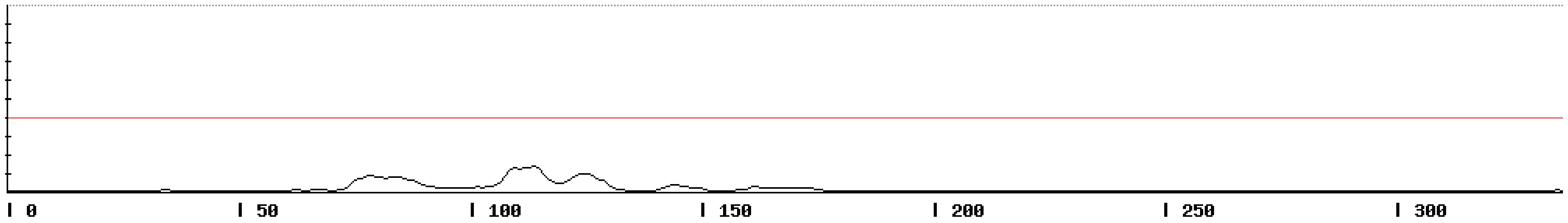

Predictions for Ipomoeatrifida_Itr_sc000587.1_g00010.1

Viterbi Path	
Viterbi Path	
Posterior @ 0.4	
Posterior @ 0.4	
Posterior Graphic. Threshold Marked @ 0.4	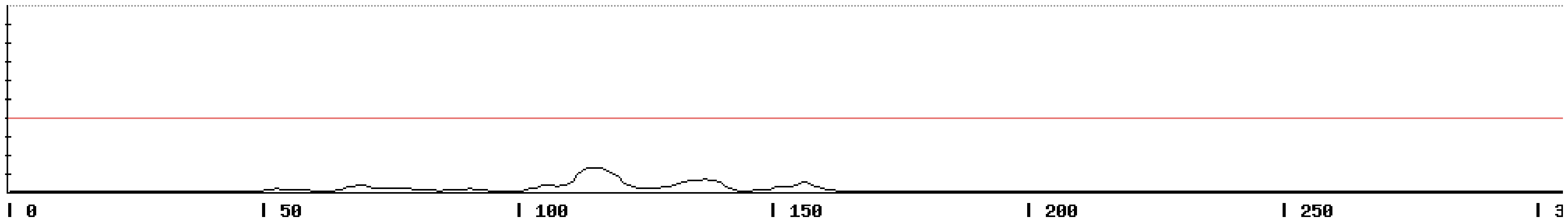

Predictions for Ipomoeatrifida_Itr_sc000727.1_g00013.1

Viterbi Path

Viterbi Path

Posterior @ 0.4

Posterior @ 0.4

Posterior Graphic. Threshold Marked @ 0.4

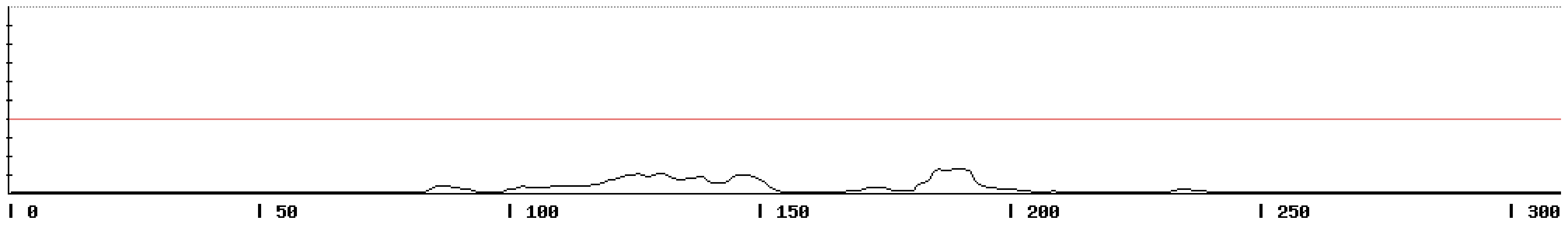

Predictions for Ipomoeatrifida_Itr_sc000740.1_g00023.1

Viterbi Path

Viterbi Path

Posterior @ 0.4

Posterior @ 0.4

Posterior Graphic. Threshold Marked @ 0.4

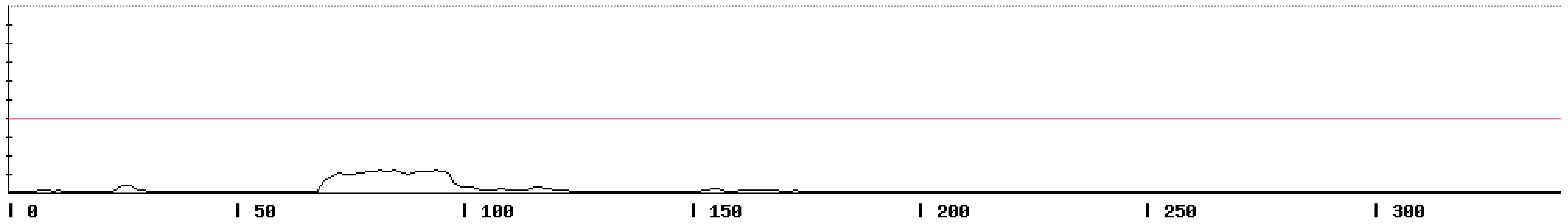

Predictions for Ipomoeatrifida_Itr_sc000781.1_g00011.1

Viterbi Path	
Viterbi Path	
Posterior @ 0.4	66 - KRKSKSSGKASSRFARRVGKSPGGTWHGQDKGK - 98
Posterior @ 0.4	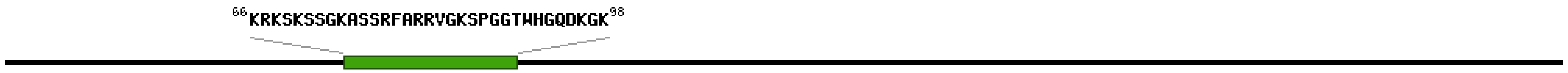
Posterior Graphic. Threshold Marked @ 0.4	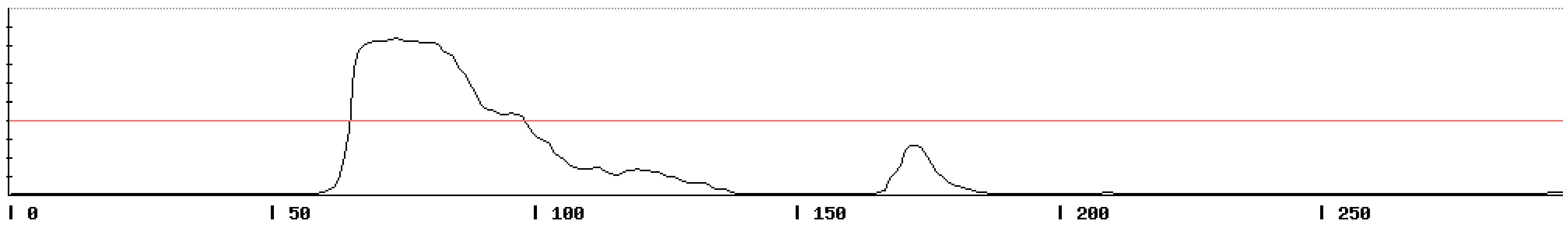

Predictions for Ipomoeatrifida_Itr_sc000793.1_g00021.1

Viterbi Path	
Viterbi Path	
Posterior @ 0.4	
Posterior @ 0.4	
Posterior Graphic. Threshold Marked @ 0.4	

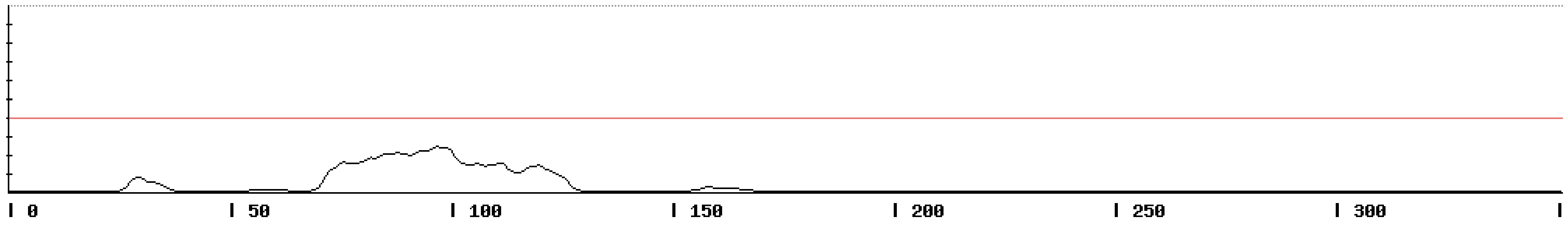

Predictions for Ipomoeatrifida_Itr_sc000803.1_g00008.1

Viterbi Path

Viterbi Path

Posterior @ 0.4

Posterior @ 0.4

Posterior Graphic. Threshold Marked @ 0.4

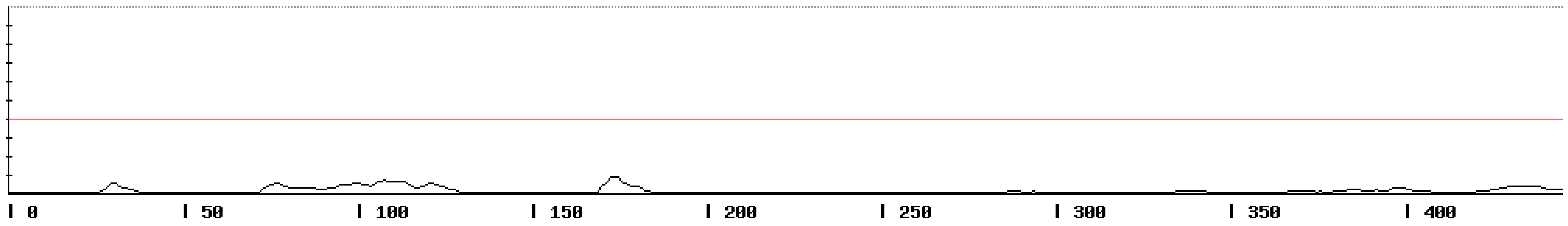

Predictions for Ipomoeatrifida_Itr_sc000828.1_g00002.1

Viterbi Path

Viterbi Path

Posterior @ 0.4

Posterior @ 0.4

Posterior Graphic. Threshold Marked @ 0.4	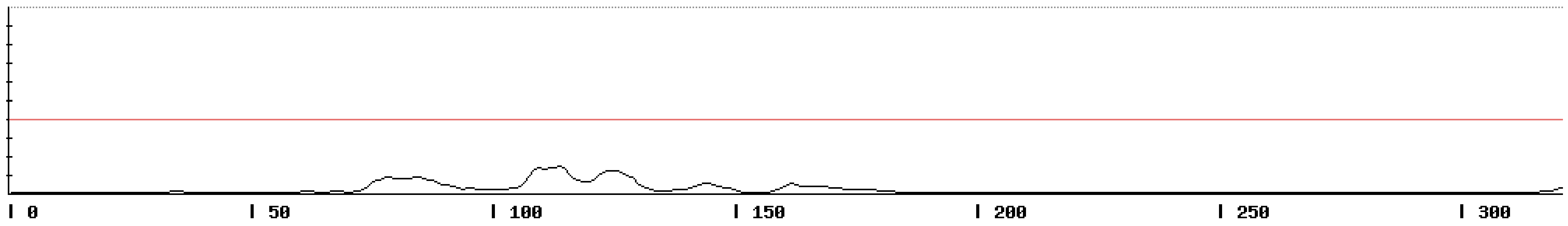

Predictions for Ipomoeatrifida_Itr_sc000868.1_g00001.1

Viterbi Path	
Viterbi Path	
Posterior @ 0.4	
Posterior @ 0.4	
Posterior Graphic. Threshold Marked @ 0.4	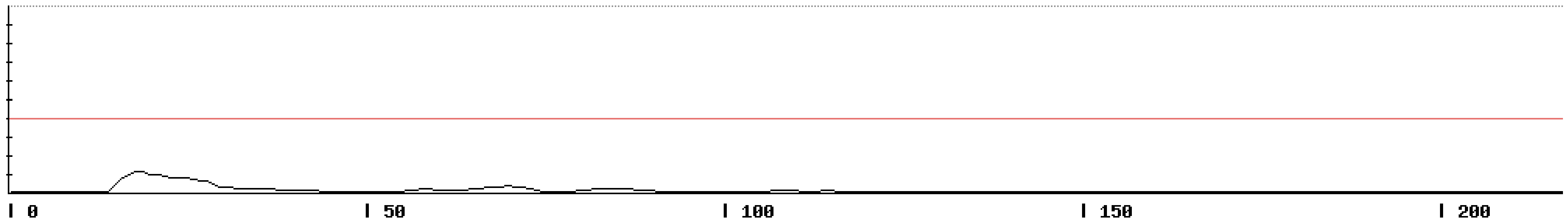

Predictions for Ipomoeatrifida_Itr_sc000875.1_g00001.1

Viterbi Path	
Viterbi Path	
Posterior @ 0.4	
Posterior @ 0.4	

Posterior Graphic. Threshold Marked @ 0.4

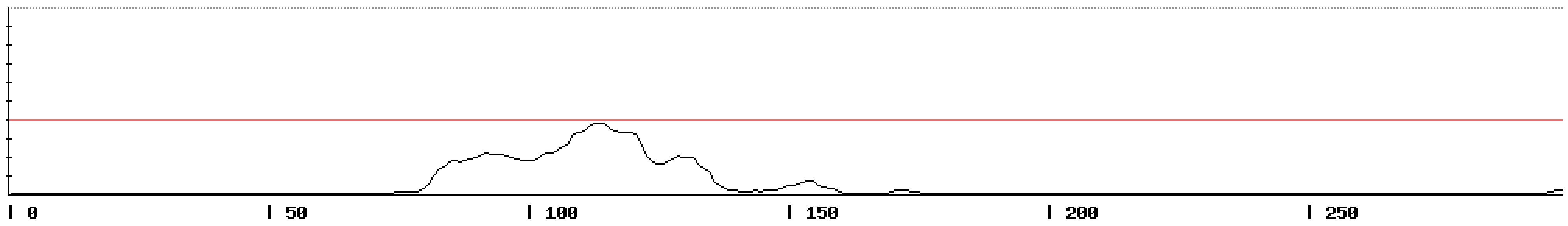

Predictions for Ipomoeatrifida_Itr_sc000875.1_g00017.1

Viterbi Path

Viterbi Path

Posterior @ 0.4

Posterior @ 0.4

Posterior Graphic. Threshold Marked @ 0.4

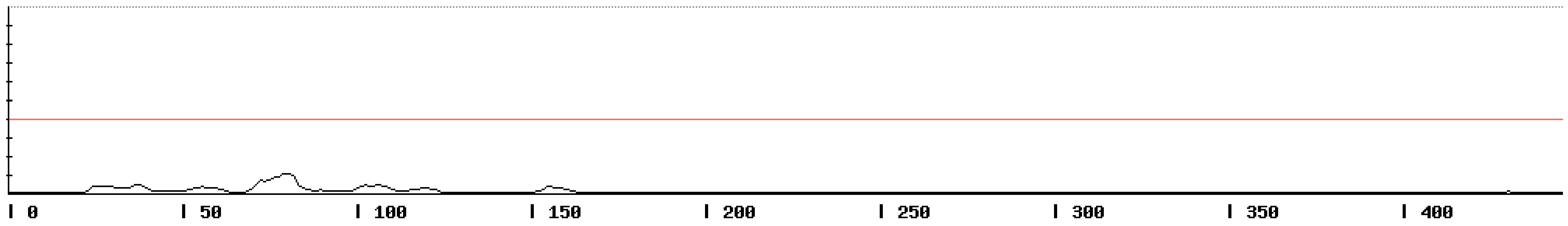

Predictions for Ipomoeatrifida_Itr_sc000880.1_g00004.1

Viterbi Path

Viterbi Path

Posterior @ 0.4	98 - VRDKVMGFRKSLKYETKKSKKSKG - 121
Posterior @ 0.4	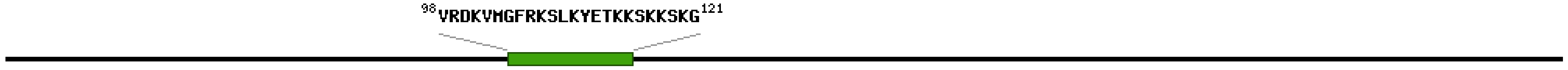
Posterior Graphic. Threshold Marked @ 0.4	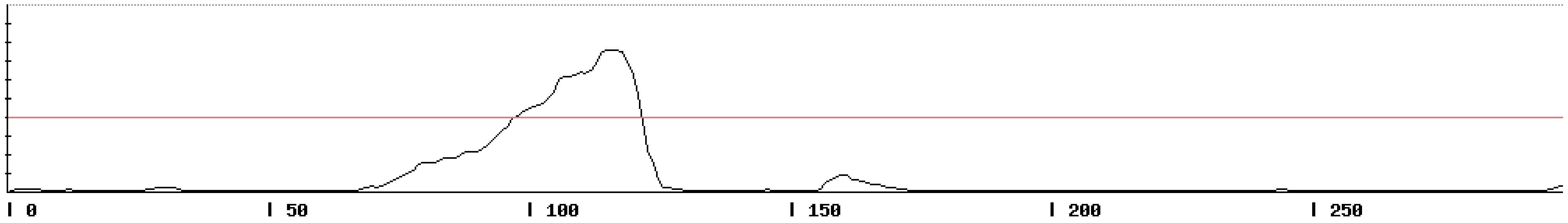

Predictions for Ipomoeatrifida_Itr_sc000887.1_g00023.1	
Viterbi Path	
Viterbi Path	
Posterior @ 0.4	
Posterior @ 0.4	
Posterior Graphic. Threshold Marked @ 0.4	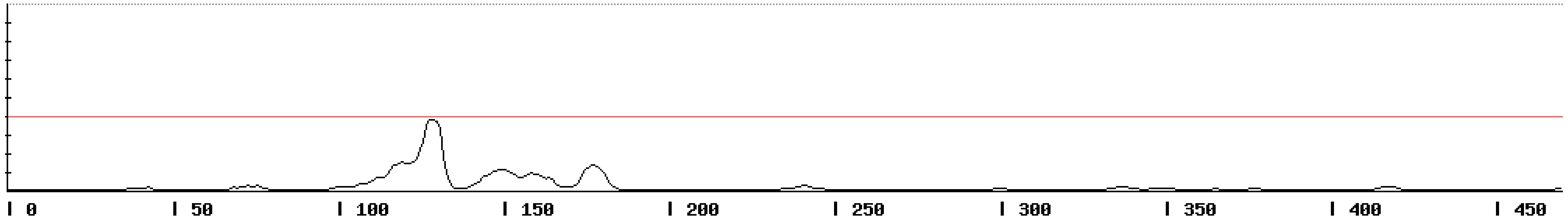

Predictions for Ipomoeatrifida_Itr_sc000894.1_g00009.1	
Viterbi Path	
Viterbi Path	

Posterior @ 0.4

Posterior @ 0.4

Posterior Graphic. Threshold Marked @ 0.4

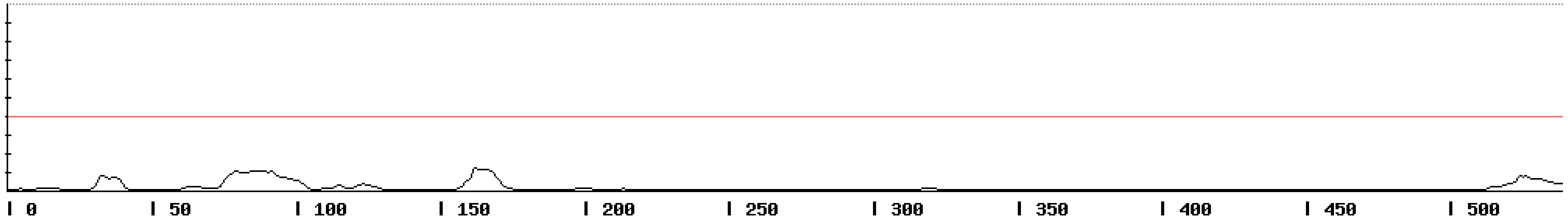

Predictions for Ipomoeatrifida_Itr_sc000973.1_g00002.1

Viterbi Path

Viterbi Path

Posterior @ 0.4

Posterior @ 0.4

Posterior Graphic. Threshold Marked @ 0.4

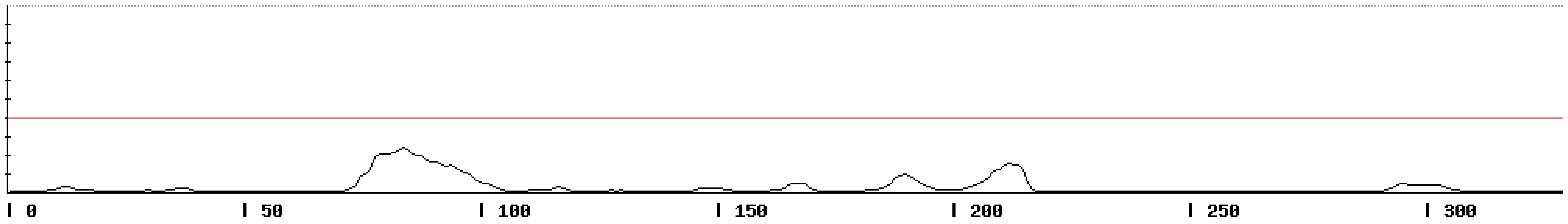

Predictions for Ipomoeatrifida_Itr_sc001001.1_g00001.1

Viterbi Path

Viterbi Path

Posterior @ 0.4

Posterior @ 0.4

Posterior Graphic. Threshold Marked @ 0.4

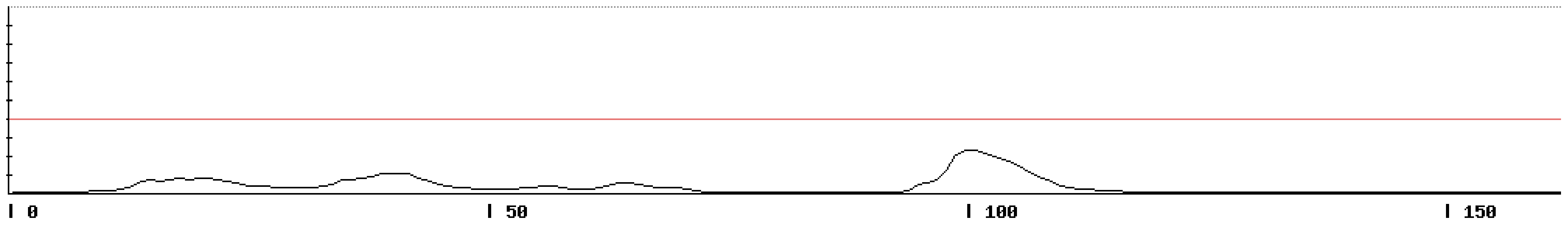

Predictions for Ipomoeatrifida_Itr_sc001001.1_g00002.1

Viterbi Path

Viterbi Path

Posterior @ 0.4

Posterior @ 0.4

Posterior Graphic. Threshold Marked @ 0.4

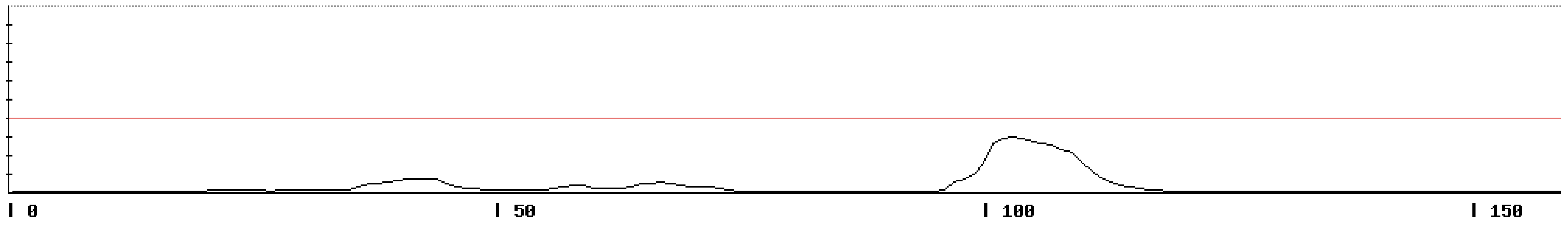

Predictions for Ipomoeatrifida_Itr_sc001001.1_g00003.1	
Viterbi Path	
Viterbi Path	
Posterior @ 0.4	
Posterior @ 0.4	
Posterior Graphic. Threshold Marked @ 0.4	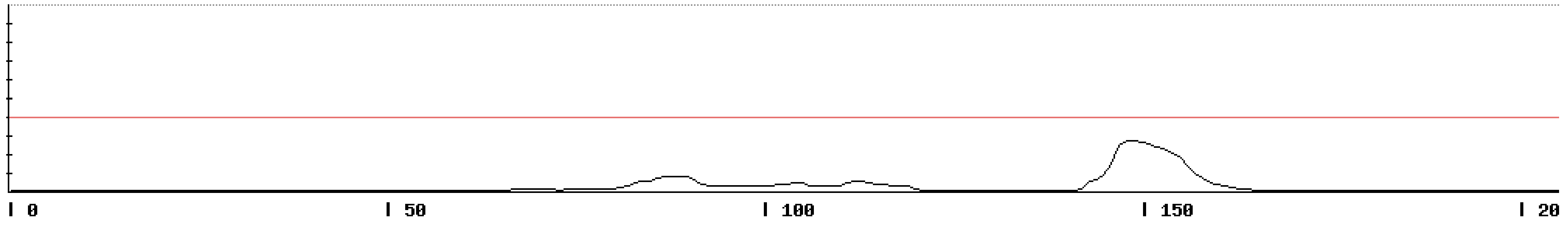

Predictions for Ipomoeatrifida_Itr_sc001052.1_g00006.1	
Viterbi Path	
Viterbi Path	
Posterior @ 0.4	
Posterior @ 0.4	
Posterior Graphic. Threshold Marked @ 0.4	

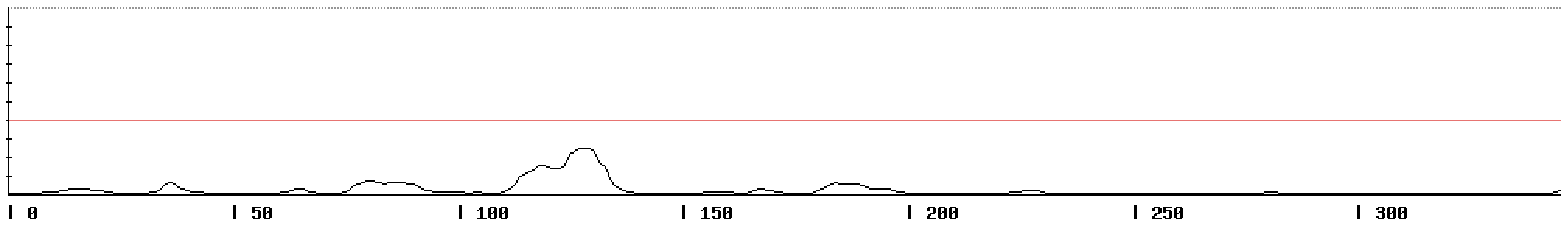

Predictions for Ipomoeatrifida_Itr_sc001123.1_g00001.1

Viterbi Path

Viterbi Path

Posterior @ 0.4

Posterior @ 0.4

Posterior Graphic. Threshold Marked @ 0.4

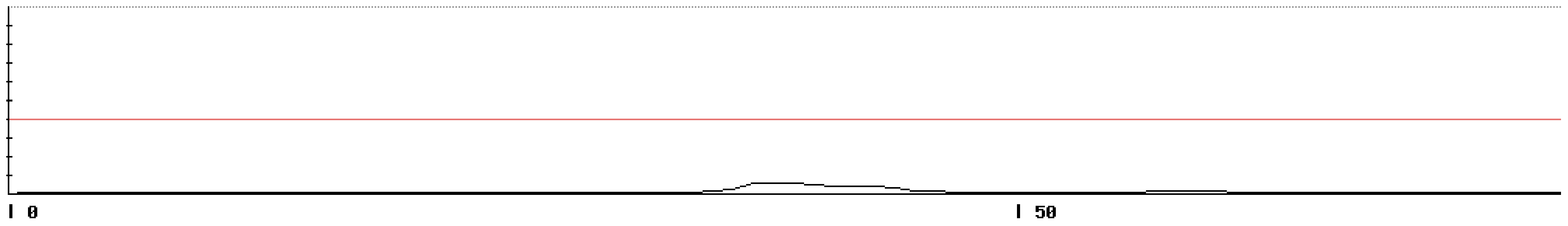

Predictions for Ipomoeatrifida_Itr_sc001159.1_g00006.1

Viterbi Path

Viterbi Path

Posterior @ 0.4

Posterior @ 0.4

Posterior Graphic. Threshold Marked @ 0.4

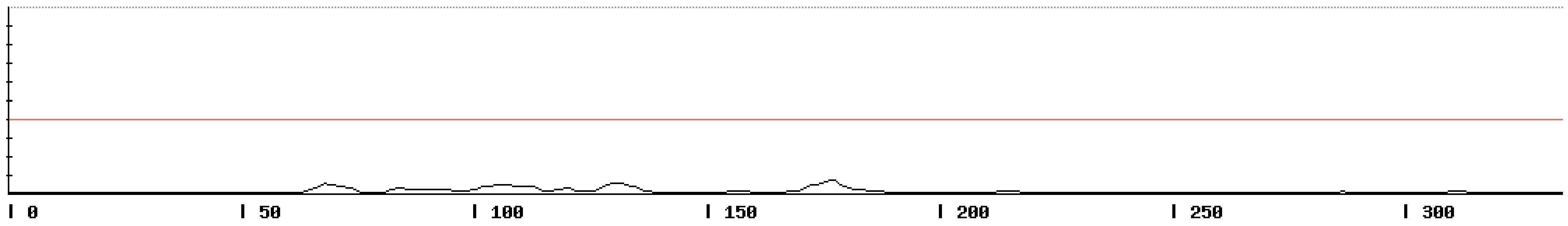

Predictions for Ipomoeatrifida_Itr_sc001238.1_g00001.1

Viterbi Path

Viterbi Path

Posterior @ 0.4

Posterior @ 0.4

Posterior Graphic. Threshold Marked @ 0.4

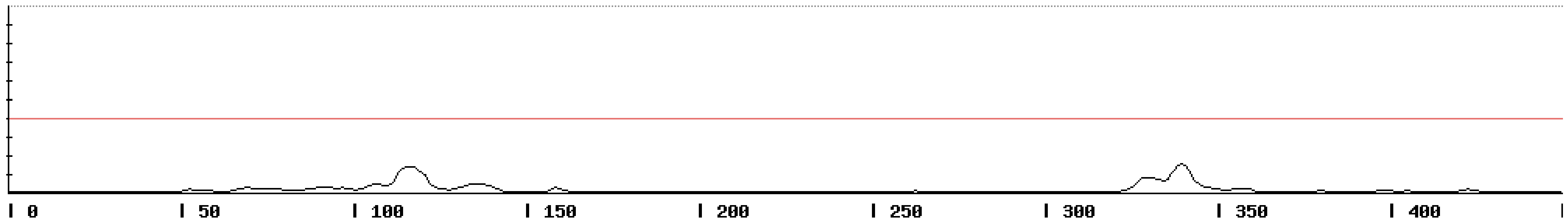

Predictions for Ipomoeatrifida_Itr_sc001346.1_g00002.1

Viterbi Path

Viterbi Path

Posterior @ 0.4

Posterior @ 0.4

Posterior Graphic. Threshold Marked @ 0.4

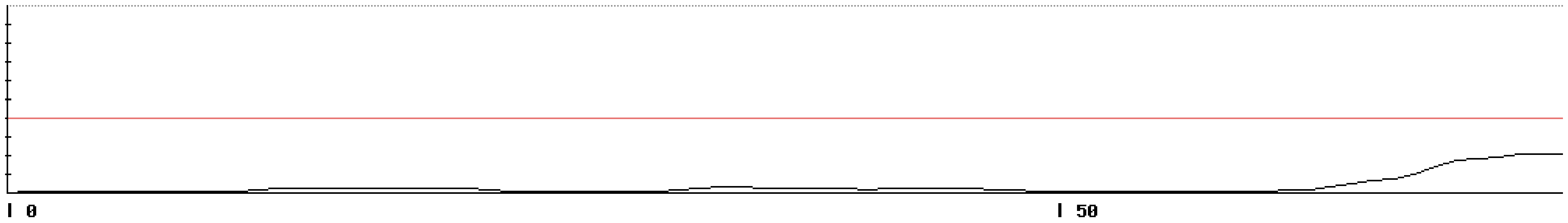

Predictions for Ipomoeatrifida_Itr_sc001380.1_g00013.1

Viterbi Path

Viterbi Path

Posterior @ 0.4 | 183 - RKRRK - 187

Posterior @ 0.4

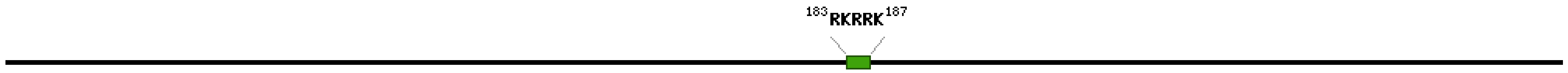

Posterior Graphic. Threshold Marked @ 0.4

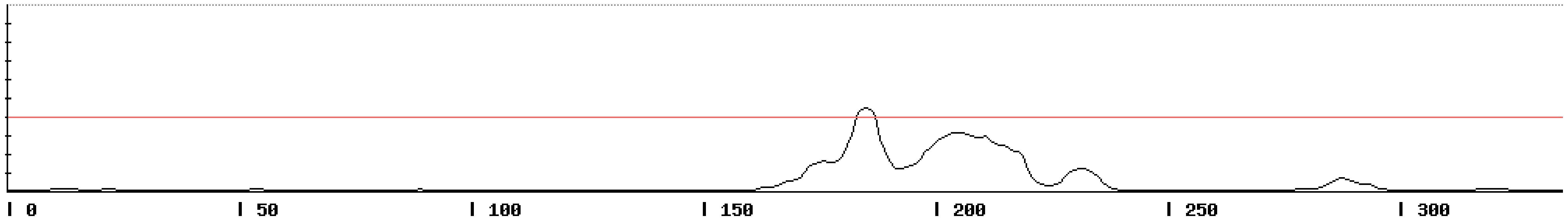

Predictions for Ipomoeatrifida_Itr_sc001565.1_g00004.1

Viterbi Path

Viterbi Path

Posterior @ 0.4

Posterior @ 0.4

Posterior Graphic. Threshold Marked @ 0.4

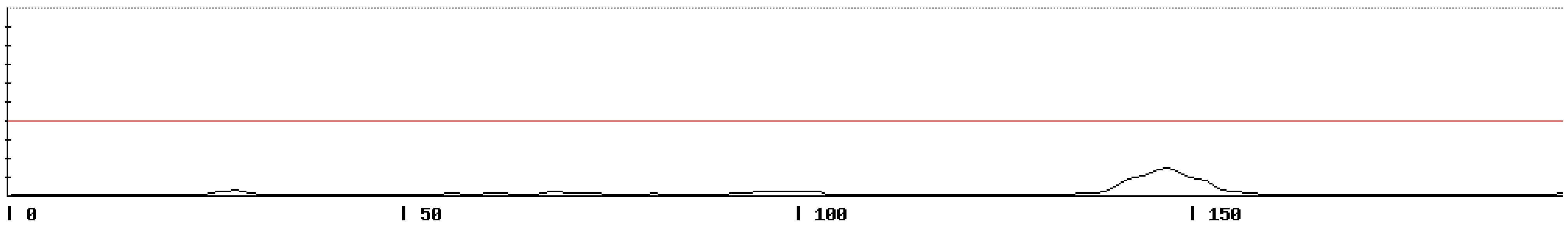

Predictions for Ipomoeatrifida_Itr_sc001584.1_g00001.1

Viterbi Path

Viterbi Path

Posterior @ 0.4 48 - FRKSLKYEMKKSKSKGG - 64

Posterior @ 0.4

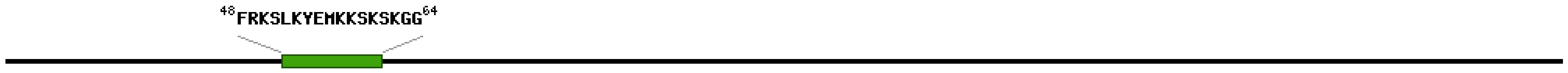

Posterior Graphic. Threshold Marked @ 0.4

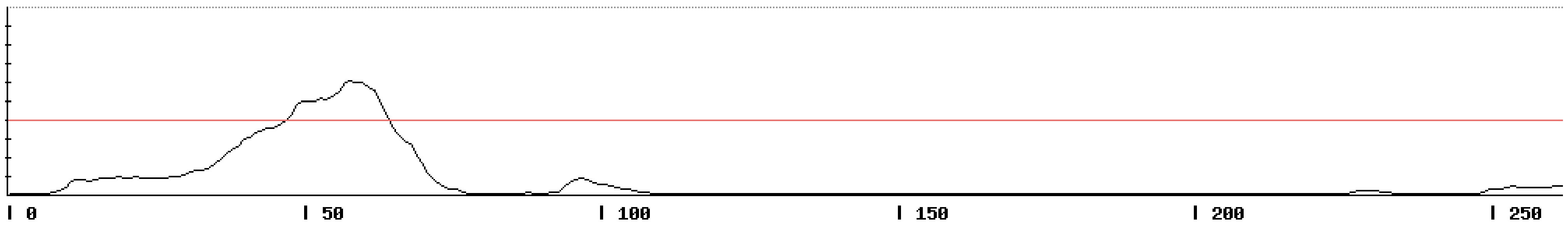

Predictions for Ipomoeatrifida_Itr_sc001584.1_g00004.1

Viterbi Path

Viterbi Path

Posterior @ 0.4 — 104 - GFRKSLKYETKKSKSKG - 120

Posterior @ 0.4

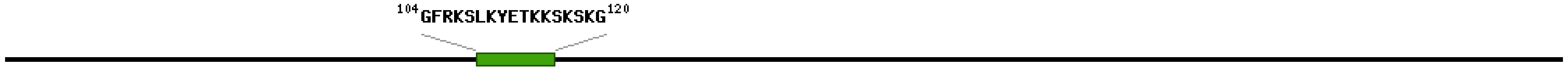

Posterior Graphic. Threshold Marked @ 0.4

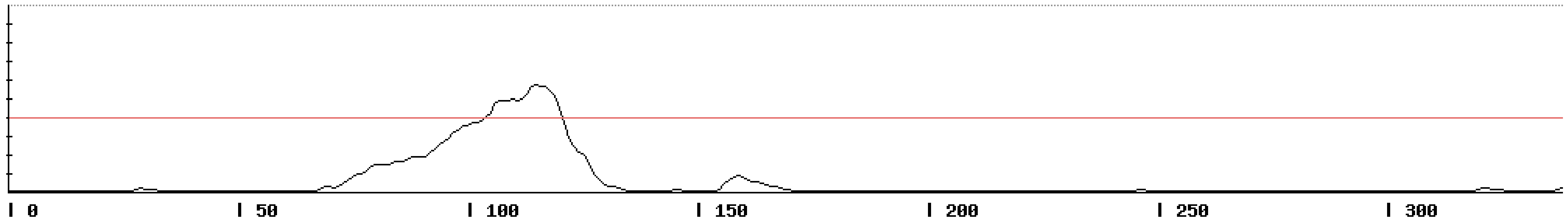

Predictions for Ipomoeatrifida_Itr_sc001700.1_g00010.1

Viterbi Path

Viterbi Path

Posterior @ 0.4

Posterior @ 0.4

Posterior Graphic. Threshold Marked @ 0.4

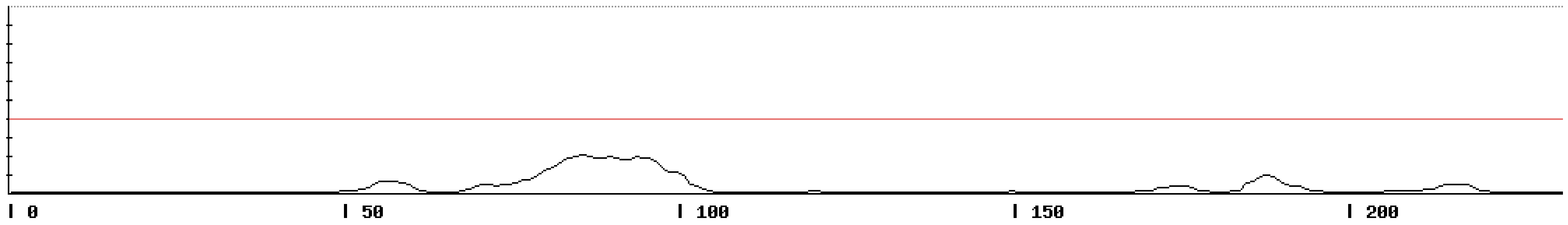

Predictions for Ipomoeatrifida_Itr_sc001767.1_g00001.1

Viterbi Path	
Viterbi Path	
Posterior @ 0.4	63 - RLKKKAKSMKGSRVHRTVGRKIM - 85
Posterior @ 0.4	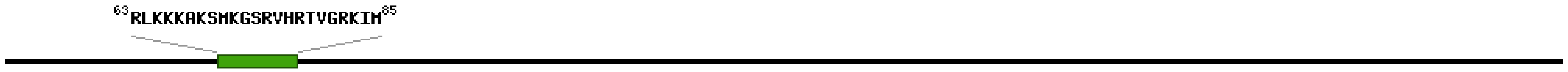
Posterior Graphic. Threshold Marked @ 0.4	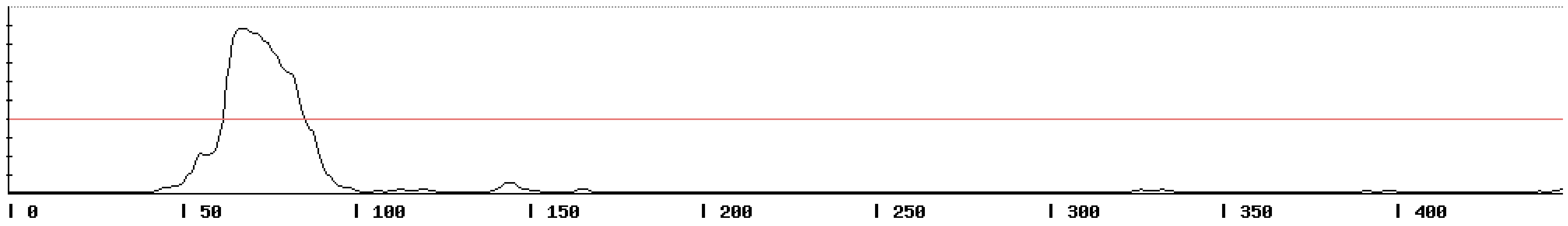

Predictions for Ipomoeatrifida_Itr_sc001767.1_g00002.1	
Viterbi Path	
Viterbi Path	
Posterior @ 0.4	62 - TRLKKKAKSMKGSRVQRTVGRKMMGKKS - 89
Posterior @ 0.4	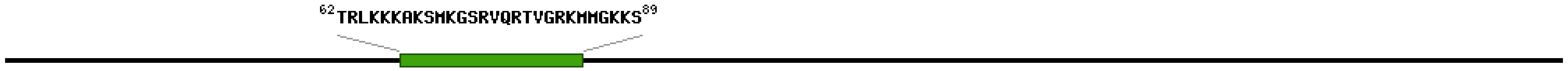
Posterior Graphic. Threshold Marked @ 0.4	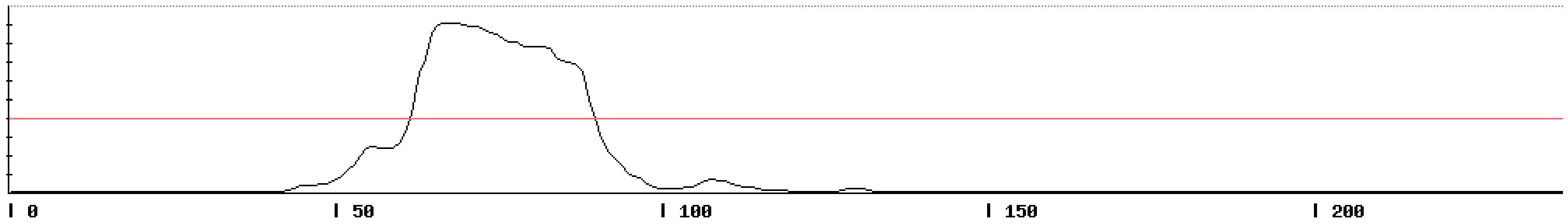

Predictions for Ipomoeatrifida_Itr_sc001859.1_g00003.1	
Viterbi Path	
Viterbi Path	
Posterior @ 0.4	
Posterior @ 0.4	
Posterior Graphic. Threshold Marked @ 0.4	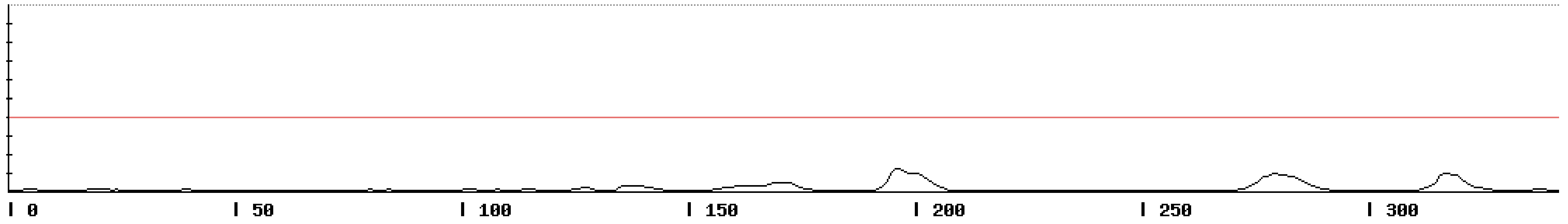

Predictions for Ipomoeatrifida_Itr_sc001868.1_g00005.1	
Viterbi Path	
Viterbi Path	
Posterior @ 0.4	
Posterior @ 0.4	
Posterior Graphic. Threshold Marked @ 0.4	

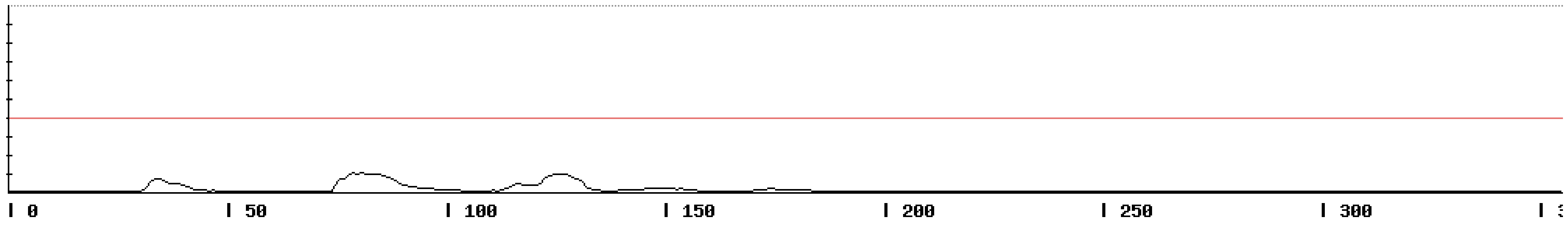

Predictions for Ipomoeatrifida_Itr_sc001875.1_g00002.1

Viterbi Path	
Viterbi Path	
Posterior @ 0.4	
Posterior @ 0.4	
Posterior Graphic. Threshold Marked @ 0.4	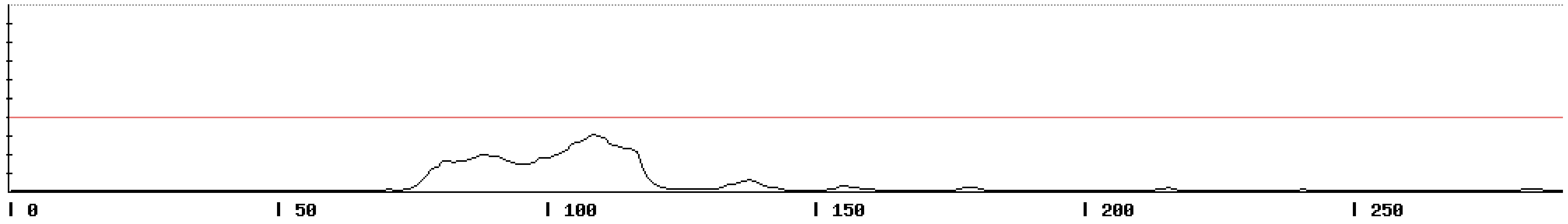

Predictions for Ipomoeatrifida_Itr_sc001890.1_g00004.1

Viterbi Path	
Viterbi Path	
Posterior @ 0.4	92 - RKRRK - 96
	318 - KEKKRK - 323
Posterior @ 0.4	

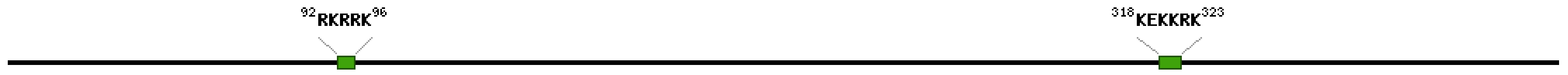

Posterior Graphic. Threshold Marked @ 0.4

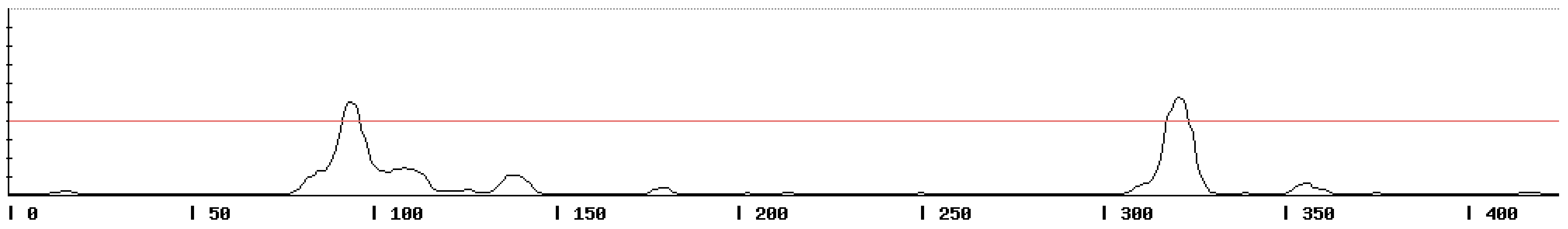

Predictions for Ipomoeatrifida_Itr_sc001891.1_g00004.1

Viterbi Path

Viterbi Path

Posterior @ 0.4

Posterior @ 0.4

Posterior Graphic. Threshold Marked @ 0.4

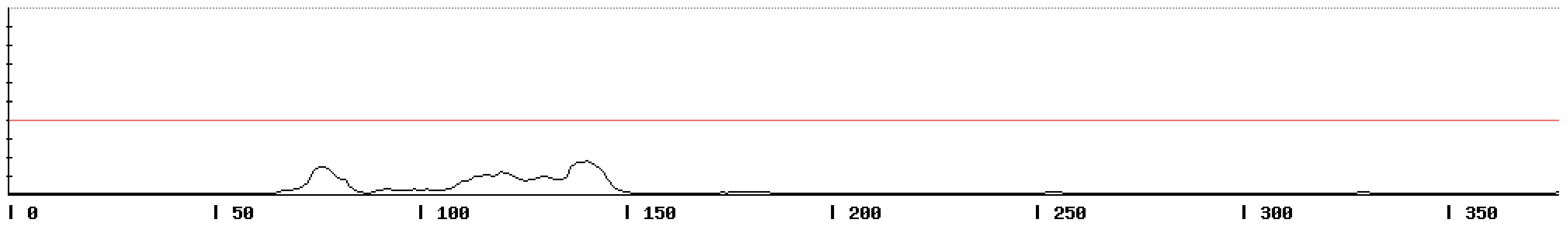

Predictions for Ipomoeatrifida_Itr_sc002075.1_g00003.1

Viterbi Path

Viterbi Path

Posterior @ 0.4

Posterior @ 0.4

Posterior Graphic. Threshold Marked @ 0.4

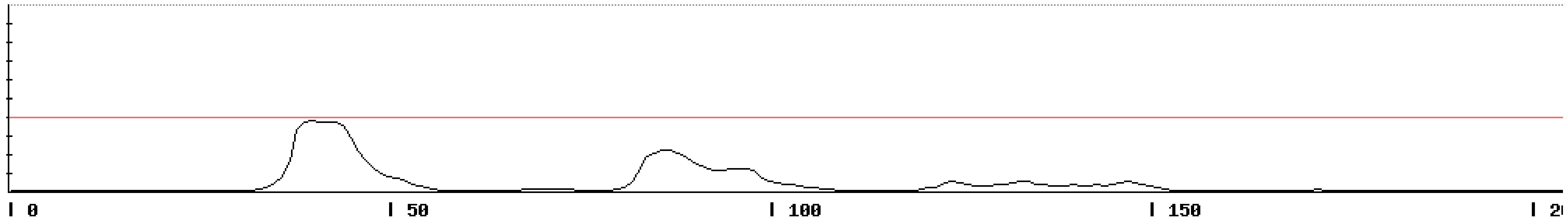

Predictions for Ipomoeatrifida_Itr_sc002135.1_g00002.1

Viterbi Path

Viterbi Path

Posterior @ 0.4

Posterior @ 0.4

Posterior Graphic. Threshold Marked @ 0.4

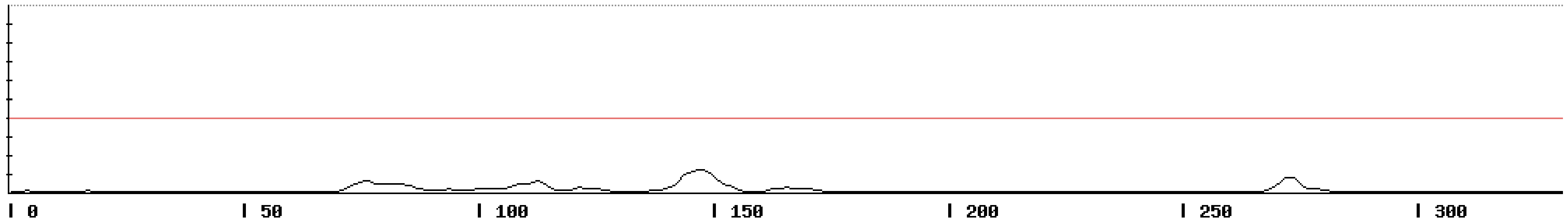

Predictions for Ipomoeatrifida_Itr_sc002135.1_g00003.1

Viterbi Path

Viterbi Path

Posterior @ 0.4

Posterior @ 0.4

Posterior Graphic. Threshold Marked @ 0.4

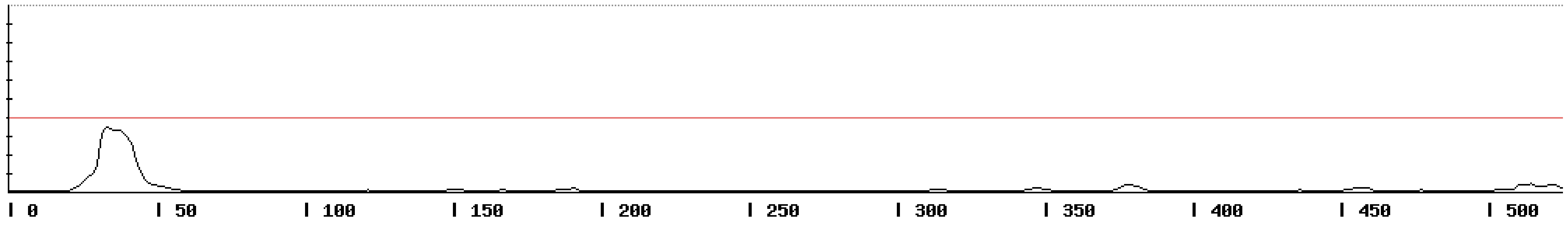

Predictions for Ipomoeatrifida_Itr_sc002146.1_g00002.1

Viterbi Path

Viterbi Path

Posterior @ 0.4 — 78 - RPNRAAGR - 85

Posterior @ 0.4

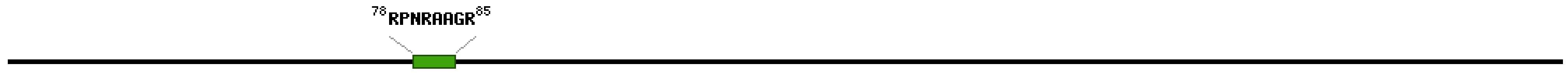

Posterior Graphic. Threshold Marked @ 0.4

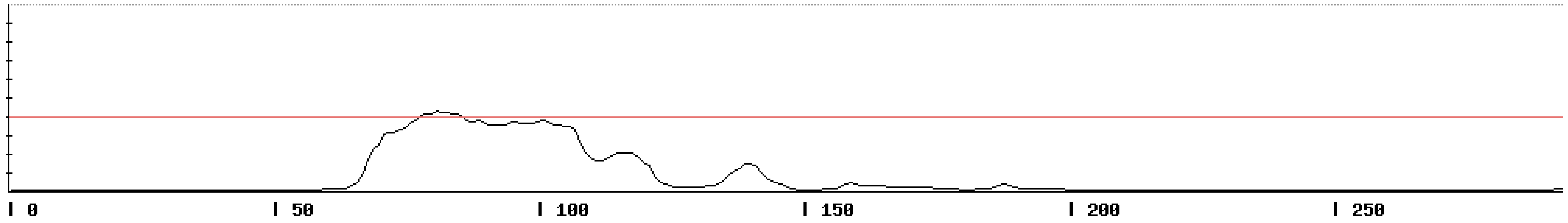

Predictions for Ipomoeatrifida_Itr_sc002200.1_g00003.1

Viterbi Path

Viterbi Path

Posterior @ 0.4

Posterior @ 0.4

Posterior Graphic. Threshold Marked @ 0.4

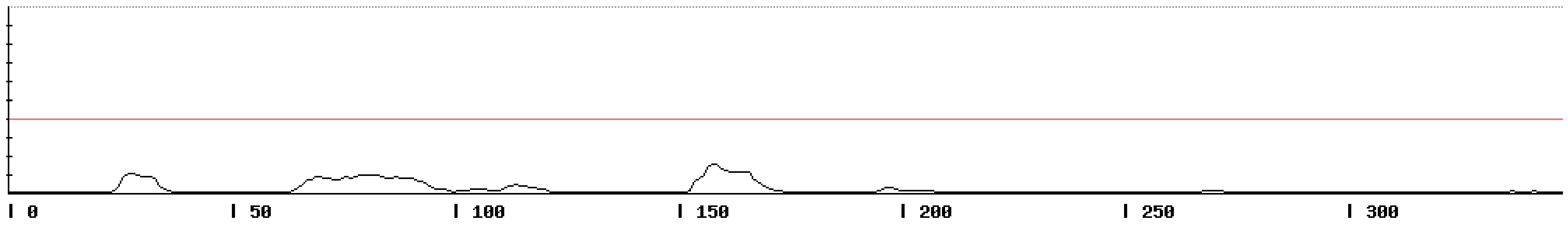

Predictions for Ipomoeatrifida_Itr_sc002231.1_g00001.1

Viterbi Path

Viterbi Path

Posterior @ 0.4

Posterior @ 0.4

Posterior Graphic. Threshold Marked @ 0.4

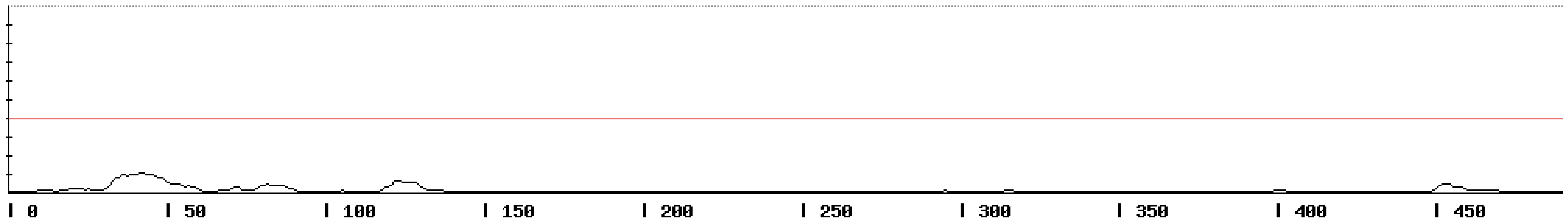

Predictions for Ipomoeatrifida_Itr_sc002231.1_g00002.1	
Viterbi Path	
Viterbi Path	
Posterior @ 0.4	
Posterior @ 0.4	
Posterior Graphic. Threshold Marked @ 0.4	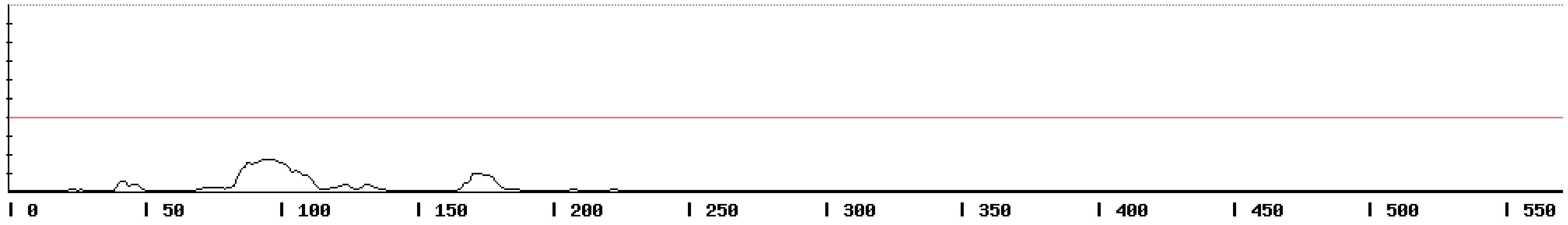

Predictions for Ipomoeatrifida_Itr_sc002366.1_g00001.1	
Viterbi Path	
Viterbi Path	
Posterior @ 0.4	
Posterior @ 0.4	
Posterior Graphic. Threshold Marked @ 0.4	

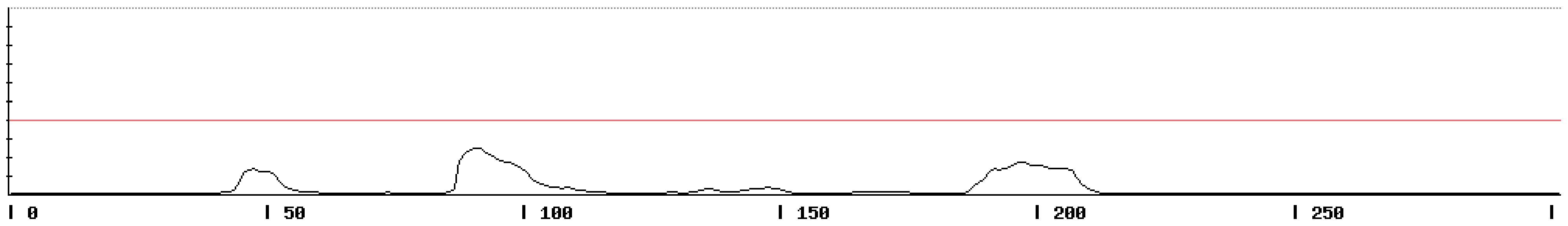

Predictions for Ipomoeatrifida_Itr_sc002521.1_g00001.1

Viterbi Path

Viterbi Path

Posterior @ 0.4

Posterior @ 0.4

Posterior Graphic. Threshold Marked @ 0.4

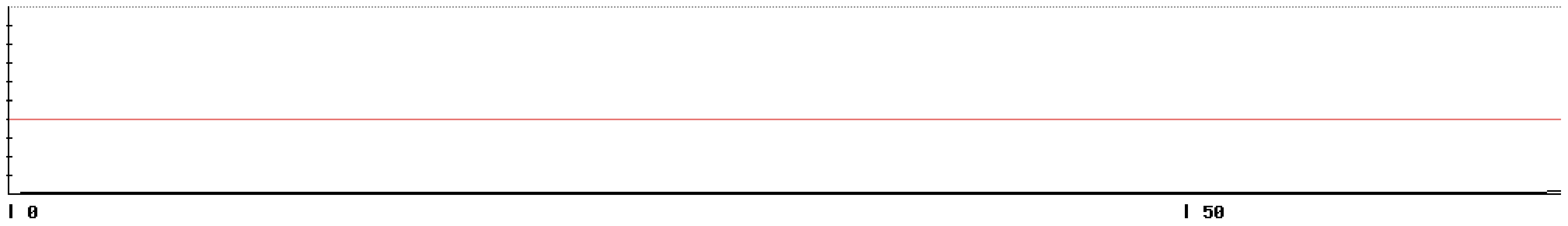

Predictions for Ipomoeatrifida_Itr_sc002521.1_g00002.1

Viterbi Path

Viterbi Path

Posterior @ 0.4

Posterior @ 0.4

Posterior Graphic. Threshold Marked @ 0.4

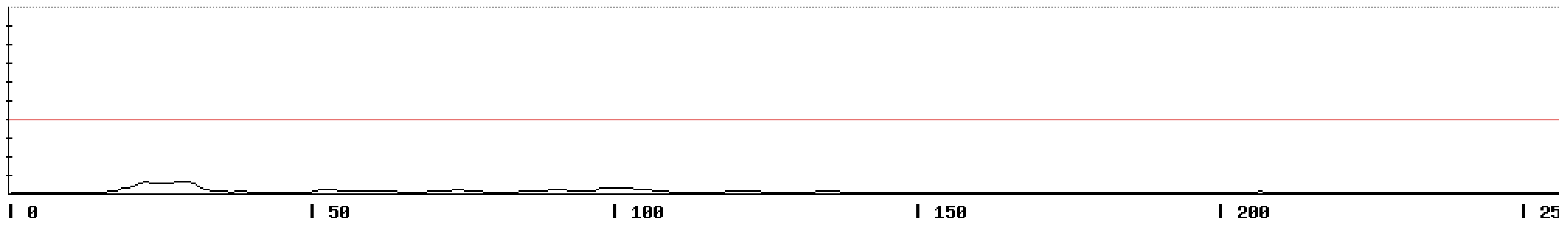

Predictions for Ipomoeatrifida_Itr_sc002567.1_g00003.1

Viterbi Path: 82 - KLKKKSKSKTGCRVERTVGKKVKGKKPGNWHGQDKGKP - 119

Viterbi Path

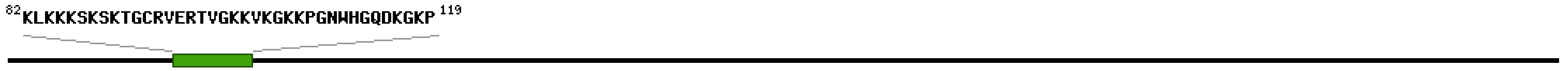

Posterior @ 0.4: 82 - KLKKKSKSKTGCRVERTVGKKVKGKKPGNWHGQDKGK - 118

Posterior @ 0.4

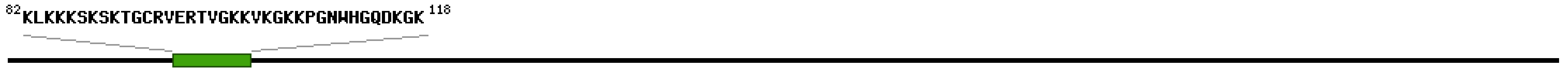

Posterior Graphic. Threshold Marked @ 0.4

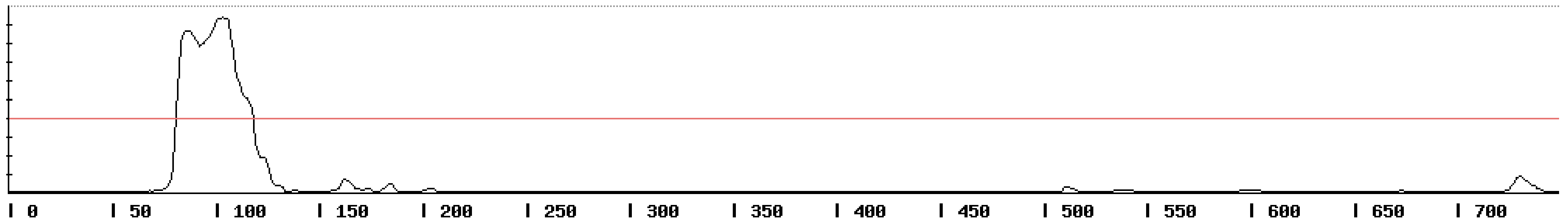

Predictions for Ipomoeatrifida_Itr_sc002692.1_g00001.1

Viterbi Path

Viterbi Path

Posterior @ 0.4

Posterior @ 0.4

Posterior Graphic. Threshold Marked @ 0.4

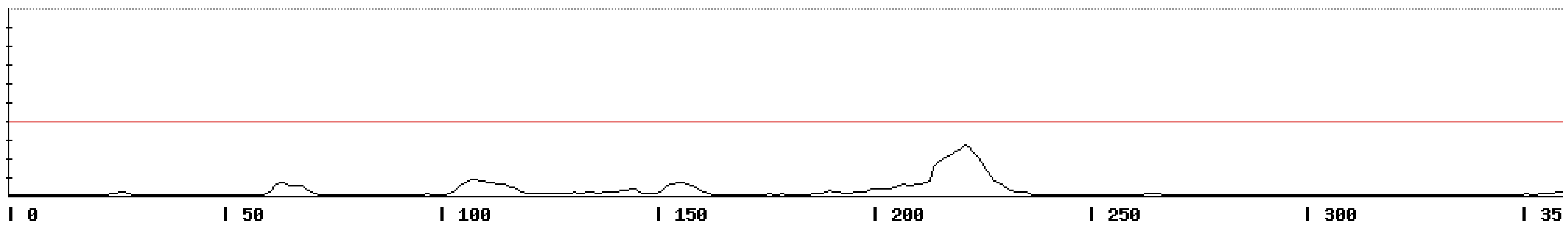

Predictions for Ipomoeatrifida_Itr_sc003030.1_g00004.1

Viterbi Path

Viterbi Path

Posterior @ 0.4 — 13 - RKVRR - 17

Posterior @ 0.4

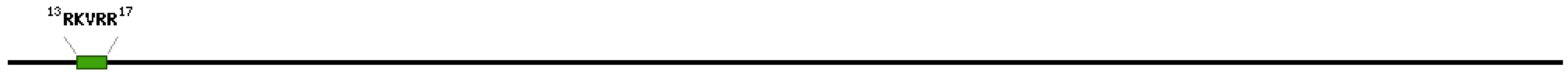

Posterior Graphic. Threshold Marked @ 0.4

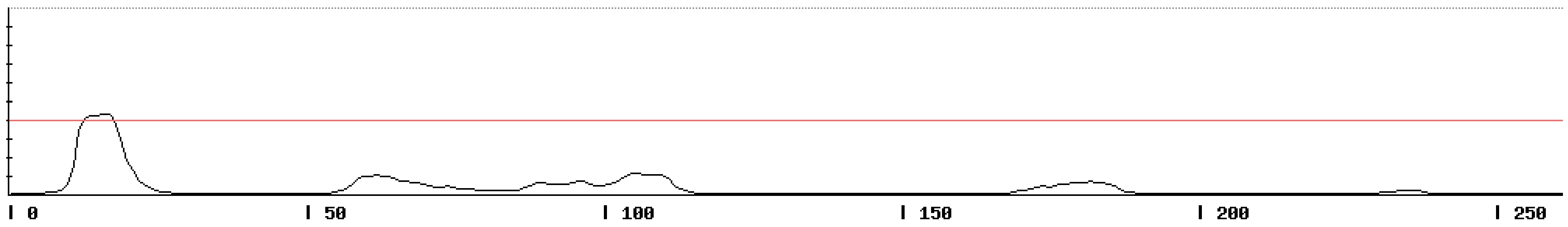

Predictions for Ipomoeatrifida_Itr_sc003080.1_g00001.1

Viterbi Path

Viterbi Path

Posterior @ 0.4	
Posterior @ 0.4	
Posterior Graphic. Threshold Marked @ 0.4	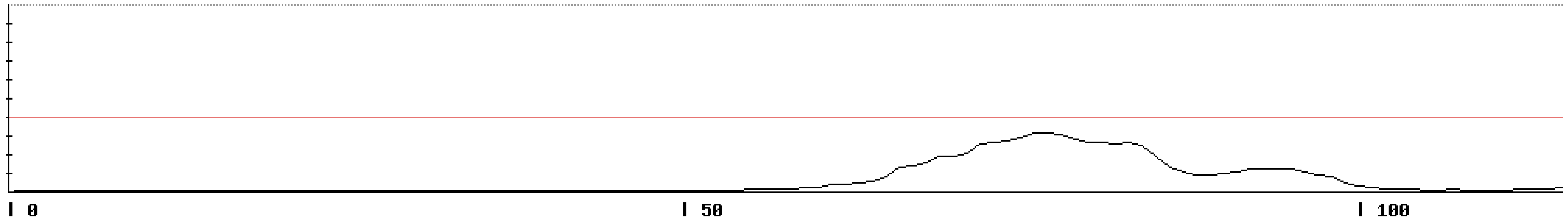

Predictions for Ipomoeatrifida_Itr_sc003094.1_g00006.1

Viterbi Path	
Viterbi Path	
Posterior @ 0.4	112 - SKKRVKIG - 119
Posterior @ 0.4	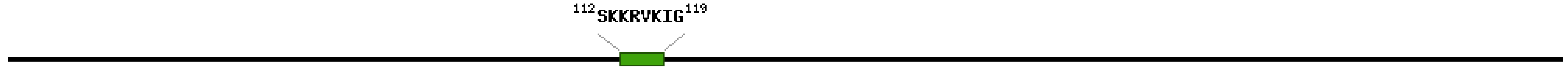
Posterior Graphic. Threshold Marked @ 0.4	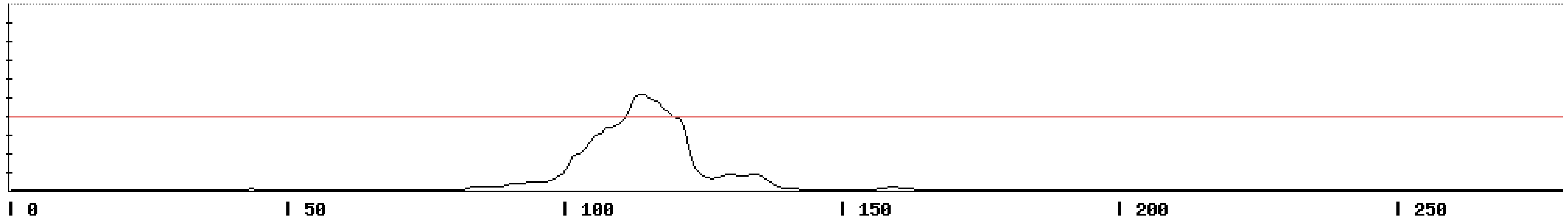

Predictions for Ipomoeatrifida_Itr_sc003352.1_g00004.1

Viterbi Path	
Viterbi Path	

Posterior @ 0.4	
Posterior @ 0.4	
Posterior Graphic. Threshold Marked @ 0.4	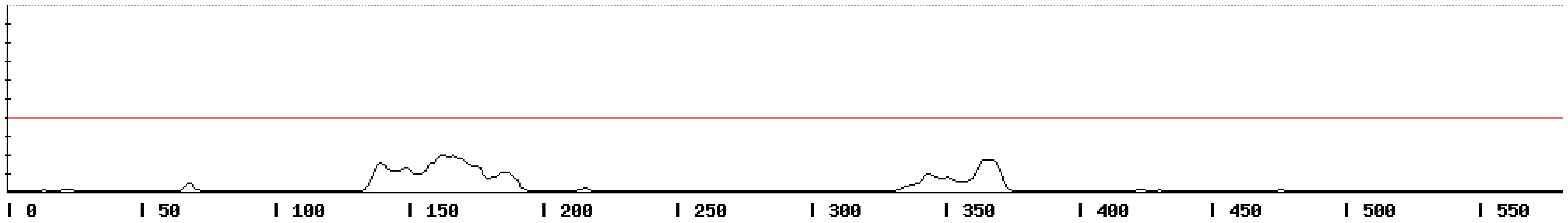

Predictions for Ipomoeatrifida_Itr_sc003587.1_g00001.1

Viterbi Path	
Viterbi Path	
Posterior @ 0.4	
Posterior @ 0.4	
Posterior Graphic. Threshold Marked @ 0.4	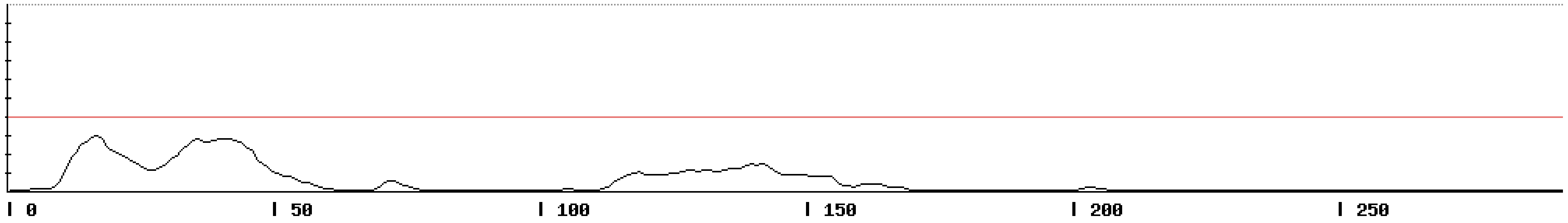

Predictions for Ipomoeatrifida_Itr_sc003700.1_g00004.1

Viterbi Path

Viterbi Path

Posterior @ 0.4

Posterior @ 0.4

Posterior Graphic. Threshold Marked @ 0.4

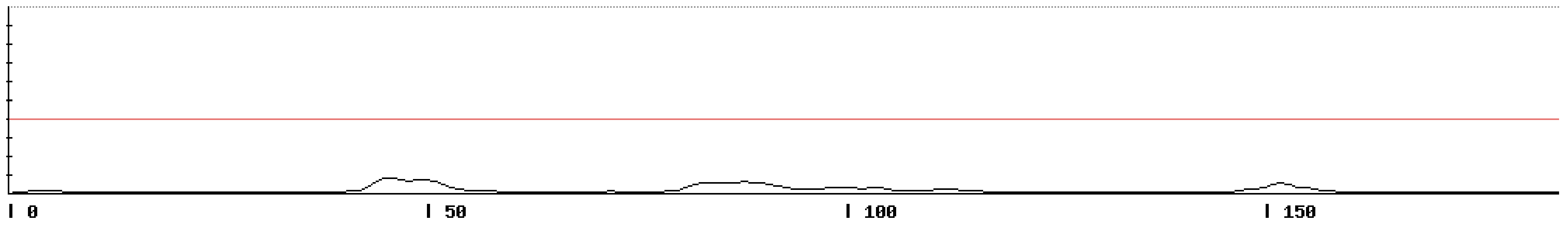

Predictions for Ipomoeatrifida_Itr_sc004105.1_g00001.1

Viterbi Path

Viterbi Path

Posterior @ 0.4

Posterior @ 0.4

Posterior Graphic. Threshold Marked @ 0.4

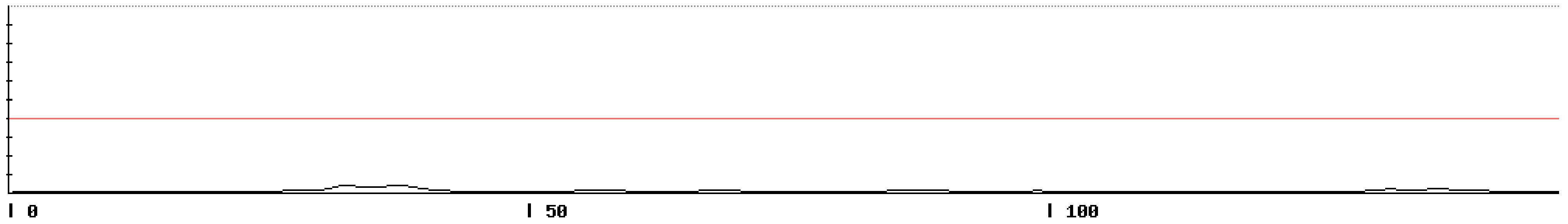

Predictions for Ipomoeatrifida_Itr_sc004223.1_g00002.1	
Viterbi Path	
Viterbi Path	
Posterior @ 0.4	
Posterior @ 0.4	
Posterior Graphic. Threshold Marked @ 0.4	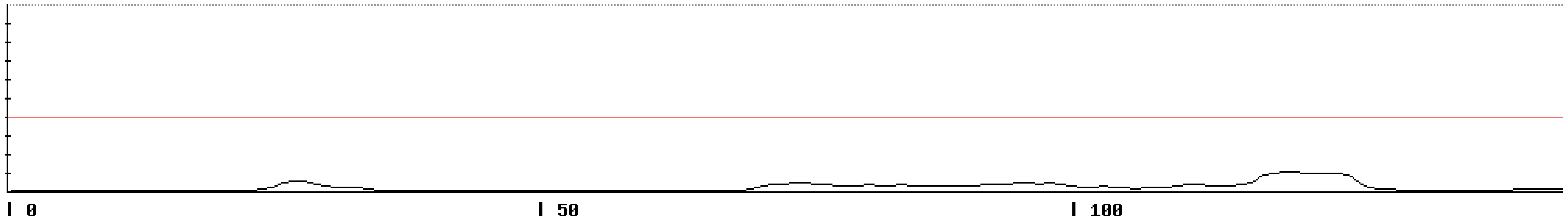

Predictions for Ipomoeatrifida_Itr_sc004419.1_g00003.1	
Viterbi Path	985 - KKKNRRRGWKKYFKRSDKEKKDKGP - 1009
Viterbi Path	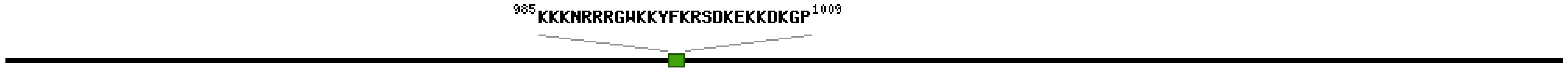
Posterior @ 0.4	729 - RAWAVRGSIREKKRSKPERR - 748<br>983 - AEKKKNRRRGWKKYFKRSDKEKKDKGPE - 1010
Posterior @ 0.4	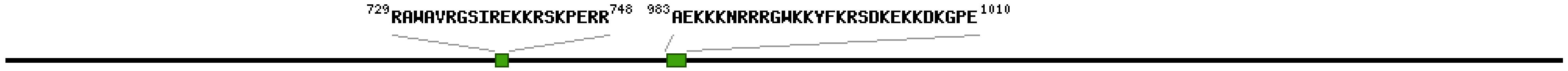
Posterior Graphic. Threshold Marked @ 0.4	

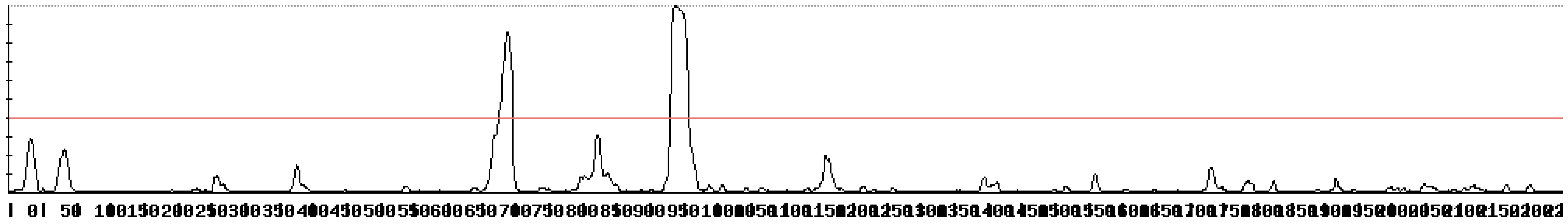

Predictions for Ipomoeatrifida_Itr_sc004616.1_g00001.1

Viterbi Path

Viterbi Path

Posterior @ 0.4

Posterior @ 0.4

Posterior Graphic. Threshold Marked @ 0.4

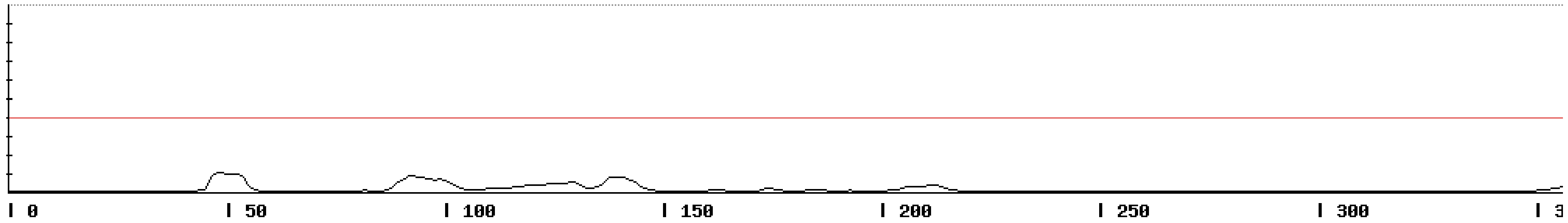

Predictions for Ipomoeatrifida_Itr_sc004671.1_g00002.1

Viterbi Path

Viterbi Path

Posterior @ 0.4

Posterior @ 0.4

Posterior Graphic. Threshold Marked @ 0.4

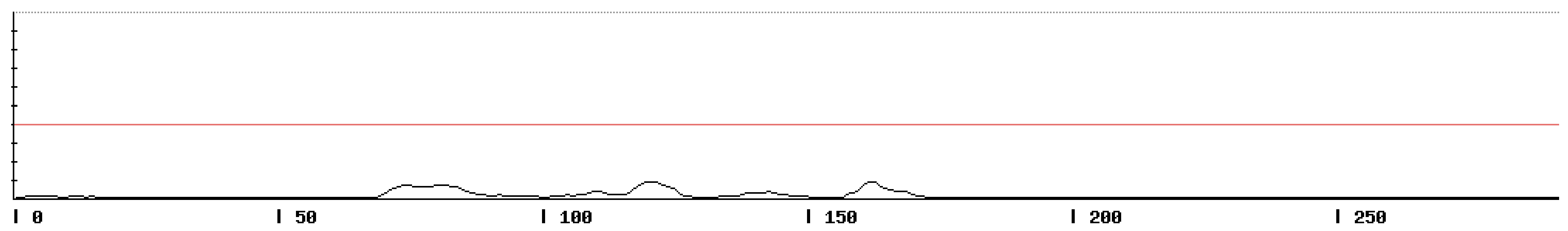

Predictions for Ipomoeatrifida_Itr_sc004877.1_g00002.1

Viterbi Path	63 - RLKKKGSRVQRTVGKKMKGKK - 83
Viterbi Path	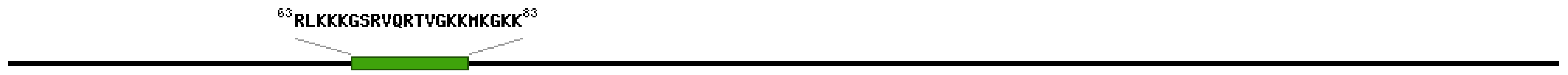
Posterior @ 0.4	62 - TRLKKKGSRVQRTVGKKMKGKKSG - 85
Posterior @ 0.4	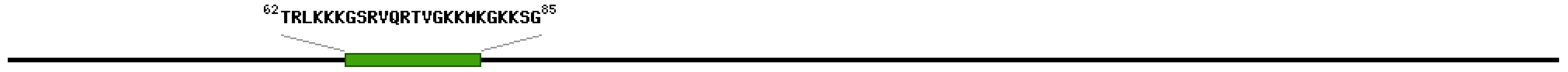
Posterior Graphic. Threshold Marked @ 0.4	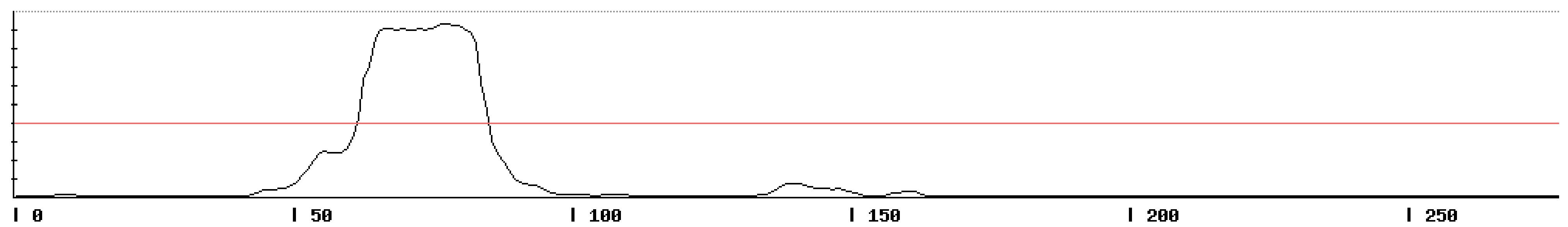

Predictions for Ipomoeatrifida_Itr_sc004968.1_g00002.1

Viterbi Path	
Viterbi Path	
Posterior @ 0.4	
Posterior @ 0.4	

Posterior Graphic. Threshold Marked @ 0.4

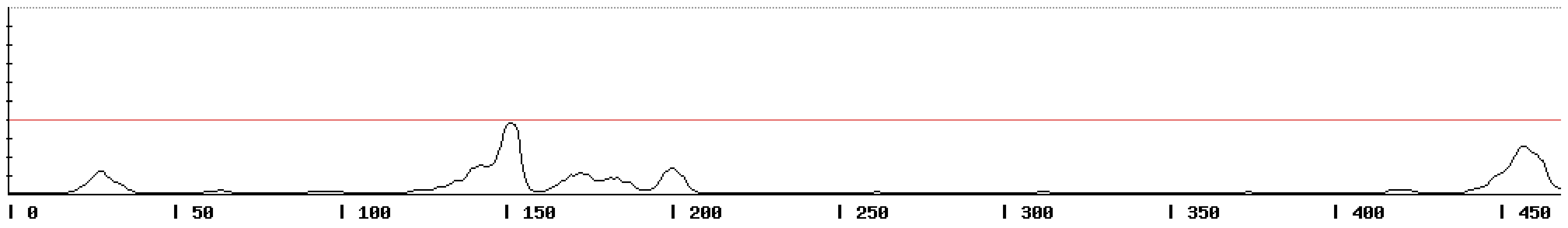

Predictions for Ipomoeatrifida_Itr_sc006031.1_g00001.1

Viterbi Path

Viterbi Path

Posterior @ 0.4 149 - KKRGGR - 154

Posterior @ 0.4

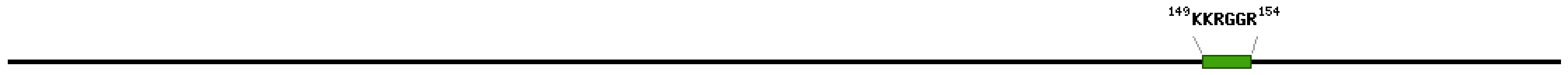

Posterior Graphic. Threshold Marked @ 0.4

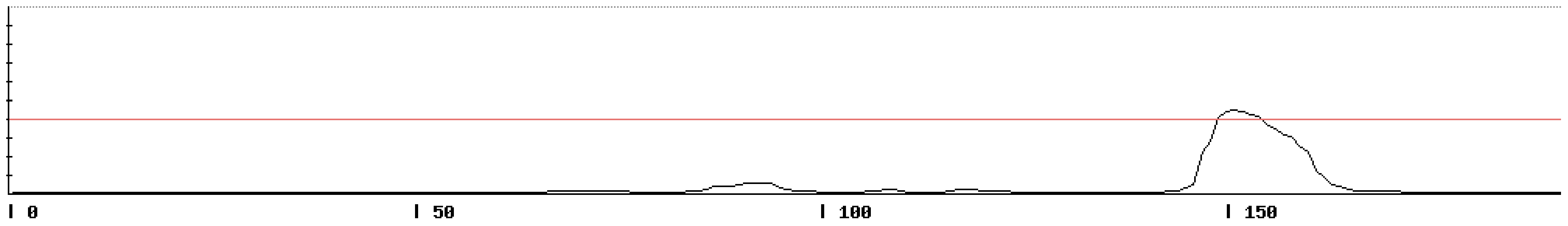

Predictions for Ipomoeatrifida_Itr_sc010236.1_g00002.1

Viterbi Path

Viterbi Path

Posterior @ 0.4

Posterior @ 0.4

Posterior Graphic. Threshold Marked @ 0.4

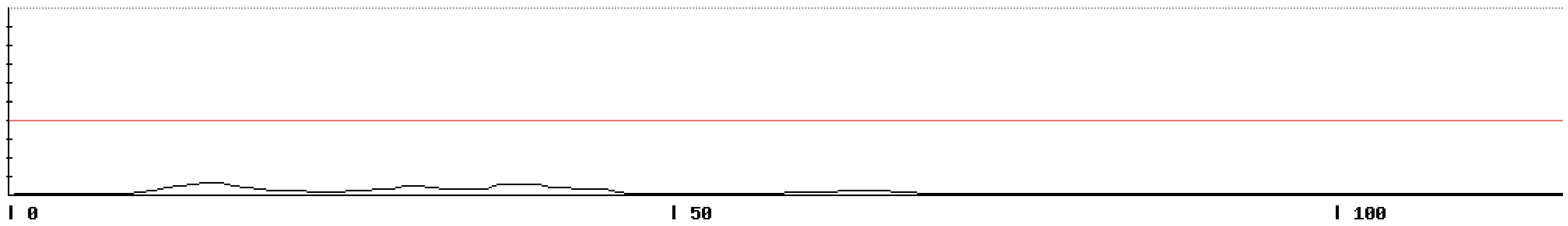

Predictions for Ipomoeatrifida_Itr_sc010894.1_g00001.1

Viterbi Path

Viterbi Path

Posterior @ 0.4

Posterior @ 0.4

Posterior Graphic. Threshold Marked @ 0.4

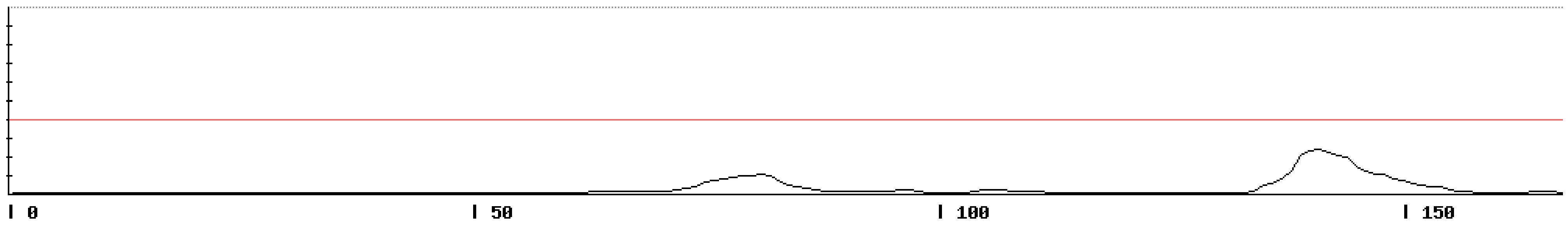

Predictions for Ipomoeatrifida_Itr_sc016143.1_g00001.1

Viterbi Path

Viterbi Path

Posterior @ 0.4	
Posterior @ 0.4	
Posterior Graphic. Threshold Marked @ 0.4	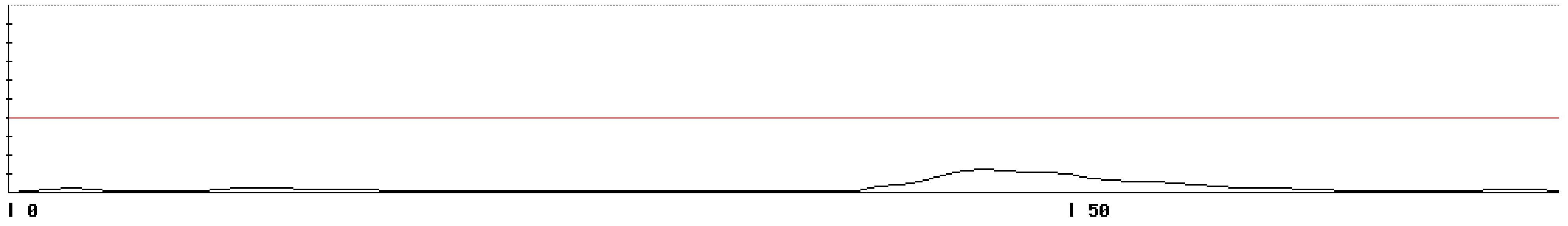

Predictions for Ipomoeatrifida_Itr_sc017881.1_g00001.1

Viterbi Path	
Viterbi Path	
Posterior @ 0.4	
Posterior @ 0.4	
Posterior Graphic. Threshold Marked @ 0.4	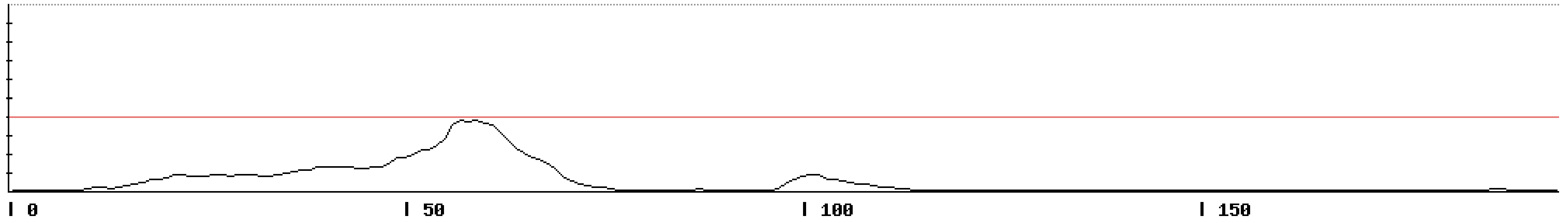

Predictions for Ipomoeatrifida_Itr_sc032464.1_g00001.1

Viterbi Path

Viterbi Path

Posterior @ 0.4

Posterior @ 0.4

Posterior Graphic. Threshold Marked @ 0.4

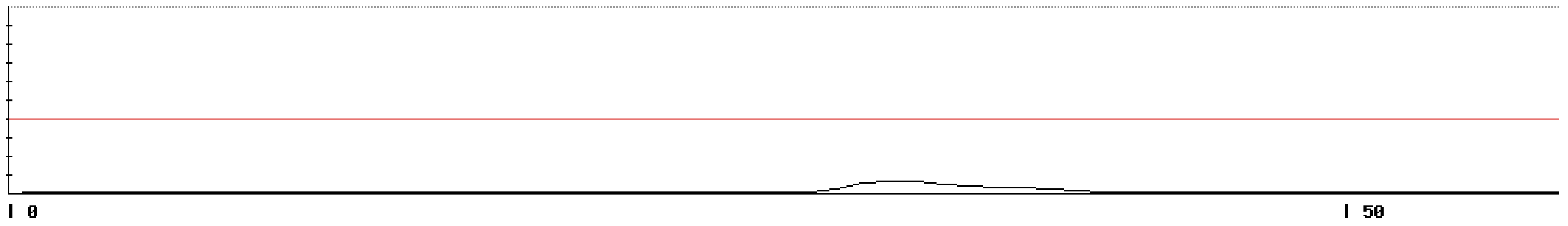

Predictions for Ipomoeatrifida_Itr_sc033226.1_g00001.1

Viterbi Path

Viterbi Path

Posterior @ 0.4

Posterior @ 0.4

Posterior Graphic. Threshold Marked @ 0.4

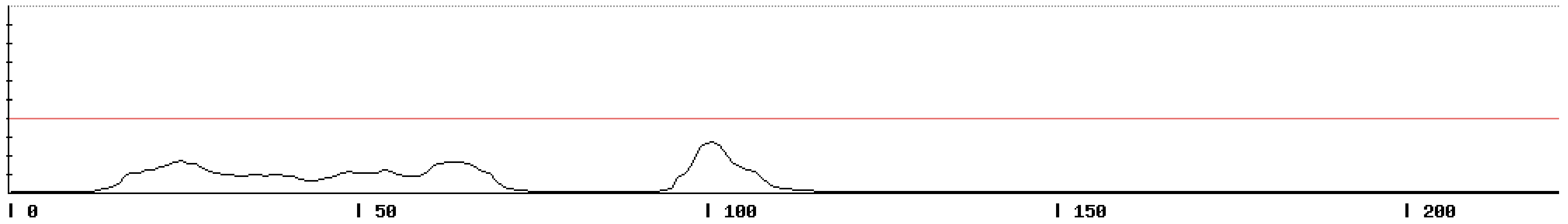

Predictions for Ipomoeatrifida_Itr_sc034048.1_g00001.1	
Viterbi Path	
Viterbi Path	
Posterior @ 0.4	
Posterior @ 0.4	
Posterior Graphic. Threshold Marked @ 0.4	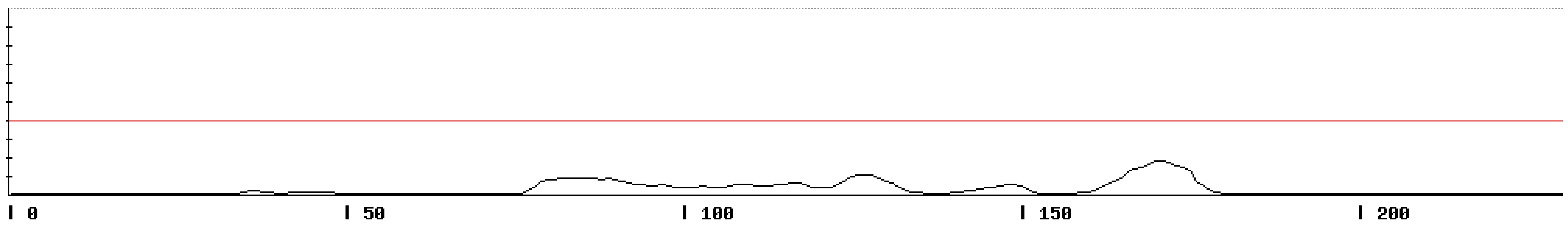

Predictions for Ipomoeatrifida_Itr_sc035183.1_g00001.1	
Viterbi Path	67 - KLKKKGKLMTARRVERTVGKKVKGKKPG - 94
Viterbi Path	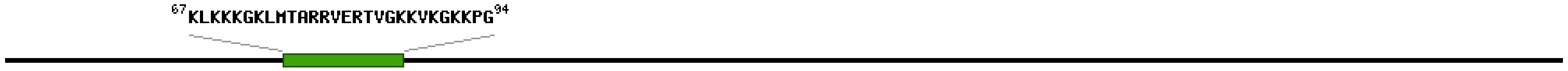
Posterior @ 0.4	67 - KLKKKGKLMTARRVERTVGKKVKGKKPGN - 95
Posterior @ 0.4	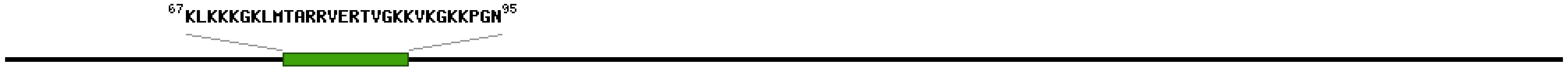
Posterior Graphic. Threshold Marked @ 0.4	

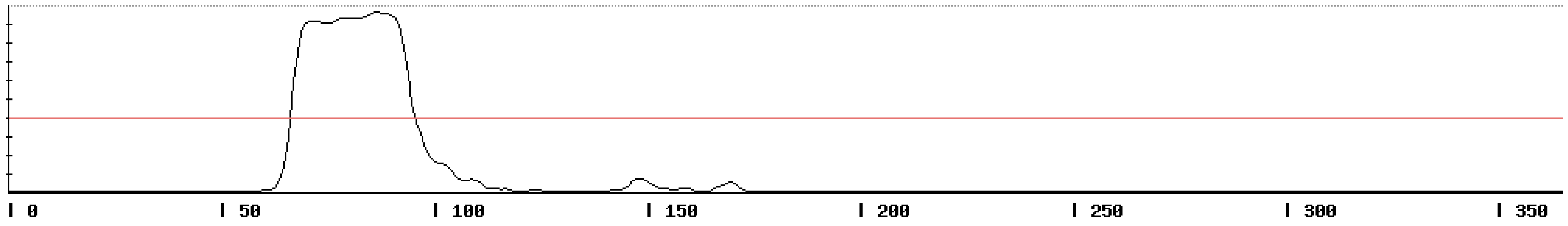

Predictions for Ipomoeatrifida_Itr_sc041614.1_g00001.1

Viterbi Path	
Viterbi Path	
Posterior @ 0.4	64 - RVRDKVMGFRKKFEVRDEKIKKQGRLGNR - 92
Posterior @ 0.4	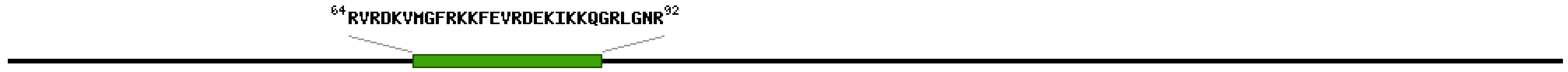
Posterior Graphic. Threshold Marked @ 0.4	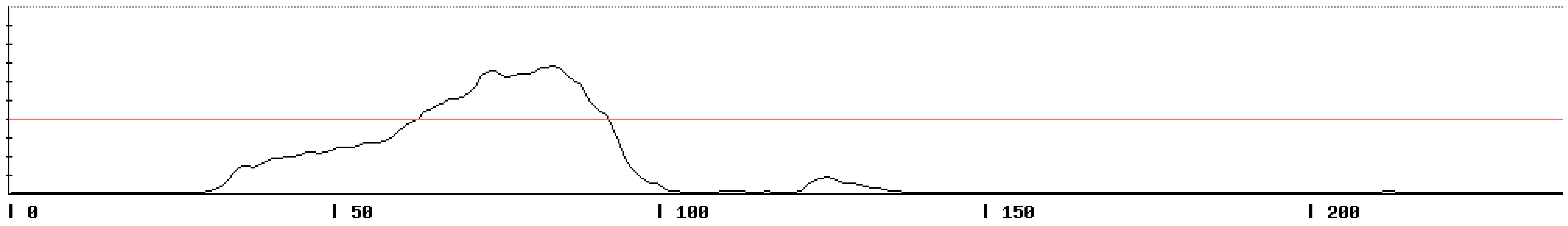

Revision r.9 - Last Updated on : February 24 2015 17:45:20. alex.nguyenba@utoronto.ca

Intended to be used with Firefox

Viterbi Path

Posterior @ 0.4

Posterior @ 0.4

Posterior Graphic. Threshold Marked @ 0.4

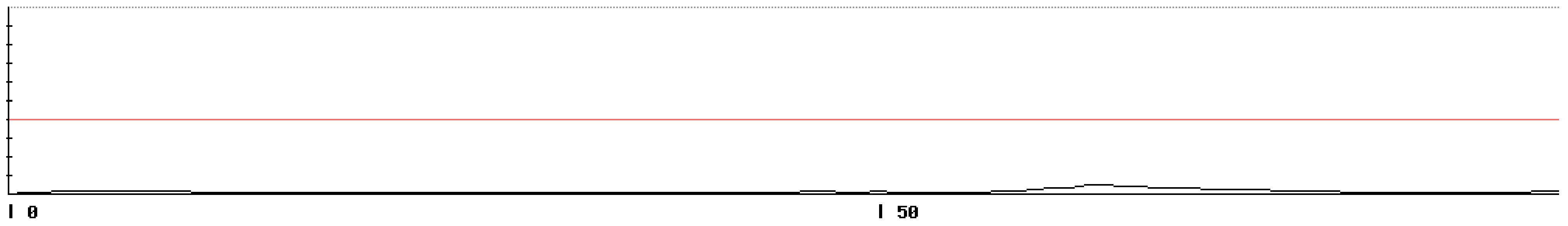

Predictions for Jatrophacurcas_Jcr4S00047.90

Viterbi Path

Viterbi Path

Posterior @ 0.4

Posterior @ 0.4

Posterior Graphic. Threshold Marked @ 0.4

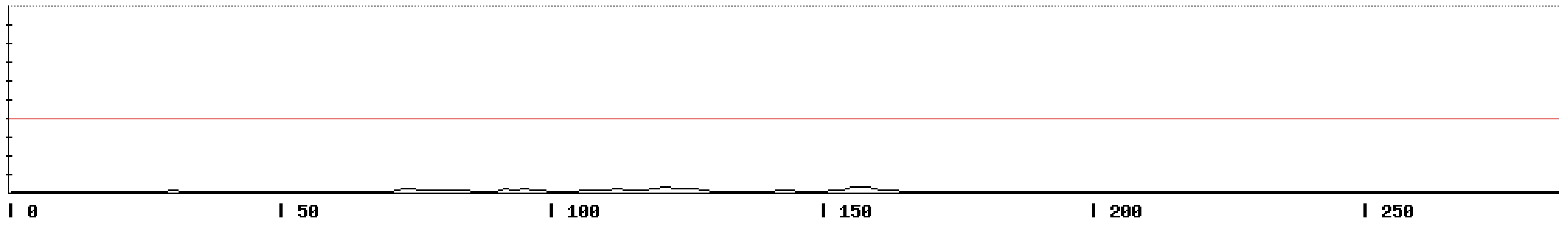

Predictions for Jatrophacurcas_Jcr4S00104.30

Viterbi Path	
Viterbi Path	
Posterior @ 0.4	166 - NGSKTGEKNKGKRK - 179
Posterior @ 0.4	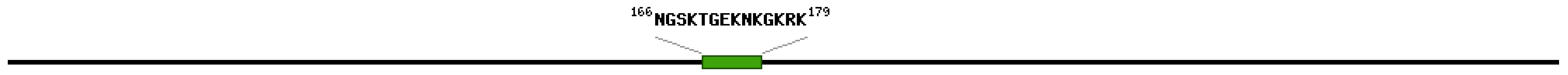
Posterior Graphic. Threshold Marked @ 0.4	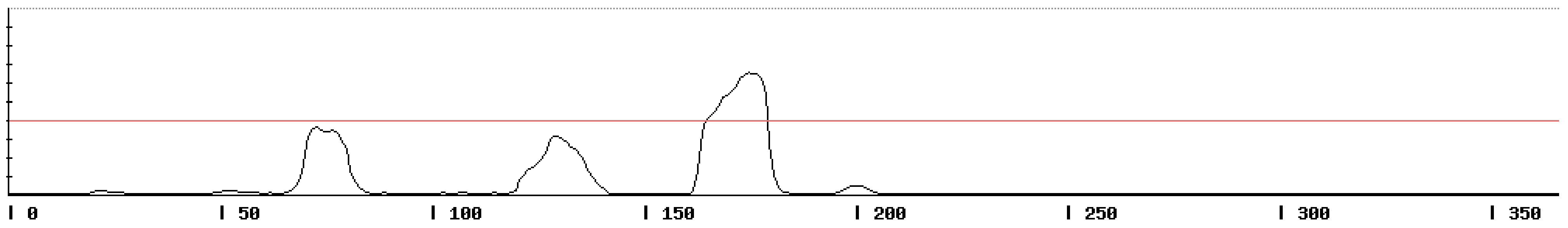

Predictions for Jatrophacurcas_Jcr4S00123.30

Viterbi Path	152 - KLKKKPNEKKKTVKRSKKEEPICKKPRAQKKARKG - 186
Viterbi Path	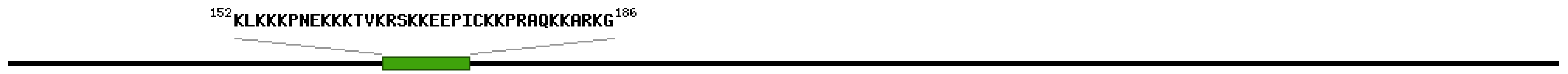
Posterior @ 0.4	152 - KLKKKPNEKKKTVKRSKKEEPICKKPRAQKKARKGE - 187
Posterior @ 0.4	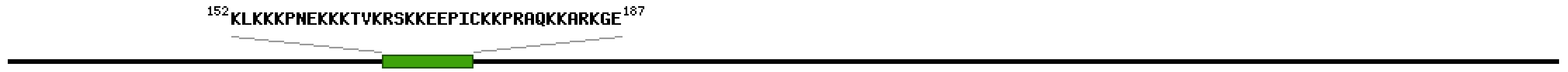
Posterior Graphic. Threshold Marked @ 0.4	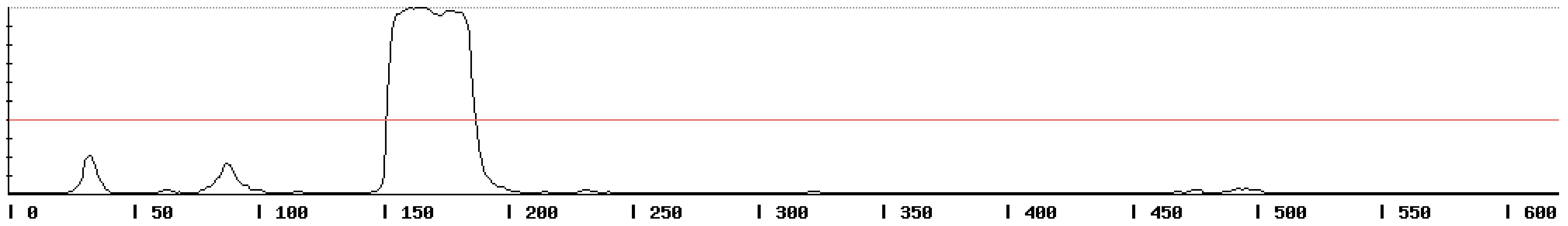

Predictions for Jatrophacurcas_Jcr4S00168.70	
Viterbi Path	
Viterbi Path	
Posterior @ 0.4	
Posterior @ 0.4	
Posterior Graphic. Threshold Marked @ 0.4	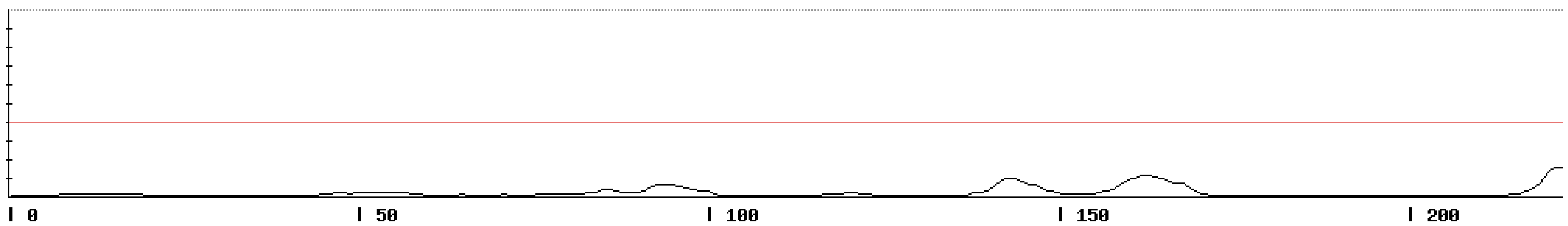

Predictions for Jatrophacurcas_Jcr4S00187.70	
Viterbi Path	
Viterbi Path	
Posterior @ 0.4	117 - LRKK - 120
	129 - GSKKR - 133
Posterior @ 0.4	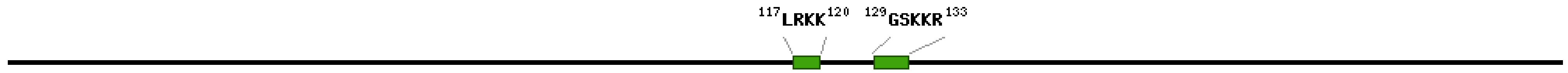
Posterior Graphic. Threshold Marked @ 0.4	

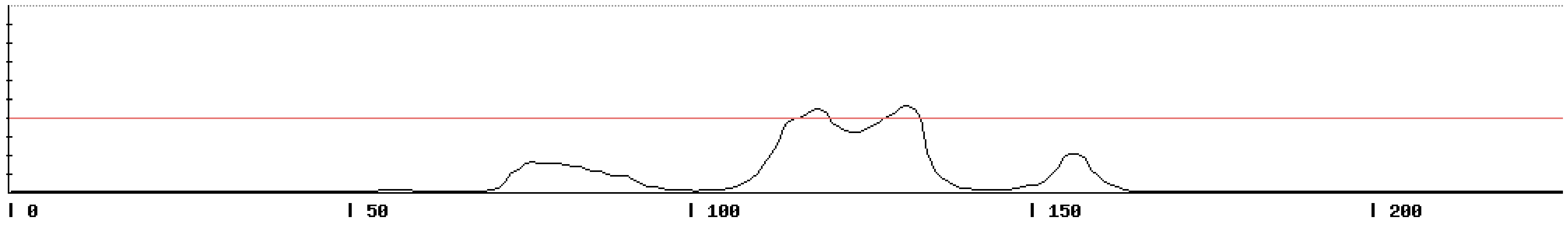

Predictions for Jatrophacurcas_Jcr4S00208.200

Viterbi Path

Viterbi Path

Posterior @ 0.4

Posterior @ 0.4

Posterior Graphic. Threshold Marked @ 0.4

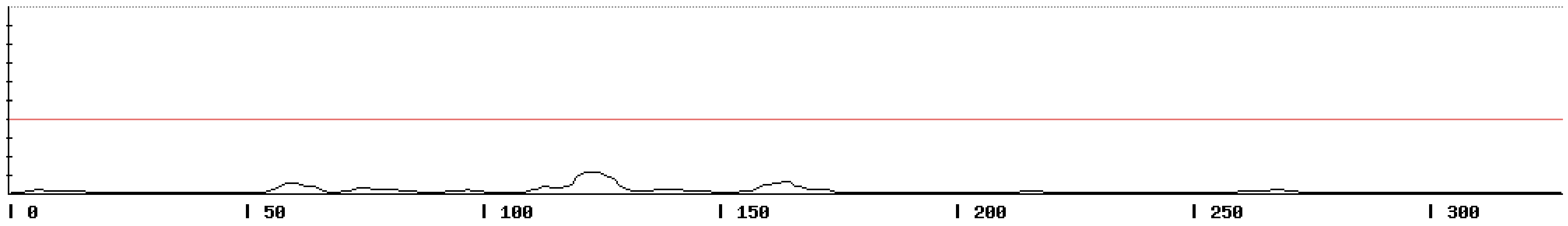

Predictions for Jatrophacurcas_Jcr4S00259.40

Viterbi Path

Viterbi Path

Posterior @ 0.4

Posterior @ 0.4

Posterior Graphic. Threshold Marked @ 0.4

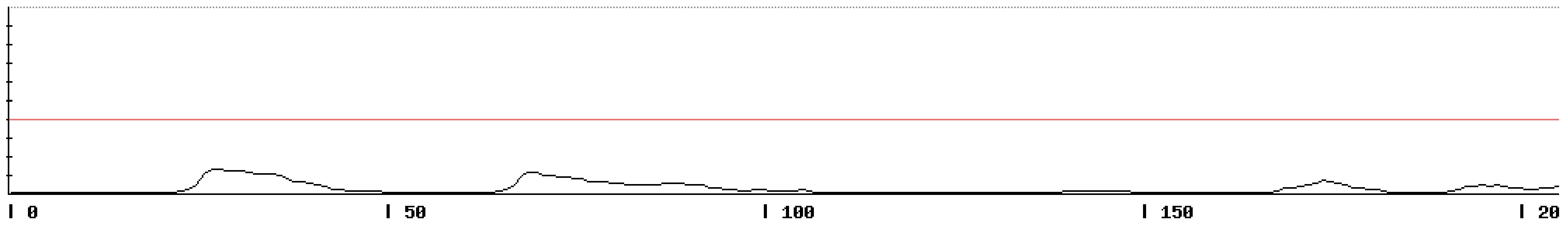

Predictions for Jatrophacurcas_Jcr4S00282.90

Viterbi Path

Viterbi Path

Posterior @ 0.4

Posterior @ 0.4

Posterior Graphic. Threshold Marked @ 0.4

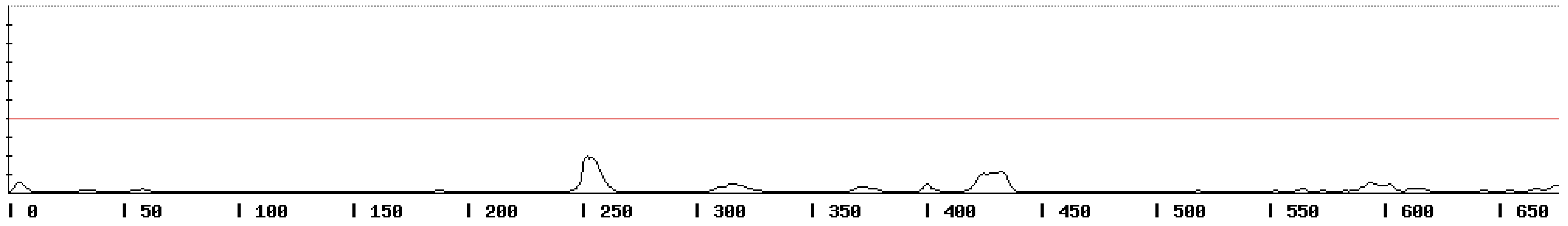

Predictions for Jatrophacurcas_Jcr4S00351.50

Viterbi Path

Viterbi Path

Posterior @ 0.4

Posterior @ 0.4

Posterior Graphic. Threshold Marked @ 0.4	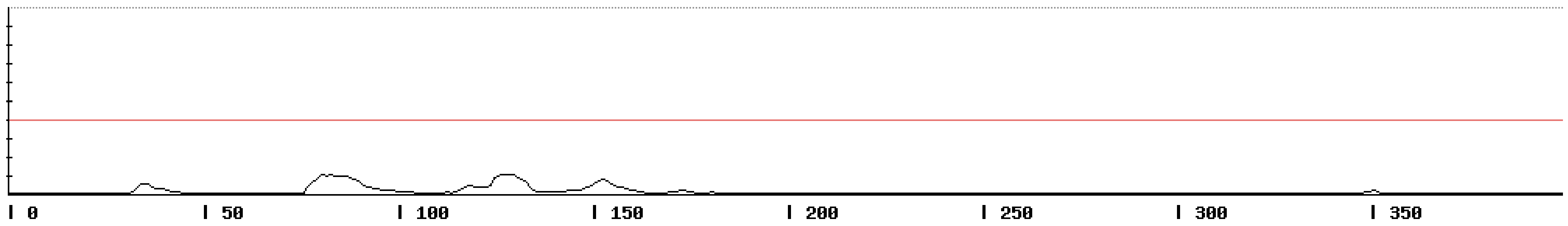

Predictions for Jatrophacurcas_Jcr4S00402.80	
Viterbi Path	
Viterbi Path	
Posterior @ 0.4	
Posterior @ 0.4	
Posterior Graphic. Threshold Marked @ 0.4	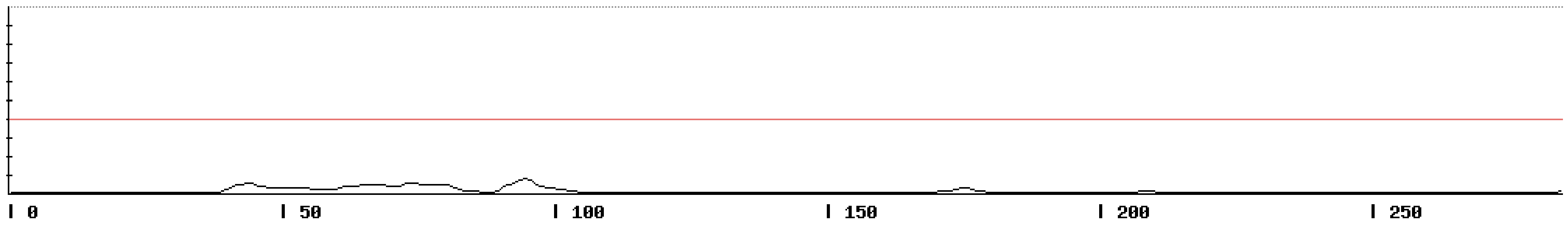

Predictions for Jatrophacurcas_Jcr4S00409.30	
Viterbi Path	
Viterbi Path	

Posterior @ 0.4

Posterior @ 0.4

Posterior Graphic. Threshold Marked @ 0.4

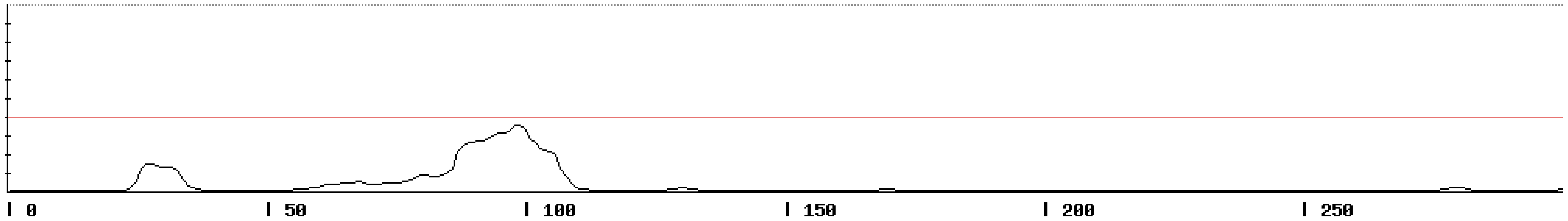

Predictions for Jatrophacurcas_Jcr4S00483.80

Viterbi Path

Viterbi Path

Posterior @ 0.4

144 - RKRRK - 148

Posterior @ 0.4

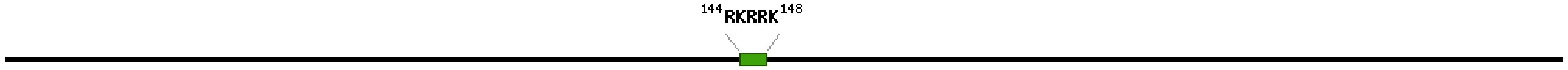

Posterior Graphic. Threshold Marked @ 0.4

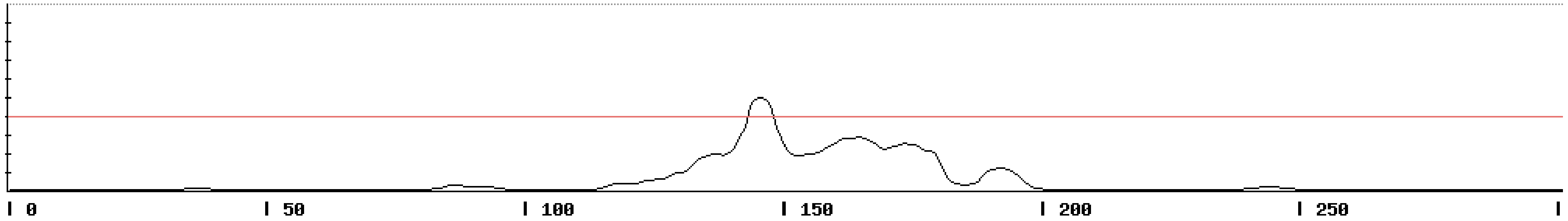

Predictions for Jatrophacurcas_Jcr4S00511.40

Viterbi Path

Viterbi Path

Posterior @ 0.4

Posterior @ 0.4

Posterior Graphic. Threshold Marked @ 0.4

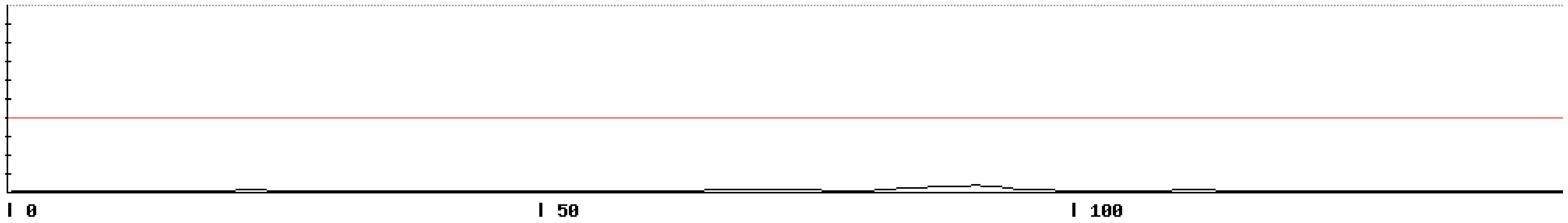

Predictions for Jatrophacurcas_Jcr4S00639.30

Viterbi Path

Viterbi Path

Posterior @ 0.4 | 160 - QKKKRK - 165

Posterior @ 0.4

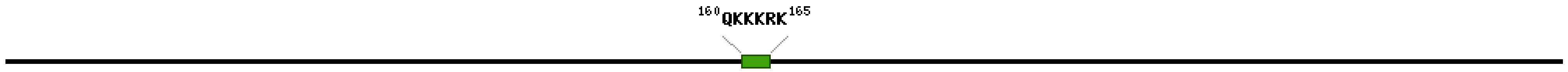

Posterior Graphic. Threshold Marked @ 0.4

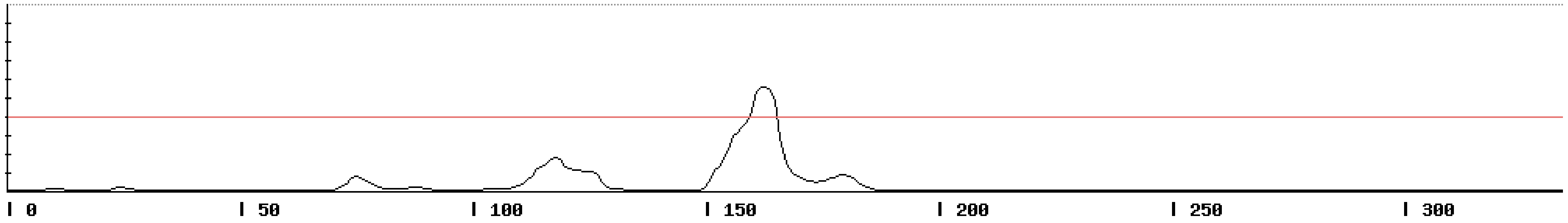

Predictions for Jatrophacurcas_Jcr4S00694.30

Viterbi Path

Viterbi Path

Posterior @ 0.4

Posterior @ 0.4

Posterior Graphic. Threshold Marked @ 0.4

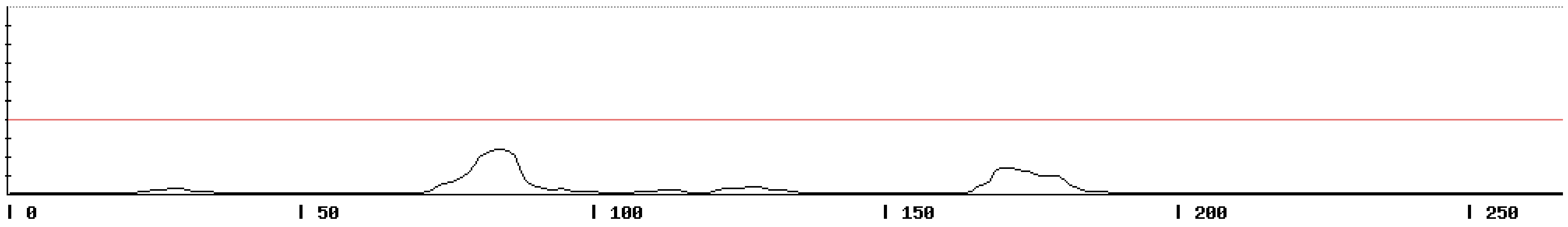

Predictions for Jatrophacurcas_Jcr4S00745.90

Viterbi Path

Viterbi Path

Posterior @ 0.4

Posterior @ 0.4

Posterior Graphic. Threshold Marked @ 0.4

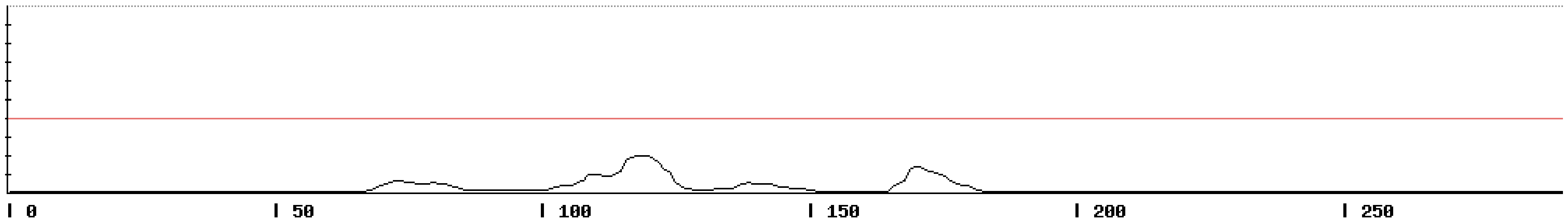

Predictions for Jatrophacurcas_Jcr4S00782.110	
Viterbi Path	
Viterbi Path	
Posterior @ 0.4	
Posterior @ 0.4	
Posterior Graphic. Threshold Marked @ 0.4	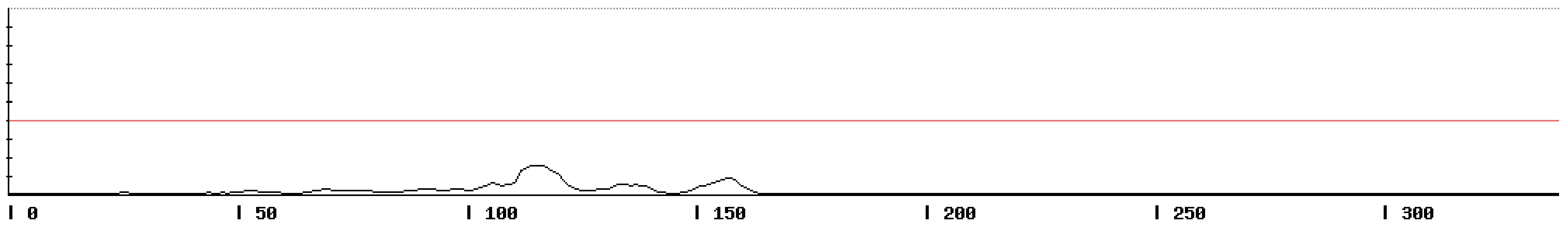

Predictions for Jatrophacurcas_Jcr4S00818.170	
Viterbi Path	
Viterbi Path	
Posterior @ 0.4	
Posterior @ 0.4	
Posterior Graphic. Threshold Marked @ 0.4	

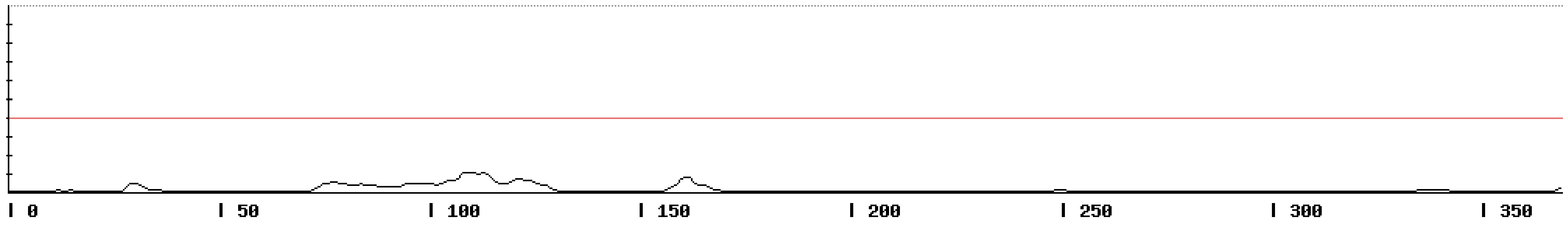

Predictions for Jatrophacurcas_Jcr4S00899.90	
Viterbi Path	
Viterbi Path	
Posterior @ 0.4	
Posterior @ 0.4	
Posterior Graphic. Threshold Marked @ 0.4	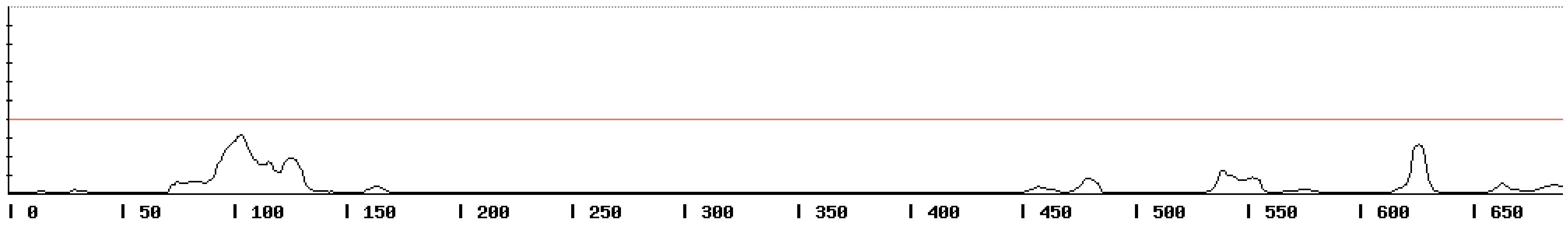

Predictions for Jatrophacurcas_Jcr4S01107.10	
Viterbi Path	
Viterbi Path	
Posterior @ 0.4	
Posterior @ 0.4	

Posterior Graphic. Threshold Marked @ 0.4	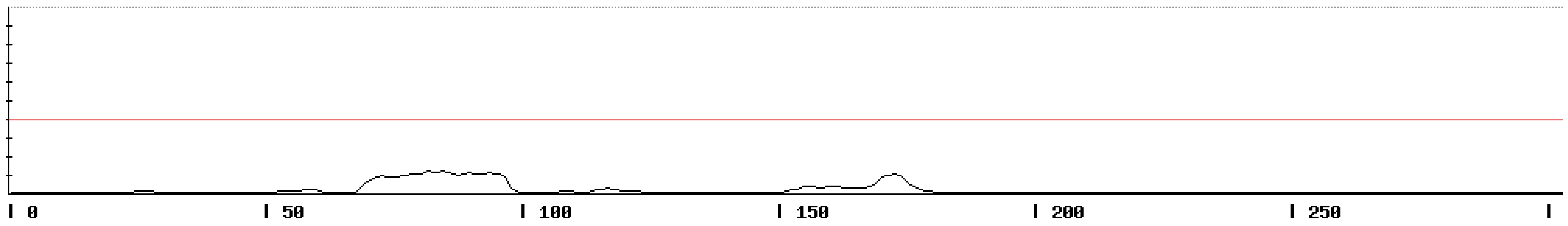

Predictions for Jatrophacurcas_Jcr4S01202.10	
Viterbi Path	
Viterbi Path	
Posterior @ 0.4	
Posterior @ 0.4	
Posterior Graphic. Threshold Marked @ 0.4	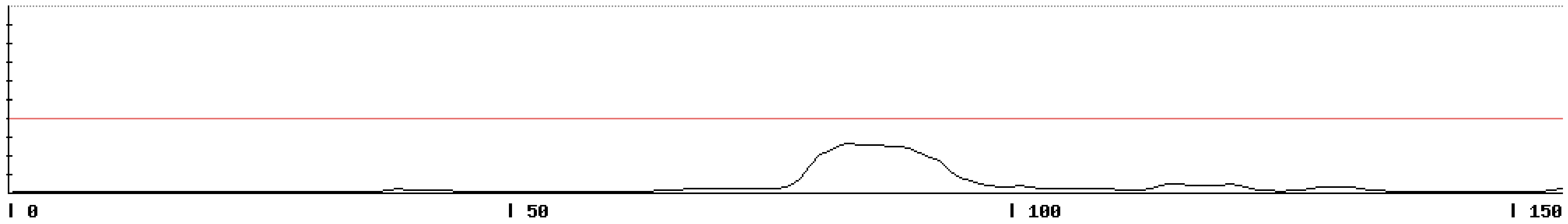

Predictions for Jatrophacurcas_Jcr4S01222.10	
Viterbi Path	
Viterbi Path	
Posterior @ 0.4	
Posterior @ 0.4	

Posterior Graphic. Threshold Marked @ 0.4

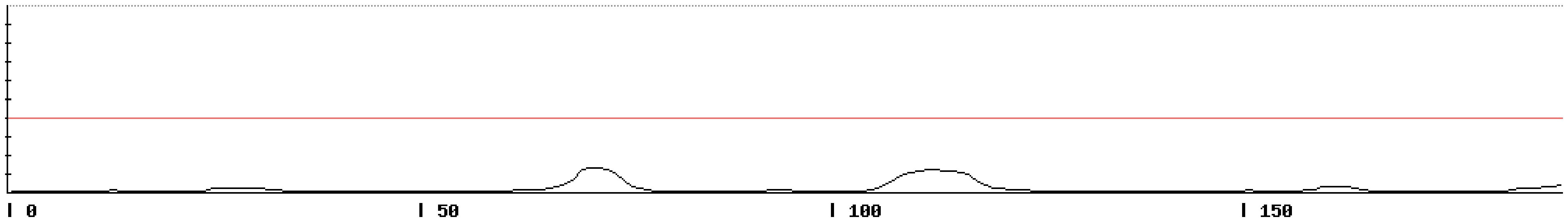

Predictions for Jatrophacurcas_Jcr4S01288.50

Viterbi Path

Viterbi Path

Posterior @ 0.4 — 88 - SRGGRSRRAKGGYWRKSGRVRDVAGPGGKAVIGRR - 122

Posterior @ 0.4

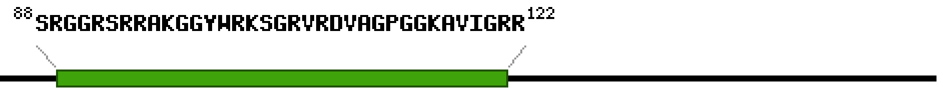

Posterior Graphic. Threshold Marked @ 0.4

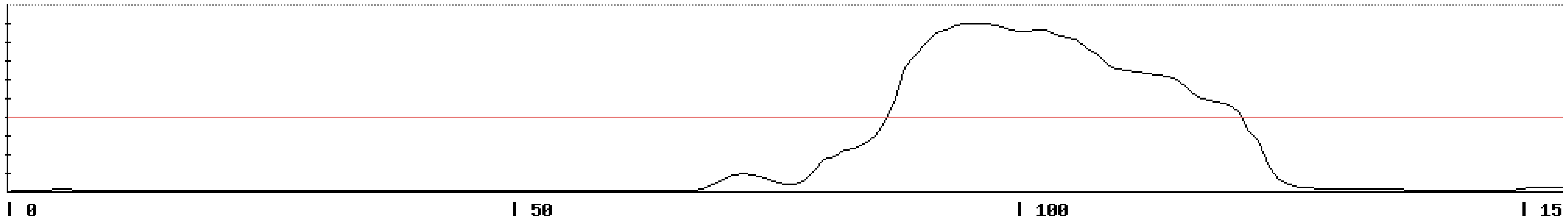

Predictions for Jatrophacurcas_Jcr4S01292.40

Viterbi Path

Viterbi Path

Posterior @ 0.4

Posterior @ 0.4

Posterior Graphic. Threshold Marked @ 0.4

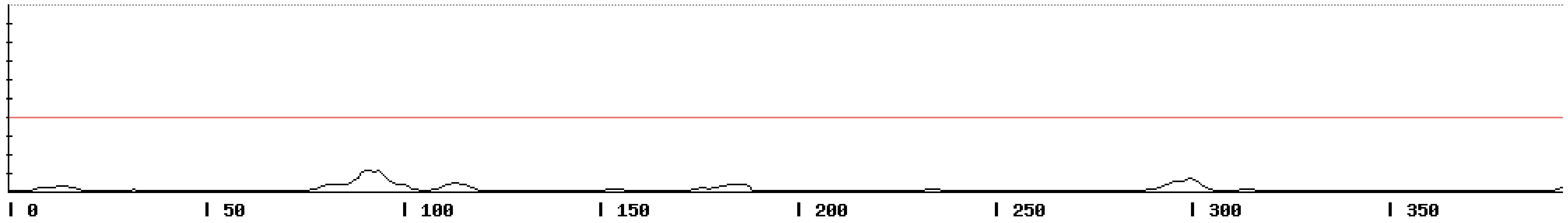

Predictions for Jatrophacurcas_Jcr4S01394.20

Viterbi Path

Viterbi Path

Posterior @ 0.4 — 70 - KLKKKHGKGMR - 80

Posterior @ 0.4

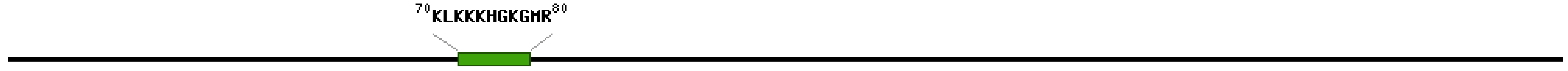

Posterior Graphic. Threshold Marked @ 0.4

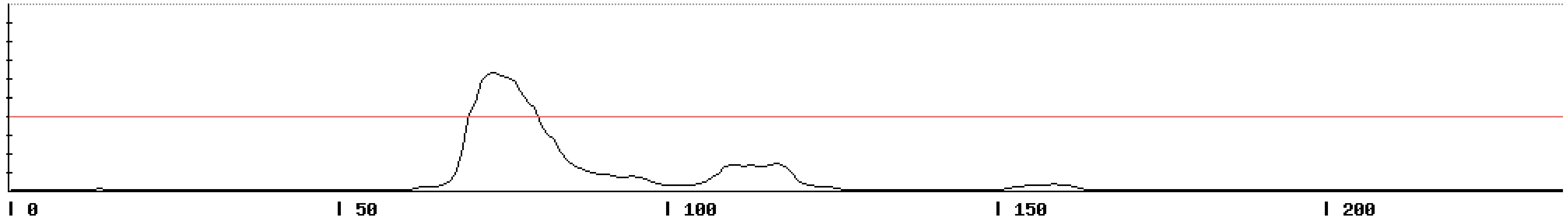

Predictions for Jatrophacurcas_Jcr4S01477.60

Viterbi Path

Viterbi Path

Posterior @ 0.4

Posterior @ 0.4

Posterior Graphic. Threshold Marked @ 0.4

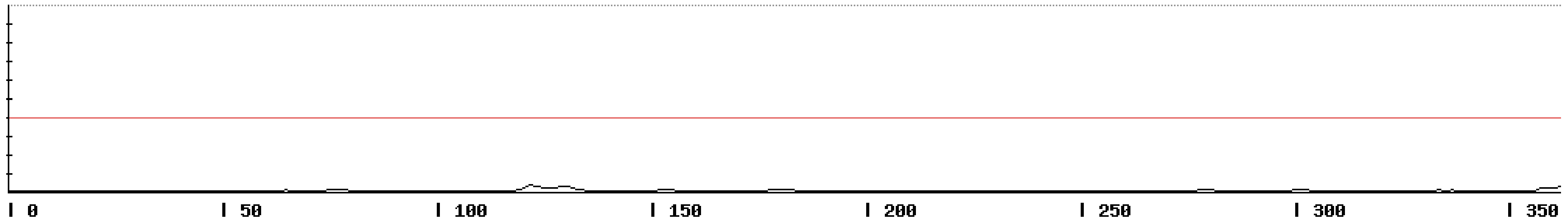

Predictions for Jatrophacurcas_Jcr4S01621.70

Viterbi Path

Viterbi Path

Posterior @ 0.4

Posterior @ 0.4

Posterior Graphic. Threshold Marked @ 0.4

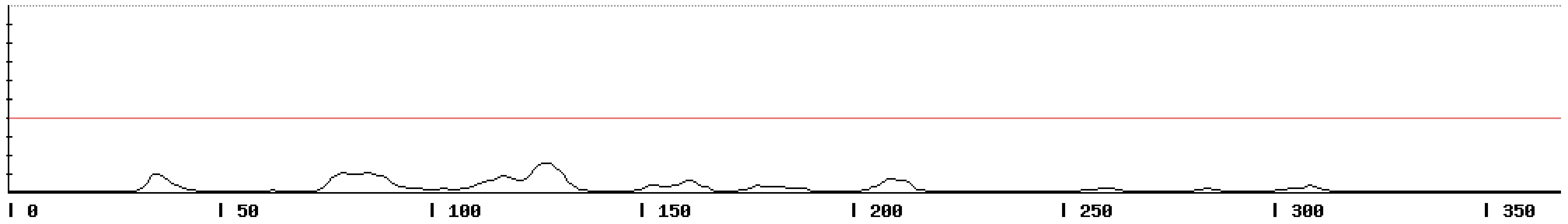

Predictions for Jatrophacurcas_Jcr4S01657.60

Viterbi Path

Viterbi Path

Posterior @ 0.4

Posterior @ 0.4

Posterior Graphic. Threshold Marked @ 0.4

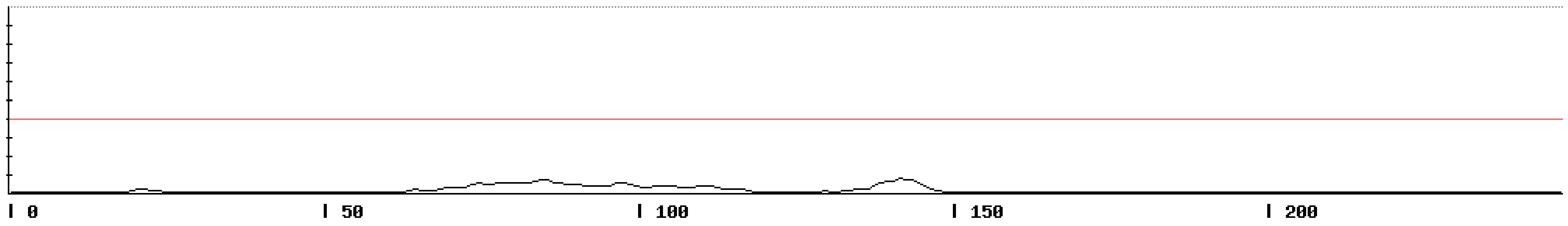

Predictions for Jatrophacurcas_Jcr4S01683.60

Viterbi Path

Viterbi Path

Posterior @ 0.4

Posterior @ 0.4

Posterior Graphic. Threshold Marked @ 0.4

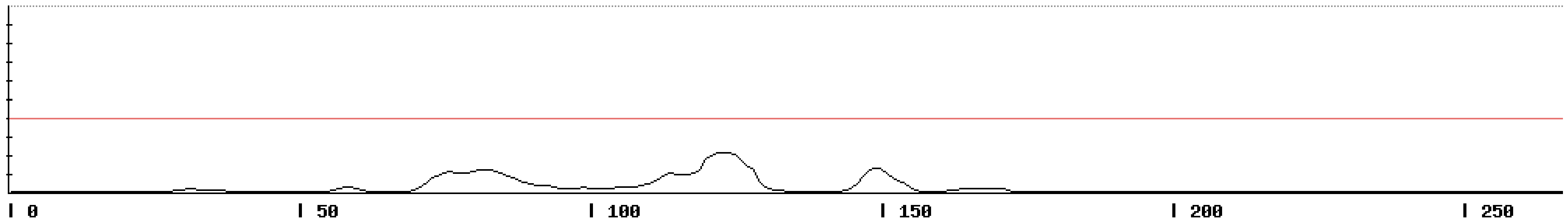

Predictions for Jatrophacurcas_Jcr4S01698.20

Viterbi Path

Viterbi Path

Posterior @ 0.4

164 - RKNGSKTGEKNKGKRK - 179

Posterior @ 0.4

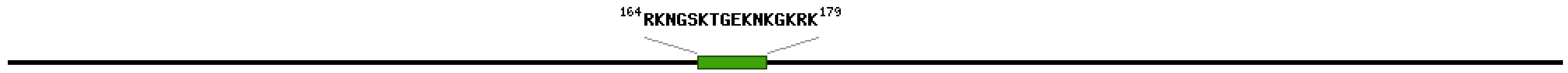

Posterior Graphic. Threshold Marked @ 0.4

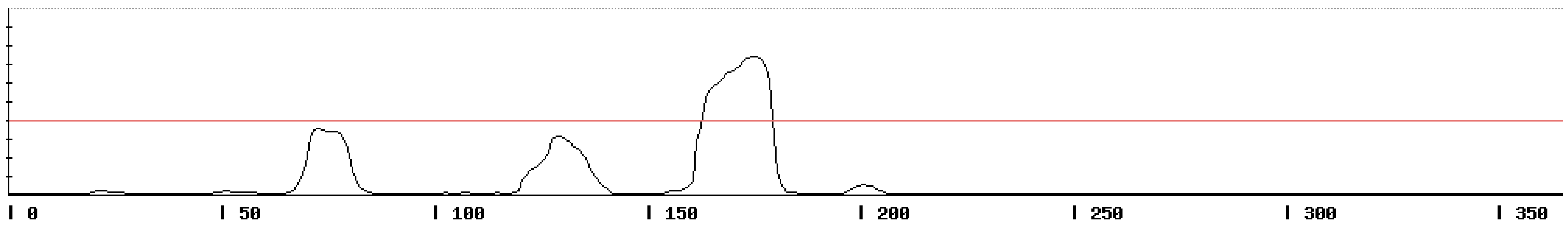

Predictions for Jatrophacurcas_Jcr4S01700.30

Viterbi Path

Viterbi Path

Posterior @ 0.4

Posterior @ 0.4

Posterior Graphic. Threshold Marked @ 0.4

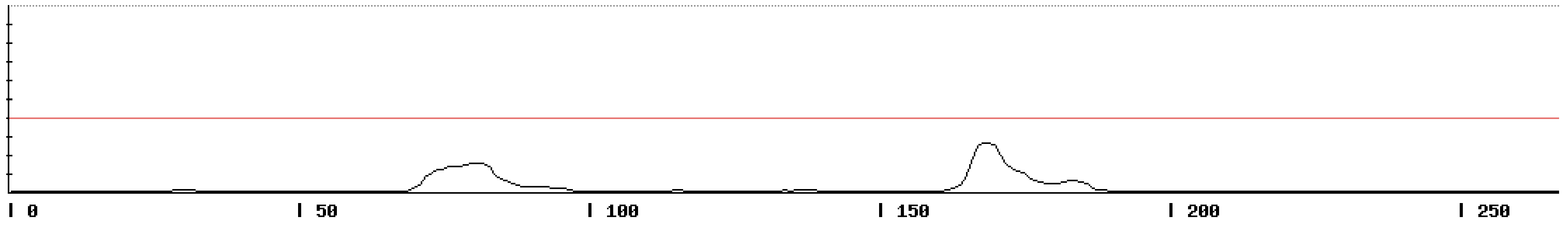

Predictions for Jatrophacurcas_Jcr4S01764.20

Viterbi Path	
Viterbi Path	
Posterior @ 0.4	
Posterior @ 0.4	
Posterior Graphic. Threshold Marked @ 0.4	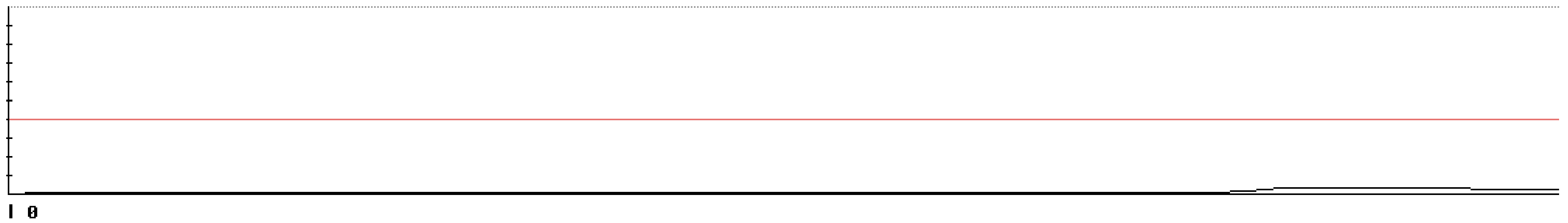

Predictions for Jatrophacurcas_Jcr4S01764.60

Viterbi Path	
Viterbi Path	
Posterior @ 0.4	132 - KKRKS - 136
Posterior @ 0.4	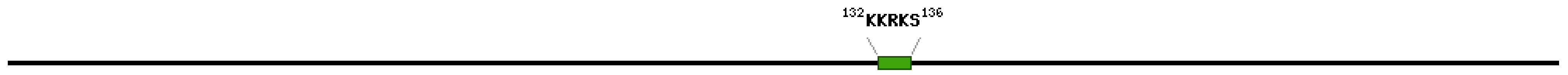

Posterior Graphic. Threshold Marked @ 0.4

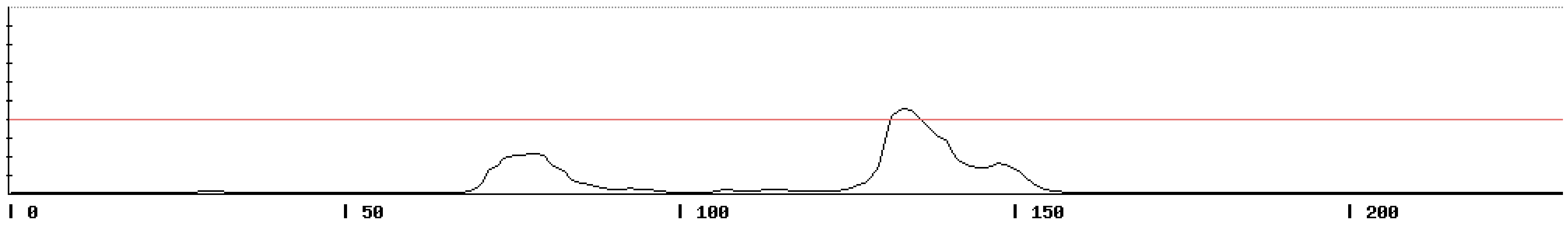

Predictions for Jatrophacurcas_Jcr4S01772.90

Viterbi Path

Viterbi Path

Posterior @ 0.4

Posterior @ 0.4

Posterior Graphic. Threshold Marked @ 0.4

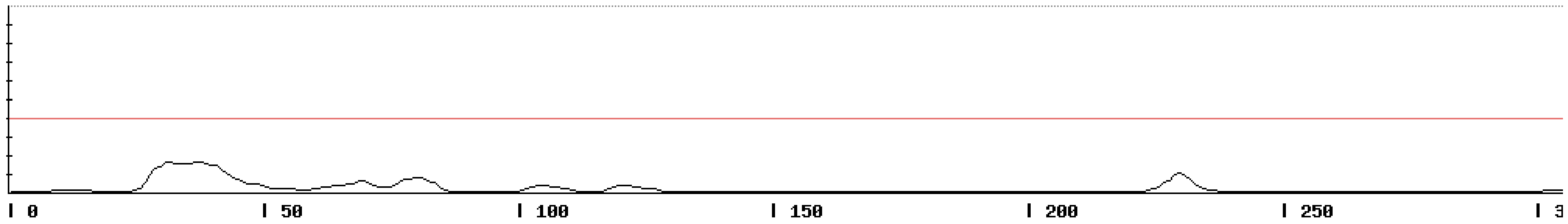

Predictions for Jatrophacurcas_Jcr4S01829.10

Viterbi Path

Viterbi Path

Posterior @ 0.4

Posterior @ 0.4

Posterior Graphic. Threshold Marked @ 0.4

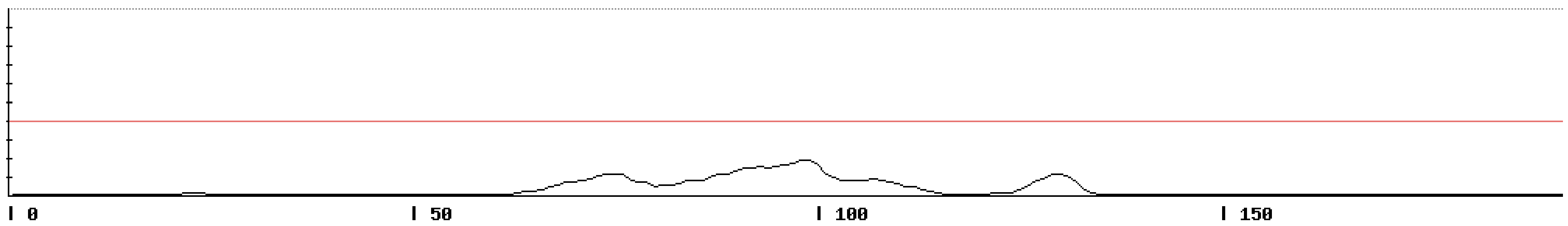

Predictions for Jatrophacurcas_Jcr4S01829.30

Viterbi Path

Viterbi Path

Posterior @ 0.4 93 - WKPTGKLRKVKKKKMVIGTKRSLVFYKKEHPKPVR - 127

Posterior @ 0.4

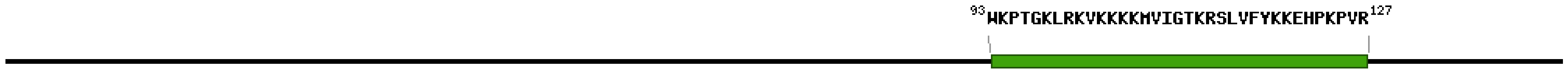

Posterior Graphic. Threshold Marked @ 0.4

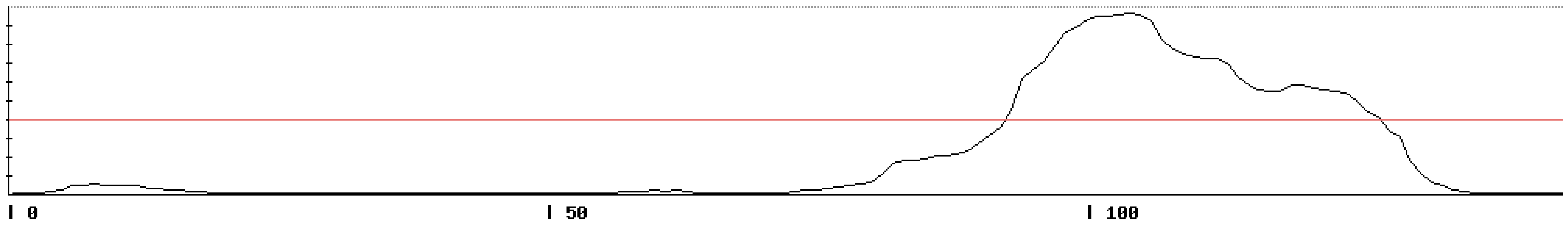

Predictions for Jatrophacurcas_Jcr4S01829.40

Viterbi Path 104 - KPTGKPRKVKDKHKKKEIGTKR - 125

Viterbi Path

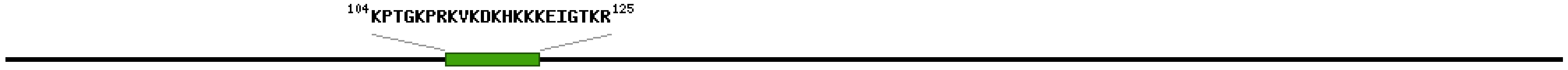

Posterior @ 0.4	100 - VGYWKPTGKPRKVKDKHKKKEIGTKRSLVFYKKD - 133
Posterior @ 0.4	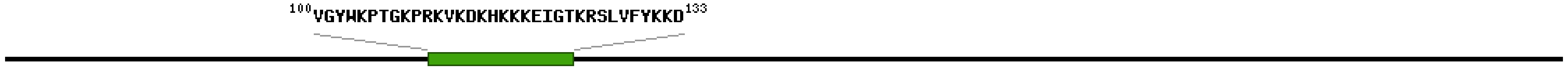
Posterior Graphic. Threshold Marked @ 0.4	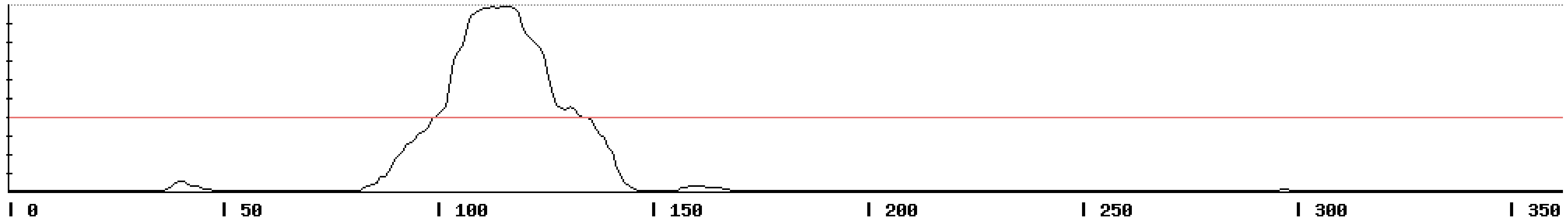

Predictions for Jatrophacurcas_Jcr4S01829.50

Viterbi Path	198 - KKKTVKRPMKAEPSCKKPRTKKKARKG - 224
Viterbi Path	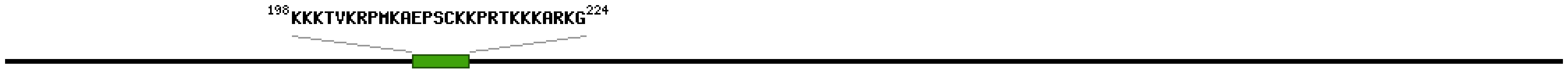
Posterior @ 0.4	197 - EKKKTVKRPMKAEPSCKKPRTKKKARKG - 224
Posterior @ 0.4	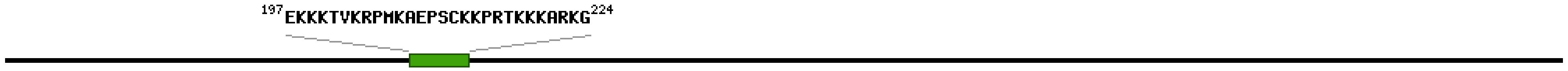
Posterior Graphic. Threshold Marked @ 0.4	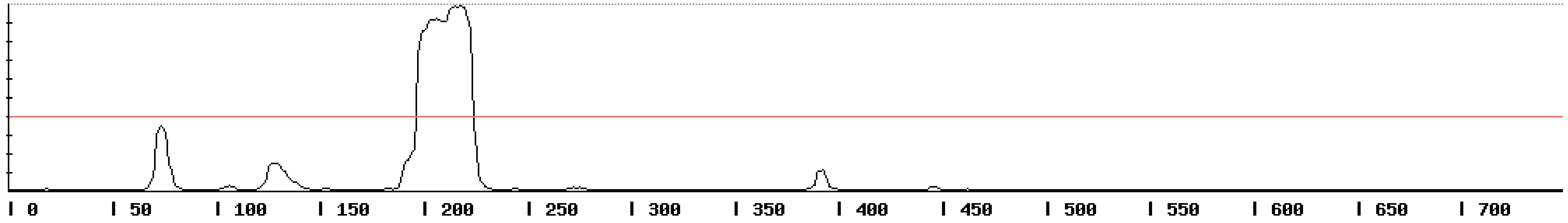

Predictions for Jatrophacurcas_Jcr4S02020.20

Viterbi Path	
Viterbi Path	

Posterior @ 0.4	
Posterior @ 0.4	
Posterior Graphic. Threshold Marked @ 0.4	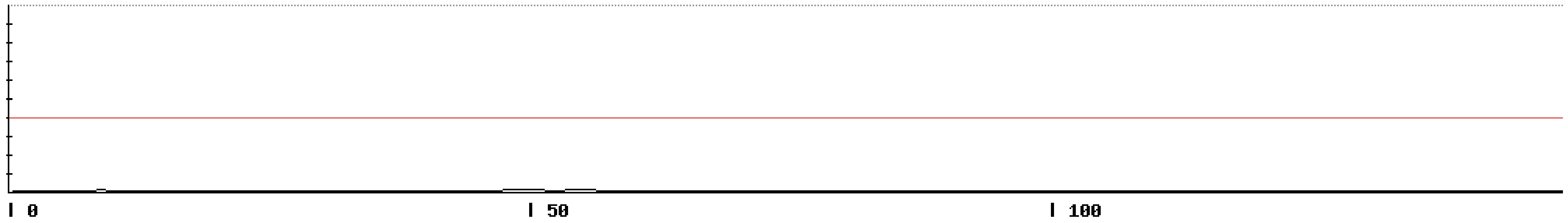

Predictions for Jatrophacurcas_Jcr4S02022.10

Viterbi Path	
Viterbi Path	
Posterior @ 0.4	165 - QRKRRPKKERIVI - 177
Posterior @ 0.4	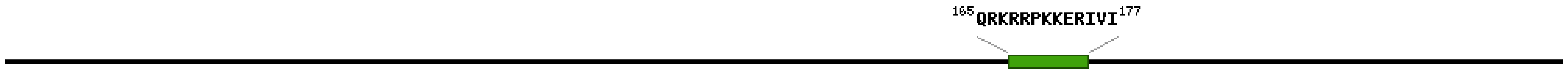
Posterior Graphic. Threshold Marked @ 0.4	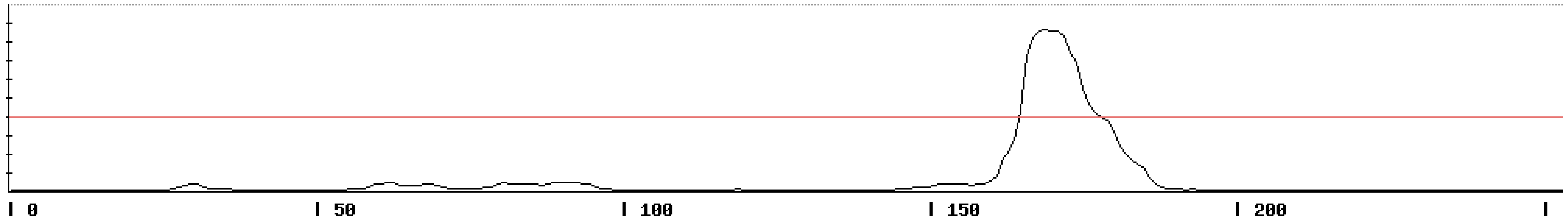

Predictions for Jatrophacurcas_Jcr4S02111.10

Viterbi Path	
Viterbi Path	
Posterior @ 0.4	77 - GRPRRTTKAGYWKGTGKKREI - 97<br>452 - KKR - 454
Posterior @ 0.4	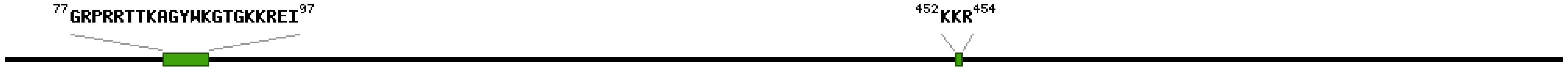
Posterior Graphic. Threshold Marked @ 0.4	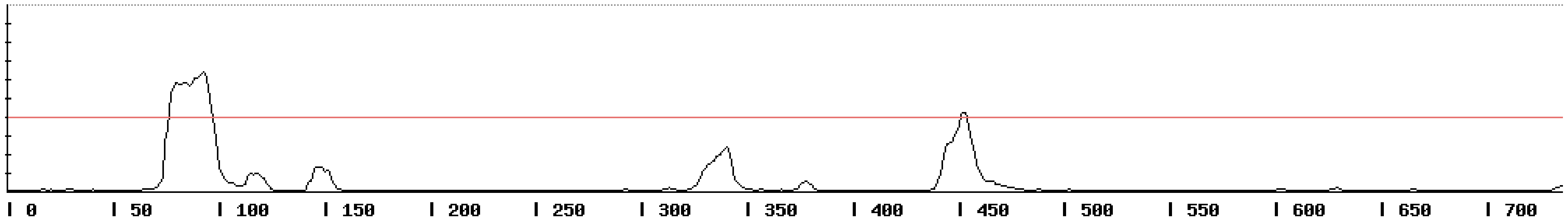

Predictions for Jatrophacurcas_Jcr4S02266.20

Viterbi Path	
Viterbi Path	
Posterior @ 0.4	
Posterior @ 0.4	
Posterior Graphic. Threshold Marked @ 0.4	

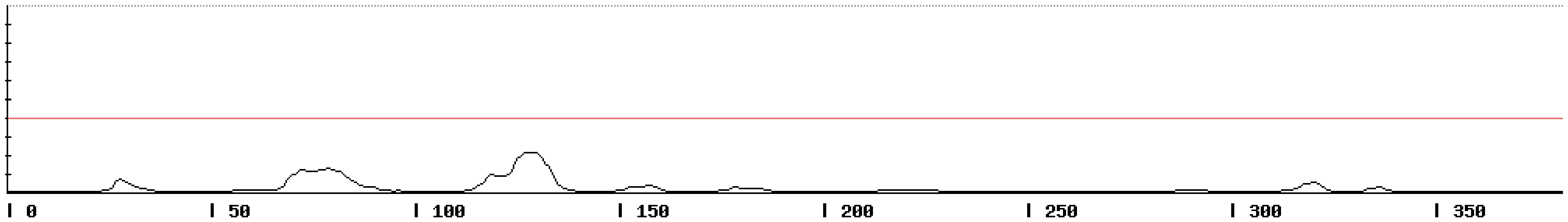

Predictions for Jatrophacurcas_Jcr4S02298.20

Viterbi Path

Viterbi Path

Posterior @ 0.4

Posterior @ 0.4

Posterior Graphic. Threshold Marked @ 0.4

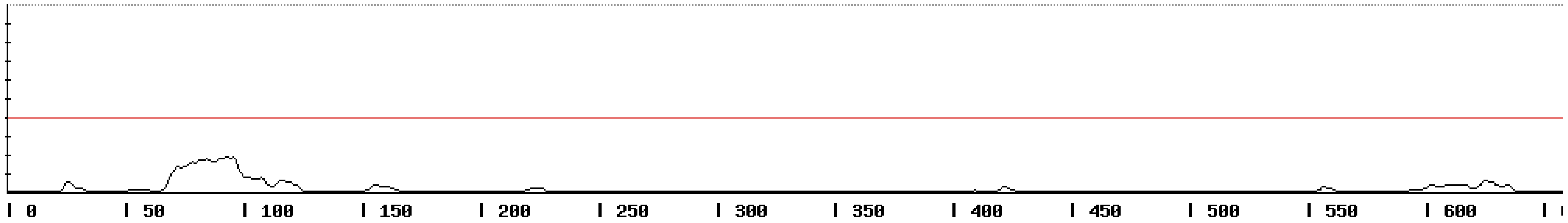

Predictions for Jatrophacurcas_Jcr4S02405.40

Viterbi Path

Viterbi Path

Posterior @ 0.4

Posterior @ 0.4

Posterior Graphic. Threshold Marked @ 0.4	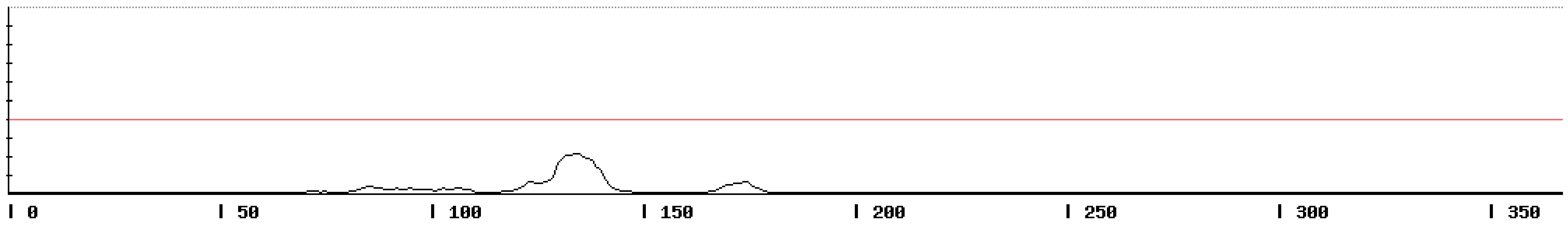

Predictions for Jatrophacurcas_Jcr4S02577.10	
Viterbi Path	
Viterbi Path	
Posterior @ 0.4	
Posterior @ 0.4	
Posterior Graphic. Threshold Marked @ 0.4	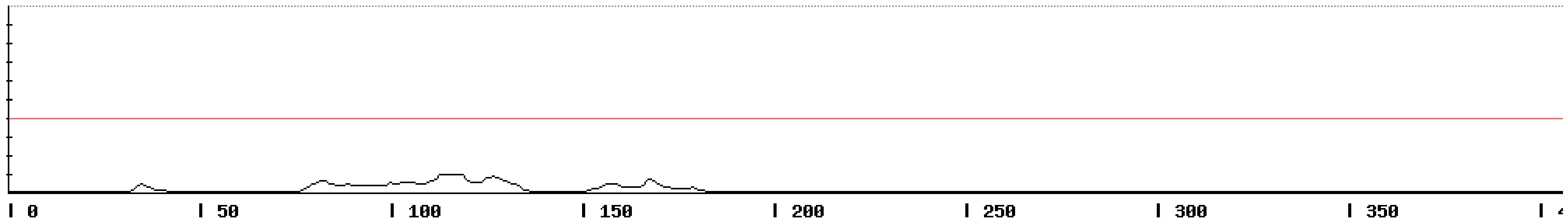

Predictions for Jatrophacurcas_Jcr4S02713.30	
Viterbi Path	
Viterbi Path	
Posterior @ 0.4	
Posterior @ 0.4	

Posterior Graphic. Threshold Marked @ 0.4	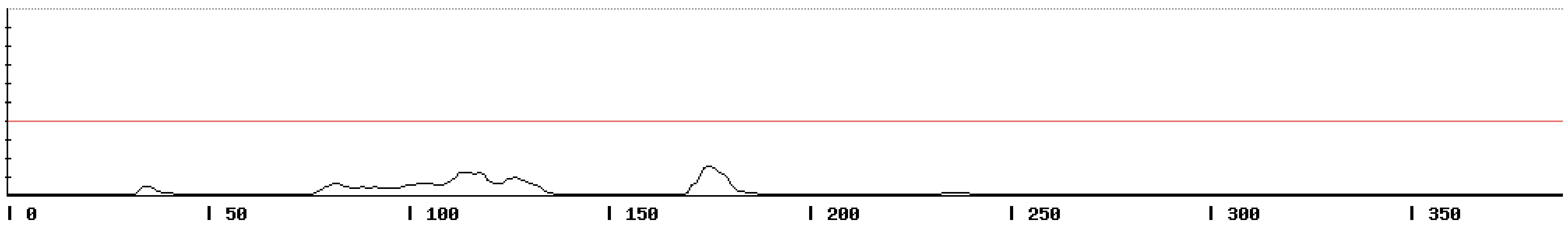

Predictions for Jatrophacurcas_Jcr4S02816.10	
Viterbi Path	
Viterbi Path	
Posterior @ 0.4	143 - RGHPK - 147
Posterior @ 0.4	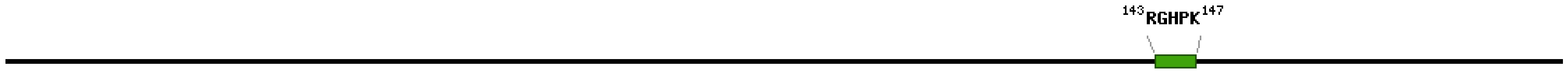
Posterior Graphic. Threshold Marked @ 0.4	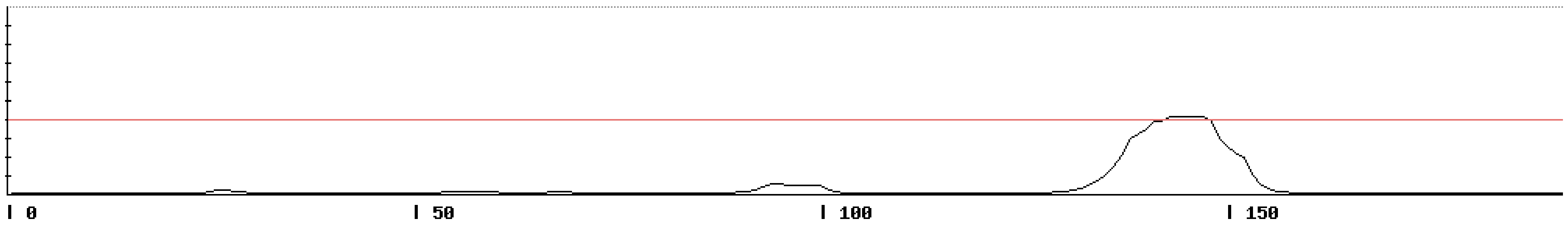

Predictions for Jatrophacurcas_Jcr4S02979.30	
Viterbi Path	
Viterbi Path	

Posterior @ 0.4

Posterior @ 0.4

Posterior Graphic. Threshold Marked @ 0.4

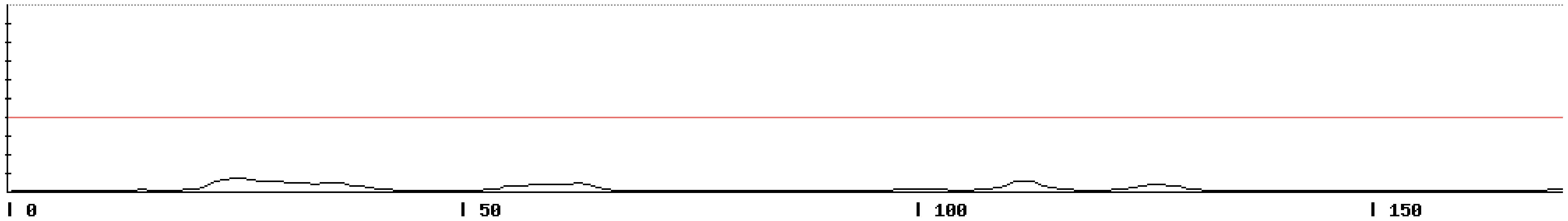

Predictions for Jatrophacurcas_Jcr4S03292.60

Viterbi Path

Viterbi Path

Posterior @ 0.4

Posterior @ 0.4

Posterior Graphic. Threshold Marked @ 0.4

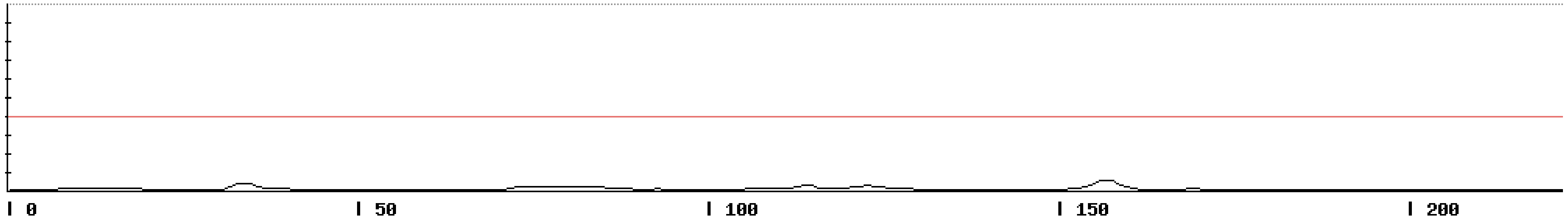

Predictions for Jatrophacurcas_Jcr4S03351.20

Viterbi Path

Viterbi Path

Posterior @ 0.4	134 - KRR - 136
Posterior @ 0.4	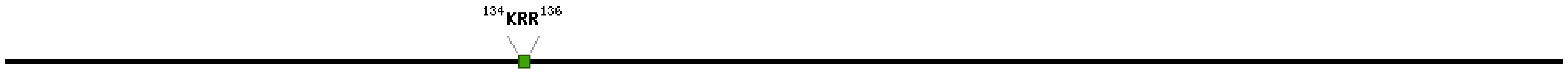
Posterior Graphic. Threshold Marked @ 0.4	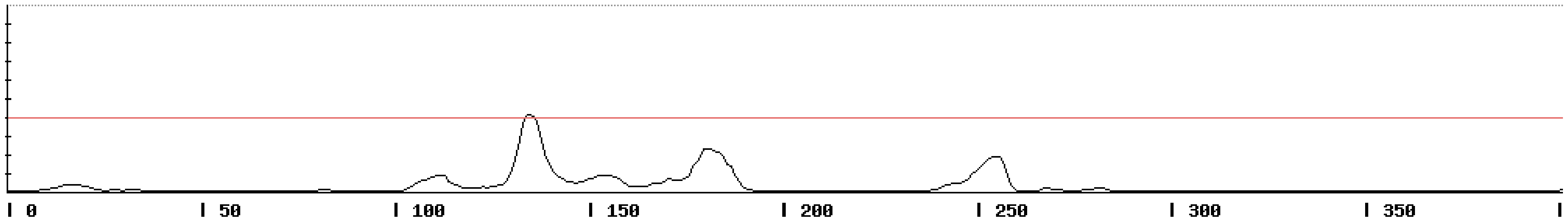

Predictions for Jatrophacurcas_Jcr4S03394.10

Viterbi Path	
Viterbi Path	
Posterior @ 0.4	
Posterior @ 0.4	
Posterior Graphic. Threshold Marked @ 0.4	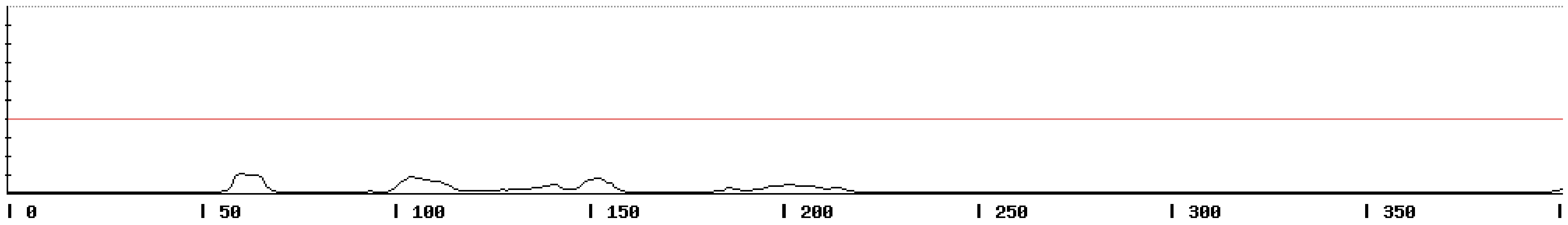

Predictions for Jatrophacurcas_Jcr4S03466.20

Viterbi Path

Viterbi Path

Posterior @ 0.4 — 93 - GADKPIGRPKPLGIKKA - 109

Posterior @ 0.4

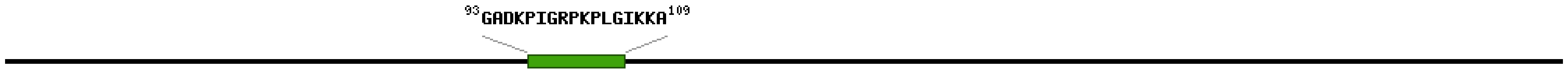

Posterior Graphic. Threshold Marked @ 0.4

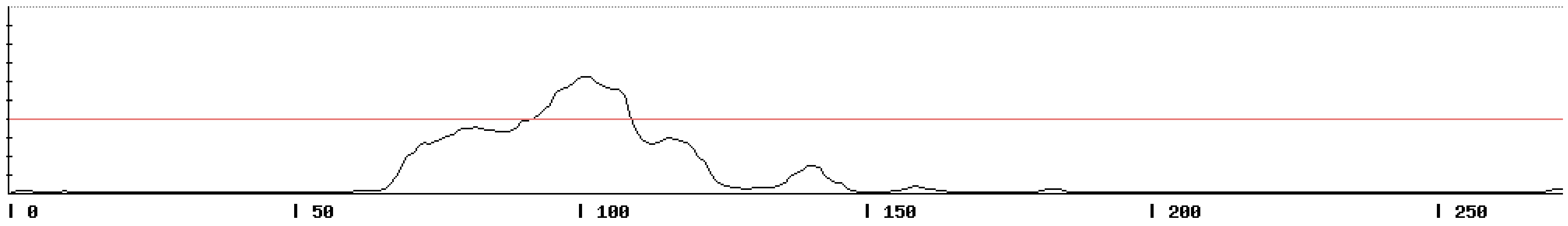

Predictions for Jatrophacurcas_Jcr4S03541.30

Viterbi Path

Viterbi Path

Posterior @ 0.4

Posterior @ 0.4

Posterior Graphic. Threshold Marked @ 0.4

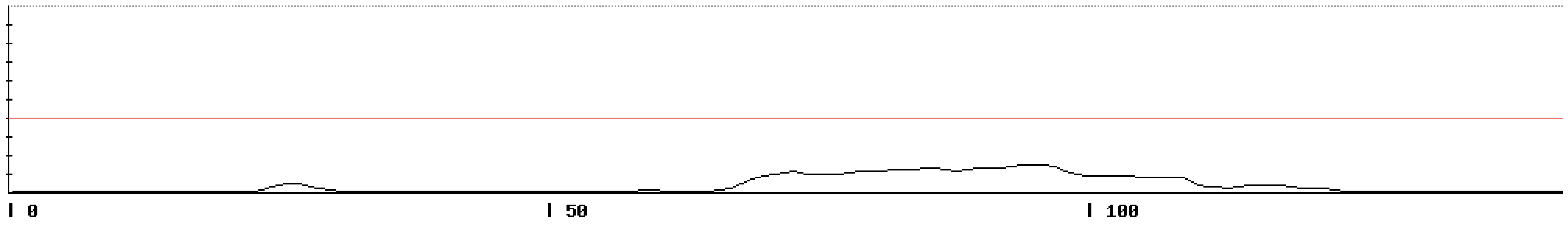

Predictions for Jatrophacurcas_Jcr4S03651.20	
Viterbi Path	
Viterbi Path	
Posterior @ 0.4	230 - RNERDRKGKEKDQNEKGNERIKK - 252
Posterior @ 0.4	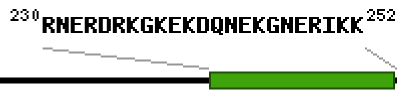
Posterior Graphic. Threshold Marked @ 0.4	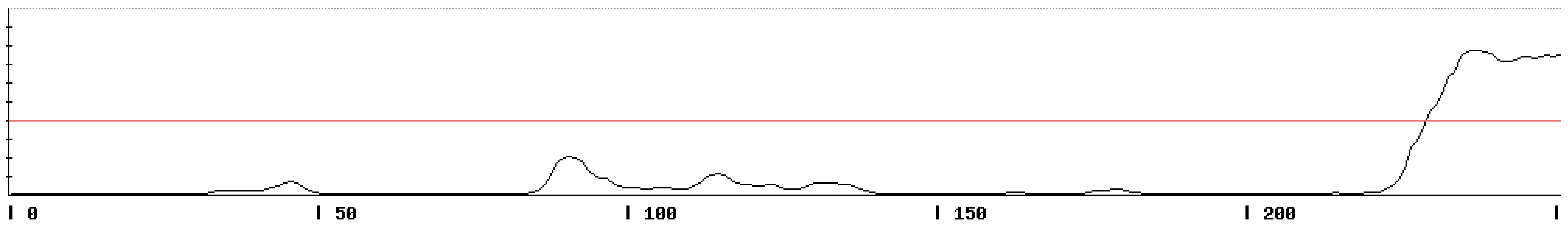

Predictions for Jatrophacurcas_Jcr4S03681.20	
Viterbi Path	
Viterbi Path	
Posterior @ 0.4	
Posterior @ 0.4	
Posterior Graphic. Threshold Marked @ 0.4	

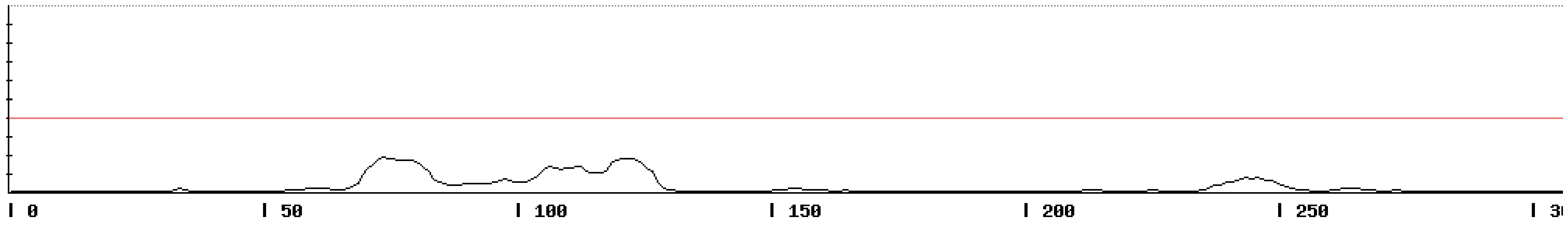

Predictions for Jatrophacurcas_Jcr4S04578.10	
Viterbi Path	
Viterbi Path	
Posterior @ 0.4	
Posterior @ 0.4	
Posterior Graphic. Threshold Marked @ 0.4	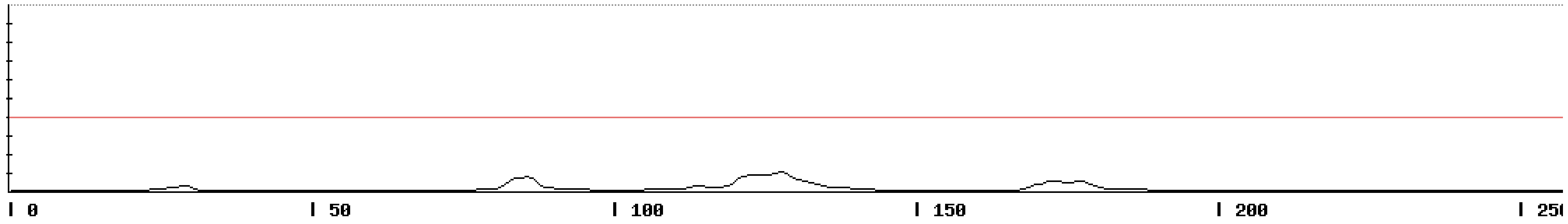

Predictions for Jatrophacurcas_Jcr4S04584.10	
Viterbi Path	
Viterbi Path	
Posterior @ 0.4	
Posterior @ 0.4	

Posterior Graphic. Threshold Marked @ 0.4

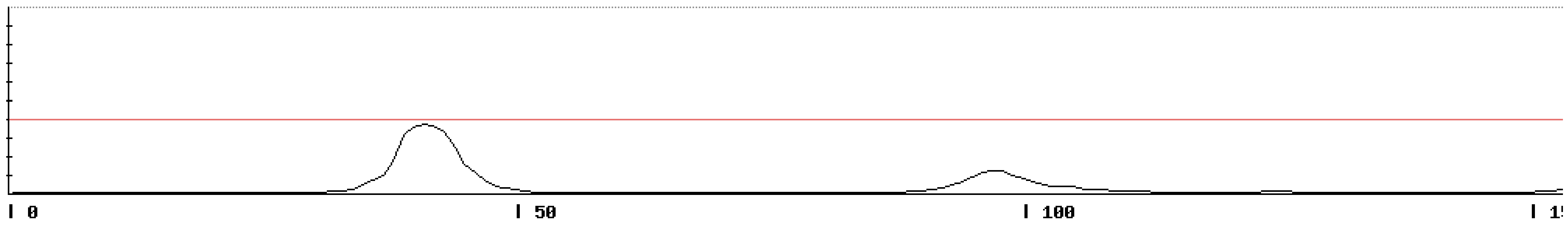

Predictions for Jatrophacurcas_Jcr4S04670.20

Viterbi Path

Viterbi Path

Posterior @ 0.4

Posterior @ 0.4

Posterior Graphic. Threshold Marked @ 0.4

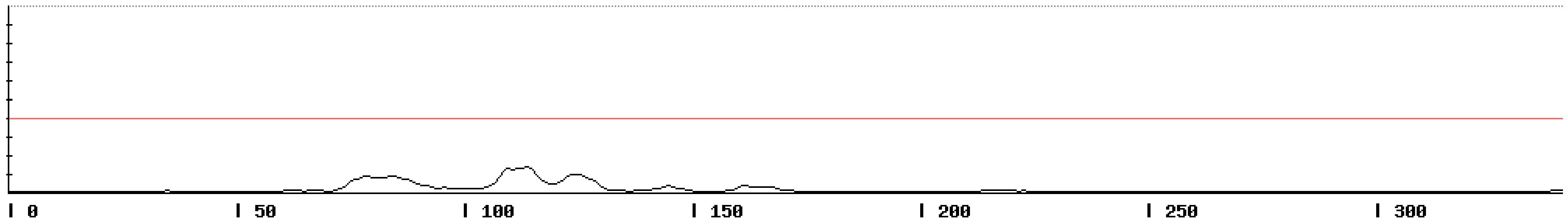

Predictions for Jatrophacurcas_Jcr4S04697.30

Viterbi Path

Viterbi Path

Posterior @ 0.4

Posterior @ 0.4

Posterior Graphic. Threshold Marked @ 0.4

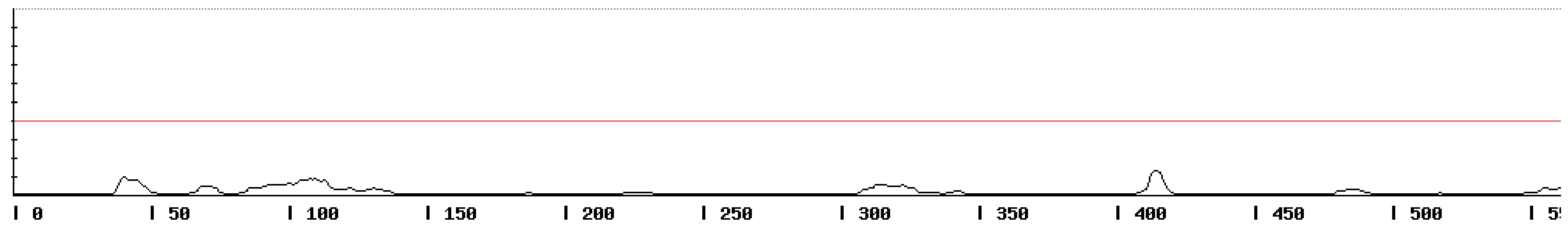

Predictions for Jatrophacurcas_Jcr4S04736.10

Viterbi Path

Viterbi Path

Posterior @ 0.4

Posterior @ 0.4

Posterior Graphic. Threshold Marked @ 0.4

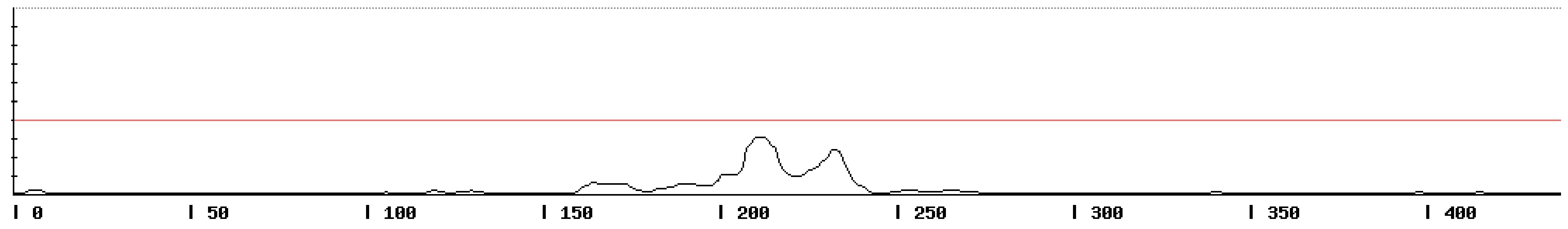

Predictions for Jatrophacurcas_Jcr4S04869.20

Viterbi Path

Viterbi Path

Posterior @ 0.4

Posterior @ 0.4

Posterior Graphic. Threshold Marked @ 0.4

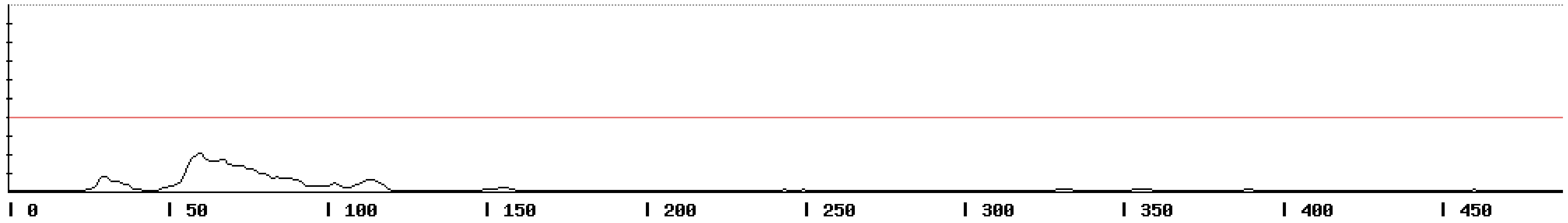

Predictions for Jatrophacurcas_Jcr4S05163.20

Viterbi Path

Viterbi Path

Posterior @ 0.4

Posterior @ 0.4

Posterior Graphic. Threshold Marked @ 0.4

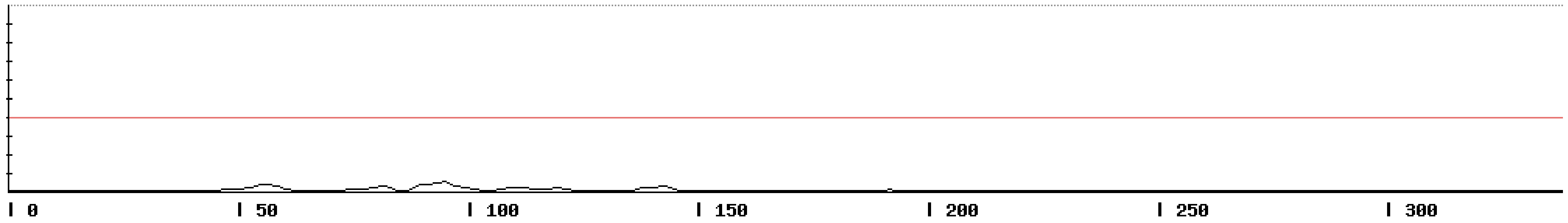

Predictions for Jatrophacurcas_Jcr4S05260.20

Viterbi Path

Viterbi Path

Posterior @ 0.4

Posterior @ 0.4

Posterior Graphic. Threshold Marked @ 0.4

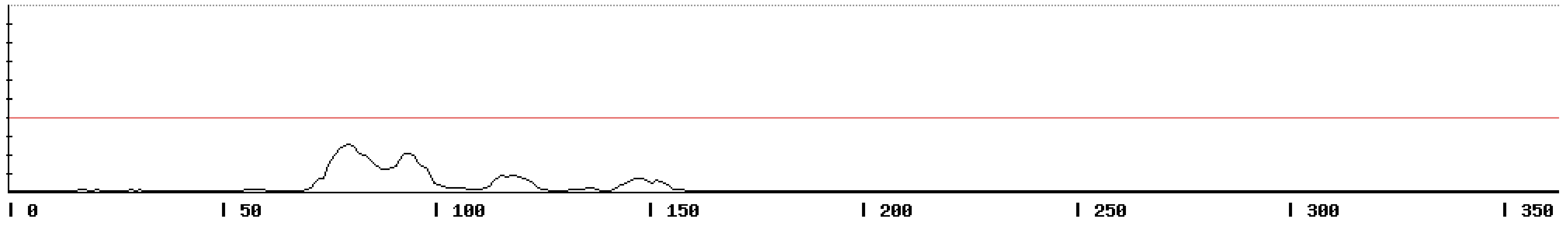

Predictions for Jatrophacurcas_Jcr4S05464.30

Viterbi Path

Viterbi Path

Posterior @ 0.4

Posterior @ 0.4

Posterior Graphic. Threshold Marked @ 0.4

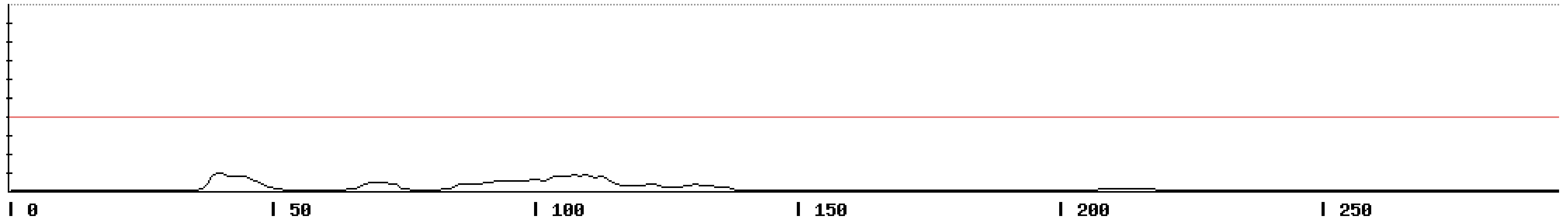

Predictions for Jatrophacurcas_Jcr4S05497.20

Viterbi Path

Viterbi Path

Posterior @ 0.4

Posterior @ 0.4

Posterior Graphic. Threshold Marked @ 0.4

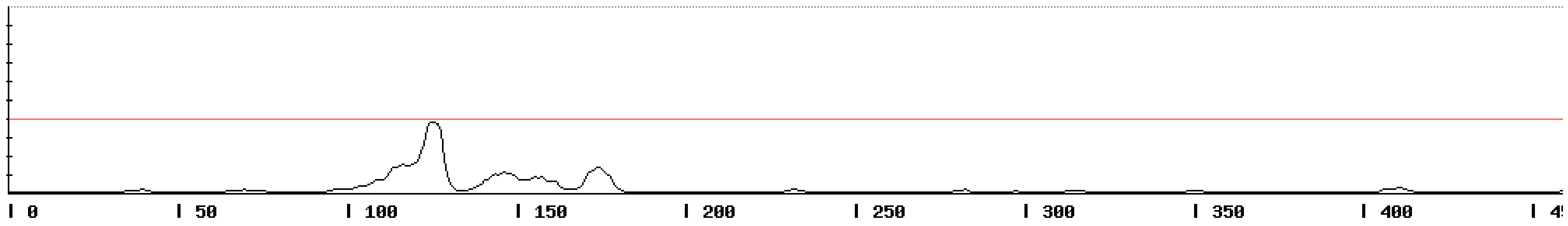

Predictions for Jatrophacurcas_Jcr4S06278.20

Viterbi Path

Viterbi Path

Posterior @ 0.4

Posterior @ 0.4

Posterior Graphic. Threshold Marked @ 0.4

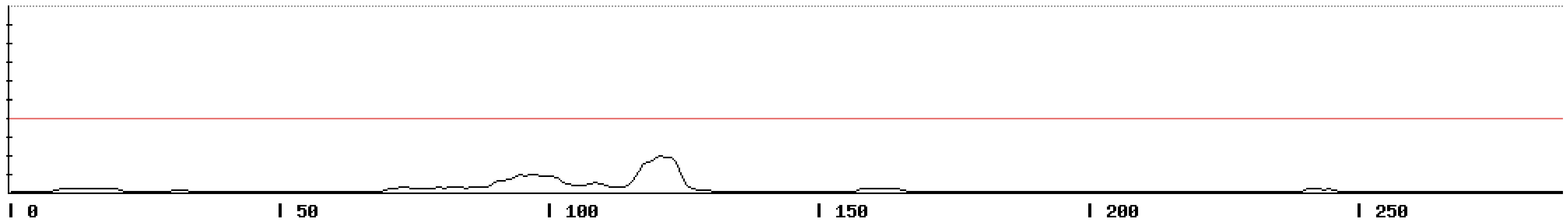

Predictions for Jatrophacurcas_Jcr4S06450.20	
Viterbi Path	
Viterbi Path	
Posterior @ 0.4	
Posterior @ 0.4	
Posterior Graphic. Threshold Marked @ 0.4	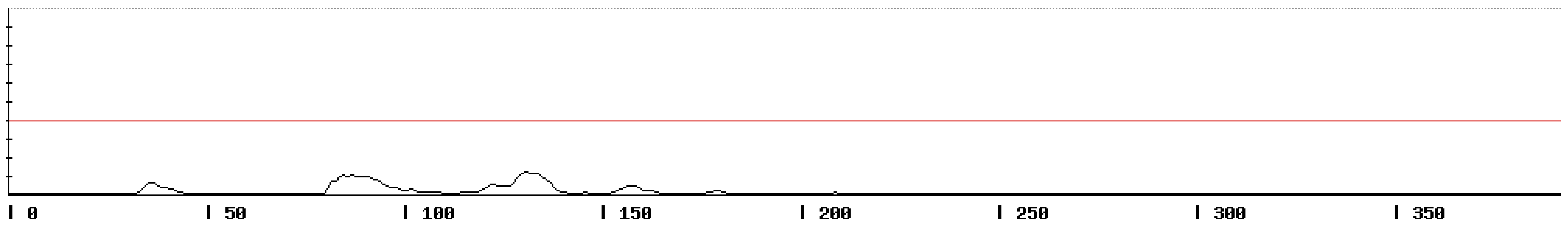

Predictions for Jatrophacurcas_Jcr4S07369.20	
Viterbi Path	
Viterbi Path	
Posterior @ 0.4	
Posterior @ 0.4	
Posterior Graphic. Threshold Marked @ 0.4	

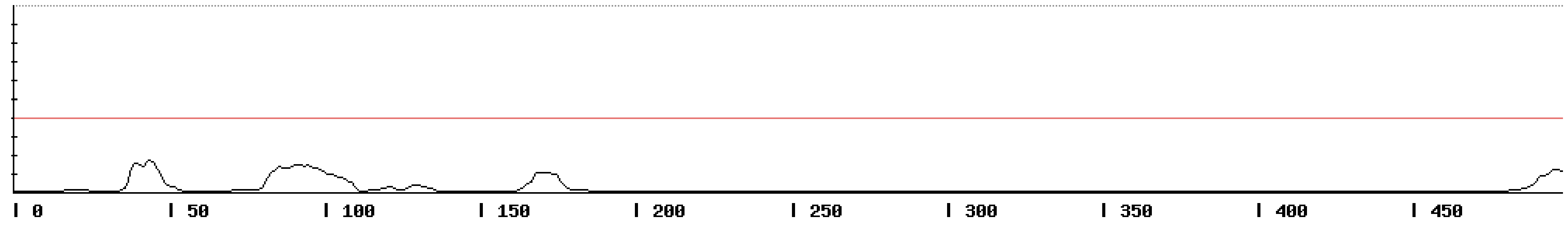

Predictions for Jatrophacurcas_Jcr4S07916.10

Viterbi Path	
Viterbi Path	
Posterior @ 0.4	
Posterior @ 0.4	
Posterior Graphic. Threshold Marked @ 0.4	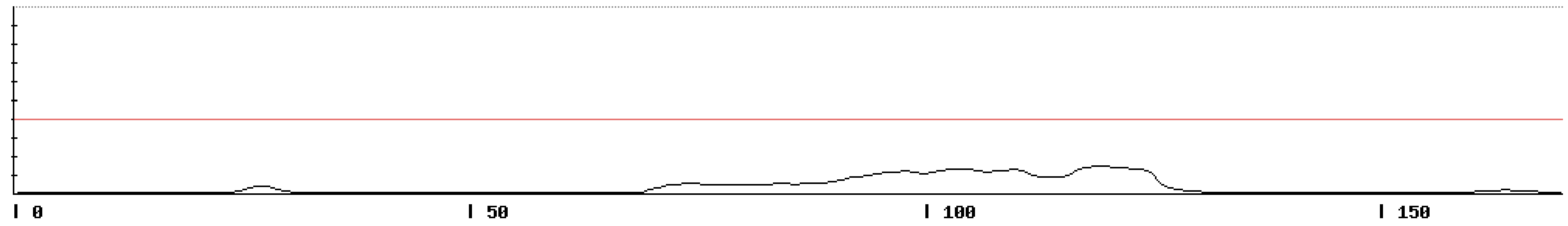

Predictions for Jatrophacurcas_Jcr4S08160.10

Viterbi Path	
Viterbi Path	
Posterior @ 0.4	
Posterior @ 0.4	

Posterior Graphic. Threshold Marked @ 0.4

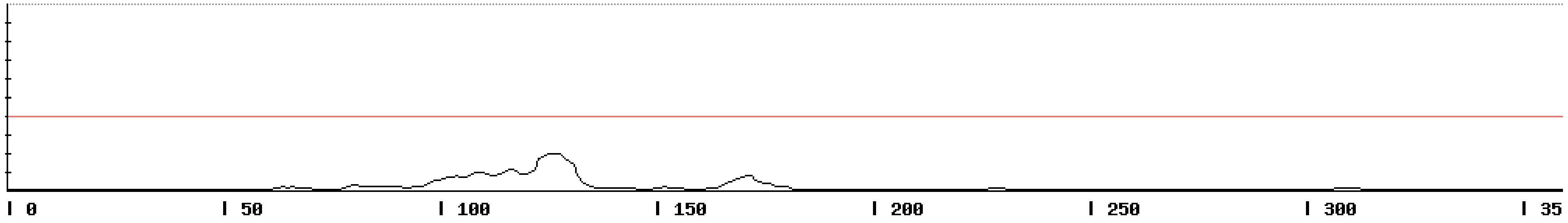

Predictions for Jatrophacurcas_Jcr4S08640.20

Viterbi Path	
Viterbi Path	
Posterior @ 0.4	
Posterior @ 0.4	
Posterior Graphic. Threshold Marked @ 0.4	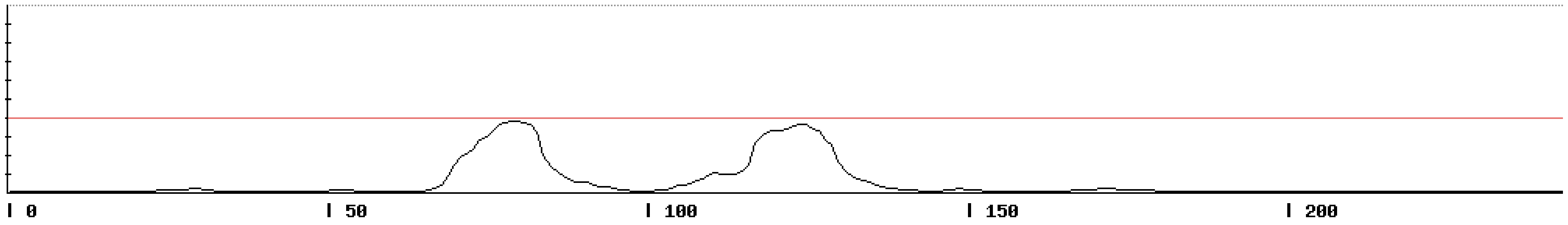

Predictions for Jatrophacurcas_Jcr4S08703.20

Viterbi Path	
Viterbi Path	
Posterior @ 0.4	29 - KRR - 31
Posterior @ 0.4	

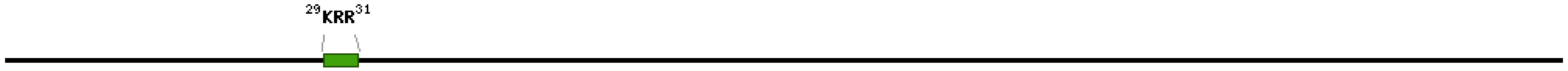

Posterior Graphic. Threshold Marked @ 0.4

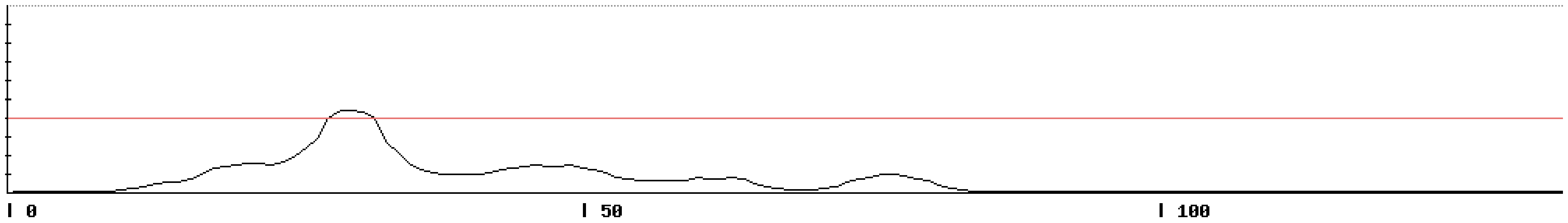

Predictions for Jatrophacurcas_Jcr4S09148.30

Viterbi Path

Viterbi Path

Posterior @ 0.4

Posterior @ 0.4

Posterior Graphic. Threshold Marked @ 0.4

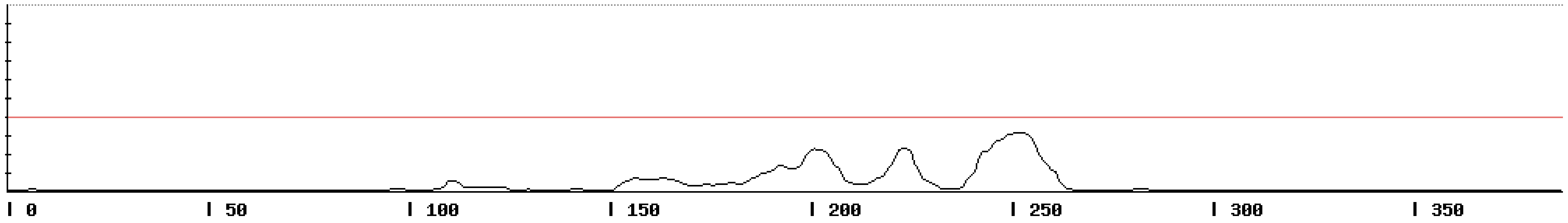

Predictions for Jatrophacurcas_Jcr4S09200.10

Viterbi Path

Viterbi Path

Posterior @ 0.4

Posterior @ 0.4

Posterior Graphic. Threshold Marked @ 0.4

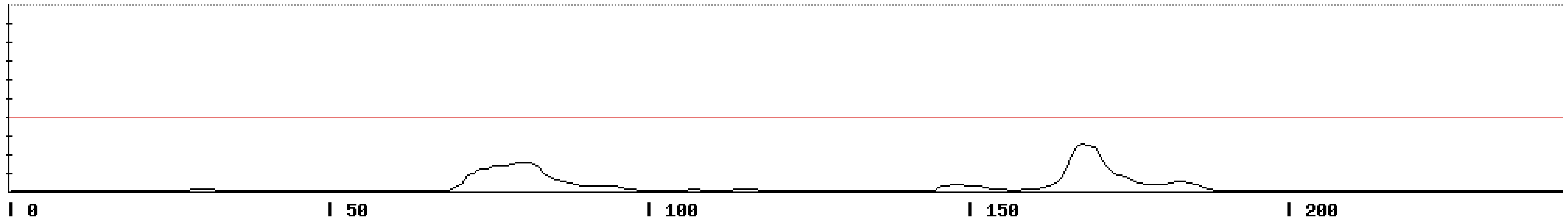

Predictions for Jatrophacurcas_Jcr4S09242.20

Viterbi Path

Viterbi Path

Posterior @ 0.4

Posterior @ 0.4

Posterior Graphic. Threshold Marked @ 0.4

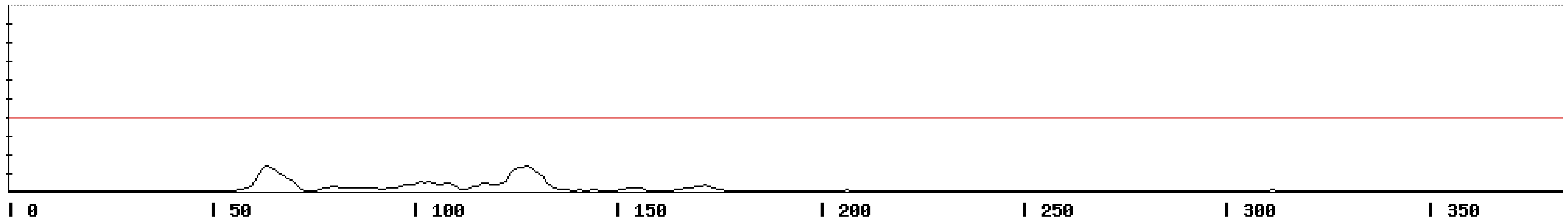

Predictions for Jatrophacurcas_Jcr4S09529.20

Viterbi Path

Viterbi Path

Posterior @ 0.4

Posterior @ 0.4

Posterior Graphic. Threshold Marked @ 0.4

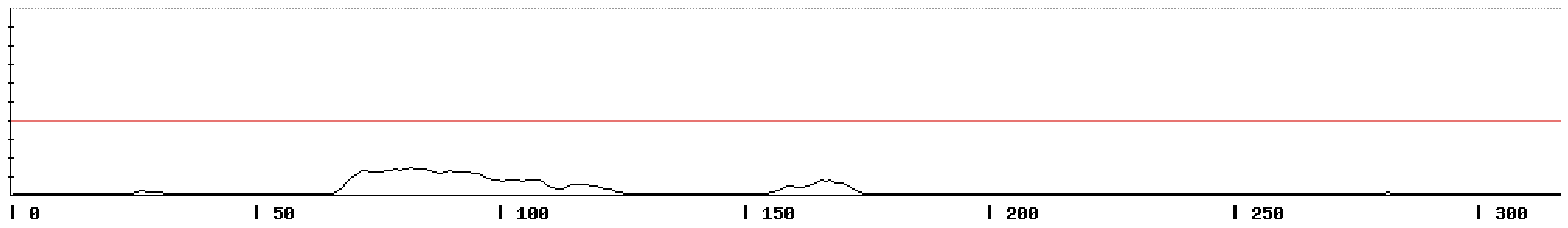

Predictions for Jatrophacurcas_Jcr4S09904.10

Viterbi Path

Viterbi Path

Posterior @ 0.4

Posterior @ 0.4

Posterior Graphic. Threshold Marked @ 0.4

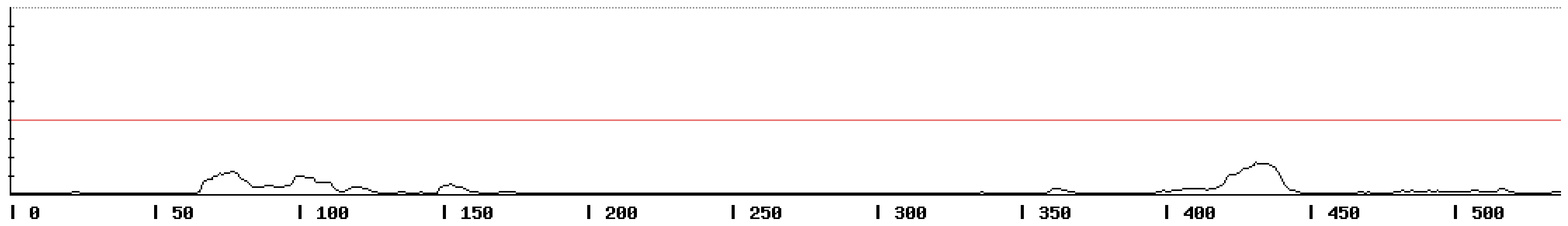

Predictions for Jatrophacurcas_Jcr4S09904.20

Viterbi Path	
Viterbi Path	
Posterior @ 0.4	91 - HSHSKRANRRTKAGFWKSTGKVRRVKAKQ - 119
Posterior @ 0.4	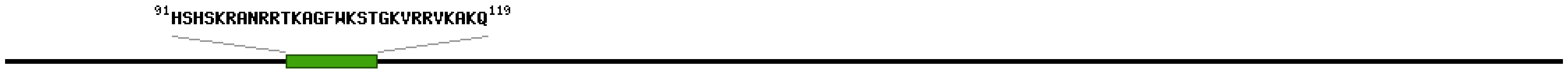
Posterior Graphic. Threshold Marked @ 0.4	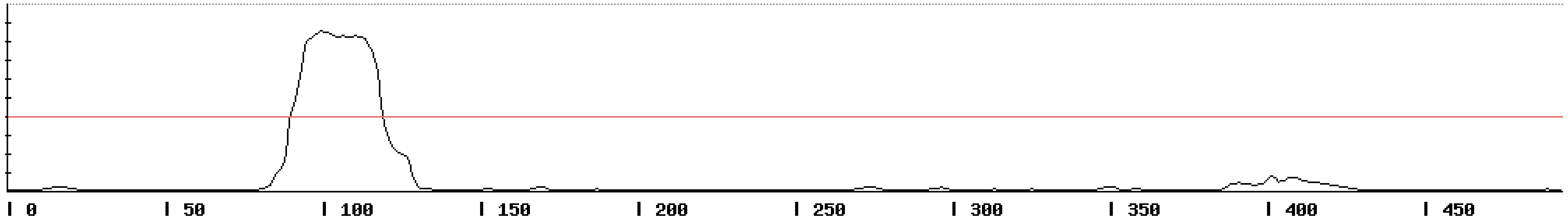

Predictions for Jatrophacurcas_Jcr4S10515.30

Viterbi Path	
Viterbi Path	
Posterior @ 0.4	
Posterior @ 0.4	
Posterior Graphic. Threshold Marked @ 0.4	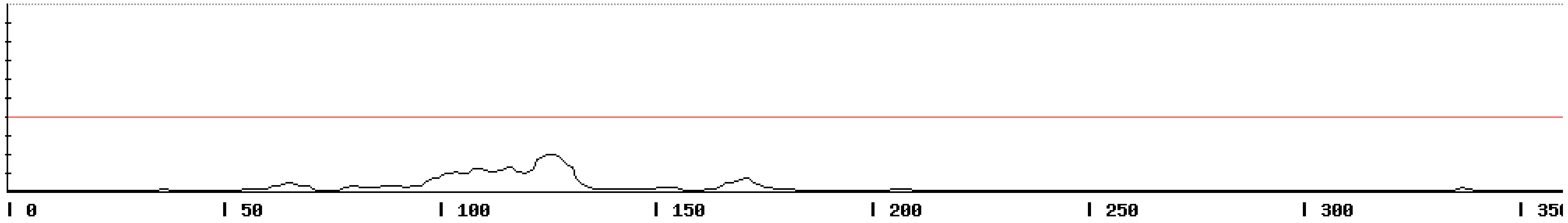

Predictions for Jatrophacurcas_Jcr4S11139.20

Viterbi Path

Viterbi Path

Posterior @ 0.4

Posterior @ 0.4

Posterior Graphic. Threshold Marked @ 0.4

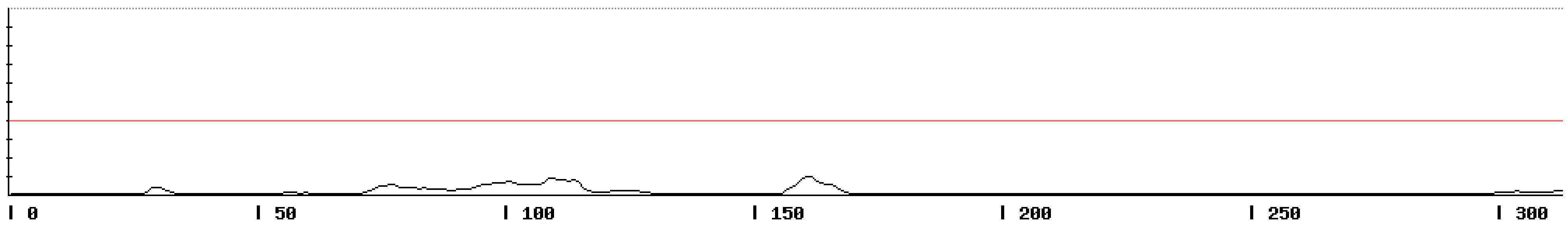

Predictions for Jatrophacurcas_Jcr4S11637.10

Viterbi Path

Viterbi Path

Posterior @ 0.4

Posterior @ 0.4

Posterior Graphic. Threshold Marked @ 0.4

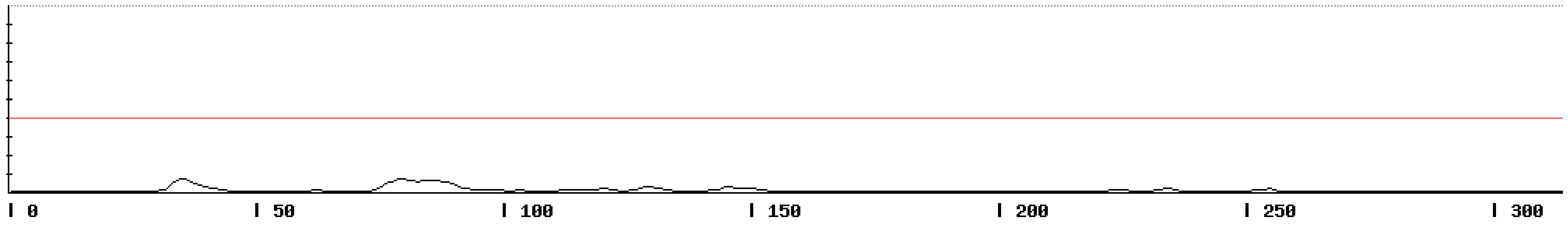

Predictions for Jatrophacurcas_Jcr4S11673.20

Viterbi Path

Viterbi Path

Posterior @ 0.4

Posterior @ 0.4

Posterior Graphic. Threshold Marked @ 0.4

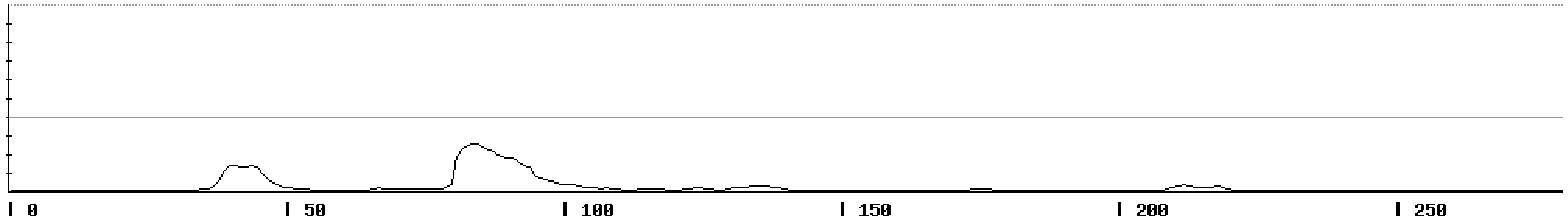

Predictions for Jatrophacurcas_Jcr4S11783.10

Viterbi Path

Viterbi Path

Posterior @ 0.4

Posterior @ 0.4

Posterior Graphic. Threshold Marked @ 0.4

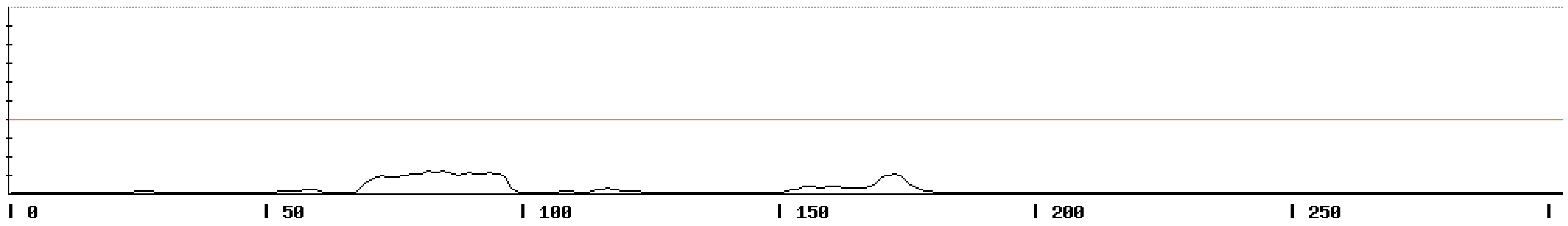

Predictions for Jatrophacurcas_Jcr4S12092.10

Viterbi Path

Viterbi Path

Posterior @ 0.4: 99 - R - 99

Posterior @ 0.4

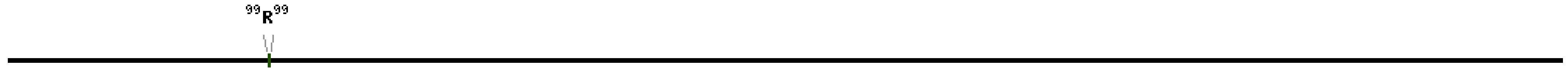

Posterior Graphic. Threshold Marked @ 0.4

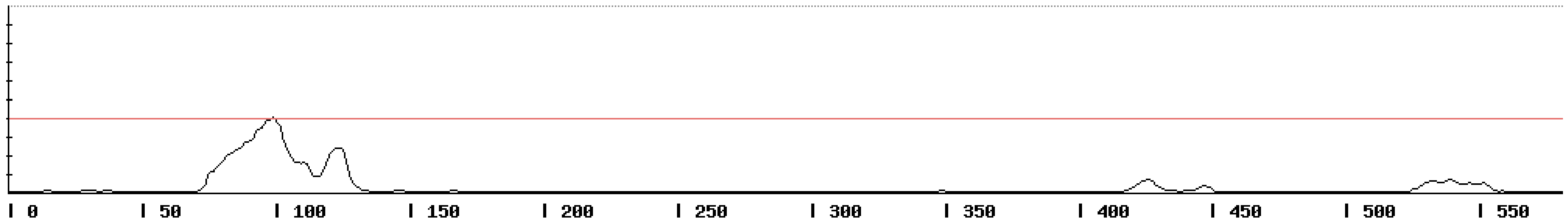

Predictions for Jatrophacurcas_Jcr4S13209.20

Viterbi Path

Viterbi Path

Posterior @ 0.4

Posterior @ 0.4

Posterior Graphic. Threshold Marked @ 0.4

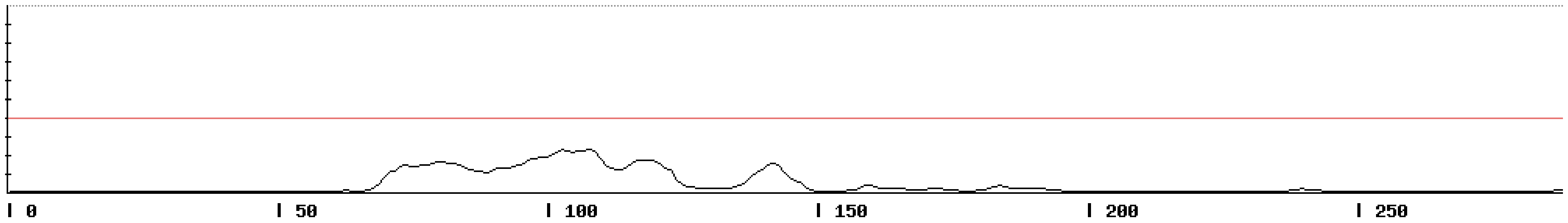

Predictions for Jatrophacurcas_Jcr4S14128.10

Viterbi Path

Viterbi Path

Posterior @ 0.4

Posterior @ 0.4

Posterior Graphic. Threshold Marked @ 0.4

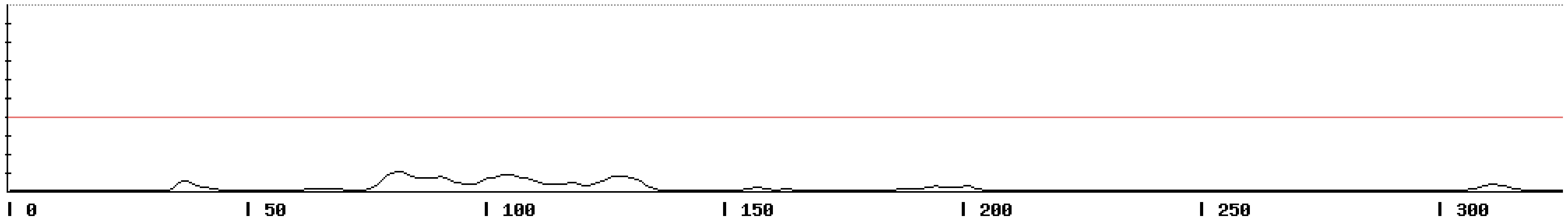

Predictions for Jatrophacurcas_Jcr4S14571.10

Viterbi Path

Viterbi Path

Posterior @ 0.4

Posterior @ 0.4

Posterior Graphic. Threshold Marked @ 0.4

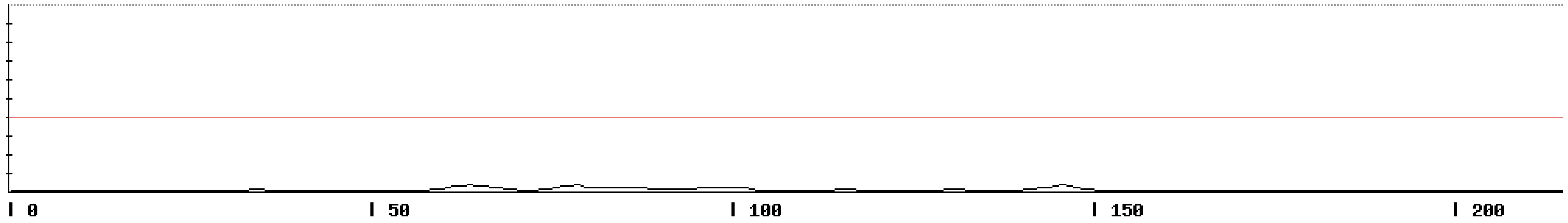

Predictions for Jatrophacurcas_Jcr4S14739.10

Viterbi Path

Viterbi Path

Posterior @ 0.4

Posterior @ 0.4

Posterior Graphic. Threshold Marked @ 0.4

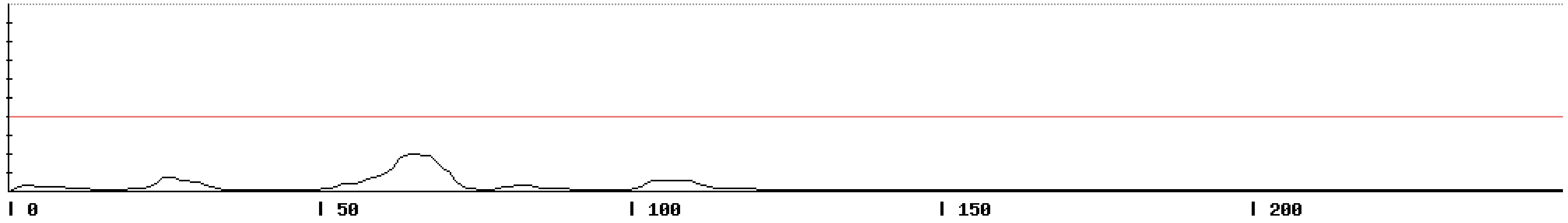

Predictions for Jatrophacurcas_Jcr4S15477.10

Viterbi Path

Viterbi Path

Posterior @ 0.4	88 - KRRDR - 92
Posterior @ 0.4	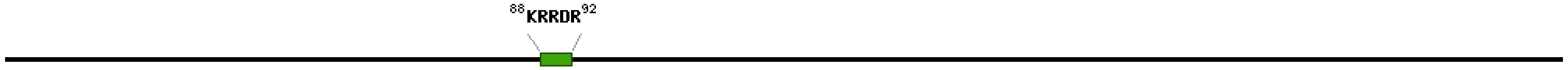
Posterior Graphic. Threshold Marked @ 0.4	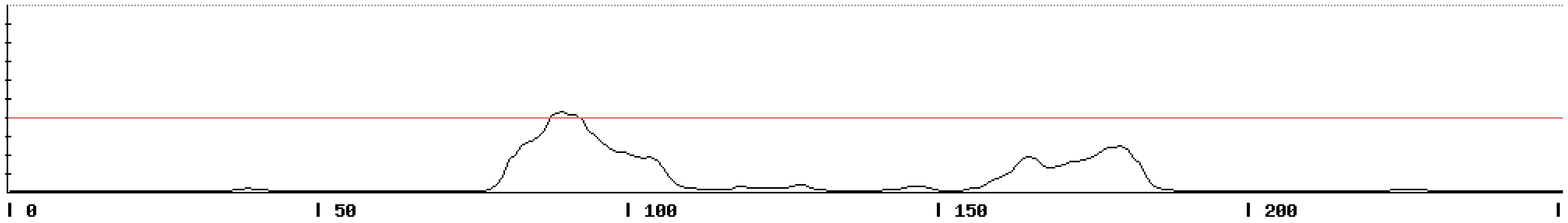

Predictions for Jatrophacurcas_Jcr4S16185.10	
Viterbi Path	
Viterbi Path	
Posterior @ 0.4	
Posterior @ 0.4	
Posterior Graphic. Threshold Marked @ 0.4	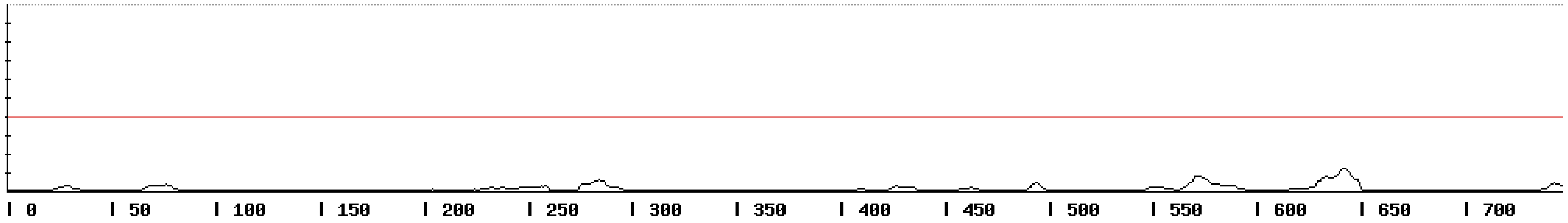

Predictions for Jatrophacurcas_Jcr4S16874.10

Viterbi Path

Viterbi Path

Posterior @ 0.4

Posterior @ 0.4

Posterior Graphic. Threshold Marked @ 0.4

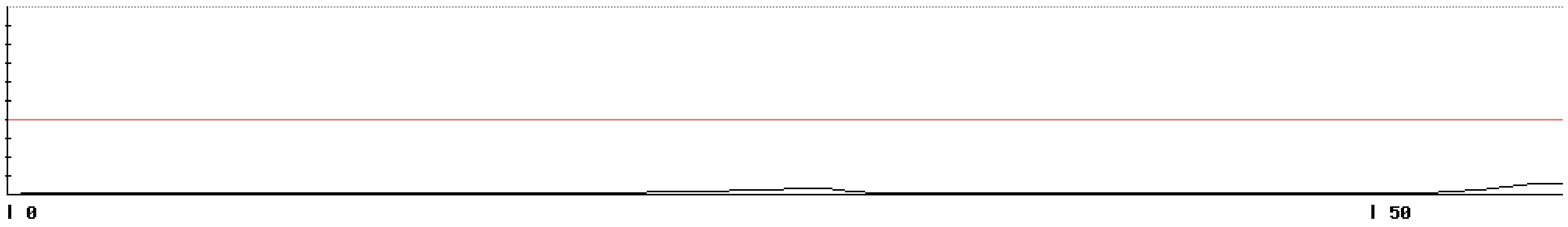

Predictions for Jatrophacurcas_Jcr4S19737.10

Viterbi Path

Viterbi Path

Posterior @ 0.4

125 - PRGR - 128

201 - KRK - 203

Posterior @ 0.4

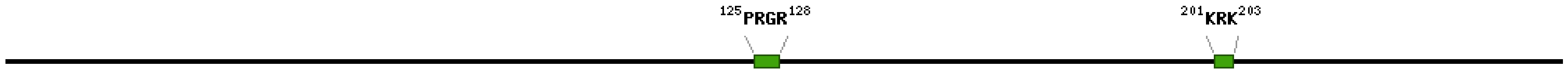

Posterior Graphic. Threshold Marked @ 0.4

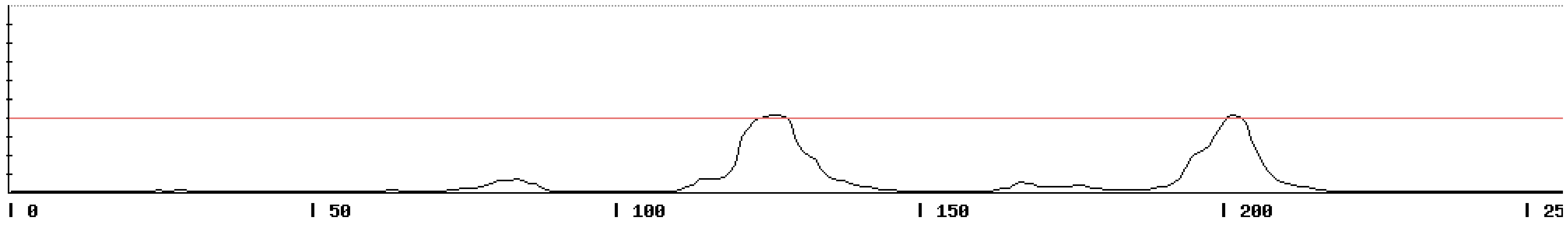

Predictions for Jatrophacurcas_Jcr4S26818.10	
Viterbi Path	
Viterbi Path	
Posterior @ 0.4	
Posterior @ 0.4	
Posterior Graphic. Threshold Marked @ 0.4	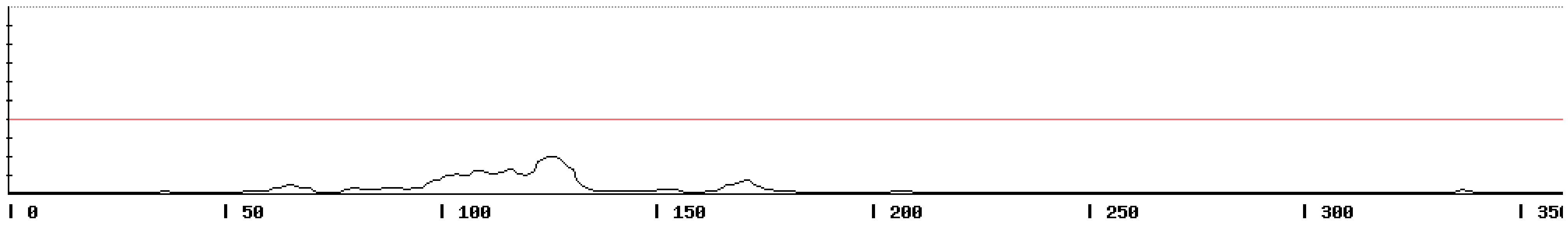

Revision r.9 - Last Updated on : February 24 2015 17:45:20. alex.nguyenba@utoronto.ca

Intended to be used with Firefox

Viterbi Path

Posterior @ 0.4 | 93 - ADKPIGKPKPLGIKKA - 108

Posterior @ 0.4

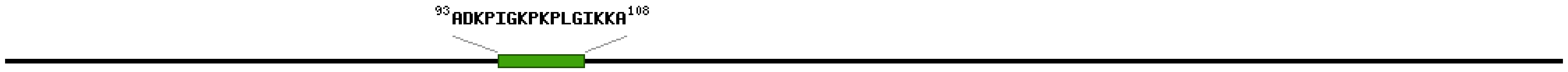

Posterior Graphic. Threshold Marked @ 0.4

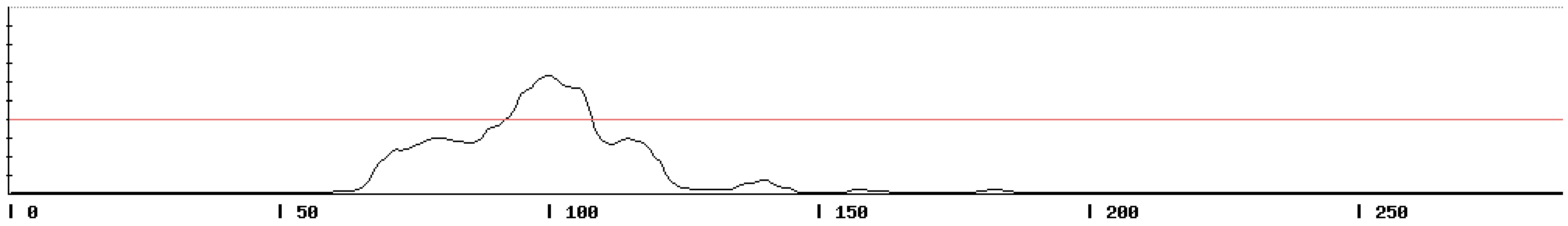

Predictions for Juglansregia_WALNUT_00000224-RA

Viterbi Path

Viterbi Path

Posterior @ 0.4

Posterior @ 0.4

Posterior Graphic. Threshold Marked @ 0.4

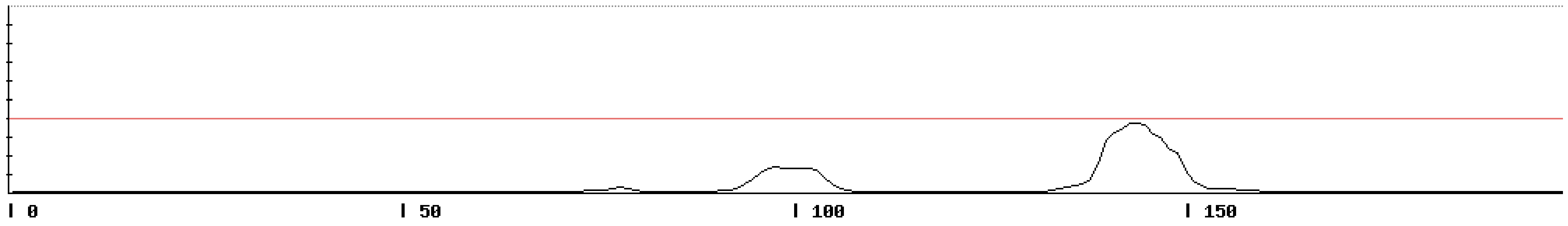

Predictions for Juglansregia_WALNUT_00000247-RA

Viterbi Path	
Viterbi Path	
Posterior @ 0.4	
Posterior @ 0.4	
Posterior Graphic. Threshold Marked @ 0.4	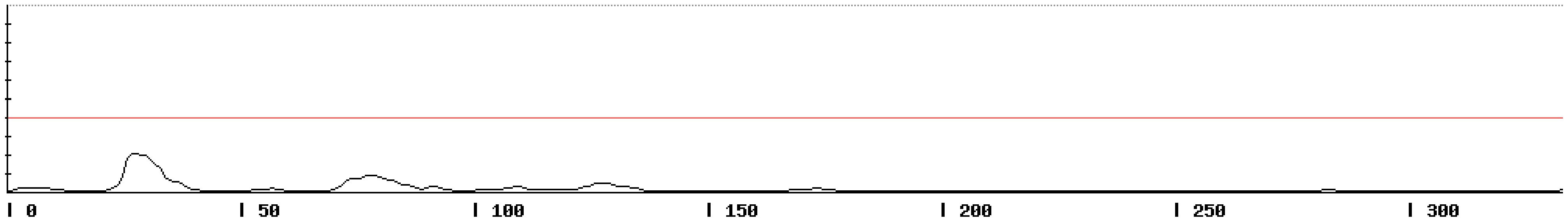

Predictions for Juglansregia_WALNUT_00000581-RA

Viterbi Path	
Viterbi Path	
Posterior @ 0.4	
Posterior @ 0.4	
Posterior Graphic. Threshold Marked @ 0.4	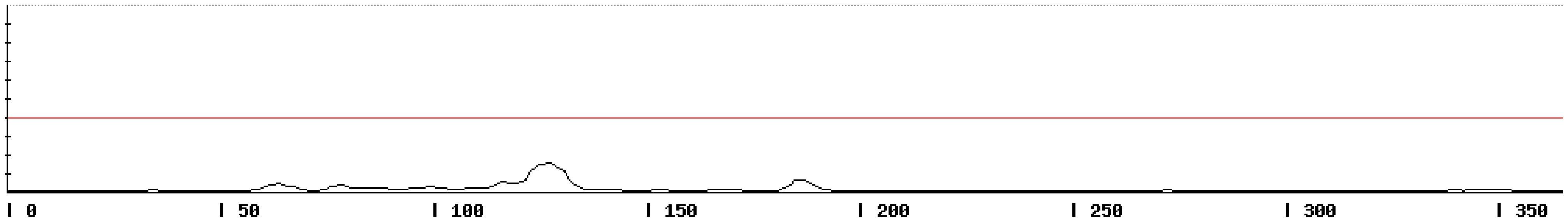

Predictions for Juglansregia_WALNUT_00000640-RA

Viterbi Path

Viterbi Path

Posterior @ 0.4

Posterior @ 0.4

Posterior Graphic. Threshold Marked @ 0.4

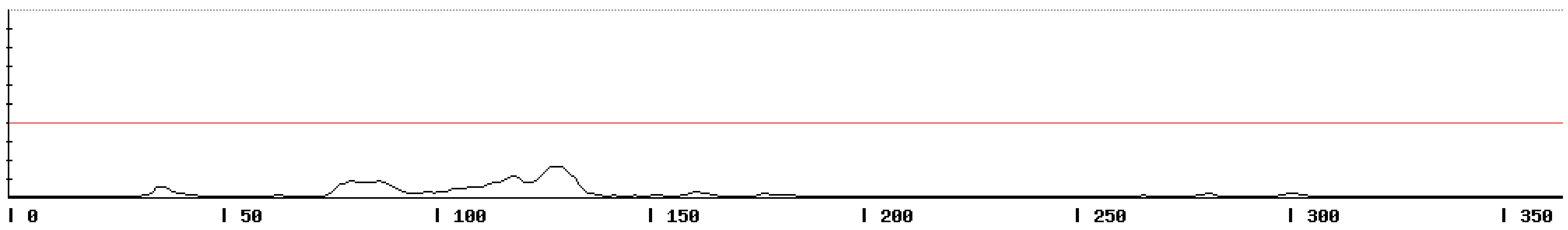

Predictions for Juglansregia_WALNUT_00000849-RA

Viterbi Path

Viterbi Path

Posterior @ 0.4

Posterior @ 0.4

Posterior Graphic. Threshold Marked @ 0.4

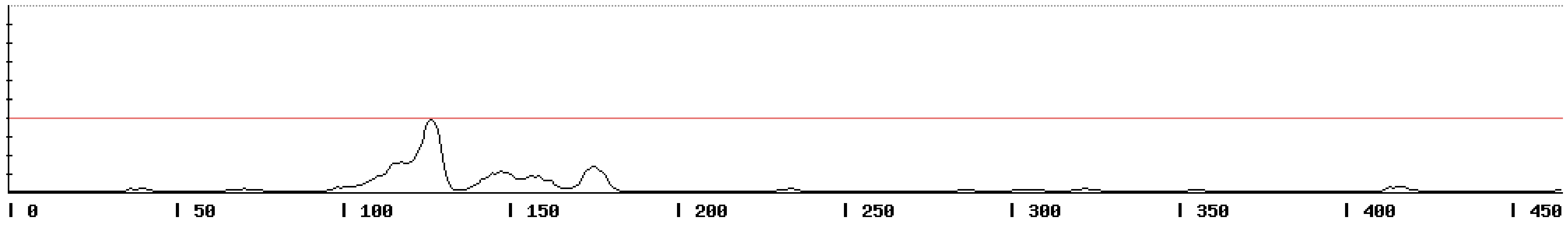

Predictions for Juglansregia_WALNUT_00001753-RA

Viterbi Path

Viterbi Path

Posterior @ 0.4

Posterior @ 0.4

Posterior Graphic. Threshold Marked @ 0.4

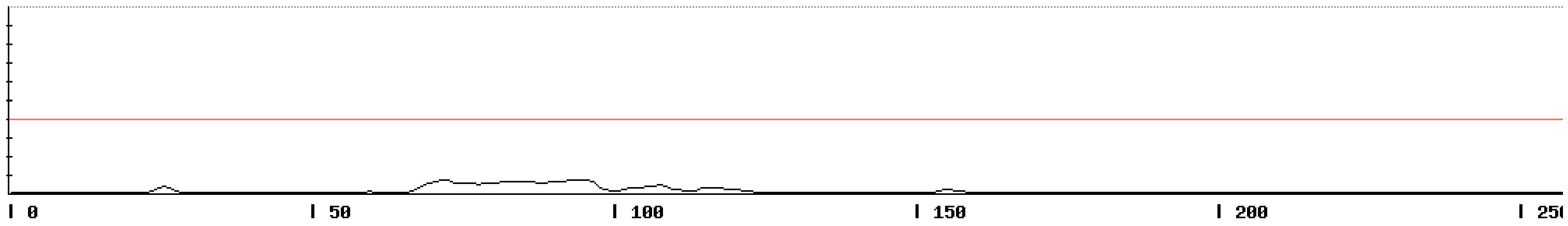

Predictions for Juglansregia_WALNUT_00001946-RA

Viterbi Path

Viterbi Path

Posterior @ 0.4

Posterior @ 0.4

Posterior Graphic. Threshold Marked @ 0.4

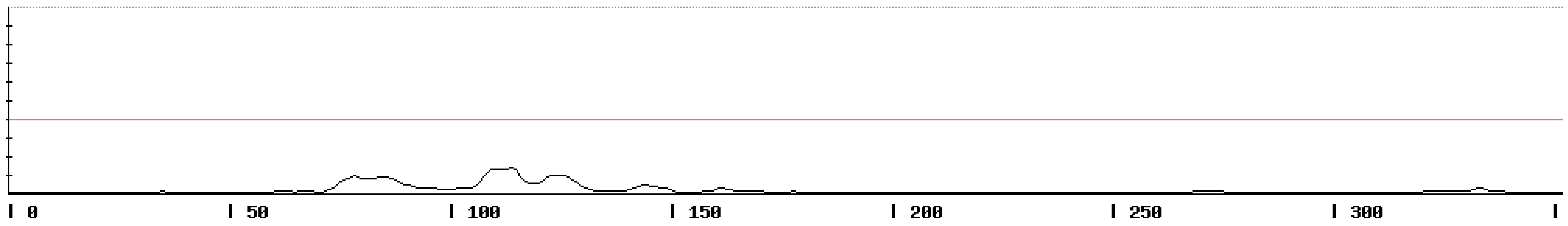

Predictions for Juglansregia_WALNUT_00001947-RA

Viterbi Path

Viterbi Path

Posterior @ 0.4

Posterior @ 0.4

Posterior Graphic. Threshold Marked @ 0.4

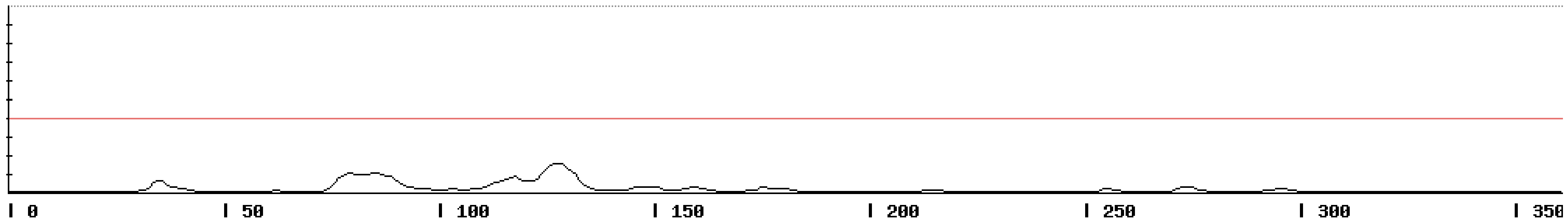

Predictions for Juglansregia_WALNUT_00003086-RA

Viterbi Path

Viterbi Path

Posterior @ 0.4

Posterior @ 0.4

Posterior Graphic. Threshold Marked @ 0.4

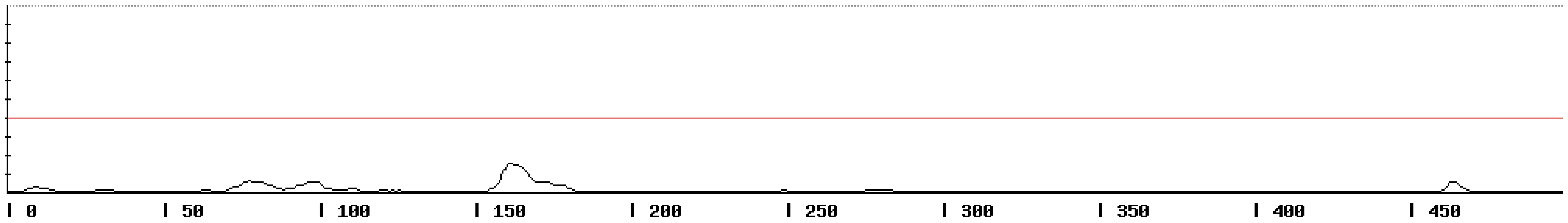

Predictions for Juglansregia_WALNUT_00003659-RA

Viterbi Path

Viterbi Path

Posterior @ 0.4

Posterior @ 0.4

Posterior Graphic. Threshold Marked @ 0.4

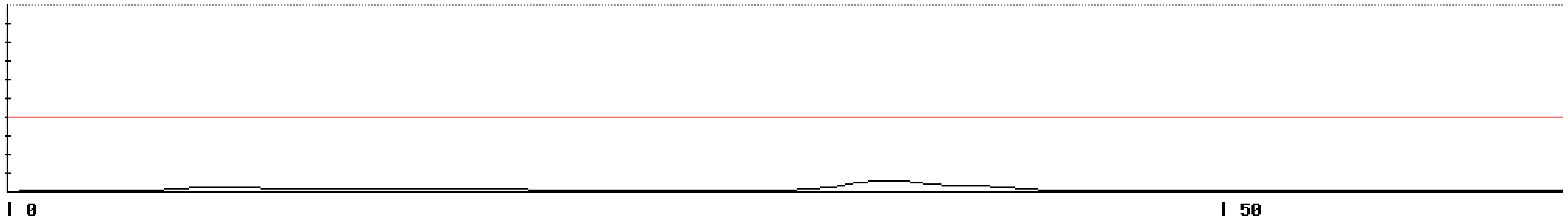

Predictions for Juglansregia_WALNUT_00003682-RA

Viterbi Path

Viterbi Path

Posterior @ 0.4	121 - PKG - 123
Posterior @ 0.4	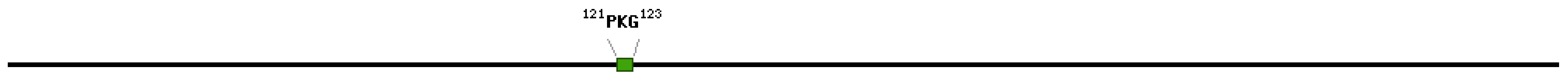
Posterior Graphic. Threshold Marked @ 0.4	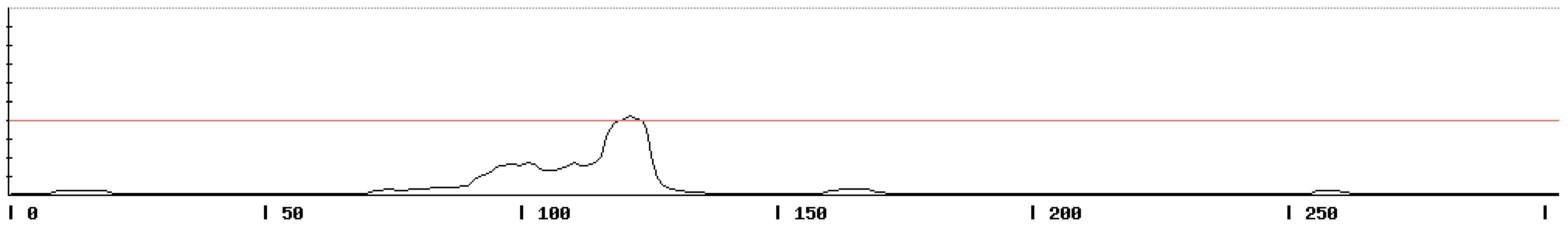

Predictions for Juglansregia_WALNUT_00003992-RA

Viterbi Path	
Viterbi Path	
Posterior @ 0.4	
Posterior @ 0.4	
Posterior Graphic. Threshold Marked @ 0.4	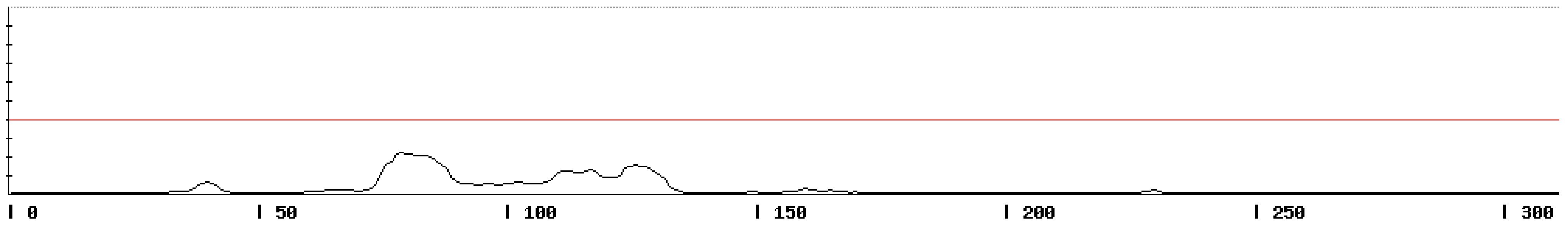

Predictions for Juglansregia_WALNUT_00004105-RA

Viterbi Path	
Viterbi Path	

Posterior @ 0.4

Posterior @ 0.4

Posterior Graphic. Threshold Marked @ 0.4

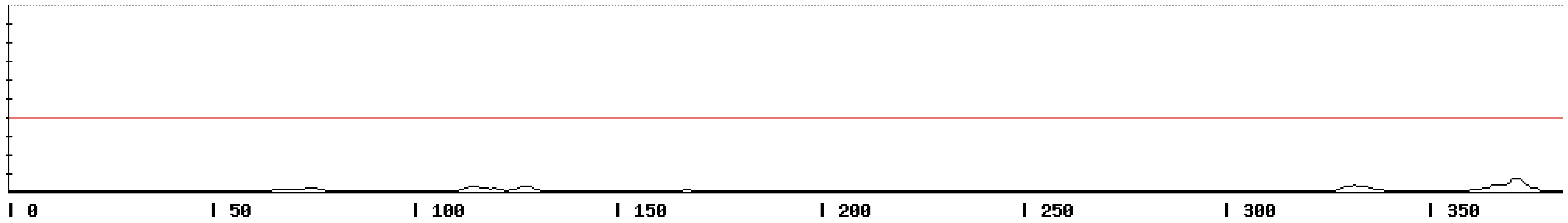

Predictions for Juglansregia_WALNUT_00004145-RA

Viterbi Path

Viterbi Path

Posterior @ 0.4

Posterior @ 0.4

Posterior Graphic. Threshold Marked @ 0.4

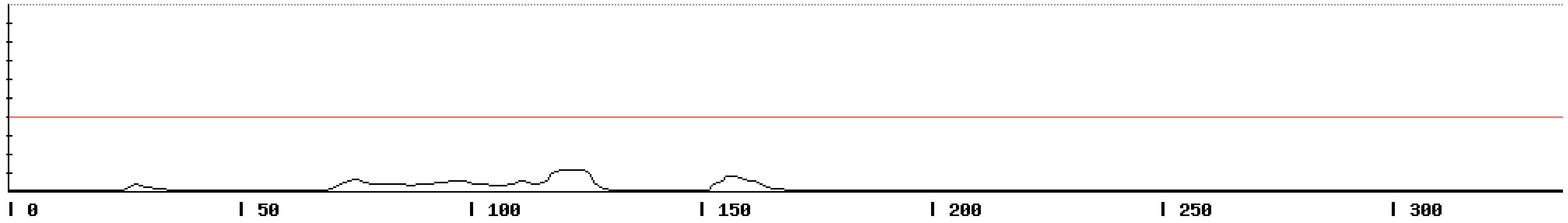

Predictions for Juglansregia_WALNUT_00004318-RA

Viterbi Path	
Viterbi Path	
Posterior @ 0.4	
Posterior @ 0.4	
Posterior Graphic. Threshold Marked @ 0.4	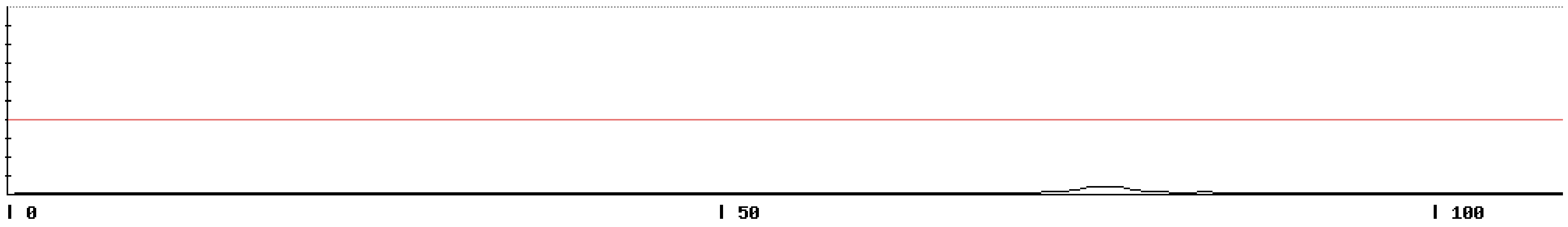

Predictions for Juglansregia_WALNUT_00004727-RA

Viterbi Path	
Viterbi Path	
Posterior @ 0.4	
Posterior @ 0.4	
Posterior Graphic. Threshold Marked @ 0.4	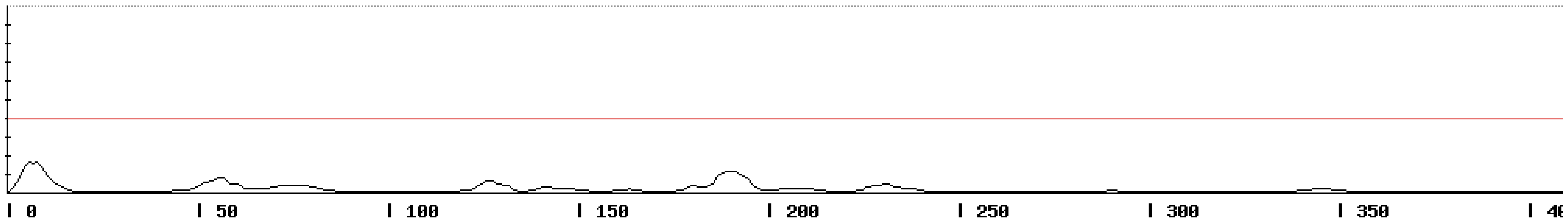

Predictions for Juglansregia_WALNUT_00005261-RA	
Viterbi Path	
Viterbi Path	
Posterior @ 0.4	
Posterior @ 0.4	
Posterior Graphic. Threshold Marked @ 0.4	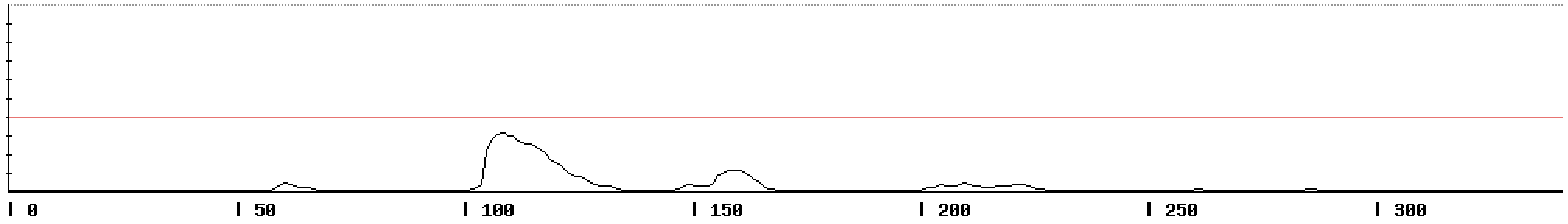

Predictions for Juglansregia_WALNUT_00006161-RA	
Viterbi Path	
Viterbi Path	
Posterior @ 0.4	158 - RKRRK - 162
Posterior @ 0.4	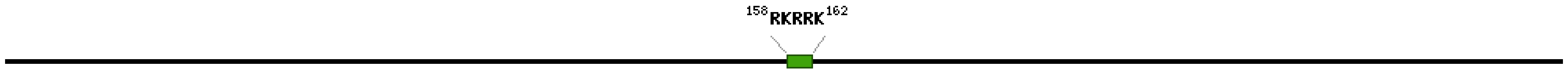
Posterior Graphic. Threshold Marked @ 0.4	

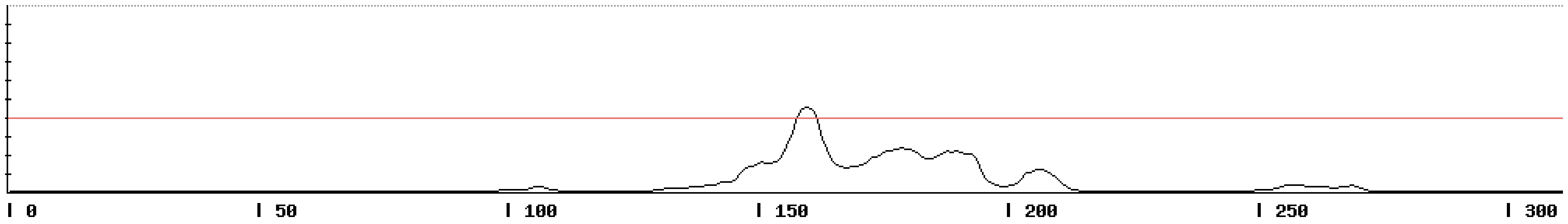

Predictions for Juglansregia_WALNUT_00006758-RA

Viterbi Path	
Viterbi Path	
Posterior @ 0.4	
Posterior @ 0.4	
Posterior Graphic. Threshold Marked @ 0.4	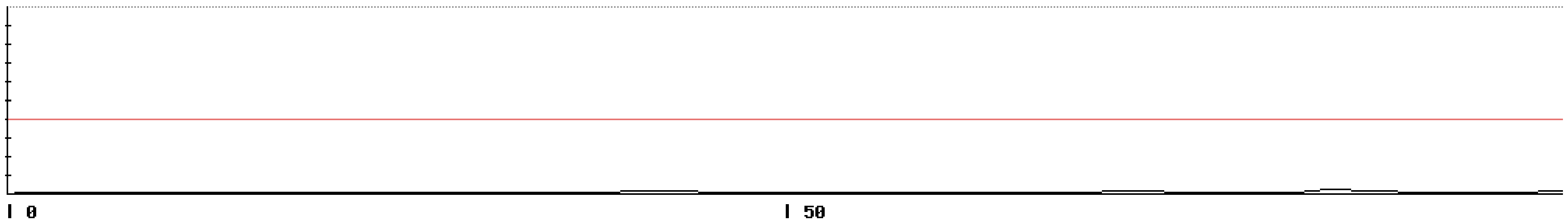

Predictions for Juglansregia_WALNUT_00006989-RA

Viterbi Path	
Viterbi Path	
Posterior @ 0.4	26 - KRKVRSRRFRPR - 37
Posterior @ 0.4	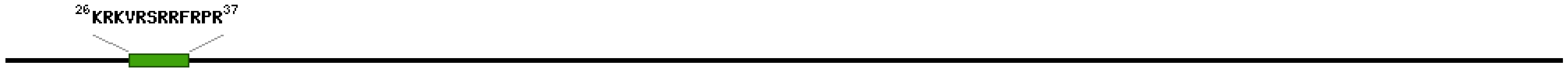

Posterior Graphic. Threshold Marked @ 0.4

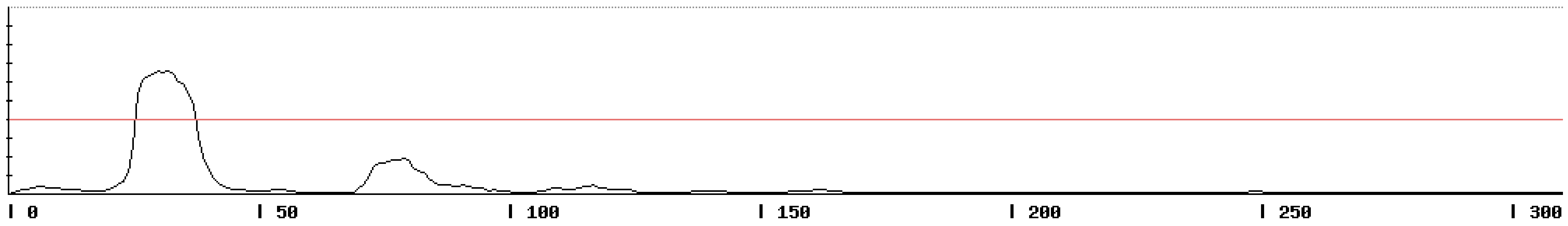

Predictions for Juglansregia_WALNUT_00007192-RA

Viterbi Path

Viterbi Path

Posterior @ 0.4

Posterior @ 0.4

Posterior Graphic. Threshold Marked @ 0.4

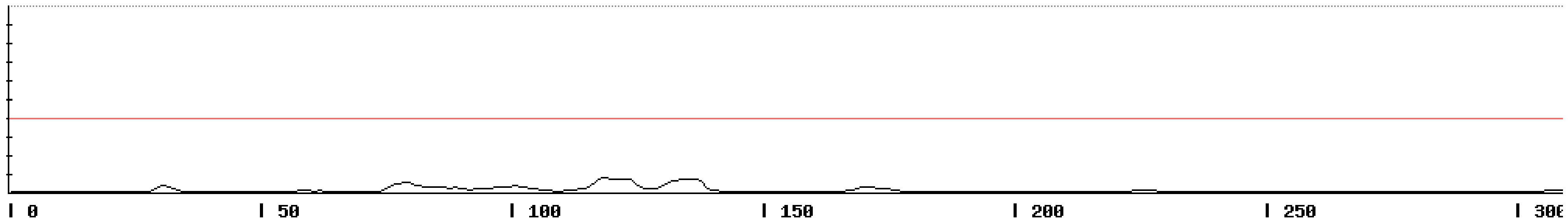

Predictions for Juglansregia_WALNUT_00007966-RA

Viterbi Path

Viterbi Path

Posterior @ 0.4

Posterior @ 0.4

Posterior Graphic. Threshold Marked @ 0.4

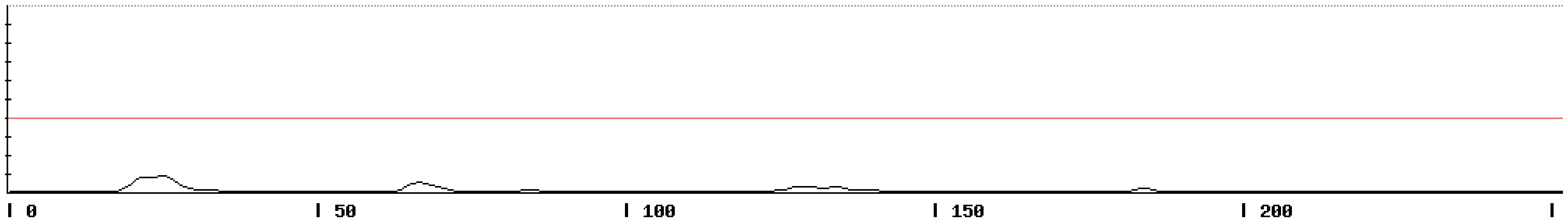

Predictions for Juglansregia_WALNUT_00008006-RA

Viterbi Path

Viterbi Path

Posterior @ 0.4 170 - KKR - 172

Posterior @ 0.4

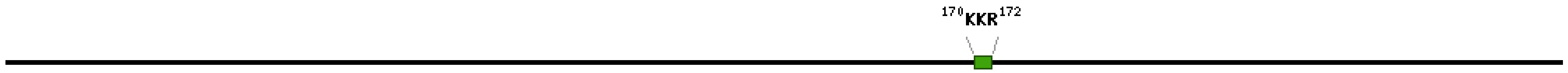

Posterior Graphic. Threshold Marked @ 0.4

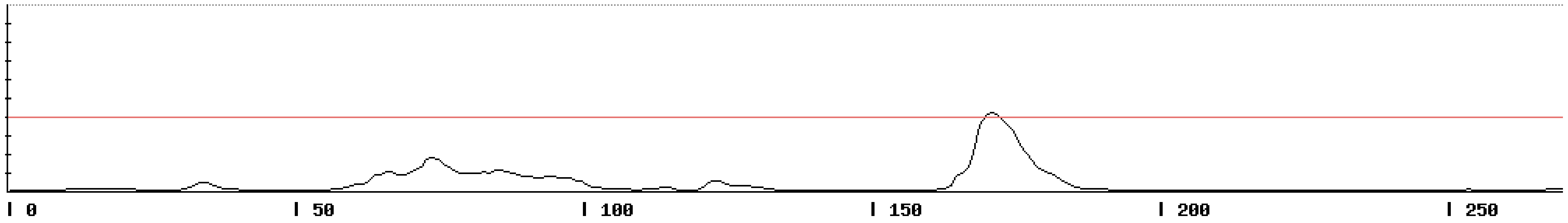

Predictions for Juglansregia_WALNUT_00008063-RA

Viterbi Path

Viterbi Path

Posterior @ 0.4

Posterior @ 0.4

Posterior Graphic. Threshold Marked @ 0.4

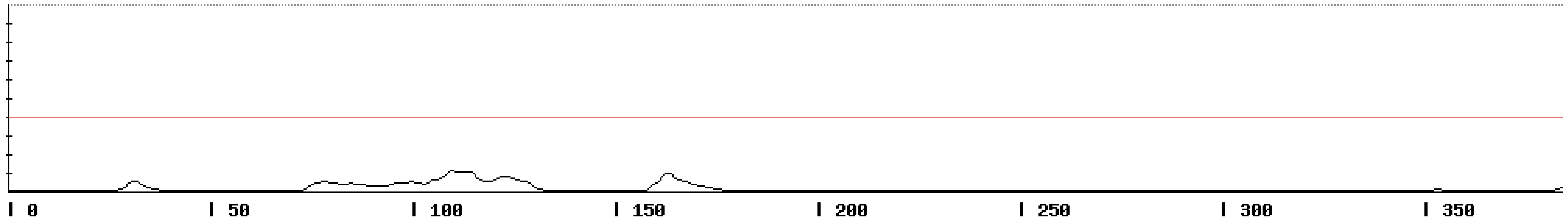

Predictions for Juglansregia_WALNUT_00008208-RA

Viterbi Path

Viterbi Path

Posterior @ 0.4

Posterior @ 0.4

Posterior Graphic. Threshold Marked @ 0.4

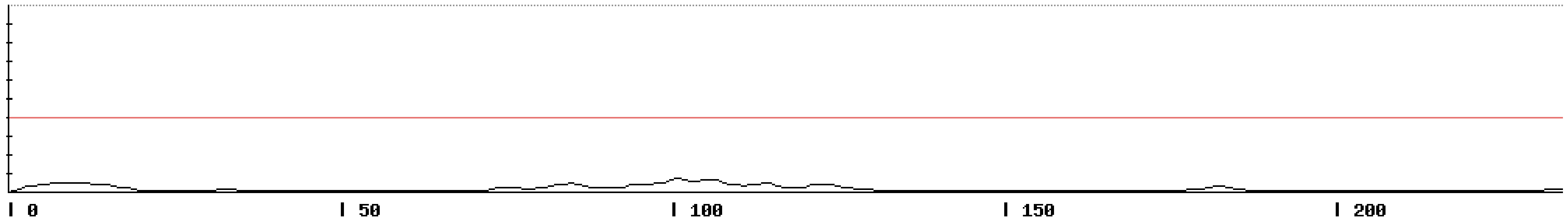

Predictions for Juglansregia_WALNUT_00009467-RA

Viterbi Path

Viterbi Path

Posterior @ 0.4

Posterior @ 0.4

Posterior Graphic. Threshold Marked @ 0.4

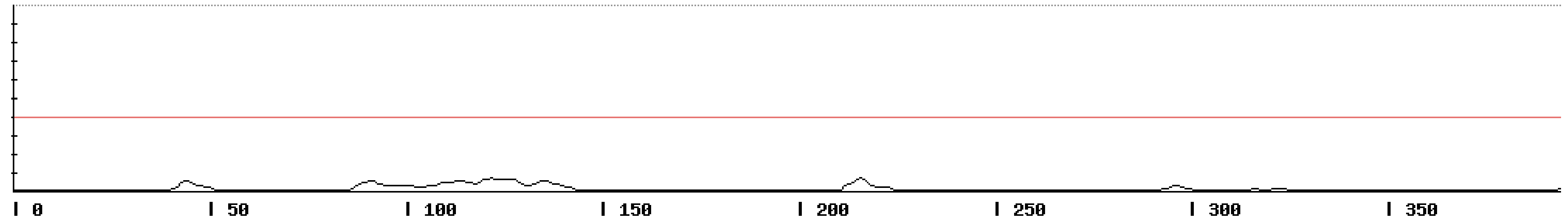

Predictions for Juglansregia_WALNUT_00009590-RA

Viterbi Path

Viterbi Path

Posterior @ 0.4

Posterior @ 0.4

Posterior Graphic. Threshold Marked @ 0.4

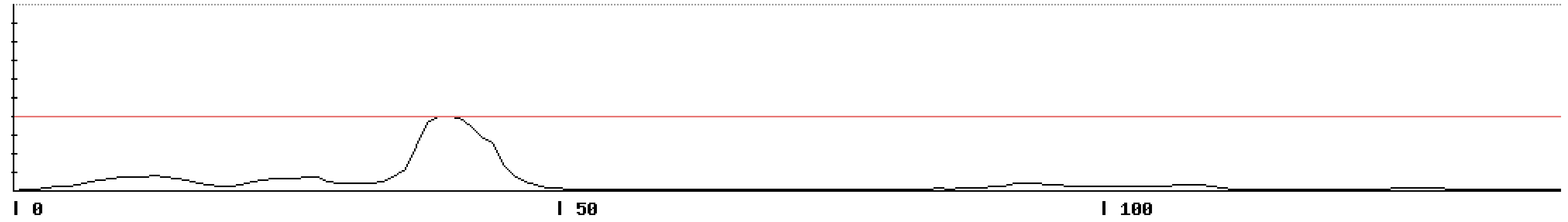

Predictions for Juglansregia_WALNUT_00009593-RA

Viterbi Path	
Viterbi Path	
Posterior @ 0.4	132 - R - 132
	178 - R - 178
Posterior @ 0.4	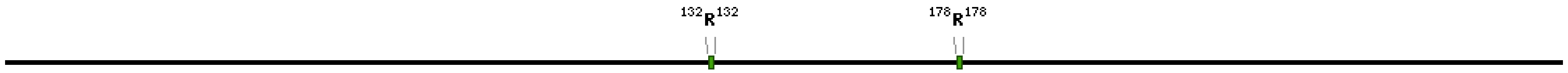
Posterior Graphic. Threshold Marked @ 0.4	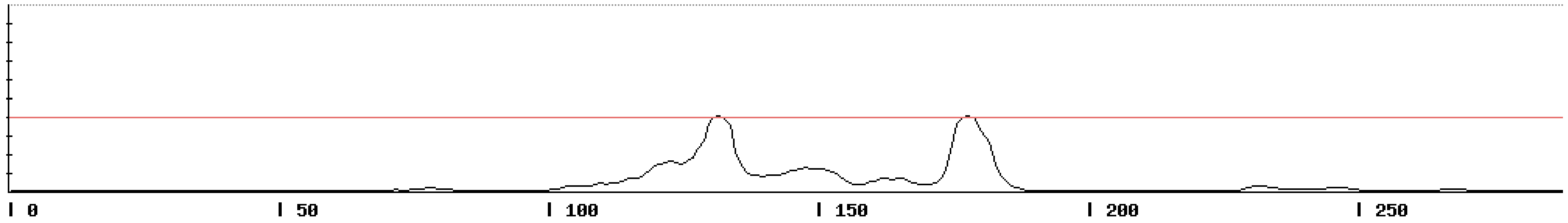

Predictions for Juglansregia_WALNUT_00010348-RA

Viterbi Path	
Viterbi Path	
Posterior @ 0.4	368 - RANSRIKPRMKKAR - 381
Posterior @ 0.4	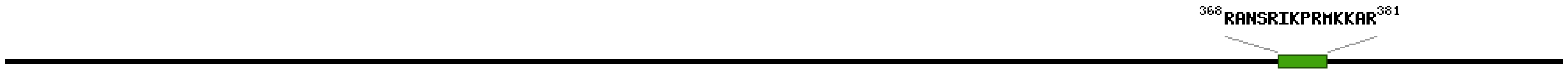
Posterior Graphic. Threshold Marked @ 0.4	

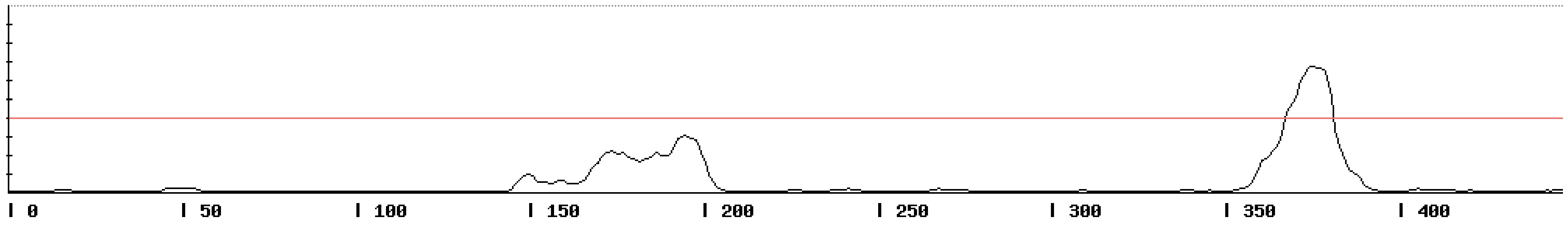

Predictions for Juglansregia_WALNUT_00010992-RA

Viterbi Path	
Viterbi Path	
Posterior @ 0.4	
Posterior @ 0.4	
Posterior Graphic. Threshold Marked @ 0.4	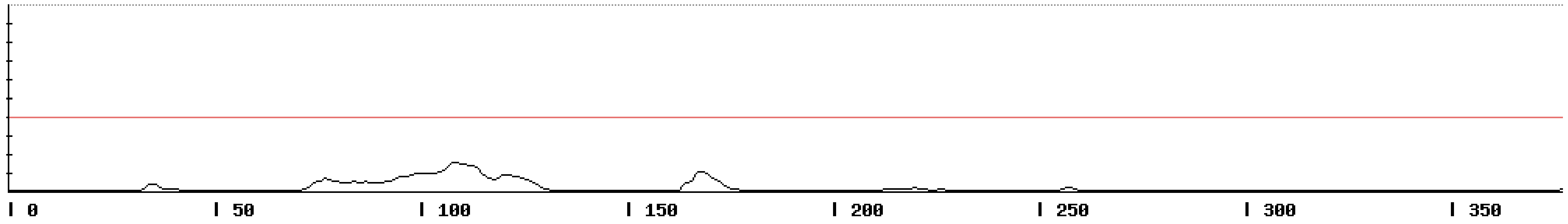

Predictions for Juglansregia_WALNUT_00011679-RA

Viterbi Path	
Viterbi Path	
Posterior @ 0.4	
Posterior @ 0.4	

Posterior Graphic. Threshold Marked @ 0.4

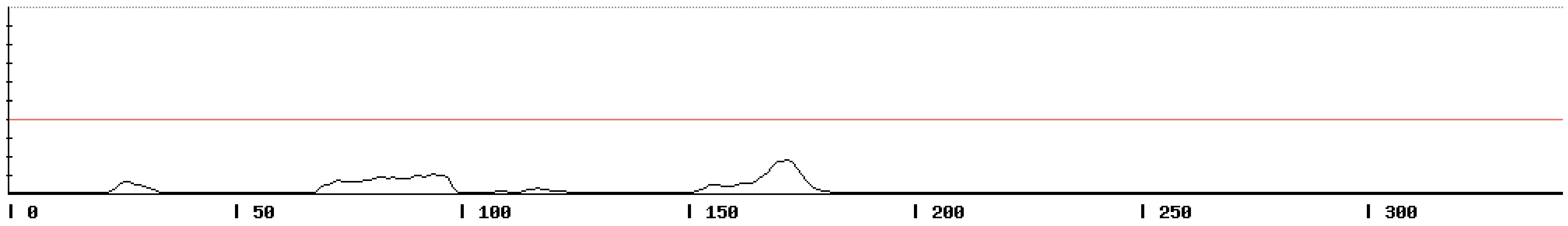

Predictions for Juglansregia_WALNUT_00012256-RA

Viterbi Path

Viterbi Path

Posterior @ 0.4

Posterior @ 0.4

Posterior Graphic. Threshold Marked @ 0.4

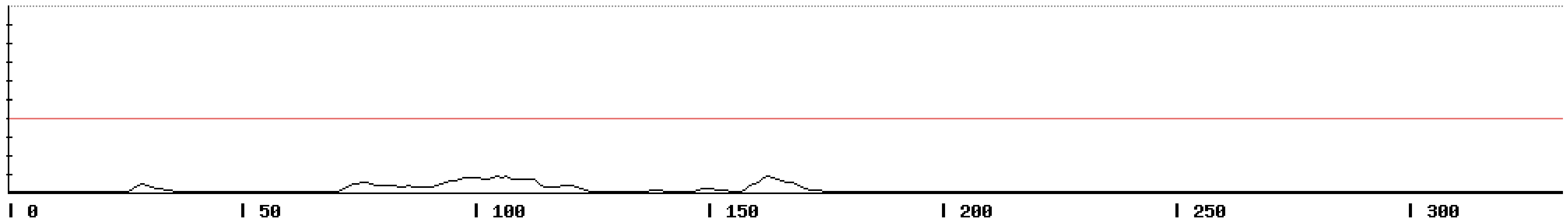

Predictions for Juglansregia_WALNUT_00013110-RA

Viterbi Path

Viterbi Path

Posterior @ 0.4

Posterior @ 0.4

Posterior Graphic. Threshold Marked @ 0.4

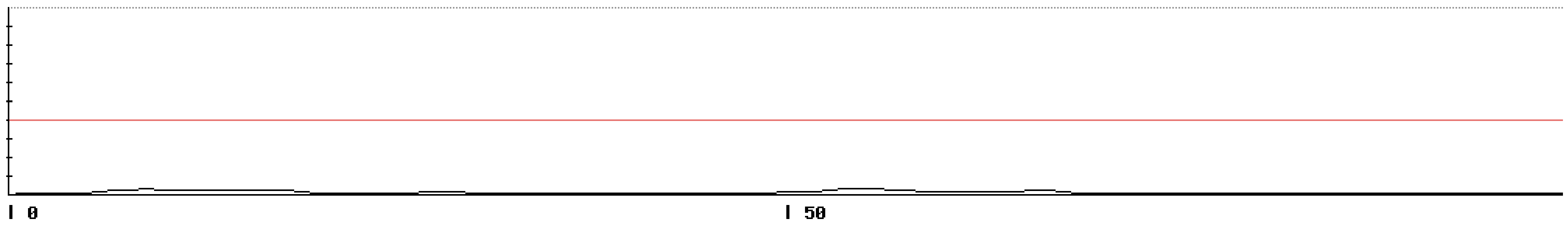

Predictions for Juglansregia_WALNUT_00013111-RA

Viterbi Path

Viterbi Path

Posterior @ 0.4

Posterior @ 0.4

Posterior Graphic. Threshold Marked @ 0.4

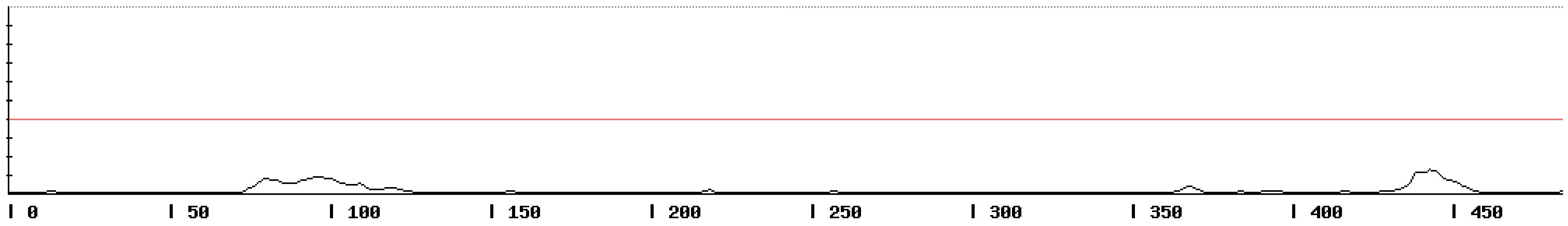

Predictions for Juglansregia_WALNUT_00013243-RA

Viterbi Path

Viterbi Path

Posterior @ 0.4

Posterior @ 0.4

Posterior Graphic. Threshold Marked @ 0.4

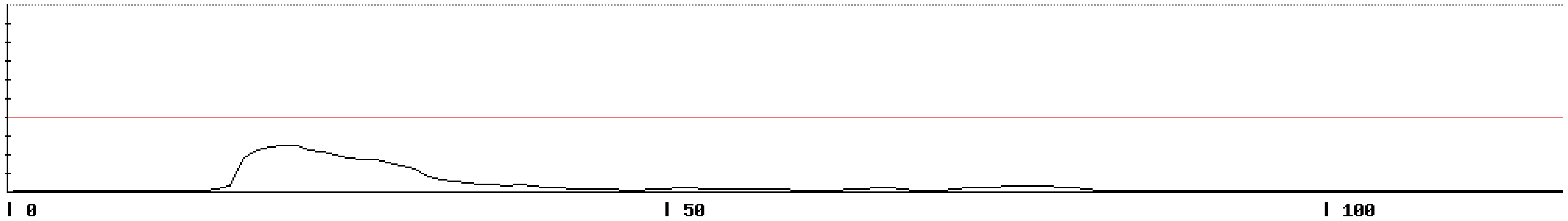

Predictions for Juglansregia_WALNUT_00013375-RA

Viterbi Path

Viterbi Path

Posterior @ 0.4

Posterior @ 0.4

Posterior Graphic. Threshold Marked @ 0.4

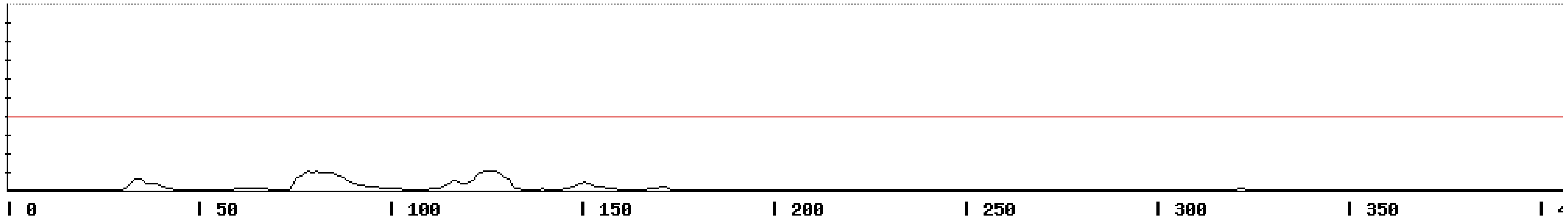

Predictions for Juglansregia_WALNUT_00013406-RA

Viterbi Path

Viterbi Path

Posterior @ 0.4	88 - RKYRNGRRPNR - 98
Posterior @ 0.4	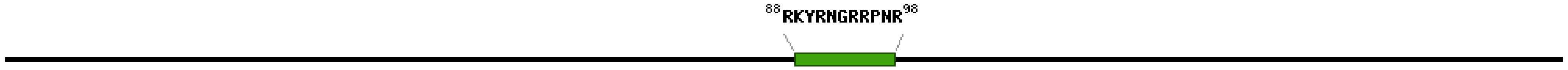
Posterior Graphic. Threshold Marked @ 0.4	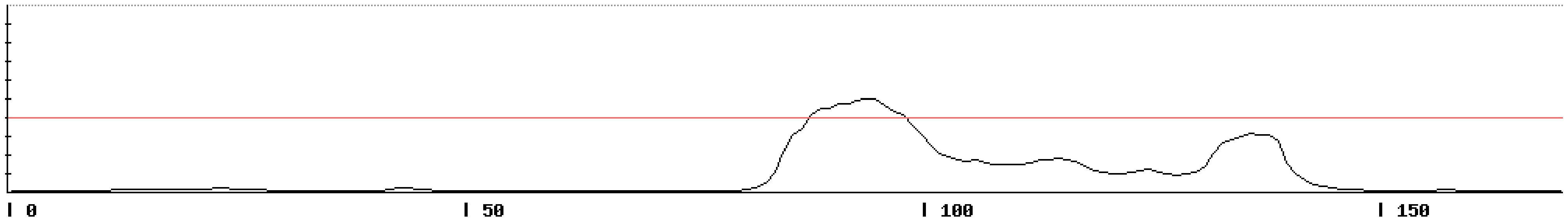

Predictions for Juglansregia_WALNUT_00013569-RA

Viterbi Path	
Viterbi Path	
Posterior @ 0.4	
Posterior @ 0.4	
Posterior Graphic. Threshold Marked @ 0.4	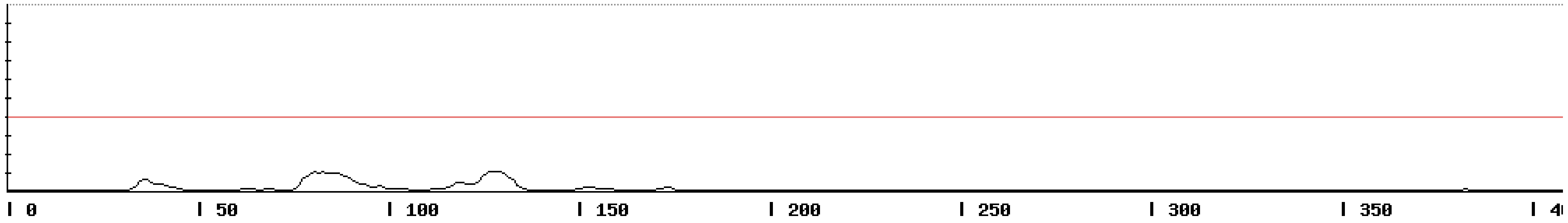

Predictions for Juglansregia_WALNUT_00013589-RA

Viterbi Path

Viterbi Path

Posterior @ 0.4

Posterior @ 0.4

Posterior Graphic. Threshold Marked @ 0.4

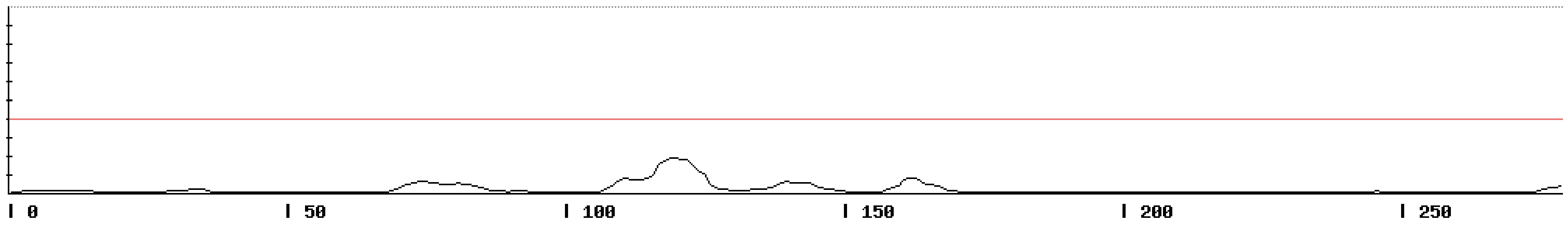

Predictions for Juglansregia_WALNUT_00013875-RA

Viterbi Path

Viterbi Path

Posterior @ 0.4

141 - KR - 142

208 - RKVRNKEPKGRYLGTPRLGKRNPR - 231

Posterior @ 0.4

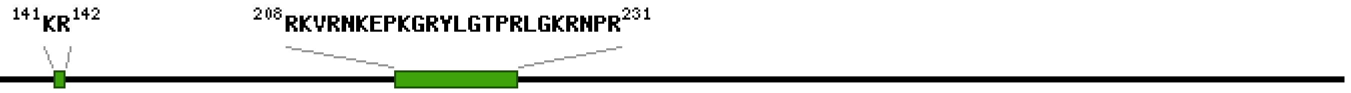

Posterior Graphic. Threshold Marked @ 0.4

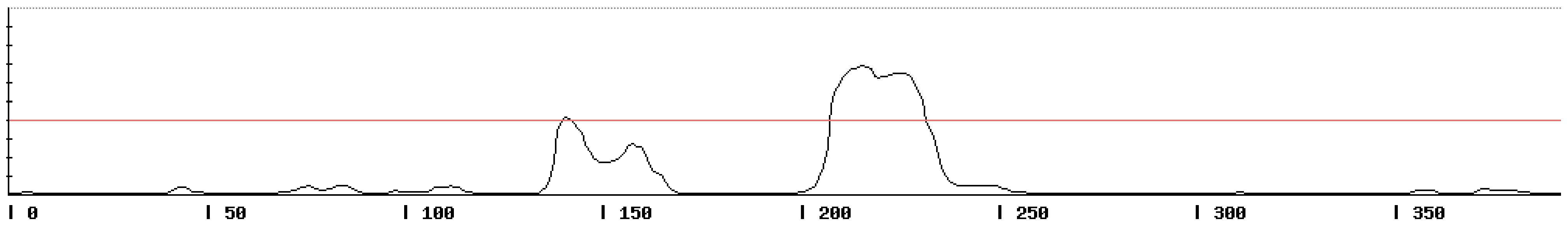

Predictions for Juglansregia_WALNUT_00014048-RA	
Viterbi Path	
Viterbi Path	
Posterior @ 0.4	
Posterior @ 0.4	
Posterior Graphic. Threshold Marked @ 0.4	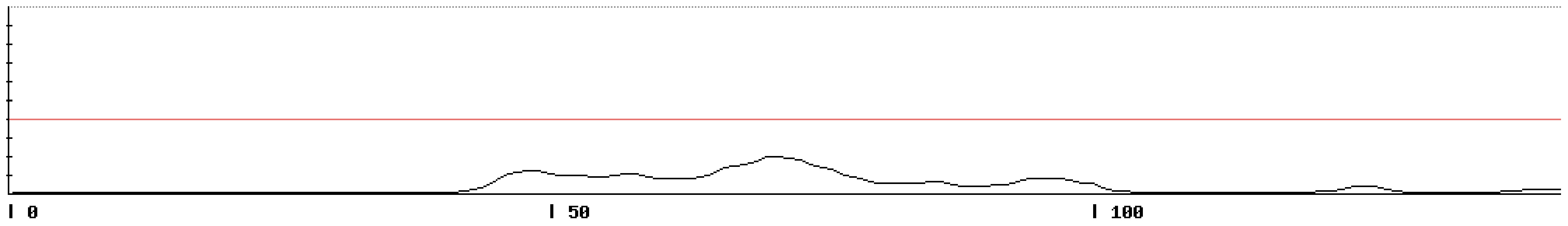

Predictions for Juglansregia_WALNUT_00014050-RA	
Viterbi Path	
Viterbi Path	
Posterior @ 0.4	269 - KLRLKEAK - 276
Posterior @ 0.4	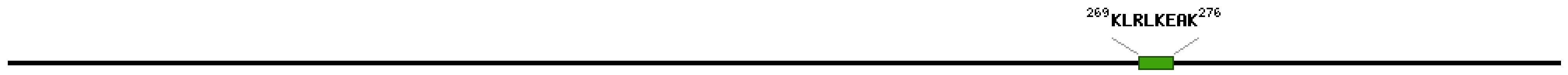

Posterior Graphic. Threshold Marked @ 0.4	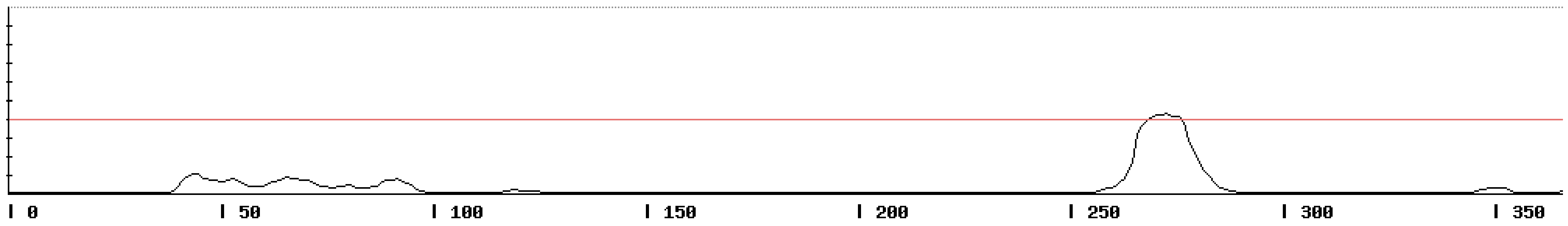

Predictions for Juglansregia_WALNUT_00014062-RA	
Viterbi Path	
Viterbi Path	
Posterior @ 0.4	
Posterior @ 0.4	
Posterior Graphic. Threshold Marked @ 0.4	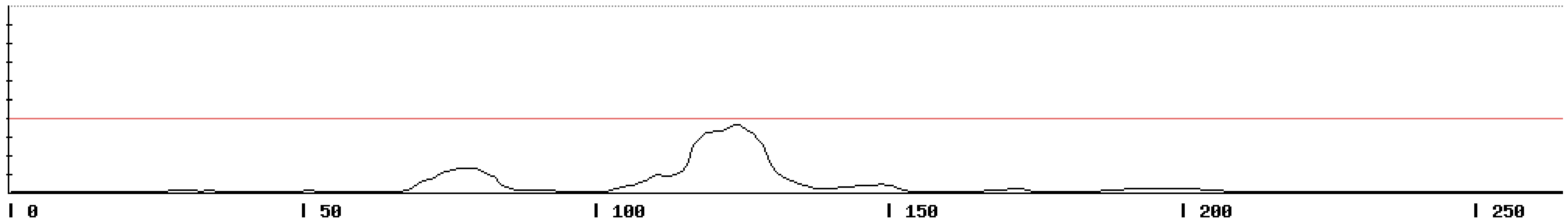

Predictions for Juglansregia_WALNUT_00014993-RA	
Viterbi Path	
Viterbi Path	
Posterior @ 0.4	88 - ATGNDRKIKAKRTNKVIGKKK - 108
Posterior @ 0.4	

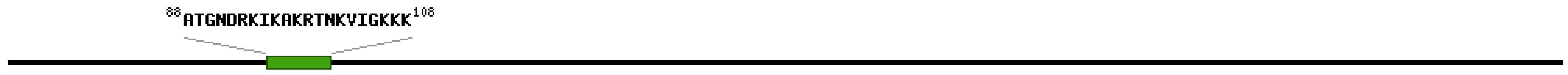

Posterior Graphic. Threshold Marked @ 0.4

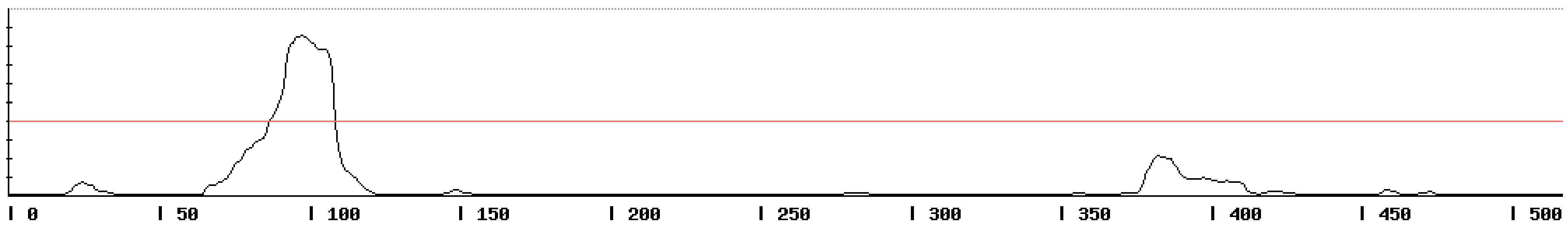

Predictions for Juglansregia_WALNUT_00015576-RA

Viterbi Path

Viterbi Path

Posterior @ 0.4

Posterior @ 0.4

Posterior Graphic. Threshold Marked @ 0.4

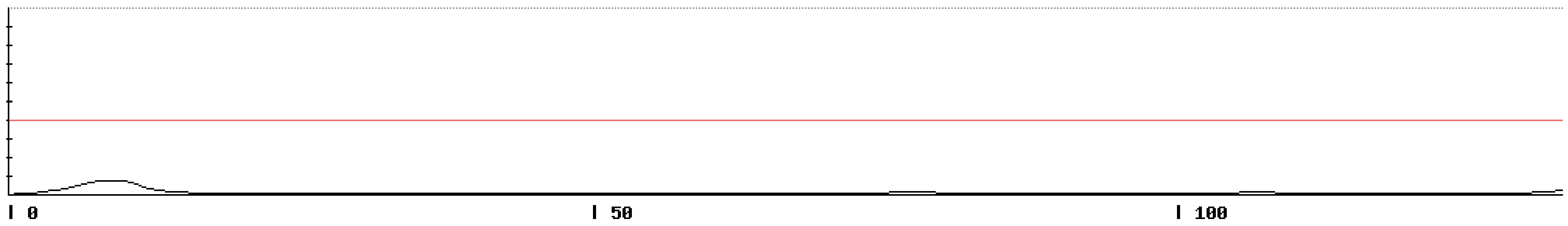

Predictions for Juglansregia_WALNUT_00015577-RA

Viterbi Path

Viterbi Path

Posterior @ 0.4	30 - RKYRNGRRPNRA - 41
Posterior @ 0.4	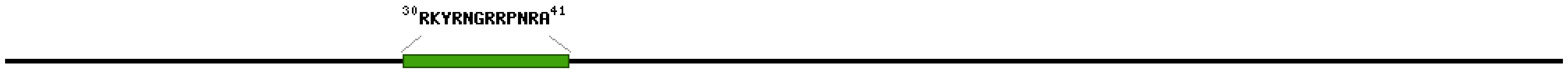
Posterior Graphic. Threshold Marked @ 0.4	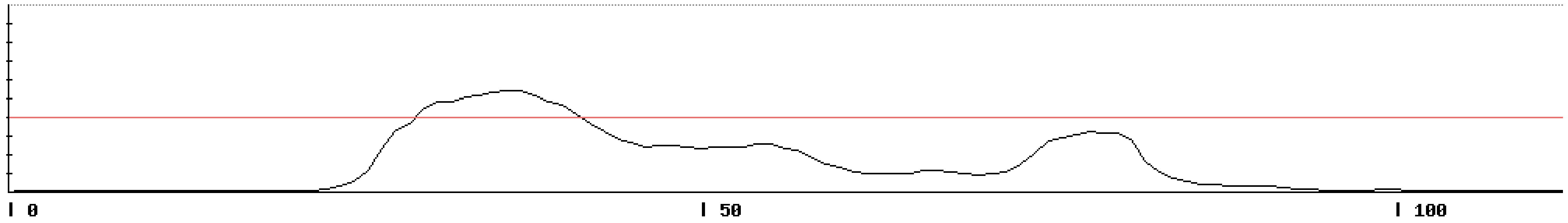

Predictions for Juglansregia_WALNUT_00015965-RA

Viterbi Path	
Viterbi Path	
Posterior @ 0.4	136 - RKRRK - 140
Posterior @ 0.4	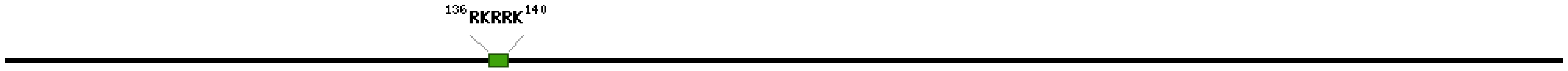
Posterior Graphic. Threshold Marked @ 0.4	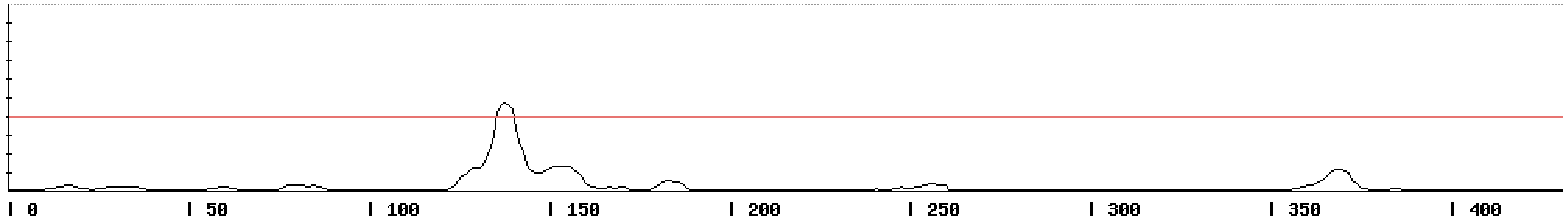

Predictions for Juglansregia_WALNUT_00016655-RA

Viterbi Path	
Viterbi Path	

Posterior @ 0.4

Posterior @ 0.4

Posterior Graphic. Threshold Marked @ 0.4

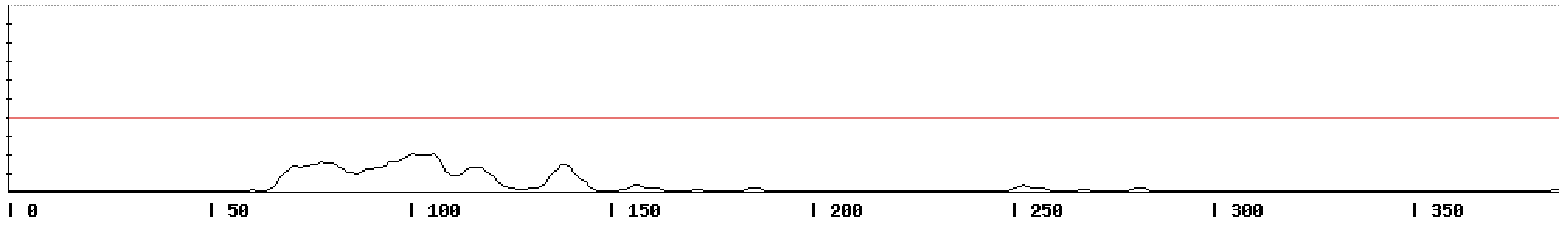

Predictions for Juglansregia_WALNUT_00016841-RA

Viterbi Path

Viterbi Path

Posterior @ 0.4

Posterior @ 0.4

Posterior Graphic. Threshold Marked @ 0.4

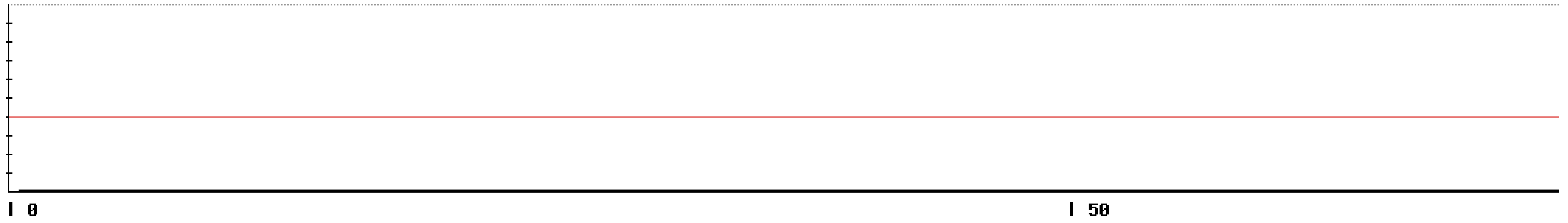

Predictions for Juglansregia_WALNUT_00017141-RA

Viterbi Path

Viterbi Path

Posterior @ 0.4

Posterior @ 0.4

Posterior Graphic. Threshold Marked @ 0.4

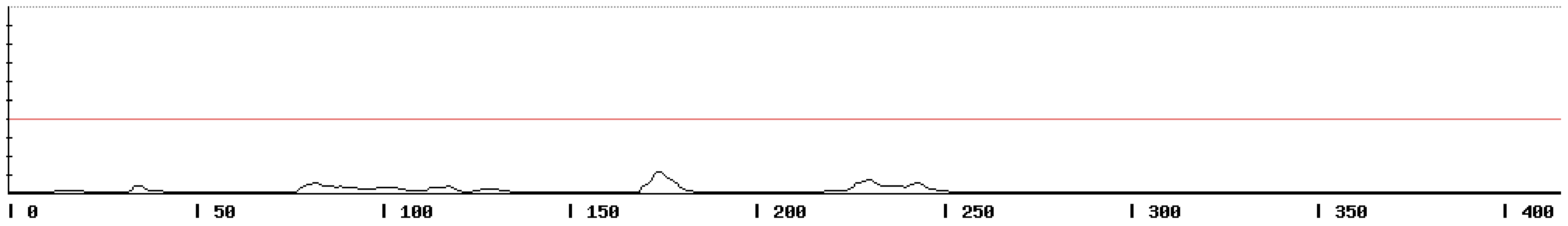

Predictions for Juglansregia_WALNUT_00017195-RA

Viterbi Path

Viterbi Path

Posterior @ 0.4

Posterior @ 0.4

Posterior Graphic. Threshold Marked @ 0.4

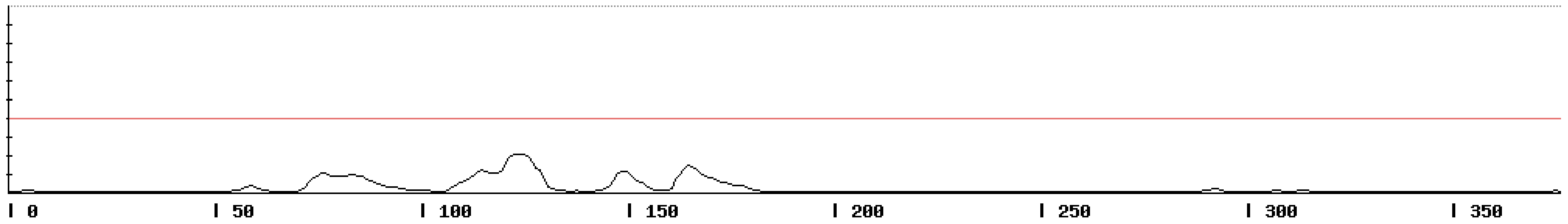

Predictions for Juglansregia_WALNUT_00017585-RA

Viterbi Path	
Viterbi Path	
Posterior @ 0.4	
Posterior @ 0.4	
Posterior Graphic. Threshold Marked @ 0.4	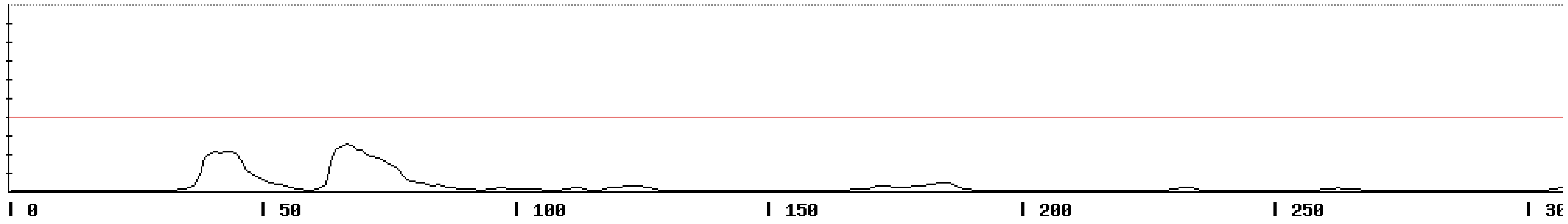

Predictions for Juglansregia_WALNUT_00017609-RA

Viterbi Path	
Viterbi Path	
Posterior @ 0.4	105 - VKESRGGRRKTKSGFWKRG - 123
Posterior @ 0.4	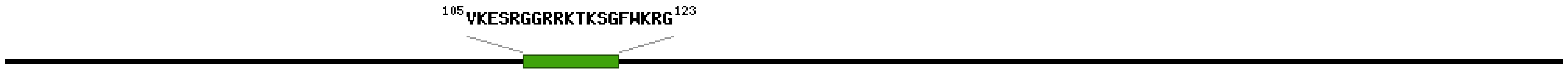
Posterior Graphic. Threshold Marked @ 0.4	

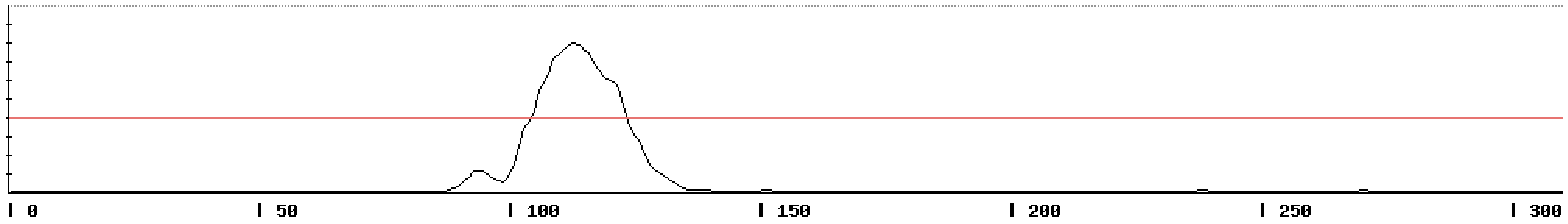

Predictions for Juglansregia_WALNUT_00018397-RA

Viterbi Path

Viterbi Path

Posterior @ 0.4

Posterior @ 0.4

Posterior Graphic. Threshold Marked @ 0.4

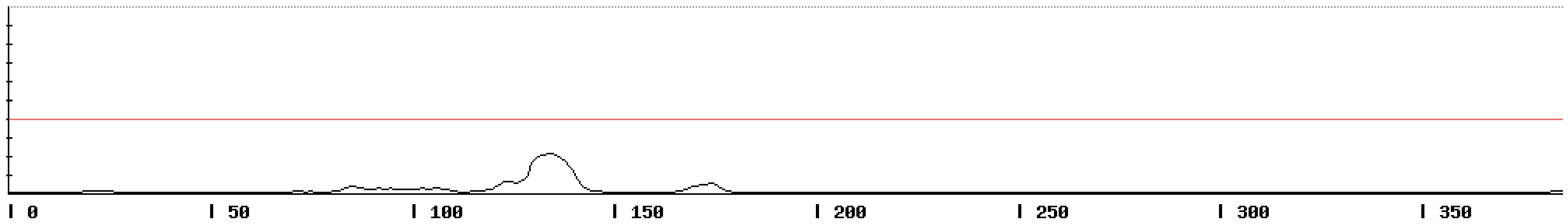

Predictions for Juglansregia_WALNUT_00018866-RA

Viterbi Path

Viterbi Path

Posterior @ 0.4

Posterior @ 0.4

Posterior Graphic. Threshold Marked @ 0.4

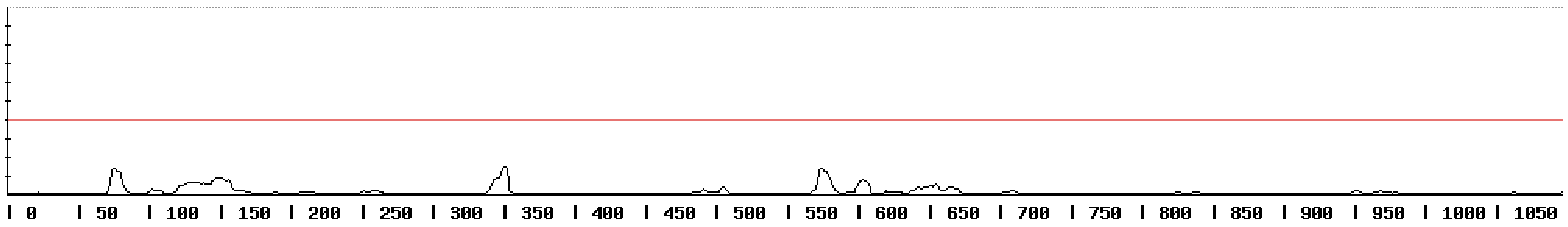

Predictions for Juglansregia_WALNUT_00019140-RA

Viterbi Path

Viterbi Path

Posterior @ 0.4

90 - ATGADKPIGKPKPLGIKKA - 108

Posterior @ 0.4

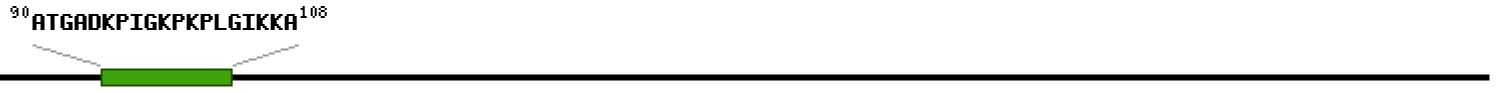

Posterior Graphic. Threshold Marked @ 0.4

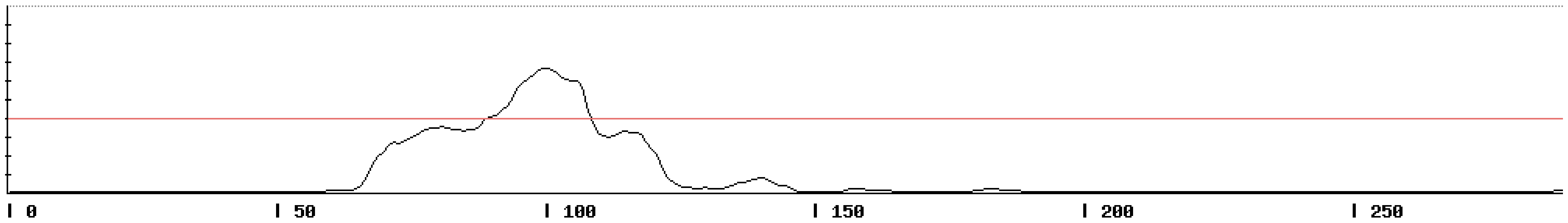

Predictions for Juglansregia_WALNUT_00019177-RA

Viterbi Path

Viterbi Path

Posterior @ 0.4

Posterior @ 0.4

Posterior Graphic. Threshold Marked @ 0.4

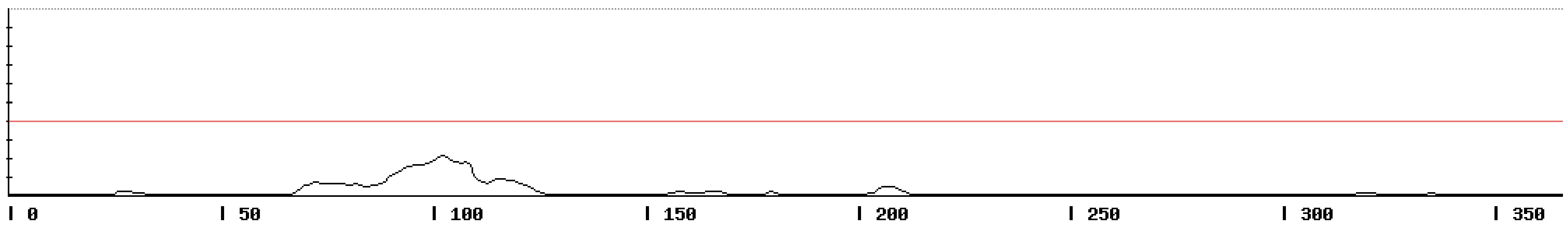

Predictions for Juglansregia_WALNUT_00019245-RA

Viterbi Path

Viterbi Path

Posterior @ 0.4

Posterior @ 0.4

Posterior Graphic. Threshold Marked @ 0.4

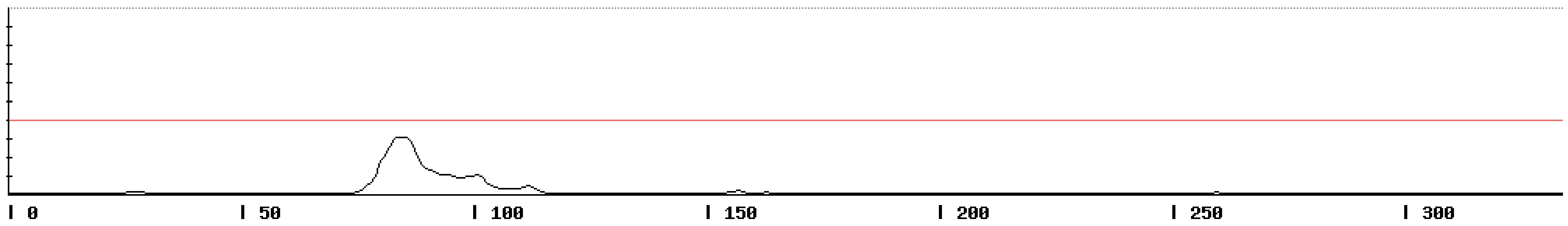

Predictions for Juglansregia_WALNUT_00019247-RA

Viterbi Path

Viterbi Path

Posterior @ 0.4

Posterior @ 0.4

Posterior Graphic. Threshold Marked @ 0.4

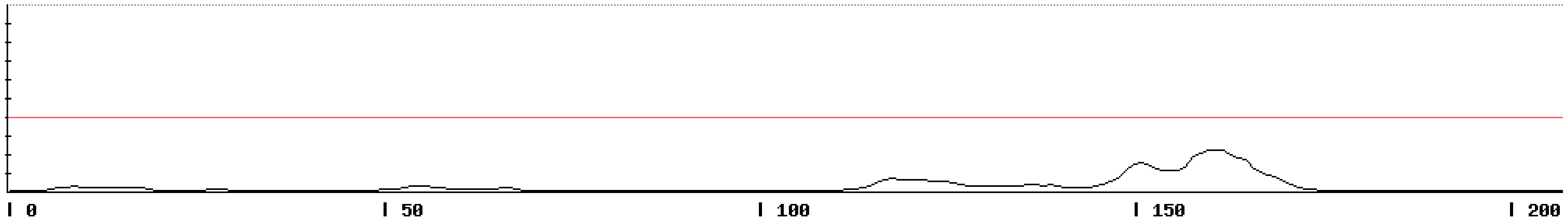

Predictions for Juglansregia_WALNUT_00019442-RA

Viterbi Path

Viterbi Path

Posterior @ 0.4

Posterior @ 0.4

Posterior Graphic. Threshold Marked @ 0.4

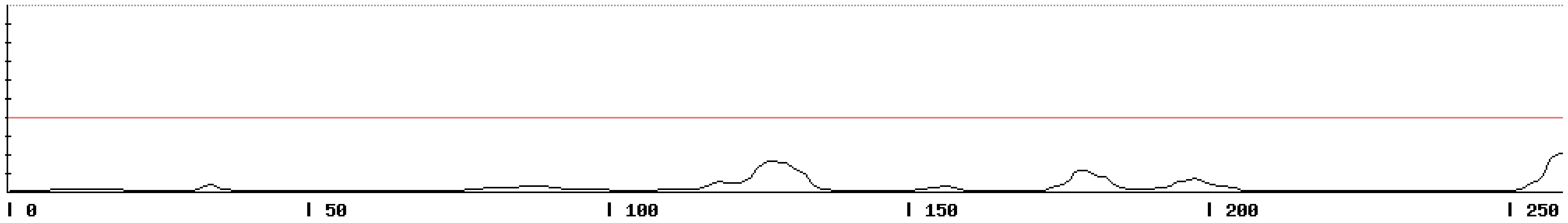

Predictions for Juglansregia_WALNUT_00019490-RA

Viterbi Path

Viterbi Path

Posterior @ 0.4

6 - KQRRGR - 11

Posterior @ 0.4

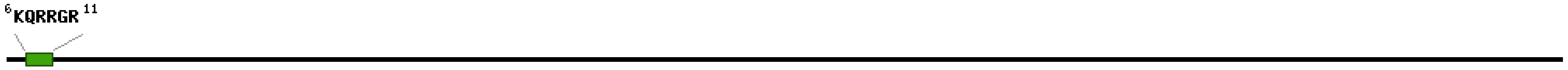

Posterior Graphic. Threshold Marked @ 0.4

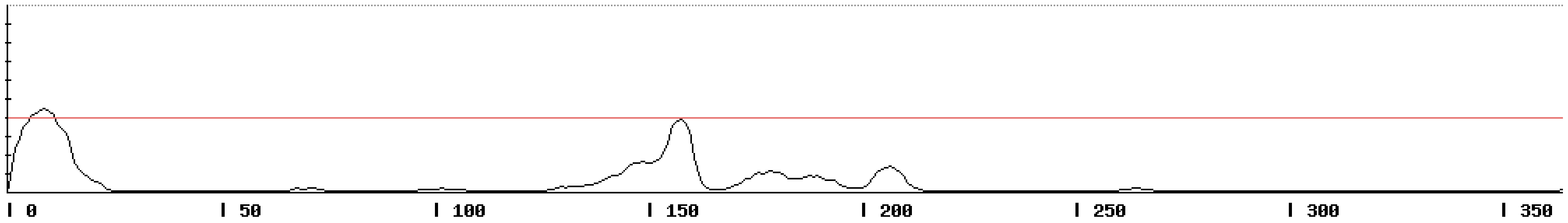

Predictions for Juglansregia_WALNUT_00019511-RA

Viterbi Path

Viterbi Path

Posterior @ 0.4

Posterior @ 0.4

Posterior Graphic. Threshold Marked @ 0.4

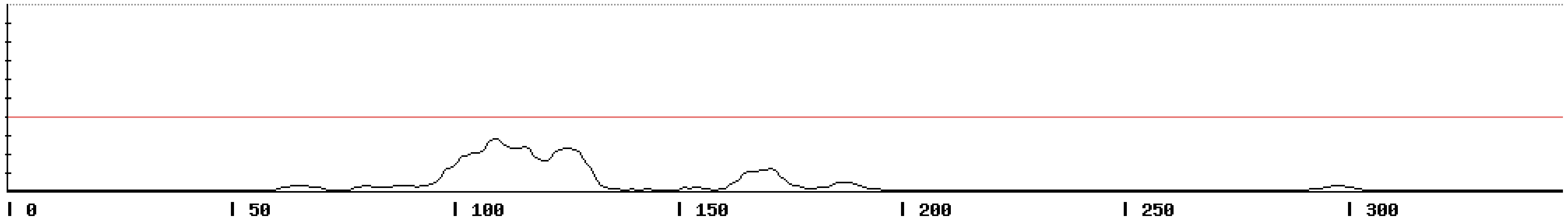

Predictions for Juglansregia_WALNUT_00019759-RA

Viterbi Path	
Viterbi Path	
Posterior @ 0.4	
Posterior @ 0.4	
Posterior Graphic. Threshold Marked @ 0.4	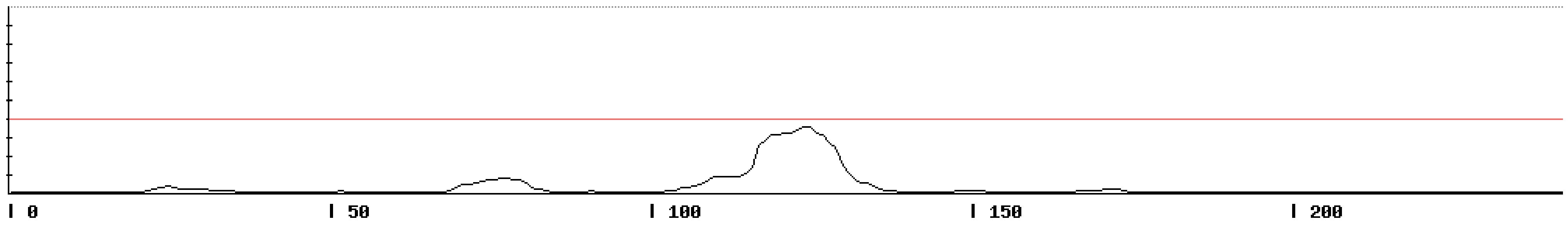

Predictions for Juglansregia_WALNUT_00019810-RA

Viterbi Path	
Viterbi Path	
Posterior @ 0.4	35 - KGKRKAKGKG - 44
Posterior @ 0.4	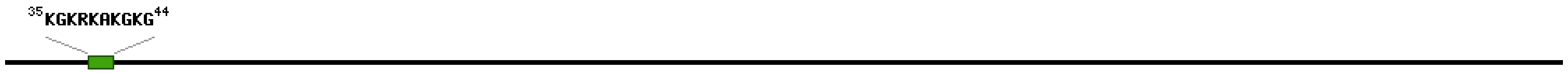
Posterior Graphic. Threshold Marked @ 0.4	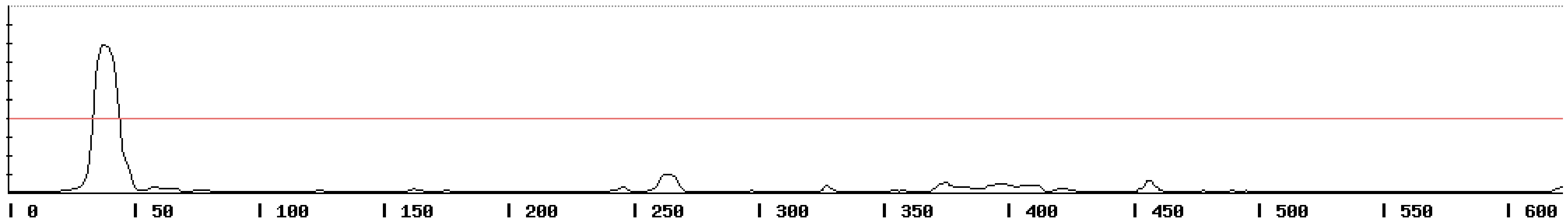

Predictions for Juglansregia_WALNUT_00021384-RA

Viterbi Path	
Viterbi Path	
Posterior @ 0.4	
Posterior @ 0.4	
Posterior Graphic. Threshold Marked @ 0.4	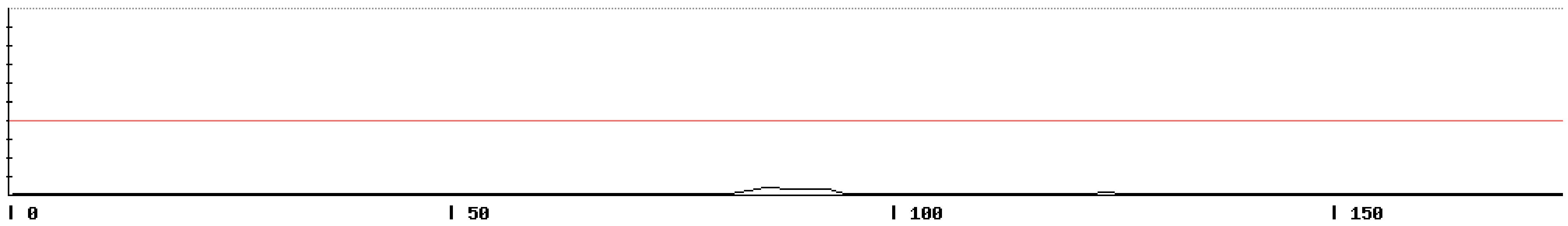

Predictions for Juglansregia_WALNUT_00021799-RA

Viterbi Path	
Viterbi Path	
Posterior @ 0.4	
Posterior @ 0.4	
Posterior Graphic. Threshold Marked @ 0.4	

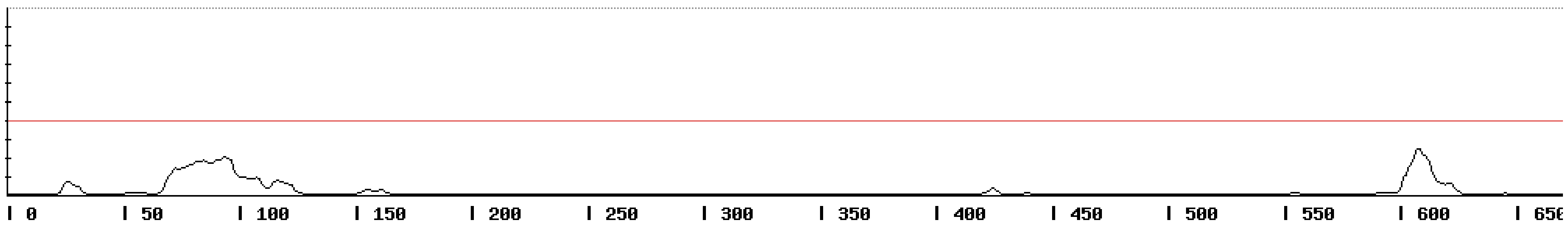

Predictions for Juglansregia_WALNUT_00023624-RA

Viterbi Path

Viterbi Path

Posterior @ 0.4

Posterior @ 0.4

Posterior Graphic. Threshold Marked @ 0.4

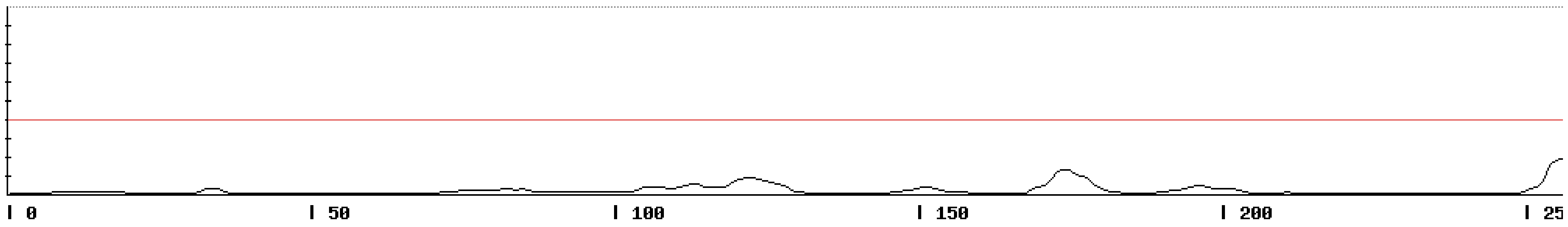

Predictions for Juglansregia_WALNUT_00024081-RA

Viterbi Path

Viterbi Path

Posterior @ 0.4

Posterior @ 0.4

Posterior Graphic. Threshold Marked @ 0.4

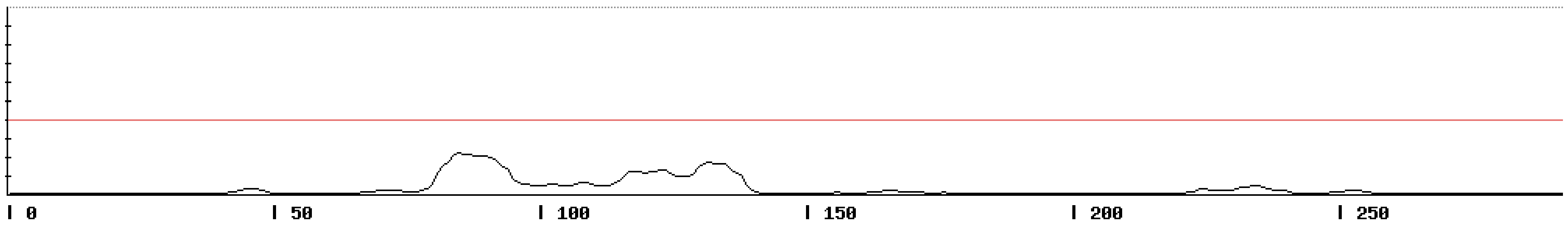

Predictions for Juglansregia_WALNUT_00025170-RA

Viterbi Path

Viterbi Path

Posterior @ 0.4

Posterior @ 0.4

Posterior Graphic. Threshold Marked @ 0.4

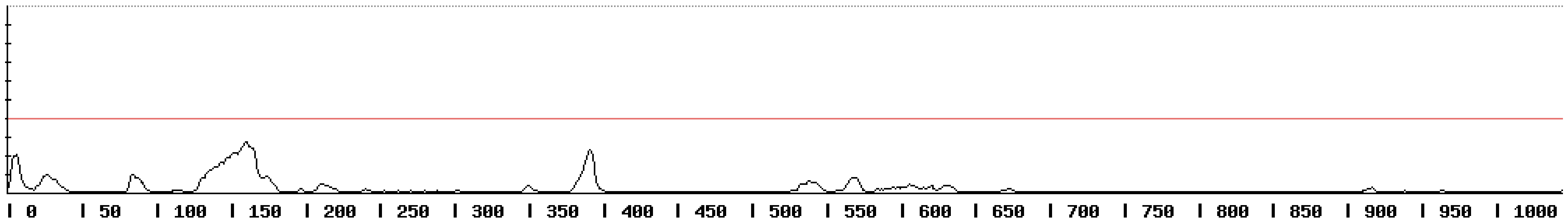

Predictions for Juglansregia_WALNUT_00025175-RA

Viterbi Path

Viterbi Path

Posterior @ 0.4

Posterior @ 0.4

Posterior Graphic. Threshold Marked @ 0.4

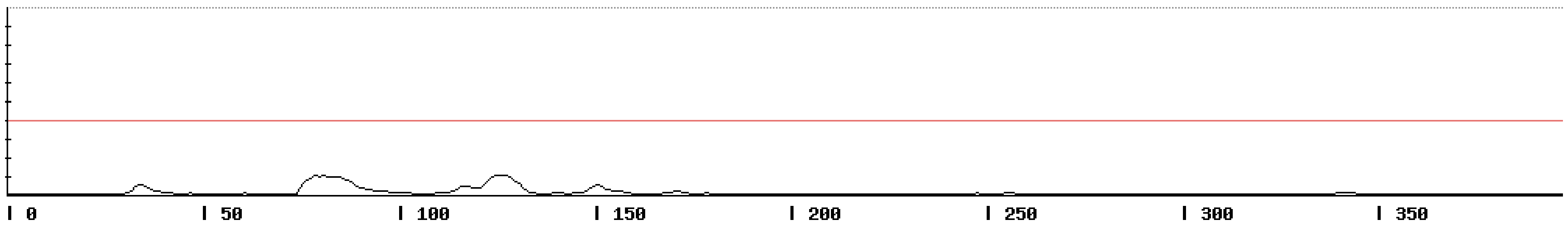

Predictions for Juglansregia_WALNUT_00025182-RA

Viterbi Path

Viterbi Path

Posterior @ 0.4

Posterior @ 0.4

Posterior Graphic. Threshold Marked @ 0.4

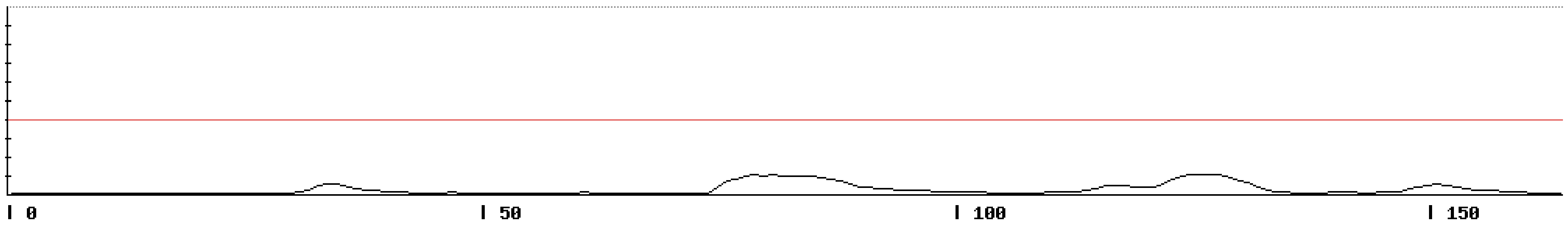

Predictions for Juglansregia_WALNUT_00025809-RA

Viterbi Path

Viterbi Path

Posterior @ 0.4

Posterior @ 0.4

Posterior Graphic. Threshold Marked @ 0.4

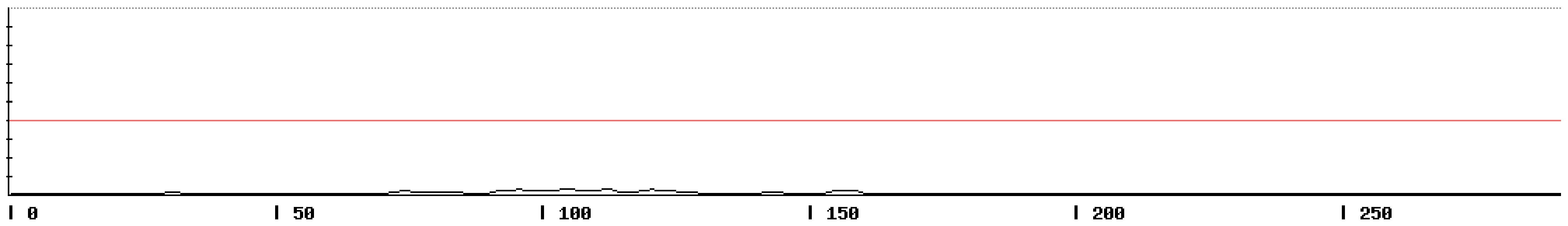

Predictions for Juglansregia_WALNUT_00025834-RA

Viterbi Path

Viterbi Path

Posterior @ 0.4

Posterior @ 0.4

Posterior Graphic. Threshold Marked @ 0.4

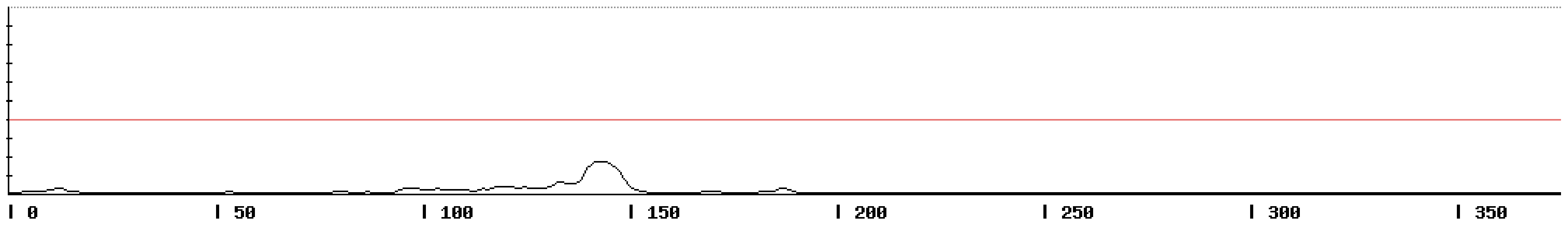

Predictions for Juglansregia_WALNUT_00026009-RA

Viterbi Path

Viterbi Path

Posterior @ 0.4

220 - GHRKRRKIHSQHSLNKEHVRWHKTGK - 245

267 - GSKKGSKP - 274

Posterior @ 0.4

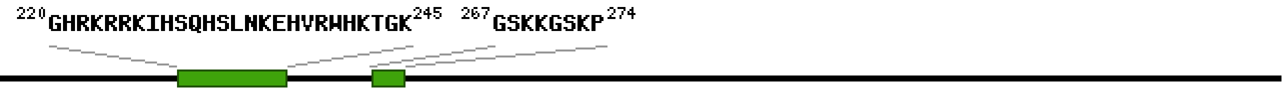

Posterior Graphic. Threshold Marked @ 0.4

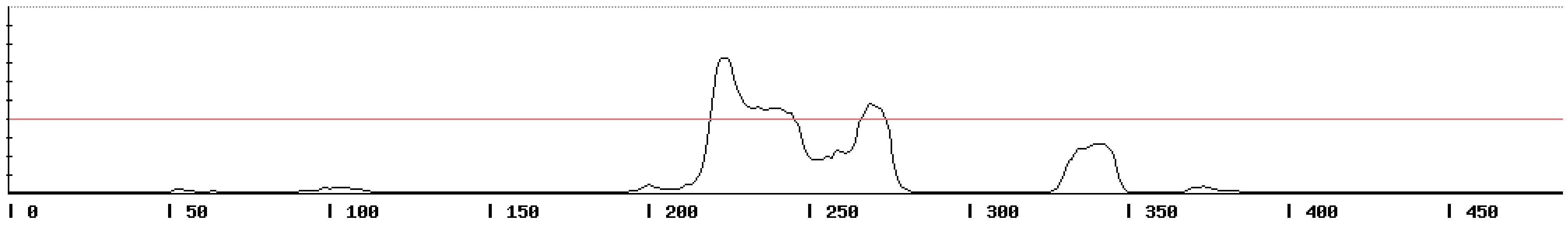

Predictions for Juglansregia_WALNUT_00026011-RA

Viterbi Path

Viterbi Path

Posterior @ 0.4

Posterior @ 0.4

Posterior Graphic. Threshold Marked @ 0.4

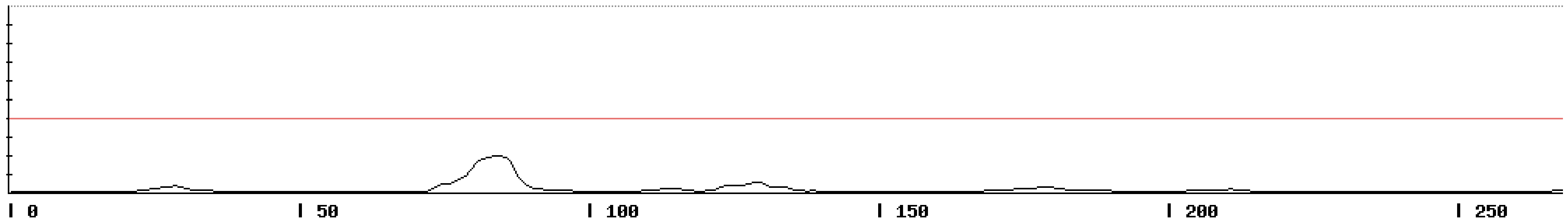

Predictions for Juglansregia_WALNUT_00026705-RA

Viterbi Path

Viterbi Path

Posterior @ 0.4

Posterior @ 0.4

Posterior Graphic. Threshold Marked @ 0.4

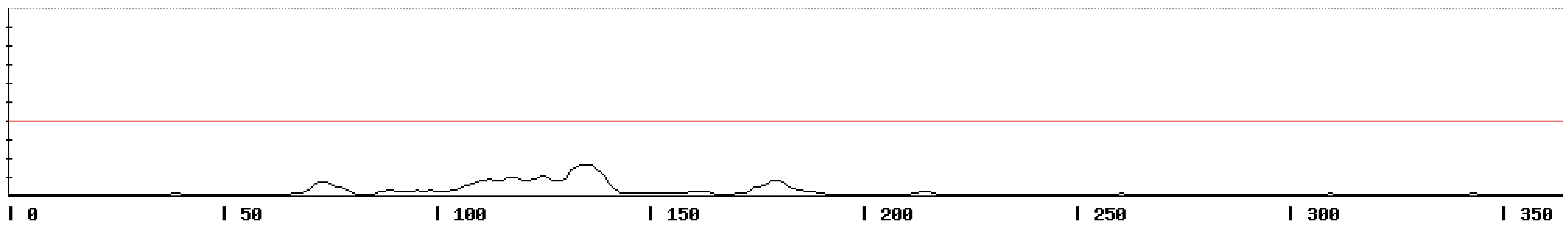

Predictions for Juglansregia_WALNUT_00027010-RA

Viterbi Path

Viterbi Path

Posterior @ 0.4

29 - KKKEKRKA - 36

Posterior @ 0.4

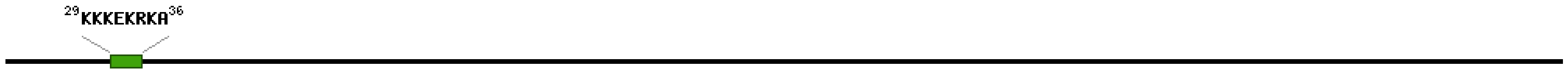

Posterior Graphic. Threshold Marked @ 0.4

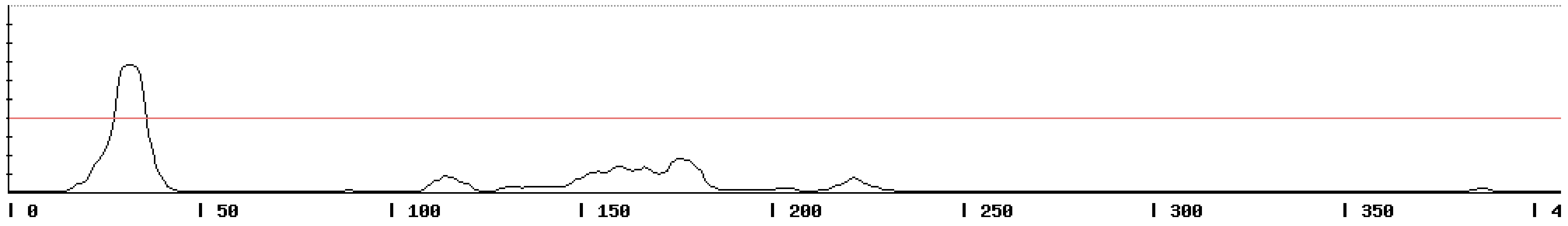

Predictions for Juglansregia_WALNUT_00027144-RA

Viterbi Path

Viterbi Path

Posterior @ 0.4

132 - HRKRRKI - 138

Posterior @ 0.4

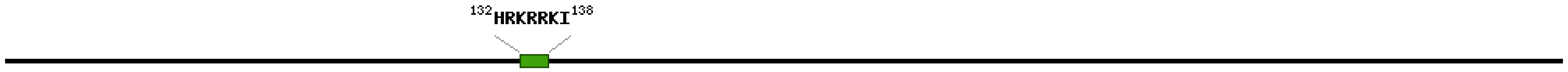

Posterior Graphic. Threshold Marked @ 0.4

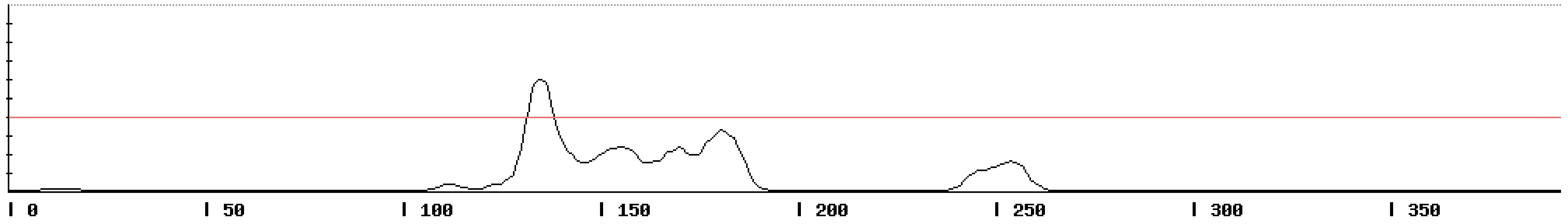

Predictions for Juglansregia_WALNUT_00028291-RA

Viterbi Path

Viterbi Path

Posterior @ 0.4

Posterior @ 0.4

Posterior Graphic. Threshold Marked @ 0.4

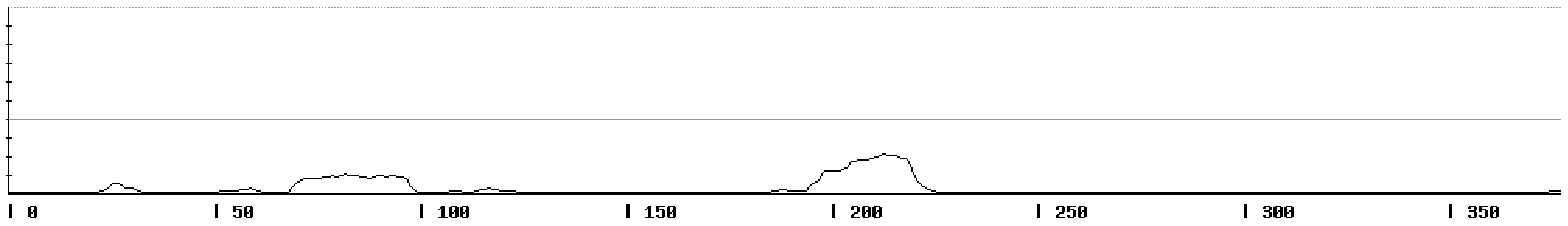

Predictions for Juglansregia_WALNUT_00028334-RA

Viterbi Path

Viterbi Path

Posterior @ 0.4

Posterior @ 0.4

Posterior Graphic. Threshold Marked @ 0.4

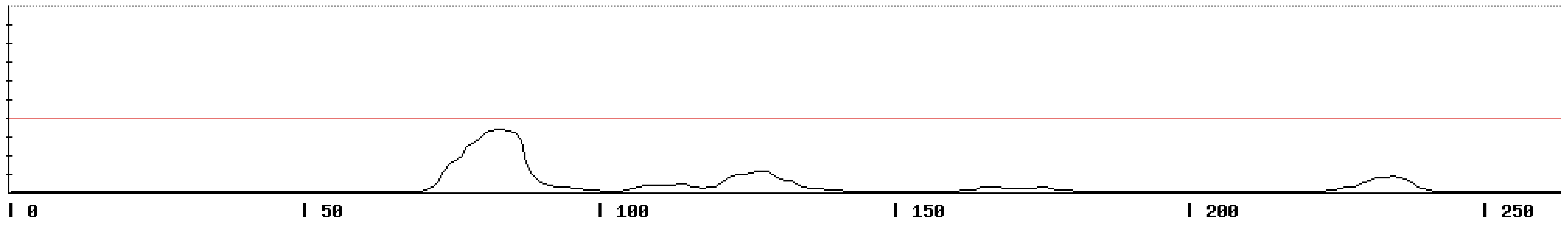

Predictions for Juglansregia_WALNUT_00028362-RA

Viterbi Path

Viterbi Path

Posterior @ 0.4

Posterior @ 0.4

Posterior Graphic. Threshold Marked @ 0.4

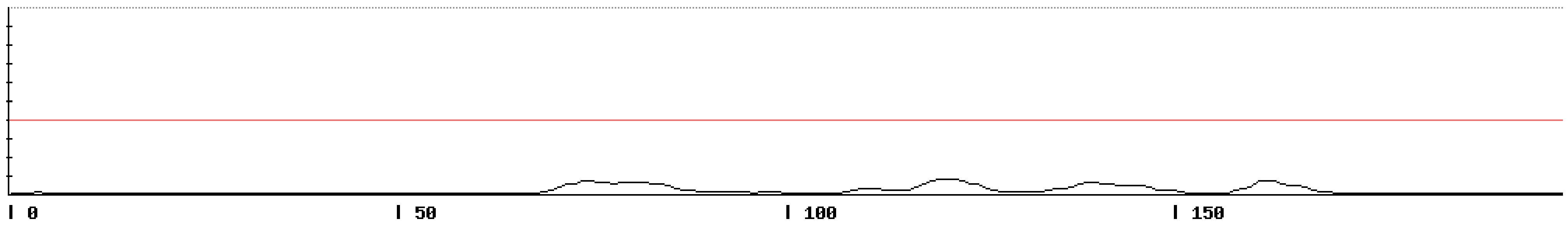

Predictions for Juglansregia_WALNUT_00029198-RA

Viterbi Path

Viterbi Path

Posterior @ 0.4

Posterior @ 0.4

Posterior Graphic. Threshold Marked @ 0.4

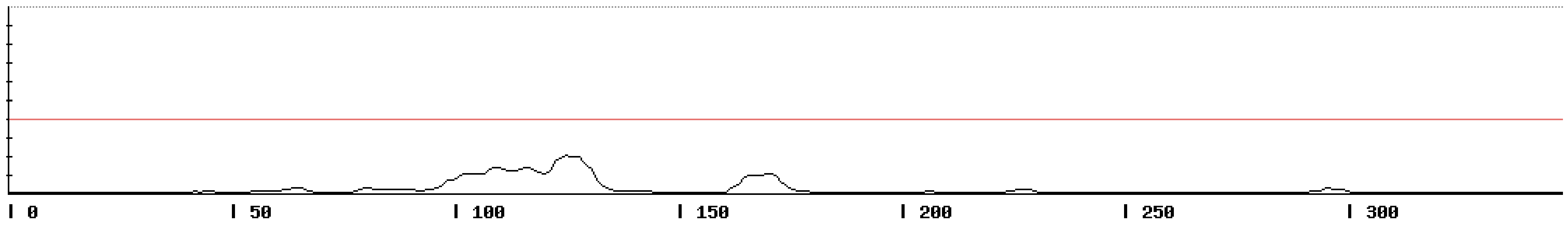

Predictions for Juglansregia_WALNUT_00029651-RA

Viterbi Path

Viterbi Path

Posterior @ 0.4

Posterior @ 0.4

Posterior Graphic. Threshold Marked @ 0.4

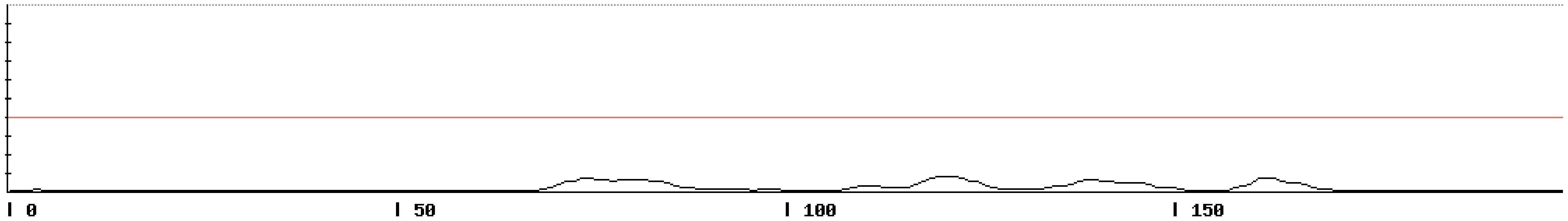

Predictions for Juglansregia_WALNUT_00030364-RA

Viterbi Path

Viterbi Path

Posterior @ 0.4

Posterior @ 0.4

Posterior Graphic. Threshold Marked @ 0.4

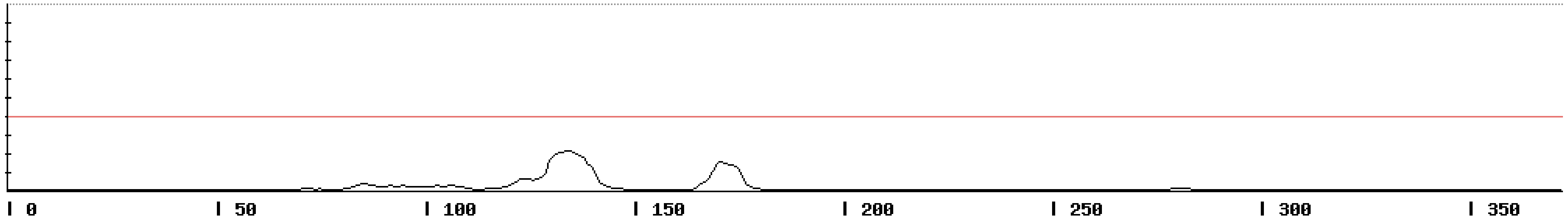

Predictions for Juglansregia_WALNUT_00030593-RA

Viterbi Path

Viterbi Path

Posterior @ 0.4

Posterior @ 0.4

Posterior Graphic. Threshold Marked @ 0.4

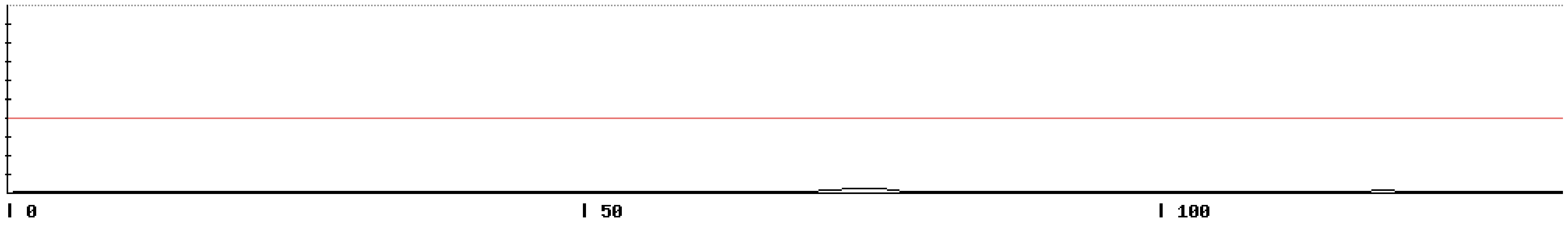

Predictions for Juglansregia_WALNUT_00030727-RA

Viterbi Path

Viterbi Path

Posterior @ 0.4

Posterior @ 0.4

Posterior Graphic. Threshold Marked @ 0.4

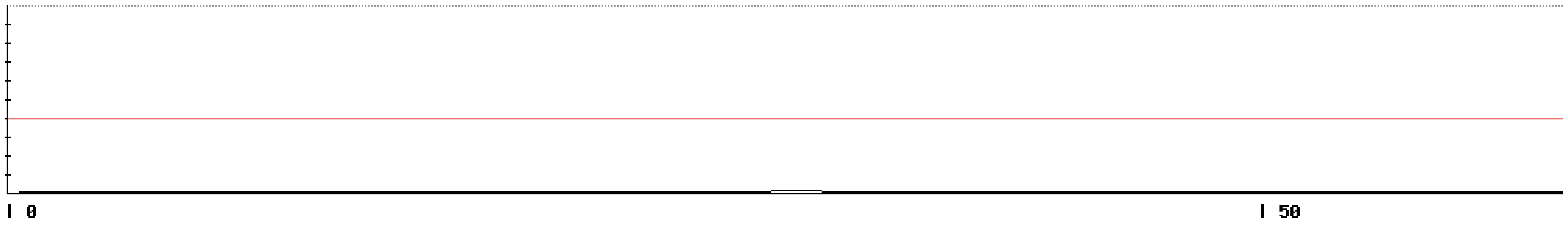

Predictions for Juglansregia_WALNUT_00031397-RA

Viterbi Path

Viterbi Path

Posterior @ 0.4

Posterior @ 0.4

Posterior Graphic. Threshold Marked @ 0.4

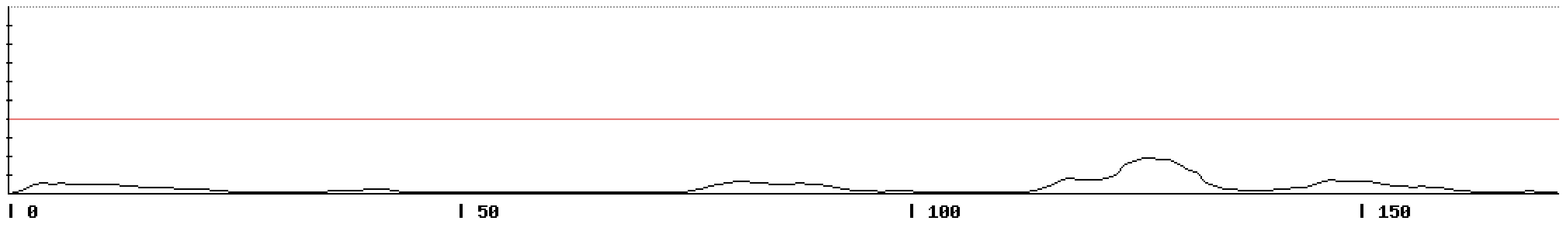

Predictions for Juglansregia_WALNUT_00031849-RA

Viterbi Path

Viterbi Path

Posterior @ 0.4

Posterior @ 0.4

Posterior Graphic. Threshold Marked @ 0.4

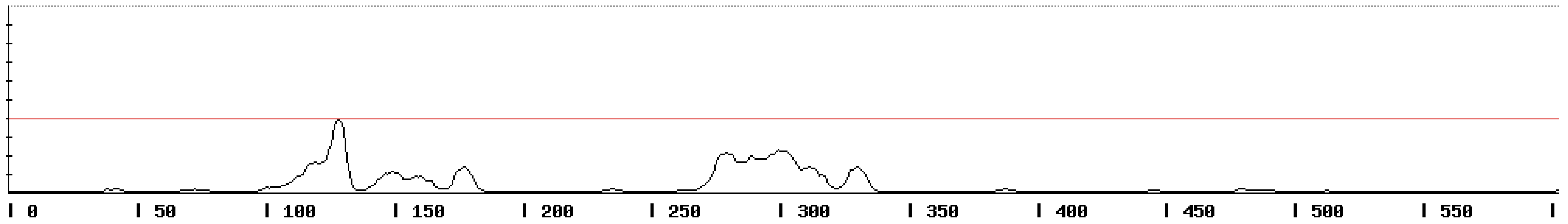

Predictions for Juglansregia_WALNUT_00032240-RA	
Viterbi Path	
Viterbi Path	
Posterior @ 0.4	
Posterior @ 0.4	
Posterior Graphic. Threshold Marked @ 0.4	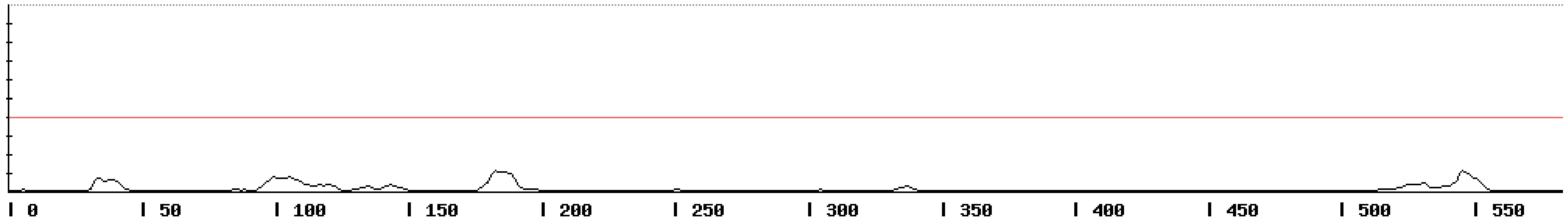

Predictions for Juglansregia_WALNUT_00032276-RA	
Viterbi Path	
Viterbi Path	
Posterior @ 0.4	
Posterior @ 0.4	
Posterior Graphic. Threshold Marked @ 0.4	

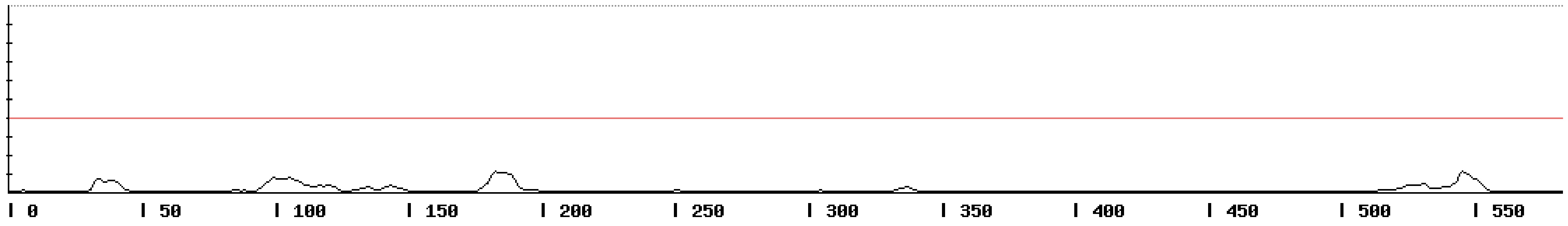

Predictions for Juglansregia_WALNUT_00032389-RA	
Viterbi Path	
Viterbi Path	
Posterior @ 0.4	
Posterior @ 0.4	
Posterior Graphic. Threshold Marked	

Revision r.9 - Last Updated on : February 24 2015 17:45:20. alex.nguyenba@utoronto.ca

Intended to be used with Firefox

Viterbi Path

Posterior @ 0.4

Posterior @ 0.4

Posterior Graphic. Threshold Marked @ 0.4

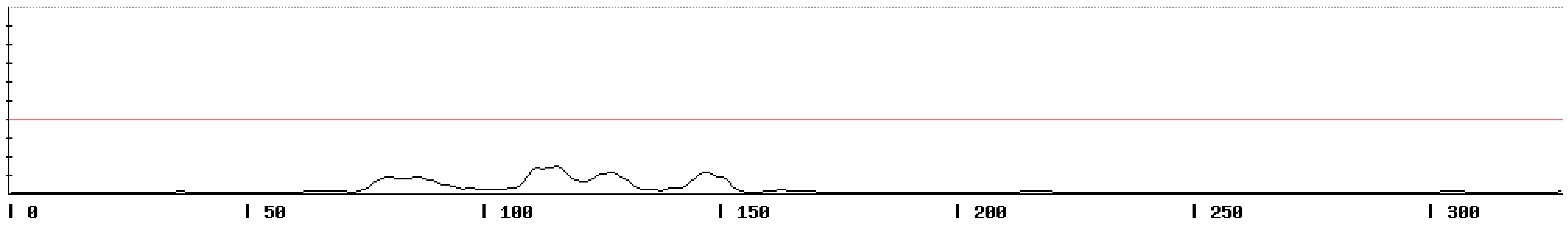

Predictions for Kalanchoelaxiflora_Kalax.0001s0118

Viterbi Path

Viterbi Path

Posterior @ 0.4

Posterior @ 0.4

Posterior Graphic. Threshold Marked @ 0.4

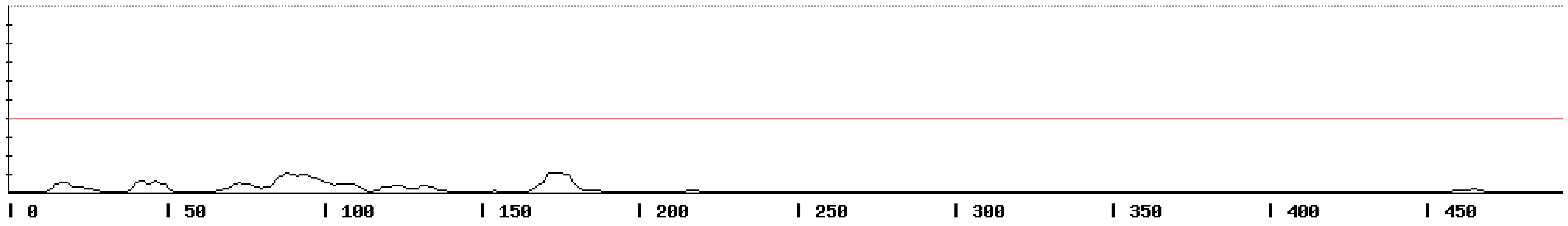

Predictions for Kalanchoelaxiflora_Kalax.0001s0186

Viterbi Path

Viterbi Path

Posterior @ 0.4

Posterior @ 0.4

Posterior Graphic. Threshold Marked @ 0.4

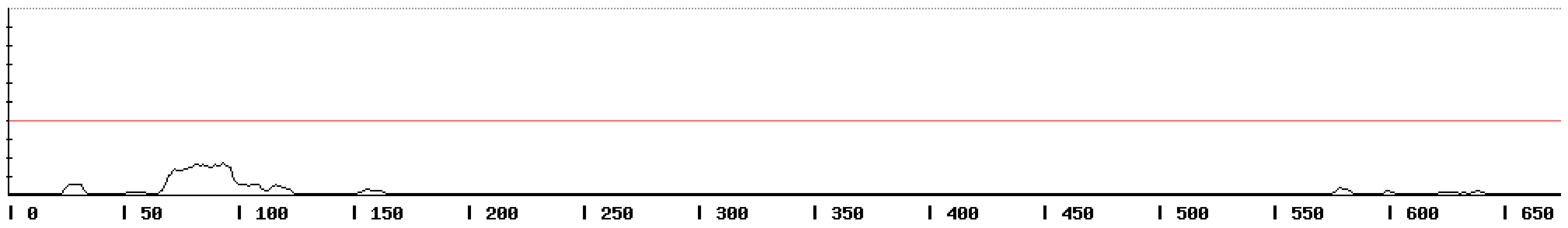

Predictions for Kalanchoelaxiflora_Kalax.0001s0201

Viterbi Path

Viterbi Path

Posterior @ 0.4

Posterior @ 0.4

Posterior Graphic. Threshold Marked @ 0.4

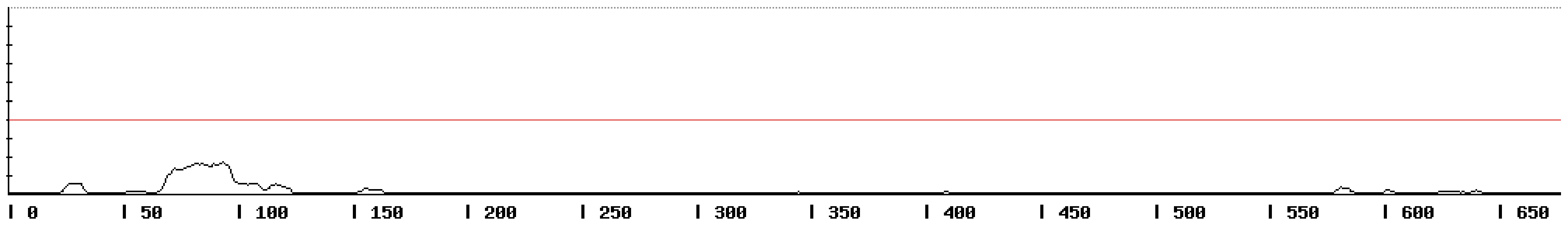

Predictions for Kalanchoelaxiflora_Kalax.0002s0023

Viterbi Path

Viterbi Path

Posterior @ 0.4

Posterior @ 0.4

Posterior Graphic. Threshold Marked @ 0.4

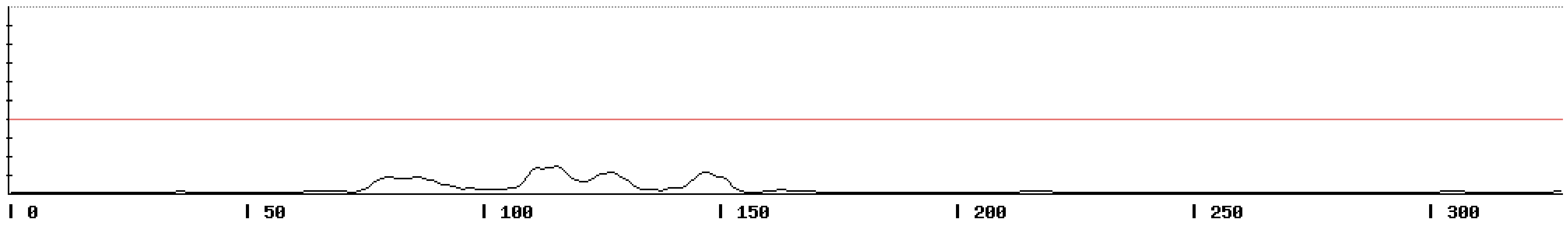

Predictions for Kalanchoelaxiflora_Kalax.0002s0024

Viterbi Path

Viterbi Path

Posterior @ 0.4

Posterior @ 0.4

Posterior Graphic. Threshold Marked @ 0.4

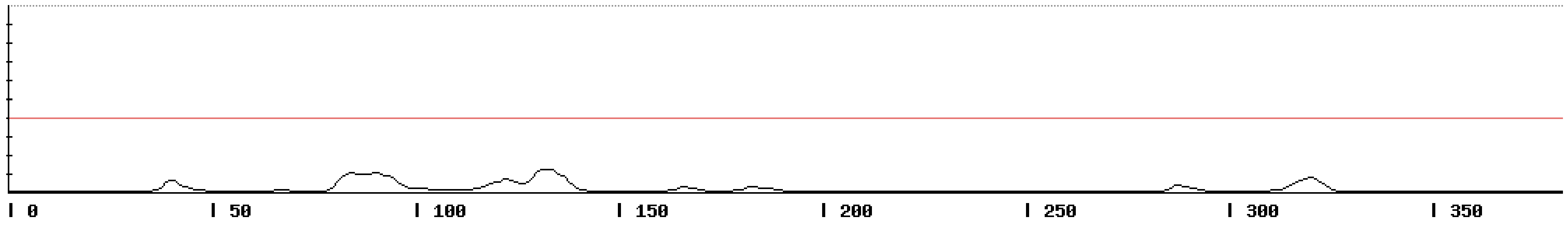

Predictions for Kalanchoelaxiflora_Kalax.0003s0029

Viterbi Path	
Viterbi Path	
Posterior @ 0.4	
Posterior @ 0.4	
Posterior Graphic. Threshold Marked @ 0.4	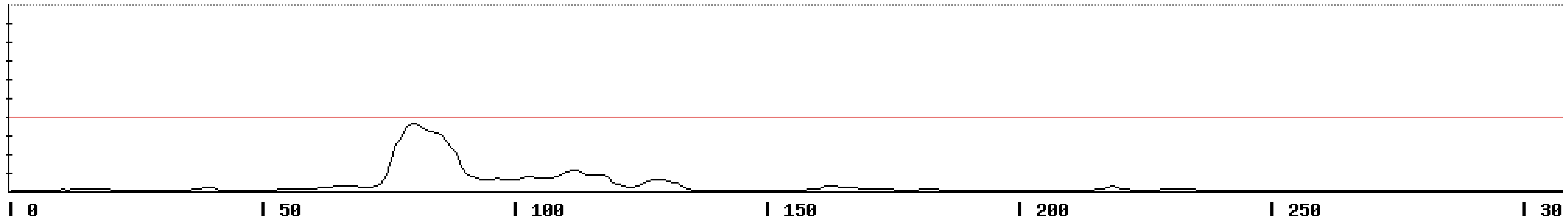

Predictions for Kalanchoelaxiflora_Kalax.0003s0239

Viterbi Path	
Viterbi Path	
Posterior @ 0.4	
Posterior @ 0.4	

Posterior Graphic. Threshold Marked @ 0.4	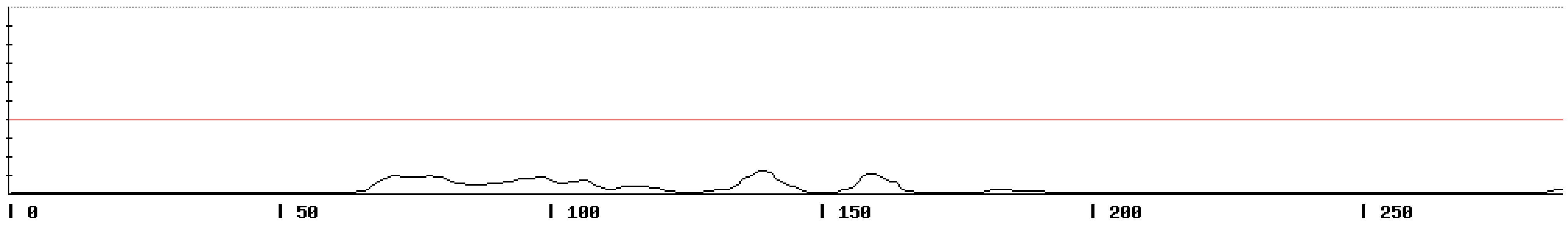

Predictions for Kalanchoelaxiflora_Kalax.0004s0214

Viterbi Path	
Viterbi Path	
Posterior @ 0.4	
Posterior @ 0.4	
Posterior Graphic. Threshold Marked @ 0.4	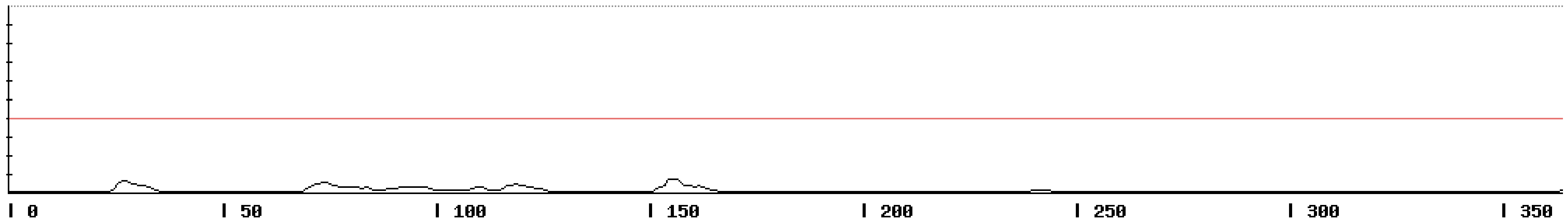

Predictions for Kalanchoelaxiflora_Kalax.0006s0245

Viterbi Path	
Viterbi Path	
Posterior @ 0.4	
Posterior @ 0.4	

Posterior Graphic. Threshold Marked @ 0.4

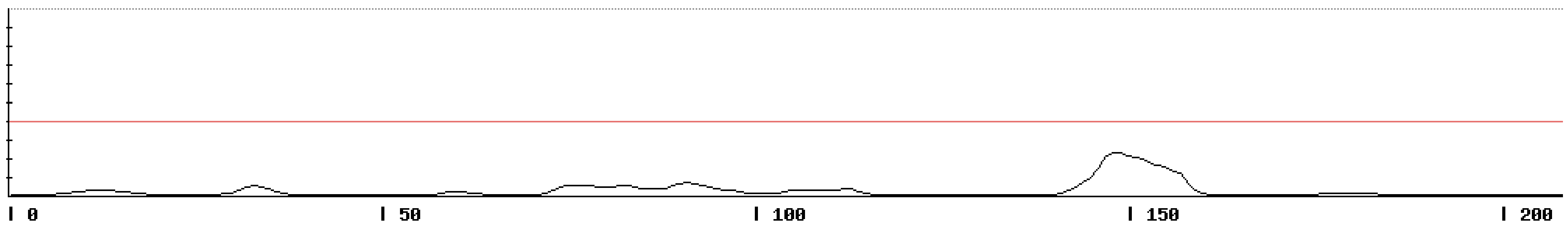

Predictions for Kalanchoelaxiflora_Kalax.0009s0083

Viterbi Path

Viterbi Path

Posterior @ 0.4

Posterior @ 0.4

Posterior Graphic. Threshold Marked @ 0.4

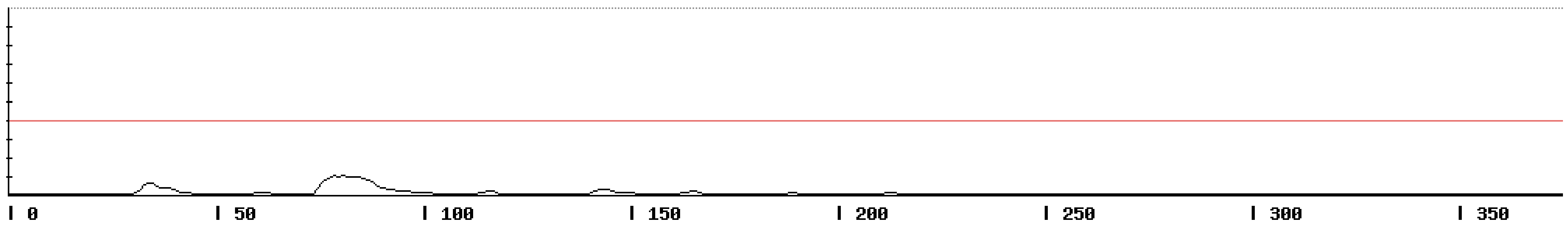

Predictions for Kalanchoelaxiflora_Kalax.0009s0097

Viterbi Path

Viterbi Path

Posterior @ 0.4

Posterior @ 0.4

Posterior Graphic. Threshold Marked @ 0.4

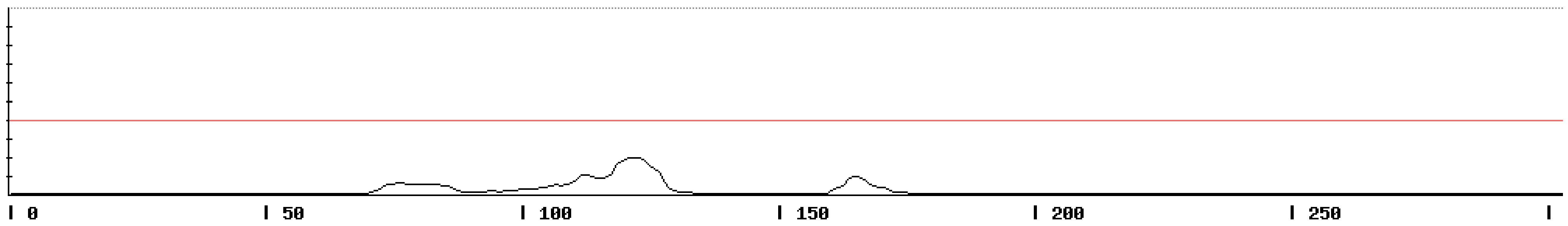

Predictions for Kalanchoelaxiflora_Kalax.0011s0164

Viterbi Path

Viterbi Path

Posterior @ 0.4

Posterior @ 0.4

Posterior Graphic. Threshold Marked @ 0.4

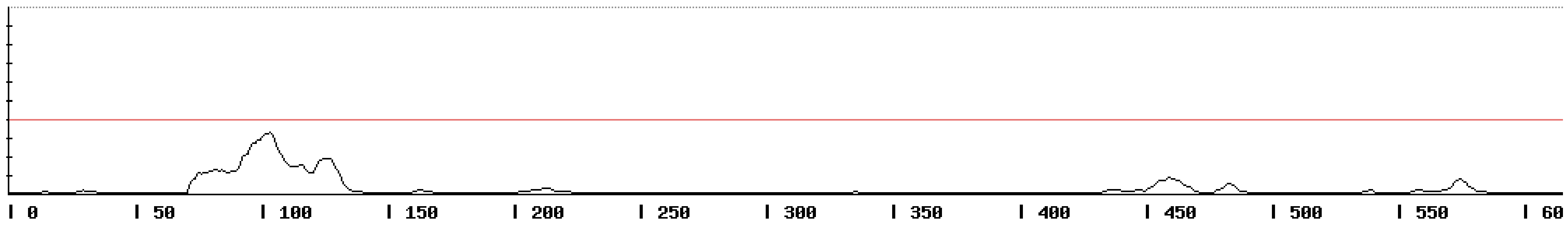

Predictions for Kalanchoelaxiflora_Kalax.0012s0219

Viterbi Path

Viterbi Path

Posterior @ 0.4

Posterior @ 0.4

Posterior Graphic. Threshold Marked @ 0.4

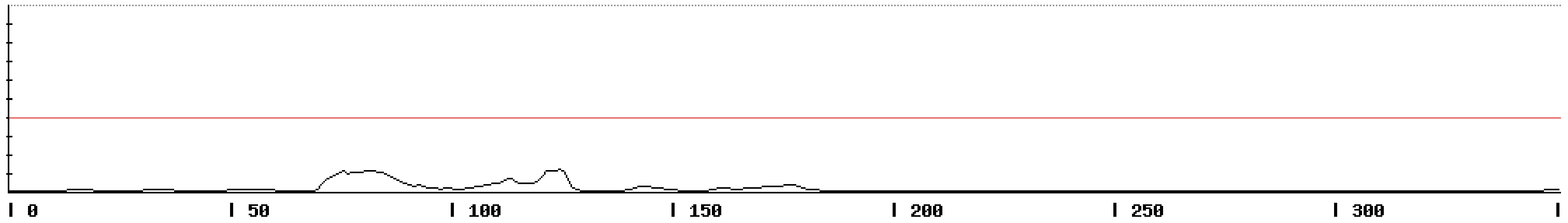

Predictions for Kalanchoelaxiflora_Kalax.0013s0167

Viterbi Path

Viterbi Path

Posterior @ 0.4

Posterior @ 0.4

Posterior Graphic. Threshold Marked @ 0.4

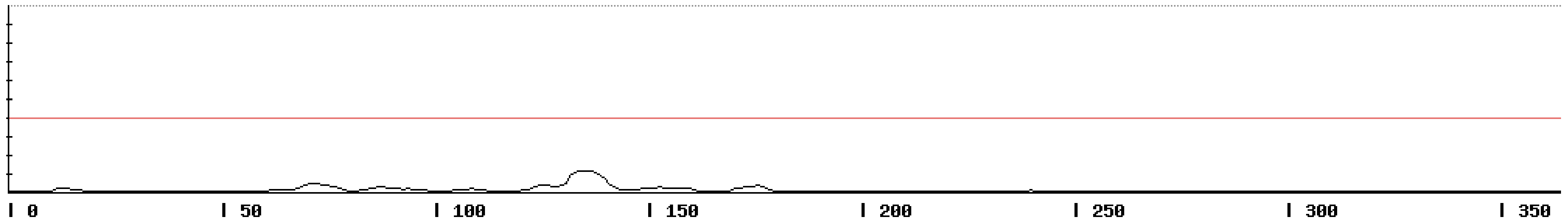

Predictions for Kalanchoelaxiflora_Kalax.0017s0102

Viterbi Path

Viterbi Path

Posterior @ 0.4

Posterior @ 0.4

Posterior Graphic. Threshold Marked @ 0.4

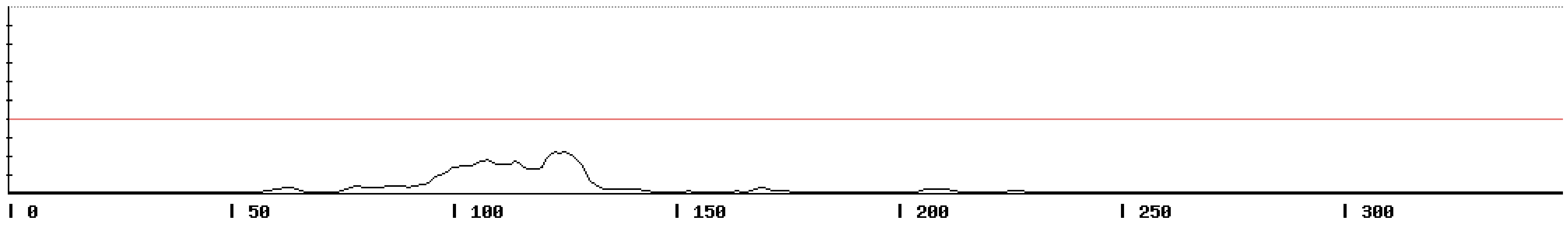

Predictions for Kalanchoelaxiflora_Kalax.0019s0108

Viterbi Path

Viterbi Path

Posterior @ 0.4

Posterior @ 0.4

Posterior Graphic. Threshold Marked @ 0.4

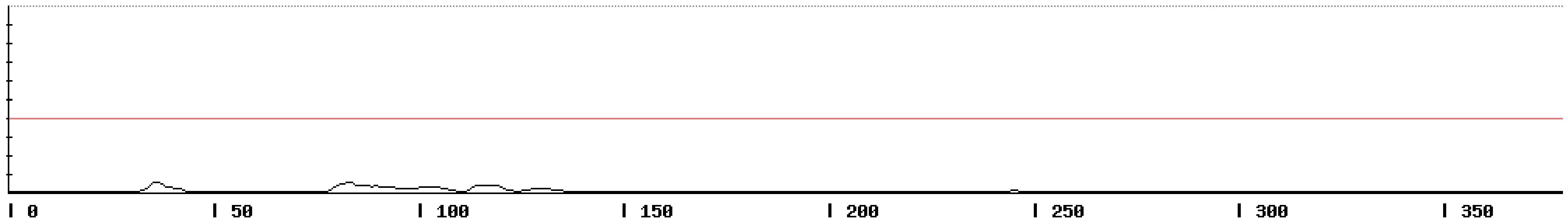

Predictions for Kalanchoelaxiflora_Kalax.0019s0114	
Viterbi Path	2 - KMIKRSTEERKSKIEKSKSKEIRRRRRRRNK - 32
Viterbi Path	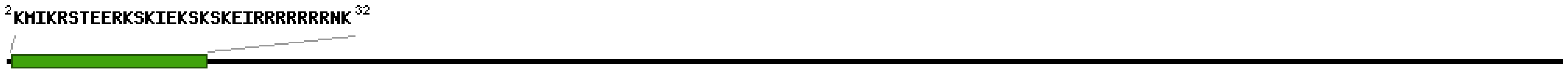
Posterior @ 0.4	5 - KRSTEERKSKIEKSKSKEIRRRRRRRNKVVGKA - 37
Posterior @ 0.4	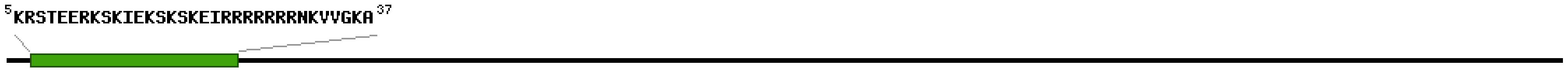
Posterior Graphic. Threshold Marked @ 0.4	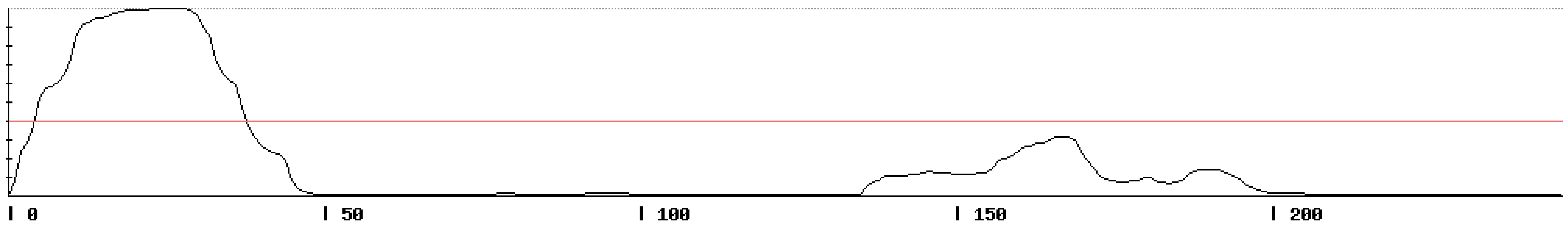

Predictions for Kalanchoelaxiflora_Kalax.0020s0089	
Viterbi Path	
Viterbi Path	
Posterior @ 0.4	
Posterior @ 0.4	
Posterior Graphic. Threshold Marked @ 0.4	

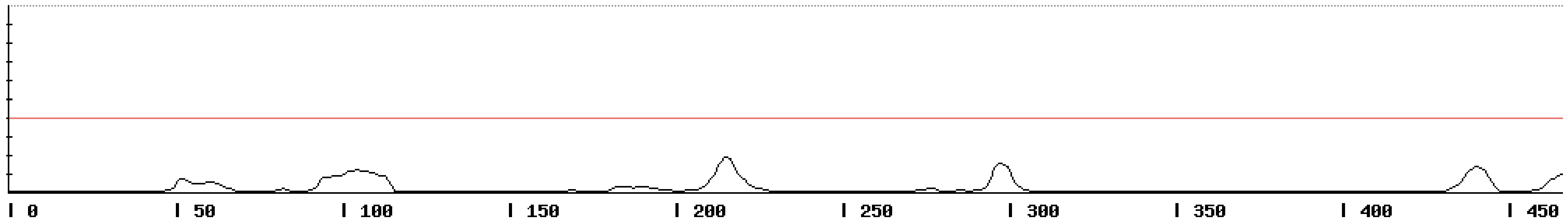

Predictions for Kalanchoelaxiflora_Kalax.0020s0092

Viterbi Path

Viterbi Path

Posterior @ 0.4

Posterior @ 0.4

Posterior Graphic. Threshold Marked @ 0.4

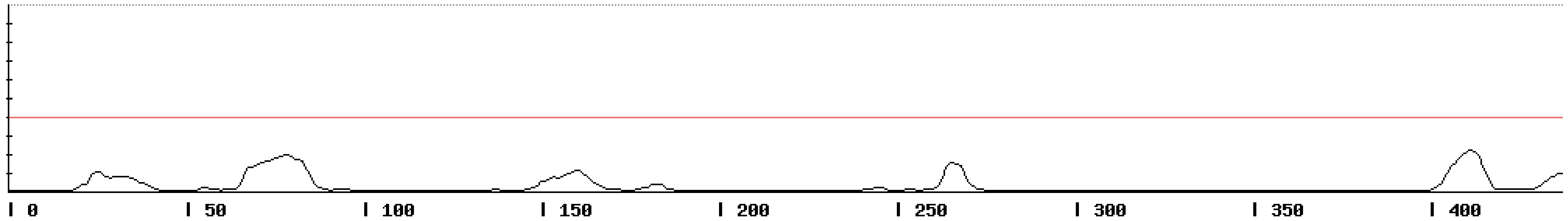

Predictions for Kalanchoelaxiflora_Kalax.0020s0093

Viterbi Path

Viterbi Path

Posterior @ 0.4

Posterior @ 0.4

Posterior Graphic. Threshold Marked @ 0.4

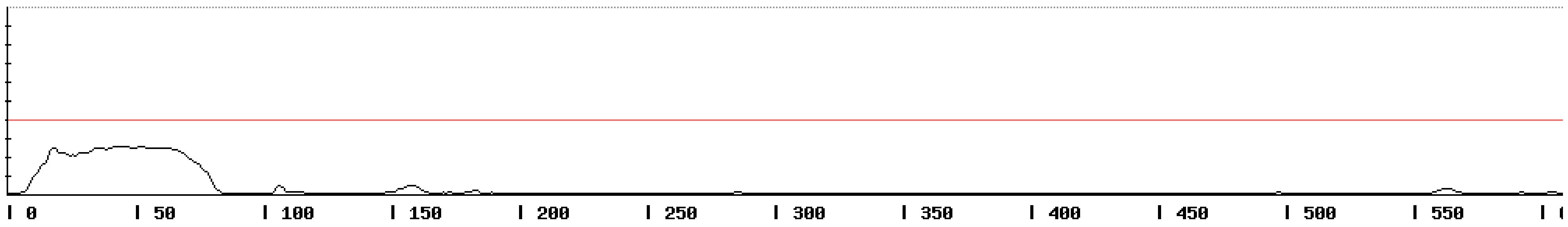

Predictions for Kalanchoelaxiflora_Kalax.0020s0094

Viterbi Path

Viterbi Path

Posterior @ 0.4

70 - RRGSARNKRRRNNAPPPPPPPPPPP - 94

Posterior @ 0.4

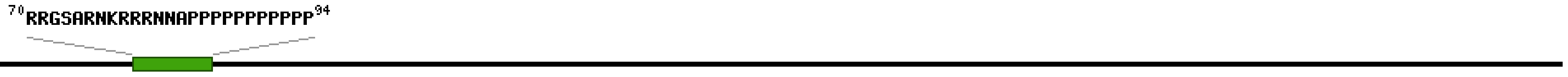

Posterior Graphic. Threshold Marked @ 0.4

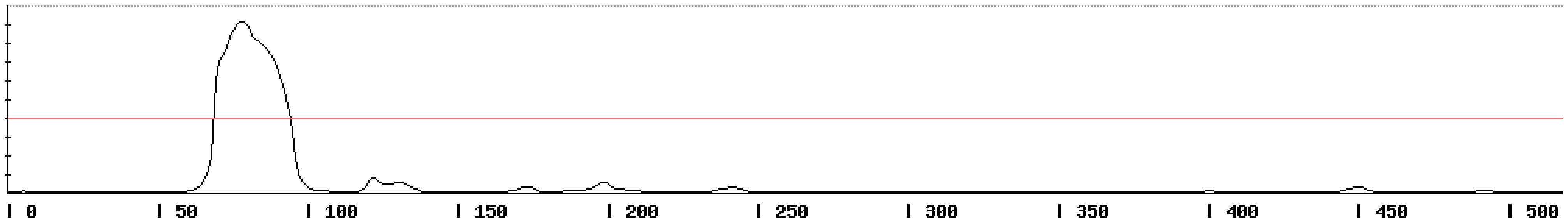

Predictions for Kalanchoelaxiflora_Kalax.0022s0004

Viterbi Path

Viterbi Path

Posterior @ 0.4

Posterior @ 0.4

Posterior Graphic. Threshold Marked @ 0.4

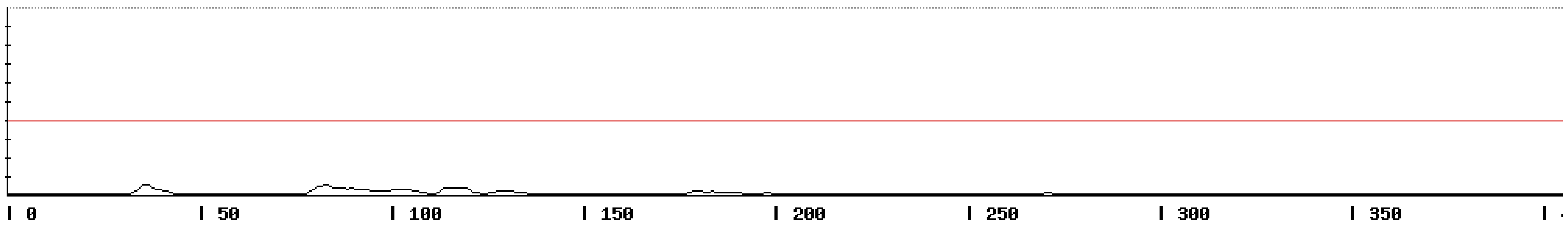

Predictions for Kalanchoelaxiflora_Kalax.0024s0136

Viterbi Path

Viterbi Path

Posterior @ 0.4

Posterior @ 0.4

Posterior Graphic. Threshold Marked @ 0.4

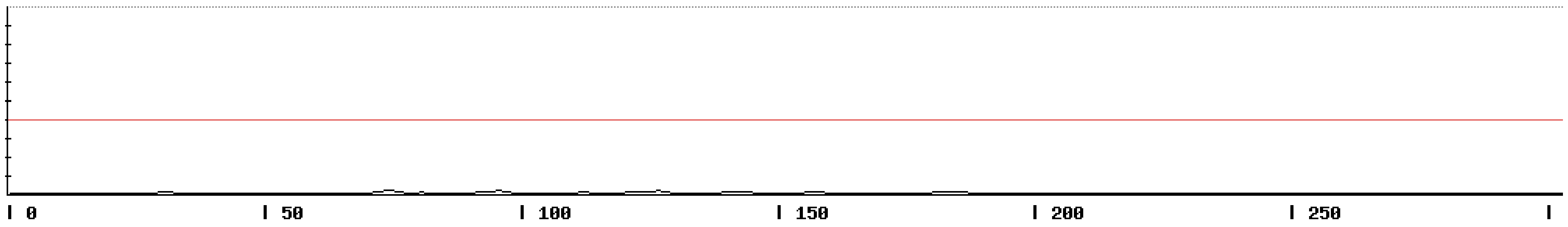

Predictions for Kalanchoelaxiflora_Kalax.0026s0117

Viterbi Path

Viterbi Path

Posterior @ 0.4

Posterior @ 0.4

Posterior Graphic. Threshold Marked @ 0.4

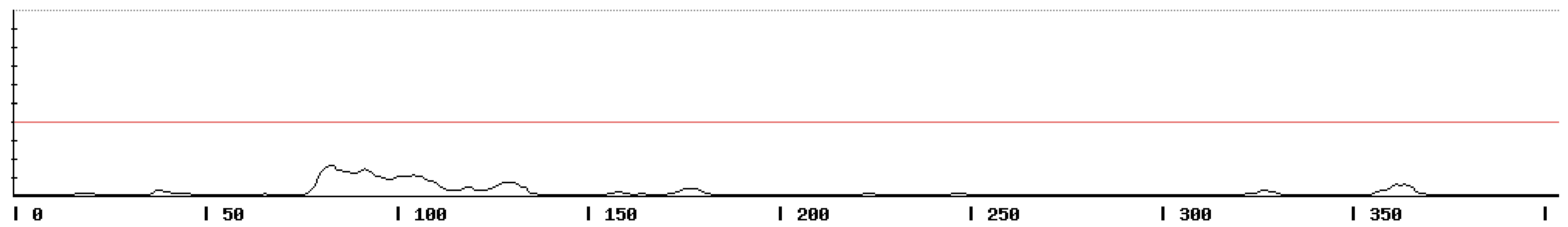

Predictions for Kalanchoelaxiflora_Kalax.0026s0128

Viterbi Path

Viterbi Path

Posterior @ 0.4

Posterior @ 0.4

Posterior Graphic. Threshold Marked @ 0.4

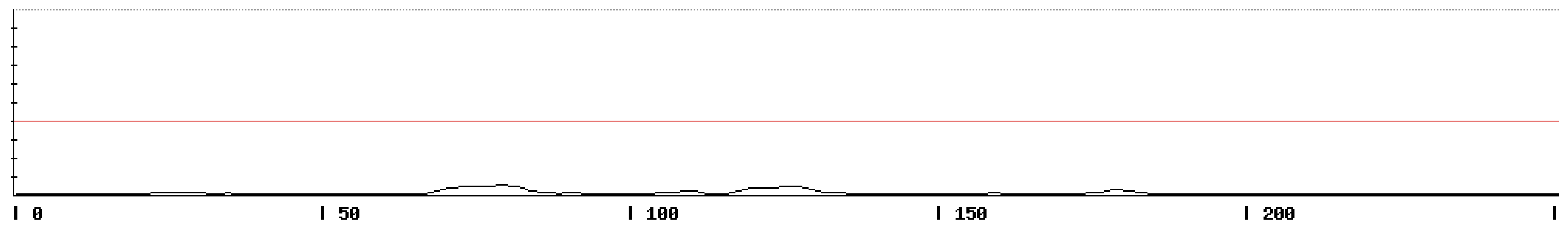

Predictions for Kalanchoelaxiflora_Kalax.0028s0113

Viterbi Path

Viterbi Path

Posterior @ 0.4

Posterior @ 0.4

Posterior Graphic. Threshold Marked @ 0.4

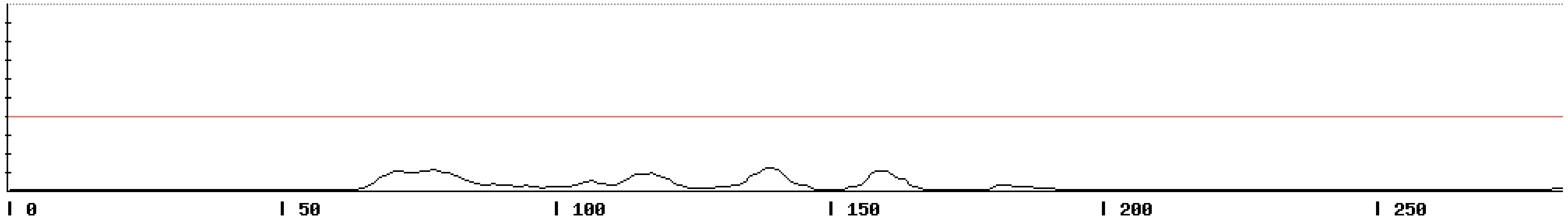

Predictions for Kalanchoelaxiflora_Kalax.0033s0014

Viterbi Path

Viterbi Path

Posterior @ 0.4

Posterior @ 0.4

Posterior Graphic. Threshold Marked @ 0.4

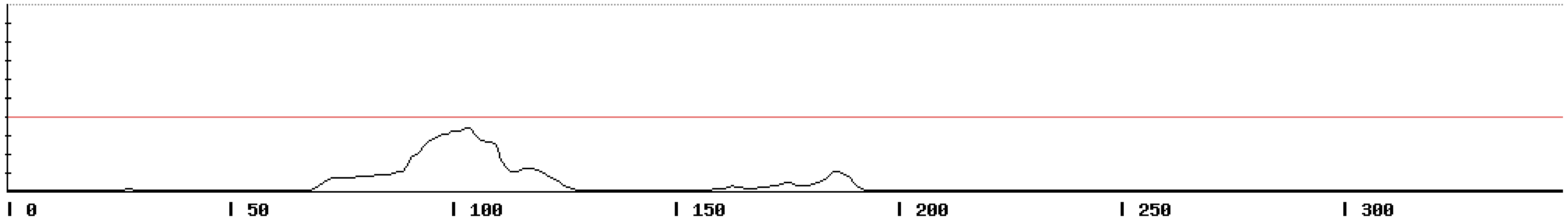

Predictions for Kalanchoelaxiflora_Kalax.0033s0042

Viterbi Path

Viterbi Path

Posterior @ 0.4

Posterior @ 0.4

Posterior Graphic. Threshold Marked @ 0.4

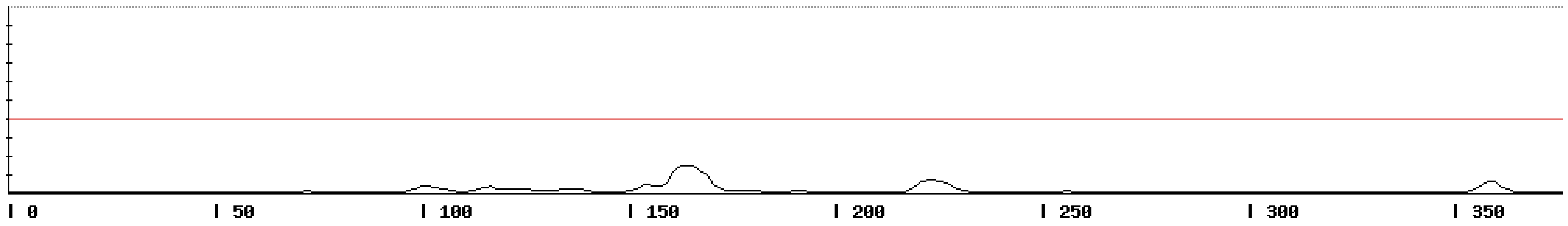

Predictions for Kalanchoelaxiflora_Kalax.0039s0055

Viterbi Path

Viterbi Path

Posterior @ 0.4

Posterior @ 0.4

Posterior Graphic. Threshold Marked @ 0.4

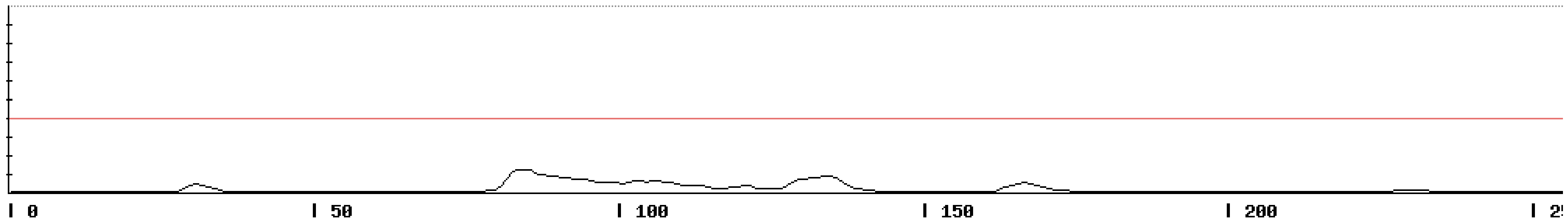

Predictions for Kalanchoelaxiflora_Kalax.0039s0145	
Viterbi Path	
Viterbi Path	
Posterior @ 0.4	136 - SRKRRKI - 142
Posterior @ 0.4	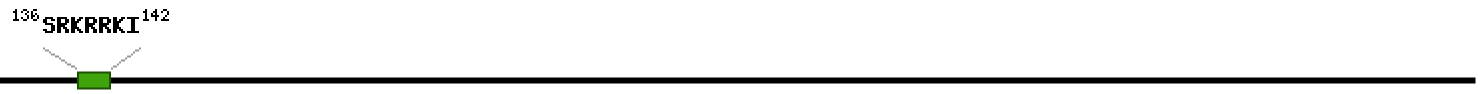
Posterior Graphic. Threshold Marked @ 0.4	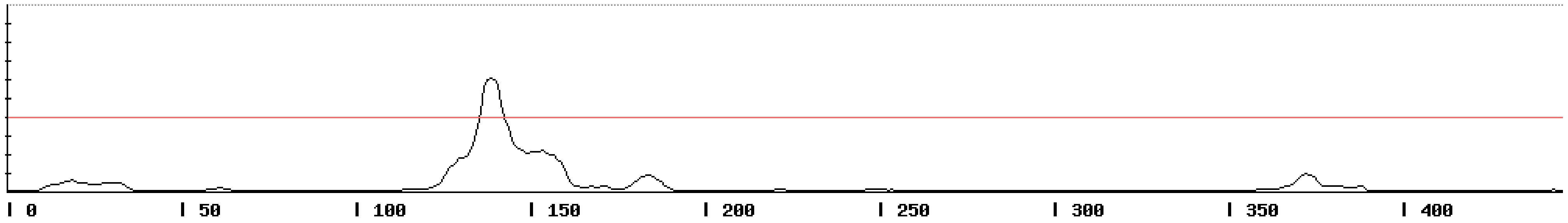

Predictions for Kalanchoelaxiflora_Kalax.0041s0099	
Viterbi Path	
Viterbi Path	
Posterior @ 0.4	
Posterior @ 0.4	
Posterior Graphic. Threshold Marked @ 0.4	

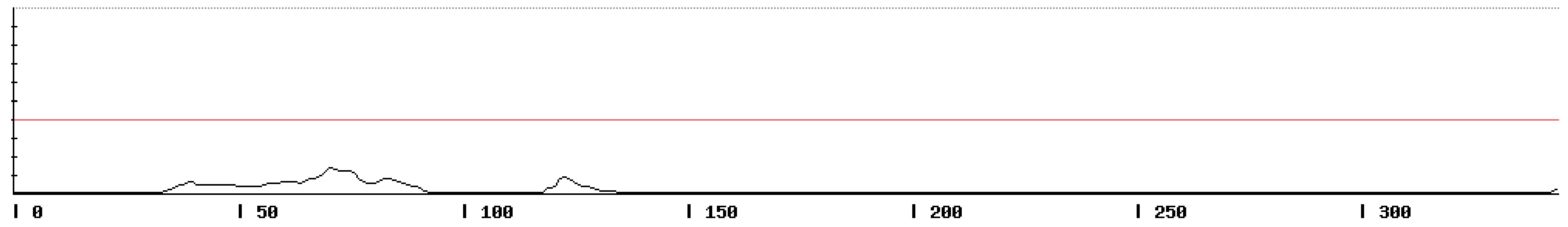

Predictions for Kalanchoelaxiflora_Kalax.0041s0116

Viterbi Path

Viterbi Path

Posterior @ 0.4

Posterior @ 0.4

Posterior Graphic. Threshold Marked @ 0.4

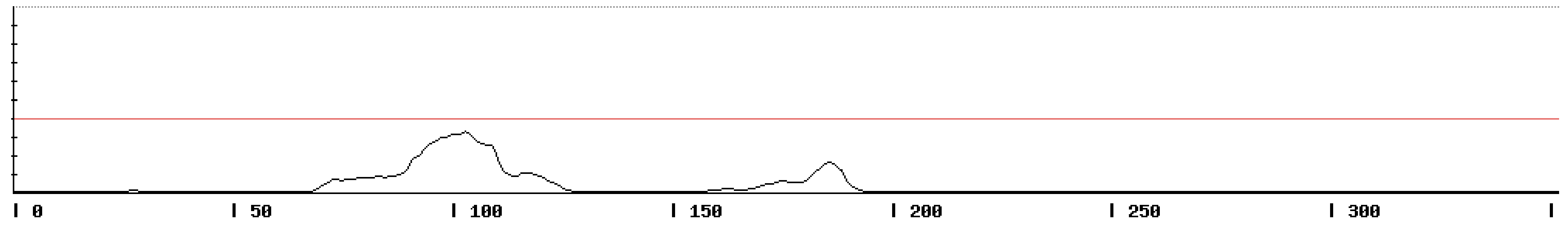

Predictions for Kalanchoelaxiflora_Kalax.0046s0014

Viterbi Path

Viterbi Path

Posterior @ 0.4

Posterior @ 0.4

Posterior Graphic. Threshold Marked @ 0.4

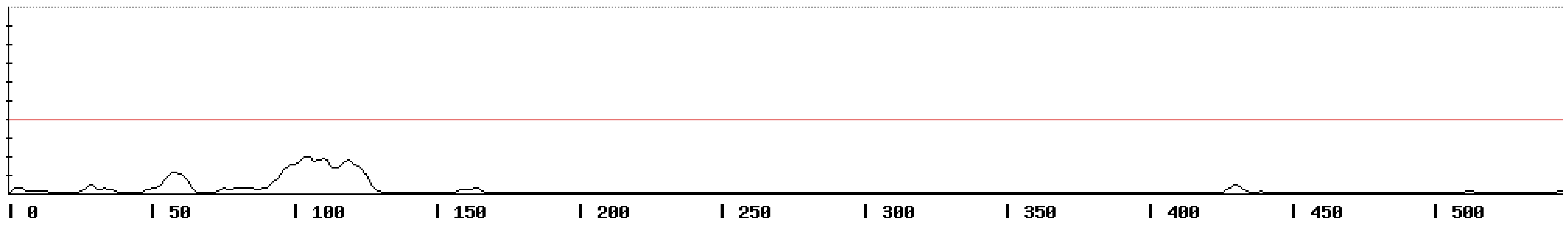

Predictions for Kalanchoelaxiflora_Kalax.0048s0059

Viterbi Path

Viterbi Path

Posterior @ 0.4

Posterior @ 0.4

Posterior Graphic. Threshold Marked @ 0.4

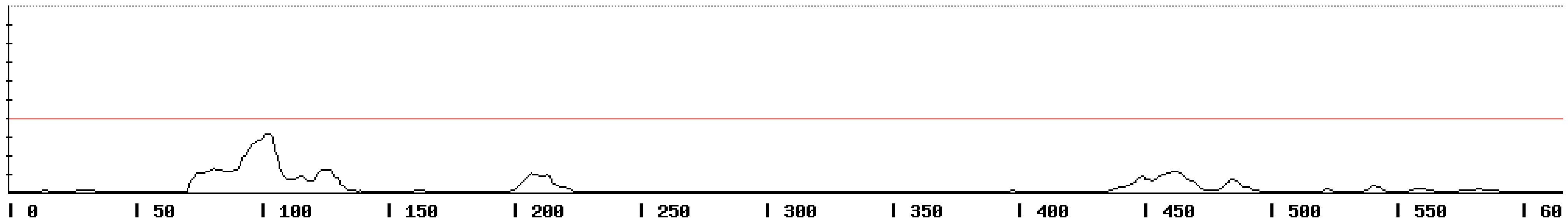

Predictions for Kalanchoelaxiflora_Kalax.0052s0074

Viterbi Path

Viterbi Path

Posterior @ 0.4

Posterior @ 0.4

Posterior Graphic. Threshold Marked @ 0.4

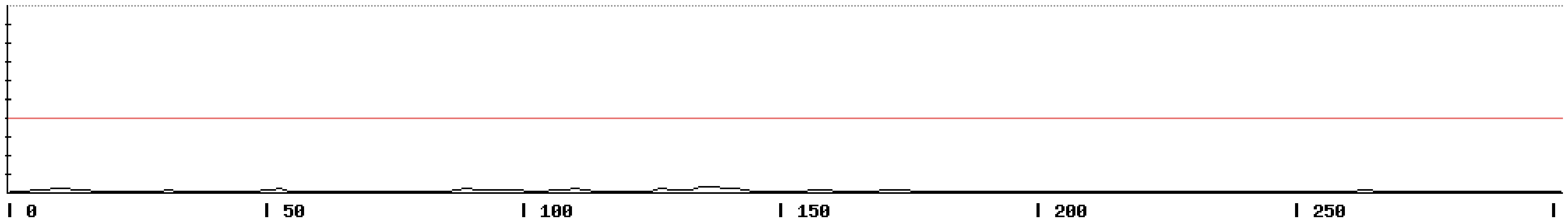

Predictions for Kalanchoelaxiflora_Kalax.0054s0134

Viterbi Path

Viterbi Path

Posterior @ 0.4

91 - R - 91

93 - R - 93

95 - IKTASKKL - 102

Posterior @ 0.4

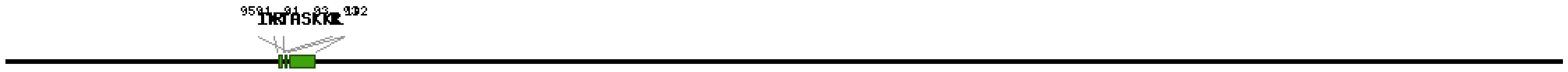

Posterior Graphic. Threshold Marked @ 0.4

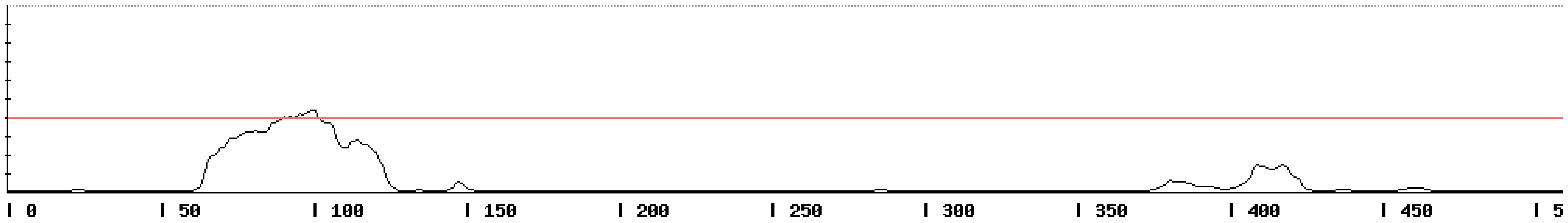

Predictions for Kalanchoelaxiflora_Kalax.0056s0128

Viterbi Path

Viterbi Path

Posterior @ 0.4

Posterior @ 0.4

Posterior Graphic. Threshold Marked @ 0.4

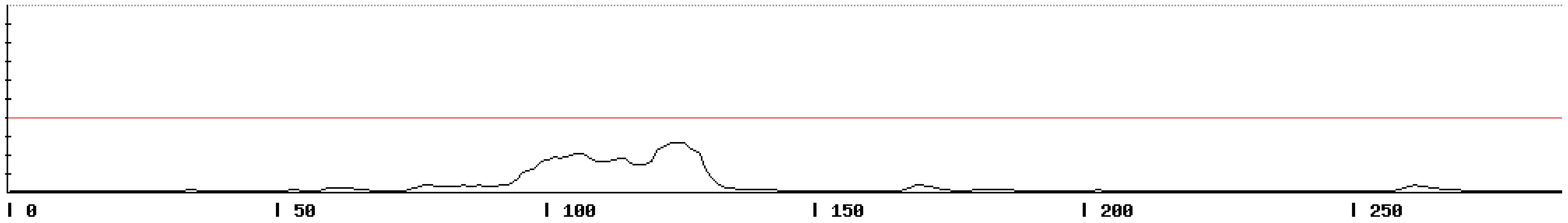

Predictions for Kalanchoelaxiflora_Kalax.0057s0052

Viterbi Path: 86 - RKRTRMRRTRGGYWKRKGKVR - 106

Viterbi Path

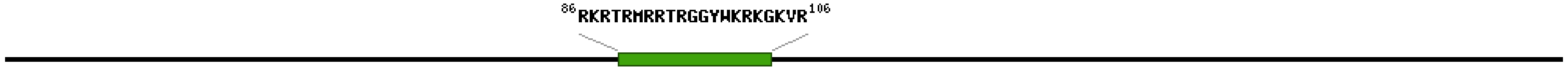

Posterior @ 0.4: 83 - RVERKRTRMRRTRGGYWKRKGKVR - 106

Posterior @ 0.4

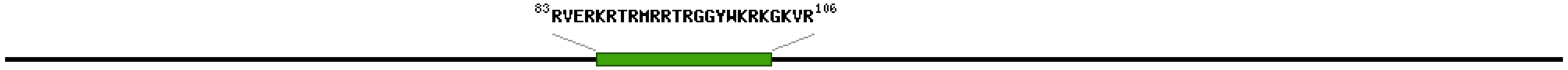

Posterior Graphic. Threshold Marked @ 0.4

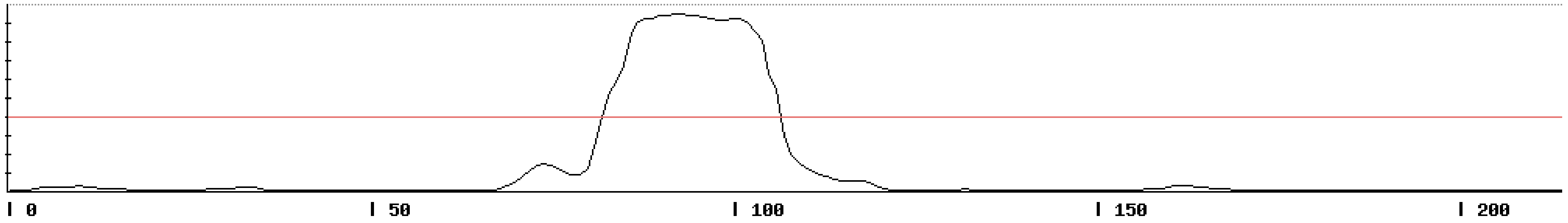

Predictions for Kalanchoelaxiflora_Kalax.0070s0067

Viterbi Path

Viterbi Path

Posterior @ 0.4

Posterior @ 0.4

Posterior Graphic. Threshold Marked @ 0.4

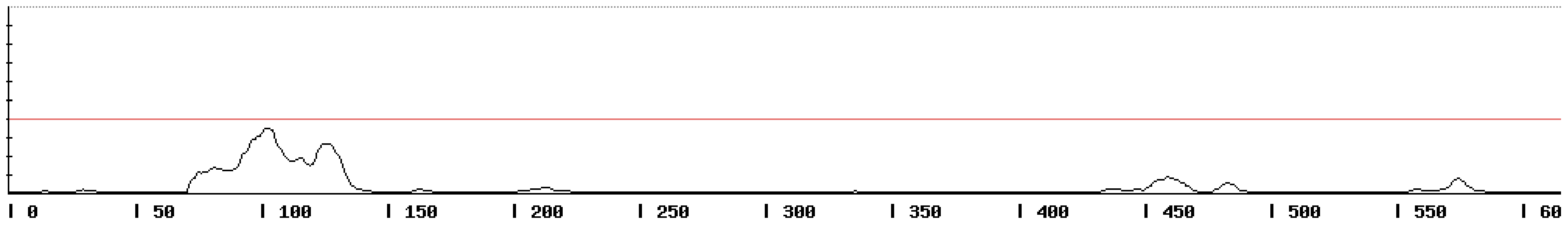

Predictions for Kalanchoelaxiflora_Kalax.0076s0001

Viterbi Path

Viterbi Path

Posterior @ 0.4

Posterior @ 0.4

Posterior Graphic. Threshold Marked @ 0.4

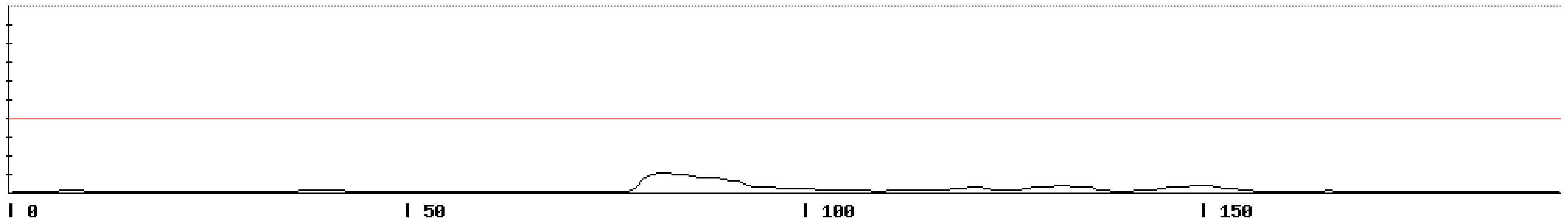

Predictions for Kalanchoelaxiflora_Kalax.0081s0007

Viterbi Path	
Viterbi Path	
Posterior @ 0.4	
Posterior @ 0.4	
Posterior Graphic. Threshold Marked @ 0.4	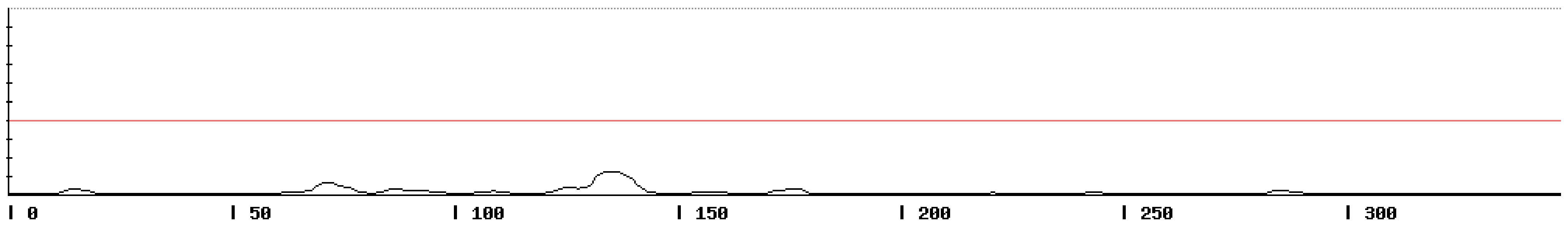

Predictions for Kalanchoelaxiflora_Kalax.0085s0072

Viterbi Path	
Viterbi Path	
Posterior @ 0.4	
Posterior @ 0.4	
Posterior Graphic. Threshold Marked @ 0.4	

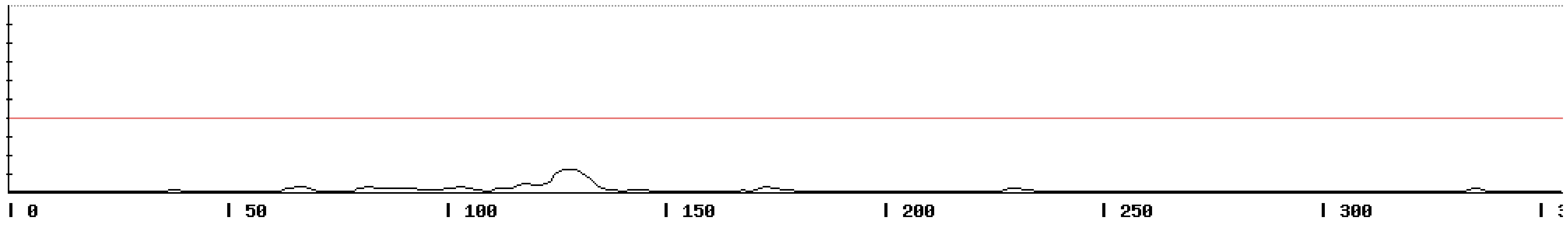

Predictions for Kalanchoelaxiflora_Kalax.0090s0019

Viterbi Path

Viterbi Path

Posterior @ 0.4

Posterior @ 0.4

Posterior Graphic. Threshold Marked @ 0.4

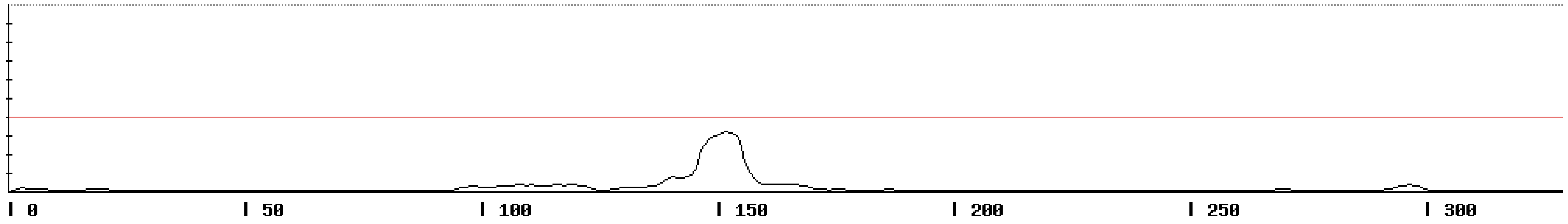

Predictions for Kalanchoelaxiflora_Kalax.0095s0061

Viterbi Path

Viterbi Path

Posterior @ 0.4

Posterior @ 0.4

Posterior Graphic. Threshold Marked @ 0.4

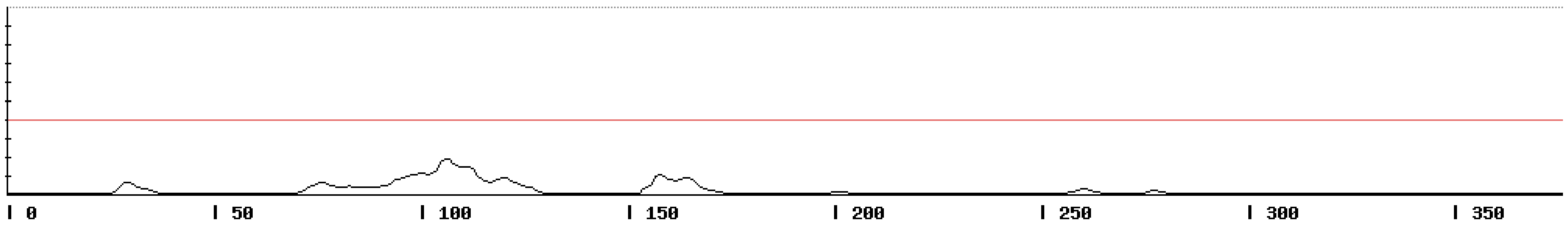

Predictions for Kalanchoelaxiflora_Kalax.0099s0031

Viterbi Path

Viterbi Path

Posterior @ 0.4

Posterior @ 0.4

Posterior Graphic. Threshold Marked @ 0.4

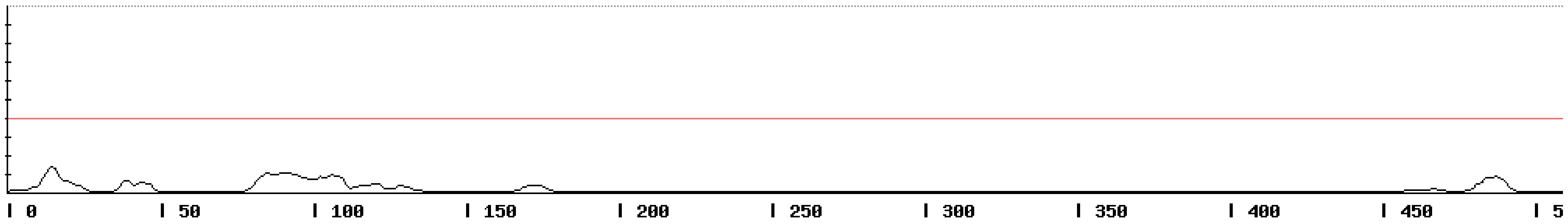

Predictions for Kalanchoelaxiflora_Kalax.0100s0047

Viterbi Path

Viterbi Path

Posterior @ 0.4

Posterior @ 0.4

Posterior Graphic. Threshold Marked @ 0.4	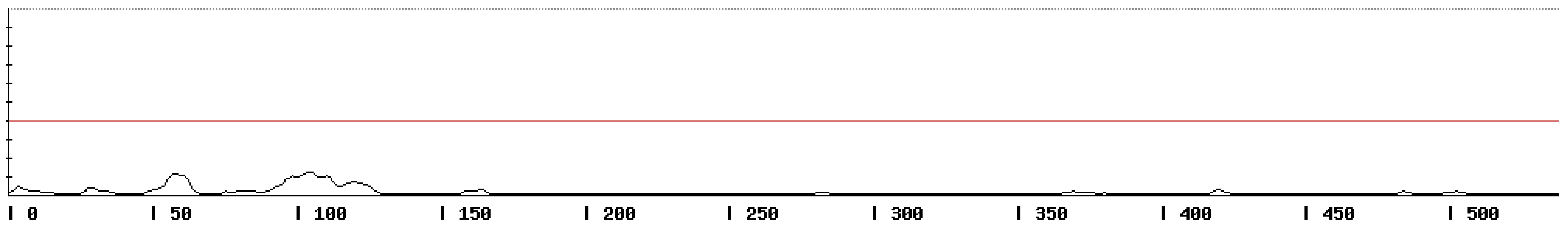

Predictions for Kalanchoelaxiflora_Kalax.0103s0071	
Viterbi Path	86 - RKRTRMRRTRGGYWKRKGKVR - 106
Viterbi Path	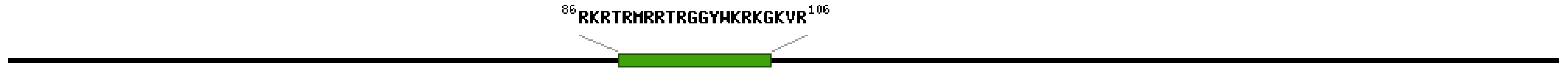
Posterior @ 0.4	83 - RVERKRTRMRRTRGGYWKRKGKVR - 106
Posterior @ 0.4	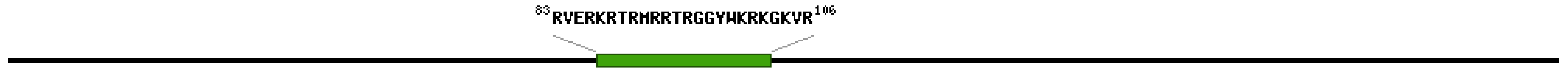
Posterior Graphic. Threshold Marked @ 0.4	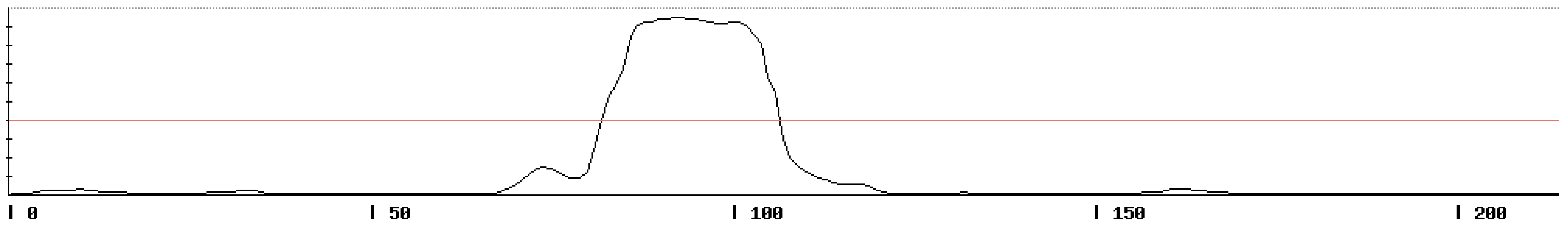

Predictions for Kalanchoelaxiflora_Kalax.0104s0078	
Viterbi Path	
Viterbi Path	

Posterior @ 0.4

Posterior @ 0.4

Posterior Graphic. Threshold Marked @ 0.4

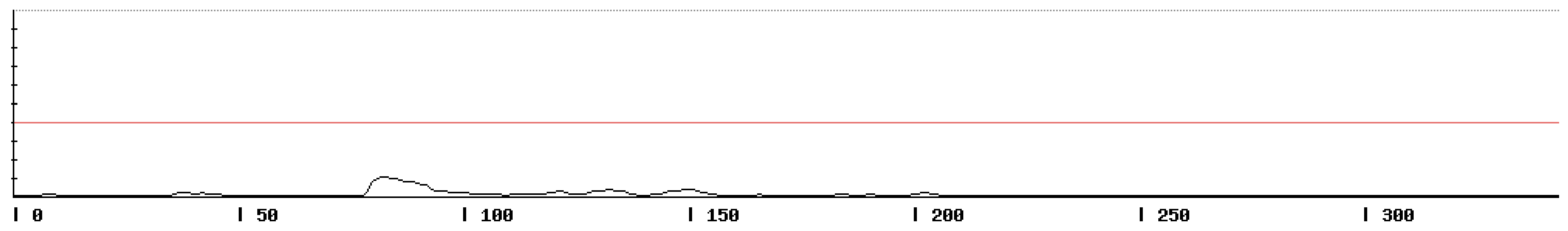

Predictions for Kalanchoelaxiflora_Kalax.0104s0092

Viterbi Path

Viterbi Path

Posterior @ 0.4

Posterior @ 0.4

Posterior Graphic. Threshold Marked @ 0.4

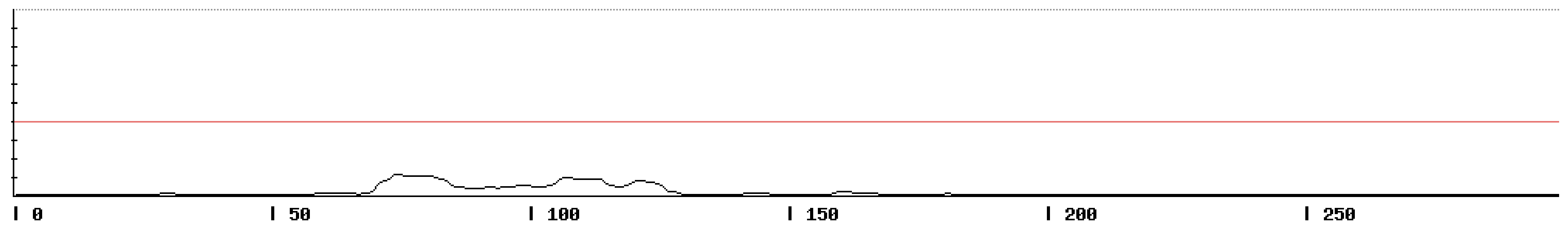

Predictions for Kalanchoelaxiflora_Kalax.0115s0080

Viterbi Path

Viterbi Path

Posterior @ 0.4

Posterior @ 0.4

Posterior Graphic. Threshold Marked @ 0.4

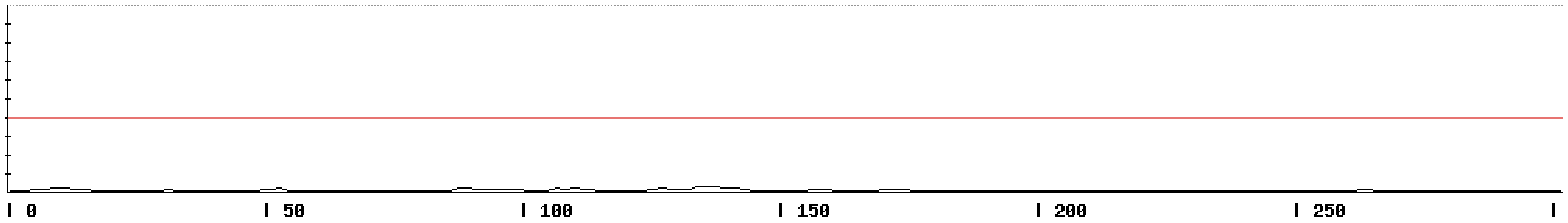

Predictions for Kalanchoelaxiflora_Kalax.0119s0023

Viterbi Path

Viterbi Path

Posterior @ 0.4

Posterior @ 0.4

Posterior Graphic. Threshold Marked @ 0.4

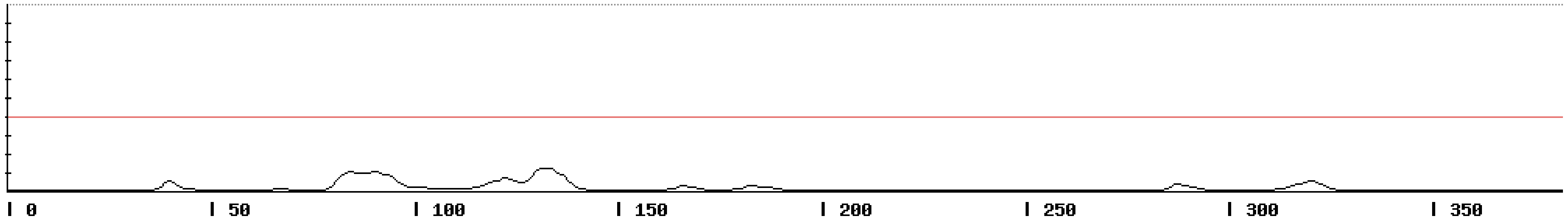

Predictions for Kalanchoelaxiflora_Kalax.0126s0052

Viterbi Path

Viterbi Path

Posterior @ 0.4

Posterior @ 0.4

Posterior Graphic. Threshold Marked @ 0.4

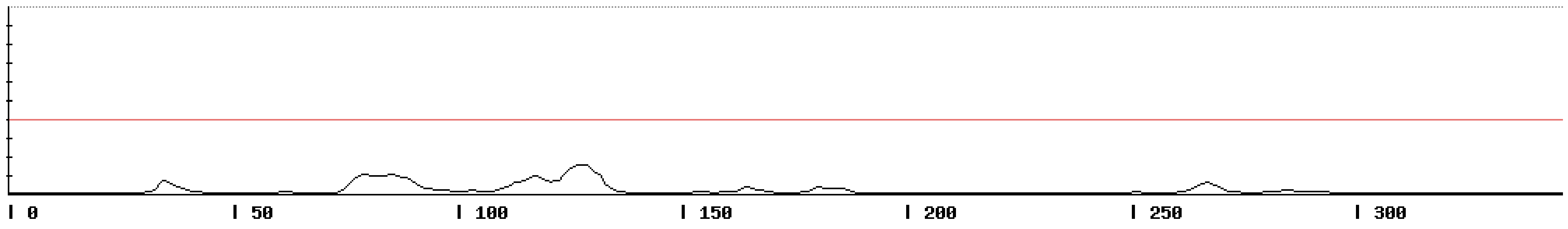

Predictions for Kalanchoelaxiflora_Kalax.0130s0010

Viterbi Path

Viterbi Path

Posterior @ 0.4

Posterior @ 0.4

Posterior Graphic. Threshold Marked @ 0.4

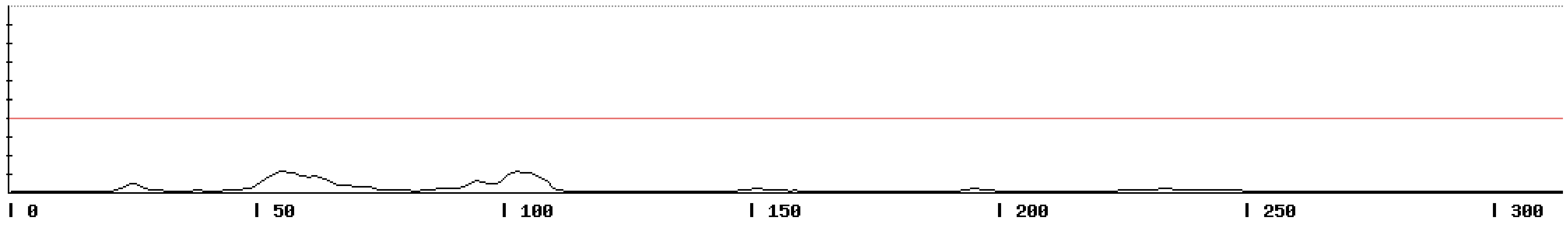

Predictions for Kalanchoelaxiflora_Kalax.0131s0011	
Viterbi Path	
Viterbi Path	
Posterior @ 0.4	
Posterior @ 0.4	
Posterior Graphic. Threshold Marked @ 0.4	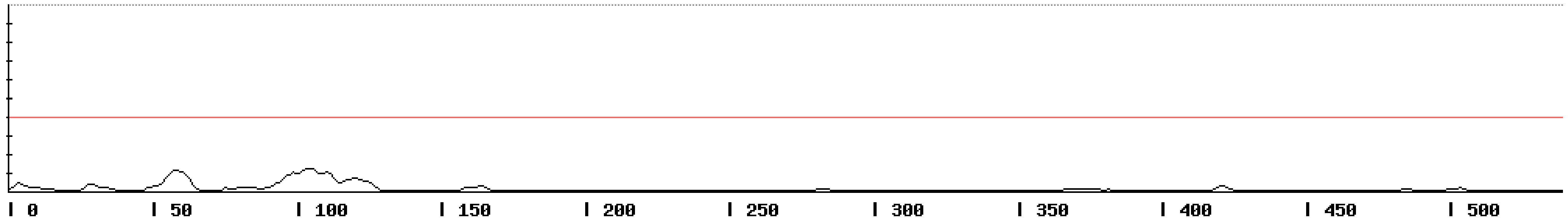

Predictions for Kalanchoelaxiflora_Kalax.0132s0094	
Viterbi Path	
Viterbi Path	
Posterior @ 0.4	
Posterior @ 0.4	
Posterior Graphic. Threshold Marked @ 0.4	

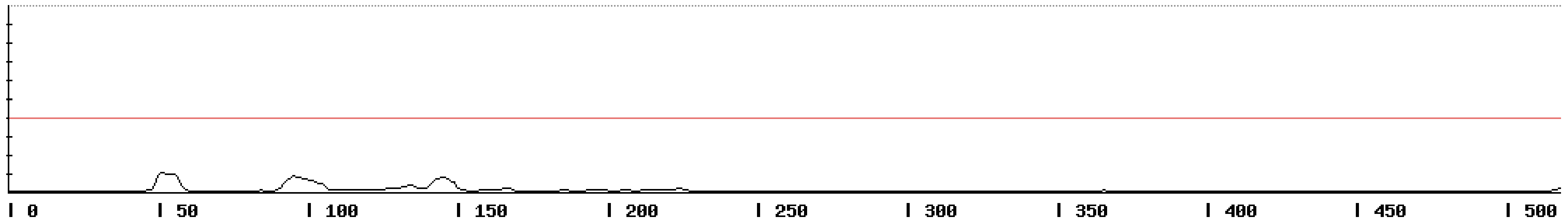

Predictions for Kalanchoelaxiflora_Kalax.0136s0013

Viterbi Path

Viterbi Path

Posterior @ 0.4

Posterior @ 0.4

Posterior Graphic. Threshold Marked @ 0.4

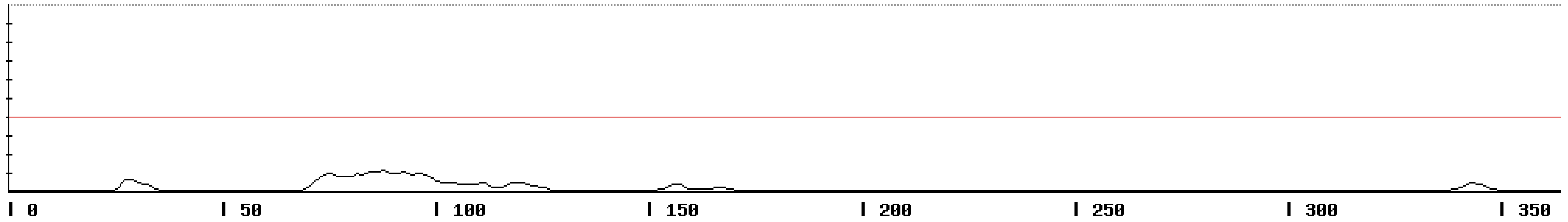

Predictions for Kalanchoelaxiflora_Kalax.0137s0047

Viterbi Path

Viterbi Path

Posterior @ 0.4

Posterior @ 0.4

Posterior Graphic. Threshold Marked @ 0.4	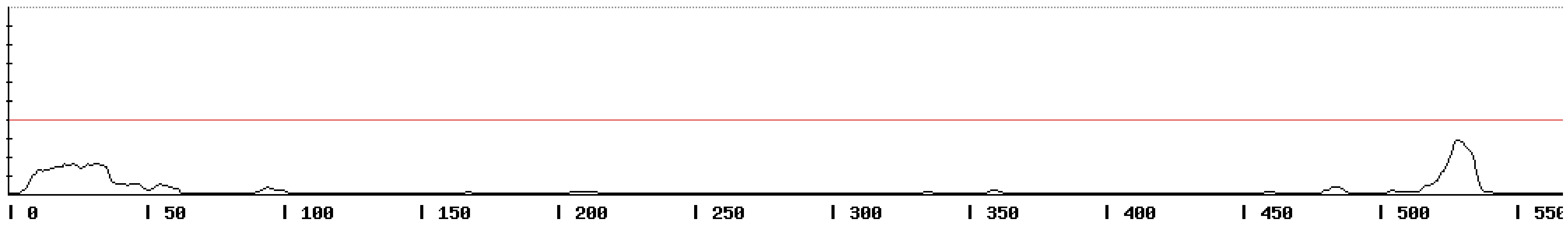

Predictions for Kalanchoelaxiflora_Kalax.0139s0008

Viterbi Path	
Viterbi Path	
Posterior @ 0.4	
Posterior @ 0.4	
Posterior Graphic. Threshold Marked @ 0.4	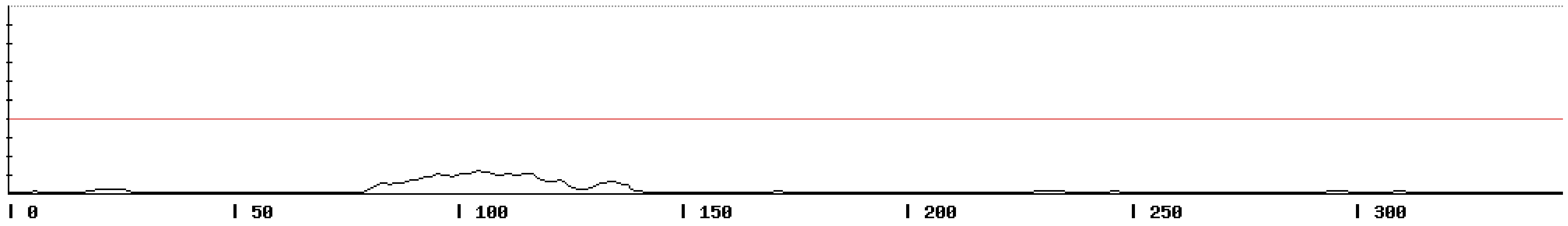

Predictions for Kalanchoelaxiflora_Kalax.0139s0009

Viterbi Path	
Viterbi Path	
Posterior @ 0.4	
Posterior @ 0.4	

Posterior Graphic. Threshold Marked @ 0.4

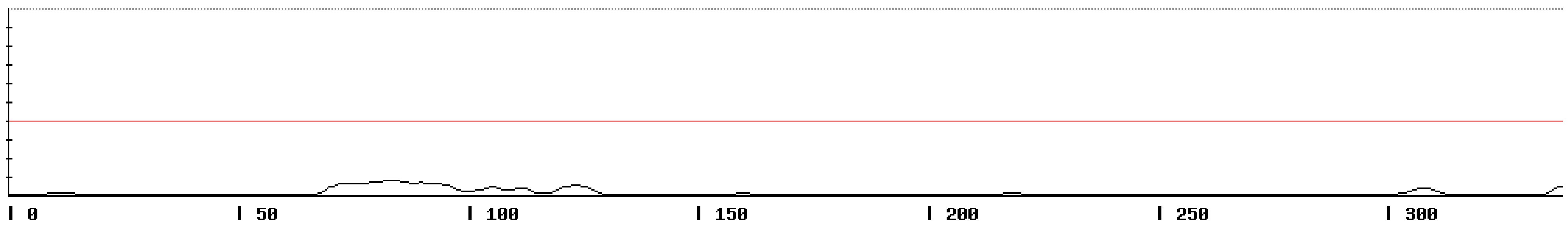

Predictions for Kalanchoelaxiflora_Kalax.0139s0010

Viterbi Path

Viterbi Path

Posterior @ 0.4

Posterior @ 0.4

Posterior Graphic. Threshold Marked @ 0.4

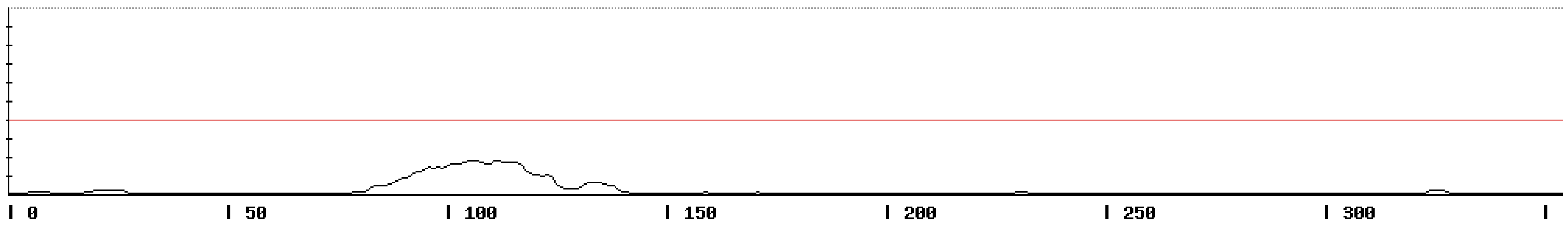

Predictions for Kalanchoelaxiflora_Kalax.0139s0060

Viterbi Path

Viterbi Path

Posterior @ 0.4	
Posterior @ 0.4	
Posterior Graphic. Threshold Marked @ 0.4	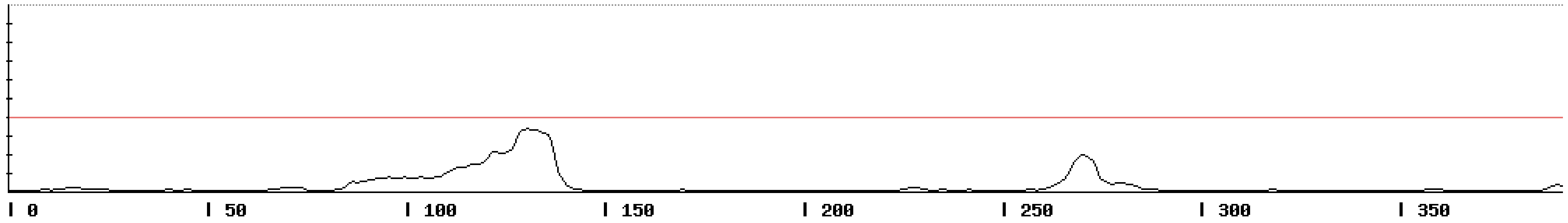

Predictions for Kalanchoelaxiflora_Kalax.0139s0061

Viterbi Path	
Viterbi Path	
Posterior @ 0.4	
Posterior @ 0.4	
Posterior Graphic. Threshold Marked @ 0.4	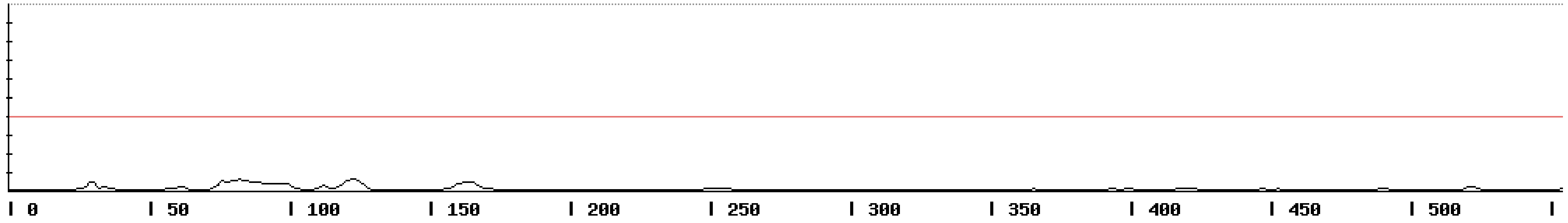

Predictions for Kalanchoelaxiflora_Kalax.0139s0062

Viterbi Path	
Viterbi Path	

Posterior @ 0.4	129 - RP - 130
Posterior @ 0.4	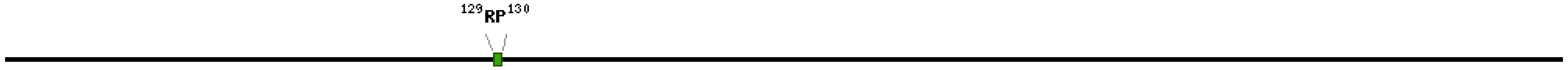
Posterior Graphic. Threshold Marked @ 0.4	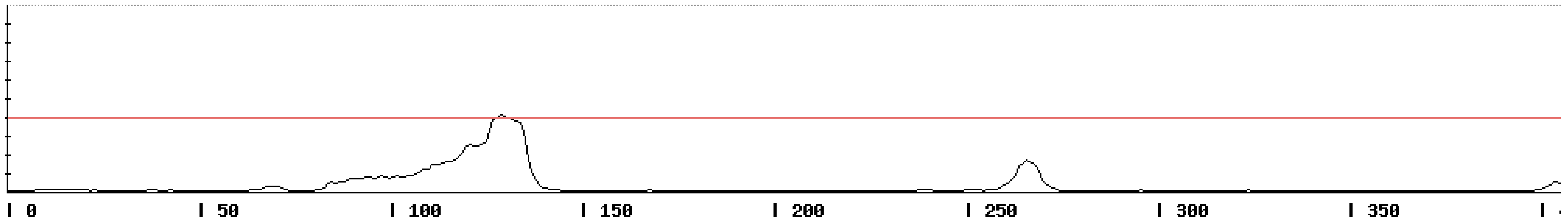

Predictions for Kalanchoelaxiflora_Kalax.0140s0019

Viterbi Path	
Viterbi Path	
Posterior @ 0.4	
Posterior @ 0.4	
Posterior Graphic. Threshold Marked @ 0.4	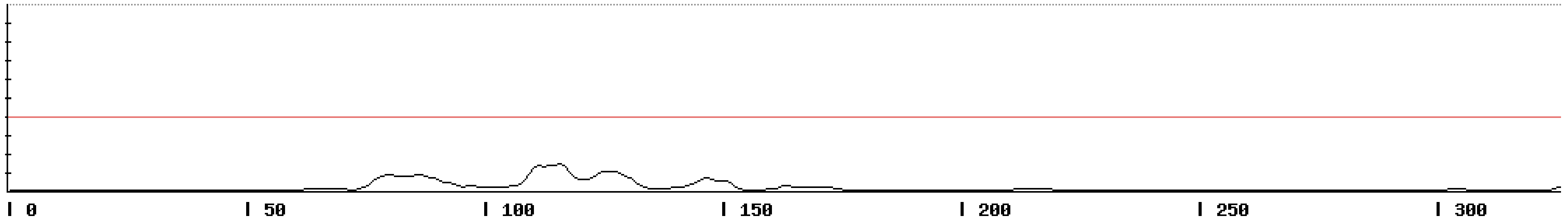

Predictions for Kalanchoelaxiflora_Kalax.0140s0020

Viterbi Path

Viterbi Path

Posterior @ 0.4

Posterior @ 0.4

Posterior Graphic. Threshold Marked @ 0.4

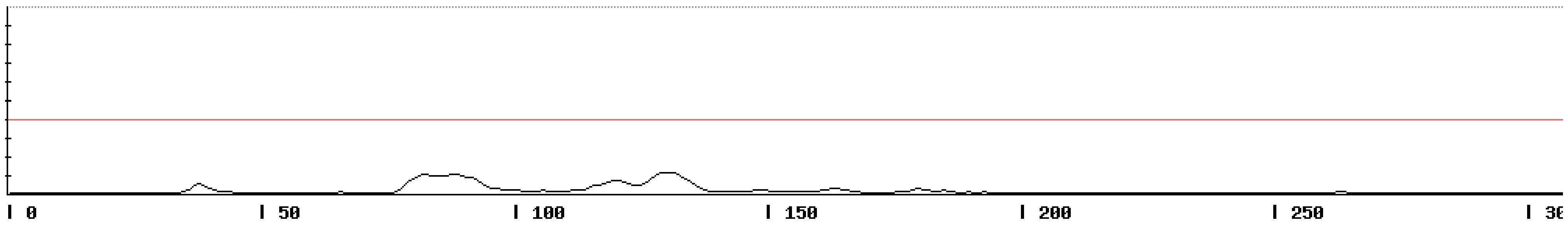

Predictions for Kalanchoelaxiflora_Kalax.0140s0052

Viterbi Path 72 - RKSPGGSRLNRATRKGYWKATGKDRKIRSKKNEIGMKK - 109

Viterbi Path

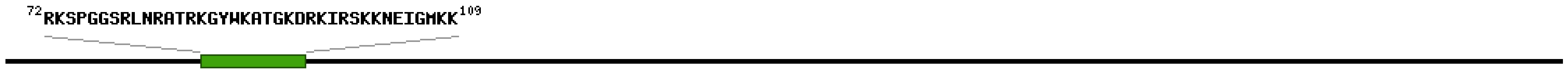

Posterior @ 0.4 72 - RKSPGGSRLNRATRKGYWKATGKDRKIRSKKNEIGMKK - 109

Posterior @ 0.4

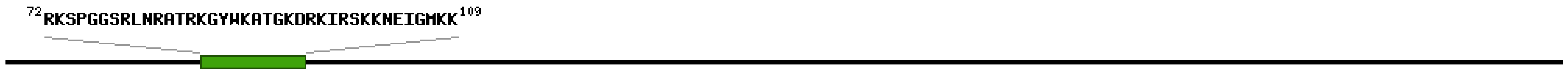

Posterior Graphic. Threshold Marked @ 0.4

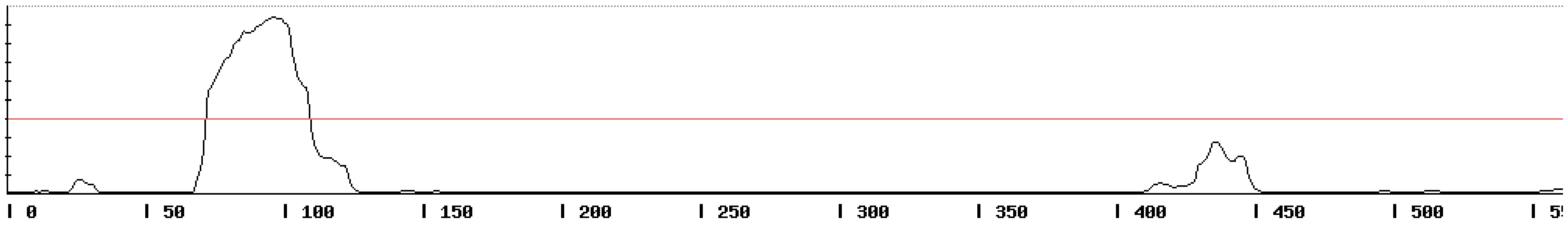

Predictions for Kalanchoelaxiflora_Kalax.0144s0050

Viterbi Path	
Viterbi Path	
Posterior @ 0.4	
Posterior @ 0.4	
Posterior Graphic. Threshold Marked @ 0.4	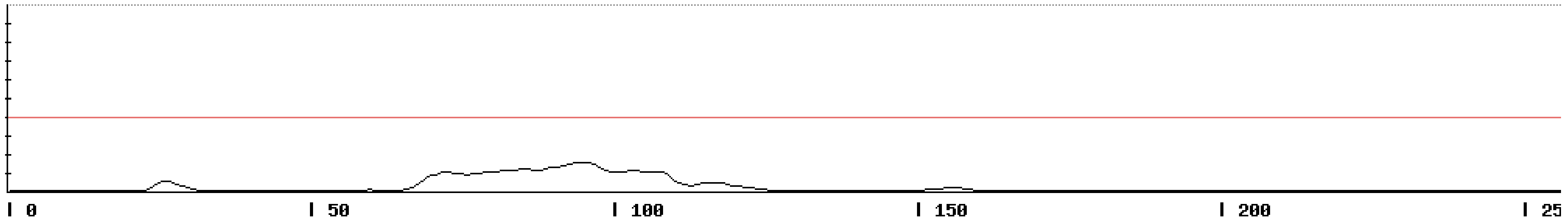

Predictions for Kalanchoelaxiflora_Kalax.0149s0055

Viterbi Path	
Viterbi Path	
Posterior @ 0.4	
Posterior @ 0.4	
Posterior Graphic. Threshold Marked @ 0.4	

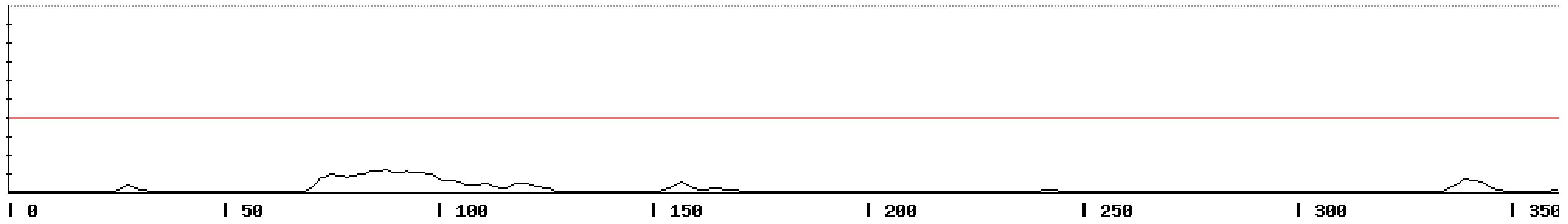

Predictions for Kalanchoelaxiflora_Kalax.0150s0017	
Viterbi Path	
Viterbi Path	
Posterior @ 0.4	
Posterior @ 0.4	
Posterior Graphic. Threshold Marked @ 0.4	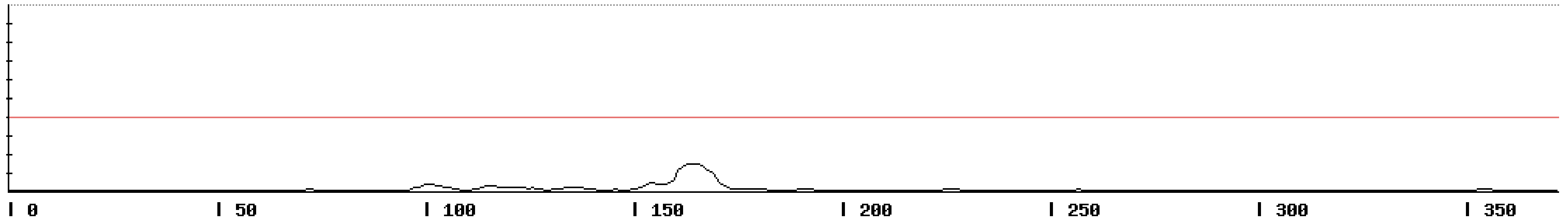

Predictions for Kalanchoelaxiflora_Kalax.0151s0058	
Viterbi Path	
Viterbi Path	
Posterior @ 0.4	
Posterior @ 0.4	

Posterior Graphic. Threshold Marked @ 0.4

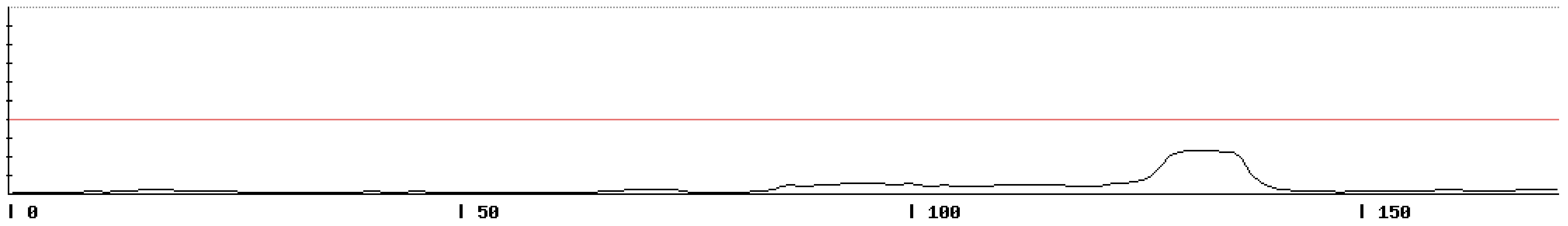

Predictions for Kalanchoelaxiflora_Kalax.0152s0060

Viterbi Path

Viterbi Path

Posterior @ 0.4

Posterior @ 0.4

Posterior Graphic. Threshold Marked @ 0.4

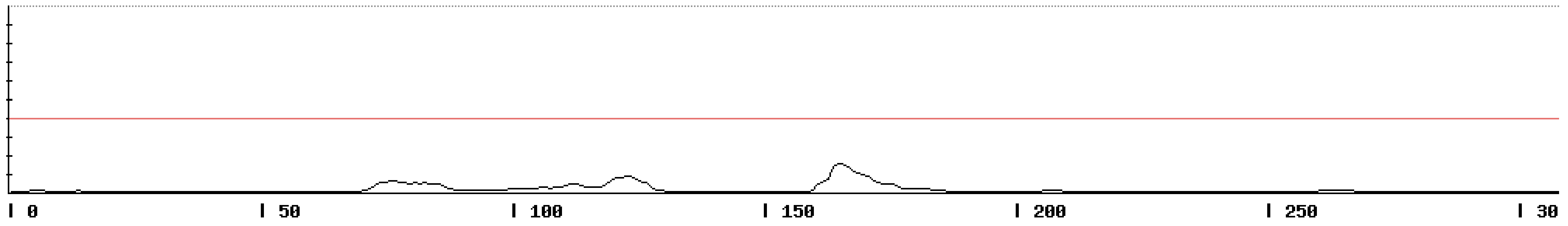

Predictions for Kalanchoelaxiflora_Kalax.0154s0005

Viterbi Path

Viterbi Path

Posterior @ 0.4

Posterior @ 0.4

Posterior Graphic. Threshold Marked @ 0.4

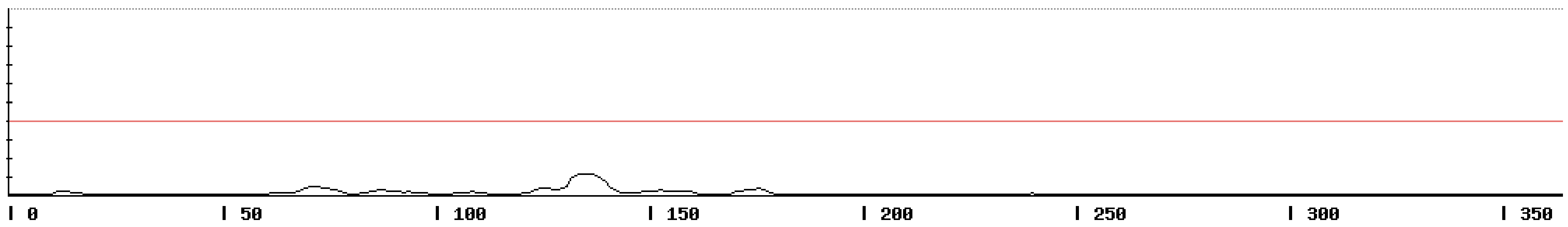

Predictions for Kalanchoelaxiflora_Kalax.0158s0076

Viterbi Path

Viterbi Path

Posterior @ 0.4

Posterior @ 0.4

Posterior Graphic. Threshold Marked @ 0.4

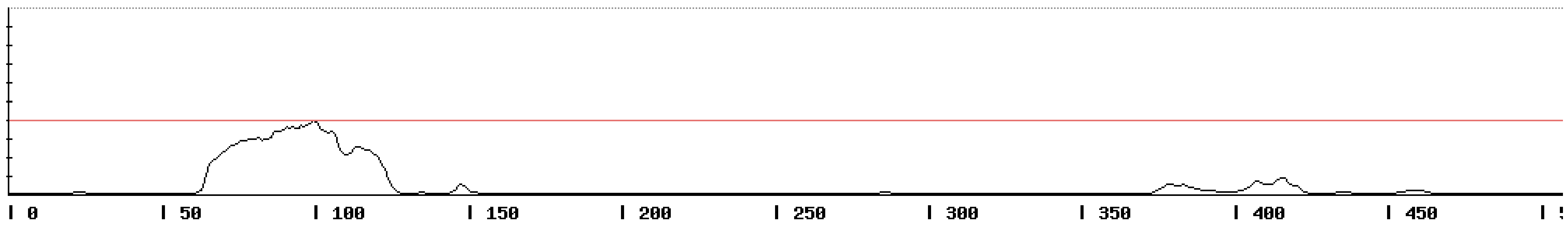

Predictions for Kalanchoelaxiflora_Kalax.0160s0031

Viterbi Path

Viterbi Path

Posterior @ 0.4	
Posterior @ 0.4	
Posterior Graphic. Threshold Marked @ 0.4	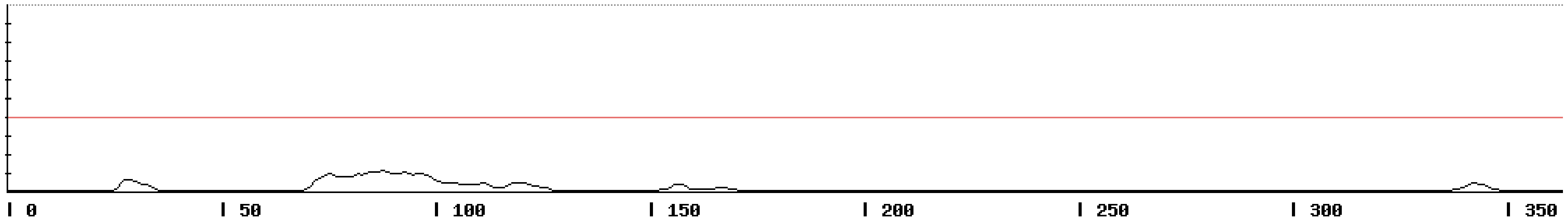

Predictions for Kalanchoelaxiflora_Kalax.0168s0017

Viterbi Path	
Viterbi Path	
Posterior @ 0.4	
Posterior @ 0.4	
Posterior Graphic. Threshold Marked @ 0.4	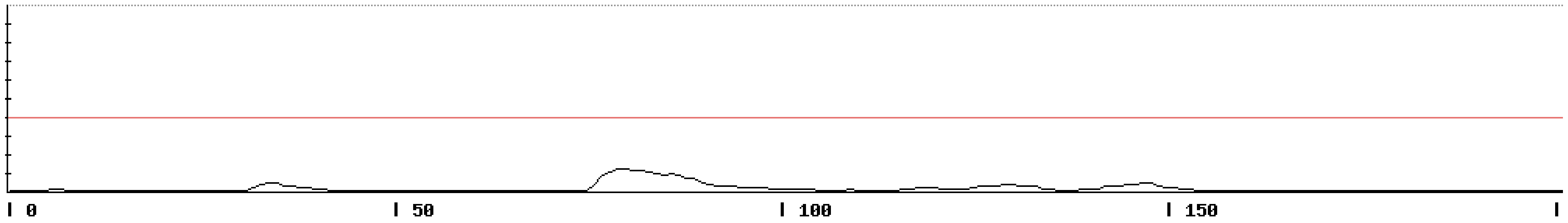

Predictions for Kalanchoelaxiflora_Kalax.0181s0046

Viterbi Path	
Viterbi Path	

Posterior @ 0.4

Posterior @ 0.4

Posterior Graphic. Threshold Marked @ 0.4

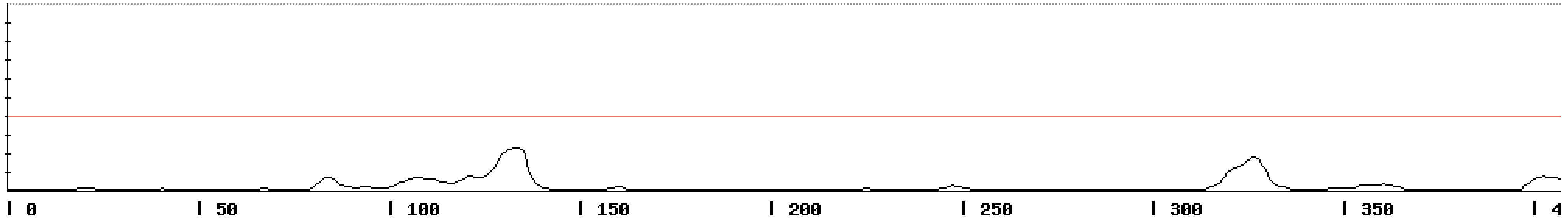

Predictions for Kalanchoelaxiflora_Kalax.0182s0012

Viterbi Path

Viterbi Path

Posterior @ 0.4

Posterior @ 0.4

Posterior Graphic. Threshold Marked @ 0.4

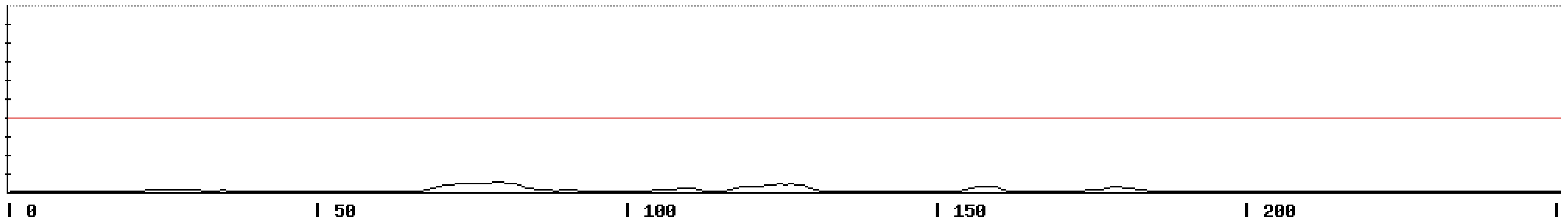

Predictions for Kalanchoelaxiflora_Kalax.0182s0022

Viterbi Path

Viterbi Path

Posterior @ 0.4

Posterior @ 0.4

Posterior Graphic. Threshold Marked @ 0.4

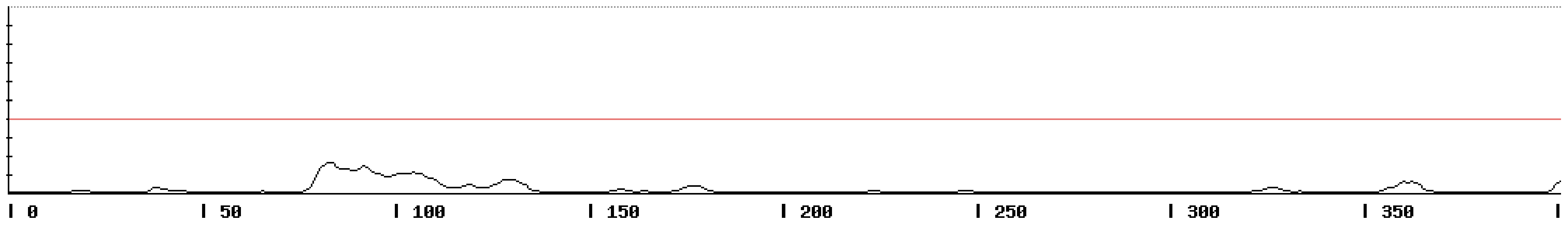

Predictions for Kalanchoelaxiflora_Kalax.0184s0004

Viterbi Path

Viterbi Path

Posterior @ 0.4

Posterior @ 0.4

Posterior Graphic. Threshold Marked @ 0.4

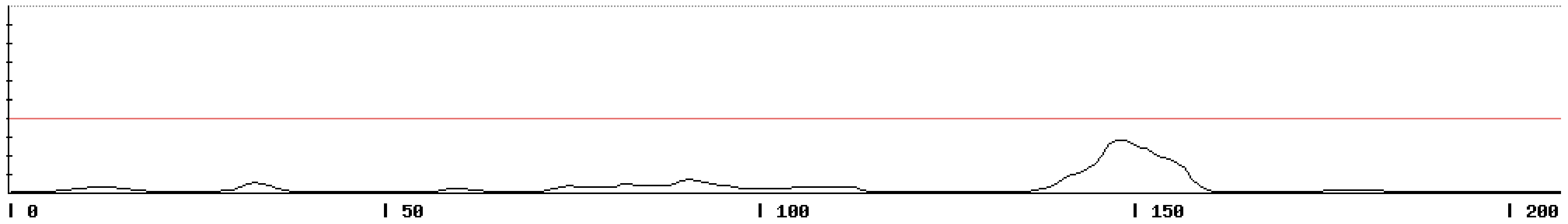

Predictions for Kalanchoelaxiflora_Kalax.0199s0022	
Viterbi Path	
Viterbi Path	
Posterior @ 0.4	
Posterior @ 0.4	
Posterior Graphic. Threshold Marked @ 0.4	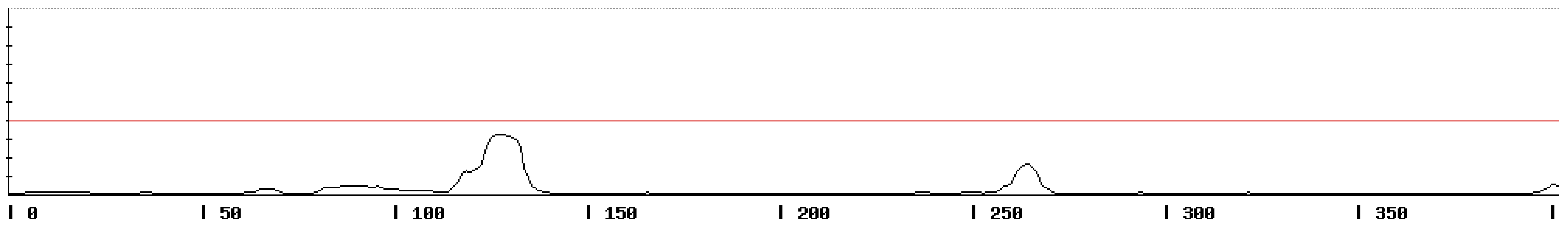

Predictions for Kalanchoelaxiflora_Kalax.0199s0023	
Viterbi Path	
Viterbi Path	
Posterior @ 0.4	
Posterior @ 0.4	
Posterior Graphic. Threshold Marked @ 0.4	

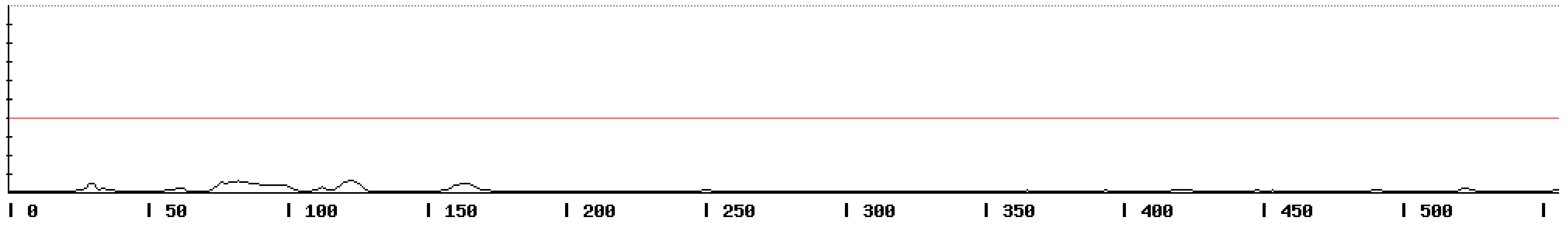

Predictions for Kalanchoelaxiflora_Kalax.0199s0024

Viterbi Path

Viterbi Path

Posterior @ 0.4

Posterior @ 0.4

Posterior Graphic. Threshold Marked @ 0.4

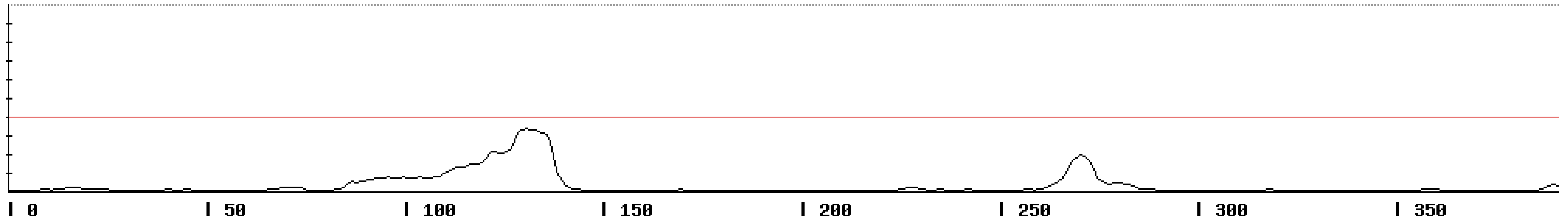

Predictions for Kalanchoelaxiflora_Kalax.0200s0025

Viterbi Path

Viterbi Path

Posterior @ 0.4

Posterior @ 0.4

Posterior Graphic. Threshold Marked @ 0.4

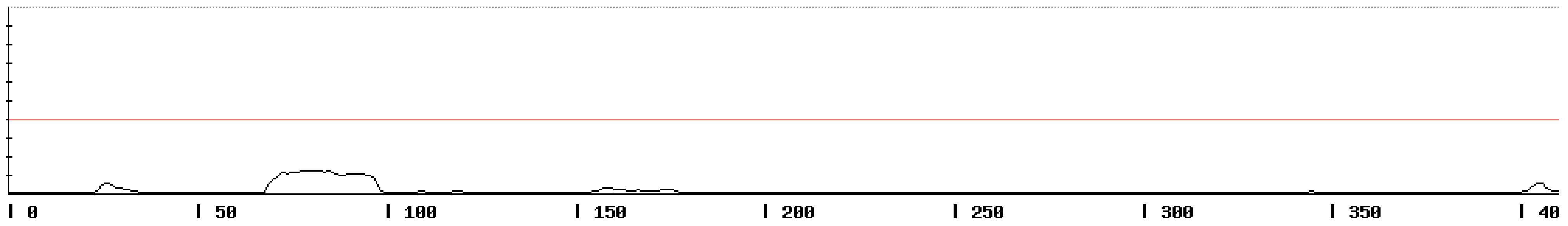

Predictions for Kalanchoelaxiflora_Kalax.0201s0025

Viterbi Path

Viterbi Path

Posterior @ 0.4

Posterior @ 0.4

Posterior Graphic. Threshold Marked @ 0.4

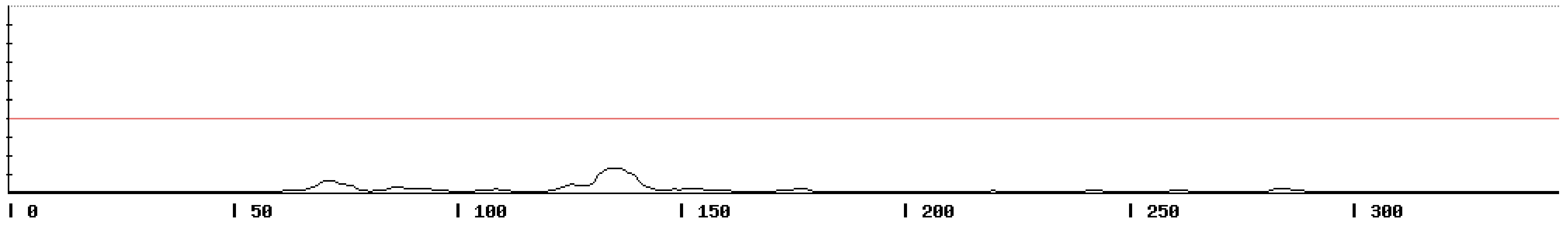

Predictions for Kalanchoelaxiflora_Kalax.0211s0006

Viterbi Path

Viterbi Path

Posterior @ 0.4

Posterior @ 0.4

Posterior Graphic. Threshold Marked @ 0.4	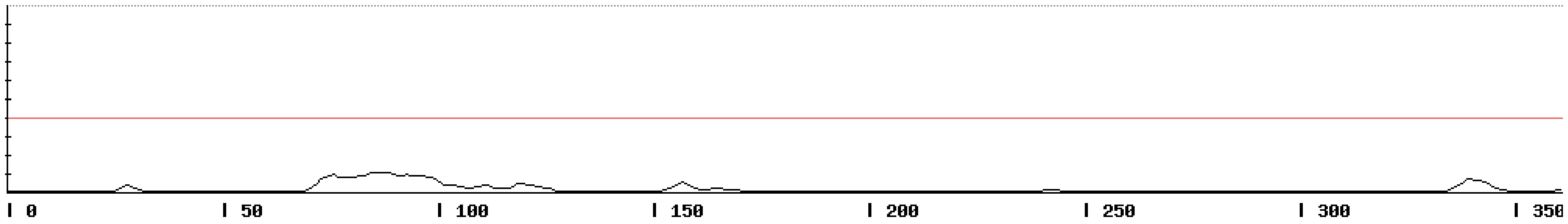

Predictions for Kalanchoelaxiflora_Kalax.0217s0043	
Viterbi Path	
Viterbi Path	
Posterior @ 0.4	
Posterior @ 0.4	
Posterior Graphic. Threshold Marked @ 0.4	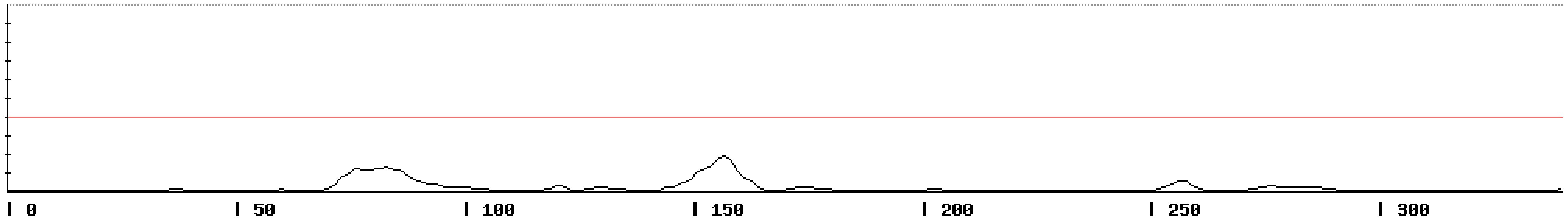

Predictions for Kalanchoelaxiflora_Kalax.0219s0006	
Viterbi Path	
Viterbi Path	

Posterior @ 0.4

Posterior @ 0.4

Posterior Graphic. Threshold Marked @ 0.4

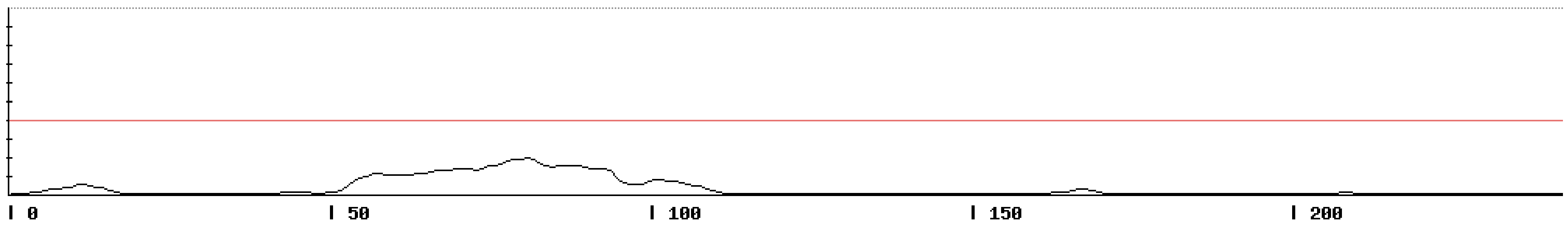

Predictions for Kalanchoelaxiflora_Kalax.0219s0017

Viterbi Path

Viterbi Path

Posterior @ 0.4

Posterior @ 0.4

Posterior Graphic. Threshold Marked @ 0.4

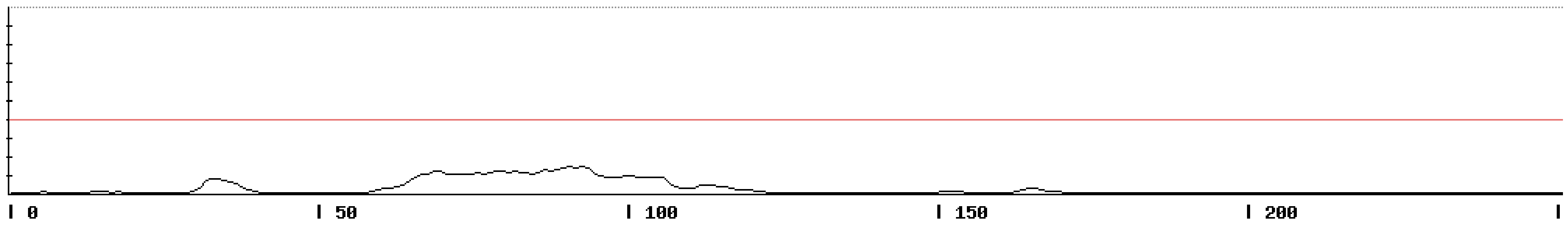

Predictions for Kalanchoelaxiflora_Kalax.0221s0022

Viterbi Path

Viterbi Path

Posterior @ 0.4

Posterior @ 0.4

Posterior Graphic. Threshold Marked @ 0.4

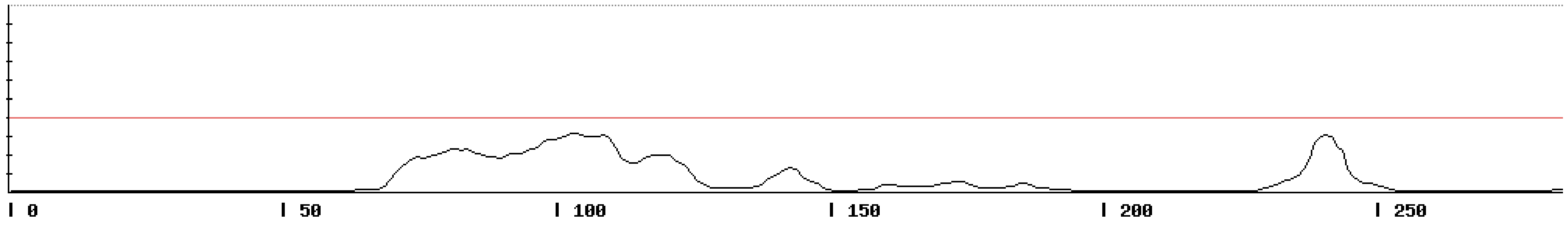

Predictions for Kalanchoelaxiflora_Kalax.0222s0012

Viterbi Path

Viterbi Path

Posterior @ 0.4

Posterior @ 0.4

Posterior Graphic. Threshold Marked @ 0.4

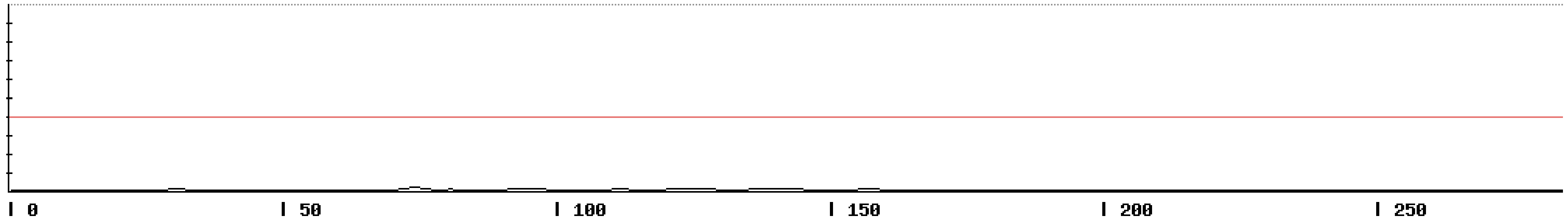

Predictions for Kalanchoelaxiflora_Kalax.0223s0043

Viterbi Path

Viterbi Path

Posterior @ 0.4

Posterior @ 0.4

Posterior Graphic. Threshold Marked @ 0.4

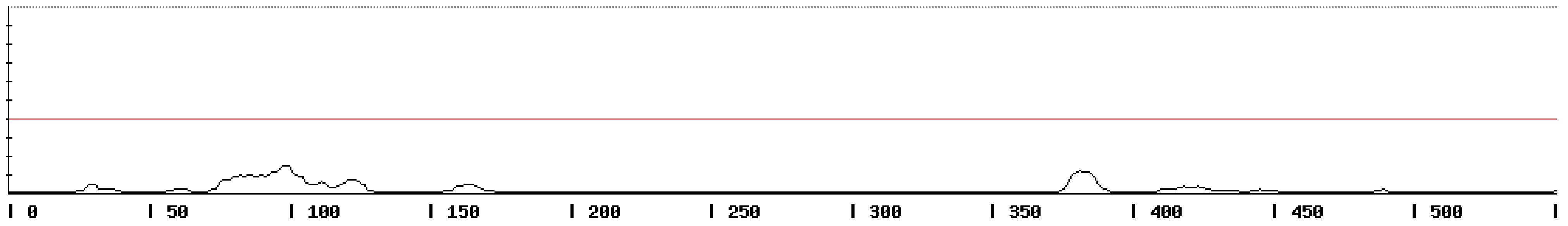

Predictions for Kalanchoelaxiflora_Kalax.0223s0044

Viterbi Path

Viterbi Path

Posterior @ 0.4

Posterior @ 0.4

Posterior Graphic. Threshold Marked @ 0.4

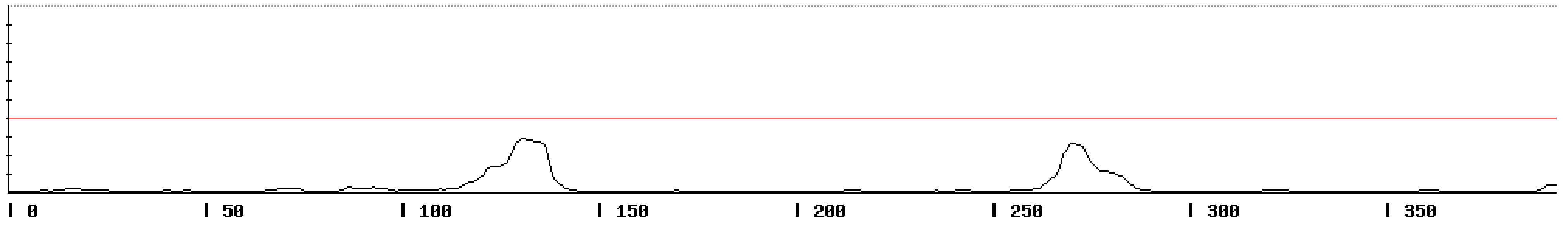

Predictions for Kalanchoelaxiflora_Kalax.0226s0021

Viterbi Path

Viterbi Path

Posterior @ 0.4

Posterior @ 0.4

Posterior Graphic. Threshold Marked @ 0.4

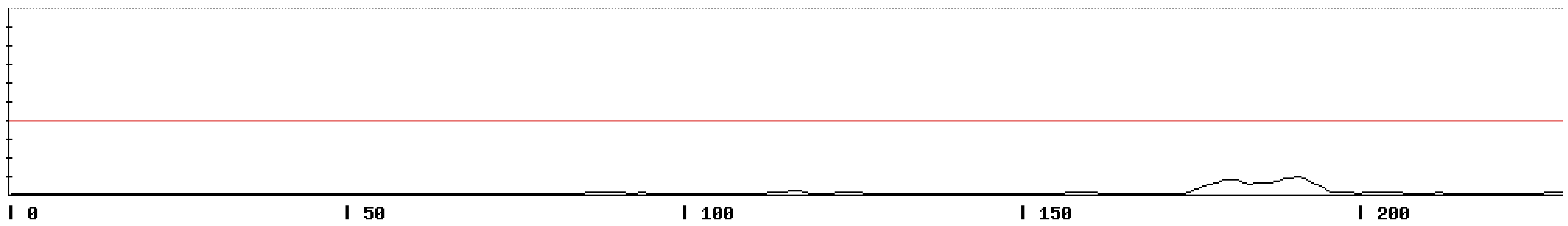

Predictions for Kalanchoelaxiflora_Kalax.0227s0023

Viterbi Path

Viterbi Path

Posterior @ 0.4

Posterior @ 0.4

Posterior Graphic. Threshold Marked @ 0.4

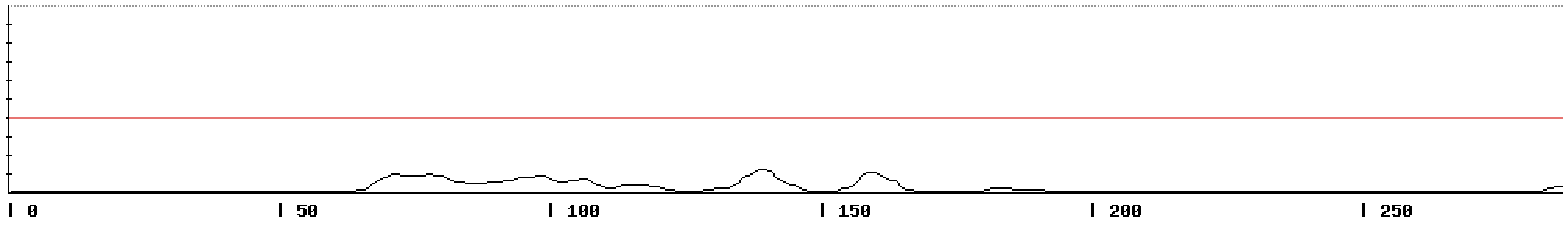

Predictions for Kalanchoelaxiflora_Kalax.0227s0051	
Viterbi Path	
Viterbi Path	
Posterior @ 0.4	
Posterior @ 0.4	
Posterior Graphic. Threshold Marked @ 0.4	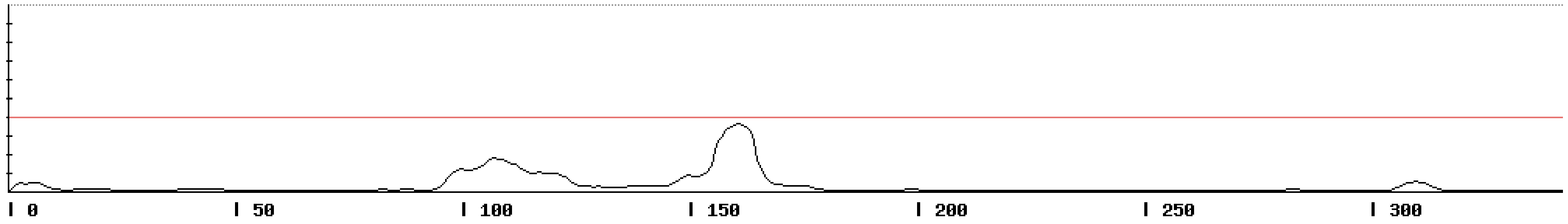

Predictions for Kalanchoelaxiflora_Kalax.0230s0011	
Viterbi Path	
Viterbi Path	
Posterior @ 0.4	
Posterior @ 0.4	

Posterior Graphic. Threshold Marked @ 0.4	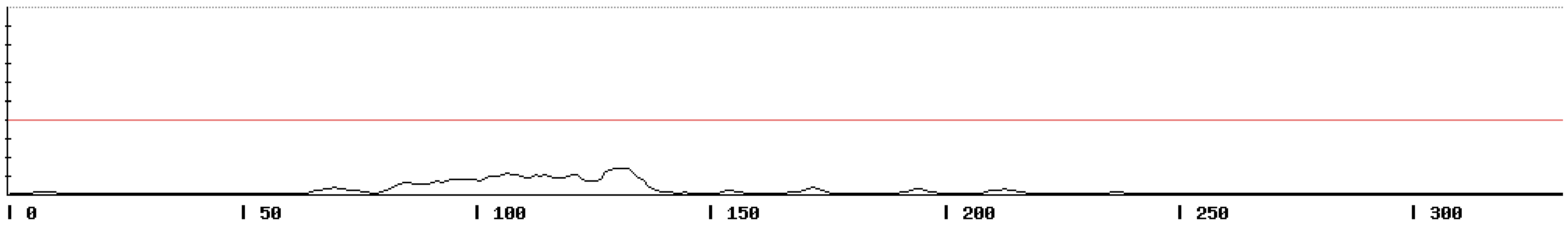

Predictions for Kalanchoelaxiflora_Kalax.0252s0019

Viterbi Path	
Viterbi Path	
Posterior @ 0.4	
Posterior @ 0.4	
Posterior Graphic. Threshold Marked @ 0.4	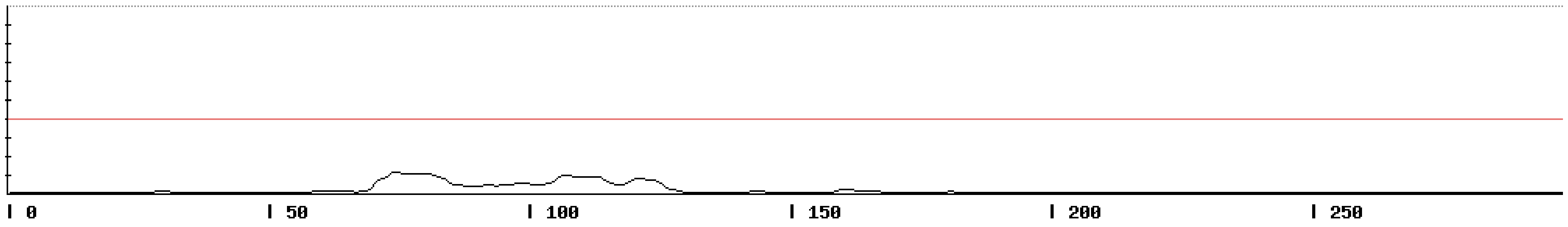

Predictions for Kalanchoelaxiflora_Kalax.0264s0036

Viterbi Path	
Viterbi Path	
Posterior @ 0.4	
Posterior @ 0.4	

Posterior Graphic. Threshold Marked @ 0.4	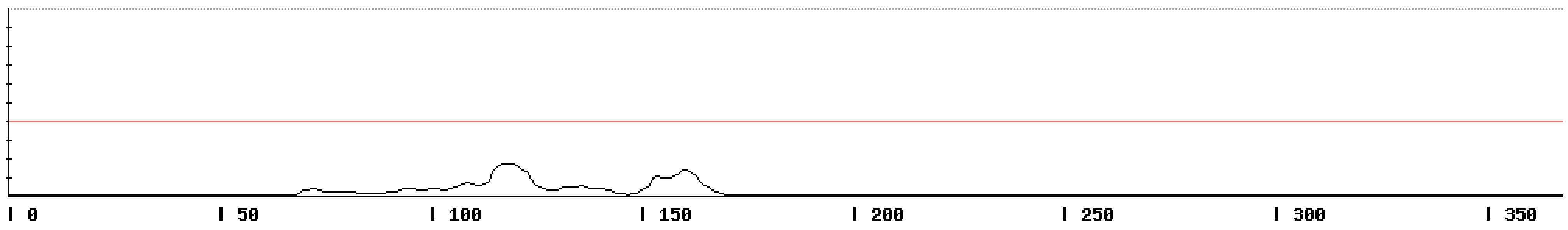

Predictions for Kalanchoelaxiflora_Kalax.0270s0004

Viterbi Path	
Viterbi Path	
Posterior @ 0.4	
Posterior @ 0.4	
Posterior Graphic. Threshold Marked @ 0.4	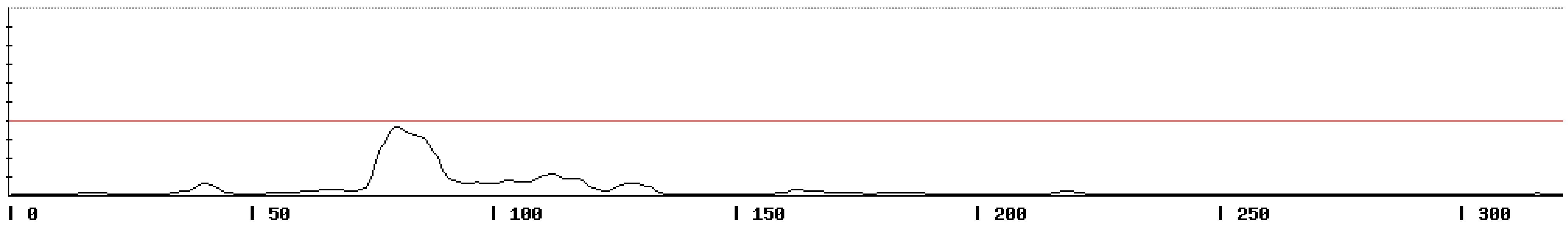

Predictions for Kalanchoelaxiflora_Kalax.0285s0054

Viterbi Path	
Viterbi Path	

Posterior @ 0.4

Posterior @ 0.4

Posterior Graphic. Threshold Marked @ 0.4

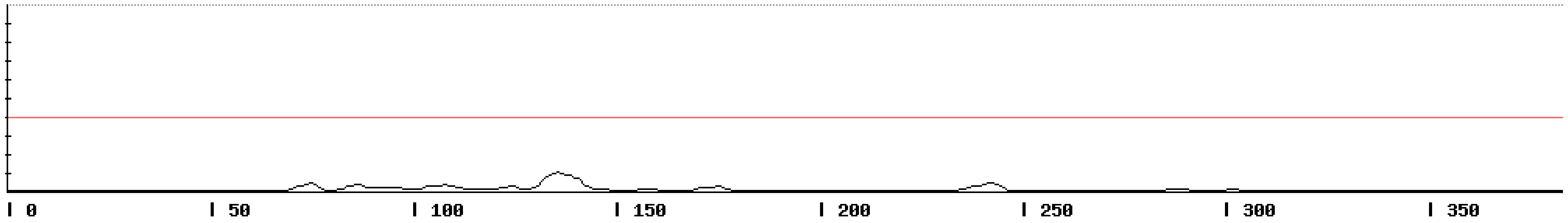

Predictions for Kalanchoelaxiflora_Kalax.0294s0015

Viterbi Path

Viterbi Path

Posterior @ 0.4

Posterior @ 0.4

Posterior Graphic. Threshold Marked @ 0.4

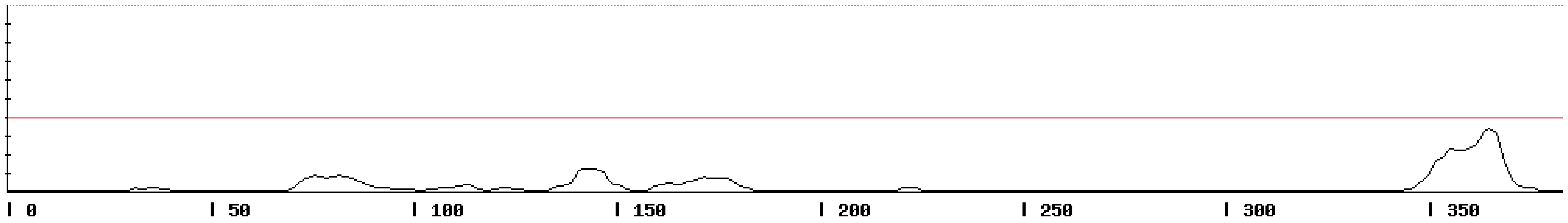

Predictions for Kalanchoelaxiflora_Kalax.0298s0056

Viterbi Path

Viterbi Path

Posterior @ 0.4	351 - KDKSKRRR - 358
	415 - RRRRSV - 420

Posterior @ 0.4

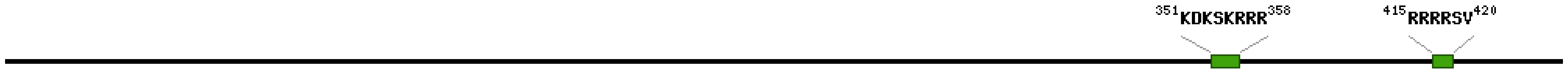

Posterior Graphic. Threshold Marked @ 0.4

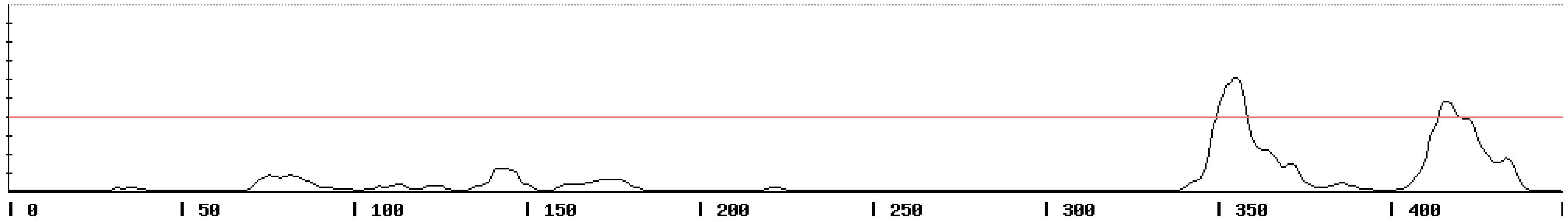

Predictions for Kalanchoelaxiflora_Kalax.0335s0038

Viterbi Path

Viterbi Path

Posterior @ 0.4	94 - GKIPR - 98

Posterior @ 0.4

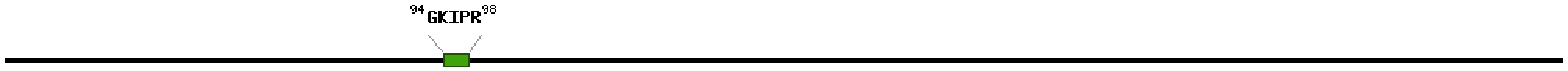

Posterior Graphic. Threshold Marked @ 0.4

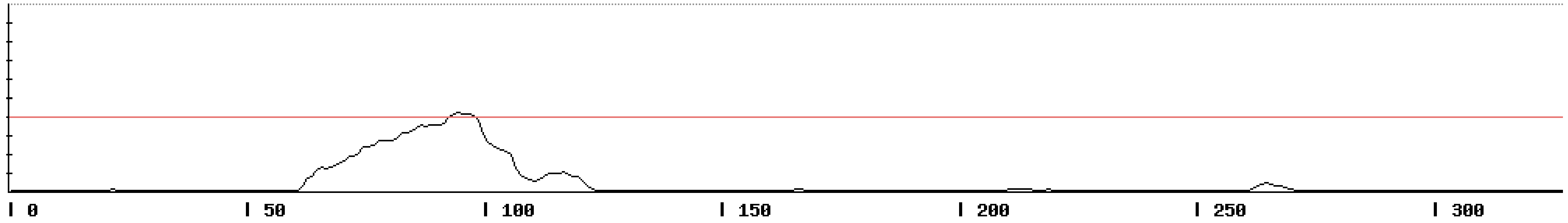

Predictions for Kalanchoelaxiflora_Kalax.0335s0039

Viterbi Path	
Viterbi Path	
Posterior @ 0.4	
Posterior @ 0.4	
Posterior Graphic. Threshold Marked @ 0.4	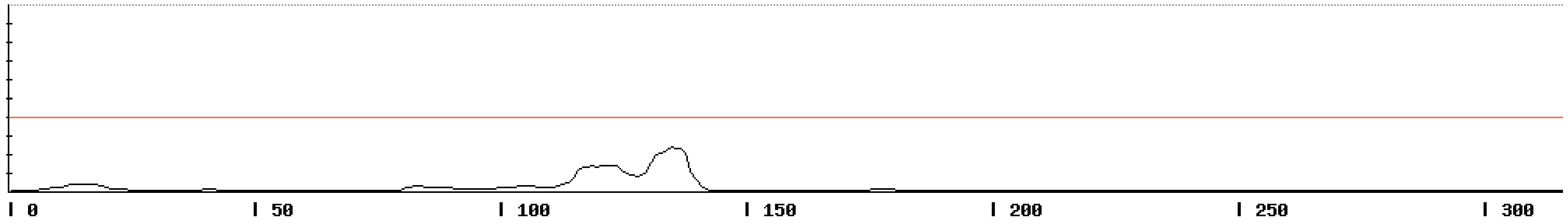

Predictions for Kalanchoelaxiflora_Kalax.0337s0011

Viterbi Path	
Viterbi Path	
Posterior @ 0.4	
Posterior @ 0.4	
Posterior Graphic. Threshold Marked @ 0.4	

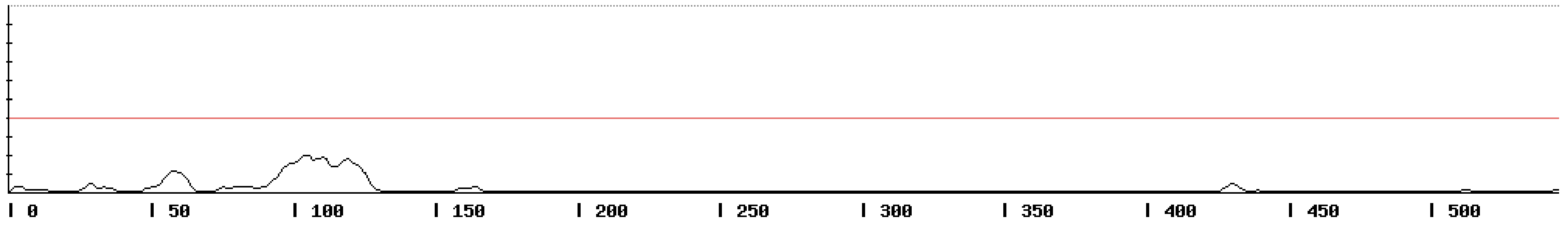

Predictions for Kalanchoelaxiflora_Kalax.0341s0035

Viterbi Path

Viterbi Path

Posterior @ 0.4

94 - GKIPR - 98

Posterior @ 0.4

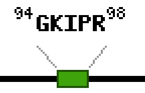

Posterior Graphic. Threshold Marked @ 0.4

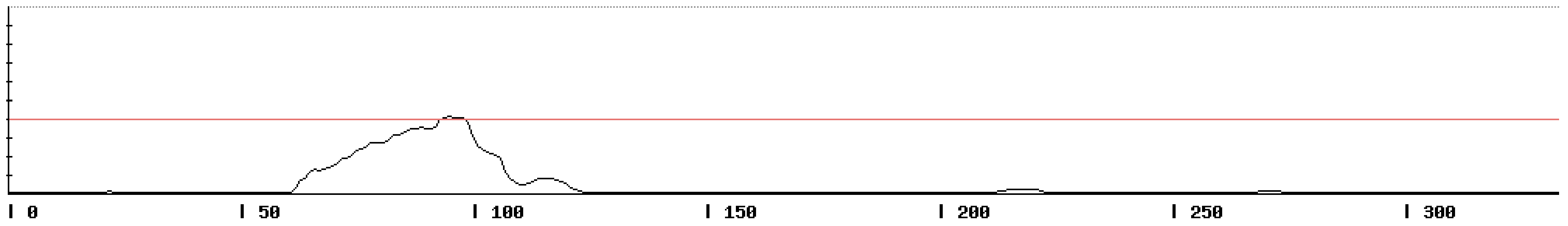

Predictions for Kalanchoelaxiflora_Kalax.0341s0036

Viterbi Path

Viterbi Path

Posterior @ 0.4

Posterior @ 0.4

Posterior Graphic. Threshold Marked @ 0.4	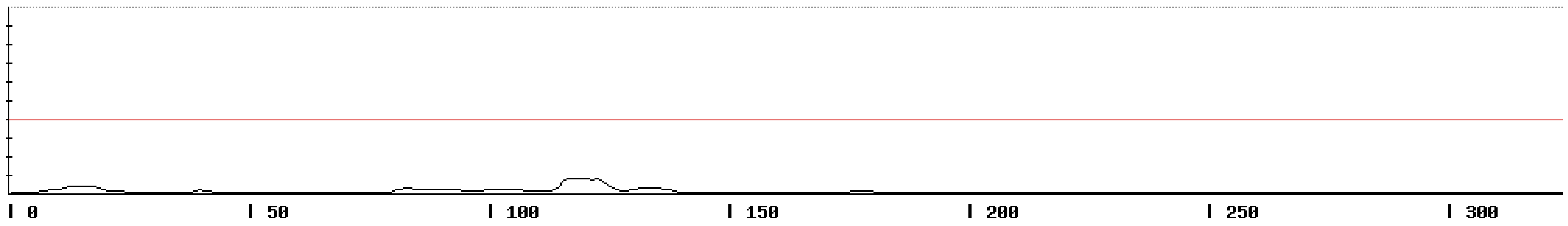

Predictions for Kalanchoelaxiflora_Kalax.0342s0019

Viterbi Path	
Viterbi Path	
Posterior @ 0.4	
Posterior @ 0.4	
Posterior Graphic. Threshold Marked @ 0.4	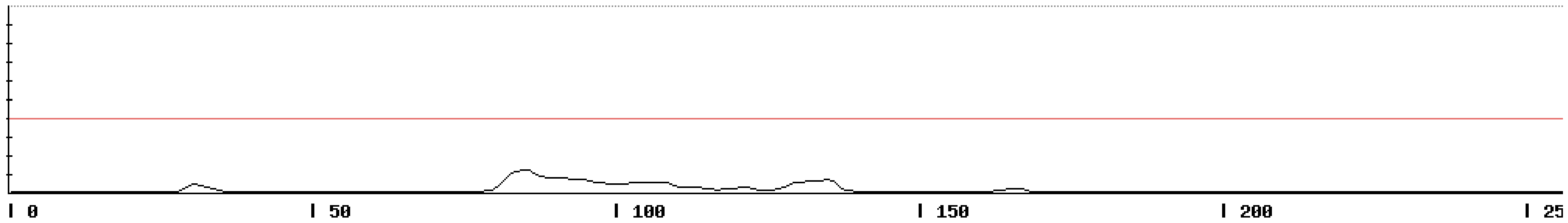

Predictions for Kalanchoelaxiflora_Kalax.0346s0002

Viterbi Path	
Viterbi Path	
Posterior @ 0.4	
Posterior @ 0.4	

Posterior Graphic. Threshold Marked @ 0.4

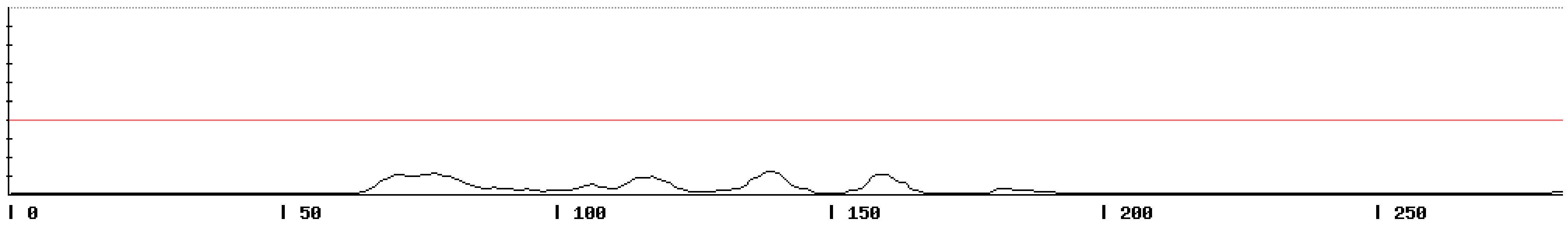

Predictions for Kalanchoelaxiflora_Kalax.0355s0003

Viterbi Path

Viterbi Path

Posterior @ 0.4

Posterior @ 0.4

Posterior Graphic. Threshold Marked @ 0.4

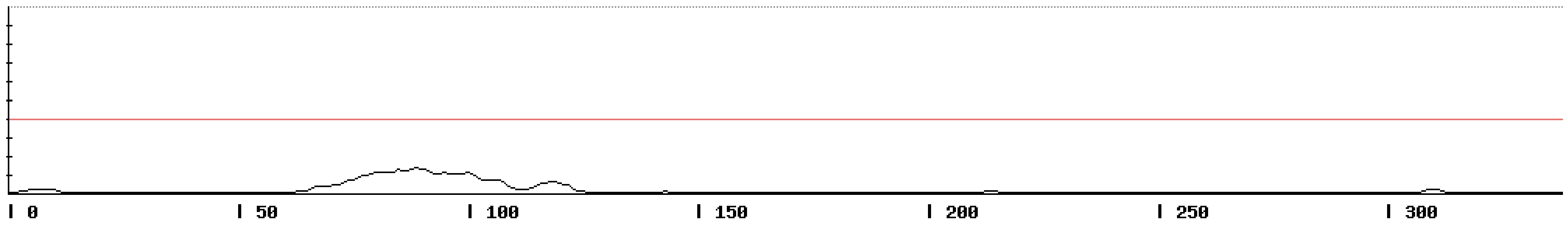

Predictions for Kalanchoelaxiflora_Kalax.0355s0004

Viterbi Path

Viterbi Path

Posterior @ 0.4

Posterior @ 0.4

Posterior Graphic. Threshold Marked @ 0.4

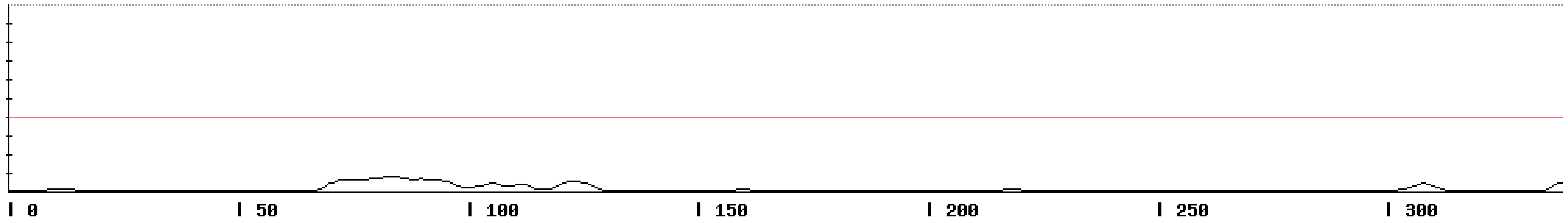

Predictions for Kalanchoelaxiflora_Kalax.0355s0005

Viterbi Path

Viterbi Path

Posterior @ 0.4

Posterior @ 0.4

Posterior Graphic. Threshold Marked @ 0.4

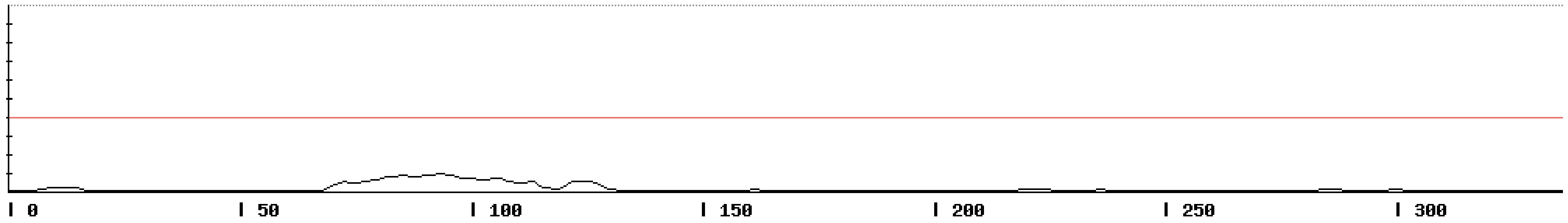

Predictions for Kalanchoelaxiflora_Kalax.0363s0038

Viterbi Path

Viterbi Path

Posterior @ 0.4

Posterior @ 0.4

Posterior Graphic. Threshold Marked @ 0.4

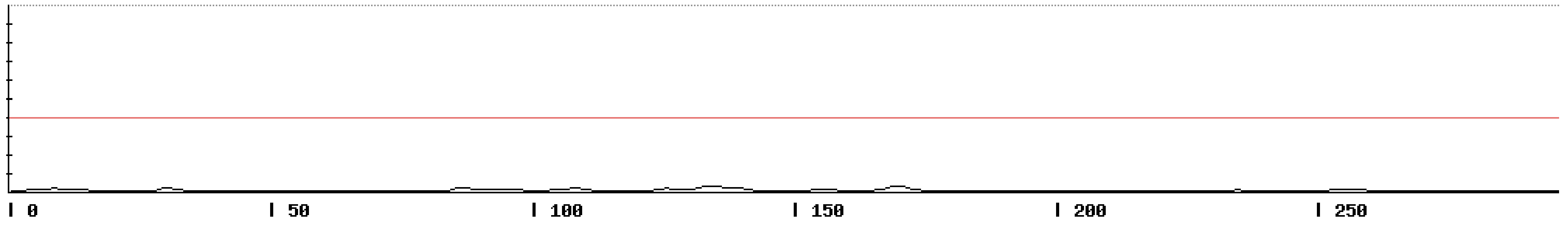

Predictions for Kalanchoelaxiflora_Kalax.0370s0001

Viterbi Path

Viterbi Path

Posterior @ 0.4

Posterior @ 0.4

Posterior Graphic. Threshold Marked @ 0.4

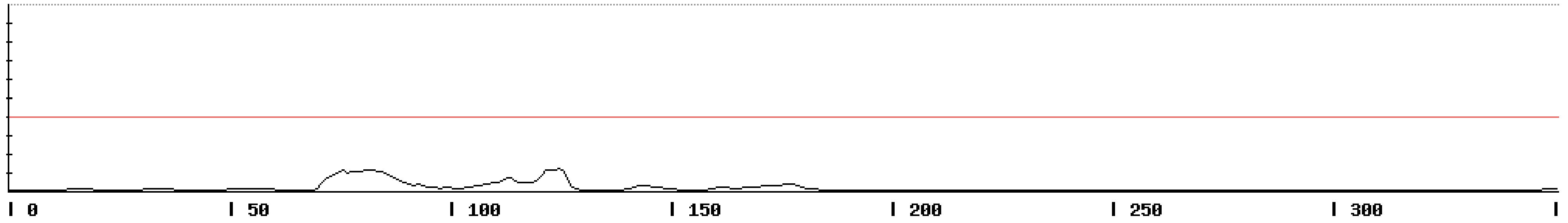

Predictions for Kalanchoelaxiflora_Kalax.0371s0043

Viterbi Path

Viterbi Path

Posterior @ 0.4

Posterior @ 0.4

Posterior Graphic. Threshold Marked @ 0.4

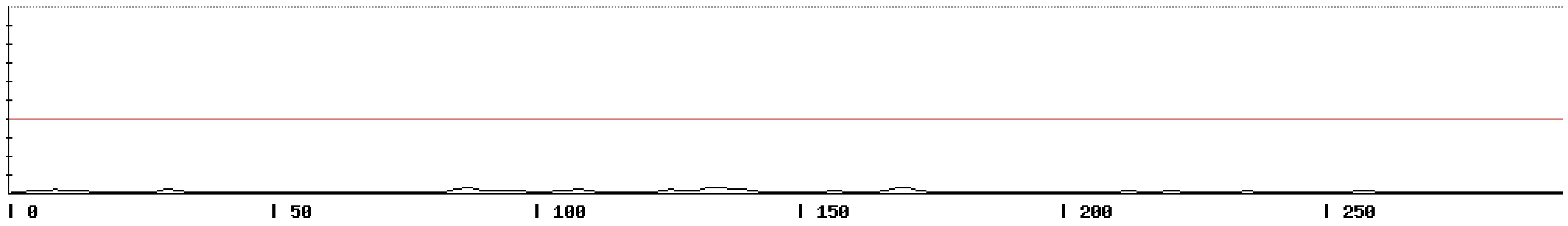

Predictions for Kalanchoelaxiflora_Kalax.0373s0029

Viterbi Path

Viterbi Path

Posterior @ 0.4

Posterior @ 0.4

Posterior Graphic. Threshold Marked @ 0.4

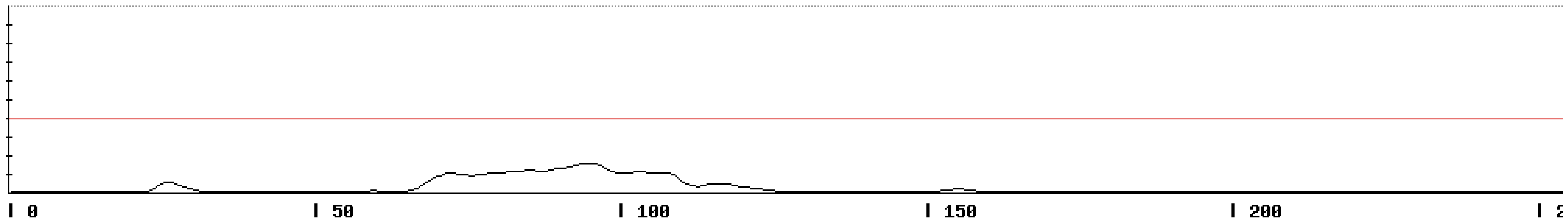

Predictions for Kalanchoelaxiflora_Kalax.0380s0025	
Viterbi Path	
Viterbi Path	
Posterior @ 0.4	9 - RRGSARNKKRRNNAPPPPPPPPPPPPPPP - 36
Posterior @ 0.4	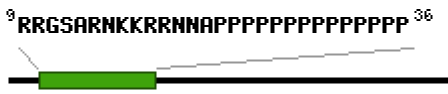
Posterior Graphic. Threshold Marked @ 0.4	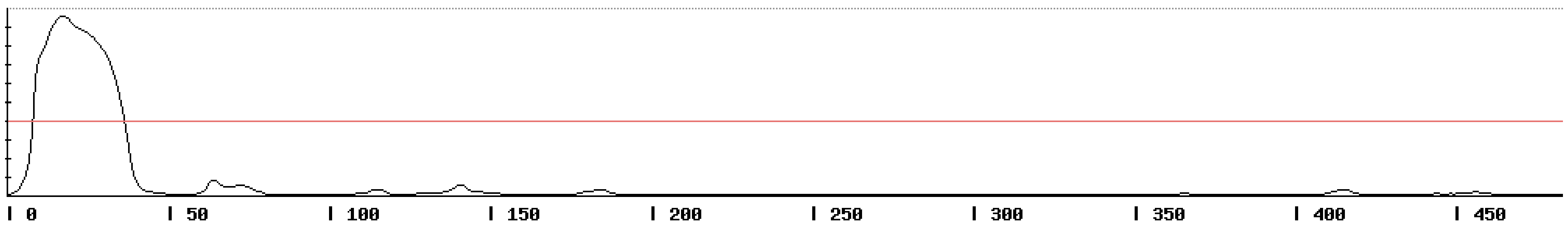

Predictions for Kalanchoelaxiflora_Kalax.0380s0026	
Viterbi Path	
Viterbi Path	
Posterior @ 0.4	
Posterior @ 0.4	
Posterior Graphic. Threshold Marked @ 0.4	

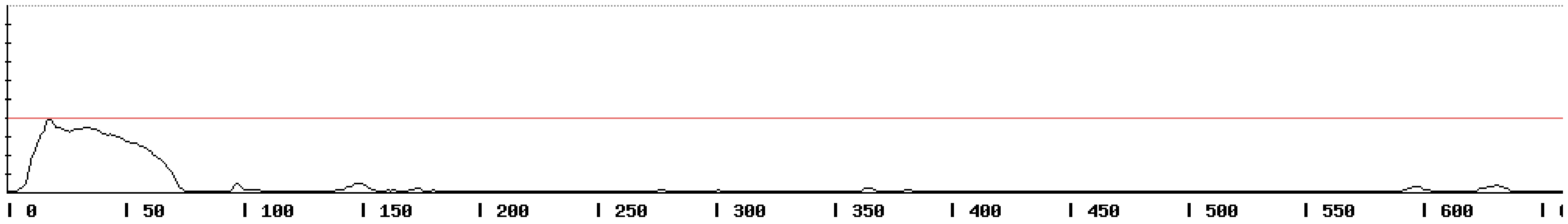

Predictions for Kalanchoelaxiflora_Kalax.0380s0027

Viterbi Path

Viterbi Path

Posterior @ 0.4

Posterior @ 0.4

Posterior Graphic. Threshold Marked @ 0.4

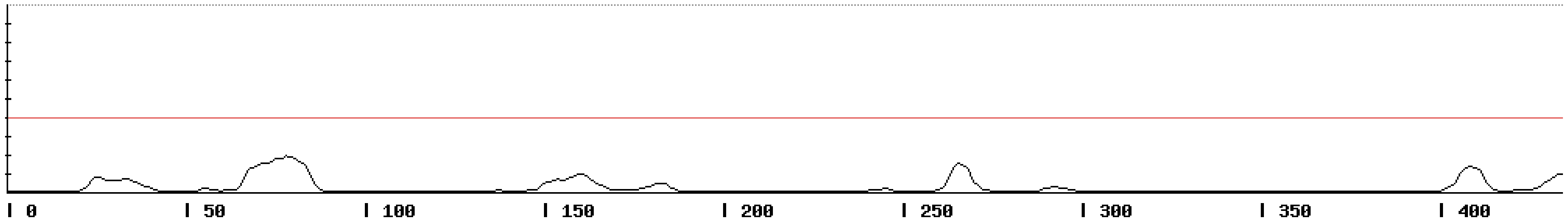

Predictions for Kalanchoelaxiflora_Kalax.0385s0005

Viterbi Path

Viterbi Path

Posterior @ 0.4

Posterior @ 0.4

Posterior Graphic. Threshold Marked @ 0.4

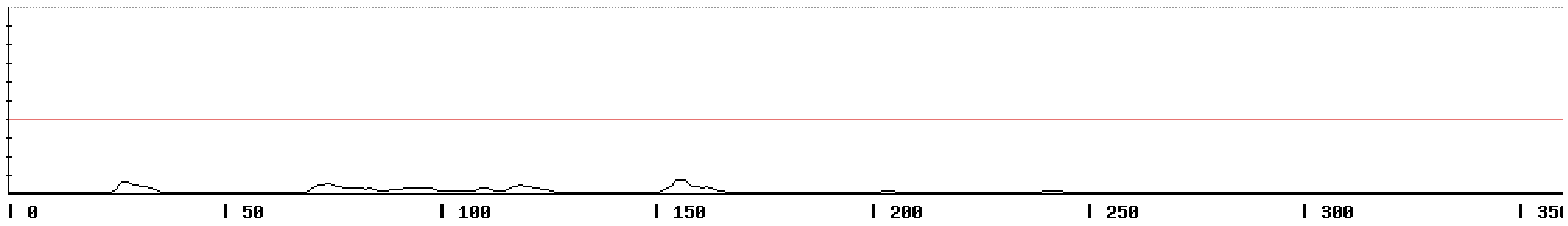

Predictions for Kalanchoelaxiflora_Kalax.0422s0023

Viterbi Path

Viterbi Path

Posterior @ 0.4

Posterior @ 0.4

Posterior Graphic. Threshold Marked @ 0.4

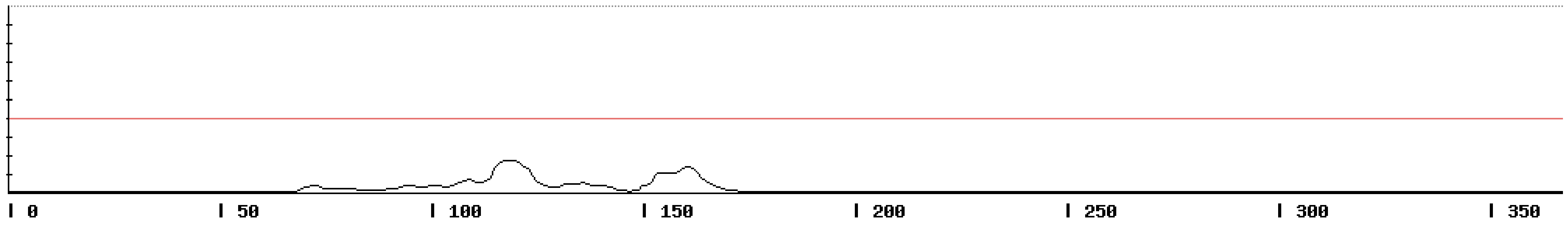

Predictions for Kalanchoelaxiflora_Kalax.0432s0034

Viterbi Path

Viterbi Path

Posterior @ 0.4

Posterior @ 0.4

Posterior Graphic. Threshold Marked @ 0.4

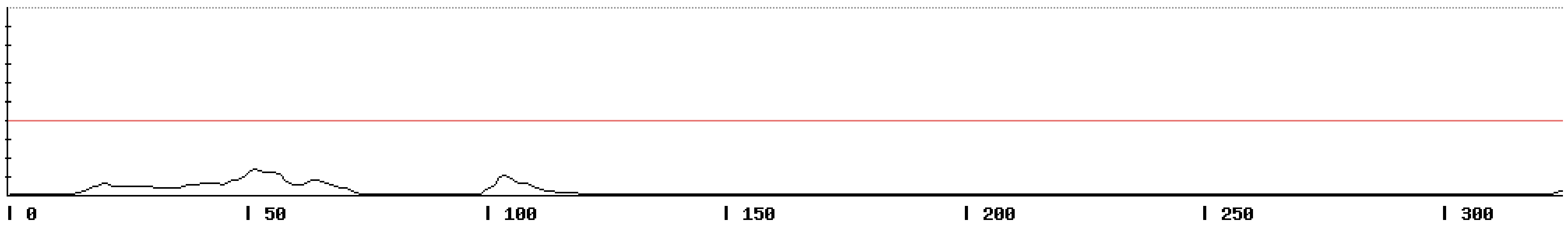

Predictions for Kalanchoelaxiflora_Kalax.0445s0023

Viterbi Path

Viterbi Path

Posterior @ 0.4

Posterior @ 0.4

Posterior Graphic. Threshold Marked @ 0.4

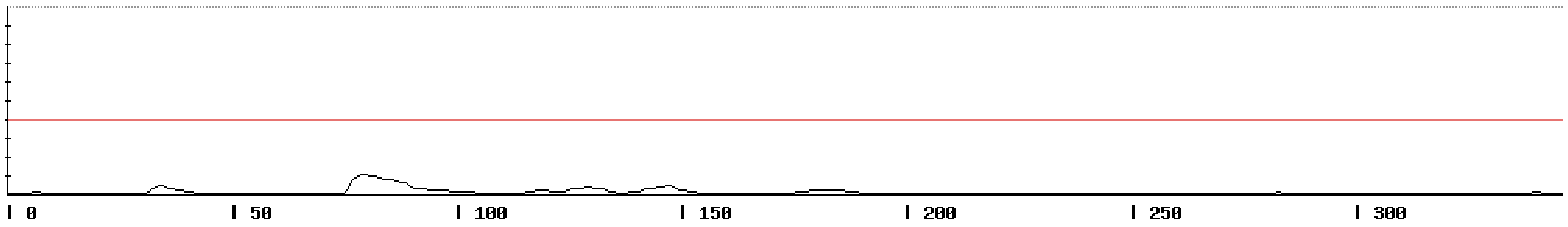

Predictions for Kalanchoelaxiflora_Kalax.0446s0004

Viterbi Path

Viterbi Path

Posterior @ 0.4	
Posterior @ 0.4	
Posterior Graphic. Threshold Marked @ 0.4	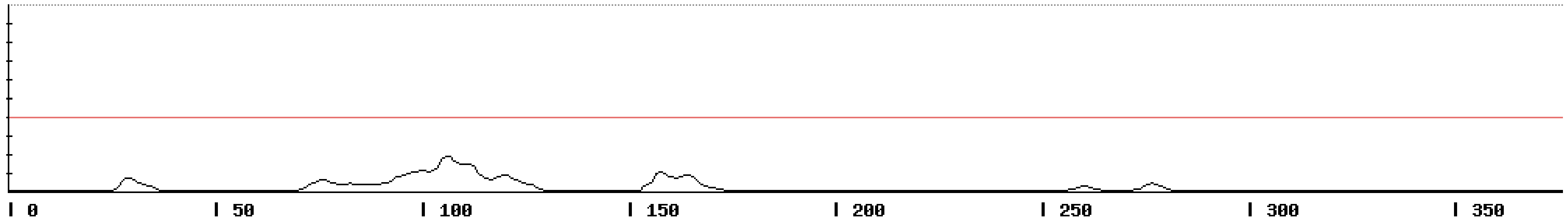

Predictions for Kalanchoelaxiflora_Kalax.0455s0006	
Viterbi Path	
Viterbi Path	
Posterior @ 0.4	
Posterior @ 0.4	
Posterior Graphic. Threshold Marked @ 0.4	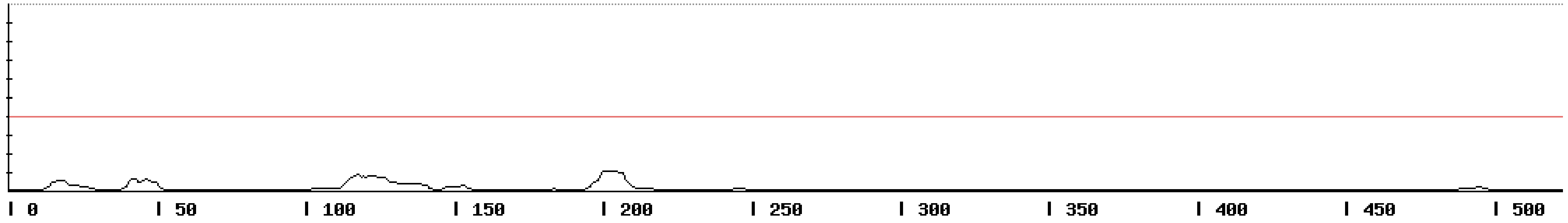

Predictions for Kalanchoelaxiflora_Kalax.0465s0024	
Viterbi Path	
Viterbi Path	

Posterior @ 0.4	
Posterior @ 0.4	
Posterior Graphic. Threshold Marked @ 0.4	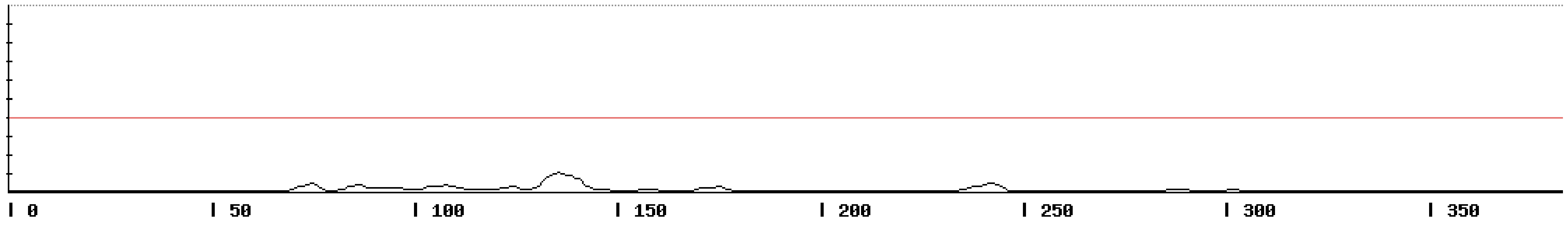

Predictions for Kalanchoelaxiflora_Kalax.0472s0007	
Viterbi Path	
Viterbi Path	
Posterior @ 0.4	
Posterior @ 0.4	
Posterior Graphic. Threshold Marked @ 0.4	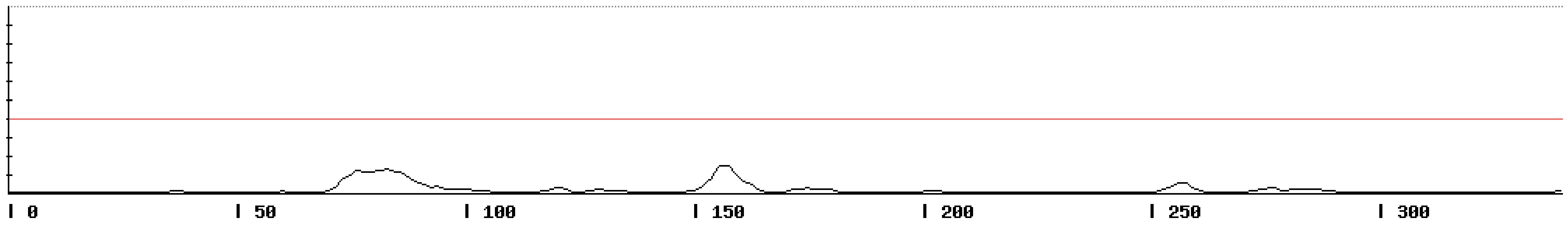

Predictions for Kalanchoelaxiflora_Kalax.0476s0030

Viterbi Path	
Viterbi Path	
Posterior @ 0.4	
Posterior @ 0.4	
Posterior Graphic. Threshold Marked @ 0.4	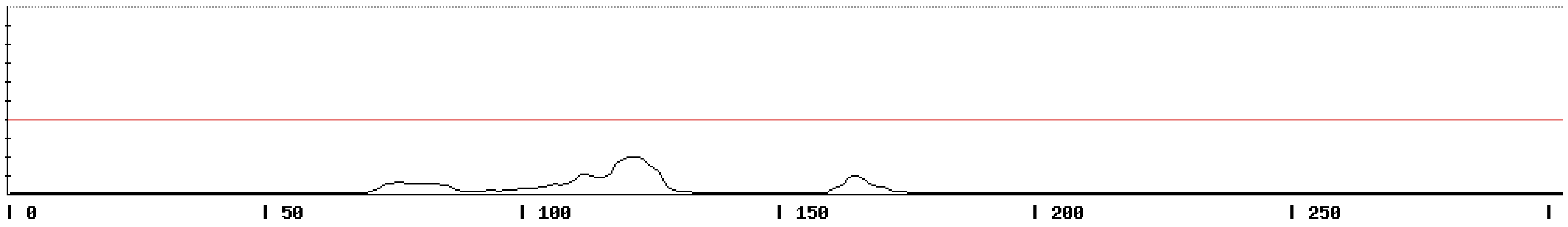

Predictions for Kalanchoelaxiflora_Kalax.0476s0044

Viterbi Path	
Viterbi Path	
Posterior @ 0.4	
Posterior @ 0.4	
Posterior Graphic. Threshold Marked @ 0.4	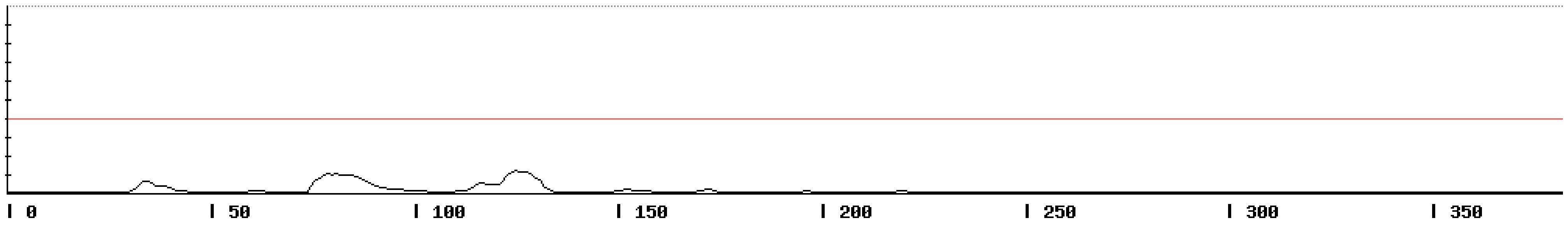

Predictions for Kalanchoelaxiflora_Kalax.0483s0011	
Viterbi Path	
Viterbi Path	
Posterior @ 0.4	129 - KGRAP - 133
Posterior @ 0.4	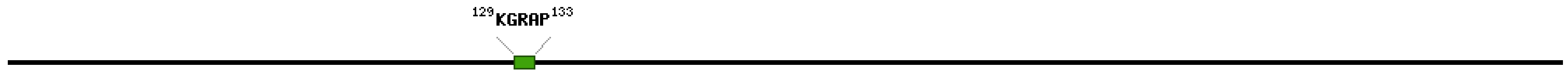
Posterior Graphic. Threshold Marked @ 0.4	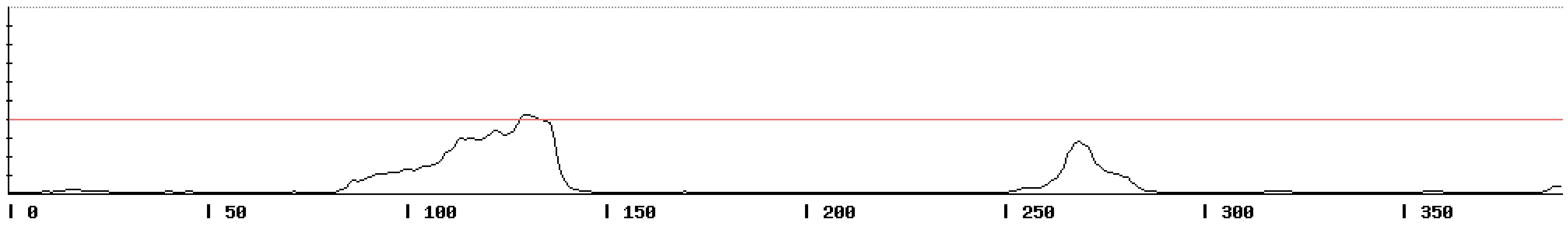

Predictions for Kalanchoelaxiflora_Kalax.0490s0030	
Viterbi Path	
Viterbi Path	
Posterior @ 0.4	
Posterior @ 0.4	
Posterior Graphic. Threshold Marked @ 0.4	

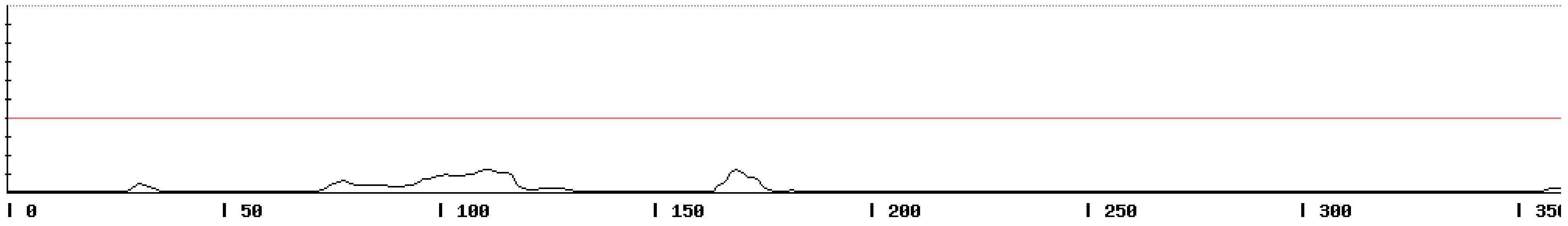

Predictions for Kalanchoelaxiflora_Kalax.0507s0019

Viterbi Path

Viterbi Path

Posterior @ 0.4

Posterior @ 0.4

Posterior Graphic. Threshold Marked @ 0.4

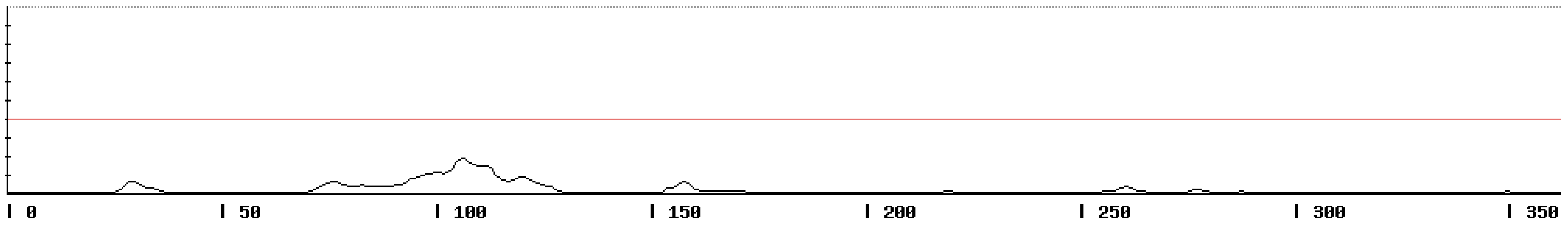

Predictions for Kalanchoelaxiflora_Kalax.0514s0014

Viterbi Path

Viterbi Path

Posterior @ 0.4

Posterior @ 0.4

Posterior Graphic. Threshold Marked @ 0.4

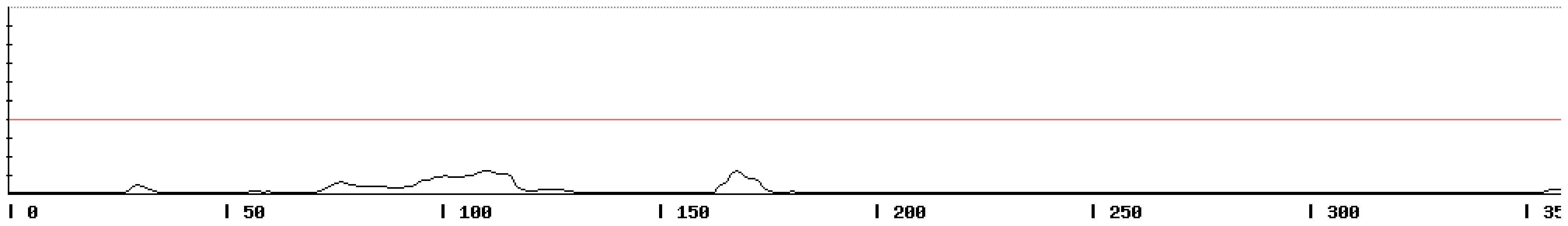

Predictions for Kalanchoelaxiflora_Kalax.0561s0017

Viterbi Path

Viterbi Path

Posterior @ 0.4

Posterior @ 0.4

Posterior Graphic. Threshold Marked @ 0.4

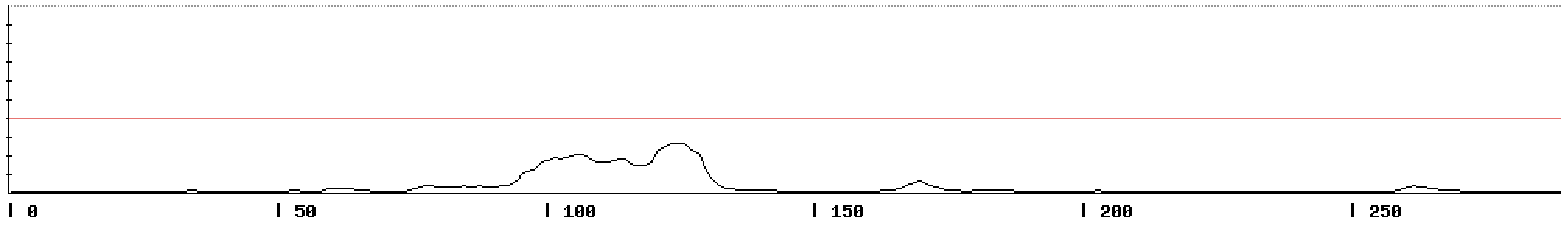

Predictions for Kalanchoelaxiflora_Kalax.0568s0017

Viterbi Path

Viterbi Path

Posterior @ 0.4

Posterior @ 0.4

Posterior Graphic. Threshold Marked @ 0.4

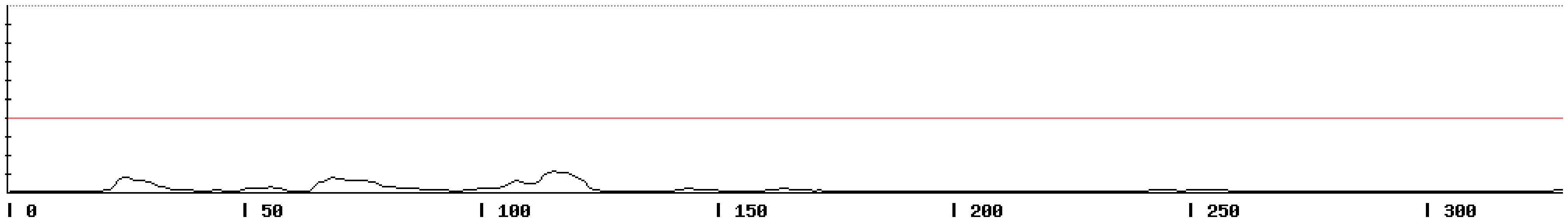

Predictions for Kalanchoelaxiflora_Kalax.0595s0009

Viterbi Path

Viterbi Path

Posterior @ 0.4

89 - WKATGKDRKIKKRGRDVGMKR - 109

497 - RKWPLFASHGKSKRRPEEFHIRLAKKHK - 524

Posterior @ 0.4

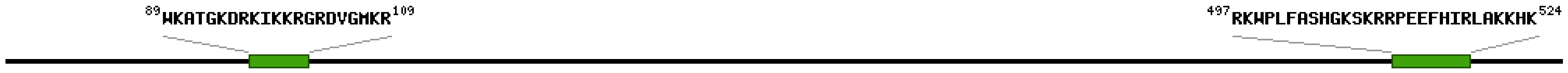

Posterior Graphic. Threshold Marked @ 0.4

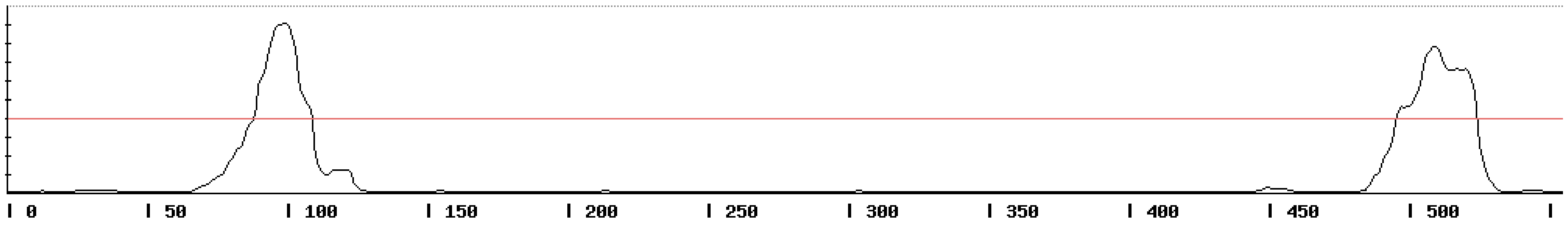

Predictions for Kalanchoelaxiflora_Kalax.0609s0006

Viterbi Path

Viterbi Path

Posterior @ 0.4	
Posterior @ 0.4	
Posterior Graphic. Threshold Marked @ 0.4	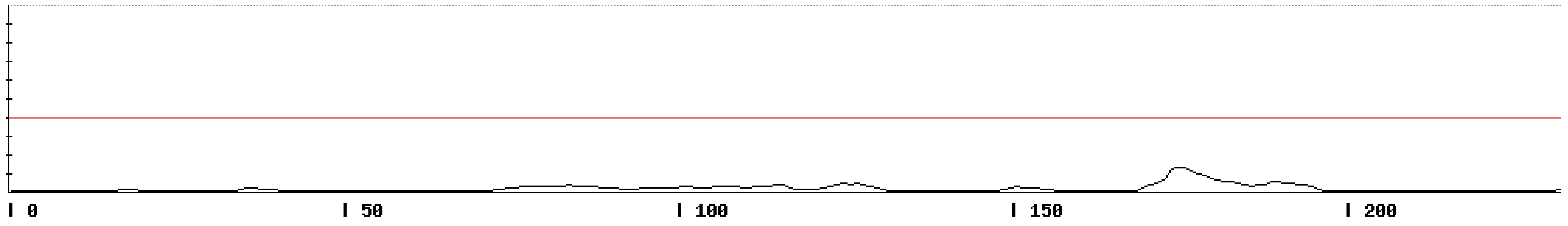

Predictions for Kalanchoelaxiflora_Kalax.0628s0003	
Viterbi Path	
Viterbi Path	
Posterior @ 0.4	89 - WKATGKDRKIKKRGRDVGMKR - 109<br>504 - SHGKSKRRPE - 513
Posterior @ 0.4	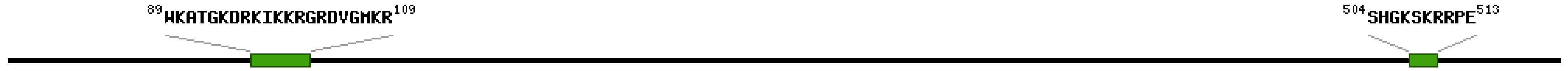
Posterior Graphic. Threshold Marked @ 0.4	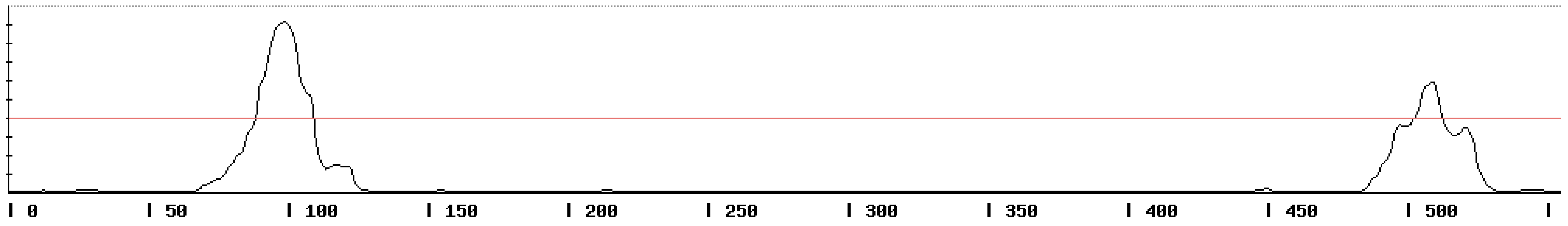

Predictions for Kalanchoelaxiflora_Kalax.0677s0017	
Viterbi Path	
Viterbi Path	
Posterior @ 0.4	
Posterior @ 0.4	
Posterior Graphic. Threshold Marked @ 0.4	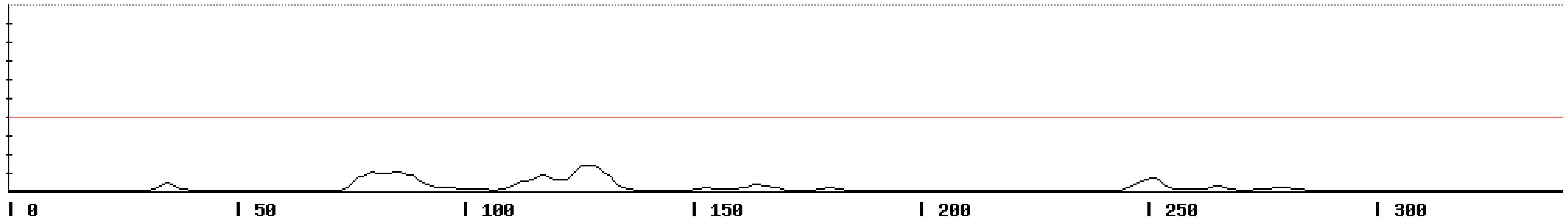

Predictions for Kalanchoelaxiflora_Kalax.0679s0010	
Viterbi Path	
Viterbi Path	
Posterior @ 0.4	
Posterior @ 0.4	
Posterior Graphic. Threshold Marked @ 0.4	

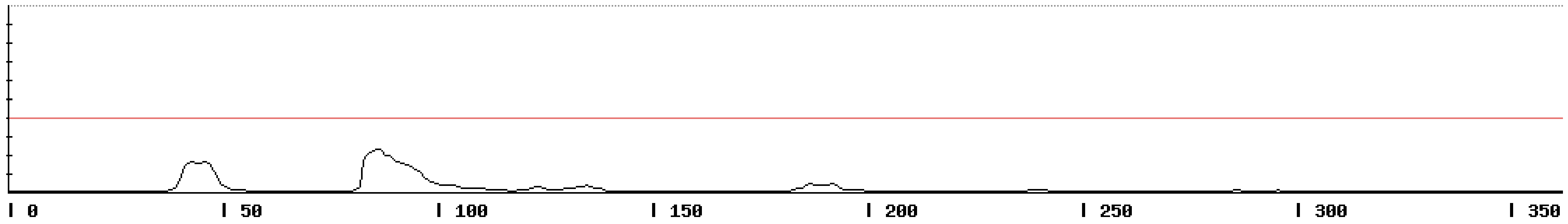

Predictions for Kalanchoelaxiflora_Kalax.0686s0005

Viterbi Path

Viterbi Path

Posterior @ 0.4

Posterior @ 0.4

Posterior Graphic. Threshold Marked @ 0.4

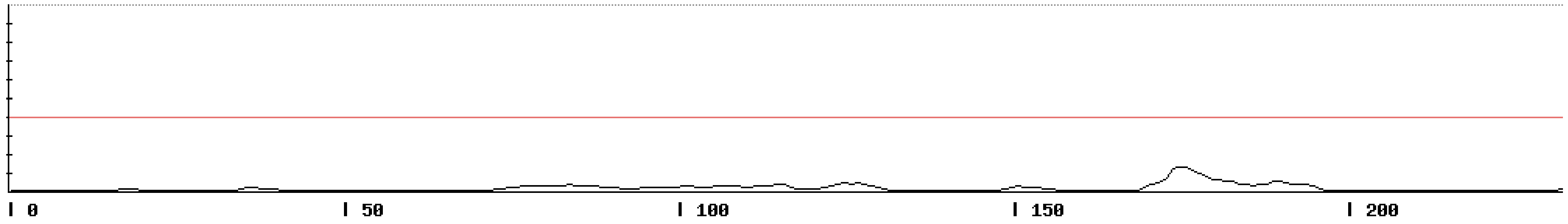

Predictions for Kalanchoelaxiflora_Kalax.0727s0009

Viterbi Path

Viterbi Path

Posterior @ 0.4

Posterior @ 0.4

Posterior Graphic. Threshold Marked @ 0.4

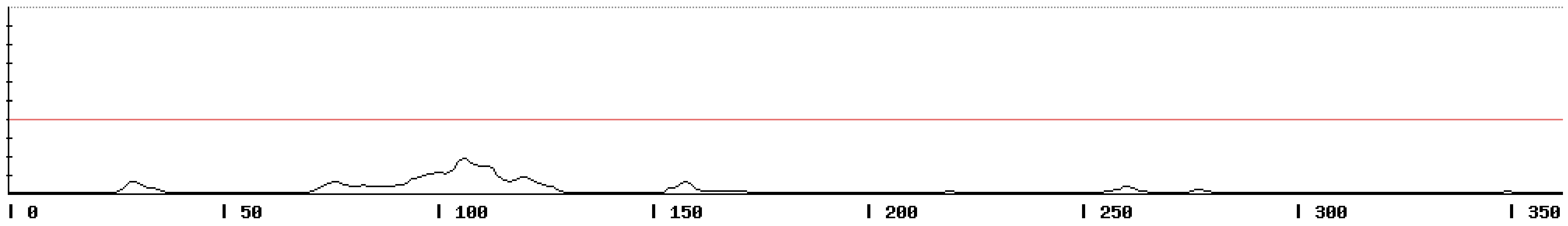

Predictions for Kalanchoelaxiflora_Kalax.0728s0011

Viterbi Path

Viterbi Path

Posterior @ 0.4

Posterior @ 0.4

Posterior Graphic. Threshold Marked @ 0.4

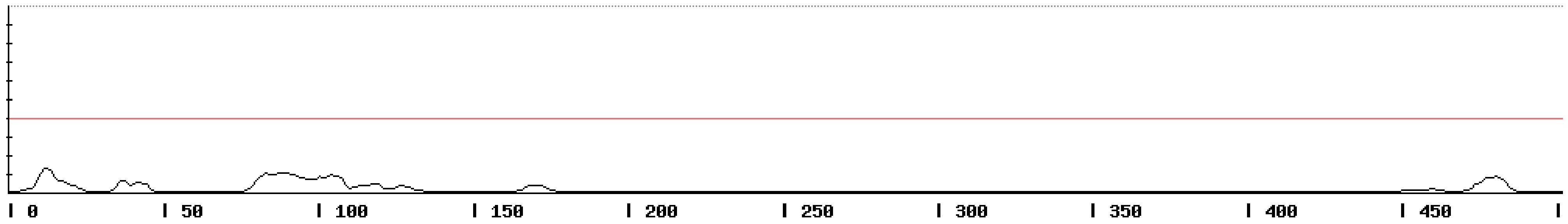

Predictions for Kalanchoelaxiflora_Kalax.0729s0019

Viterbi Path

Viterbi Path

Posterior @ 0.4

Posterior @ 0.4

Posterior Graphic. Threshold Marked @ 0.4	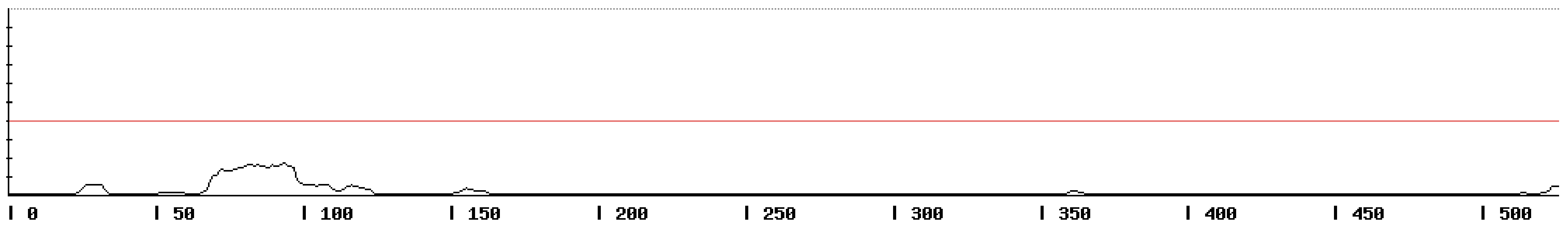

Predictions for Kalanchoelaxiflora_Kalax.0743s0006

Viterbi Path	83 - RKRVGGRRRTRGGYWKRKGRVR - 104
Viterbi Path	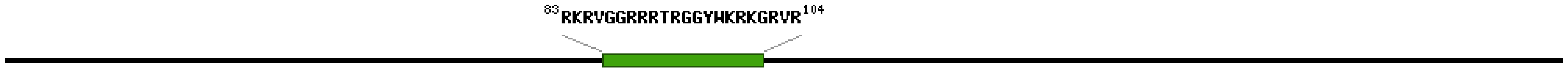
Posterior @ 0.4	81 - AERKRVGGRRRTRGGYWKRKGRVR - 104
Posterior @ 0.4	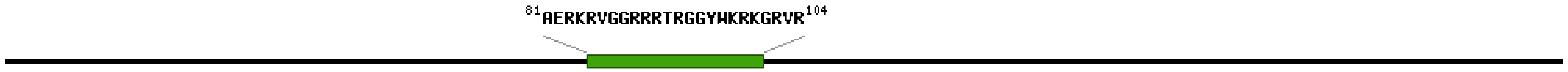
Posterior Graphic. Threshold Marked @ 0.4	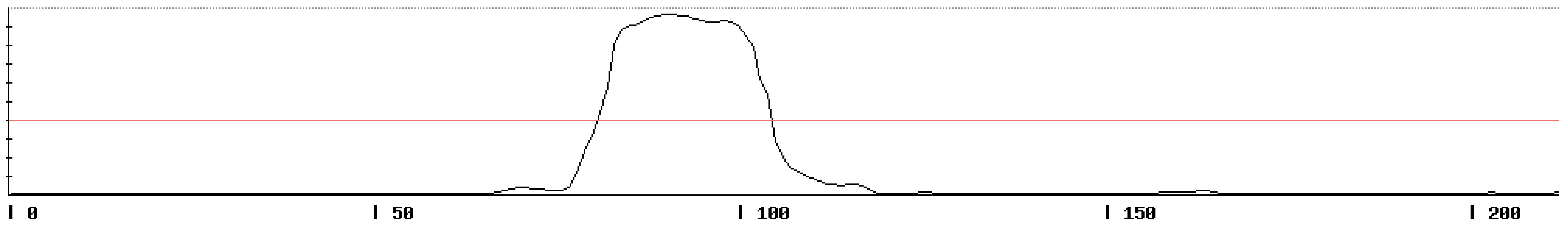

Predictions for Kalanchoelaxiflora_Kalax.0748s0009

Viterbi Path	
Viterbi Path	

Posterior @ 0.4	
Posterior @ 0.4	
Posterior Graphic. Threshold Marked @ 0.4	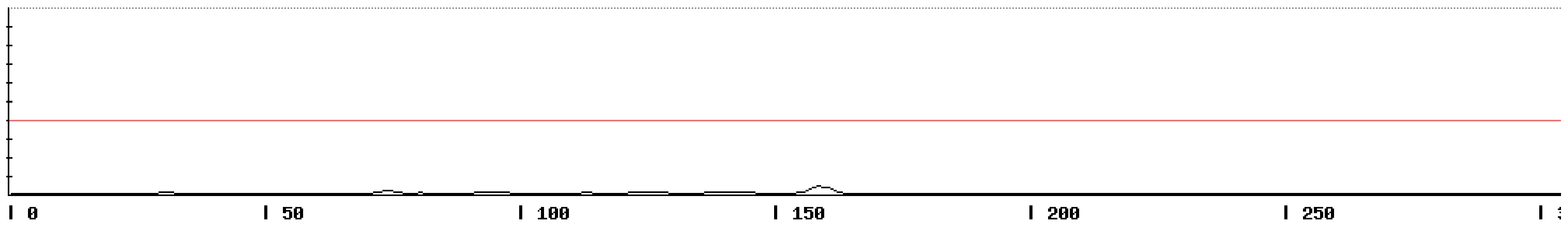

Predictions for Kalanchoelaxiflora_Kalax.0756s0005

Viterbi Path	
Viterbi Path	
Posterior @ 0.4	
Posterior @ 0.4	
Posterior Graphic. Threshold Marked @ 0.4	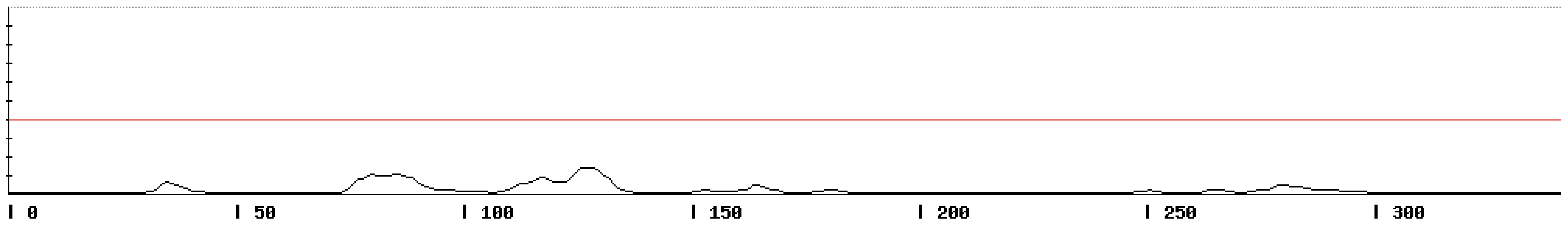

Predictions for Kalanchoelaxiflora_Kalax.0757s0019

Viterbi Path	
Viterbi Path	

Posterior @ 0.4

Posterior @ 0.4

Posterior Graphic. Threshold Marked @ 0.4

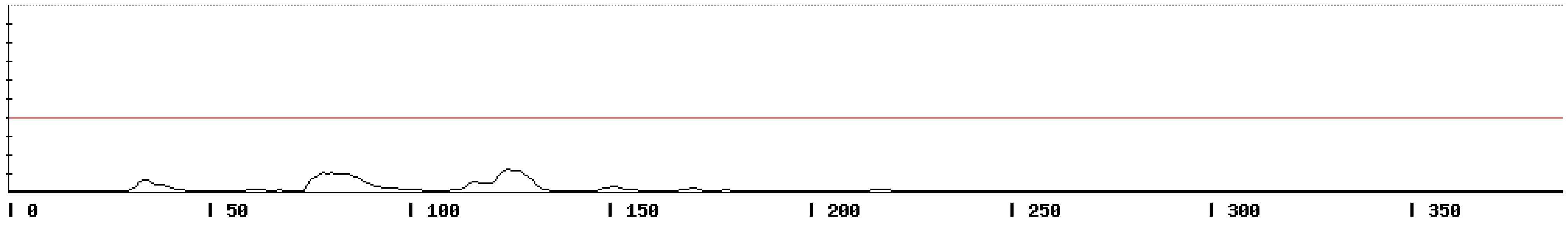

Predictions for Kalanchoelaxiflora_Kalax.0854s0001

Viterbi Path

Viterbi Path

Posterior @ 0.4

Posterior @ 0.4

Posterior Graphic. Threshold Marked @ 0.4

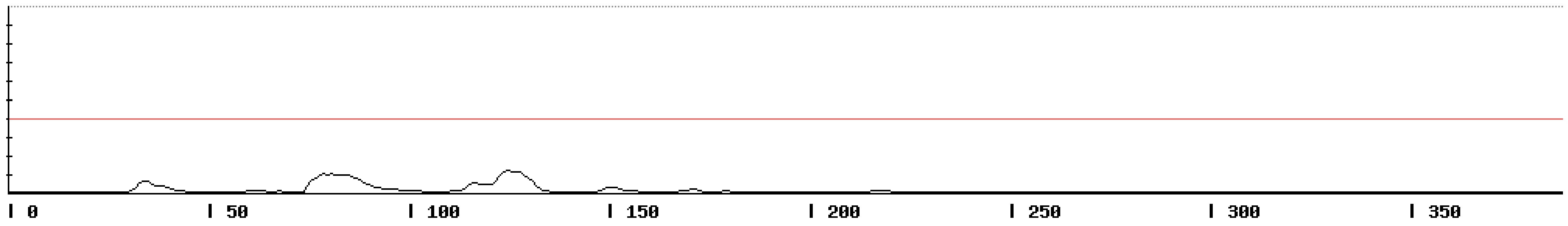

Predictions for Kalanchoelaxiflora_Kalax.0854s0016

Viterbi Path

Viterbi Path

Posterior @ 0.4

Posterior @ 0.4

Posterior Graphic. Threshold Marked @ 0.4

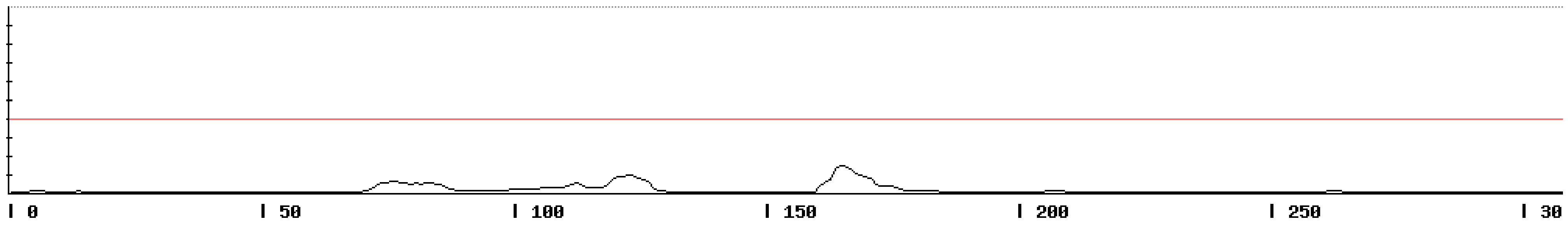

Predictions for Kalanchoelaxiflora_Kalax.0870s0004

Viterbi Path

Viterbi Path

Posterior @ 0.4

Posterior @ 0.4

Posterior Graphic. Threshold Marked @ 0.4

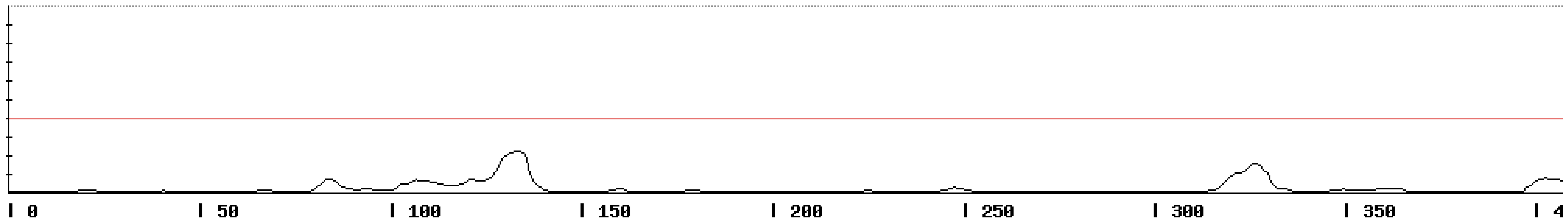

Predictions for Kalanchoelaxiflora_Kalax.0896s0001	
Viterbi Path	
Viterbi Path	
Posterior @ 0.4	
Posterior @ 0.4	
Posterior Graphic. Threshold Marked @ 0.4	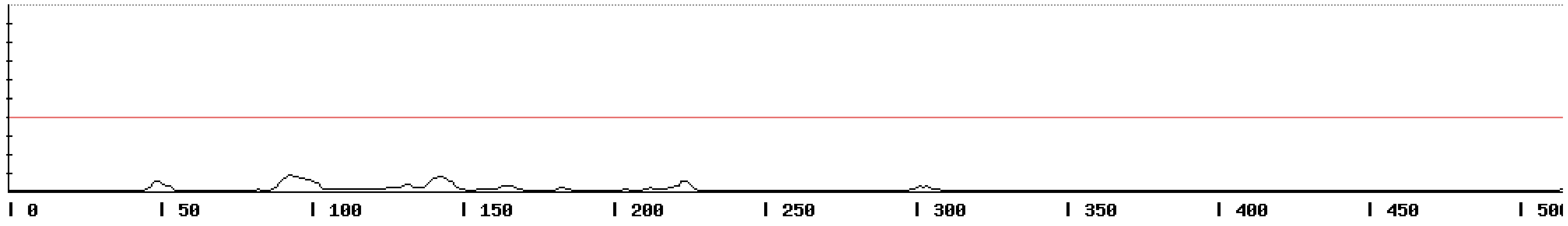

Predictions for Kalanchoelaxiflora_Kalax.0901s0011	
Viterbi Path	
Viterbi Path	
Posterior @ 0.4	
Posterior @ 0.4	
Posterior Graphic. Threshold Marked @ 0.4	

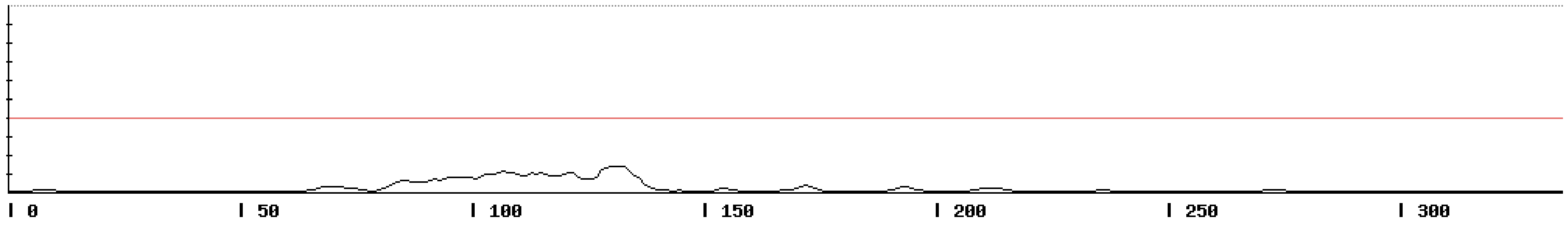

Predictions for Kalanchoelaxiflora_Kalax.0935s0008	
Viterbi Path	
Viterbi Path	
Posterior @ 0.4	
Posterior @ 0.4	
Posterior Graphic. Threshold Marked @ 0.4	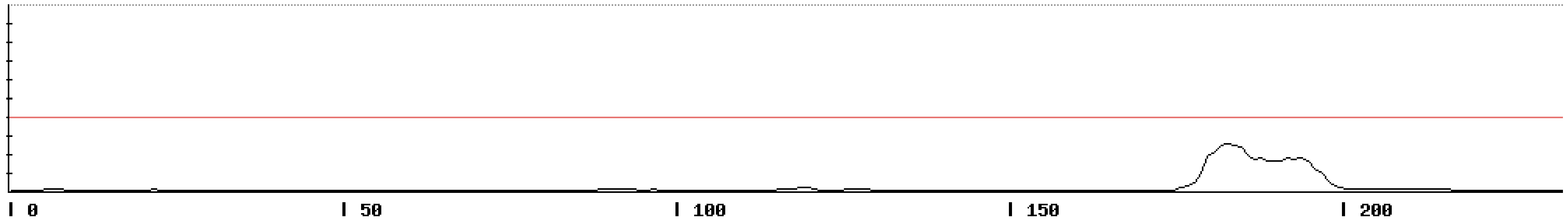

Predictions for Kalanchoelaxiflora_Kalax.0939s0002	
Viterbi Path	
Viterbi Path	
Posterior @ 0.4	
Posterior @ 0.4	

Posterior Graphic. Threshold Marked @ 0.4

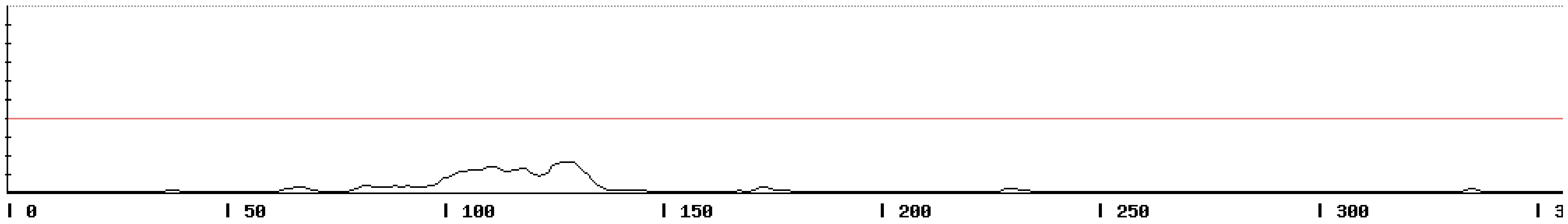

Predictions for Kalanchoelaxiflora_Kalax.0941s0002

Viterbi Path

Viterbi Path

Posterior @ 0.4

Posterior @ 0.4

Posterior Graphic. Threshold Marked @ 0.4

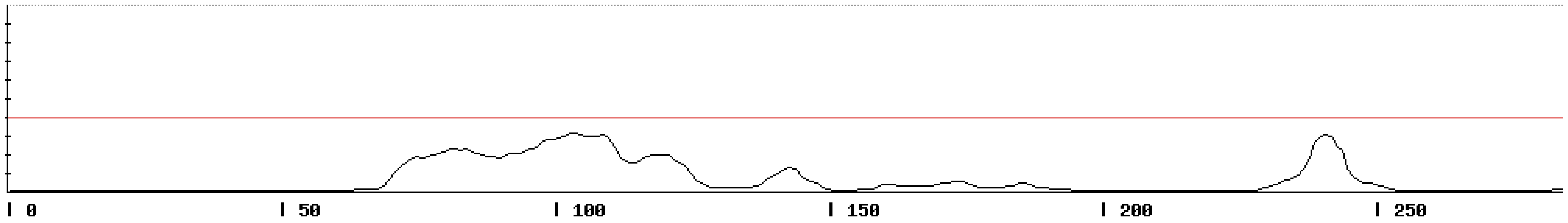

Predictions for Kalanchoelaxiflora_Kalax.1035s0002

Viterbi Path

Viterbi Path

Posterior @ 0.4

Posterior @ 0.4

Posterior Graphic. Threshold Marked @ 0.4

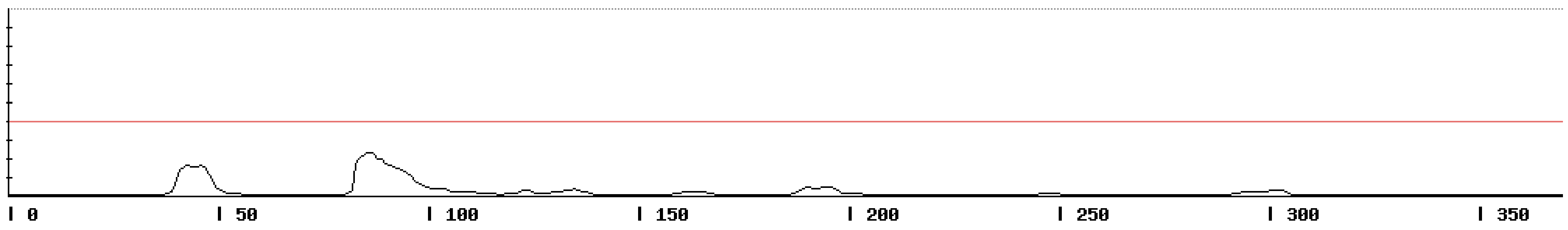

Predictions for Kalanchoelaxiflora_Kalax.1115s0008

Viterbi Path

Viterbi Path

Posterior @ 0.4

Posterior @ 0.4

Posterior Graphic. Threshold Marked @ 0.4

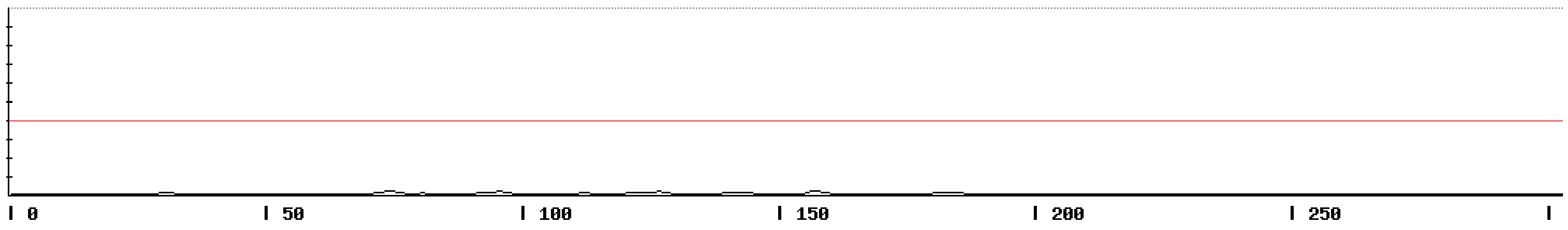

Predictions for Kalanchoelaxiflora_Kalax.1234s0006

Viterbi Path

Viterbi Path

Posterior @ 0.4

Posterior @ 0.4

Posterior Graphic. Threshold Marked @ 0.4

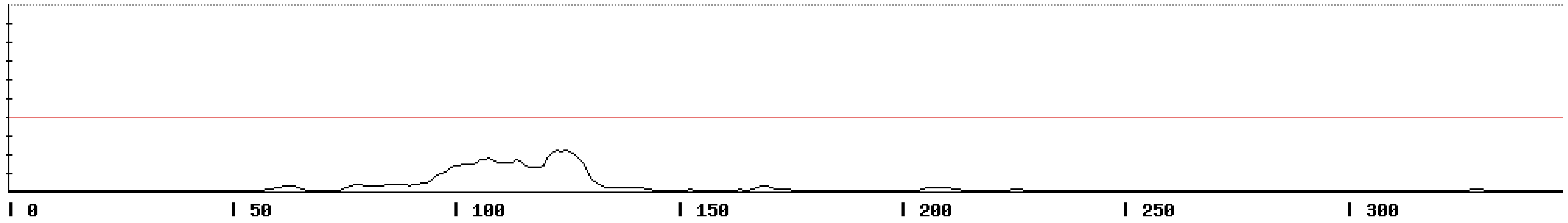

Predictions for Kalanchoelaxiflora_Kalax.1240s0007

Viterbi Path

Viterbi Path

Posterior @ 0.4

Posterior @ 0.4

Posterior Graphic. Threshold Marked @ 0.4

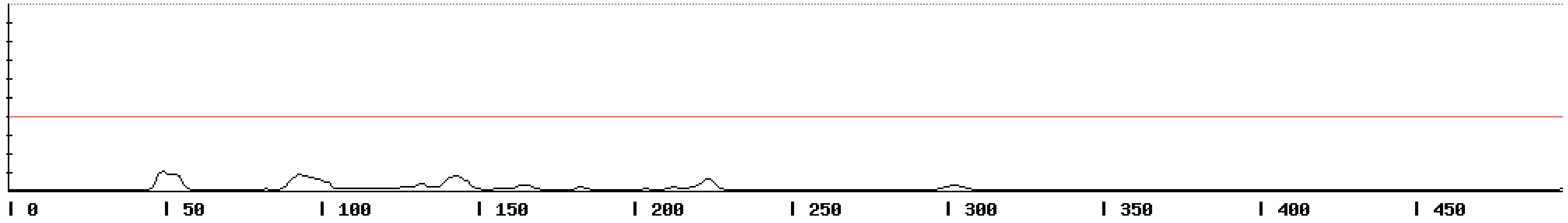

Predictions for Kalanchoelaxiflora_Kalax.1261s0008

Viterbi Path

Viterbi Path

Posterior @ 0.4	
Posterior @ 0.4	
Posterior Graphic. Threshold Marked @ 0.4	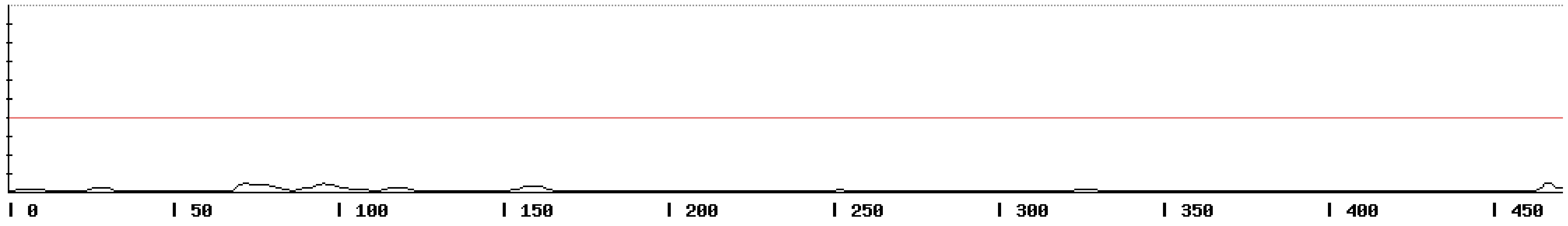

**Predictions for Kalanchoelaxiflora_Kalax.1284s0006**	
Viterbi Path	
Viterbi Path	
Posterior @ 0.4	
Posterior @ 0.4	
Posterior Graphic. Threshold Marked @ 0.4	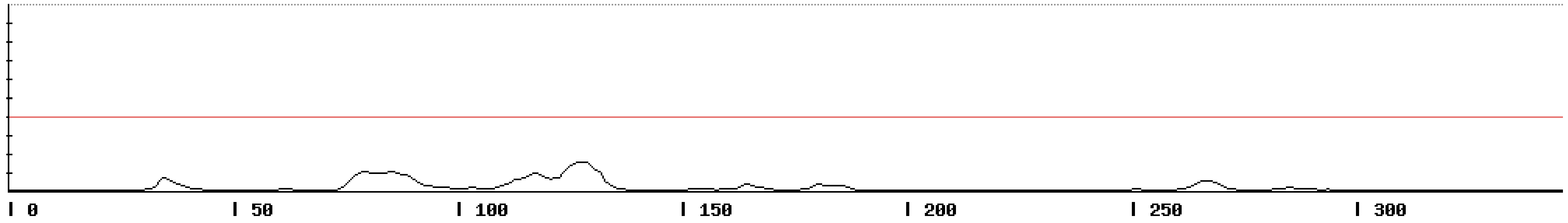

**Predictions for Kalanchoelaxiflora_Kalax.1411s0004**

Viterbi Path	
Viterbi Path	
Posterior @ 0.4	
Posterior @ 0.4	
Posterior Graphic. Threshold Marked @ 0.4	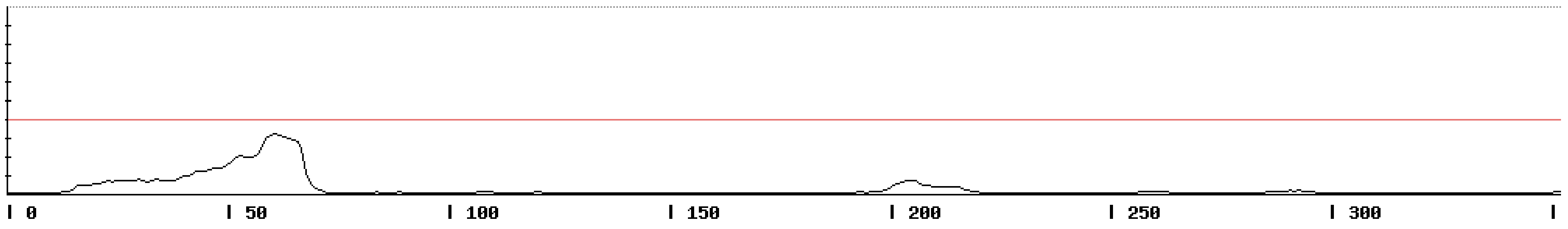

Predictions for Kalanchoelaxiflora_Kalax.1420s0002

Viterbi Path	
Viterbi Path	
Posterior @ 0.4	
Posterior @ 0.4	
Posterior Graphic. Threshold Marked @ 0.4	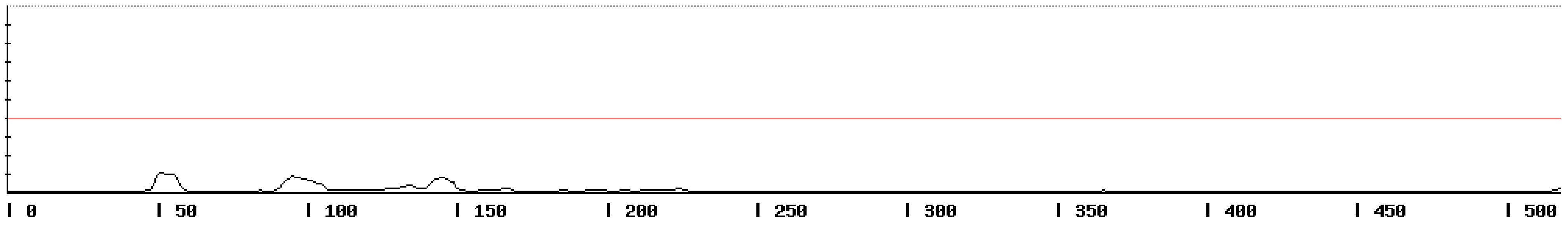

Predictions for Kalanchoelaxiflora_Kalax.1717s0001	
Viterbi Path	
Viterbi Path	
Posterior @ 0.4	
Posterior @ 0.4	

Revision r.9 - Last Updated on : February 24 2015 17:45:20. alex.nguyenba@utoronto.ca

Intended to be used with Firefox

Viterbi Path	
Posterior @ 0.4	
Posterior @ 0.4	
Posterior Graphic. Threshold Marked @ 0.4	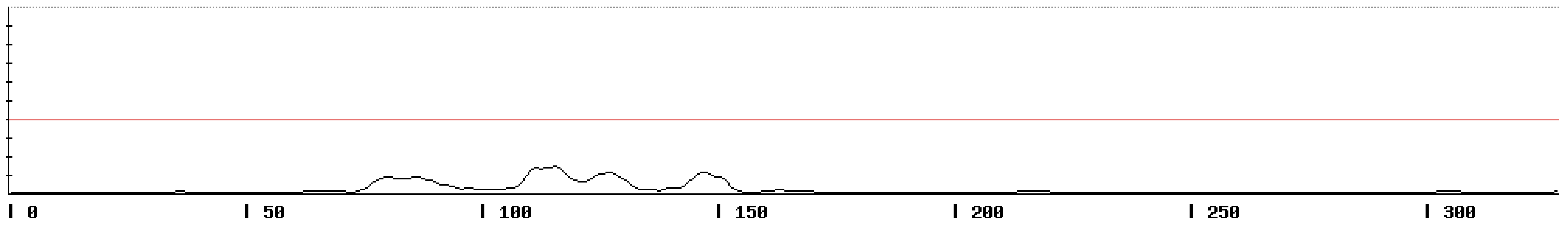

Predictions for Kalanchoemarnieriana_Kalax.0001s0118.1.p	
Viterbi Path	
Viterbi Path	
Posterior @ 0.4	
Posterior @ 0.4	
Posterior Graphic. Threshold Marked @ 0.4	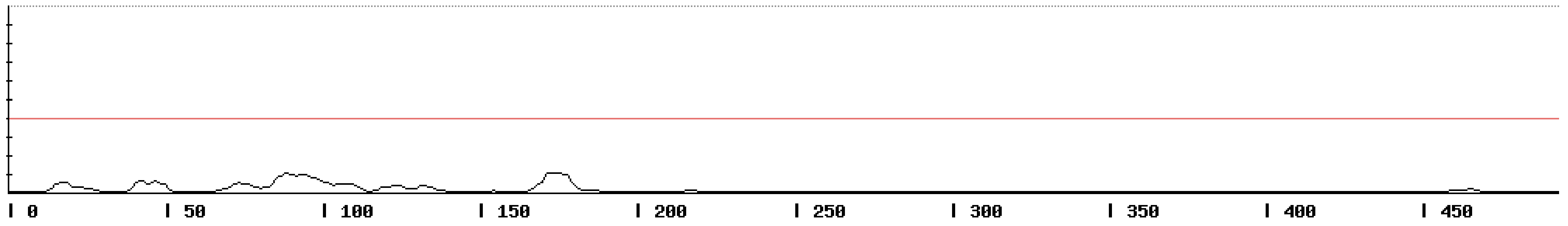

Predictions for Kalanchoemarnieriana_Kalax.0001s0186.1.p

Viterbi Path

Viterbi Path

Posterior @ 0.4

Posterior @ 0.4

Posterior Graphic. Threshold Marked @ 0.4

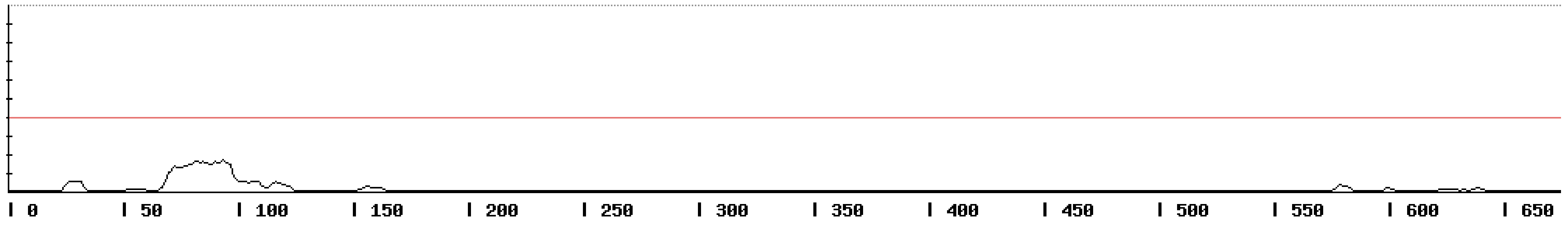

Predictions for Kalanchoemarnieriana_Kalax.0001s0201.1.p

Viterbi Path

Viterbi Path

Posterior @ 0.4

Posterior @ 0.4

Posterior Graphic. Threshold Marked @ 0.4

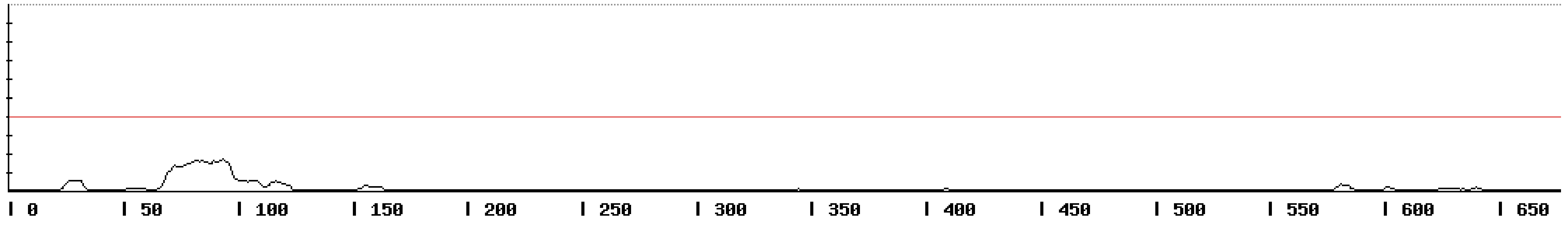

Predictions for Kalanchoemarnieriana_Kalax.0002s0023.1.p	
Viterbi Path	
Viterbi Path	
Posterior @ 0.4	
Posterior @ 0.4	
Posterior Graphic. Threshold Marked @ 0.4	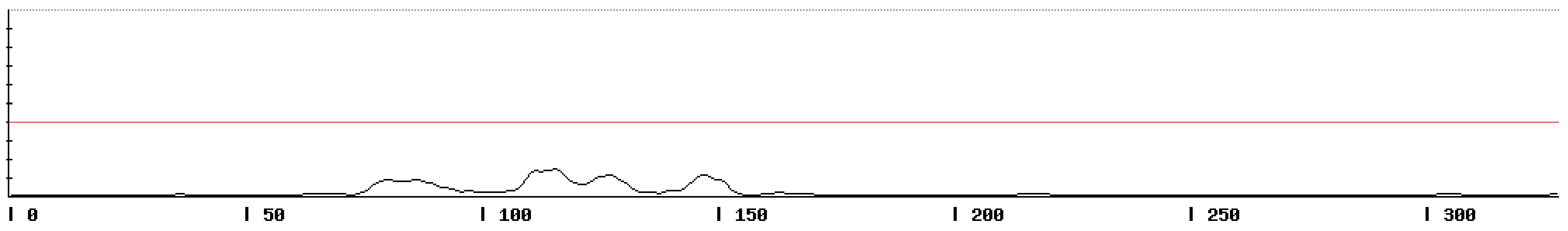

Predictions for Kalanchoemarnieriana_Kalax.0002s0024.1.p	
Viterbi Path	
Viterbi Path	
Posterior @ 0.4	
Posterior @ 0.4	
Posterior Graphic. Threshold Marked @ 0.4	

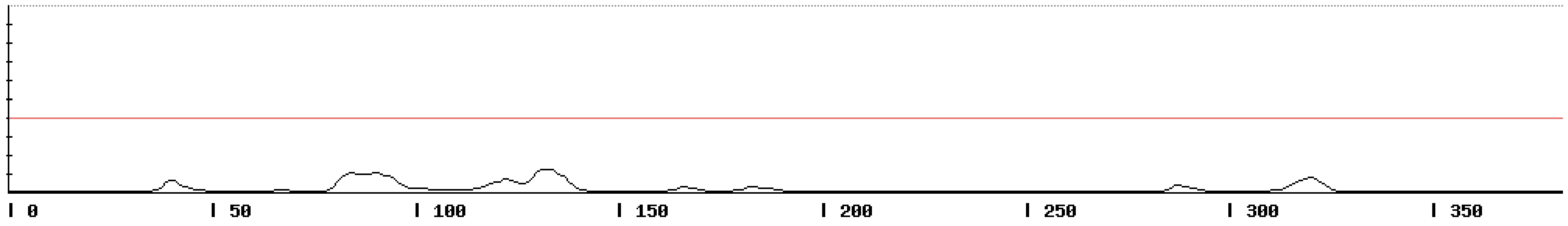

Predictions for Kalanchoemarnieriana_Kalax.0003s0029.1.p

Viterbi Path	
Viterbi Path	
Posterior @ 0.4	
Posterior @ 0.4	
Posterior Graphic. Threshold Marked @ 0.4	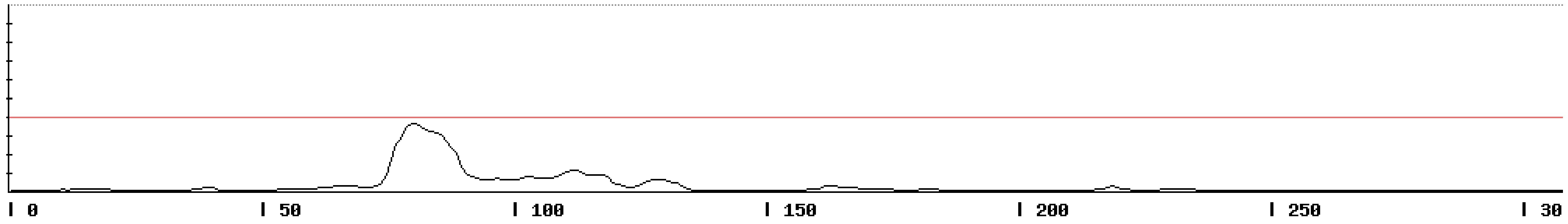

Predictions for Kalanchoemarnieriana_Kalax.0003s0239.1.p

Viterbi Path	
Viterbi Path	
Posterior @ 0.4	
Posterior @ 0.4	

Posterior Graphic. Threshold Marked @ 0.4

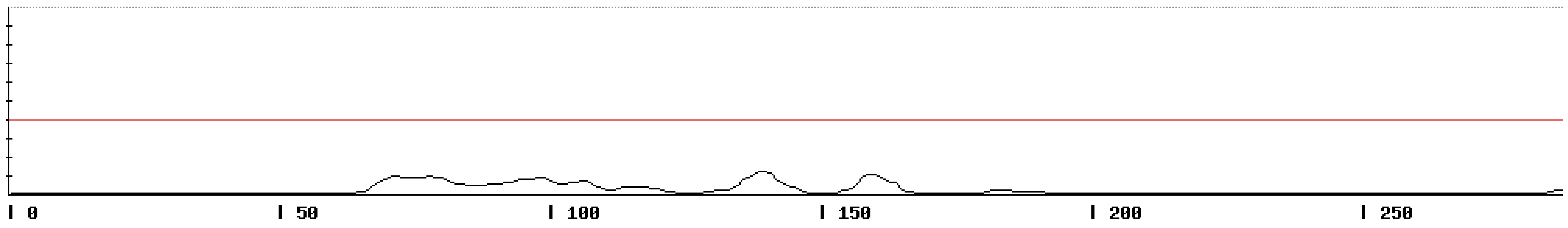

Predictions for Kalanchoemarnieriana_Kalax.0004s0214.1.p

Viterbi Path

Viterbi Path

Posterior @ 0.4

Posterior @ 0.4

Posterior Graphic. Threshold Marked @ 0.4

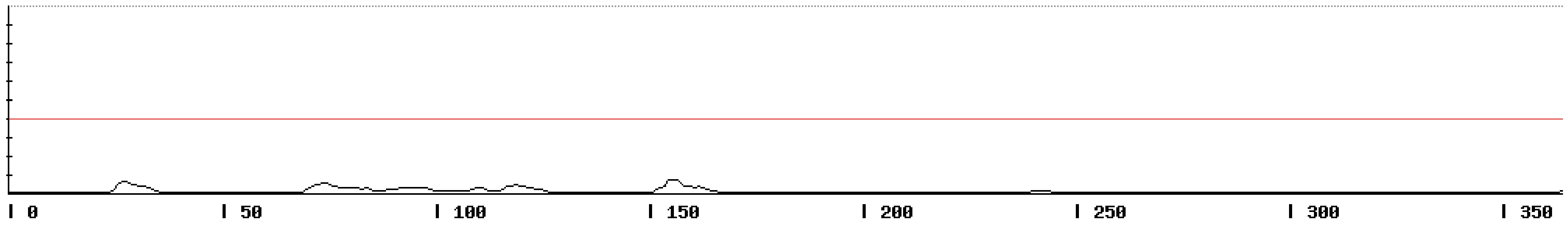

Predictions for Kalanchoemarnieriana_Kalax.0006s0245.1.p

Viterbi Path

Viterbi Path

Posterior @ 0.4

Posterior @ 0.4

Posterior Graphic. Threshold Marked @ 0.4

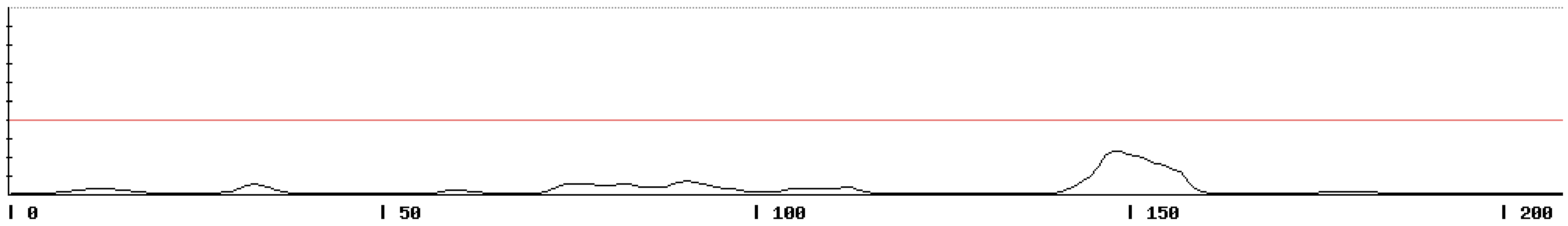

Predictions for Kalanchoemarnieriana_Kalax.0009s0083.1.p

Viterbi Path

Viterbi Path

Posterior @ 0.4

Posterior @ 0.4

Posterior Graphic. Threshold Marked @ 0.4

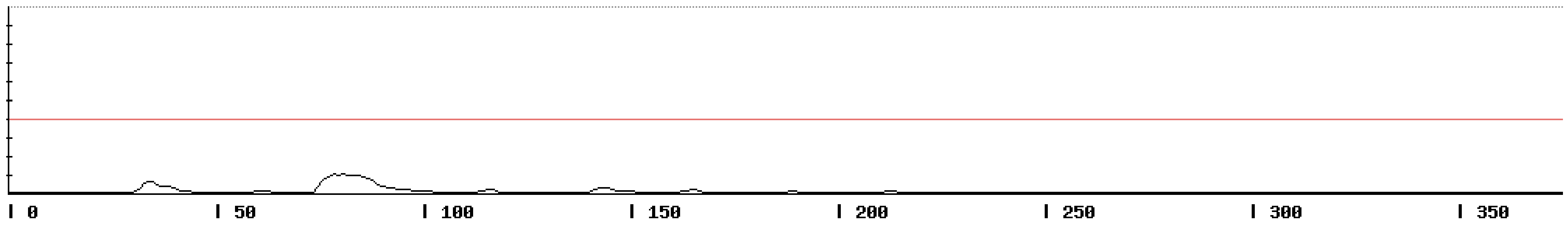

Predictions for Kalanchoemarnieriana_Kalax.0009s0097.1.p

Viterbi Path

Viterbi Path

Posterior @ 0.4

Posterior @ 0.4

Posterior Graphic. Threshold Marked @ 0.4

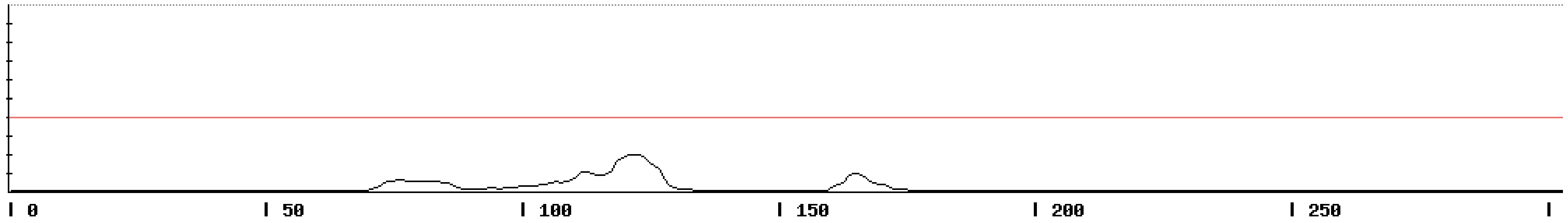

Predictions for Kalanchoemarnieriana_Kalax.0011s0164.1.p

Viterbi Path

Viterbi Path

Posterior @ 0.4

Posterior @ 0.4

Posterior Graphic. Threshold Marked @ 0.4

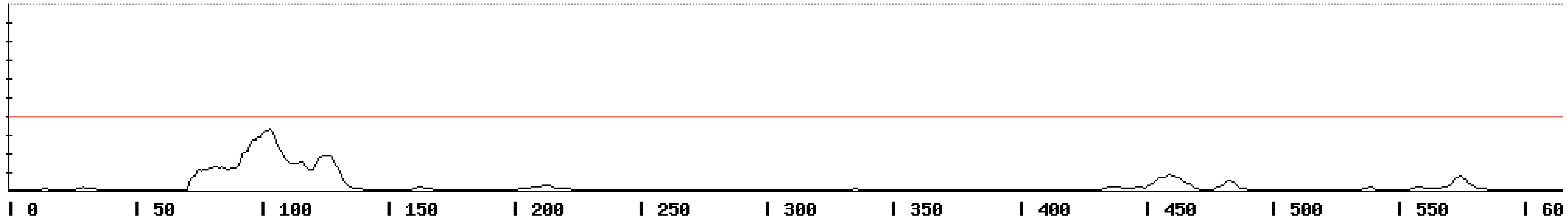

Predictions for Kalanchoemarnieriana_Kalax.0012s0219.1.p

Viterbi Path

Viterbi Path

Posterior @ 0.4

Posterior @ 0.4

Posterior Graphic. Threshold Marked @ 0.4

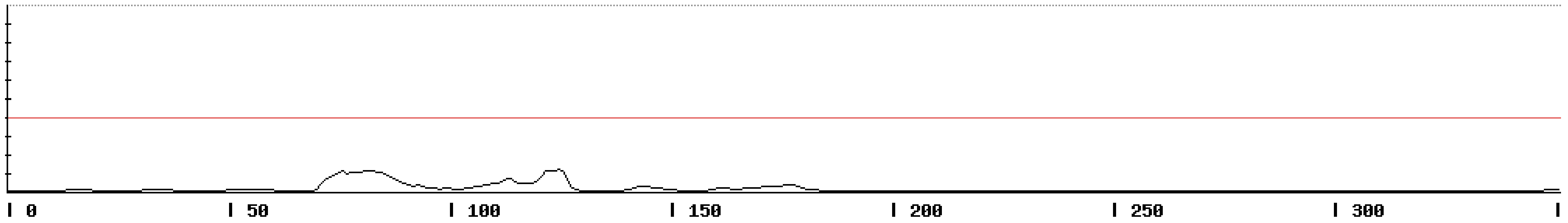

Predictions for Kalanchoemarnieriana_Kalax.0013s0167.1.p

Viterbi Path

Viterbi Path

Posterior @ 0.4

Posterior @ 0.4

Posterior Graphic. Threshold Marked @ 0.4

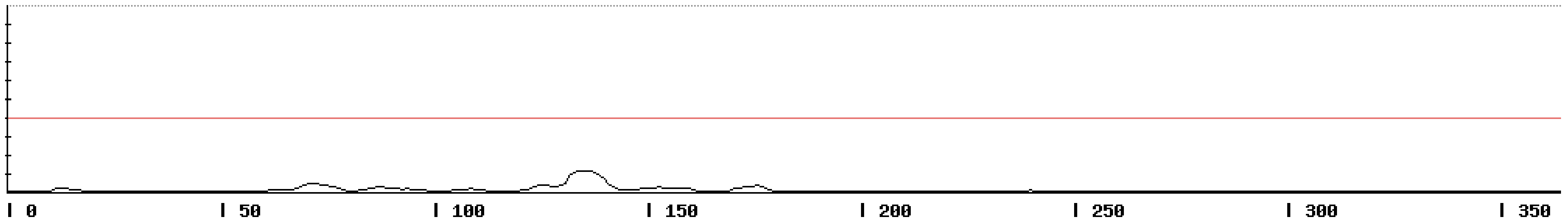

Predictions for Kalanchoemarnieriana_Kalax.0017s0102.1.p

Viterbi Path

Viterbi Path

Posterior @ 0.4

Posterior @ 0.4

Posterior Graphic. Threshold Marked @ 0.4

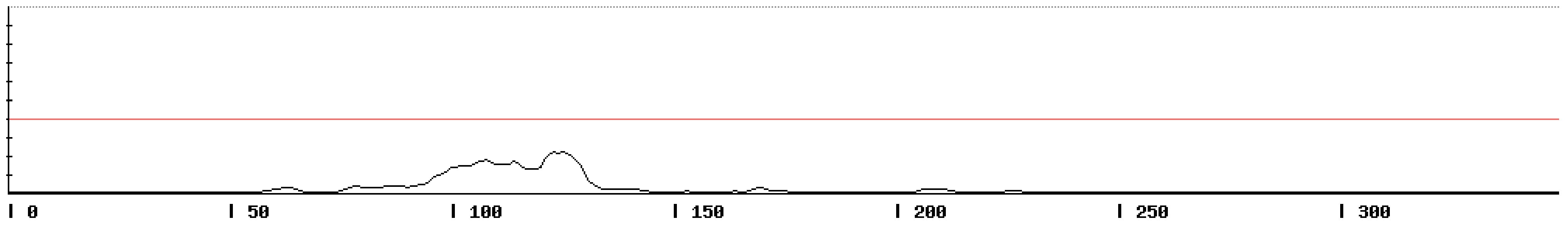

Predictions for Kalanchoemarnieriana_Kalax.0019s0108.1.p

Viterbi Path

Viterbi Path

Posterior @ 0.4

Posterior @ 0.4

Posterior Graphic. Threshold Marked @ 0.4

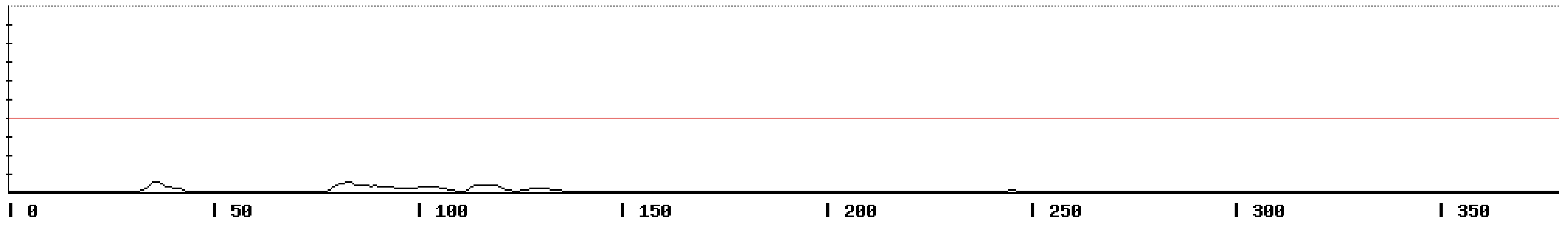

Predictions for Kalanchoemarnieriana_Kalax.0019s0114.1.p	
Viterbi Path	2 - KMIKRSTEERKSKIEKSKSKEIRRRRRRRNK - 32
Viterbi Path	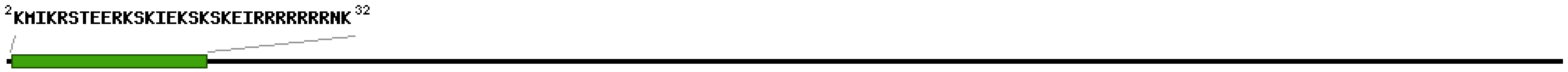
Posterior @ 0.4	5 - KRSTEERKSKIEKSKSKEIRRRRRRRNKVVGKA - 37
Posterior @ 0.4	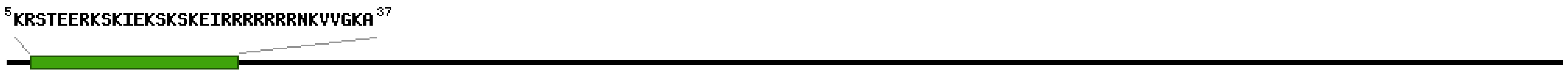
Posterior Graphic. Threshold Marked @ 0.4	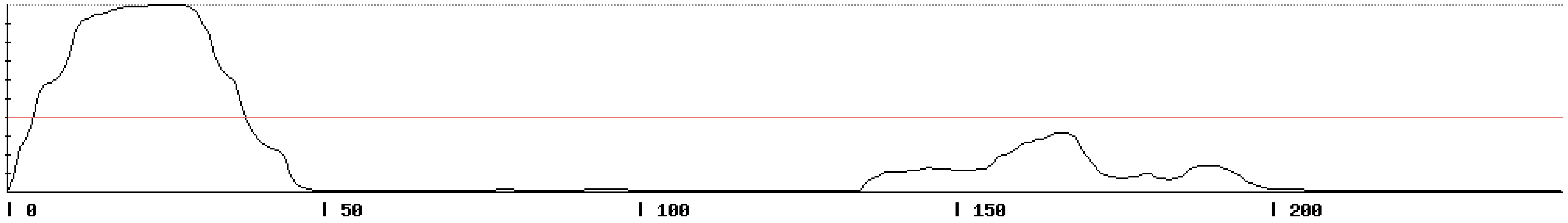

Predictions for Kalanchoemarnieriana_Kalax.0020s0089.1.p	
Viterbi Path	
Viterbi Path	
Posterior @ 0.4	
Posterior @ 0.4	
Posterior Graphic. Threshold Marked @ 0.4	

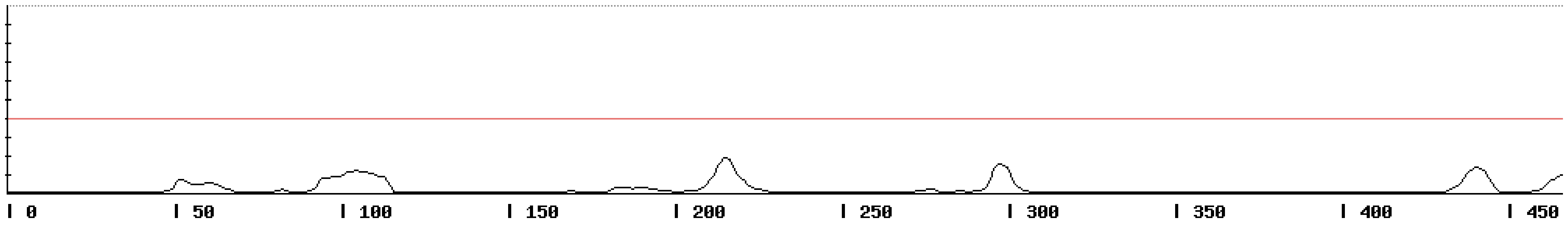

Predictions for Kalanchoemarnieriana_Kalax.0020s0091.1.p

Viterbi Path

Viterbi Path

Posterior @ 0.4

Posterior @ 0.4

Posterior Graphic. Threshold Marked @ 0.4

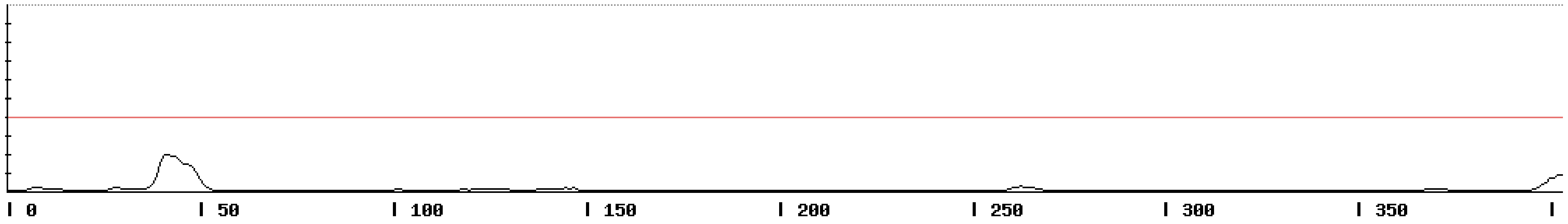

Predictions for Kalanchoemarnieriana_Kalax.0020s0092.1.p

Viterbi Path

Viterbi Path

Posterior @ 0.4

Posterior @ 0.4

Posterior Graphic. Threshold Marked @ 0.4

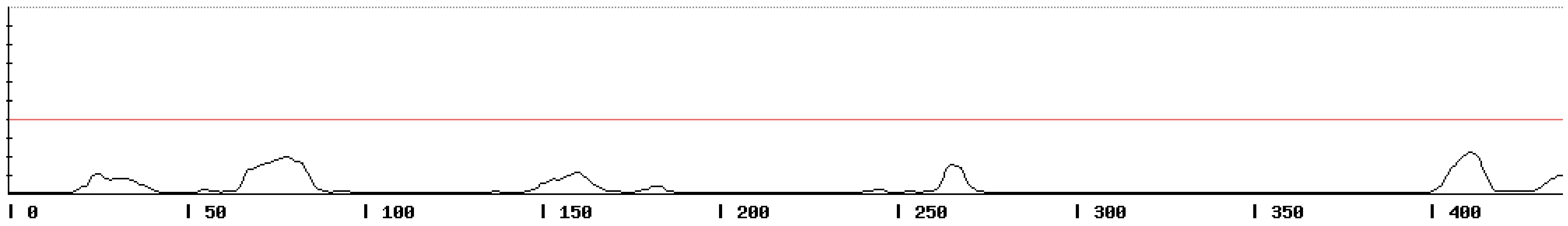

Predictions for Kalanchoemarnieriana_Kalax.0020s0093.1.p

Viterbi Path

Viterbi Path

Posterior @ 0.4

Posterior @ 0.4

Posterior Graphic. Threshold Marked @ 0.4

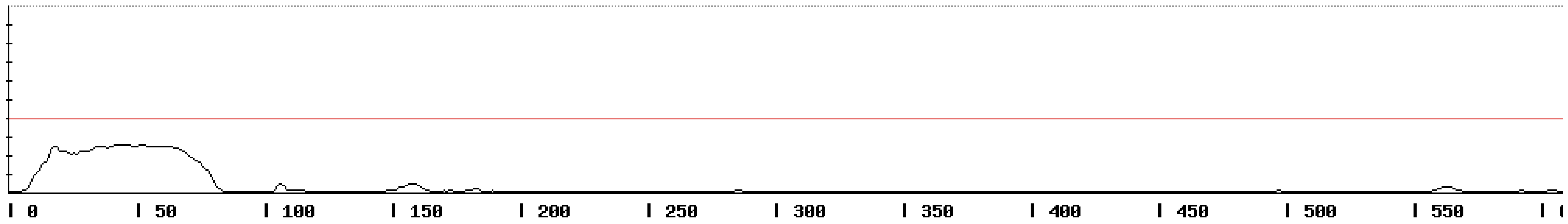

Predictions for Kalanchoemarnieriana_Kalax.0020s0094.1.p

Viterbi Path

Viterbi Path

Posterior @ 0.4

70 - RRGSARNKRRRNNAPPPPPPPPPPP - 94

Posterior @ 0.4

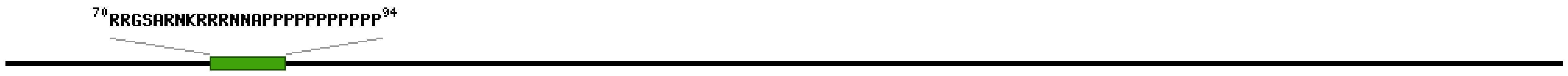

Posterior Graphic. Threshold Marked @ 0.4

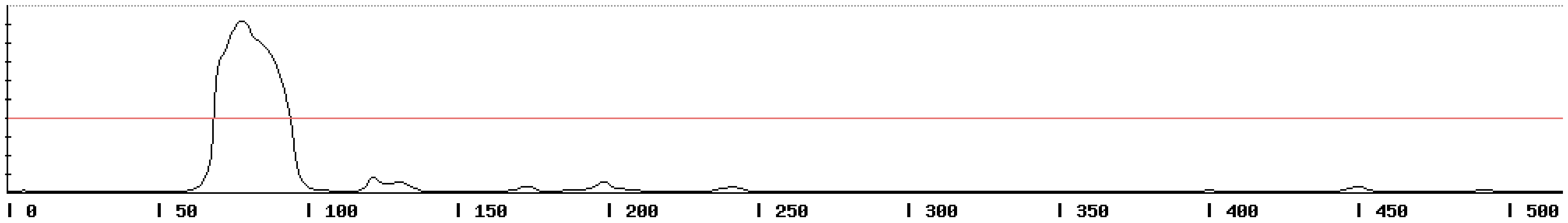

Predictions for Kalanchoemarnieriana_Kalax.0022s0004.1.p

Viterbi Path

Viterbi Path

Posterior @ 0.4

Posterior @ 0.4

Posterior Graphic. Threshold Marked @ 0.4

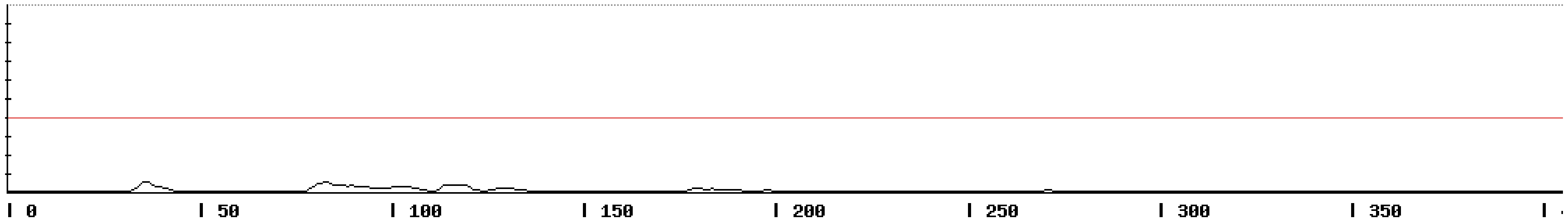

Predictions for Kalanchoemarnieriana_Kalax.0022s0146.1.p

Viterbi Path

Viterbi Path

Posterior @ 0.4

Posterior @ 0.4

Posterior Graphic. Threshold Marked @ 0.4

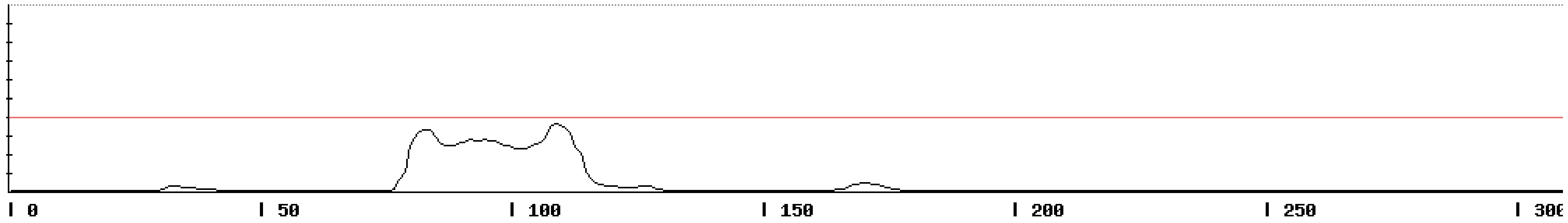

Predictions for Kalanchoemarnieriana_Kalax.0024s0136.1.p

Viterbi Path

Viterbi Path

Posterior @ 0.4

Posterior @ 0.4

Posterior Graphic. Threshold Marked @ 0.4

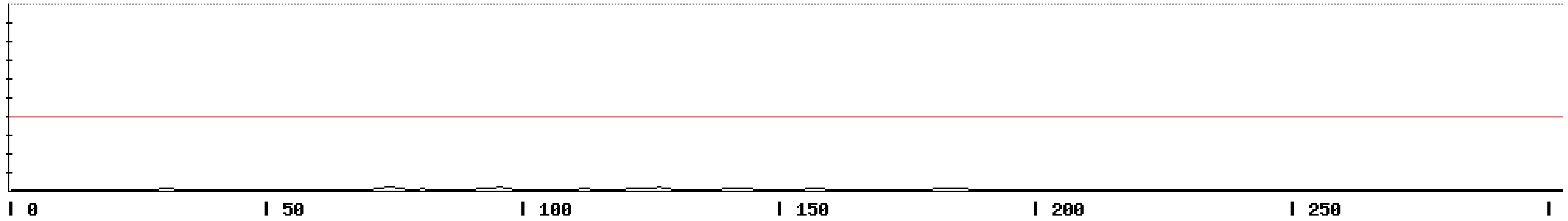

Predictions for Kalanchoemarnieriana_Kalax.0026s0117.1.p

Viterbi Path

Viterbi Path

Posterior @ 0.4	
Posterior @ 0.4	
Posterior Graphic. Threshold Marked @ 0.4	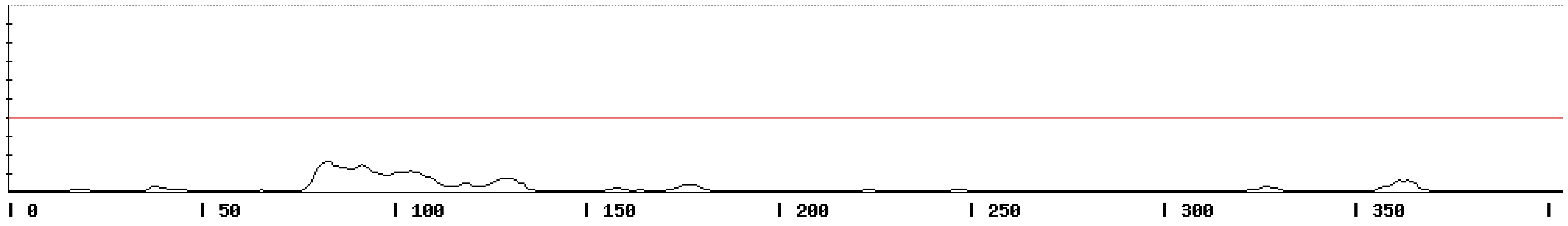

Predictions for Kalanchoemarnieriana_Kalax.0026s0128.1.p

Viterbi Path	
Viterbi Path	
Posterior @ 0.4	
Posterior @ 0.4	
Posterior Graphic. Threshold Marked @ 0.4	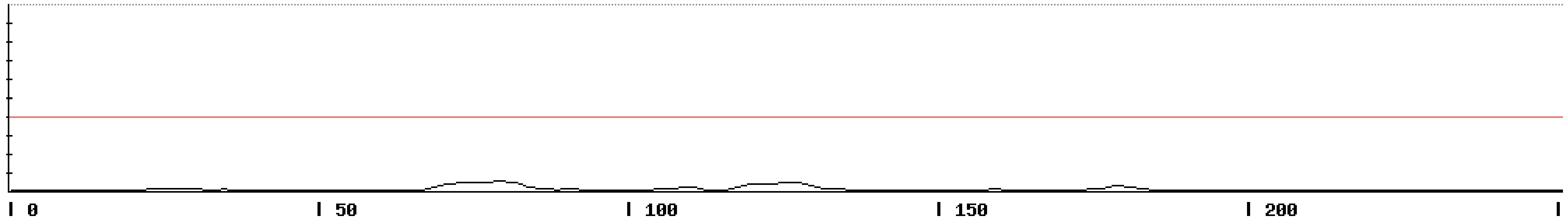

Predictions for Kalanchoemarnieriana_Kalax.0028s0113.1.p

Viterbi Path	
Viterbi Path	
Posterior @ 0.4	
Posterior @ 0.4	
Posterior Graphic. Threshold Marked @ 0.4	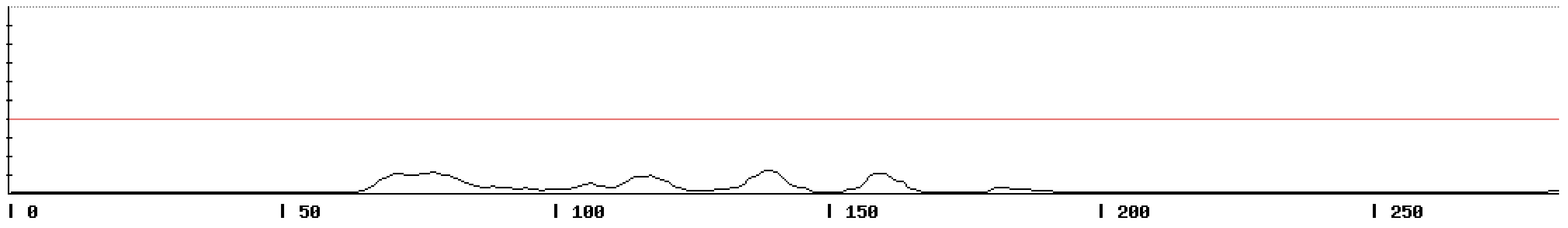

Predictions for Kalanchoemarnieriana_Kalax.0033s0014.1.p

Viterbi Path	
Viterbi Path	
Posterior @ 0.4	
Posterior @ 0.4	
Posterior Graphic. Threshold Marked @ 0.4	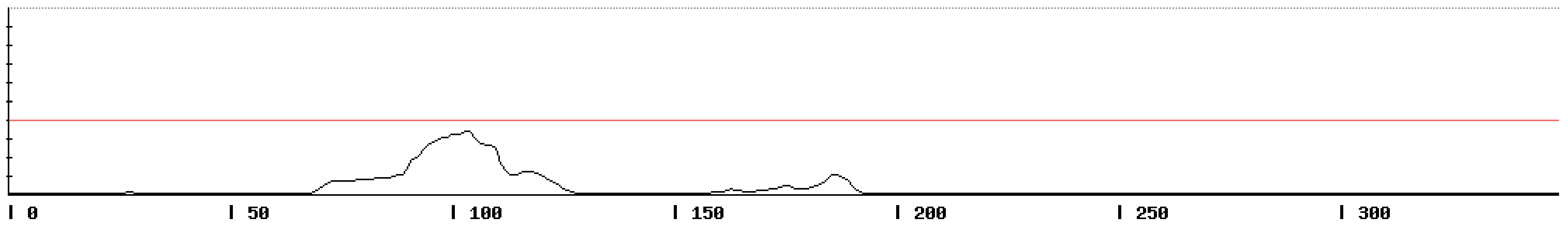

Predictions for Kalanchoemarnieriana_Kalax.0033s0042.1.p	
Viterbi Path	
Viterbi Path	
Posterior @ 0.4	
Posterior @ 0.4	
Posterior Graphic. Threshold Marked @ 0.4	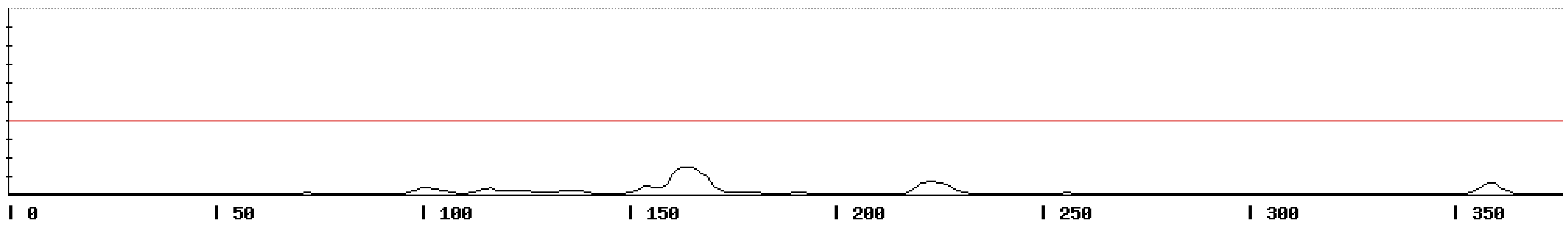

Predictions for Kalanchoemarnieriana_Kalax.0033s0124.1.p	
Viterbi Path	
Viterbi Path	
Posterior @ 0.4	168 - RKRRK - 172
Posterior @ 0.4	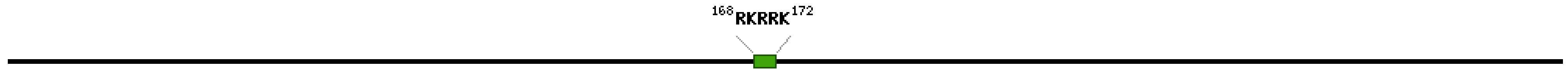
Posterior Graphic. Threshold Marked @ 0.4	

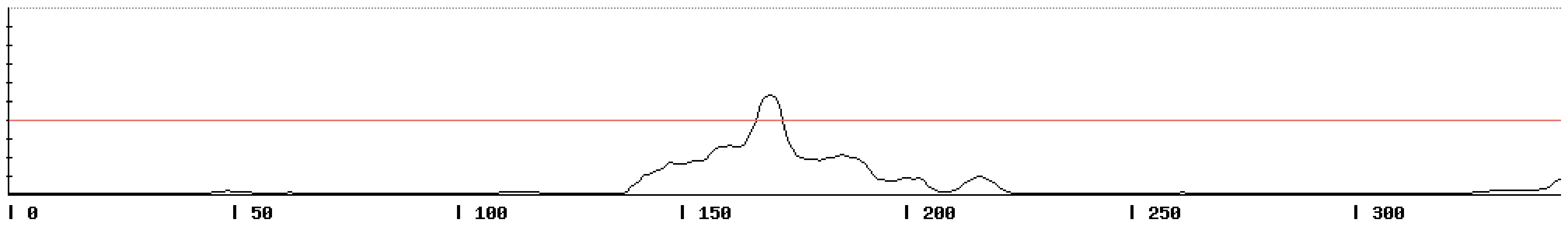

Predictions for Kalanchoemarnieriana_Kalax.0039s0055.1.p

Viterbi Path	
Viterbi Path	
Posterior @ 0.4	
Posterior @ 0.4	
Posterior Graphic. Threshold Marked @ 0.4	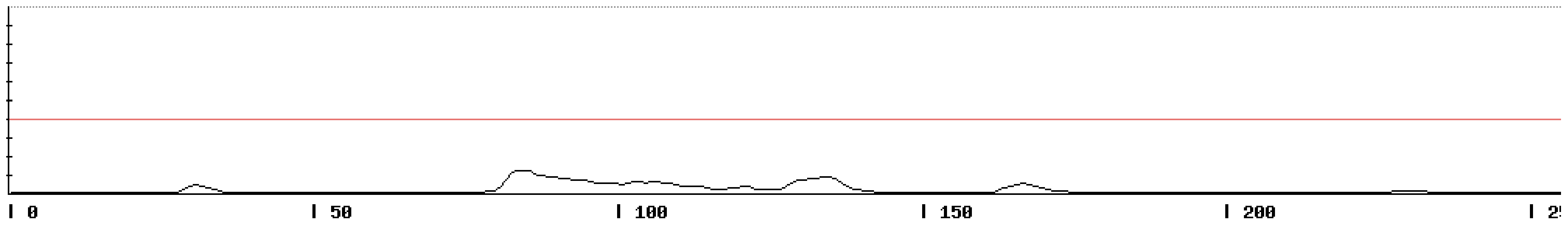

Predictions for Kalanchoemarnieriana_Kalax.0039s0145.1.p

Viterbi Path	
Viterbi Path	
Posterior @ 0.4	136 - SRKRRKI - 142
Posterior @ 0.4	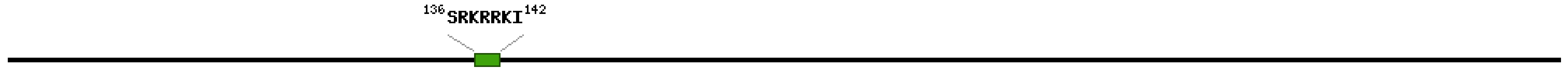

Posterior Graphic. Threshold Marked @ 0.4

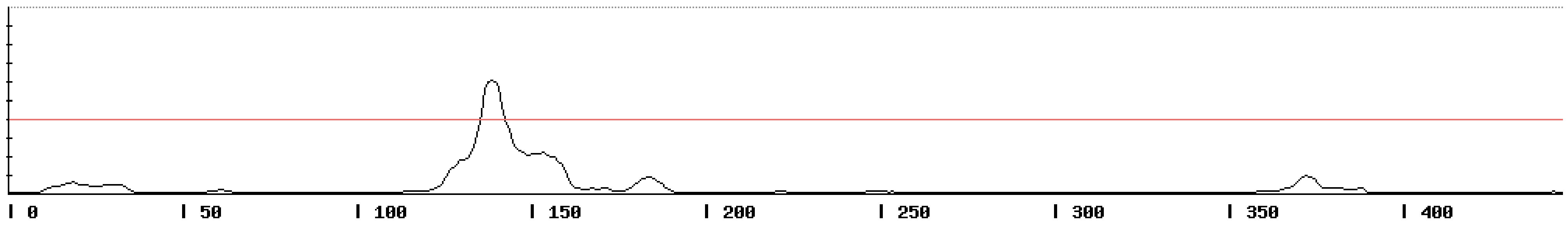

Predictions for Kalanchoemarnieriana_Kalax.0041s0099.1.p

Viterbi Path

Viterbi Path

Posterior @ 0.4

Posterior @ 0.4

Posterior Graphic. Threshold Marked @ 0.4

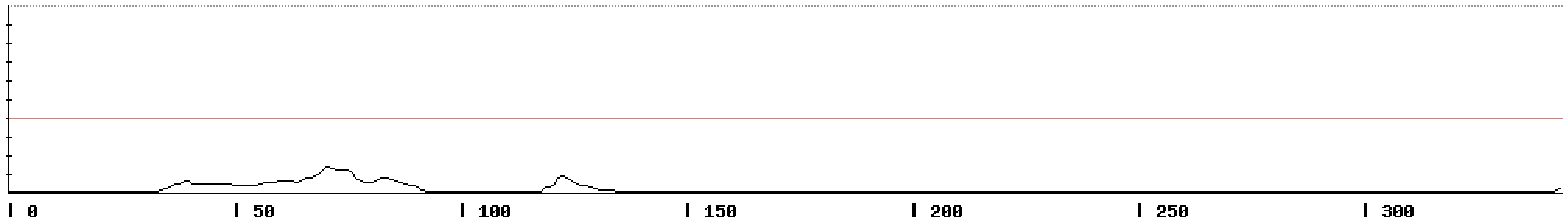

Predictions for Kalanchoemarnieriana_Kalax.0041s0116.1.p

Viterbi Path

Viterbi Path

Posterior @ 0.4

Posterior @ 0.4

Posterior Graphic. Threshold Marked @ 0.4	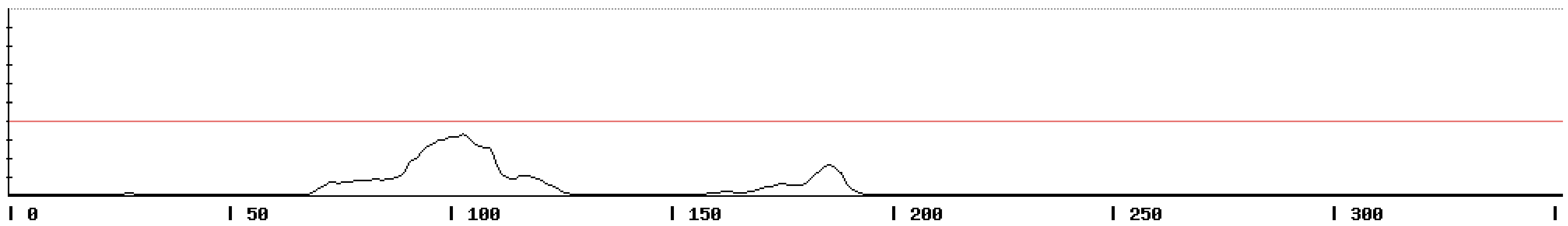

Predictions for Kalanchoemarnieriana_Kalax.0046s0014.1.p

Viterbi Path	
Viterbi Path	
Posterior @ 0.4	
Posterior @ 0.4	
Posterior Graphic. Threshold Marked @ 0.4	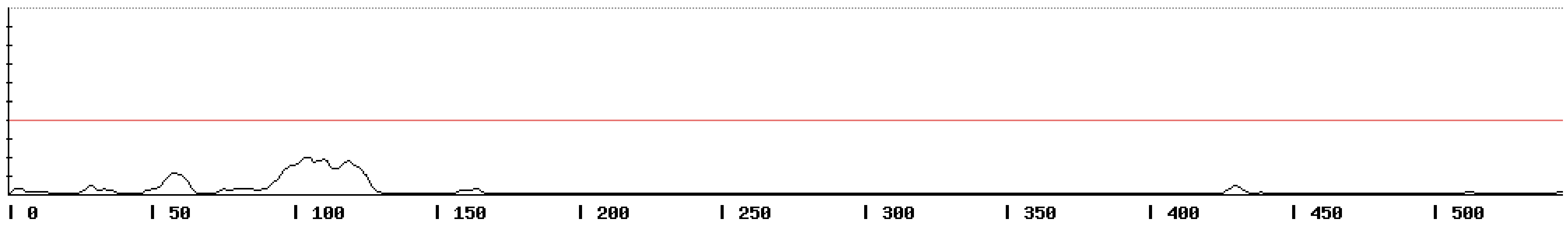

Predictions for Kalanchoemarnieriana_Kalax.0048s0059.1.p

Viterbi Path	
Viterbi Path	

Posterior @ 0.4

Posterior @ 0.4

Posterior Graphic. Threshold Marked @ 0.4

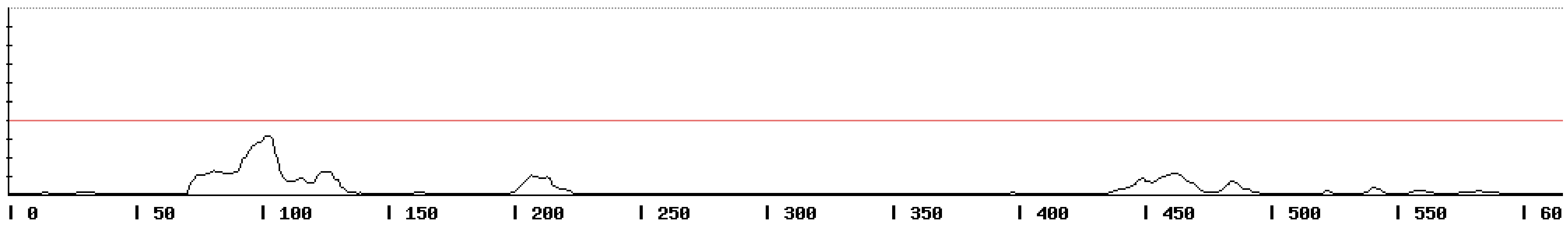

Predictions for Kalanchoemarnieriana_Kalax.0052s0074.1.p

Viterbi Path

Viterbi Path

Posterior @ 0.4

Posterior @ 0.4

Posterior Graphic. Threshold Marked @ 0.4

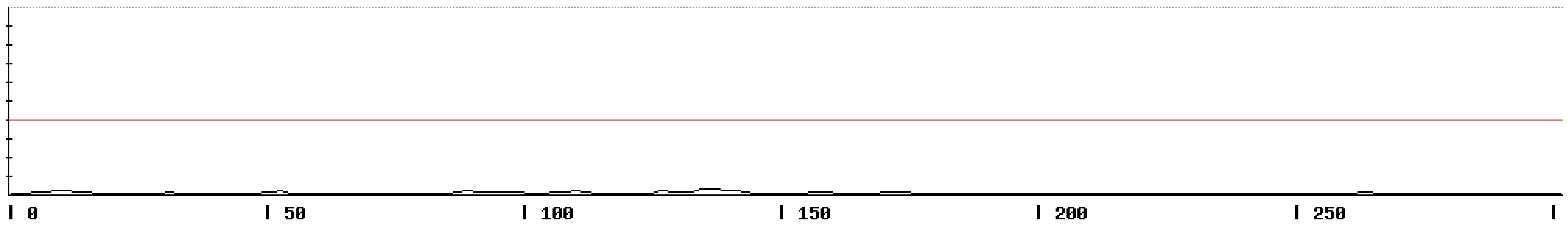

Predictions for Kalanchoemarnieriana_Kalax.0054s0134.1.p

Viterbi Path

Viterbi Path

Posterior @ 0.4	91 - R - 91
	93 - R - 93
	95 - IKTASKKL - 102
Posterior @ 0.4	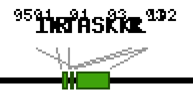
Posterior Graphic. Threshold Marked @ 0.4	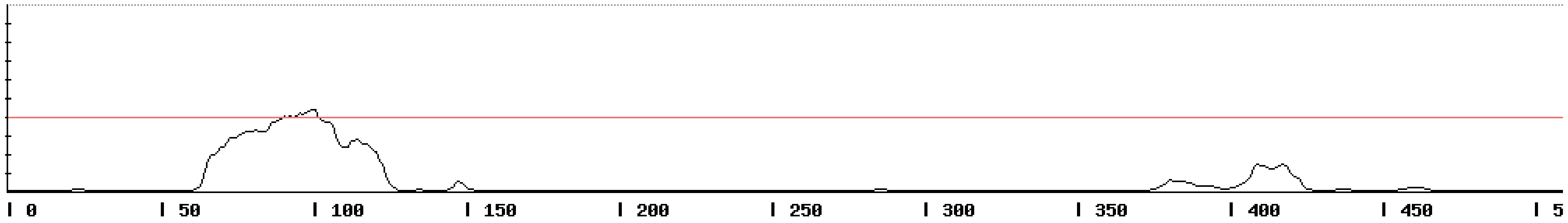

Predictions for Kalanchoemarnieriana_Kalax.0056s0128.1.p

Viterbi Path	
Viterbi Path	
Posterior @ 0.4	
Posterior @ 0.4	
Posterior Graphic. Threshold Marked @ 0.4	

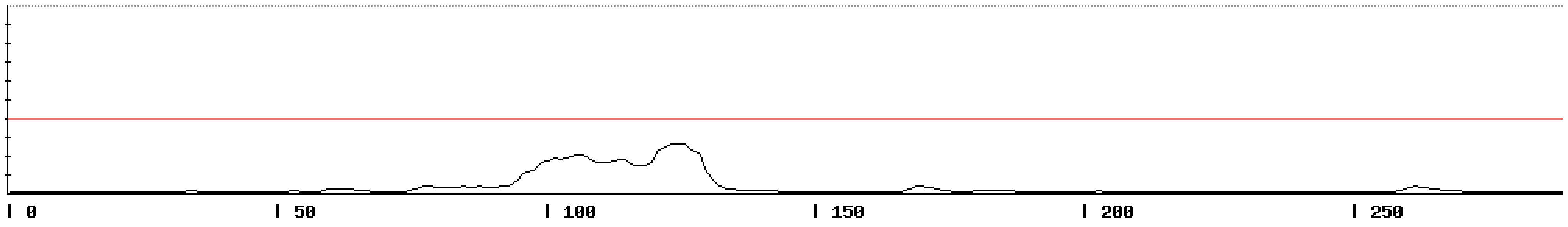

Predictions for Kalanchoemarnieriana_Kalax.0057s0052.1.p	
Viterbi Path	86 - RKRTRMRRTRGGYWKRKGKVR - 106
Viterbi Path	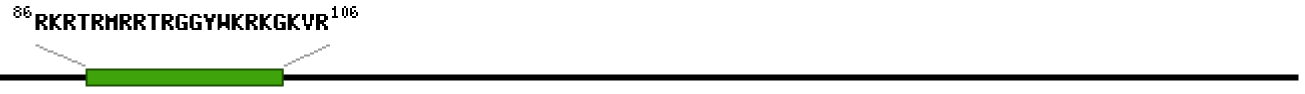
Posterior @ 0.4	83 - RVERKRTRMRRTRGGYWKRKGKVR - 106
Posterior @ 0.4	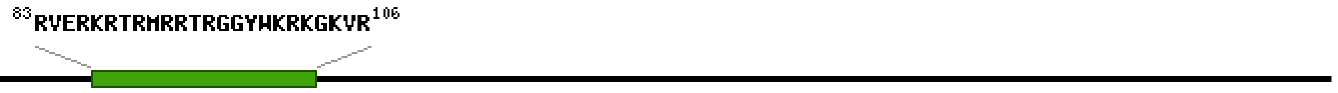
Posterior Graphic. Threshold Marked @ 0.4	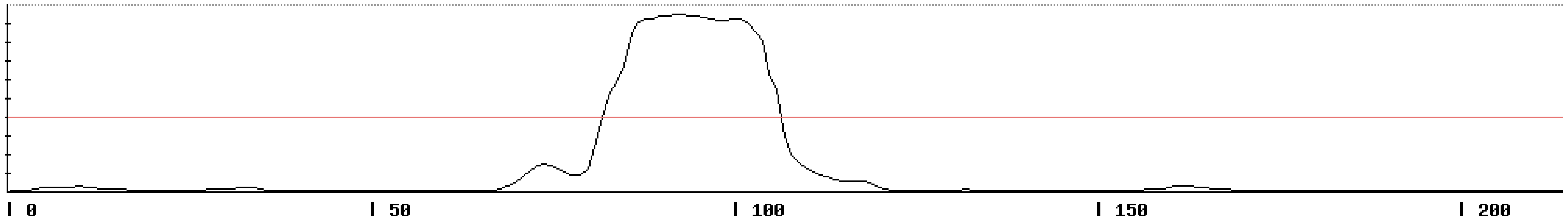

Predictions for Kalanchoemarnieriana_Kalax.0059s0070.1.p	
Viterbi Path	
Viterbi Path	
Posterior @ 0.4	
Posterior @ 0.4	

Posterior Graphic. Threshold Marked @ 0.4

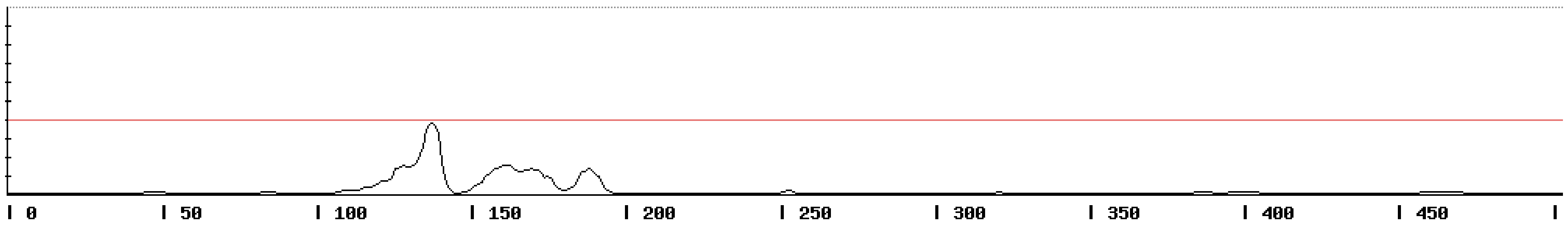

Predictions for Kalanchoemarnieriana_Kalax.0069s0007.1.p

Viterbi Path

Viterbi Path

Posterior @ 0.4 — 135 - TRKRRKIH - 142

Posterior @ 0.4

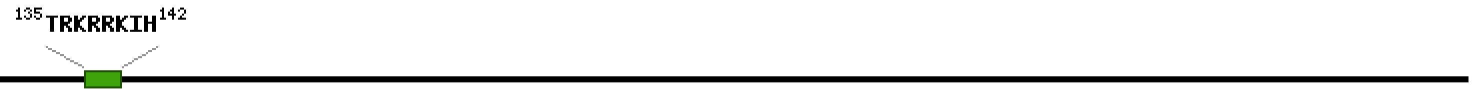

Posterior Graphic. Threshold Marked @ 0.4

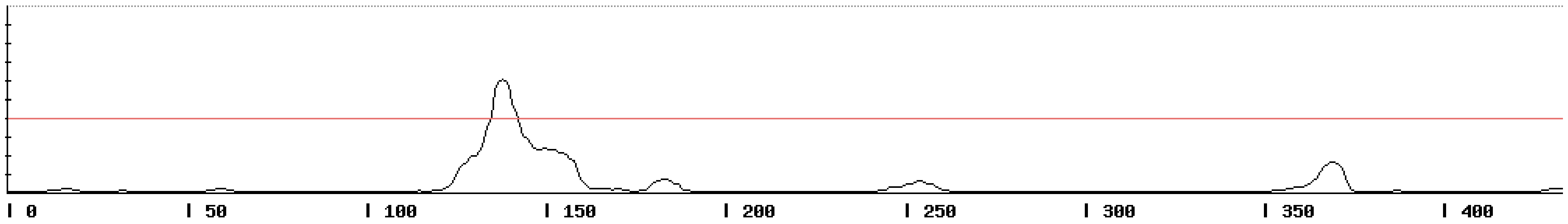

Predictions for Kalanchoemarnieriana_Kalax.0070s0067.1.p

Viterbi Path

Viterbi Path

Posterior @ 0.4

Posterior @ 0.4

Posterior Graphic. Threshold Marked @ 0.4

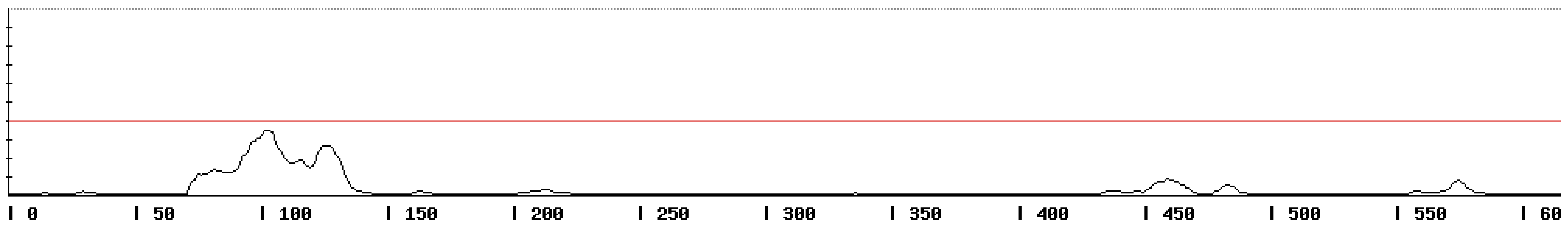

Predictions for Kalanchoemarnieriana_Kalax.0076s0001.1.p

Viterbi Path

Viterbi Path

Posterior @ 0.4

Posterior @ 0.4

Posterior Graphic. Threshold Marked @ 0.4

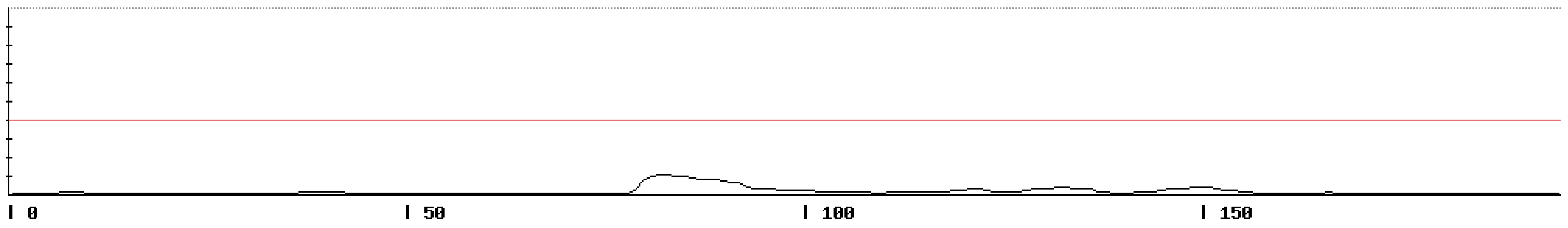

Predictions for Kalanchoemarnieriana_Kalax.0081s0007.1.p

Viterbi Path

Viterbi Path

Posterior @ 0.4	
Posterior @ 0.4	
Posterior Graphic. Threshold Marked @ 0.4	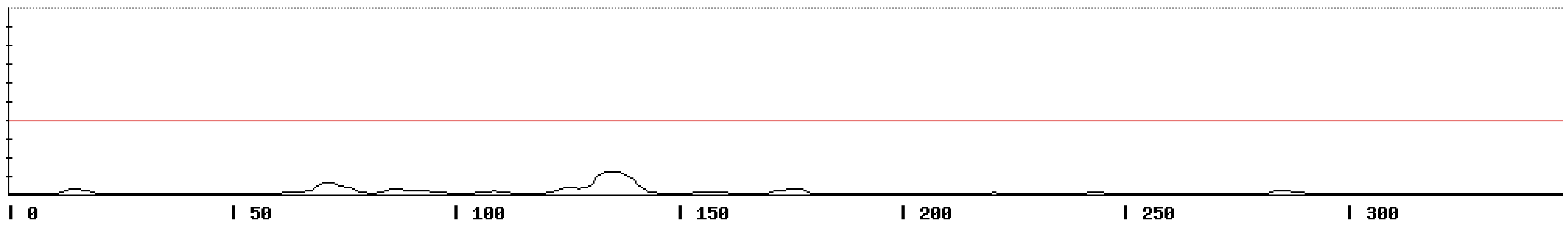

Predictions for Kalanchoemarnieriana_Kalax.0085s0072.1.p	
Viterbi Path	
Viterbi Path	
Posterior @ 0.4	
Posterior @ 0.4	
Posterior Graphic. Threshold Marked @ 0.4	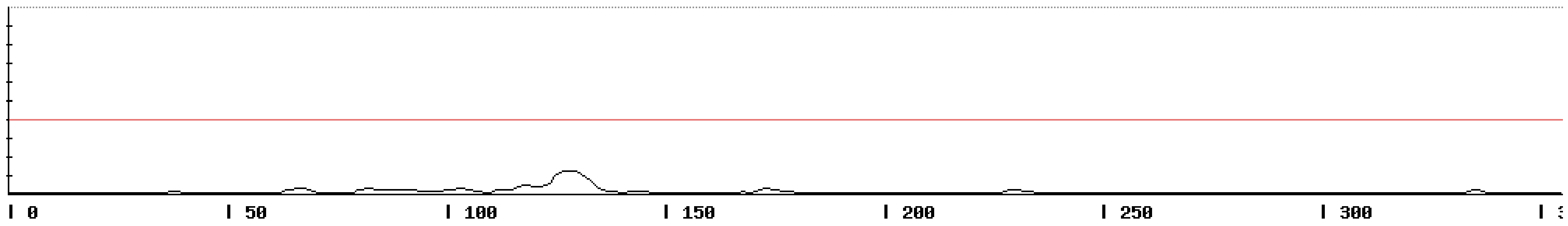

Predictions for Kalanchoemarnieriana_Kalax.0090s0019.1.p	
Viterbi Path	
Viterbi Path	

Posterior @ 0.4

Posterior @ 0.4

Posterior Graphic. Threshold Marked @ 0.4

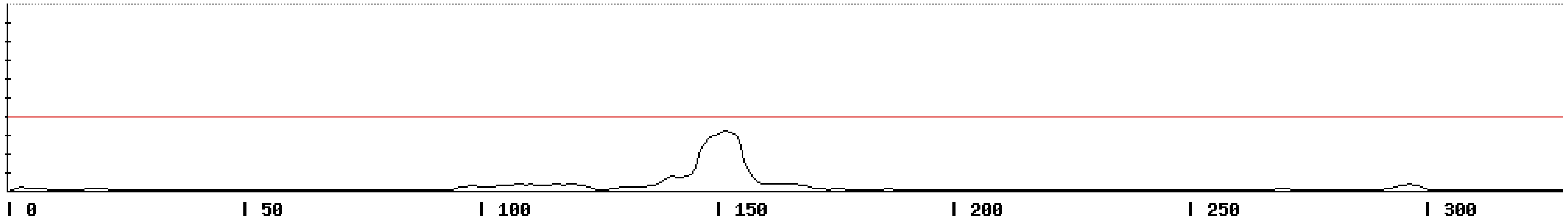

Predictions for Kalanchoemarnieriana_Kalax.0095s0061.1.p

Viterbi Path

Viterbi Path

Posterior @ 0.4

Posterior @ 0.4

Posterior Graphic. Threshold Marked @ 0.4

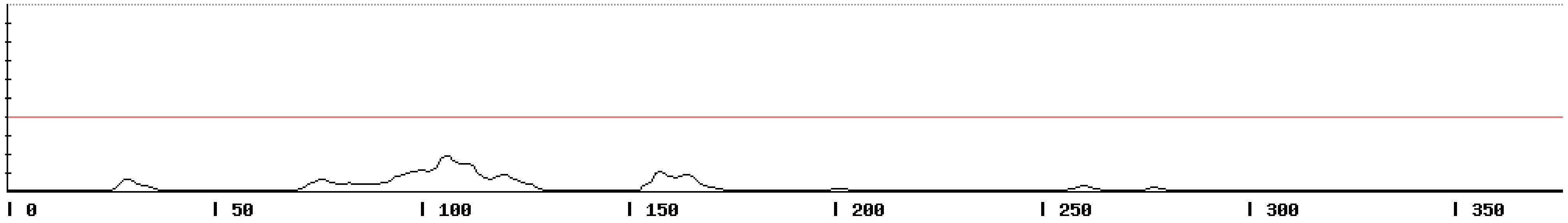

Predictions for Kalanchoemarnieriana_Kalax.0099s0031.1.p

Viterbi Path

Viterbi Path

Posterior @ 0.4

Posterior @ 0.4

Posterior Graphic. Threshold Marked @ 0.4

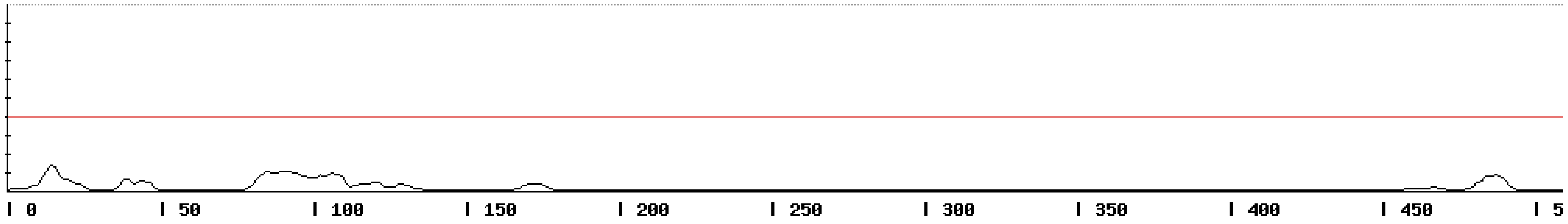

Predictions for Kalanchoemarnieriana_Kalax.0100s0047.1.p

Viterbi Path

Viterbi Path

Posterior @ 0.4

Posterior @ 0.4

Posterior Graphic. Threshold Marked @ 0.4

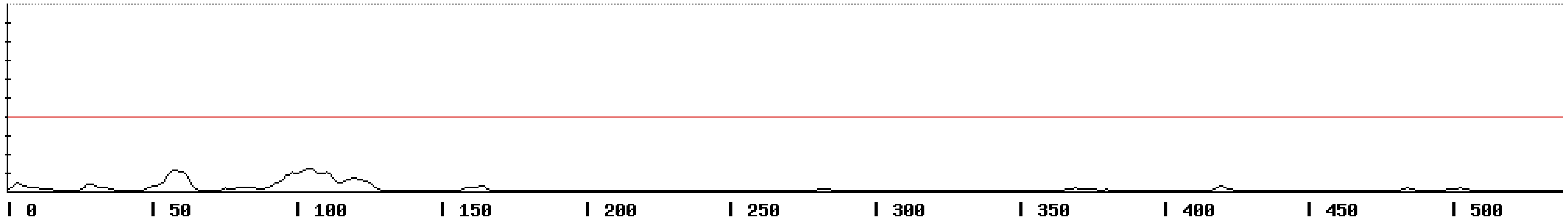

Predictions for Kalanchoemarnieriana_Kalax.0103s0071.1.p	
Viterbi Path	86 - RKRTRMRRTRGGYWKRKGKVR - 106
Viterbi Path	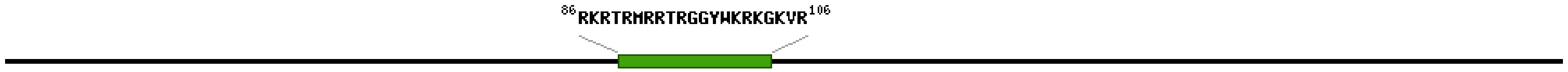
Posterior @ 0.4	83 - RVERKRTRMRRTRGGYWKRKGKVR - 106
Posterior @ 0.4	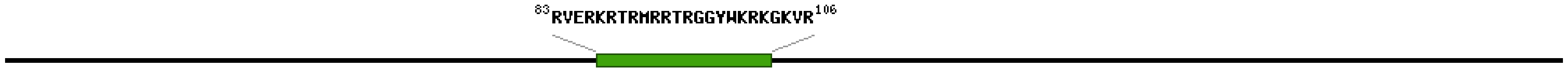
Posterior Graphic. Threshold Marked @ 0.4	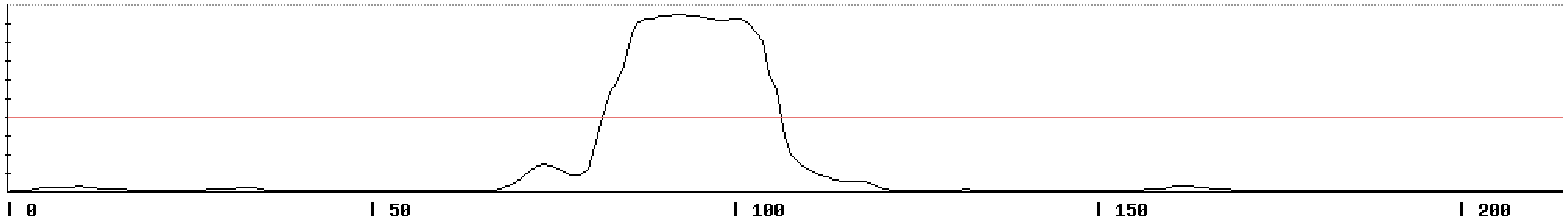

Predictions for Kalanchoemarnieriana_Kalax.0104s0078.1.p	
Viterbi Path	
Viterbi Path	
Posterior @ 0.4	
Posterior @ 0.4	
Posterior Graphic. Threshold Marked @ 0.4	

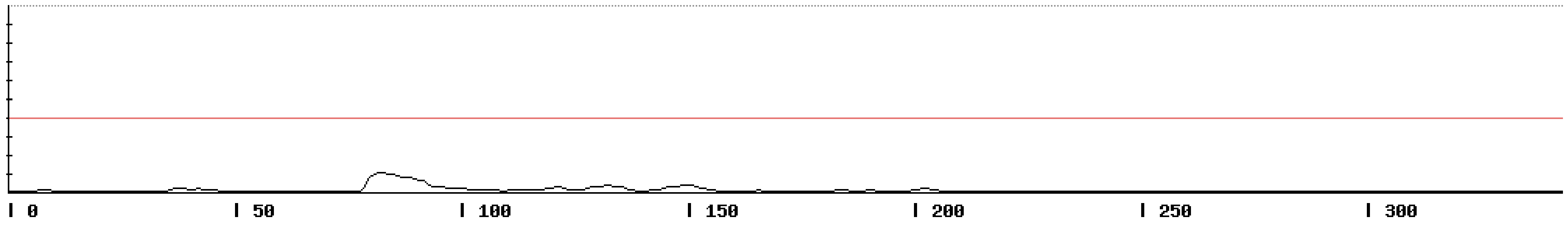

Predictions for Kalanchoemarnieriana_Kalax.0104s0092.1.p

Viterbi Path

Viterbi Path

Posterior @ 0.4

Posterior @ 0.4

Posterior Graphic. Threshold Marked @ 0.4

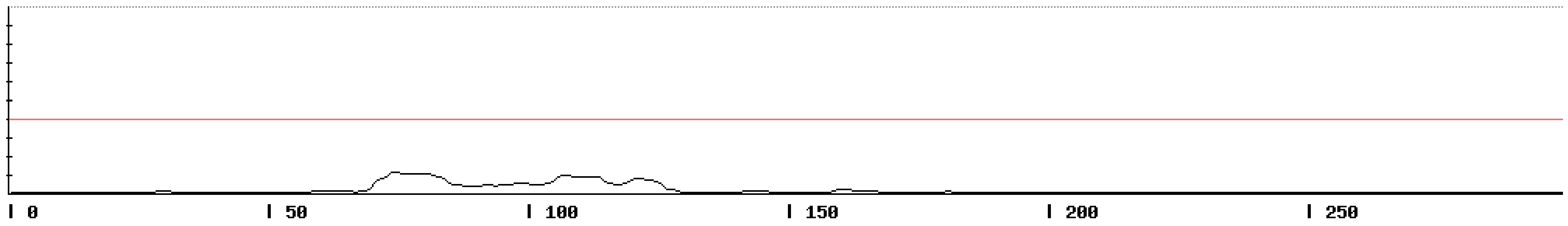

Predictions for Kalanchoemarnieriana_Kalax.0115s0080.1.p

Viterbi Path

Viterbi Path

Posterior @ 0.4

Posterior @ 0.4

Posterior Graphic. Threshold Marked @ 0.4

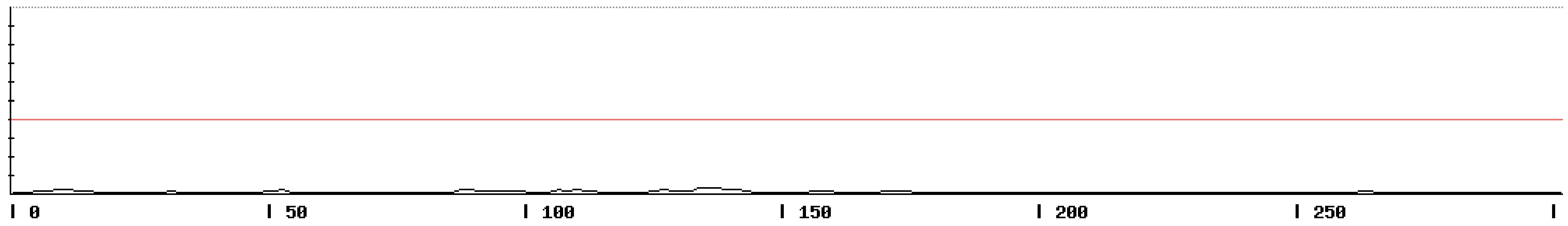

Predictions for Kalanchoemarnieriana_Kalax.0119s0023.1.p

Viterbi Path

Viterbi Path

Posterior @ 0.4

Posterior @ 0.4

Posterior Graphic. Threshold Marked @ 0.4

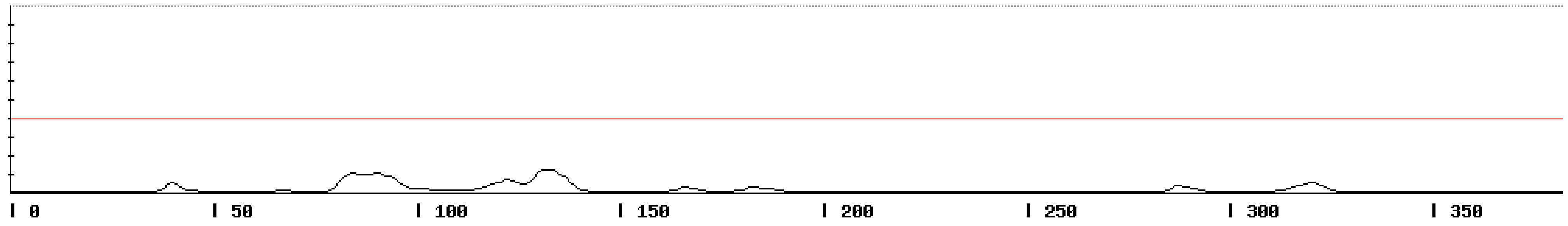

Predictions for Kalanchoemarnieriana_Kalax.0126s0052.1.p

Viterbi Path

Viterbi Path

Posterior @ 0.4

Posterior @ 0.4

Posterior Graphic. Threshold Marked @ 0.4

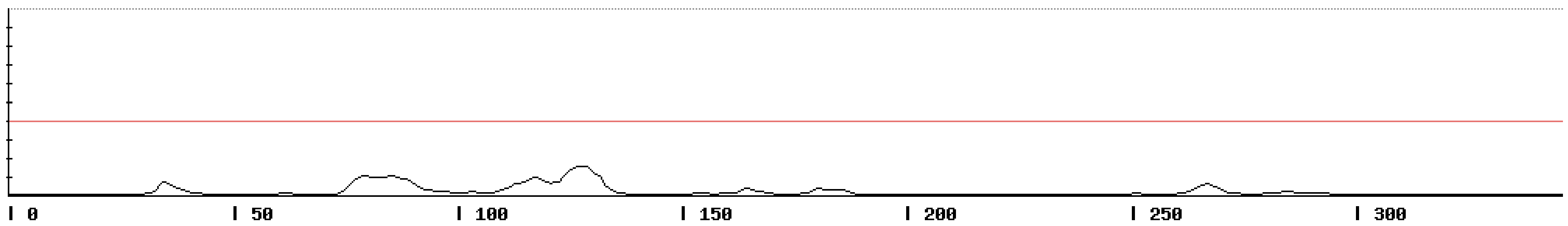

Predictions for Kalanchoemarnieriana_Kalax.0130s0010.1.p

Viterbi Path

Viterbi Path

Posterior @ 0.4

Posterior @ 0.4

Posterior Graphic. Threshold Marked @ 0.4

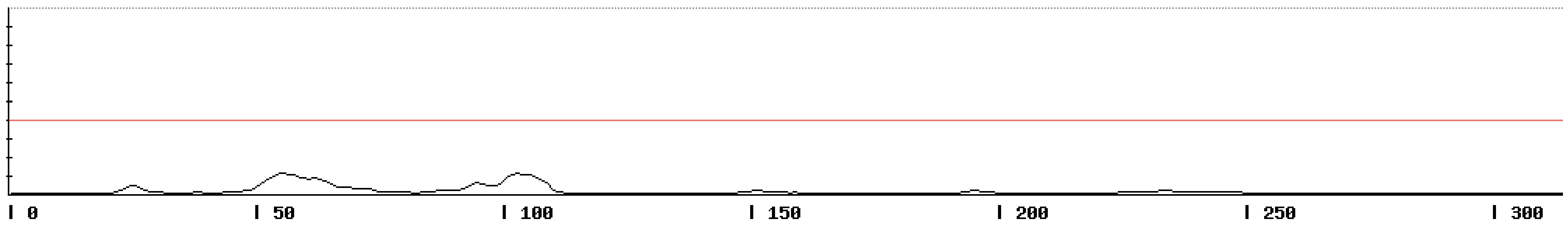

Predictions for Kalanchoemarnieriana_Kalax.0131s0011.1.p

Viterbi Path

Viterbi Path

Posterior @ 0.4

Posterior @ 0.4

Posterior Graphic. Threshold Marked @ 0.4

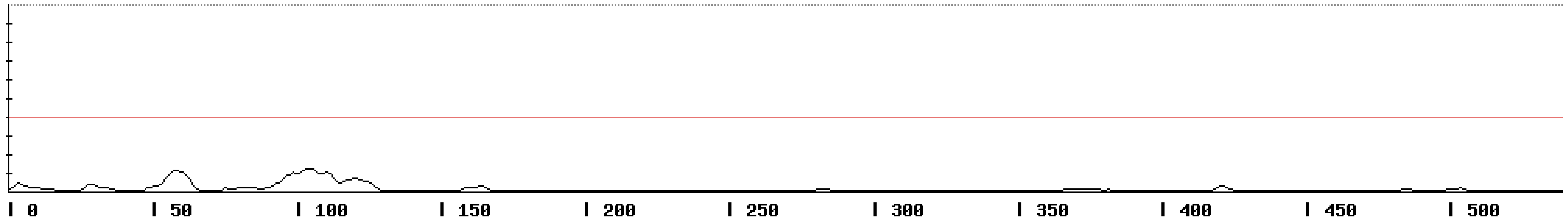

Predictions for Kalanchoemarnieriana_Kalax.0132s0094.1.p

Viterbi Path

Viterbi Path

Posterior @ 0.4

Posterior @ 0.4

Posterior Graphic. Threshold Marked @ 0.4

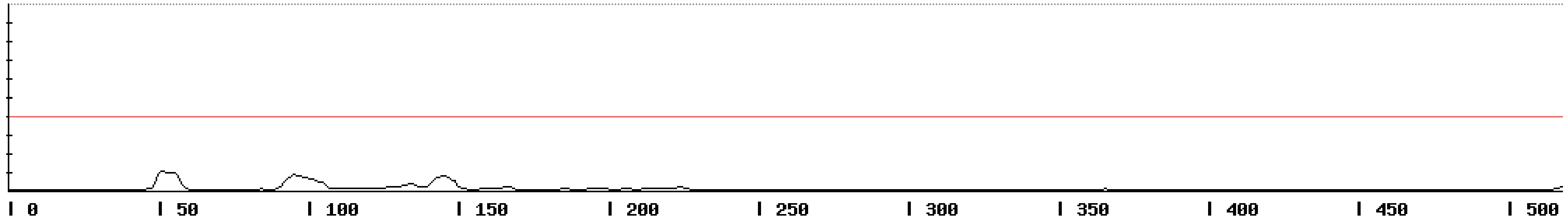

Predictions for Kalanchoemarnieriana_Kalax.0136s0013.1.p

Viterbi Path

Viterbi Path

Posterior @ 0.4

Posterior @ 0.4

Posterior Graphic. Threshold Marked @ 0.4

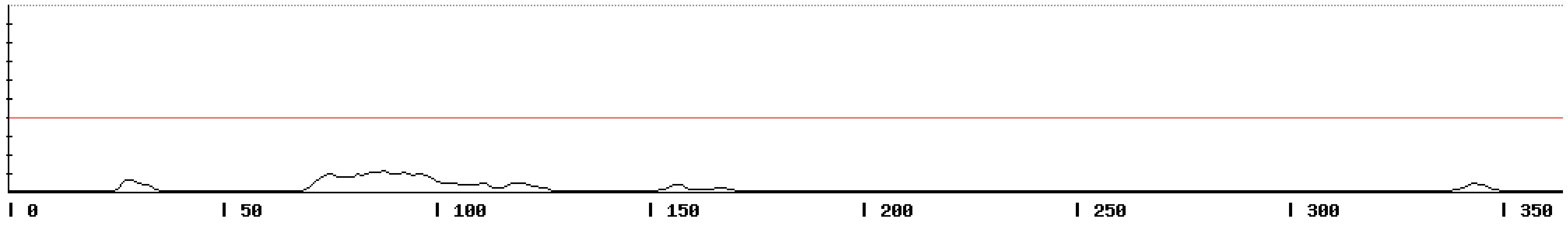

Predictions for Kalanchoemarnieriana_Kalax.0137s0047.1.p

Viterbi Path

Viterbi Path

Posterior @ 0.4

Posterior @ 0.4

Posterior Graphic. Threshold Marked @ 0.4

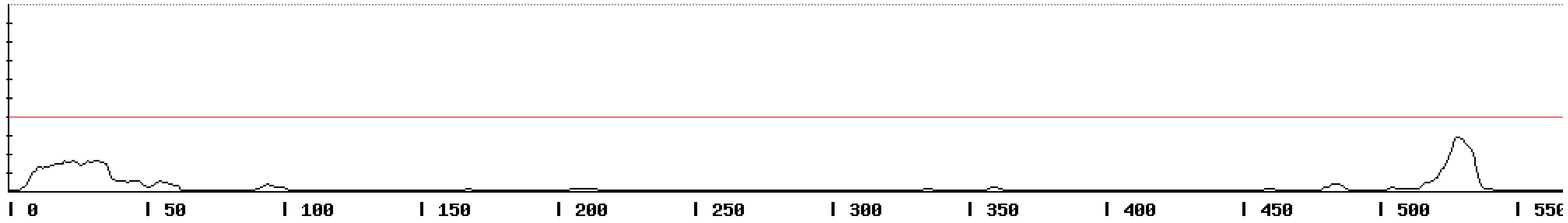

Predictions for Kalanchoemarnieriana_Kalax.0139s0008.1.p

Viterbi Path

Viterbi Path

Posterior @ 0.4

Posterior @ 0.4

Posterior Graphic. Threshold Marked @ 0.4

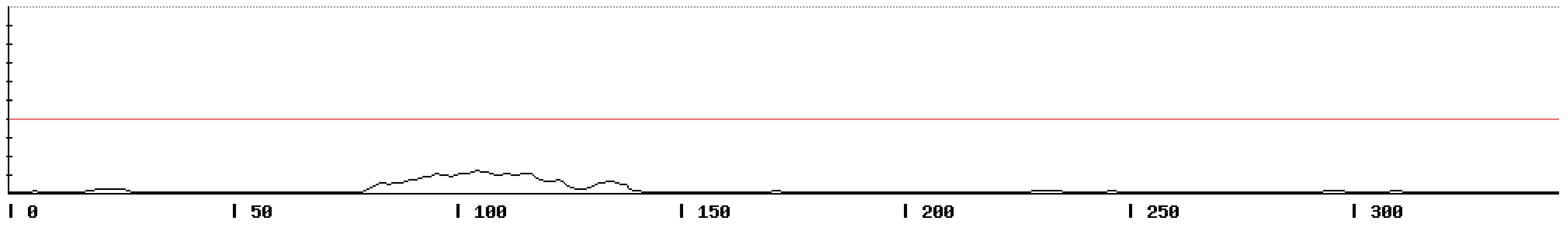

Predictions for Kalanchoemarnieriana_Kalax.0139s0009.1.p

Viterbi Path

Viterbi Path

Posterior @ 0.4

Posterior @ 0.4

Posterior Graphic. Threshold Marked @ 0.4

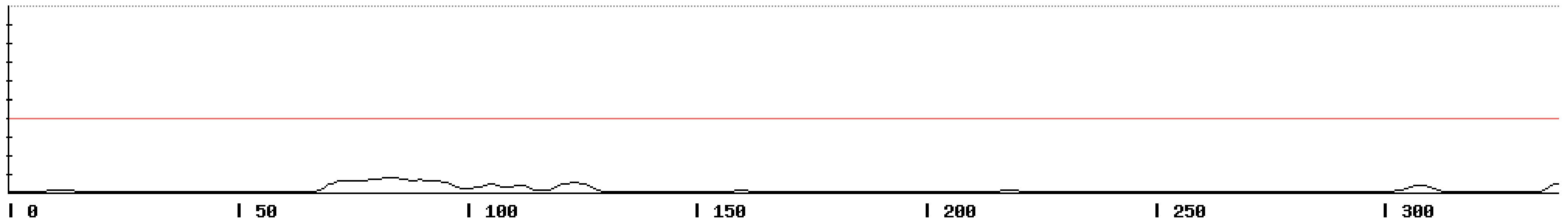

Predictions for Kalanchoemarnieriana_Kalax.0139s0010.1.p

Viterbi Path

Viterbi Path

Posterior @ 0.4

Posterior @ 0.4

Posterior Graphic. Threshold Marked @ 0.4

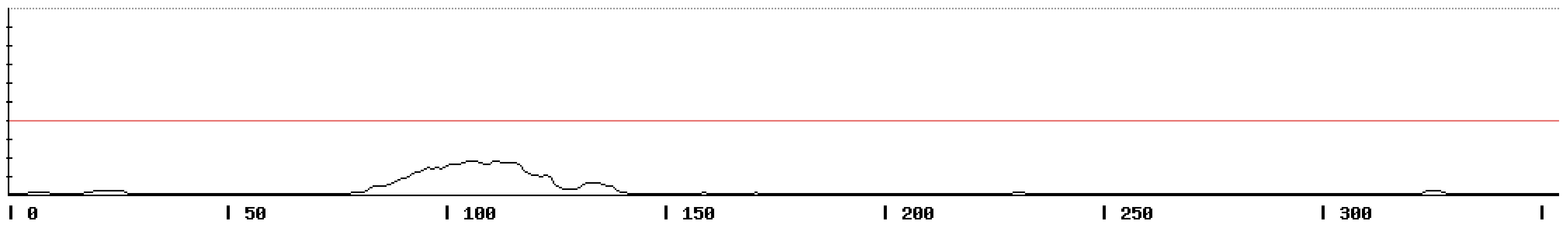

Predictions for Kalanchoemarnieriana_Kalax.0139s0060.1.p

Viterbi Path

Viterbi Path

Posterior @ 0.4

Posterior @ 0.4

Posterior Graphic. Threshold Marked @ 0.4

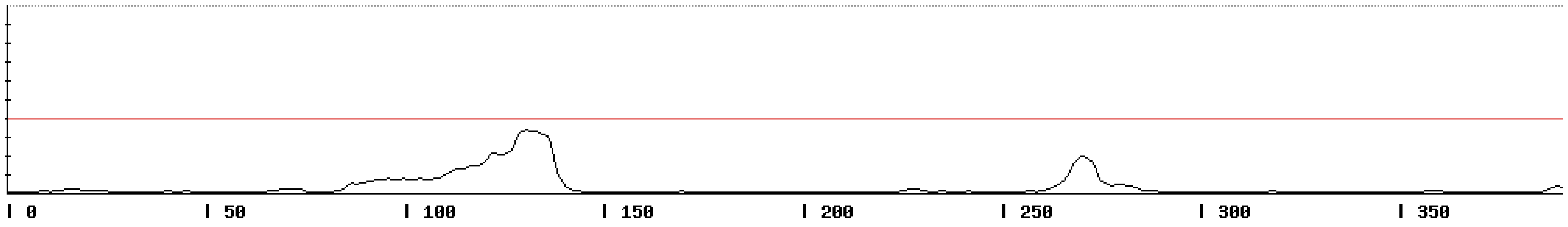

Predictions for Kalanchoemarnieriana_Kalax.0139s0061.1.p

Viterbi Path	
Viterbi Path	
Posterior @ 0.4	
Posterior @ 0.4	
Posterior Graphic. Threshold Marked @ 0.4	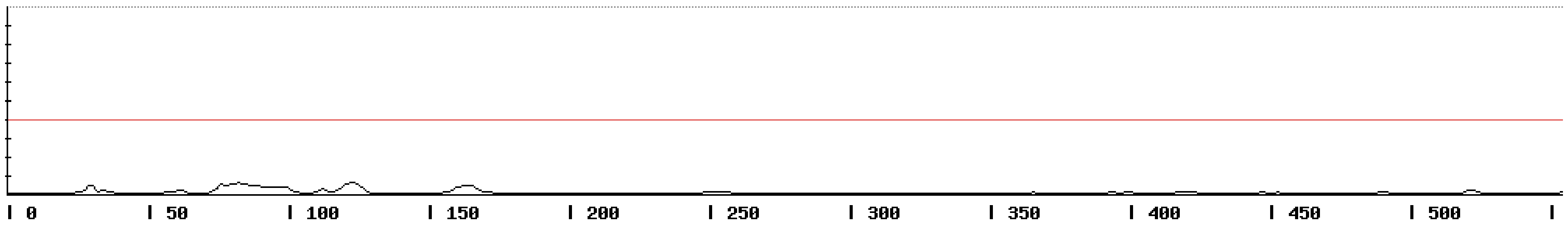

Predictions for Kalanchoemarnieriana_Kalax.0139s0062.1.p

Viterbi Path	
Viterbi Path	
Posterior @ 0.4	129 - RP - 130
Posterior @ 0.4	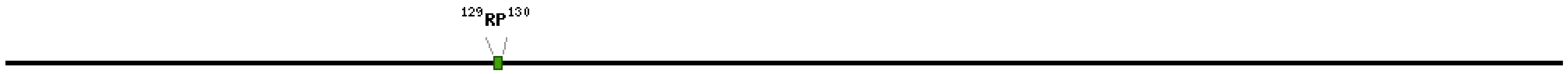

Posterior Graphic. Threshold Marked @ 0.4

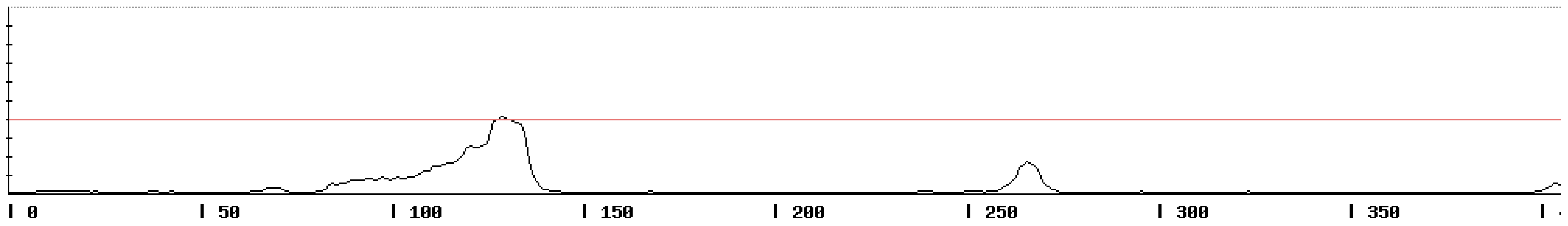

Predictions for Kalanchoemarnieriana_Kalax.0140s0019.1.p

Viterbi Path

Viterbi Path

Posterior @ 0.4

Posterior @ 0.4

Posterior Graphic. Threshold Marked @ 0.4

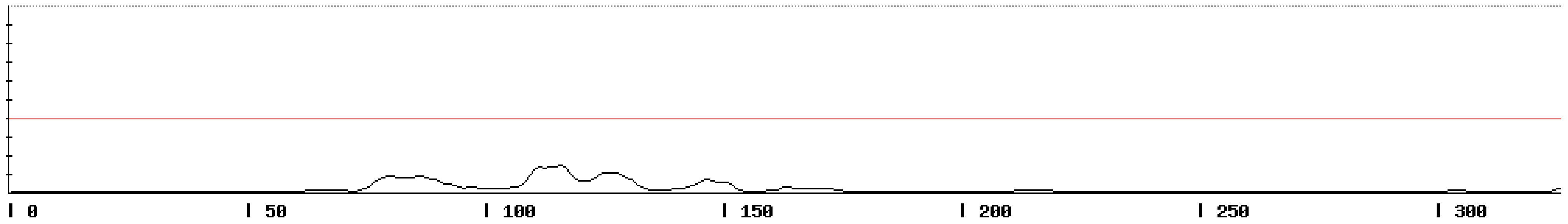

Predictions for Kalanchoemarnieriana_Kalax.0140s0020.1.p

Viterbi Path

Viterbi Path

Posterior @ 0.4

Posterior @ 0.4

Posterior Graphic. Threshold Marked @ 0.4

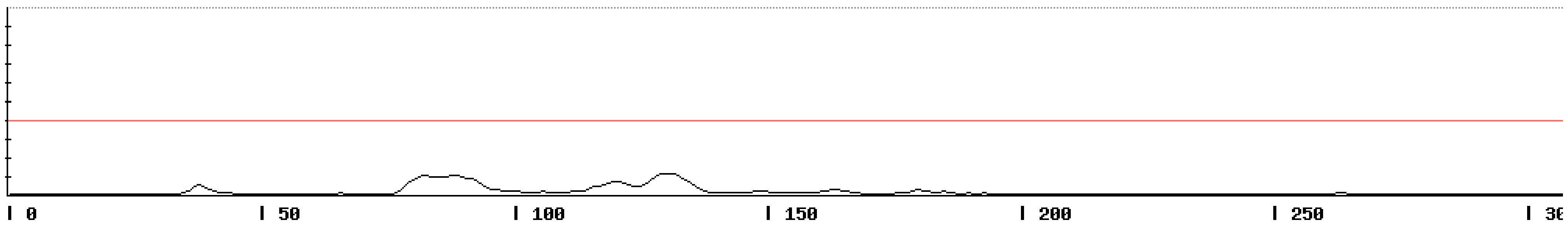

Predictions for Kalanchoemarnieriana_Kalax.0140s0052.1.p

Viterbi Path	72 - RKSPGGSRLNRATRKGYWKATGKDRKIRSKKNEIGMKK - 109

Viterbi Path

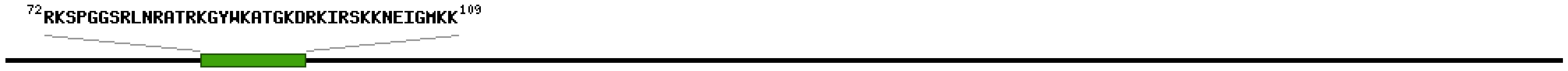

Posterior @ 0.4	72 - RKSPGGSRLNRATRKGYWKATGKDRKIRSKKNEIGMKK - 109

Posterior @ 0.4

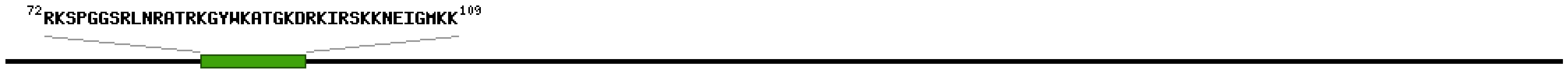

Posterior Graphic. Threshold Marked @ 0.4

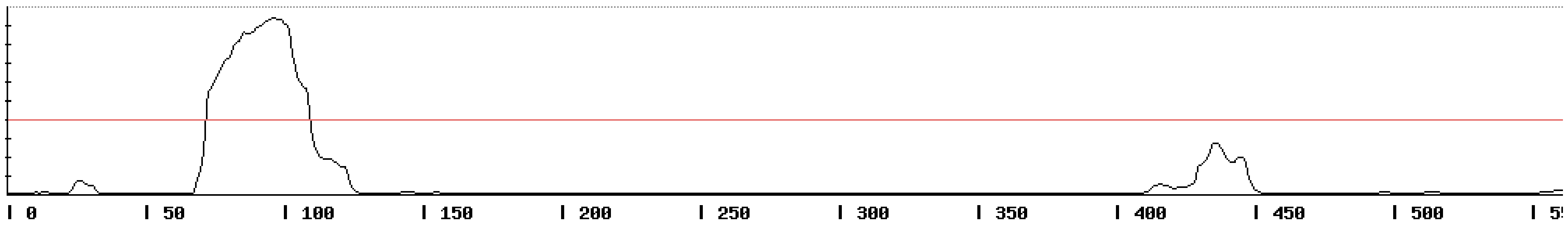

Predictions for Kalanchoemarnieriana_Kalax.0144s0050.1.p

Viterbi Path

Viterbi Path

Posterior @ 0.4

Posterior @ 0.4

Posterior Graphic. Threshold Marked @ 0.4

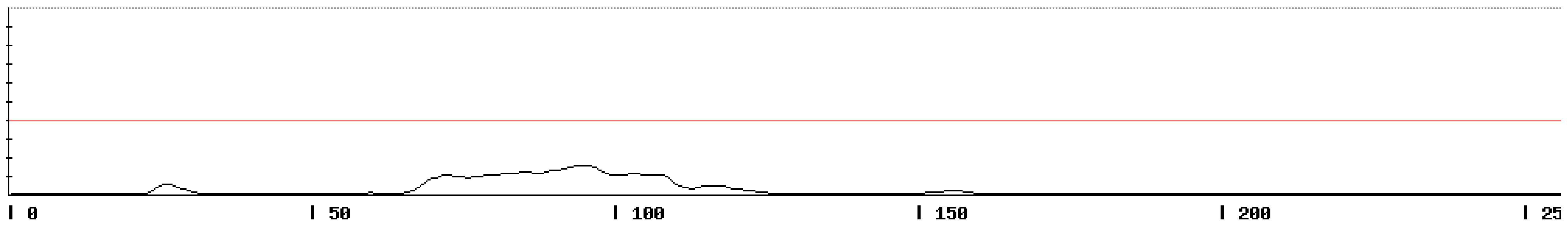

Predictions for Kalanchoemarnieriana_Kalax.0149s0055.1.p

Viterbi Path

Viterbi Path

Posterior @ 0.4

Posterior @ 0.4

Posterior Graphic. Threshold Marked @ 0.4

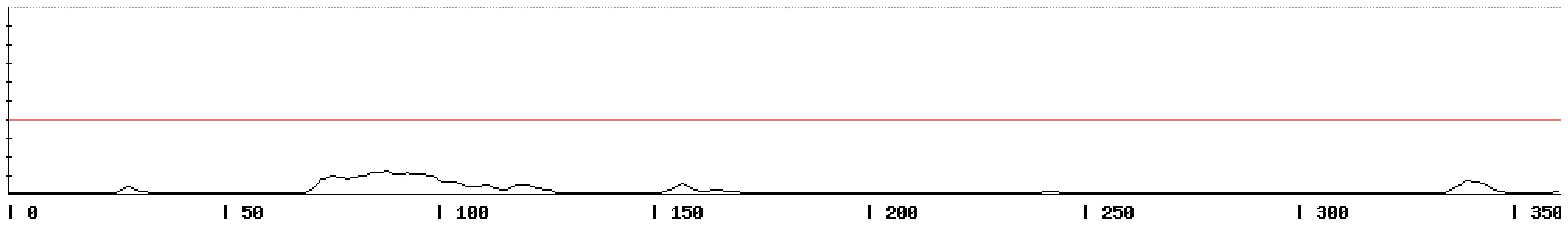

Predictions for Kalanchoemarnieriana_Kalax.0150s0017.1.p

Viterbi Path

Viterbi Path

Posterior @ 0.4	
Posterior @ 0.4	
Posterior Graphic. Threshold Marked @ 0.4	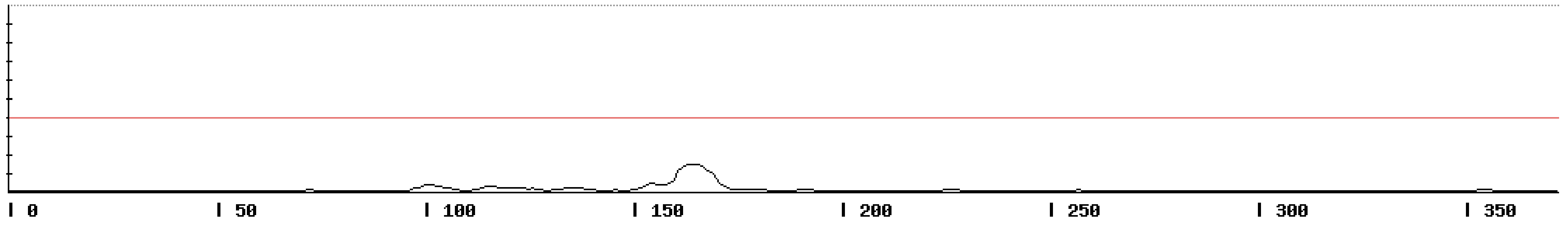

Predictions for Kalanchoemarnieriana_Kalax.0151s0058.1.p

Viterbi Path	
Viterbi Path	
Posterior @ 0.4	
Posterior @ 0.4	
Posterior Graphic. Threshold Marked @ 0.4	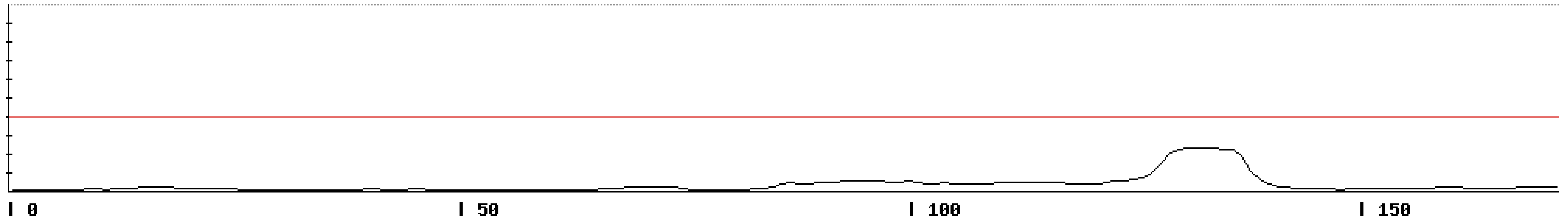

Predictions for Kalanchoemarnieriana_Kalax.0152s0060.1.p

Viterbi Path

Viterbi Path

Posterior @ 0.4

Posterior @ 0.4

Posterior Graphic. Threshold Marked @ 0.4

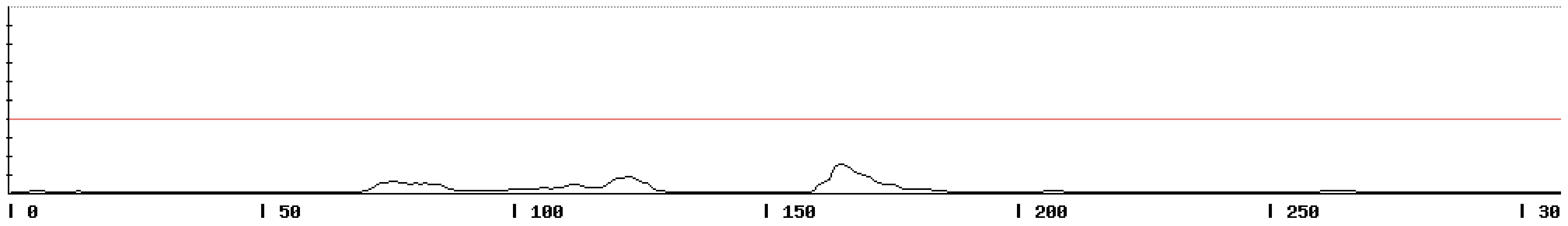

Predictions for Kalanchoemarnieriana_Kalax.0154s0005.1.p

Viterbi Path

Viterbi Path

Posterior @ 0.4

Posterior @ 0.4

Posterior Graphic. Threshold Marked @ 0.4

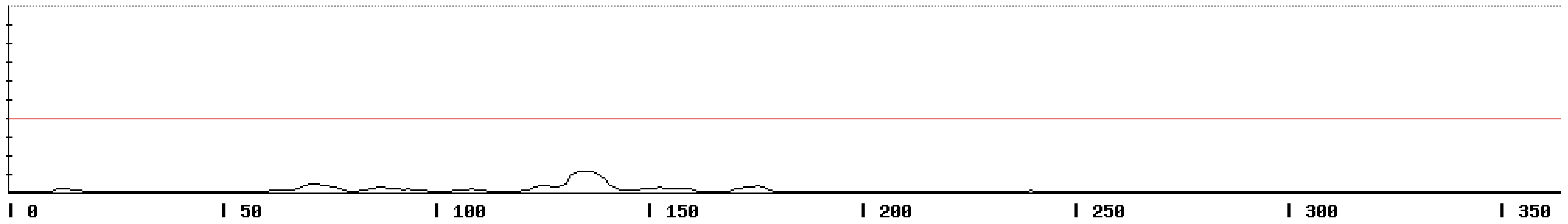

Predictions for Kalanchoemarnieriana_Kalax.0158s0076.1.p

Viterbi Path	
Viterbi Path	
Posterior @ 0.4	
Posterior @ 0.4	
Posterior Graphic. Threshold Marked @ 0.4	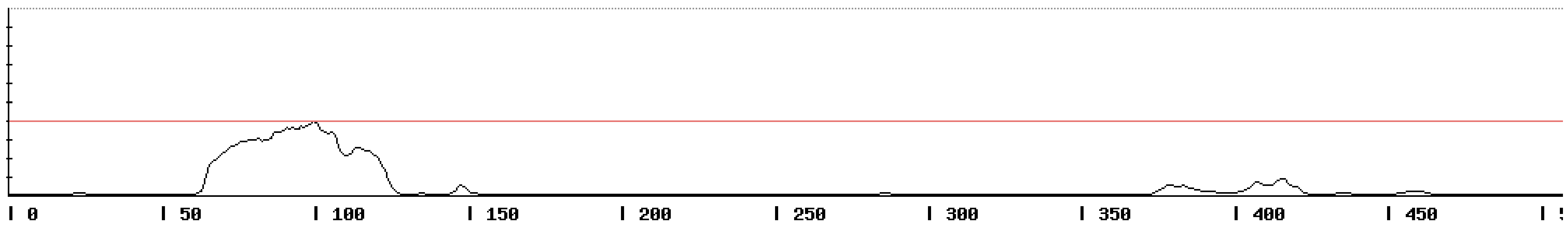

Predictions for Kalanchoemarnieriana_Kalax.0160s0031.1.p

Viterbi Path	
Viterbi Path	
Posterior @ 0.4	
Posterior @ 0.4	
Posterior Graphic. Threshold Marked @ 0.4	

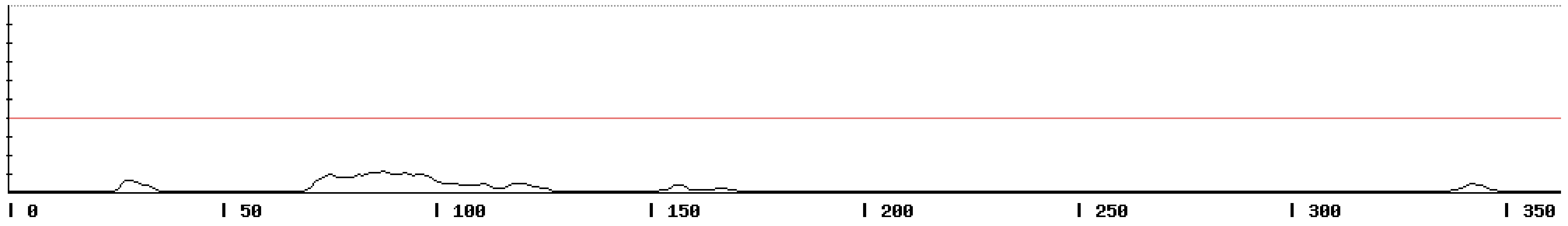

Predictions for Kalanchoemarnieriana_Kalax.0168s0017.1.p

Viterbi Path	
Viterbi Path	
Posterior @ 0.4	
Posterior @ 0.4	
Posterior Graphic. Threshold Marked @ 0.4	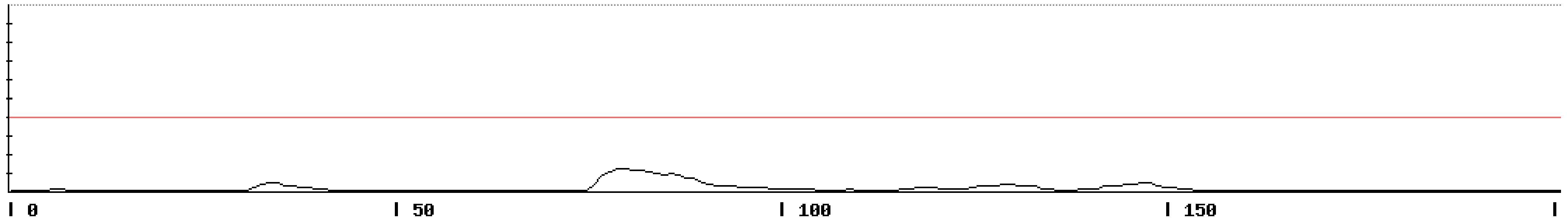

Predictions for Kalanchoemarnieriana_Kalax.0181s0046.1.p

Viterbi Path	
Viterbi Path	
Posterior @ 0.4	
Posterior @ 0.4	

Posterior Graphic. Threshold Marked @ 0.4

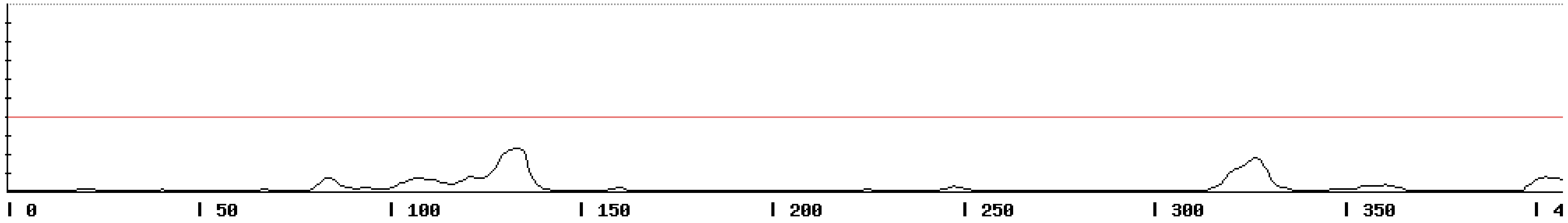

Predictions for Kalanchoemarnieriana_Kalax.0182s0012.1.p

Viterbi Path

Viterbi Path

Posterior @ 0.4

Posterior @ 0.4

Posterior Graphic. Threshold Marked @ 0.4

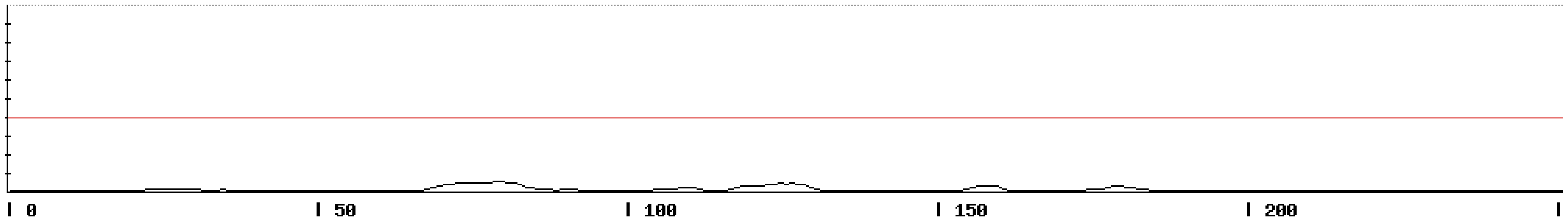

Predictions for Kalanchoemarnieriana_Kalax.0182s0022.1.p

Viterbi Path

Viterbi Path

Posterior @ 0.4

Posterior @ 0.4

Posterior Graphic. Threshold Marked @ 0.4

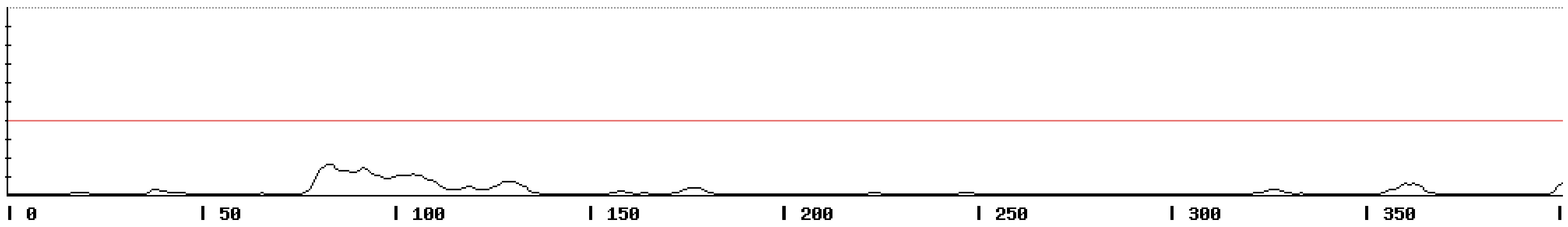

Predictions for Kalanchoemarnieriana_Kalax.0184s0004.1.p

Viterbi Path

Viterbi Path

Posterior @ 0.4

Posterior @ 0.4

Posterior Graphic. Threshold Marked @ 0.4

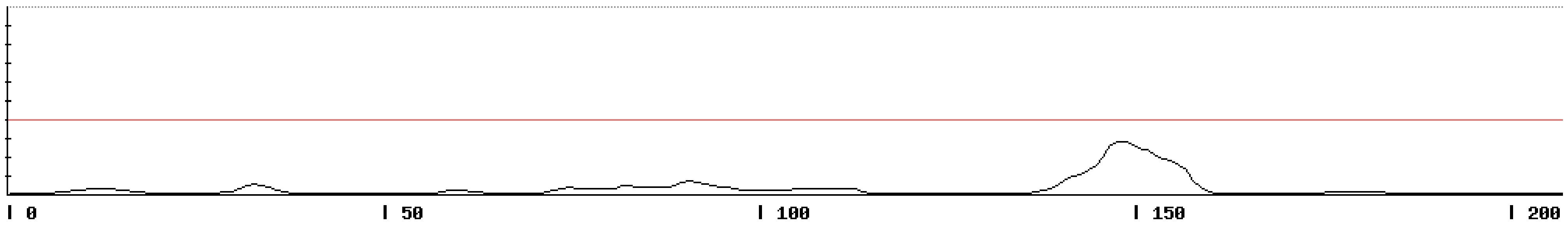

Predictions for Kalanchoemarnieriana_Kalax.0199s0022.1.p

Viterbi Path

Viterbi Path

Posterior @ 0.4

Posterior @ 0.4

Posterior Graphic. Threshold Marked @ 0.4

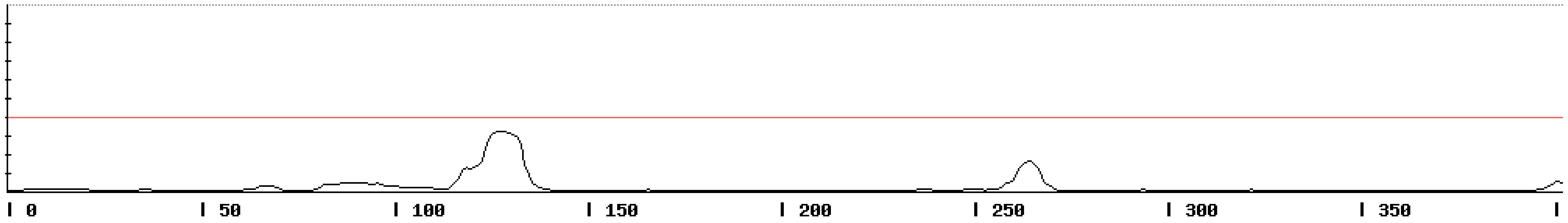

Predictions for Kalanchoemarnieriana_Kalax.0199s0023.1.p

Viterbi Path

Viterbi Path

Posterior @ 0.4

Posterior @ 0.4

Posterior Graphic. Threshold Marked @ 0.4

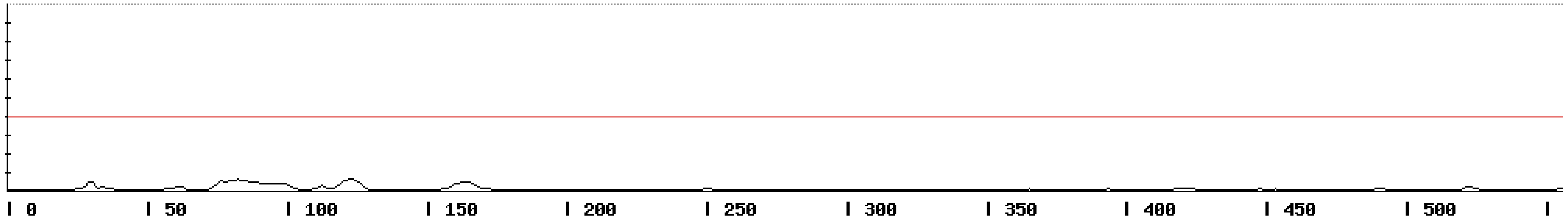

Predictions for Kalanchoemarnieriana_Kalax.0199s0024.1.p

Viterbi Path

Viterbi Path

Posterior @ 0.4

Posterior @ 0.4

Posterior Graphic. Threshold Marked @ 0.4

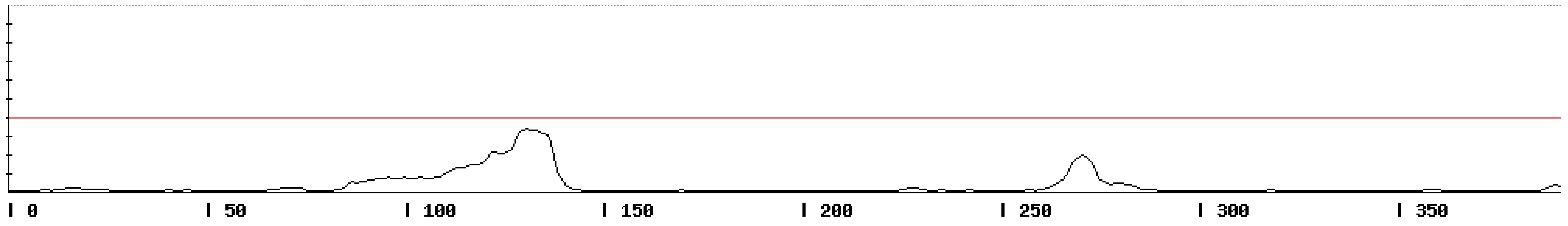

Predictions for Kalanchoemarnieriana_Kalax.0200s0025.1.p

Viterbi Path

Viterbi Path

Posterior @ 0.4

Posterior @ 0.4

Posterior Graphic. Threshold Marked @ 0.4

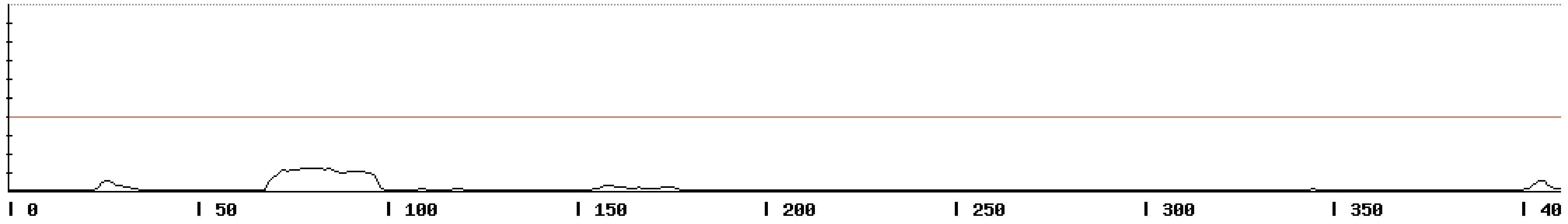

Predictions for Kalanchoemarnieriana_Kalax.0201s0025.1.p

Viterbi Path

Viterbi Path

Posterior @ 0.4

Posterior @ 0.4

Posterior Graphic. Threshold Marked @ 0.4

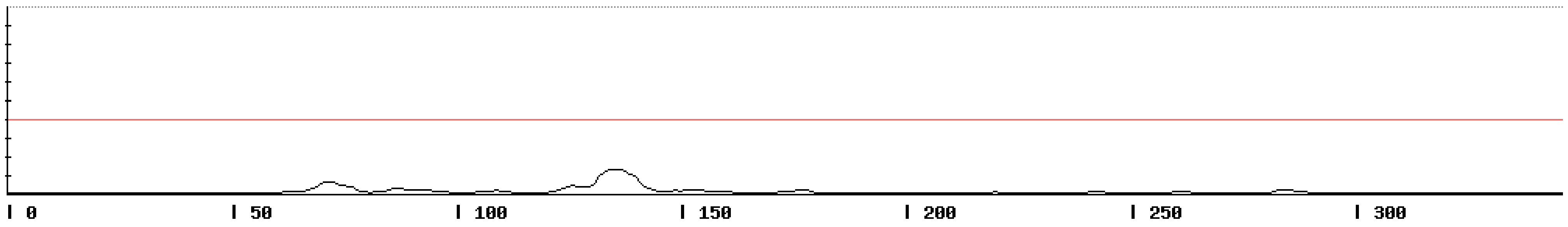

Predictions for Kalanchoemarnieriana_Kalax.0211s0006.1.p

Viterbi Path

Viterbi Path

Posterior @ 0.4

Posterior @ 0.4

Posterior Graphic. Threshold Marked @ 0.4

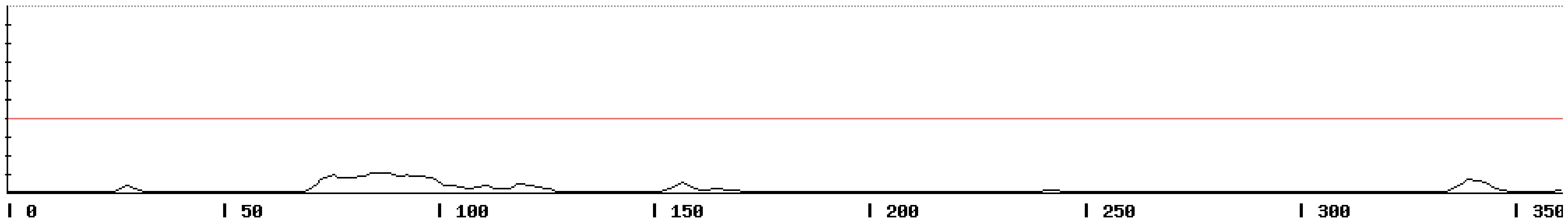

Predictions for Kalanchoemarnieriana_Kalax.0217s0004.1.p	
Viterbi Path	
Viterbi Path	
Posterior @ 0.4	137 - RKRRK - 141
Posterior @ 0.4	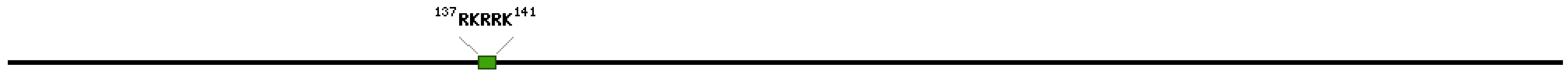
Posterior Graphic. Threshold Marked @ 0.4	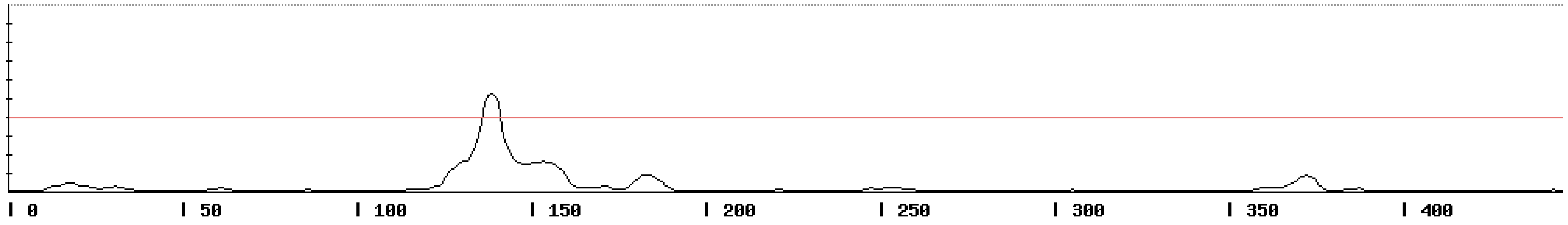

Predictions for Kalanchoemarnieriana_Kalax.0217s0043.1.p	
Viterbi Path	
Viterbi Path	
Posterior @ 0.4	
Posterior @ 0.4	
Posterior Graphic. Threshold Marked @ 0.4	

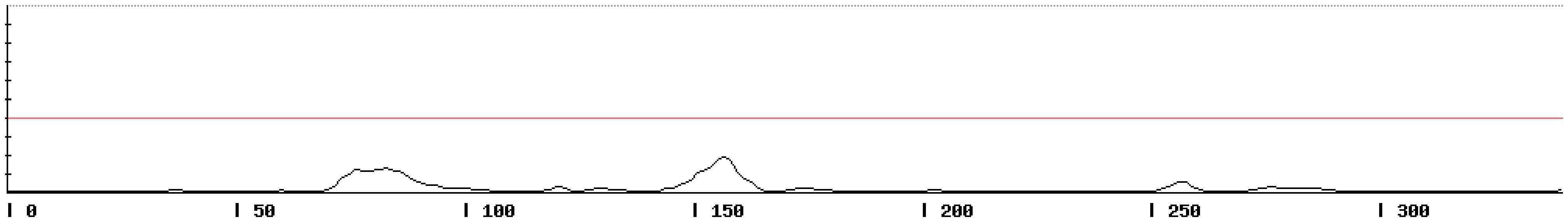

Predictions for Kalanchoemarnieriana_Kalax.0219s0006.1.p

Viterbi Path

Viterbi Path

Posterior @ 0.4

Posterior @ 0.4

Posterior Graphic. Threshold Marked @ 0.4

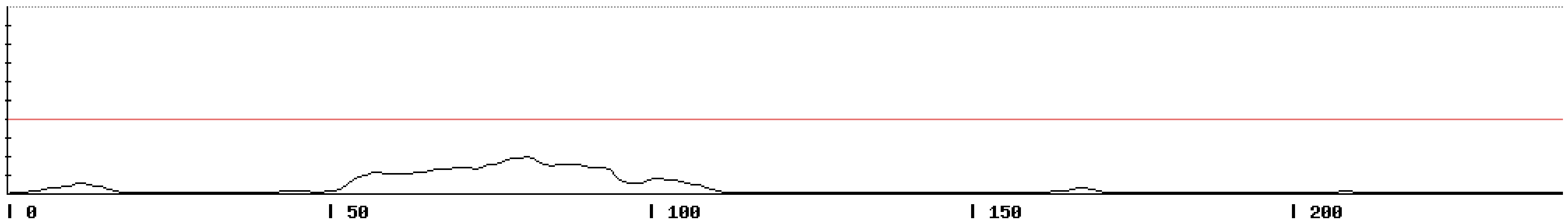

Predictions for Kalanchoemarnieriana_Kalax.0219s0017.1.p

Viterbi Path

Viterbi Path

Posterior @ 0.4

Posterior @ 0.4

Posterior Graphic. Threshold Marked @ 0.4

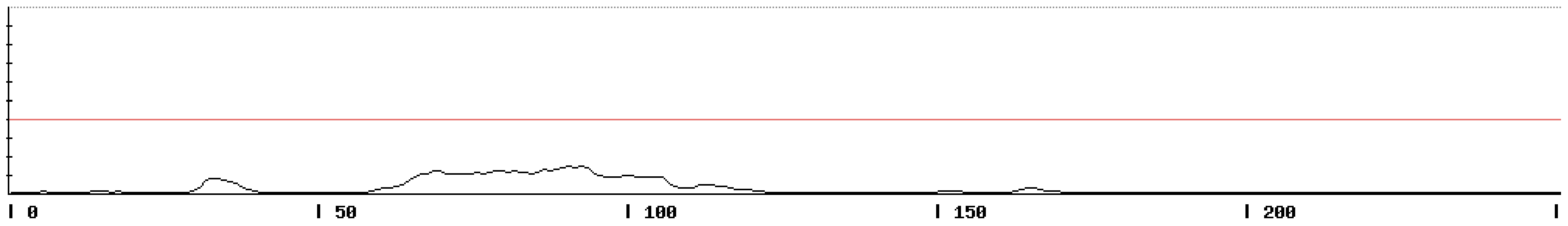

Predictions for Kalanchoemarnieriana_Kalax.0221s0022.1.p

Viterbi Path

Viterbi Path

Posterior @ 0.4

Posterior @ 0.4

Posterior Graphic. Threshold Marked @ 0.4

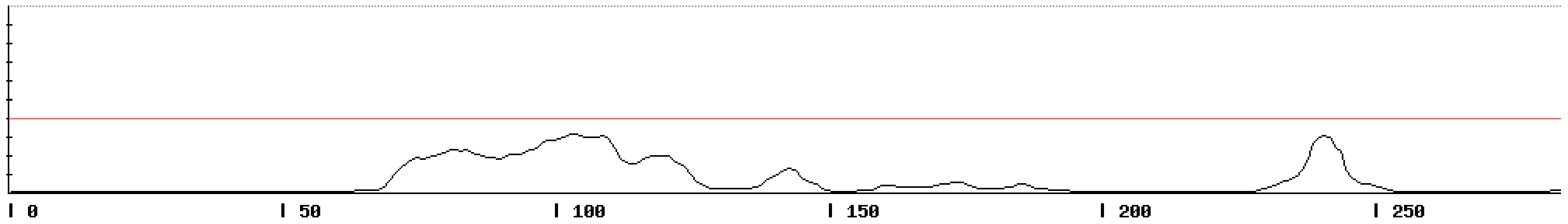

Predictions for Kalanchoemarnieriana_Kalax.0222s0012.1.p

Viterbi Path

Viterbi Path

Posterior @ 0.4

Posterior @ 0.4

Posterior Graphic. Threshold Marked @ 0.4

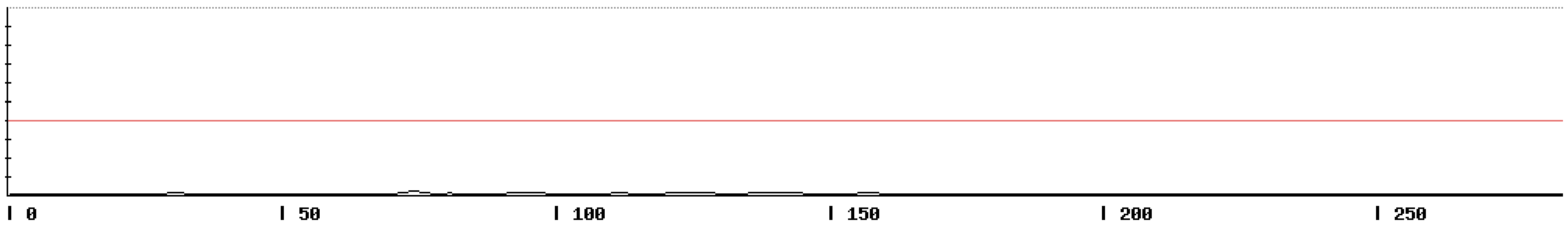

Predictions for Kalanchoemarnieriana_Kalax.0223s0043.1.p

Viterbi Path

Viterbi Path

Posterior @ 0.4

Posterior @ 0.4

Posterior Graphic. Threshold Marked @ 0.4

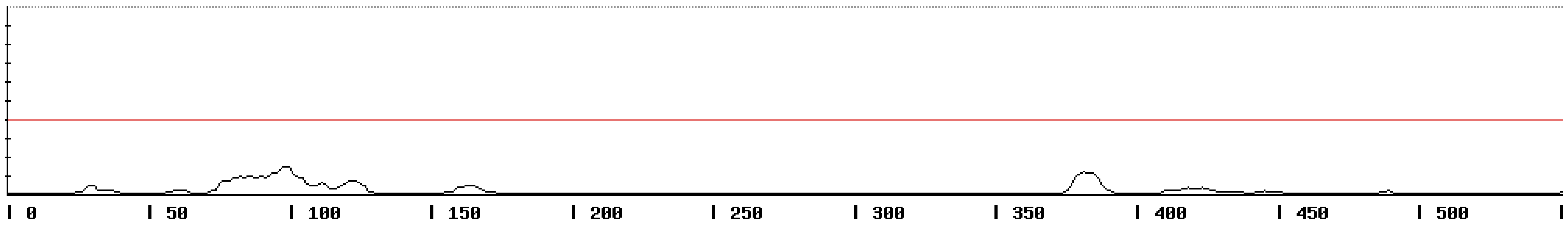

Predictions for Kalanchoemarnieriana_Kalax.0223s0044.1.p

Viterbi Path

Viterbi Path

Posterior @ 0.4

Posterior @ 0.4

Posterior Graphic. Threshold Marked @ 0.4

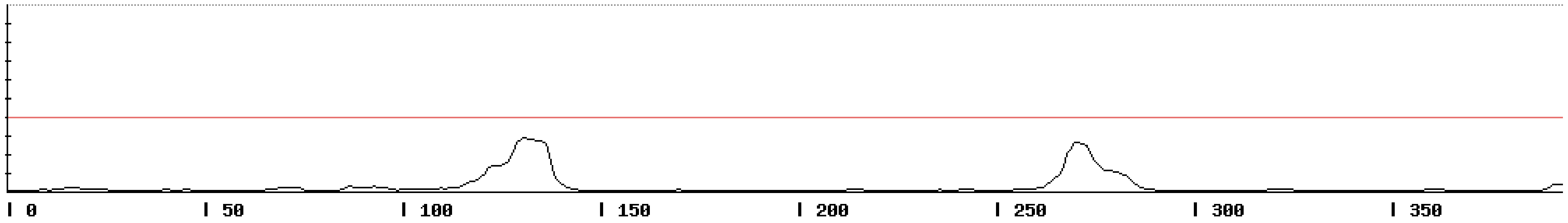

Predictions for Kalanchoemarnieriana_Kalax.0226s0021.1.p

Viterbi Path

Viterbi Path

Posterior @ 0.4

Posterior @ 0.4

Posterior Graphic. Threshold Marked @ 0.4

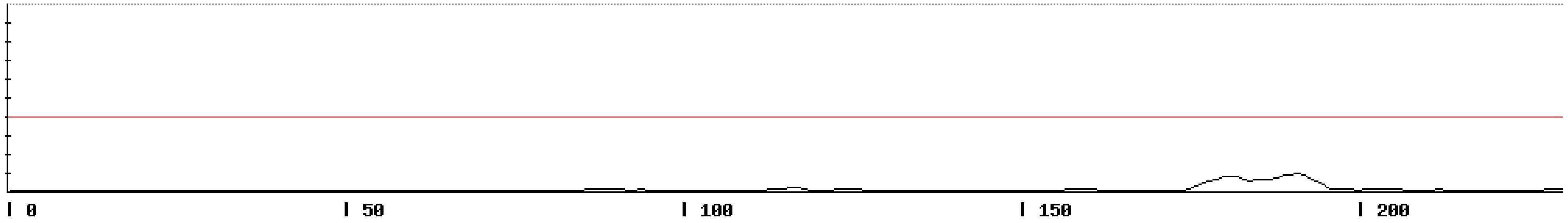

Predictions for Kalanchoemarnieriana_Kalax.0227s0023.1.p

Viterbi Path

Viterbi Path

Posterior @ 0.4

Posterior @ 0.4

Posterior Graphic. Threshold Marked @ 0.4

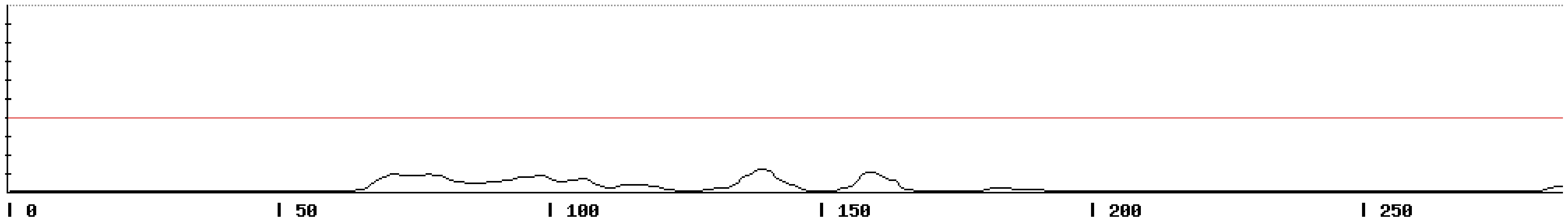

Predictions for Kalanchoemarnieriana_Kalax.0227s0051.1.p

Viterbi Path

Viterbi Path

Posterior @ 0.4

Posterior @ 0.4

Posterior Graphic. Threshold Marked @ 0.4

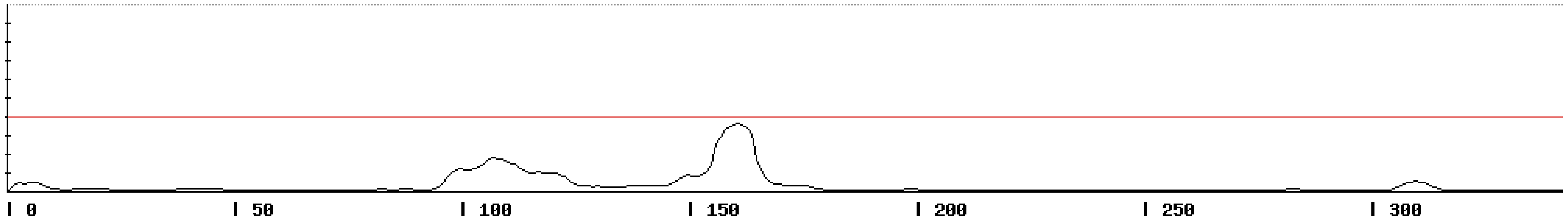

Predictions for Kalanchoemarnieriana_Kalax.0230s0011.1.p

Viterbi Path

Viterbi Path

Posterior @ 0.4

Posterior @ 0.4

Posterior Graphic. Threshold Marked @ 0.4

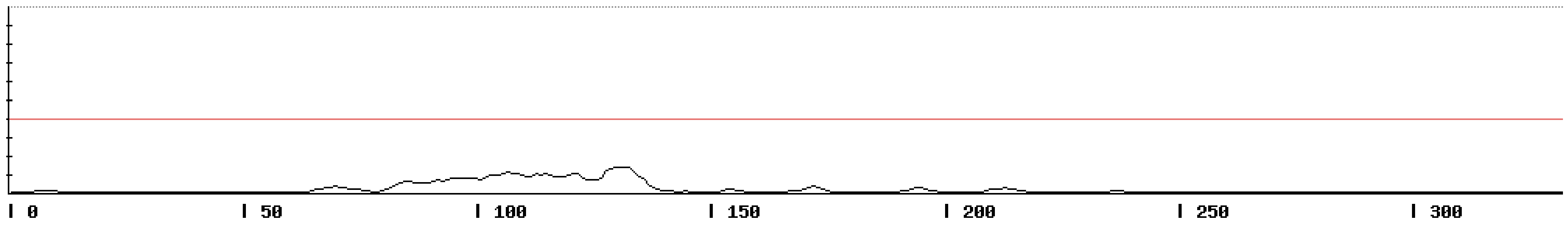

Predictions for Kalanchoemarnieriana_Kalax.0252s0019.1.p

Viterbi Path

Viterbi Path

Posterior @ 0.4

Posterior @ 0.4

Posterior Graphic. Threshold Marked @ 0.4

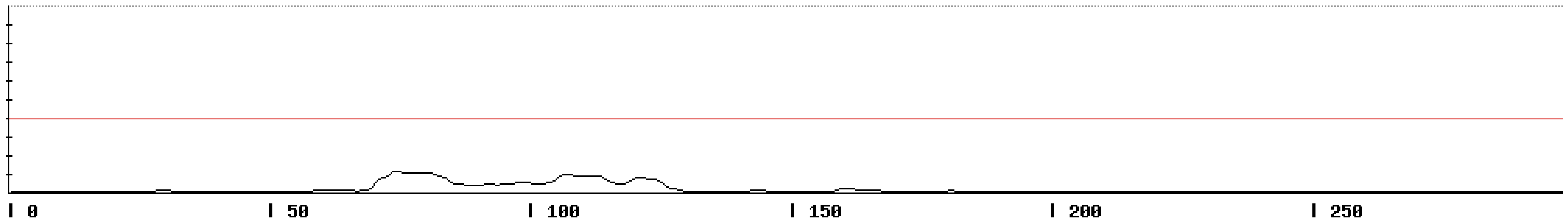

Predictions for Kalanchoemarnieriana_Kalax.0264s0036.1.p	
Viterbi Path	
Viterbi Path	
Posterior @ 0.4	
Posterior @ 0.4	
Posterior Graphic. Threshold Marked @ 0.4	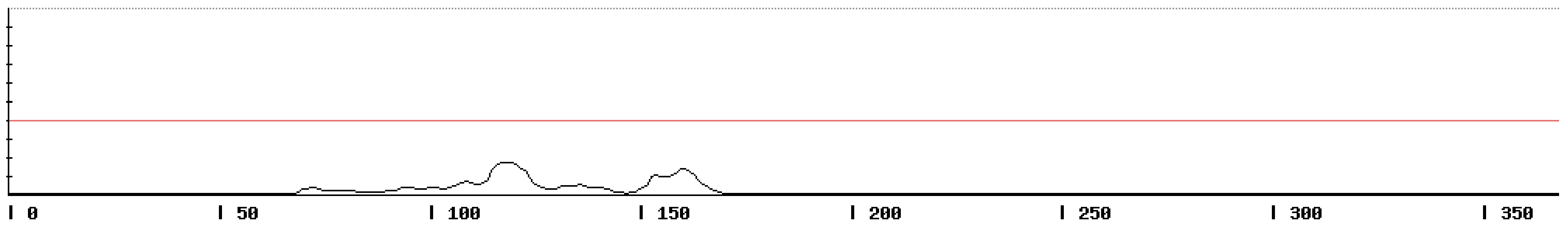

Predictions for Kalanchoemarnieriana_Kalax.0270s0004.1.p	
Viterbi Path	
Viterbi Path	
Posterior @ 0.4	
Posterior @ 0.4	
Posterior Graphic. Threshold Marked @ 0.4	

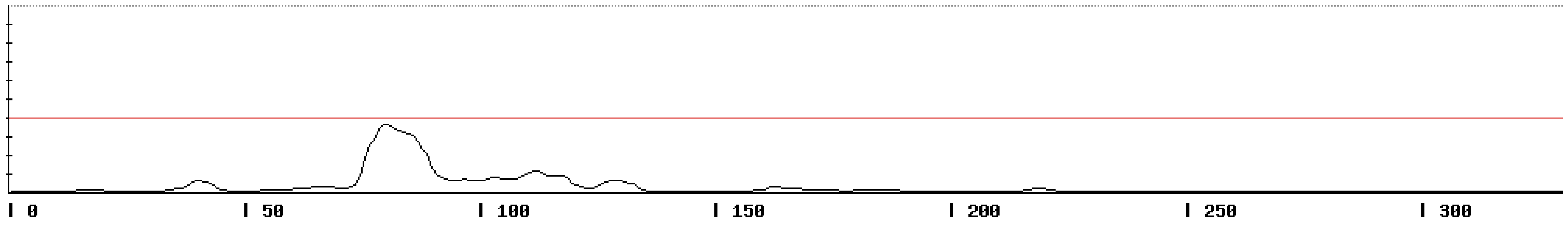

Predictions for Kalanchoemarnieriana_Kalax.0285s0054.1.p

Viterbi Path

Viterbi Path

Posterior @ 0.4

Posterior @ 0.4

Posterior Graphic. Threshold Marked @ 0.4

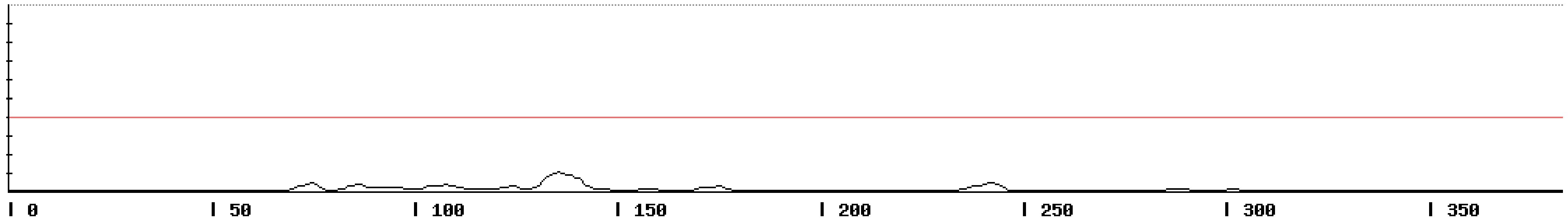

Predictions for Kalanchoemarnieriana_Kalax.0294s0015.1.p

Viterbi Path

Viterbi Path

Posterior @ 0.4

Posterior @ 0.4

Posterior Graphic. Threshold Marked @ 0.4

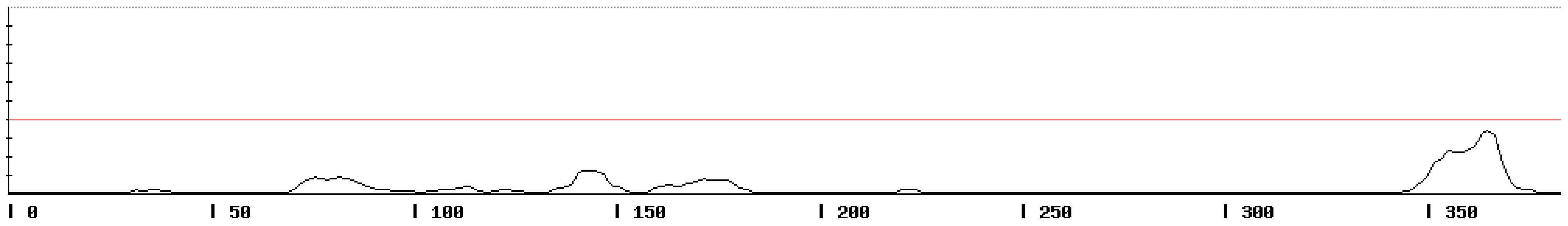

Predictions for Kalanchoemarnieriana_Kalax.0298s0056.1.p

Viterbi Path

Viterbi Path

Posterior @ 0.4

351 - KDKSKRRR - 358

415 - RRRRSV - 420

Posterior @ 0.4

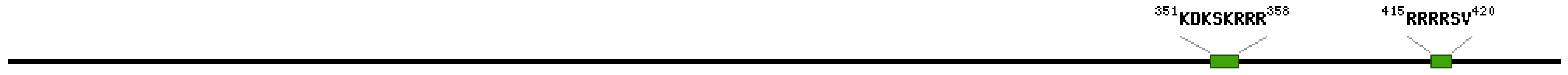

Posterior Graphic. Threshold Marked @ 0.4

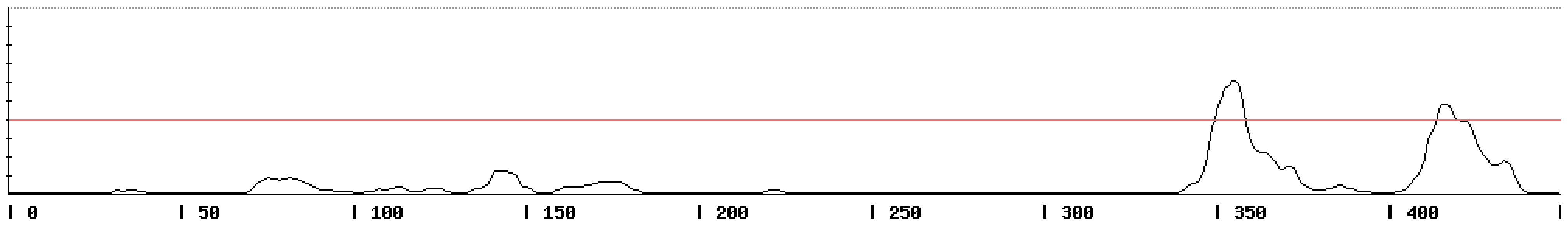

Predictions for Kalanchoemarnieriana_Kalax.0316s0029.1.p

Viterbi Path

Viterbi Path

Posterior @ 0.4

169 - RKRRK - 173

Posterior @ 0.4

	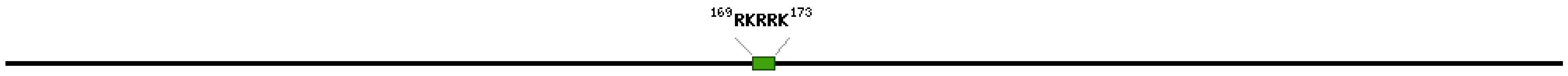
Posterior Graphic. Threshold Marked @ 0.4	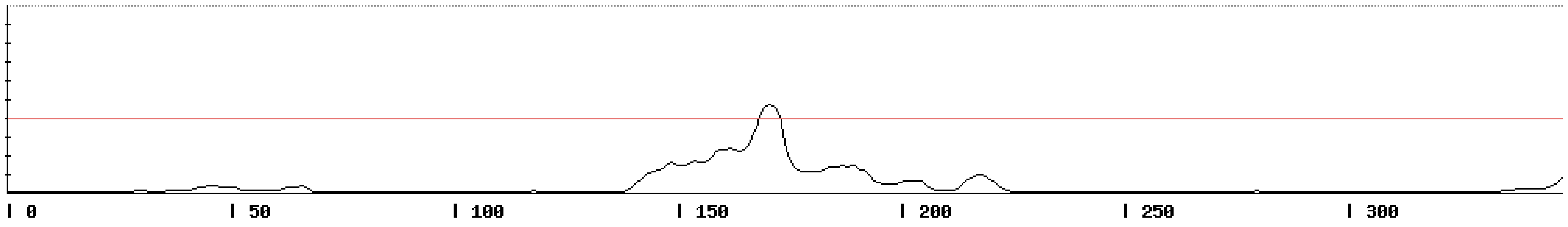

Predictions for Kalanchoemarnieriana_Kalax.0335s0038.1.p	
Viterbi Path	
Viterbi Path	
Posterior @ 0.4	94 - GKIPR - 98
Posterior @ 0.4	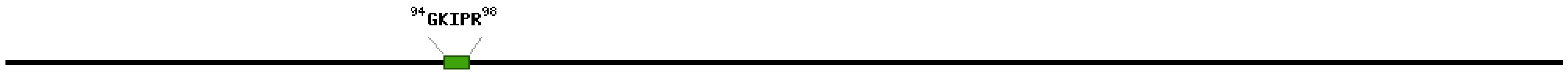
Posterior Graphic. Threshold Marked @ 0.4	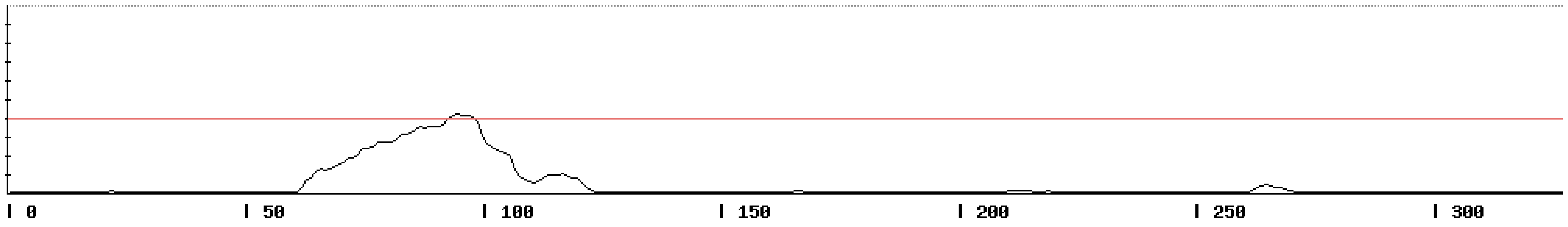

Predictions for Kalanchoemarnieriana_Kalax.0335s0039.1.p	
Viterbi Path	
Viterbi Path	

Posterior @ 0.4

Posterior @ 0.4

Posterior Graphic. Threshold Marked @ 0.4

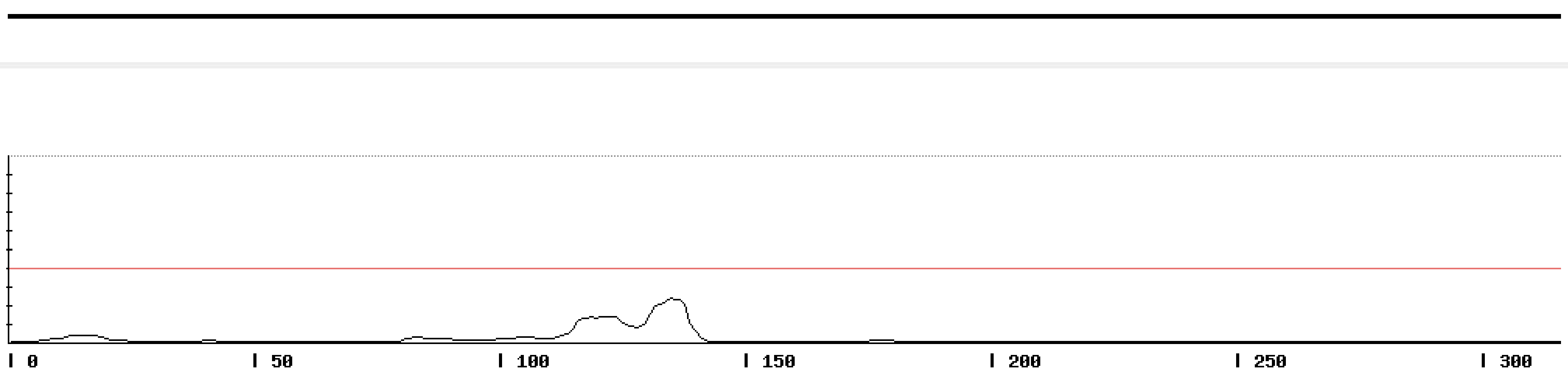

Predictions for Kalanchoemarnieriana_Kalax.0337s0011.1.p

Viterbi Path

Viterbi Path

Posterior @ 0.4

Posterior @ 0.4

Posterior Graphic. Threshold Marked @ 0.4

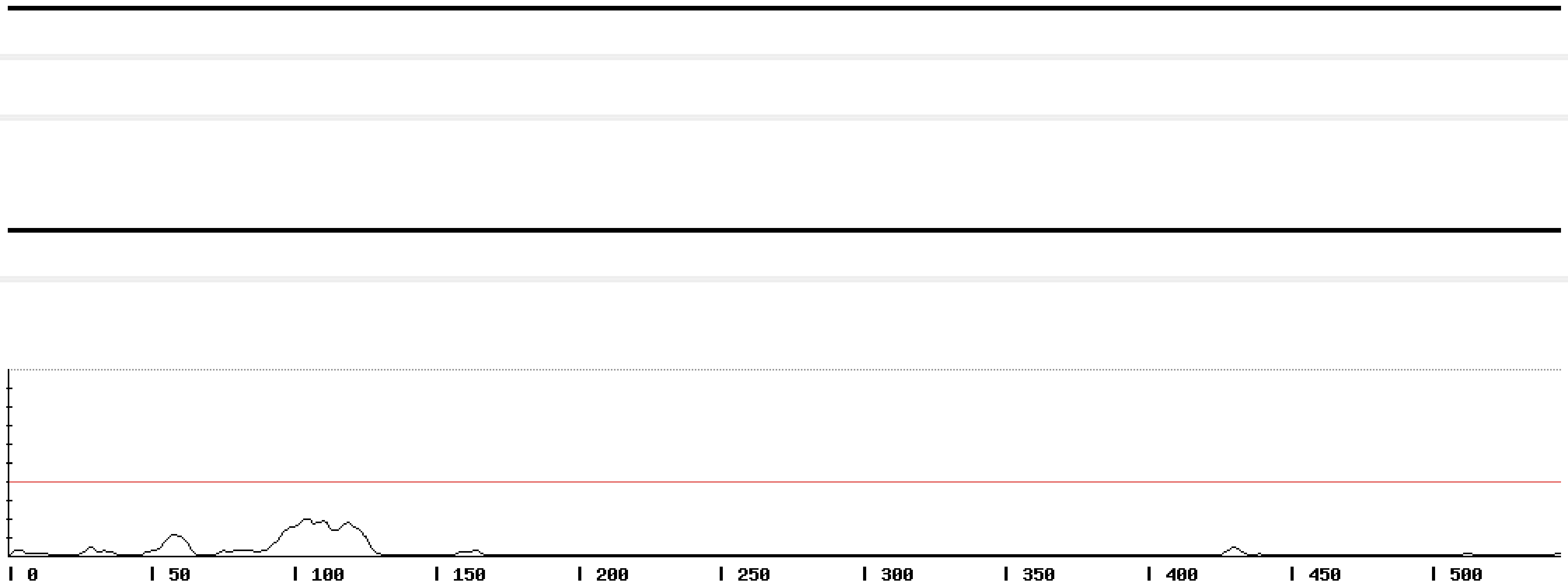

Predictions for Kalanchoemarnieriana_Kalax.0341s0035.1.p

Viterbi Path

Viterbi Path

Posterior @ 0.4	94 - GKIPR - 98
Posterior @ 0.4	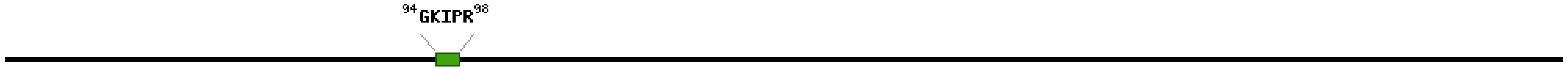
Posterior Graphic. Threshold Marked @ 0.4	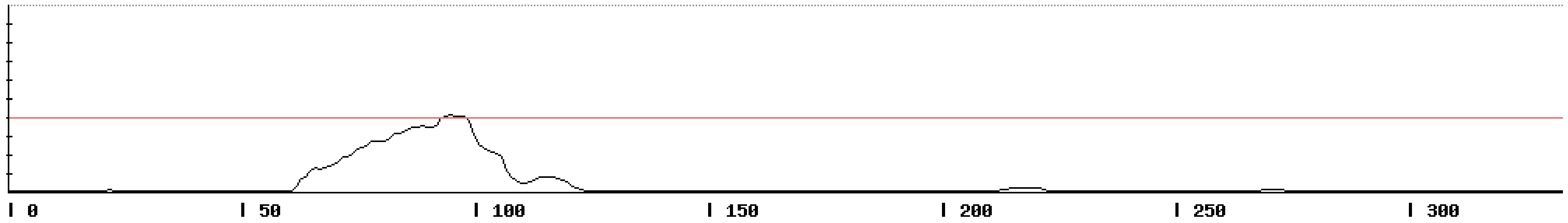

Predictions for Kalanchoemarnieriana_Kalax.0341s0036.1.p

Viterbi Path	
Viterbi Path	
Posterior @ 0.4	
Posterior @ 0.4	
Posterior Graphic. Threshold Marked @ 0.4	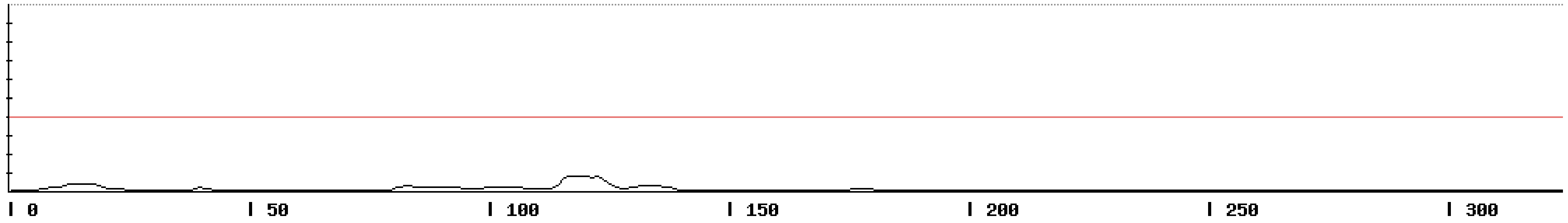

Predictions for Kalanchoemarnieriana_Kalax.0342s0019.1.p

Viterbi Path	
Viterbi Path	
Posterior @ 0.4	
Posterior @ 0.4	
Posterior Graphic. Threshold Marked @ 0.4	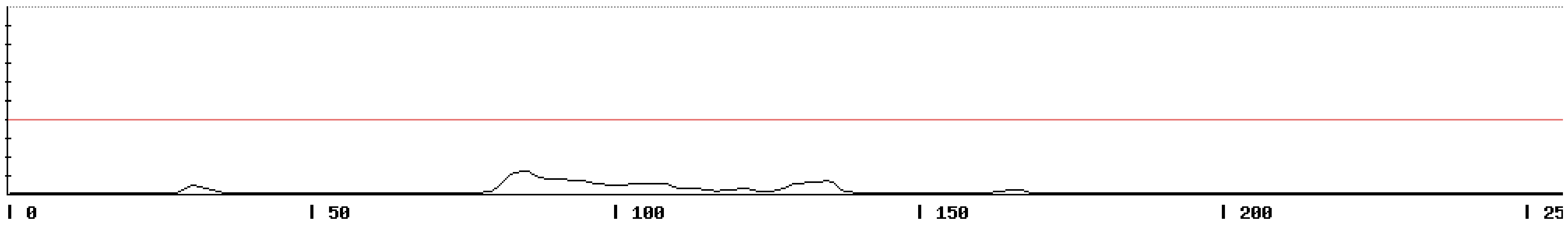

Predictions for Kalanchoemarnieriana_Kalax.0343s0007.1.p

Viterbi Path	
Viterbi Path	
Posterior @ 0.4	134 - KRR - 136
Posterior @ 0.4	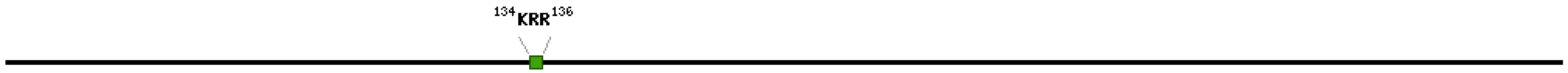
Posterior Graphic. Threshold Marked @ 0.4	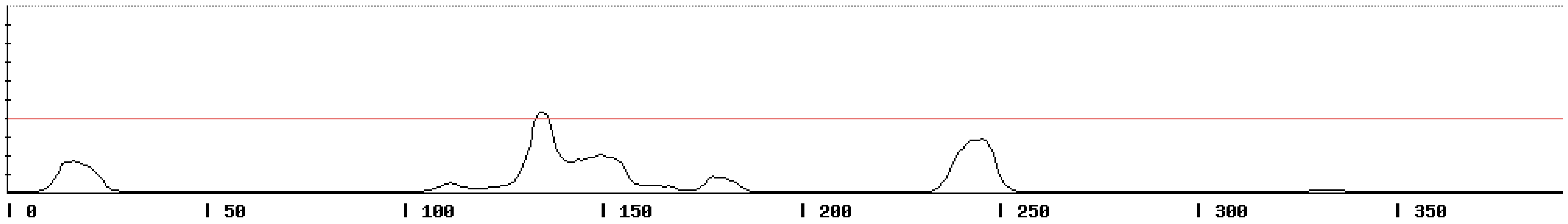

Predictions for Kalanchoemarnieriana_Kalax.0344s0007.1.p	
Viterbi Path	
Viterbi Path	
Posterior @ 0.4	
Posterior @ 0.4	
Posterior Graphic. Threshold Marked @ 0.4	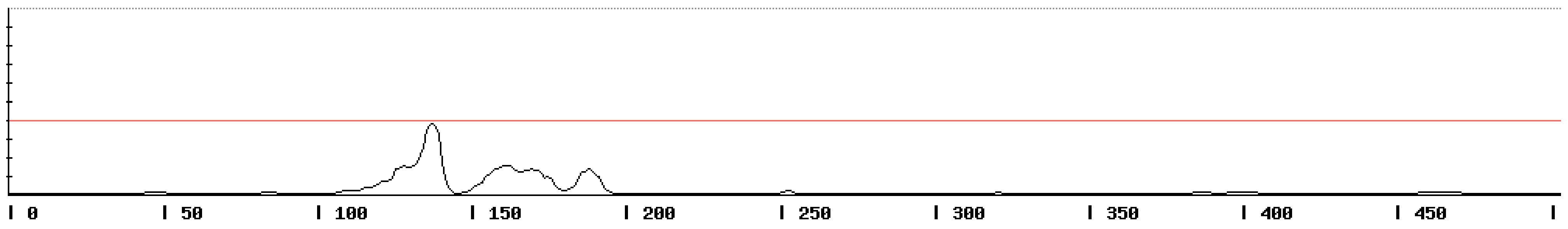

Predictions for Kalanchoemarnieriana_Kalax.0346s0002.1.p	
Viterbi Path	
Viterbi Path	
Posterior @ 0.4	
Posterior @ 0.4	
Posterior Graphic. Threshold Marked @ 0.4	

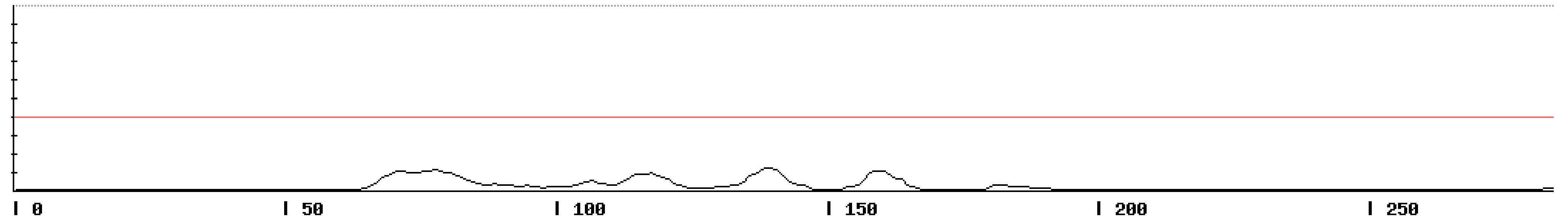

Predictions for Kalanchoemarnieriana_Kalax.0355s0003.1.p

Viterbi Path	
Viterbi Path	
Posterior @ 0.4	
Posterior @ 0.4	
Posterior Graphic. Threshold Marked @ 0.4	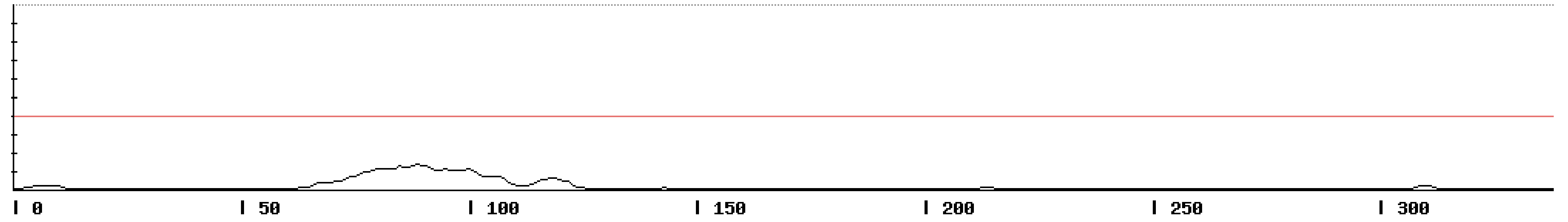

Predictions for Kalanchoemarnieriana_Kalax.0355s0004.1.p

Viterbi Path	
Viterbi Path	
Posterior @ 0.4	
Posterior @ 0.4	

Posterior Graphic. Threshold Marked @ 0.4

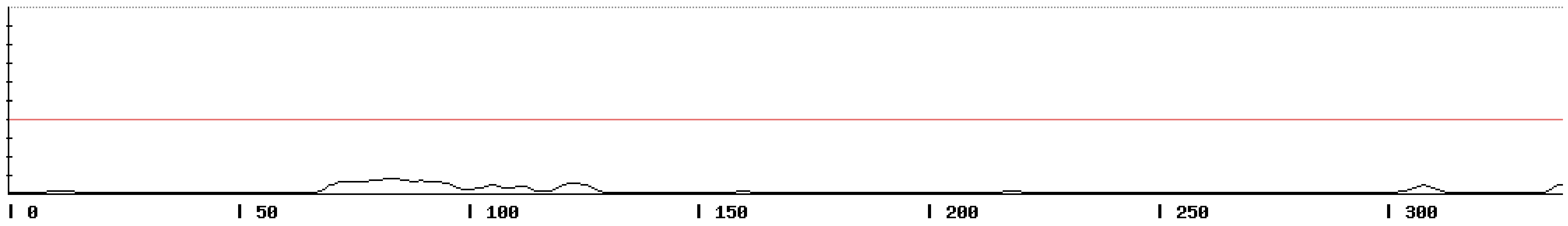

Predictions for Kalanchoemarnieriana_Kalax.0355s0005.1.p

Viterbi Path

Viterbi Path

Posterior @ 0.4

Posterior @ 0.4

Posterior Graphic. Threshold Marked @ 0.4

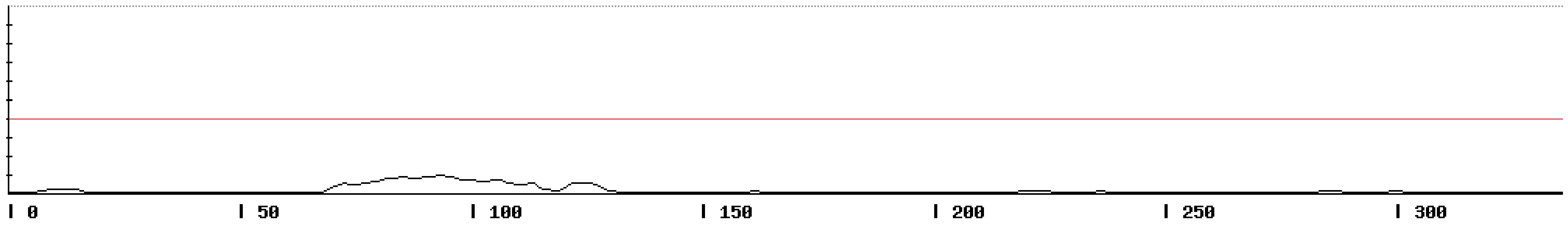

Predictions for Kalanchoemarnieriana_Kalax.0363s0038.1.p

Viterbi Path

Viterbi Path

Posterior @ 0.4

Posterior @ 0.4

Posterior Graphic. Threshold Marked @ 0.4

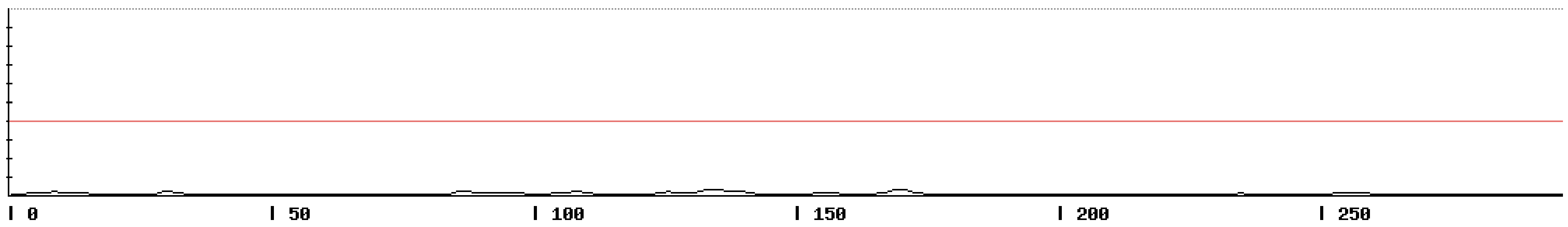

Predictions for Kalanchoemarnieriana_Kalax.0370s0001.1.p

Viterbi Path

Viterbi Path

Posterior @ 0.4

Posterior @ 0.4

Posterior Graphic. Threshold Marked @ 0.4

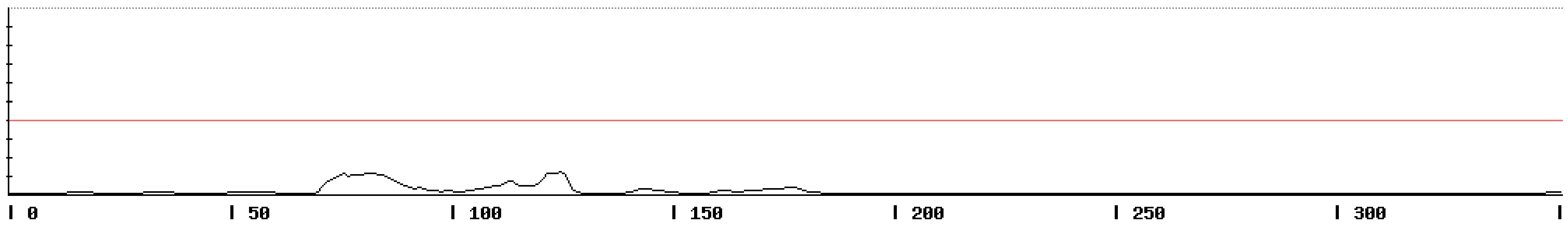

Predictions for Kalanchoemarnieriana_Kalax.0371s0043.1.p

Viterbi Path

Viterbi Path

Posterior @ 0.4

Posterior @ 0.4

Posterior Graphic. Threshold Marked @ 0.4

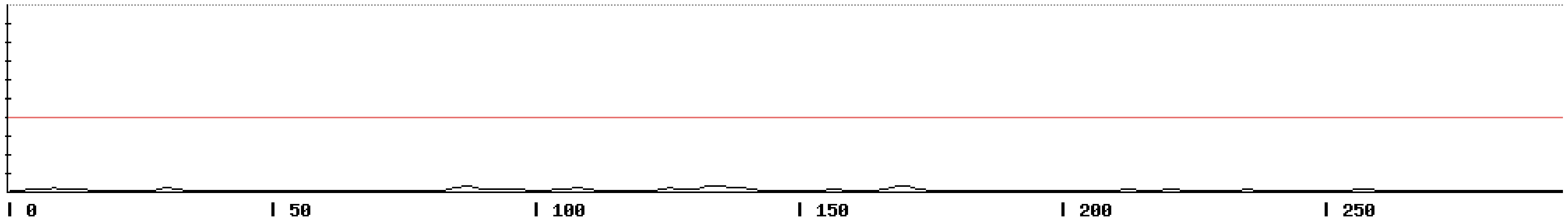

Predictions for Kalanchoemarnieriana_Kalax.0373s0029.1.p

Viterbi Path

Viterbi Path

Posterior @ 0.4

Posterior @ 0.4

Posterior Graphic. Threshold Marked @ 0.4

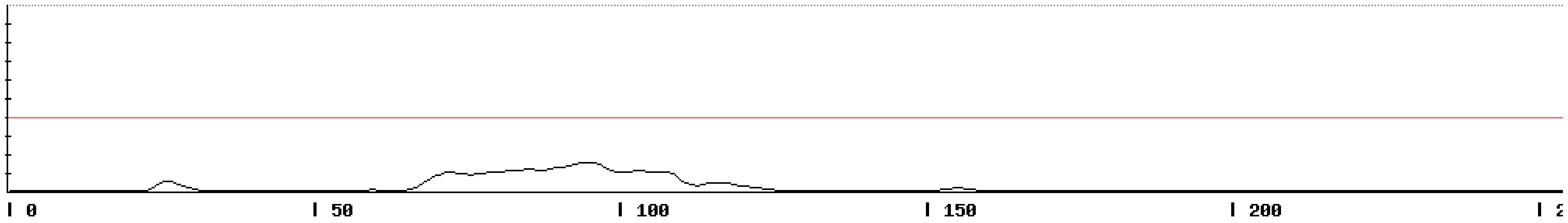

Predictions for Kalanchoemarnieriana_Kalax.0380s0025.1.p

Viterbi Path

Viterbi Path

Posterior @ 0.4: 9 - RRGSARNKKRRNNAPPPPPPPPPPPPPP - 36

Posterior @ 0.4

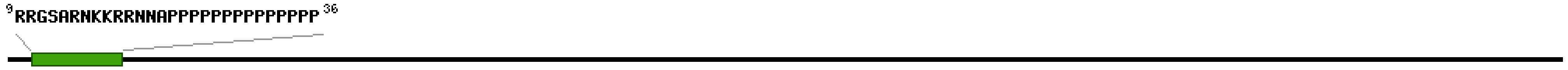

Posterior Graphic. Threshold Marked @ 0.4

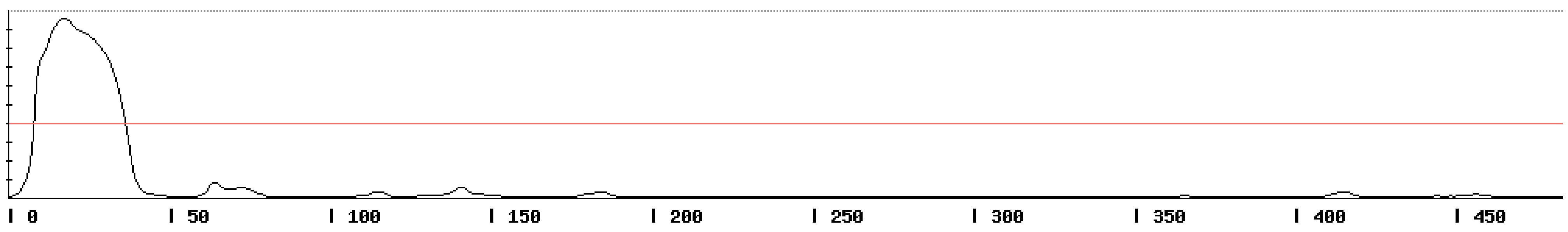

Predictions for Kalanchoemarnieriana_Kalax.0380s0026.1.p

Viterbi Path

Viterbi Path

Posterior @ 0.4

Posterior @ 0.4

Posterior Graphic. Threshold Marked @ 0.4

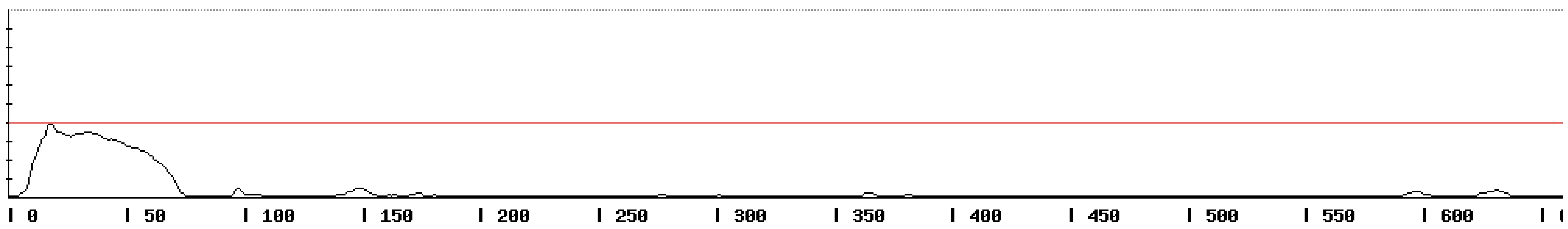

Predictions for Kalanchoemarnieriana_Kalax.0380s0027.1.p

Viterbi Path

Viterbi Path

Posterior @ 0.4

Posterior @ 0.4

Posterior Graphic. Threshold Marked @ 0.4

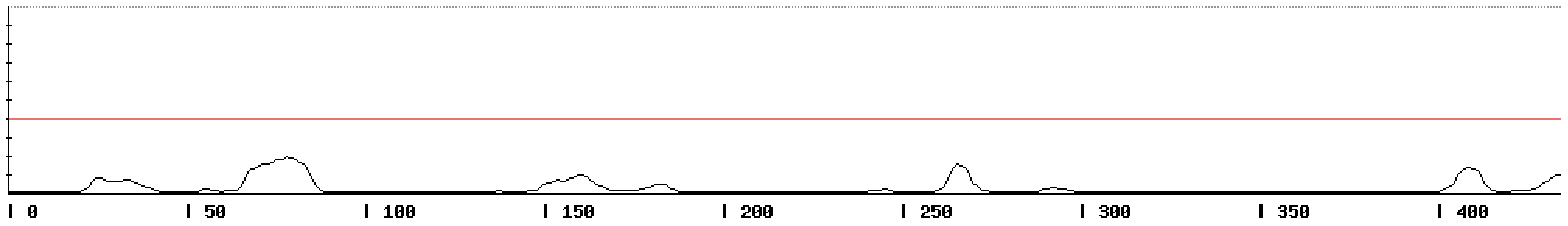

Predictions for Kalanchoemarnieriana_Kalax.0385s0005.1.p

Viterbi Path

Viterbi Path

Posterior @ 0.4

Posterior @ 0.4

Posterior Graphic. Threshold Marked @ 0.4

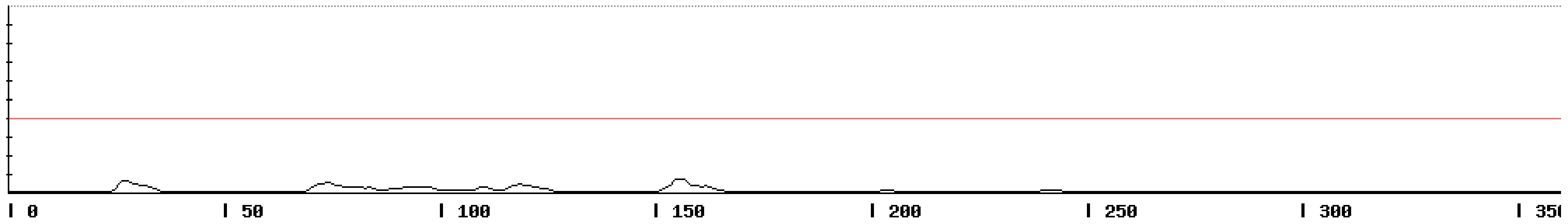

Predictions for Kalanchoemarnieriana_Kalax.0422s0023.1.p	
Viterbi Path	
Viterbi Path	
Posterior @ 0.4	
Posterior @ 0.4	
Posterior Graphic. Threshold Marked @ 0.4	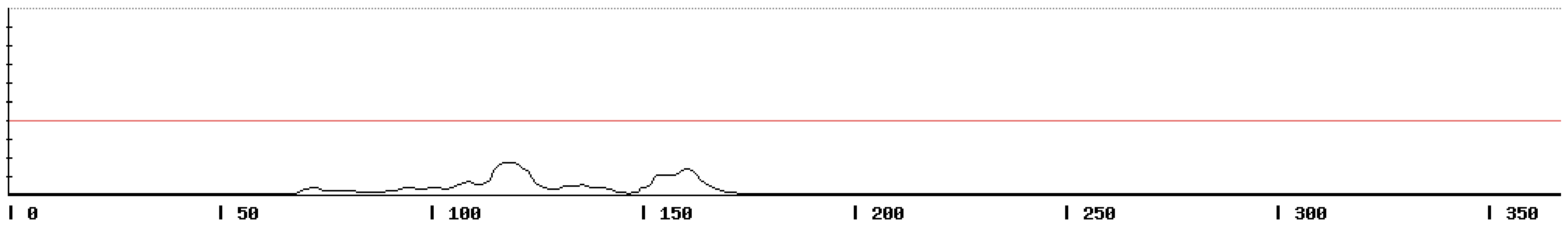

Predictions for Kalanchoemarnieriana_Kalax.0432s0034.1.p	
Viterbi Path	
Viterbi Path	
Posterior @ 0.4	
Posterior @ 0.4	
Posterior Graphic. Threshold Marked @ 0.4	

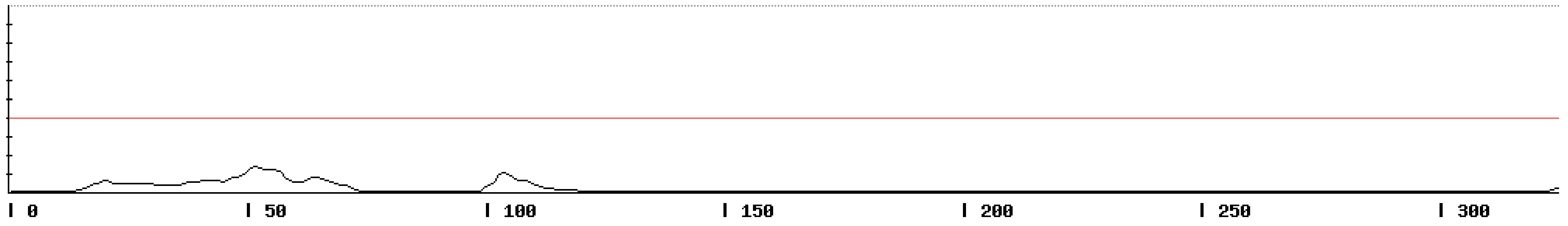

Predictions for Kalanchoemarnieriana_Kalax.0445s0023.1.p	
Viterbi Path	
Viterbi Path	
Posterior @ 0.4	
Posterior @ 0.4	
Posterior Graphic. Threshold Marked @ 0.4	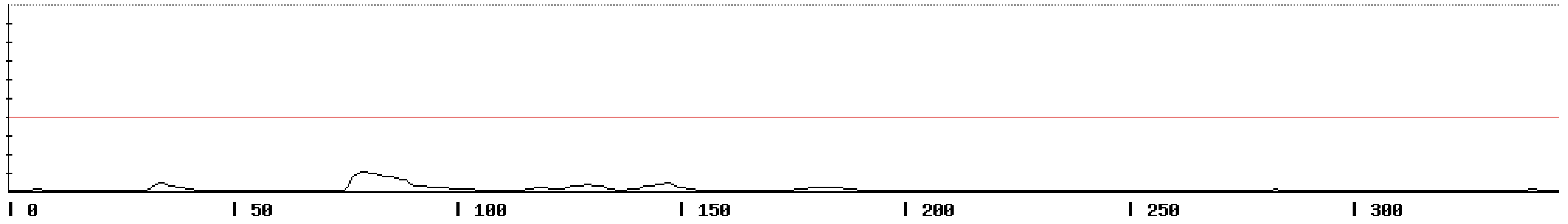

Predictions for Kalanchoemarnieriana_Kalax.0446s0004.1.p	
Viterbi Path	
Viterbi Path	
Posterior @ 0.4	
Posterior @ 0.4	

Posterior Graphic. Threshold Marked @ 0.4

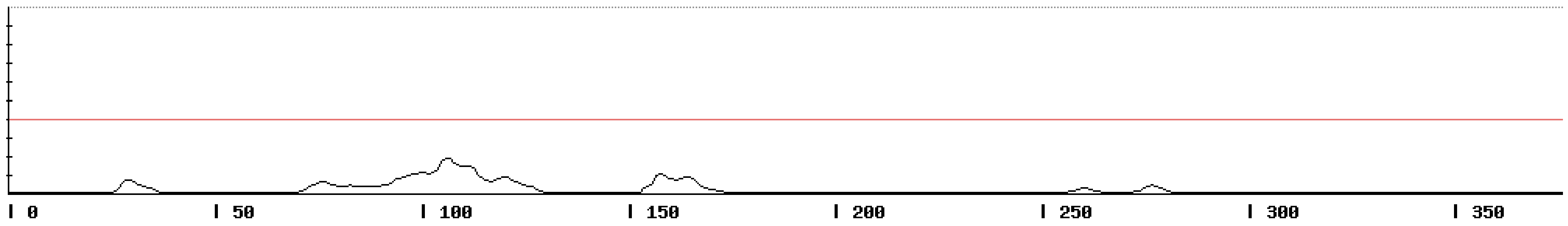

Predictions for Kalanchoemarnieriana_Kalax.0455s0006.1.p

Viterbi Path

Viterbi Path

Posterior @ 0.4

Posterior @ 0.4

Posterior Graphic. Threshold Marked @ 0.4

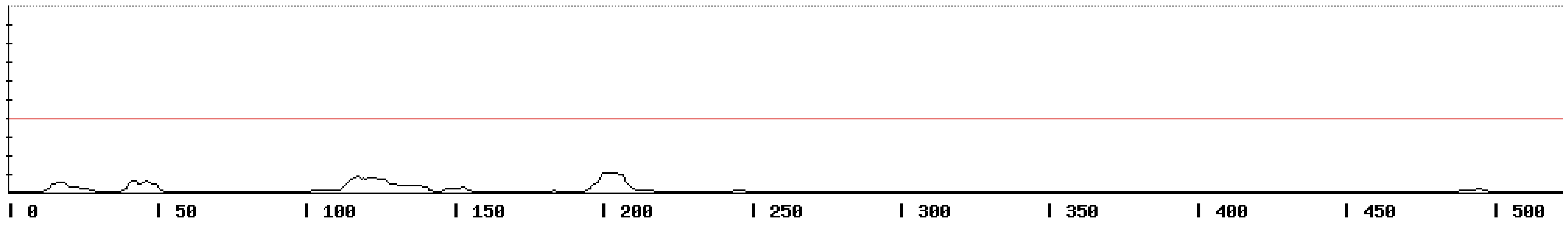

Predictions for Kalanchoemarnieriana_Kalax.0465s0024.1.p

Viterbi Path

Viterbi Path

Posterior @ 0.4

Posterior @ 0.4

Posterior Graphic. Threshold Marked @ 0.4

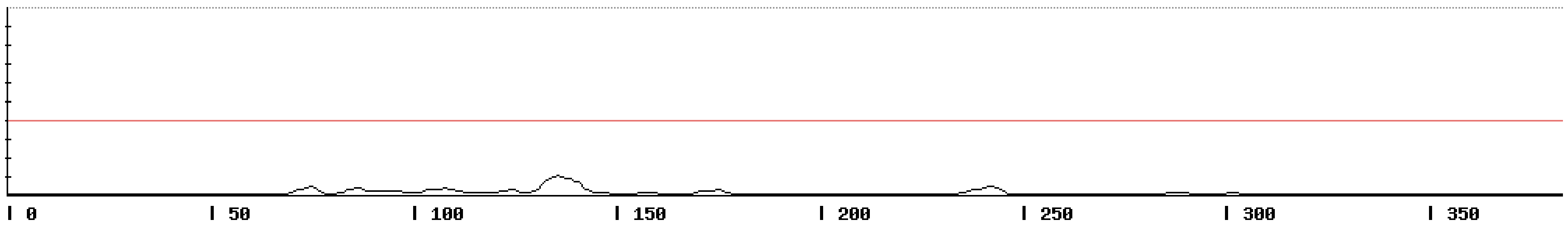

Predictions for Kalanchoemarnieriana_Kalax.0472s0007.1.p

Viterbi Path

Viterbi Path

Posterior @ 0.4

Posterior @ 0.4

Posterior Graphic. Threshold Marked @ 0.4

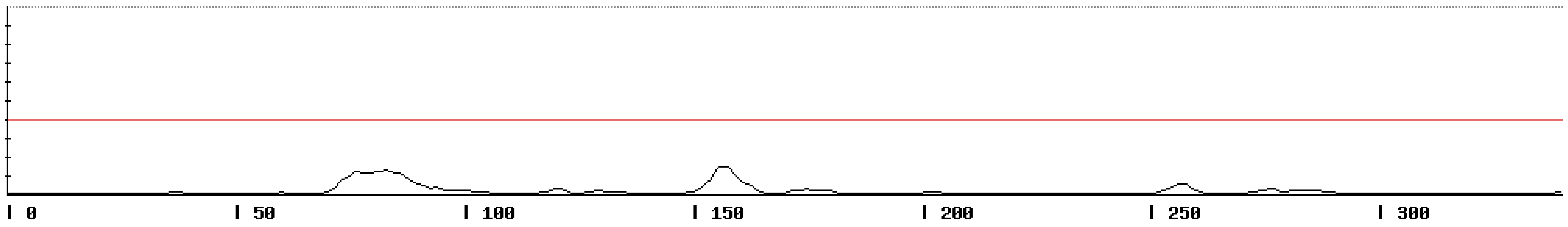

Predictions for Kalanchoemarnieriana_Kalax.0476s0030.1.p

Viterbi Path

Viterbi Path

Posterior @ 0.4

Posterior @ 0.4

Posterior Graphic. Threshold Marked @ 0.4

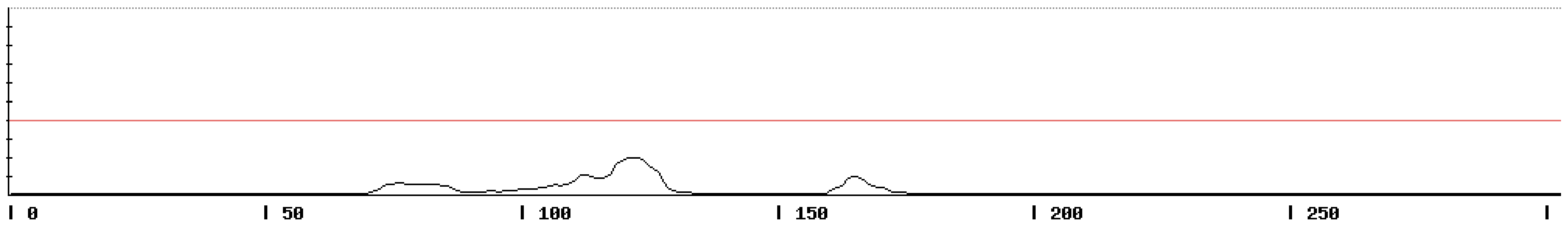

Predictions for Kalanchoemarnieriana_Kalax.0476s0044.1.p

Viterbi Path

Viterbi Path

Posterior @ 0.4

Posterior @ 0.4

Posterior Graphic. Threshold Marked @ 0.4

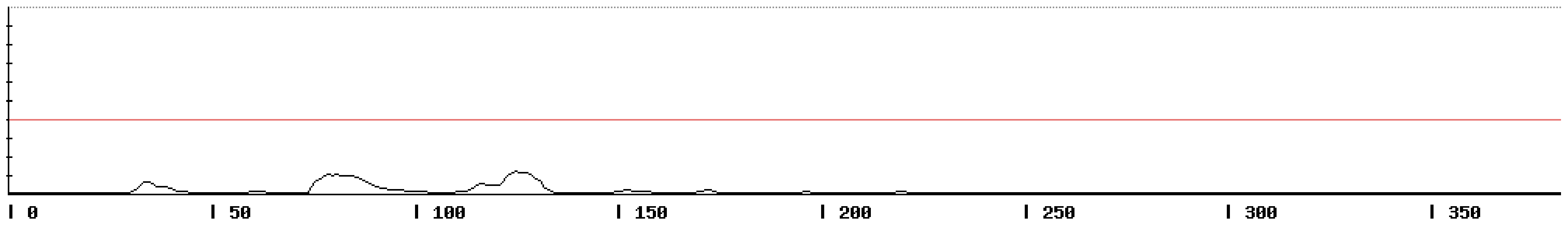

Predictions for Kalanchoemarnieriana_Kalax.0483s0011.1.p

Viterbi Path

Viterbi Path

Posterior @ 0.4 | 129 - KGRAP - 133

Posterior @ 0.4

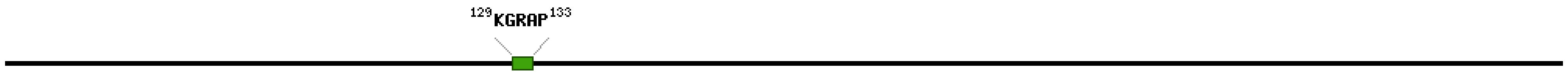

Posterior Graphic. Threshold Marked @ 0.4

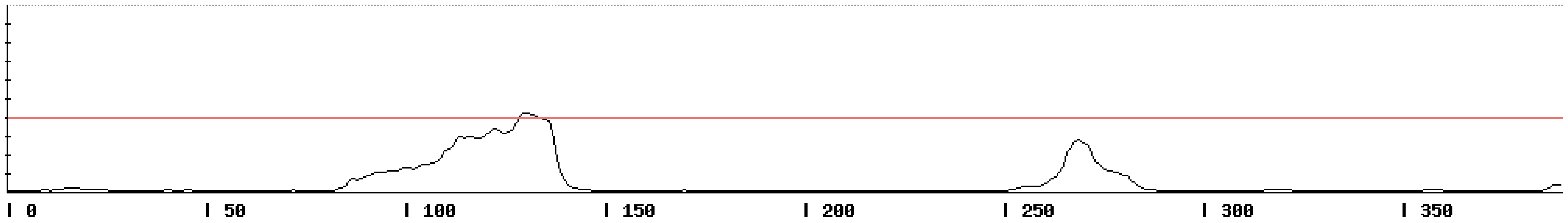

Predictions for Kalanchoemarnieriana_Kalax.0490s0030.1.p

Viterbi Path

Viterbi Path

Posterior @ 0.4

Posterior @ 0.4

Posterior Graphic. Threshold Marked @ 0.4

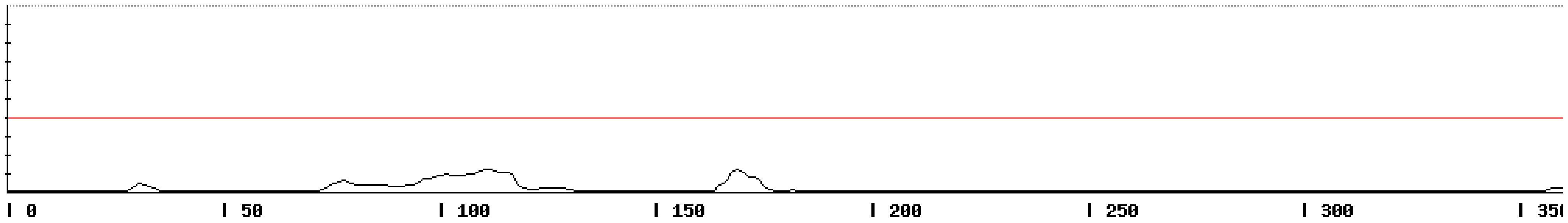

Predictions for Kalanchoemarnieriana_Kalax.0495s0022.1.p

Viterbi Path	83 - RKRIGGRRRTRGGYWKRKGRVR - 104
Viterbi Path	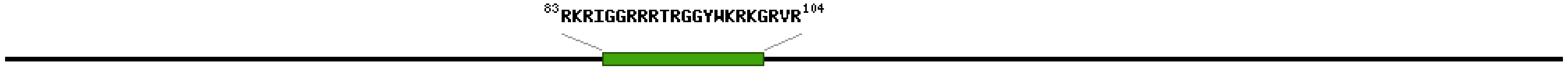
Posterior @ 0.4	81 - AERKRIGGRRRTRGGYWKRKGRVR - 104
Posterior @ 0.4	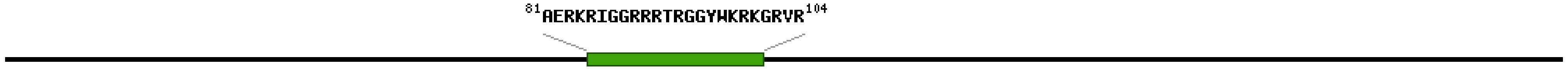
Posterior Graphic. Threshold Marked @ 0.4	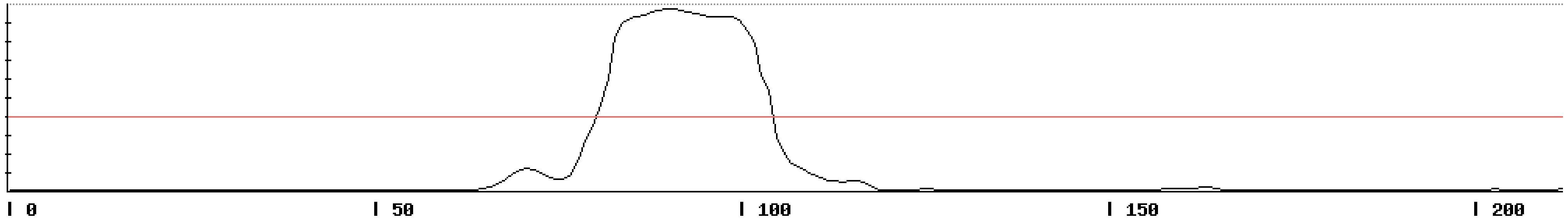

Predictions for Kalanchoemarnieriana_Kalax.0507s0019.1.p

Viterbi Path	
Viterbi Path	
Posterior @ 0.4	
Posterior @ 0.4	
Posterior Graphic. Threshold Marked @ 0.4	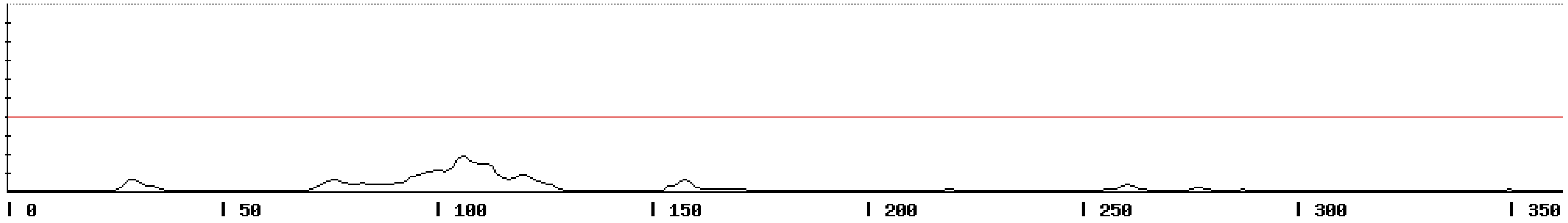

Predictions for Kalanchoemarnieriana_Kalax.0509s0026.1.p	
Viterbi Path	
Viterbi Path	
Posterior @ 0.4	136 - RKRRK - 140
Posterior @ 0.4	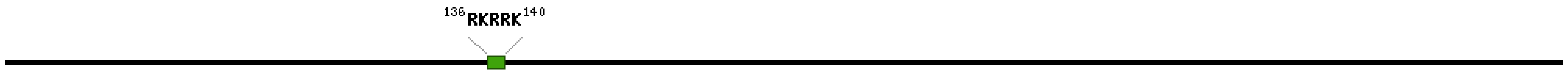
Posterior Graphic. Threshold Marked @ 0.4	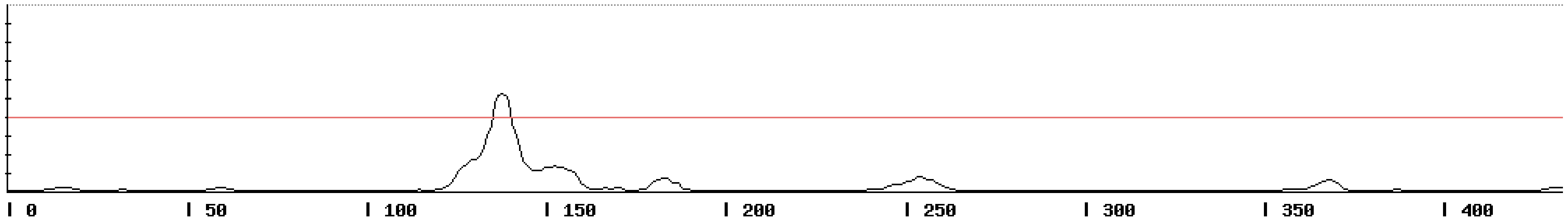

Predictions for Kalanchoemarnieriana_Kalax.0514s0014.1.p	
Viterbi Path	
Viterbi Path	
Posterior @ 0.4	
Posterior @ 0.4	
Posterior Graphic. Threshold Marked @ 0.4	

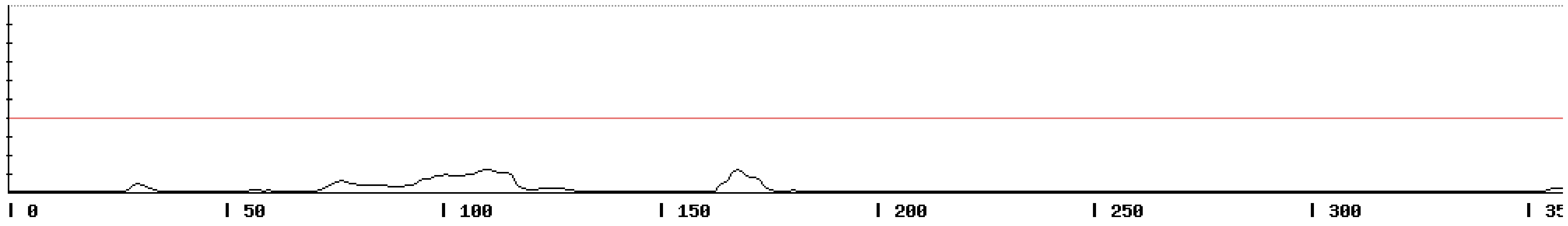

Predictions for Kalanchoemarnieriana_Kalax.0561s0017.1.p	
Viterbi Path	
Viterbi Path	
Posterior @ 0.4	
Posterior @ 0.4	
Posterior Graphic. Threshold Marked @ 0.4	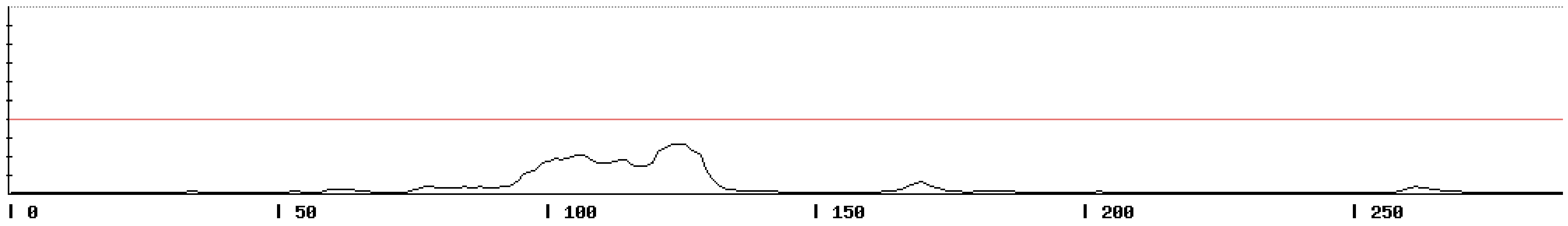

Predictions for Kalanchoemarnieriana_Kalax.0568s0017.1.p	
Viterbi Path	
Viterbi Path	
Posterior @ 0.4	
Posterior @ 0.4	

Posterior Graphic. Threshold Marked @ 0.4

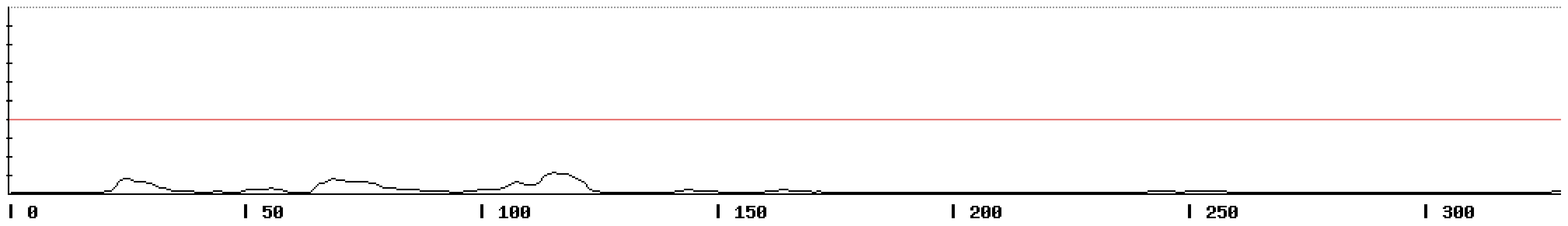

Predictions for Kalanchoemarnieriana_Kalax.0595s0009.1.p

Viterbi Path

Viterbi Path

Posterior @ 0.4

89 - WKATGKDRKIKKRGRDVGMKR - 109

497 - RKWPLFASHGKSKRRPEEFHIRLAKKHK - 524

Posterior @ 0.4

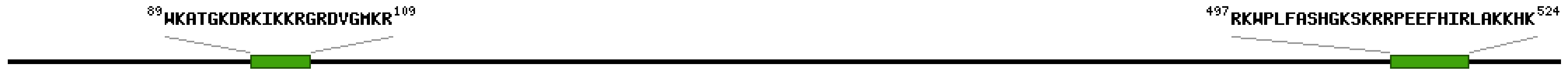

Posterior Graphic. Threshold Marked @ 0.4

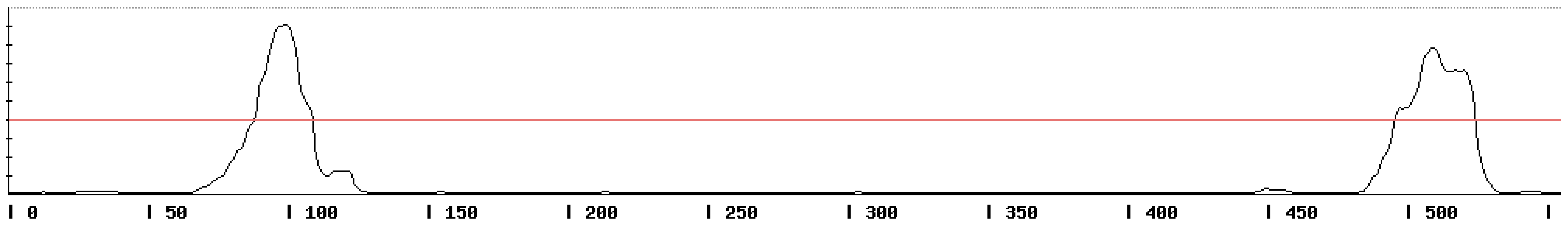

Predictions for Kalanchoemarnieriana_Kalax.0609s0006.1.p

Viterbi Path

Viterbi Path

Posterior @ 0.4

Posterior @ 0.4

Posterior Graphic. Threshold Marked @ 0.4

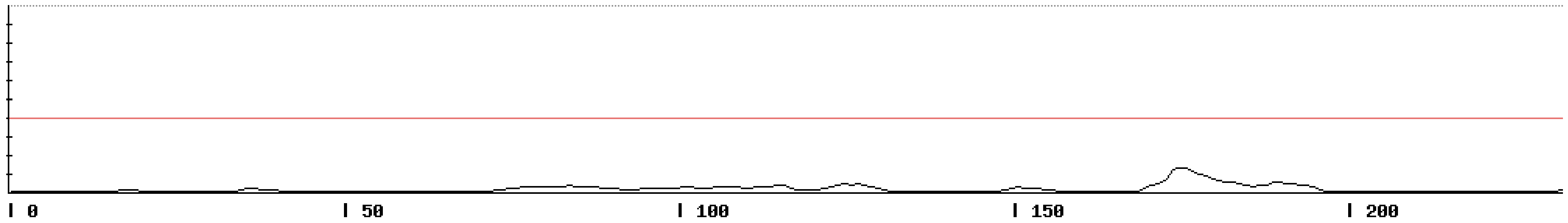

Predictions for Kalanchoemarnieriana_Kalax.0628s0003.1.p

Viterbi Path

Viterbi Path

Posterior @ 0.4

89 - WKATGKDRKIKKRGRDVGMKR - 109

504 - SHGKSKRRPE - 513

Posterior @ 0.4

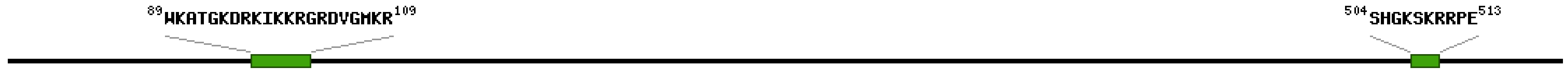

Posterior Graphic. Threshold Marked @ 0.4

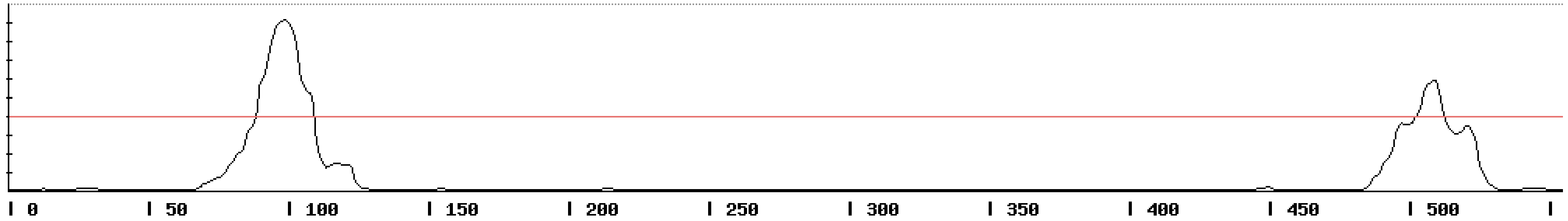

Predictions for Kalanchoemarnieriana_Kalax.0677s0017.1.p

Viterbi Path

Viterbi Path

Posterior @ 0.4

Posterior @ 0.4

Posterior Graphic. Threshold Marked @ 0.4

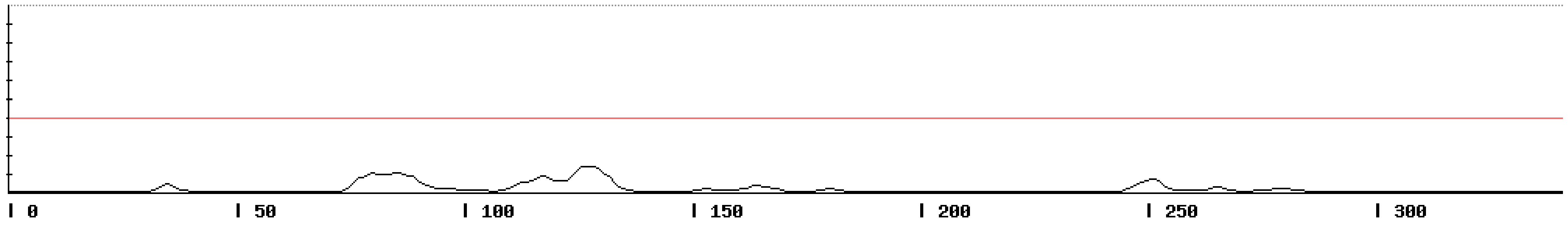

Predictions for Kalanchoemarnieriana_Kalax.0679s0010.1.p

Viterbi Path

Viterbi Path

Posterior @ 0.4

Posterior @ 0.4

Posterior Graphic. Threshold Marked @ 0.4

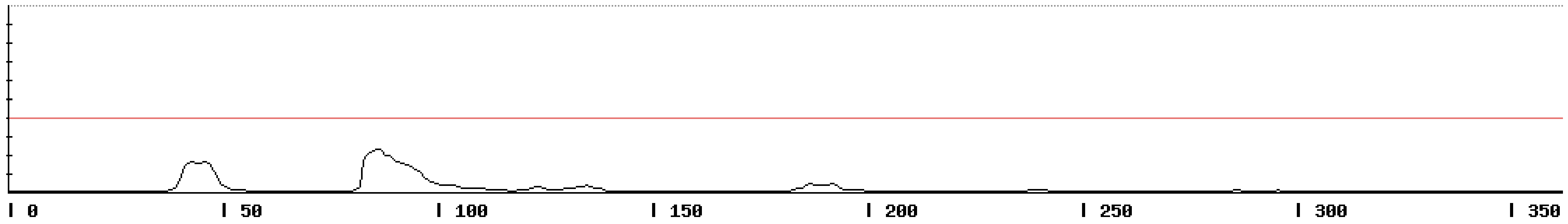

Predictions for Kalanchoemarnieriana_Kalax.0686s0005.1.p

Viterbi Path

Viterbi Path

Posterior @ 0.4

Posterior @ 0.4

Posterior Graphic. Threshold Marked @ 0.4

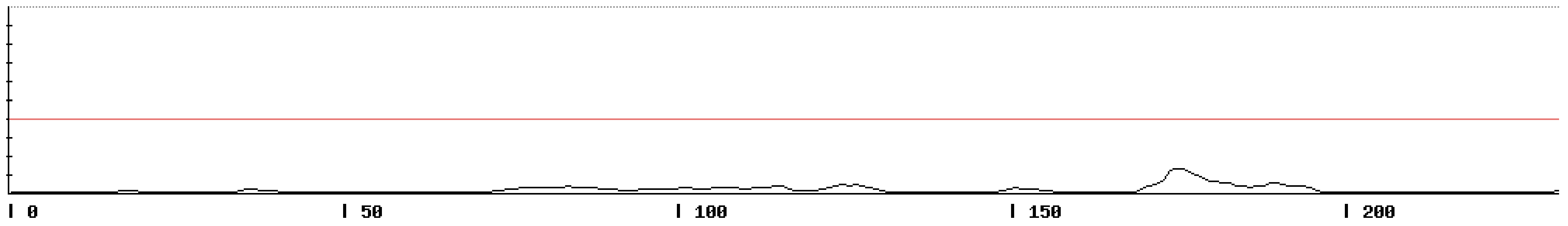

Predictions for Kalanchoemarnieriana_Kalax.0711s0024.1.p

Viterbi Path

Viterbi Path

Posterior @ 0.4

168 - RKRRK - 172

Posterior @ 0.4

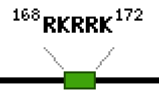

Posterior Graphic. Threshold Marked @ 0.4

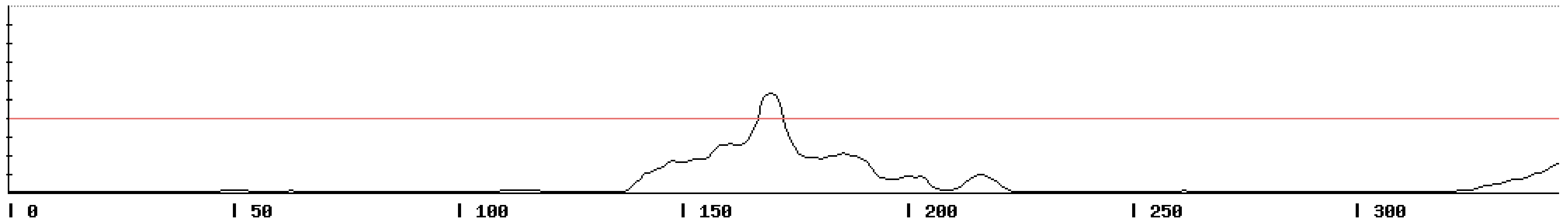

Predictions for Kalanchoemarnieriana_Kalax.0727s0009.1.p

Viterbi Path	
Viterbi Path	
Posterior @ 0.4	
Posterior @ 0.4	
Posterior Graphic. Threshold Marked @ 0.4	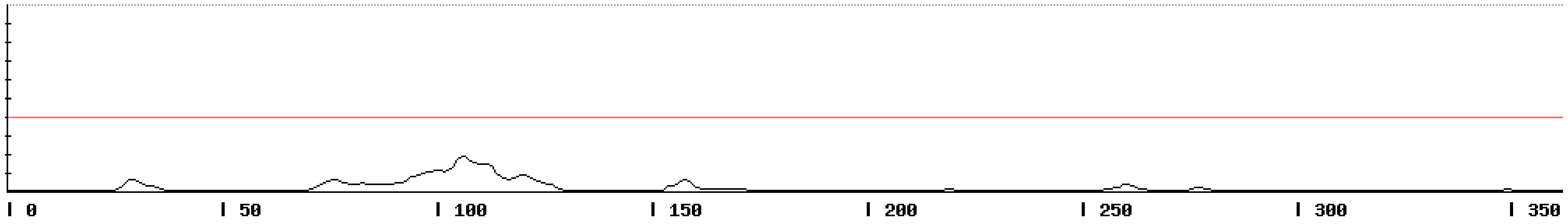

Predictions for Kalanchoemarnieriana_Kalax.0728s0011.1.p

Viterbi Path	
Viterbi Path	
Posterior @ 0.4	
Posterior @ 0.4	
Posterior Graphic. Threshold Marked @ 0.4	

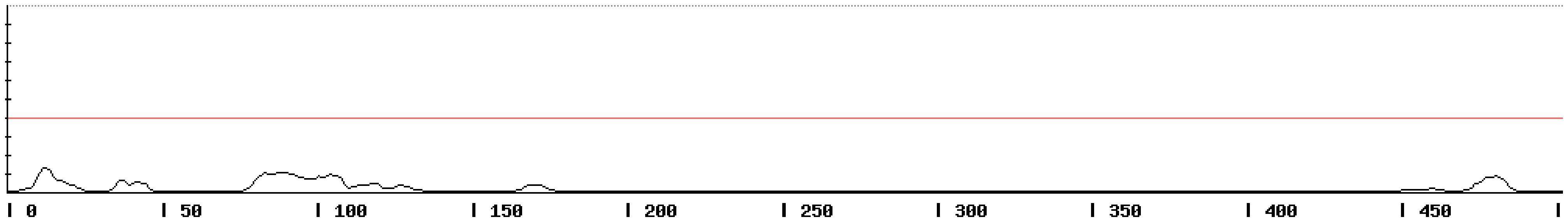

Predictions for Kalanchoemarnieriana_Kalax.0729s0019.1.p

Viterbi Path

Viterbi Path

Posterior @ 0.4

Posterior @ 0.4

Posterior Graphic. Threshold Marked @ 0.4

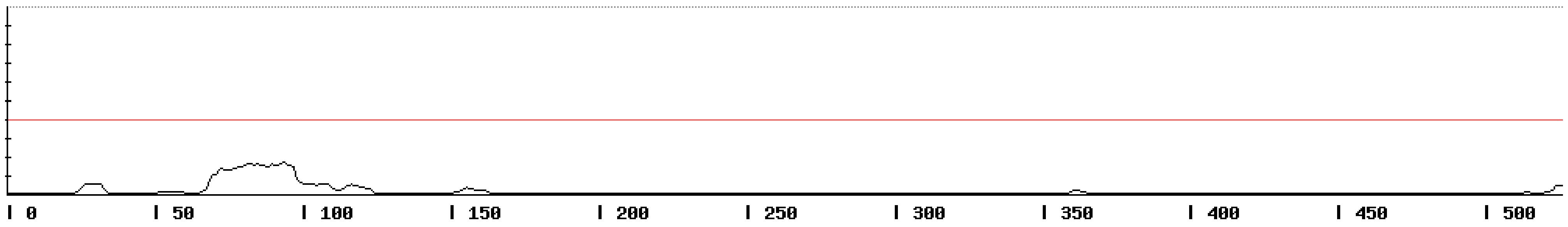

Predictions for Kalanchoemarnieriana_Kalax.0743s0006.1.p

Viterbi Path: 83 - RKRVGGRRRTRGGYWKRKGRVR - 104

Viterbi Path

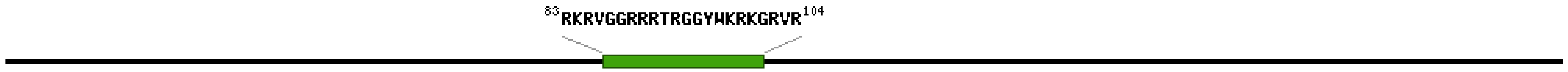

Posterior @ 0.4: 81 - AERKRVGGRRRTRGGYWKRKGRVR - 104

Posterior @ 0.4

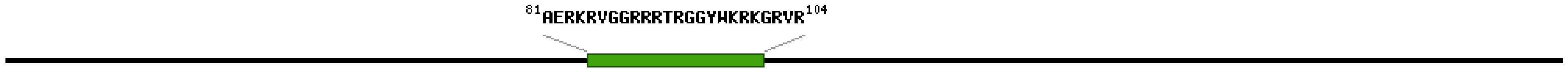

Posterior Graphic. Threshold Marked @ 0.4

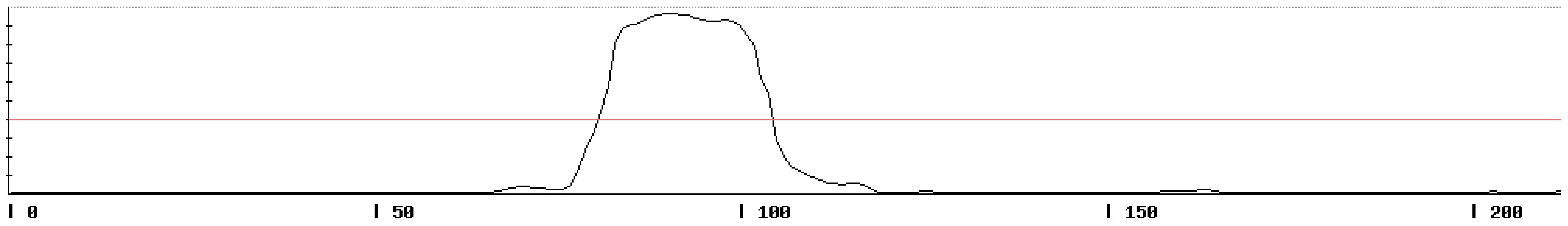

Predictions for Kalanchoemarnieriana_Kalax.0748s0009.1.p

Viterbi Path

Viterbi Path

Posterior @ 0.4

Posterior @ 0.4

Posterior Graphic. Threshold Marked @ 0.4

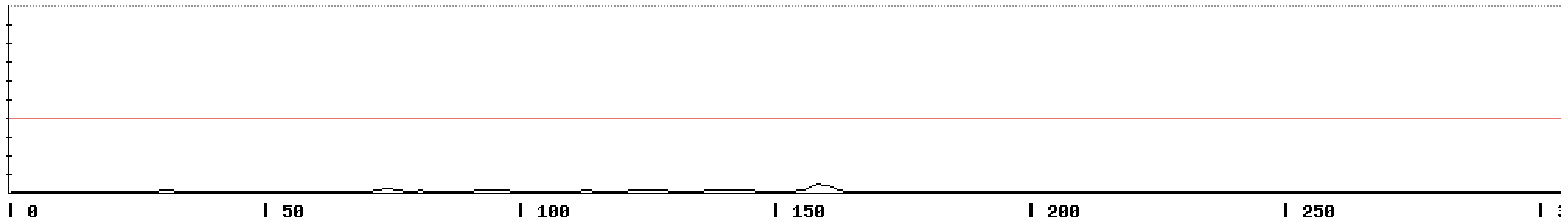

Predictions for Kalanchoemarnieriana_Kalax.0756s0005.1.p

Viterbi Path

Viterbi Path

Posterior @ 0.4

Posterior @ 0.4

Posterior Graphic. Threshold Marked @ 0.4

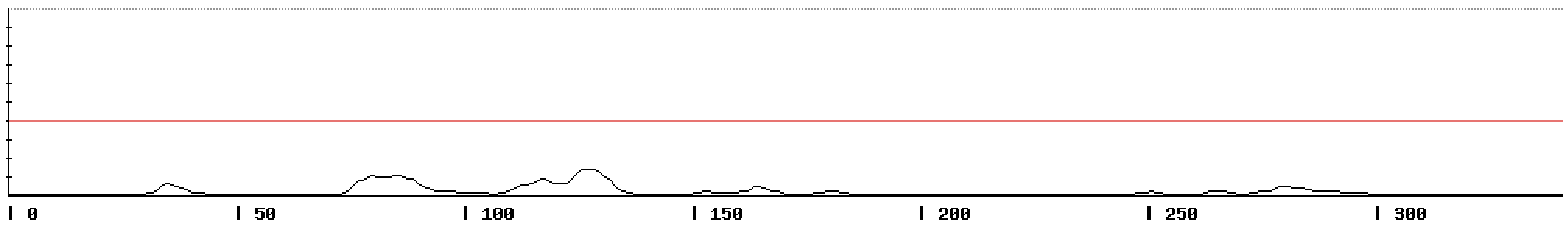

Predictions for Kalanchoemarnieriana_Kalax.0757s0019.1.p

Viterbi Path

Viterbi Path

Posterior @ 0.4

Posterior @ 0.4

Posterior Graphic. Threshold Marked @ 0.4

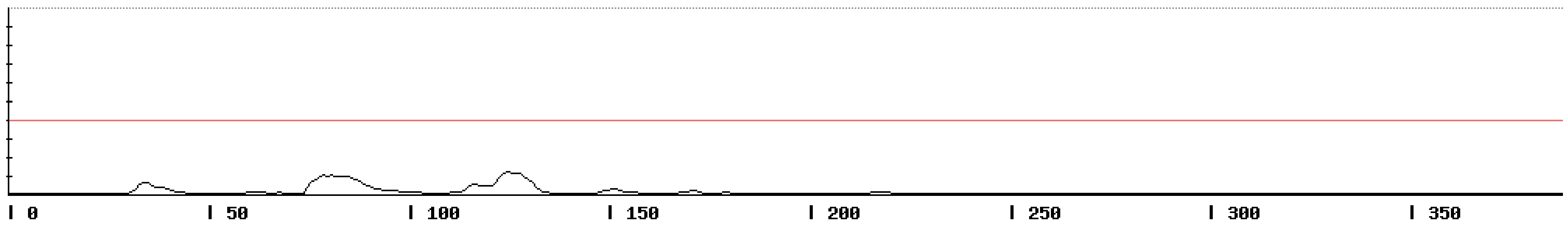

Predictions for Kalanchoemarnieriana_Kalax.0854s0001.1.p

Viterbi Path

Viterbi Path

Posterior @ 0.4	
Posterior @ 0.4	
Posterior Graphic. Threshold Marked @ 0.4	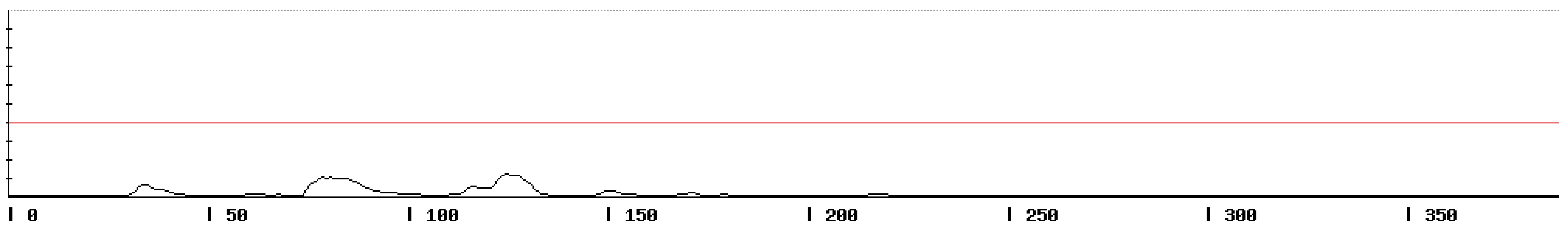

Predictions for Kalanchoemarnieriana_Kalax.0854s0016.1.p	
Viterbi Path	
Viterbi Path	
Posterior @ 0.4	
Posterior @ 0.4	
Posterior Graphic. Threshold Marked @ 0.4	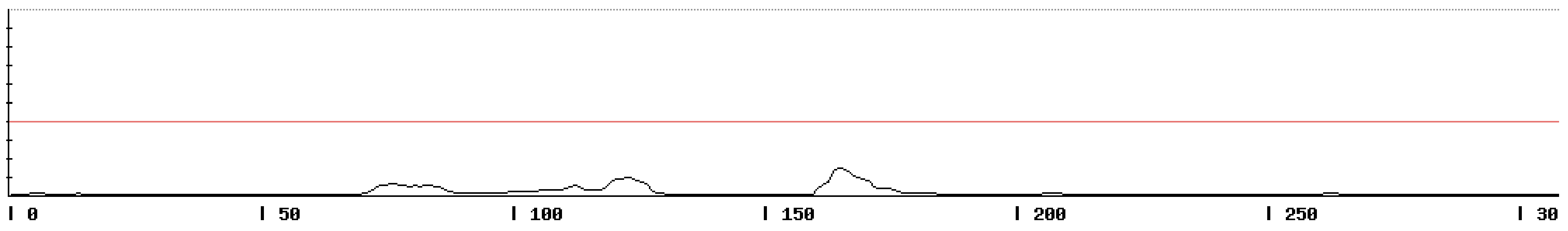

Predictions for Kalanchoemarnieriana_Kalax.0870s0004.1.p	
Viterbi Path	
Viterbi Path	

Posterior @ 0.4

Posterior @ 0.4

Posterior Graphic. Threshold Marked @ 0.4

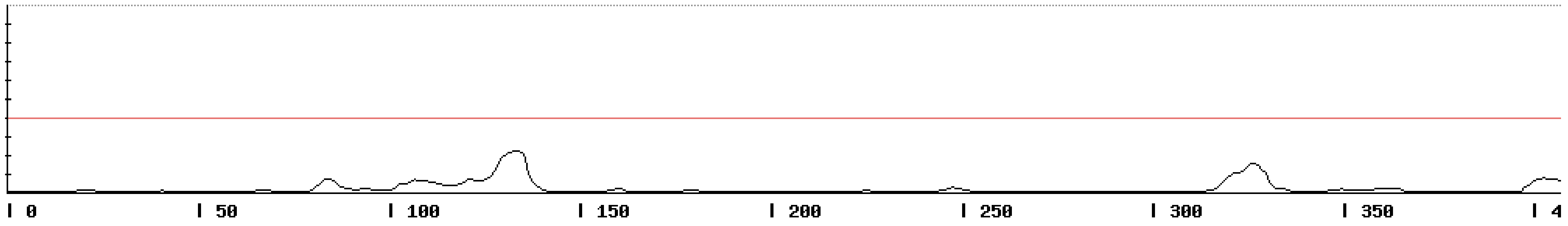

Predictions for Kalanchoemarnieriana_Kalax.0896s0001.1.p

Viterbi Path

Viterbi Path

Posterior @ 0.4

Posterior @ 0.4

Posterior Graphic. Threshold Marked @ 0.4

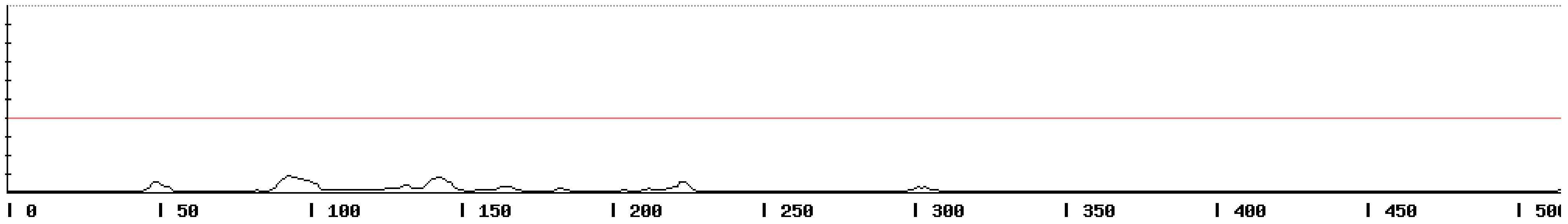

Predictions for Kalanchoemarnieriana_Kalax.0901s0011.1.p

Viterbi Path

Viterbi Path

Posterior @ 0.4

Posterior @ 0.4

Posterior Graphic. Threshold Marked @ 0.4

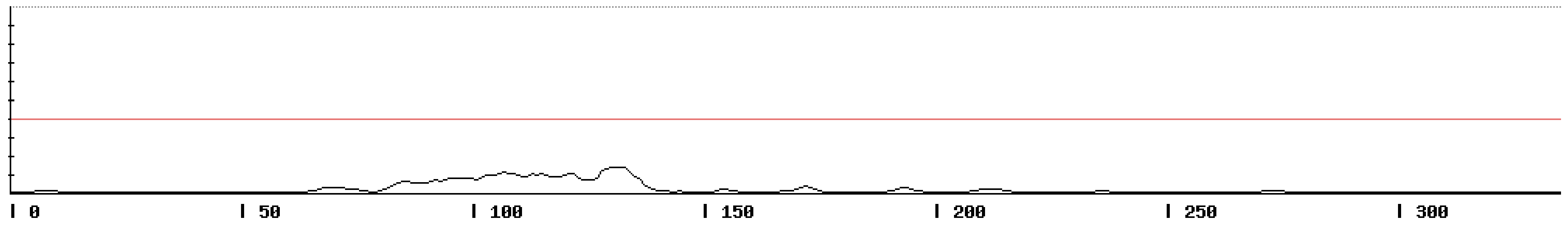

Predictions for Kalanchoemarnieriana_Kalax.0935s0008.1.p

Viterbi Path

Viterbi Path

Posterior @ 0.4

Posterior @ 0.4

Posterior Graphic. Threshold Marked @ 0.4

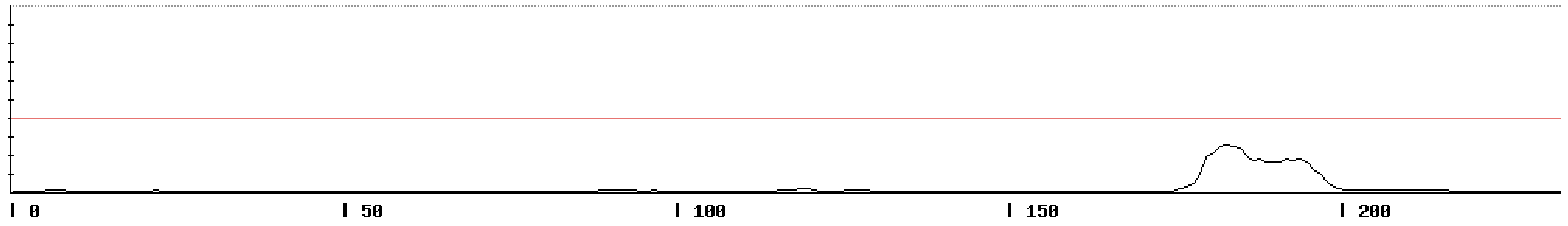

Predictions for Kalanchoemarnieriana_Kalax.0939s0002.1.p

Viterbi Path

Viterbi Path

Posterior @ 0.4

Posterior @ 0.4

Posterior Graphic. Threshold Marked @ 0.4

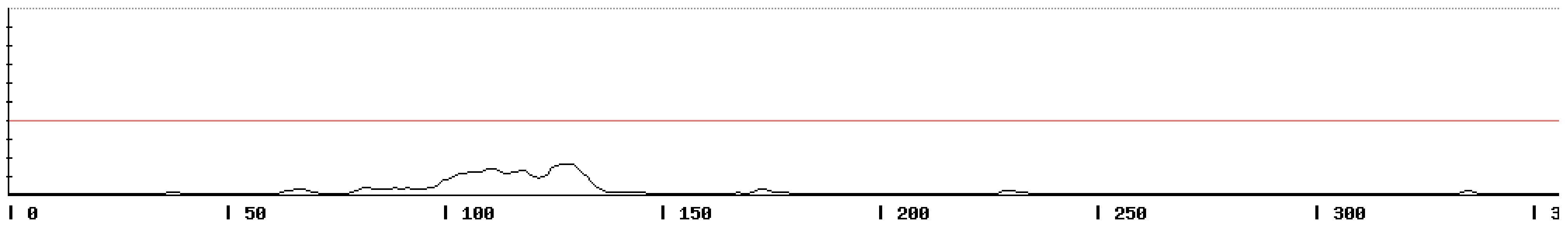

Predictions for Kalanchoemarnieriana_Kalax.0941s0002.1.p

Viterbi Path

Viterbi Path

Posterior @ 0.4

Posterior @ 0.4

Posterior Graphic. Threshold Marked @ 0.4

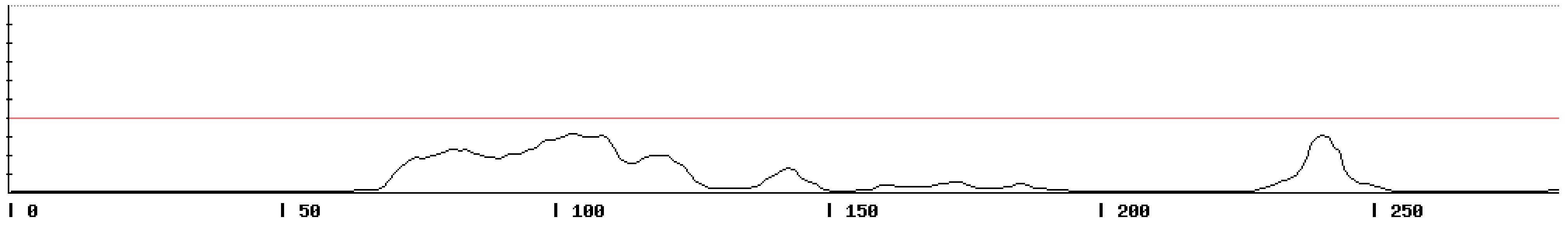

Predictions for Kalanchoemarnieriana_Kalax.1030s0012.1.p

Viterbi Path

Viterbi Path

Posterior @ 0.4 168 - RKRRK - 172

Posterior @ 0.4

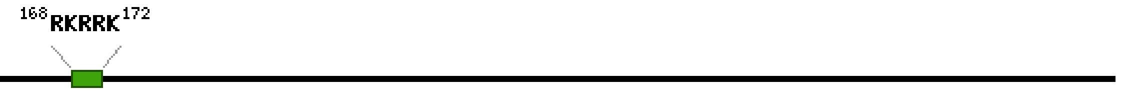

Posterior Graphic. Threshold Marked @ 0.4

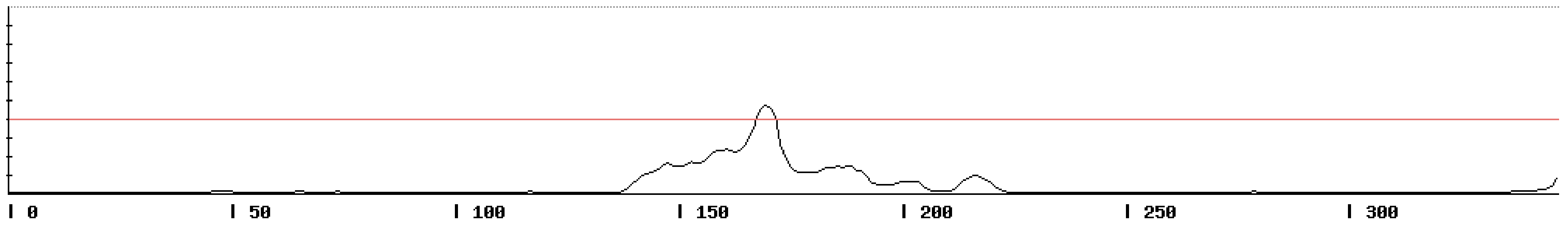

Predictions for Kalanchoemarnieriana_Kalax.1035s0002.1.p

Viterbi Path

Viterbi Path

Posterior @ 0.4

Posterior @ 0.4

Posterior Graphic. Threshold Marked @ 0.4

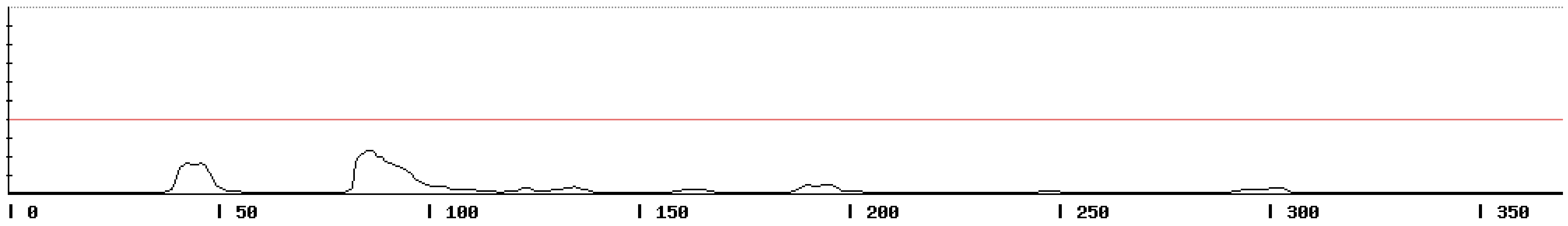

Predictions for Kalanchoemarnieriana_Kalax.1115s0008.1.p

Viterbi Path

Viterbi Path

Posterior @ 0.4

Posterior @ 0.4

Posterior Graphic. Threshold Marked @ 0.4

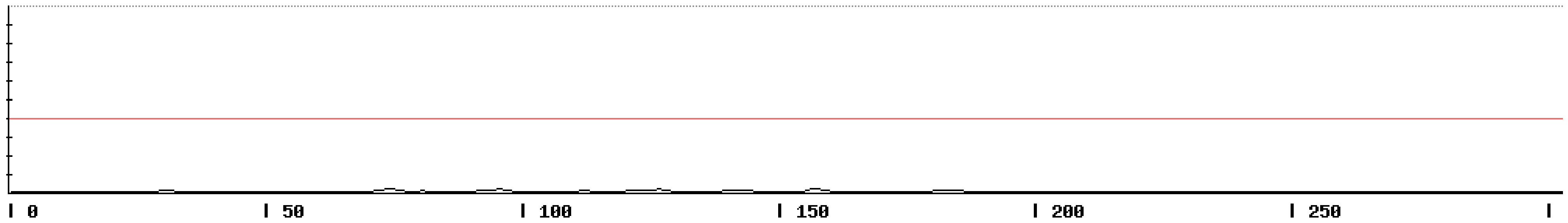

Predictions for Kalanchoemarnieriana_Kalax.1234s0006.1.p

Viterbi Path

Viterbi Path

Posterior @ 0.4

Posterior @ 0.4

Posterior Graphic. Threshold Marked @ 0.4

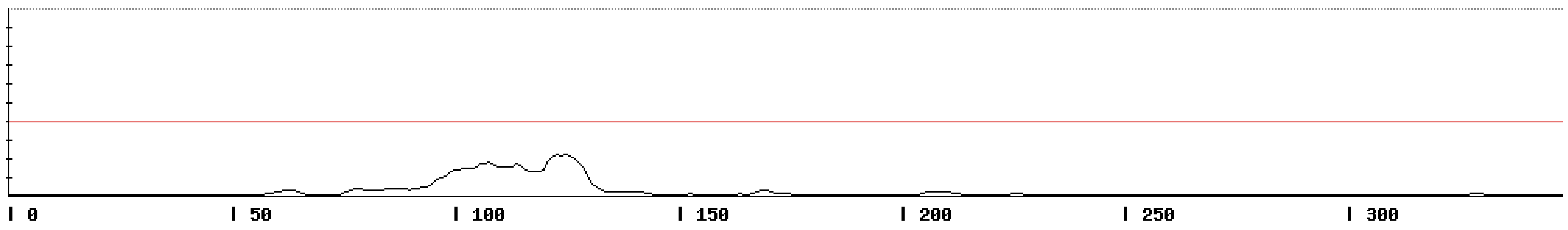

Predictions for Kalanchoemarnieriana_Kalax.1240s0007.1.p

Viterbi Path

Viterbi Path

Posterior @ 0.4

Posterior @ 0.4

Posterior Graphic. Threshold Marked @ 0.4

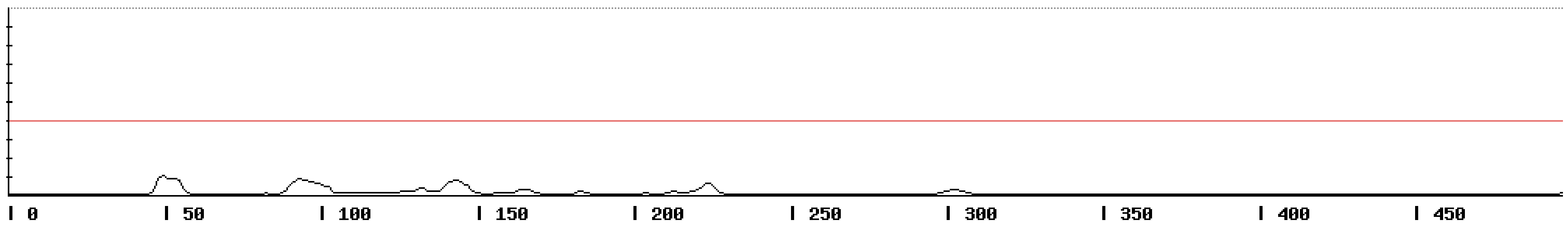

Predictions for Kalanchoemarnieriana_Kalax.1261s0008.1.p

Viterbi Path

Viterbi Path

Posterior @ 0.4

Posterior @ 0.4

Posterior Graphic. Threshold Marked @ 0.4

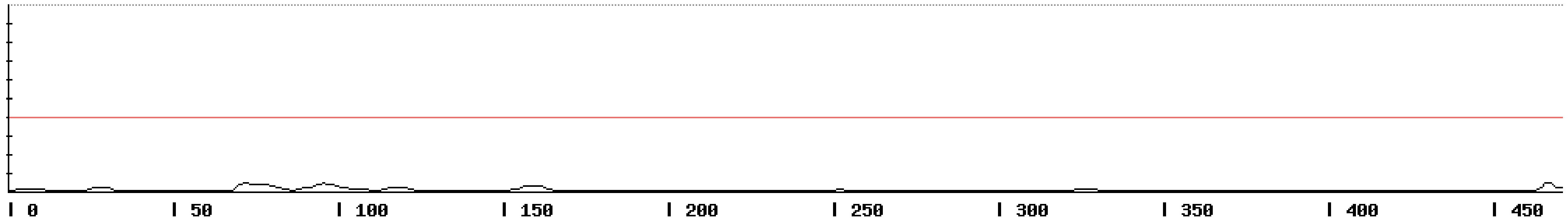

Predictions for Kalanchoemarnieriana_Kalax.1284s0006.1.p

Viterbi Path

Viterbi Path

Posterior @ 0.4

Posterior @ 0.4

Posterior Graphic. Threshold Marked @ 0.4

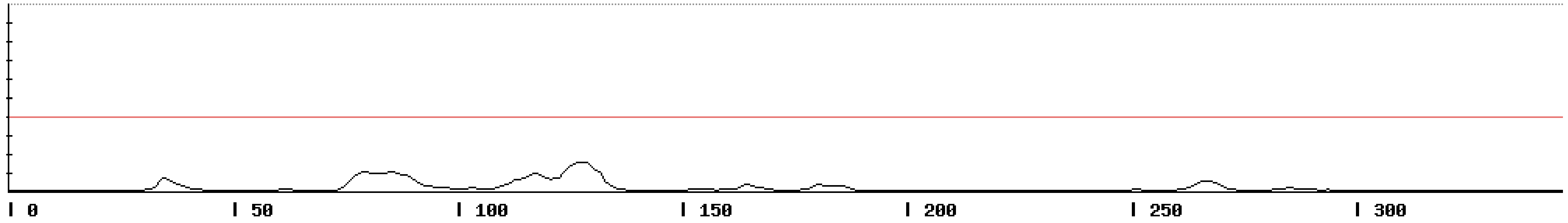

Predictions for Kalanchoemarnieriana_Kalax.1411s0004.1.p

Viterbi Path

Viterbi Path

Posterior @ 0.4

Posterior @ 0.4

Posterior Graphic. Threshold Marked @ 0.4

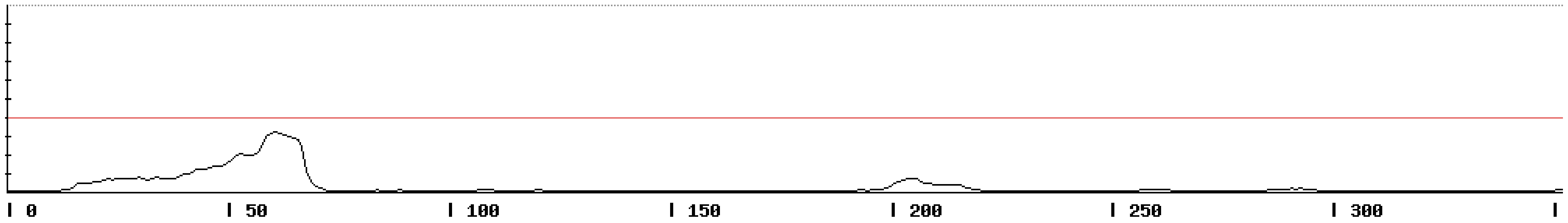

Predictions for Kalanchoemarnieriana_Kalax.1420s0002.1.p

Viterbi Path

Viterbi Path

Posterior @ 0.4

Posterior @ 0.4

Posterior Graphic. Threshold Marked @ 0.4

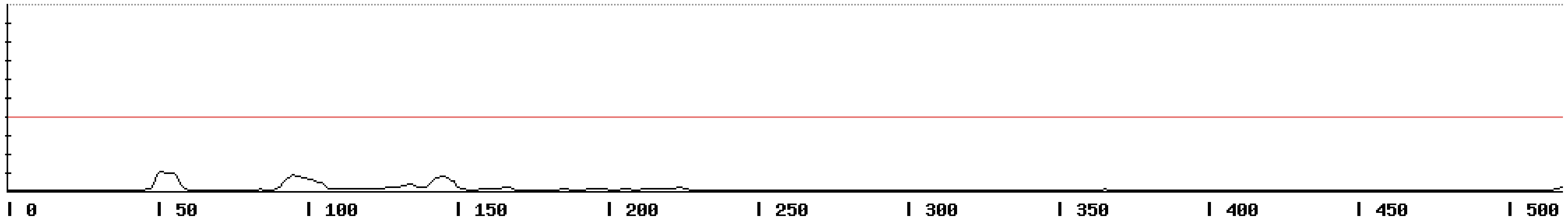

Predictions for Kalanchoemarnieriana_Kalax.1717s0001.1.p

Viterbi Path	
Viterbi Path	
Posterior @ 0.4	
Posterior @ 0.4	
Posterior Graphic. Threshold Marked @ 0.4	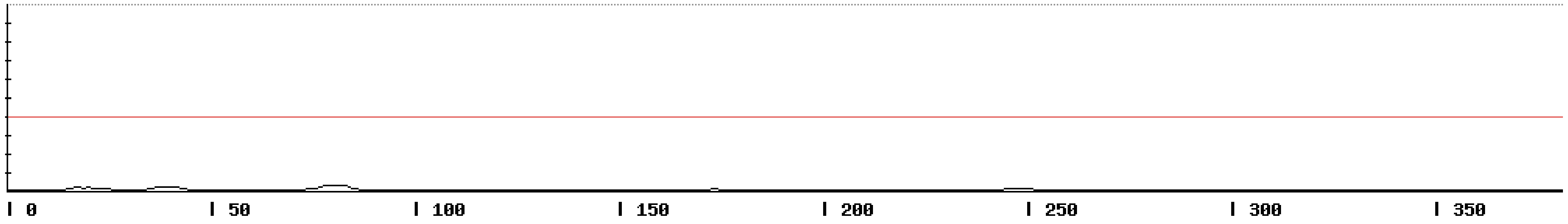

Revision r.9 - Last Updated on : February 24 2015 17:45:20. alex.nguyenba@utoronto.ca

Intended to be used with Firefox

Viterbi Path

Posterior @ 0.2

Posterior @ 0.2

Posterior Graphic. Threshold Marked @ 0.2

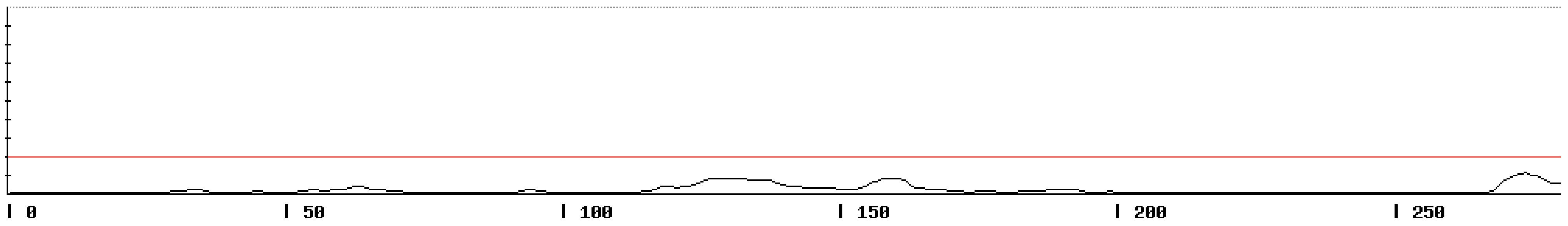

Predictions for Klebsormidiumflaccidum_kfl00333_0190

Viterbi Path

Viterbi Path

Posterior @ 0.2

186 - QPRRKPVKRQRSPAEQAESGVPDKPLGSRIRPRRPES - 222

Posterior @ 0.2

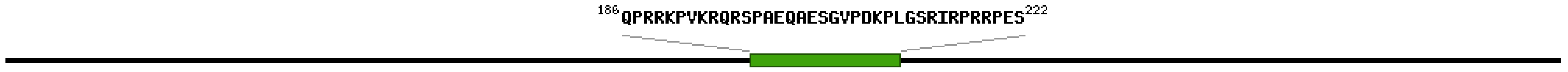

Posterior Graphic. Threshold Marked @ 0.2

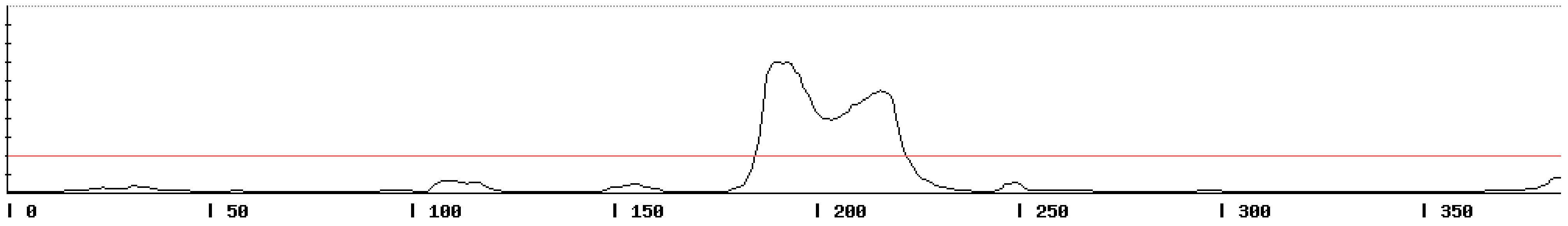

Predictions for Klebsormidiumflaccidum_kfl00817_0040

Viterbi Path	
Viterbi Path	
Posterior @ 0.2	309 - DRPRRKGITGVKREPVTATTPPPEKVRKLHYKRPRV - 344
Posterior @ 0.2	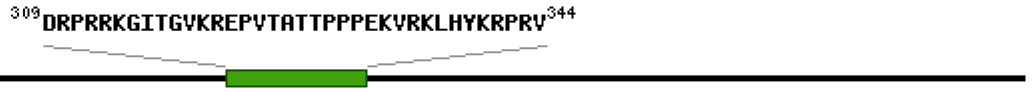
Posterior Graphic. Threshold Marked @ 0.2	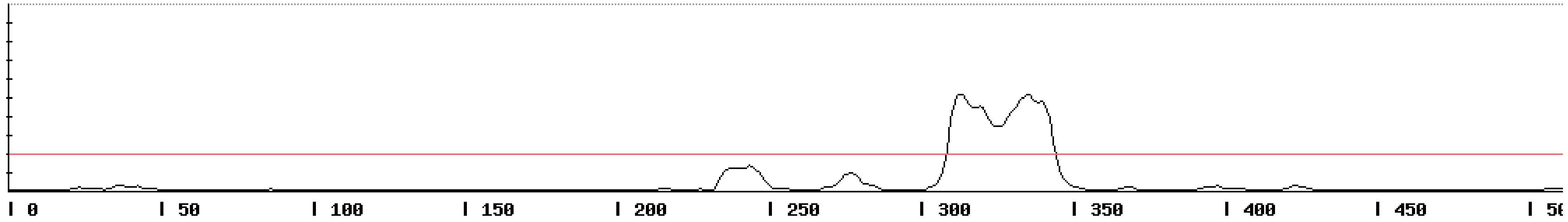

Revision r.9 - Last Updated on : February 24 2015 17:45:20.

alex.nguyenba@utoronto.ca

Intended to be used with Firefox

Viterbi Path

Posterior @ 0.2 — 116 - GTRKRRK - 122

Posterior @ 0.2

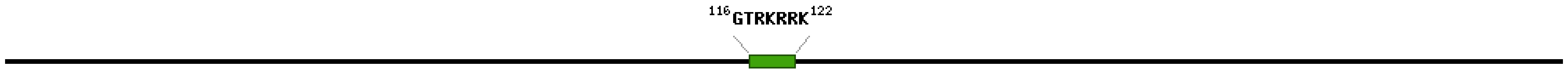

Posterior Graphic. Threshold Marked @ 0.2

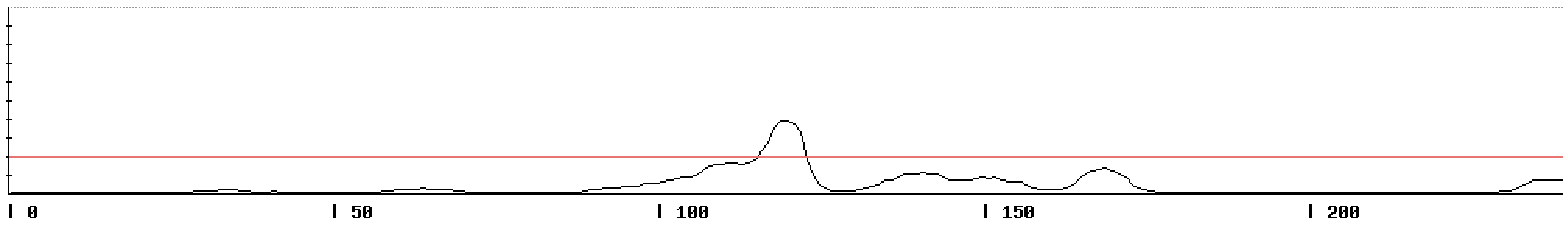

Predictions for Lactucasativa_Lsa000820

Viterbi Path

Viterbi Path

Posterior @ 0.2

Posterior @ 0.2

Posterior Graphic. Threshold Marked @ 0.2

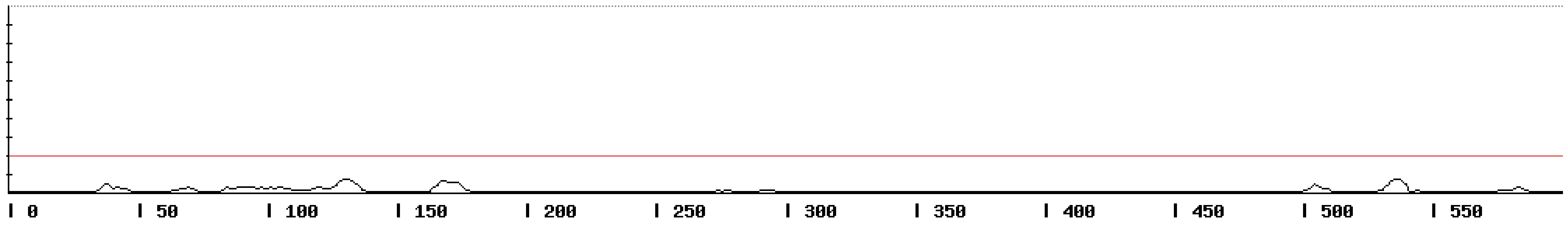

Predictions for Lactucasativa_Lsa001397

Viterbi Path	
Viterbi Path	
Posterior @ 0.2	81 - NRATKGGYWKATGKDRK - 97
Posterior @ 0.2	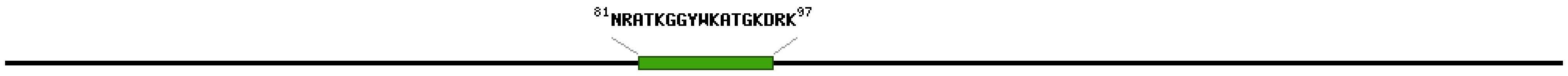
Posterior Graphic. Threshold Marked @ 0.2	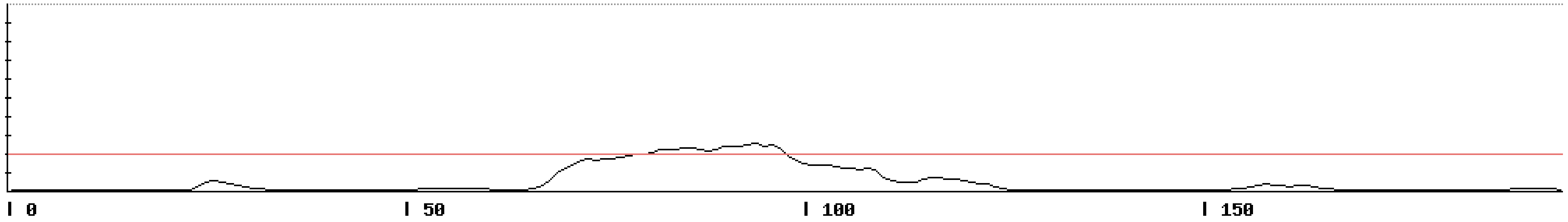

Predictions for Lactucasativa_Lsa001658

Viterbi Path	
Viterbi Path	
Posterior @ 0.2	113 - K - 113<br>116 - WKATGKDRSVRHK - 128
Posterior @ 0.2	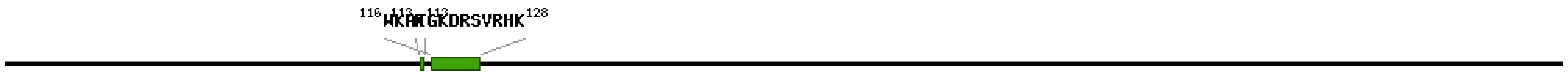
Posterior Graphic. Threshold Marked @ 0.2	

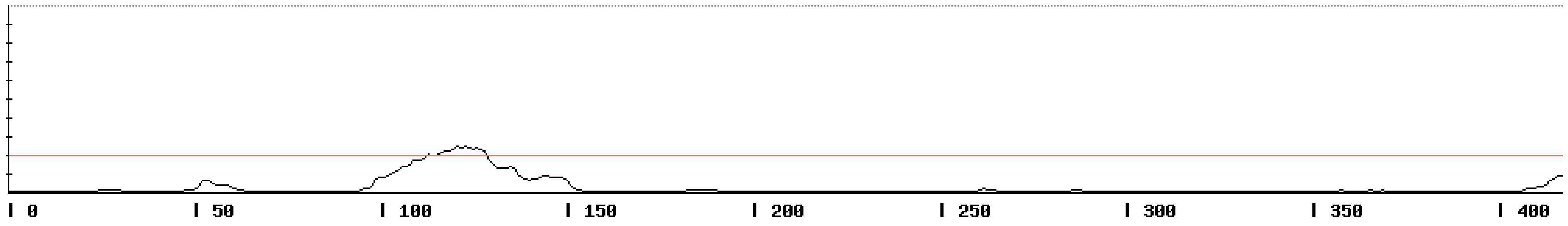

Predictions for Lactucasativa_Lsa002776

Viterbi Path

Viterbi Path

Posterior @ 0.2

Posterior @ 0.2

Posterior Graphic. Threshold Marked @ 0.2

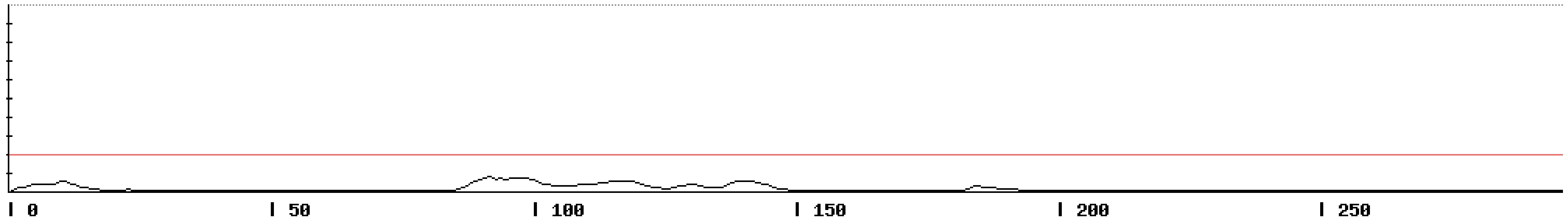

Predictions for Lactucasativa_Lsa002920

Viterbi Path

Viterbi Path

Posterior @ 0.2

Posterior @ 0.2

Posterior Graphic. Threshold Marked @ 0.2

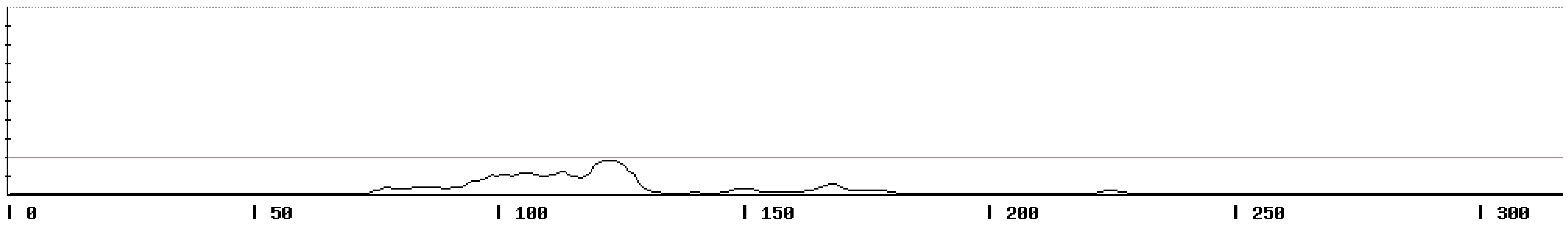

Predictions for Lactucasativa_Lsa003012

Viterbi Path

Viterbi Path

Posterior @ 0.2

Posterior @ 0.2

Posterior Graphic. Threshold Marked @ 0.2

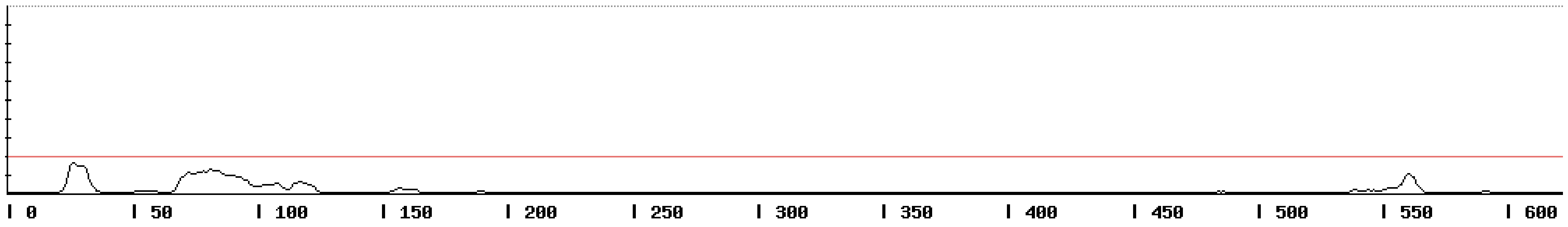

Predictions for Lactucasativa_Lsa003093

Viterbi Path

Viterbi Path

Posterior @ 0.2 171 - KPIKRK - 176

Posterior @ 0.2

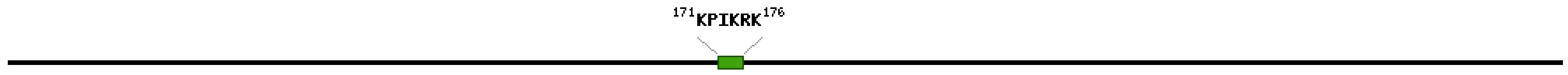

Posterior Graphic. Threshold Marked @ 0.2

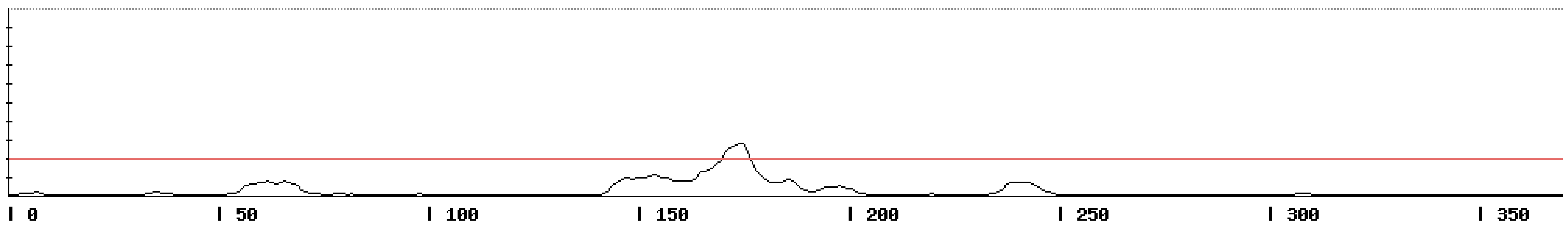

Predictions for Lactucasativa_Lsa003627

Viterbi Path

Viterbi Path

Posterior @ 0.2 184 - SGRSKRKGHSK - 194

Posterior @ 0.2

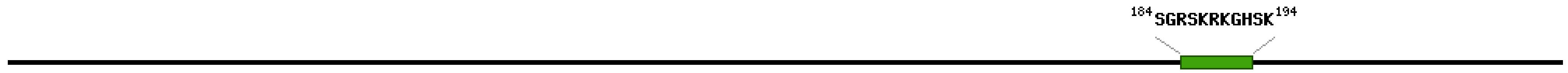

Posterior Graphic. Threshold Marked @ 0.2

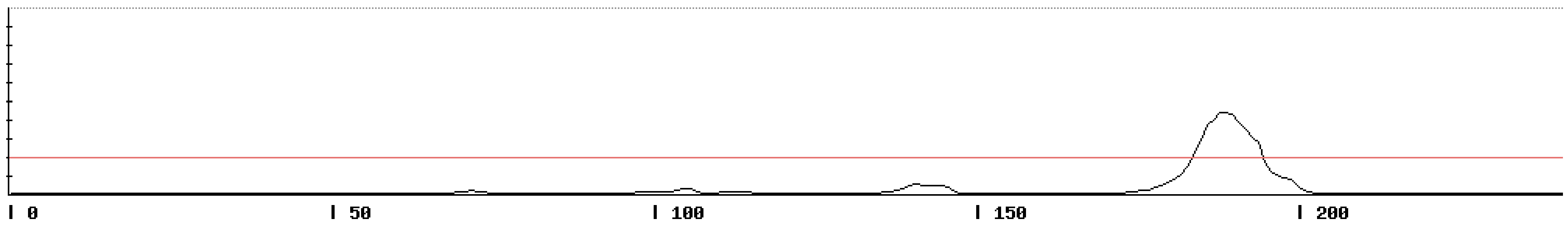

Predictions for Lactucasativa_Lsa003641

Viterbi Path

Viterbi Path

Posterior @ 0.2	
Posterior @ 0.2	
Posterior Graphic. Threshold Marked @ 0.2	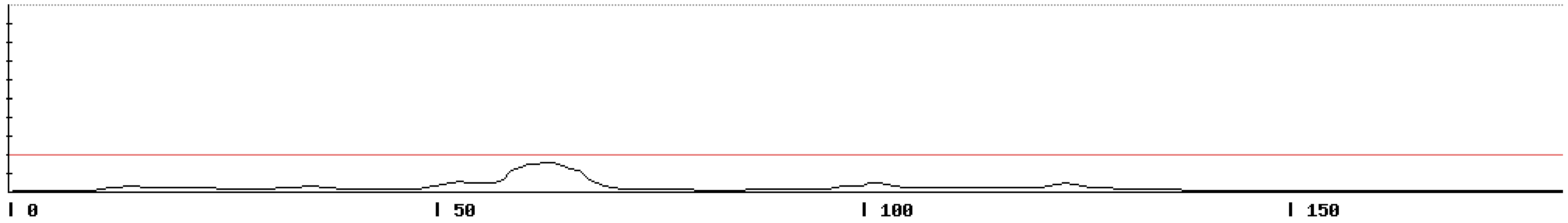

Predictions for Lactucasativa_Lsa003913

Viterbi Path	
Viterbi Path	
Posterior @ 0.2	88 - KATGKDRTIKTRGRSDAKIIGMKKTLVFYRGRAPKG - 123
	432 - RSKKK - 436
Posterior @ 0.2	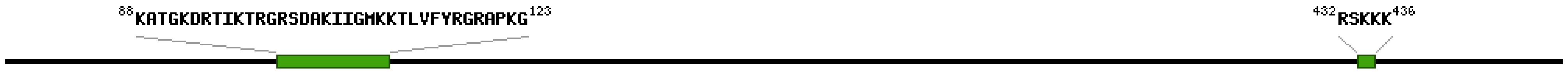
Posterior Graphic. Threshold Marked @ 0.2	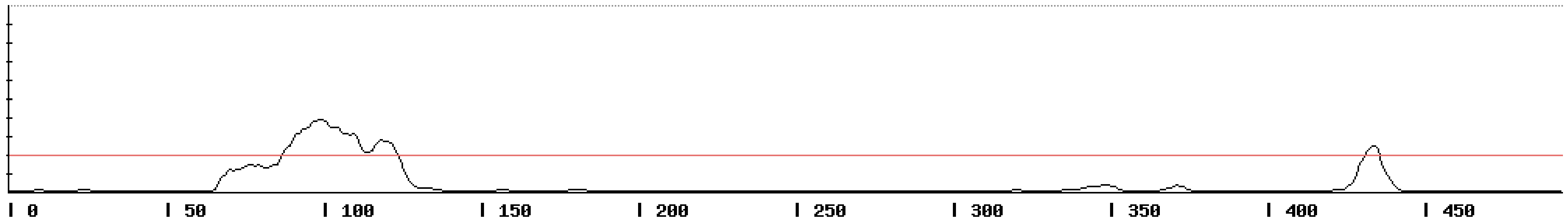

Predictions for Lactucasativa_Lsa004975

Viterbi Path	
Viterbi Path	

Posterior @ 0.2	72 - RKYPNGSRPNRAAGTGYWKATGADKPIGKPKAVGIKKALVF - 112
	114 - AGKAPR - 119
Posterior @ 0.2	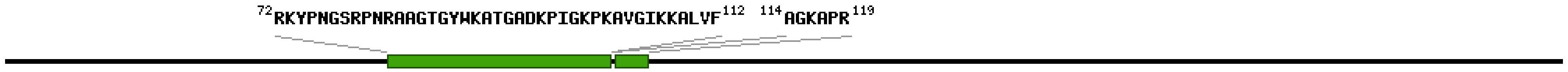
Posterior Graphic. Threshold Marked @ 0.2	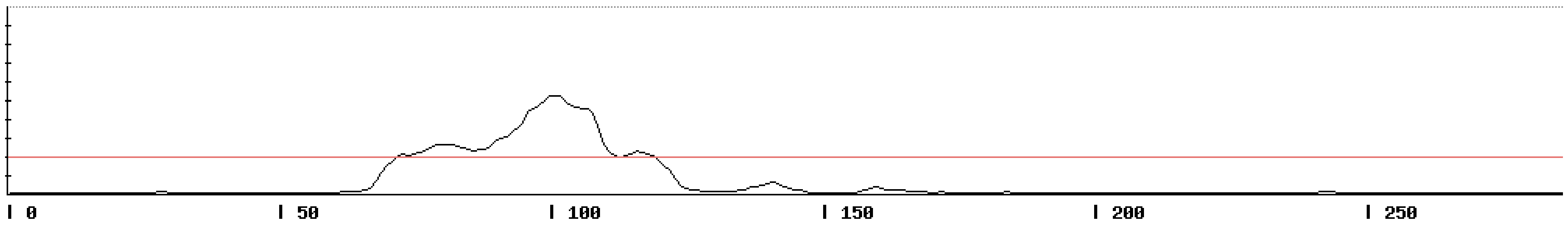

Predictions for Lactucasativa_Lsa005206

Viterbi Path	
Viterbi Path	
Posterior @ 0.2	
Posterior @ 0.2	
Posterior Graphic. Threshold Marked @ 0.2	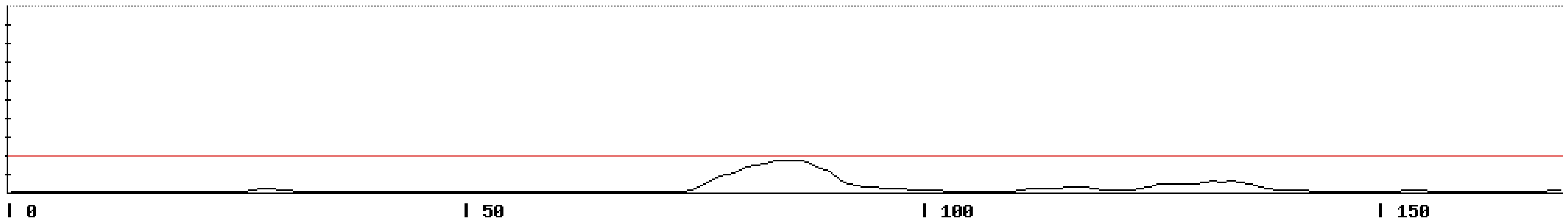

Predictions for Lactucasativa_Lsa005319	
Viterbi Path	
Viterbi Path	
Posterior @ 0.2	
Posterior @ 0.2	
Posterior Graphic. Threshold Marked @ 0.2	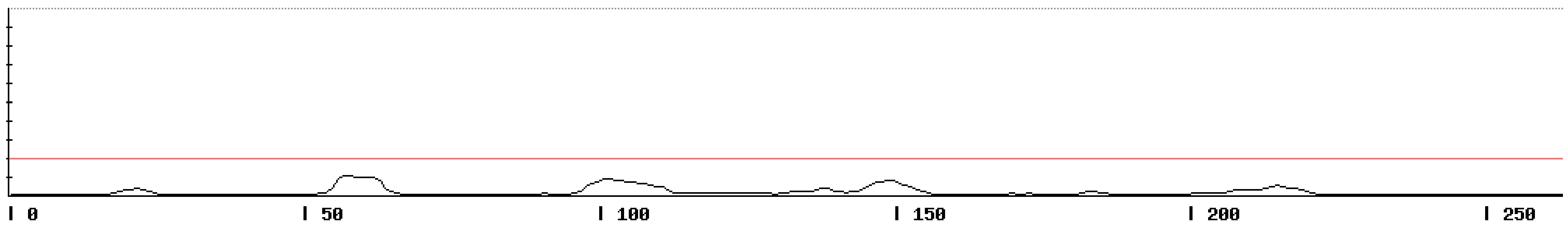

Predictions for Lactucasativa_Lsa005481	
Viterbi Path	
Viterbi Path	
Posterior @ 0.2	74 - RKHGSGGR - 81
	107 - KKHLGLKK - 114
	120 - KGRAPR - 125
Posterior @ 0.2	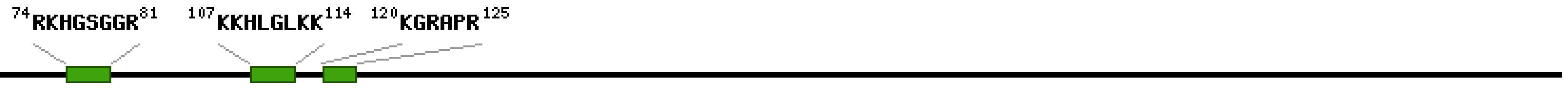
Posterior Graphic. Threshold Marked @ 0.2	

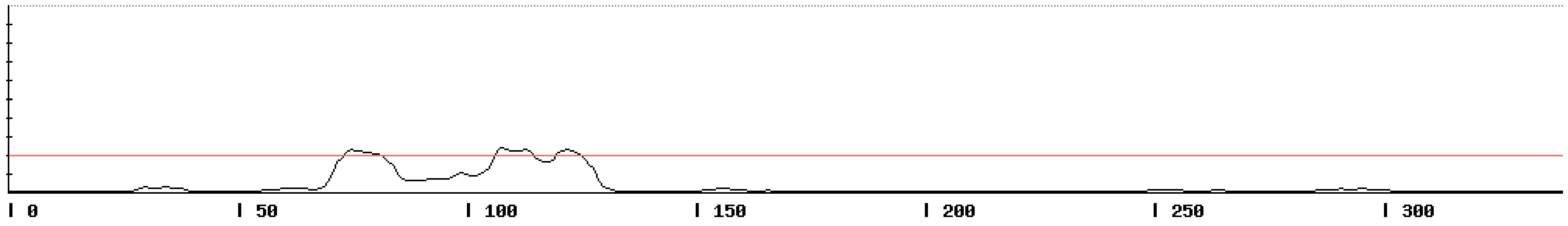

Predictions for Lactucasativa_Lsa005632

Viterbi Path

Viterbi Path

Posterior @ 0.2 — 100 - KATGKDKEIHNKKGKSTGKLVGMKKTLVFYKGRAPKGEK - 138

Posterior @ 0.2

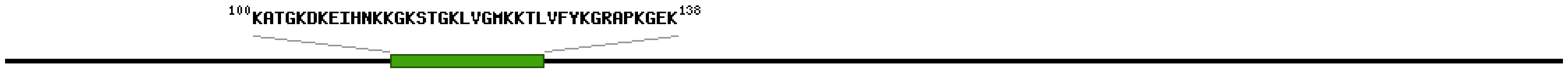

Posterior Graphic. Threshold Marked @ 0.2

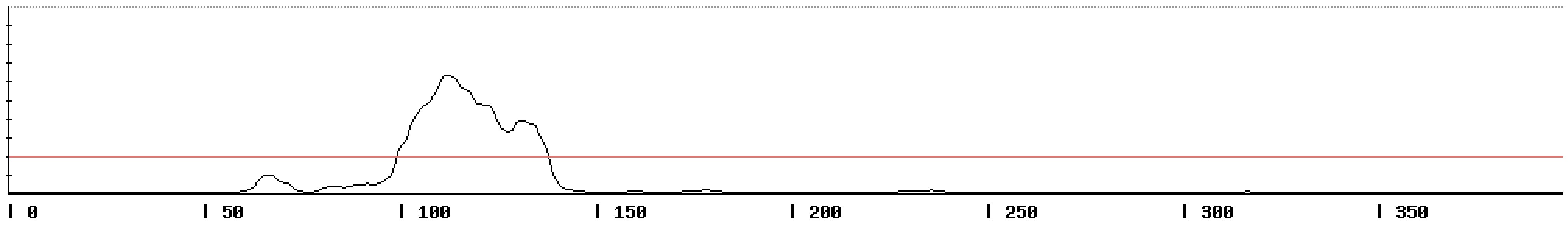

Predictions for Lactucasativa_Lsa006253

Viterbi Path

Viterbi Path

Posterior @ 0.2

Posterior @ 0.2

Posterior Graphic. Threshold Marked @ 0.2

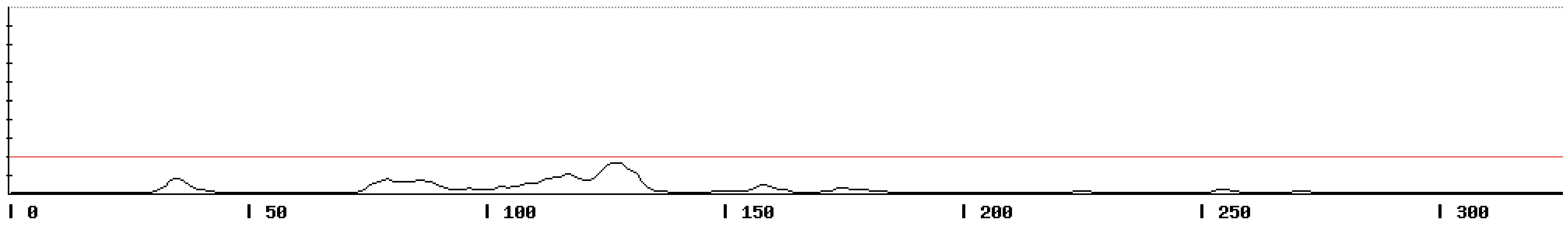

Predictions for Lactucasativa_Lsa006424

Viterbi Path	
Viterbi Path	
Posterior @ 0.2	134 - GTRKRRKIH - 142
Posterior @ 0.2	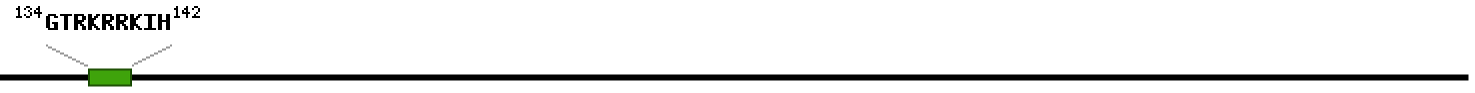
Posterior Graphic. Threshold Marked @ 0.2	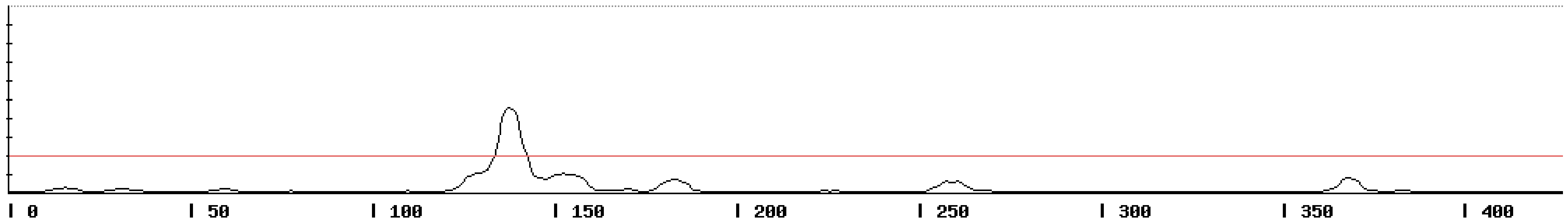

Predictions for Lactucasativa_Lsa006465

Viterbi Path	
Viterbi Path	
Posterior @ 0.2	46 - RKIEKK - 51
	88 - RGRKY - 92
Posterior @ 0.2	

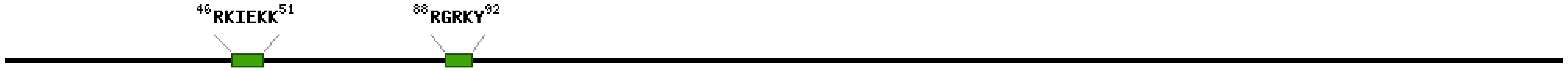

Posterior Graphic. Threshold Marked @ 0.2

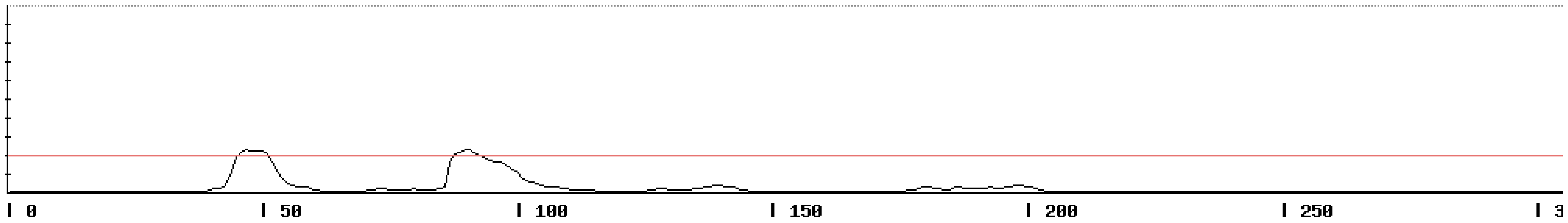

Predictions for Lactucasativa_Lsa006615

Viterbi Path

Viterbi Path

Posterior @ 0.2

Posterior @ 0.2

Posterior Graphic. Threshold Marked @ 0.2

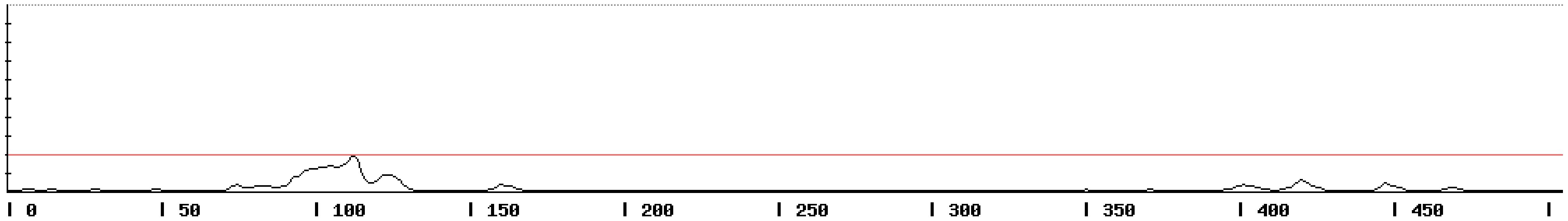

Predictions for Lactucasativa_Lsa006959

Viterbi Path

Viterbi Path

Posterior @ 0.2	
Posterior @ 0.2	
Posterior Graphic. Threshold Marked @ 0.2	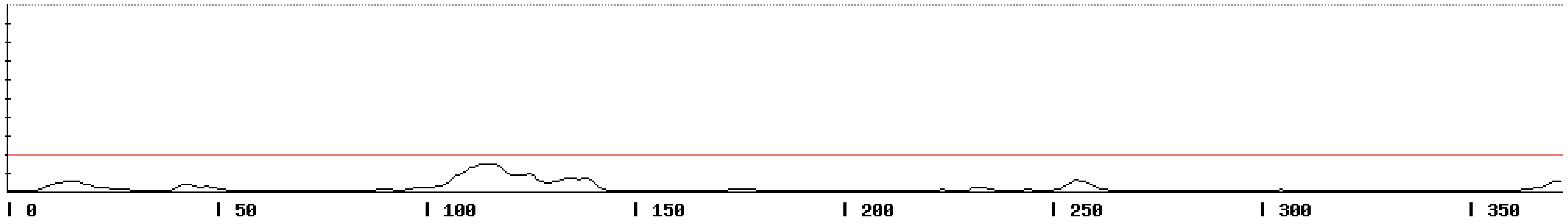

Predictions for Lactucasativa_Lsa007052

Viterbi Path	
Viterbi Path	
Posterior @ 0.2	
Posterior @ 0.2	
Posterior Graphic. Threshold Marked @ 0.2	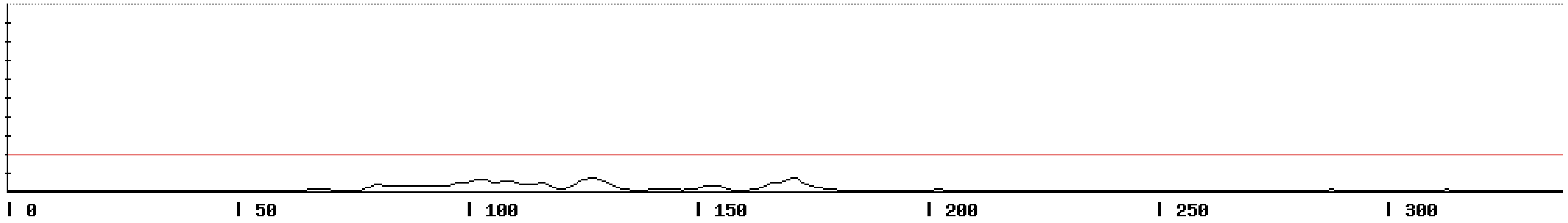

Predictions for Lactucasativa_Lsa007133

Viterbi Path	
Viterbi Path	

Posterior @ 0.2 | 156 - DKPIKRKS - 163

Posterior @ 0.2

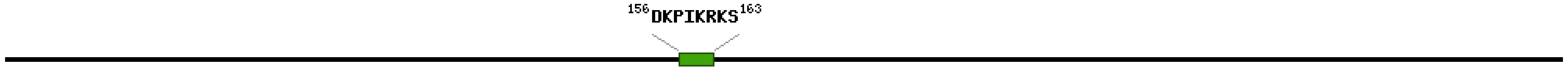

Posterior Graphic. Threshold Marked @ 0.2

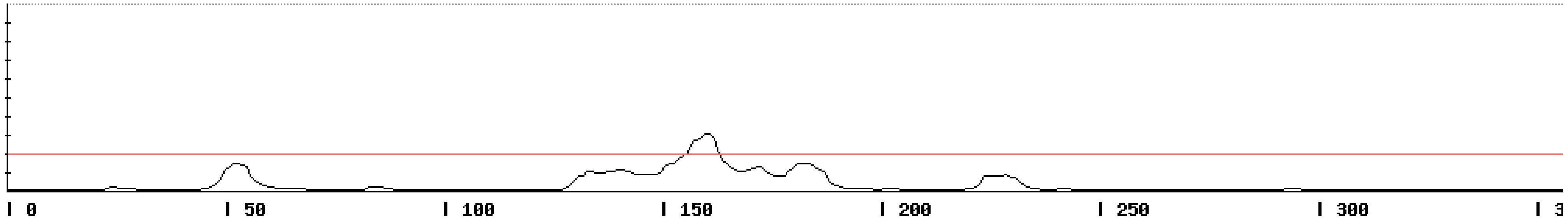

Predictions for Lactucasativa_Lsa007562

Viterbi Path

Viterbi Path

Posterior @ 0.2

Posterior @ 0.2

Posterior Graphic. Threshold Marked @ 0.2

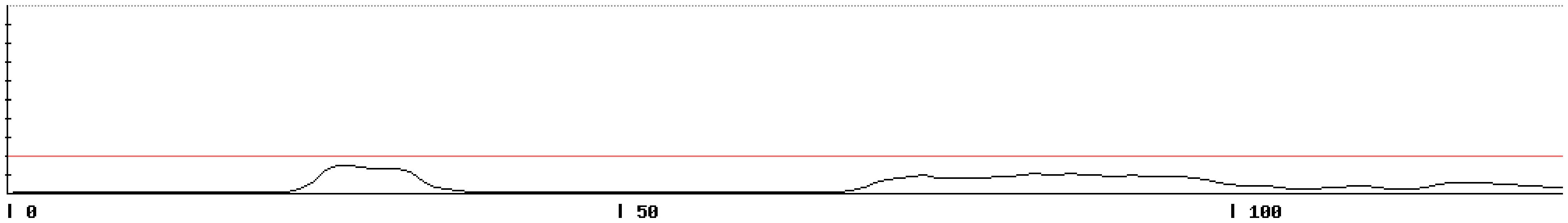

Predictions for Lactucasativa_Lsa007688

Viterbi Path	
Viterbi Path	
Posterior @ 0.2	93 - TGKDRTIKTSRGSRVIGKKKT - 113
Posterior @ 0.2	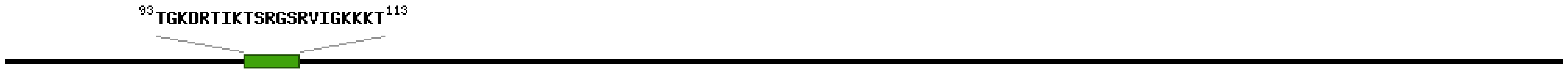
Posterior Graphic. Threshold Marked @ 0.2	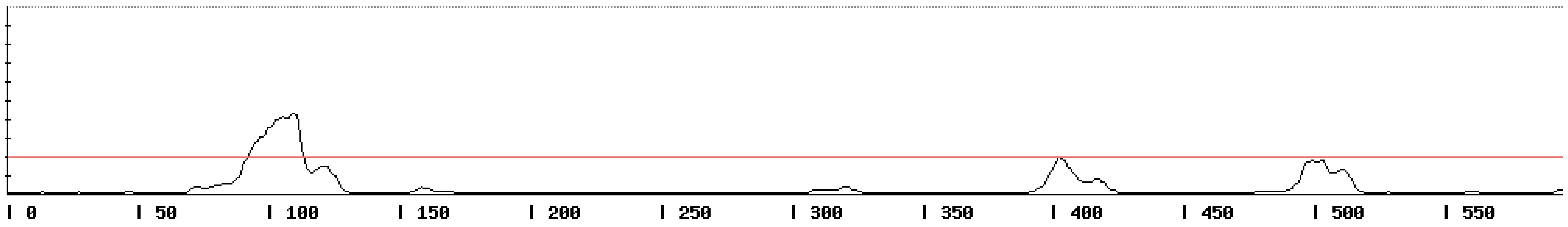

Predictions for Lactucasativa_Lsa008217

Viterbi Path	
Viterbi Path	
Posterior @ 0.2	
Posterior @ 0.2	
Posterior Graphic. Threshold Marked @ 0.2	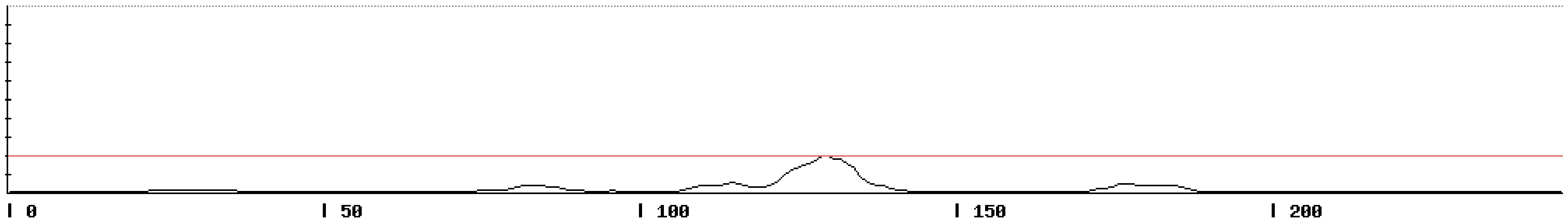

Predictions for Lactucasativa_Lsa008320	
Viterbi Path	
Viterbi Path	
Posterior @ 0.2	
Posterior @ 0.2	
Posterior Graphic. Threshold Marked @ 0.2	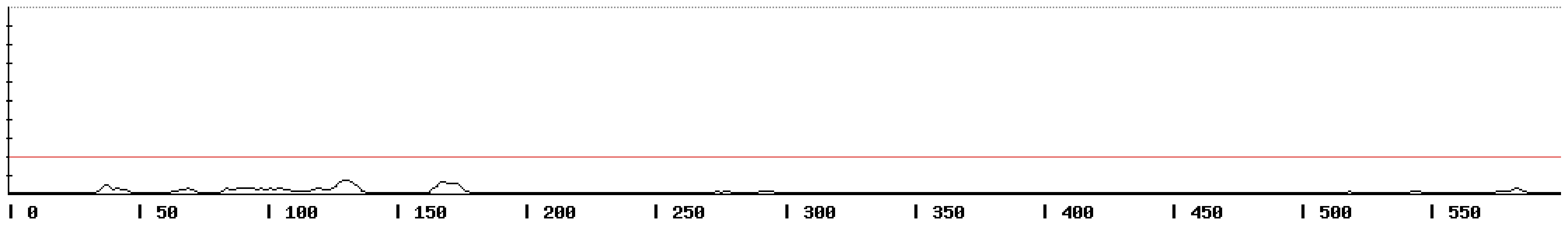

Predictions for Lactucasativa_Lsa008324	
Viterbi Path	
Viterbi Path	
Posterior @ 0.2	
Posterior @ 0.2	
Posterior Graphic. Threshold Marked @ 0.2	

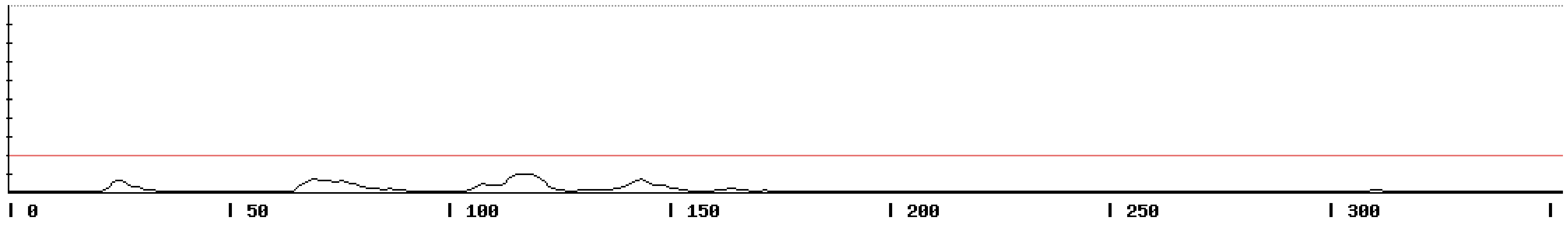

Predictions for Lactucasativa_Lsa008924

Viterbi Path	
Viterbi Path	
Posterior @ 0.2	108 - KATGKDRIIKRKSFPVGIKK - 127
	460 - KP - 461
Posterior @ 0.2	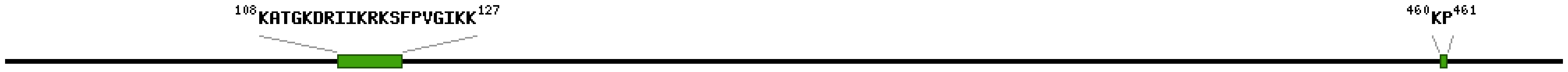
Posterior Graphic. Threshold Marked @ 0.2	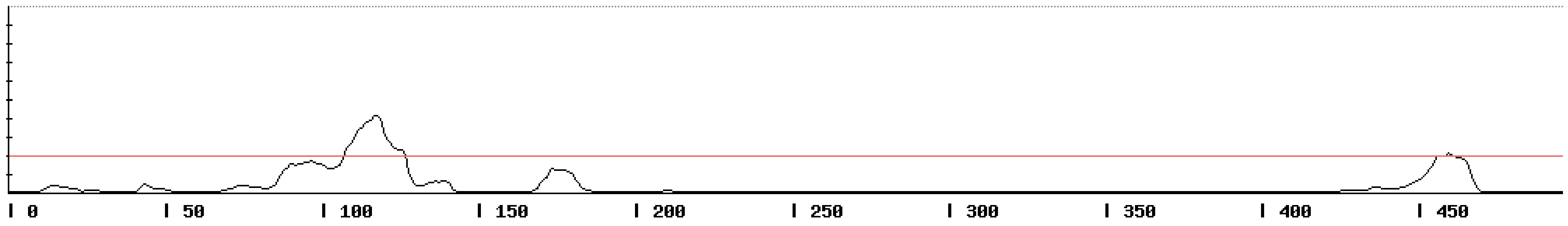

Predictions for Lactucasativa_Lsa009990

Viterbi Path	
Viterbi Path	
Posterior @ 0.2	280 - VRRIKLRRQKGRTH - 293
Posterior @ 0.2	

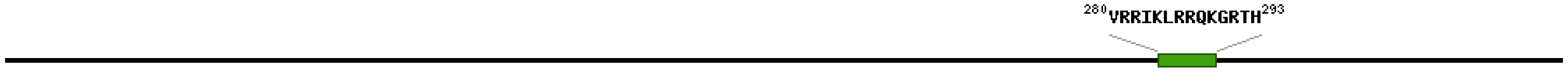

Posterior Graphic. Threshold Marked @ 0.2

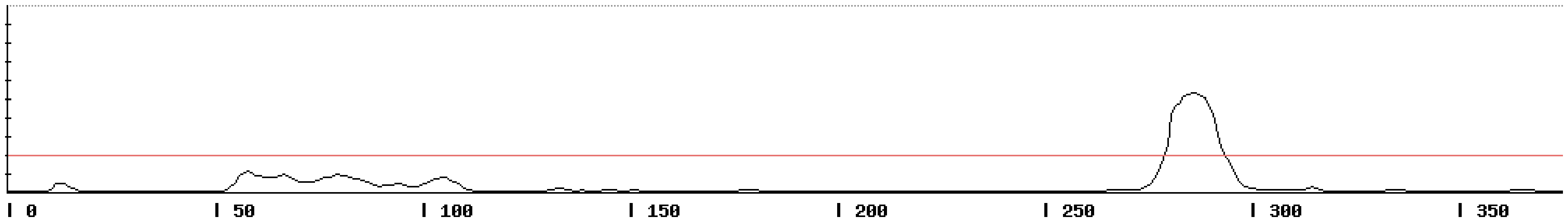

Predictions for Lactucasativa_Lsa010115

Viterbi Path

Viterbi Path

Posterior @ 0.2

Posterior @ 0.2

Posterior Graphic. Threshold Marked @ 0.2

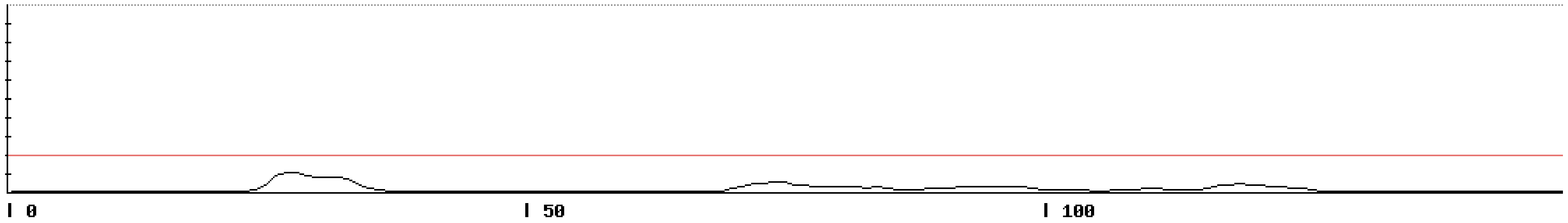

Predictions for Lactucasativa_Lsa010199

Viterbi Path

Viterbi Path

Posterior @ 0.2

Posterior @ 0.2

Posterior Graphic. Threshold Marked @ 0.2

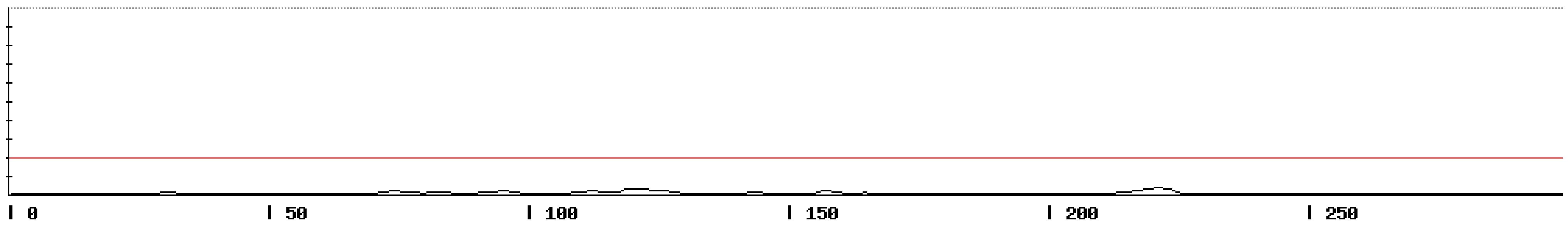

Predictions for Lactucasativa_Lsa011065

Viterbi Path

Viterbi Path

Posterior @ 0.2

Posterior @ 0.2

Posterior Graphic. Threshold Marked @ 0.2

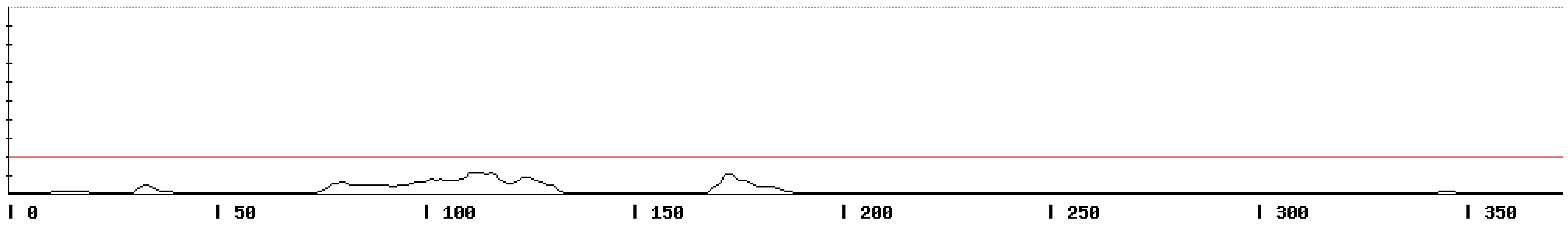

Predictions for Lactucasativa_Lsa011669

Viterbi Path

Viterbi Path

Posterior @ 0.2

Posterior @ 0.2

Posterior Graphic. Threshold Marked @ 0.2

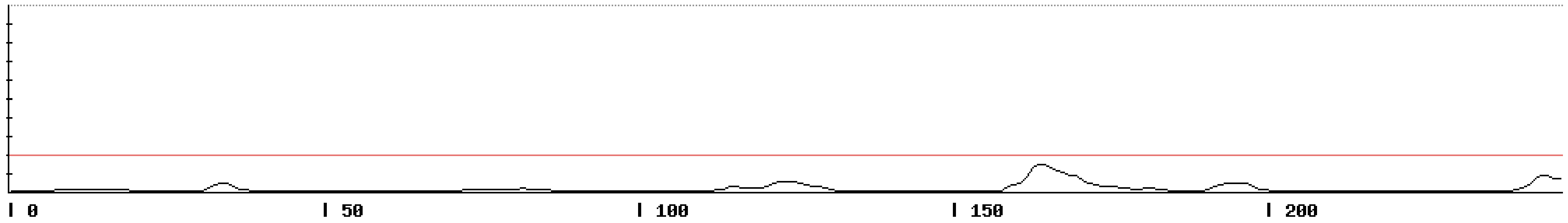

Predictions for Lactucasativa_Lsa011674

Viterbi Path

Viterbi Path

Posterior @ 0.2

94 - GGRSNRGTKHGYWKA - 108

110 - GK - 111

458 - RRVKRSLRPK - 467

Posterior @ 0.2

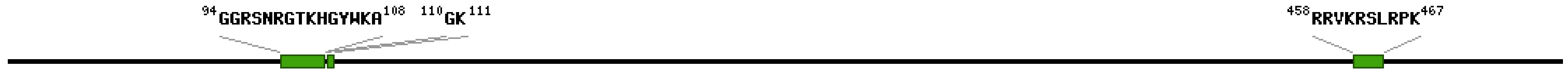

Posterior Graphic. Threshold Marked @ 0.2

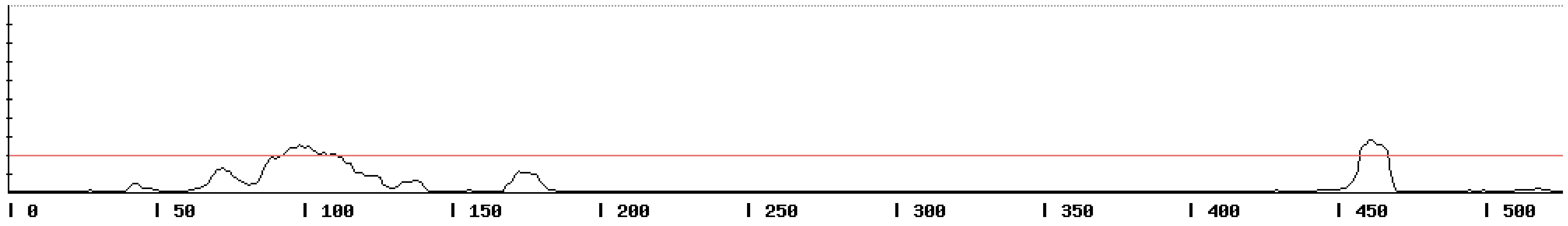

Predictions for Lactucasativa_Lsa014287

Viterbi Path	
Viterbi Path	
Posterior @ 0.2	
Posterior @ 0.2	
Posterior Graphic. Threshold Marked @ 0.2	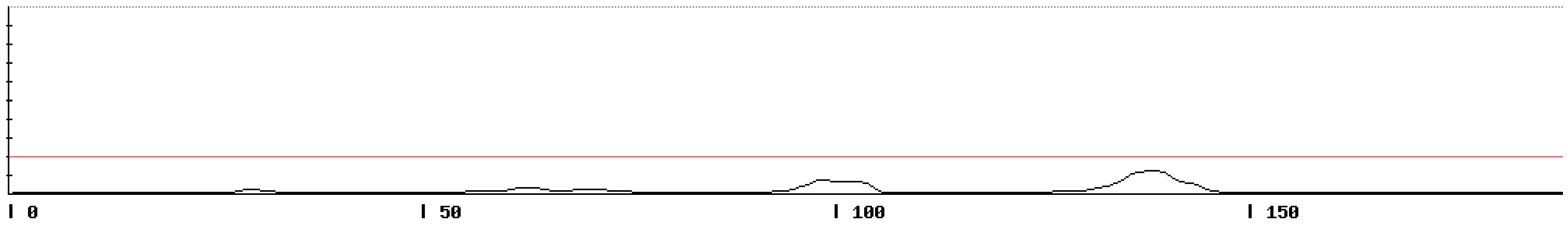

Predictions for Lactucasativa_Lsa014343

Viterbi Path	
Viterbi Path	
Posterior @ 0.2	
Posterior @ 0.2	

Posterior Graphic. Threshold Marked @ 0.2

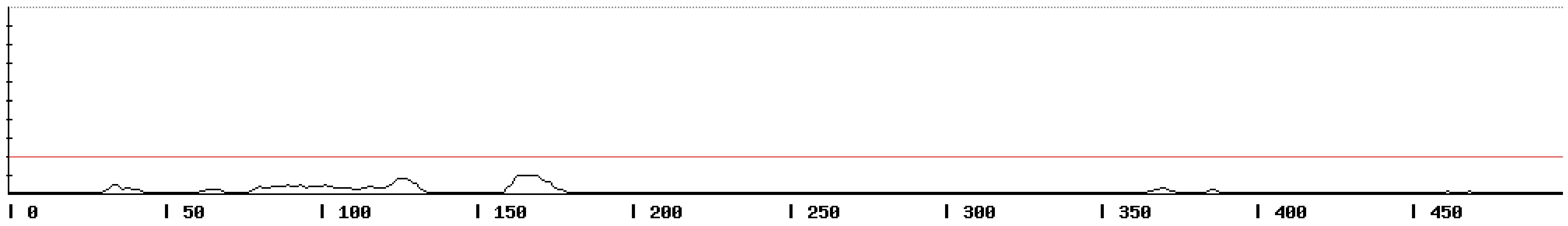

Predictions for Lactucasativa_Lsa014378

Viterbi Path

Viterbi Path

Posterior @ 0.2

Posterior @ 0.2

Posterior Graphic. Threshold Marked @ 0.2

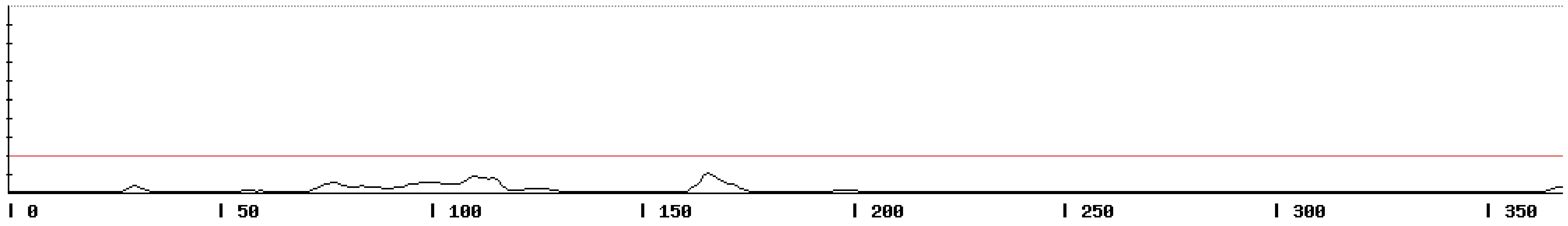

Predictions for Lactucasativa_Lsa014428

Viterbi Path

Viterbi Path

Posterior @ 0.2

Posterior @ 0.2

Posterior Graphic. Threshold Marked @ 0.2

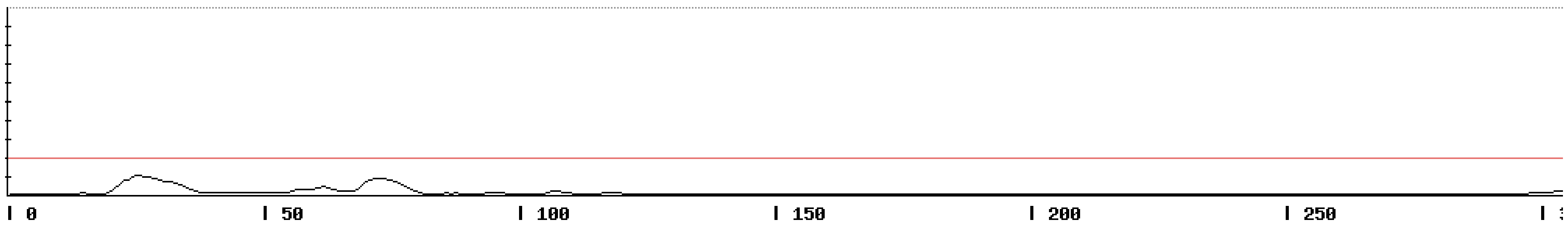

Predictions for Lactucasativa_Lsa014722

Viterbi Path

Viterbi Path

Posterior @ 0.2 — 131 - GQRKRRKISKESG - 143

Posterior @ 0.2

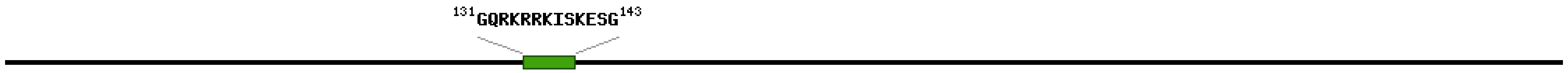

Posterior Graphic. Threshold Marked @ 0.2

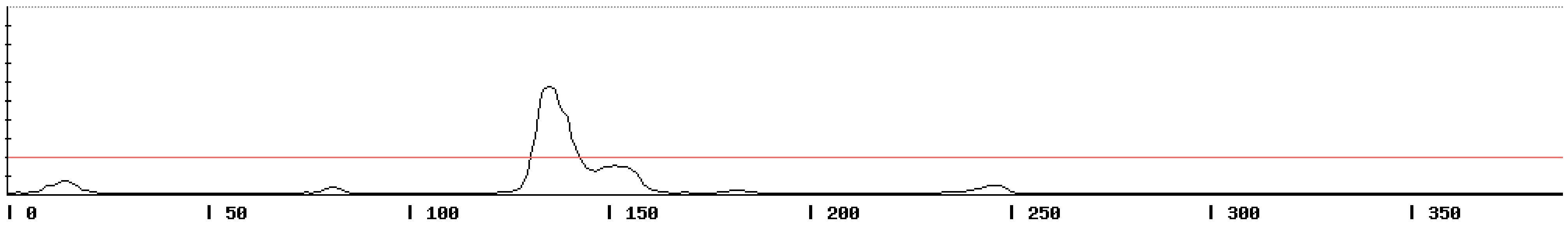

Predictions for Lactucasativa_Lsa014934

Viterbi Path

Viterbi Path

Posterior @ 0.2

Posterior @ 0.2

Posterior Graphic. Threshold Marked @ 0.2

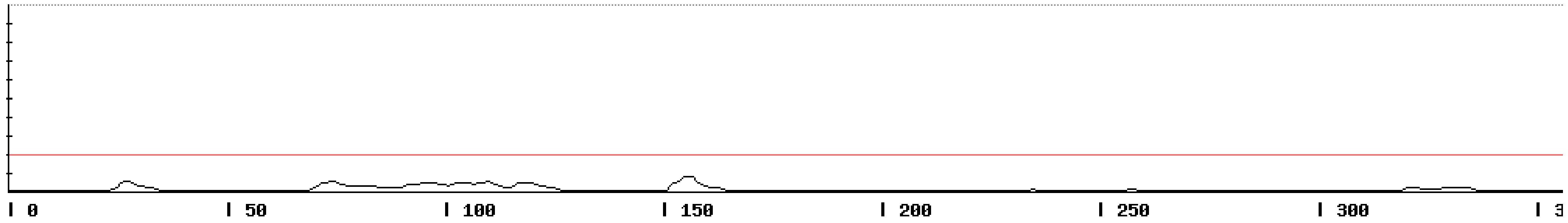

Predictions for Lactucasativa_Lsa015455

Viterbi Path

Viterbi Path

Posterior @ 0.2

Posterior @ 0.2

Posterior Graphic. Threshold Marked @ 0.2

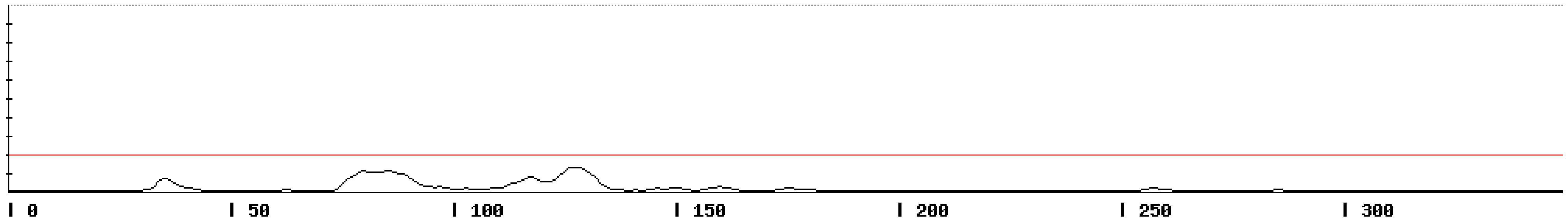

Predictions for Lactucasativa_Lsa015516

Viterbi Path

Viterbi Path

Posterior @ 0.2

Posterior @ 0.2

Posterior Graphic. Threshold Marked @ 0.2

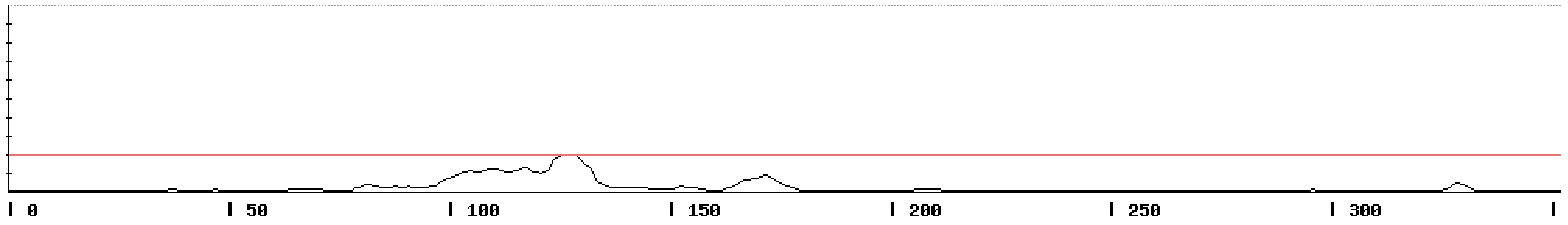

Predictions for Lactucasativa_Lsa015559

Viterbi Path

Viterbi Path

Posterior @ 0.2 | 72 - RKYPNGSRPNRAAGTGYWKATGADKPIGKPKPVGIKKALVFYAGKAPR - 119

Posterior @ 0.2

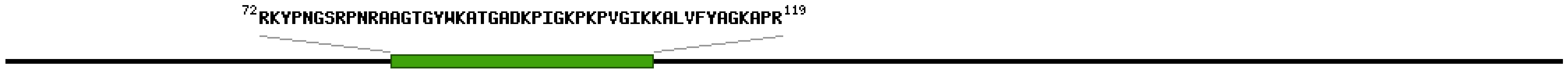

Posterior Graphic. Threshold Marked @ 0.2

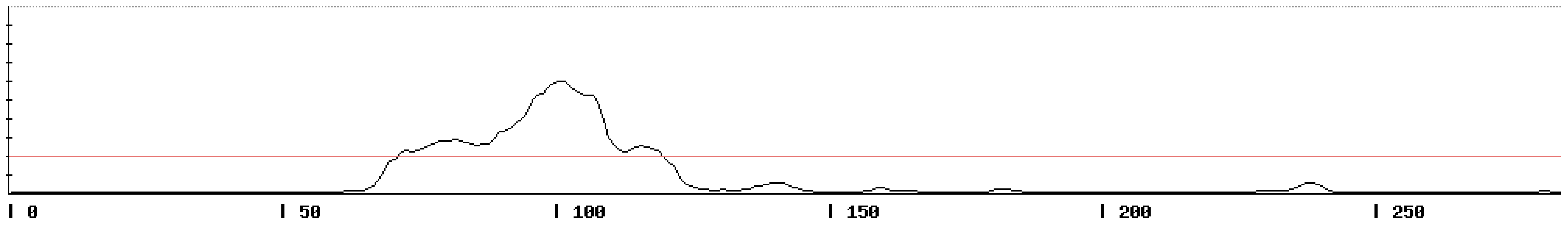

Predictions for Lactucasativa_Lsa016181

Viterbi Path

Viterbi Path

Posterior @ 0.2

Posterior @ 0.2

Posterior Graphic. Threshold Marked @ 0.2

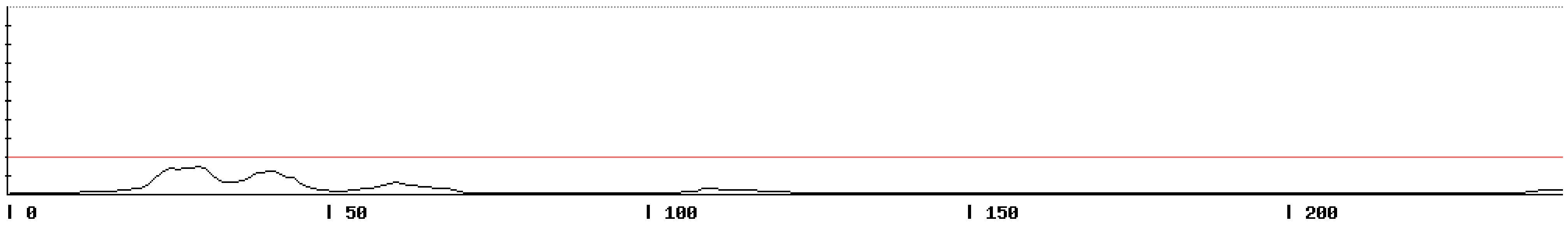

Predictions for Lactucasativa_Lsa016324

Viterbi Path

Viterbi Path

Posterior @ 0.2

Posterior @ 0.2

Posterior Graphic. Threshold Marked @ 0.2

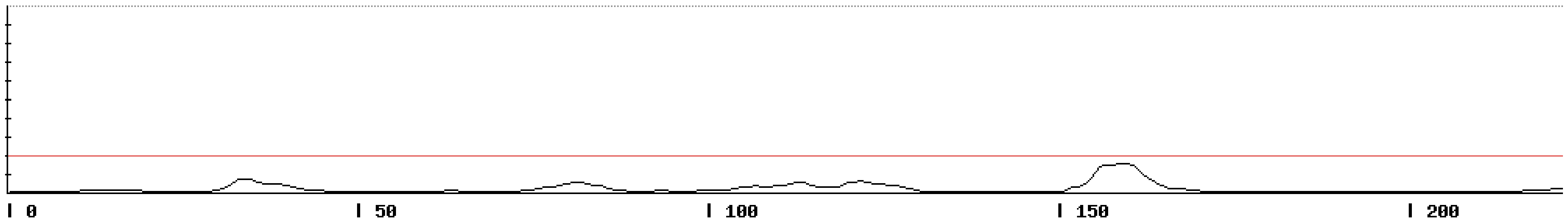

Predictions for Lactucasativa_Lsa016895	
Viterbi Path	
Viterbi Path	
Posterior @ 0.2	
Posterior @ 0.2	
Posterior Graphic. Threshold Marked @ 0.2	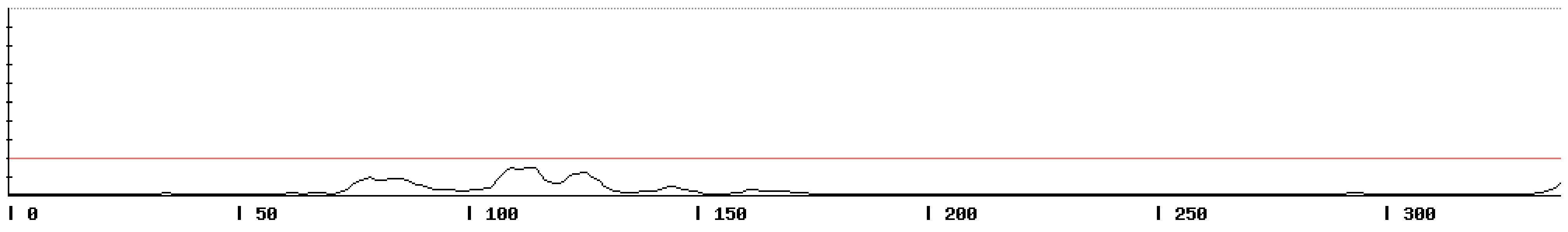

Predictions for Lactucasativa_Lsa017437	
Viterbi Path	
Viterbi Path	
Posterior @ 0.2	
Posterior @ 0.2	
Posterior Graphic. Threshold Marked @ 0.2	

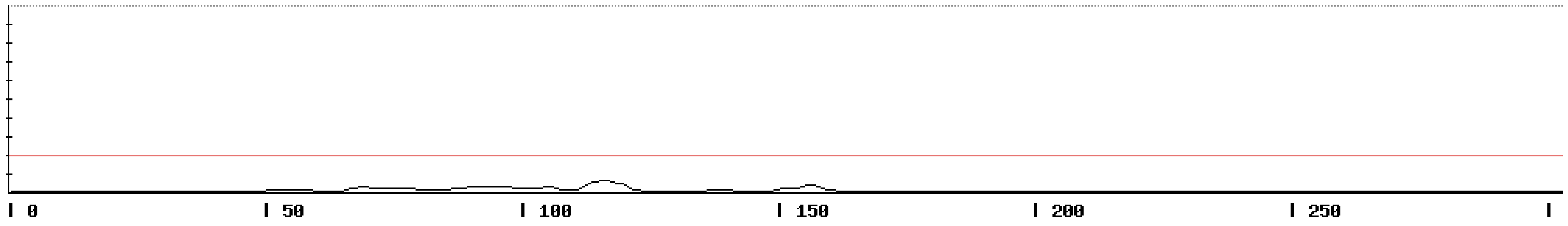

Predictions for Lactucasativa_Lsa017607

Viterbi Path	
Viterbi Path	
Posterior @ 0.2	128 - K - 128
	131 - TTGTRKRRKVHMDAERGETRWHKTGKTR - 158
Posterior @ 0.2	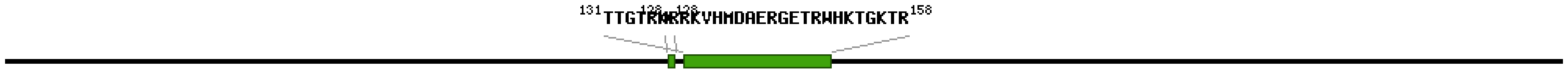
Posterior Graphic. Threshold Marked @ 0.2	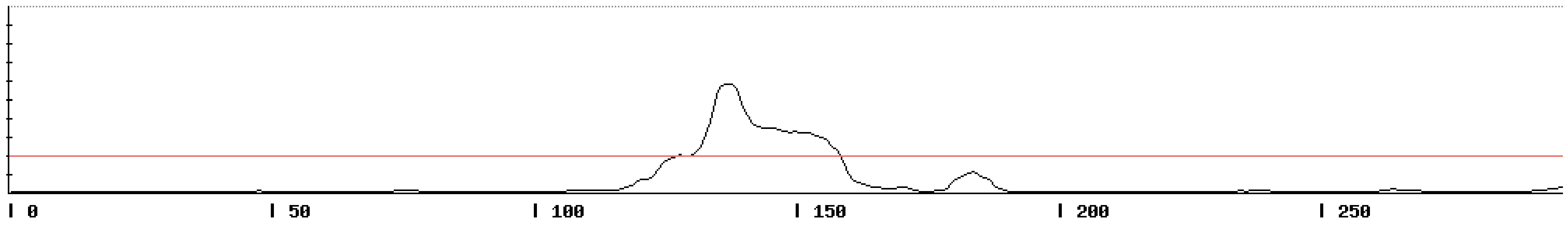

Predictions for Lactucasativa_Lsa017657

Viterbi Path	
Viterbi Path	
Posterior @ 0.2	106 - K - 106
	109 - WKATGKDRSVRHK - 121
Posterior @ 0.2	

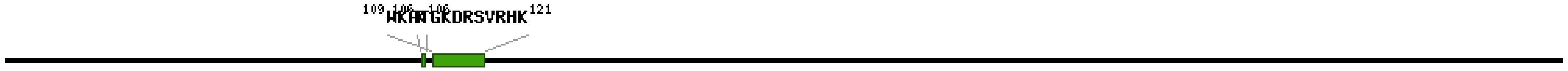

Posterior Graphic. Threshold Marked @ 0.2

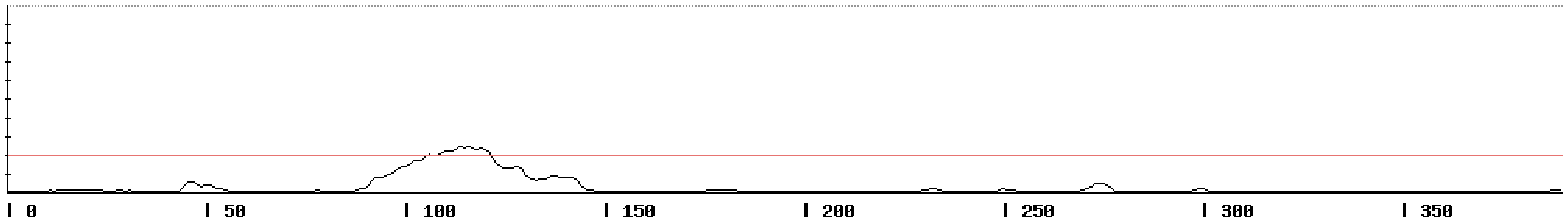

Predictions for Lactucasativa_Lsa018128

Viterbi Path

Viterbi Path

Posterior @ 0.2

128 - KAYTTGTRKRRKVHSEIDGSETRWHKTGKTRPVFLKTKVKGYKK - 171

Posterior @ 0.2

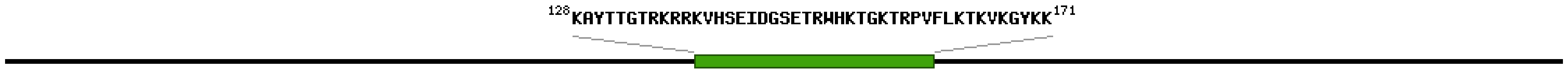

Posterior Graphic. Threshold Marked @ 0.2

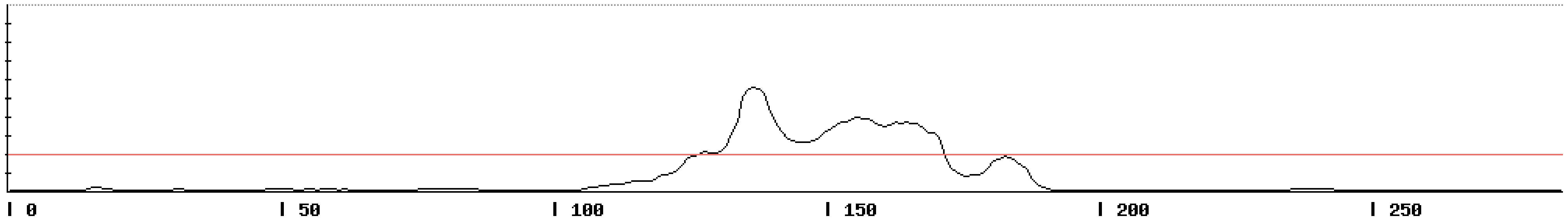

Predictions for Lactucasativa_Lsa018582

Viterbi Path

Viterbi Path

Posterior @ 0.2	
Posterior @ 0.2	
Posterior Graphic. Threshold Marked @ 0.2	

Revision r.9 - Last Updated on : February 24 2015 17:45:20.
alex.nguyenba@utoronto.ca

Intended to be used with Firefox

Viterbi Path

Posterior @ 0.2

Posterior @ 0.2

Posterior Graphic. Threshold Marked @ 0.2

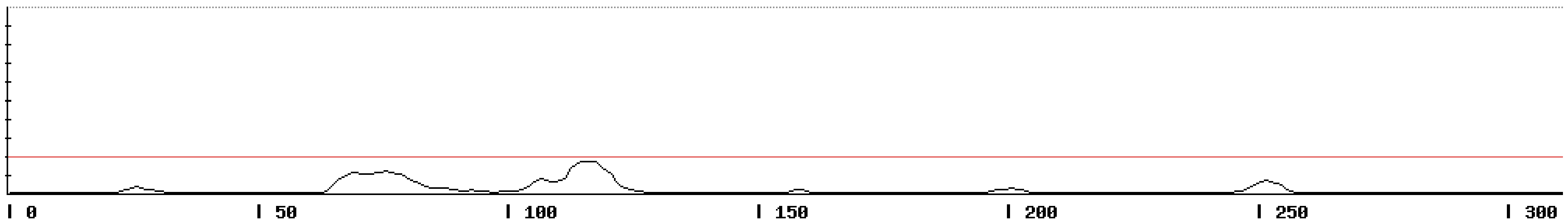

Predictions for Leersiaperrieri_LPERR01G00270.1

Viterbi Path

Viterbi Path

Posterior @ 0.2

Posterior @ 0.2

Posterior Graphic. Threshold Marked @ 0.2

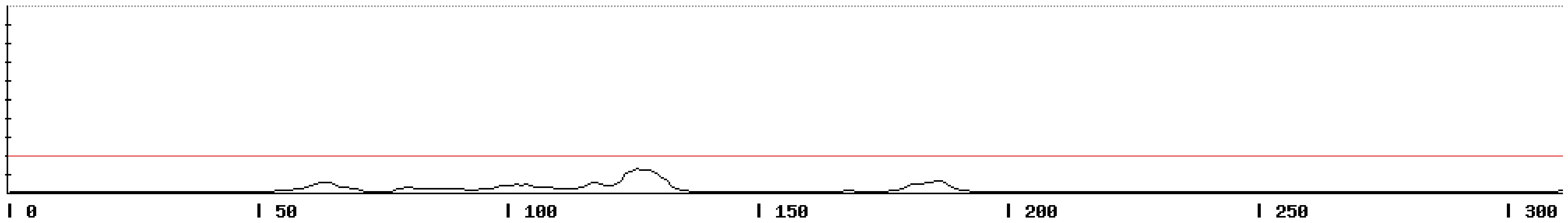

Predictions for Leersiaperrieri_LPERR01G05530.1

Viterbi Path

Viterbi Path

Posterior @ 0.2 168 - GTRKRRK - 174

Posterior @ 0.2

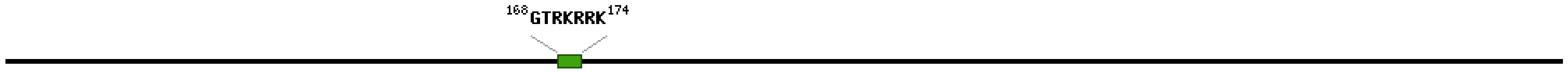

Posterior Graphic. Threshold Marked @ 0.2

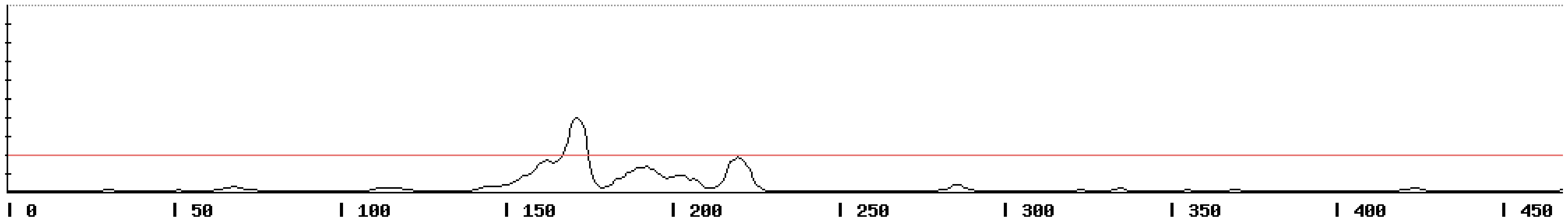

Predictions for Leersiaperrieri_LPERR01G08400.1

Viterbi Path

Viterbi Path

Posterior @ 0.2

Posterior @ 0.2

Posterior Graphic. Threshold Marked @ 0.2

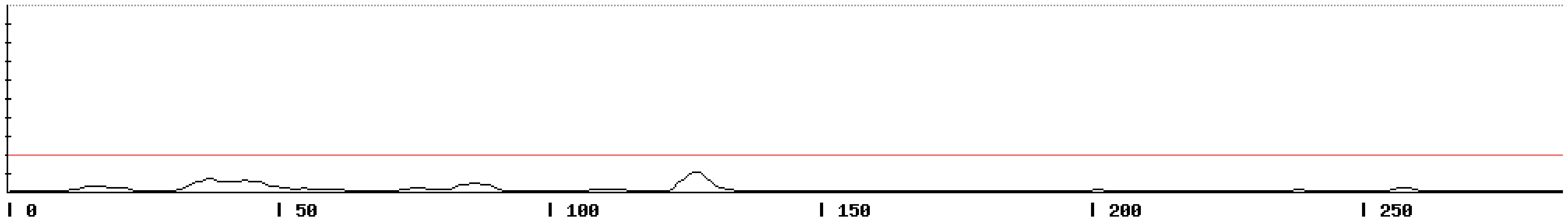

Predictions for Leersiaperrieri_LPERR01G08530.1

Viterbi Path	
Viterbi Path	
Posterior @ 0.2	
Posterior @ 0.2	
Posterior Graphic. Threshold Marked @ 0.2	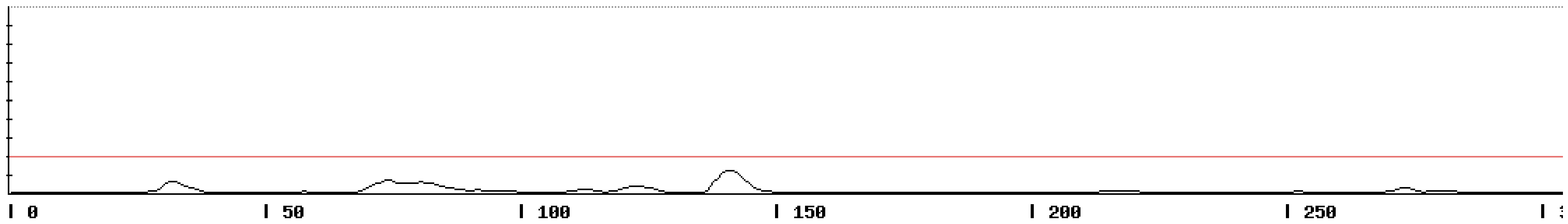

Predictions for Leersiaperrieri_LPERR01G09570.1

Viterbi Path	
Viterbi Path	
Posterior @ 0.2	99 - RAIKVDKR - 106
Posterior @ 0.2	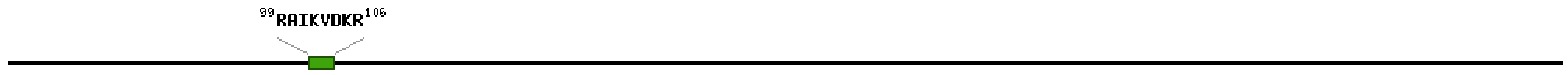
Posterior Graphic. Threshold Marked @ 0.2	

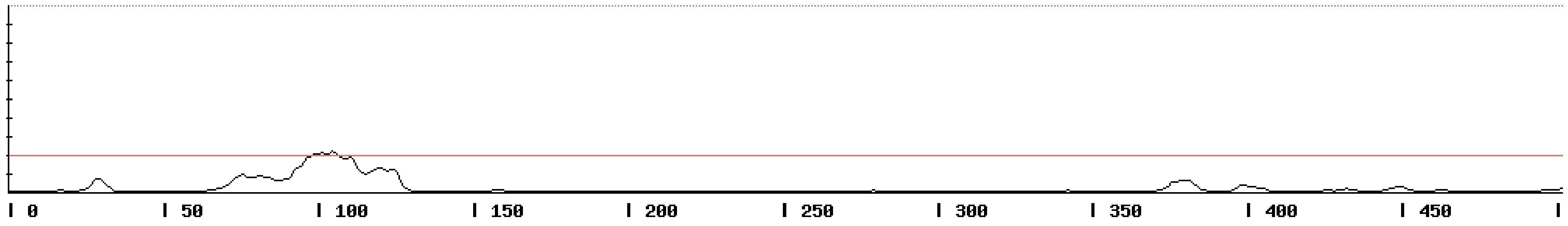

Predictions for Leersiaperrieri_LPERR01G14420.1

Viterbi Path	
Viterbi Path	
Posterior @ 0.2	96 - LVARHGGRADRRRGEAFYFAPRWRRRRHVLRT - 127
	184 - RSRSRRLTGAPTRRRR - 199
Posterior @ 0.2	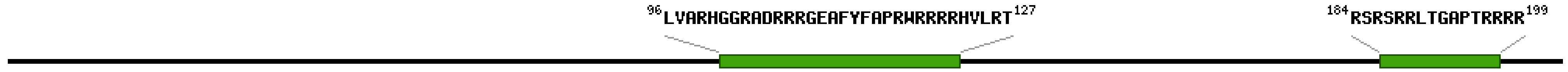
Posterior Graphic. Threshold Marked @ 0.2	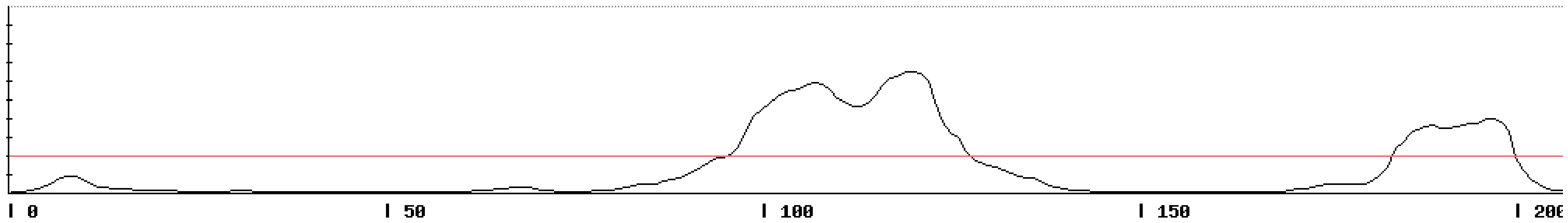

Predictions for Leersiaperrieri_LPERR01G22040.1

Viterbi Path	
Viterbi Path	
Posterior @ 0.2	5 - SSRPRRRCRAPRRRE - 19
	97 - KGGGQRK - 103
	182 - KNHVTAKKRKNLATTASPPPRHRRRRAPSPPEEEEEKK - 219

Posterior @ 0.2	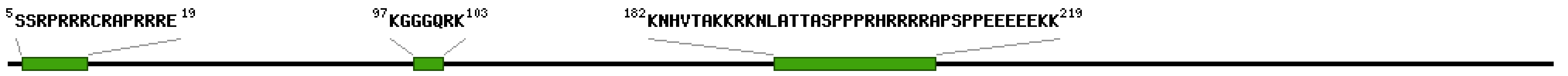
Posterior Graphic. Threshold Marked @ 0.2	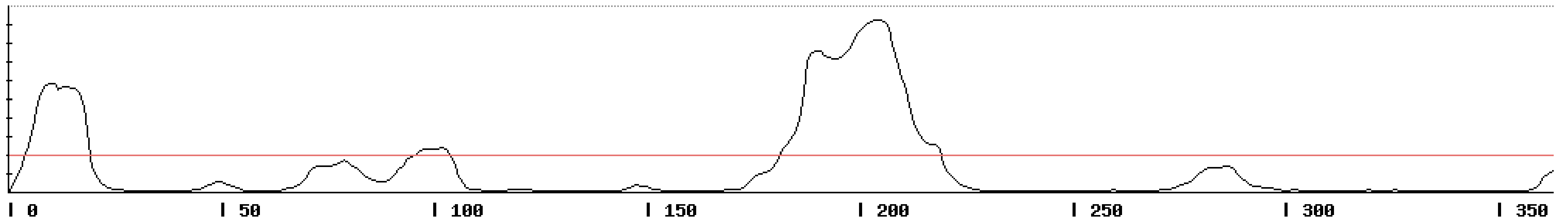

Predictions for Leersiaperrieri_LPERR01G22300.1	
Viterbi Path	
Viterbi Path	
Posterior @ 0.2	153 - K - 153
	156 - TTGTRKRRKVHTDEQGGETRWHKTGKTRPVFTGGKLKGYKK - 196
Posterior @ 0.2	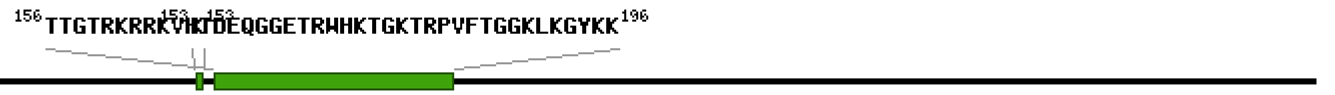
Posterior Graphic. Threshold Marked @ 0.2	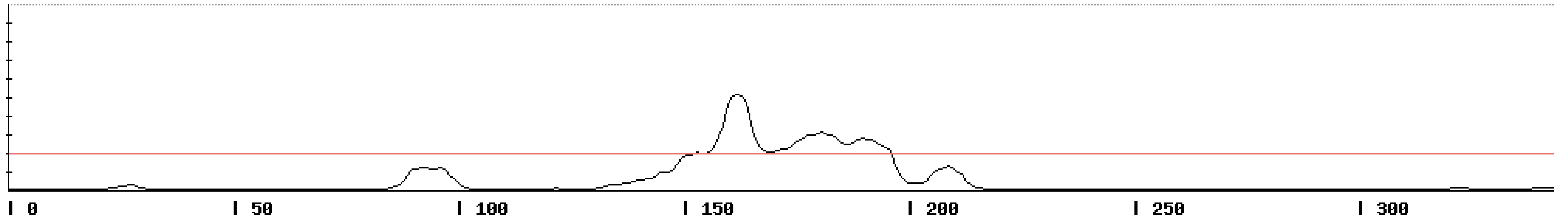

Predictions for Leersiaperrieri_LPERR01G22560.1	
Viterbi Path	
Viterbi Path	

Posterior @ 0.2 — 223 - AAKEAAAAATGCKRKADGKSSGARRGKRL - 251

Posterior @ 0.2

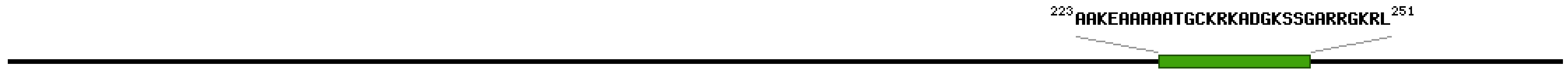

Posterior Graphic. Threshold Marked @ 0.2

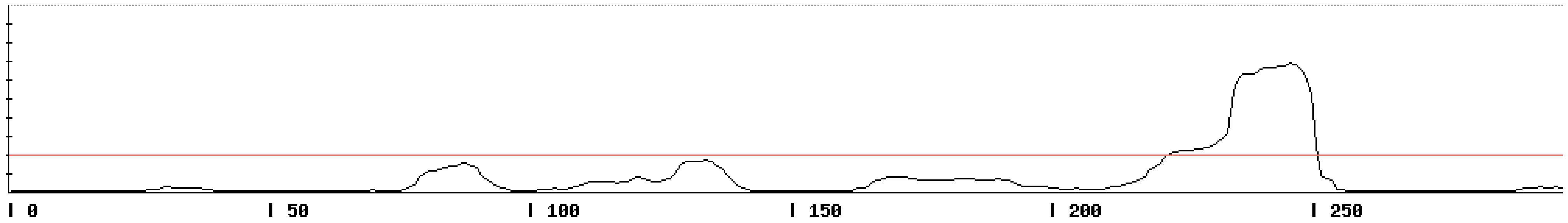

Predictions for Leersiaperrieri_LPERR01G29910.1

Viterbi Path

Viterbi Path

Posterior @ 0.2 — 205 - SPRNRKYPRGKRPSRN - 220

Posterior @ 0.2

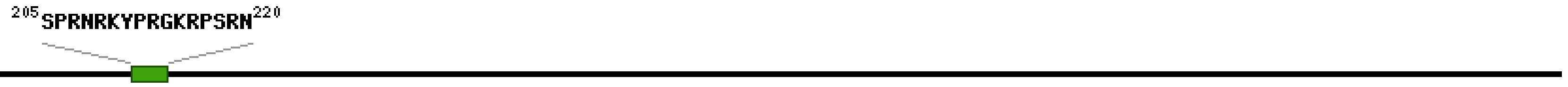

Posterior Graphic. Threshold Marked @ 0.2

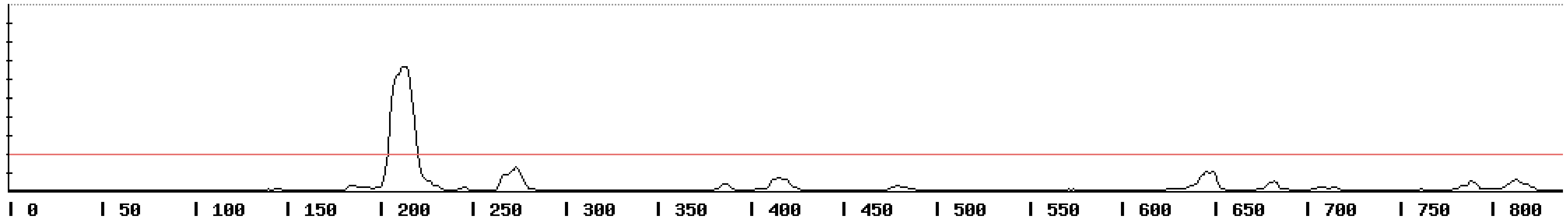

Predictions for Leersiaperrieri_LPERR01G30200.1

Viterbi Path	
Viterbi Path	
Posterior @ 0.2	83 - RDRKYPNGSRPNRAAGRGYWKATGADKPVAPKGSARTVGIKKA - 125
Posterior @ 0.2	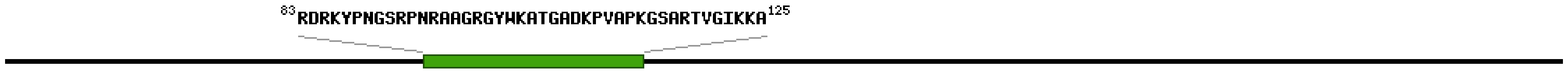
Posterior Graphic. Threshold Marked @ 0.2	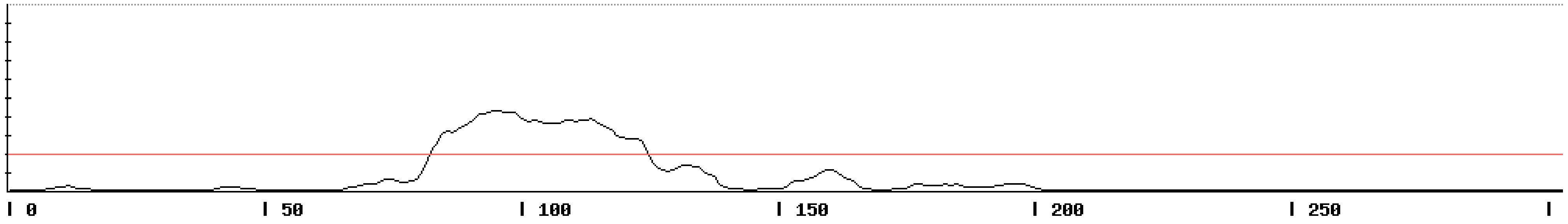

Predictions for Leersiaperrieri_LPERR01G32790.1

Viterbi Path	
Viterbi Path	
Posterior @ 0.2	
Posterior @ 0.2	
Posterior Graphic. Threshold Marked @ 0.2	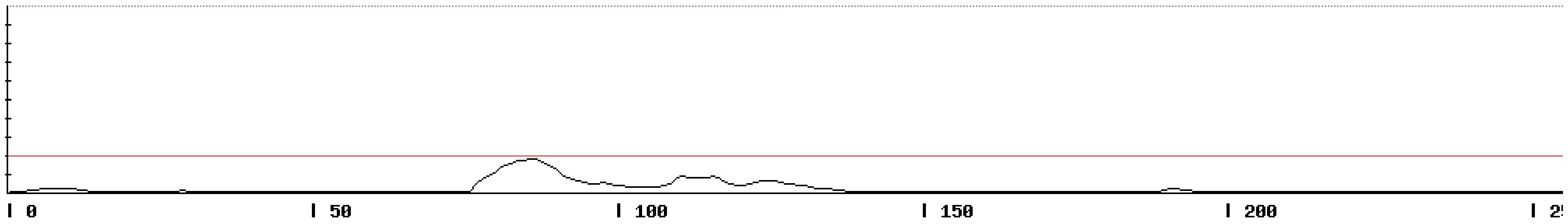

Predictions for Leersiaperrieri_LPERR01G34090.1	
Viterbi Path	
Viterbi Path	
Posterior @ 0.2	147 - GSPKPVAIKK - 156
Posterior @ 0.2	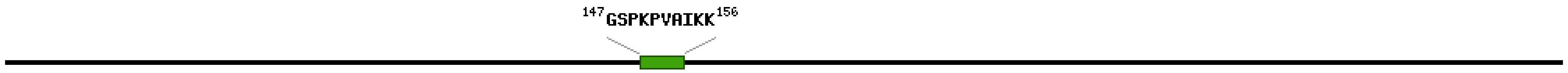
Posterior Graphic. Threshold Marked @ 0.2	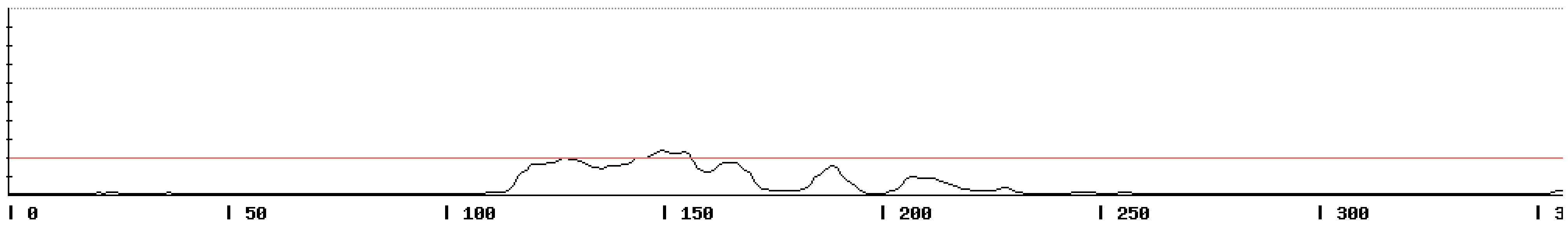

Predictions for Leersiaperrieri_LPERR01G34330.1	
Viterbi Path	
Viterbi Path	
Posterior @ 0.2	
Posterior @ 0.2	
Posterior Graphic. Threshold Marked @ 0.2	

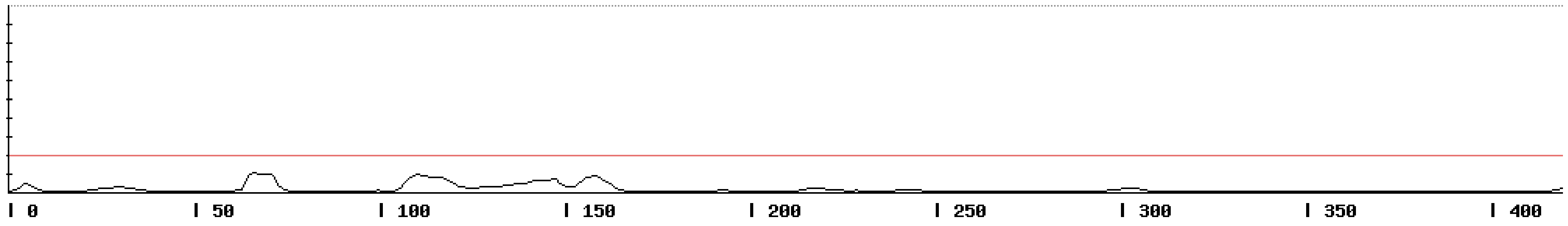

Predictions for Leersiaperrieri_LPERR01G36990.1

Viterbi Path

Viterbi Path

Posterior @ 0.2

87 - RKGGGGGRAGGGGGGGGVWRASGKEKL - 113

176 - RRSPPRVRRR - 185

Posterior @ 0.2

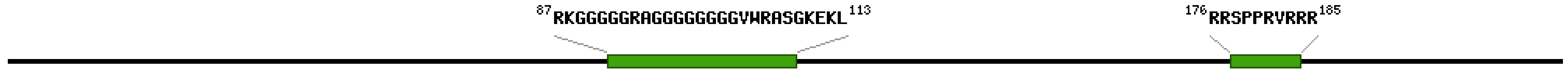

Posterior Graphic. Threshold Marked @ 0.2

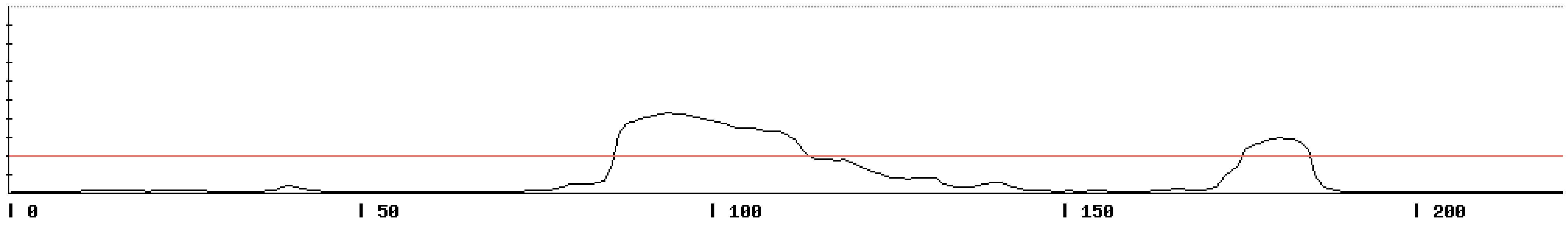

Predictions for Leersiaperrieri_LPERR01G38080.1

Viterbi Path

Viterbi Path

Posterior @ 0.2

72 - PRARASRRKQSVRRTI - 87

172 - RSPAVRDSTSAAAPAKSRRRKAAVA - 196

Posterior @ 0.2

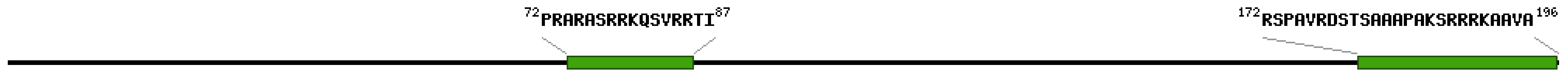

Posterior Graphic. Threshold Marked @ 0.2

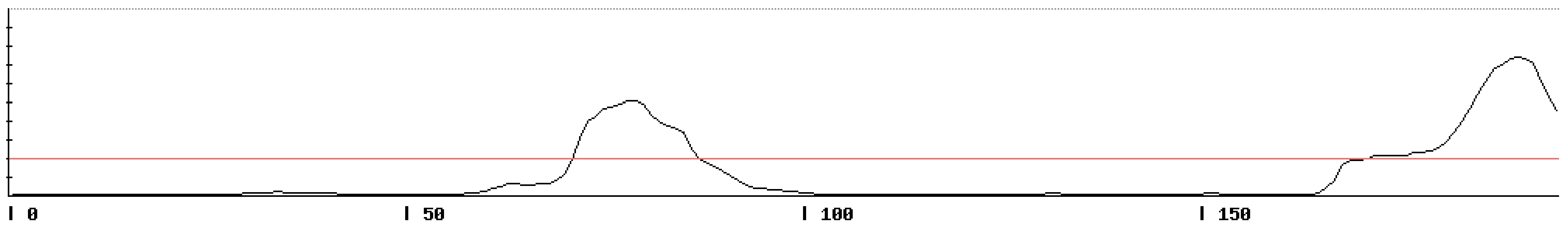

Predictions for Leersiaperrieri_LPERR02G04310.1

Viterbi Path

Viterbi Path

Posterior @ 0.2

Posterior @ 0.2

Posterior Graphic. Threshold Marked @ 0.2

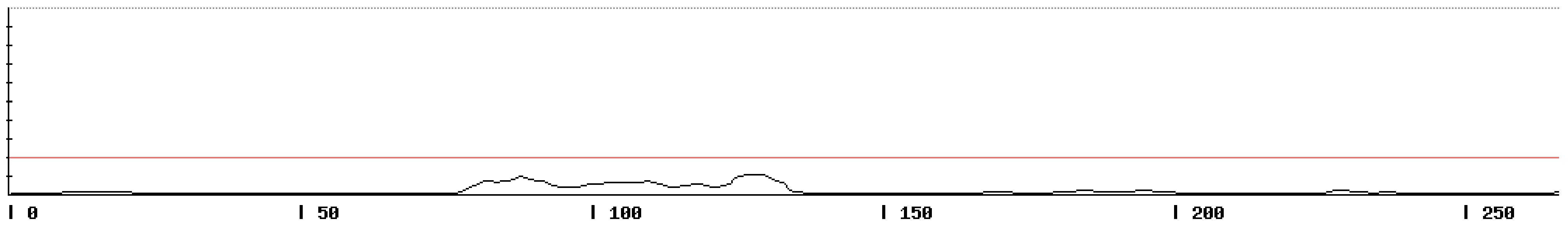

Predictions for Leersiaperrieri_LPERR02G09170.1

Viterbi Path

Viterbi Path

Posterior @ 0.2 | 114 - RR - 115

Posterior @ 0.2

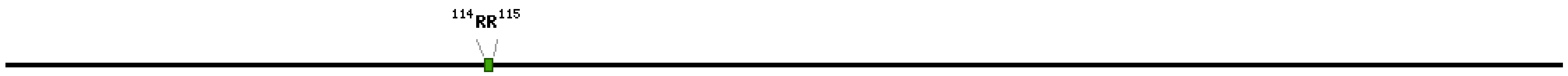

Posterior Graphic. Threshold Marked @ 0.2

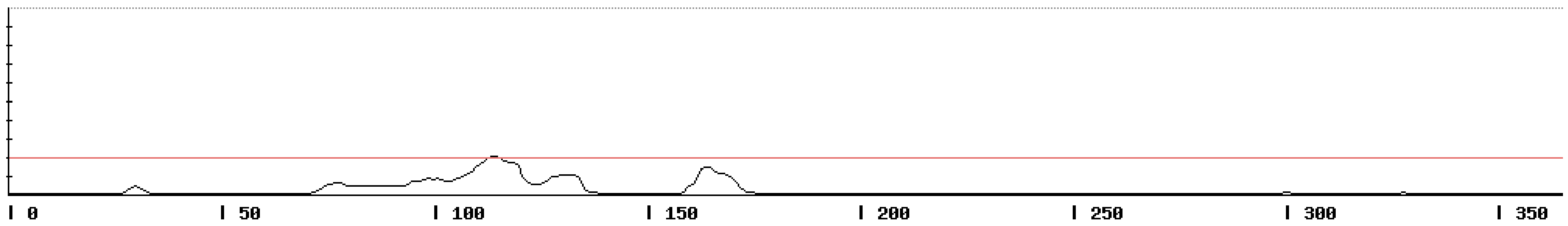

Predictions for Leersiaperrieri_LPERR02G10500.1

Viterbi Path

Viterbi Path

Posterior @ 0.2 | 77 - GGKGRQPRSVAGGGRWVK - 94

Posterior @ 0.2

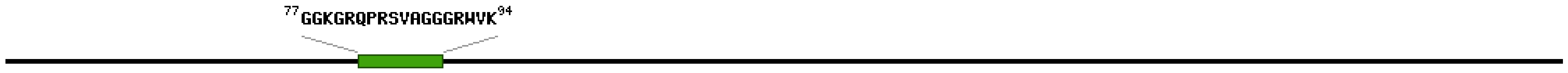

Posterior Graphic. Threshold Marked @ 0.2

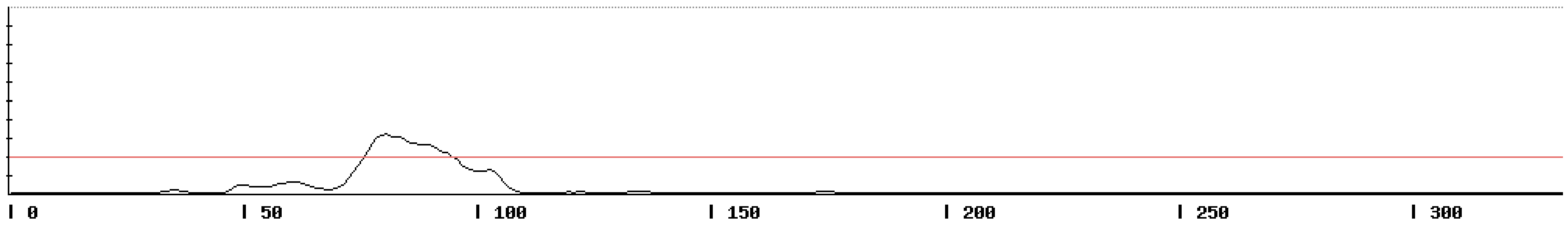

Predictions for Leersiaperrieri_LPERR02G10510.1

Viterbi Path

Viterbi Path

Posterior @ 0.2	76 - PASGRGGRRQVSRSVAGGGKWIKQRRRKAFAWEKYSLSFHRRARGG - 121

Posterior @ 0.2

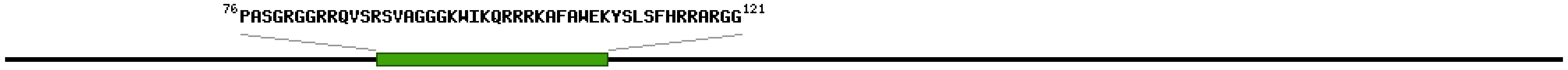

Posterior Graphic. Threshold Marked @ 0.2

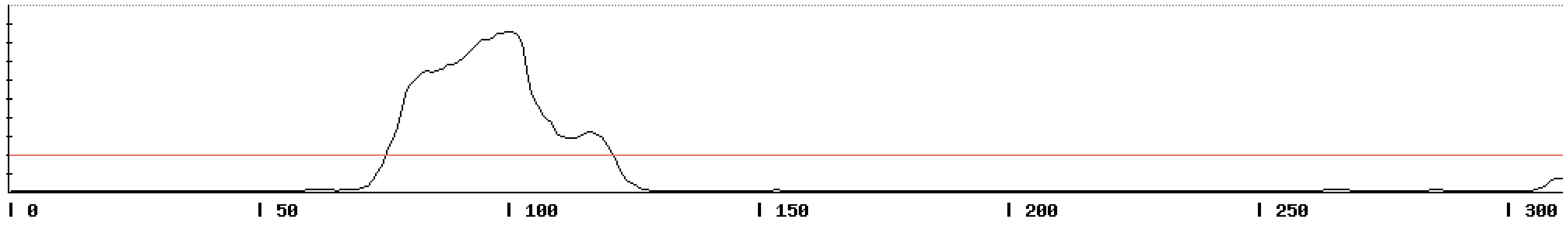

Predictions for Leersiaperrieri_LPERR02G10910.1

Viterbi Path

Viterbi Path

Posterior @ 0.2	160 - GKGRNRKR - 167

Posterior @ 0.2

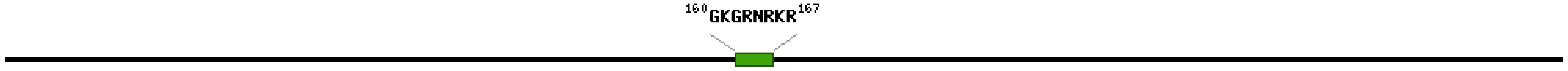

Posterior Graphic. Threshold Marked @ 0.2

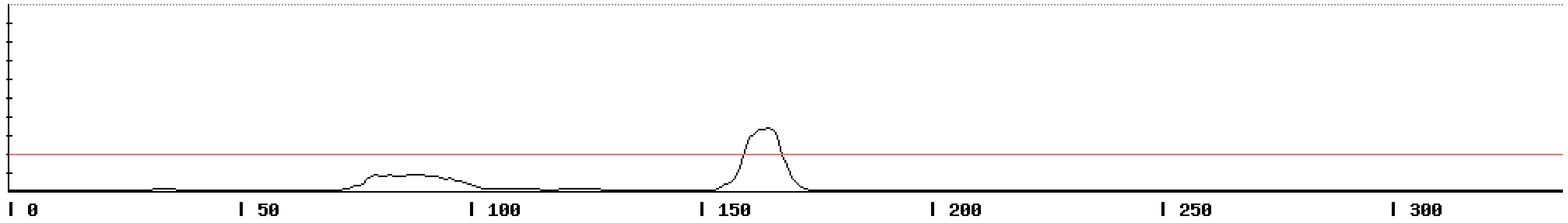

Predictions for Leersiaperrieri_LPERR02G10920.1

Viterbi Path

Viterbi Path

Posterior @ 0.2

Posterior @ 0.2

Posterior Graphic. Threshold Marked @ 0.2

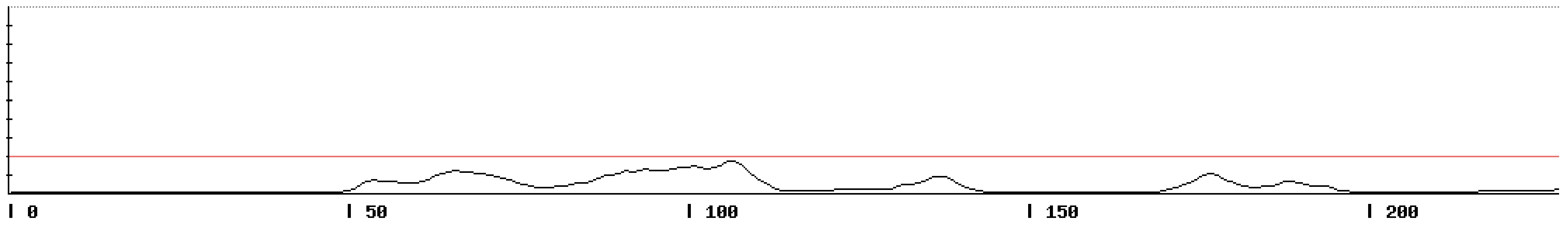

Predictions for Leersiaperrieri_LPERR02G16450.1

Viterbi Path

Viterbi Path

Posterior @ 0.2 | 143 - NRKSHRKRGH - 152

Posterior @ 0.2

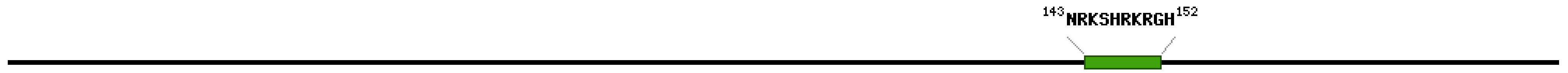

Posterior Graphic. Threshold Marked @ 0.2

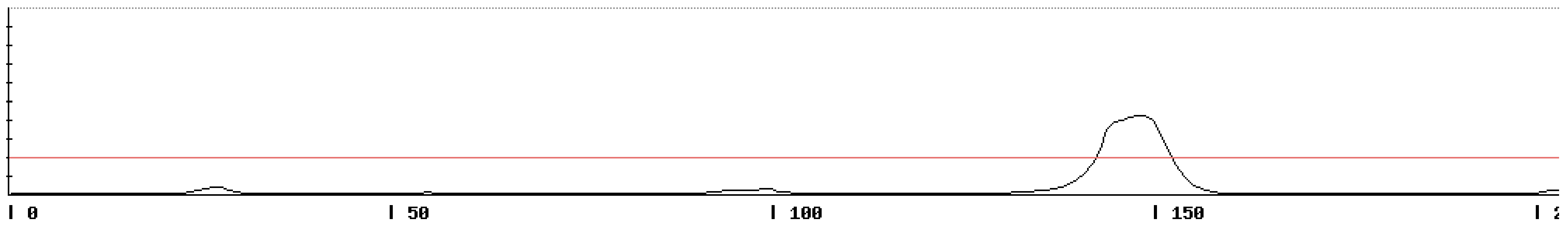

Predictions for Leersiaperrieri_LPERR02G17680.1

Viterbi Path	
Viterbi Path	
Posterior @ 0.2	
Posterior @ 0.2	
Posterior Graphic. Threshold Marked @ 0.2	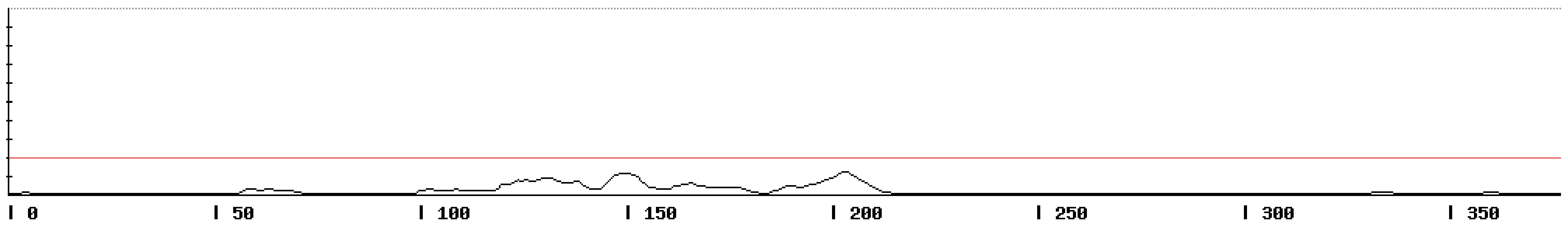

Predictions for Leersiaperrieri_LPERR02G18380.1

Viterbi Path	
Viterbi Path	
Posterior @ 0.2	164 - RKRRK - 168
	187 - GKSKA - 191
	199 - KG - 200
Posterior @ 0.2	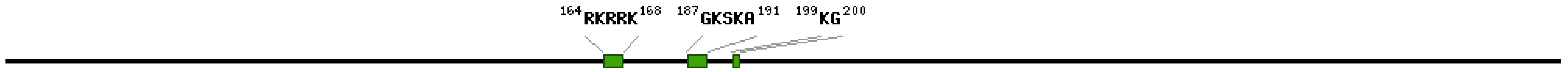
Posterior Graphic. Threshold Marked @ 0.2	

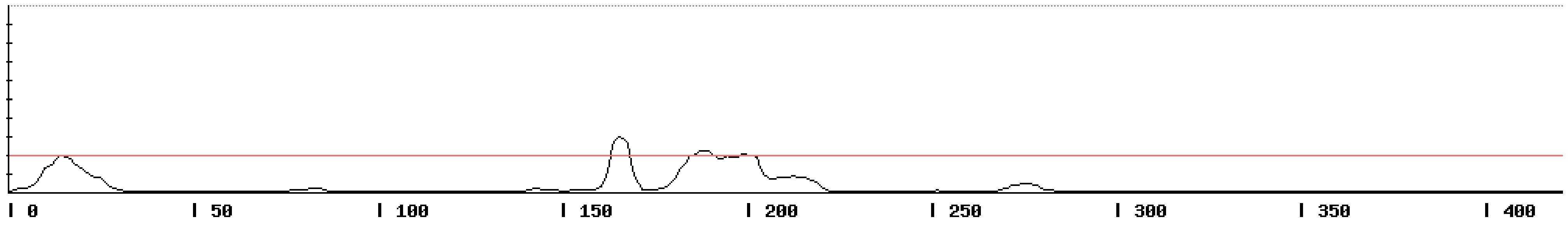

Predictions for Leersiaperrieri_LPERR02G20100.1

Viterbi Path

Viterbi Path

Posterior @ 0.2

Posterior @ 0.2

Posterior Graphic. Threshold Marked @ 0.2

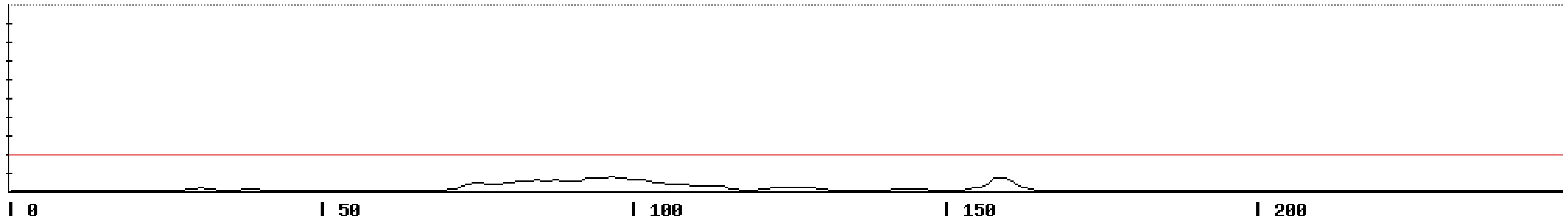

Predictions for Leersiaperrieri_LPERR02G21010.1

Viterbi Path

Viterbi Path

Posterior @ 0.2

Posterior @ 0.2

Posterior Graphic. Threshold Marked @ 0.2	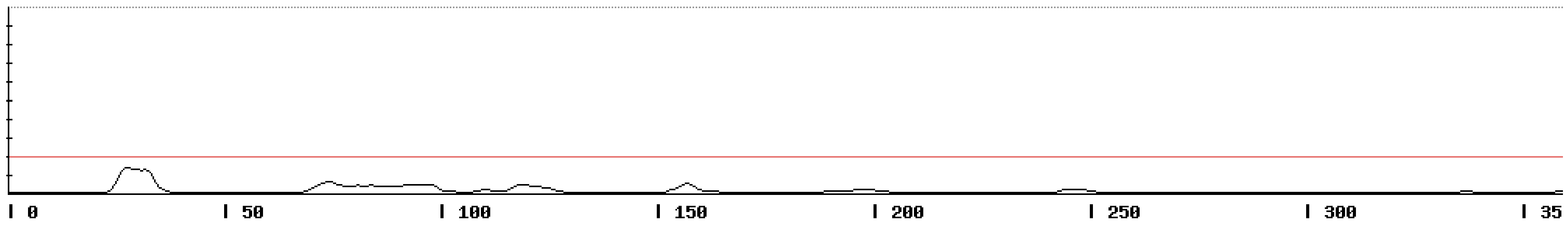

Predictions for Leersiaperrieri_LPERR02G26510.1	
Viterbi Path	
Viterbi Path	
Posterior @ 0.2	
Posterior @ 0.2	
Posterior Graphic. Threshold Marked @ 0.2	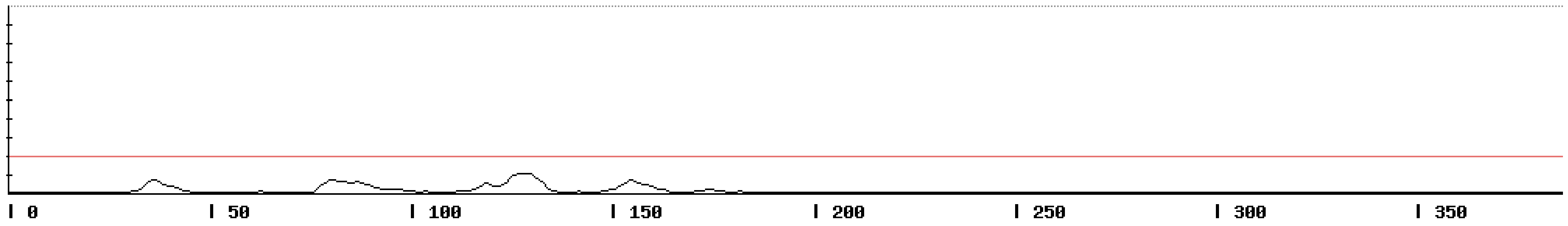

Predictions for Leersiaperrieri_LPERR02G30240.1	
Viterbi Path	
Viterbi Path	
Posterior @ 0.2	
Posterior @ 0.2	

Posterior Graphic. Threshold Marked @ 0.2	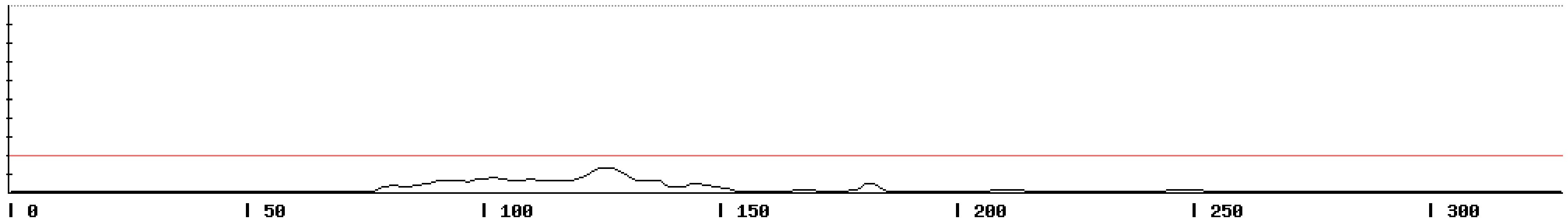

Predictions for Leersiaperrieri_LPERR02G31180.1

Viterbi Path	
Viterbi Path	
Posterior @ 0.2	85 - GAGAGGRGGPGNRTNRATPRGYWKTTGKDRDVHHRGKLVGMKK - 127
Posterior @ 0.2	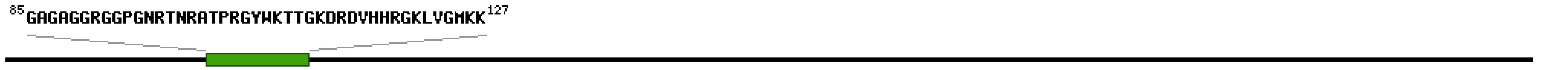
Posterior Graphic. Threshold Marked @ 0.2	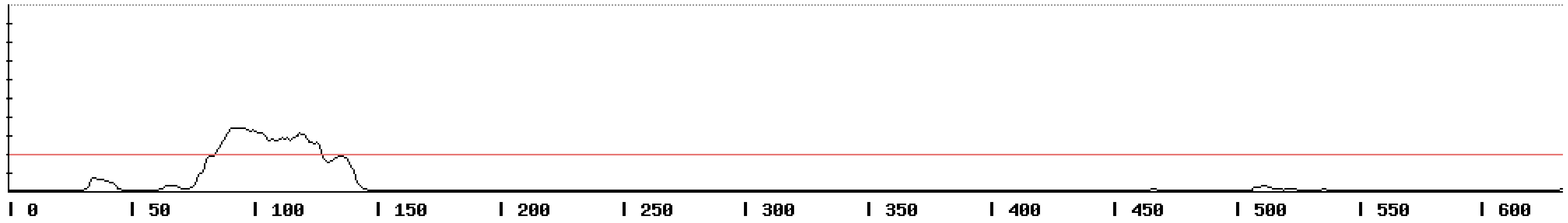

Predictions for Leersiaperrieri_LPERR03G00700.1

Viterbi Path	
Viterbi Path	

Posterior @ 0.2 — 91 - RRNRAT - 96

Posterior @ 0.2

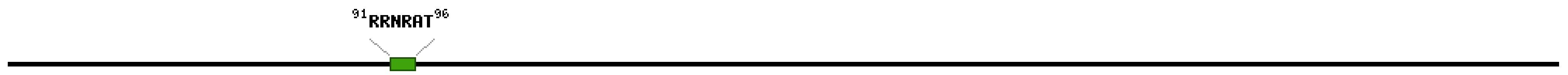

Posterior Graphic. Threshold Marked @ 0.2

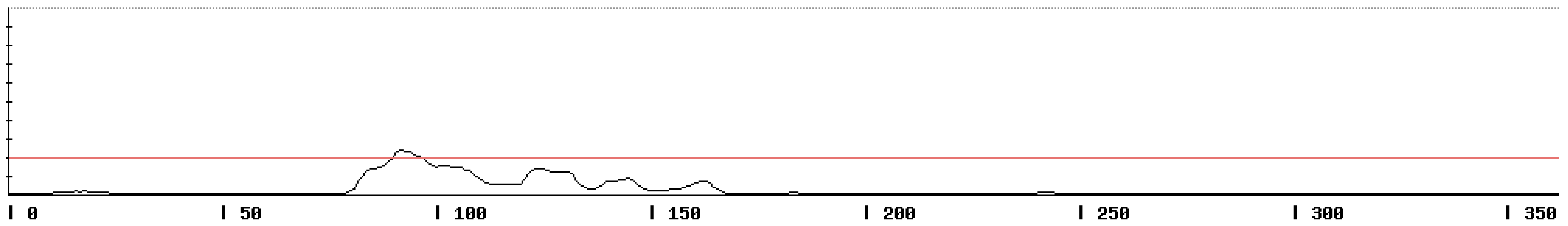

Predictions for Leersiaperrieri_LPERR03G01480.1

Viterbi Path

Viterbi Path

Posterior @ 0.2 — 72 - RKYPNGSRTNRATKAGYWKATGKDRKVNSQRRAVGMKK - 109

594 - VKKKAKEVMKKPGWGRDGGNKLWLPIPKGKG - 624

Posterior @ 0.2

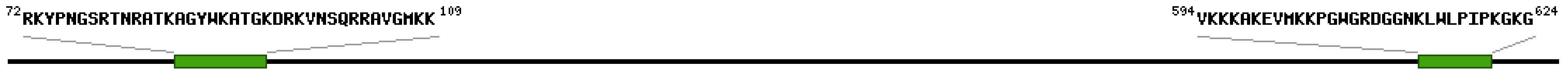

Posterior Graphic. Threshold Marked @ 0.2

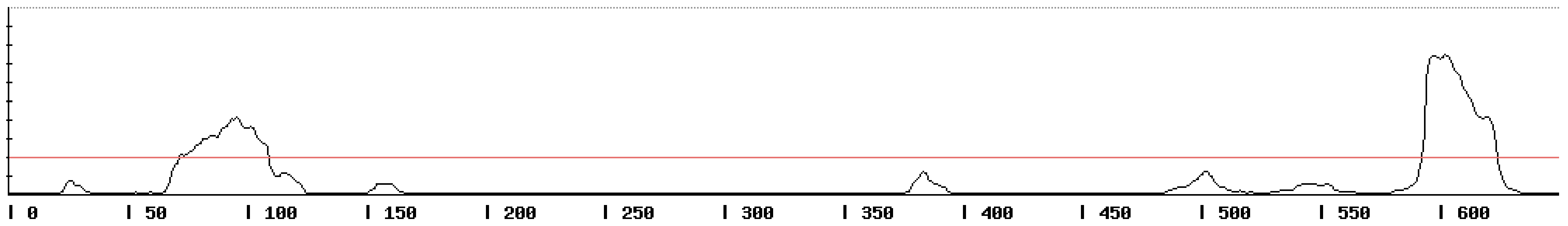

Predictions for Leersiaperrieri_LPERR03G01990.1

Viterbi Path

Viterbi Path

Posterior @ 0.2	268 - RRKAKKARAA - 277
Posterior @ 0.2	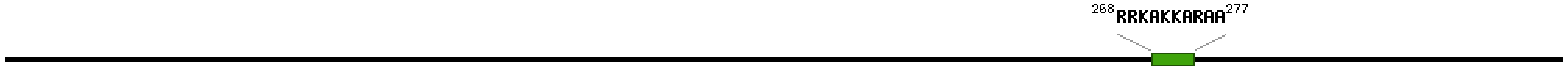
Posterior Graphic. Threshold Marked @ 0.2	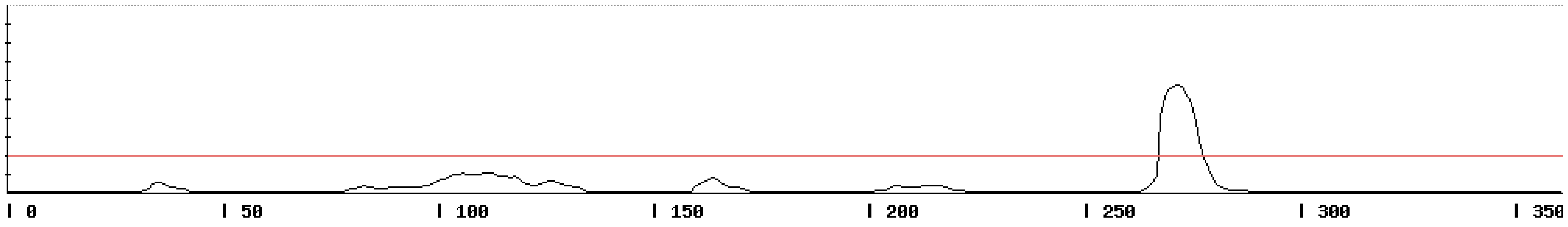

Predictions for Leersiaperrieri_LPERR03G02450.1	
Viterbi Path	
Viterbi Path	
Posterior @ 0.2	73 - RDRKAGNGGRPNRTTERG - 90
	108 - KR - 109
Posterior @ 0.2	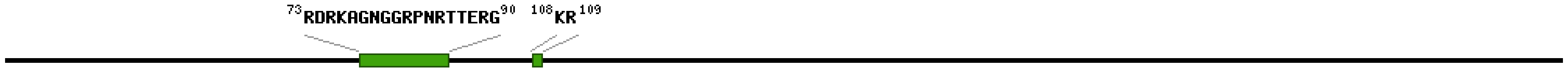
Posterior Graphic. Threshold Marked @ 0.2	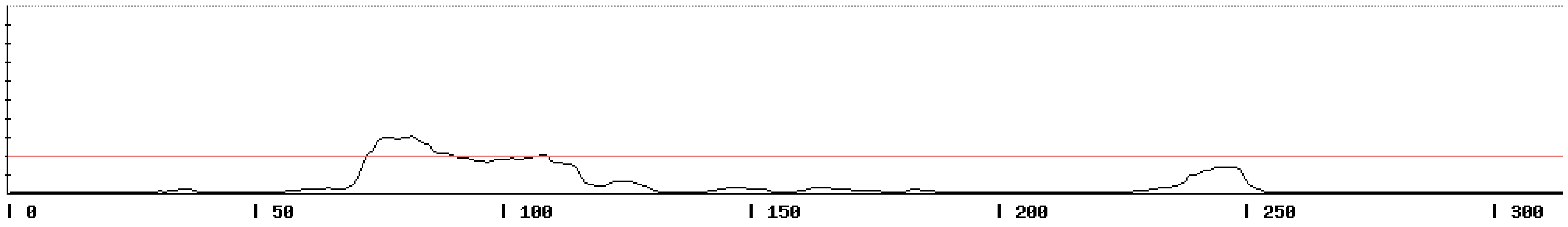

Predictions for Leersiaperrieri_LPERR03G07880.1	
Viterbi Path	
Viterbi Path	
Posterior @ 0.2	27 - RRRRLLRPQ - 35<br>517 - QKRKGRKTVRNPS - 529<br>548 - SRRKRRRKTAT - 558<br>844 - IPGRMRK - 850
Posterior @ 0.2	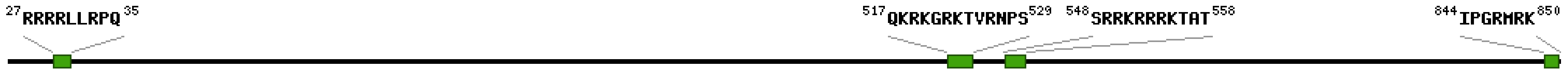
Posterior Graphic. Threshold Marked @ 0.2	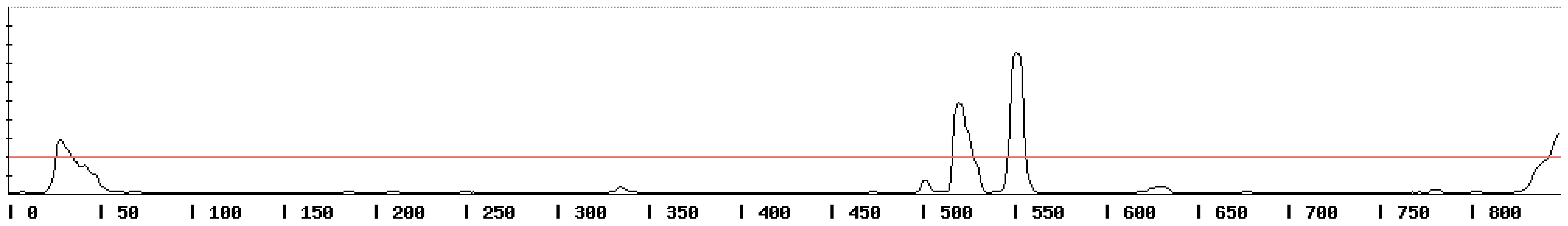

Predictions for Leersiaperrieri_LPERR03G13930.1	
Viterbi Path	
Viterbi Path	
Posterior @ 0.2	
Posterior @ 0.2	
Posterior Graphic. Threshold Marked @ 0.2	

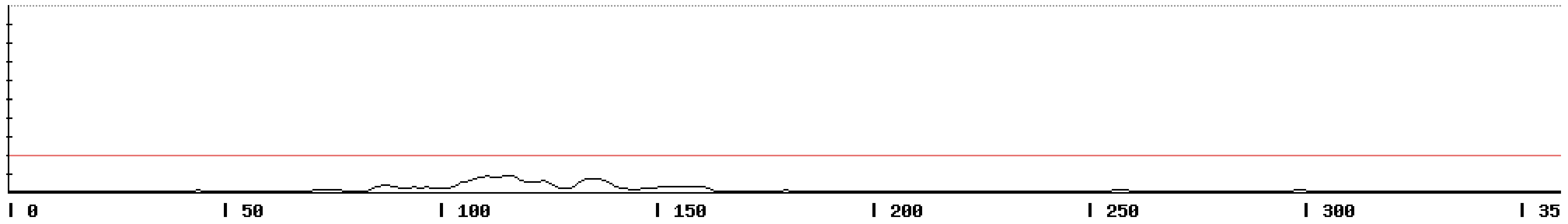

Predictions for Leersiaperrieri_LPERR03G13950.1

Viterbi Path	
Viterbi Path	
Posterior @ 0.2	124 - KGRPPK - 129
	331 - KRKR - 334
Posterior @ 0.2	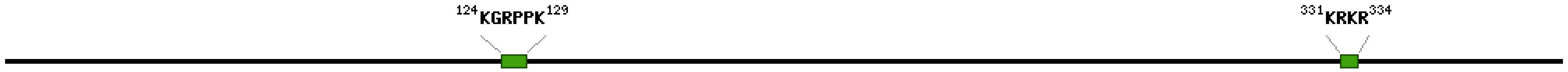
Posterior Graphic. Threshold Marked @ 0.2	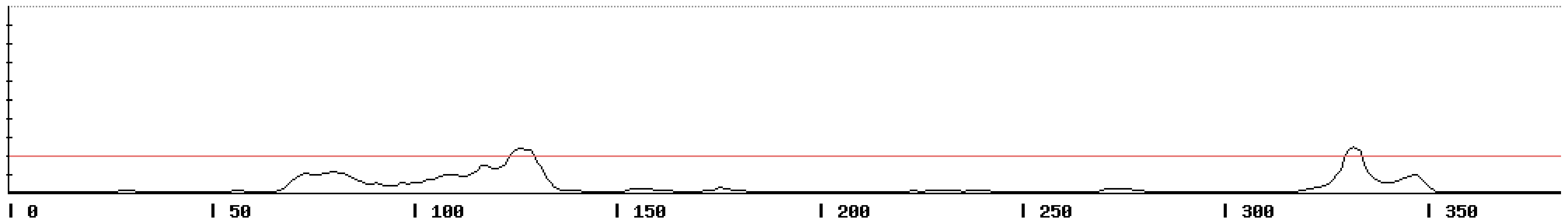

Predictions for Leersiaperrieri_LPERR03G16120.1

Viterbi Path	233 - KEGRKQRKGRKKGKK - 247
Viterbi Path	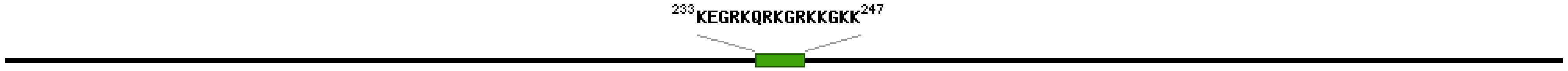
Posterior @ 0.2	188 - NCGKNGVRKGKKGMKRKEPSPD - 209
	231 - GDKEGRKQRKGRKKGKKAN - 249
Posterior @ 0.2	

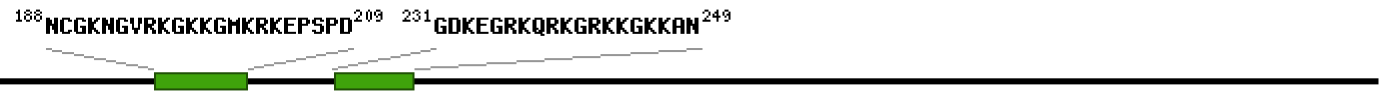

Posterior Graphic. Threshold Marked @ 0.2

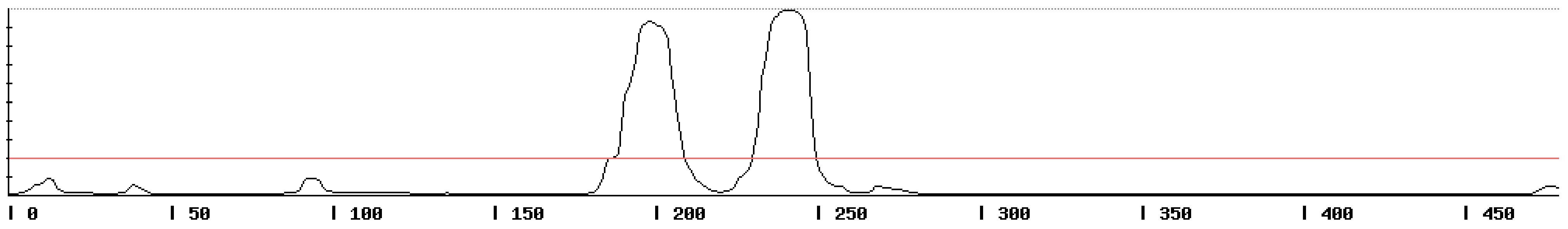

Predictions for Leersiaperrieri_LPERR03G21900.1

Viterbi Path

Viterbi Path

Posterior @ 0.2

Posterior @ 0.2

Posterior Graphic. Threshold Marked @ 0.2

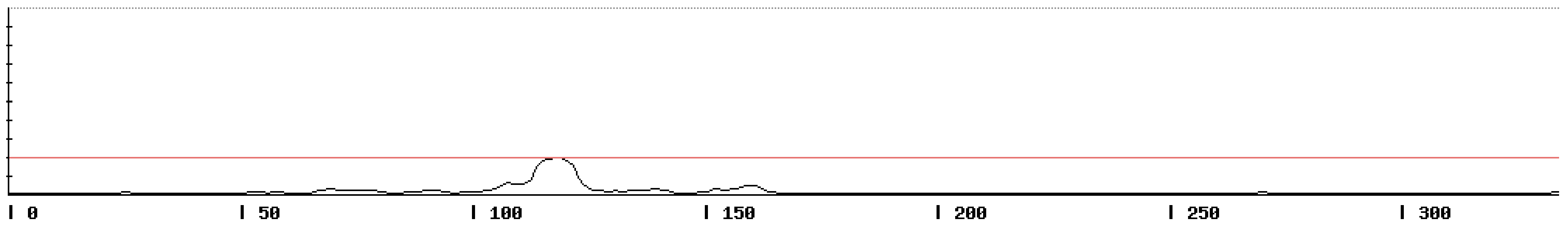

Predictions for Leersiaperrieri_LPERR03G30390.1

Viterbi Path

Viterbi Path

Posterior @ 0.2

Posterior @ 0.2

Posterior Graphic. Threshold Marked @ 0.2

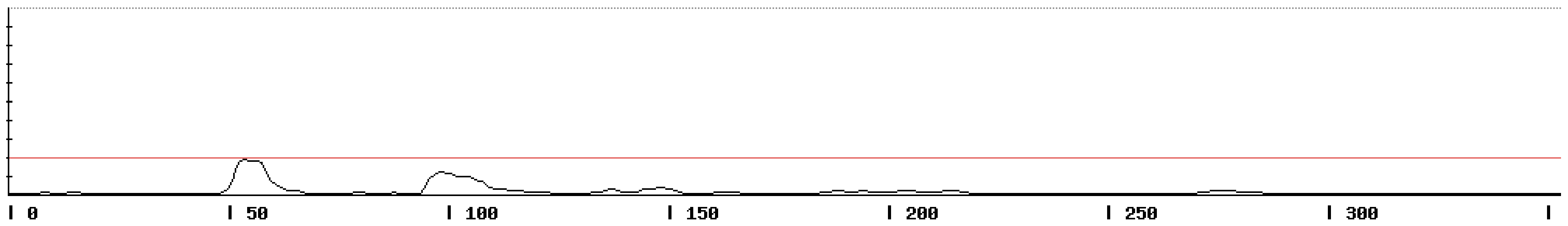

Predictions for Leersiaperrieri_LPERR03G32860.1

Viterbi Path

Viterbi Path

Posterior @ 0.2

86 - RPNRA - 90

97 - KATGADKPVAPKGRTLGIKKA - 117

Posterior @ 0.2

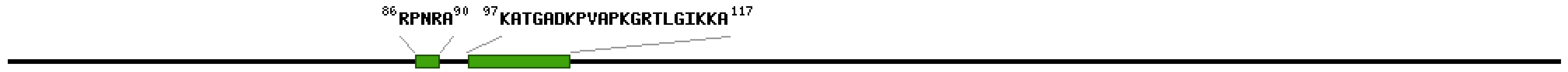

Posterior Graphic. Threshold Marked @ 0.2

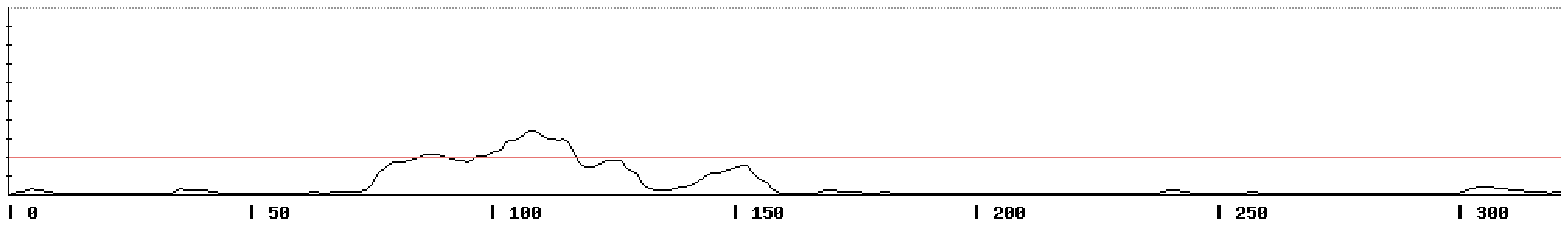

Predictions for Leersiaperrieri_LPERR03G33770.1

Viterbi Path

Viterbi Path

Posterior @ 0.2 | 82 - SKRVSSGRRQRHPAWMITRRMRHPACGRGKRPPPAASKISSRR - 124

Posterior @ 0.2

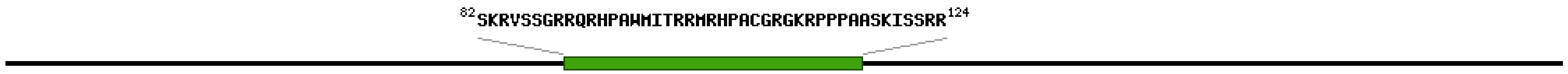

Posterior Graphic. Threshold Marked @ 0.2

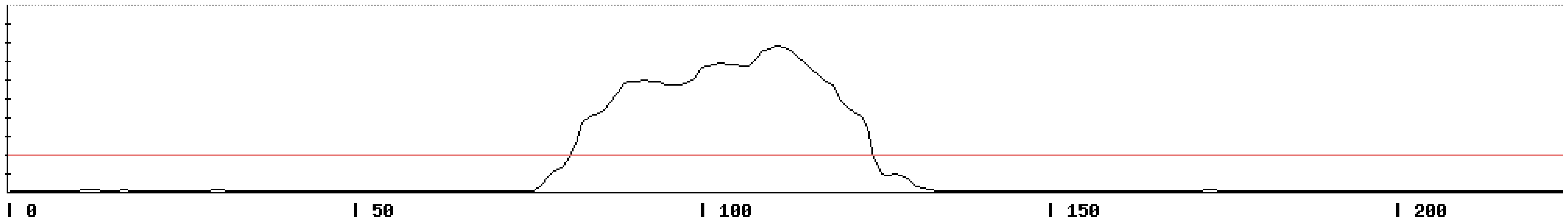

Predictions for Leersiaperrieri_LPERR03G33790.1

Viterbi Path

Viterbi Path

Posterior @ 0.2 | 88 - GRRQR - 92

Posterior @ 0.2

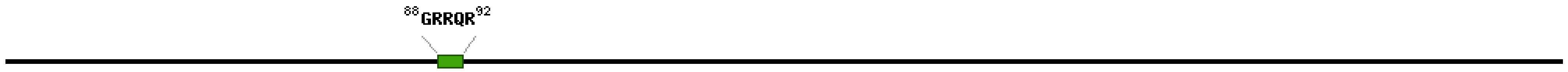

Posterior Graphic. Threshold Marked @ 0.2

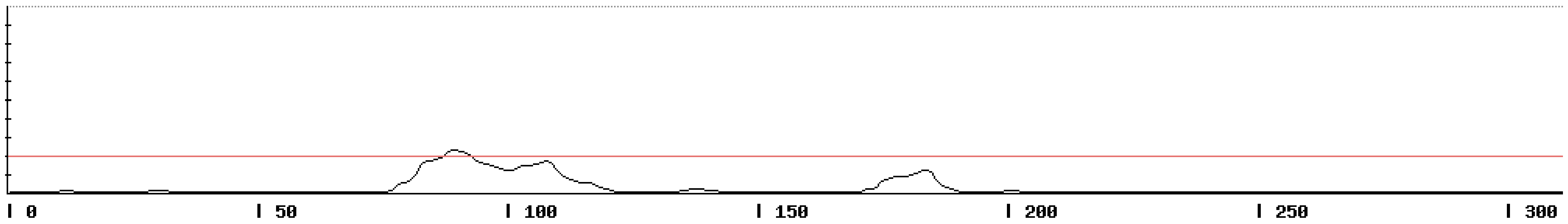

Predictions for Leersiaperrieri_LPERR03G33950.1

Viterbi Path	
Viterbi Path	
Posterior @ 0.2	82 - LGGKIARRRKDR - 93
Posterior @ 0.2	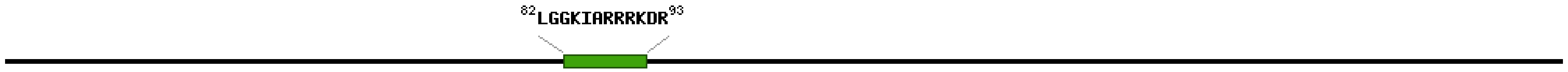
Posterior Graphic. Threshold Marked @ 0.2	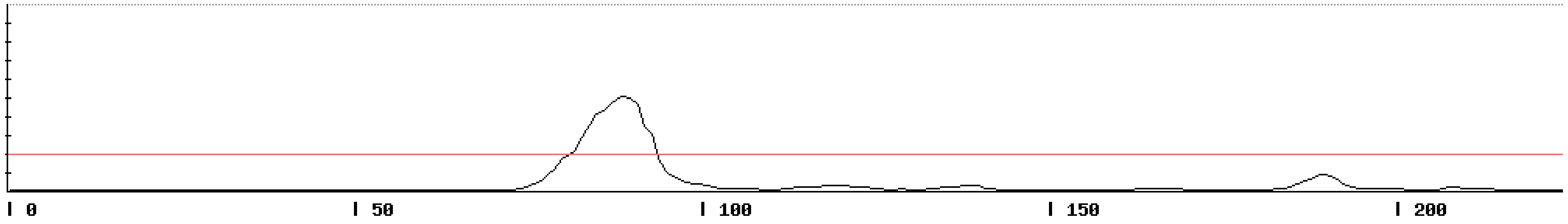

Predictions for Leersiaperrieri_LPERR03G34130.1

Viterbi Path	
Viterbi Path	
Posterior @ 0.2	88 - GRRQR - 92
Posterior @ 0.2	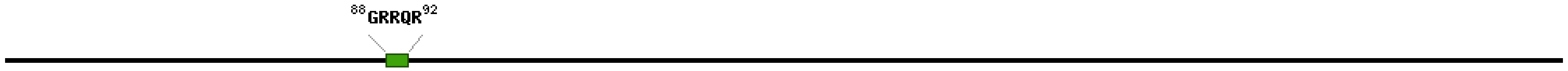
Posterior Graphic. Threshold Marked @ 0.2	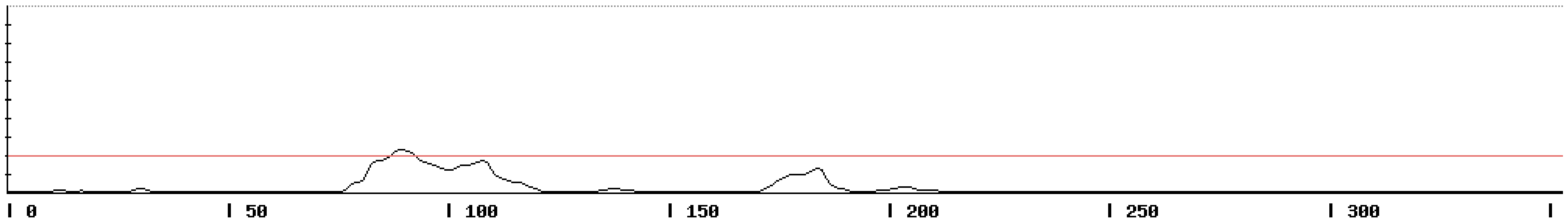

Predictions for Leersiaperrieri_LPERR03G34140.1	
Viterbi Path	
Viterbi Path	
Posterior @ 0.2	82 - SKRVSSGRRQRHPAWMITRRMRHPACGRGKRPPPAASKISSRRS - 125
Posterior @ 0.2	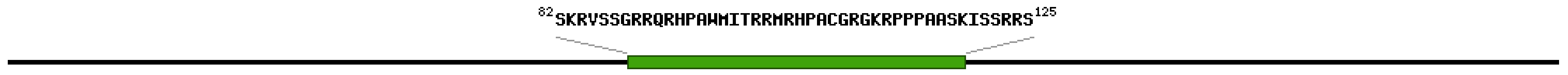
Posterior Graphic. Threshold Marked @ 0.2	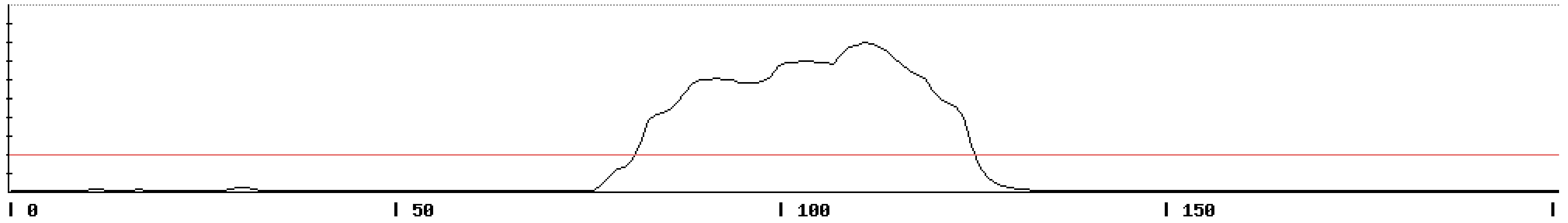

Predictions for Leersiaperrieri_LPERR03G34530.1	
Viterbi Path	
Viterbi Path	
Posterior @ 0.2	
Posterior @ 0.2	
Posterior Graphic. Threshold Marked @ 0.2	

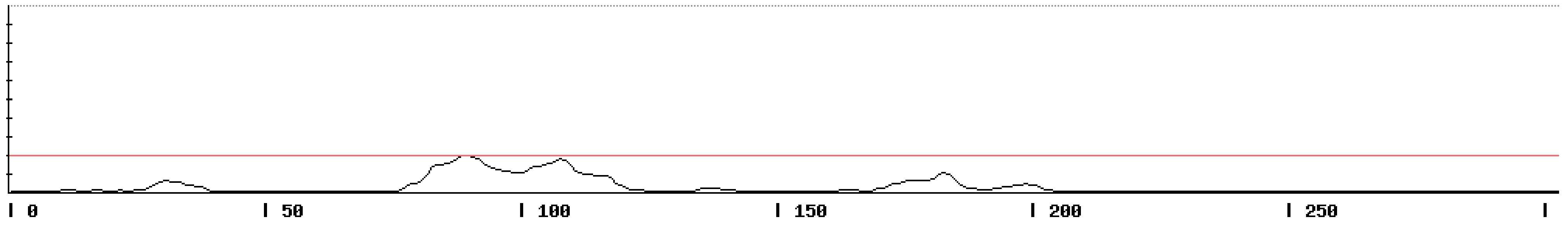

Predictions for Leersiaperrieri_LPERR04G10110.1

Viterbi Path	
Viterbi Path	
Posterior @ 0.2	141 - SKRSHKKKGH - 150
Posterior @ 0.2	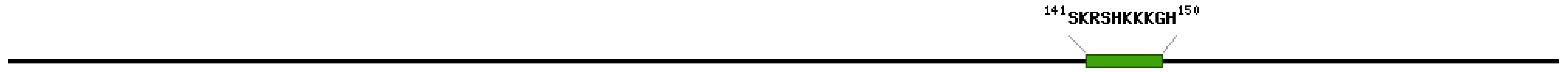
Posterior Graphic. Threshold Marked @ 0.2	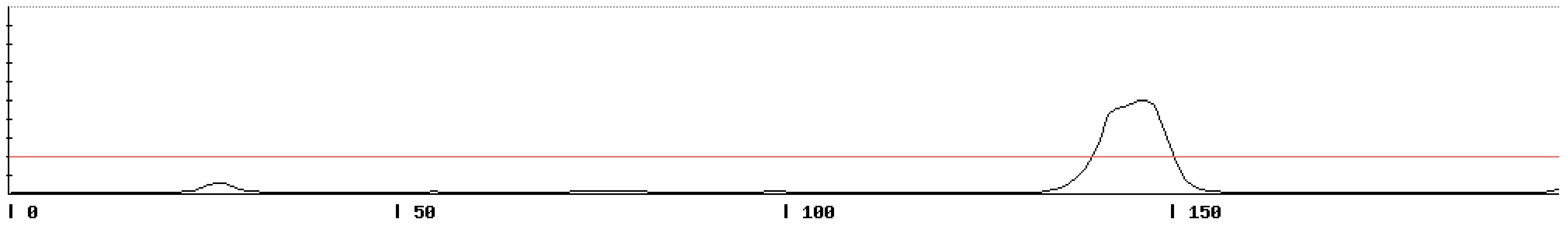

Predictions for Leersiaperrieri_LPERR04G10720.1

Viterbi Path	
Viterbi Path	
Posterior @ 0.2	424 - KALKRKANF - 432
	436 - KK - 437
Posterior @ 0.2	

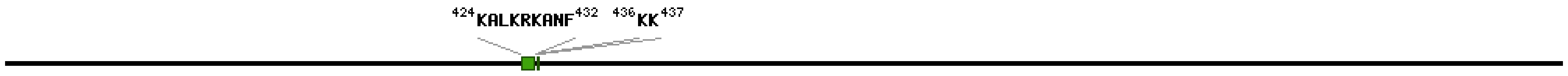

Posterior Graphic. Threshold Marked @ 0.2

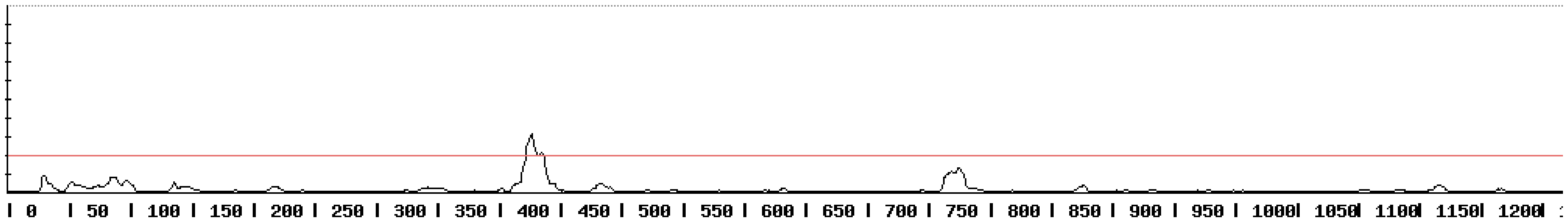

Predictions for Leersiaperrieri_LPERR04G11740.1

Viterbi Path

Viterbi Path

Posterior @ 0.2

Posterior @ 0.2

Posterior Graphic. Threshold Marked @ 0.2

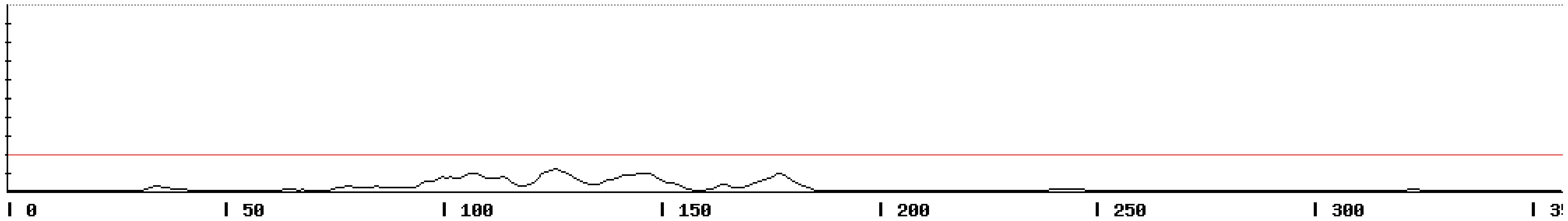

Predictions for Leersiaperrieri_LPERR04G12670.1

Viterbi Path

Viterbi Path

Posterior @ 0.2	129 - TGGRRRGPRWRMKEFRLNKGAAAFRRAHARPNPKA - 163

Posterior @ 0.2

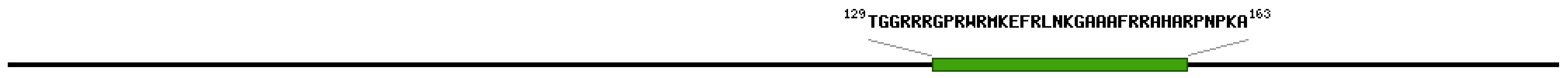

Posterior Graphic. Threshold Marked @ 0.2

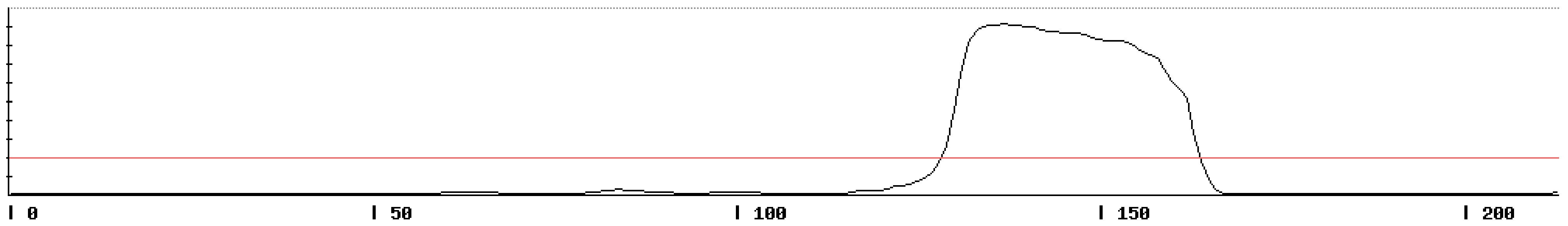

Predictions for Leersiaperrieri_LPERR04G12800.1

Viterbi Path

Viterbi Path

Posterior @ 0.2	183 - GKRKRRKIR - 191
	206 - KTGRSKEIHENNGVKKGLKK - 225

Posterior @ 0.2

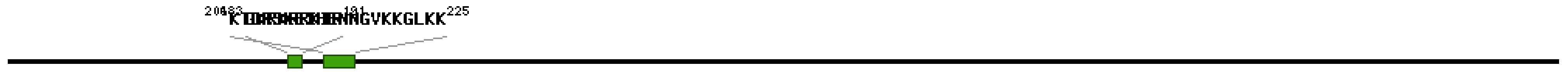

Posterior Graphic. Threshold Marked @ 0.2

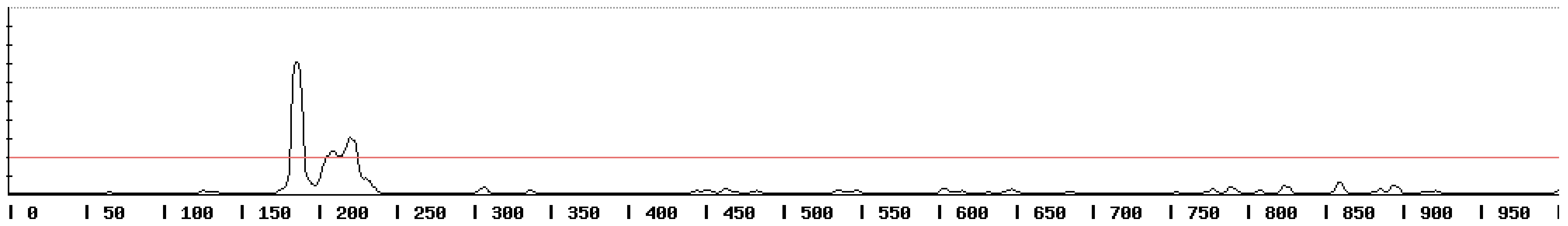

Predictions for Leersiaperrieri_LPERR04G14690.1

Viterbi Path

Viterbi Path

Posterior @ 0.2	120 - FRRRRCPGGRRRAGPGEYR - 138
	280 - AVRQRRRGEHGAARPRKAGPG - 300
Posterior @ 0.2	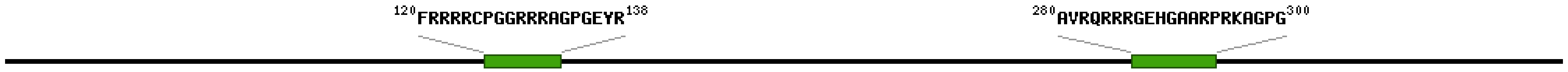
Posterior Graphic. Threshold Marked @ 0.2	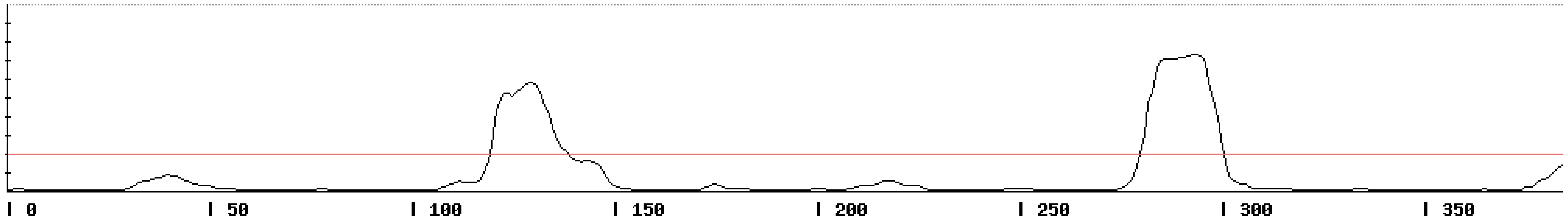

Predictions for Leersiaperrieri_LPERR04G15170.1

Viterbi Path	
Viterbi Path	
Posterior @ 0.2	
Posterior @ 0.2	
Posterior Graphic. Threshold Marked @ 0.2	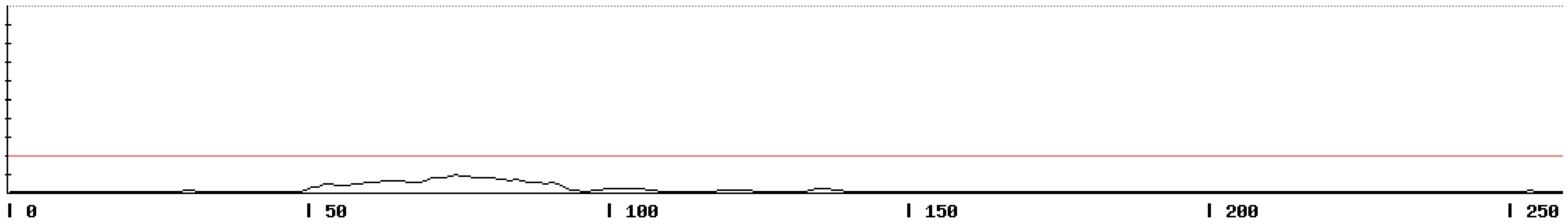

Predictions for Leersiaperrieri_LPERR04G16200.1

Viterbi Path	
Viterbi Path	
Posterior @ 0.2	
Posterior @ 0.2	
Posterior Graphic. Threshold Marked @ 0.2	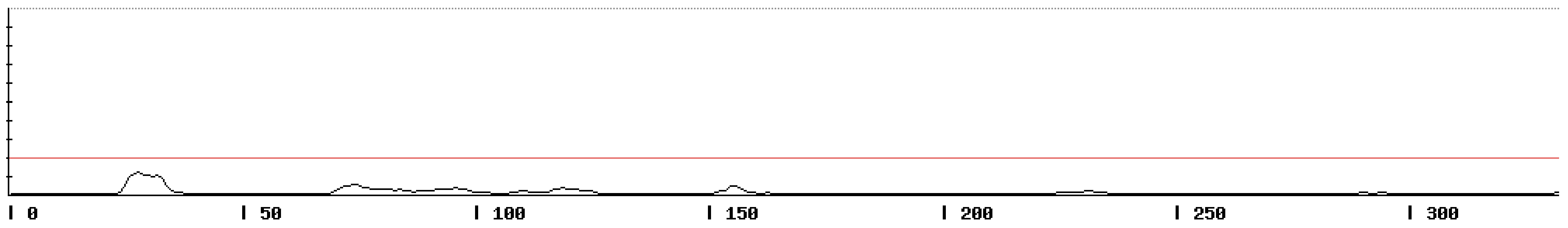

Predictions for Leersiaperrieri_LPERR04G17350.1

Viterbi Path	
Viterbi Path	
Posterior @ 0.2	20 - SYRRLGSRRHGKGEEKGAGGGGDKIPPVFR - 49
	108 - PRARISRGGTRPTRSTEDRRGRWKASTGNKKA - 139
Posterior @ 0.2	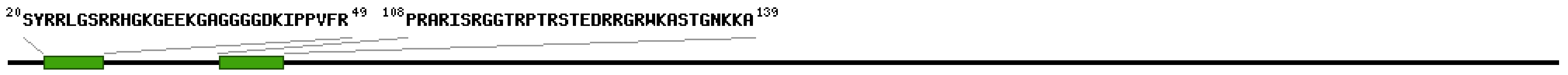
Posterior Graphic. Threshold Marked @ 0.2	

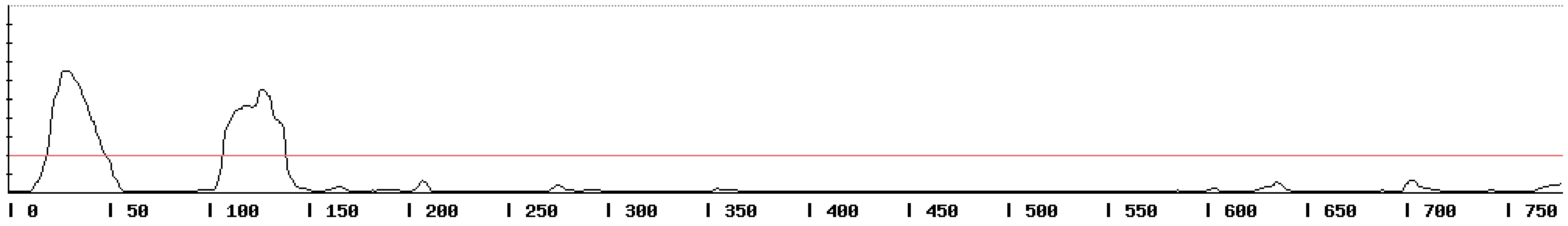

Predictions for Leersiaperrieri_LPERR04G20380.1

Viterbi Path

Viterbi Path

Posterior @ 0.2

Posterior @ 0.2

Posterior Graphic. Threshold Marked @ 0.2

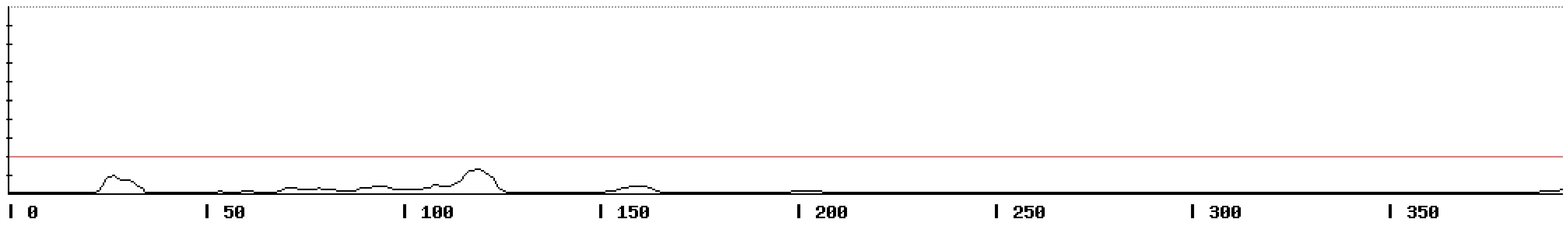

Predictions for Leersiaperrieri_LPERR04G21110.5

Viterbi Path

Viterbi Path

Posterior @ 0.2

Posterior @ 0.2

Posterior Graphic. Threshold Marked @ 0.2

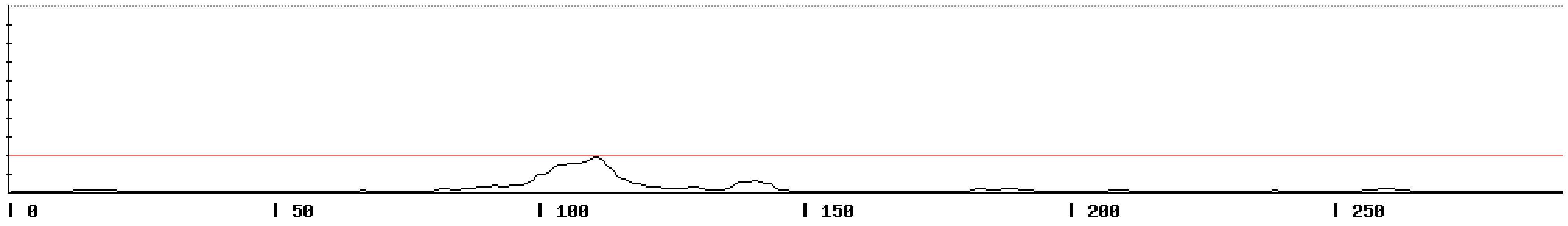

Predictions for Leersiaperrieri_LPERR04G26130.1

Viterbi Path

Viterbi Path

Posterior @ 0.2

Posterior @ 0.2

Posterior Graphic. Threshold Marked @ 0.2

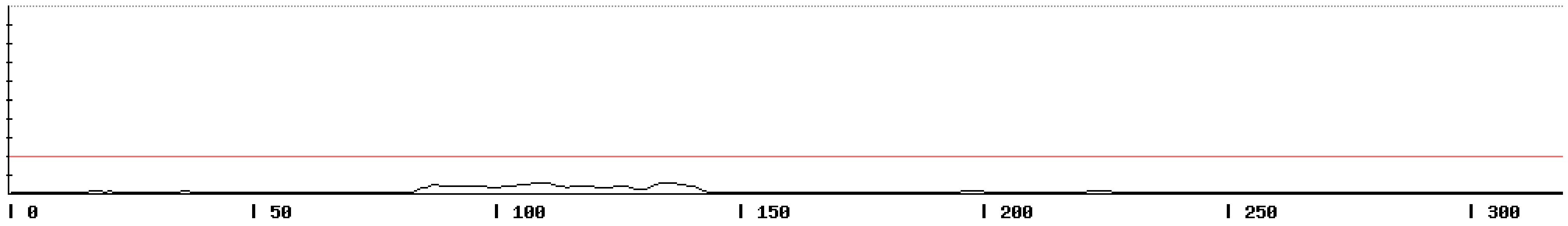

Predictions for Leersiaperrieri_LPERR04G26370.1

Viterbi Path

Viterbi Path

Posterior @ 0.2 281 - RTPKGKPRPPG - 291

Posterior @ 0.2

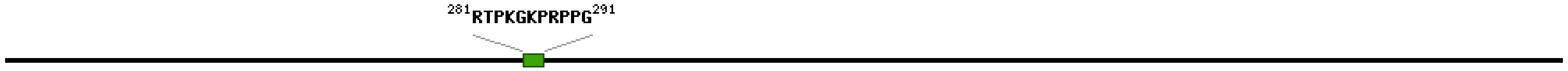

Posterior Graphic. Threshold Marked @ 0.2

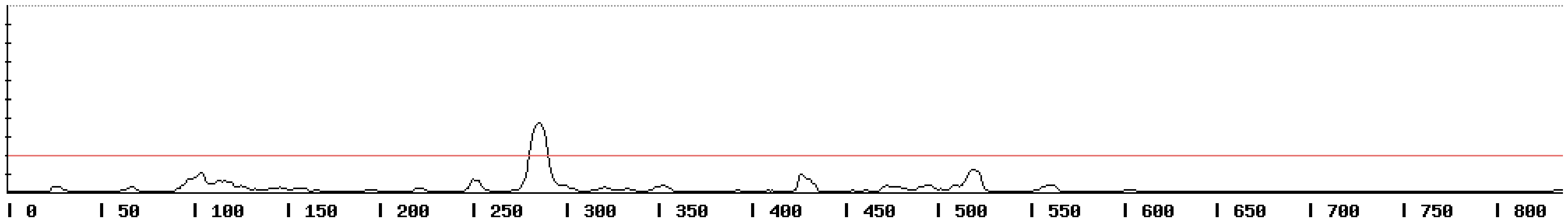

Predictions for Leersiaperrieri_LPERR05G00120.1

Viterbi Path

Viterbi Path

Posterior @ 0.2

Posterior @ 0.2

Posterior Graphic. Threshold Marked @ 0.2

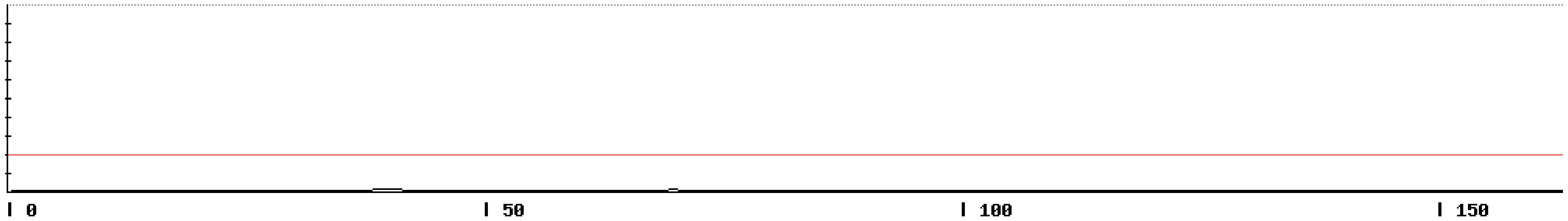

Predictions for Leersiaperrieri_LPERR05G05170.1

Viterbi Path

Viterbi Path

Posterior @ 0.2	148 - GKKRKSE - 154
Posterior @ 0.2	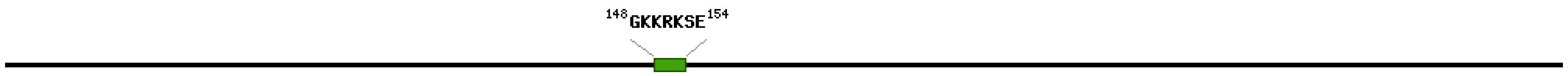
Posterior Graphic. Threshold Marked @ 0.2	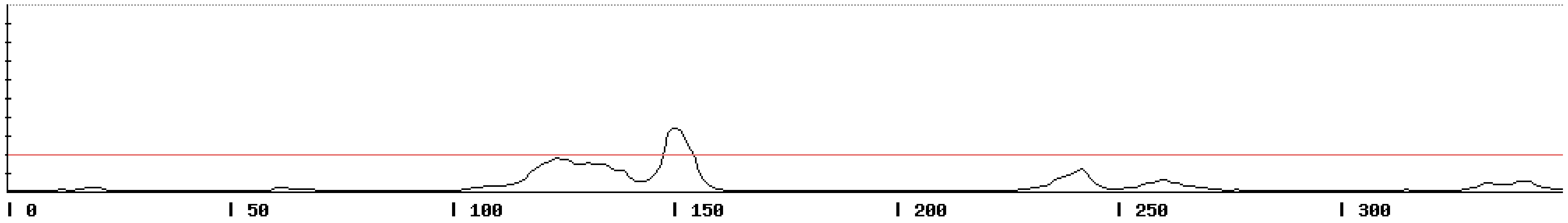

Predictions for Leersiaperrieri_LPERR05G09480.1

Viterbi Path	
Viterbi Path	
Posterior @ 0.2	73 - EARAKNGKGKRQKRTV - 88
	158 - RVRFSGHGKKRKREP - 172
Posterior @ 0.2	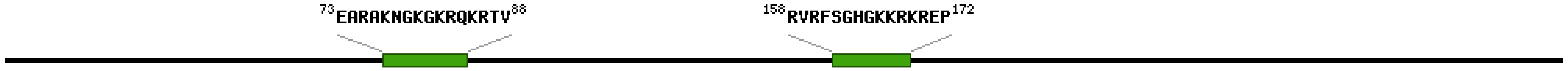
Posterior Graphic. Threshold Marked @ 0.2	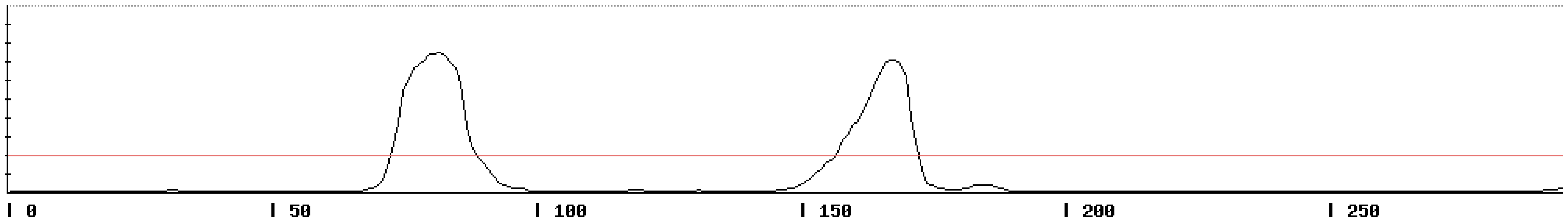

Predictions for Leersiaperrieri_LPERR05G09540.1

Viterbi Path	
Viterbi Path	

Posterior @ 0.2

73 - EARAKNGKGKRQKRTV - 88

158 - RVRFSGHGKKRKREP - 172

Posterior @ 0.2

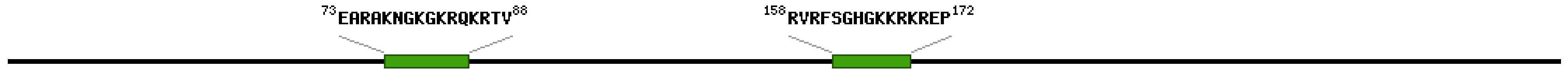

Posterior Graphic. Threshold Marked @ 0.2

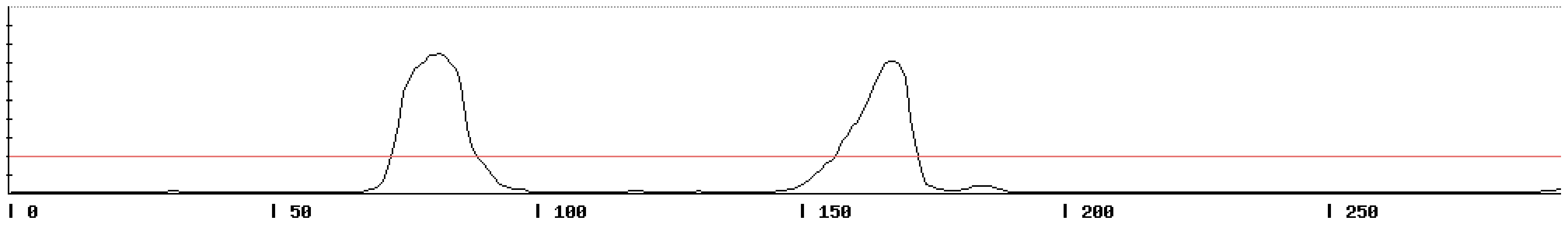

Predictions for Leersiaperrieri_LPERR05G13140.1

Viterbi Path

Viterbi Path

Posterior @ 0.2

Posterior @ 0.2

Posterior Graphic. Threshold Marked @ 0.2

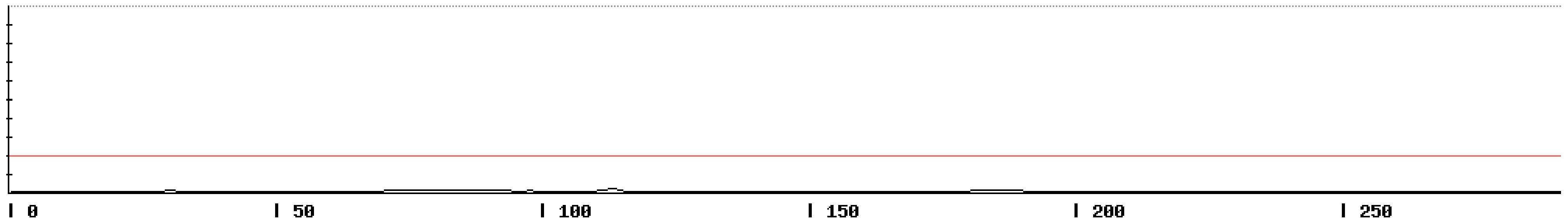

Predictions for Leersiaperrieri_LPERR05G13320.1

Viterbi Path

Viterbi Path

Posterior @ 0.2

Posterior @ 0.2

Posterior Graphic. Threshold Marked @ 0.2

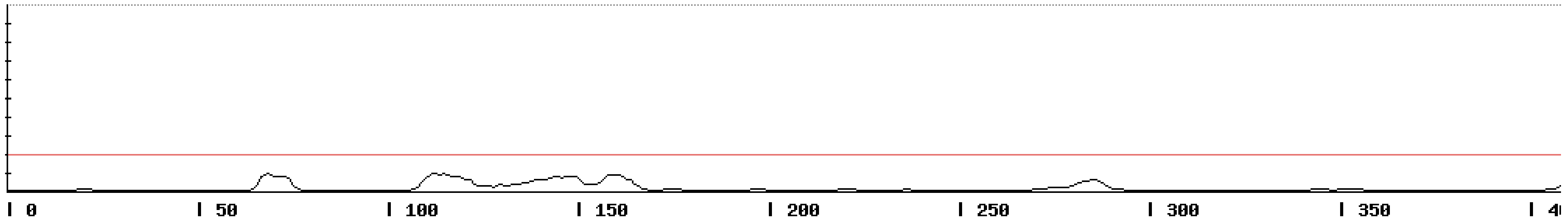

Predictions for Leersiaperrieri_LPERR05G13490.1

Viterbi Path

Viterbi Path

Posterior @ 0.2

96 - RP - 97

119 - R - 119

Posterior @ 0.2

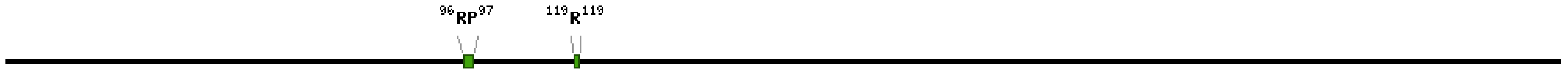

Posterior Graphic. Threshold Marked @ 0.2

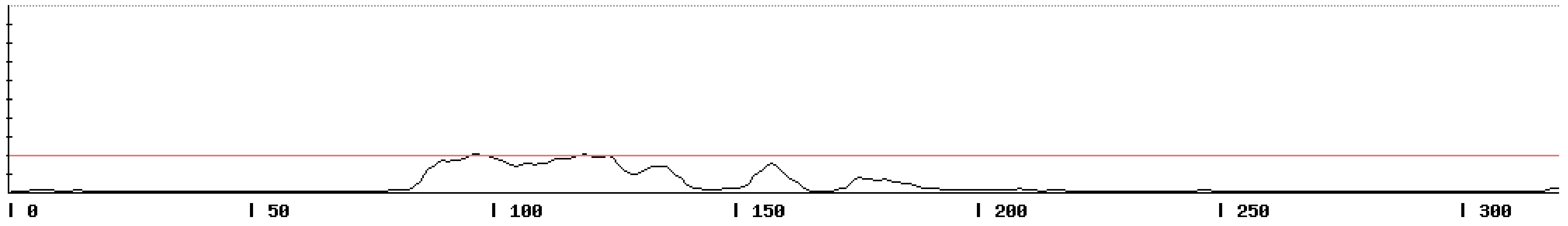

Predictions for Leersiaperrieri_LPERR05G13650.1

Viterbi Path	
Viterbi Path	
Posterior @ 0.2	
Posterior @ 0.2	
Posterior Graphic. Threshold Marked @ 0.2	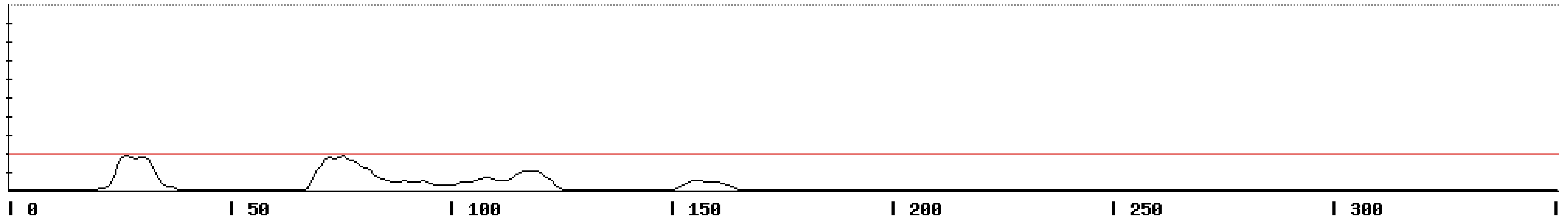

Predictions for Leersiaperrieri_LPERR05G14390.1

Viterbi Path	
Viterbi Path	
Posterior @ 0.2	215 - KRKR - 218
Posterior @ 0.2	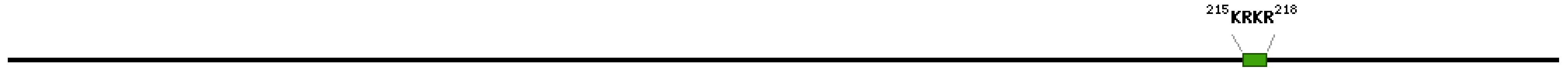

Posterior Graphic. Threshold Marked @ 0.2

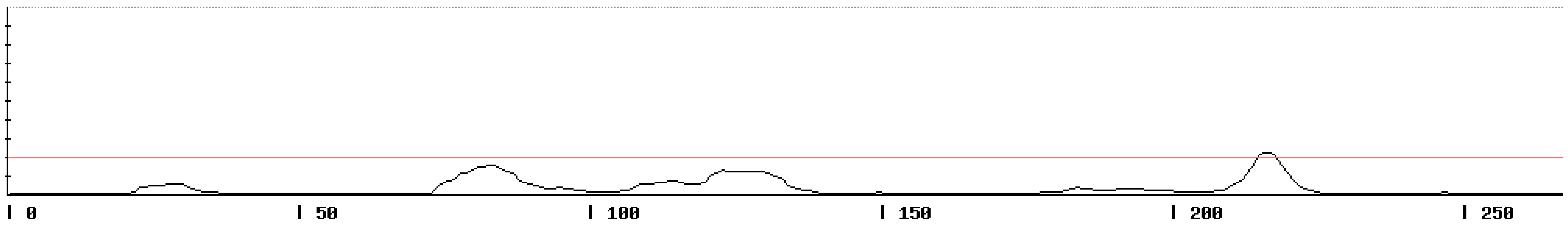

Predictions for Leersiaperrieri_LPERR05G18690.1

Viterbi Path

Viterbi Path

Posterior @ 0.2

19 - GGEKRRPVGFRFKPR - 33

92 - DRKYKNGSRPSRN - 104

Posterior @ 0.2

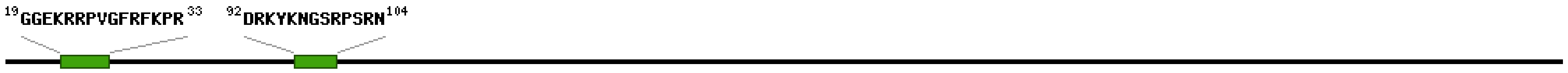

Posterior Graphic. Threshold Marked @ 0.2

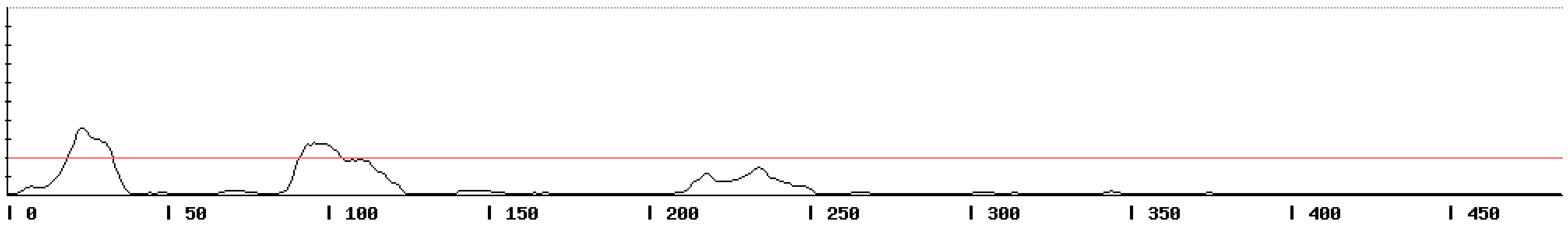

Predictions for Leersiaperrieri_LPERR05G21400.1

Viterbi Path

Viterbi Path

Posterior @ 0.2

141 - GTRKRRKV - 148

Posterior @ 0.2

	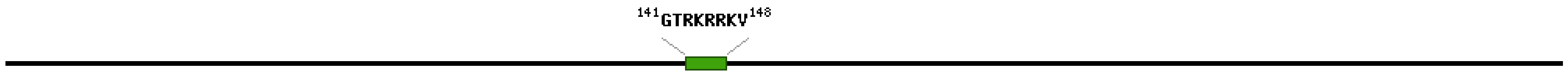
Posterior Graphic. Threshold Marked @ 0.2	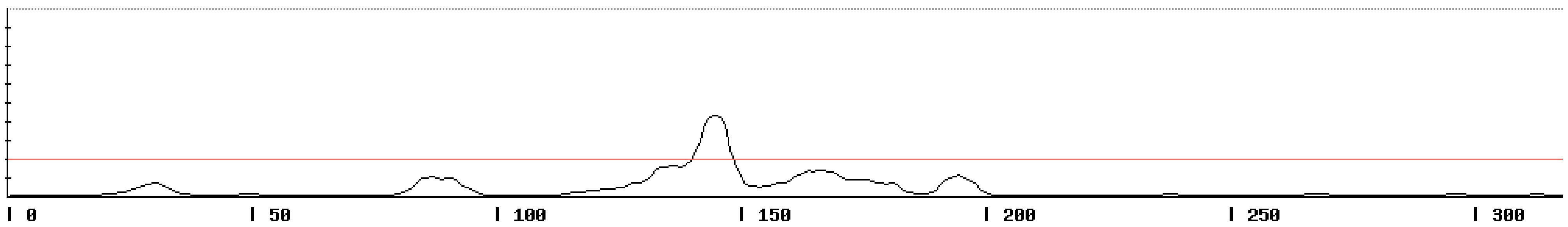

Predictions for Leersiaperrieri_LPERR06G00060.1	
Viterbi Path	
Viterbi Path	
Posterior @ 0.2	110 - RLIRHAGALIGIKKTLVFHRGRAPRGHR - 137
Posterior @ 0.2	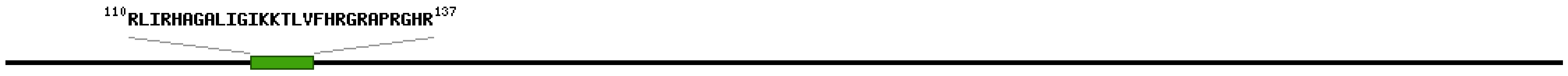
Posterior Graphic. Threshold Marked @ 0.2	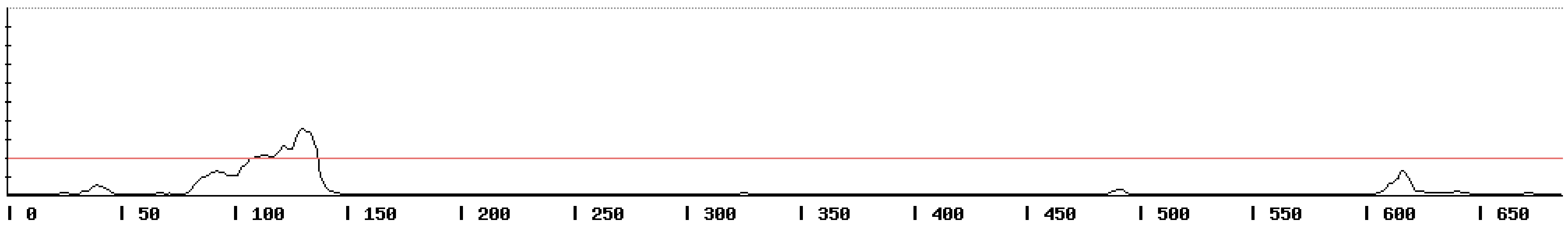

Predictions for Leersiaperrieri_LPERR06G00240.1	
Viterbi Path	
Viterbi Path	

Posterior @ 0.2	
Posterior @ 0.2	
Posterior Graphic. Threshold Marked @ 0.2	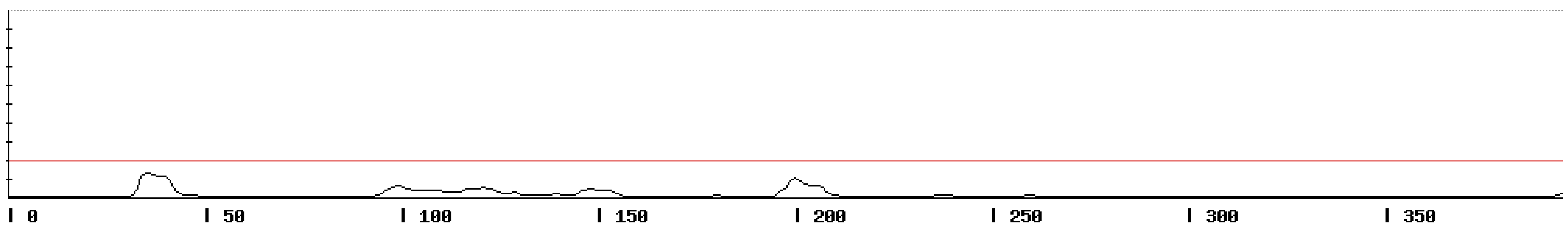

Predictions for Leersiaperrieri_LPERR06G01790.1	
Viterbi Path	
Viterbi Path	
Posterior @ 0.2	
Posterior @ 0.2	
Posterior Graphic. Threshold Marked @ 0.2	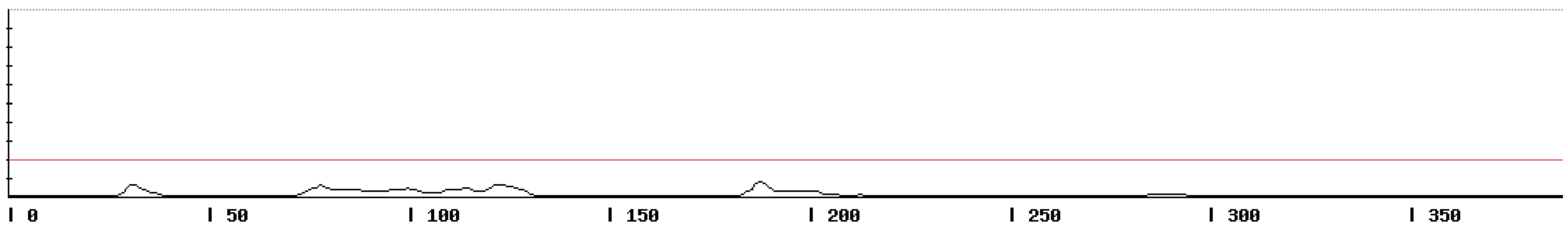

Predictions for Leersiaperrieri_LPERR06G09130.1	
Viterbi Path	
Viterbi Path	

Posterior @ 0.2	14 - VNSGSKKKKS - 23<br>200 - IRKRRKI - 206
Posterior @ 0.2	
Posterior Graphic. Threshold Marked @ 0.2	

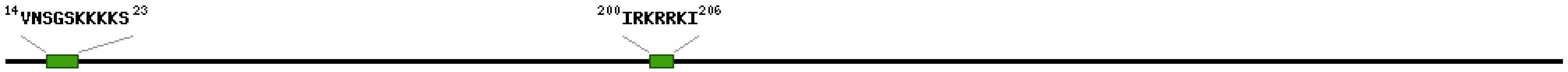

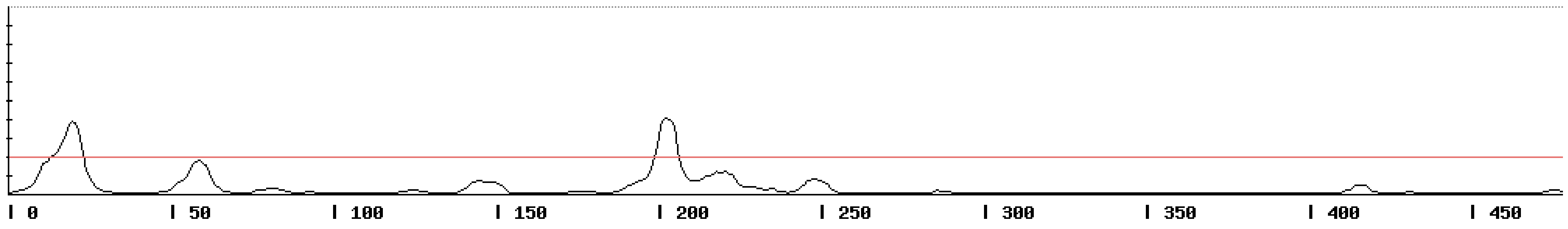

Predictions for Leersiaperrieri_LPERR06G11390.1

Viterbi Path	
Viterbi Path	
Posterior @ 0.2	72 - RAKNGKGSSRQKR - 84<br>159 - RFSGYGKKRKREPE - 172
Posterior @ 0.2	
Posterior Graphic. Threshold Marked @ 0.2	

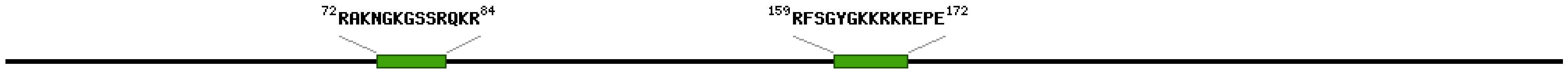

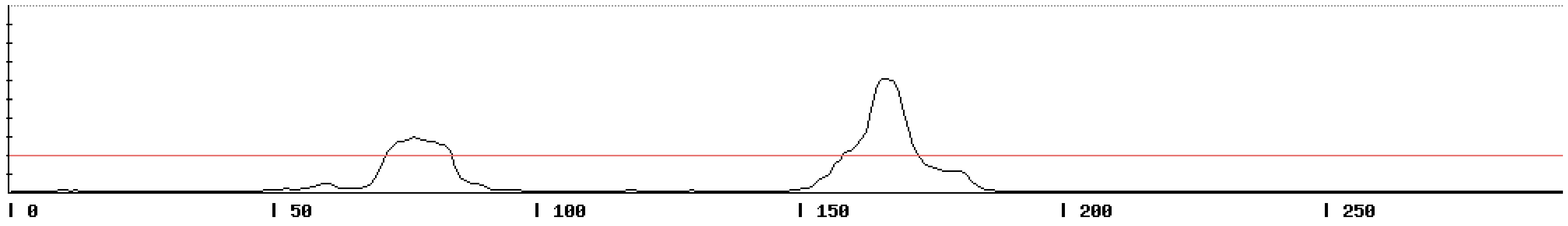

Predictions for Leersiaperrieri_LPERR06G12840.1

Viterbi Path

Viterbi Path

Posterior @ 0.2

Posterior @ 0.2

Posterior Graphic. Threshold Marked @ 0.2

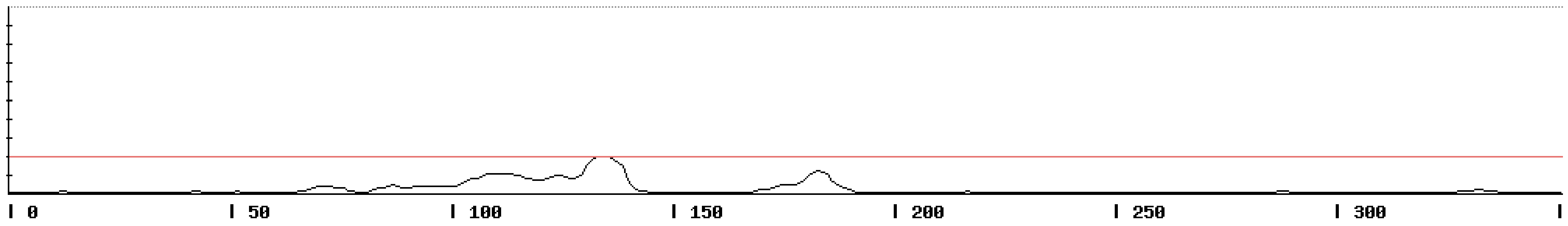

Predictions for Leersiaperrieri_LPERR06G14120.1

Viterbi Path

Viterbi Path

Posterior @ 0.2

Posterior @ 0.2

Posterior Graphic. Threshold Marked @ 0.2

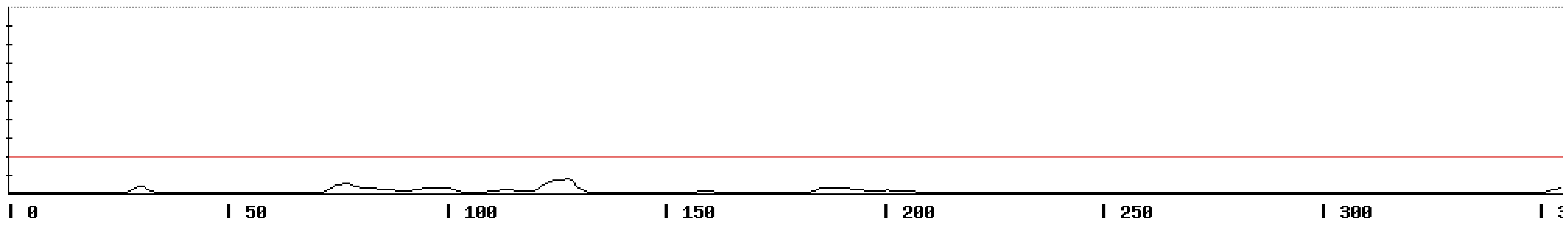

Predictions for Leersiaperrieri_LPERR06G15260.1

Viterbi Path

Viterbi Path

Posterior @ 0.2

Posterior @ 0.2

Posterior Graphic. Threshold Marked @ 0.2

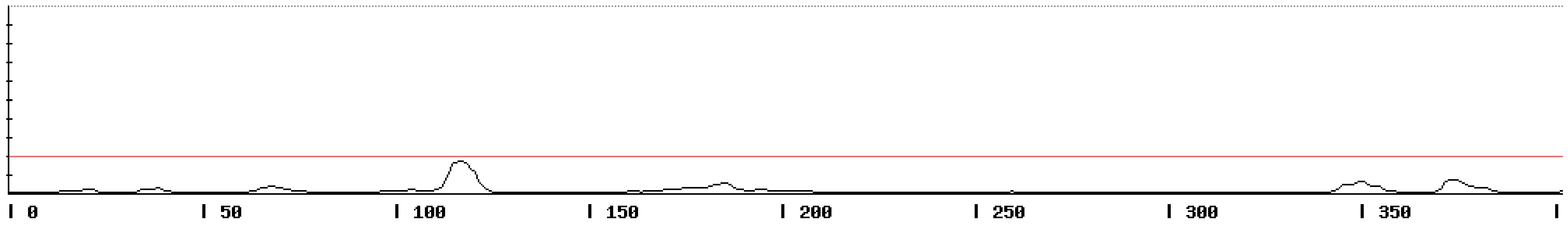

Predictions for Leersiaperrieri_LPERR06G20430.1

Viterbi Path

Viterbi Path

Posterior @ 0.2 — 109 - RSISRKGLLVGMRKTLVFYKGRAPKGKKT - 137

Posterior @ 0.2

	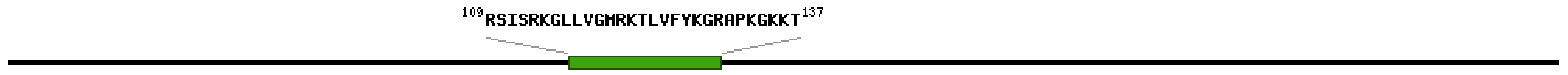
Posterior Graphic. Threshold Marked @ 0.2	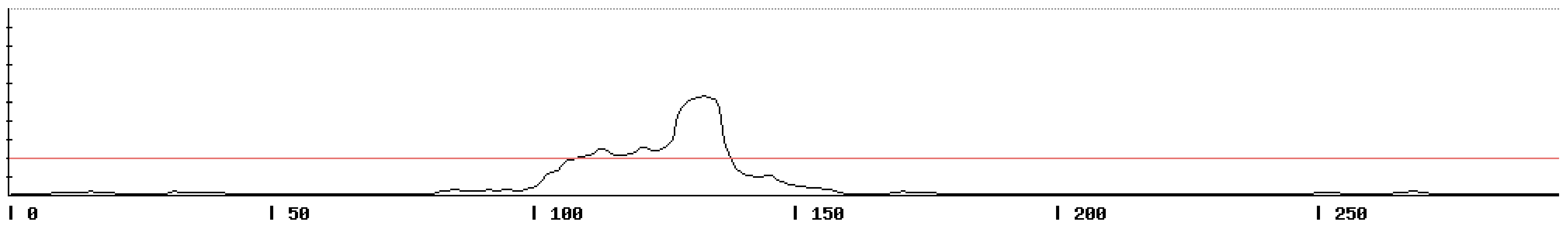

Predictions for Leersiaperrieri_LPERR06G23420.1	
Viterbi Path	
Viterbi Path	
Posterior @ 0.2	
Posterior @ 0.2	
Posterior Graphic. Threshold Marked @ 0.2	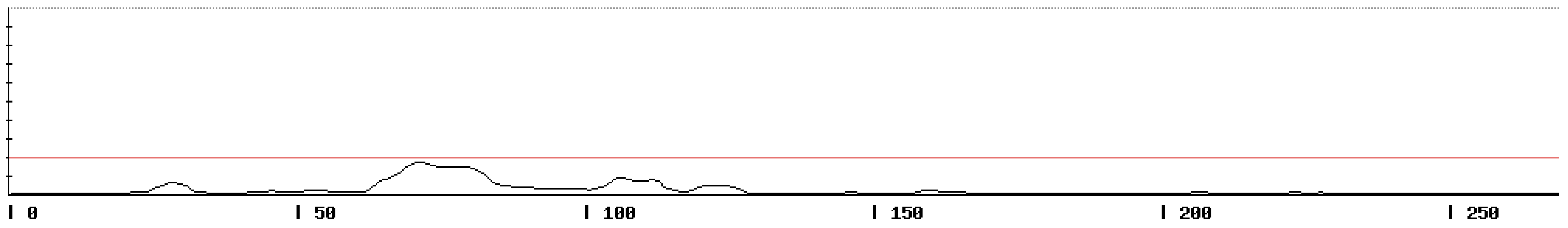

Predictions for Leersiaperrieri_LPERR07G02250.1	
Viterbi Path	
Viterbi Path	

Posterior @ 0.2

Posterior @ 0.2

Posterior Graphic. Threshold Marked @ 0.2

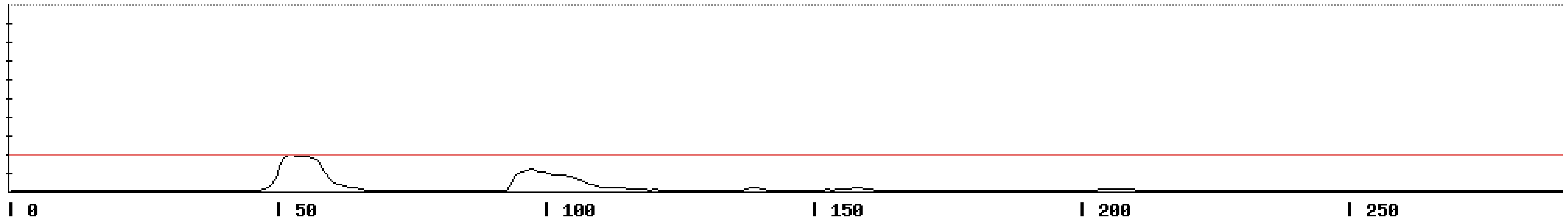

Predictions for Leersiaperrieri_LPERR07G03950.1

Viterbi Path

Viterbi Path

Posterior @ 0.2

75 - ARAKNNKGKRQKRTV - 89

149 - YRVRFSGHGKKRRRQPESEEGEPKRTR - 175

Posterior @ 0.2

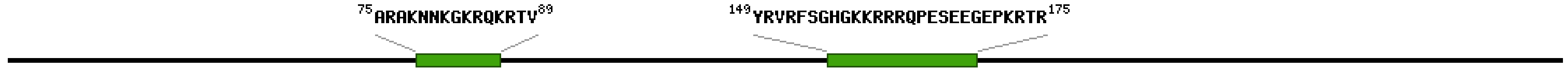

Posterior Graphic. Threshold Marked @ 0.2

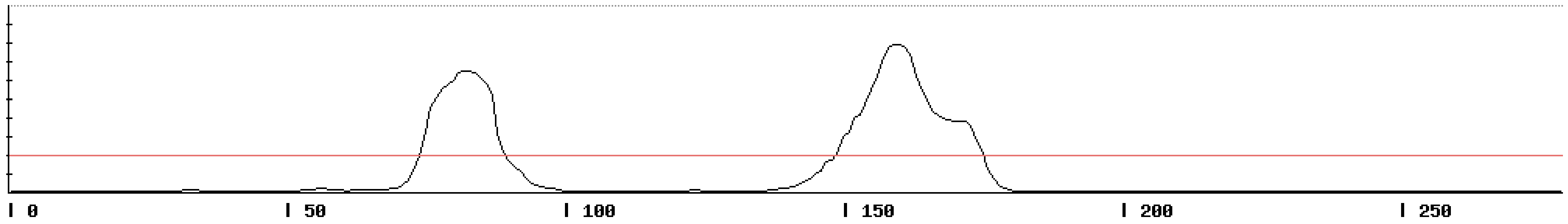

Predictions for Leersiaperrieri_LPERR07G05500.1

Viterbi Path

Viterbi Path

Posterior @ 0.2	72 - ARAKNGKGRRQKRTV - 86
	148 - KRLYRIRFSGHGRKRKREPEN - 168
	303 - AKNGKGSRQRR - 313
	385 - RIRFSGHGKKRKREPENQSSSHDDGGGRARRK - 416

Posterior @ 0.2

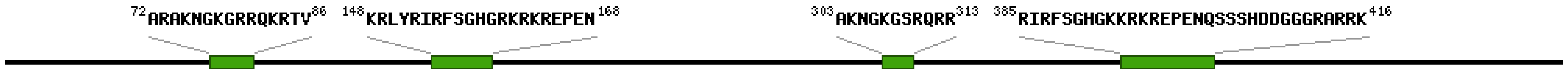

Posterior Graphic. Threshold Marked @ 0.2

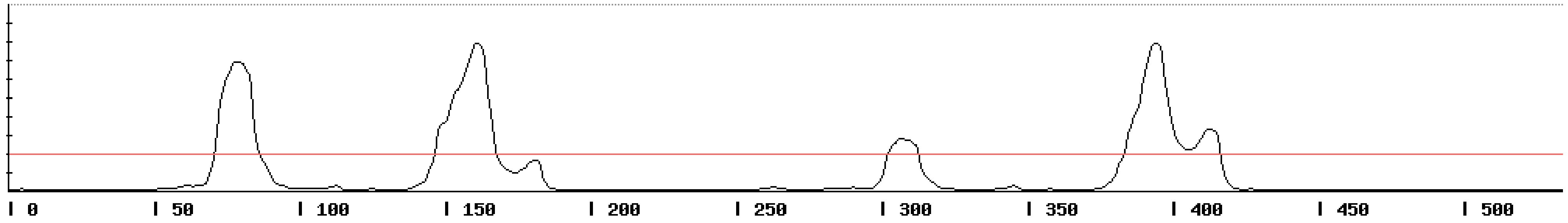

Predictions for Leersiaperrieri_LPERR07G05530.1

Viterbi Path

Viterbi Path

Posterior @ 0.2	70 - FGKARAKNGKGSRQKRTV - 87
	157 - RIRFSGHGKKRKREPENQ - 174
	182 - GGGRARRK - 189
	392 - ARAKNGKGRRQRRTV - 406
	467 - TKRLYRIRFSGHGKKRKREPENQS - 490
	499 - RARRK - 503

Posterior @ 0.2

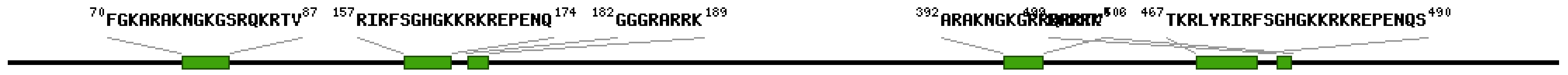

Posterior Graphic. Threshold Marked @ 0.2

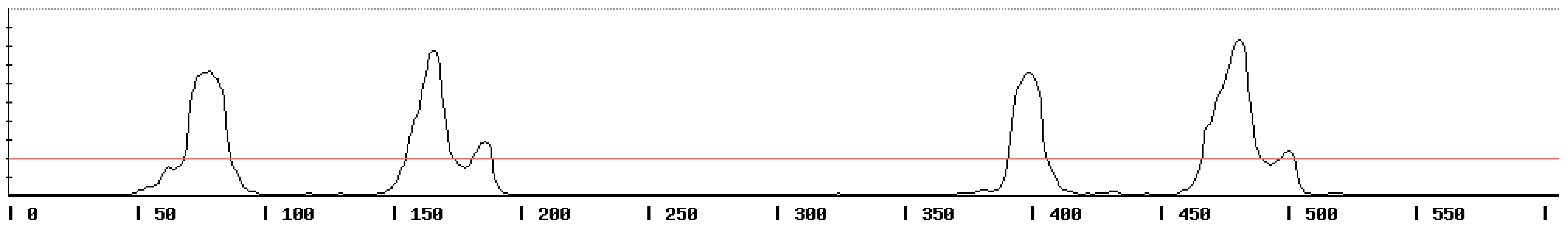

Predictions for Leersiaperrieri_LPERR07G06440.1

Viterbi Path

Viterbi Path

Posterior @ 0.2

Posterior @ 0.2

Posterior Graphic. Threshold Marked @ 0.2

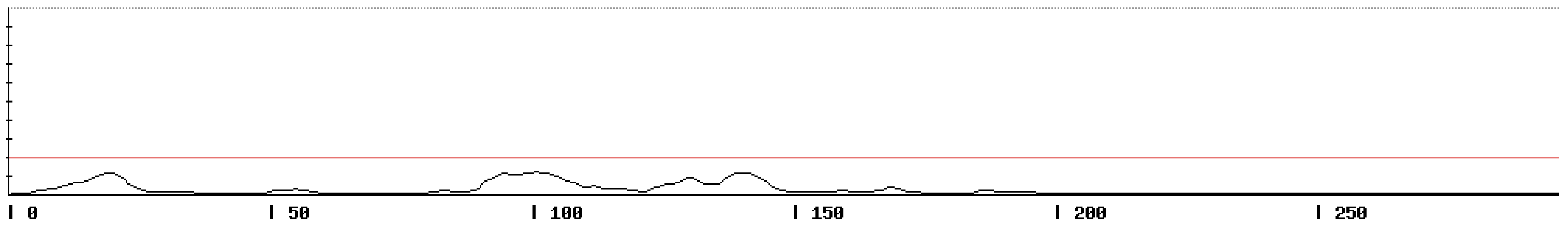

Predictions for Leersiaperrieri_LPERR07G07240.1

Viterbi Path

Viterbi Path

Posterior @ 0.2

69 - HAKNGKGKRQKRTV - 82

149 - GYGKKRKREP - 158

Posterior @ 0.2

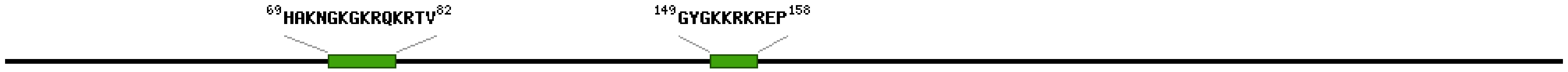

Posterior Graphic. Threshold Marked @ 0.2

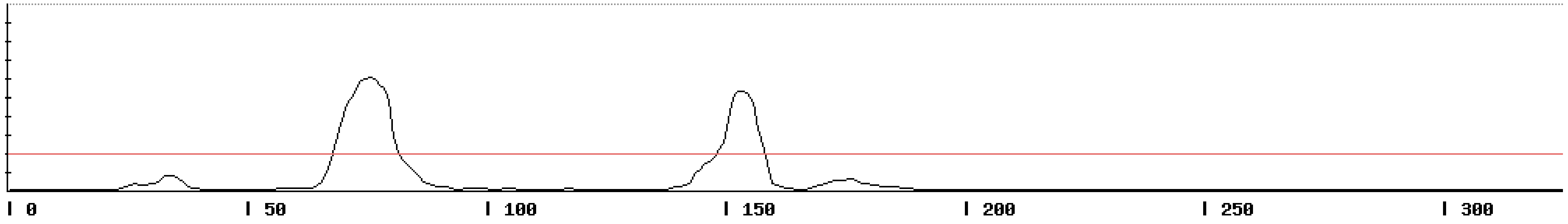

Predictions for Leersiaperrieri_LPERR07G08560.1

Viterbi Path

Viterbi Path

Posterior @ 0.2

73 - EARAKNNKGKREKRTV - 88

150 - RVRFSGHGKKRRREPES - 166

337 - RSKNTKGKRQKRT - 349

Posterior @ 0.2

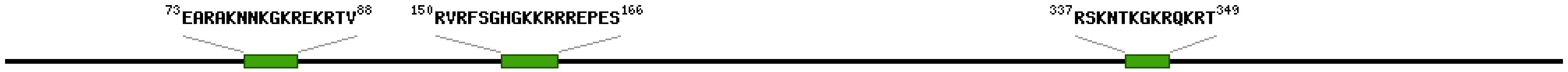

Posterior Graphic. Threshold Marked @ 0.2

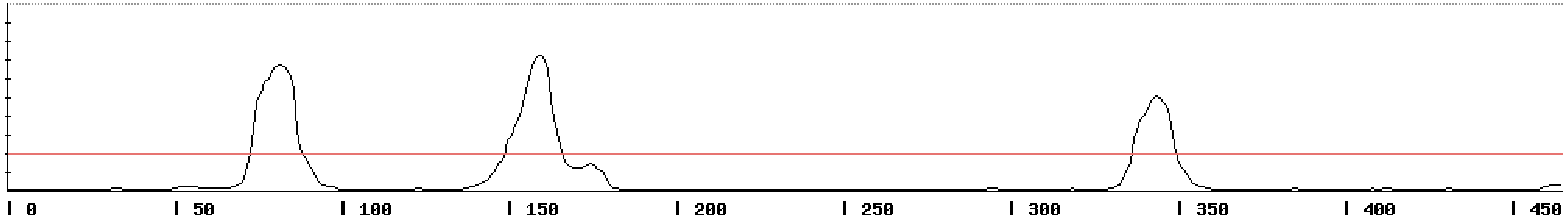

Predictions for Leersiaperrieri_LPERR07G08570.1

Viterbi Path

Viterbi Path

Posterior @ 0.2 144 - VRFSGHGKKPRREP - 157

Posterior @ 0.2

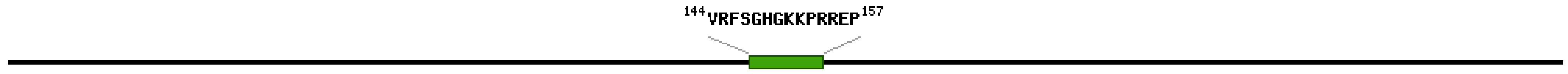

Posterior Graphic. Threshold Marked @ 0.2

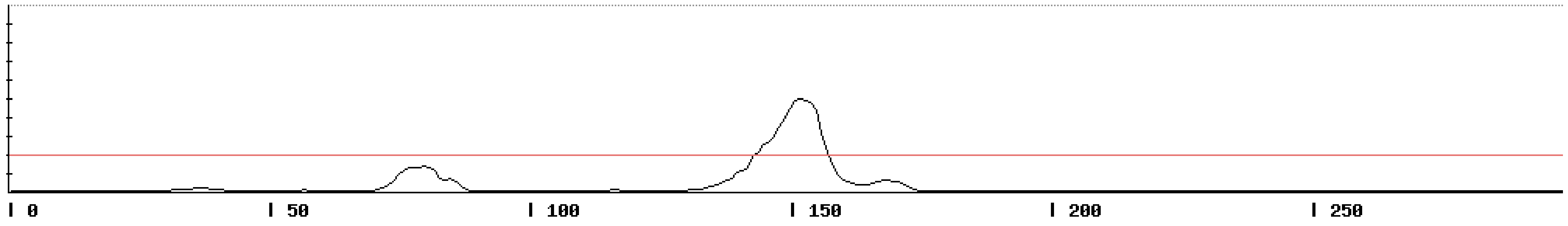

Predictions for Leersiaperrieri_LPERR07G08580.1

Viterbi Path

Viterbi Path

Posterior @ 0.2

74 - IRAKNTKGKRQKRT - 87

147 - RLYRVRFSGHGKKRRREPQ - 165

Posterior @ 0.2

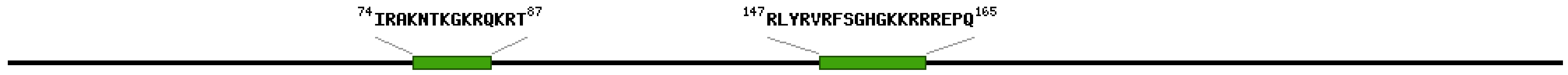

Posterior Graphic. Threshold Marked @ 0.2

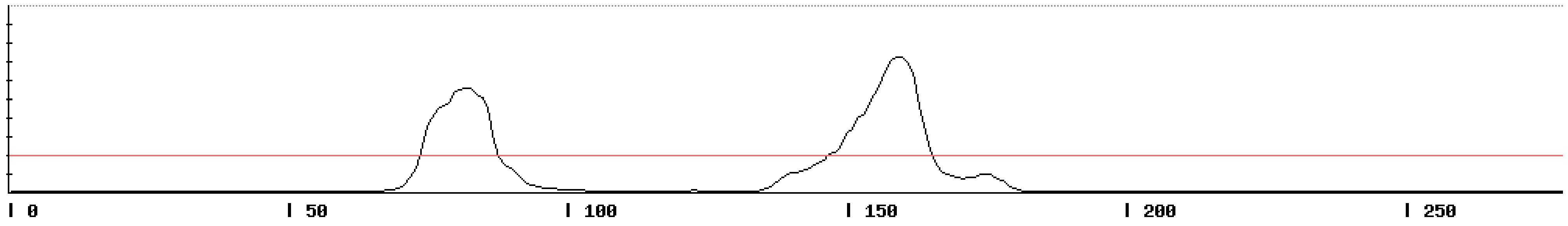

Predictions for Leersiaperrieri_LPERR07G10030.1

Viterbi Path

Viterbi Path

Posterior @ 0.2

73 - EARAKNGKGKRQKRTV - 88

150 - RVRFSGHGRKRRREPES - 166

Posterior @ 0.2

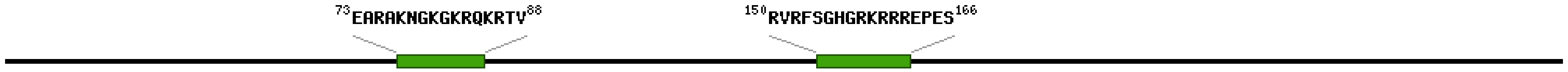

Posterior Graphic. Threshold Marked @ 0.2

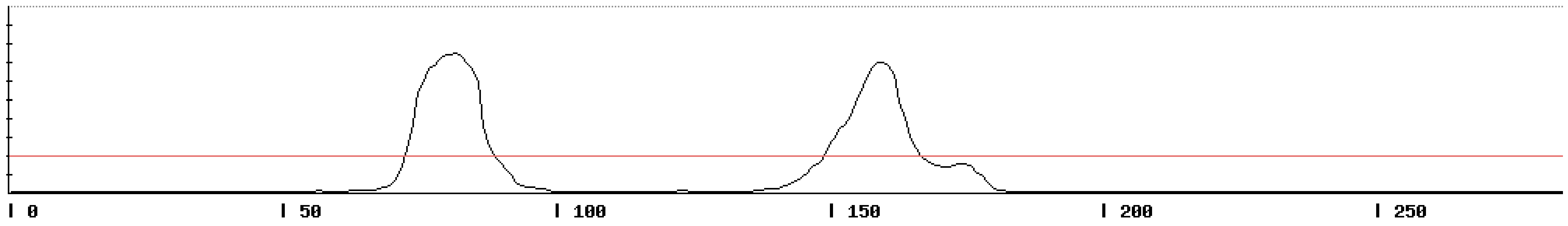

Predictions for Leersiaperrieri_LPERR07G10040.1

Viterbi Path

Viterbi Path

Posterior @ 0.2

72 - AKARAKNSKGKRQNR - 86

147 - SGHGKKRRREPE - 158

Posterior @ 0.2

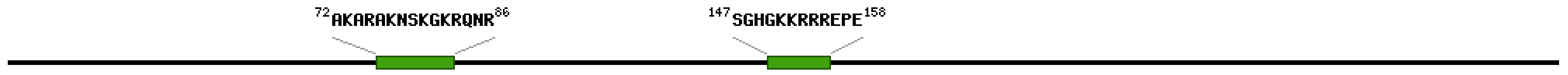

Posterior Graphic. Threshold Marked @ 0.2

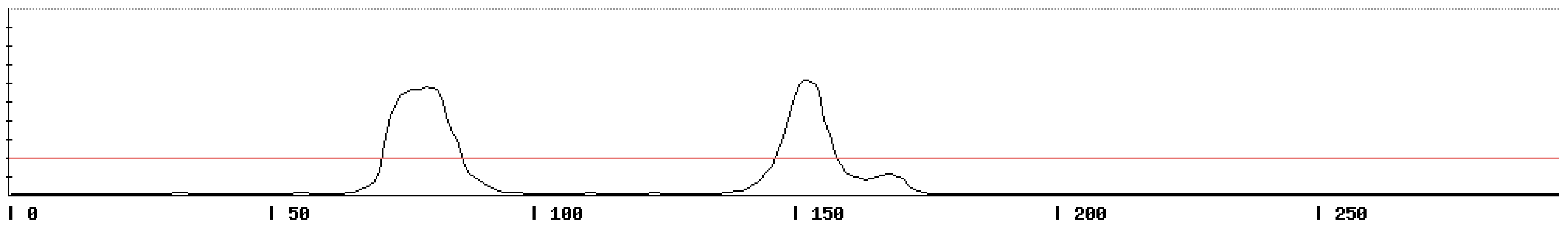

Predictions for Leersiaperrieri_LPERR07G10050.1

Viterbi Path

Viterbi Path

Posterior @ 0.2

73 - EARAKNGKGKRQKRTV - 88

150 - RVRFSGHGRKRRREPES - 166

Posterior @ 0.2

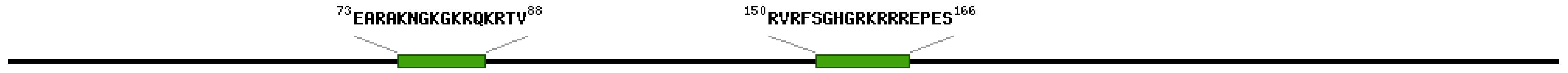

Posterior Graphic. Threshold Marked @ 0.2

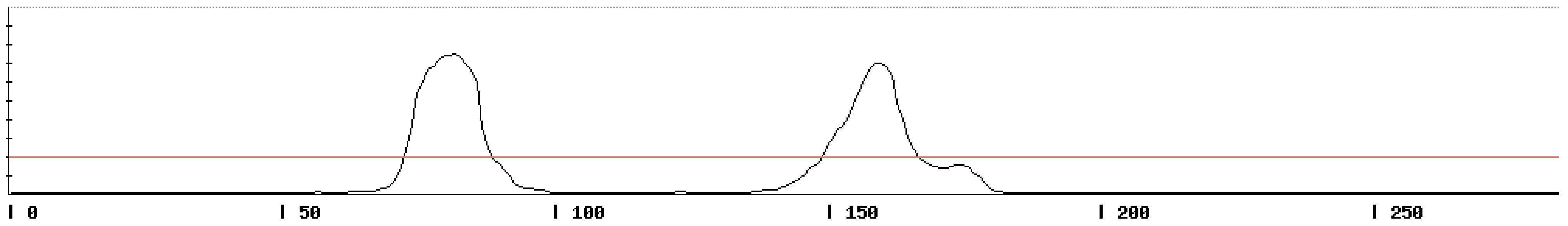

Predictions for Leersiaperrieri_LPERR07G12300.1

Viterbi Path

Viterbi Path

Posterior @ 0.2	84 - RGRR - 87<br>181 - GRKRK - 185
Posterior @ 0.2	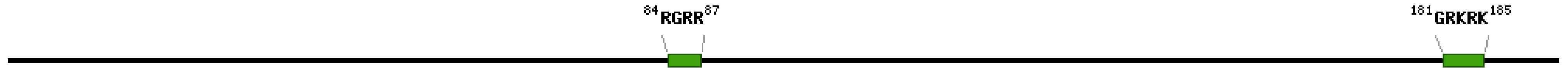
Posterior Graphic. Threshold Marked @ 0.2	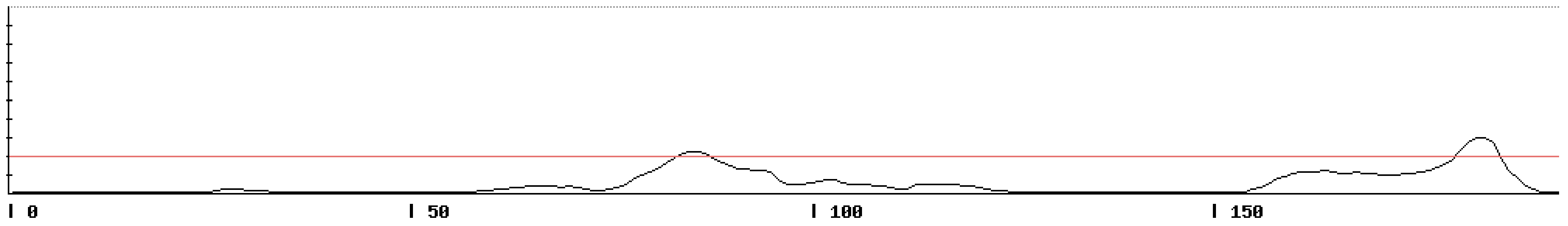

Predictions for Leersiaperrieri_LPERR07G12470.1

Viterbi Path	
Viterbi Path	
Posterior @ 0.2	83 - GRGRRVGG - 90<br>172 - IGRKRK - 177
Posterior @ 0.2	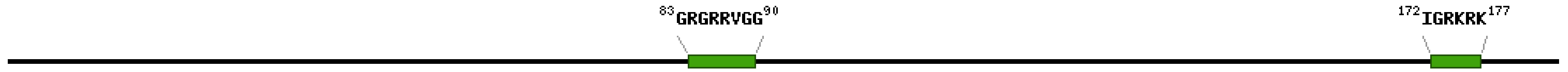
Posterior Graphic. Threshold Marked @ 0.2	

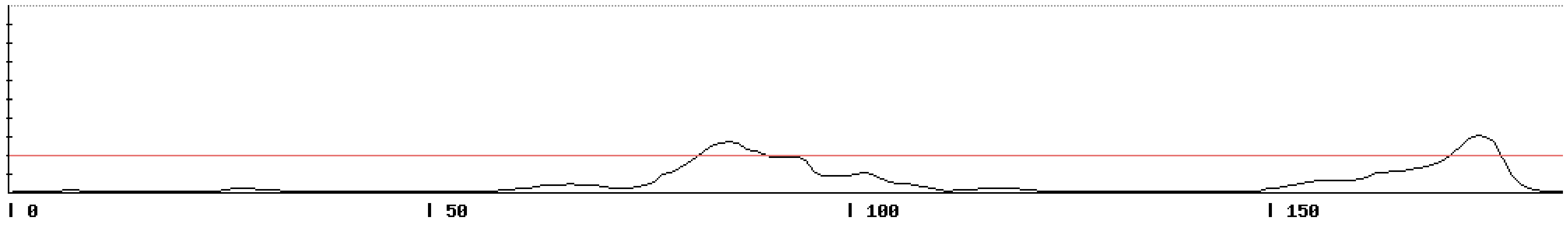

Predictions for Leersiaperrieri_LPERR07G12620.1

Viterbi Path	
Viterbi Path	
Posterior @ 0.2	116 - LRRK - 119
	325 - GRKRKAG - 331
Posterior @ 0.2	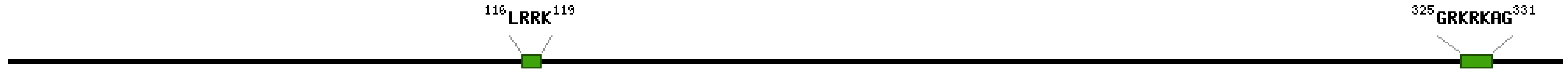
Posterior Graphic. Threshold Marked @ 0.2	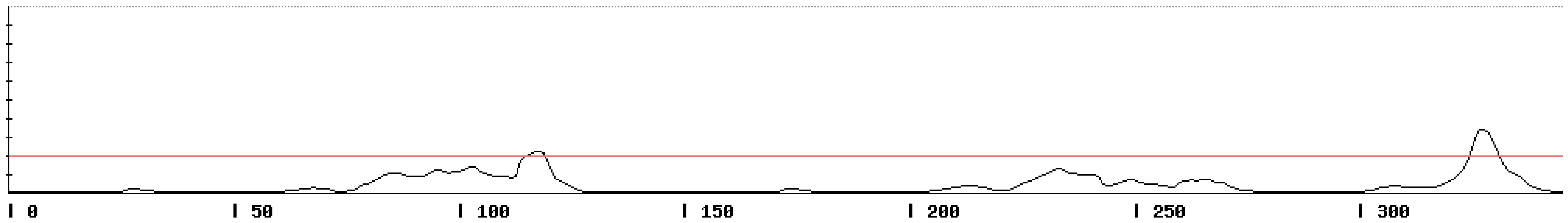

Predictions for Leersiaperrieri_LPERR07G12690.1

Viterbi Path	
Viterbi Path	
Posterior @ 0.2	178 - RKRK - 181
Posterior @ 0.2	

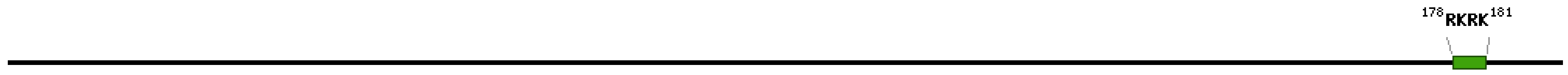

Posterior Graphic. Threshold Marked @ 0.2

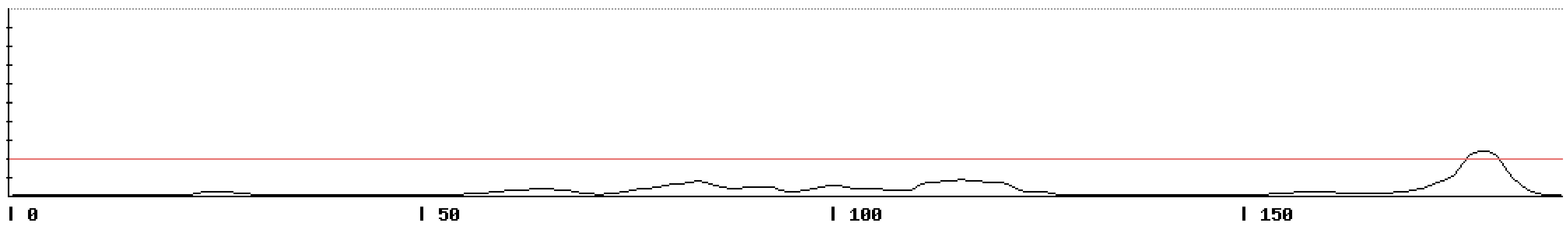

Predictions for Leersiaperrieri_LPERR07G12700.1

Viterbi Path

Viterbi Path

Posterior @ 0.2

80 - GGRGRRVGGDGSK - 92

170 - AGRKRK - 175

Posterior @ 0.2

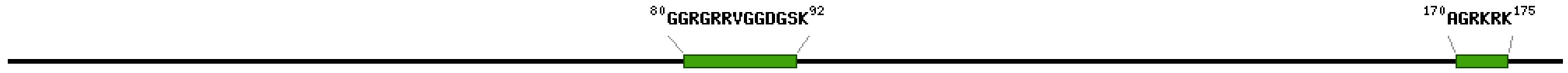

Posterior Graphic. Threshold Marked @ 0.2

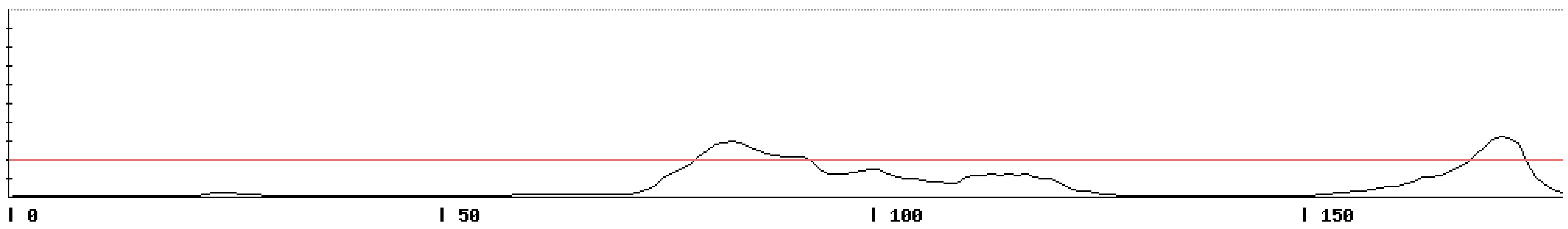

Predictions for Leersiaperrieri_LPERR07G13460.1

Viterbi Path

Viterbi Path

Posterior @ 0.2

79 - KPKNGRTARRS - 89

163 - PRAPSRKRKAA - 173

Posterior @ 0.2

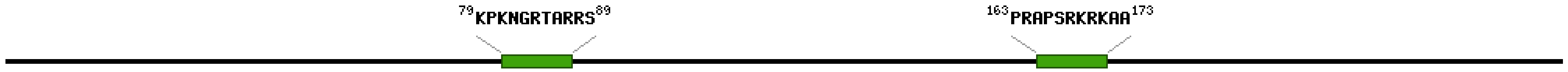

Posterior Graphic. Threshold Marked @ 0.2

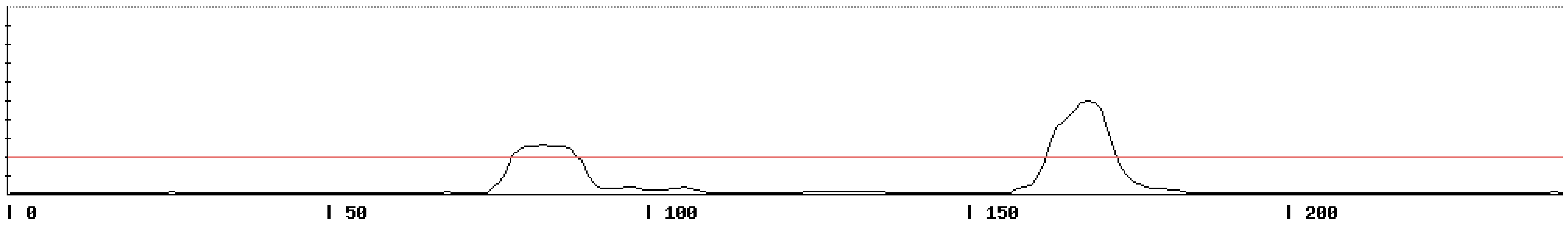

Predictions for Leersiaperrieri_LPERR07G13470.1

Viterbi Path

Viterbi Path

Posterior @ 0.2

12 - DRRKKL - 17

202 - KPKNGRTARRSR - 213

320 - PRAPSRKRKAA - 330

387 - DSRKKLAVKKAPAPAP - 402

Posterior @ 0.2

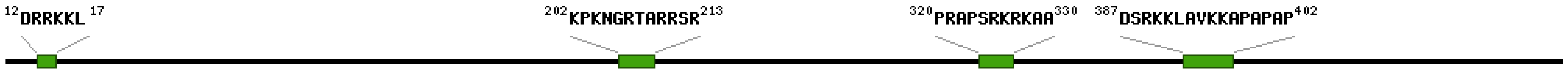

Posterior Graphic. Threshold Marked @ 0.2

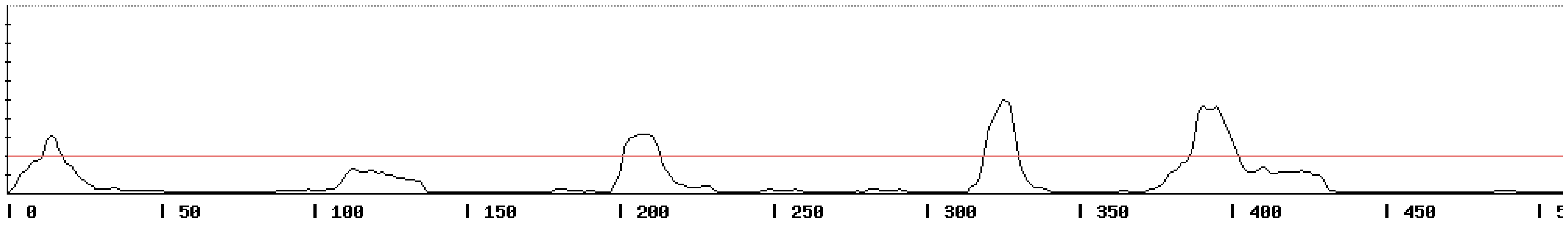

Predictions for Leersiaperrieri_LPERR07G13540.1

Viterbi Path

Viterbi Path

Posterior @ 0.2

79 - KPKNAGKGTIRRSRTIGG - 96

165 - PRAPSRKRKAA - 175

Posterior @ 0.2

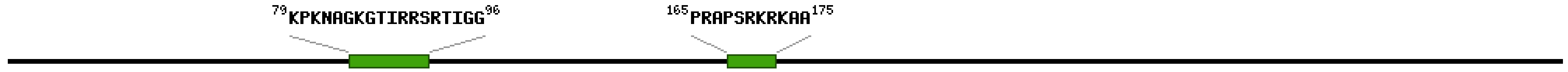

Posterior Graphic. Threshold Marked @ 0.2

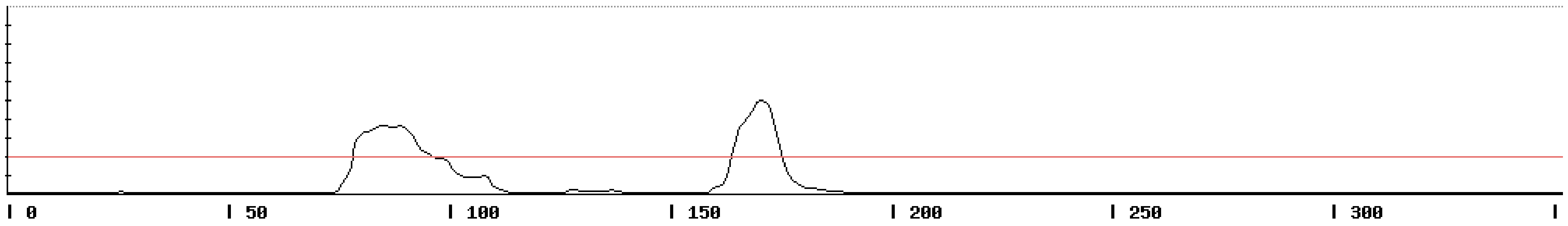

Predictions for Leersiaperrieri_LPERR07G13560.1

Viterbi Path

Viterbi Path

Posterior @ 0.2

44 - KNGRTTRR - 51

126 - PRAPSRKRKAA - 136

Posterior @ 0.2

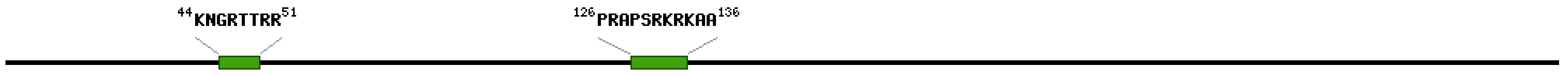

Posterior Graphic. Threshold Marked @ 0.2

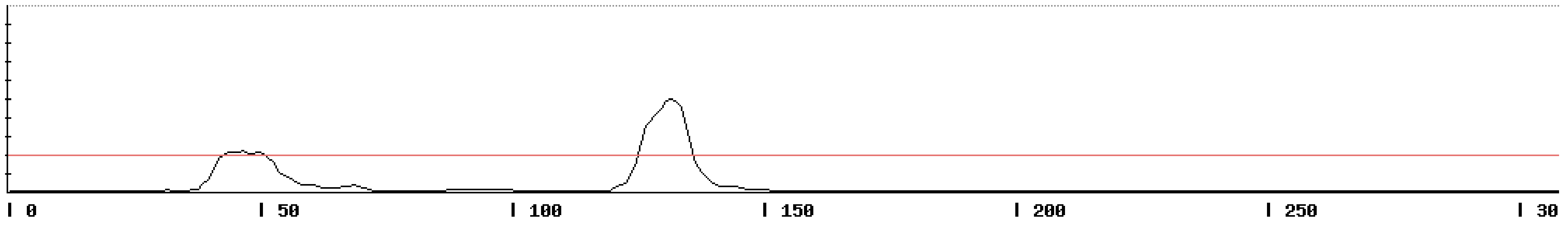

Predictions for Leersiaperrieri_LPERR07G16320.1

Viterbi Path

Viterbi Path

Posterior @ 0.2

130 - VKKAL - 134

136 - FYRGKPPKGI - 145

Posterior @ 0.2

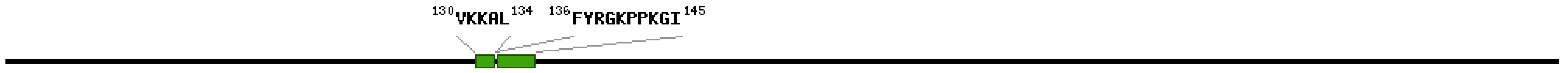

Posterior Graphic. Threshold Marked @ 0.2

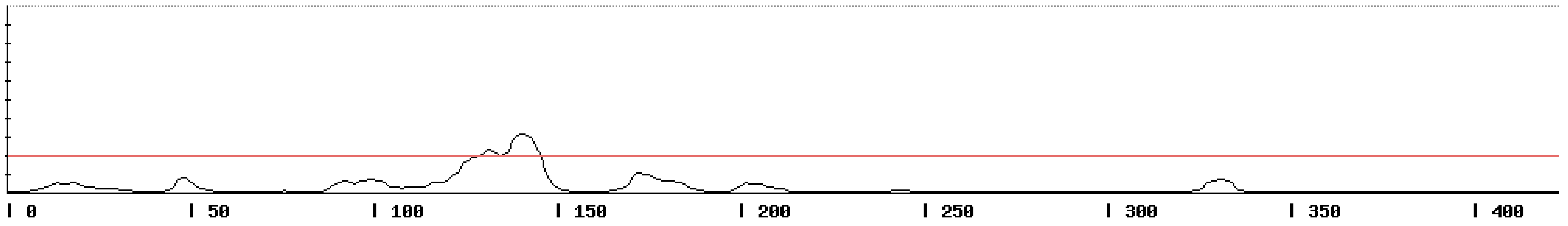

Predictions for Leersiaperrieri_LPERR07G23420.1

Viterbi Path

Viterbi Path

Posterior @ 0.2	312 - IKRKAS - 317
Posterior @ 0.2	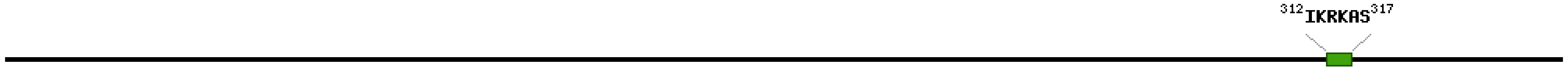
Posterior Graphic. Threshold Marked @ 0.2	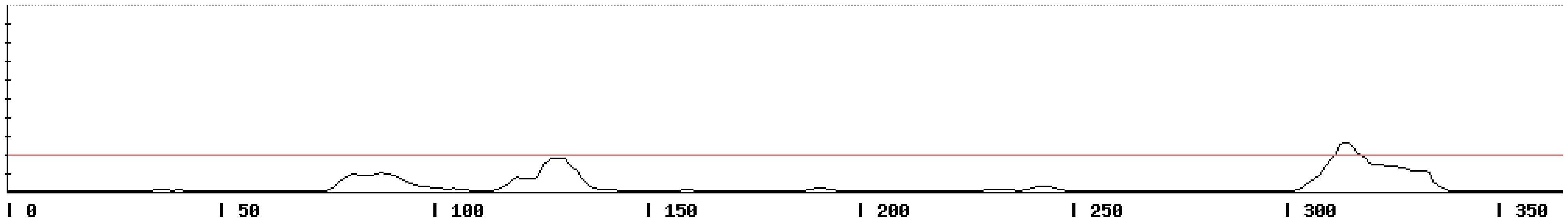

Predictions for Leersiaperrieri_LPERR07G23450.1

Viterbi Path	
Viterbi Path	
Posterior @ 0.2	
Posterior @ 0.2	
Posterior Graphic. Threshold Marked @ 0.2	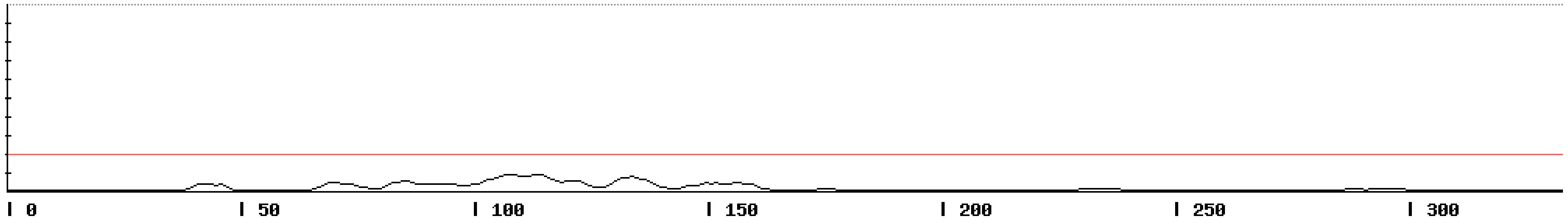

Predictions for Leersiaperrieri_LPERR08G00380.1

Viterbi Path

Viterbi Path

Posterior @ 0.2

Posterior @ 0.2

Posterior Graphic. Threshold Marked @ 0.2

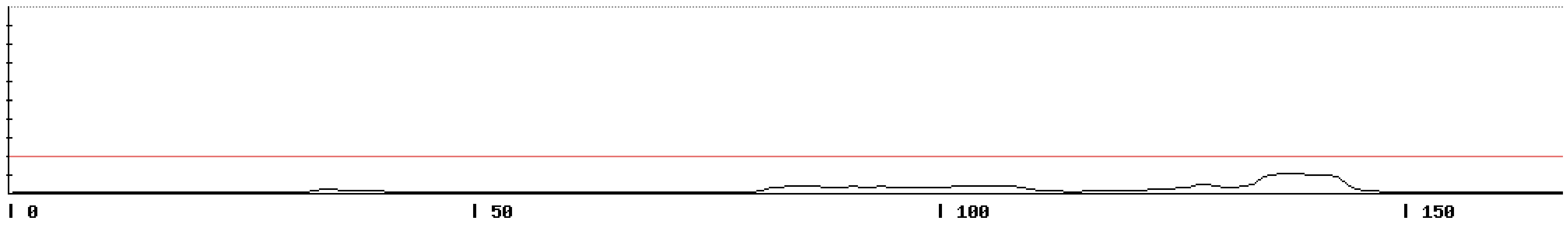

Predictions for Leersiaperrieri_LPERR08G01330.1

Viterbi Path

Viterbi Path

Posterior @ 0.2

Posterior @ 0.2

Posterior Graphic. Threshold Marked @ 0.2

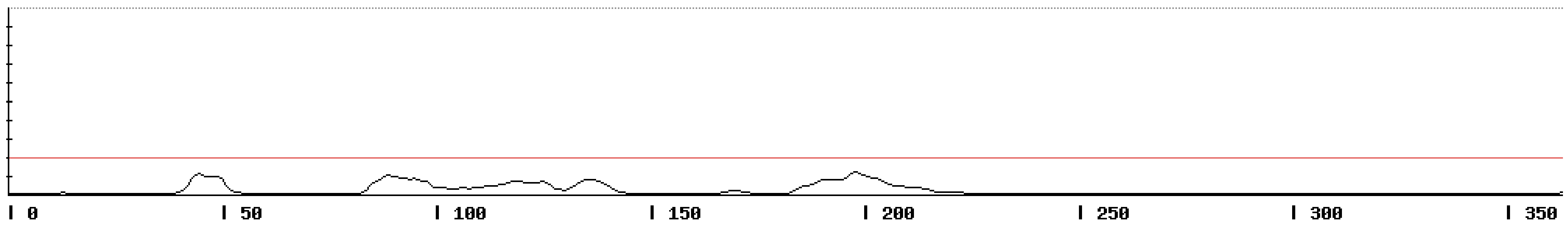

Predictions for Leersiaperrieri_LPERR08G01440.1	
Viterbi Path	
Viterbi Path	
Posterior @ 0.2	173 - KKKHHHKEYSGGGKN - 187
Posterior @ 0.2	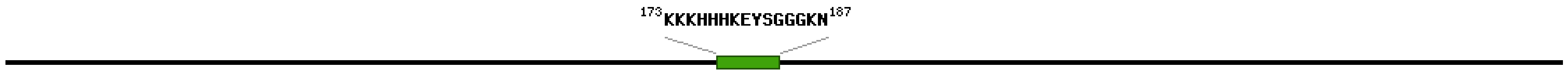
Posterior Graphic. Threshold Marked @ 0.2	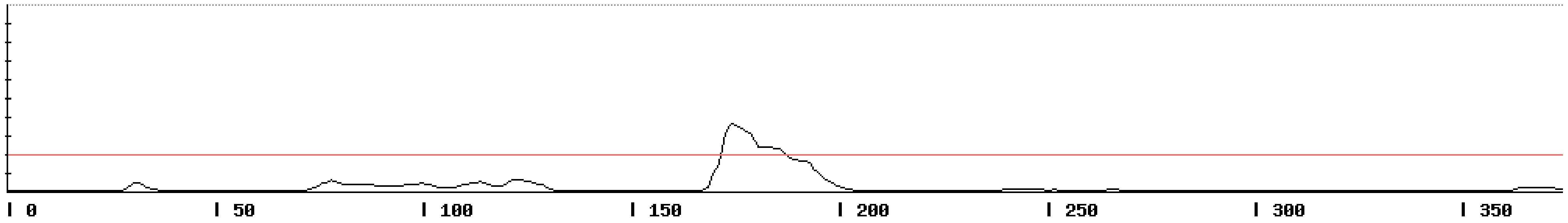

Predictions for Leersiaperrieri_LPERR08G03510.1	
Viterbi Path	
Viterbi Path	
Posterior @ 0.2	76 - RKYPNGSRSNRATEAGYWKATGKDRIIKSKGDKKKQHMIGMKKTLVFHRGRAPKGERT - 133
Posterior @ 0.2	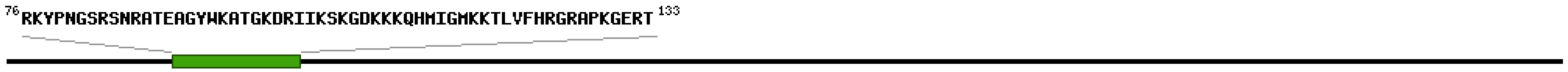
Posterior Graphic. Threshold Marked @ 0.2	

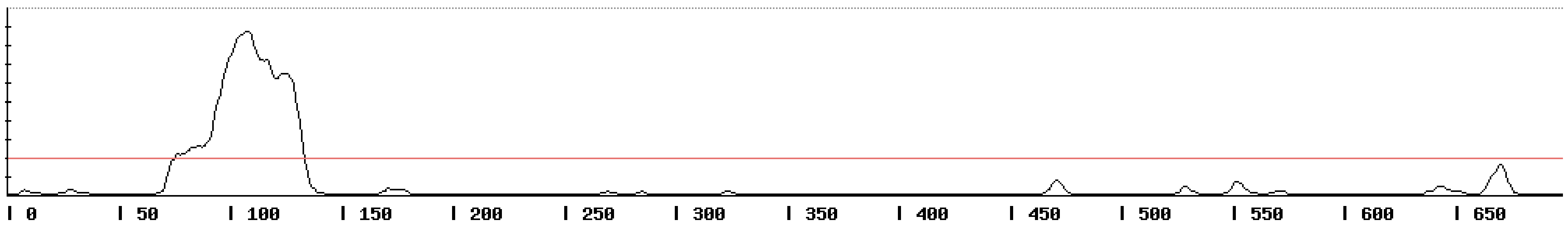

Predictions for Leersiaperrieri_LPERR08G05360.1

Viterbi Path

Viterbi Path

Posterior @ 0.2 122 - YRGRAPKGRK - 131

Posterior @ 0.2

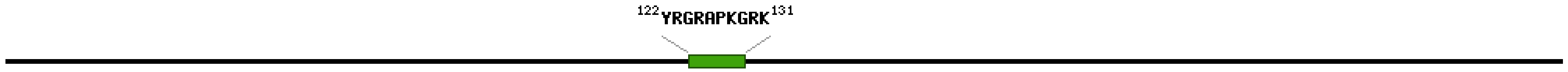

Posterior Graphic. Threshold Marked @ 0.2

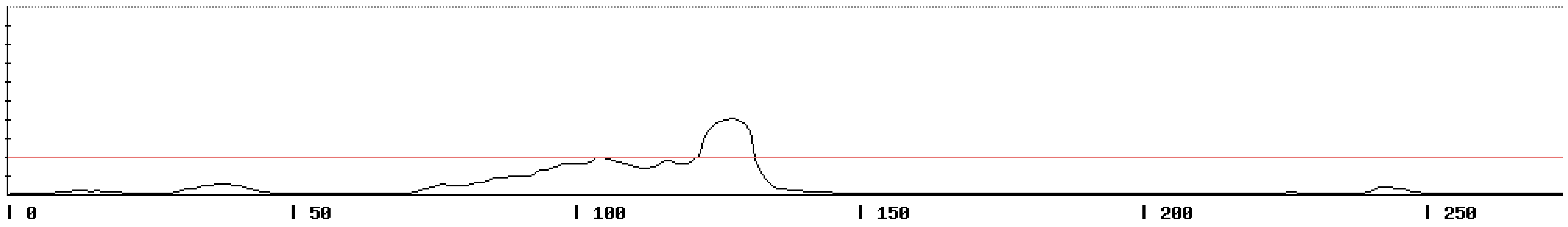

Predictions for Leersiaperrieri_LPERR08G06210.1

Viterbi Path

Viterbi Path

Posterior @ 0.2

Posterior @ 0.2

Posterior Graphic. Threshold Marked @ 0.2

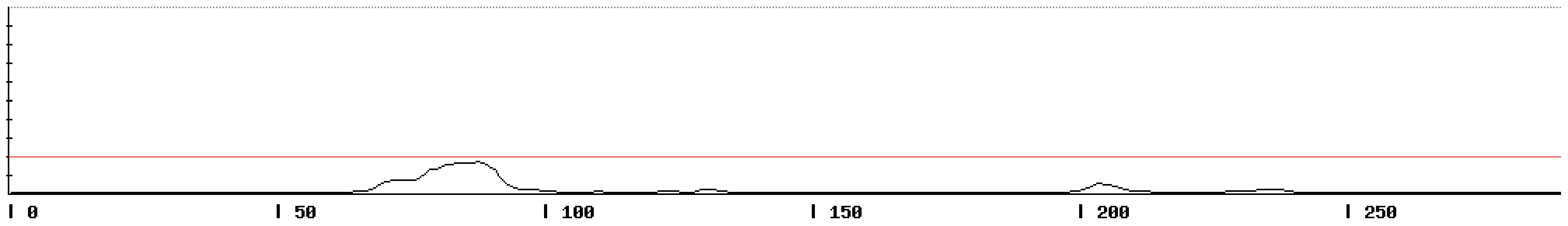

Predictions for Leersiaperrieri_LPERR08G06220.1

Viterbi Path

Viterbi Path

Posterior @ 0.2

Posterior @ 0.2

Posterior Graphic. Threshold Marked @ 0.2

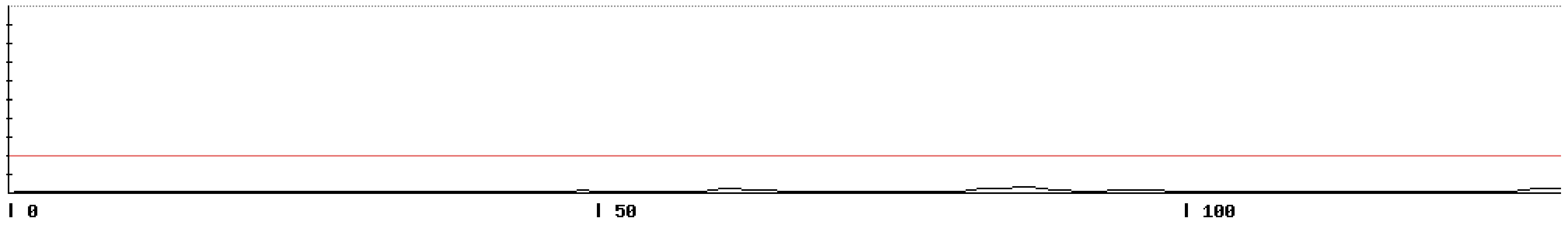

Predictions for Leersiaperrieri_LPERR08G06230.1

Viterbi Path

Viterbi Path

Posterior @ 0.2

69 - KKNGKHFFTRKENKNPGDQRRNR - 91

Posterior @ 0.2

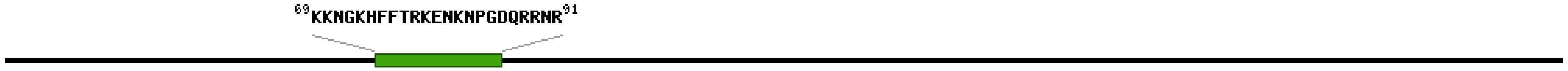

Posterior Graphic. Threshold Marked @ 0.2

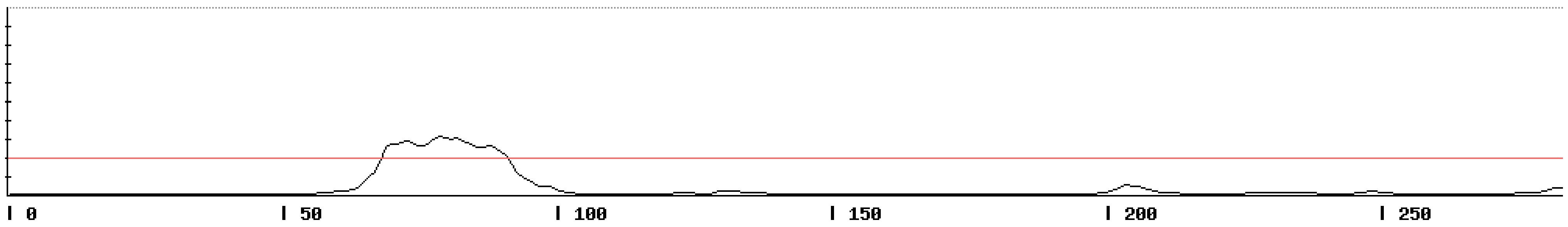

Predictions for Leersiaperrieri_LPERR08G09130.1

Viterbi Path

Viterbi Path

Posterior @ 0.2

Posterior @ 0.2

Posterior Graphic. Threshold Marked @ 0.2

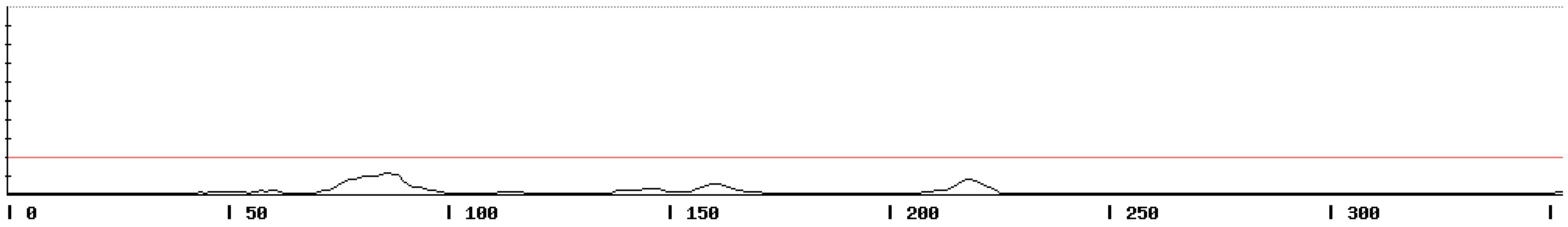

Predictions for Leersiaperrieri_LPERR08G12970.1

Viterbi Path

Viterbi Path

Posterior @ 0.2

Posterior @ 0.2

Posterior Graphic. Threshold Marked @ 0.2

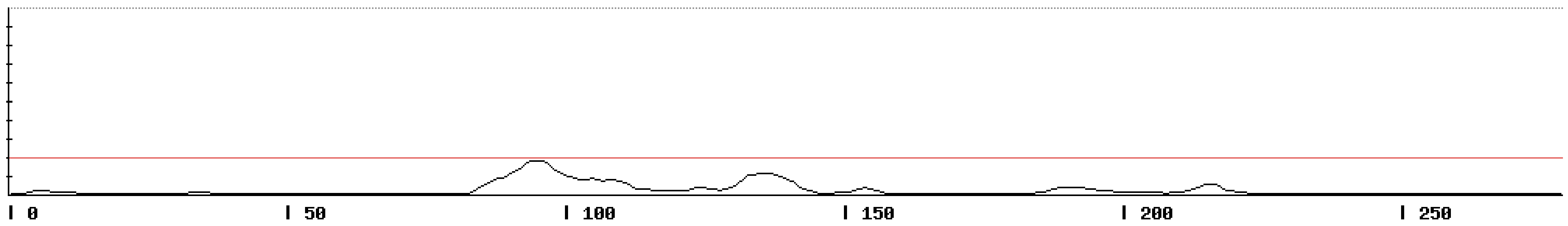

Predictions for Leersiaperrieri_LPERR08G13170.1

Viterbi Path

Viterbi Path

Posterior @ 0.2

Posterior @ 0.2

Posterior Graphic. Threshold Marked @ 0.2

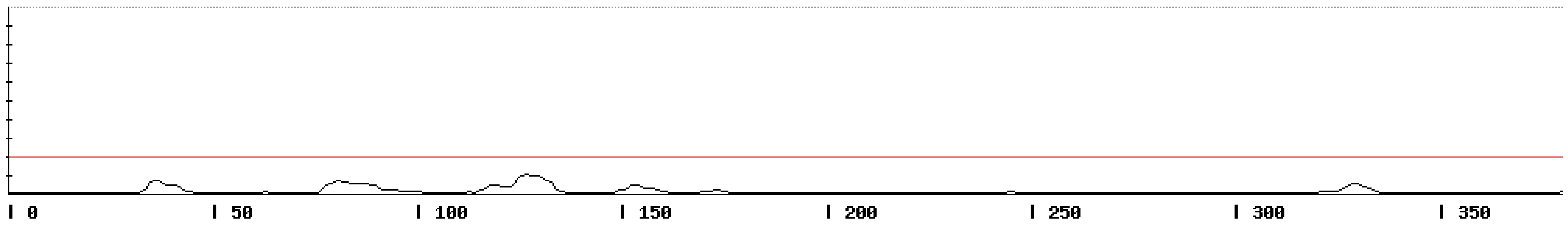

Predictions for Leersiaperrieri_LPERR08G17020.1

Viterbi Path

Viterbi Path

Posterior @ 0.2	145 - KGRAPRGEK - 153
Posterior @ 0.2	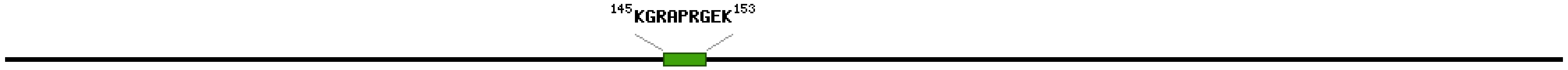
Posterior Graphic. Threshold Marked @ 0.2	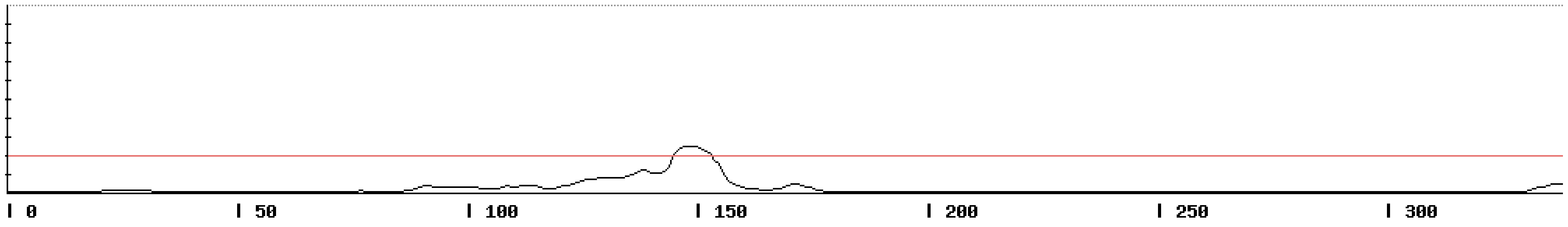

Predictions for Leersiaperrieri_LPERR08G18520.1

Viterbi Path	
Viterbi Path	
Posterior @ 0.2	87 - GGGKNRR - 93
Posterior @ 0.2	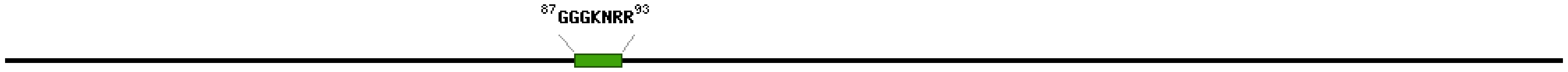
Posterior Graphic. Threshold Marked @ 0.2	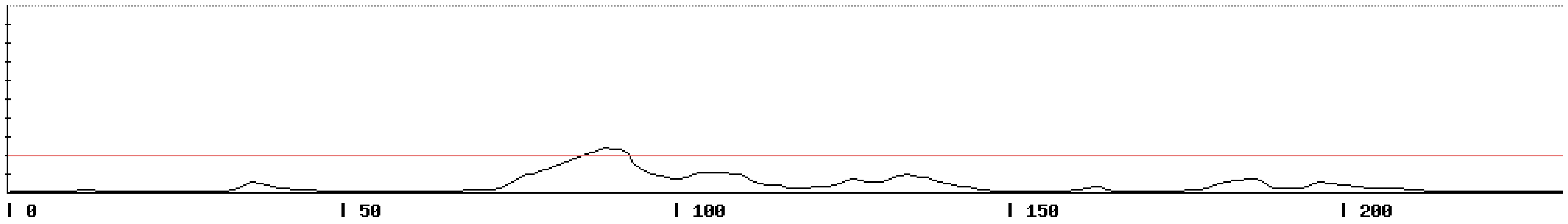

Predictions for Leersiaperrieri_LPERR08G20450.1

Viterbi Path

Viterbi Path

Posterior @ 0.2
97 - RKHANRARTNR - 107
119 - KDREVRHGPRT - 129

Posterior @ 0.2

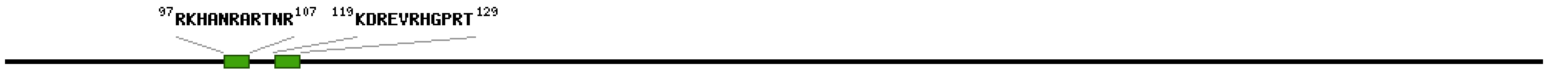

Posterior Graphic. Threshold Marked @ 0.2

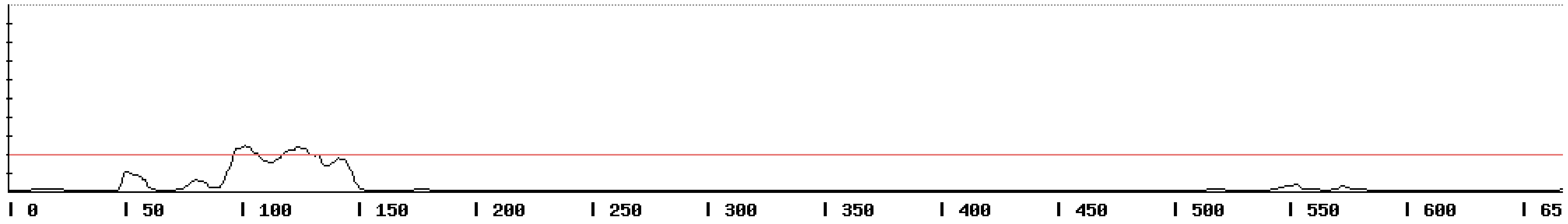

Predictions for Leersiaperrieri_LPERR09G02990.1

Viterbi Path

Viterbi Path

Posterior @ 0.2
79 - RRR - 81

Posterior @ 0.2

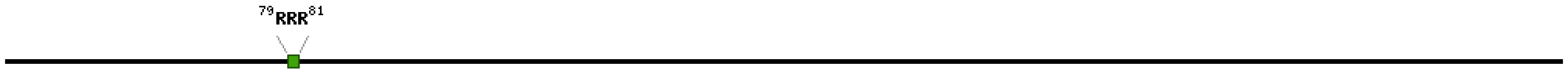

Posterior Graphic. Threshold Marked @ 0.2

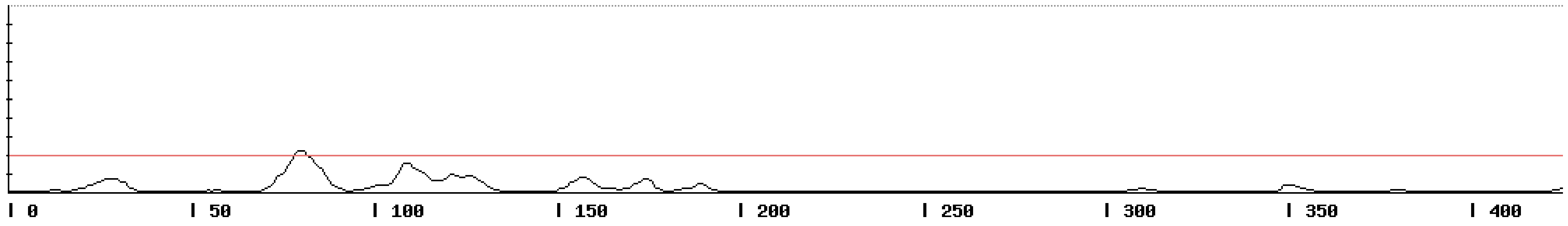

Predictions for Leersiaperrieri_LPERR09G06940.1

Viterbi Path

Viterbi Path

Posterior @ 0.2

Posterior @ 0.2

Posterior Graphic. Threshold Marked @ 0.2

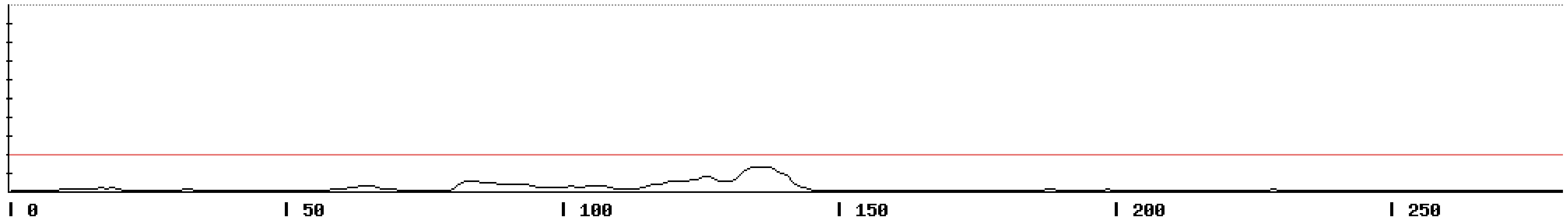

Predictions for Leersiaperrieri_LPERR09G07020.1

Viterbi Path

Viterbi Path

Posterior @ 0.2

Posterior @ 0.2

Posterior Graphic. Threshold Marked @ 0.2	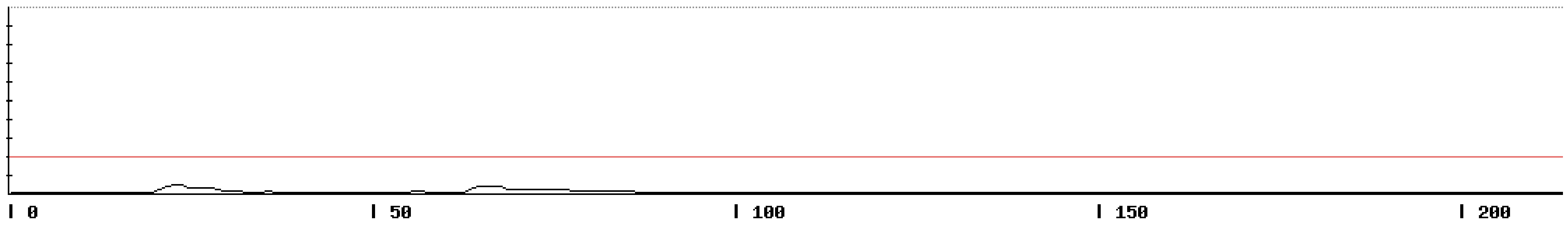

Predictions for Leersiaperrieri_LPERR09G11150.1	
Viterbi Path	
Viterbi Path	
Posterior @ 0.2	
Posterior @ 0.2	
Posterior Graphic. Threshold Marked @ 0.2	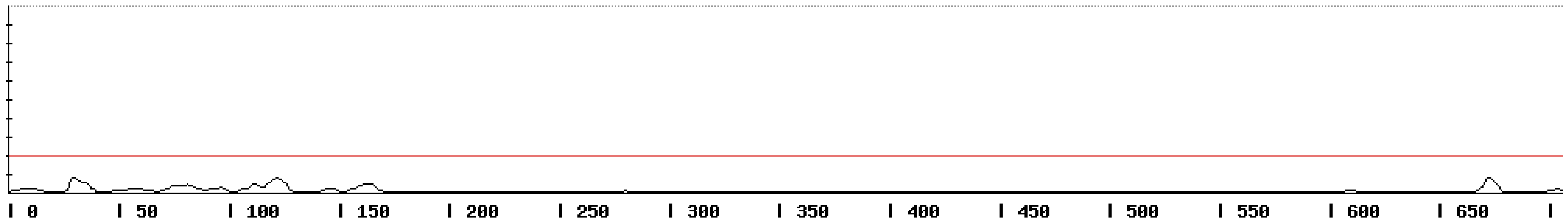

Predictions for Leersiaperrieri_LPERR09G11330.1	
Viterbi Path	
Viterbi Path	
Posterior @ 0.2	
Posterior @ 0.2	

Posterior Graphic. Threshold Marked @ 0.2

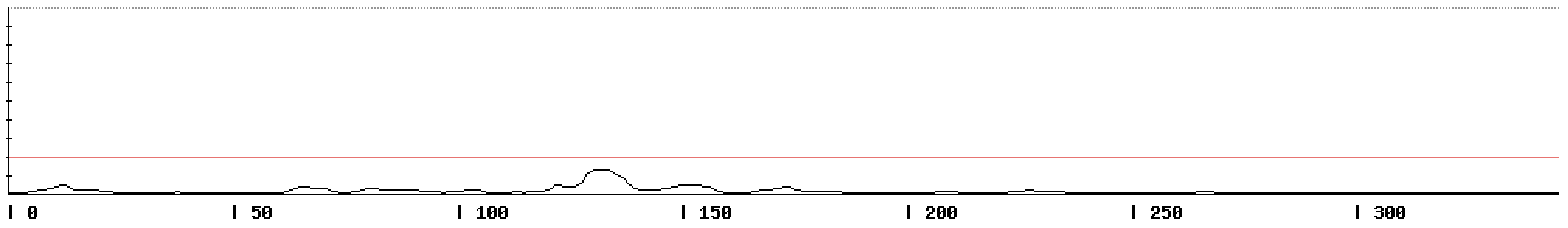

Predictions for Leersiaperrieri_LPERR09G14720.1

Viterbi Path

Viterbi Path

Posterior @ 0.2

Posterior @ 0.2

Posterior Graphic. Threshold Marked @ 0.2

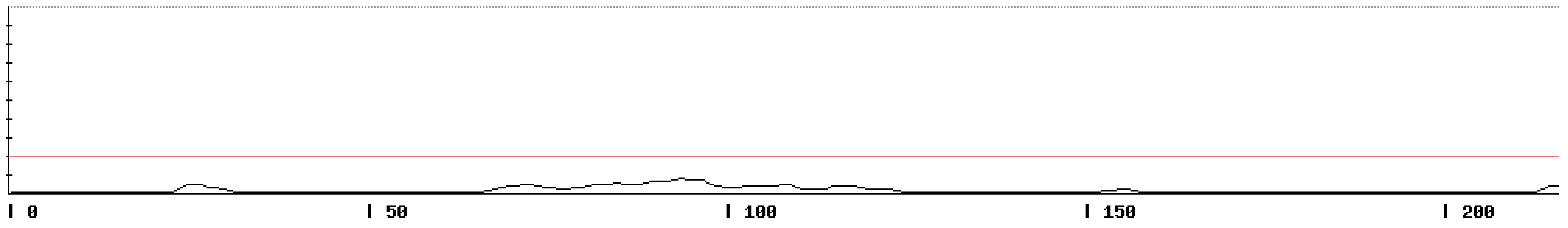

Predictions for Leersiaperrieri_LPERR09G14740.1

Viterbi Path

Viterbi Path

Posterior @ 0.2

Posterior @ 0.2

Posterior Graphic. Threshold Marked @ 0.2

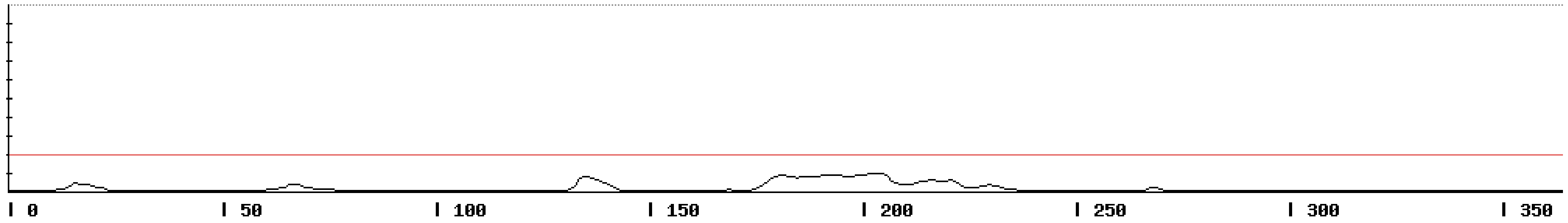

Predictions for Leersiaperrieri_LPERR10G02920.1

Viterbi Path: 187 - KKAQEDGKIRGNKRKRRATRKGMK - 210

Viterbi Path

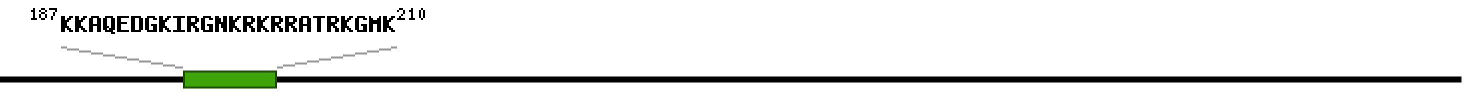

Posterior @ 0.2: 186 - EKKAQEDGKIRGNKRKRRATRKGMKNM - 212

Posterior @ 0.2

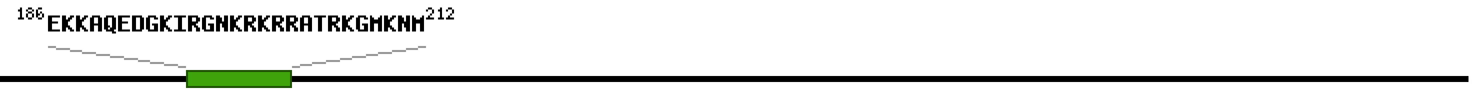

Posterior Graphic. Threshold Marked @ 0.2

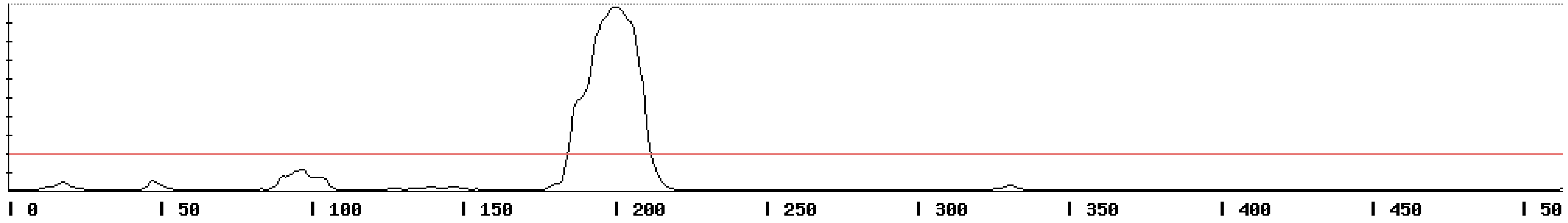

Predictions for Leersiaperrieri_LPERR10G04870.1

Viterbi Path

Viterbi Path

Posterior @ 0.2	
Posterior @ 0.2	
Posterior Graphic. Threshold Marked @ 0.2	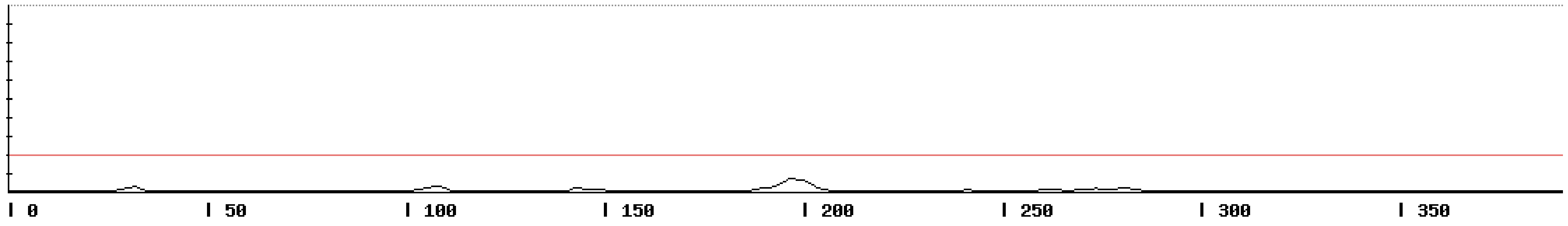

Predictions for Leersiaperrieri_LPERR10G06290.1

Viterbi Path	
Viterbi Path	
Posterior @ 0.2	83 - TGNKKRPIRVARGGTWKASGGSKTVRSKKVGG - 114
Posterior @ 0.2	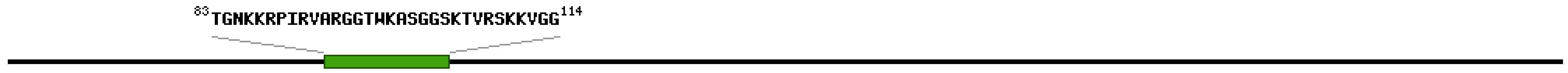
Posterior Graphic. Threshold Marked @ 0.2	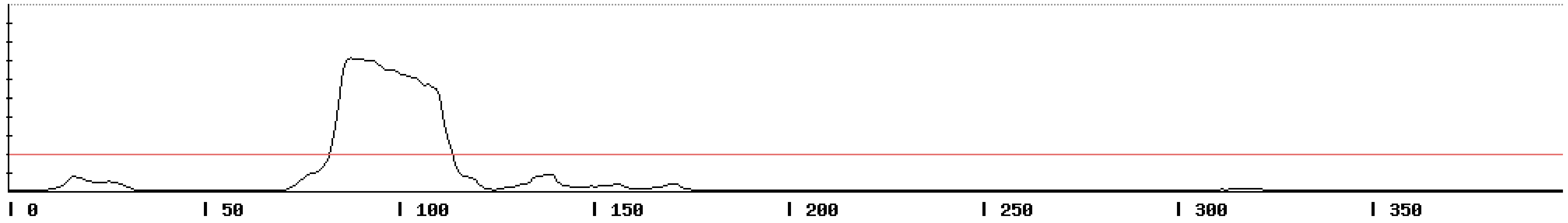

Predictions for Leersiaperrieri_LPERR10G09740.1

Viterbi Path

Viterbi Path

Posterior @ 0.2

Posterior @ 0.2

Posterior Graphic. Threshold Marked @ 0.2

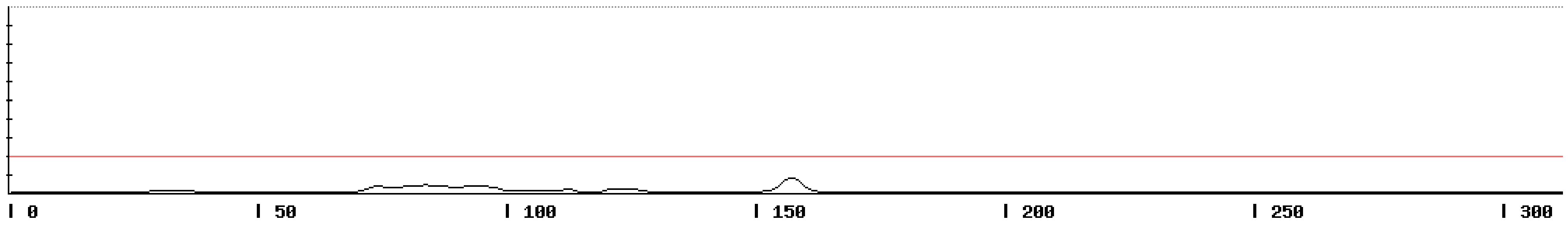

Predictions for Leersiaperrieri_LPERR10G15090.1

Viterbi Path

Viterbi Path

Posterior @ 0.2

Posterior @ 0.2

Posterior Graphic. Threshold Marked @ 0.2

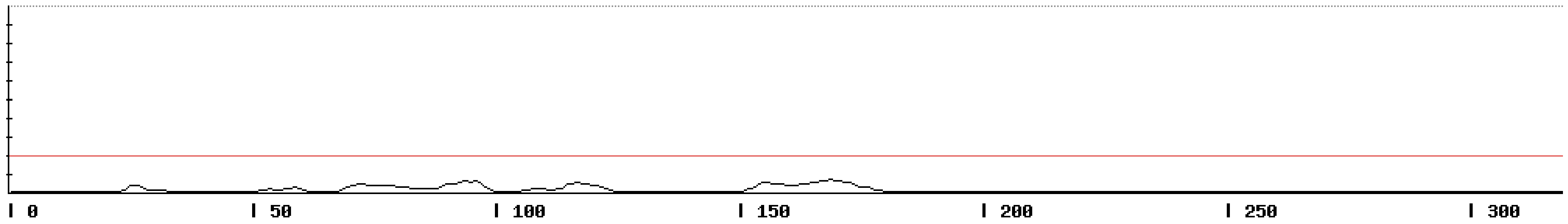

Predictions for Leersiaperrieri_LPERR11G01300.1

Viterbi Path	
Viterbi Path	
Posterior @ 0.2	124 - GKPPK - 128
Posterior @ 0.2	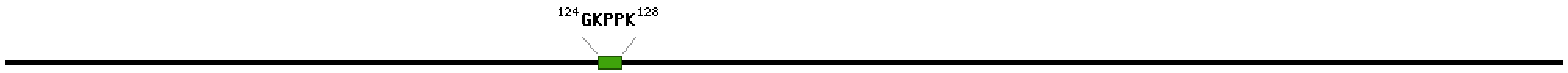
Posterior Graphic. Threshold Marked @ 0.2	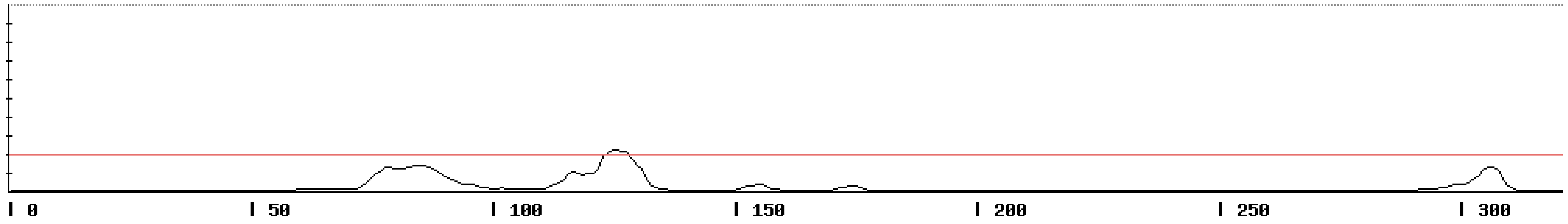

Predictions for Leersiaperrieri_LPERR11G02100.1

Viterbi Path	
Viterbi Path	
Posterior @ 0.2	
Posterior @ 0.2	
Posterior Graphic. Threshold Marked @ 0.2	

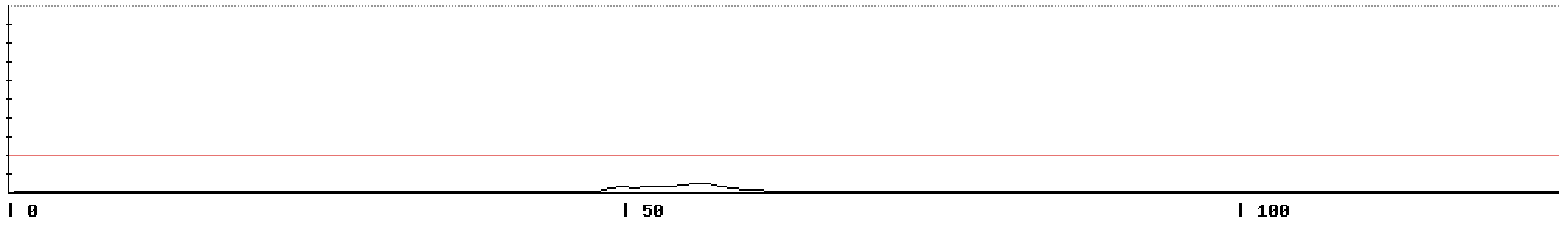

Predictions for Leersiaperrieri_LPERR11G02180.1

Viterbi Path	
Viterbi Path	
Posterior @ 0.2	76 - KWKGNKRGNA - 85
	157 - KSPRKNKGDENVVGKKRLR - 175
Posterior @ 0.2	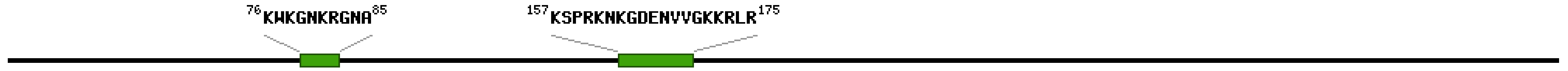
Posterior Graphic. Threshold Marked @ 0.2	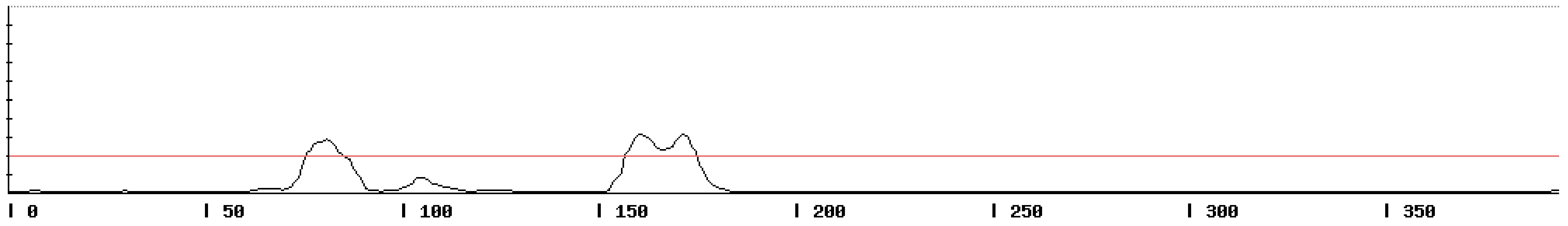

Predictions for Leersiaperrieri_LPERR11G02510.1

Viterbi Path	189 - KKAAEEKSSTNKRNRKAARKGRR - 211
Viterbi Path	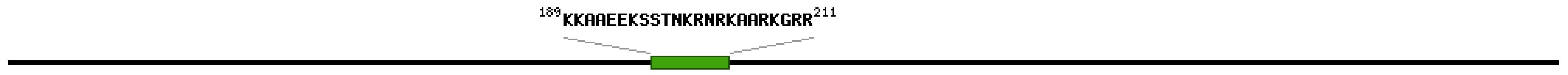
Posterior @ 0.2	95 - PSKTKKKGEPNRK - 107
	188 - EKKAAEEKSSTNKRNRKAARKGRRDM - 213
Posterior @ 0.2	

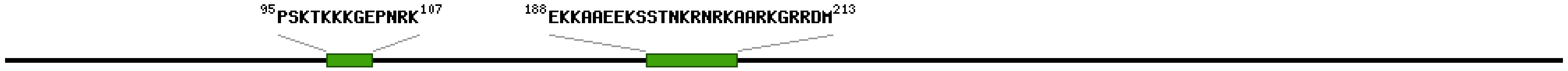

Posterior Graphic. Threshold Marked @ 0.2

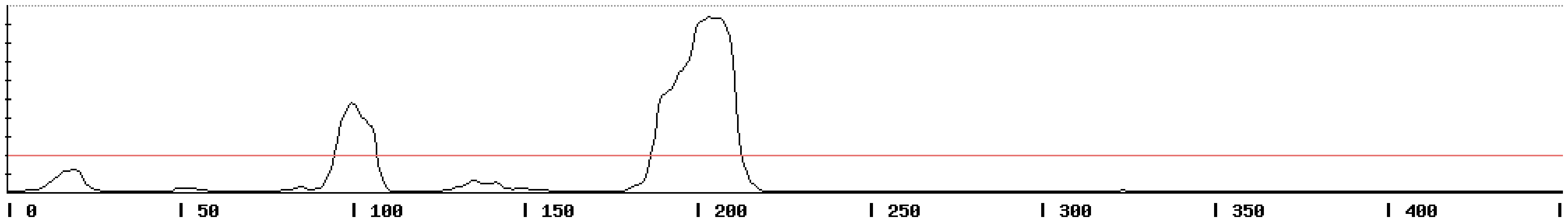

Predictions for Leersiaperrieri_LPERR11G02950.1

Viterbi Path

Viterbi Path

Posterior @ 0.2

Posterior @ 0.2

Posterior Graphic. Threshold Marked @ 0.2

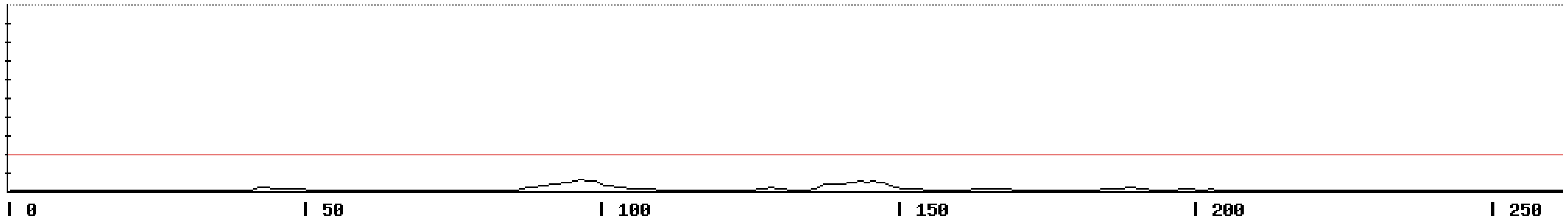

Predictions for Leersiaperrieri_LPERR11G04670.1

Viterbi Path

Viterbi Path

Posterior @ 0.2	
Posterior @ 0.2	
Posterior Graphic. Threshold Marked @ 0.2	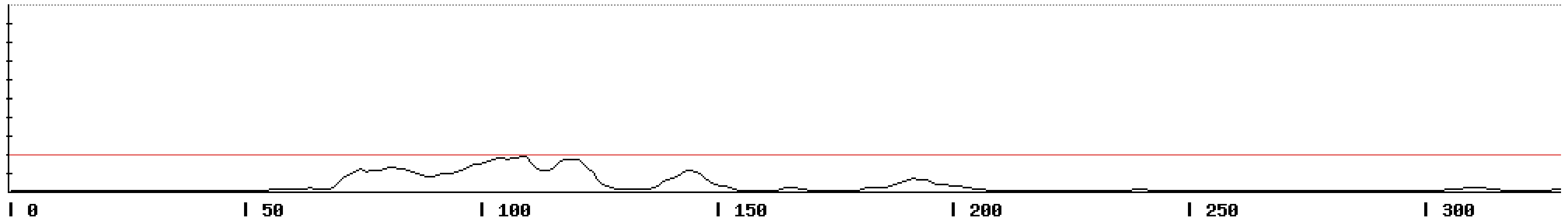

Predictions for Leersiaperrieri_LPERR11G09550.1

Viterbi Path	
Viterbi Path	
Posterior @ 0.2	217 - KR - 218
Posterior @ 0.2	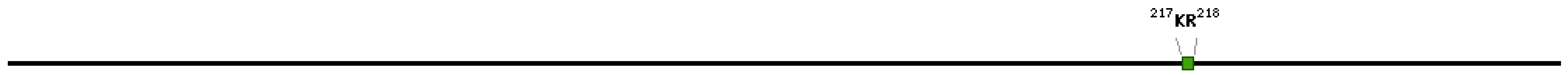
Posterior Graphic. Threshold Marked @ 0.2	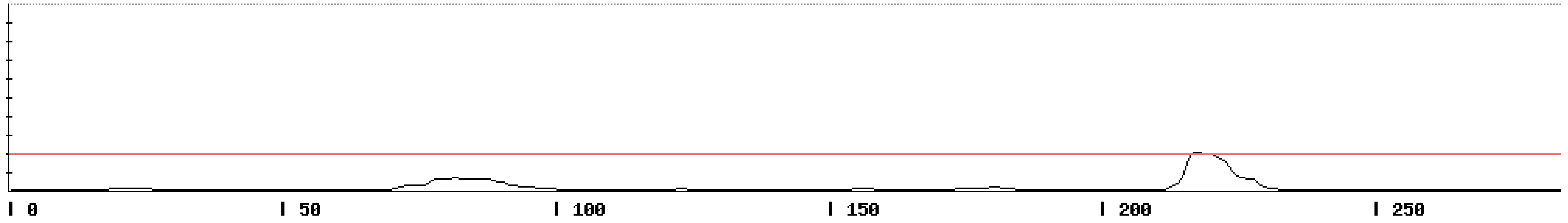

Predictions for Leersiaperrieri_LPERR11G09560.1

Viterbi Path	
Viterbi Path	

Posterior @ 0.2	78 - RKKIKYPGGHR - 88
Posterior @ 0.2	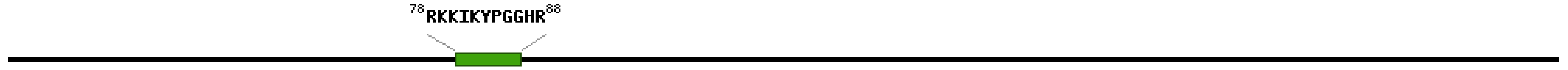
Posterior Graphic. Threshold Marked @ 0.2	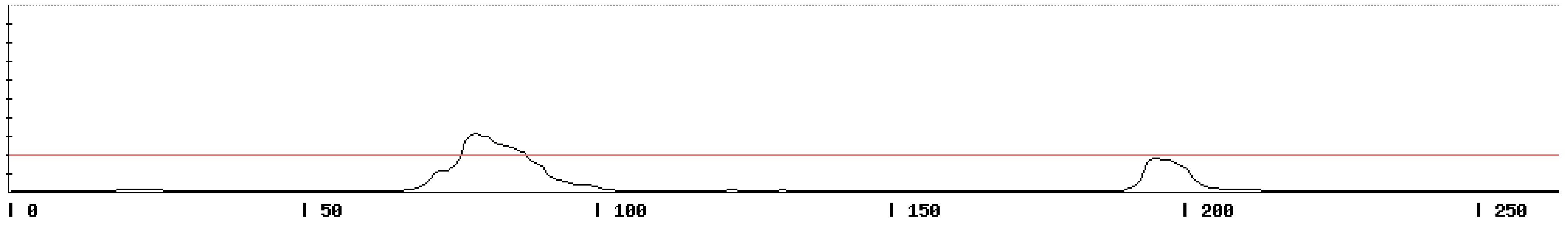

Predictions for Leersiaperrieri_LPERR11G10970.2

Viterbi Path	
Viterbi Path	
Posterior @ 0.2	79 - TRKENKNPGDKHRNRA - 94
Posterior @ 0.2	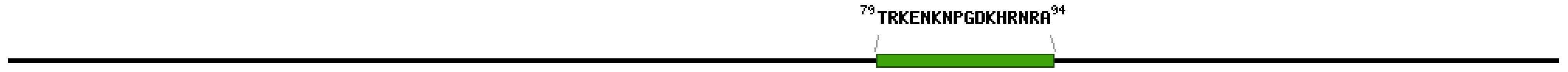
Posterior Graphic. Threshold Marked @ 0.2	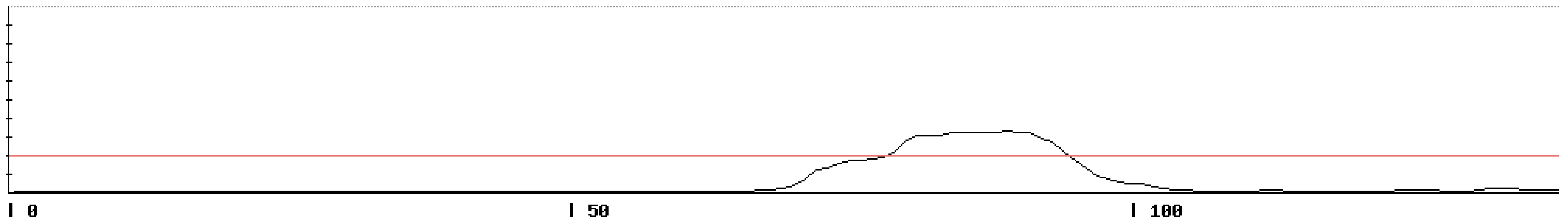

Predictions for Leersiaperrieri_LPERR11G11560.1

Viterbi Path	
Viterbi Path	
Posterior @ 0.2	
Posterior @ 0.2	
Posterior Graphic. Threshold Marked @ 0.2	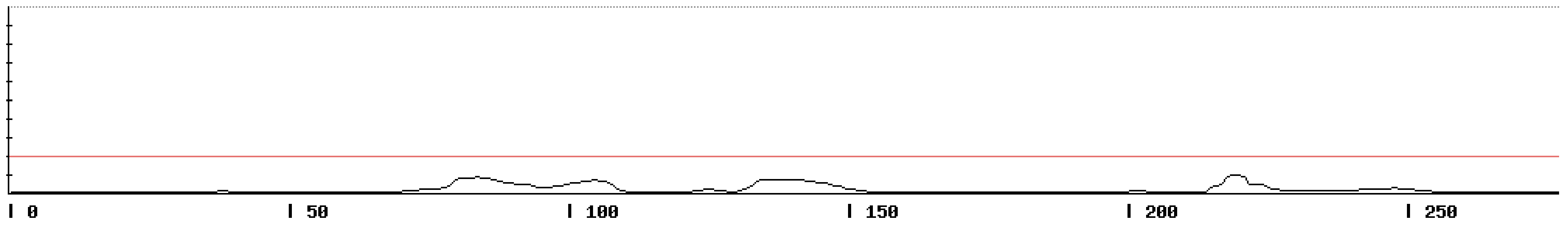

Predictions for Leersiaperrieri_LPERR11G11570.1

Viterbi Path	
Viterbi Path	
Posterior @ 0.2	82 - PGGRR - 86
Posterior @ 0.2	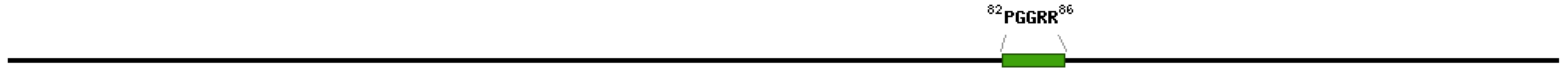
Posterior Graphic. Threshold Marked @ 0.2	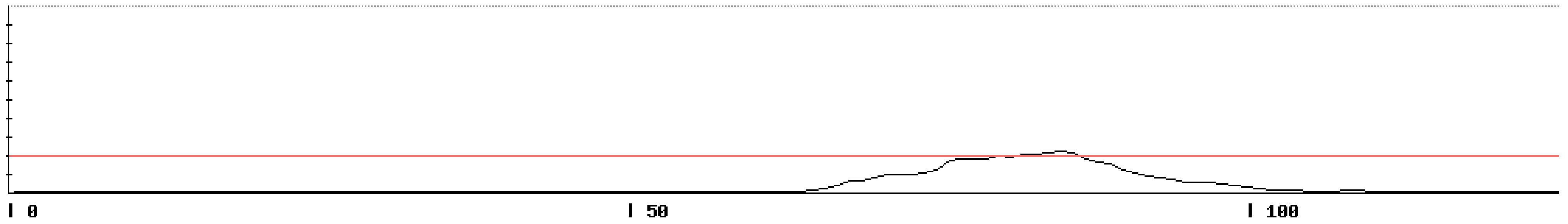

Predictions for Leersiaperrieri_LPERR11G11610.1	
Viterbi Path	
Viterbi Path	
Posterior @ 0.2	
Posterior @ 0.2	
Posterior Graphic. Threshold Marked @ 0.2	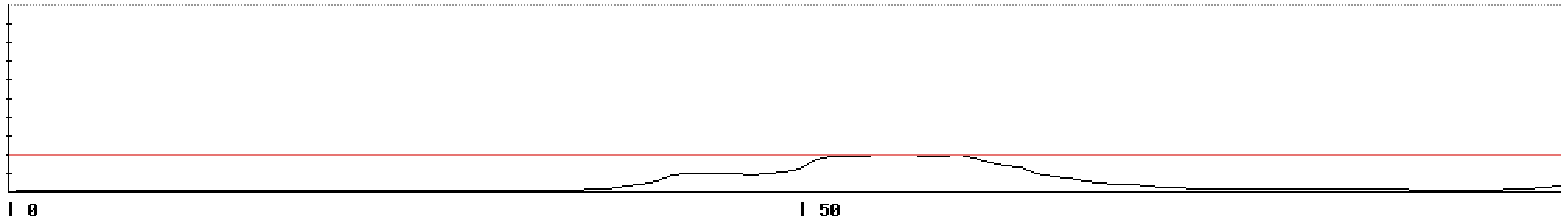

Predictions for Leersiaperrieri_LPERR11G11620.1	
Viterbi Path	
Viterbi Path	
Posterior @ 0.2	
Posterior @ 0.2	
Posterior Graphic. Threshold Marked @ 0.2	

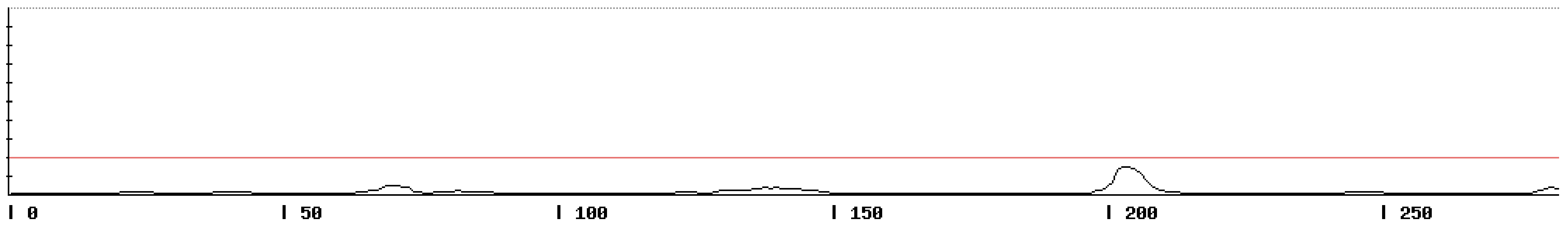

Predictions for Leersiaperrieri_LPERR11G11960.1

Viterbi Path

Viterbi Path

Posterior @ 0.2 — 220 - TRRTPGGVGPGVRQGGHTPQKGFRPPEATRHAPEEGTPRGLRGGH - 264

Posterior @ 0.2

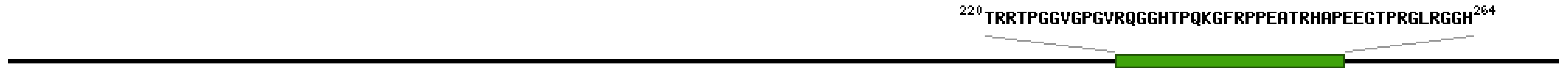

Posterior Graphic. Threshold Marked @ 0.2

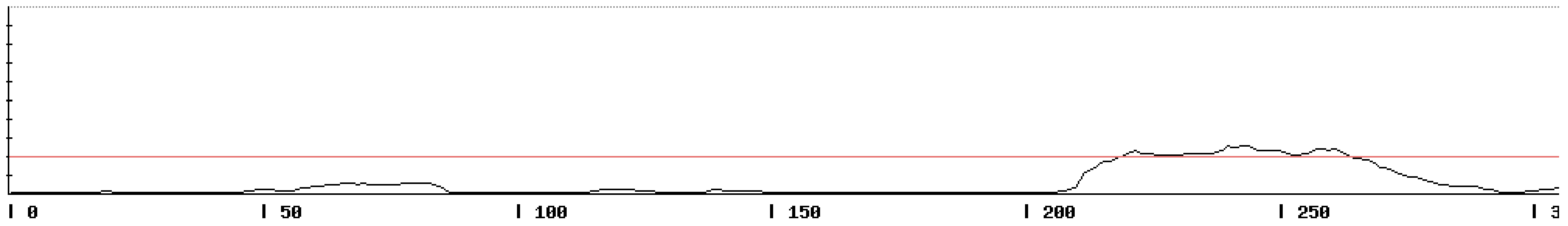

Predictions for Leersiaperrieri_LPERR11G11970.1

Viterbi Path

Viterbi Path

Posterior @ 0.2 — 69 - KKNGKHFFTRKEYKHPGYRRRNRA - 92

Posterior @ 0.2

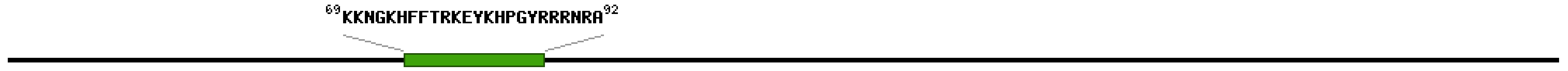

Posterior Graphic. Threshold Marked @ 0.2

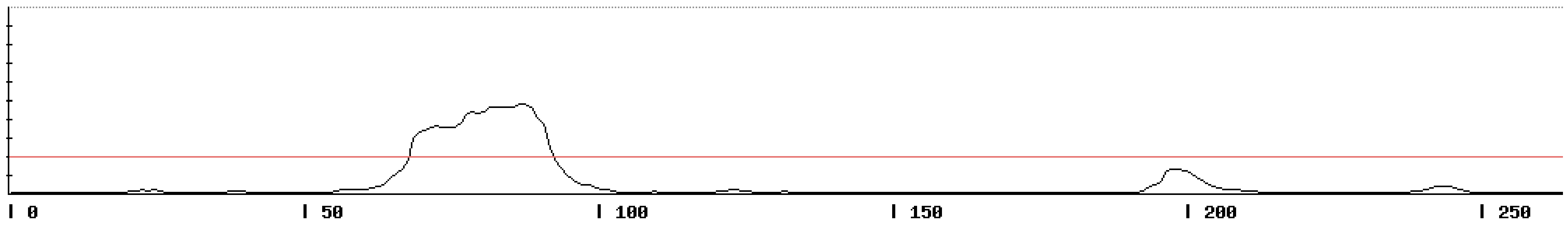

Predictions for Leersiaperrieri_LPERR11G11980.1

Viterbi Path

Viterbi Path

Posterior @ 0.2 69 - KKNGKHFFTRKEYKHPGYRRRNRA - 92

Posterior @ 0.2

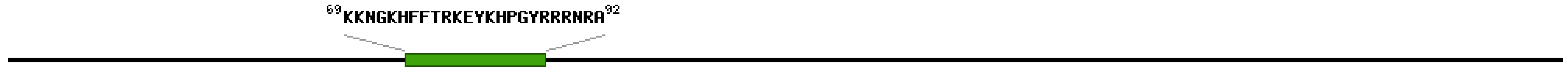

Posterior Graphic. Threshold Marked @ 0.2

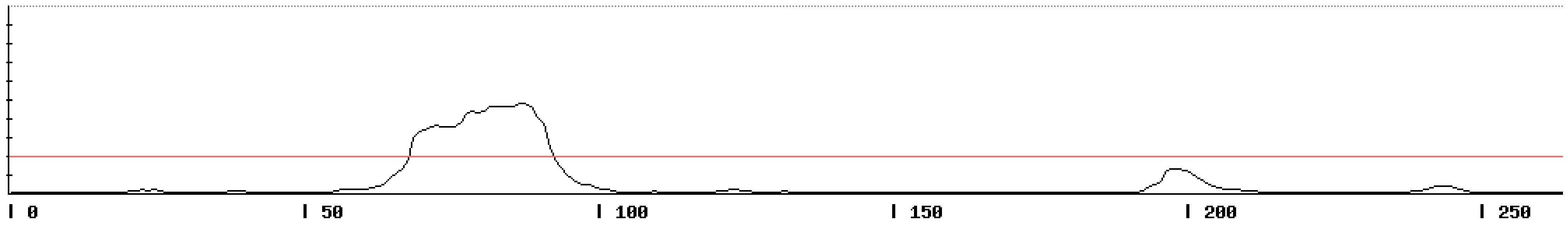

Predictions for Leersiaperrieri_LPERR12G01440.1

Viterbi Path

Viterbi Path

Posterior @ 0.2 120 - GKPPK - 124

Posterior @ 0.2

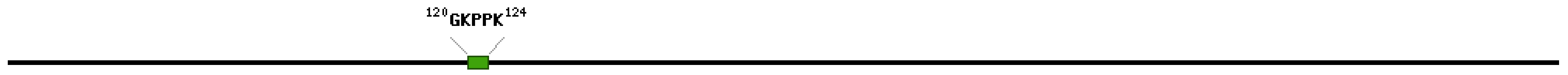

Posterior Graphic. Threshold Marked @ 0.2

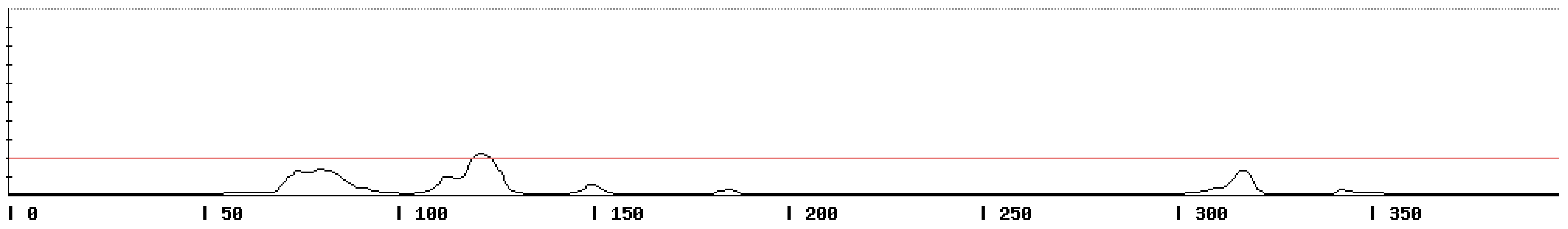

Predictions for Leersiaperrieri_LPERR12G01450.1

Viterbi Path

Viterbi Path

Posterior @ 0.2

Posterior @ 0.2

Posterior Graphic. Threshold Marked @ 0.2

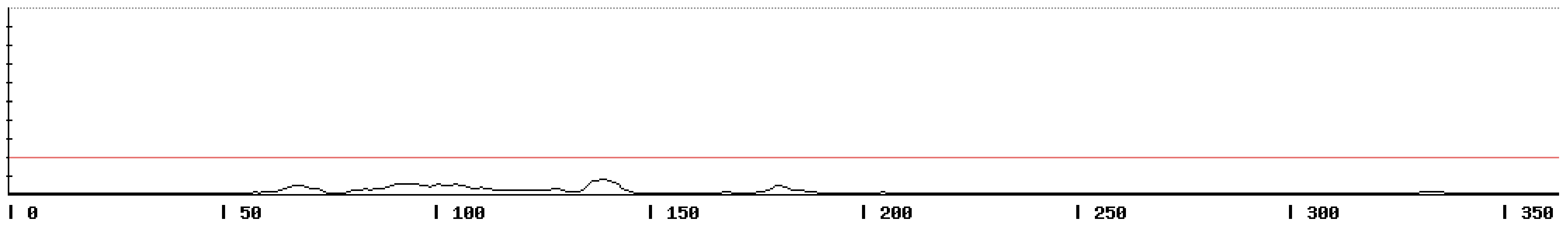

Predictions for Leersiaperrieri_LPERR12G02200.1

Viterbi Path

Viterbi Path

Posterior @ 0.2

Posterior @ 0.2

Posterior Graphic. Threshold Marked @ 0.2

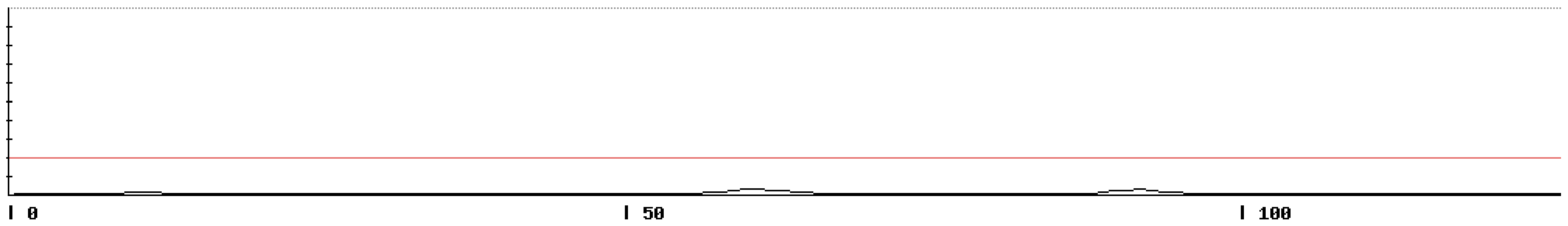

Predictions for Leersiaperrieri_LPERR12G02290.1

Viterbi Path

Viterbi Path

Posterior @ 0.2

74 - VAKRKGNKRGNAGRN - 88

157 - KSPRKNKGDENVVGKKRLR - 175

Posterior @ 0.2

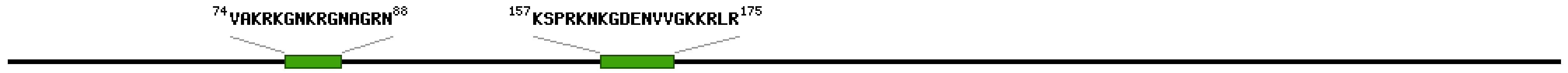

Posterior Graphic. Threshold Marked @ 0.2

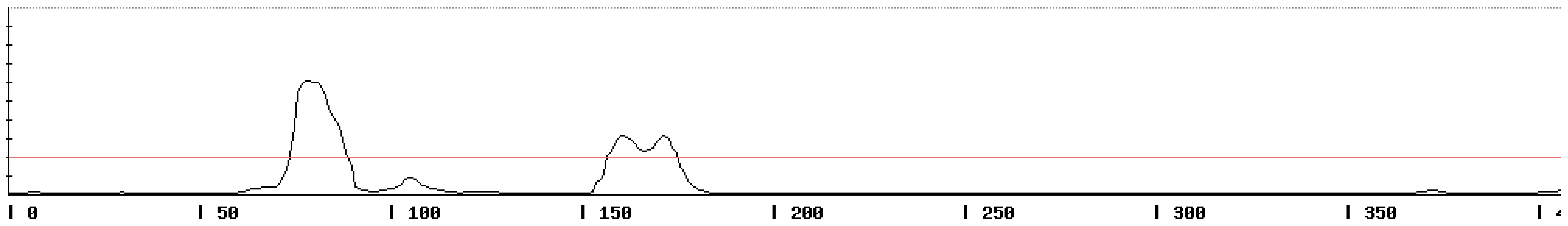

Predictions for Leersiaperrieri_LPERR12G02630.1

Viterbi Path

Viterbi Path

Posterior @ 0.2 — 9 - PAARPPPATGRSRKHKM - 25

Posterior @ 0.2

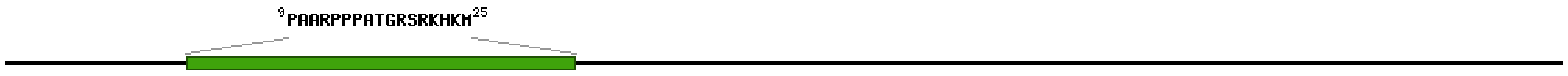

Posterior Graphic. Threshold Marked @ 0.2

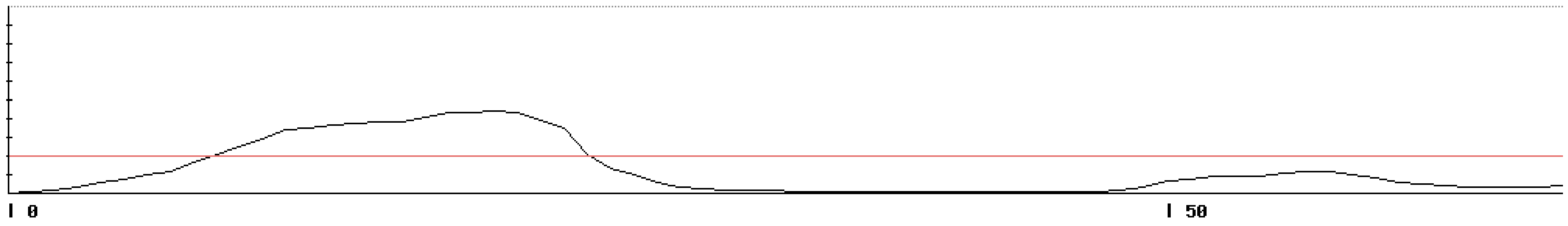

Predictions for Leersiaperrieri_LPERR12G03220.1

Viterbi Path

Viterbi Path

Posterior @ 0.2

Posterior @ 0.2

Posterior Graphic. Threshold Marked @ 0.2

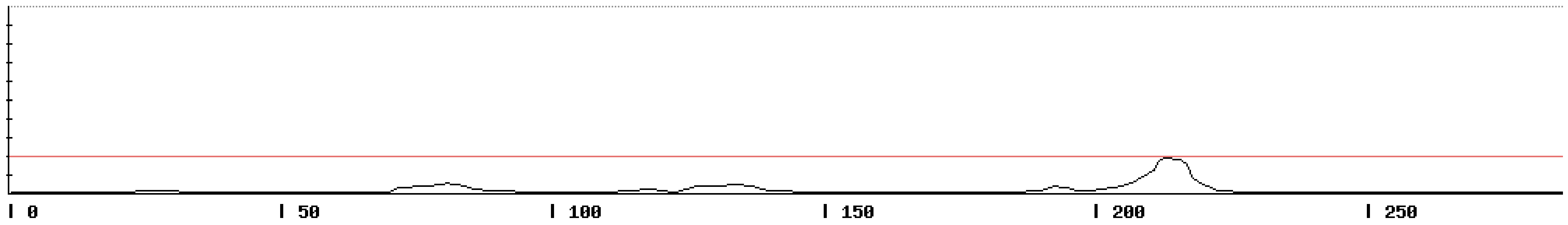

Predictions for Leersiaperrieri_LPERR12G08370.1

Viterbi Path

Viterbi Path

Posterior @ 0.2

Posterior @ 0.2

Posterior Graphic. Threshold Marked @ 0.2

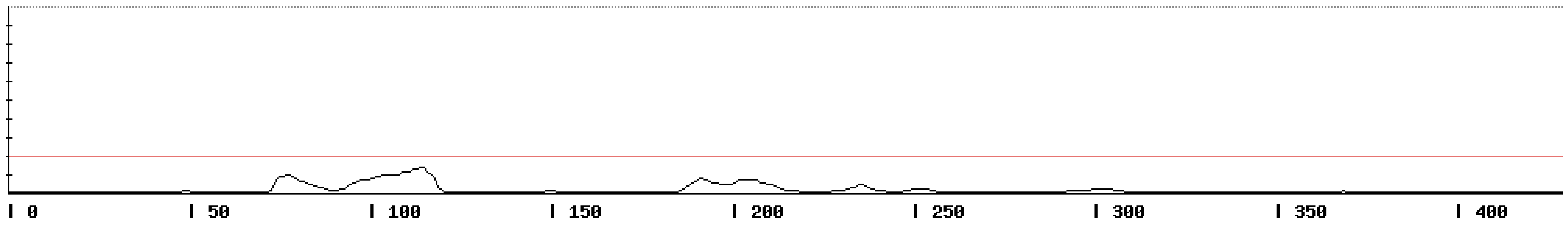

Predictions for Leersiaperrieri_LPERR12G08380.1

Viterbi Path

Viterbi Path

Posterior @ 0.2

Posterior @ 0.2

Posterior Graphic. Threshold Marked @ 0.2

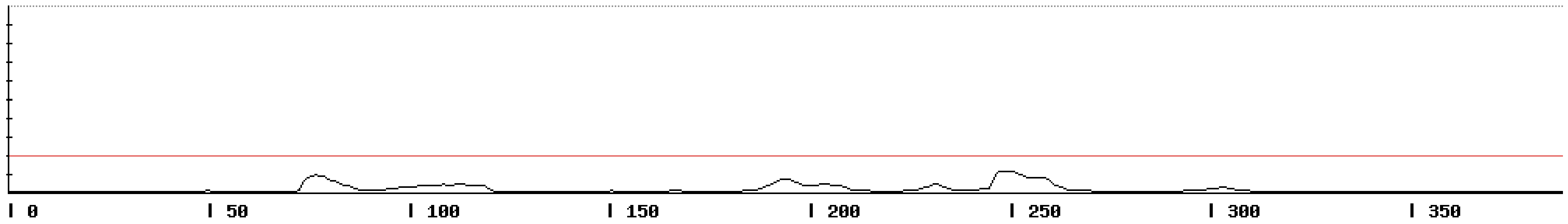

Predictions for Leersiaperrieri_LPERR12G08390.1

Viterbi Path

Viterbi Path

Posterior @ 0.2

75 - RVKGKVSRAAG - 85

93 - SSKDIKNREGKKIGETKNFRFKKGANK - 119

211 - KKMRRPVSAKRV - 222

Posterior @ 0.2

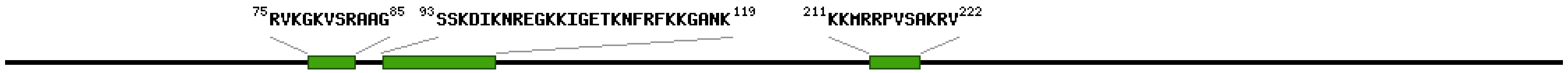

Posterior Graphic. Threshold Marked @ 0.2

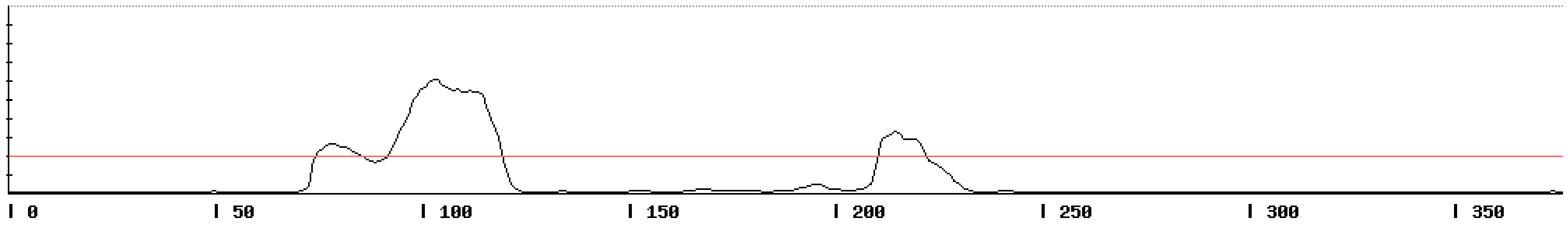

Predictions for Leersiaperrieri_LPERR12G08400.1

Viterbi Path

Viterbi Path

Posterior @ 0.2

177 - PPSPPPPPSPEEPEPPNRDPTPPPPPPSPQASPQKRSASPPPPSSAKIRGPTSRRVMA - 234

Posterior @ 0.2

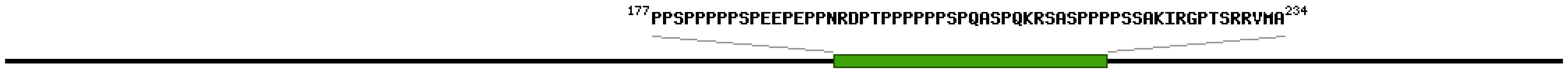

Posterior Graphic. Threshold Marked @ 0.2

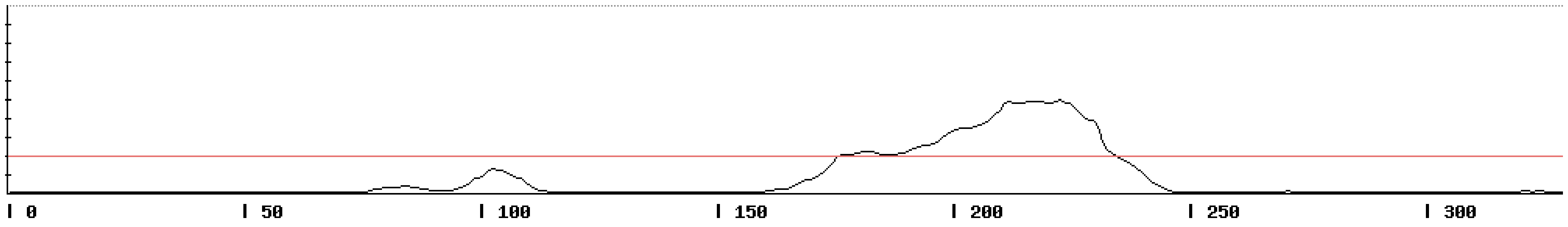

Predictions for Leersiaperrieri_LPERR12G08420.1	
Viterbi Path	
Viterbi Path	
Posterior @ 0.2	194 - PNRDPTPPPAPKASPLKRSASPPPSSAKKIRGPTSRRL - 231
Posterior @ 0.2	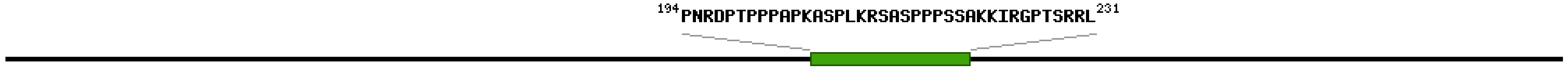
Posterior Graphic. Threshold Marked @ 0.2	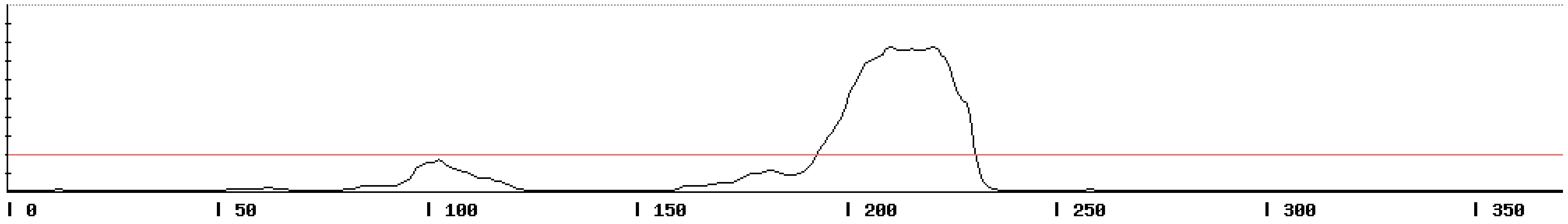

Predictions for Leersiaperrieri_LPERR12G08470.1	
Viterbi Path	
Viterbi Path	
Posterior @ 0.2	114 - KNVTKKGRKIGEVKSFRFQLRKGK - 137
Posterior @ 0.2	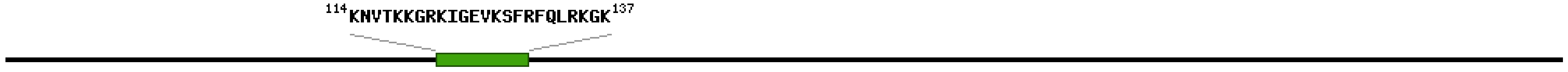

Posterior Graphic. Threshold Marked @ 0.2

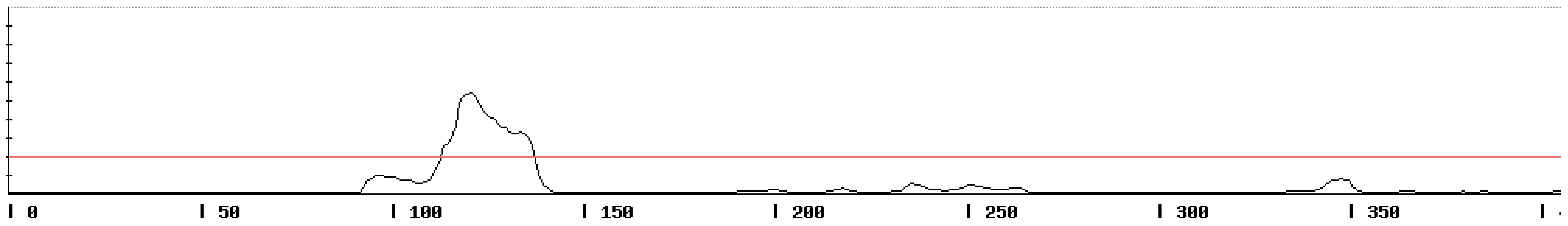

Predictions for Leersiaperrieri_LPERR12G09780.1

Viterbi Path

Viterbi Path

Posterior @ 0.2

83 - KGRRA - 87

132 - KPPAGK - 137

Posterior @ 0.2

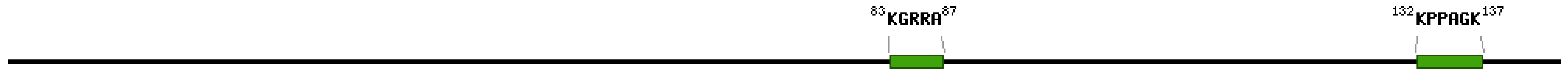

Posterior Graphic. Threshold Marked @ 0.2

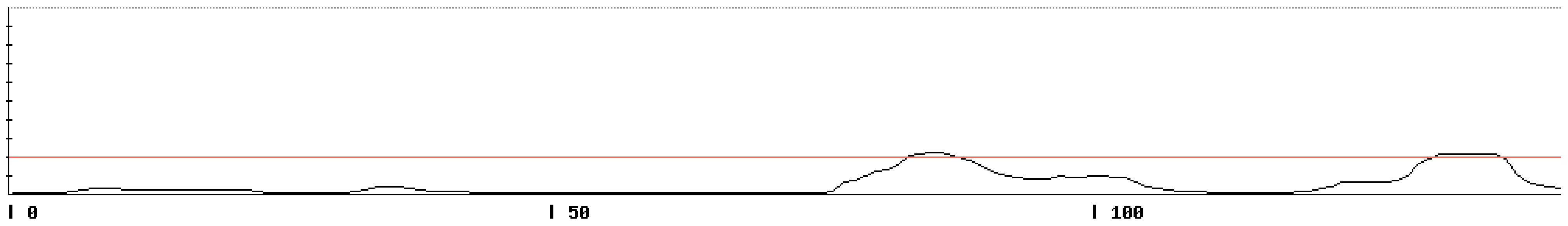

Predictions for Leersiaperrieri_LPERR12G15510.1

Viterbi Path

Viterbi Path

Posterior @ 0.2

10 - ERKGKTETKEKKKLAKGEEEIERR - 33

172 - PKGR - 175

Posterior @ 0.2	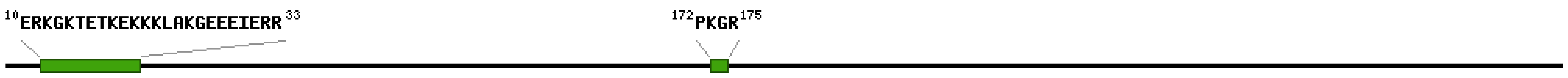
Posterior Graphic. Threshold Marked @ 0.2	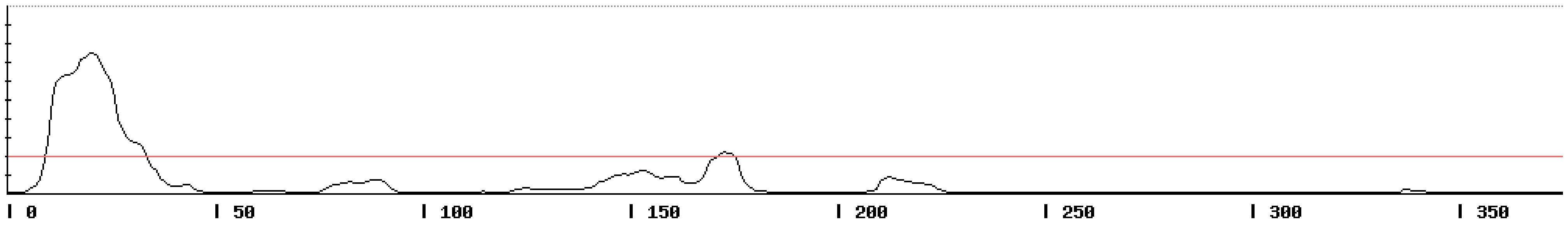

Revision r.9 - Last Updated on : February 24 2015 17:45:20.

alex.nguyenba@utoronto.ca

Intended to be used with Firefox

Viterbi Path	
Posterior @ 0.2	
Posterior @ 0.2	
Posterior Graphic. Threshold Marked @ 0.2	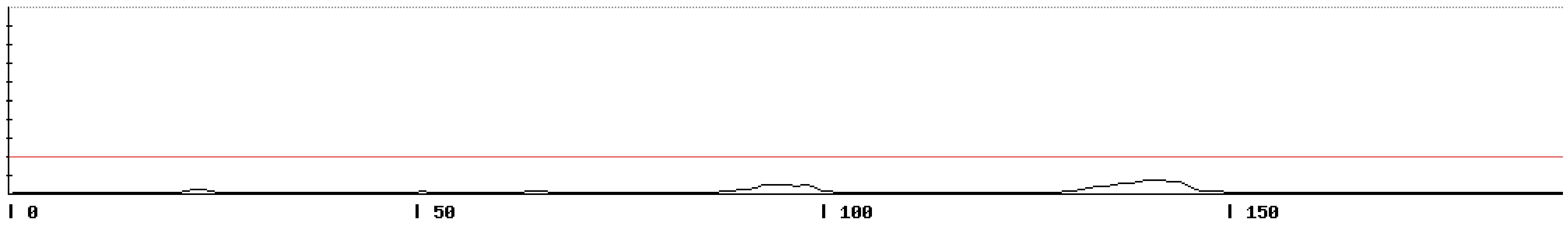

Predictions for Linumusitatissimum_Lus10001648

Viterbi Path	
Viterbi Path	
Posterior @ 0.2	130 - GRAPK - 134
Posterior @ 0.2	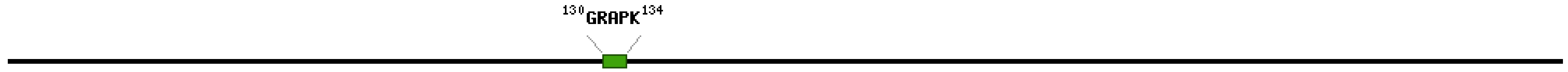
Posterior Graphic. Threshold Marked @ 0.2	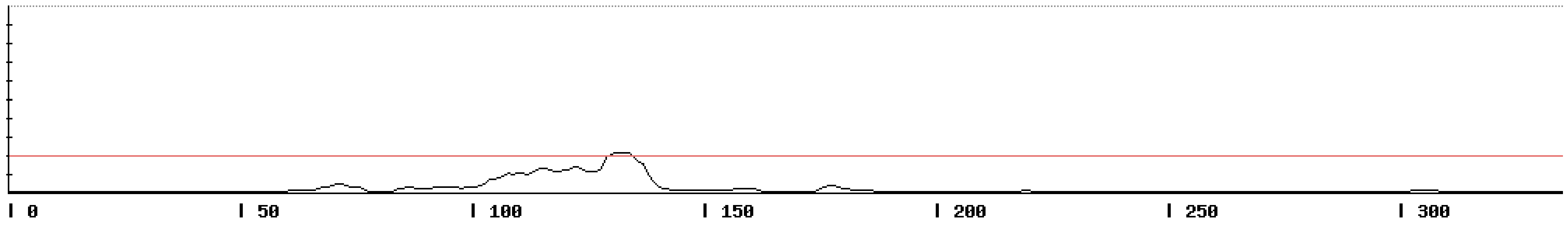

Predictions for Linumusitatissimum_Lus10001664

Viterbi Path	
Viterbi Path	
Posterior @ 0.2	
Posterior @ 0.2	
Posterior Graphic. Threshold Marked @ 0.2	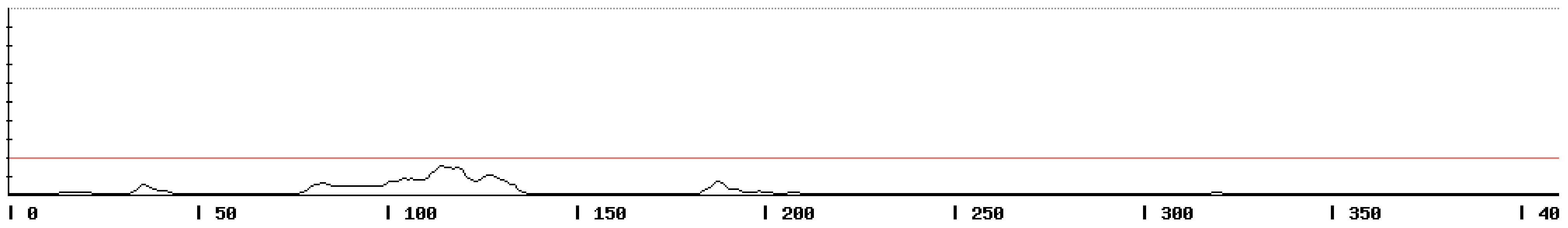

Predictions for Linumusitatissimum_Lus10001809

Viterbi Path	
Viterbi Path	
Posterior @ 0.2	243 - RRKP - 246
Posterior @ 0.2	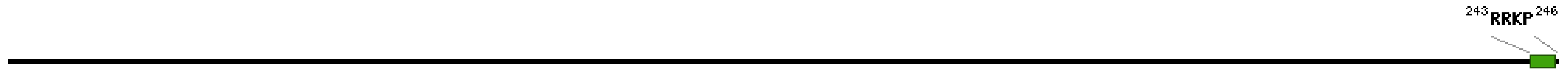
Posterior Graphic. Threshold Marked @ 0.2	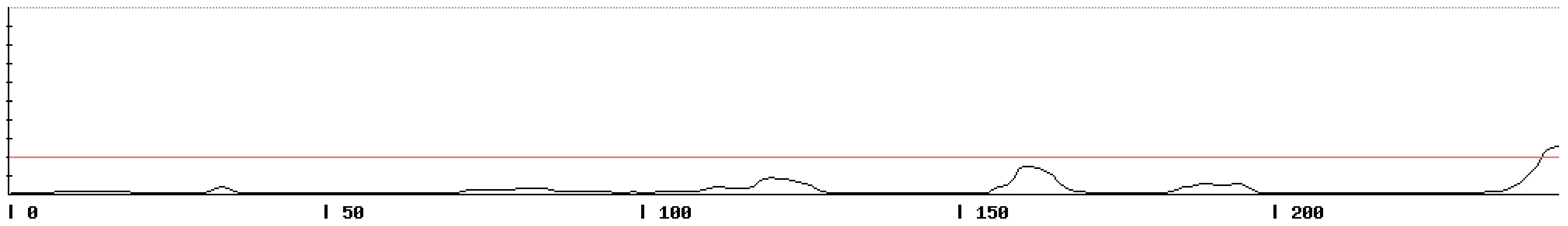

Predictions for Linumusitatissimum_Lus10001876

Viterbi Path	
Viterbi Path	
Posterior @ 0.2	
Posterior @ 0.2	
Posterior Graphic. Threshold Marked @ 0.2	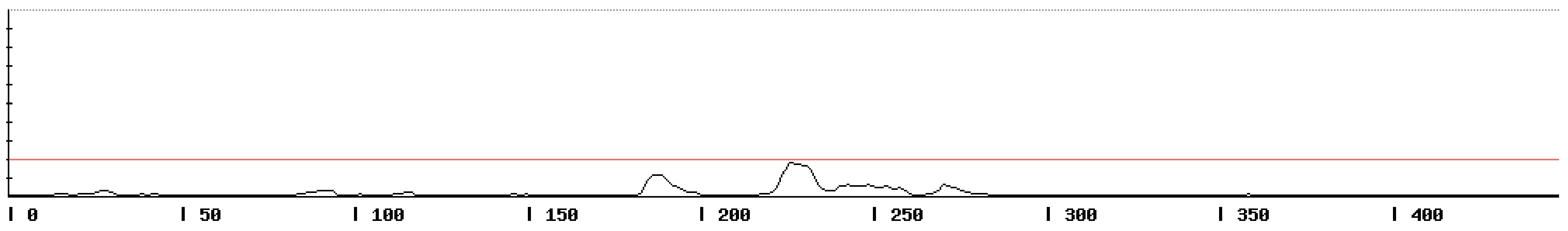

Predictions for Linumusitatissimum_Lus10002083

Viterbi Path	
Viterbi Path	
Posterior @ 0.2	21 - NGSRPNRAAGA - 31
	35 - KATGVDKPIGKPKVMGIKKS - 54
Posterior @ 0.2	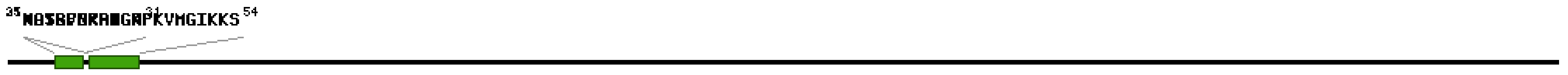
Posterior Graphic. Threshold Marked @ 0.2	

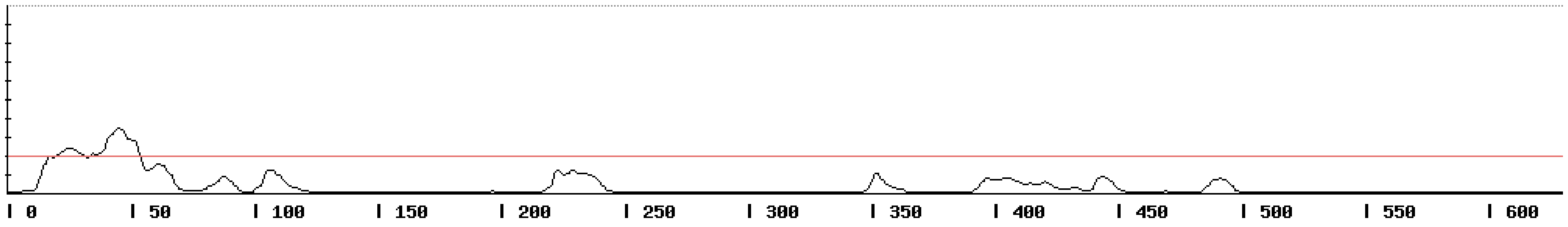

Predictions for Linumusitatissimum_Lus10002581

Viterbi Path

Viterbi Path

Posterior @ 0.2 — 247 - RRKP - 250

Posterior @ 0.2

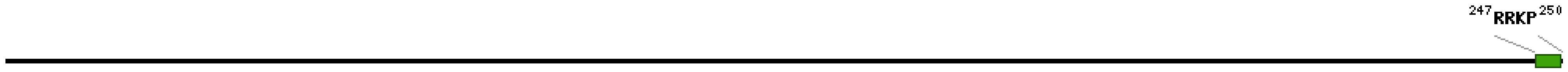

Posterior Graphic. Threshold Marked @ 0.2

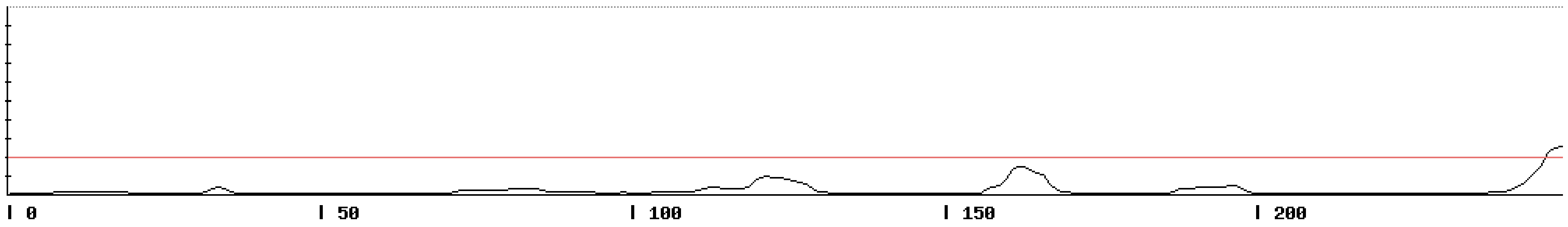

Predictions for Linumusitatissimum_Lus10002687

Viterbi Path

Viterbi Path

Posterior @ 0.2 — 108 - GRRIGMR - 114

Posterior @ 0.2

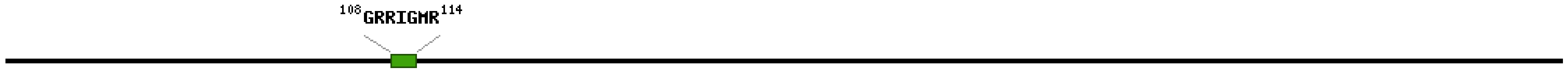

Posterior Graphic. Threshold Marked @ 0.2

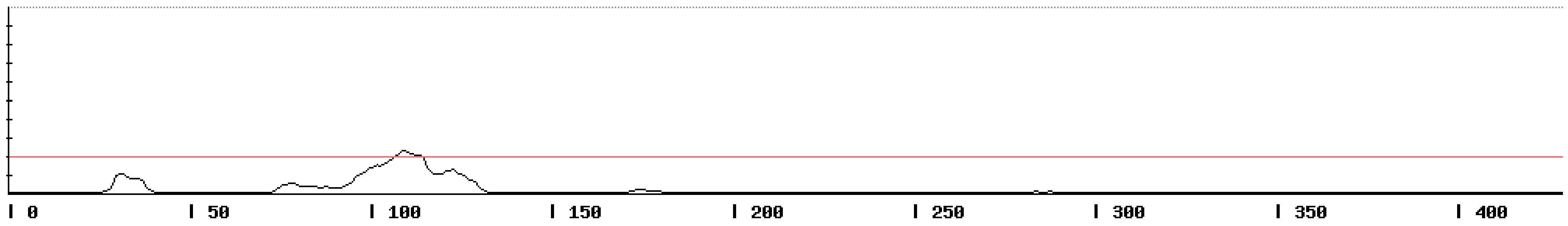

Predictions for Linumusitatissimum_Lus10003269

Viterbi Path

Viterbi Path

Posterior @ 0.2 — 124 - KGKPPKG - 130

Posterior @ 0.2

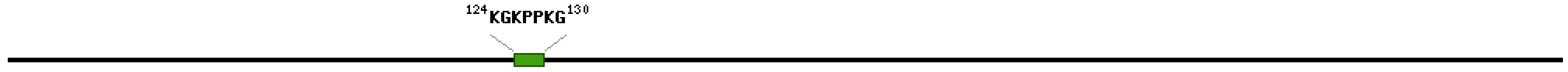

Posterior Graphic. Threshold Marked @ 0.2

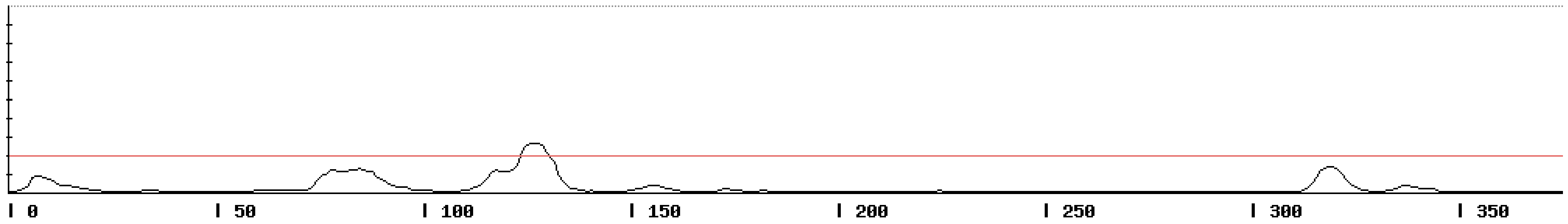

Predictions for Linumusitatissimum_Lus10003333

Viterbi Path

Viterbi Path

Posterior @ 0.2 — 78 - PRDRKHGGNGRPNRTTEKGFWKATGSDRKIWRLSEPKRQIGLRK - 121

Posterior @ 0.2

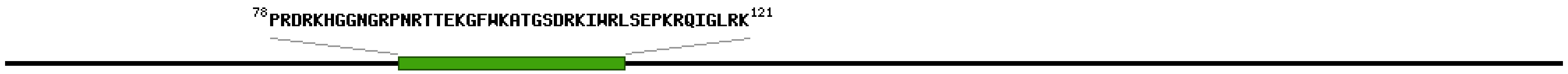

Posterior Graphic. Threshold Marked @ 0.2

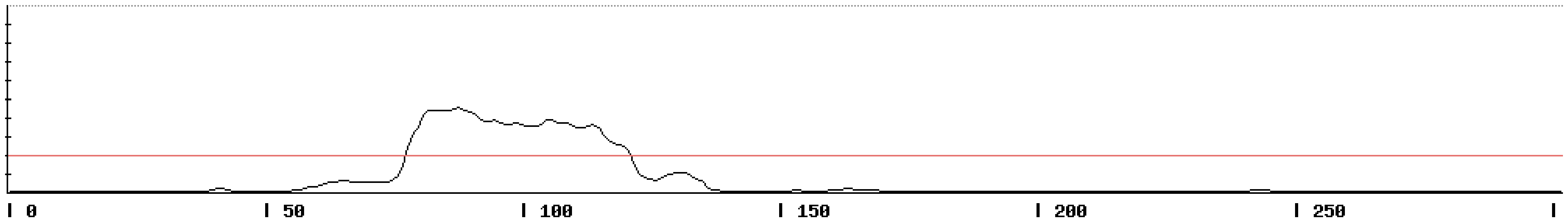

Predictions for Linumusitatissimum_Lus10003334

Viterbi Path

Viterbi Path

Posterior @ 0.2

127 - KRFR - 130

135 - SCTEKKRKRKNNGGP - 149

Posterior @ 0.2

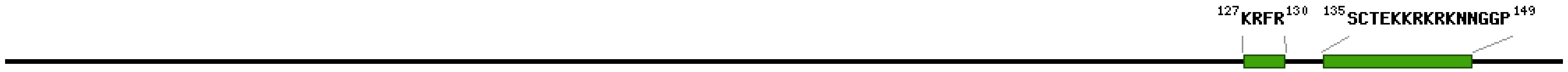

Posterior Graphic. Threshold Marked @ 0.2

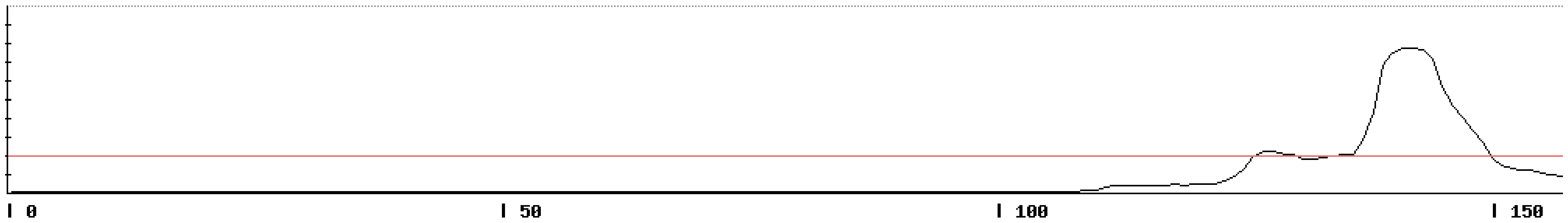

Predictions for Linumusitatissimum_Lus10003366

Viterbi Path

Viterbi Path

Posterior @ 0.2

Posterior @ 0.2

Posterior Graphic. Threshold Marked @ 0.2

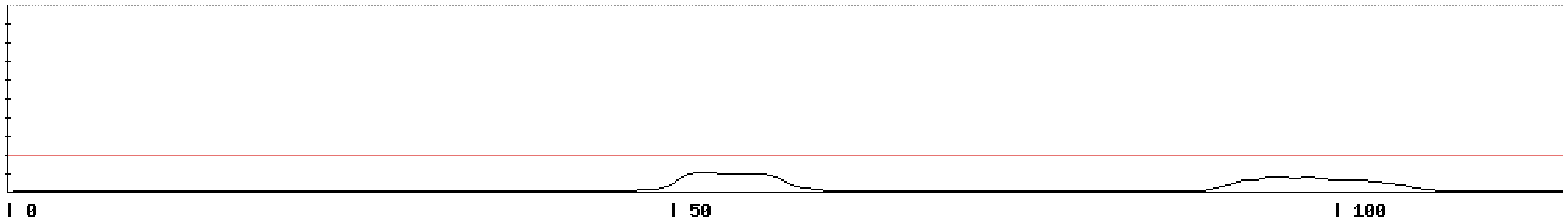

Predictions for Linumusitatissimum_Lus10003367

Viterbi Path

Viterbi Path

Posterior @ 0.2

Posterior @ 0.2

Posterior Graphic. Threshold Marked @ 0.2

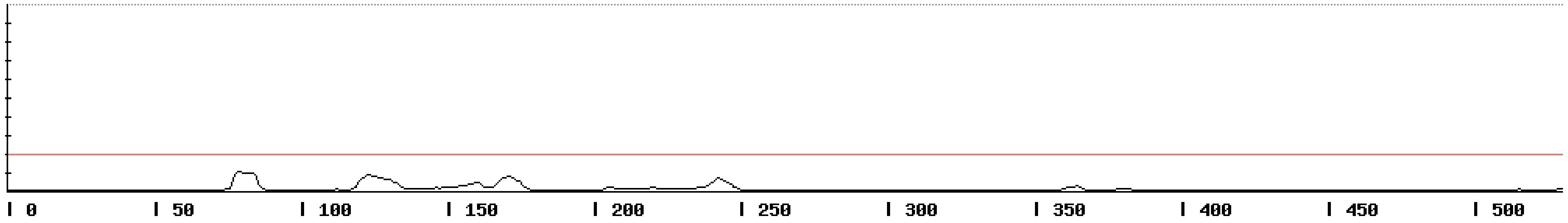

Predictions for Linumusitatissimum_Lus10003435

Viterbi Path

Viterbi Path

Posterior @ 0.2

Posterior @ 0.2

Posterior Graphic. Threshold Marked @ 0.2

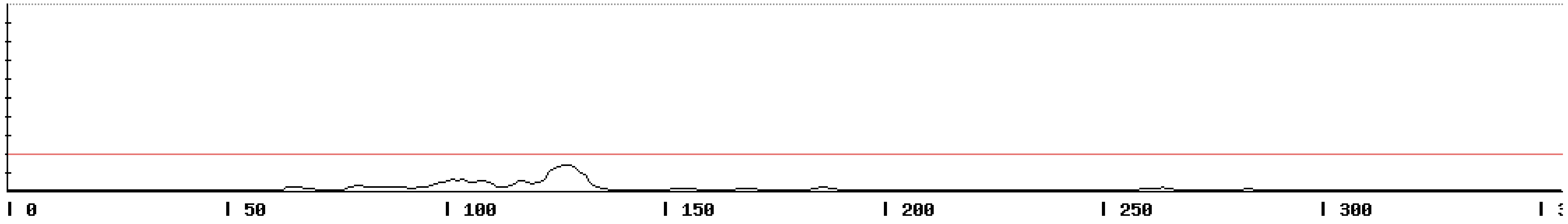

Predictions for Linumusitatissimum_Lus10003458

Viterbi Path

Viterbi Path

Posterior @ 0.2

Posterior @ 0.2

Posterior Graphic. Threshold Marked @ 0.2

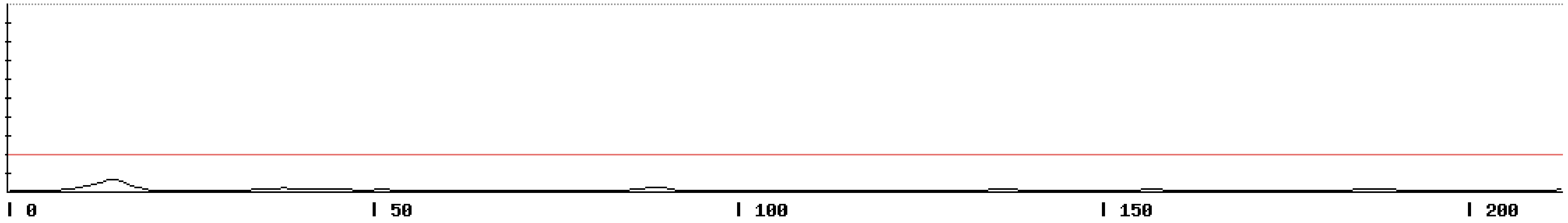

Predictions for Linumusitatissimum_Lus10003548	
Viterbi Path	
Viterbi Path	
Posterior @ 0.2	97 - KATGKERTGGENKKKKSDGGG - 117
	698 - RKYSKTR - 704
	890 - RPK - 892
Posterior @ 0.2	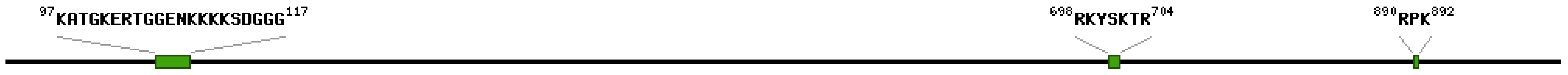
Posterior Graphic. Threshold Marked @ 0.2	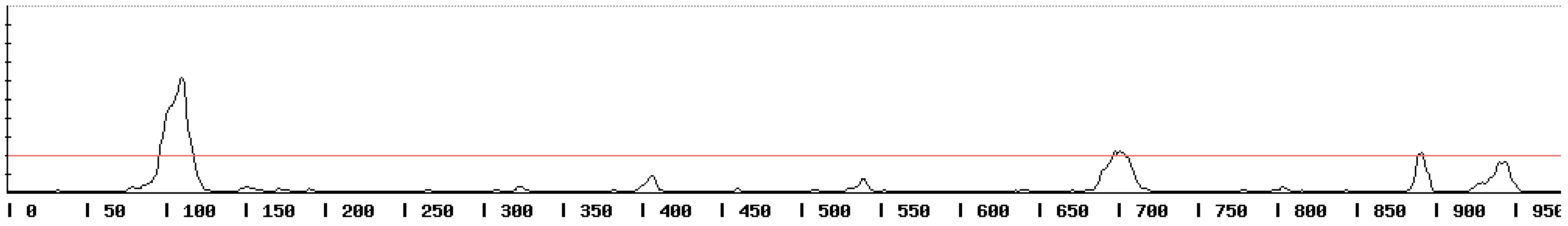

Predictions for Linumusitatissimum_Lus10003668	
Viterbi Path	
Viterbi Path	
Posterior @ 0.2	186 - GHRKRRKIL - 194
Posterior @ 0.2	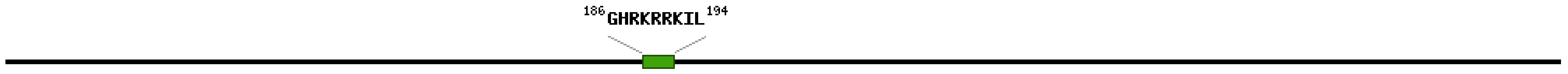
Posterior Graphic. Threshold Marked @ 0.2	

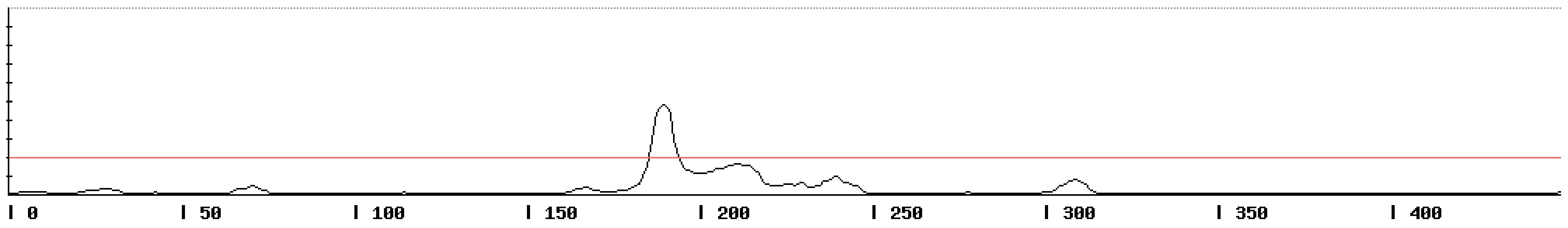

Predictions for Linumusitatissimum_Lus10003847

Viterbi Path

Viterbi Path

Posterior @ 0.2

Posterior @ 0.2

Posterior Graphic. Threshold Marked @ 0.2

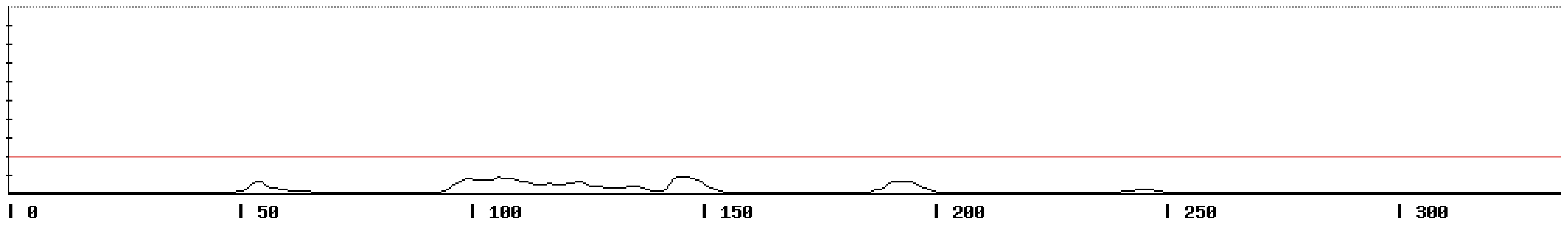

Predictions for Linumusitatissimum_Lus10003848

Viterbi Path

Viterbi Path

Posterior @ 0.2

Posterior @ 0.2

Posterior Graphic. Threshold Marked @ 0.2

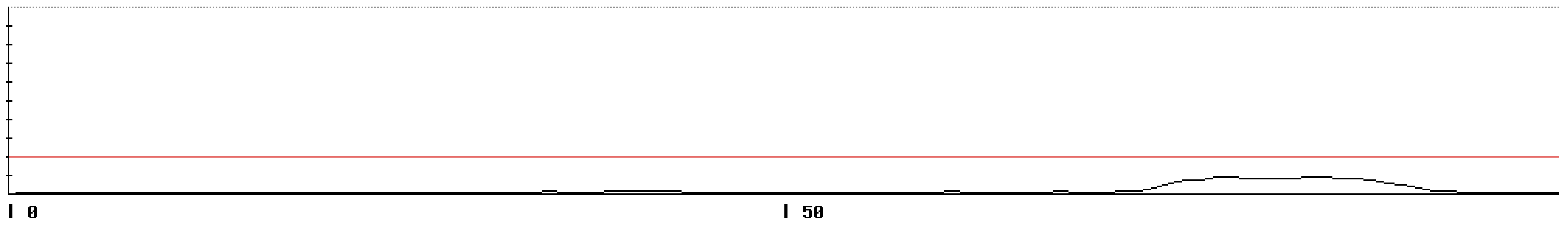

Predictions for Linumusitatissimum_Lus10004206

Viterbi Path

Viterbi Path

Posterior @ 0.2

16 - GRRWRKGKISSGSSLGGCKRGKEEEGGR - 43

84 - YKGKAPKGRKTHWKI - 98

Posterior @ 0.2

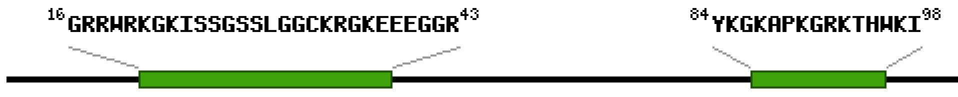

Posterior Graphic. Threshold Marked @ 0.2

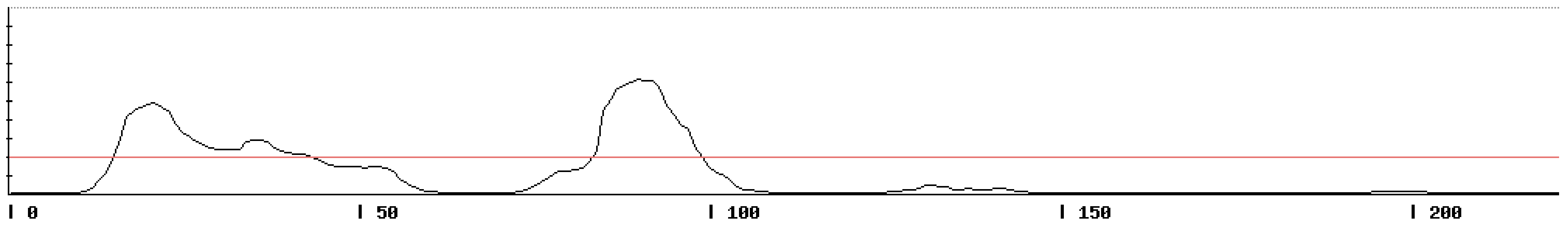

Predictions for Linumusitatissimum_Lus10004338

Viterbi Path

Viterbi Path

Posterior @ 0.2

Posterior @ 0.2

Posterior Graphic. Threshold Marked @ 0.2

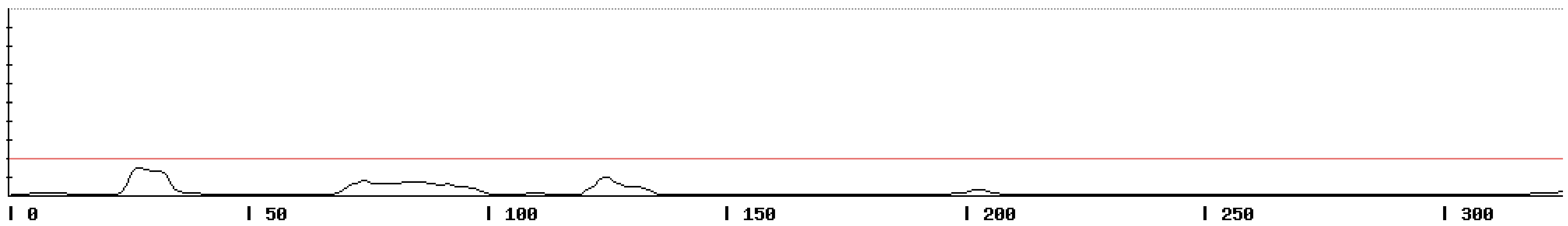

Predictions for Linumusitatissimum_Lus10004531

Viterbi Path	
Viterbi Path	
Posterior @ 0.2	88 - KLKQKVSGKQLKQKKKMAG - 106
Posterior @ 0.2	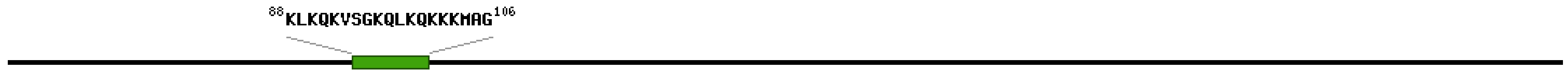
Posterior Graphic. Threshold Marked @ 0.2	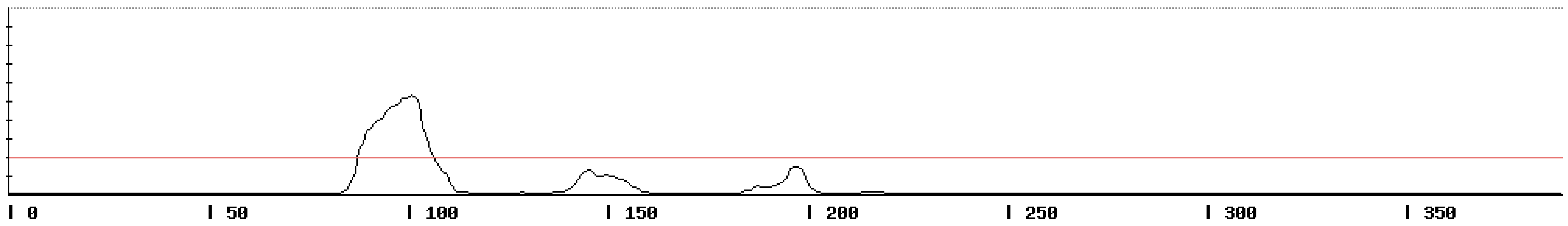

Predictions for Linumusitatissimum_Lus10004846

Viterbi Path	
Viterbi Path	

Posterior @ 0.2	119 - KGKAPAGRKTKWKM - 132
Posterior @ 0.2	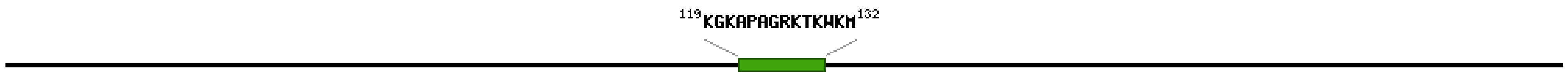
Posterior Graphic. Threshold Marked @ 0.2	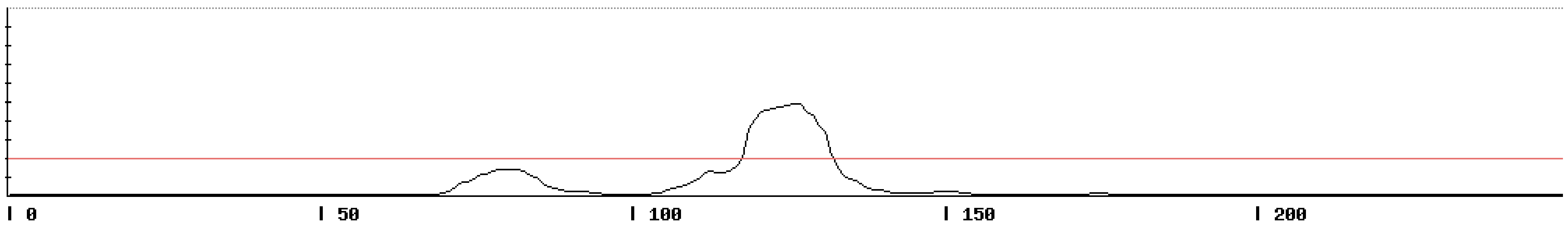

Predictions for Linumusitatissimum_Lus10005143

Viterbi Path	
Viterbi Path	
Posterior @ 0.2	
Posterior @ 0.2	
Posterior Graphic. Threshold Marked @ 0.2	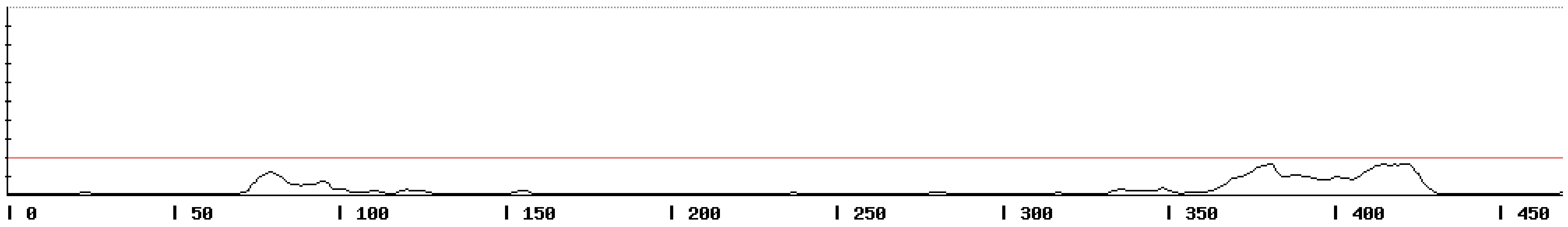

Predictions for Linumusitatissimum_Lus10005144

Viterbi Path	
Viterbi Path	

Posterior @ 0.2

80 - KRSNRTTKKGYWKPTGRE - 97

Posterior @ 0.2

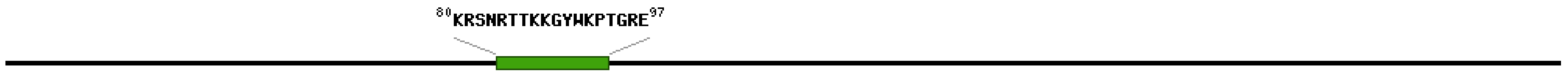

Posterior Graphic. Threshold Marked @ 0.2

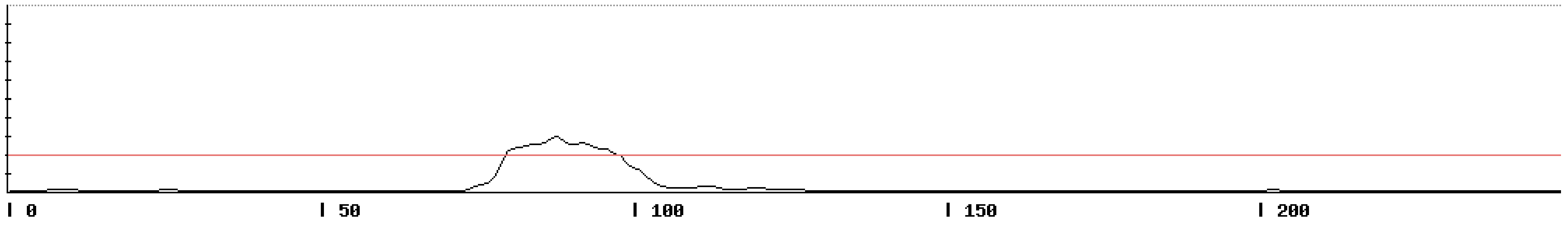

Predictions for Linumusitatissimum_Lus10005204

Viterbi Path

Viterbi Path

Posterior @ 0.2

291 - AAPSNRRVKLESRRRKKKIDQ - 311

323 - AEKERRSKSKKV - 334

Posterior @ 0.2

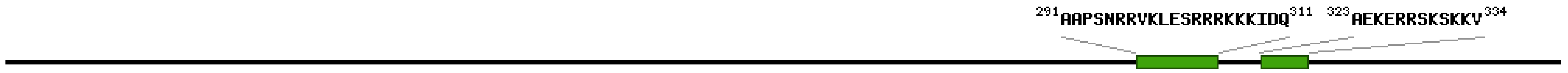

Posterior Graphic. Threshold Marked @ 0.2

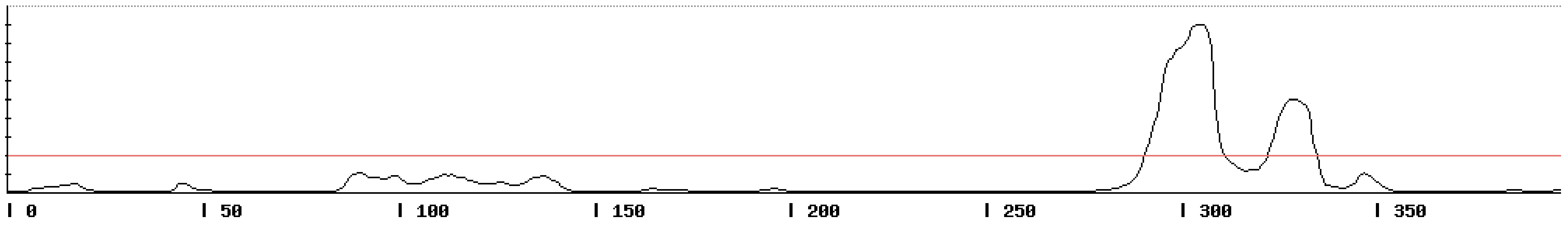

Predictions for Linumusitatissimum_Lus10005537

Viterbi Path

Viterbi Path

Posterior @ 0.2

Posterior @ 0.2

Posterior Graphic. Threshold Marked @ 0.2

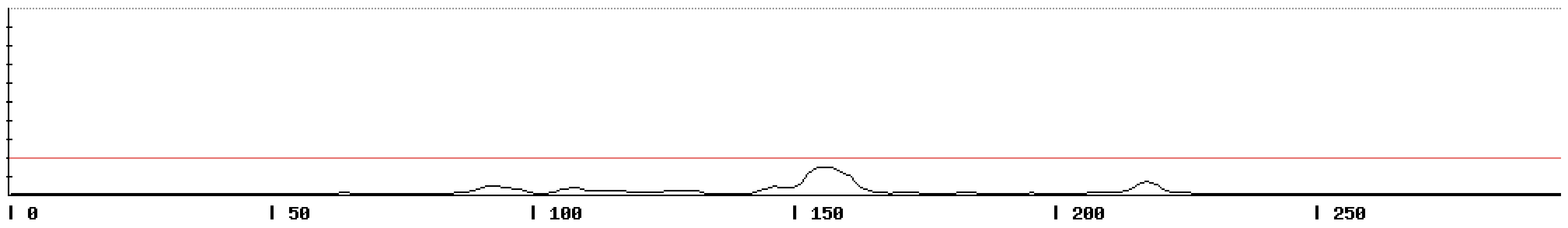

Predictions for Linumusitatissimum_Lus10005917

Viterbi Path

Viterbi Path

Posterior @ 0.2

Posterior @ 0.2

Posterior Graphic. Threshold Marked @ 0.2

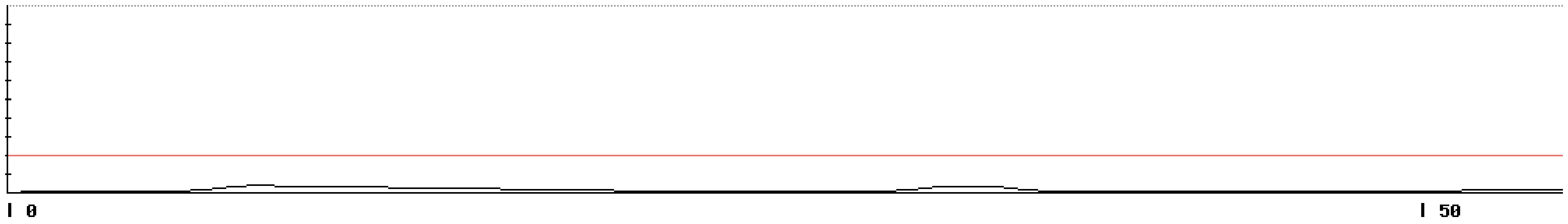

Predictions for Linumusitatissimum_Lus10006054

Viterbi Path

Viterbi Path

Posterior @ 0.2 — 528 - ANARRAGGKKG - 538

Posterior @ 0.2

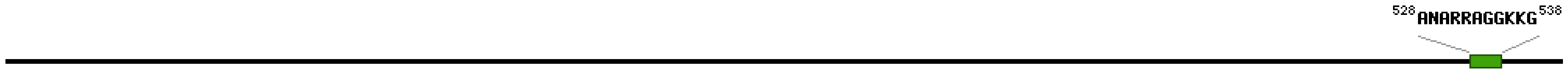

Posterior Graphic. Threshold Marked @ 0.2

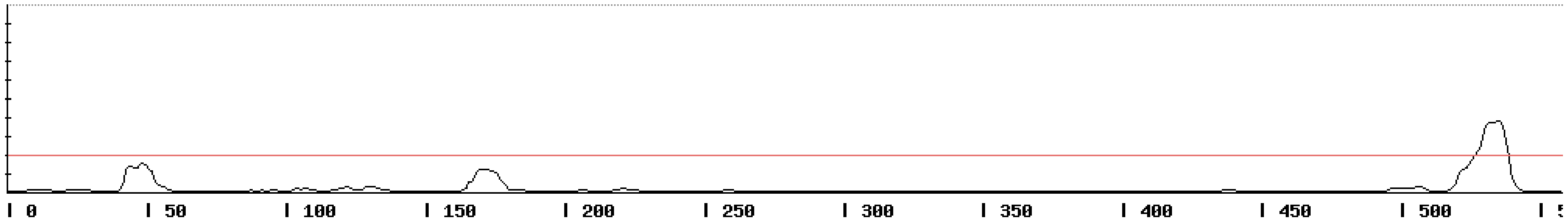

Predictions for Linumusitatissimum_Lus10006119

Viterbi Path

Viterbi Path

Posterior @ 0.2

Posterior @ 0.2

Posterior Graphic. Threshold Marked @ 0.2

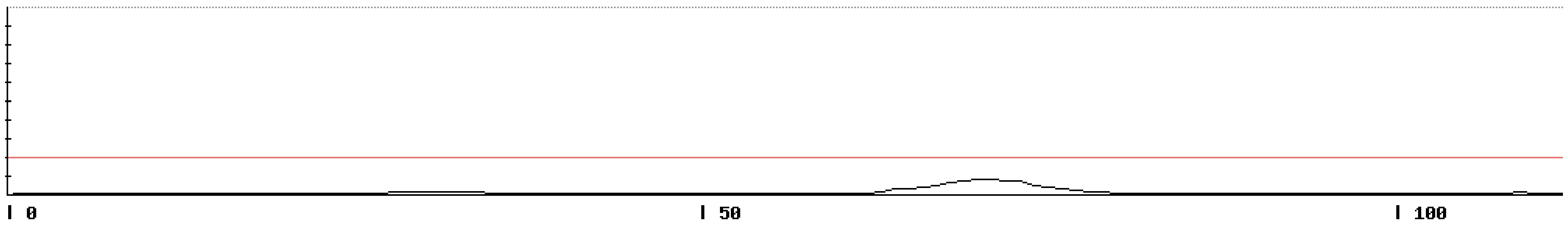

Predictions for Linumusitatissimum_Lus10006547

Viterbi Path	
Viterbi Path	
Posterior @ 0.2	124 - KGKPPKG - 130
Posterior @ 0.2	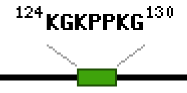
Posterior Graphic. Threshold Marked @ 0.2	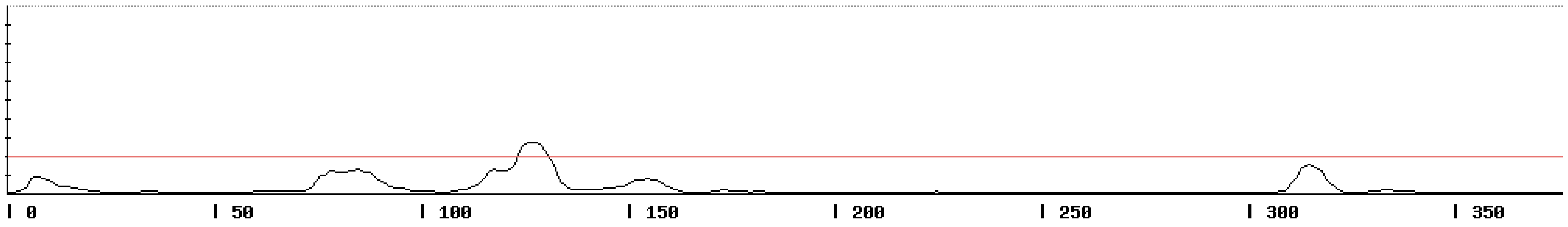

Predictions for Linumusitatissimum_Lus10007204

Viterbi Path	
Viterbi Path	
Posterior @ 0.2	88 - KLKQKVSGKQLKQKKKMAGS - 107
	197 - KR - 198
Posterior @ 0.2	

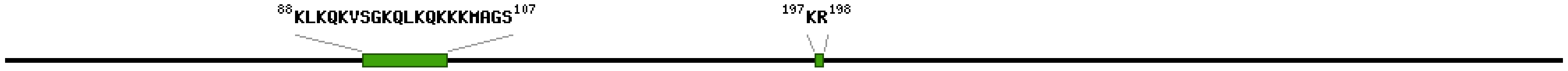

Posterior Graphic. Threshold Marked @ 0.2

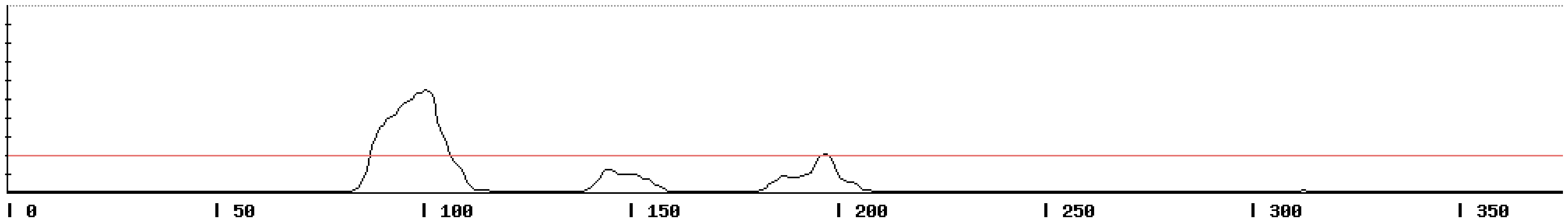

Predictions for Linumusitatissimum_Lus10007216

Viterbi Path

98 - KPTGKIRDVKGKKTKKKIGTKK - 119

Viterbi Path

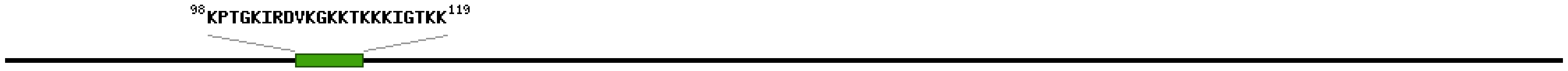

Posterior @ 0.2

93 - KNGYWKPTGKIRDVKGKKTKKKIGTKKTLVFYKKDKEFPK - 132

161 - KLKRKRDKRIPKN - 173

Posterior @ 0.2

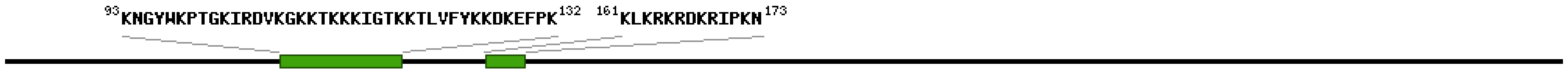

Posterior Graphic. Threshold Marked @ 0.2

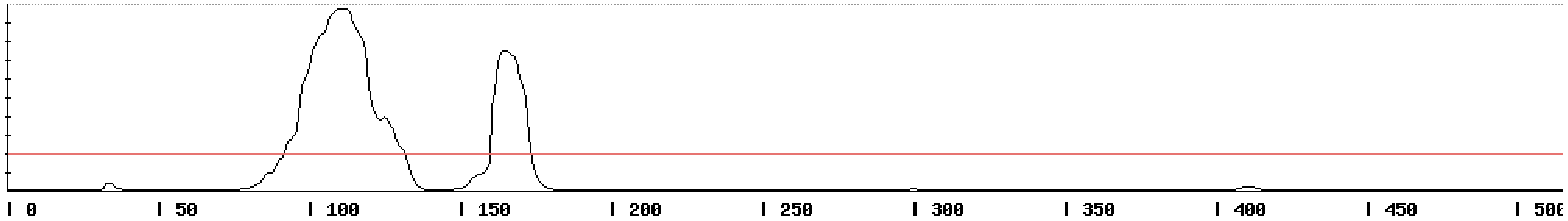

Predictions for Linumusitatissimum_Lus10007263

Viterbi Path

Viterbi Path

Posterior @ 0.2

Posterior @ 0.2

Posterior Graphic. Threshold Marked @ 0.2

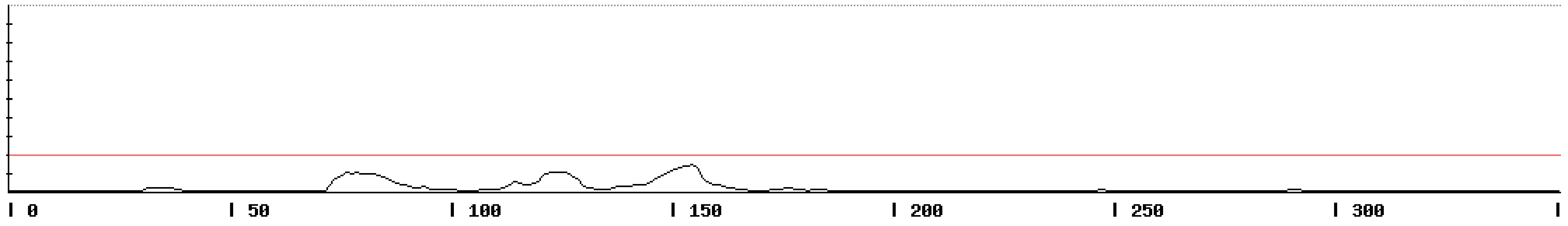

Predictions for Linumusitatissimum_Lus10007377

Viterbi Path

Viterbi Path

Posterior @ 0.2

Posterior @ 0.2

Posterior Graphic. Threshold Marked @ 0.2

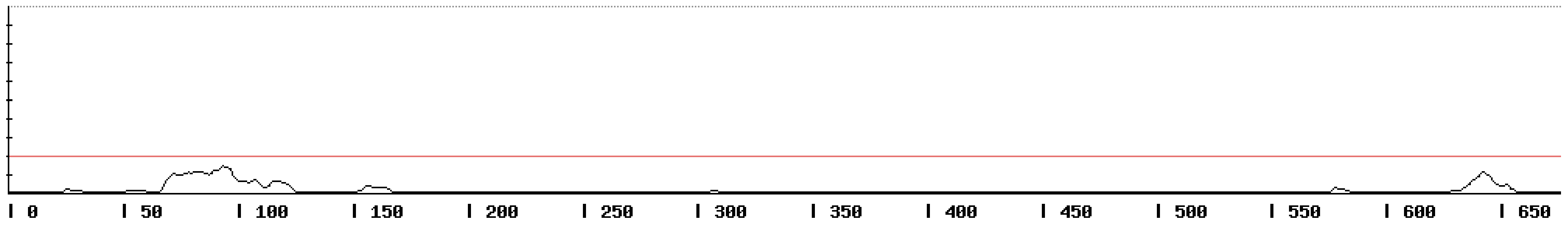

Predictions for Linumusitatissimum_Lus10007410

Viterbi Path

Viterbi Path

Posterior @ 0.2

Posterior @ 0.2

Posterior Graphic. Threshold Marked @ 0.2

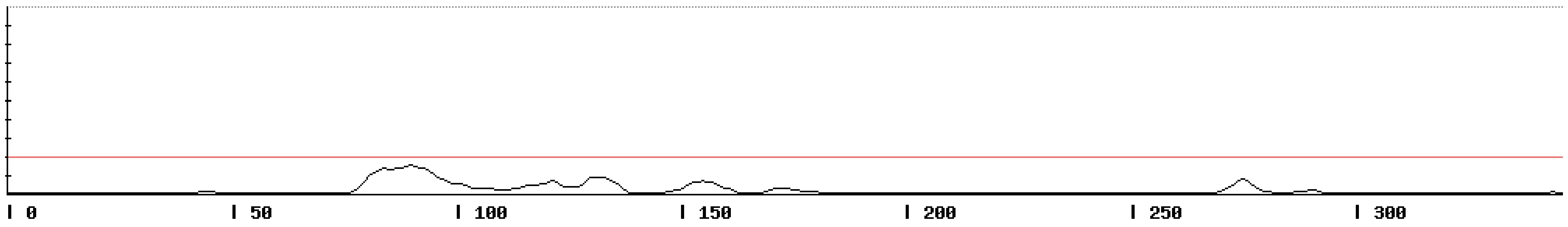

Predictions for Linumusitatissimum_Lus10008200

Viterbi Path

Viterbi Path

Posterior @ 0.2

91 - TKLKHKKVAGKQLKQKKMAGSS - 112

200 - KR - 201

Posterior @ 0.2

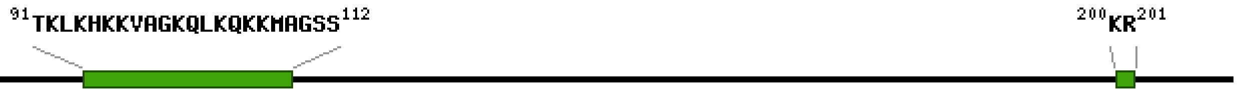

Posterior Graphic. Threshold Marked @ 0.2

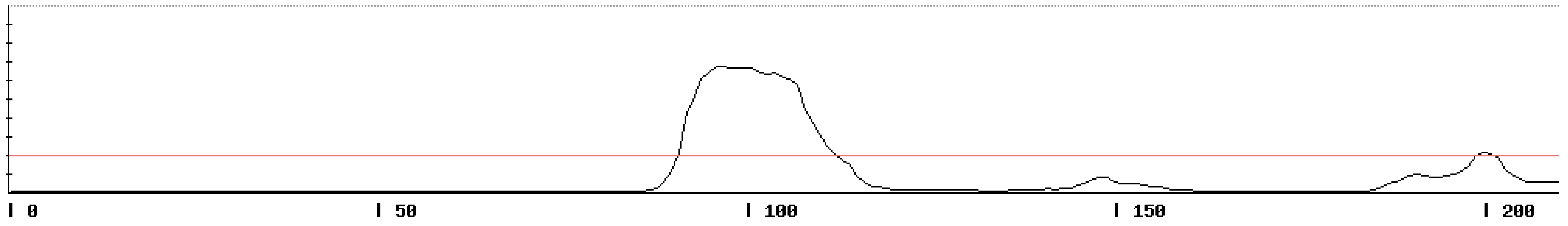

Predictions for Linumusitatissimum_Lus10008240

Viterbi Path	
Viterbi Path	
Posterior @ 0.2	91 - TKLKHKKVAGKQLKQKKMAGKSNKQ - 115
	199 - KKRK - 202
Posterior @ 0.2	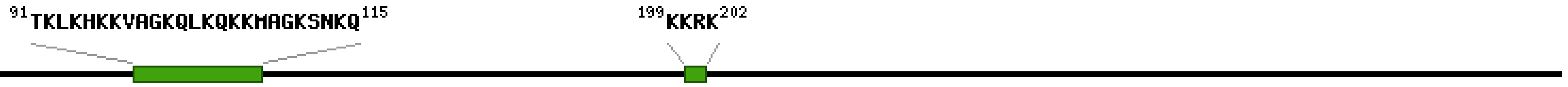
Posterior Graphic. Threshold Marked @ 0.2	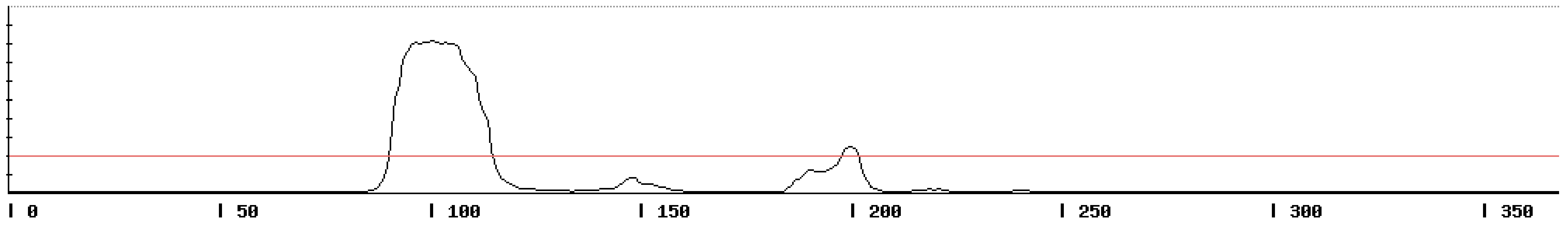

Predictions for Linumusitatissimum_Lus10008271

Viterbi Path	
Viterbi Path	
Posterior @ 0.2	115 - RR - 116
Posterior @ 0.2	

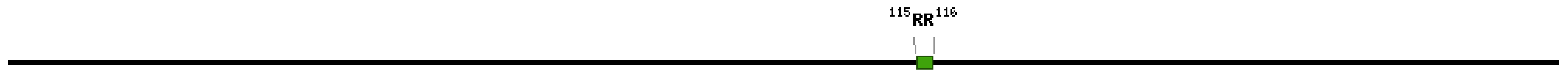

Posterior Graphic. Threshold Marked @ 0.2

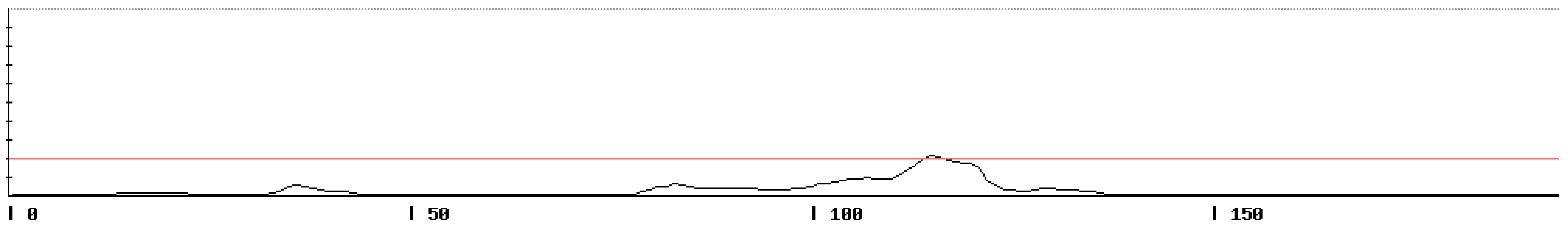

Predictions for Linumusitatissimum_Lus10008285

Viterbi Path	
Viterbi Path	
Posterior @ 0.2	95 - TGKDRTIRSRRPG - 107
Posterior @ 0.2	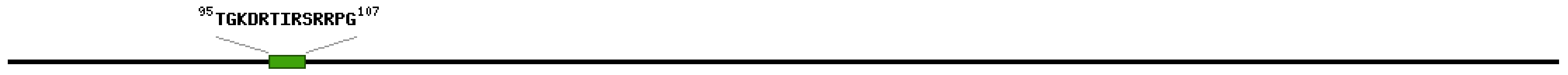
Posterior Graphic. Threshold Marked @ 0.2	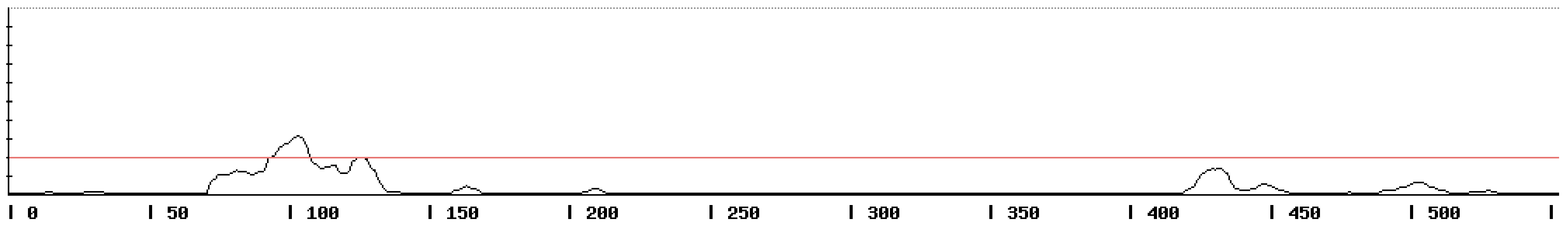

Predictions for Linumusitatissimum_Lus10008419

Viterbi Path	
Viterbi Path	

Posterior @ 0.2

Posterior @ 0.2

Posterior Graphic. Threshold Marked @ 0.2

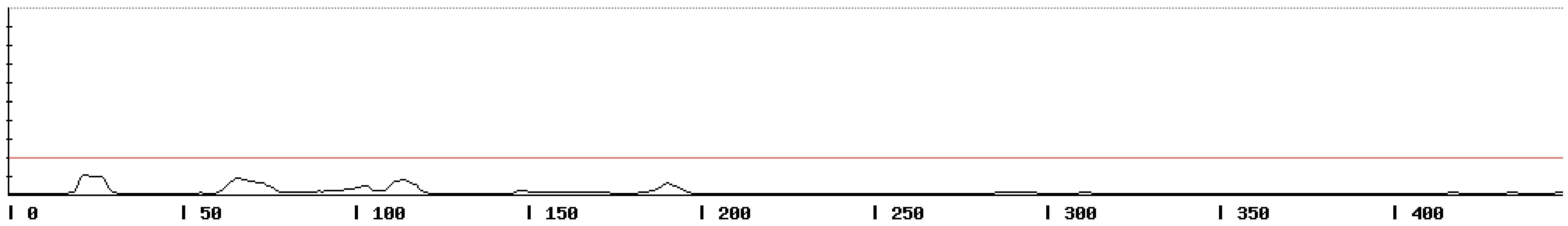

Predictions for Linumusitatissimum_Lus10008420

Viterbi Path

Viterbi Path

Posterior @ 0.2

Posterior @ 0.2

Posterior Graphic. Threshold Marked @ 0.2

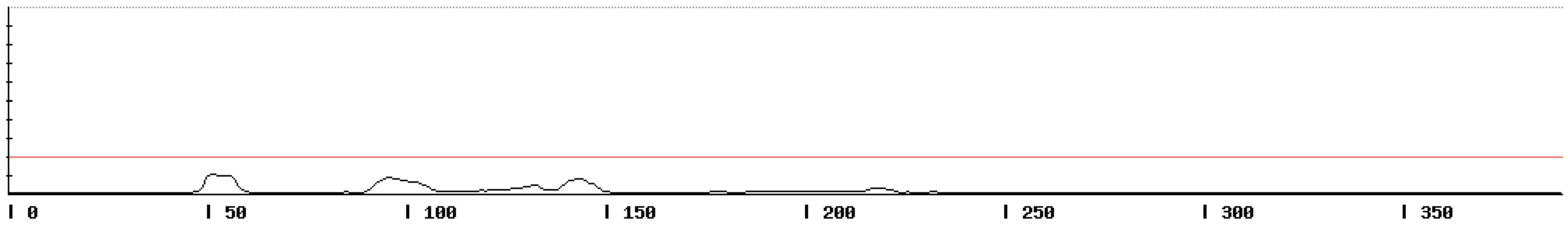

Predictions for Linumusitatissimum_Lus10008897

Viterbi Path

Viterbi Path

Posterior @ 0.2

Posterior @ 0.2

Posterior Graphic. Threshold Marked @ 0.2

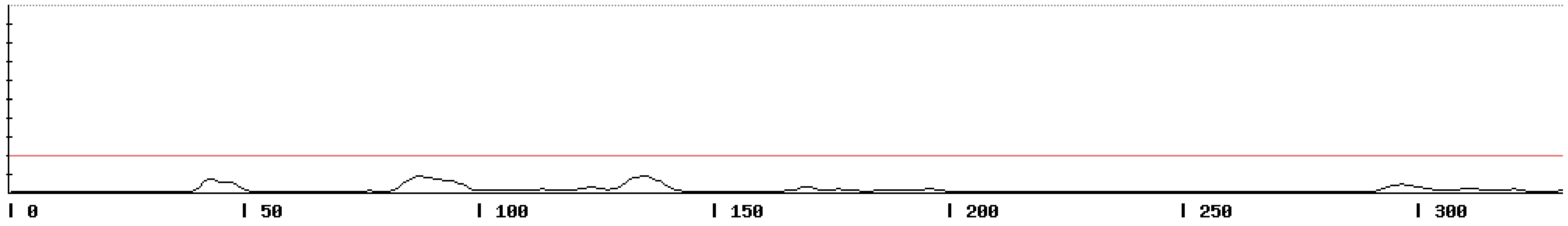

Predictions for Linumusitatissimum_Lus10009029

Viterbi Path

Viterbi Path

Posterior @ 0.2

Posterior @ 0.2

Posterior Graphic. Threshold Marked @ 0.2

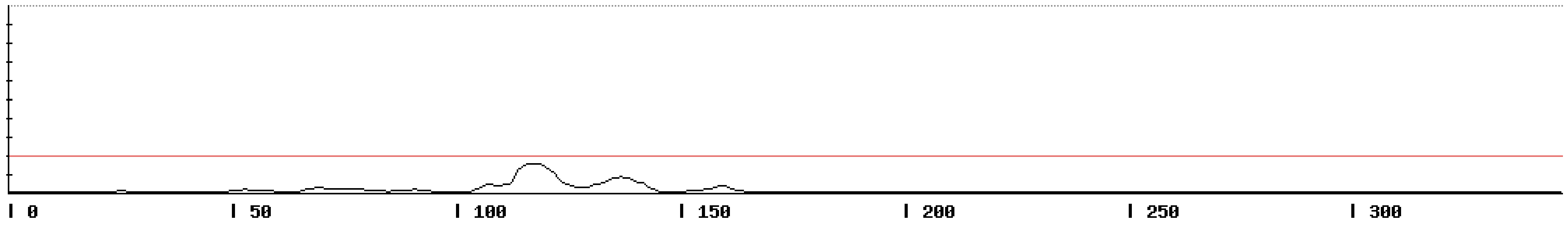

Predictions for Linumusitatissimum_Lus10009669

Viterbi Path	
Viterbi Path	
Posterior @ 0.2	
Posterior @ 0.2	
Posterior Graphic. Threshold Marked @ 0.2	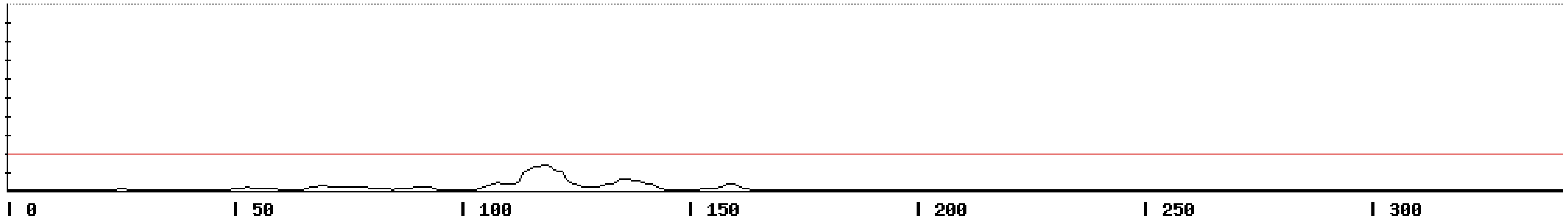

Predictions for Linumusitatissimum_Lus10009858

Viterbi Path	
Viterbi Path	
Posterior @ 0.2	126 - RR - 127<br>170 - EKRSVNRMRRRK - 181
Posterior @ 0.2	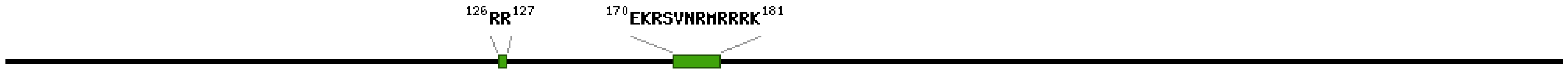
Posterior Graphic. Threshold Marked @ 0.2	

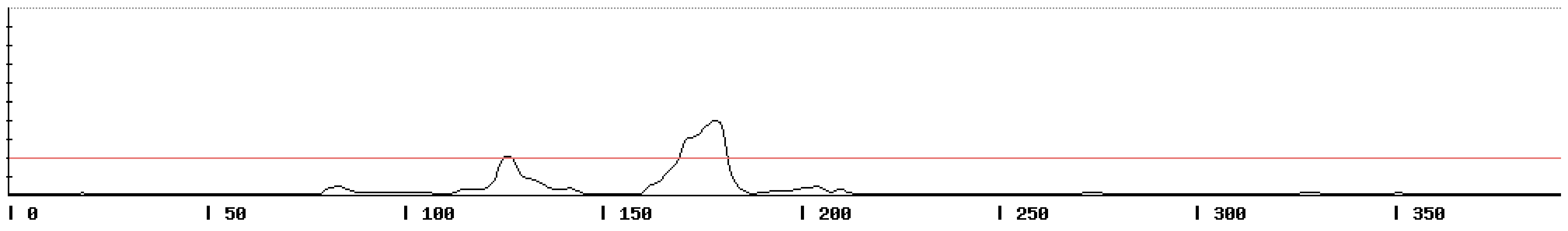

Predictions for Linumusitatissimum_Lus10009924

Viterbi Path

Viterbi Path

Posterior @ 0.2

66 - KLKRKKTEKNGHHMERGYG - 84

104 - KKTKMRSRR - 112

Posterior @ 0.2

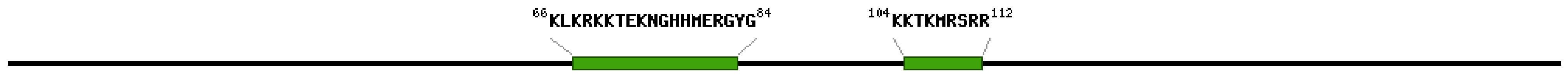

Posterior Graphic. Threshold Marked @ 0.2

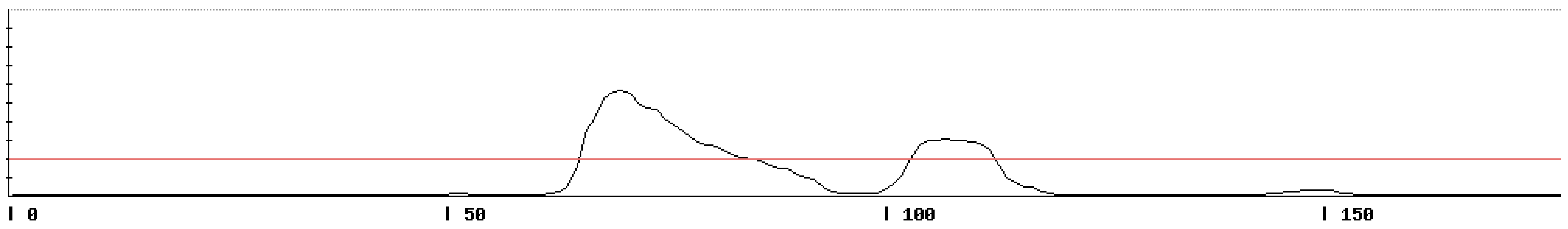

Predictions for Linumusitatissimum_Lus10009939

Viterbi Path

Viterbi Path

Posterior @ 0.2

235 - RRRSSSSRS - 243

Posterior @ 0.2

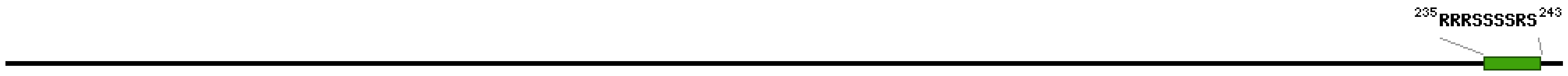

Posterior Graphic. Threshold Marked @ 0.2

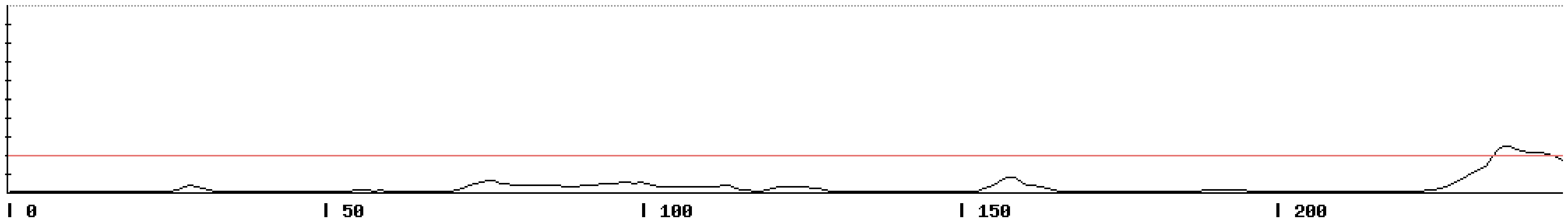

Predictions for Linumusitatissimum_Lus10010037

Viterbi Path

Viterbi Path

Posterior @ 0.2 — 68 - GPKGRKKHWFCYAKIRVSKEENRGNRRRAKGGYWRRLGKVR - 108

Posterior @ 0.2

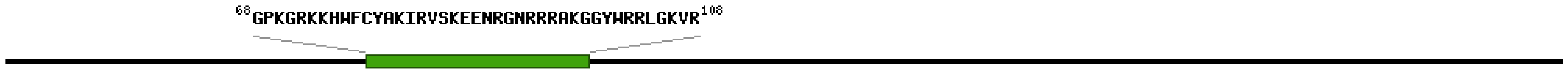

Posterior Graphic. Threshold Marked @ 0.2

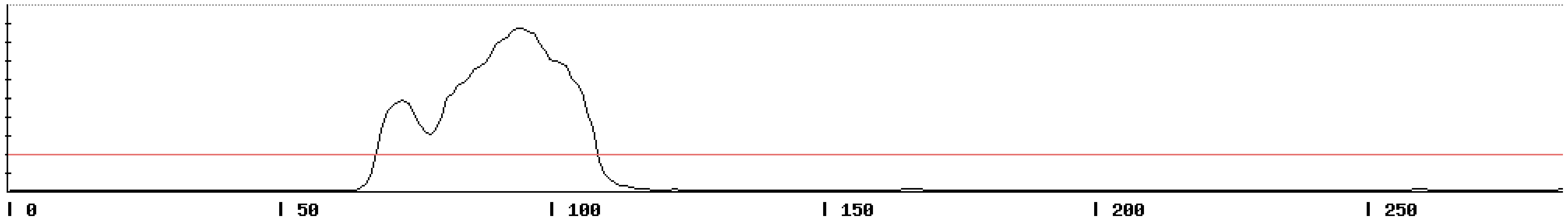

Predictions for Linumusitatissimum_Lus10010096

Viterbi Path — 159 - KPTGKIREVKGKKTKKKIGTKK - 180

Viterbi Path

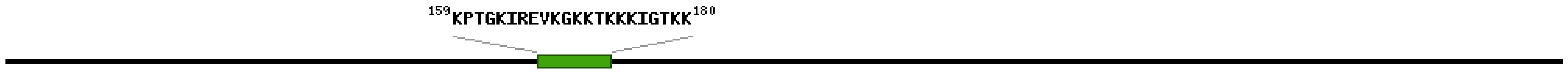

Posterior @ 0.2 — 153 - TKNGYWKPTGKIREVKGKKTKKKIGTKKTLVFYKKDKEFPK - 193

222 - KLKRKRDKRIPKN - 234

Posterior @ 0.2

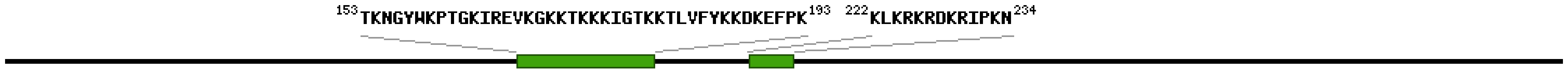

Posterior Graphic. Threshold Marked @ 0.2

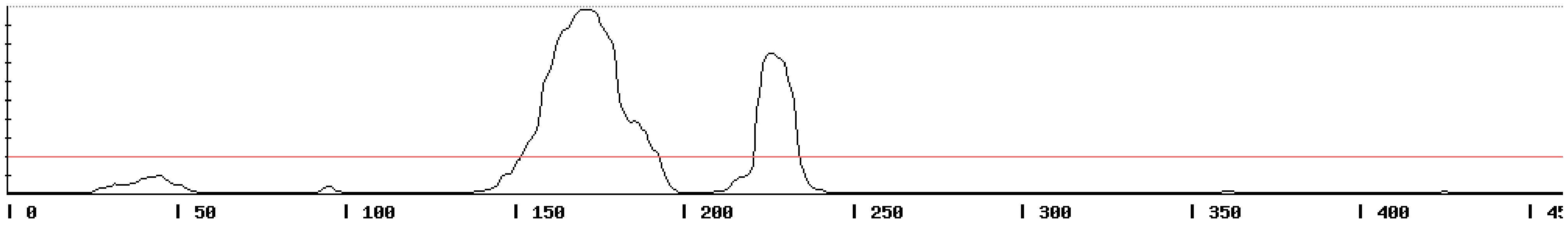

Predictions for Linumusitatissimum_Lus10010098

Viterbi Path

Viterbi Path

Posterior @ 0.2 — 53 - KQVNRKTKAGYWKPTGKARIVRAKSSRNQIGTKKTLVFYKGRVRKGV - 99

Posterior @ 0.2

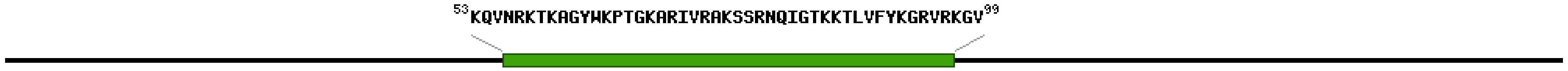

Posterior Graphic. Threshold Marked @ 0.2

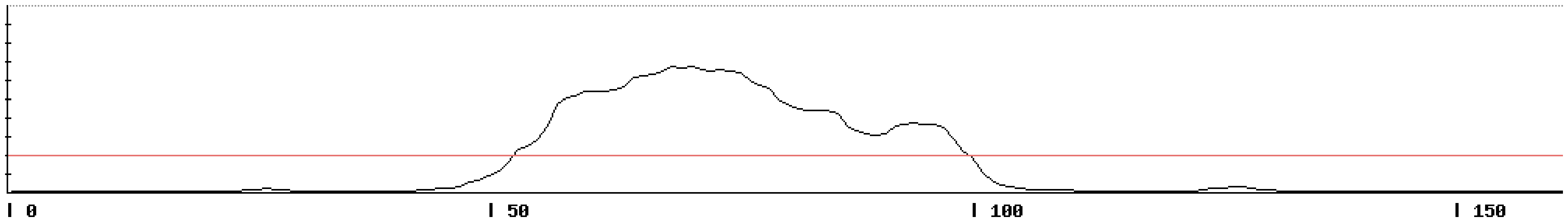

Predictions for Linumusitatissimum_Lus10010148

Viterbi Path

Viterbi Path

Posterior @ 0.2	91 - YWKATGKDRTIKSRRAGFSPKSIGMKKTLVFYKGRAPRGER - 131
	460 - RRK - 462

Posterior @ 0.2

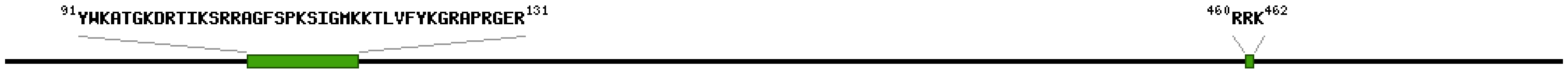

Posterior Graphic. Threshold Marked @ 0.2

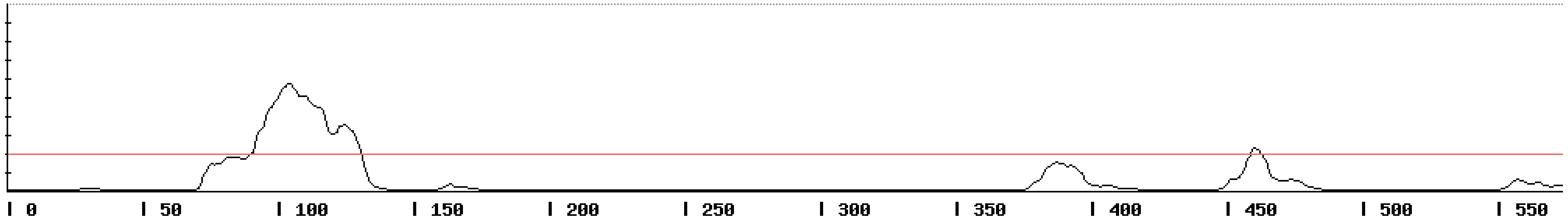

Predictions for Linumusitatissimum_Lus10010294

Viterbi Path

Viterbi Path

Posterior @ 0.2 — 183 - RKRK - 186

Posterior @ 0.2

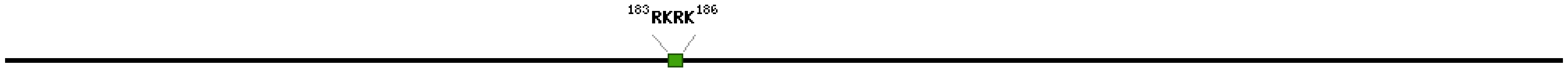

Posterior Graphic. Threshold Marked @ 0.2

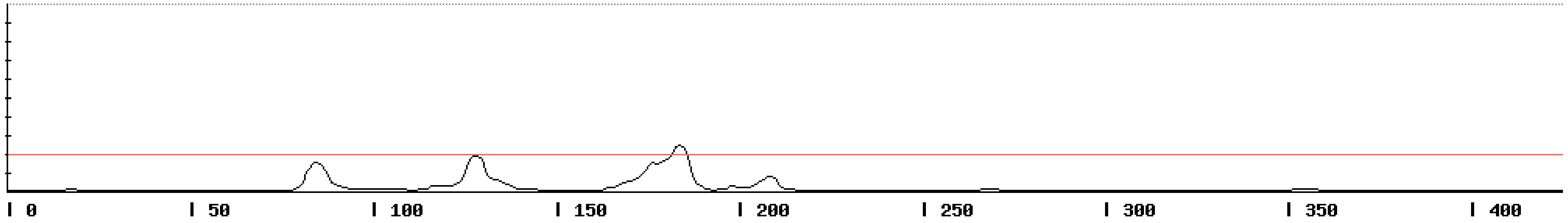

Predictions for Linumusitatissimum_Lus10010371	
Viterbi Path	
Viterbi Path	
Posterior @ 0.2	81 - KIRVSKEENRGNRSRAKGGYWRRLGK - 106
Posterior @ 0.2	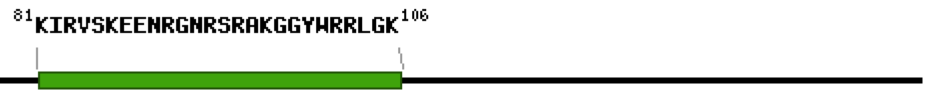
Posterior Graphic. Threshold Marked @ 0.2	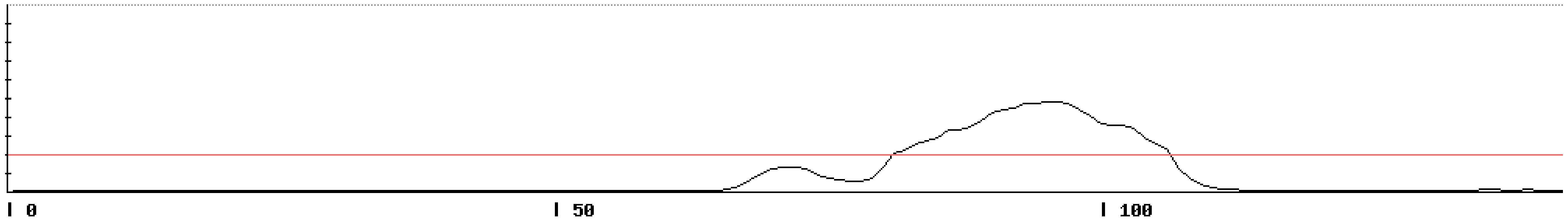

Predictions for Linumusitatissimum_Lus10010747	
Viterbi Path	
Viterbi Path	
Posterior @ 0.2	
Posterior @ 0.2	
Posterior Graphic. Threshold Marked @ 0.2	

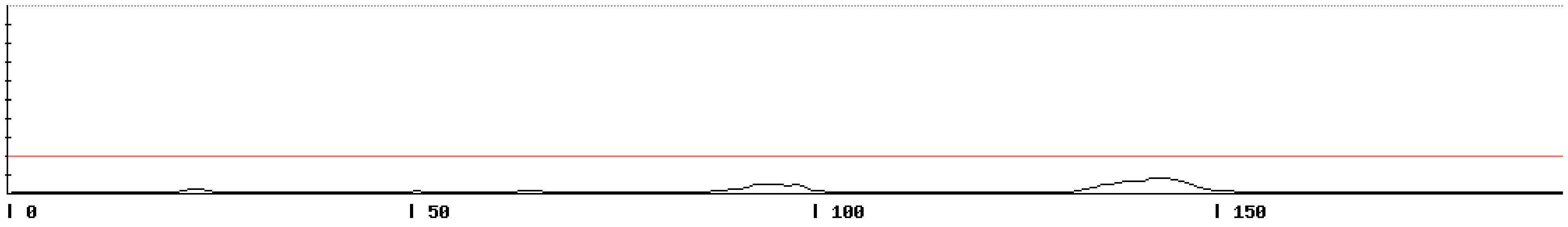

Predictions for Linumusitatissimum_Lus10010959

Viterbi Path	
Viterbi Path	
Posterior @ 0.2	564 - ISIRRRRRRKVS - 575
Posterior @ 0.2	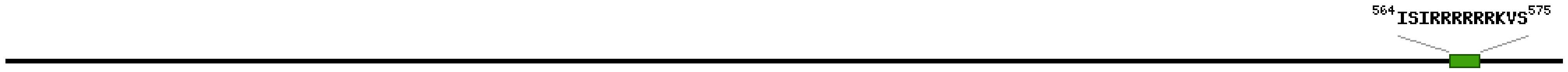
Posterior Graphic. Threshold Marked @ 0.2	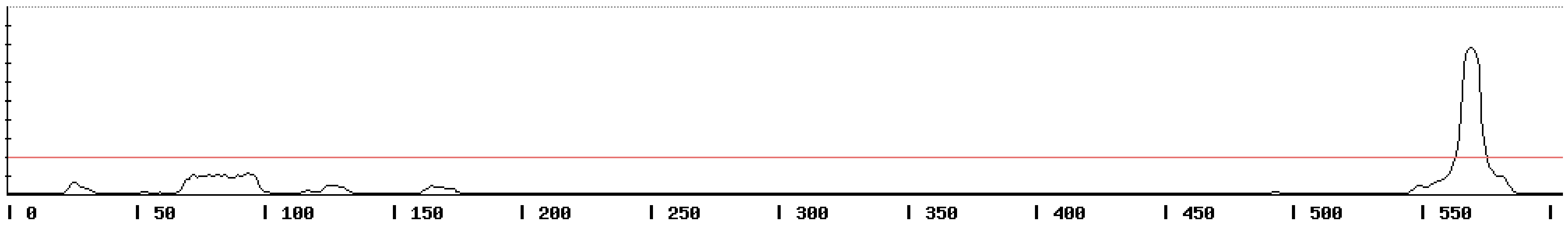

Predictions for Linumusitatissimum_Lus10011215

Viterbi Path	
Viterbi Path	
Posterior @ 0.2	
Posterior @ 0.2	

Posterior Graphic. Threshold Marked @ 0.2

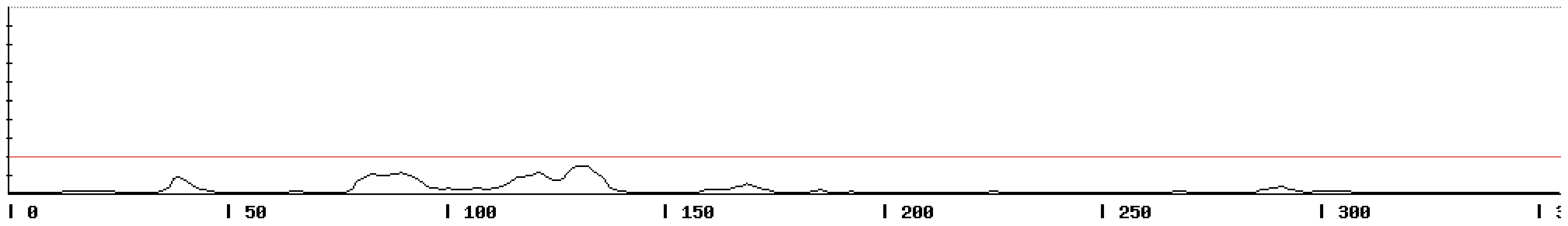

Predictions for Linumusitatissimum_Lus10012557

Viterbi Path

Viterbi Path

Posterior @ 0.2 176 - QKKKRPSYNNRKKPPNP - 192

Posterior @ 0.2

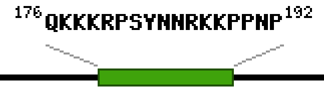

Posterior Graphic. Threshold Marked @ 0.2

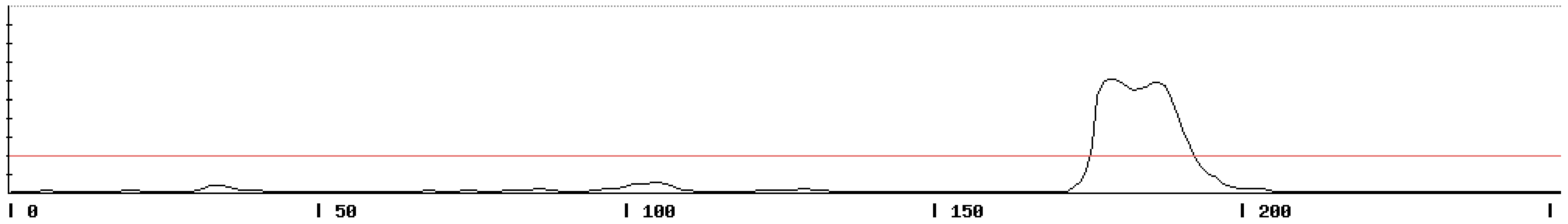

Predictions for Linumusitatissimum_Lus10012927

Viterbi Path

Viterbi Path

Posterior @ 0.2 85 - GTRKRRK - 91

Posterior @ 0.2

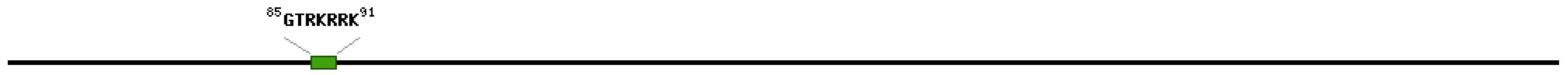

Posterior Graphic. Threshold Marked @ 0.2

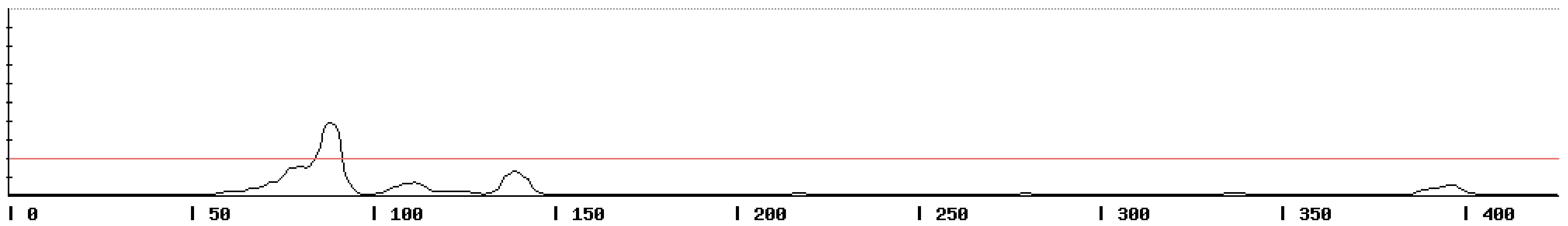

Predictions for Linumusitatissimum_Lus10013205

Viterbi Path	
Viterbi Path	
Posterior @ 0.2	152 - RAPRG - 156
Posterior @ 0.2	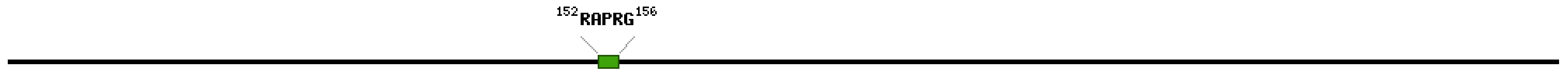
Posterior Graphic. Threshold Marked @ 0.2	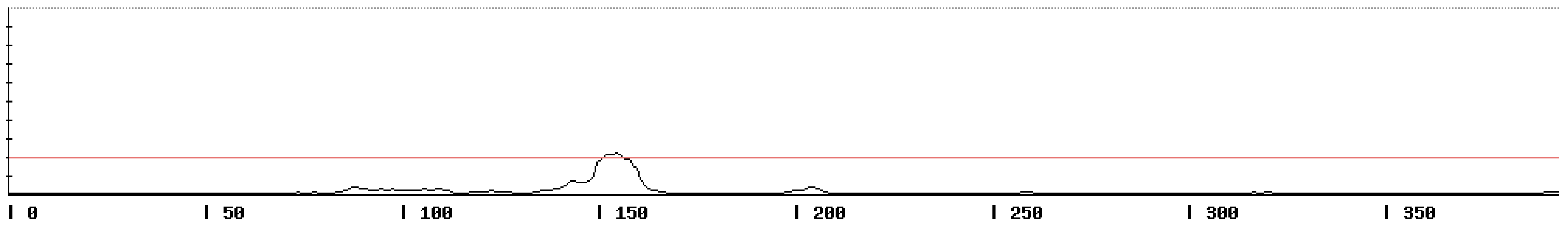

Predictions for Linumusitatissimum_Lus10013316

Viterbi Path	301 - RRVKLESRRRKKKIDQVAAPVEGKDEKAEKERRSKSKK - 338
Viterbi Path	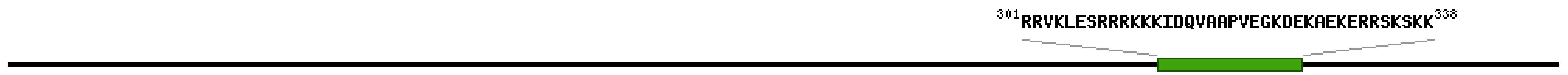

Posterior @ 0.2	296 - AAPSNRRVKLESRRRKKKIDQVAAPVEGKDEKAEKERRSKSKKVPS - 341
	351 - RKKKRGN - 356
Posterior @ 0.2	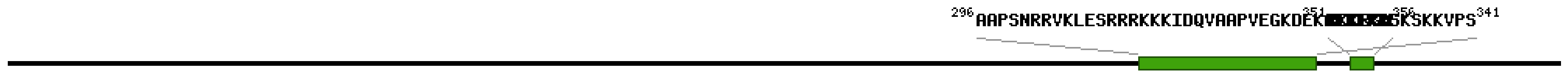
Posterior Graphic. Threshold Marked @ 0.2	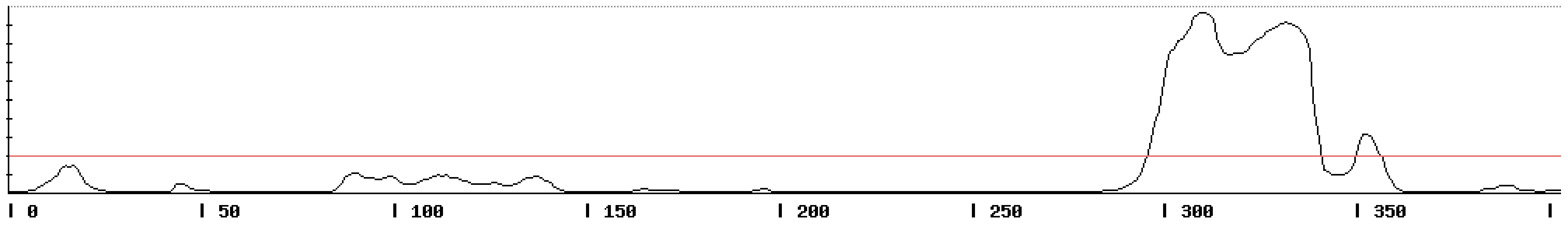

Predictions for Linumusitatissimum_Lus10013782

Viterbi Path	
Viterbi Path	
Posterior @ 0.2	
Posterior @ 0.2	
Posterior Graphic. Threshold Marked @ 0.2	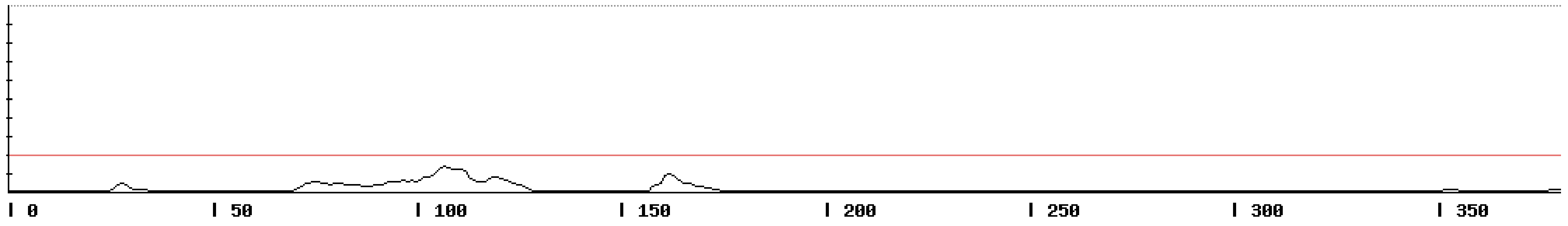

Predictions for Linumusitatissimum_Lus10013964

Viterbi Path	
Viterbi Path	

Posterior @ 0.2

Posterior @ 0.2

Posterior Graphic. Threshold Marked @ 0.2

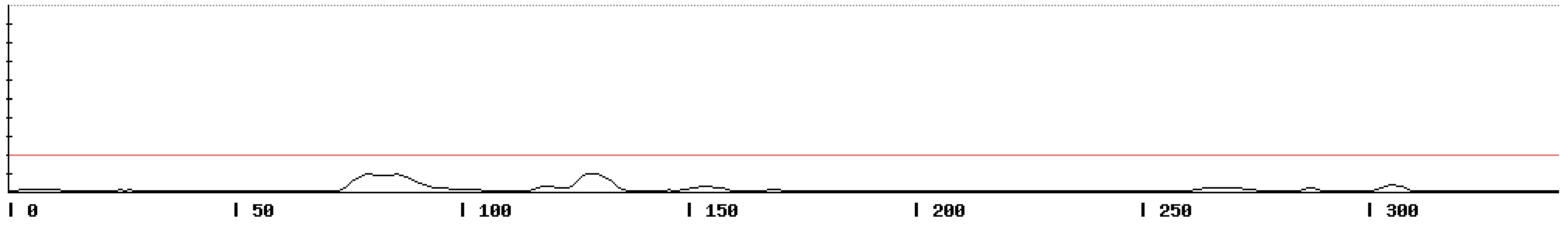

Predictions for Linumusitatissimum_Lus10013967

Viterbi Path

Viterbi Path

Posterior @ 0.2 141 - GTRKRRKV - 148

Posterior @ 0.2

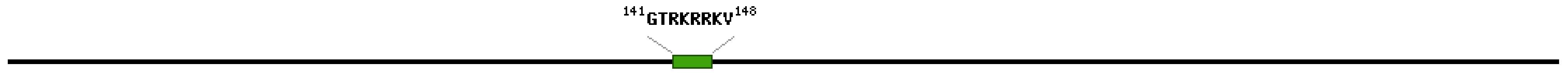

Posterior Graphic. Threshold Marked @ 0.2

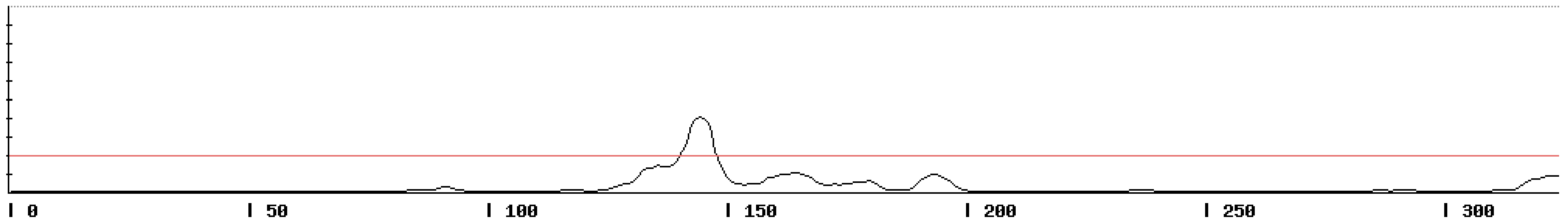

Predictions for Linumusitatissimum_Lus10014342

Viterbi Path	
Viterbi Path	
Posterior @ 0.2	90 - LKRKSSKV - 97
	178 - KTPEKTQRSR - 187
Posterior @ 0.2	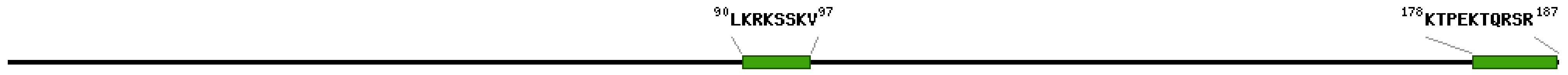
Posterior Graphic. Threshold Marked @ 0.2	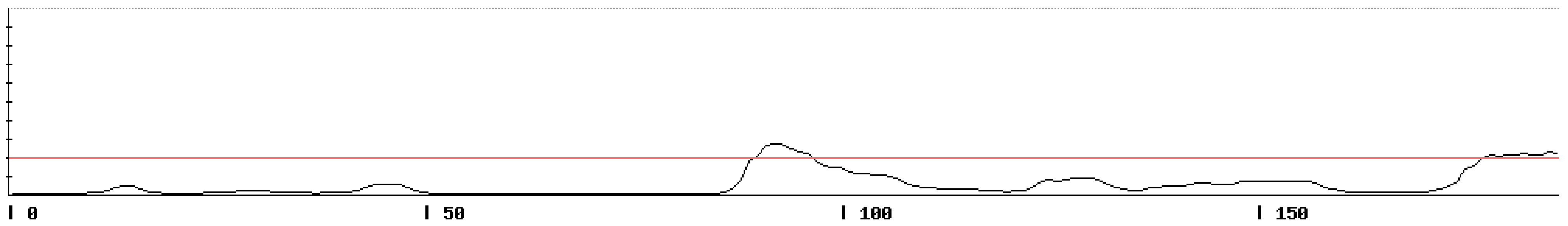

Predictions for Linumusitatissimum_Lus10014911

Viterbi Path	
Viterbi Path	
Posterior @ 0.2	110 - RKYKNGERPNRA - 121
Posterior @ 0.2	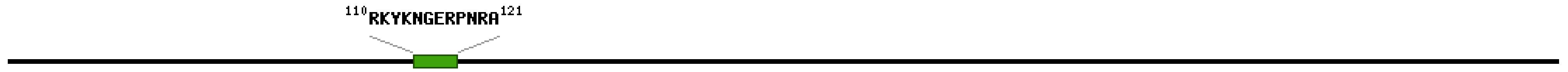
Posterior Graphic. Threshold Marked @ 0.2	

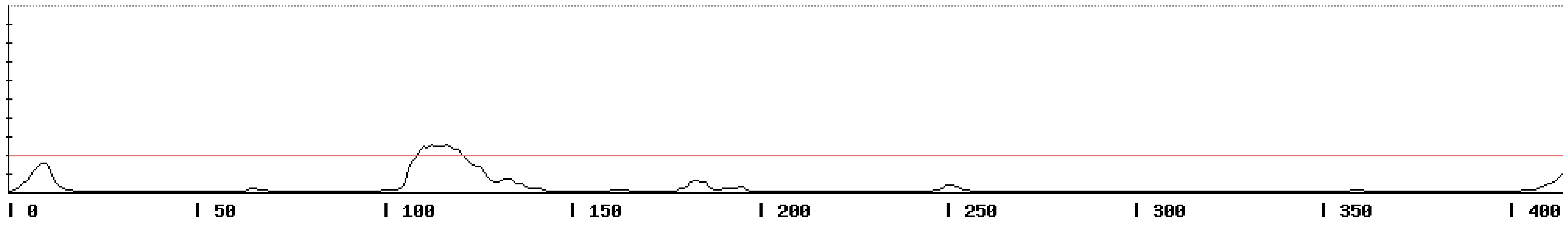

Predictions for Linumusitatissimum_Lus10015076

Viterbi Path	
Viterbi Path	
Posterior @ 0.2	122 - RPP - 124
Posterior @ 0.2	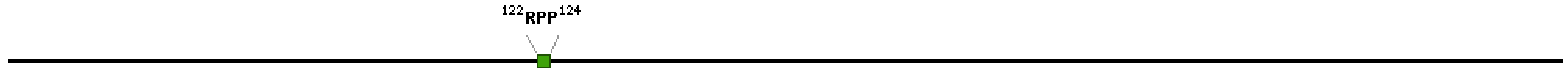
Posterior Graphic. Threshold Marked @ 0.2	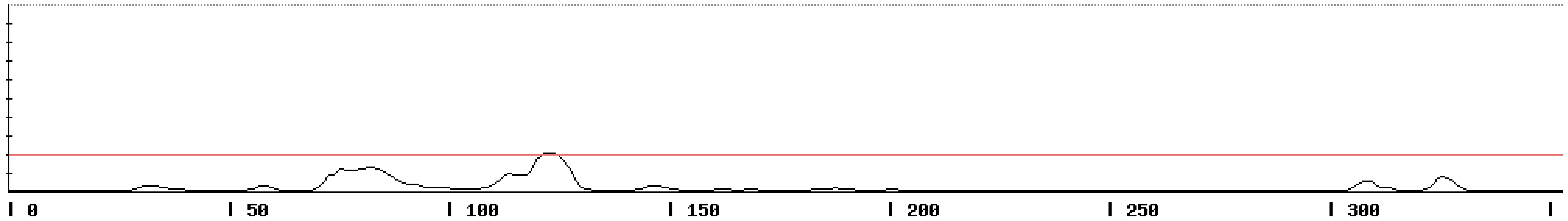

Predictions for Linumusitatissimum_Lus10015312

Viterbi Path	190 - KTKPVIKKPKKKKKVK - 205
Viterbi Path	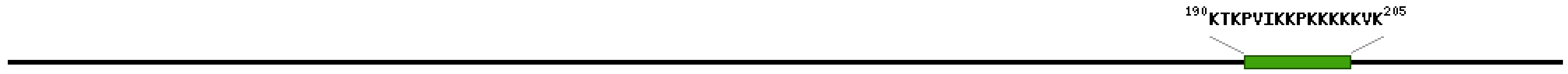
Posterior @ 0.2	149 - GRRVPKRRQ - 157
	190 - KTKPVIKKPKKKKKVKTG - 207
Posterior @ 0.2	

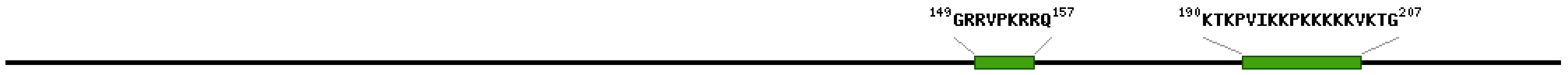

Posterior Graphic. Threshold Marked @ 0.2

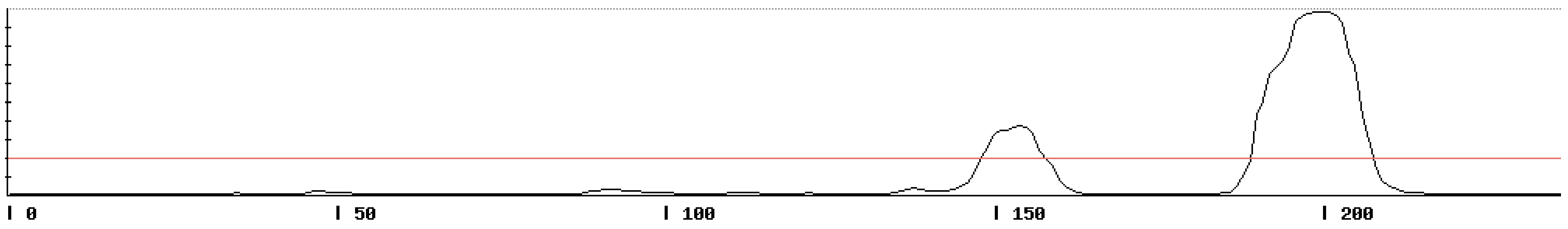

Predictions for Linumusitatissimum_Lus10015367

Viterbi Path

Viterbi Path

Posterior @ 0.2 — 152 - PPK - 154

Posterior @ 0.2

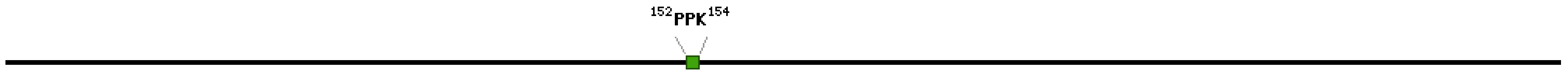

Posterior Graphic. Threshold Marked @ 0.2

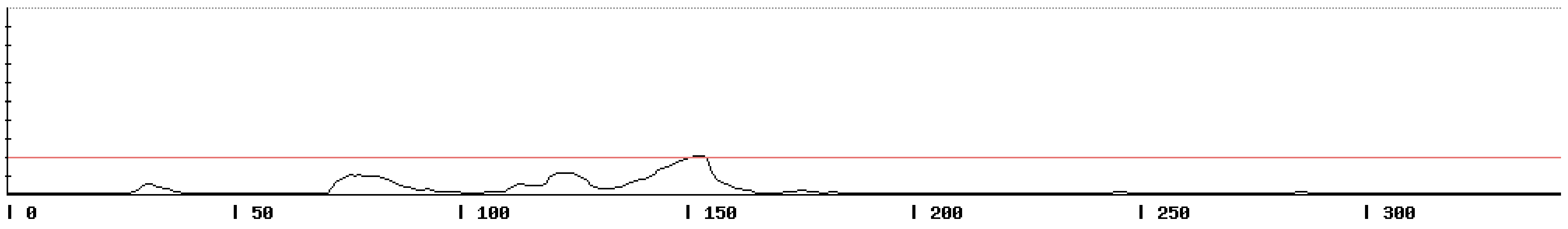

Predictions for Linumusitatissimum_Lus10015389

Viterbi Path

Viterbi Path

Posterior @ 0.2

Posterior @ 0.2

Posterior Graphic. Threshold Marked @ 0.2

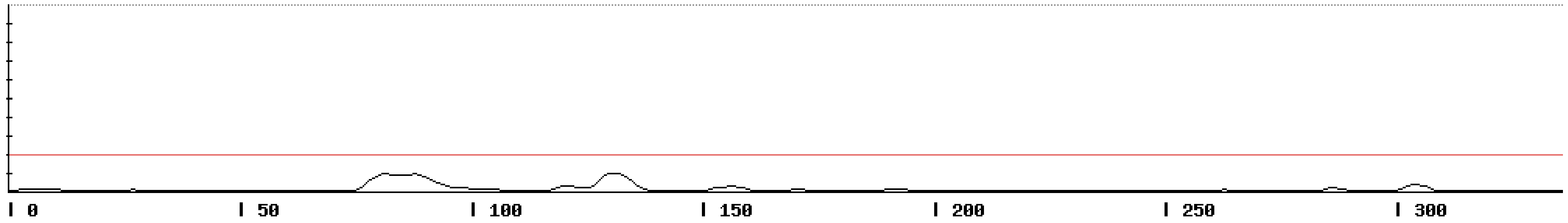

Predictions for Linumusitatissimum_Lus10015392

Viterbi Path

Viterbi Path

Posterior @ 0.2: 100 - GTRKRRKV - 107

Posterior @ 0.2

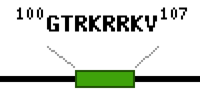

Posterior Graphic. Threshold Marked @ 0.2

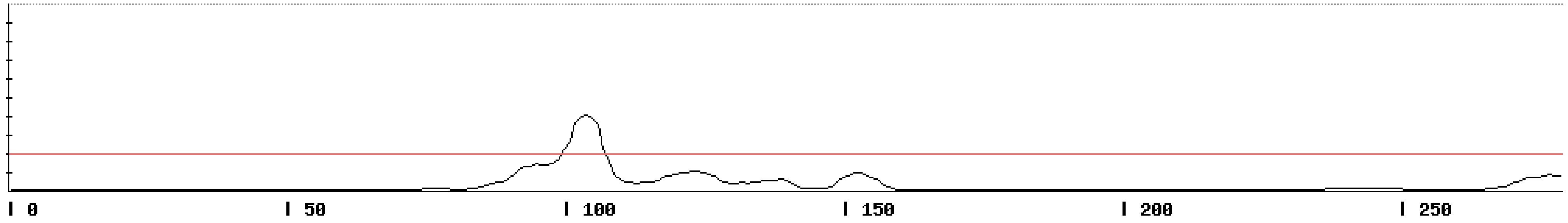

Predictions for Linumusitatissimum_Lus10015554

Viterbi Path

Viterbi Path

Posterior @ 0.2	
Posterior @ 0.2	
Posterior Graphic. Threshold Marked @ 0.2	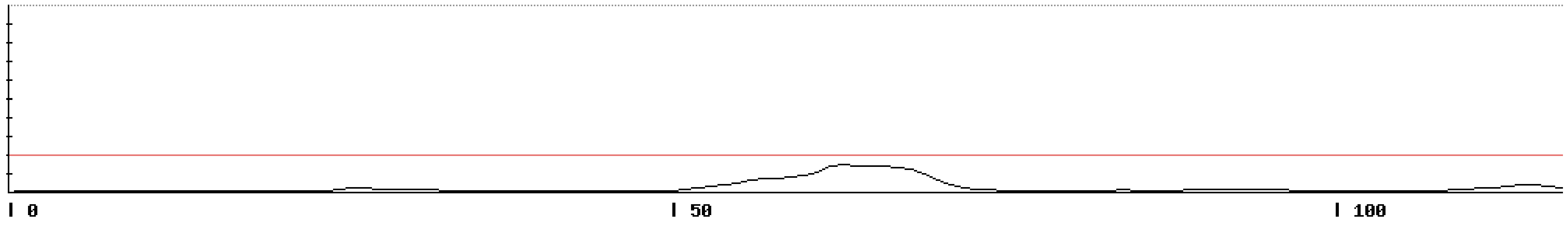

Predictions for Linumusitatissimum_Lus10015587	
Viterbi Path	
Viterbi Path	
Posterior @ 0.2	93 - KATGKDRRIK - 102
Posterior @ 0.2	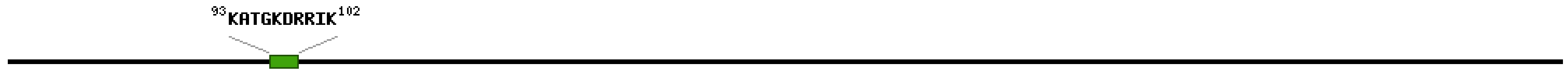
Posterior Graphic. Threshold Marked @ 0.2	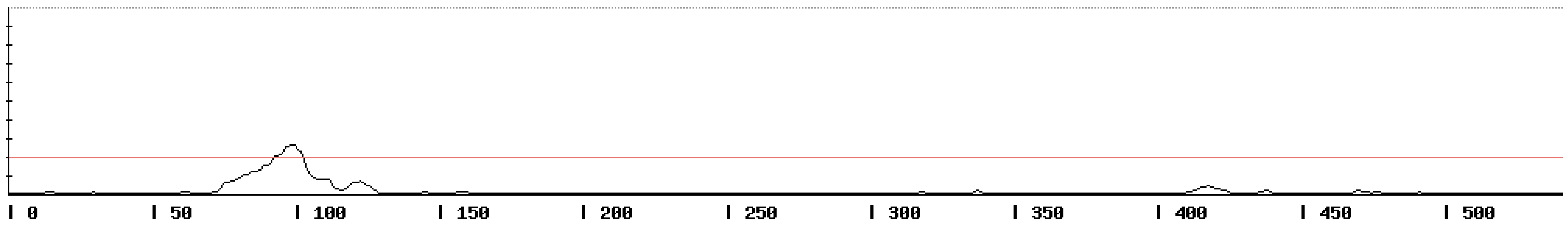

Predictions for Linumusitatissimum_Lus10015743

Viterbi Path	
Viterbi Path	
Posterior @ 0.2	
Posterior @ 0.2	
Posterior Graphic. Threshold Marked @ 0.2	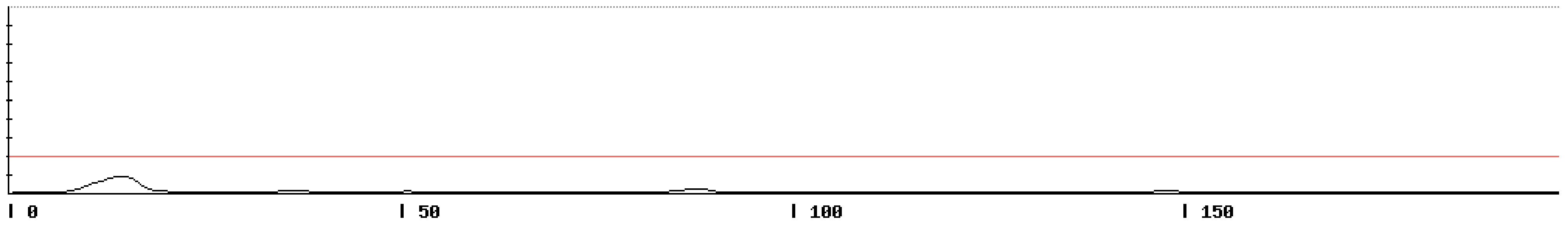

Predictions for Linumusitatissimum_Lus10017340

Viterbi Path	
Viterbi Path	
Posterior @ 0.2	
Posterior @ 0.2	
Posterior Graphic. Threshold Marked @ 0.2	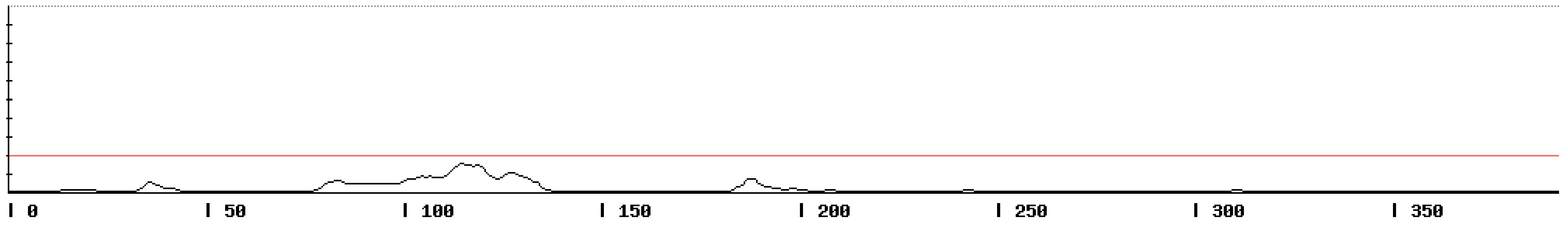

Predictions for Linumusitatissimum_Lus10017353

Viterbi Path

Viterbi Path

Posterior @ 0.2

69 - KSRK - 72

Posterior @ 0.2

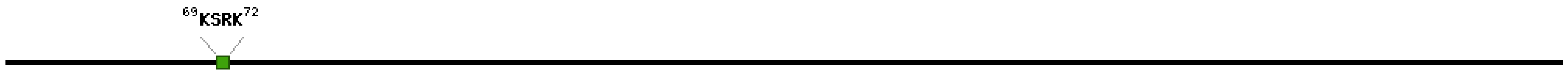

Posterior Graphic. Threshold Marked @ 0.2

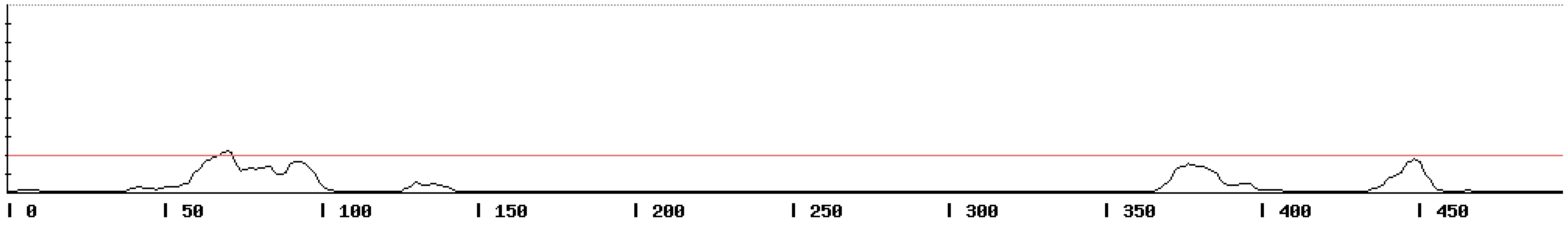

Predictions for Linumusitatissimum_Lus10017458

Viterbi Path

Viterbi Path

Posterior @ 0.2

Posterior @ 0.2

Posterior Graphic. Threshold Marked @ 0.2

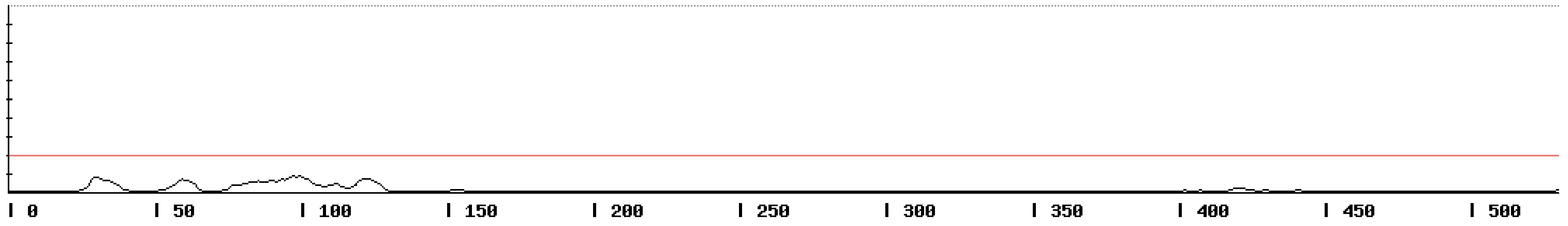

Predictions for Linumusitatissimum_Lus10017807

Viterbi Path

Viterbi Path

Posterior @ 0.2

44 - RRRKNSSIITSRVKPKGR - 61

94 - K - 94

Posterior @ 0.2

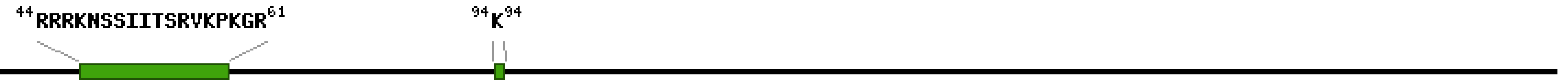

Posterior Graphic. Threshold Marked @ 0.2

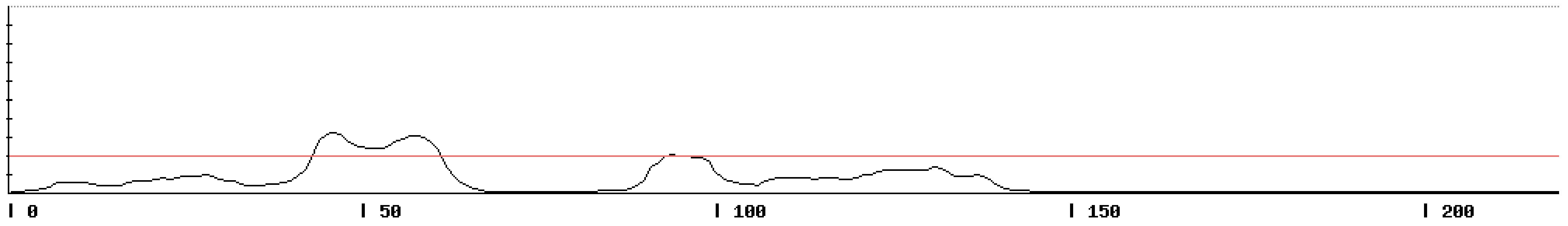

Predictions for Linumusitatissimum_Lus10017915

Viterbi Path

Viterbi Path

Posterior @ 0.2

Posterior @ 0.2

Posterior Graphic. Threshold Marked @ 0.2	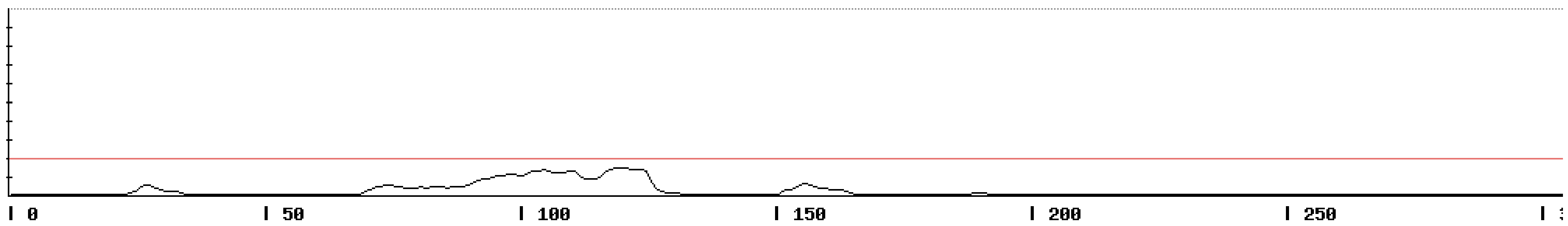

Predictions for Linumusitatissimum_Lus10018142

Viterbi Path	
Viterbi Path	
Posterior @ 0.2	
Posterior @ 0.2	
Posterior Graphic. Threshold Marked @ 0.2	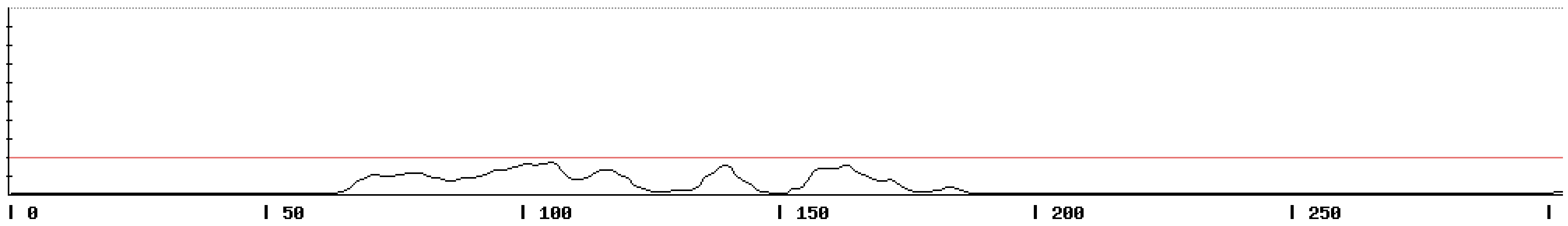

Predictions for Linumusitatissimum_Lus10018469

Viterbi Path	
Viterbi Path	

Posterior @ 0.2

Posterior @ 0.2

Posterior Graphic. Threshold Marked @ 0.2

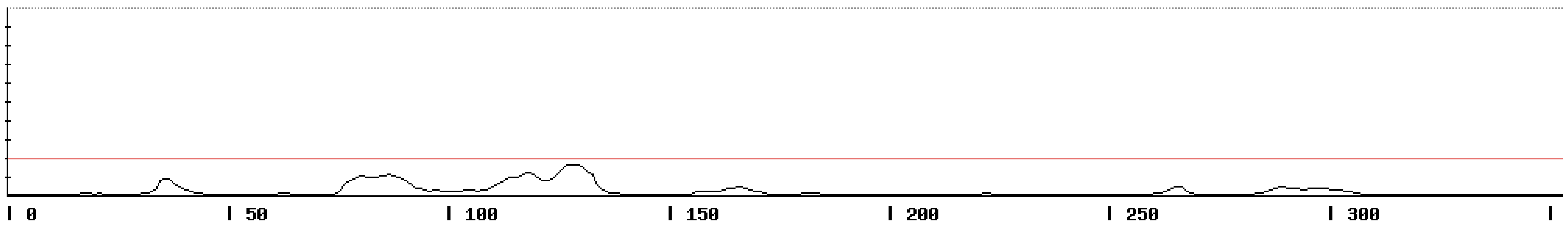

Predictions for Linumusitatissimum_Lus10018591

Viterbi Path

Viterbi Path

Posterior @ 0.2

Posterior @ 0.2

Posterior Graphic. Threshold Marked @ 0.2

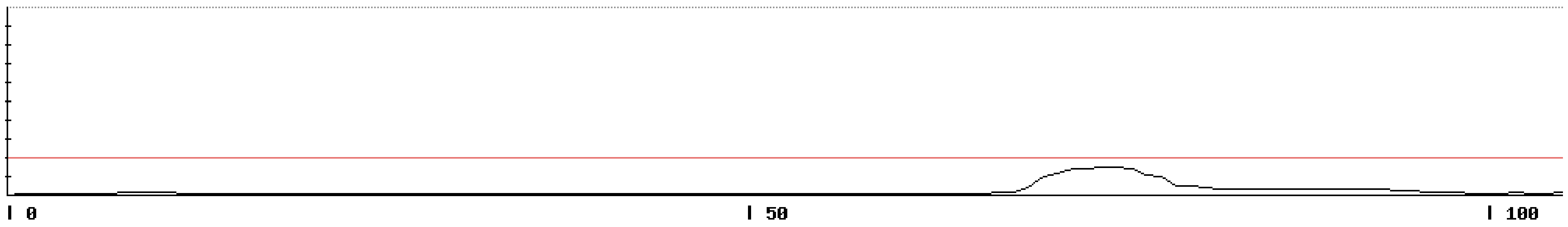

Predictions for Linumusitatissimum_Lus10018637

Viterbi Path

Viterbi Path

Posterior @ 0.2

141 - GTRKRRKV - 148

164 - KTGKTR - 169

174 - GGKTKG - 179

Posterior @ 0.2

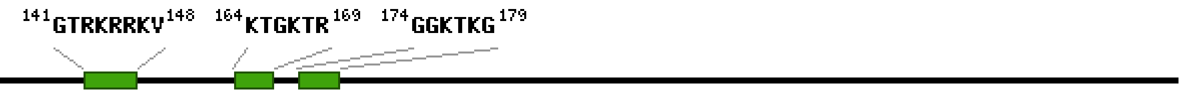

Posterior Graphic. Threshold Marked @ 0.2

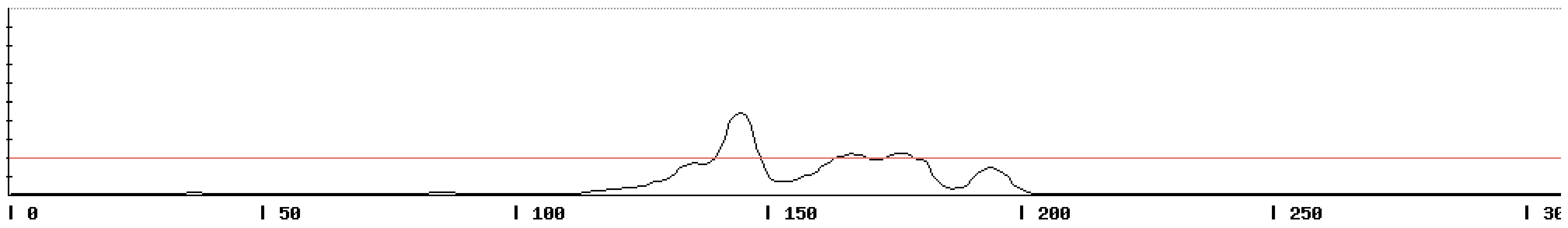

Predictions for Linumusitatissimum_Lus10018810

Viterbi Path

Viterbi Path

Posterior @ 0.2

326 - ASRKHKLADKDKG - 338

347 - GSSSGASRDVEKKGSKGILKKRKNK - 371

Posterior @ 0.2

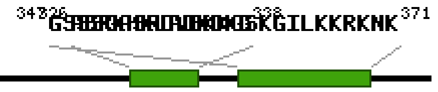

Posterior Graphic. Threshold Marked @ 0.2

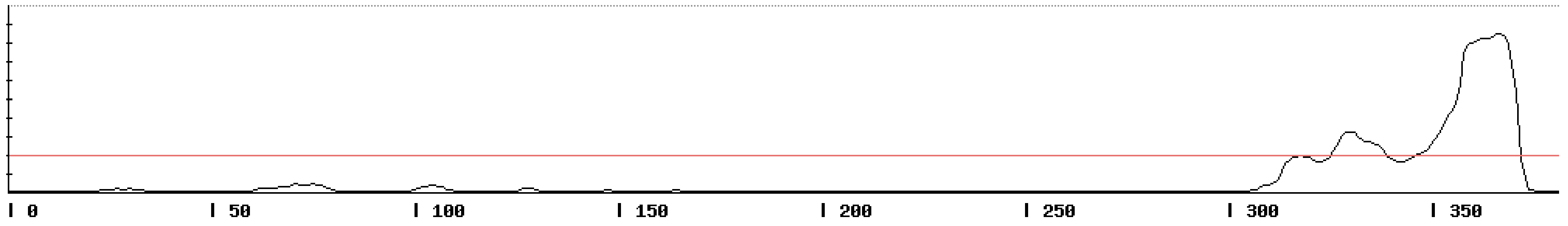

Predictions for Linumusitatissimum_Lus10019638

Viterbi Path

Viterbi Path

Posterior @ 0.2 | 86 - KLKRKPSNNKIDRGVAGGKGRWQ - 108

Posterior @ 0.2

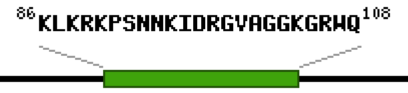

Posterior Graphic. Threshold Marked @ 0.2

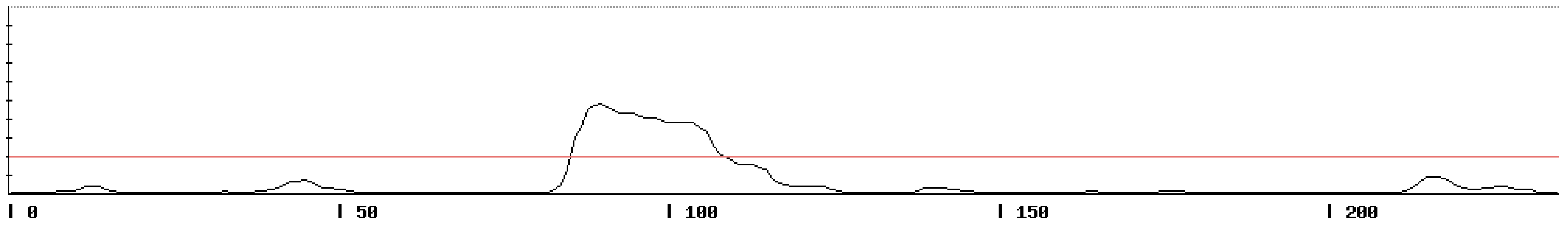

Predictions for Linumusitatissimum_Lus10019926

Viterbi Path

Viterbi Path

Posterior @ 0.2 | 122 - RP - 123
322 - PPRKKPKT - 329

Posterior @ 0.2

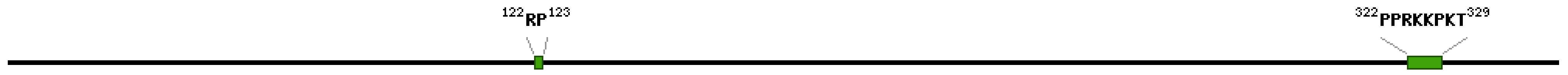

Posterior Graphic. Threshold Marked @ 0.2

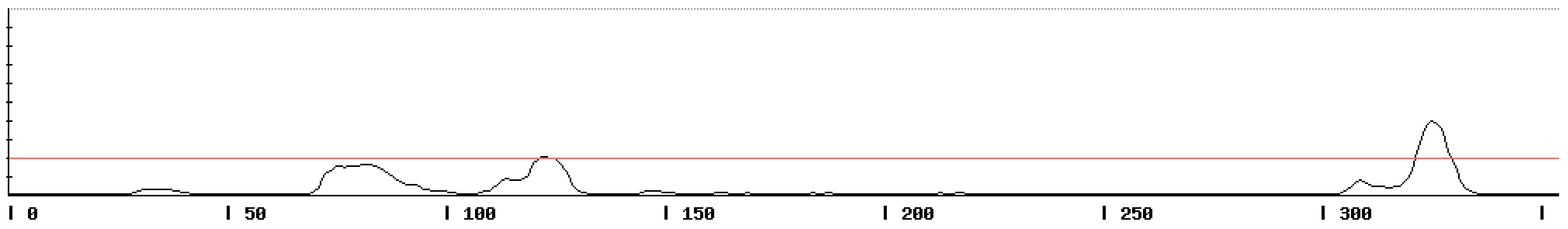

Predictions for Linumusitatissimum_Lus10020165

Viterbi Path

Viterbi Path

Posterior @ 0.2

Posterior @ 0.2

Posterior Graphic. Threshold Marked @ 0.2

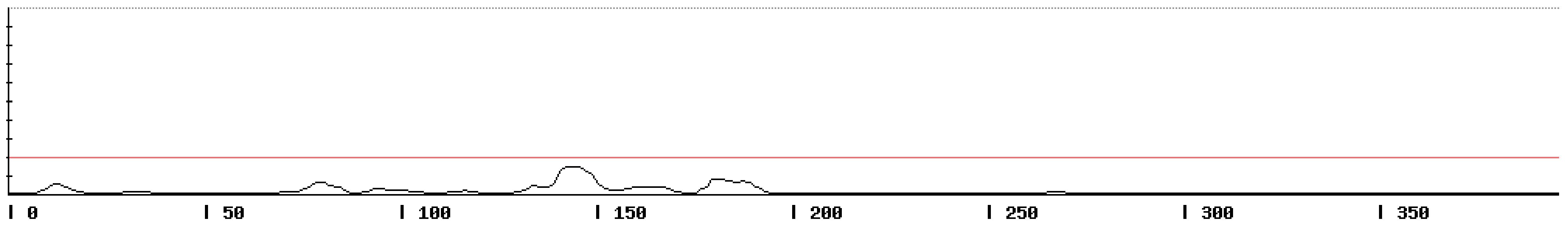

Predictions for Linumusitatissimum_Lus10020643

Viterbi Path

Viterbi Path

Posterior @ 0.2 — 118 - YKGKAPAGRKTKWKM - 132

Posterior @ 0.2

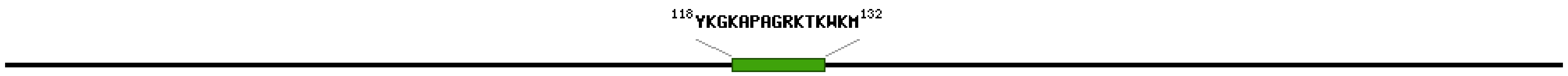

Posterior Graphic. Threshold Marked @ 0.2

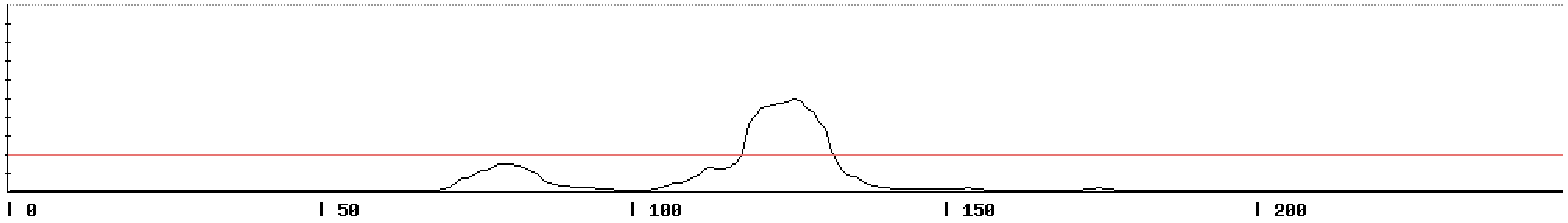

Predictions for Linumusitatissimum_Lus10020794

Viterbi Path

Viterbi Path

Posterior @ 0.2

748 - GYGRRNGERRRKSVR - 762

792 - EKPRMRGPKRASKIRKLFNL - 811

831 - KNGKEVSKAPKIQRLVTPVTLQRKRARLAEKKRRVEKANSEAAEYQKLLASRLKEQRERRSESLAKKRSRLSTASKP - 907

Posterior @ 0.2

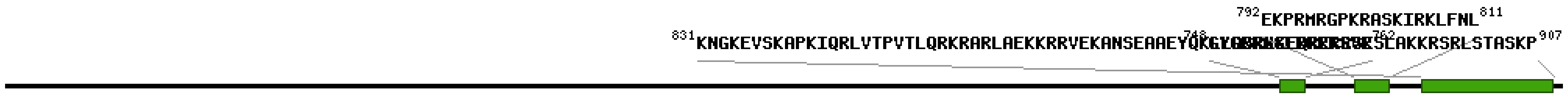

Posterior Graphic. Threshold Marked @ 0.2

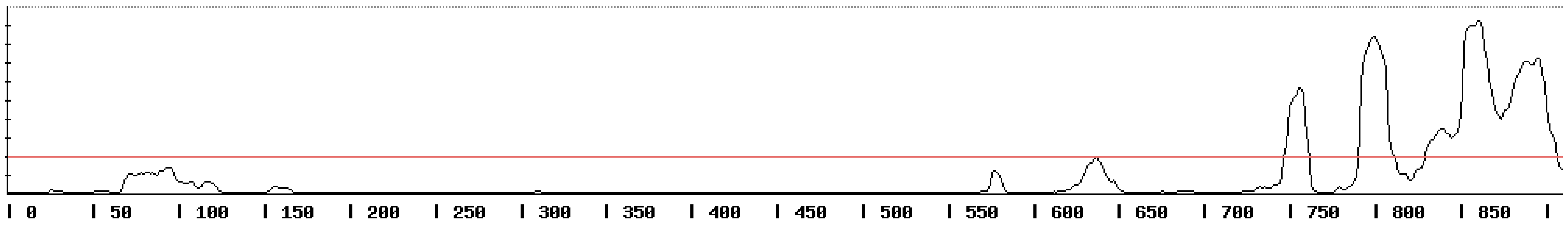

Predictions for Linumusitatissimum_Lus10020883

Viterbi Path

Viterbi Path	
Posterior @ 0.2	78 - RKYPNGSRPNRAAGTGYWKATGADKPIGKPKALGIKKALVFYAGKAPR - 125
Posterior @ 0.2	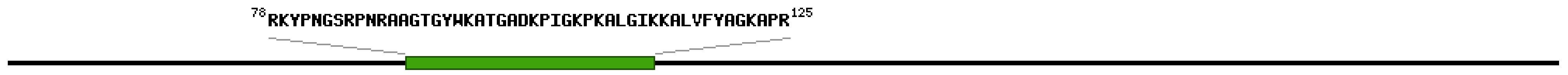
Posterior Graphic. Threshold Marked @ 0.2	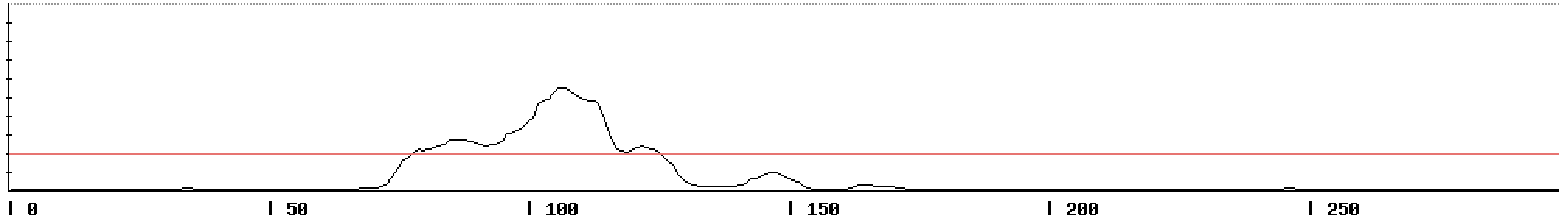

Predictions for Linumusitatissimum_Lus10020896

Viterbi Path	
Viterbi Path	
Posterior @ 0.2	73 - KKYAIGPRMKRAA - 85
Posterior @ 0.2	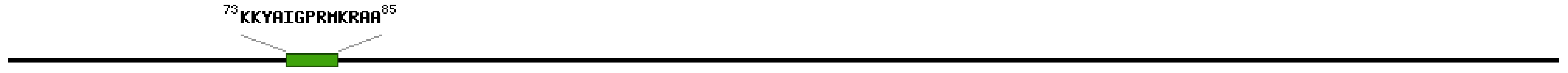
Posterior Graphic. Threshold Marked @ 0.2	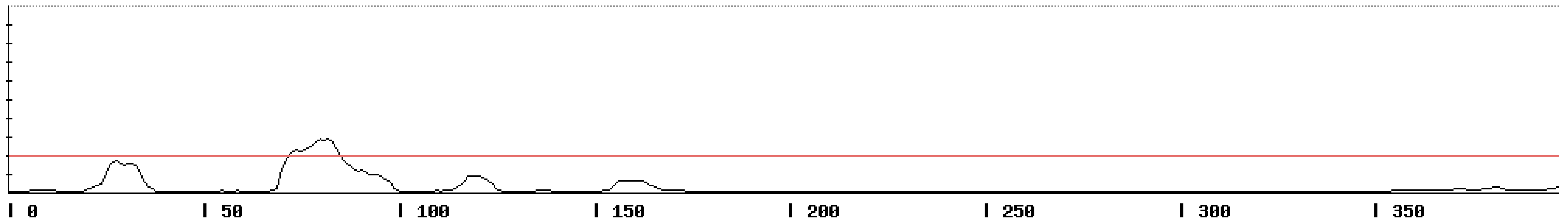

Predictions for Linumusitatissimum_Lus10021659

Viterbi Path	
Viterbi Path	
Posterior @ 0.2	130 - GRAPK - 134
Posterior @ 0.2	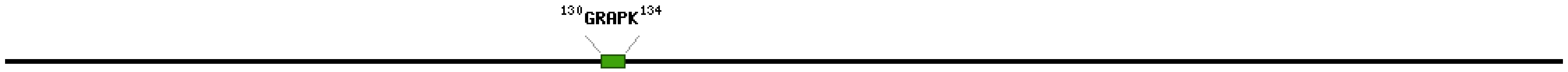
Posterior Graphic. Threshold Marked @ 0.2	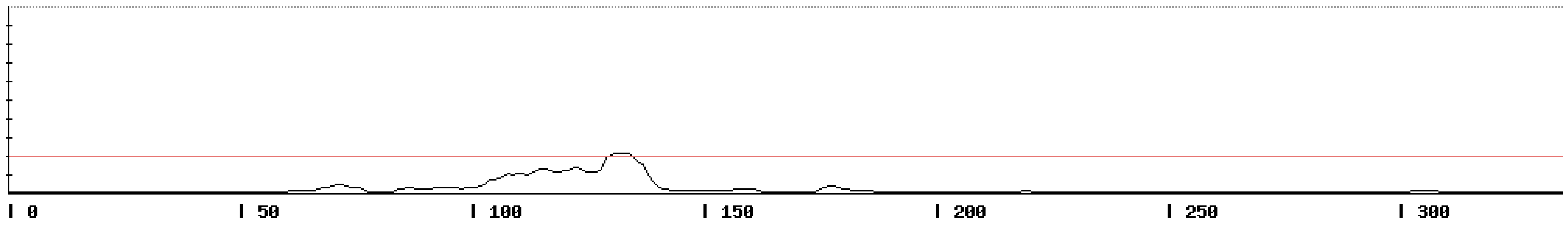

Predictions for Linumusitatissimum_Lus10021708

Viterbi Path	
Viterbi Path	
Posterior @ 0.2	138 - GTRKRRKIQ - 146
Posterior @ 0.2	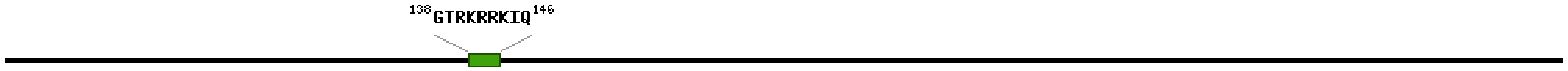
Posterior Graphic. Threshold Marked @ 0.2	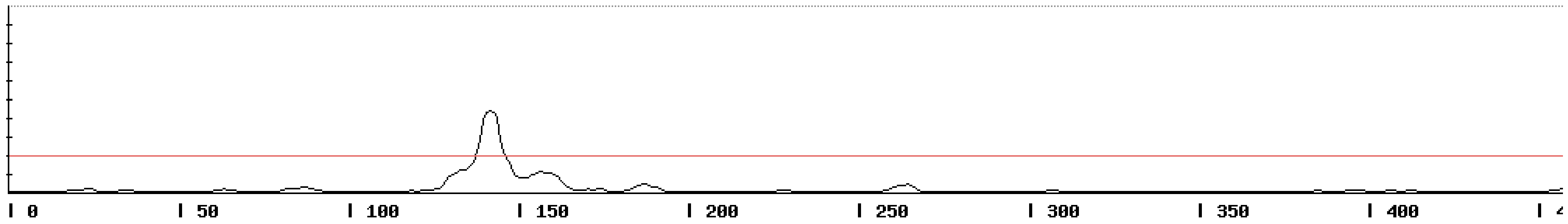

Predictions for Linumusitatissimum_Lus10021992	
Viterbi Path	
Viterbi Path	
Posterior @ 0.2	83 - ARPNR - 87
	131 - RPPK - 134
Posterior @ 0.2	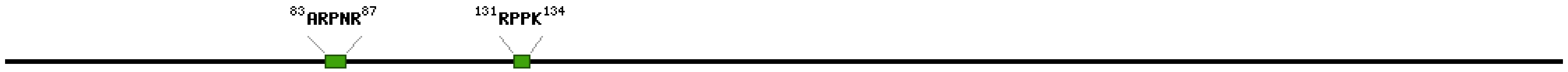
Posterior Graphic. Threshold Marked @ 0.2	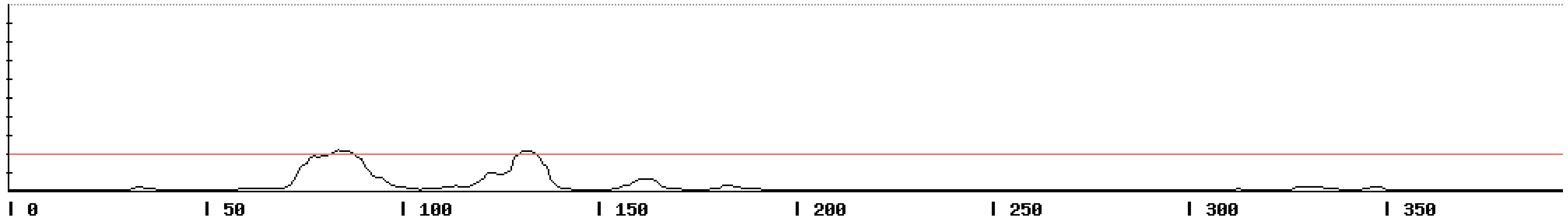

Predictions for Linumusitatissimum_Lus10022018	
Viterbi Path	
Viterbi Path	
Posterior @ 0.2	58 - GNKRIKDARERQIGIKKTFSFKVTSEKAKVKGK - 90
Posterior @ 0.2	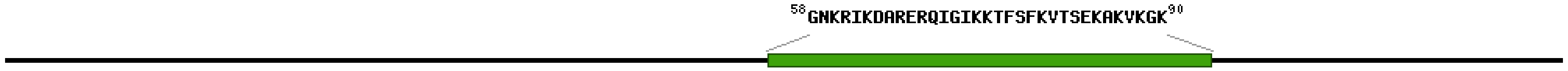
Posterior Graphic. Threshold Marked @ 0.2	

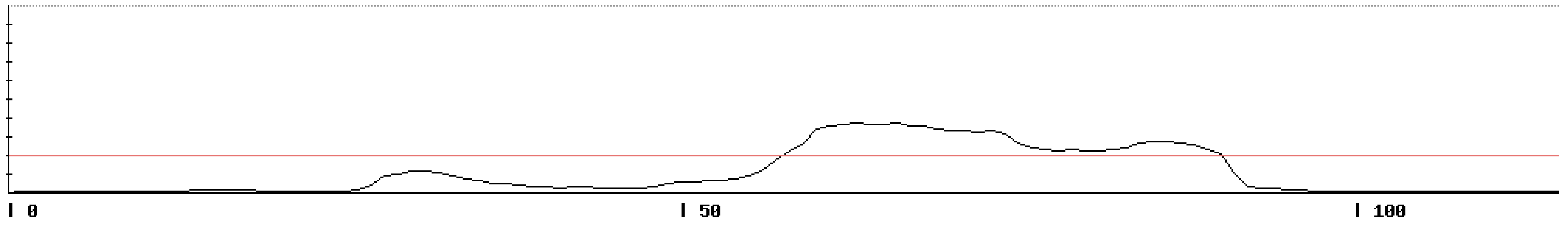

Predictions for Linumusitatissimum_Lus10022636

Viterbi Path

Viterbi Path

Posterior @ 0.2 — 77 - PRDRKHGGNGRPNRTTEKGFWKATGSDRKIWRLSEPKRQIGLRK - 120

Posterior @ 0.2

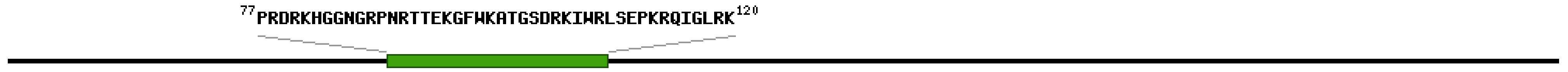

Posterior Graphic. Threshold Marked @ 0.2

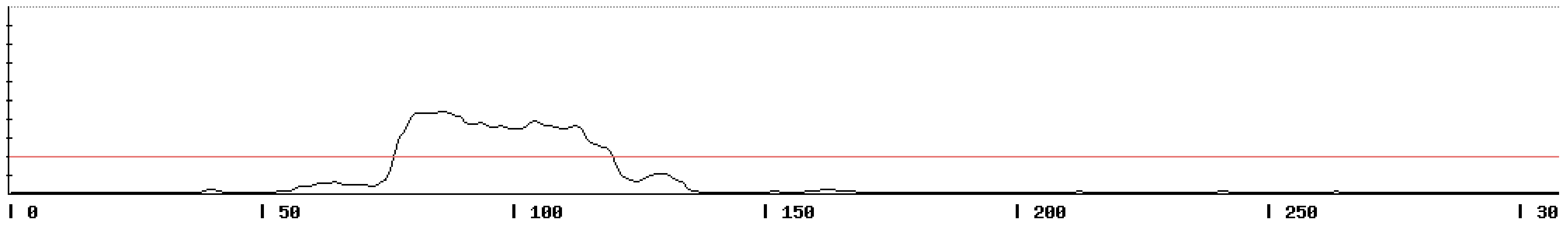

Predictions for Linumusitatissimum_Lus10022914

Viterbi Path

Viterbi Path

Posterior @ 0.2 — 65 - KLPKKKKKGGIRVARTVGT - 83

Posterior @ 0.2

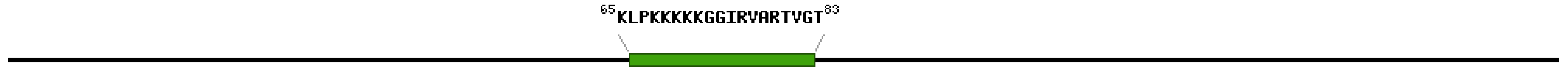

Posterior Graphic. Threshold Marked @ 0.2	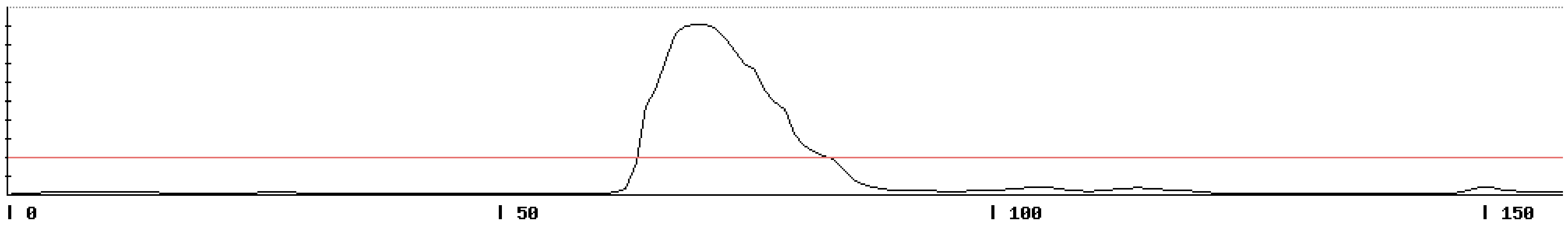

Predictions for Linumusitatissimum_Lus10022915	
Viterbi Path	
Viterbi Path	
Posterior @ 0.2	
Posterior @ 0.2	
Posterior Graphic. Threshold Marked @ 0.2	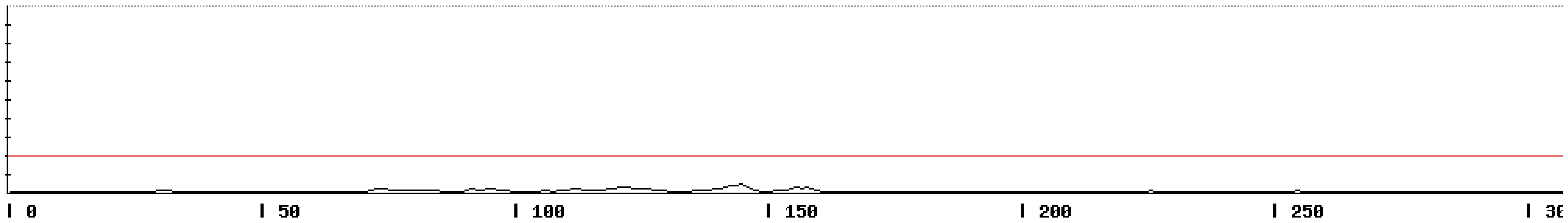

Predictions for Linumusitatissimum_Lus10022965	
Viterbi Path	
Viterbi Path	
Posterior @ 0.2	
Posterior @ 0.2	

Posterior Graphic. Threshold Marked @ 0.2

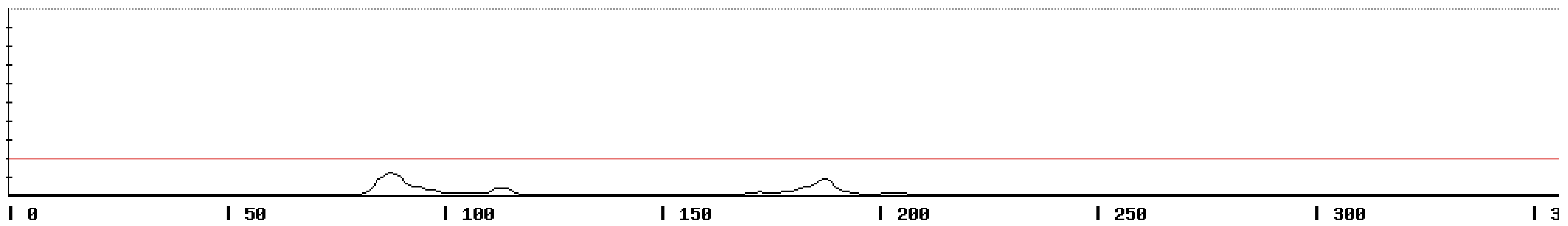

Predictions for Linumusitatissimum_Lus10023179

Viterbi Path

Viterbi Path

Posterior @ 0.2 122 - RPP - 124

Posterior @ 0.2

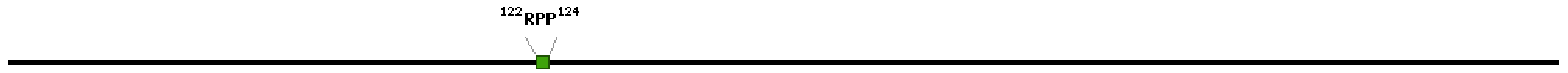

Posterior Graphic. Threshold Marked @ 0.2

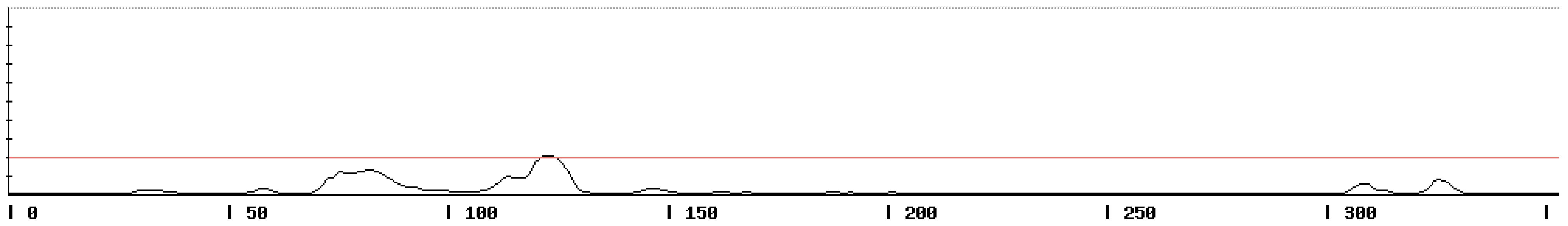

Predictions for Linumusitatissimum_Lus10023208

Viterbi Path

Viterbi Path

Posterior @ 0.2	
Posterior @ 0.2	
Posterior Graphic. Threshold Marked @ 0.2	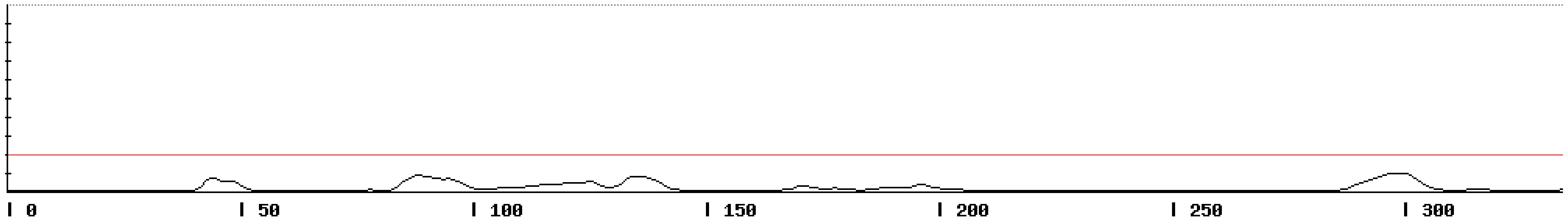

Predictions for Linumusitatissimum_Lus10023537

Viterbi Path	
Viterbi Path	
Posterior @ 0.2	305 - RRPP - 308
Posterior @ 0.2	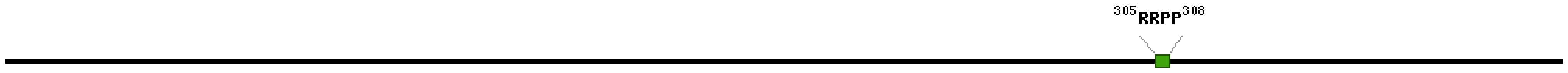
Posterior Graphic. Threshold Marked @ 0.2	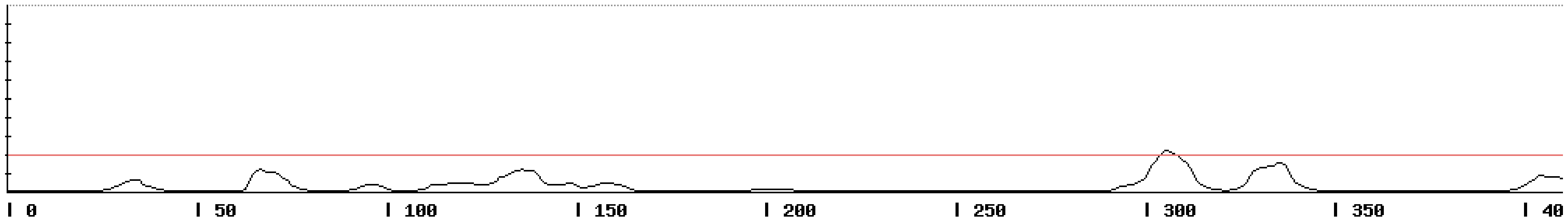

Predictions for Linumusitatissimum_Lus10023966

Viterbi Path	
Viterbi Path	

Posterior @ 0.2 | 84 - RERKNGSRPNRVTEKGFWK - 102

Posterior @ 0.2

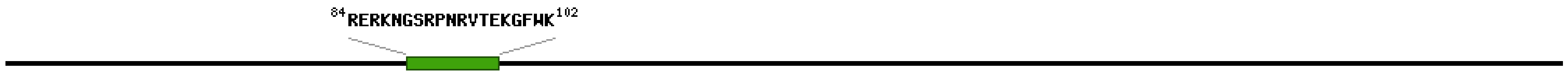

Posterior Graphic. Threshold Marked @ 0.2

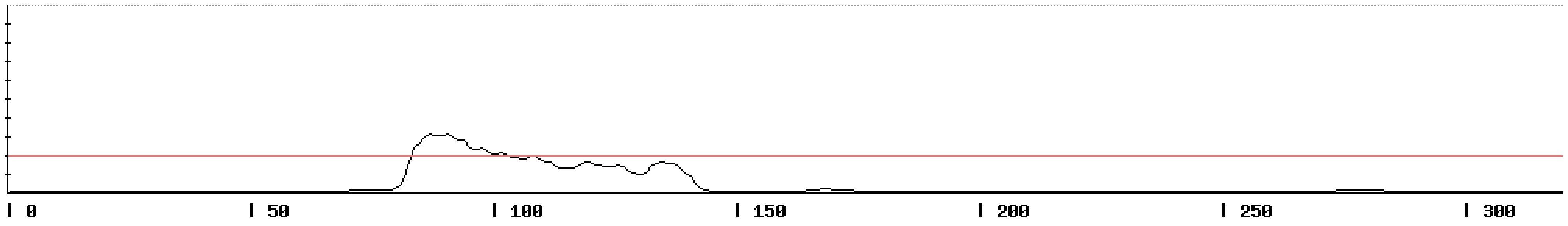

Predictions for Linumusitatissimum_Lus10024006

Viterbi Path

Viterbi Path

Posterior @ 0.2 | 89 - LKKKRDTDKNHGRKIGKGGGNWHGEDRGKKL - 119

Posterior @ 0.2

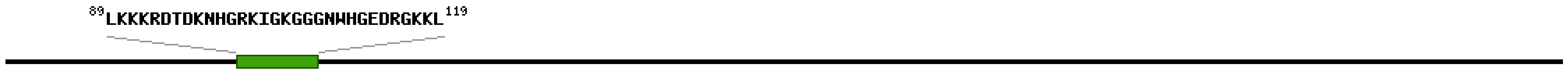

Posterior Graphic. Threshold Marked @ 0.2

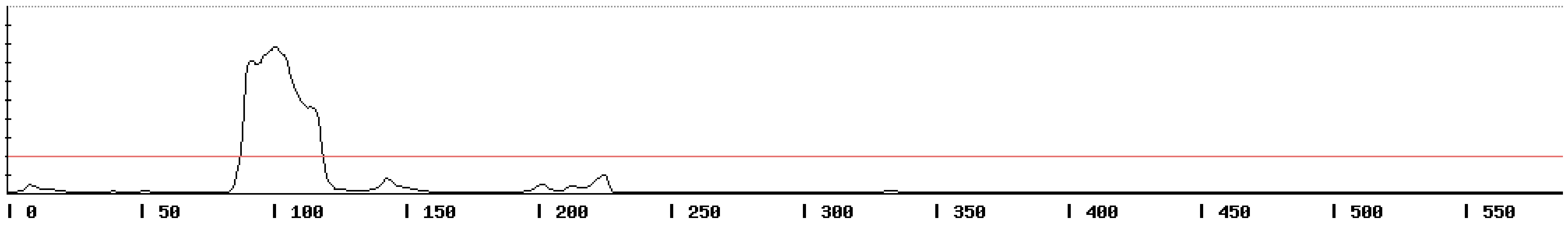

Predictions for Linumusitatissimum_Lus10024601

Viterbi Path

Viterbi Path

Posterior @ 0.2

Posterior @ 0.2

Posterior Graphic. Threshold Marked @ 0.2

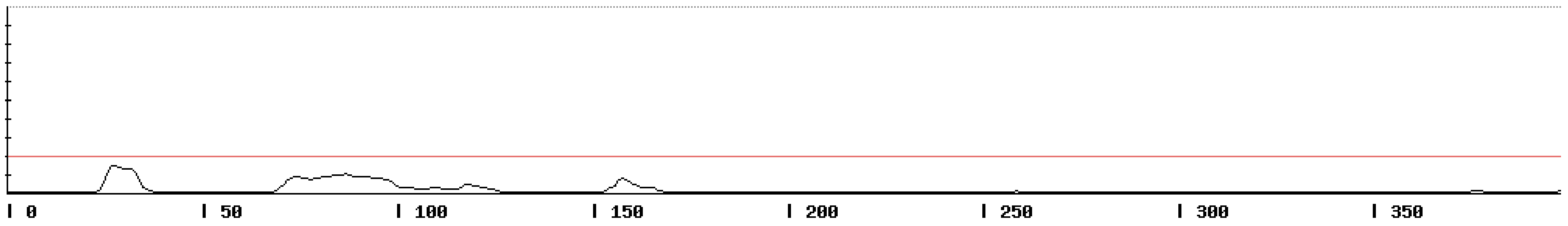

Predictions for Linumusitatissimum_Lus10024907

Viterbi Path

Viterbi Path

Posterior @ 0.2

65 - KLLKKKKKDGKRVVRKVGTNG - 85

149 - RKKLDKKMGKSNRYRL - 164

Posterior @ 0.2

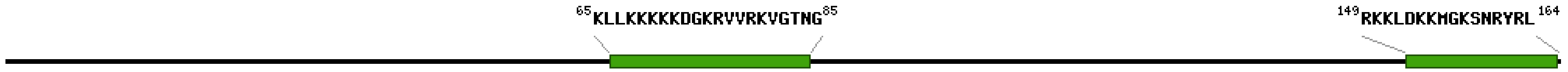

Posterior Graphic. Threshold Marked @ 0.2

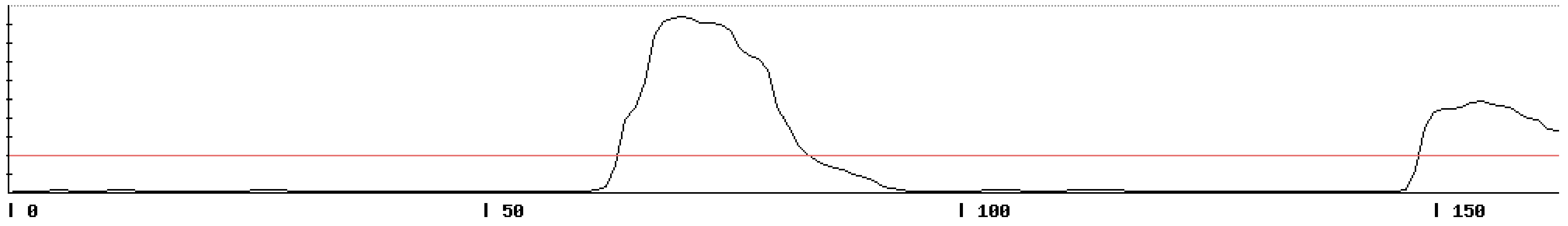

Predictions for Linumusitatissimum_Lus10024908

Viterbi Path

Viterbi Path

Posterior @ 0.2

Posterior @ 0.2

Posterior Graphic. Threshold Marked @ 0.2

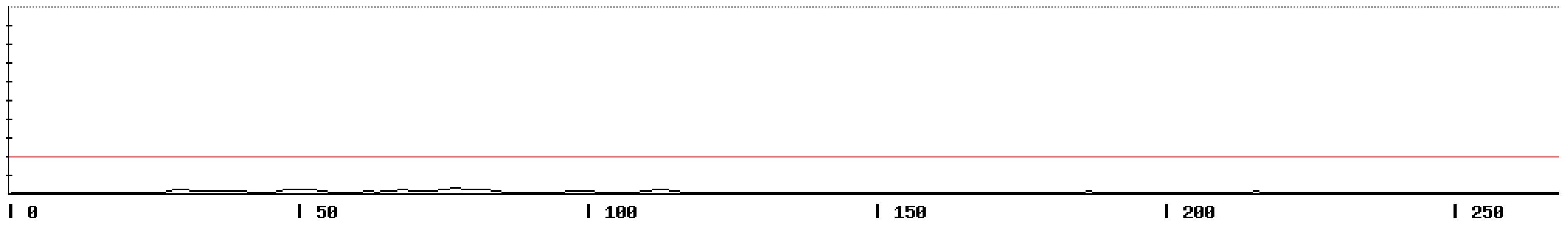

Predictions for Linumusitatissimum_Lus10025078

Viterbi Path

Viterbi Path

Posterior @ 0.2

92 - KTRYTDKNHGRKIGKGGGHWHGEDRGKK - 119

Posterior @ 0.2

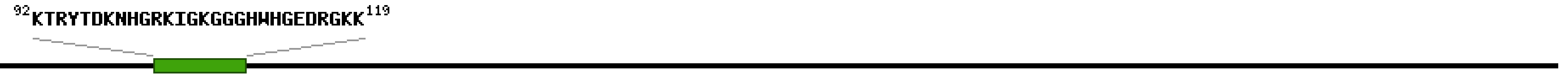

Posterior Graphic. Threshold Marked @ 0.2

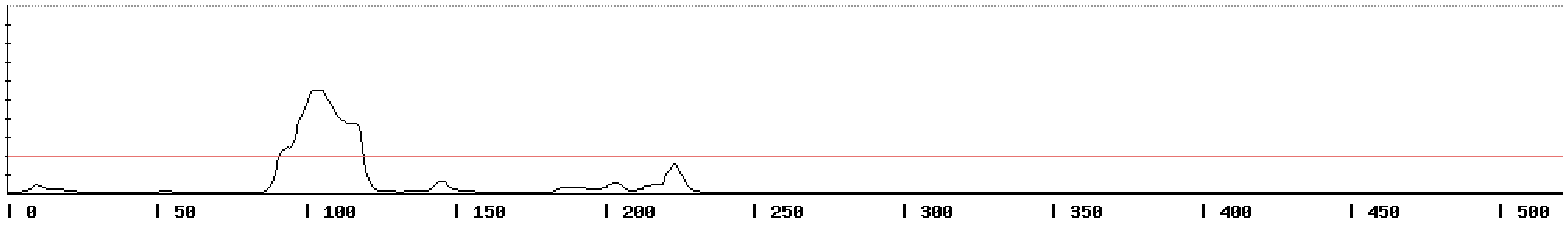

Predictions for Linumusitatissimum_Lus10025118

Viterbi Path

Viterbi Path

Posterior @ 0.2

78 - RERKNGSRPNRVTEKGFWK - 96

Posterior @ 0.2

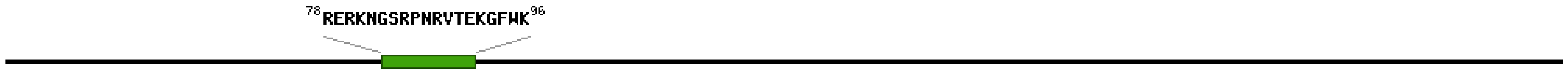

Posterior Graphic. Threshold Marked @ 0.2

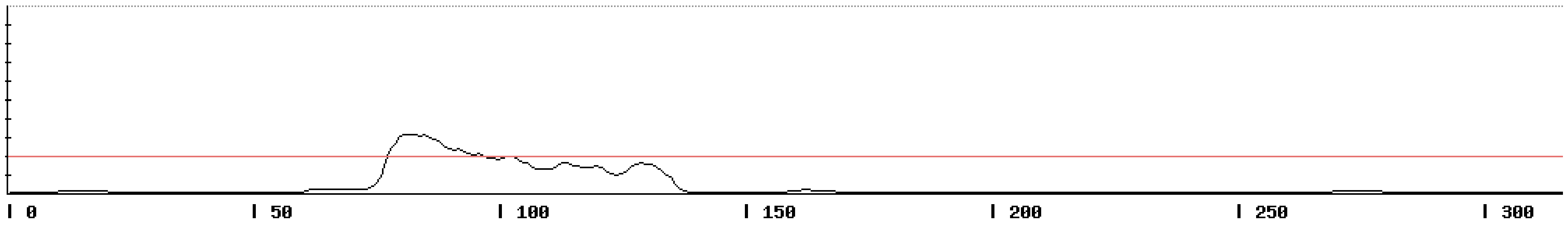

Predictions for Linumusitatissimum_Lus10025690

Viterbi Path

Viterbi Path

Posterior @ 0.2

Posterior @ 0.2

Posterior Graphic. Threshold Marked @ 0.2

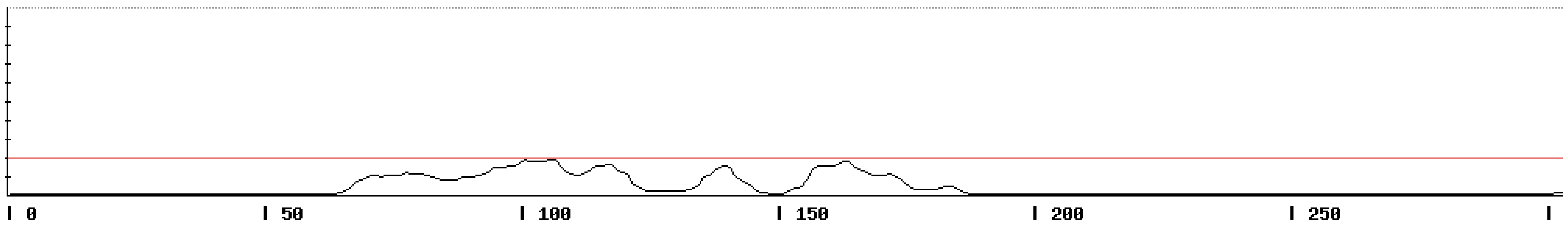

Predictions for Linumusitatissimum_Lus10026200

Viterbi Path

Viterbi Path

Posterior @ 0.2

Posterior @ 0.2

Posterior Graphic. Threshold Marked @ 0.2

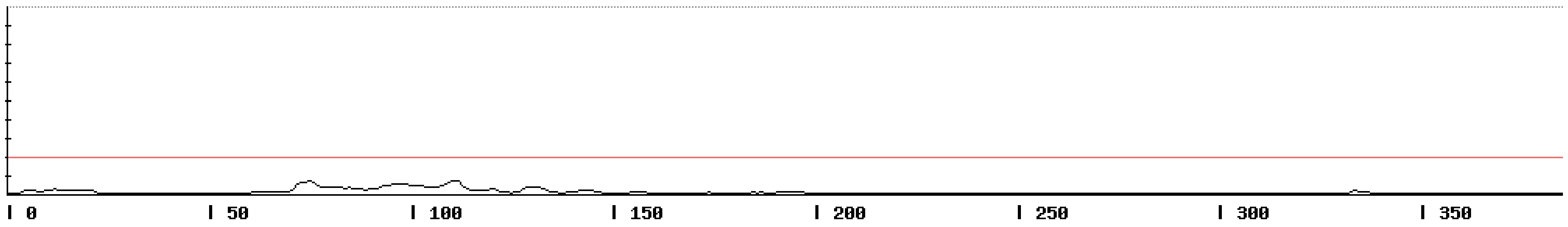

Predictions for Linumusitatissimum_Lus10026373

Viterbi Path

Viterbi Path

Posterior @ 0.2	
Posterior @ 0.2	
Posterior Graphic. Threshold Marked @ 0.2	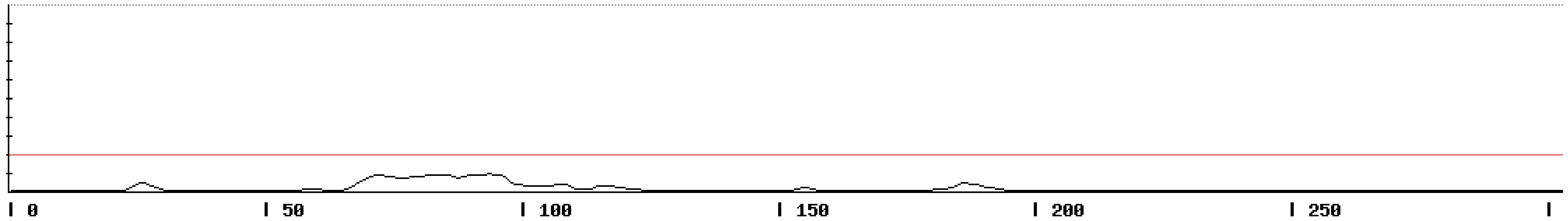

Predictions for Linumusitatissimum_Lus10026496

Viterbi Path	
Viterbi Path	
Posterior @ 0.2	122 - RP - 123
	323 - RKKPK - 327
Posterior @ 0.2	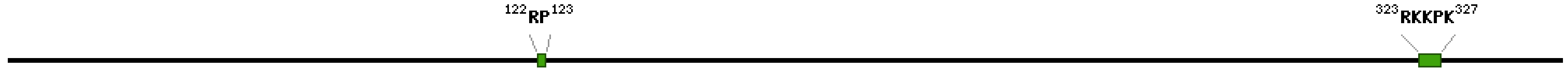
Posterior Graphic. Threshold Marked @ 0.2	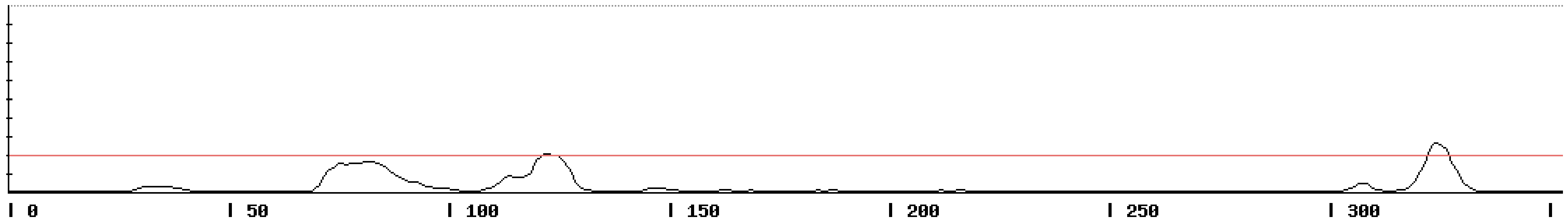

Predictions for Linumusitatissimum_Lus10026588

Viterbi Path	
Viterbi Path	

Posterior @ 0.2

Posterior @ 0.2

Posterior Graphic. Threshold Marked @ 0.2

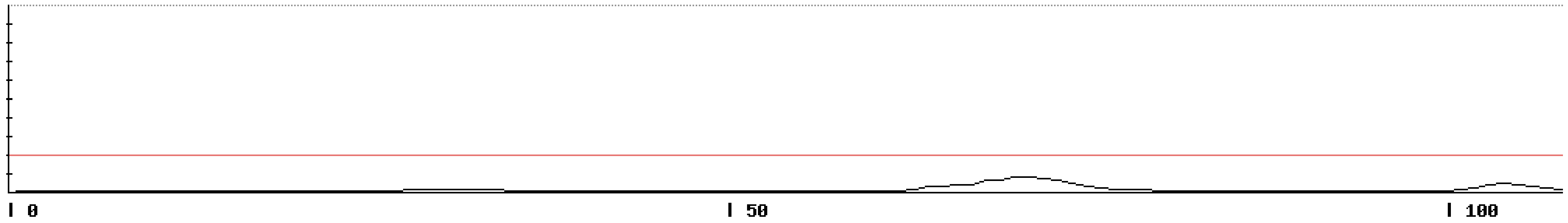

Predictions for Linumusitatissimum_Lus10026617

Viterbi Path

Viterbi Path

Posterior @ 0.2 134 - GKPPK - 138

Posterior @ 0.2

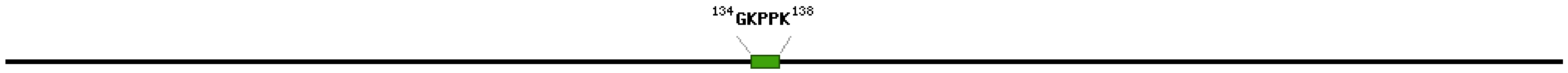

Posterior Graphic. Threshold Marked @ 0.2

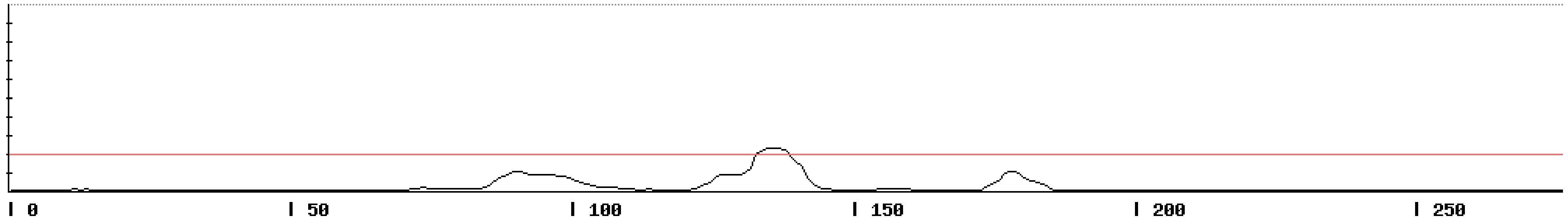

Predictions for Linumusitatissimum_Lus10026879

Viterbi Path

Viterbi Path

Posterior @ 0.2

Posterior @ 0.2

Posterior Graphic. Threshold Marked @ 0.2

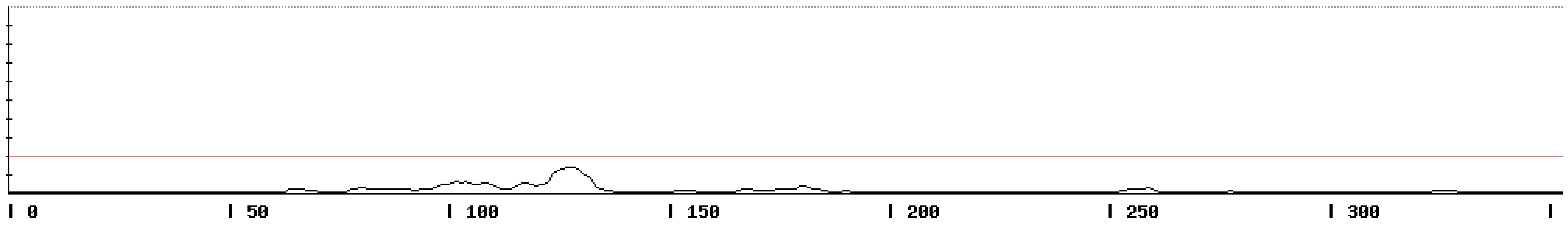

Predictions for Linumusitatissimum_Lus10026966

Viterbi Path

Viterbi Path

Posterior @ 0.2

Posterior @ 0.2

Posterior Graphic. Threshold Marked @ 0.2

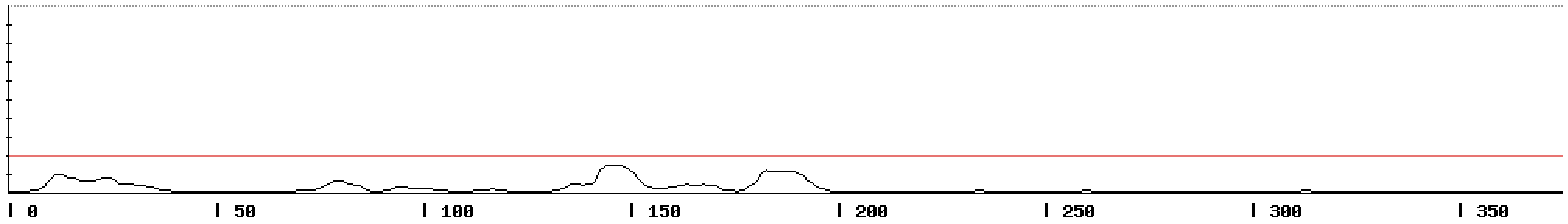

Predictions for Linumusitatissimum_Lus10027227	
Viterbi Path	
Viterbi Path	
Posterior @ 0.2	
Posterior @ 0.2	
Posterior Graphic. Threshold Marked @ 0.2	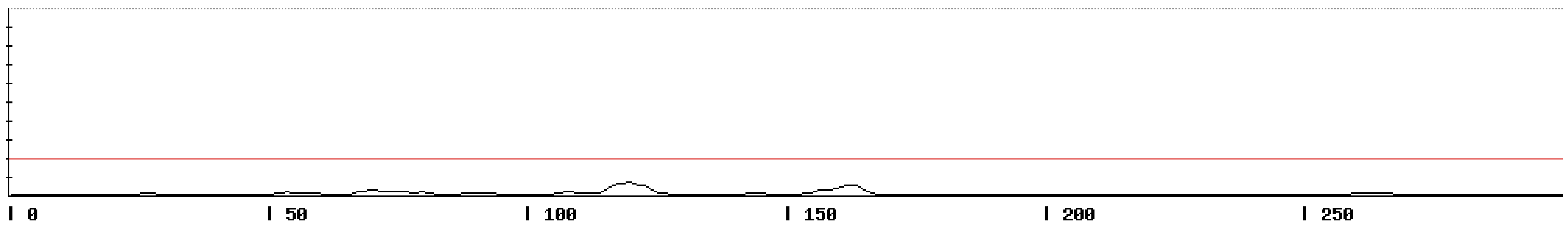

Predictions for Linumusitatissimum_Lus10027357	
Viterbi Path	
Viterbi Path	
Posterior @ 0.2	222 - PRK - 224
Posterior @ 0.2	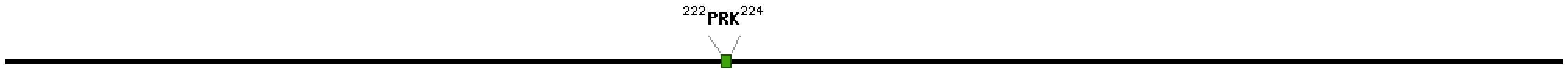
Posterior Graphic. Threshold Marked @ 0.2	

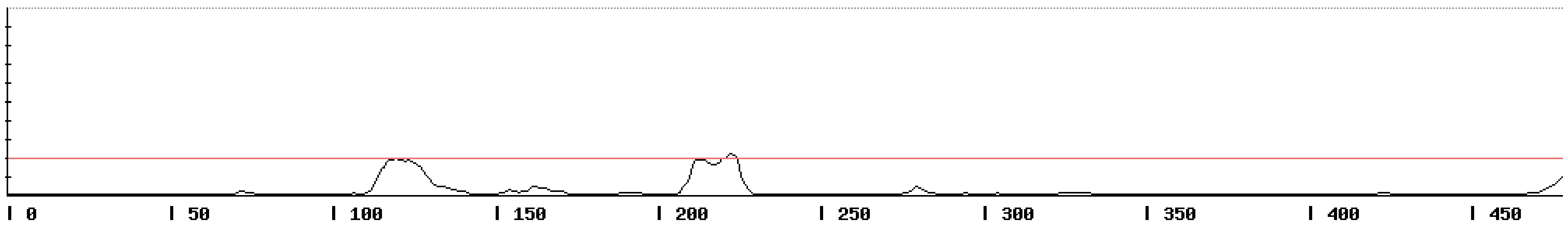

Predictions for Linumusitatissimum_Lus10028372

Viterbi Path

Viterbi Path

Posterior @ 0.2 158 - KR - 159

Posterior @ 0.2

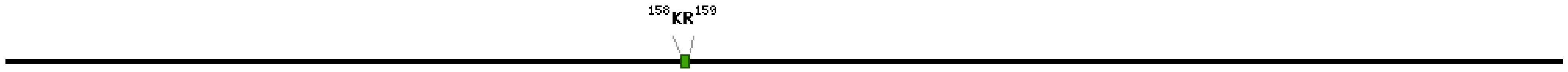

Posterior Graphic. Threshold Marked @ 0.2

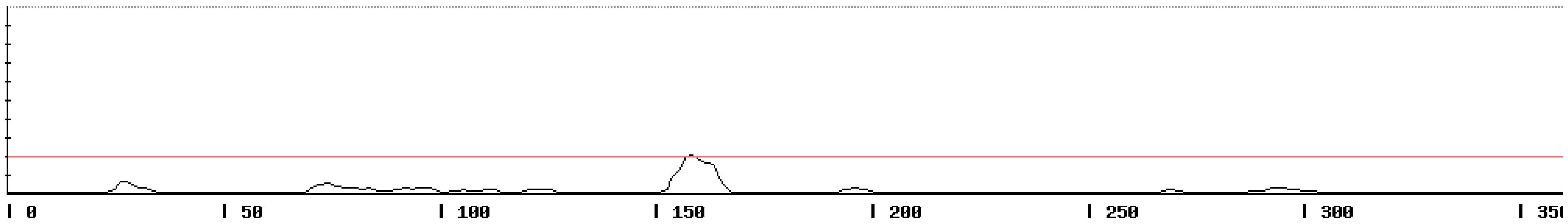

Predictions for Linumusitatissimum_Lus10028713

Viterbi Path

Viterbi Path

Posterior @ 0.2 536 - NARRAGGKKG - 545

Posterior @ 0.2

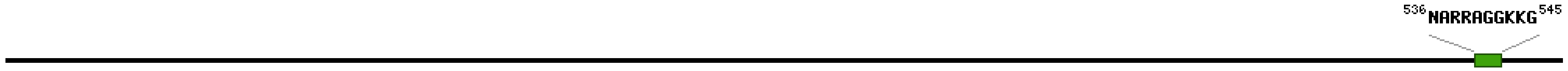

Posterior Graphic. Threshold Marked @ 0.2

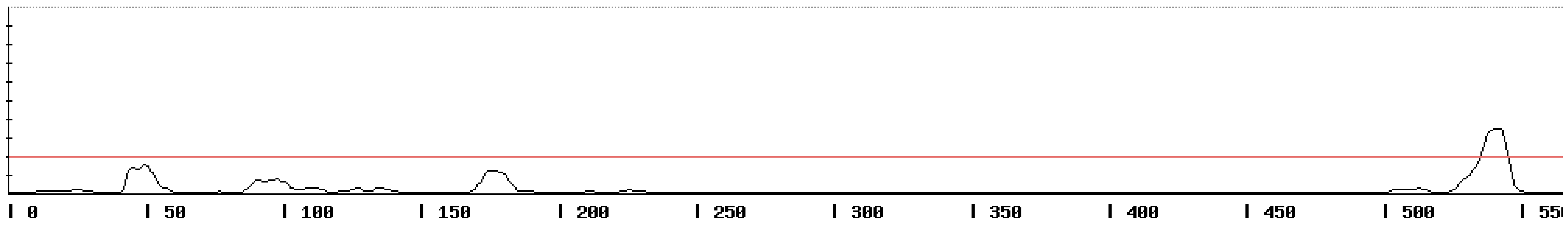

Predictions for Linumusitatissimum_Lus10028824

Viterbi Path

Viterbi Path

Posterior @ 0.2

Posterior @ 0.2

Posterior Graphic. Threshold Marked @ 0.2

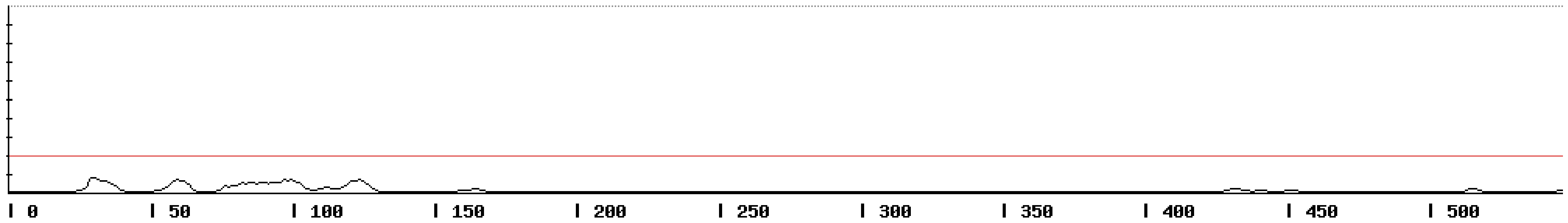

Predictions for Linumusitatissimum_Lus10029410

Viterbi Path

Viterbi Path

Posterior @ 0.2

81 - FRRMKEREGRGGRPKRVA - 98

139 - YKGKAPRGRKTHWKI - 153

Posterior @ 0.2

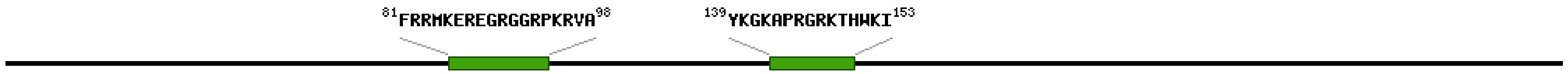

Posterior Graphic. Threshold Marked @ 0.2

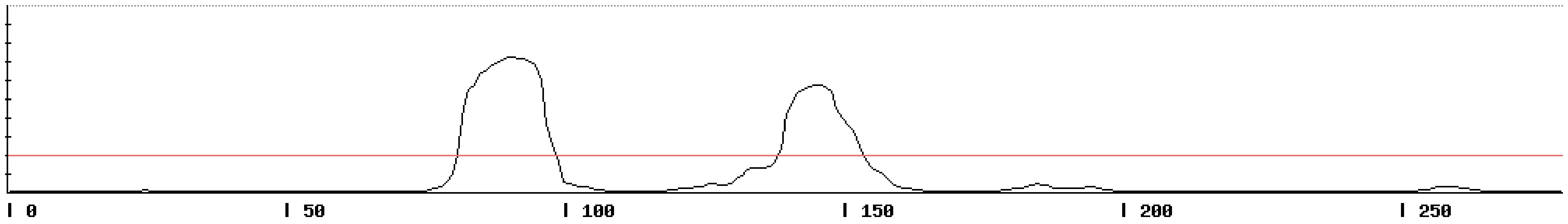

Predictions for Linumusitatissimum_Lus10029692

Viterbi Path

Viterbi Path

Posterior @ 0.2

Posterior @ 0.2

Posterior Graphic. Threshold Marked @ 0.2

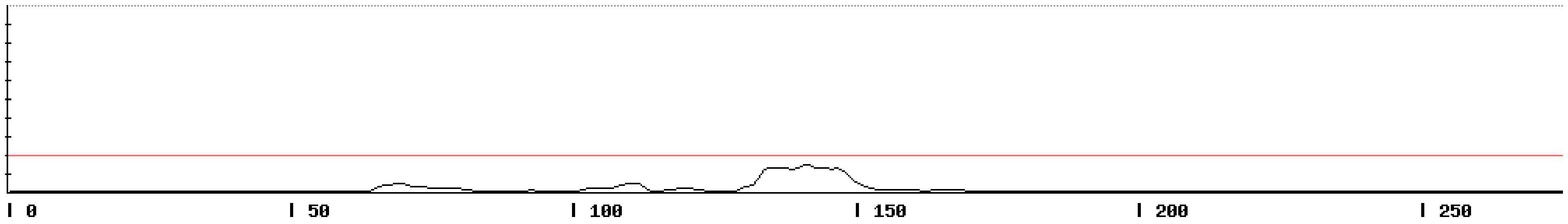

Predictions for Linumusitatissimum_Lus10030143

Viterbi Path

Viterbi Path

Posterior @ 0.2

Posterior @ 0.2

Posterior Graphic. Threshold Marked @ 0.2

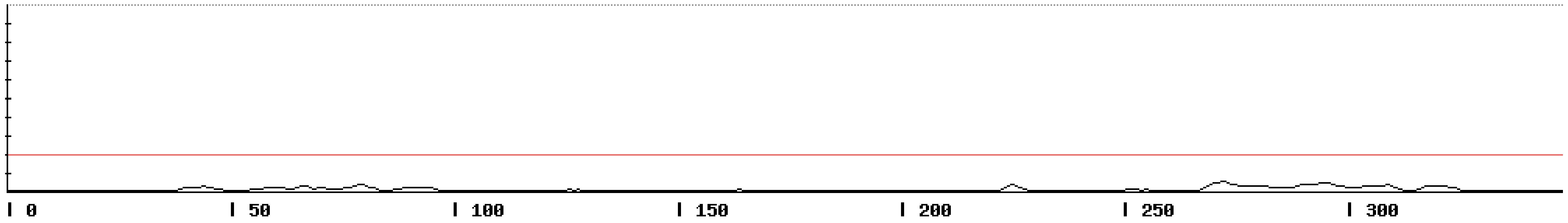

Predictions for Linumusitatissimum_Lus10030174

Viterbi Path

Viterbi Path

Posterior @ 0.2 — 80 - KRSNRTTKKGYWKPTGKER - 98

Posterior @ 0.2

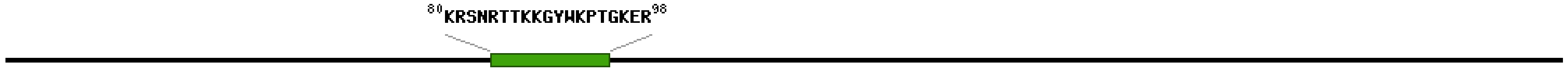

Posterior Graphic. Threshold Marked @ 0.2

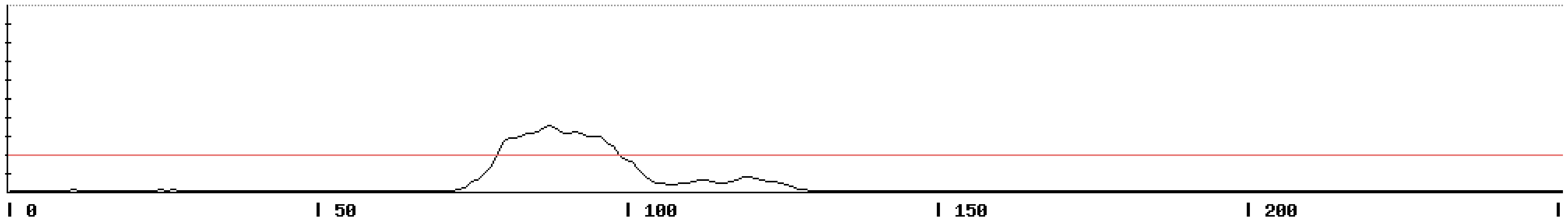

Predictions for Linumusitatissimum_Lus10030175

Viterbi Path

Viterbi Path

Posterior @ 0.2

Posterior @ 0.2

Posterior Graphic. Threshold Marked @ 0.2

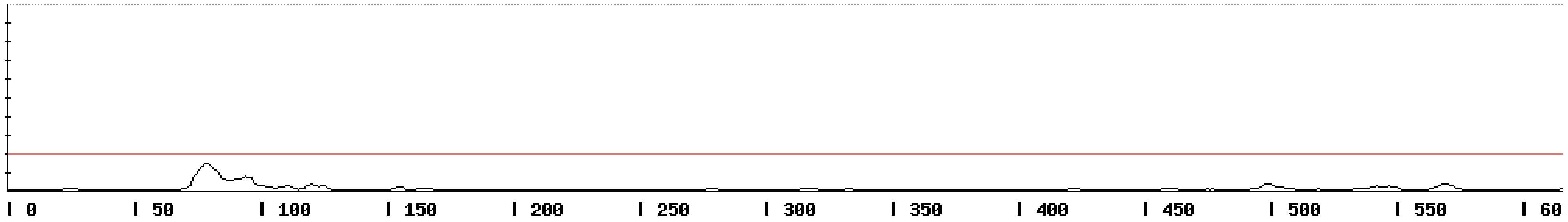

Predictions for Linumusitatissimum_Lus10030205

Viterbi Path

Viterbi Path

Posterior @ 0.2 108 - GRRIGMR - 114

Posterior @ 0.2

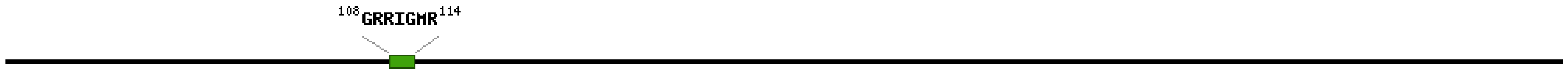

Posterior Graphic. Threshold Marked @ 0.2

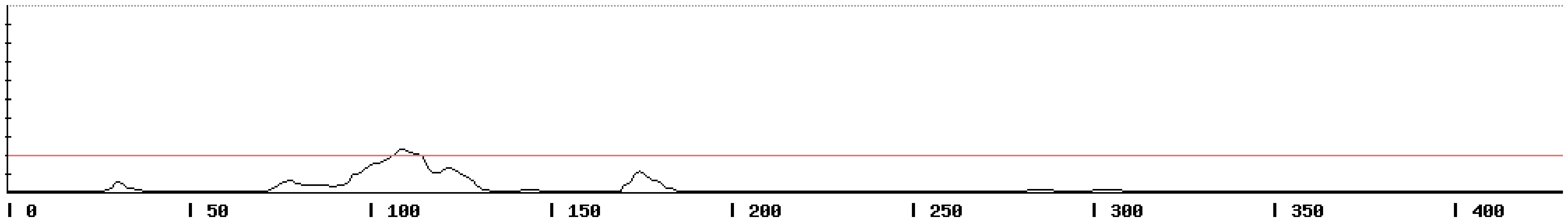

Predictions for Linumusitatissimum_Lus10030446

Viterbi Path	
Viterbi Path	
Posterior @ 0.2	132 - GKPPK - 136
Posterior @ 0.2	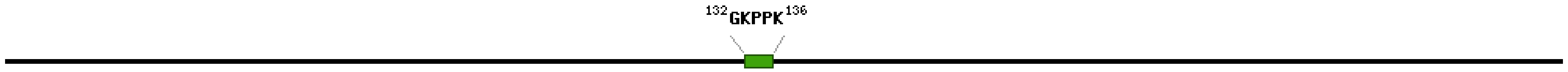
Posterior Graphic. Threshold Marked @ 0.2	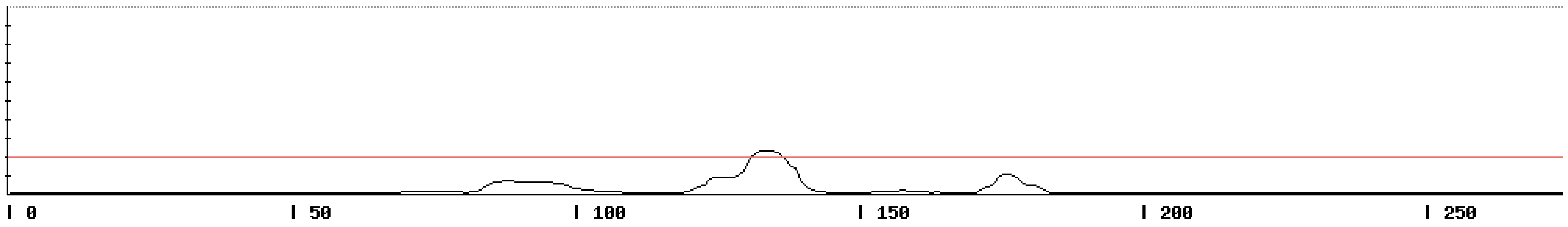

Predictions for Linumusitatissimum_Lus10030478

Viterbi Path	
Viterbi Path	
Posterior @ 0.2	
Posterior @ 0.2	
Posterior Graphic. Threshold Marked @ 0.2	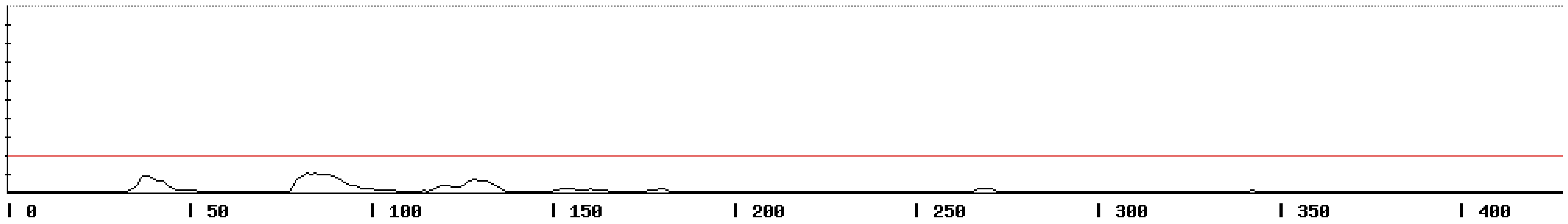

Predictions for Linumusitatissimum_Lus10030723	
Viterbi Path	
Viterbi Path	
Posterior @ 0.2	155 - RAPRG - 159
Posterior @ 0.2	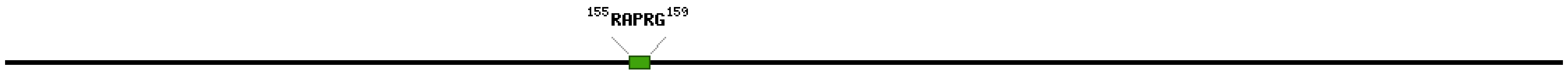
Posterior Graphic. Threshold Marked @ 0.2	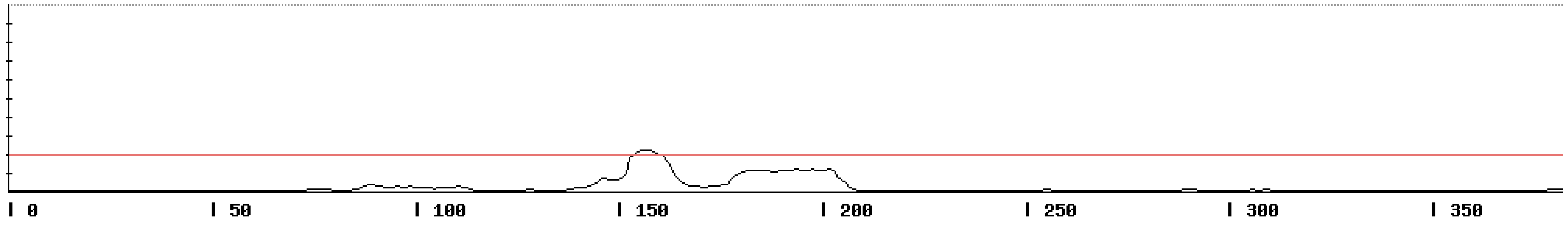

Predictions for Linumusitatissimum_Lus10030978	
Viterbi Path	
Viterbi Path	
Posterior @ 0.2	183 - RSRRGSRK - 190
Posterior @ 0.2	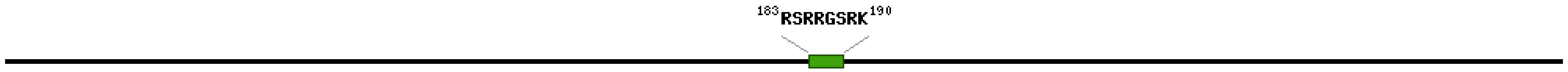
Posterior Graphic. Threshold Marked @ 0.2	

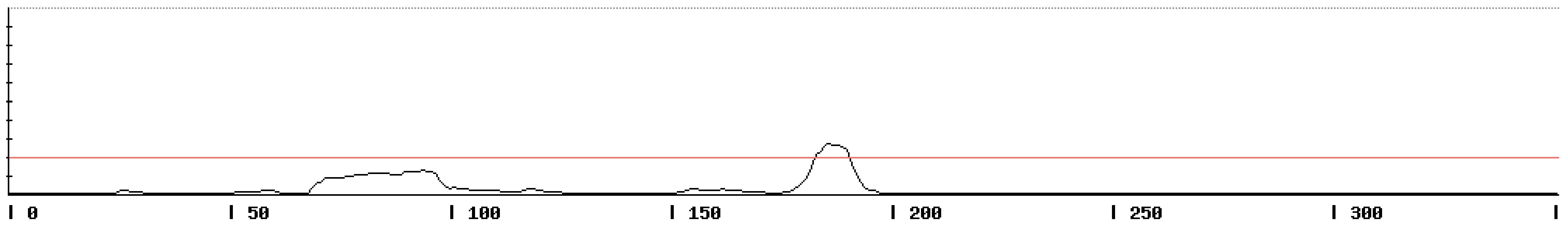

Predictions for Linumusitatissimum_Lus10031142

Viterbi Path

Viterbi Path

Posterior @ 0.2

Posterior @ 0.2

Posterior Graphic. Threshold Marked @ 0.2

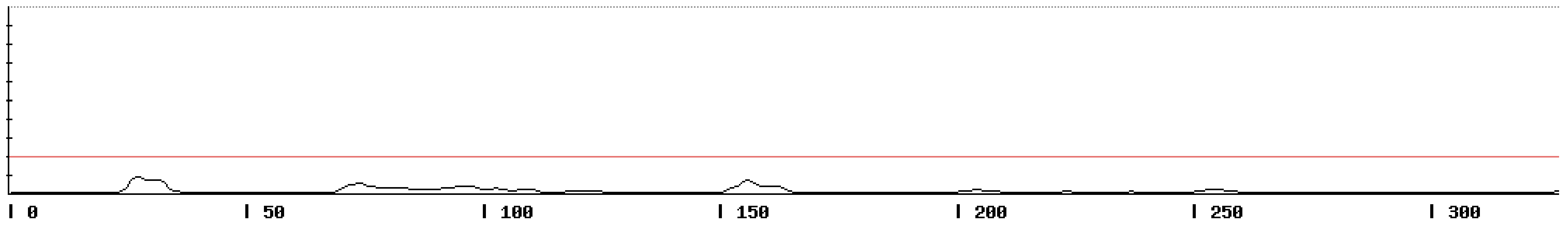

Predictions for Linumusitatissimum_Lus10031189

Viterbi Path

Viterbi Path

Posterior @ 0.2

94 - RGRKY - 98

Posterior @ 0.2

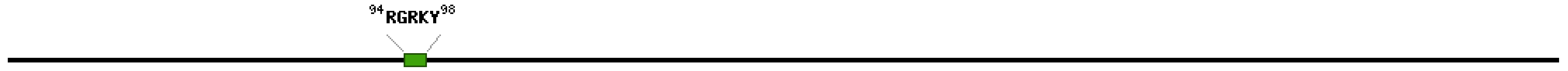

Posterior Graphic. Threshold Marked @ 0.2

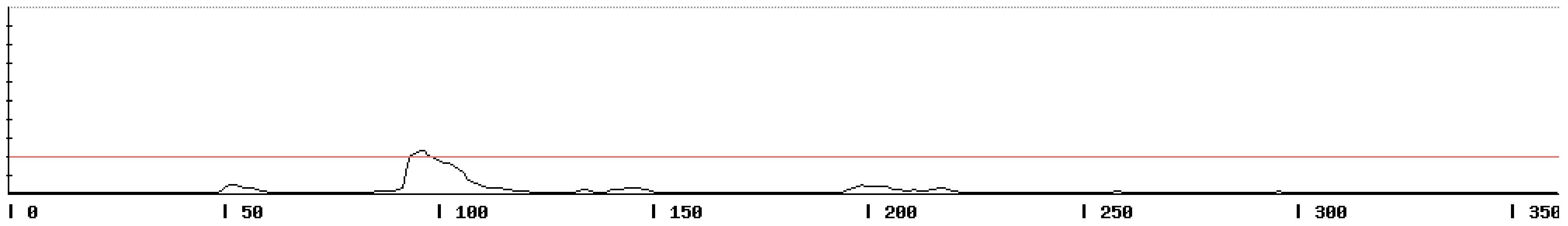

Predictions for Linumusitatissimum_Lus10031595

Viterbi Path	
Viterbi Path	
Posterior @ 0.2	43 - TKLKKKSKNAKNFERRI - 59
	84 - SMWAGKKRKFKYENKKSEAEH - 104
	143 - GKMMPRKRK - 151

Posterior @ 0.2

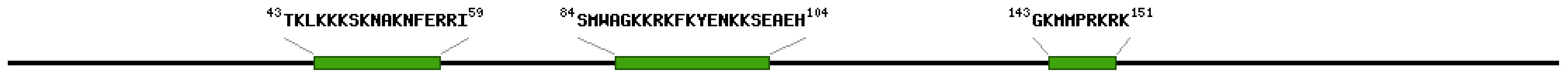

Posterior Graphic. Threshold Marked @ 0.2

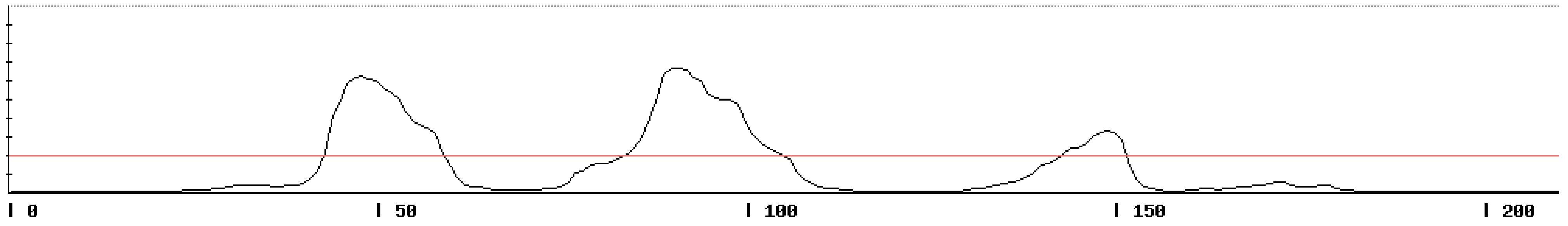

Predictions for Linumusitatissimum_Lus10031639

Viterbi Path	
Viterbi Path	
Posterior @ 0.2	84 - KLKKKSKNAKYFER - 97

127 - AGKKRKFK - 134

Posterior @ 0.2

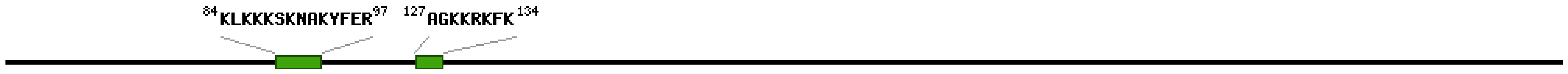

Posterior Graphic. Threshold Marked @ 0.2

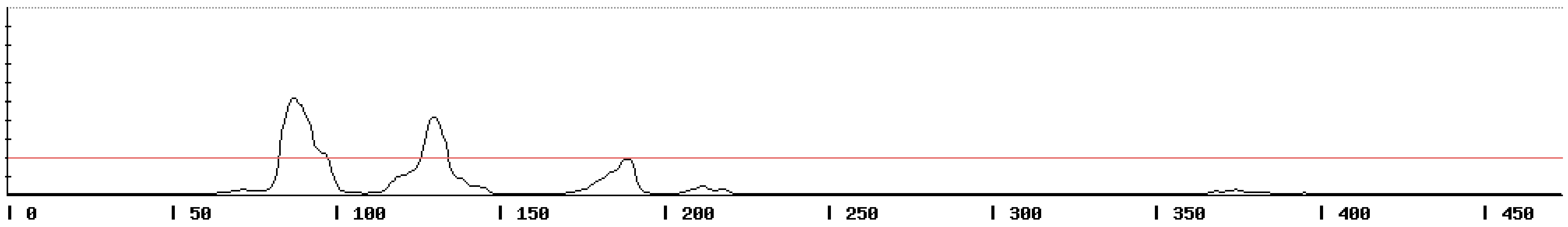

Predictions for Linumusitatissimum_Lus10031721

Viterbi Path

Viterbi Path

Posterior @ 0.2

Posterior @ 0.2

Posterior Graphic. Threshold Marked @ 0.2

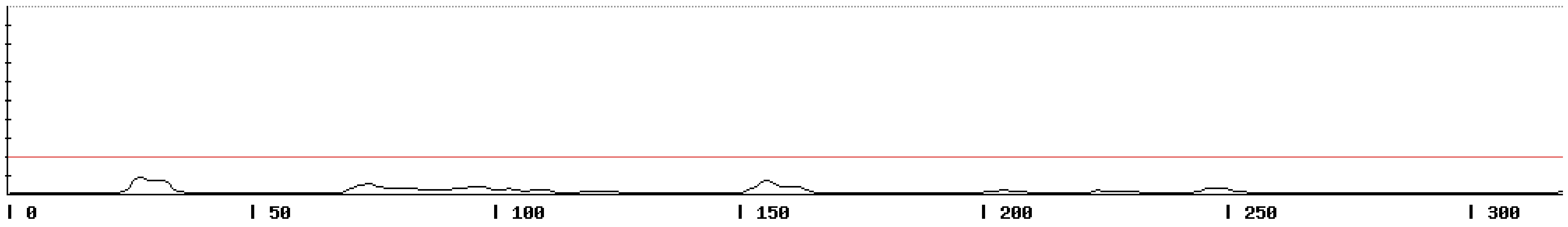

Predictions for Linumusitatissimum_Lus10031767

Viterbi Path

Viterbi Path

Posterior @ 0.2	94 - RGRKY - 98
Posterior @ 0.2	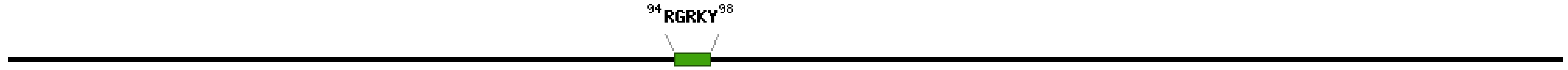
Posterior Graphic. Threshold Marked @ 0.2	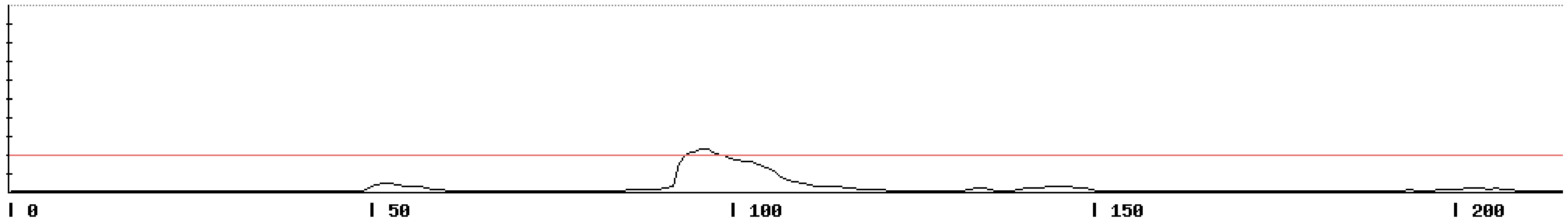

Predictions for Linumusitatissimum_Lus10031937

Viterbi Path	119 - KRYKYLSKKDSEKKKKRKRG - 138
	189 - RLRKAKEDSKKKKNGKKRRK - 208
Viterbi Path	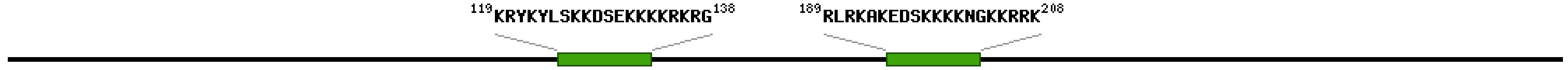
Posterior @ 0.2	77 - LKKKKQNFKRF - 87
	118 - LKRYKYLSKKDSEKKKKRKRGQ - 139
	189 - RLRKAKEDSKKKKNGKKRRKVIR - 211
Posterior @ 0.2	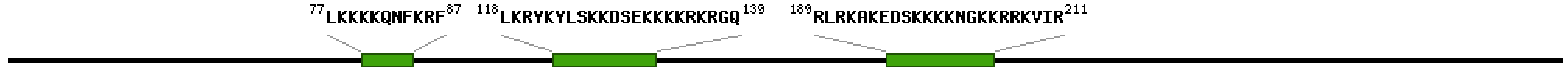
Posterior Graphic. Threshold Marked @ 0.2	

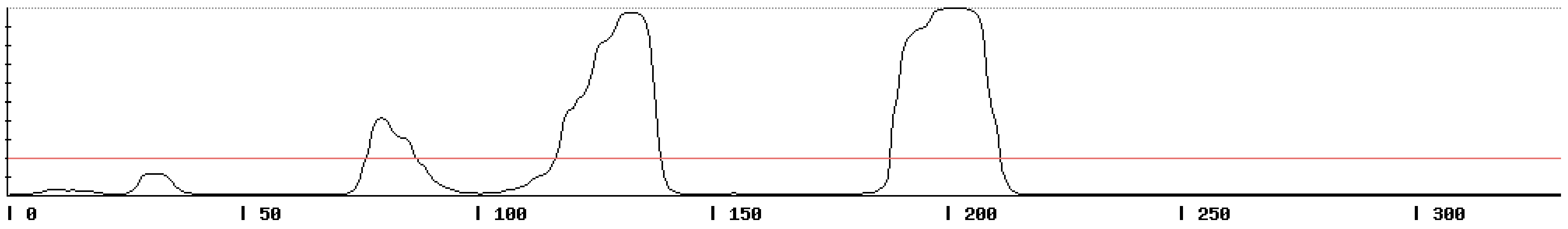

Predictions for Linumusitatissimum_Lus10031951

Viterbi Path

Viterbi Path

Posterior @ 0.2

Posterior @ 0.2

Posterior Graphic. Threshold Marked @ 0.2

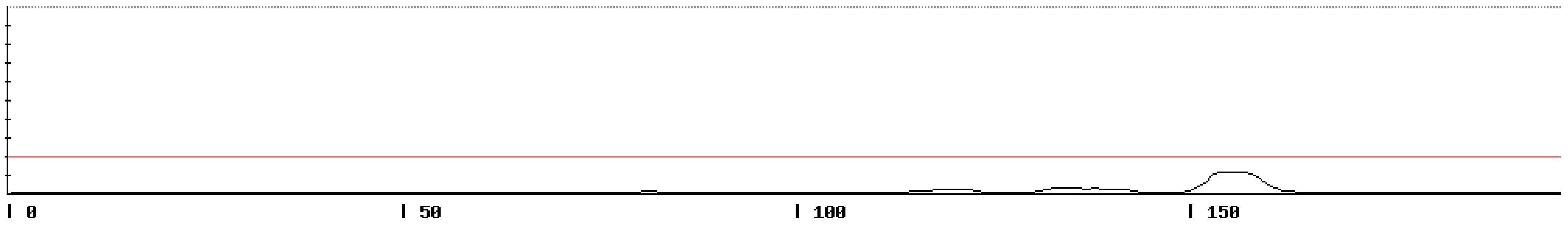

Predictions for Linumusitatissimum_Lus10032004

Viterbi Path

Viterbi Path

Posterior @ 0.2 166 - KKRRASTNANKKKL - 179

Posterior @ 0.2

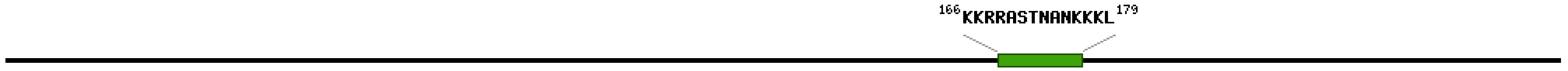

Posterior Graphic. Threshold Marked @ 0.2	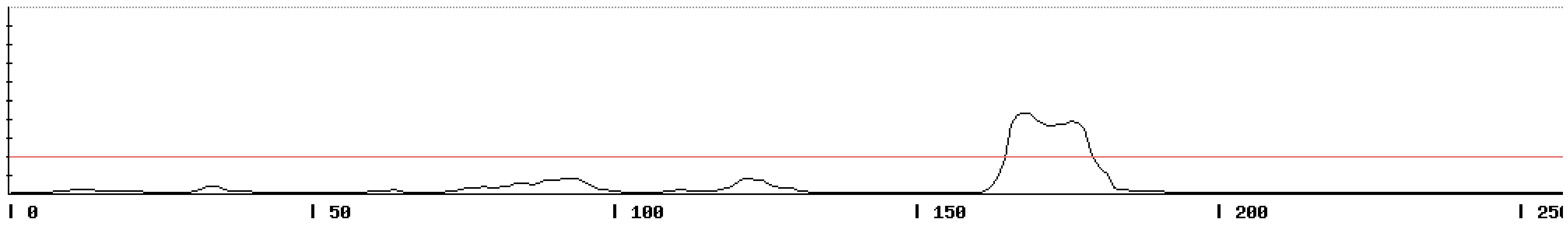

Predictions for Linumusitatissimum_Lus10032238	
Viterbi Path	
Viterbi Path	
Posterior @ 0.2	
Posterior @ 0.2	
Posterior Graphic. Threshold Marked @ 0.2	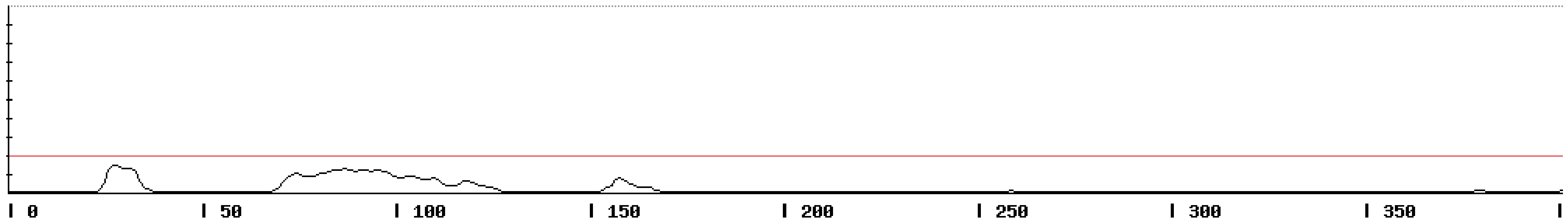

Predictions for Linumusitatissimum_Lus10032653	
Viterbi Path	
Viterbi Path	
Posterior @ 0.2	
Posterior @ 0.2	

Posterior Graphic. Threshold Marked @ 0.2

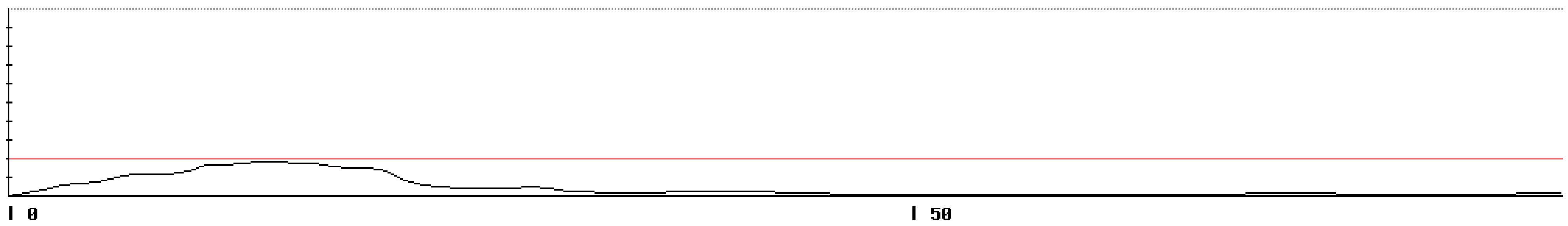

Predictions for Linumusitatissimum_Lus10032657

Viterbi Path

Viterbi Path

Posterior @ 0.2

Posterior @ 0.2

Posterior Graphic. Threshold Marked @ 0.2

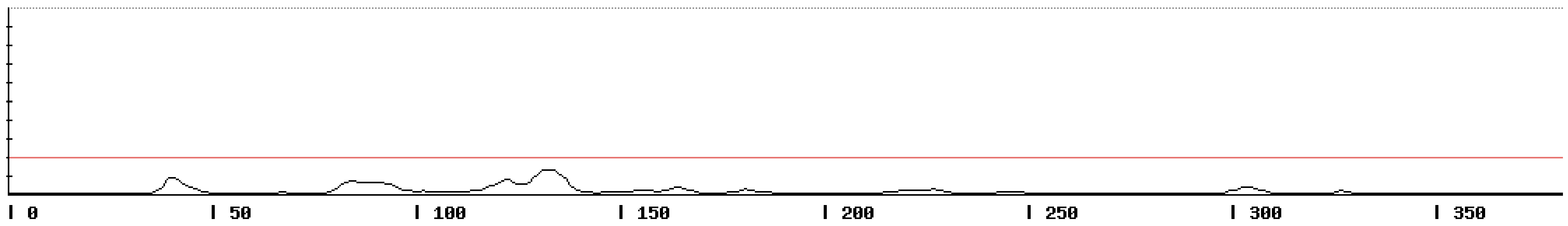

Predictions for Linumusitatissimum_Lus10032724

Viterbi Path

Viterbi Path

Posterior @ 0.2 | 315 - KAKQKQKQQQVSRKHKLADKDKGSSSDSSHGGSSSGASRDVKKEGSKGTMKKRKNK - 370

Posterior @ 0.2

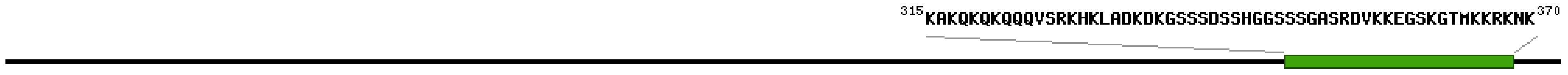

Posterior Graphic. Threshold Marked @ 0.2

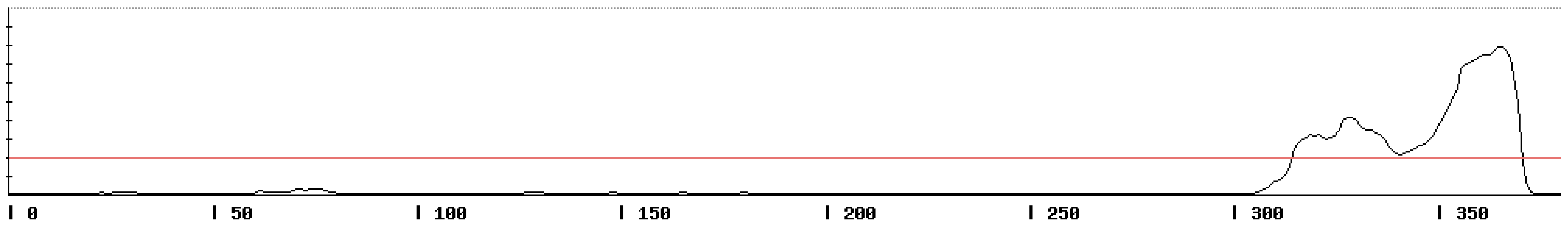

Predictions for Linumusitatissimum_Lus10032919

Viterbi Path

Viterbi Path

Posterior @ 0.2 | 93 - KATGKDRKIKS - 103

478 - AGKQMKGSKDLGRKNAT - 494

Posterior @ 0.2

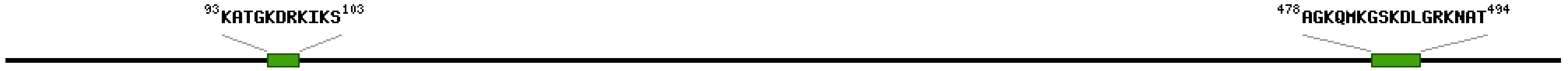

Posterior Graphic. Threshold Marked @ 0.2

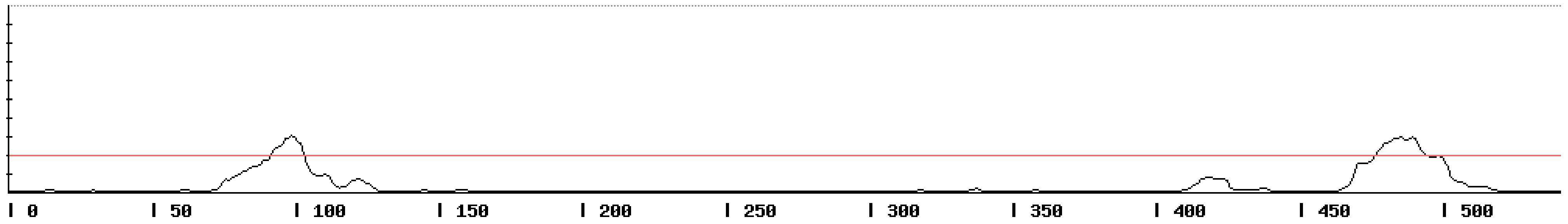

Predictions for Linumusitatissimum_Lus10033239

Viterbi Path

Viterbi Path

Posterior @ 0.2	115 - RR - 116
Posterior @ 0.2	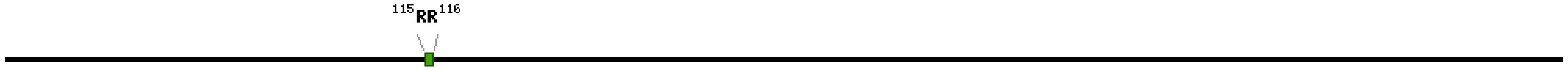
Posterior Graphic. Threshold Marked @ 0.2	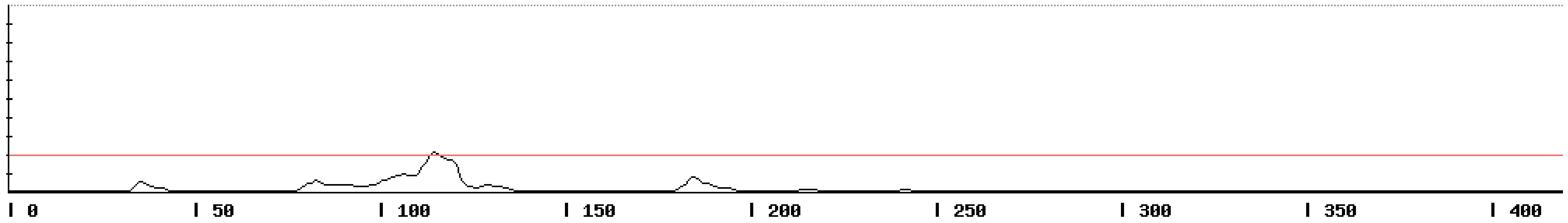

Predictions for Linumusitatissimum_Lus10033251

Viterbi Path	
Viterbi Path	
Posterior @ 0.2	95 - TGKDRTIRSRRPG - 107
Posterior @ 0.2	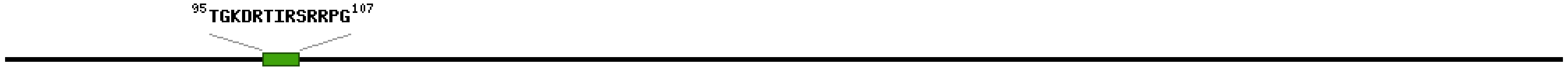
Posterior Graphic. Threshold Marked @ 0.2	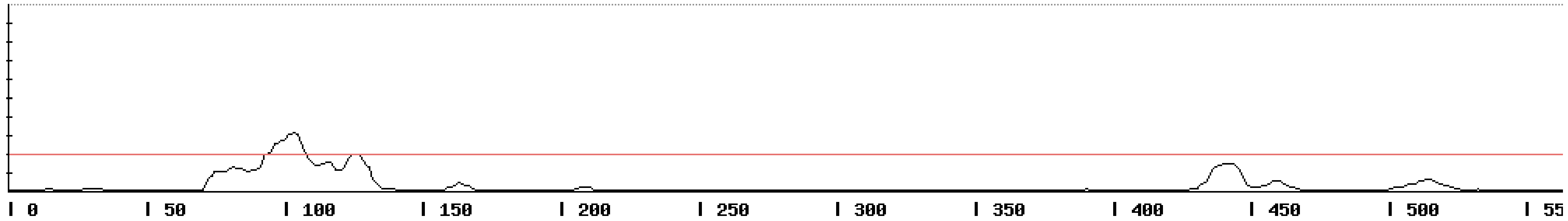

Predictions for Linumusitatissimum_Lus10033279

Viterbi Path

Viterbi Path

Posterior @ 0.2

Posterior @ 0.2

Posterior Graphic. Threshold Marked @ 0.2

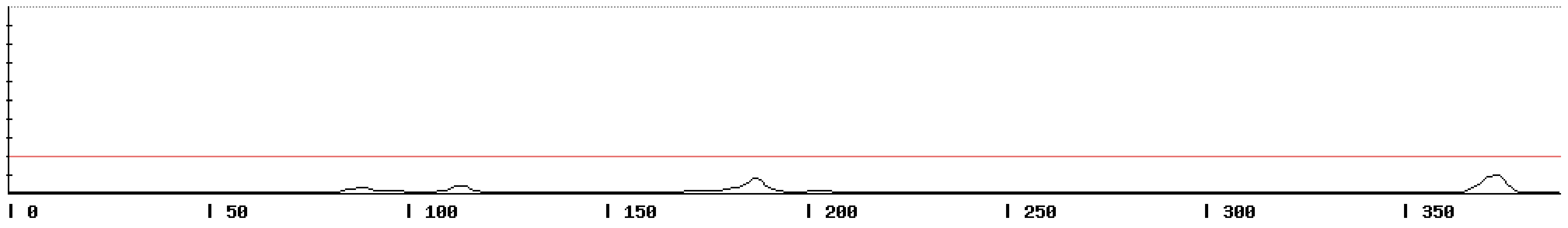

Predictions for Linumusitatissimum_Lus10033281

Viterbi Path

Viterbi Path

Posterior @ 0.2

Posterior @ 0.2

Posterior Graphic. Threshold Marked @ 0.2

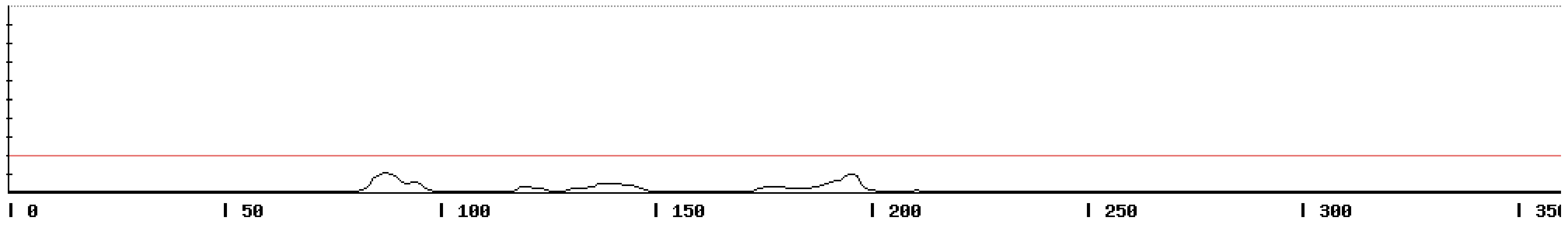

Predictions for Linumusitatissimum_Lus10033493

Viterbi Path

Viterbi Path

Posterior @ 0.2 — 78 - RKYPNGSRPNRAAGTGYWKATGADKPIGKPKALGIKKALVFYAGKAPR - 125

Posterior @ 0.2

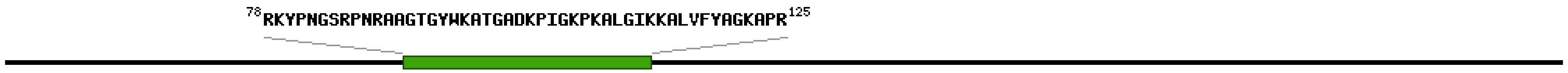

Posterior Graphic. Threshold Marked @ 0.2

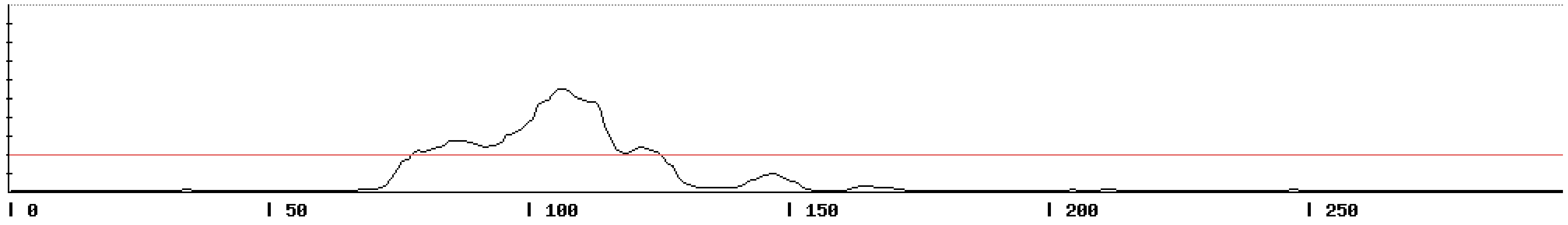

Predictions for Linumusitatissimum_Lus10033650

Viterbi Path

Viterbi Path

Posterior @ 0.2 — 92 - KLKSKKLSNGERKSSRI - 108

138 - KK - 139

Posterior @ 0.2

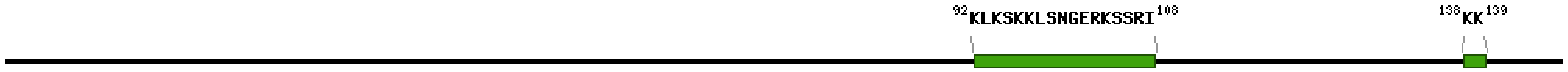

Posterior Graphic. Threshold Marked @ 0.2

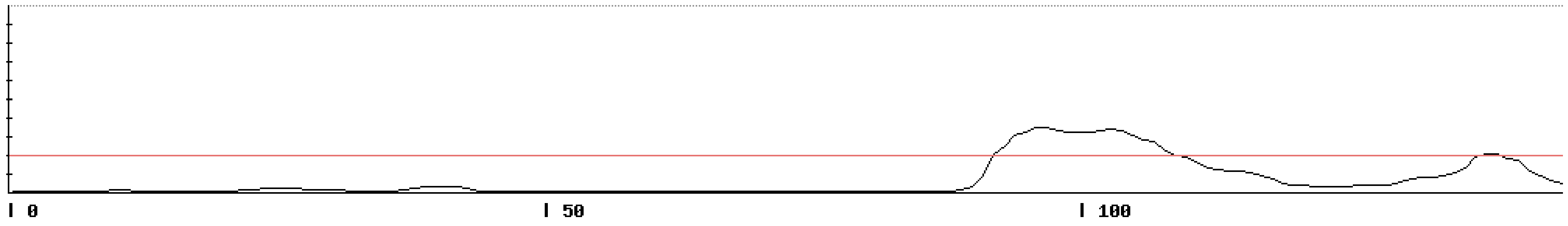

Predictions for Linumusitatissimum_Lus10033652

Viterbi Path

Viterbi Path

Posterior @ 0.2

92 - KLKSKKLSNGERKSSRI - 108

138 - K - 138

Posterior @ 0.2

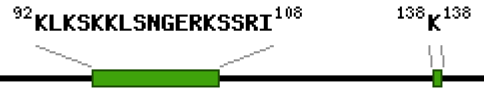

Posterior Graphic. Threshold Marked @ 0.2

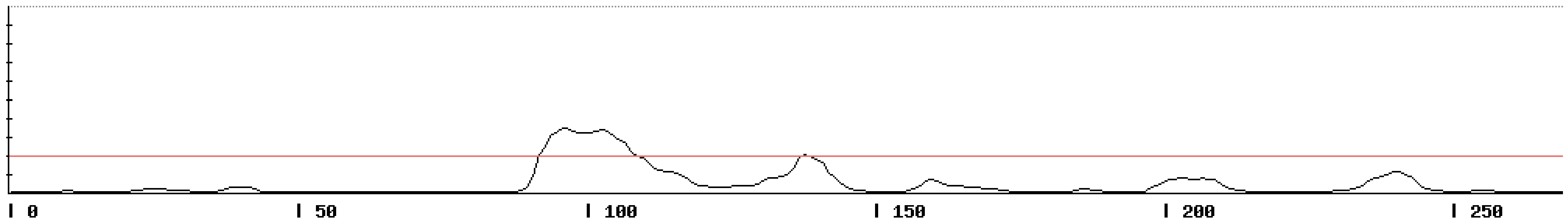

Predictions for Linumusitatissimum_Lus10033676

Viterbi Path

Viterbi Path

Posterior @ 0.2

92 - GKRILRRV - 99

329 - KKK - 331

395 - QAKGKKIYEKAKSGREM - 411

	416 - RKGK - 419
	439 - KPLPKTGRGHGKRP - 452
	468 - KGKKPR - 473
Posterior @ 0.2	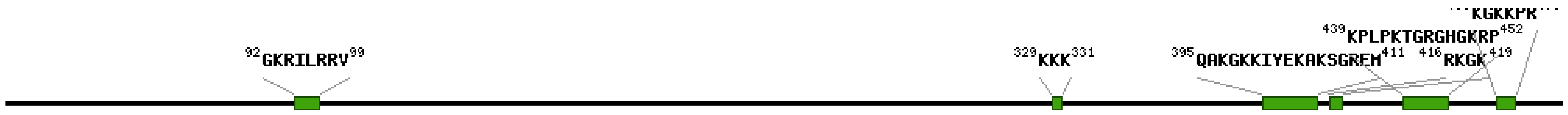
Posterior Graphic. Threshold Marked @ 0.2	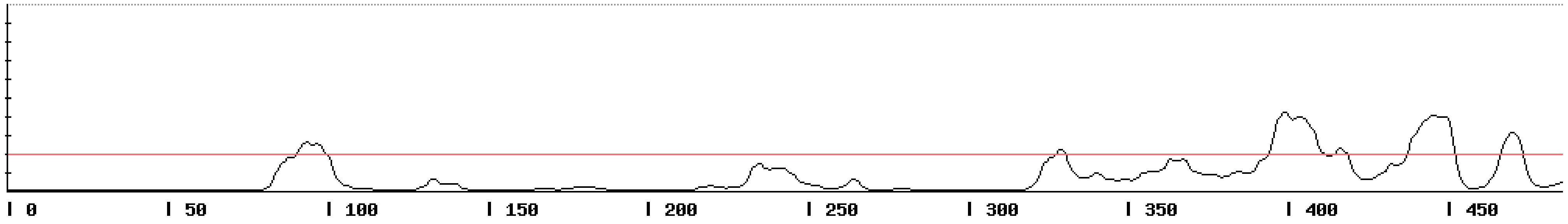

Predictions for Linumusitatissimum_Lus10033699

Viterbi Path	
Viterbi Path	
Posterior @ 0.2	83 - TKLKKKSKNAKYFERR - 98
	126 - WAGKKRKFKYENKKS - 140
	188 - RKR - 190
Posterior @ 0.2	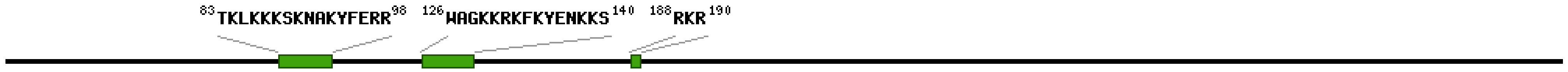
Posterior Graphic. Threshold Marked @ 0.2	

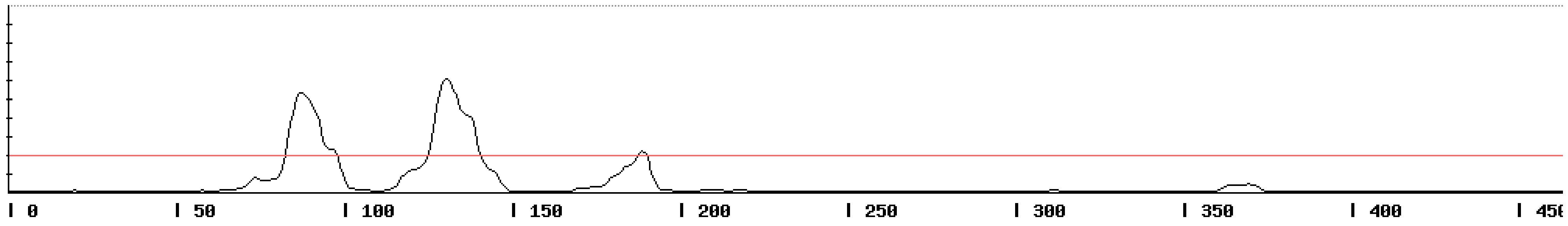

Predictions for Linumusitatissimum_Lus10033905	
Viterbi Path	
Viterbi Path	
Posterior @ 0.2	
Posterior @ 0.2	
Posterior Graphic. Threshold Marked @ 0.2	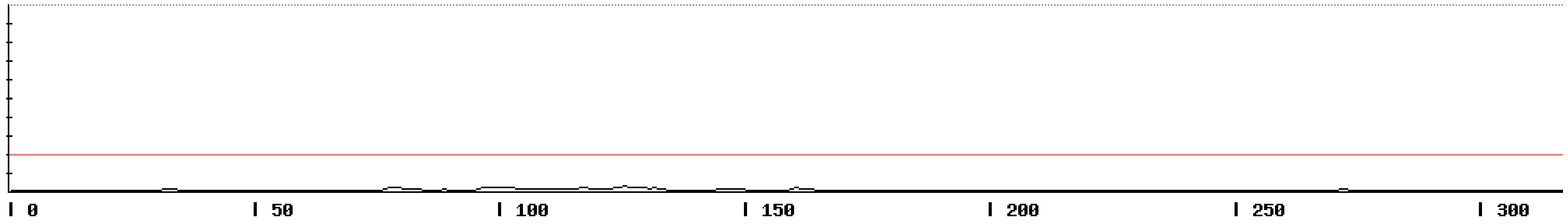

Predictions for Linumusitatissimum_Lus10034183	
Viterbi Path	
Viterbi Path	
Posterior @ 0.2	330 - RRVKLGNRMRVGSVVVAPGAAGKKKGKKKWD - 360
Posterior @ 0.2	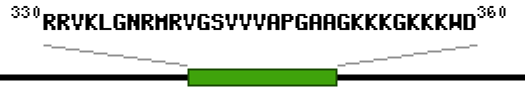

Posterior Graphic. Threshold Marked @ 0.2

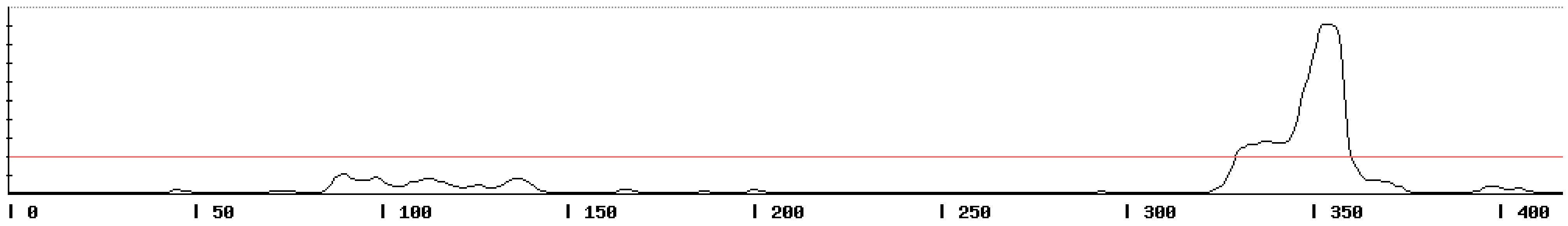

Predictions for Linumusitatissimum_Lus10034700

Viterbi Path

Viterbi Path

Posterior @ 0.2 — 123 - KRAGKK - 128

Posterior @ 0.2

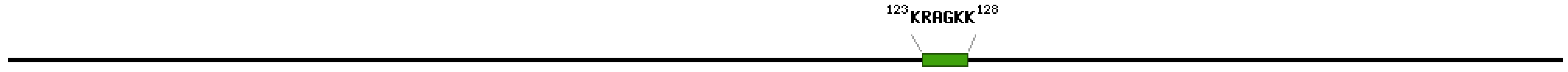

Posterior Graphic. Threshold Marked @ 0.2

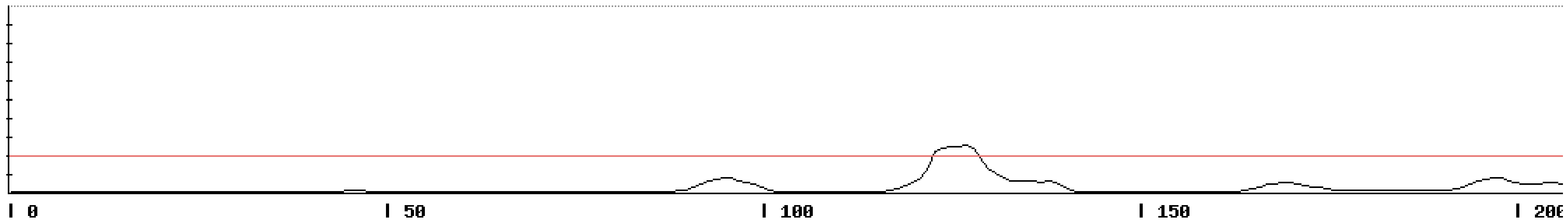

Predictions for Linumusitatissimum_Lus10034999

Viterbi Path

Viterbi Path

Posterior @ 0.2 — 140 - GTRKRRK - 146

Posterior @ 0.2

	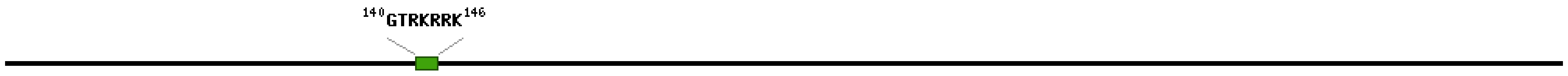
Posterior Graphic. Threshold Marked @ 0.2	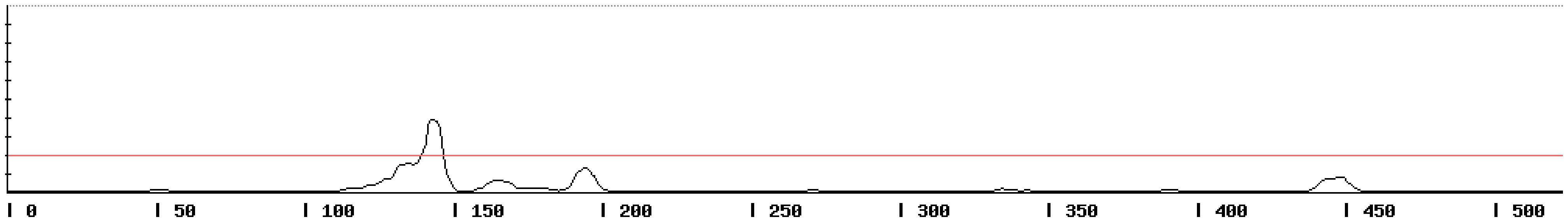

Predictions for Linumusitatissimum_Lus10035174

Viterbi Path	
Viterbi Path	
Posterior @ 0.2	123 - GKRKK - 127
	168 - KKRRA - 172
Posterior @ 0.2	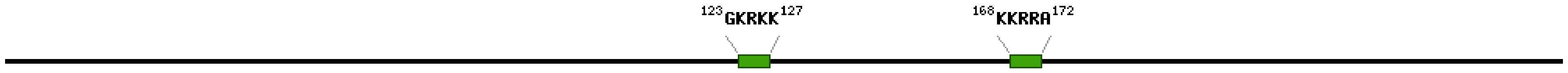
Posterior Graphic. Threshold Marked @ 0.2	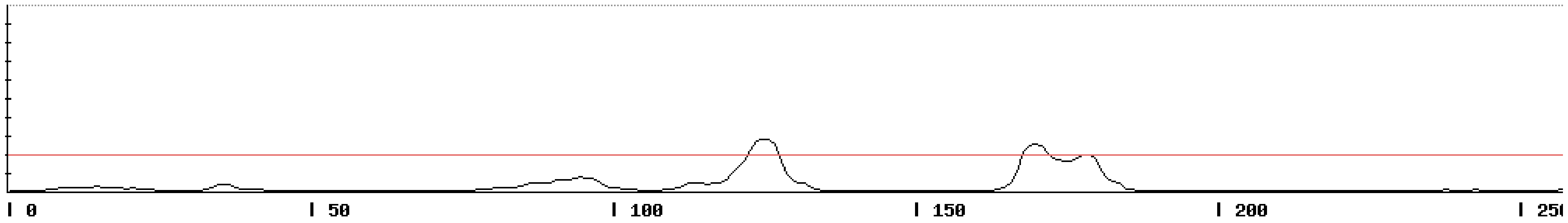

Predictions for Linumusitatissimum_Lus10035373

Viterbi Path	
Viterbi Path	

Posterior @ 0.2 | 184 - SRRASRK - 190

Posterior @ 0.2

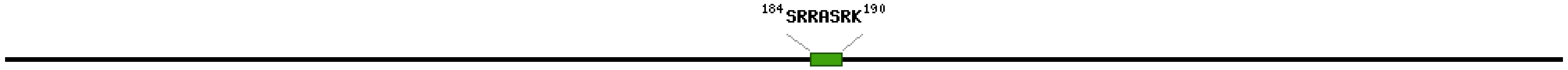

Posterior Graphic. Threshold Marked @ 0.2

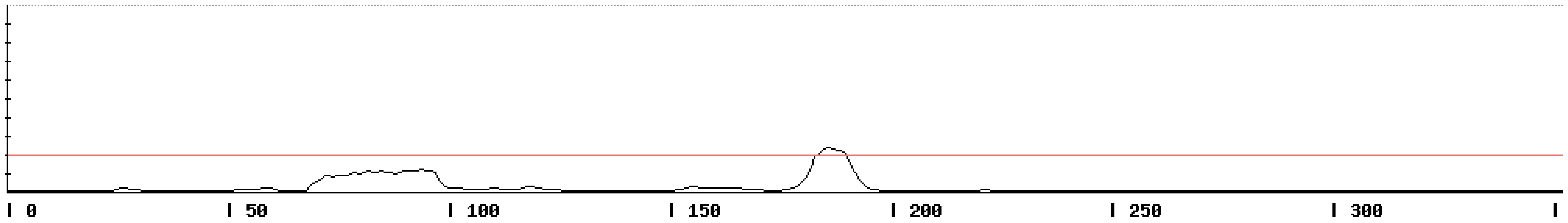

Predictions for Linumusitatissimum_Lus10035400

Viterbi Path

Viterbi Path

Posterior @ 0.2 | 85 - KLKRKPSNNKIDRG - 98

Posterior @ 0.2

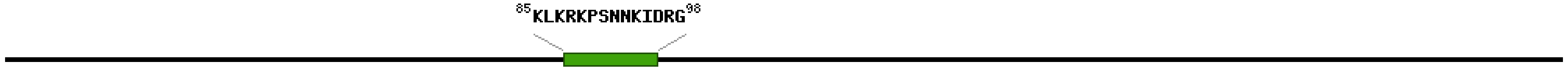

Posterior Graphic. Threshold Marked @ 0.2

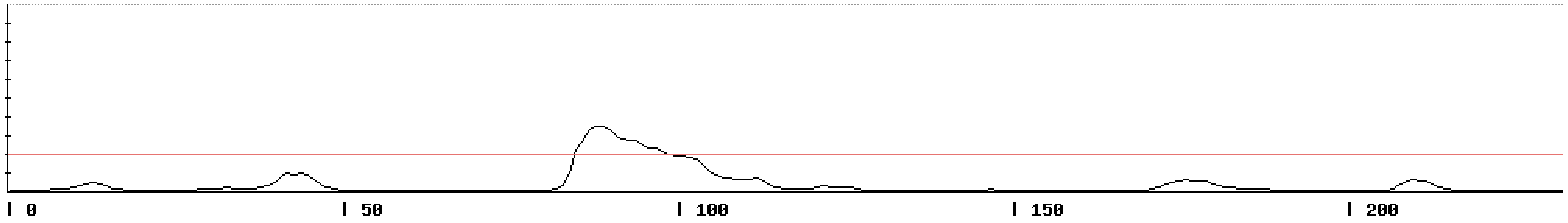

Predictions for Linumusitatissimum_Lus10035647

Viterbi Path

Viterbi Path

Posterior @ 0.2

Posterior @ 0.2

Posterior Graphic. Threshold Marked @ 0.2

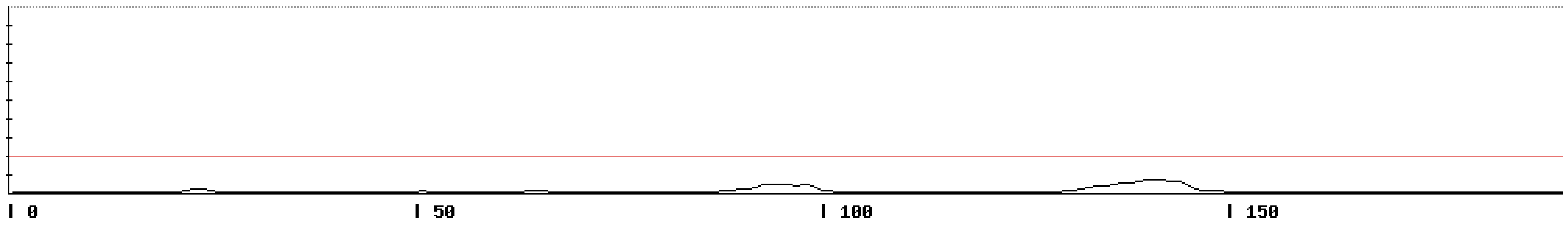

Predictions for Linumusitatissimum_Lus10035648

Viterbi Path

Viterbi Path

Posterior @ 0.2

Posterior @ 0.2

Posterior Graphic. Threshold Marked @ 0.2

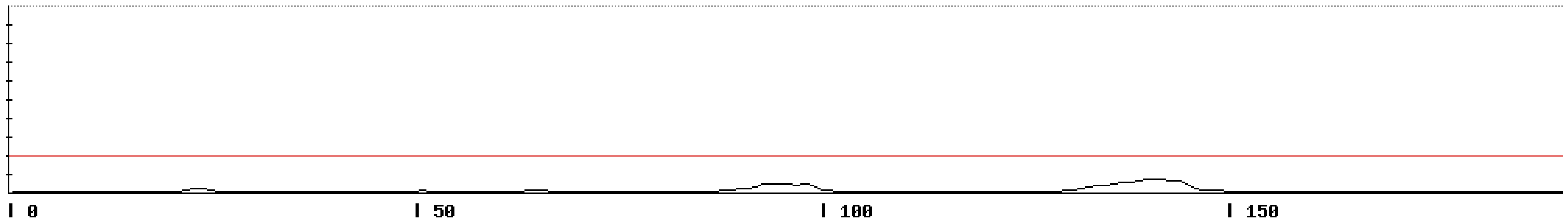

Predictions for Linumusitatissimum_Lus10036117

Viterbi Path

Viterbi Path

Posterior @ 0.2

Posterior @ 0.2

Posterior Graphic. Threshold Marked @ 0.2

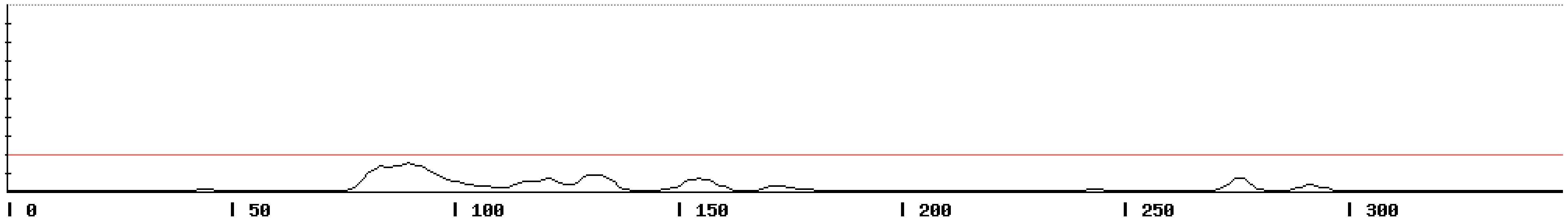

Predictions for Linumusitatissimum_Lus10036194

Viterbi Path

Viterbi Path

Posterior @ 0.2

45 - RKVEKK - 50

88 - RK - 89

Posterior @ 0.2

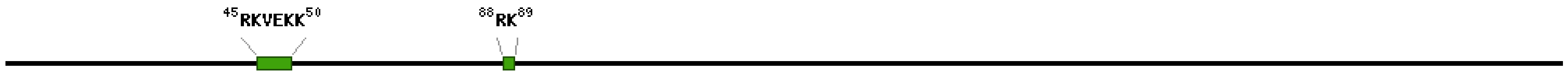

Posterior Graphic. Threshold Marked @ 0.2

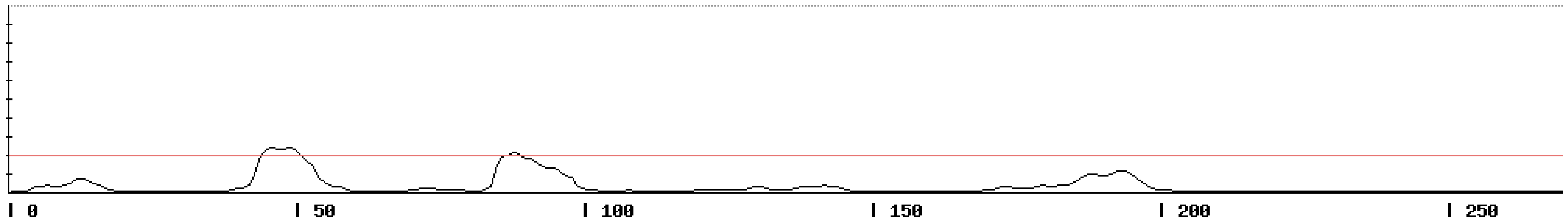

Predictions for Linumusitatissimum_Lus10036749	
Viterbi Path	
Viterbi Path	
Posterior @ 0.2	
Posterior @ 0.2	
Posterior Graphic. Threshold Marked @ 0.2	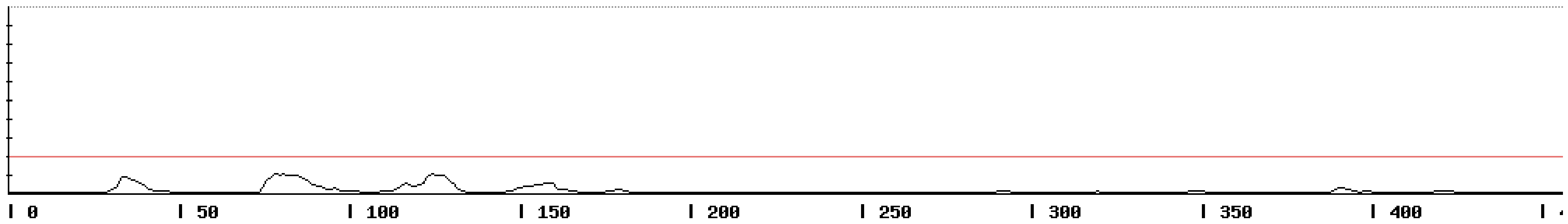

Predictions for Linumusitatissimum_Lus10036773	
Viterbi Path	
Viterbi Path	
Posterior @ 0.2	
Posterior @ 0.2	

Posterior Graphic. Threshold Marked @ 0.2

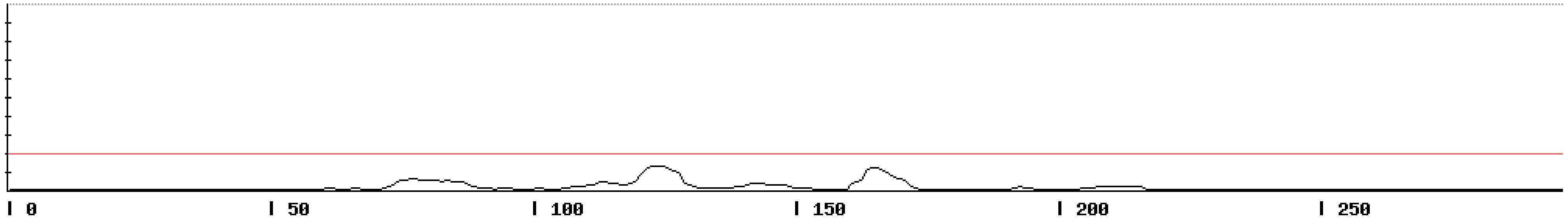

Predictions for Linumusitatissimum_Lus10036955

Viterbi Path

Viterbi Path

Posterior @ 0.2 | 74 - TKQKKKKNEKGKRVDRS - 90

Posterior @ 0.2

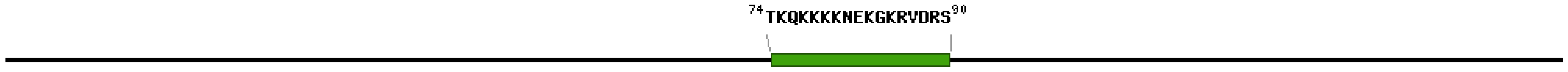

Posterior Graphic. Threshold Marked @ 0.2

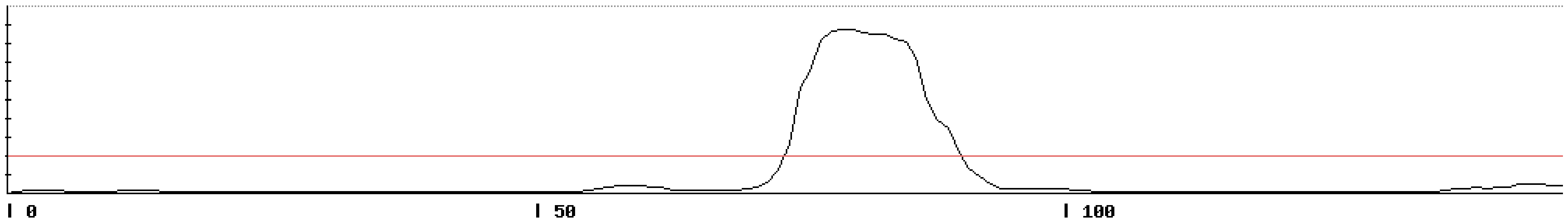

Predictions for Linumusitatissimum_Lus10036959

Viterbi Path | 75 - KRKKKNEKGKR - 85

Viterbi Path

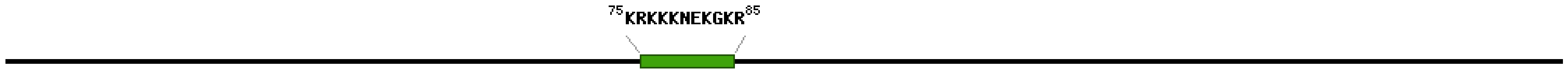

Posterior @ 0.2 | 74 - TKRKKKNEKGKRVERS - 89

170 - RKKEGSKKS - 178

Posterior @ 0.2

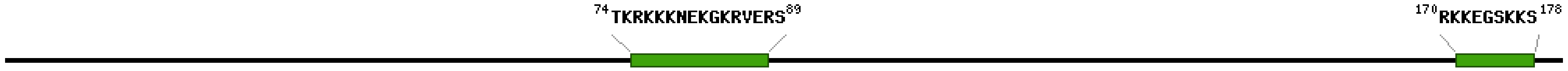

Posterior Graphic. Threshold Marked @ 0.2

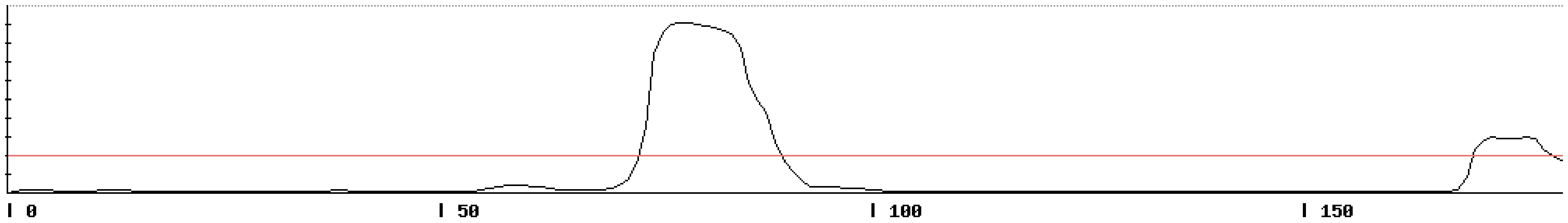

Predictions for Linumusitatissimum_Lus10037106

Viterbi Path

Viterbi Path

Posterior @ 0.2 — 74 - TKQKKKKNEKGKRVERSF - 91

Posterior @ 0.2

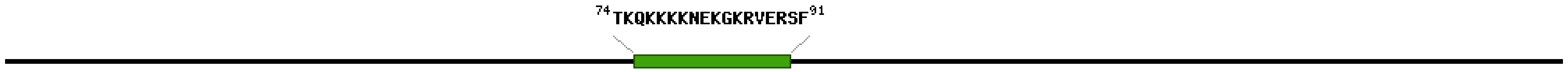

Posterior Graphic. Threshold Marked @ 0.2

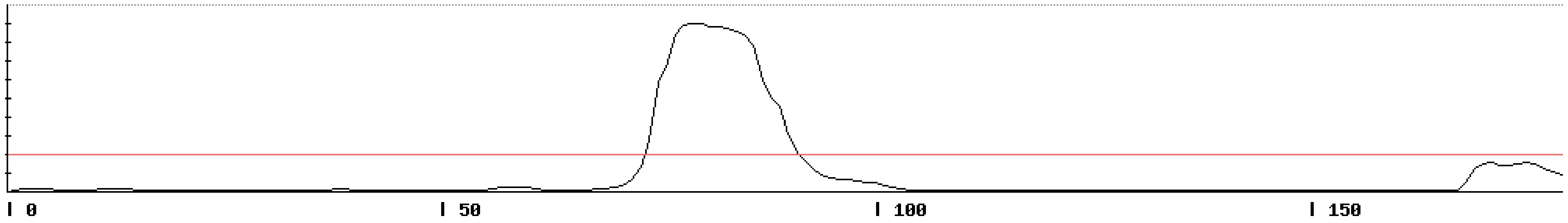

Predictions for Linumusitatissimum_Lus10037156

Viterbi Path

Viterbi Path

Posterior @ 0.2

Posterior @ 0.2

Posterior Graphic. Threshold Marked @ 0.2

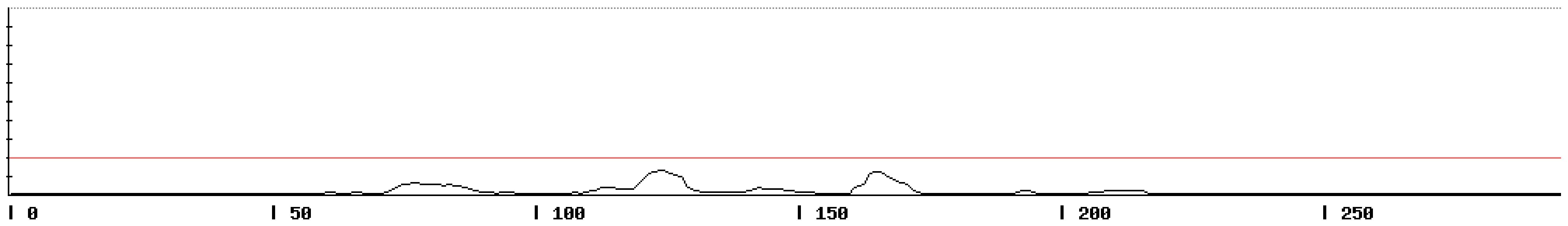

Predictions for Linumusitatissimum_Lus10037178

Viterbi Path

Viterbi Path

Posterior @ 0.2

Posterior @ 0.2

Posterior Graphic. Threshold Marked @ 0.2

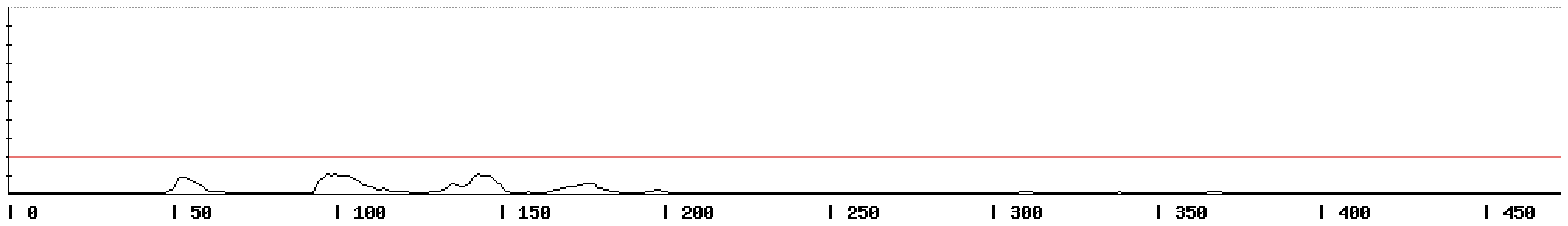

Predictions for Linumusitatissimum_Lus10037939

Viterbi Path

Viterbi Path

Posterior @ 0.2	
Posterior @ 0.2	
Posterior Graphic. Threshold Marked @ 0.2	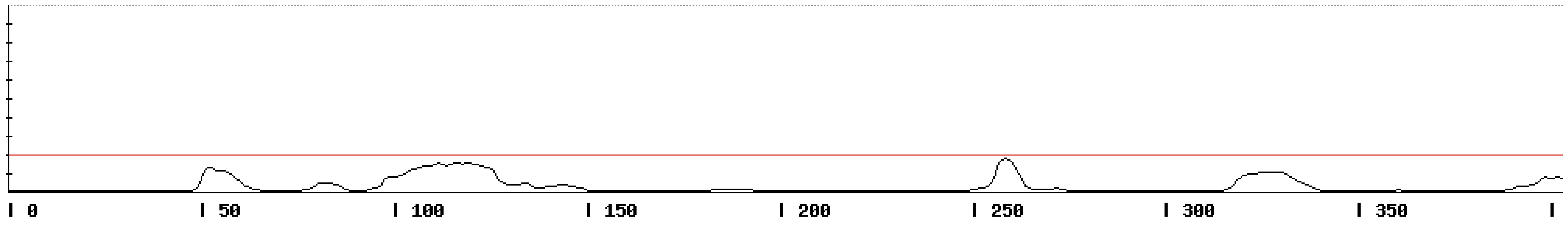

Predictions for Linumusitatissimum_Lus10038332

Viterbi Path	
Viterbi Path	
Posterior @ 0.2	54 - RKVEKKA - 60
	98 - GRK - 100
Posterior @ 0.2	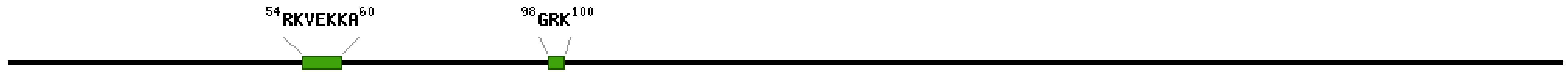
Posterior Graphic. Threshold Marked @ 0.2	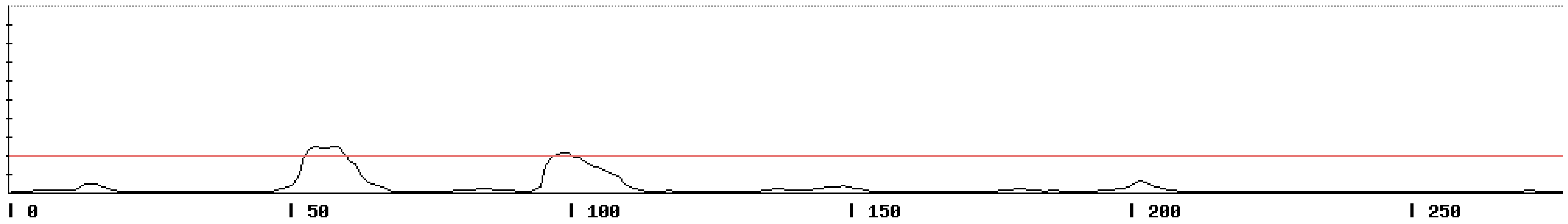

Predictions for Linumusitatissimum_Lus10038670

Viterbi Path	
Viterbi Path	
Posterior @ 0.2	
Posterior @ 0.2	
Posterior Graphic. Threshold Marked @ 0.2	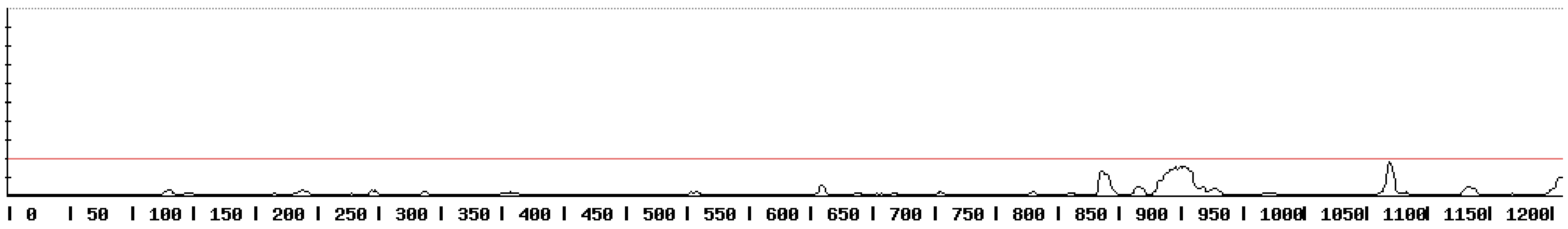

Predictions for Linumusitatissimum_Lus10038937

Viterbi Path	
Viterbi Path	
Posterior @ 0.2	
Posterior @ 0.2	
Posterior Graphic. Threshold Marked @ 0.2	

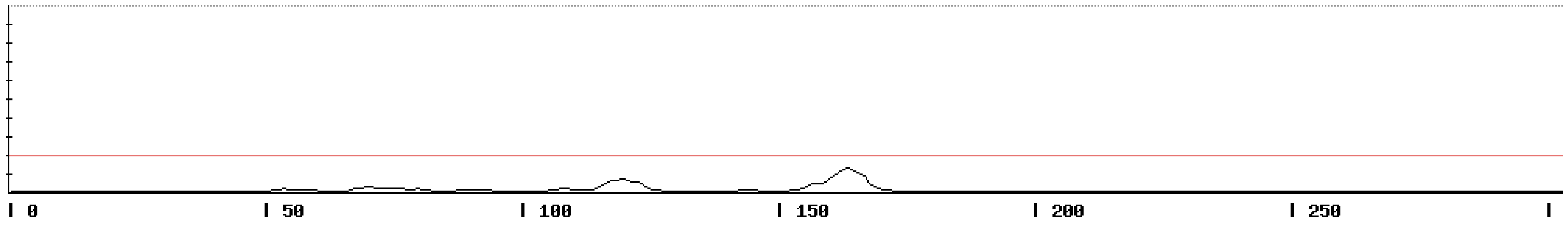

Predictions for Linumusitatissimum_Lus10039153

Viterbi Path	
Viterbi Path	
Posterior @ 0.2	
Posterior @ 0.2	
Posterior Graphic. Threshold Marked @ 0.2	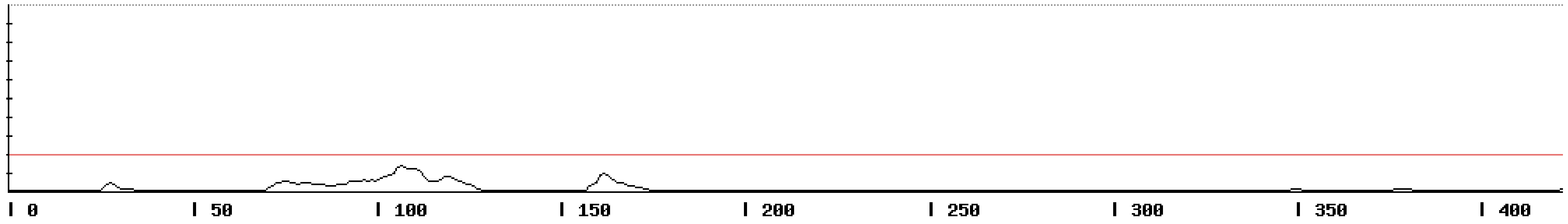

Predictions for Linumusitatissimum_Lus10039873

Viterbi Path	
Viterbi Path	
Posterior @ 0.2	148 - GTRKRRKV - 155
	171 - KTGKTR - 176
	181 - GGKTKG - 186
Posterior @ 0.2	

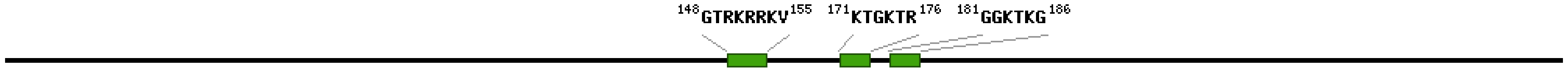

Posterior Graphic. Threshold Marked @ 0.2

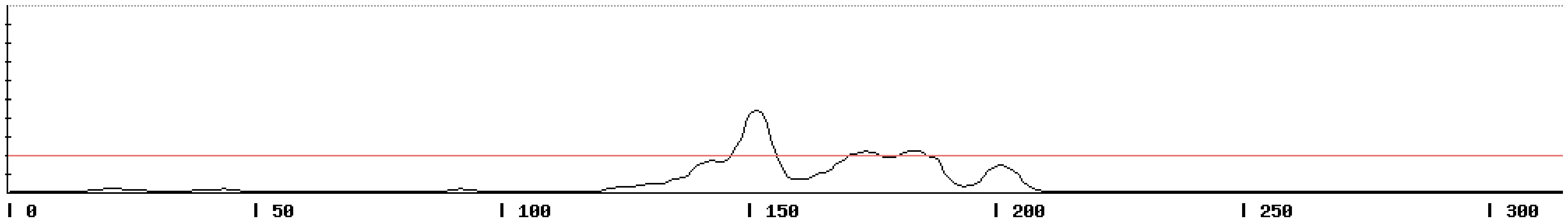

Predictions for Linumusitatissimum_Lus10040422

Viterbi Path

Viterbi Path

Posterior @ 0.2

Posterior @ 0.2

Posterior Graphic. Threshold Marked @ 0.2

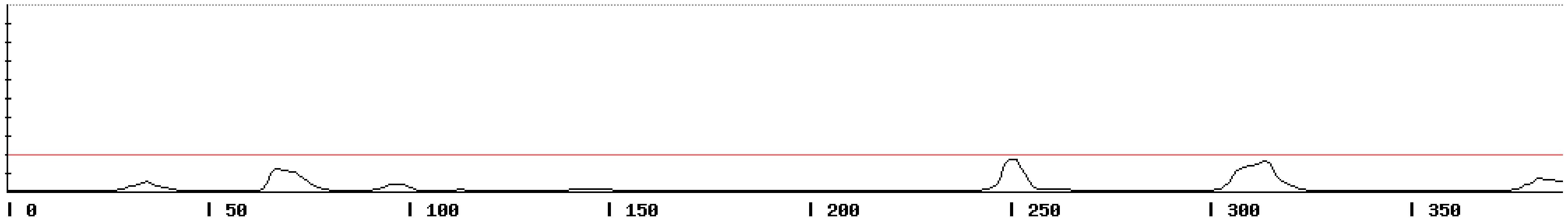

Predictions for Linumusitatissimum_Lus10041492

Viterbi Path

Viterbi Path

Posterior @ 0.2 — 150 - GTRKRRKI - 157

Posterior @ 0.2

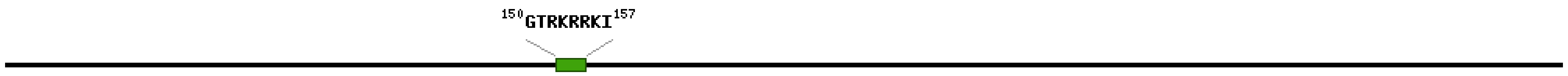

Posterior Graphic. Threshold Marked @ 0.2

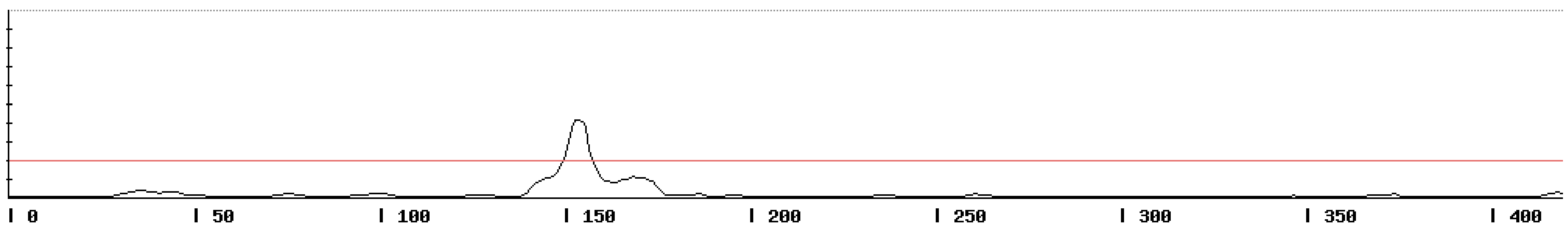

Predictions for Linumusitatissimum_Lus10041534

Viterbi Path

Viterbi Path

Posterior @ 0.2 — 176 - KKKRHSNSNMKKPP - 189

Posterior @ 0.2

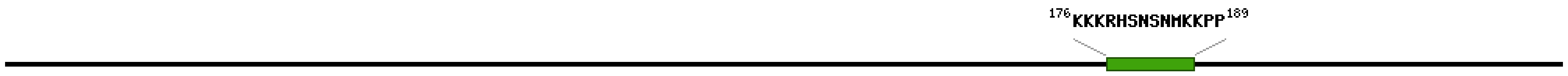

Posterior Graphic. Threshold Marked @ 0.2

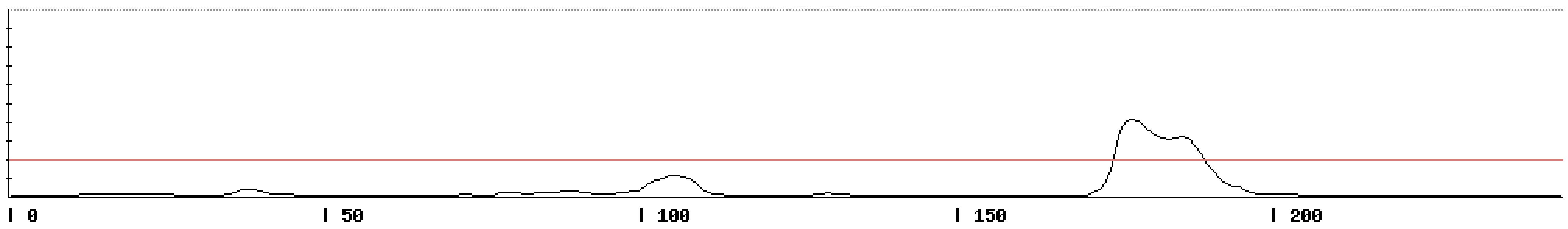

Predictions for Linumusitatissimum_Lus10041822

Viterbi Path

Viterbi Path

Posterior @ 0.2

Posterior @ 0.2

Posterior Graphic. Threshold Marked @ 0.2

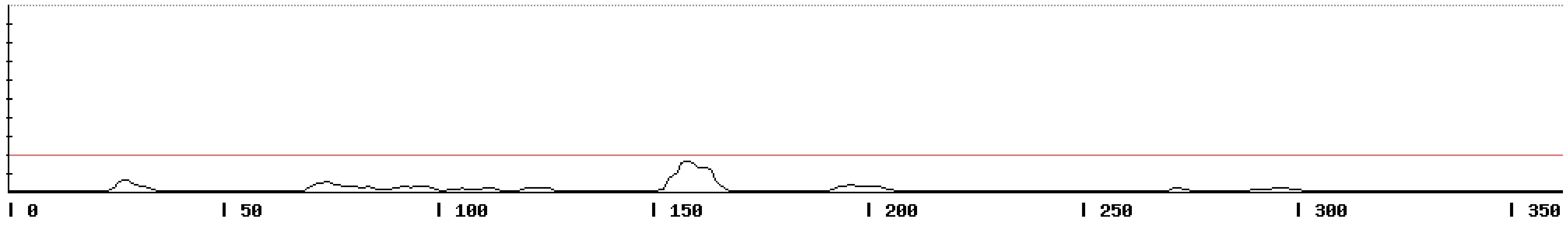

Predictions for Linumusitatissimum_Lus10041924

Viterbi Path

Viterbi Path

Posterior @ 0.2

Posterior @ 0.2

Posterior Graphic. Threshold Marked @ 0.2

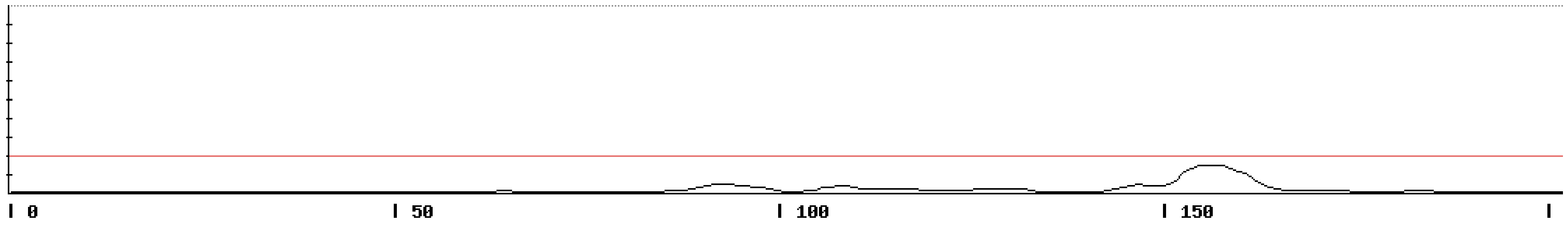

Predictions for Linumusitatissimum_Lus10042284

Viterbi Path

Viterbi Path

Posterior @ 0.2

Posterior @ 0.2

Posterior Graphic. Threshold Marked @ 0.2

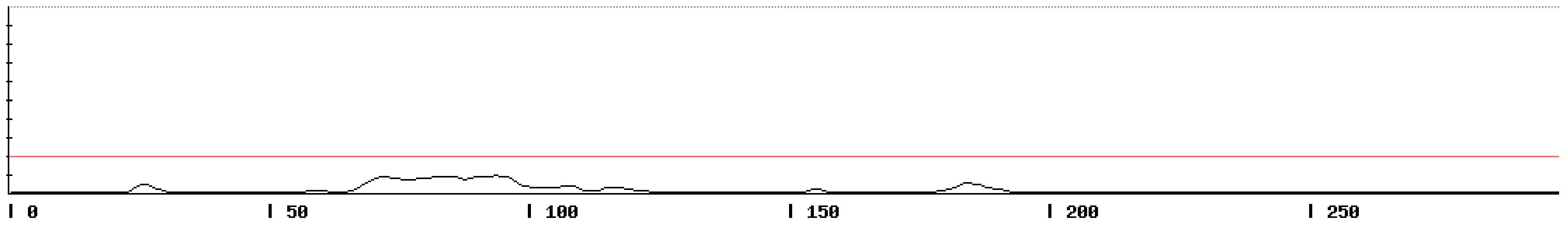

Predictions for Linumusitatissimum_Lus10042466

Viterbi Path

Viterbi Path

Posterior @ 0.2

Posterior @ 0.2

Posterior Graphic. Threshold Marked @ 0.2

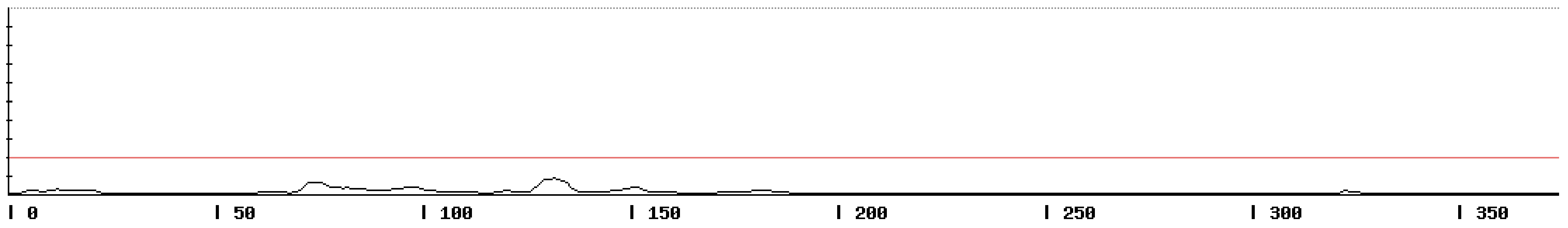

Predictions for Linumusitatissimum_Lus10042518

Viterbi Path	
Viterbi Path	
Posterior @ 0.2	304 - KDRYKR - 309
	317 - KPTGKPRHIK - 326
	378 - VMKKSNKTKAATKRKAKT - 395
Posterior @ 0.2	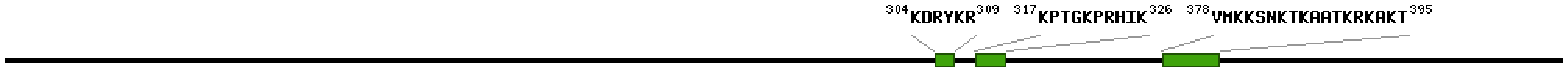
Posterior Graphic. Threshold Marked @ 0.2	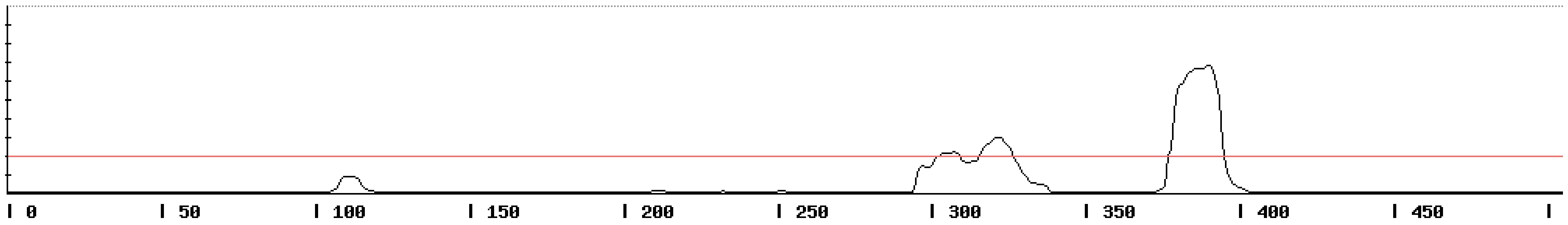

Predictions for Linumusitatissimum_Lus10042531

Viterbi Path	
Viterbi Path	
Posterior @ 0.2	82 - ARPNR - 86
	129 - RPPK - 132
Posterior @ 0.2	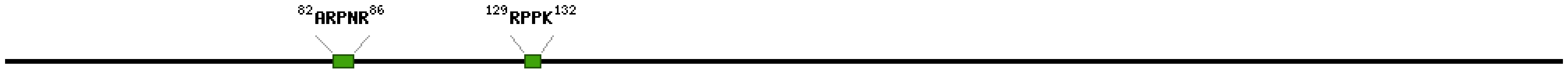
Posterior Graphic. Threshold Marked @ 0.2	

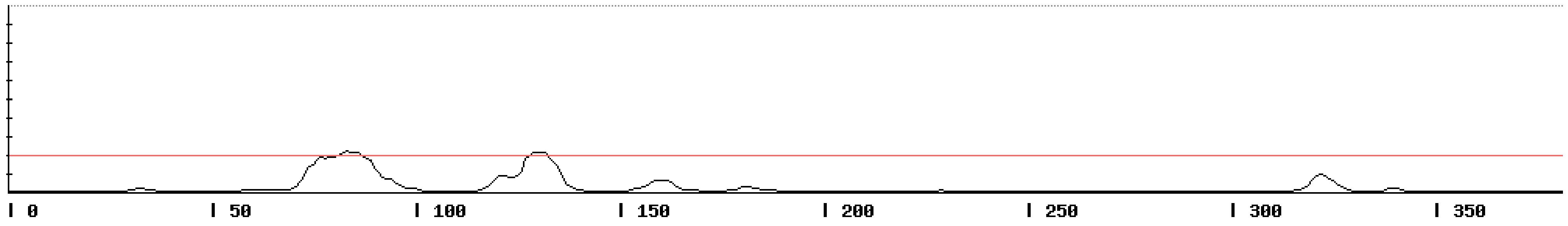

Predictions for Linumusitatissimum_Lus10042731	
Viterbi Path	
Viterbi Path	
Posterior @ 0.2	
Posterior @ 0.2	
Posterior Graphic. Threshold Marked @ 0.2	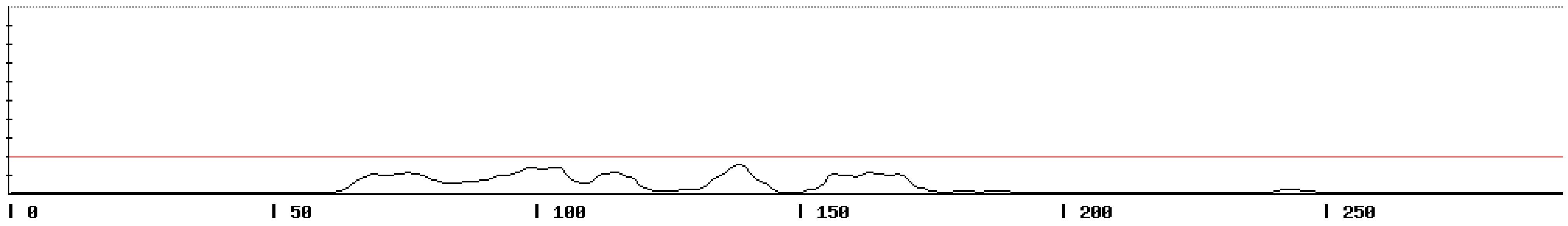

Predictions for Linumusitatissimum_Lus10043095	
Viterbi Path	
Viterbi Path	
Posterior @ 0.2	
Posterior @ 0.2	

Posterior Graphic. Threshold Marked @ 0.2

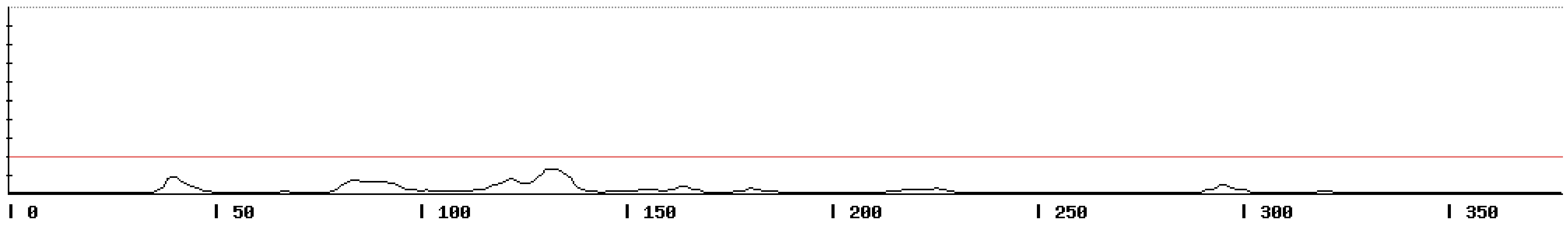

Predictions for Linumusitatissimum_Lus10043402

Viterbi Path

Viterbi Path

Posterior @ 0.2

Posterior @ 0.2

Posterior Graphic. Threshold Marked @ 0.2

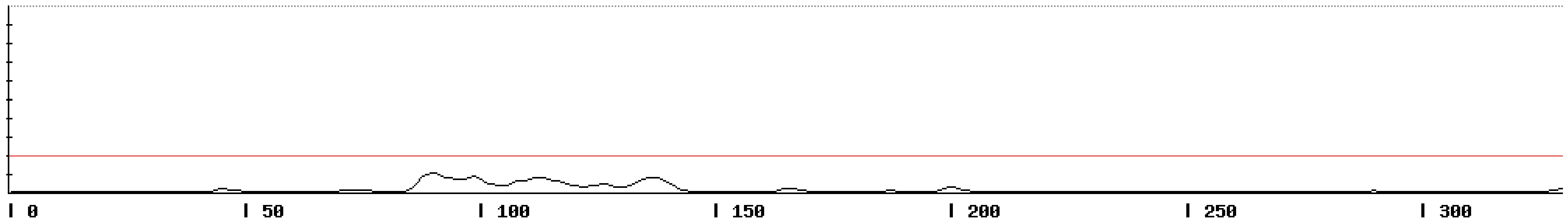

Revision r.9 - Last Updated on : February 24 2015 17:45:20.
alex.nguyenba@utoronto.ca

Intended to be used with Firefox

Viterbi Path

Posterior @ 0.2

74 - DRKYPKGVRPNR - 85

120 - KGRPPKG - 126

Posterior @ 0.2

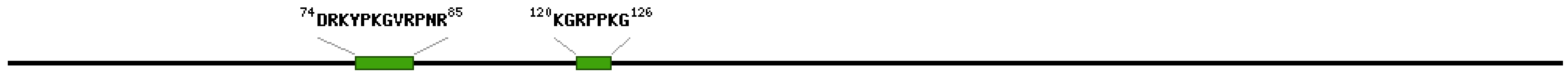

Posterior Graphic. Threshold Marked @ 0.2

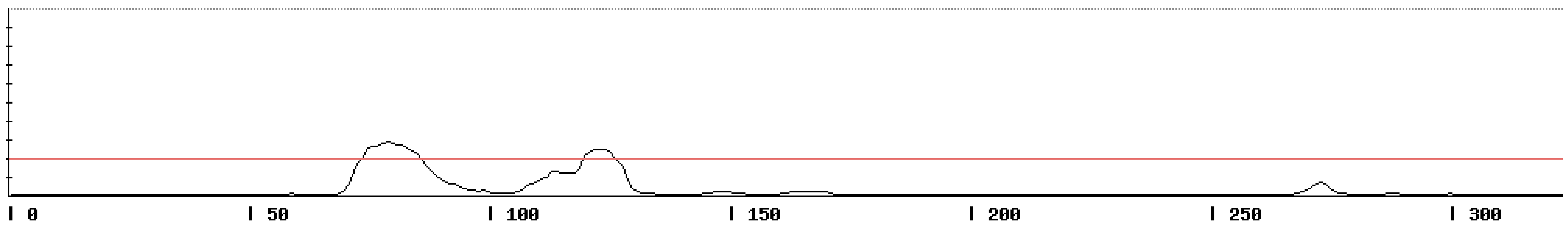

Predictions for Lotusjaponicus_chr1.CM0017.910.r2.m

Viterbi Path

Viterbi Path

Posterior @ 0.2

Posterior @ 0.2

Posterior Graphic. Threshold Marked @ 0.2

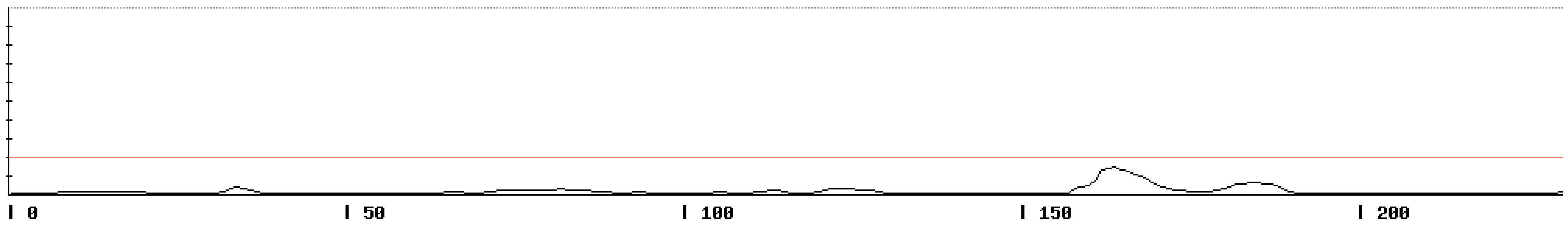

Predictions for Lotusjaponicus_chr1.CM0104.1970.r2.m

Viterbi Path	
Viterbi Path	
Posterior @ 0.2	120 - KGKPPKG - 126
Posterior @ 0.2	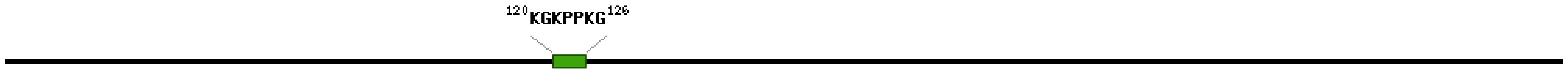
Posterior Graphic. Threshold Marked @ 0.2	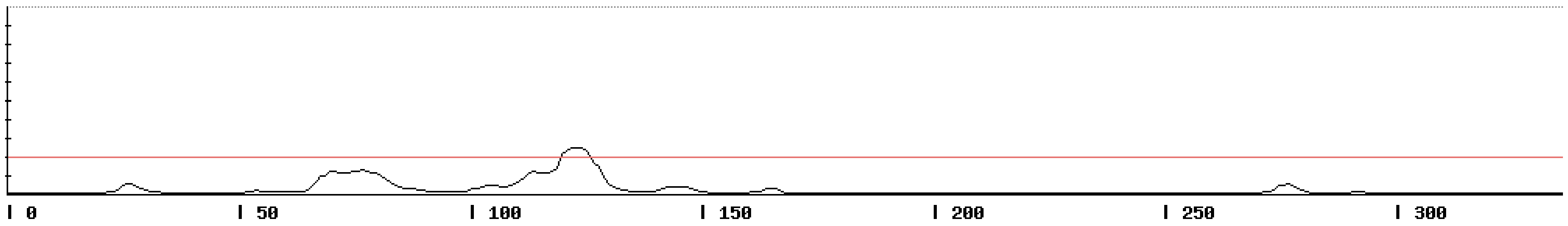

Predictions for Lotusjaponicus_chr1.CM0104.1990.r2.m

Viterbi Path	
Viterbi Path	
Posterior @ 0.2	
Posterior @ 0.2	
Posterior Graphic. Threshold Marked @ 0.2	

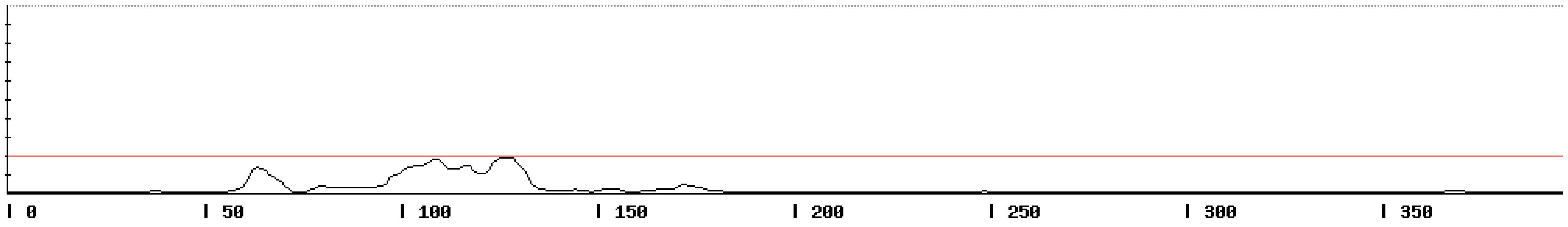

Predictions for Lotusjaponicus_chr1.CM0113.1590.r2.d

Viterbi Path	
Viterbi Path	
Posterior @ 0.2	141 - R - 141
Posterior @ 0.2	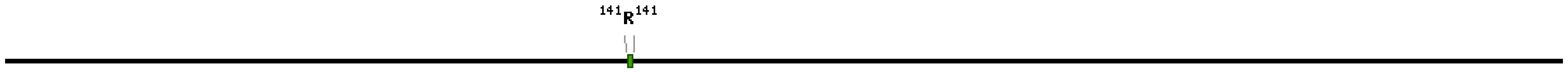
Posterior Graphic. Threshold Marked @ 0.2	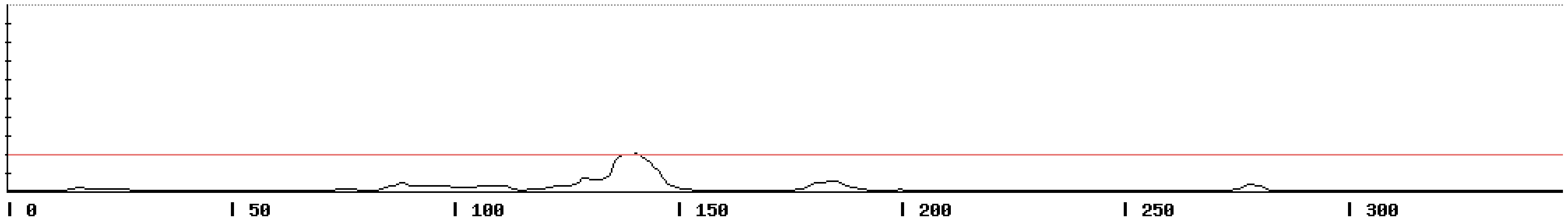

Predictions for Lotusjaponicus_chr1.CM0141.310.r2.m

Viterbi Path	
Viterbi Path	
Posterior @ 0.2	
Posterior @ 0.2	

Posterior Graphic. Threshold Marked @ 0.2

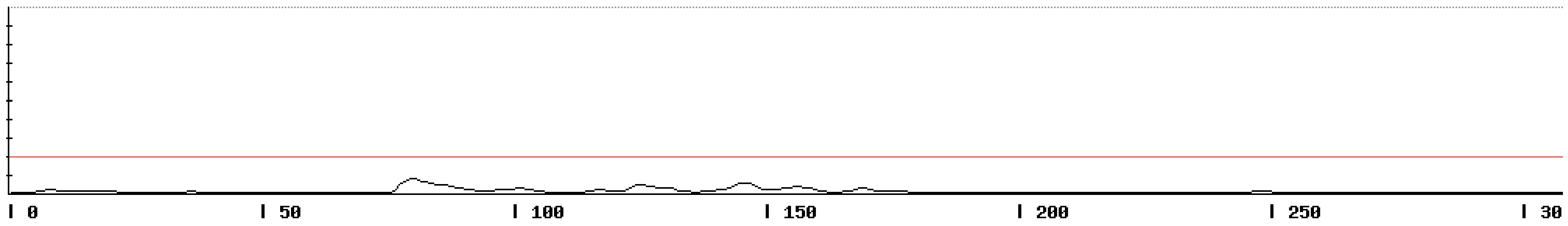

Predictions for Lotusjaponicus_chr1.CM0207.190.r2.m

Viterbi Path

Viterbi Path

Posterior @ 0.2

127 - RAPKGKR - 133

329 - DRKVK - 333

353 - PRGK - 356

Posterior @ 0.2

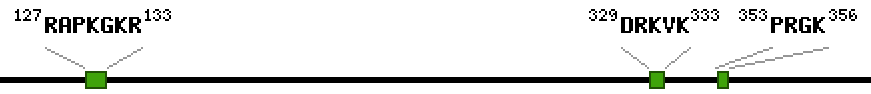

Posterior Graphic. Threshold Marked @ 0.2

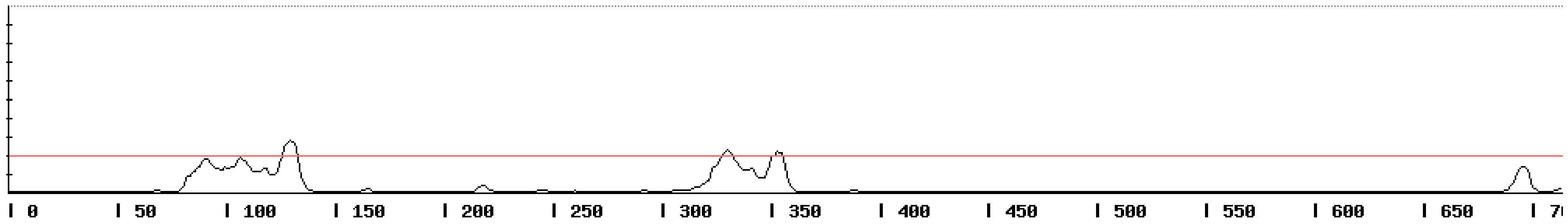

Predictions for Lotusjaponicus_chr1.CM0207.220.r2.m

Viterbi Path

Viterbi Path

Posterior @ 0.2

103 - GKDRKIKSG - 111

	126 - GRAPKGKR - 133
	472 - RRIRLLKSAHGSSRVVKGGRRAQEDRNSK - 500
Posterior @ 0.2	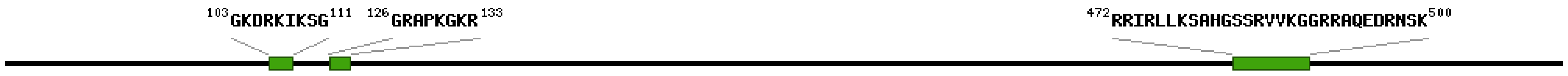
Posterior Graphic. Threshold Marked @ 0.2	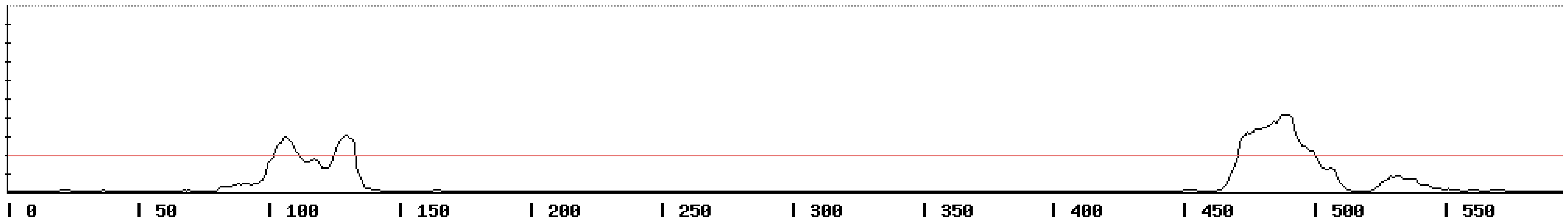

Predictions for Lotusjaponicus_chr1.CM1852.210.r2.m

Viterbi Path	
Viterbi Path	
Posterior @ 0.2	
Posterior @ 0.2	
Posterior Graphic. Threshold Marked @ 0.2	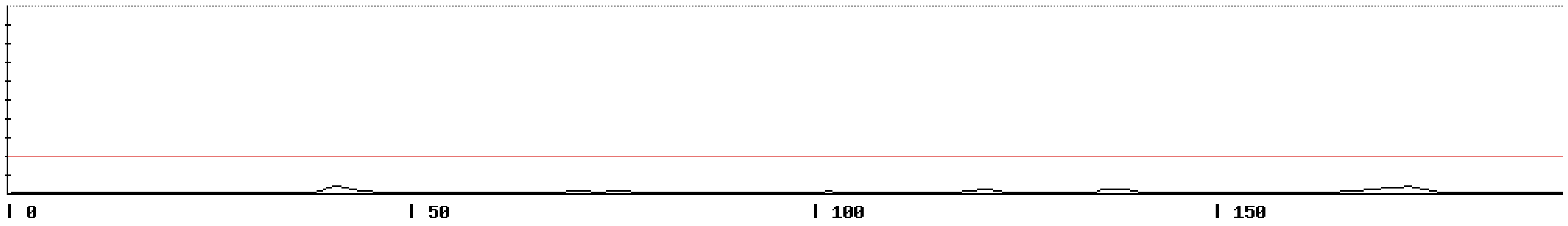

Predictions for Lotusjaponicus_chr1.CM1852.230.r2.m

Viterbi Path	
Viterbi Path	

Posterior @ 0.2

Posterior @ 0.2

Posterior Graphic. Threshold Marked @ 0.2

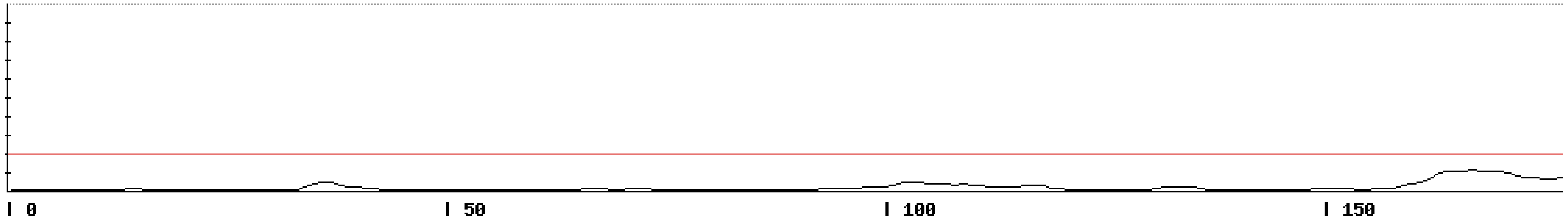

Predictions for Lotusjaponicus_chr1.CM1852.250.r2.m

Viterbi Path

Viterbi Path

Posterior @ 0.2

Posterior @ 0.2

Posterior Graphic. Threshold Marked @ 0.2

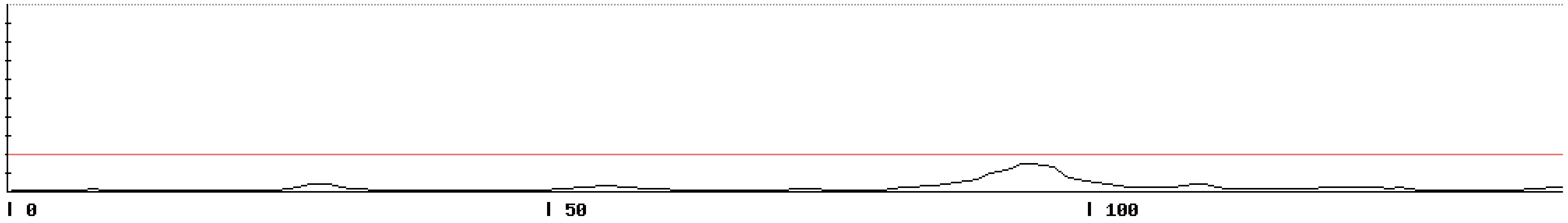

Predictions for Lotusjaponicus_chr1.LjT04N09.160.r2.d

Viterbi Path

Viterbi Path

Posterior @ 0.2 93 - ADKPIGHPKPVGIKKA - 108

Posterior @ 0.2

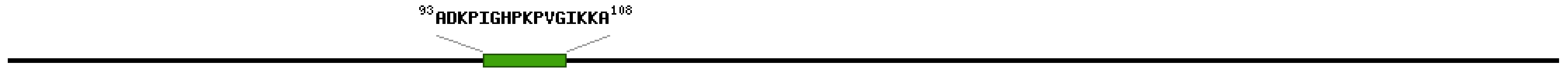

Posterior Graphic. Threshold Marked @ 0.2

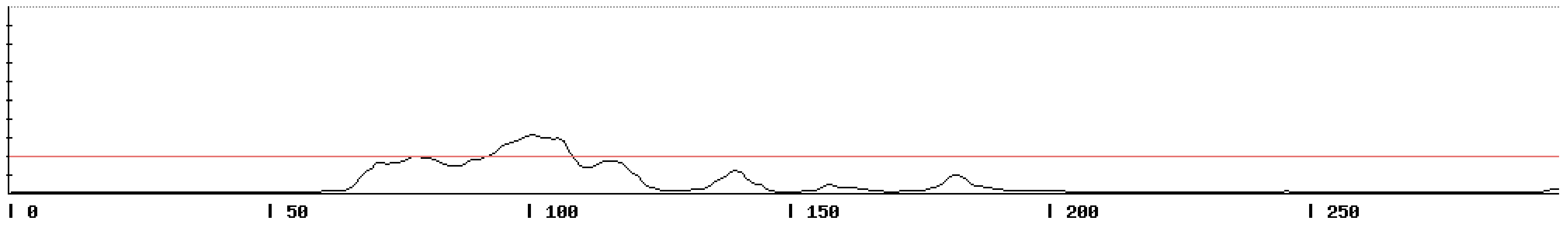

Predictions for Lotusjaponicus_chr1.LjT35C22.100.r2.m

Viterbi Path

Viterbi Path

Posterior @ 0.2

Posterior @ 0.2

Posterior Graphic. Threshold Marked @ 0.2

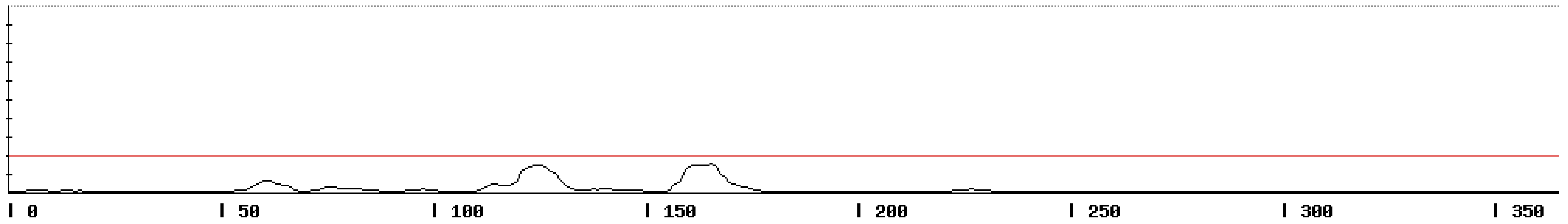

Predictions for Lotusjaponicus_chr1.LjT38B21.180.r2.m	
Viterbi Path	
Viterbi Path	
Posterior @ 0.2	163 - KNDEKKVKKTRGSKEGNSNGGK - 184
Posterior @ 0.2	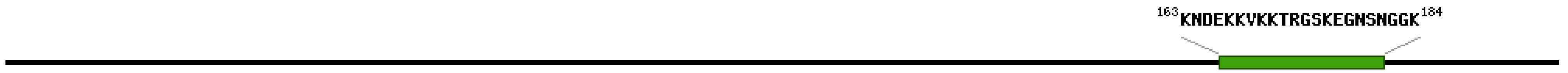
Posterior Graphic. Threshold Marked @ 0.2	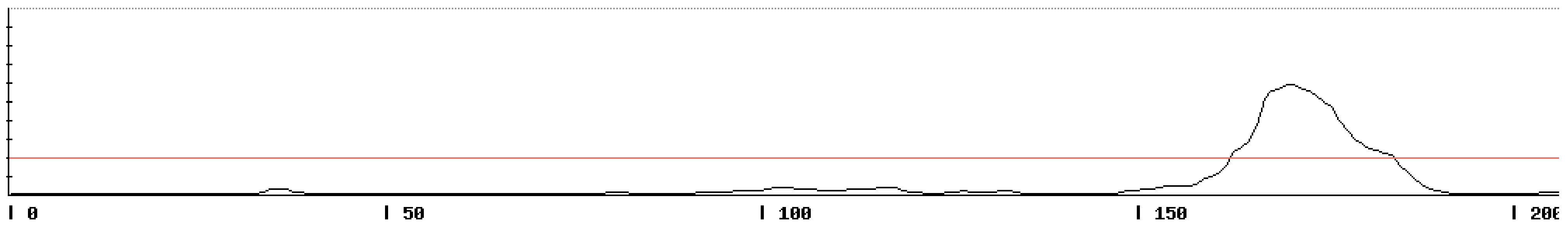

Predictions for Lotusjaponicus_chr1.LjT43D18.120.r2.d	
Viterbi Path	
Viterbi Path	
Posterior @ 0.2	
Posterior @ 0.2	
Posterior Graphic. Threshold Marked @ 0.2	

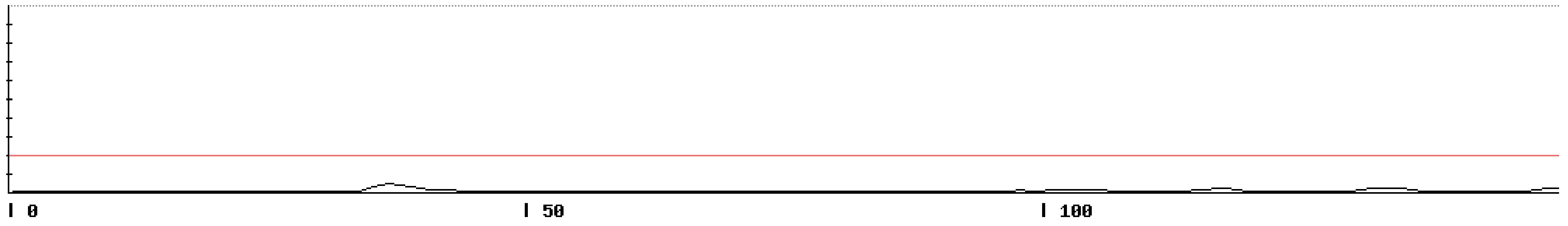

Predictions for Lotusjaponicus_chr2.CM0021.2310.r2.m

Viterbi Path

Viterbi Path

Posterior @ 0.2

Posterior @ 0.2

Posterior Graphic. Threshold Marked @ 0.2

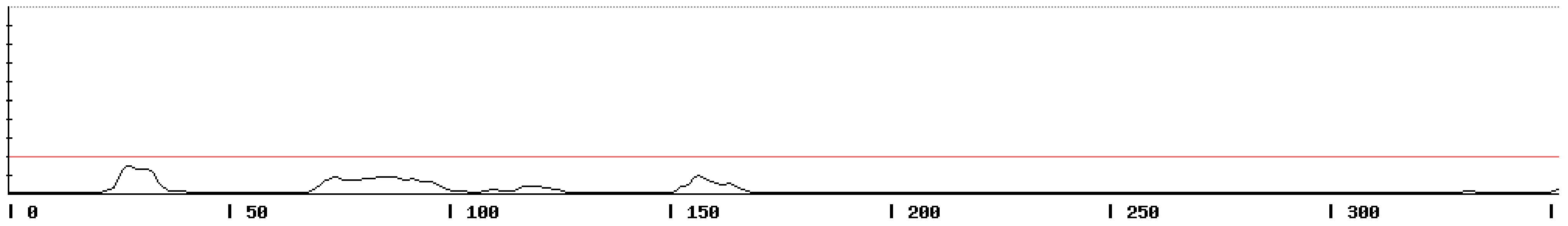

Predictions for Lotusjaponicus_chr2.CM0021.660.r2.m

Viterbi Path

Viterbi Path

Posterior @ 0.2 — 156 - MKKNEKANEKGKGRAG - 171

Posterior @ 0.2

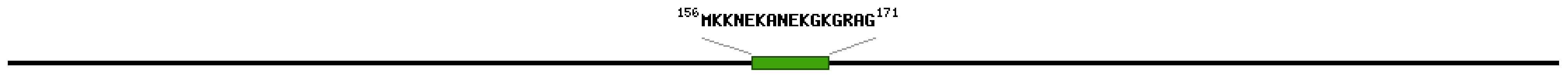

Posterior Graphic. Threshold Marked @ 0.2

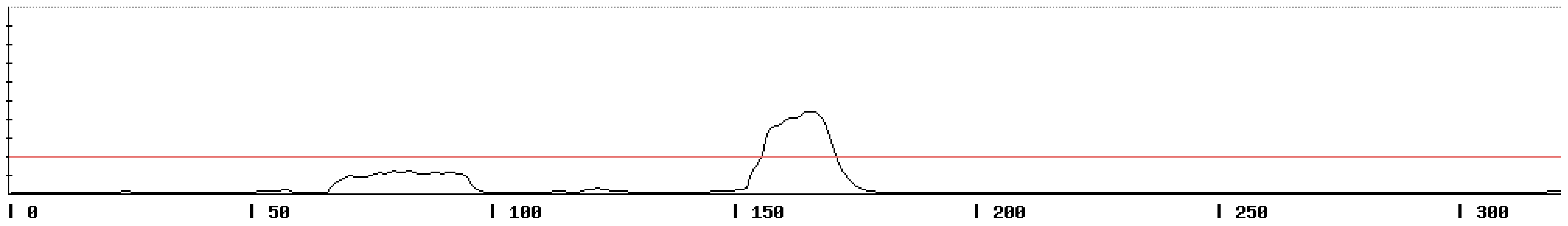

Predictions for Lotusjaponicus_chr2.CM0124.10.r2.m

Viterbi Path	
Viterbi Path	
Posterior @ 0.2	134 - GTRKRRKIHG - 143
Posterior @ 0.2	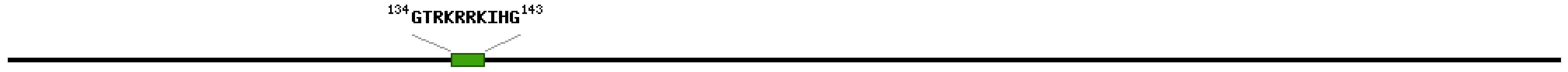
Posterior Graphic. Threshold Marked @ 0.2	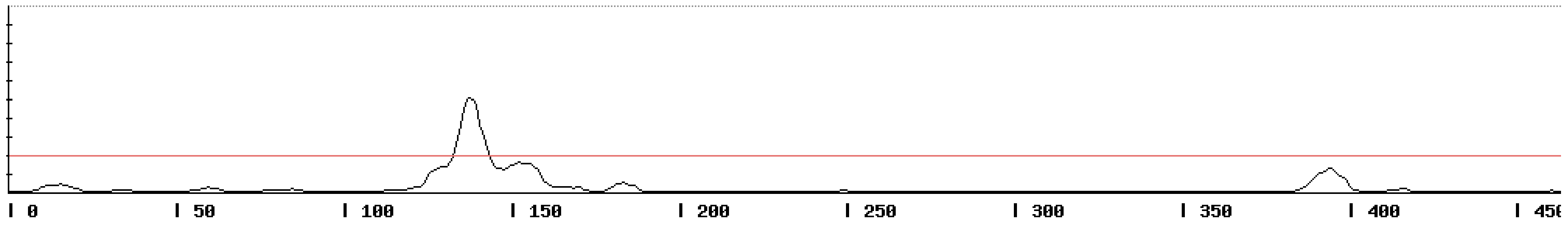

Predictions for Lotusjaponicus_chr2.CM0435.760.r2.m

Viterbi Path	
Viterbi Path	
Posterior @ 0.2	
Posterior @ 0.2	

Posterior Graphic. Threshold Marked @ 0.2

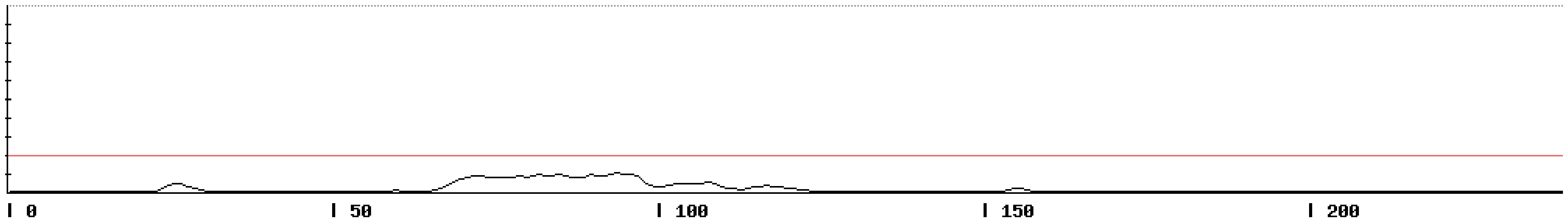

Predictions for Lotusjaponicus_chr2.CM0608.320.r2.m

Viterbi Path

Viterbi Path

Posterior @ 0.2 — 117 - GKPPK - 121

Posterior @ 0.2

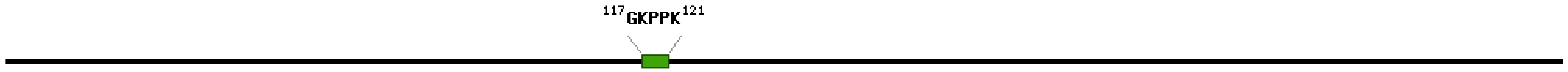

Posterior Graphic. Threshold Marked @ 0.2

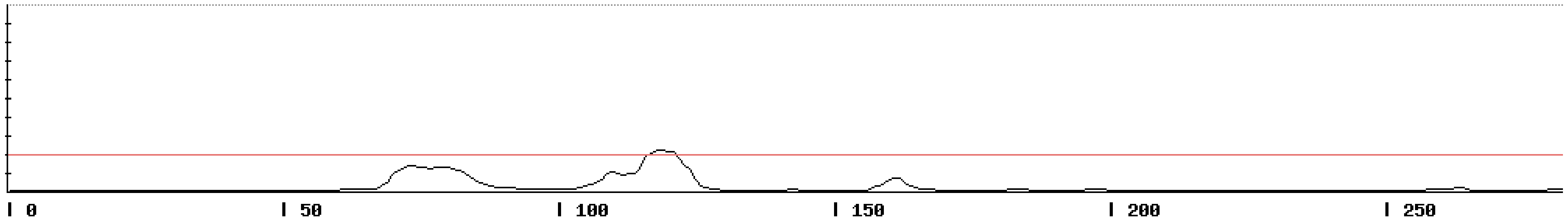

Predictions for Lotusjaponicus_chr2.CM0608.640.r2.d

Viterbi Path

Viterbi Path

Posterior @ 0.2

Posterior @ 0.2

Posterior Graphic. Threshold Marked @ 0.2

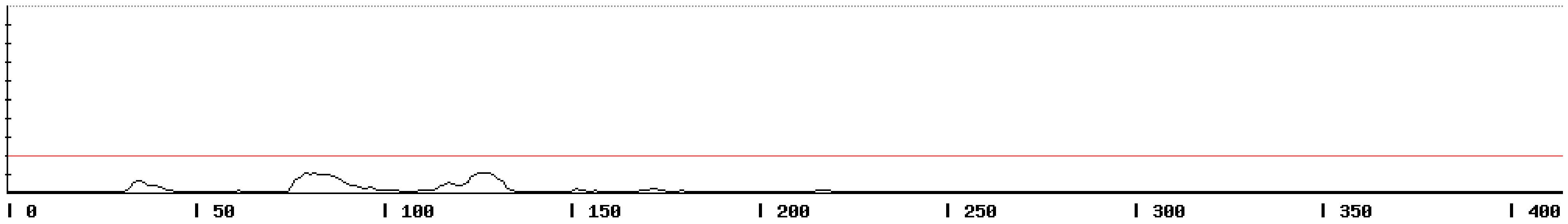

Predictions for Lotusjaponicus_chr2.CM1835.10.r2.m

Viterbi Path

Viterbi Path

Posterior @ 0.2

Posterior @ 0.2

Posterior Graphic. Threshold Marked @ 0.2

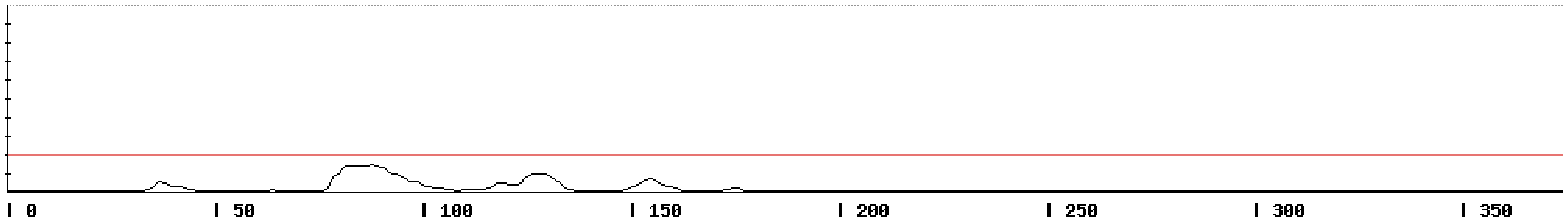

Predictions for Lotusjaponicus_chr2.CM1835.270.r2.d

Viterbi Path

Viterbi Path

Posterior @ 0.2

Posterior @ 0.2

Posterior Graphic. Threshold Marked @ 0.2

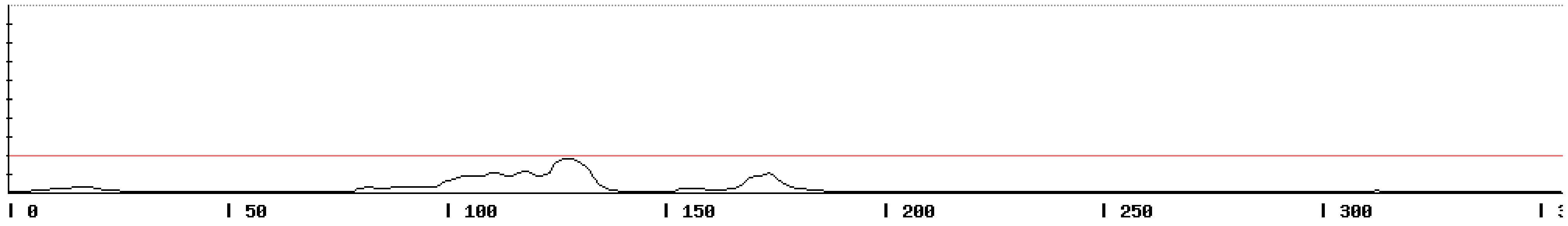

Predictions for Lotusjaponicus_chr2.CM2143.140.r2.d

Viterbi Path

Viterbi Path

Posterior @ 0.2

Posterior @ 0.2

Posterior Graphic. Threshold Marked @ 0.2

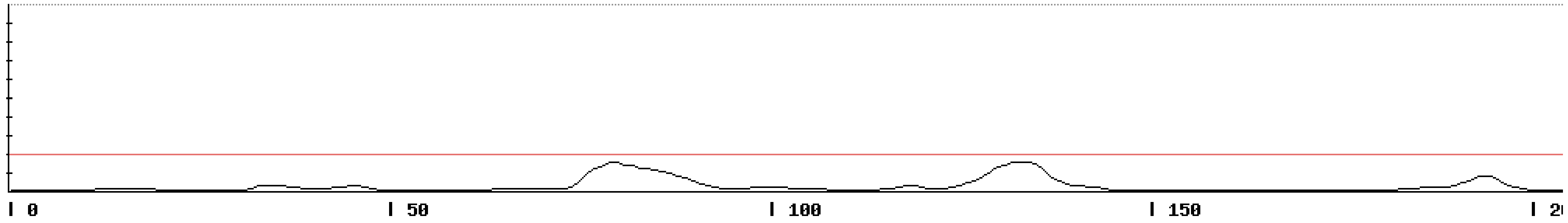

Predictions for Lotusjaponicus_chr2.LjT04L10.100.r2.m

Viterbi Path	
Viterbi Path	
Posterior @ 0.2	37 - RRKVEKKPL - 45
Posterior @ 0.2	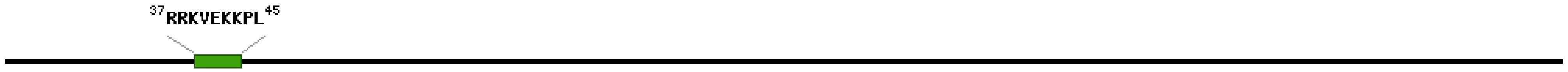
Posterior Graphic. Threshold Marked @ 0.2	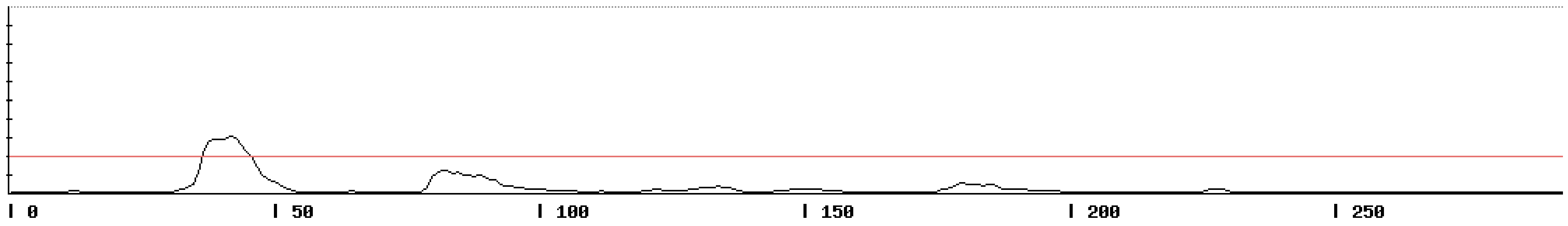

Predictions for Lotusjaponicus_chr2.LjT04L10.120.r2.m

Viterbi Path	
Viterbi Path	
Posterior @ 0.2	
Posterior @ 0.2	
Posterior Graphic. Threshold Marked @ 0.2	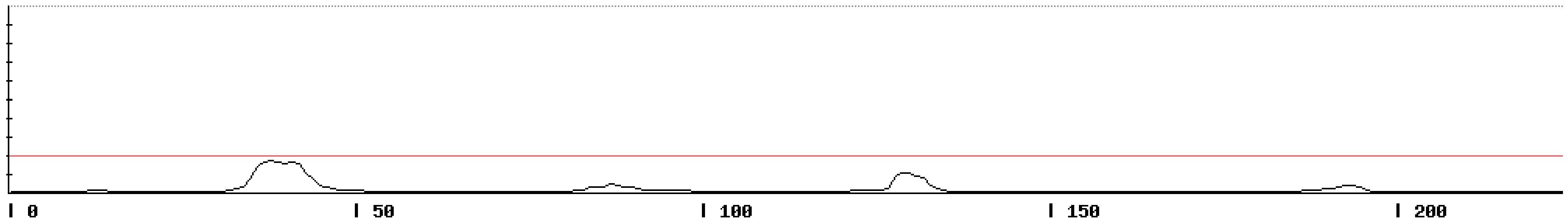

Predictions for Lotusjaponicus_chr2.LjT36E17.140.r2.d	
Viterbi Path	
Viterbi Path	
Posterior @ 0.2	
Posterior @ 0.2	
Posterior Graphic. Threshold Marked @ 0.2	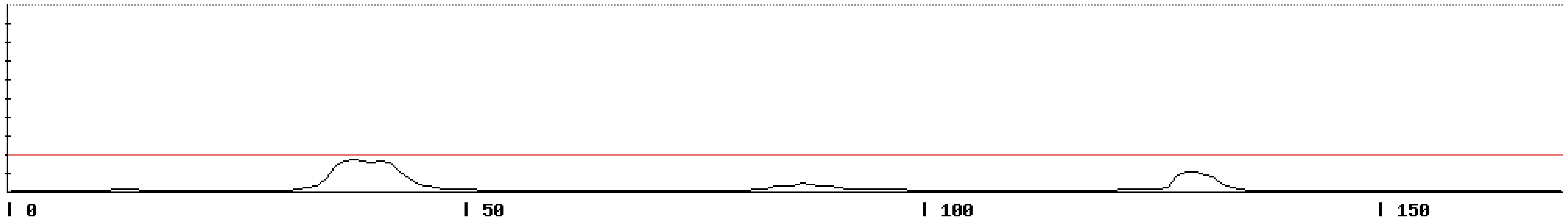

Predictions for Lotusjaponicus_chr3.CM0111.130.r2.d	
Viterbi Path	
Viterbi Path	
Posterior @ 0.2	
Posterior @ 0.2	
Posterior Graphic. Threshold Marked @ 0.2	

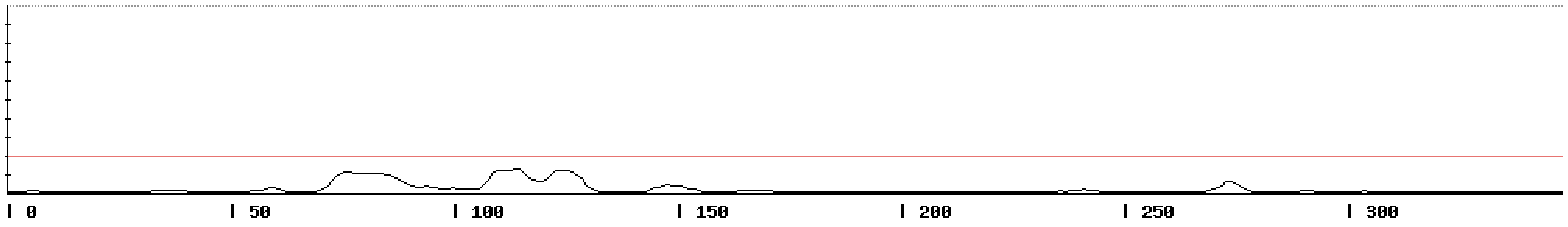

Predictions for Lotusjaponicus_chr3.CM0160.820.r2.d

Viterbi Path	
Viterbi Path	
Posterior @ 0.2	124 - TGTRKRRKVH - 133
	148 - K - 148
	175 - RK - 176
Posterior @ 0.2	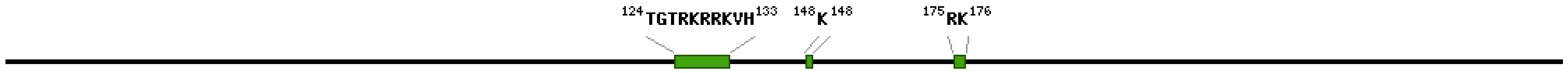
Posterior Graphic. Threshold Marked @ 0.2	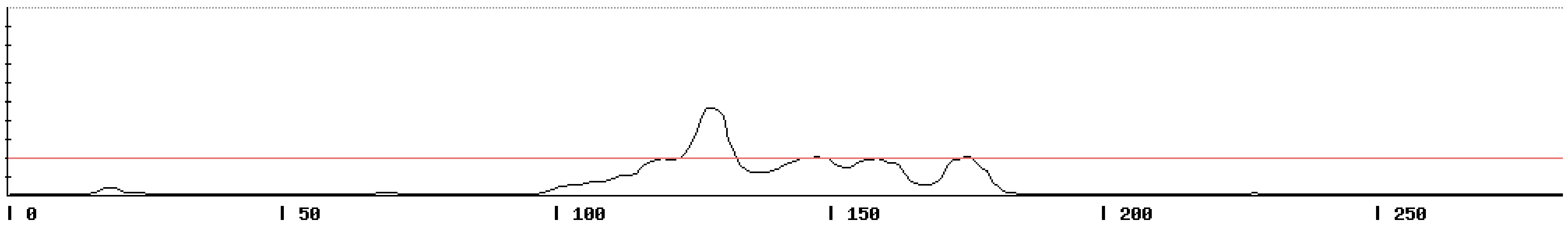

Predictions for Lotusjaponicus_chr3.CM0176.150.r2.m

Viterbi Path	
Viterbi Path	
Posterior @ 0.2	85 - RGRKYR - 90
Posterior @ 0.2	

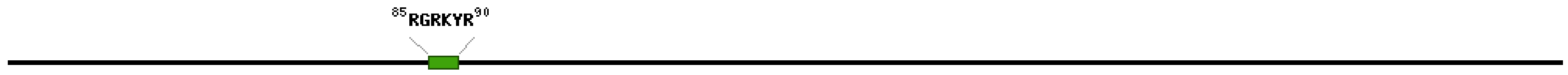

Posterior Graphic. Threshold Marked @ 0.2

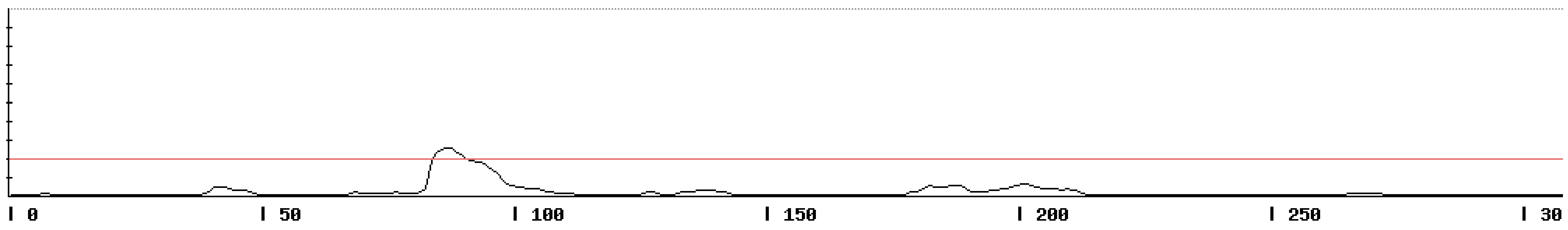

Predictions for Lotusjaponicus_chr3.CM0190.100.r2.d

Viterbi Path	
Viterbi Path	
Posterior @ 0.2	166 - FKKKDEKKVK - 175
Posterior @ 0.2	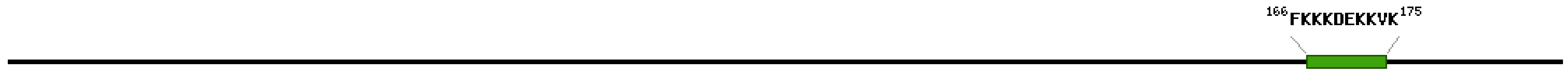
Posterior Graphic. Threshold Marked @ 0.2	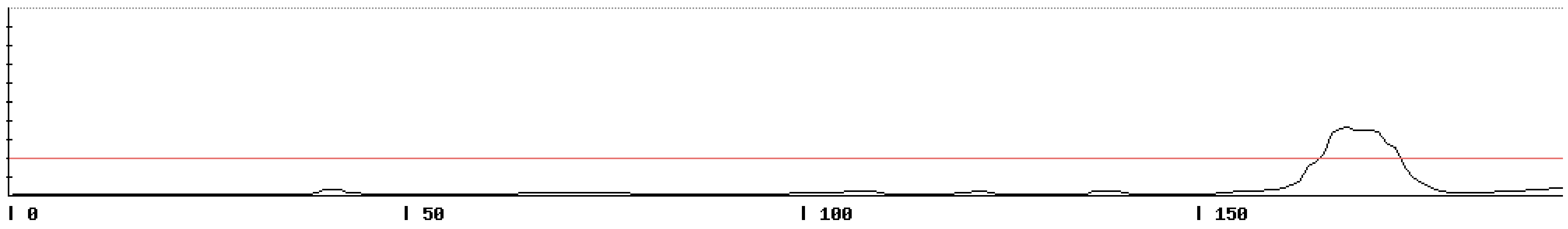

Predictions for Lotusjaponicus_chr3.CM0208.570.r2.m

Viterbi Path	
Viterbi Path	

Posterior @ 0.2	310 - GKSKRVKRE - 318
	693 - TRKMKKPEKDHVI - 705

Posterior @ 0.2

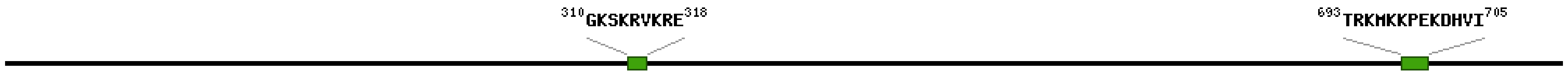

Posterior Graphic. Threshold Marked @ 0.2

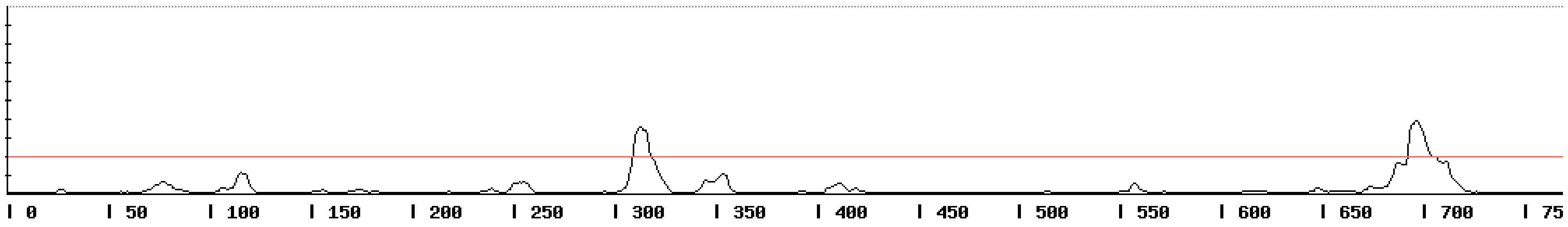

Predictions for Lotusjaponicus_chr3.CM0208.580.r2.m

Viterbi Path

Viterbi Path

Posterior @ 0.2

Posterior @ 0.2

Posterior Graphic. Threshold Marked @ 0.2

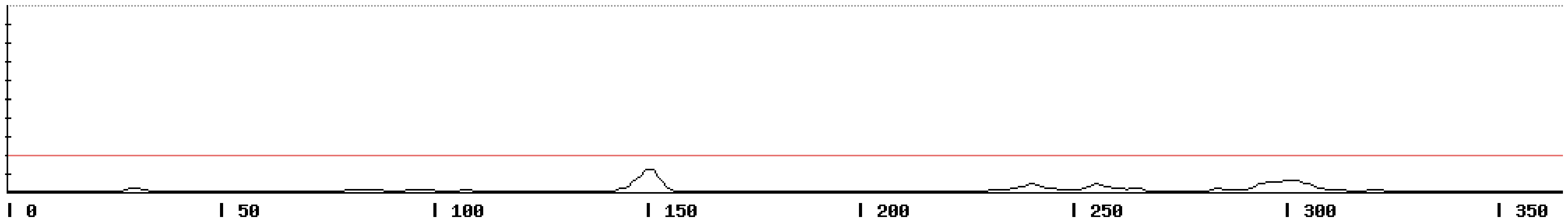

Predictions for Lotusjaponicus_chr3.CM0279.160.r2.d

Viterbi Path

Viterbi Path

Posterior @ 0.2 — 83 - RGRK - 86

Posterior @ 0.2

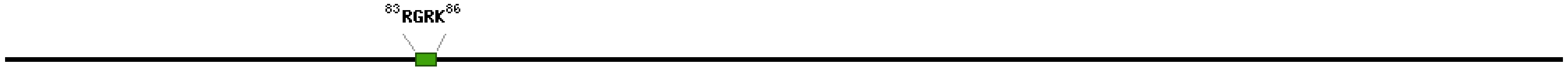

Posterior Graphic. Threshold Marked @ 0.2

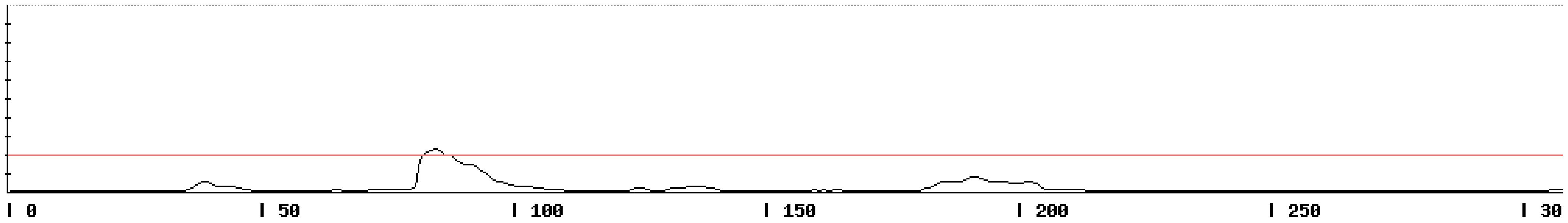

Predictions for Lotusjaponicus_chr3.CM0279.790.r2.a

Viterbi Path

Viterbi Path

Posterior @ 0.2 — 119 - APKGRK - 124

Posterior @ 0.2

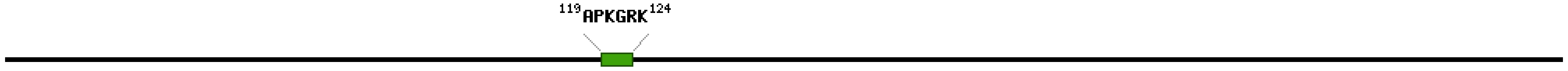

Posterior Graphic. Threshold Marked @ 0.2

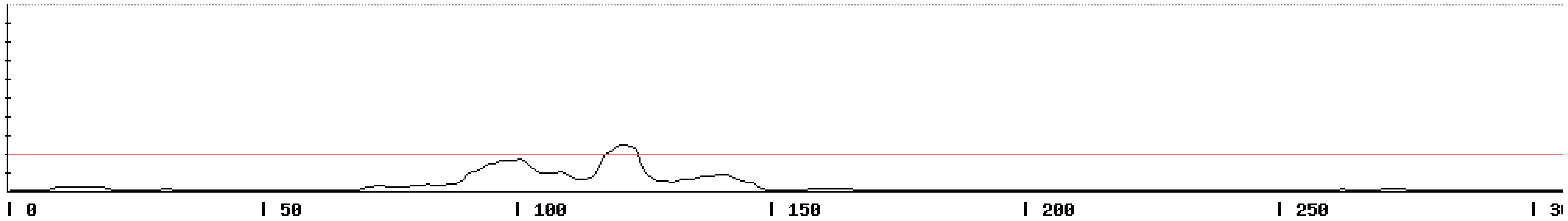

Predictions for Lotusjaponicus_chr3.CM0282.670.r2.m

Viterbi Path

Viterbi Path

Posterior @ 0.2

Posterior @ 0.2

Posterior Graphic. Threshold Marked @ 0.2

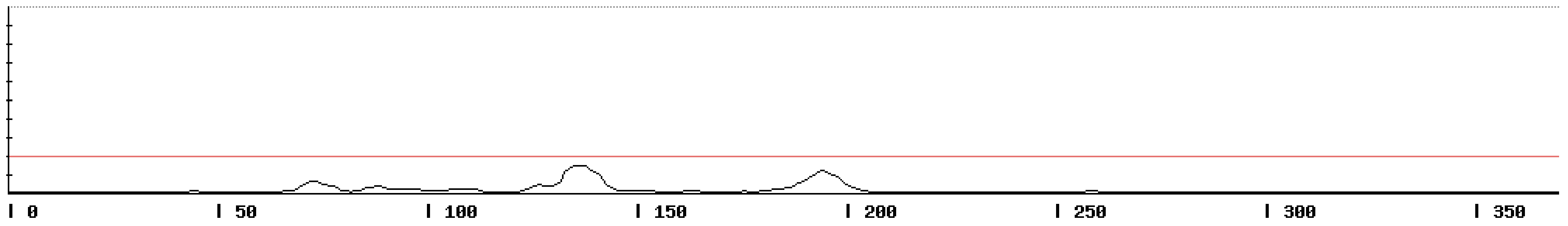

Predictions for Lotusjaponicus_chr3.CM0426.100.r2.a

Viterbi Path

Viterbi Path

Posterior @ 0.2 151 - SKNKDAKKVKNAR - 163

Posterior @ 0.2

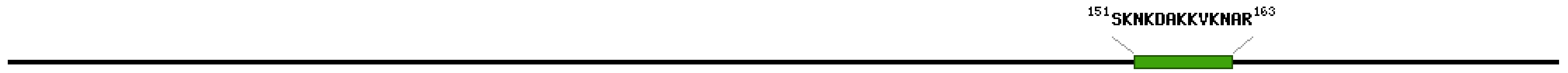

Posterior Graphic. Threshold Marked @ 0.2

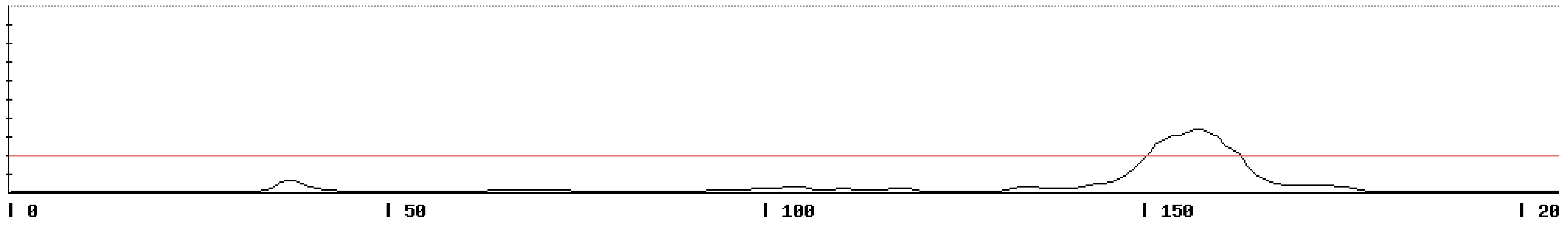

Predictions for Lotusjaponicus_chr3.CM0426.120.r2.a	
Viterbi Path	
Viterbi Path	
Posterior @ 0.2	
Posterior @ 0.2	
Posterior Graphic. Threshold Marked @ 0.2	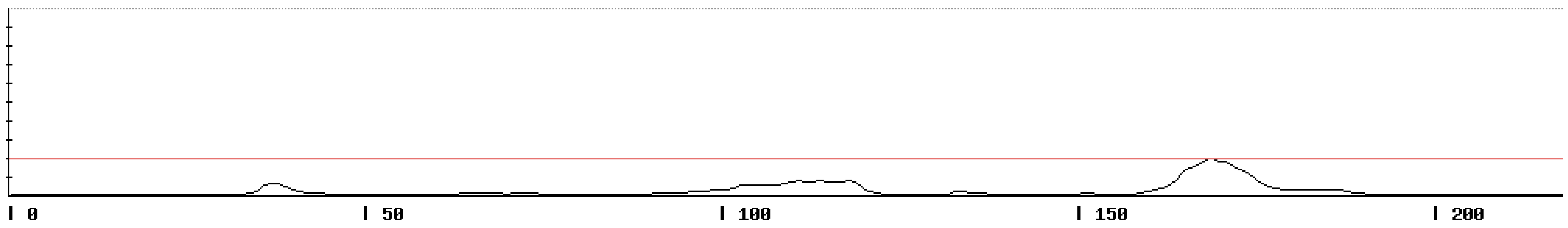

Predictions for Lotusjaponicus_chr3.CM0460.20.r2.d	
Viterbi Path	
Viterbi Path	
Posterior @ 0.2	71 - RDKKHGSGGRPNRTTEKG - 88
Posterior @ 0.2	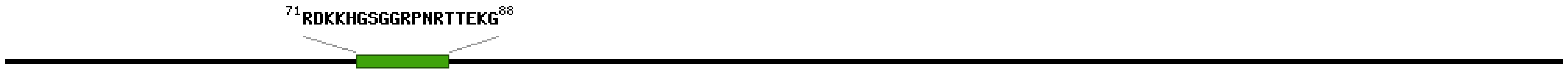
Posterior Graphic. Threshold Marked @ 0.2	

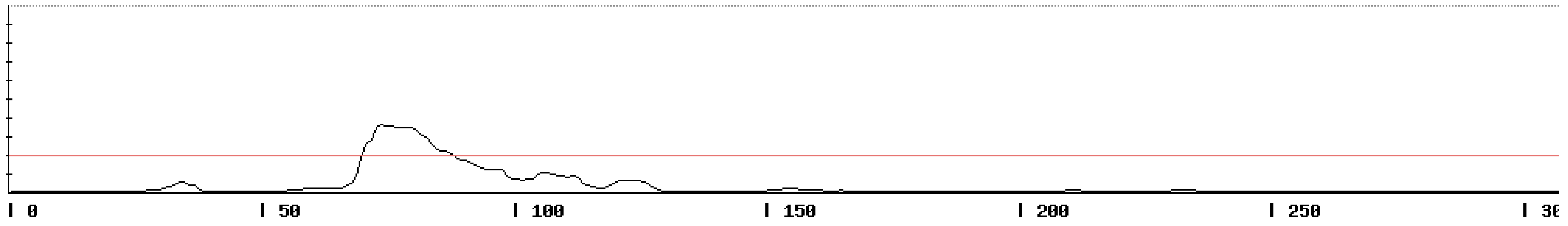

Predictions for Lotusjaponicus_chr3.CM0590.350.r2.d

Viterbi Path	
Viterbi Path	
Posterior @ 0.2	
Posterior @ 0.2	
Posterior Graphic. Threshold Marked @ 0.2	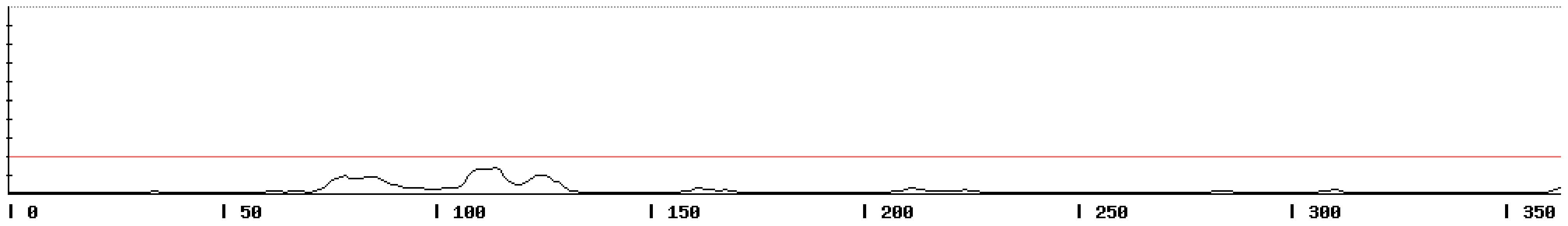

Predictions for Lotusjaponicus_chr3.CM0590.550.r2.a

Viterbi Path	
Viterbi Path	
Posterior @ 0.2	
Posterior @ 0.2	

Posterior Graphic. Threshold Marked @ 0.2

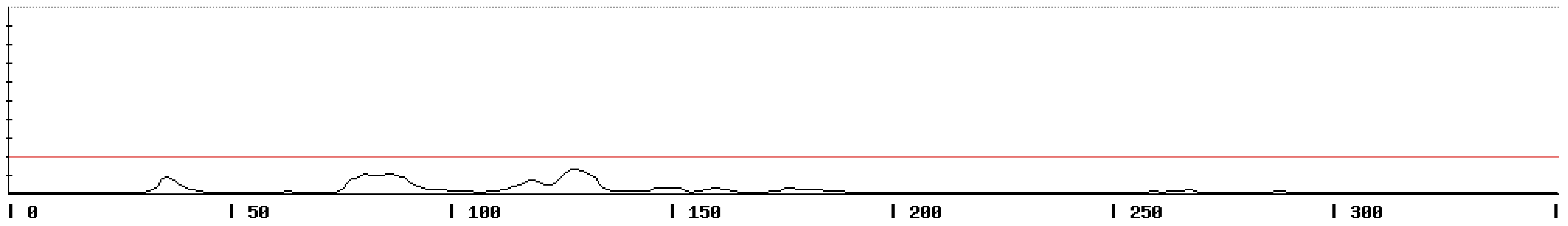

Predictions for Lotusjaponicus_chr3.CM0996.110.r2.a

Viterbi Path

Viterbi Path

Posterior @ 0.2

Posterior @ 0.2

Posterior Graphic. Threshold Marked @ 0.2

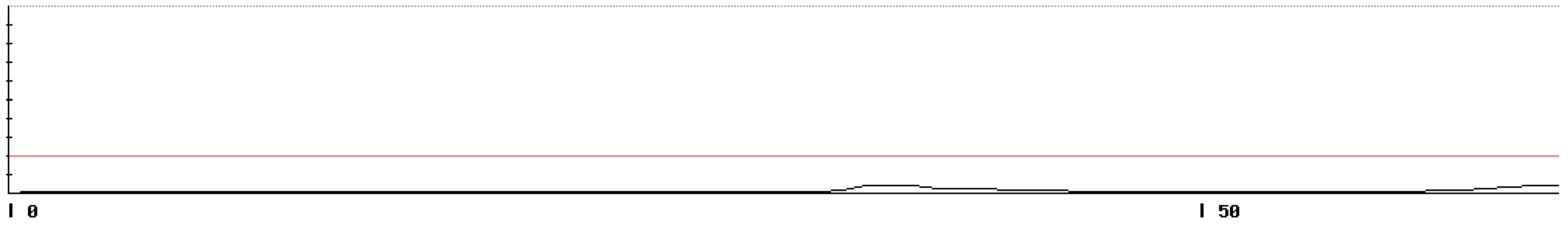

Predictions for Lotusjaponicus_chr3.CM0996.530.r2.a

Viterbi Path

Viterbi Path

Posterior @ 0.2

Posterior @ 0.2

Posterior Graphic. Threshold Marked @ 0.2

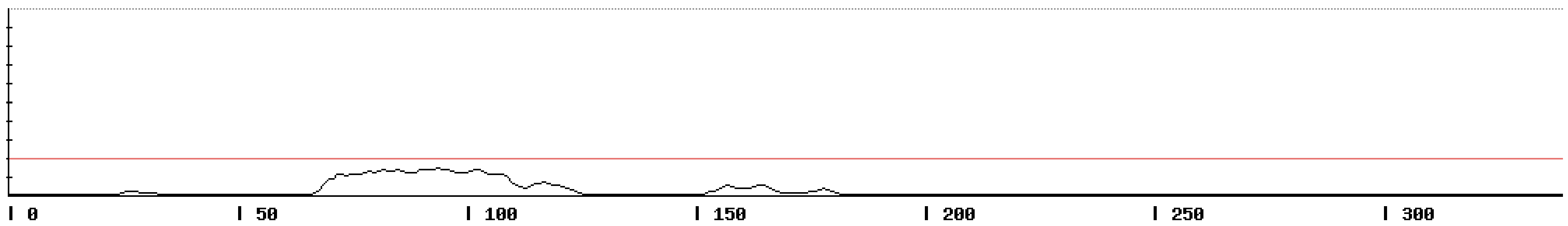

Predictions for Lotusjaponicus_chr3.LjB13E21.110.r2.m

Viterbi Path

Viterbi Path

Posterior @ 0.2

Posterior @ 0.2

Posterior Graphic. Threshold Marked @ 0.2

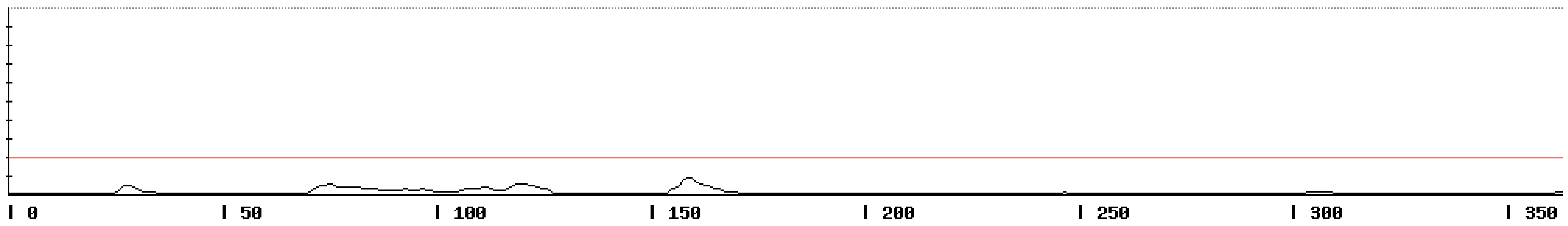

Predictions for Lotusjaponicus_chr3.LjT33C23.90.r2.d

Viterbi Path

Viterbi Path

Posterior @ 0.2	283 - AQRRIRLRVKRPRRSAEGAR - 302
Posterior @ 0.2	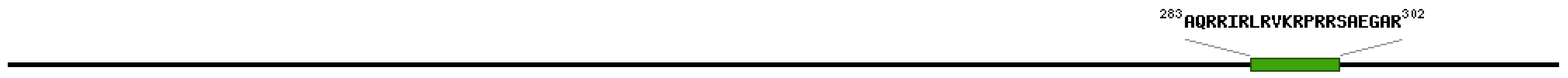
Posterior Graphic. Threshold Marked @ 0.2	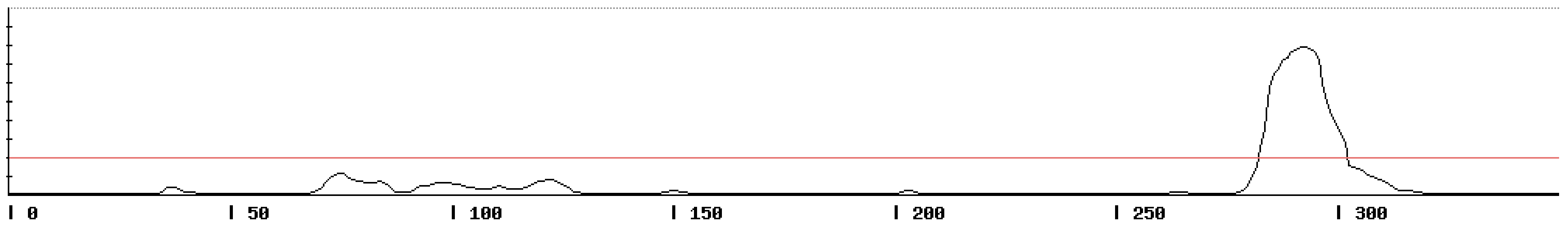

Predictions for Lotusjaponicus_chr4.CM0087.290.r2.d

Viterbi Path	
Viterbi Path	
Posterior @ 0.2	123 - KGRAPKG - 129
Posterior @ 0.2	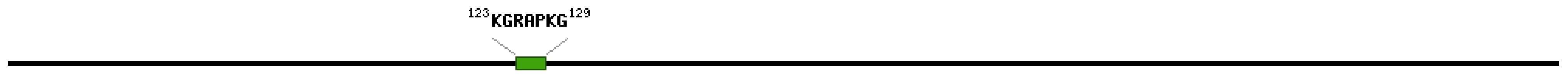
Posterior Graphic. Threshold Marked @ 0.2	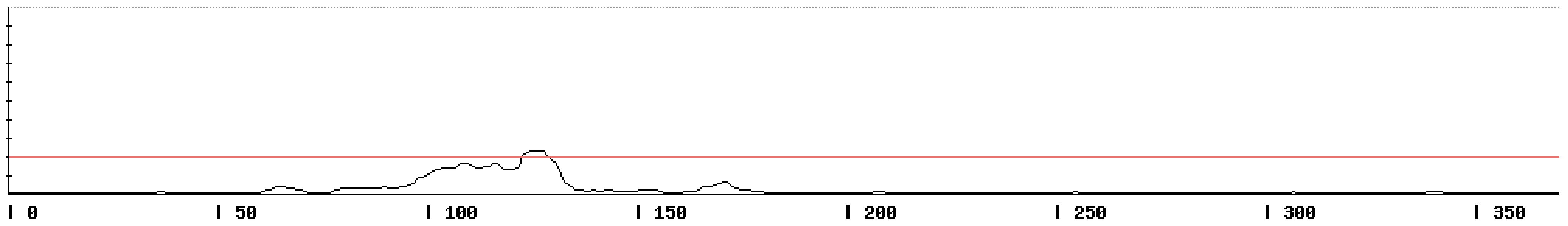

Predictions for Lotusjaponicus_chr4.CM0175.500.r2.m

Viterbi Path	
Viterbi Path	

Posterior @ 0.2

Posterior @ 0.2

Posterior Graphic. Threshold Marked @ 0.2

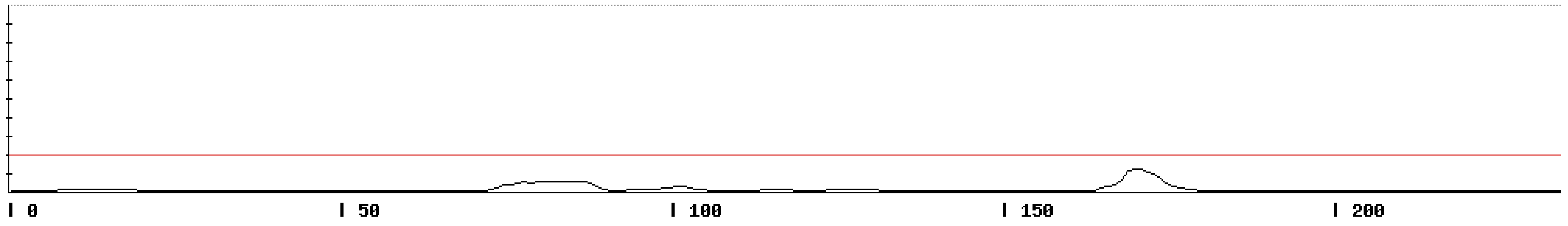

Predictions for Lotusjaponicus_chr4.CM0337.760.r2.m

Viterbi Path

Viterbi Path

Posterior @ 0.2

Posterior @ 0.2

Posterior Graphic. Threshold Marked @ 0.2

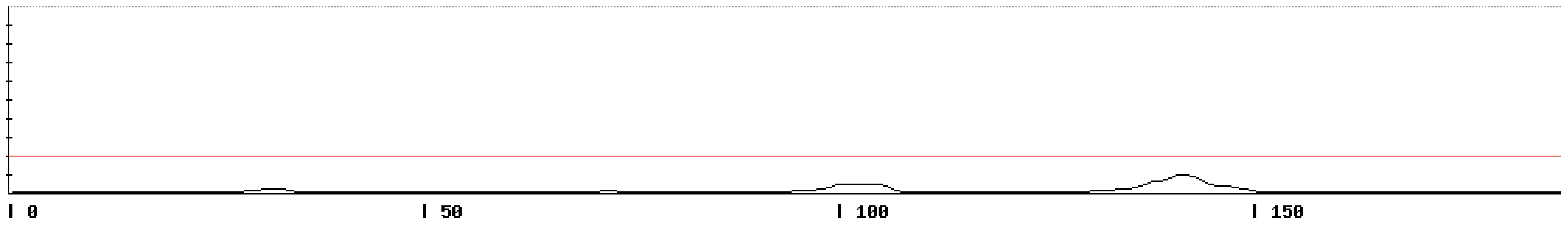

Predictions for Lotusjaponicus_chr4.CM0432.2580.r2.a

Viterbi Path	
Viterbi Path	
Posterior @ 0.2	134 - TRKRRKVH - 141<br>309 - RK - 310
Posterior @ 0.2	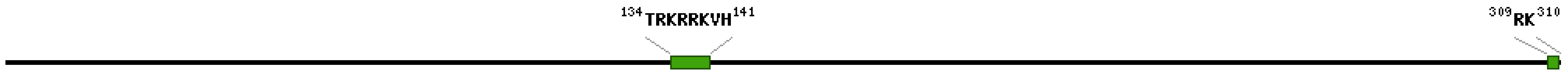
Posterior Graphic. Threshold Marked @ 0.2	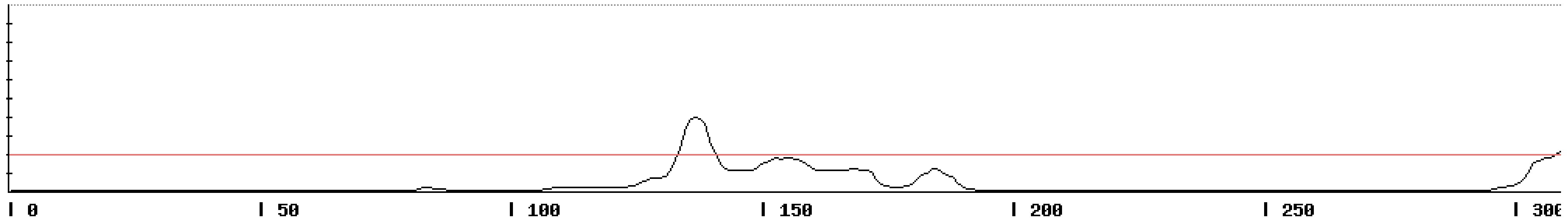

Predictions for Lotusjaponicus_chr4.CM0432.810.r2.m

Viterbi Path	
Viterbi Path	
Posterior @ 0.2	
Posterior @ 0.2	
Posterior Graphic. Threshold Marked @ 0.2	

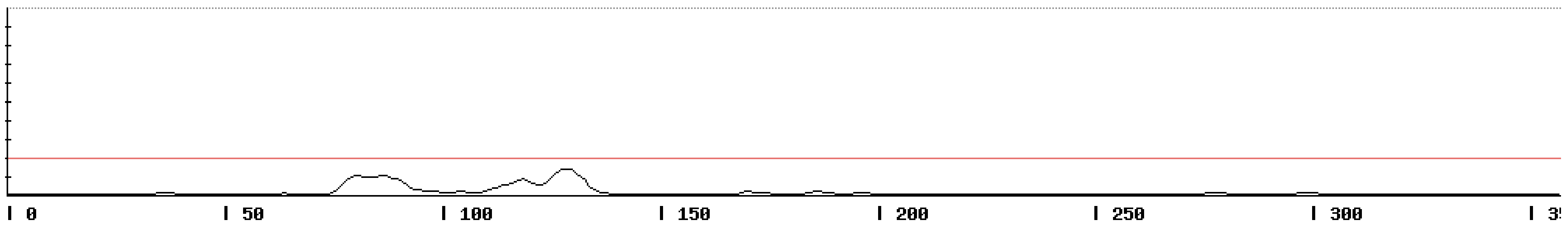

Predictions for Lotusjaponicus_chr4.CM0525.80.r2.d

Viterbi Path

Viterbi Path

Posterior @ 0.2

Posterior @ 0.2

Posterior Graphic. Threshold Marked @ 0.2

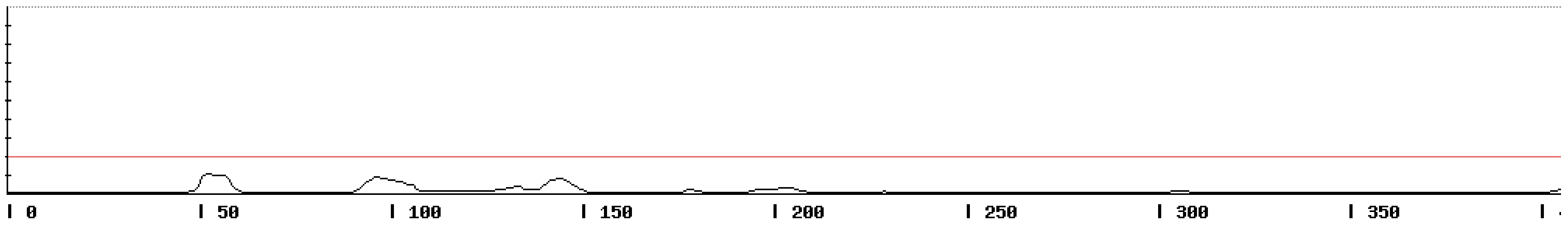

Predictions for Lotusjaponicus_chr4.CM0617.60.r2.d

Viterbi Path

Viterbi Path

Posterior @ 0.2

121 - GTRKRRK - 127

Posterior @ 0.2

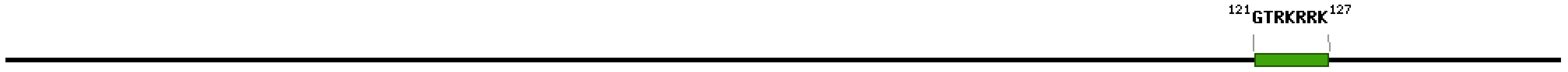

Posterior Graphic. Threshold Marked @ 0.2

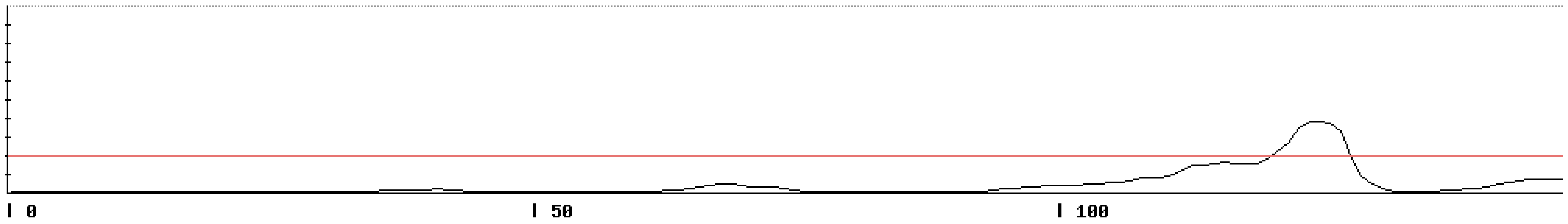

Predictions for Lotusjaponicus_chr4.CM0680.120.r2.m

Viterbi Path

Viterbi Path

Posterior @ 0.2

Posterior @ 0.2

Posterior Graphic. Threshold Marked @ 0.2

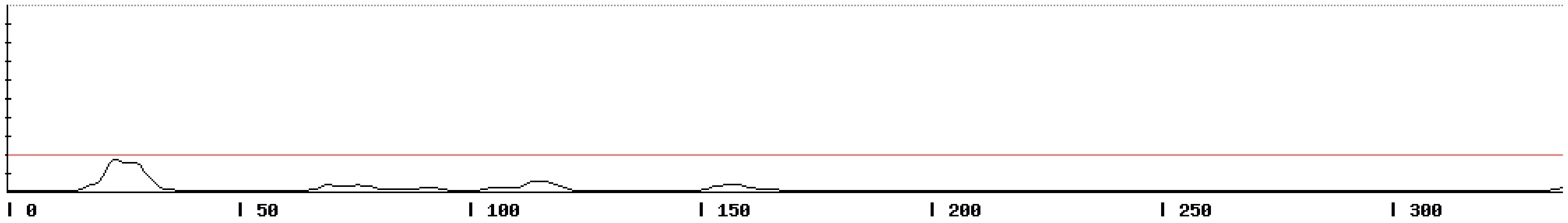

Predictions for Lotusjaponicus_chr4.LjT06B21.210.r2.d

Viterbi Path

Viterbi Path

Posterior @ 0.2	
	77 - SRPNRAA - 83
	88 - WKATGADKPIGKPKTLGIKKAL - 109
	115 - KAP - 117

Posterior @ 0.2	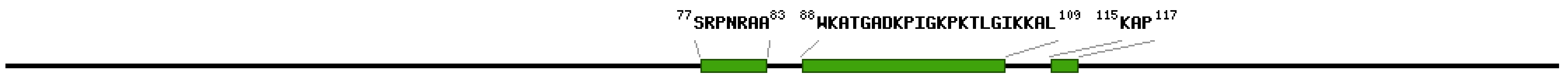
Posterior Graphic. Threshold Marked @ 0.2	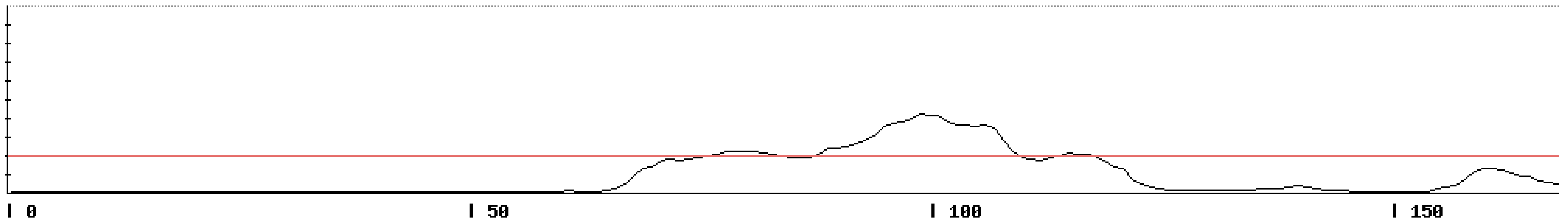

Predictions for Lotusjaponicus_chr4.LjT10K15.60.r2.m

Viterbi Path	
Viterbi Path	
Posterior @ 0.2	
Posterior @ 0.2	
Posterior Graphic. Threshold Marked @ 0.2	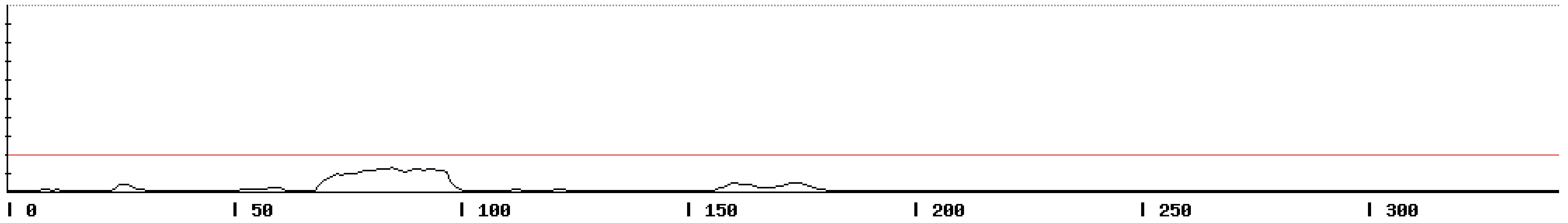

Predictions for Lotusjaponicus_chr5.CM0148.180.r2.a

Viterbi Path	
Viterbi Path	

Posterior @ 0.2

Posterior @ 0.2

Posterior Graphic. Threshold Marked @ 0.2

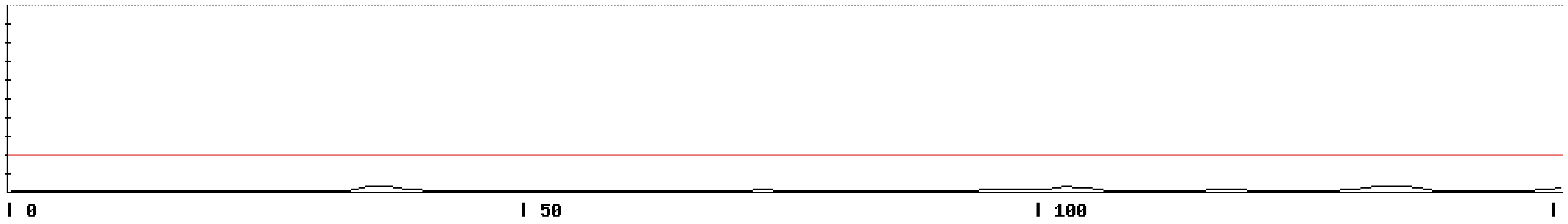

Predictions for Lotusjaponicus_chr5.CM0325.90.r2.m

Viterbi Path

Viterbi Path

Posterior @ 0.2

Posterior @ 0.2

Posterior Graphic. Threshold Marked @ 0.2

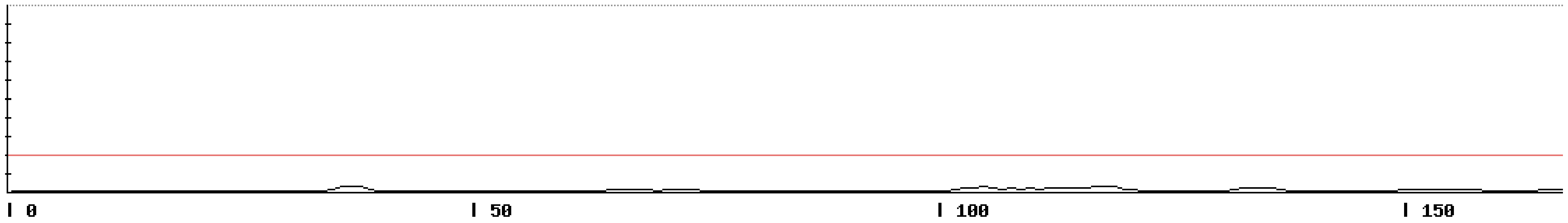

Predictions for Lotusjaponicus_chr5.CM0909.750.r2.m

Viterbi Path

Viterbi Path

Posterior @ 0.2	
Posterior @ 0.2	
Posterior Graphic. Threshold Marked @ 0.2	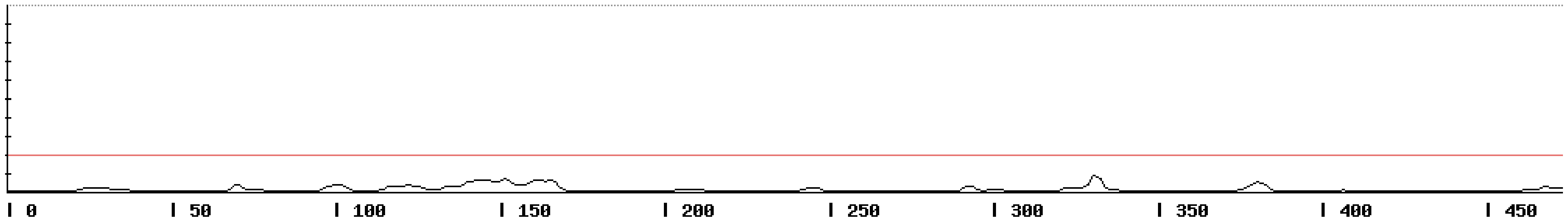

Predictions for Lotusjaponicus_chr5.CM0909.760.r2.m	
Viterbi Path	
Viterbi Path	
Posterior @ 0.2	
Posterior @ 0.2	
Posterior Graphic. Threshold Marked @ 0.2	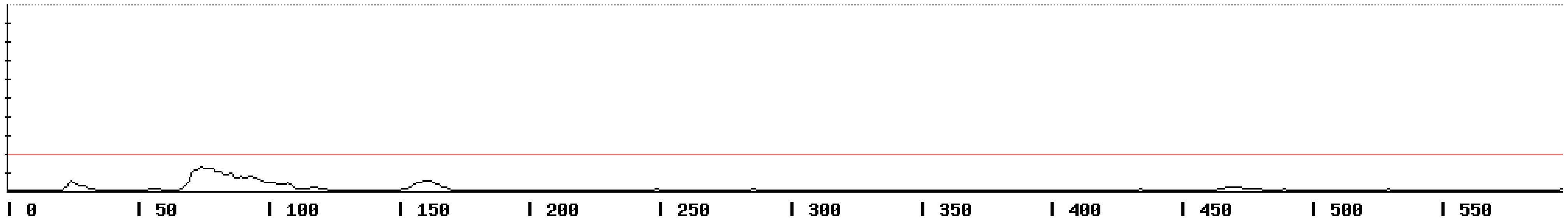

Predictions for Lotusjaponicus_chr6.CM0114.370.r2.m

Viterbi Path

Viterbi Path

Posterior @ 0.2 | 132 - GTRKRRKV - 139

Posterior @ 0.2

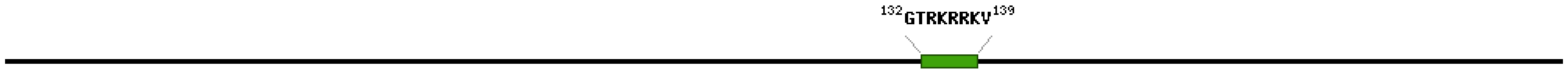

Posterior Graphic. Threshold Marked @ 0.2

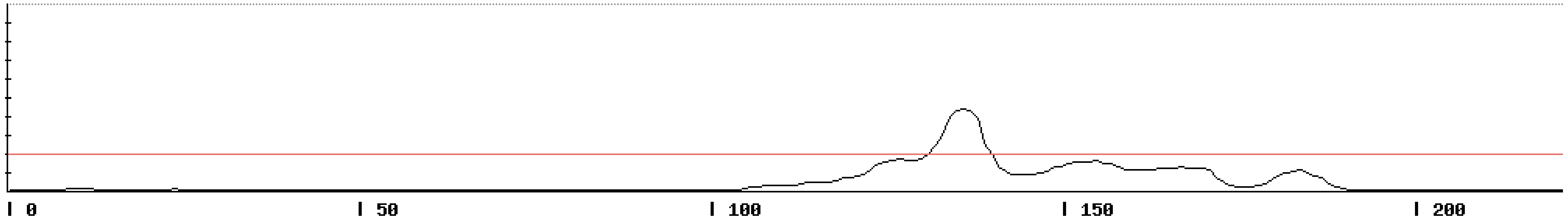

Predictions for Lotusjaponicus_chr6.CM0314.510.r2.d

Viterbi Path

Viterbi Path

Posterior @ 0.2

Posterior @ 0.2

Posterior Graphic. Threshold Marked @ 0.2

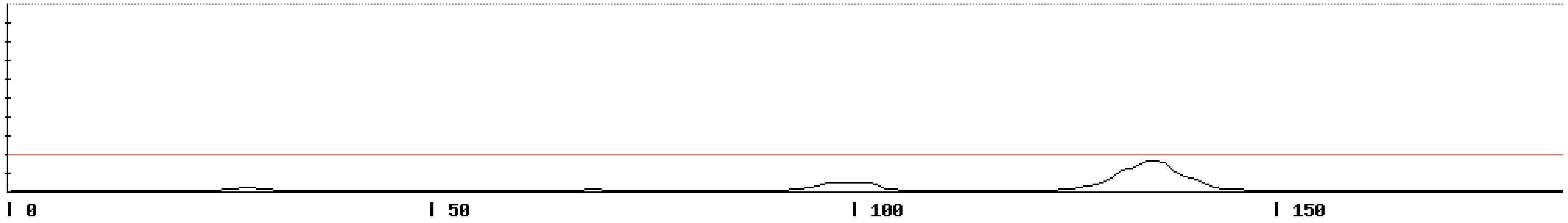

Predictions for Lotusjaponicus_chr6.CM0336.30.r2.m	
Viterbi Path	
Viterbi Path	
Posterior @ 0.2	132 - GTRKRRKV - 139
Posterior @ 0.2	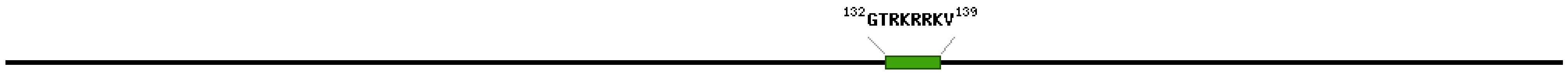
Posterior Graphic. Threshold Marked @ 0.2	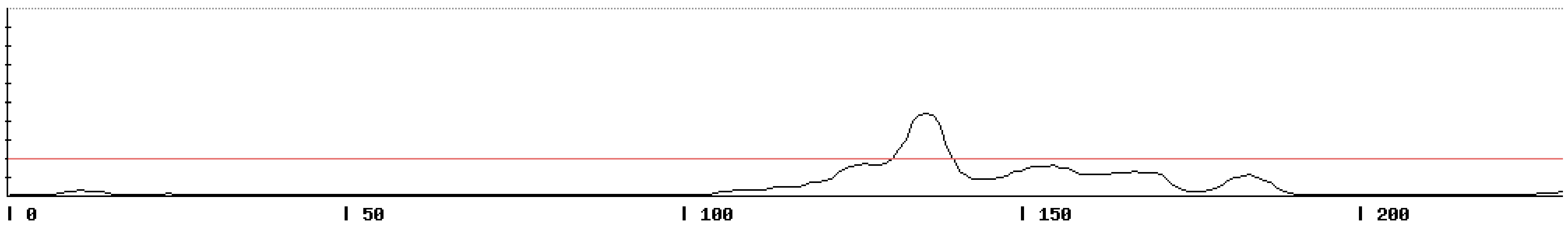

Predictions for Lotusjaponicus_chr6.CM0508.820.r2.a	
Viterbi Path	
Viterbi Path	
Posterior @ 0.2	
Posterior @ 0.2	
Posterior Graphic. Threshold Marked @ 0.2	

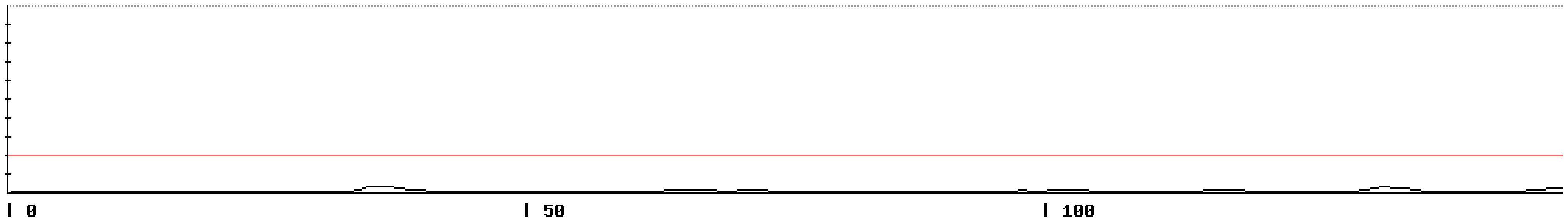

Predictions for Lotusjaponicus_LjSGA_010925.1	
Viterbi Path	
Viterbi Path	
Posterior @ 0.2	86 - RATKSGYWKATGKDRKVH - 103
Posterior @ 0.2	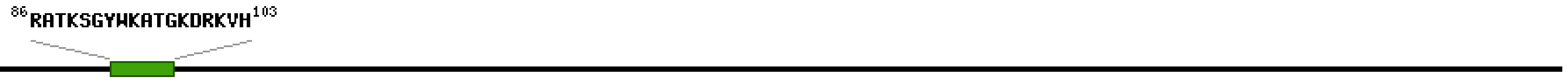
Posterior Graphic. Threshold Marked @ 0.2	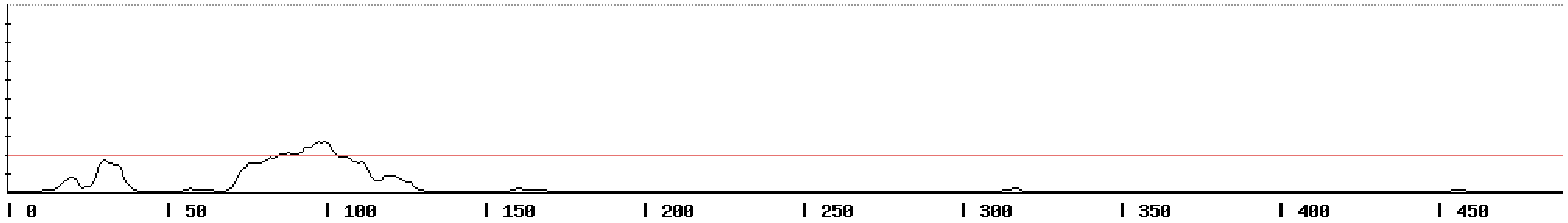

Predictions for Lotusjaponicus_LjSGA_013316.1	
Viterbi Path	
Viterbi Path	
Posterior @ 0.2	143 - KK - 144
Posterior @ 0.2	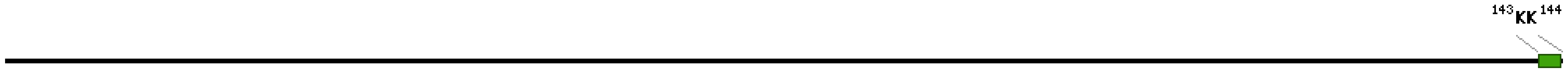

Posterior Graphic. Threshold Marked @ 0.2

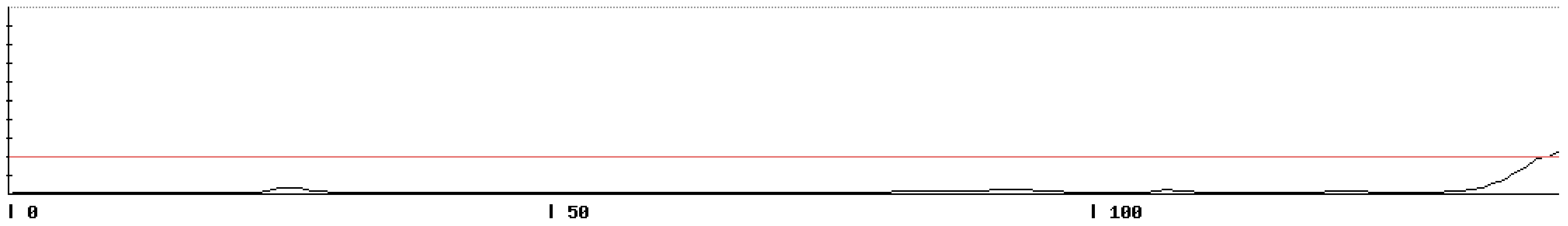

Predictions for Lotusjaponicus_LjSGA_014778.2

Viterbi Path

Viterbi Path

Posterior @ 0.2

77 - RGGRPKR - 83

120 - APRGKK - 125

Posterior @ 0.2

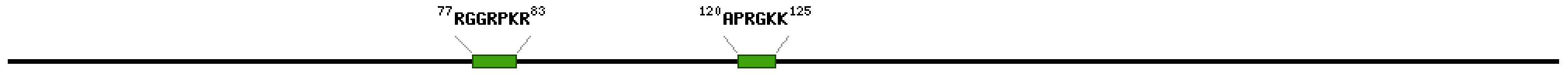

Posterior Graphic. Threshold Marked @ 0.2

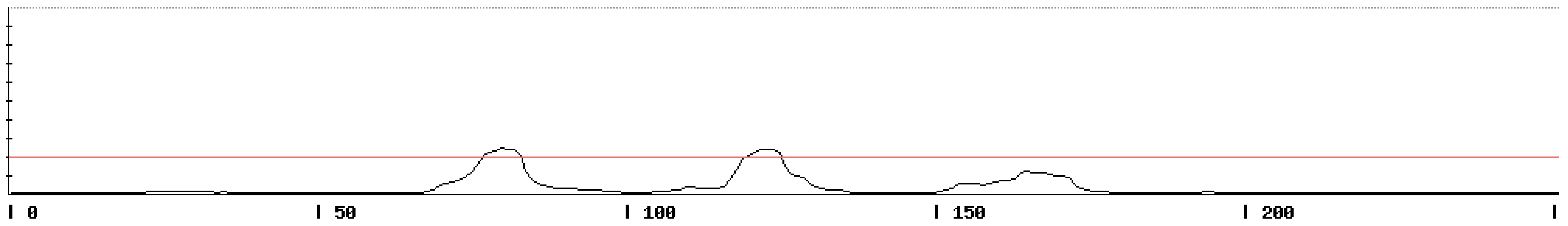

Predictions for Lotusjaponicus_LjSGA_014921.1

Viterbi Path

Viterbi Path

Posterior @ 0.2

Posterior @ 0.2

Posterior Graphic. Threshold Marked @ 0.2

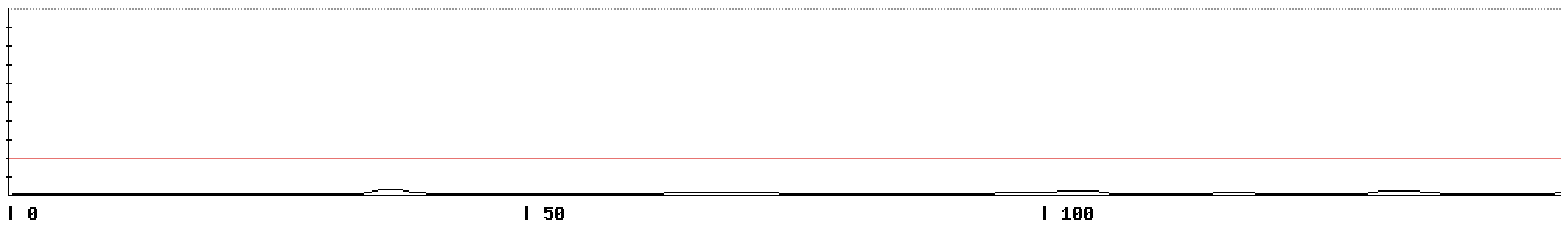

Predictions for Lotusjaponicus_LjSGA_018440.1

Viterbi Path

Viterbi Path

Posterior @ 0.2

Posterior @ 0.2

Posterior Graphic. Threshold Marked @ 0.2

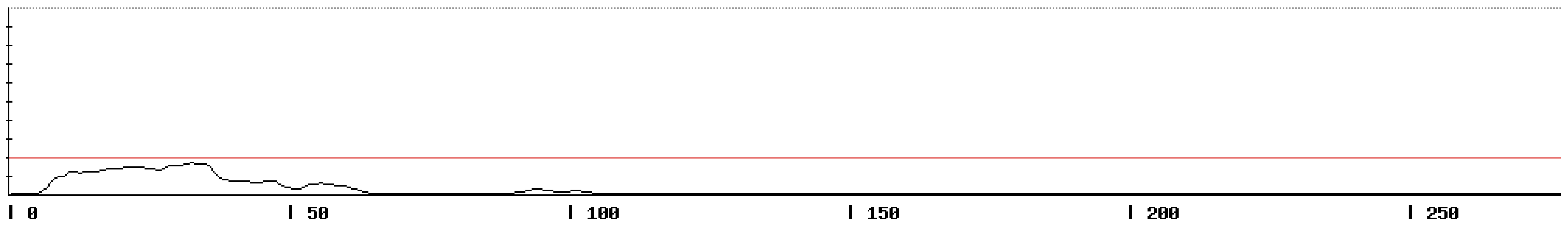

Predictions for Lotusjaponicus_LjSGA_024981.1

Viterbi Path

Viterbi Path

Posterior @ 0.2

Posterior @ 0.2

Posterior Graphic. Threshold Marked @ 0.2

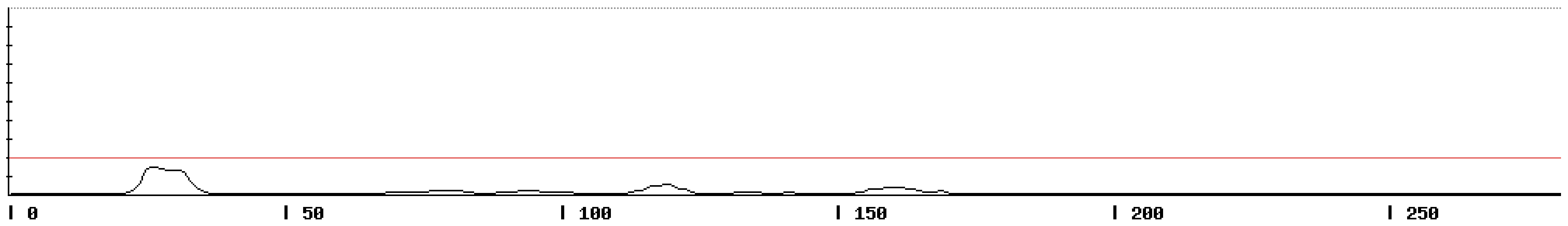

Predictions for Lotusjaponicus_LjSGA_027787.1

Viterbi Path

Viterbi Path

Posterior @ 0.2

Posterior @ 0.2

Posterior Graphic. Threshold Marked @ 0.2

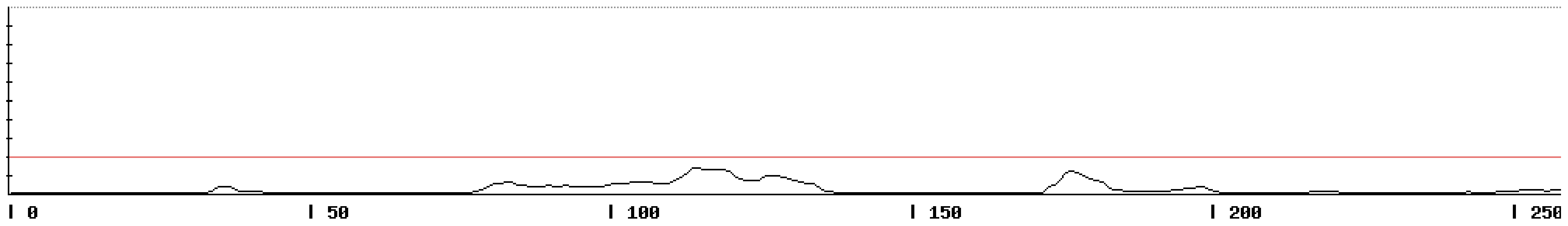

Predictions for Lotusjaponicus_LjSGA_028569.1

Viterbi Path

Viterbi Path

Posterior @ 0.2

Posterior @ 0.2

Posterior Graphic. Threshold Marked @ 0.2

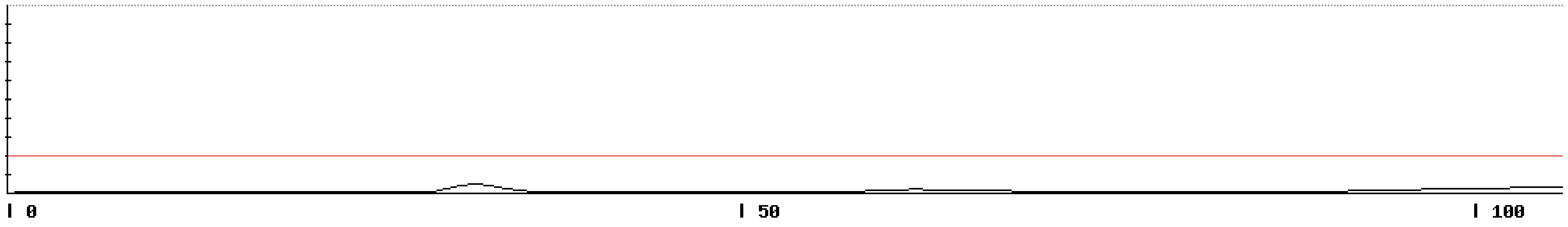

Predictions for Lotusjaponicus_LjSGA_034671.1

Viterbi Path

Viterbi Path

Posterior @ 0.2

Posterior @ 0.2

Posterior Graphic. Threshold Marked @ 0.2

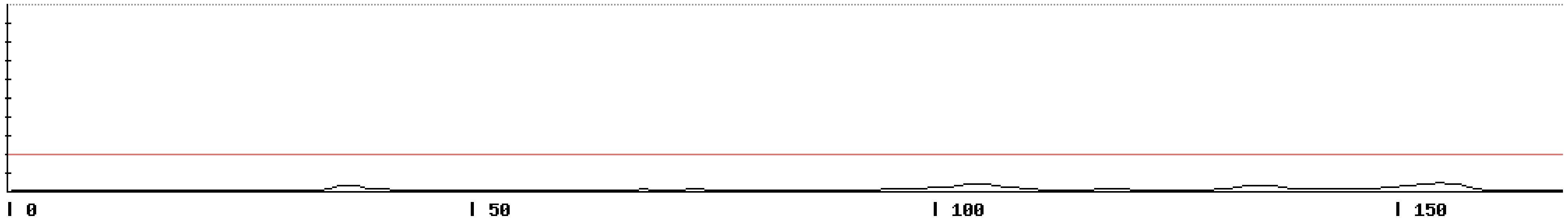

Predictions for Lotusjaponicus_LjSGA_035655.2

Viterbi Path

Viterbi Path

Posterior @ 0.2

Posterior @ 0.2

Posterior Graphic. Threshold Marked @ 0.2

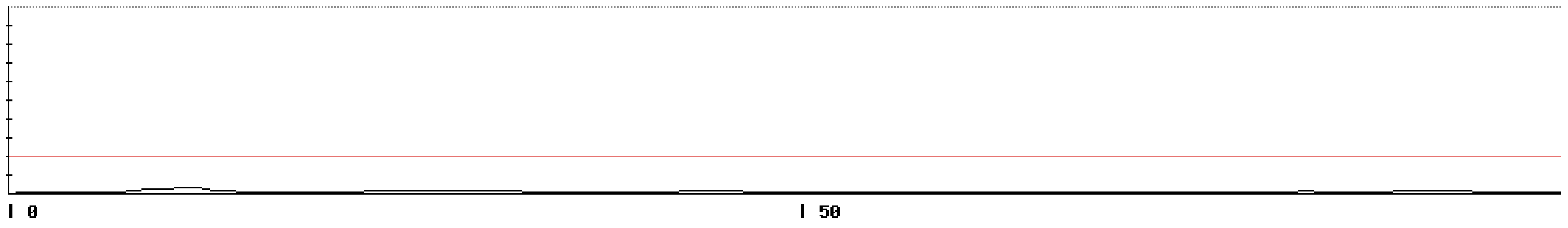

Predictions for Lotusjaponicus_LjSGA_036303.1

Viterbi Path

Viterbi Path

Posterior @ 0.2

Posterior @ 0.2

Posterior Graphic. Threshold Marked @ 0.2

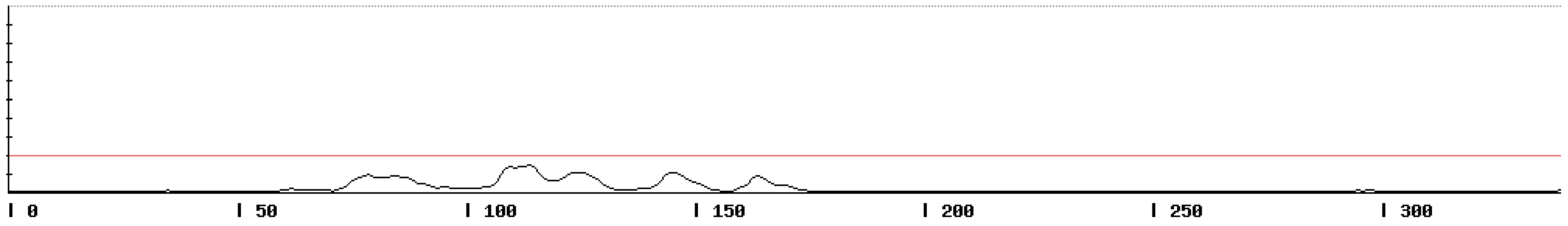

Predictions for Lotusjaponicus_LjSGA_037917.1	
Viterbi Path	
Viterbi Path	
Posterior @ 0.2	
Posterior @ 0.2	
Posterior Graphic. Threshold Marked @ 0.2	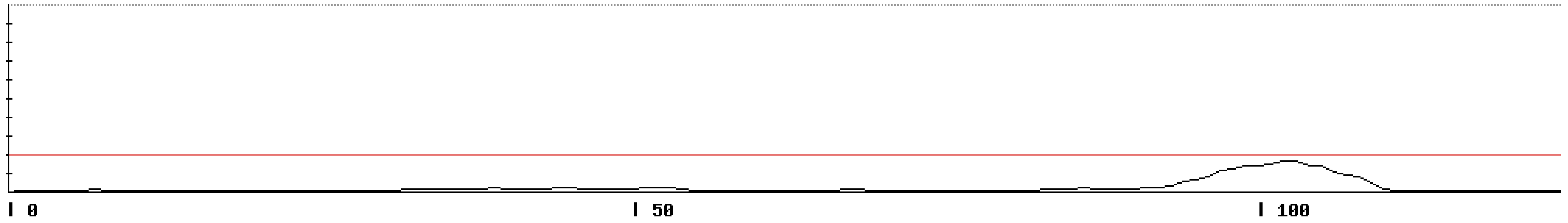

Predictions for Lotusjaponicus_LjSGA_040439.1	
Viterbi Path	
Viterbi Path	
Posterior @ 0.2	
Posterior @ 0.2	
Posterior Graphic. Threshold Marked @ 0.2	

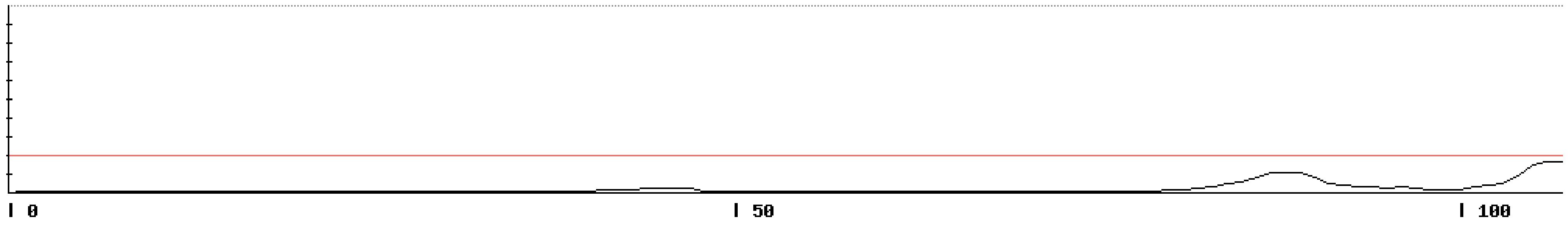

Predictions for Lotusjaponicus_LjSGA_040638.1

Viterbi Path	
Viterbi Path	
Posterior @ 0.2	
Posterior @ 0.2	
Posterior Graphic. Threshold Marked @ 0.2	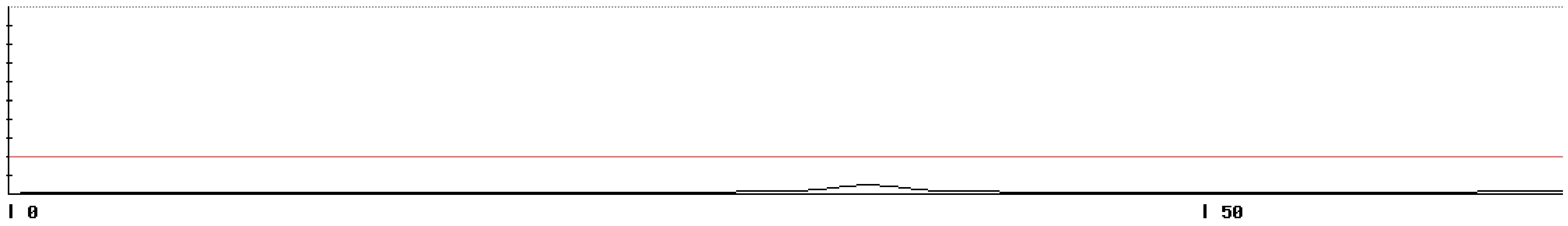

Predictions for Lotusjaponicus_LjSGA_042770.1

Viterbi Path	
Viterbi Path	
Posterior @ 0.2	
Posterior @ 0.2	

Posterior Graphic. Threshold Marked @ 0.2

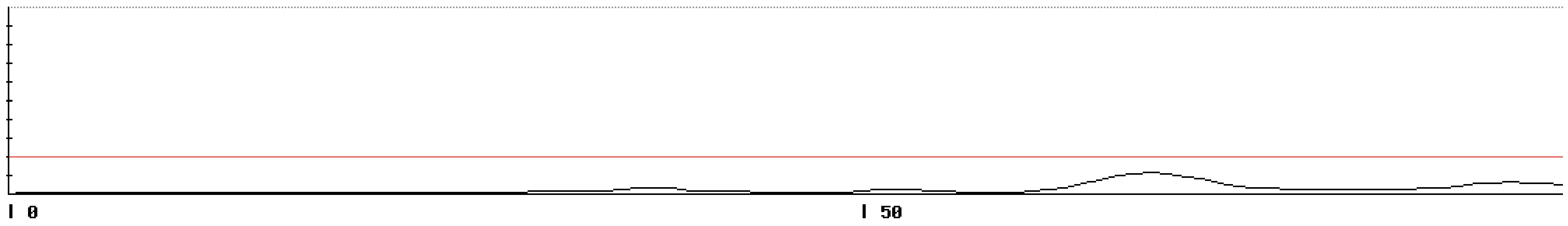

Predictions for Lotusjaponicus_LjSGA_043084.1

Viterbi Path

Viterbi Path

Posterior @ 0.2

Posterior @ 0.2

Posterior Graphic. Threshold Marked @ 0.2

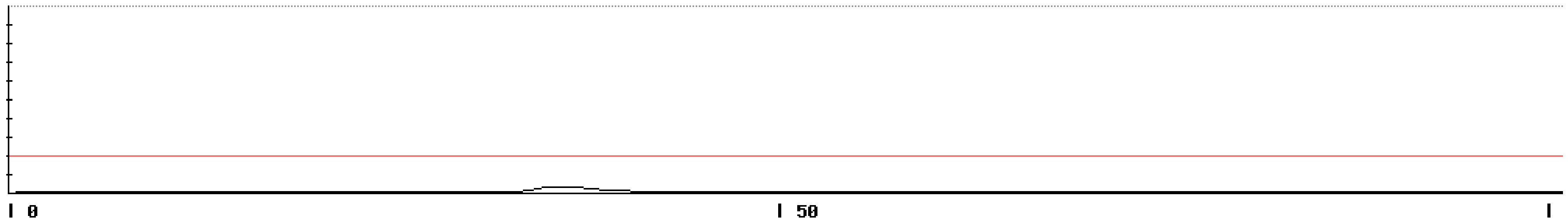

Predictions for Lotusjaponicus_LjSGA_045720.1

Viterbi Path

Viterbi Path

Posterior @ 0.2

Posterior @ 0.2

Posterior Graphic. Threshold Marked @ 0.2

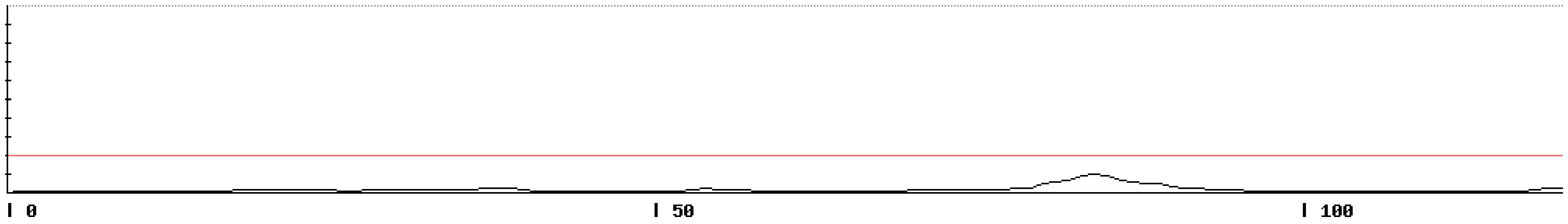

Predictions for Lotusjaponicus_LjSGA_051579.1

Viterbi Path

Viterbi Path

Posterior @ 0.2

Posterior @ 0.2

Posterior Graphic. Threshold Marked @ 0.2

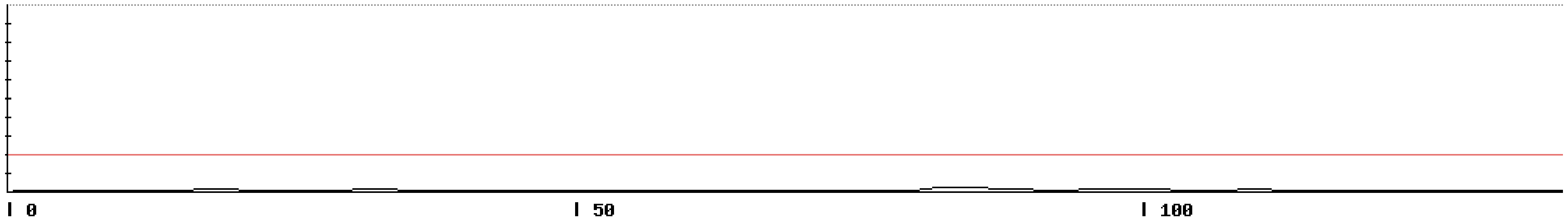

Predictions for Lotusjaponicus_LjSGA_051773.3

Viterbi Path

Viterbi Path

Posterior @ 0.2

Posterior @ 0.2

Posterior Graphic. Threshold Marked @ 0.2

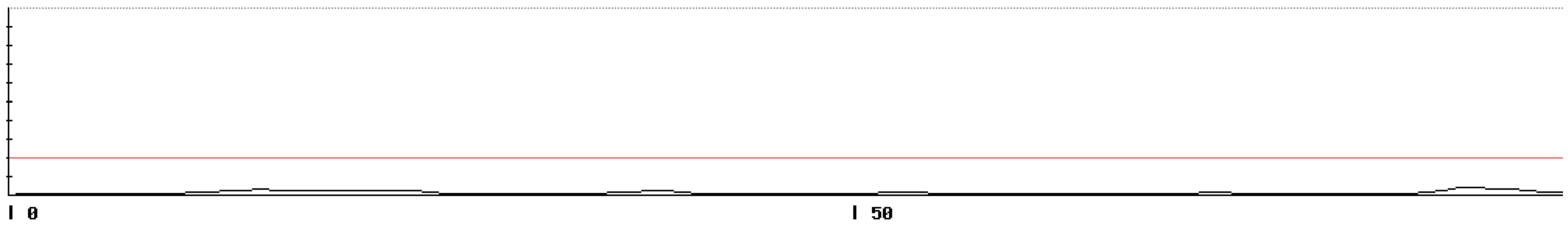

Predictions for Lotusjaponicus_LjSGA_053302.1

Viterbi Path

Viterbi Path

Posterior @ 0.2

Posterior @ 0.2

Posterior Graphic. Threshold Marked @ 0.2

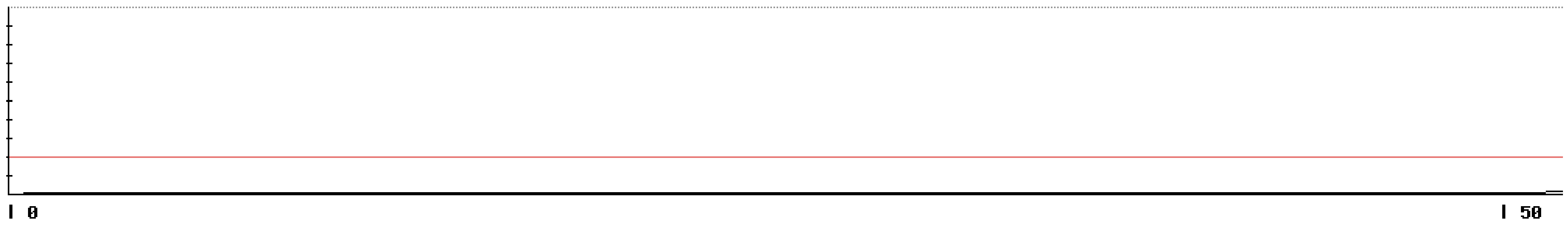

Predictions for Lotusjaponicus_LjSGA_056309.1

Viterbi Path

Viterbi Path

Posterior @ 0.2

Posterior @ 0.2

Posterior Graphic. Threshold Marked @ 0.2

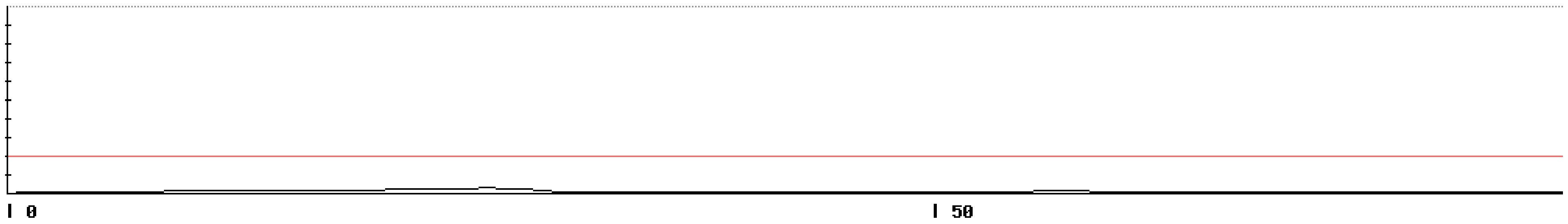

Predictions for Lotusjaponicus_LjSGA_059487.1

Viterbi Path

Viterbi Path

Posterior @ 0.2

Posterior @ 0.2

Posterior Graphic. Threshold Marked @ 0.2

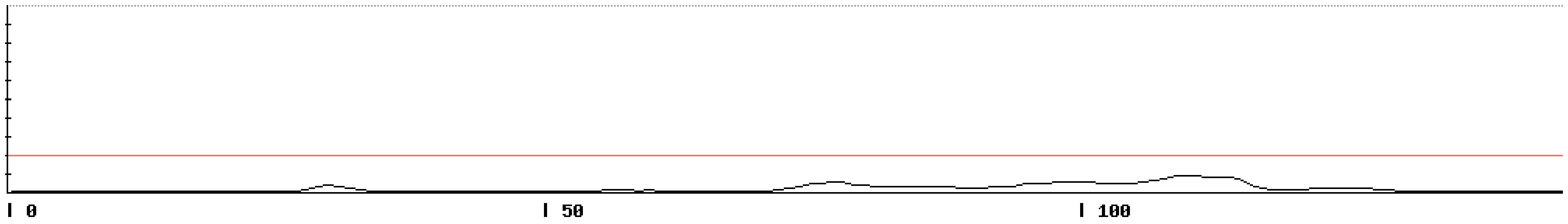

Predictions for Lotusjaponicus_LjSGA_062695.1

Viterbi Path	
Viterbi Path	
Posterior @ 0.2	
Posterior @ 0.2	
Posterior Graphic. Threshold Marked @ 0.2	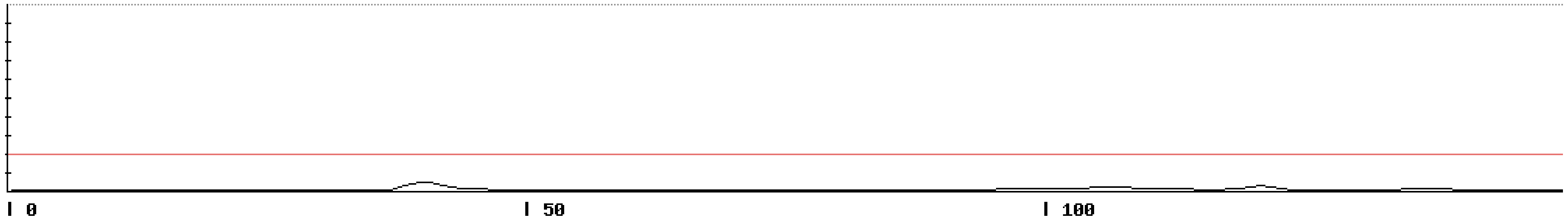

Predictions for Lotusjaponicus_LjSGA_066344.1	
Viterbi Path	
Viterbi Path	
Posterior @ 0.2	
Posterior @ 0.2	
Posterior Graphic. Threshold Marked @ 0.2	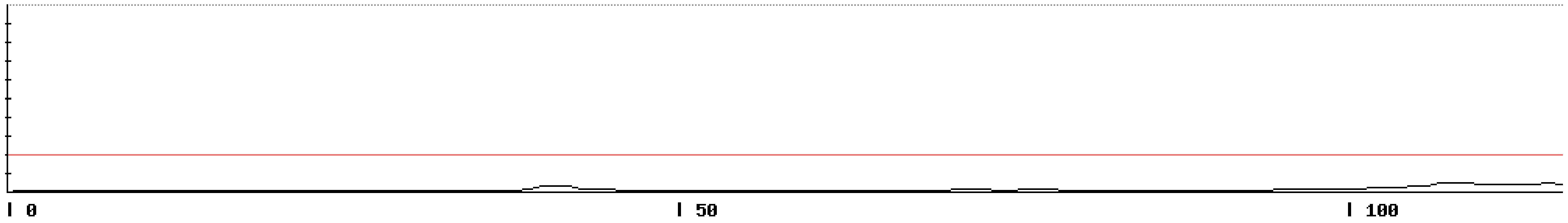

Predictions for Lotusjaponicus_LjSGA_068911.1	
Viterbi Path	
Viterbi Path	
Posterior @ 0.2	
Posterior @ 0.2	
Posterior Graphic. Threshold Marked @ 0.2	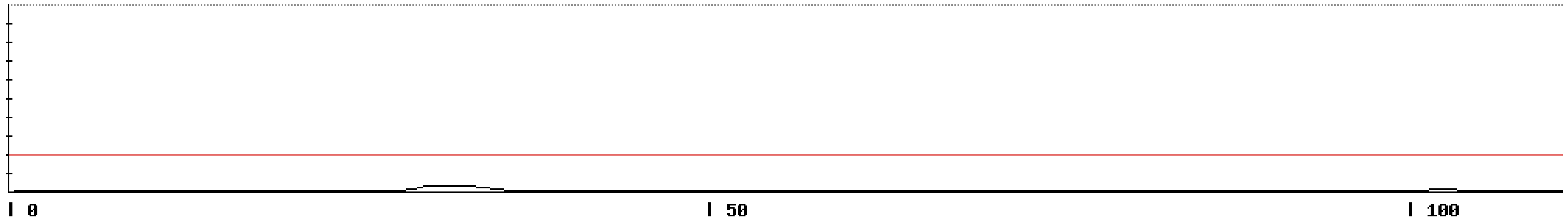

Predictions for Lotusjaponicus_LjSGA_069569.1	
Viterbi Path	
Viterbi Path	
Posterior @ 0.2	
Posterior @ 0.2	
Posterior Graphic. Threshold Marked @ 0.2	

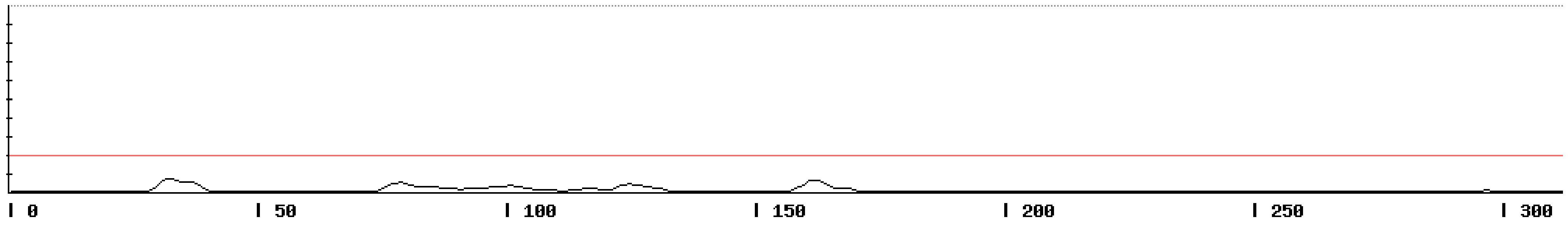

Predictions for Lotusjaponicus_LjSGA_072789.1

Viterbi Path	
Viterbi Path	
Posterior @ 0.2	
Posterior @ 0.2	
Posterior Graphic. Threshold Marked @ 0.2	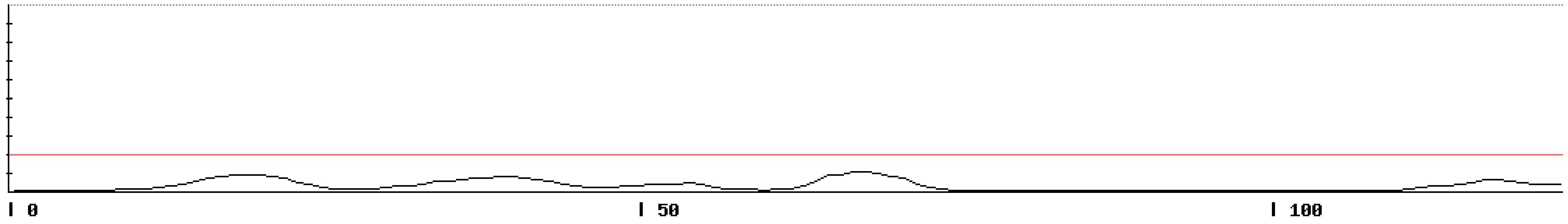

Predictions for Lotusjaponicus_LjSGA_075859.1

Viterbi Path	
Viterbi Path	
Posterior @ 0.2	117 - KGRAPNGRKT - 126
Posterior @ 0.2	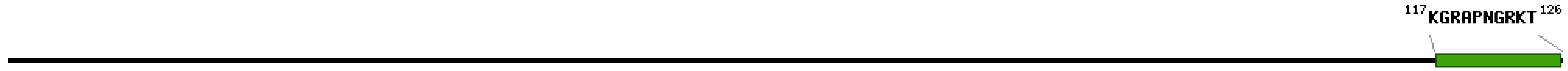

Posterior Graphic. Threshold Marked @ 0.2

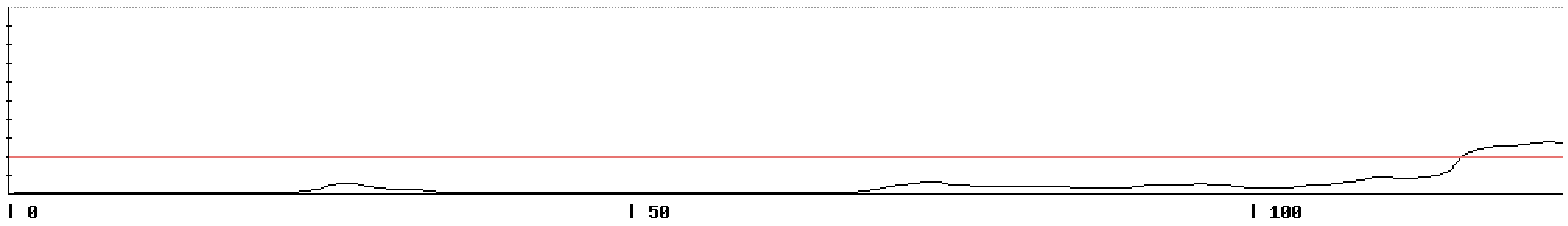

Predictions for Lotusjaponicus_LjSGA_078266.1

Viterbi Path	
Viterbi Path	
Posterior @ 0.2	
Posterior @ 0.2	
Posterior Graphic. Threshold Marked @ 0.2	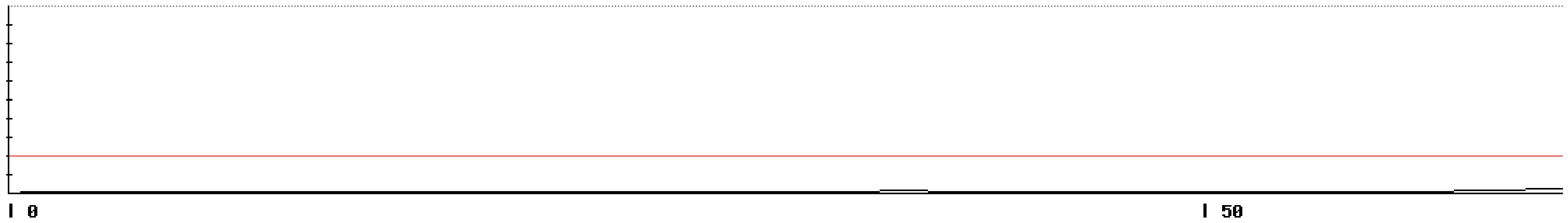

Predictions for Lotusjaponicus_LjSGA_090586.1

Viterbi Path	
Viterbi Path	
Posterior @ 0.2	72 - KVKK - 75
Posterior @ 0.2	

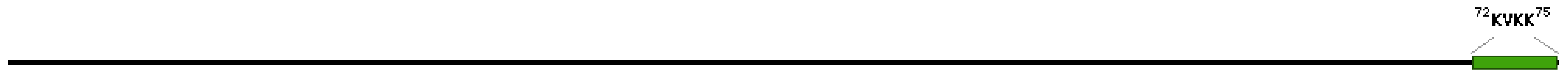

Posterior Graphic. Threshold Marked @ 0.2

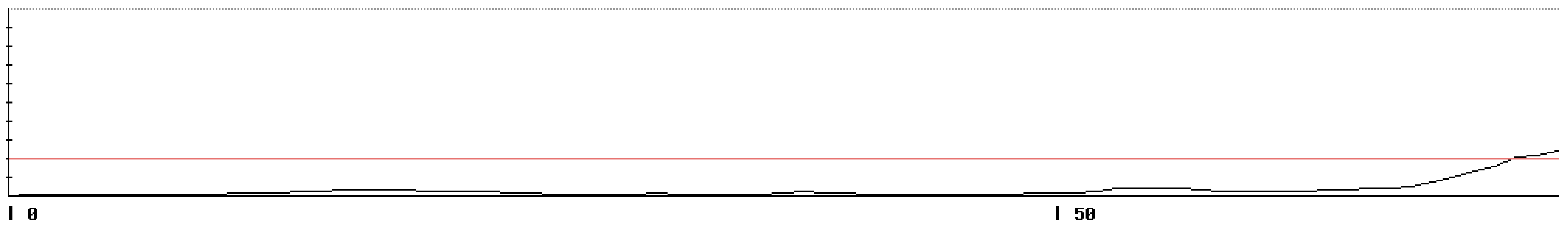

Predictions for Lotusjaponicus_LjSGA_110417.1

Viterbi Path

Viterbi Path

Posterior @ 0.2

Posterior @ 0.2

Posterior Graphic. Threshold Marked @ 0.2

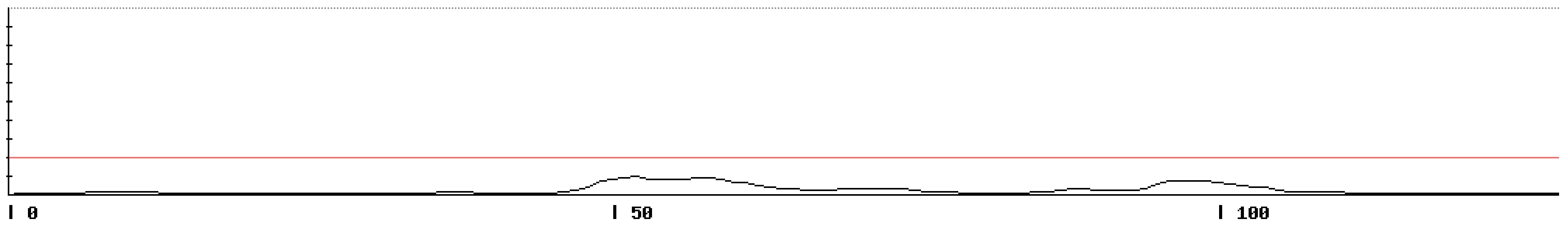

Predictions for Lotusjaponicus_LjSGA_127686.1

Viterbi Path

Viterbi Path

Posterior @ 0.2	36 - K - 36
	39 - KG - 40
Posterior @ 0.2	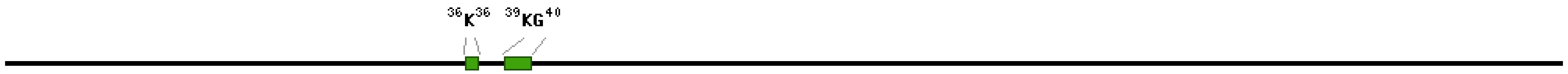
Posterior Graphic. Threshold Marked @ 0.2	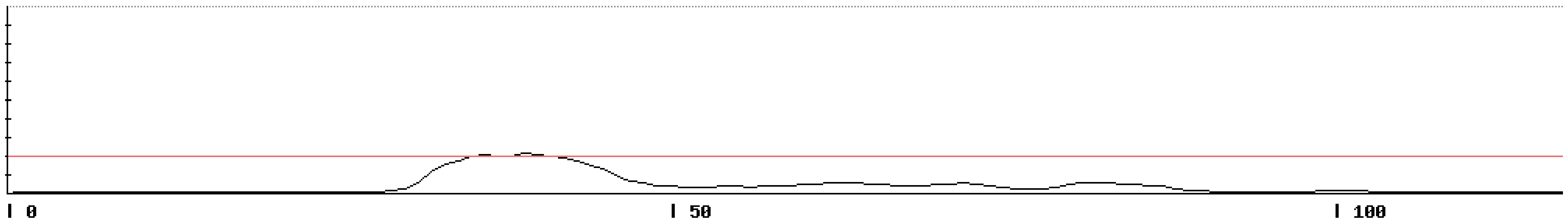

Predictions for Lotusjaponicus_LjSGA_130728.1

Viterbi Path	
Viterbi Path	
Posterior @ 0.2	
Posterior @ 0.2	
Posterior Graphic. Threshold Marked @ 0.2	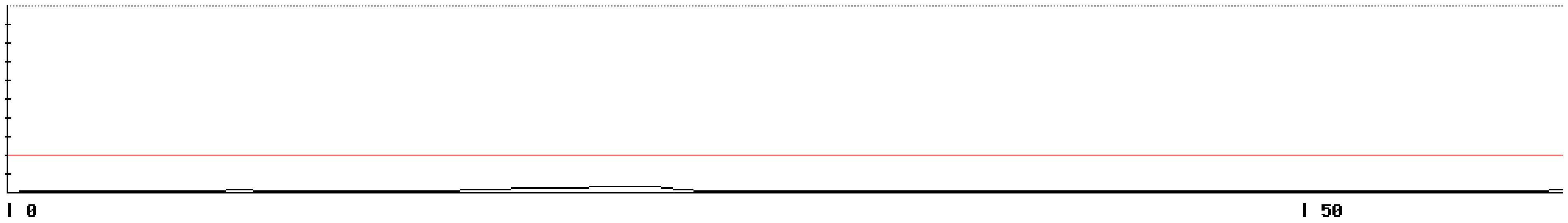

Predictions for Lotusjaponicus_LjSGA_137192.1

Viterbi Path	

Revision r.9 - Last Updated on : February 24 2015 17:45:20.

alex.nguyenba@utoronto.ca

Intended to be used with Firefox

Viterbi Path

Posterior @ 0.4

Posterior @ 0.4

Posterior Graphic. Threshold Marked @ 0.4

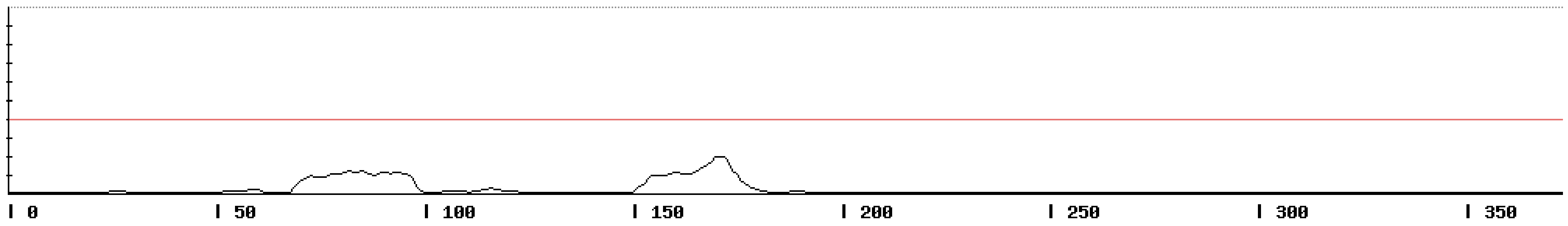

Predictions for Malusdomestica_MDP0000120881

Viterbi Path

Viterbi Path

Posterior @ 0.4

Posterior @ 0.4

Posterior Graphic. Threshold Marked @ 0.4

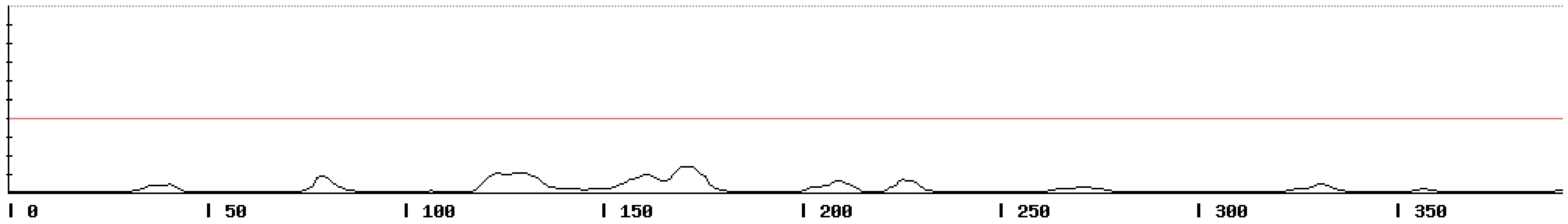

Predictions for Malusdomestica_MDP0000121265

Viterbi Path

Viterbi Path

Posterior @ 0.4

Posterior @ 0.4

Posterior Graphic. Threshold Marked @ 0.4

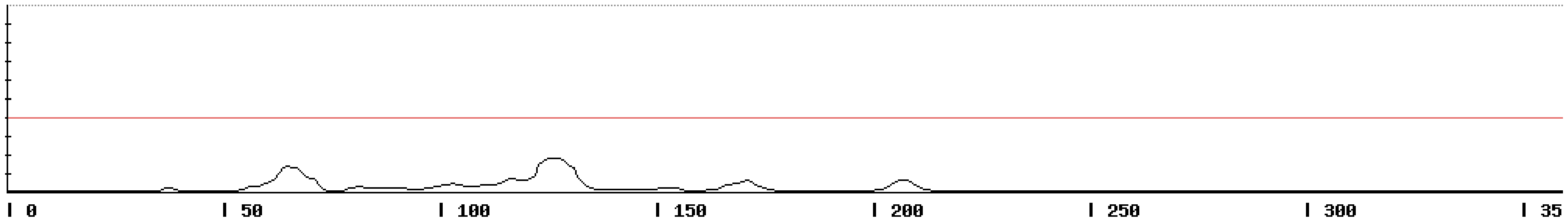

Predictions for Malusdomestica_MDP0000124509

Viterbi Path

Viterbi Path

Posterior @ 0.4

Posterior @ 0.4

Posterior Graphic. Threshold Marked @ 0.4

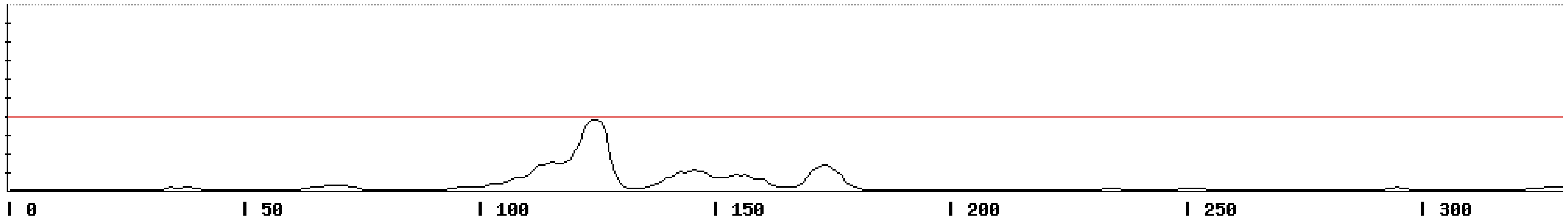

Predictions for Malusdomestica_MDP0000125980	
Viterbi Path	
Viterbi Path	
Posterior @ 0.4	156 - RKNRRSCGKKR - 166
Posterior @ 0.4	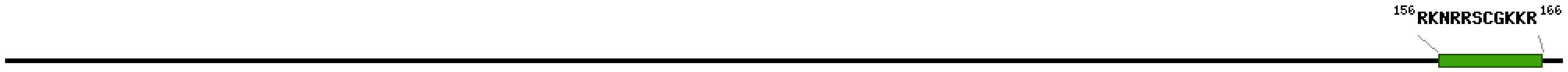
Posterior Graphic. Threshold Marked @ 0.4	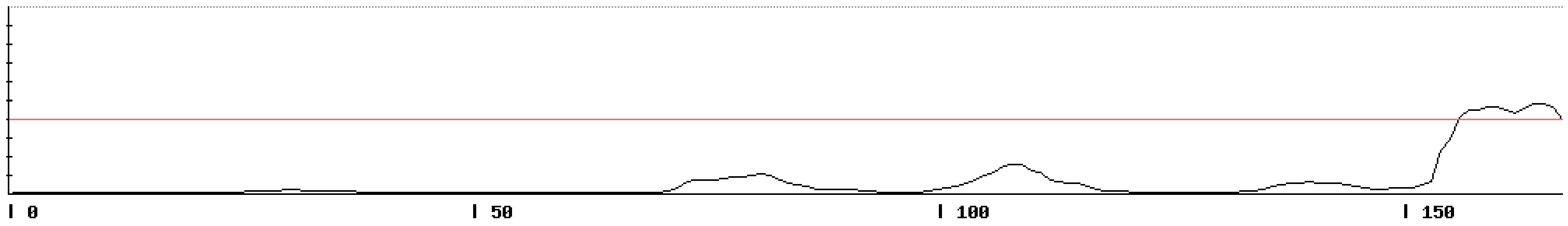

Predictions for Malusdomestica_MDP0000126009	
Viterbi Path	205 - KKRARKEVKEKPVKKRARKEVKEKP - 229
Viterbi Path	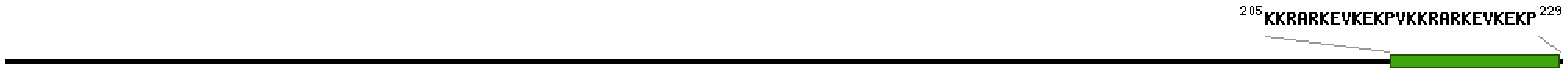
Posterior @ 0.4	204 - AKKRARKEVKEKPVKKRARKEVKEKP - 229
Posterior @ 0.4	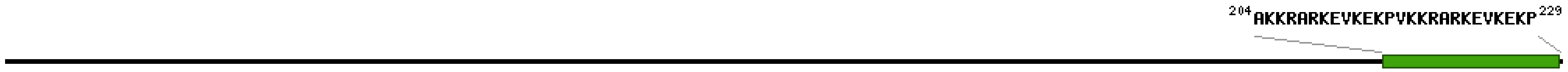
Posterior Graphic. Threshold Marked @ 0.4	

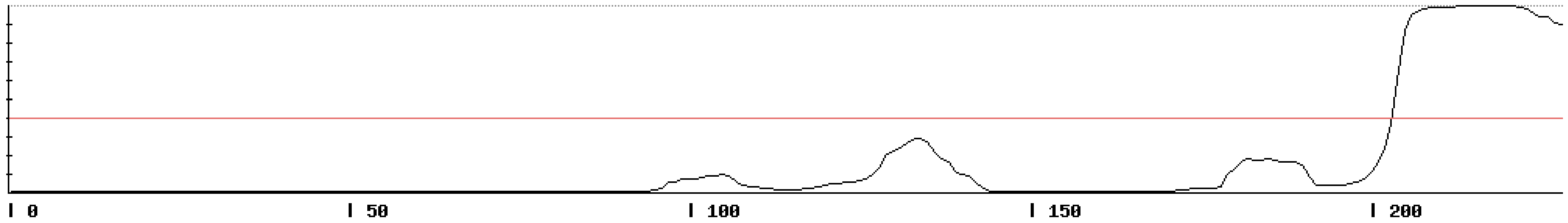

Predictions for Malusdomestica_MDP0000126141

Viterbi Path	
Viterbi Path	
Posterior @ 0.4	
Posterior @ 0.4	
Posterior Graphic. Threshold Marked @ 0.4	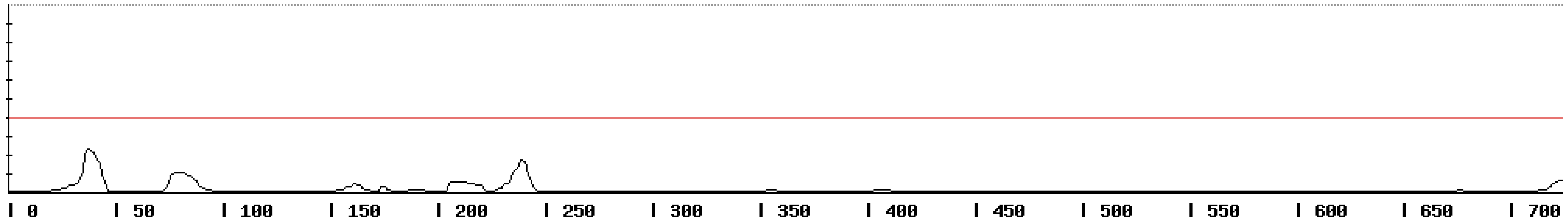

Predictions for Malusdomestica_MDP0000126517

Viterbi Path	
Viterbi Path	
Posterior @ 0.4	
Posterior @ 0.4	

Posterior Graphic. Threshold Marked @ 0.4

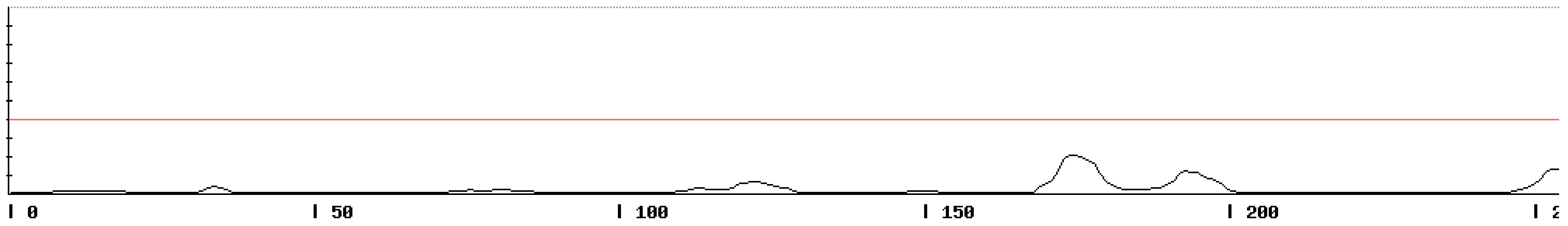

Predictions for Malusdomestica_MDP0000128461

Viterbi Path

Viterbi Path

Posterior @ 0.4

Posterior @ 0.4

Posterior Graphic. Threshold Marked @ 0.4

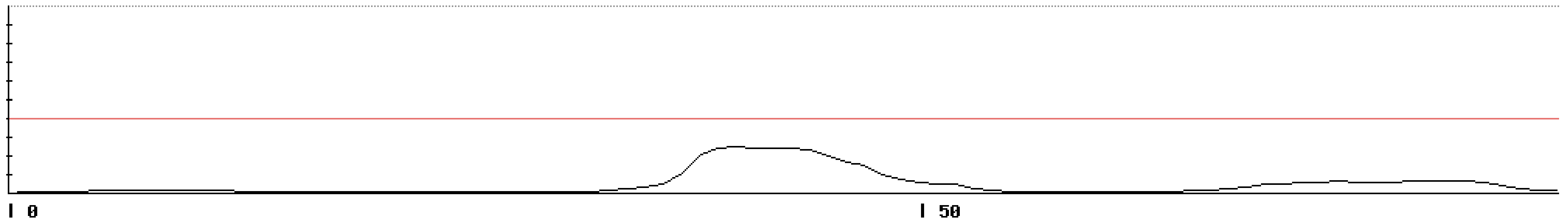

Predictions for Malusdomestica_MDP0000129335

Viterbi Path

Viterbi Path

Posterior @ 0.4

Posterior @ 0.4

Posterior Graphic. Threshold Marked @ 0.4	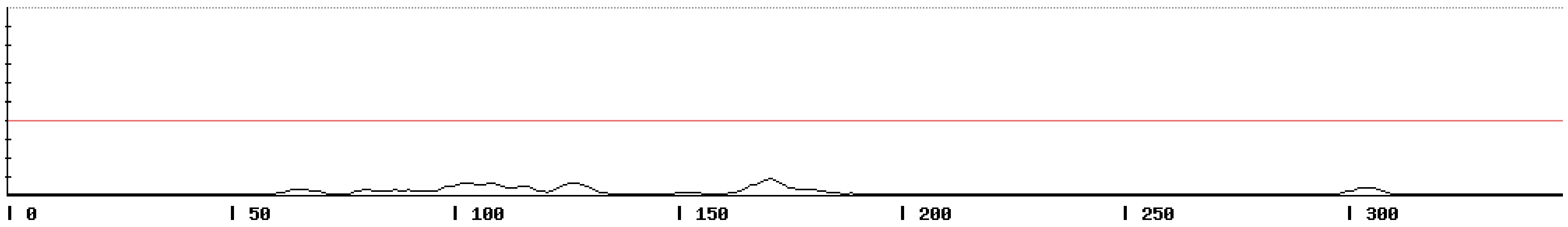

Predictions for Malusdomestica_MDP0000130123

Viterbi Path	
Viterbi Path	
Posterior @ 0.4	
Posterior @ 0.4	
Posterior Graphic. Threshold Marked @ 0.4	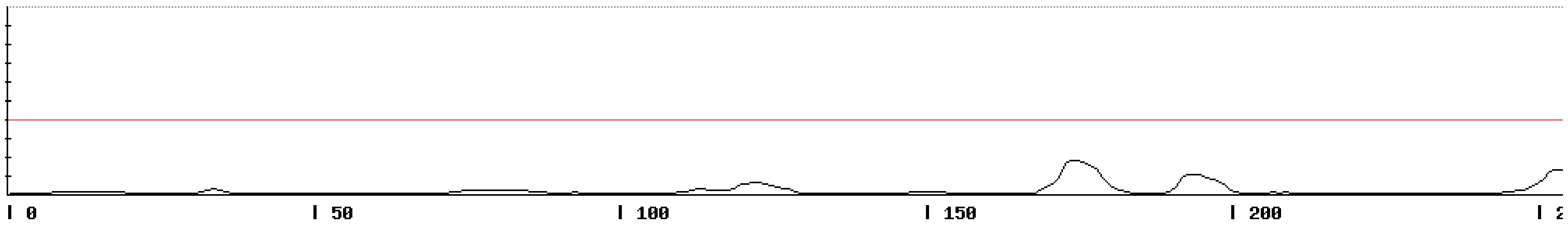

Predictions for Malusdomestica_MDP0000130686

Viterbi Path	
Viterbi Path	

Posterior @ 0.4

Posterior @ 0.4

Posterior Graphic. Threshold Marked @ 0.4

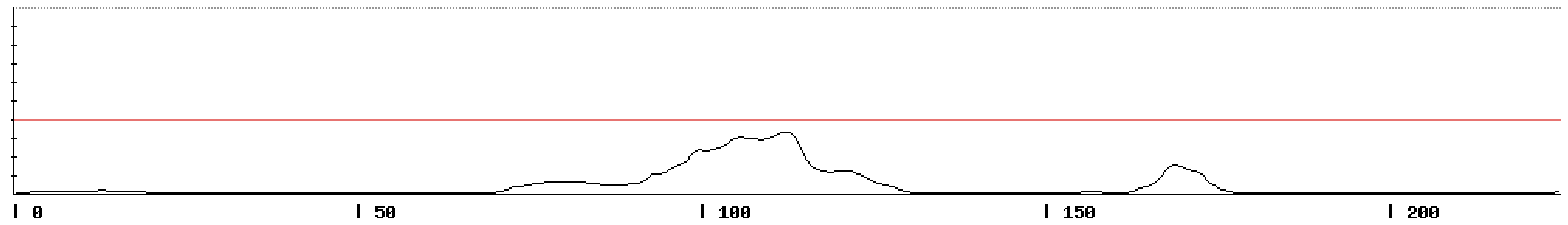

Predictions for Malusdomestica_MDP0000130785

Viterbi Path

Viterbi Path

Posterior @ 0.4

Posterior @ 0.4

Posterior Graphic. Threshold Marked @ 0.4

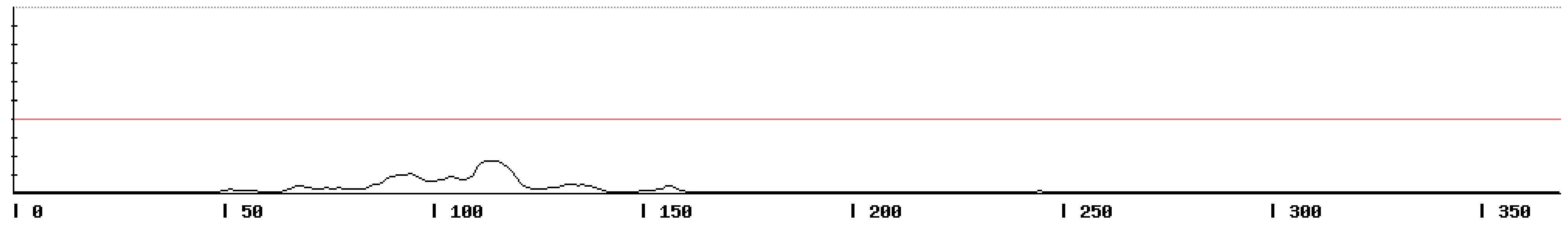

Predictions for Malusdomestica_MDP0000130797

Viterbi Path

Viterbi Path

Posterior @ 0.4

Posterior @ 0.4

Posterior Graphic. Threshold Marked @ 0.4

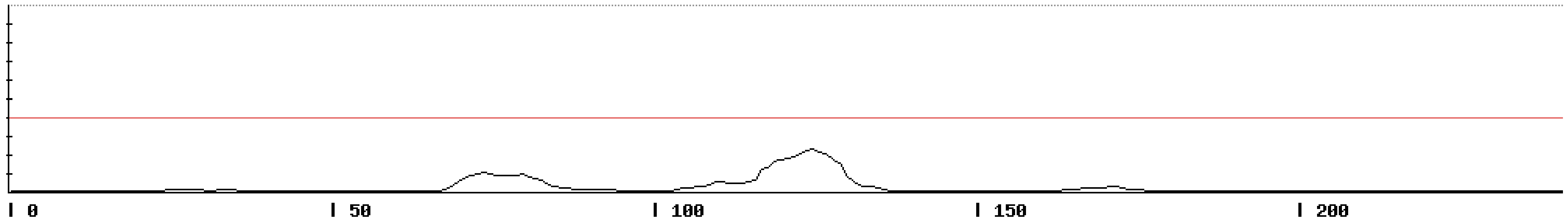

Predictions for Malusdomestica_MDP0000131126

Viterbi Path

Viterbi Path

Posterior @ 0.4

Posterior @ 0.4

Posterior Graphic. Threshold Marked @ 0.4

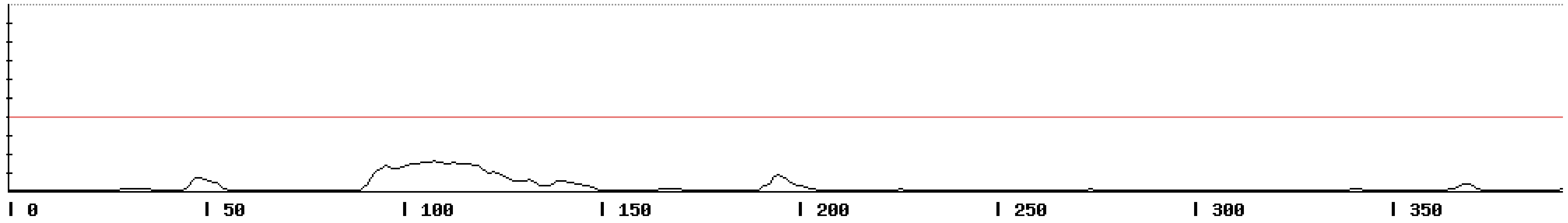

Predictions for Malusdomestica_MDP0000132623

Viterbi Path	
Viterbi Path	
Posterior @ 0.4	
Posterior @ 0.4	
Posterior Graphic. Threshold Marked @ 0.4	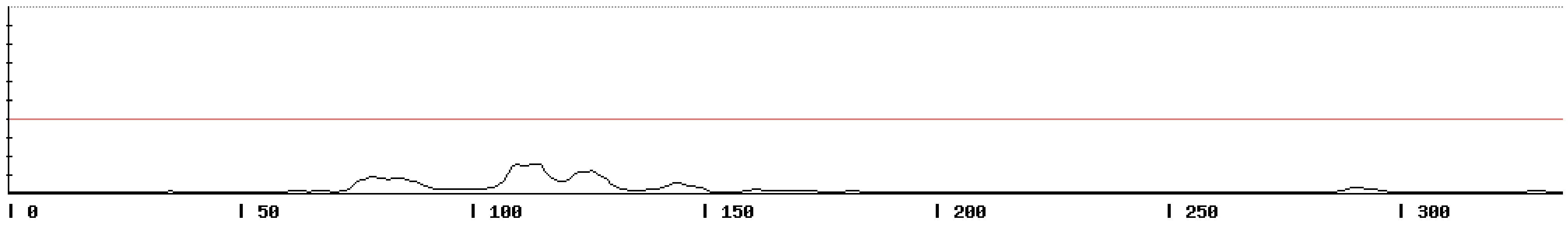

Predictions for Malusdomestica_MDP0000132627

Viterbi Path	
Viterbi Path	
Posterior @ 0.4	154 - RKYRNGSRPKRA - 165
Posterior @ 0.4	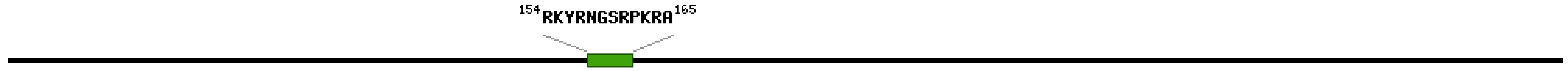
Posterior Graphic. Threshold Marked @ 0.4	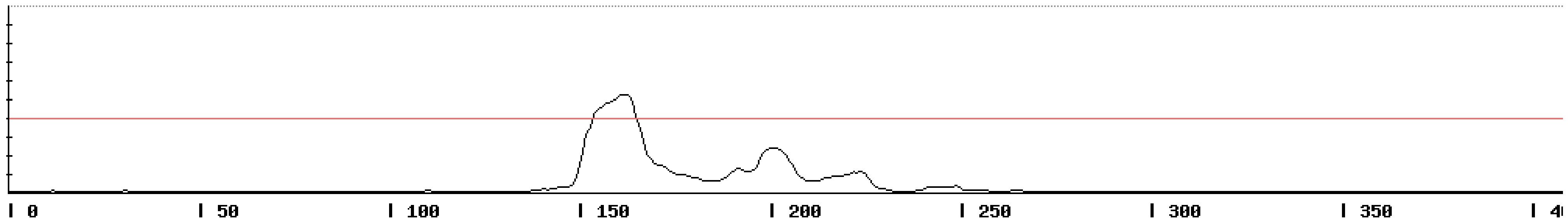

Predictions for Malusdomestica_MDP0000133636	
Viterbi Path	
Viterbi Path	
Posterior @ 0.4	306 - RRISLRKRKEKFRAE - 320
Posterior @ 0.4	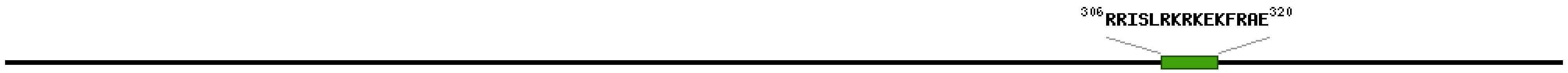
Posterior Graphic. Threshold Marked @ 0.4	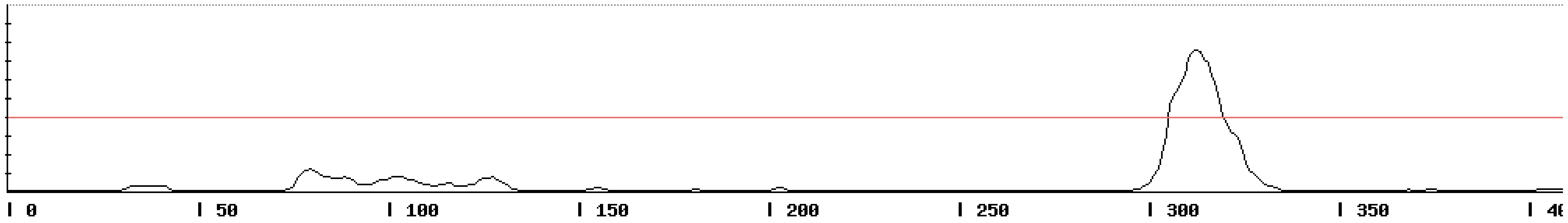

Predictions for Malusdomestica_MDP0000135374	
Viterbi Path	
Viterbi Path	
Posterior @ 0.4	
Posterior @ 0.4	
Posterior Graphic. Threshold Marked @ 0.4	

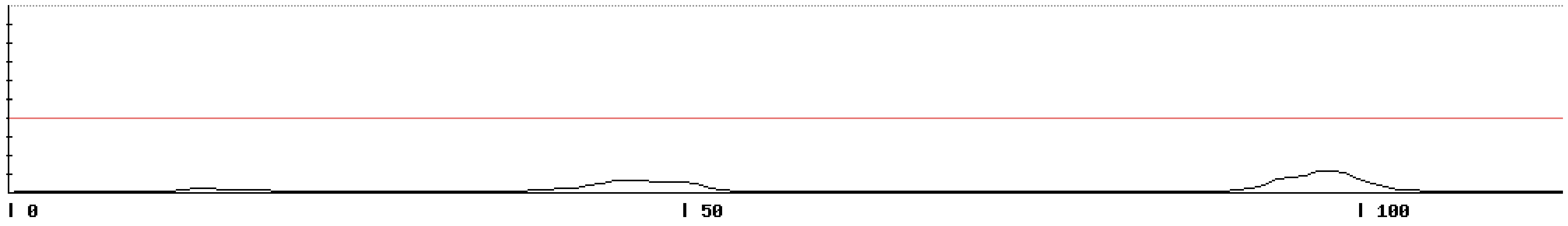

Predictions for Malusdomestica_MDP0000135860

Viterbi Path	
Viterbi Path	
Posterior @ 0.4	
Posterior @ 0.4	
Posterior Graphic. Threshold Marked @ 0.4	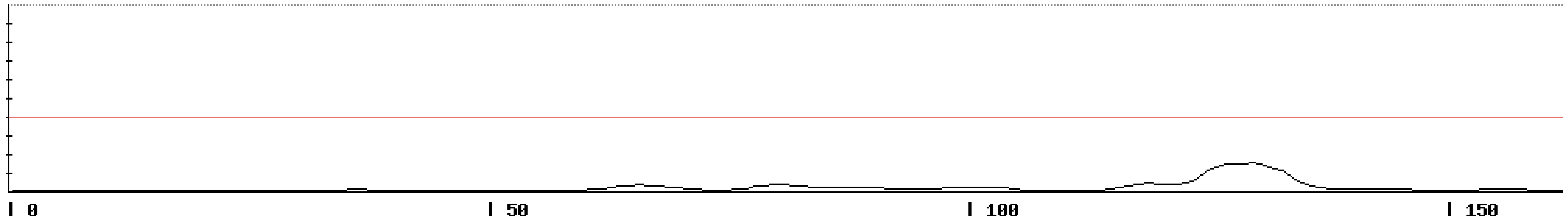

Predictions for Malusdomestica_MDP0000136180

Viterbi Path	
Viterbi Path	
Posterior @ 0.4	
Posterior @ 0.4	

Posterior Graphic. Threshold Marked @ 0.4

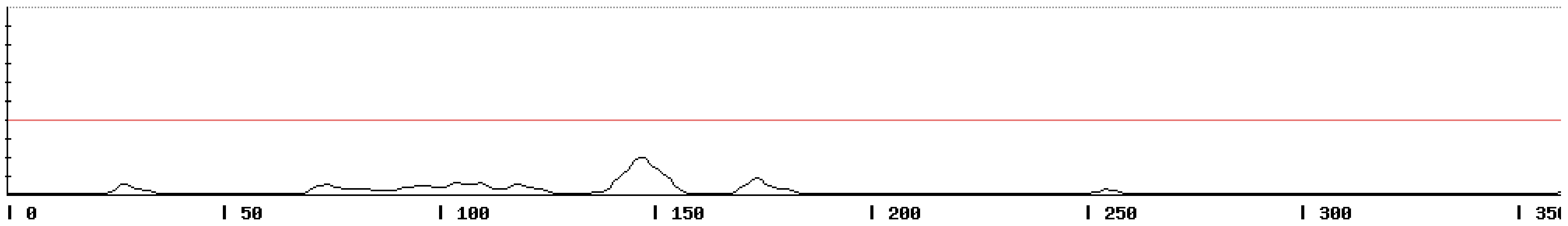

Predictions for Malusdomestica_MDP0000136652

Viterbi Path	
Viterbi Path	
Posterior @ 0.4	166 - KKRSRSYKKPKG - 177
	393 - KRKR - 396

Posterior @ 0.4

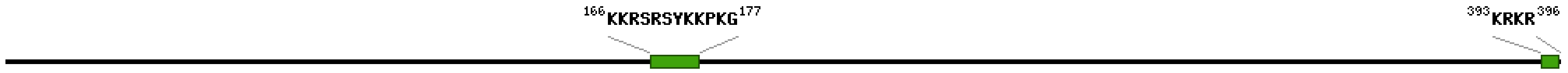

Posterior Graphic. Threshold Marked @ 0.4

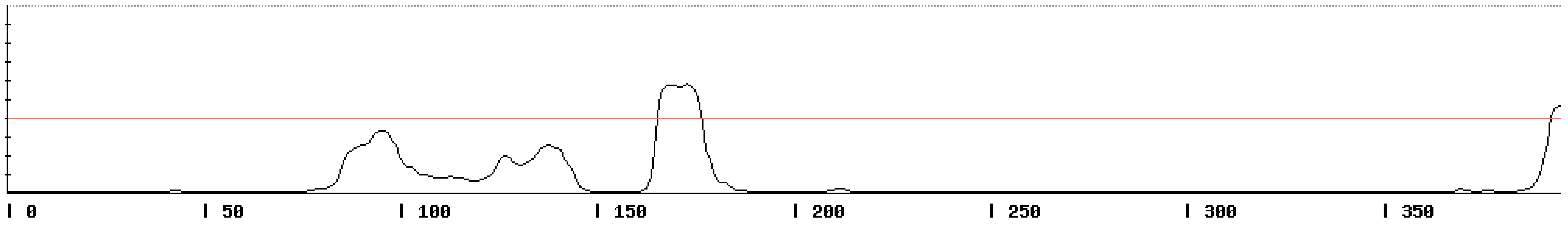

Predictions for Malusdomestica_MDP0000137733

Viterbi Path	
Viterbi Path	
Posterior @ 0.4	
Posterior @ 0.4	

Posterior Graphic. Threshold Marked @ 0.4

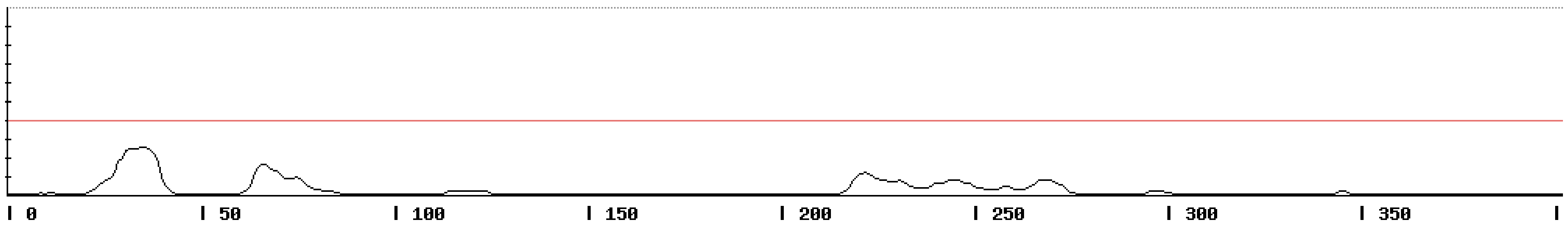

Predictions for Malusdomestica_MDP0000138340

Viterbi Path

Viterbi Path

Posterior @ 0.4

Posterior @ 0.4

Posterior Graphic. Threshold Marked @ 0.4

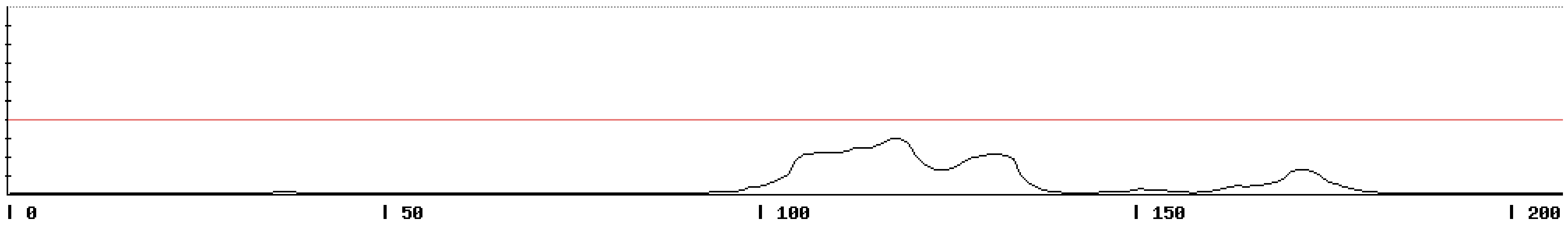

Predictions for Malusdomestica_MDP0000139773

Viterbi Path

Viterbi Path

Posterior @ 0.4	
Posterior @ 0.4	
Posterior Graphic. Threshold Marked @ 0.4	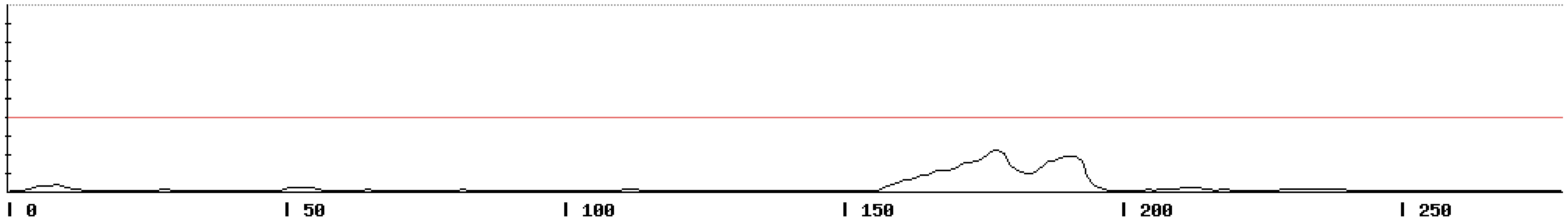

Predictions for Malusdomestica_MDP0000140229	
Viterbi Path	
Viterbi Path	
Posterior @ 0.4	
Posterior @ 0.4	
Posterior Graphic. Threshold Marked @ 0.4	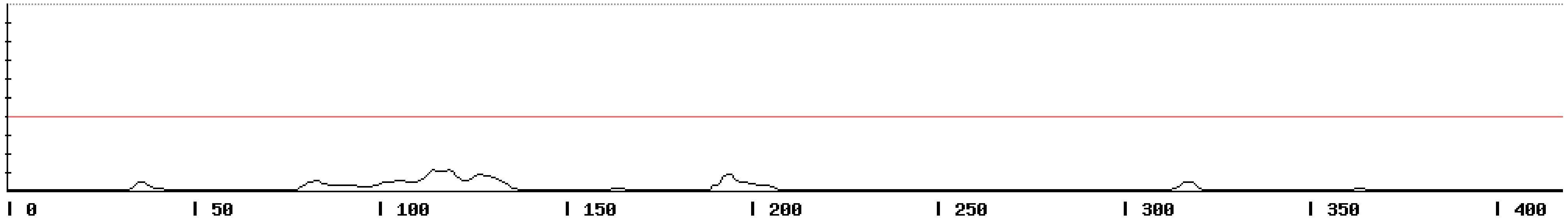

Predictions for Malusdomestica_MDP0000140493	
Viterbi Path	
Viterbi Path	

Posterior @ 0.4

Posterior @ 0.4

Posterior Graphic. Threshold Marked @ 0.4

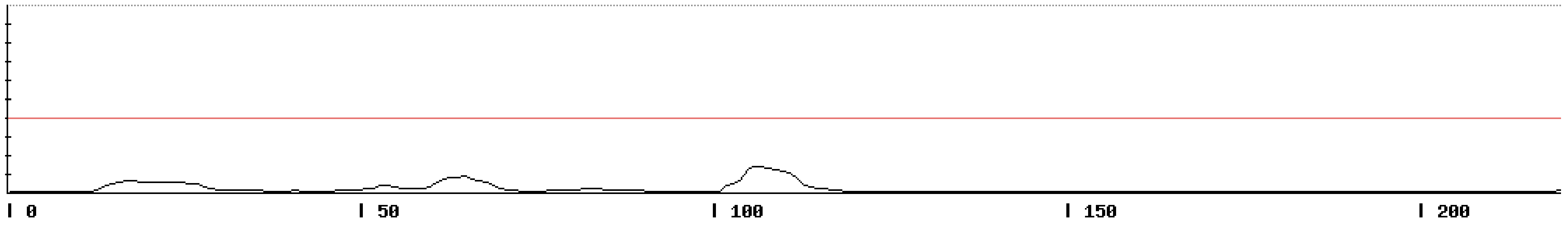

Predictions for Malusdomestica_MDP0000146969

Viterbi Path

Viterbi Path

Posterior @ 0.4

Posterior @ 0.4

Posterior Graphic. Threshold Marked @ 0.4

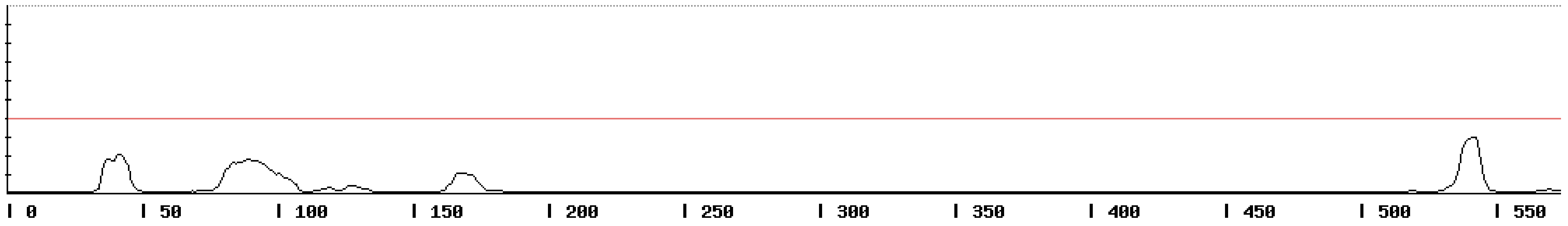

Predictions for Malusdomestica_MDP0000147080

Viterbi Path

Viterbi Path

Posterior @ 0.4

Posterior @ 0.4

Posterior Graphic. Threshold Marked @ 0.4

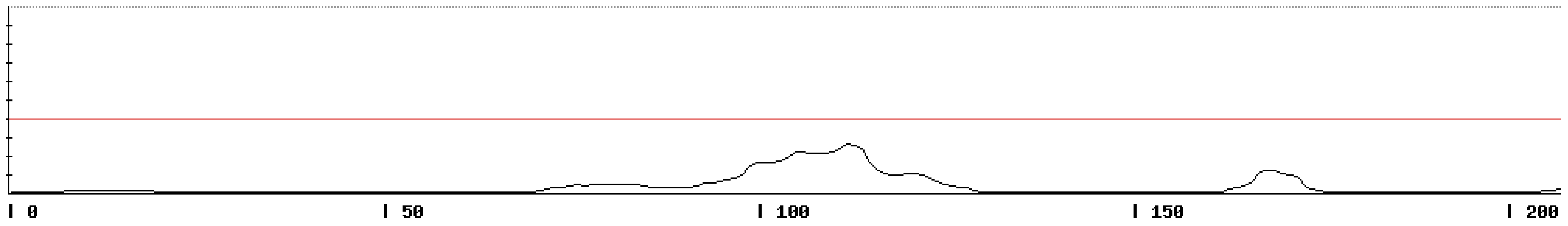

Predictions for Malusdomestica_MDP0000148746

Viterbi Path

Viterbi Path

Posterior @ 0.4

Posterior @ 0.4

Posterior Graphic. Threshold Marked @ 0.4

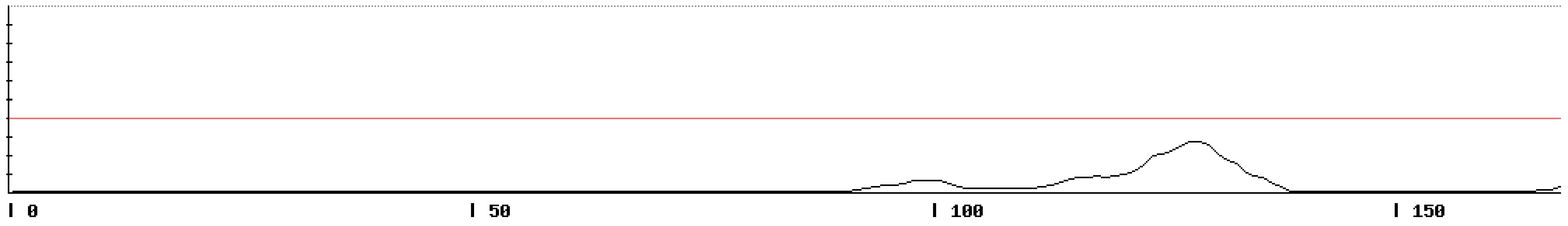

Predictions for Malusdomestica_MDP0000152400	
Viterbi Path	
Viterbi Path	
Posterior @ 0.4	
Posterior @ 0.4	
Posterior Graphic. Threshold Marked @ 0.4	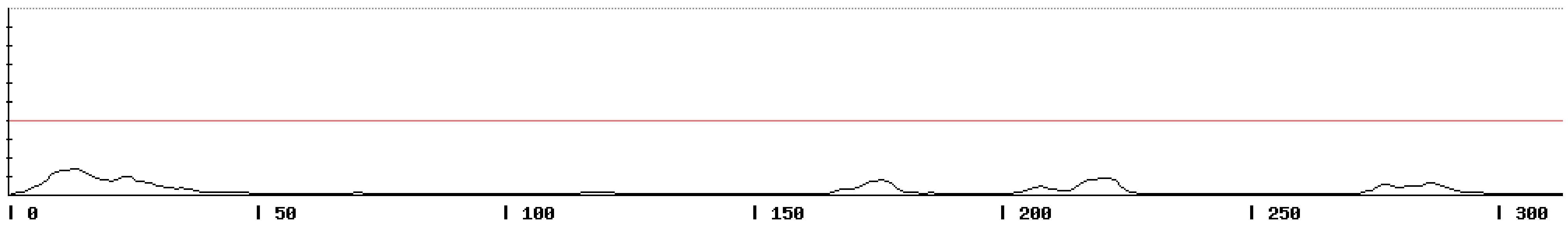

Predictions for Malusdomestica_MDP0000152774	
Viterbi Path	
Viterbi Path	
Posterior @ 0.4	142 - LKERRAKSGYWRRKGRVRDVVGRGGK - 167
Posterior @ 0.4	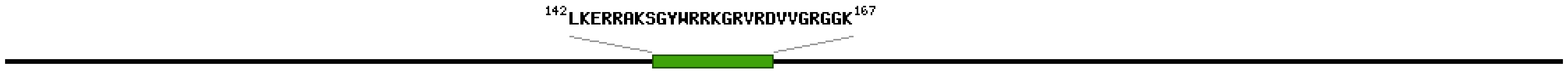
Posterior Graphic. Threshold Marked @ 0.4	

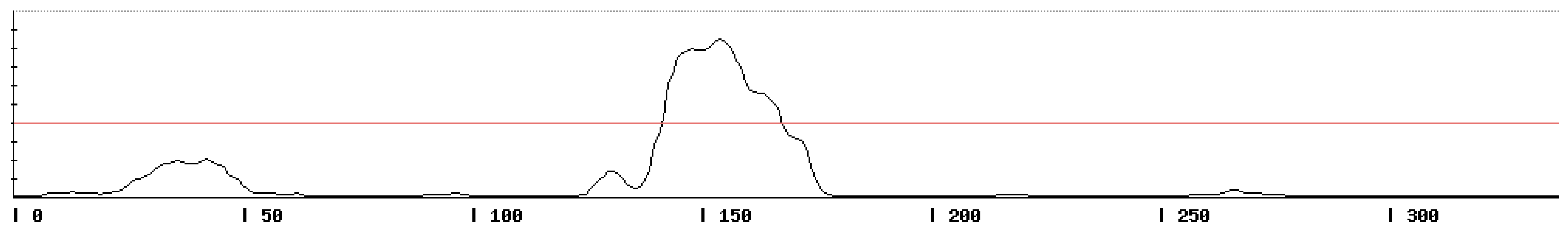

Predictions for Malusdomestica_MDP0000153949

Viterbi Path	
Viterbi Path	
Posterior @ 0.4	
Posterior @ 0.4	
Posterior Graphic. Threshold Marked @ 0.4	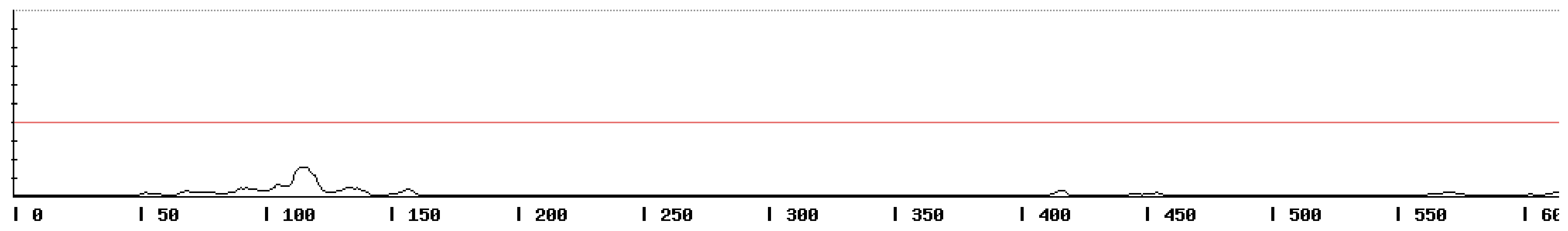

Predictions for Malusdomestica_MDP0000157070

Viterbi Path	
Viterbi Path	
Posterior @ 0.4	
Posterior @ 0.4	

Posterior Graphic. Threshold Marked @ 0.4

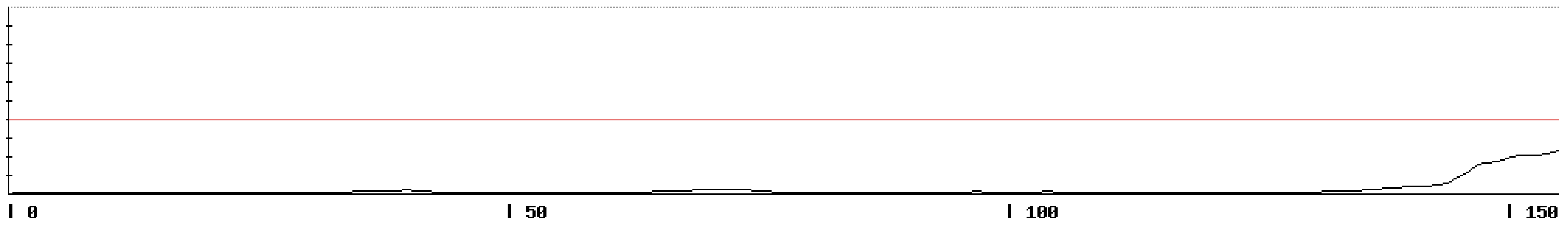

Predictions for Malusdomestica_MDP0000165845

Viterbi Path

Viterbi Path

Posterior @ 0.4 136 - RKRRK - 140

Posterior @ 0.4

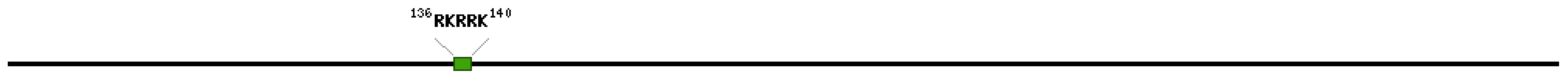

Posterior Graphic. Threshold Marked @ 0.4

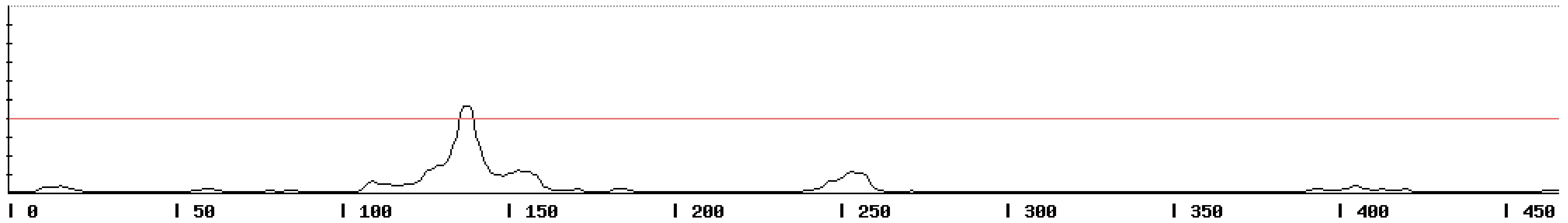

Predictions for Malusdomestica_MDP0000167208

Viterbi Path

Viterbi Path

Posterior @ 0.4

Posterior @ 0.4

Posterior Graphic. Threshold Marked @ 0.4

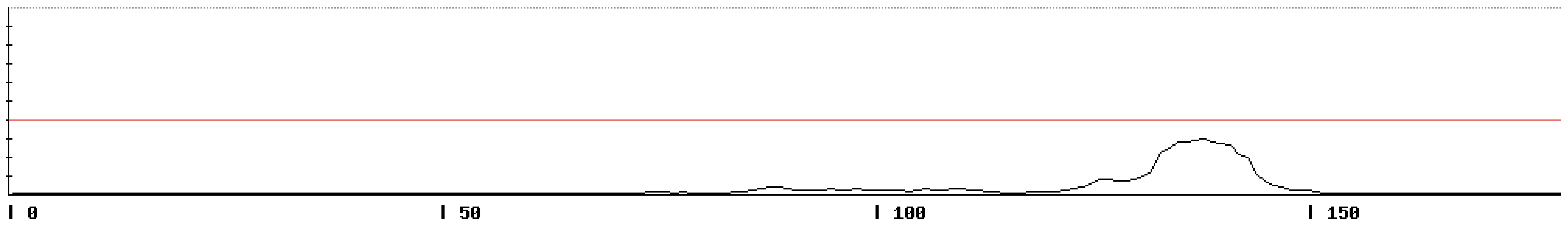

Predictions for Malusdomestica_MDP0000168681

Viterbi Path

Viterbi Path

Posterior @ 0.4

Posterior @ 0.4

Posterior Graphic. Threshold Marked @ 0.4

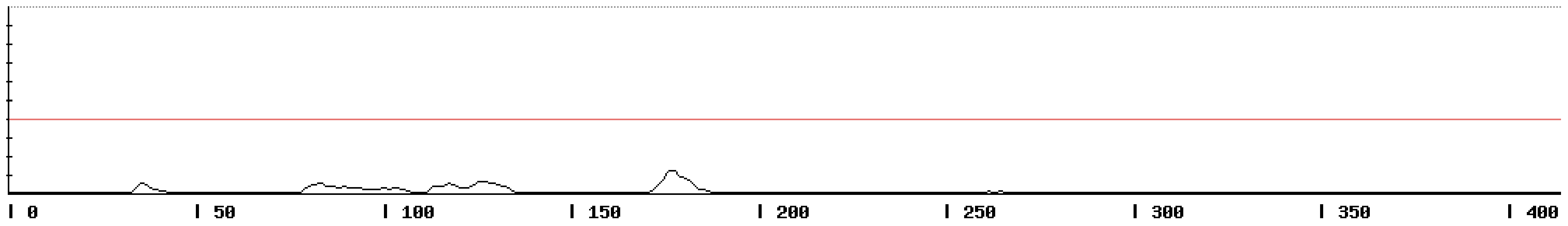

Predictions for Malusdomestica_MDP0000169485

Viterbi Path

Viterbi Path

Posterior @ 0.4

Posterior @ 0.4

Posterior Graphic. Threshold Marked @ 0.4

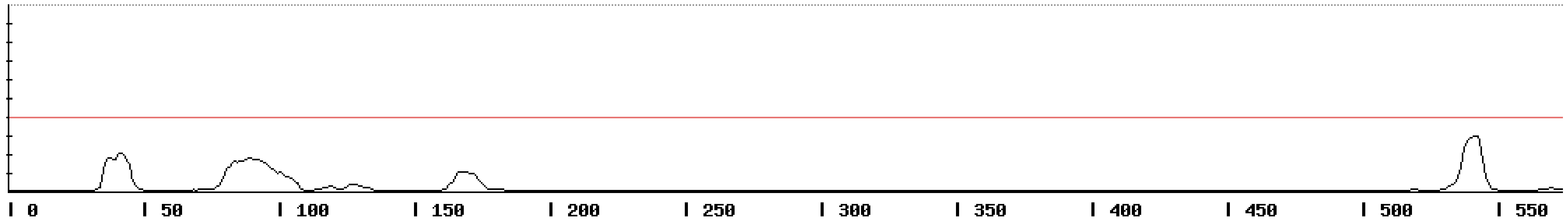

Predictions for Malusdomestica_MDP0000171771

Viterbi Path	195 - KQPAKKRARKEVMEKPAKKRARKEVKEKPAKTKARNK - 231

Viterbi Path

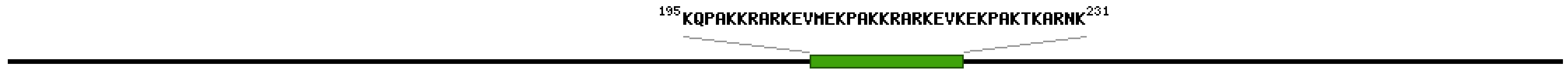

Posterior @ 0.4	195 - KQPAKKRARKEVMEKPAKKRARKEVKEKPAKTKARNKV - 232

Posterior @ 0.4

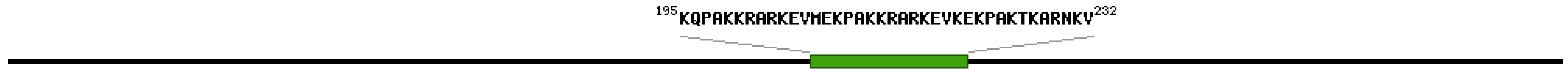

Posterior Graphic. Threshold Marked @ 0.4

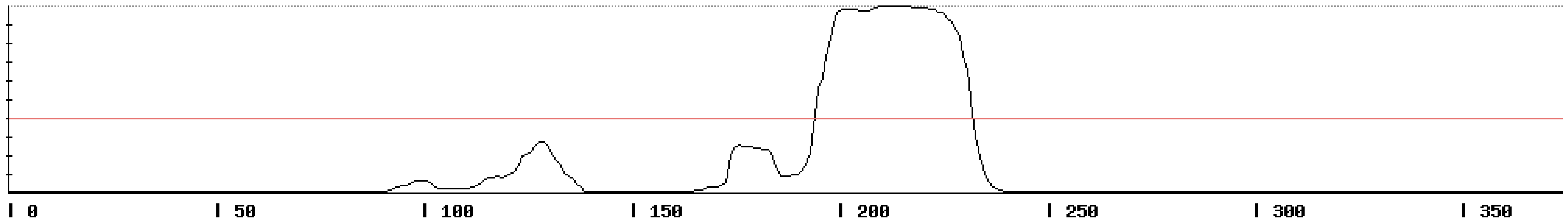

Predictions for Malusdomestica_MDP0000172303

Viterbi Path

Viterbi Path

Posterior @ 0.4

Posterior @ 0.4

Posterior Graphic. Threshold Marked @ 0.4

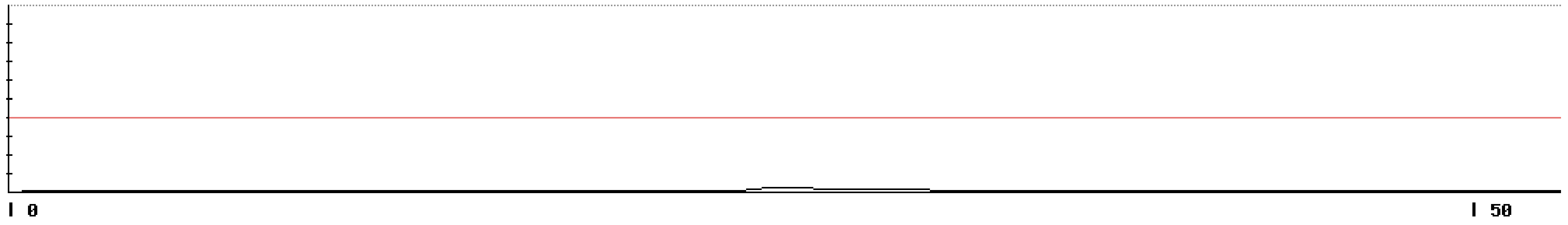

Predictions for Malusdomestica_MDP0000173636

Viterbi Path

Viterbi Path

Posterior @ 0.4

Posterior @ 0.4

Posterior Graphic. Threshold Marked @ 0.4

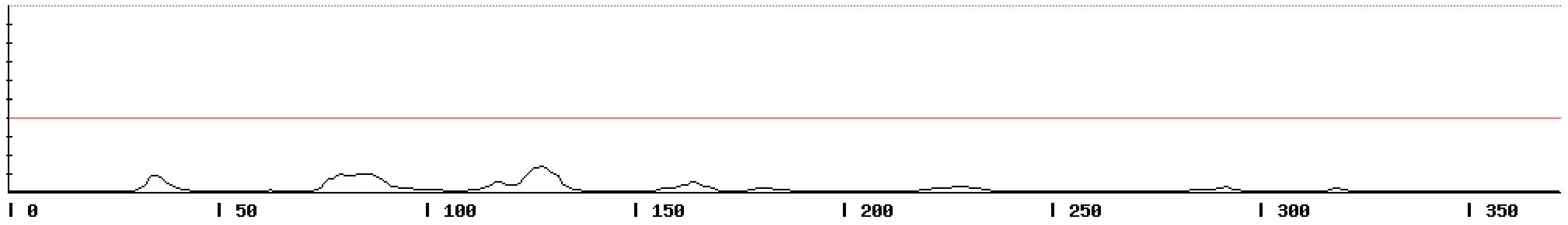

Predictions for Malusdomestica_MDP0000174734

Viterbi Path

Viterbi Path

Posterior @ 0.4

Posterior @ 0.4

Posterior Graphic. Threshold Marked @ 0.4

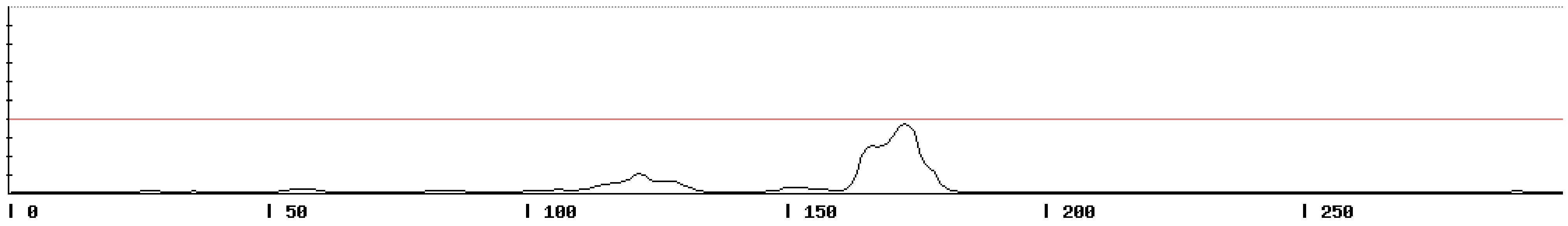

Predictions for Malusdomestica_MDP0000175446

Viterbi Path

Viterbi Path

Posterior @ 0.4

Posterior @ 0.4

Posterior Graphic. Threshold Marked @ 0.4

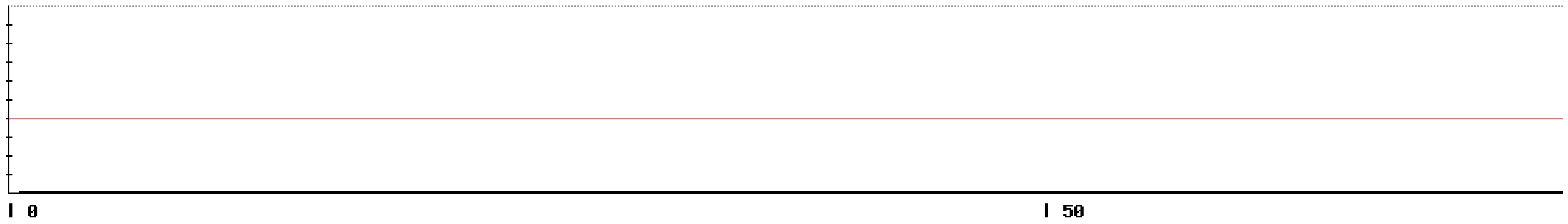

Predictions for Malusdomestica_MDP0000176447

Viterbi Path	
Viterbi Path	
Posterior @ 0.4	
Posterior @ 0.4	
Posterior Graphic. Threshold Marked @ 0.4	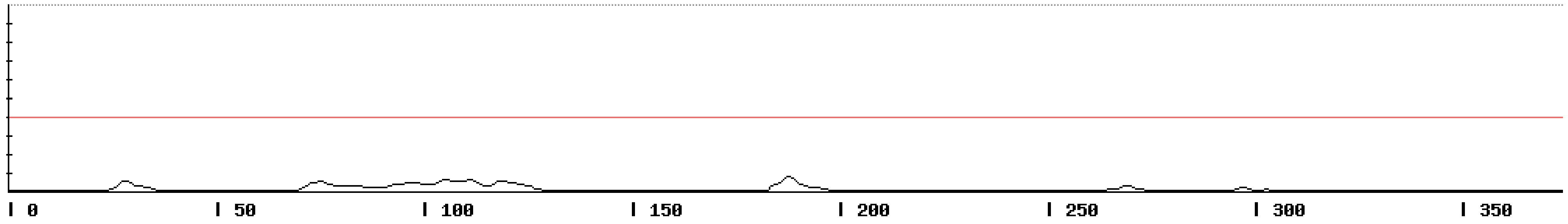

Predictions for Malusdomestica_MDP0000177041

Viterbi Path	
Viterbi Path	
Posterior @ 0.4	140 - KPPIKRAIKEVSNEKLLIKRKRARKEPGSQK - 170
Posterior @ 0.4	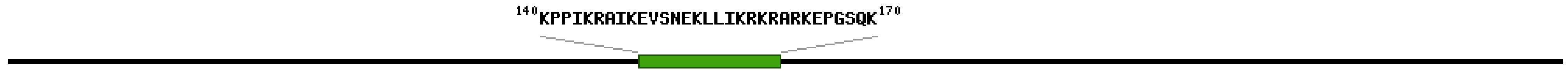
Posterior Graphic. Threshold Marked @ 0.4	

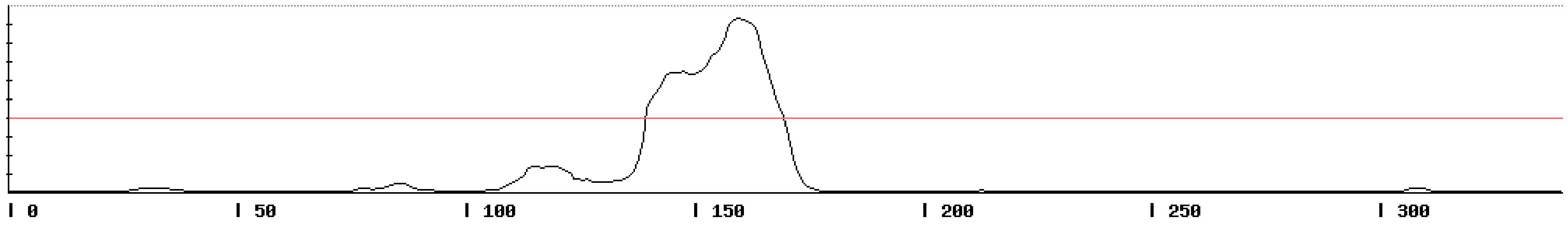

Predictions for Malusdomestica_MDP0000180046

Viterbi Path

Viterbi Path

Posterior @ 0.4

Posterior @ 0.4

Posterior Graphic. Threshold Marked @ 0.4

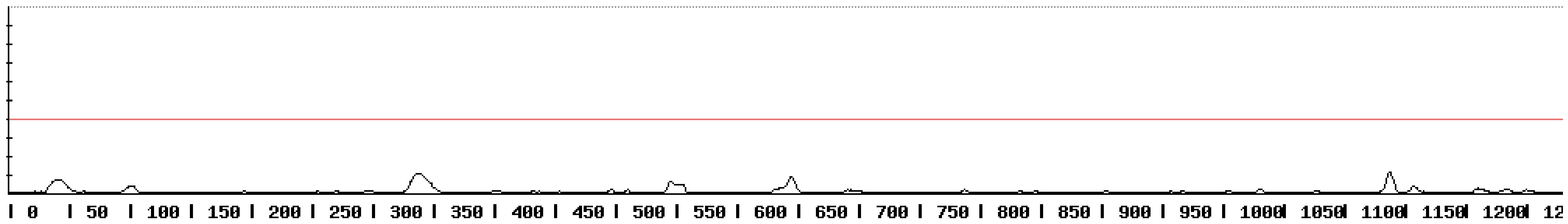

Predictions for Malusdomestica_MDP0000180343

Viterbi Path

Viterbi Path

Posterior @ 0.4

Posterior @ 0.4

Posterior Graphic. Threshold Marked @ 0.4	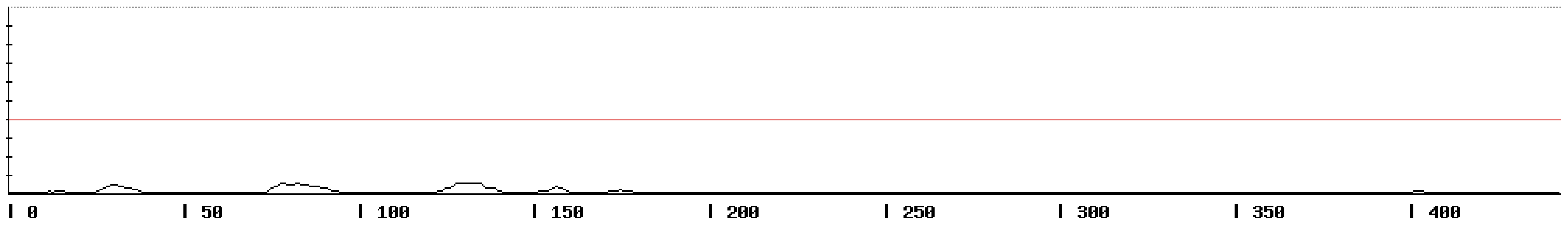

Predictions for Malusdomestica_MDP0000180605

Viterbi Path	
Viterbi Path	
Posterior @ 0.4	
Posterior @ 0.4	
Posterior Graphic. Threshold Marked @ 0.4	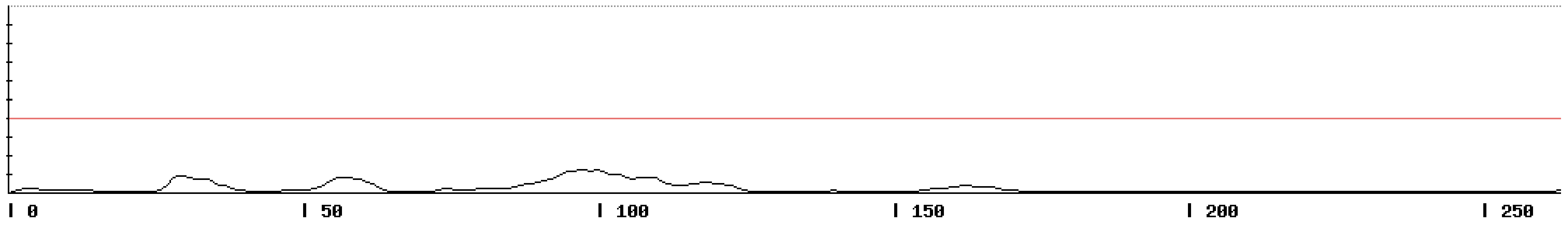

Predictions for Malusdomestica_MDP0000180683

Viterbi Path	
Viterbi Path	
Posterior @ 0.4	
Posterior @ 0.4	

Posterior Graphic. Threshold Marked @ 0.4

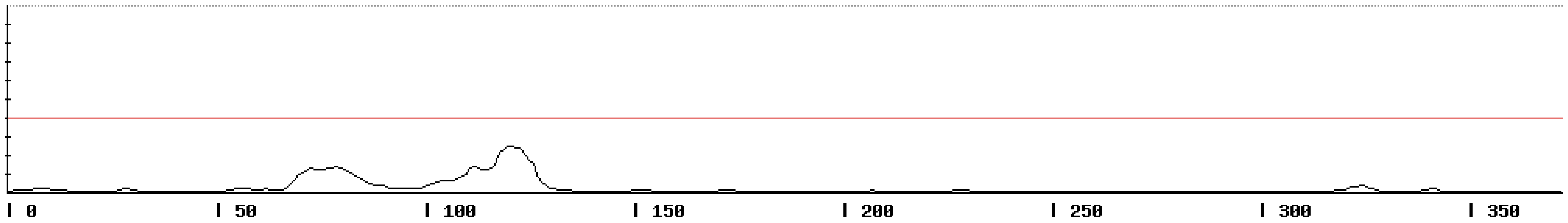

Predictions for Malusdomestica_MDP0000182344

Viterbi Path

Viterbi Path

Posterior @ 0.4

166 - KKRSRSYKKPKG - 177

466 - RKR - 468

Posterior @ 0.4

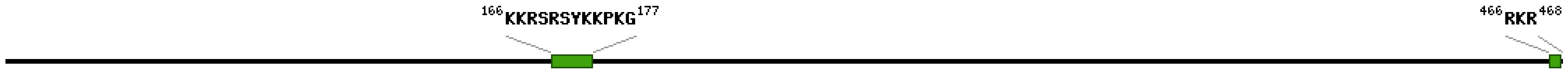

Posterior Graphic. Threshold Marked @ 0.4

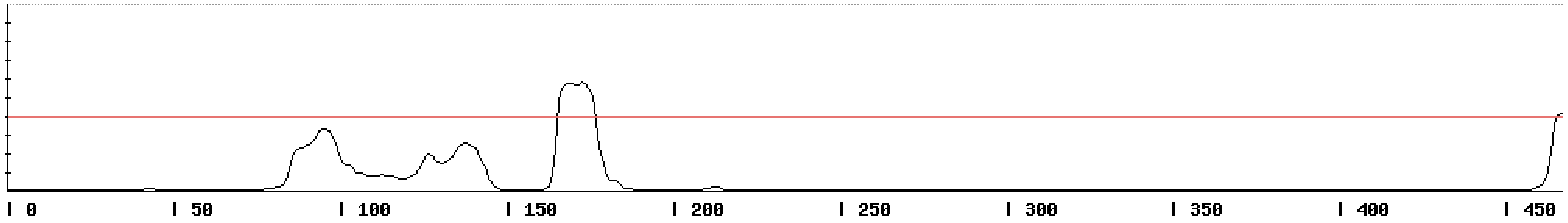

Predictions for Malusdomestica_MDP0000183558

Viterbi Path

Viterbi Path

Posterior @ 0.4	
Posterior @ 0.4	
Posterior Graphic. Threshold Marked @ 0.4	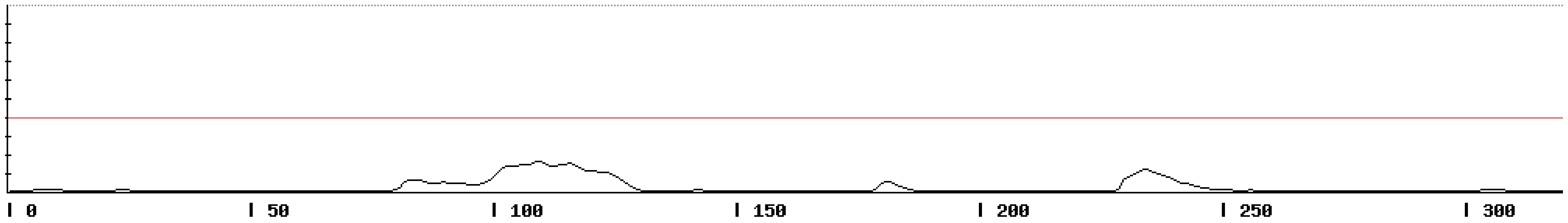

Predictions for Malusdomestica_MDP0000183754

Viterbi Path	
Viterbi Path	
Posterior @ 0.4	46 - NRWGLRRPPRH - 56
Posterior @ 0.4	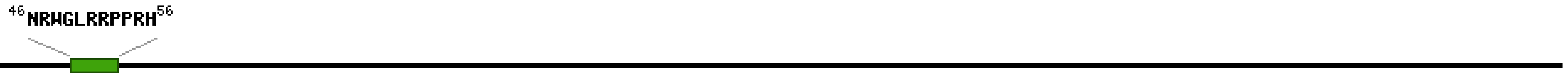
Posterior Graphic. Threshold Marked @ 0.4	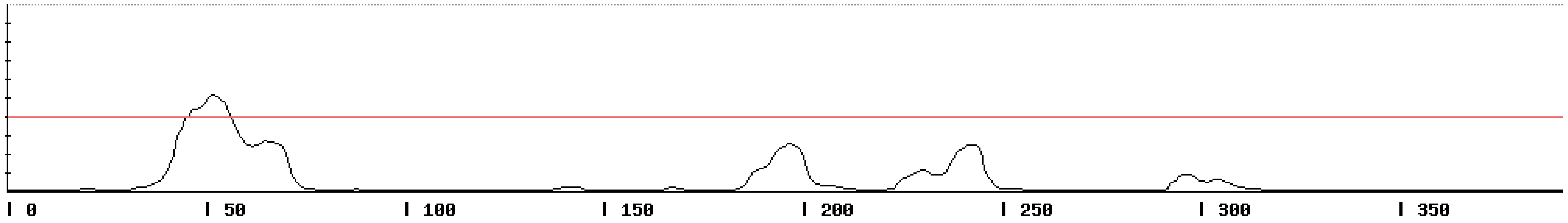

Predictions for Malusdomestica_MDP0000184069

Viterbi Path

Viterbi Path

Posterior @ 0.4

Posterior @ 0.4

Posterior Graphic. Threshold Marked @ 0.4

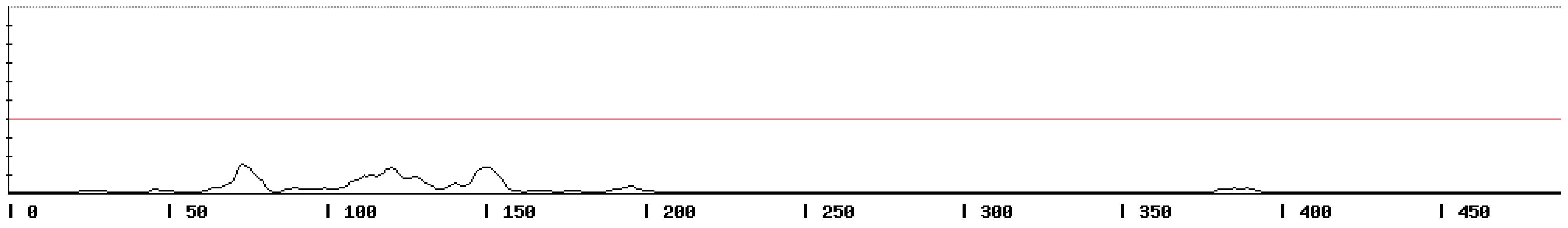

Predictions for Malusdomestica_MDP0000184706

Viterbi Path

Viterbi Path

Posterior @ 0.4

Posterior @ 0.4

Posterior Graphic. Threshold Marked @ 0.4

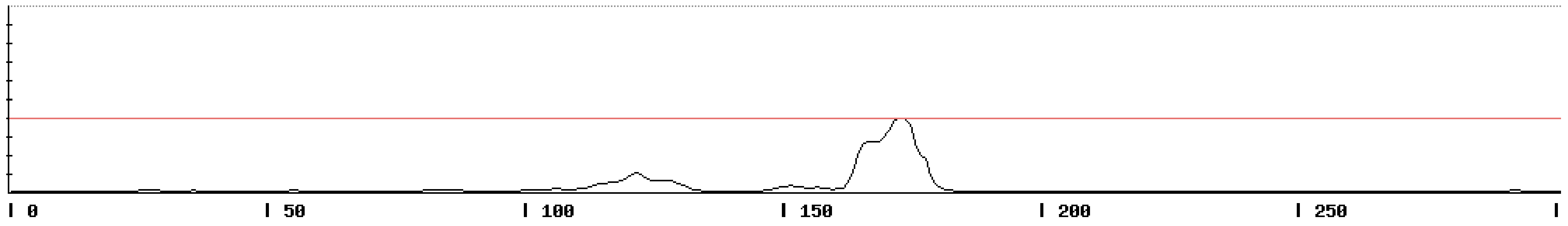

Predictions for Malusdomestica_MDP0000186765	
Viterbi Path	
Viterbi Path	
Posterior @ 0.4	
Posterior @ 0.4	
Posterior Graphic. Threshold Marked @ 0.4	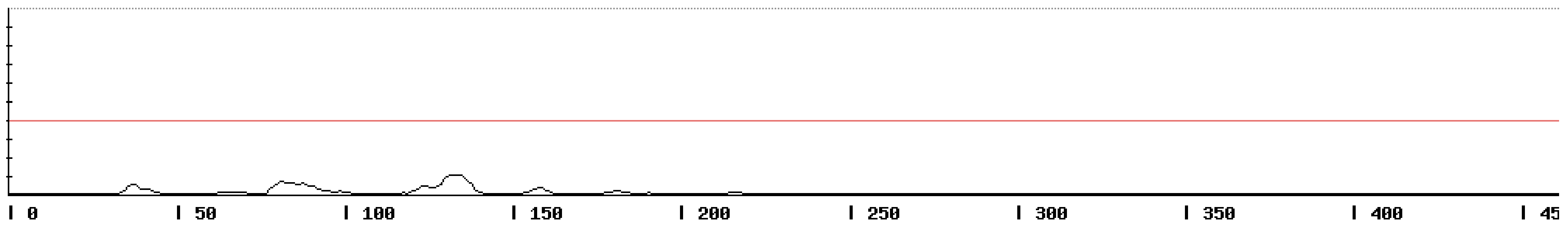

Predictions for Malusdomestica_MDP0000189394	
Viterbi Path	
Viterbi Path	
Posterior @ 0.4	
Posterior @ 0.4	
Posterior Graphic. Threshold Marked @ 0.4	

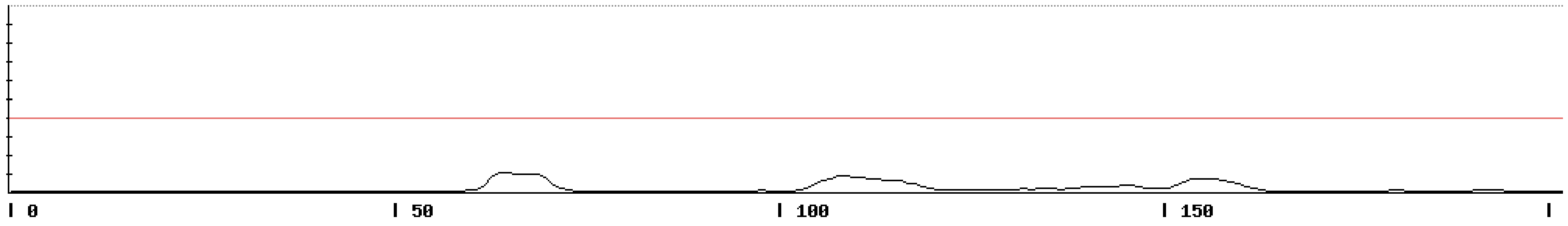

Predictions for Malusdomestica_MDP0000191344

Viterbi Path	
Viterbi Path	
Posterior @ 0.4	
Posterior @ 0.4	
Posterior Graphic. Threshold Marked @ 0.4	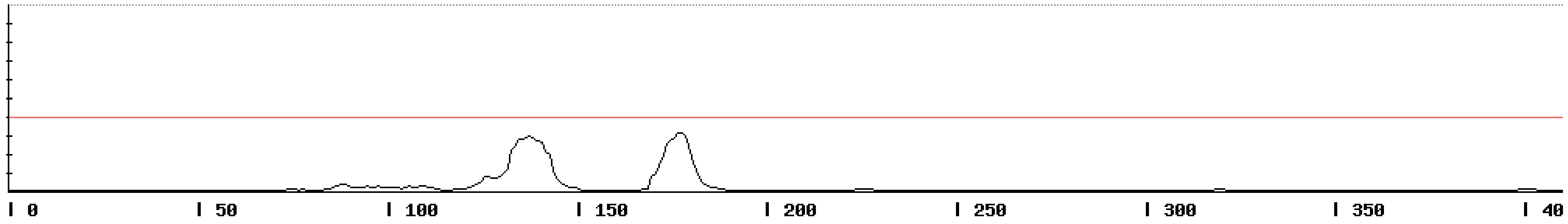

Predictions for Malusdomestica_MDP0000191614

Viterbi Path	
Viterbi Path	
Posterior @ 0.4	5 - EKTERAKERREKRR - 18
Posterior @ 0.4	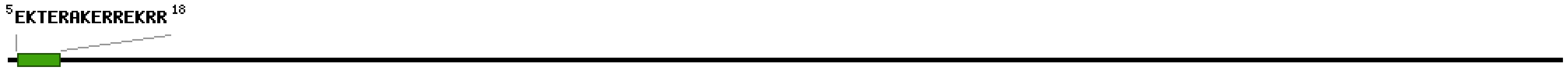

Posterior Graphic. Threshold Marked @ 0.4	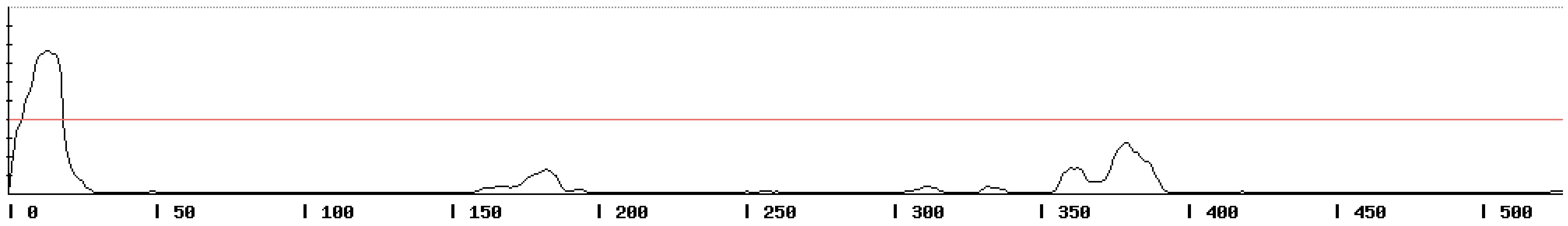

Predictions for Malusdomestica_MDP0000198108	
Viterbi Path	
Viterbi Path	
Posterior @ 0.4	
Posterior @ 0.4	
Posterior Graphic. Threshold Marked @ 0.4	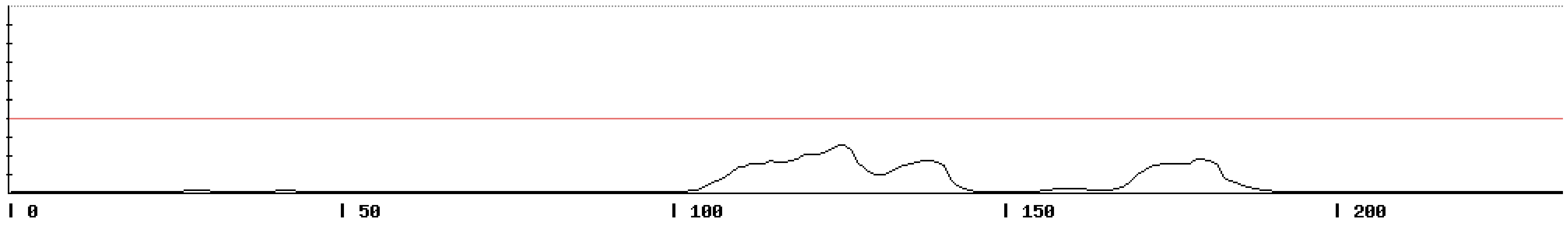

Predictions for Malusdomestica_MDP0000198235	
Viterbi Path	
Viterbi Path	
Posterior @ 0.4	
Posterior @ 0.4	

Posterior Graphic. Threshold Marked @ 0.4

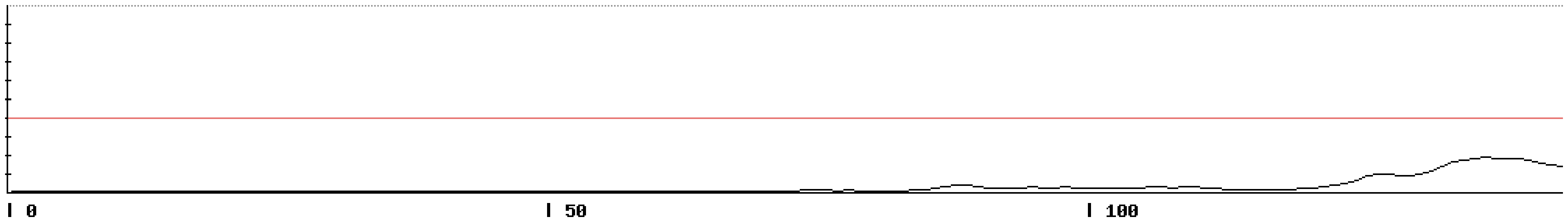

Predictions for Malusdomestica_MDP0000199840

Viterbi Path

Viterbi Path

Posterior @ 0.4

Posterior @ 0.4

Posterior Graphic. Threshold Marked @ 0.4

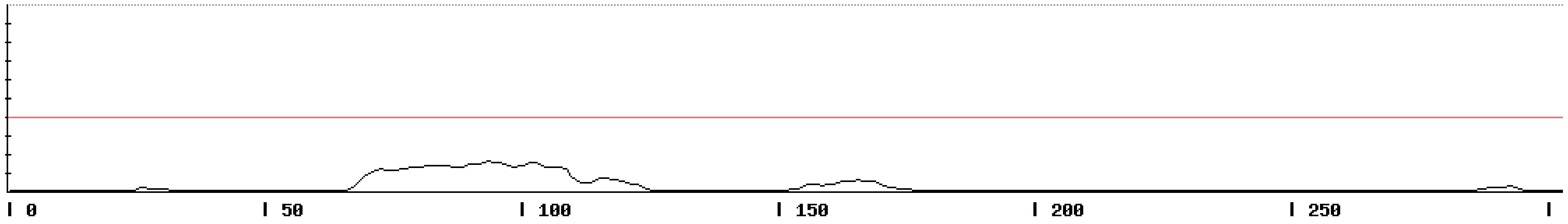

Predictions for Malusdomestica_MDP0000200646

Viterbi Path

Viterbi Path

Posterior @ 0.4	
Posterior @ 0.4	
Posterior Graphic. Threshold Marked @ 0.4	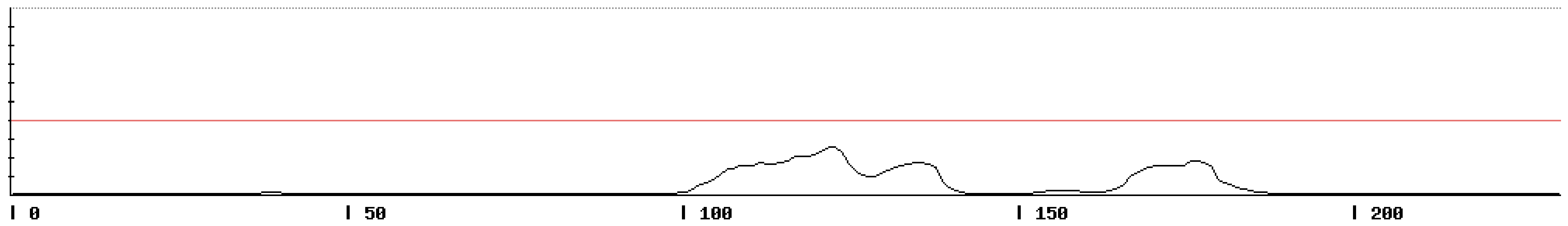

Predictions for Malusdomestica_MDP0000204582

Viterbi Path	
Viterbi Path	
Posterior @ 0.4	
Posterior @ 0.4	
Posterior Graphic. Threshold Marked @ 0.4	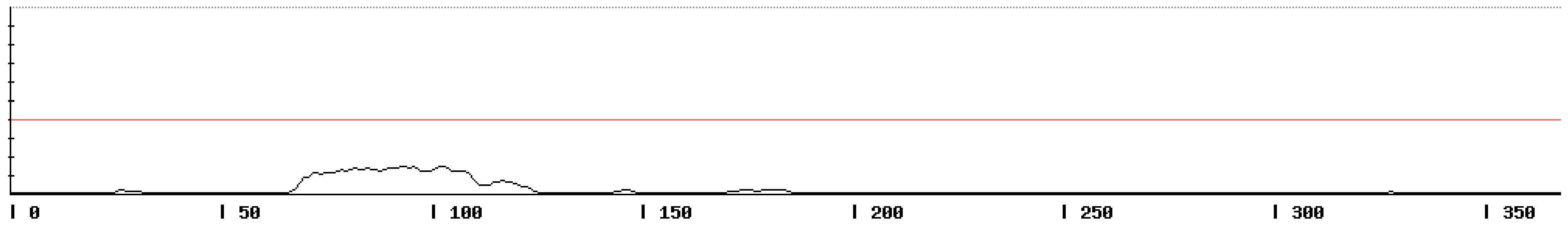

Predictions for Malusdomestica_MDP0000205938

Viterbi Path	
Viterbi Path	

Posterior @ 0.4	
Posterior @ 0.4	
Posterior Graphic. Threshold Marked @ 0.4	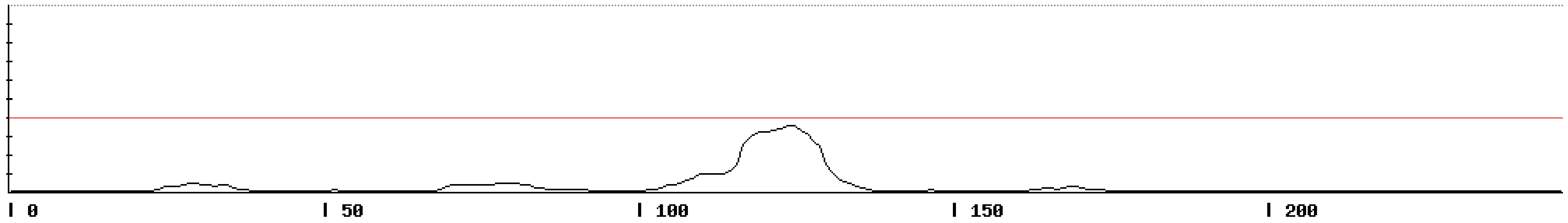

Predictions for Malusdomestica_MDP0000206199

Viterbi Path	
Viterbi Path	
Posterior @ 0.4	
Posterior @ 0.4	
Posterior Graphic. Threshold Marked @ 0.4	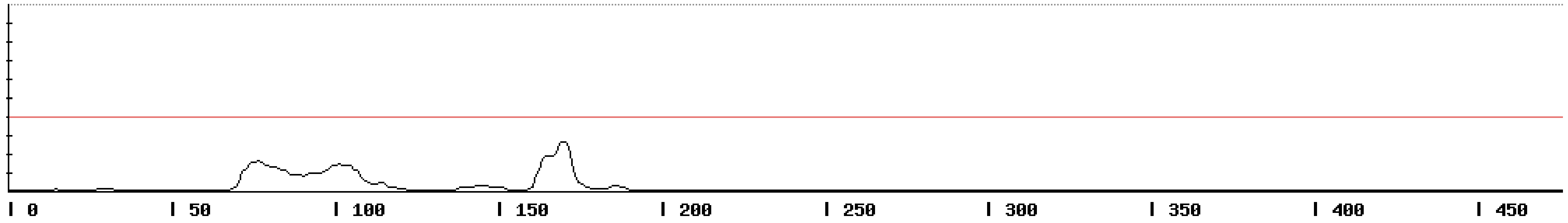

Predictions for Malusdomestica_MDP0000206212

Viterbi Path

Viterbi Path

Posterior @ 0.4

Posterior @ 0.4

Posterior Graphic. Threshold Marked @ 0.4

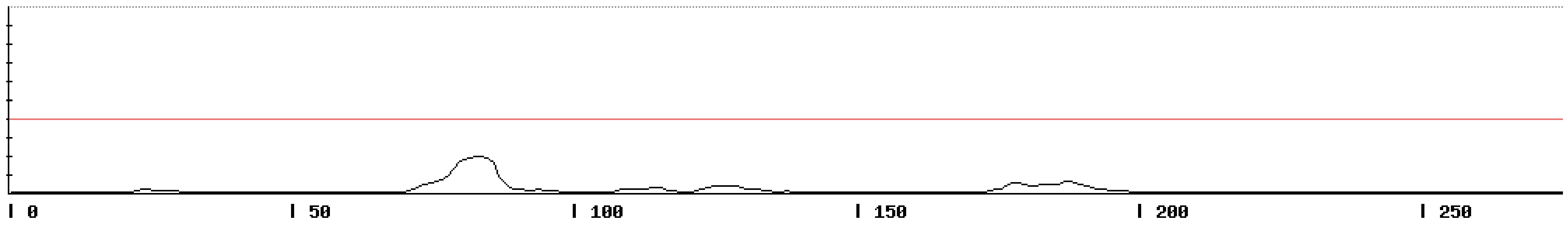

Predictions for Malusdomestica_MDP0000206868

Viterbi Path

Viterbi Path

Posterior @ 0.4

Posterior @ 0.4

Posterior Graphic. Threshold Marked @ 0.4

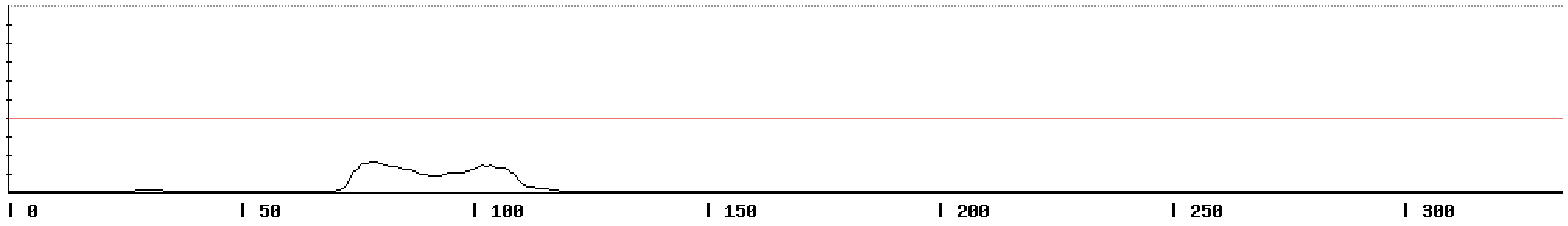

Predictions for Malusdomestica_MDP0000213555	
Viterbi Path	
Viterbi Path	
Posterior @ 0.4	79 - AGRIRKNYDRKRKADDR - 95<br>106 - K - 106
Posterior @ 0.4	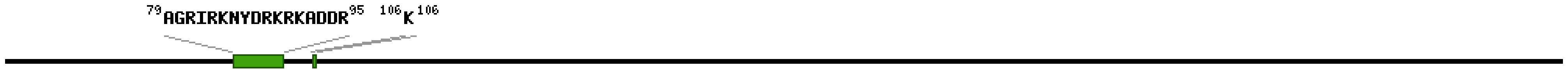
Posterior Graphic. Threshold Marked @ 0.4	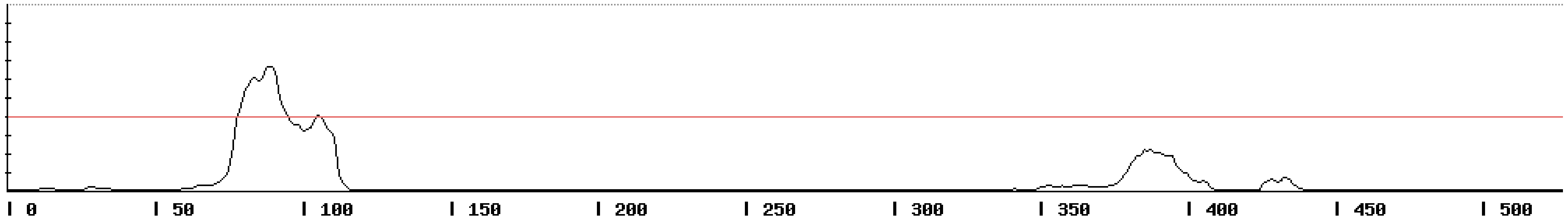

Predictions for Malusdomestica_MDP0000214515	
Viterbi Path	
Viterbi Path	
Posterior @ 0.4	108 - KIVKDRDNKIIGKKRI - 123
Posterior @ 0.4	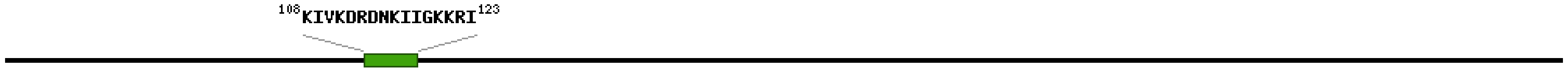
Posterior Graphic. Threshold Marked @ 0.4	

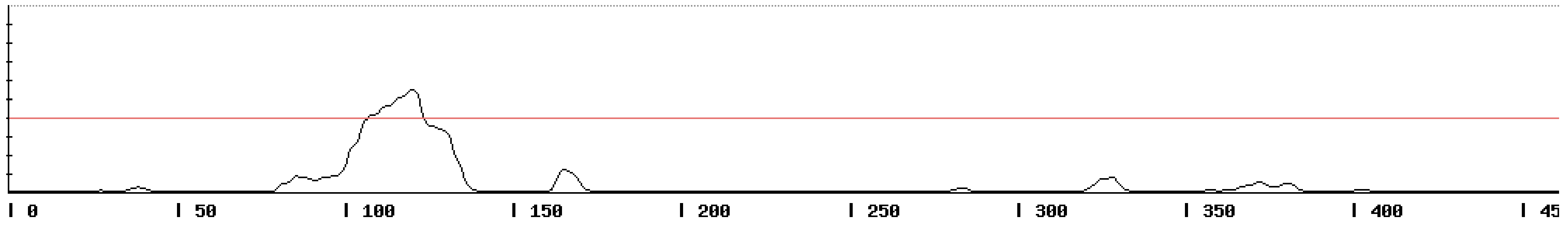

Predictions for Malusdomestica_MDP0000215351

Viterbi Path	
Viterbi Path	
Posterior @ 0.4	
Posterior @ 0.4	
Posterior Graphic. Threshold Marked @ 0.4	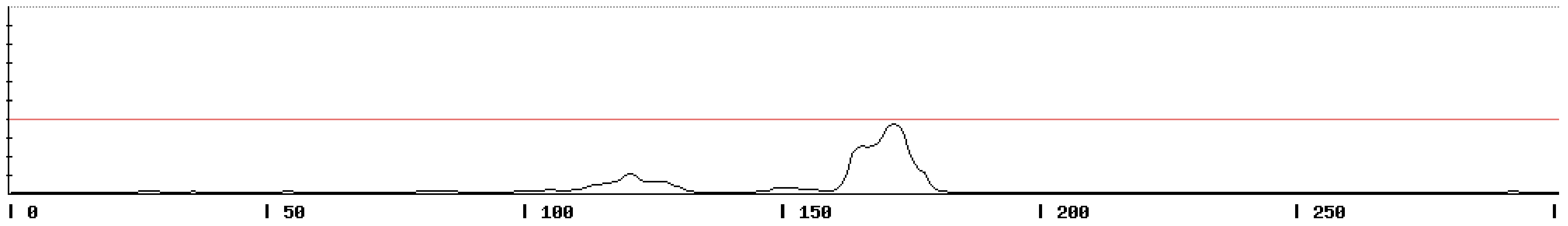

Predictions for Malusdomestica_MDP0000215904

Viterbi Path	218 - KRKNKRKSSKHDCLLSAAEKKRGRR - 242
Viterbi Path	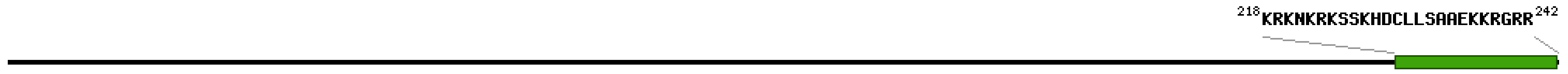
Posterior @ 0.4	217 - SKRKNKRKSSKHDCLLSAAEKKRGRR - 242
Posterior @ 0.4	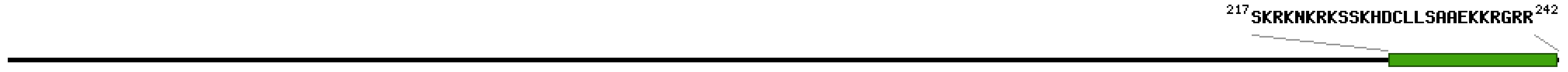

Posterior Graphic. Threshold Marked @ 0.4

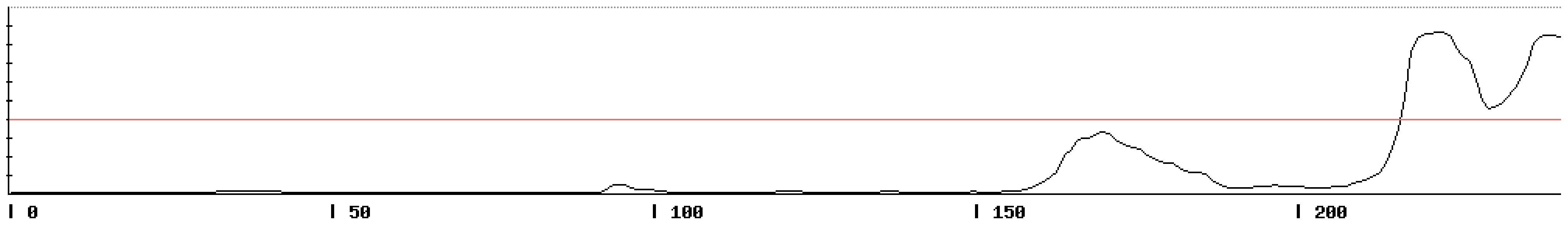

Predictions for Malusdomestica_MDP0000216205

Viterbi Path

Viterbi Path

Posterior @ 0.4 | 150 - RKRRK - 154

Posterior @ 0.4

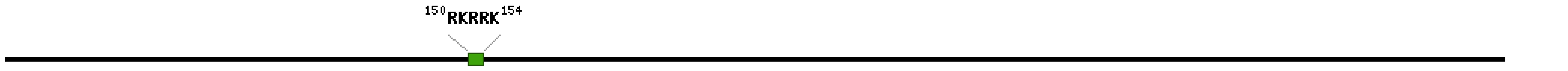

Posterior Graphic. Threshold Marked @ 0.4

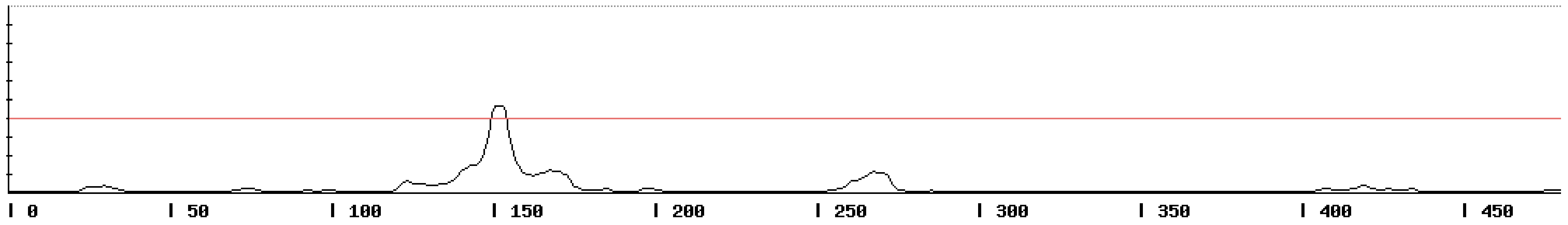

Predictions for Malusdomestica_MDP0000216357

Viterbi Path

Viterbi Path

Posterior @ 0.4

Posterior @ 0.4

Posterior Graphic. Threshold Marked @ 0.4

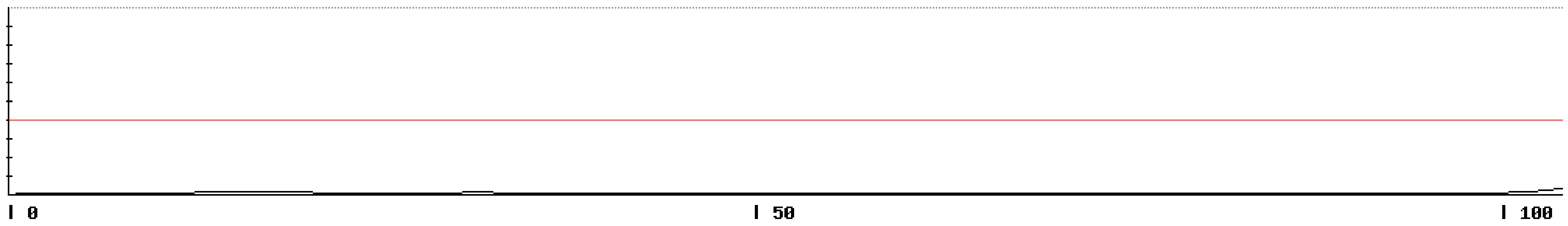

Predictions for Malusdomestica_MDP0000216714

Viterbi Path

Viterbi Path

Posterior @ 0.4

Posterior @ 0.4

Posterior Graphic. Threshold Marked @ 0.4

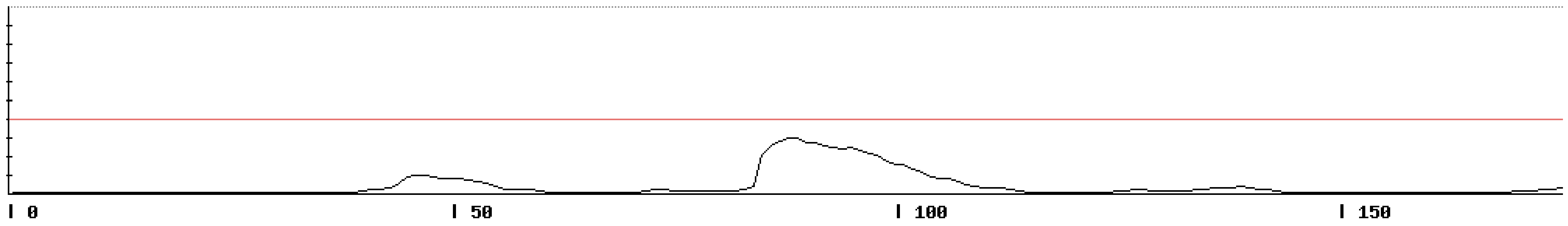

Predictions for Malusdomestica_MDP0000219488

Viterbi Path

Viterbi Path

Posterior @ 0.4

Posterior @ 0.4

Posterior Graphic. Threshold Marked @ 0.4

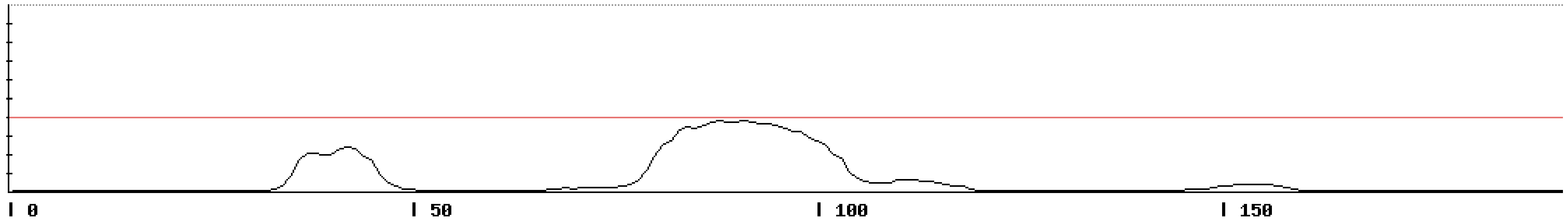

Predictions for Malusdomestica_MDP0000221977

Viterbi Path

Viterbi Path

Posterior @ 0.4

Posterior @ 0.4

Posterior Graphic. Threshold Marked @ 0.4

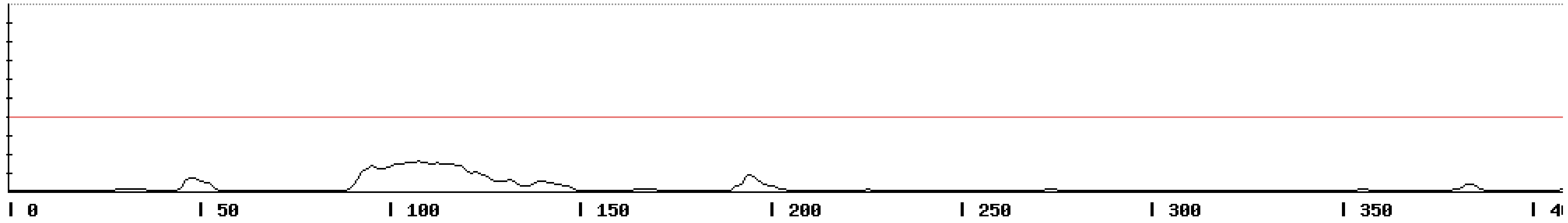

Predictions for Malusdomestica_MDP0000222045

Viterbi Path

Viterbi Path

Posterior @ 0.4

Posterior @ 0.4

Posterior Graphic. Threshold Marked @ 0.4

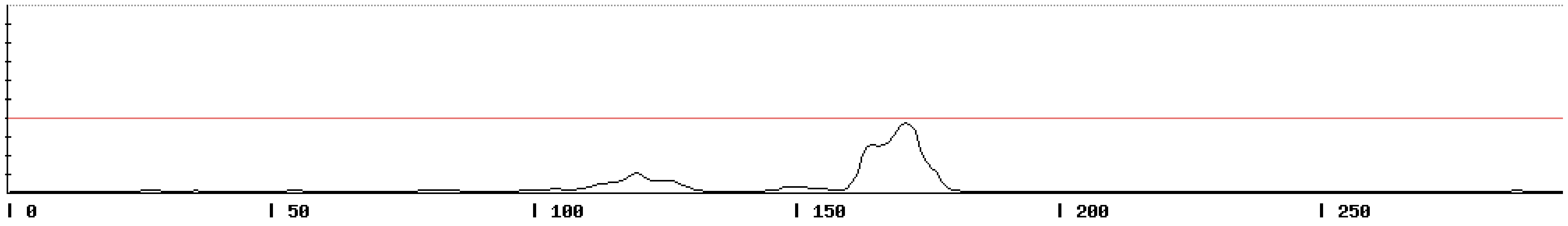

Predictions for Malusdomestica_MDP0000224592

Viterbi Path

Viterbi Path

Posterior @ 0.4 135 - RKRRK - 139

Posterior @ 0.4

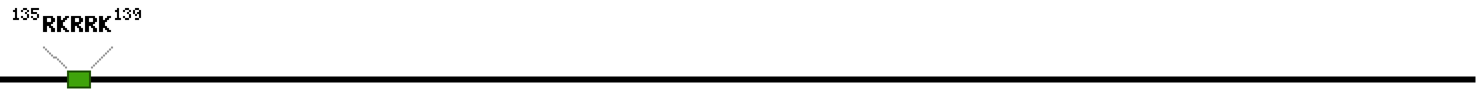

Posterior Graphic. Threshold Marked @ 0.4

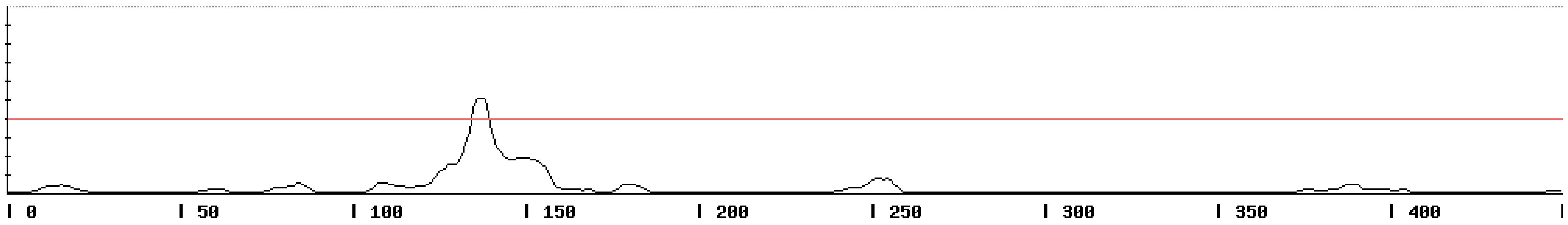

Predictions for Malusdomestica_MDP0000225852

Viterbi Path

Viterbi Path

Posterior @ 0.4

Posterior @ 0.4

Posterior Graphic. Threshold Marked @ 0.4

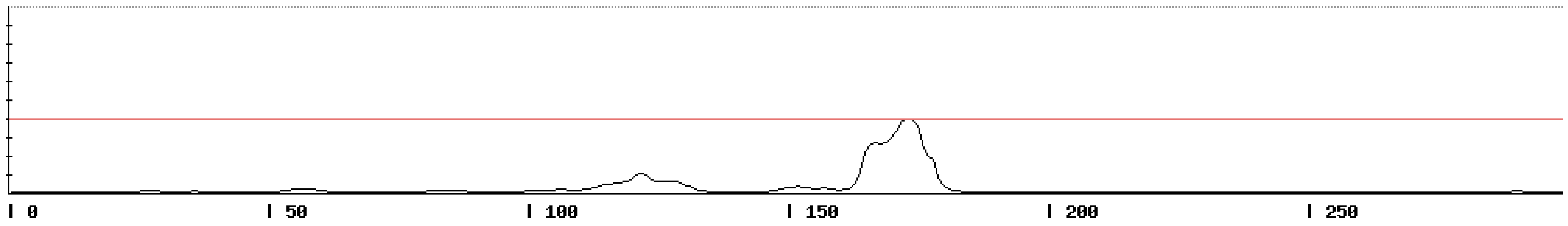

Predictions for Malusdomestica_MDP0000226701

Viterbi Path

Viterbi Path

Posterior @ 0.4

Posterior @ 0.4

Posterior Graphic. Threshold Marked @ 0.4

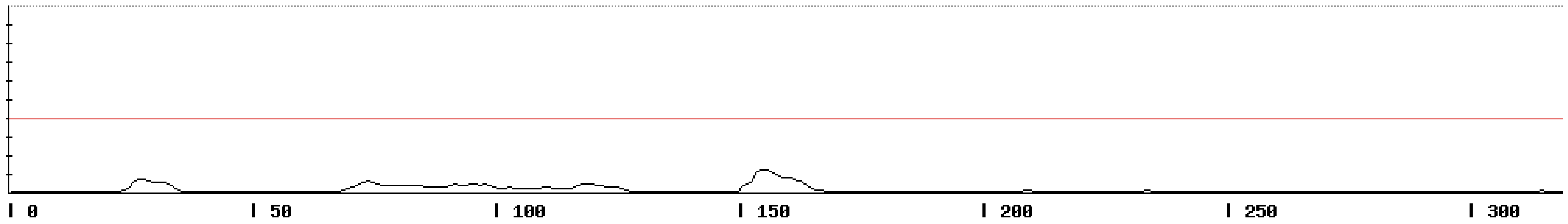

Predictions for Malusdomestica_MDP0000231843	
Viterbi Path	
Viterbi Path	
Posterior @ 0.4	
Posterior @ 0.4	
Posterior Graphic. Threshold Marked @ 0.4	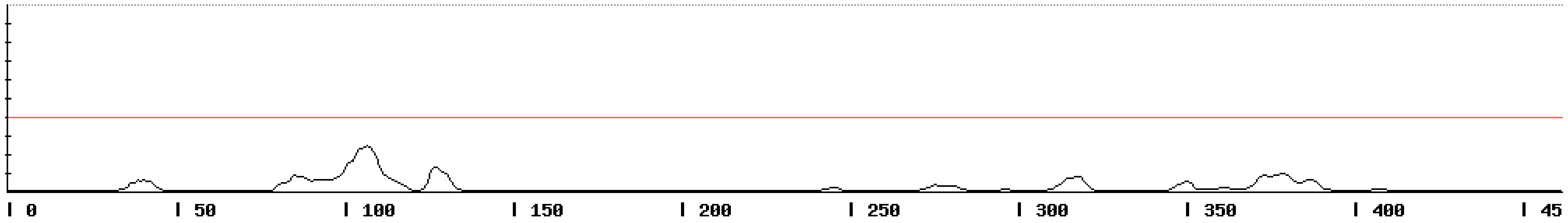

Predictions for Malusdomestica_MDP0000231845	
Viterbi Path	
Viterbi Path	
Posterior @ 0.4	5 - GRKRKTRG - 12
	82 - KYKNSKRSNRTTPGGYWKITGKERDIKRRSKAVIGKKRTL - 121
	471 - LKRRYRRRTKRSNGRLAKPPPPA - 493
Posterior @ 0.4	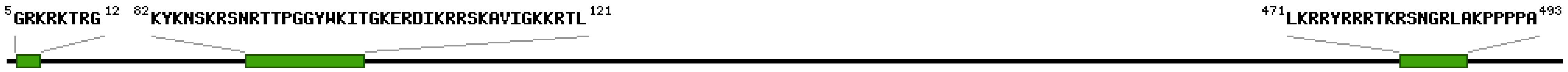
Posterior Graphic. Threshold Marked @ 0.4	

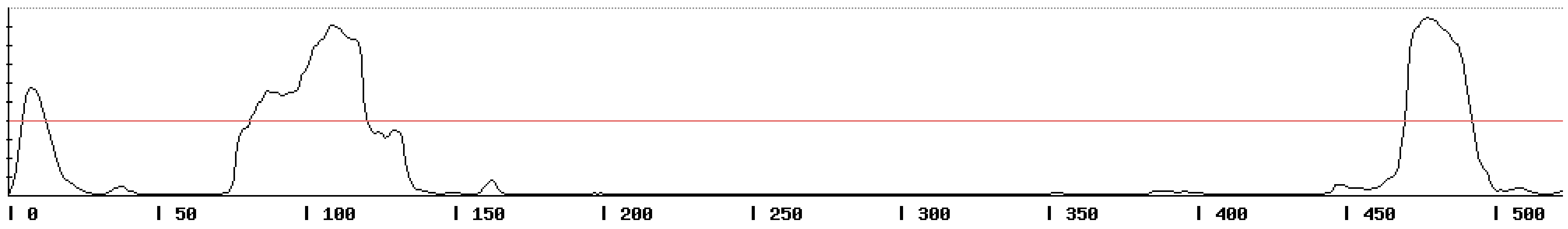

Predictions for Malusdomestica_MDP0000232008

Viterbi Path	
Viterbi Path	
Posterior @ 0.4	
Posterior @ 0.4	
Posterior Graphic. Threshold Marked @ 0.4	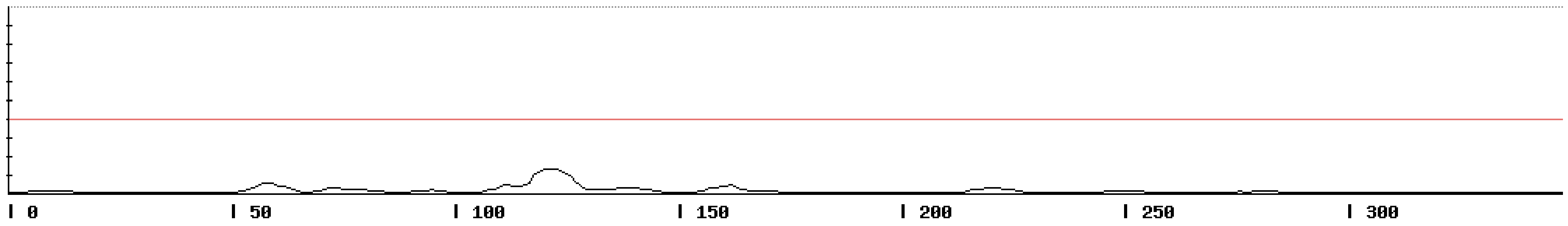

Predictions for Malusdomestica_MDP0000232050

Viterbi Path	
Viterbi Path	
Posterior @ 0.4	
Posterior @ 0.4	

Posterior Graphic. Threshold Marked @ 0.4

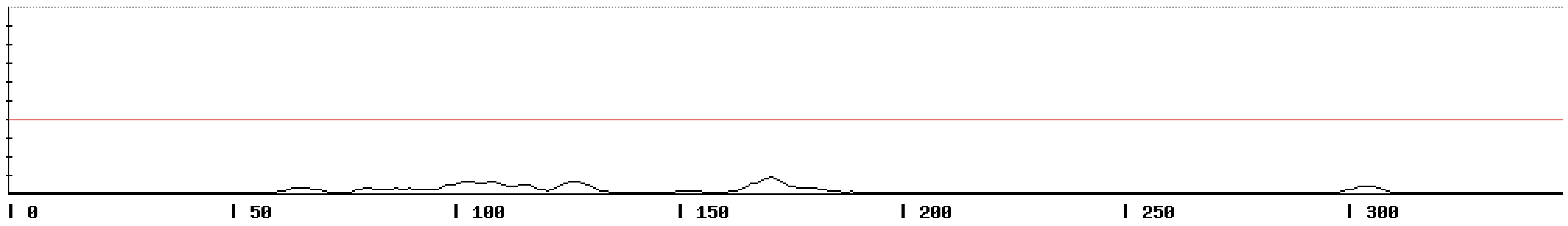

Predictions for Malusdomestica_MDP0000233010

Viterbi Path	
Viterbi Path	
Posterior @ 0.4	164 - RKYRNGSRPKRA - 175
Posterior @ 0.4	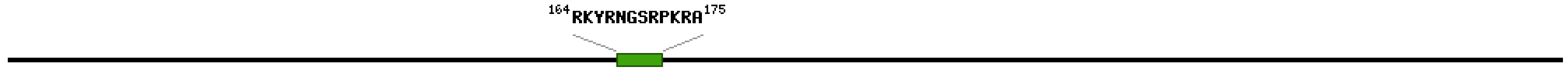
Posterior Graphic. Threshold Marked @ 0.4	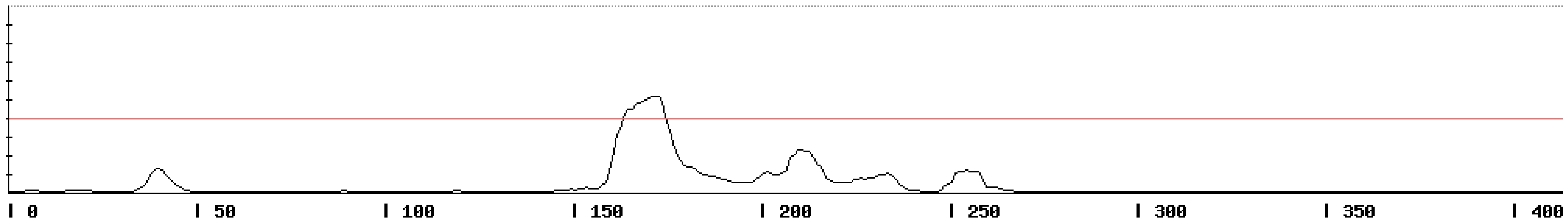

Predictions for Malusdomestica_MDP0000233614

Viterbi Path	
Viterbi Path	
Posterior @ 0.4	
Posterior @ 0.4	

Posterior Graphic. Threshold Marked @ 0.4

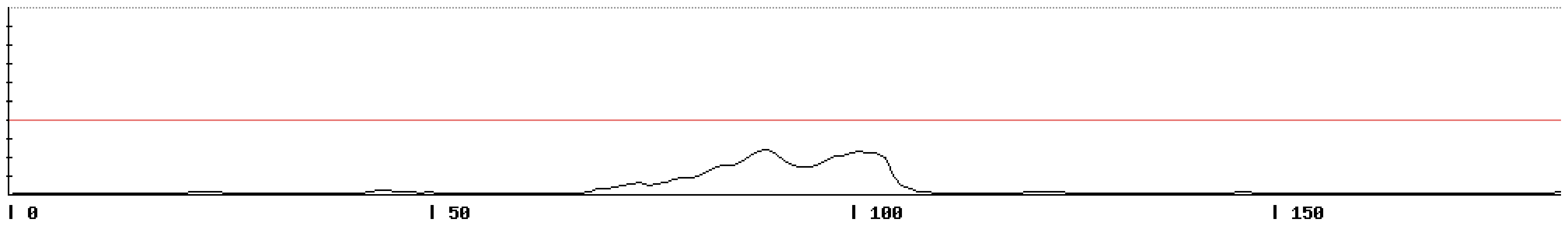

Predictions for Malusdomestica_MDP0000235213

Viterbi Path

Viterbi Path

Posterior @ 0.4

Posterior @ 0.4

Posterior Graphic. Threshold Marked @ 0.4

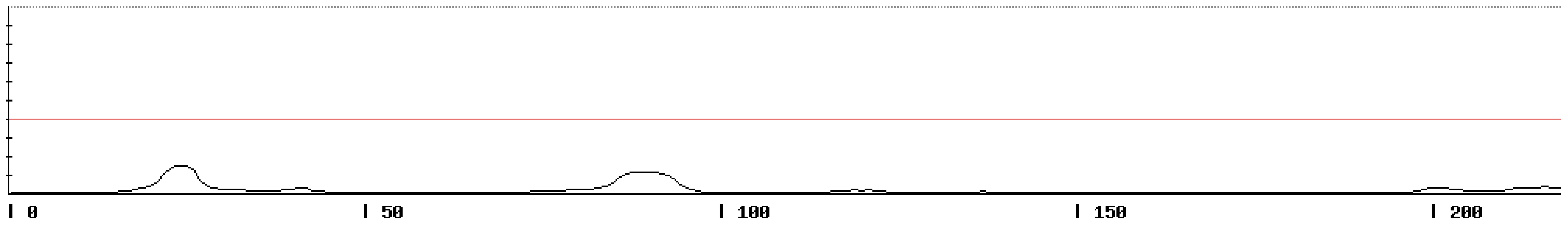

Predictions for Malusdomestica_MDP0000236349

Viterbi Path

Viterbi Path

Posterior @ 0.4

Posterior @ 0.4

Posterior Graphic. Threshold Marked @ 0.4

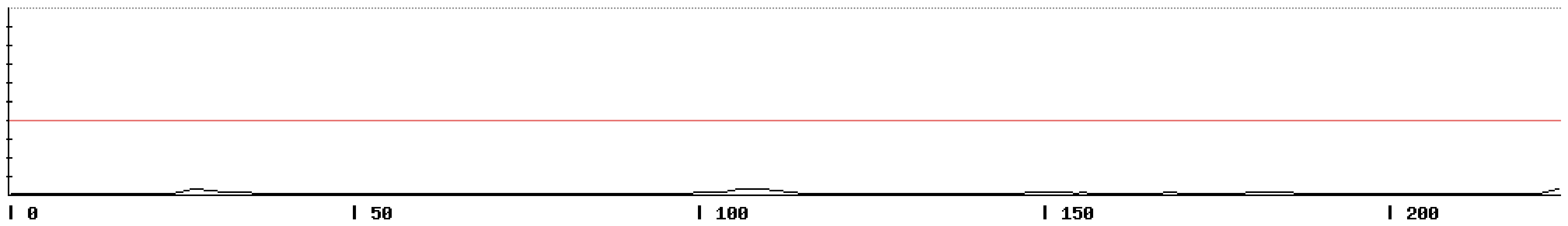

Predictions for Malusdomestica_MDP0000238035

Viterbi Path

Viterbi Path

Posterior @ 0.4 — 143 - RKRKVMG - 149

Posterior @ 0.4

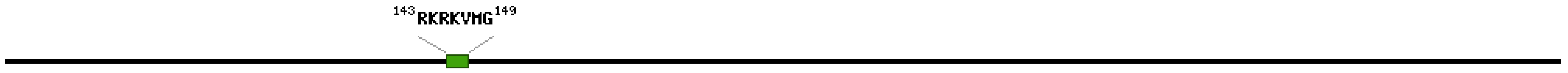

Posterior Graphic. Threshold Marked @ 0.4

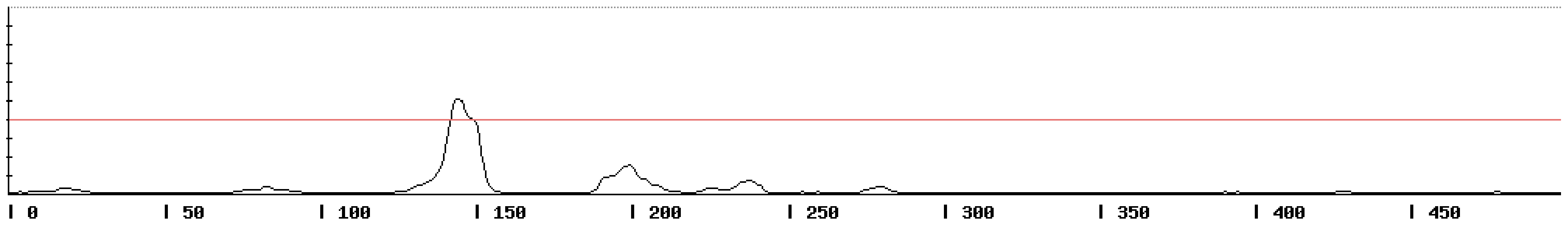

Predictions for Malusdomestica_MDP0000238773

Viterbi Path

Viterbi Path

Posterior @ 0.4	351 - KKLKRKK - 357
Posterior @ 0.4	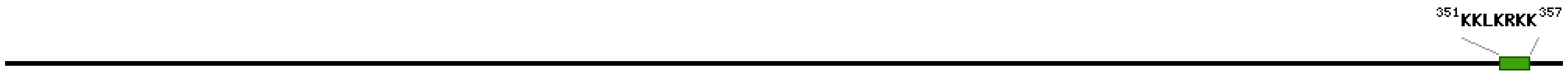
Posterior Graphic. Threshold Marked @ 0.4	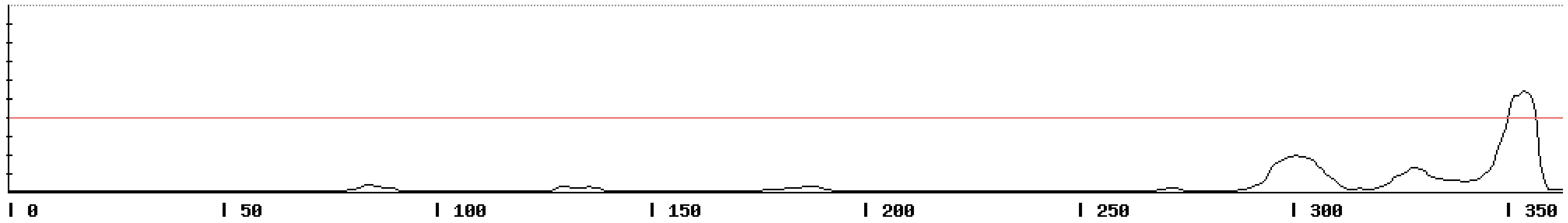

Predictions for Malusdomestica_MDP0000239596

Viterbi Path	
Viterbi Path	
Posterior @ 0.4	
Posterior @ 0.4	
Posterior Graphic. Threshold Marked @ 0.4	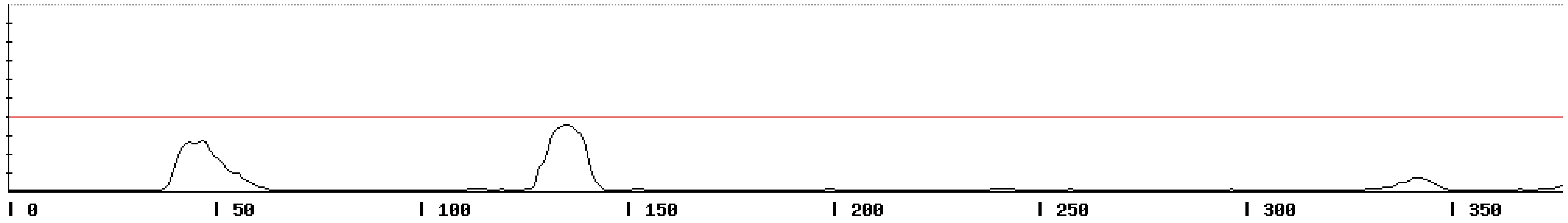

Predictions for Malusdomestica_MDP0000240094

Viterbi Path	
Viterbi Path	
Posterior @ 0.4	
Posterior @ 0.4	
Posterior Graphic. Threshold Marked @ 0.4	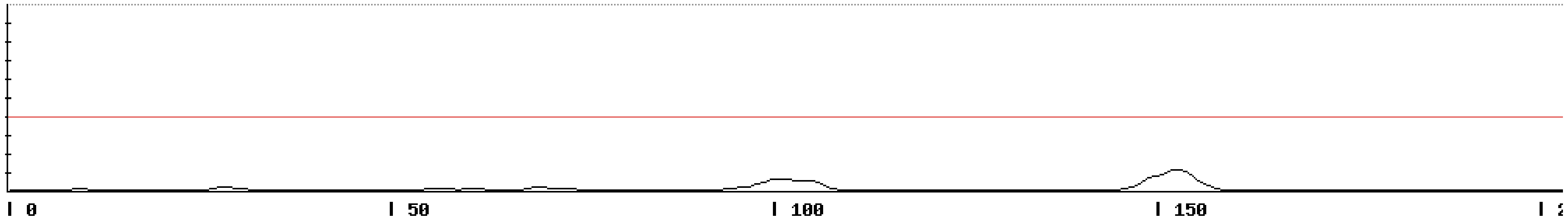

**Predictions for Malusdomestica_MDP0000240855**	
Viterbi Path	
Viterbi Path	
Posterior @ 0.4	
Posterior @ 0.4	
Posterior Graphic. Threshold Marked @ 0.4	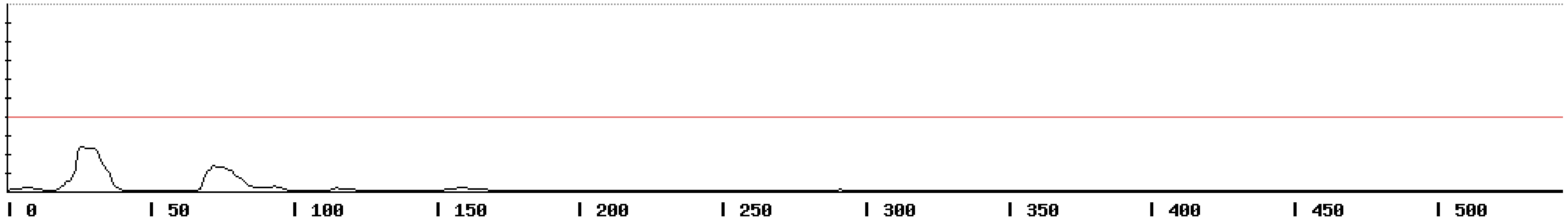

Predictions for Malusdomestica_MDP0000241063	
Viterbi Path	
Viterbi Path	
Posterior @ 0.4	
Posterior @ 0.4	
Posterior Graphic. Threshold Marked @ 0.4	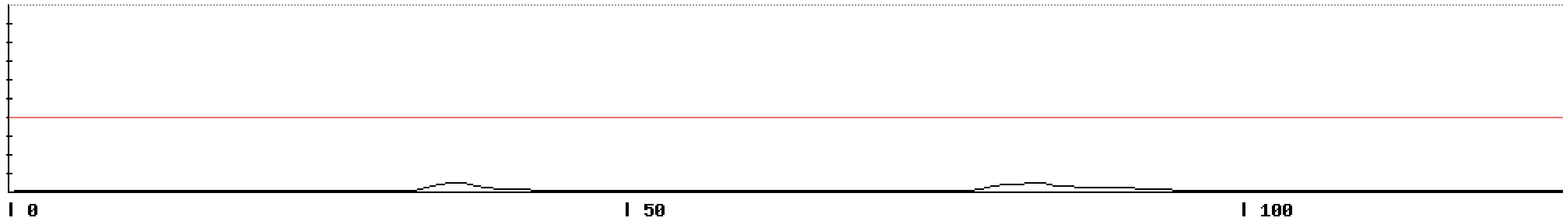

Predictions for Malusdomestica_MDP0000241821	
Viterbi Path	
Viterbi Path	
Posterior @ 0.4	
Posterior @ 0.4	
Posterior Graphic. Threshold Marked @ 0.4	

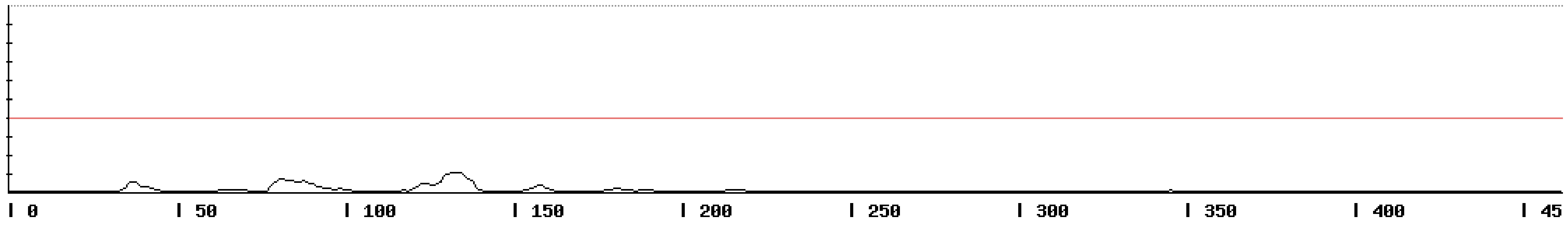

Predictions for Malusdomestica_MDP0000242297

Viterbi Path	
Viterbi Path	
Posterior @ 0.4	192 - GKKKSTRQRR - 201
Posterior @ 0.4	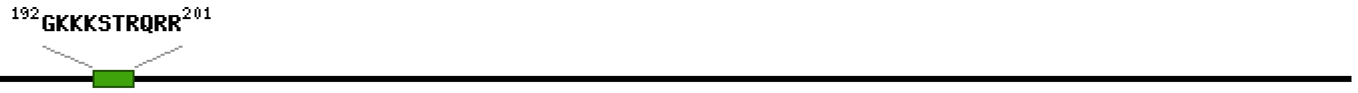
Posterior Graphic. Threshold Marked @ 0.4	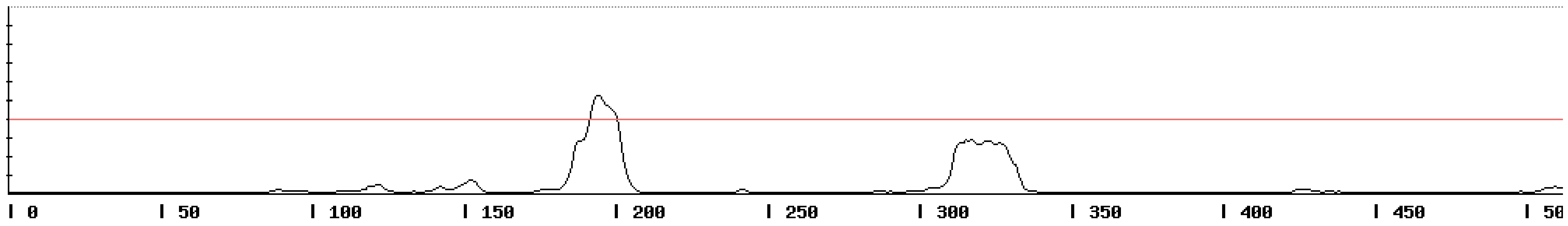

Predictions for Malusdomestica_MDP0000242656

Viterbi Path	
Viterbi Path	
Posterior @ 0.4	
Posterior @ 0.4	

Posterior Graphic. Threshold Marked @ 0.4	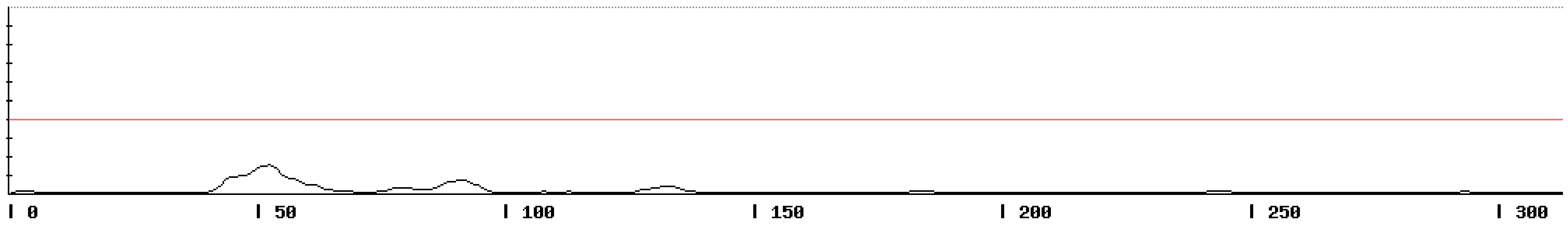

Predictions for Malusdomestica_MDP0000246470	
Viterbi Path	
Viterbi Path	
Posterior @ 0.4	
Posterior @ 0.4	
Posterior Graphic. Threshold Marked @ 0.4	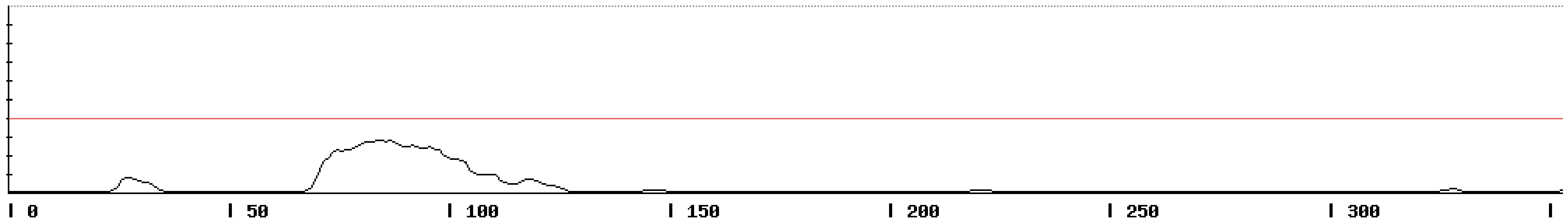

Predictions for Malusdomestica_MDP0000246482	
Viterbi Path	
Viterbi Path	
Posterior @ 0.4	
Posterior @ 0.4	

Posterior Graphic. Threshold Marked @ 0.4

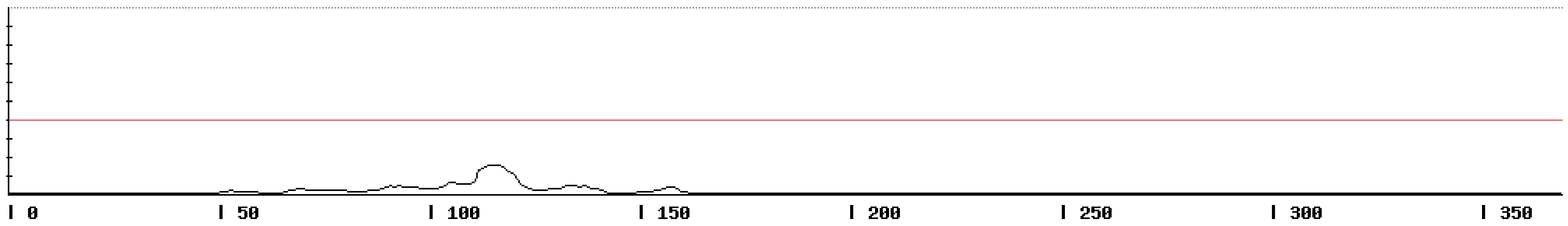

Predictions for Malusdomestica_MDP0000249947

Viterbi Path

Viterbi Path

Posterior @ 0.4

Posterior @ 0.4

Posterior Graphic. Threshold Marked @ 0.4

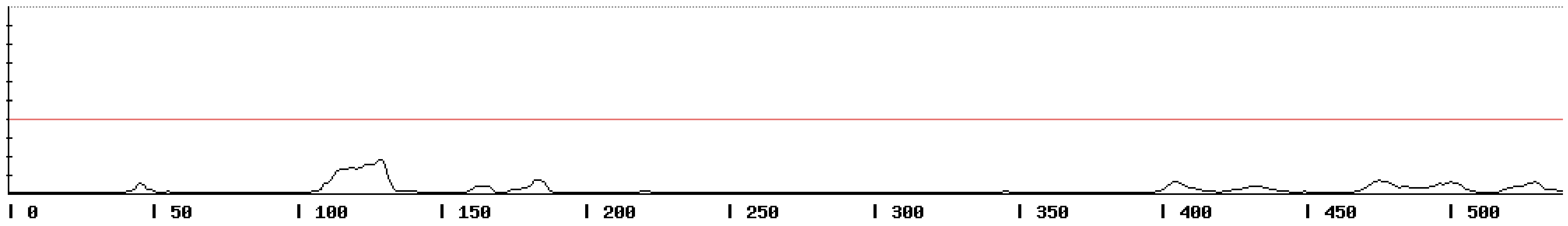

Predictions for Malusdomestica_MDP0000250487

Viterbi Path

Viterbi Path

Posterior @ 0.4

Posterior @ 0.4

Posterior Graphic. Threshold Marked @ 0.4

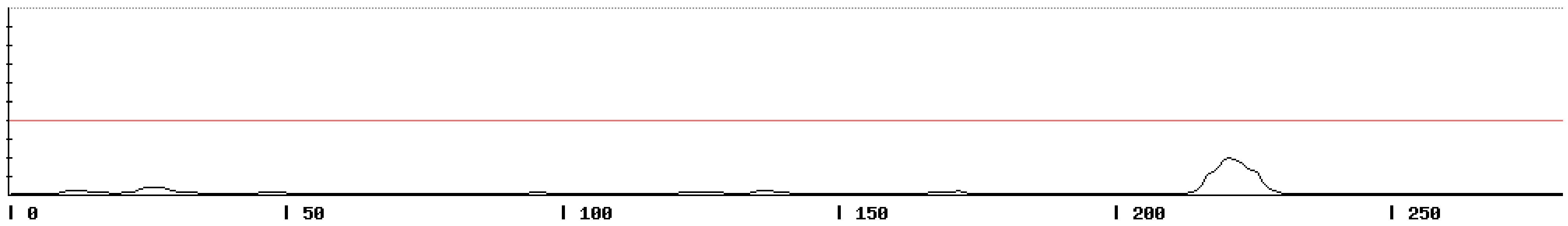

Predictions for Malusdomestica_MDP0000252435

Viterbi Path

Viterbi Path

Posterior @ 0.4

Posterior @ 0.4

Posterior Graphic. Threshold Marked @ 0.4

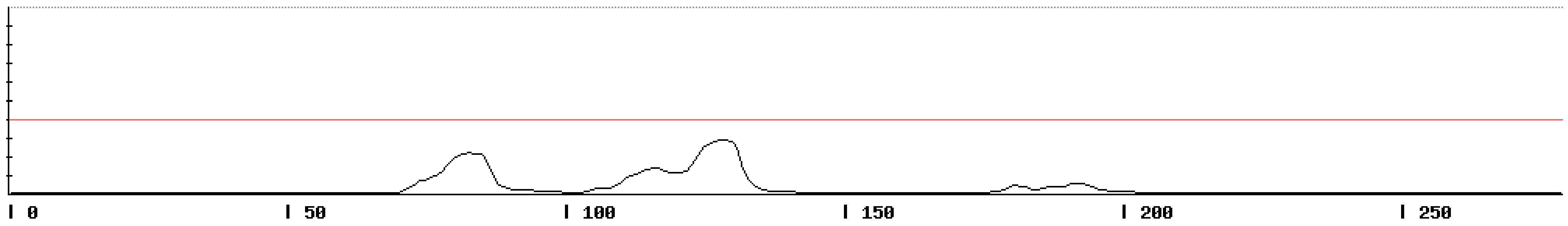

Predictions for Malusdomestica_MDP0000252746

Viterbi Path

Viterbi Path

Posterior @ 0.4	
Posterior @ 0.4	
Posterior Graphic. Threshold Marked @ 0.4	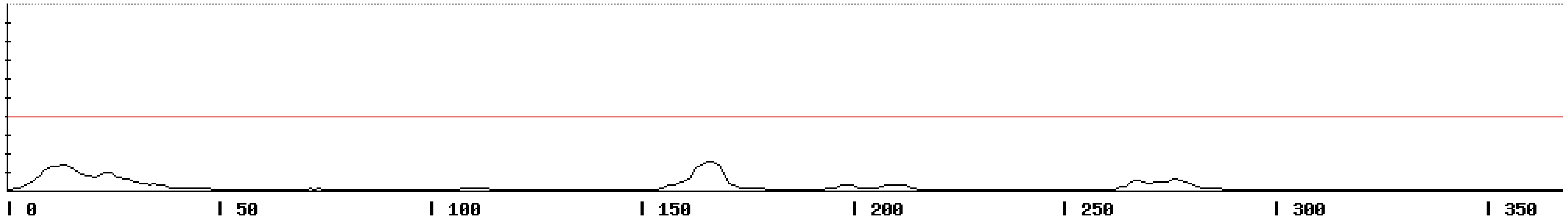

Predictions for Malusdomestica_MDP0000254112

Viterbi Path	
Viterbi Path	
Posterior @ 0.4	
Posterior @ 0.4	
Posterior Graphic. Threshold Marked @ 0.4	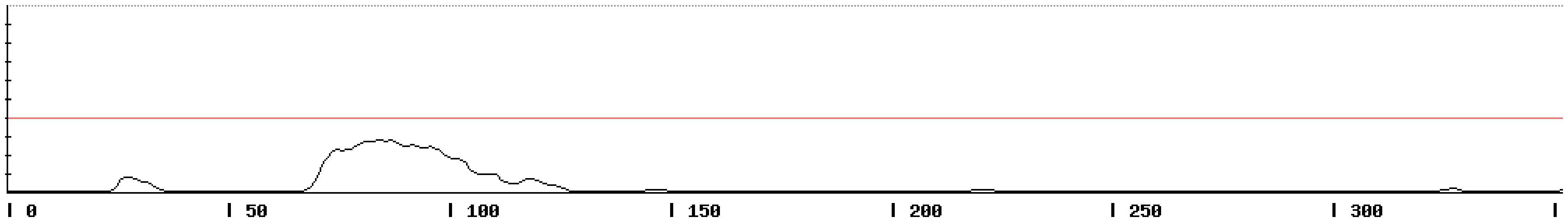

Predictions for Malusdomestica_MDP0000255565

Viterbi Path	
Viterbi Path	
Posterior @ 0.4	
Posterior @ 0.4	
Posterior Graphic. Threshold Marked @ 0.4	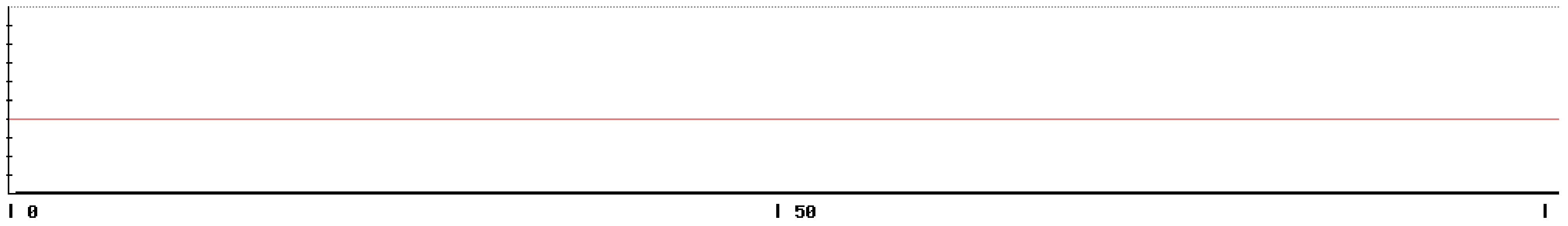

Predictions for Malusdomestica_MDP0000255884

Viterbi Path	
Viterbi Path	
Posterior @ 0.4	
Posterior @ 0.4	
Posterior Graphic. Threshold Marked @ 0.4	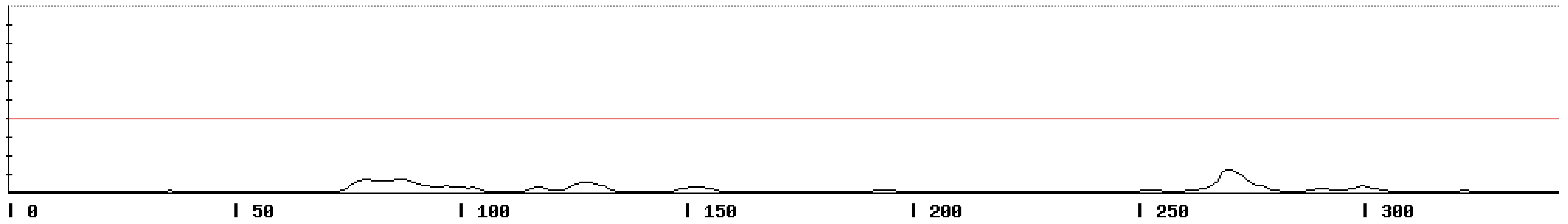

Predictions for Malusdomestica_MDP0000256730

Viterbi Path

Viterbi Path

Posterior @ 0.4

Posterior @ 0.4

Posterior Graphic. Threshold Marked @ 0.4

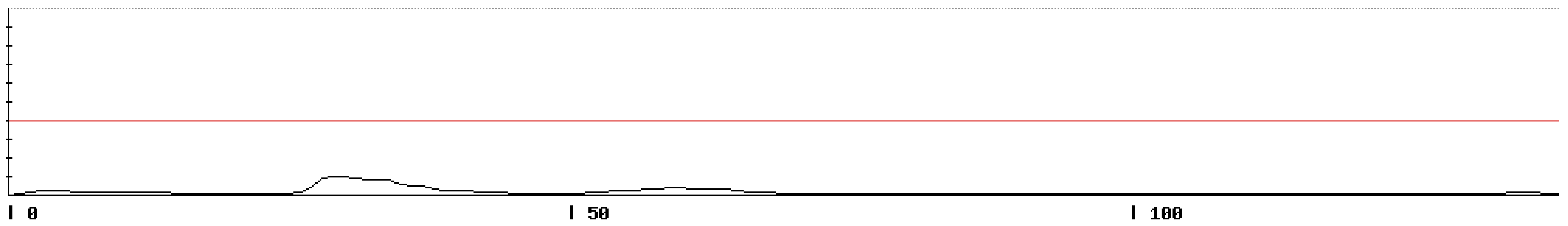

Predictions for Malusdomestica_MDP0000256806

Viterbi Path

Viterbi Path

Posterior @ 0.4

Posterior @ 0.4

Posterior Graphic. Threshold Marked @ 0.4

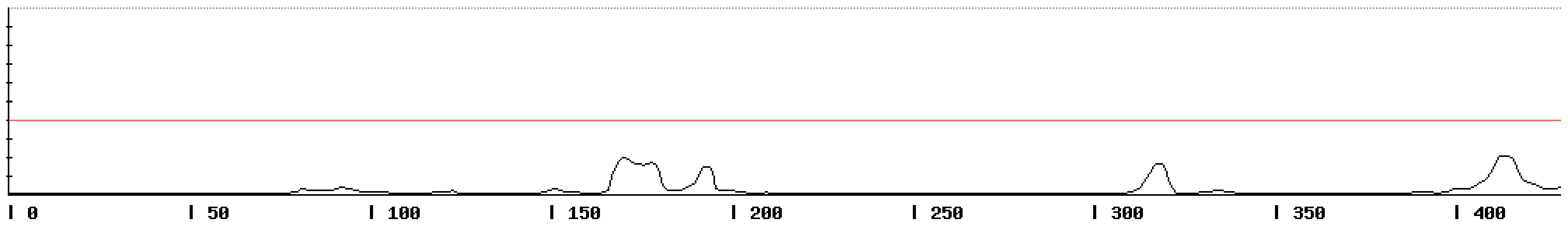

Predictions for Malusdomestica_MDP0000257674

Viterbi Path

Viterbi Path

Posterior @ 0.4

Posterior @ 0.4

Posterior Graphic. Threshold Marked @ 0.4

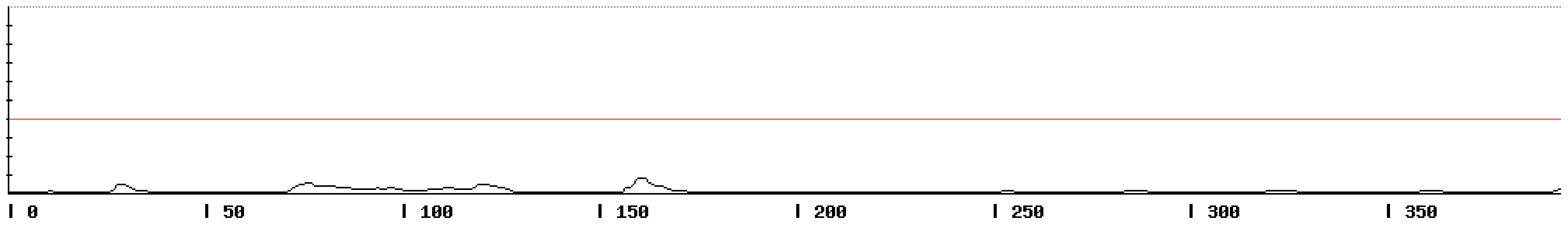

Predictions for Malusdomestica_MDP0000258167

Viterbi Path

Viterbi Path

Posterior @ 0.4

Posterior @ 0.4

Posterior Graphic. Threshold Marked @ 0.4	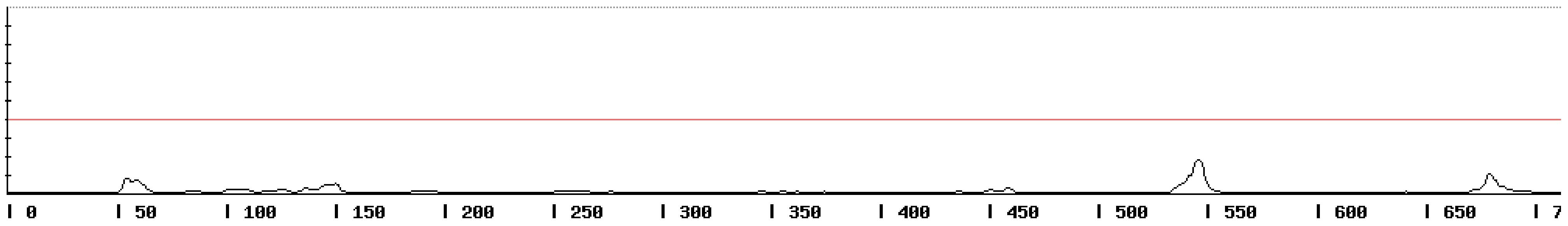

Predictions for Malusdomestica_MDP0000258530

Viterbi Path	
Viterbi Path	
Posterior @ 0.4	14 - KTSPHGLPKKRYRPR - 28
	145 - GRPEAATGRRPG - 156
Posterior @ 0.4	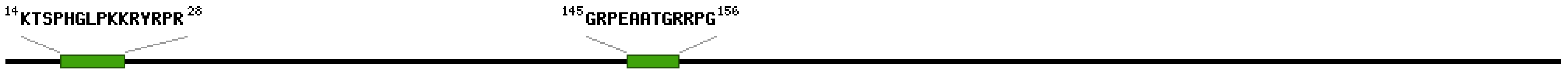
Posterior Graphic. Threshold Marked @ 0.4	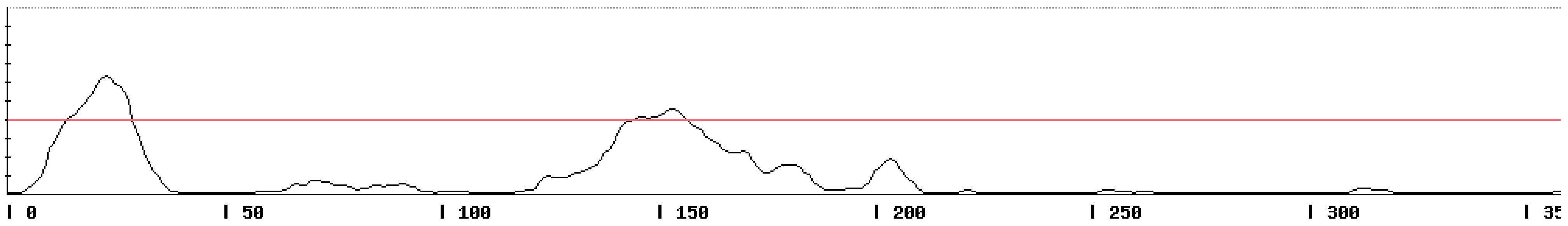

Predictions for Malusdomestica_MDP0000259556

Viterbi Path	175 - KPPVKRAGKQVLNEKPPVKRKRARTEELNEKPSVKKEKKKWDRK - 218
Viterbi Path	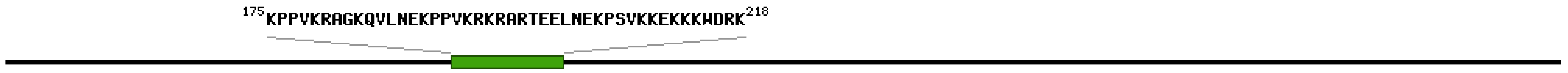
Posterior @ 0.4	175 - KPPVKRAGKQVLNEKPPVKRKRARTEELNEKPSVKKEKKKWDRKQ - 219
Posterior @ 0.4	

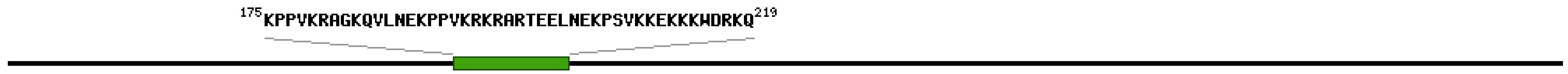

Posterior Graphic. Threshold Marked @ 0.4

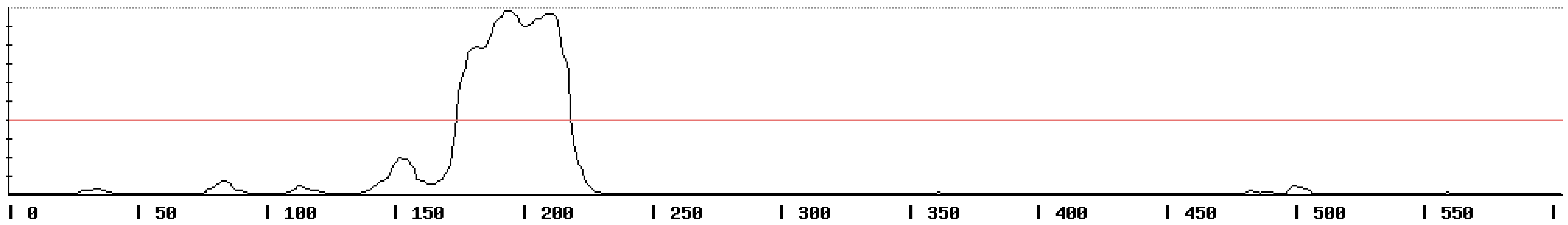

Predictions for Malusdomestica_MDP0000262032

Viterbi Path

Viterbi Path

Posterior @ 0.4

Posterior @ 0.4

Posterior Graphic. Threshold Marked @ 0.4

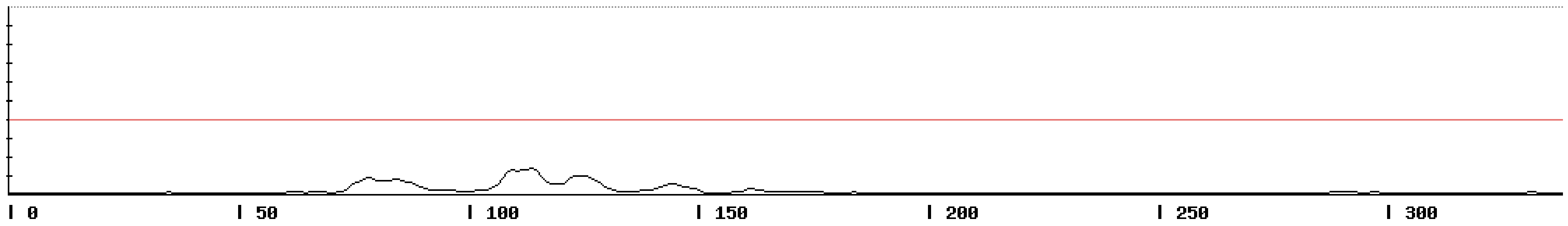

Predictions for Malusdomestica_MDP0000262990

Viterbi Path

Viterbi Path

Posterior @ 0.4	
Posterior @ 0.4	
Posterior Graphic. Threshold Marked @ 0.4	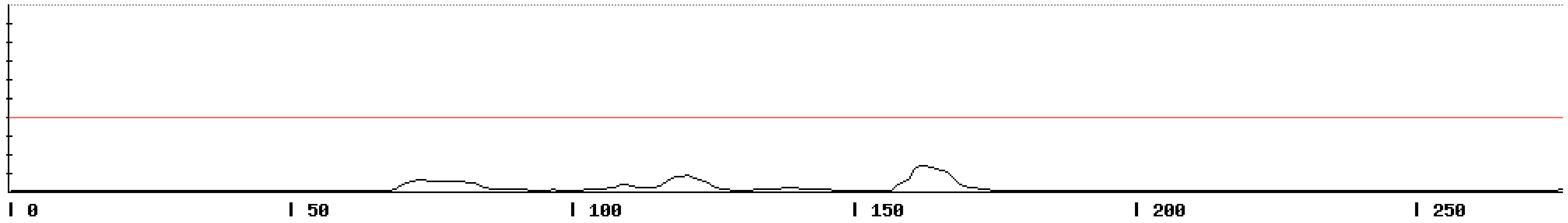

Predictions for Malusdomestica_MDP0000265073

Viterbi Path	
Viterbi Path	
Posterior @ 0.4	379 - RKYRNGSRPKRA - 390
Posterior @ 0.4	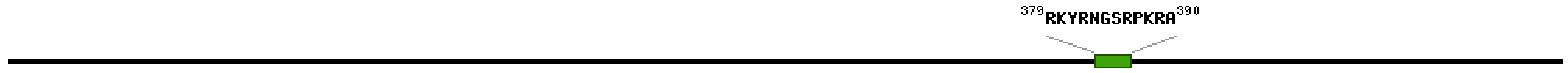
Posterior Graphic. Threshold Marked @ 0.4	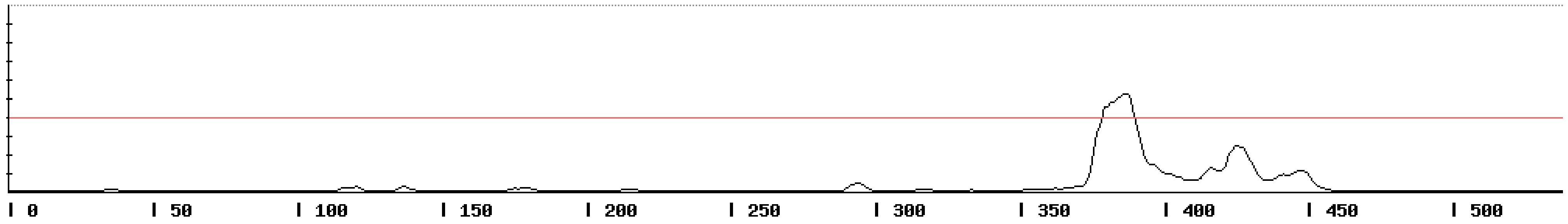

Predictions for Malusdomestica_MDP0000265416

Viterbi Path	
Viterbi Path	

Posterior @ 0.4	
Posterior @ 0.4	
Posterior Graphic. Threshold Marked @ 0.4	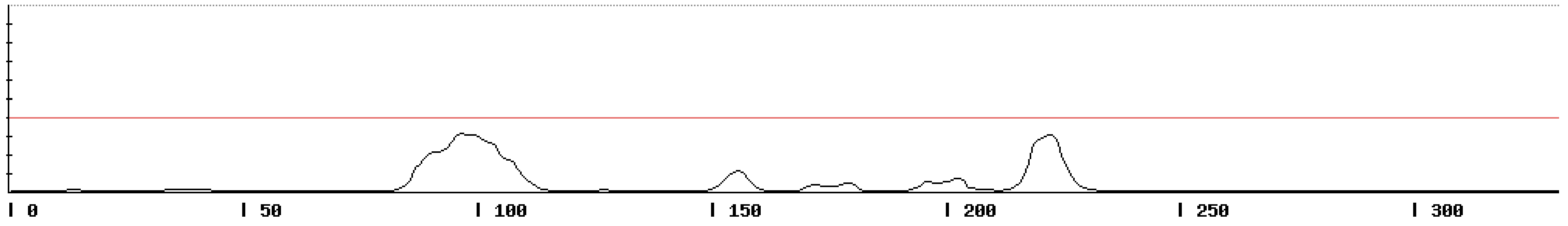

Predictions for Malusdomestica_MDP0000265616

Viterbi Path	324 - RLRKNDKSGGKKKRNN - 339
Viterbi Path	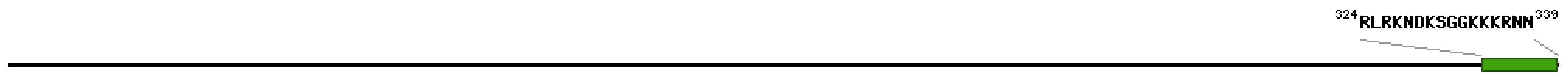
Posterior @ 0.4	64 - KKLKKSHKKGQ - 74<br>325 - LRKNDKSGGKKKRNN - 339
Posterior @ 0.4	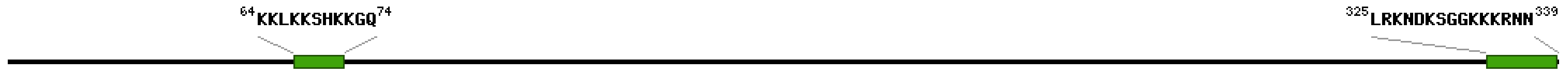
Posterior Graphic. Threshold Marked @ 0.4	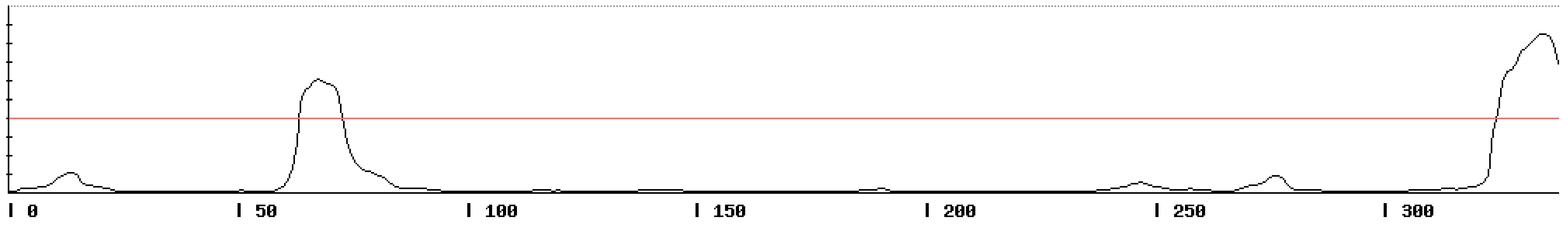

Predictions for Malusdomestica_MDP0000266267	
Viterbi Path	785 - KPPIKRARKEVSKEKPLIKRKTARK - 809
Viterbi Path	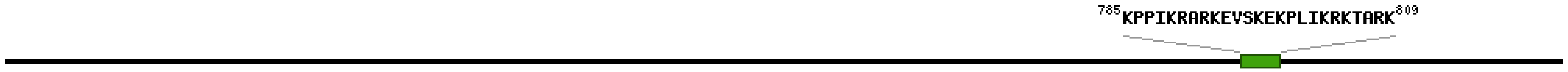
Posterior @ 0.4	273 - KKRRKQQSKRWKGK - 286<br>784 - EKPPIKRARKEVSKEKPLIKRKTARKEPGSQK - 815
Posterior @ 0.4	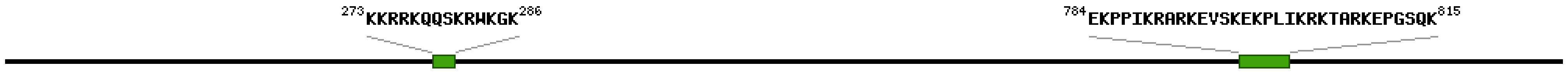
Posterior Graphic. Threshold Marked @ 0.4	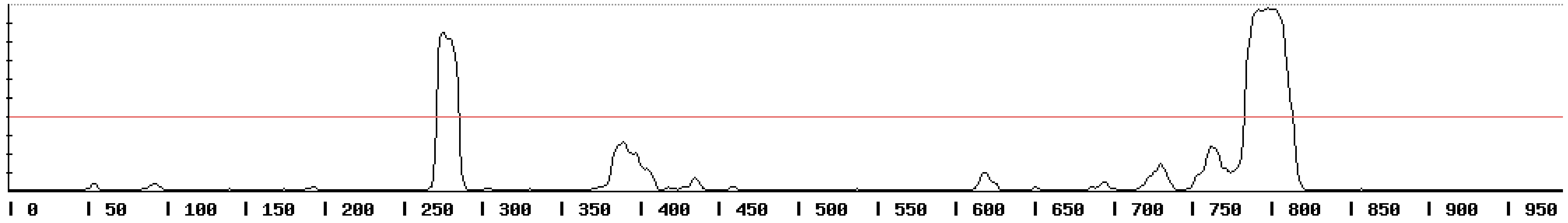

Predictions for Malusdomestica_MDP0000266908	
Viterbi Path	
Viterbi Path	
Posterior @ 0.4	
Posterior @ 0.4	
Posterior Graphic. Threshold Marked @ 0.4	

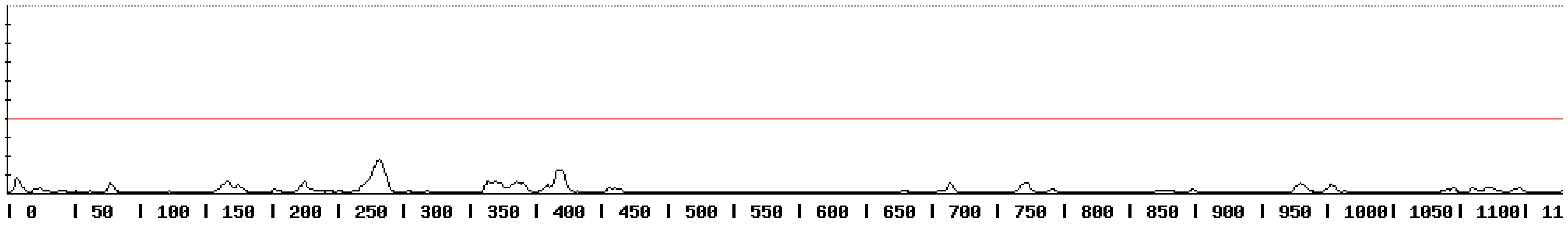

Predictions for Malusdomestica_MDP0000267678

Viterbi Path

Viterbi Path

Posterior @ 0.4: 199 - KK - 200

Posterior @ 0.4

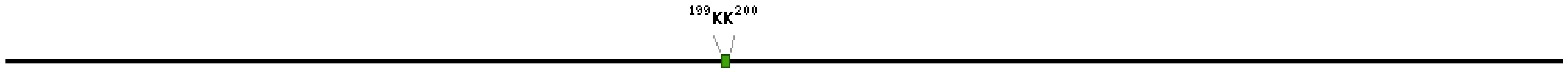

Posterior Graphic. Threshold Marked @ 0.4

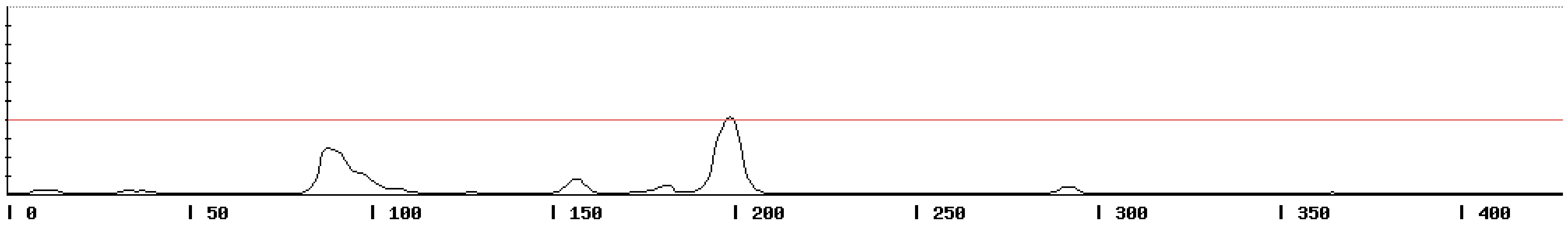

Predictions for Malusdomestica_MDP0000267681

Viterbi Path

Viterbi Path

Posterior @ 0.4:
168 - RKKEGSRINKRK - 179
193 - SKKQRGTNKKMKK - 205

Posterior @ 0.4

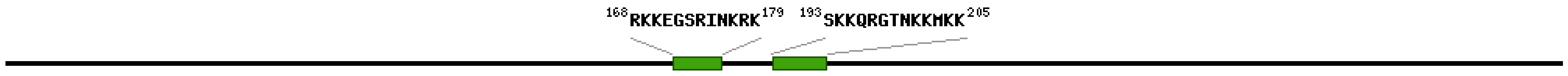

Posterior Graphic. Threshold Marked @ 0.4

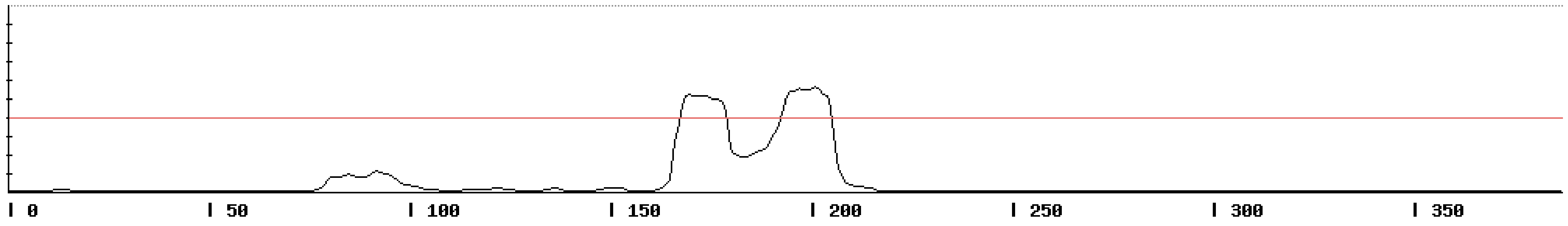

Predictions for Malusdomestica_MDP0000267984

Viterbi Path	158 - KPPIKRARKEVSEKPLIKRKRARK - 181
Viterbi Path	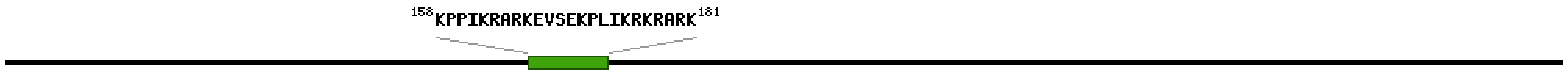
Posterior @ 0.4	157 - EKPPIKRARKEVSEKPLIKRKRARKEPGSQK - 187
Posterior @ 0.4	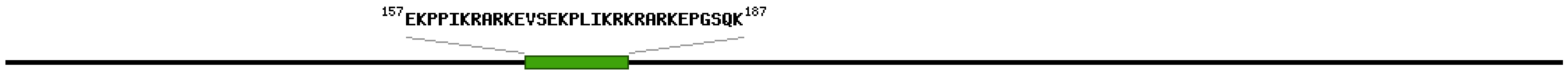
Posterior Graphic. Threshold Marked @ 0.4	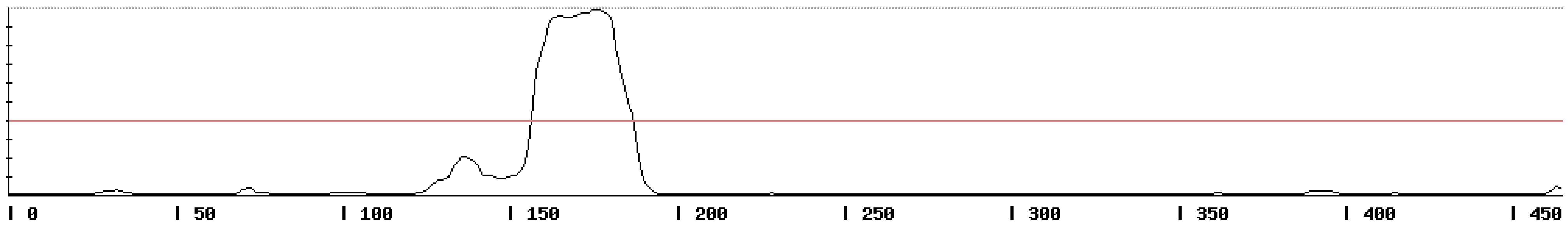

Predictions for Malusdomestica_MDP0000272164

Viterbi Path	
Viterbi Path	

Posterior @ 0.4

Posterior @ 0.4

Posterior Graphic. Threshold Marked @ 0.4

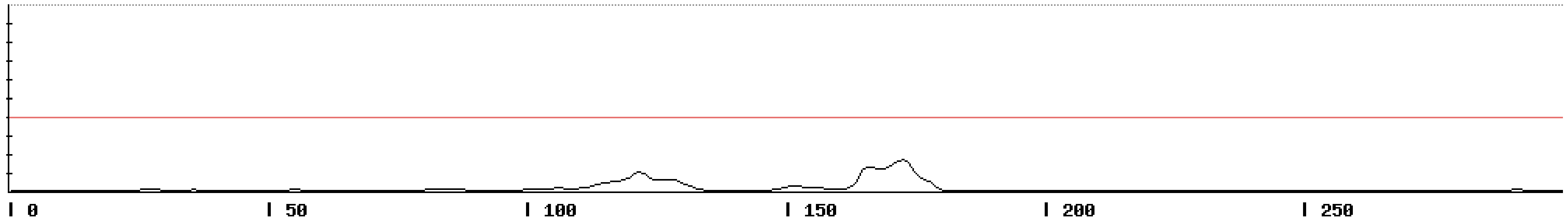

Predictions for Malusdomestica_MDP0000272823

Viterbi Path

Viterbi Path

Posterior @ 0.4

Posterior @ 0.4

Posterior Graphic. Threshold Marked @ 0.4

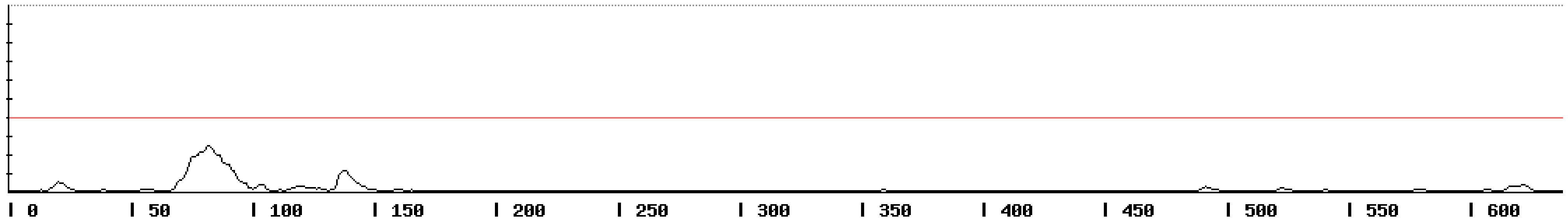

Predictions for Malusdomestica_MDP0000274989

Viterbi Path

Viterbi Path

Posterior @ 0.4	
Posterior @ 0.4	
Posterior Graphic. Threshold Marked @ 0.4	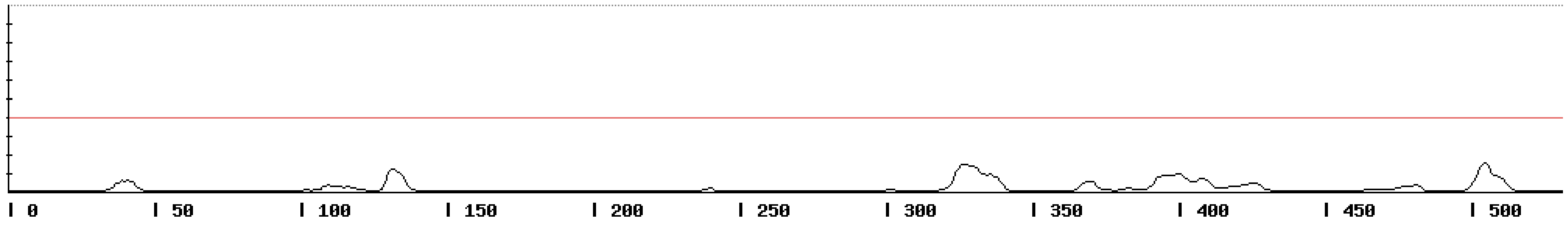

Predictions for Malusdomestica_MDP0000276278

Viterbi Path	206 - PREERPIGMRGRRVLGRWGRRRRRRRRLQGERRVR - 240
Viterbi Path	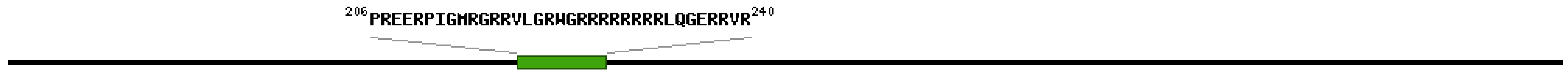
Posterior @ 0.4	207 - REERPIGMRGRRVLGRWGRRRRRRRRLQGERRVR - 240<br>430 - RKRRK - 434
Posterior @ 0.4	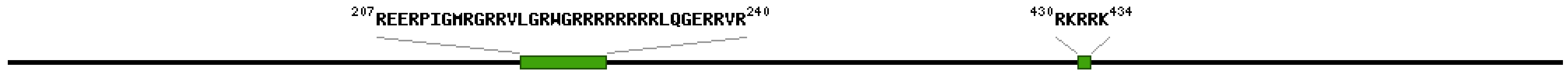
Posterior Graphic. Threshold Marked @ 0.4	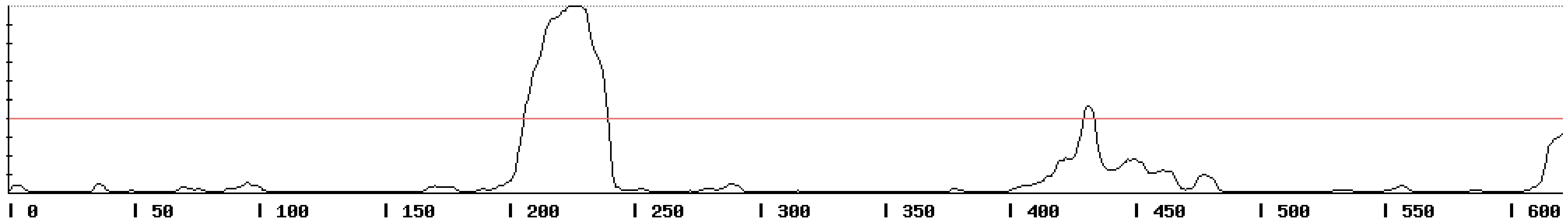

Predictions for Malusdomestica_MDP0000276765	
Viterbi Path	
Viterbi Path	
Posterior @ 0.4	
Posterior @ 0.4	
Posterior Graphic. Threshold Marked @ 0.4	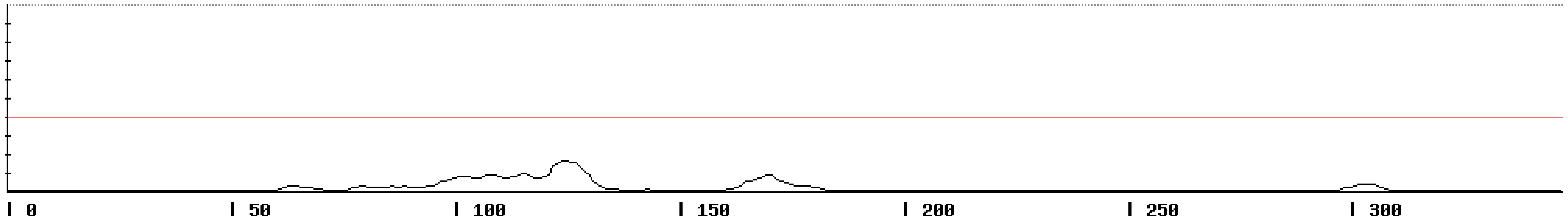

Predictions for Malusdomestica_MDP0000276982	
Viterbi Path	
Viterbi Path	
Posterior @ 0.4	
Posterior @ 0.4	
Posterior Graphic. Threshold Marked @ 0.4	

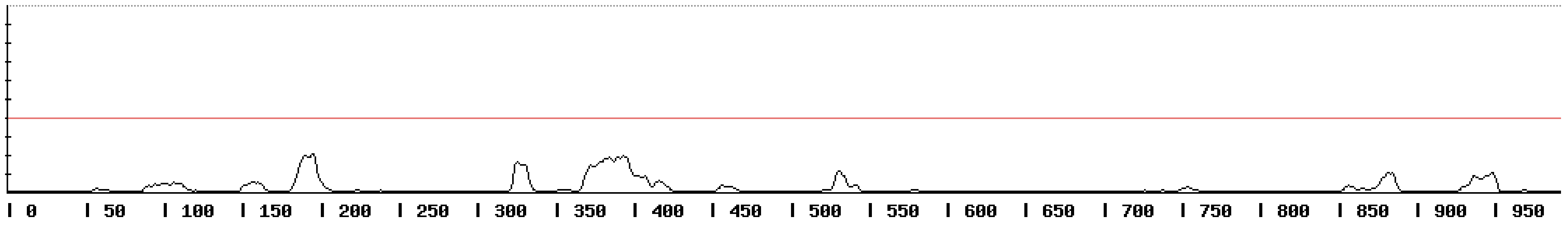

Predictions for Malusdomestica_MDP0000278387

Viterbi Path

Viterbi Path

Posterior @ 0.4

98 - WKITGKERAIKARRSKALIGKKRTLTF - 124

126 - RR - 127

130 - PKPKK - 134

Posterior @ 0.4

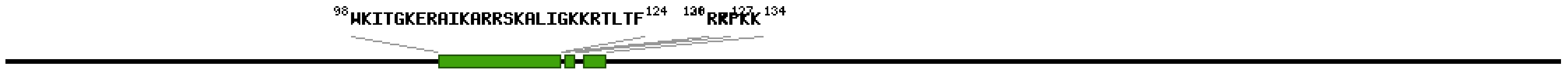

Posterior Graphic. Threshold Marked @ 0.4

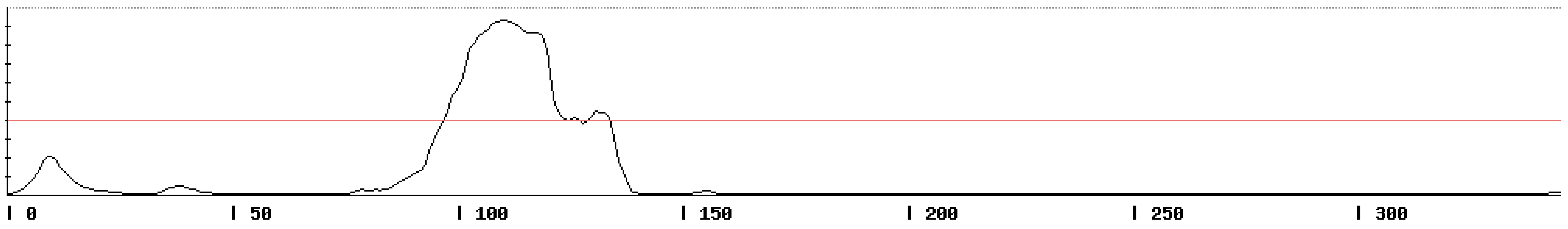

Predictions for Malusdomestica_MDP0000279197

Viterbi Path

Viterbi Path

Posterior @ 0.4

90 - GHWKITGKERAIKARRSKALIGKKR - 114

Posterior @ 0.4

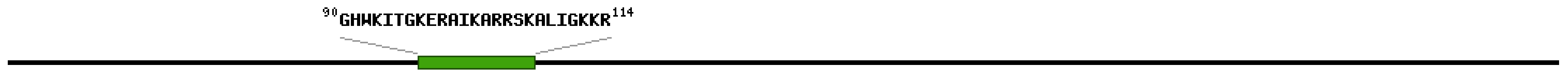

Posterior Graphic. Threshold Marked @ 0.4

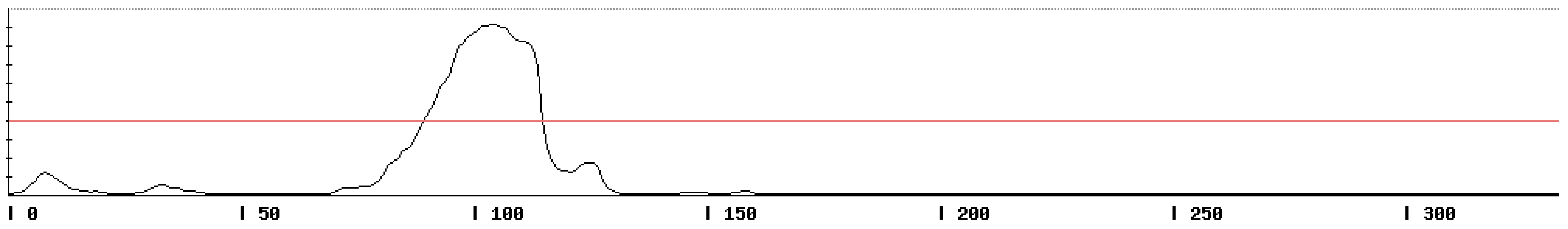

Predictions for Malusdomestica_MDP0000280486

Viterbi Path

Viterbi Path

Posterior @ 0.4: 97 - WKITGKERAIKARRSKALIGKKRT - 120

Posterior @ 0.4

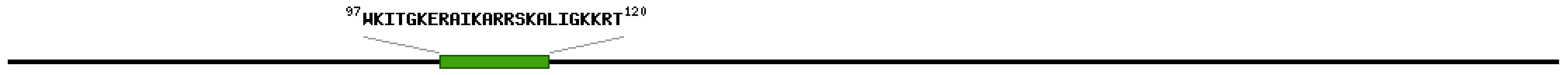

Posterior Graphic. Threshold Marked @ 0.4

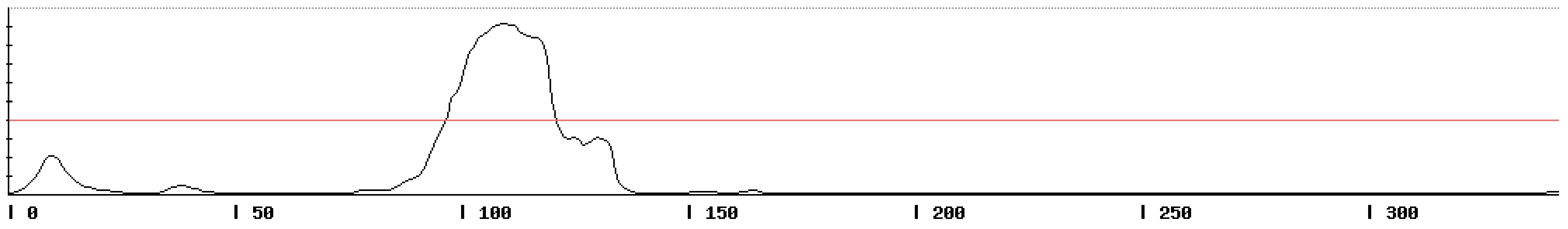

Predictions for Malusdomestica_MDP0000282616

Viterbi Path

Viterbi Path

Posterior @ 0.4	
Posterior @ 0.4	
Posterior Graphic. Threshold Marked @ 0.4	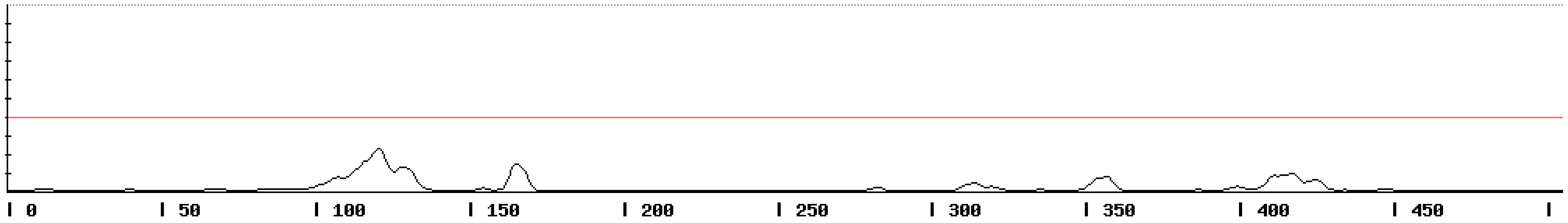

**Predictions for Malusdomestica_MDP0000283092**	
Viterbi Path	
Viterbi Path	
Posterior @ 0.4	100 - TGKERAIRSKAVIGKKR - 116
Posterior @ 0.4	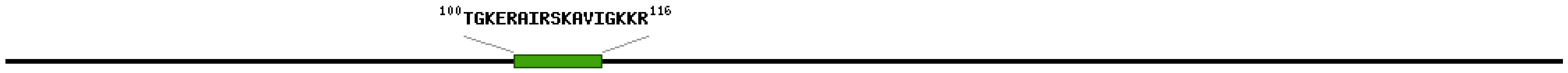
Posterior Graphic. Threshold Marked @ 0.4	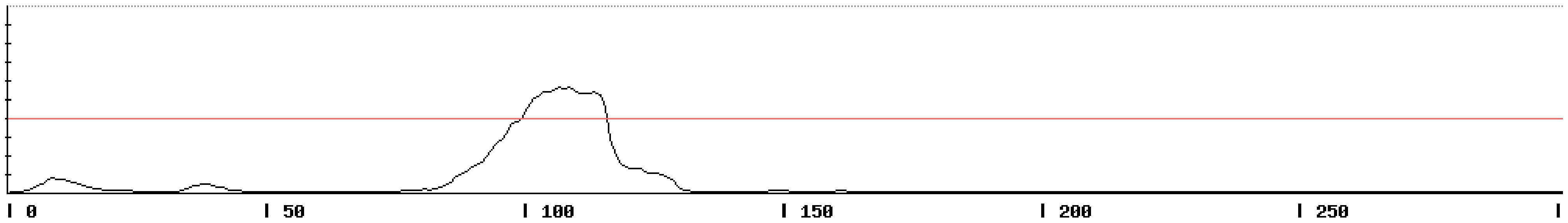

**Predictions for Malusdomestica_MDP0000283975**	
Viterbi Path	
Viterbi Path	

Posterior @ 0.4	921 - KRR - 923
Posterior @ 0.4	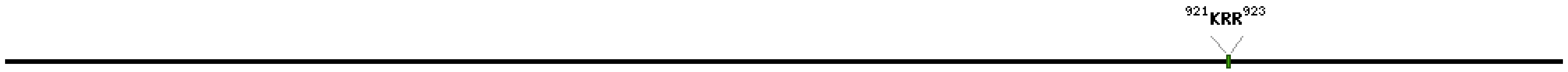
Posterior Graphic. Threshold Marked @ 0.4	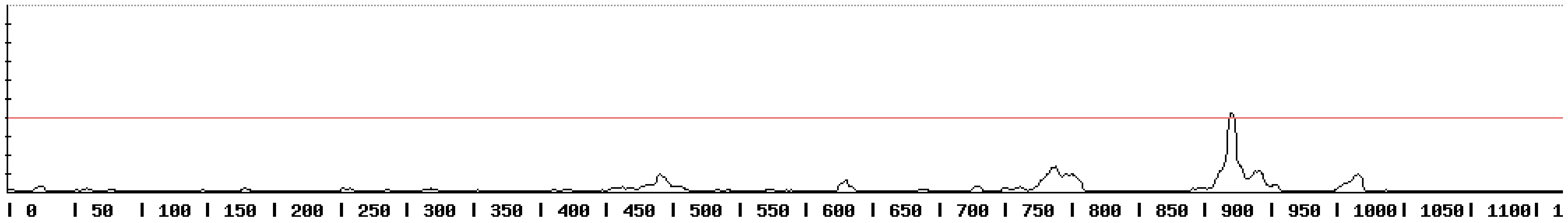

Predictions for Malusdomestica_MDP0000285555	
Viterbi Path	
Viterbi Path	
Posterior @ 0.4	173 - KKRK - 176
Posterior @ 0.4	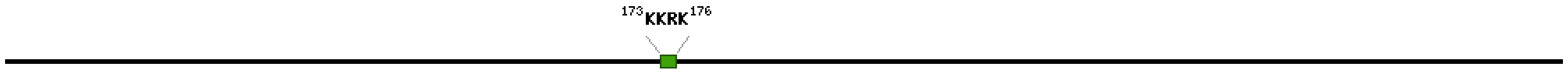
Posterior Graphic. Threshold Marked @ 0.4	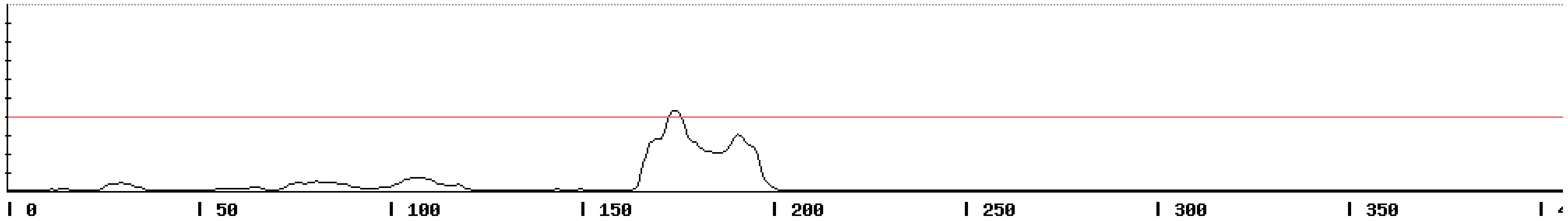

Predictions for Malusdomestica_MDP0000286960

Viterbi Path

Viterbi Path

Posterior @ 0.4

Posterior @ 0.4

Posterior Graphic. Threshold Marked @ 0.4

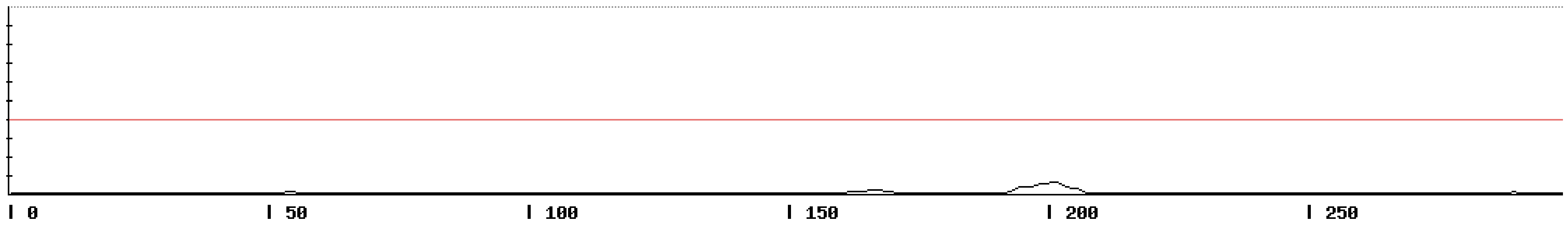

Predictions for Malusdomestica_MDP0000288624

Viterbi Path

Viterbi Path

Posterior @ 0.4

Posterior @ 0.4

Posterior Graphic. Threshold Marked @ 0.4

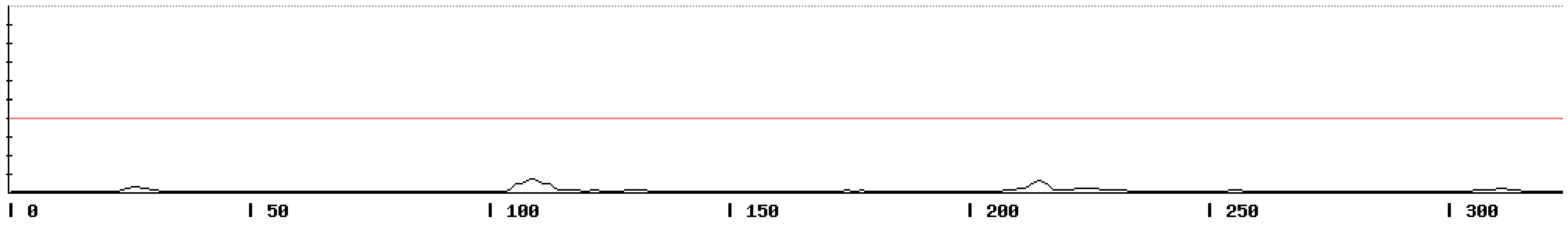

Predictions for Malusdomestica_MDP0000289955	
Viterbi Path	194 - PAKKRARKEVKEKPAKKKARKEVKQKPAKKKAR - 226
Viterbi Path	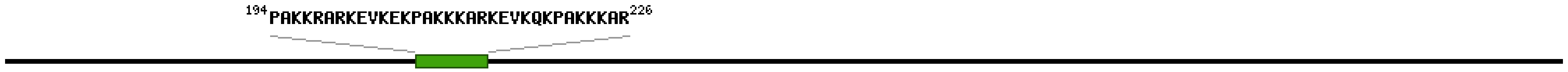
Posterior @ 0.4	194 - PAKKRARKEVKEKPAKKKARKEVKQKPAKKKARN - 227
Posterior @ 0.4	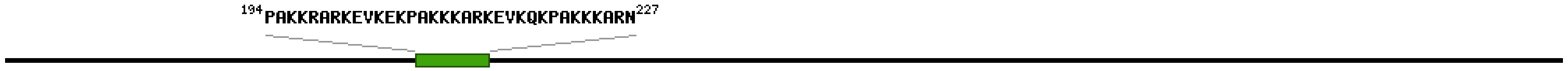
Posterior Graphic. Threshold Marked @ 0.4	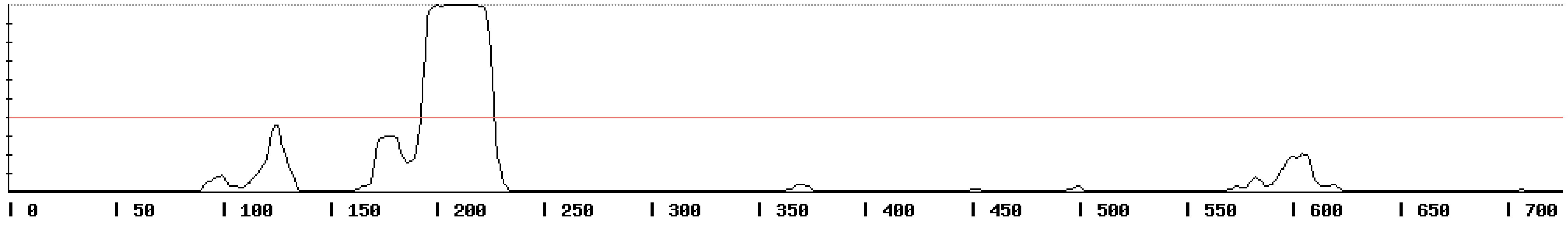

Predictions for Malusdomestica_MDP0000291838	
Viterbi Path	
Viterbi Path	
Posterior @ 0.4	
Posterior @ 0.4	
Posterior Graphic. Threshold Marked @ 0.4	

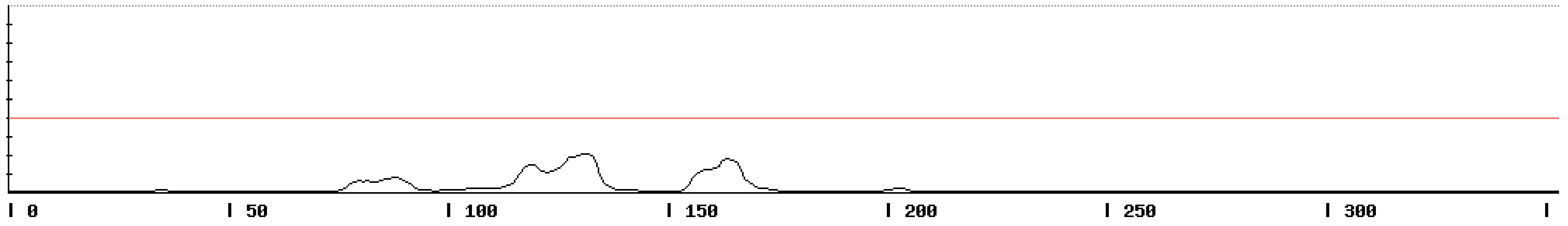

Predictions for Malusdomestica_MDP0000296292

Viterbi Path

Viterbi Path

Posterior @ 0.4

Posterior @ 0.4

Posterior Graphic. Threshold Marked @ 0.4

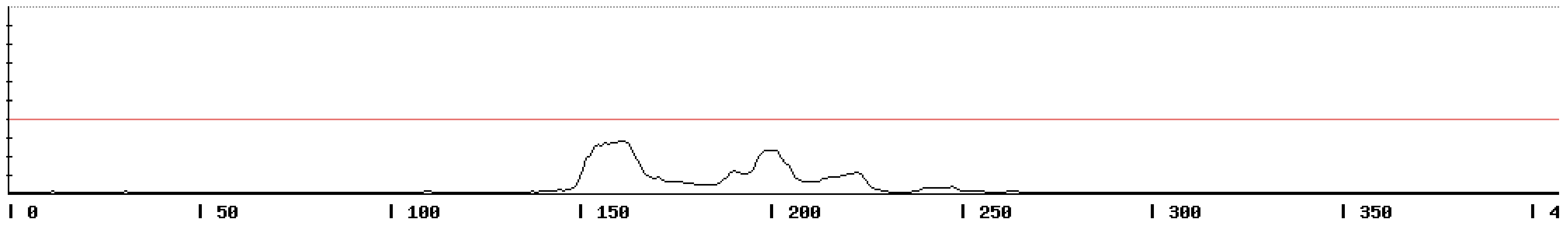

Predictions for Malusdomestica_MDP0000298182

Viterbi Path

Viterbi Path

Posterior @ 0.4

100 - RRK - 102

Posterior @ 0.4

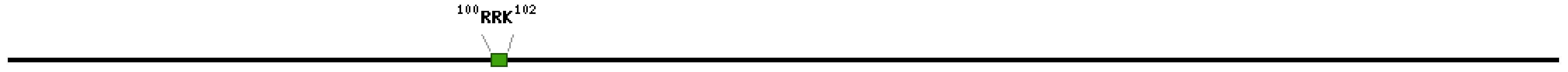

Posterior Graphic. Threshold Marked @ 0.4

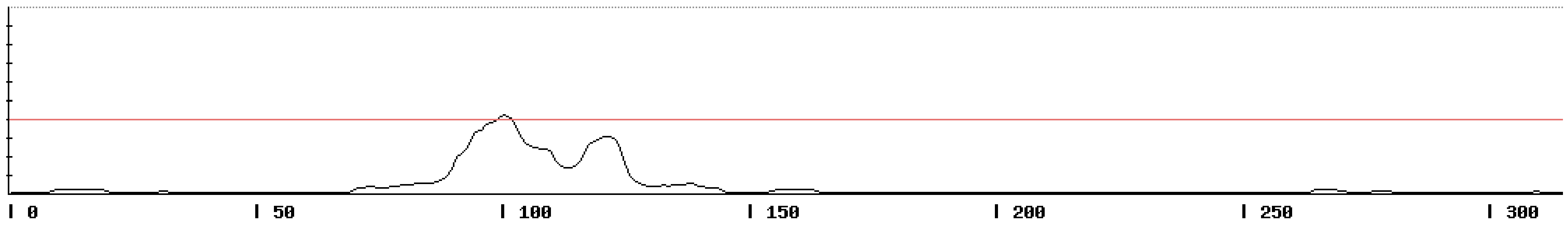

Predictions for Malusdomestica_MDP0000298965

Viterbi Path

Viterbi Path

Posterior @ 0.4

Posterior @ 0.4

Posterior Graphic. Threshold Marked @ 0.4

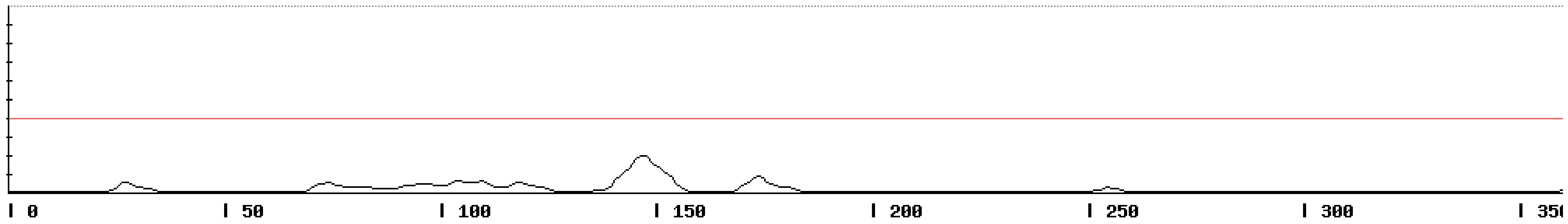

Predictions for Malusdomestica_MDP0000299944

Viterbi Path

Viterbi Path

Posterior @ 0.4 154 - RKYRNGSRPKRA - 165

Posterior @ 0.4

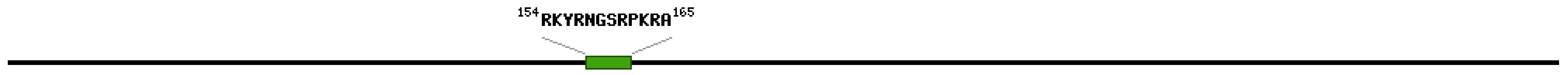

Posterior Graphic. Threshold Marked @ 0.4

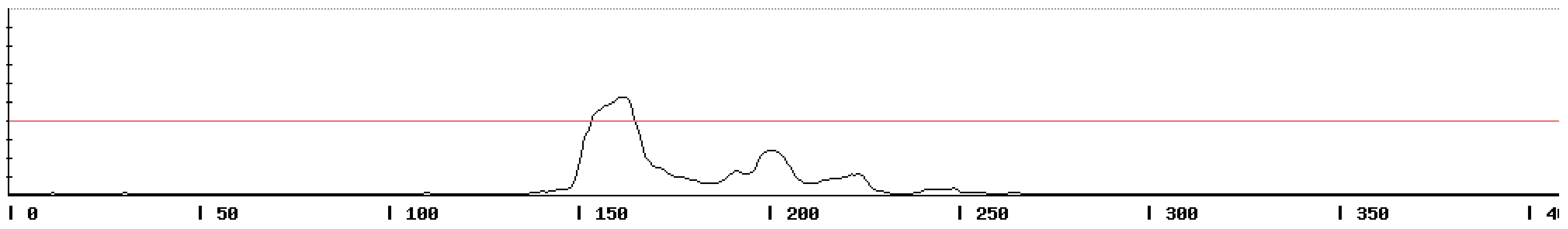

Predictions for Malusdomestica_MDP0000299959

Viterbi Path

Viterbi Path

Posterior @ 0.4

Posterior @ 0.4

Posterior Graphic. Threshold Marked @ 0.4

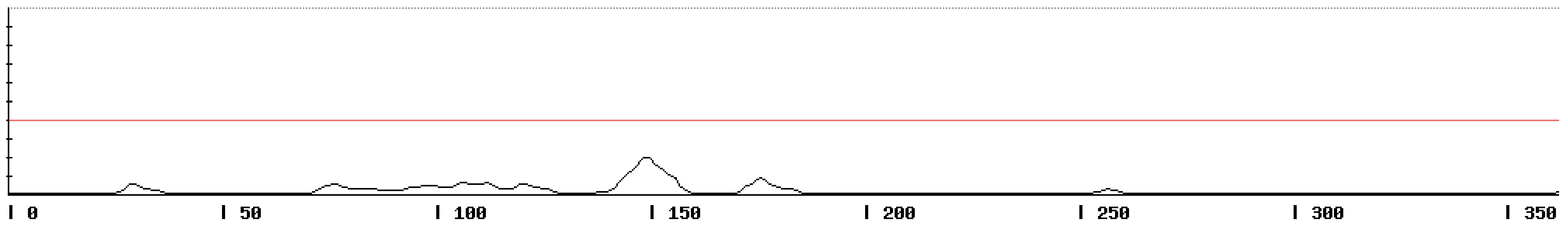

Predictions for Malusdomestica_MDP0000300393

Viterbi Path

Viterbi Path

Posterior @ 0.4

Posterior @ 0.4

Posterior Graphic. Threshold Marked @ 0.4

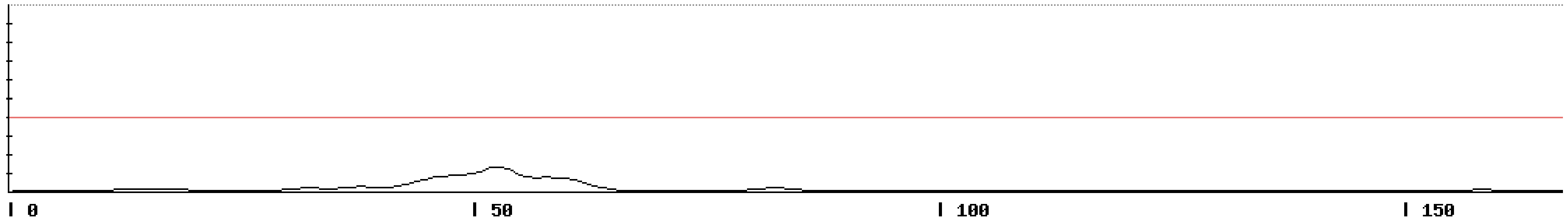

Predictions for Malusdomestica_MDP0000301561

Viterbi Path

Viterbi Path

Posterior @ 0.4

183 - LRNIVKRQRKDLKARMLKVSRAEAERKR - 210

Posterior @ 0.4

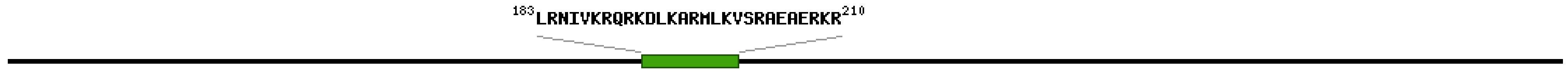

Posterior Graphic. Threshold Marked @ 0.4

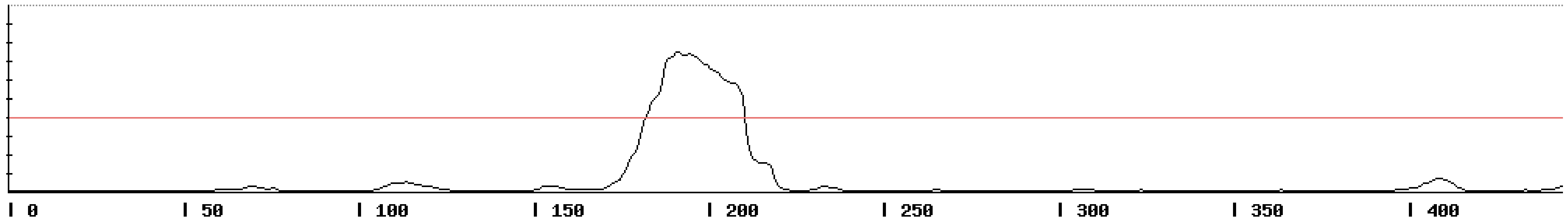

Predictions for Malusdomestica_MDP0000303041

Viterbi Path

Viterbi Path

Posterior @ 0.4

Posterior @ 0.4

Posterior Graphic. Threshold Marked @ 0.4

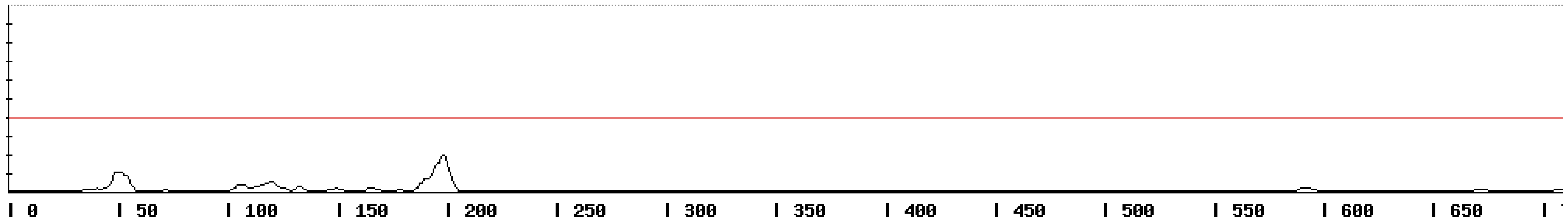

Predictions for Malusdomestica_MDP0000307020

Viterbi Path

Viterbi Path

Posterior @ 0.4 — 267 - KK - 268

Posterior @ 0.4

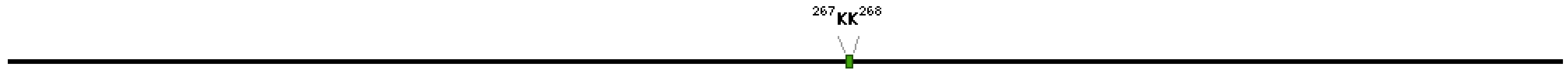

Posterior Graphic. Threshold Marked @ 0.4

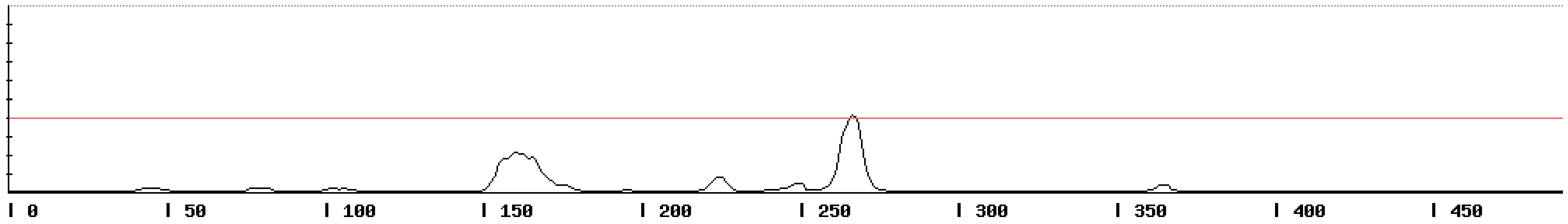

Predictions for Malusdomestica_MDP0000309351

Viterbi Path

Viterbi Path

Posterior @ 0.4 | 471 - RKRRK - 475

Posterior @ 0.4

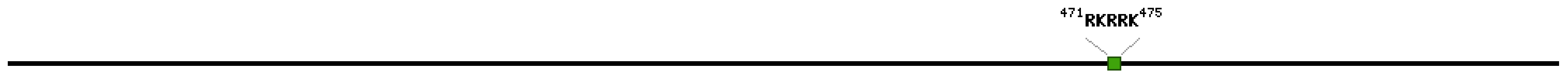

Posterior Graphic. Threshold Marked @ 0.4

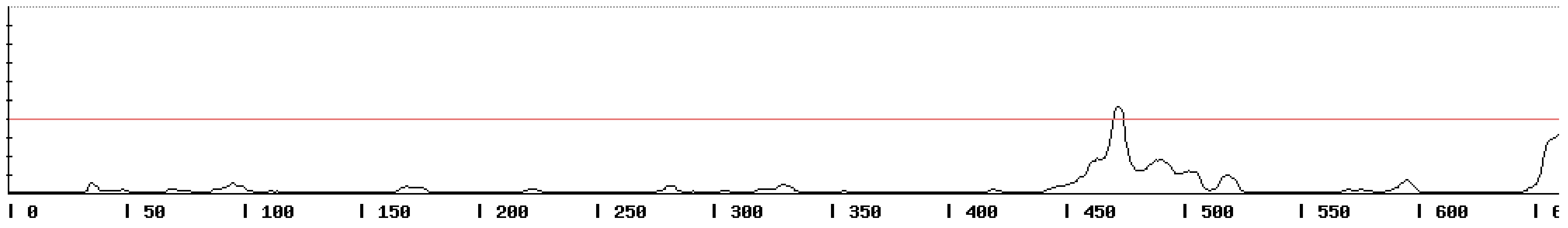

Predictions for Malusdomestica_MDP0000309382

Viterbi Path

Viterbi Path

Posterior @ 0.4

Posterior @ 0.4

Posterior Graphic. Threshold Marked @ 0.4

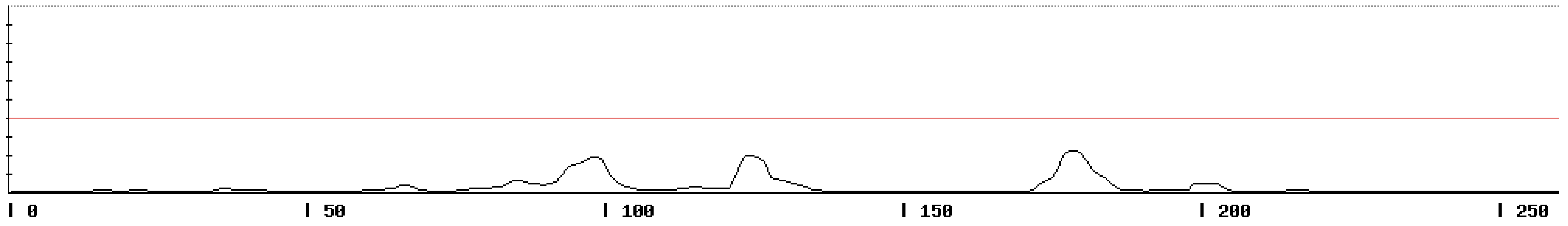

Predictions for Malusdomestica_MDP0000309445	
Viterbi Path	
Viterbi Path	
Posterior @ 0.4	30 - ITGKERAIKDRHSKAVIGKKR - 50
Posterior @ 0.4	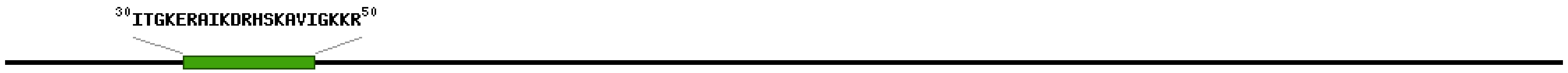
Posterior Graphic. Threshold Marked @ 0.4	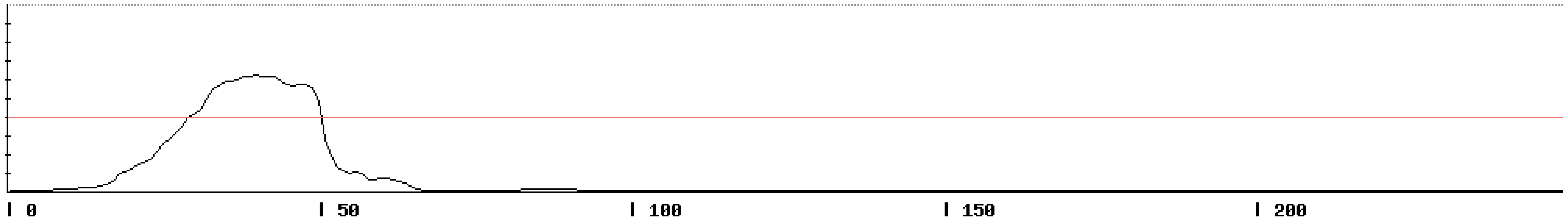

Predictions for Malusdomestica_MDP0000310981	
Viterbi Path	
Viterbi Path	
Posterior @ 0.4	
Posterior @ 0.4	
Posterior Graphic. Threshold Marked @ 0.4	

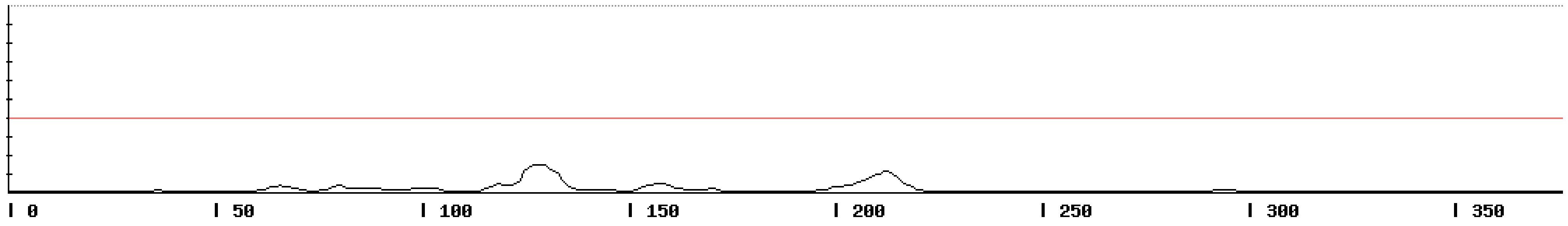

Predictions for Malusdomestica_MDP0000311190

Viterbi Path	
Viterbi Path	
Posterior @ 0.4	105 - GKEKIVKRDNKIIGKKRILTFRQGR - 129
Posterior @ 0.4	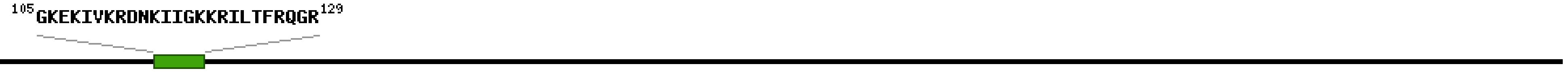
Posterior Graphic. Threshold Marked @ 0.4	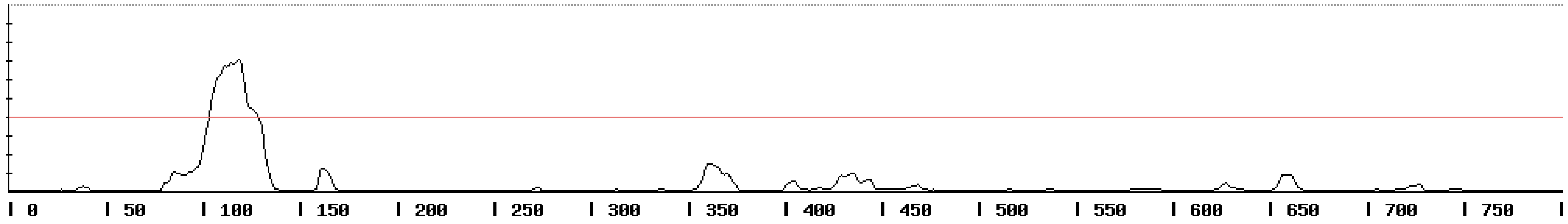

Predictions for Malusdomestica_MDP0000315947

Viterbi Path	
Viterbi Path	
Posterior @ 0.4	162 - KNDRKRK - 168
Posterior @ 0.4	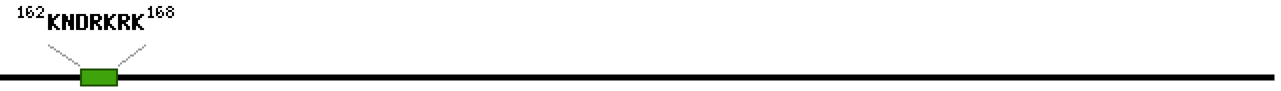

Posterior Graphic. Threshold Marked @ 0.4

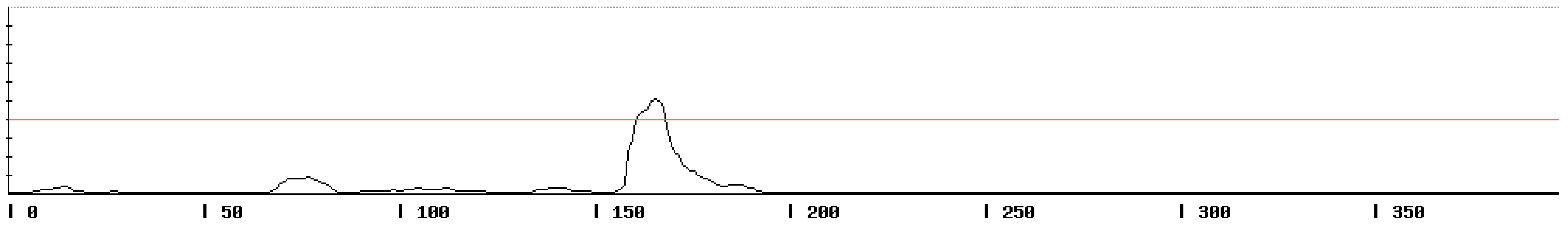

Predictions for Malusdomestica_MDP0000320563

Viterbi Path

Viterbi Path

Posterior @ 0.4

31 - TGKERAIRSKAVIGKKR - 47

Posterior @ 0.4

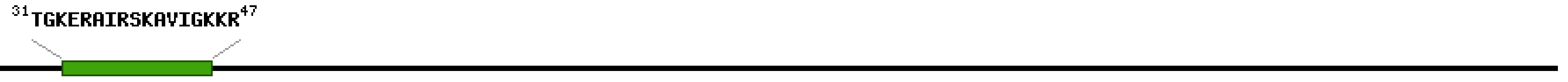

Posterior Graphic. Threshold Marked @ 0.4

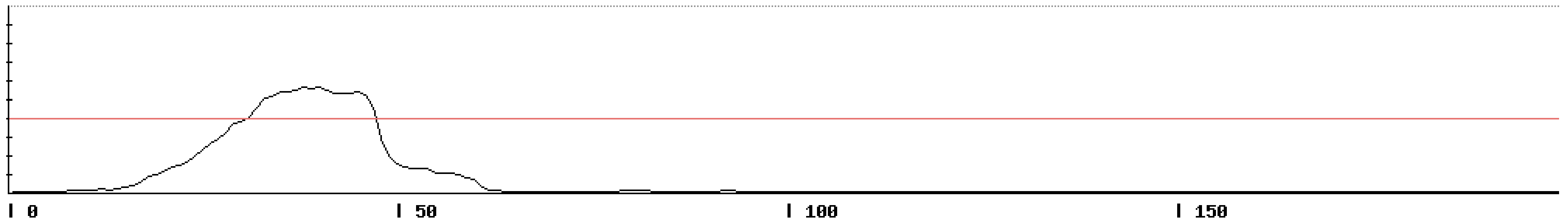

Predictions for Malusdomestica_MDP0000320566

Viterbi Path

Viterbi Path

Posterior @ 0.4

Posterior @ 0.4

Posterior Graphic. Threshold Marked @ 0.4

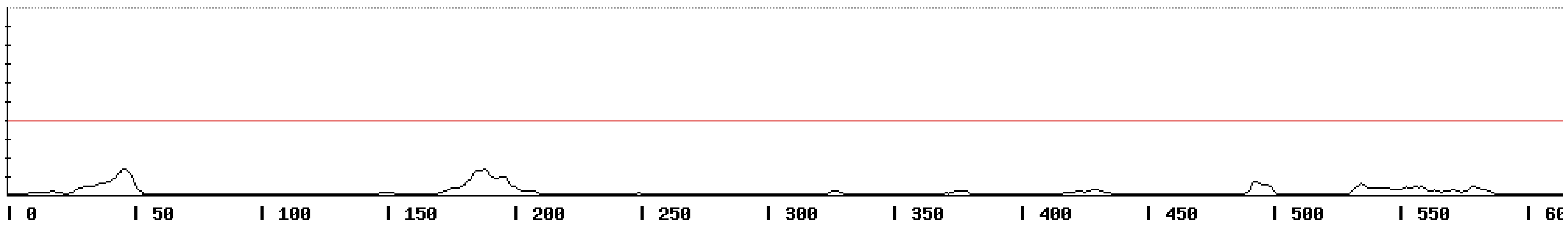

Predictions for Malusdomestica_MDP0000323779

Viterbi Path

Viterbi Path

Posterior @ 0.4

Posterior @ 0.4

Posterior Graphic. Threshold Marked @ 0.4

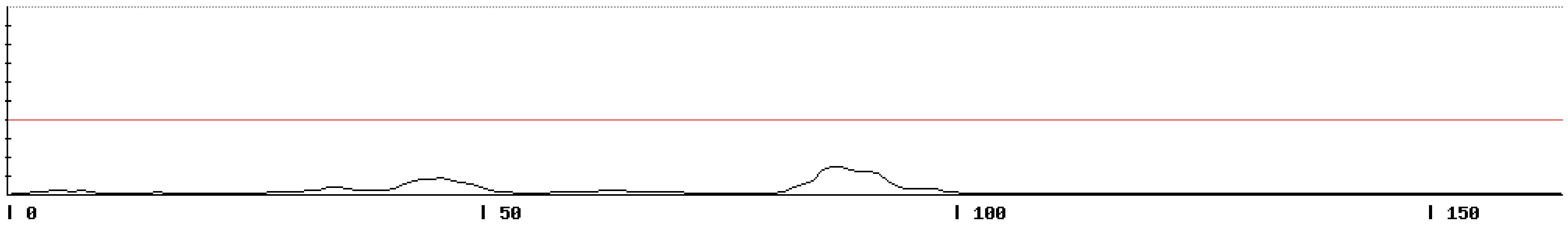

Predictions for Malusdomestica_MDP0000334047

Viterbi Path

Viterbi Path

Posterior @ 0.4 | 102 - KHIGKPKALGIKK - 114

Posterior @ 0.4

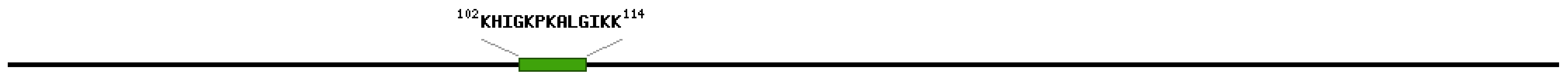

Posterior Graphic. Threshold Marked @ 0.4

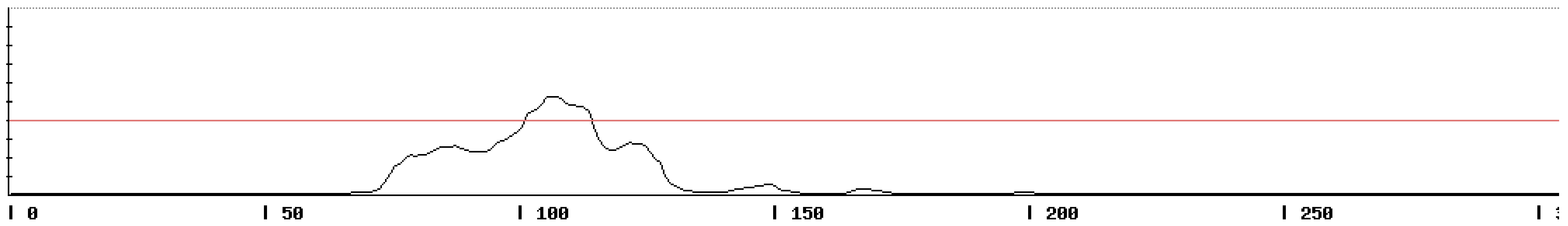

Predictions for Malusdomestica_MDP0000364750

Viterbi Path

Viterbi Path

Posterior @ 0.4

Posterior @ 0.4

Posterior Graphic. Threshold Marked @ 0.4

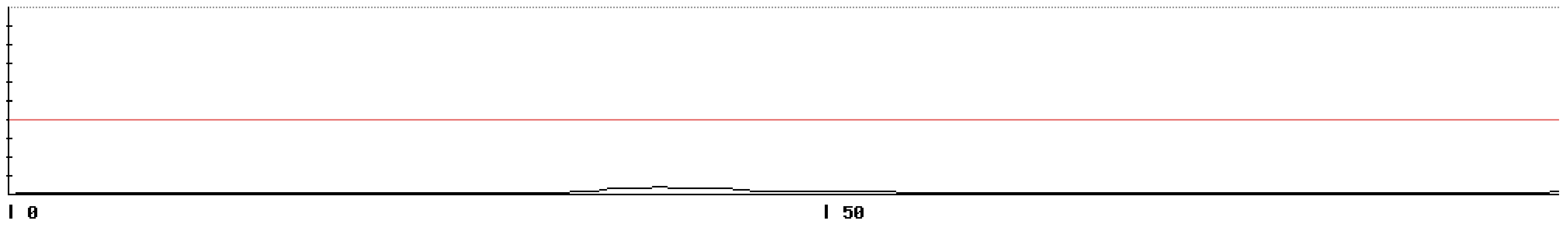

Predictions for Malusdomestica_MDP0000381514

Viterbi Path

Viterbi Path

Posterior @ 0.4

Posterior @ 0.4

Posterior Graphic. Threshold Marked @ 0.4

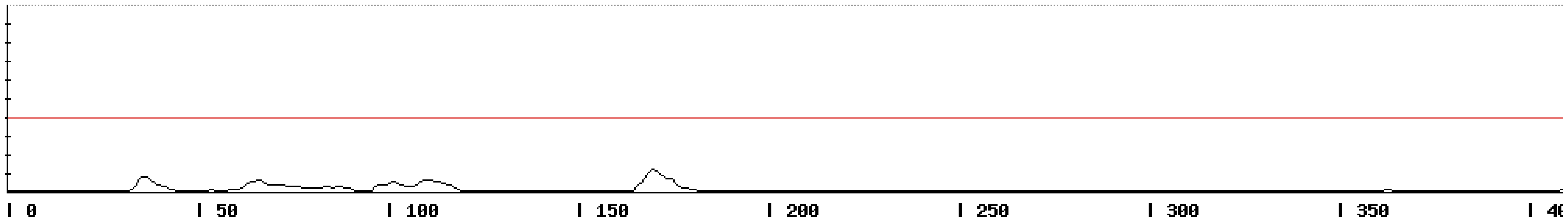

Predictions for Malusdomestica_MDP0000382720

Viterbi Path

Viterbi Path

Posterior @ 0.4
224 - K - 224

Posterior @ 0.4

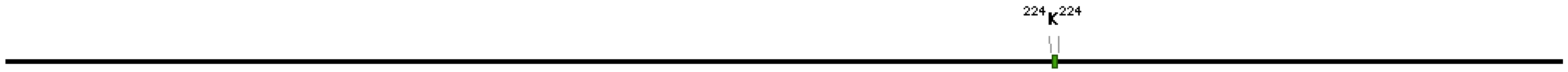

Posterior Graphic. Threshold Marked @ 0.4

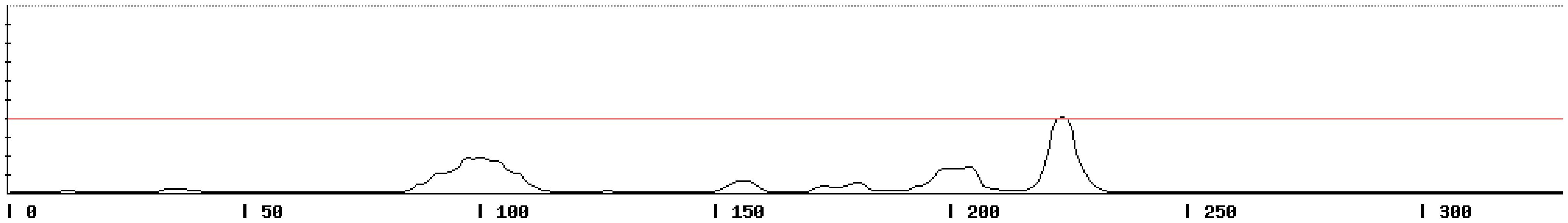

Predictions for Malusdomestica_MDP0000387787

Viterbi Path

Viterbi Path

Posterior @ 0.4

Posterior @ 0.4

Posterior Graphic. Threshold Marked @ 0.4

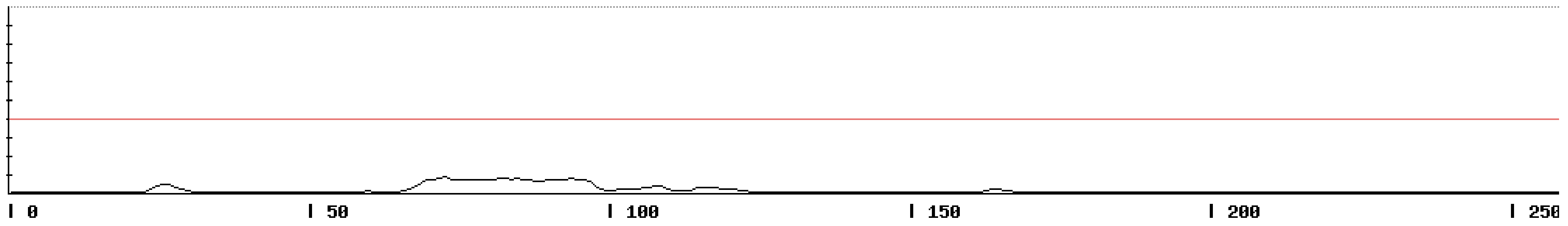

Predictions for Malusdomestica_MDP0000392668

Viterbi Path

Viterbi Path

Posterior @ 0.4

Posterior @ 0.4

Posterior Graphic. Threshold Marked @ 0.4

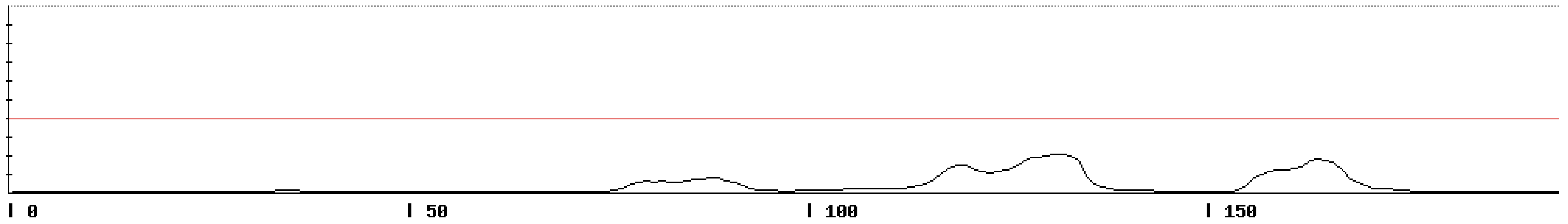

Predictions for Malusdomestica_MDP0000394116

Viterbi Path	189 - KQPAKKRARKEVMEKPAKKRARKEVKEKPAKTKARNK - 225
Viterbi Path	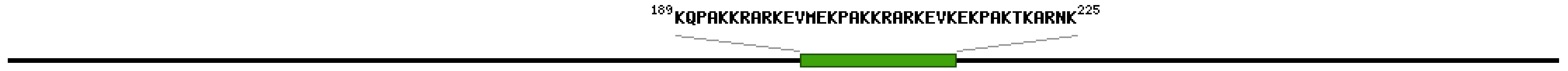
Posterior @ 0.4	122 - KK - 123<br>189 - KQPAKKRARKEVMEKPAKKRARKEVKEKPAKTKARNKV - 226
Posterior @ 0.4	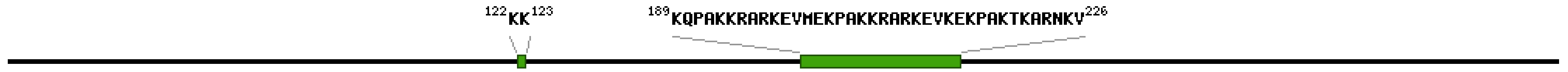
Posterior Graphic. Threshold Marked @ 0.4	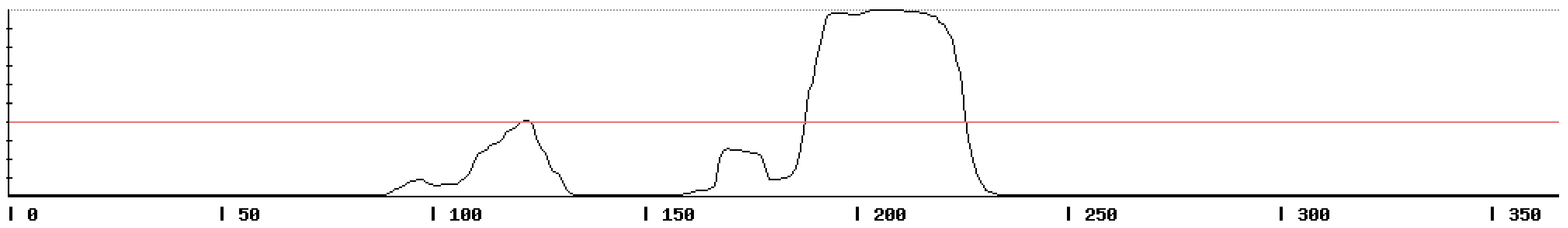

Predictions for Malusdomestica_MDP0000404409

Viterbi Path	
Viterbi Path	
Posterior @ 0.4	
Posterior @ 0.4	
Posterior Graphic. Threshold Marked @ 0.4	

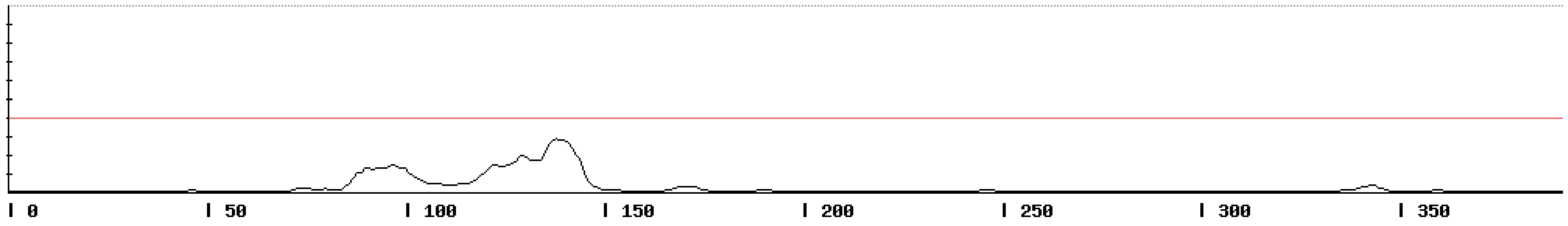

Predictions for Malusdomestica_MDP0000428265

Viterbi Path

Viterbi Path

Posterior @ 0.4

Posterior @ 0.4

Posterior Graphic. Threshold Marked @ 0.4

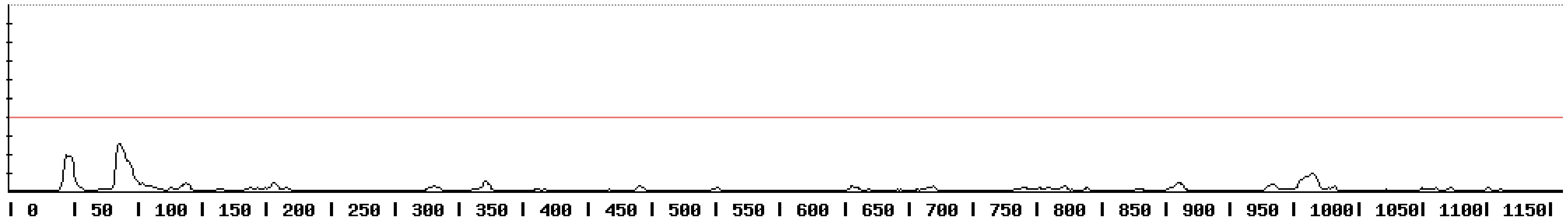

Predictions for Malusdomestica_MDP0000437676

Viterbi Path

Viterbi Path

Posterior @ 0.4

Posterior @ 0.4

Posterior Graphic. Threshold Marked @ 0.4

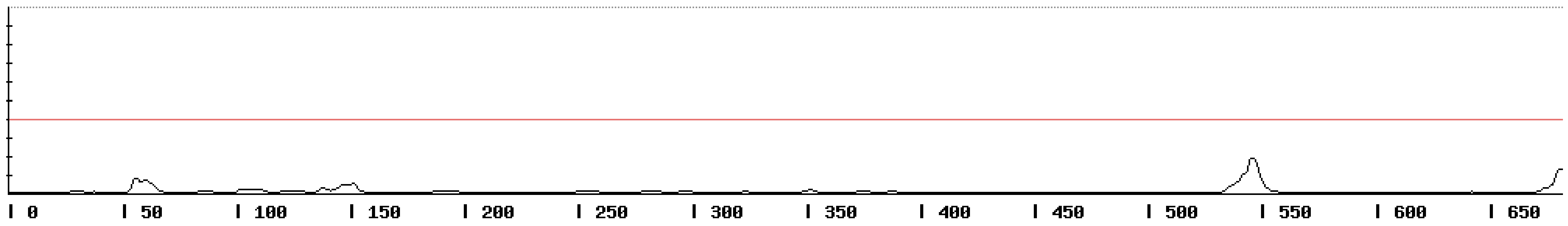

Predictions for Malusdomestica_MDP0000440829

Viterbi Path	
Viterbi Path	
Posterior @ 0.4	95 - RITGKERPVKARRSKALIGKKR - 116
Posterior @ 0.4	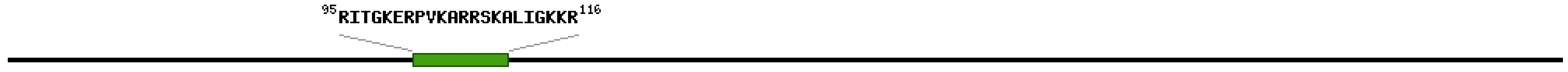
Posterior Graphic. Threshold Marked @ 0.4	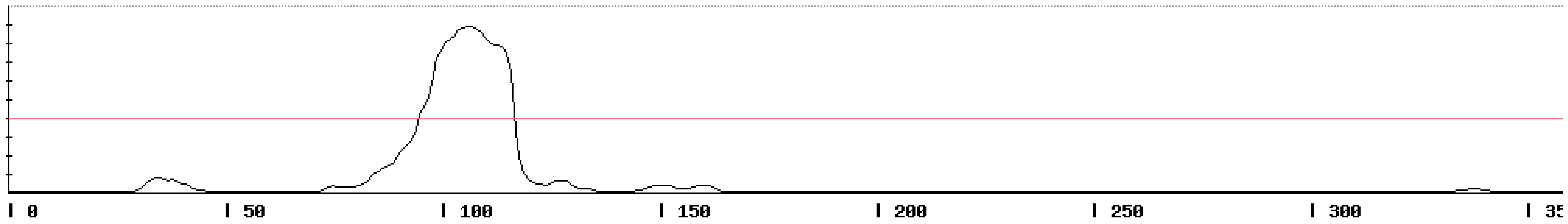

Predictions for Malusdomestica_MDP0000457996

Viterbi Path	
Viterbi Path	
Posterior @ 0.4	
Posterior @ 0.4	

Posterior Graphic. Threshold Marked @ 0.4

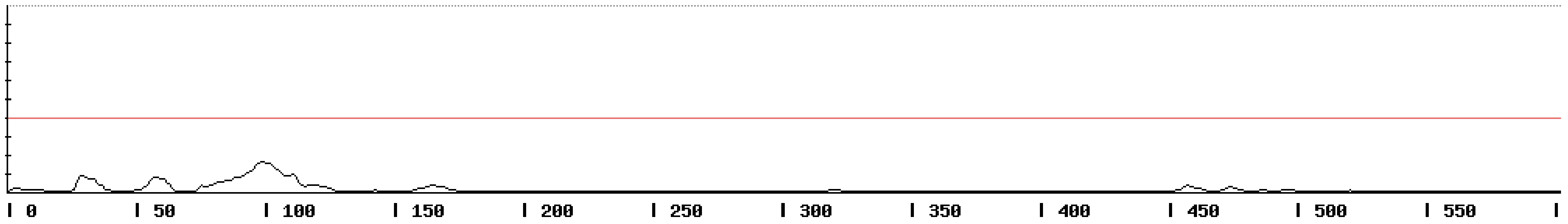

Predictions for Malusdomestica_MDP0000458799

Viterbi Path

Viterbi Path

Posterior @ 0.4

Posterior @ 0.4

Posterior Graphic. Threshold Marked @ 0.4

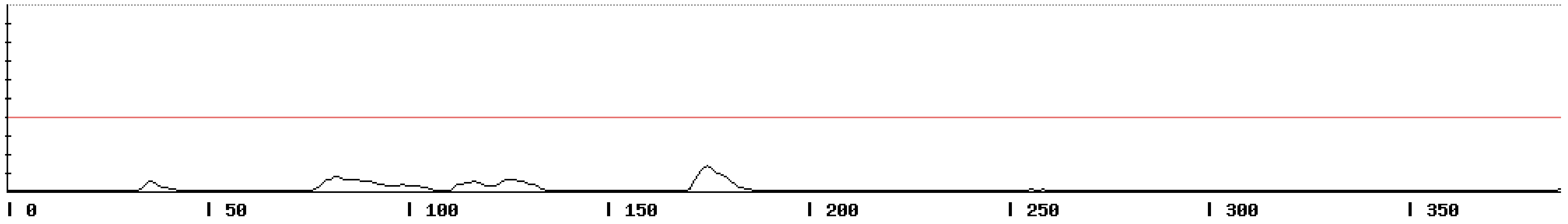

Predictions for Malusdomestica_MDP0000460430

Viterbi Path

Viterbi Path

Posterior @ 0.4	
Posterior @ 0.4	
Posterior Graphic. Threshold Marked @ 0.4	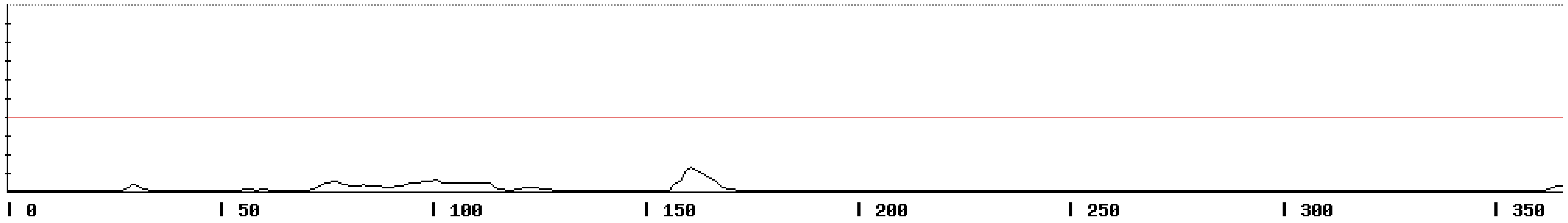

Predictions for Malusdomestica_MDP0000468411

Viterbi Path	
Viterbi Path	
Posterior @ 0.4	77 - GKPRALIKK - 85
Posterior @ 0.4	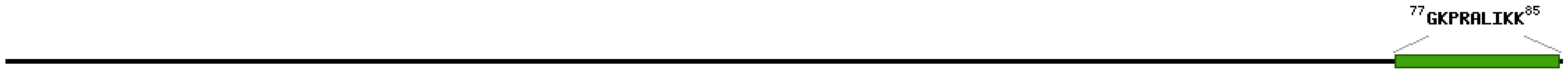
Posterior Graphic. Threshold Marked @ 0.4	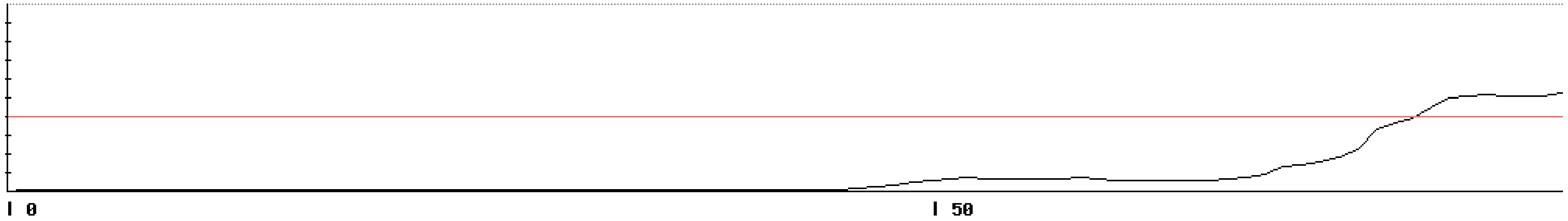

Predictions for Malusdomestica_MDP0000480581

Viterbi Path	
Viterbi Path	

Posterior @ 0.4

Posterior @ 0.4

Posterior Graphic. Threshold Marked @ 0.4

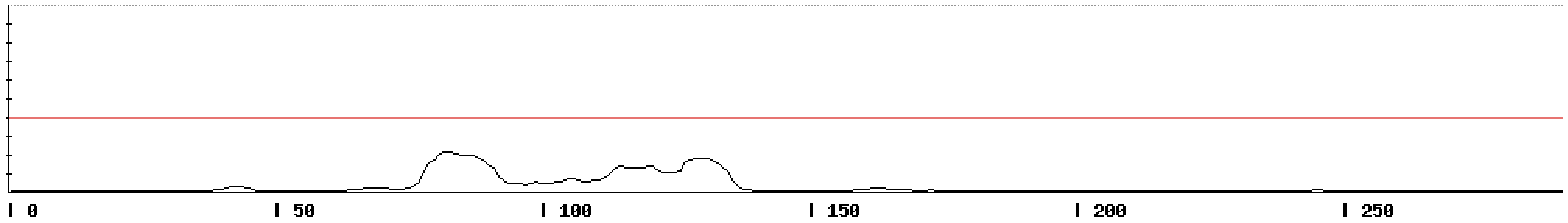

Predictions for Malusdomestica_MDP0000481448

Viterbi Path

Viterbi Path

Posterior @ 0.4

Posterior @ 0.4

Posterior Graphic. Threshold Marked @ 0.4

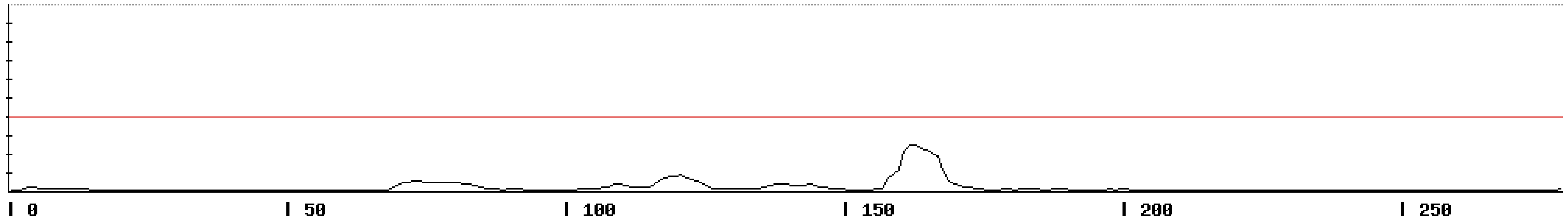

Predictions for Malusdomestica_MDP0000497016

Viterbi Path

Viterbi Path

Posterior @ 0.4

Posterior @ 0.4

Posterior Graphic. Threshold Marked @ 0.4

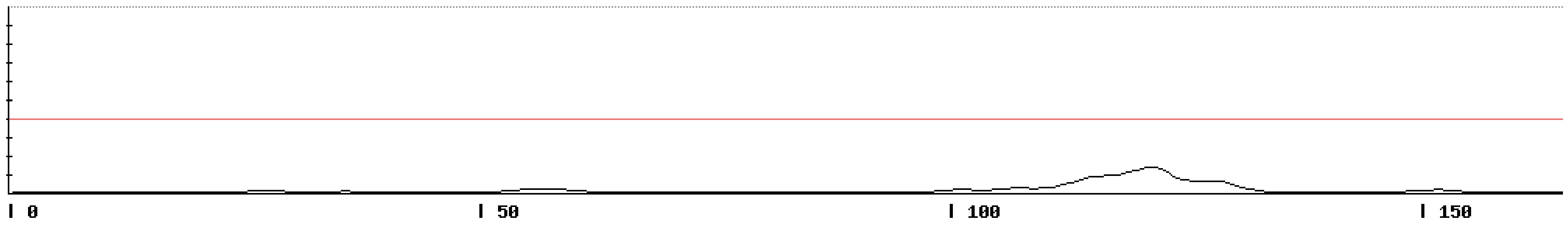

Predictions for Malusdomestica_MDP0000499023

Viterbi Path

Viterbi Path

Posterior @ 0.4

Posterior @ 0.4

Posterior Graphic. Threshold Marked @ 0.4

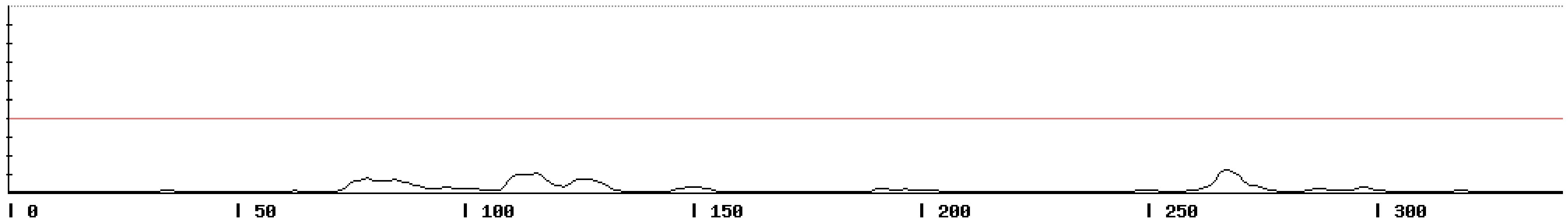

Predictions for Malusdomestica_MDP0000501518	
Viterbi Path	
Viterbi Path	
Posterior @ 0.4	
Posterior @ 0.4	
Posterior Graphic. Threshold Marked @ 0.4	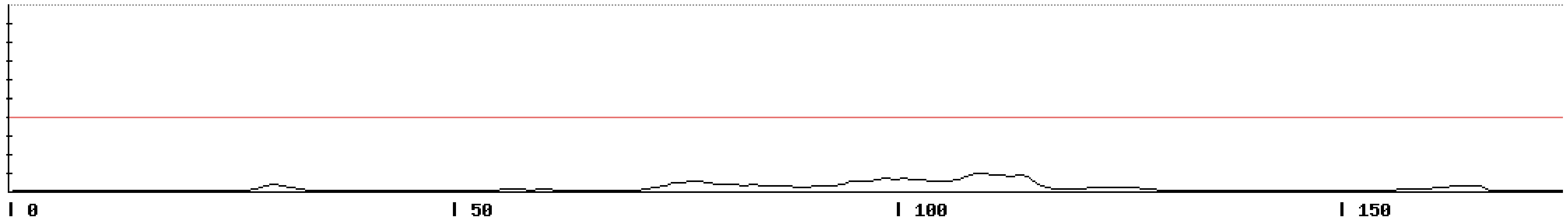

Predictions for Malusdomestica_MDP0000506233	
Viterbi Path	
Viterbi Path	
Posterior @ 0.4	
Posterior @ 0.4	
Posterior Graphic. Threshold Marked @ 0.4	

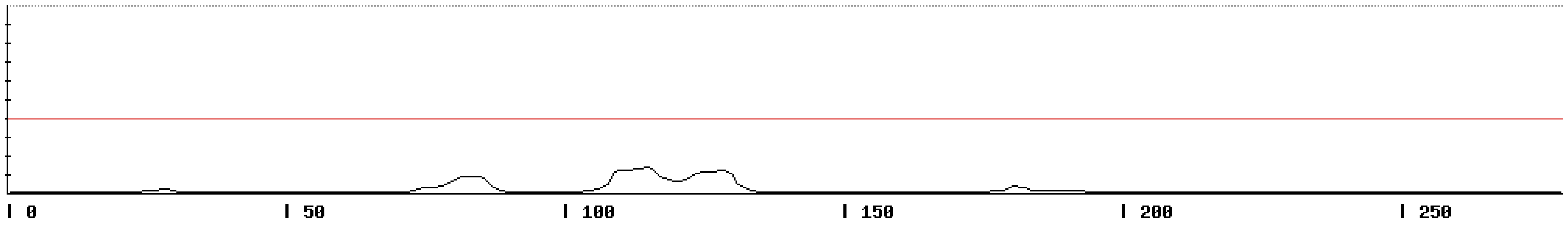

Predictions for Malusdomestica_MDP0000516159

Viterbi Path	
Viterbi Path	
Posterior @ 0.4	
Posterior @ 0.4	
Posterior Graphic. Threshold Marked @ 0.4	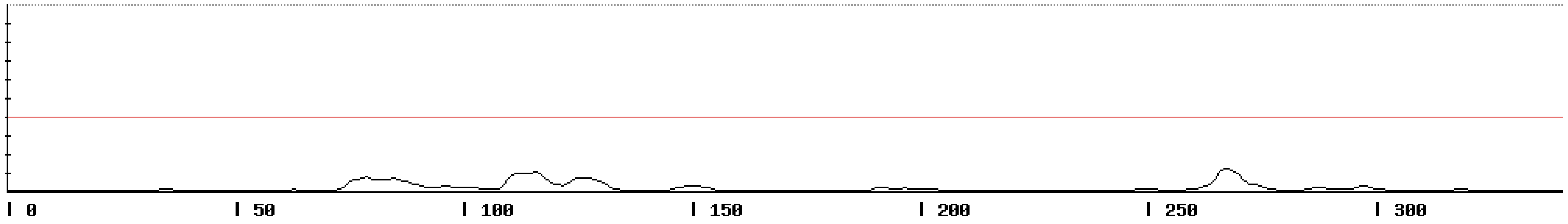

Predictions for Malusdomestica_MDP0000520807

Viterbi Path	
Viterbi Path	
Posterior @ 0.4	
Posterior @ 0.4	

Posterior Graphic. Threshold Marked @ 0.4

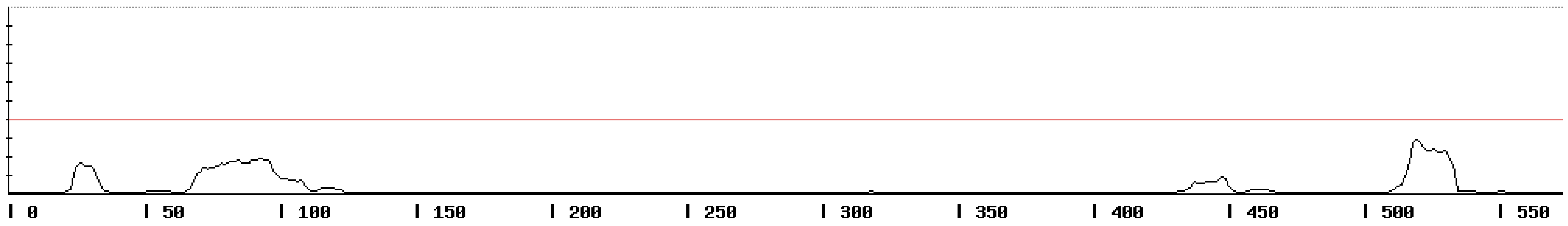

Predictions for Malusdomestica_MDP0000528658

Viterbi Path

Viterbi Path

Posterior @ 0.4

Posterior @ 0.4

Posterior Graphic. Threshold Marked @ 0.4

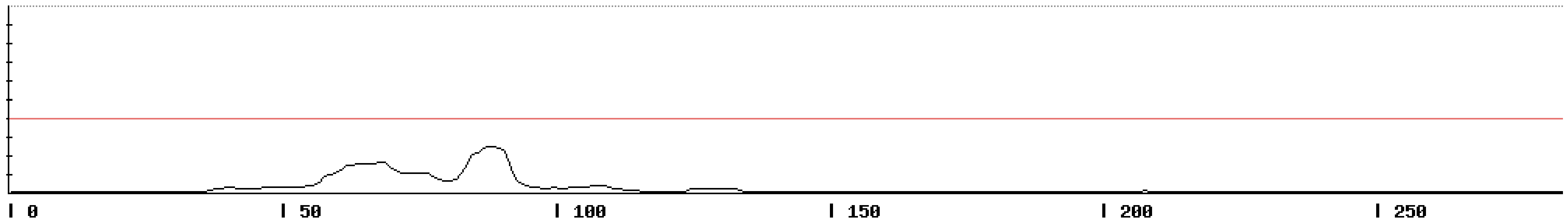

Predictions for Malusdomestica_MDP0000528857

Viterbi Path

Viterbi Path

Posterior @ 0.4

Posterior @ 0.4

Posterior Graphic. Threshold Marked @ 0.4

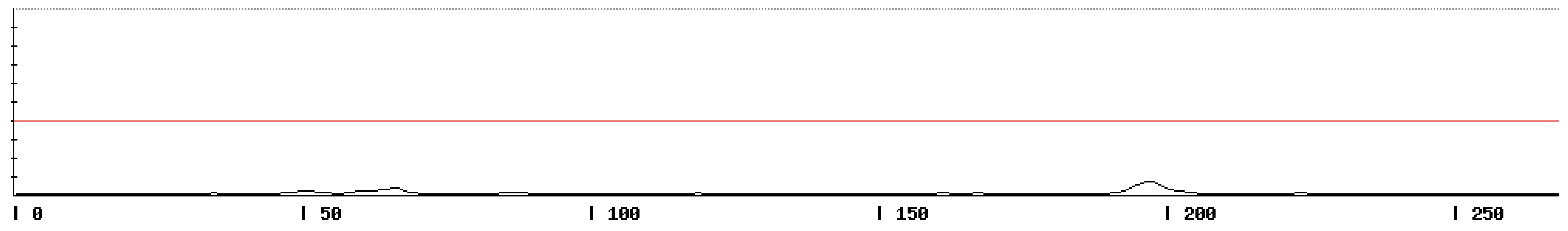

Predictions for Malusdomestica_MDP0000552812

Viterbi Path

Viterbi Path

Posterior @ 0.4

Posterior @ 0.4

Posterior Graphic. Threshold Marked @ 0.4

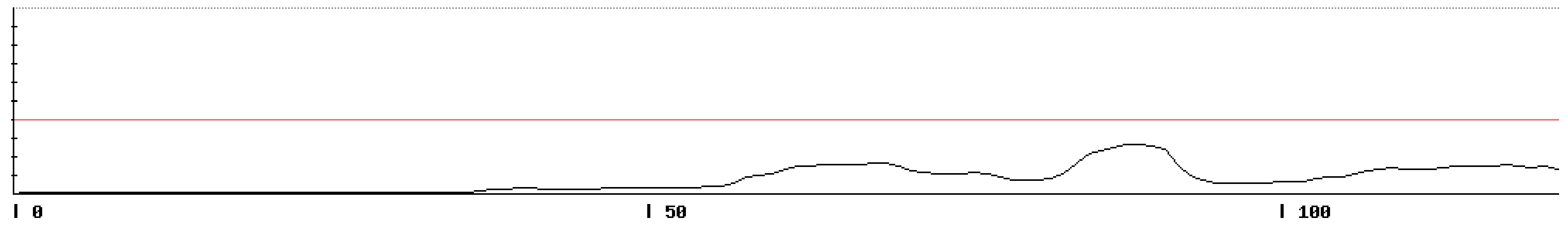

Predictions for Malusdomestica_MDP0000557836

Viterbi Path

Viterbi Path

Posterior @ 0.4

Posterior @ 0.4

Posterior Graphic. Threshold Marked @ 0.4

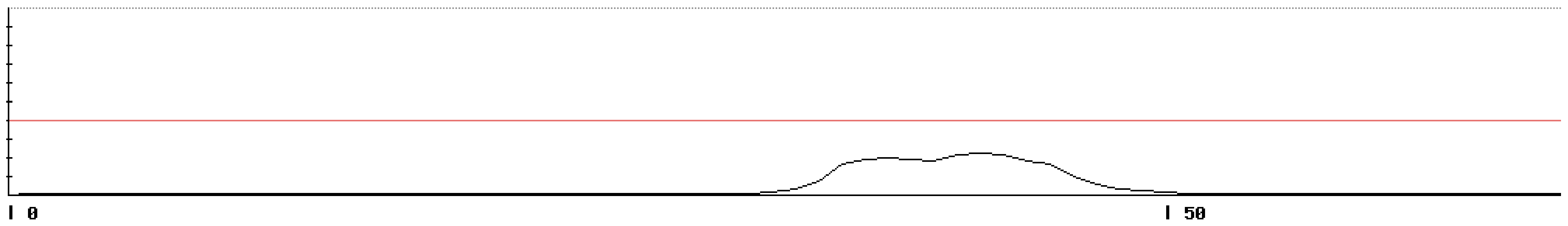

Predictions for Malusdomestica_MDP0000558493

Viterbi Path

Viterbi Path

Posterior @ 0.4

Posterior @ 0.4

Posterior Graphic. Threshold Marked @ 0.4

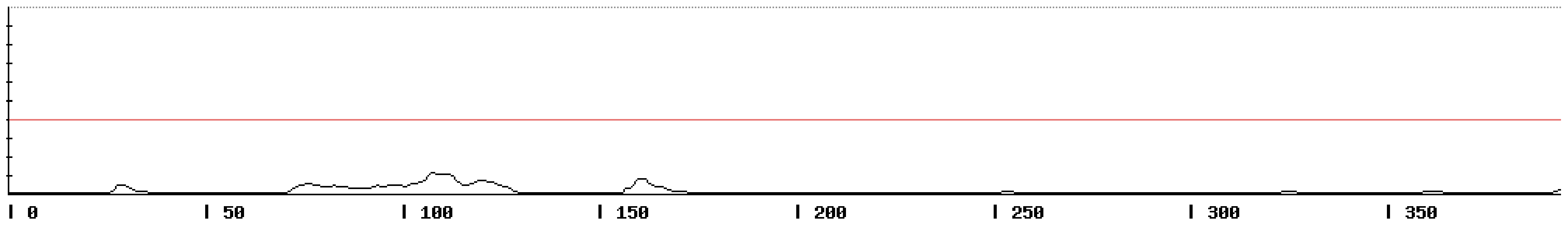

Predictions for Malusdomestica_MDP0000561788

Viterbi Path

Viterbi Path

Posterior @ 0.4	
Posterior @ 0.4	
Posterior Graphic. Threshold Marked @ 0.4	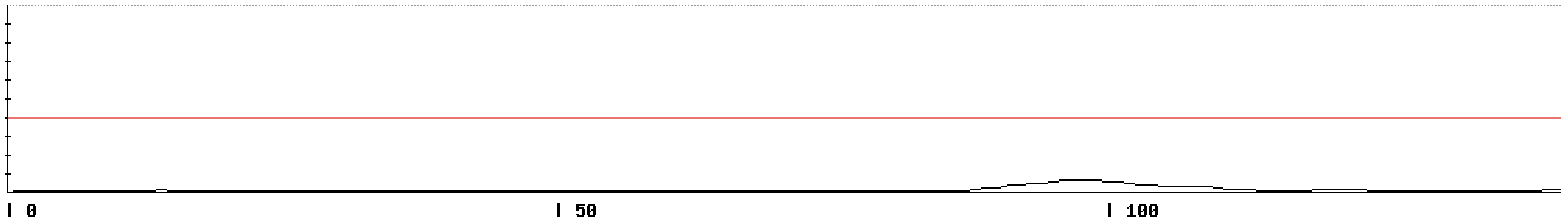

Predictions for Malusdomestica_MDP0000563165

Viterbi Path	
Viterbi Path	
Posterior @ 0.4	
Posterior @ 0.4	
Posterior Graphic. Threshold Marked @ 0.4	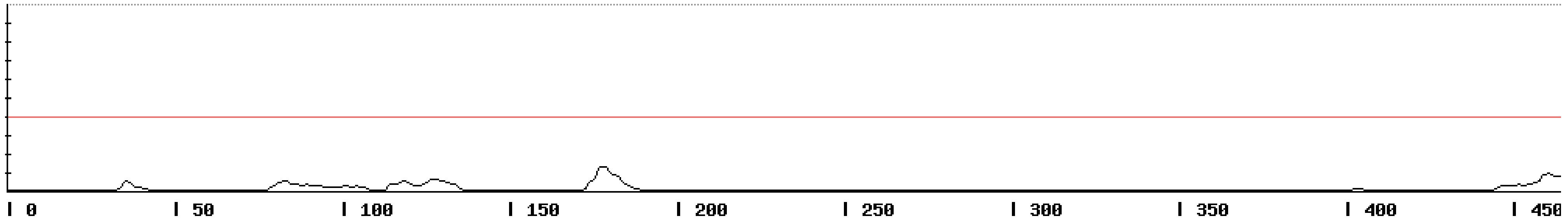

Predictions for Malusdomestica_MDP0000564592

Viterbi Path

Viterbi Path

Posterior @ 0.4

Posterior @ 0.4

Posterior Graphic. Threshold Marked @ 0.4

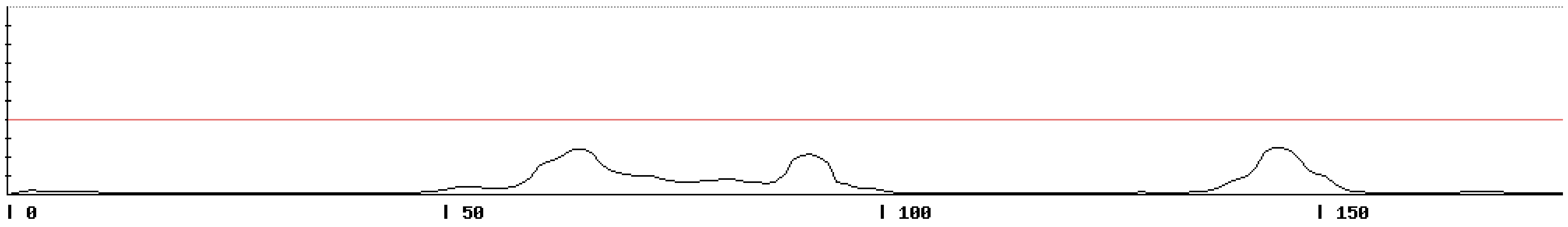

Predictions for Malusdomestica_MDP0000575835

Viterbi Path

Viterbi Path

Posterior @ 0.4

105 - GKEKIVKARDNKIIGKKRILTFRQGR - 130

Posterior @ 0.4

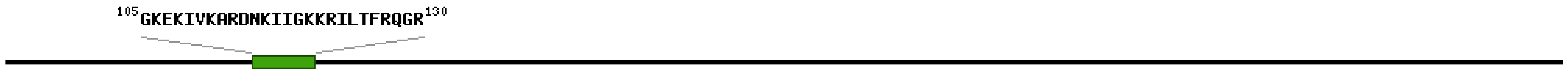

Posterior Graphic. Threshold Marked @ 0.4

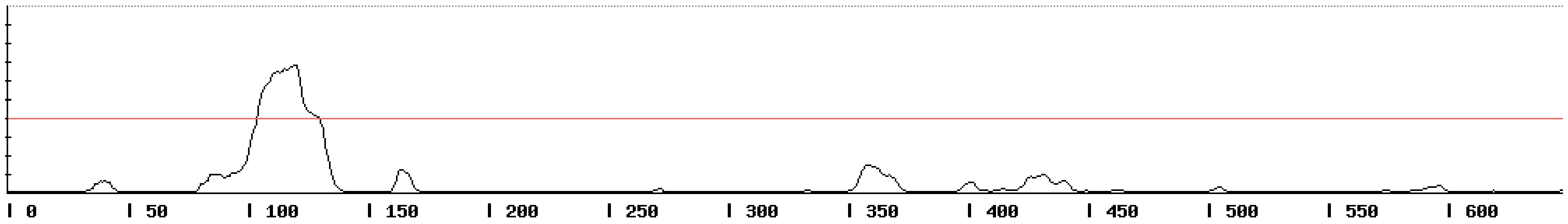

Predictions for Malusdomestica_MDP0000587003	
Viterbi Path	
Viterbi Path	
Posterior @ 0.4	
Posterior @ 0.4	
Posterior Graphic. Threshold Marked @ 0.4	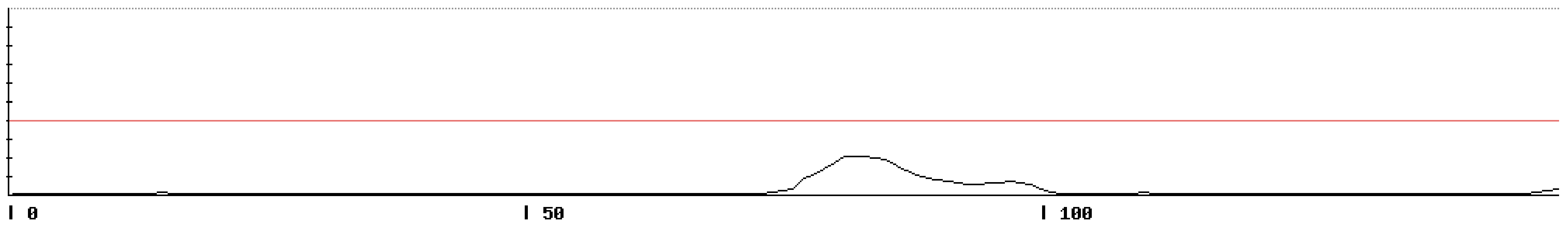

Predictions for Malusdomestica_MDP0000595295	
Viterbi Path	
Viterbi Path	
Posterior @ 0.4	74 - KRRKNISRKMGHAGTWSESGSKIEGHRNPNPIGRKRKFRFENK - 116
Posterior @ 0.4	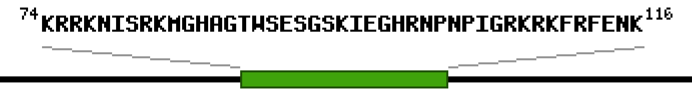
Posterior Graphic. Threshold Marked @ 0.4	

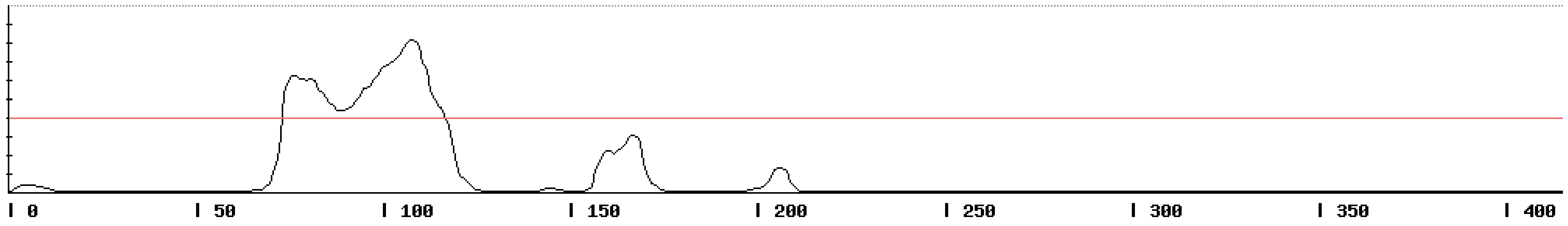

Predictions for Malusdomestica_MDP0000599388

Viterbi Path

Viterbi Path

Posterior @ 0.4

Posterior @ 0.4

Posterior Graphic. Threshold Marked @ 0.4

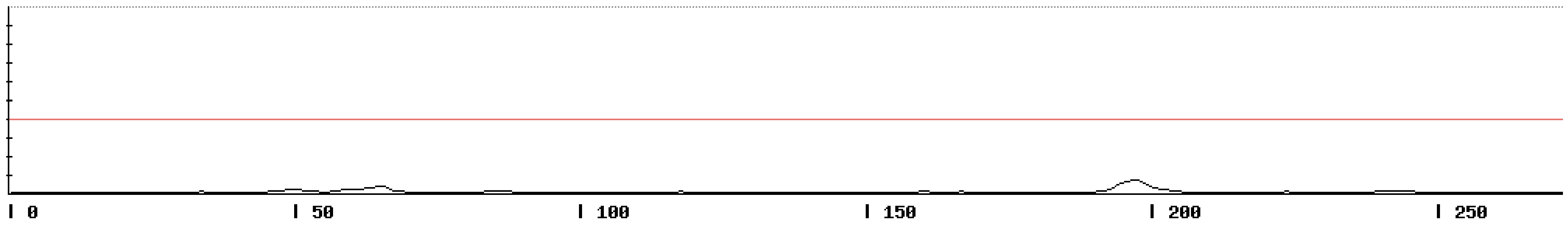

Predictions for Malusdomestica_MDP0000618650

Viterbi Path

Viterbi Path

Posterior @ 0.4

Posterior @ 0.4

Posterior Graphic. Threshold Marked @ 0.4

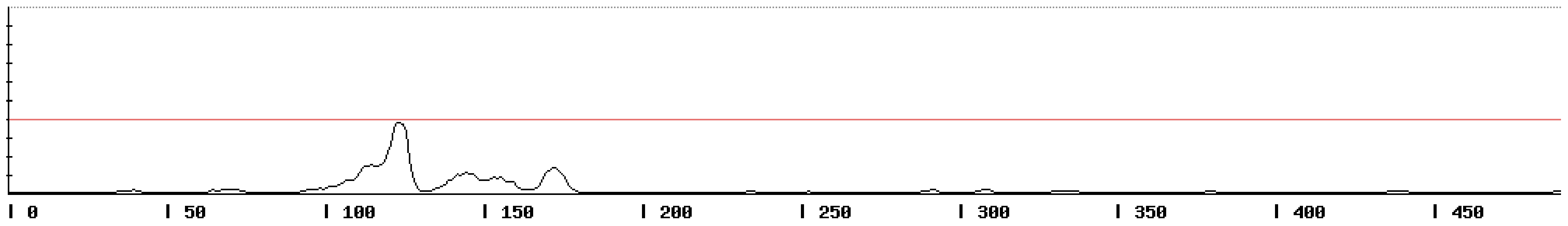

Predictions for Malusdomestica_MDP0000619501

Viterbi Path

Viterbi Path

Posterior @ 0.4

Posterior @ 0.4

Posterior Graphic. Threshold Marked @ 0.4

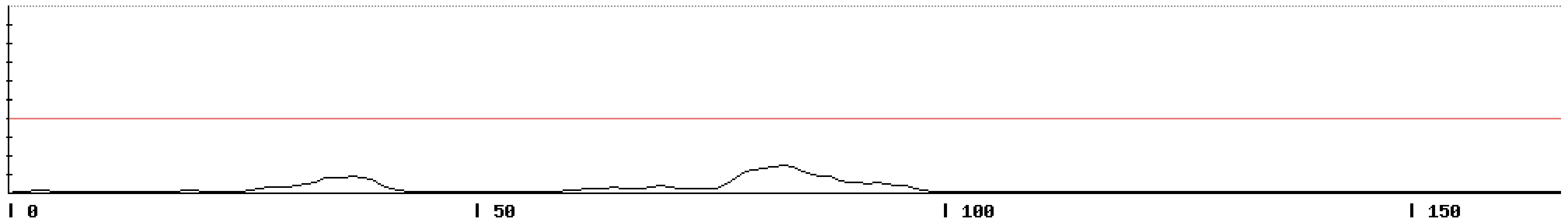

Predictions for Malusdomestica_MDP0000626215

Viterbi Path

Viterbi Path

Posterior @ 0.4

Posterior @ 0.4

Posterior Graphic. Threshold Marked @ 0.4

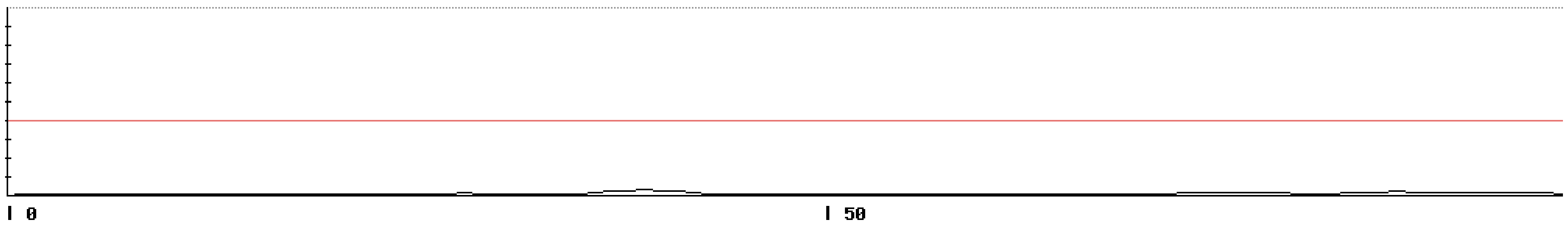

Predictions for Malusdomestica_MDP0000634006

Viterbi Path

Viterbi Path

Posterior @ 0.4

Posterior @ 0.4

Posterior Graphic. Threshold Marked @ 0.4

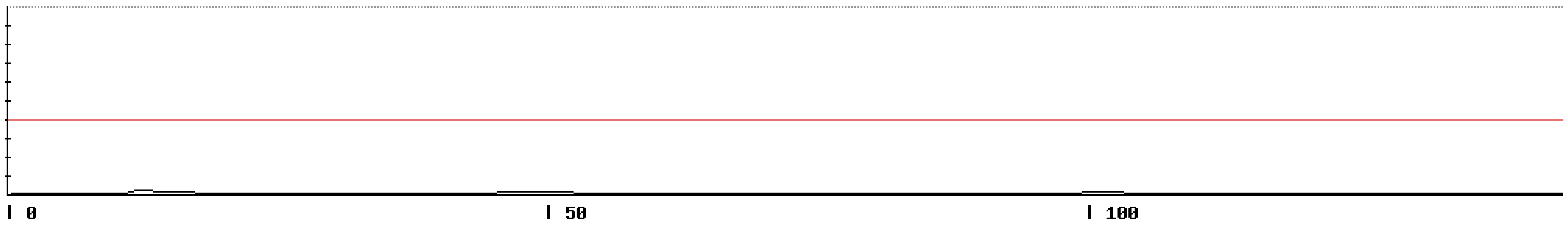

Predictions for Malusdomestica_MDP0000652584

Viterbi Path

Viterbi Path

Posterior @ 0.4	
Posterior @ 0.4	
Posterior Graphic. Threshold Marked @ 0.4	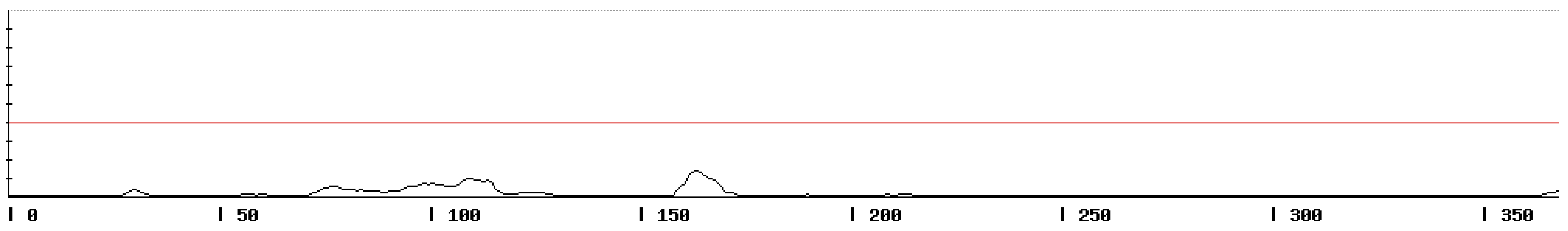

Predictions for Malusdomestica_MDP0000655623	
Viterbi Path	
Viterbi Path	
Posterior @ 0.4	
Posterior @ 0.4	
Posterior Graphic. Threshold Marked @ 0.4	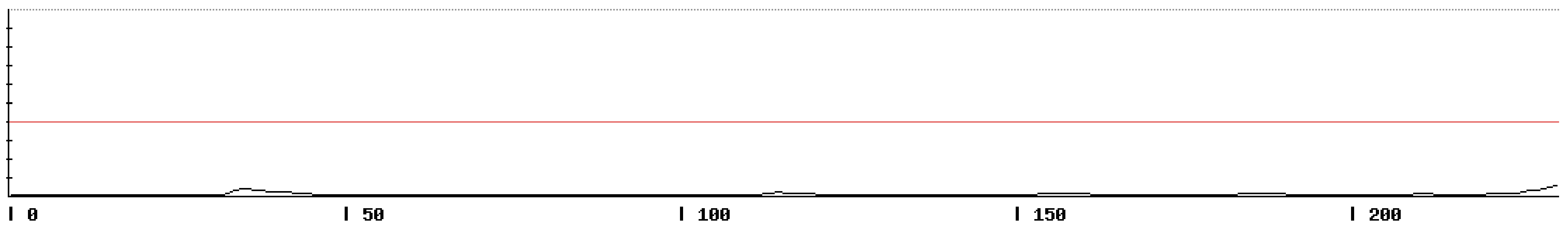

Predictions for Malusdomestica_MDP0000655666	
Viterbi Path	
Viterbi Path	

Posterior @ 0.4 | 162 - KKVNGSEKKRK - 172

Posterior @ 0.4

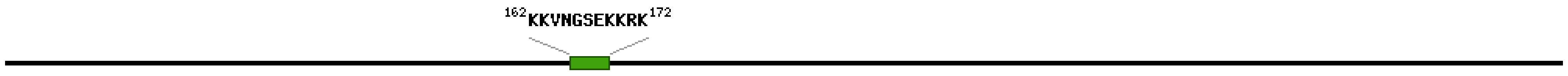

Posterior Graphic. Threshold Marked @ 0.4

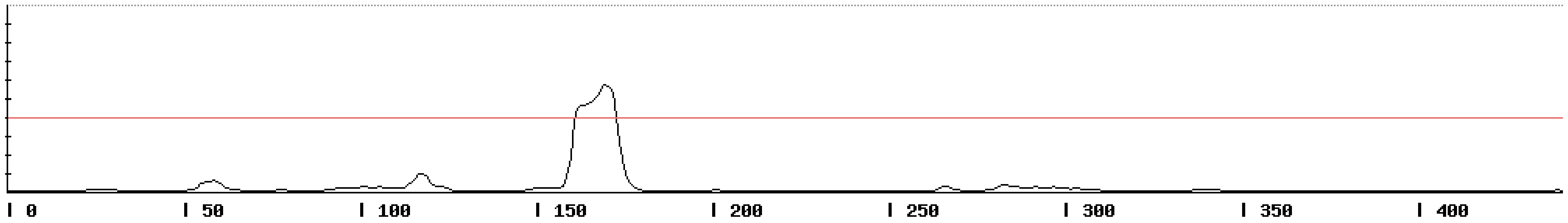

Predictions for Malusdomestica_MDP0000656113

Viterbi Path

Viterbi Path

Posterior @ 0.4

Posterior @ 0.4

Posterior Graphic. Threshold Marked @ 0.4

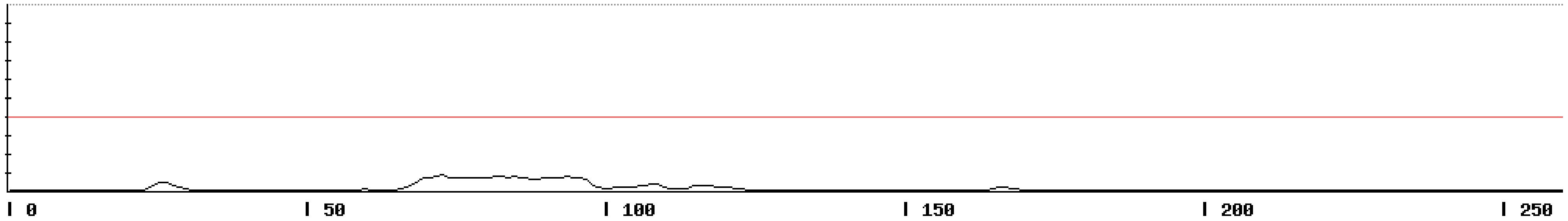

Predictions for Malusdomestica_MDP0000658409

Viterbi Path	
Viterbi Path	
Posterior @ 0.4	
Posterior @ 0.4	
Posterior Graphic. Threshold Marked @ 0.4	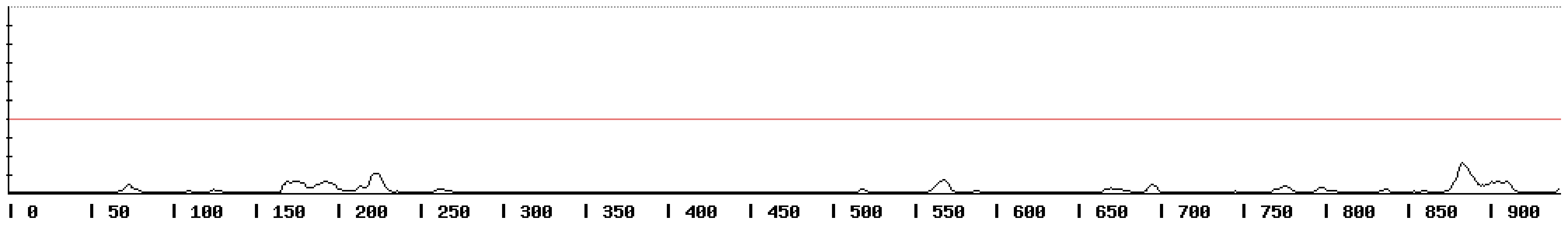

Predictions for Malusdomestica_MDP0000663564

Viterbi Path	
Viterbi Path	
Posterior @ 0.4	104 - RKRRK - 108
	150 - KKG - 152
	512 - MRSKVAKRMQKESGKTLREVRRAKKLQKKLMTENERLIYNLKRAKRKVALL - 562
	565 - K - 565
Posterior @ 0.4	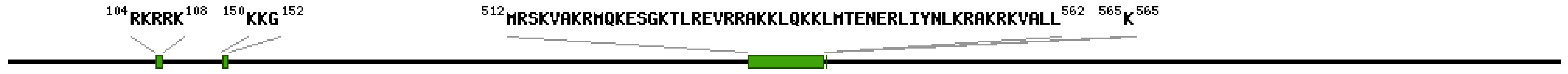
Posterior Graphic. Threshold Marked @ 0.4	

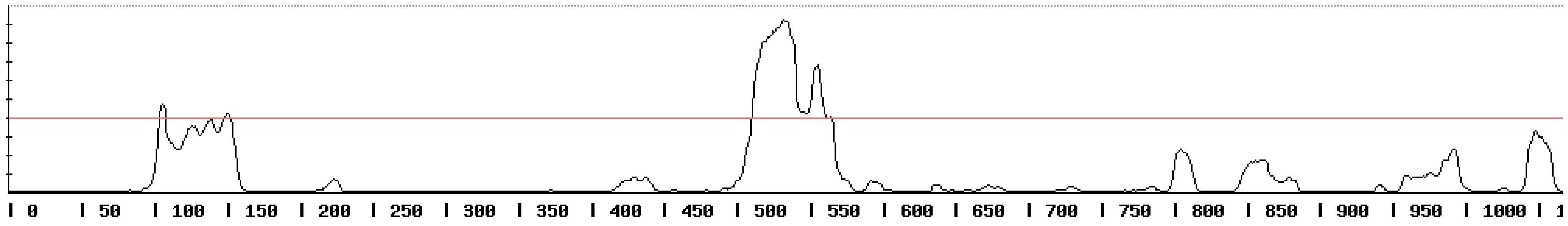

Predictions for Malusdomestica_MDP0000667743

Viterbi Path

Viterbi Path

Posterior @ 0.4

Posterior @ 0.4

Posterior Graphic. Threshold Marked @ 0.4

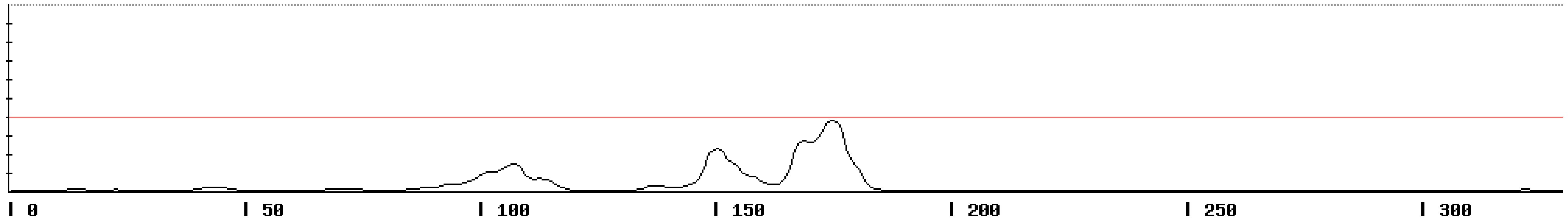

Predictions for Malusdomestica_MDP0000674514

Viterbi Path

Viterbi Path

Posterior @ 0.4

Posterior @ 0.4

Posterior Graphic. Threshold Marked @ 0.4

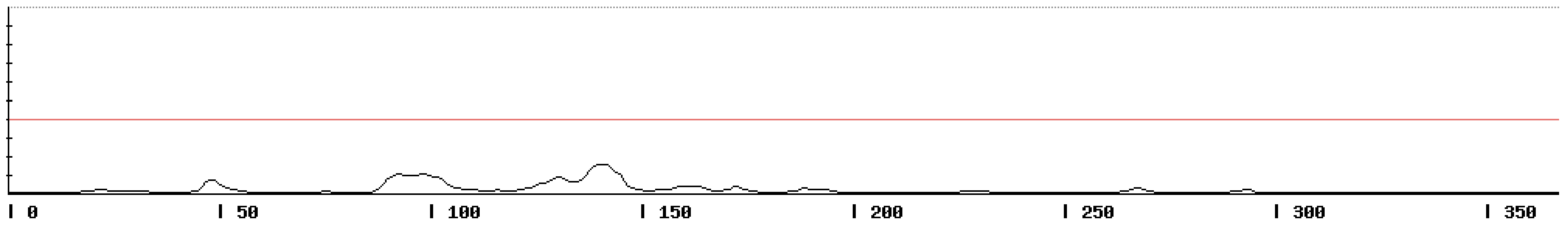

Predictions for Malusdomestica_MDP0000679946

Viterbi Path

Viterbi Path

Posterior @ 0.4

Posterior @ 0.4

Posterior Graphic. Threshold Marked @ 0.4

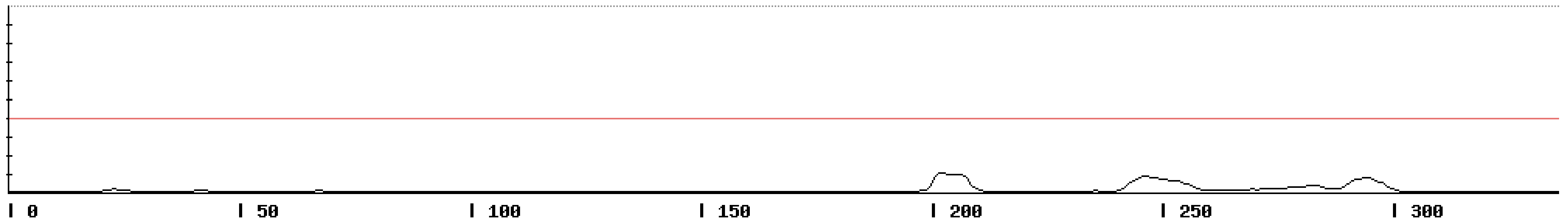

Predictions for Malusdomestica_MDP0000688878

Viterbi Path

Viterbi Path

Posterior @ 0.4

Posterior @ 0.4

Posterior Graphic. Threshold Marked @ 0.4

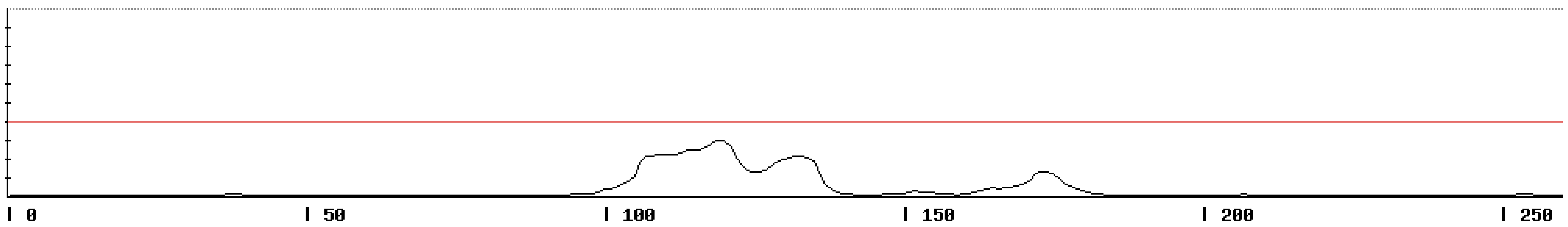

Predictions for Malusdomestica_MDP0000689991

Viterbi Path

Viterbi Path

Posterior @ 0.4

Posterior @ 0.4

Posterior Graphic. Threshold Marked @ 0.4

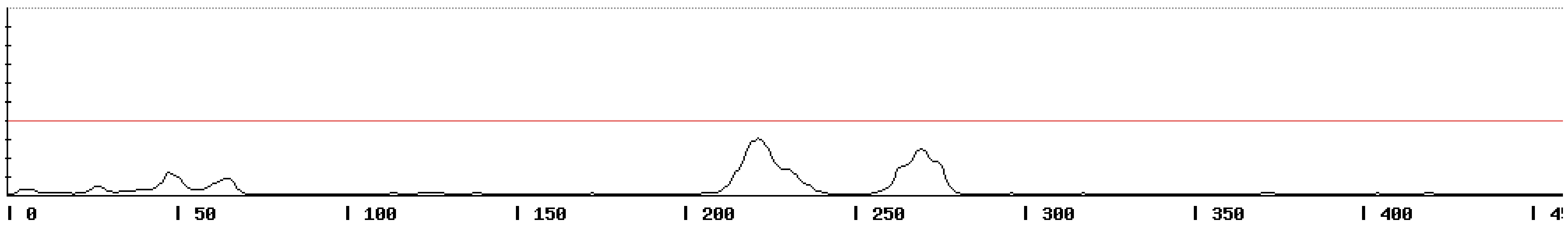

Predictions for Malusdomestica_MDP0000690168

Viterbi Path

Viterbi Path

Posterior @ 0.4

Posterior @ 0.4

Posterior Graphic. Threshold Marked @ 0.4

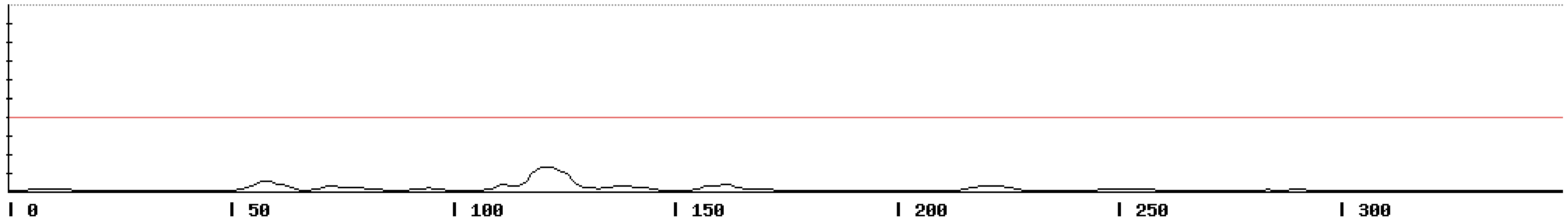

Predictions for Malusdomestica_MDP0000693024

Viterbi Path: 128 - KPPIKRARKEVSEKPLIKRKARK - 150

Viterbi Path

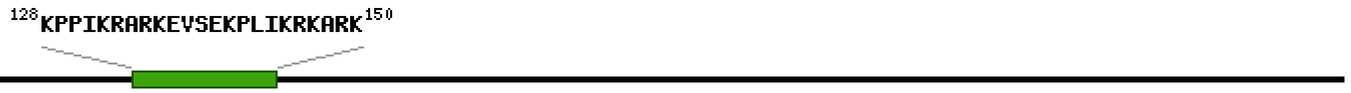

Posterior @ 0.4: 127 - EKPPIKRARKEVSEKPLIKRKARKEPGSQK - 156

Posterior @ 0.4

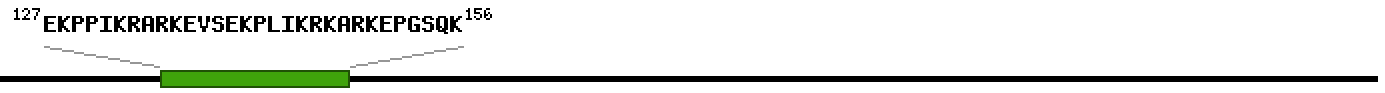

Posterior Graphic. Threshold Marked @ 0.4

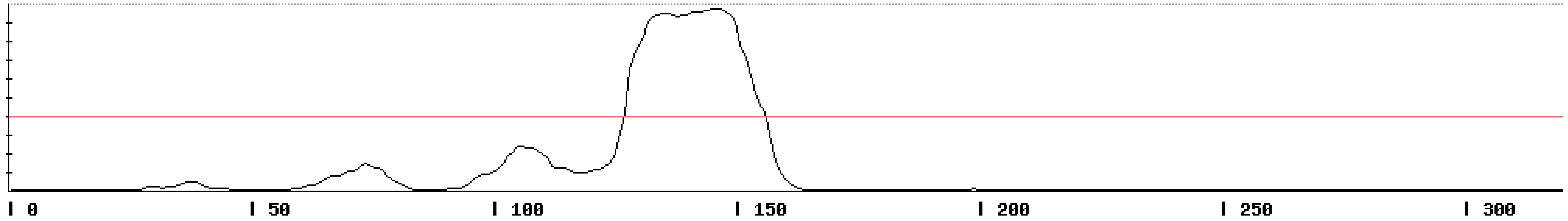

Predictions for Malusdomestica_MDP0000695714

Viterbi Path

Viterbi Path

Posterior @ 0.4

Posterior @ 0.4

Posterior Graphic. Threshold Marked @ 0.4

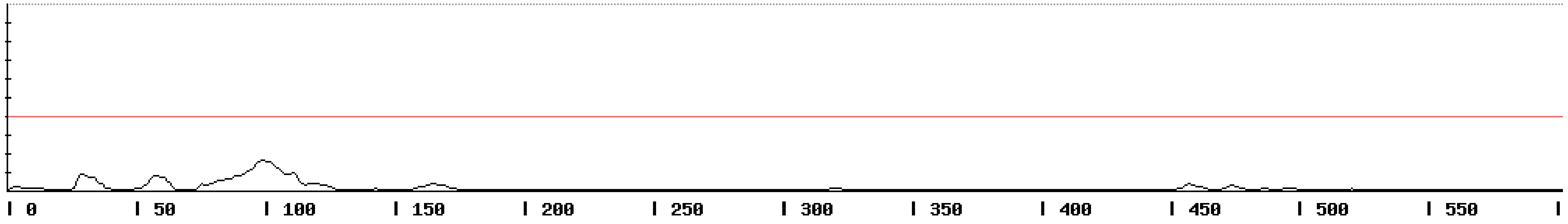

Predictions for Malusdomestica_MDP0000697030

Viterbi Path

Viterbi Path

Posterior @ 0.4

Posterior @ 0.4

Posterior Graphic. Threshold Marked @ 0.4

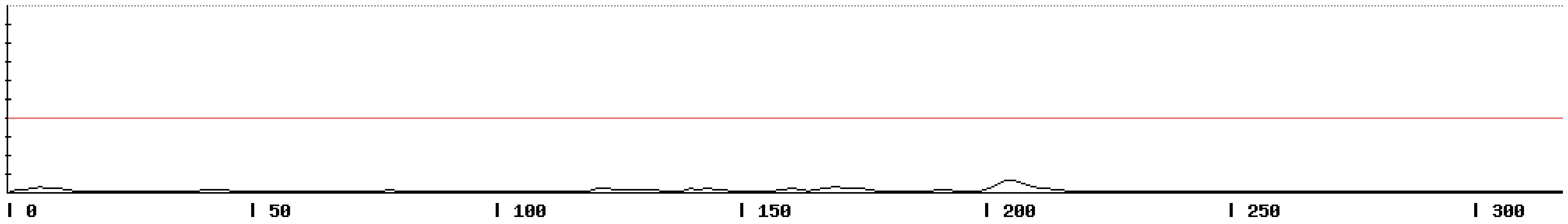

Predictions for Malusdomestica_MDP0000703244

Viterbi Path	
Viterbi Path	
Posterior @ 0.4	
Posterior @ 0.4	
Posterior Graphic. Threshold Marked @ 0.4	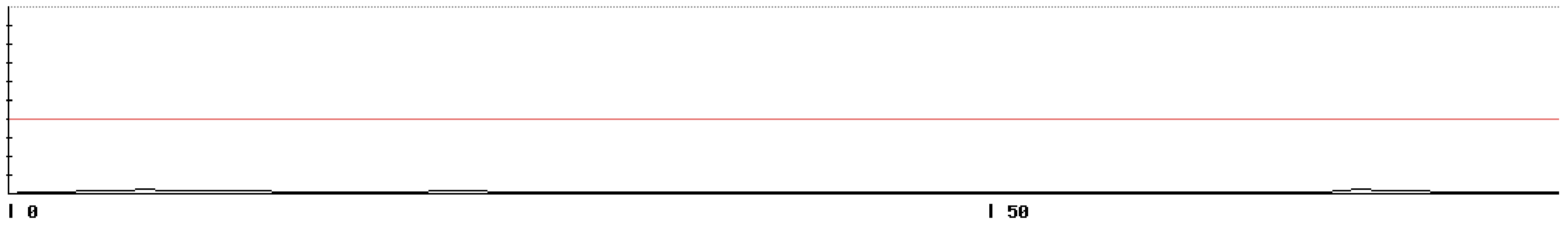

Predictions for Malusdomestica_MDP0000705510	
Viterbi Path	
Viterbi Path	
Posterior @ 0.4	
Posterior @ 0.4	
Posterior Graphic. Threshold Marked @ 0.4	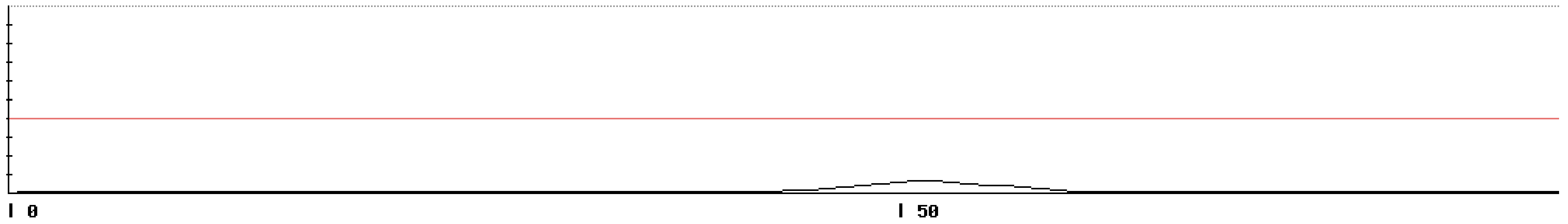

Predictions for Malusdomestica_MDP0000705786

Viterbi Path	
Viterbi Path	
Posterior @ 0.4	100 - KRKTK - 104
Posterior @ 0.4	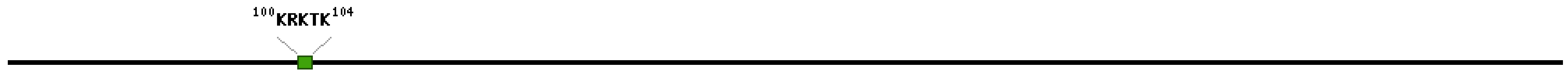
Posterior Graphic. Threshold Marked @ 0.4	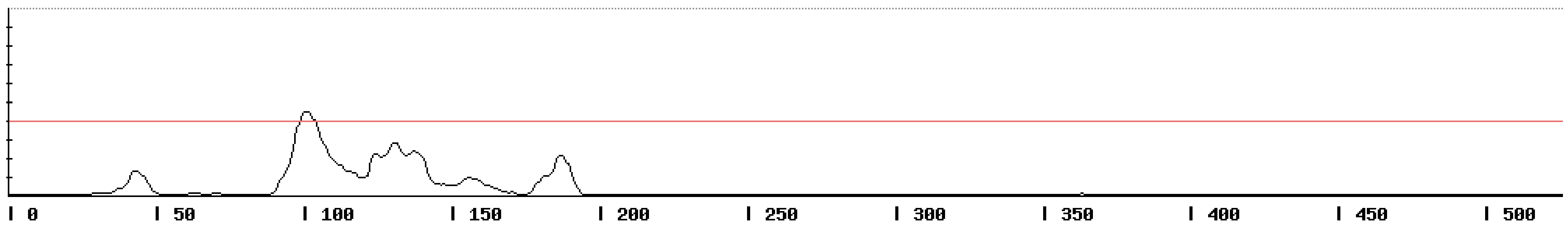

Predictions for Malusdomestica_MDP0000718993

Viterbi Path	
Viterbi Path	
Posterior @ 0.4	
Posterior @ 0.4	
Posterior Graphic. Threshold Marked @ 0.4	

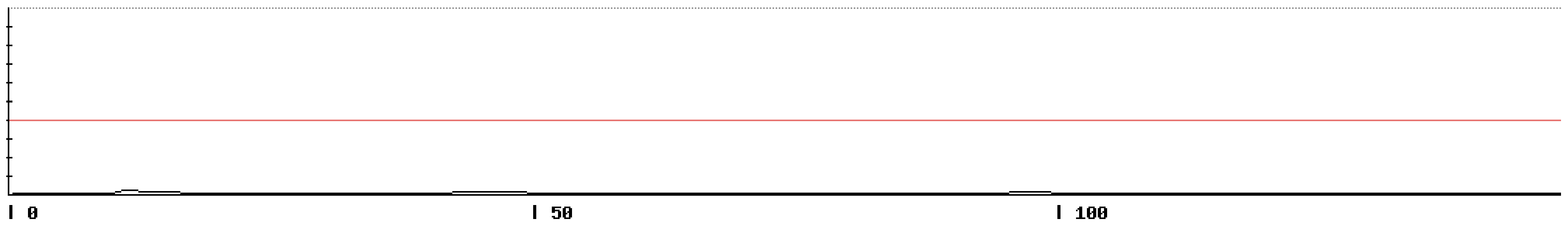

Predictions for Malusdomestica_MDP0000719421

Viterbi Path

Viterbi Path

Posterior @ 0.4

Posterior @ 0.4

Posterior Graphic. Threshold Marked @ 0.4

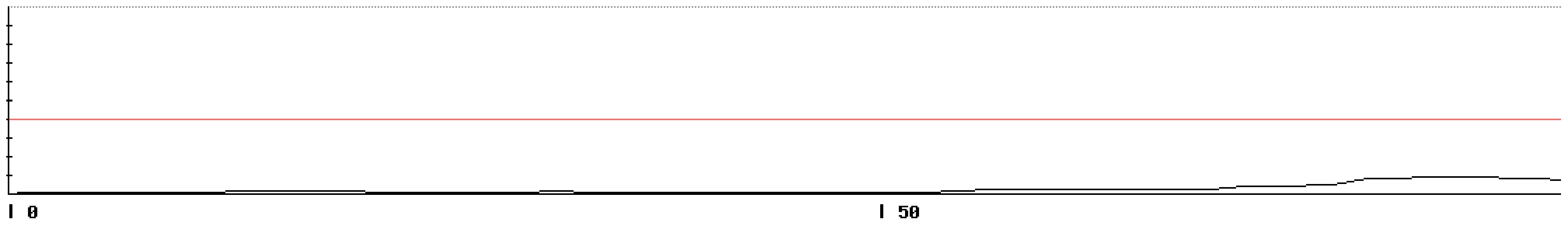

Predictions for Malusdomestica_MDP0000722092

Viterbi Path

Viterbi Path

Posterior @ 0.4 221 - KKSKKNRK - 228

Posterior @ 0.4

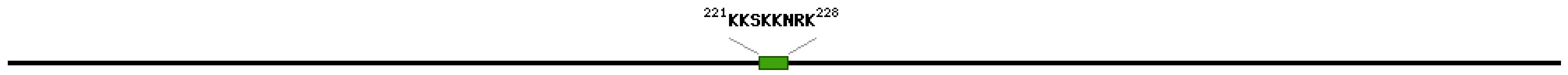

Posterior Graphic. Threshold Marked @ 0.4

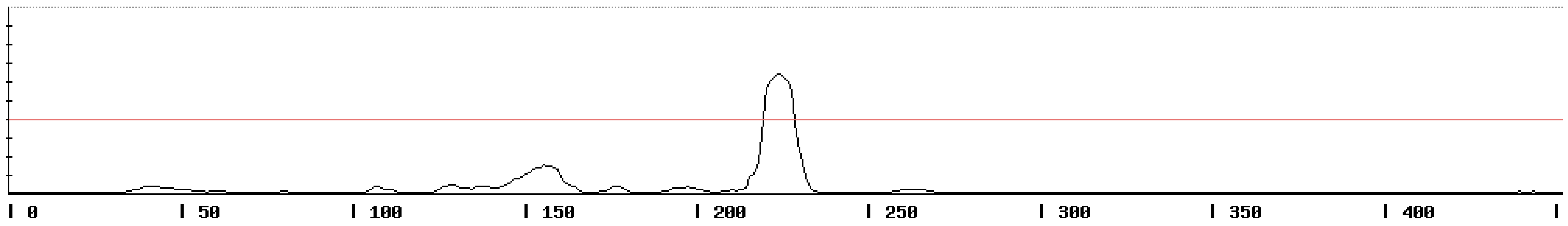

Predictions for Malusdomestica_MDP0000723277

Viterbi Path

Viterbi Path

Posterior @ 0.4

Posterior @ 0.4

Posterior Graphic. Threshold Marked @ 0.4

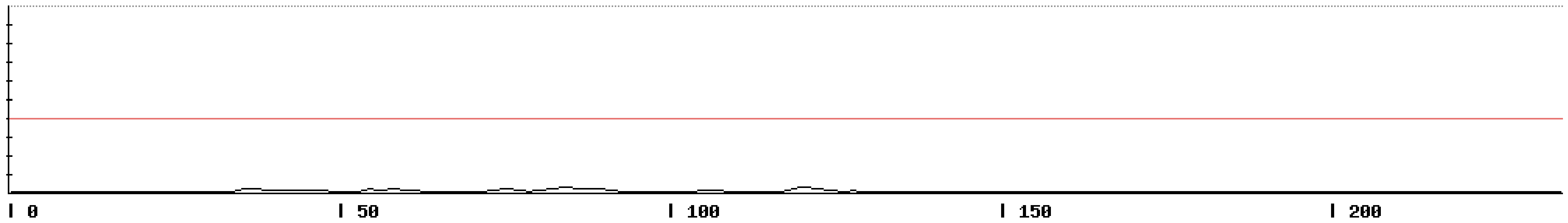

Predictions for Malusdomestica_MDP0000723762

Viterbi Path

Viterbi Path

Posterior @ 0.4

Posterior @ 0.4

Posterior Graphic. Threshold Marked @ 0.4

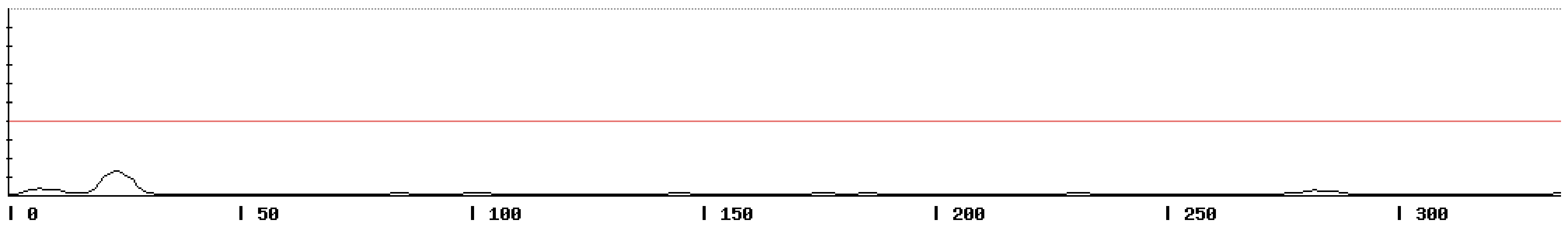

Predictions for Malusdomestica_MDP0000725651

Viterbi Path

Viterbi Path

Posterior @ 0.4

Posterior @ 0.4

Posterior Graphic. Threshold Marked @ 0.4

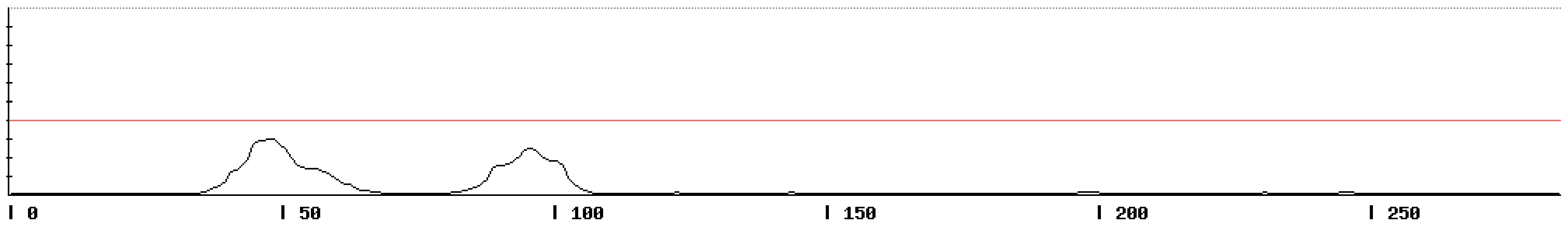

Predictions for Malusdomestica_MDP0000740927

Viterbi Path

Viterbi Path

Posterior @ 0.4	
Posterior @ 0.4	
Posterior Graphic. Threshold Marked @ 0.4	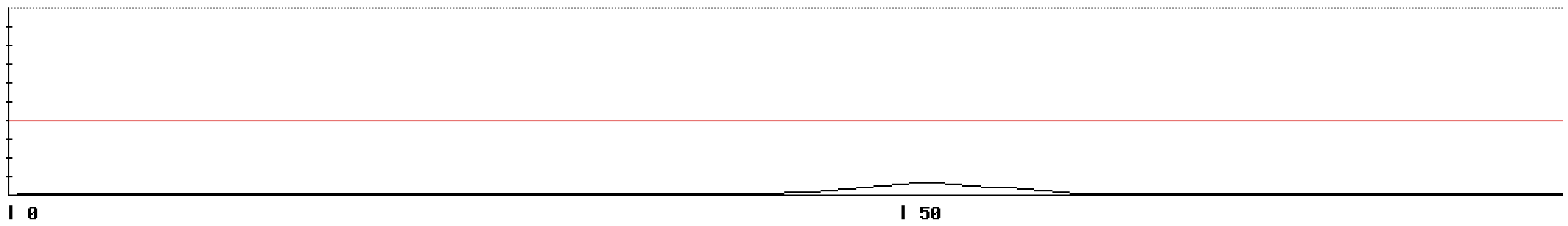

Predictions for Malusdomestica_MDP0000759504	
Viterbi Path	
Viterbi Path	
Posterior @ 0.4	
Posterior @ 0.4	
Posterior Graphic. Threshold Marked @ 0.4	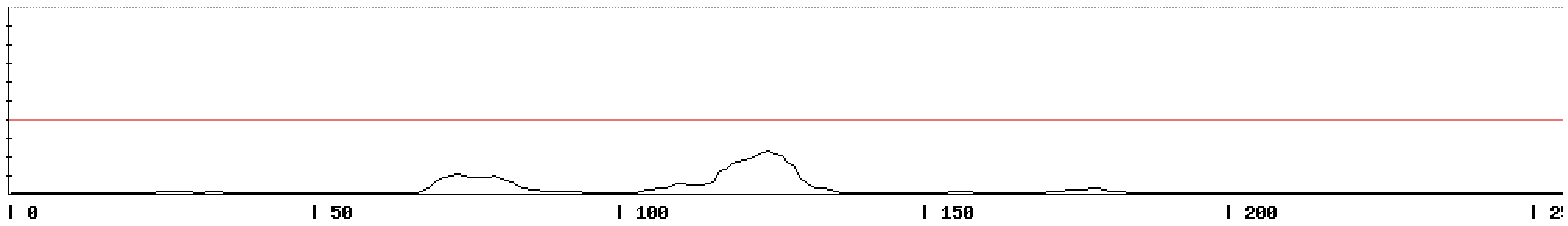

Predictions for Malusdomestica_MDP0000759612	
Viterbi Path	205 - KKRARKEVKEKPVKKRARKEVKEKPVKKRARKEANEKPAKTKAR - 248
Viterbi Path	

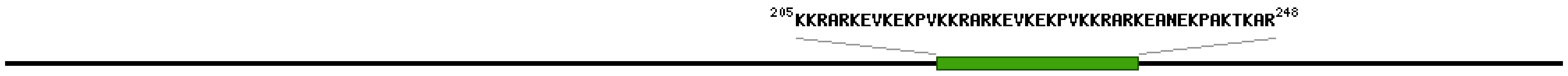

Posterior @ 0.4 — 204 - AKKRARKEVKEKPVKKRARKEVKEKPVKKRARKEANEKPAKTKAR - 248

Posterior @ 0.4

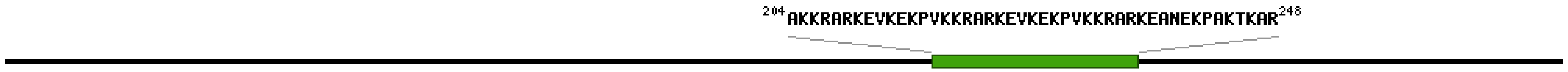

Posterior Graphic. Threshold Marked @ 0.4

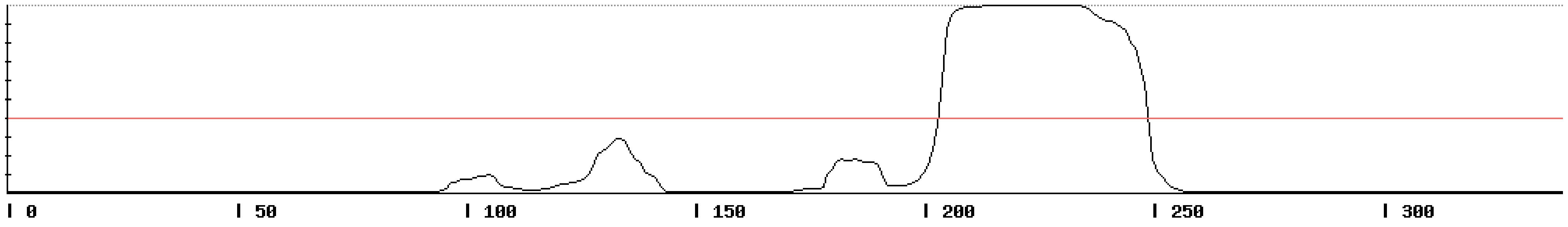

Predictions for Malusdomestica_MDP0000762302

Viterbi Path

Viterbi Path

Posterior @ 0.4

Posterior @ 0.4

Posterior Graphic. Threshold Marked @ 0.4

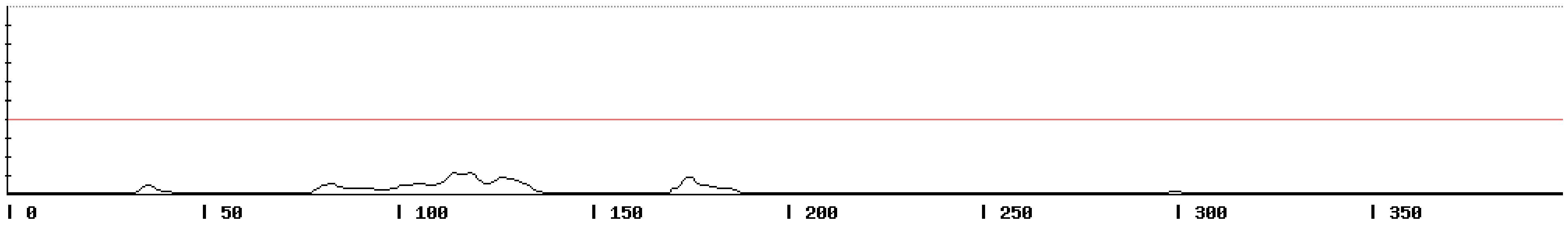

Predictions for Malusdomestica_MDP0000772731

Viterbi Path

Viterbi Path

Posterior @ 0.4

Posterior @ 0.4

Posterior Graphic. Threshold Marked @ 0.4

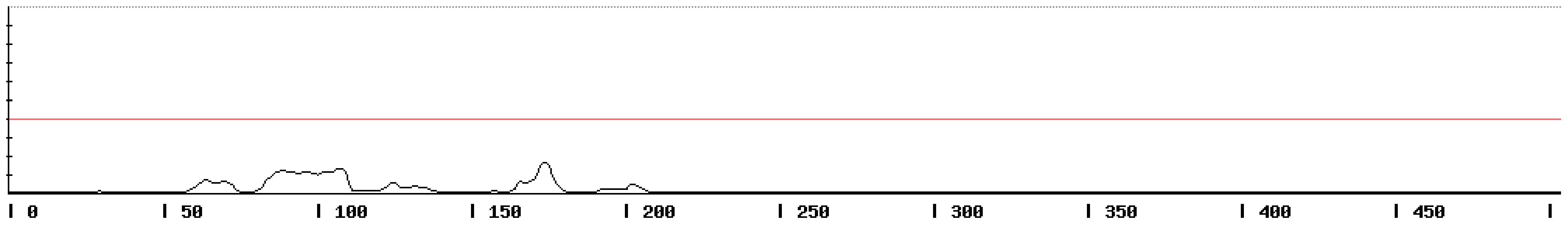

Predictions for Malusdomestica_MDP0000776583

Viterbi Path

Viterbi Path

Posterior @ 0.4

Posterior @ 0.4

Posterior Graphic. Threshold Marked @ 0.4

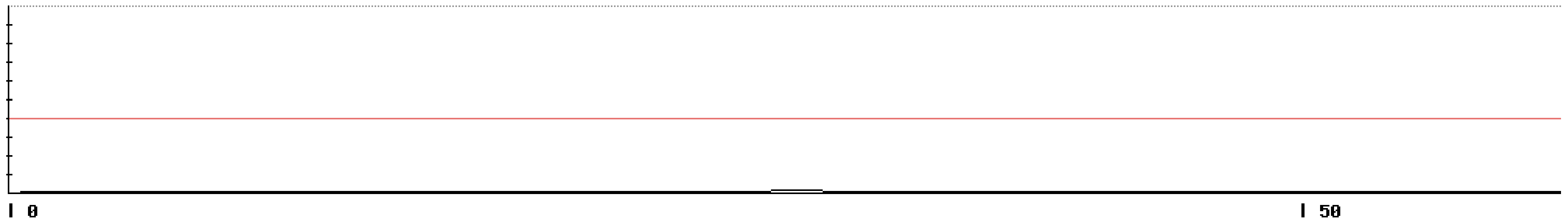

Predictions for Malusdomestica_MDP0000788655	
Viterbi Path	
Viterbi Path	
Posterior @ 0.4	
Posterior @ 0.4	
Posterior Graphic. Threshold Marked @ 0.4	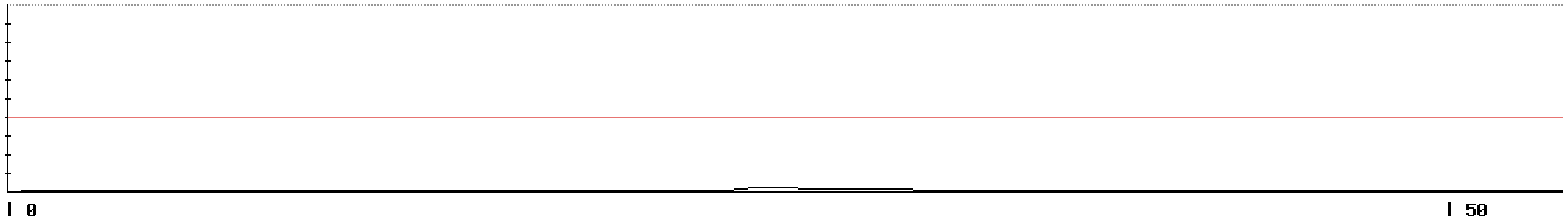

Predictions for Malusdomestica_MDP0000800550	
Viterbi Path	171 - KPPIKKRARKEILNEEPAIKRARKDLLNEKSPVKKTQRKELLNEKSPVKKTQRKELLNEKPSIKKRARK - 239
Viterbi Path	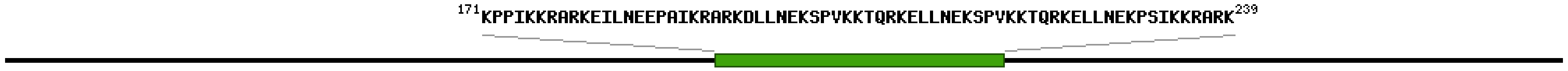
Posterior @ 0.4	171 - KPPIKKRARKEILNEEPAIKRARKDLLNEKSPVKKTQRKELLNEKSPVKKTQRKELLNEKPSIKKRARKE - 240
Posterior @ 0.4	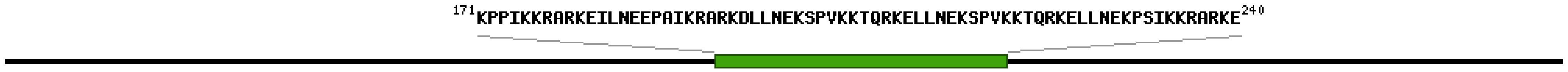
Posterior Graphic. Threshold Marked @ 0.4	

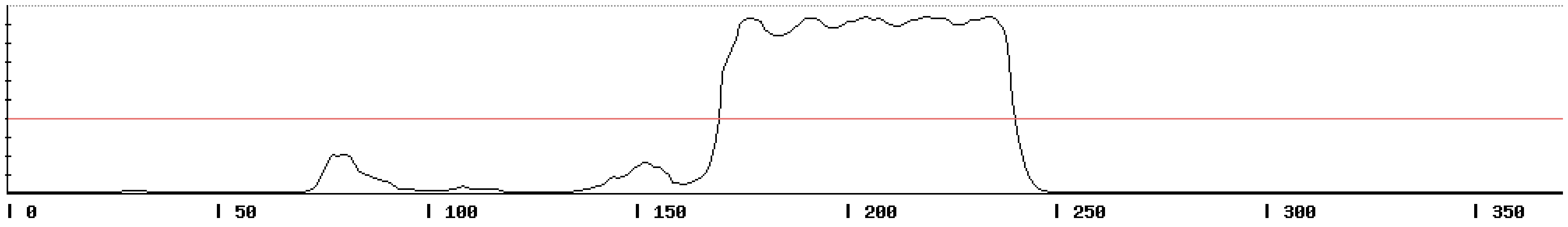

Predictions for Malusdomestica_MDP0000802924

Viterbi Path

Viterbi Path

Posterior @ 0.4

Posterior @ 0.4

Posterior Graphic. Threshold Marked @ 0.4

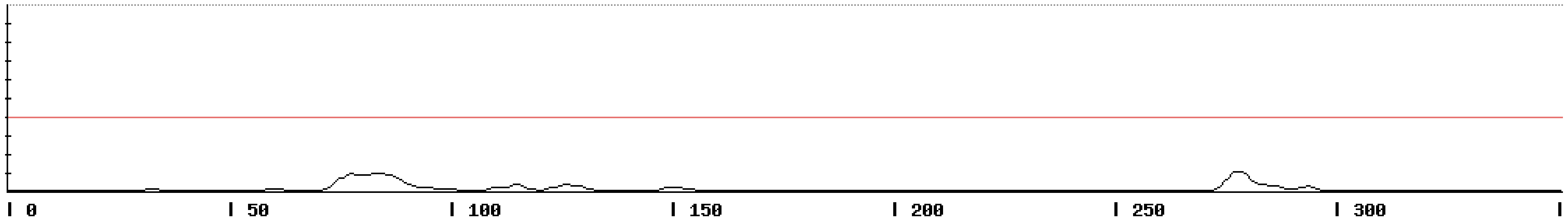

Predictions for Malusdomestica_MDP0000807087

Viterbi Path

Viterbi Path

Posterior @ 0.4

Posterior @ 0.4

Posterior Graphic. Threshold Marked @ 0.4

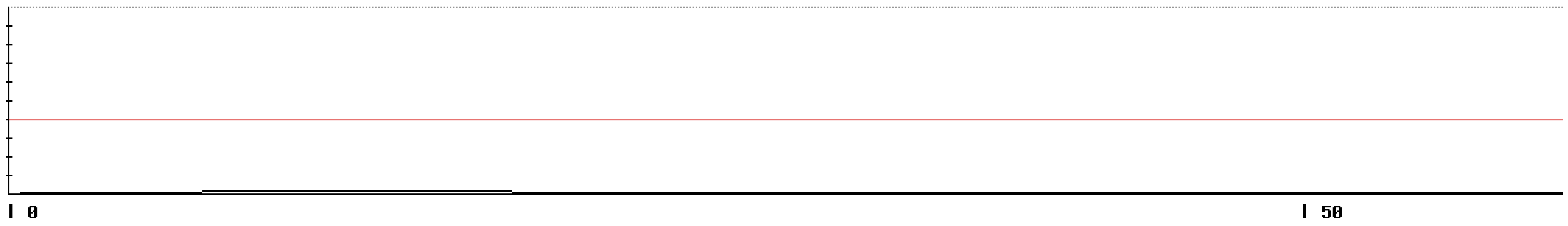

Predictions for Malusdomestica_MDP0000807984

Viterbi Path

Viterbi Path

Posterior @ 0.4 — 156 - RKNRRSCGKKR - 166

Posterior @ 0.4

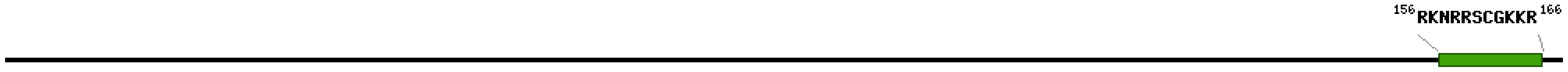

Posterior Graphic. Threshold Marked @ 0.4

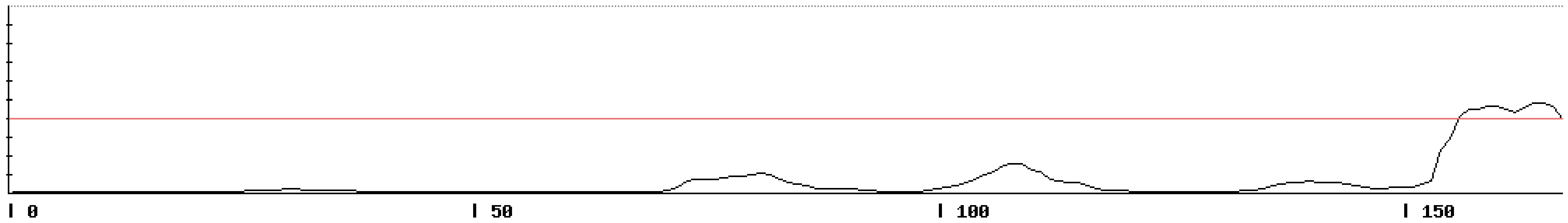

Predictions for Malusdomestica_MDP0000835180

Viterbi Path

Viterbi Path

Posterior @ 0.4

Posterior @ 0.4

Posterior Graphic. Threshold Marked @ 0.4

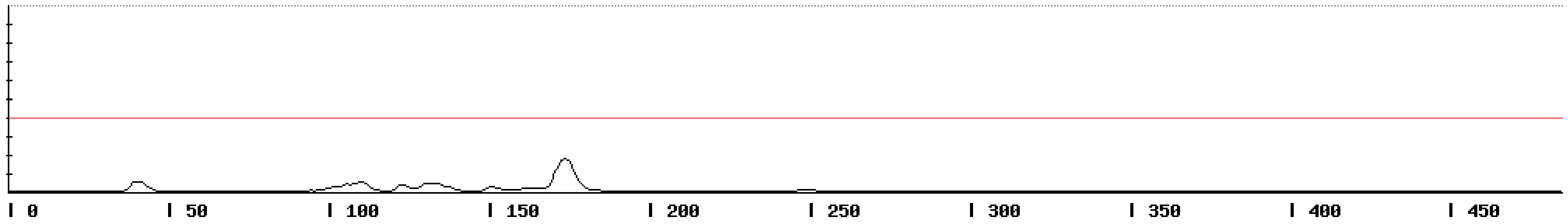

Predictions for Malusdomestica_MDP0000836293

Viterbi Path

Viterbi Path

Posterior @ 0.4

Posterior @ 0.4

Posterior Graphic. Threshold Marked @ 0.4

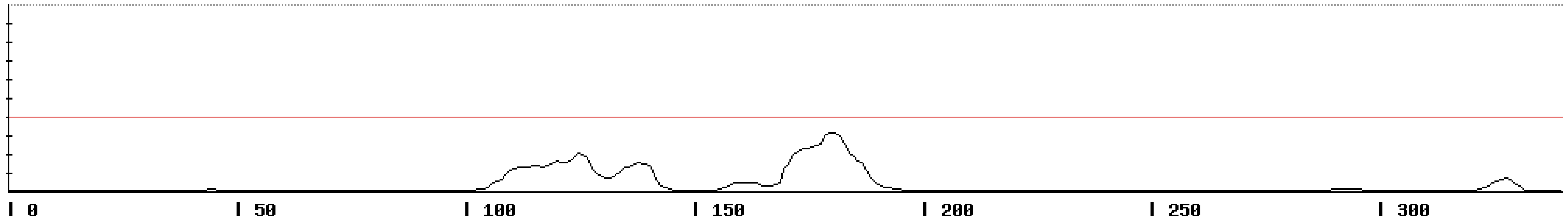

Predictions for Malusdomestica_MDP0000842702

Viterbi Path

Viterbi Path

Posterior @ 0.4	
Posterior @ 0.4	
Posterior Graphic. Threshold Marked @ 0.4	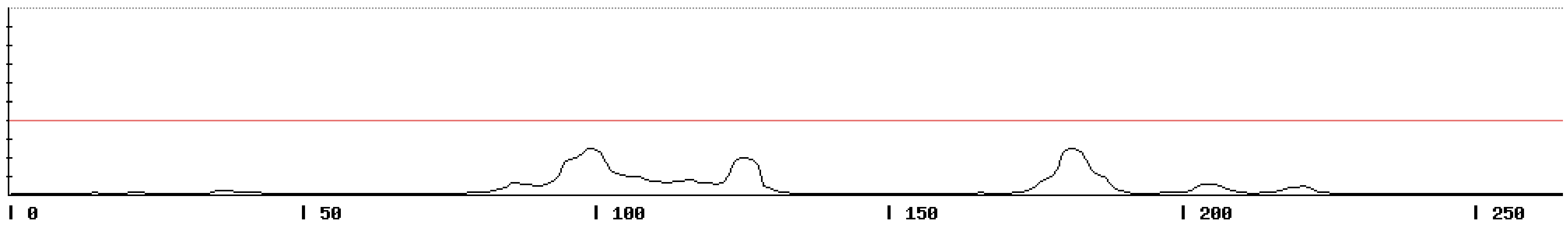

Predictions for Malusdomestica_MDP0000844682

Viterbi Path	
Viterbi Path	
Posterior @ 0.4	
Posterior @ 0.4	
Posterior Graphic. Threshold Marked @ 0.4	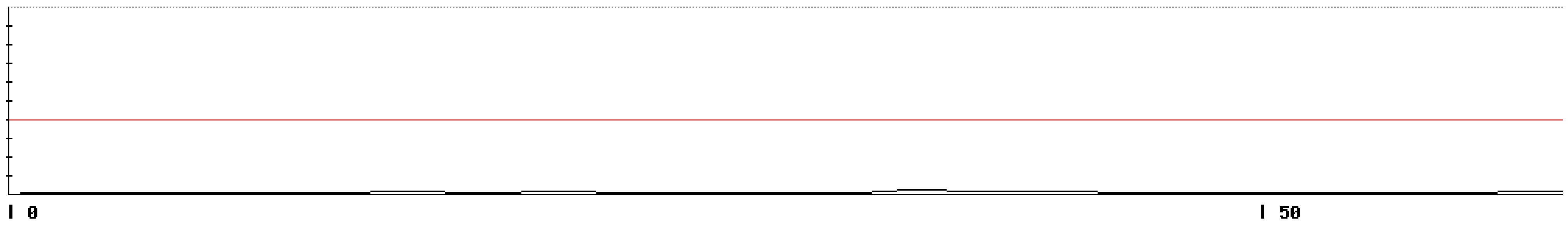

Predictions for Malusdomestica_MDP0000849944

Viterbi Path	
Viterbi Path	

Posterior @ 0.4

Posterior @ 0.4

Posterior Graphic. Threshold Marked @ 0.4

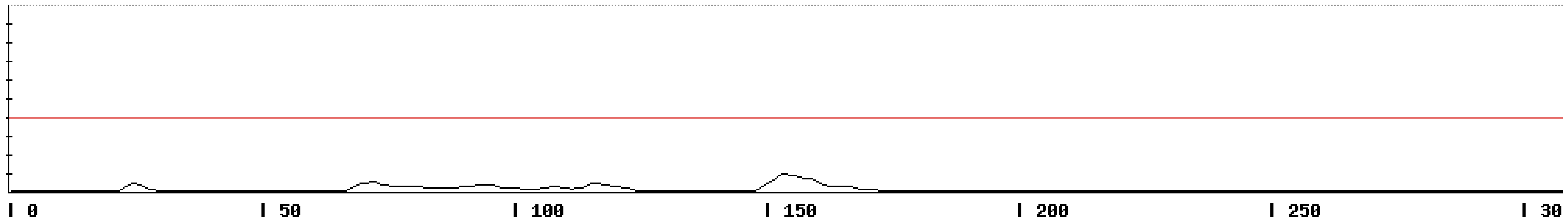

Predictions for Malusdomestica_MDP0000852271

Viterbi Path

Viterbi Path

Posterior @ 0.4

Posterior @ 0.4

Posterior Graphic. Threshold Marked @ 0.4

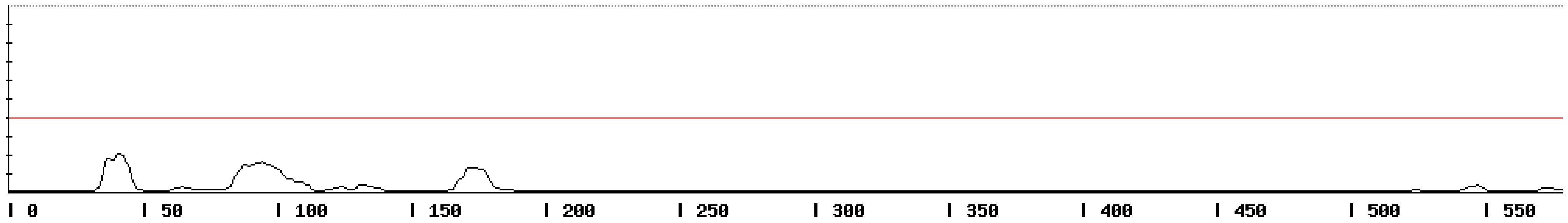

Predictions for Malusdomestica_MDP0000868419

Viterbi Path

Viterbi Path

Posterior @ 0.4

Posterior @ 0.4

Posterior Graphic. Threshold Marked @ 0.4

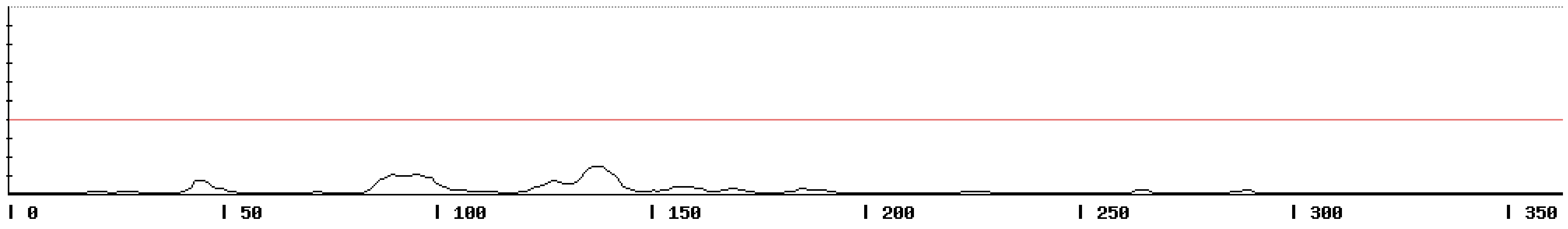

Predictions for Malusdomestica_MDP0000868556

Viterbi Path

Viterbi Path

Posterior @ 0.4

Posterior @ 0.4

Posterior Graphic. Threshold Marked @ 0.4

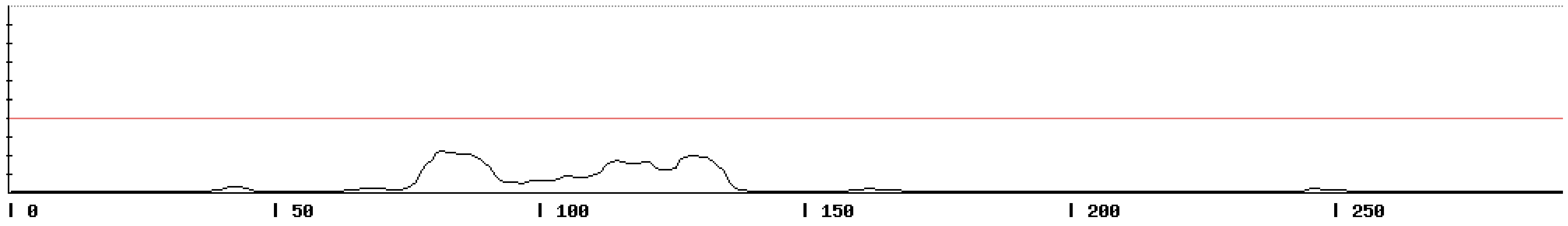

Predictions for Malusdomestica_MDP0000882033	
Viterbi Path	
Viterbi Path	
Posterior @ 0.4	
Posterior @ 0.4	
Posterior Graphic. Threshold Marked @ 0.4	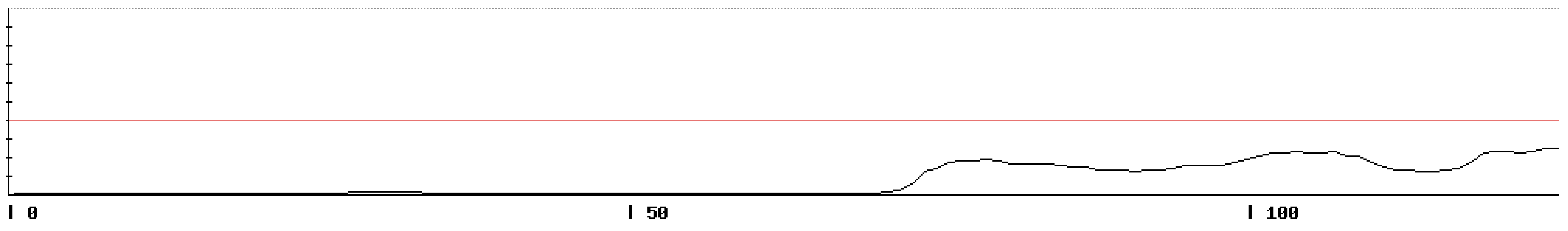

Predictions for Malusdomestica_MDP0000882983	
Viterbi Path	
Viterbi Path	
Posterior @ 0.4	
Posterior @ 0.4	
Posterior Graphic. Threshold Marked @ 0.4	

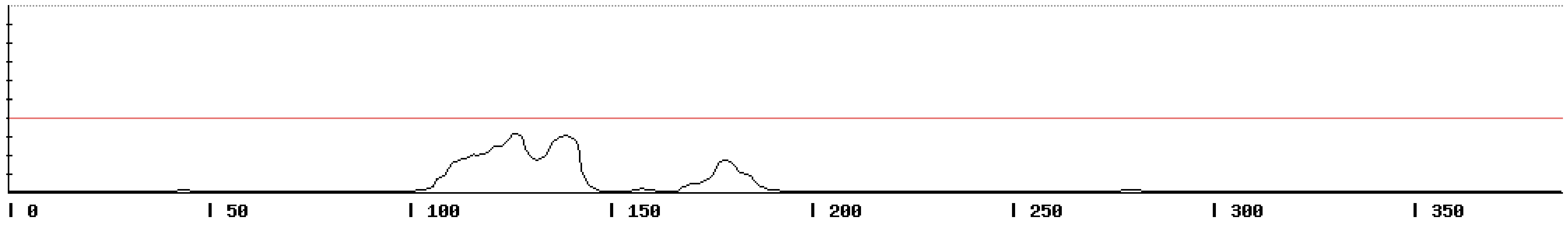

Predictions for Malusdomestica_MDP0000902822

Viterbi Path	
Viterbi Path	
Posterior @ 0.4	
Posterior @ 0.4	
Posterior Graphic. Threshold Marked @ 0.4	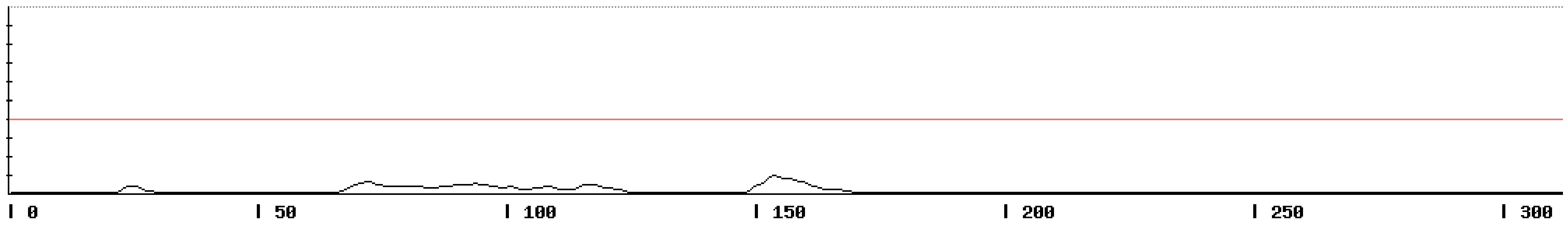

Predictions for Malusdomestica_MDP0000907178

Viterbi Path	
Viterbi Path	
Posterior @ 0.4	
Posterior @ 0.4	

Posterior Graphic. Threshold Marked @ 0.4

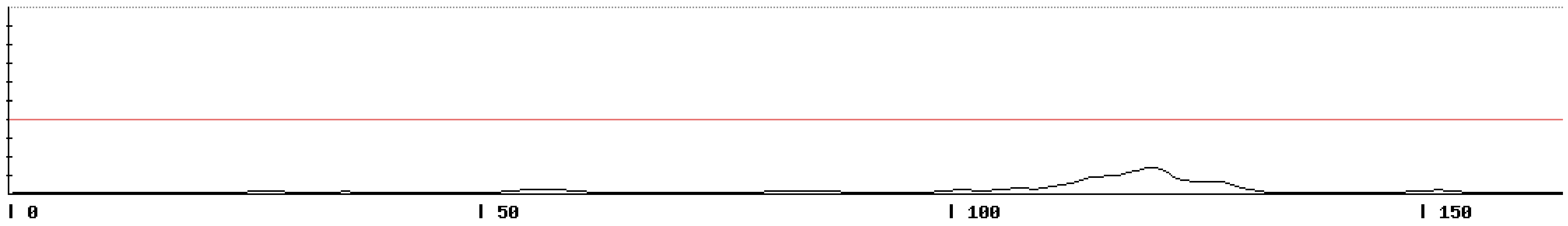

Predictions for Malusdomestica_MDP0000907489

Viterbi Path

Viterbi Path

Posterior @ 0.4

Posterior @ 0.4

Posterior Graphic. Threshold Marked @ 0.4

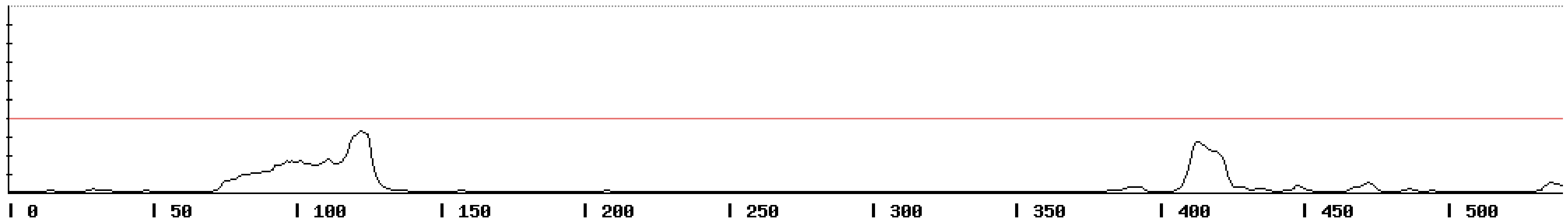

Predictions for Malusdomestica_MDP0000911336

Viterbi Path

Viterbi Path

Posterior @ 0.4

Posterior @ 0.4

Posterior Graphic. Threshold Marked @ 0.4	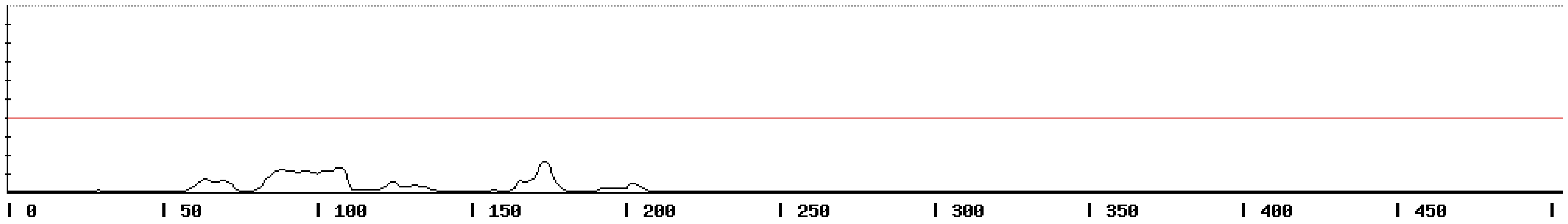

Predictions for Malusdomestica_MDP0000911724	
Viterbi Path	
Viterbi Path	
Posterior @ 0.4	
Posterior @ 0.4	
Posterior Graphic. Threshold Marked @ 0.4	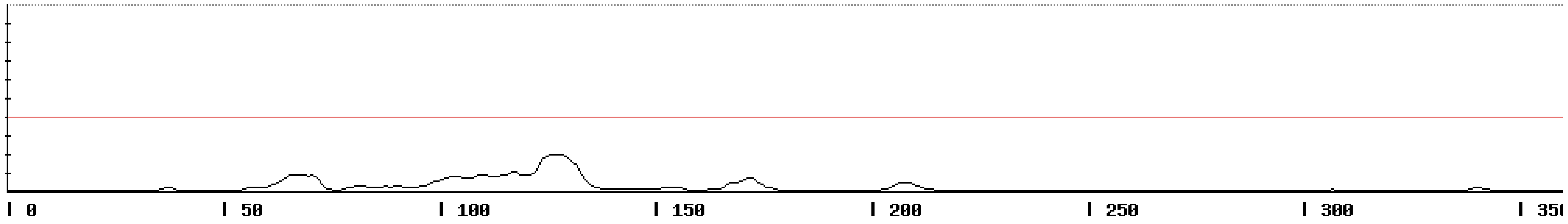

Predictions for Malusdomestica_MDP0000918566	
Viterbi Path	
Viterbi Path	

Posterior @ 0.4

Posterior @ 0.4

Posterior Graphic. Threshold Marked @ 0.4

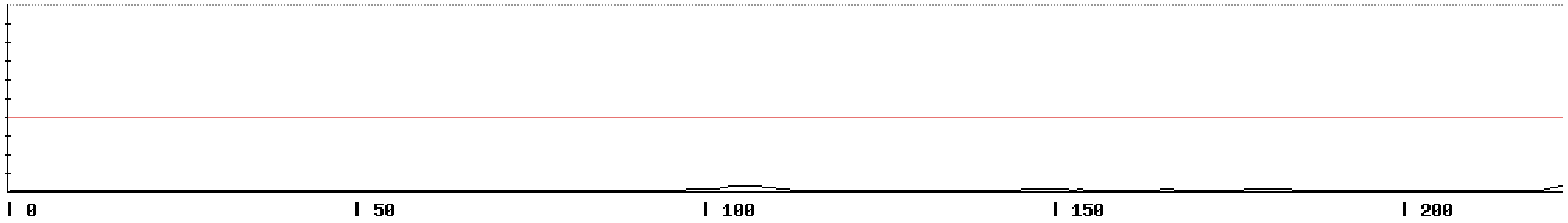

Predictions for Malusdomestica_MDP0000921016

Viterbi Path

Viterbi Path

Posterior @ 0.4 199 - K - 199

Posterior @ 0.4

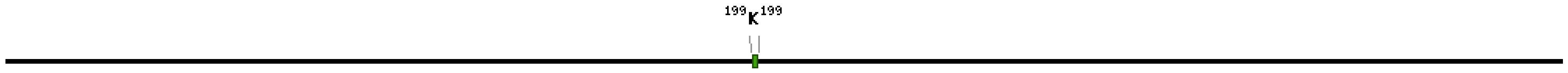

Posterior Graphic. Threshold Marked @ 0.4

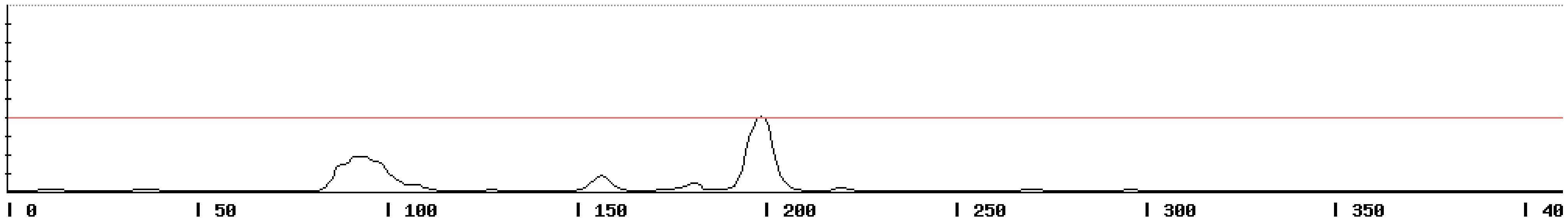

Predictions for Malusdomestica_MDP0000936269

Viterbi Path

Viterbi Path

Posterior @ 0.4

Posterior @ 0.4

Posterior Graphic. Threshold Marked @ 0.4

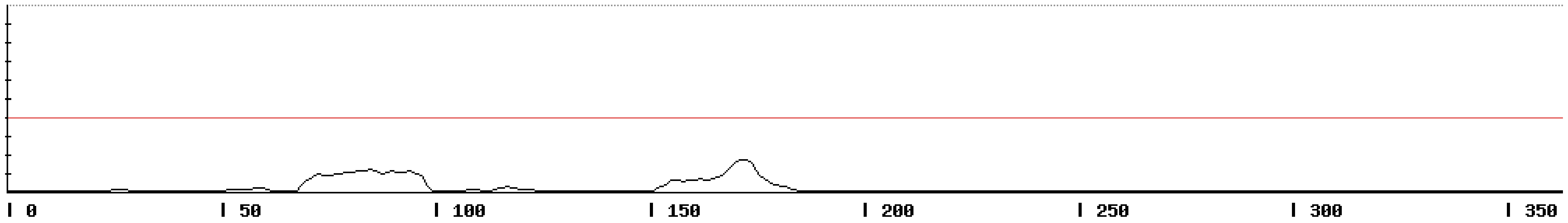

Predictions for Malusdomestica_MDP0000936441

Viterbi Path

Viterbi Path

Posterior @ 0.4 82 - RRKYPR - 87

Posterior @ 0.4

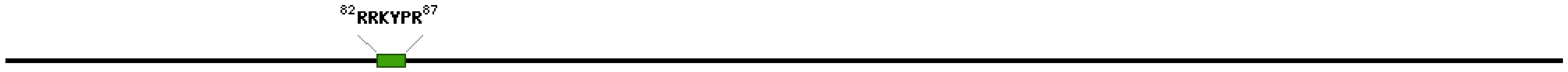

Revision r.9 - Last Updated on : February 24 2015 17:45:20. alex.nguyenba@utoronto.ca

Intended to be used with Firefox

Viterbi Path	
Posterior @ 0.4	598 - RPKKKE - 603
Posterior @ 0.4	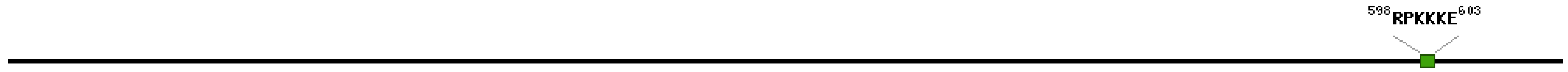
Posterior Graphic. Threshold Marked @ 0.4	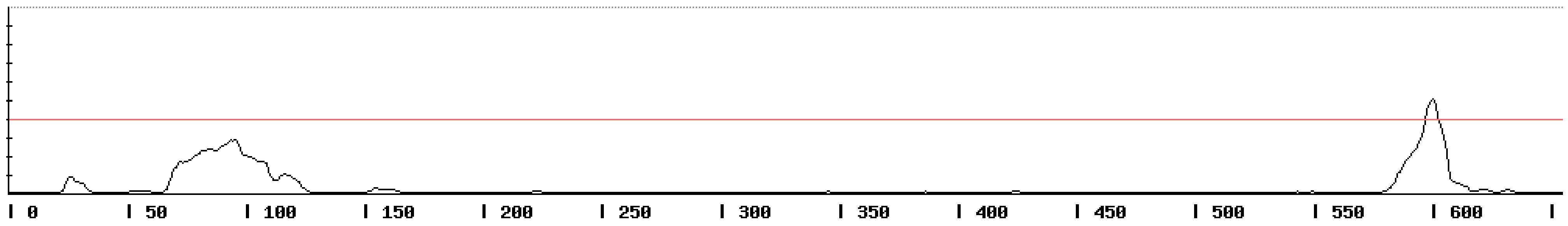

Predictions for Manihotesculenta_cassava4.1_004148m

Viterbi Path	
Viterbi Path	
Posterior @ 0.4	
Posterior @ 0.4	
Posterior Graphic. Threshold Marked @ 0.4	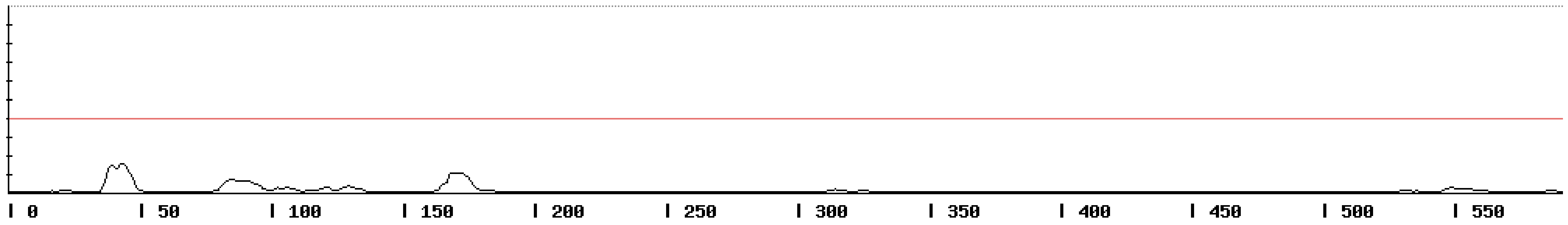

Predictions for Manihotesculenta_cassava4.1_004348m

Viterbi Path

Viterbi Path

Posterior @ 0.4

Posterior @ 0.4

Posterior Graphic. Threshold Marked @ 0.4

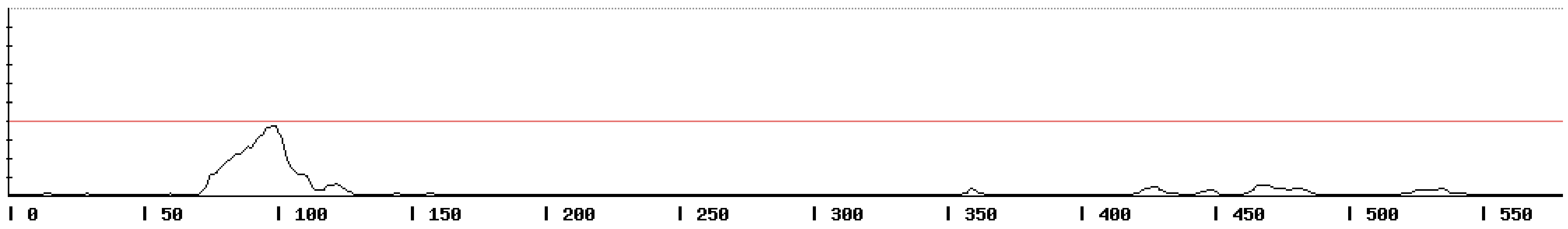

Predictions for Manihotesculenta_cassava4.1_004386m

Viterbi Path

Viterbi Path

Posterior @ 0.4

Posterior @ 0.4

Posterior Graphic. Threshold Marked @ 0.4

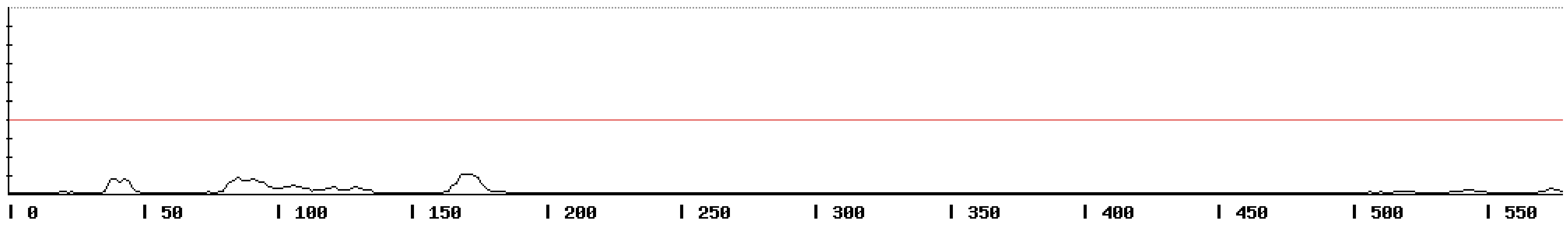

Predictions for Manihotesculenta_cassava4.1_004618m

Viterbi Path	
Viterbi Path	
Posterior @ 0.4	
Posterior @ 0.4	
Posterior Graphic. Threshold Marked @ 0.4	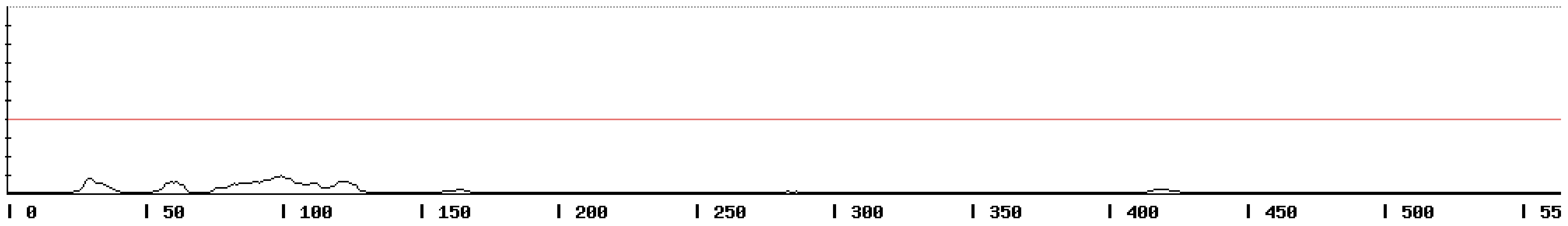

Predictions for Manihotesculenta_cassava4.1_005571m

Viterbi Path	154 - KKQTGNKKSTKIQPSSKNKKANQKLKDIKLGCKKGKPSKKAR - 195
Viterbi Path	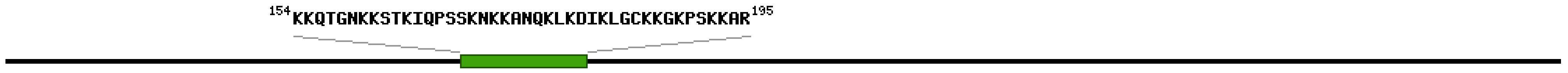
Posterior @ 0.4	79 - GRRKTKRKARTGFWK - 93
	154 - KKQTGNKKSTKIQPSSKNKKANQKLKDIKLGCKKGKPSKKAR - 195
Posterior @ 0.4	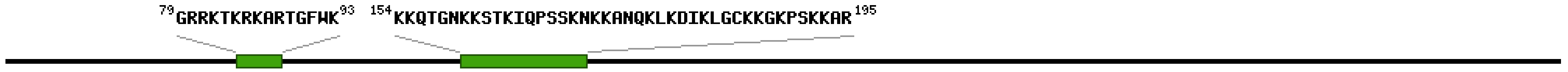
Posterior Graphic. Threshold Marked @ 0.4	

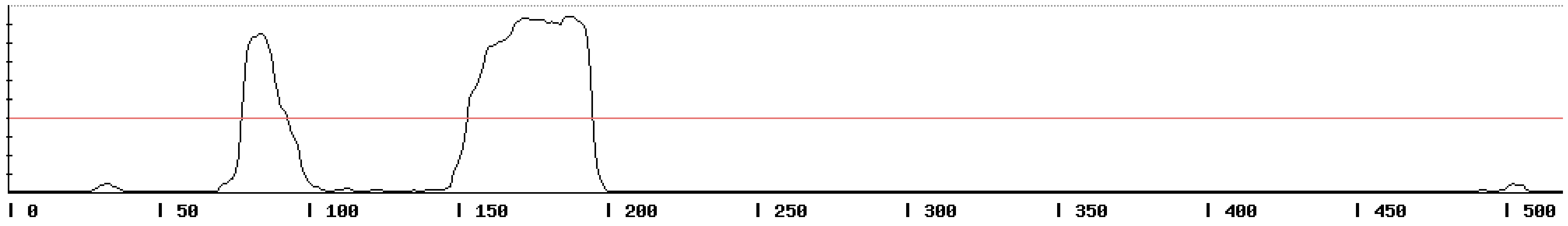

Predictions for Manihotesculenta_cassava4.1_007528m	
Viterbi Path	
Viterbi Path	
Posterior @ 0.4	
Posterior @ 0.4	
Posterior Graphic. Threshold Marked @ 0.4	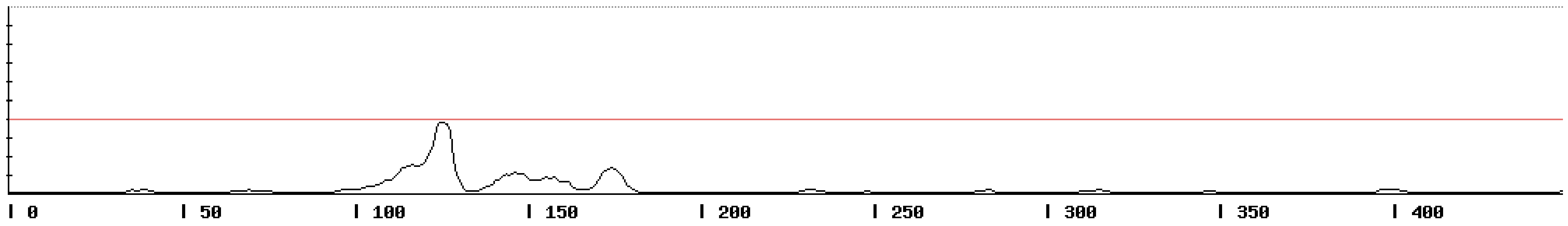

Predictions for Manihotesculenta_cassava4.1_008059m	
Viterbi Path	
Viterbi Path	
Posterior @ 0.4	134 - KRR - 136
Posterior @ 0.4	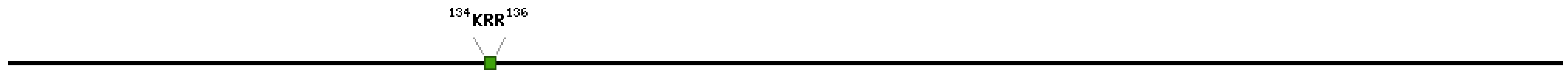

Posterior Graphic. Threshold Marked @ 0.4	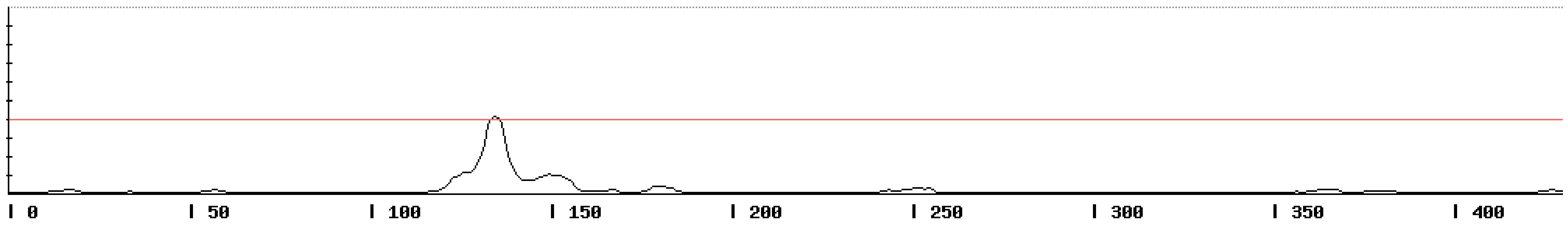

Predictions for Manihotesculenta_cassava4.1_008093m

Viterbi Path	
Viterbi Path	
Posterior @ 0.4	
Posterior @ 0.4	
Posterior Graphic. Threshold Marked @ 0.4	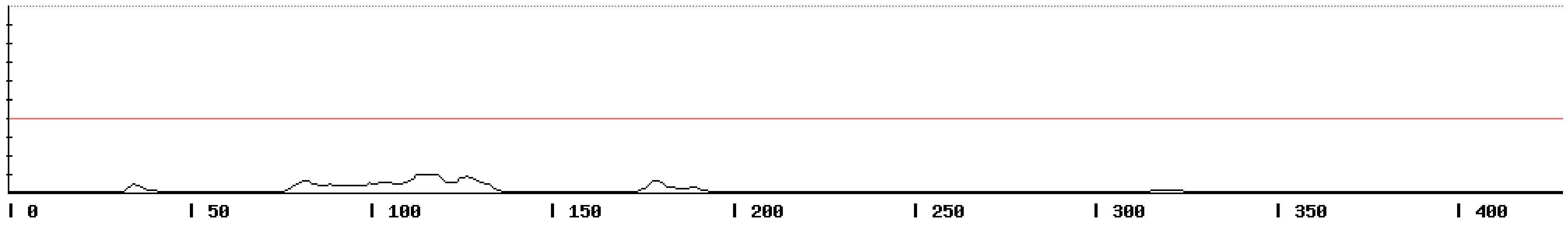

Predictions for Manihotesculenta_cassava4.1_008493m

Viterbi Path	
Viterbi Path	
Posterior @ 0.4	
Posterior @ 0.4	

Posterior Graphic. Threshold Marked @ 0.4	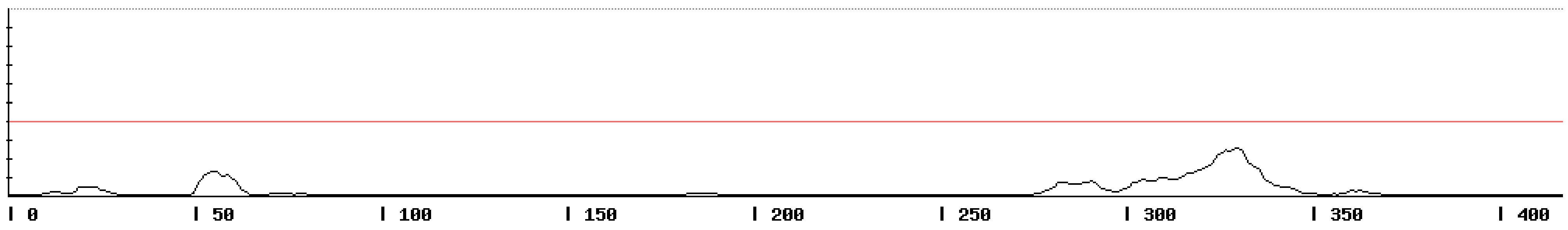

Predictions for Manihotesculenta_cassava4.1_009151m	
Viterbi Path	
Viterbi Path	
Posterior @ 0.4	
Posterior @ 0.4	
Posterior Graphic. Threshold Marked @ 0.4	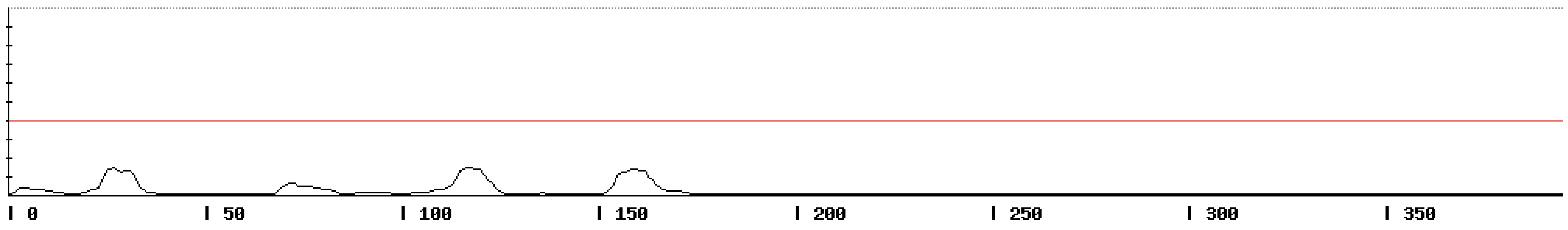

Predictions for Manihotesculenta_cassava4.1_009166m	
Viterbi Path	
Viterbi Path	

Posterior @ 0.4

Posterior @ 0.4

Posterior Graphic. Threshold Marked @ 0.4

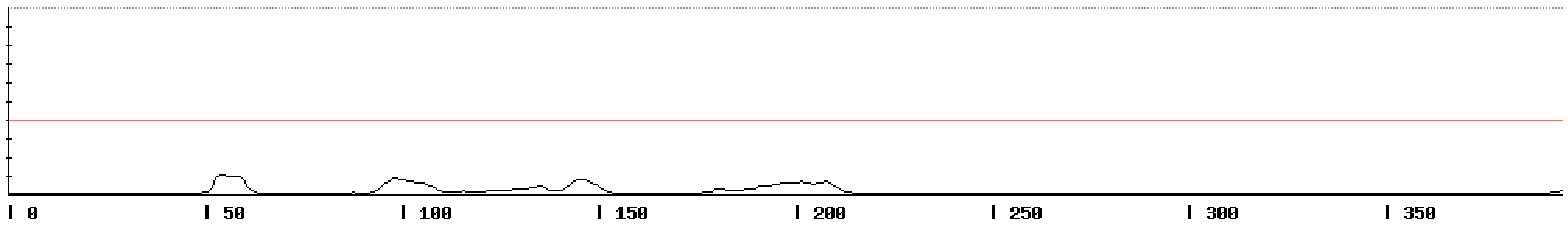

Predictions for Manihotesculenta_cassava4.1_009201m

Viterbi Path

Viterbi Path

Posterior @ 0.4

Posterior @ 0.4

Posterior Graphic. Threshold Marked @ 0.4

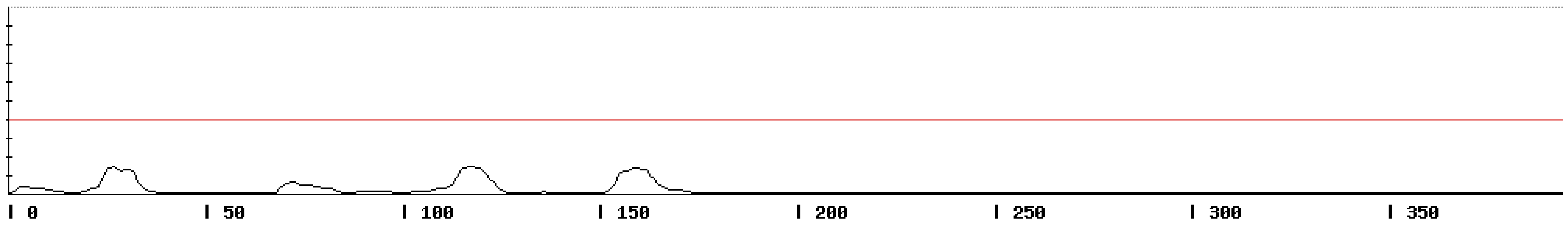

Predictions for Manihotesculenta_cassava4.1_009209m

Viterbi Path

Viterbi Path

Posterior @ 0.4	367 - RDGKRKVSKLRTKKHKK - 383
Posterior @ 0.4	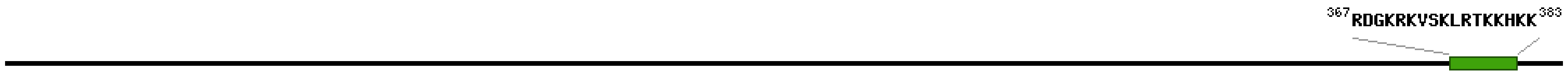
Posterior Graphic. Threshold Marked @ 0.4	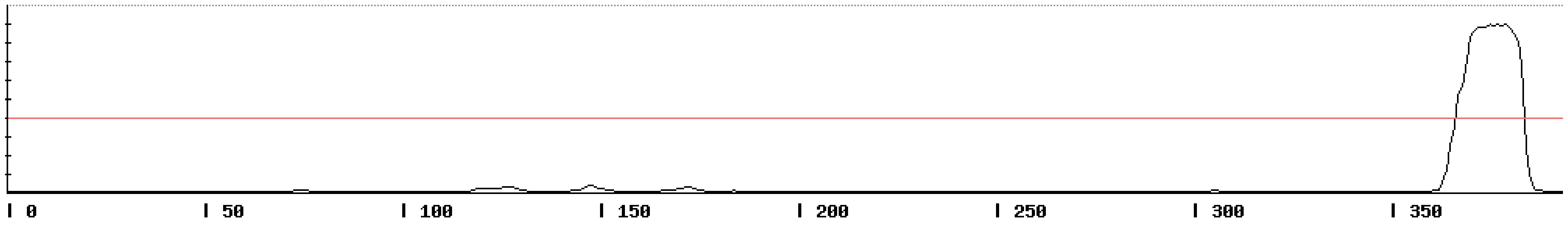

Predictions for Manihotesculenta_cassava4.1_009225m

Viterbi Path	
Viterbi Path	
Posterior @ 0.4	
Posterior @ 0.4	
Posterior Graphic. Threshold Marked @ 0.4	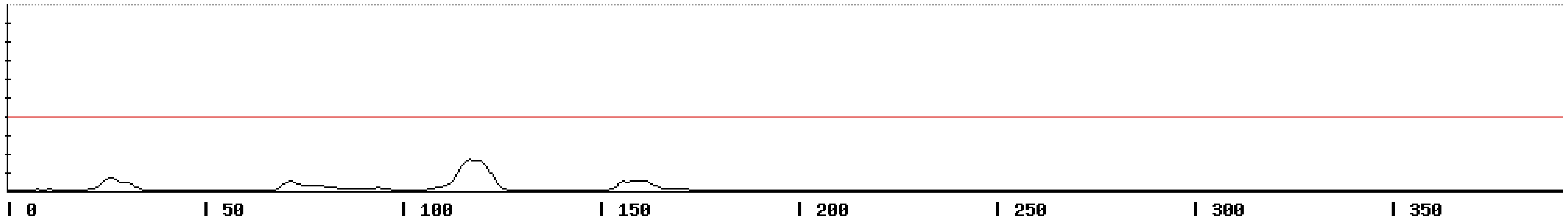

Predictions for Manihotesculenta_cassava4.1_009240m

Viterbi Path

Viterbi Path

Posterior @ 0.4

Posterior @ 0.4

Posterior Graphic. Threshold Marked @ 0.4

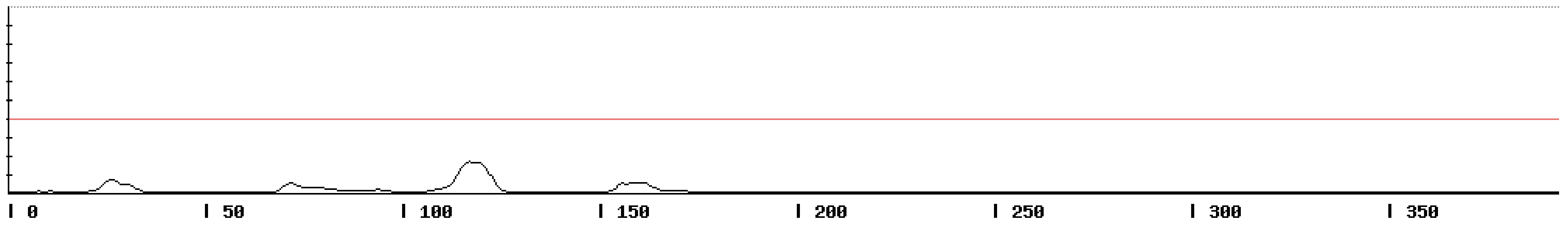

Predictions for Manihotesculenta_cassava4.1_009914m

Viterbi Path

Viterbi Path

Posterior @ 0.4 — 94 - WKPTGKPRKVKGIGTKKPIGTKRTLVFYKKEHPKPVR - 130

Posterior @ 0.4

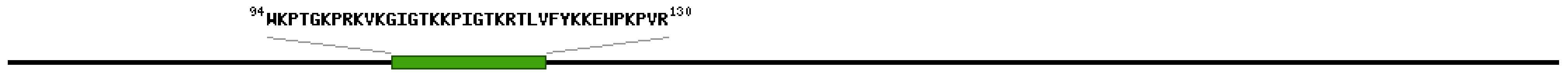

Posterior Graphic. Threshold Marked @ 0.4

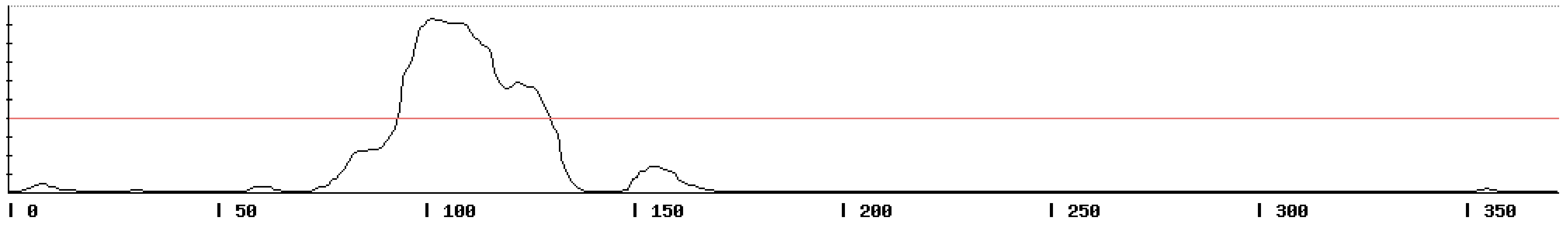

Predictions for Manihotesculenta_cassava4.1_010010m

Viterbi Path	
Viterbi Path	
Posterior @ 0.4	
Posterior @ 0.4	
Posterior Graphic. Threshold Marked @ 0.4	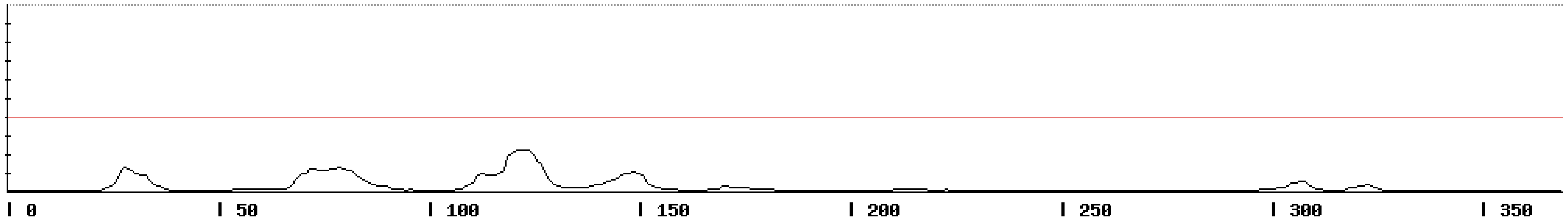

Predictions for Manihotesculenta_cassava4.1_010179m

Viterbi Path	
Viterbi Path	
Posterior @ 0.4	
Posterior @ 0.4	
Posterior Graphic. Threshold Marked @ 0.4	

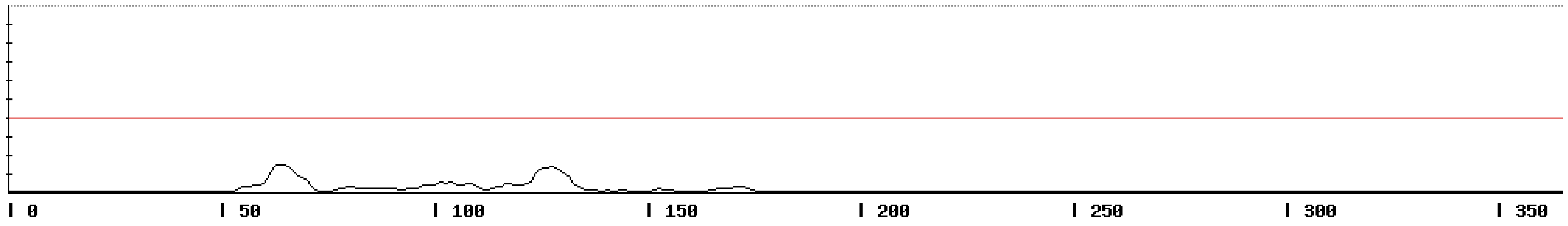

Predictions for Manihotesculenta_cassava4.1_010191m

Viterbi Path

Viterbi Path

Posterior @ 0.4

Posterior @ 0.4

Posterior Graphic. Threshold Marked @ 0.4

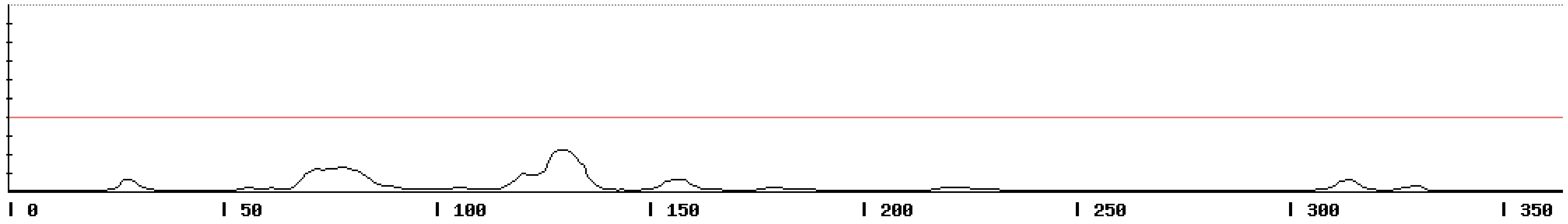

Predictions for Manihotesculenta_cassava4.1_010323m

Viterbi Path

Viterbi Path

Posterior @ 0.4

Posterior @ 0.4

Posterior Graphic. Threshold Marked @ 0.4

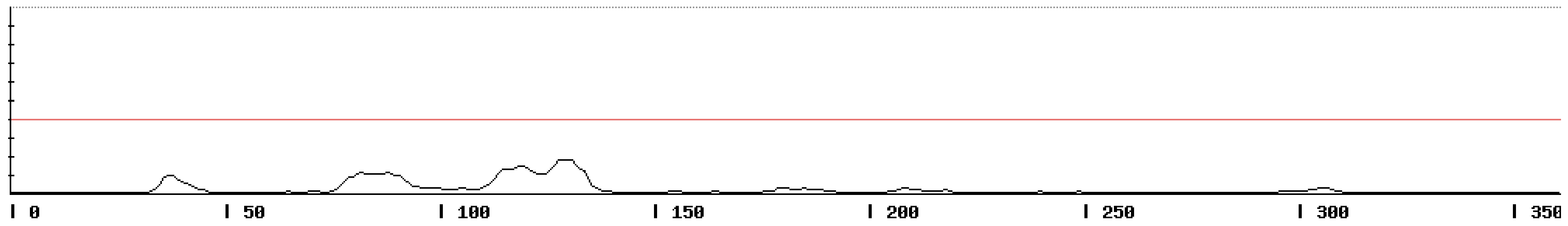

Predictions for Manihotesculenta_cassava4.1_010528m

Viterbi Path

Viterbi Path

Posterior @ 0.4

Posterior @ 0.4

Posterior Graphic. Threshold Marked @ 0.4

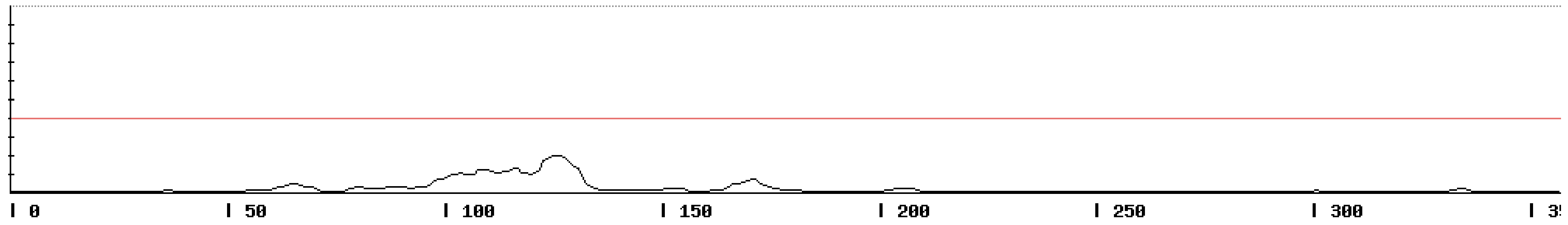

Predictions for Manihotesculenta_cassava4.1_010869m

Viterbi Path

Viterbi Path

Posterior @ 0.4

Posterior @ 0.4

Posterior Graphic. Threshold Marked @ 0.4

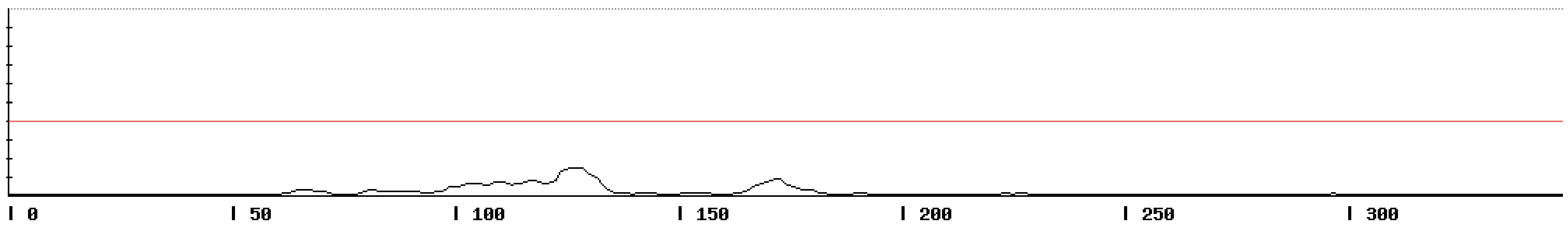

Predictions for Manihotesculenta_cassava4.1_010885m

Viterbi Path

Viterbi Path

Posterior @ 0.4

Posterior @ 0.4

Posterior Graphic. Threshold Marked @ 0.4

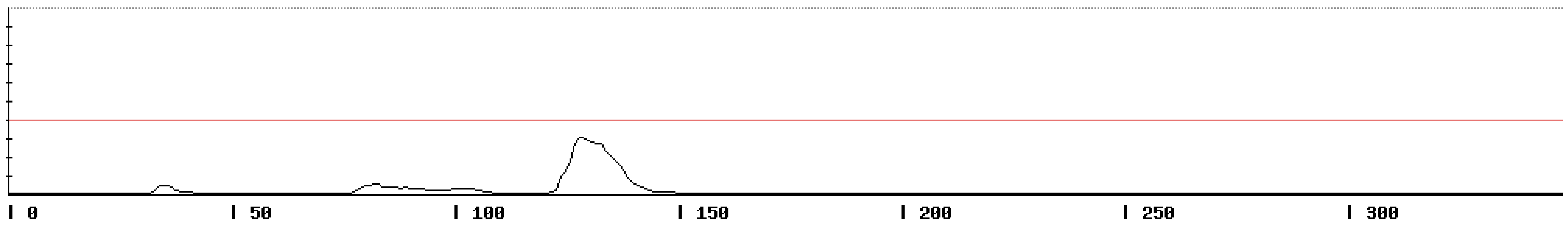

Predictions for Manihotesculenta_cassava4.1_010958m

Viterbi Path

Viterbi Path

Posterior @ 0.4

Posterior @ 0.4

Posterior Graphic. Threshold Marked @ 0.4

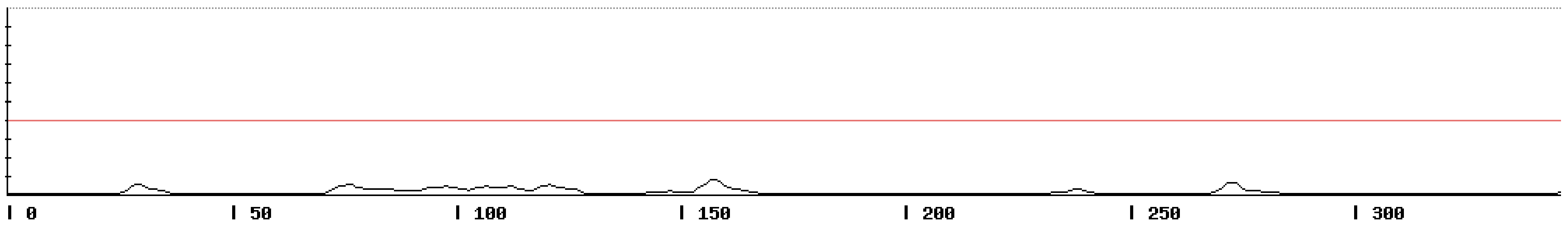

Predictions for Manihotesculenta_cassava4.1_010968m

Viterbi Path

Viterbi Path

Posterior @ 0.4

Posterior @ 0.4

Posterior Graphic. Threshold Marked @ 0.4

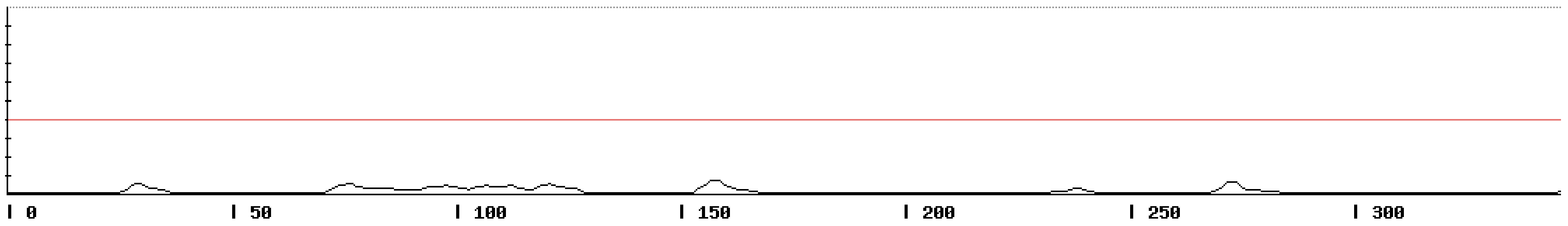

Predictions for Manihotesculenta_cassava4.1_010976m

Viterbi Path

Viterbi Path

Posterior @ 0.4

Posterior @ 0.4

Posterior Graphic. Threshold Marked @ 0.4

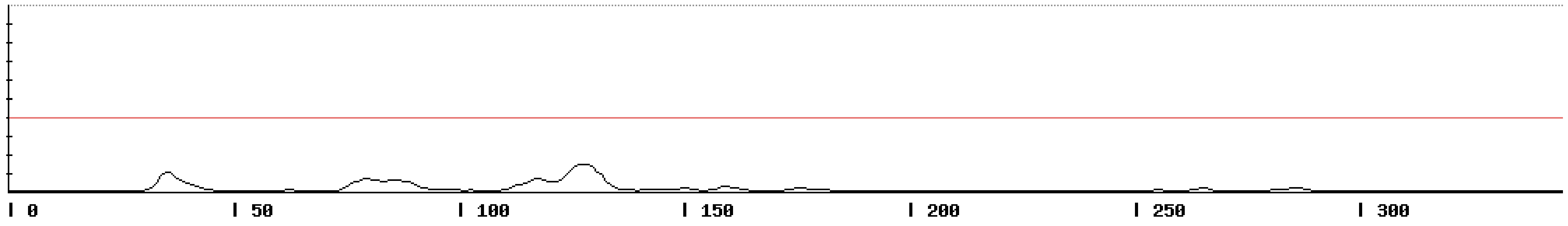

Predictions for Manihotesculenta_cassava4.1_010991m

Viterbi Path

Viterbi Path

Posterior @ 0.4

Posterior @ 0.4

Posterior Graphic. Threshold Marked @ 0.4

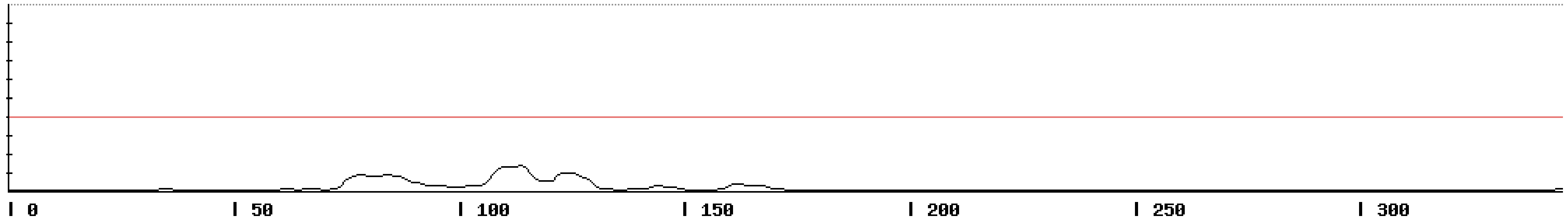

Predictions for Manihotesculenta_cassava4.1_010999m

Viterbi Path

Viterbi Path

Posterior @ 0.4

Posterior @ 0.4

Posterior Graphic. Threshold Marked @ 0.4

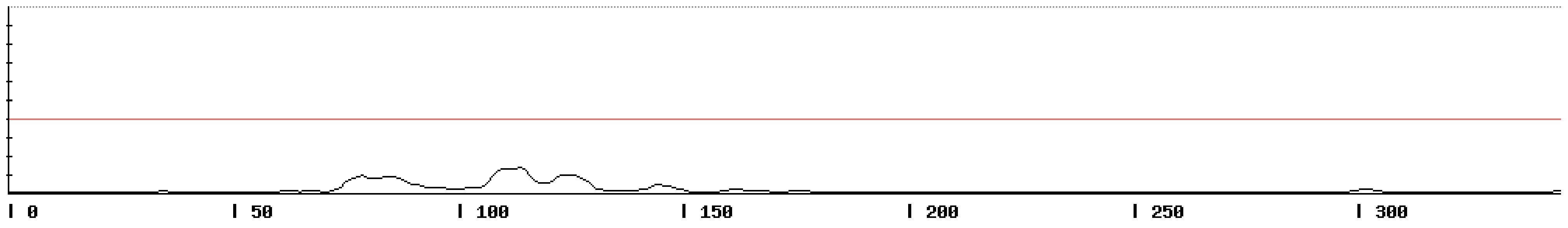

Predictions for Manihotesculenta_cassava4.1_011029m

Viterbi Path

Viterbi Path

Posterior @ 0.4

Posterior @ 0.4

Posterior Graphic. Threshold Marked @ 0.4

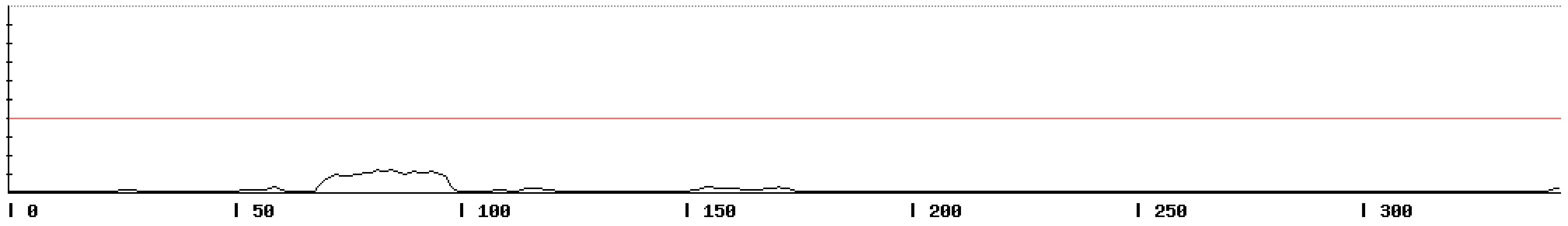

Predictions for Manihotesculenta_cassava4.1_011070m

Viterbi Path	
Viterbi Path	
Posterior @ 0.4	94 - WKPTGKPRKVKGIGTKKPIGTKRTLVFYKKEHPKPVR - 130
Posterior @ 0.4	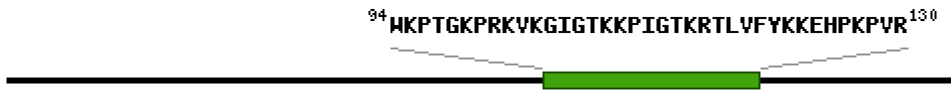
Posterior Graphic. Threshold Marked @ 0.4	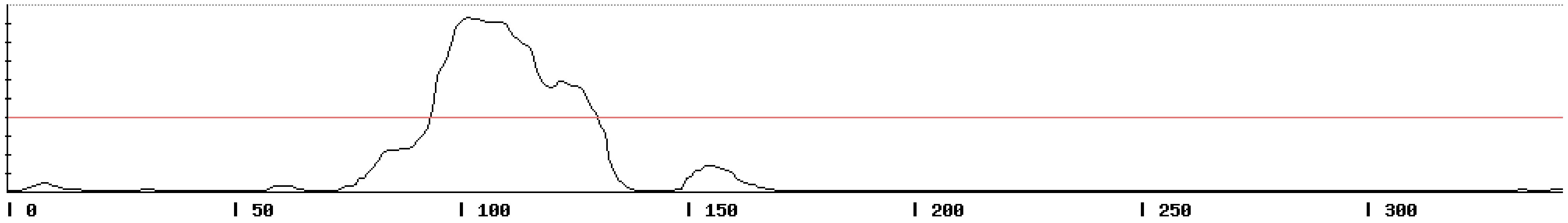

Predictions for Manihotesculenta_cassava4.1_011262m

Viterbi Path	
Viterbi Path	
Posterior @ 0.4	
Posterior @ 0.4	
Posterior Graphic. Threshold Marked @ 0.4	

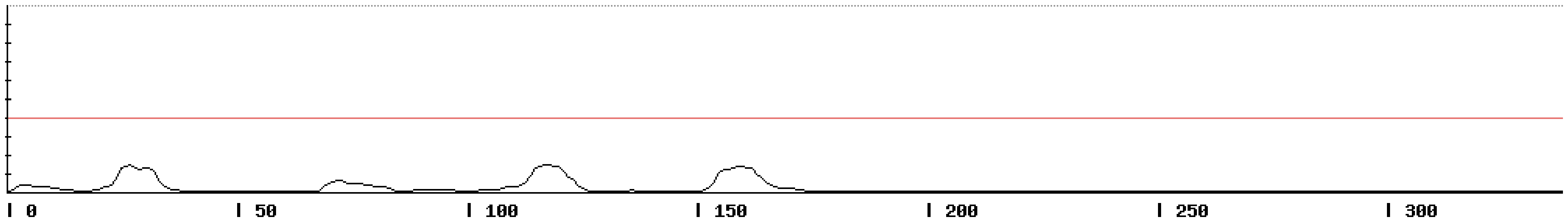

Predictions for Manihotesculenta_cassava4.1_011442m

Viterbi Path	
Viterbi Path	
Posterior @ 0.4	
Posterior @ 0.4	
Posterior Graphic. Threshold Marked @ 0.4	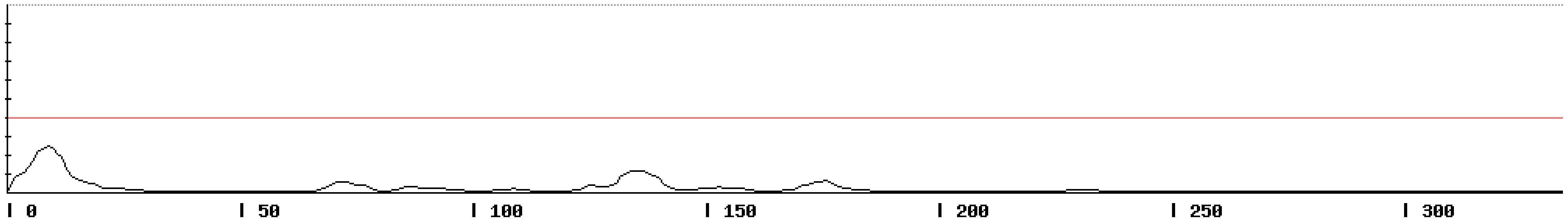

Predictions for Manihotesculenta_cassava4.1_011537m

Viterbi Path	55 - KPTGKPRKVKDKRSKEEIGTKRSLVFHVKDHPKPKRTK - 92
Viterbi Path	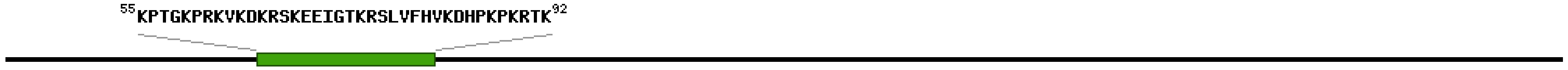
Posterior @ 0.4	55 - KPTGKPRKVKDKRSKEEIGTKRSLVFHVKDHPKPKRTK - 92
Posterior @ 0.4	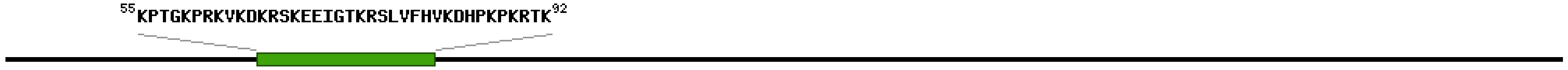

Posterior Graphic. Threshold Marked @ 0.4

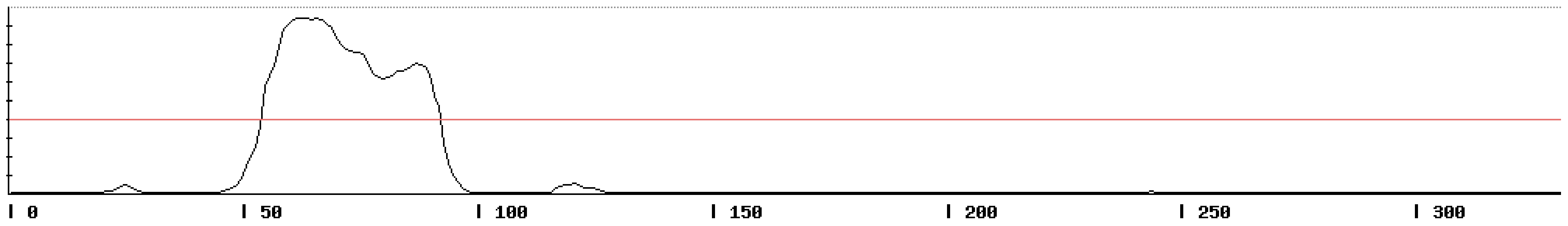

Predictions for Manihotesculenta_cassava4.1_012314m

Viterbi Path

Viterbi Path

Posterior @ 0.4

142 - KRR - 144

Posterior @ 0.4

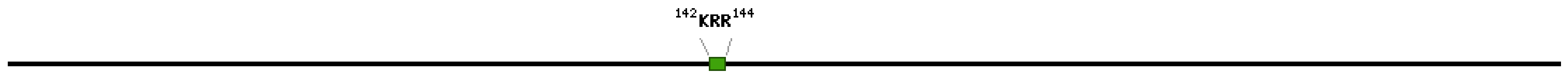

Posterior Graphic. Threshold Marked @ 0.4

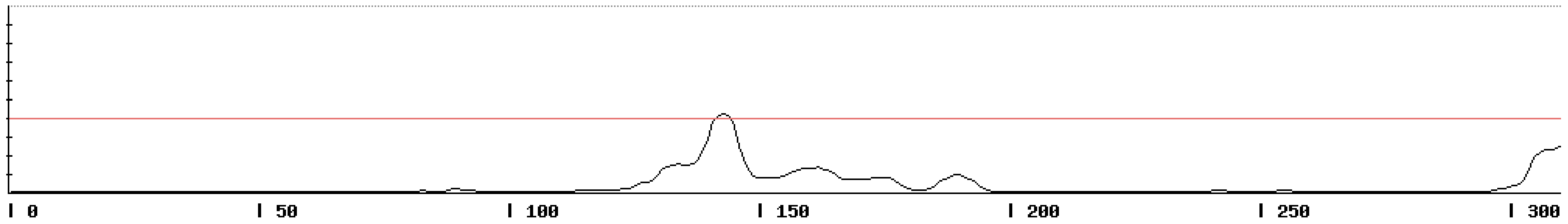

Predictions for Manihotesculenta_cassava4.1_012939m

Viterbi Path

Viterbi Path

Posterior @ 0.4

Posterior @ 0.4

Posterior Graphic. Threshold Marked @ 0.4

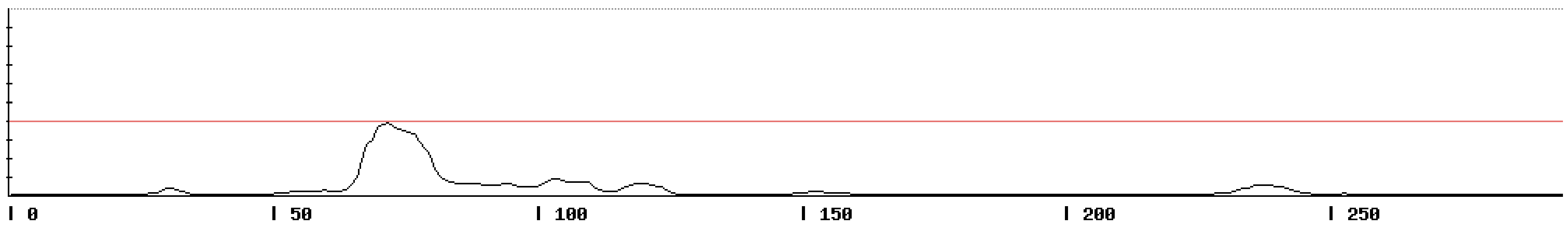

Predictions for Manihotesculenta_cassava4.1_012943m

Viterbi Path

Viterbi Path

Posterior @ 0.4

Posterior @ 0.4

Posterior Graphic. Threshold Marked @ 0.4

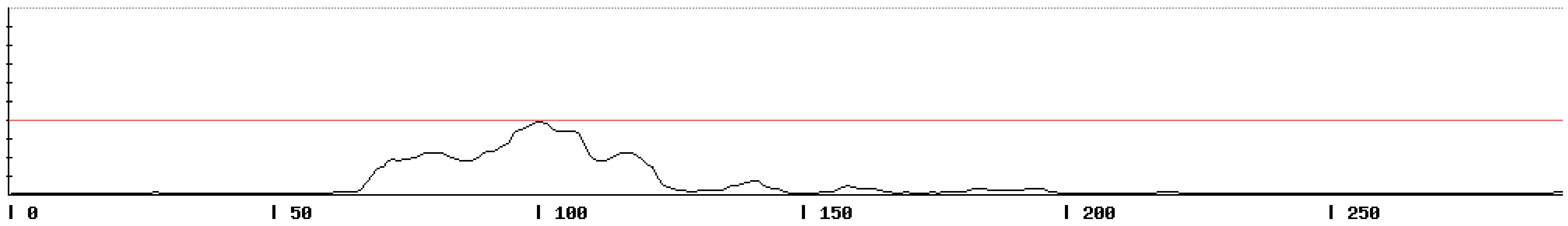

Predictions for Manihotesculenta_cassava4.1_013132m

Viterbi Path

Viterbi Path

Posterior @ 0.4

Posterior @ 0.4

Posterior Graphic. Threshold Marked @ 0.4

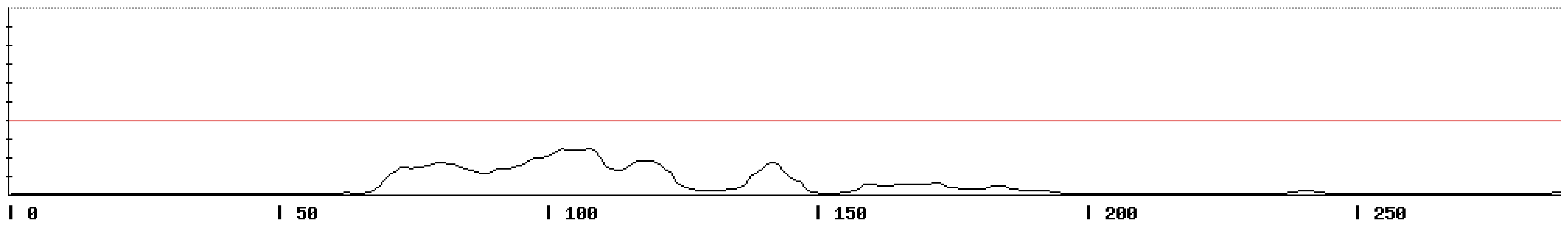

Predictions for Manihotesculenta_cassava4.1_013334m

Viterbi Path

Viterbi Path

Posterior @ 0.4

Posterior @ 0.4

Posterior Graphic. Threshold Marked @ 0.4

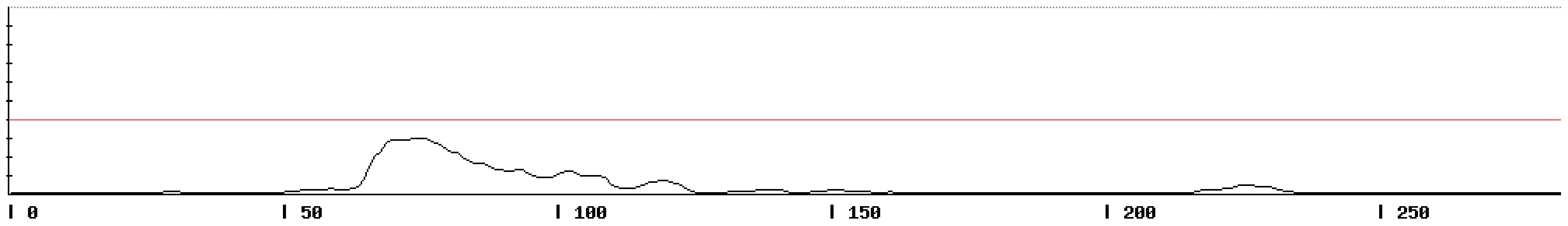

Predictions for Manihotesculenta_cassava4.1_013467m

Viterbi Path

Viterbi Path

Posterior @ 0.4

Posterior @ 0.4

Posterior Graphic. Threshold Marked @ 0.4

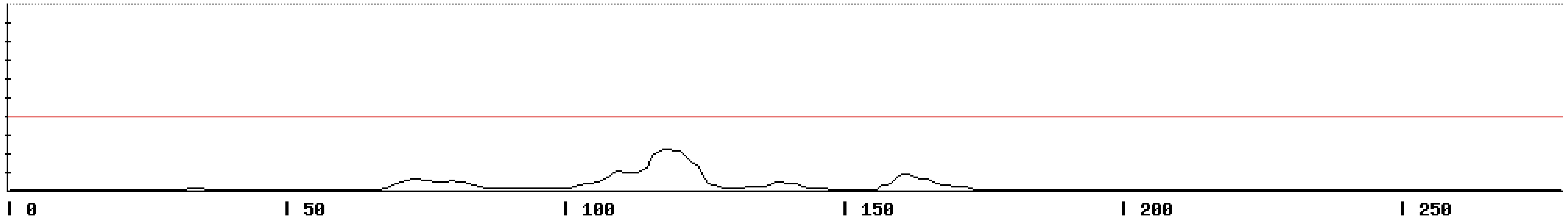

Predictions for Manihotesculenta_cassava4.1_013664m

Viterbi Path

Viterbi Path

Posterior @ 0.4

Posterior @ 0.4

Posterior Graphic. Threshold Marked @ 0.4

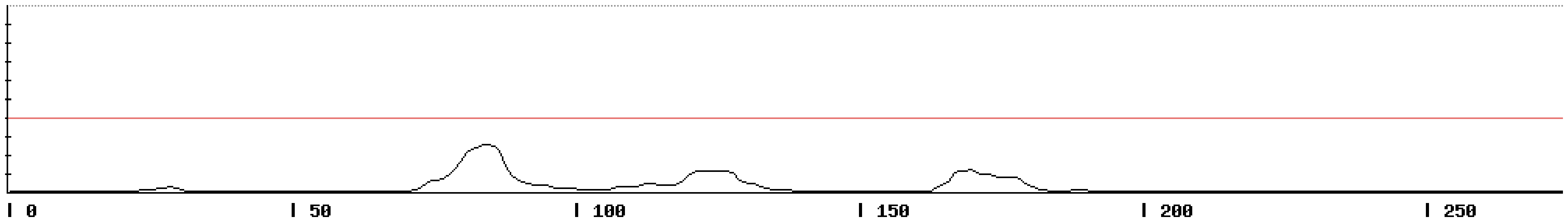

Predictions for Manihotesculenta_cassava4.1_014491m

Viterbi Path

Viterbi Path

Posterior @ 0.4

Posterior @ 0.4

Posterior Graphic. Threshold Marked @ 0.4

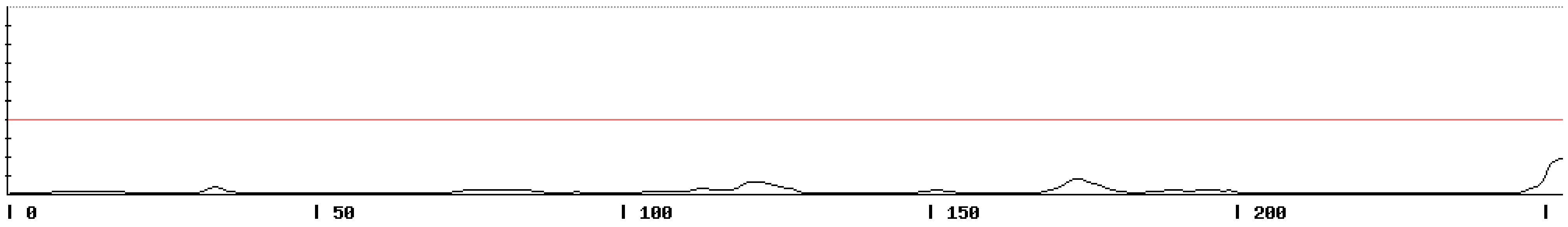

Predictions for Manihotesculenta_cassava4.1_014492m

Viterbi Path

Viterbi Path

Posterior @ 0.4

Posterior @ 0.4

Posterior Graphic. Threshold Marked @ 0.4

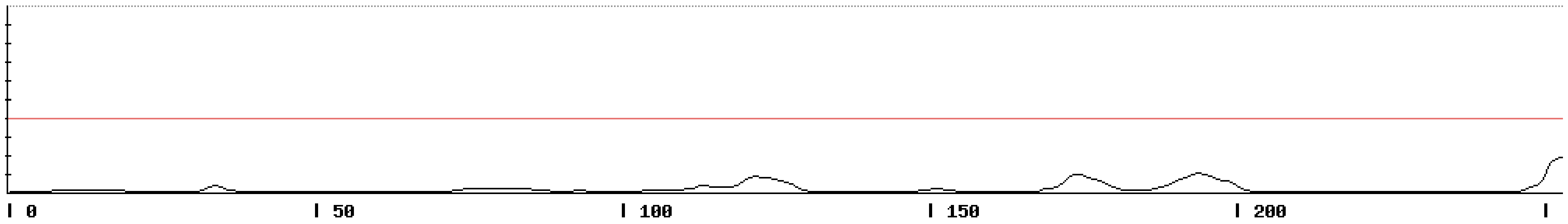

Predictions for Manihotesculenta_cassava4.1_014914m	
Viterbi Path	
Viterbi Path	
Posterior @ 0.4	119 - KAPAGRKTK - 127
Posterior @ 0.4	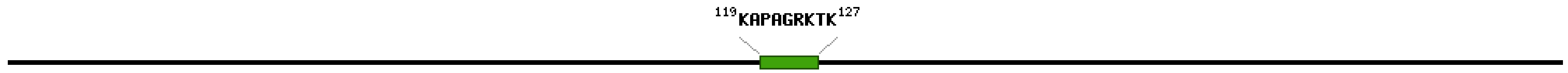
Posterior Graphic. Threshold Marked @ 0.4	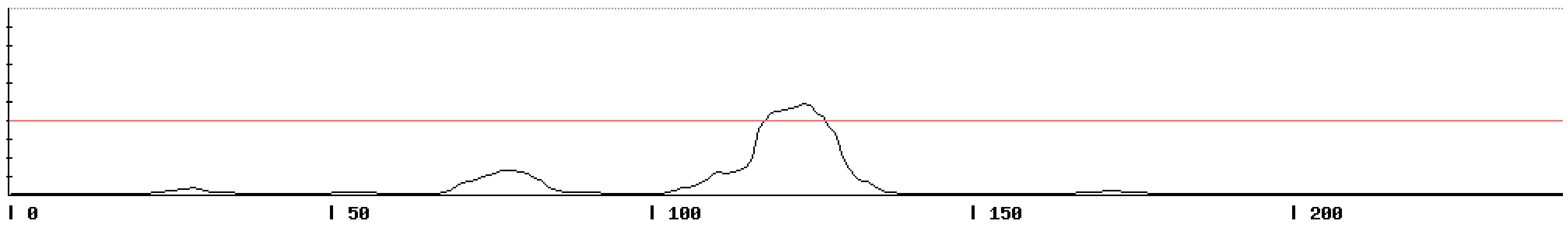

Predictions for Manihotesculenta_cassava4.1_015927m	
Viterbi Path	
Viterbi Path	
Posterior @ 0.4	
Posterior @ 0.4	
Posterior Graphic. Threshold Marked @ 0.4	

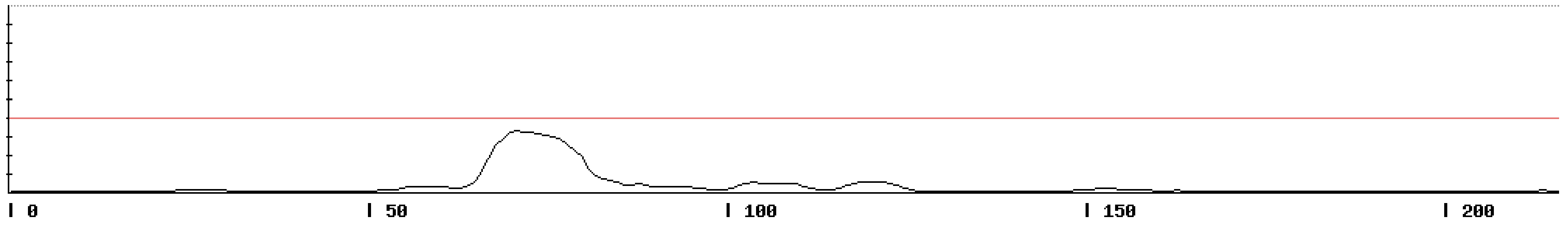

Predictions for Manihotesculenta_cassava4.1_015961m	
Viterbi Path	
Viterbi Path	
Posterior @ 0.4	
Posterior @ 0.4	
Posterior Graphic. Threshold Marked @ 0.4	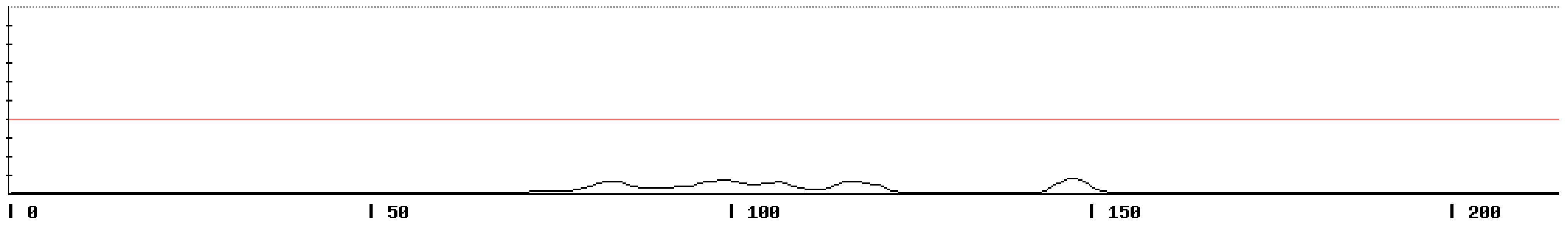

Predictions for Manihotesculenta_cassava4.1_016624m	
Viterbi Path	
Viterbi Path	
Posterior @ 0.4	
Posterior @ 0.4	

Posterior Graphic. Threshold Marked @ 0.4

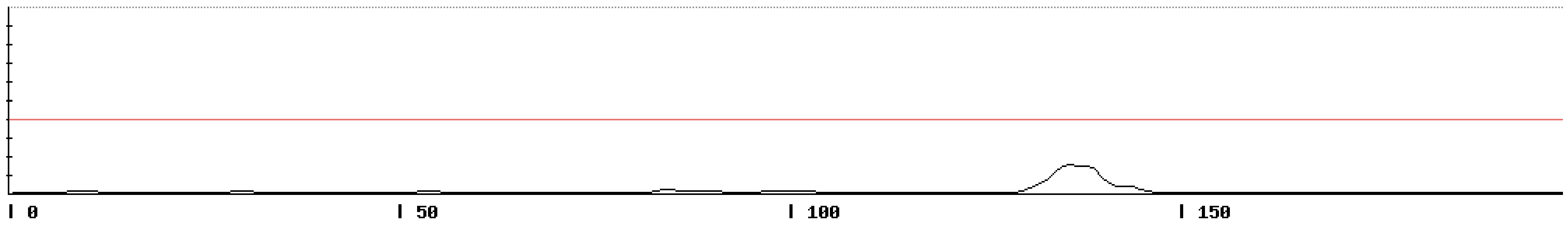

Predictions for Manihotesculenta_cassava4.1_019103m

Viterbi Path

Viterbi Path

Posterior @ 0.4

Posterior @ 0.4

Posterior Graphic. Threshold Marked @ 0.4

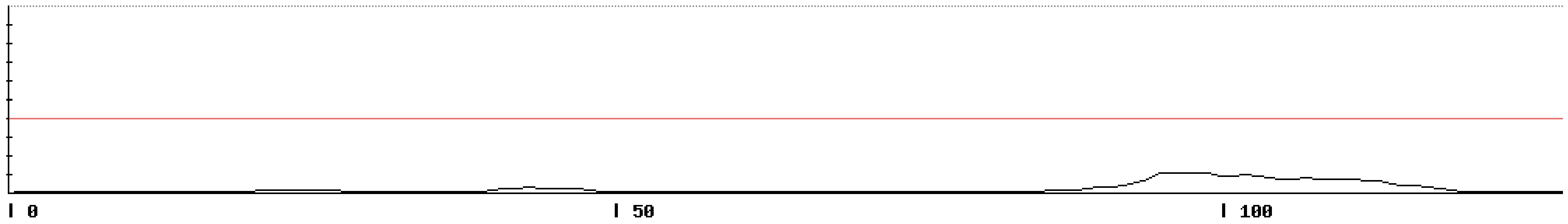

Predictions for Manihotesculenta_cassava4.1_021102m

Viterbi Path

Viterbi Path

Posterior @ 0.4

Posterior @ 0.4

Posterior Graphic. Threshold Marked @ 0.4

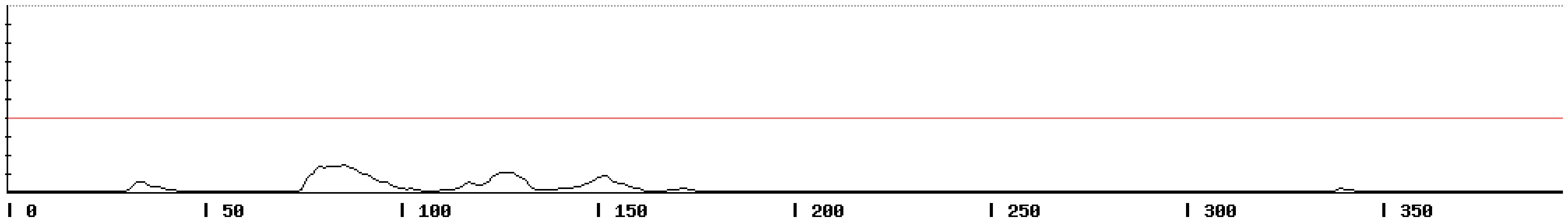

Predictions for Manihotesculenta_cassava4.1_021360m

Viterbi Path

Viterbi Path

Posterior @ 0.4

Posterior @ 0.4

Posterior Graphic. Threshold Marked @ 0.4

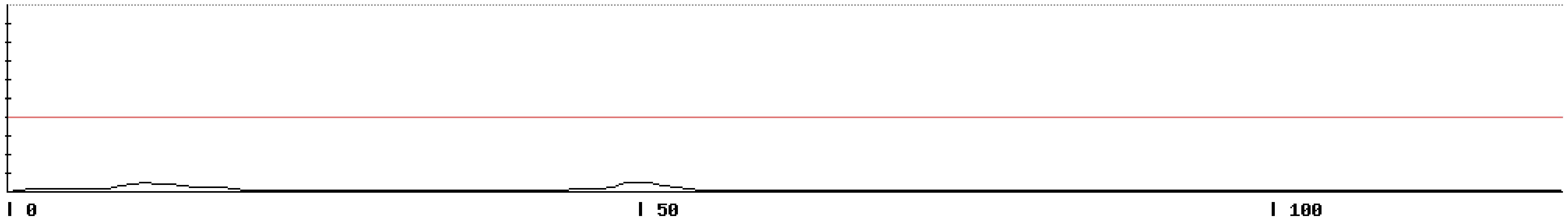

Predictions for Manihotesculenta_cassava4.1_021507m

Viterbi Path

Viterbi Path

Posterior @ 0.4

Posterior @ 0.4

Posterior Graphic. Threshold Marked @ 0.4

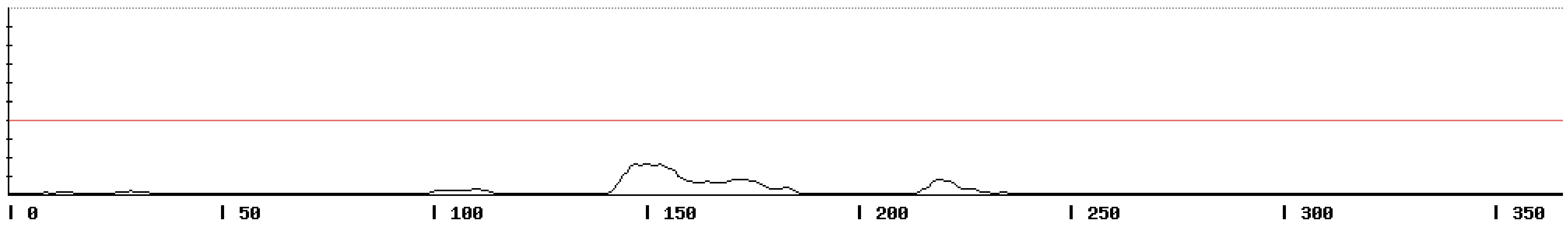

Predictions for Manihotesculenta_cassava4.1_021626m

Viterbi Path

Viterbi Path

Posterior @ 0.4

Posterior @ 0.4

Posterior Graphic. Threshold Marked @ 0.4

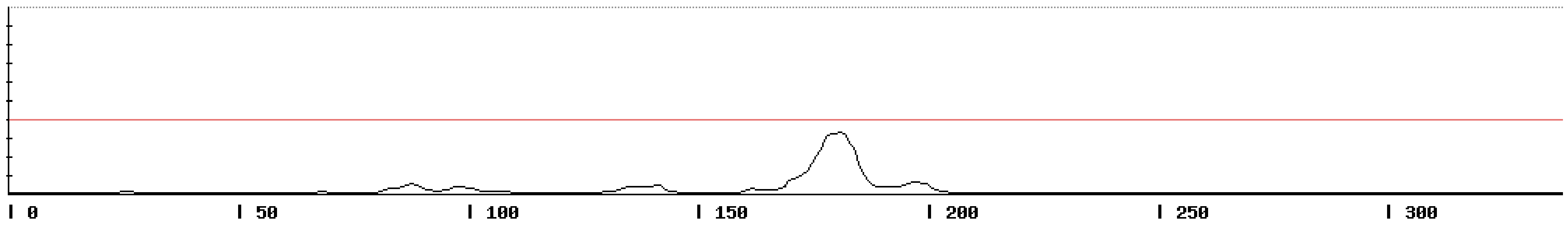

Predictions for Manihotesculenta_cassava4.1_022025m

Viterbi Path

Viterbi Path

Posterior @ 0.4	133 - K - 133
Posterior @ 0.4	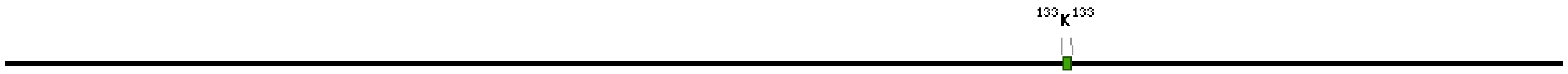
Posterior Graphic. Threshold Marked @ 0.4	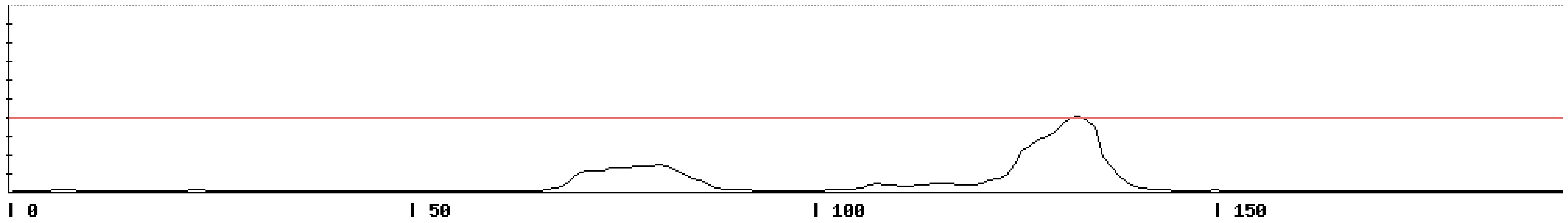

Predictions for Manihotesculenta_cassava4.1_022242m

Viterbi Path	
Viterbi Path	
Posterior @ 0.4	69 - KLKKKSKAK - 77
Posterior @ 0.4	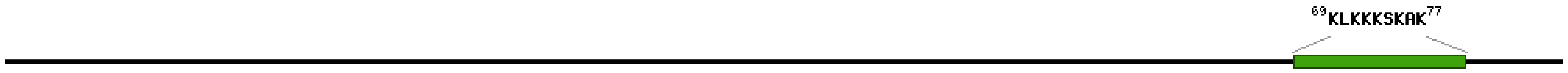
Posterior Graphic. Threshold Marked @ 0.4	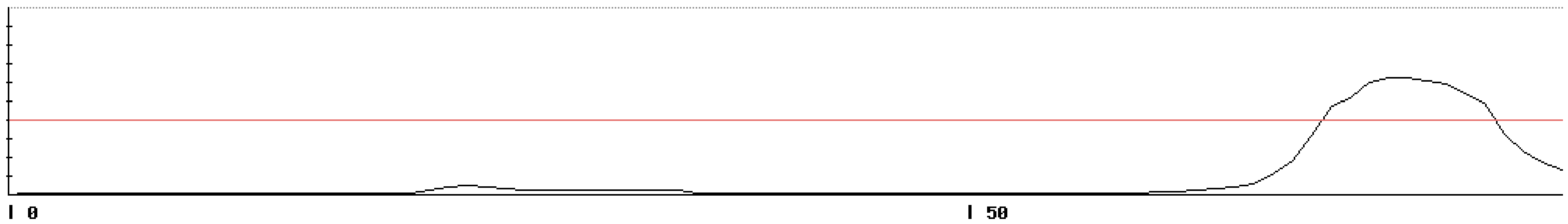

Predictions for Manihotesculenta_cassava4.1_022395m

Viterbi Path	
Viterbi Path	
Posterior @ 0.4	
Posterior @ 0.4	
Posterior Graphic. Threshold Marked @ 0.4	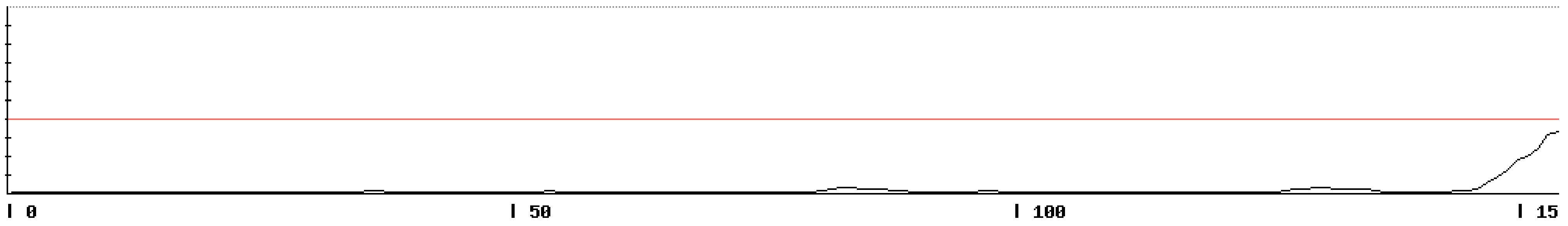

Predictions for Manihotesculenta_cassava4.1_022415m

Viterbi Path	
Viterbi Path	
Posterior @ 0.4	138 - RKRRK - 142
Posterior @ 0.4	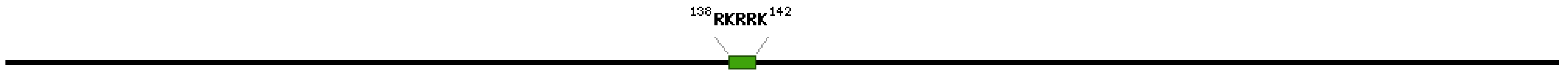
Posterior Graphic. Threshold Marked @ 0.4	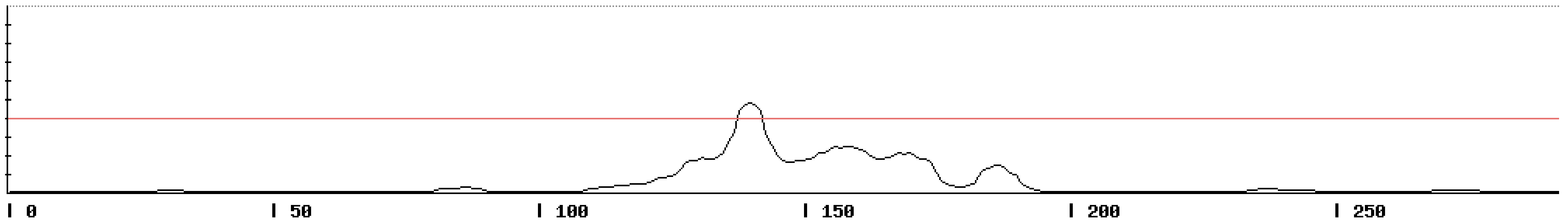

Predictions for Manihotesculenta_cassava4.1_022751m	
Viterbi Path	
Viterbi Path	
Posterior @ 0.4	
Posterior @ 0.4	
Posterior Graphic. Threshold Marked @ 0.4	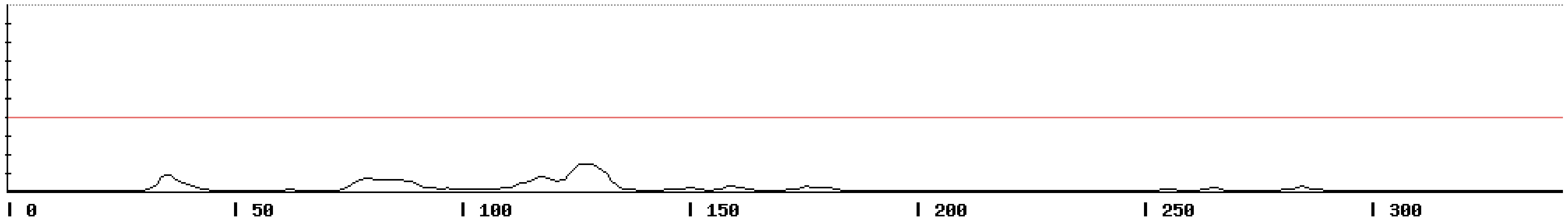

Predictions for Manihotesculenta_cassava4.1_022893m	
Viterbi Path	
Viterbi Path	
Posterior @ 0.4	119 - KAPAGRKTK - 127
Posterior @ 0.4	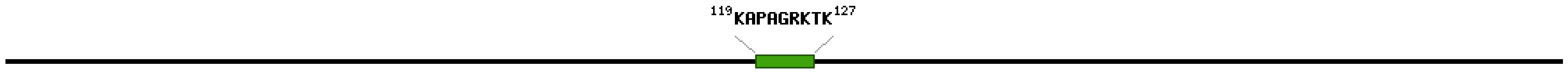
Posterior Graphic. Threshold Marked @ 0.4	

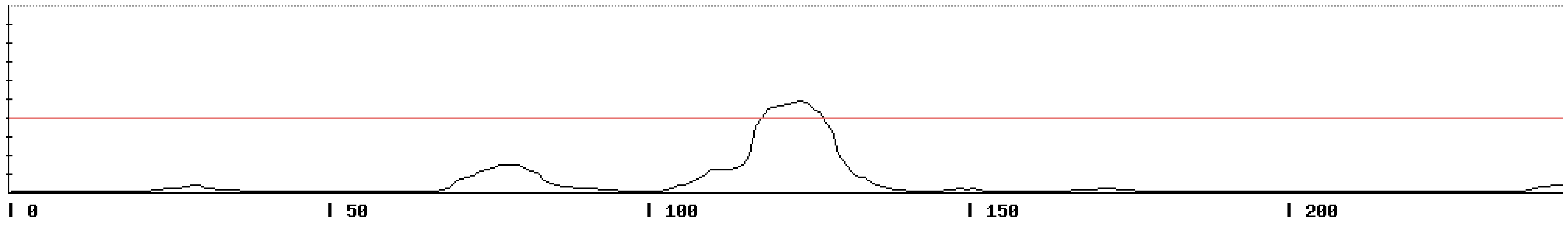

Predictions for Manihotesculenta_cassava4.1_022922m

Viterbi Path	
Viterbi Path	
Posterior @ 0.4	
Posterior @ 0.4	
Posterior Graphic. Threshold Marked @ 0.4	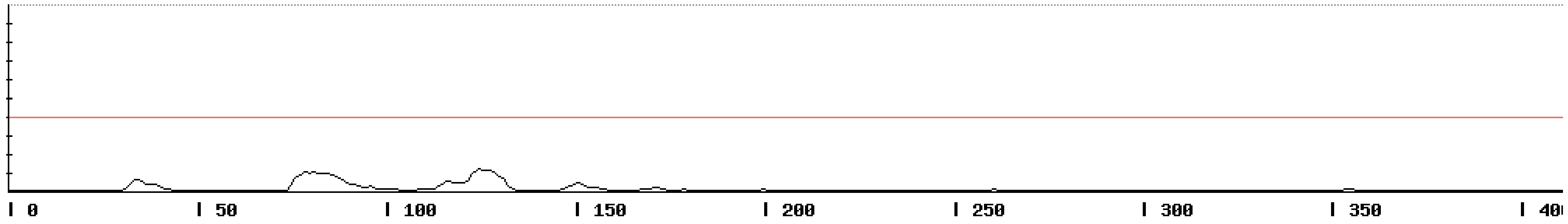

Predictions for Manihotesculenta_cassava4.1_023225m

Viterbi Path	
Viterbi Path	
Posterior @ 0.4	
Posterior @ 0.4	

Posterior Graphic. Threshold Marked @ 0.4	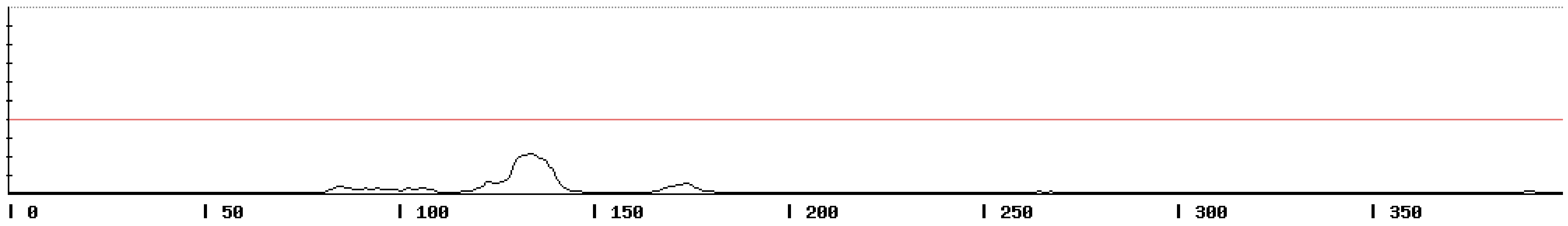

Predictions for Manihotesculenta_cassava4.1_023521m	
Viterbi Path	
Viterbi Path	
Posterior @ 0.4	
Posterior @ 0.4	
Posterior Graphic. Threshold Marked @ 0.4	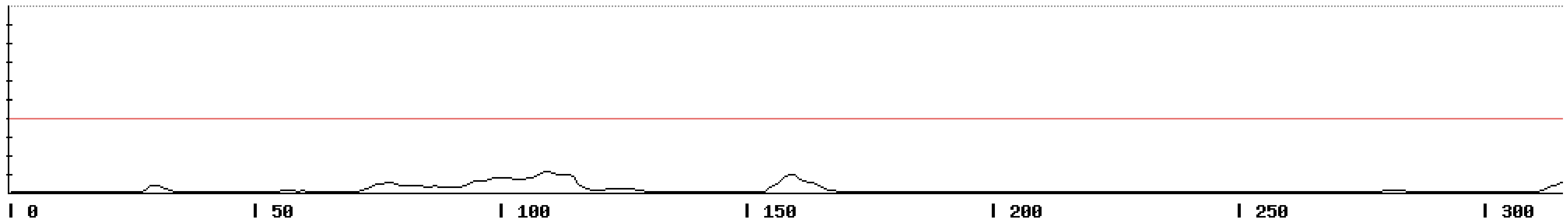

Predictions for Manihotesculenta_cassava4.1_023555m	
Viterbi Path	
Viterbi Path	
Posterior @ 0.4	
Posterior @ 0.4	

Posterior Graphic. Threshold Marked @ 0.4

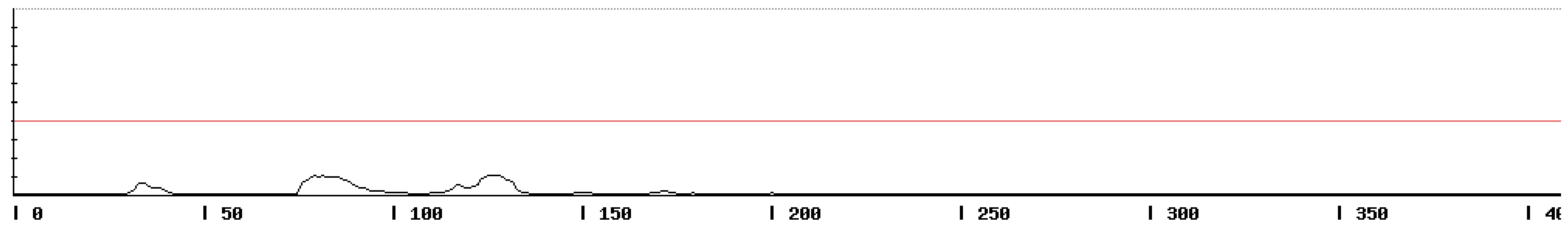

Predictions for Manihotesculenta_cassava4.1_023803m

Viterbi Path

Viterbi Path

Posterior @ 0.4

Posterior @ 0.4

Posterior Graphic. Threshold Marked @ 0.4

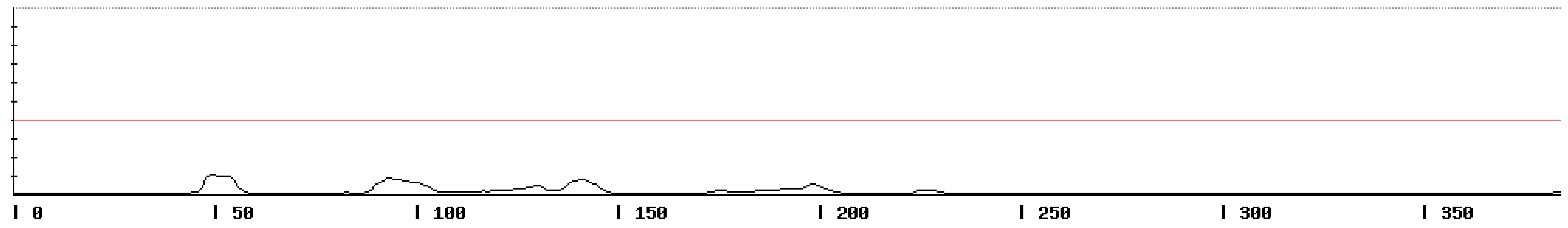

Predictions for Manihotesculenta_cassava4.1_023870m

Viterbi Path

Viterbi Path

Posterior @ 0.4

Posterior @ 0.4

Posterior Graphic. Threshold Marked @ 0.4

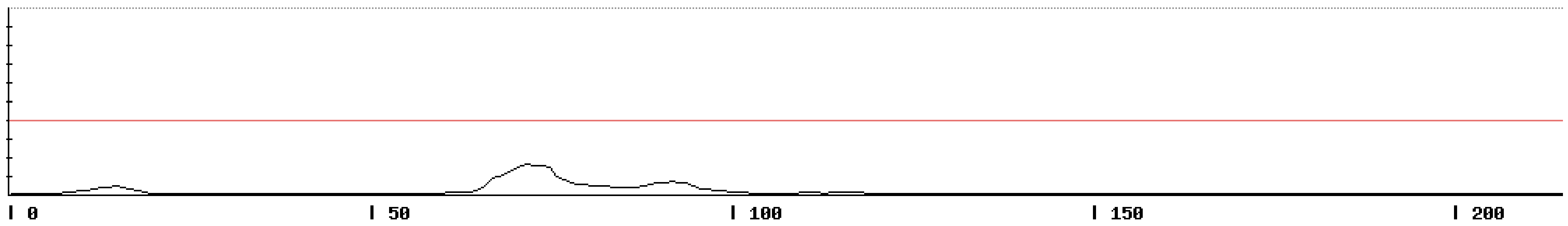

Predictions for Manihotesculenta_cassava4.1_023875m

Viterbi Path

Viterbi Path

Posterior @ 0.4

Posterior @ 0.4

Posterior Graphic. Threshold Marked @ 0.4

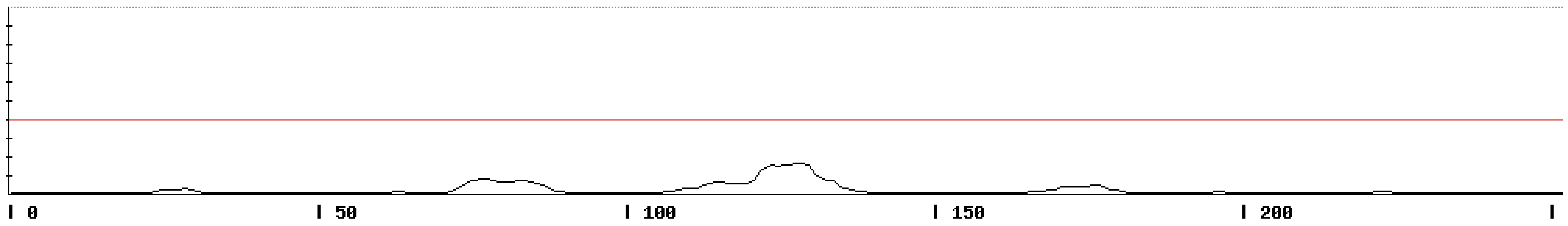

Predictions for Manihotesculenta_cassava4.1_023947m

Viterbi Path

Viterbi Path

Posterior @ 0.4	
Posterior @ 0.4	
Posterior Graphic. Threshold Marked @ 0.4	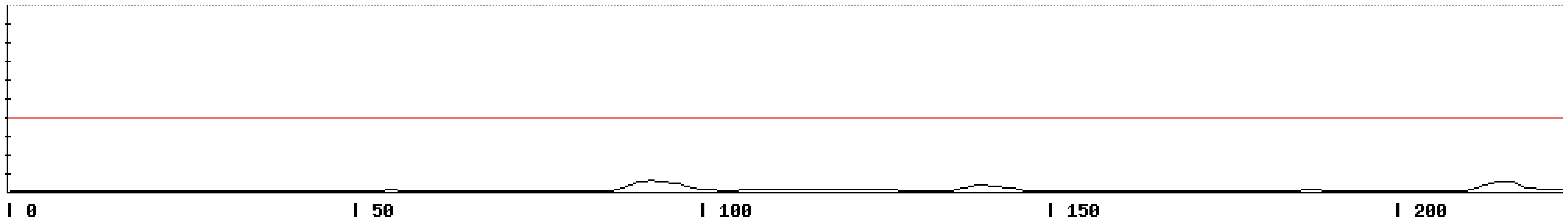

Predictions for Manihotesculenta_cassava4.1_023969m	
Viterbi Path	
Viterbi Path	
Posterior @ 0.4	
Posterior @ 0.4	
Posterior Graphic. Threshold Marked @ 0.4	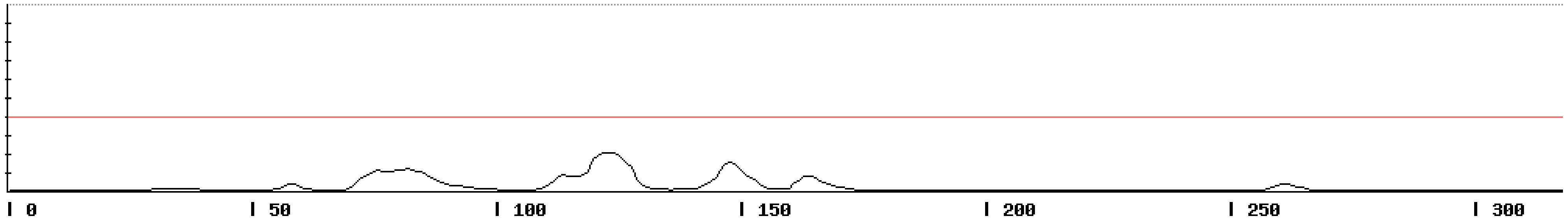

Predictions for Manihotesculenta_cassava4.1_024467m

Viterbi Path

Viterbi Path

Posterior @ 0.4

Posterior @ 0.4

Posterior Graphic. Threshold Marked @ 0.4

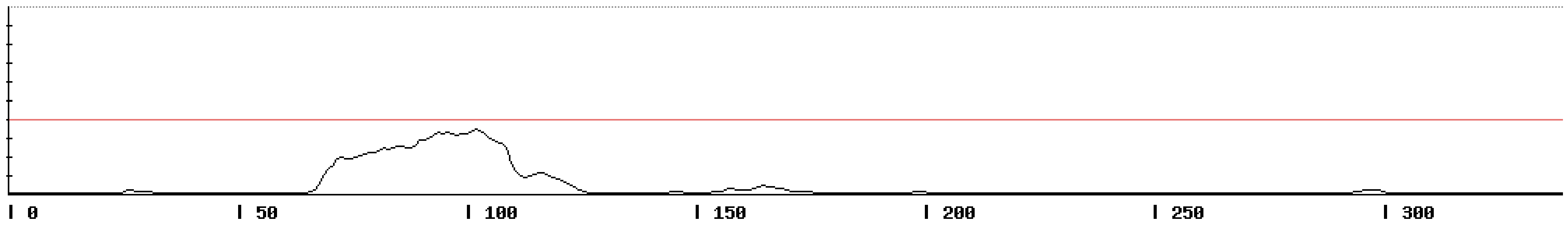

Predictions for Manihotesculenta_cassava4.1_024488m

Viterbi Path

Viterbi Path

Posterior @ 0.4

Posterior @ 0.4

Posterior Graphic. Threshold Marked @ 0.4

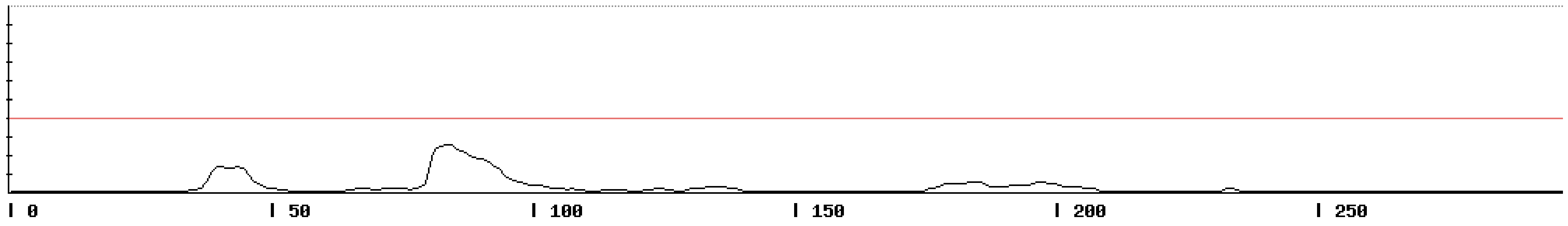

Predictions for Manihotesculenta_cassava4.1_025056m	
Viterbi Path	
Viterbi Path	
Posterior @ 0.4	
Posterior @ 0.4	
Posterior Graphic. Threshold Marked @ 0.4	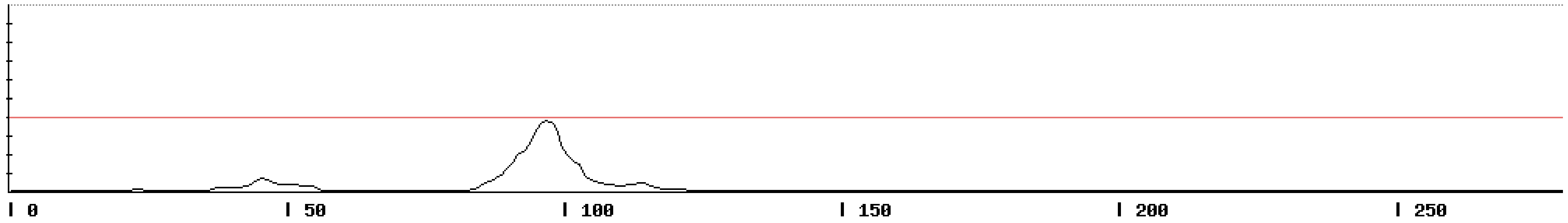

Predictions for Manihotesculenta_cassava4.1_025277m	
Viterbi Path	
Viterbi Path	
Posterior @ 0.4	
Posterior @ 0.4	
Posterior Graphic. Threshold Marked @ 0.4	

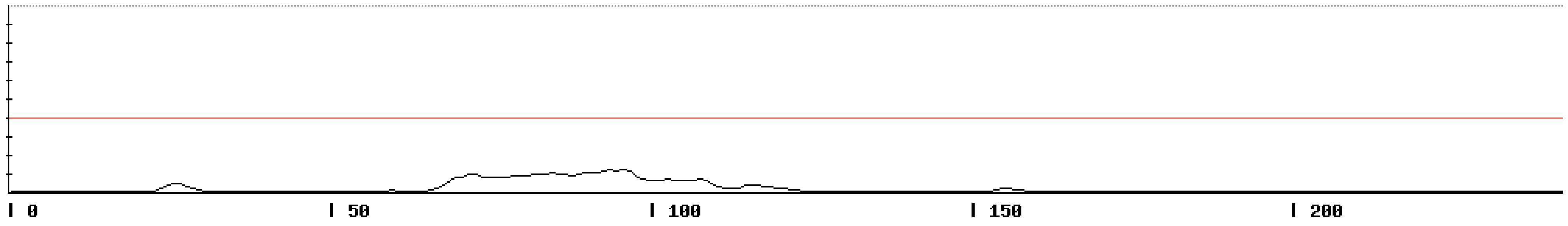

Predictions for Manihotesculenta_cassava4.1_025371m

Viterbi Path	
Viterbi Path	
Posterior @ 0.4	131 - GRRKRRKV - 138
Posterior @ 0.4	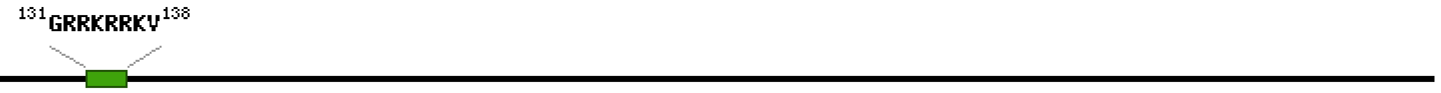
Posterior Graphic. Threshold Marked @ 0.4	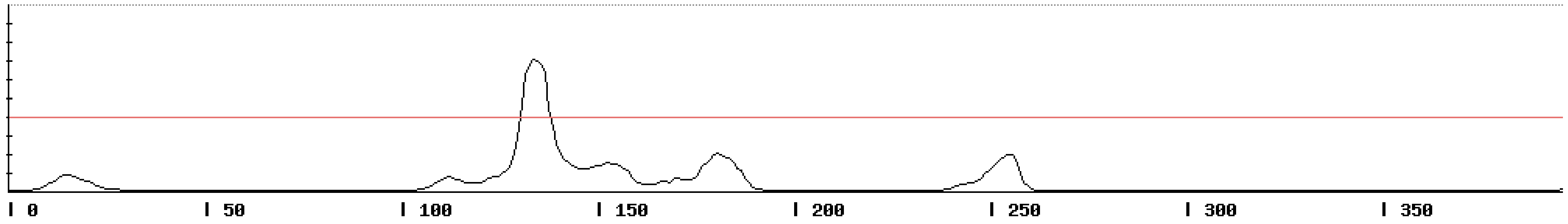

Predictions for Manihotesculenta_cassava4.1_025545m

Viterbi Path	
Viterbi Path	
Posterior @ 0.4	
Posterior @ 0.4	

Posterior Graphic. Threshold Marked @ 0.4	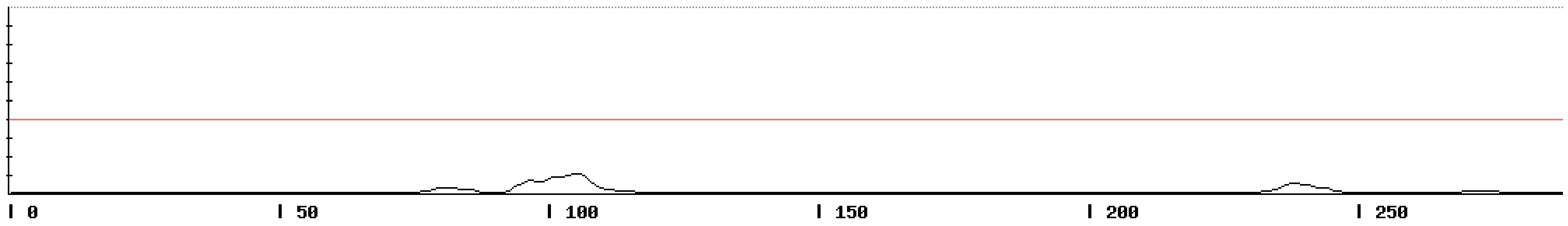

Predictions for Manihotesculenta_cassava4.1_025554m	
Viterbi Path	171 - KSFRRKAGKKNRKFK - 185
Viterbi Path	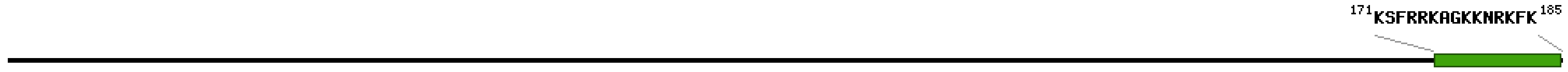
Posterior @ 0.4	170 - SKSFRRKAGKKNRKFK - 185
Posterior @ 0.4	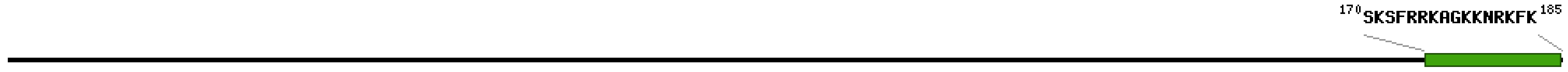
Posterior Graphic. Threshold Marked @ 0.4	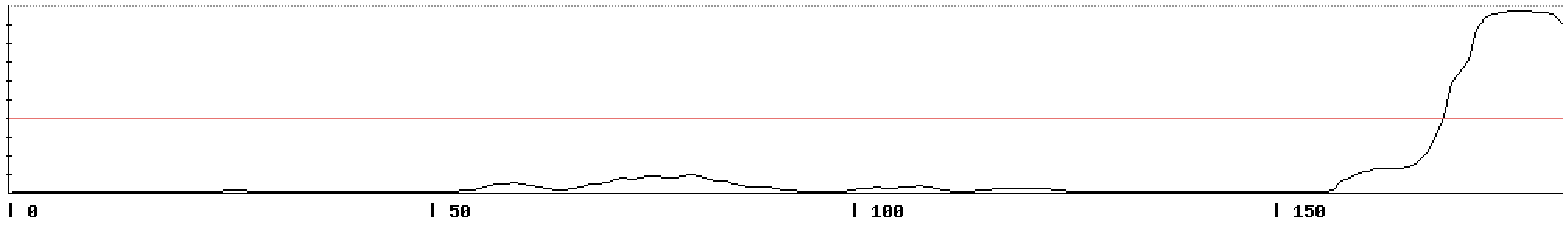

Predictions for Manihotesculenta_cassava4.1_025821m	
Viterbi Path	
Viterbi Path	
Posterior @ 0.4	
Posterior @ 0.4	

Posterior Graphic. Threshold Marked @ 0.4

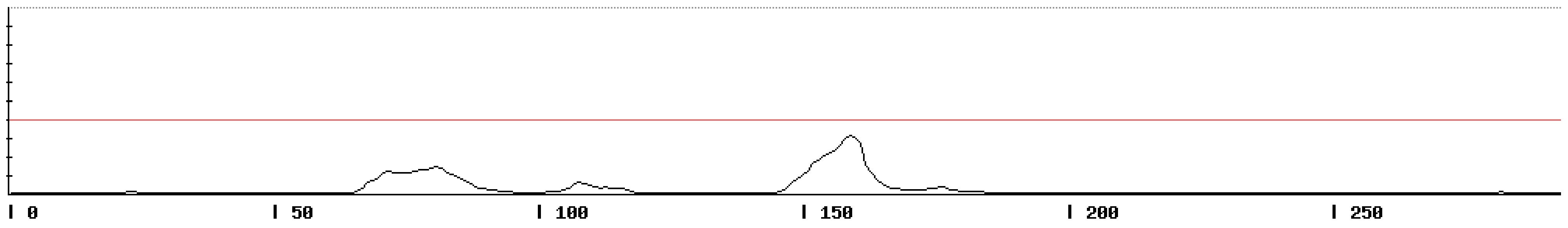

Predictions for Manihotesculenta_cassava4.1_025977m

Viterbi Path

Viterbi Path

Posterior @ 0.4

Posterior @ 0.4

Posterior Graphic. Threshold Marked @ 0.4

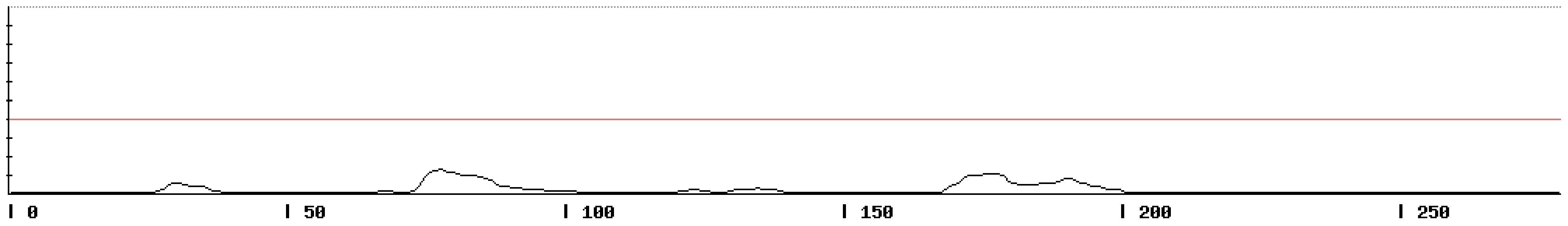

Predictions for Manihotesculenta_cassava4.1_026167m

Viterbi Path

Viterbi Path

Posterior @ 0.4	165 - KKSKRLRSFDRRPIGAEIGR - 184
Posterior @ 0.4	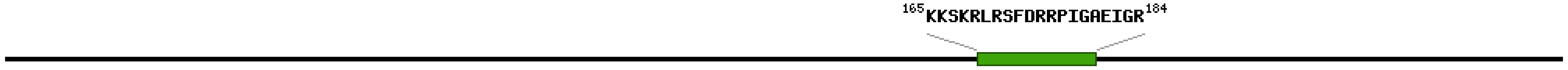
Posterior Graphic. Threshold Marked @ 0.4	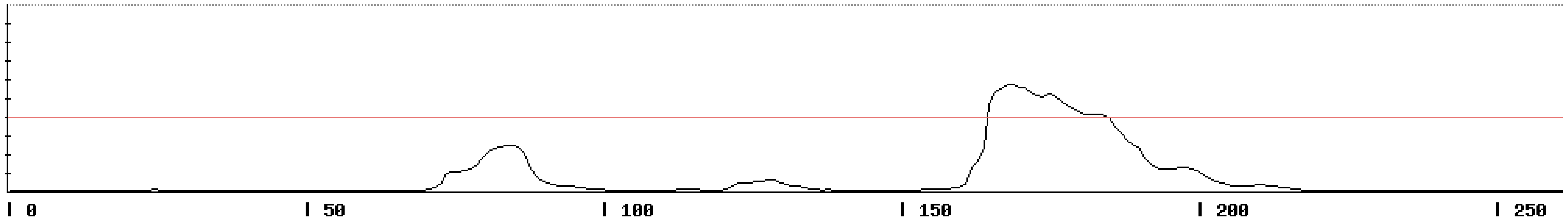

Predictions for Manihotesculenta_cassava4.1_026446m

Viterbi Path	
Viterbi Path	
Posterior @ 0.4	
Posterior @ 0.4	
Posterior Graphic. Threshold Marked @ 0.4	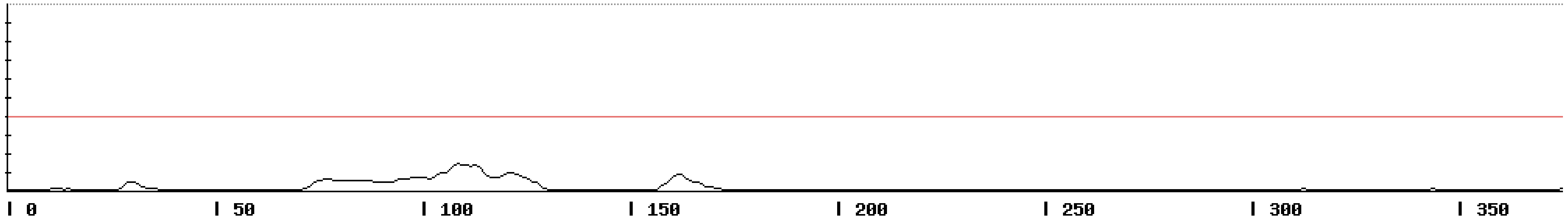

Predictions for Manihotesculenta_cassava4.1_026590m

Viterbi Path	
Viterbi Path	

Posterior @ 0.4

Posterior @ 0.4

Posterior Graphic. Threshold Marked @ 0.4

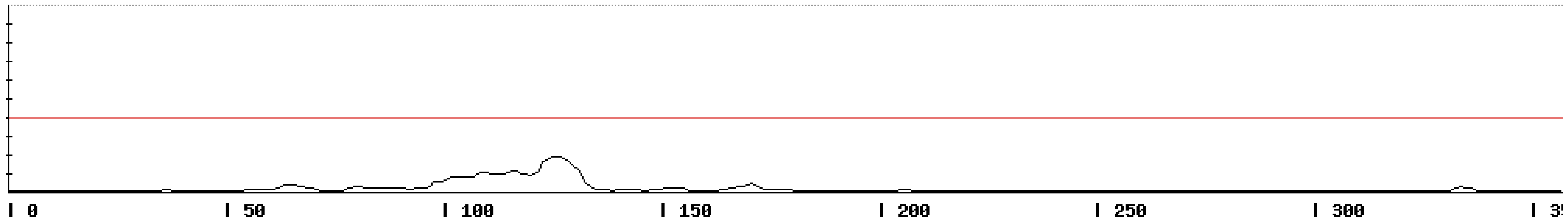

Predictions for Manihotesculenta_cassava4.1_026710m

Viterbi Path

Viterbi Path

Posterior @ 0.4 138 - KRR - 140

Posterior @ 0.4

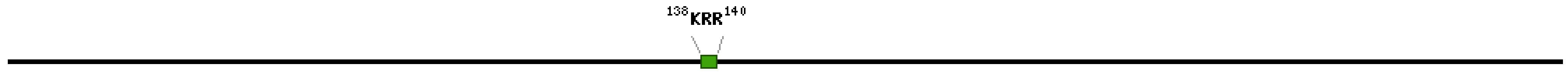

Posterior Graphic. Threshold Marked @ 0.4

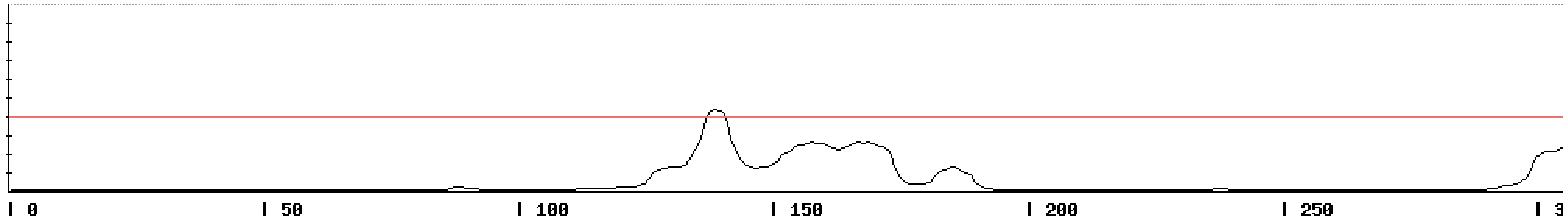

Predictions for Manihotesculenta_cassava4.1_026759m

Viterbi Path

Viterbi Path

Posterior @ 0.4

Posterior @ 0.4

Posterior Graphic. Threshold Marked @ 0.4

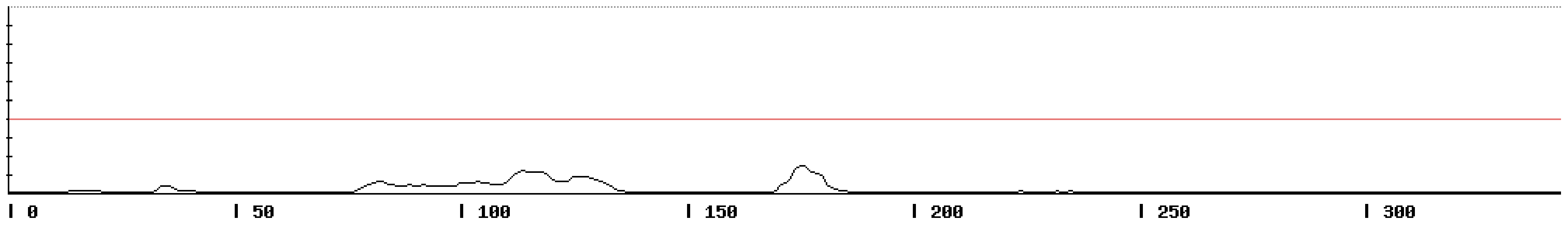

Predictions for Manihotesculenta_cassava4.1_026876m

Viterbi Path

Viterbi Path

Posterior @ 0.4

Posterior @ 0.4

Posterior Graphic. Threshold Marked @ 0.4

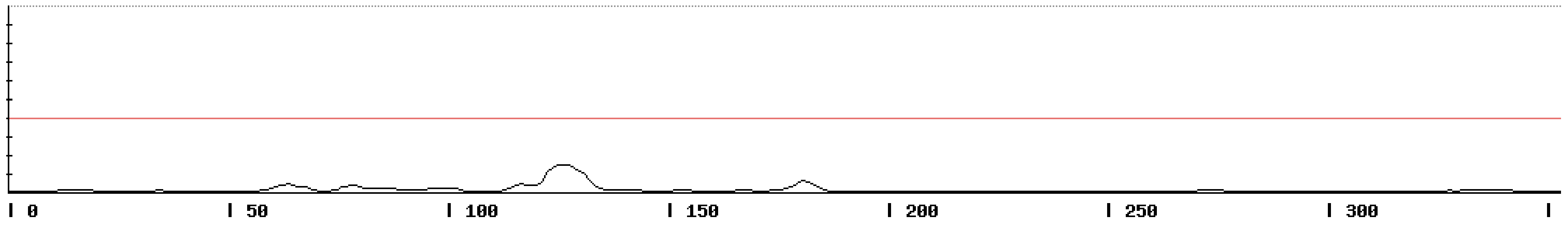

Predictions for Manihotesculenta_cassava4.1_026948m

Viterbi Path	
Viterbi Path	
Posterior @ 0.4	
Posterior @ 0.4	
Posterior Graphic. Threshold Marked @ 0.4	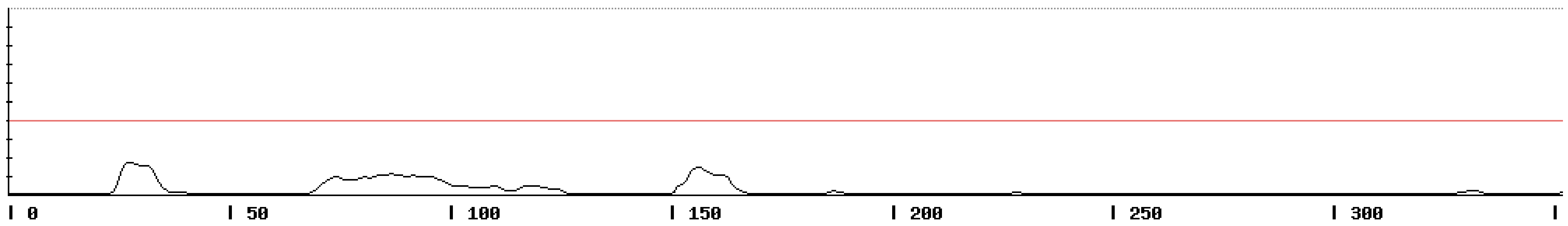

Predictions for Manihotesculenta_cassava4.1_026973m

Viterbi Path	
Viterbi Path	
Posterior @ 0.4	
Posterior @ 0.4	
Posterior Graphic. Threshold Marked @ 0.4	

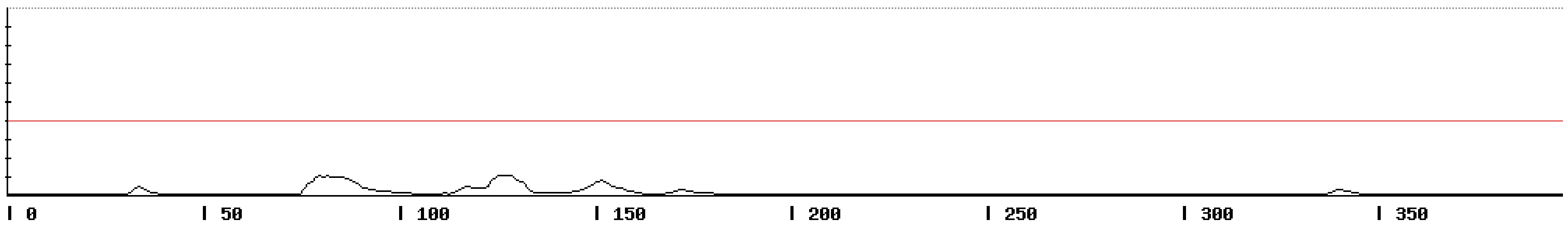

Predictions for Manihotesculenta_cassava4.1_027253m

Viterbi Path	
Viterbi Path	
Posterior @ 0.4	
Posterior @ 0.4	
Posterior Graphic. Threshold Marked @ 0.4	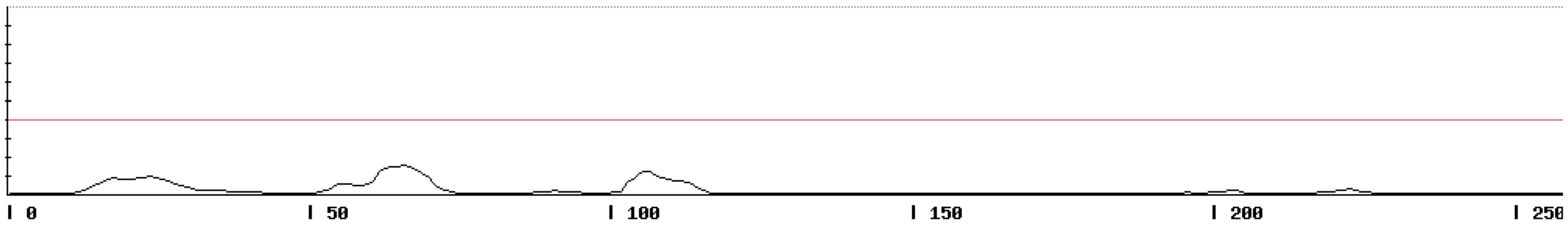

Predictions for Manihotesculenta_cassava4.1_027291m

Viterbi Path	
Viterbi Path	
Posterior @ 0.4	
Posterior @ 0.4	

Posterior Graphic. Threshold Marked @ 0.4

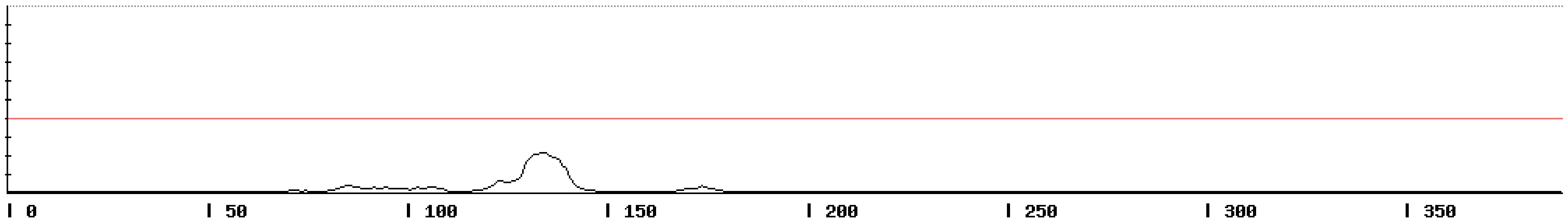

Predictions for Manihotesculenta_cassava4.1_027454m

Viterbi Path

Viterbi Path

Posterior @ 0.4

Posterior @ 0.4

Posterior Graphic. Threshold Marked @ 0.4

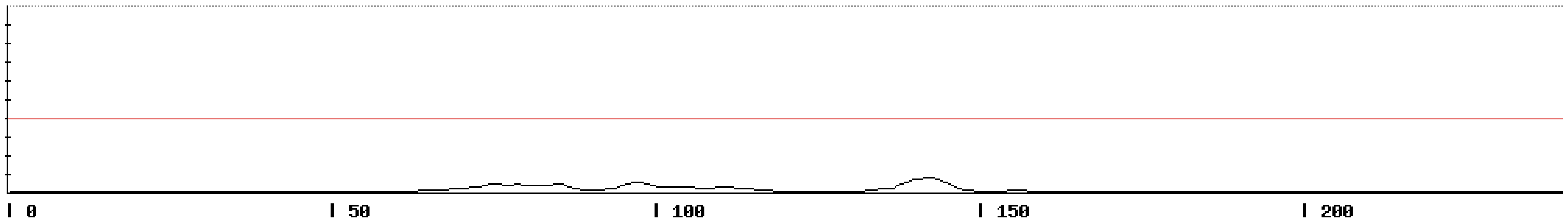

Predictions for Manihotesculenta_cassava4.1_027587m

Viterbi Path

Viterbi Path

Posterior @ 0.4

Posterior @ 0.4

Posterior Graphic. Threshold Marked @ 0.4

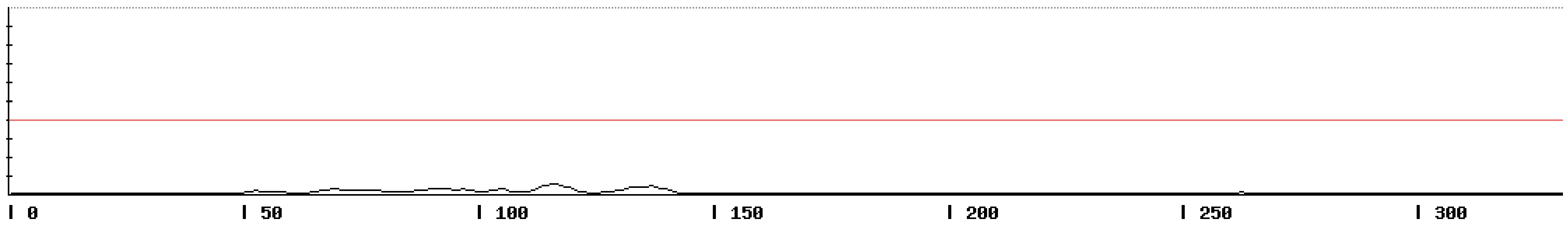

Predictions for Manihotesculenta_cassava4.1_027686m

Viterbi Path

Viterbi Path

Posterior @ 0.4

Posterior @ 0.4

Posterior Graphic. Threshold Marked @ 0.4

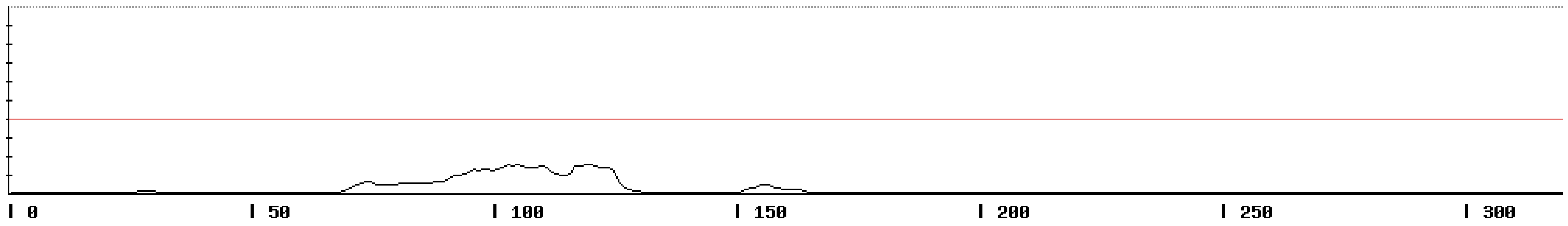

Predictions for Manihotesculenta_cassava4.1_027756m

Viterbi Path

Viterbi Path

Posterior @ 0.4

Posterior @ 0.4

Posterior Graphic. Threshold Marked @ 0.4

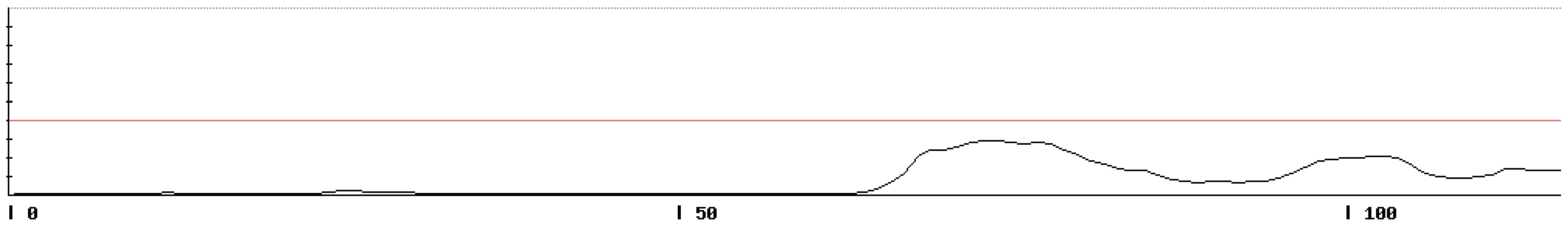

Predictions for Manihotesculenta_cassava4.1_027836m

Viterbi Path

Viterbi Path

Posterior @ 0.4

Posterior @ 0.4

Posterior Graphic. Threshold Marked @ 0.4

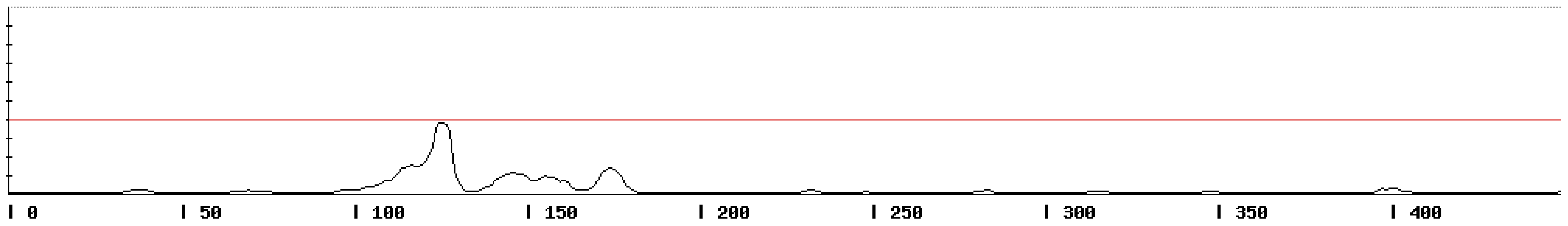

Predictions for Manihotesculenta_cassava4.1_027882m

Viterbi Path

Viterbi Path

Posterior @ 0.4	134 - KRR - 136
Posterior @ 0.4	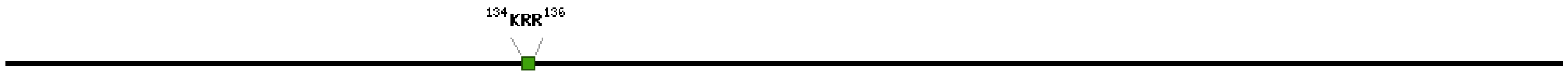
Posterior Graphic. Threshold Marked @ 0.4	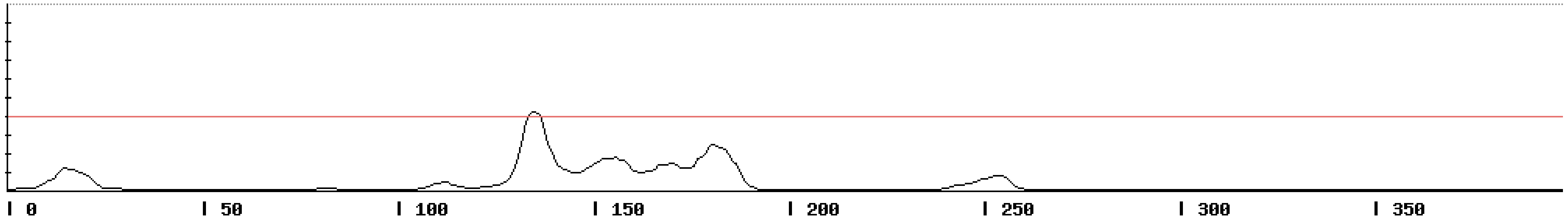

Predictions for Manihotesculenta_cassava4.1_027890m

Viterbi Path	
Viterbi Path	
Posterior @ 0.4	182 - RKKK - 185
Posterior @ 0.4	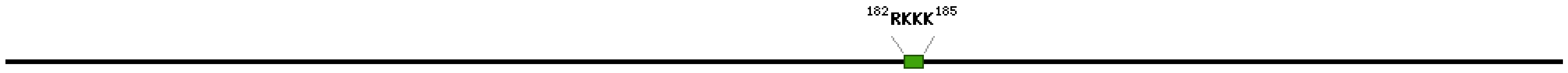
Posterior Graphic. Threshold Marked @ 0.4	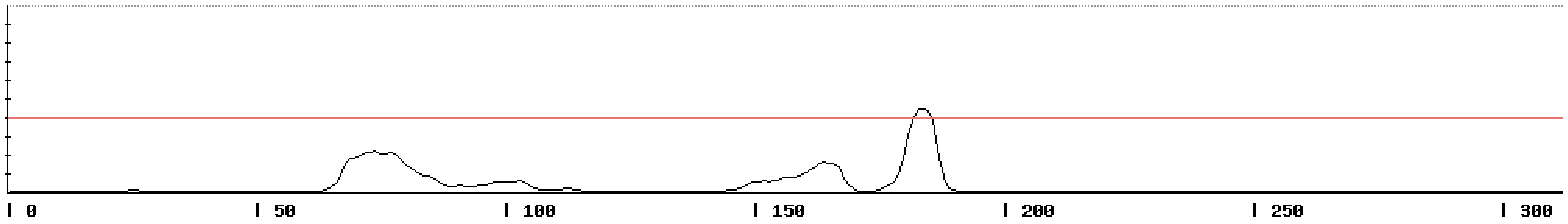

Predictions for Manihotesculenta_cassava4.1_028212m

Viterbi Path

Viterbi Path

Posterior @ 0.4

Posterior @ 0.4

Posterior Graphic. Threshold Marked @ 0.4

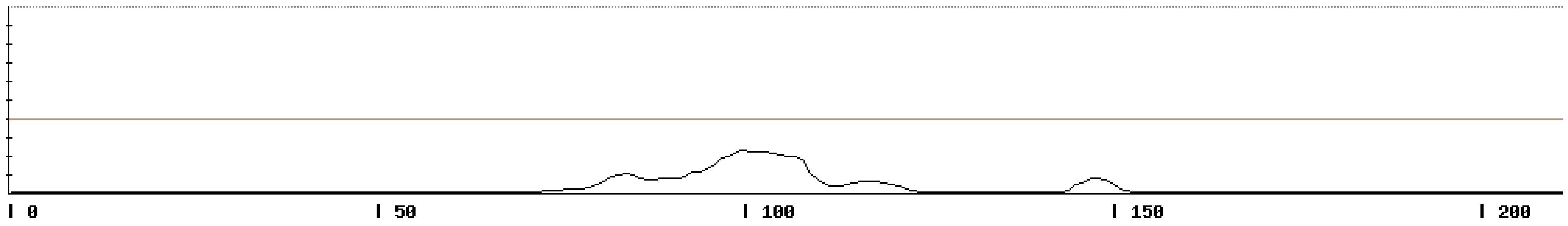

Predictions for Manihotesculenta_cassava4.1_028254m

Viterbi Path

Viterbi Path

Posterior @ 0.4

Posterior @ 0.4

Posterior Graphic. Threshold Marked @ 0.4

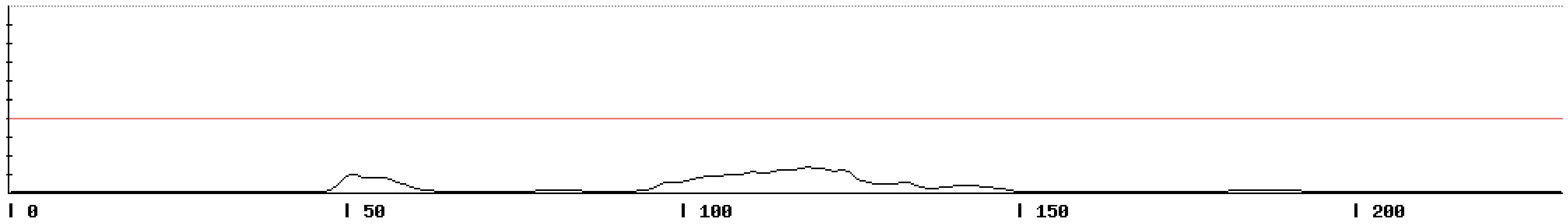

Predictions for Manihotesculenta_cassava4.1_028258m	
Viterbi Path	
Viterbi Path	
Posterior @ 0.4	
Posterior @ 0.4	
Posterior Graphic. Threshold Marked @ 0.4	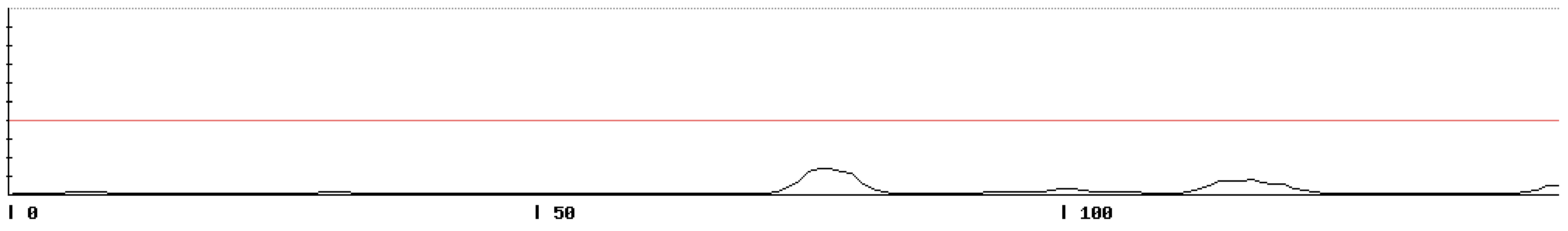

Predictions for Manihotesculenta_cassava4.1_028269m	
Viterbi Path	
Viterbi Path	
Posterior @ 0.4	
Posterior @ 0.4	
Posterior Graphic. Threshold Marked @ 0.4	

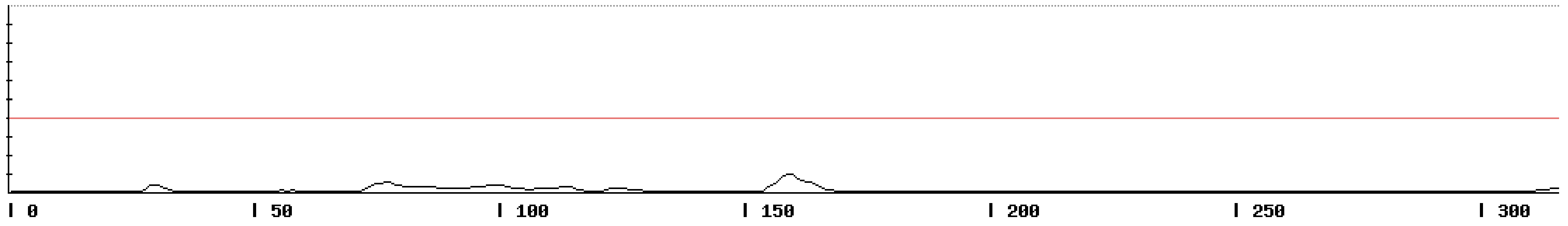

Predictions for Manihotesculenta_cassava4.1_028980m

Viterbi Path

Viterbi Path

Posterior @ 0.4

Posterior @ 0.4

Posterior Graphic. Threshold Marked @ 0.4

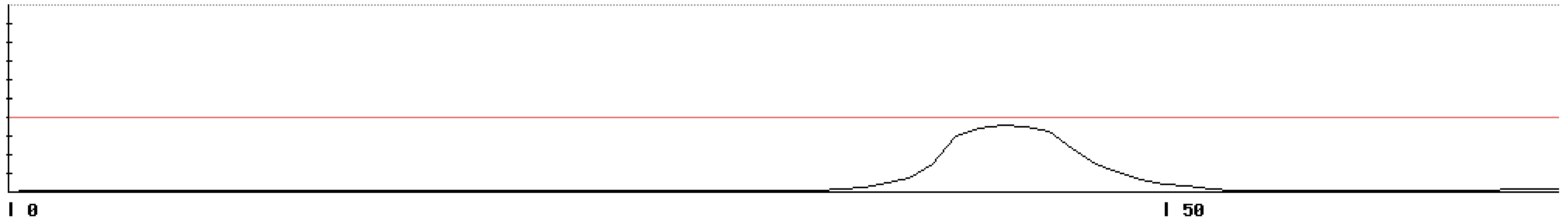

Predictions for Manihotesculenta_cassava4.1_029154m

Viterbi Path

Viterbi Path

Posterior @ 0.4

Posterior @ 0.4

Posterior Graphic. Threshold Marked @ 0.4

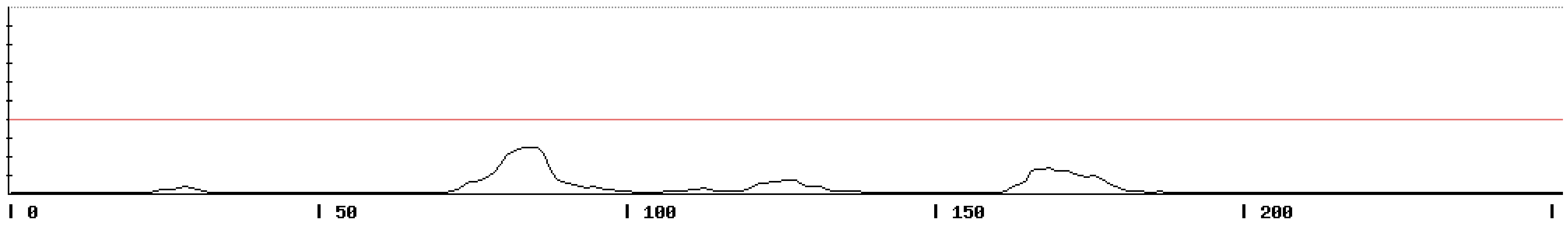

Predictions for Manihotesculenta_cassava4.1_029170m

Viterbi Path

Viterbi Path

Posterior @ 0.4 — 78 - PKGAR - 82

Posterior @ 0.4

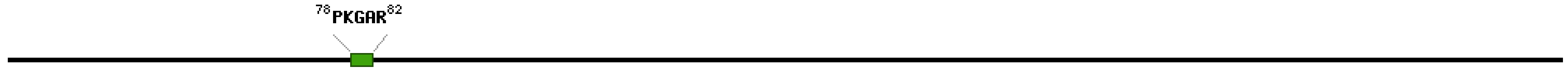

Posterior Graphic. Threshold Marked @ 0.4

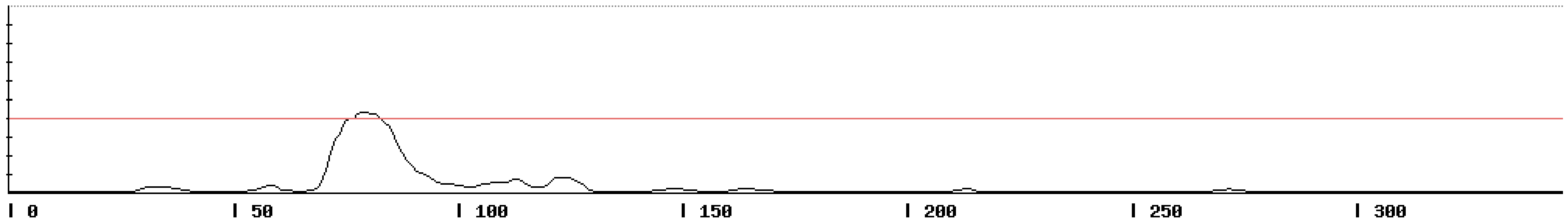

Predictions for Manihotesculenta_cassava4.1_029418m

Viterbi Path

Viterbi Path

Posterior @ 0.4

Posterior @ 0.4

Posterior Graphic. Threshold Marked @ 0.4

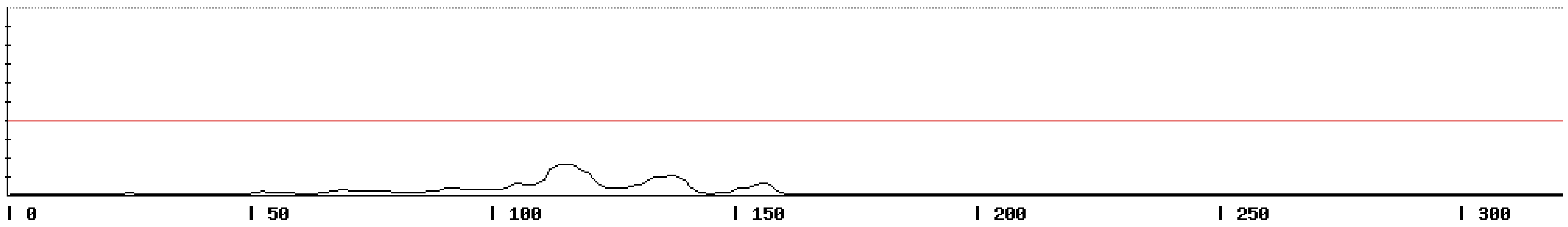

Predictions for Manihotesculenta_cassava4.1_029539m

Viterbi Path

Viterbi Path

Posterior @ 0.4

Posterior @ 0.4

Posterior Graphic. Threshold Marked @ 0.4

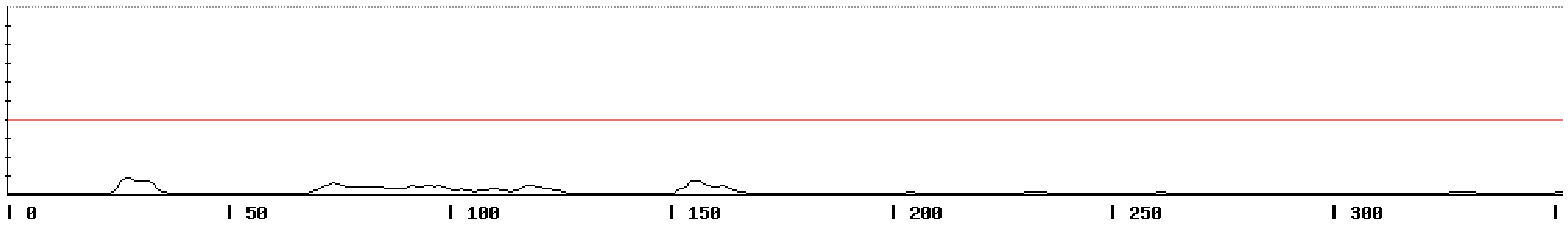

Predictions for Manihotesculenta_cassava4.1_029652m

Viterbi Path

Viterbi Path

Posterior @ 0.4

Posterior @ 0.4

Posterior Graphic. Threshold Marked @ 0.4

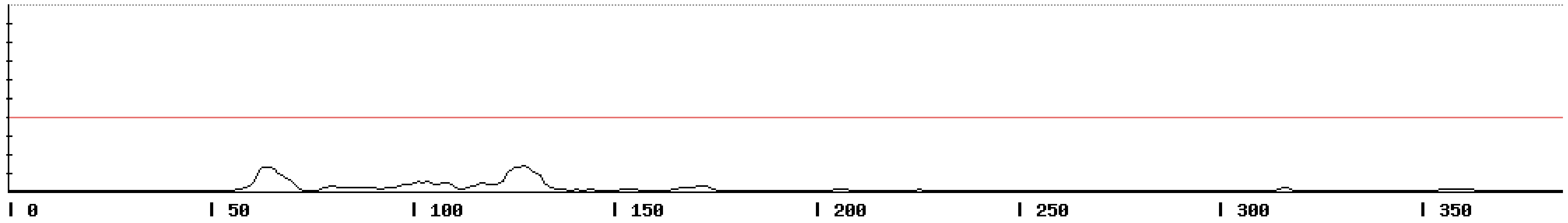

Predictions for Manihotesculenta_cassava4.1_029733m

Viterbi Path

Viterbi Path

Posterior @ 0.4 163 - KKYPNGSRPRRAAR - 176

Posterior @ 0.4

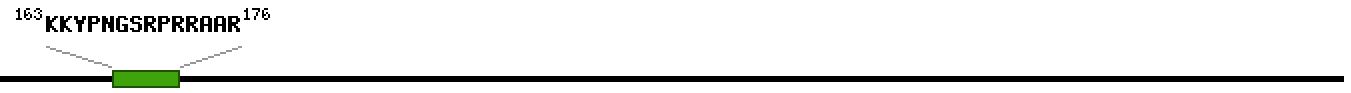

Posterior Graphic. Threshold Marked @ 0.4

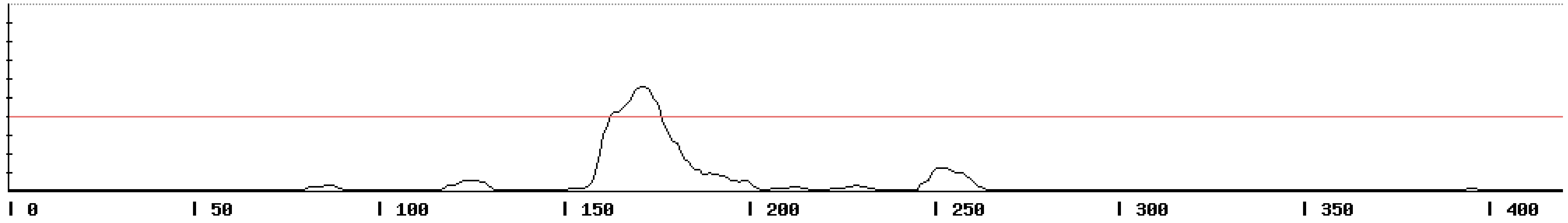

Predictions for Manihotesculenta_cassava4.1_029889m

Viterbi Path

Viterbi Path

Posterior @ 0.4

Posterior @ 0.4

Posterior Graphic. Threshold Marked @ 0.4

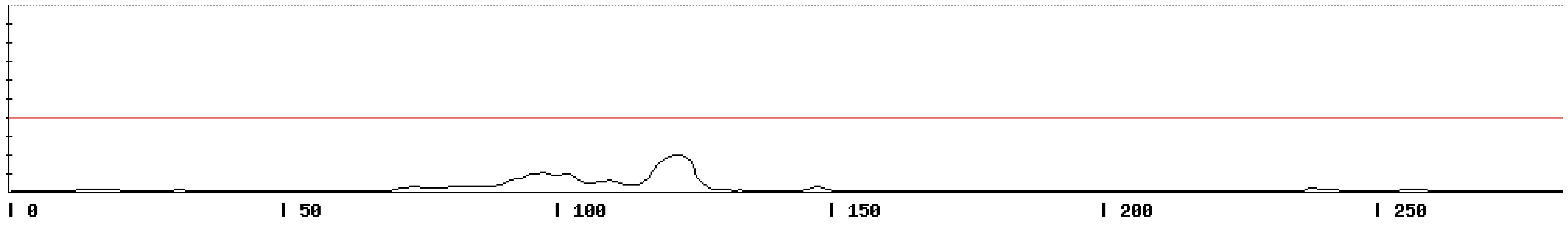

Predictions for Manihotesculenta_cassava4.1_029980m

Viterbi Path

Viterbi Path

Posterior @ 0.4

78 - KGGKPK - 83

165 - KKSAKRLRTFGRRP - 178

Posterior @ 0.4

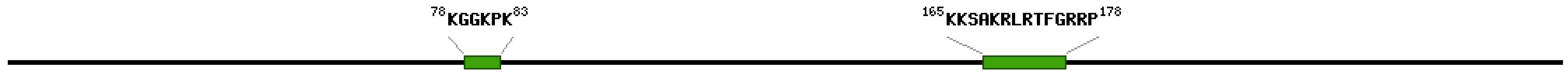

Posterior Graphic. Threshold Marked @ 0.4

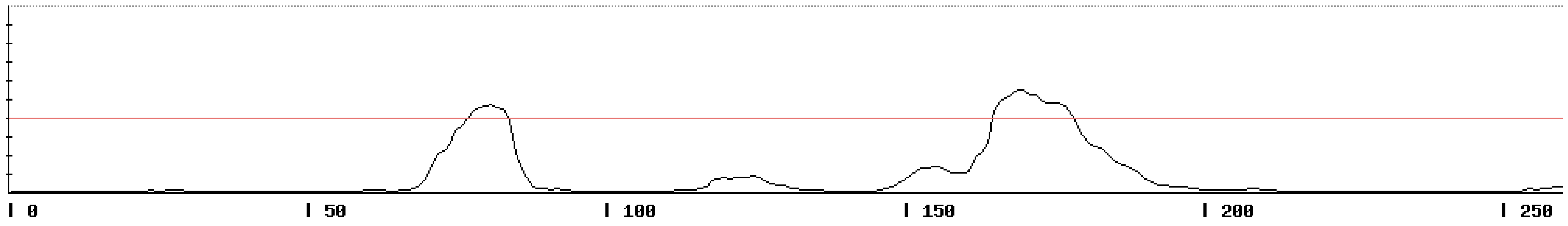

Predictions for Manihotesculenta_cassava4.1_030134m	
Viterbi Path	
Viterbi Path	
Posterior @ 0.4	
Posterior @ 0.4	
Posterior Graphic. Threshold Marked @ 0.4	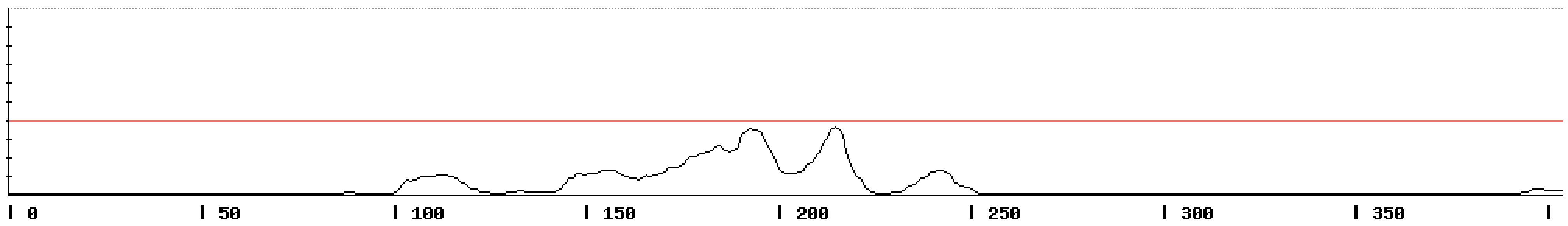

Predictions for Manihotesculenta_cassava4.1_030482m	
Viterbi Path	
Viterbi Path	
Posterior @ 0.4	
Posterior @ 0.4	
Posterior Graphic. Threshold Marked @ 0.4	

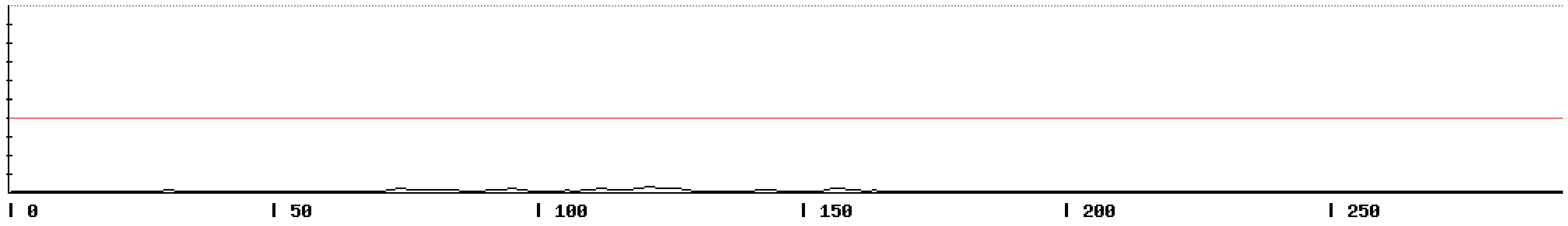

Predictions for Manihotesculenta_cassava4.1_030551m

Viterbi Path

Viterbi Path

Posterior @ 0.4

Posterior @ 0.4

Posterior Graphic. Threshold Marked @ 0.4

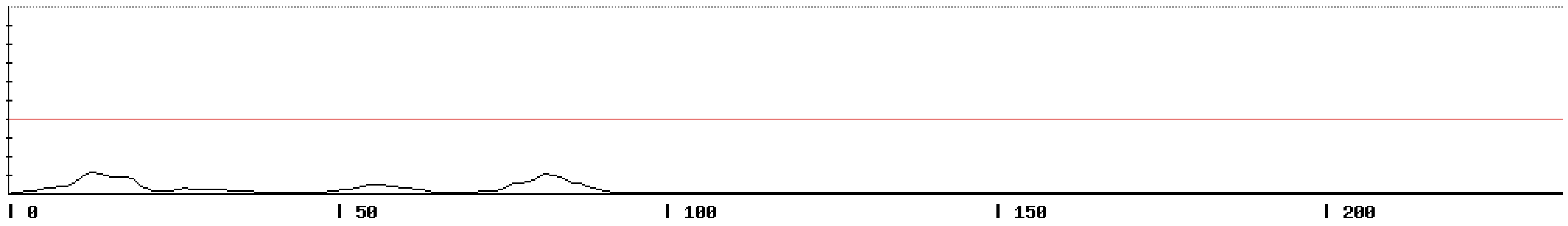

Predictions for Manihotesculenta_cassava4.1_030726m

Viterbi Path

Viterbi Path

Posterior @ 0.4

Posterior @ 0.4

Posterior Graphic. Threshold Marked @ 0.4	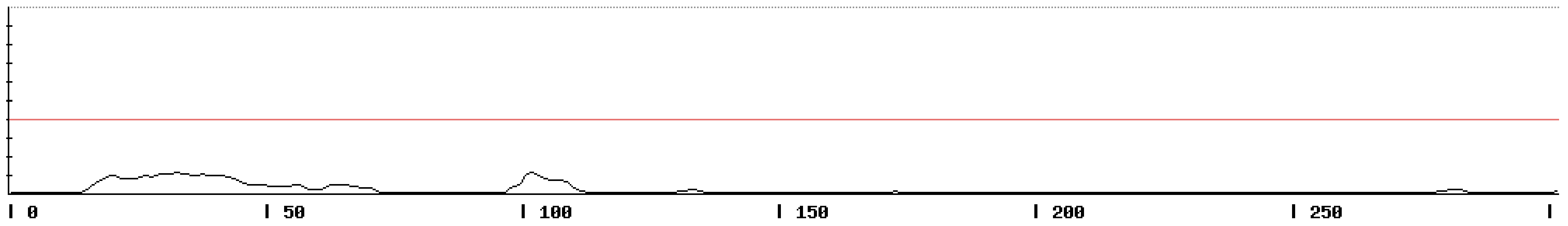

Predictions for Manihotesculenta_cassava4.1_030875m

Viterbi Path	
Viterbi Path	
Posterior @ 0.4	
Posterior @ 0.4	
Posterior Graphic. Threshold Marked @ 0.4	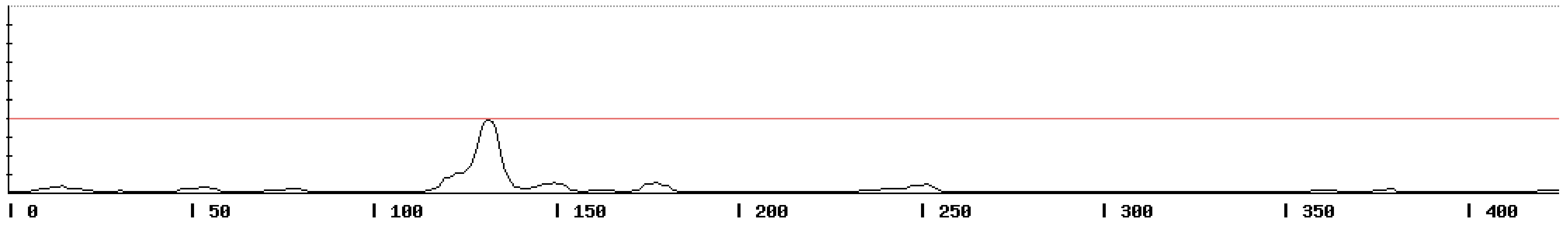

Predictions for Manihotesculenta_cassava4.1_031228m

Viterbi Path	
Viterbi Path	
Posterior @ 0.4	
Posterior @ 0.4	

Posterior Graphic. Threshold Marked @ 0.4

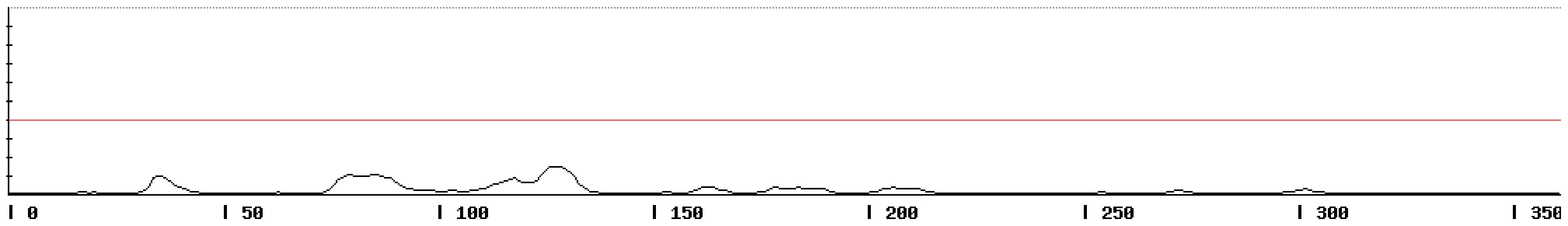

Predictions for Manihotesculenta_cassava4.1_031247m

Viterbi Path

Viterbi Path

Posterior @ 0.4

Posterior @ 0.4

Posterior Graphic. Threshold Marked @ 0.4

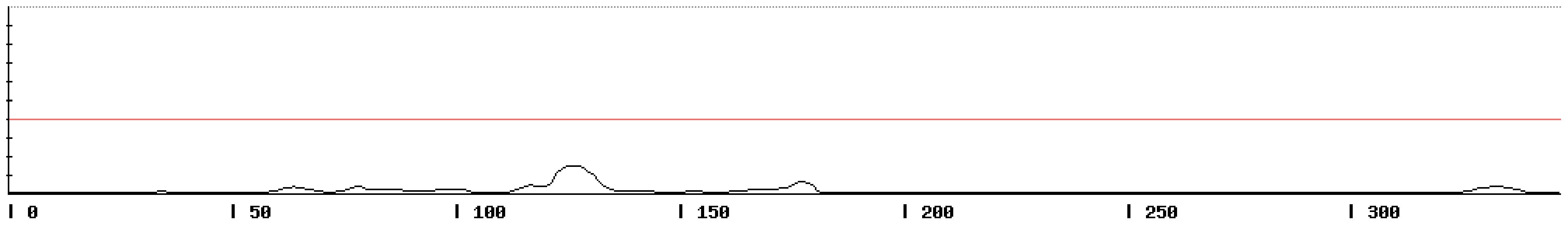

Predictions for Manihotesculenta_cassava4.1_031351m

Viterbi Path

Viterbi Path

Posterior @ 0.4	
Posterior @ 0.4	
Posterior Graphic. Threshold Marked @ 0.4	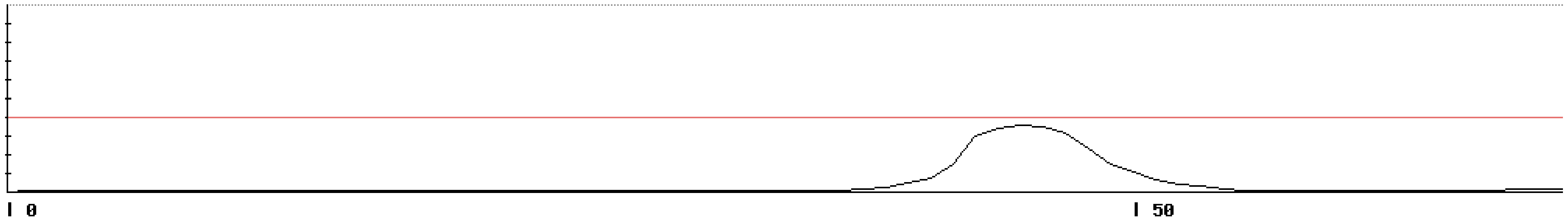

Predictions for Manihotesculenta_cassava4.1_031698m

Viterbi Path	
Viterbi Path	
Posterior @ 0.4	
Posterior @ 0.4	
Posterior Graphic. Threshold Marked @ 0.4	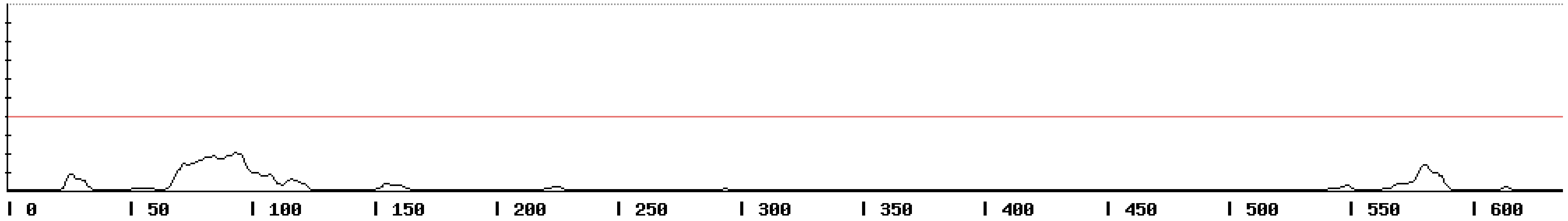

Predictions for Manihotesculenta_cassava4.1_031758m

Viterbi Path	
Viterbi Path	

Posterior @ 0.4	165 - RKRRPRKQGN - 174
Posterior @ 0.4	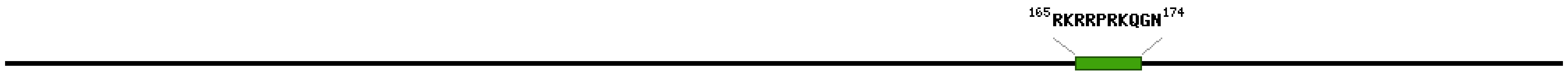
Posterior Graphic. Threshold Marked @ 0.4	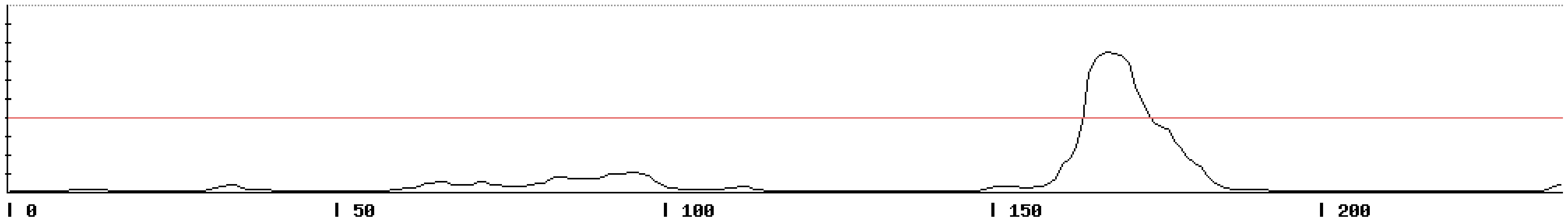

Predictions for Manihotesculenta_cassava4.1_031776m

Viterbi Path	
Viterbi Path	
Posterior @ 0.4	
Posterior @ 0.4	
Posterior Graphic. Threshold Marked @ 0.4	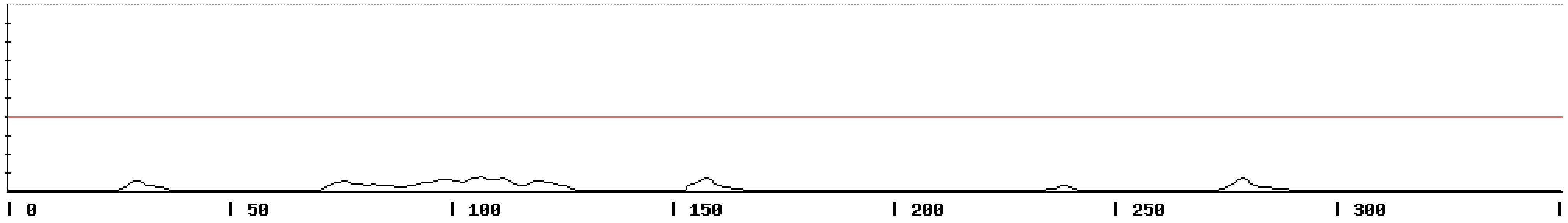

Predictions for Manihotesculenta_cassava4.1_031849m

Viterbi Path

Viterbi Path

Posterior @ 0.4

Posterior @ 0.4

Posterior Graphic. Threshold Marked @ 0.4

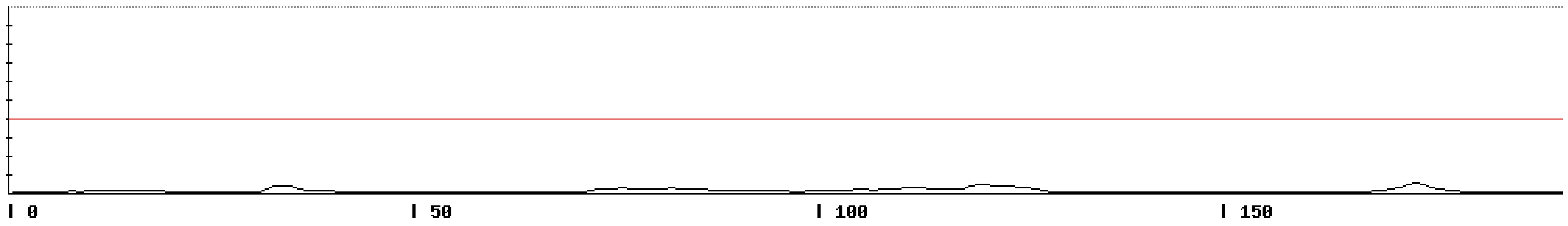

Predictions for Manihotesculenta_cassava4.1_032341m

Viterbi Path

Viterbi Path

Posterior @ 0.4

Posterior @ 0.4

Posterior Graphic. Threshold Marked @ 0.4

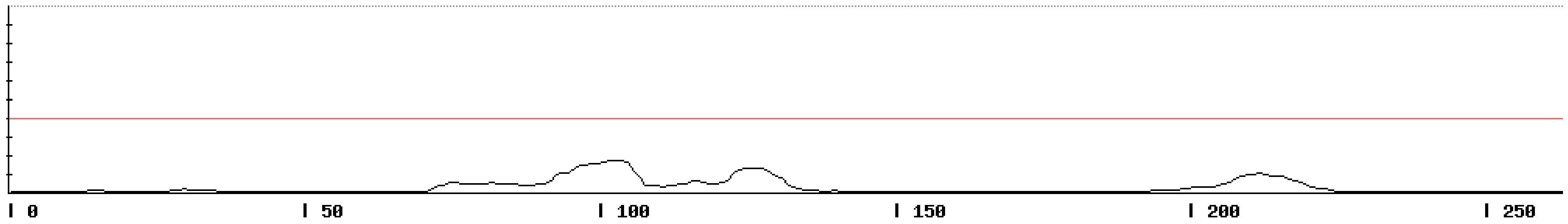

Predictions for Manihotesculenta_cassava4.1_032497m	
Viterbi Path	
Viterbi Path	
Posterior @ 0.4	
Posterior @ 0.4	
Posterior Graphic. Threshold Marked @ 0.4	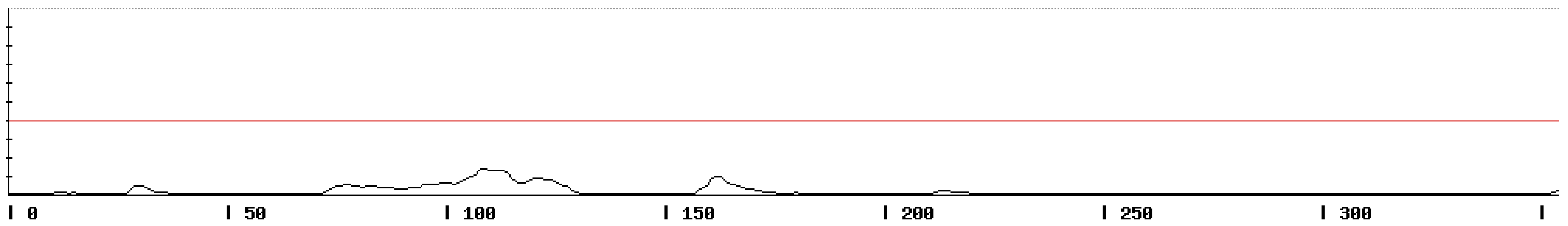

Predictions for Manihotesculenta_cassava4.1_032633m	
Viterbi Path	
Viterbi Path	
Posterior @ 0.4	
Posterior @ 0.4	
Posterior Graphic. Threshold Marked @ 0.4	

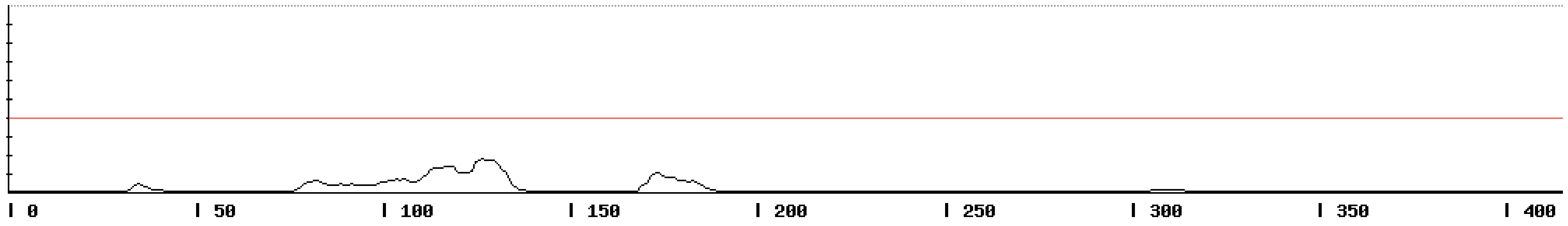

Predictions for Manihotesculenta_cassava4.1_032712m

Viterbi Path	
Viterbi Path	
Posterior @ 0.4	94 - RKRKIR - 99
Posterior @ 0.4	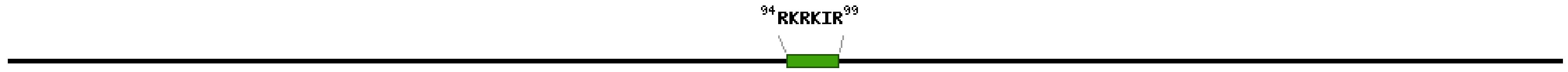
Posterior Graphic. Threshold Marked @ 0.4	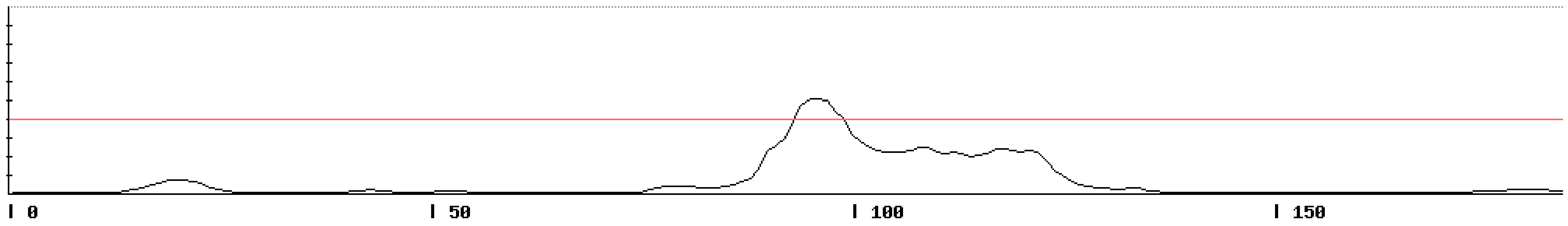

Predictions for Manihotesculenta_cassava4.1_032739m

Viterbi Path	
Viterbi Path	
Posterior @ 0.4	139 - RKRRKV - 144
Posterior @ 0.4	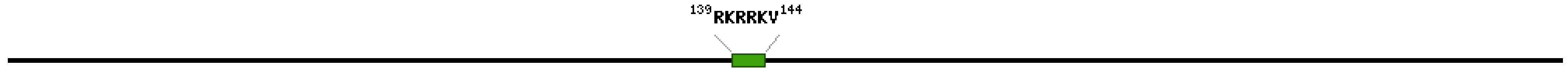

Posterior Graphic. Threshold Marked @ 0.4

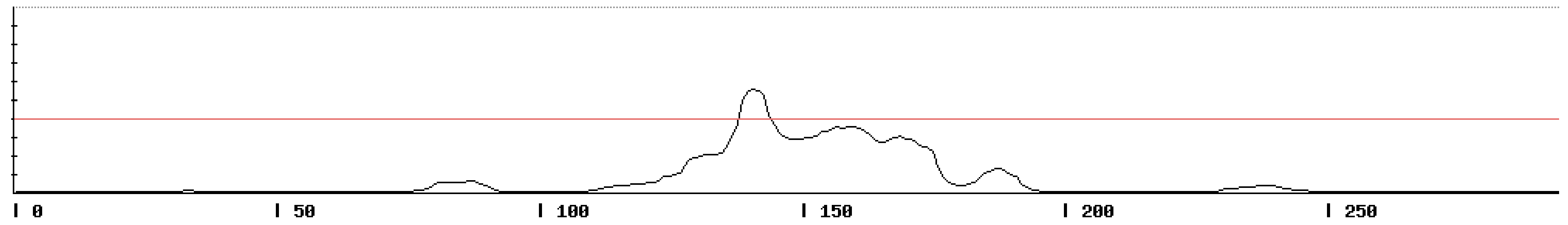

Predictions for Manihotesculenta_cassava4.1_032848m

Viterbi Path

Viterbi Path

Posterior @ 0.4

Posterior @ 0.4

Posterior Graphic. Threshold Marked @ 0.4

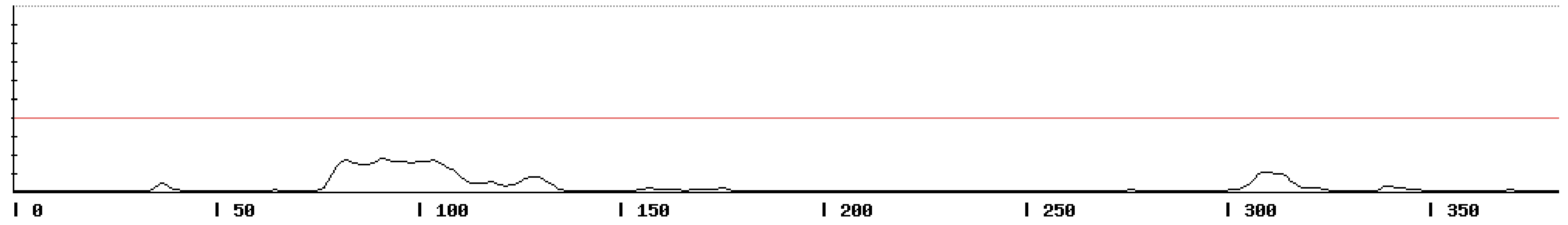

Predictions for Manihotesculenta_cassava4.1_032971m

Viterbi Path

Viterbi Path

Posterior @ 0.4 136 - RRKSSKTRANPK - 147

Posterior @ 0.4

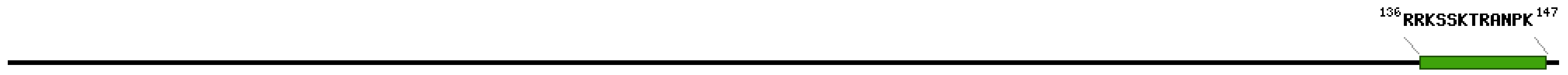

Posterior Graphic. Threshold Marked @ 0.4

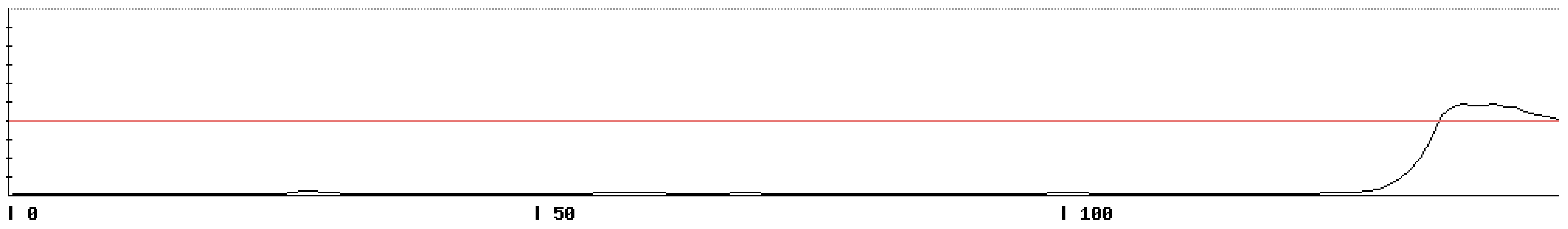

Predictions for Manihotesculenta_cassava4.1_033023m

Viterbi Path

Viterbi Path

Posterior @ 0.4

Posterior @ 0.4

Posterior Graphic. Threshold Marked @ 0.4

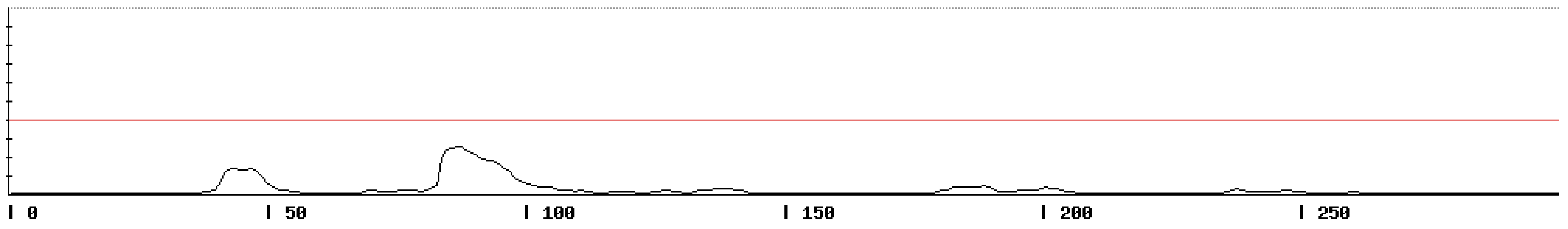

Predictions for Manihotesculenta_cassava4.1_033050m

Viterbi Path

Viterbi Path

Posterior @ 0.4

Posterior @ 0.4

Posterior Graphic. Threshold Marked @ 0.4

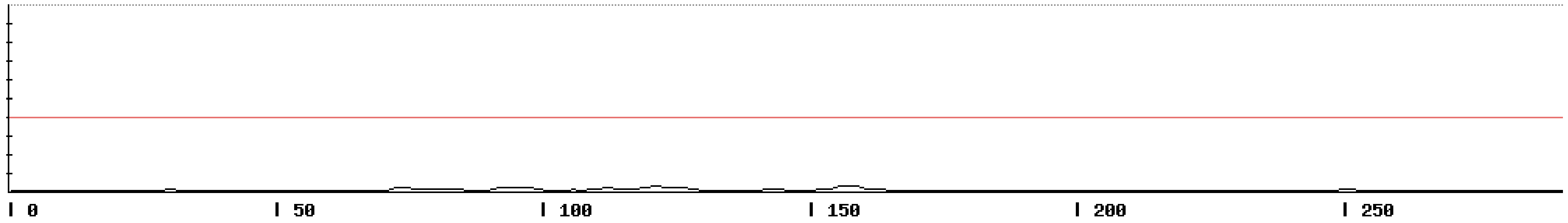

Predictions for Manihotesculenta_cassava4.1_033374m

Viterbi Path

Viterbi Path

Posterior @ 0.4

Posterior @ 0.4

Posterior Graphic. Threshold Marked @ 0.4

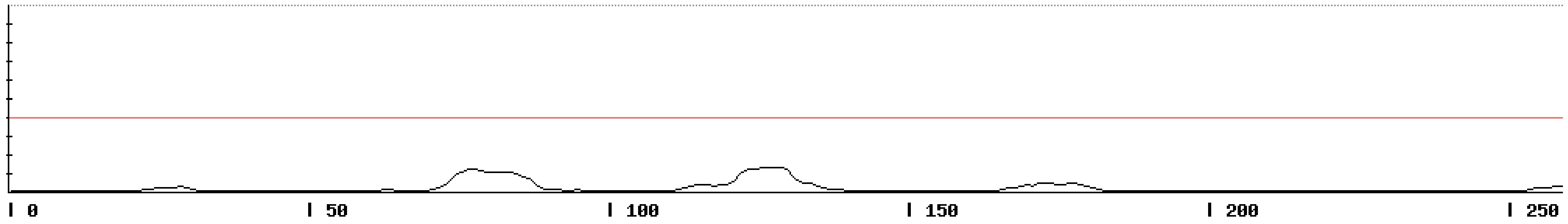

Revision r.9 - Last Updated on : February 24 2015 17:45:20.

alex.nguyenba@utoronto.ca

Intended to be used with Firefox

Viterbi Path

Posterior @ 0.4

Posterior @ 0.4

Posterior Graphic. Threshold Marked @ 0.4

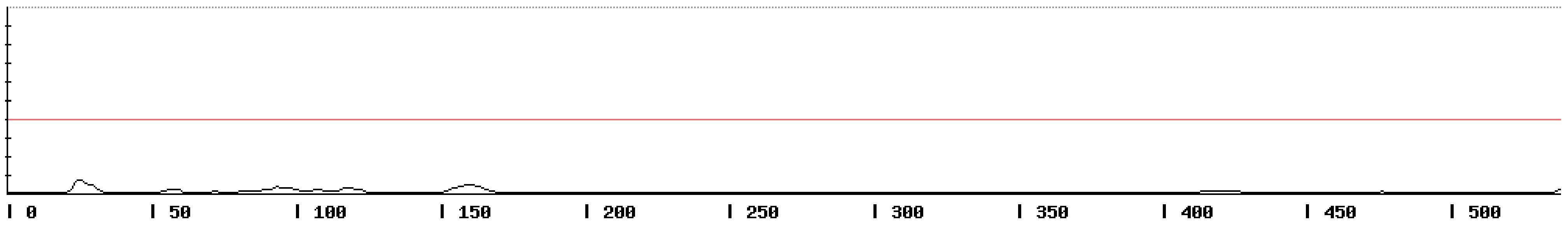

Predictions for Medicagotruncatula_Medtr1g008740.1

Viterbi Path

Viterbi Path

Posterior @ 0.4

Posterior @ 0.4

Posterior Graphic. Threshold Marked @ 0.4

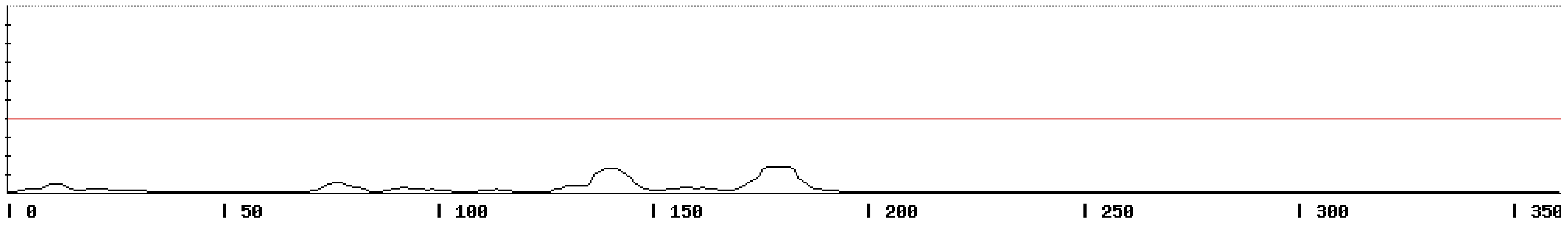

Predictions for Medicagotruncatula_Medtr1g045470.1

Viterbi Path

Viterbi Path

Posterior @ 0.4

Posterior @ 0.4

Posterior Graphic. Threshold Marked @ 0.4

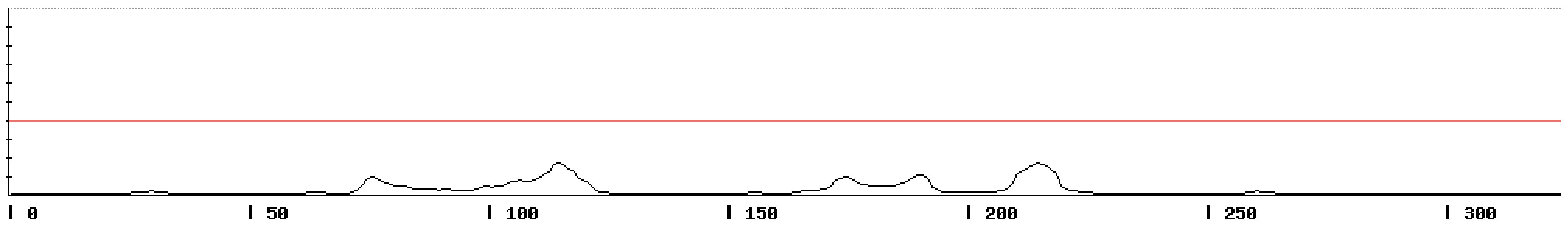

Predictions for Medicagotruncatula_Medtr1g053575.1

Viterbi Path

Viterbi Path

Posterior @ 0.4

Posterior @ 0.4

Posterior Graphic. Threshold Marked @ 0.4

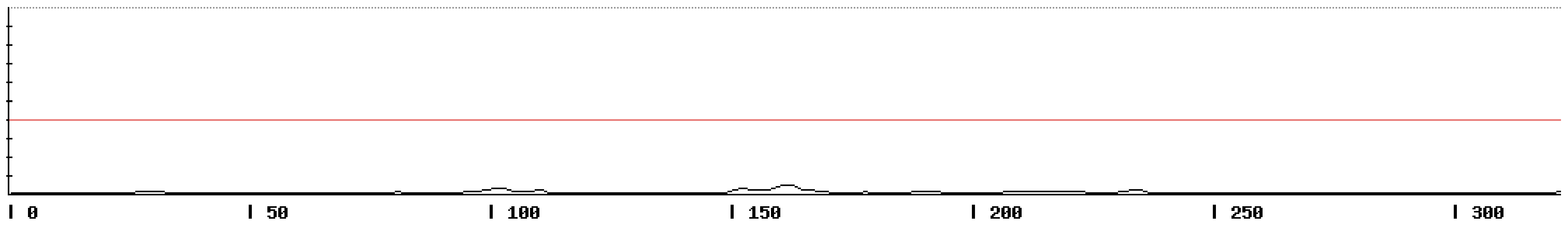

Predictions for Medicagotruncatula_Medtr1g069805.1

Viterbi Path

Viterbi Path

Posterior @ 0.4

Posterior @ 0.4

Posterior Graphic. Threshold Marked @ 0.4

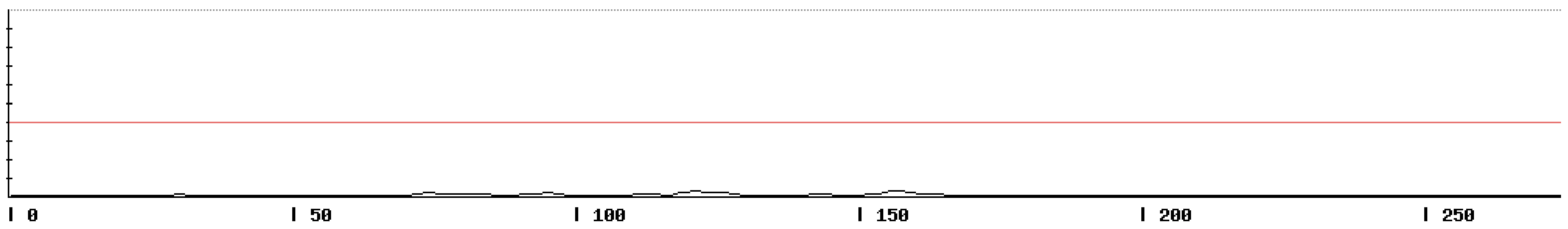

Predictions for Medicagotruncatula_Medtr1g087190.1

Viterbi Path

Viterbi Path

Posterior @ 0.4

Posterior @ 0.4

Posterior Graphic. Threshold Marked @ 0.4

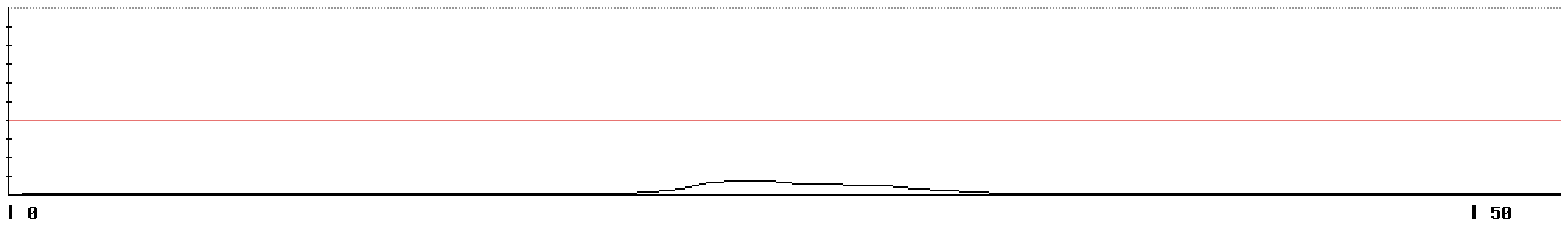

Predictions for Medicagotruncatula_Medtr1g090720.1

Viterbi Path

Viterbi Path

Posterior @ 0.4

Posterior @ 0.4

Posterior Graphic. Threshold Marked @ 0.4

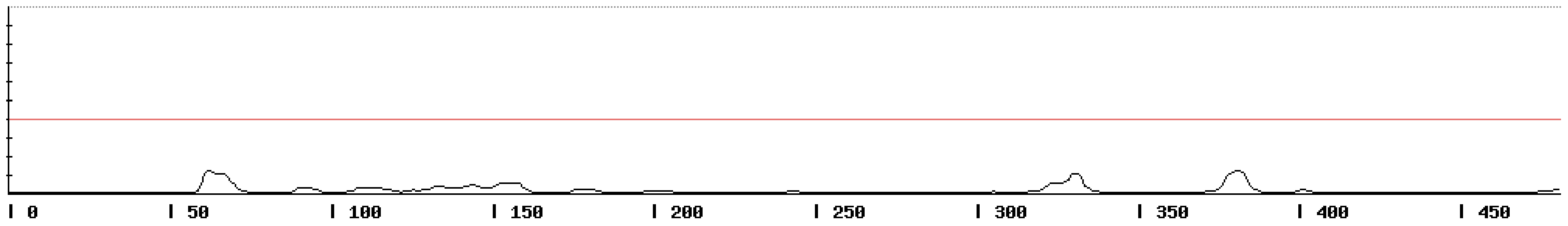

Predictions for Medicagotruncatula_Medtr1g090723.1

Viterbi Path

Viterbi Path

Posterior @ 0.4

Posterior @ 0.4

Posterior Graphic. Threshold Marked @ 0.4

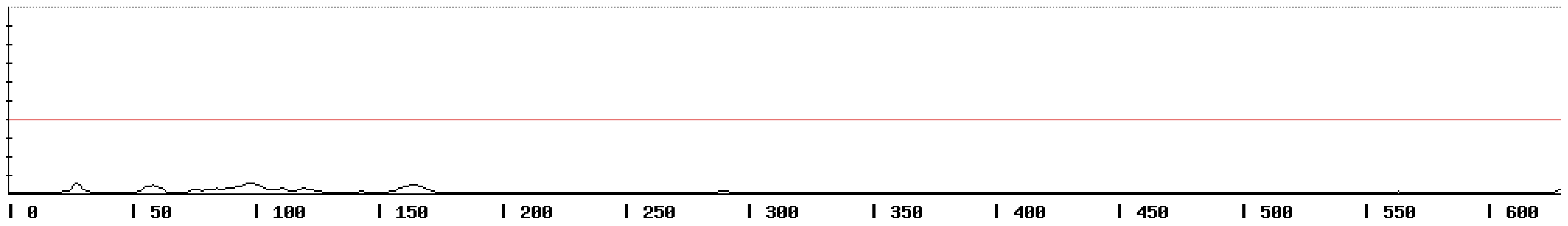

Predictions for Medicagotruncatula_Medtr1g093670.1

Viterbi Path

Viterbi Path

Posterior @ 0.4

102 - KR - 103

Posterior @ 0.4

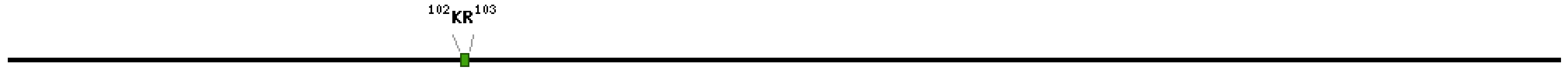

Posterior Graphic. Threshold Marked @ 0.4

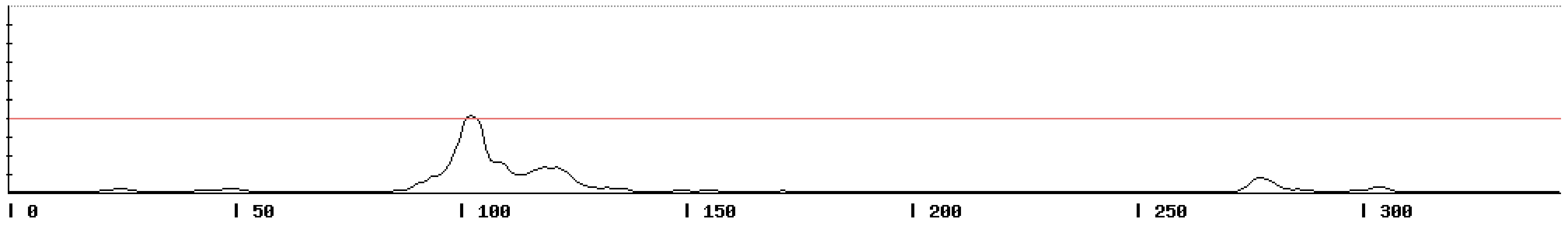

Predictions for Medicagotruncatula_Medtr1g093680.1

Viterbi Path

Viterbi Path

Posterior @ 0.4

Posterior @ 0.4

Posterior Graphic. Threshold Marked @ 0.4

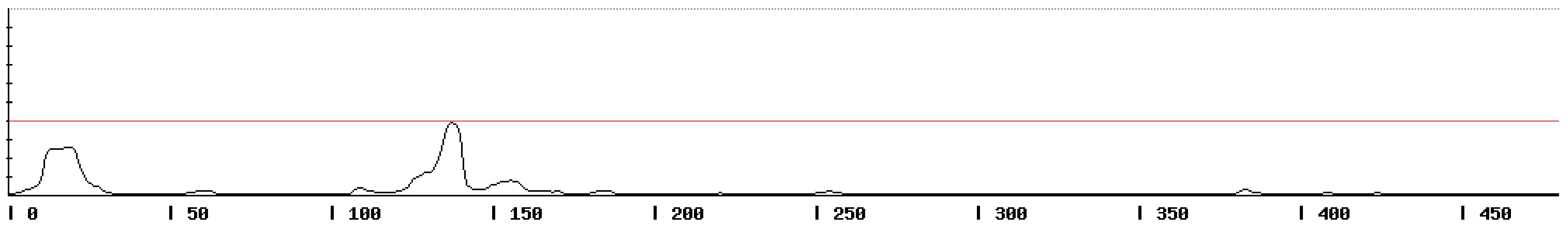

Predictions for Medicagotruncatula_Medtr1g096430.1

Viterbi Path

Viterbi Path

Posterior @ 0.4

Posterior @ 0.4

Posterior Graphic. Threshold Marked @ 0.4

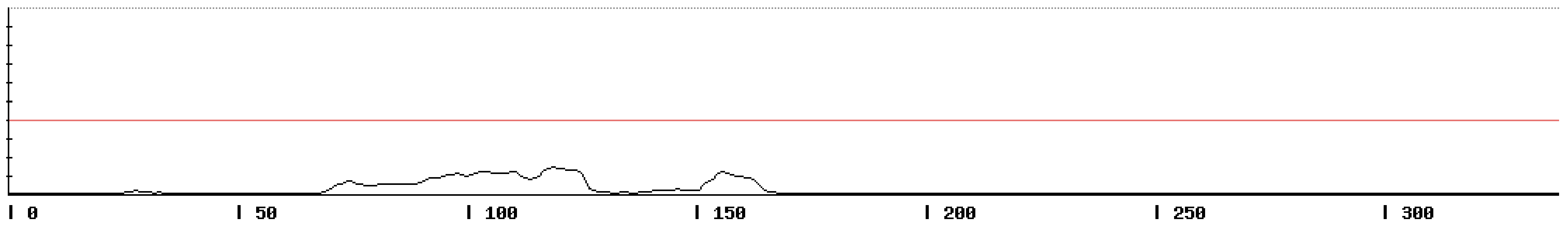

Predictions for Medicagotruncatula_Medtr1g097300.1

Viterbi Path

Viterbi Path

Posterior @ 0.4	
Posterior @ 0.4	
Posterior Graphic. Threshold Marked @ 0.4	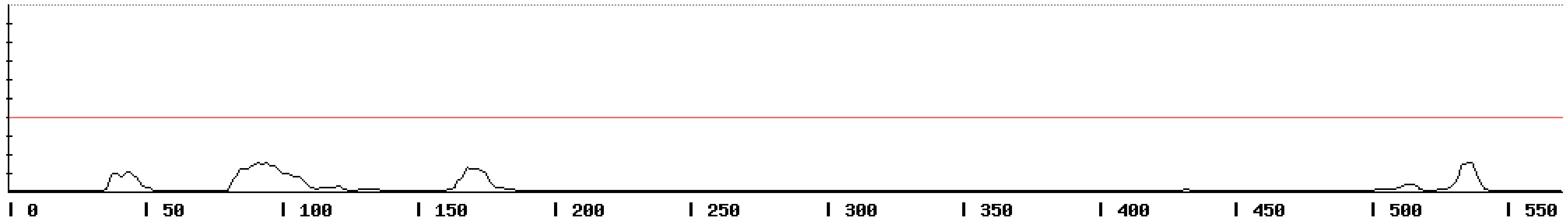

Predictions for Medicagotruncatula_Medtr2157s0010.1

Viterbi Path	
Viterbi Path	
Posterior @ 0.4	96 - KPIRASRSRKENIGVKKALVFYKGKPPKG - 124
Posterior @ 0.4	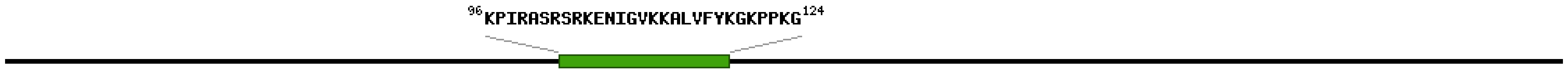
Posterior Graphic. Threshold Marked @ 0.4	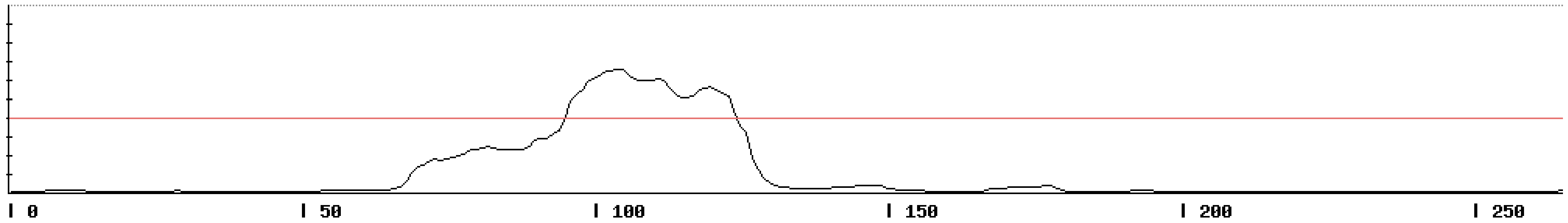

Predictions for Medicagotruncatula_Medtr2g014680.1

Viterbi Path	
Viterbi Path	

Posterior @ 0.4 — 132 - KRR - 134

Posterior @ 0.4

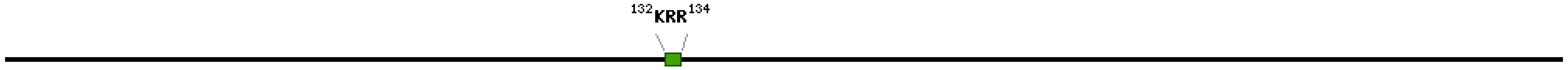

Posterior Graphic. Threshold Marked @ 0.4

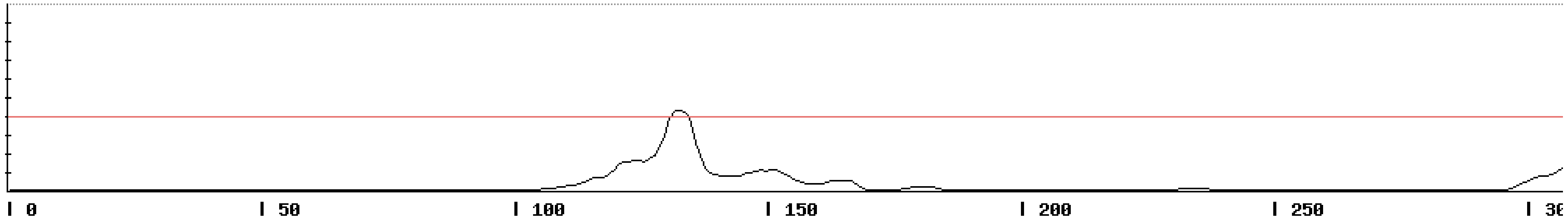

Predictions for Medicagotruncatula_Medtr2g062730.1

Viterbi Path

Viterbi Path

Posterior @ 0.4

Posterior @ 0.4

Posterior Graphic. Threshold Marked @ 0.4

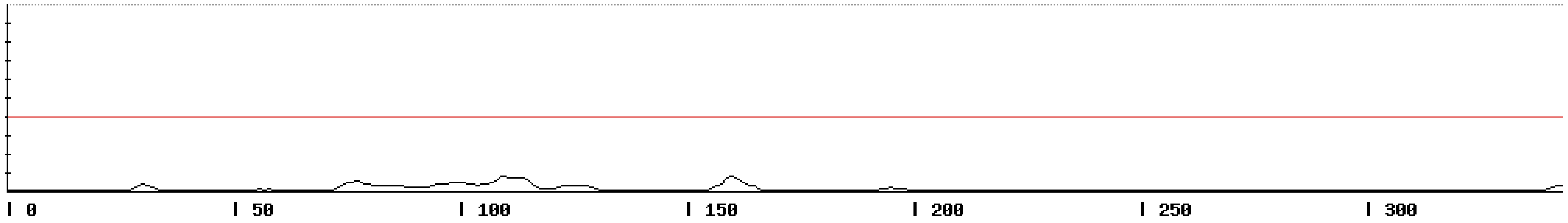

Predictions for Medicagotruncatula_Medtr2g064090.1

Viterbi Path

Viterbi Path

Posterior @ 0.4

Posterior @ 0.4

Posterior Graphic. Threshold Marked @ 0.4

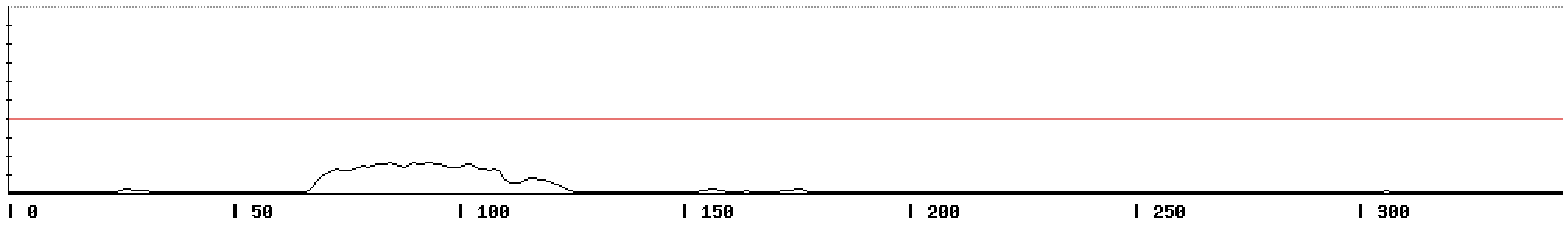

Predictions for Medicagotruncatula_Medtr2g064470.1

Viterbi Path

Viterbi Path

Posterior @ 0.4

Posterior @ 0.4

Posterior Graphic. Threshold Marked @ 0.4

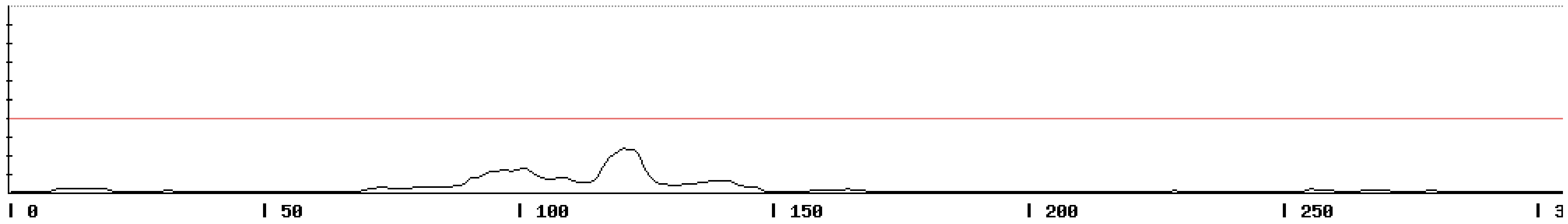

Predictions for Medicagotruncatula_Medtr2g068880.1	
Viterbi Path	
Viterbi Path	
Posterior @ 0.4	
Posterior @ 0.4	
Posterior Graphic. Threshold Marked @ 0.4	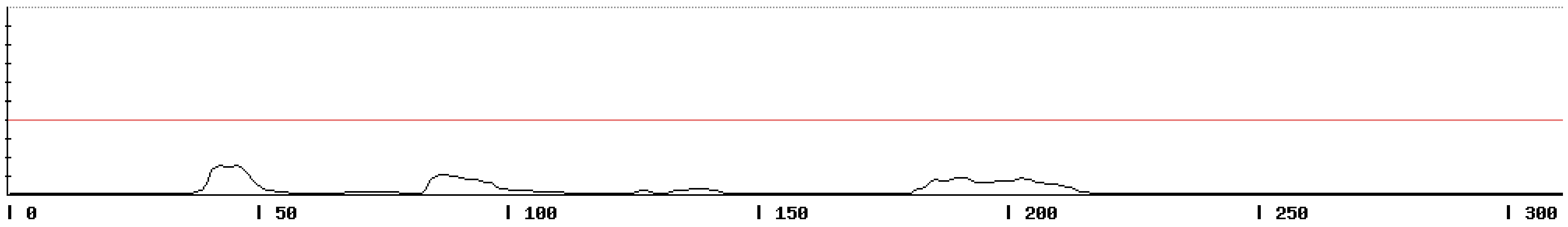

Predictions for Medicagotruncatula_Medtr2g068920.1	
Viterbi Path	
Viterbi Path	
Posterior @ 0.4	
Posterior @ 0.4	
Posterior Graphic. Threshold Marked @ 0.4	

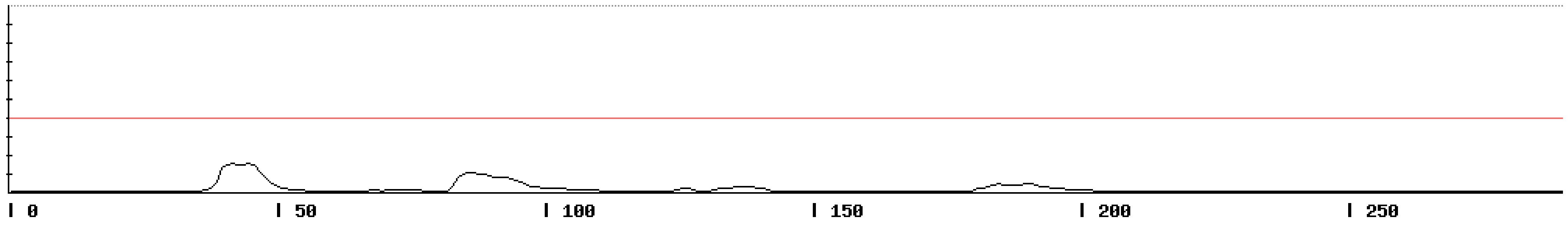

Predictions for Medicagotruncatula_Medtr2g078700.1

Viterbi Path

Viterbi Path

Posterior @ 0.4

Posterior @ 0.4

Posterior Graphic. Threshold Marked @ 0.4

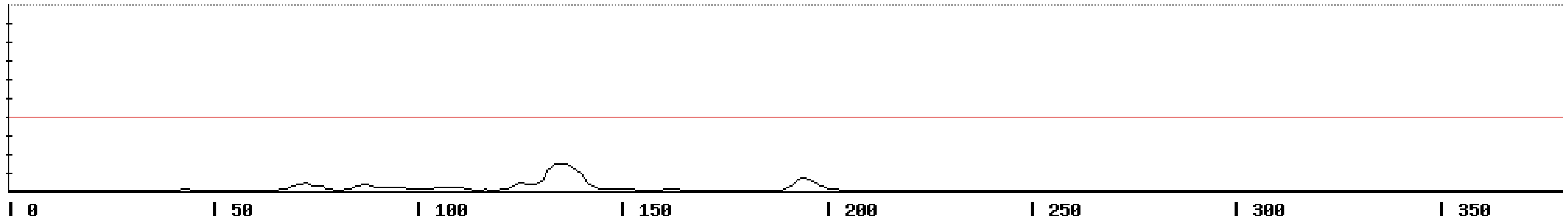

Predictions for Medicagotruncatula_Medtr2g079990.1

Viterbi Path

Viterbi Path

Posterior @ 0.4

Posterior @ 0.4

Posterior Graphic. Threshold Marked @ 0.4

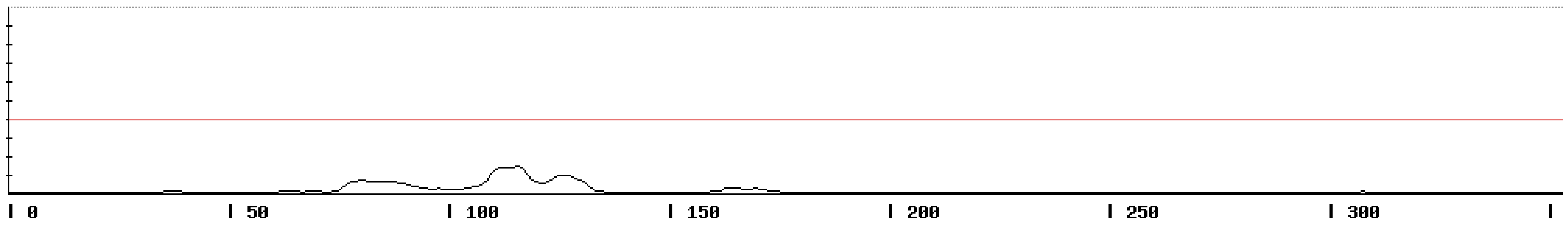

Predictions for Medicagotruncatula_Medtr2g080010.1

Viterbi Path

Viterbi Path

Posterior @ 0.4

Posterior @ 0.4

Posterior Graphic. Threshold Marked @ 0.4

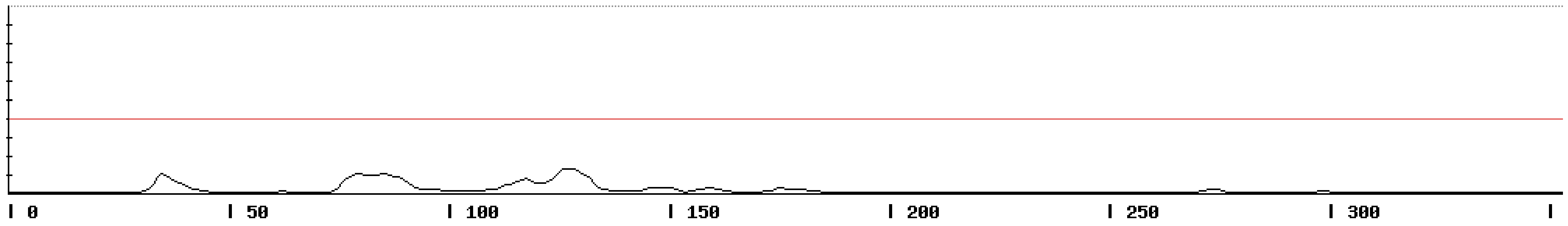

Predictions for Medicagotruncatula_Medtr2g086690.1

Viterbi Path

Viterbi Path

Posterior @ 0.4

Posterior @ 0.4

Posterior Graphic. Threshold Marked @ 0.4

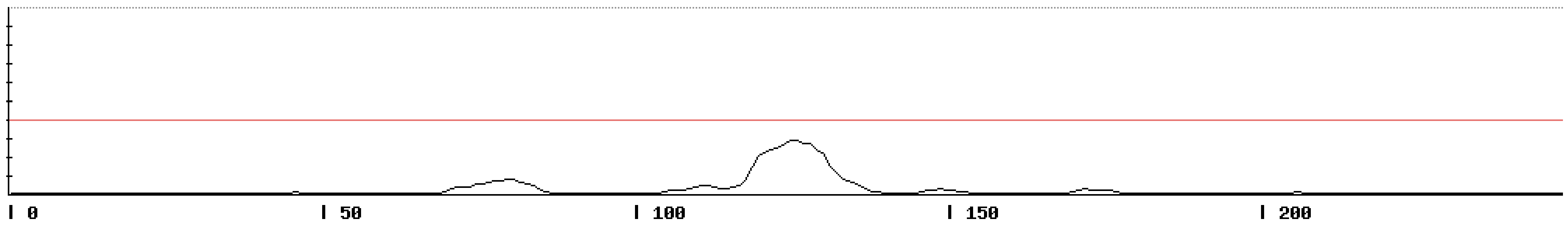

Predictions for Medicagotruncatula_Medtr2g086880.1

Viterbi Path

Viterbi Path

Posterior @ 0.4

Posterior @ 0.4

Posterior Graphic. Threshold Marked @ 0.4

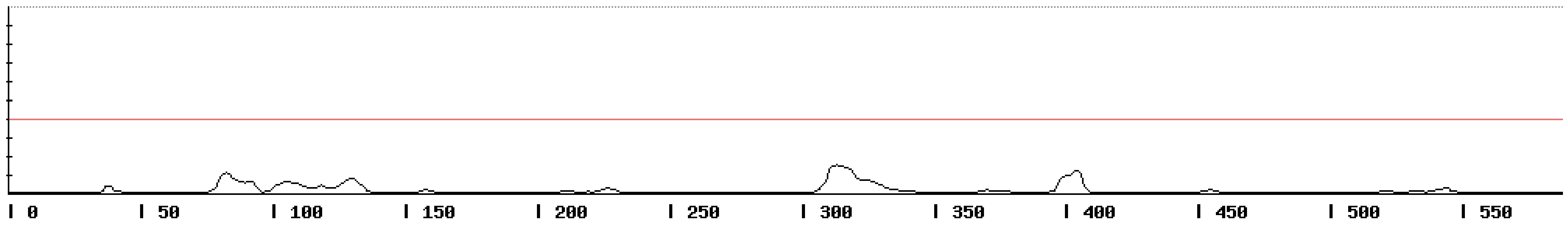

Predictions for Medicagotruncatula_Medtr2g090735.1

Viterbi Path

Viterbi Path

Posterior @ 0.4	127 - RKRRK - 131
Posterior @ 0.4	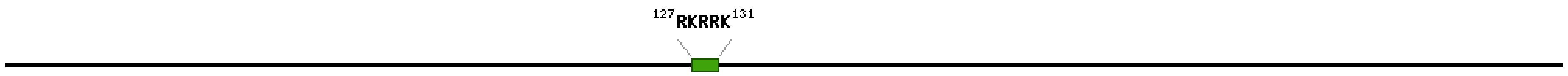
Posterior Graphic. Threshold Marked @ 0.4	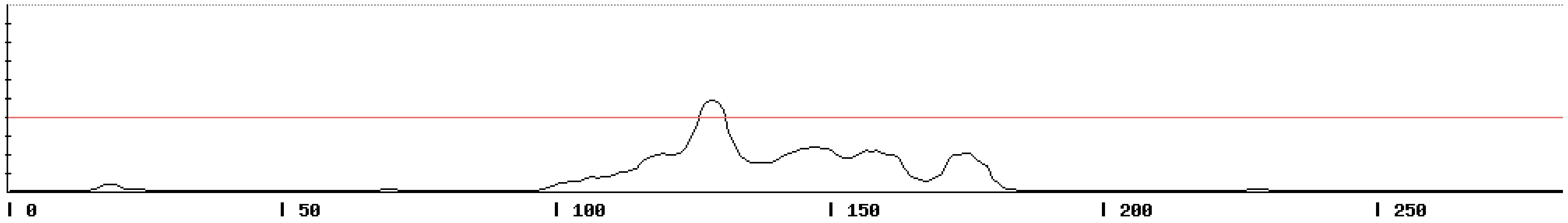

Predictions for Medicagotruncatula_Medtr2g093810.1

Viterbi Path	
Viterbi Path	
Posterior @ 0.4	
Posterior @ 0.4	
Posterior Graphic. Threshold Marked @ 0.4	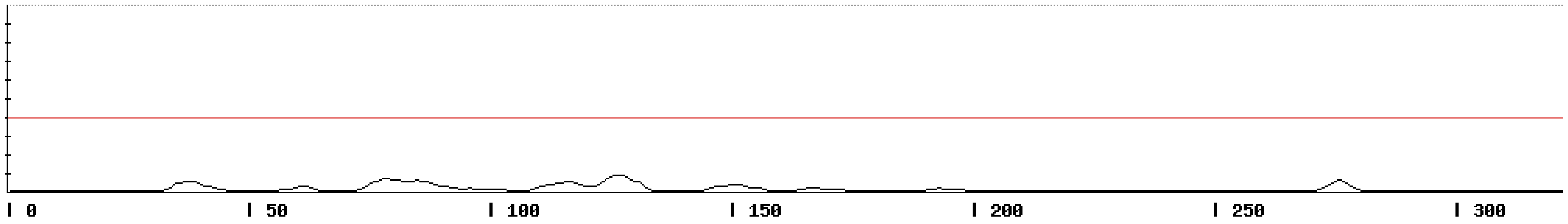

Predictions for Medicagotruncatula_Medtr3g064580.1

Viterbi Path	
Viterbi Path	

Posterior @ 0.4

Posterior @ 0.4

Posterior Graphic. Threshold Marked @ 0.4

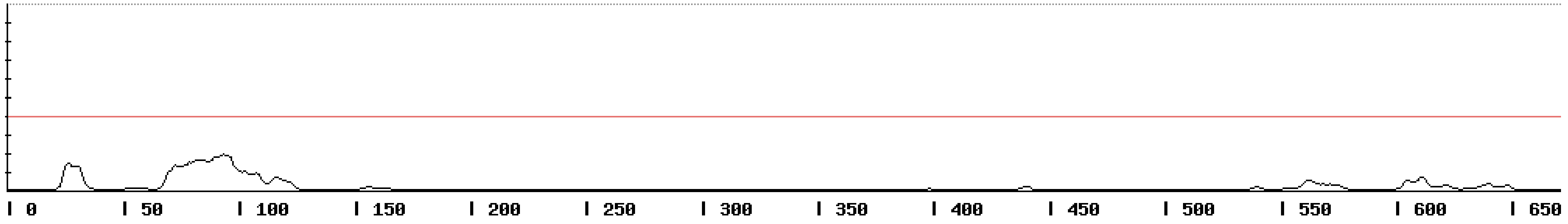

Predictions for Medicagotruncatula_Medtr3g070030.1

Viterbi Path

Viterbi Path

Posterior @ 0.4

Posterior @ 0.4

Posterior Graphic. Threshold Marked @ 0.4

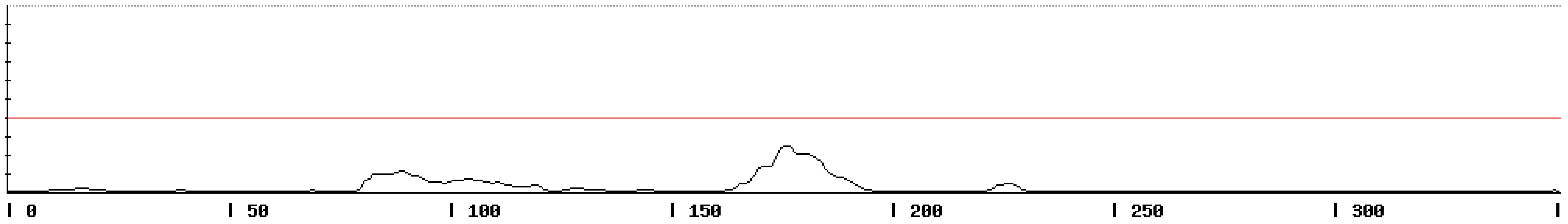

Predictions for Medicagotruncatula_Medtr3g070040.1

Viterbi Path	
Viterbi Path	
Posterior @ 0.4	163 - KK - 164
Posterior @ 0.4	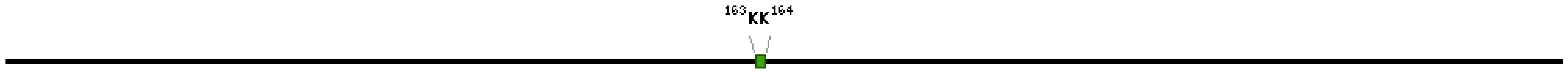
Posterior Graphic. Threshold Marked @ 0.4	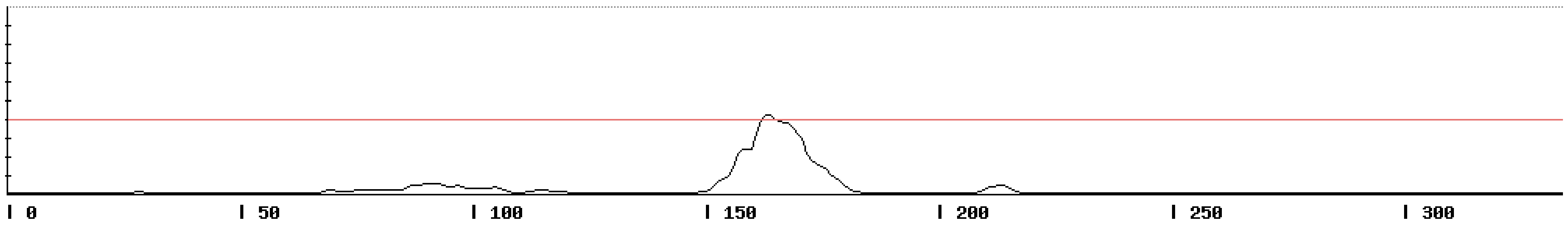

Predictions for Medicagotruncatula_Medtr3g088110.1

Viterbi Path	
Viterbi Path	
Posterior @ 0.4	
Posterior @ 0.4	
Posterior Graphic. Threshold Marked @ 0.4	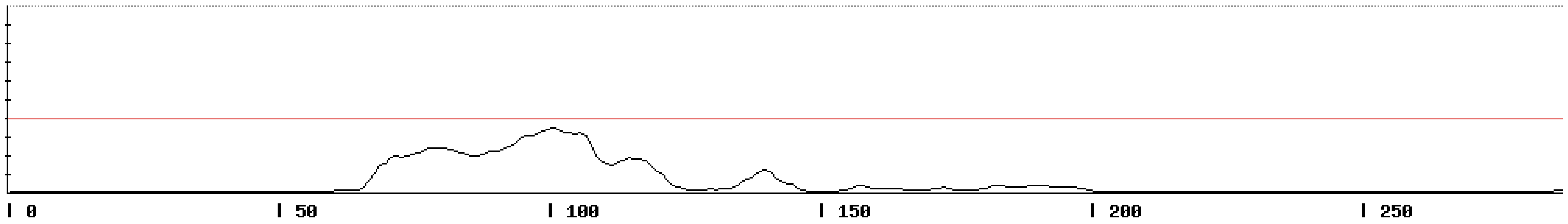

Predictions for Medicagotruncatula_Medtr3g093040.1	
Viterbi Path	
Viterbi Path	
Posterior @ 0.4	23 - RKNK - 26
Posterior @ 0.4	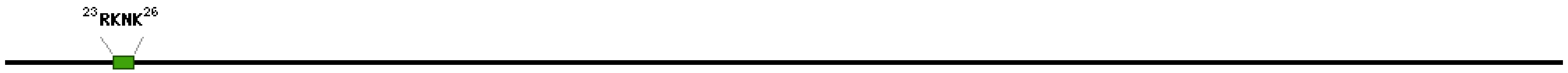
Posterior Graphic. Threshold Marked @ 0.4	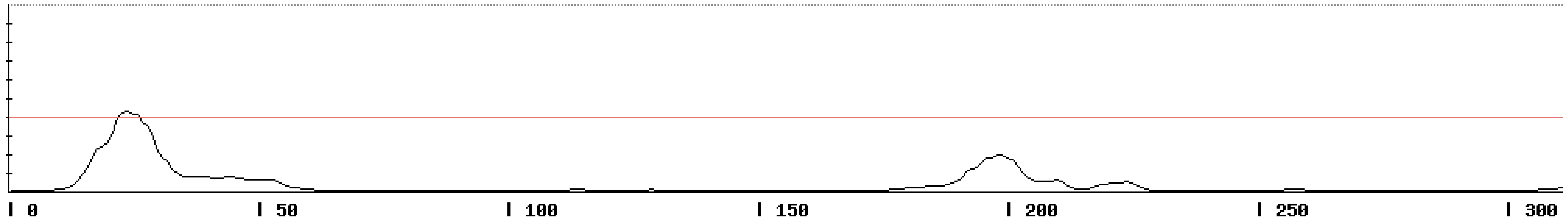

Predictions for Medicagotruncatula_Medtr3g093050.1	
Viterbi Path	
Viterbi Path	
Posterior @ 0.4	
Posterior @ 0.4	
Posterior Graphic. Threshold Marked @ 0.4	

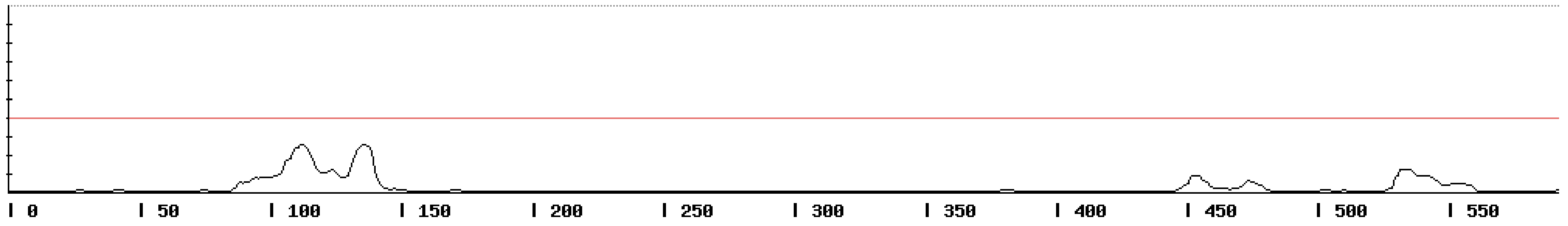

Predictions for Medicagotruncatula_Medtr3g096140.1

Viterbi Path	
Viterbi Path	
Posterior @ 0.4	
Posterior @ 0.4	
Posterior Graphic. Threshold Marked @ 0.4	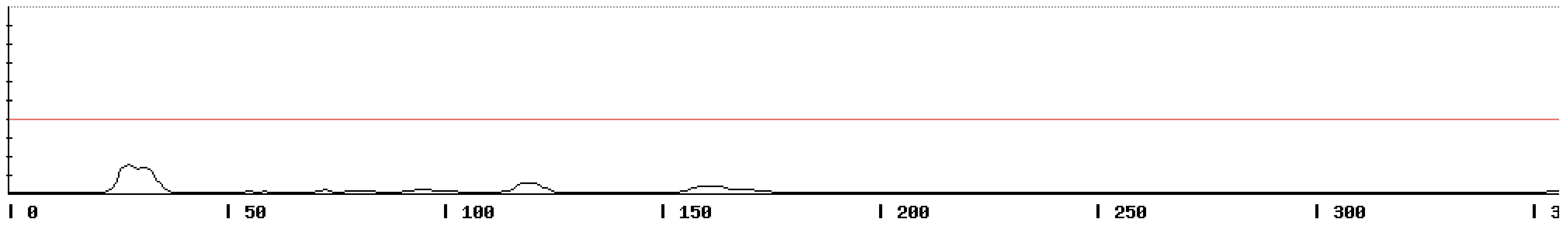

Predictions for Medicagotruncatula_Medtr3g096400.1

Viterbi Path	
Viterbi Path	
Posterior @ 0.4	
Posterior @ 0.4	

Posterior Graphic. Threshold Marked @ 0.4

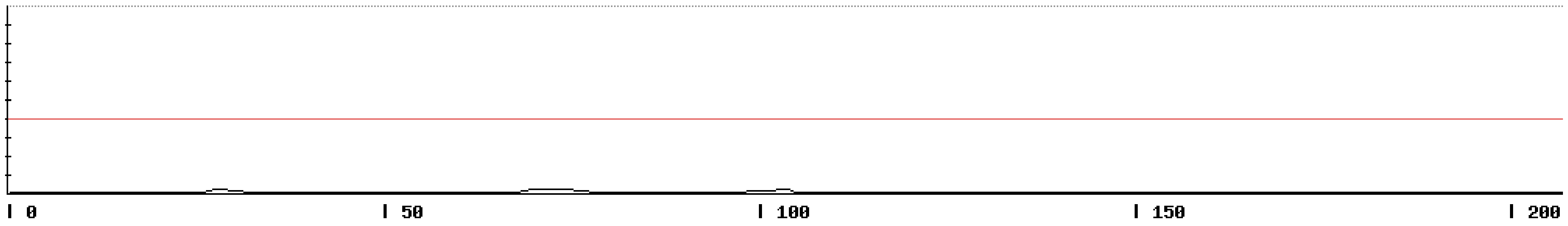

Predictions for Medicagotruncatula_Medtr3g096920.1

Viterbi Path

Viterbi Path

Posterior @ 0.4

95 - KPIGKLKPMGIK - 106

Posterior @ 0.4

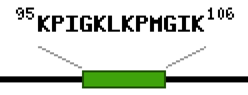

Posterior Graphic. Threshold Marked @ 0.4

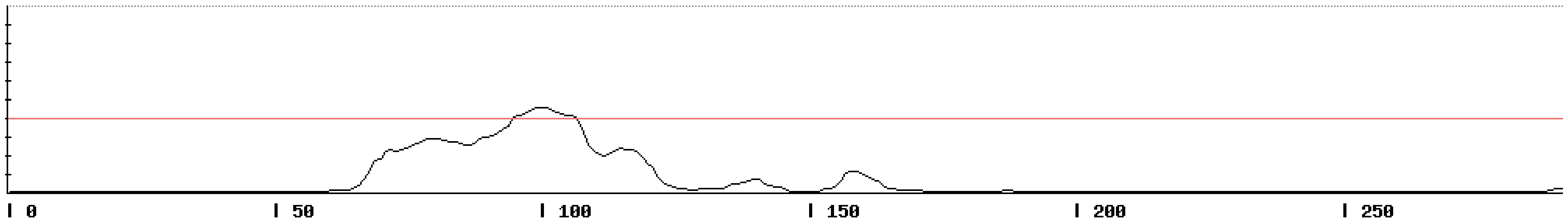

Predictions for Medicagotruncatula_Medtr3g098810.1

Viterbi Path

Viterbi Path

Posterior @ 0.4

Posterior @ 0.4

Posterior Graphic. Threshold Marked @ 0.4

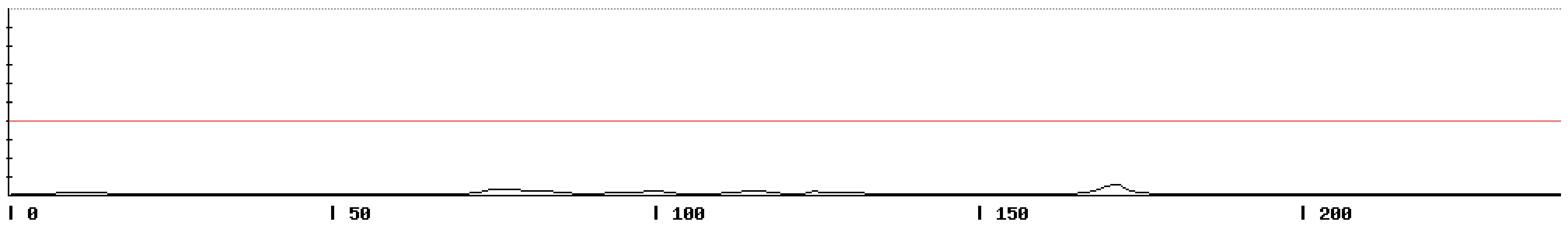

Predictions for Medicagotruncatula_Medtr3g109340.1

Viterbi Path

Viterbi Path

Posterior @ 0.4

Posterior @ 0.4

Posterior Graphic. Threshold Marked @ 0.4

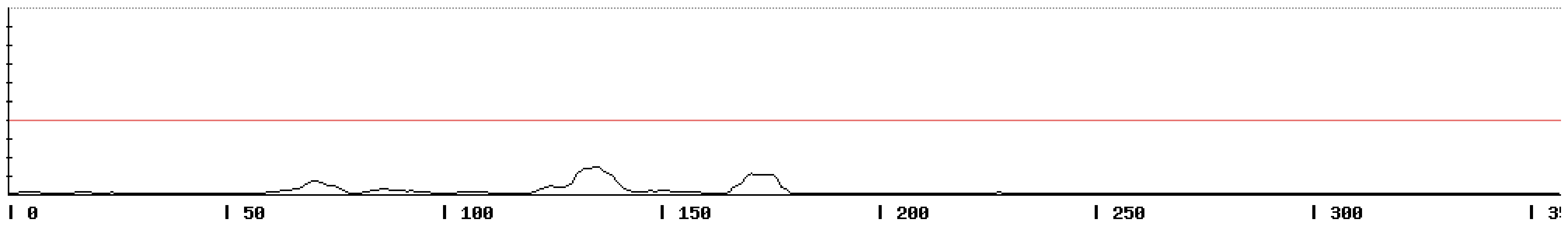

Predictions for Medicagotruncatula_Medtr3g116070.1

Viterbi Path

Viterbi Path

Posterior @ 0.4

Posterior @ 0.4

Posterior Graphic. Threshold Marked @ 0.4

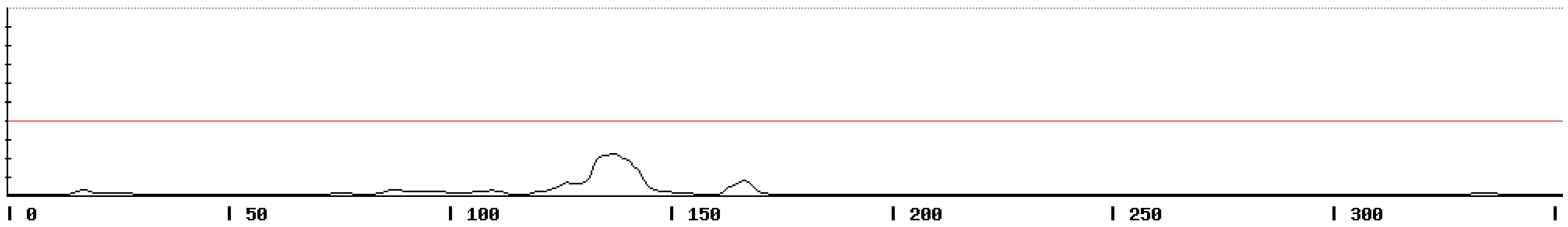

Predictions for Medicagotruncatula_Medtr3g435150.1

Viterbi Path

Viterbi Path

Posterior @ 0.4

Posterior @ 0.4

Posterior Graphic. Threshold Marked @ 0.4

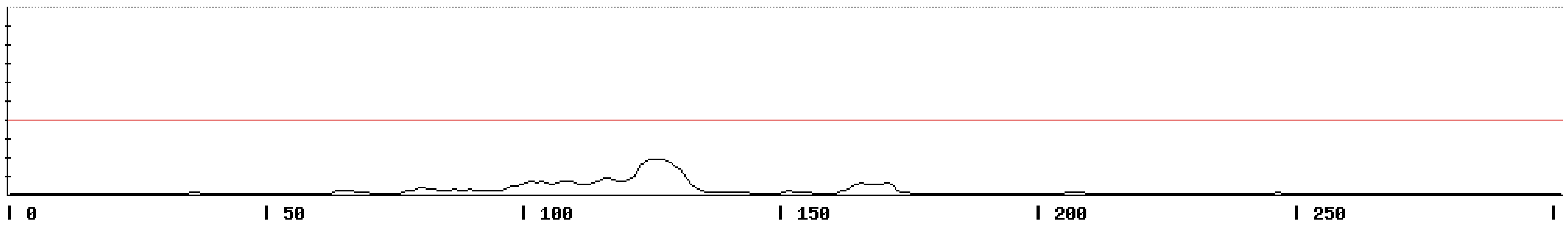

Predictions for Medicagotruncatula_Medtr4g035590.1

Viterbi Path

Viterbi Path

Posterior @ 0.4	
Posterior @ 0.4	
Posterior Graphic. Threshold Marked @ 0.4	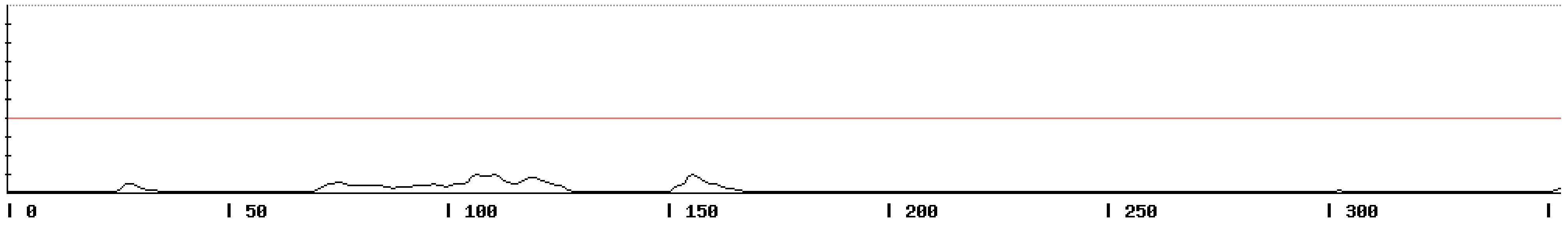

Predictions for Medicagotruncatula_Medtr4g036030.1

Viterbi Path	
Viterbi Path	
Posterior @ 0.4	
Posterior @ 0.4	
Posterior Graphic. Threshold Marked @ 0.4	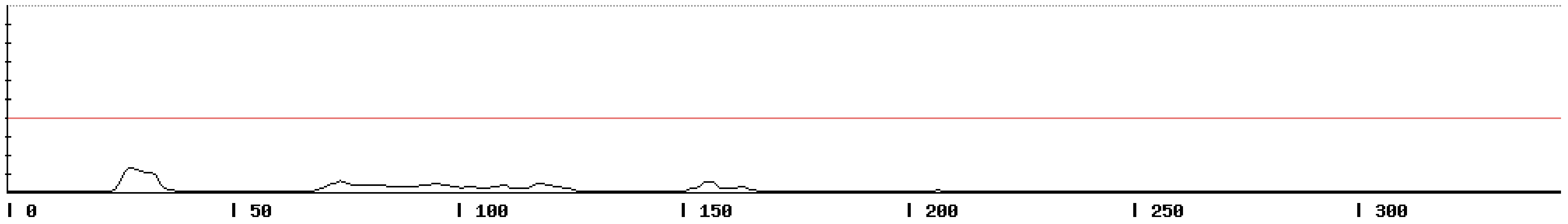

Predictions for Medicagotruncatula_Medtr4g052620.1

Viterbi Path	
Viterbi Path	
Posterior @ 0.4	122 - PSGRK - 126
Posterior @ 0.4	(graphic below)
Posterior Graphic. Threshold Marked @ 0.4	(graphic below)

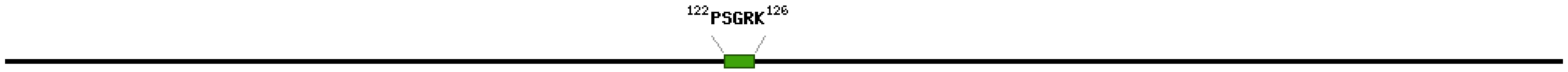

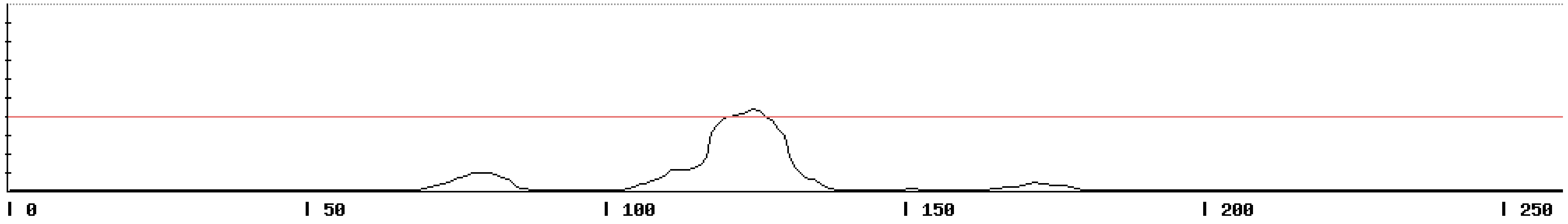

Predictions for Medicagotruncatula_Medtr4g075980.1

Viterbi Path	
Viterbi Path	
Posterior @ 0.4	
Posterior @ 0.4	
Posterior Graphic. Threshold Marked @ 0.4	

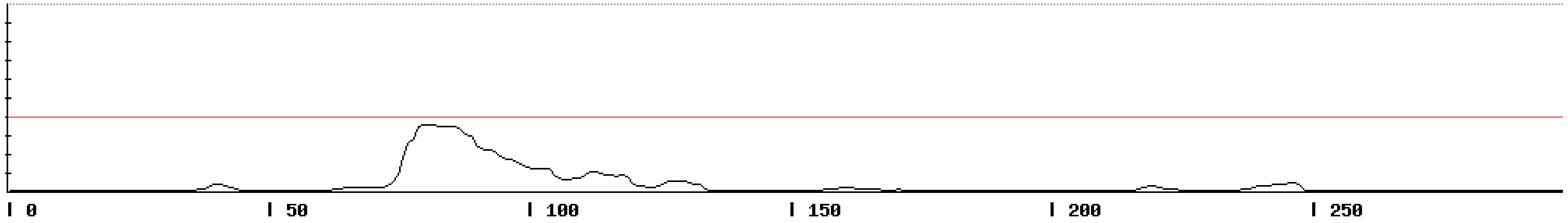

Predictions for Medicagotruncatula_Medtr4g078875.1	
Viterbi Path	
Viterbi Path	
Posterior @ 0.4	126 - RKRRK - 130
Posterior @ 0.4	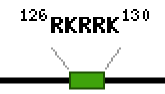
Posterior Graphic. Threshold Marked @ 0.4	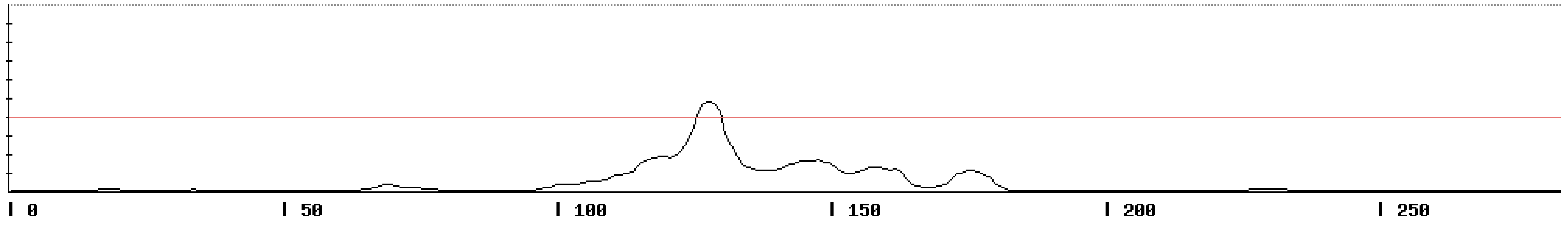

Predictions for Medicagotruncatula_Medtr4g081870.1	
Viterbi Path	
Viterbi Path	
Posterior @ 0.4	
Posterior @ 0.4	
Posterior Graphic. Threshold Marked @ 0.4	

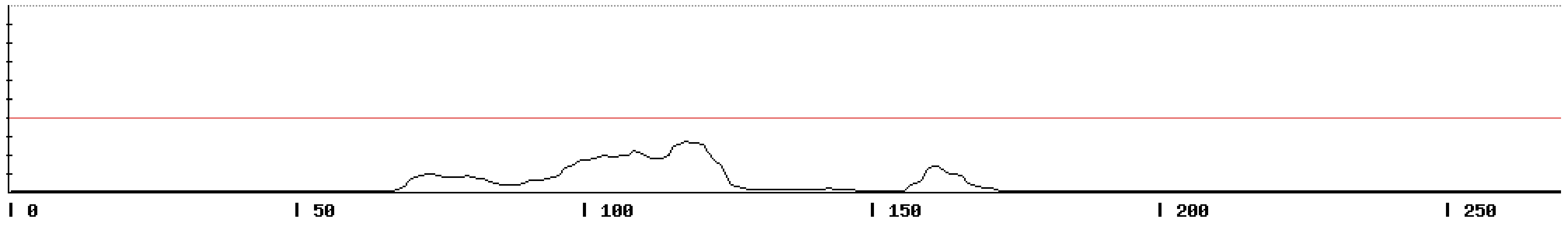

Predictions for Medicagotruncatula_Medtr4g088245.1

Viterbi Path	
Viterbi Path	
Posterior @ 0.4	146 - RKRR - 149
Posterior @ 0.4	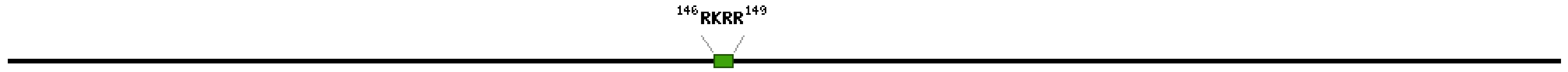
Posterior Graphic. Threshold Marked @ 0.4	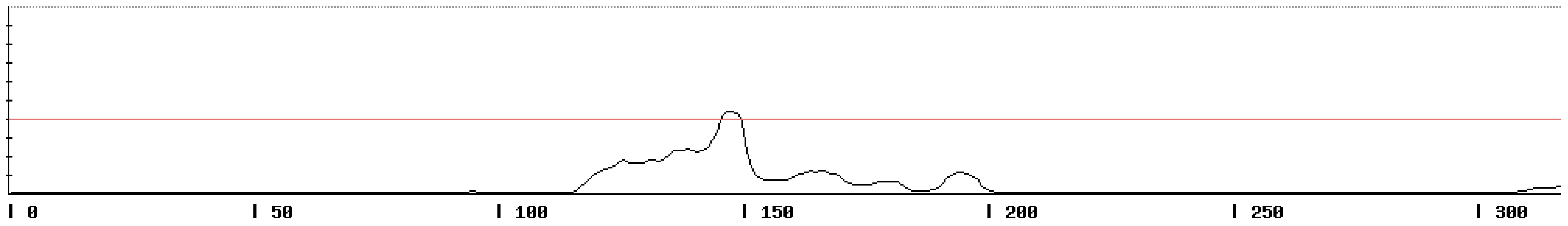

Predictions for Medicagotruncatula_Medtr4g089135.1

Viterbi Path	
Viterbi Path	
Posterior @ 0.4	
Posterior @ 0.4	

Posterior Graphic. Threshold Marked @ 0.4

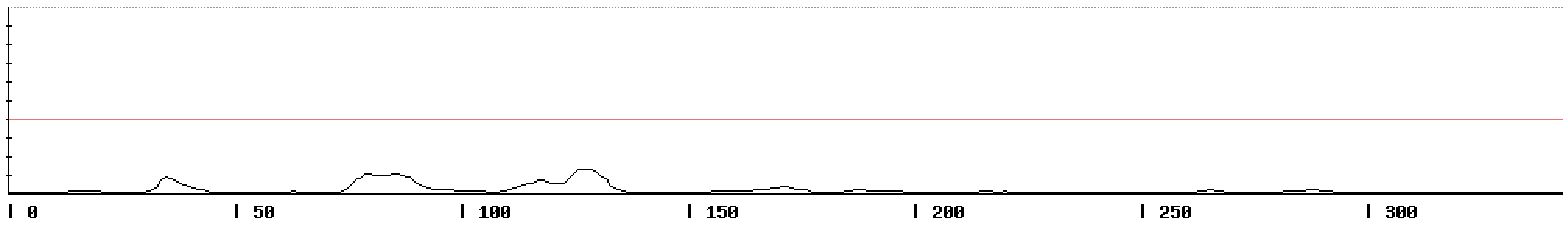

Predictions for Medicagotruncatula_Medtr4g094302.1

Viterbi Path

Viterbi Path

Posterior @ 0.4

Posterior @ 0.4

Posterior Graphic. Threshold Marked @ 0.4

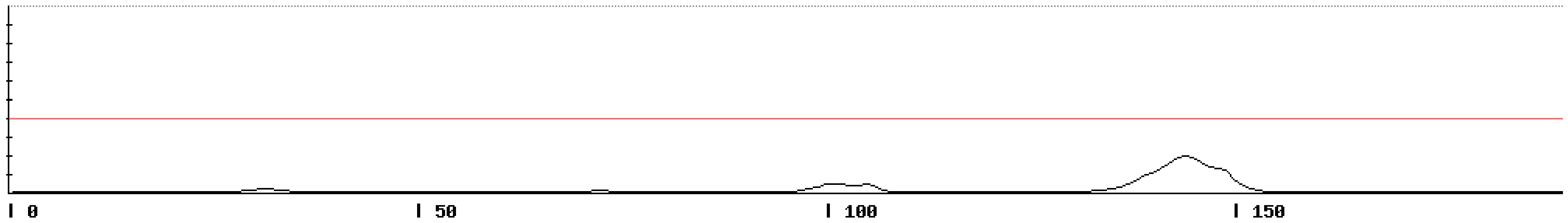

Predictions for Medicagotruncatula_Medtr4g098630.1

Viterbi Path

Viterbi Path

Posterior @ 0.4

Posterior @ 0.4

Posterior Graphic. Threshold Marked @ 0.4

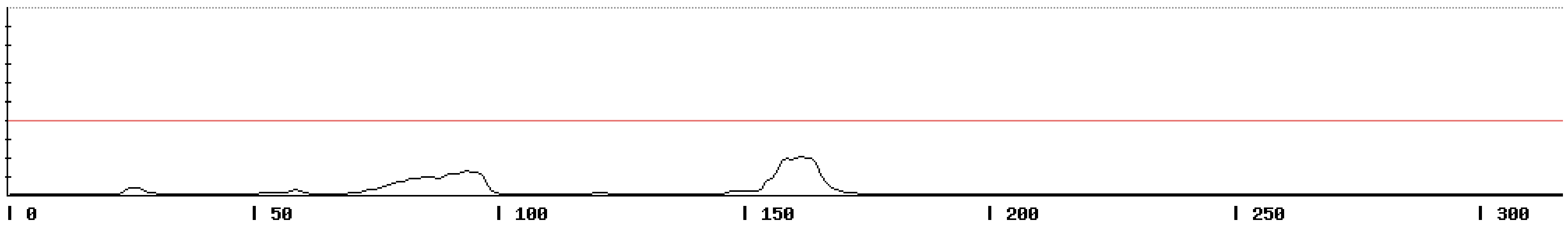

Predictions for Medicagotruncatula_Medtr4g101680.1

Viterbi Path

Viterbi Path

Posterior @ 0.4

Posterior @ 0.4

Posterior Graphic. Threshold Marked @ 0.4

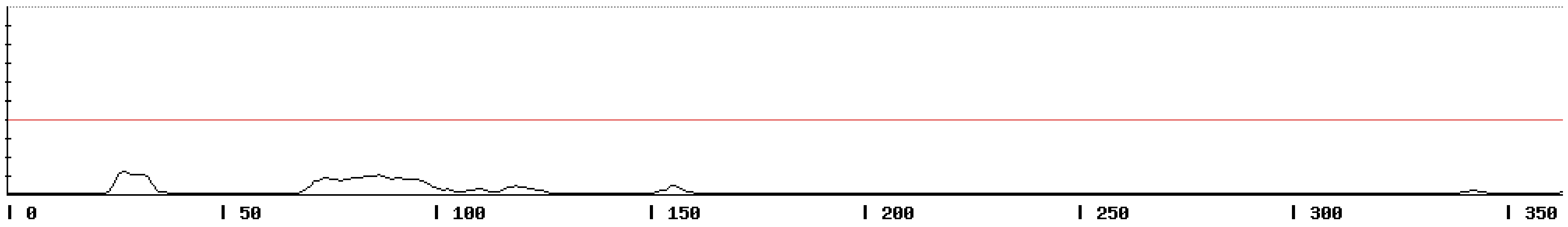

Predictions for Medicagotruncatula_Medtr4g108760.1

Viterbi Path

Viterbi Path

Posterior @ 0.4

Posterior @ 0.4

Posterior Graphic. Threshold Marked @ 0.4

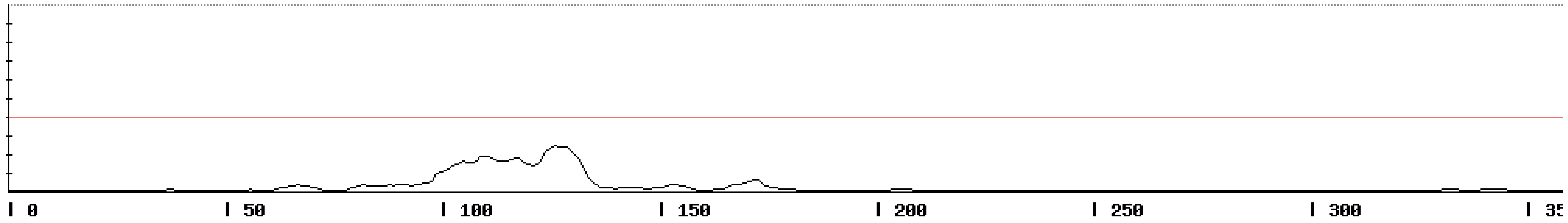

Predictions for Medicagotruncatula_Medtr4g134460.1

Viterbi Path

Viterbi Path

Posterior @ 0.4

Posterior @ 0.4

Posterior Graphic. Threshold Marked @ 0.4

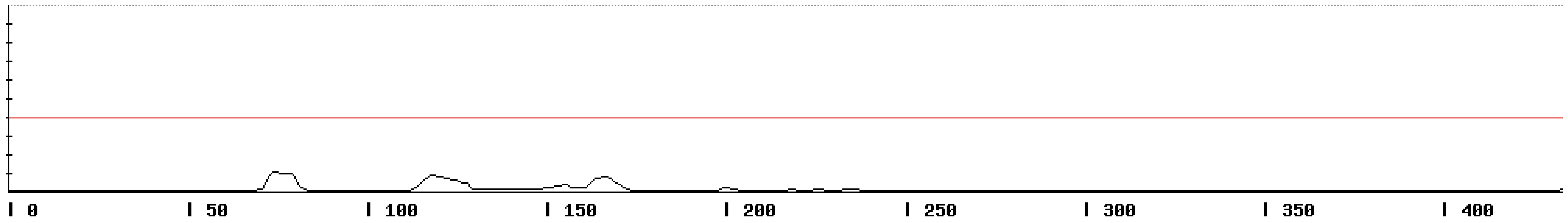

Predictions for Medicagotruncatula_Medtr5g012080.1

Viterbi Path

Viterbi Path

Posterior @ 0.4	
Posterior @ 0.4	
Posterior Graphic. Threshold Marked @ 0.4	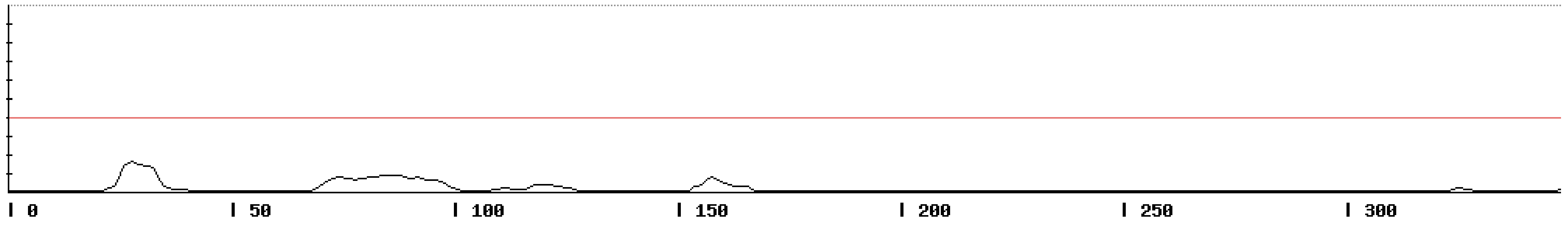

Predictions for Medicagotruncatula_Medtr5g014300.1

Viterbi Path	
Viterbi Path	
Posterior @ 0.4	
Posterior @ 0.4	
Posterior Graphic. Threshold Marked @ 0.4	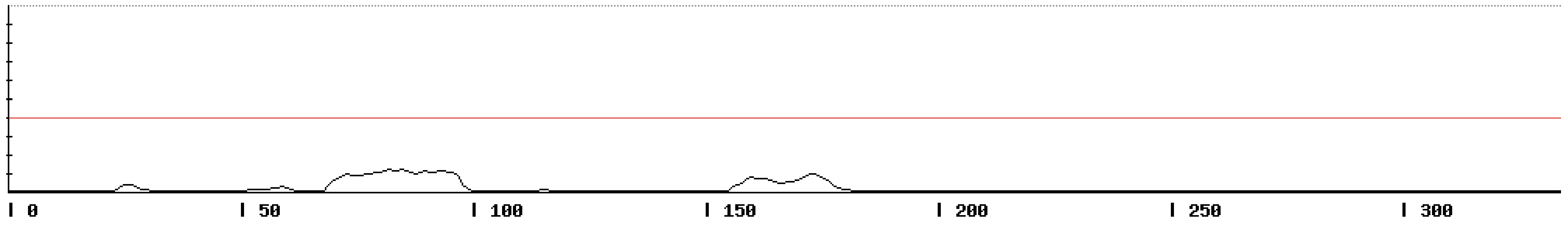

Predictions for Medicagotruncatula_Medtr5g021710.1

Viterbi Path	
Viterbi Path	
Posterior @ 0.4	
Posterior @ 0.4	
Posterior Graphic. Threshold Marked @ 0.4	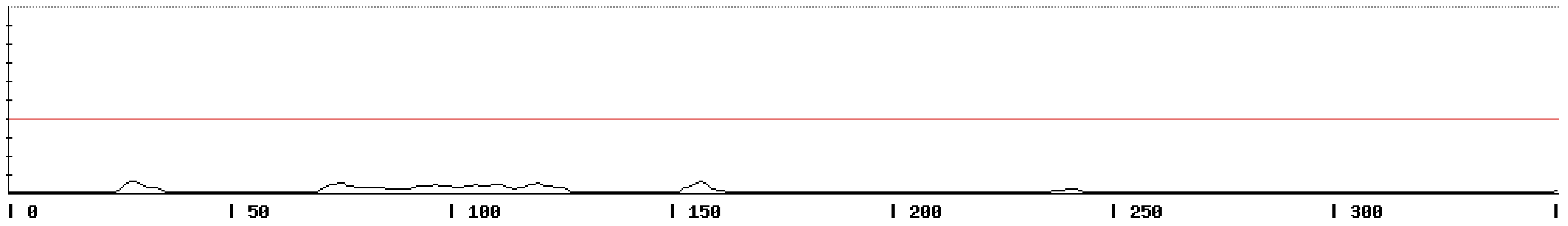

Predictions for Medicagotruncatula_Medtr5g040420.1

Viterbi Path	
Viterbi Path	
Posterior @ 0.4	
Posterior @ 0.4	
Posterior Graphic. Threshold Marked @ 0.4	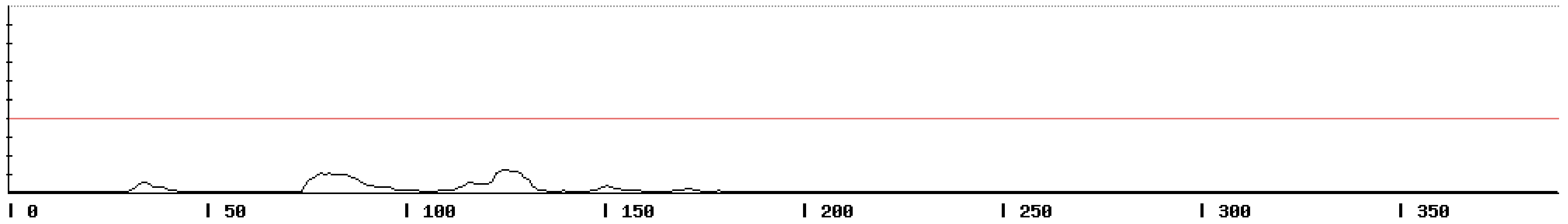

Predictions for Medicagotruncatula_Medtr5g041940.1	
Viterbi Path	
Viterbi Path	
Posterior @ 0.4	
Posterior @ 0.4	
Posterior Graphic. Threshold Marked @ 0.4	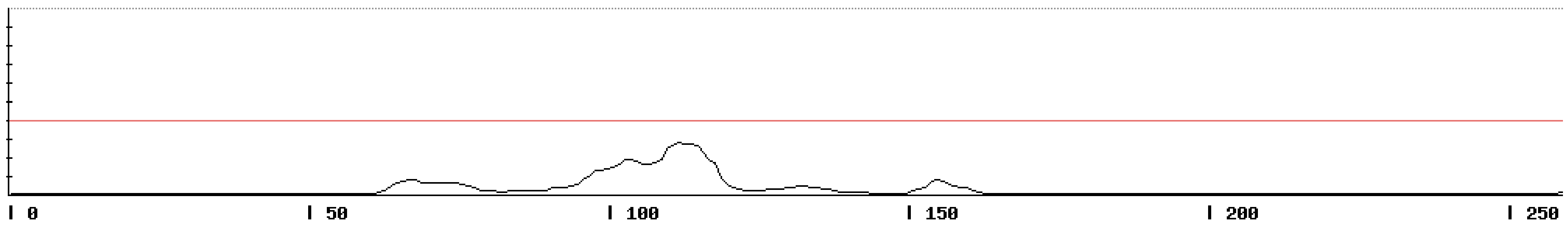

Predictions for Medicagotruncatula_Medtr5g053430.1	
Viterbi Path	
Viterbi Path	
Posterior @ 0.4	137 - KRR - 139
Posterior @ 0.4	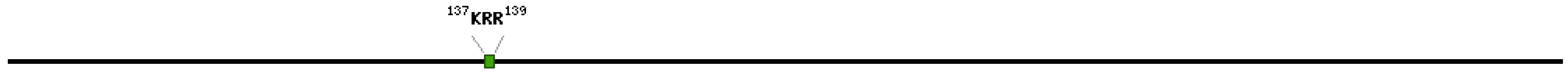
Posterior Graphic. Threshold Marked @ 0.4	

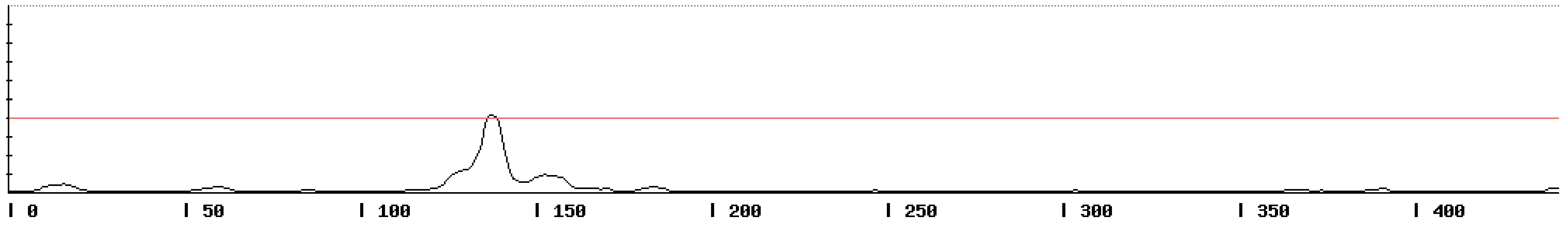

Predictions for Medicagotruncatula_Medtr5g069030.1

Viterbi Path	
Viterbi Path	
Posterior @ 0.4	
Posterior @ 0.4	
Posterior Graphic. Threshold Marked @ 0.4	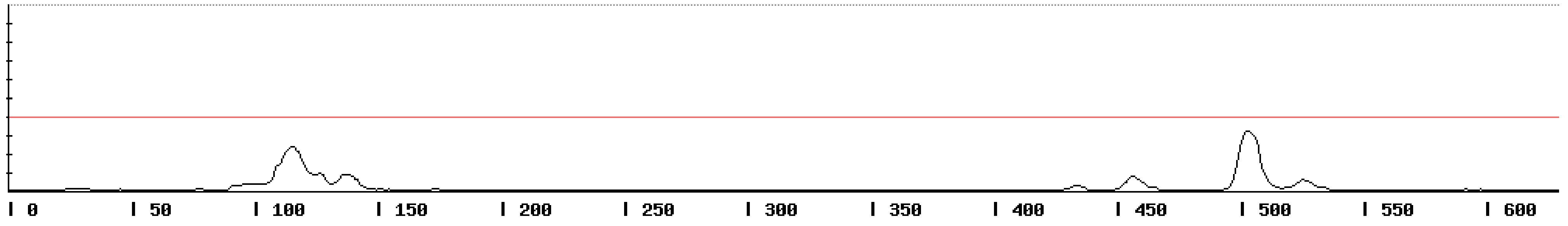

Predictions for Medicagotruncatula_Medtr5g076850.1

Viterbi Path	
Viterbi Path	
Posterior @ 0.4	
Posterior @ 0.4	

Posterior Graphic. Threshold Marked @ 0.4

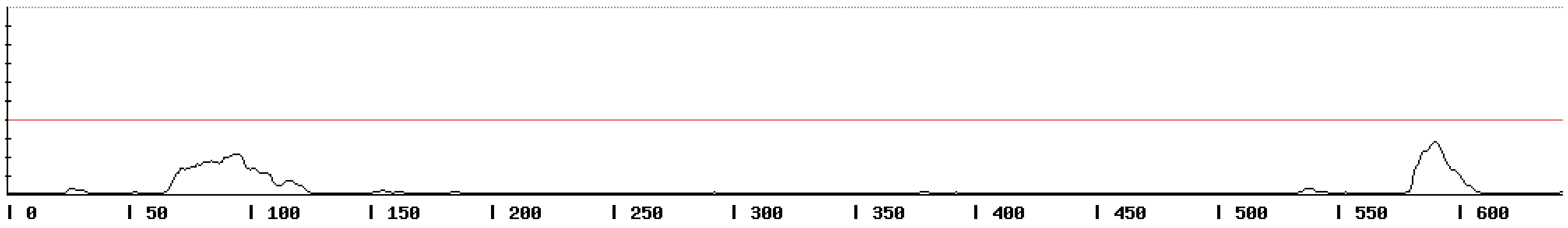

Predictions for Medicagotruncatula_Medtr5g090970.1

Viterbi Path

Viterbi Path

Posterior @ 0.4

Posterior @ 0.4

Posterior Graphic. Threshold Marked @ 0.4

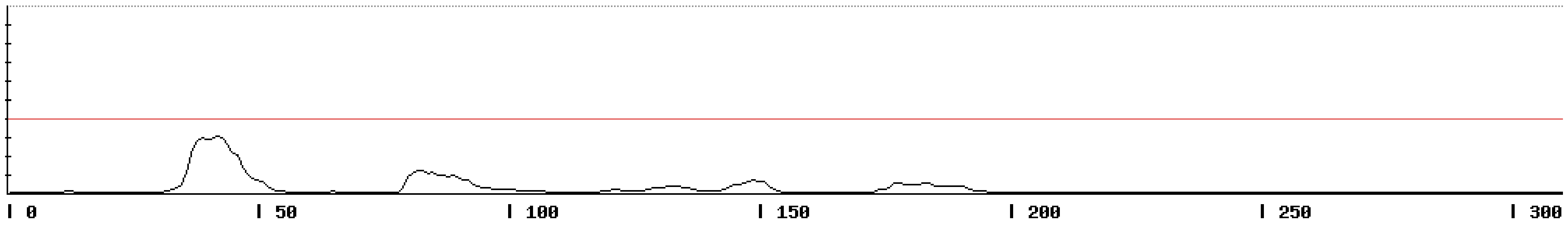

Predictions for Medicagotruncatula_Medtr6g011860.1

Viterbi Path

Viterbi Path

Posterior @ 0.4

Posterior @ 0.4

Posterior Graphic. Threshold Marked @ 0.4

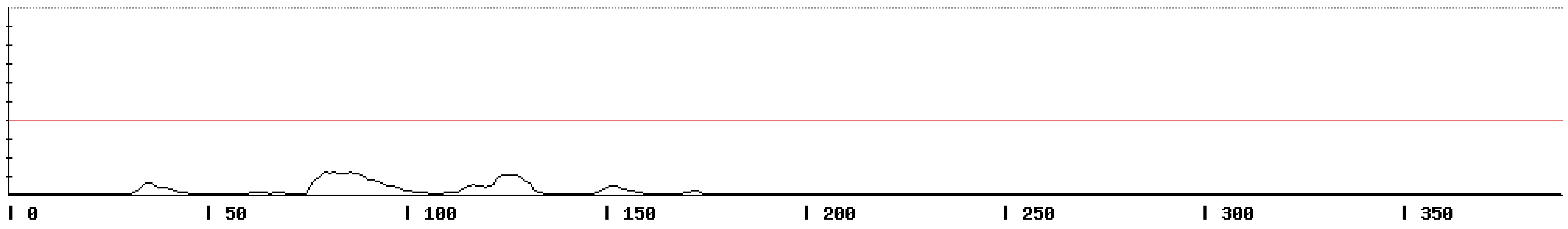

Predictions for Medicagotruncatula_Medtr6g012670.1

Viterbi Path

Viterbi Path

Posterior @ 0.4

Posterior @ 0.4

Posterior Graphic. Threshold Marked @ 0.4

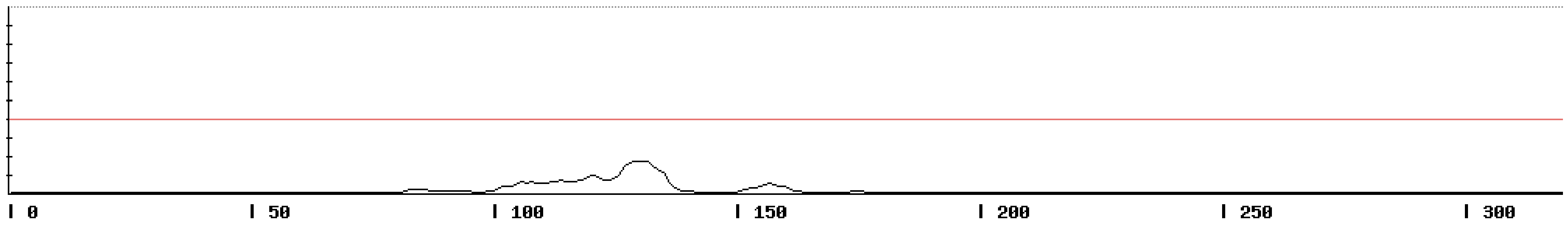

Predictions for Medicagotruncatula_Medtr6g032770.1

Viterbi Path

Viterbi Path

Posterior @ 0.4

Posterior @ 0.4

Posterior Graphic. Threshold Marked @ 0.4

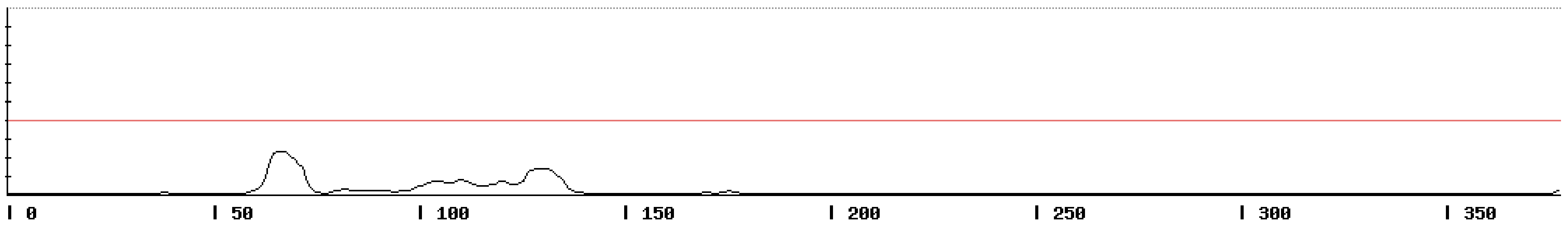

Predictions for Medicagotruncatula_Medtr6g084430.1

Viterbi Path

Viterbi Path

Posterior @ 0.4

Posterior @ 0.4

Posterior Graphic. Threshold Marked @ 0.4

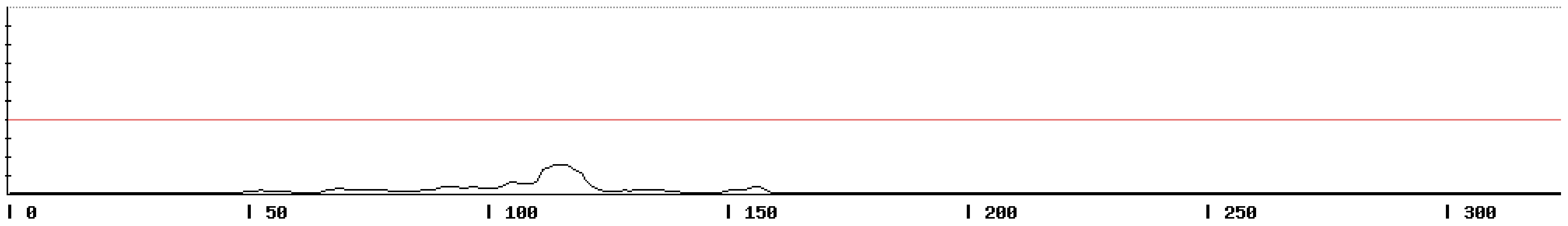

Predictions for Medicagotruncatula_Medtr6g477900.1

Viterbi Path

Viterbi Path

Posterior @ 0.4

Posterior @ 0.4

Posterior Graphic. Threshold Marked @ 0.4

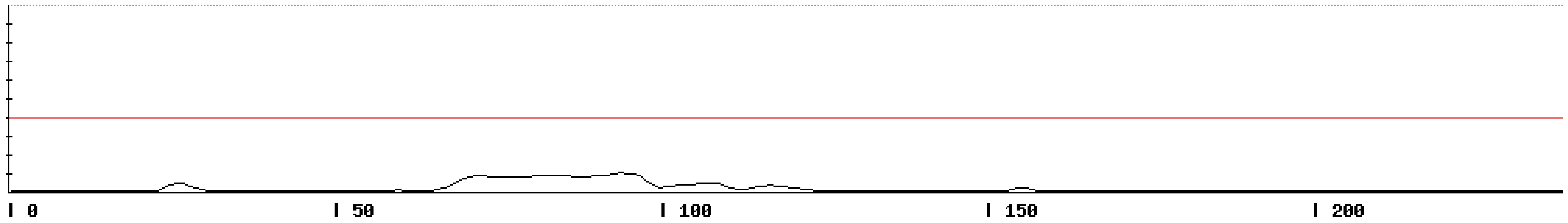

Predictions for Medicagotruncatula_Medtr7g005280.1

Viterbi Path

Viterbi Path

Posterior @ 0.4

Posterior @ 0.4

Posterior Graphic. Threshold Marked @ 0.4

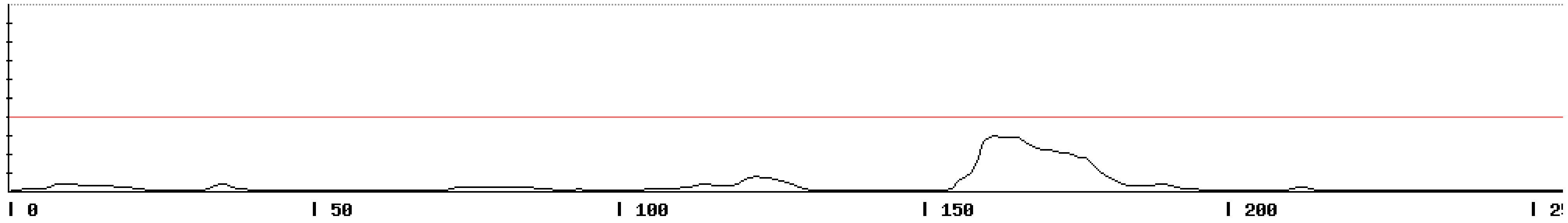

Predictions for Medicagotruncatula_Medtr7g011120.1

Viterbi Path

Viterbi Path

Posterior @ 0.4

Posterior @ 0.4

Posterior Graphic. Threshold Marked @ 0.4

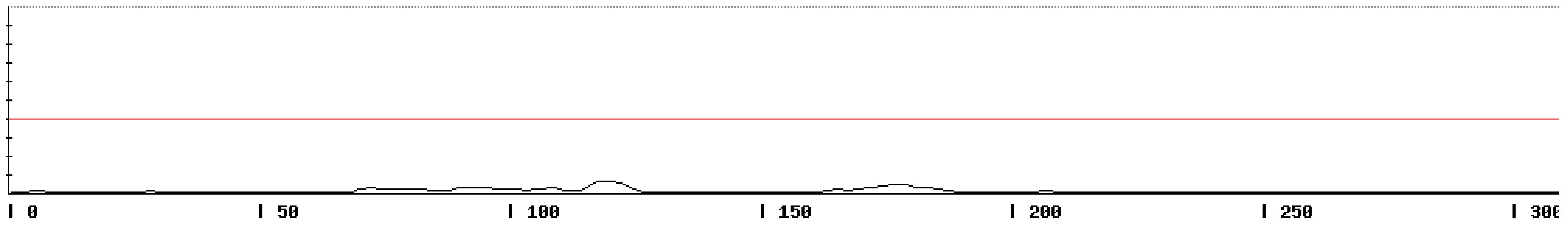

Predictions for Medicagotruncatula_Medtr7g011130.1

Viterbi Path

Viterbi Path

Posterior @ 0.4

Posterior @ 0.4

Posterior Graphic. Threshold Marked @ 0.4

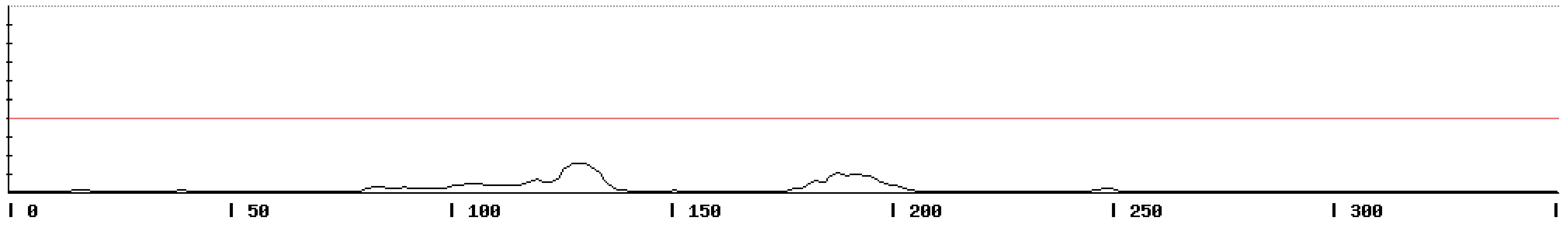

Predictions for Medicagotruncatula_Medtr7g033320.1	
Viterbi Path	
Viterbi Path	
Posterior @ 0.4	132 - QRKRRKIHHRGSTEDHVRWHKTGKTK - 157
Posterior @ 0.4	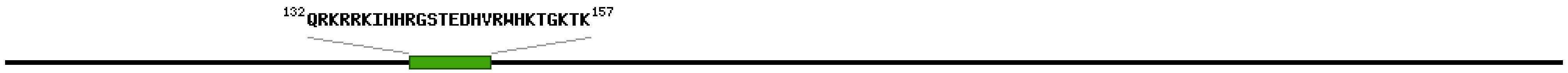
Posterior Graphic. Threshold Marked @ 0.4	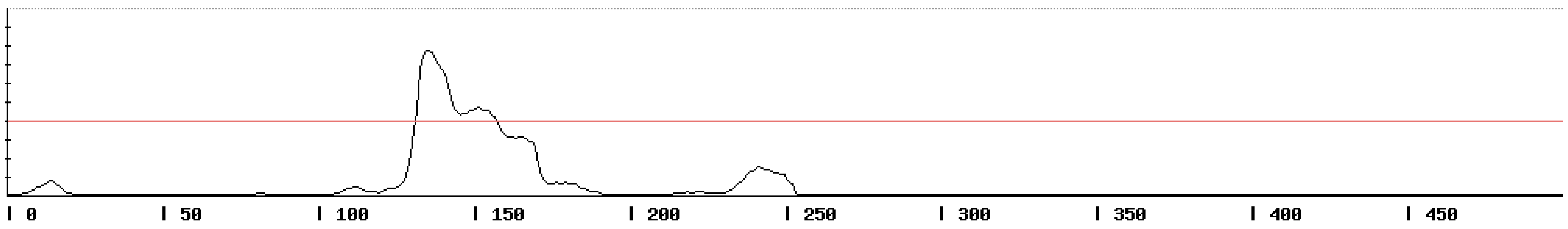

Predictions for Medicagotruncatula_Medtr7g070140.1	
Viterbi Path	
Viterbi Path	
Posterior @ 0.4	
Posterior @ 0.4	
Posterior Graphic. Threshold Marked @ 0.4	

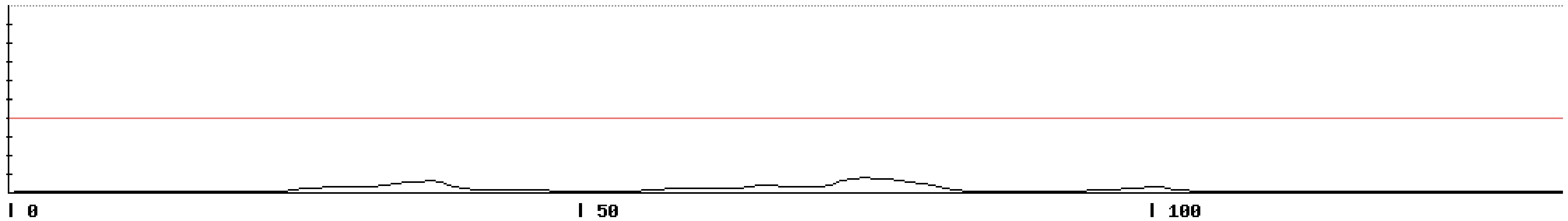

Predictions for Medicagotruncatula_Medtr7g070150.1

Viterbi Path	
Viterbi Path	
Posterior @ 0.4	
Posterior @ 0.4	
Posterior Graphic. Threshold Marked @ 0.4	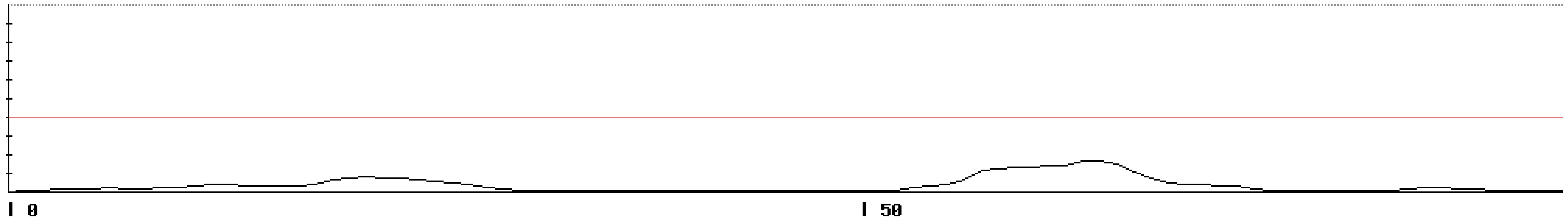

Predictions for Medicagotruncatula_Medtr7g083330.1

Viterbi Path	
Viterbi Path	
Posterior @ 0.4	
Posterior @ 0.4	

Posterior Graphic. Threshold Marked @ 0.4

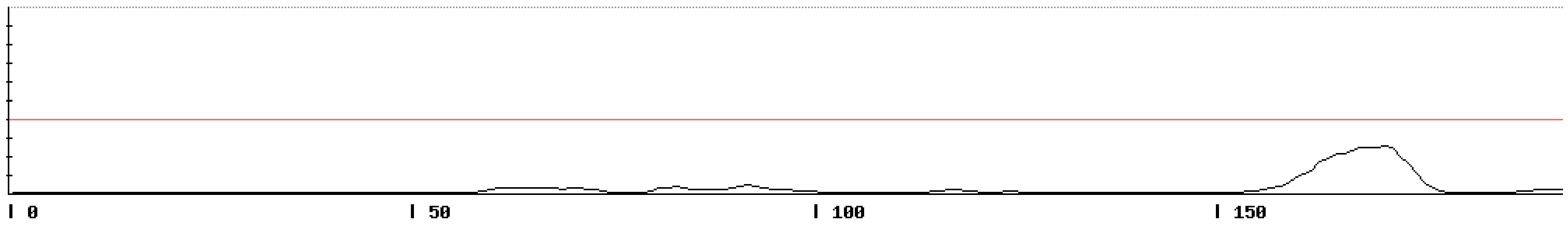

Predictions for Medicagotruncatula_Medtr7g083360.1

Viterbi Path

Viterbi Path

Posterior @ 0.4

Posterior @ 0.4

Posterior Graphic. Threshold Marked @ 0.4

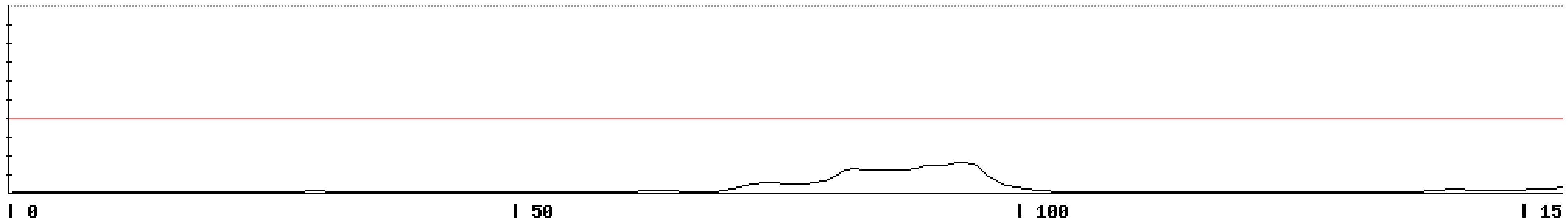

Predictions for Medicagotruncatula_Medtr7g083370.1

Viterbi Path

Viterbi Path

Posterior @ 0.4

Posterior @ 0.4

Posterior Graphic. Threshold Marked @ 0.4

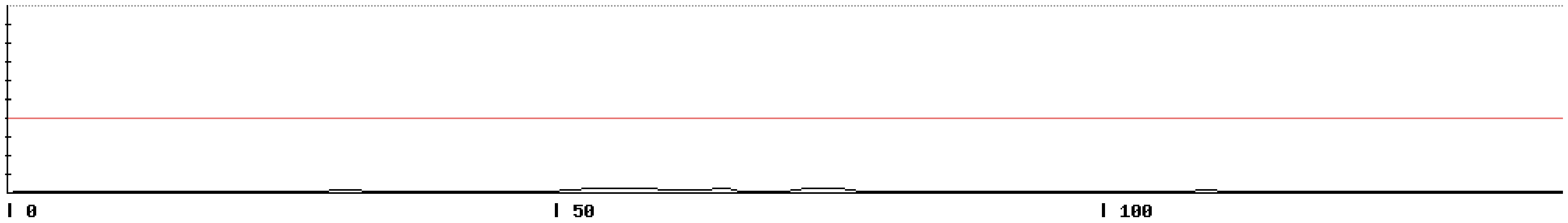

Predictions for Medicagotruncatula_Medtr7g085220.1

Viterbi Path

Viterbi Path

Posterior @ 0.4

Posterior @ 0.4

Posterior Graphic. Threshold Marked @ 0.4

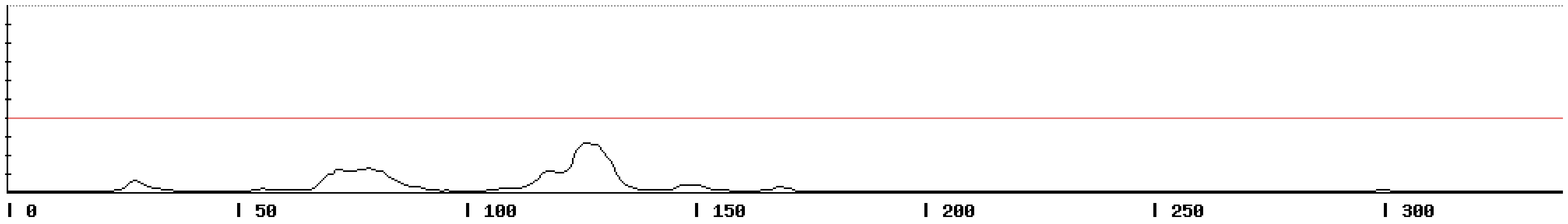

Predictions for Medicagotruncatula_Medtr7g085260.1

Viterbi Path

Viterbi Path

Posterior @ 0.4

Posterior @ 0.4

Posterior Graphic. Threshold Marked @ 0.4

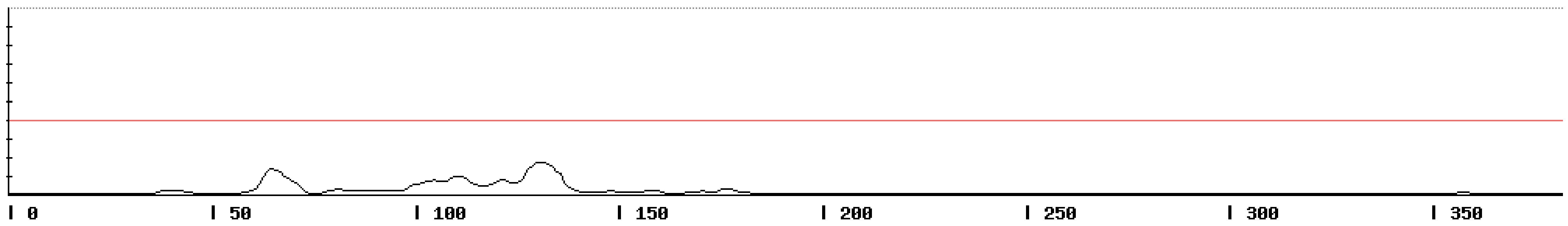

Predictions for Medicagotruncatula_Medtr7g097090.1

Viterbi Path

Viterbi Path

Posterior @ 0.4

Posterior @ 0.4

Posterior Graphic. Threshold Marked @ 0.4

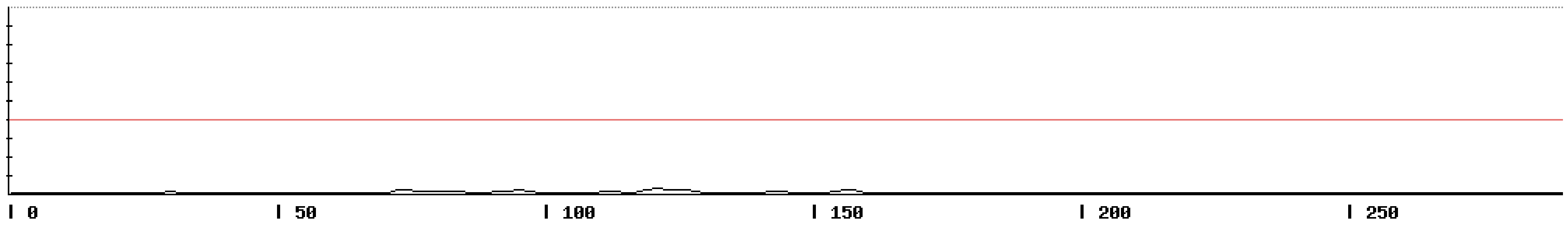

Predictions for Medicagotruncatula_Medtr7g100990.1

Viterbi Path

Viterbi Path

Posterior @ 0.4

Posterior @ 0.4

Posterior Graphic. Threshold Marked @ 0.4

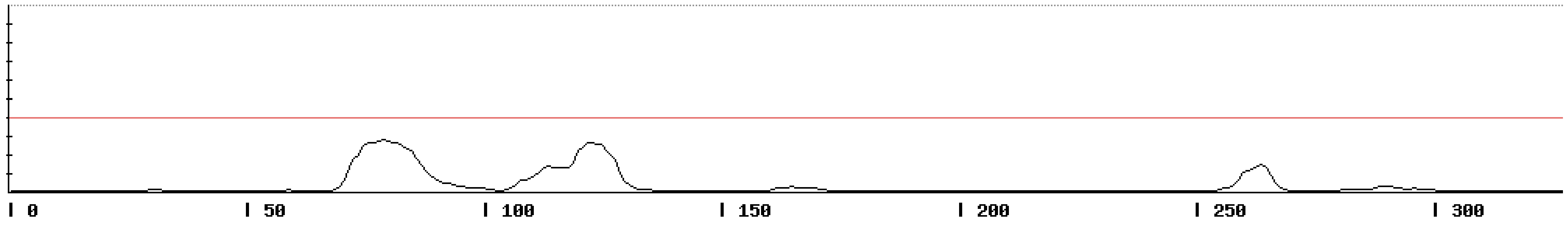

Predictions for Medicagotruncatula_Medtr7g105170.1

Viterbi Path

Viterbi Path

Posterior @ 0.4

Posterior @ 0.4

Posterior Graphic. Threshold Marked @ 0.4

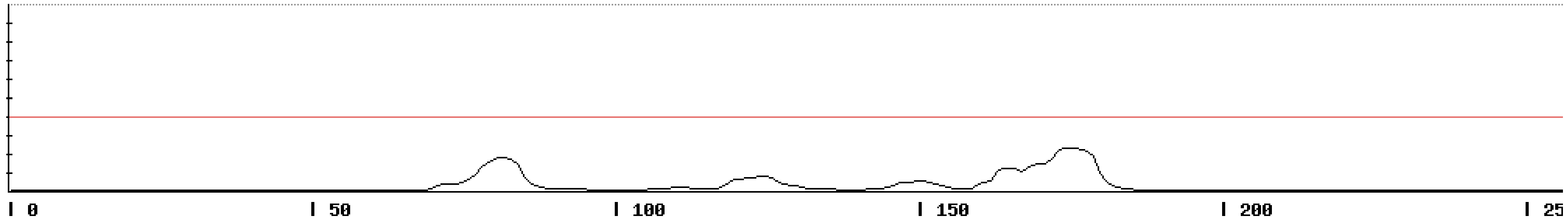

Predictions for Medicagotruncatula_Medtr7g116460.1

Viterbi Path

Viterbi Path

Posterior @ 0.4

Posterior @ 0.4

Posterior Graphic. Threshold Marked @ 0.4

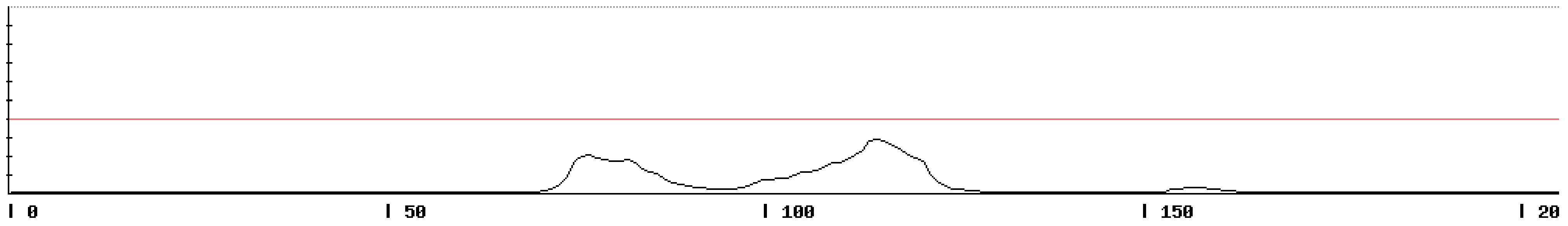

Predictions for Medicagotruncatula_Medtr8g023840.1

Viterbi Path

Viterbi Path

Posterior @ 0.4

Posterior @ 0.4

Posterior Graphic. Threshold Marked @ 0.4

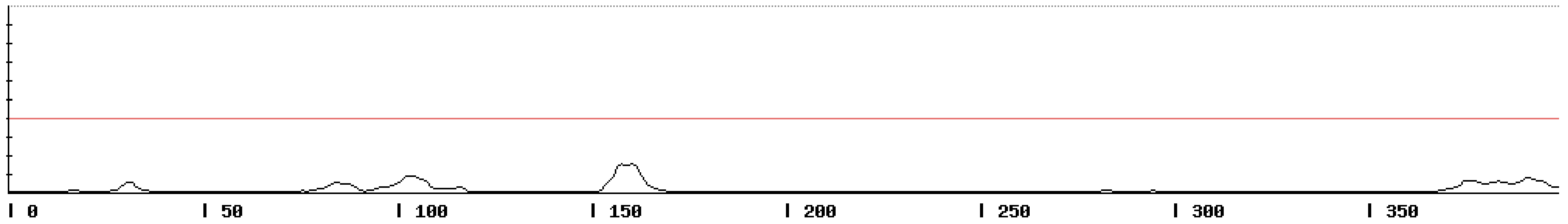

Predictions for Medicagotruncatula_Medtr8g023860.1

Viterbi Path

Viterbi Path

Posterior @ 0.4

85 - KRFKR - 89

98 - ATGKDRKIKV - 107

412 - KRAASKR - 418

Posterior @ 0.4

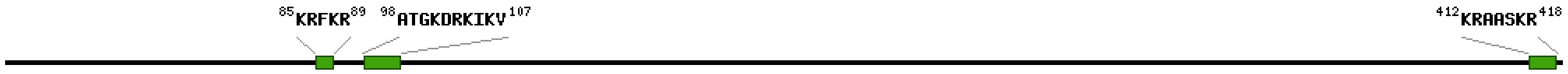

Posterior Graphic. Threshold Marked @ 0.4

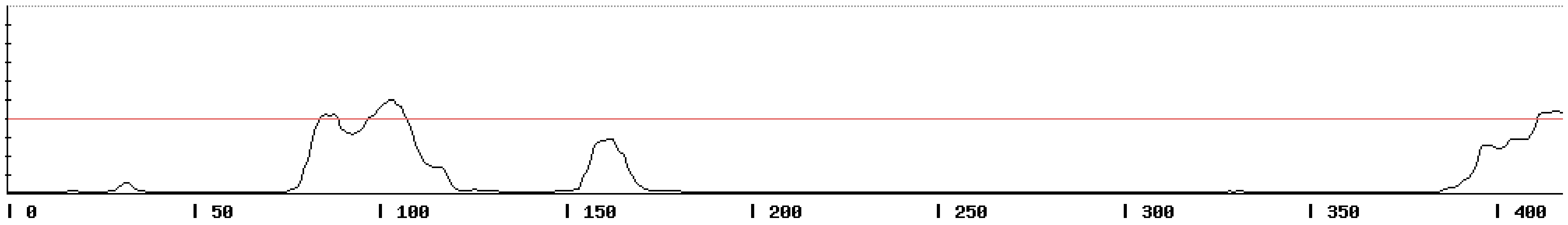

Predictions for Medicagotruncatula_Medtr8g023880.1

Viterbi Path

Viterbi Path

Posterior @ 0.4

Posterior @ 0.4

Posterior Graphic. Threshold Marked @ 0.4

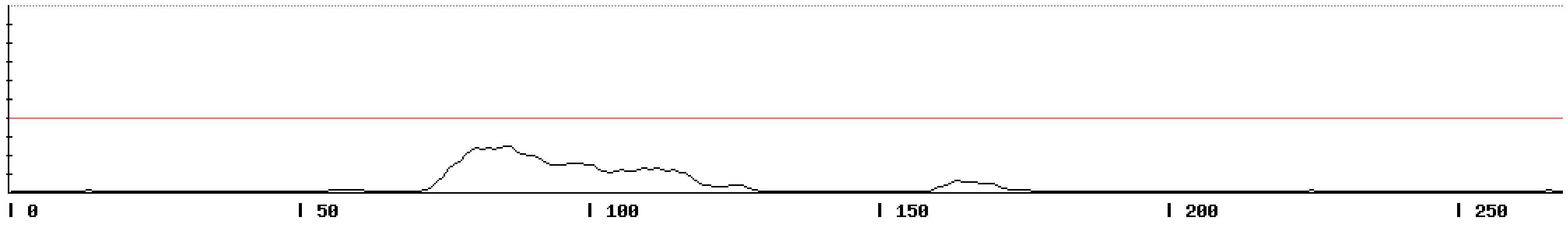

Predictions for Medicagotruncatula_Medtr8g023900.1	
Viterbi Path	
Viterbi Path	
Posterior @ 0.4	
Posterior @ 0.4	
Posterior Graphic. Threshold Marked @ 0.4	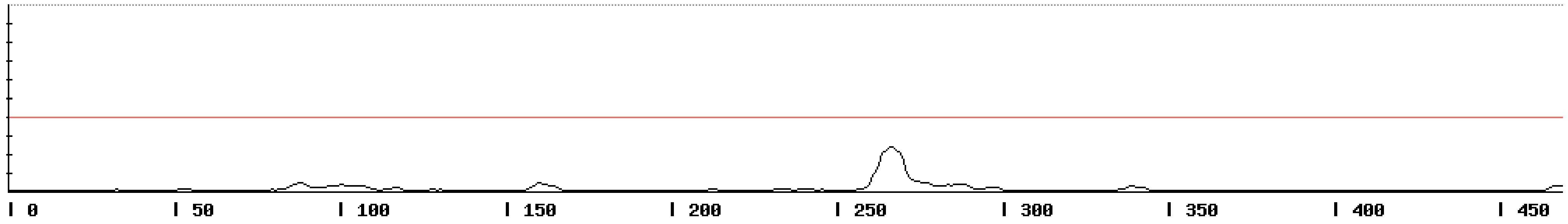

Predictions for Medicagotruncatula_Medtr8g023930.1	
Viterbi Path	
Viterbi Path	
Posterior @ 0.4	
Posterior @ 0.4	

Posterior Graphic. Threshold Marked @ 0.4	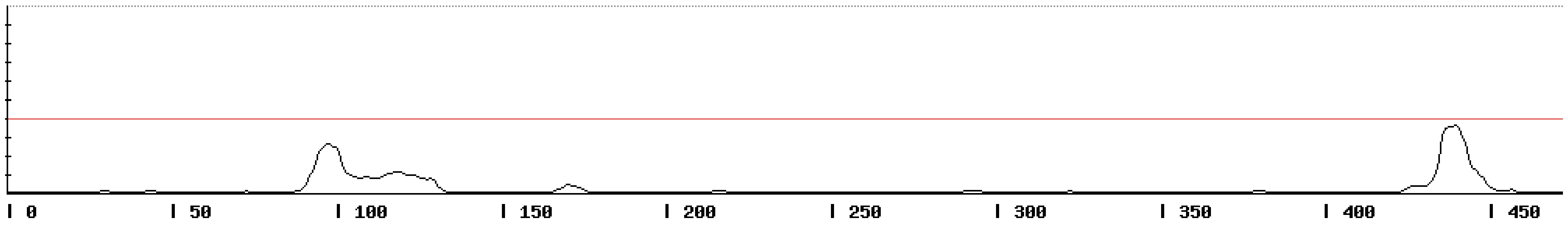

Predictions for Medicagotruncatula_Medtr8g024480.1	
Viterbi Path	
Viterbi Path	
Posterior @ 0.4	
Posterior @ 0.4	
Posterior Graphic. Threshold Marked @ 0.4	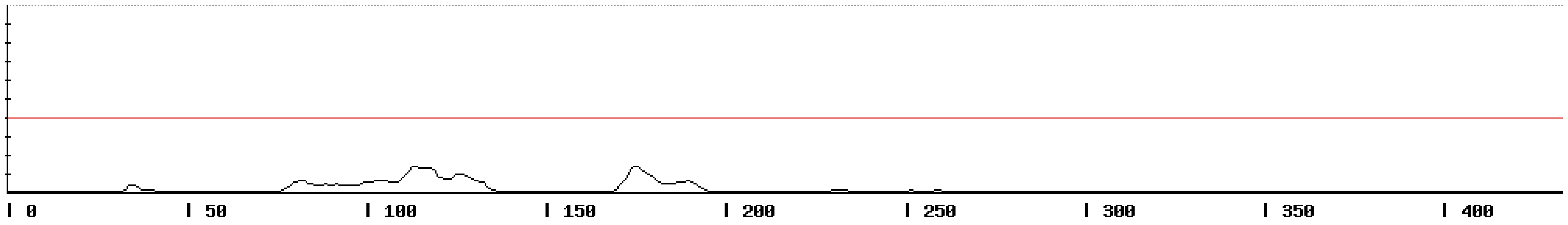

Predictions for Medicagotruncatula_Medtr8g059170.1	
Viterbi Path	
Viterbi Path	
Posterior @ 0.4	
Posterior @ 0.4	

Posterior Graphic. Threshold Marked @ 0.4

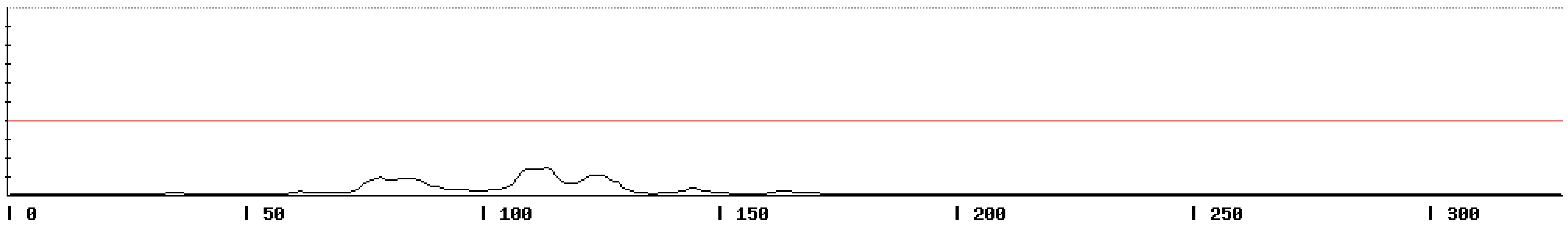

Predictions for Medicagotruncatula_Medtr8g063550.1

Viterbi Path

Viterbi Path

Posterior @ 0.4

Posterior @ 0.4

Posterior Graphic. Threshold Marked @ 0.4

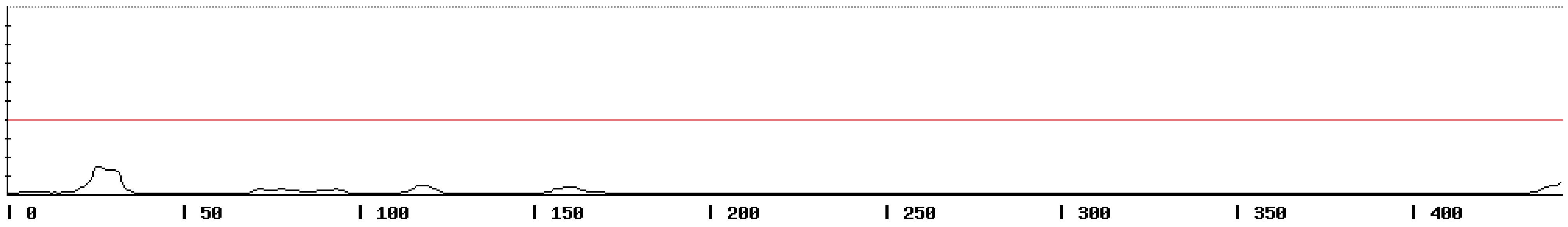

Predictions for Medicagotruncatula_Medtr8g069160.1

Viterbi Path

Viterbi Path

Posterior @ 0.4	146 - RKRR - 149
Posterior @ 0.4	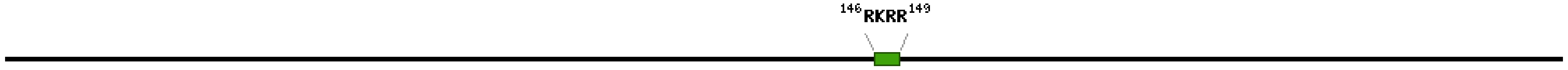
Posterior Graphic. Threshold Marked @ 0.4	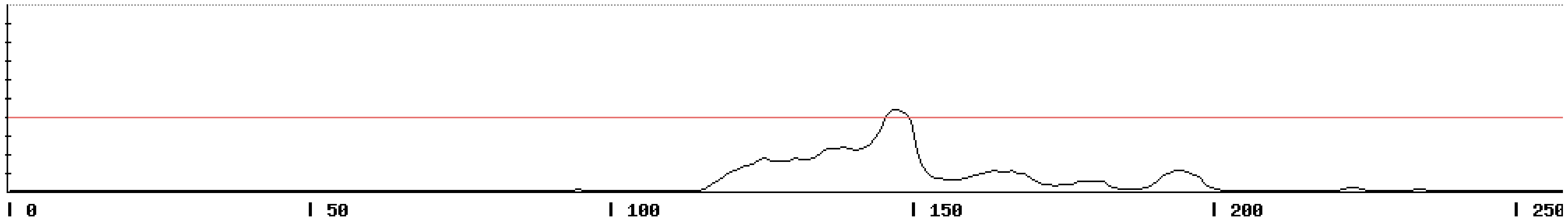

Predictions for Medicagotruncatula_Medtr8g076110.1

Viterbi Path	
Viterbi Path	
Posterior @ 0.4	
Posterior @ 0.4	
Posterior Graphic. Threshold Marked @ 0.4	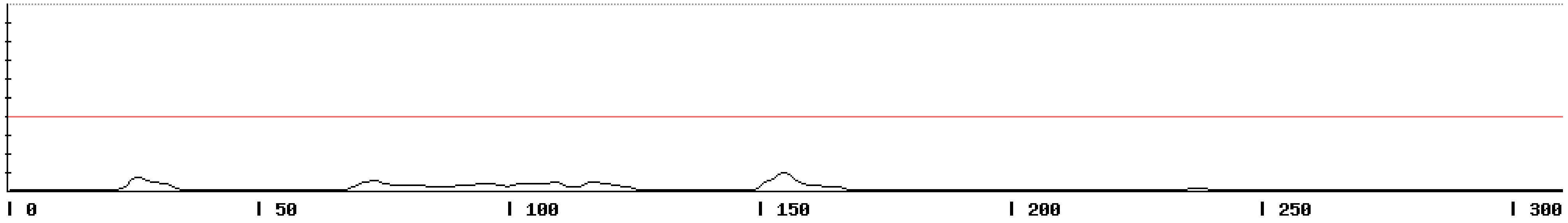

Predictions for Medicagotruncatula_Medtr8g093580.1

Viterbi Path	
Viterbi Path	

Posterior @ 0.4	
Posterior @ 0.4	
Posterior Graphic. Threshold Marked @ 0.4	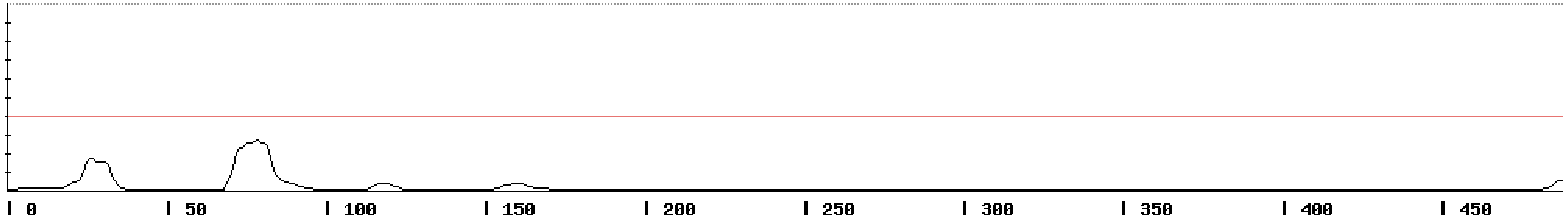

Predictions for Medicagotruncatula_Medtr8g093790.1	
Viterbi Path	
Viterbi Path	
Posterior @ 0.4	
Posterior @ 0.4	
Posterior Graphic. Threshold Marked @ 0.4	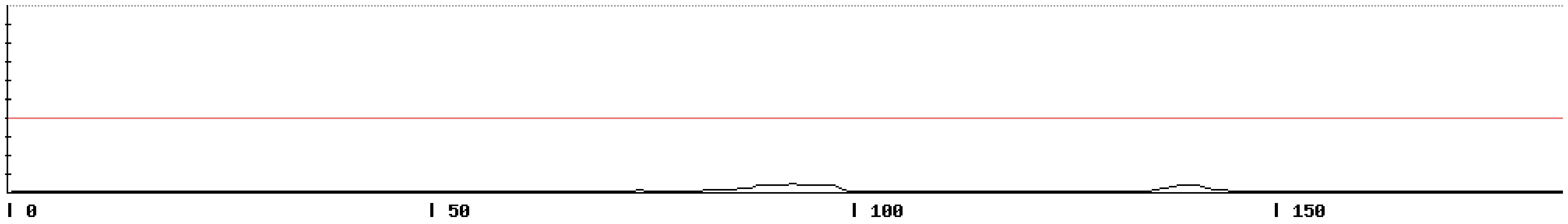

Predictions for Medicagotruncatula_Medtr8g094580.1

Viterbi Path	
Viterbi Path	
Posterior @ 0.4	94 - DKPIGKPKALGIKK - 107
Posterior @ 0.4	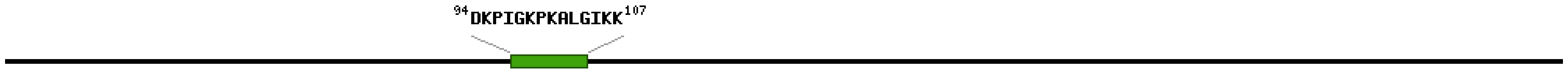
Posterior Graphic. Threshold Marked @ 0.4	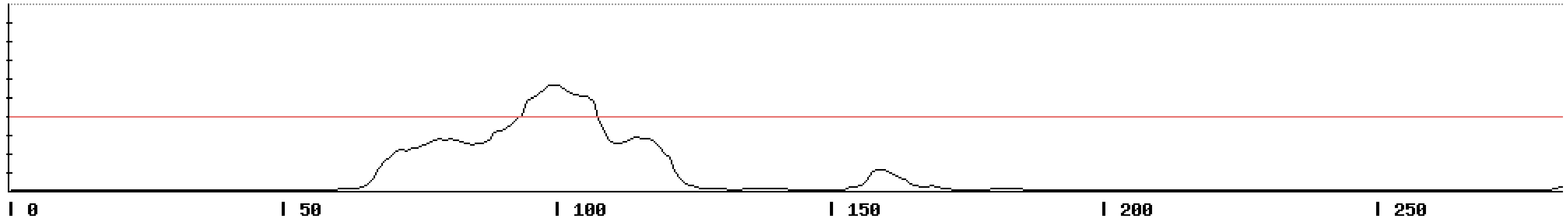

Predictions for Medicagotruncatula_Medtr8g099750.1

Viterbi Path	
Viterbi Path	
Posterior @ 0.4	

alex.nguyenba@utoronto.ca

Intended to be used with Firefox

Viterbi Path	
Posterior @ 0.4	
Posterior @ 0.4	
Posterior Graphic. Threshold Marked @ 0.4	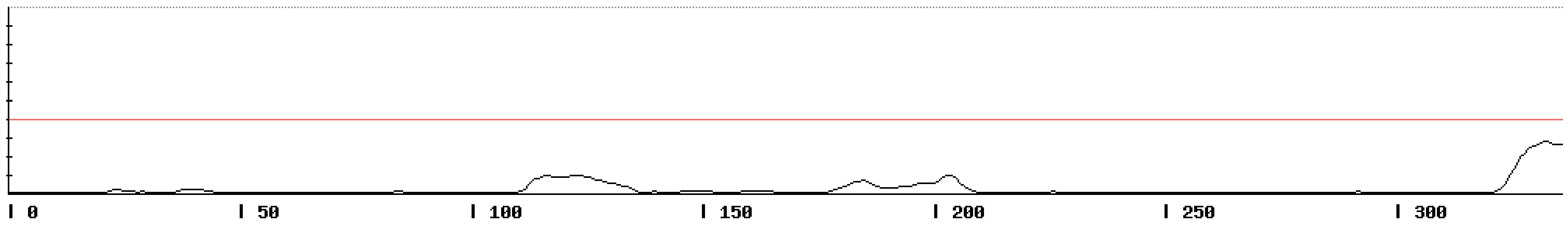

Predictions for Mimulusguttatus_mgv1a002437m

Viterbi Path	
Viterbi Path	
Posterior @ 0.4	
Posterior @ 0.4	
Posterior Graphic. Threshold Marked @ 0.4	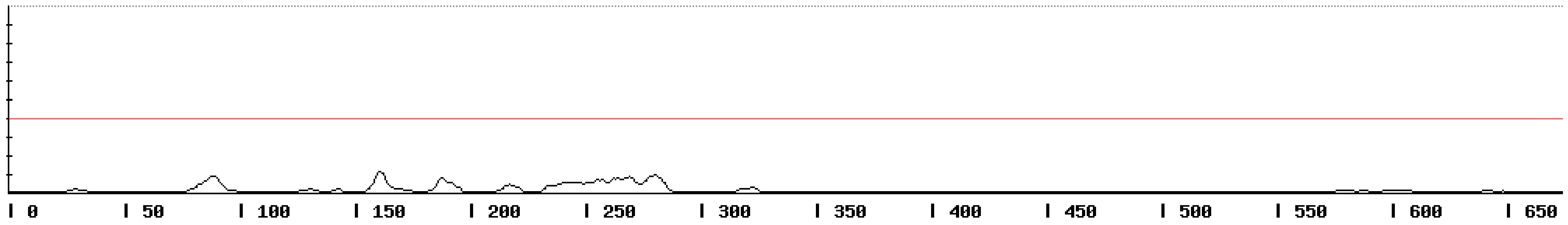

Predictions for Mimulusguttatus_mgv1a003123m

Viterbi Path	
Viterbi Path	
Posterior @ 0.4	104 - RKGAK - 108
Posterior @ 0.4	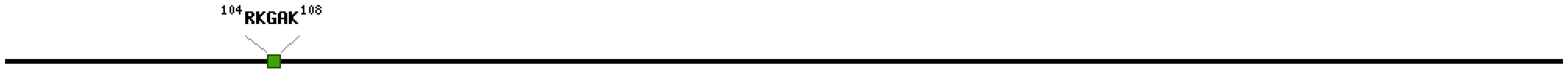
Posterior Graphic. Threshold Marked @ 0.4	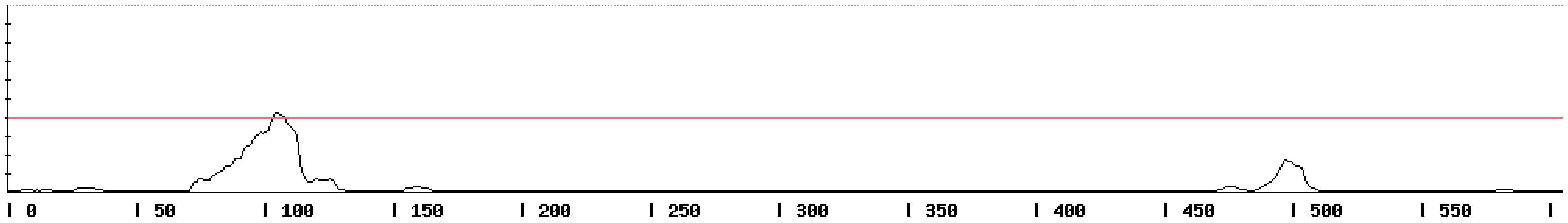

Predictions for Mimulusguttatus_mgv1a003672m

Viterbi Path	
Viterbi Path	
Posterior @ 0.4	
Posterior @ 0.4	
Posterior Graphic. Threshold Marked @ 0.4	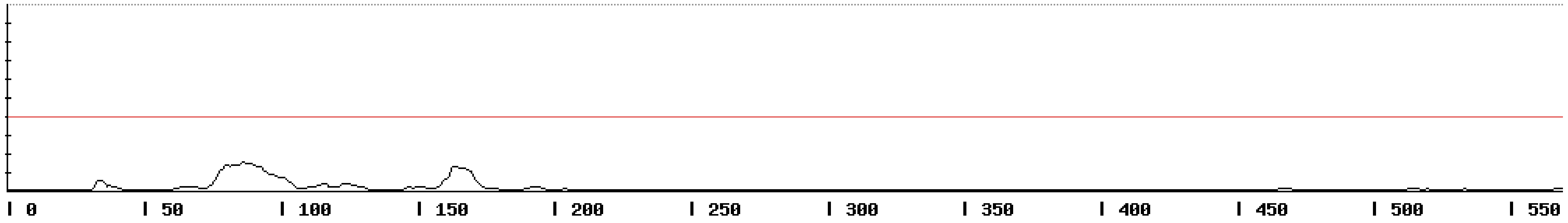

Predictions for Mimulusguttatus_mgv1a003896m

Viterbi Path

Viterbi Path

Posterior @ 0.4

Posterior @ 0.4

Posterior Graphic. Threshold Marked @ 0.4

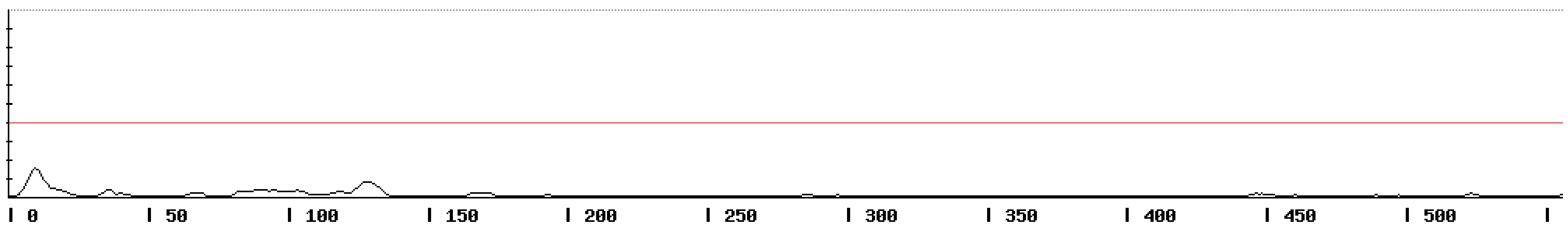

Predictions for Mimulusguttatus_mgv1a005759m

Viterbi Path

Viterbi Path

Posterior @ 0.4

Posterior @ 0.4

Posterior Graphic. Threshold Marked @ 0.4

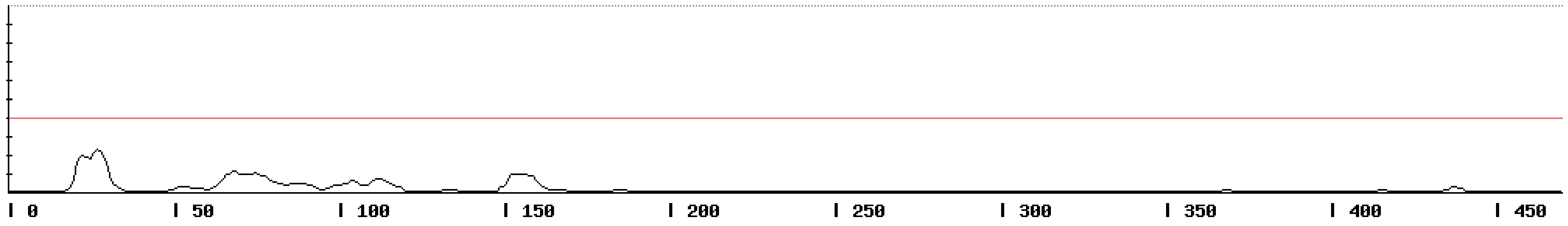

Predictions for Mimulusguttatus_mgv1a007138m

Viterbi Path

Viterbi Path

Posterior @ 0.4

Posterior @ 0.4

Posterior Graphic. Threshold Marked @ 0.4

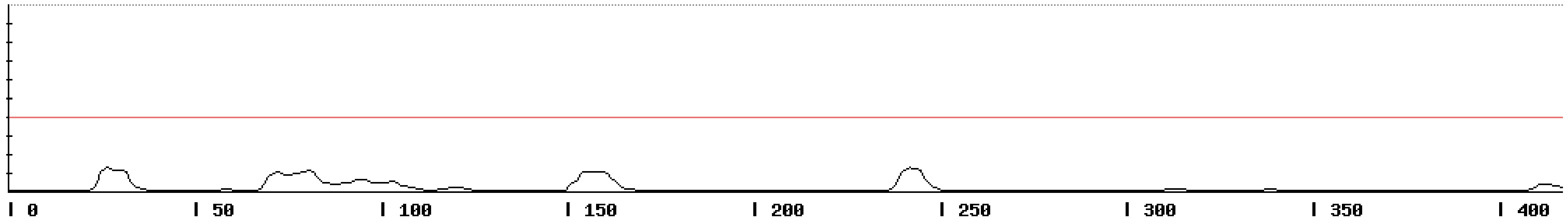

Predictions for Mimulusguttatus_mgv1a007298m

Viterbi Path

Viterbi Path

Posterior @ 0.4

Posterior @ 0.4

Posterior Graphic. Threshold Marked @ 0.4

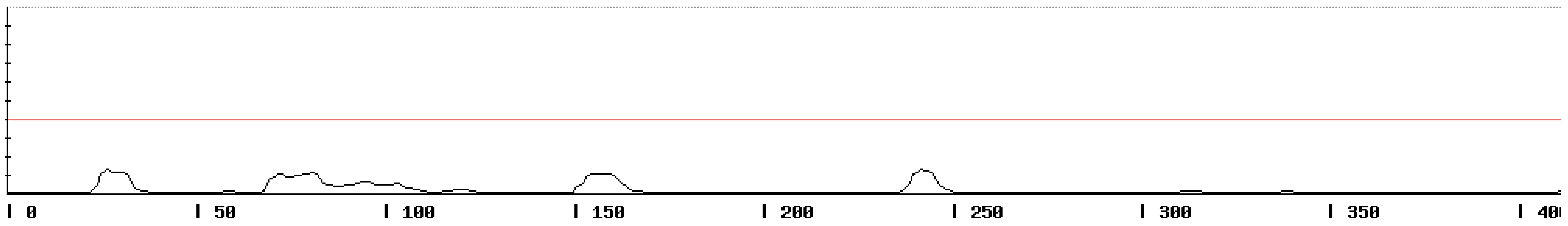

Predictions for Mimulusguttatus_mgv1a007422m

Viterbi Path

Viterbi Path

Posterior @ 0.4 137 - KRR - 139

Posterior @ 0.4

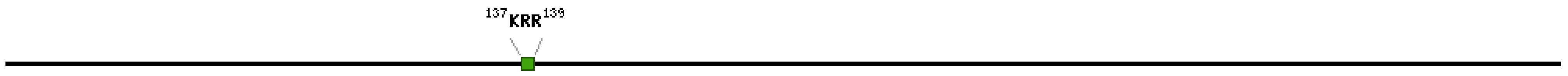

Posterior Graphic. Threshold Marked @ 0.4

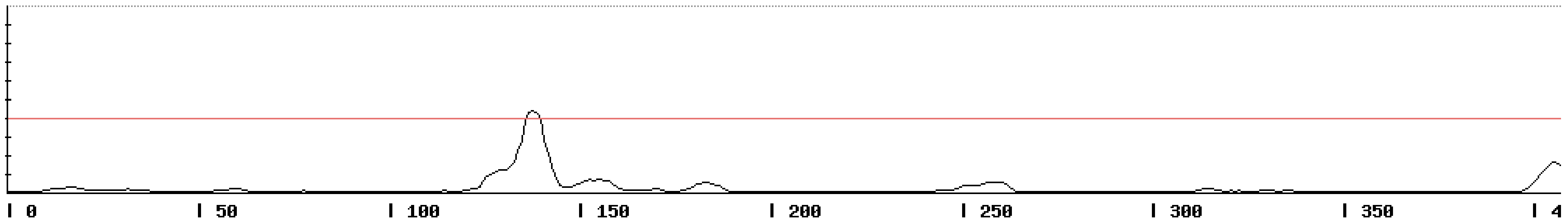

Predictions for Mimulusguttatus_mgv1a007979m

Viterbi Path

Viterbi Path

Posterior @ 0.4

Posterior @ 0.4

Posterior Graphic. Threshold Marked @ 0.4

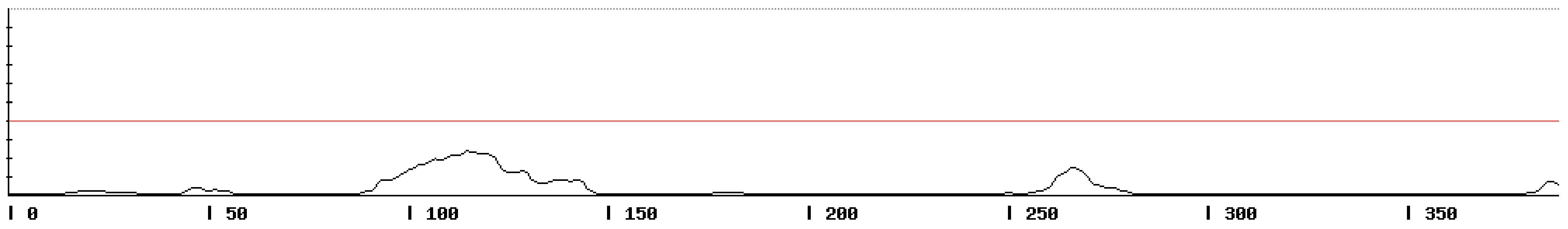

Predictions for Mimulusguttatus_mgv1a008377m

Viterbi Path

Viterbi Path

Posterior @ 0.4

Posterior @ 0.4

Posterior Graphic. Threshold Marked @ 0.4

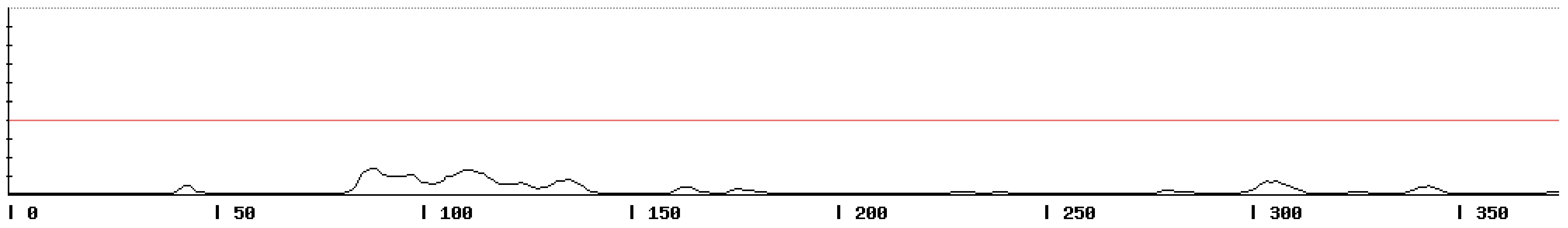

Predictions for Mimulusguttatus_mgv1a009074m

Viterbi Path

Viterbi Path

Posterior @ 0.4

Posterior @ 0.4

Posterior Graphic. Threshold Marked @ 0.4

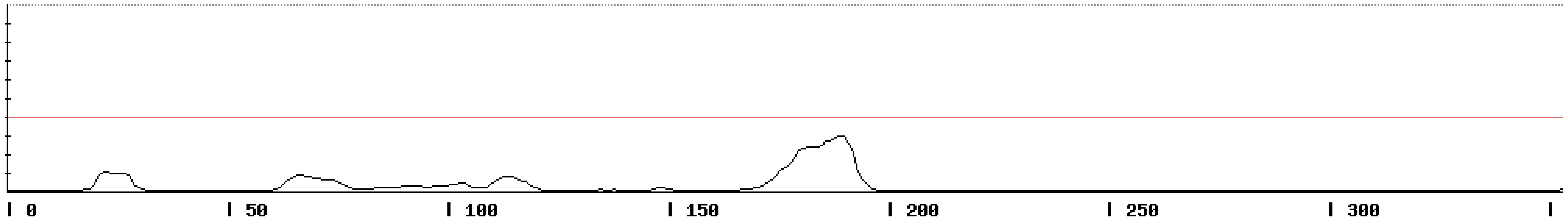

Predictions for Mimulusguttatus_mgv1a009356m

Viterbi Path

Viterbi Path

Posterior @ 0.4

Posterior @ 0.4

Posterior Graphic. Threshold Marked @ 0.4

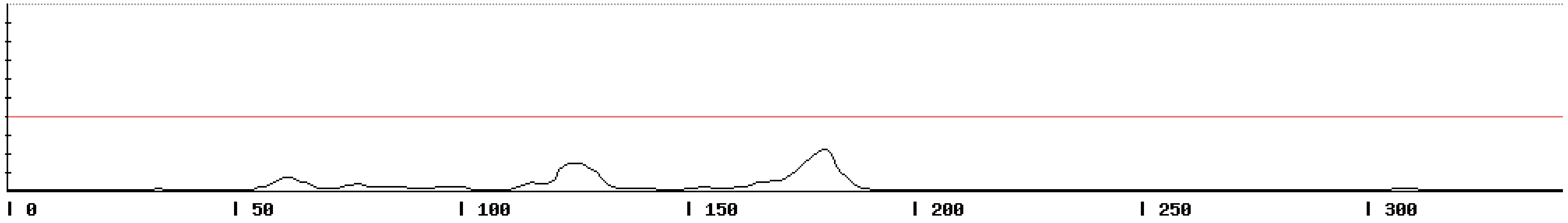

Predictions for Mimulusguttatus_mgv1a009375m

Viterbi Path

Viterbi Path

Posterior @ 0.4

Posterior @ 0.4

Posterior Graphic. Threshold Marked @ 0.4

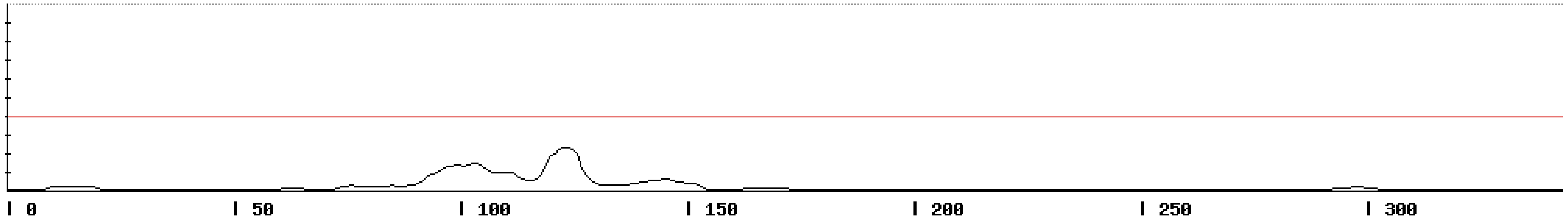

Predictions for Mimulusguttatus_mgv1a009589m

Viterbi Path

Viterbi Path

Posterior @ 0.4

Posterior @ 0.4

Posterior Graphic. Threshold Marked @ 0.4

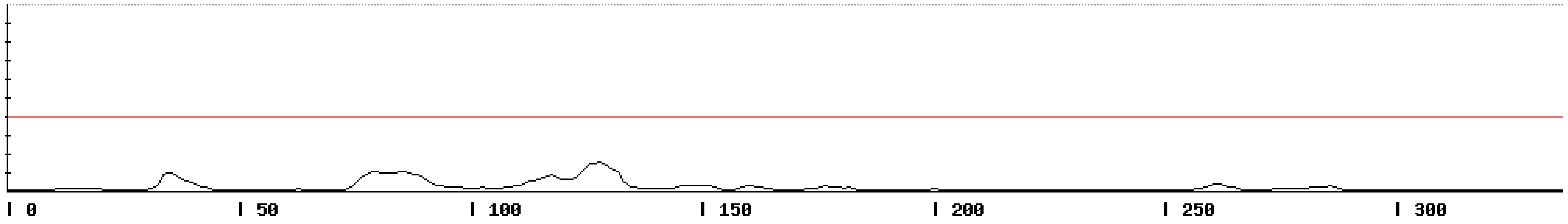

Predictions for Mimulusguttatus_mgv1a009604m

Viterbi Path

Viterbi Path

Posterior @ 0.4

Posterior @ 0.4

Posterior Graphic. Threshold Marked @ 0.4

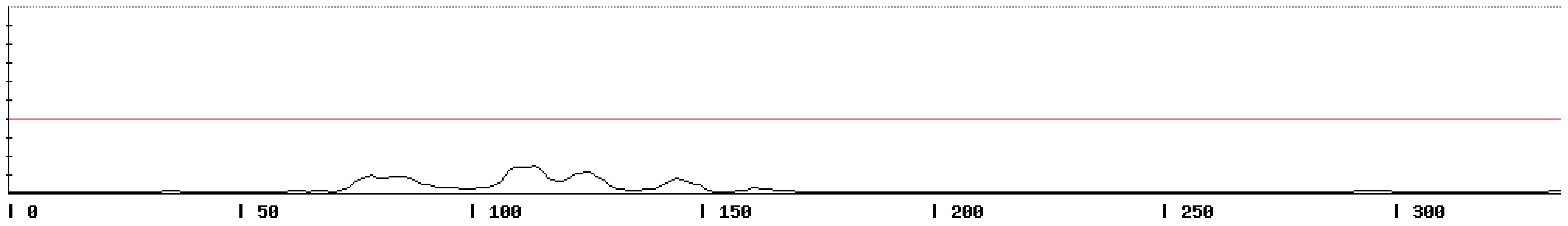

Predictions for Mimulusguttatus_mgv1a010134m

Viterbi Path

Viterbi Path

Posterior @ 0.4

Posterior @ 0.4

Posterior Graphic. Threshold Marked @ 0.4

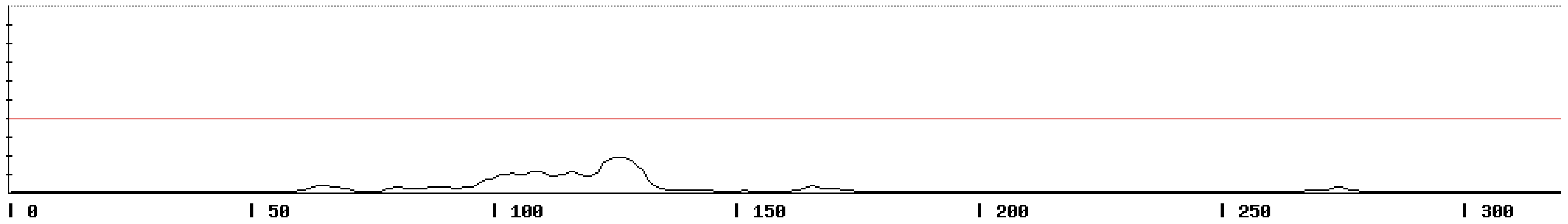

Predictions for Mimulusguttatus_mgv1a010297m	
Viterbi Path	
Viterbi Path	
Posterior @ 0.4	
Posterior @ 0.4	
Posterior Graphic. Threshold Marked @ 0.4	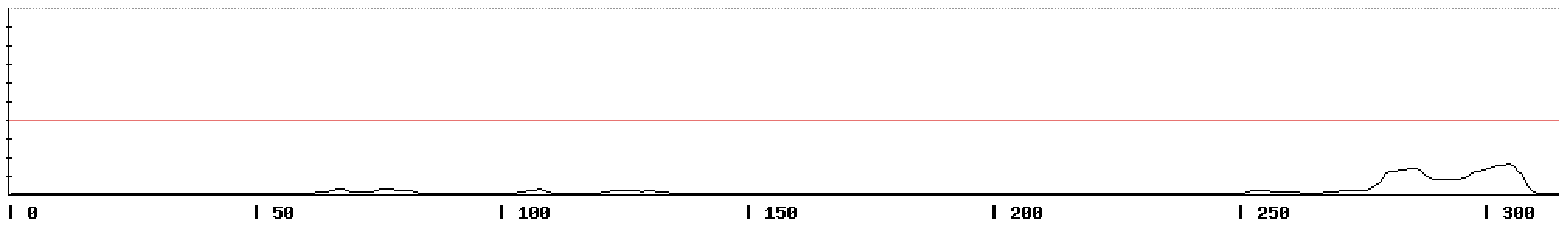

Predictions for Mimulusguttatus_mgv1a010607m	
Viterbi Path	
Viterbi Path	
Posterior @ 0.4	
Posterior @ 0.4	
Posterior Graphic. Threshold Marked @ 0.4	

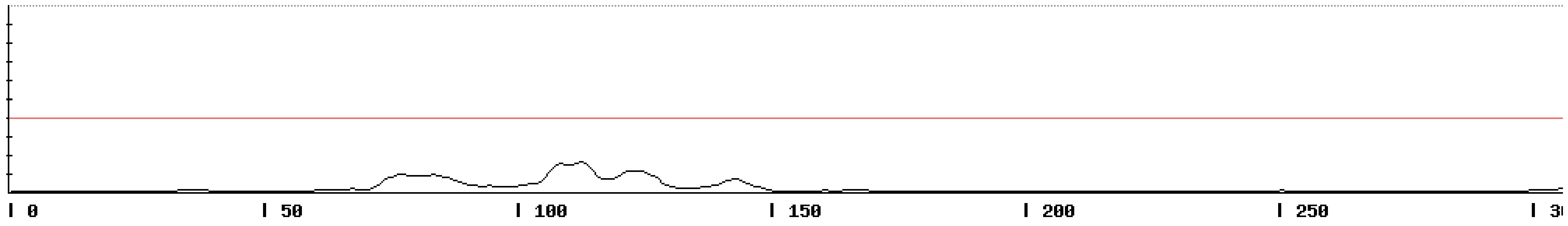

Predictions for Mimulusguttatus_mgv1a010993m

Viterbi Path

Viterbi Path

Posterior @ 0.4

Posterior @ 0.4

Posterior Graphic. Threshold Marked @ 0.4

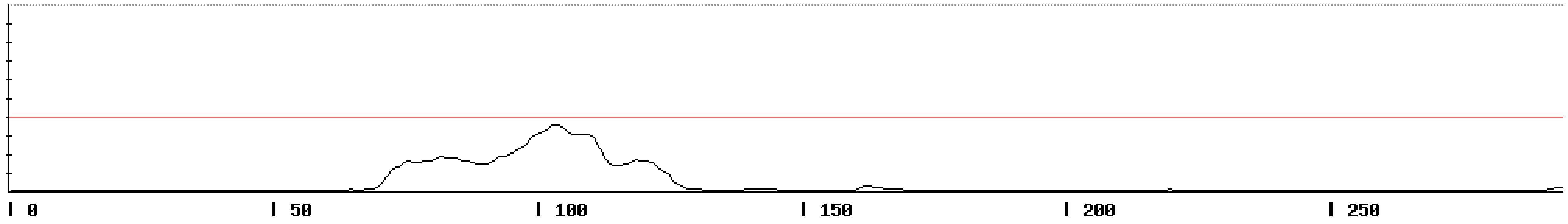

Predictions for Mimulusguttatus_mgv1a011129m

Viterbi Path

Viterbi Path

Posterior @ 0.4

Posterior @ 0.4

Posterior Graphic. Threshold Marked @ 0.4	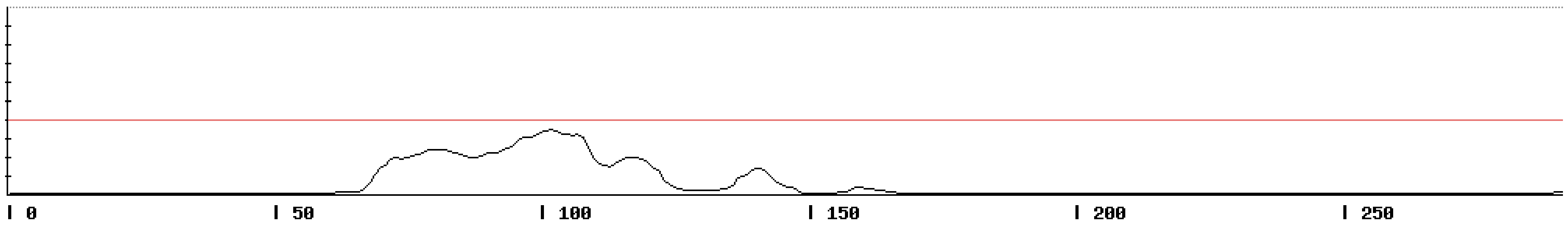

**Predictions for Mimulusguttatus_mgv1a011270m**	
Viterbi Path	
Viterbi Path	
Posterior @ 0.4	
Posterior @ 0.4	
Posterior Graphic. Threshold Marked @ 0.4	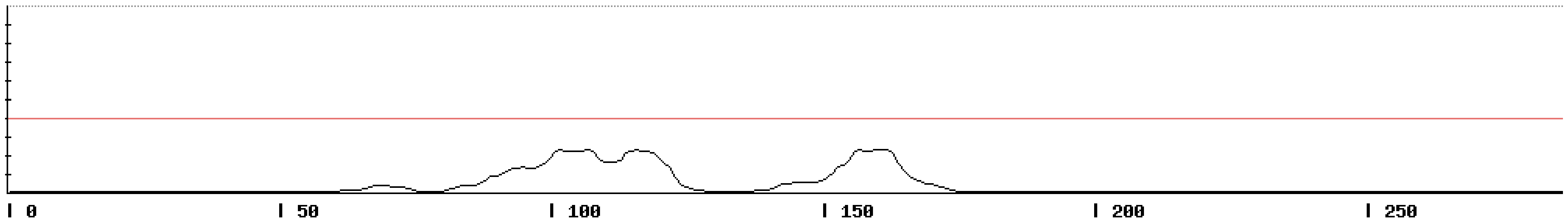

**Predictions for Mimulusguttatus_mgv1a011503m**	
Viterbi Path	
Viterbi Path	
Posterior @ 0.4	
Posterior @ 0.4	

Posterior Graphic. Threshold Marked @ 0.4

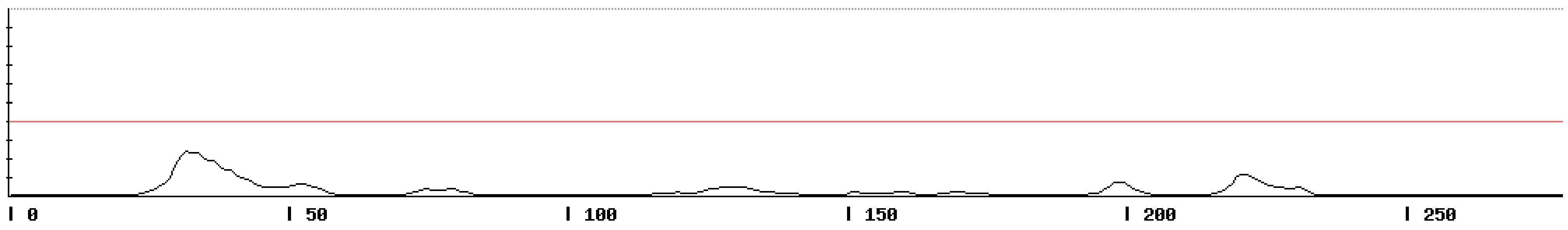

Predictions for Mimulusguttatus_mgv1a011510m

Viterbi Path

Viterbi Path

Posterior @ 0.4

Posterior @ 0.4

Posterior Graphic. Threshold Marked @ 0.4

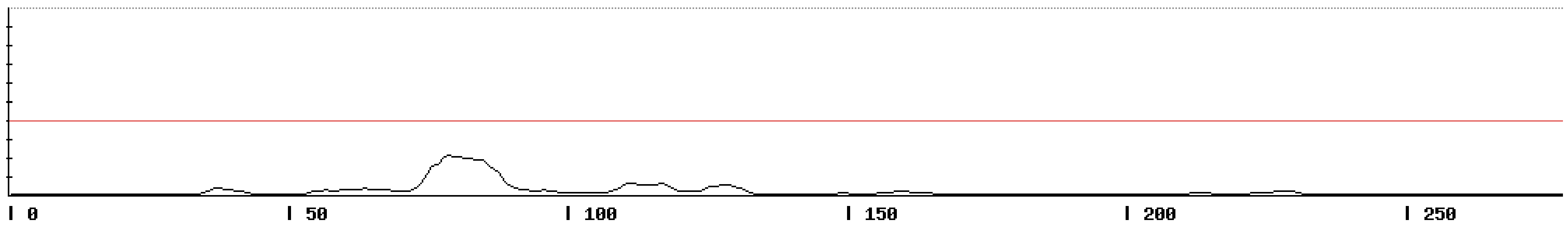

Predictions for Mimulusguttatus_mgv1a011880m

Viterbi Path

Viterbi Path

Posterior @ 0.4

Posterior @ 0.4

Posterior Graphic. Threshold Marked @ 0.4

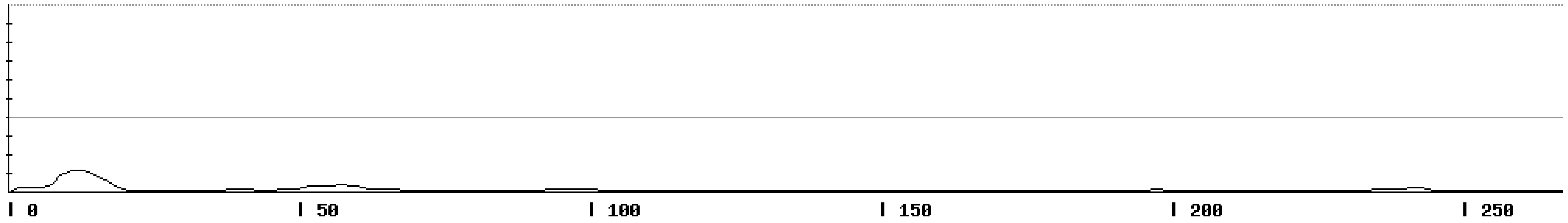

Predictions for Mimulusguttatus_mgv1a011938m

Viterbi Path

Viterbi Path

Posterior @ 0.4

Posterior @ 0.4

Posterior Graphic. Threshold Marked @ 0.4

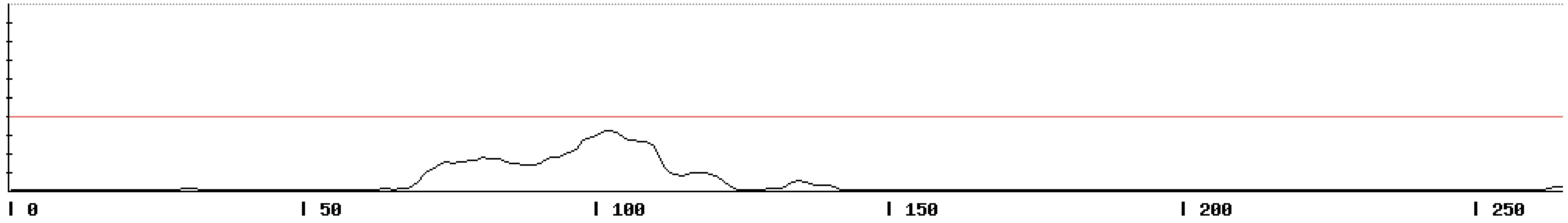

Predictions for Mimulusguttatus_mgv1a012130m

Viterbi Path

Viterbi Path

Posterior @ 0.4	99 - PVGKPKT - 105
Posterior @ 0.4	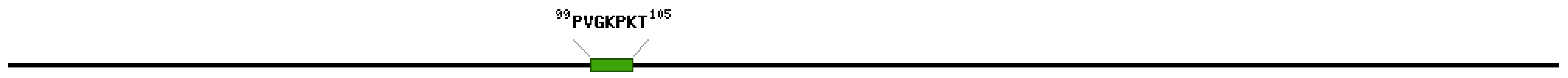
Posterior Graphic. Threshold Marked @ 0.4	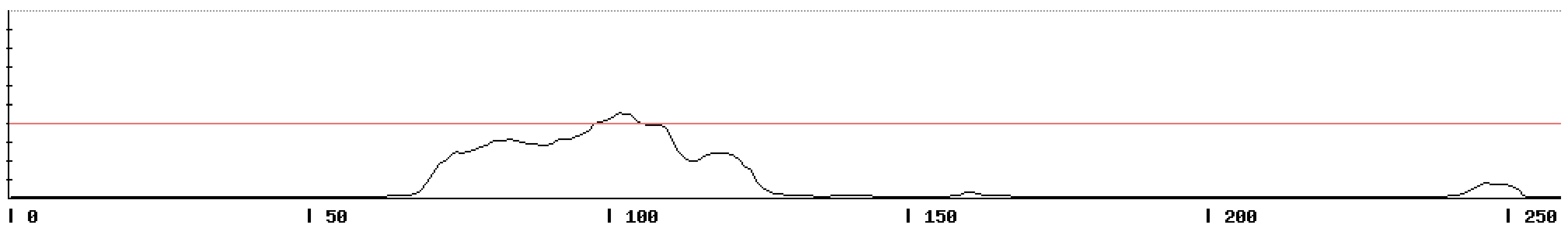

Predictions for Mimulusguttatus_mgv1a012155m

Viterbi Path	
Viterbi Path	
Posterior @ 0.4	118 - KRR - 120
Posterior @ 0.4	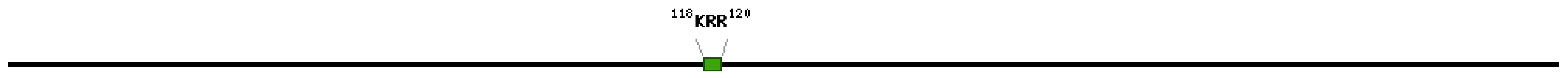
Posterior Graphic. Threshold Marked @ 0.4	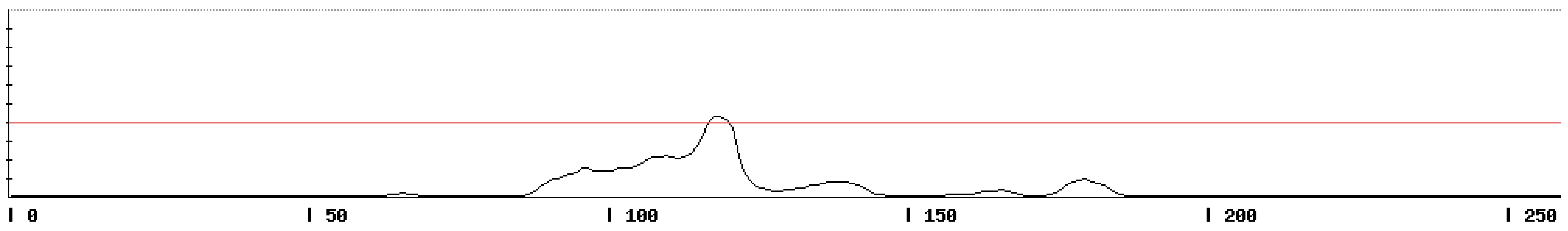

Predictions for Mimulusguttatus_mgv1a012157m

Viterbi Path	
Viterbi Path	
Posterior @ 0.4	81 - ERKRGSGGR - 89
Posterior @ 0.4	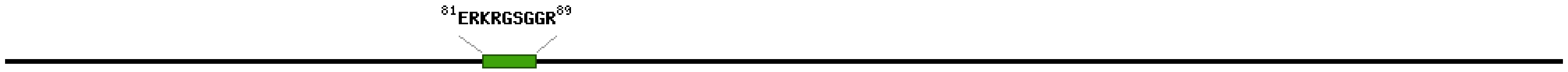
Posterior Graphic. Threshold Marked @ 0.4	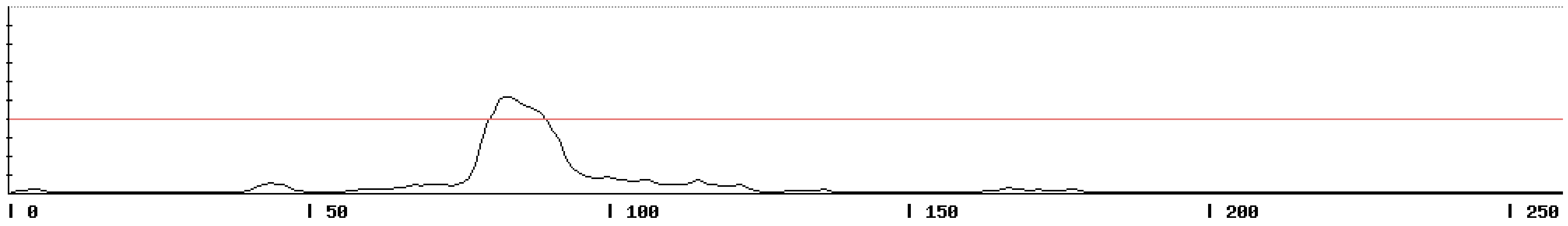

Predictions for Mimulusguttatus_mgv1a012228m

Viterbi Path	
Viterbi Path	
Posterior @ 0.4	
Posterior @ 0.4	
Posterior Graphic. Threshold Marked @ 0.4	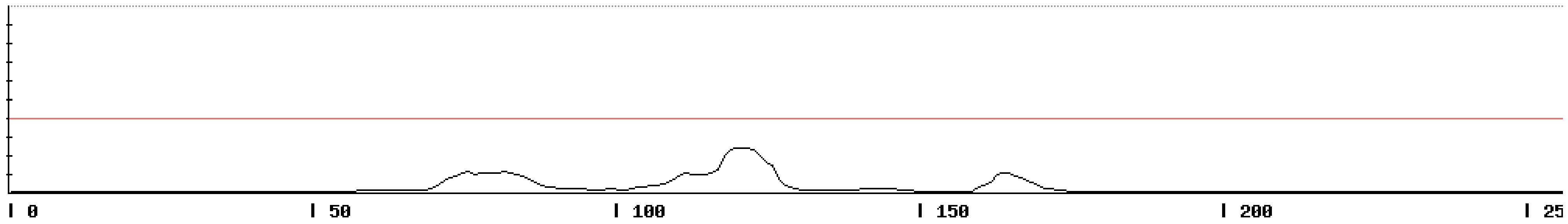

Predictions for Mimulusguttatus_mgv1a012693m	
Viterbi Path	
Viterbi Path	
Posterior @ 0.4	
Posterior @ 0.4	
Posterior Graphic. Threshold Marked @ 0.4	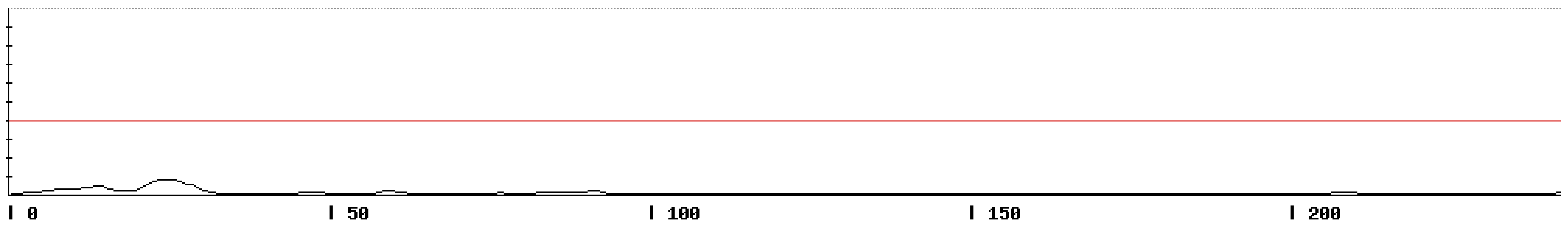

Predictions for Mimulusguttatus_mgv1a012899m	
Viterbi Path	
Viterbi Path	
Posterior @ 0.4	
Posterior @ 0.4	
Posterior Graphic. Threshold Marked @ 0.4	

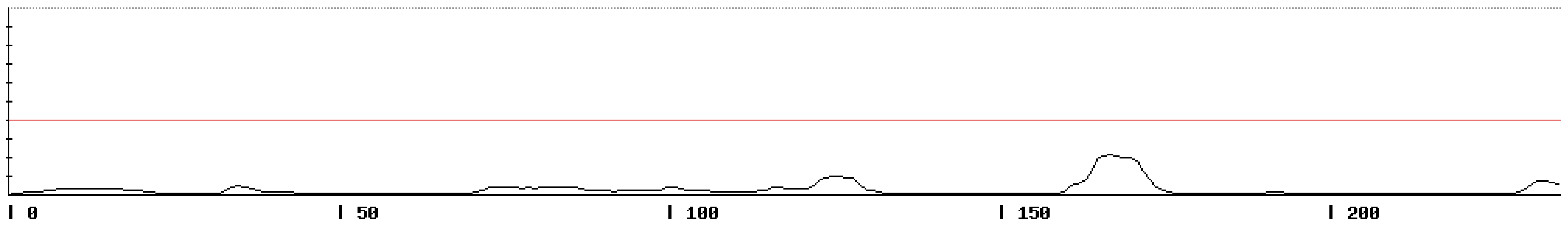

Predictions for Mimulusguttatus_mgv1a013180m

Viterbi Path	
Viterbi Path	
Posterior @ 0.4	165 - QRKRKARNHGKLVDSKKS - 182
Posterior @ 0.4	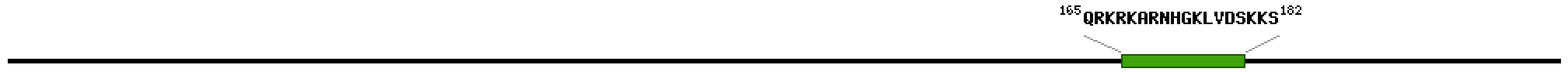
Posterior Graphic. Threshold Marked @ 0.4	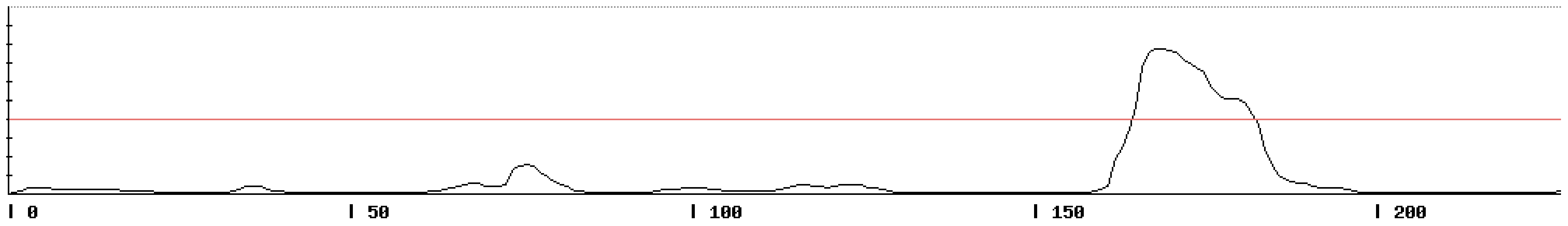

Predictions for Mimulusguttatus_mgv1a017147m

Viterbi Path	
Viterbi Path	
Posterior @ 0.4	
Posterior @ 0.4	

Posterior Graphic. Threshold Marked @ 0.4

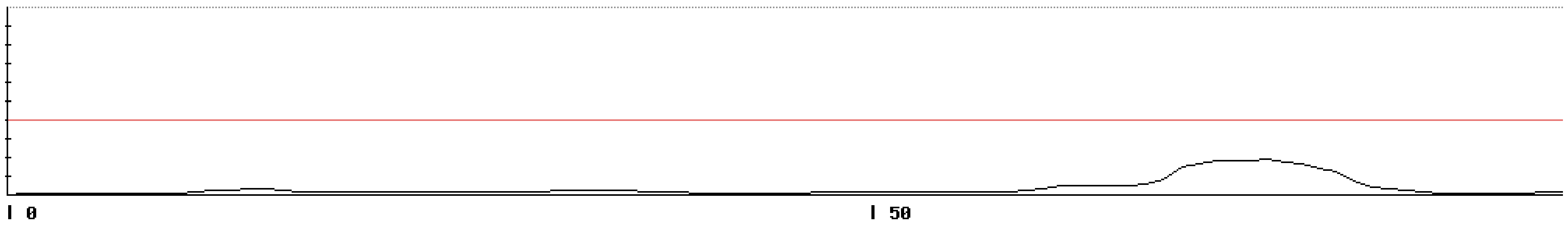

Predictions for Mimulusguttatus_mgv1a017649m

Viterbi Path

Viterbi Path

Posterior @ 0.4: 73 - K - 73

Posterior @ 0.4

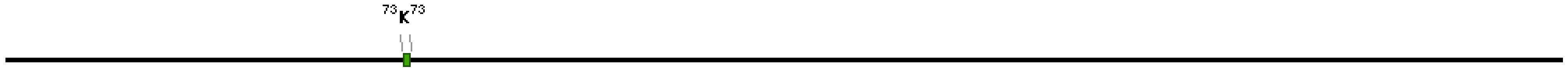

Posterior Graphic. Threshold Marked @ 0.4

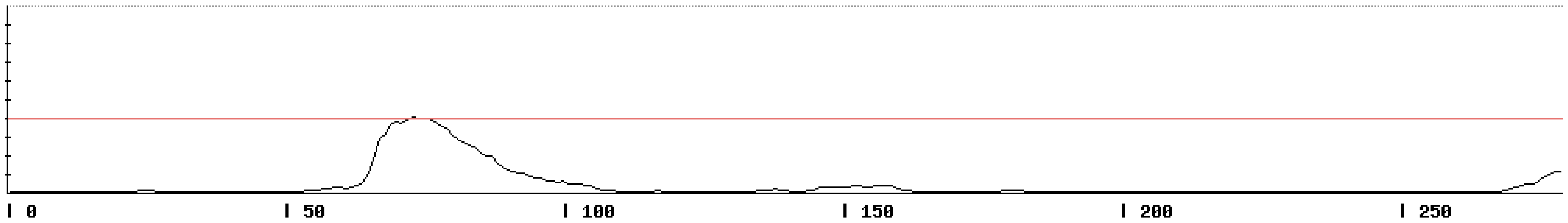

Predictions for Mimulusguttatus_mgv1a017707m

Viterbi Path

Viterbi Path

Posterior @ 0.4

Posterior @ 0.4

Posterior Graphic. Threshold Marked @ 0.4

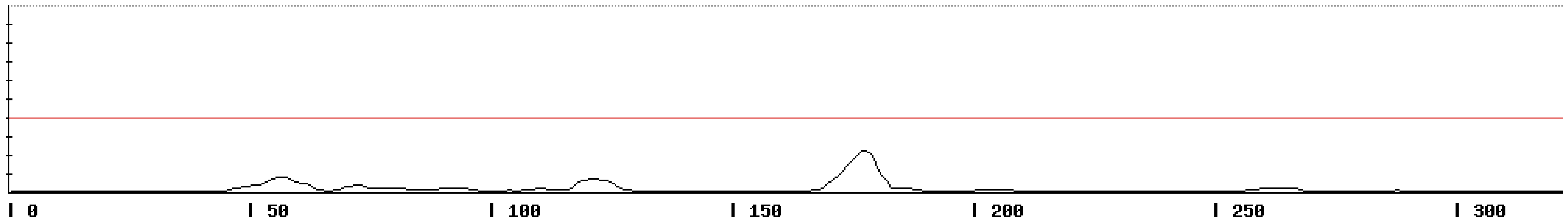

Predictions for Mimulusguttatus_mgv1a017724m

Viterbi Path

Viterbi Path

Posterior @ 0.4

Posterior @ 0.4

Posterior Graphic. Threshold Marked @ 0.4

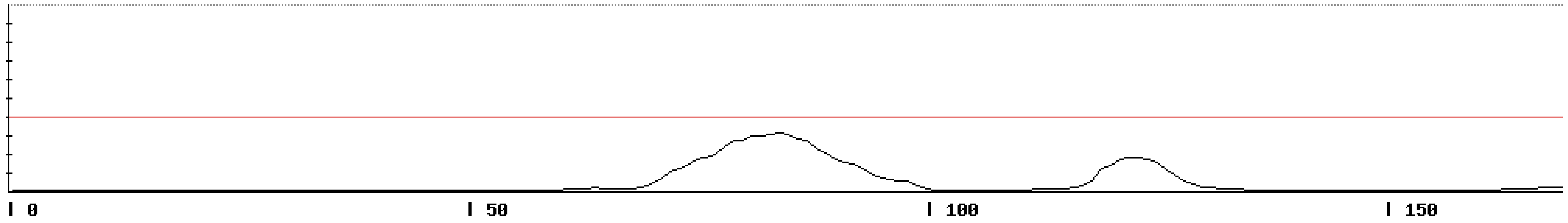

Predictions for Mimulusguttatus_mgv1a017775m

Viterbi Path

Viterbi Path

Posterior @ 0.4

Posterior @ 0.4

Posterior Graphic. Threshold Marked @ 0.4

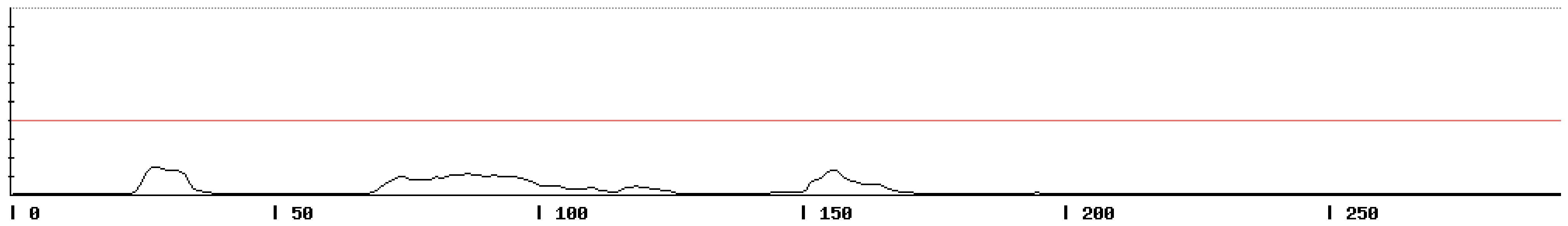

Predictions for Mimulusguttatus_mgv1a017785m

Viterbi Path

Viterbi Path

Posterior @ 0.4

Posterior @ 0.4

Posterior Graphic. Threshold Marked @ 0.4

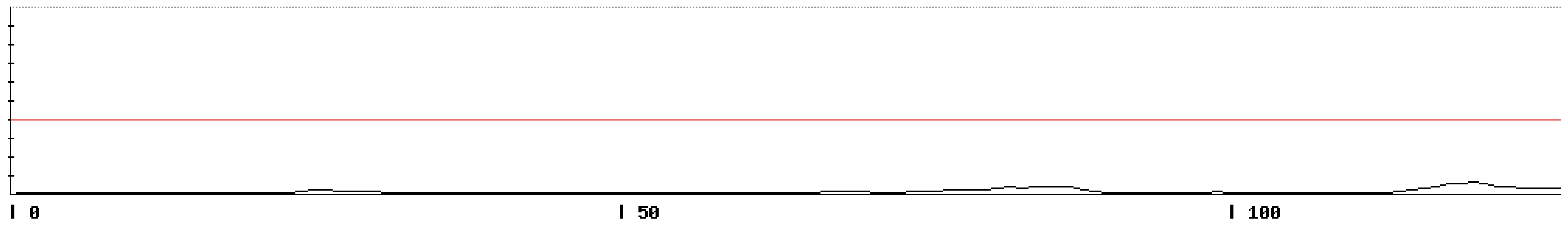

Predictions for Mimulusguttatus_mgv1a017839m

Viterbi Path 178 - KEKTKKQPKESKKIVRNRRRS - 198

Viterbi Path

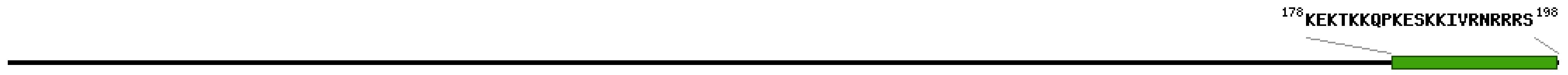

Posterior @ 0.4 | 175 - ENLKEKTKKQPKESKKIVRNRRRS - 198

Posterior @ 0.4

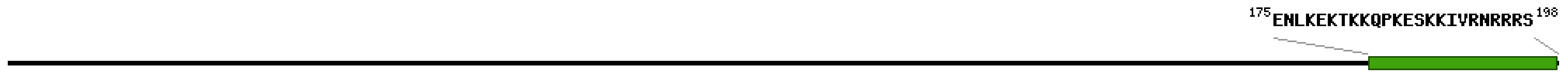

Posterior Graphic. Threshold Marked @ 0.4

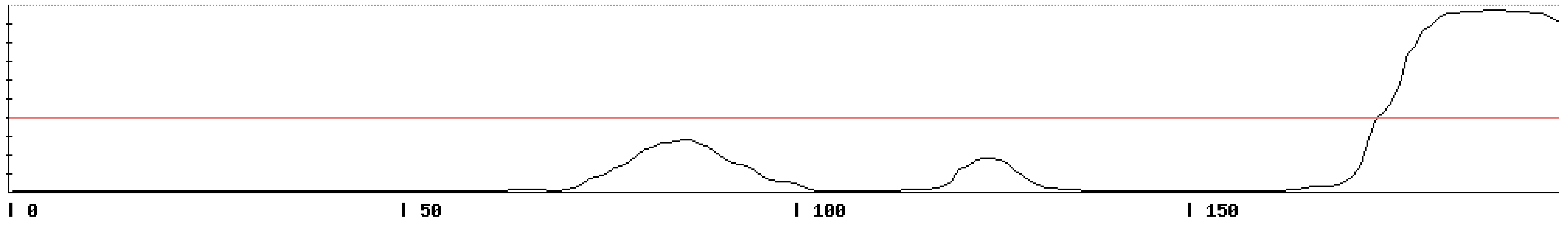

Predictions for Mimulusguttatus_mgv1a017974m

Viterbi Path

Viterbi Path

Posterior @ 0.4

Posterior @ 0.4

Posterior Graphic. Threshold Marked @ 0.4

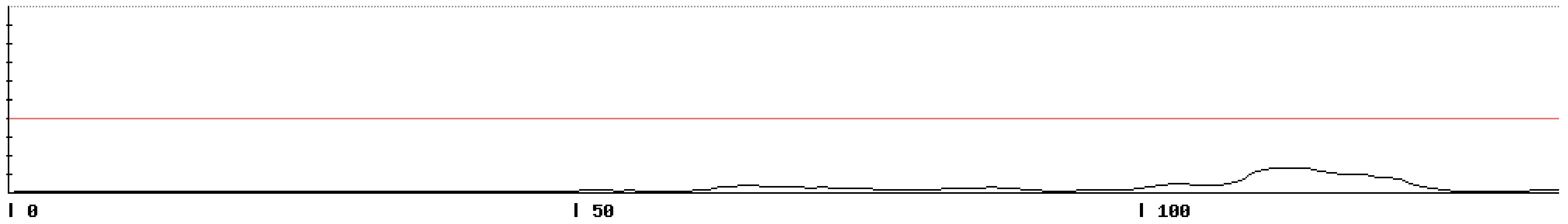

Predictions for Mimulusguttatus_mgv1a018136m

Viterbi Path	
Viterbi Path	
Posterior @ 0.4	
Posterior @ 0.4	
Posterior Graphic. Threshold Marked @ 0.4	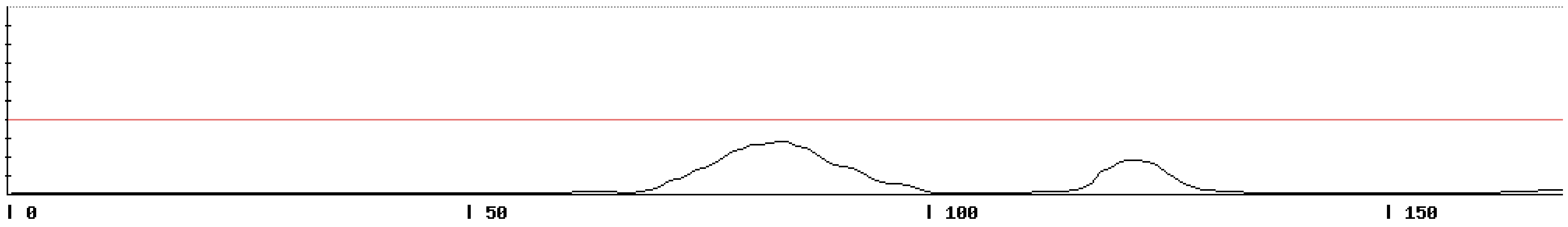

Predictions for Mimulusguttatus_mgv1a018141m

Viterbi Path	
Viterbi Path	
Posterior @ 0.4	
Posterior @ 0.4	
Posterior Graphic. Threshold Marked @ 0.4	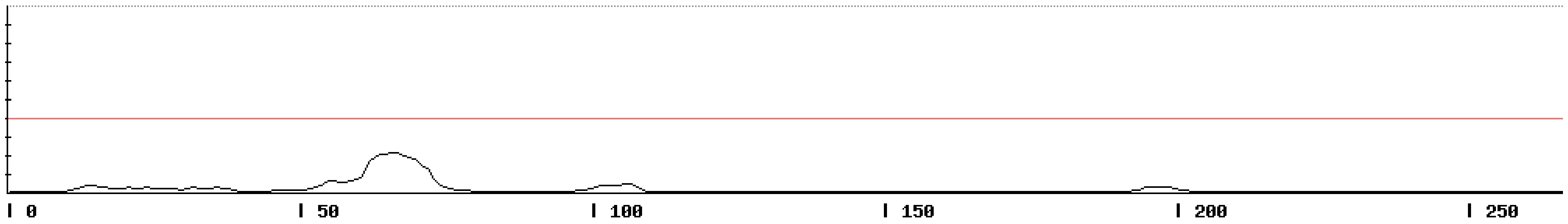

Predictions for Mimulusguttatus_mgv1a018160m

Viterbi Path	
Viterbi Path	
Posterior @ 0.4	
Posterior @ 0.4	
Posterior Graphic. Threshold Marked @ 0.4	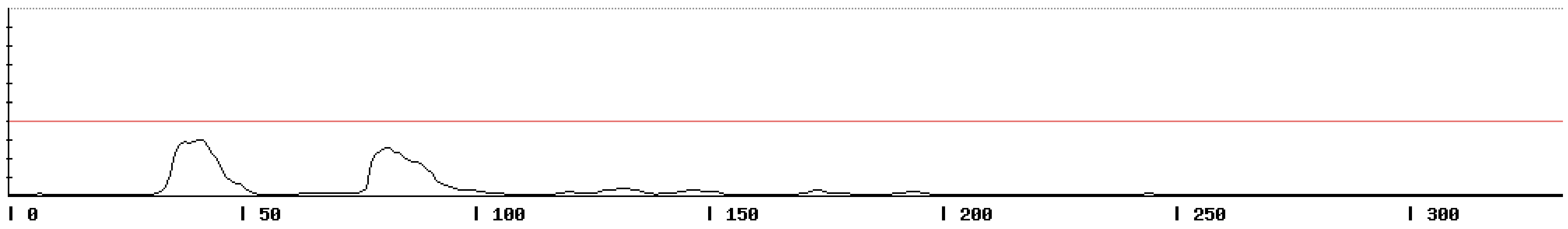

Predictions for Mimulusguttatus_mgv1a018459m

Viterbi Path	
Viterbi Path	
Posterior @ 0.4	
Posterior @ 0.4	
Posterior Graphic. Threshold Marked @ 0.4	

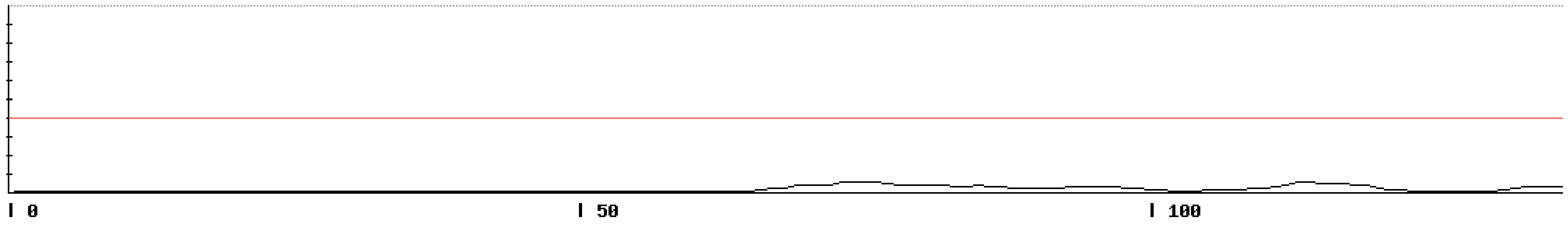

Predictions for Mimulusguttatus_mgv1a018697m

Viterbi Path	
Viterbi Path	
Posterior @ 0.4	
Posterior @ 0.4	
Posterior Graphic. Threshold Marked @ 0.4	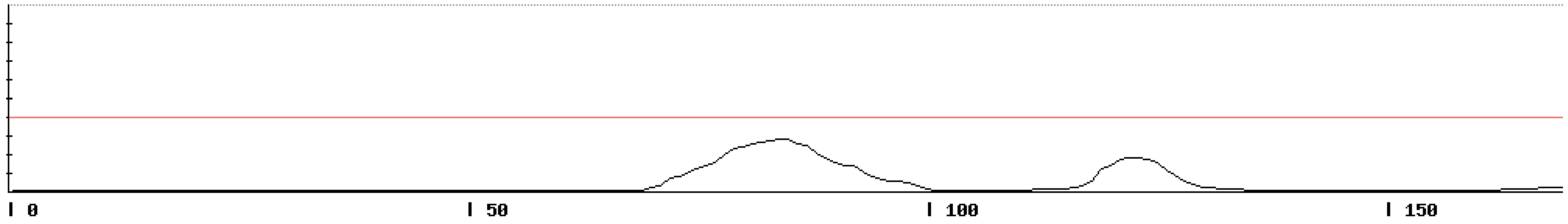

Predictions for Mimulusguttatus_mgv1a018754m

Viterbi Path	
Viterbi Path	
Posterior @ 0.4	
Posterior @ 0.4	

Posterior Graphic. Threshold Marked @ 0.4

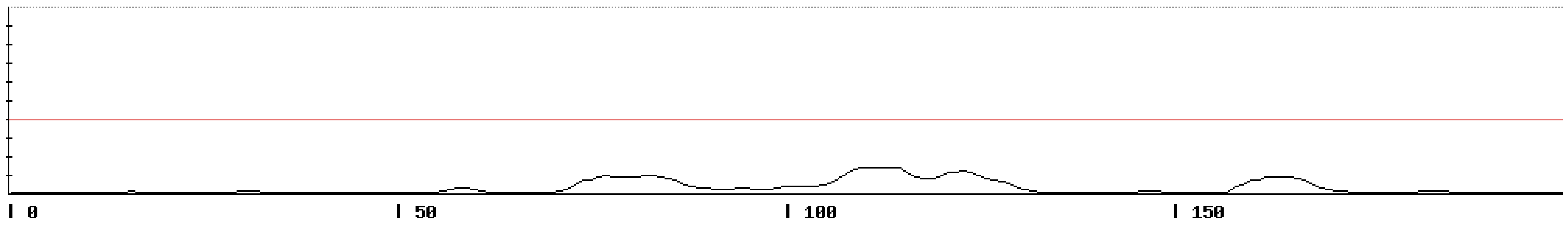

Predictions for Mimulusguttatus_mgv1a018900m

Viterbi Path

Viterbi Path

Posterior @ 0.4 188 - RRA - 190

Posterior @ 0.4

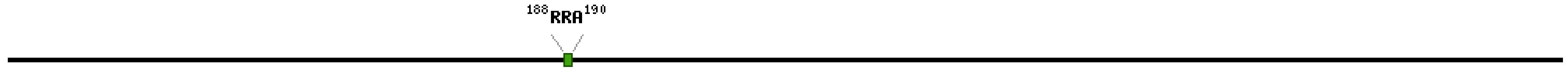

Posterior Graphic. Threshold Marked @ 0.4

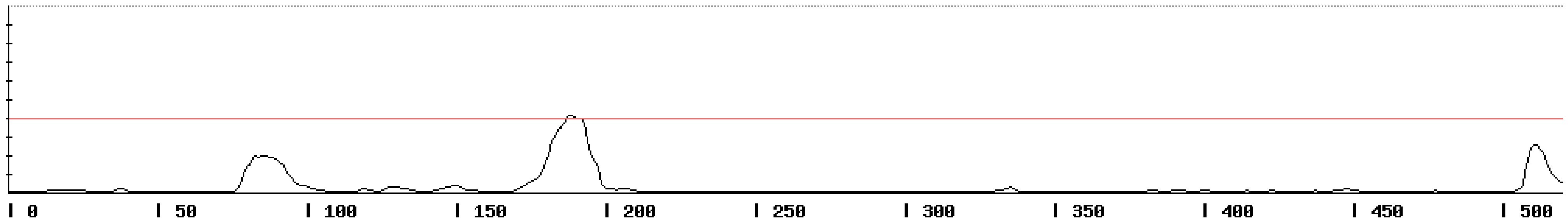

Predictions for Mimulusguttatus_mgv1a018985m

Viterbi Path

Viterbi Path

Posterior @ 0.4 174 - KEK - 176

Posterior @ 0.4

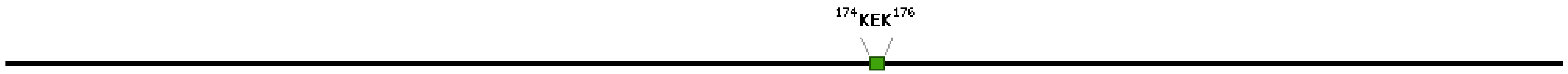

Posterior Graphic. Threshold Marked @ 0.4

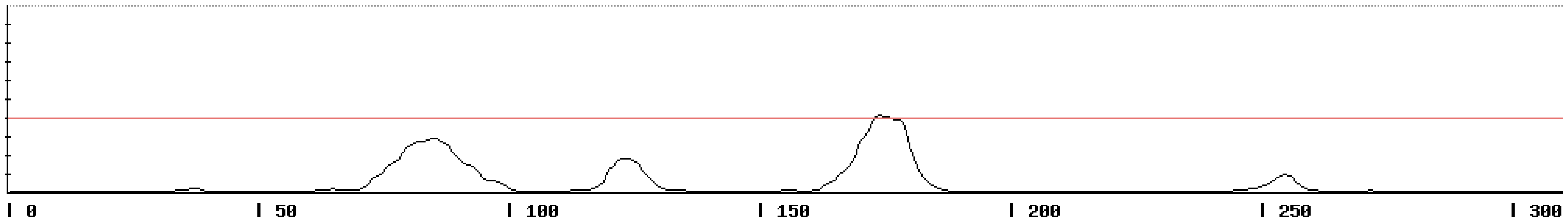

Predictions for Mimulusguttatus_mgv1a019064m

Viterbi Path

Viterbi Path

Posterior @ 0.4

Posterior @ 0.4

Posterior Graphic. Threshold Marked @ 0.4

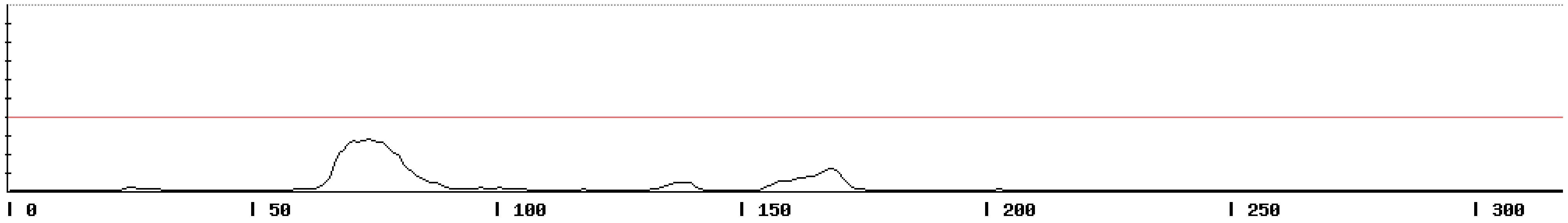

Predictions for Mimulusguttatus_mgv1a019069m

Viterbi Path

Viterbi Path

Posterior @ 0.4

Posterior @ 0.4

Posterior Graphic. Threshold Marked @ 0.4

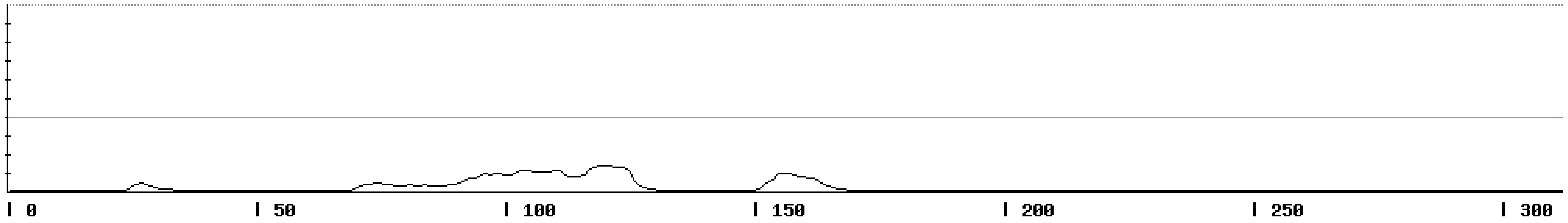

Predictions for Mimulusguttatus_mgv1a019307m

Viterbi Path

Viterbi Path

Posterior @ 0.4

Posterior @ 0.4

Posterior Graphic. Threshold Marked @ 0.4

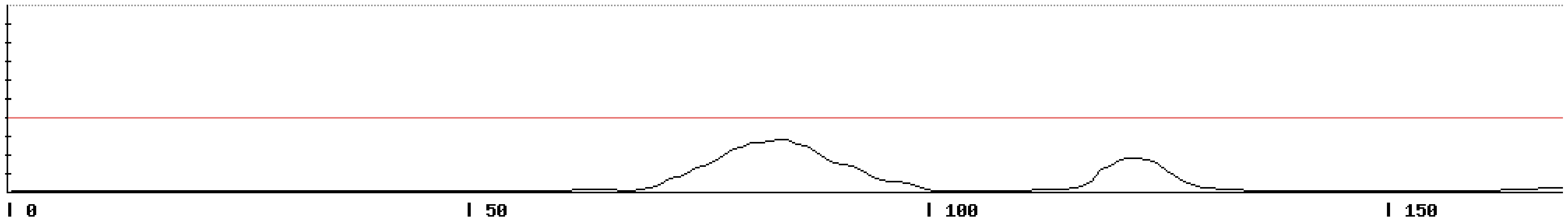

Predictions for Mimulusguttatus_mgv1a019349m

Viterbi Path

Viterbi Path

Posterior @ 0.4

Posterior @ 0.4

Posterior Graphic. Threshold Marked @ 0.4

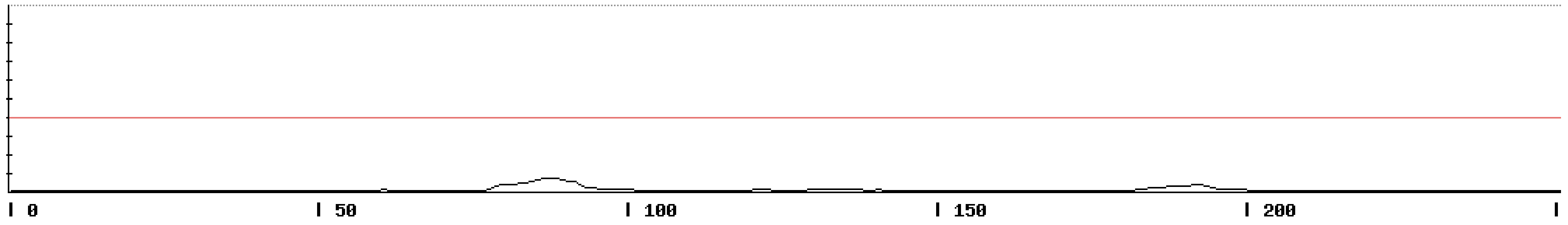

Predictions for Mimulusguttatus_mgv1a019489m

Viterbi Path

Viterbi Path

Posterior @ 0.4

Posterior @ 0.4

Posterior Graphic. Threshold Marked @ 0.4

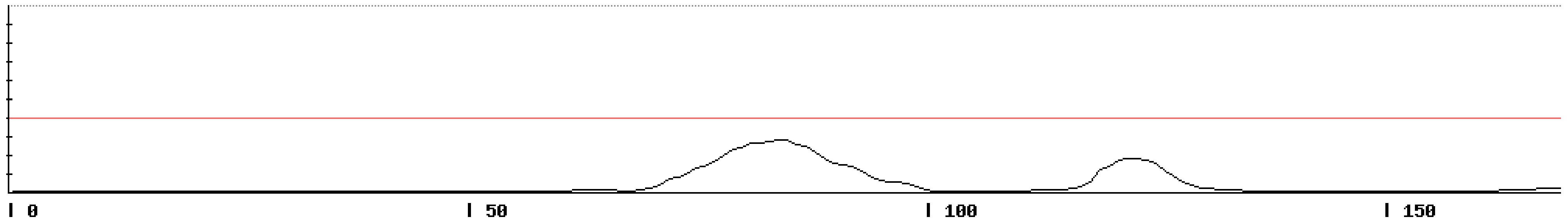

Predictions for Mimulusguttatus_mgv1a019868m

Viterbi Path	
Viterbi Path	
Posterior @ 0.4	
Posterior @ 0.4	
Posterior Graphic. Threshold Marked @ 0.4	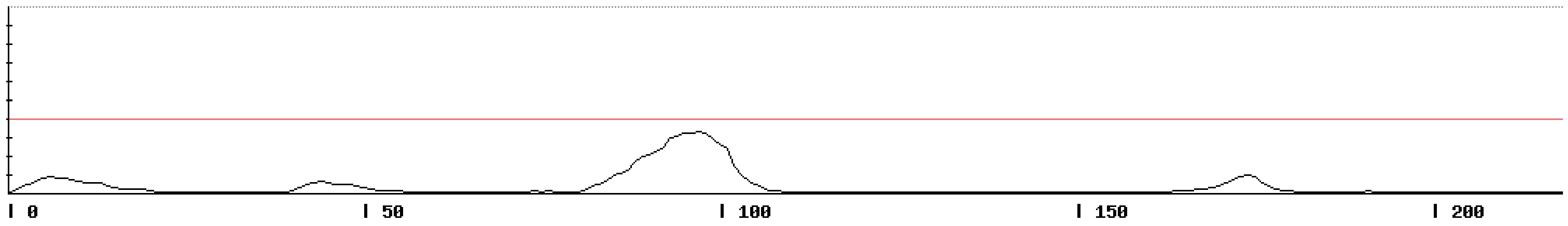

Predictions for Mimulusguttatus_mgv1a020209m

Viterbi Path	
Viterbi Path	
Posterior @ 0.4	
Posterior @ 0.4	
Posterior Graphic. Threshold Marked @ 0.4	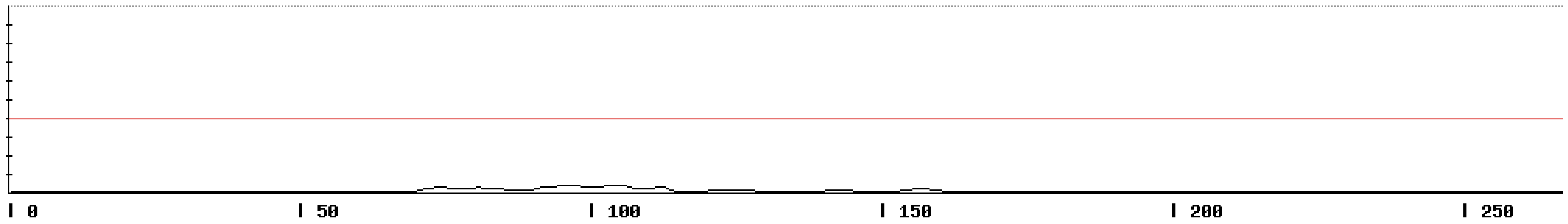

Predictions for Mimulusguttatus_mgv1a020224m	
Viterbi Path	
Viterbi Path	
Posterior @ 0.4	
Posterior @ 0.4	
Posterior Graphic. Threshold Marked @ 0.4	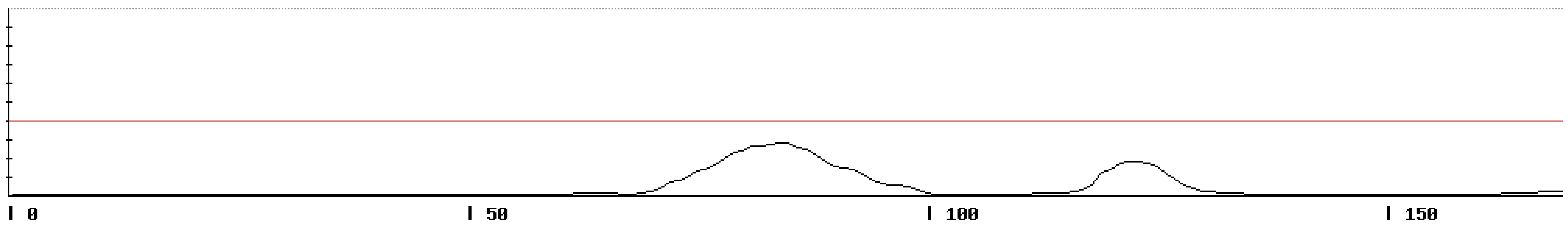

Predictions for Mimulusguttatus_mgv1a020269m	
Viterbi Path	
Viterbi Path	
Posterior @ 0.4	
Posterior @ 0.4	
Posterior Graphic. Threshold Marked @ 0.4	

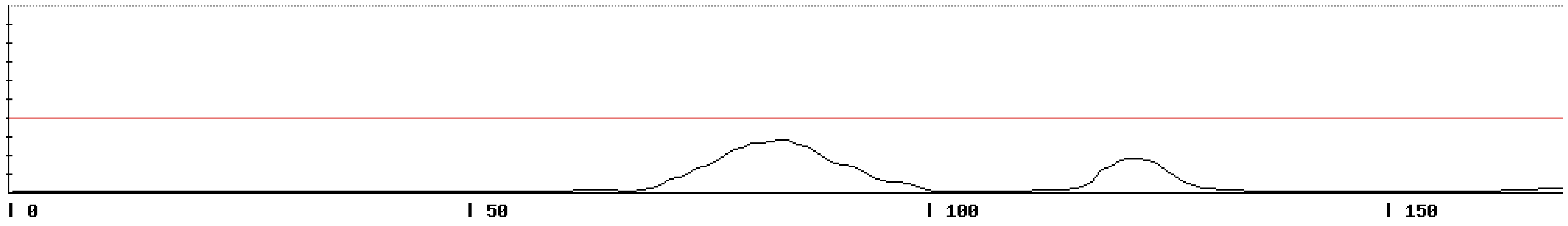

Predictions for Mimulusguttatus_mgv1a020345m

Viterbi Path

Viterbi Path

Posterior @ 0.4

Posterior @ 0.4

Posterior Graphic. Threshold Marked @ 0.4

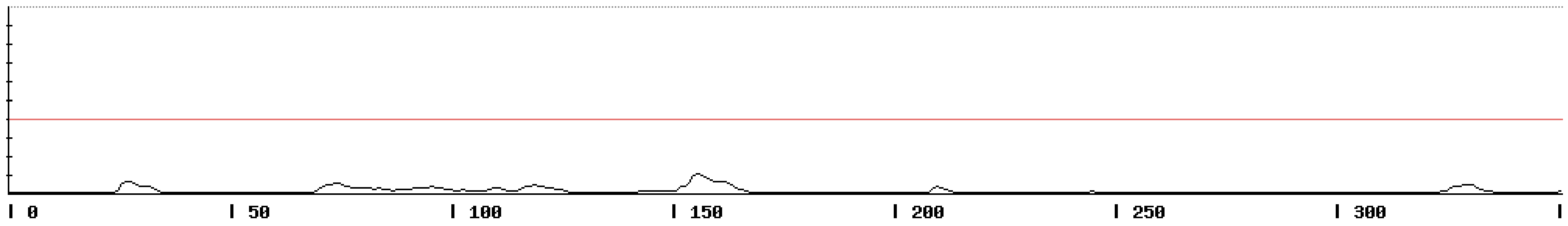

Predictions for Mimulusguttatus_mgv1a020465m

Viterbi Path

Viterbi Path

Posterior @ 0.4

Posterior @ 0.4

Posterior Graphic. Threshold Marked @ 0.4

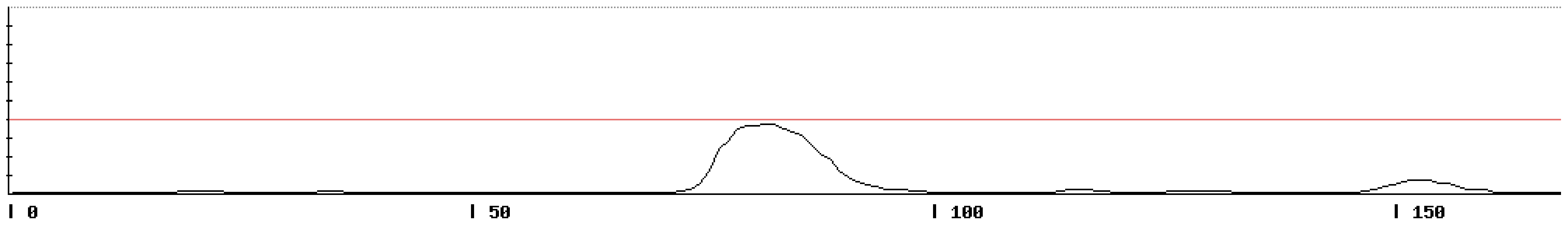

Predictions for Mimulusguttatus_mgv1a020467m

Viterbi Path	
Viterbi Path	
Posterior @ 0.4	143 - RKRRK - 147
Posterior @ 0.4	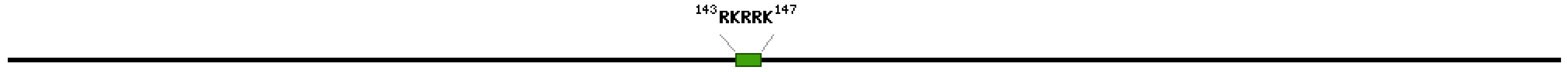
Posterior Graphic. Threshold Marked @ 0.4	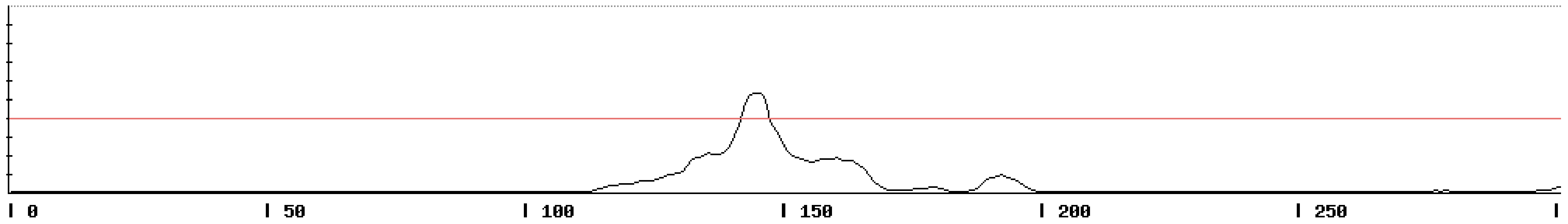

Predictions for Mimulusguttatus_mgv1a020476m

Viterbi Path	
Viterbi Path	
Posterior @ 0.4	
Posterior @ 0.4	

Posterior Graphic. Threshold Marked @ 0.4

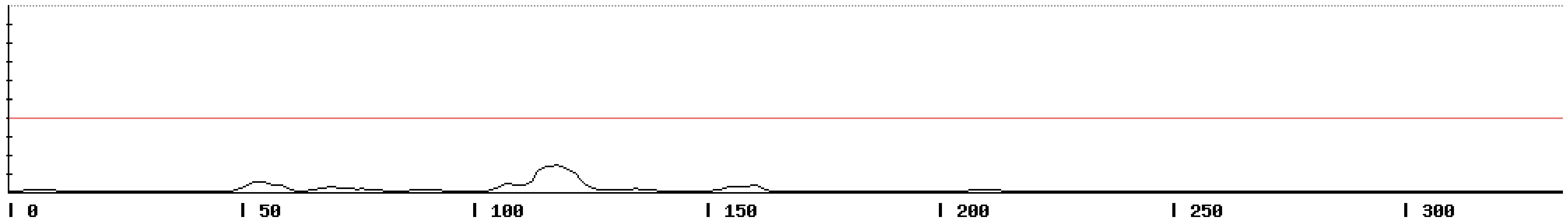

Predictions for Mimulusguttatus_mgv1a020477m

Viterbi Path

Viterbi Path

Posterior @ 0.4

Posterior @ 0.4

Posterior Graphic. Threshold Marked @ 0.4

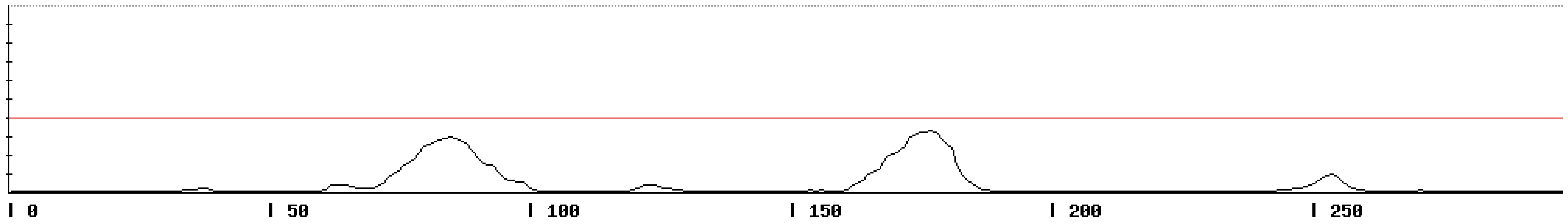

Predictions for Mimulusguttatus_mgv1a020591m

Viterbi Path

Viterbi Path

Posterior @ 0.4

Posterior @ 0.4

Posterior Graphic. Threshold Marked @ 0.4

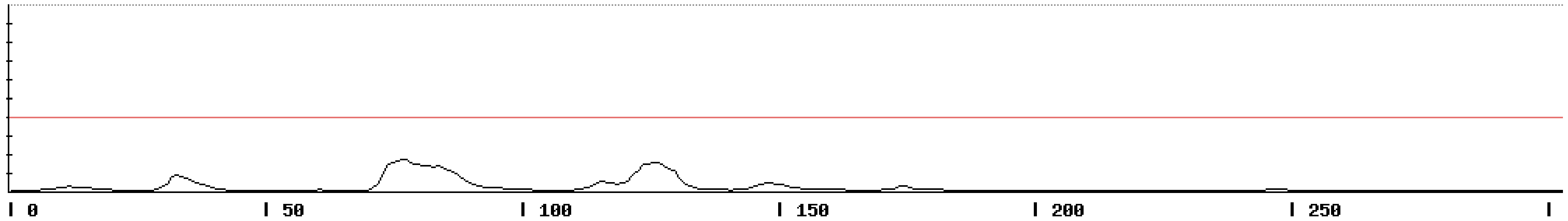

Predictions for Mimulusguttatus_mgv1a020617m

Viterbi Path

Viterbi Path

Posterior @ 0.4

Posterior @ 0.4

Posterior Graphic. Threshold Marked @ 0.4

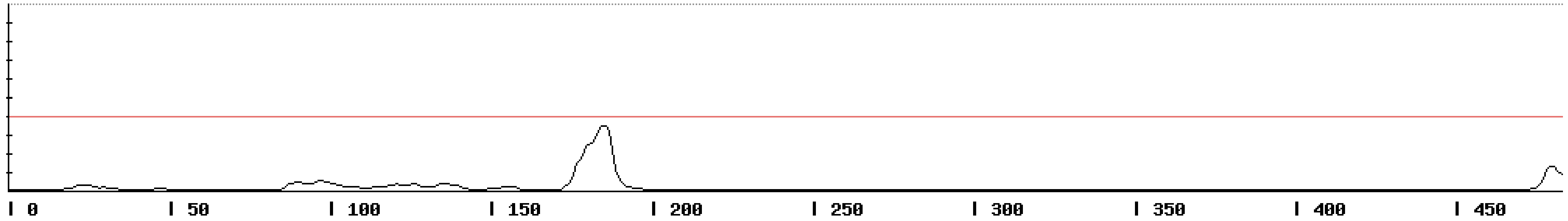

Predictions for Mimulusguttatus_mgv1a020627m

Viterbi Path

Viterbi Path

Posterior @ 0.4

Posterior @ 0.4

Posterior Graphic. Threshold Marked @ 0.4

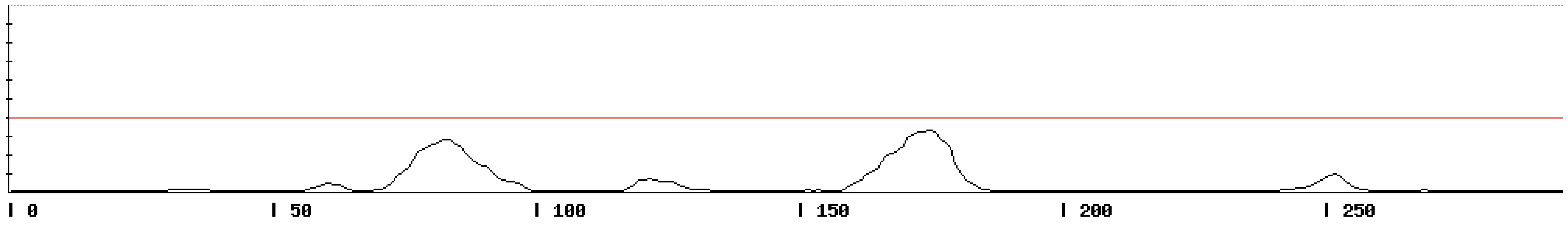

Predictions for Mimulusguttatus_mgv1a020759m

Viterbi Path

Viterbi Path

Posterior @ 0.4

Posterior @ 0.4

Posterior Graphic. Threshold Marked @ 0.4

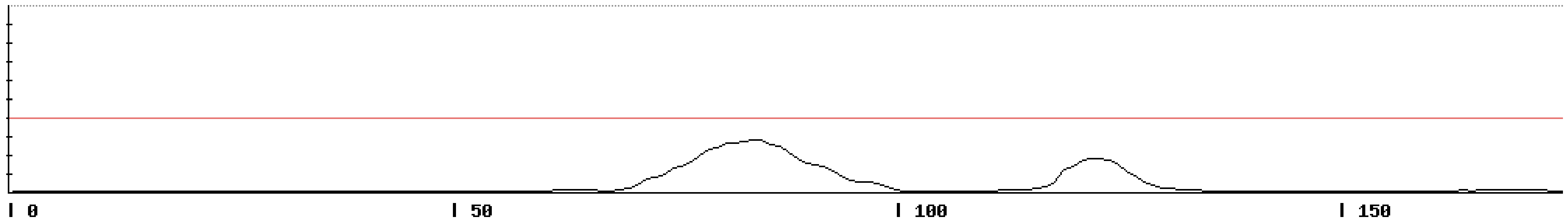

Predictions for Mimulusguttatus_mgv1a020840m

Viterbi Path

Viterbi Path

Posterior @ 0.4

Posterior @ 0.4

Posterior Graphic. Threshold Marked @ 0.4

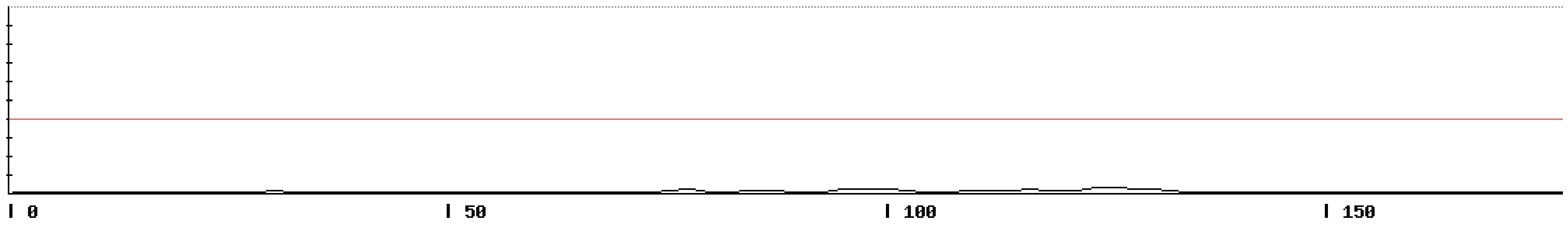

Predictions for Mimulusguttatus_mgv1a021075m

Viterbi Path

Viterbi Path

Posterior @ 0.4

Posterior @ 0.4

Posterior Graphic. Threshold Marked @ 0.4

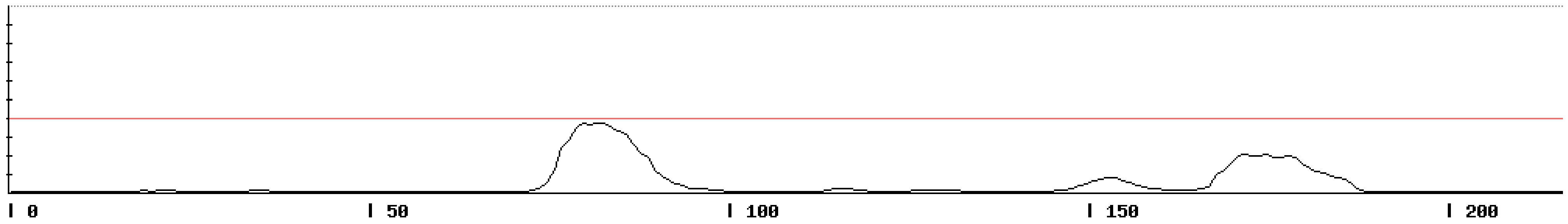

Predictions for Mimulusguttatus_mgv1a021471m

Viterbi Path

Viterbi Path

Posterior @ 0.4

Posterior @ 0.4

Posterior Graphic. Threshold Marked @ 0.4

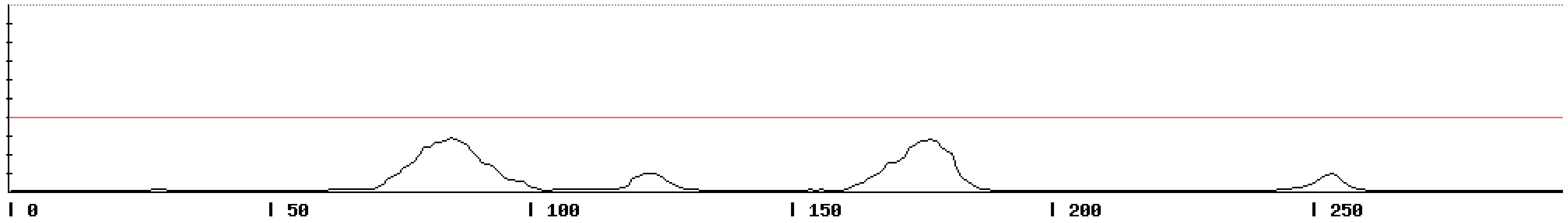

Predictions for Mimulusguttatus_mgv1a021525m

Viterbi Path

Viterbi Path

Posterior @ 0.4

Posterior @ 0.4

Posterior Graphic. Threshold Marked @ 0.4

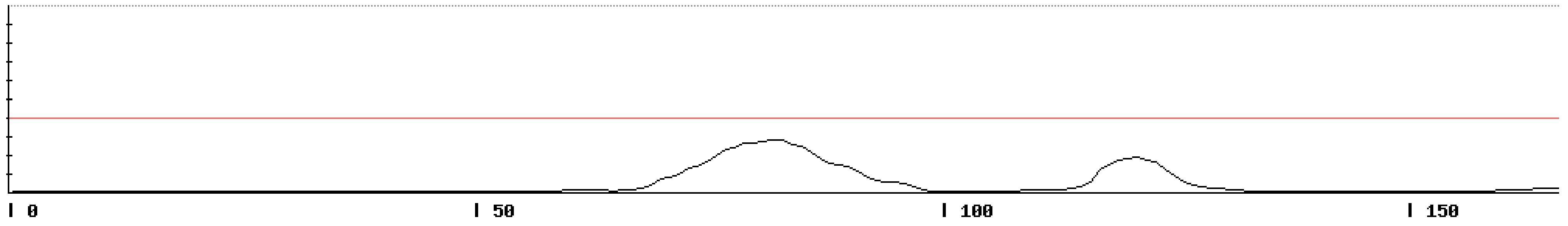

Predictions for Mimulusguttatus_mgv1a021552m

Viterbi Path

Viterbi Path

Posterior @ 0.4

Posterior @ 0.4

Posterior Graphic. Threshold Marked @ 0.4

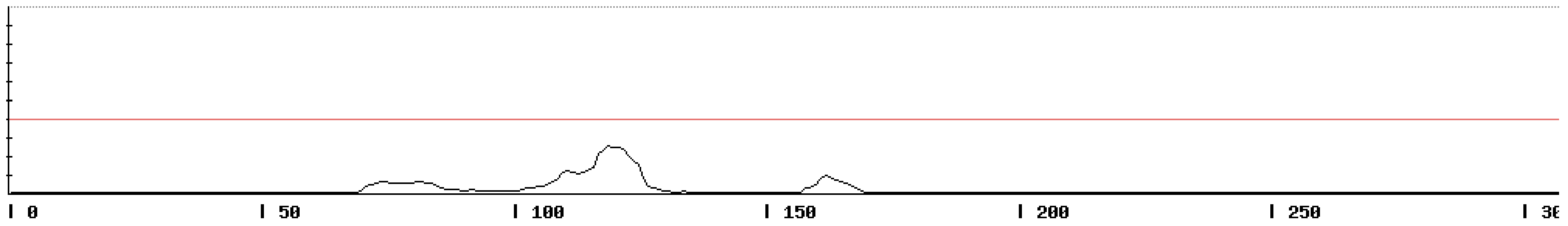

Predictions for Mimulusguttatus_mgv1a021980m

Viterbi Path

Viterbi Path

Posterior @ 0.4

Posterior @ 0.4

Posterior Graphic. Threshold Marked @ 0.4	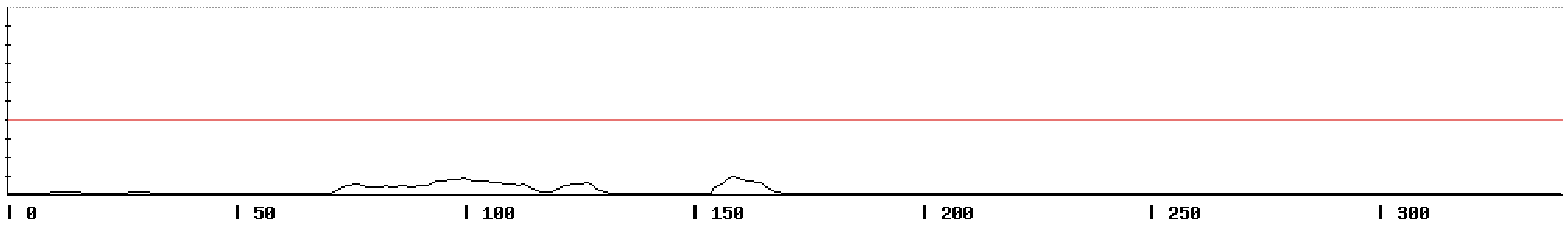

Predictions for Mimulusguttatus_mgv1a022092m	
Viterbi Path	
Viterbi Path	
Posterior @ 0.4	467 - RKRRH - 471
Posterior @ 0.4	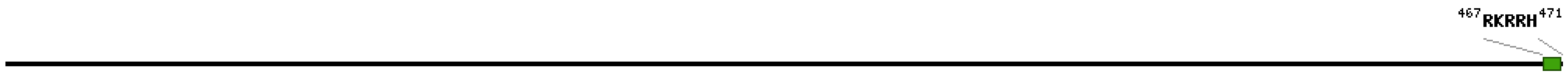
Posterior Graphic. Threshold Marked @ 0.4	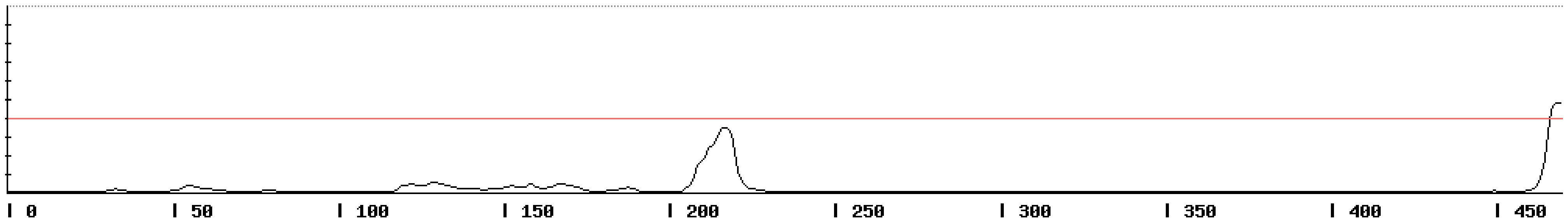

Predictions for Mimulusguttatus_mgv1a022233m	
Viterbi Path	
Viterbi Path	
Posterior @ 0.4	326 - RSPKRRH - 332
Posterior @ 0.4	

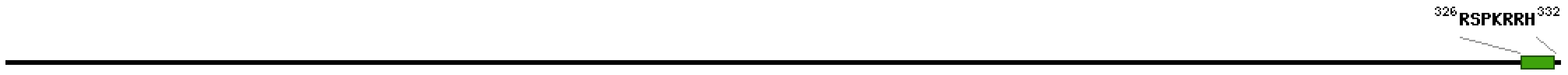

Posterior Graphic. Threshold Marked @ 0.4

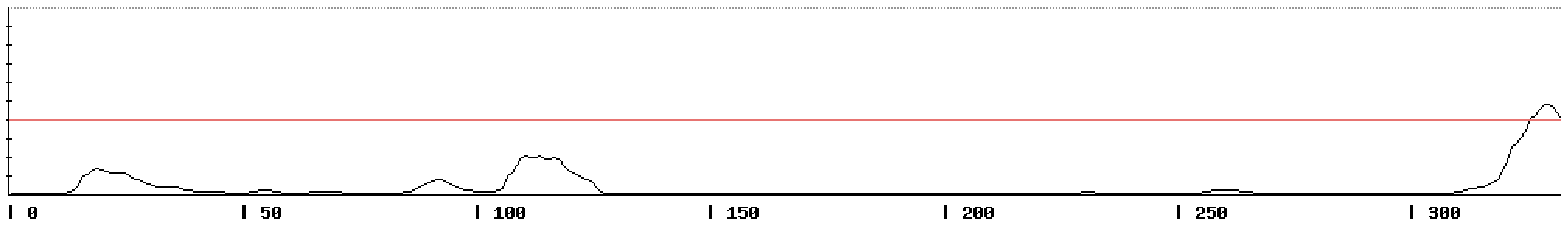

Predictions for Mimulusguttatus_mgv1a022295m

Viterbi Path

Viterbi Path

Posterior @ 0.4

Posterior @ 0.4

Posterior Graphic. Threshold Marked @ 0.4

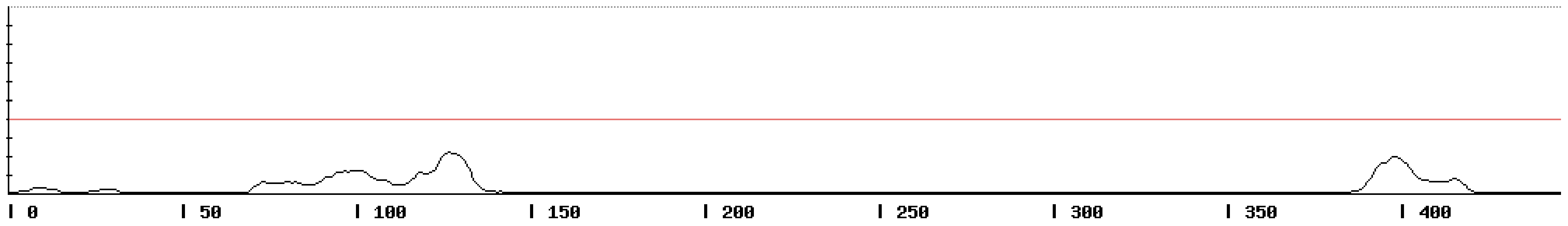

Predictions for Mimulusguttatus_mgv1a022481m

Viterbi Path

Viterbi Path

Posterior @ 0.4

Posterior @ 0.4

Posterior Graphic. Threshold Marked @ 0.4

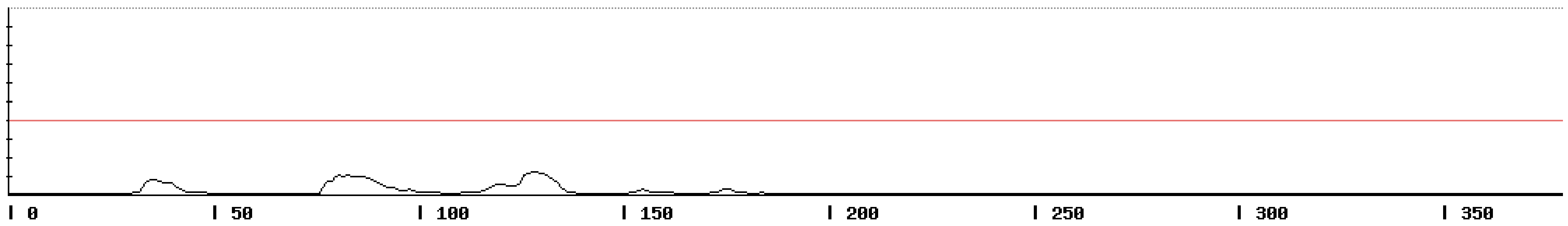

Predictions for Mimulusguttatus_mgv1a022548m

Viterbi Path

Viterbi Path

Posterior @ 0.4

Posterior @ 0.4

Posterior Graphic. Threshold Marked @ 0.4

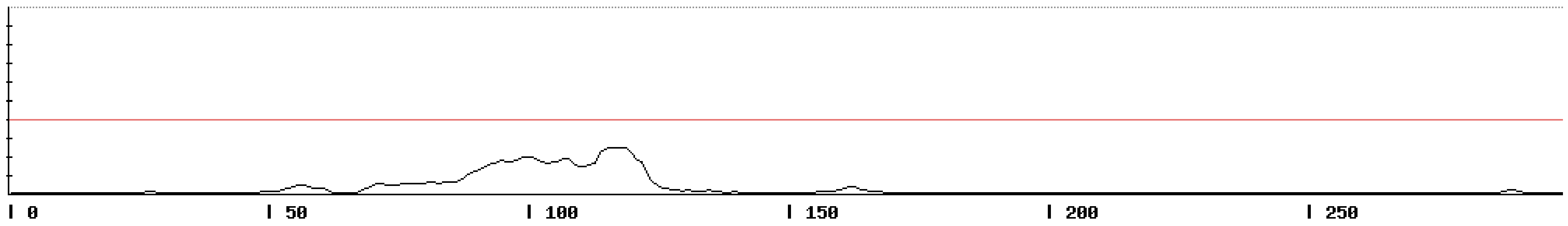

Predictions for Mimulusguttatus_mgv1a022601m

Viterbi Path

Viterbi Path

Posterior @ 0.4

Posterior @ 0.4

Posterior Graphic. Threshold Marked @ 0.4

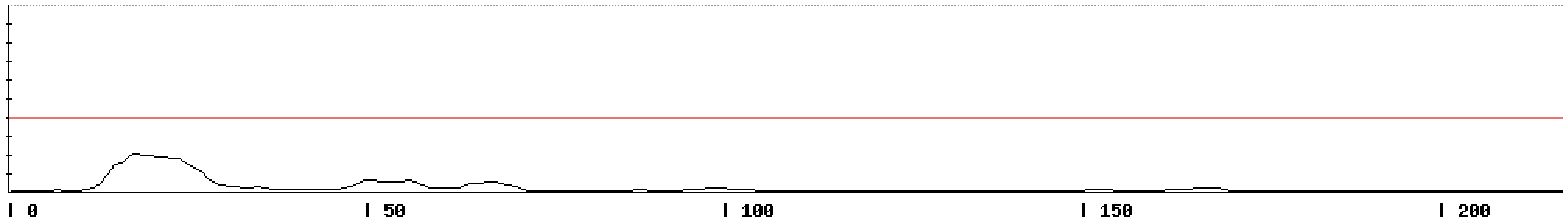

Predictions for Mimulusguttatus_mgv1a022636m

Viterbi Path

Viterbi Path

Posterior @ 0.4

Posterior @ 0.4

Posterior Graphic. Threshold Marked @ 0.4

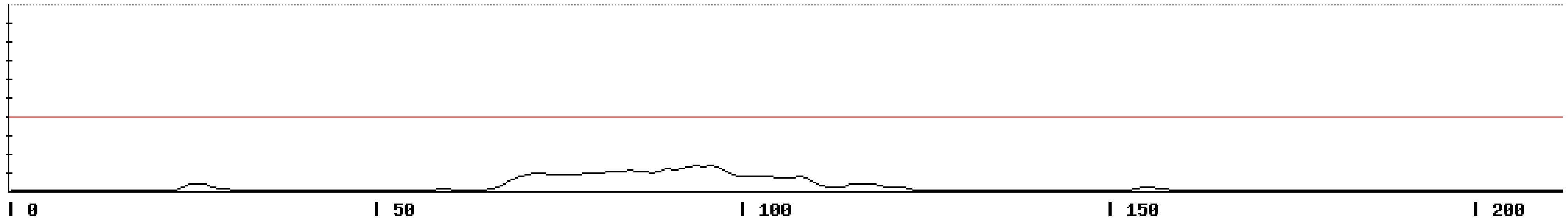

Predictions for Mimulusguttatus_mgv1a022798m

Viterbi Path	
Viterbi Path	
Posterior @ 0.4	
Posterior @ 0.4	
Posterior Graphic. Threshold Marked @ 0.4	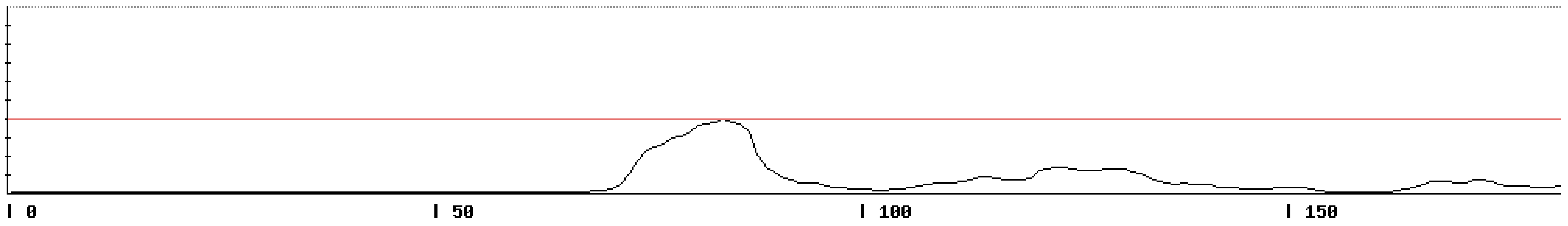

Predictions for Mimulusguttatus_mgv1a022938m

Viterbi Path	
Viterbi Path	
Posterior @ 0.4	
Posterior @ 0.4	
Posterior Graphic. Threshold Marked @ 0.4	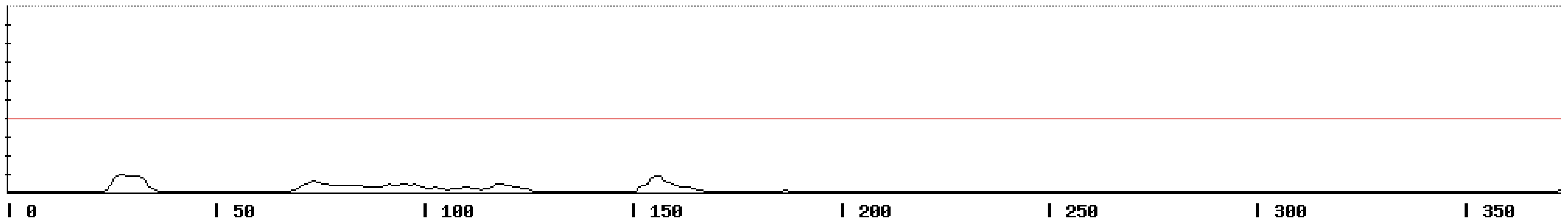

Predictions for Mimulusguttatus_mgv1a022991m

Viterbi Path

Viterbi Path

Posterior @ 0.4

Posterior @ 0.4

Posterior Graphic. Threshold Marked @ 0.4

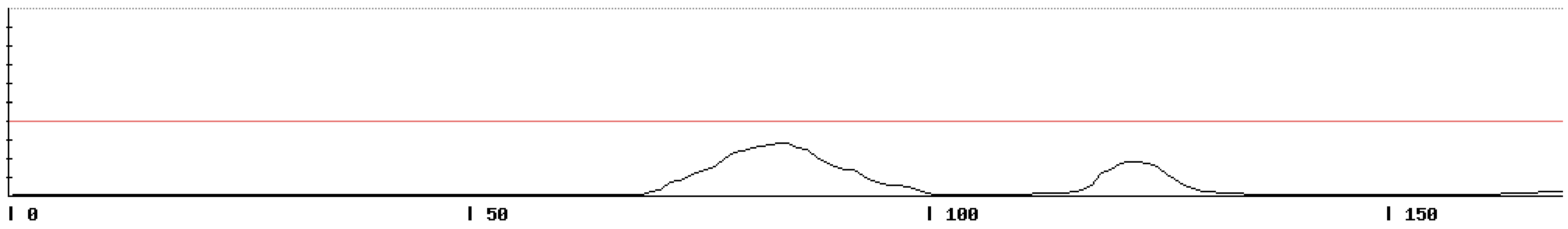

Predictions for Mimulusguttatus_mgv1a023230m

Viterbi Path

Viterbi Path

Posterior @ 0.4

Posterior @ 0.4

Posterior Graphic. Threshold Marked @ 0.4

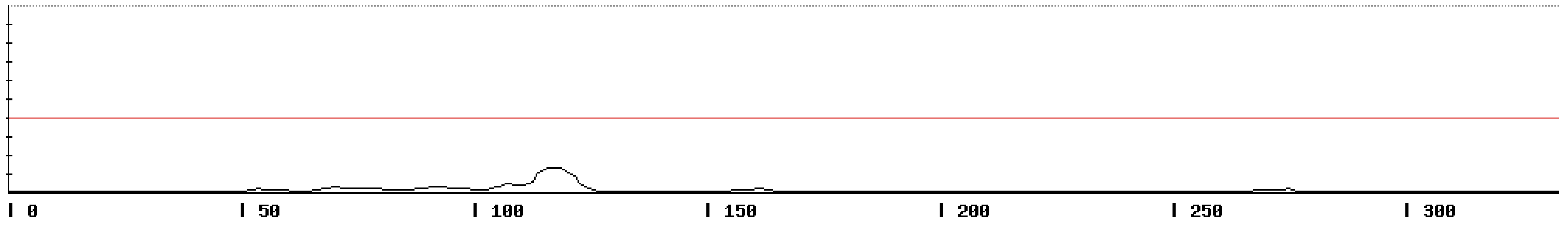

Predictions for Mimulusguttatus_mgv1a023618m

Viterbi Path	
Viterbi Path	
Posterior @ 0.4	
Posterior @ 0.4	
Posterior Graphic. Threshold Marked @ 0.4	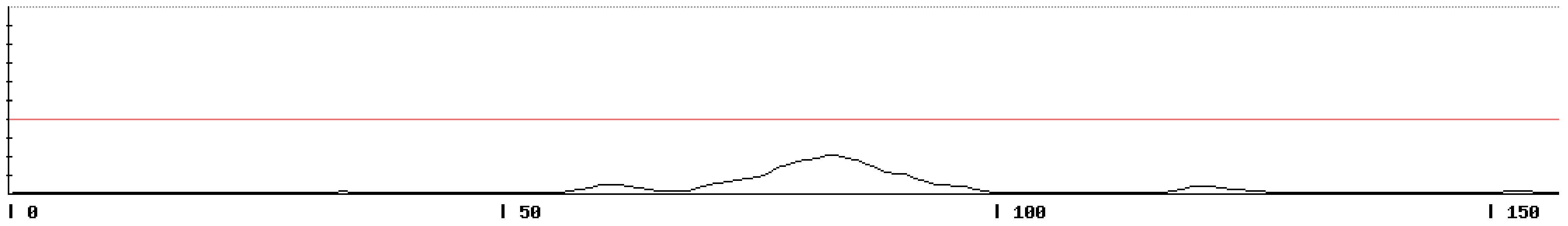

Predictions for Mimulusguttatus_mgv1a023741m

Viterbi Path	
Viterbi Path	
Posterior @ 0.4	
Posterior @ 0.4	

Posterior Graphic. Threshold Marked @ 0.4	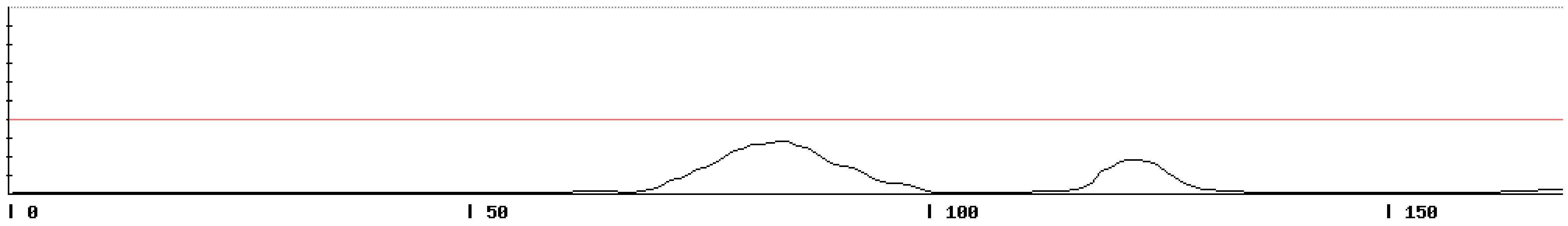

Predictions for Mimulusguttatus_mgv1a023743m	
Viterbi Path	
Viterbi Path	
Posterior @ 0.4	
Posterior @ 0.4	
Posterior Graphic. Threshold Marked @ 0.4	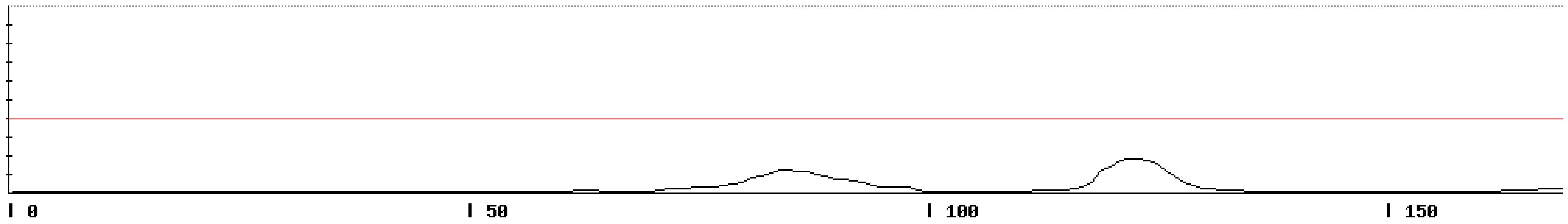

Predictions for Mimulusguttatus_mgv1a023855m	
Viterbi Path	
Viterbi Path	
Posterior @ 0.4	134 - RKRRRIEDQERTIKAAHVRWHKTGKTKSVK - 163
Posterior @ 0.4	

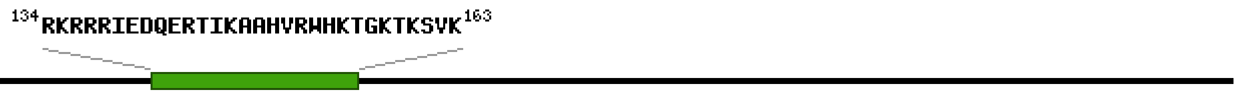

Posterior Graphic. Threshold Marked @ 0.4

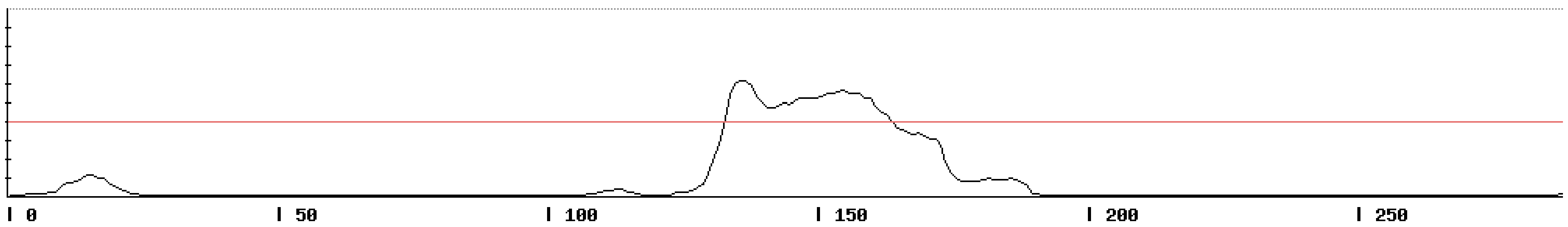

Predictions for Mimulusguttatus_mgv1a023923m

Viterbi Path

Viterbi Path

Posterior @ 0.4

Posterior @ 0.4

Posterior Graphic. Threshold Marked @ 0.4

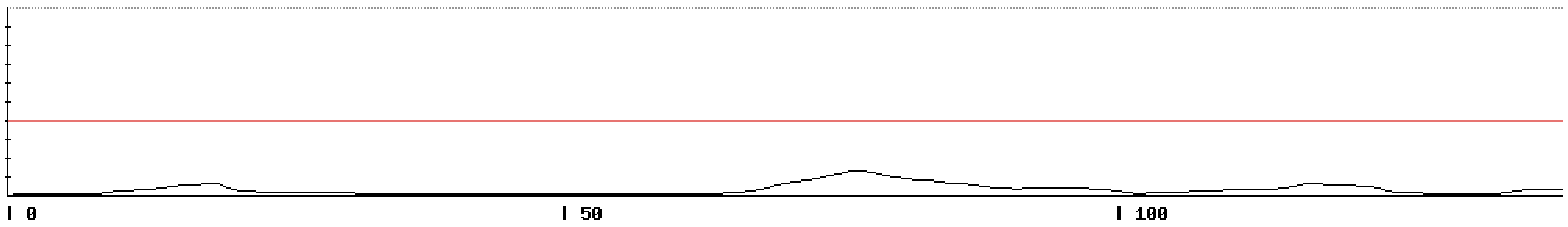

Predictions for Mimulusguttatus_mgv1a023944m

Viterbi Path

Viterbi Path

Posterior @ 0.4

Posterior @ 0.4

Posterior Graphic. Threshold Marked @ 0.4

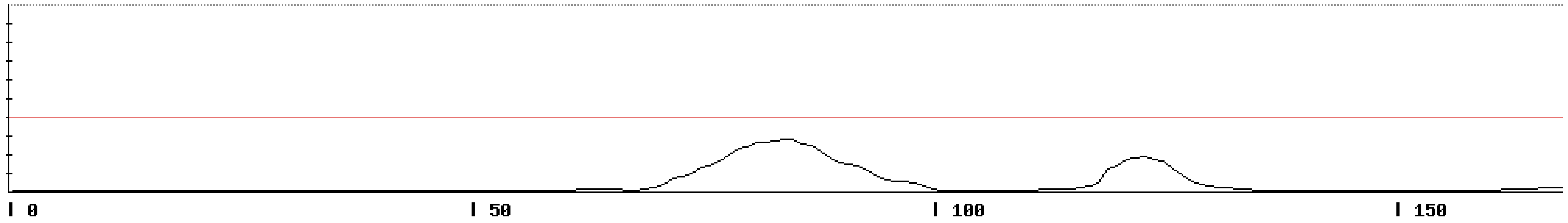

Predictions for Mimulusguttatus_mgv1a024039m

Viterbi Path

Viterbi Path

Posterior @ 0.4

Posterior @ 0.4

Posterior Graphic. Threshold Marked @ 0.4

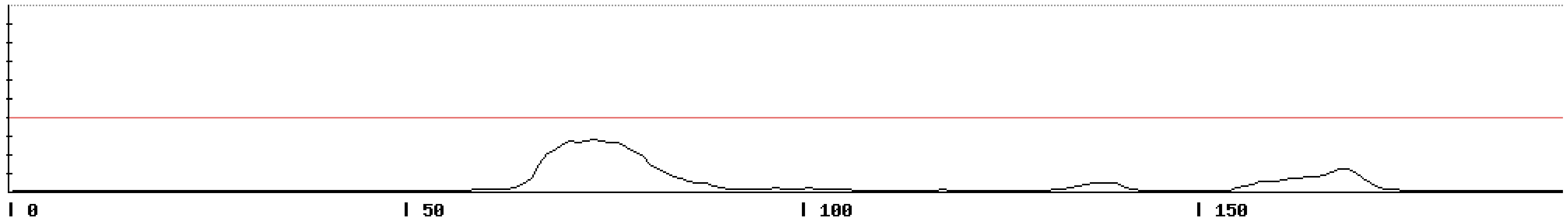

Predictions for Mimulusguttatus_mgv1a024673m

Viterbi Path

Viterbi Path

Posterior @ 0.4	
Posterior @ 0.4	
Posterior Graphic. Threshold Marked @ 0.4	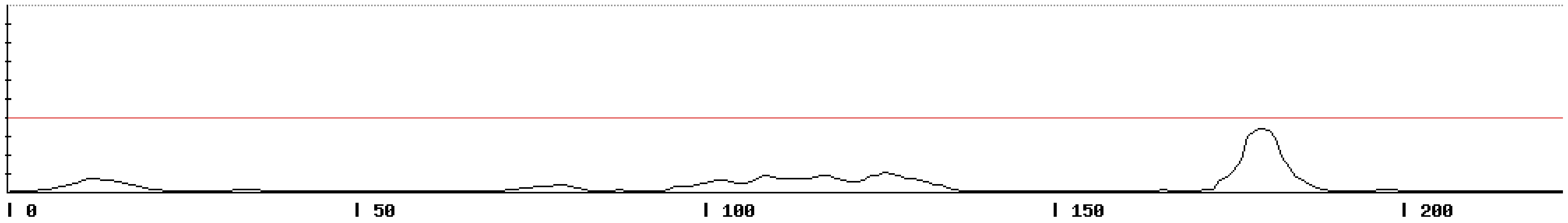

Predictions for Mimulusguttatus_mgv1a024753m

Viterbi Path	
Viterbi Path	
Posterior @ 0.4	170 - PRR - 172
Posterior @ 0.4	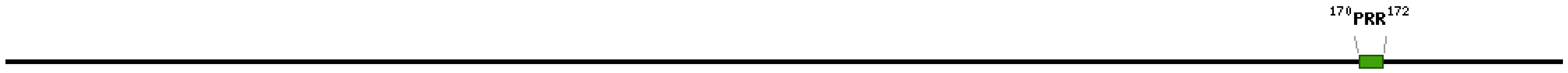
Posterior Graphic. Threshold Marked @ 0.4	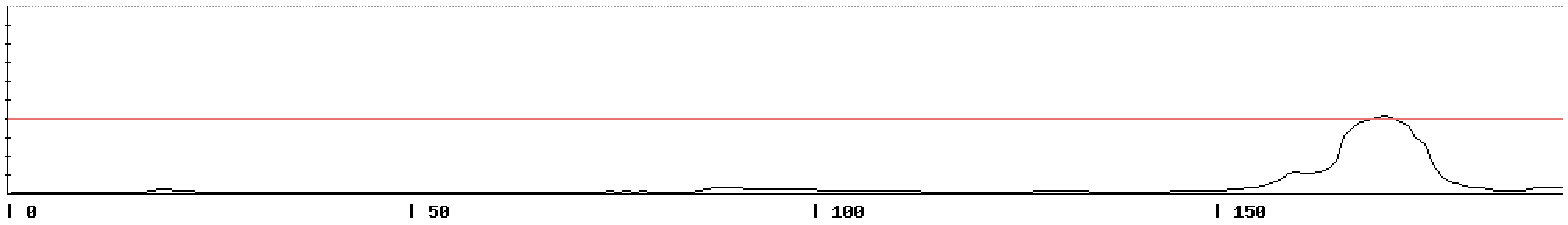

Predictions for Mimulusguttatus_mgv1a024889m

Viterbi Path	
Viterbi Path	
Posterior @ 0.4	
Posterior @ 0.4	
Posterior Graphic. Threshold Marked @ 0.4	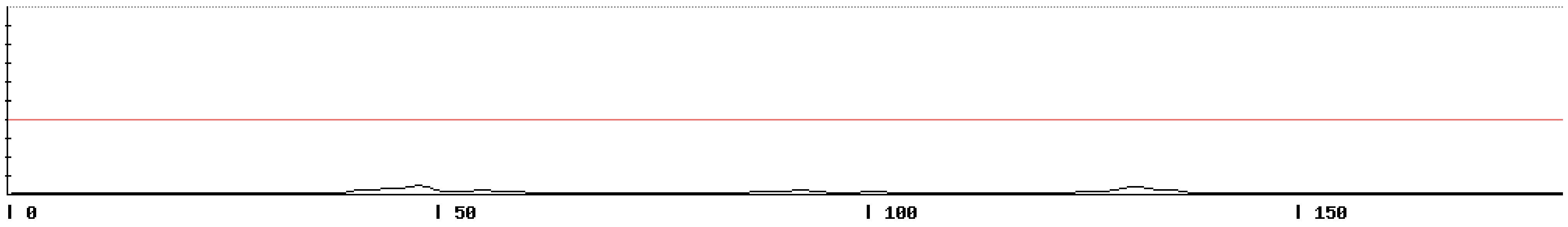

Predictions for Mimulusguttatus_mgv1a024959m

Viterbi Path	
Viterbi Path	
Posterior @ 0.4	
Posterior @ 0.4	
Posterior Graphic. Threshold Marked @ 0.4	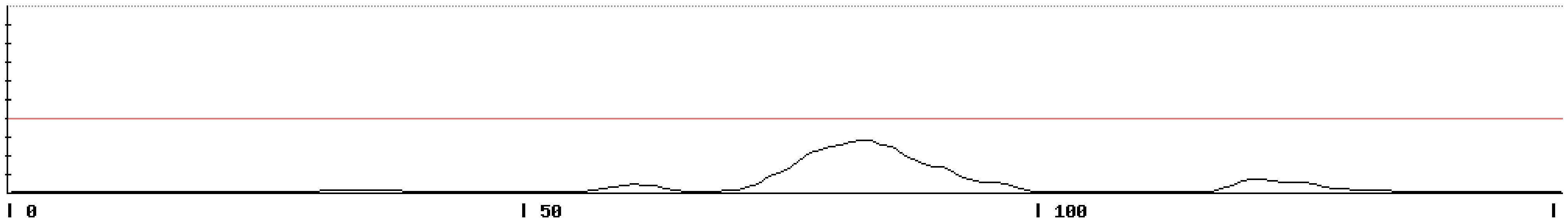

Predictions for Mimulusguttatus_mgv1a025009m	
Viterbi Path	
Viterbi Path	
Posterior @ 0.4	178 - RKTKRKRQRSSRR - 190
Posterior @ 0.4	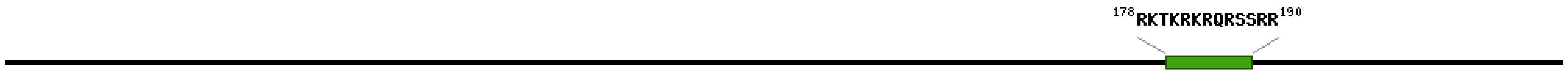
Posterior Graphic. Threshold Marked @ 0.4	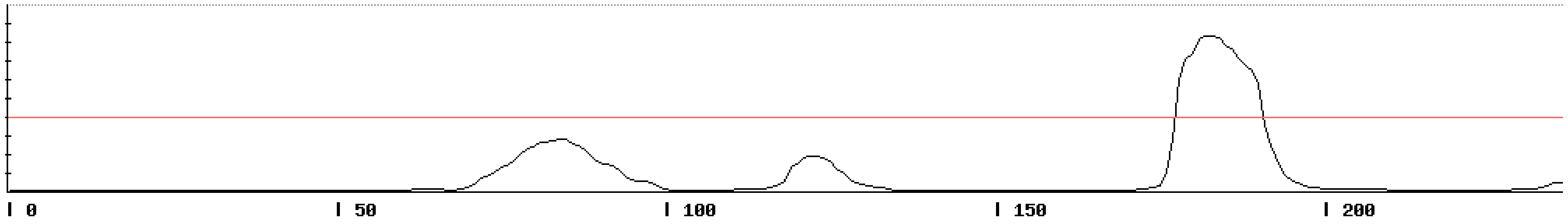

Predictions for Mimulusguttatus_mgv1a025077m	
Viterbi Path	
Viterbi Path	
Posterior @ 0.4	
Posterior @ 0.4	
Posterior Graphic. Threshold Marked @ 0.4	

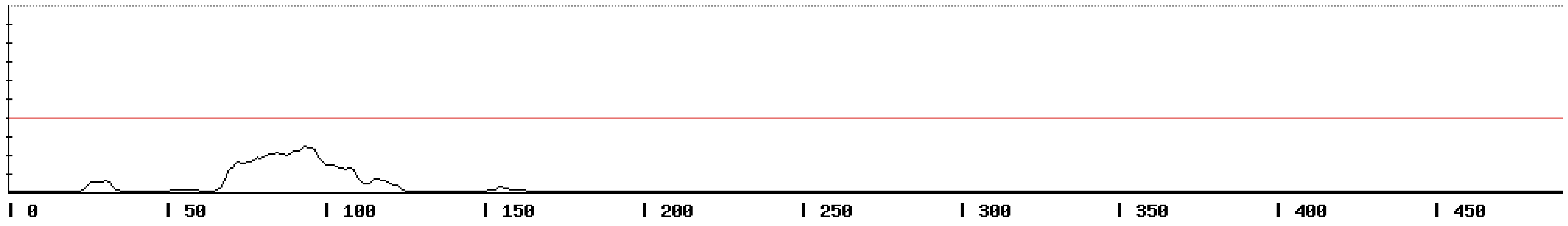

Predictions for Mimulusguttatus_mgv1a025081m

Viterbi Path

Viterbi Path

Posterior @ 0.4

Posterior @ 0.4

Posterior Graphic. Threshold Marked @ 0.4

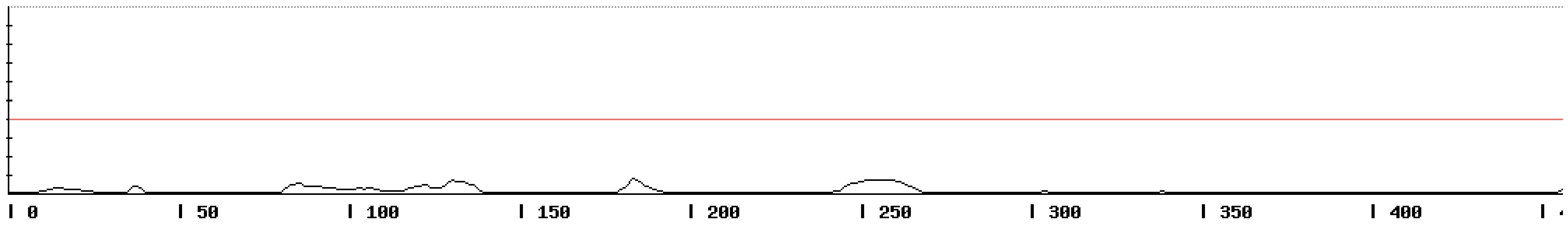

Predictions for Mimulusguttatus_mgv1a025108m

Viterbi Path

Viterbi Path

Posterior @ 0.4

Posterior @ 0.4

Posterior Graphic. Threshold Marked @ 0.4

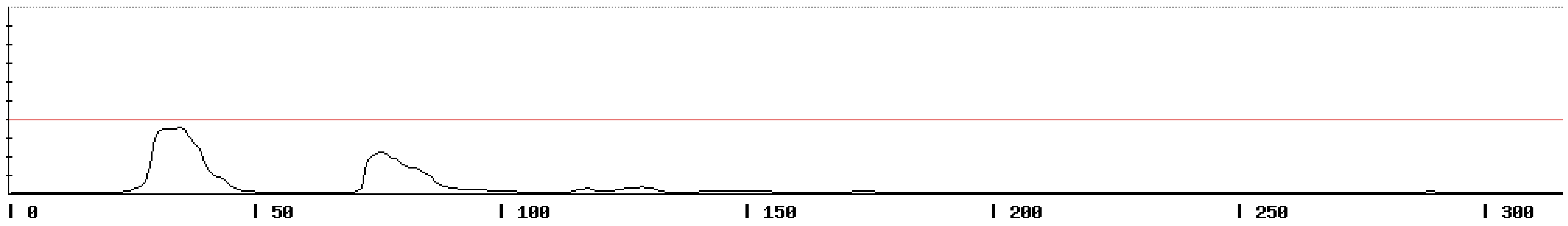

Predictions for Mimulusguttatus_mgv1a025187m

Viterbi Path

Viterbi Path

Posterior @ 0.4

Posterior @ 0.4

Posterior Graphic. Threshold Marked @ 0.4

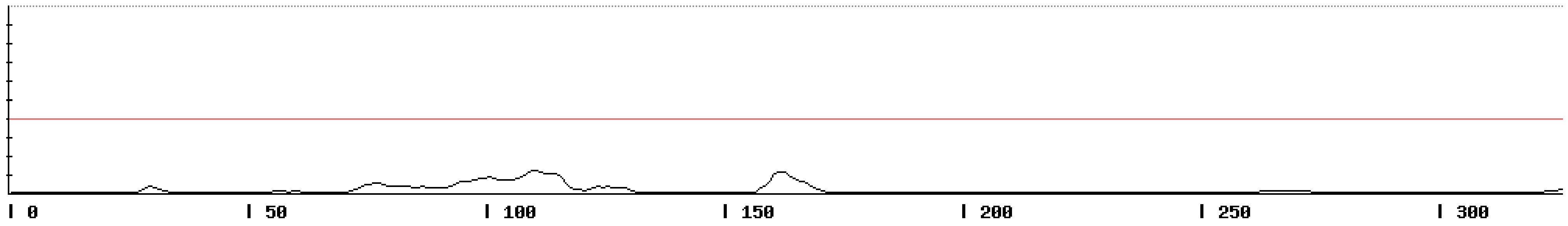

Predictions for Mimulusguttatus_mgv1a025388m

Viterbi Path

Viterbi Path

Posterior @ 0.4

Posterior @ 0.4

Posterior Graphic. Threshold Marked @ 0.4

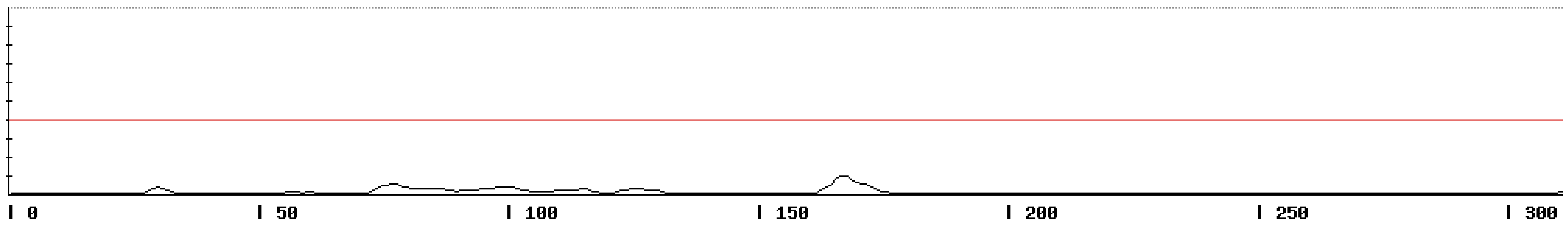

Predictions for Mimulusguttatus_mgv1a025425m

Viterbi Path

Viterbi Path

Posterior @ 0.4

Posterior @ 0.4

Posterior Graphic. Threshold Marked @ 0.4

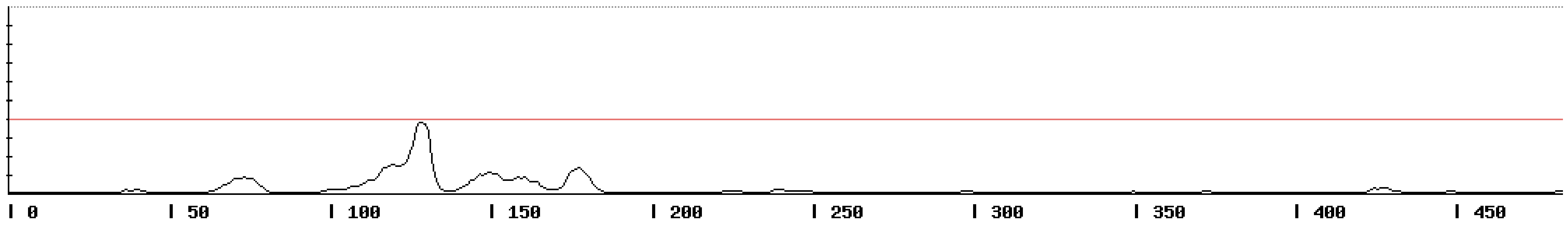

Predictions for Mimulusguttatus_mgv1a025443m

Viterbi Path

Viterbi Path

Posterior @ 0.4

Posterior @ 0.4

Posterior Graphic. Threshold Marked @ 0.4

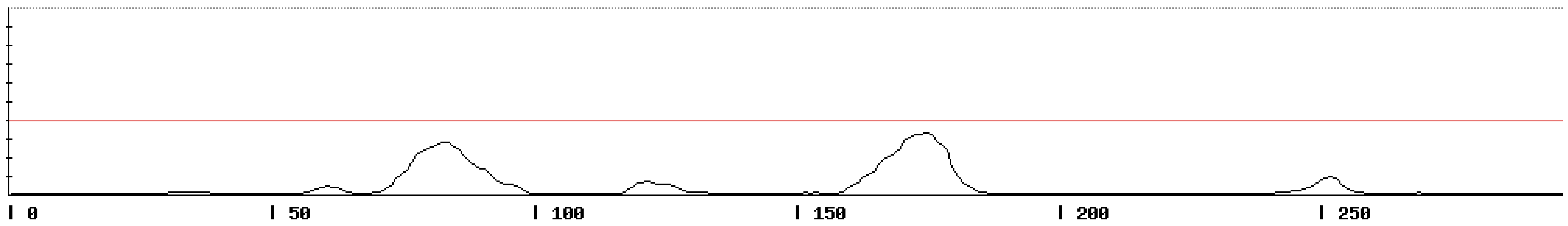

Predictions for Mimulusguttatus_mgv1a025627m

Viterbi Path

Viterbi Path

Posterior @ 0.4

Posterior @ 0.4

Posterior Graphic. Threshold Marked @ 0.4

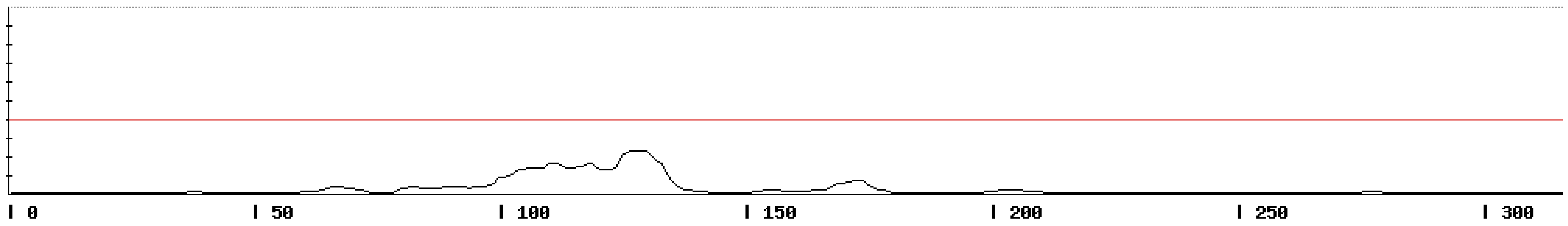

Predictions for Mimulusguttatus_mgv1a025709m

Viterbi Path

Viterbi Path

Posterior @ 0.4	
Posterior @ 0.4	
Posterior Graphic. Threshold Marked @ 0.4	

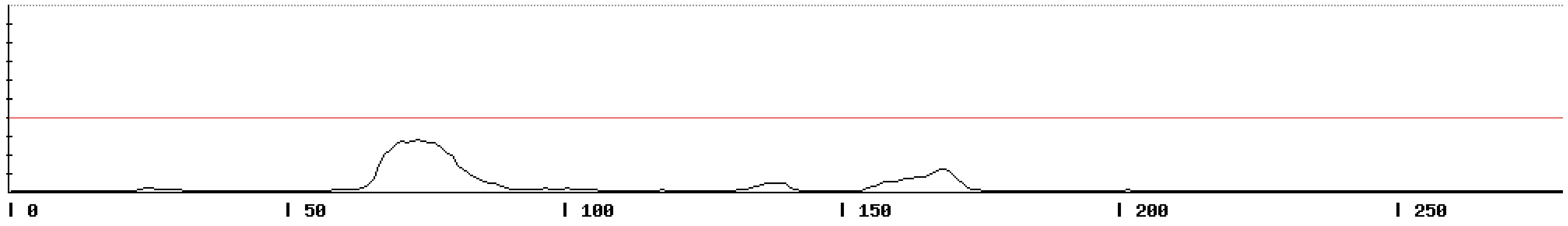

Predictions for Mimulusguttatus_mgv1a025738m	
Viterbi Path	
Viterbi Path	
Posterior @ 0.4	51 - K - 51
	60 - FWRRDKKYPRGKRTNRA - 76
Posterior @ 0.4	
Posterior Graphic. Threshold Marked @ 0.4	

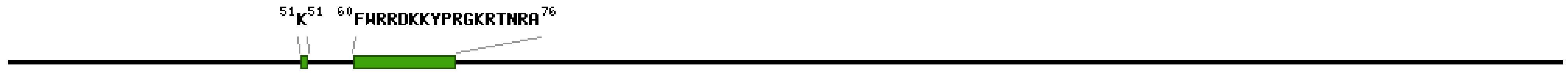

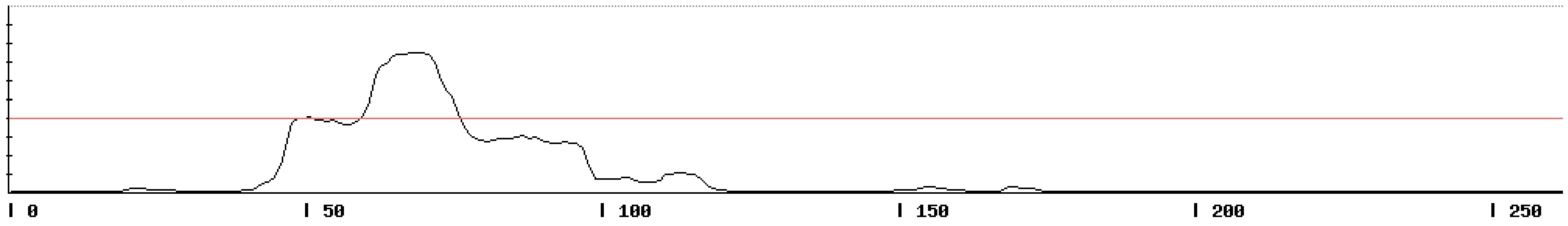

Predictions for Mimulusguttatus_mgv1a025891m

Viterbi Path	
Viterbi Path	
Posterior @ 0.4	
Posterior @ 0.4	
Posterior Graphic. Threshold Marked @ 0.4	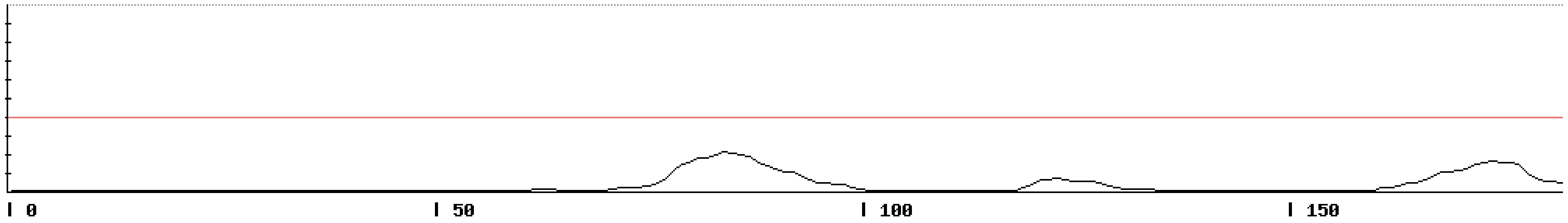

Predictions for Mimulusguttatus_mgv1a026064m

Viterbi Path	
Viterbi Path	
Posterior @ 0.4	
Posterior @ 0.4	
Posterior Graphic. Threshold Marked @ 0.4	

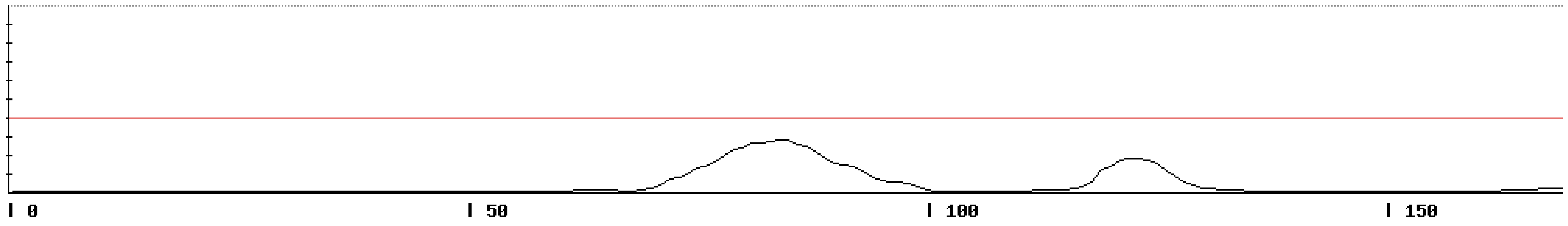

Predictions for Mimulusguttatus_mgv1a026129m

Viterbi Path

Viterbi Path

Posterior @ 0.4

Posterior @ 0.4

Posterior Graphic. Threshold Marked @ 0.4

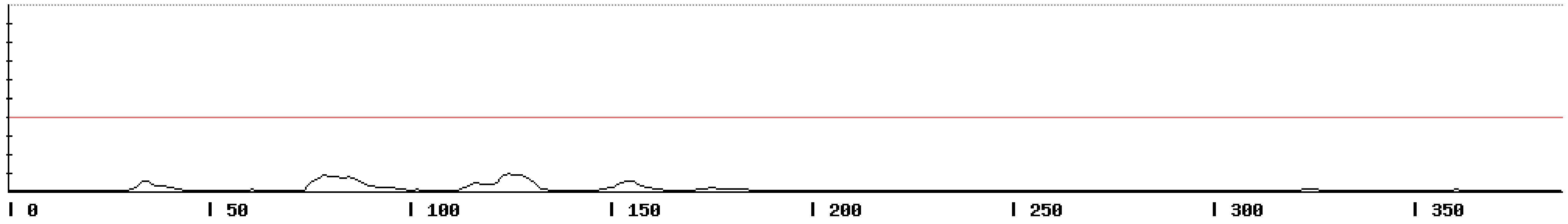

Predictions for Mimulusguttatus_mgv1a026208m

Viterbi Path

Viterbi Path

Posterior @ 0.4

Posterior @ 0.4

Posterior Graphic. Threshold Marked @ 0.4	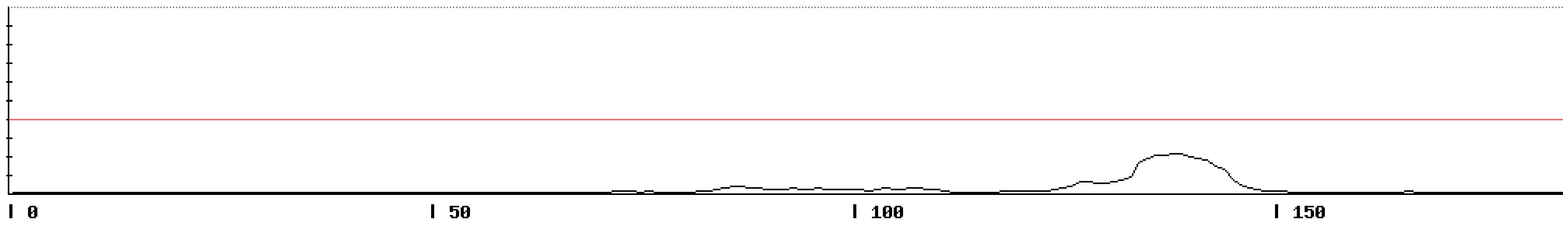

Predictions for Mimulusguttatus_mgv1a026435m

Viterbi Path	
Viterbi Path	
Posterior @ 0.4	
Posterior @ 0.4	
Posterior Graphic. Threshold Marked @ 0.4	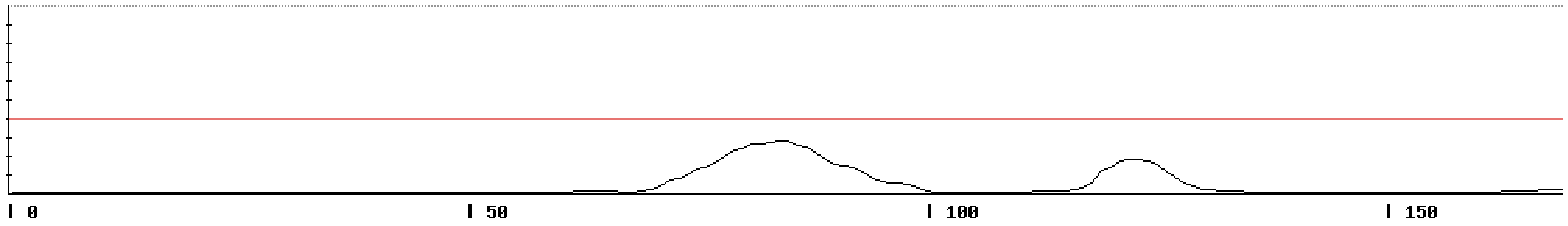

Predictions for Mimulusguttatus_mgv1a026489m

Revision r.9 - Last Updated on : February 24 2015 17:45:20.

alex.nguyenba@utoronto.ca

Intended to be used with Firefox

Viterbi Path

Posterior @ 0.4

Posterior @ 0.4

Posterior Graphic. Threshold Marked @ 0.4

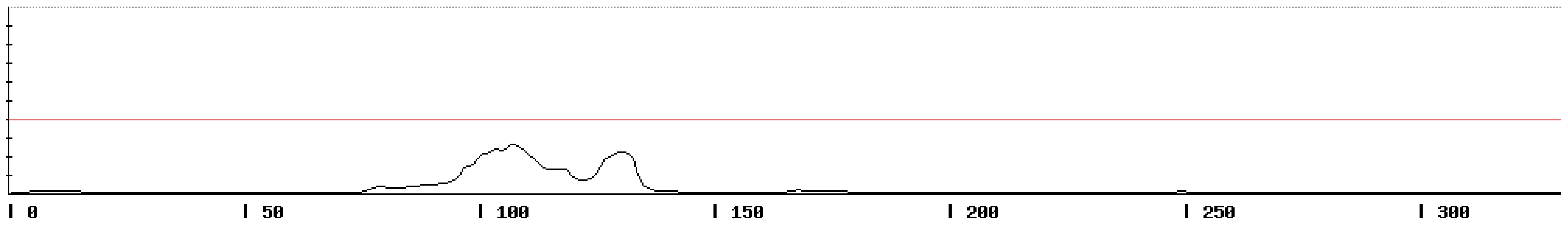

Predictions for Morusnotabilis_XP_010088106.1

Viterbi Path

Viterbi Path

Posterior @ 0.4

Posterior @ 0.4

Posterior Graphic. Threshold Marked @ 0.4

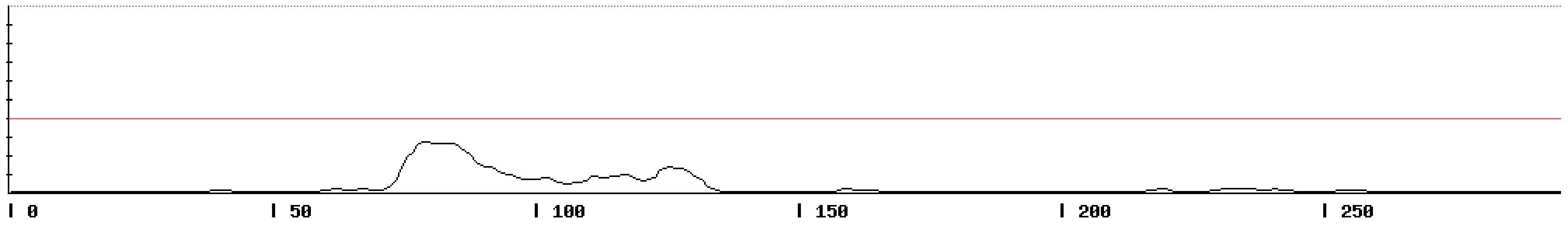

Predictions for Morusnotabilis_XP_010088131.1

Viterbi Path	
Viterbi Path	
Posterior @ 0.4	
Posterior @ 0.4	
Posterior Graphic. Threshold Marked @ 0.4	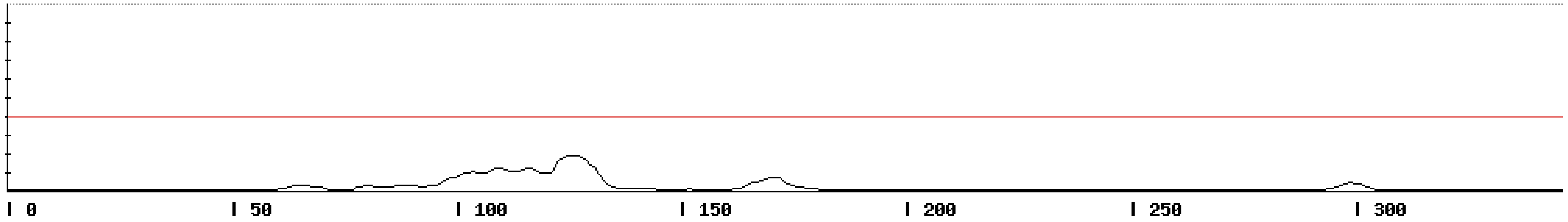

Predictions for Morusnotabilis_XP_010088481.1

Viterbi Path	
Viterbi Path	
Posterior @ 0.4	
Posterior @ 0.4	
Posterior Graphic. Threshold Marked @ 0.4	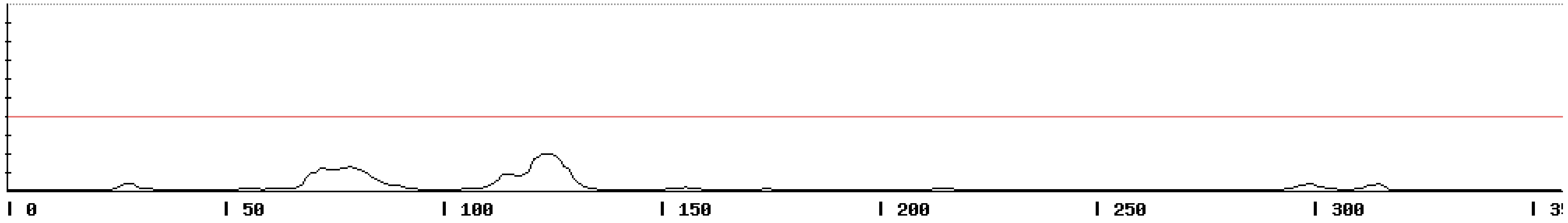

Predictions for Morusnotabilis_XP_010088484.1

Viterbi Path	
Viterbi Path	
Posterior @ 0.4	
Posterior @ 0.4	
Posterior Graphic. Threshold Marked @ 0.4	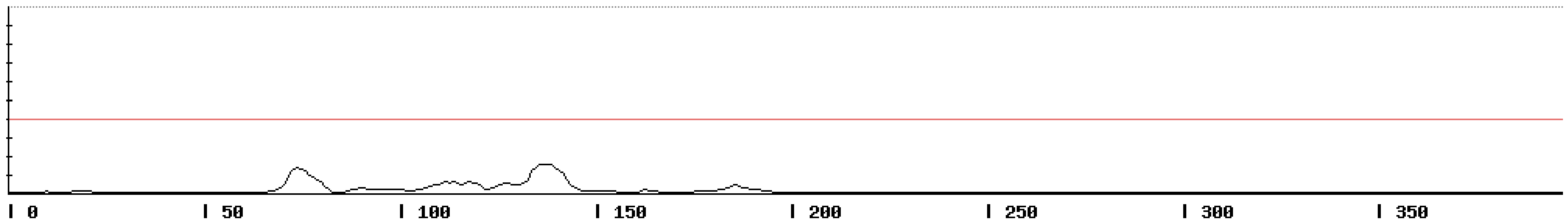

Predictions for Morusnotabilis_XP_010088702.1

Viterbi Path	
Viterbi Path	
Posterior @ 0.4	
Posterior @ 0.4	
Posterior Graphic. Threshold Marked @ 0.4	

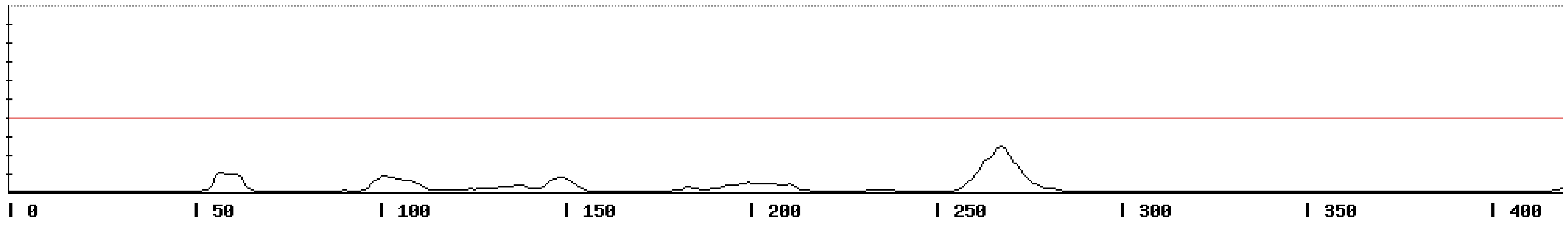

Predictions for Morusnotabilis_XP_010089502.1

Viterbi Path

Viterbi Path

Posterior @ 0.4

Posterior @ 0.4

Posterior Graphic. Threshold Marked @ 0.4

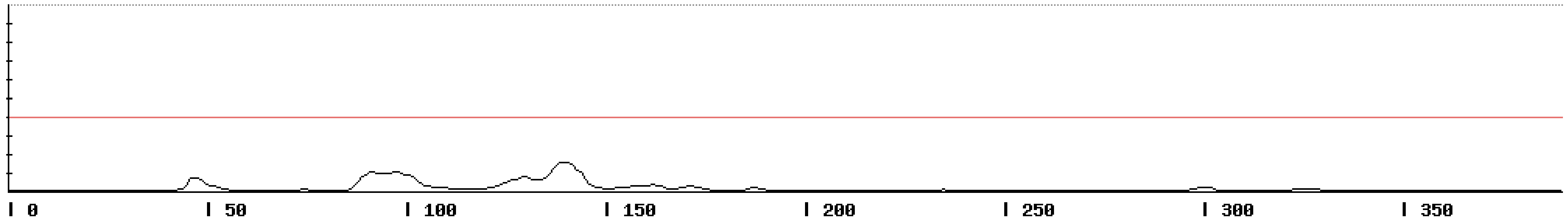

Predictions for Morusnotabilis_XP_010089503.1

Viterbi Path

Viterbi Path

Posterior @ 0.4

Posterior @ 0.4

Posterior Graphic. Threshold Marked @ 0.4

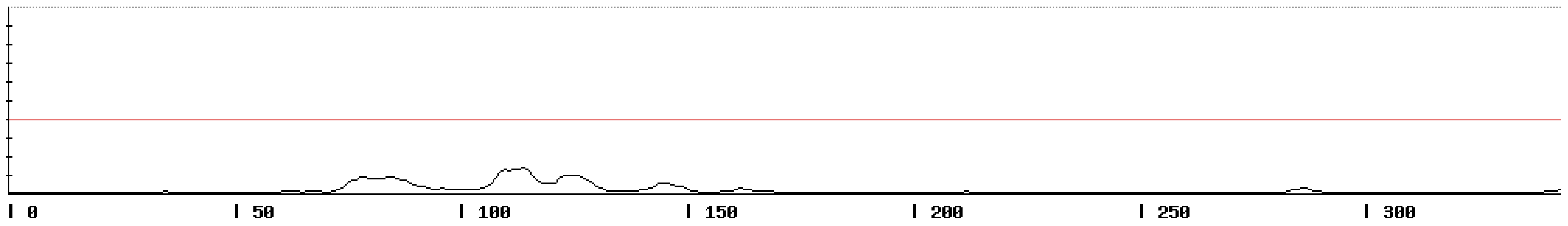

Predictions for Morusnotabilis_XP_010089907.1

Viterbi Path

Viterbi Path

Posterior @ 0.4 77 - KHQKGKRL - 84

Posterior @ 0.4

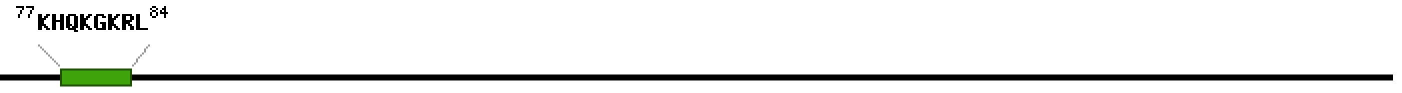

Posterior Graphic. Threshold Marked @ 0.4

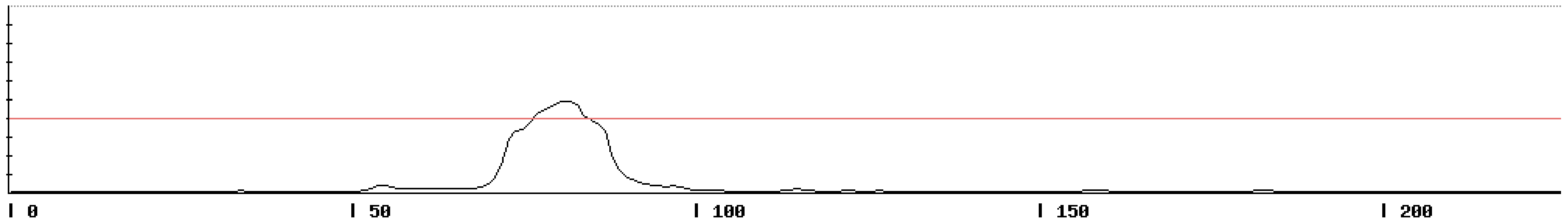

Predictions for Morusnotabilis_XP_010089914.1

Viterbi Path

Viterbi Path

Posterior @ 0.4 74 - KKVLKHRKGKRLERR - 88

Posterior @ 0.4

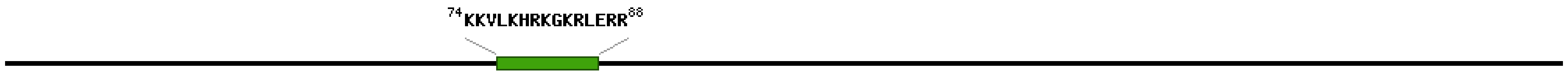

Posterior Graphic. Threshold Marked @ 0.4

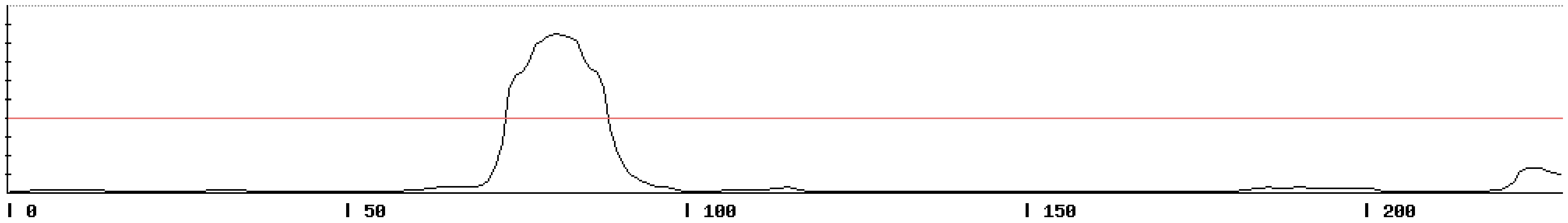

Predictions for Morusnotabilis_XP_010090439.1

Viterbi Path

Viterbi Path

Posterior @ 0.4

Posterior @ 0.4

Posterior Graphic. Threshold Marked @ 0.4

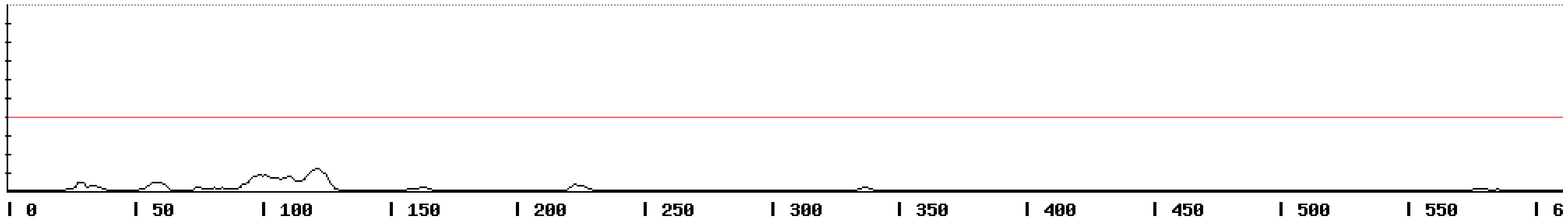

Predictions for Morusnotabilis_XP_010090441.1

Viterbi Path

Viterbi Path

Posterior @ 0.4

Posterior @ 0.4

Posterior Graphic. Threshold Marked @ 0.4

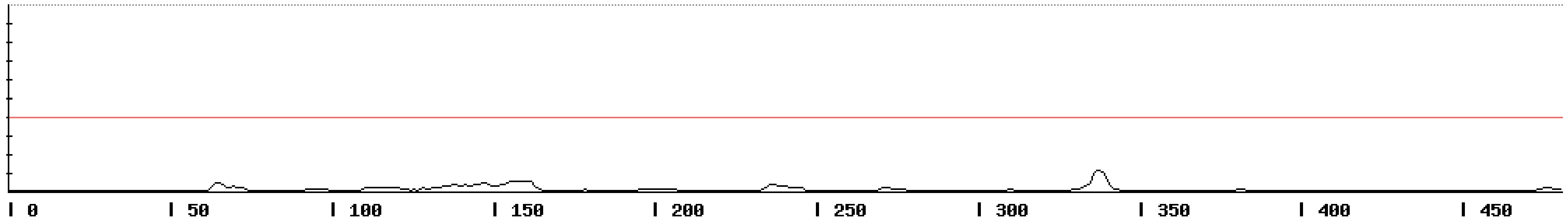

Predictions for Morusnotabilis_XP_010090688.1

Viterbi Path

Viterbi Path

Posterior @ 0.4

Posterior @ 0.4

Posterior Graphic. Threshold Marked @ 0.4

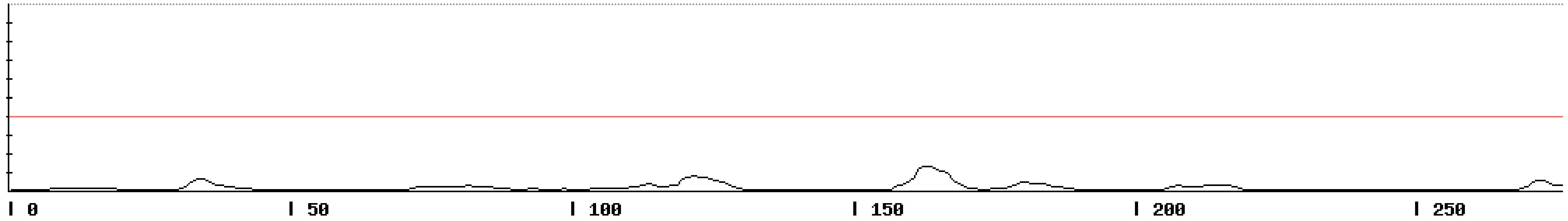

Predictions for Morusnotabilis_XP_010090830.1

Viterbi Path

Viterbi Path

Posterior @ 0.4

Posterior @ 0.4

Posterior Graphic. Threshold Marked @ 0.4

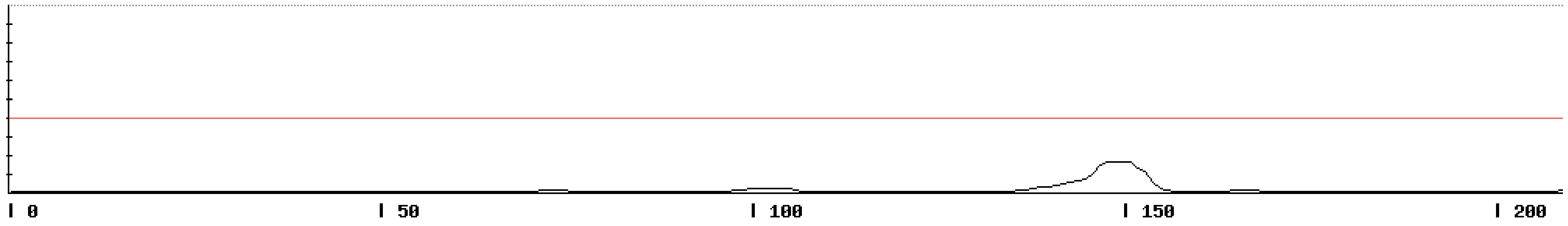

Predictions for Morusnotabilis_XP_010090859.1

Viterbi Path

Viterbi Path

Posterior @ 0.4

Posterior @ 0.4

Posterior Graphic. Threshold Marked @ 0.4

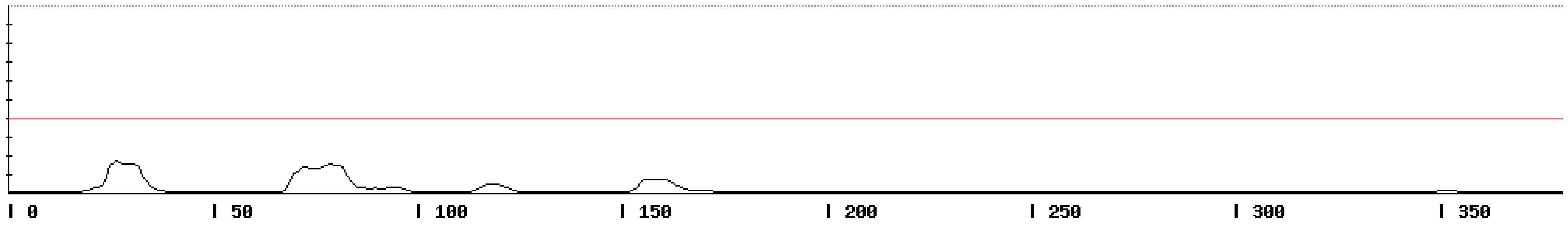

Predictions for Morusnotabilis_XP_010091280.1

Viterbi Path

Viterbi Path

Posterior @ 0.4

Posterior @ 0.4

Posterior Graphic. Threshold Marked @ 0.4

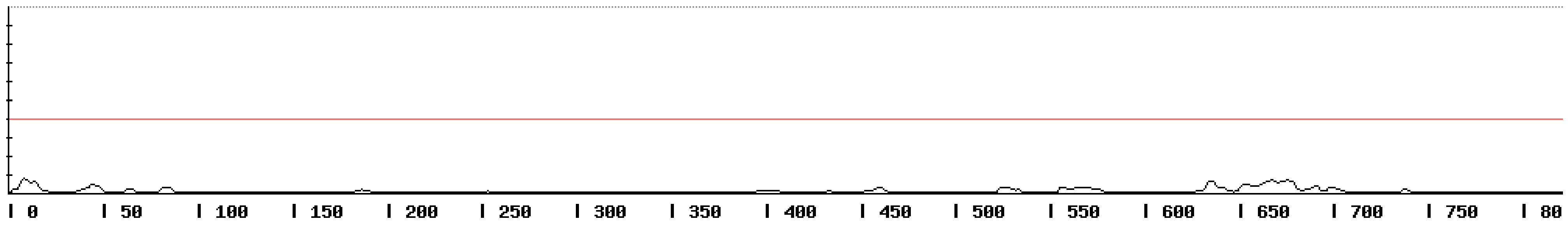

Predictions for Morusnotabilis_XP_010092473.1

Viterbi Path

Viterbi Path

Posterior @ 0.4

Posterior @ 0.4

Posterior Graphic. Threshold Marked @ 0.4

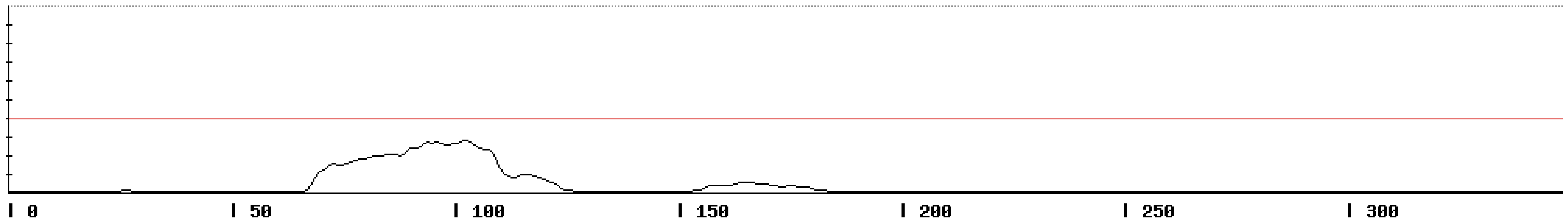

Predictions for Morusnotabilis_XP_010093055.1

Viterbi Path

Viterbi Path

Posterior @ 0.4

Posterior @ 0.4

Posterior Graphic. Threshold Marked @ 0.4

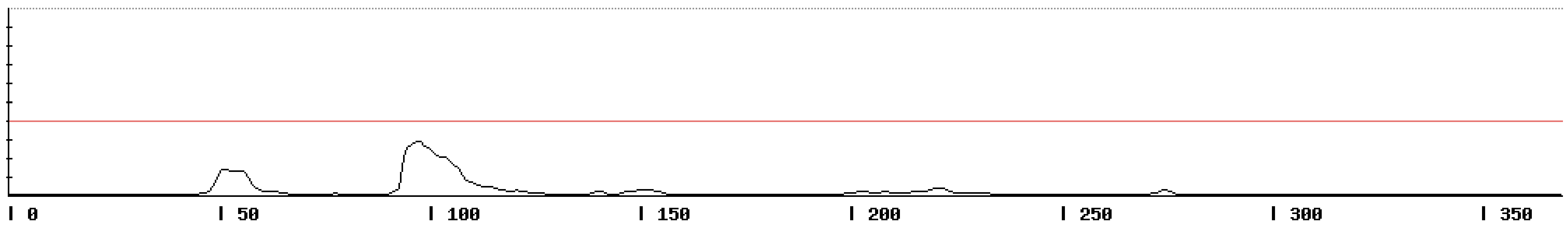

Predictions for Morusnotabilis_XP_010093108.1

Viterbi Path

Viterbi Path

Posterior @ 0.4

73 - RKYPNGSRPNRAAGRGYWKATGADKPIGKPKPLGIKKA - 110

Posterior @ 0.4

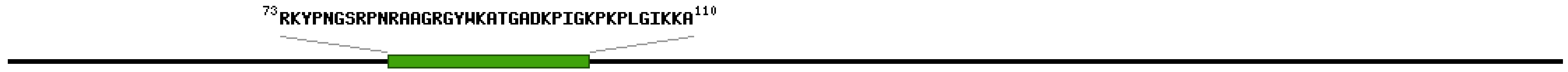

Posterior Graphic. Threshold Marked @ 0.4

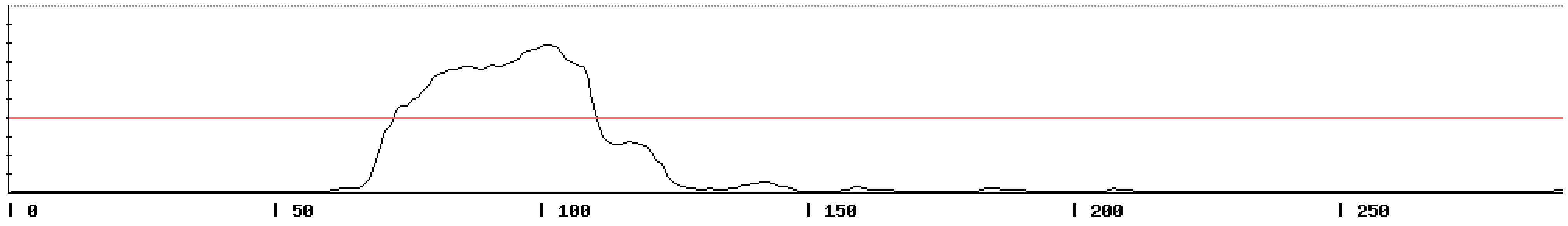

Predictions for Morusnotabilis_XP_010093172.1

Viterbi Path	
Viterbi Path	
Posterior @ 0.4	
Posterior @ 0.4	
Posterior Graphic. Threshold Marked @ 0.4	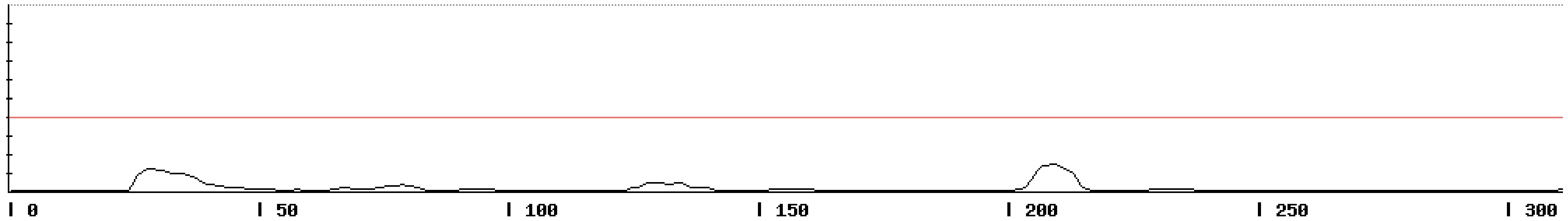

Predictions for Morusnotabilis_XP_010093173.1

Viterbi Path	
Viterbi Path	
Posterior @ 0.4	
Posterior @ 0.4	

Posterior Graphic. Threshold Marked @ 0.4

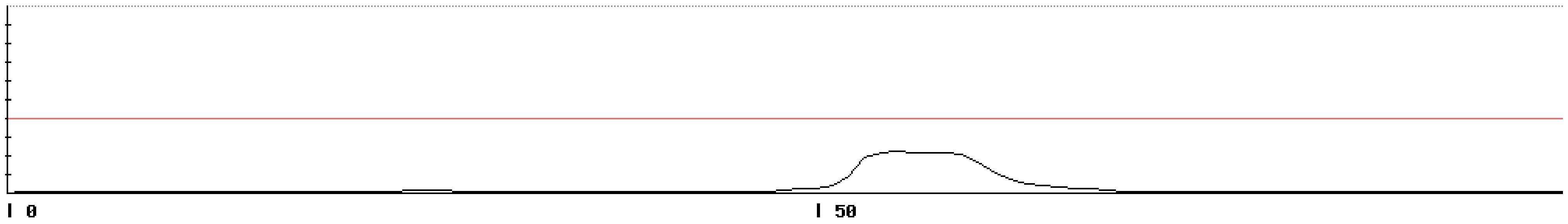

Predictions for Morusnotabilis_XP_010093594.1

Viterbi Path

Viterbi Path

Posterior @ 0.4

Posterior @ 0.4

Posterior Graphic. Threshold Marked @ 0.4

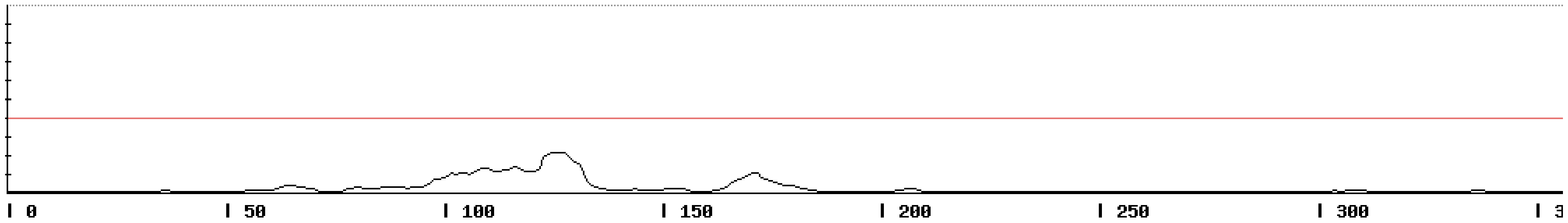

Predictions for Morusnotabilis_XP_010093604.1

Viterbi Path

Viterbi Path

Posterior @ 0.4 77 - KHQKGKRL - 84

Posterior @ 0.4

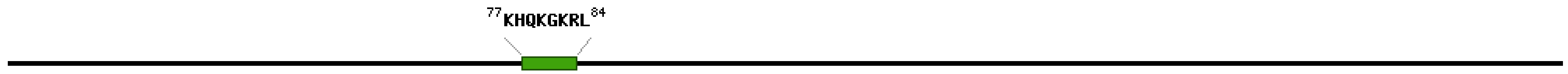

Posterior Graphic. Threshold Marked @ 0.4

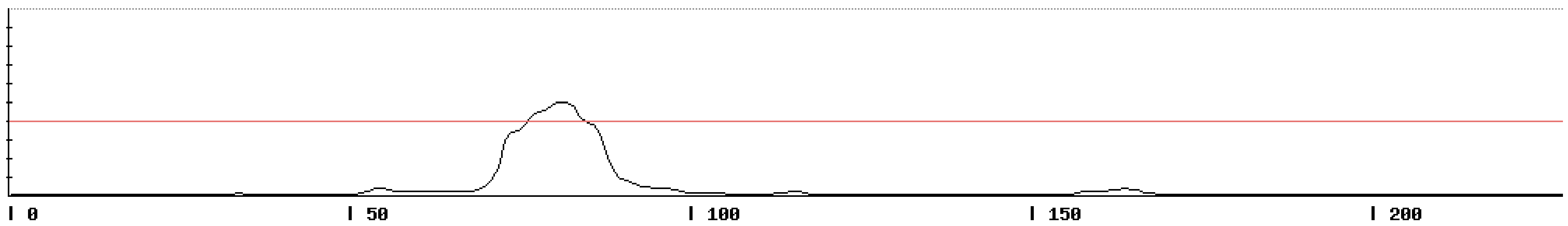

Predictions for Morusnotabilis_XP_010093669.1

Viterbi Path

Viterbi Path

Posterior @ 0.4

Posterior @ 0.4

Posterior Graphic. Threshold Marked @ 0.4

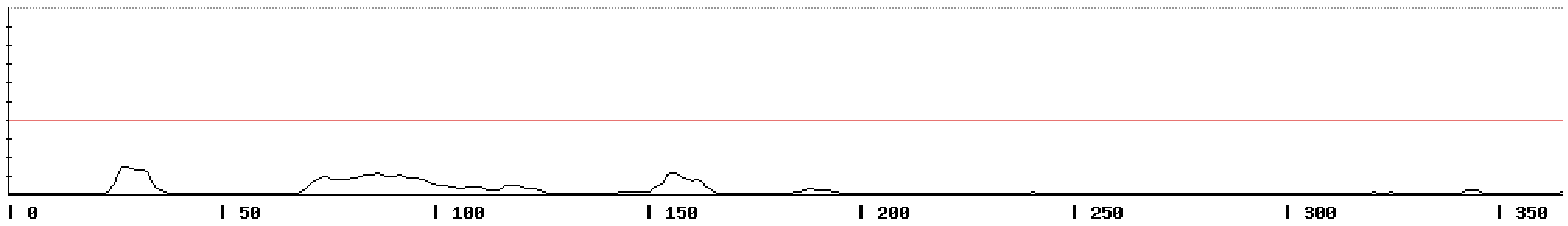

Predictions for Morusnotabilis_XP_010093764.1

Viterbi Path

Viterbi Path

Posterior @ 0.4

Posterior @ 0.4

Posterior Graphic. Threshold Marked @ 0.4

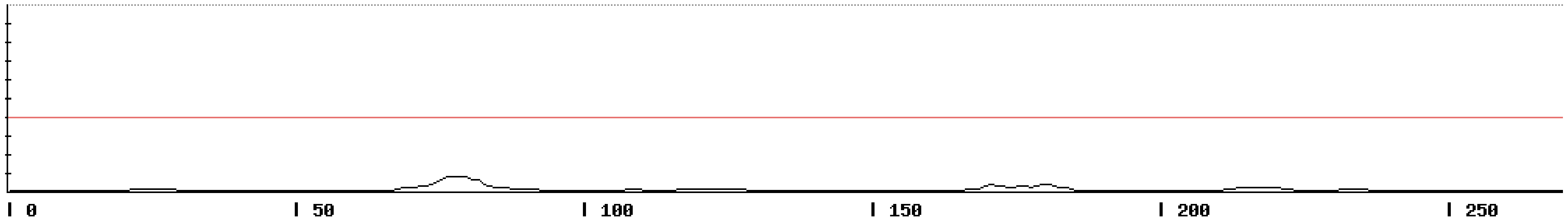

Predictions for Morusnotabilis_XP_010093938.1

Viterbi Path

Viterbi Path

Posterior @ 0.4

Posterior @ 0.4

Posterior Graphic. Threshold Marked @ 0.4

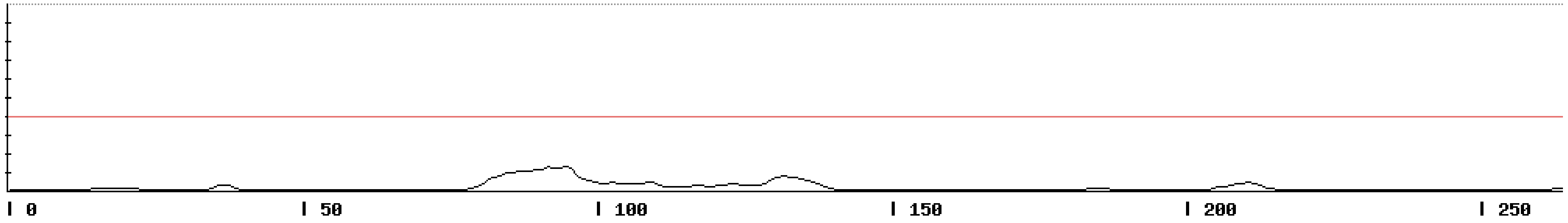

Predictions for Morusnotabilis_XP_010093961.1

Viterbi Path

Viterbi Path

Posterior @ 0.4	135 - RKRRK - 139
Posterior @ 0.4	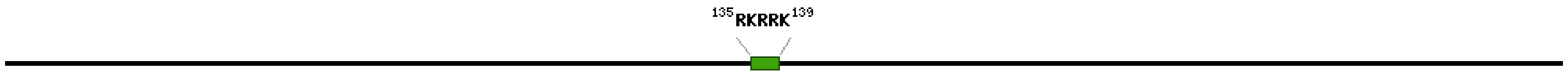
Posterior Graphic. Threshold Marked @ 0.4	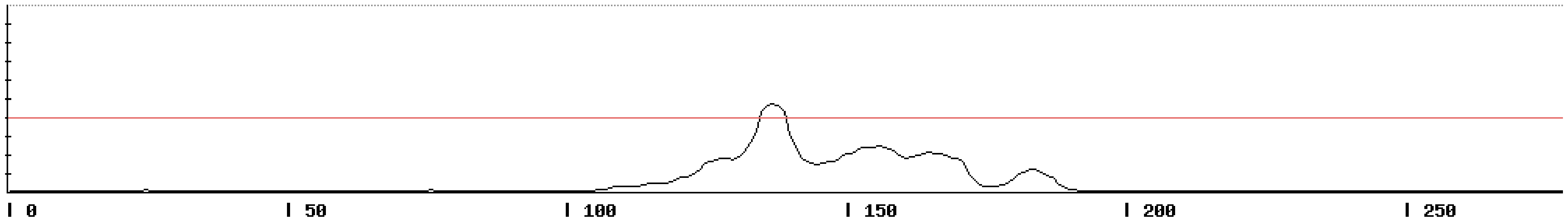

Predictions for Morusnotabilis_XP_010094111.1

Viterbi Path	
Viterbi Path	
Posterior @ 0.4	
Posterior @ 0.4	
Posterior Graphic. Threshold Marked @ 0.4	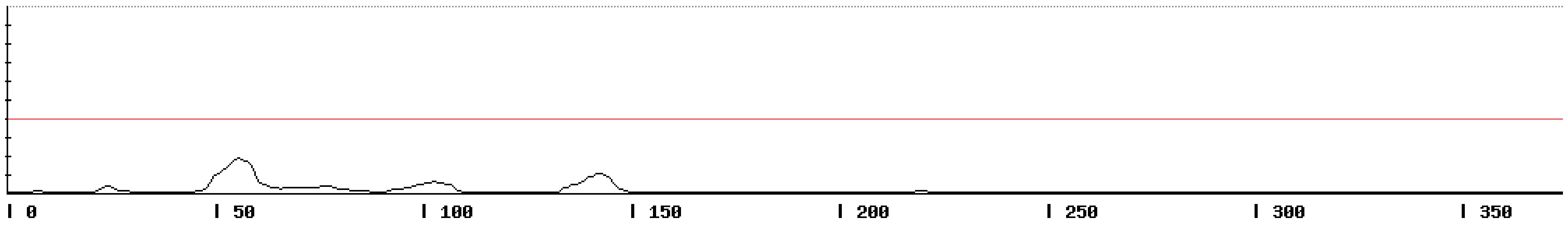

Predictions for Morusnotabilis_XP_010094187.1

Viterbi Path	
Viterbi Path	
Posterior @ 0.4	
Posterior @ 0.4	
Posterior Graphic. Threshold Marked @ 0.4	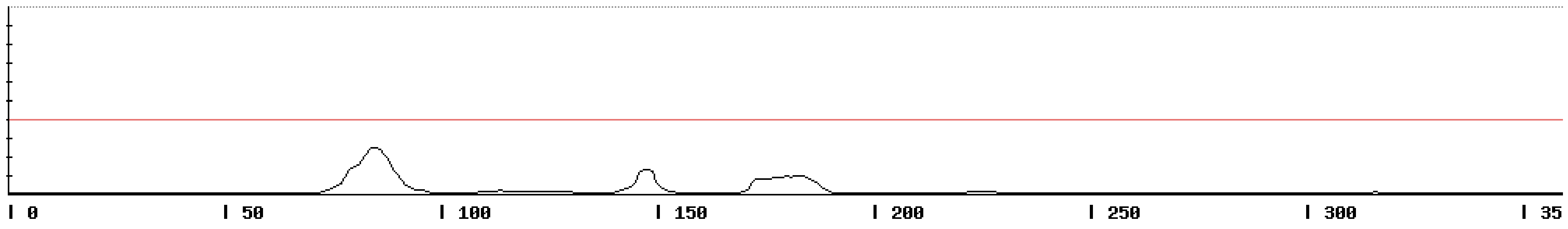

Predictions for Morusnotabilis_XP_010094524.1

Viterbi Path	
Viterbi Path	
Posterior @ 0.4	
Posterior @ 0.4	
Posterior Graphic. Threshold Marked @ 0.4	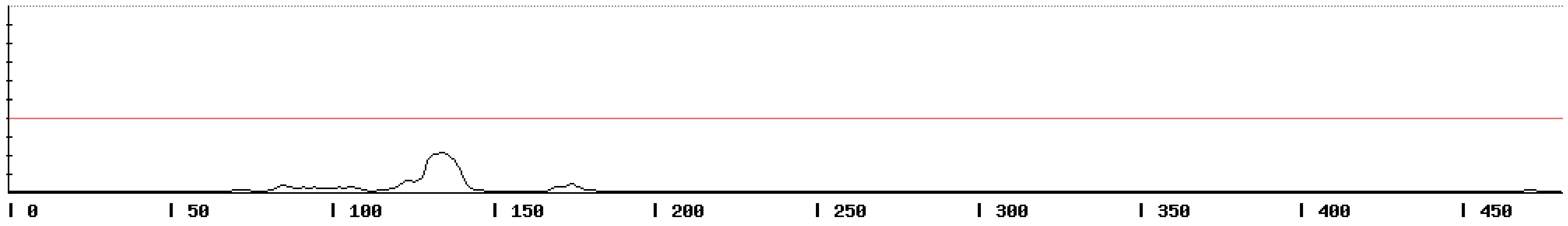

Predictions for Morusnotabilis_XP_010094751.1	
Viterbi Path	
Viterbi Path	
Posterior @ 0.4	
Posterior @ 0.4	
Posterior Graphic. Threshold Marked @ 0.4	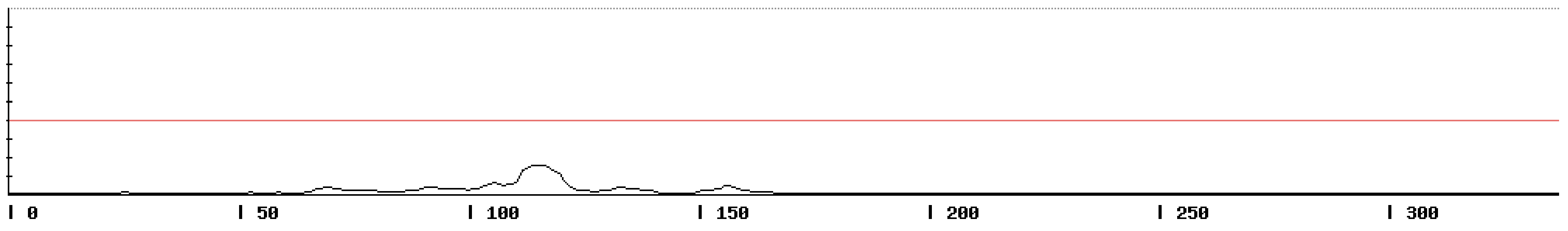

Predictions for Morusnotabilis_XP_010094888.1	
Viterbi Path	
Viterbi Path	
Posterior @ 0.4	
Posterior @ 0.4	
Posterior Graphic. Threshold Marked @ 0.4	

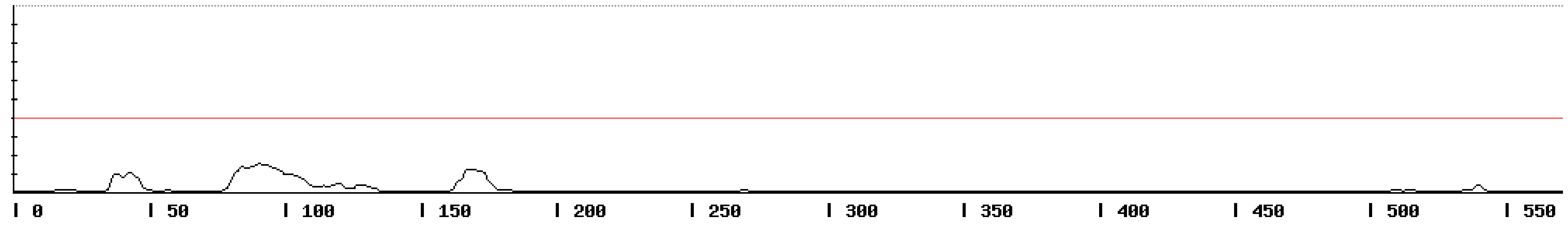

Predictions for Morusnotabilis_XP_010094969.1

Viterbi Path	
Viterbi Path	
Posterior @ 0.4	
Posterior @ 0.4	
Posterior Graphic. Threshold Marked @ 0.4	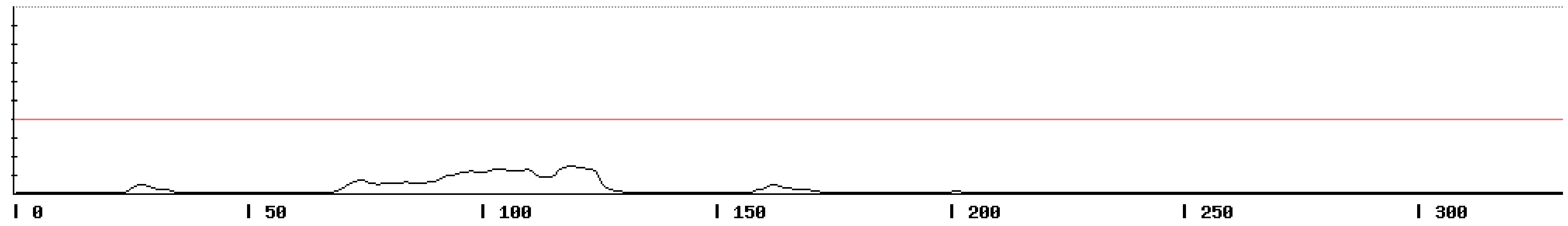

Predictions for Morusnotabilis_XP_010095416.1

Viterbi Path	
Viterbi Path	
Posterior @ 0.4	
Posterior @ 0.4	

Posterior Graphic. Threshold Marked @ 0.4

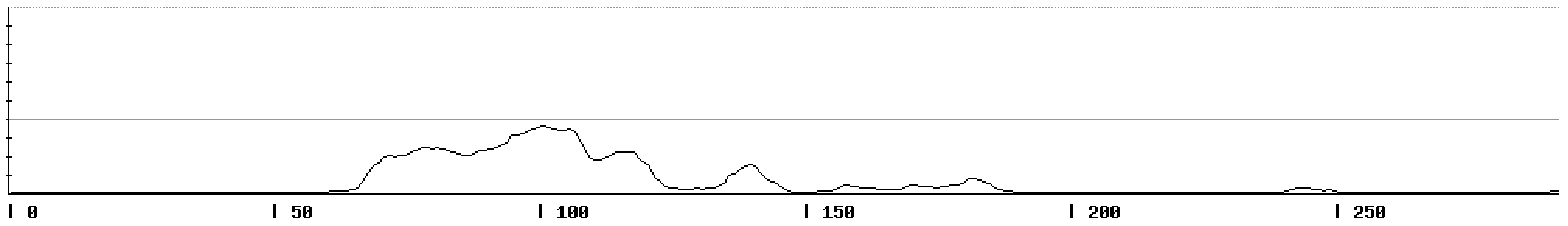

Predictions for Morusnotabilis_XP_010096002.1

Viterbi Path

Viterbi Path

Posterior @ 0.4

Posterior @ 0.4

Posterior Graphic. Threshold Marked @ 0.4

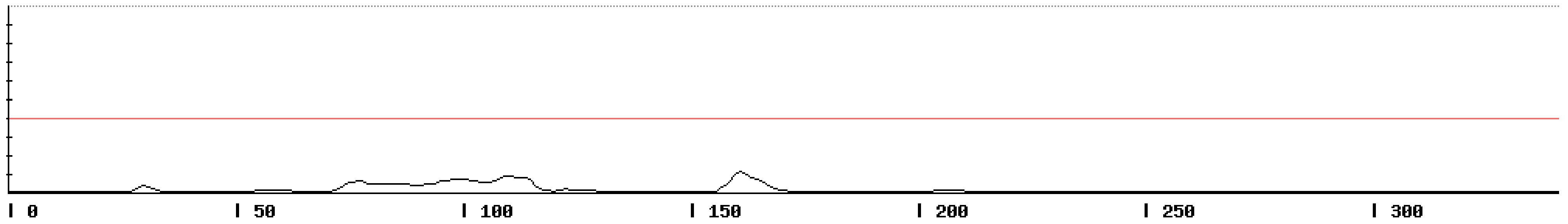

Predictions for Morusnotabilis_XP_010096550.1

Viterbi Path

Viterbi Path

Posterior @ 0.4

Posterior @ 0.4

Posterior Graphic. Threshold Marked @ 0.4

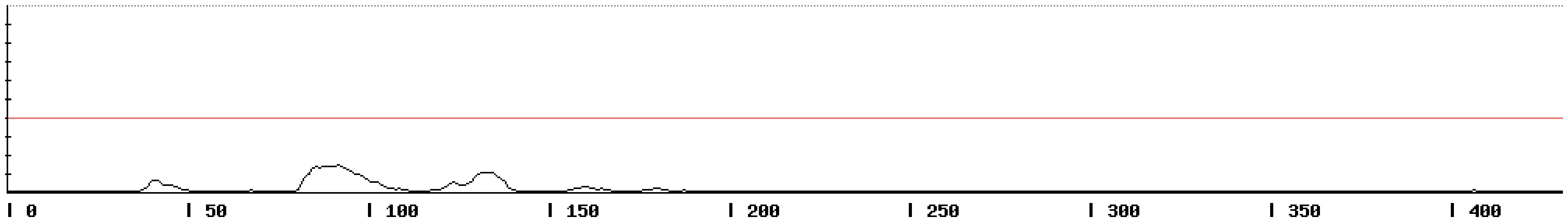

Predictions for Morusnotabilis_XP_010096921.1

Viterbi Path

Viterbi Path

Posterior @ 0.4: 99 - GKRR - 102

Posterior @ 0.4

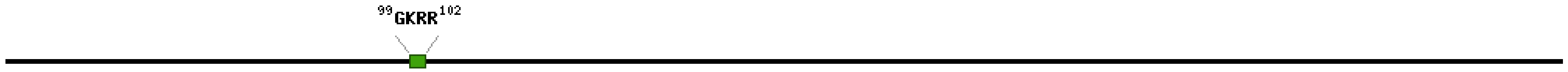

Posterior Graphic. Threshold Marked @ 0.4

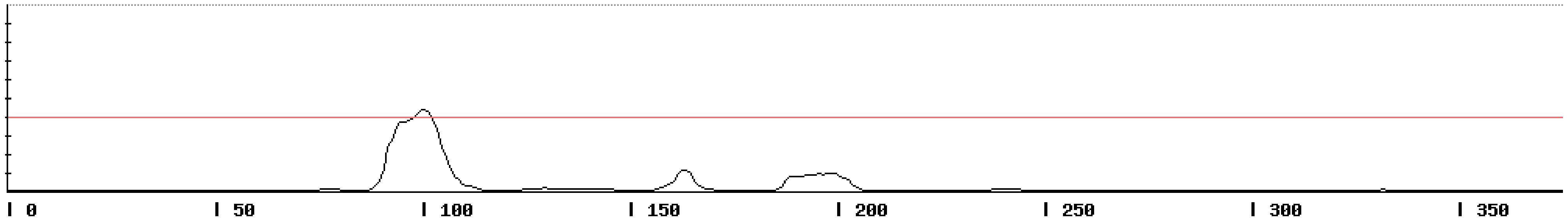

Predictions for Morusnotabilis_XP_010098434.1

Viterbi Path

Viterbi Path

Posterior @ 0.4

Posterior @ 0.4

Posterior Graphic. Threshold Marked @ 0.4

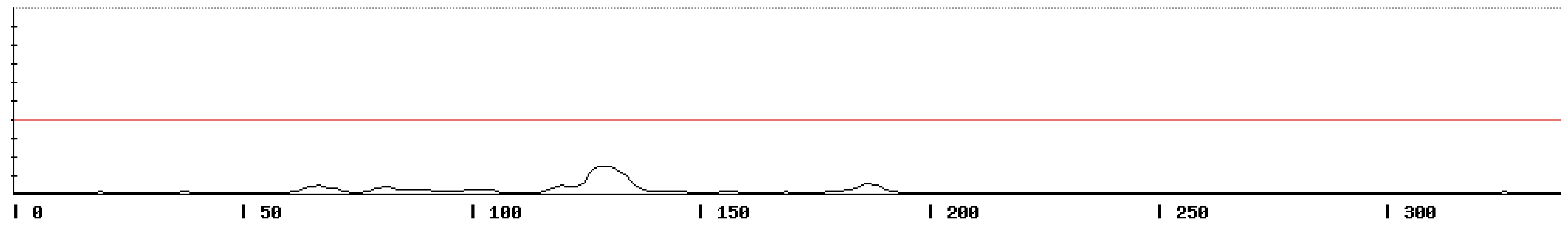

Predictions for Morusnotabilis_XP_010099580.1

Viterbi Path

Viterbi Path

Posterior @ 0.4

Posterior @ 0.4

Posterior Graphic. Threshold Marked @ 0.4

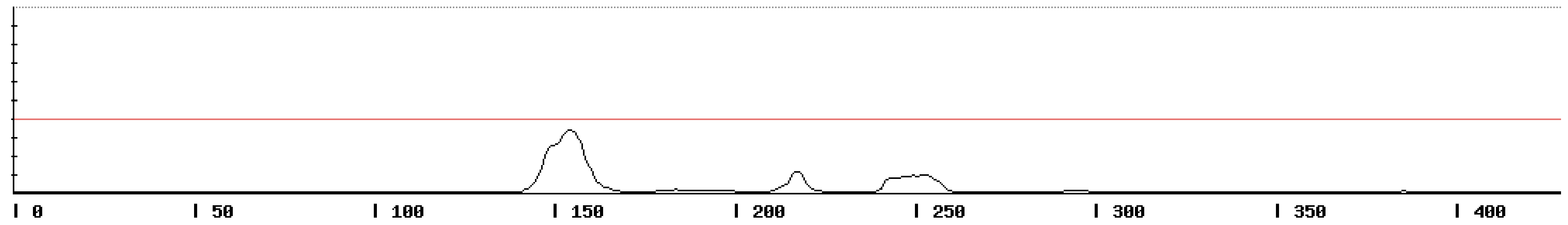

Predictions for Morusnotabilis_XP_010099671.1

Viterbi Path

Viterbi Path

Posterior @ 0.4

Posterior @ 0.4

Posterior Graphic. Threshold Marked @ 0.4

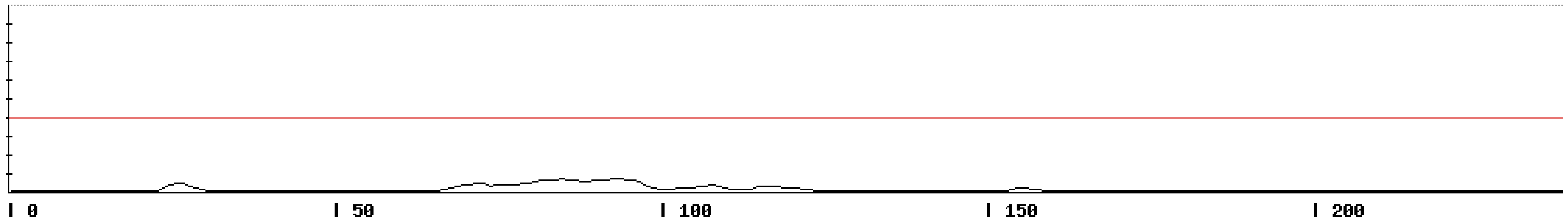

Predictions for Morusnotabilis_XP_010100216.1

Viterbi Path

Viterbi Path

Posterior @ 0.4

Posterior @ 0.4

Posterior Graphic. Threshold Marked @ 0.4

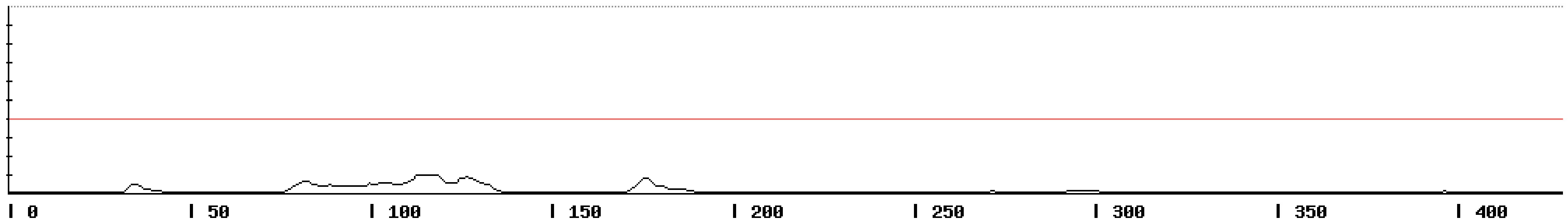

Predictions for Morusnotabilis_XP_010100577.1

Viterbi Path

Viterbi Path

Posterior @ 0.4

Posterior @ 0.4

Posterior Graphic. Threshold Marked @ 0.4

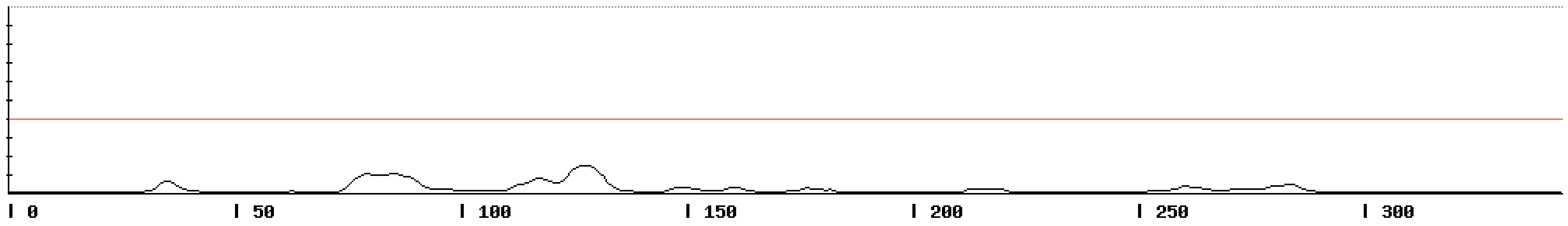

Predictions for Morusnotabilis_XP_010100656.1

Viterbi Path

Viterbi Path

Posterior @ 0.4

Posterior @ 0.4

Posterior Graphic. Threshold Marked @ 0.4

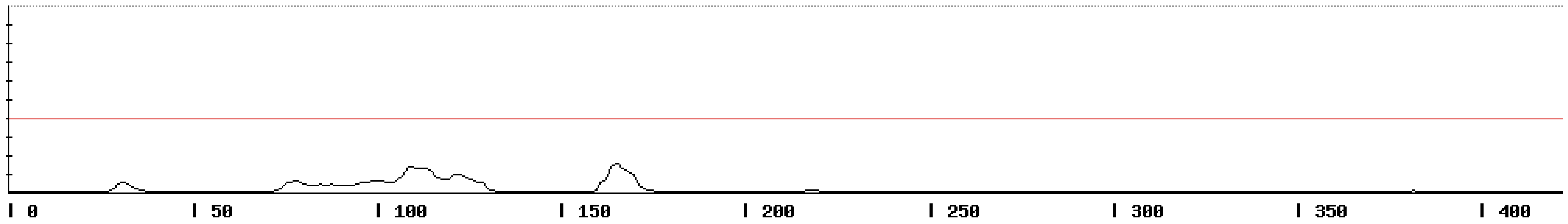

Predictions for Morusnotabilis_XP_010101086.1

Viterbi Path

Viterbi Path

Posterior @ 0.4

Posterior @ 0.4

Posterior Graphic. Threshold Marked @ 0.4

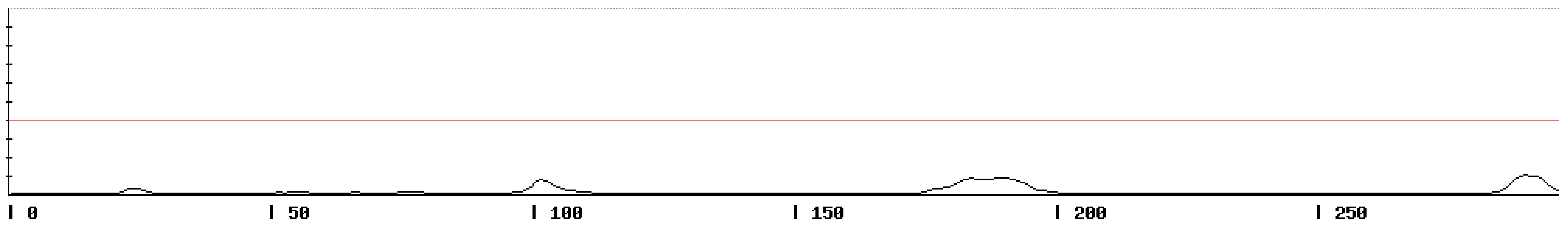

Predictions for Morusnotabilis_XP_010101093.1

Viterbi Path

Viterbi Path

Posterior @ 0.4

Posterior @ 0.4

Posterior Graphic. Threshold Marked @ 0.4

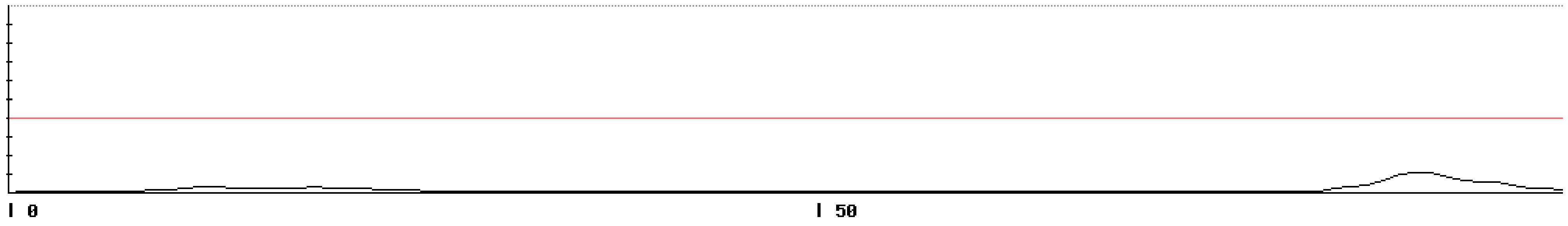

Predictions for Morusnotabilis_XP_010101412.1

Viterbi Path

Viterbi Path

Posterior @ 0.4

Posterior @ 0.4

Posterior Graphic. Threshold Marked @ 0.4

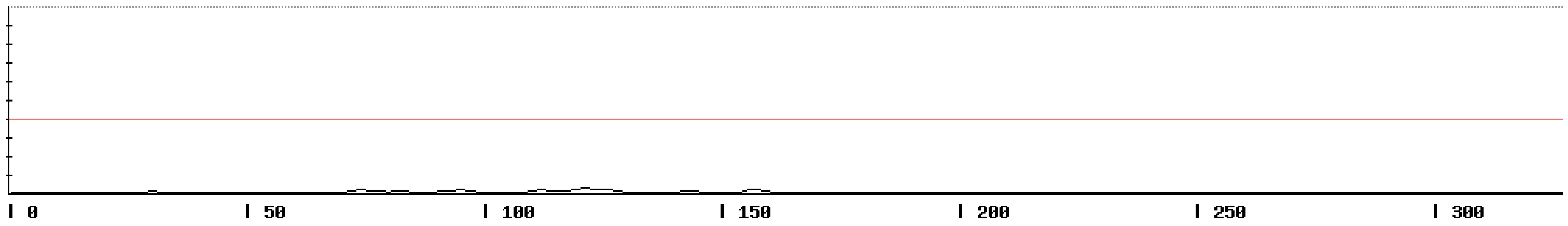

Predictions for Morusnotabilis_XP_010101882.1

Viterbi Path

Viterbi Path

Posterior @ 0.4

Posterior @ 0.4

Posterior Graphic. Threshold Marked @ 0.4

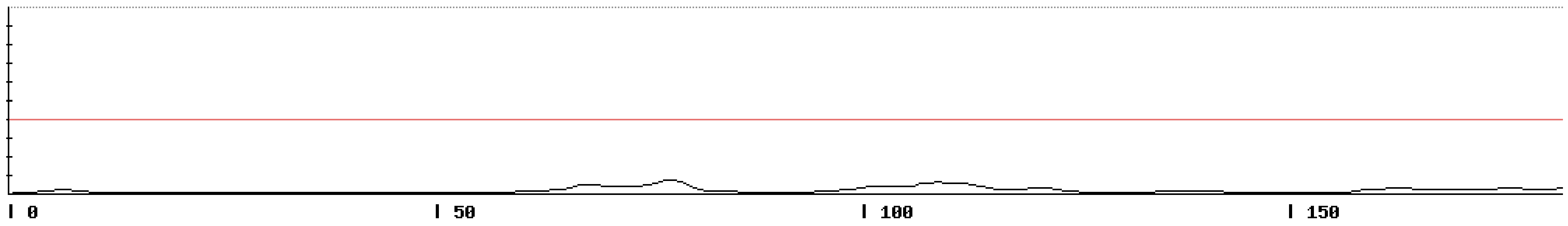

Predictions for Morusnotabilis_XP_010102010.1

Viterbi Path

Viterbi Path

Posterior @ 0.4

Posterior @ 0.4

Posterior Graphic. Threshold Marked @ 0.4

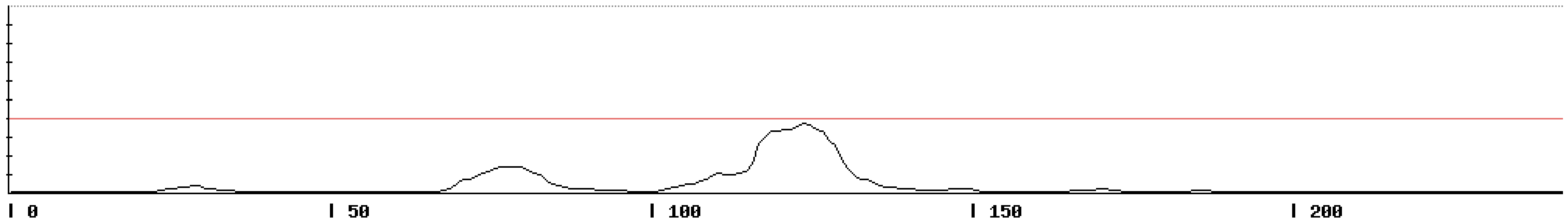

Predictions for Morusnotabilis_XP_010102025.1

Viterbi Path

Viterbi Path

Posterior @ 0.4

Posterior @ 0.4

Posterior Graphic. Threshold Marked @ 0.4

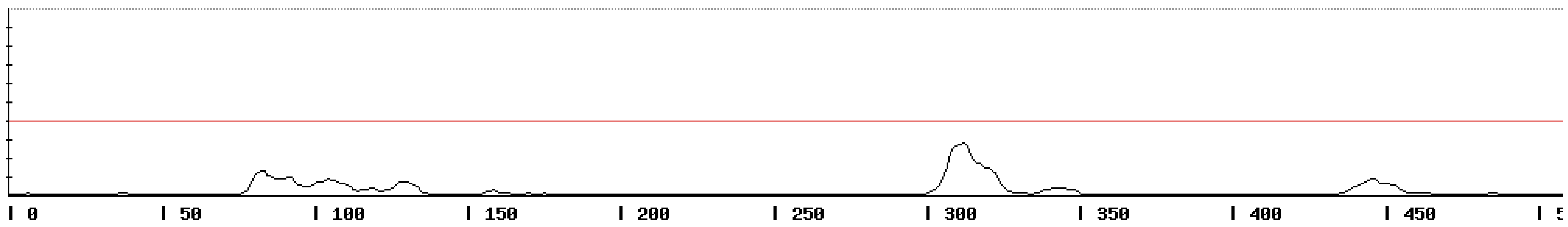

Predictions for Morusnotabilis_XP_010103069.1

Viterbi Path

Viterbi Path

Posterior @ 0.4

Posterior @ 0.4

Posterior Graphic. Threshold Marked @ 0.4

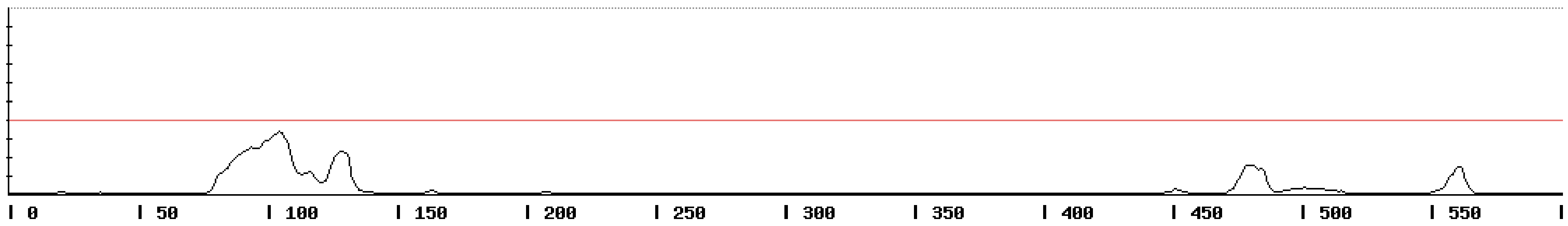

Predictions for Morusnotabilis_XP_010103318.1

Viterbi Path

Viterbi Path

Posterior @ 0.4

Posterior @ 0.4

Posterior Graphic. Threshold Marked @ 0.4

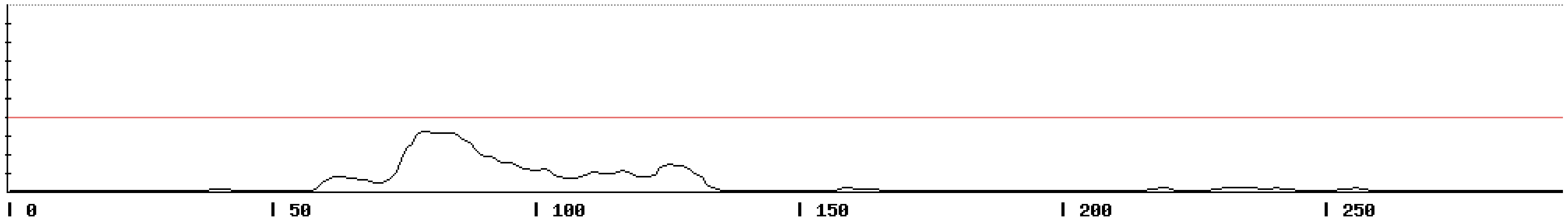

Predictions for Morusnotabilis_XP_010103818.1

Viterbi Path

Viterbi Path

Posterior @ 0.4

39 - KGQRR - 43

139 - KKARRRSNK - 147

Posterior @ 0.4

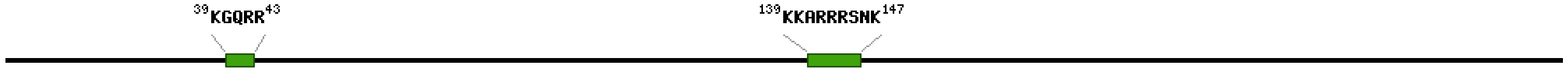

Posterior Graphic. Threshold Marked @ 0.4

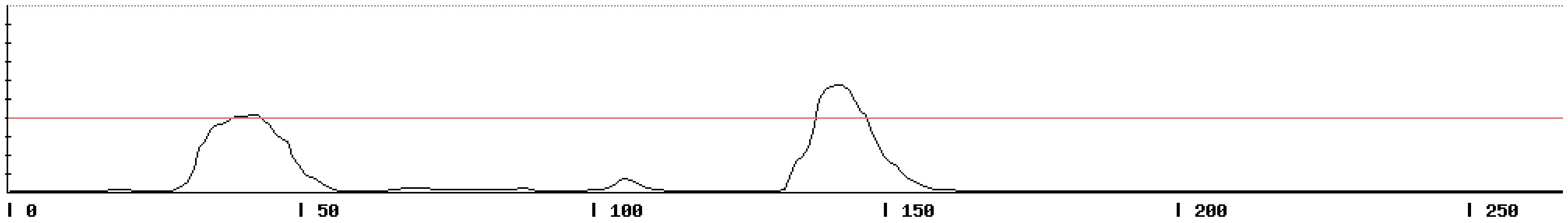

Predictions for Morusnotabilis_XP_010103879.1

Viterbi Path

Viterbi Path

Posterior @ 0.4	144 - SKRRAPKK - 151
Posterior @ 0.4	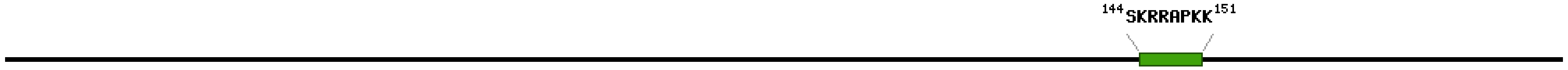
Posterior Graphic. Threshold Marked @ 0.4	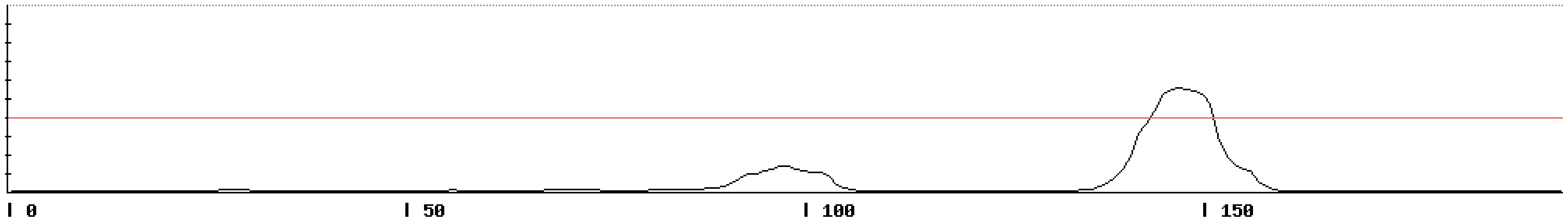

Predictions for Morusnotabilis_XP_010104110.1

Viterbi Path	
Viterbi Path	
Posterior @ 0.4	
Posterior @ 0.4	
Posterior Graphic. Threshold Marked @ 0.4	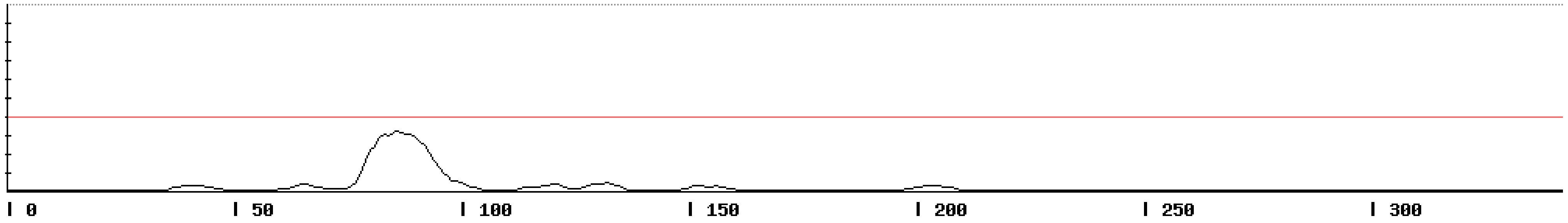

Predictions for Morusnotabilis_XP_010104346.1

Viterbi Path

Viterbi Path

Posterior @ 0.4

Posterior @ 0.4

Posterior Graphic. Threshold Marked @ 0.4

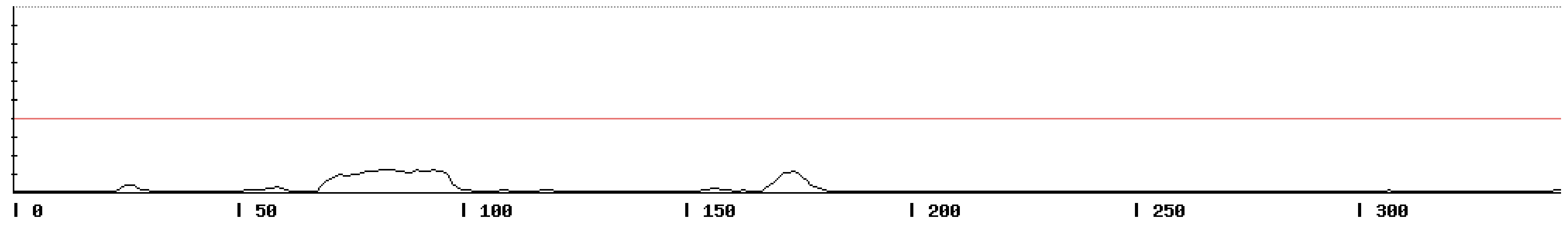

Predictions for Morusnotabilis_XP_010104895.1

Viterbi Path

Viterbi Path

Posterior @ 0.4

Posterior @ 0.4

Posterior Graphic. Threshold Marked @ 0.4

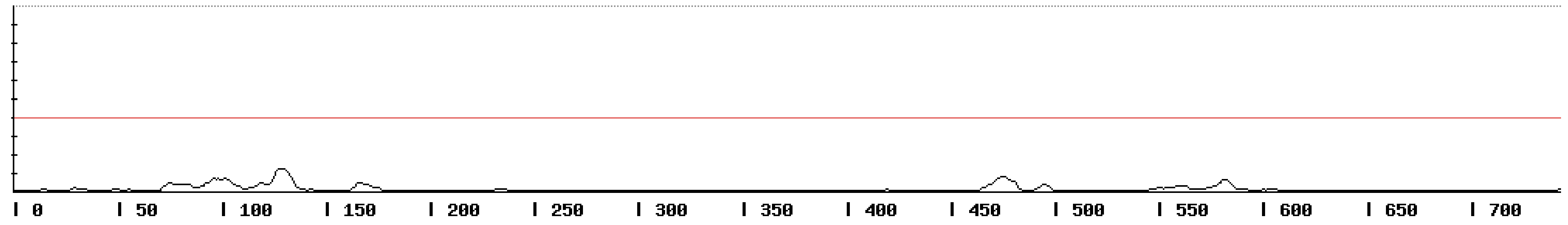

Predictions for Morusnotabilis_XP_010105605.1	
Viterbi Path	
Viterbi Path	
Posterior @ 0.4	
Posterior @ 0.4	
Posterior Graphic. Threshold Marked @ 0.4	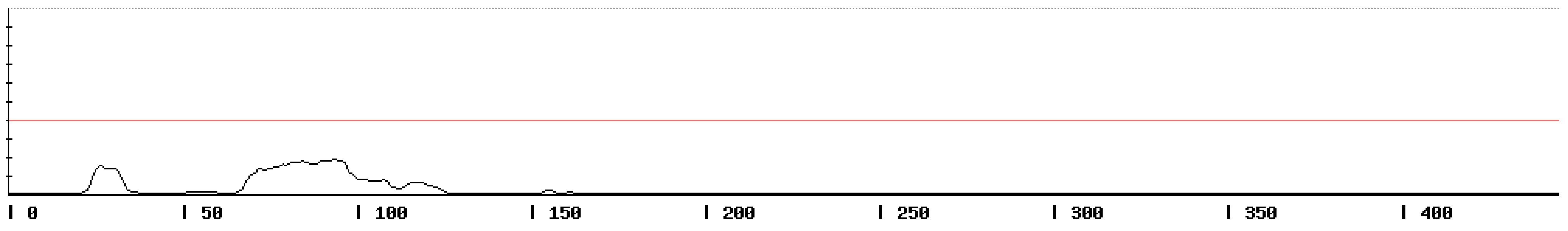

Predictions for Morusnotabilis_XP_010105636.1	
Viterbi Path	
Viterbi Path	
Posterior @ 0.4	
Posterior @ 0.4	
Posterior Graphic. Threshold Marked @ 0.4	

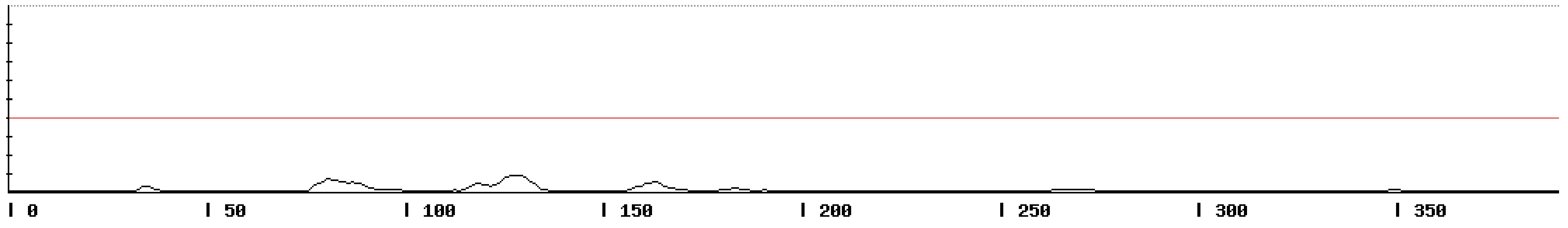

Predictions for Morusnotabilis_XP_010106034.1

Viterbi Path: 373 - RKAARGRMKKLKKK - 386

Viterbi Path

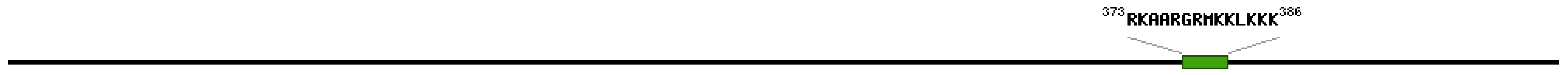

Posterior @ 0.4: 372 - DRKAARGRMKKLKKK - 386

Posterior @ 0.4

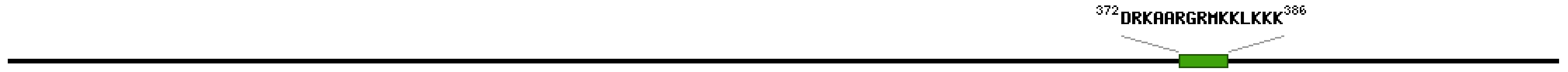

Posterior Graphic. Threshold Marked @ 0.4

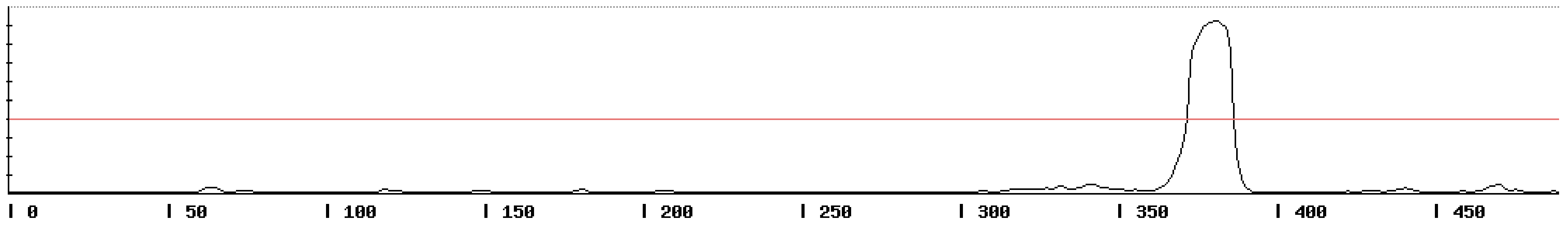

Predictions for Morusnotabilis_XP_010106645.1

Viterbi Path:

Viterbi Path

Posterior @ 0.4: 74 - KVLKHQKGKRLERR - 87

Posterior @ 0.4

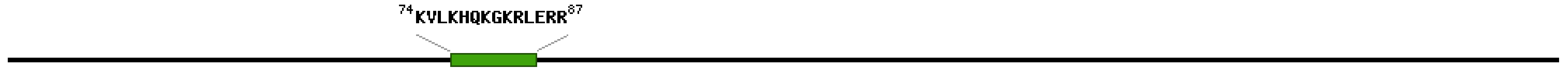

Posterior Graphic. Threshold Marked @ 0.4

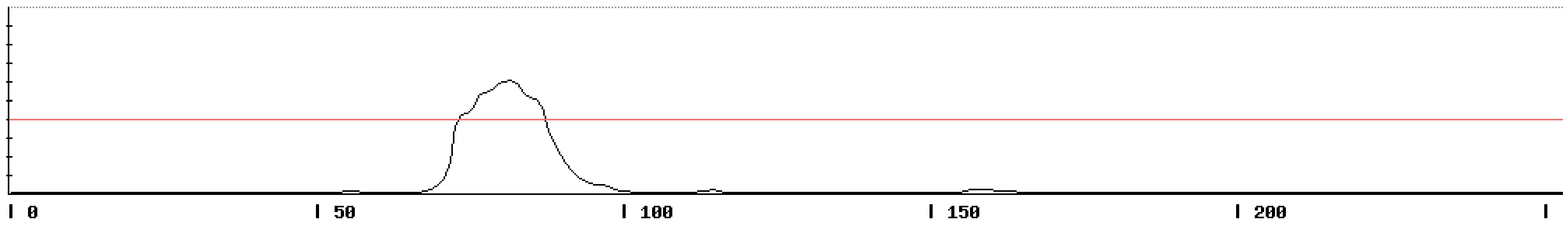

Predictions for Morusnotabilis_XP_010107113.1

Viterbi Path

Viterbi Path

Posterior @ 0.4 — 74 - KRSKRTTK - 81

Posterior @ 0.4

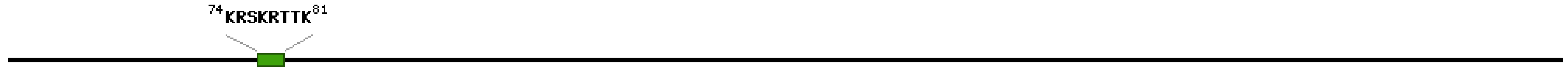

Posterior Graphic. Threshold Marked @ 0.4

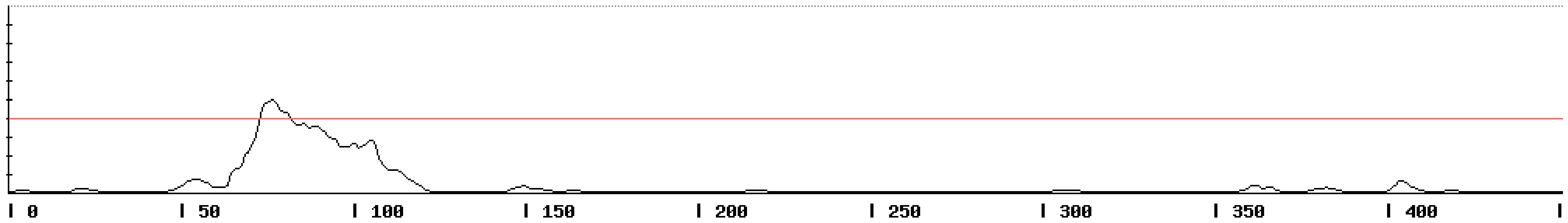

Predictions for Morusnotabilis_XP_010107114.1

Viterbi Path

Viterbi Path

Posterior @ 0.4 — 82 - SKRSKRTTKEG - 92

Posterior @ 0.4

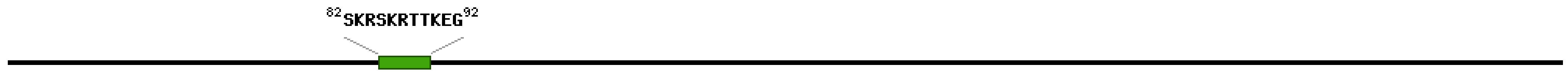

Posterior Graphic. Threshold Marked @ 0.4

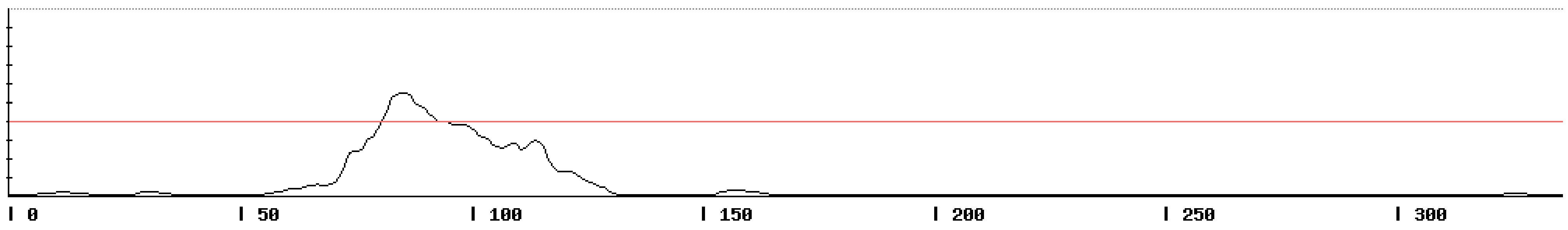

Predictions for Morusnotabilis_XP_010107116.1

Viterbi Path

Viterbi Path

Posterior @ 0.4 — 88 - KKPRSKRLKRSTKTG - 102

Posterior @ 0.4

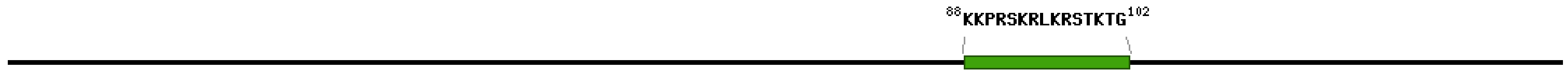

Posterior Graphic. Threshold Marked @ 0.4

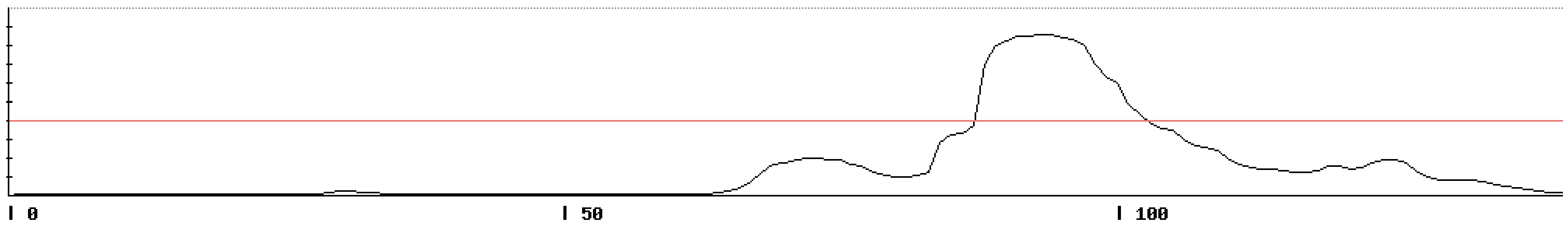

Predictions for Morusnotabilis_XP_010107118.1

Viterbi Path

Viterbi Path

Posterior @ 0.4 — 77 - PERKTTKRKTK - 87

Posterior @ 0.4

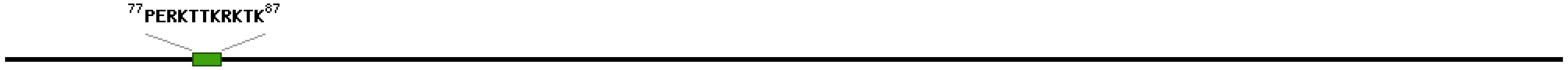

Posterior Graphic. Threshold Marked @ 0.4

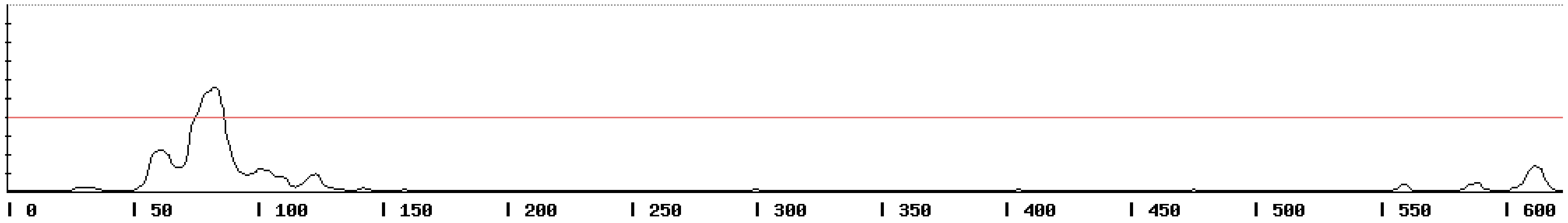

Predictions for Morusnotabilis_XP_010107122.1

Viterbi Path — 74 - RRDPKNKRTKRKTKTGFWKITGKEQEIKGRDGRVIGKKK - 112

Viterbi Path

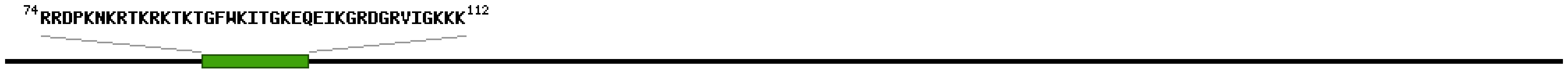

Posterior @ 0.4 — 74 - RRDPKNKRTKRKTKTGFWKITGKEQEIKGRDGRVIGKKK - 112

Posterior @ 0.4

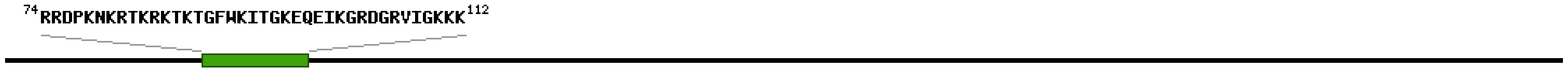

Posterior Graphic. Threshold Marked @ 0.4

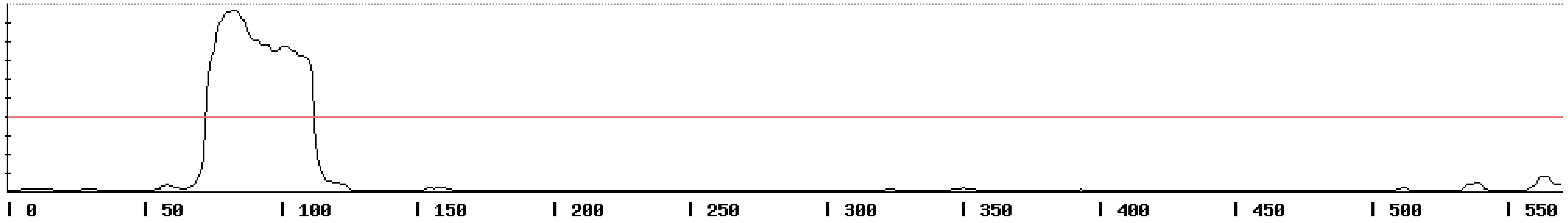

Predictions for Morusnotabilis_XP_010107124.1

Viterbi Path

Viterbi Path

Posterior @ 0.4

Posterior @ 0.4

Posterior Graphic. Threshold Marked @ 0.4

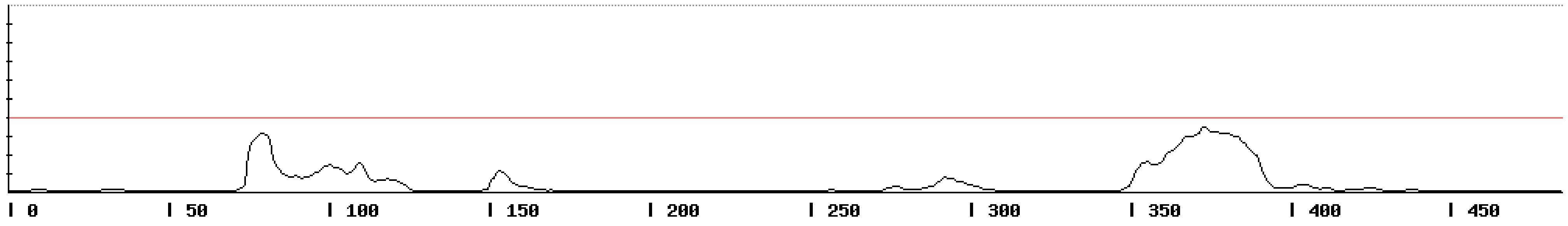

Predictions for Morusnotabilis_XP_010107555.1

Viterbi Path

Viterbi Path

Posterior @ 0.4

Posterior @ 0.4

Posterior Graphic. Threshold Marked @ 0.4

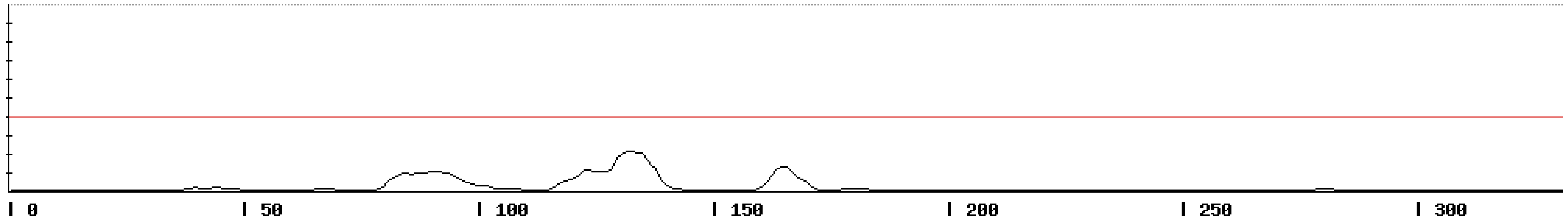

Predictions for Morusnotabilis_XP_010108028.1

Viterbi Path	
Viterbi Path	
Posterior @ 0.4	
Posterior @ 0.4	
Posterior Graphic. Threshold Marked @ 0.4	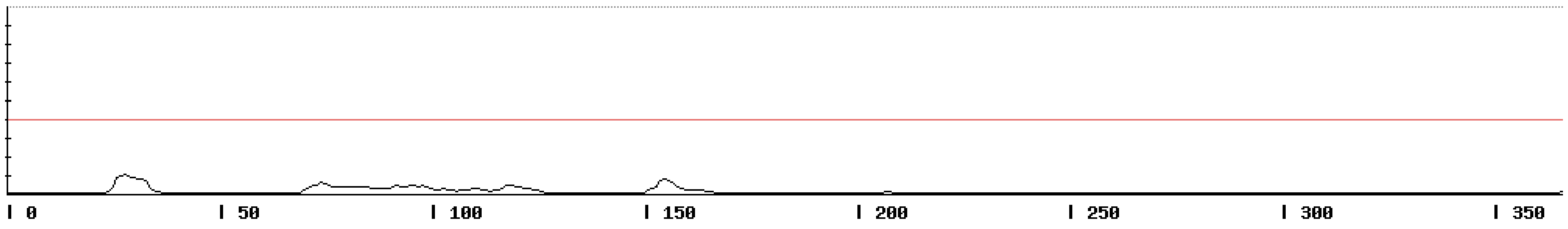

Predictions for Morusnotabilis_XP_010108694.1

Viterbi Path	
Viterbi Path	
Posterior @ 0.4	
Posterior @ 0.4	
Posterior Graphic. Threshold Marked @ 0.4	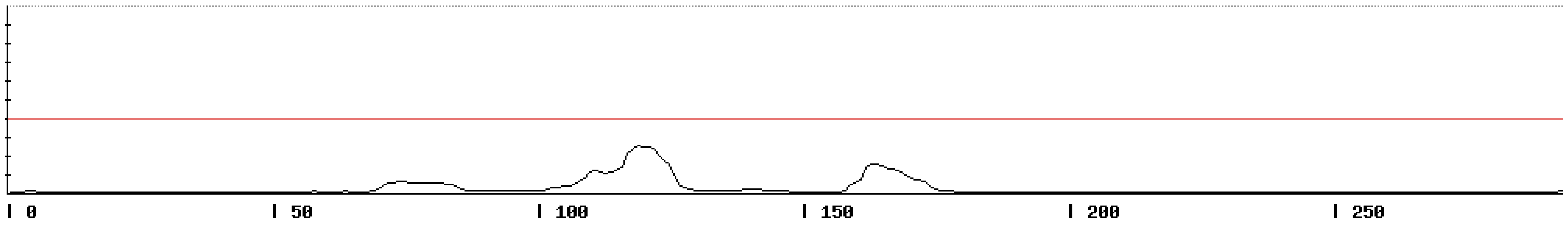

Predictions for Morusnotabilis_XP_010108843.1	
Viterbi Path	538 - RGGSMRSKVEKRMKKESGKTLREIRRAKKIQKKLMTESERLIYNLKRAKKK - 588
Viterbi Path	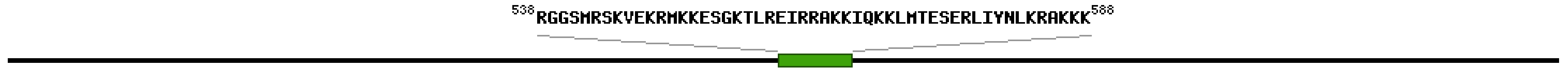
Posterior @ 0.4	161 - KRR - 163<br>537 - ARGGSMRSKVEKRMKKESGKTLREIRRAKKIQKKLMTESERLIYNLKRAKKKVA - 590
Posterior @ 0.4	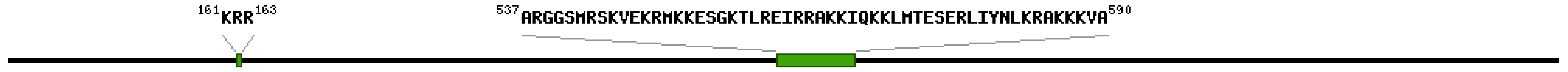
Posterior Graphic. Threshold Marked @ 0.4	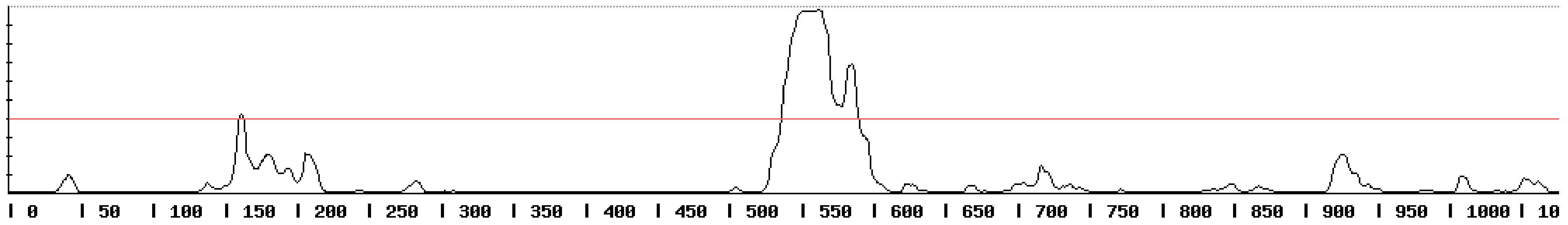

Predictions for Morusnotabilis_XP_010109365.1	
Viterbi Path	
Viterbi Path	
Posterior @ 0.4	
Posterior @ 0.4	
Posterior Graphic. Threshold Marked @ 0.4	

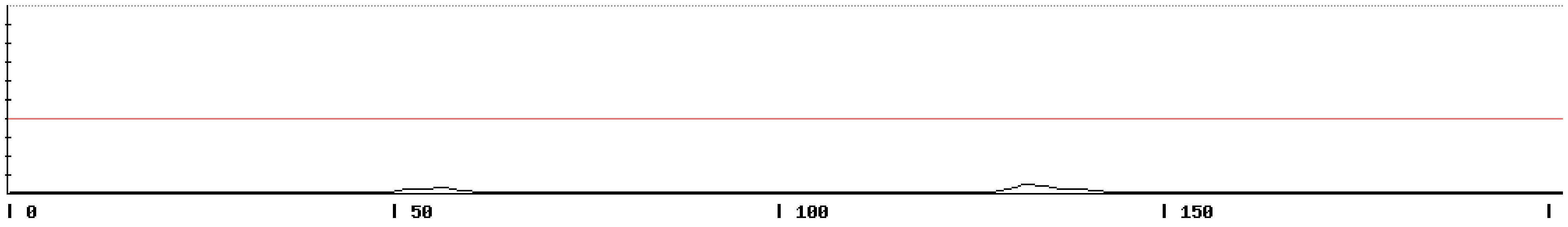

Predictions for Morusnotabilis_XP_010110814.1

Viterbi Path

Viterbi Path

Posterior @ 0.4

Posterior @ 0.4

Posterior Graphic. Threshold Marked @ 0.4

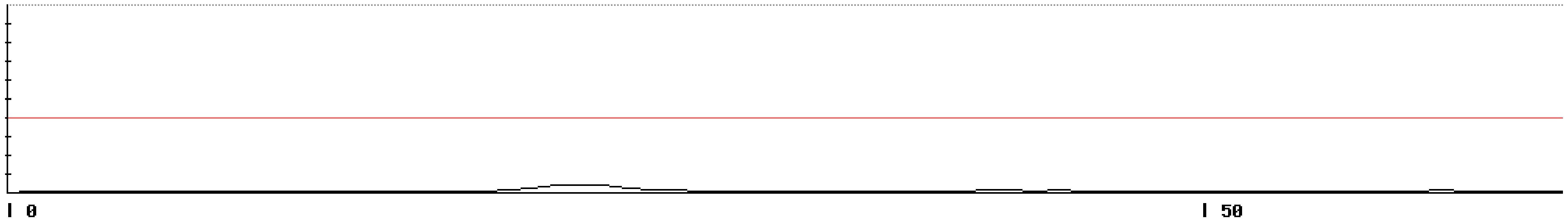

Predictions for Morusnotabilis_XP_010110815.1

Viterbi Path: 87 - RERGGKRRARSGFWRRKGRMR - 107

Viterbi Path

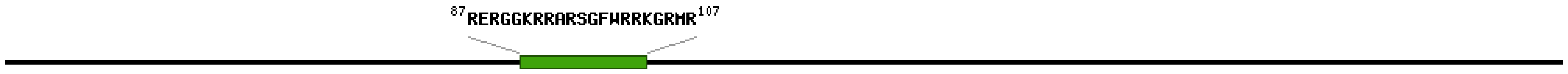

Posterior @ 0.4: 85 - AVRERGGKRRARSGFWRRKGRMRI - 108

Posterior @ 0.4

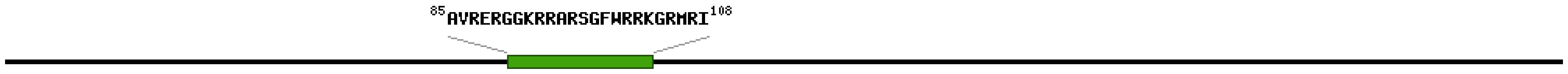

Posterior Graphic. Threshold Marked @ 0.4

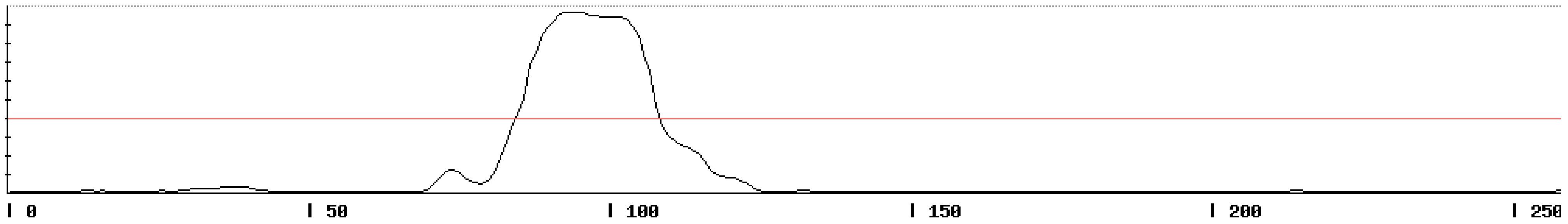

Predictions for Morusnotabilis_XP_010110863.1

Viterbi Path

Viterbi Path

Posterior @ 0.4

Posterior @ 0.4

Posterior Graphic. Threshold Marked @ 0.4

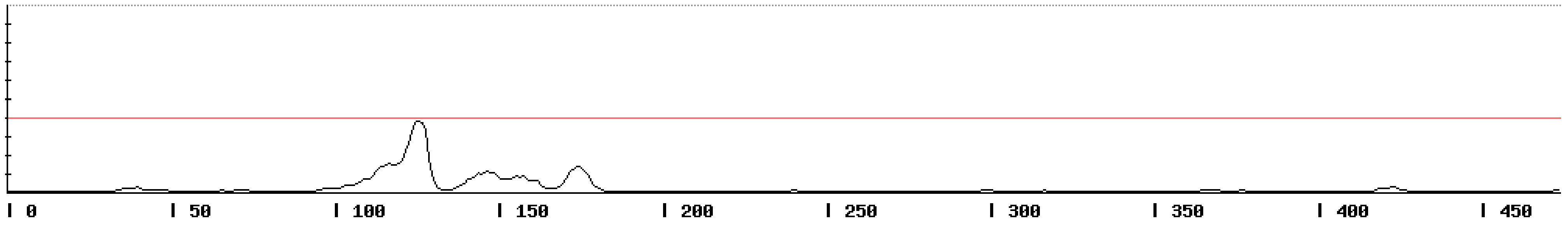

Predictions for Morusnotabilis_XP_010110935.1

Viterbi Path

Viterbi Path

Posterior @ 0.4 174 - KKRRPKKKNGTNSER - 188

Posterior @ 0.4

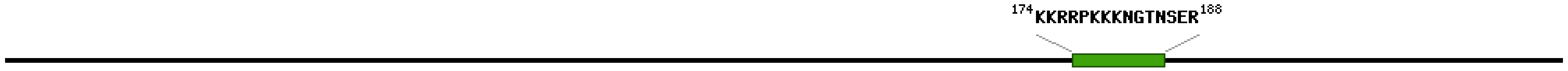

Posterior Graphic. Threshold Marked @ 0.4	

Revision r.9 - Last Updated on : February 24 2015 17:45:20. alex.nguyenba@utoronto.ca

Intended to be used with Firefox

Viterbi Path

Posterior @ 0.6

Posterior @ 0.6

Posterior Graphic. Threshold Marked @ 0.6

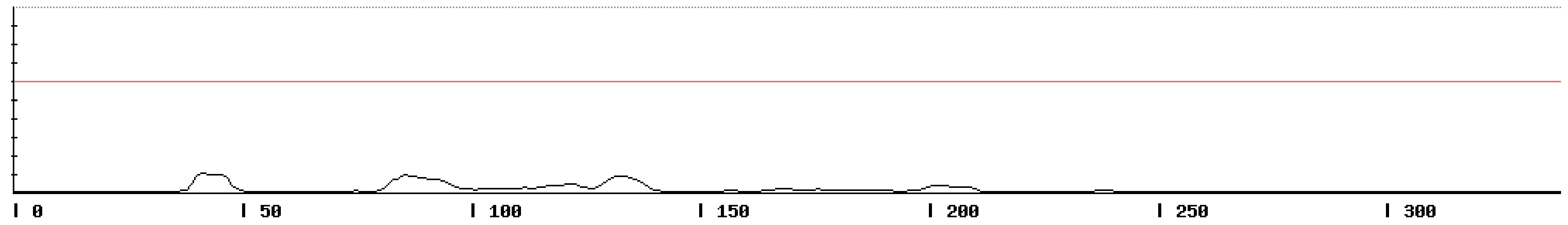

Predictions for Musaacuminata_GSMUA_Achr10P04530_001

Viterbi Path

Viterbi Path

Posterior @ 0.6

Posterior @ 0.6

Posterior Graphic. Threshold Marked @ 0.6

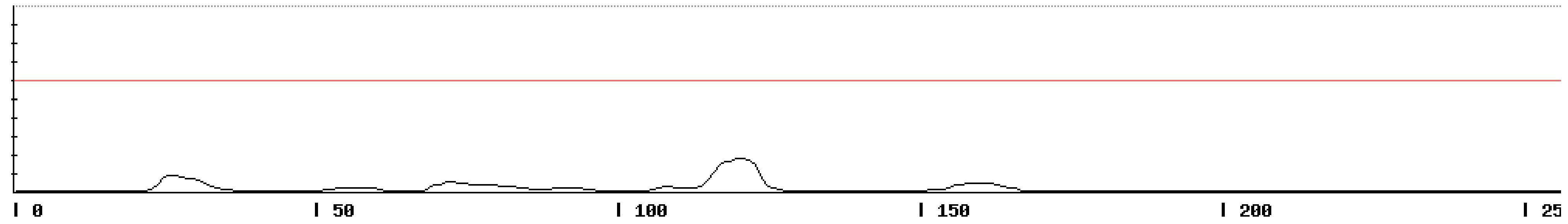

Predictions for Musaacuminata_GSMUA_Achr10P04570_001

Viterbi Path	
Viterbi Path	
Posterior @ 0.6	
Posterior @ 0.6	
Posterior Graphic. Threshold Marked @ 0.6	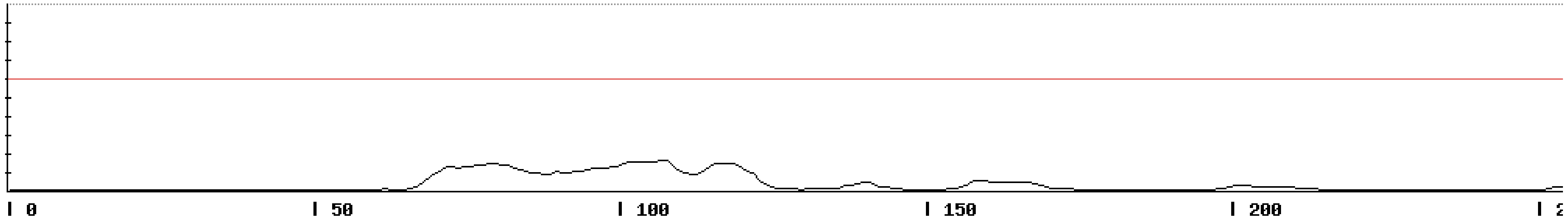

Predictions for Musaacuminata_GSMUA_Achr10P04720_001

Viterbi Path	
Viterbi Path	
Posterior @ 0.6	
Posterior @ 0.6	
Posterior Graphic. Threshold Marked @ 0.6	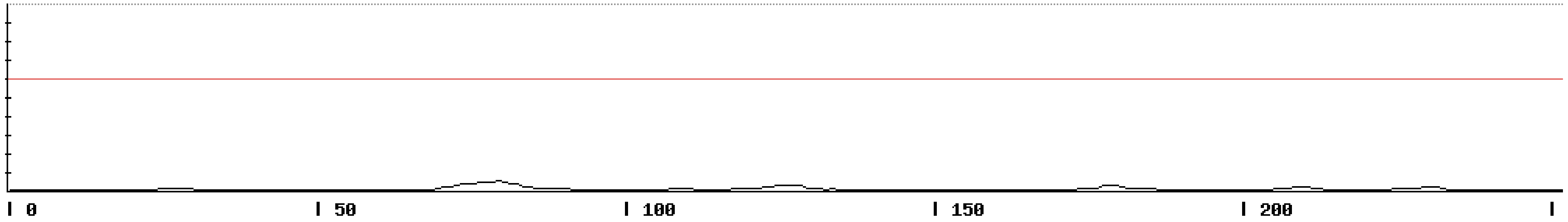

Predictions for Musaacuminata_GSMUA_Achr10P05070_001

Viterbi Path

Viterbi Path

Posterior @ 0.6

Posterior @ 0.6

Posterior Graphic. Threshold Marked @ 0.6

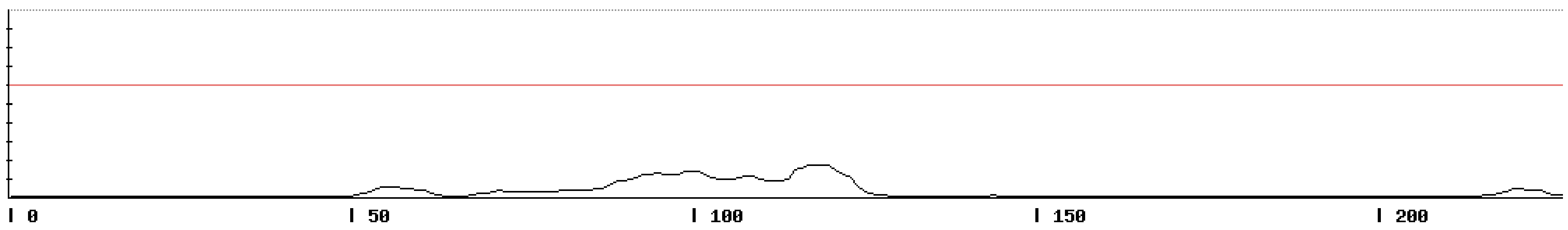

Predictions for Musaacuminata_GSMUA_Achr10P08120_001

Viterbi Path

Viterbi Path

Posterior @ 0.6

Posterior @ 0.6

Posterior Graphic. Threshold Marked @ 0.6

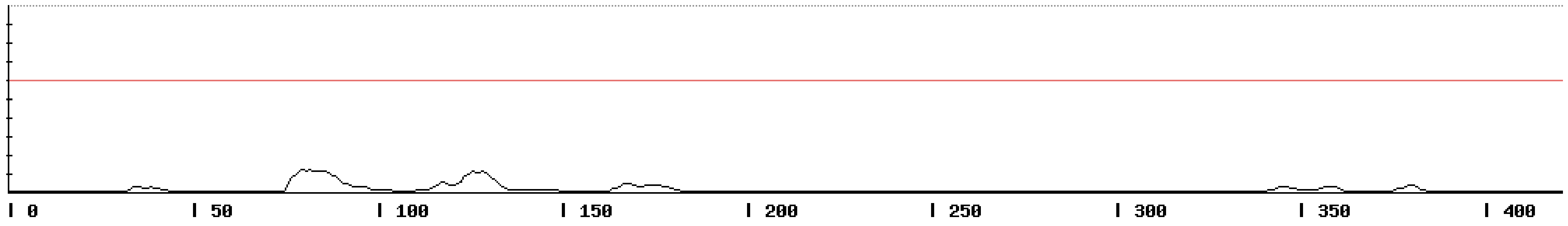

Predictions for Musaacuminata_GSMUA_Achr10P08420_001

Viterbi Path

Viterbi Path

Posterior @ 0.6

Posterior @ 0.6

Posterior Graphic. Threshold Marked @ 0.6

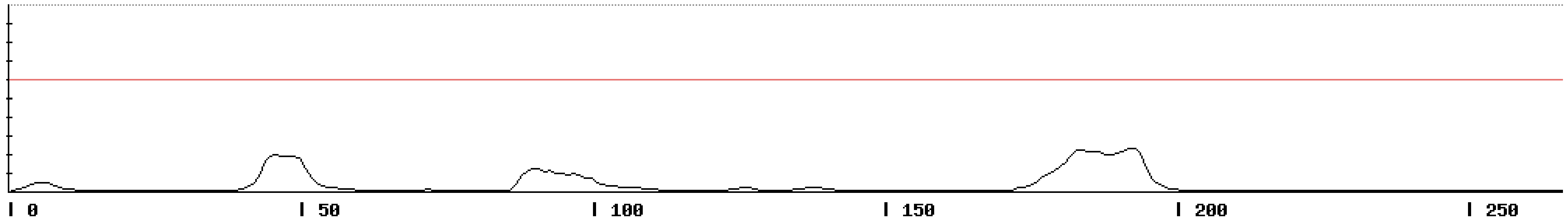

Predictions for Musaacuminata_GSMUA_Achr10P10690_001

Viterbi Path

Viterbi Path

Posterior @ 0.6

Posterior @ 0.6

Posterior Graphic. Threshold Marked @ 0.6

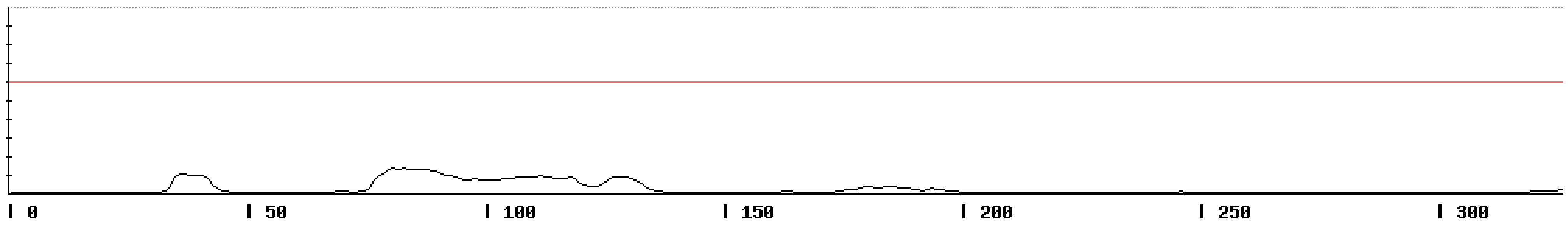

Predictions for Musaacuminata_GSMUA_Achr10P10790_001

Viterbi Path

Viterbi Path

Posterior @ 0.6

Posterior @ 0.6

Posterior Graphic. Threshold Marked @ 0.6

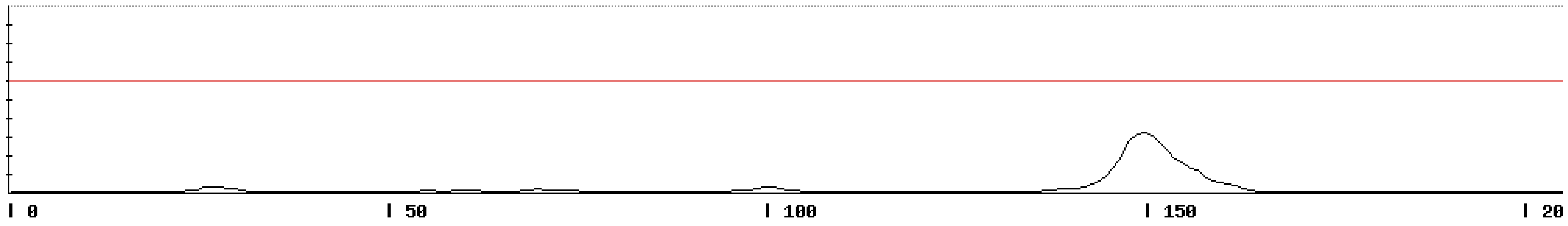

Predictions for Musaacuminata_GSMUA_Achr10P11910_001

Viterbi Path

Viterbi Path

Posterior @ 0.6

Posterior @ 0.6

Posterior Graphic. Threshold Marked @ 0.6

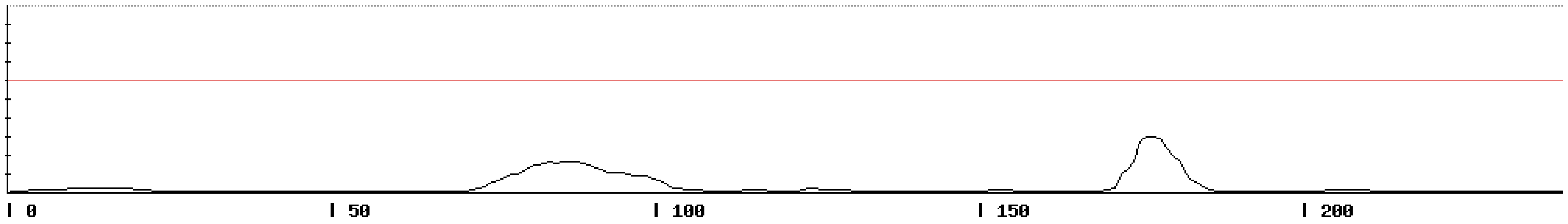

Predictions for Musaacuminata_GSMUA_Achr10P12860_001

Viterbi Path

Viterbi Path

Posterior @ 0.6

Posterior @ 0.6

Posterior Graphic. Threshold Marked @ 0.6

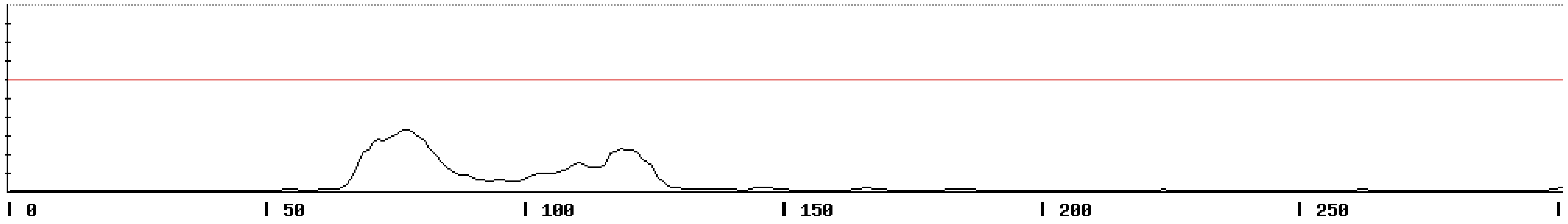

Predictions for Musaacuminata_GSMUA_Achr10P14400_001

Viterbi Path

Viterbi Path

Posterior @ 0.6

Posterior @ 0.6

Posterior Graphic. Threshold Marked @ 0.6

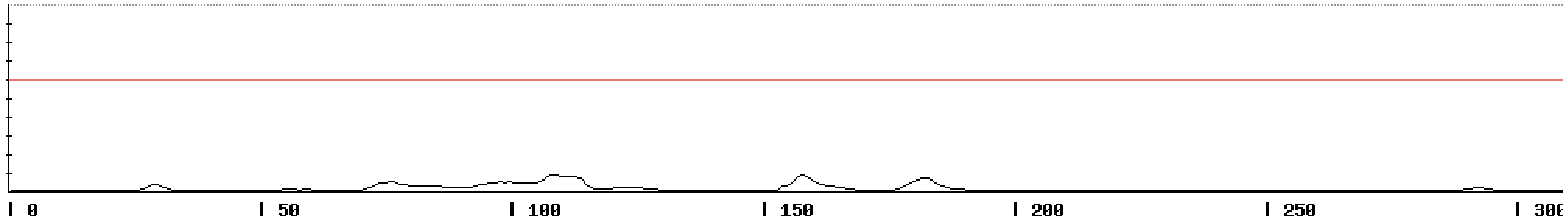

Predictions for Musaacuminata_GSMUA_Achr10P16940_001

Viterbi Path

Viterbi Path

Posterior @ 0.6

Posterior @ 0.6

Posterior Graphic. Threshold Marked @ 0.6

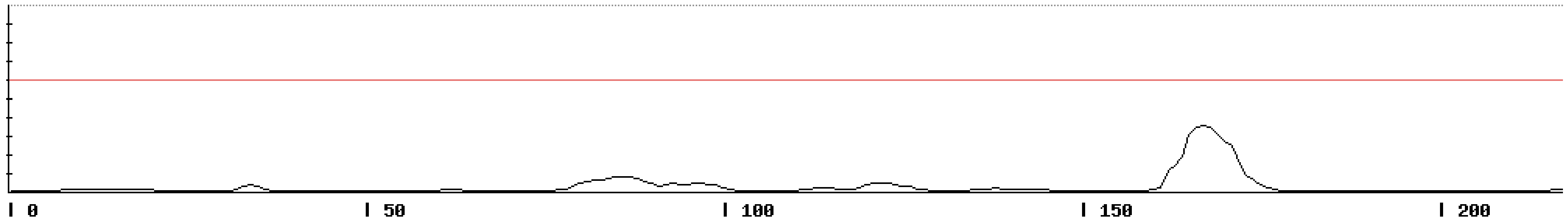

Predictions for Musaacuminata_GSMUA_Achr10P19900_001

Viterbi Path

Viterbi Path

Posterior @ 0.6

Posterior @ 0.6

Posterior Graphic. Threshold Marked @ 0.6

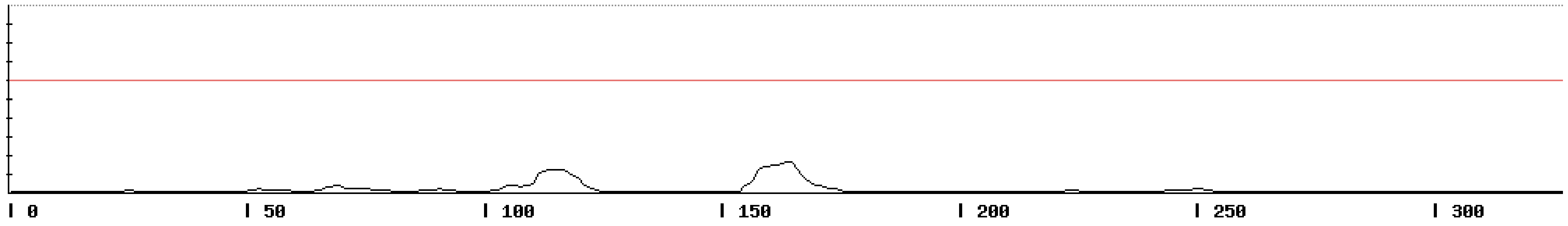

Predictions for Musaacuminata_GSMUA_Achr10P21750_001

Viterbi Path

Viterbi Path

Posterior @ 0.6

Posterior @ 0.6

Posterior Graphic. Threshold Marked @ 0.6

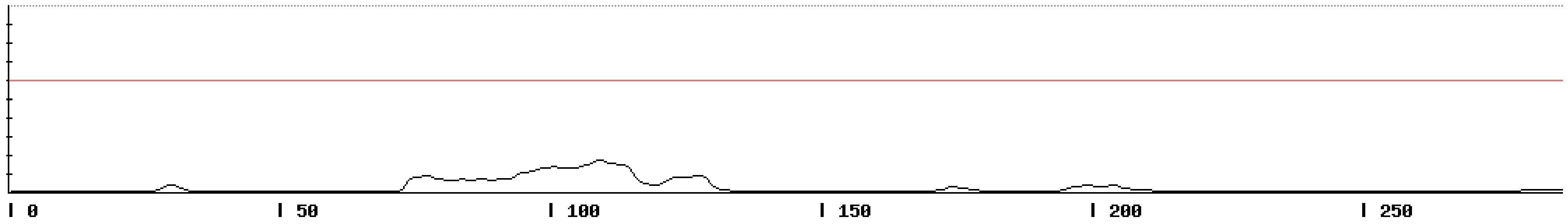

Predictions for Musaacuminata_GSMUA_Achr10P22350_001

Viterbi Path

Viterbi Path

Posterior @ 0.6

Posterior @ 0.6

Posterior Graphic. Threshold Marked @ 0.6

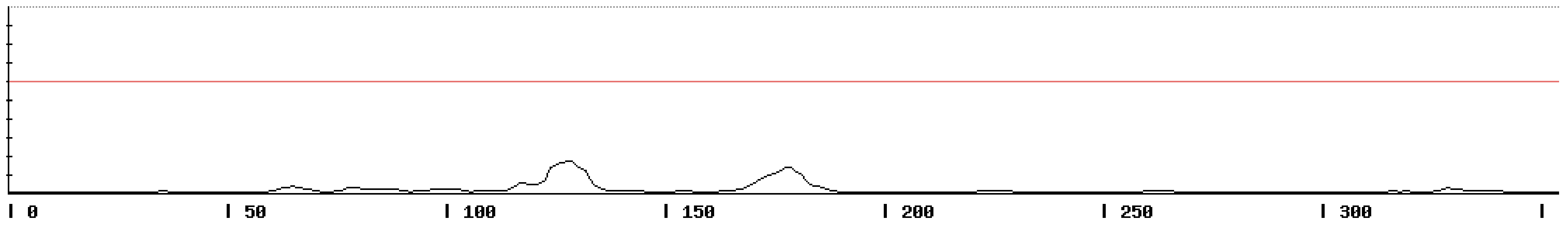

Predictions for Musaacuminata_GSMUA_Achr10P26180_001

Viterbi Path

Viterbi Path

Posterior @ 0.6

Posterior @ 0.6

Posterior Graphic. Threshold Marked @ 0.6

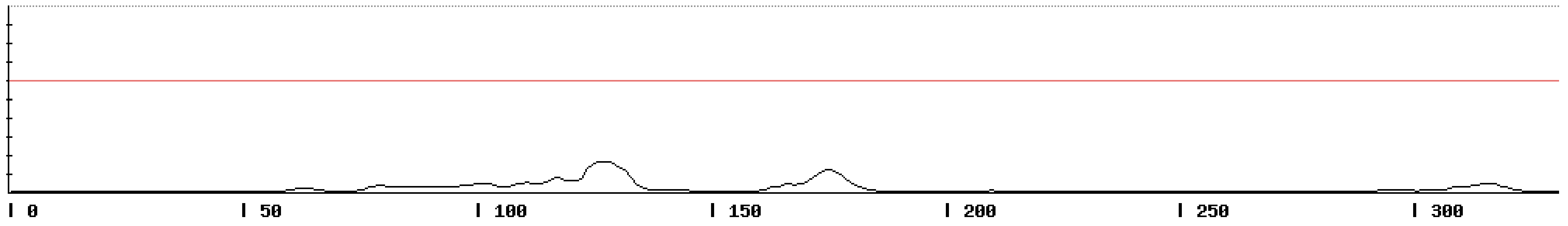

Predictions for Musaacuminata_GSMUA_Achr10P29200_001	
Viterbi Path	
Viterbi Path	
Posterior @ 0.6	
Posterior @ 0.6	
Posterior Graphic. Threshold Marked @ 0.6	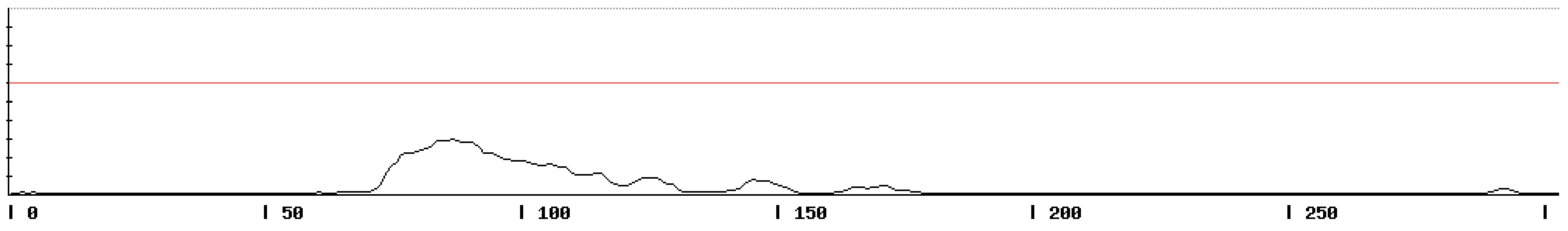

Predictions for Musaacuminata_GSMUA_Achr11P00880_001	
Viterbi Path	
Viterbi Path	
Posterior @ 0.6	
Posterior @ 0.6	
Posterior Graphic. Threshold Marked @ 0.6	

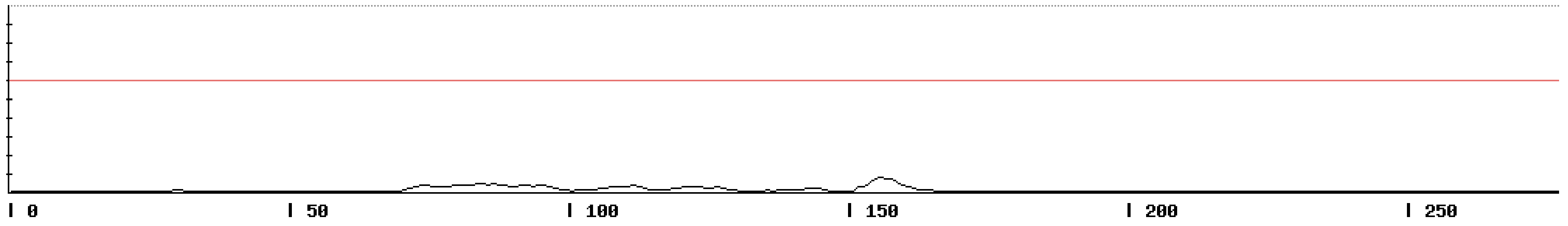

Predictions for Musaacuminata_GSMUA_Achr11P01320_001

Viterbi Path

Viterbi Path

Posterior @ 0.6

Posterior @ 0.6

Posterior Graphic. Threshold Marked @ 0.6

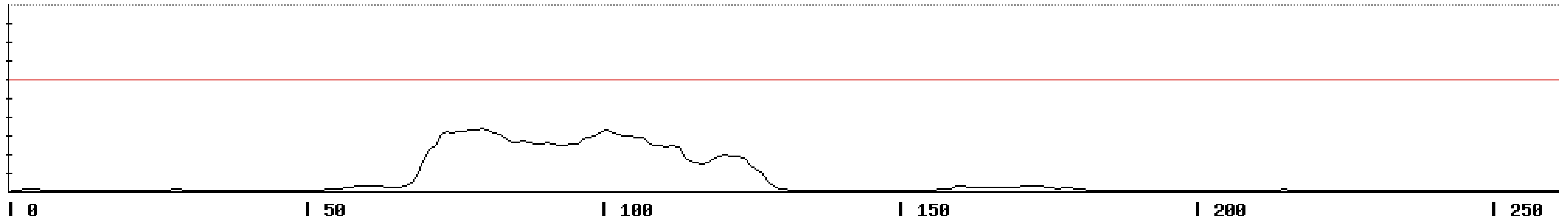

Predictions for Musaacuminata_GSMUA_Achr11P01400_001

Viterbi Path

Viterbi Path

Posterior @ 0.6

Posterior @ 0.6

Posterior Graphic. Threshold Marked @ 0.6

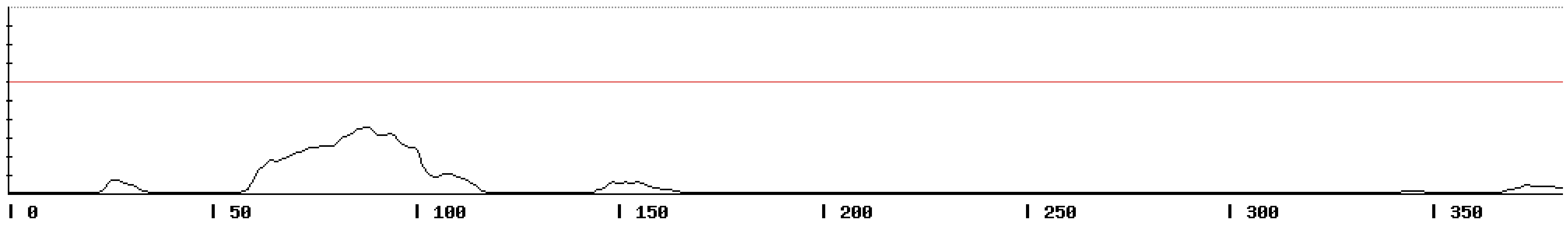

Predictions for Musaacuminata_GSMUA_Achr11P03040_001

Viterbi Path

Viterbi Path

Posterior @ 0.6

Posterior @ 0.6

Posterior Graphic. Threshold Marked @ 0.6

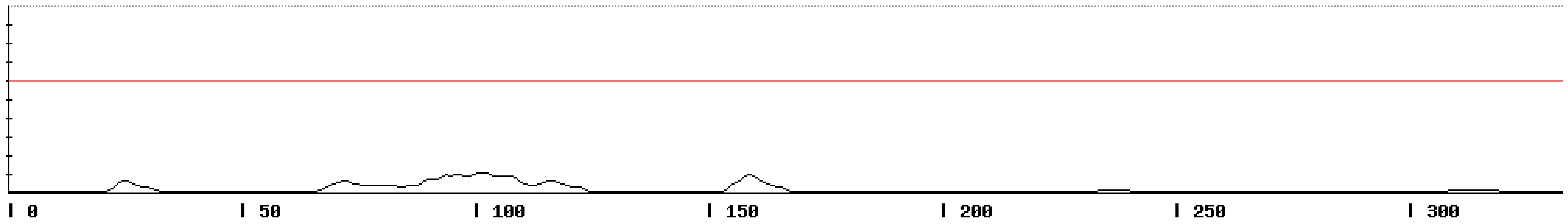

Predictions for Musaacuminata_GSMUA_Achr11P03780_001

Viterbi Path

Viterbi Path

Posterior @ 0.6

Posterior @ 0.6

Posterior Graphic. Threshold Marked @ 0.6

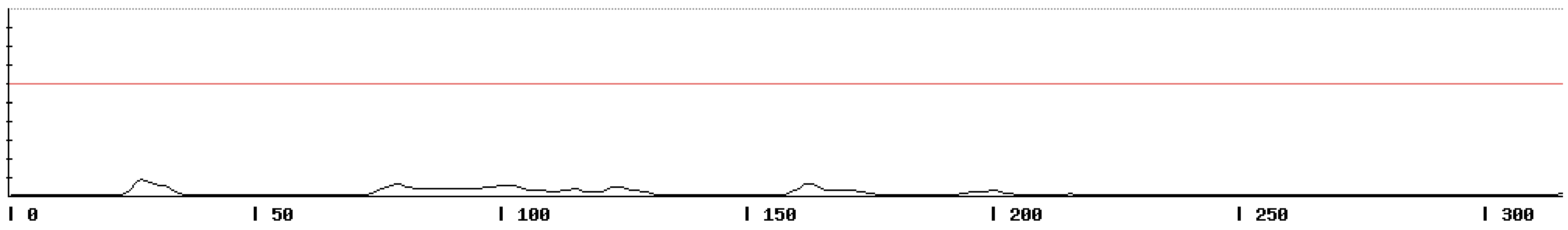

Predictions for Musaacuminata_GSMUA_Achr11P07800_001

Viterbi Path

Viterbi Path

Posterior @ 0.6

Posterior @ 0.6

Posterior Graphic. Threshold Marked @ 0.6

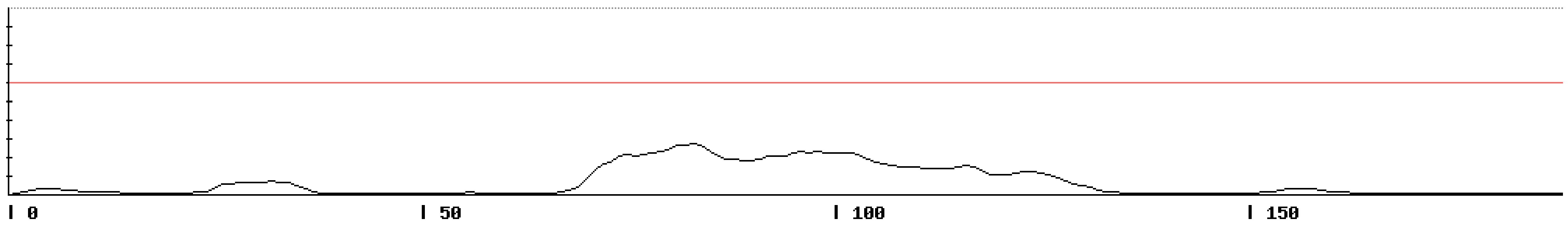

Predictions for Musaacuminata_GSMUA_Achr11P08970_001

Viterbi Path

Viterbi Path

Posterior @ 0.6

Posterior @ 0.6

Posterior Graphic. Threshold Marked @ 0.6

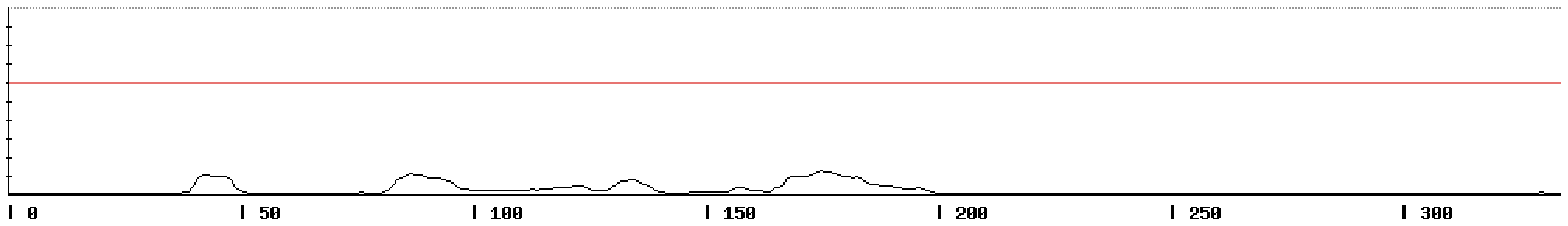

Predictions for Musaacuminata_GSMUA_Achr11P09000_001

Viterbi Path

Viterbi Path

Posterior @ 0.6

Posterior @ 0.6

Posterior Graphic. Threshold Marked @ 0.6

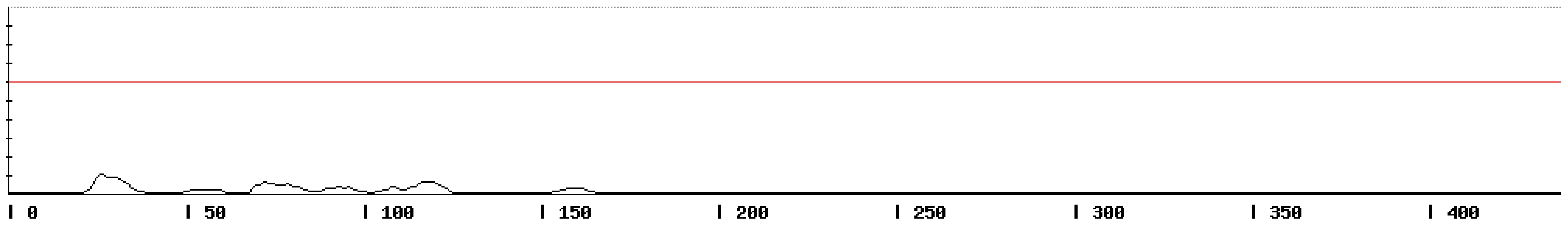

Predictions for Musaacuminata_GSMUA_Achr11P16860_001

Viterbi Path 504 - KKKKKKKAK - 512

Viterbi Path

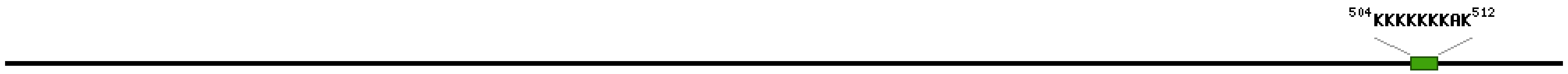

Posterior @ 0.6 — 504 - KKKKKKKAK - 512

Posterior @ 0.6

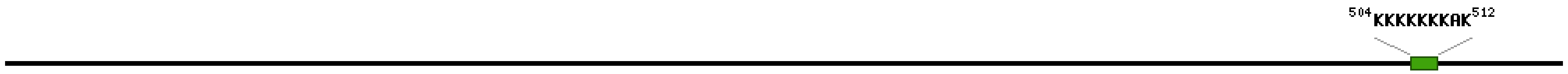

Posterior Graphic. Threshold Marked @ 0.6

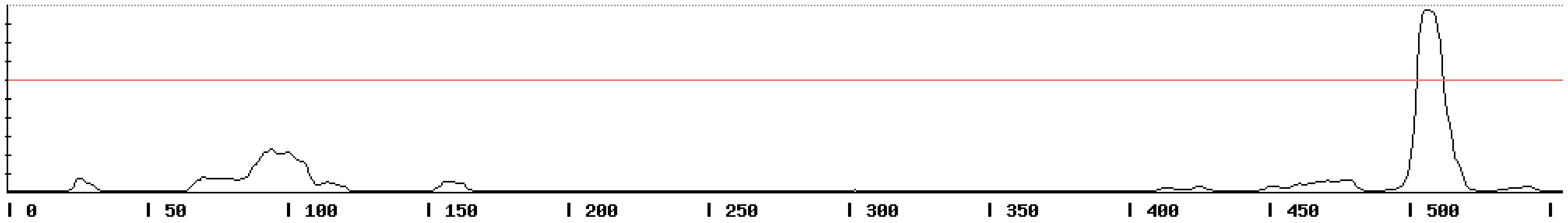

Predictions for Musaacuminata_GSMUA_Achr11P17510_001

Viterbi Path

Viterbi Path

Posterior @ 0.6

Posterior @ 0.6

Posterior Graphic. Threshold Marked @ 0.6

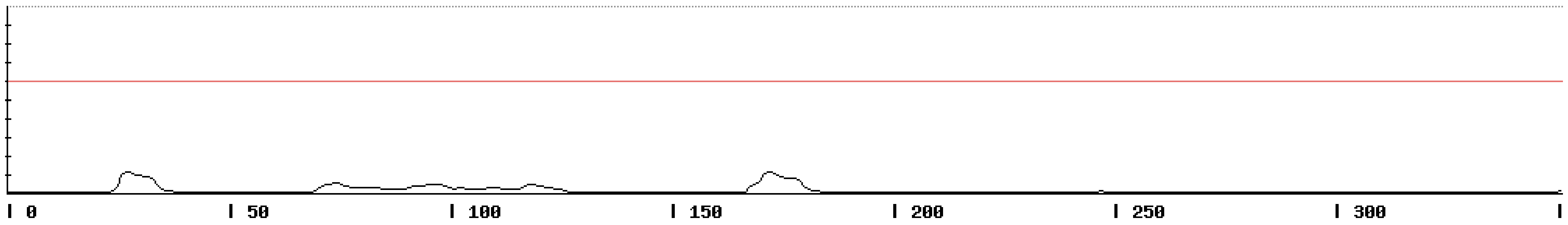

Predictions for Musaacuminata_GSMUA_Achr11P17780_001

Viterbi Path

Viterbi Path

Posterior @ 0.6

Posterior @ 0.6

Posterior Graphic. Threshold Marked @ 0.6

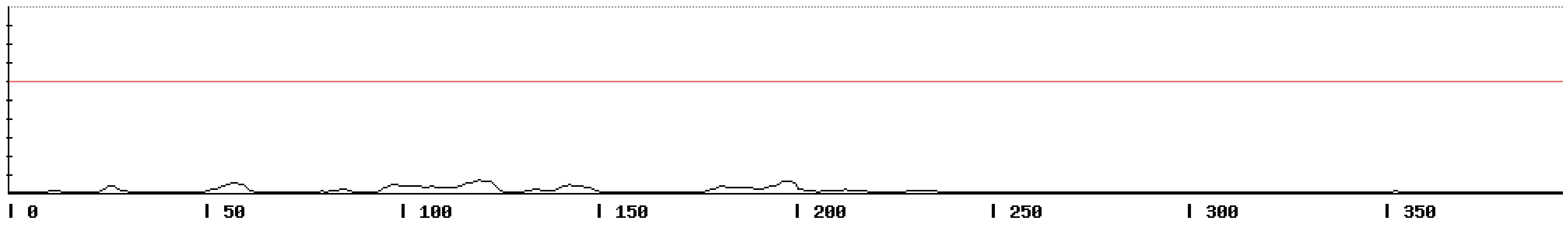

Predictions for Musaacuminata_GSMUA_Achr11P22590_001

Viterbi Path

Viterbi Path

Posterior @ 0.6

Posterior @ 0.6

Posterior Graphic. Threshold Marked @ 0.6

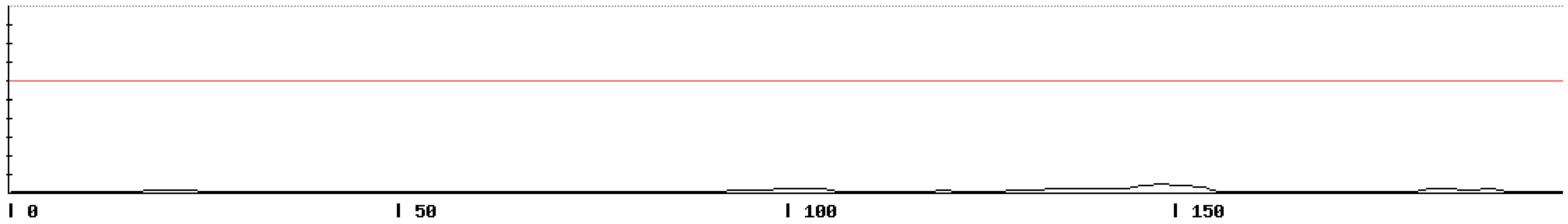

Predictions for Musaacuminata_GSMUA_Achr11P22760_001

Viterbi Path

Viterbi Path

Posterior @ 0.6

Posterior @ 0.6

Posterior Graphic. Threshold Marked @ 0.6

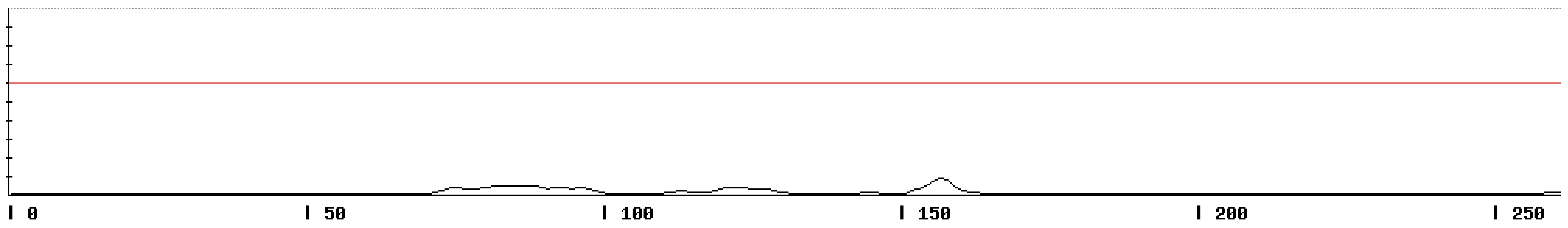

Predictions for Musaacuminata_GSMUA_Achr11P25720_001

Viterbi Path

Viterbi Path

Posterior @ 0.6

Posterior @ 0.6

Posterior Graphic. Threshold Marked @ 0.6

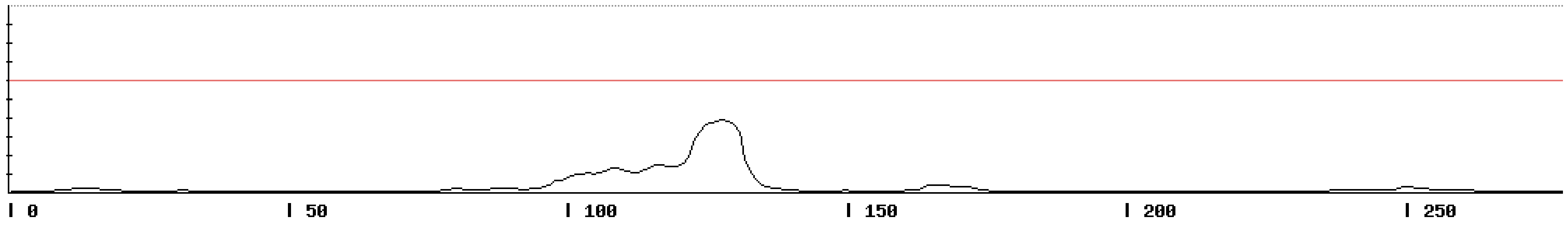

Predictions for Musaacuminata_GSMUA_Achr11P26450_001

Viterbi Path	
Viterbi Path	
Posterior @ 0.6	
Posterior @ 0.6	
Posterior Graphic. Threshold Marked @ 0.6	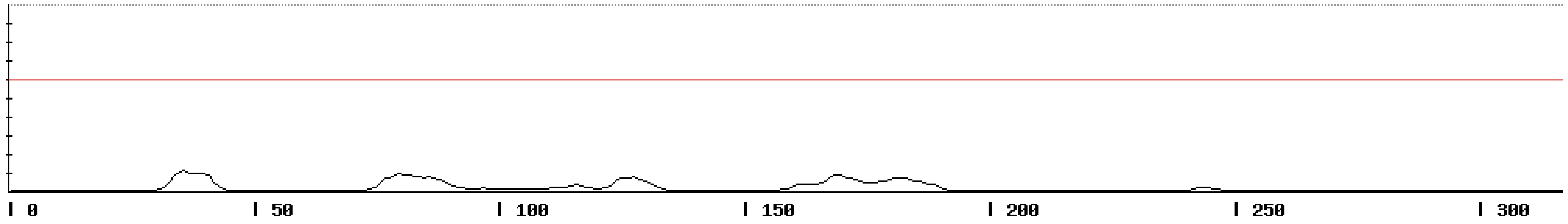

Predictions for Musaacuminata_GSMUA_Achr1P02710_001

Viterbi Path	
Viterbi Path	
Posterior @ 0.6	
Posterior @ 0.6	

Posterior Graphic. Threshold Marked @ 0.6

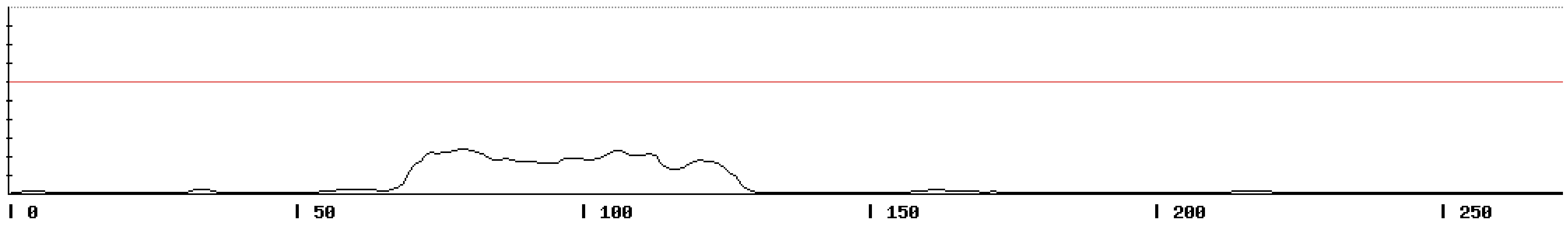

Predictions for Musaacuminata_GSMUA_Achr1P02820_001

Viterbi Path

Viterbi Path

Posterior @ 0.6

Posterior @ 0.6

Posterior Graphic. Threshold Marked @ 0.6

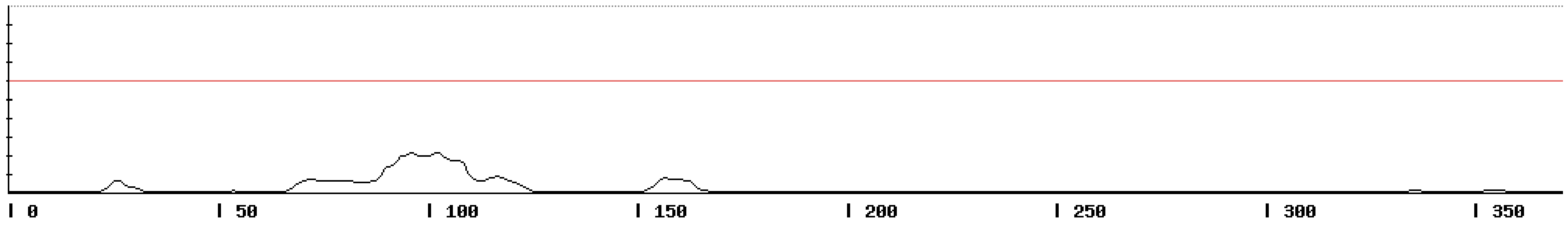

Predictions for Musaacuminata_GSMUA_Achr1P03560_001

Viterbi Path

Viterbi Path

Posterior @ 0.6

Posterior @ 0.6

Posterior Graphic. Threshold Marked @ 0.6	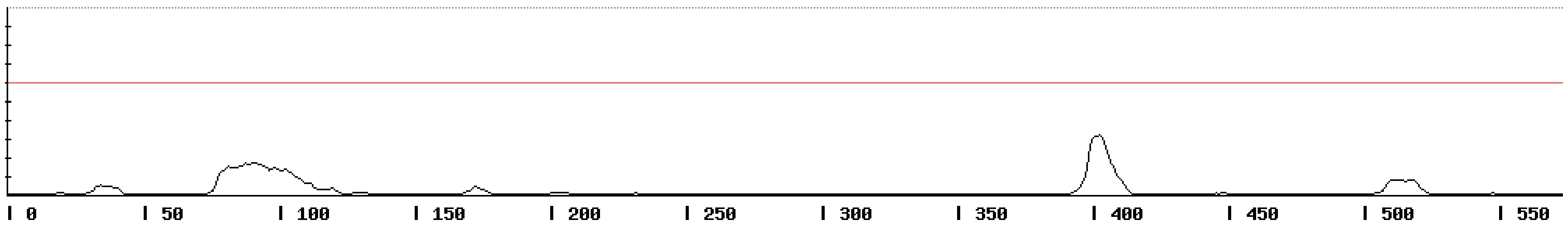

Predictions for Musaacuminata_GSMUA_Achr1P08860_001	
Viterbi Path	
Viterbi Path	
Posterior @ 0.6	
Posterior @ 0.6	
Posterior Graphic. Threshold Marked @ 0.6	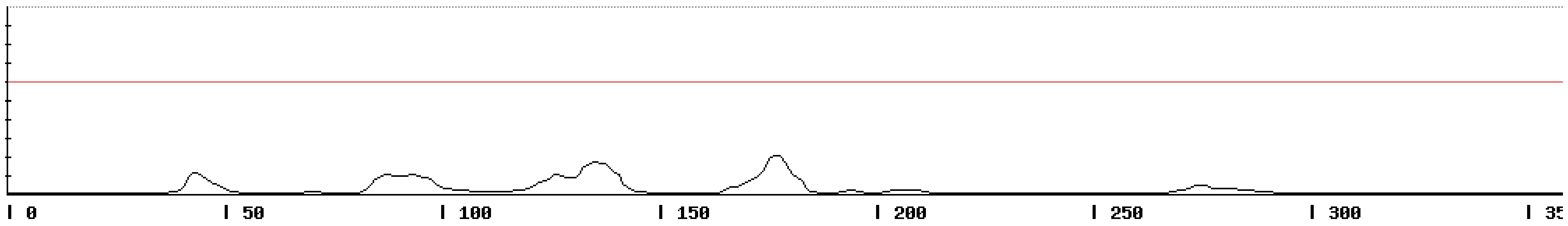

Predictions for Musaacuminata_GSMUA_Achr1P10860_001	
Viterbi Path	
Viterbi Path	

Posterior @ 0.6

Posterior @ 0.6

Posterior Graphic. Threshold Marked @ 0.6

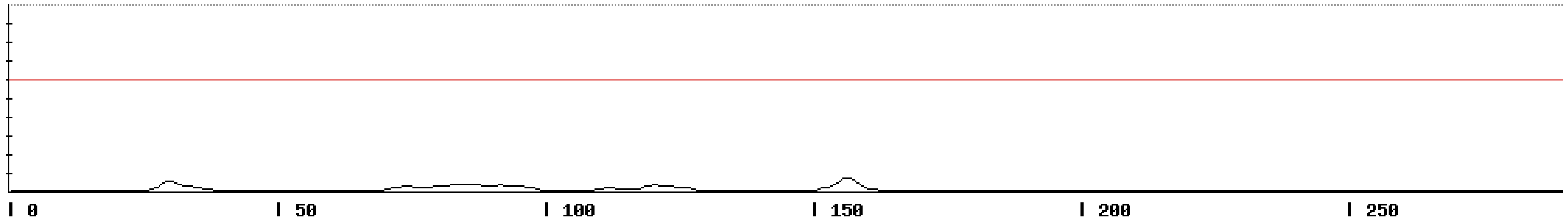

Predictions for Musaacuminata_GSMUA_Achr1P18110_001

Viterbi Path

Viterbi Path

Posterior @ 0.6

Posterior @ 0.6

Posterior Graphic. Threshold Marked @ 0.6

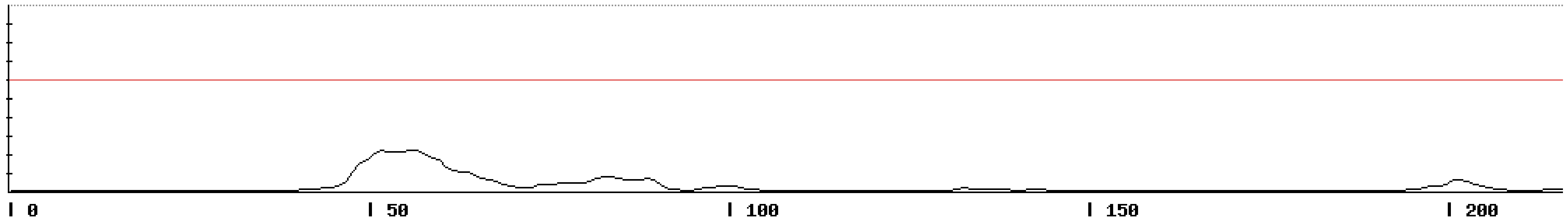

Predictions for Musaacuminata_GSMUA_Achr1P20530_001

Viterbi Path

Viterbi Path

Posterior @ 0.6	
Posterior @ 0.6	
Posterior Graphic. Threshold Marked @ 0.6	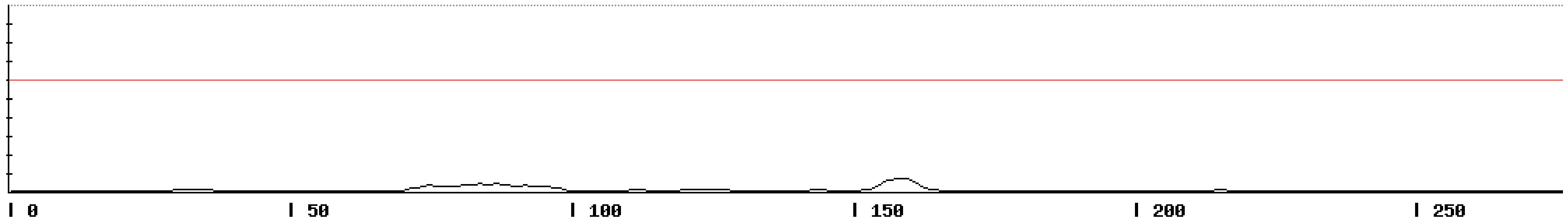

Predictions for Musaacuminata_GSMUA_Achr2P04010_001	
Viterbi Path	
Viterbi Path	
Posterior @ 0.6	
Posterior @ 0.6	
Posterior Graphic. Threshold Marked @ 0.6	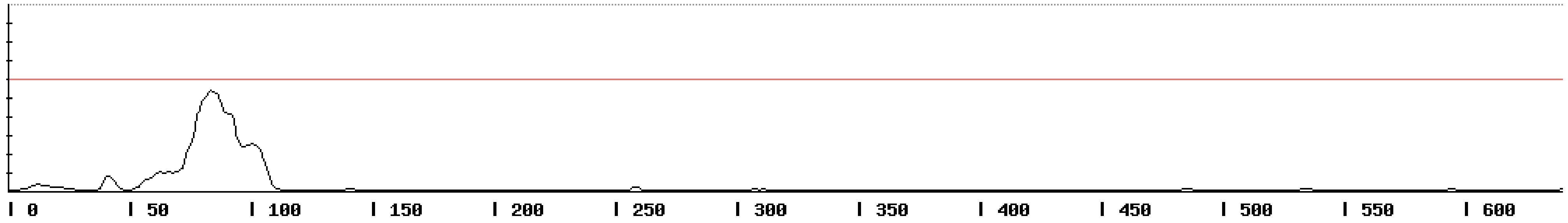

Predictions for Musaacuminata_GSMUA_Achr2P05640_001

Viterbi Path

Viterbi Path

Posterior @ 0.6

Posterior @ 0.6

Posterior Graphic. Threshold Marked @ 0.6

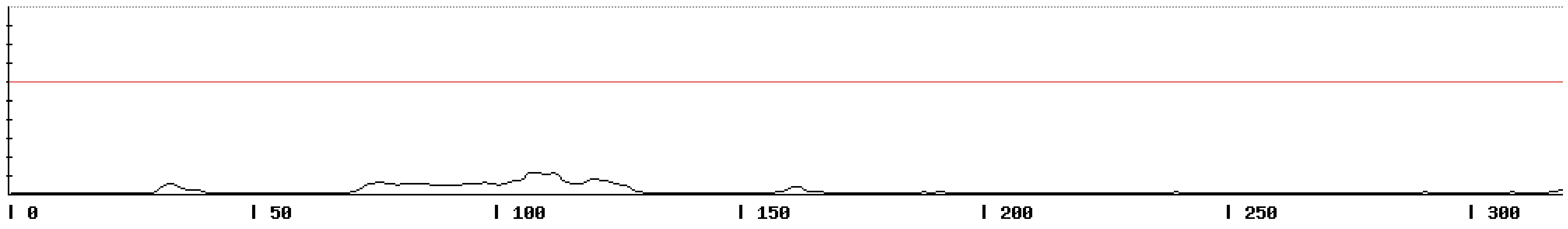

Predictions for Musaacuminata_GSMUA_Achr2P09080_001

Viterbi Path

Viterbi Path

Posterior @ 0.6

Posterior @ 0.6

Posterior Graphic. Threshold Marked @ 0.6

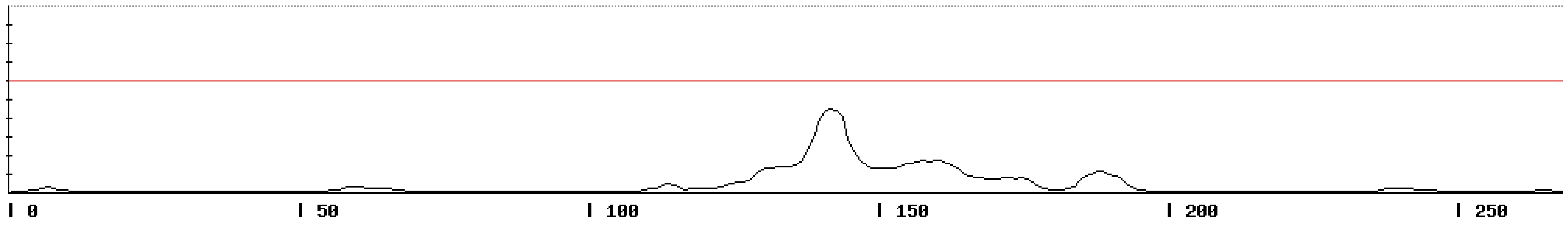

Predictions for Musaacuminata_GSMUA_Achr2P11810_001

Viterbi Path

Viterbi Path

Posterior @ 0.6

Posterior @ 0.6

Posterior Graphic. Threshold Marked @ 0.6

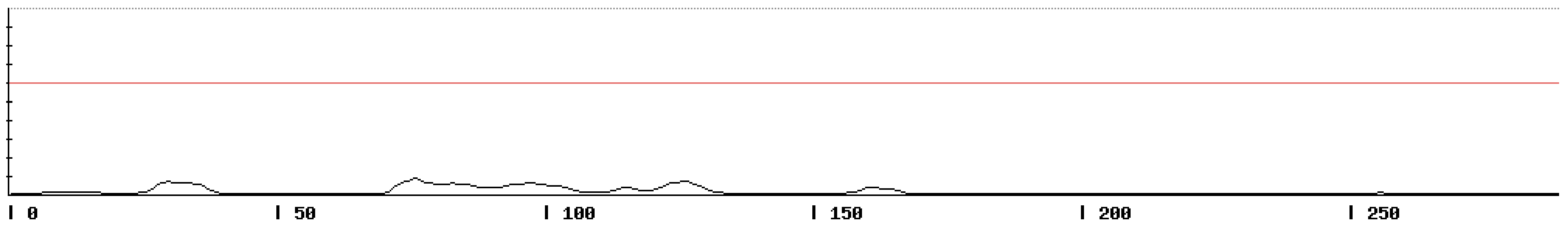

Predictions for Musaacuminata_GSMUA_Achr2P16590_001

Viterbi Path

Viterbi Path

Posterior @ 0.6

Posterior @ 0.6

Posterior Graphic. Threshold Marked @ 0.6

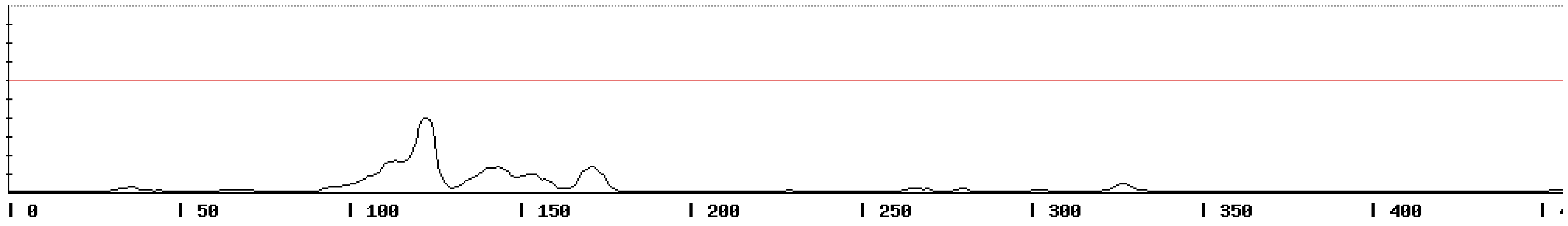

Predictions for Musaacuminata_GSMUA_Achr2P20020_001	
Viterbi Path	
Viterbi Path	
Posterior @ 0.6	
Posterior @ 0.6	
Posterior Graphic. Threshold Marked @ 0.6	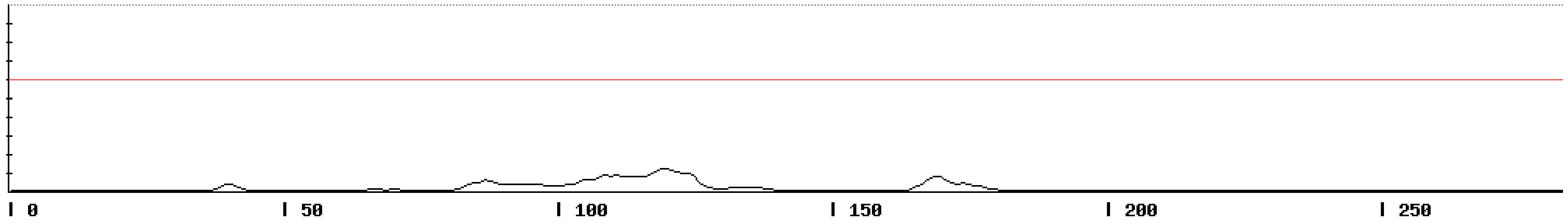

Predictions for Musaacuminata_GSMUA_Achr2P21110_001	
Viterbi Path	
Viterbi Path	
Posterior @ 0.6	
Posterior @ 0.6	

Posterior Graphic. Threshold Marked @ 0.6

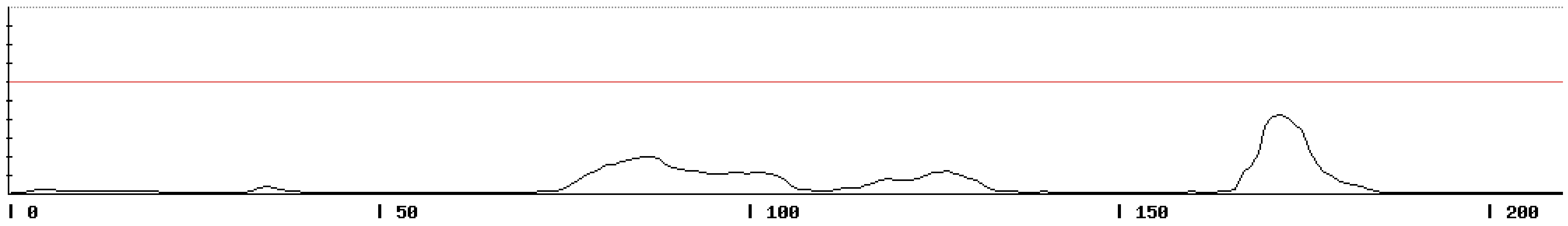

Predictions for Musaacuminata_GSMUA_Achr3P00330_001

Viterbi Path	
Viterbi Path	
Posterior @ 0.6	759 - RLRKRR - 764
Posterior @ 0.6	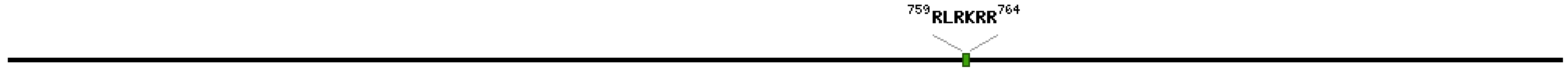
Posterior Graphic. Threshold Marked @ 0.6	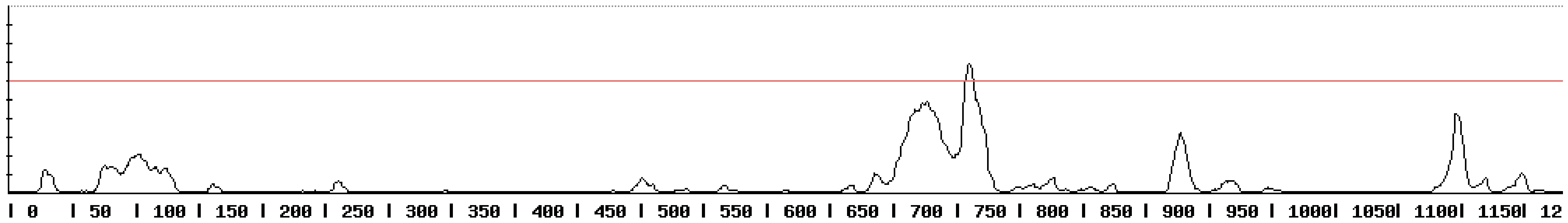

Predictions for Musaacuminata_GSMUA_Achr3P00560_001

Viterbi Path	
Viterbi Path	
Posterior @ 0.6	
Posterior @ 0.6	

Posterior Graphic. Threshold Marked @ 0.6

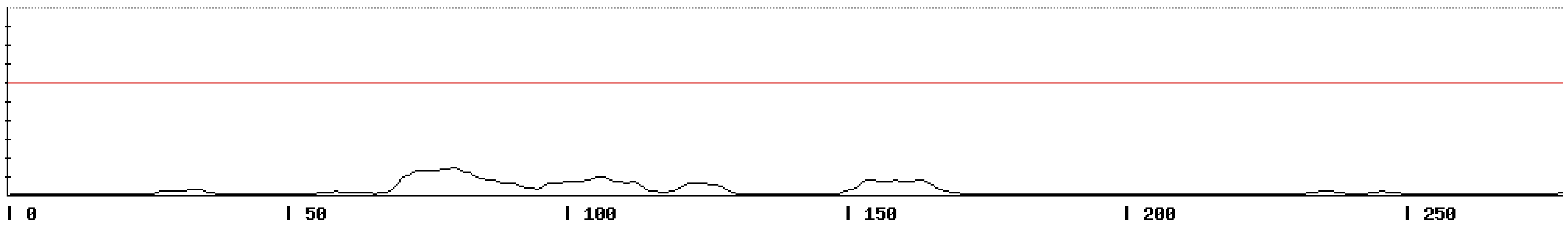

Predictions for Musaacuminata_GSMUA_Achr3P03500_001

Viterbi Path

Viterbi Path

Posterior @ 0.6

Posterior @ 0.6

Posterior Graphic. Threshold Marked @ 0.6

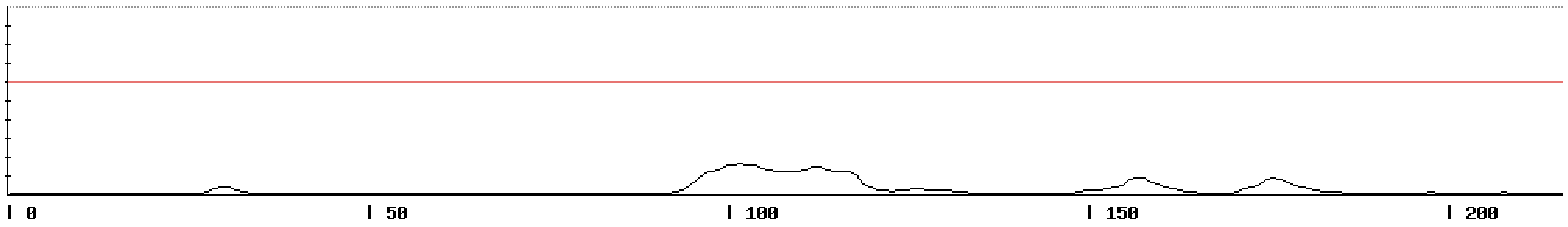

Predictions for Musaacuminata_GSMUA_Achr3P07330_001

Viterbi Path

Viterbi Path

Posterior @ 0.6	
Posterior @ 0.6	
Posterior Graphic. Threshold Marked @ 0.6	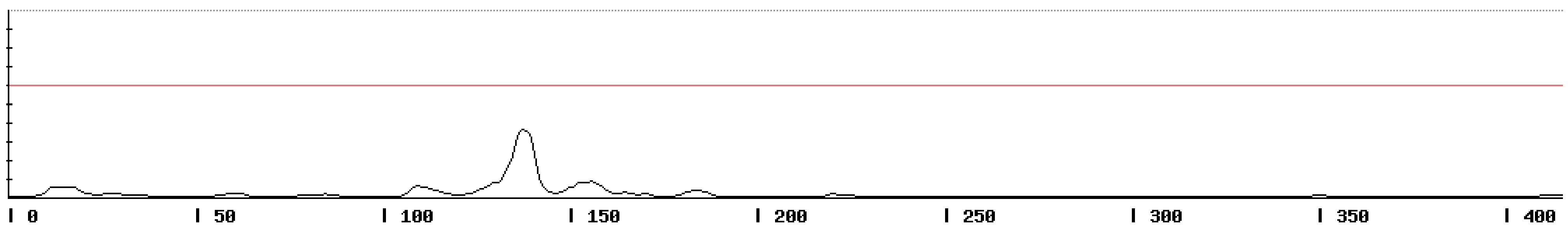

Predictions for Musaacuminata_GSMUA_Achr3P09520_001

Viterbi Path	
Viterbi Path	
Posterior @ 0.6	
Posterior @ 0.6	
Posterior Graphic. Threshold Marked @ 0.6	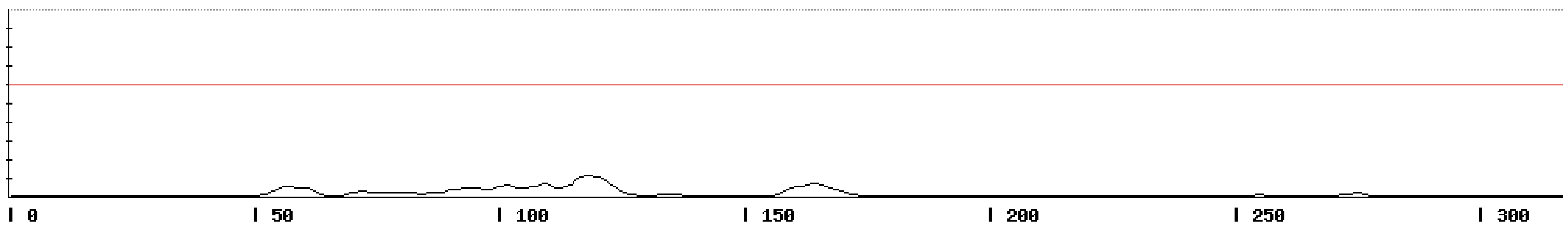

Predictions for Musaacuminata_GSMUA_Achr3P12230_001

Viterbi Path	
Viterbi Path	

Posterior @ 0.6

Posterior @ 0.6

Posterior Graphic. Threshold Marked @ 0.6

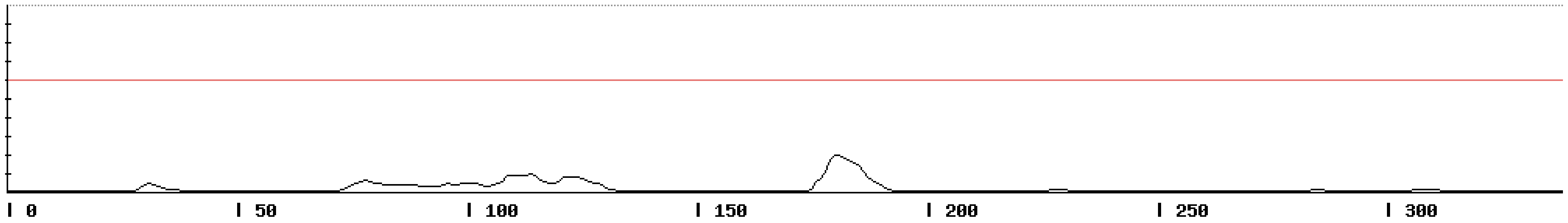

Predictions for Musaacuminata_GSMUA_Achr3P13880_001

Viterbi Path

Viterbi Path

Posterior @ 0.6

Posterior @ 0.6

Posterior Graphic. Threshold Marked @ 0.6

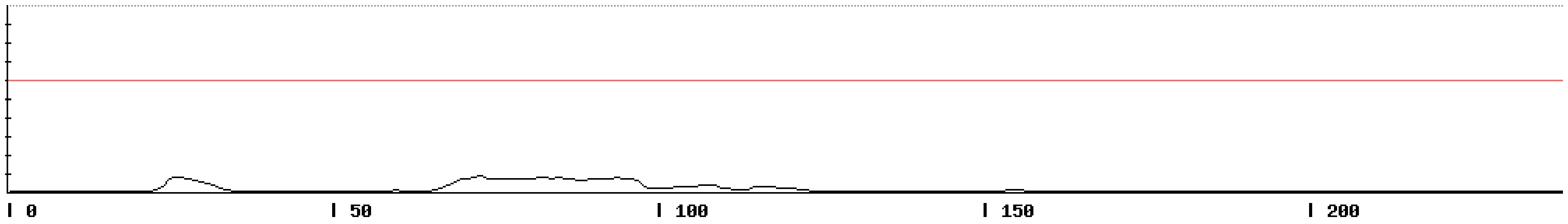

Predictions for Musaacuminata_GSMUA_Achr3P14720_001

Viterbi Path

Viterbi Path

Posterior @ 0.6

Posterior @ 0.6

Posterior Graphic. Threshold Marked @ 0.6

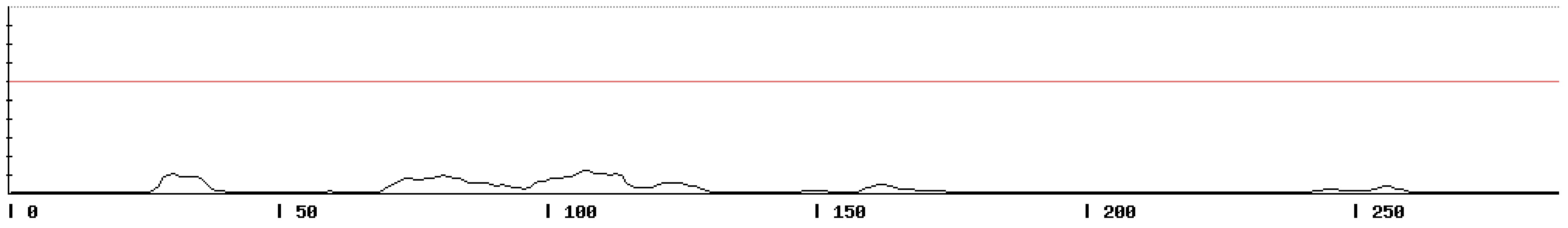

Predictions for Musaacuminata_GSMUA_Achr3P17390_001

Viterbi Path

Viterbi Path

Posterior @ 0.6

Posterior @ 0.6

Posterior Graphic. Threshold Marked @ 0.6

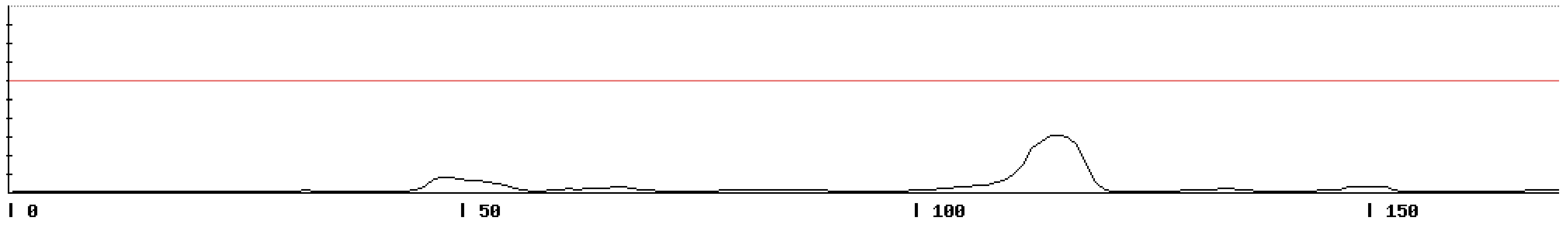

Predictions for Musaacuminata_GSMUA_Achr3P17400_001

Viterbi Path	
Viterbi Path	
Posterior @ 0.6	
Posterior @ 0.6	
Posterior Graphic. Threshold Marked @ 0.6	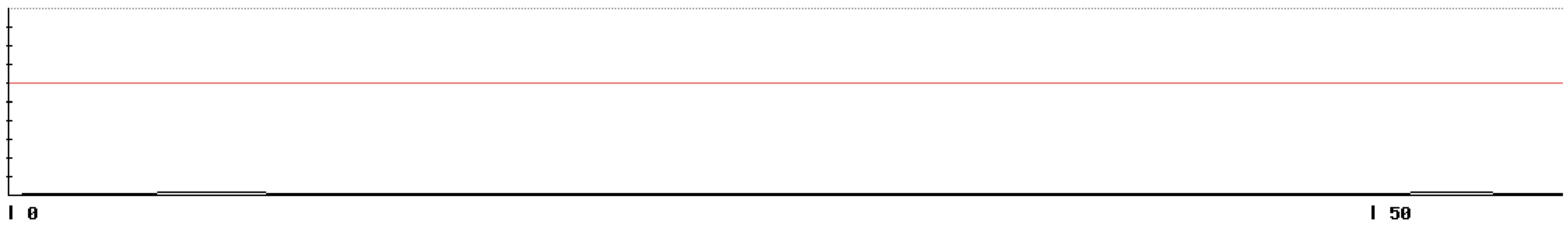

Predictions for Musaacuminata_GSMUA_Achr3P18010_001

Viterbi Path	
Viterbi Path	
Posterior @ 0.6	
Posterior @ 0.6	
Posterior Graphic. Threshold Marked @ 0.6	

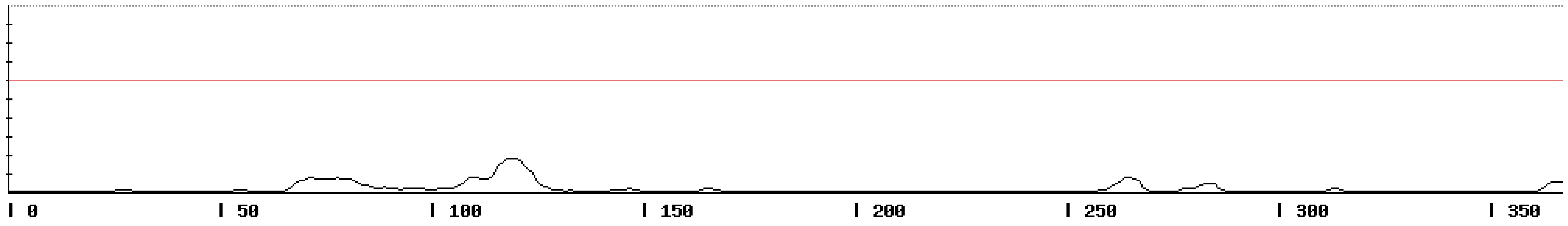

Predictions for Musaacuminata_GSMUA_Achr3P18020_001

Viterbi Path	
Viterbi Path	
Posterior @ 0.6	
Posterior @ 0.6	
Posterior Graphic. Threshold Marked @ 0.6	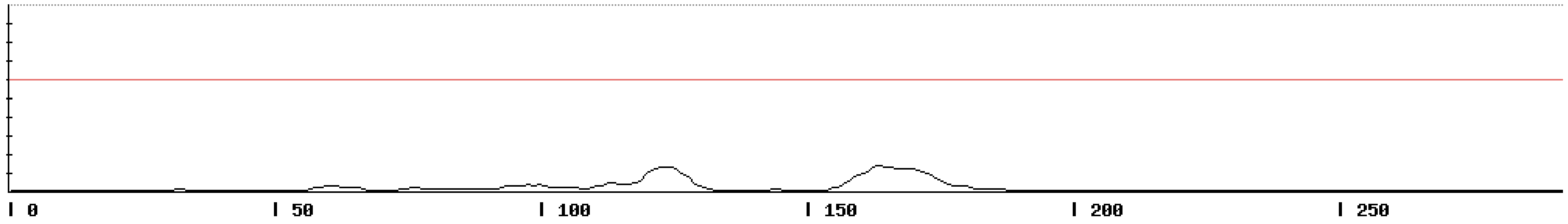

Predictions for Musaacuminata_GSMUA_Achr3P18070_001

Viterbi Path	
Viterbi Path	
Posterior @ 0.6	
Posterior @ 0.6	

Posterior Graphic. Threshold Marked @ 0.6	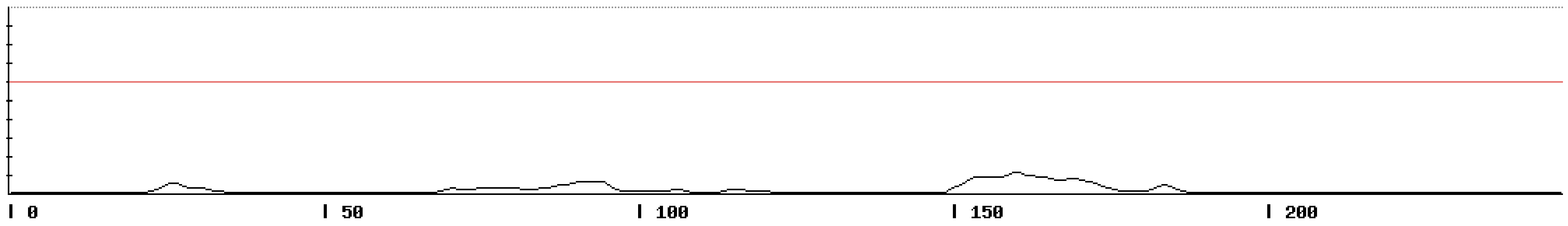

Predictions for Musaacuminata_GSMUA_Achr3P18680_001

Viterbi Path	
Viterbi Path	
Posterior @ 0.6	
Posterior @ 0.6	
Posterior Graphic. Threshold Marked @ 0.6	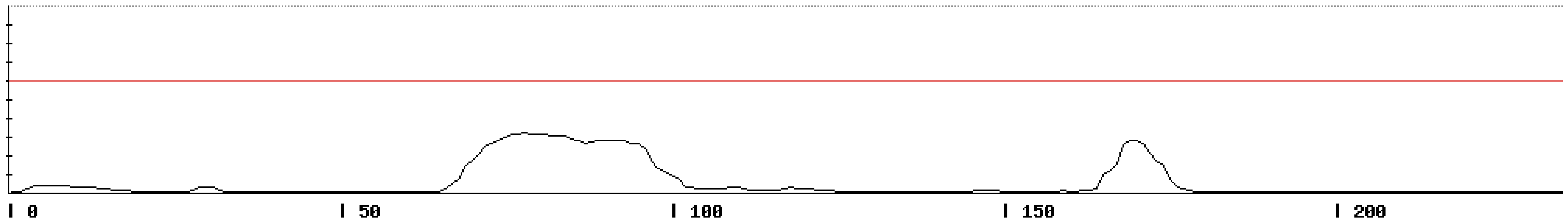

Predictions for Musaacuminata_GSMUA_Achr3P18990_001

Viterbi Path	
Viterbi Path	
Posterior @ 0.6	202 - RKPAGP - 207
Posterior @ 0.6	

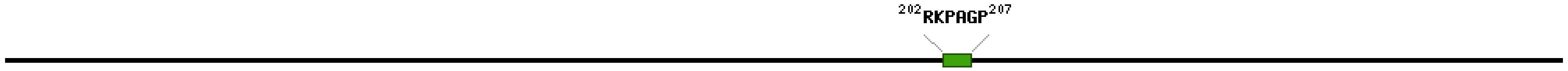

Posterior Graphic. Threshold Marked @ 0.6

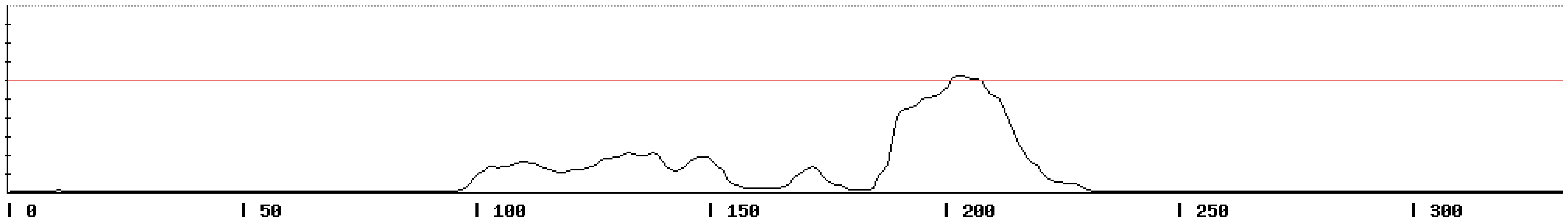

Predictions for Musaacuminata_GSMUA_Achr3P21690_001

Viterbi Path

Viterbi Path

Posterior @ 0.6

Posterior @ 0.6

Posterior Graphic. Threshold Marked @ 0.6

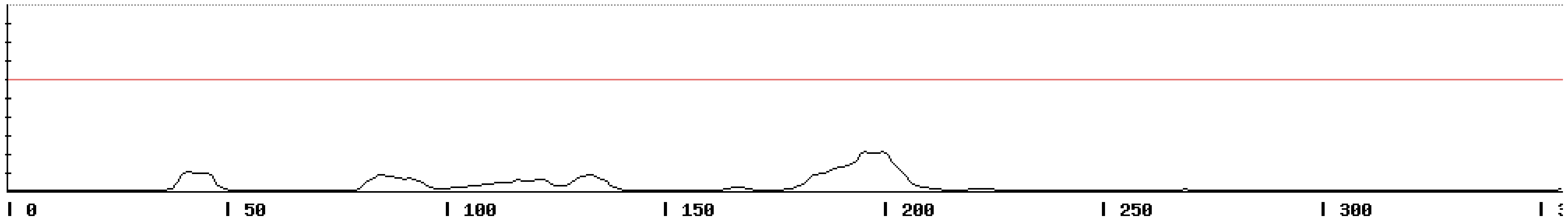

Predictions for Musaacuminata_GSMUA_Achr3P22360_001

Viterbi Path

Viterbi Path

Posterior @ 0.6

Posterior @ 0.6

Posterior Graphic. Threshold Marked @ 0.6

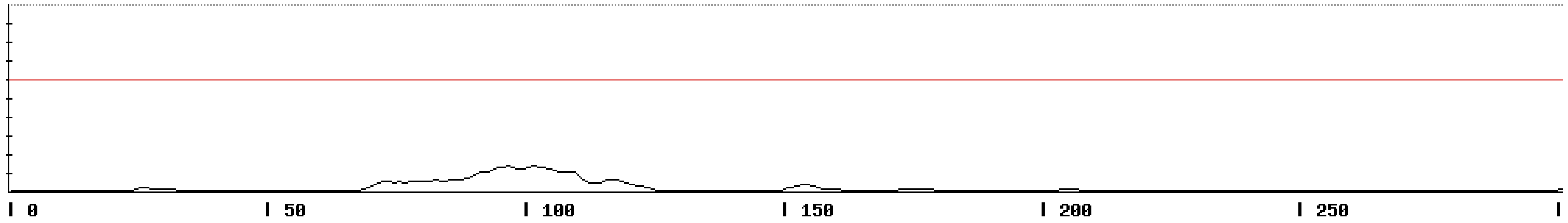

Predictions for Musaacuminata_GSMUA_Achr3P23360_001

Viterbi Path

Viterbi Path

Posterior @ 0.6

Posterior @ 0.6

Posterior Graphic. Threshold Marked @ 0.6

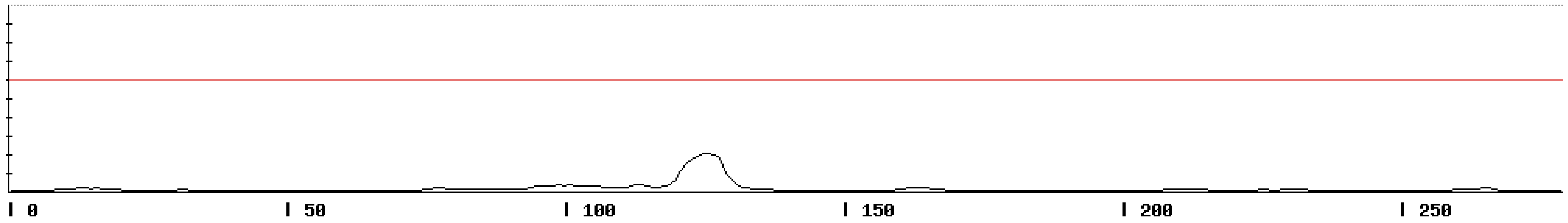

Predictions for Musaacuminata_GSMUA_Achr4P02380_001

Viterbi Path

Viterbi Path

Posterior @ 0.6

Posterior @ 0.6

Posterior Graphic. Threshold Marked @ 0.6

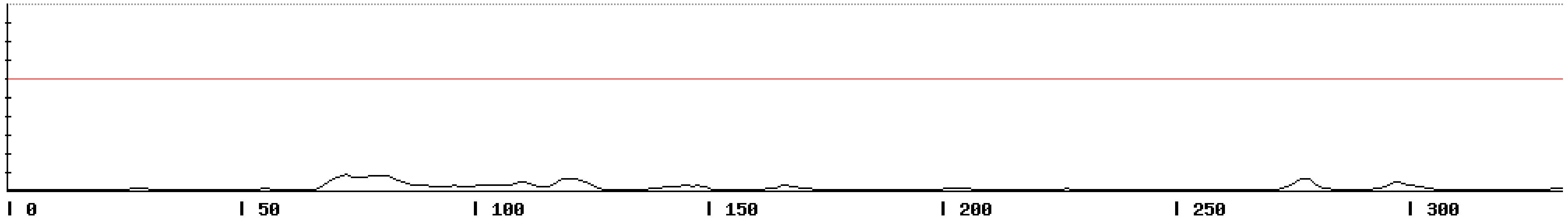

Predictions for Musaacuminata_GSMUA_Achr4P02390_001

Viterbi Path

Viterbi Path

Posterior @ 0.6

Posterior @ 0.6

Posterior Graphic. Threshold Marked @ 0.6

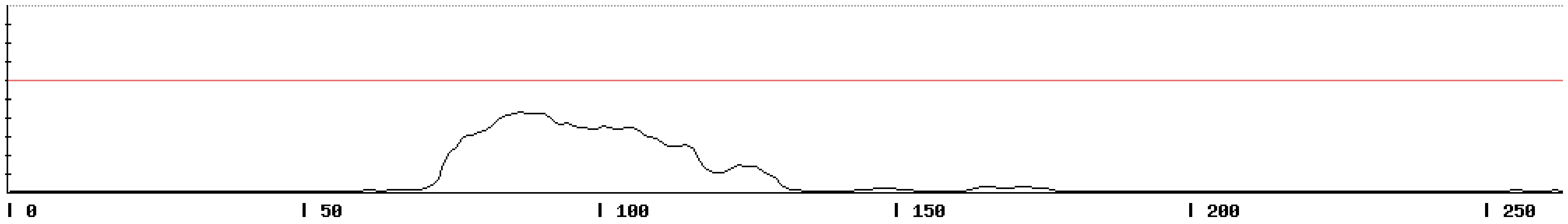

Predictions for Musaacuminata_GSMUA_Achr4P02730_001

Viterbi Path

Viterbi Path

Posterior @ 0.6

Posterior @ 0.6

Posterior Graphic. Threshold Marked @ 0.6

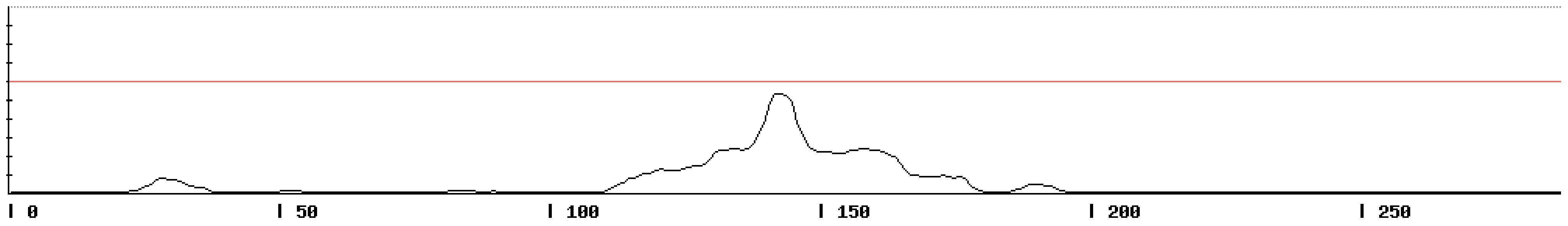

Predictions for Musaacuminata_GSMUA_Achr4P07150_001

Viterbi Path

Viterbi Path

Posterior @ 0.6

Posterior @ 0.6

Posterior Graphic. Threshold Marked @ 0.6

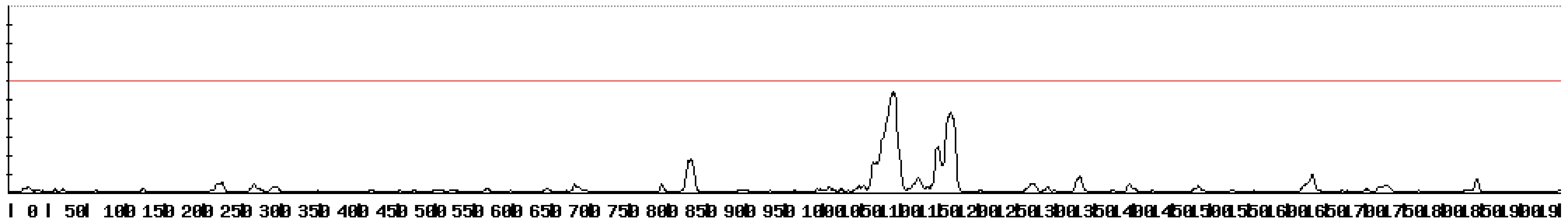

Predictions for Musaacuminata_GSMUA_Achr4P10310_001	
Viterbi Path	
Viterbi Path	
Posterior @ 0.6	
Posterior @ 0.6	
Posterior Graphic. Threshold Marked @ 0.6	

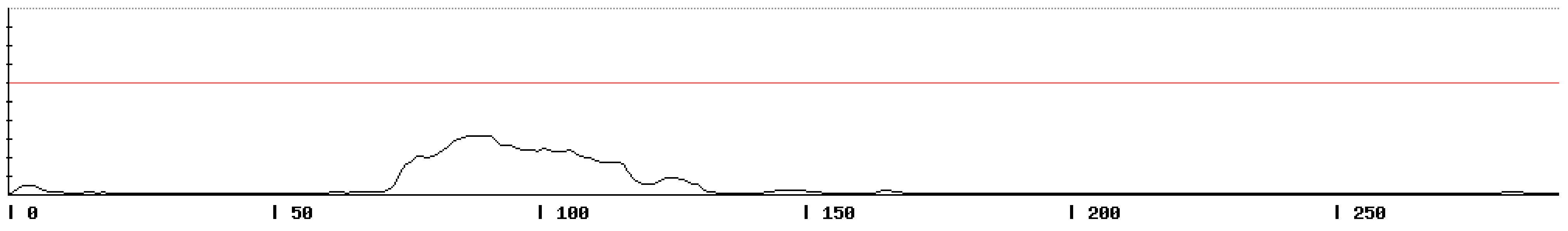

Predictions for Musaacuminata_GSMUA_Achr4P21900_001	
Viterbi Path	
Viterbi Path	
Posterior @ 0.6	
Posterior @ 0.6	
Posterior Graphic. Threshold Marked @ 0.6	

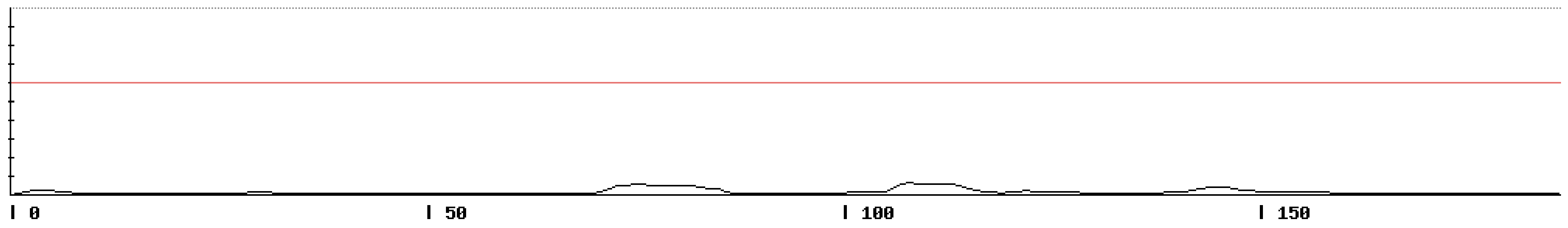

Predictions for Musaacuminata_GSMUA_Achr4P23030_001

Viterbi Path

Viterbi Path

Posterior @ 0.6

Posterior @ 0.6

Posterior Graphic. Threshold Marked @ 0.6

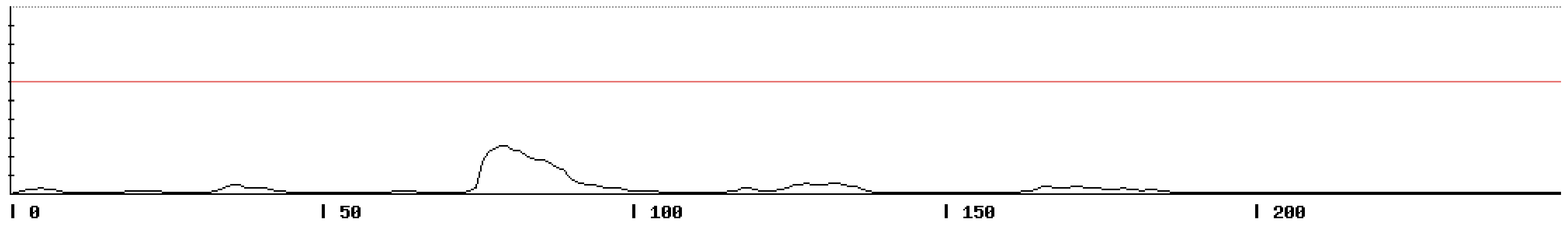

Predictions for Musaacuminata_GSMUA_Achr4P29170_001

Viterbi Path

Viterbi Path

Posterior @ 0.6

Posterior @ 0.6

Posterior Graphic. Threshold Marked @ 0.6	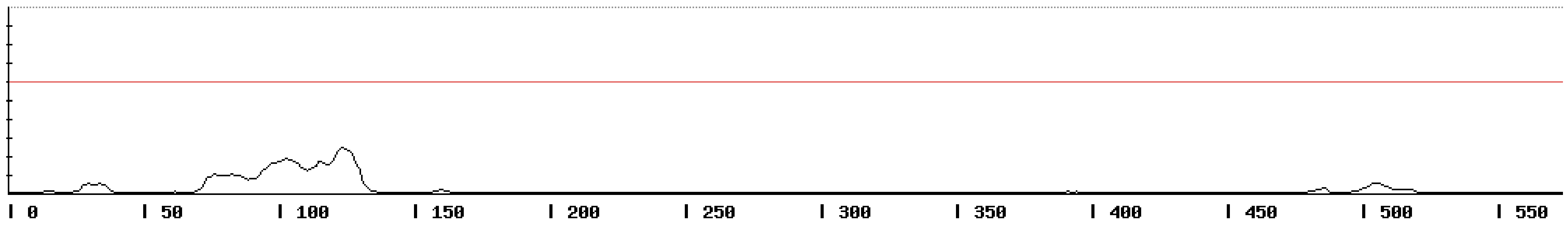

Predictions for Musaacuminata_GSMUA_Achr4P30940_001	
Viterbi Path	
Viterbi Path	
Posterior @ 0.6	
Posterior @ 0.6	
Posterior Graphic. Threshold Marked @ 0.6	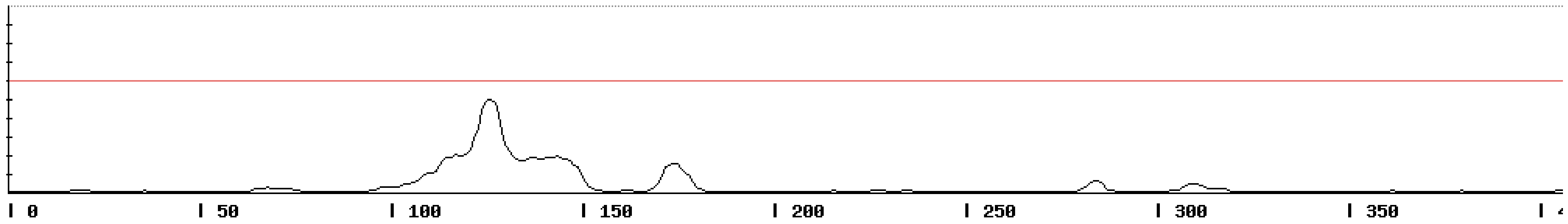

Predictions for Musaacuminata_GSMUA_Achr4P32010_001	
Viterbi Path	
Viterbi Path	
Posterior @ 0.6	
Posterior @ 0.6	

Posterior Graphic. Threshold Marked @ 0.6	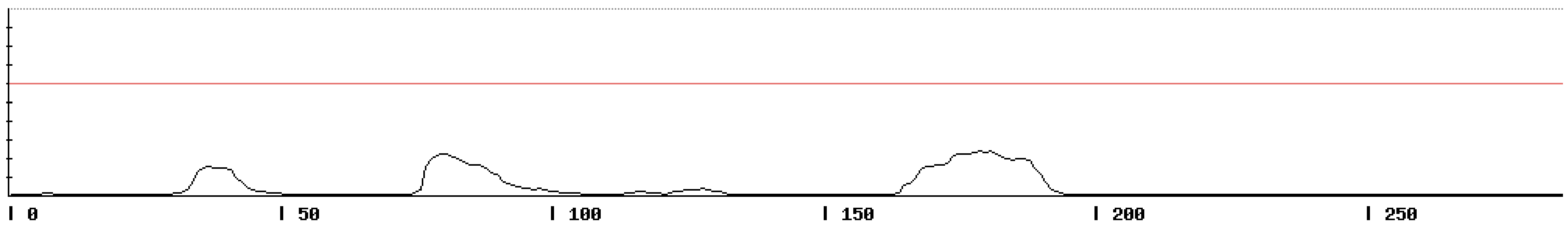

Predictions for Musaacuminata_GSMUA_Achr5P00500_001

Viterbi Path	
Viterbi Path	
Posterior @ 0.6	
Posterior @ 0.6	
Posterior Graphic. Threshold Marked @ 0.6	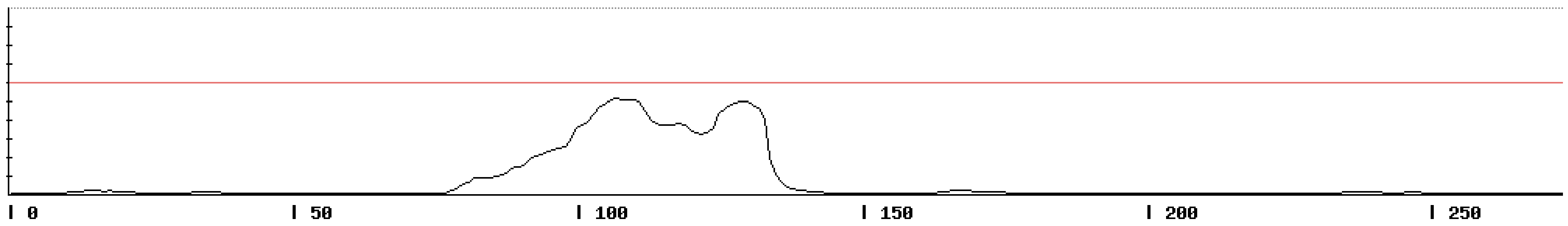

Predictions for Musaacuminata_GSMUA_Achr5P02170_001

Viterbi Path	
Viterbi Path	

Posterior @ 0.6	
Posterior @ 0.6	
Posterior Graphic. Threshold Marked @ 0.6	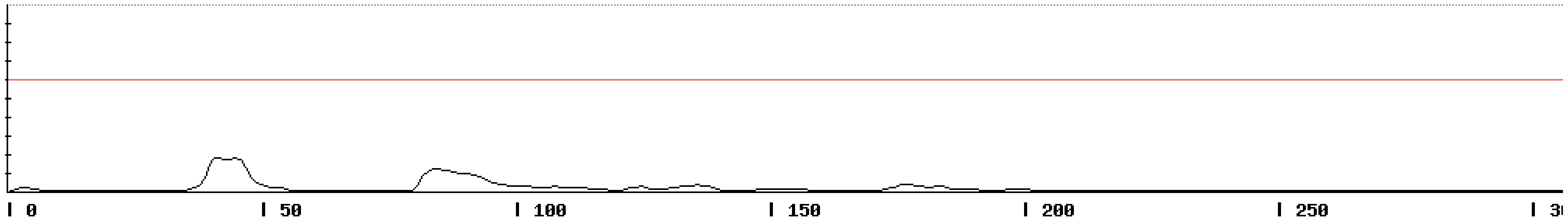

Predictions for Musaacuminata_GSMUA_Achr5P03360_001	
Viterbi Path	
Viterbi Path	
Posterior @ 0.6	
Posterior @ 0.6	
Posterior Graphic. Threshold Marked @ 0.6	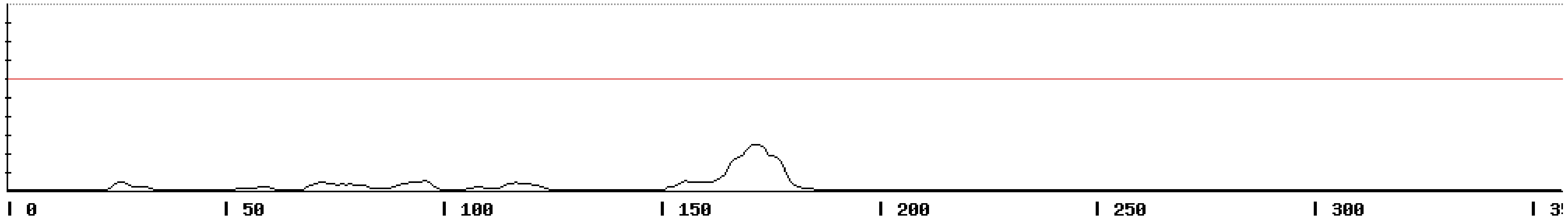

Predictions for Musaacuminata_GSMUA_Achr5P07590_001	
Viterbi Path	
Viterbi Path	

Posterior @ 0.6

Posterior @ 0.6

Posterior Graphic. Threshold Marked @ 0.6

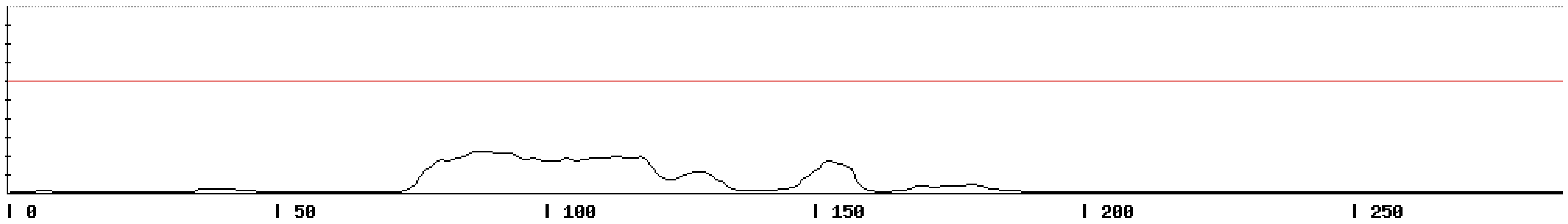

Predictions for Musaacuminata_GSMUA_Achr5P07600_001

Viterbi Path

Viterbi Path

Posterior @ 0.6

Posterior @ 0.6

Posterior Graphic. Threshold Marked @ 0.6

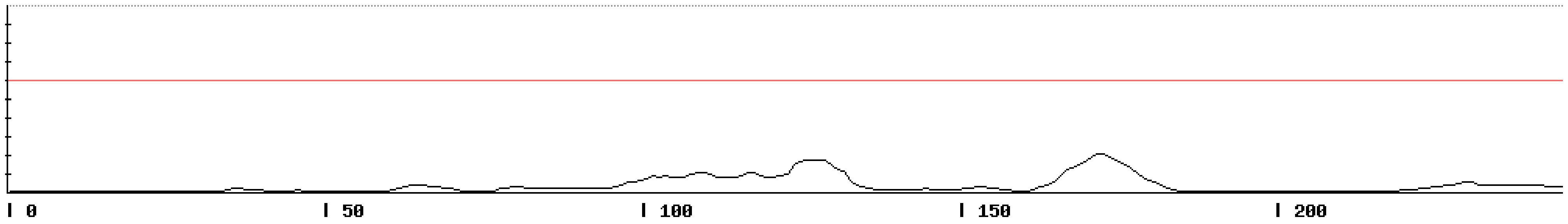

Predictions for Musaacuminata_GSMUA_Achr5P17060_001

Viterbi Path

Viterbi Path

Posterior @ 0.6

Posterior @ 0.6

Posterior Graphic. Threshold Marked @ 0.6

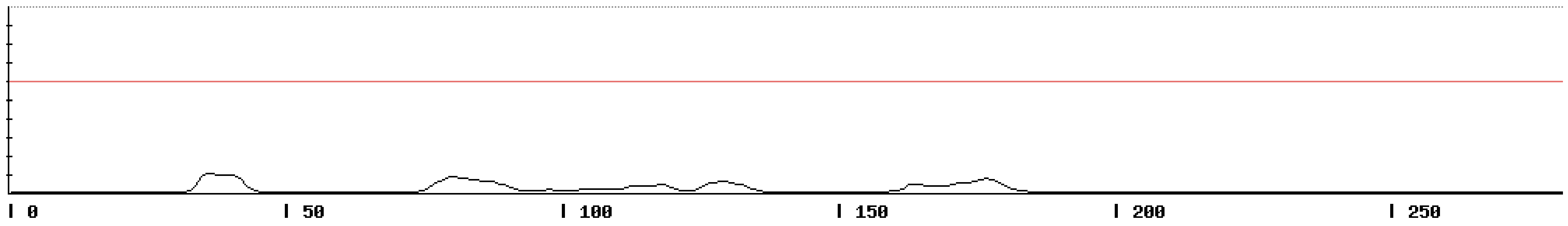

Predictions for Musaacuminata_GSMUA_Achr5P18140_001

Viterbi Path

Viterbi Path

Posterior @ 0.6

Posterior @ 0.6

Posterior Graphic. Threshold Marked @ 0.6

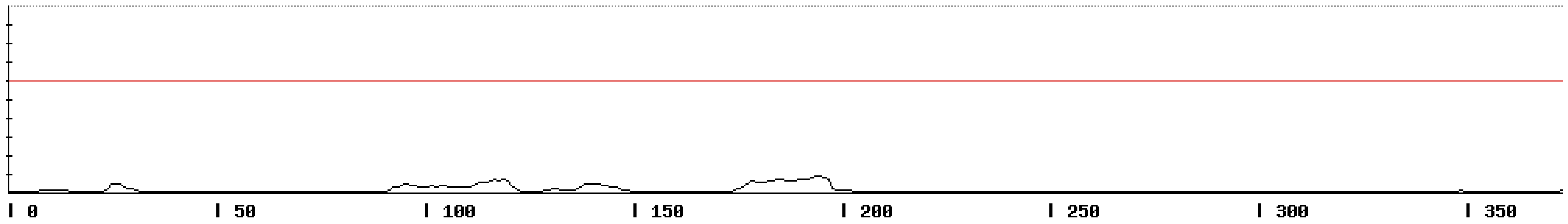

Predictions for Musaacuminata_GSMUA_Achr5P18670_001	
Viterbi Path	
Viterbi Path	
Posterior @ 0.6	
Posterior @ 0.6	
Posterior Graphic. Threshold Marked @ 0.6	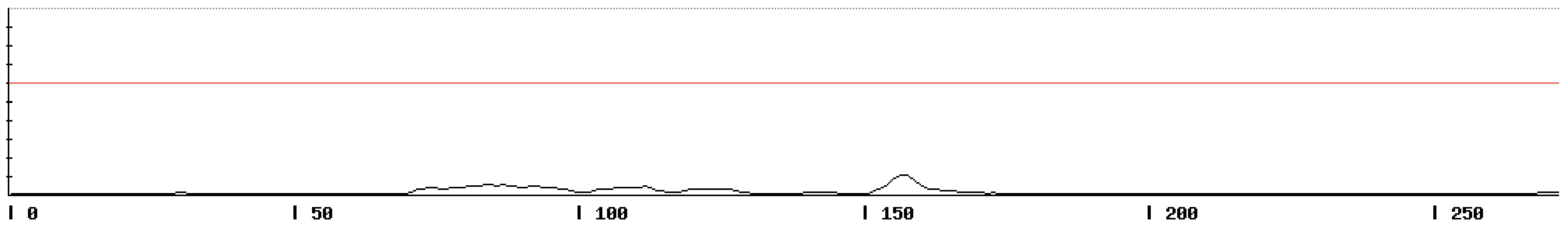

Predictions for Musaacuminata_GSMUA_Achr5P19060_001	
Viterbi Path	
Viterbi Path	
Posterior @ 0.6	73 - RKSRSGGRPSRR - 84
Posterior @ 0.6	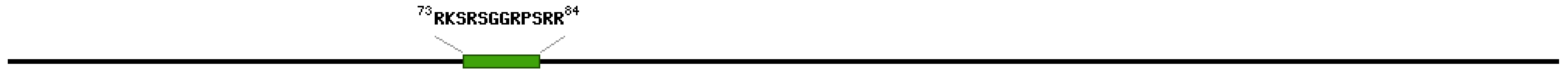
Posterior Graphic. Threshold Marked @ 0.6	

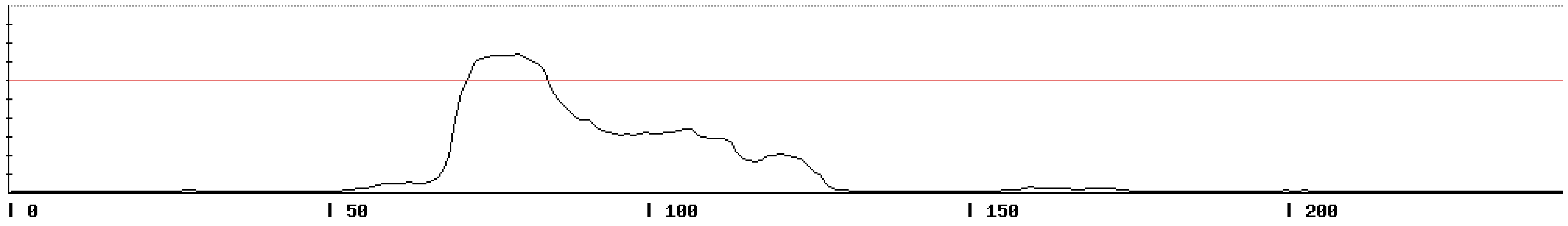

Predictions for Musaacuminata_GSMUA_Achr5P23620_001

Viterbi Path	
Viterbi Path	
Posterior @ 0.6	
Posterior @ 0.6	
Posterior Graphic. Threshold Marked @ 0.6	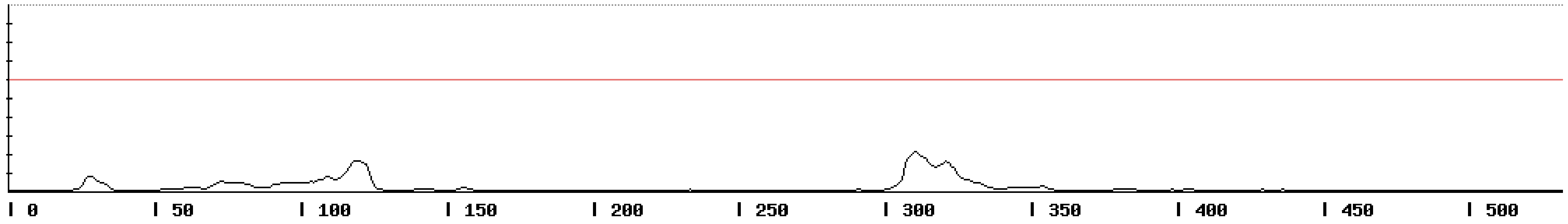

Predictions for Musaacuminata_GSMUA_Achr5P26640_001

Viterbi Path	
Viterbi Path	
Posterior @ 0.6	
Posterior @ 0.6	

Posterior Graphic. Threshold Marked @ 0.6

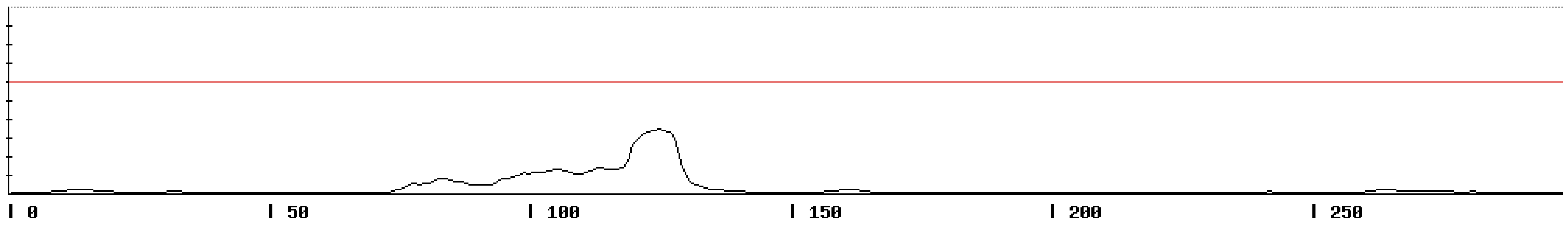

Predictions for Musaacuminata_GSMUA_Achr6P01770_001

Viterbi Path

Viterbi Path

Posterior @ 0.6

Posterior @ 0.6

Posterior Graphic. Threshold Marked @ 0.6

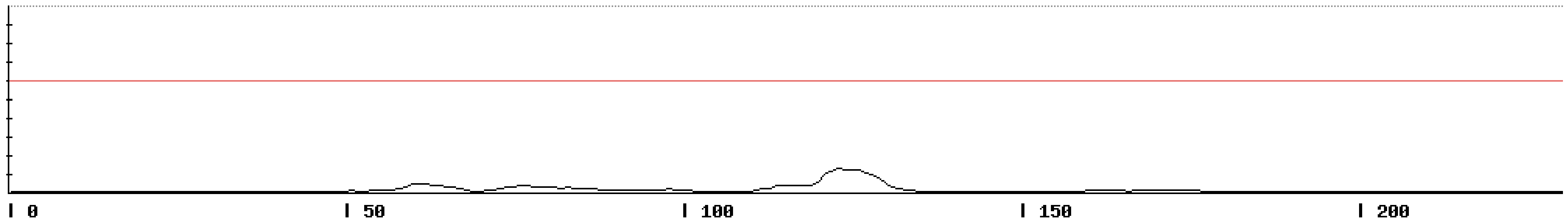

Predictions for Musaacuminata_GSMUA_Achr6P02680_001

Viterbi Path

Viterbi Path

Posterior @ 0.6

Posterior @ 0.6

Posterior Graphic. Threshold Marked @ 0.6

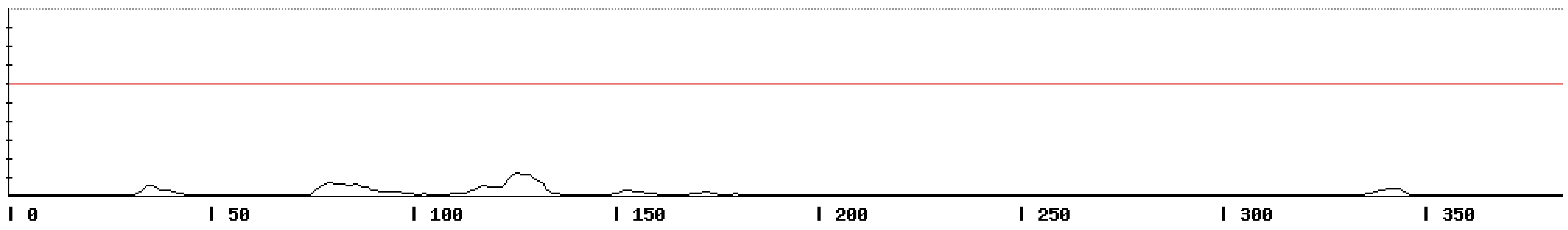

Predictions for Musaacuminata_GSMUA_Achr6P03200_001

Viterbi Path

Viterbi Path

Posterior @ 0.6

Posterior @ 0.6

Posterior Graphic. Threshold Marked @ 0.6

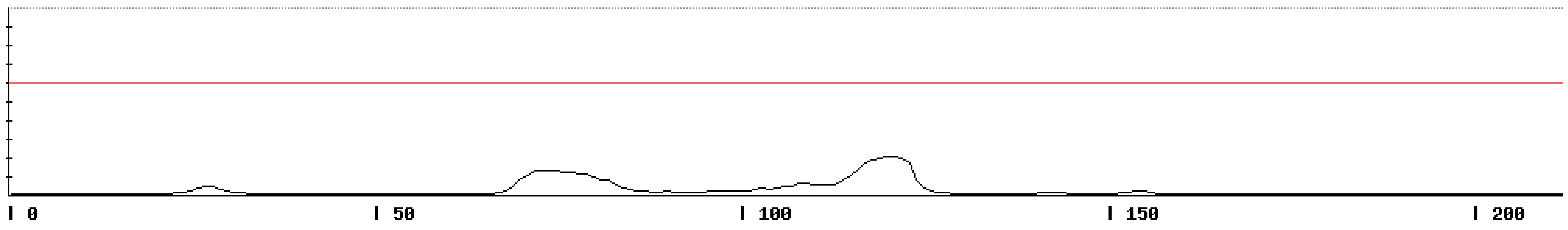

Predictions for Musaacuminata_GSMUA_Achr6P05480_001

Viterbi Path

Viterbi Path

Posterior @ 0.6	
Posterior @ 0.6	
Posterior Graphic. Threshold Marked @ 0.6	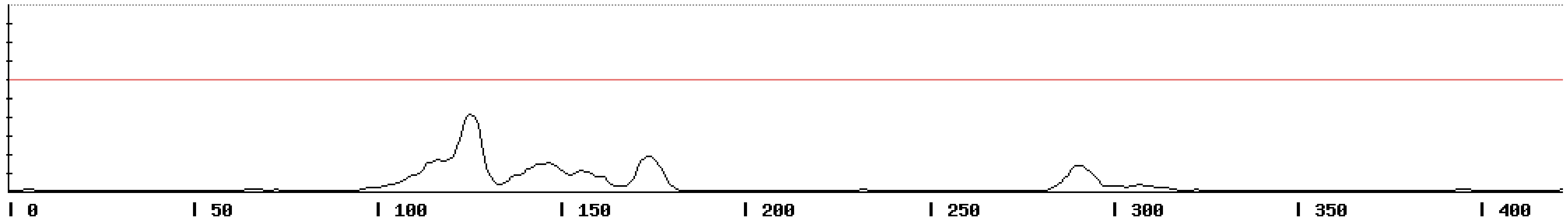

Predictions for Musaacuminata_GSMUA_Achr6P08600_001

Viterbi Path	
Viterbi Path	
Posterior @ 0.6	
Posterior @ 0.6	
Posterior Graphic. Threshold Marked @ 0.6	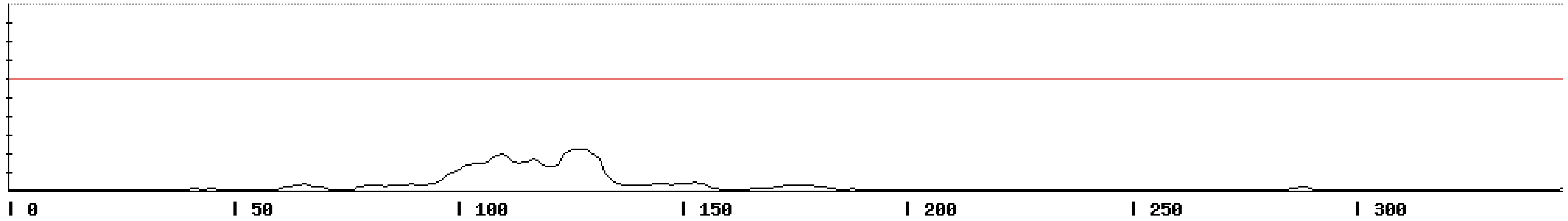

Predictions for Musaacuminata_GSMUA_Achr6P11230_001

Viterbi Path	
Viterbi Path	

Posterior @ 0.6	
Posterior @ 0.6	
Posterior Graphic. Threshold Marked @ 0.6	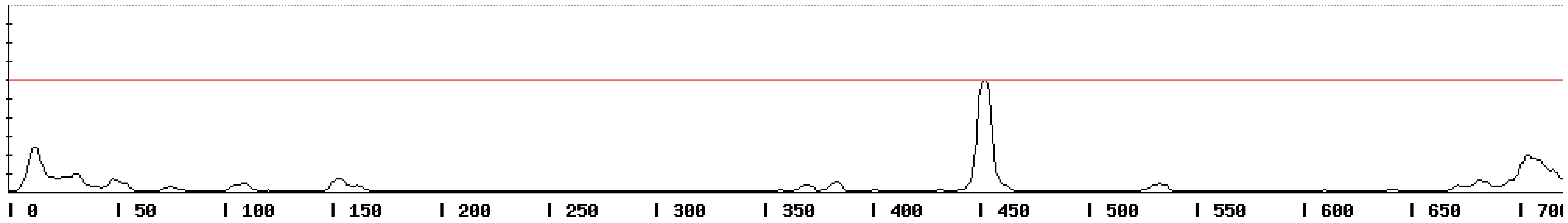

Predictions for Musaacuminata_GSMUA_Achr6P16560_001	
Viterbi Path	
Viterbi Path	
Posterior @ 0.6	
Posterior @ 0.6	
Posterior Graphic. Threshold Marked @ 0.6	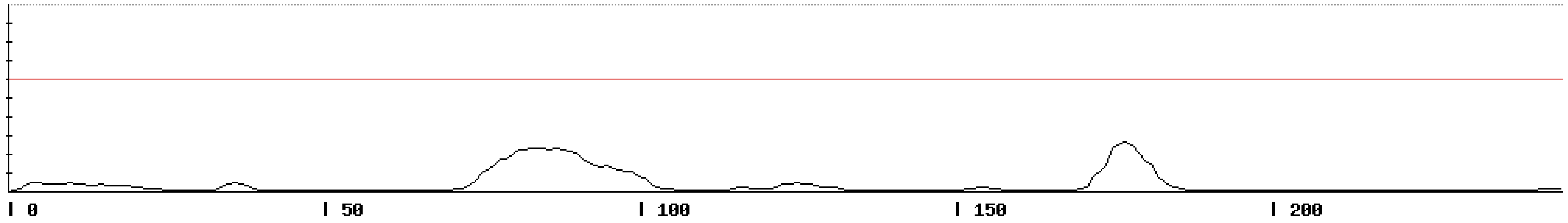

Predictions for Musaacuminata_GSMUA_Achr6P17670_001

Viterbi Path

Viterbi Path

Posterior @ 0.6

Posterior @ 0.6

Posterior Graphic. Threshold Marked @ 0.6

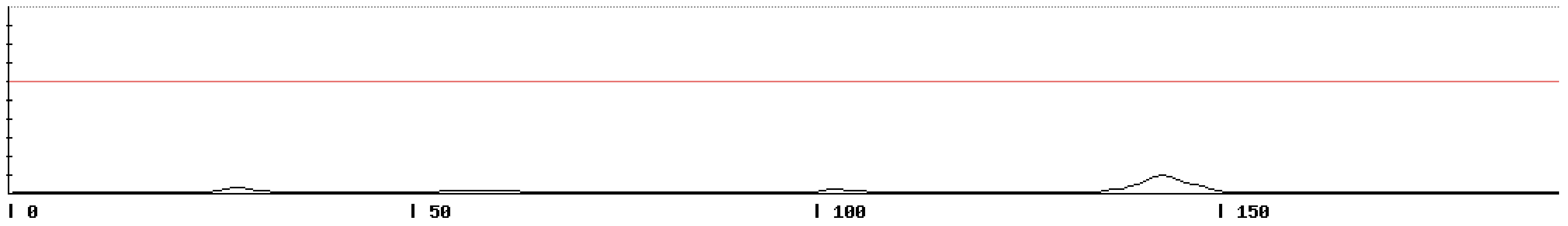

Predictions for Musaacuminata_GSMUA_Achr6P17720_001

Viterbi Path

Viterbi Path

Posterior @ 0.6

163 - GKQANPGRRAAGPKKTR - 179

Posterior @ 0.6

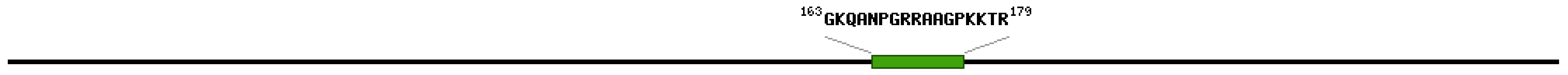

Posterior Graphic. Threshold Marked @ 0.6

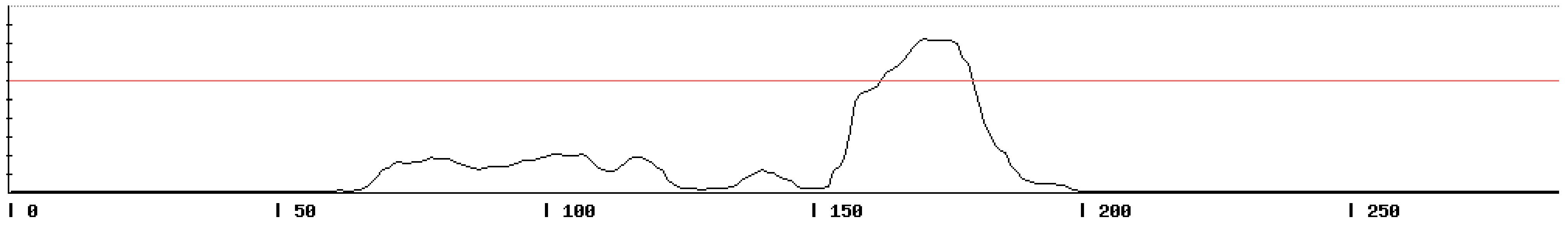

Predictions for Musaacuminata_GSMUA_Achr6P18640_001	
Viterbi Path	
Viterbi Path	
Posterior @ 0.6	
Posterior @ 0.6	
Posterior Graphic. Threshold Marked @ 0.6	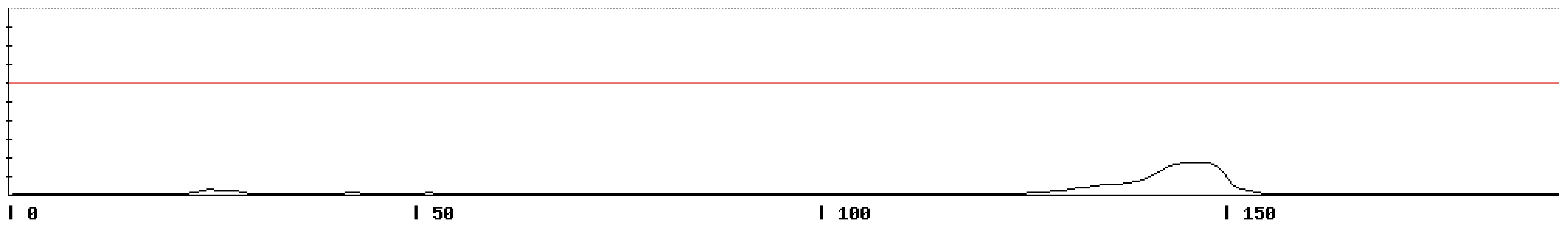

Predictions for Musaacuminata_GSMUA_Achr6P18720_001	
Viterbi Path	
Viterbi Path	
Posterior @ 0.6	
Posterior @ 0.6	
Posterior Graphic. Threshold Marked @ 0.6	

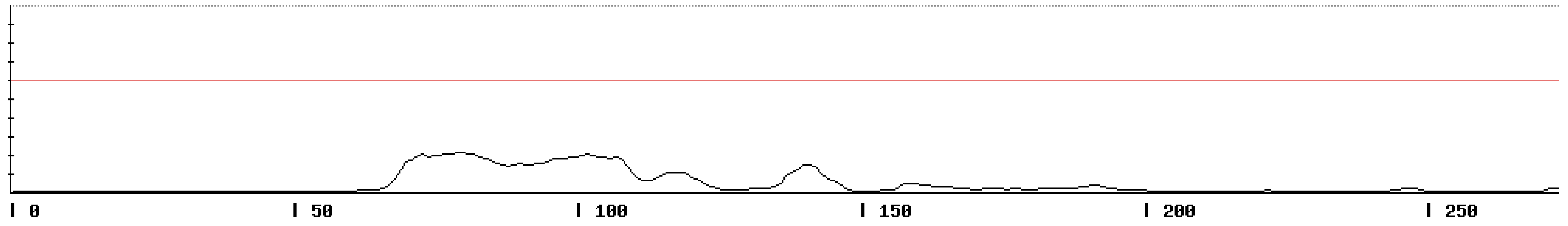

Predictions for Musaacuminata_GSMUA_Achr6P18860_001

Viterbi Path

Viterbi Path

Posterior @ 0.6

Posterior @ 0.6

Posterior Graphic. Threshold Marked @ 0.6

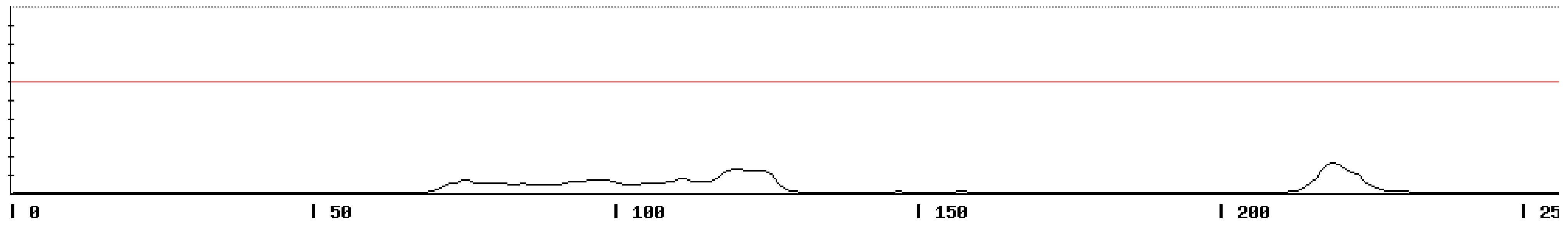

Predictions for Musaacuminata_GSMUA_Achr6P19400_001

Viterbi Path

Viterbi Path

Posterior @ 0.6

Posterior @ 0.6

Posterior Graphic. Threshold Marked @ 0.6

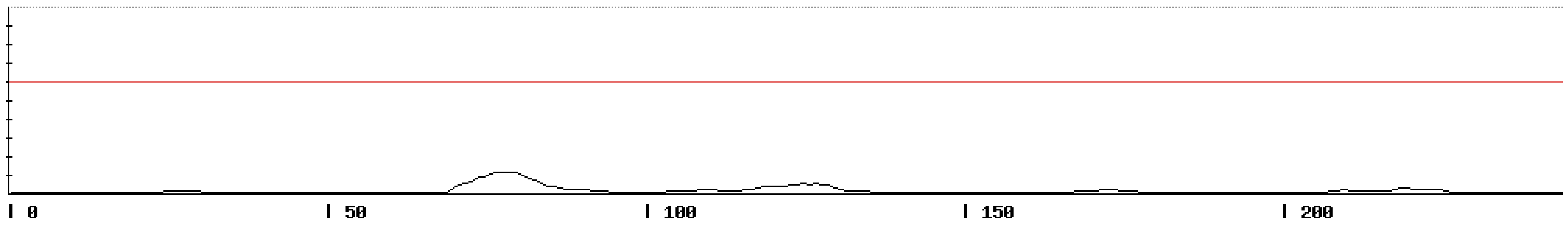

Predictions for Musaacuminata_GSMUA_Achr6P20870_001

Viterbi Path

Viterbi Path

Posterior @ 0.6 — 79 - KRSNRAAR - 86

Posterior @ 0.6

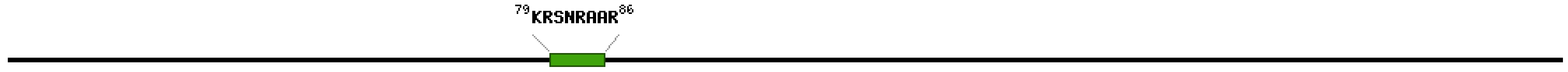

Posterior Graphic. Threshold Marked @ 0.6

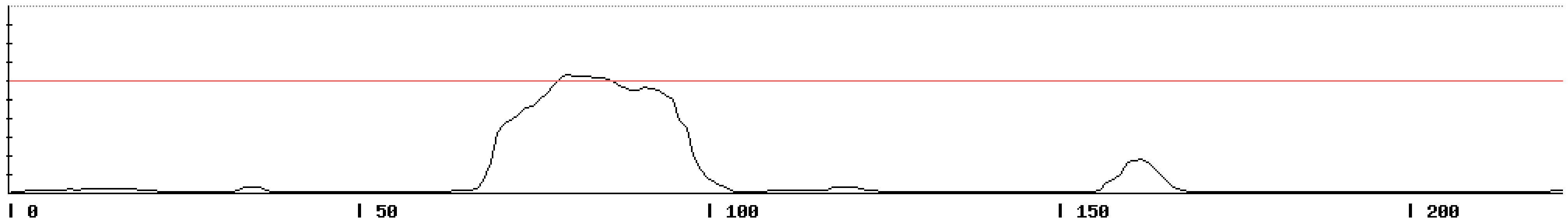

Predictions for Musaacuminata_GSMUA_Achr6P23840_001

Viterbi Path

Viterbi Path

Posterior @ 0.6

Posterior @ 0.6

Posterior Graphic. Threshold Marked @ 0.6

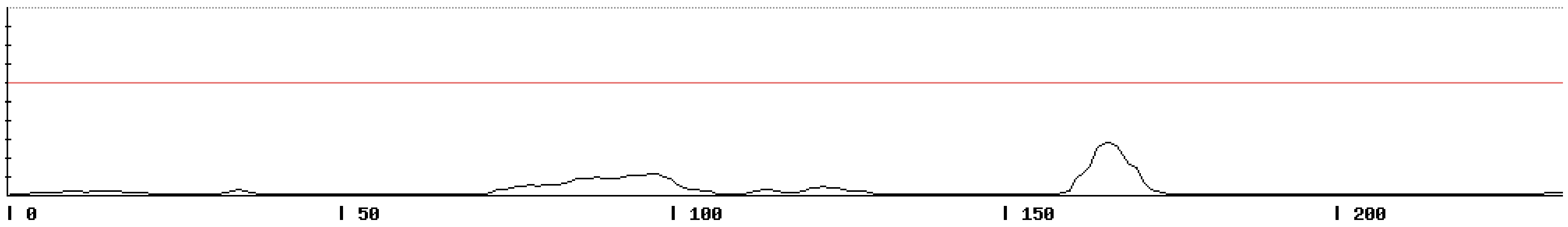

Predictions for Musaacuminata_GSMUA_Achr6P25380_001

Viterbi Path

Viterbi Path

Posterior @ 0.6

Posterior @ 0.6

Posterior Graphic. Threshold Marked @ 0.6

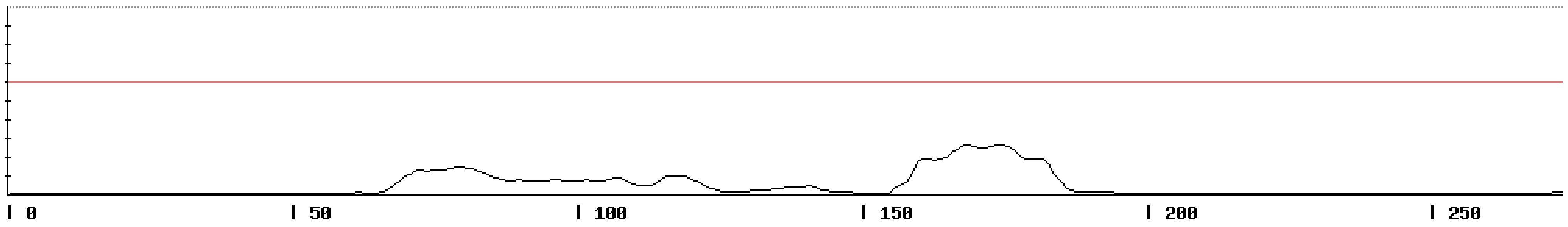

Predictions for Musaacuminata_GSMUA_Achr6P25790_001

Viterbi Path

Viterbi Path

Posterior @ 0.6

Posterior @ 0.6

Posterior Graphic. Threshold Marked @ 0.6

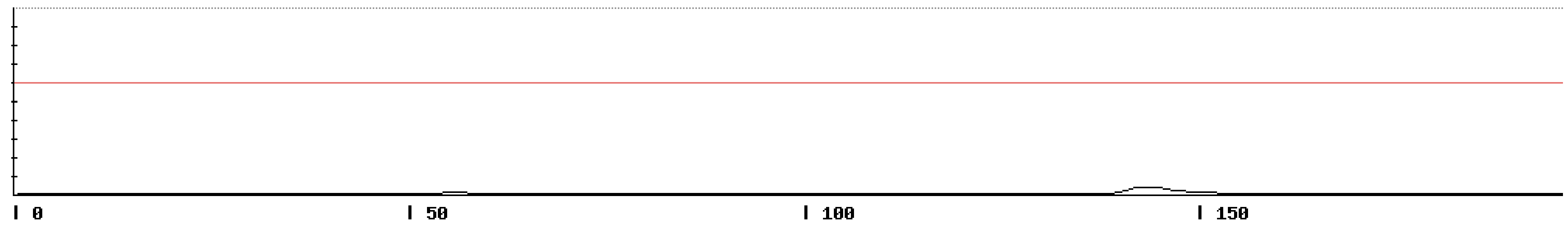

Predictions for Musaacuminata_GSMUA_Achr6P27000_001

Viterbi Path

Viterbi Path

Posterior @ 0.6

118 - RKRRKVHTDARGGETRWHKTGKTRPV - 143

Posterior @ 0.6

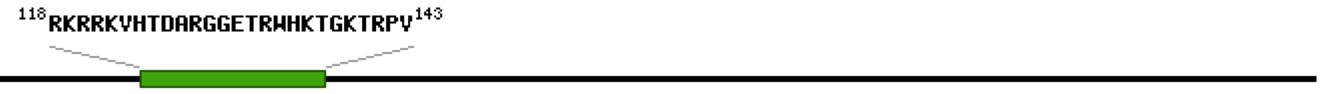

Posterior Graphic. Threshold Marked @ 0.6

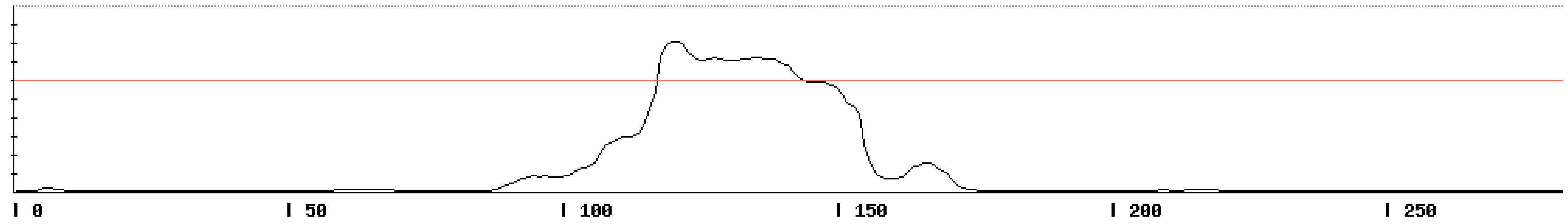

Predictions for Musaacuminata_GSMUA_Achr6P28890_001

Viterbi Path

Viterbi Path

Posterior @ 0.6

Posterior @ 0.6

Posterior Graphic. Threshold Marked @ 0.6

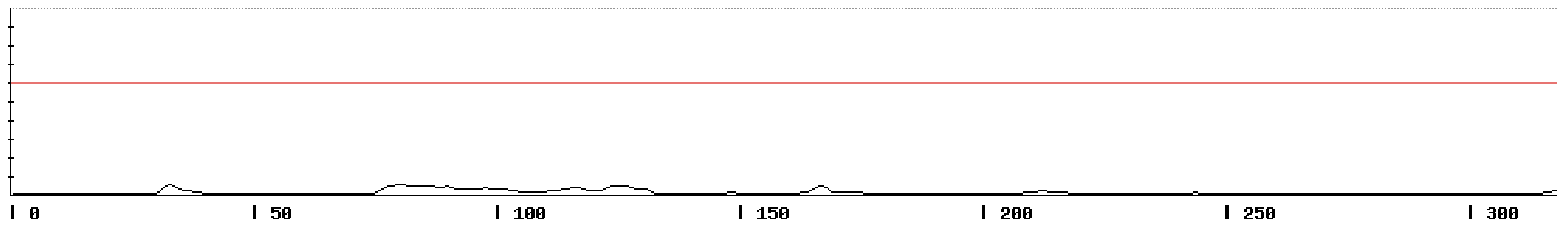

Predictions for Musaacuminata_GSMUA_Achr6P30050_001

Viterbi Path

Viterbi Path

Posterior @ 0.6

Posterior @ 0.6

Posterior Graphic. Threshold Marked @ 0.6

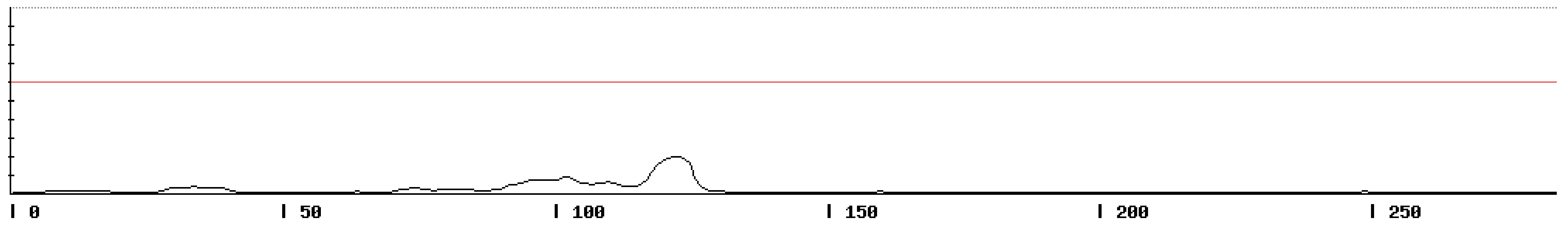

Predictions for Musaacuminata_GSMUA_Achr6P30570_001

Viterbi Path	
Viterbi Path	
Posterior @ 0.6	
Posterior @ 0.6	
Posterior Graphic. Threshold Marked @ 0.6	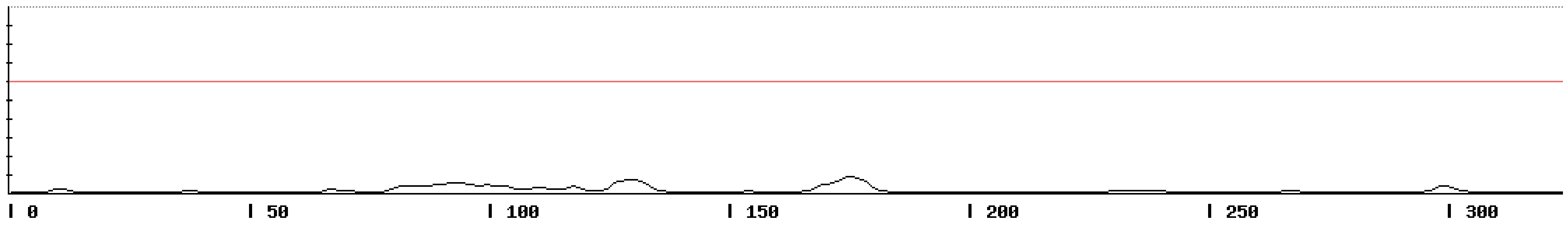

Predictions for Musaacuminata_GSMUA_Achr6P31350_001

Viterbi Path	
Viterbi Path	
Posterior @ 0.6	
Posterior @ 0.6	
Posterior Graphic. Threshold Marked @ 0.6	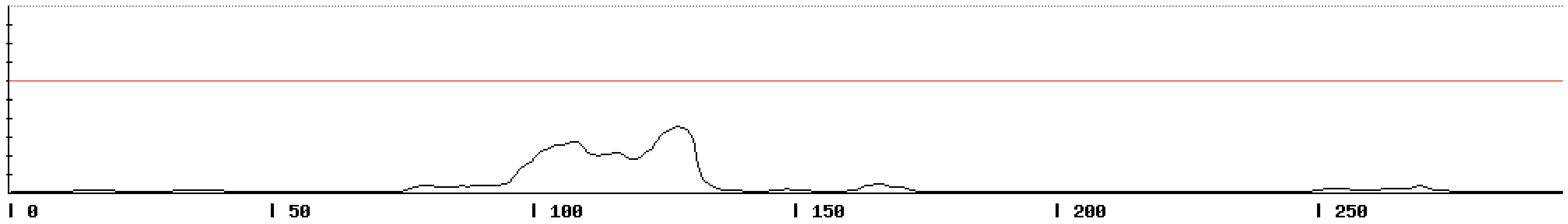

Predictions for Musaacuminata_GSMUA_Achr6P31580_001	
Viterbi Path	
Viterbi Path	
Posterior @ 0.6	
Posterior @ 0.6	
Posterior Graphic. Threshold Marked @ 0.6	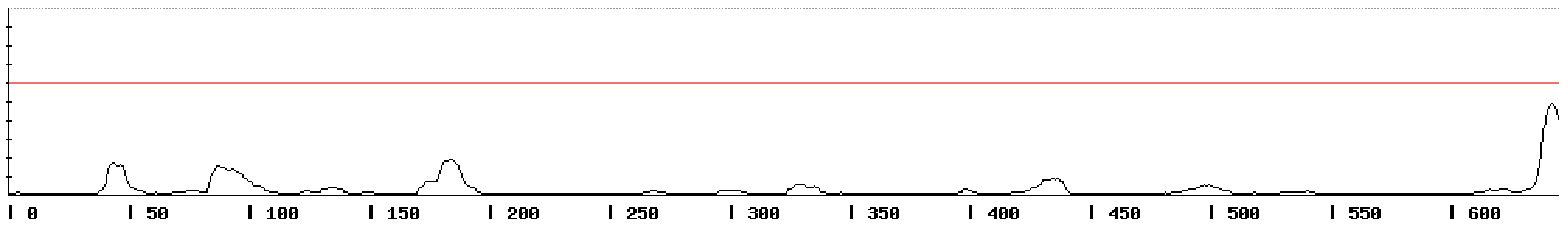

Predictions for Musaacuminata_GSMUA_Achr6P32290_001	
Viterbi Path	
Viterbi Path	
Posterior @ 0.6	
Posterior @ 0.6	
Posterior Graphic. Threshold Marked @ 0.6	

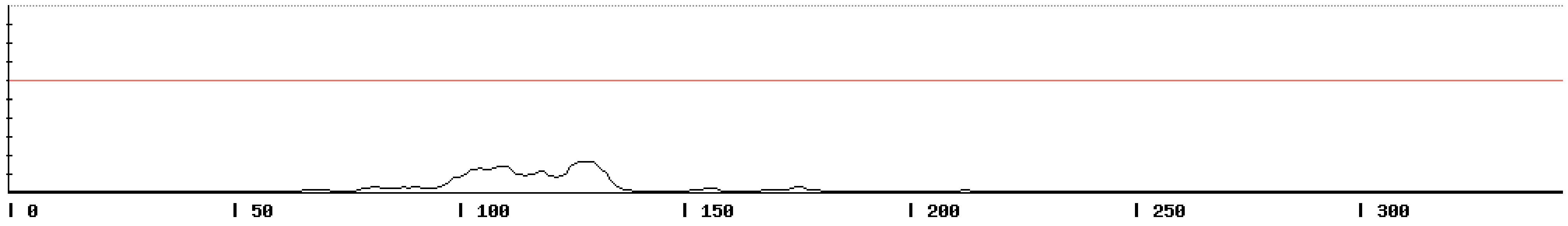

Predictions for Musaacuminata_GSMUA_Achr6P32320_001

Viterbi Path

Viterbi Path

Posterior @ 0.6

Posterior @ 0.6

Posterior Graphic. Threshold Marked @ 0.6

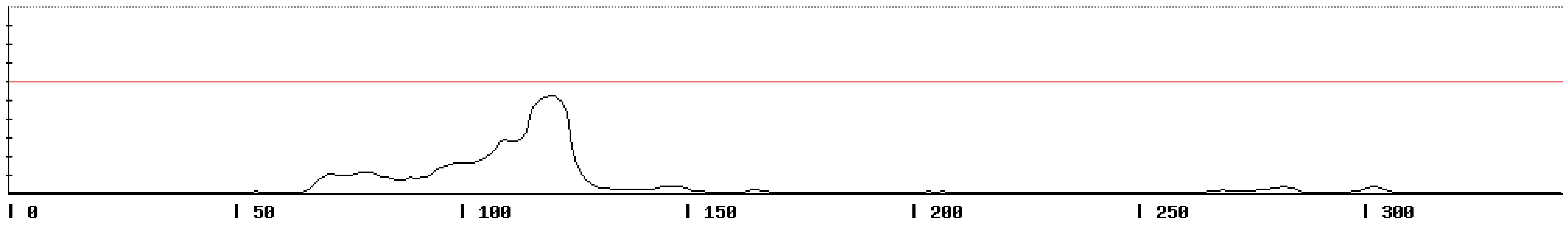

Predictions for Musaacuminata_GSMUA_Achr6P32330_001

Viterbi Path

Viterbi Path

Posterior @ 0.6

Posterior @ 0.6

Posterior Graphic. Threshold Marked @ 0.6

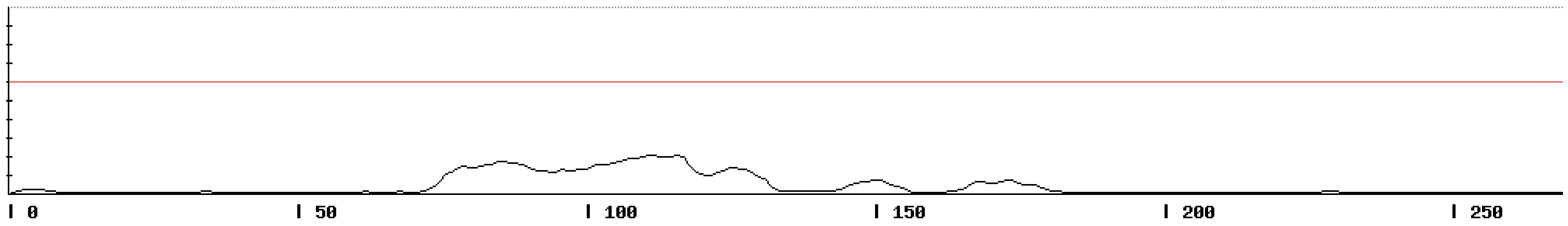

Predictions for Musaacuminata_GSMUA_Achr6P36550_001

Viterbi Path

Viterbi Path

Posterior @ 0.6

Posterior @ 0.6

Posterior Graphic. Threshold Marked @ 0.6

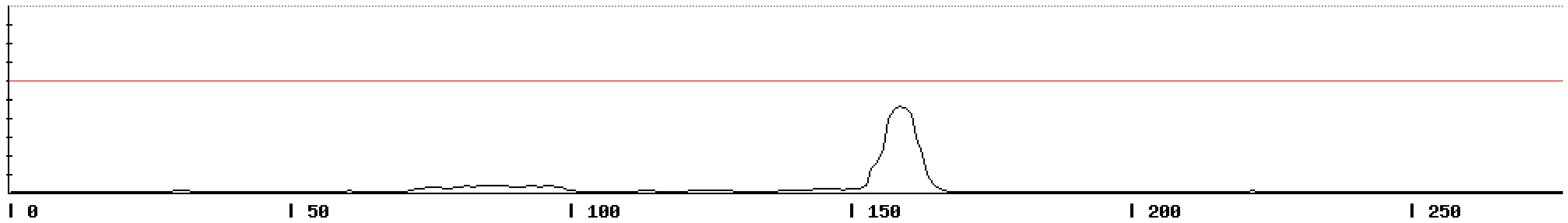

Predictions for Musaacuminata_GSMUA_Achr6P36840_001

Viterbi Path

Viterbi Path

Posterior @ 0.6

Posterior @ 0.6

Posterior Graphic. Threshold Marked @ 0.6

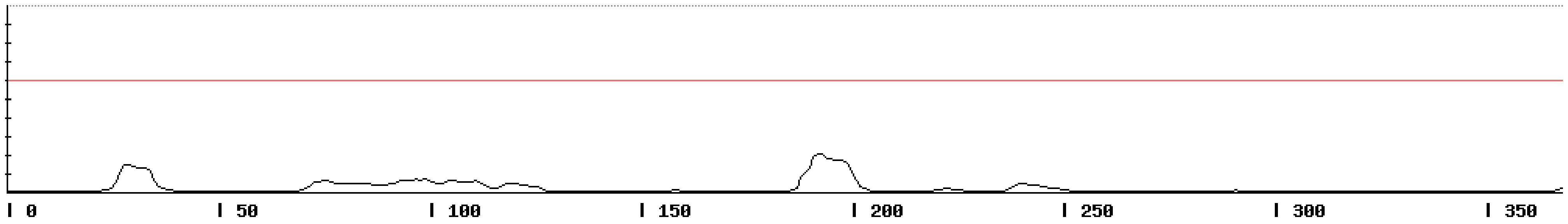

Predictions for Musaacuminata_GSMUA_Achr7P00860_001

Viterbi Path

Viterbi Path

Posterior @ 0.6

Posterior @ 0.6

Posterior Graphic. Threshold Marked @ 0.6

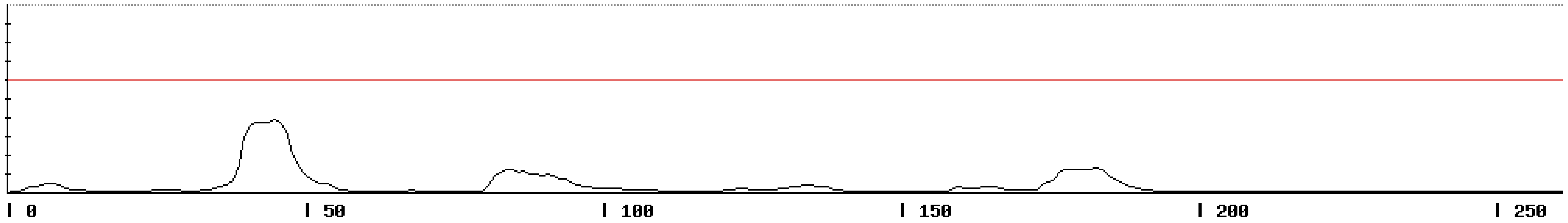

Predictions for Musaacuminata_GSMUA_Achr7P04030_001

Viterbi Path

Viterbi Path

Posterior @ 0.6

Posterior @ 0.6

Posterior Graphic. Threshold Marked @ 0.6

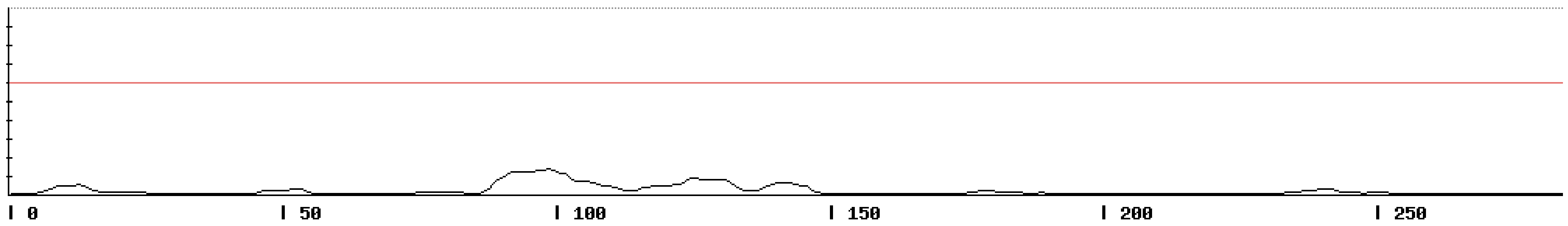

Predictions for Musaacuminata_GSMUA_Achr7P05980_001

Viterbi Path

Viterbi Path

Posterior @ 0.6

Posterior @ 0.6

Posterior Graphic. Threshold Marked @ 0.6

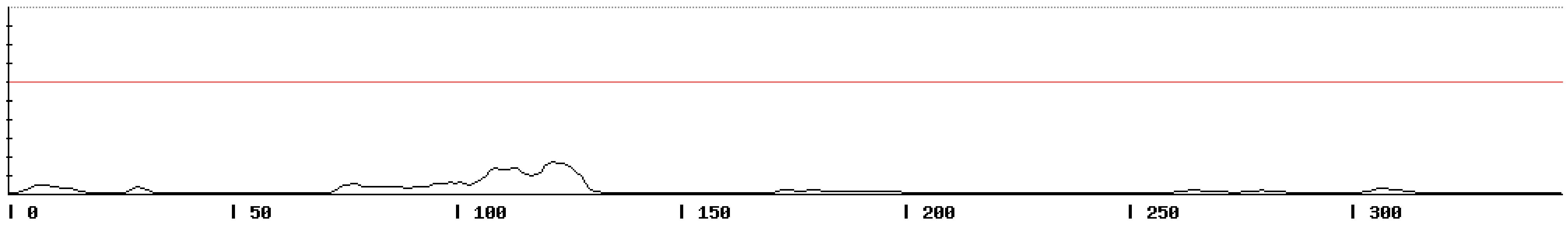

Predictions for Musaacuminata_GSMUA_Achr7P06640_001

Viterbi Path

Viterbi Path

Posterior @ 0.6

Posterior @ 0.6

Posterior Graphic. Threshold Marked @ 0.6

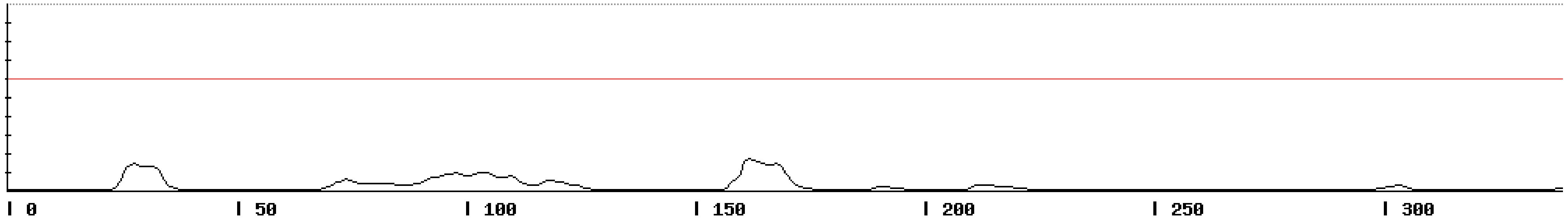

Predictions for Musaacuminata_GSMUA_Achr7P09510_001

Viterbi Path

Viterbi Path

Posterior @ 0.6

Posterior @ 0.6

Posterior Graphic. Threshold Marked @ 0.6

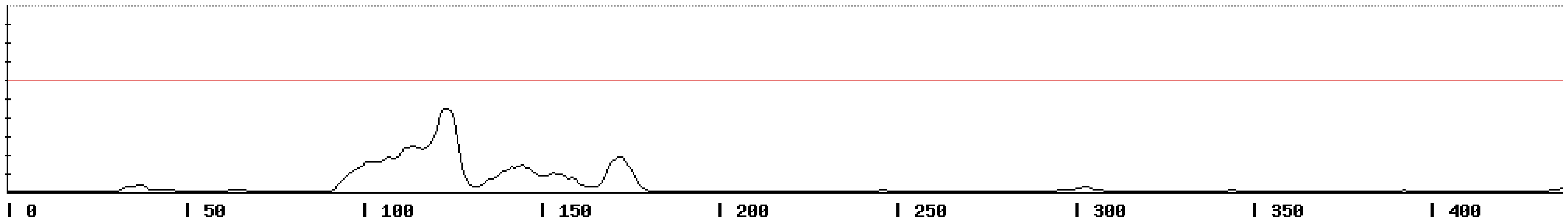

Predictions for Musaacuminata_GSMUA_Achr7P11500_001

Viterbi Path

Viterbi Path

Posterior @ 0.6

Posterior @ 0.6

Posterior Graphic. Threshold Marked @ 0.6

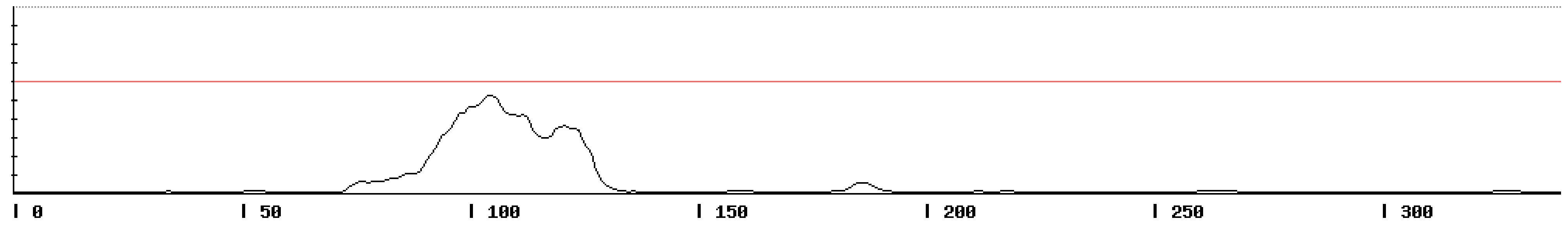

Predictions for Musaacuminata_GSMUA_Achr7P18330_001

Viterbi Path

Viterbi Path

Posterior @ 0.6

Posterior @ 0.6

Posterior Graphic. Threshold Marked @ 0.6

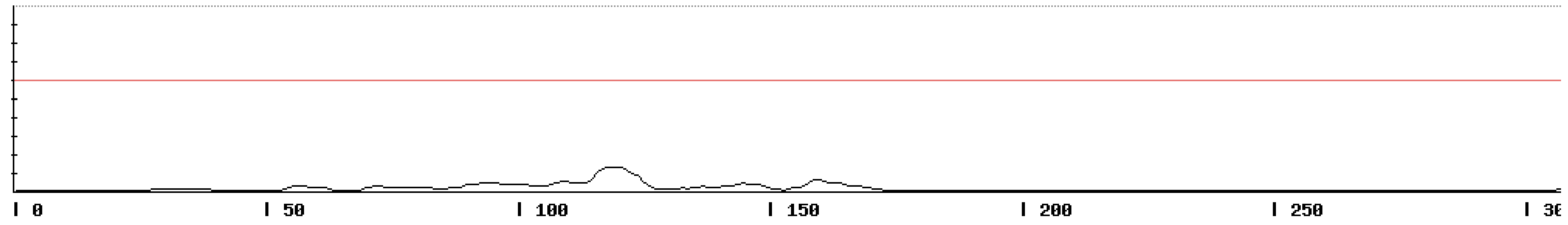

Predictions for Musaacuminata_GSMUA_Achr7P19680_001	
Viterbi Path	
Viterbi Path	
Posterior @ 0.6	
Posterior @ 0.6	
Posterior Graphic. Threshold Marked @ 0.6	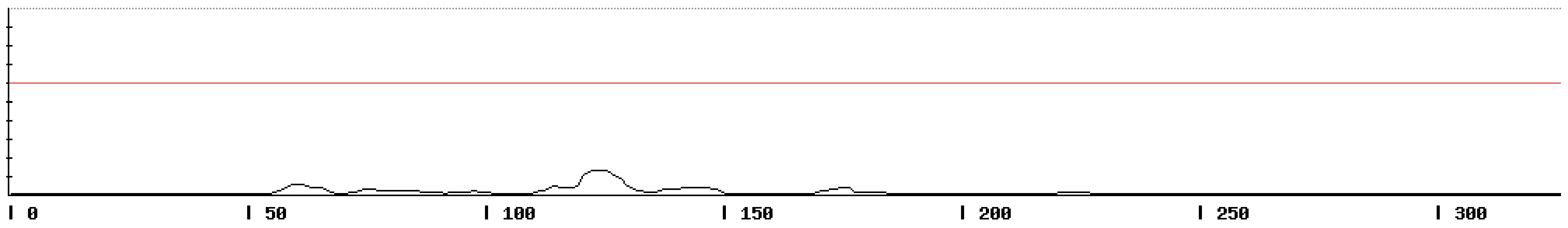

Predictions for Musaacuminata_GSMUA_Achr7P21770_001	
Viterbi Path	
Viterbi Path	
Posterior @ 0.6	
Posterior @ 0.6	
Posterior Graphic. Threshold Marked @ 0.6	

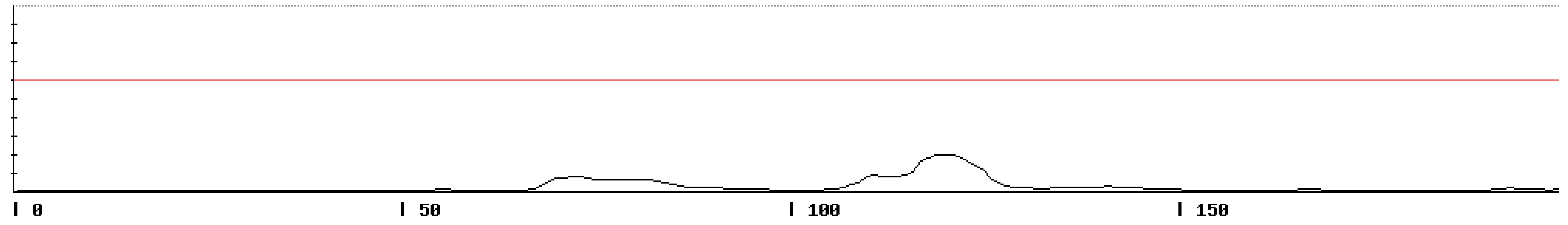

Predictions for Musaacuminata_GSMUA_Achr7P21780_001

Viterbi Path	
Viterbi Path	
Posterior @ 0.6	
Posterior @ 0.6	
Posterior Graphic. Threshold Marked @ 0.6	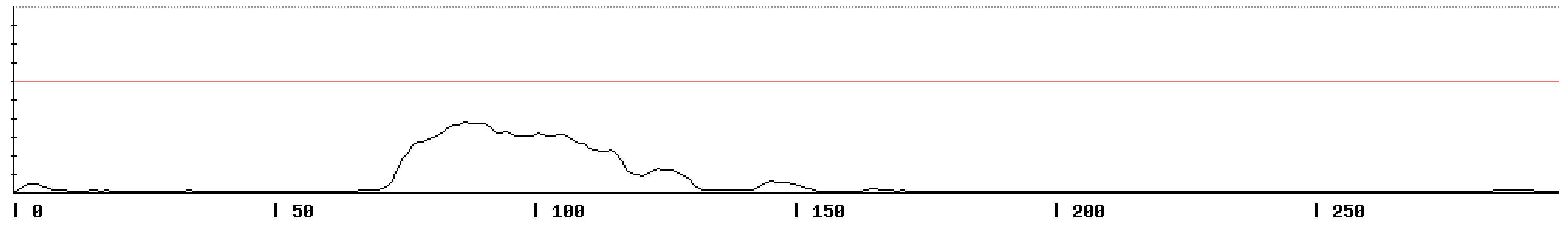

Predictions for Musaacuminata_GSMUA_Achr7P22480_001

Viterbi Path	
Viterbi Path	
Posterior @ 0.6	
Posterior @ 0.6	

Posterior Graphic. Threshold Marked @ 0.6

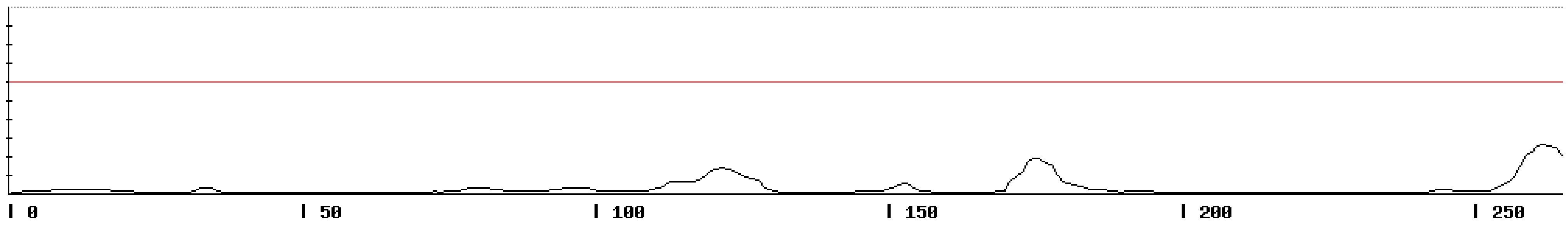

Predictions for Musaacuminata_GSMUA_Achr7P23170_001

Viterbi Path

Viterbi Path

Posterior @ 0.6

Posterior @ 0.6

Posterior Graphic. Threshold Marked @ 0.6

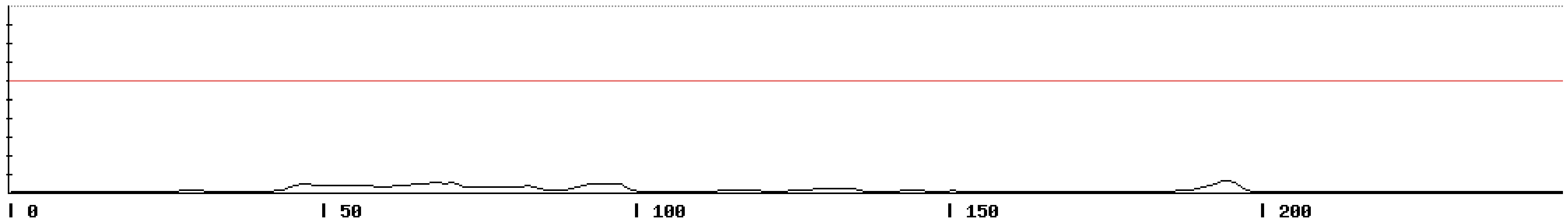

Predictions for Musaacuminata_GSMUA_Achr7P23250_001

Viterbi Path

Viterbi Path

Posterior @ 0.6

Posterior @ 0.6

Posterior Graphic. Threshold Marked @ 0.6

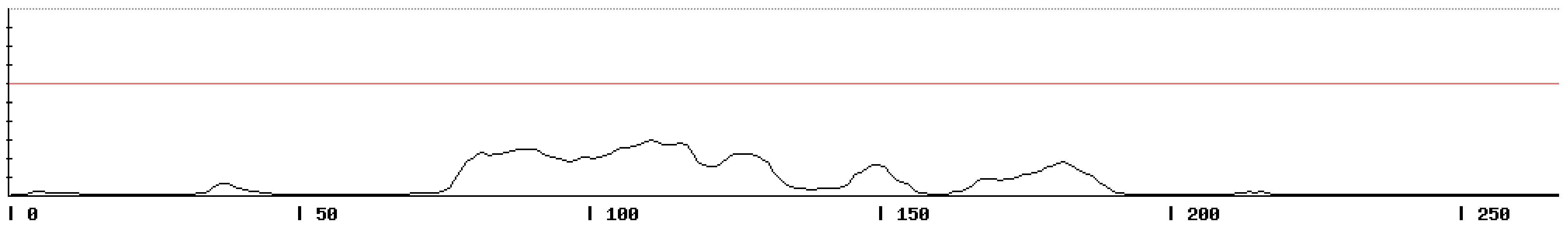

Predictions for Musaacuminata_GSMUA_Achr7P23330_001

Viterbi Path

Viterbi Path

Posterior @ 0.6

Posterior @ 0.6

Posterior Graphic. Threshold Marked @ 0.6

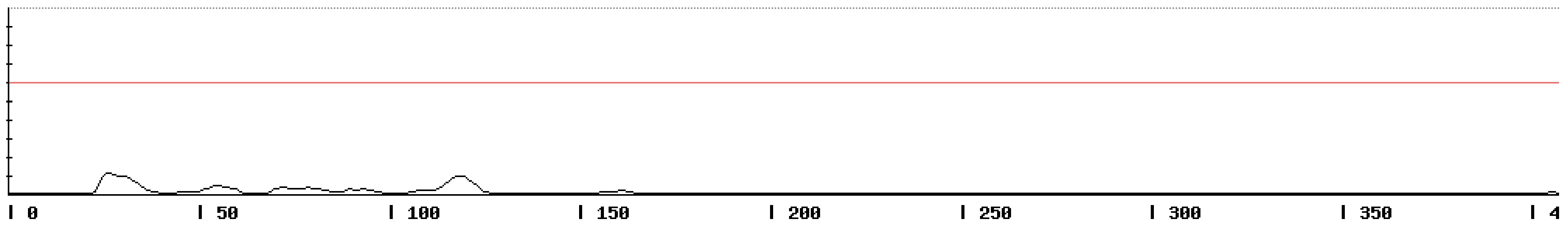

Predictions for Musaacuminata_GSMUA_Achr7P23650_001

Viterbi Path

Viterbi Path

Posterior @ 0.6

Posterior @ 0.6

Posterior Graphic. Threshold Marked @ 0.6

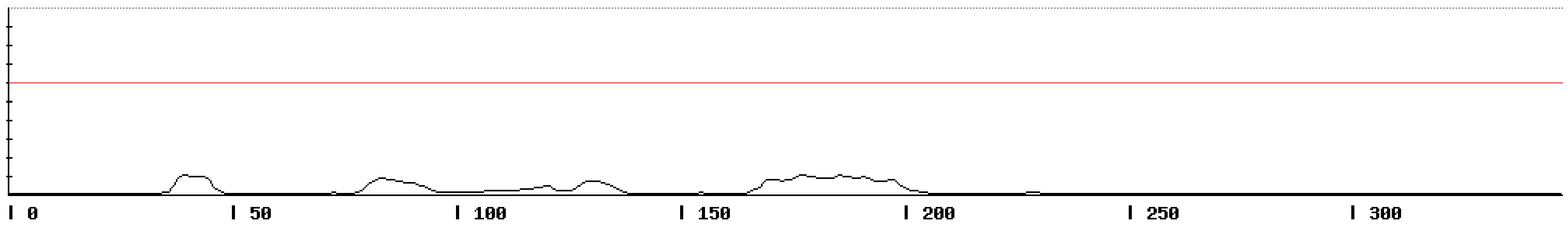

Predictions for Musaacuminata_GSMUA_Achr7P26050_001

Viterbi Path

Viterbi Path

Posterior @ 0.6

Posterior @ 0.6

Posterior Graphic. Threshold Marked @ 0.6

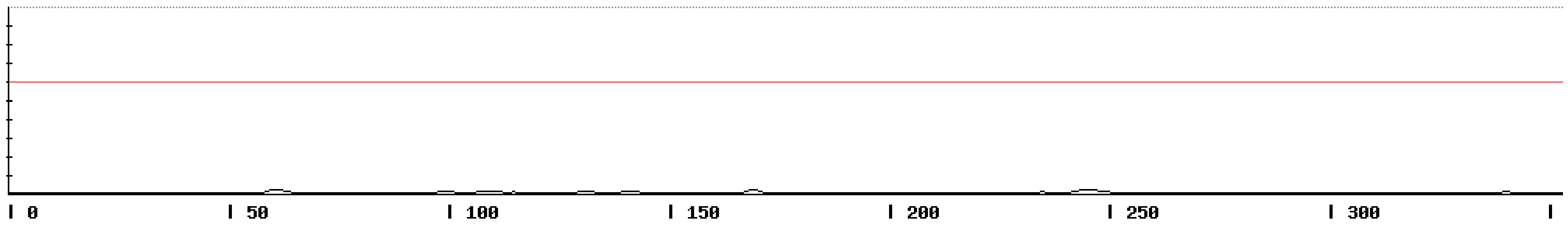

Predictions for Musaacuminata_GSMUA_Achr8P01410_001

Viterbi Path

Viterbi Path

Posterior @ 0.6

Posterior @ 0.6

Posterior Graphic. Threshold Marked @ 0.6

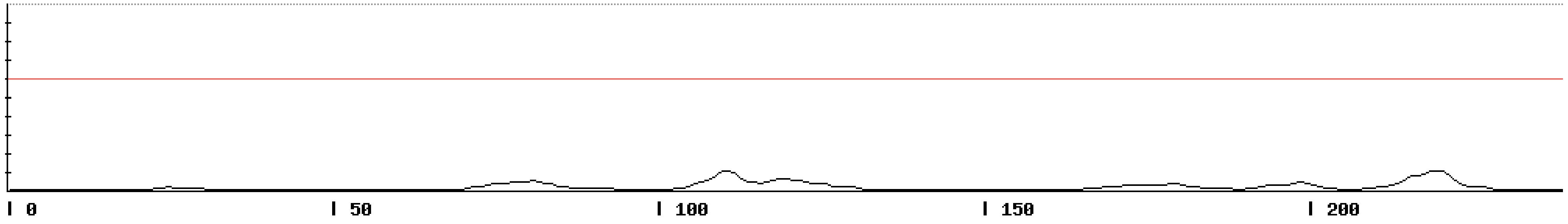

Predictions for Musaacuminata_GSMUA_Achr8P07120_001

Viterbi Path

Viterbi Path

Posterior @ 0.6

Posterior @ 0.6

Posterior Graphic. Threshold Marked @ 0.6

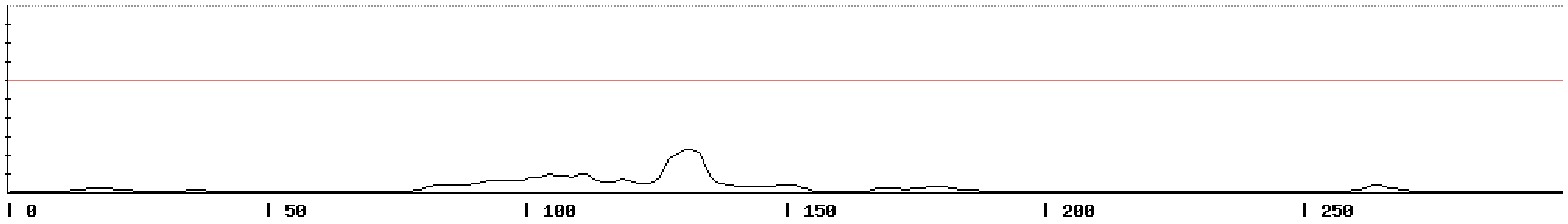

Predictions for Musaacuminata_GSMUA_Achr8P11590_001

Viterbi Path	
Viterbi Path	
Posterior @ 0.6	
Posterior @ 0.6	
Posterior Graphic. Threshold Marked @ 0.6	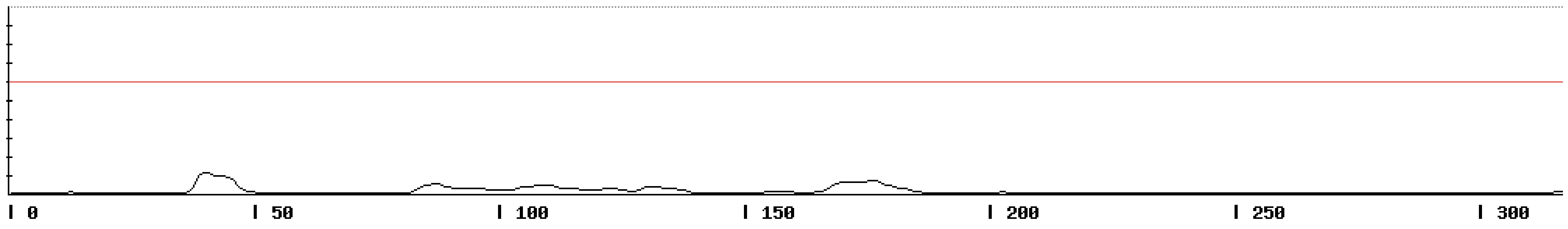

Predictions for Musaacuminata_GSMUA_Achr8P12100_001

Viterbi Path	
Viterbi Path	
Posterior @ 0.6	
Posterior @ 0.6	
Posterior Graphic. Threshold Marked @ 0.6	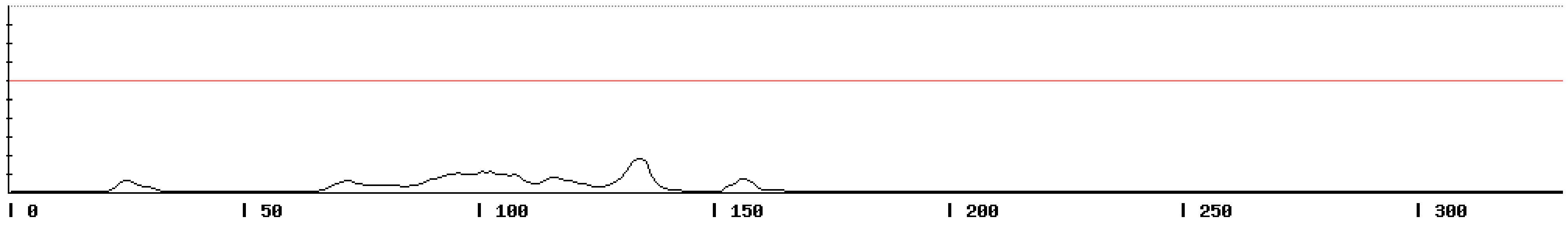

Predictions for Musaacuminata_GSMUA_Achr8P13430_001

Viterbi Path	
Viterbi Path	
Posterior @ 0.6	
Posterior @ 0.6	
Posterior Graphic. Threshold Marked @ 0.6	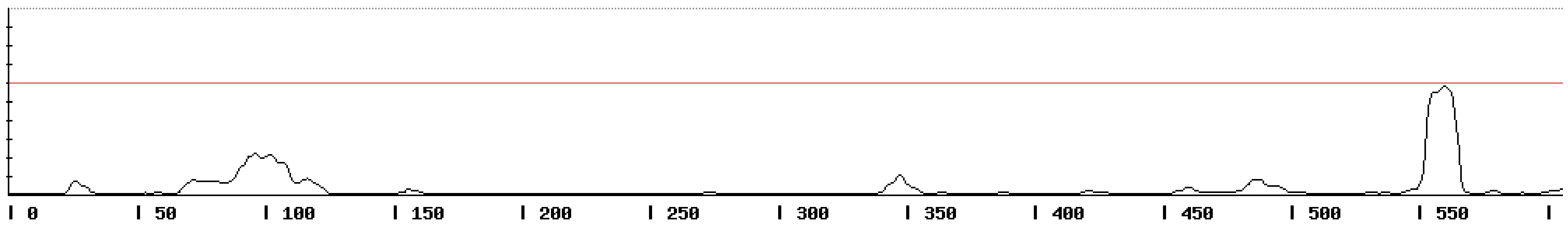

Predictions for Musaacuminata_GSMUA_Achr8P18420_001

Viterbi Path	
Viterbi Path	
Posterior @ 0.6	
Posterior @ 0.6	
Posterior Graphic. Threshold Marked @ 0.6	

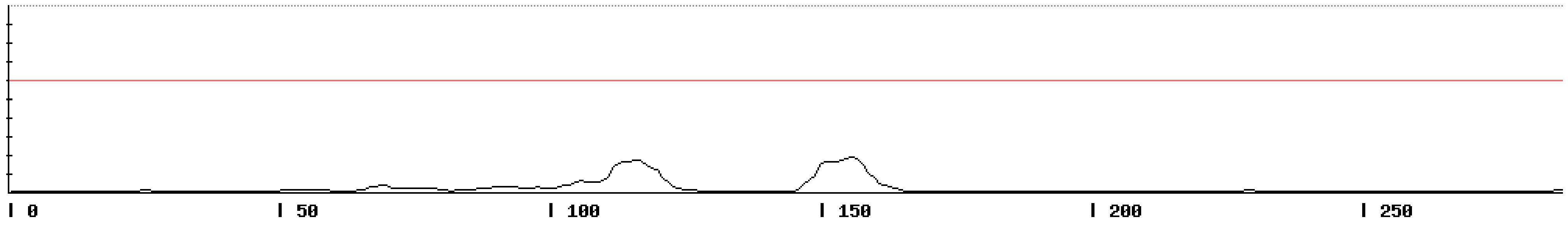

Predictions for Musaacuminata_GSMUA_Achr8P18980_001

Viterbi Path

Viterbi Path

Posterior @ 0.6

Posterior @ 0.6

Posterior Graphic. Threshold Marked @ 0.6

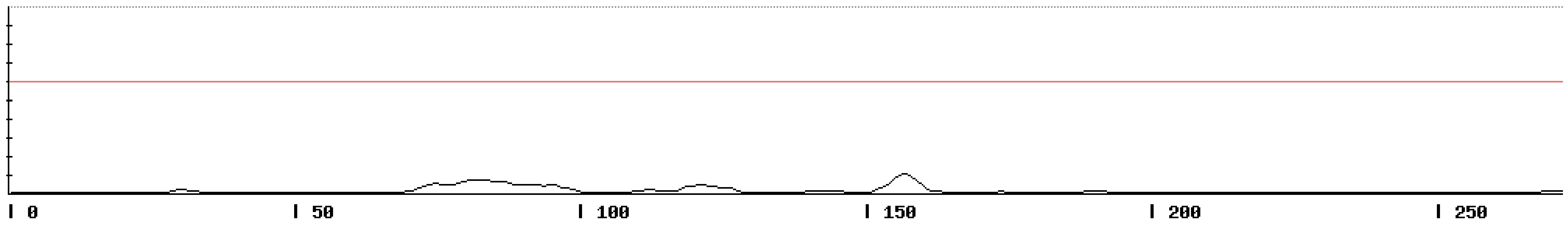

Predictions for Musaacuminata_GSMUA_Achr8P21470_001

Viterbi Path

Viterbi Path

Posterior @ 0.6

Posterior @ 0.6

Posterior Graphic. Threshold Marked @ 0.6

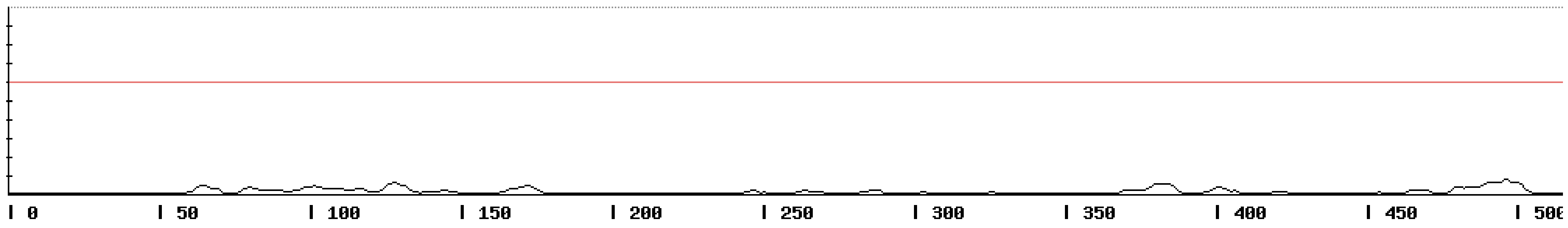

Predictions for Musaacuminata_GSMUA_Achr8P24280_001

Viterbi Path

Viterbi Path

Posterior @ 0.6

Posterior @ 0.6

Posterior Graphic. Threshold Marked @ 0.6

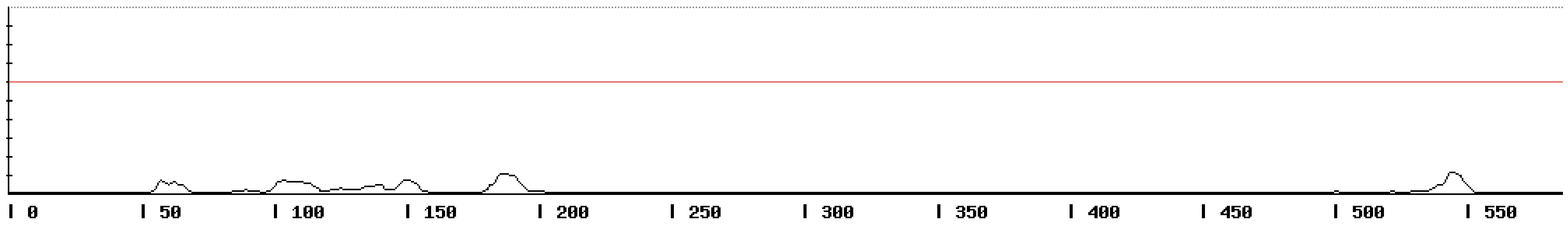

Predictions for Musaacuminata_GSMUA_Achr8P24680_001

Viterbi Path

Viterbi Path

Posterior @ 0.6

Posterior @ 0.6

Posterior Graphic. Threshold Marked @ 0.6

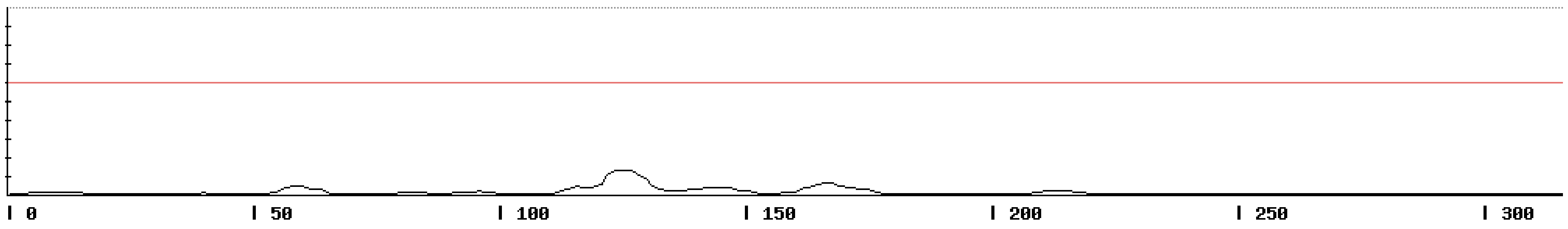

Predictions for Musaacuminata_GSMUA_Achr8P33310_001

Viterbi Path

Viterbi Path

Posterior @ 0.6

Posterior @ 0.6

Posterior Graphic. Threshold Marked @ 0.6

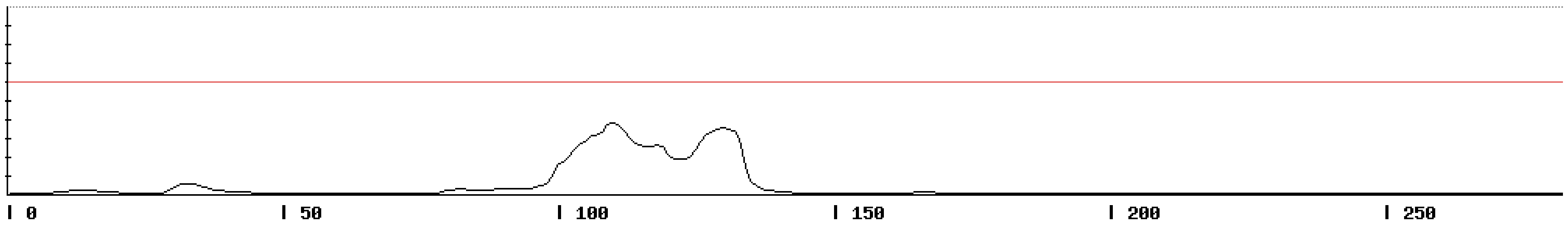

Predictions for Musaacuminata_GSMUA_Achr9P00570_001

Viterbi Path

Viterbi Path

Posterior @ 0.6

Posterior @ 0.6

Posterior Graphic. Threshold Marked @ 0.6

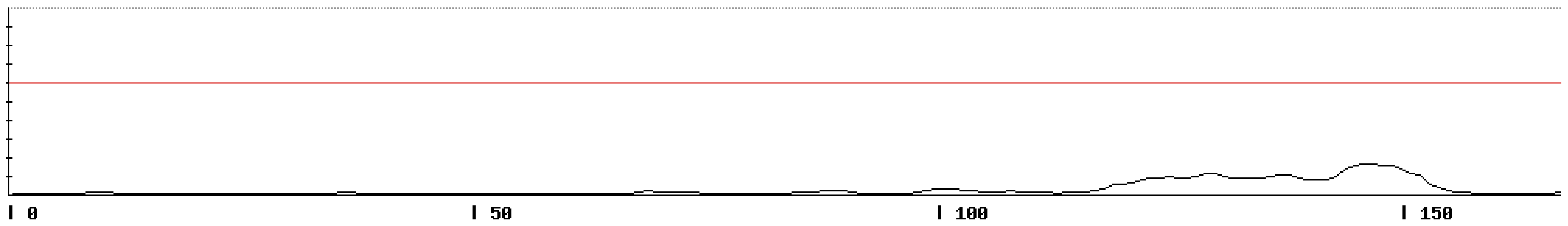

Predictions for Musaacuminata_GSMUA_Achr9P01880_001

Viterbi Path

Viterbi Path

Posterior @ 0.6

Posterior @ 0.6

Posterior Graphic. Threshold Marked @ 0.6

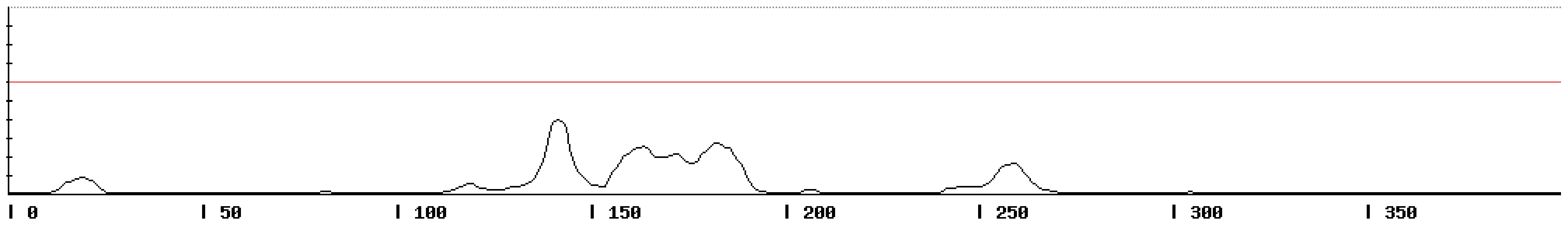

Predictions for Musaacuminata_GSMUA_Achr9P04960_001

Viterbi Path

Viterbi Path

Posterior @ 0.6

Posterior @ 0.6

Posterior Graphic. Threshold Marked @ 0.6

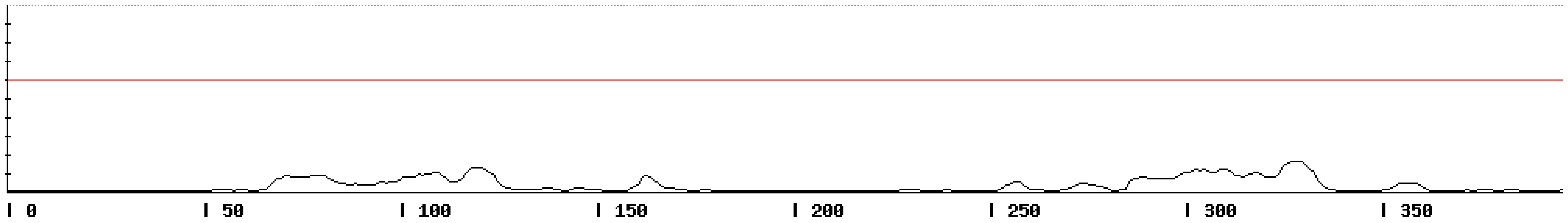

Predictions for Musaacuminata_GSMUA_Achr9P10040_001

Viterbi Path

Viterbi Path

Posterior @ 0.6

Posterior @ 0.6

Posterior Graphic. Threshold Marked @ 0.6

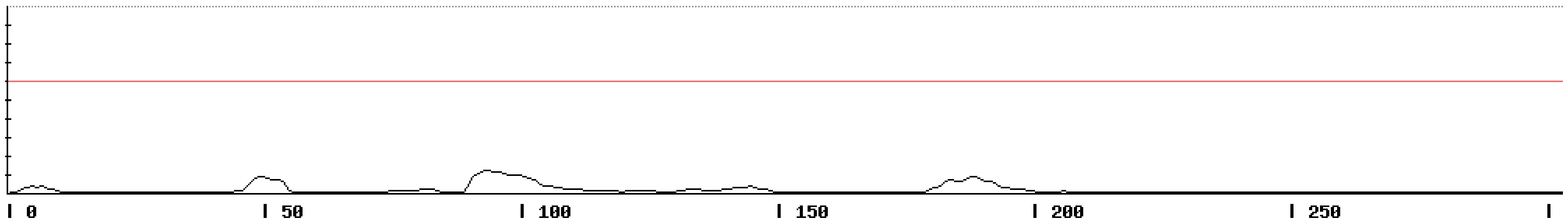

Predictions for Musaacuminata_GSMUA_Achr9P10210_001

Viterbi Path

Viterbi Path

Posterior @ 0.6

Posterior @ 0.6

Posterior Graphic. Threshold Marked @ 0.6

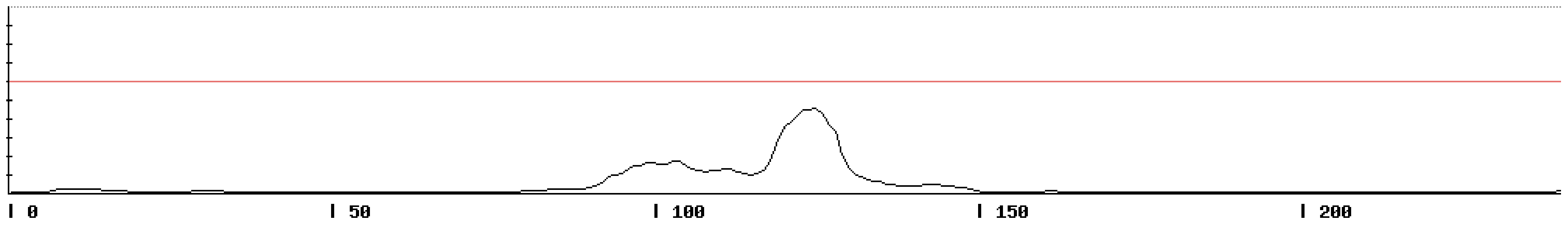

Predictions for Musaacuminata_GSMUA_Achr9P16920_001

Viterbi Path

Viterbi Path

Posterior @ 0.6

Posterior @ 0.6

Posterior Graphic. Threshold Marked @ 0.6

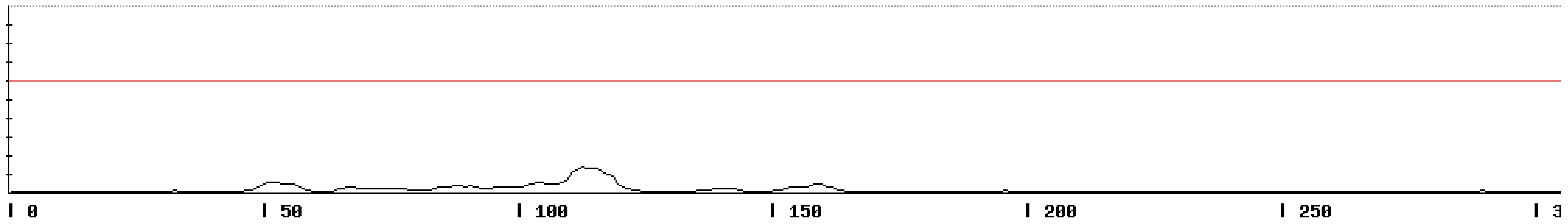

Predictions for Musaacuminata_GSMUA_Achr9P19520_001	
Viterbi Path	
Viterbi Path	
Posterior @ 0.6	
Posterior @ 0.6	
Posterior Graphic. Threshold Marked @ 0.6	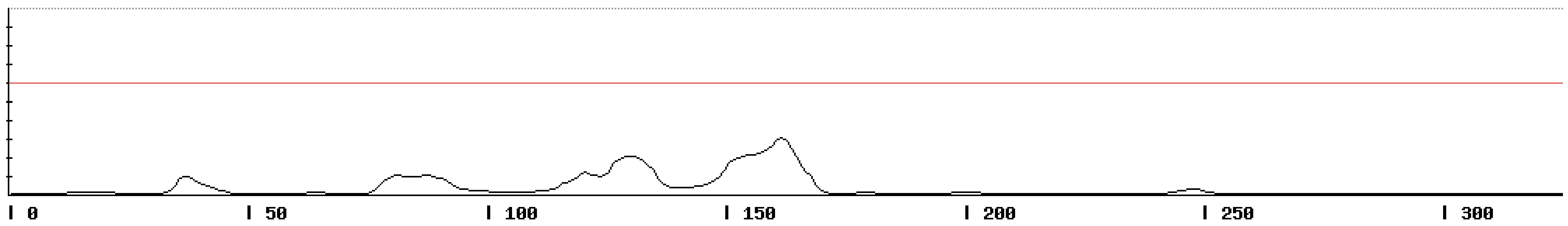

Predictions for Musaacuminata_GSMUA_Achr9P20090_001	
Viterbi Path	
Viterbi Path	
Posterior @ 0.6	
Posterior @ 0.6	
Posterior Graphic. Threshold Marked @ 0.6	

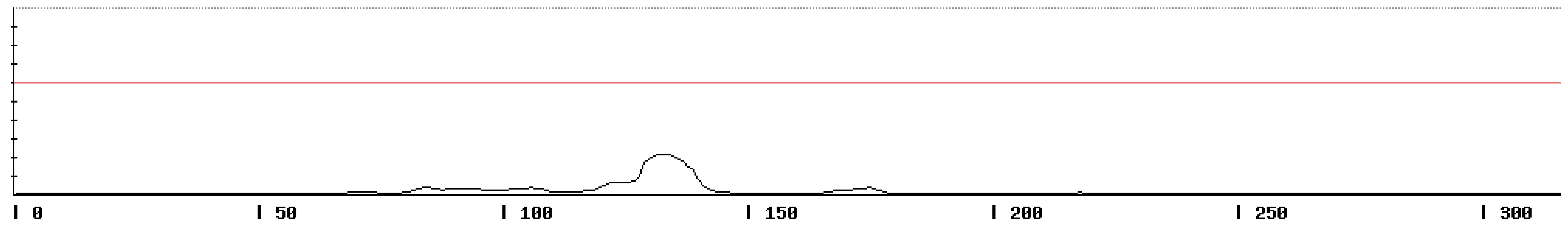

Predictions for Musaacuminata_GSMUA_Achr9P20400_001

Viterbi Path

Viterbi Path

Posterior @ 0.6

Posterior @ 0.6

Posterior Graphic. Threshold Marked @ 0.6

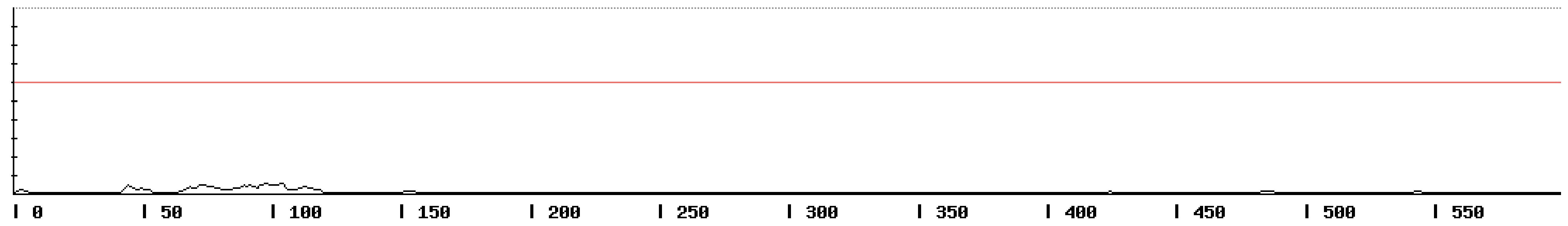

Predictions for Musaacuminata_GSMUA_Achr9P23580_001

Viterbi Path

Viterbi Path

Posterior @ 0.6

Posterior @ 0.6

Viterbi Path	
Posterior @ 0.4	
Posterior @ 0.4	
Posterior Graphic. Threshold Marked @ 0.4	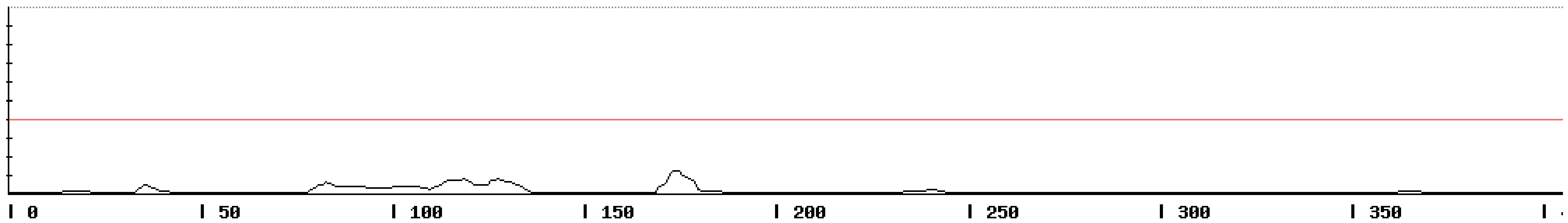

Predictions for Nelumbonucifera_NNU_000815-RA	
Viterbi Path	
Viterbi Path	
Posterior @ 0.4	
Posterior @ 0.4	
Posterior Graphic. Threshold Marked @ 0.4	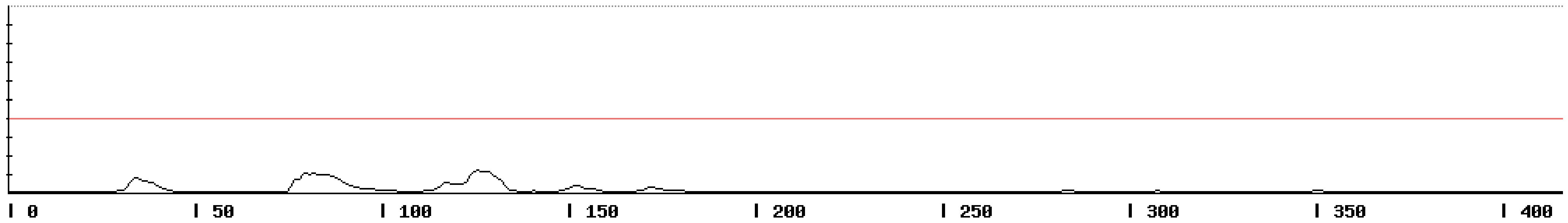

Predictions for Nelumbonucifera_NNU_000863-RA

Viterbi Path

Viterbi Path

Posterior @ 0.4

Posterior @ 0.4

Posterior Graphic. Threshold Marked @ 0.4

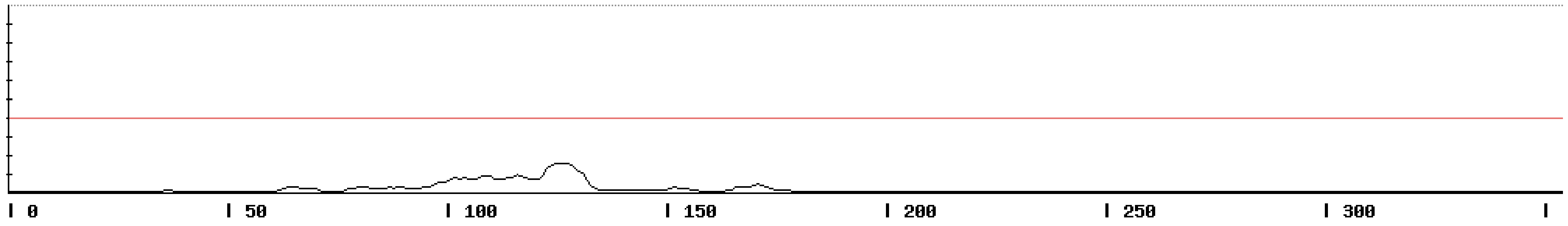

Predictions for Nelumbonucifera_NNU_000864-RA

Viterbi Path

Viterbi Path

Posterior @ 0.4

Posterior @ 0.4

Posterior Graphic. Threshold Marked @ 0.4

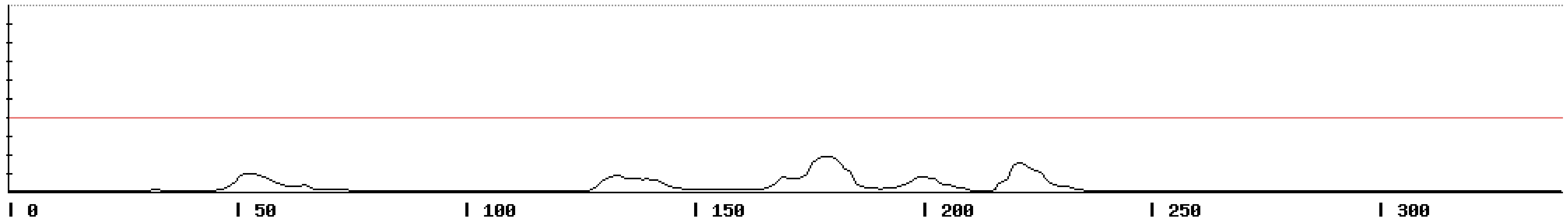

Predictions for Nelumbonucifera_NNU_001248-RA

Viterbi Path	
Viterbi Path	
Posterior @ 0.4	
Posterior @ 0.4	
Posterior Graphic. Threshold Marked @ 0.4	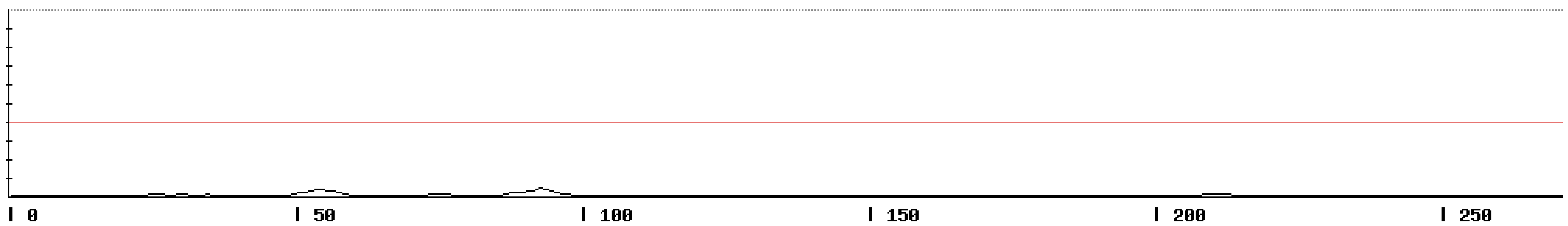

Predictions for Nelumbonucifera_NNU_002063-RA

Viterbi Path	
Viterbi Path	
Posterior @ 0.4	105 - SERAKRKTRSGFWKRRGQARD - 125
Posterior @ 0.4	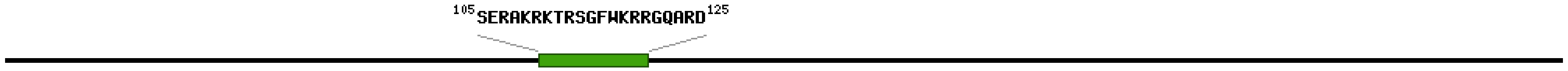
Posterior Graphic. Threshold Marked @ 0.4	

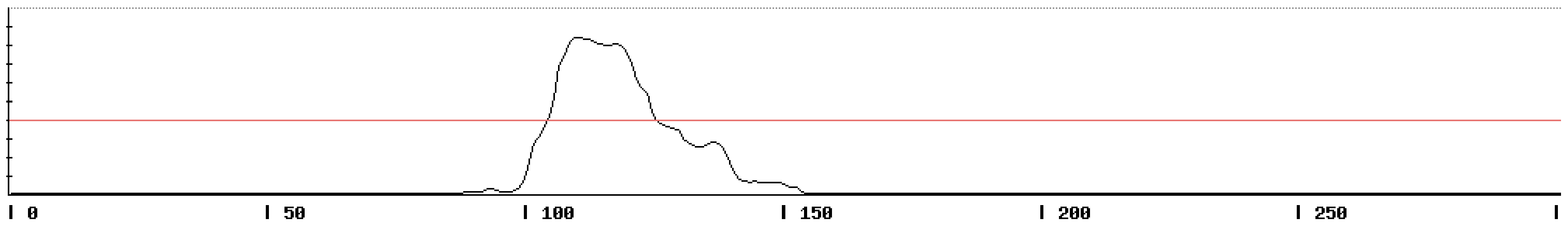

Predictions for Nelumbonucifera_NNU_002530-RA

Viterbi Path	
Viterbi Path	
Posterior @ 0.4	
Posterior @ 0.4	
Posterior Graphic. Threshold Marked @ 0.4	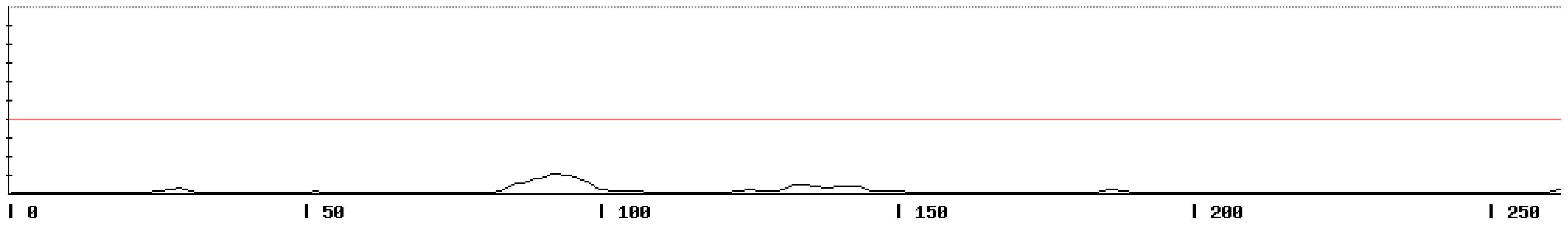

Predictions for Nelumbonucifera_NNU_002531-RA

Viterbi Path	
Viterbi Path	
Posterior @ 0.4	
Posterior @ 0.4	

Posterior Graphic. Threshold Marked @ 0.4

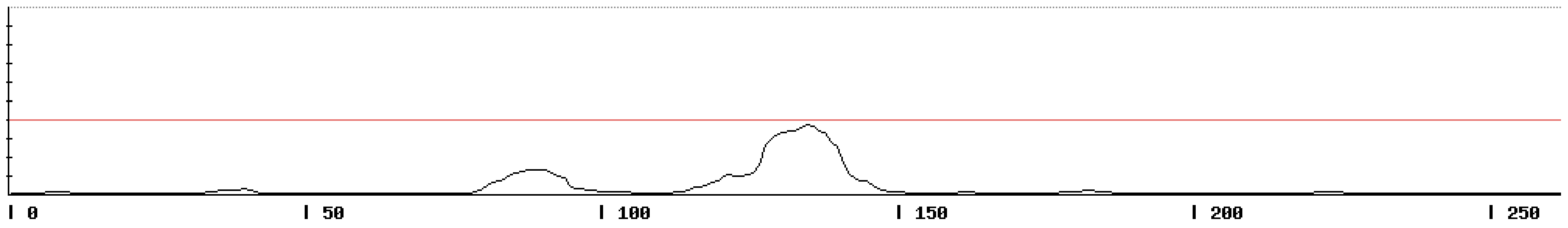

Predictions for Nelumbonucifera_NNU_002554-RA

Viterbi Path	
Viterbi Path	
Posterior @ 0.4	
Posterior @ 0.4	
Posterior Graphic. Threshold Marked @ 0.4	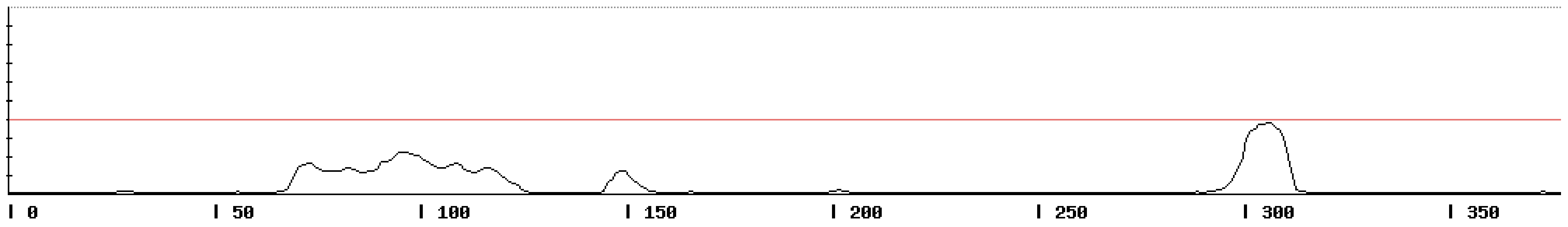

Predictions for Nelumbonucifera_NNU_002898-RA

Viterbi Path	
Viterbi Path	
Posterior @ 0.4	132 - HRKRRKI - 138
Posterior @ 0.4	

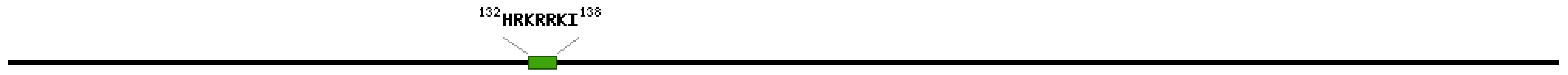

Posterior Graphic. Threshold Marked @ 0.4

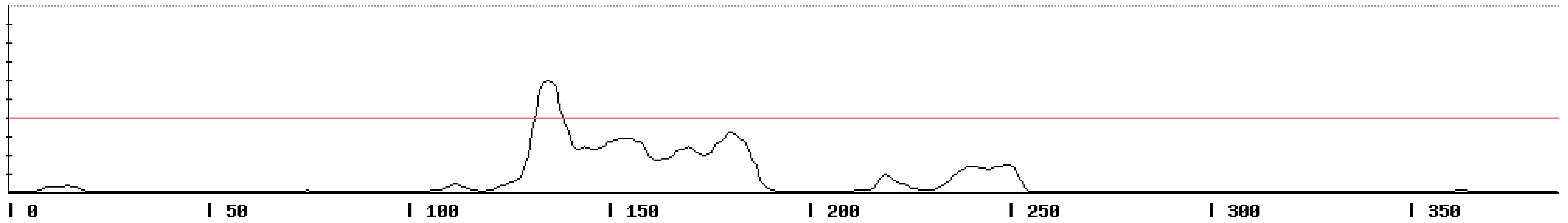

Predictions for Nelumbonucifera_NNU_003181-RA

Viterbi Path

Viterbi Path

Posterior @ 0.4

Posterior @ 0.4

Posterior Graphic. Threshold Marked @ 0.4

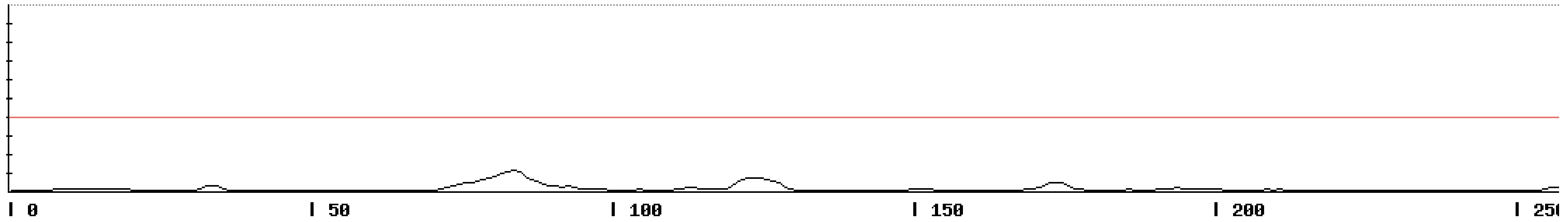

Predictions for Nelumbonucifera_NNU_004001-RA

Viterbi Path

Viterbi Path

Posterior @ 0.4

Posterior @ 0.4

Posterior Graphic. Threshold Marked @ 0.4

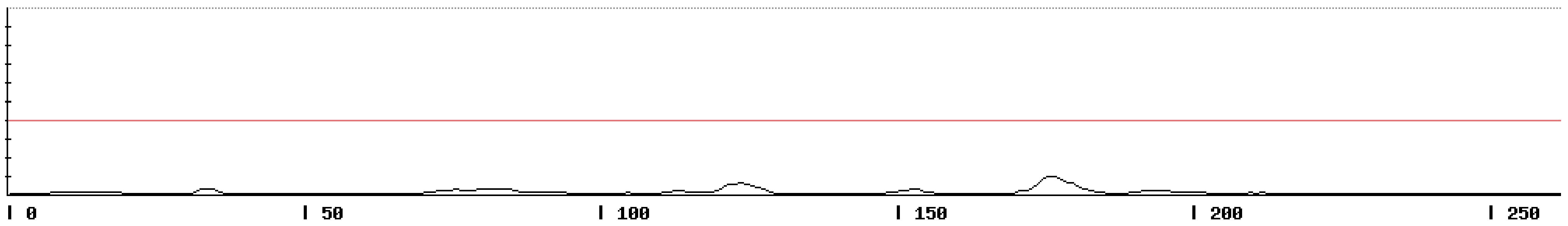

Predictions for Nelumbonucifera_NNU_004270-RA

Viterbi Path

Viterbi Path

Posterior @ 0.4 132 - QRKRRKIHGQGGKIEEHVRWHKTGKT - 157

Posterior @ 0.4

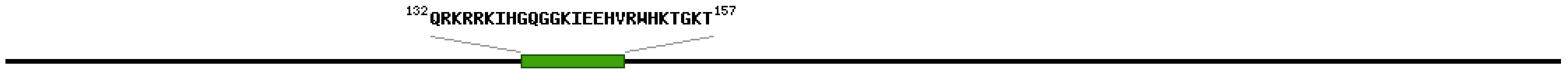

Posterior Graphic. Threshold Marked @ 0.4

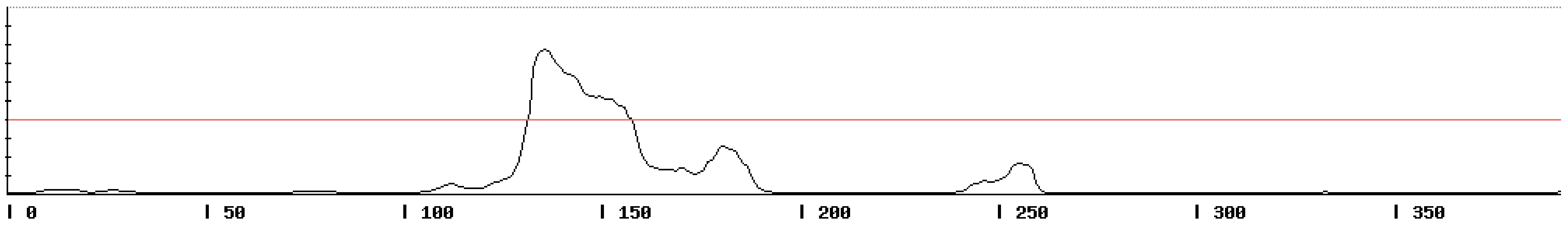

Predictions for Nelumbonucifera_NNU_004655-RA

Viterbi Path

Viterbi Path

Posterior @ 0.4	
Posterior @ 0.4	
Posterior Graphic. Threshold Marked @ 0.4	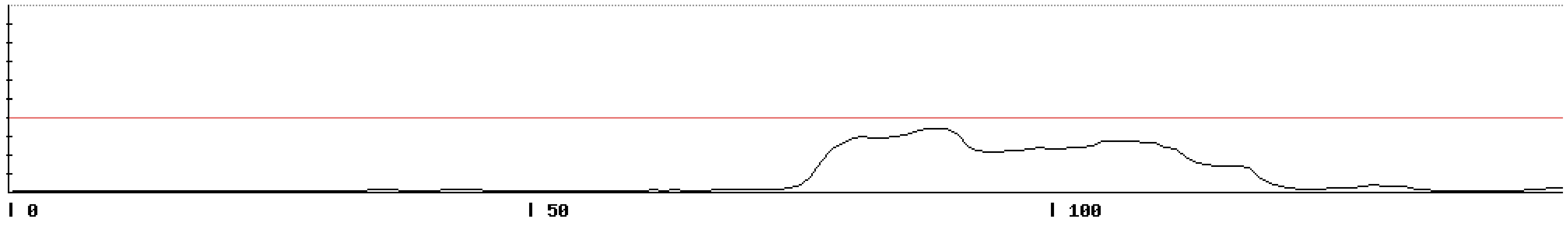

Predictions for Nelumbonucifera_NNU_004688-RA

Viterbi Path	
Viterbi Path	
Posterior @ 0.4	140 - RKYPKGNRPKR - 150
Posterior @ 0.4	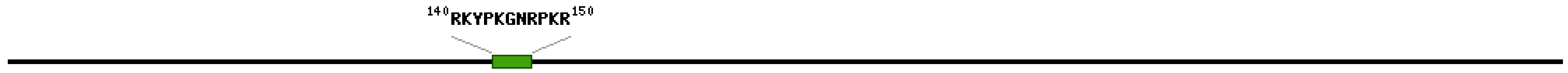
Posterior Graphic. Threshold Marked @ 0.4	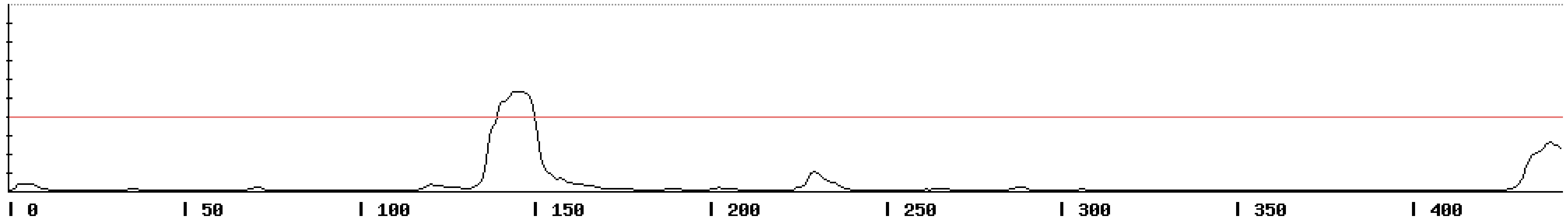

Predictions for Nelumbonucifera_NNU_004689-RA

Viterbi Path	
Viterbi Path	
Posterior @ 0.4	
Posterior @ 0.4	
Posterior Graphic. Threshold Marked @ 0.4	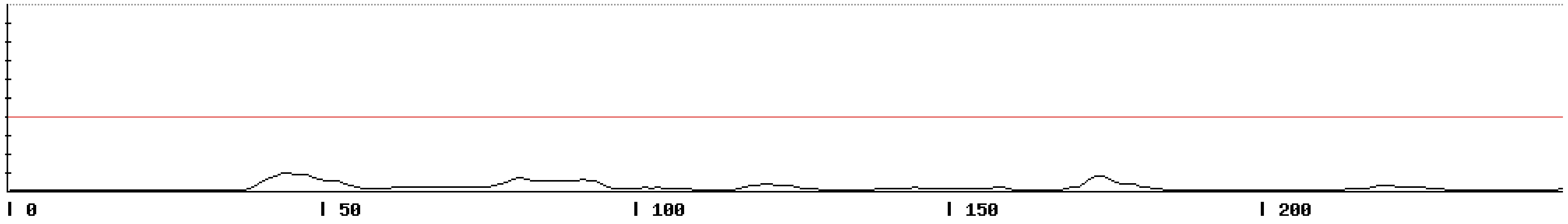

Predictions for Nelumbonucifera_NNU_004690-RA

Viterbi Path	
Viterbi Path	
Posterior @ 0.4	
Posterior @ 0.4	
Posterior Graphic. Threshold Marked @ 0.4	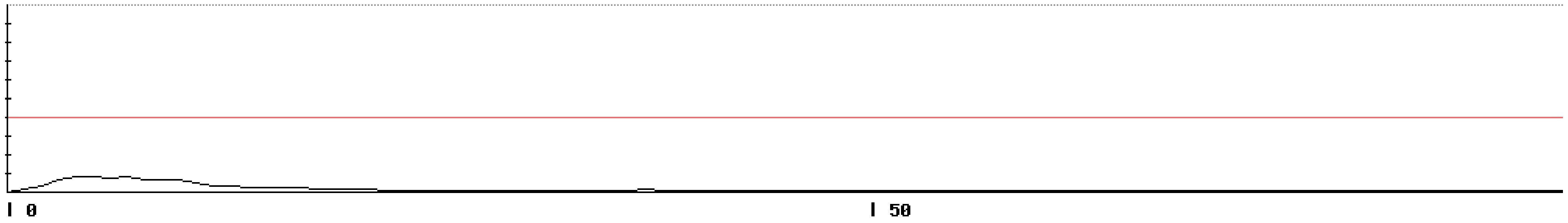

Predictions for Nelumbonucifera_NNU_004901-RA	
Viterbi Path	
Viterbi Path	
Posterior @ 0.4	
Posterior @ 0.4	
Posterior Graphic. Threshold Marked @ 0.4	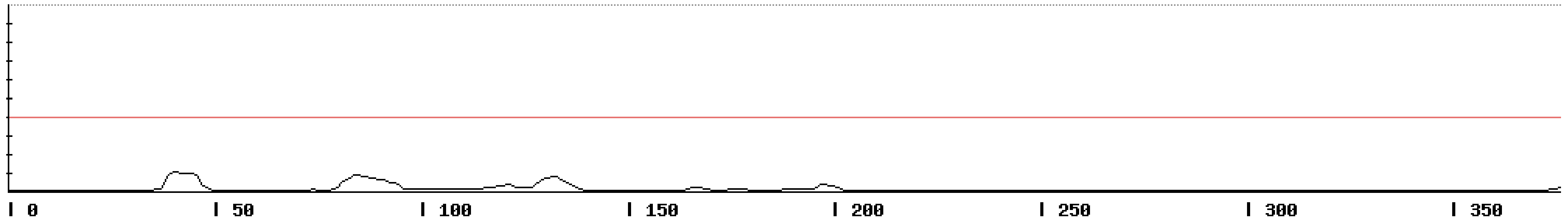

Predictions for Nelumbonucifera_NNU_004940-RA	
Viterbi Path	
Viterbi Path	
Posterior @ 0.4	
Posterior @ 0.4	
Posterior Graphic. Threshold Marked @ 0.4	

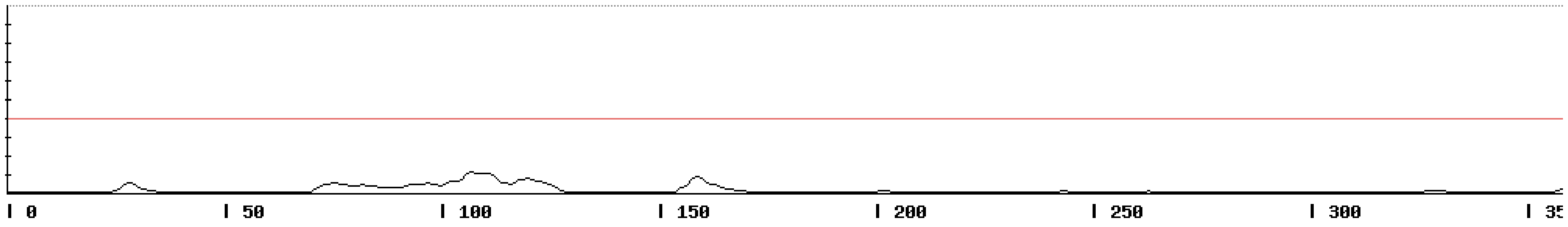

Predictions for Nelumbonucifera_NNU_006481-RA

Viterbi Path	
Viterbi Path	
Posterior @ 0.4	385 - SRGKGKAVSRMSKLKKK - 401
Posterior @ 0.4	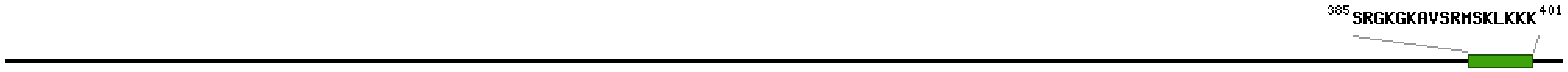
Posterior Graphic. Threshold Marked @ 0.4	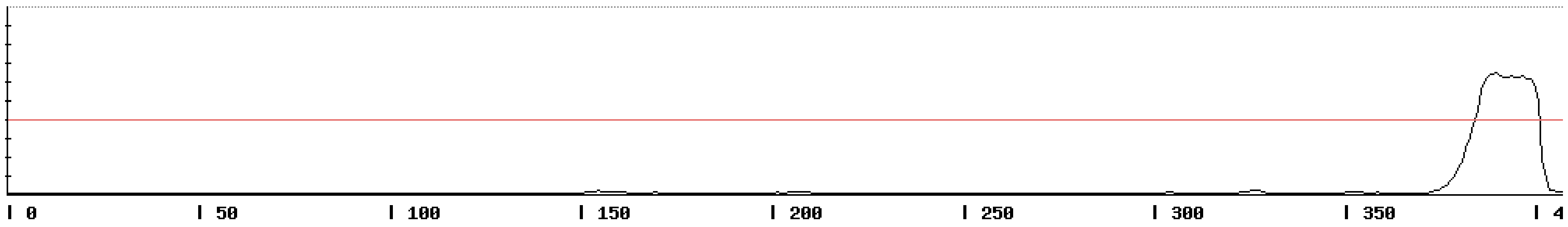

Predictions for Nelumbonucifera_NNU_007358-RA

Viterbi Path	
Viterbi Path	
Posterior @ 0.4	
Posterior @ 0.4	

Posterior Graphic. Threshold Marked @ 0.4

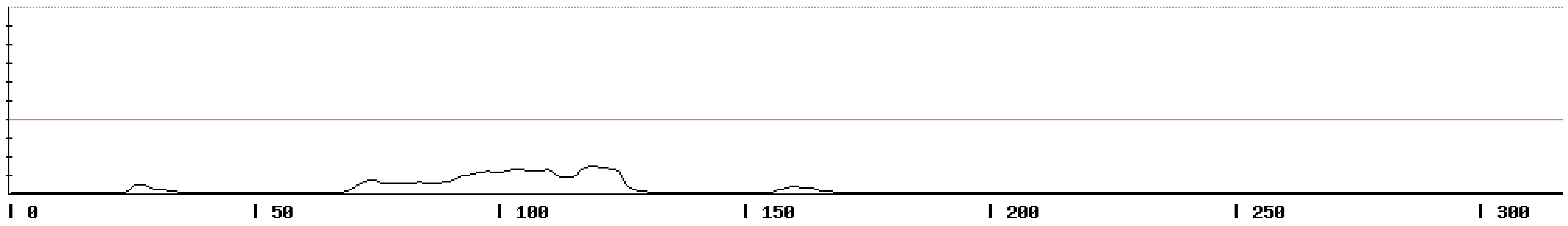

Predictions for Nelumbonucifera_NNU_008163-RA

Viterbi Path	
Viterbi Path	
Posterior @ 0.4	87 - R - 87
Posterior @ 0.4	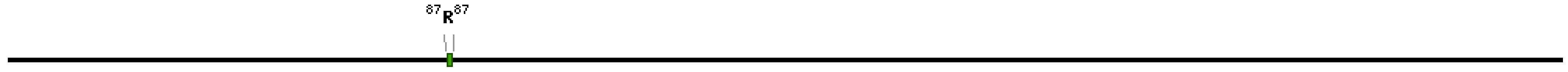
Posterior Graphic. Threshold Marked @ 0.4	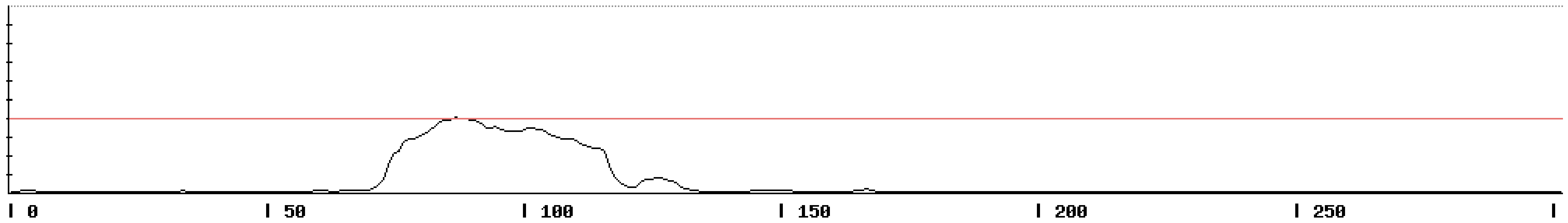

Predictions for Nelumbonucifera_NNU_008352-RA

Viterbi Path	
Viterbi Path	
Posterior @ 0.4	135 - RSRGRR - 140
Posterior @ 0.4	

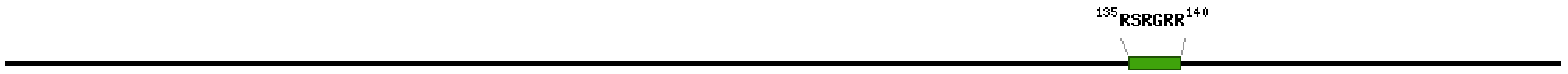

Posterior Graphic. Threshold Marked @ 0.4

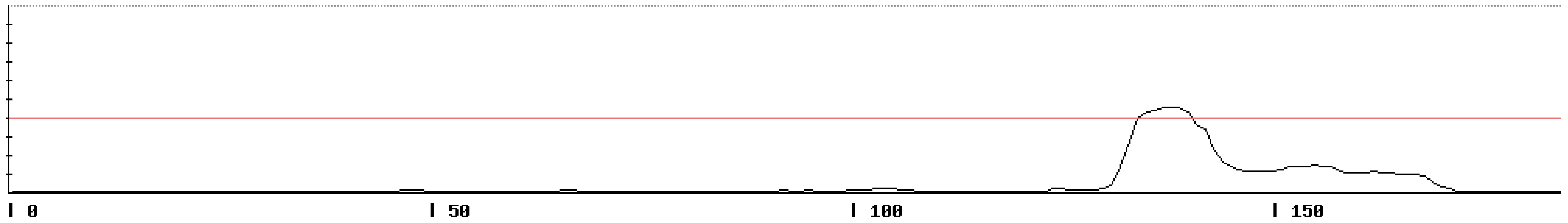

Predictions for Nelumbonucifera_NNU_008461-RA

Viterbi Path

Viterbi Path

Posterior @ 0.4

Posterior @ 0.4

Posterior Graphic. Threshold Marked @ 0.4

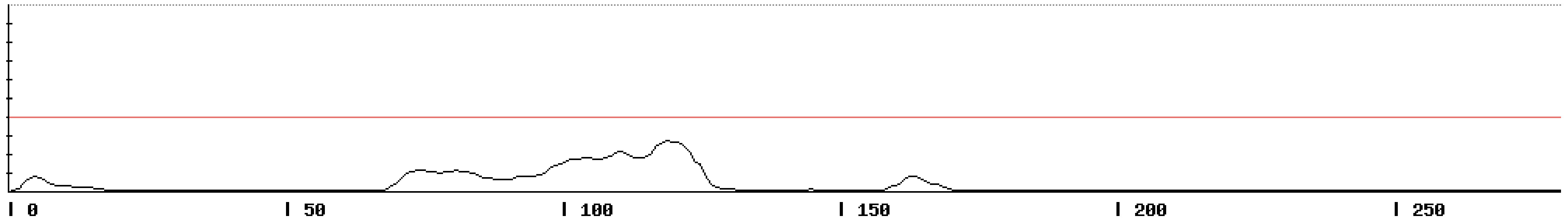

Predictions for Nelumbonucifera_NNU_008468-RA

Viterbi Path

Viterbi Path

Posterior @ 0.4

Posterior @ 0.4

Posterior Graphic. Threshold Marked @ 0.4

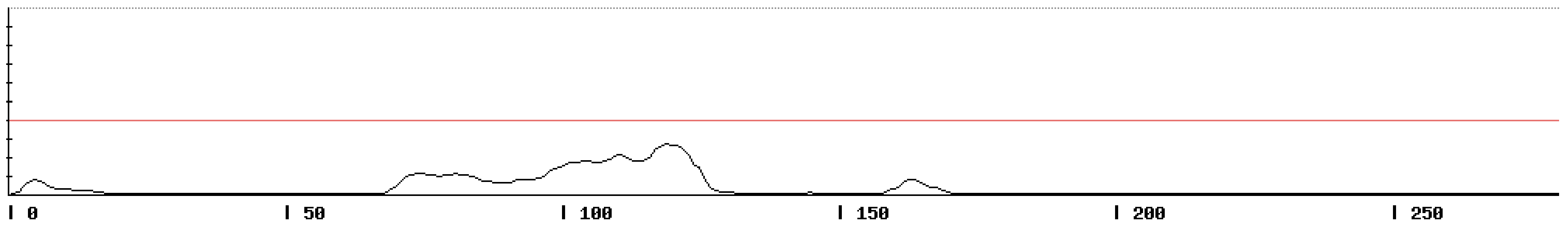

Predictions for Nelumbonucifera_NNU_008469-RA

Viterbi Path

Viterbi Path

Posterior @ 0.4

Posterior @ 0.4

Posterior Graphic. Threshold Marked @ 0.4

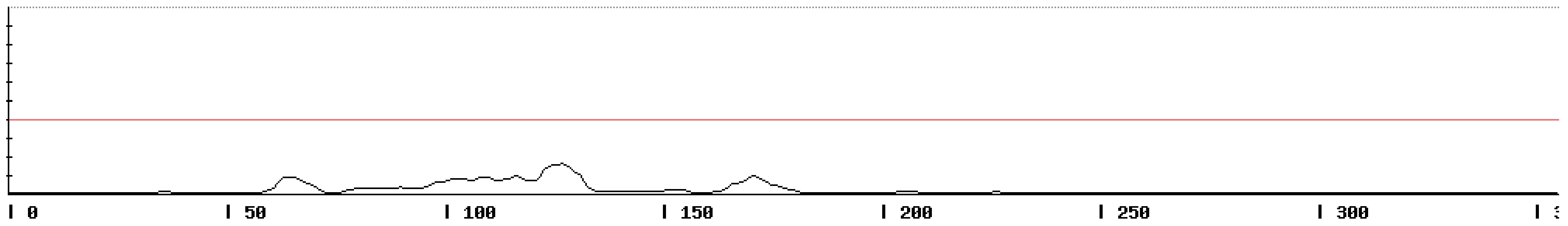

Predictions for Nelumbonucifera_NNU_008524-RA

Viterbi Path

Viterbi Path

Posterior @ 0.4

Posterior @ 0.4

Posterior Graphic. Threshold Marked @ 0.4

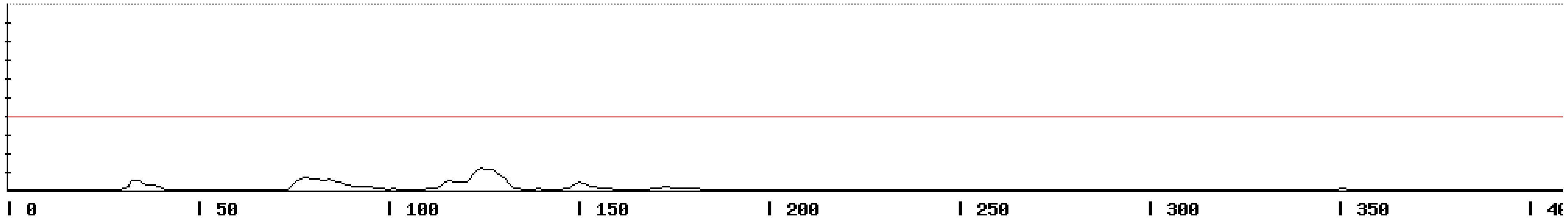

Predictions for Nelumbonucifera_NNU_008679-RA

Viterbi Path

Viterbi Path

Posterior @ 0.4

Posterior @ 0.4

Posterior Graphic. Threshold Marked @ 0.4

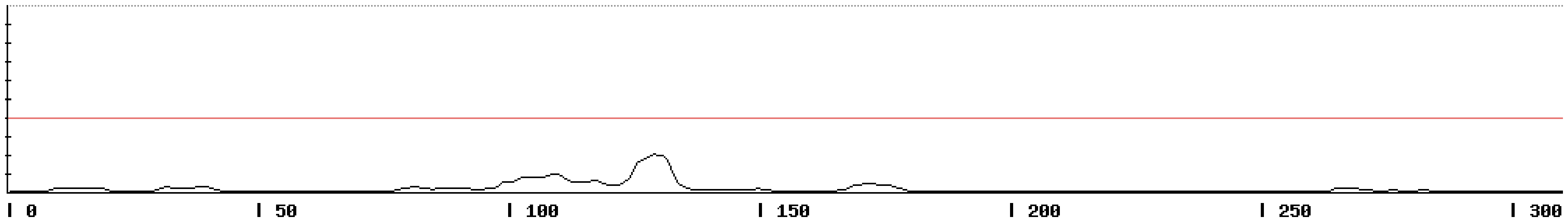

Predictions for Nelumbonucifera_NNU_008894-RA

Viterbi Path

Viterbi Path

Posterior @ 0.4

Posterior @ 0.4

Posterior Graphic. Threshold Marked @ 0.4

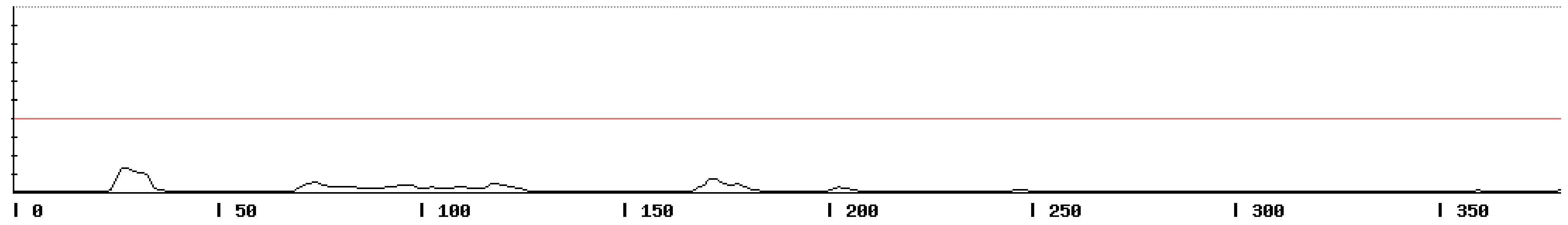

Predictions for Nelumbonucifera_NNU_009408-RA

Viterbi Path

Viterbi Path

Posterior @ 0.4

Posterior @ 0.4

Posterior Graphic. Threshold Marked @ 0.4

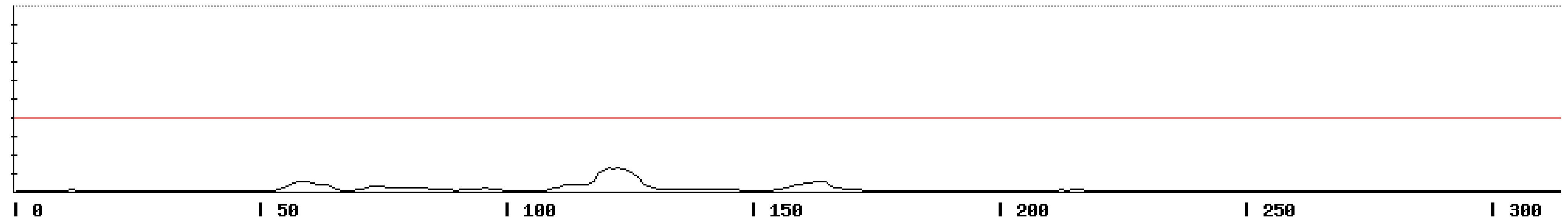

Predictions for Nelumbonucifera_NNU_009645-RA

Viterbi Path

Viterbi Path

Posterior @ 0.4

Posterior @ 0.4

Posterior Graphic. Threshold Marked @ 0.4

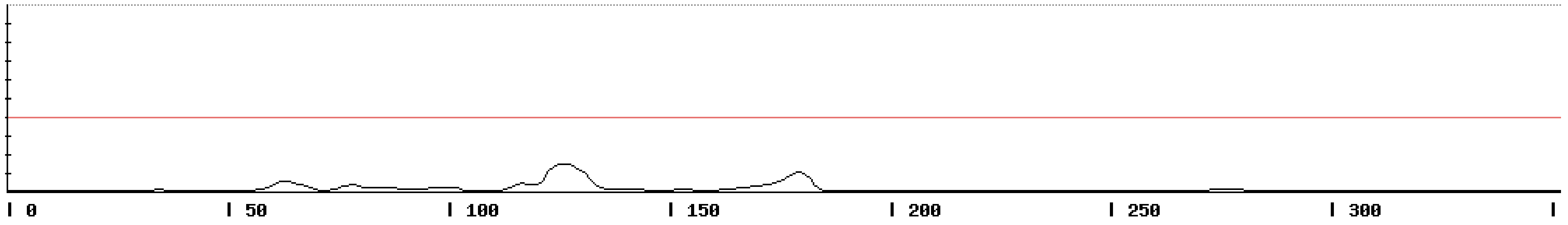

Predictions for Nelumbonucifera_NNU_009671-RA

Viterbi Path

Viterbi Path

Posterior @ 0.4

139 - RKRRKV - 144

159 - GKTR - 162

Posterior @ 0.4

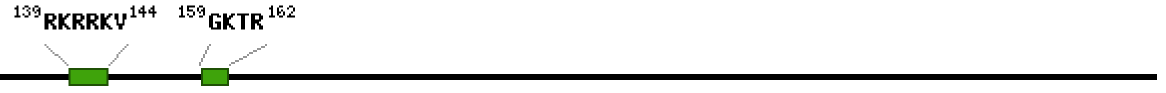

Posterior Graphic. Threshold Marked @ 0.4

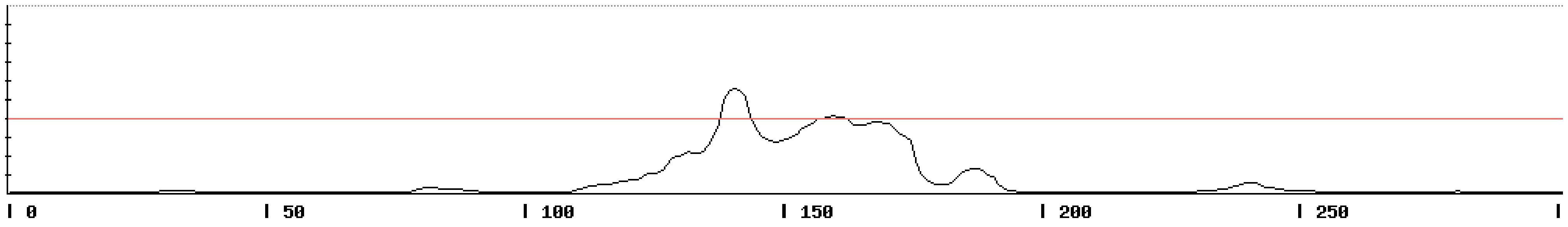

Predictions for Nelumbonucifera_NNU_009756-RA

Viterbi Path	
Viterbi Path	
Posterior @ 0.4	133 - RKK - 135
Posterior @ 0.4	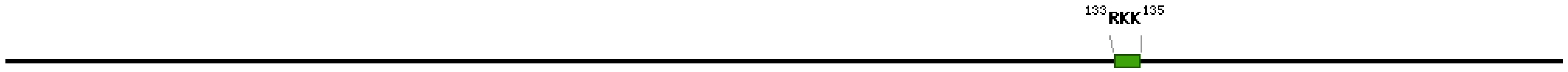
Posterior Graphic. Threshold Marked @ 0.4	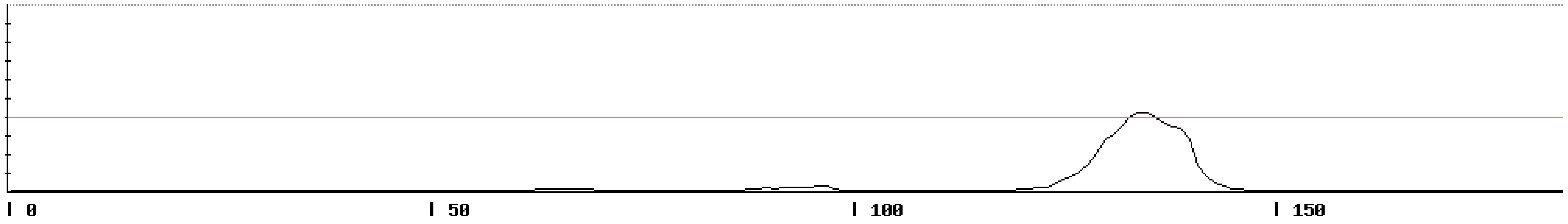

Predictions for Nelumbonucifera_NNU_009790-RA

Viterbi Path	
Viterbi Path	
Posterior @ 0.4	
Posterior @ 0.4	

Posterior Graphic. Threshold Marked @ 0.4

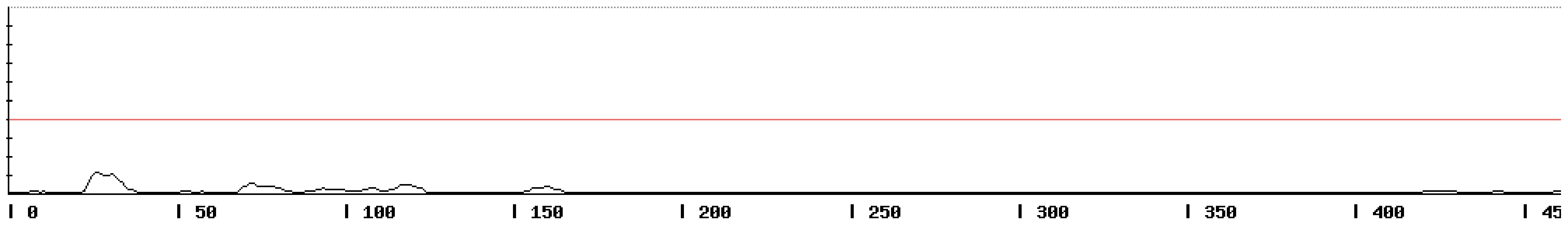

Predictions for Nelumbonucifera_NNU_009951-RA

Viterbi Path	
Viterbi Path	
Posterior @ 0.4	
Posterior @ 0.4	
Posterior Graphic. Threshold Marked @ 0.4	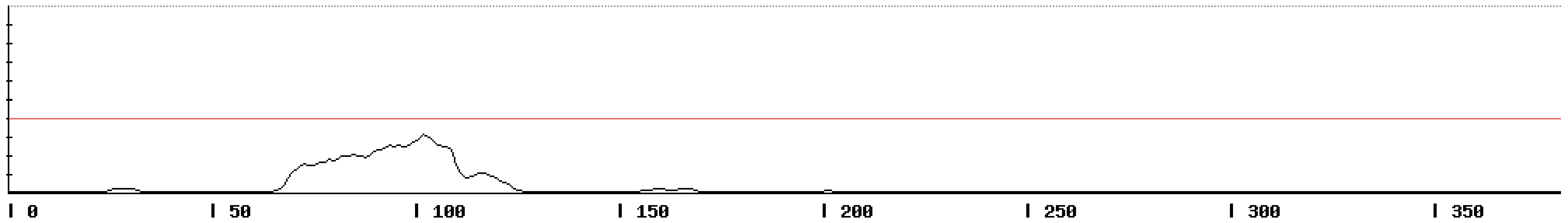

Predictions for Nelumbonucifera_NNU_010152-RA

Viterbi Path	
Viterbi Path	
Posterior @ 0.4	
Posterior @ 0.4	

Posterior Graphic. Threshold Marked @ 0.4

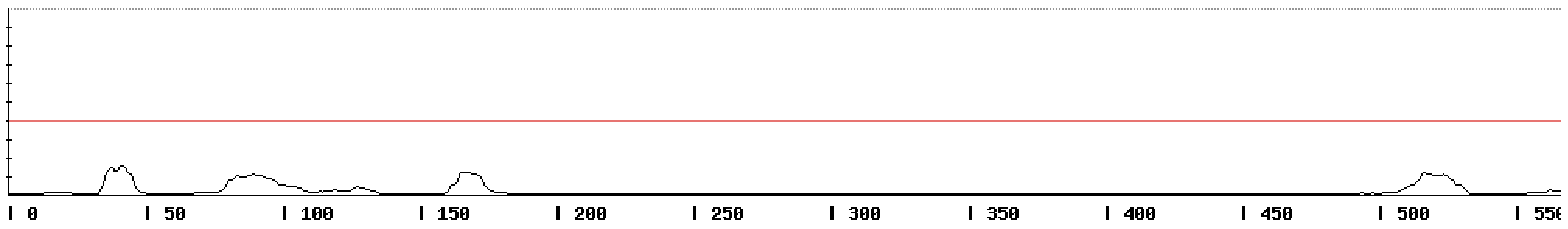

Predictions for Nelumbonucifera_NNU_010292-RA

Viterbi Path

Viterbi Path

Posterior @ 0.4

Posterior @ 0.4

Posterior Graphic. Threshold Marked @ 0.4

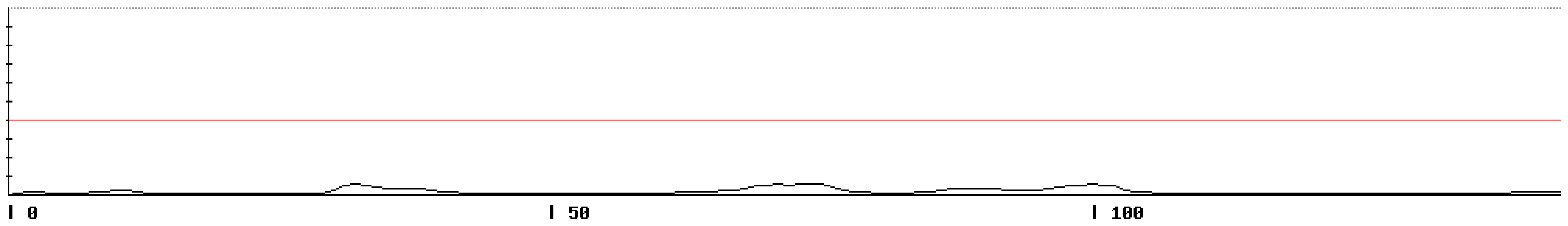

Predictions for Nelumbonucifera_NNU_010910-RA

Viterbi Path

Viterbi Path

Posterior @ 0.4

Posterior @ 0.4

Posterior Graphic. Threshold Marked @ 0.4

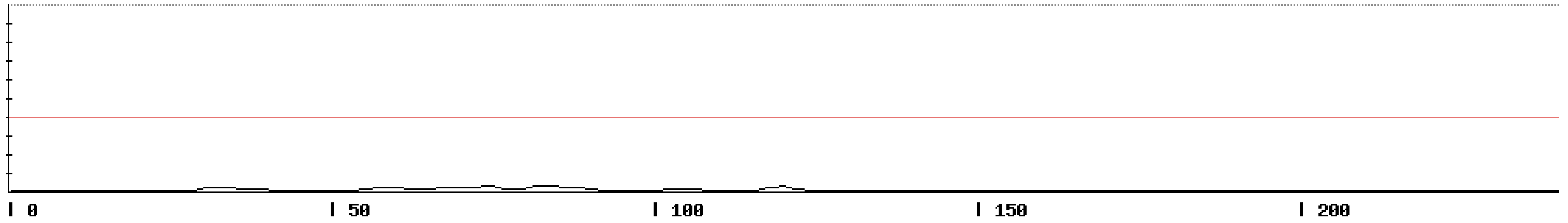

Predictions for Nelumbonucifera_NNU_011308-RA

Viterbi Path

Viterbi Path

Posterior @ 0.4 — 34 - KKSKNGSR - 41

Posterior @ 0.4

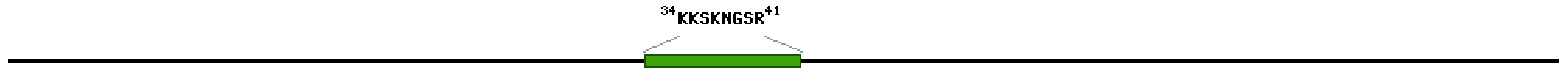

Posterior Graphic. Threshold Marked @ 0.4

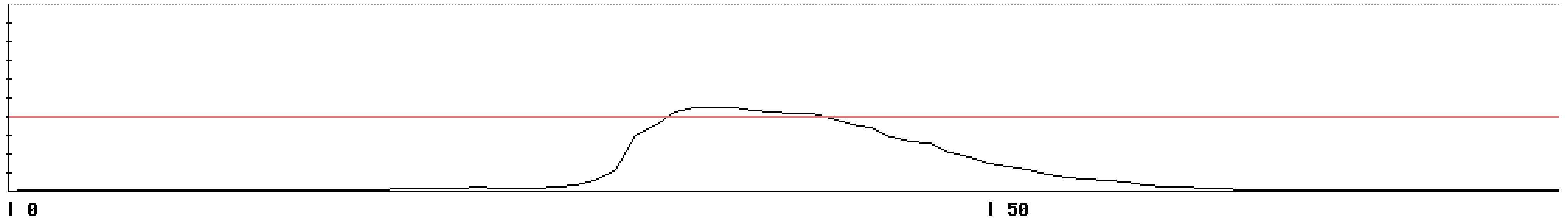

Predictions for Nelumbonucifera_NNU_011383-RA

Viterbi Path

Viterbi Path

Posterior @ 0.4	
Posterior @ 0.4	
Posterior Graphic. Threshold Marked @ 0.4	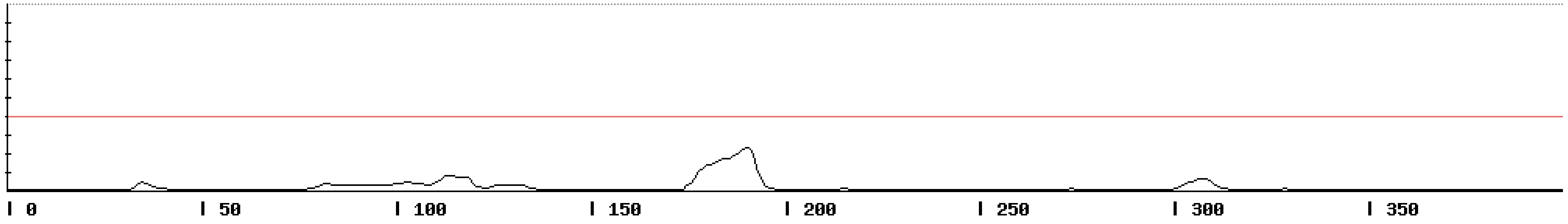

Predictions for Nelumbonucifera_NNU_011384-RA	
Viterbi Path	
Viterbi Path	
Posterior @ 0.4	
Posterior @ 0.4	
Posterior Graphic. Threshold Marked @ 0.4	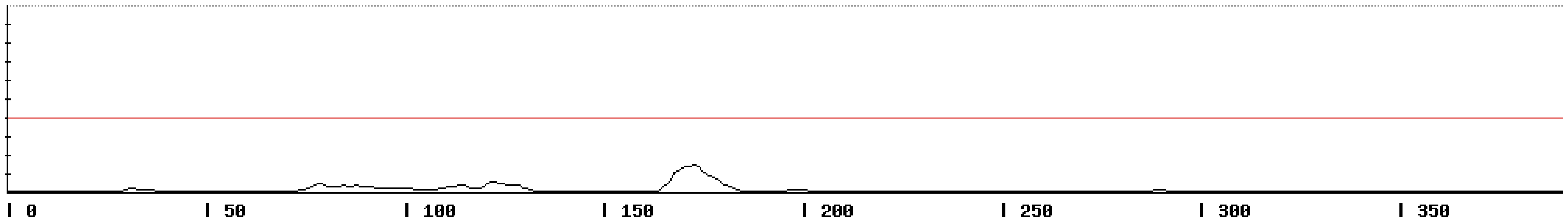

Predictions for Nelumbonucifera_NNU_011890-RA

Viterbi Path	
Viterbi Path	
Posterior @ 0.4	
Posterior @ 0.4	
Posterior Graphic. Threshold Marked @ 0.4	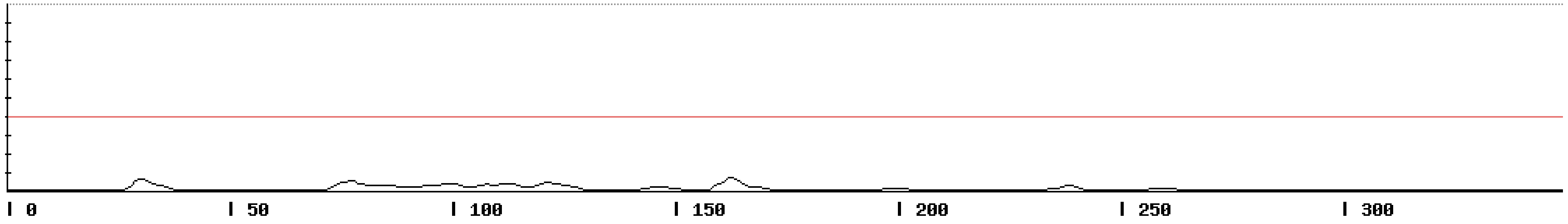

Predictions for Nelumbonucifera_NNU_012069-RA

Viterbi Path	
Viterbi Path	
Posterior @ 0.4	
Posterior @ 0.4	
Posterior Graphic. Threshold Marked @ 0.4	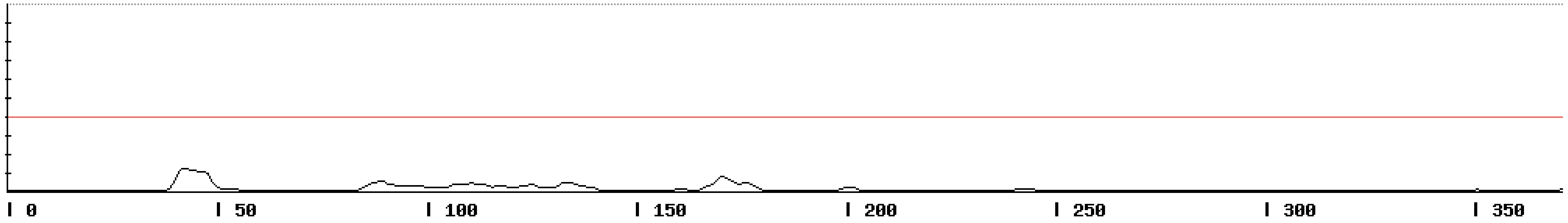

Predictions for Nelumbonucifera_NNU_012630-RA

Viterbi Path

Viterbi Path

Posterior @ 0.4

Posterior @ 0.4

Posterior Graphic. Threshold Marked @ 0.4

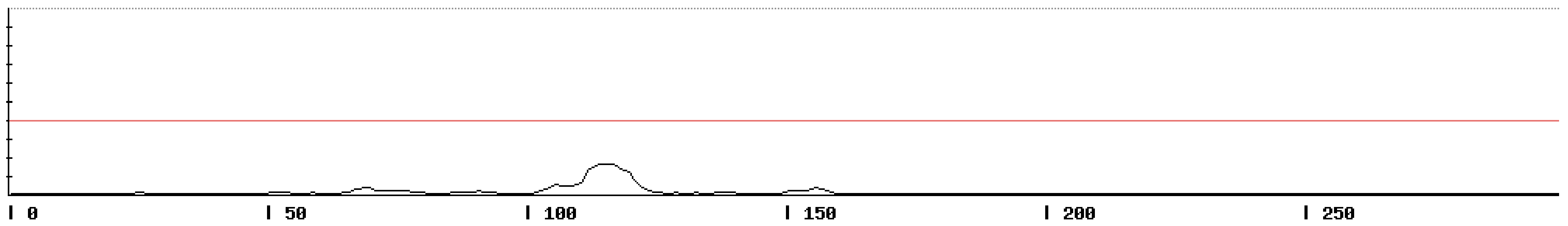

Predictions for Nelumbonucifera_NNU_013288-RA

Viterbi Path

Viterbi Path

Posterior @ 0.4

Posterior @ 0.4

Posterior Graphic. Threshold Marked @ 0.4

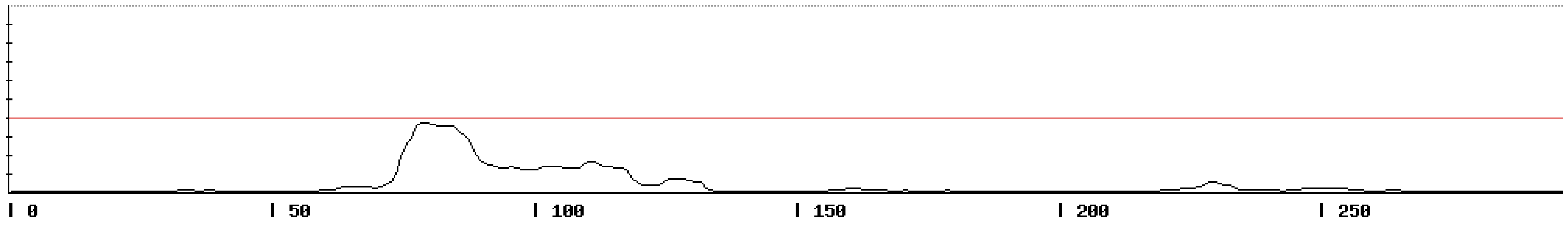

Predictions for Nelumbonucifera_NNU_013383-RA	
Viterbi Path	
Viterbi Path	
Posterior @ 0.4	
Posterior @ 0.4	
Posterior Graphic. Threshold Marked @ 0.4	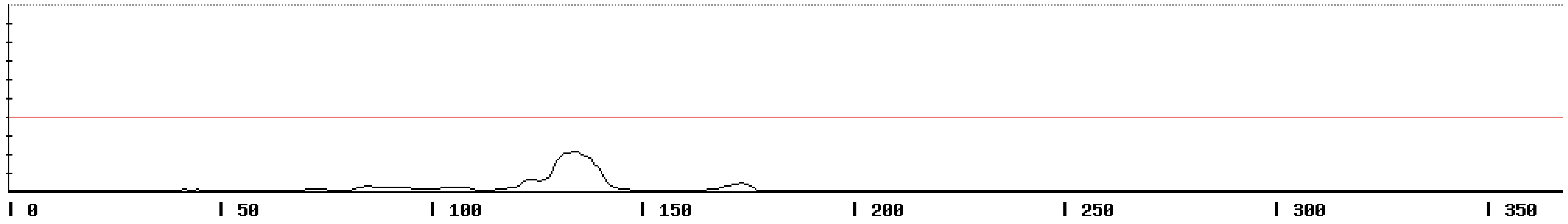

Predictions for Nelumbonucifera_NNU_013929-RA	
Viterbi Path	
Viterbi Path	
Posterior @ 0.4	
Posterior @ 0.4	

Posterior Graphic. Threshold Marked @ 0.4

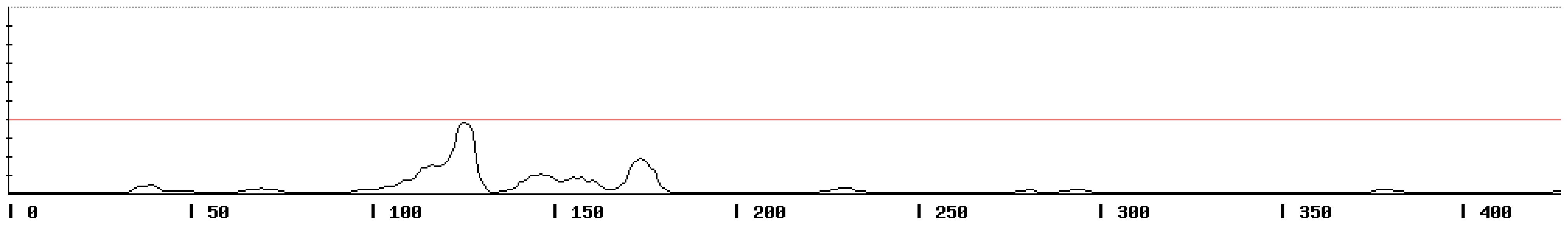

Predictions for Nelumbonucifera_NNU_014031-RA

Viterbi Path

Viterbi Path

Posterior @ 0.4

Posterior @ 0.4

Posterior Graphic. Threshold Marked @ 0.4

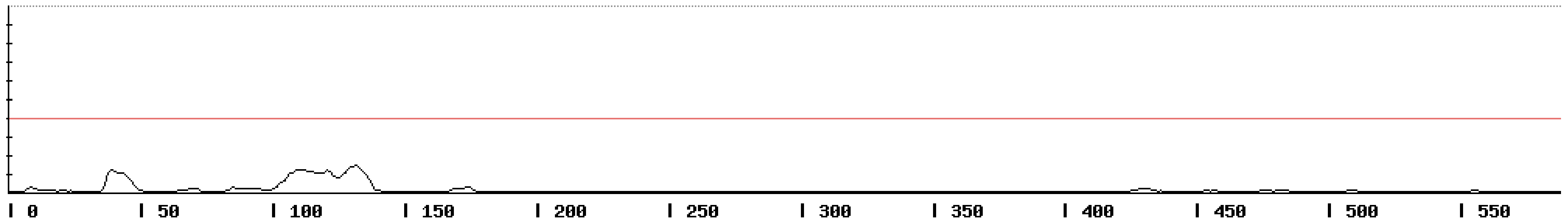

Predictions for Nelumbonucifera_NNU_014110-RA

Viterbi Path

Viterbi Path

Posterior @ 0.4

Posterior @ 0.4

Posterior Graphic. Threshold Marked @ 0.4

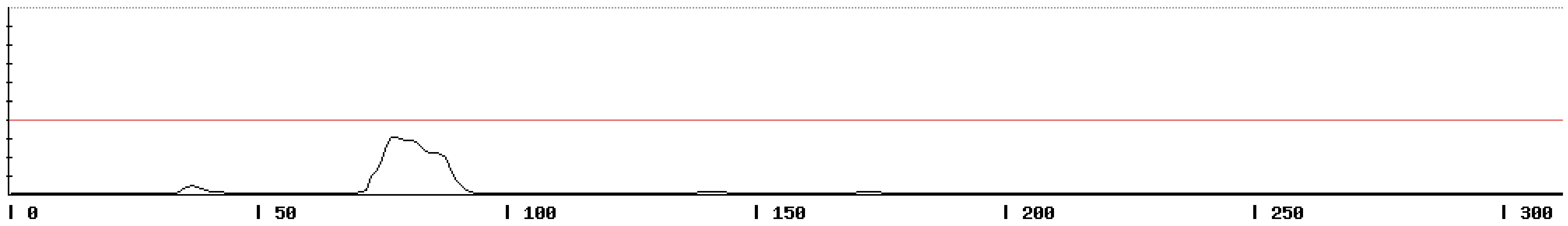

Predictions for Nelumbonucifera_NNU_014940-RA

Viterbi Path

Viterbi Path

Posterior @ 0.4

Posterior @ 0.4

Posterior Graphic. Threshold Marked @ 0.4

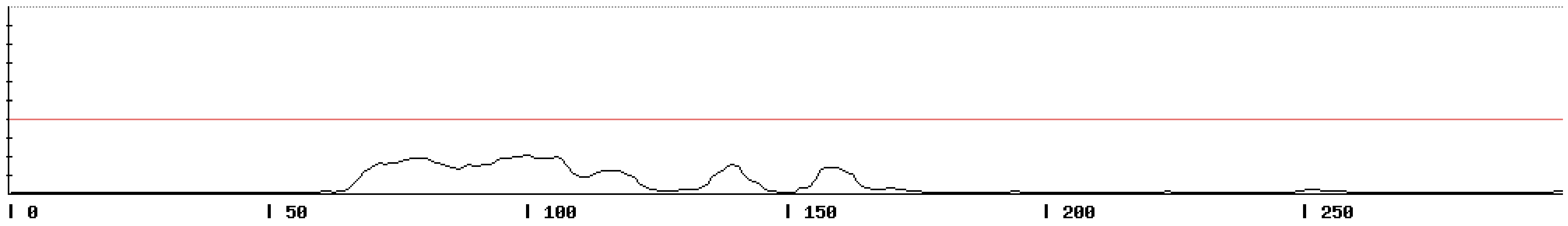

Predictions for Nelumbonucifera_NNU_015012-RA

Viterbi Path

Viterbi Path

Posterior @ 0.4

Posterior @ 0.4

Posterior Graphic. Threshold Marked @ 0.4

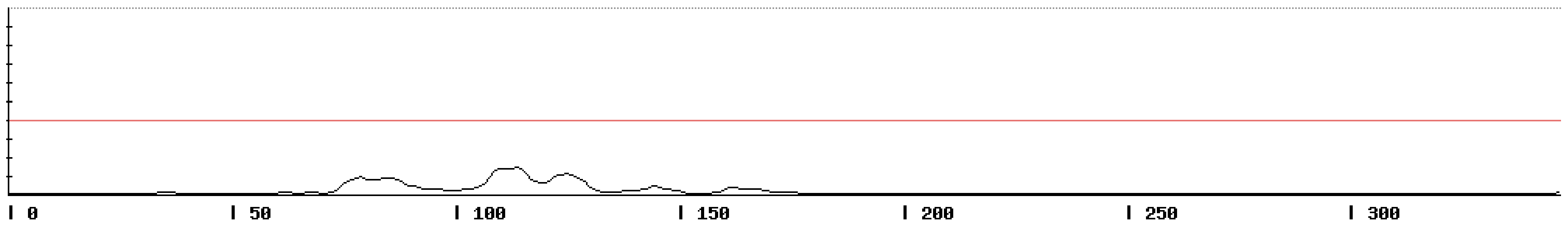

Predictions for Nelumbonucifera_NNU_015017-RA

Viterbi Path

Viterbi Path

Posterior @ 0.4

Posterior @ 0.4

Posterior Graphic. Threshold Marked @ 0.4

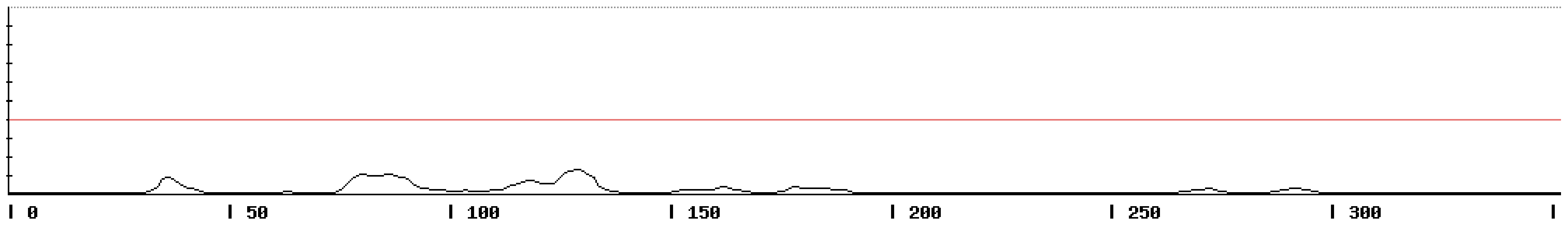

Predictions for Nelumbonucifera_NNU_015672-RA

Viterbi Path

Viterbi Path

Posterior @ 0.4

Posterior @ 0.4

Posterior Graphic. Threshold Marked @ 0.4

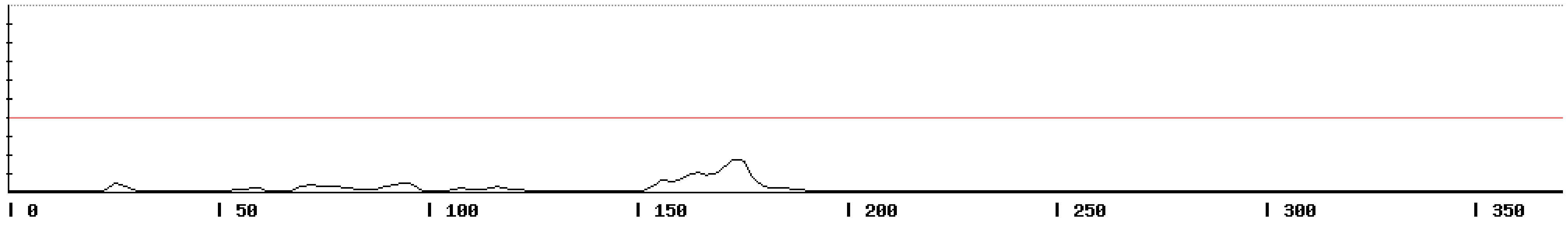

Predictions for Nelumbonucifera_NNU_015908-RA

Viterbi Path

Viterbi Path

Posterior @ 0.4

Posterior @ 0.4

Posterior Graphic. Threshold Marked @ 0.4

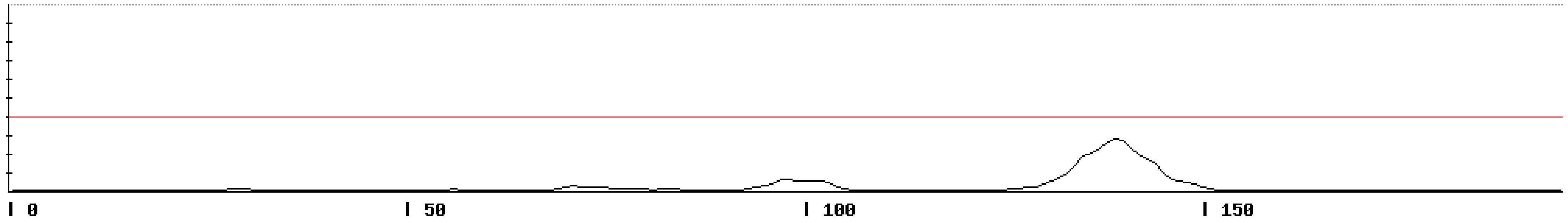

Predictions for Nelumbonucifera_NNU_015961-RA

Viterbi Path

Viterbi Path

Posterior @ 0.4

Posterior @ 0.4

Posterior Graphic. Threshold Marked @ 0.4

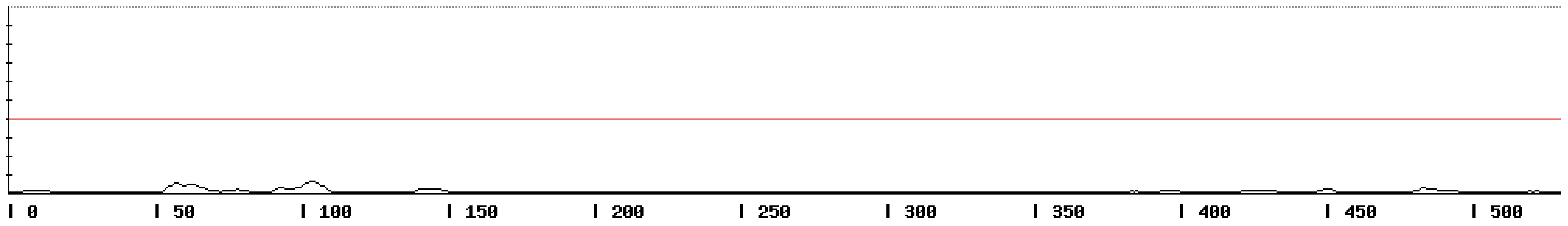

Predictions for Nelumbonucifera_NNU_016007-RA

Viterbi Path

Viterbi Path

Posterior @ 0.4

Posterior @ 0.4

Posterior Graphic. Threshold Marked @ 0.4

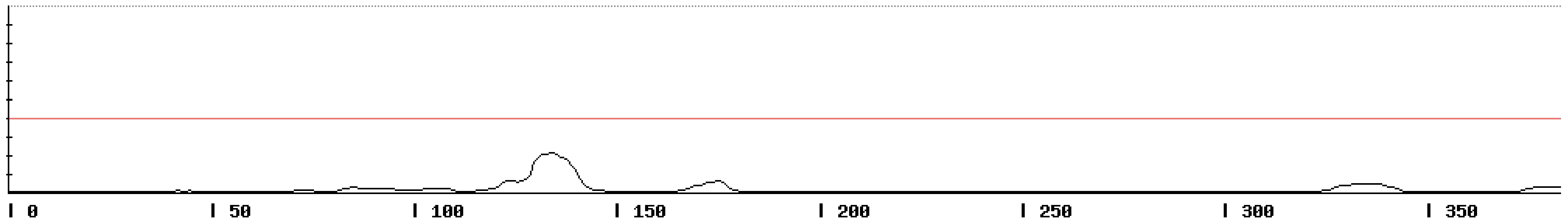

Predictions for Nelumbonucifera_NNU_016252-RA	
Viterbi Path	
Viterbi Path	
Posterior @ 0.4	
Posterior @ 0.4	
Posterior Graphic. Threshold Marked @ 0.4	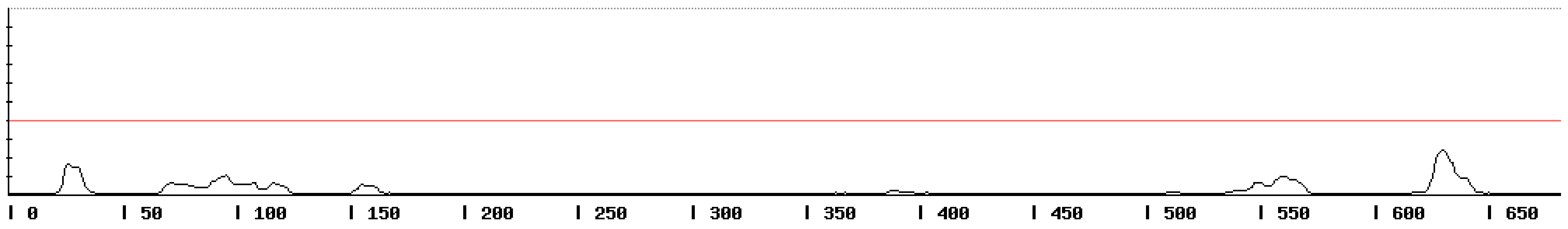

Predictions for Nelumbonucifera_NNU_016253-RA	
Viterbi Path	
Viterbi Path	
Posterior @ 0.4	
Posterior @ 0.4	
Posterior Graphic. Threshold Marked @ 0.4	

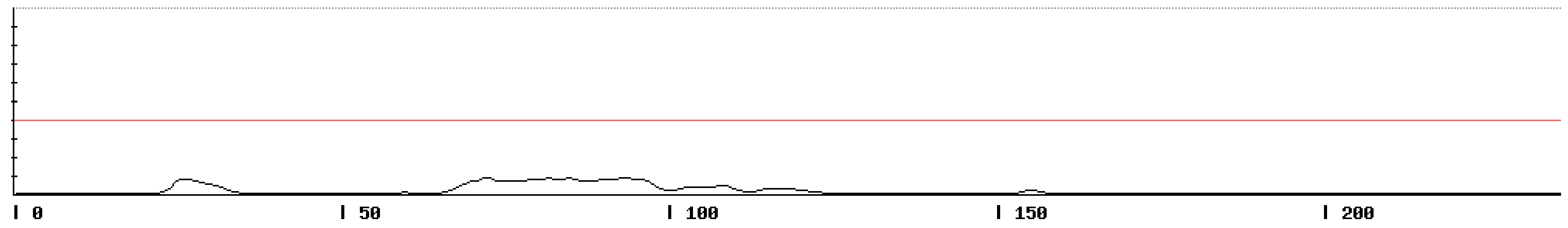

Predictions for Nelumbonucifera_NNU_016572-RA

Viterbi Path	
Viterbi Path	
Posterior @ 0.4	
Posterior @ 0.4	
Posterior Graphic. Threshold Marked @ 0.4	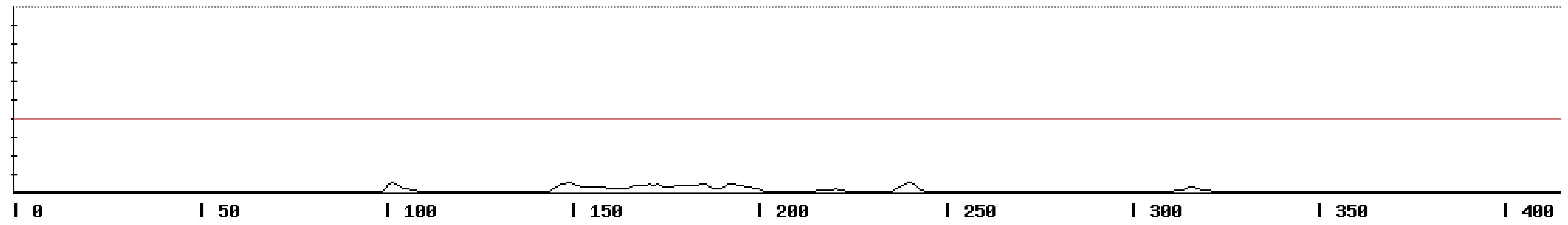

Predictions for Nelumbonucifera_NNU_016870-RA

Viterbi Path	
Viterbi Path	
Posterior @ 0.4	
Posterior @ 0.4	

Posterior Graphic. Threshold Marked @ 0.4

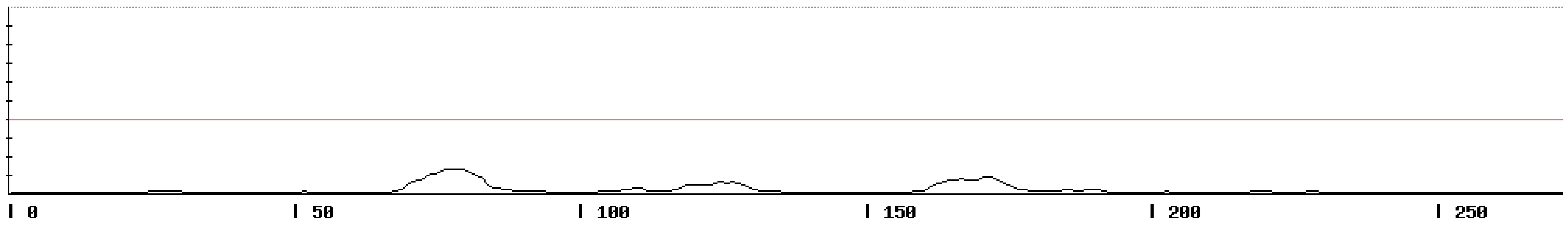

Predictions for Nelumbonucifera_NNU_016873-RA

Viterbi Path

Viterbi Path

Posterior @ 0.4

Posterior @ 0.4

Posterior Graphic. Threshold Marked @ 0.4

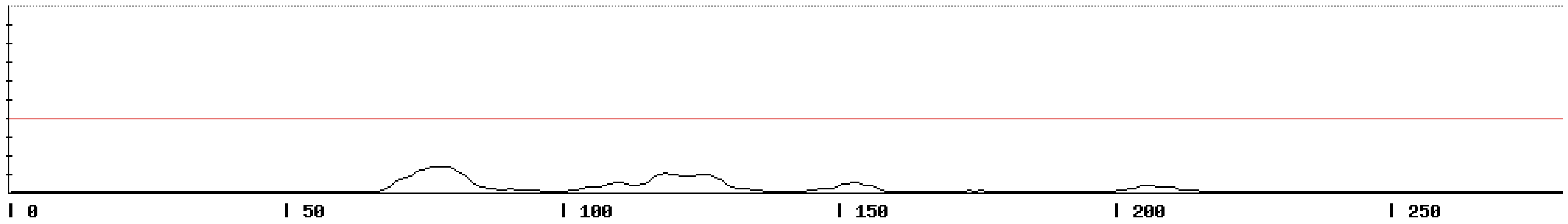

Predictions for Nelumbonucifera_NNU_017147-RA

Viterbi Path

Viterbi Path

Posterior @ 0.4

Posterior @ 0.4

Posterior Graphic. Threshold Marked @ 0.4	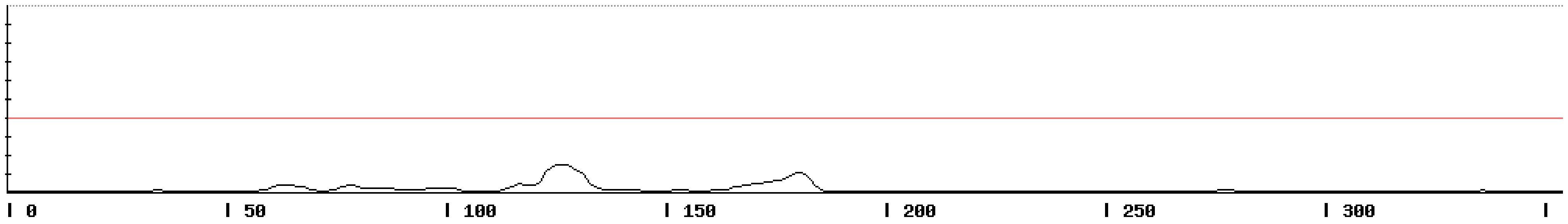

Predictions for Nelumbonucifera_NNU_017245-RA	
Viterbi Path	
Viterbi Path	
Posterior @ 0.4	
Posterior @ 0.4	
Posterior Graphic. Threshold Marked @ 0.4	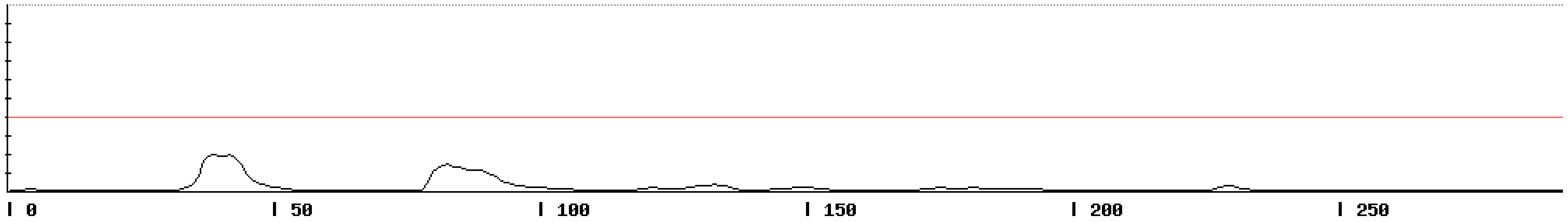

Predictions for Nelumbonucifera_NNU_017396-RA	
Viterbi Path	
Viterbi Path	

Posterior @ 0.4

Posterior @ 0.4

Posterior Graphic. Threshold Marked @ 0.4

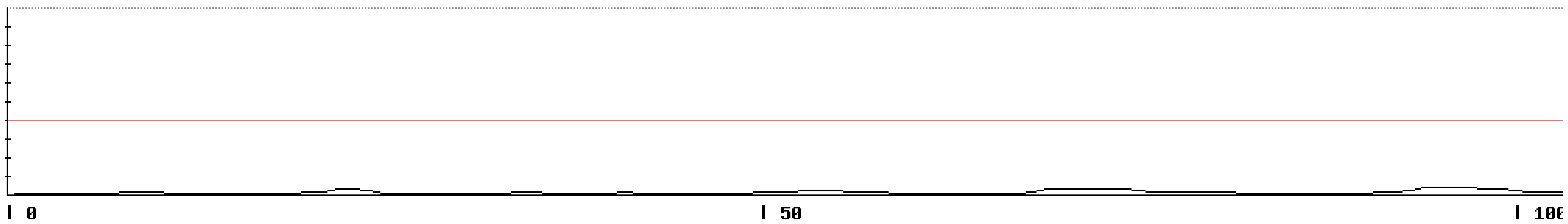

Predictions for Nelumbonucifera_NNU_019157-RA

Viterbi Path

Viterbi Path

Posterior @ 0.4

Posterior @ 0.4

Posterior Graphic. Threshold Marked @ 0.4

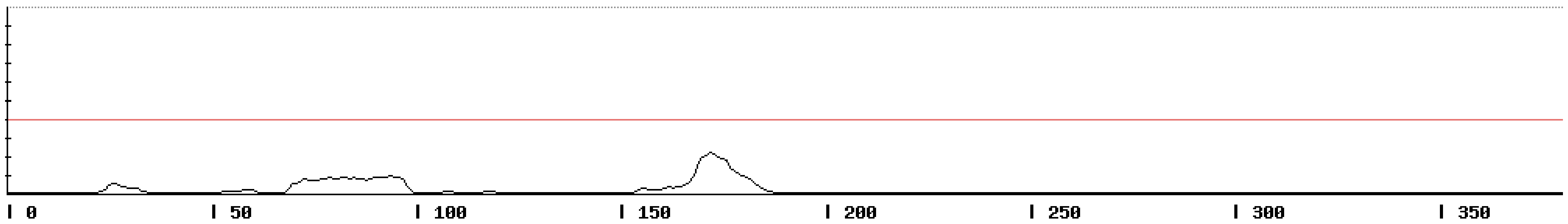

Predictions for Nelumbonucifera_NNU_019341-RA

Viterbi Path

Viterbi Path

Posterior @ 0.4

Posterior @ 0.4

Posterior Graphic. Threshold Marked @ 0.4

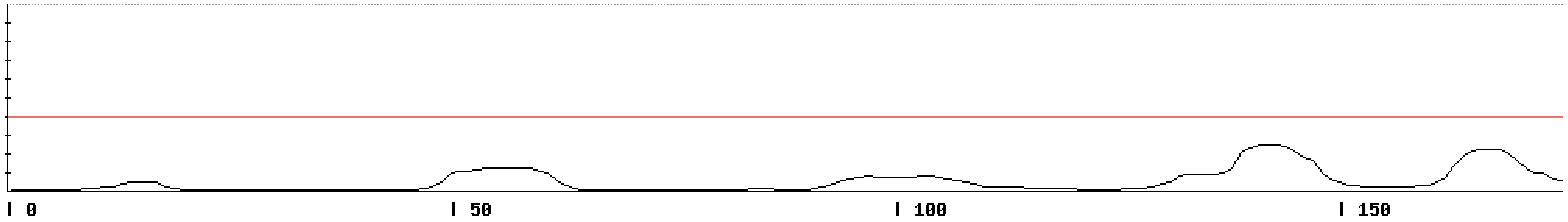

Predictions for Nelumbonucifera_NNU_019375-RA

Viterbi Path

Viterbi Path

Posterior @ 0.4

Posterior @ 0.4

Posterior Graphic. Threshold Marked @ 0.4

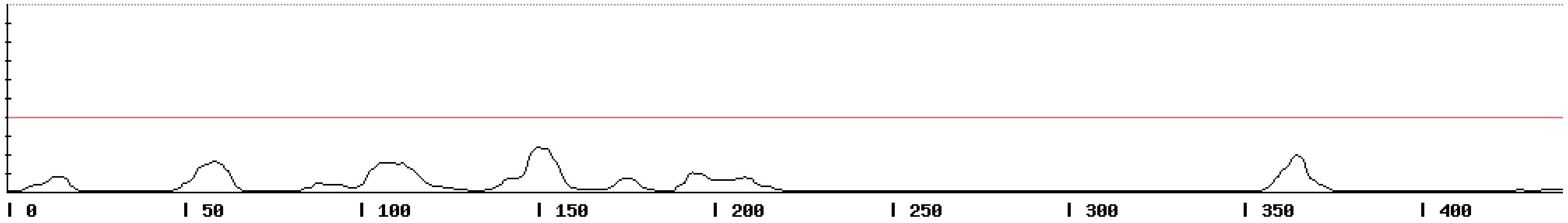

Predictions for Nelumbonucifera_NNU_019386-RA

Viterbi Path	
Viterbi Path	
Posterior @ 0.4	
Posterior @ 0.4	
Posterior Graphic. Threshold Marked @ 0.4	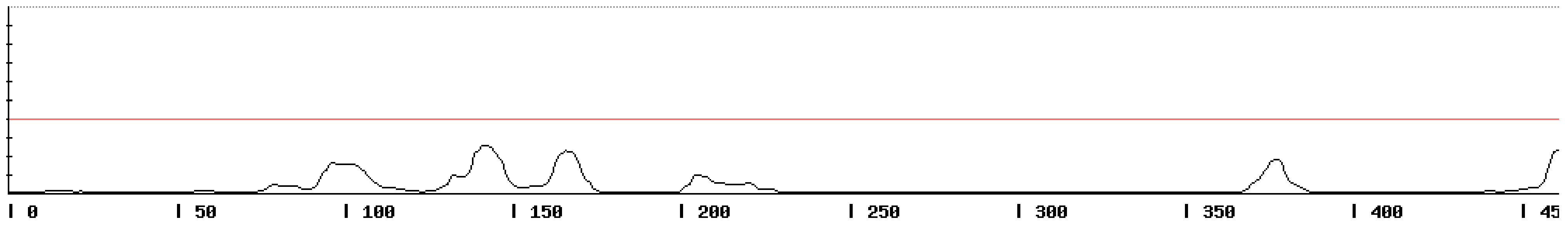

Predictions for Nelumbonucifera_NNU_019622-RA

Viterbi Path	
Viterbi Path	
Posterior @ 0.4	
Posterior @ 0.4	
Posterior Graphic. Threshold Marked @ 0.4	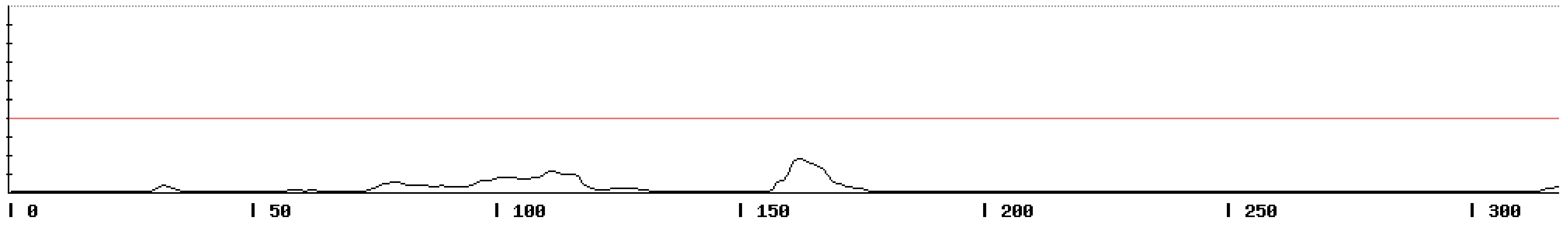

Predictions for Nelumbonucifera_NNU_019757-RA	
Viterbi Path	
Viterbi Path	
Posterior @ 0.4	
Posterior @ 0.4	
Posterior Graphic. Threshold Marked @ 0.4	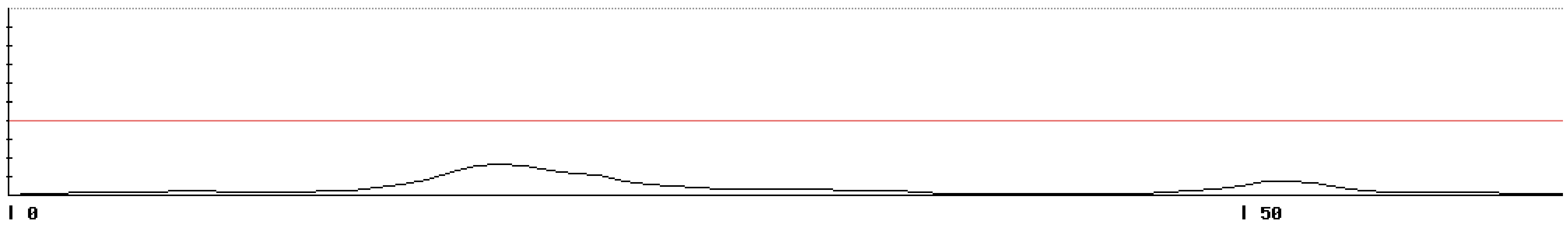

Predictions for Nelumbonucifera_NNU_019843-RA	
Viterbi Path	
Viterbi Path	
Posterior @ 0.4	
Posterior @ 0.4	
Posterior Graphic. Threshold Marked @ 0.4	

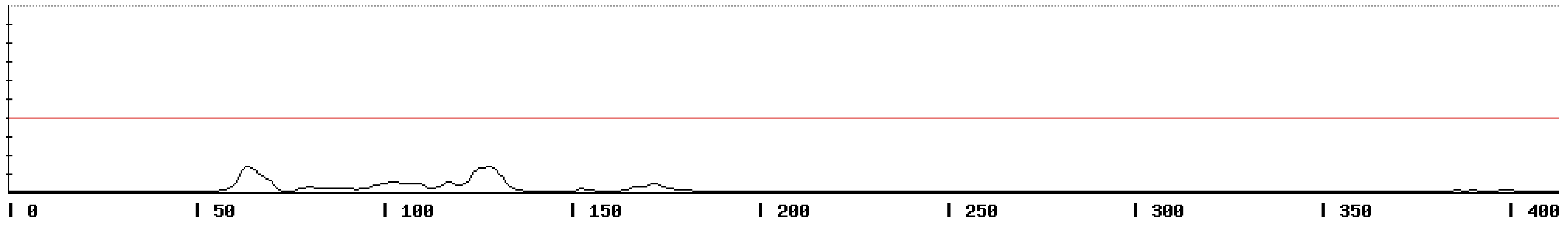

Predictions for Nelumbonucifera_NNU_019848-RA

Viterbi Path

Viterbi Path

Posterior @ 0.4

Posterior @ 0.4

Posterior Graphic. Threshold Marked @ 0.4

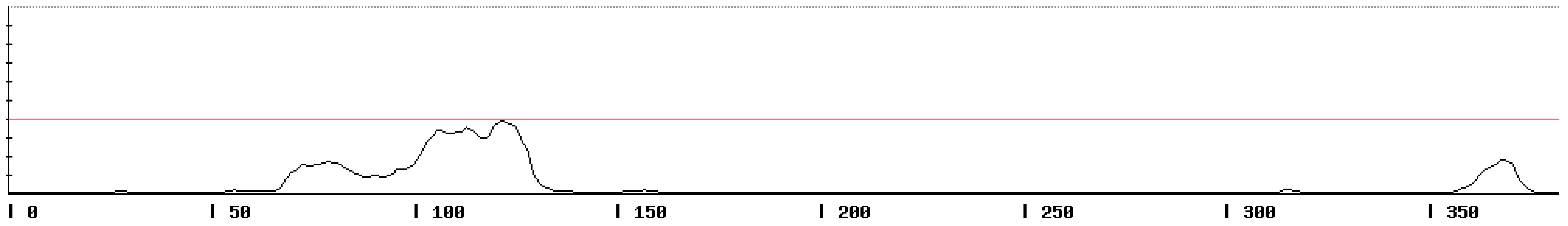

Predictions for Nelumbonucifera_NNU_020244-RA

Viterbi Path

Viterbi Path

Posterior @ 0.4

Posterior @ 0.4

Posterior Graphic. Threshold Marked @ 0.4	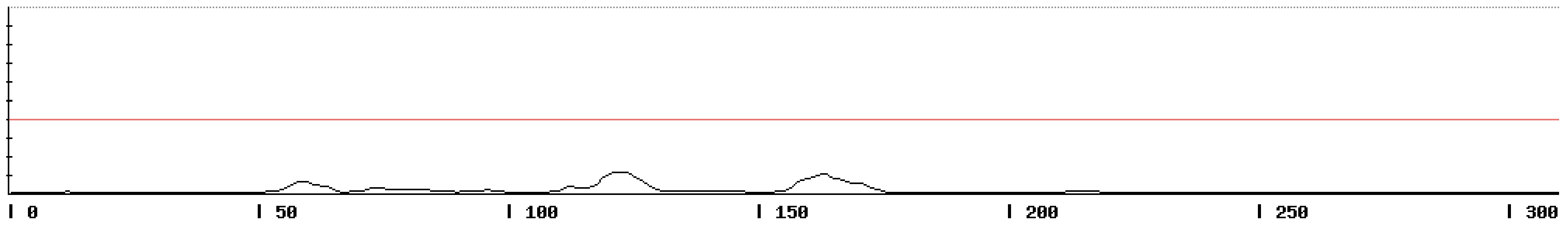

Predictions for Nelumbonucifera_NNU_021044-RA

Viterbi Path	
Viterbi Path	
Posterior @ 0.4	
Posterior @ 0.4	
Posterior Graphic. Threshold Marked @ 0.4	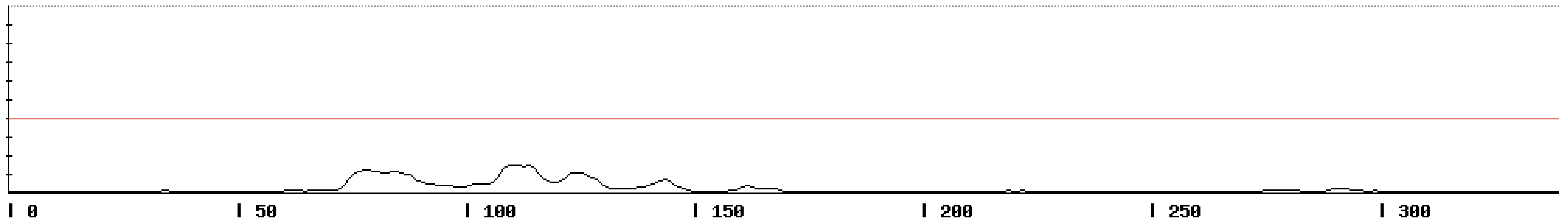

Predictions for Nelumbonucifera_NNU_021046-RA

Viterbi Path	
Viterbi Path	
Posterior @ 0.4	
Posterior @ 0.4	

Posterior Graphic. Threshold Marked @ 0.4

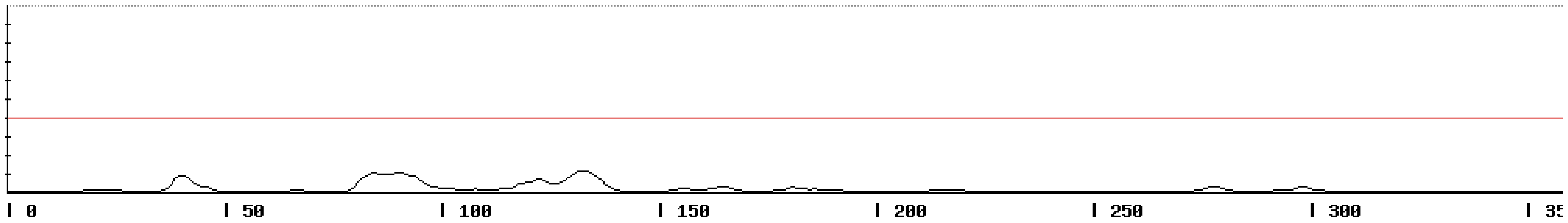

Predictions for Nelumbonucifera_NNU_022098-RA

Viterbi Path

Viterbi Path

Posterior @ 0.4

Posterior @ 0.4

Posterior Graphic. Threshold Marked @ 0.4

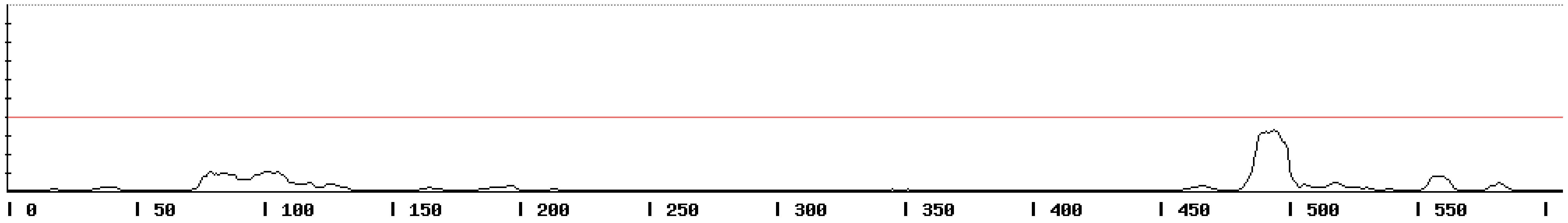

Predictions for Nelumbonucifera_NNU_022570-RA

Viterbi Path

Viterbi Path

Posterior @ 0.4

Posterior @ 0.4

Posterior Graphic. Threshold Marked @ 0.4

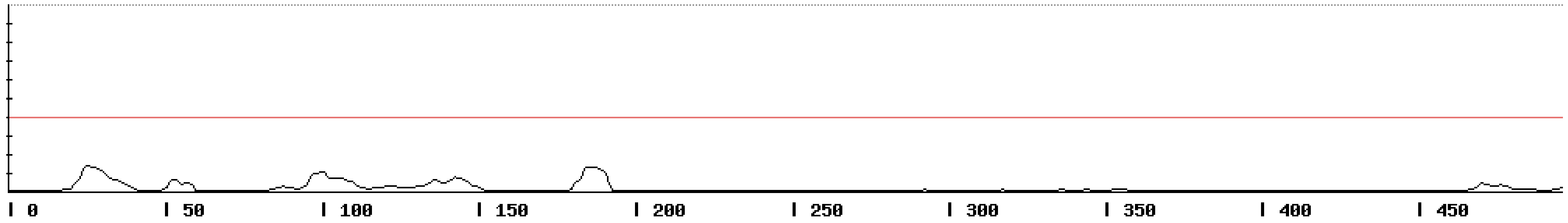

Predictions for Nelumbonucifera_NNU_023112-RA

Viterbi Path

Viterbi Path

Posterior @ 0.4

Posterior @ 0.4

Posterior Graphic. Threshold Marked @ 0.4

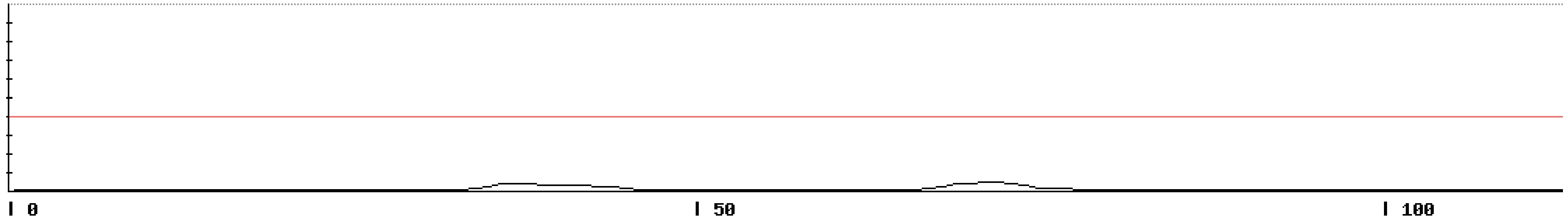

Predictions for Nelumbonucifera_NNU_023337-RA

Viterbi Path

Viterbi Path

Posterior @ 0.4

Posterior @ 0.4

Posterior Graphic. Threshold Marked @ 0.4

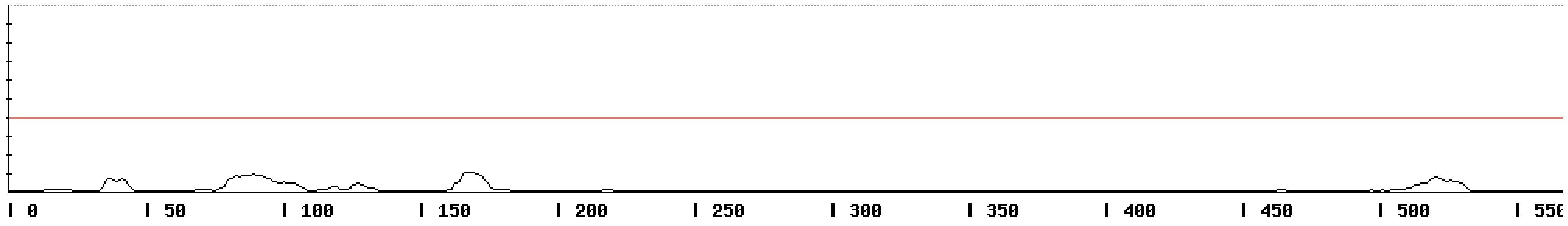

Predictions for Nelumbonucifera_NNU_023364-RA

Viterbi Path

Viterbi Path

Posterior @ 0.4

Posterior @ 0.4

Posterior Graphic. Threshold Marked @ 0.4

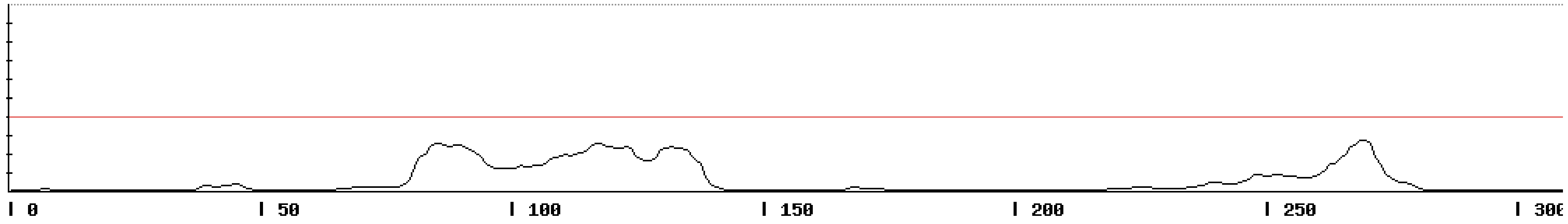

Predictions for Nelumbonucifera_NNU_023472-RA

Viterbi Path

Viterbi Path

Posterior @ 0.4

Posterior @ 0.4

Posterior Graphic. Threshold Marked @ 0.4

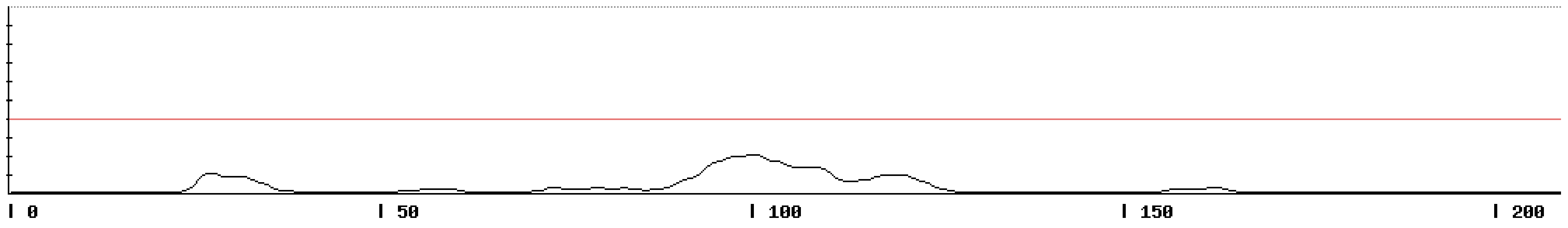

Predictions for Nelumbonucifera_NNU_023668-RA

Viterbi Path

Viterbi Path

Posterior @ 0.4

Posterior @ 0.4

Posterior Graphic. Threshold Marked @ 0.4

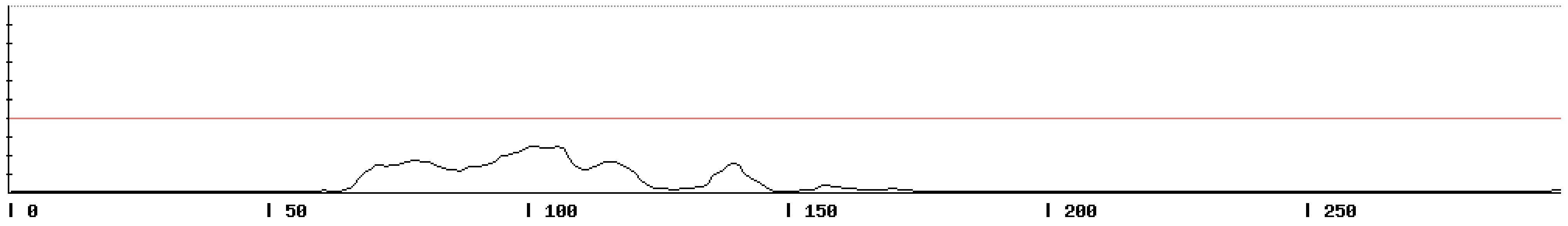

Predictions for Nelumbonucifera_NNU_024112-RA	
Viterbi Path	
Viterbi Path	
Posterior @ 0.4	165 - PWKKSIHKKKSLPRN - 179
Posterior @ 0.4	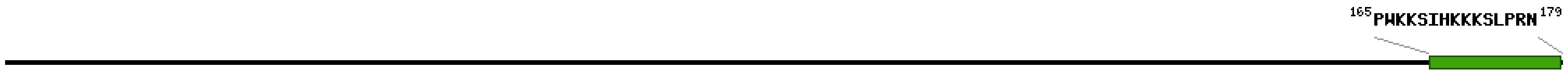
Posterior Graphic. Threshold Marked @ 0.4	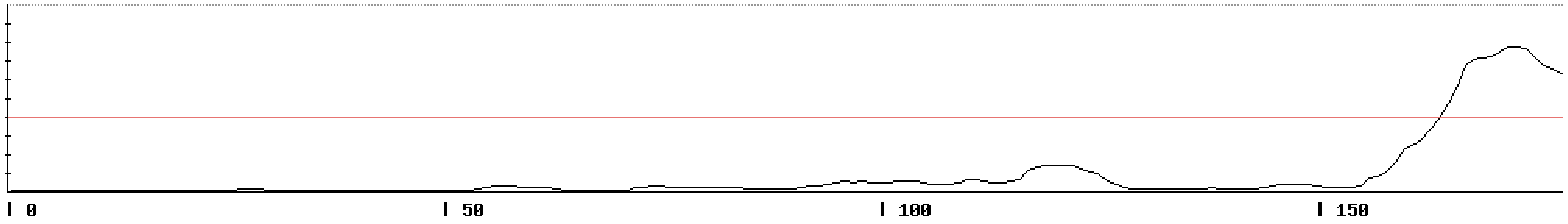

Predictions for Nelumbonucifera_NNU_024330-RA	
Viterbi Path	
Viterbi Path	
Posterior @ 0.4	
Posterior @ 0.4	
Posterior Graphic. Threshold Marked @ 0.4	

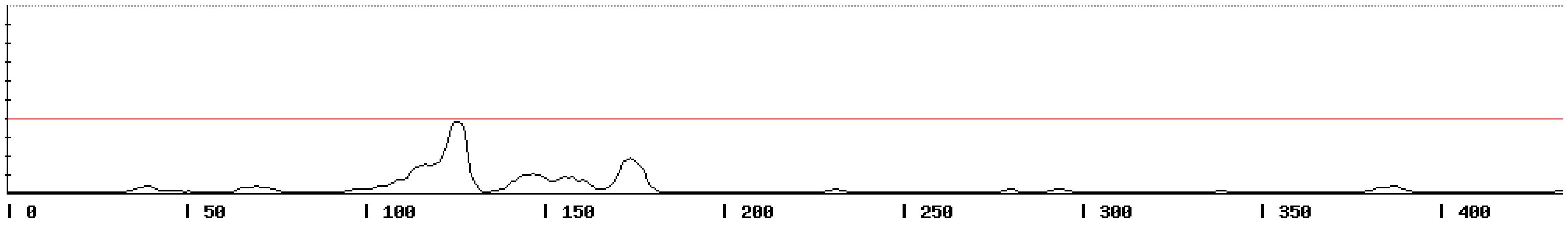

Predictions for Nelumbonucifera_NNU_024939-RA

Viterbi Path	
Viterbi Path	
Posterior @ 0.4	140 - RKRRK - 144
Posterior @ 0.4	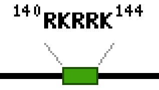
Posterior Graphic. Threshold Marked @ 0.4	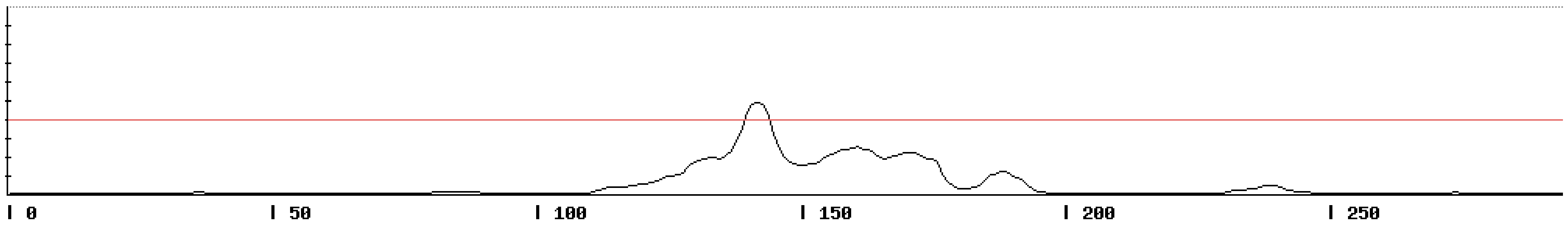

Predictions for Nelumbonucifera_NNU_025210-RA

Viterbi Path	
Viterbi Path	
Posterior @ 0.4	
Posterior @ 0.4	

Posterior Graphic. Threshold Marked @ 0.4

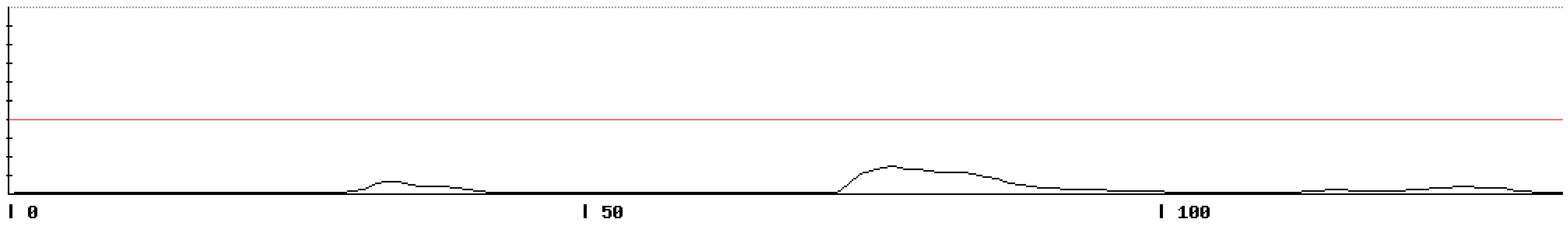

Predictions for Nelumbonucifera_NNU_025766-RA

Viterbi Path

Viterbi Path

Posterior @ 0.4

Posterior @ 0.4

Posterior Graphic. Threshold Marked @ 0.4

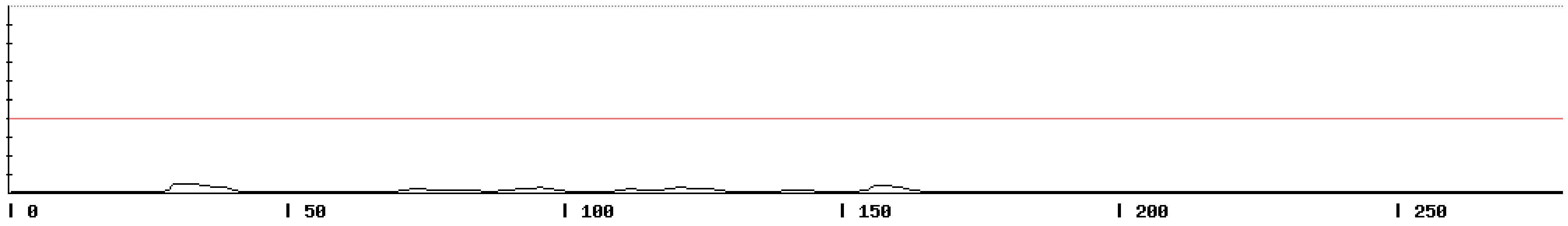

Predictions for Nelumbonucifera_NNU_025861-RA

Viterbi Path

Viterbi Path

Posterior @ 0.4

Posterior @ 0.4

Posterior Graphic. Threshold Marked @ 0.4

Revision r.9 - Last Updated on : February 24 2015 17:45:20.

alex.nguyenba@utoronto.ca

Intended to be used with Firefox

Viterbi Path	
Posterior @ 0.4	82 - QKKEKSRRKSVTRTIGKGIWK - 102
Posterior @ 0.4	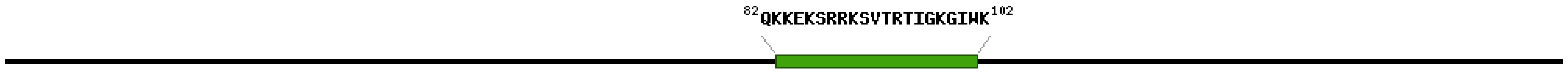
Posterior Graphic. Threshold Marked @ 0.4	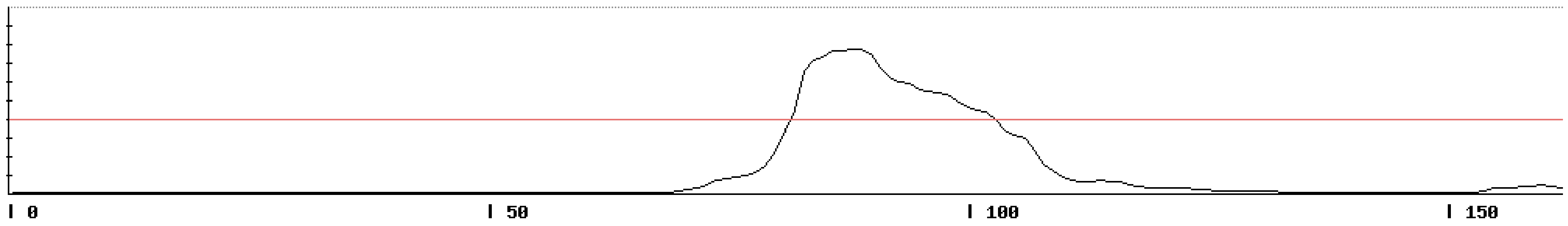

Predictions for Nicotianabenthamiana_Niben101Ctg08087g00001.1

Viterbi Path	
Viterbi Path	
Posterior @ 0.4	69 - RKKNKGR - 75
Posterior @ 0.4	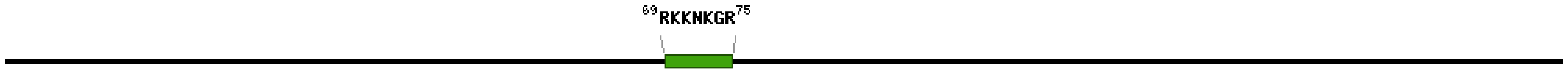
Posterior Graphic. Threshold Marked @ 0.4	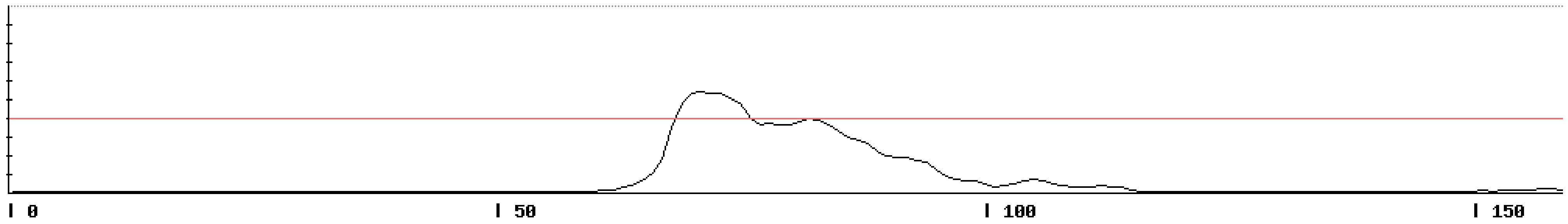

Predictions for Nicotianabenthamiana_Niben101Ctg12301g00003.1

Viterbi Path

Viterbi Path

Posterior @ 0.4

Posterior @ 0.4

Posterior Graphic. Threshold Marked @ 0.4

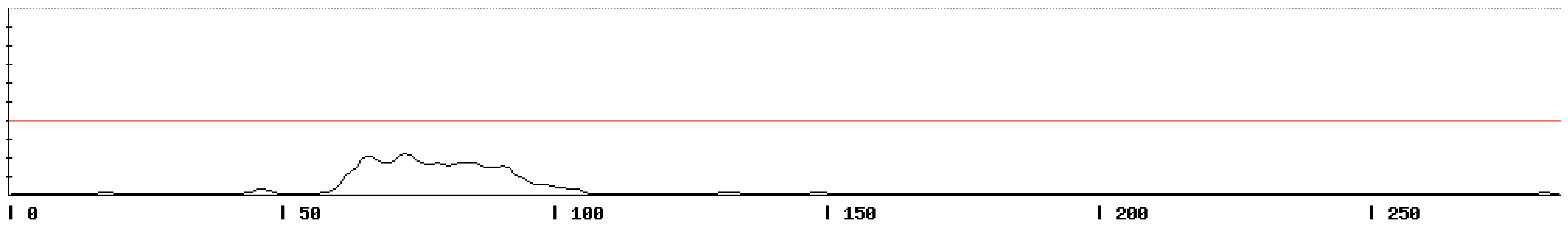

Predictions for Nicotianabenthamiana_Niben101Ctg13774g00001.1

Viterbi Path

Viterbi Path

Posterior @ 0.4

Posterior @ 0.4

Posterior Graphic. Threshold Marked @ 0.4

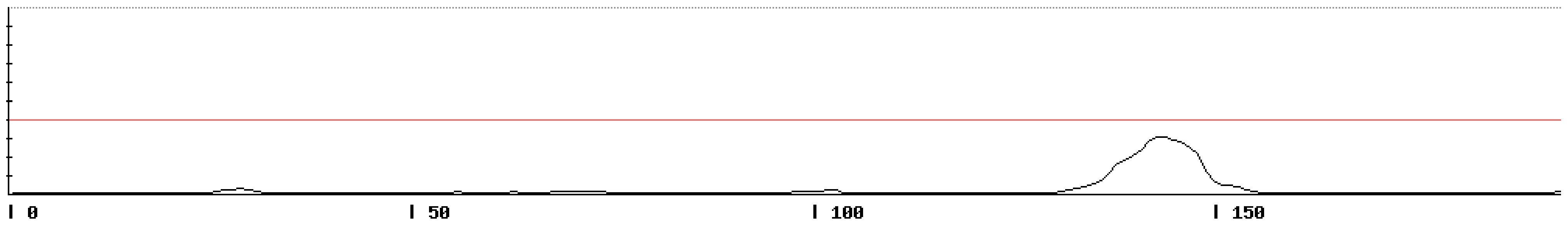

Predictions for Nicotianabenthamiana_Niben101Ctg15807g00002.1

Viterbi Path

Viterbi Path

Posterior @ 0.4

Posterior @ 0.4

Posterior Graphic. Threshold Marked @ 0.4

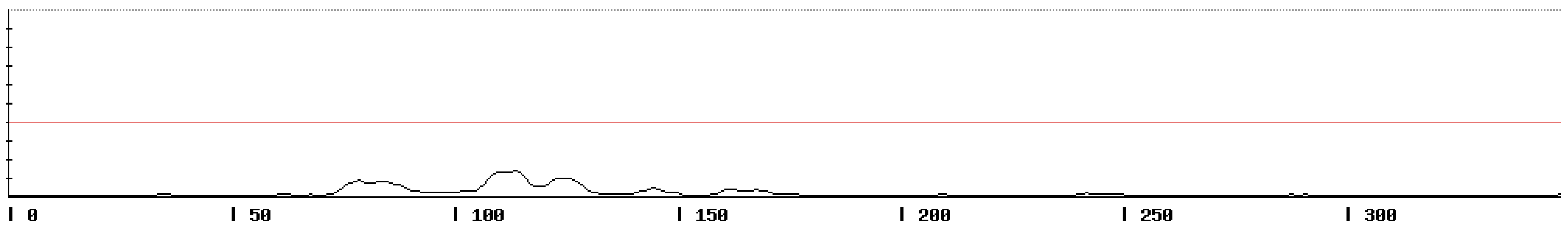

Predictions for Nicotianabenthamiana_Niben101Ctg15860g00004.1

Viterbi Path

Viterbi Path

Posterior @ 0.4

Posterior @ 0.4

Posterior Graphic. Threshold Marked @ 0.4

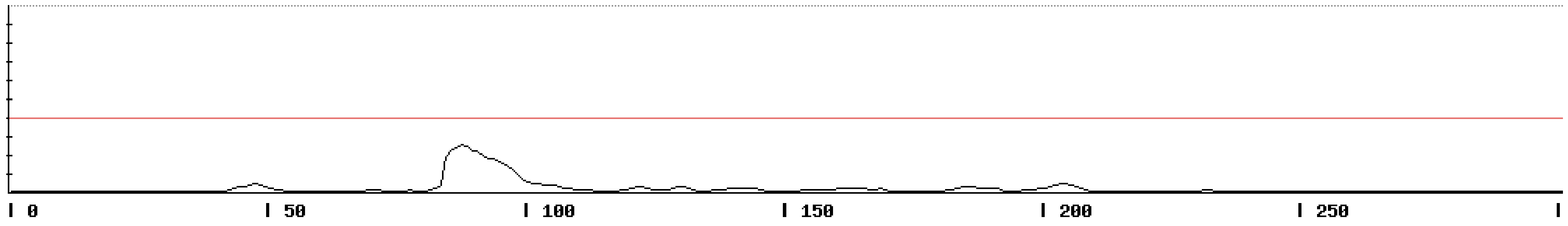

Predictions for Nicotianabenthamiana_Niben101Ctg15918g00002.1

Viterbi Path	
Viterbi Path	
Posterior @ 0.4	
Posterior @ 0.4	
Posterior Graphic. Threshold Marked @ 0.4	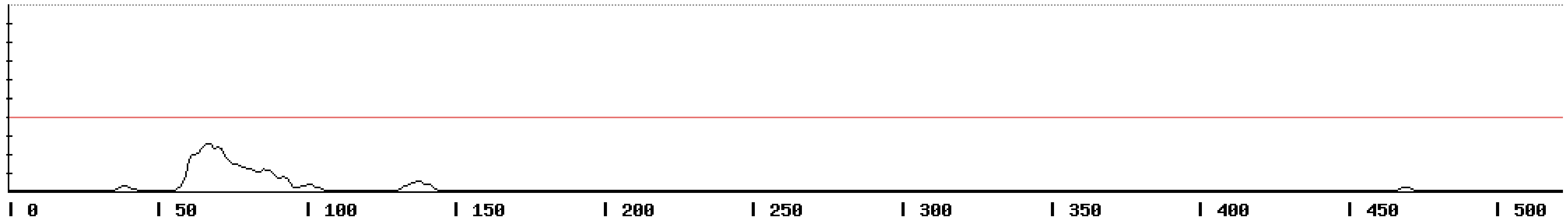

Predictions for Nicotianabenthamiana_Niben101Scf00010g04002.1

Viterbi Path	
Viterbi Path	
Posterior @ 0.4	
Posterior @ 0.4	

Posterior Graphic. Threshold Marked @ 0.4

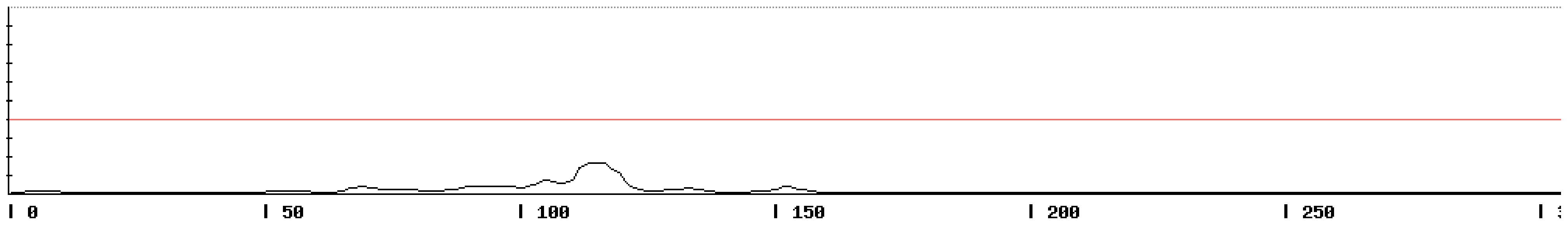

Predictions for Nicotianabenthamiana_Niben101Scf00046g02008.1

Viterbi Path

Viterbi Path

Posterior @ 0.4 130 - GKRKRRKI - 137

Posterior @ 0.4

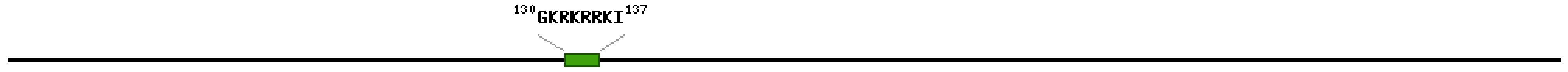

Posterior Graphic. Threshold Marked @ 0.4

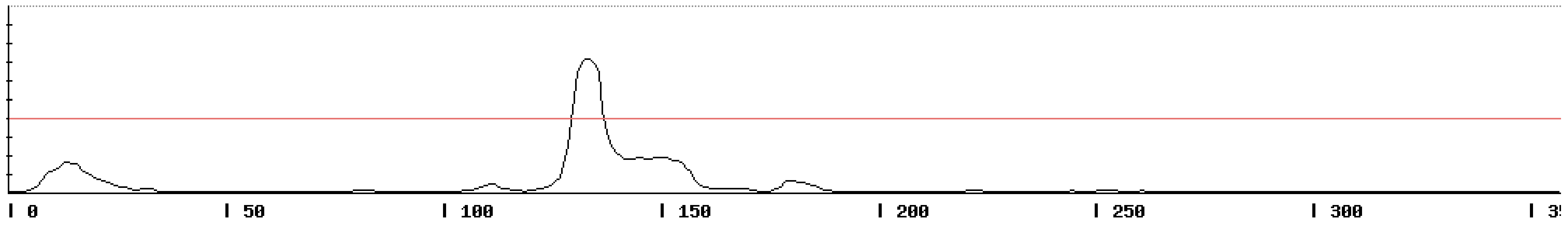

Predictions for Nicotianabenthamiana_Niben101Scf00048g00003.1

Viterbi Path

Viterbi Path

Posterior @ 0.4

Posterior @ 0.4

Posterior Graphic. Threshold Marked @ 0.4

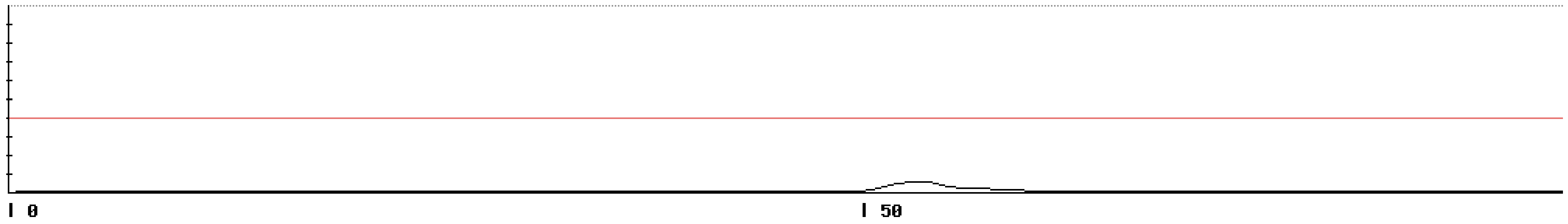

Predictions for Nicotianabenthamiana_Niben101Scf00048g00004.1

Viterbi Path

Viterbi Path

Posterior @ 0.4

Posterior @ 0.4

Posterior Graphic. Threshold Marked @ 0.4

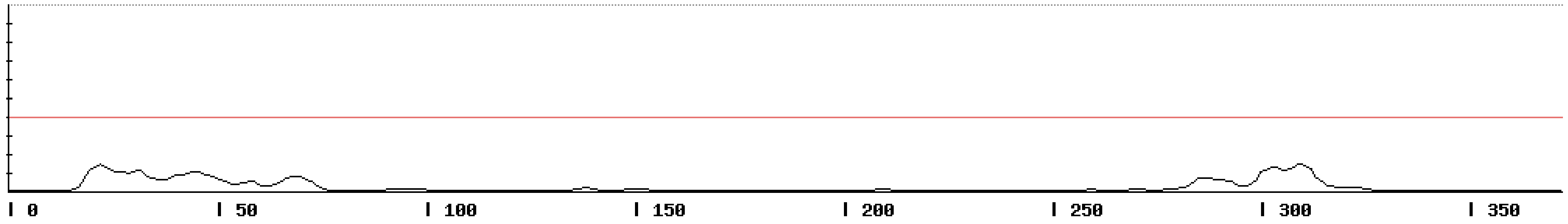

Predictions for Nicotianabenthamiana_Niben101Scf00063g07011.1

Viterbi Path

Viterbi Path

Posterior @ 0.4

Posterior @ 0.4

Posterior Graphic. Threshold Marked @ 0.4

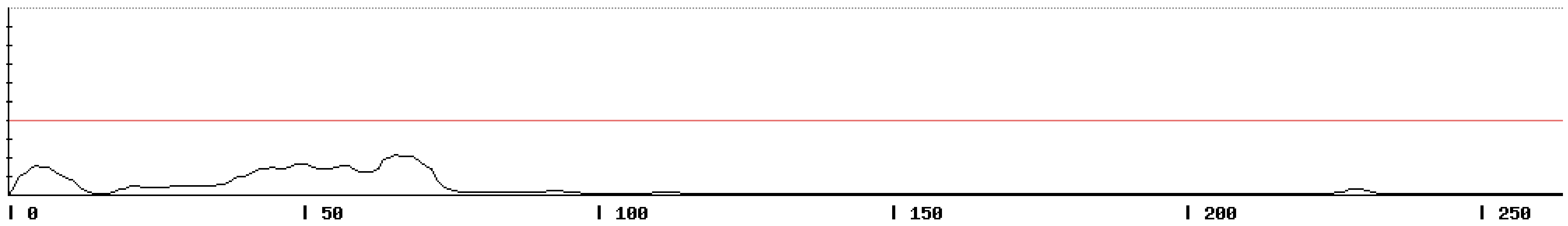

Predictions for Nicotianabenthamiana_Niben101Scf00109g02027.1

Viterbi Path

Viterbi Path

Posterior @ 0.4

Posterior @ 0.4

Posterior Graphic. Threshold Marked @ 0.4

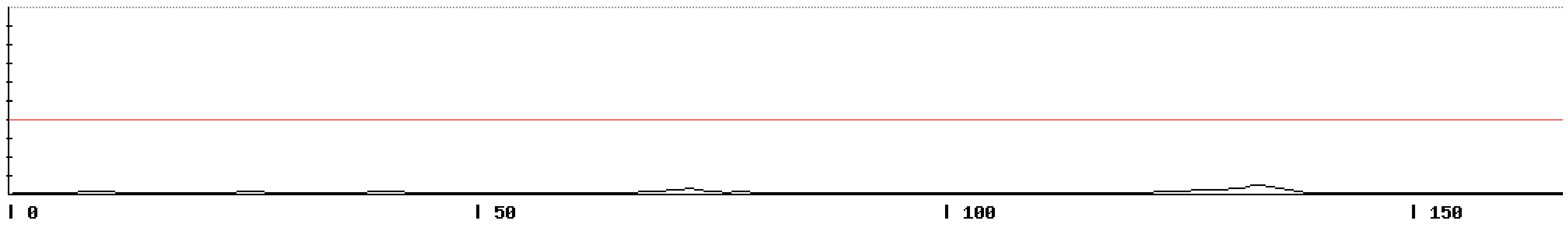

Predictions for Nicotianabenthamiana_Niben101Scf00136g01010.1

Viterbi Path

Viterbi Path

Posterior @ 0.4

Posterior @ 0.4

Posterior Graphic. Threshold Marked @ 0.4

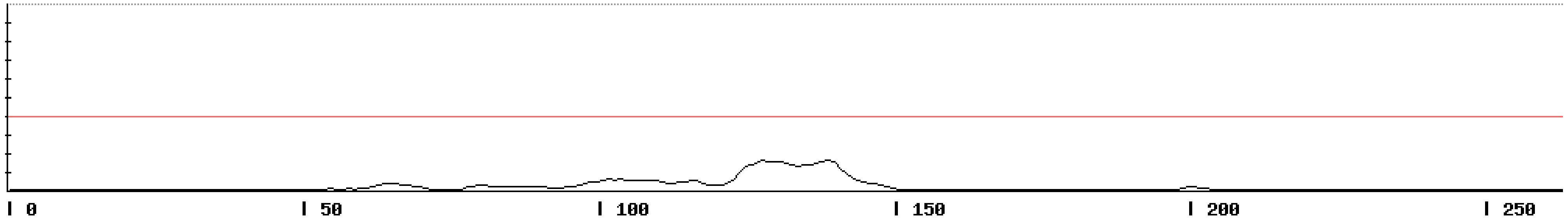

Predictions for Nicotianabenthamiana_Niben101Scf00138g04001.1

Viterbi Path

Viterbi Path

Posterior @ 0.4

Posterior @ 0.4

Posterior Graphic. Threshold Marked @ 0.4

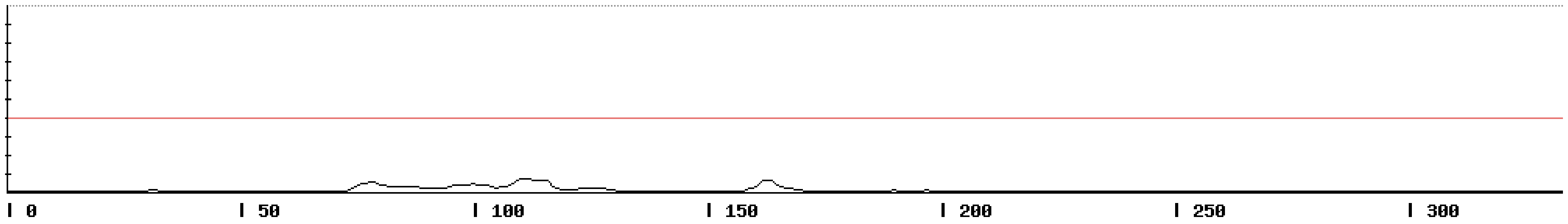

Predictions for Nicotianabenthamiana_Niben101Scf00146g03007.1

Viterbi Path

Viterbi Path

Posterior @ 0.4

77 - RLKKKKREHTRIRR - 90

Posterior @ 0.4

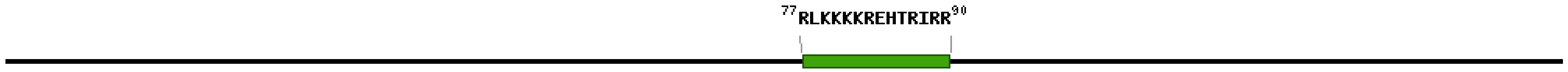

Posterior Graphic. Threshold Marked @ 0.4

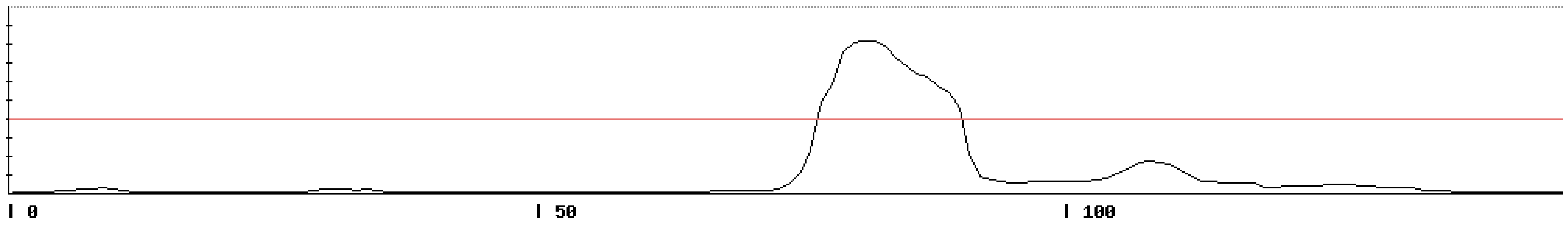

Predictions for Nicotianabenthamiana_Niben101Scf00193g01009.1

Viterbi Path

Viterbi Path

Posterior @ 0.4

Posterior @ 0.4

Posterior Graphic. Threshold Marked @ 0.4

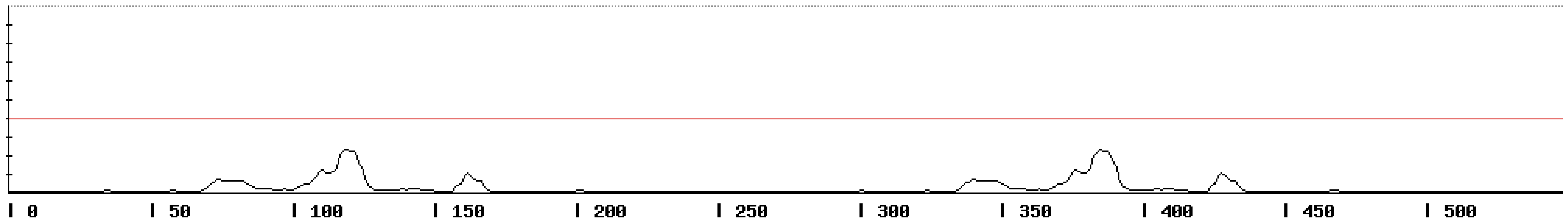

Predictions for Nicotianabenthamiana_Niben101Scf00245g02010.1	
Viterbi Path	
Viterbi Path	
Posterior @ 0.4	
Posterior @ 0.4	
Posterior Graphic. Threshold Marked @ 0.4	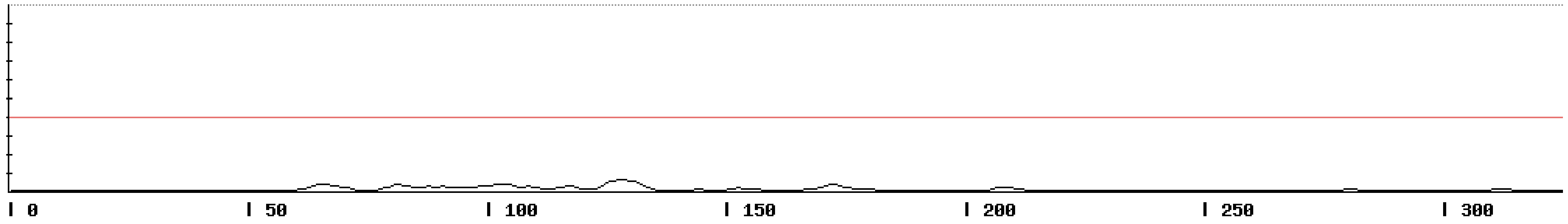

Predictions for Nicotianabenthamiana_Niben101Scf00272g08005.1	
Viterbi Path	
Viterbi Path	
Posterior @ 0.4	
Posterior @ 0.4	
Posterior Graphic. Threshold Marked @ 0.4	

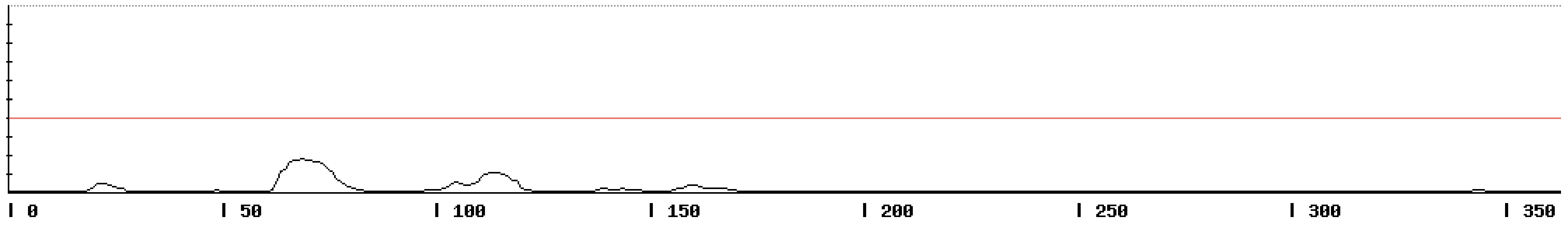

Predictions for Nicotianabenthamiana_Niben101Scf00290g01019.1

Viterbi Path

Viterbi Path

Posterior @ 0.4

Posterior @ 0.4

Posterior Graphic. Threshold Marked @ 0.4

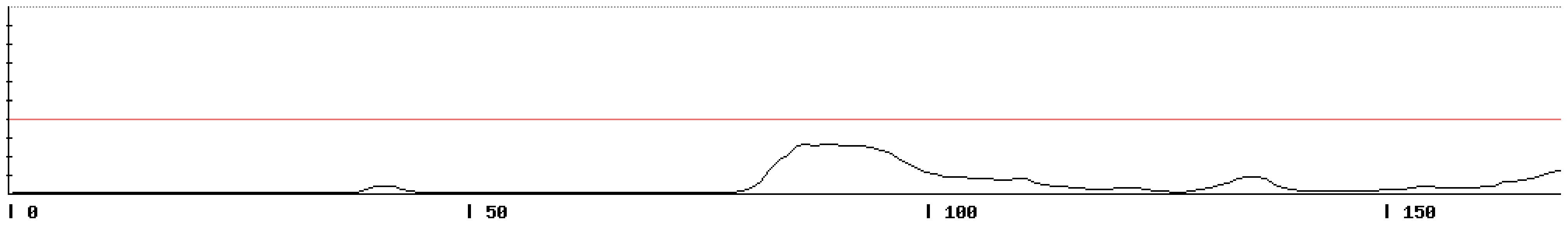

Predictions for Nicotianabenthamiana_Niben101Scf00308g01003.1

Viterbi Path

Viterbi Path

Posterior @ 0.4

121 - ATKKGAKIG - 129

Posterior @ 0.4

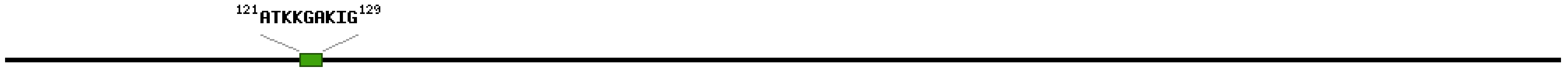

Posterior Graphic. Threshold Marked @ 0.4

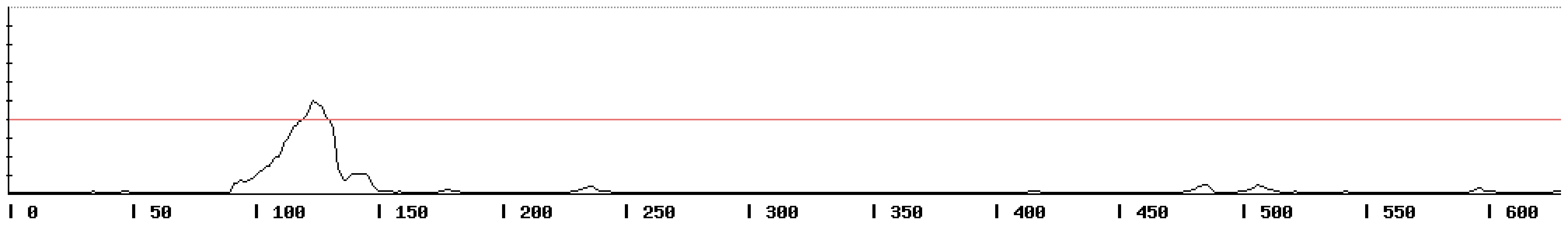

Predictions for Nicotianabenthamiana_Niben101Scf00320g02010.1

Viterbi Path

Viterbi Path

Posterior @ 0.4

37 - PRKKNKGRFRRTVGERGGT - 55

Posterior @ 0.4

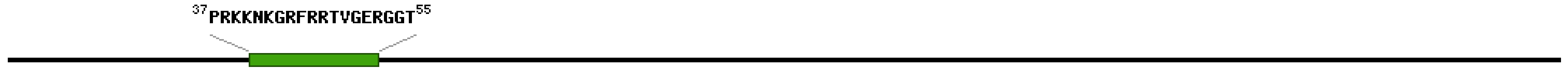

Posterior Graphic. Threshold Marked @ 0.4

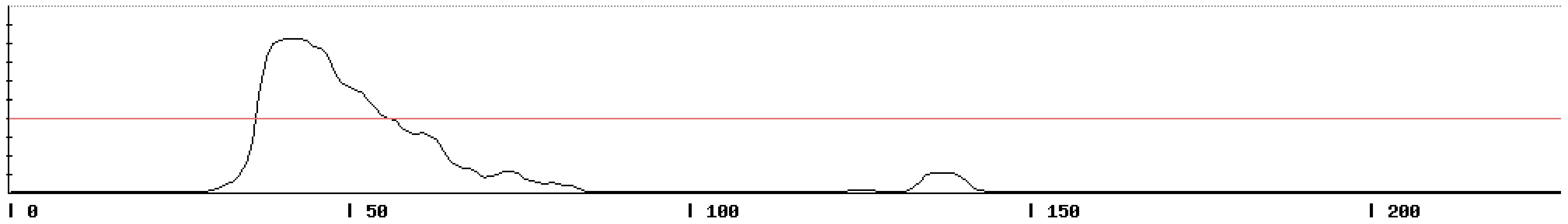

Predictions for Nicotianabenthamiana_Niben101Scf00416g07004.1

Viterbi Path

Viterbi Path

Posterior @ 0.4

Posterior @ 0.4

Posterior Graphic. Threshold Marked @ 0.4

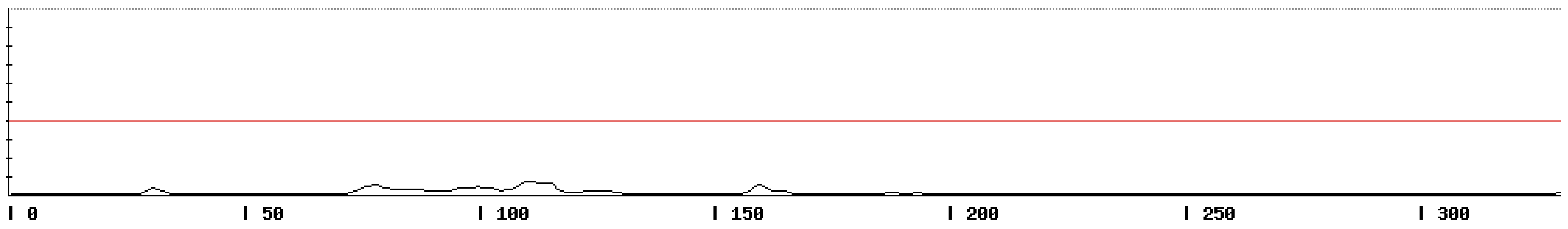

Predictions for Nicotianabenthamiana_Niben101Scf00445g02005.1

Viterbi Path

Viterbi Path

Posterior @ 0.4

Posterior @ 0.4

Posterior Graphic. Threshold Marked @ 0.4

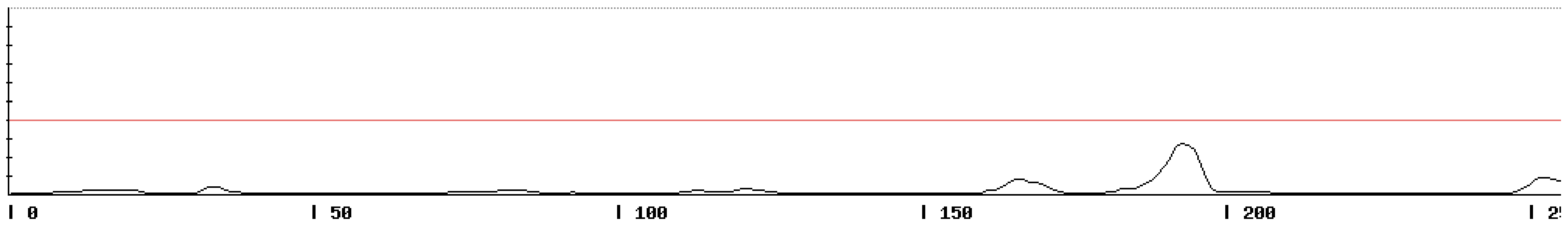

Predictions for Nicotianabenthamiana_Niben101Scf00453g07008.1

Viterbi Path

Viterbi Path

Posterior @ 0.4	78 - RTKAGGSR - 85
Posterior @ 0.4	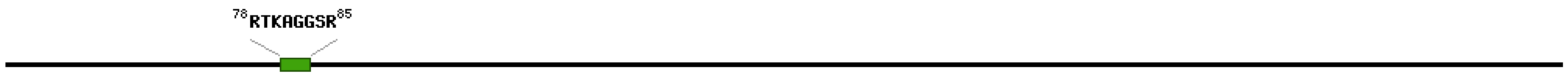
Posterior Graphic. Threshold Marked @ 0.4	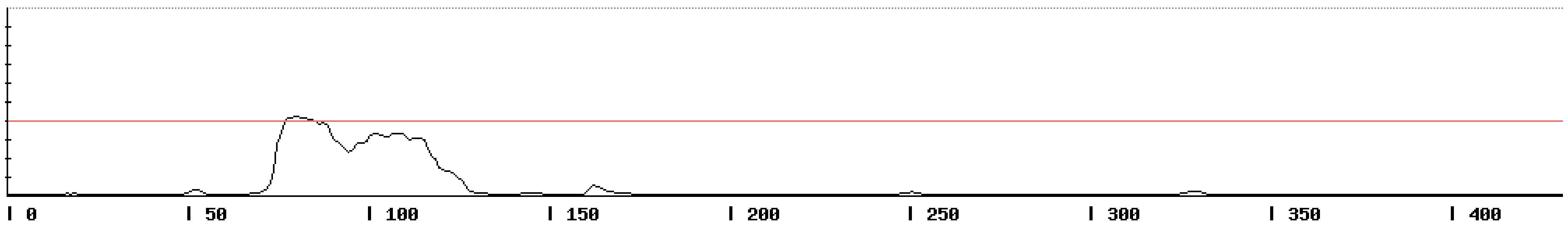

Predictions for Nicotianabenthamiana_Niben101Scf00491g00001.1

Viterbi Path	
Viterbi Path	
Posterior @ 0.4	
Posterior @ 0.4	
Posterior Graphic. Threshold Marked @ 0.4	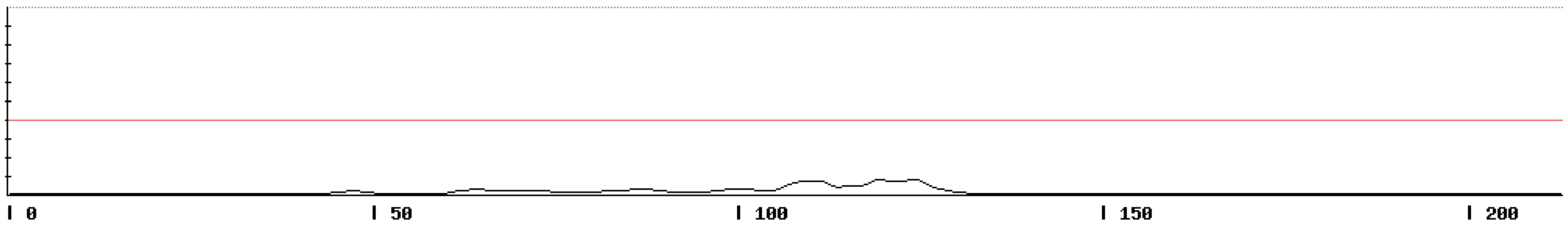

Predictions for Nicotianabenthamiana_Niben101Scf00526g00018.1

Viterbi Path	
Viterbi Path	

Posterior @ 0.4

Posterior @ 0.4

Posterior Graphic. Threshold Marked @ 0.4

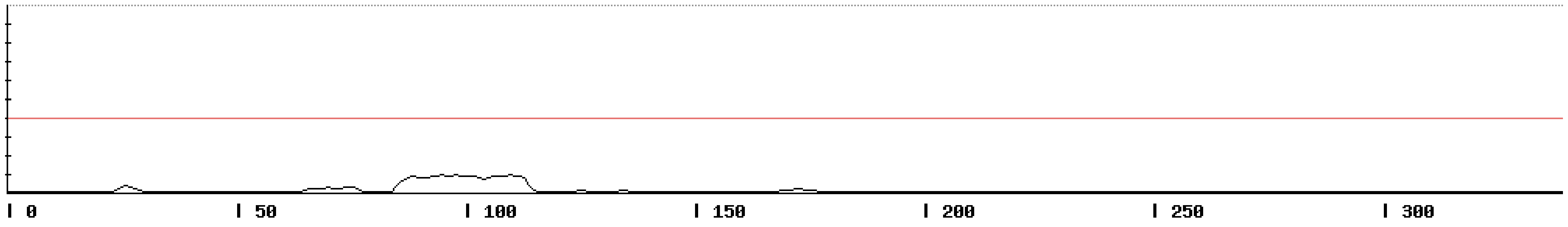

Predictions for Nicotianabenthamiana_Niben101Scf00553g04007.1

Viterbi Path

Viterbi Path

Posterior @ 0.4

Posterior @ 0.4

Posterior Graphic. Threshold Marked @ 0.4

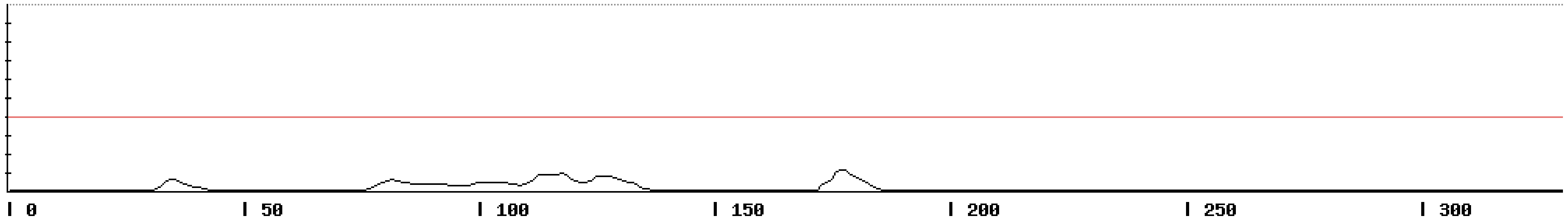

Predictions for Nicotianabenthamiana_Niben101Scf00563g01001.1

Viterbi Path

Viterbi Path

Posterior @ 0.4

Posterior @ 0.4

Posterior Graphic. Threshold Marked @ 0.4

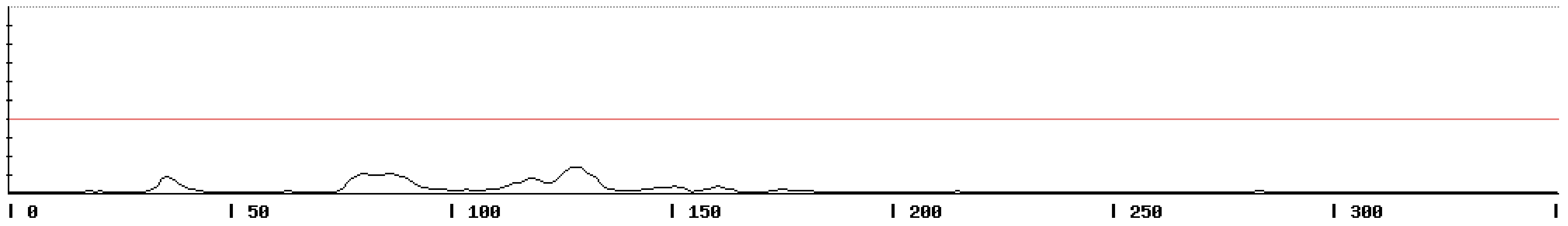

Predictions for Nicotianabenthamiana_Niben101Scf00712g03003.1

Viterbi Path

Viterbi Path

Posterior @ 0.4

Posterior @ 0.4

Posterior Graphic. Threshold Marked @ 0.4

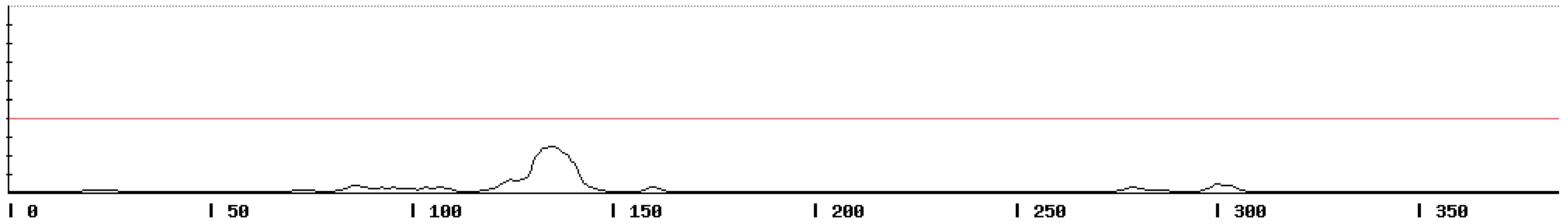

Predictions for Nicotianabenthamiana_Niben101Scf00792g03002.1

Viterbi Path

Viterbi Path

Posterior @ 0.4

Posterior @ 0.4

Posterior Graphic. Threshold Marked @ 0.4

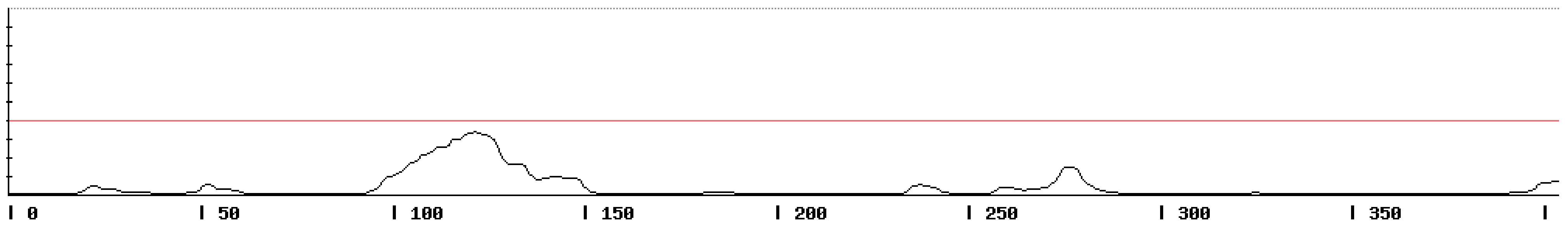

Predictions for Nicotianabenthamiana_Niben101Scf00792g04006.1

Viterbi Path

Viterbi Path

Posterior @ 0.4

Posterior @ 0.4

Posterior Graphic. Threshold Marked @ 0.4

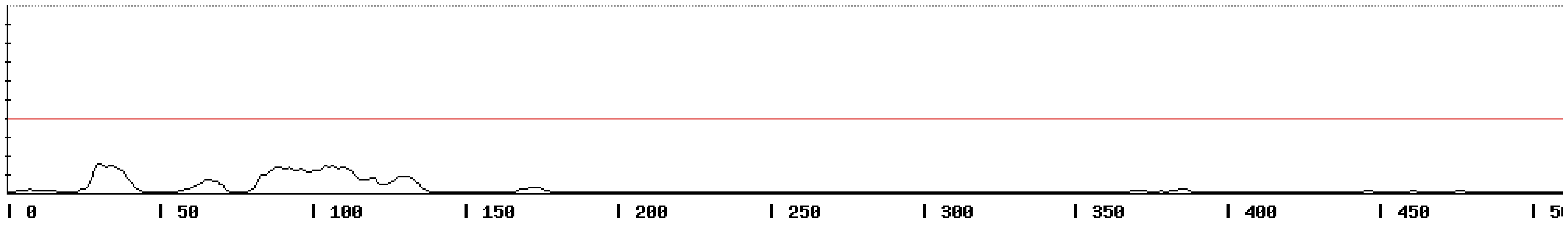

Predictions for Nicotianabenthamiana_Niben101Scf00799g00011.1

Viterbi Path	
Viterbi Path	
Posterior @ 0.4	
Posterior @ 0.4	
Posterior Graphic. Threshold Marked @ 0.4	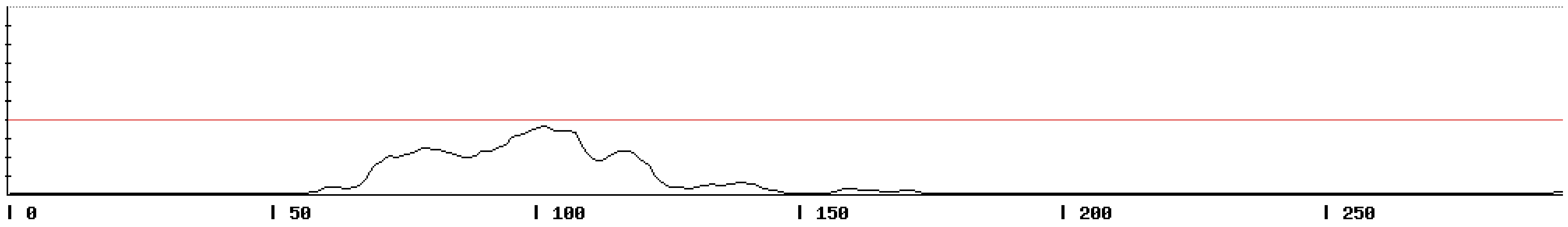

Predictions for Nicotianabenthamiana_Niben101Scf00929g02007.1

Viterbi Path	
Viterbi Path	
Posterior @ 0.4	443 - REEKRKKKLKK - 453
Posterior @ 0.4	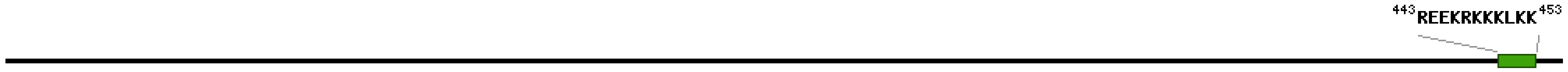

Posterior Graphic. Threshold Marked @ 0.4

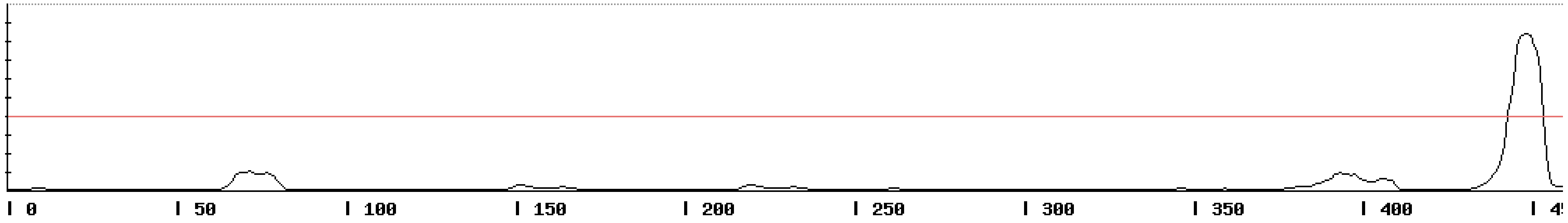

Predictions for Nicotianabenthamiana_Niben101Scf00944g04003.1

Viterbi Path

Viterbi Path

Posterior @ 0.4

Posterior @ 0.4

Posterior Graphic. Threshold Marked @ 0.4

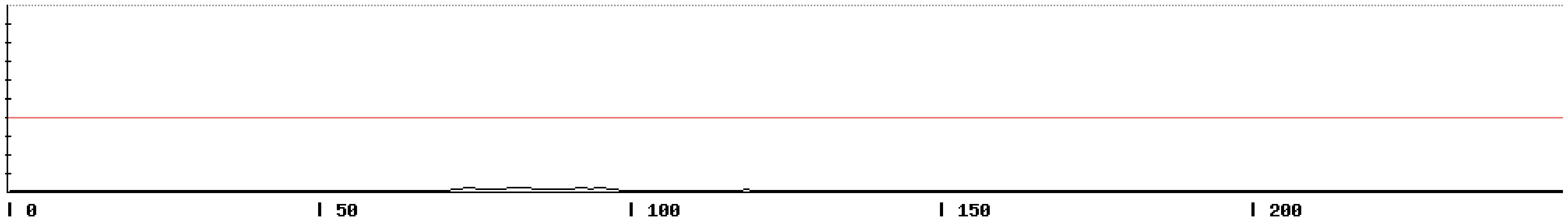

Predictions for Nicotianabenthamiana_Niben101Scf00966g02001.1

Viterbi Path

Viterbi Path

Posterior @ 0.4

Posterior @ 0.4

Posterior Graphic. Threshold Marked @ 0.4	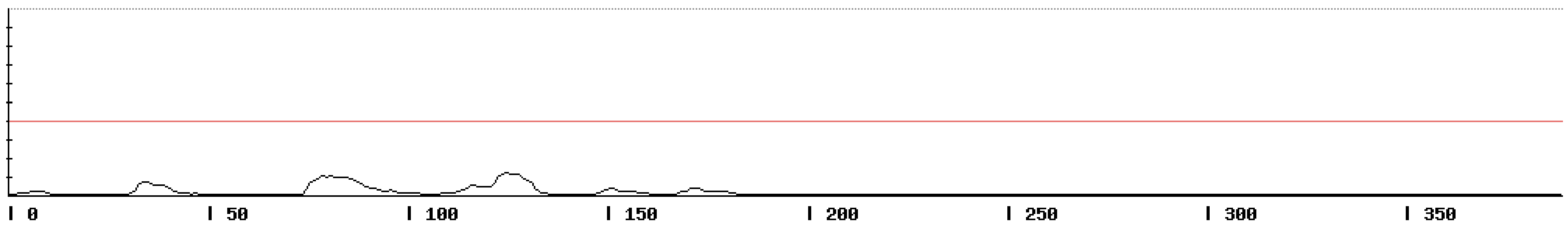

Predictions for Nicotianabenthamiana_Niben101Scf01055g00004.1

Viterbi Path	
Viterbi Path	
Posterior @ 0.4	216 - KTRKRPLNRVLKDK - 229
Posterior @ 0.4	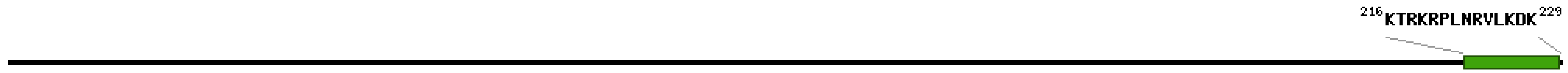
Posterior Graphic. Threshold Marked @ 0.4	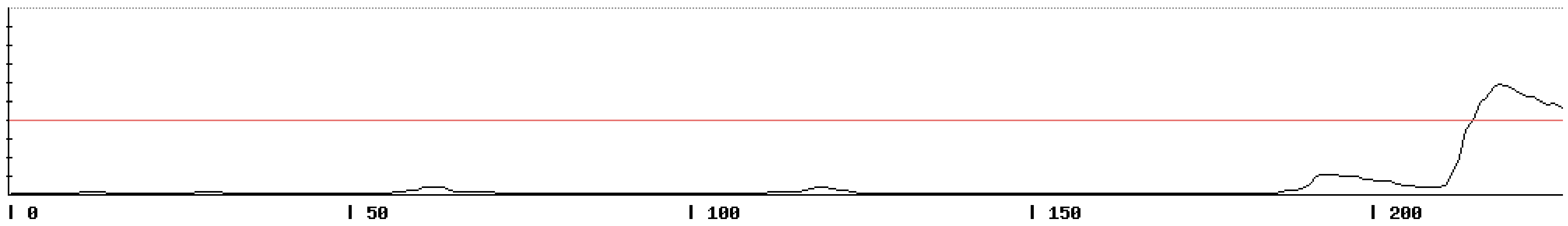

Predictions for Nicotianabenthamiana_Niben101Scf01062g01005.1

Viterbi Path	76 - RLKKKKKREHSRVRR - 90
Viterbi Path	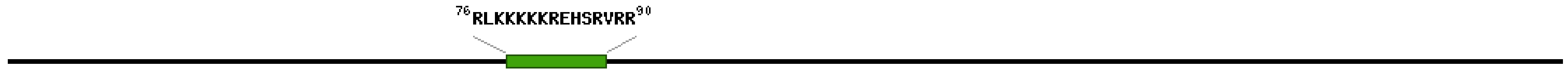

Posterior @ 0.4	76 - RLKKKKKREHSRVRR - 90
Posterior @ 0.4	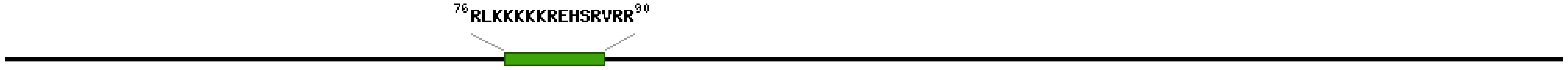
Posterior Graphic. Threshold Marked @ 0.4	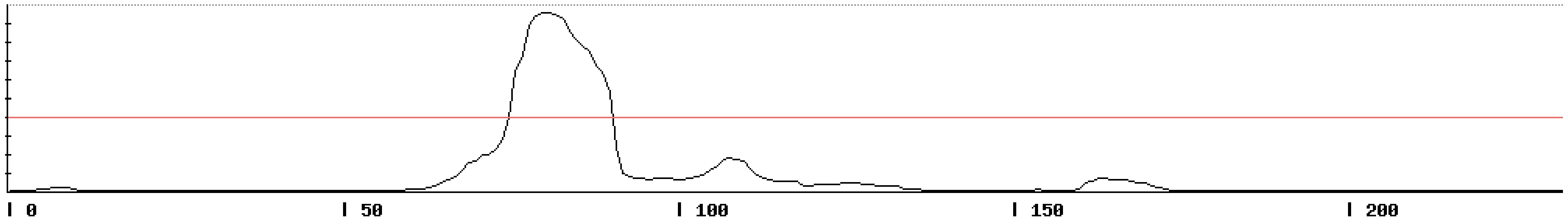

Predictions for Nicotianabenthamiana_Niben101Scf01146g07001.1

Viterbi Path	
Viterbi Path	
Posterior @ 0.4	
Posterior @ 0.4	
Posterior Graphic. Threshold Marked @ 0.4	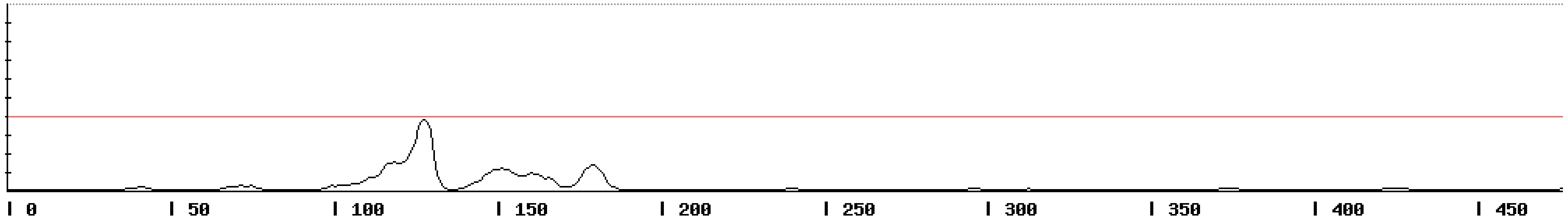

Predictions for Nicotianabenthamiana_Niben101Scf01153g00009.1

Viterbi Path	
Viterbi Path	

Posterior @ 0.4

Posterior @ 0.4

Posterior Graphic. Threshold Marked @ 0.4

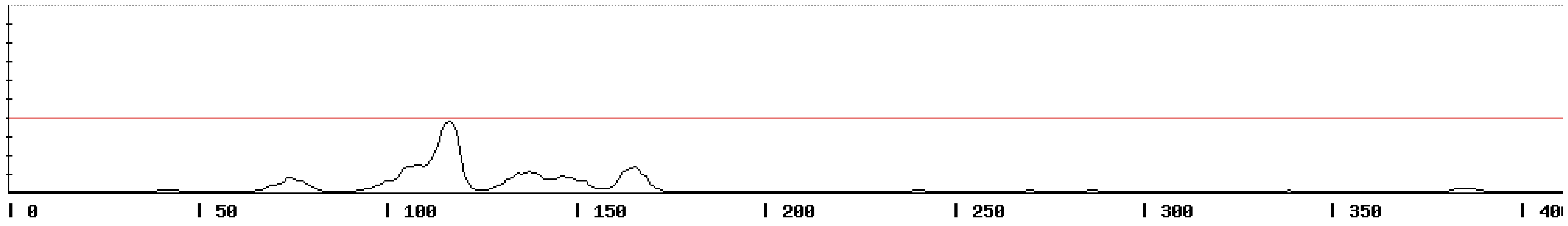

Predictions for Nicotianabenthamiana_Niben101Scf01166g16008.1

Viterbi Path

Viterbi Path

Posterior @ 0.4

Posterior @ 0.4

Posterior Graphic. Threshold Marked @ 0.4

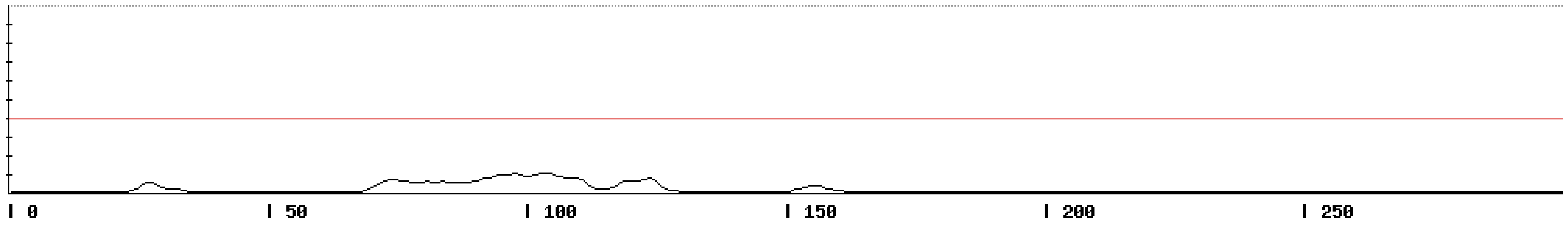

Predictions for Nicotianabenthamiana_Niben101Scf01218g01003.1

Viterbi Path

Viterbi Path

Posterior @ 0.4

Posterior @ 0.4

Posterior Graphic. Threshold Marked @ 0.4

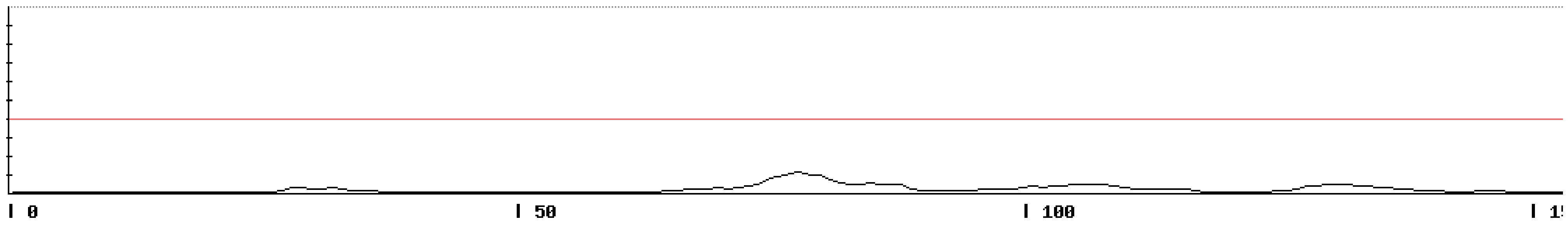

Predictions for Nicotianabenthamiana_Niben101Scf01277g00002.1

Viterbi Path

Viterbi Path

Posterior @ 0.4

Posterior @ 0.4

Posterior Graphic. Threshold Marked @ 0.4

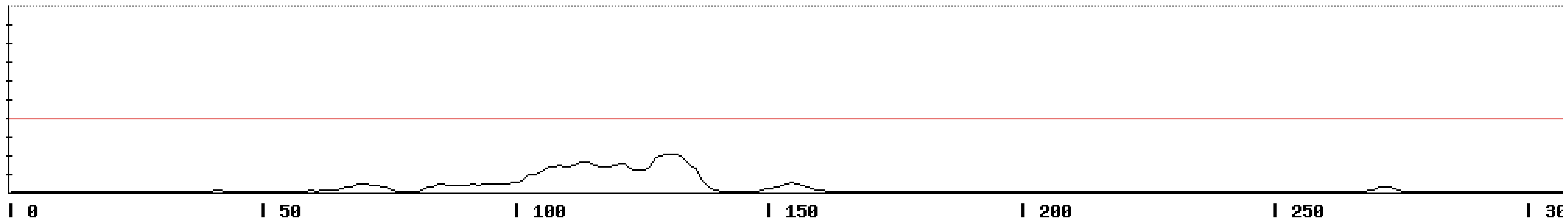

Predictions for Nicotianabenthamiana_Niben101Scf01293g05014.1	
Viterbi Path	
Viterbi Path	
Posterior @ 0.4	151 - K - 151
Posterior @ 0.4	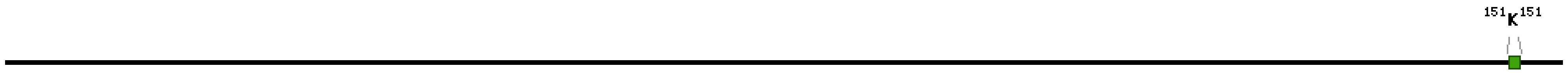
Posterior Graphic. Threshold Marked @ 0.4	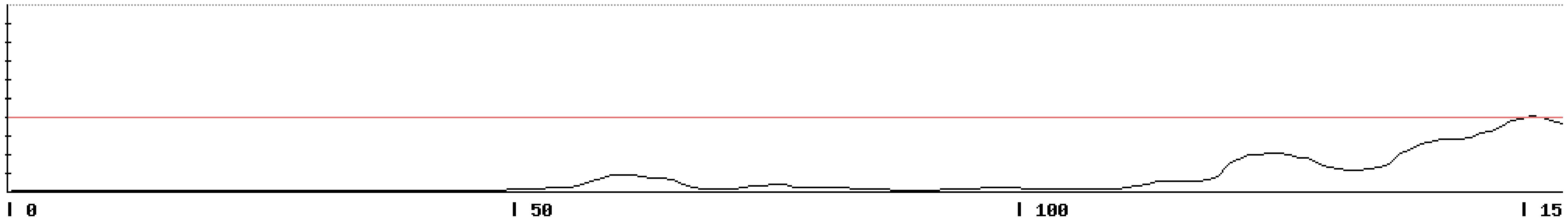

Predictions for Nicotianabenthamiana_Niben101Scf01320g01003.1	
Viterbi Path	
Viterbi Path	
Posterior @ 0.4	
Posterior @ 0.4	
Posterior Graphic. Threshold Marked @ 0.4	

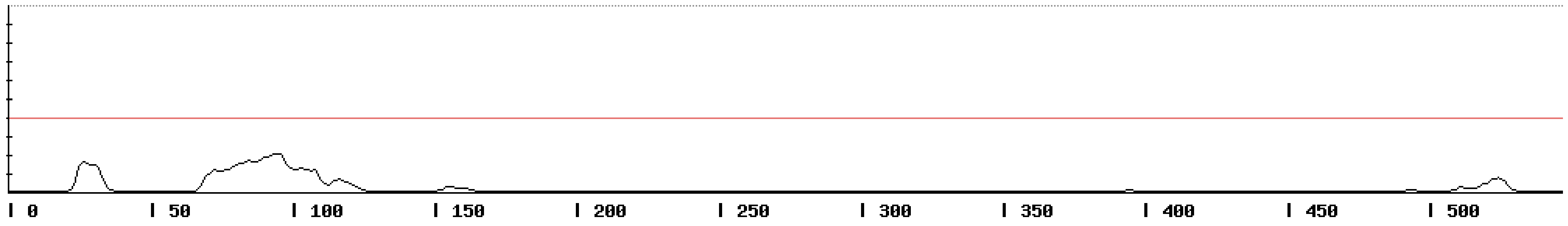

Predictions for Nicotianabenthamiana_Niben101Scf01326g04010.1

Viterbi Path

Viterbi Path

Posterior @ 0.4

Posterior @ 0.4

Posterior Graphic. Threshold Marked @ 0.4

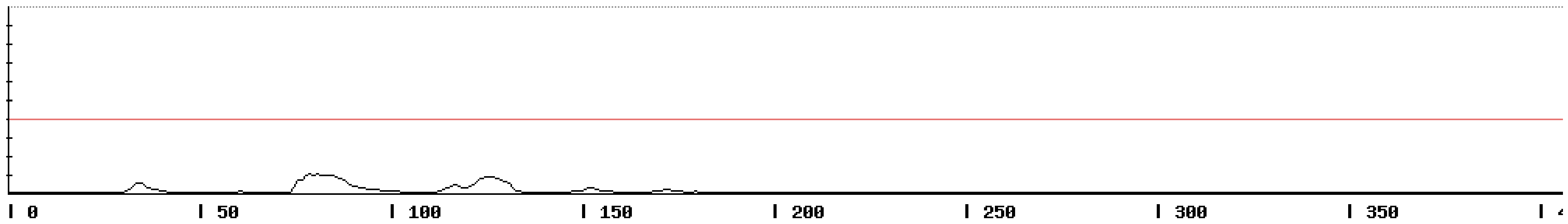

Predictions for Nicotianabenthamiana_Niben101Scf01330g01003.1

Viterbi Path

Viterbi Path

Posterior @ 0.4

Posterior @ 0.4

Posterior Graphic. Threshold Marked @ 0.4

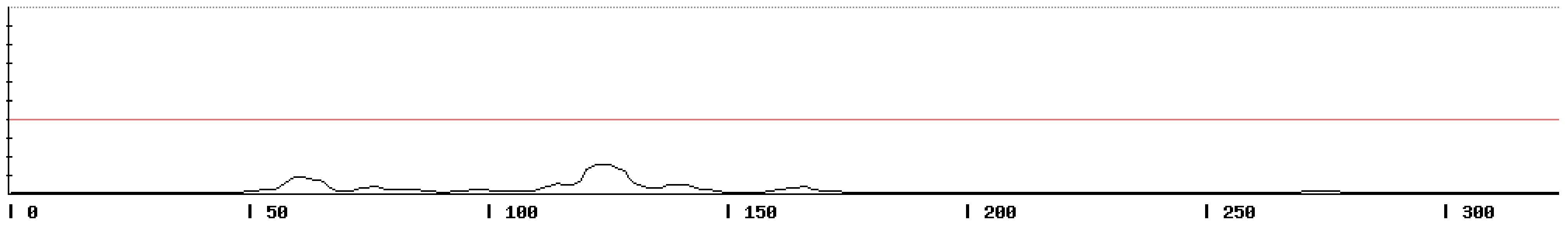

Predictions for Nicotianabenthamiana_Niben101Scf01337g00001.1

Viterbi Path

Viterbi Path

Posterior @ 0.4 — 82 - QKKEKSRRKSVTRTIGKGIWK - 102

Posterior @ 0.4

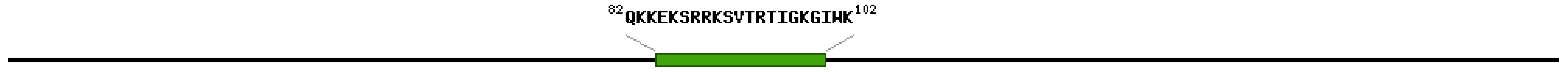

Posterior Graphic. Threshold Marked @ 0.4

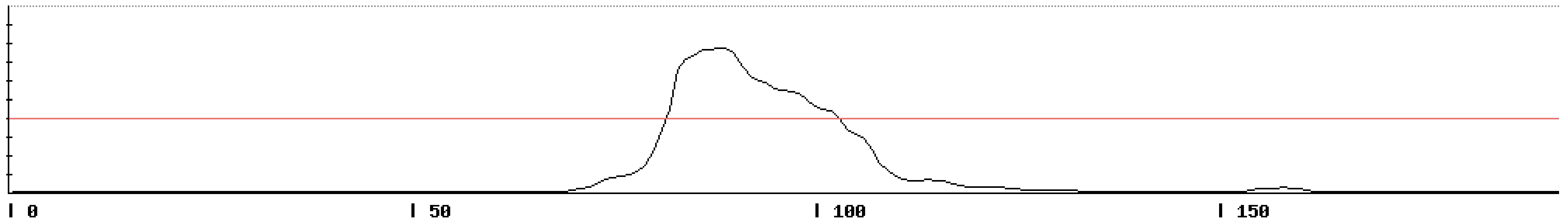

Predictions for Nicotianabenthamiana_Niben101Scf01369g02001.1

Viterbi Path

Viterbi Path

Posterior @ 0.4

Posterior @ 0.4

Posterior Graphic. Threshold Marked @ 0.4

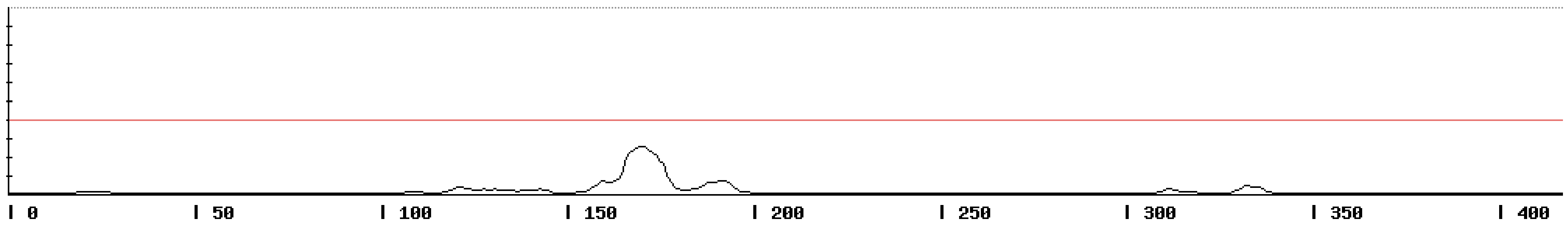

Predictions for Nicotianabenthamiana_Niben101Scf01435g02002.1

Viterbi Path

Viterbi Path

Posterior @ 0.4

Posterior @ 0.4

Posterior Graphic. Threshold Marked @ 0.4

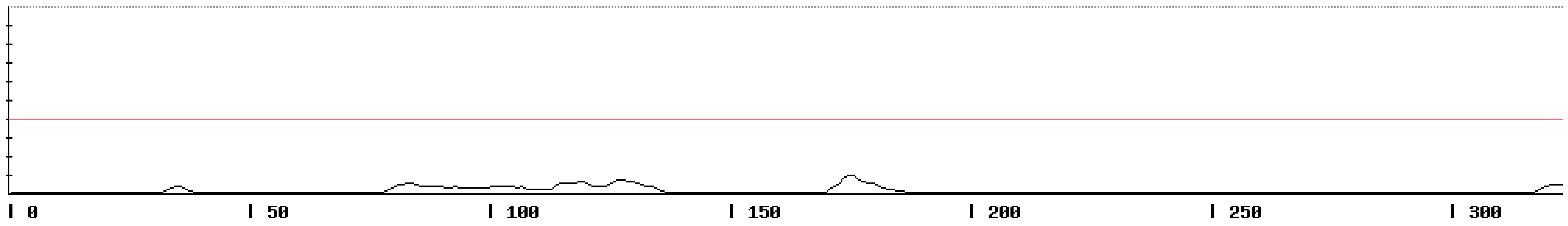

Predictions for Nicotianabenthamiana_Niben101Scf01450g05003.1

Viterbi Path

Viterbi Path

Posterior @ 0.4

Posterior @ 0.4

Posterior Graphic. Threshold Marked @ 0.4

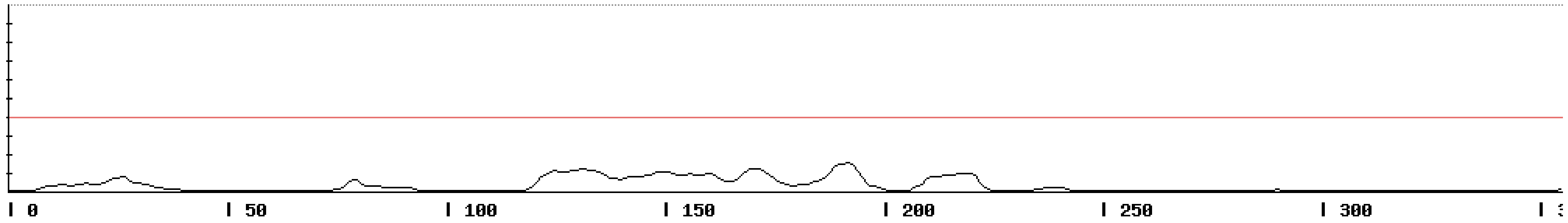

Predictions for Nicotianabenthamiana_Niben101Scf01463g08015.1

Viterbi Path

Viterbi Path

Posterior @ 0.4 — 67 - LKKRKESHKRFRR - 79

Posterior @ 0.4

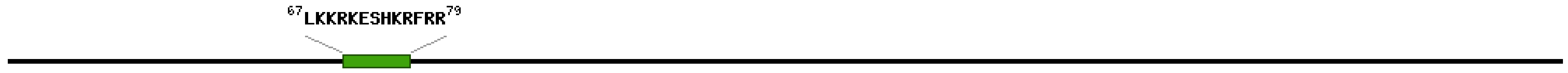

Posterior Graphic. Threshold Marked @ 0.4

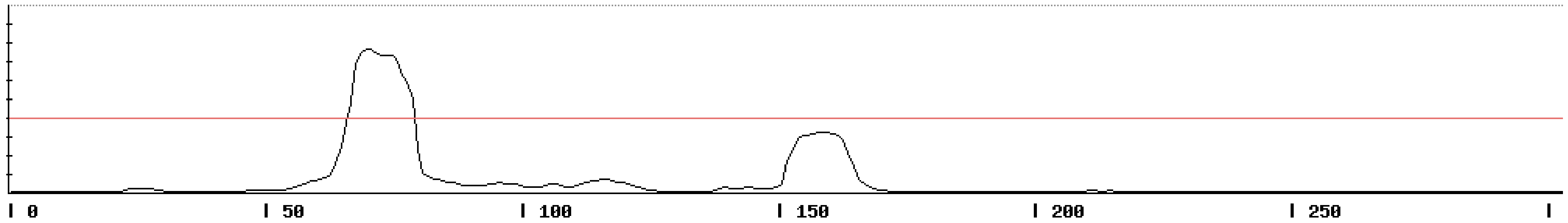

Predictions for Nicotianabenthamiana_Niben101Scf01479g01004.1

Viterbi Path

Viterbi Path

Posterior @ 0.4

Posterior @ 0.4

Posterior Graphic. Threshold Marked @ 0.4

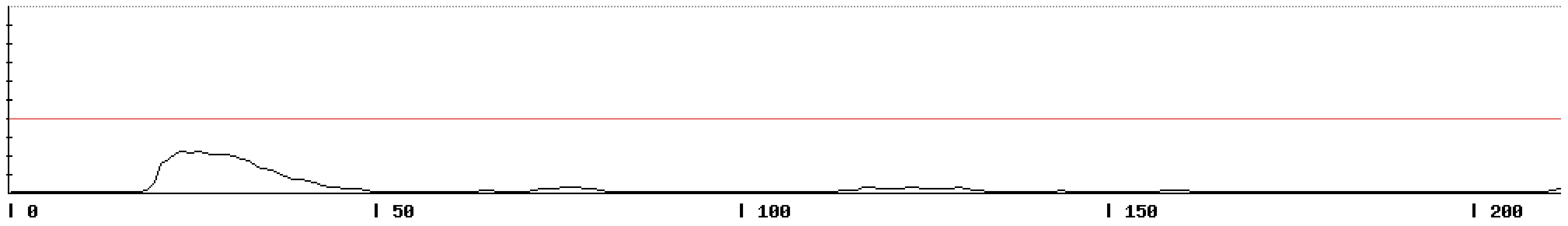

Predictions for Nicotianabenthamiana_Niben101Scf01479g01006.1

Viterbi Path

Viterbi Path

Posterior @ 0.4

Posterior @ 0.4

Posterior Graphic. Threshold Marked @ 0.4

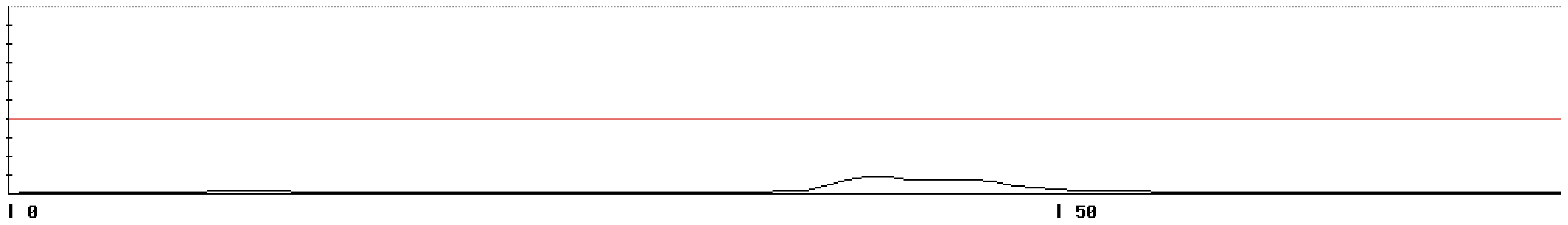

Predictions for Nicotianabenthamiana_Niben101Scf01481g02006.1

Viterbi Path

Viterbi Path

Posterior @ 0.4

Posterior @ 0.4

Posterior Graphic. Threshold Marked @ 0.4

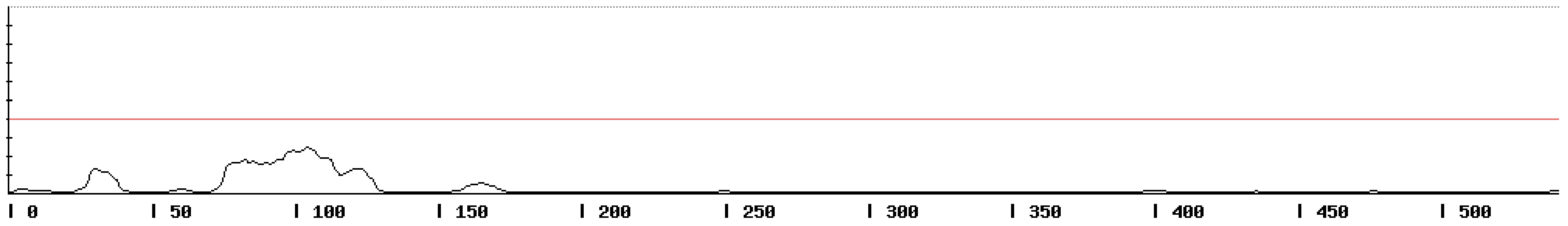

Predictions for Nicotianabenthamiana_Niben101Scf01481g03011.1

Viterbi Path

Viterbi Path

Posterior @ 0.4

Posterior @ 0.4

Posterior Graphic. Threshold Marked @ 0.4

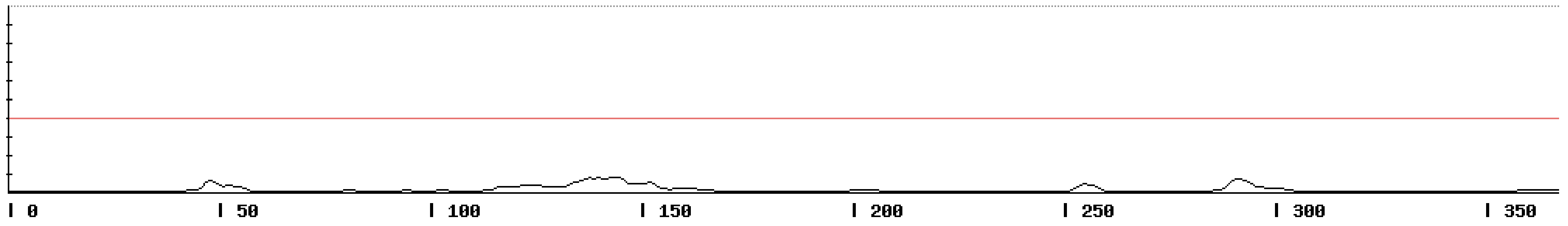

Predictions for Nicotianabenthamiana_Niben101Scf01498g04003.1

Viterbi Path

Viterbi Path

Posterior @ 0.4

Posterior @ 0.4

Posterior Graphic. Threshold Marked @ 0.4

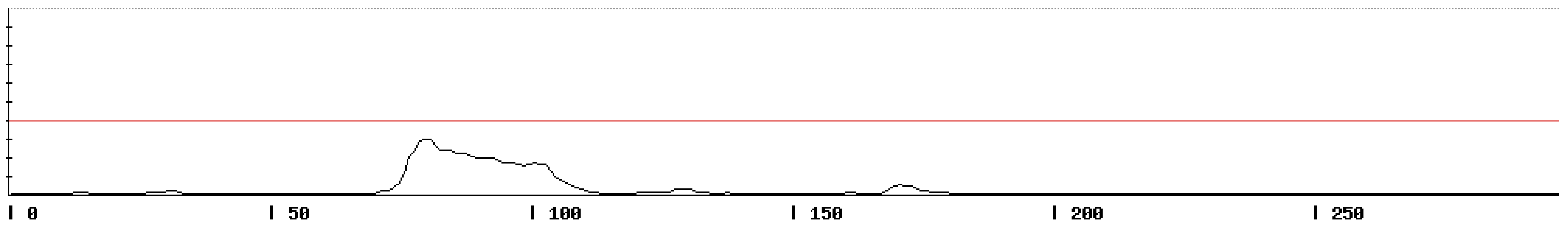

Predictions for Nicotianabenthamiana_Niben101Scf01498g06001.1

Viterbi Path

Viterbi Path

Posterior @ 0.4

Posterior @ 0.4

Posterior Graphic. Threshold Marked @ 0.4

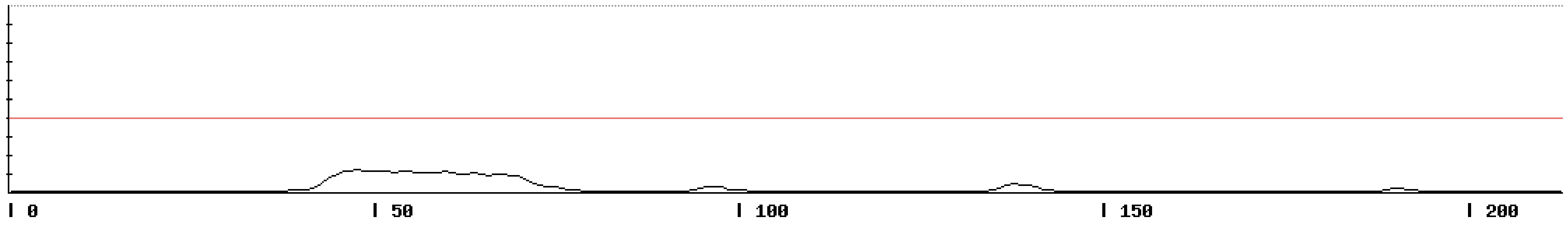

Predictions for Nicotianabenthamiana_Niben101Scf01526g04017.1

Viterbi Path

Viterbi Path

Posterior @ 0.4

Posterior @ 0.4

Posterior Graphic. Threshold Marked @ 0.4

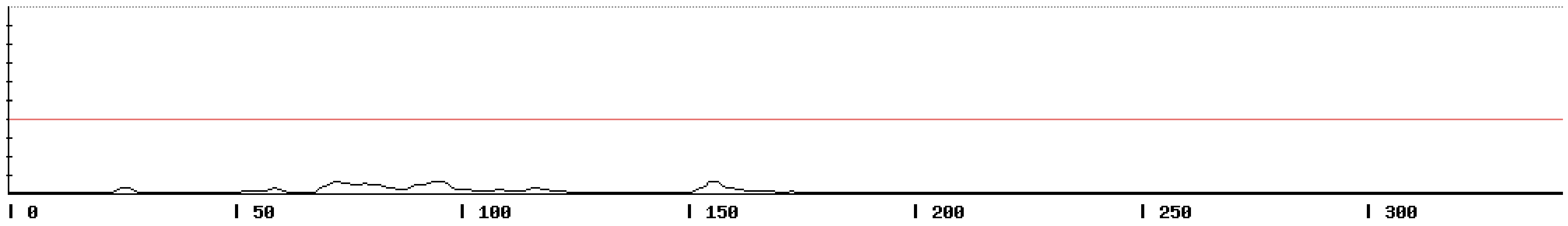

Predictions for Nicotianabenthamiana_Niben101Scf01591g00001.1

Viterbi Path

Viterbi Path

Posterior @ 0.4

Posterior @ 0.4

Posterior Graphic. Threshold Marked @ 0.4

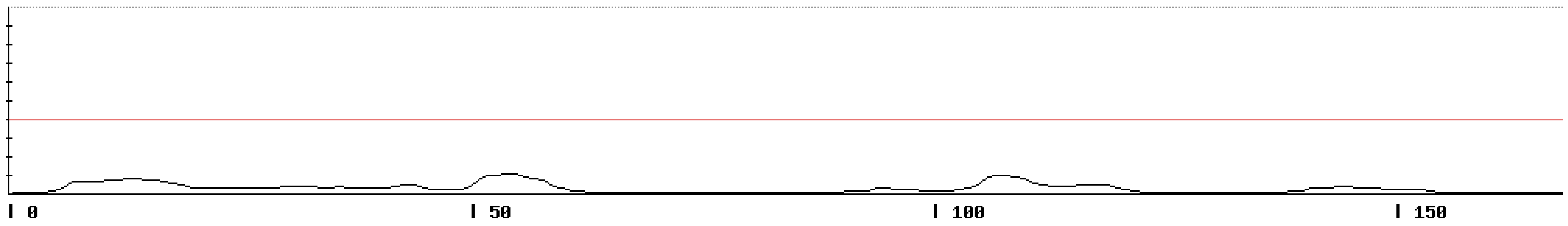

Predictions for Nicotianabenthamiana_Niben101Scf01594g02012.1

Viterbi Path	
Viterbi Path	
Posterior @ 0.4	
Posterior @ 0.4	
Posterior Graphic. Threshold Marked @ 0.4	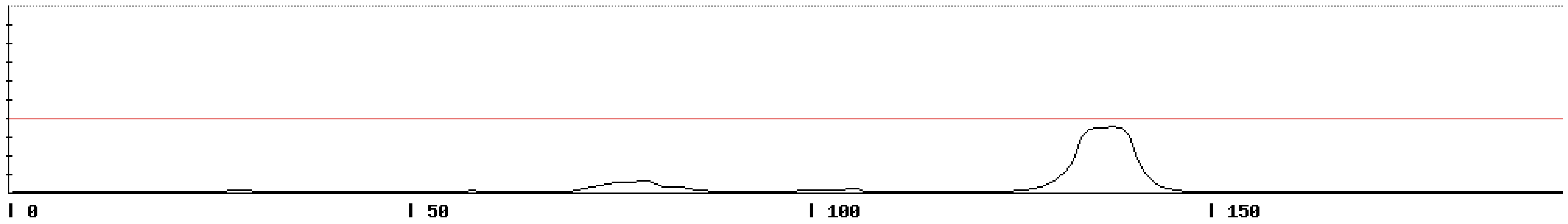

Predictions for Nicotianabenthamiana_Niben101Scf01627g02012.1

Viterbi Path	
Viterbi Path	
Posterior @ 0.4	134 - RKRRK - 138
Posterior @ 0.4	

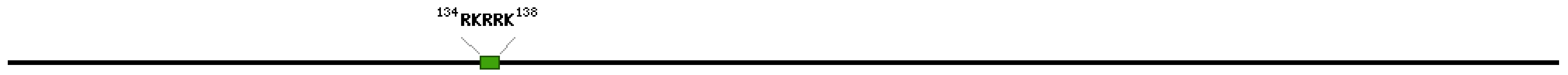

Posterior Graphic. Threshold Marked @ 0.4

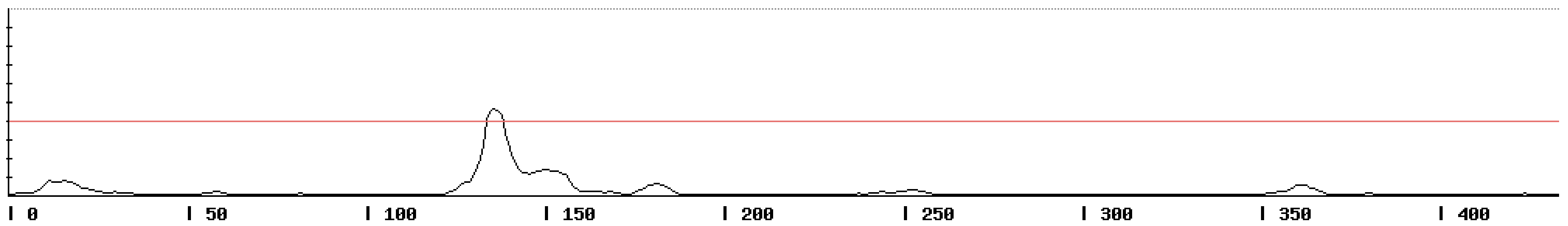

Predictions for Nicotianabenthamiana_Niben101Scf01750g12001.1	
Viterbi Path	
Viterbi Path	
Posterior @ 0.4	
Posterior @ 0.4	
Posterior Graphic. Threshold Marked @ 0.4	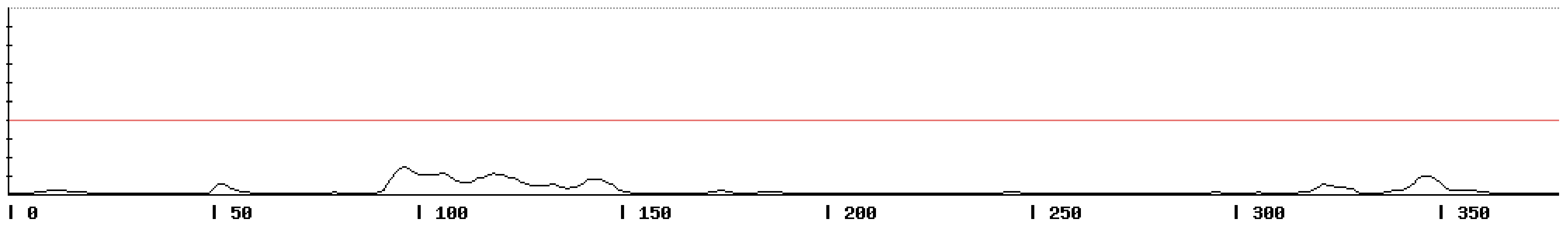

Predictions for Nicotianabenthamiana_Niben101Scf01779g04012.1	
Viterbi Path	
Viterbi Path	

Posterior @ 0.4

Posterior @ 0.4

Posterior Graphic. Threshold Marked @ 0.4

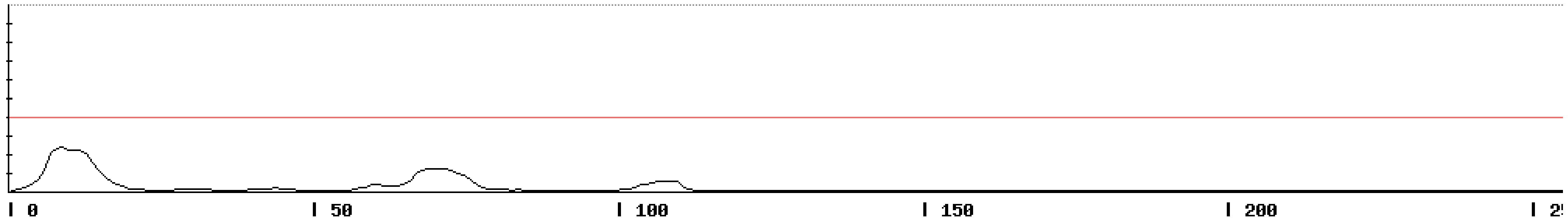

Predictions for Nicotianabenthamiana_Niben101Scf01795g17007.1

Viterbi Path

Viterbi Path

Posterior @ 0.4

Posterior @ 0.4

Posterior Graphic. Threshold Marked @ 0.4

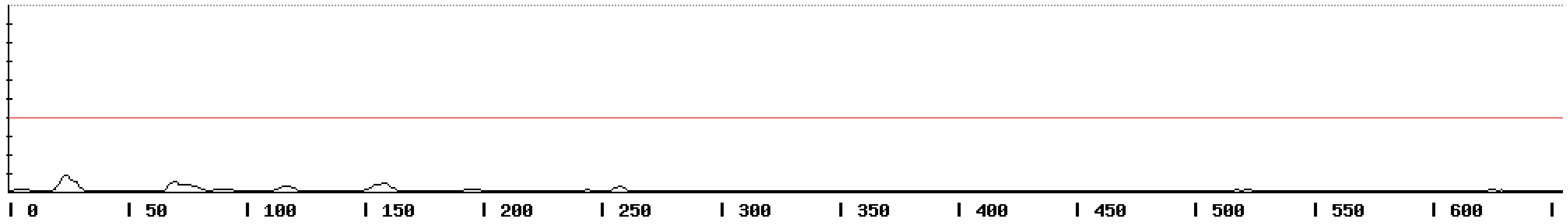

Predictions for Nicotianabenthamiana_Niben101Scf01818g03019.1

Viterbi Path

Viterbi Path

Posterior @ 0.4

Posterior @ 0.4

Posterior Graphic. Threshold Marked @ 0.4

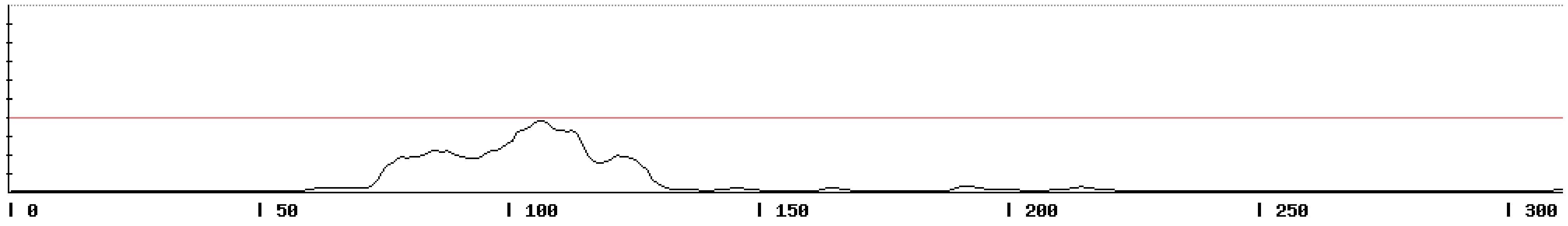

Predictions for Nicotianabenthamiana_Niben101Scf01830g01027.1

Viterbi Path

Viterbi Path

Posterior @ 0.4

Posterior @ 0.4

Posterior Graphic. Threshold Marked @ 0.4

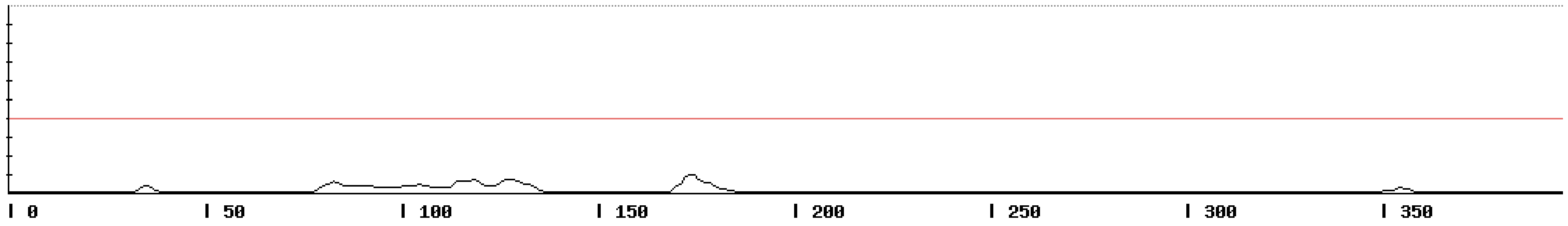

Predictions for Nicotianabenthamiana_Niben101Scf01887g00004.1

Viterbi Path

Viterbi Path

Posterior @ 0.4

Posterior @ 0.4

Posterior Graphic. Threshold Marked @ 0.4

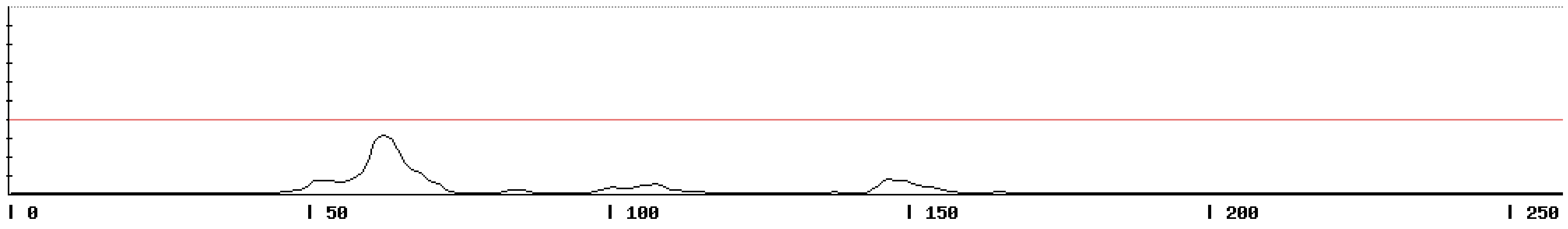

Predictions for Nicotianabenthamiana_Niben101Scf01894g03001.1

Viterbi Path

Viterbi Path

Posterior @ 0.4

Posterior @ 0.4

Posterior Graphic. Threshold Marked @ 0.4

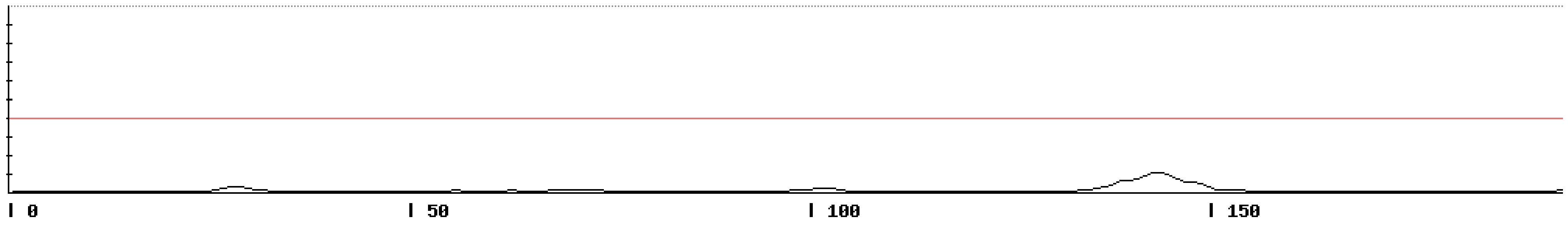

Predictions for Nicotianabenthamiana_Niben101Scf02044g08007.1	
Viterbi Path	
Viterbi Path	
Posterior @ 0.4	
Posterior @ 0.4	
Posterior Graphic. Threshold Marked @ 0.4	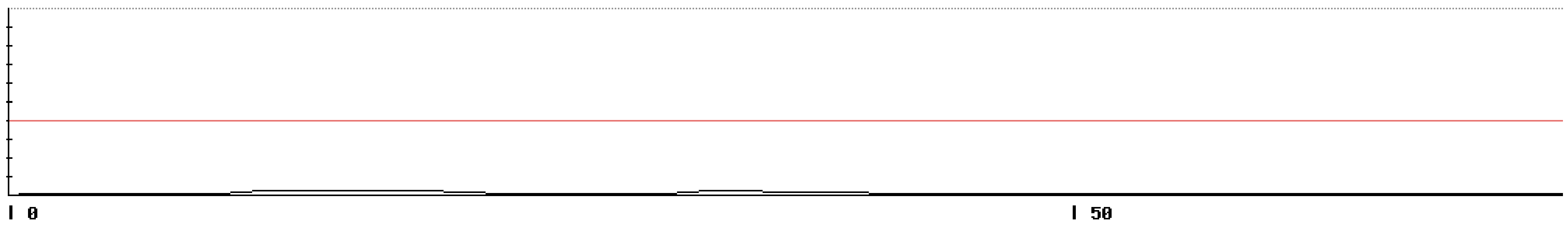

Predictions for Nicotianabenthamiana_Niben101Scf02044g08008.1	
Viterbi Path	
Viterbi Path	
Posterior @ 0.4	
Posterior @ 0.4	
Posterior Graphic. Threshold Marked @ 0.4	

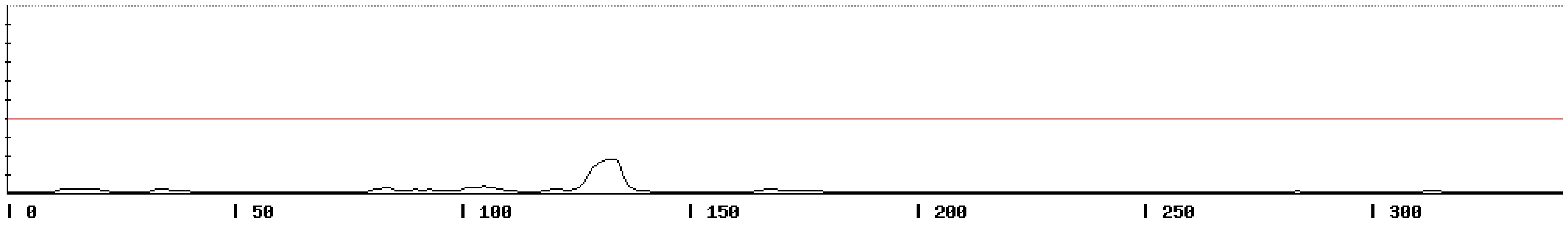

Predictions for Nicotianabenthamiana_Niben101Scf02081g03009.1

Viterbi Path	
Viterbi Path	
Posterior @ 0.4	75 - LKKKSNARYHRSVRNKGT - 92
Posterior @ 0.4	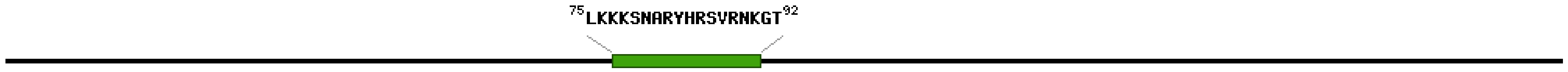
Posterior Graphic. Threshold Marked @ 0.4	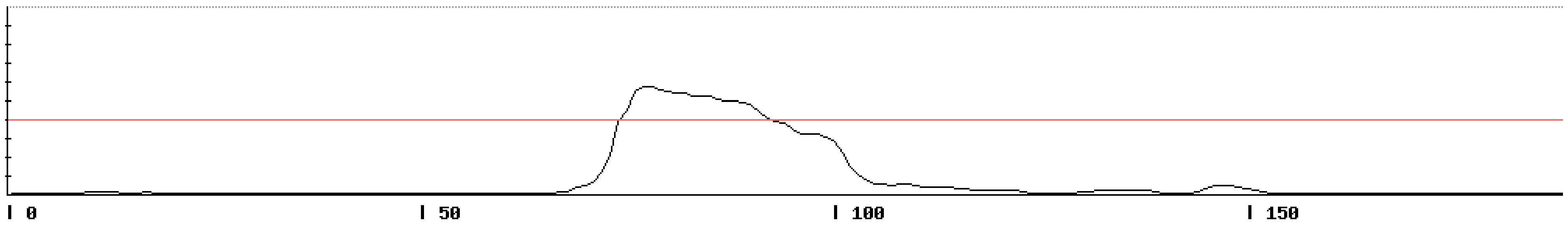

Predictions for Nicotianabenthamiana_Niben101Scf02090g06012.1

Viterbi Path	
Viterbi Path	
Posterior @ 0.4	
Posterior @ 0.4	

Posterior Graphic. Threshold Marked @ 0.4

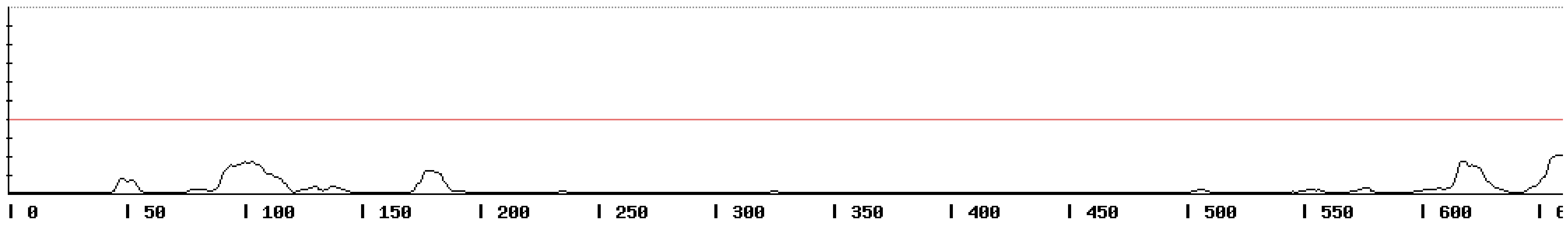

Predictions for Nicotianabenthamiana_Niben101Scf02108g01025.1

Viterbi Path

Viterbi Path

Posterior @ 0.4

Posterior @ 0.4

Posterior Graphic. Threshold Marked @ 0.4

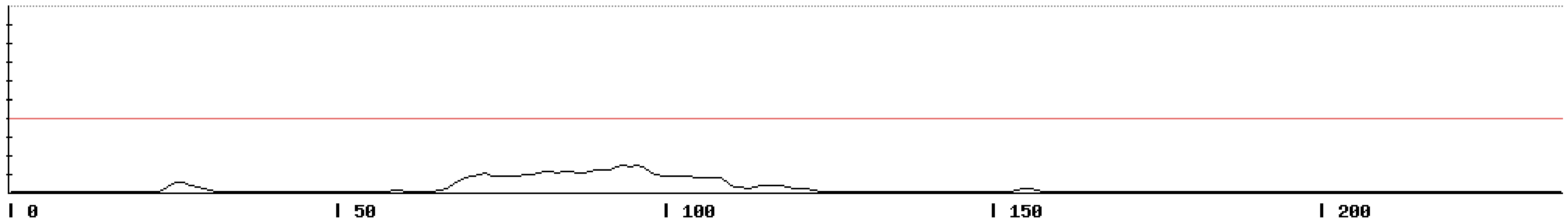

Predictions for Nicotianabenthamiana_Niben101Scf02210g01002.1

Viterbi Path

Viterbi Path

Posterior @ 0.4

Posterior @ 0.4

Posterior Graphic. Threshold Marked @ 0.4

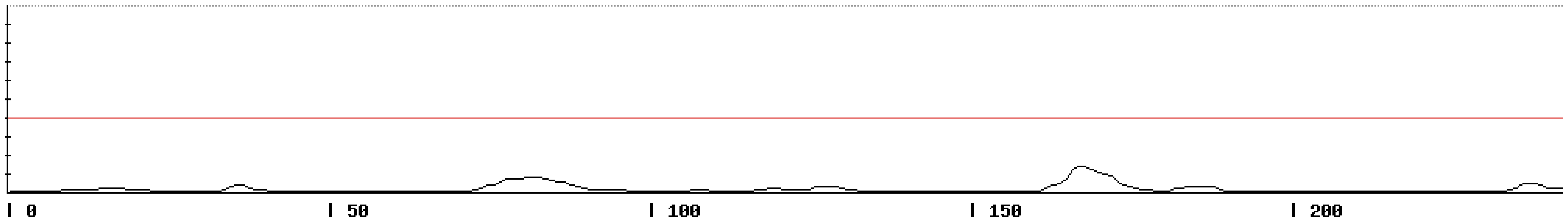

Predictions for Nicotianabenthamiana_Niben101Scf02243g03001.1

Viterbi Path

Viterbi Path

Posterior @ 0.4

Posterior @ 0.4

Posterior Graphic. Threshold Marked @ 0.4

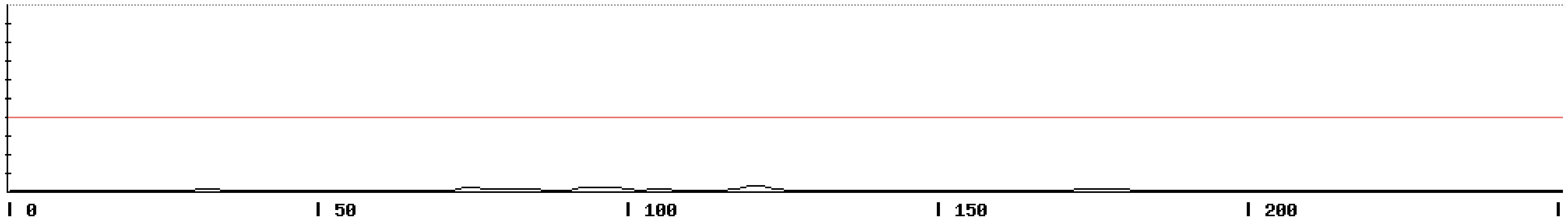

Predictions for Nicotianabenthamiana_Niben101Scf02318g03012.1

Viterbi Path

Viterbi Path

Posterior @ 0.4	
Posterior @ 0.4	
Posterior Graphic. Threshold Marked @ 0.4	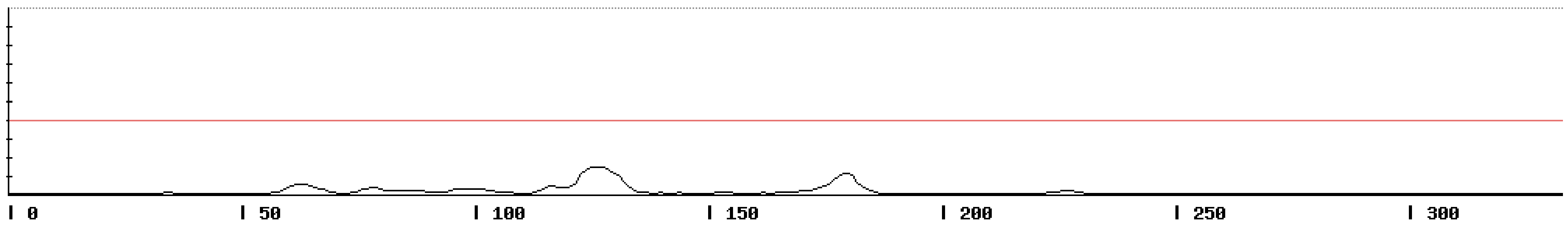

Predictions for Nicotianabenthamiana_Niben101Scf02340g06003.1

Viterbi Path	
Viterbi Path	
Posterior @ 0.4	
Posterior @ 0.4	
Posterior Graphic. Threshold Marked @ 0.4	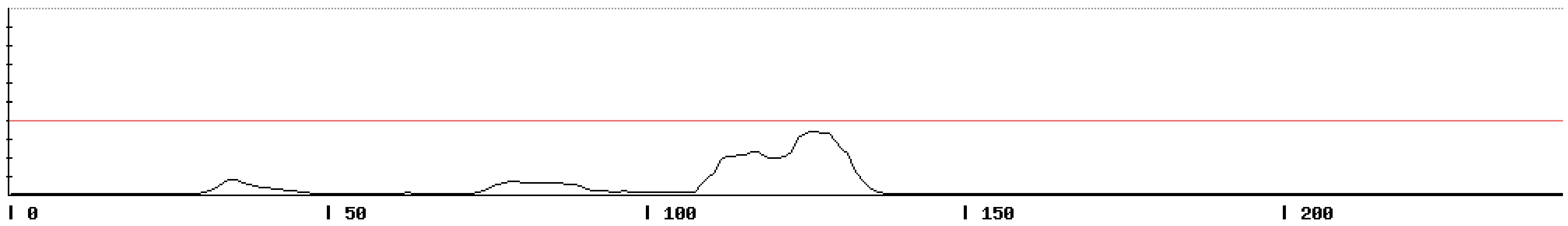

Predictions for Nicotianabenthamiana_Niben101Scf02413g02012.1

Viterbi Path	
Viterbi Path	

Posterior @ 0.4	
Posterior @ 0.4	
Posterior Graphic. Threshold Marked @ 0.4	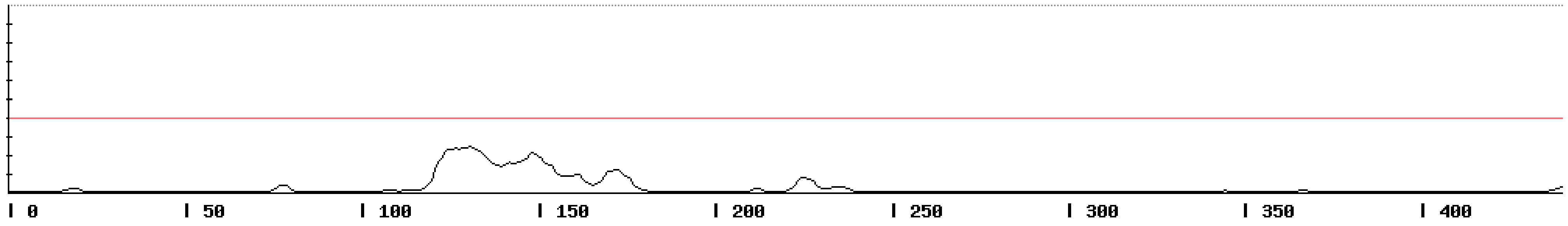

Predictions for Nicotianabenthamiana_Niben101Scf02420g03014.1

Viterbi Path	
Viterbi Path	
Posterior @ 0.4	
Posterior @ 0.4	
Posterior Graphic. Threshold Marked @ 0.4	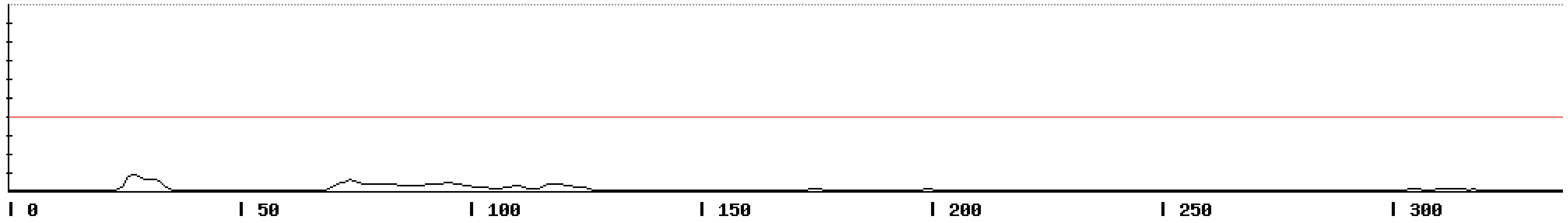

Predictions for Nicotianabenthamiana_Niben101Scf02423g00006.1

Viterbi Path

Viterbi Path

Posterior @ 0.4

Posterior @ 0.4

Posterior Graphic. Threshold Marked @ 0.4

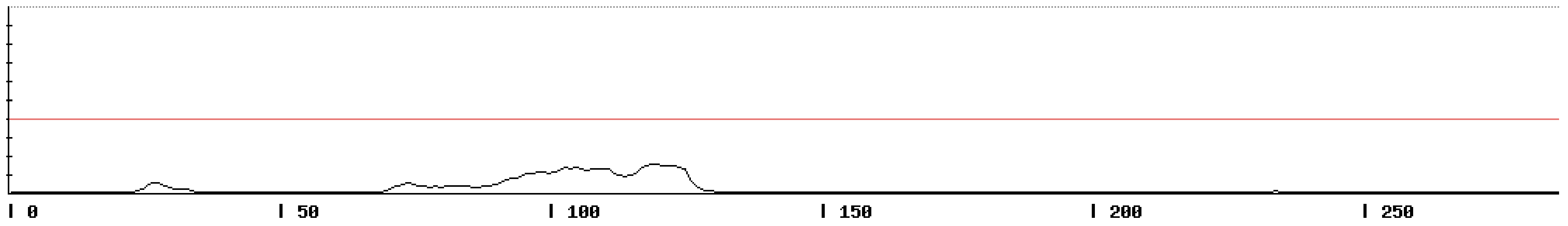

Predictions for Nicotianabenthamiana_Niben101Scf02433g00011.1

Viterbi Path

Viterbi Path

Posterior @ 0.4

Posterior @ 0.4

Posterior Graphic. Threshold Marked @ 0.4

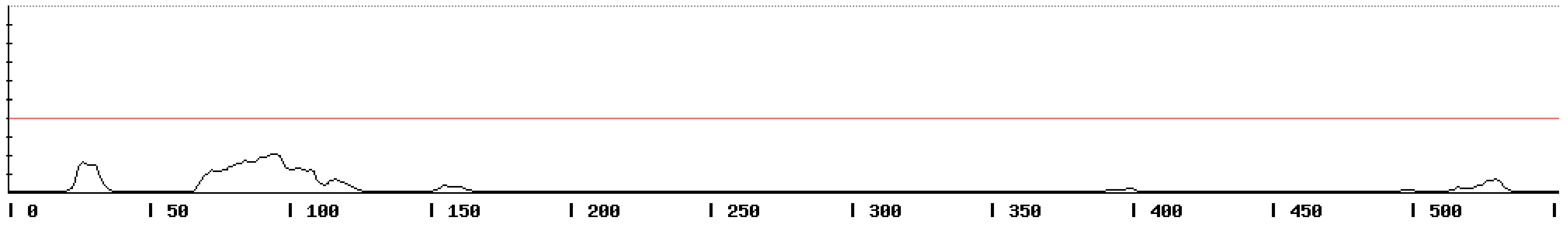

Predictions for Nicotianabenthamiana_Niben101Scf02459g02011.1	
Viterbi Path	
Viterbi Path	
Posterior @ 0.4	
Posterior @ 0.4	
Posterior Graphic. Threshold Marked @ 0.4	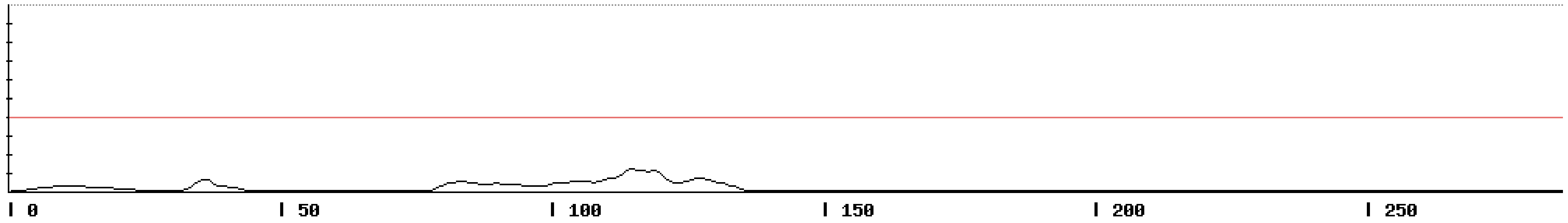

Predictions for Nicotianabenthamiana_Niben101Scf02464g00002.1	
Viterbi Path	
Viterbi Path	
Posterior @ 0.4	83 - KRR - 85
	89 - GRK - 91
	181 - NKRRKR - 186
Posterior @ 0.4	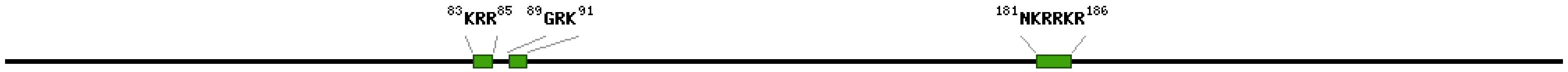
Posterior Graphic. Threshold Marked @ 0.4	

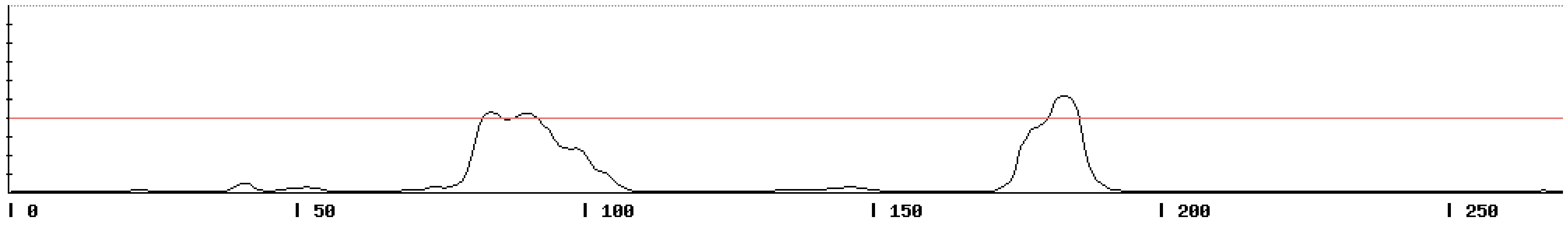

Predictions for Nicotianabenthamiana_Niben101Scf02475g08001.1

Viterbi Path	
Viterbi Path	
Posterior @ 0.4	
Posterior @ 0.4	
Posterior Graphic. Threshold Marked @ 0.4	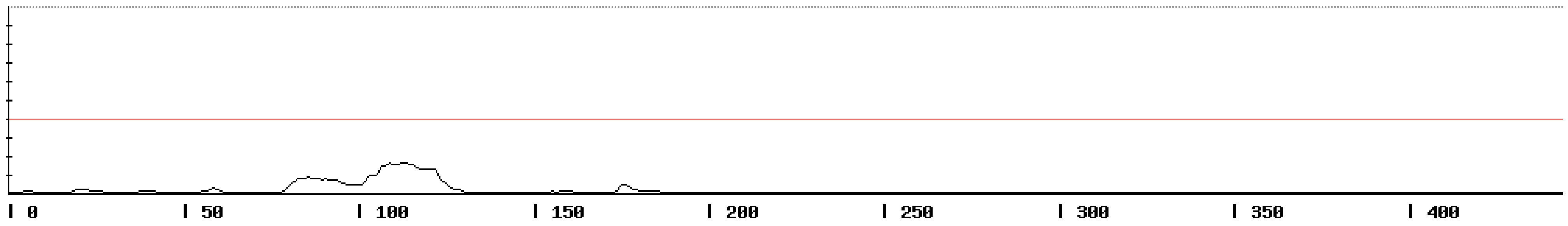

Predictions for Nicotianabenthamiana_Niben101Scf02664g01004.1

Viterbi Path	
Viterbi Path	
Posterior @ 0.4	
Posterior @ 0.4	

Posterior Graphic. Threshold Marked @ 0.4	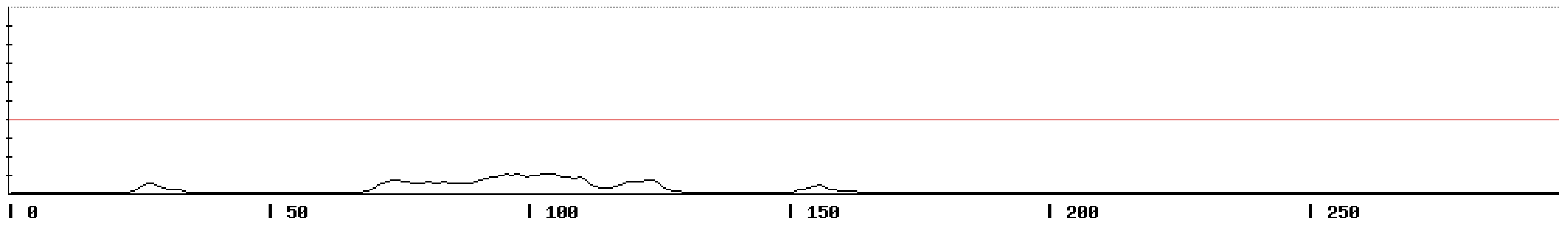

Predictions for Nicotianabenthamiana_Niben101Scf02735g01004.1

Viterbi Path	
Viterbi Path	
Posterior @ 0.4	
Posterior @ 0.4	
Posterior Graphic. Threshold Marked @ 0.4	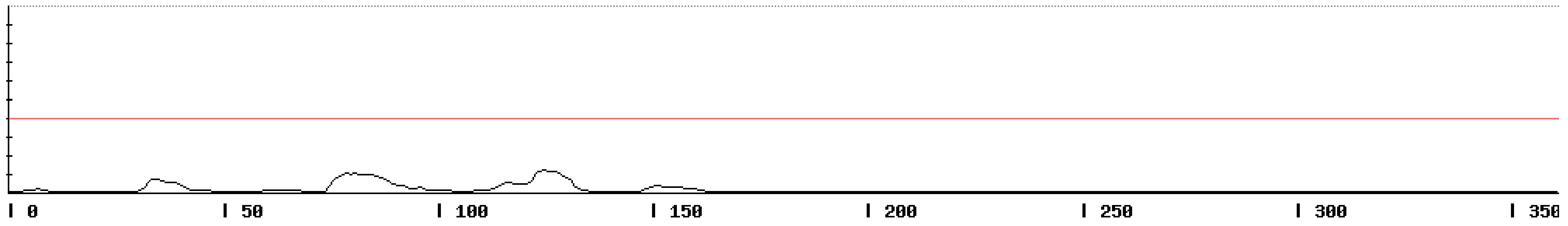

Predictions for Nicotianabenthamiana_Niben101Scf02762g01001.1

Viterbi Path	
Viterbi Path	
Posterior @ 0.4	
Posterior @ 0.4	

Posterior Graphic. Threshold Marked @ 0.4	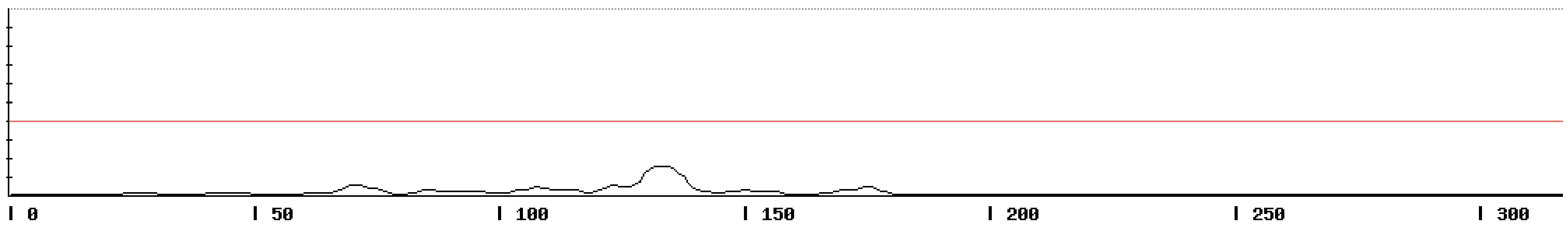

Predictions for Nicotianabenthamiana_Niben101Scf02840g00004.1	
Viterbi Path	
Viterbi Path	
Posterior @ 0.4	
Posterior @ 0.4	
Posterior Graphic. Threshold Marked @ 0.4	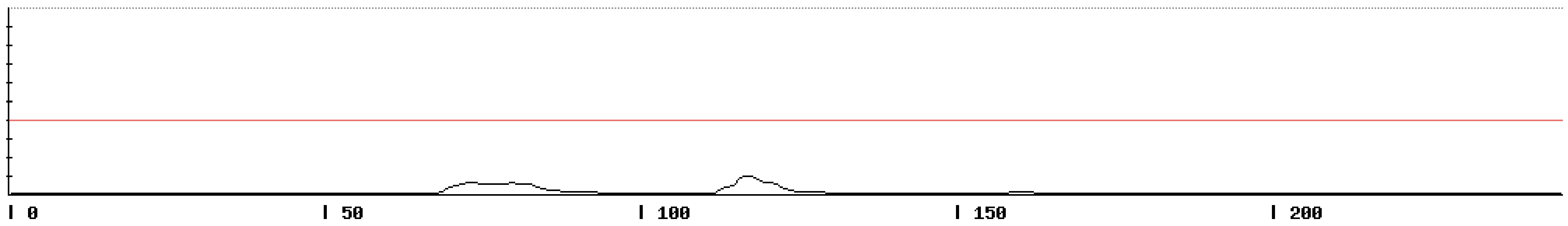

Predictions for Nicotianabenthamiana_Niben101Scf02862g01006.1	
Viterbi Path	
Viterbi Path	

Posterior @ 0.4

Posterior @ 0.4

Posterior Graphic. Threshold Marked @ 0.4

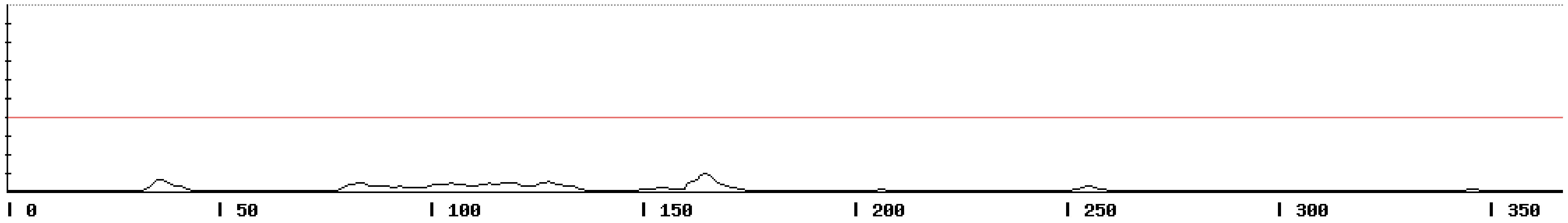

Predictions for Nicotianabenthamiana_Niben101Scf02864g06007.1

Viterbi Path

Viterbi Path

Posterior @ 0.4

Posterior @ 0.4

Posterior Graphic. Threshold Marked @ 0.4

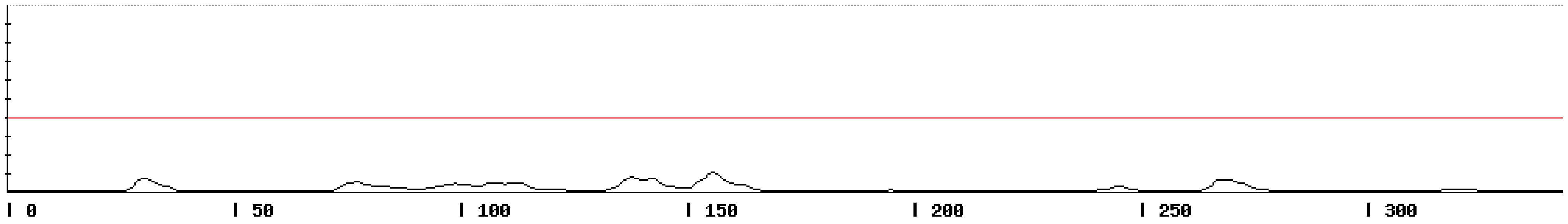

Predictions for Nicotianabenthamiana_Niben101Scf02881g01001.1

Viterbi Path

Viterbi Path

Posterior @ 0.4

Posterior @ 0.4

Posterior Graphic. Threshold Marked @ 0.4

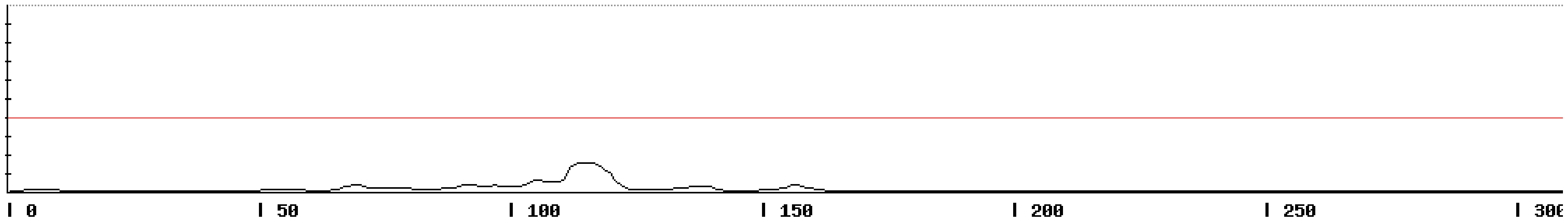

Predictions for Nicotianabenthamiana_Niben101Scf02904g00012.1

Viterbi Path

Viterbi Path

Posterior @ 0.4

Posterior @ 0.4

Posterior Graphic. Threshold Marked @ 0.4

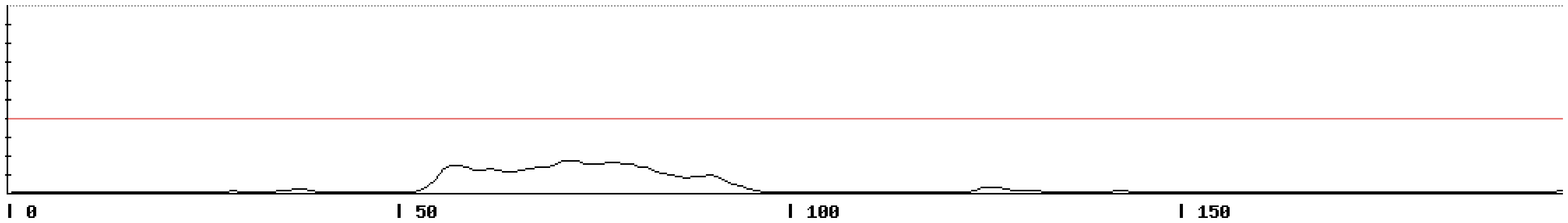

Predictions for Nicotianabenthamiana_Niben101Scf02904g00016.1

Viterbi Path

Viterbi Path

Posterior @ 0.4

188 - RNKRPRRKRDL - 198

414 - KK - 415

Posterior @ 0.4

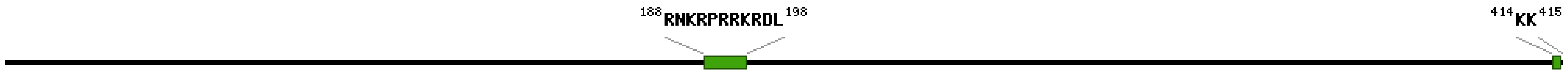

Posterior Graphic. Threshold Marked @ 0.4

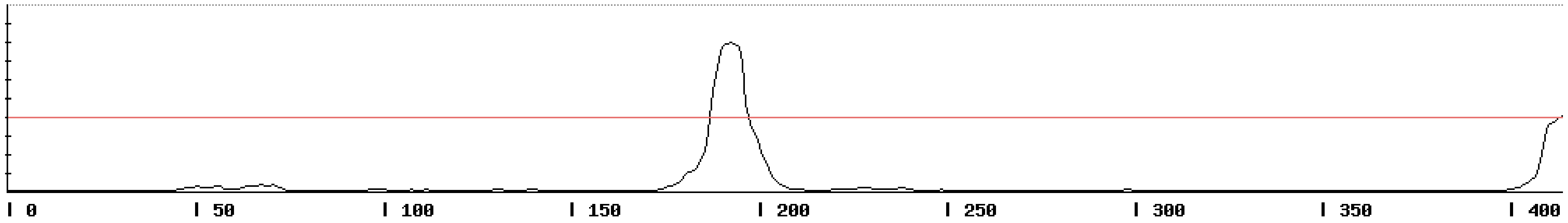

Predictions for Nicotianabenthamiana_Niben101Scf02947g00003.1

Viterbi Path

Viterbi Path

Posterior @ 0.4

Posterior @ 0.4

Posterior Graphic. Threshold Marked @ 0.4

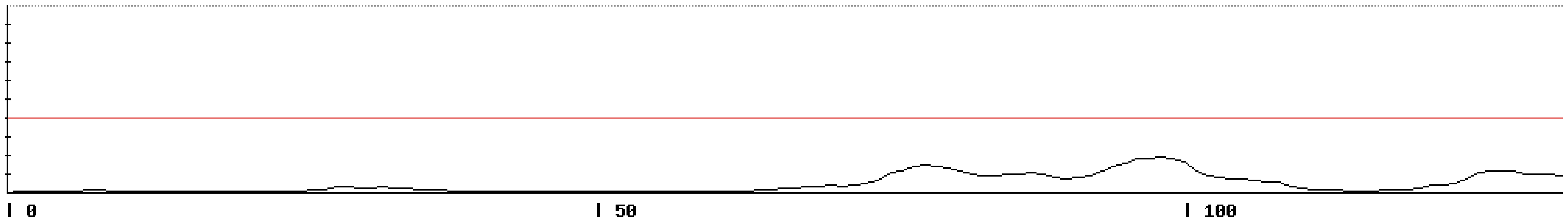

Predictions for Nicotianabenthamiana_Niben101Scf02979g04005.1	
Viterbi Path	
Viterbi Path	
Posterior @ 0.4	133 - RAPKGK - 138
Posterior @ 0.4	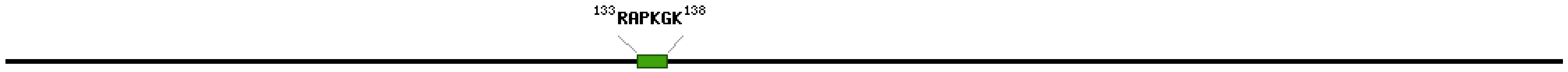
Posterior Graphic. Threshold Marked @ 0.4	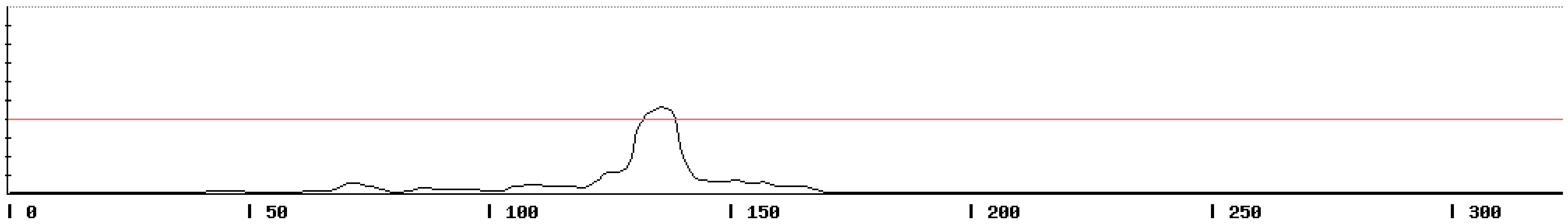

Predictions for Nicotianabenthamiana_Niben101Scf02983g01003.1	
Viterbi Path	
Viterbi Path	
Posterior @ 0.4	121 - ATKKGAKIG - 129
Posterior @ 0.4	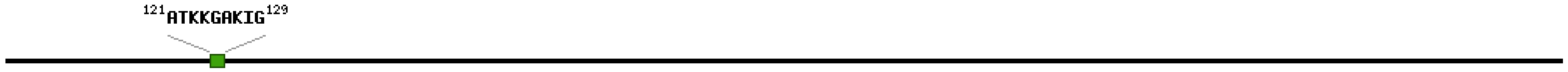

Posterior Graphic. Threshold Marked @ 0.4

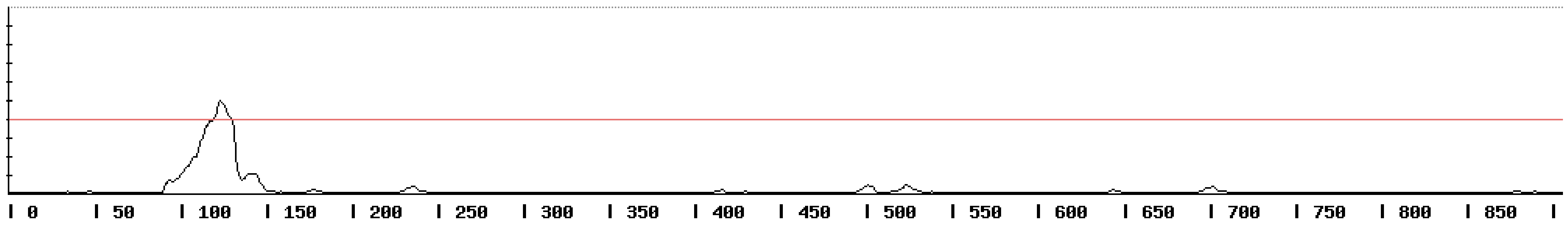

Predictions for Nicotianabenthamiana_Niben101Scf02996g08008.1

Viterbi Path

Viterbi Path

Posterior @ 0.4

Posterior @ 0.4

Posterior Graphic. Threshold Marked @ 0.4

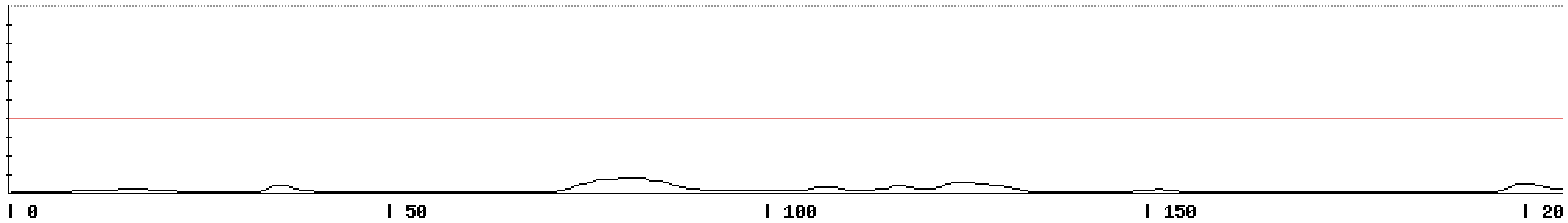

Predictions for Nicotianabenthamiana_Niben101Scf03028g01011.1

Viterbi Path

Viterbi Path

Posterior @ 0.4

Posterior @ 0.4

Posterior Graphic. Threshold Marked @ 0.4

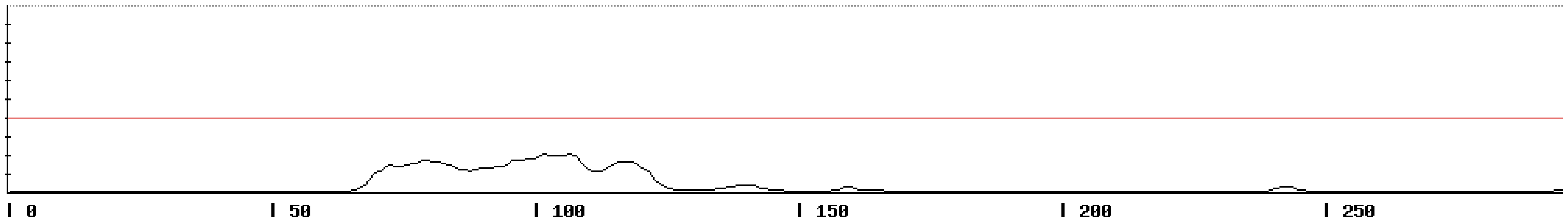

Predictions for Nicotianabenthamiana_Niben101Scf03160g01004.1

Viterbi Path

Viterbi Path

Posterior @ 0.4

Posterior @ 0.4

Posterior Graphic. Threshold Marked @ 0.4

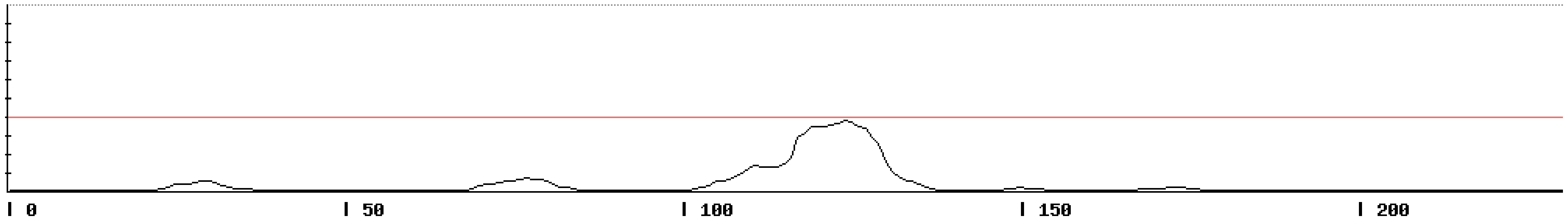

Predictions for Nicotianabenthamiana_Niben101Scf03169g04009.1

Viterbi Path

Viterbi Path

Posterior @ 0.4 — 140 - RKRRK - 144

Posterior @ 0.4

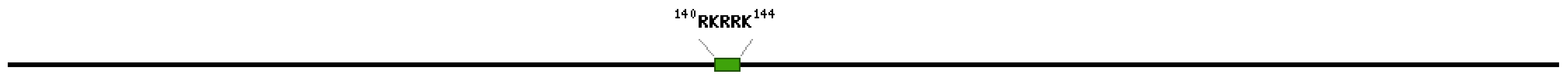

Posterior Graphic. Threshold Marked @ 0.4

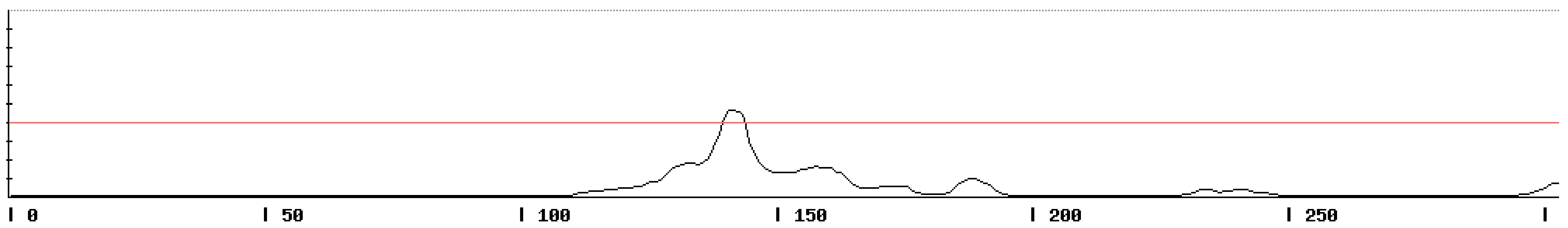

Predictions for Nicotianabenthamiana_Niben101Scf03204g02006.1

Viterbi Path

Viterbi Path

Posterior @ 0.4

Posterior @ 0.4

Posterior Graphic. Threshold Marked @ 0.4

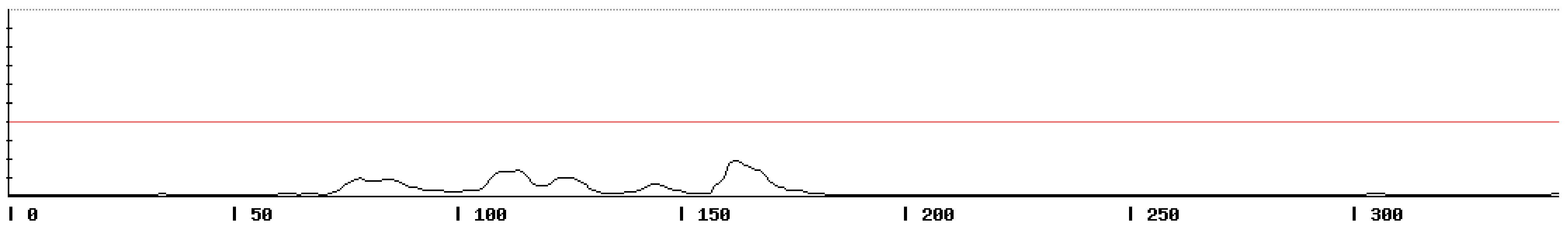

Predictions for Nicotianabenthamiana_Niben101Scf03283g02003.1

Viterbi Path

Viterbi Path

Posterior @ 0.4

Posterior @ 0.4

Posterior Graphic. Threshold Marked @ 0.4

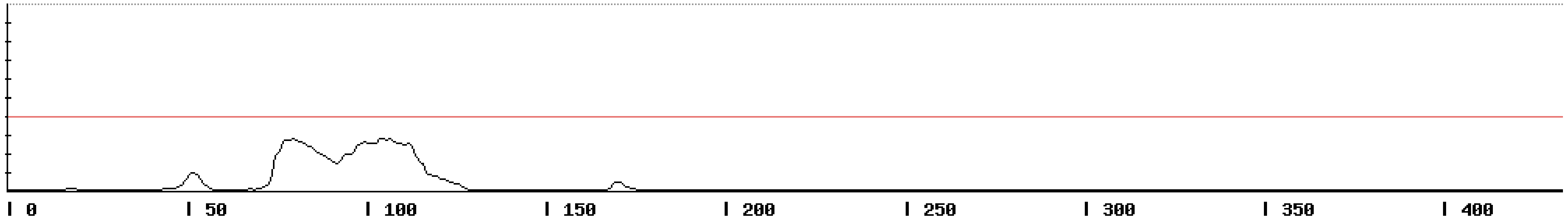

Predictions for Nicotianabenthamiana_Niben101Scf03307g05001.1

Viterbi Path

Viterbi Path

Posterior @ 0.4

Posterior @ 0.4

Posterior Graphic. Threshold Marked @ 0.4

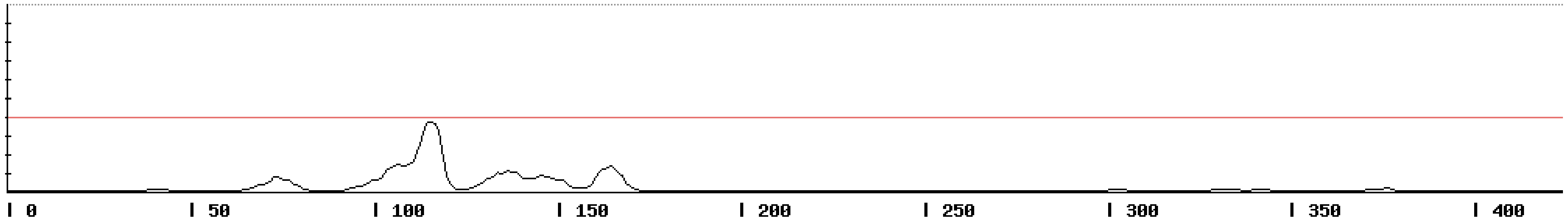

Predictions for Nicotianabenthamiana_Niben101Scf03366g00002.1

Viterbi Path	
Viterbi Path	
Posterior @ 0.4	
Posterior @ 0.4	
Posterior Graphic. Threshold Marked @ 0.4	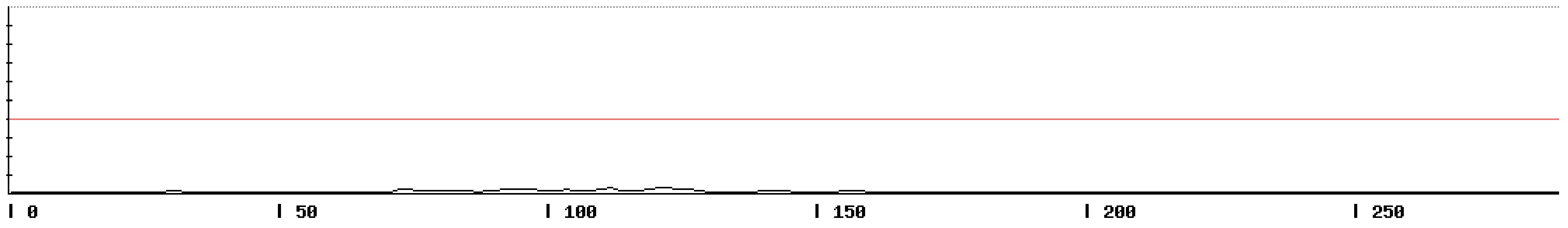

Predictions for Nicotianabenthamiana_Niben101Scf03407g02002.1

Viterbi Path	
Viterbi Path	
Posterior @ 0.4	
Posterior @ 0.4	
Posterior Graphic. Threshold Marked @ 0.4	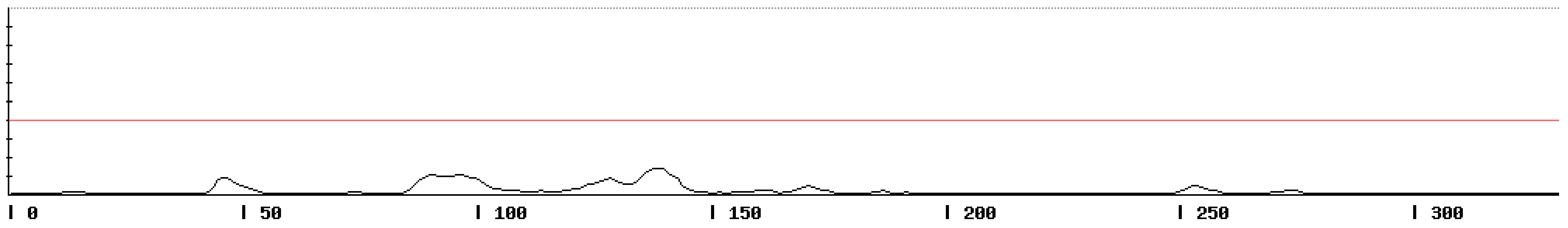

Predictions for Nicotianabenthamiana_Niben101Scf03438g06002.1

Viterbi Path	
Viterbi Path	
Posterior @ 0.4	78 - RTKAGGSR - 85
Posterior @ 0.4	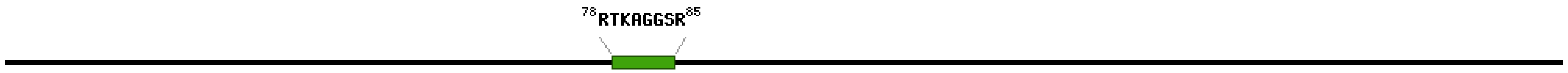
Posterior Graphic. Threshold Marked @ 0.4	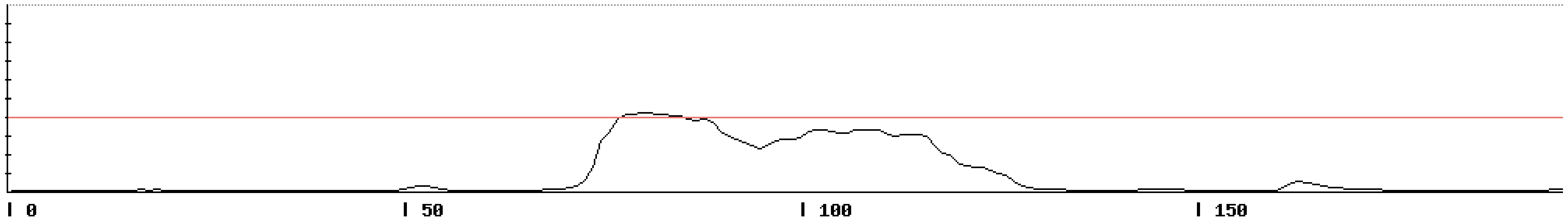

Predictions for Nicotianabenthamiana_Niben101Scf03438g06011.1

Viterbi Path	
Viterbi Path	
Posterior @ 0.4	
Posterior @ 0.4	
Posterior Graphic. Threshold Marked @ 0.4	

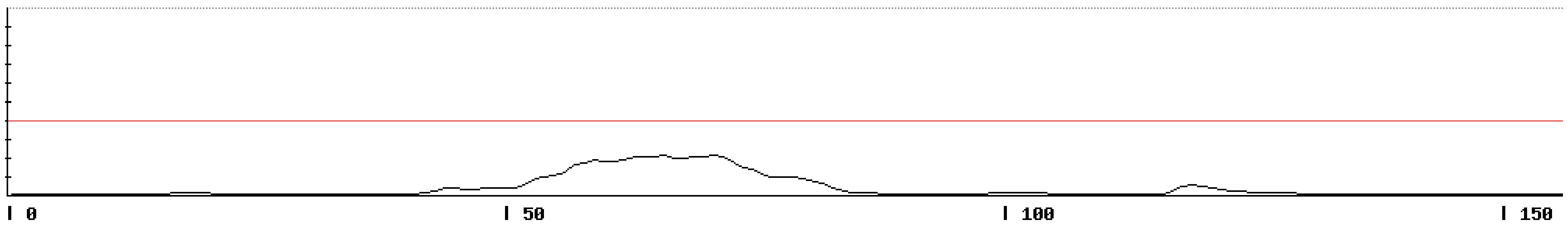

Predictions for Nicotianabenthamiana_Niben101Scf03528g03001.1

Viterbi Path	
Viterbi Path	
Posterior @ 0.4	
Posterior @ 0.4	
Posterior Graphic. Threshold Marked @ 0.4	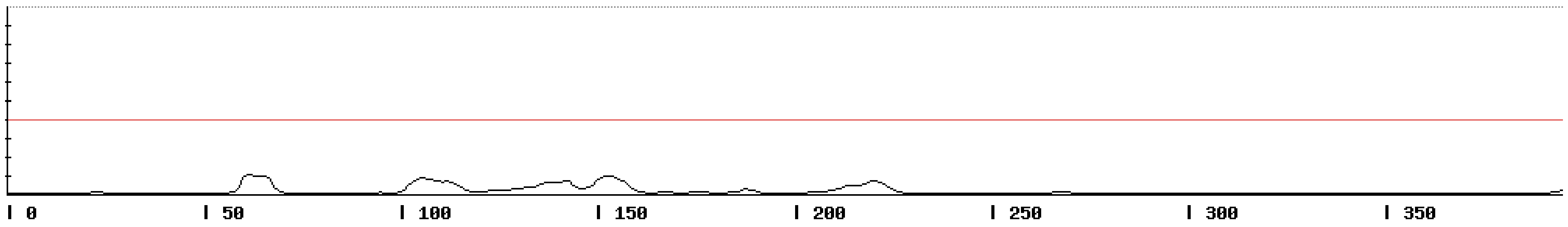

Predictions for Nicotianabenthamiana_Niben101Scf03695g01008.1

Viterbi Path	
Viterbi Path	
Posterior @ 0.4	76 - KKK - 78
Posterior @ 0.4	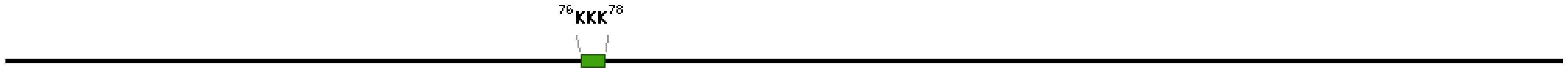

Posterior Graphic. Threshold Marked @ 0.4	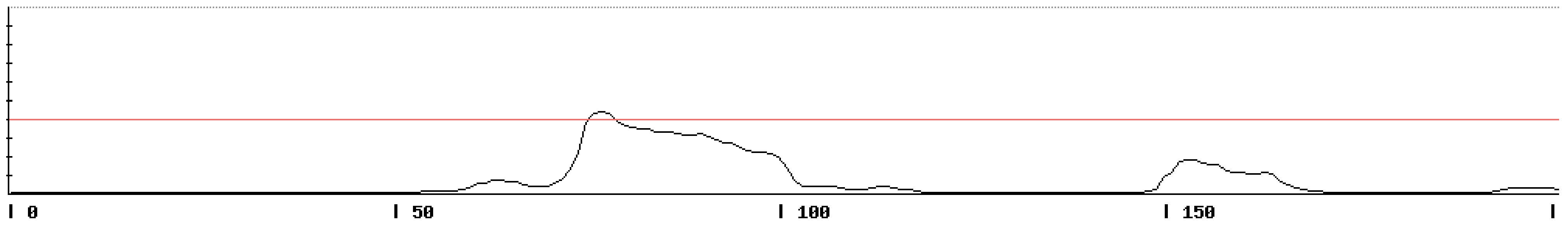

Predictions for Nicotianabenthamiana_Niben101Scf03735g07020.1

Viterbi Path	
Viterbi Path	
Posterior @ 0.4	
Posterior @ 0.4	
Posterior Graphic. Threshold Marked @ 0.4	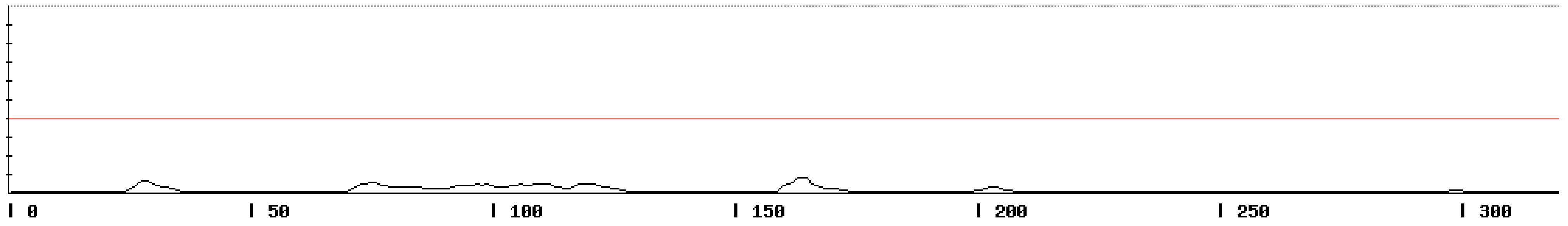

Predictions for Nicotianabenthamiana_Niben101Scf03766g14009.1

Viterbi Path	
Viterbi Path	
Posterior @ 0.4	
Posterior @ 0.4	

Posterior Graphic. Threshold Marked @ 0.4

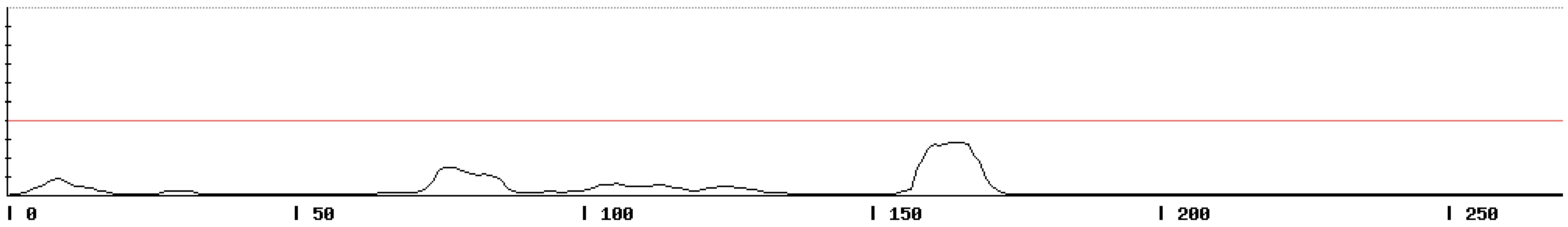

Predictions for Nicotianabenthamiana_Niben101Scf03810g07006.1

Viterbi Path

Viterbi Path

Posterior @ 0.4

Posterior @ 0.4

Posterior Graphic. Threshold Marked @ 0.4

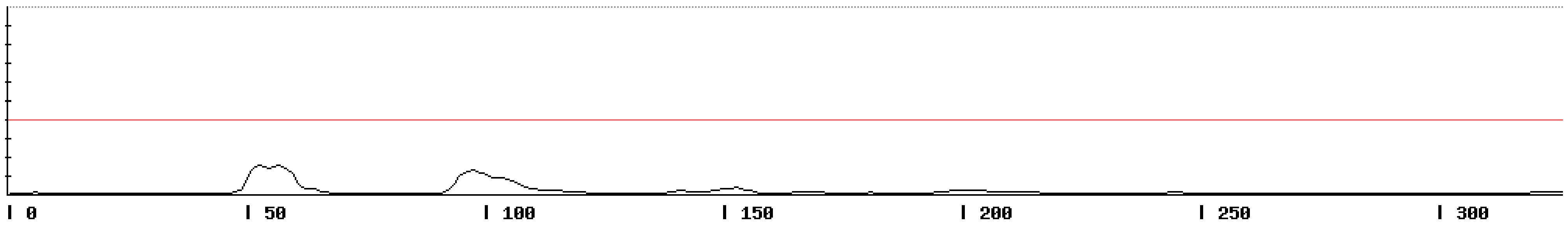

Predictions for Nicotianabenthamiana_Niben101Scf03853g00002.1

Viterbi Path

Viterbi Path

Posterior @ 0.4

Posterior @ 0.4

Posterior Graphic. Threshold Marked @ 0.4

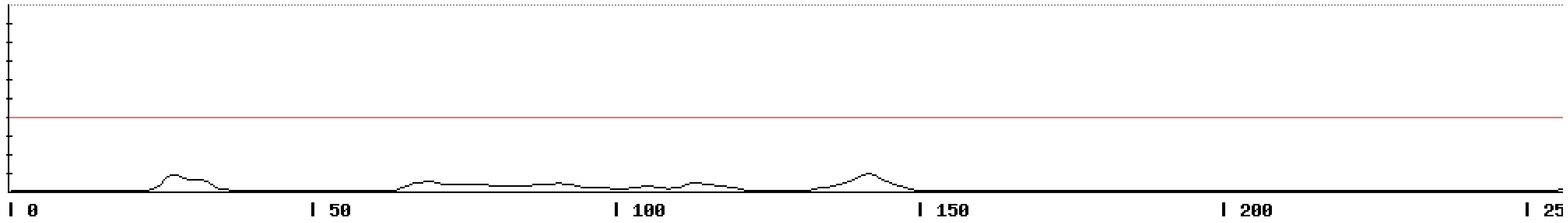

Predictions for Nicotianabenthamiana_Niben101Scf03885g02001.1

Viterbi Path

Viterbi Path

Posterior @ 0.4

Posterior @ 0.4

Posterior Graphic. Threshold Marked @ 0.4

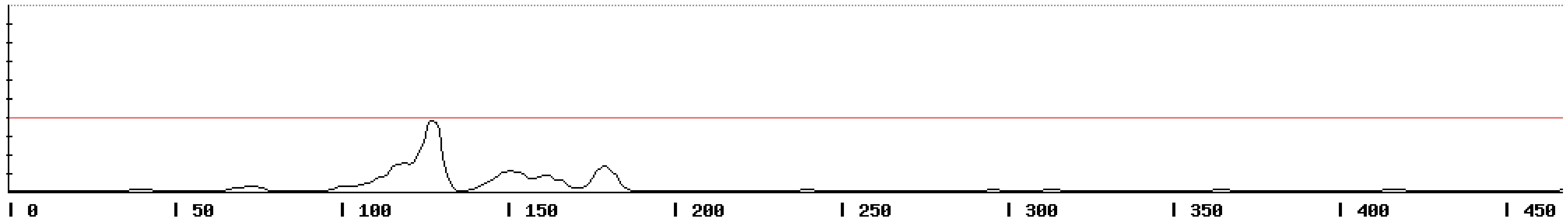

Predictions for Nicotianabenthamiana_Niben101Scf03915g02001.1

Viterbi Path

Viterbi Path

Posterior @ 0.4

Posterior @ 0.4

Posterior Graphic. Threshold Marked @ 0.4

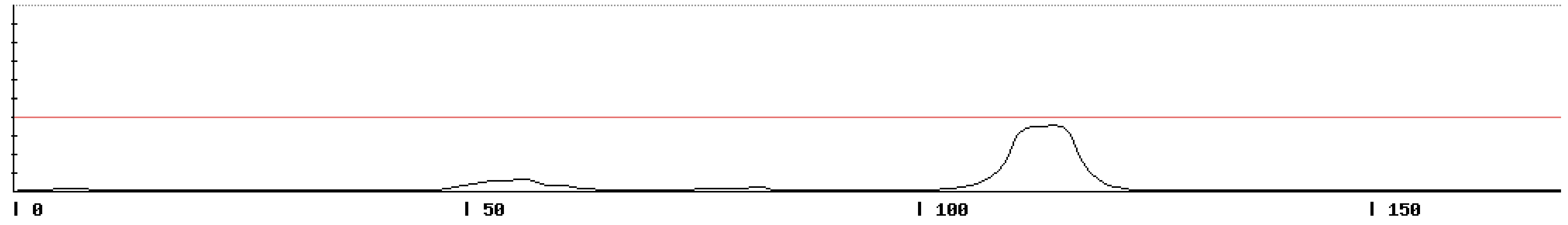

Predictions for Nicotianabenthamiana_Niben101Scf03941g01012.1

Viterbi Path

Viterbi Path

Posterior @ 0.4

Posterior @ 0.4

Posterior Graphic. Threshold Marked @ 0.4

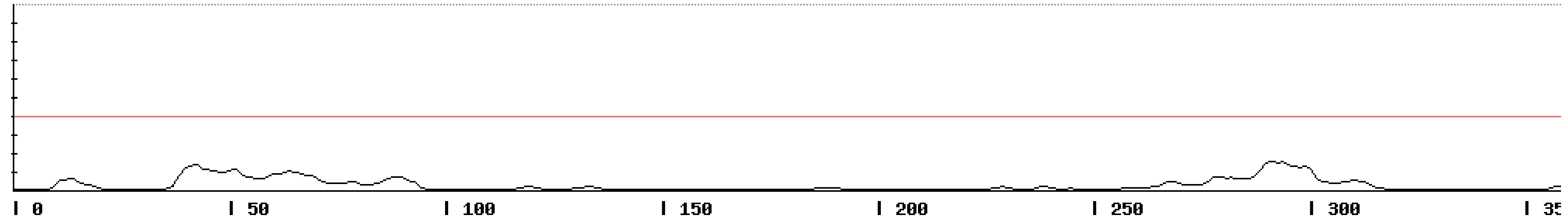

Predictions for Nicotianabenthamiana_Niben101Scf03949g02007.1

Viterbi Path

Viterbi Path

Posterior @ 0.4

Posterior @ 0.4

Posterior Graphic. Threshold Marked @ 0.4

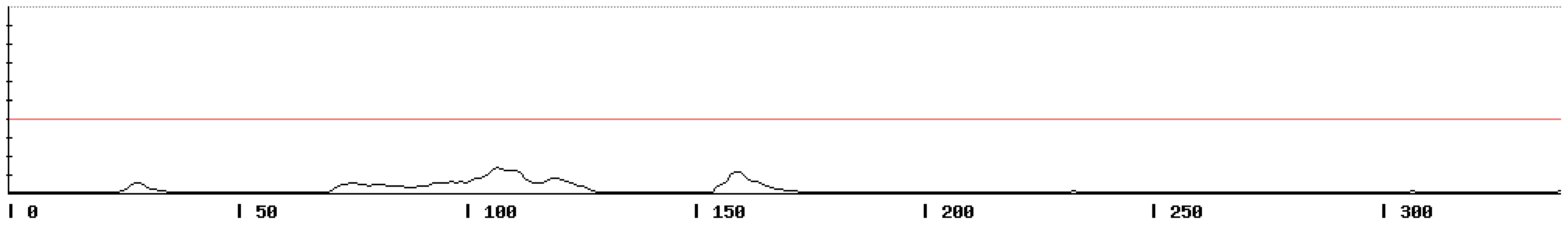

Predictions for Nicotianabenthamiana_Niben101Scf03978g09014.1

Viterbi Path

Viterbi Path

Posterior @ 0.4

Posterior @ 0.4

Posterior Graphic. Threshold Marked @ 0.4

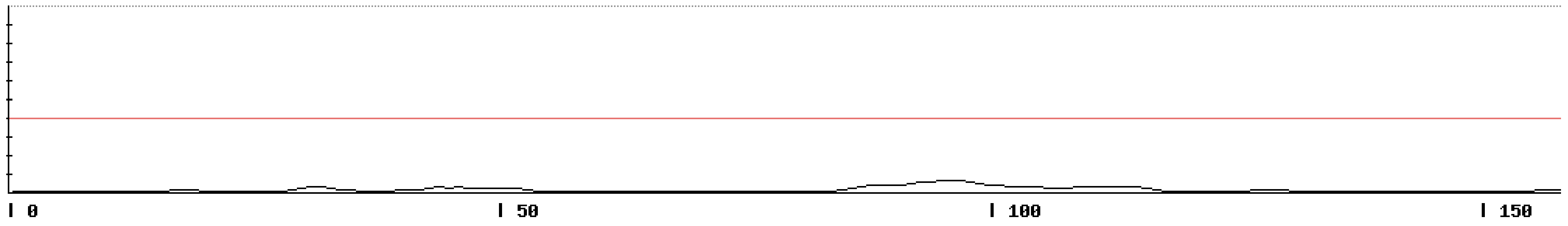

Predictions for Nicotianabenthamiana_Niben101Scf03987g00003.1

Viterbi Path	
Viterbi Path	
Posterior @ 0.4	
Posterior @ 0.4	
Posterior Graphic. Threshold Marked @ 0.4	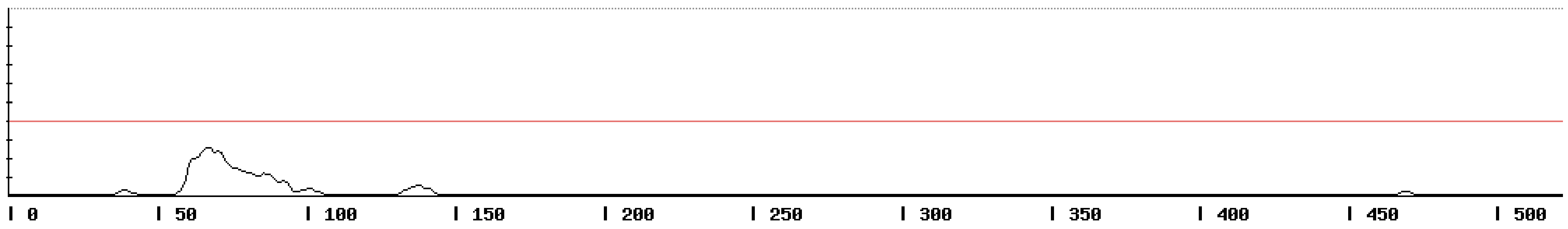

Predictions for Nicotianabenthamiana_Niben101Scf03987g00013.1

Viterbi Path	
Viterbi Path	
Posterior @ 0.4	326 - VRNKRPRRKRDSHPPP - 341<br>552 - KK - 553
Posterior @ 0.4	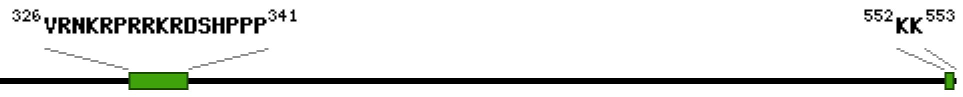
Posterior Graphic. Threshold Marked @ 0.4	

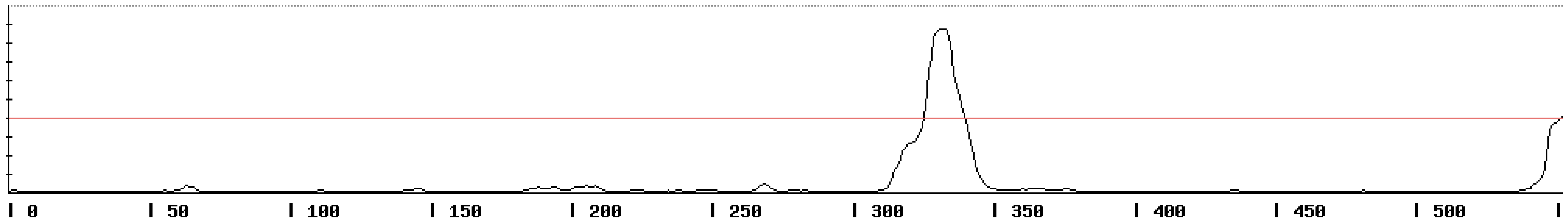

Predictions for Nicotianabenthamiana_Niben101Scf04099g08003.1

Viterbi Path	
Viterbi Path	
Posterior @ 0.4	
Posterior @ 0.4	
Posterior Graphic. Threshold Marked @ 0.4	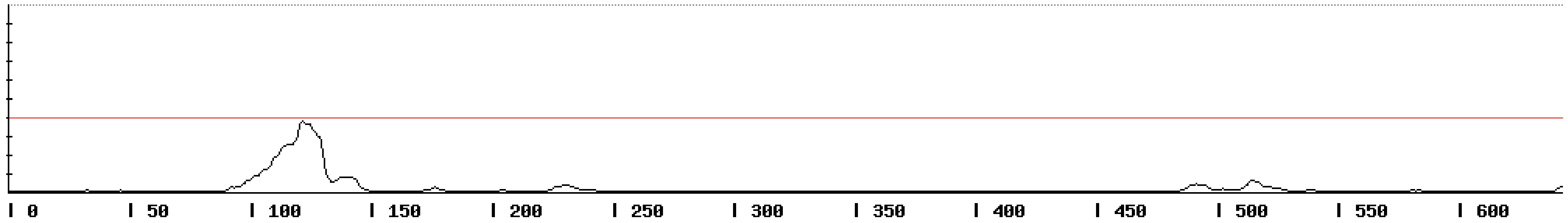

Predictions for Nicotianabenthamiana_Niben101Scf04133g02029.1

Viterbi Path	
Viterbi Path	
Posterior @ 0.4	
Posterior @ 0.4	

Posterior Graphic. Threshold Marked @ 0.4

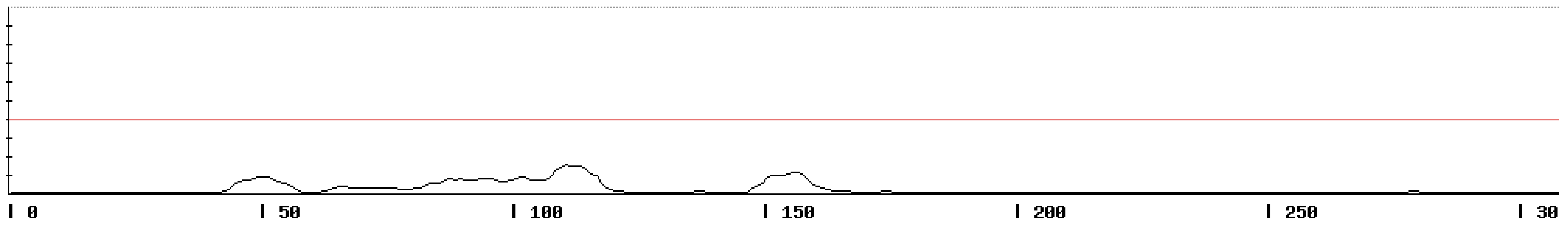

Predictions for Nicotianabenthamiana_Niben101Scf04173g03011.1

Viterbi Path

Viterbi Path

Posterior @ 0.4 122 - RKRRK - 126

Posterior @ 0.4

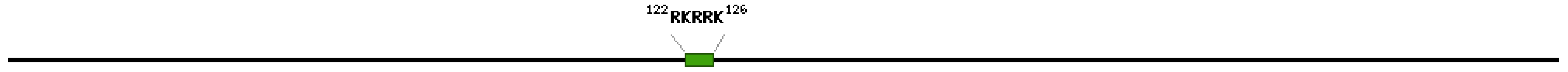

Posterior Graphic. Threshold Marked @ 0.4

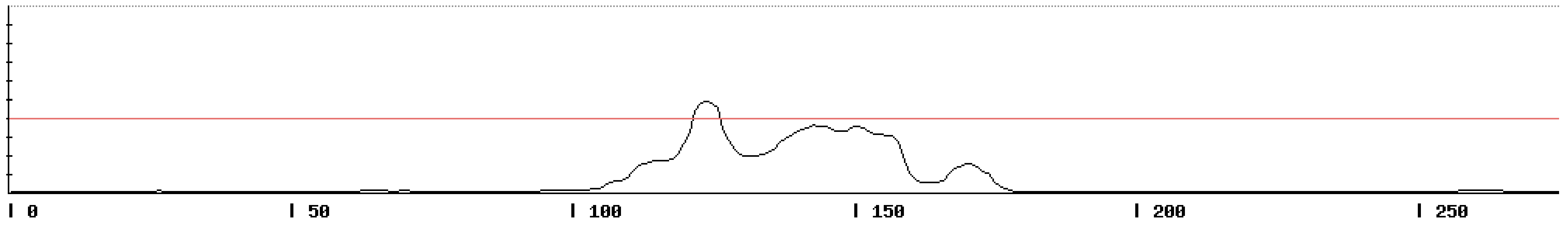

Predictions for Nicotianabenthamiana_Niben101Scf04216g06004.1

Viterbi Path

Viterbi Path

Posterior @ 0.4

Posterior @ 0.4

Posterior Graphic. Threshold Marked @ 0.4

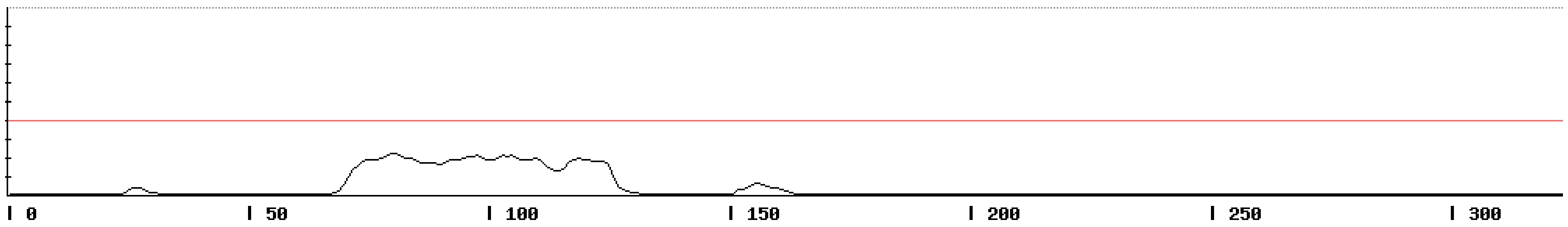

Predictions for Nicotianabenthamiana_Niben101Scf04217g02012.1

Viterbi Path

Viterbi Path

Posterior @ 0.4

Posterior @ 0.4

Posterior Graphic. Threshold Marked @ 0.4

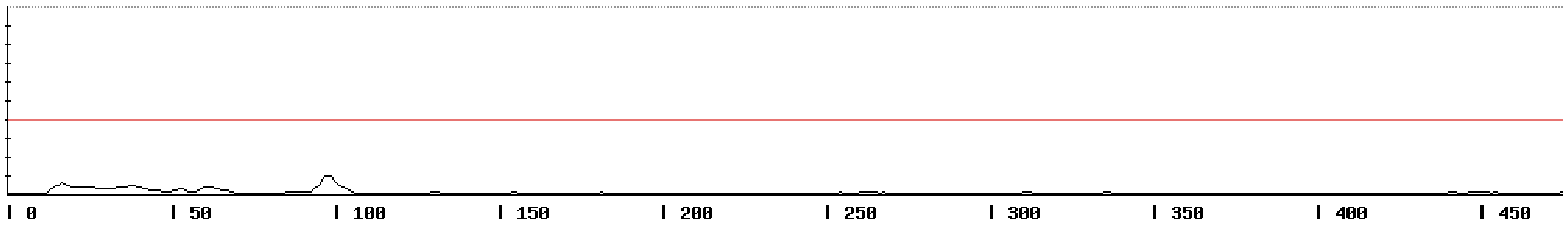

Predictions for Nicotianabenthamiana_Niben101Scf04296g00012.1

Viterbi Path

Viterbi Path

Posterior @ 0.4	
Posterior @ 0.4	
Posterior Graphic. Threshold Marked @ 0.4	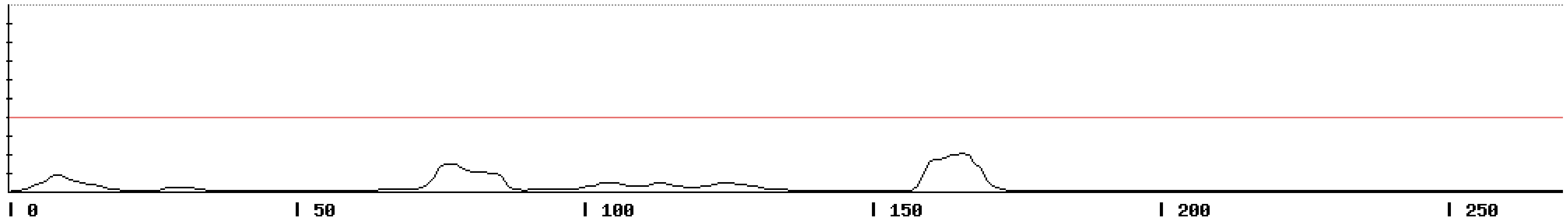

Predictions for Nicotianabenthamiana_Niben101Scf04323g01009.1	
Viterbi Path	
Viterbi Path	
Posterior @ 0.4	
Posterior @ 0.4	
Posterior Graphic. Threshold Marked @ 0.4	

Predictions for Nicotianabenthamiana_Niben101Scf04496g00016.1	
Viterbi Path	
Viterbi Path	
Posterior @ 0.4	
Posterior @ 0.4	
Posterior Graphic. Threshold Marked @ 0.4	

Predictions for Nicotianabenthamiana_Niben101Scf04654g03022.1	
Viterbi Path	
Viterbi Path	
Posterior @ 0.4	76 - RLKKKKKEHSRVRR - 89
Posterior @ 0.4	

Posterior Graphic. Threshold Marked @ 0.4	

Predictions for Nicotianabenthamiana_Niben101Scf04664g01015.1	
Viterbi Path	
Viterbi Path	
Posterior @ 0.4	79 - LKKKSKGG - 86
Posterior @ 0.4	
Posterior Graphic. Threshold Marked @ 0.4	

Predictions for Nicotianabenthamiana_Niben101Scf04745g02009.1	
Viterbi Path	
Viterbi Path	
Posterior @ 0.4	
Posterior @ 0.4	
Posterior Graphic. Threshold Marked @ 0.4	

Predictions for Nicotianabenthamiana_Niben101Scf04895g00003.1	
Viterbi Path	
Viterbi Path	
Posterior @ 0.4	78 - KKEKPTWKRVSRTVGKGTWKPQGKG - 102
	161 - KIKKKGKPGDKIK - 173
Posterior @ 0.4	
Posterior Graphic. Threshold Marked @ 0.4	

Predictions for Nicotianabenthamiana_Niben101Scf04963g01001.1	
Viterbi Path	
Viterbi Path	
Posterior @ 0.4	
Posterior @ 0.4	

Posterior Graphic. Threshold Marked @ 0.4	

Predictions for Nicotianabenthamiana_Niben101Scf05030g04002.1	
Viterbi Path	
Viterbi Path	
Posterior @ 0.4	
Posterior @ 0.4	
Posterior Graphic. Threshold Marked @ 0.4	

Predictions for Nicotianabenthamiana_Niben101Scf05036g01002.1	
Viterbi Path	
Viterbi Path	
Posterior @ 0.4	
Posterior @ 0.4	
Posterior Graphic. Threshold Marked @ 0.4	

Predictions for Nicotianabenthamiana_Niben101Scf05060g01009.1	
Viterbi Path	
Viterbi Path	
Posterior @ 0.4	
Posterior @ 0.4	
Posterior Graphic. Threshold Marked @ 0.4	

Predictions for Nicotianabenthamiana_Niben101Scf05060g08015.1	
Viterbi Path	
Viterbi Path	
Posterior @ 0.4	
Posterior @ 0.4	
Posterior Graphic. Threshold Marked @ 0.4	

Predictions for Nicotianabenthamiana_Niben101Scf05078g00004.1	
Viterbi Path	
Viterbi Path	
Posterior @ 0.4	
Posterior @ 0.4	
Posterior Graphic. Threshold Marked @ 0.4	

Predictions for Nicotianabenthamiana_Niben101Scf05175g03008.1	
Viterbi Path	
Viterbi Path	
Posterior @ 0.4	134 - RKRRK - 138
Posterior @ 0.4	
Posterior Graphic. Threshold Marked @ 0.4	

Predictions for Nicotianabenthamiana_Niben101Scf05186g00001.1	
Viterbi Path	
Viterbi Path	
Posterior @ 0.4	
Posterior @ 0.4	
Posterior Graphic. Threshold Marked @ 0.4	

Predictions for Nicotianabenthamiana_Niben101Scf05190g01001.1	
Viterbi Path	
Viterbi Path	
Posterior @ 0.4	136 - RKRRK - 140<br>152 - WHKTGKTRPVVVKGKVKGHKK - 172
Posterior @ 0.4	
Posterior Graphic. Threshold Marked @ 0.4	

Predictions for Nicotianabenthamiana_Niben101Scf05349g02002.1	
Viterbi Path	
Viterbi Path	
Posterior @ 0.4	
Posterior @ 0.4	
Posterior Graphic. Threshold Marked @ 0.4	

Predictions for Nicotianabenthamiana_Niben101Scf05363g03006.1	
Viterbi Path	
Viterbi Path	
Posterior @ 0.4	
Posterior @ 0.4	
Posterior Graphic. Threshold Marked @ 0.4	

Predictions for Nicotianabenthamiana_Niben101Scf05391g03014.1	
Viterbi Path	
Viterbi Path	
Posterior @ 0.4	99 - RKRRK - 103
Posterior @ 0.4	
Posterior Graphic. Threshold Marked @ 0.4	

Predictions for Nicotianabenthamiana_Niben101Scf05461g00003.1	
Viterbi Path	
Viterbi Path	
Posterior @ 0.4	
Posterior @ 0.4	
Posterior Graphic. Threshold Marked @ 0.4	

Predictions for Nicotianabenthamiana_Niben101Scf05629g00007.1

Viterbi Path	
Viterbi Path	
Posterior @ 0.4	
Posterior @ 0.4	
Posterior Graphic. Threshold Marked @ 0.4	

**Predictions for Nicotianabenthamiana_Niben101Scf05699g00009.1**	
Viterbi Path	
Viterbi Path	
Posterior @ 0.4	
Posterior @ 0.4	
Posterior Graphic. Threshold Marked @ 0.4	

**Predictions for Nicotianabenthamiana_Niben101Scf05750g01016.1**	
Viterbi Path	
Viterbi Path	
Posterior @ 0.4	
Posterior @ 0.4	
Posterior Graphic. Threshold Marked @ 0.4	

**Predictions for Nicotianabenthamiana_Niben101Scf05809g01004.1**	
Viterbi Path	
Viterbi Path	
Posterior @ 0.4	
Posterior @ 0.4	
Posterior Graphic. Threshold Marked @ 0.4	

**Predictions for Nicotianabenthamiana_Niben101Scf05821g02003.1**	
Viterbi Path	

Viterbi Path	
Posterior @ 0.4	
Posterior @ 0.4	
Posterior Graphic. Threshold Marked @ 0.4	

Predictions for Nicotianabenthamiana_Niben101Scf06023g00003.1	
Viterbi Path	
Viterbi Path	
Posterior @ 0.4	58 - KK - 59
Posterior @ 0.4	
Posterior Graphic. Threshold Marked @ 0.4	

Predictions for Nicotianabenthamiana_Niben101Scf06096g01013.1	
Viterbi Path	
Viterbi Path	
Posterior @ 0.4	135 - KKRKES - 140
Posterior @ 0.4	
Posterior Graphic. Threshold Marked @ 0.4	

Predictions for Nicotianabenthamiana_Niben101Scf06153g01005.1	
Viterbi Path	
Viterbi Path	
Posterior @ 0.4	
Posterior @ 0.4	
Posterior Graphic. Threshold Marked @ 0.4	

Predictions for Nicotianabenthamiana_Niben101Scf06223g00023.1	
Viterbi Path	
Viterbi Path	

Posterior @ 0.4	
Posterior @ 0.4	
Posterior Graphic. Threshold Marked @ 0.4	

**Predictions for Nicotianabenthamiana_Niben101Scf06245g01014.1**	
Viterbi Path	
Viterbi Path	
Posterior @ 0.4	
Posterior @ 0.4	
Posterior Graphic. Threshold Marked @ 0.4	

**Predictions for Nicotianabenthamiana_Niben101Scf06245g01015.1**	
Viterbi Path	
Viterbi Path	
Posterior @ 0.4	
Posterior @ 0.4	
Posterior Graphic. Threshold Marked @ 0.4	

**Predictions for Nicotianabenthamiana_Niben101Scf06588g01002.1**	
Viterbi Path	
Viterbi Path	
Posterior @ 0.4	
Posterior @ 0.4	
Posterior Graphic. Threshold Marked @ 0.4	

**Predictions for Nicotianabenthamiana_Niben101Scf06749g01004.1**	
Viterbi Path	
Viterbi Path	
Posterior @ 0.4	78 - RTKAGGSR - 85

Posterior @ 0.4	
Posterior Graphic. Threshold Marked @ 0.4	

Predictions for Nicotianabenthamiana_Niben101Scf06757g00006.1	
Viterbi Path	
Viterbi Path	
Posterior @ 0.4	
Posterior @ 0.4	
Posterior Graphic. Threshold Marked @ 0.4	

Predictions for Nicotianabenthamiana_Niben101Scf06846g01004.1	
Viterbi Path	
Viterbi Path	
Posterior @ 0.4	
Posterior @ 0.4	
Posterior Graphic. Threshold Marked @ 0.4	

Predictions for Nicotianabenthamiana_Niben101Scf06862g00001.1	
Viterbi Path	
Viterbi Path	
Posterior @ 0.4	
Posterior @ 0.4	
Posterior Graphic. Threshold Marked @ 0.4	

Predictions for Nicotianabenthamiana_Niben101Scf06916g01011.1	
Viterbi Path	
Viterbi Path	
Posterior @ 0.4	72 - KHGPKGRPN - 80
Posterior @ 0.4	

Posterior Graphic. Threshold Marked @ 0.4	

Predictions for Nicotianabenthamiana_Niben101Scf07152g04032.1	
Viterbi Path	
Viterbi Path	
Posterior @ 0.4	
Posterior @ 0.4	
Posterior Graphic. Threshold Marked @ 0.4	

Predictions for Nicotianabenthamiana_Niben101Scf07162g03025.1	
Viterbi Path	
Viterbi Path	
Posterior @ 0.4	
Posterior @ 0.4	
Posterior Graphic. Threshold Marked @ 0.4	

Predictions for Nicotianabenthamiana_Niben101Scf07162g05018.1	
Viterbi Path	
Viterbi Path	
Posterior @ 0.4	
Posterior @ 0.4	
Posterior Graphic. Threshold Marked @ 0.4	

Predictions for Nicotianabenthamiana_Niben101Scf07162g05028.1	
Viterbi Path	
Viterbi Path	
Posterior @ 0.4	77 - LKKKKREHTRVR - 88
Posterior @ 0.4	
Posterior Graphic. Threshold Marked @ 0.4	

Predictions for Nicotianabenthamiana_Niben101Scf07168g02012.1	
Viterbi Path	
Viterbi Path	
Posterior @ 0.4	78 - KKEKPTWKRVSRTVGKGTWKPQGKG - 102
	161 - KIKKKGKPGDKIK - 173
	307 - KKEKPTWKRVSRTVGKGTWKPQGKG - 331
	390 - KIKKKGKPGDKIK - 402
	568 - KKEKPTWKRVSRTVGKGTWKPQGKG - 592
	651 - KIKKKGKPGDKIK - 663
Posterior @ 0.4	
Posterior Graphic. Threshold Marked @ 0.4	

Predictions for Nicotianabenthamiana_Niben101Scf07231g03006.1	
Viterbi Path	
Viterbi Path	
Posterior @ 0.4	
Posterior @ 0.4	
Posterior Graphic. Threshold Marked @ 0.4	

Predictions for Nicotianabenthamiana_Niben101Scf07491g00006.1	
Viterbi Path	
Viterbi Path	
Posterior @ 0.4	46 - R - 46
Posterior @ 0.4	
Posterior Graphic. Threshold Marked @ 0.4	

Predictions for Nicotianabenthamiana_Niben101Scf07508g02004.1

Viterbi Path	
Viterbi Path	
Posterior @ 0.4	
Posterior @ 0.4	
Posterior Graphic. Threshold Marked @ 0.4	

Predictions for Nicotianabenthamiana_Niben101Scf07673g04009.1	
Viterbi Path	
Viterbi Path	
Posterior @ 0.4	
Posterior @ 0.4	
Posterior Graphic. Threshold Marked @ 0.4	

Predictions for Nicotianabenthamiana_Niben101Scf07764g00002.1	
Viterbi Path	
Viterbi Path	
Posterior @ 0.4	
Posterior @ 0.4	
Posterior Graphic. Threshold Marked @ 0.4	

Predictions for Nicotianabenthamiana_Niben101Scf07788g04022.1	
Viterbi Path	
Viterbi Path	
Posterior @ 0.4	133 - HKRRK - 137
Posterior @ 0.4	
Posterior Graphic. Threshold Marked @ 0.4	

Predictions for Nicotianabenthamiana_Niben101Scf07825g04013.1	
Viterbi Path	

Viterbi Path	
Posterior @ 0.4	
Posterior @ 0.4	
Posterior Graphic. Threshold Marked @ 0.4	

Predictions for Nicotianabenthamiana_Niben101Scf07850g08004.1	
Viterbi Path	
Viterbi Path	
Posterior @ 0.4	
Posterior @ 0.4	
Posterior Graphic. Threshold Marked @ 0.4	

Predictions for Nicotianabenthamiana_Niben101Scf08028g00005.1	
Viterbi Path	
Viterbi Path	
Posterior @ 0.4	
Posterior @ 0.4	
Posterior Graphic. Threshold Marked @ 0.4	

Predictions for Nicotianabenthamiana_Niben101Scf08032g00005.1	
Viterbi Path	
Viterbi Path	
Posterior @ 0.4	
Posterior @ 0.4	
Posterior Graphic. Threshold Marked @ 0.4	

Predictions for Nicotianabenthamiana_Niben101Scf08137g05017.1	
Viterbi Path	
Viterbi Path	

Posterior @ 0.4	
Posterior @ 0.4	
Posterior Graphic. Threshold Marked @ 0.4	

**Predictions for Nicotianabenthamiana_Niben101Scf08364g00014.1**	
Viterbi Path	
Viterbi Path	
Posterior @ 0.4	
Posterior @ 0.4	
Posterior Graphic. Threshold Marked @ 0.4	

**Predictions for Nicotianabenthamiana_Niben101Scf08526g04001.1**	
Viterbi Path	
Viterbi Path	
Posterior @ 0.4	
Posterior @ 0.4	
Posterior Graphic. Threshold Marked @ 0.4	

**Predictions for Nicotianabenthamiana_Niben101Scf08531g02001.1**	
Viterbi Path	
Viterbi Path	
Posterior @ 0.4	73 - KKKSSAR - 79
Posterior @ 0.4	
Posterior Graphic. Threshold Marked @ 0.4	

**Predictions for Nicotianabenthamiana_Niben101Scf08531g03002.1**	
Viterbi Path	
Viterbi Path	
Posterior @ 0.4	

Posterior @ 0.4	
Posterior Graphic. Threshold Marked @ 0.4	

Predictions for Nicotianabenthamiana_Niben101Scf08531g03004.1	
Viterbi Path	
Viterbi Path	
Posterior @ 0.4	
Posterior @ 0.4	
Posterior Graphic. Threshold Marked @ 0.4	

Predictions for Nicotianabenthamiana_Niben101Scf08665g00007.1	
Viterbi Path	118 - KIKKKGKPGDKKKG - 131
Viterbi Path	
Posterior @ 0.4	35 - KKEKPTWKRVSRTVGKGT - 52
	118 - KIKKKGKPGDKKKG - 131
Posterior @ 0.4	
Posterior Graphic. Threshold Marked @ 0.4	

Predictions for Nicotianabenthamiana_Niben101Scf09065g00002.1	
Viterbi Path	
Viterbi Path	
Posterior @ 0.4	298 - RLKKKKKEHSRVRR - 311
Posterior @ 0.4	
Posterior Graphic. Threshold Marked @ 0.4	

Predictions for Nicotianabenthamiana_Niben101Scf09078g00010.1	
Viterbi Path	
Viterbi Path	
Posterior @ 0.4	

Posterior @ 0.4	
Posterior Graphic. Threshold Marked @ 0.4	

Predictions for Nicotianabenthamiana_Niben101Scf09296g01002.1	
Viterbi Path	
Viterbi Path	
Posterior @ 0.4	
Posterior @ 0.4	
Posterior Graphic. Threshold Marked @ 0.4	

Predictions for Nicotianabenthamiana_Niben101Scf09387g05005.1	
Viterbi Path	
Viterbi Path	
Posterior @ 0.4	
Posterior @ 0.4	
Posterior Graphic. Threshold Marked @ 0.4	

Predictions for Nicotianabenthamiana_Niben101Scf09555g04002.1	
Viterbi Path	
Viterbi Path	
Posterior @ 0.4	
Posterior @ 0.4	
Posterior Graphic. Threshold Marked @ 0.4	

Predictions for Nicotianabenthamiana_Niben101Scf09658g00029.1	
Viterbi Path	
Viterbi Path	
Posterior @ 0.4	
Posterior @ 0.4	

Posterior Graphic. Threshold Marked @ 0.4	

Predictions for Nicotianabenthamiana_Niben101Scf09792g03016.1	
Viterbi Path	
Viterbi Path	
Posterior @ 0.4	
Posterior @ 0.4	
Posterior Graphic. Threshold Marked @ 0.4	

Predictions for Nicotianabenthamiana_Niben101Scf10322g01012.1	
Viterbi Path	
Viterbi Path	
Posterior @ 0.4	
Posterior @ 0.4	
Posterior Graphic. Threshold Marked @ 0.4	

Predictions for Nicotianabenthamiana_Niben101Scf10466g00016.1	
Viterbi Path	
Viterbi Path	
Posterior @ 0.4	
Posterior @ 0.4	
Posterior Graphic. Threshold Marked @ 0.4	

Predictions for Nicotianabenthamiana_Niben101Scf10713g00006.1	
Viterbi Path	
Viterbi Path	
Posterior @ 0.4	69 - RKKNKGR - 75
	166 - KKLPKRKK - 173
Posterior @ 0.4	

Posterior Graphic. Threshold Marked @ 0.4

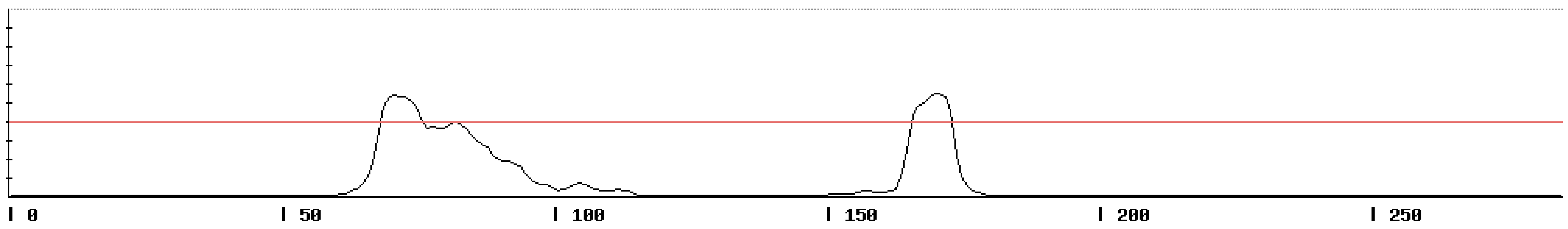

Predictions for Nicotianabenthamiana_Niben101Scf10713g00008.1

Viterbi Path

Viterbi Path

Posterior @ 0.4

69 - RKKNKGR - 75

166 - KKLPKRKK - 173

Posterior @ 0.4

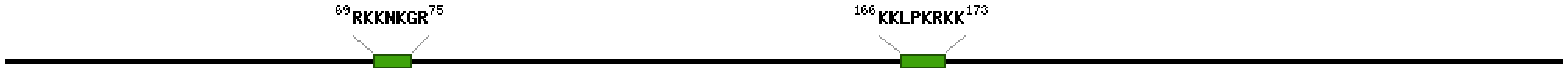

Posterior Graphic. Threshold Marked @ 0.4

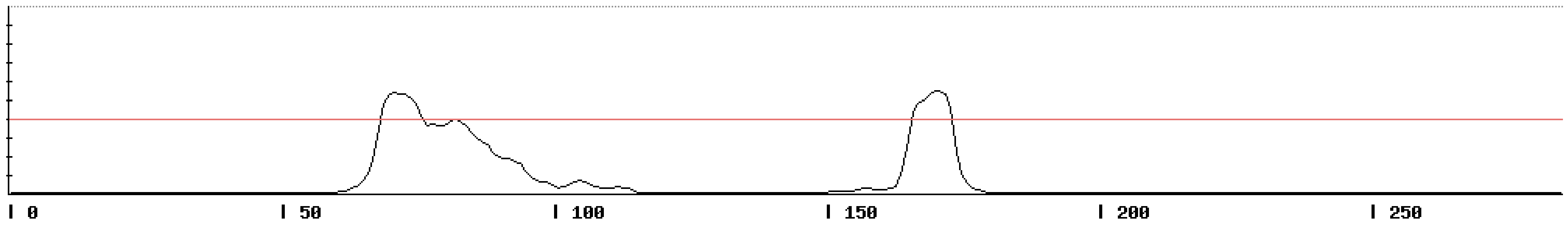

Predictions for Nicotianabenthamiana_Niben101Scf10713g00040.1

Viterbi Path

Viterbi Path

Posterior @ 0.4

38 - RKKNKGR - 44

Posterior @ 0.4

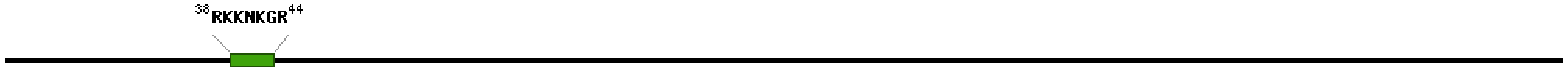

Posterior Graphic. Threshold Marked @ 0.4

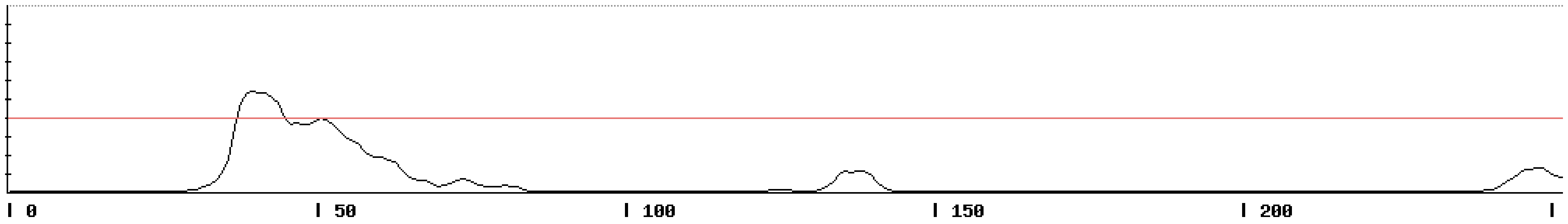

Predictions for Nicotianabenthamiana_Niben101Scf11208g00002.1	
Viterbi Path	
Viterbi Path	
Posterior @ 0.4	
Posterior @ 0.4	
Posterior Graphic. Threshold Marked @ 0.4	

Predictions for Nicotianabenthamiana_Niben101Scf11334g03005.1	
Viterbi Path	
Viterbi Path	
Posterior @ 0.4	
Posterior @ 0.4	
Posterior Graphic. Threshold Marked @ 0.4	

Predictions for Nicotianabenthamiana_Niben101Scf11334g03006.1	
Viterbi Path	
Viterbi Path	

Posterior @ 0.4	
Posterior @ 0.4	
Posterior Graphic. Threshold Marked @ 0.4	

**Predictions for Nicotianabenthamiana_Niben101Scf11410g00003.1**	
Viterbi Path	
Viterbi Path	
Posterior @ 0.4	
Posterior @ 0.4	
Posterior Graphic. Threshold Marked @ 0.4	

**Predictions for Nicotianabenthamiana_Niben101Scf11508g01003.1**	
Viterbi Path	
Viterbi Path	
Posterior @ 0.4	
Posterior @ 0.4	
Posterior Graphic. Threshold Marked @ 0.4	

**Predictions for Nicotianabenthamiana_Niben101Scf11552g02001.1**	
Viterbi Path	
Viterbi Path	
Posterior @ 0.4	139 - TRKRRKVR - 146
Posterior @ 0.4	
Posterior Graphic. Threshold Marked @ 0.4	

**Predictions for Nicotianabenthamiana_Niben101Scf11670g03002.1**	
Viterbi Path	
Viterbi Path	
Posterior @ 0.4	

Posterior @ 0.4	
Posterior Graphic. Threshold Marked @ 0.4	

**Predictions for Nicotianabenthamiana_Niben101Scf11773g01003.1**	
Viterbi Path	
Viterbi Path	
Posterior @ 0.4	
Posterior @ 0.4	
Posterior Graphic. Threshold Marked @ 0.4	

**Predictions for Nicotianabenthamiana_Niben101Scf12141g00009.1**	
Viterbi Path	
Viterbi Path	
Posterior @ 0.4	
Posterior @ 0.4	
Posterior Graphic. Threshold Marked @ 0.4	

**Predictions for Nicotianabenthamiana_Niben101Scf12245g00002.1**	
Viterbi Path	
Viterbi Path	
Posterior @ 0.4	
Posterior @ 0.4	
Posterior Graphic. Threshold Marked @ 0.4	

**Predictions for Nicotianabenthamiana_Niben101Scf12314g00005.1**	
Viterbi Path	
Viterbi Path	
Posterior @ 0.4	
Posterior @ 0.4	

Posterior Graphic. Threshold Marked @ 0.4	

Predictions for Nicotianabenthamiana_Niben101Scf12394g02003.1	
Viterbi Path	
Viterbi Path	
Posterior @ 0.4	
Posterior @ 0.4	
Posterior Graphic. Threshold Marked @ 0.4	

Predictions for Nicotianabenthamiana_Niben101Scf12714g00012.1	
Viterbi Path	
Viterbi Path	
Posterior @ 0.4	
Posterior @ 0.4	
Posterior Graphic. Threshold Marked @ 0.4	

Predictions for Nicotianabenthamiana_Niben101Scf13103g00009.1	
Viterbi Path	
Viterbi Path	
Posterior @ 0.4	
Posterior @ 0.4	
Posterior Graphic. Threshold Marked @ 0.4	

Predictions for Nicotianabenthamiana_Niben101Scf13438g00001.1	
Viterbi Path	
Viterbi Path	
Posterior @ 0.4	78 - KKEKPTWKRVSRTVGKGTWKPQGKG - 102
	161 - KIKKKGKPGDKIK - 173
Posterior @ 0.4	

Posterior Graphic. Threshold Marked @ 0.4	

Predictions for Nicotianabenthamiana_Niben101Scf14516g00017.1	
Viterbi Path	
Viterbi Path	
Posterior @ 0.4	
Posterior @ 0.4	
Posterior Graphic. Threshold Marked @ 0.4	

Predictions for Nicotianabenthamiana_Niben101Scf14516g00019.1	
Viterbi Path	
Viterbi Path	
Posterior @ 0.4	
Posterior @ 0.4	
Posterior Graphic. Threshold Marked @ 0.4	

Predictions for Nicotianabenthamiana_Niben101Scf15279g00002.1	
Viterbi Path	
Viterbi Path	
Posterior @ 0.4	78 - KKEKPTWKRVSRTVGKGTWKPQGKG - 102
	161 - KIKKKGKPGDKIK - 173
Posterior @ 0.4	
Posterior Graphic. Threshold Marked @ 0.4	

Predictions for Nicotianabenthamiana_Niben101Scf15435g00012.1	
Viterbi Path	
Viterbi Path	
Posterior @ 0.4	
Posterior @ 0.4	

Posterior Graphic. Threshold Marked @ 0.4

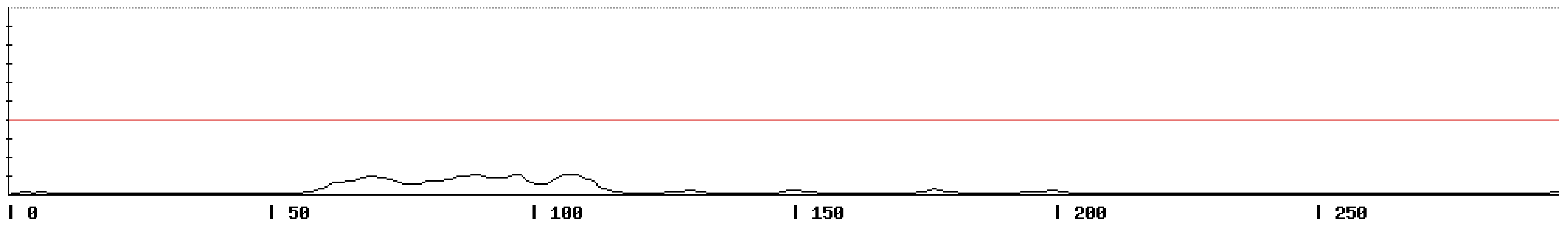

Predictions for Nicotianabenthamiana_Niben101Scf15752g00020.1

Viterbi Path

Viterbi Path

Posterior @ 0.4

Posterior @ 0.4

Posterior Graphic. Threshold Marked @ 0.4

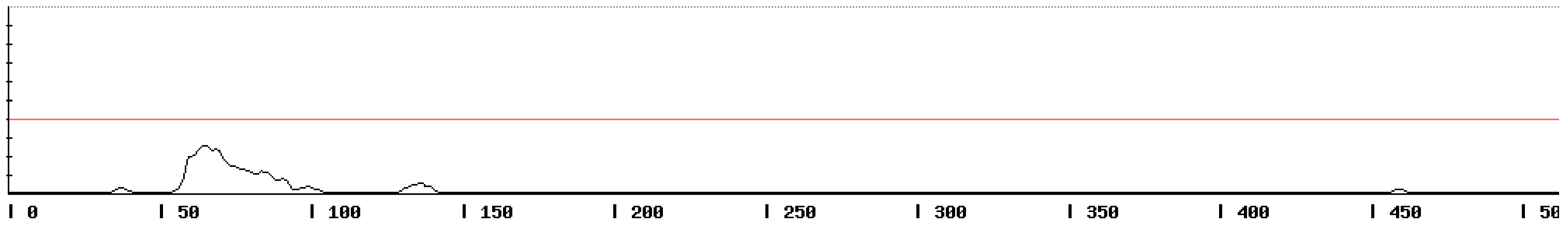

Predictions for Nicotianabenthamiana_Niben101Scf16114g00002.1

Viterbi Path

Viterbi Path

Posterior @ 0.4

Posterior @ 0.4

Posterior Graphic. Threshold Marked @ 0.4

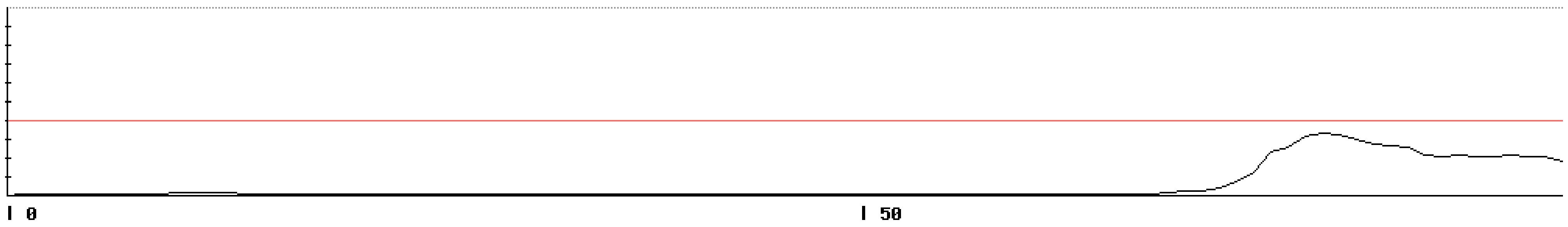

Predictions for Nicotianabenthamiana_Niben101Scf16469g01001.1

Viterbi Path

Viterbi Path

Posterior @ 0.4

Posterior @ 0.4

Posterior Graphic. Threshold Marked @ 0.4

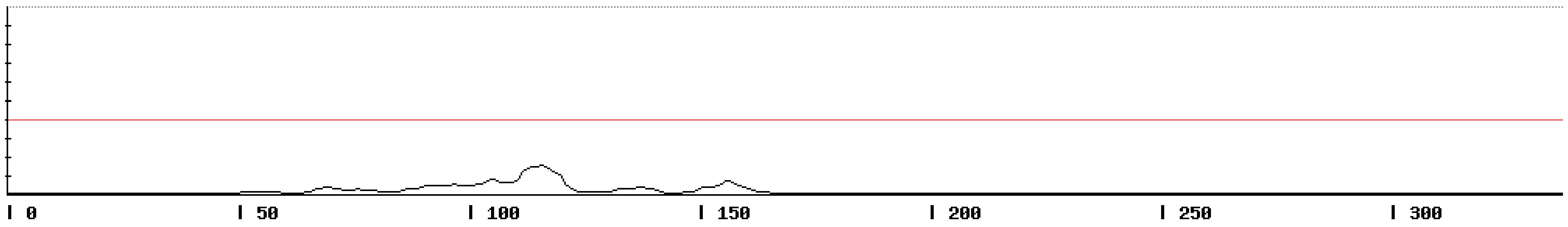

Predictions for Nicotianabenthamiana_Niben101Scf16613g03001.1

Viterbi Path

Viterbi Path

Posterior @ 0.4

Posterior @ 0.4

Posterior Graphic. Threshold Marked @ 0.4

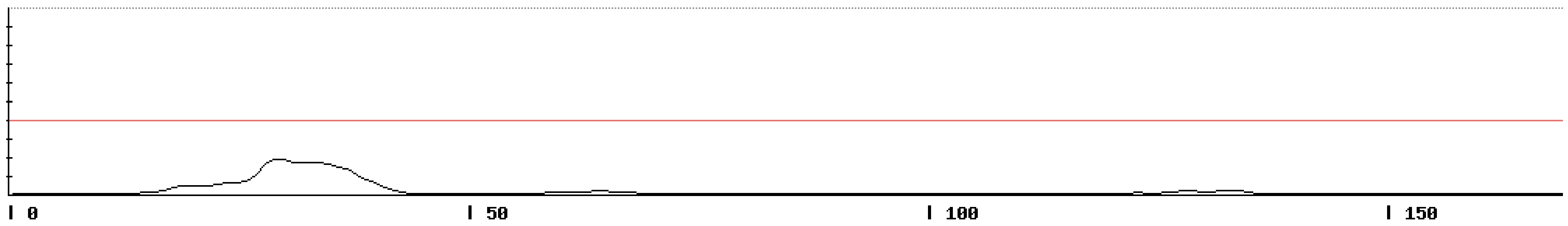

Predictions for Nicotianabenthamiana_Niben101Scf17701g01018.1

Viterbi Path

Viterbi Path

Posterior @ 0.4

Posterior @ 0.4

Posterior Graphic. Threshold Marked @ 0.4

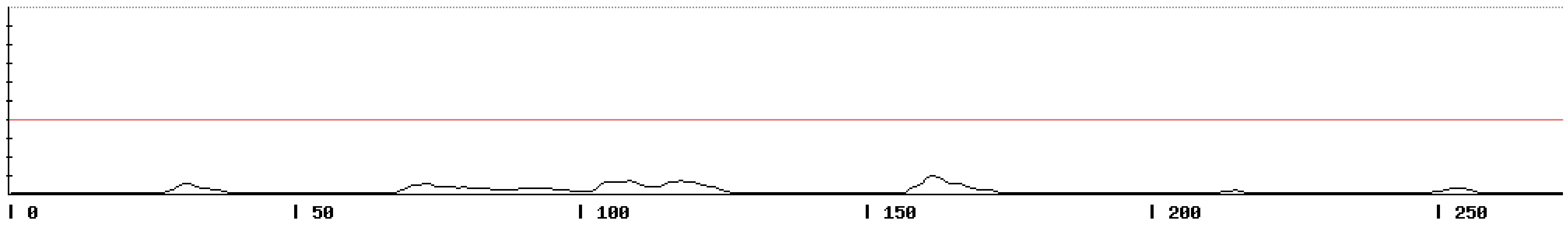

Predictions for Nicotianabenthamiana_Niben101Scf19016g00005.1

Viterbi Path

Viterbi Path

Posterior @ 0.4

Posterior @ 0.4

Posterior Graphic. Threshold Marked @ 0.4

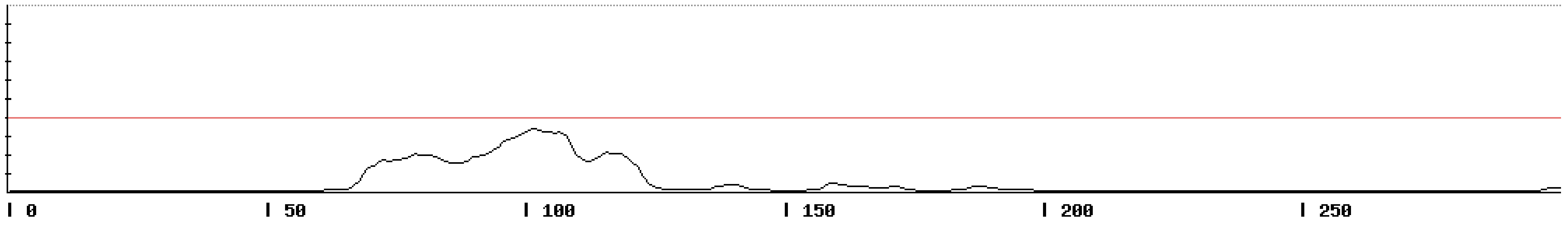

Predictions for Nicotianabenthamiana_Niben101Scf19383g00001.1

Viterbi Path

Viterbi Path

Posterior @ 0.4 — 78 - RTKAGGSR - 85

Posterior @ 0.4

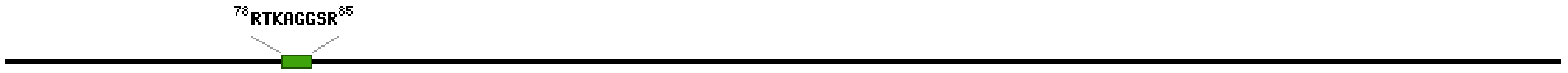

Posterior Graphic. Threshold Marked @ 0.4

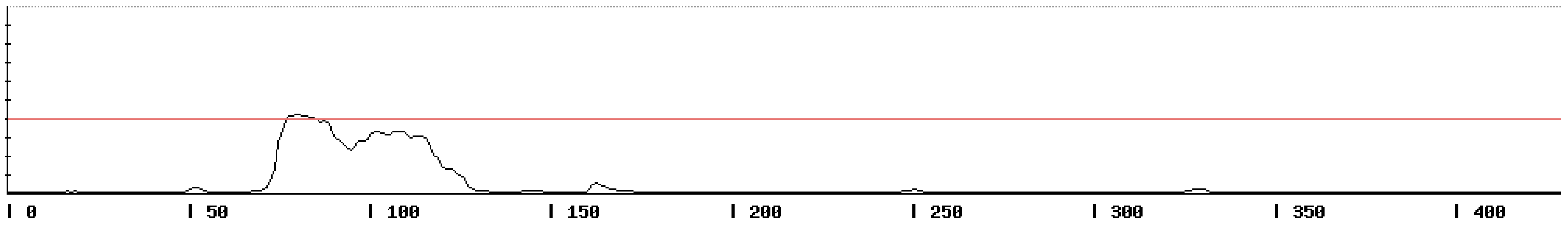

Predictions for Nicotianabenthamiana_Niben101Scf20506g01027.1

Viterbi Path	
Posterior @ 0.4	71 - RKHGPKGKPNR - 81
Posterior @ 0.4	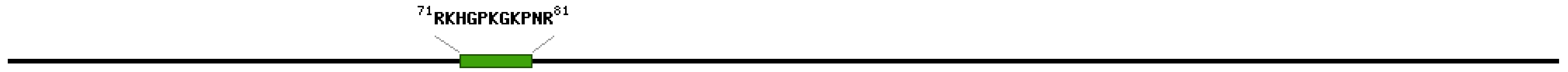
Posterior Graphic. Threshold Marked @ 0.4	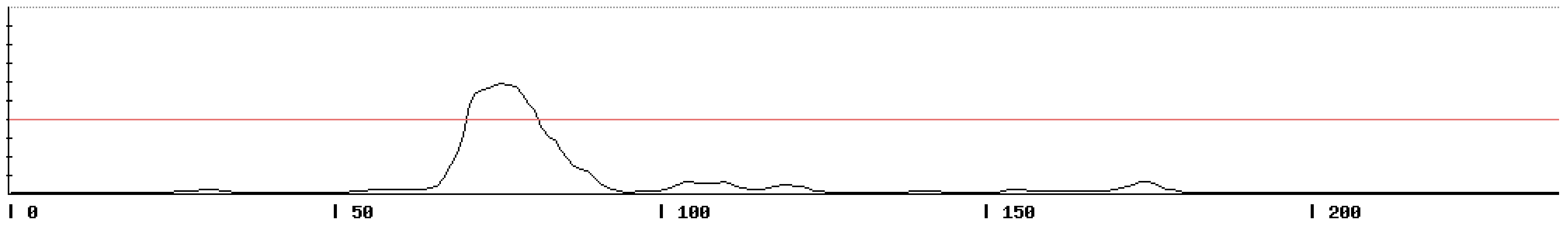

Predictions for Nicotianasylvestris_XP_009757635.1

Viterbi Path	
Viterbi Path	
Posterior @ 0.4	
Posterior @ 0.4	
Posterior Graphic. Threshold Marked @ 0.4	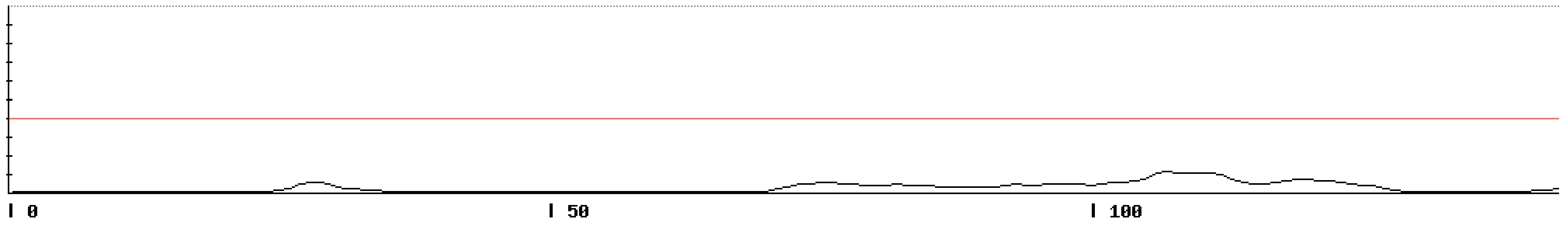

Predictions for Nicotianasylvestris_XP_009758643.1

Viterbi Path

Viterbi Path

Posterior @ 0.4

Posterior @ 0.4

Posterior Graphic. Threshold Marked @ 0.4

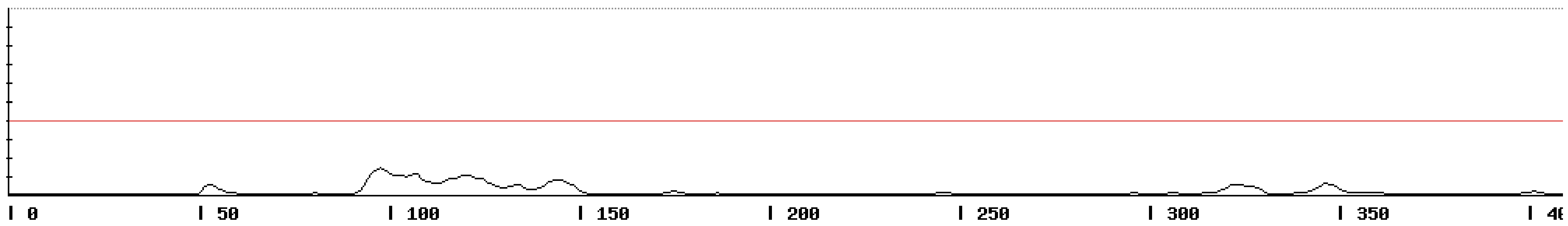

Predictions for Nicotianasylvestris_XP_009758709.1

Viterbi Path

Viterbi Path

Posterior @ 0.4

370 - REEKRKKKLKK - 380

Posterior @ 0.4

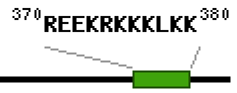

Posterior Graphic. Threshold Marked @ 0.4

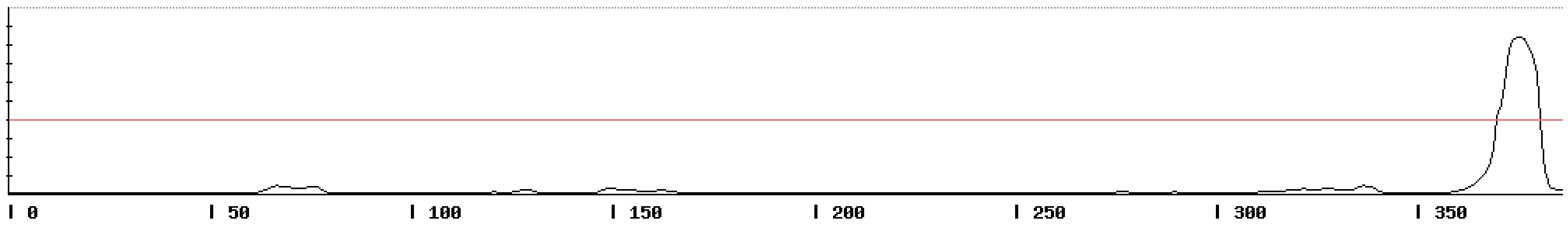

Predictions for Nicotianasylvestris_XP_009759087.1	
Viterbi Path	
Viterbi Path	
Posterior @ 0.4	
Posterior @ 0.4	
Posterior Graphic. Threshold Marked @ 0.4	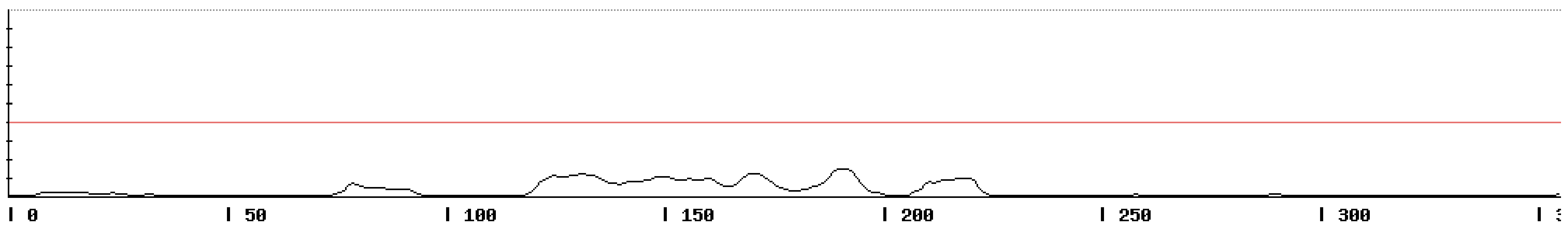

Predictions for Nicotianasylvestris_XP_009759475.1	
Viterbi Path	
Viterbi Path	
Posterior @ 0.4	
Posterior @ 0.4	
Posterior Graphic. Threshold Marked @ 0.4	

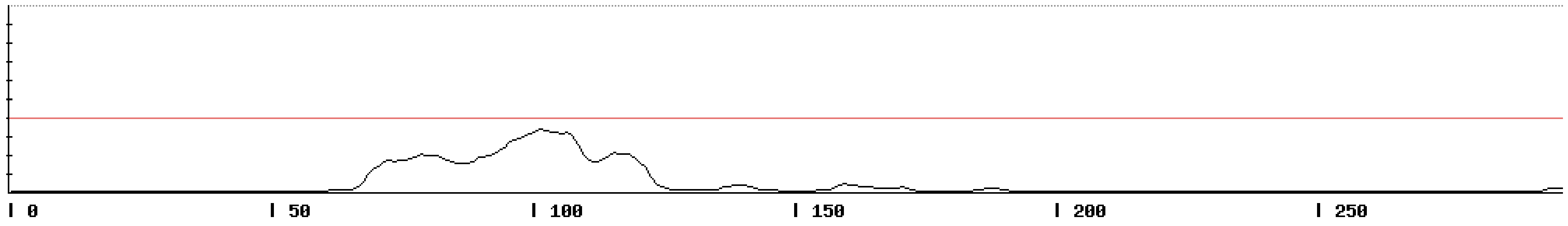

Predictions for Nicotianasylvestris_XP_009759500.1	
Viterbi Path	
Viterbi Path	
Posterior @ 0.4	
Posterior @ 0.4	
Posterior Graphic. Threshold Marked @ 0.4	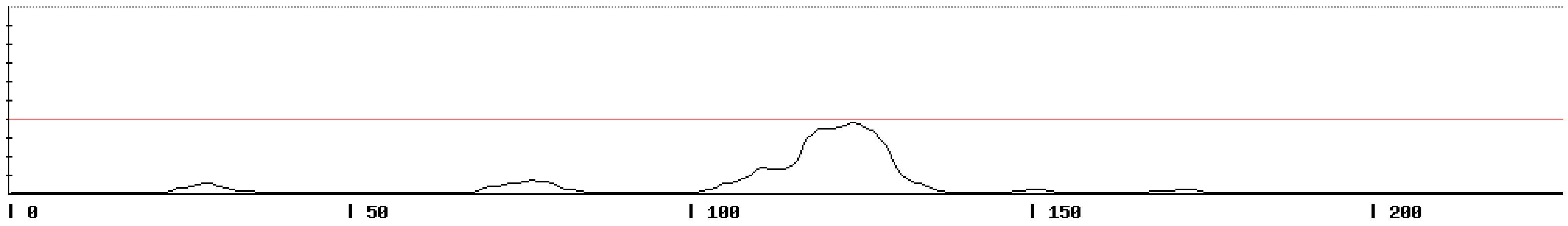

Predictions for Nicotianasylvestris_XP_009759753.1	
Viterbi Path	
Viterbi Path	
Posterior @ 0.4	
Posterior @ 0.4	

Posterior Graphic. Threshold Marked @ 0.4

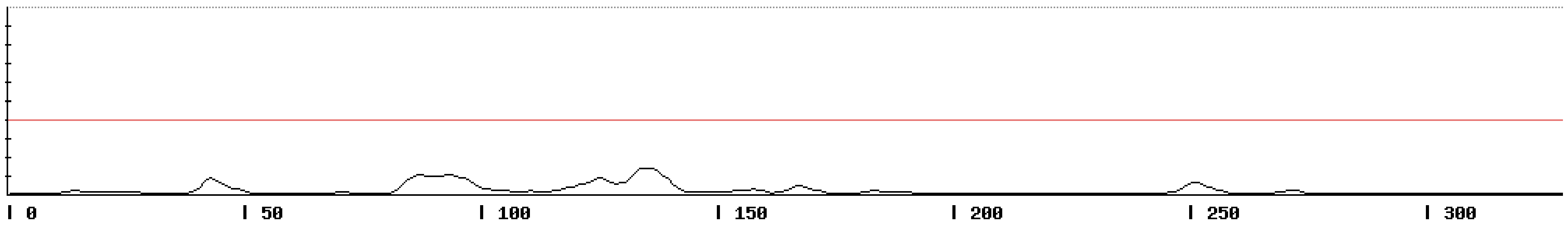

Predictions for Nicotianasylvestris_XP_009759914.1

Viterbi Path

Viterbi Path

Posterior @ 0.4

121 - ATKKGAKIG - 129

Posterior @ 0.4

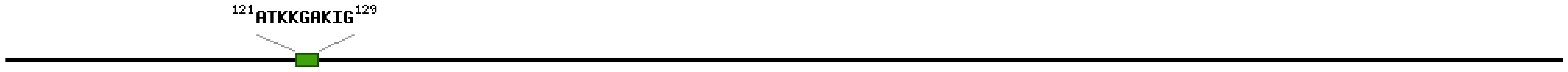

Posterior Graphic. Threshold Marked @ 0.4

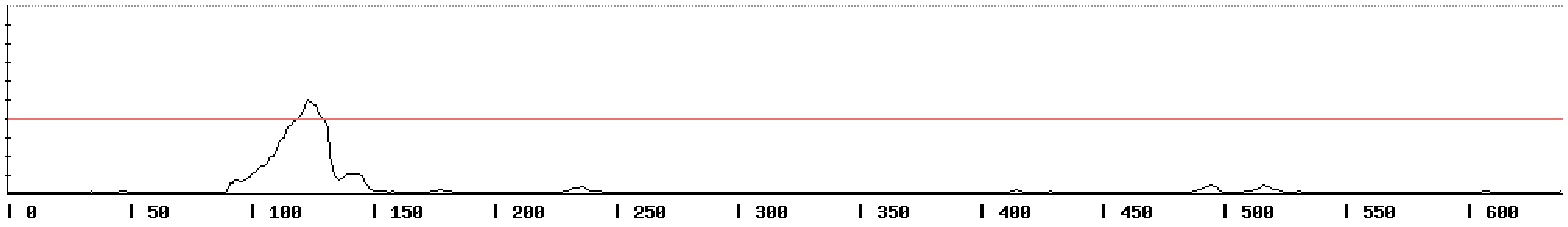

Predictions for Nicotianasylvestris_XP_009760064.1

Viterbi Path

Viterbi Path

Posterior @ 0.4

Posterior @ 0.4

Posterior Graphic. Threshold Marked @ 0.4

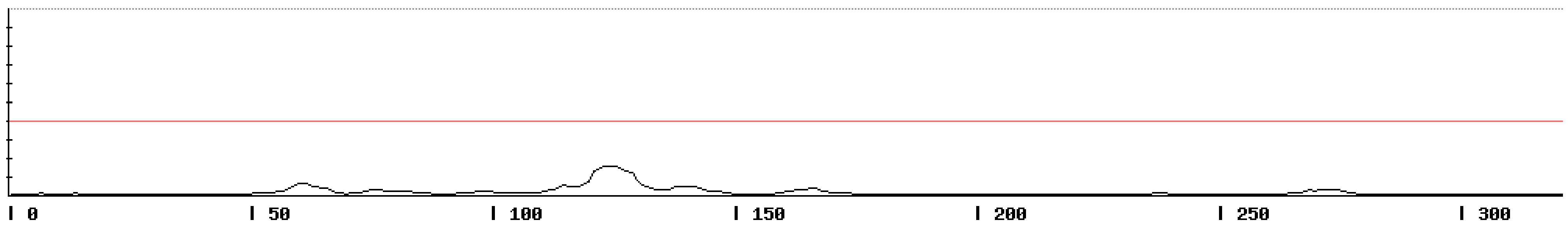

Predictions for Nicotianasylvestris_XP_009760677.1

Viterbi Path

Viterbi Path

Posterior @ 0.4

Posterior @ 0.4

Posterior Graphic. Threshold Marked @ 0.4

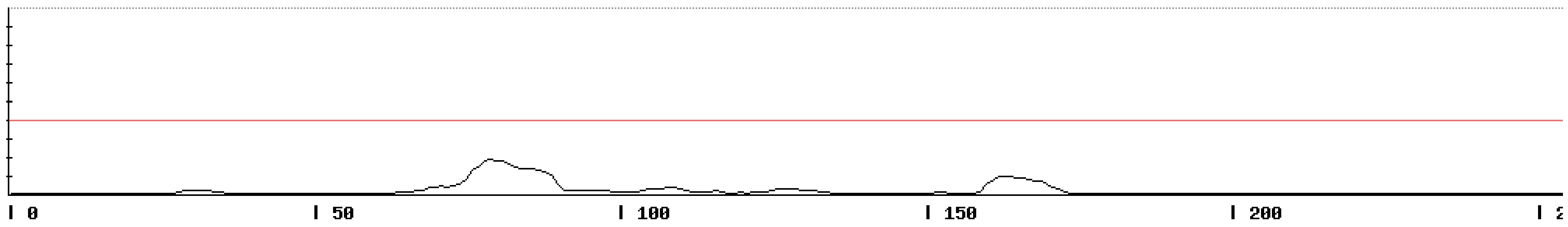

Predictions for Nicotianasylvestris_XP_009760718.1

Viterbi Path

Viterbi Path

Posterior @ 0.4

Posterior @ 0.4

Posterior Graphic. Threshold Marked @ 0.4

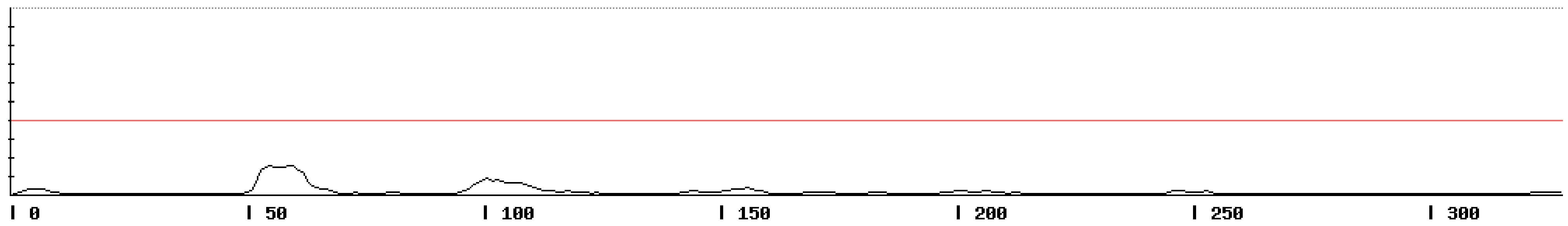

Predictions for Nicotianasylvestris_XP_009760719.1

Viterbi Path

Viterbi Path

Posterior @ 0.4

Posterior @ 0.4

Posterior Graphic. Threshold Marked @ 0.4

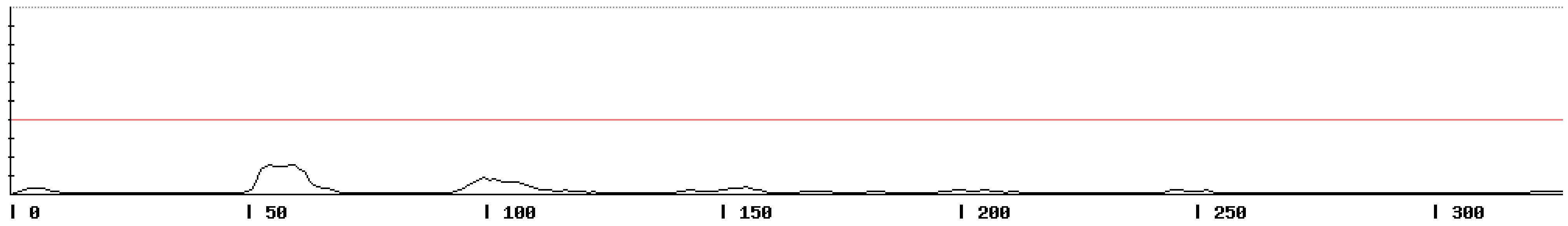

Predictions for Nicotianasylvestris_XP_009760809.1

Viterbi Path

Viterbi Path

Posterior @ 0.4	
Posterior @ 0.4	
Posterior Graphic. Threshold Marked @ 0.4	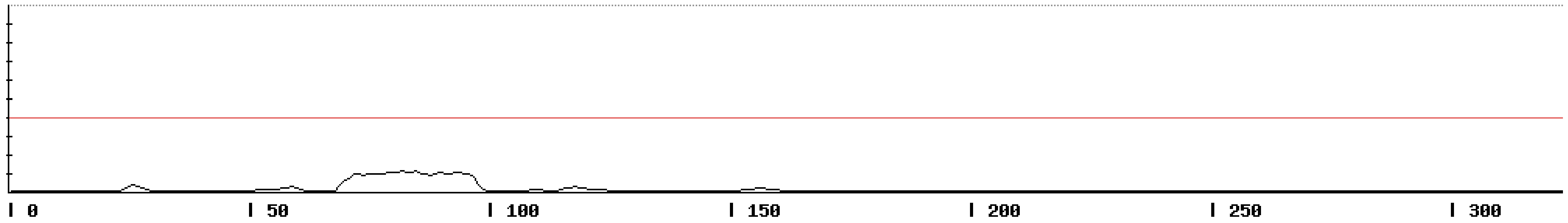

Predictions for Nicotianasylvestris_XP_009760820.1	
Viterbi Path	
Viterbi Path	
Posterior @ 0.4	
Posterior @ 0.4	
Posterior Graphic. Threshold Marked @ 0.4	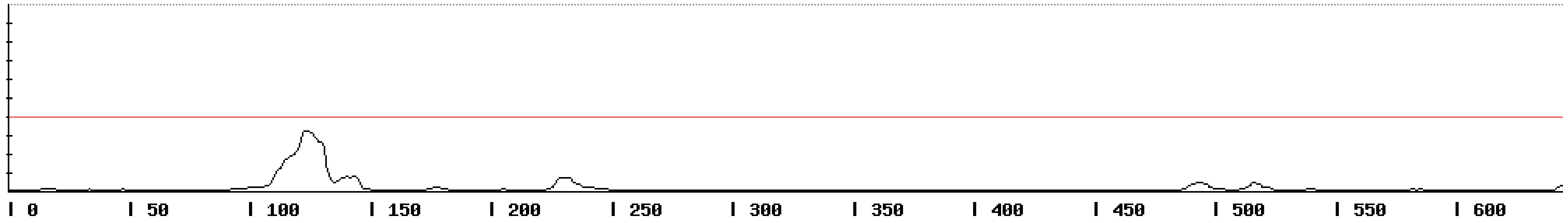

Predictions for Nicotianasylvestris_XP_009760821.1

Viterbi Path

Viterbi Path

Posterior @ 0.4

Posterior @ 0.4

Posterior Graphic. Threshold Marked @ 0.4

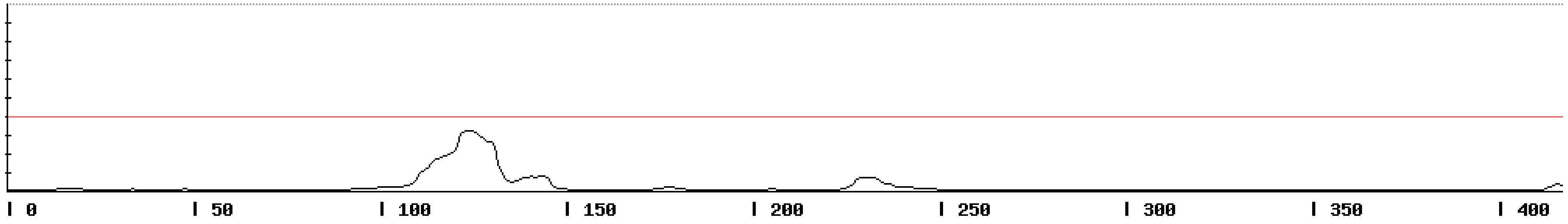

Predictions for Nicotianasylvestris_XP_009761286.1

Viterbi Path

Viterbi Path

Posterior @ 0.4

70 - KRFKNSTKK - 78

Posterior @ 0.4

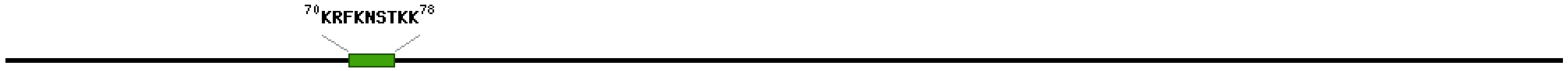

Posterior Graphic. Threshold Marked @ 0.4

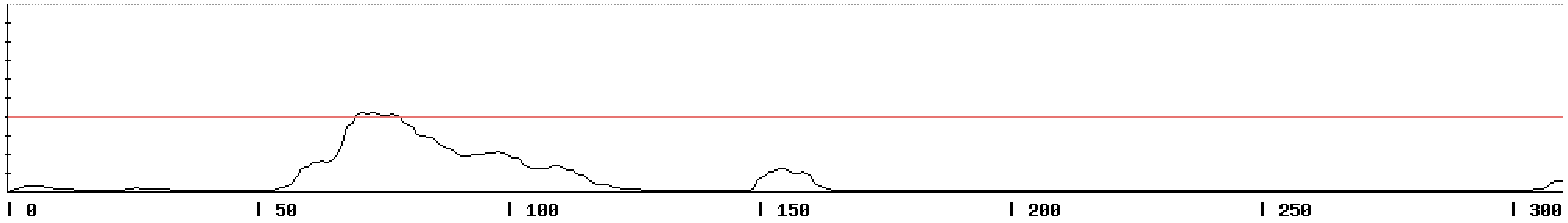

Predictions for Nicotianasylvestris_XP_009761611.1	
Viterbi Path	
Viterbi Path	
Posterior @ 0.4	
Posterior @ 0.4	
Posterior Graphic. Threshold Marked @ 0.4	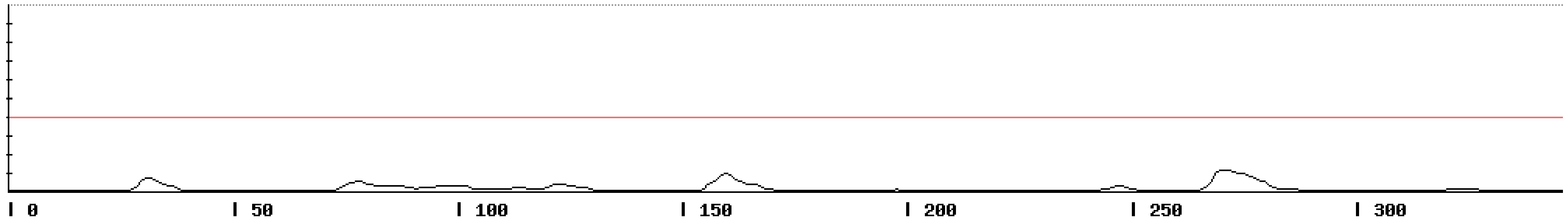

Predictions for Nicotianasylvestris_XP_009761612.1	
Viterbi Path	
Viterbi Path	
Posterior @ 0.4	
Posterior @ 0.4	
Posterior Graphic. Threshold Marked @ 0.4	

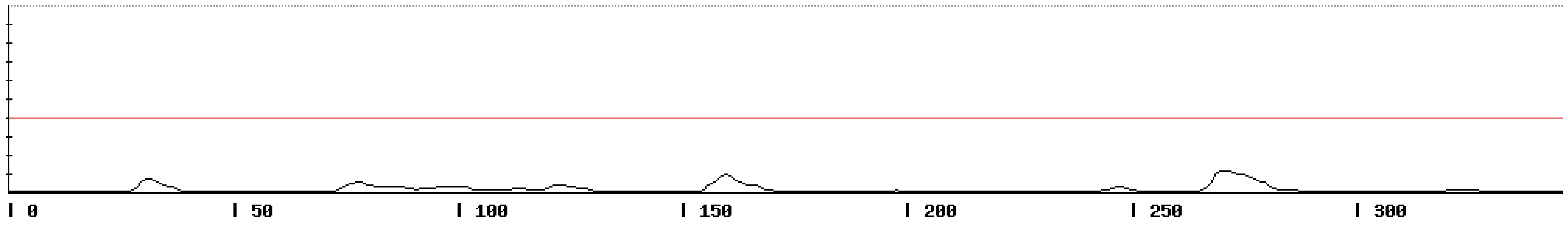

Predictions for Nicotianasylvestris_XP_009761662.1

Viterbi Path

Viterbi Path

Posterior @ 0.4

Posterior @ 0.4

Posterior Graphic. Threshold Marked @ 0.4

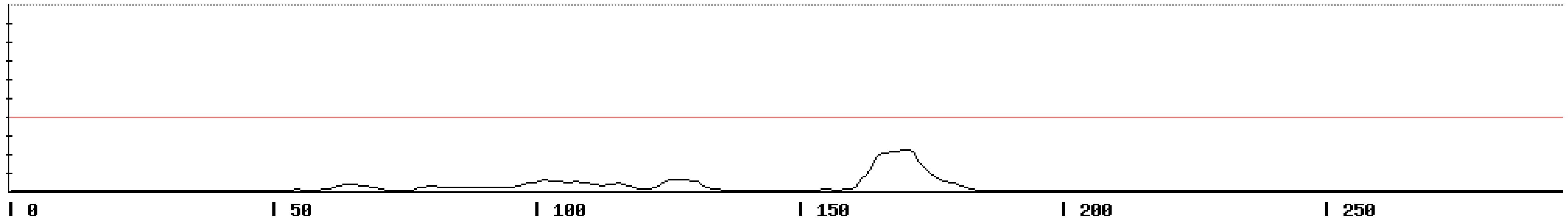

Predictions for Nicotianasylvestris_XP_009761820.1

Viterbi Path

Viterbi Path

Posterior @ 0.4

Posterior @ 0.4

Posterior Graphic. Threshold Marked @ 0.4

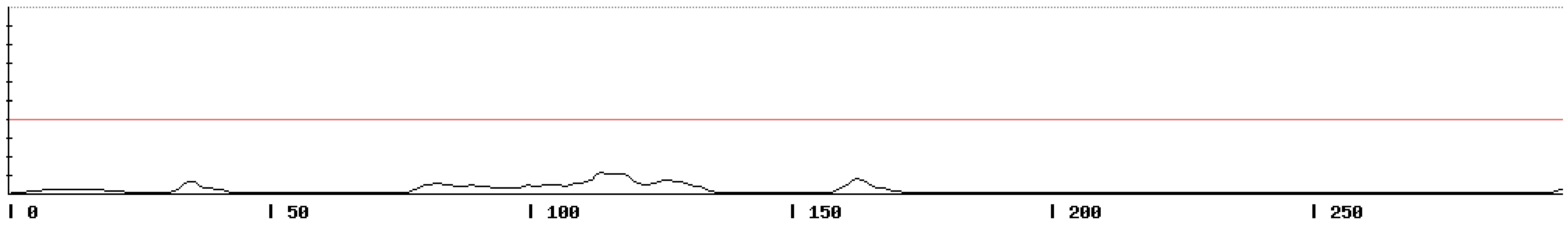

Predictions for Nicotianasylvestris_XP_009762029.1

Viterbi Path

Viterbi Path

Posterior @ 0.4

Posterior @ 0.4

Posterior Graphic. Threshold Marked @ 0.4

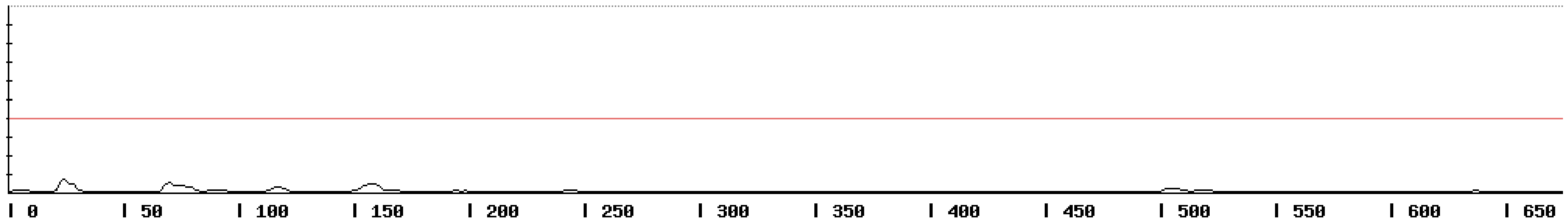

Predictions for Nicotianasylvestris_XP_009762030.1

Viterbi Path

Viterbi Path

Posterior @ 0.4

Posterior @ 0.4

Posterior Graphic. Threshold Marked @ 0.4

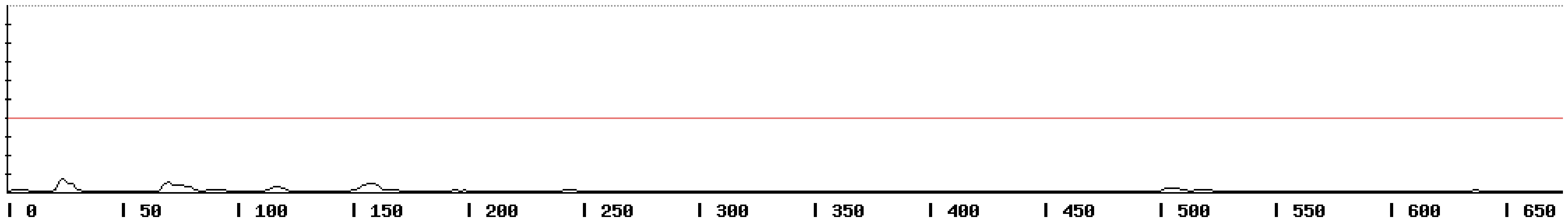

Predictions for Nicotianasylvestris_XP_009762400.1

Viterbi Path

Viterbi Path

Posterior @ 0.4

Posterior @ 0.4

Posterior Graphic. Threshold Marked @ 0.4

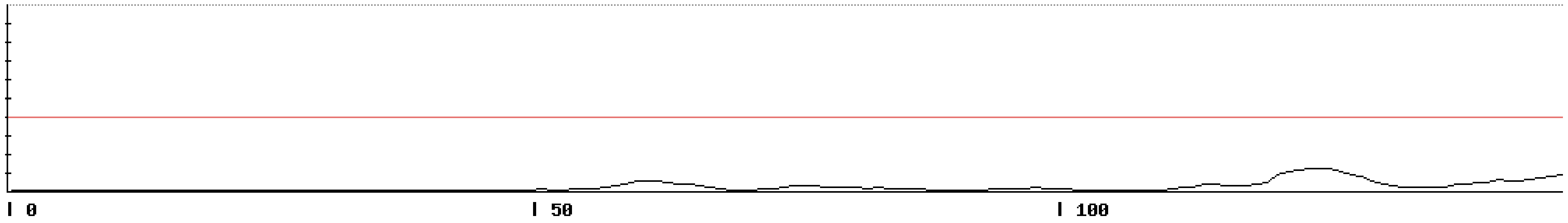

Predictions for Nicotianasylvestris_XP_009762857.1

Viterbi Path

Viterbi Path

Posterior @ 0.4	
Posterior @ 0.4	
Posterior Graphic. Threshold Marked @ 0.4	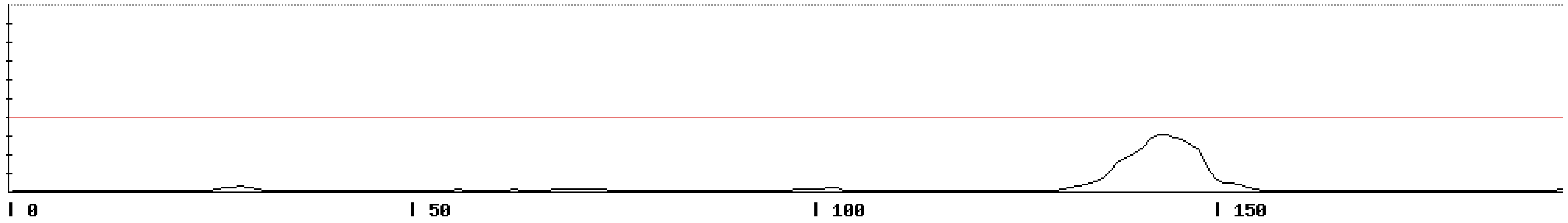

Predictions for Nicotianasylvestris_XP_009762859.1	
Viterbi Path	
Viterbi Path	
Posterior @ 0.4	
Posterior @ 0.4	
Posterior Graphic. Threshold Marked @ 0.4	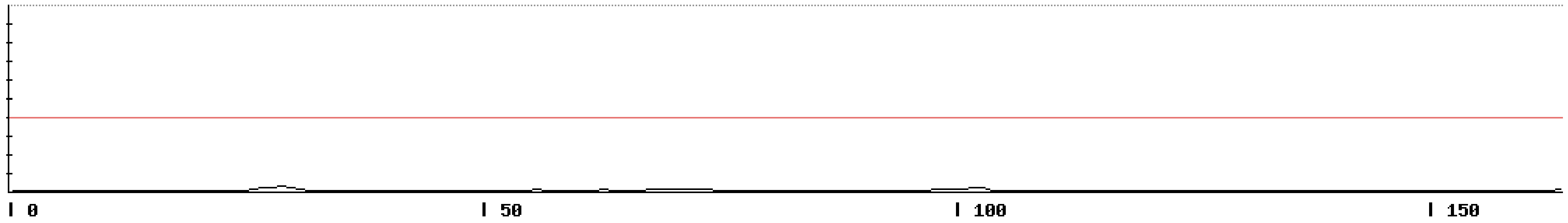

Predictions for Nicotianasylvestris_XP_009762991.1	
Viterbi Path	162 - KIKKKGKPGDKKKG - 175
Viterbi Path	

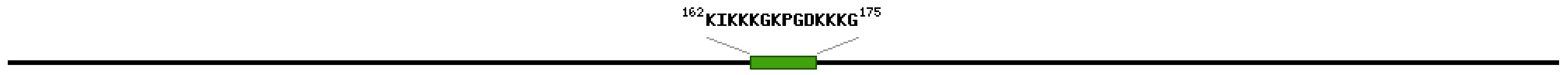

Posterior @ 0.4

79 - KKEKPTWKRVSRTVGKGTWKPQGKG - 103

162 - KIKKKGKPGDKKKG - 175

Posterior @ 0.4

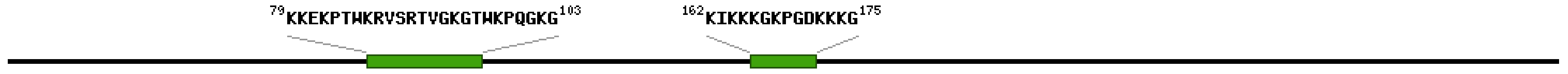

Posterior Graphic. Threshold Marked @ 0.4

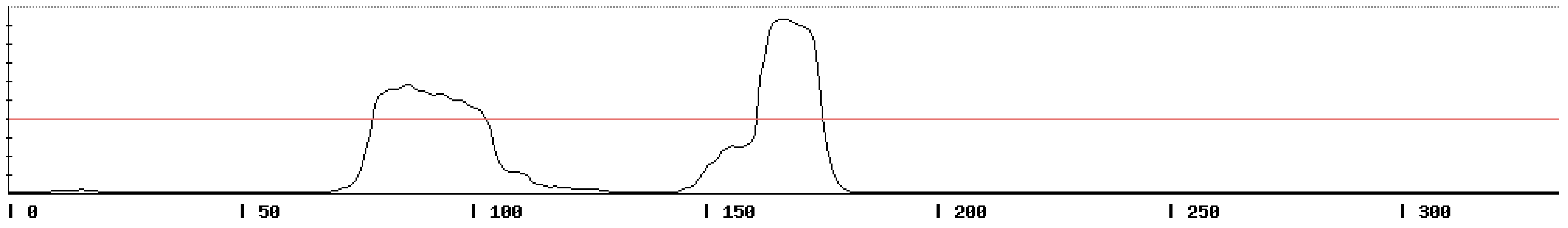

Predictions for Nicotianasylvestris_XP_009763090.1

Viterbi Path

162 - KIKKKGKPGDKKKG - 175

Viterbi Path

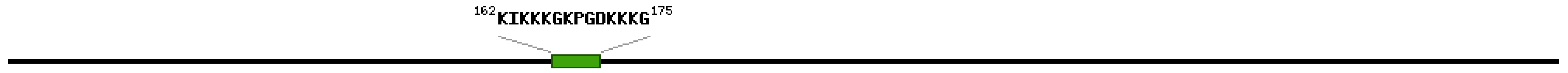

Posterior @ 0.4

79 - KKEKPTWKRVSRTVGKGTWKPQGKG - 103

162 - KIKKKGKPGDKKKG - 175

Posterior @ 0.4

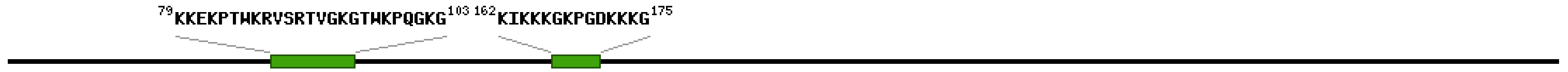

Posterior Graphic. Threshold Marked @ 0.4

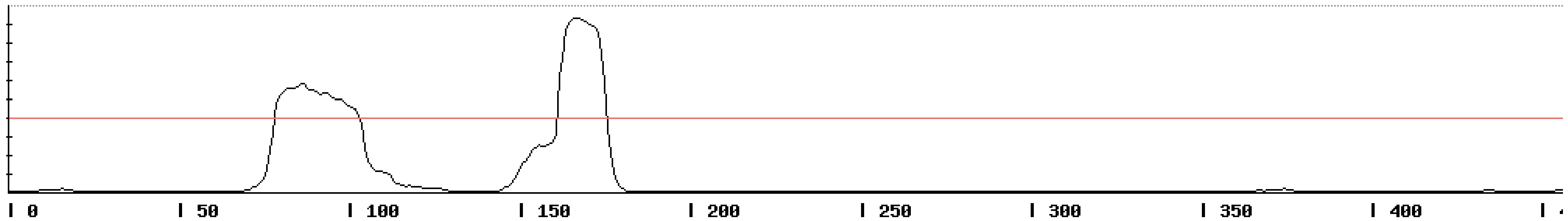

Predictions for Nicotianasylvestris_XP_009763274.1

Viterbi Path

Viterbi Path

Posterior @ 0.4

Posterior @ 0.4

Posterior Graphic. Threshold Marked @ 0.4

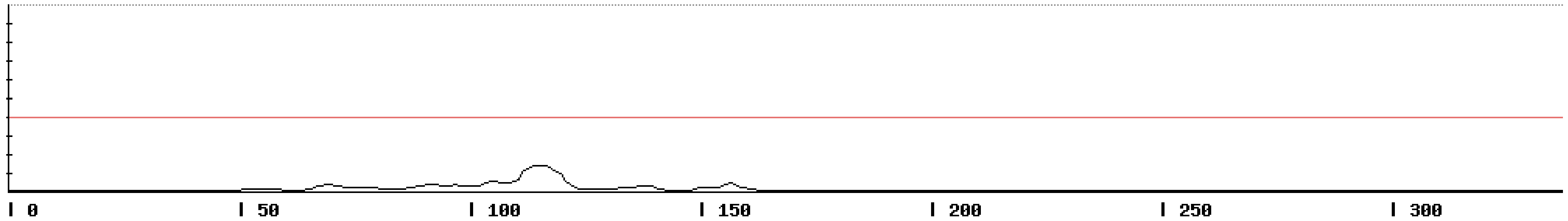

Predictions for Nicotianasylvestris_XP_009763275.1

Viterbi Path

Viterbi Path

Posterior @ 0.4

Posterior @ 0.4

Posterior Graphic. Threshold Marked @ 0.4

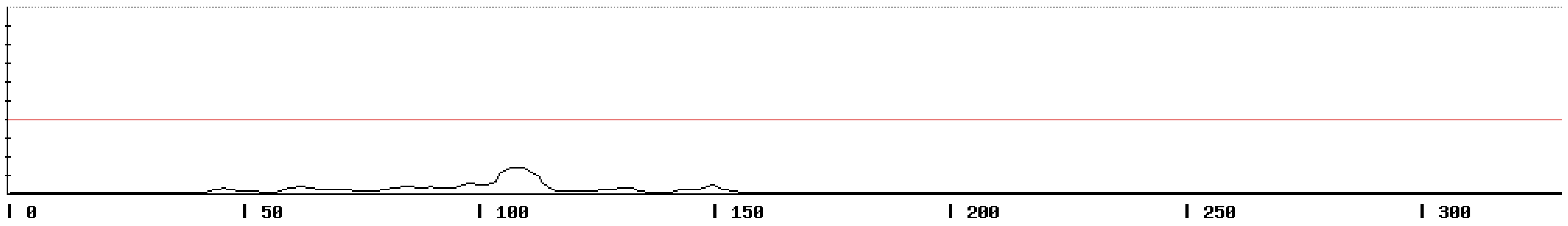

Predictions for Nicotianasylvestris_XP_009764398.1

Viterbi Path

Viterbi Path

Posterior @ 0.4

80 - NGAKTNRATKRGYWKTTGKDRAVHHKSK - 107

Posterior @ 0.4

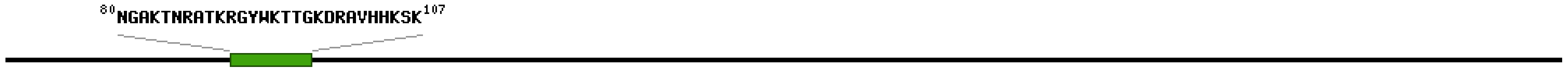

Posterior Graphic. Threshold Marked @ 0.4

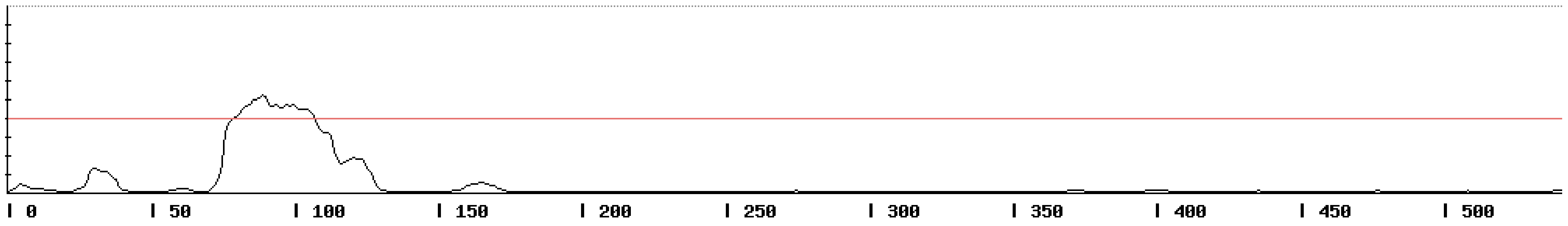

Predictions for Nicotianasylvestris_XP_009764955.1

Viterbi Path

Viterbi Path

Posterior @ 0.4

Posterior @ 0.4

Posterior Graphic. Threshold Marked @ 0.4

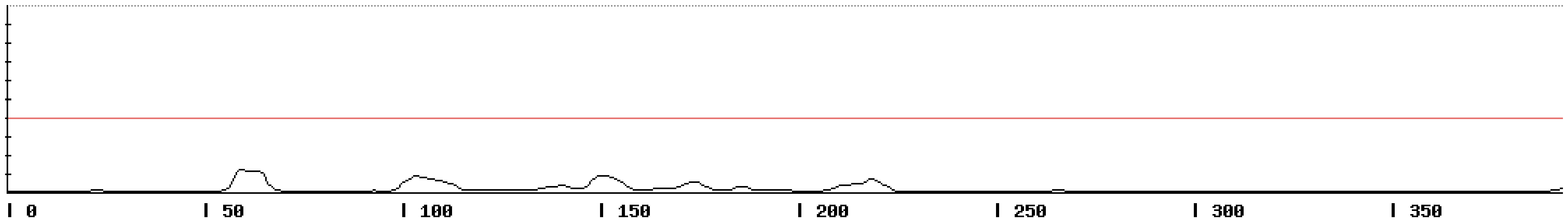

Predictions for Nicotianasylvestris_XP_009765145.1

Viterbi Path

Viterbi Path

Posterior @ 0.4

Posterior @ 0.4

Posterior Graphic. Threshold Marked @ 0.4

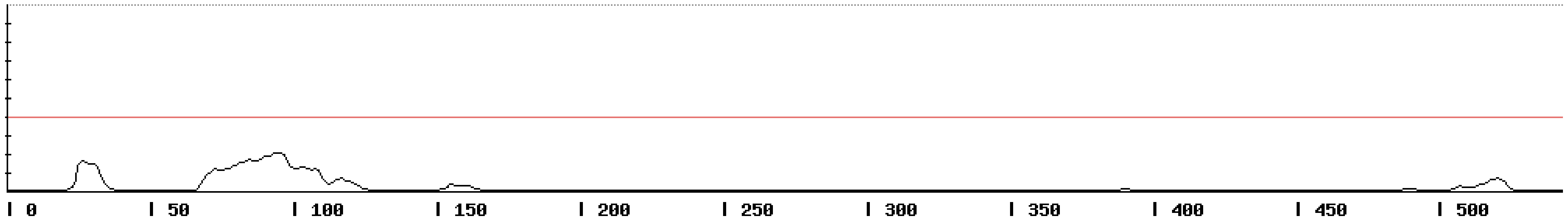

Predictions for Nicotianasylvestris_XP_009766553.1

Viterbi Path

Viterbi Path

Posterior @ 0.4	80 - KKKSKA - 85
Posterior @ 0.4	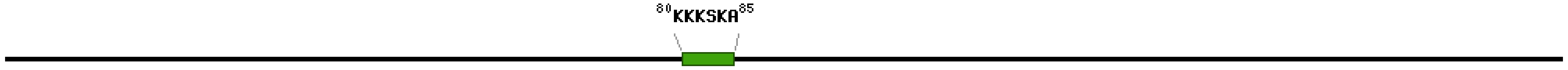
Posterior Graphic. Threshold Marked @ 0.4	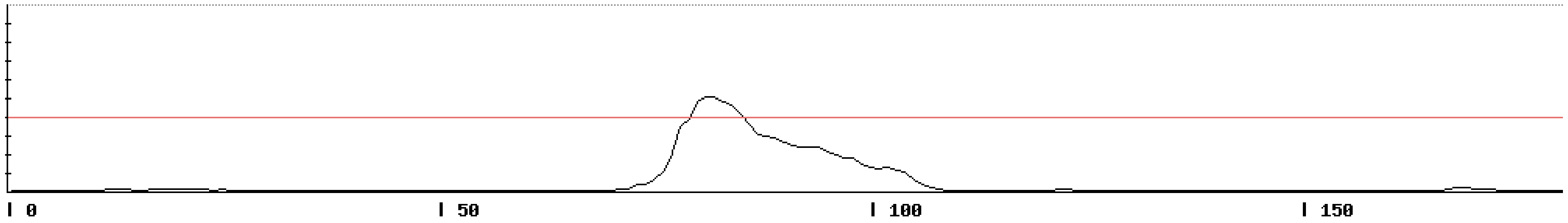

Predictions for Nicotianasylvestris_XP_009767002.1

Viterbi Path	
Viterbi Path	
Posterior @ 0.4	
Posterior @ 0.4	
Posterior Graphic. Threshold Marked @ 0.4	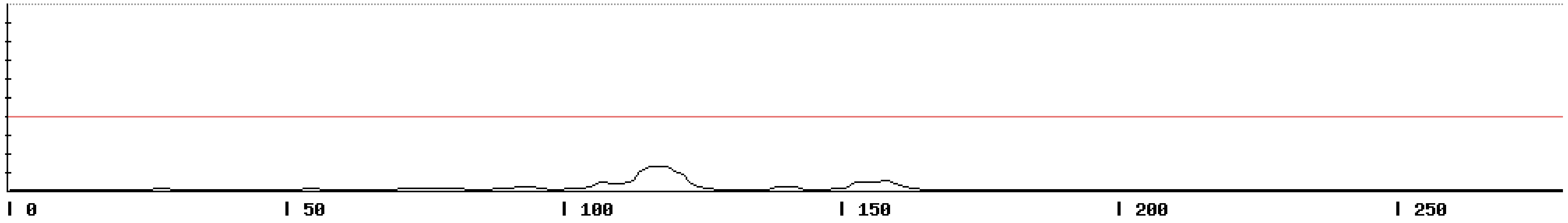

Predictions for Nicotianasylvestris_XP_009767273.1

Viterbi Path	
Viterbi Path	

Posterior @ 0.4

Posterior @ 0.4

Posterior Graphic. Threshold Marked @ 0.4

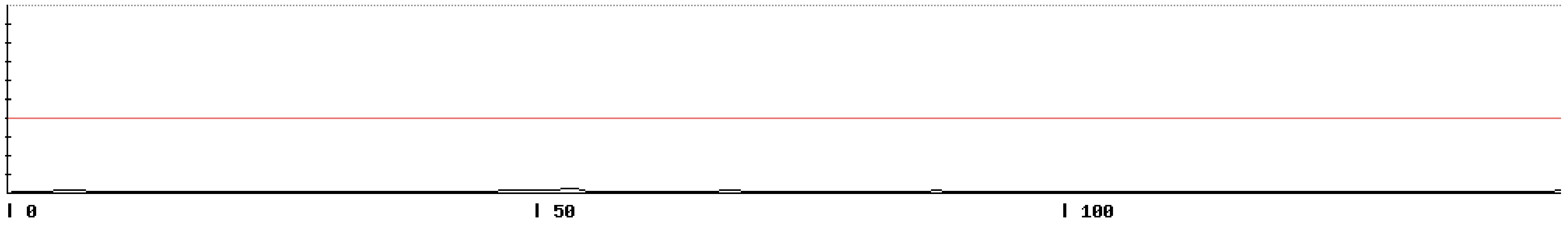

Predictions for Nicotianasylvestris_XP_009767292.1

Viterbi Path

Viterbi Path

Posterior @ 0.4

Posterior @ 0.4

Posterior Graphic. Threshold Marked @ 0.4

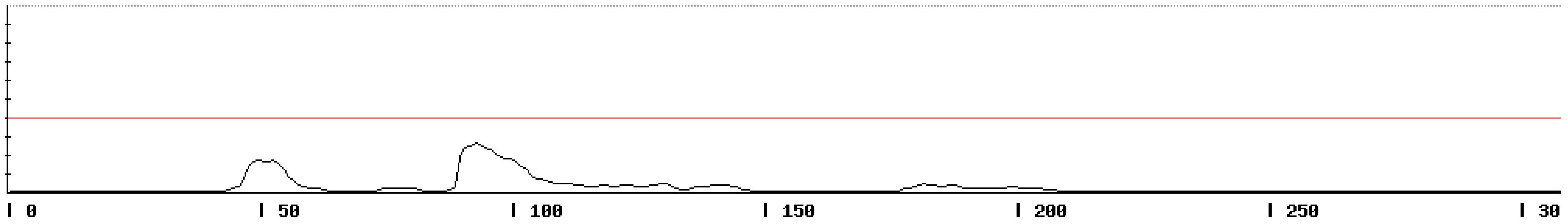

Predictions for Nicotianasylvestris_XP_009767337.1

Viterbi Path

Viterbi Path

Posterior @ 0.4

Posterior @ 0.4

Posterior Graphic. Threshold Marked @ 0.4

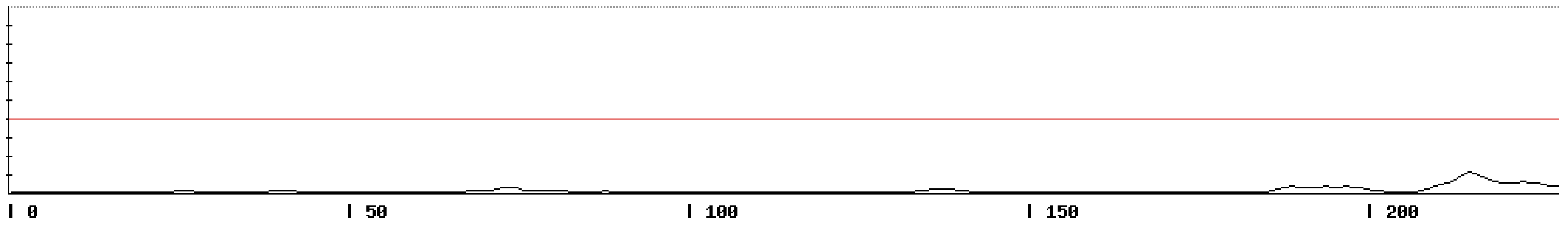

Predictions for Nicotianasylvestris_XP_009767338.1

Viterbi Path

Viterbi Path

Posterior @ 0.4

Posterior @ 0.4

Posterior Graphic. Threshold Marked @ 0.4

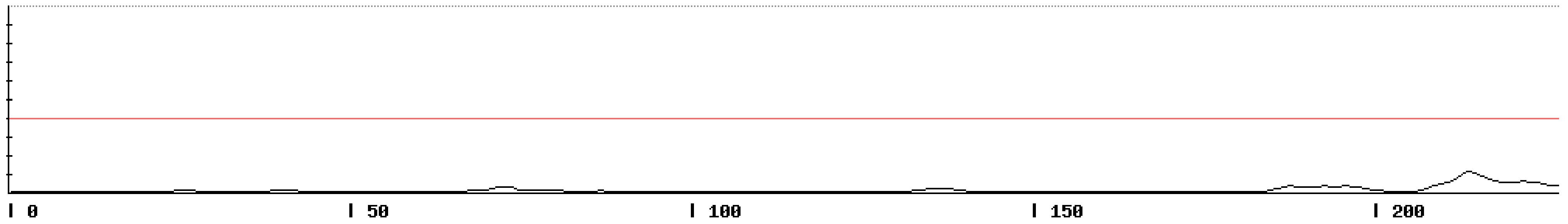

Predictions for Nicotianasylvestris_XP_009767422.1	
Viterbi Path	
Viterbi Path	
Posterior @ 0.4	
Posterior @ 0.4	
Posterior Graphic. Threshold Marked @ 0.4	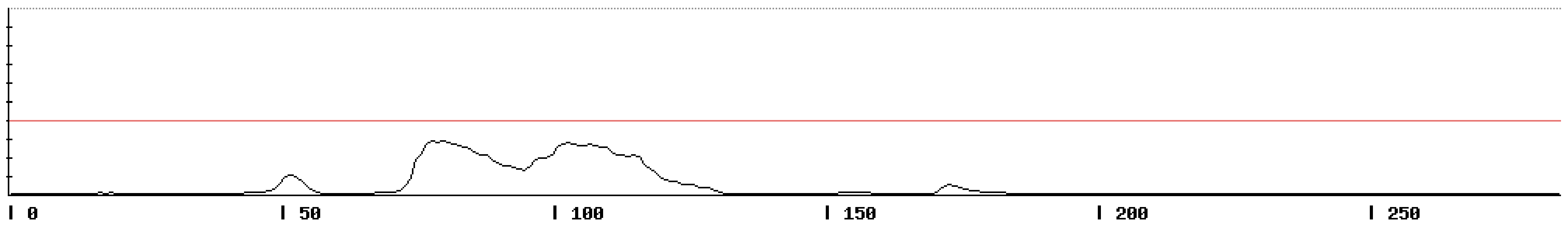

Predictions for Nicotianasylvestris_XP_009767926.1	
Viterbi Path	
Viterbi Path	
Posterior @ 0.4	
Posterior @ 0.4	
Posterior Graphic. Threshold Marked @ 0.4	

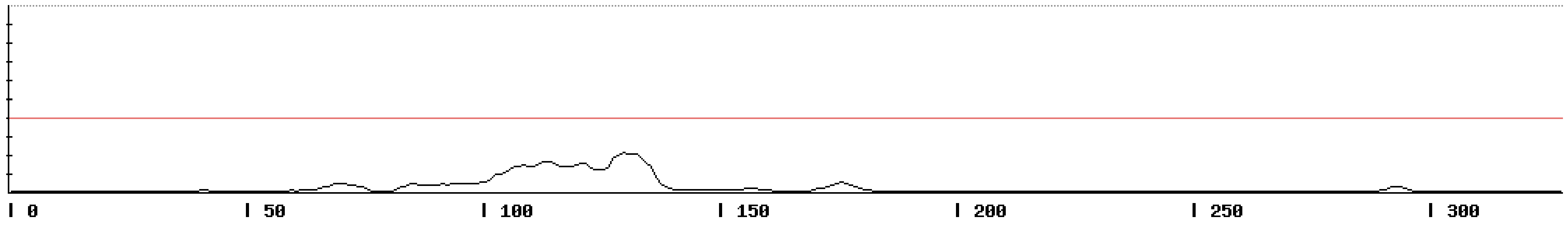

Predictions for Nicotianasylvestris_XP_009768516.1

Viterbi Path	
Viterbi Path	
Posterior @ 0.4	
Posterior @ 0.4	
Posterior Graphic. Threshold Marked @ 0.4	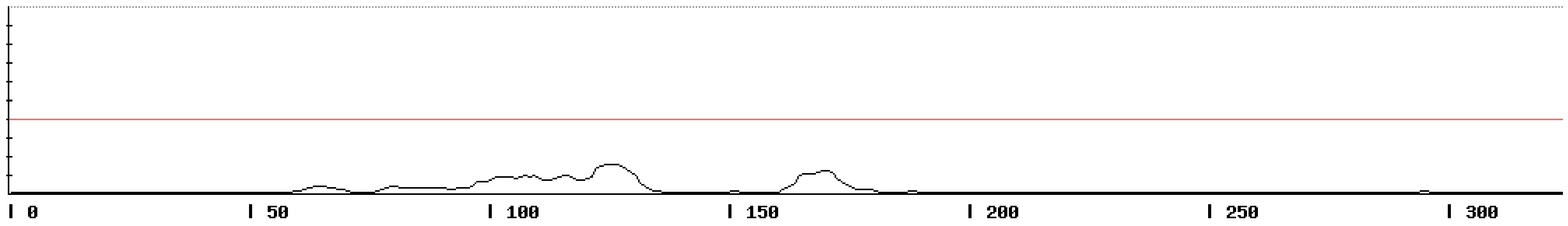

Predictions for Nicotianasylvestris_XP_009768847.1

Viterbi Path	
Viterbi Path	
Posterior @ 0.4	
Posterior @ 0.4	

Posterior Graphic. Threshold Marked @ 0.4

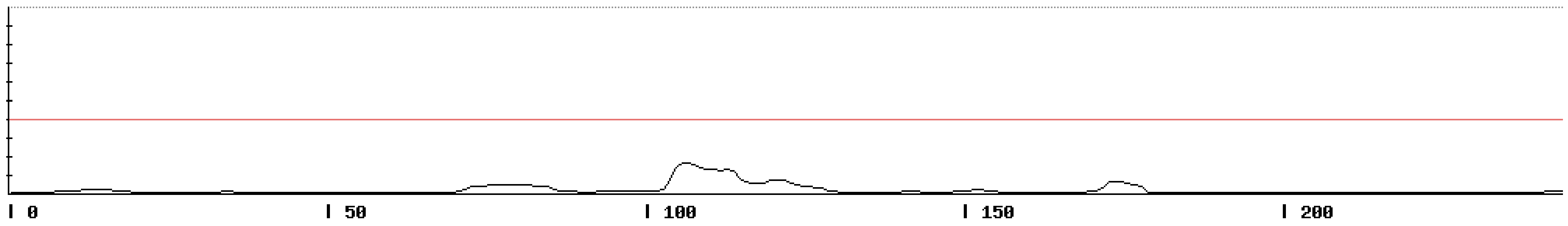

Predictions for Nicotianasylvestris_XP_009769005.1

Viterbi Path	
Viterbi Path	
Posterior @ 0.4	91 - KKK - 93
Posterior @ 0.4	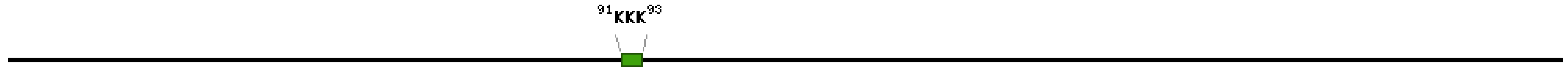
Posterior Graphic. Threshold Marked @ 0.4	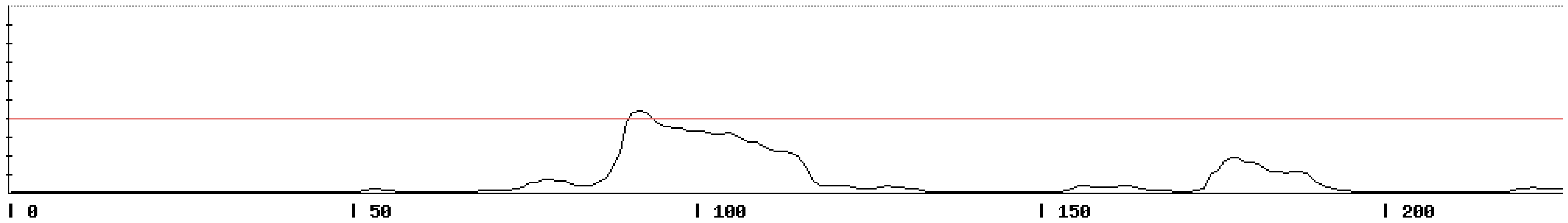

Predictions for Nicotianasylvestris_XP_009769046.1

Viterbi Path	
Viterbi Path	
Posterior @ 0.4	
Posterior @ 0.4	

Posterior Graphic. Threshold Marked @ 0.4

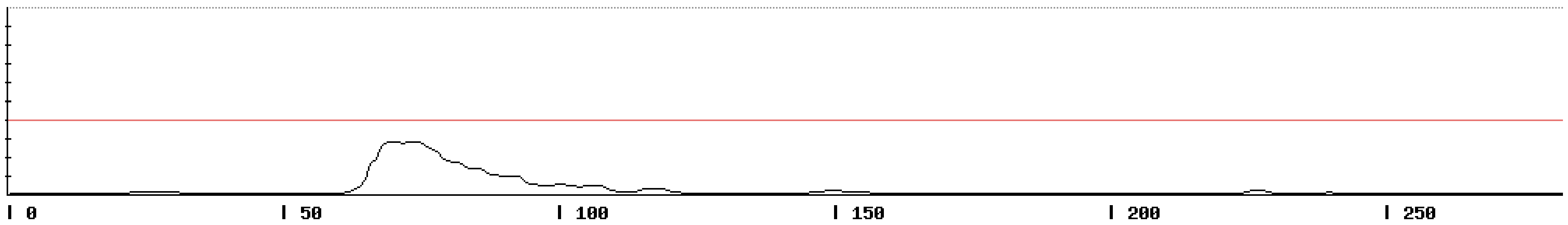

Predictions for Nicotianasylvestris_XP_009769770.1

Viterbi Path

Viterbi Path

Posterior @ 0.4

Posterior @ 0.4

Posterior Graphic. Threshold Marked @ 0.4

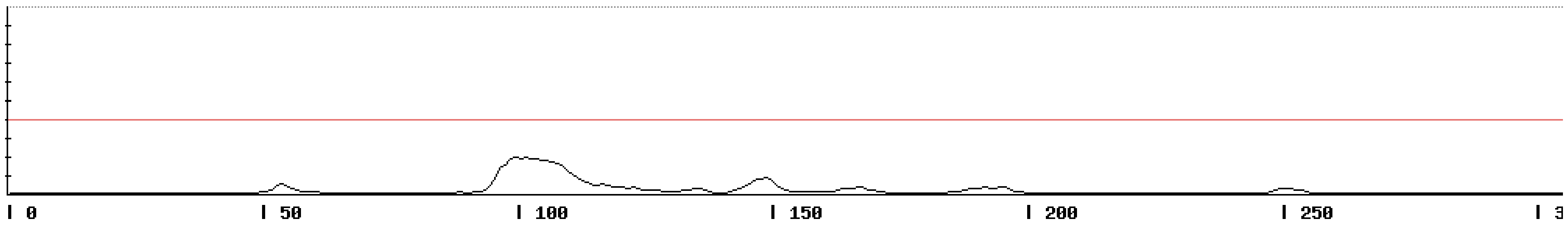

Predictions for Nicotianasylvestris_XP_009769986.1

Viterbi Path

Viterbi Path

Posterior @ 0.4

Posterior @ 0.4

Posterior Graphic. Threshold Marked @ 0.4

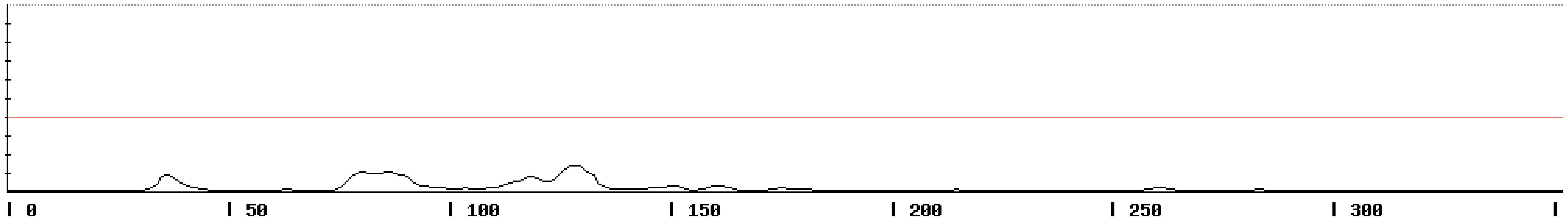

Predictions for Nicotianasylvestris_XP_009770385.1

Viterbi Path

Viterbi Path

Posterior @ 0.4 137 - RKRRK - 141

Posterior @ 0.4

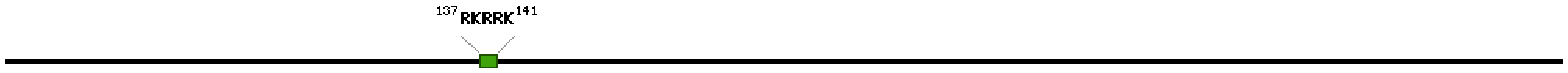

Posterior Graphic. Threshold Marked @ 0.4

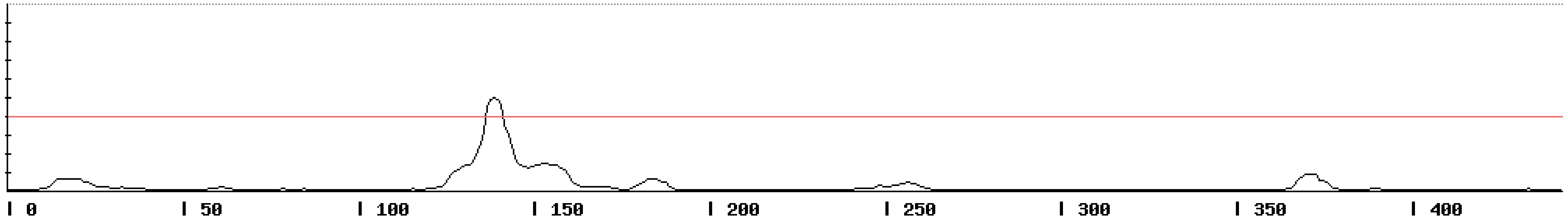

Predictions for Nicotianasylvestris_XP_009770386.1

Viterbi Path

Viterbi Path

Posterior @ 0.4	134 - RKRRK - 138
Posterior @ 0.4	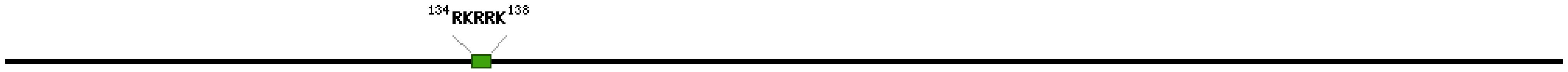
Posterior Graphic. Threshold Marked @ 0.4	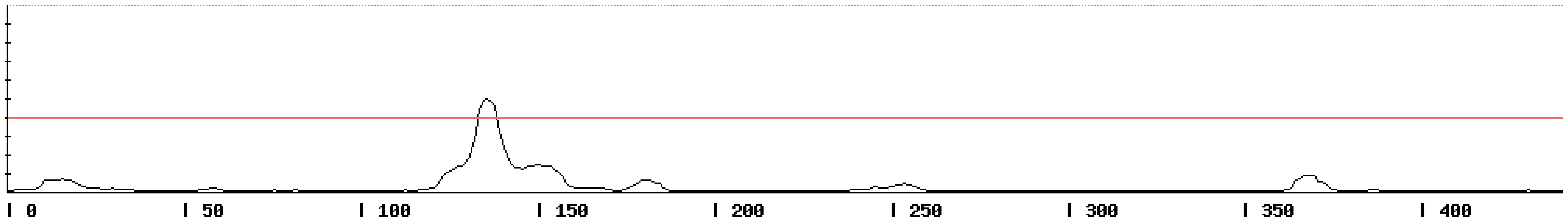

Predictions for Nicotianasylvestris_XP_009770387.1

Viterbi Path	
Viterbi Path	
Posterior @ 0.4	133 - RKRRK - 137
Posterior @ 0.4	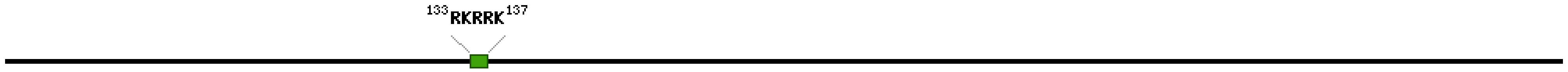
Posterior Graphic. Threshold Marked @ 0.4	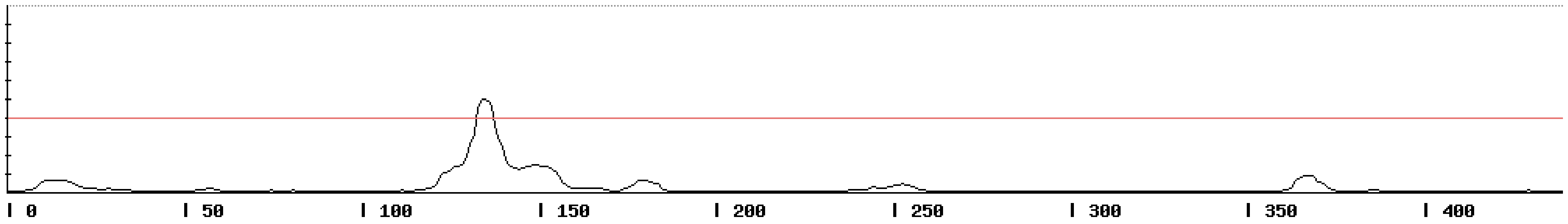

Predictions for Nicotianasylvestris_XP_009770805.1

Viterbi Path

Viterbi Path

Posterior @ 0.4

Posterior @ 0.4

Posterior Graphic. Threshold Marked @ 0.4

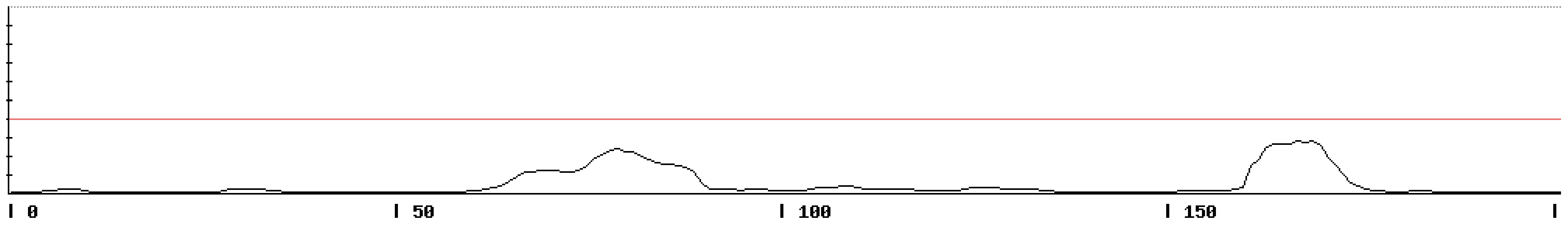

Predictions for Nicotianasylvestris_XP_009770806.1

Viterbi Path

Viterbi Path

Posterior @ 0.4

Posterior @ 0.4

Posterior Graphic. Threshold Marked @ 0.4

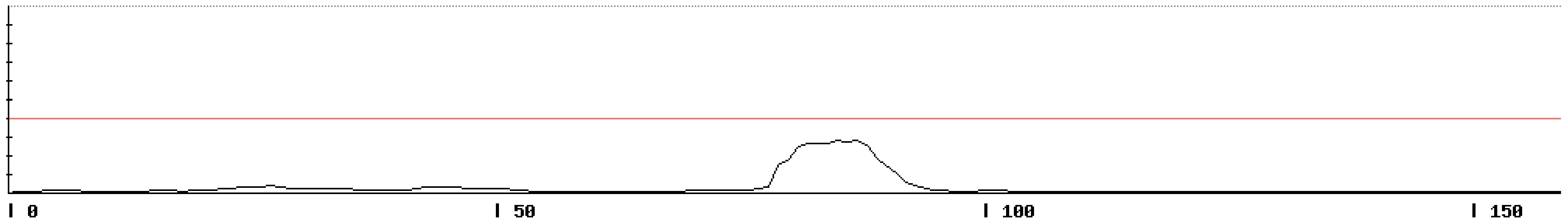

Predictions for Nicotianasylvestris_XP_009771077.1	
Viterbi Path	
Viterbi Path	
Posterior @ 0.4	82 - RVLKGGRRRAGGGYWKKRGRVR - 103
Posterior @ 0.4	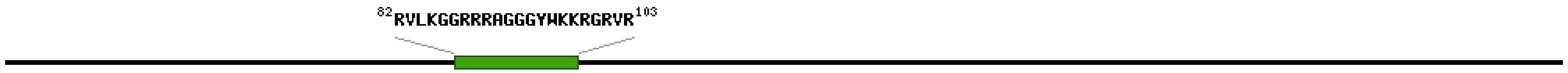
Posterior Graphic. Threshold Marked @ 0.4	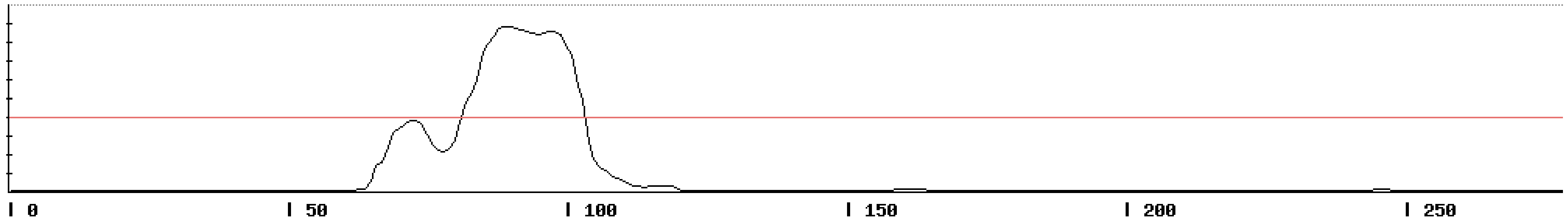

Predictions for Nicotianasylvestris_XP_009771078.1	
Viterbi Path	
Viterbi Path	
Posterior @ 0.4	82 - RVLKGGRRRAGGGYWKKRGRVR - 103
Posterior @ 0.4	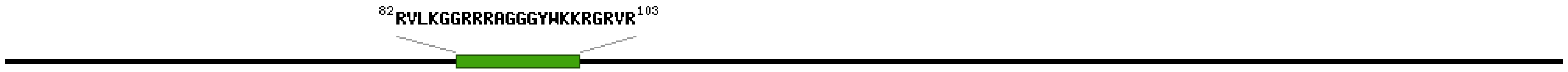
Posterior Graphic. Threshold Marked @ 0.4	

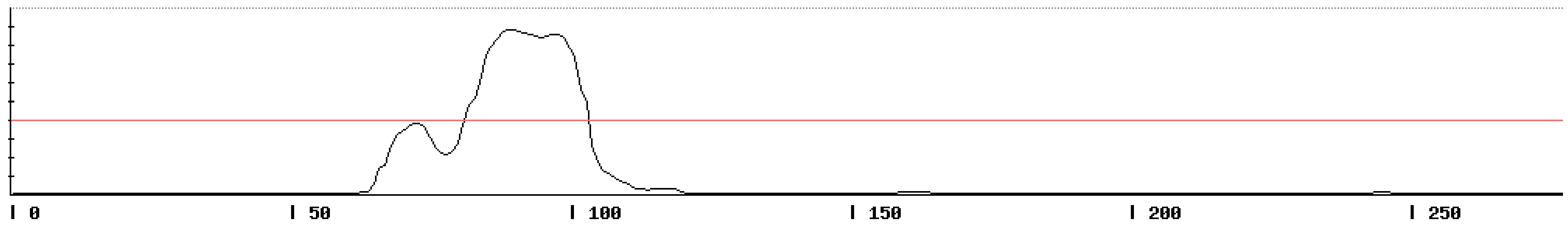

Predictions for Nicotianasylvestris_XP_009771991.1

Viterbi Path

Viterbi Path

Posterior @ 0.4

Posterior @ 0.4

Posterior Graphic. Threshold Marked @ 0.4

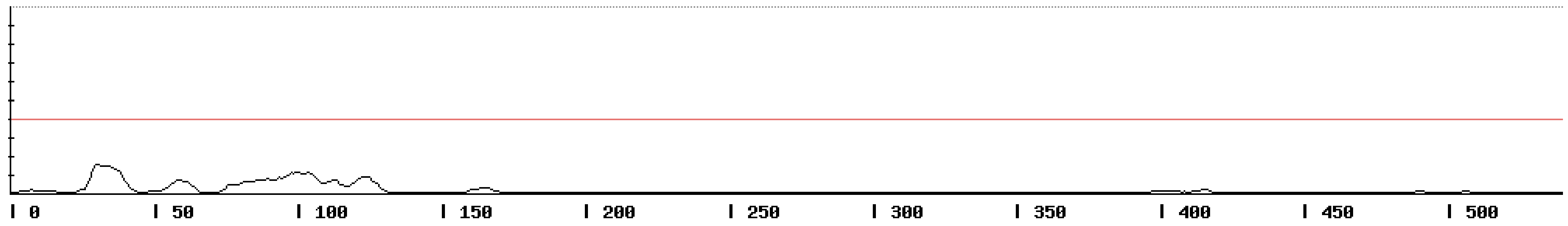

Predictions for Nicotianasylvestris_XP_009771992.1

Viterbi Path

Viterbi Path

Posterior @ 0.4

Posterior @ 0.4

Posterior Graphic. Threshold Marked @ 0.4

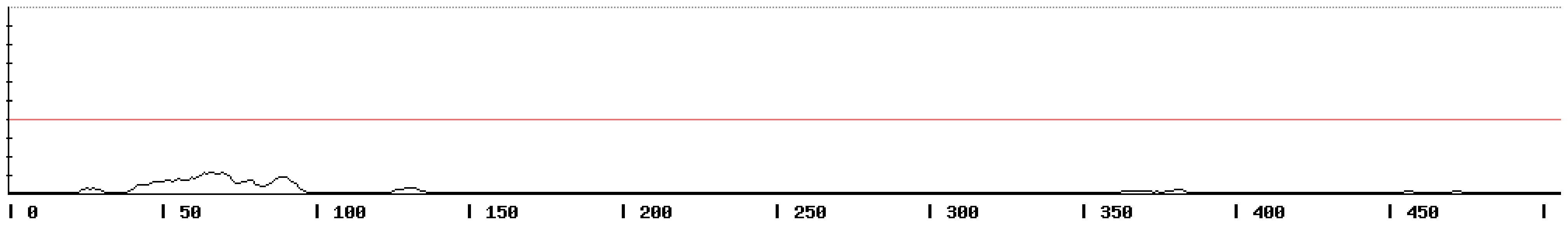

Predictions for Nicotianasylvestris_XP_009772246.1

Viterbi Path

Viterbi Path

Posterior @ 0.4

266 - VRNKRPRRKRDSHPPP - 281

492 - KK - 493

Posterior @ 0.4

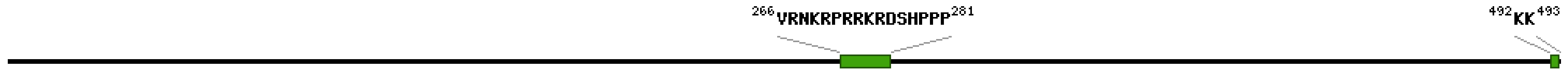

Posterior Graphic. Threshold Marked @ 0.4

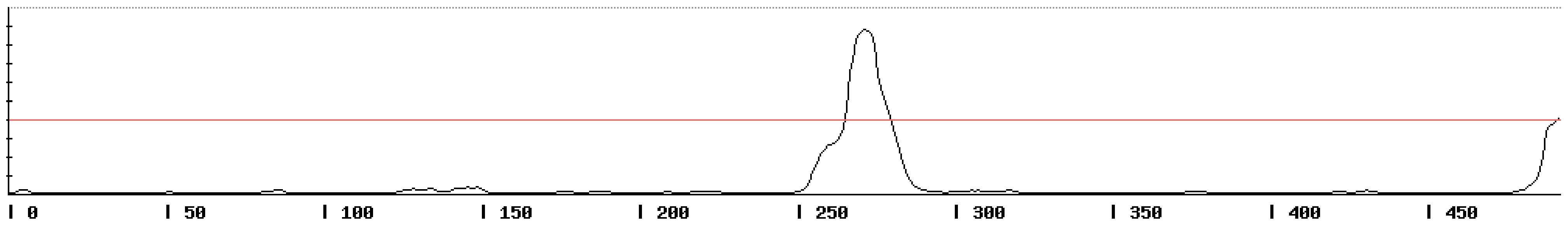

Predictions for Nicotianasylvestris_XP_009772247.1

Viterbi Path

Viterbi Path

Posterior @ 0.4

266 - VRNKRPRRKRDSHPPP - 281

492 - KK - 493

Posterior @ 0.4	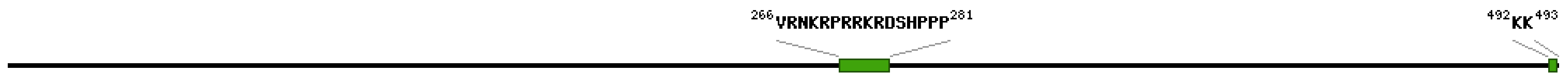
Posterior Graphic. Threshold Marked @ 0.4	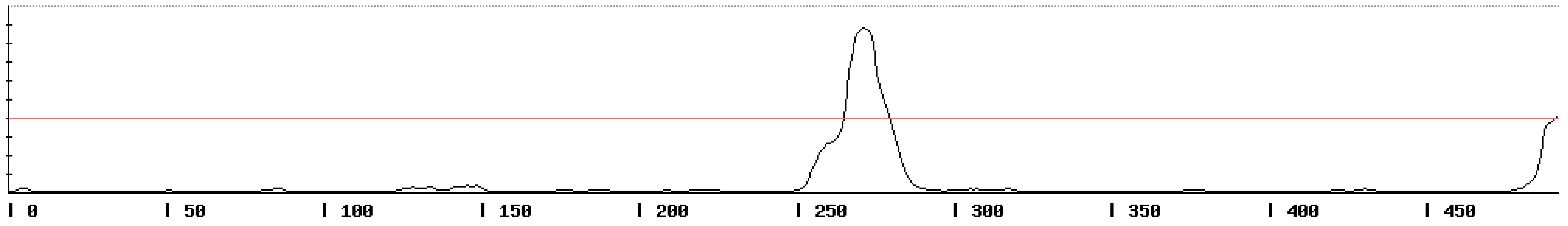

Predictions for Nicotianasylvestris_XP_009772248.1

Viterbi Path	
Viterbi Path	
Posterior @ 0.4	188 - VRNKRPRRKRDSHPPP - 203
	414 - KK - 415
Posterior @ 0.4	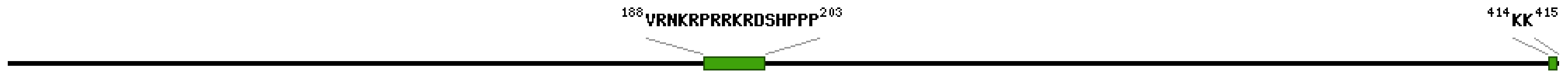
Posterior Graphic. Threshold Marked @ 0.4	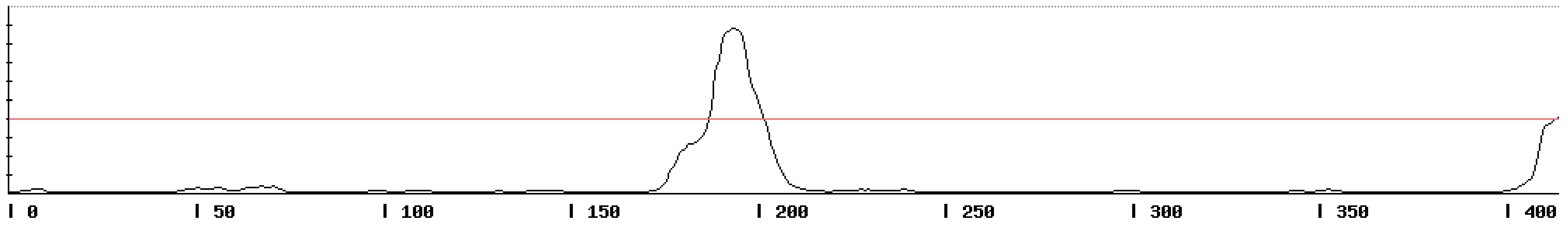

Predictions for Nicotianasylvestris_XP_009772249.1

Viterbi Path	
Viterbi Path	

Posterior @ 0.4 — 266 - VRNKRPRRKRDSHPP - 280

Posterior @ 0.4

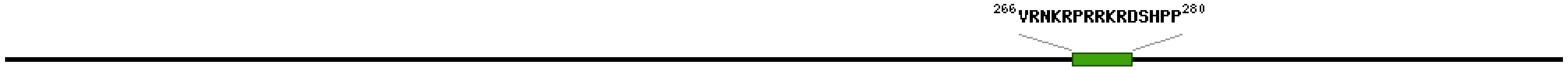

Posterior Graphic. Threshold Marked @ 0.4

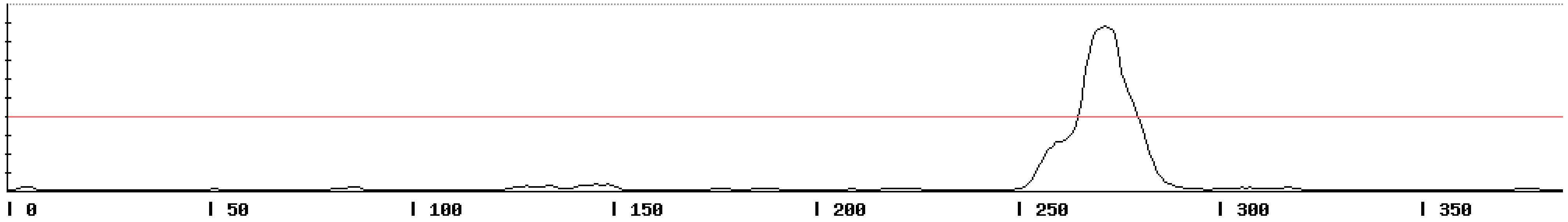

Predictions for Nicotianasylvestris_XP_009772253.1

Viterbi Path

Viterbi Path

Posterior @ 0.4

Posterior @ 0.4

Posterior Graphic. Threshold Marked @ 0.4

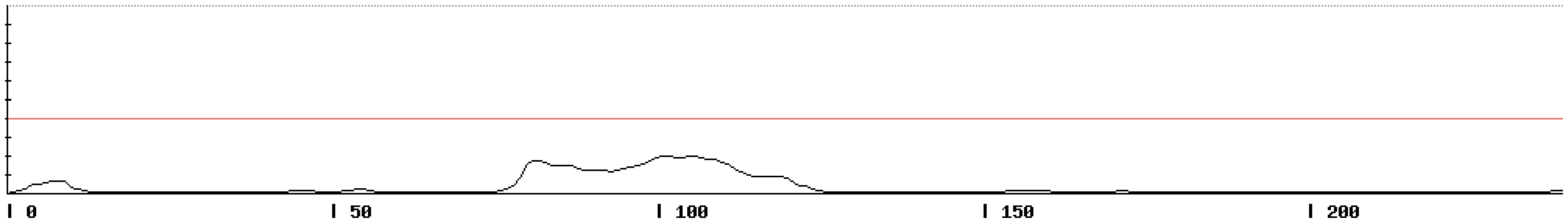

Predictions for Nicotianasylvestris_XP_009772560.1

Viterbi Path	
Viterbi Path	
Posterior @ 0.4	
Posterior @ 0.4	
Posterior Graphic. Threshold Marked @ 0.4	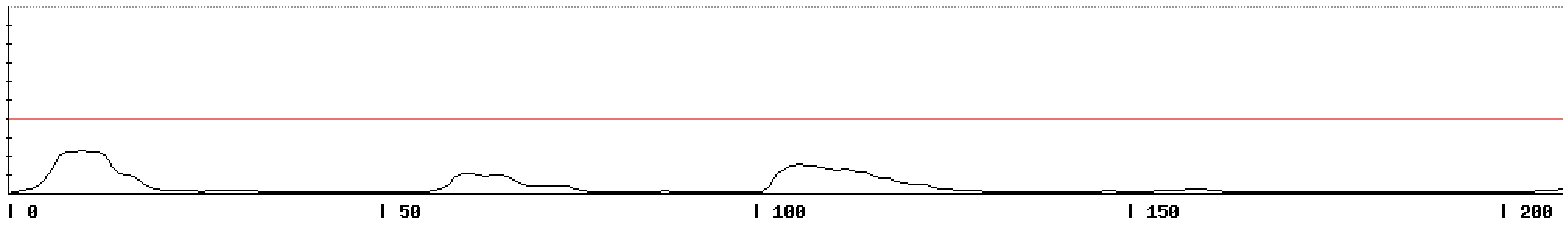

Predictions for Nicotianasylvestris_XP_009773823.1

Viterbi Path	
Viterbi Path	
Posterior @ 0.4	131 - KRR - 133<br>147 - HKTGKTRPVVVKGKVKGHKK - 166
Posterior @ 0.4	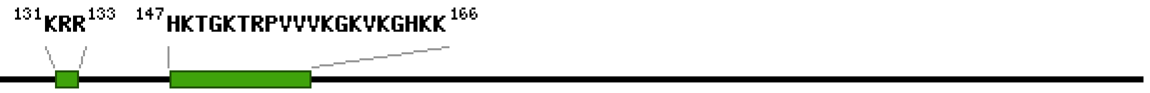
Posterior Graphic. Threshold Marked @ 0.4	

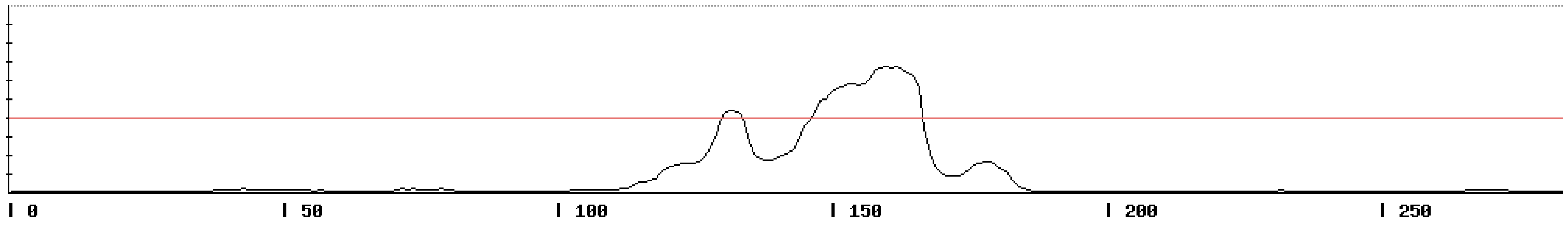

Predictions for Nicotianasylvestris_XP_009773837.1

Viterbi Path	
Viterbi Path	
Posterior @ 0.4	
Posterior @ 0.4	
Posterior Graphic. Threshold Marked @ 0.4	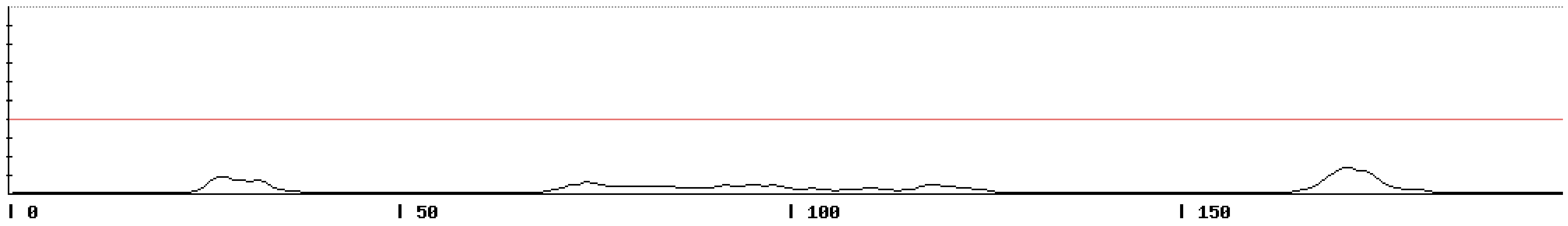

Predictions for Nicotianasylvestris_XP_009773865.1

Viterbi Path	
Viterbi Path	
Posterior @ 0.4	
Posterior @ 0.4	

Posterior Graphic. Threshold Marked @ 0.4

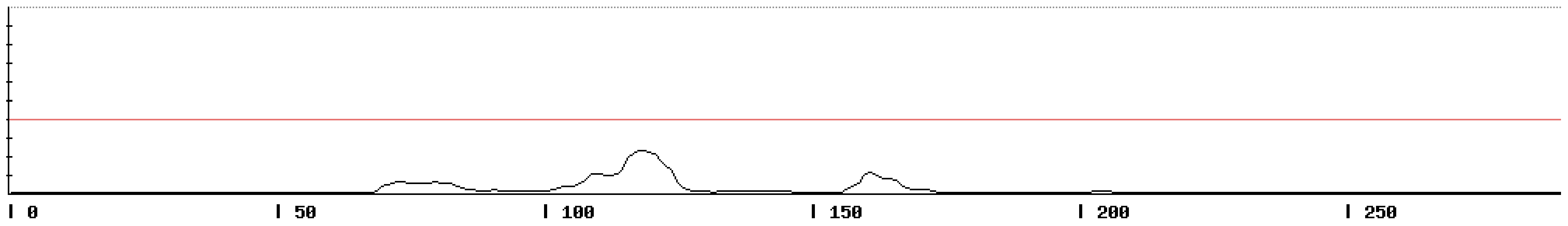

Predictions for Nicotianasylvestris_XP_009774438.1

Viterbi Path

Viterbi Path

Posterior @ 0.4

Posterior @ 0.4

Posterior Graphic. Threshold Marked @ 0.4

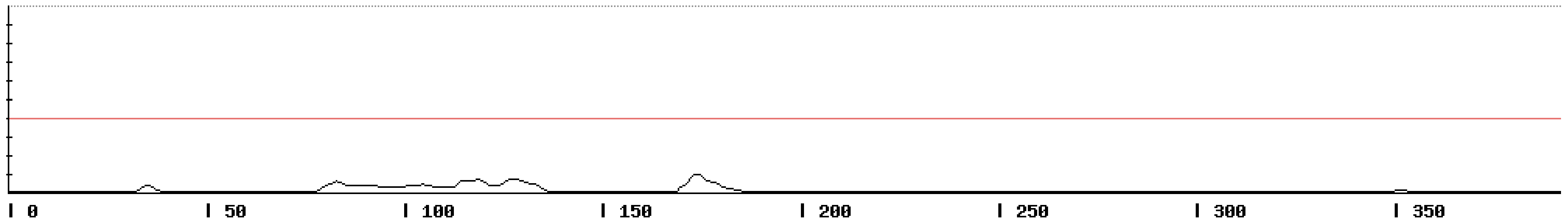

Predictions for Nicotianasylvestris_XP_009774486.1

Viterbi Path

Viterbi Path

Posterior @ 0.4

Posterior @ 0.4

Posterior Graphic. Threshold Marked @ 0.4

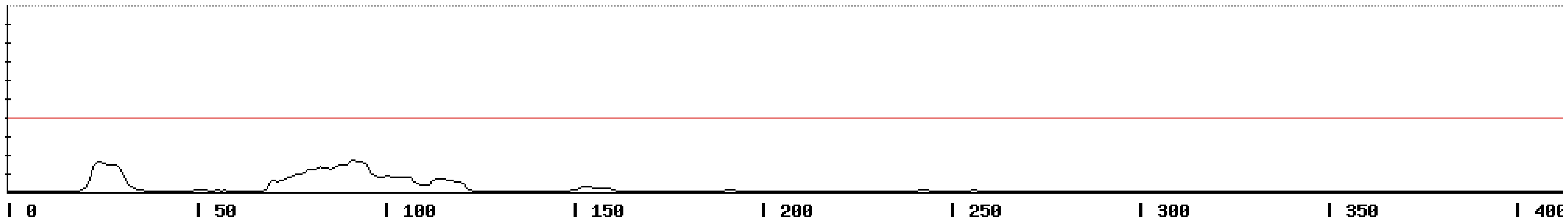

Predictions for Nicotianasylvestris_XP_009774531.1

Viterbi Path

Viterbi Path

Posterior @ 0.4

Posterior @ 0.4

Posterior Graphic. Threshold Marked @ 0.4

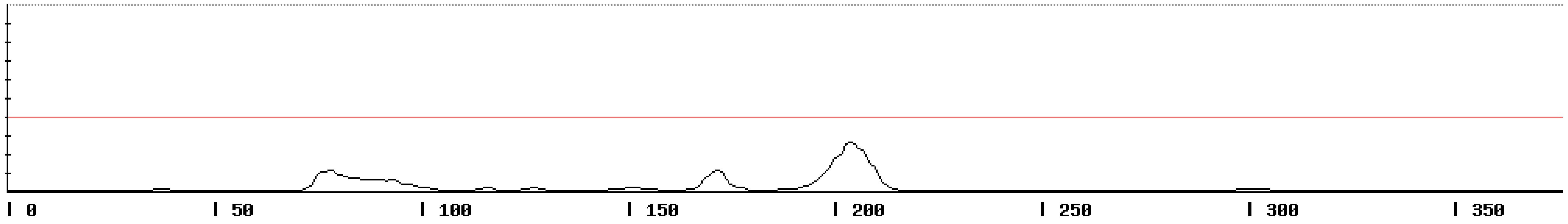

Predictions for Nicotianasylvestris_XP_009774570.1

Viterbi Path

Viterbi Path

Posterior @ 0.4	
Posterior @ 0.4	
Posterior Graphic. Threshold Marked @ 0.4	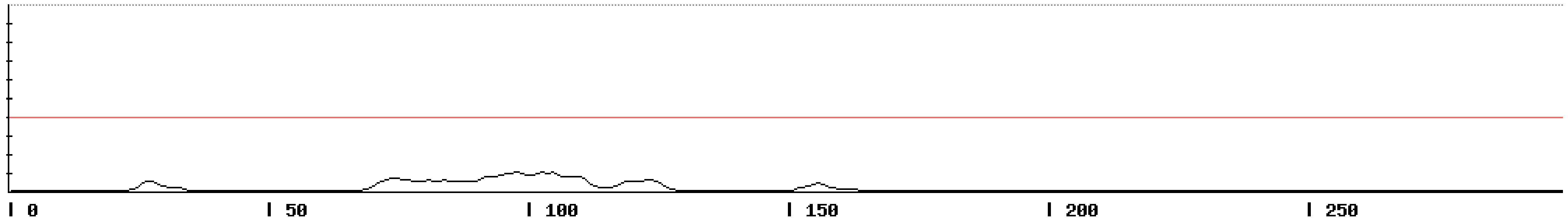

Predictions for Nicotianasylvestris_XP_009774958.1

Viterbi Path	
Viterbi Path	
Posterior @ 0.4	77 - KKKS - 80
Posterior @ 0.4	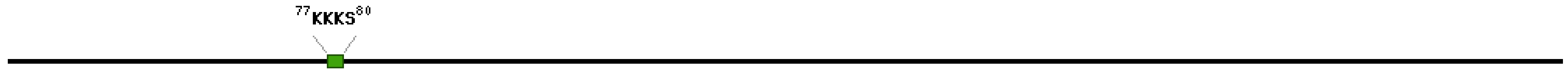
Posterior Graphic. Threshold Marked @ 0.4	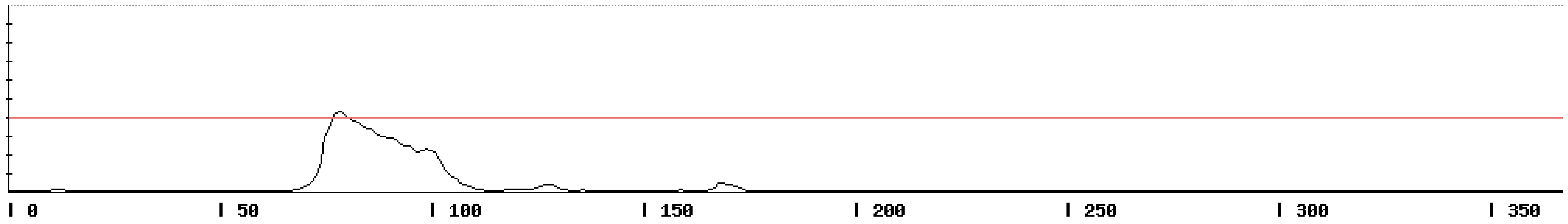

Predictions for Nicotianasylvestris_XP_009775198.1

Viterbi Path	
Viterbi Path	

Posterior @ 0.4

Posterior @ 0.4

Posterior Graphic. Threshold Marked @ 0.4

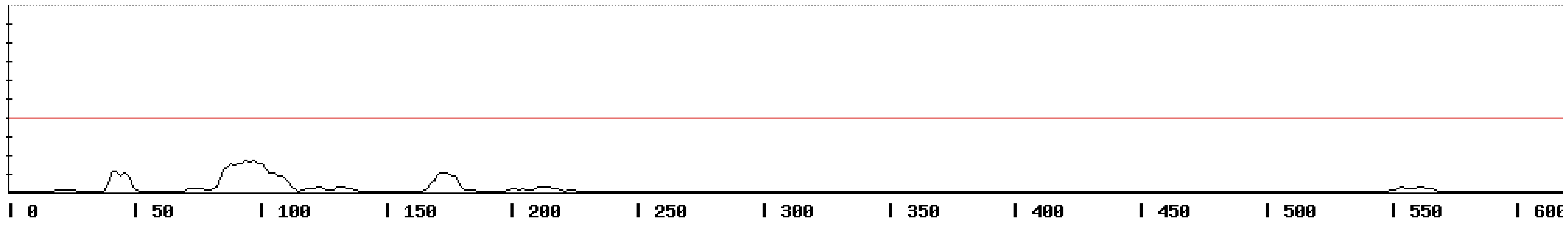

Predictions for Nicotianasylvestris_XP_009775199.1

Viterbi Path

Viterbi Path

Posterior @ 0.4

Posterior @ 0.4

Posterior Graphic. Threshold Marked @ 0.4

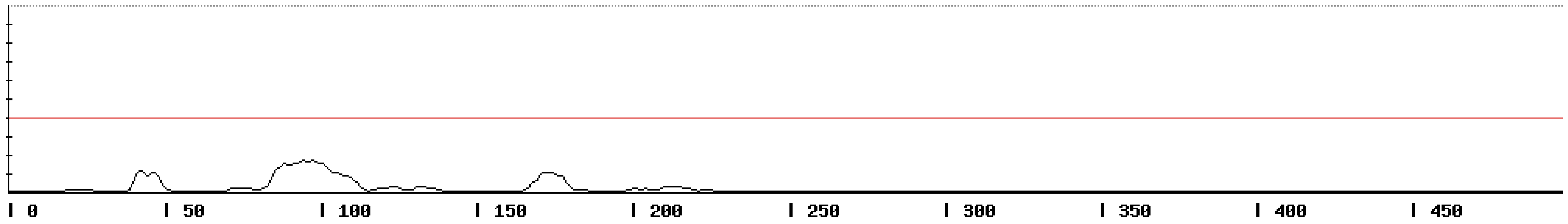

Predictions for Nicotianasylvestris_XP_009775469.1

Viterbi Path	
Viterbi Path	
Posterior @ 0.4	81 - GRKRRYKNGRKPNRQ - 95
Posterior @ 0.4	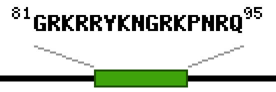
Posterior Graphic. Threshold Marked @ 0.4	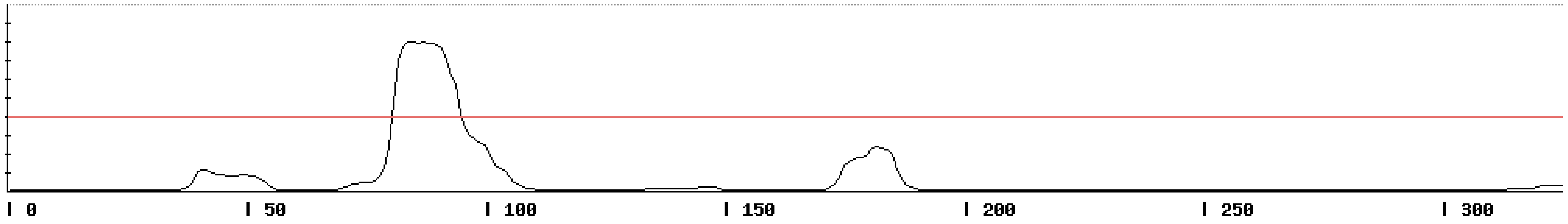

Predictions for Nicotianasylvestris_XP_009778771.1

Viterbi Path	
Viterbi Path	
Posterior @ 0.4	
Posterior @ 0.4	
Posterior Graphic. Threshold Marked @ 0.4	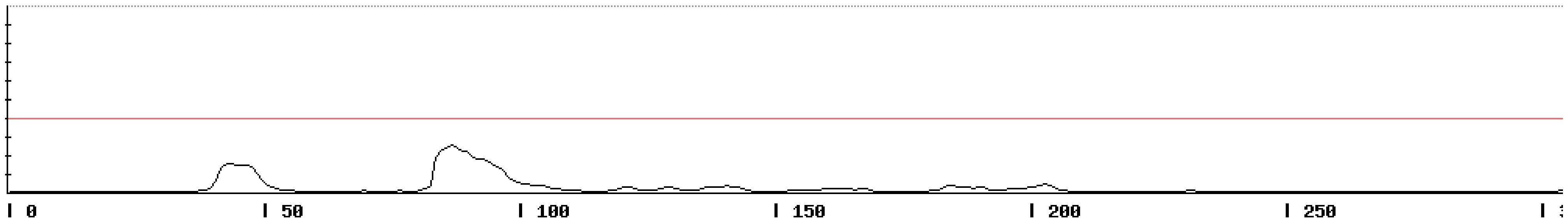

Predictions for Nicotianasylvestris_XP_009779378.1	
Viterbi Path	
Viterbi Path	
Posterior @ 0.4	
Posterior @ 0.4	
Posterior Graphic. Threshold Marked @ 0.4	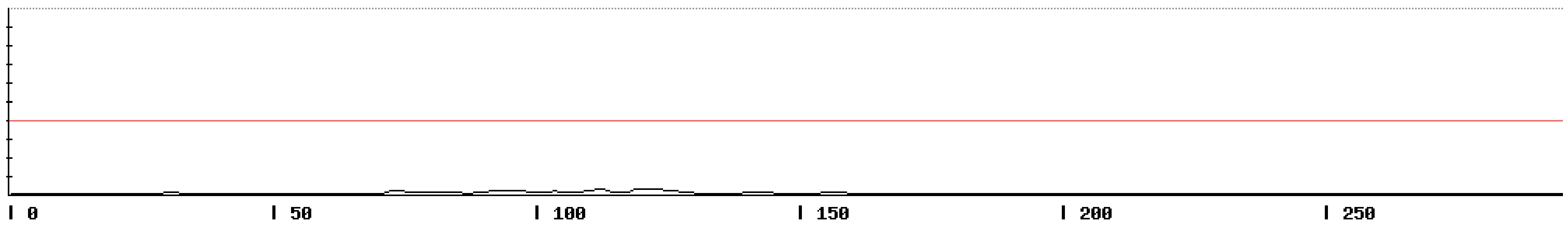

Predictions for Nicotianasylvestris_XP_009779881.1	
Viterbi Path	
Viterbi Path	
Posterior @ 0.4	
Posterior @ 0.4	
Posterior Graphic. Threshold Marked @ 0.4	

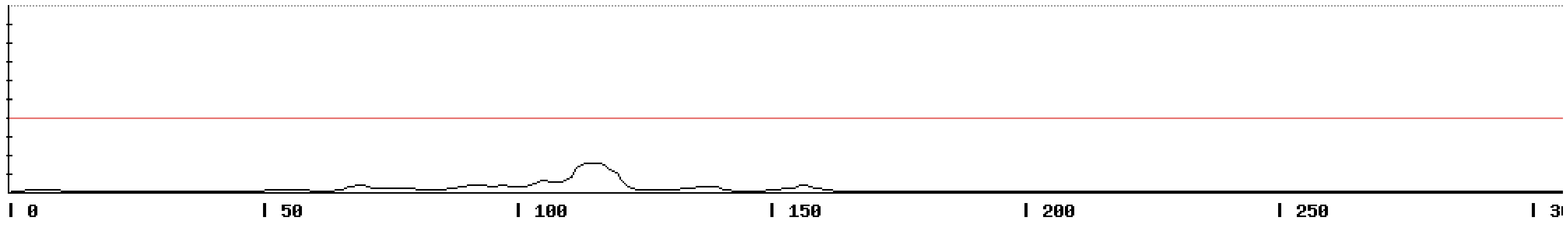

Predictions for Nicotianasylvestris_XP_009780447.1	
Viterbi Path	
Viterbi Path	
Posterior @ 0.4	
Posterior @ 0.4	
Posterior Graphic. Threshold Marked @ 0.4	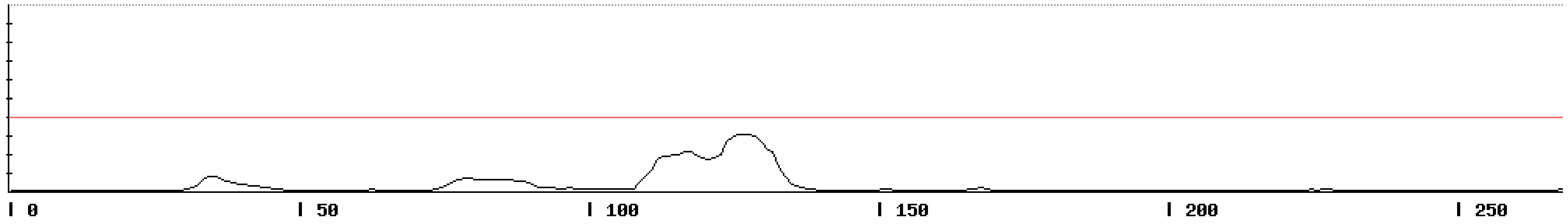

Predictions for Nicotianasylvestris_XP_009780872.1	
Viterbi Path	
Viterbi Path	
Posterior @ 0.4	
Posterior @ 0.4	

Posterior Graphic. Threshold Marked @ 0.4

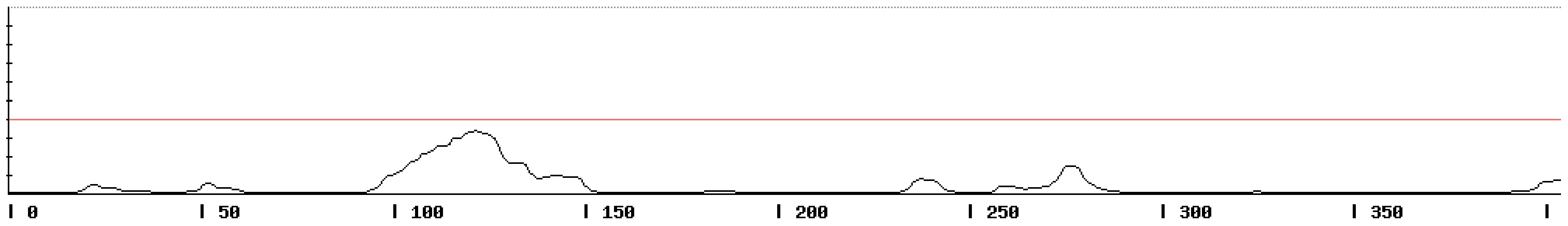

Predictions for Nicotianasylvestris_XP_009781251.1

Viterbi Path

Viterbi Path

Posterior @ 0.4

Posterior @ 0.4

Posterior Graphic. Threshold Marked @ 0.4

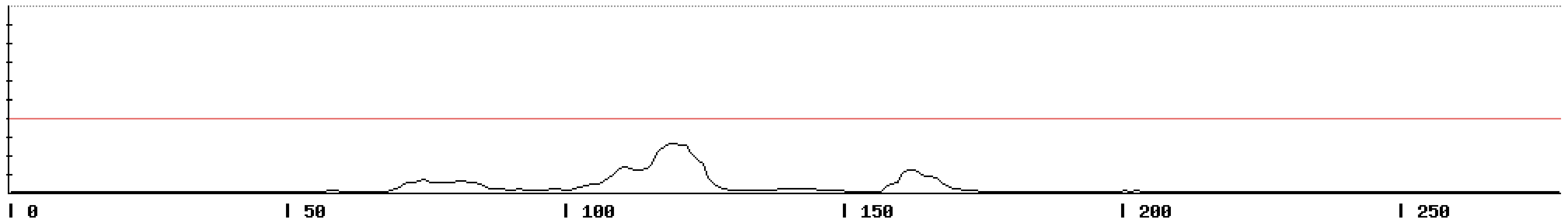

Predictions for Nicotianasylvestris_XP_009782166.1

Viterbi Path

Viterbi Path

Posterior @ 0.4

Posterior @ 0.4

Posterior Graphic. Threshold Marked @ 0.4

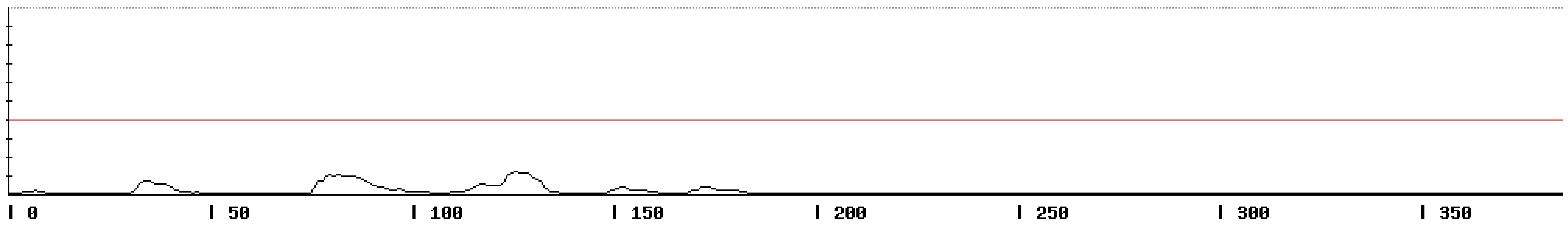

Predictions for Nicotianasylvestris_XP_009782167.1

Viterbi Path

Viterbi Path

Posterior @ 0.4

Posterior @ 0.4

Posterior Graphic. Threshold Marked @ 0.4

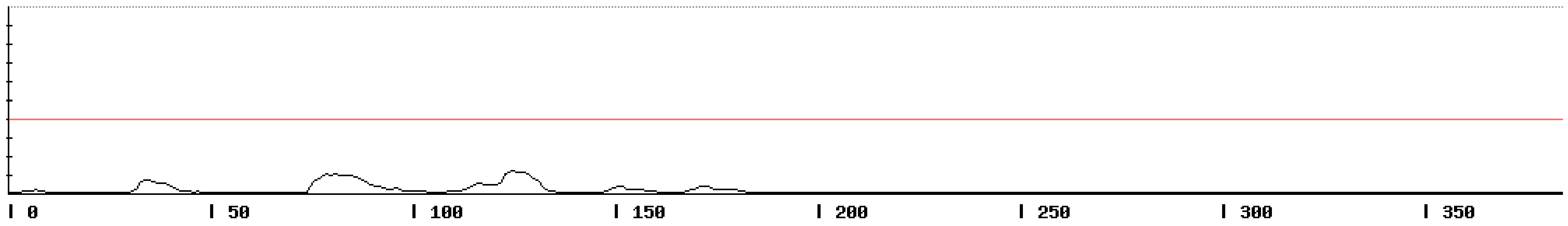

Predictions for Nicotianasylvestris_XP_009782234.1

Viterbi Path

Viterbi Path

Posterior @ 0.4

Posterior @ 0.4

Posterior Graphic. Threshold Marked @ 0.4

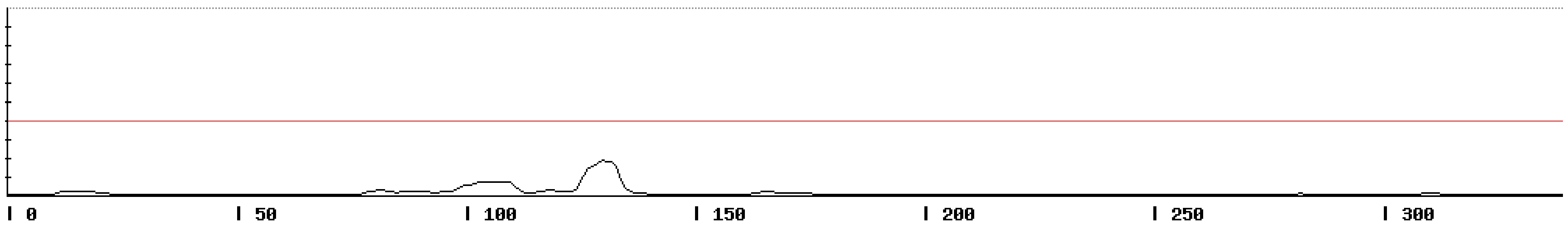

Predictions for Nicotianasylvestris_XP_009782335.1

Viterbi Path

Viterbi Path

Posterior @ 0.4

Posterior @ 0.4

Posterior Graphic. Threshold Marked @ 0.4

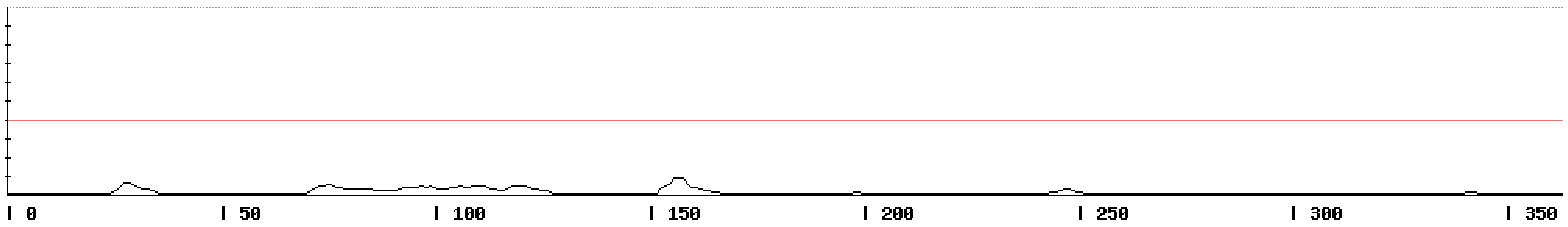

Predictions for Nicotianasylvestris_XP_009782736.1

Viterbi Path

Viterbi Path

Posterior @ 0.4 — 76 - KKKS - 79

Posterior @ 0.4

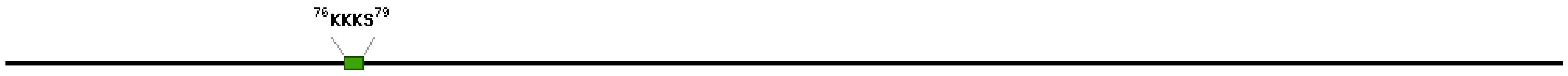

Posterior Graphic. Threshold Marked @ 0.4

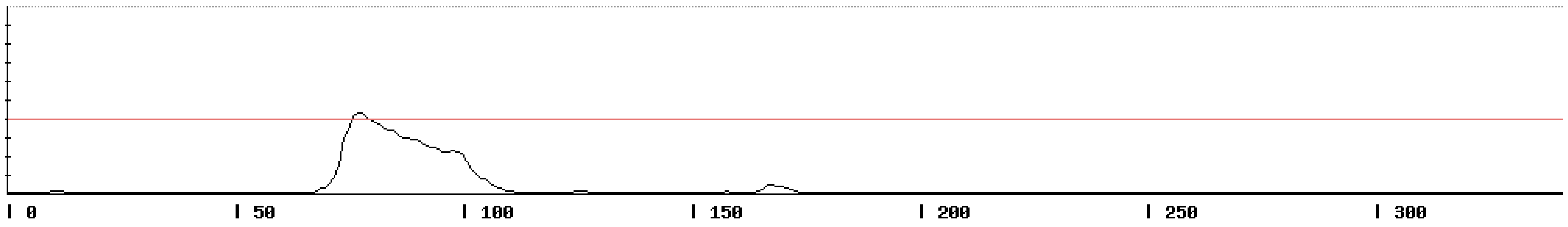

Predictions for Nicotianasylvestris_XP_009783061.1

Viterbi Path

Viterbi Path

Posterior @ 0.4

Posterior @ 0.4

Posterior Graphic. Threshold Marked @ 0.4

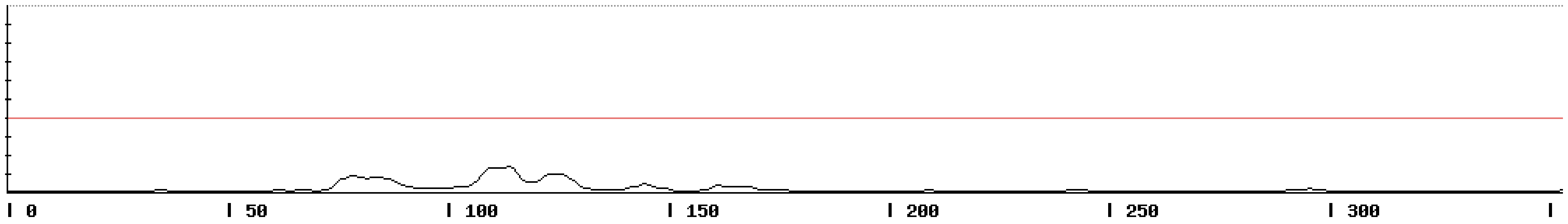

Predictions for Nicotianasylvestris_XP_009784097.1

Viterbi Path

Viterbi Path

Posterior @ 0.4

Posterior @ 0.4

Posterior Graphic. Threshold Marked @ 0.4

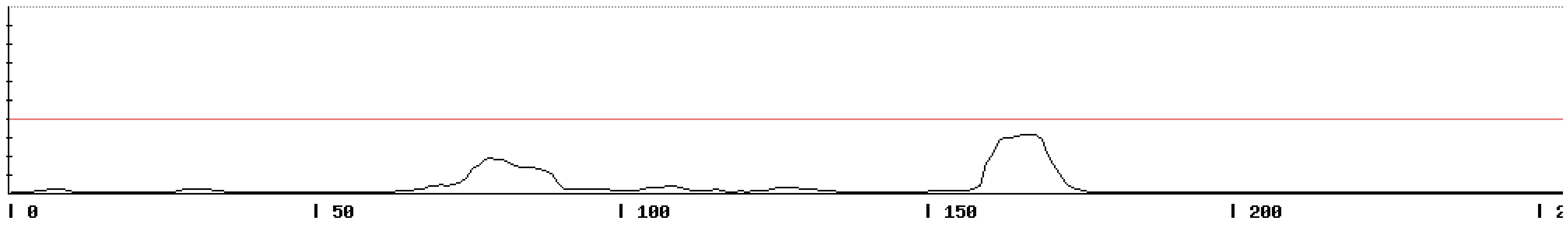

Predictions for Nicotianasylvestris_XP_009784261.1

Viterbi Path

Viterbi Path

Posterior @ 0.4

Posterior @ 0.4

Posterior Graphic. Threshold Marked @ 0.4

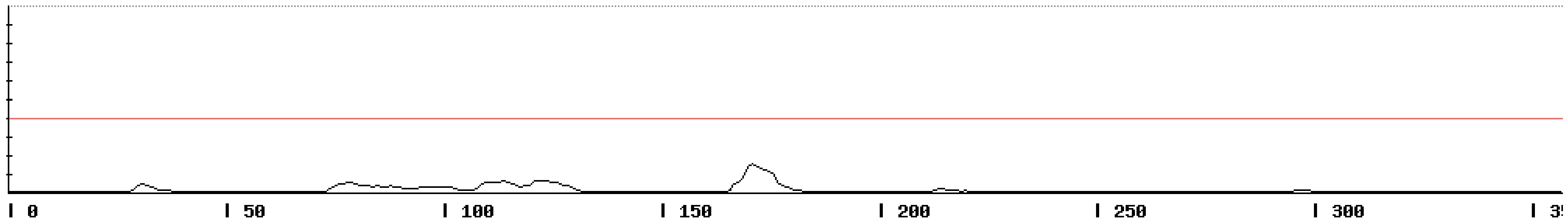

Predictions for Nicotianasylvestris_XP_009785299.1

Viterbi Path

Viterbi Path

Posterior @ 0.4

Posterior @ 0.4

Posterior Graphic. Threshold Marked @ 0.4

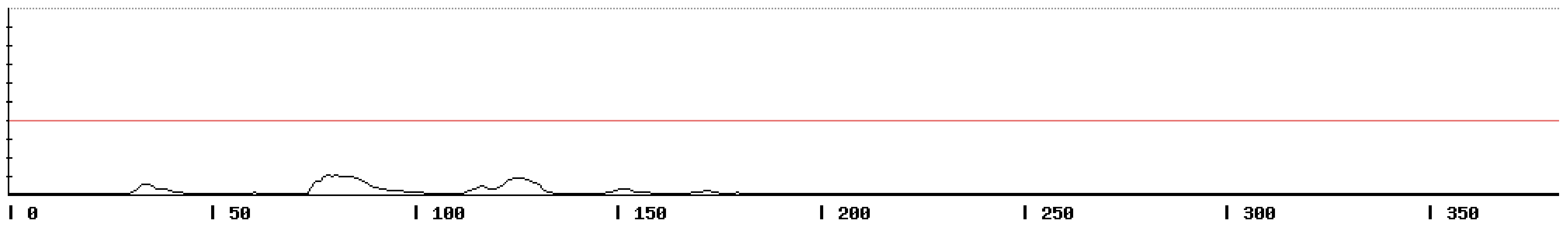

Predictions for Nicotianasylvestris_XP_009786013.1

Viterbi Path

Viterbi Path

Posterior @ 0.4

Posterior @ 0.4

Posterior Graphic. Threshold Marked @ 0.4

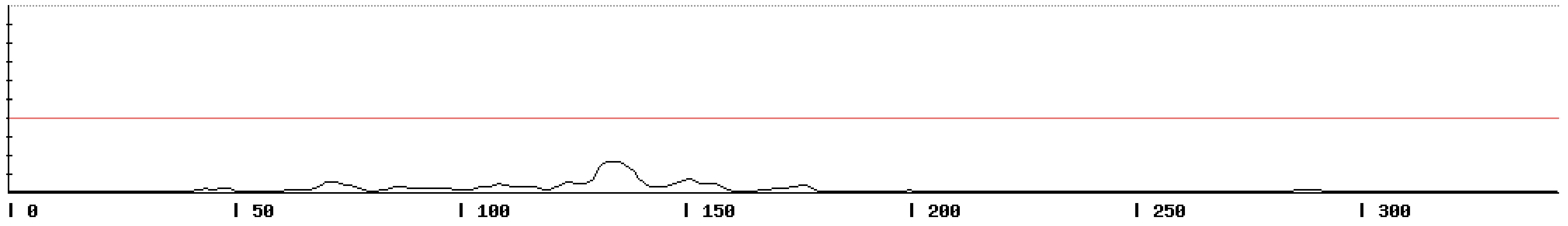

Predictions for Nicotianasylvestris_XP_009786294.1

Viterbi Path	
Viterbi Path	
Posterior @ 0.4	
Posterior @ 0.4	
Posterior Graphic. Threshold Marked @ 0.4	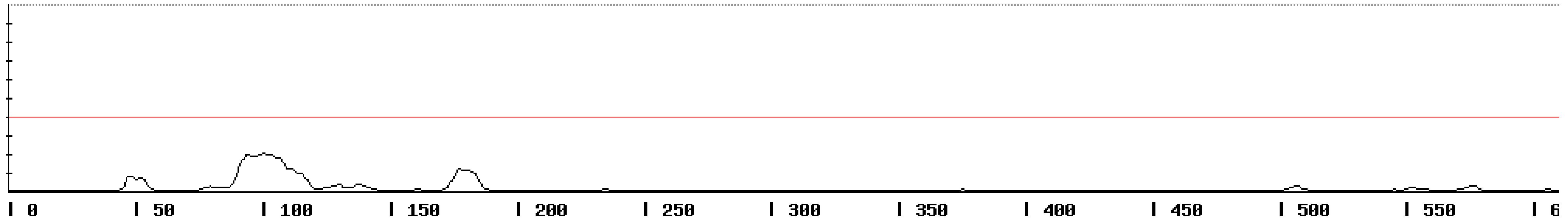

Predictions for Nicotianasylvestris_XP_009786309.1

Viterbi Path	
Viterbi Path	
Posterior @ 0.4	104 - VGKPK - 108
Posterior @ 0.4	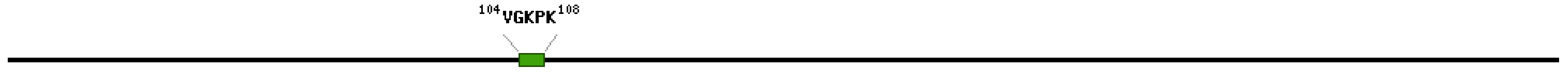

Posterior Graphic. Threshold Marked @ 0.4

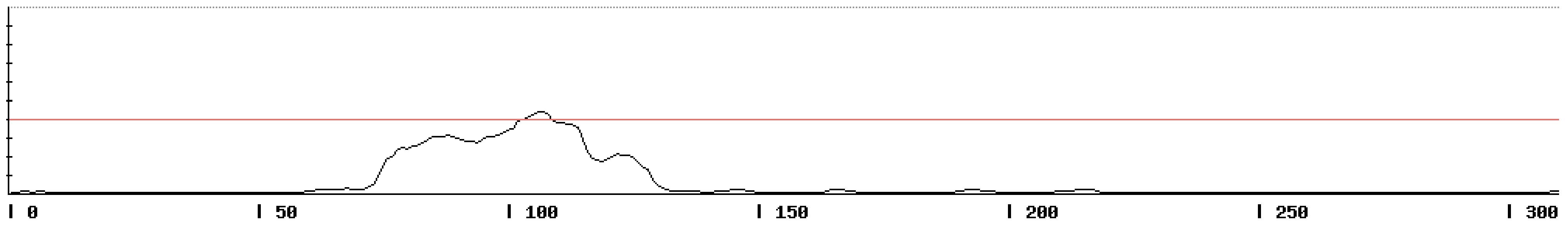

Predictions for Nicotianasylvestris_XP_009786835.1

Viterbi Path	
Viterbi Path	
Posterior @ 0.4	
Posterior @ 0.4	
Posterior Graphic. Threshold Marked @ 0.4	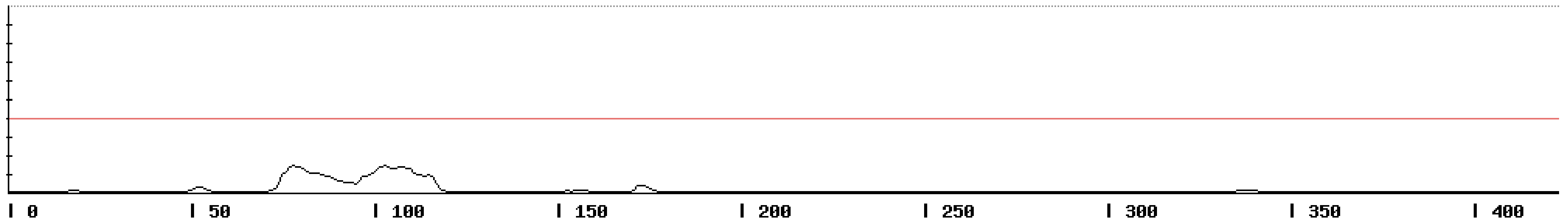

Predictions for Nicotianasylvestris_XP_009787362.1

Viterbi Path	
Viterbi Path	
Posterior @ 0.4	67 - LKKRKESHKRFRR - 79
Posterior @ 0.4	

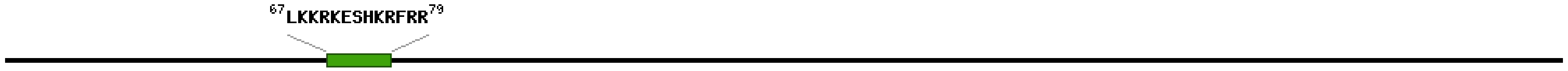

Posterior Graphic. Threshold Marked @ 0.4

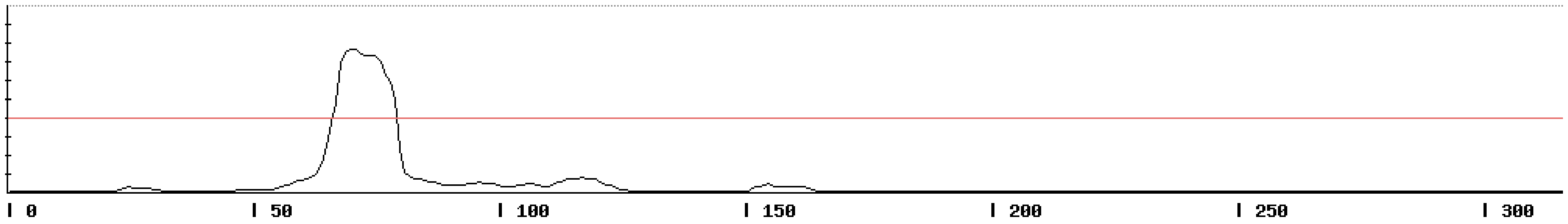

Predictions for Nicotianasylvestris_XP_009787917.1

Viterbi Path

Viterbi Path

Posterior @ 0.4

Posterior @ 0.4

Posterior Graphic. Threshold Marked @ 0.4

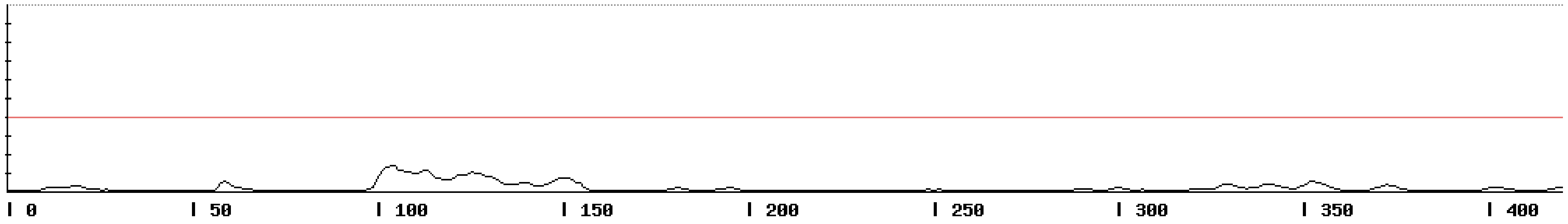

Predictions for Nicotianasylvestris_XP_009788540.1

Viterbi Path

Viterbi Path

Posterior @ 0.4

Posterior @ 0.4

Posterior Graphic. Threshold Marked @ 0.4

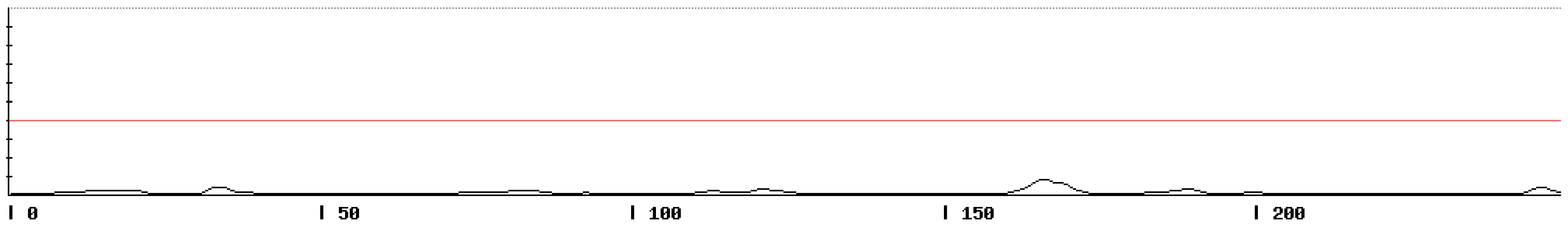

Predictions for Nicotianasylvestris_XP_009789697.1

Viterbi Path

Viterbi Path

Posterior @ 0.4

Posterior @ 0.4

Posterior Graphic. Threshold Marked @ 0.4

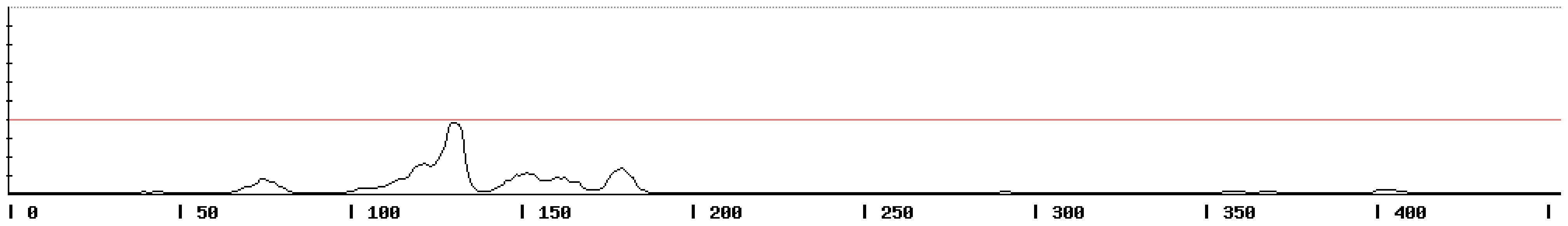

Predictions for Nicotianasylvestris_XP_009789698.1

Viterbi Path

Viterbi Path

Posterior @ 0.4

Posterior @ 0.4

Posterior Graphic. Threshold Marked @ 0.4

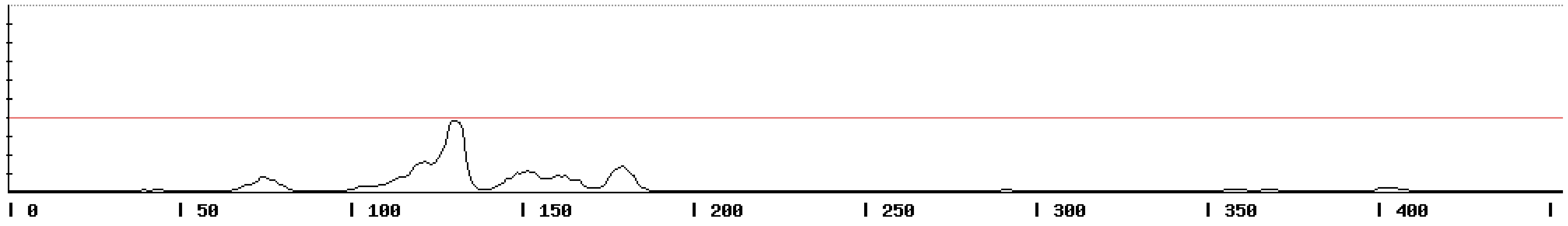

Predictions for Nicotianasylvestris_XP_009789699.1

Viterbi Path

Viterbi Path

Posterior @ 0.4

Posterior @ 0.4

Posterior Graphic. Threshold Marked @ 0.4

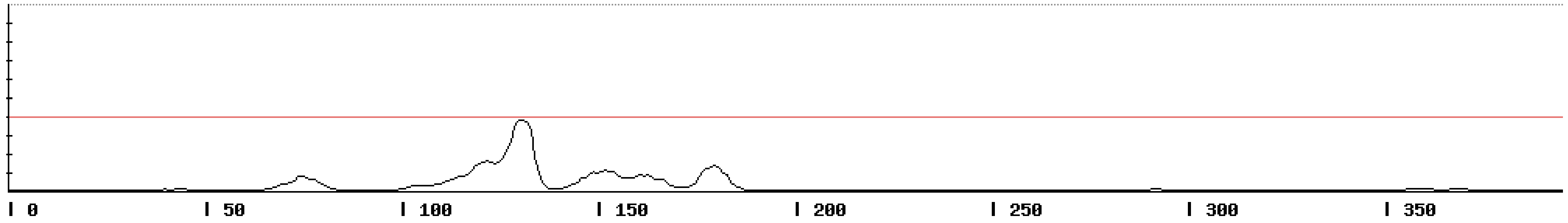

Predictions for Nicotianasylvestris_XP_009789986.1

Viterbi Path

Viterbi Path

Posterior @ 0.4

Posterior @ 0.4

Posterior Graphic. Threshold Marked @ 0.4

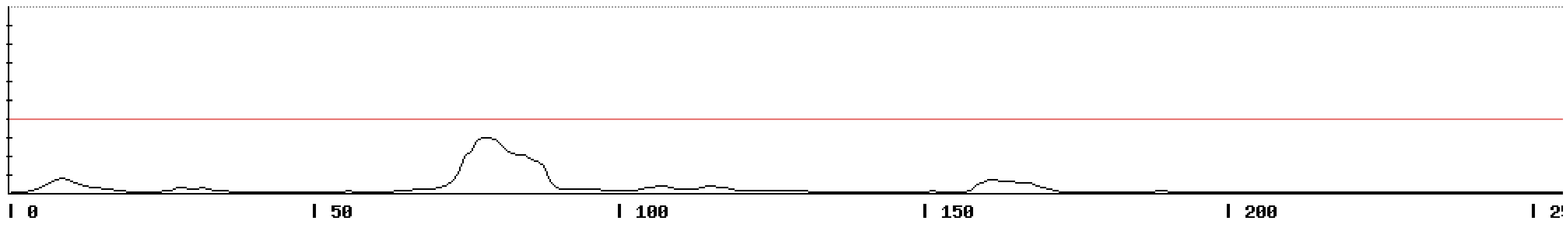

Predictions for Nicotianasylvestris_XP_009790203.1

Viterbi Path

Viterbi Path

Posterior @ 0.4

Posterior @ 0.4

Posterior Graphic. Threshold Marked @ 0.4

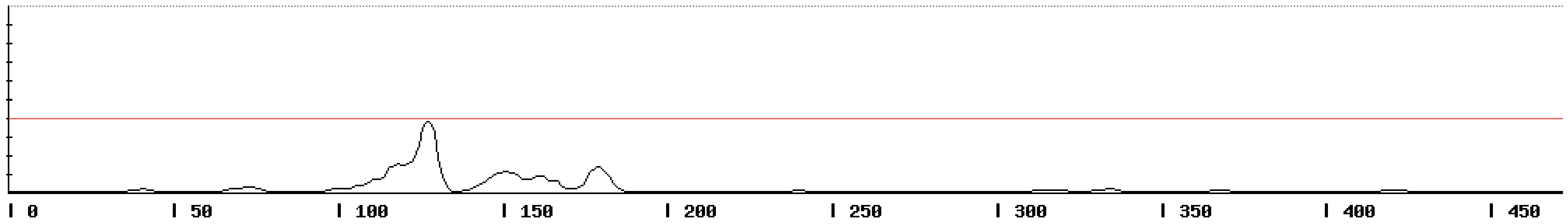

Predictions for Nicotianasylvestris_XP_009790204.1	
Viterbi Path	
Viterbi Path	
Posterior @ 0.4	
Posterior @ 0.4	
Posterior Graphic. Threshold Marked @ 0.4	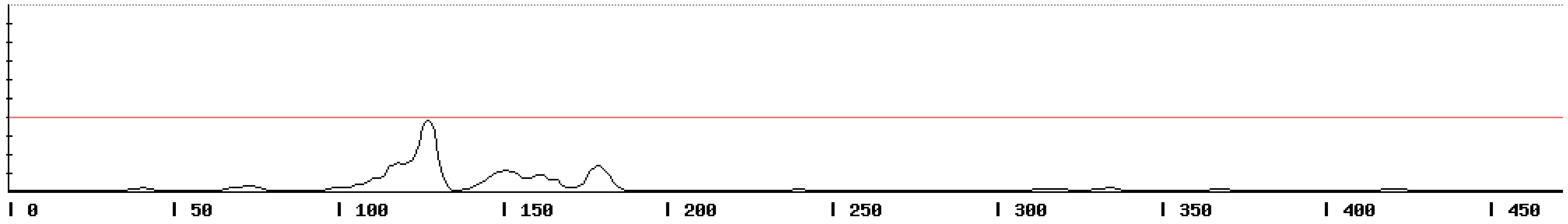

Predictions for Nicotianasylvestris_XP_009790205.1	
Viterbi Path	
Viterbi Path	
Posterior @ 0.4	
Posterior @ 0.4	
Posterior Graphic. Threshold Marked @ 0.4	

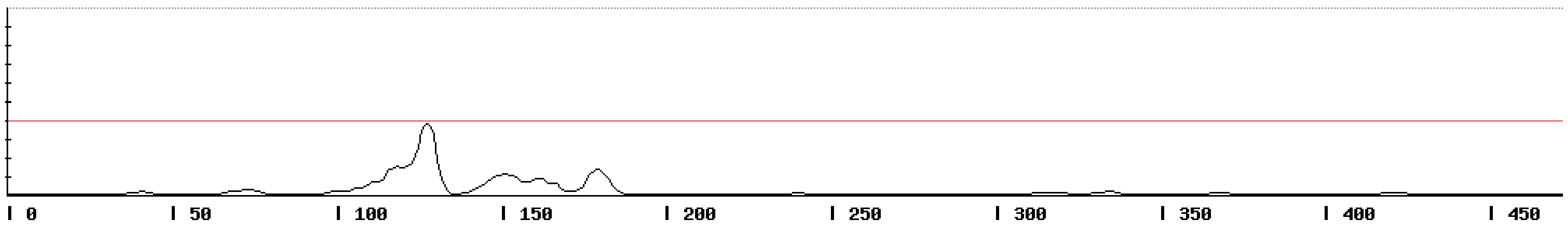

Predictions for Nicotianasylvestris_XP_009790206.1

Viterbi Path	
Viterbi Path	
Posterior @ 0.4	
Posterior @ 0.4	
Posterior Graphic. Threshold Marked @ 0.4	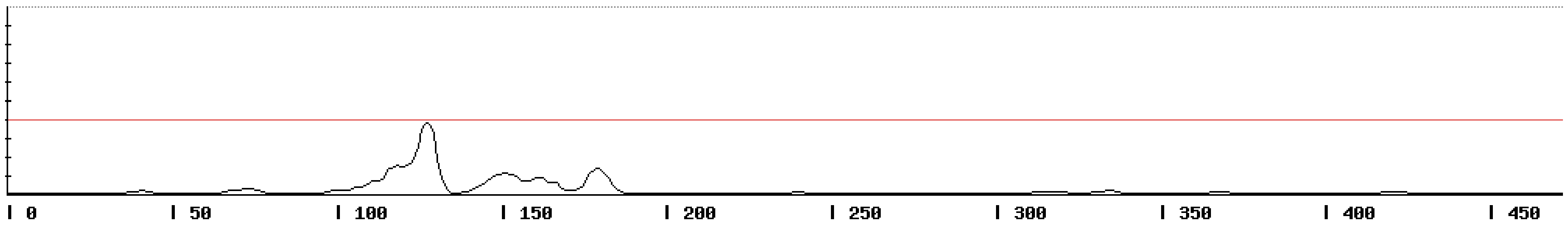

Predictions for Nicotianasylvestris_XP_009790208.1

Viterbi Path	
Viterbi Path	
Posterior @ 0.4	
Posterior @ 0.4	

Posterior Graphic. Threshold Marked @ 0.4

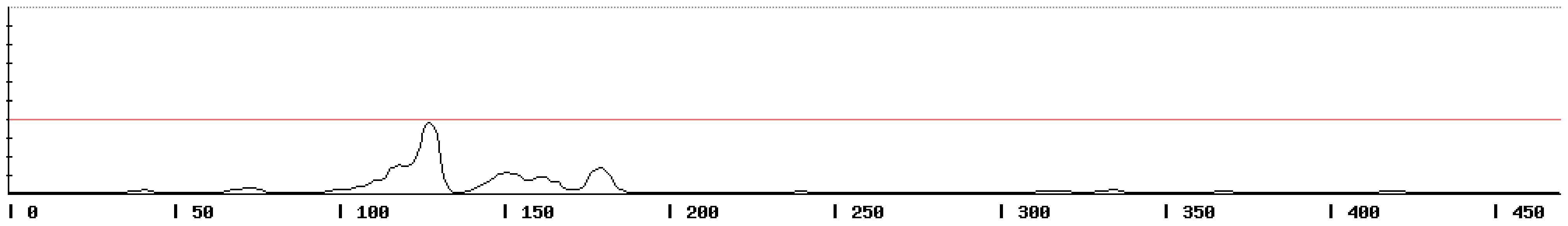

Predictions for Nicotianasylvestris_XP_009790209.1

Viterbi Path

Viterbi Path

Posterior @ 0.4

Posterior @ 0.4

Posterior Graphic. Threshold Marked @ 0.4

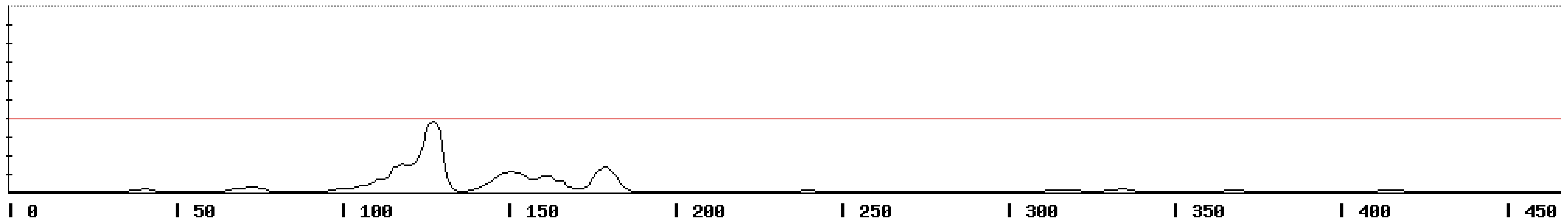

Predictions for Nicotianasylvestris_XP_009790210.1

Viterbi Path

Viterbi Path

Posterior @ 0.4

Posterior @ 0.4

Posterior Graphic. Threshold Marked @ 0.4

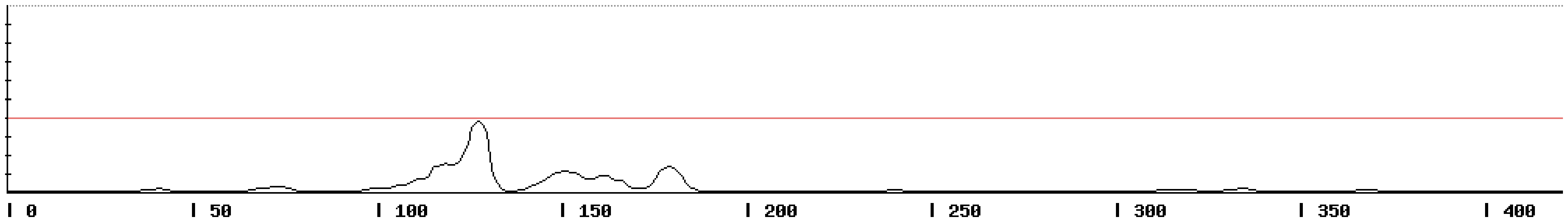

Predictions for Nicotianasylvestris_XP_009790484.1

Viterbi Path

Viterbi Path

Posterior @ 0.4

Posterior @ 0.4

Posterior Graphic. Threshold Marked @ 0.4

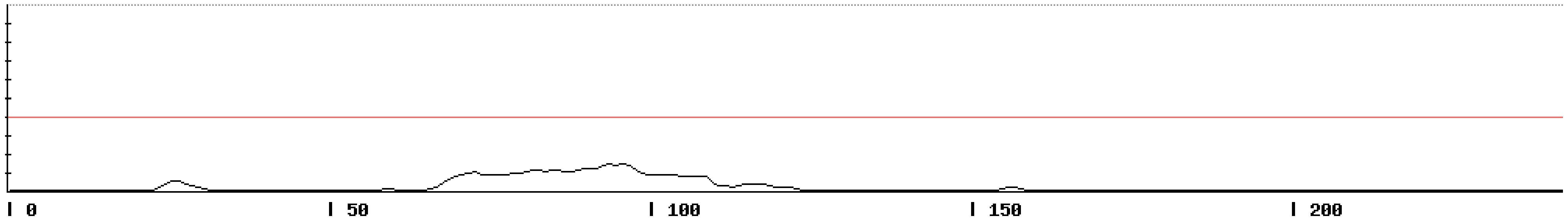

Predictions for Nicotianasylvestris_XP_009790485.1

Viterbi Path

Viterbi Path

Posterior @ 0.4

Posterior @ 0.4

Posterior Graphic. Threshold Marked @ 0.4

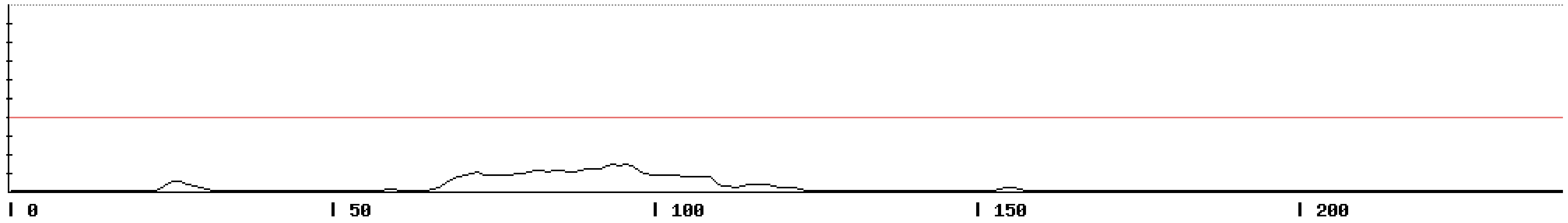

Predictions for Nicotianasylvestris_XP_009790763.1

Viterbi Path

Viterbi Path

Posterior @ 0.4

Posterior @ 0.4

Posterior Graphic. Threshold Marked @ 0.4

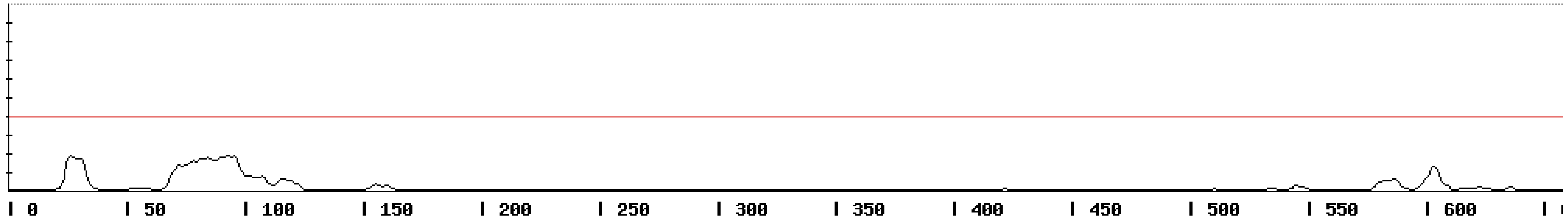

Predictions for Nicotianasylvestris_XP_009791743.1

Viterbi Path

Viterbi Path

Posterior @ 0.4

Posterior @ 0.4

Posterior Graphic. Threshold Marked @ 0.4

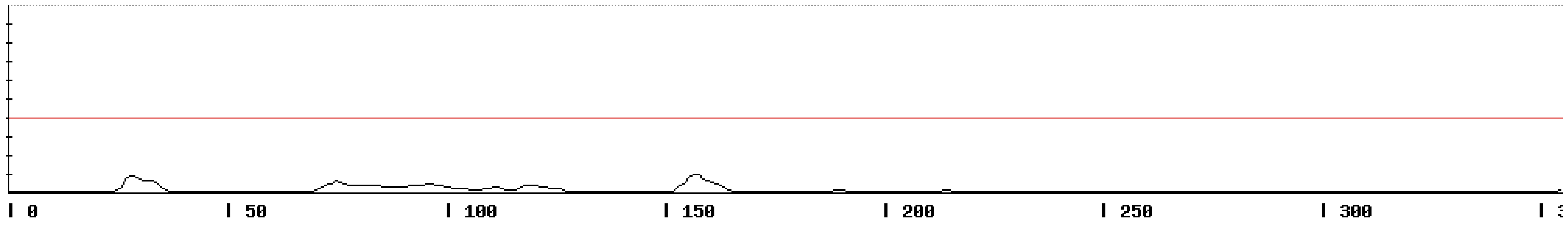

Predictions for Nicotianasylvestris_XP_009791966.1

Viterbi Path

Viterbi Path

Posterior @ 0.4

Posterior @ 0.4

Posterior Graphic. Threshold Marked @ 0.4

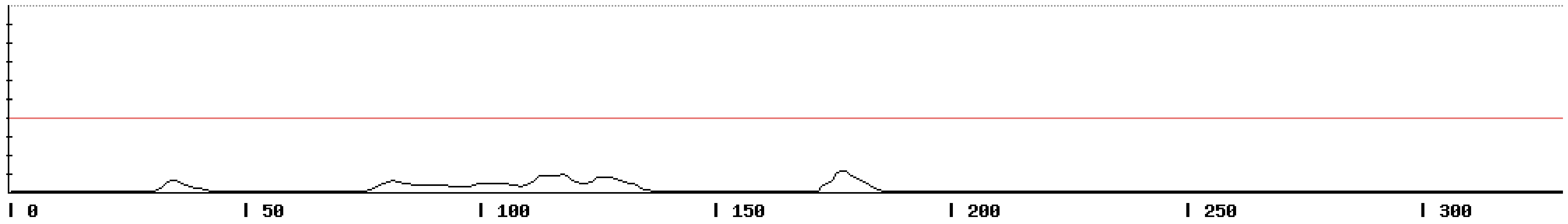

Predictions for Nicotianasylvestris_XP_009792222.1

Viterbi Path	151 - KIKKKGKPGDKKKG - 164
Viterbi Path	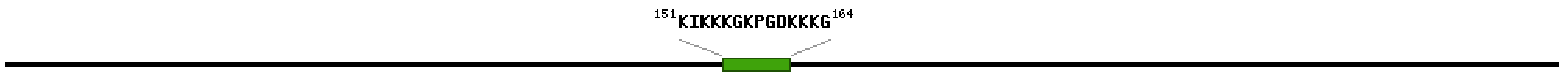
Posterior @ 0.4	68 - KKEKPTWKRVSRTVGKGTWKPQGKG - 92<br>151 - KIKKKGKPGDKKKG - 164
Posterior @ 0.4	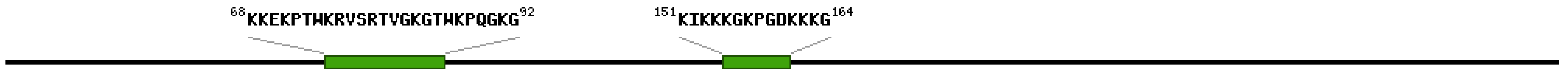
Posterior Graphic. Threshold Marked @ 0.4	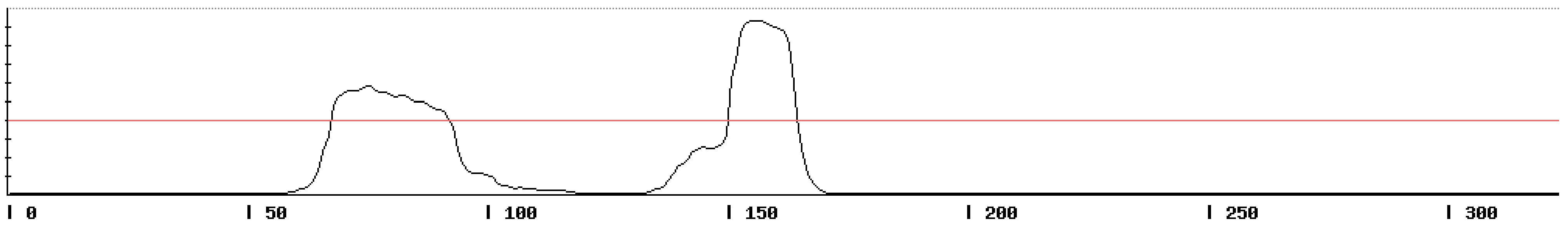

Predictions for Nicotianasylvestris_XP_009792377.1

Viterbi Path	
Viterbi Path	
Posterior @ 0.4	
Posterior @ 0.4	
Posterior Graphic. Threshold Marked @ 0.4	

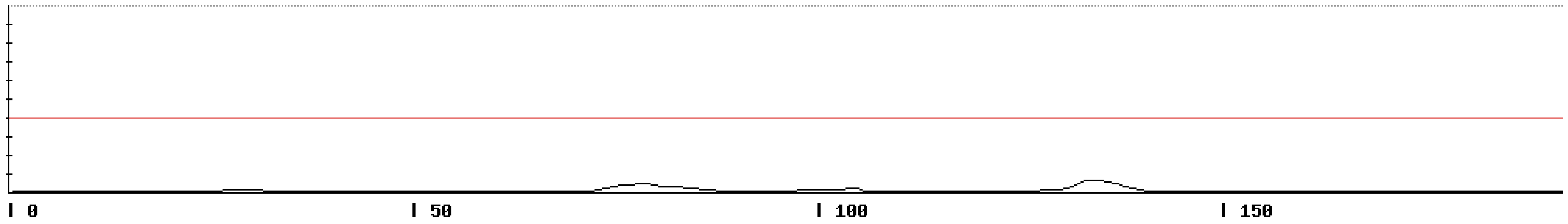

Predictions for Nicotianasylvestris_XP_009793105.1

Viterbi Path

Viterbi Path

Posterior @ 0.4

Posterior @ 0.4

Posterior Graphic. Threshold Marked @ 0.4

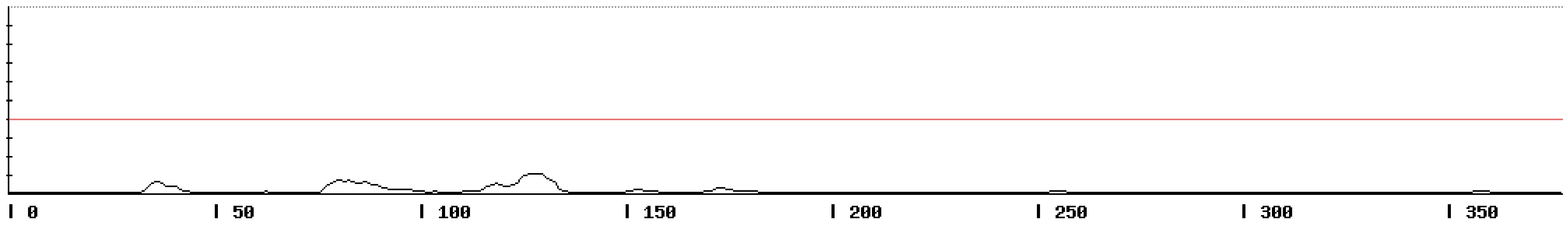

Predictions for Nicotianasylvestris_XP_009793522.1

Viterbi Path

Viterbi Path

Posterior @ 0.4

Posterior @ 0.4

Posterior Graphic. Threshold Marked @ 0.4

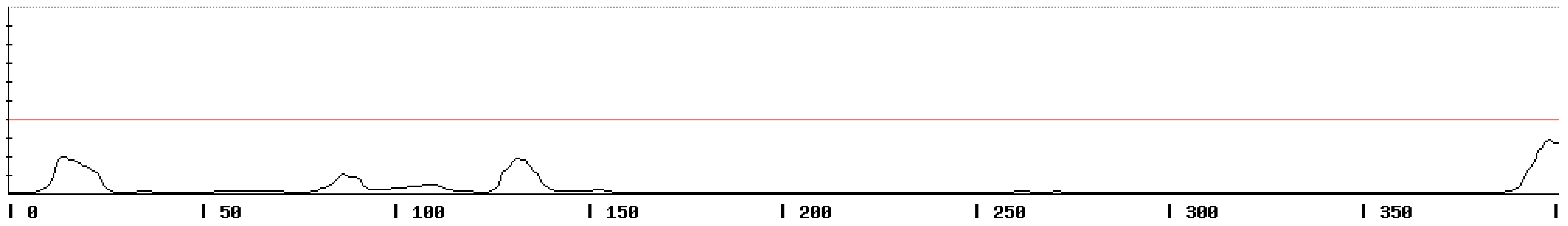

Predictions for Nicotianasylvestris_XP_009793650.1

Viterbi Path

Viterbi Path

Posterior @ 0.4

Posterior @ 0.4

Posterior Graphic. Threshold Marked @ 0.4

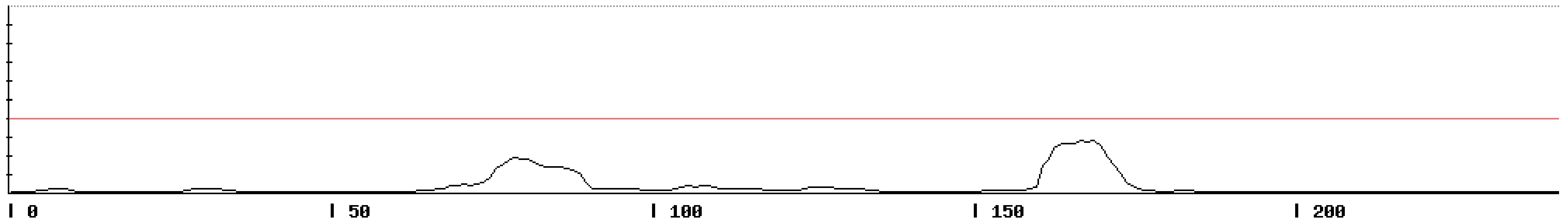

Predictions for Nicotianasylvestris_XP_009793862.1

Viterbi Path

Viterbi Path

Posterior @ 0.4

Posterior @ 0.4

Posterior Graphic. Threshold Marked @ 0.4

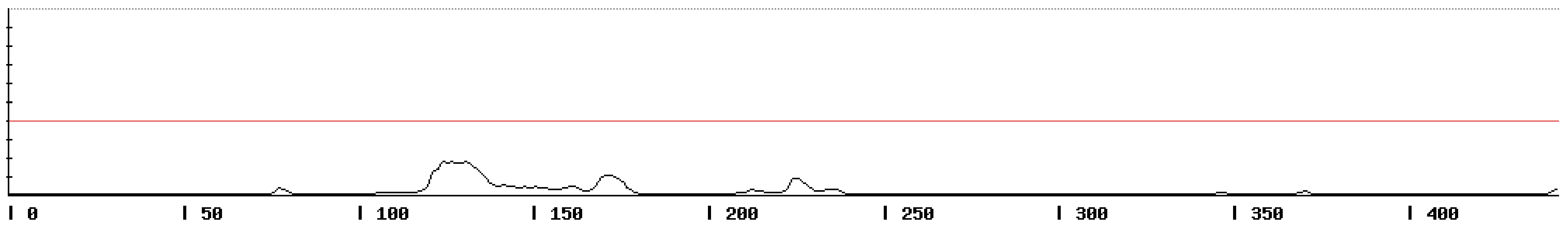

Predictions for Nicotianasylvestris_XP_009794036.1

Viterbi Path

Viterbi Path

Posterior @ 0.4

Posterior @ 0.4

Posterior Graphic. Threshold Marked @ 0.4

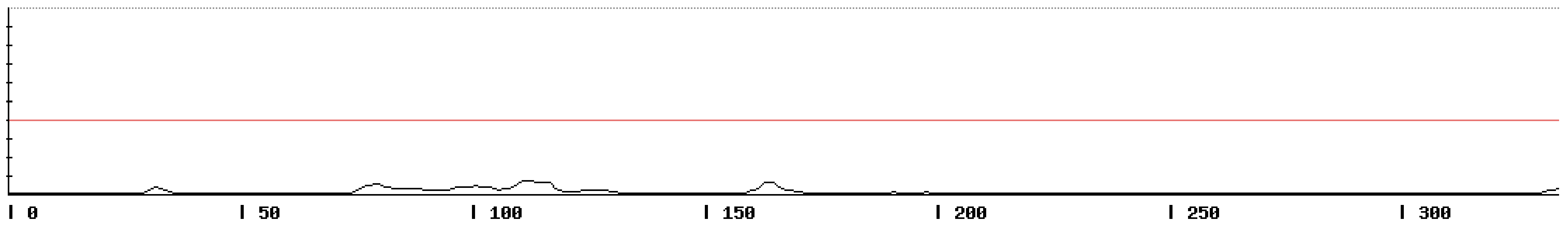

Predictions for Nicotianasylvestris_XP_009794037.1

Viterbi Path

Viterbi Path

Viterbi Path

Posterior @ 0.4

Posterior @ 0.4

Posterior Graphic. Threshold Marked @ 0.4

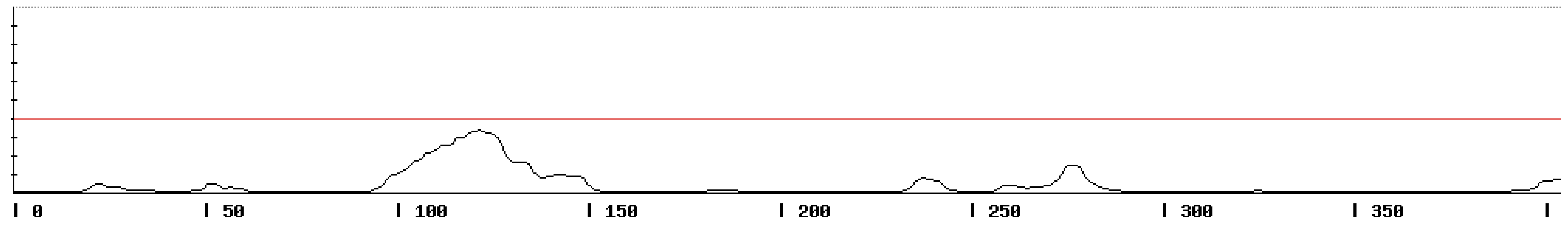

Predictions for Nicotianatabacum_XP_016433226.1

Viterbi Path

Viterbi Path

Posterior @ 0.4

Posterior @ 0.4

Posterior Graphic. Threshold Marked @ 0.4

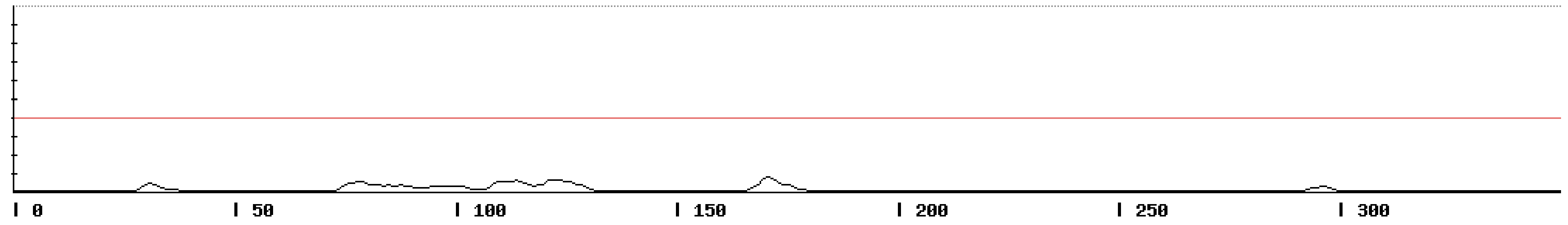

Predictions for Nicotianatabacum_XP_016433985.1

Viterbi Path

Viterbi Path

Posterior @ 0.4

Posterior @ 0.4

Posterior Graphic. Threshold Marked @ 0.4

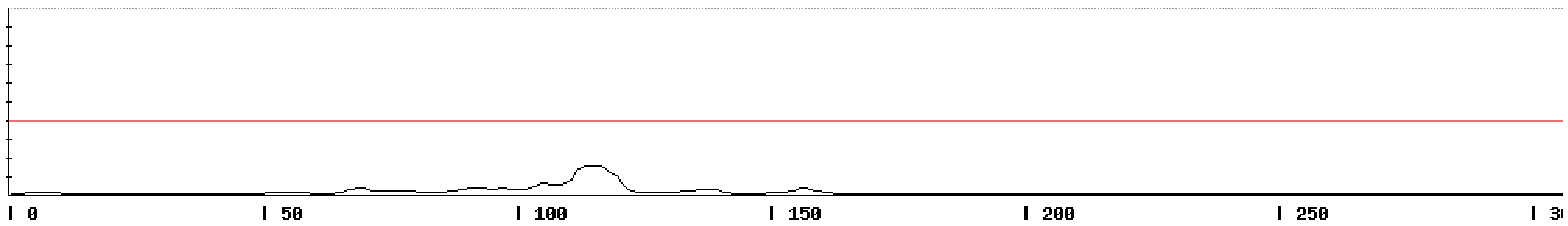

Predictions for Nicotianatabacum_XP_016434051.1

Viterbi Path

Viterbi Path

Posterior @ 0.4

138 - RKRRK - 142

Posterior @ 0.4

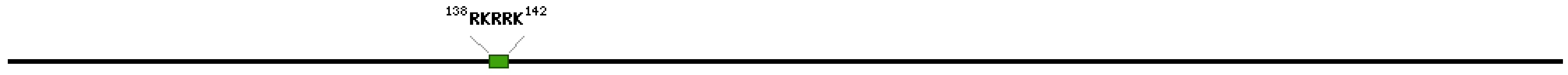

Posterior Graphic. Threshold Marked @ 0.4

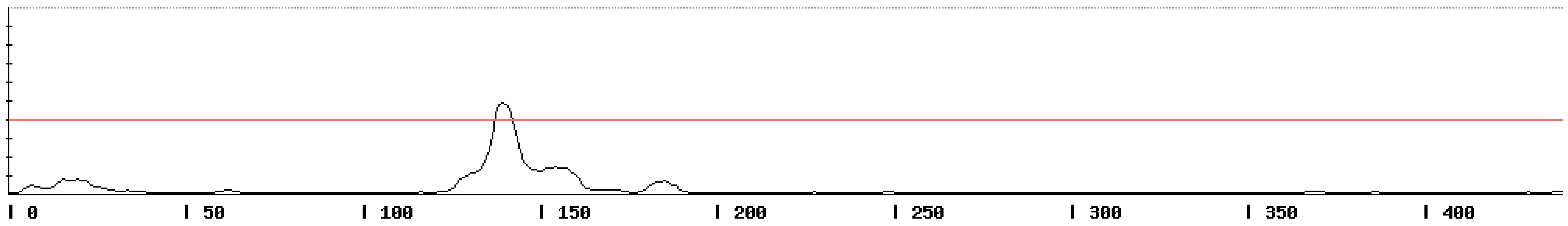

Predictions for Nicotianatabacum_XP_016434052.1	
Viterbi Path	
Viterbi Path	
Posterior @ 0.4	134 - RKRRK - 138
Posterior @ 0.4	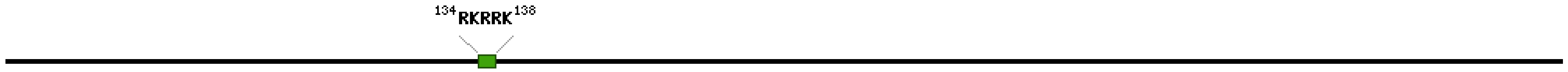
Posterior Graphic. Threshold Marked @ 0.4	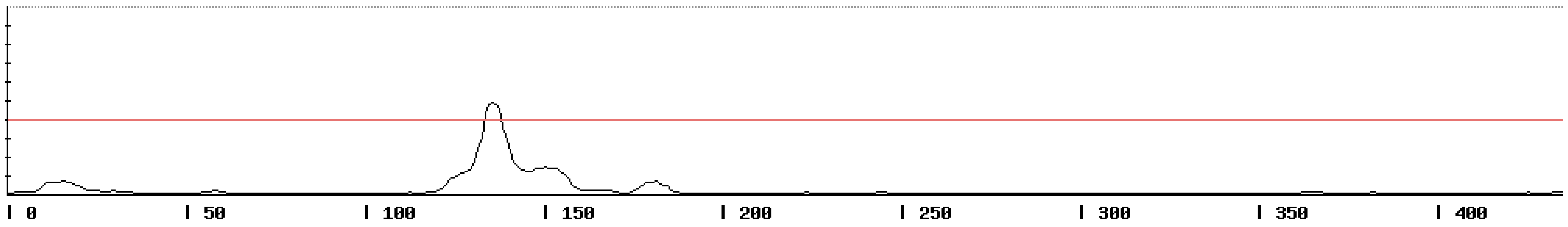

Predictions for Nicotianatabacum_XP_016434183.1	
Viterbi Path	
Viterbi Path	
Posterior @ 0.4	
Posterior @ 0.4	
Posterior Graphic. Threshold Marked @ 0.4	

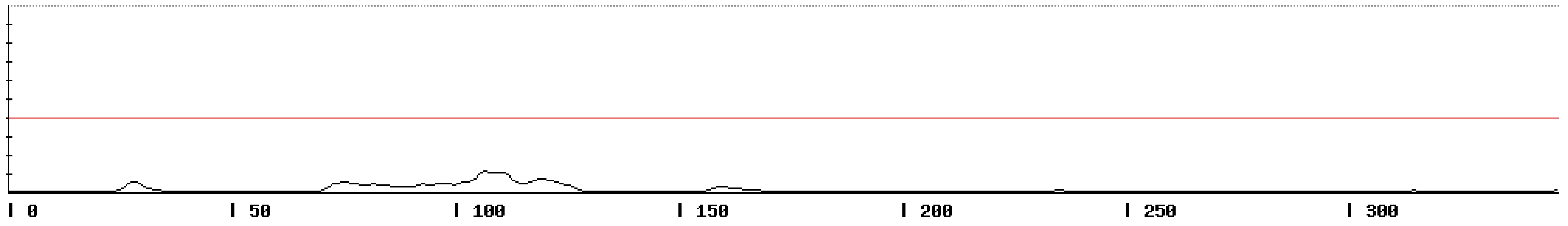

Predictions for Nicotianatabacum_XP_016434644.1	
Viterbi Path	
Viterbi Path	
Posterior @ 0.4	
Posterior @ 0.4	
Posterior Graphic. Threshold Marked @ 0.4	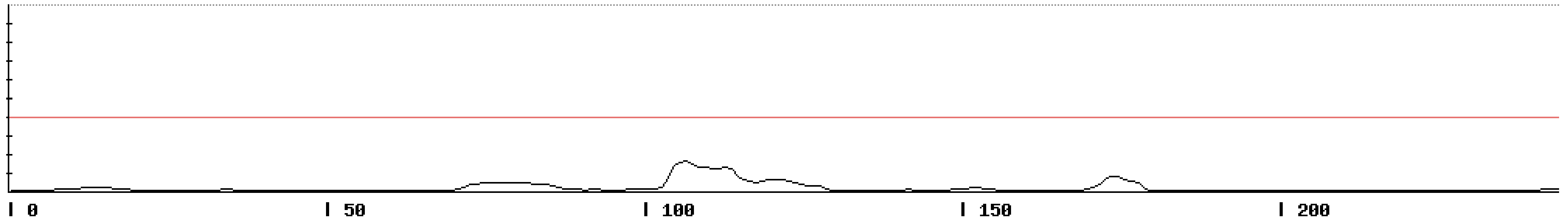

Predictions for Nicotianatabacum_XP_016435084.1	
Viterbi Path	
Viterbi Path	
Posterior @ 0.4	
Posterior @ 0.4	

Posterior Graphic. Threshold Marked @ 0.4

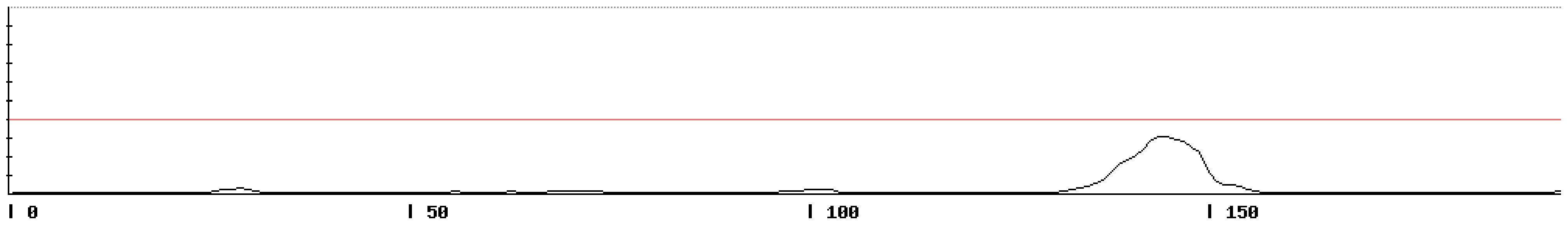

Predictions for Nicotianatabacum_XP_016435090.1

Viterbi Path

Viterbi Path

Posterior @ 0.4

Posterior @ 0.4

Posterior Graphic. Threshold Marked @ 0.4

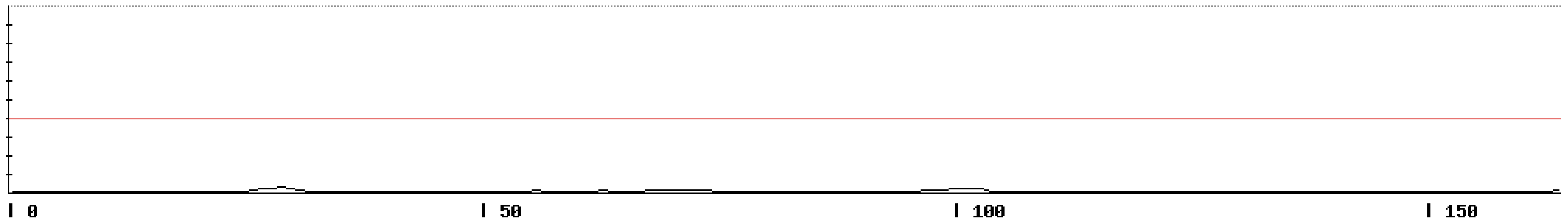

Predictions for Nicotianatabacum_XP_016436636.1

Viterbi Path

Viterbi Path

Posterior @ 0.4

Posterior @ 0.4

Posterior Graphic. Threshold Marked @ 0.4

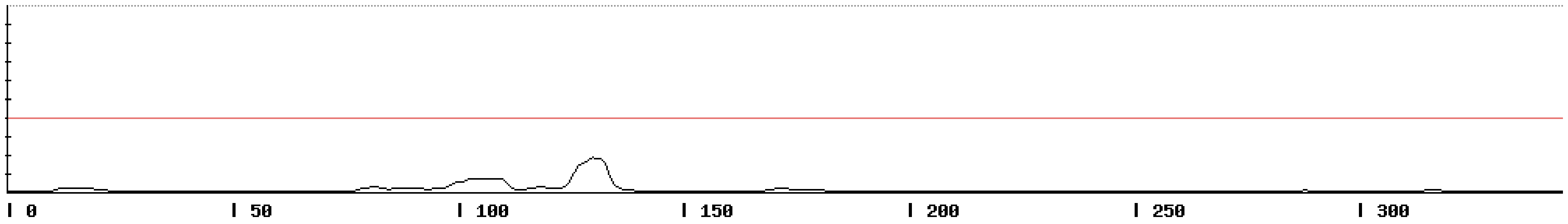

Predictions for Nicotianatabacum_XP_016436667.1

Viterbi Path

Viterbi Path

Posterior @ 0.4 — 70 - KRFKNSTKK - 78

Posterior @ 0.4

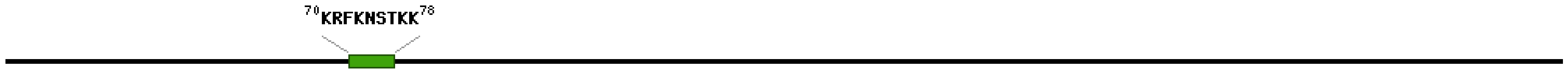

Posterior Graphic. Threshold Marked @ 0.4

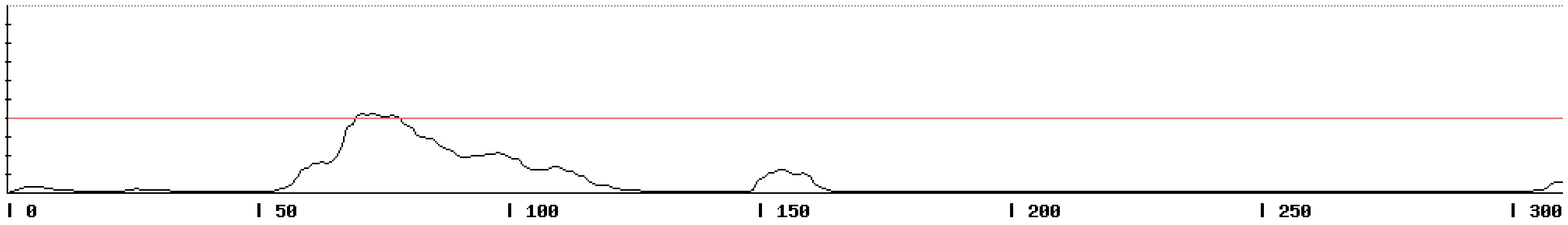

Predictions for Nicotianatabacum_XP_016436697.1

Viterbi Path

Viterbi Path

Posterior @ 0.4

Posterior @ 0.4

Posterior Graphic. Threshold Marked @ 0.4

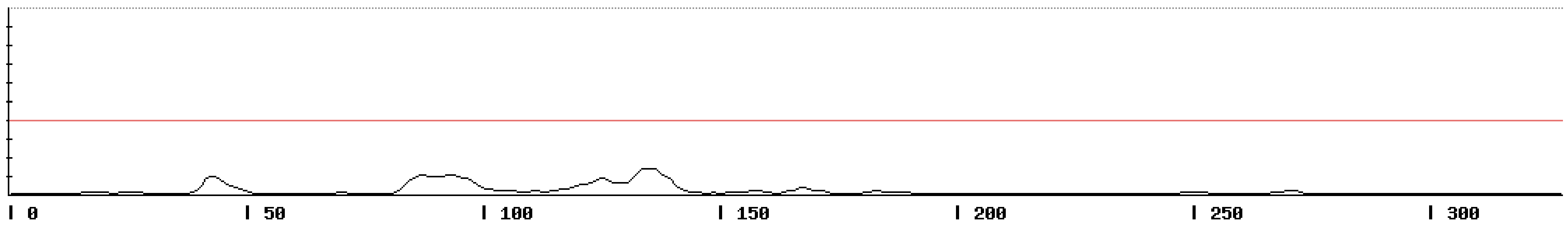

Predictions for Nicotianatabacum_XP_016436915.1

Viterbi Path

Viterbi Path

Posterior @ 0.4

Posterior @ 0.4

Posterior Graphic. Threshold Marked @ 0.4

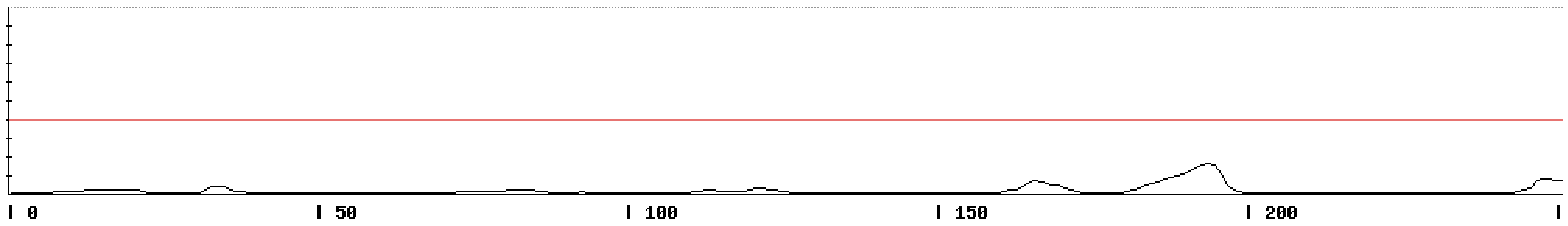

Predictions for Nicotianatabacum_XP_016437382.1

Viterbi Path

Viterbi Path

Posterior @ 0.4	
Posterior @ 0.4	
Posterior Graphic. Threshold Marked @ 0.4	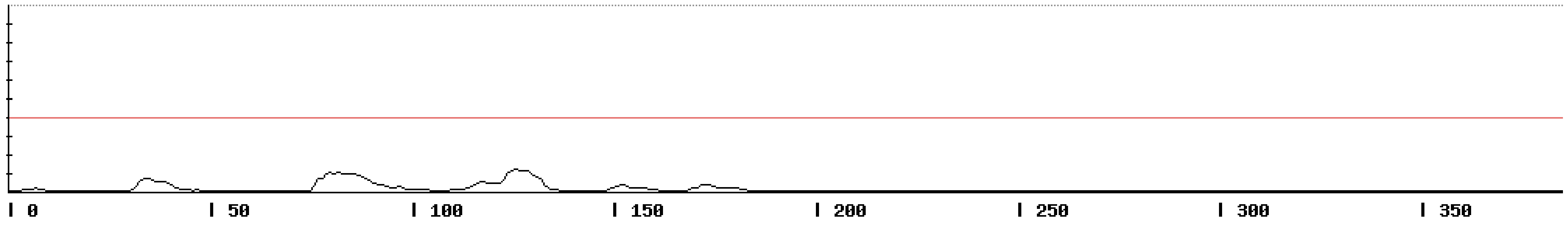

Predictions for Nicotianatabacum_XP_016437383.1	
Viterbi Path	
Viterbi Path	
Posterior @ 0.4	
Posterior @ 0.4	
Posterior Graphic. Threshold Marked @ 0.4	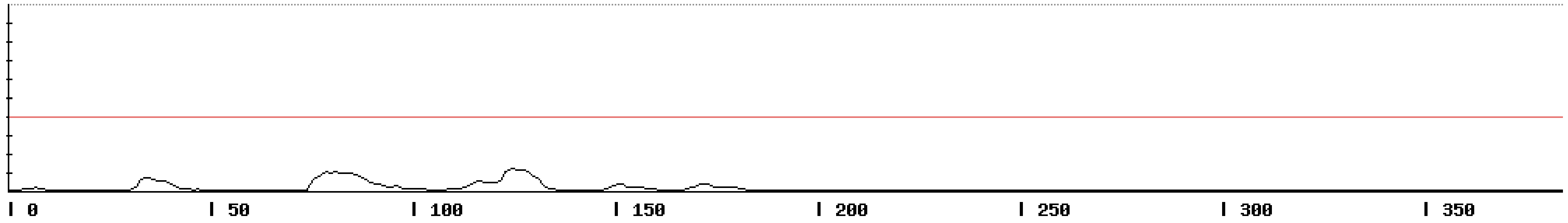

Predictions for Nicotianatabacum_XP_016437736.1

Viterbi Path

Viterbi Path

Posterior @ 0.4

Posterior @ 0.4

Posterior Graphic. Threshold Marked @ 0.4

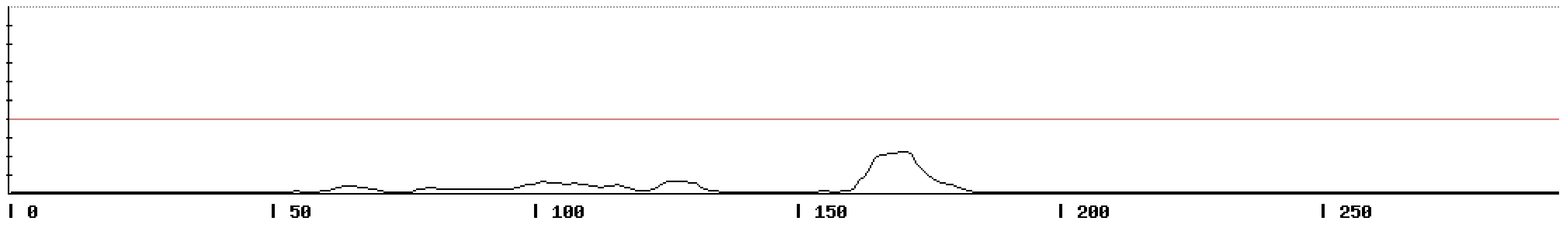

Predictions for Nicotianatabacum_XP_016438196.1

Viterbi Path

Viterbi Path

Posterior @ 0.4

Posterior @ 0.4

Posterior Graphic. Threshold Marked @ 0.4

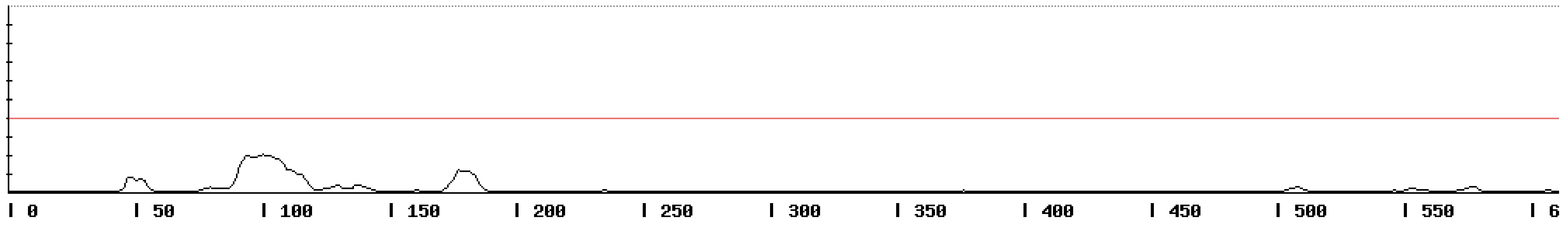

Predictions for Nicotianatabacum_XP_016438197.1	
Viterbi Path	
Viterbi Path	
Posterior @ 0.4	
Posterior @ 0.4	
Posterior Graphic. Threshold Marked @ 0.4	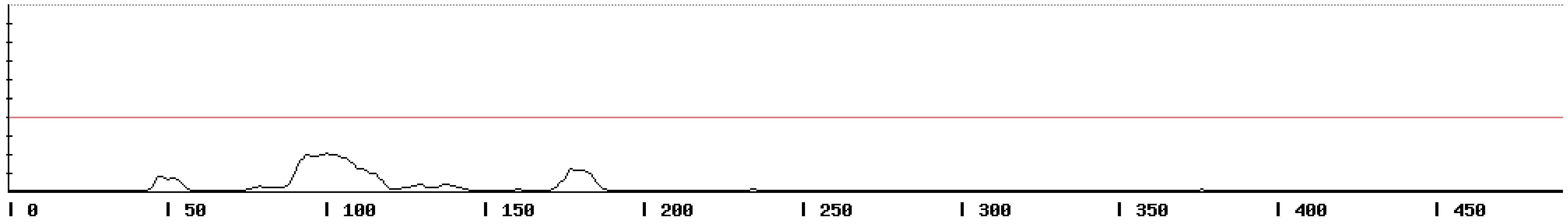

Predictions for Nicotianatabacum_XP_016438875.1	
Viterbi Path	
Viterbi Path	
Posterior @ 0.4	
Posterior @ 0.4	
Posterior Graphic. Threshold Marked @ 0.4	

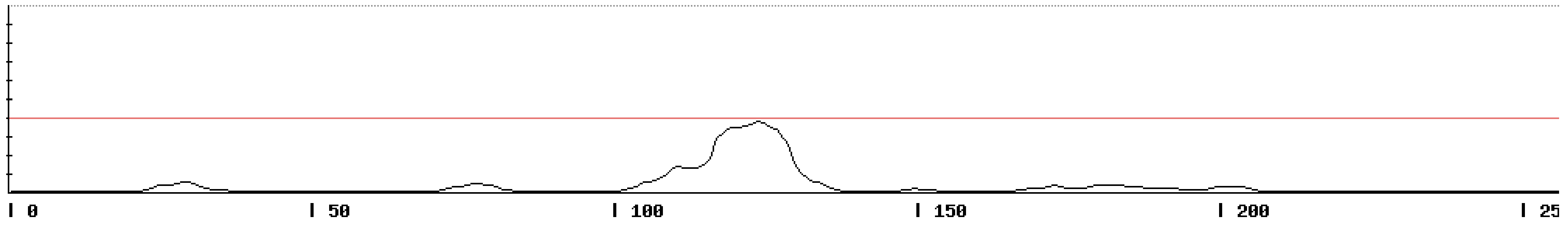

Predictions for Nicotianatabacum_XP_016438996.1

Viterbi Path	
Viterbi Path	
Posterior @ 0.4	121 - ATKKGAKIG - 129
Posterior @ 0.4	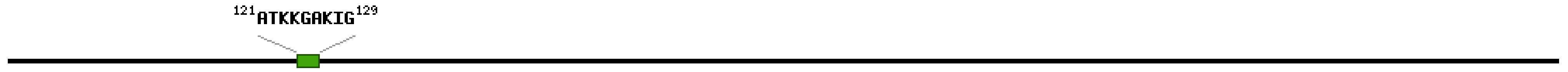
Posterior Graphic. Threshold Marked @ 0.4	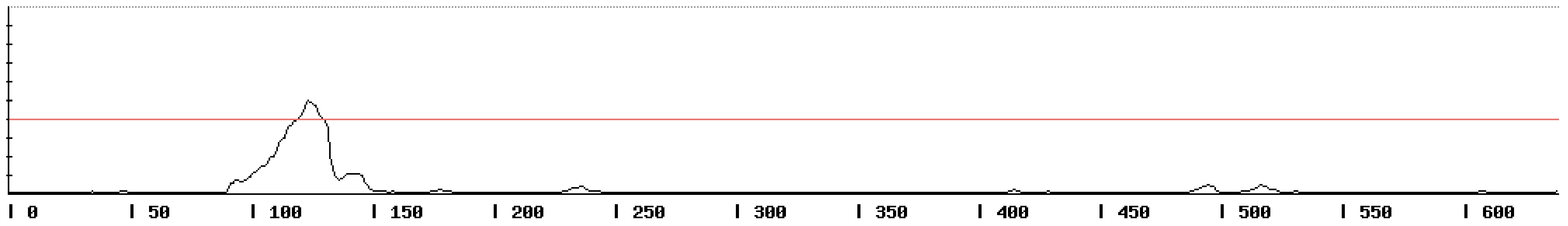

Predictions for Nicotianatabacum_XP_016439609.1

Viterbi Path	
Viterbi Path	
Posterior @ 0.4	
Posterior @ 0.4	

Posterior Graphic. Threshold Marked @ 0.4

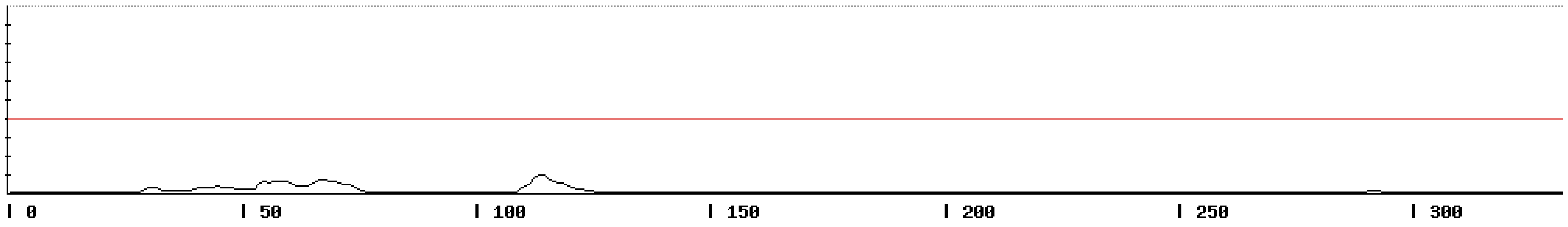

Predictions for Nicotianatabacum_XP_016439711.1

Viterbi Path

Viterbi Path

Posterior @ 0.4

Posterior @ 0.4

Posterior Graphic. Threshold Marked @ 0.4

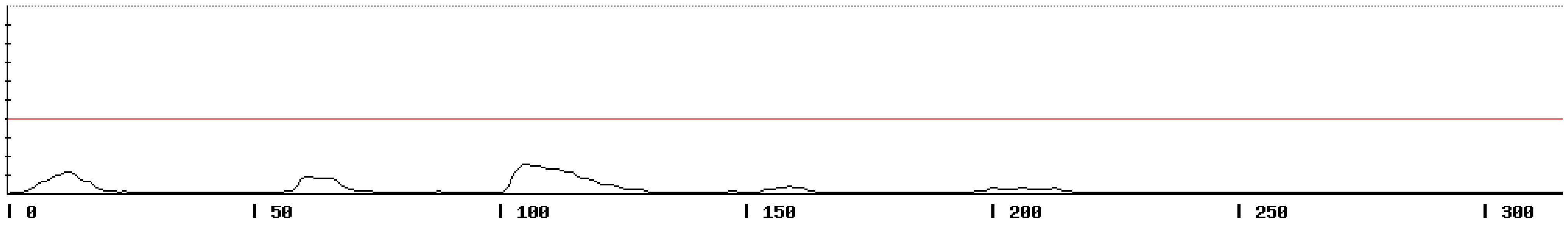

Predictions for Nicotianatabacum_XP_016439814.1

Viterbi Path

Viterbi Path

Posterior @ 0.4

Posterior @ 0.4

Posterior Graphic. Threshold Marked @ 0.4

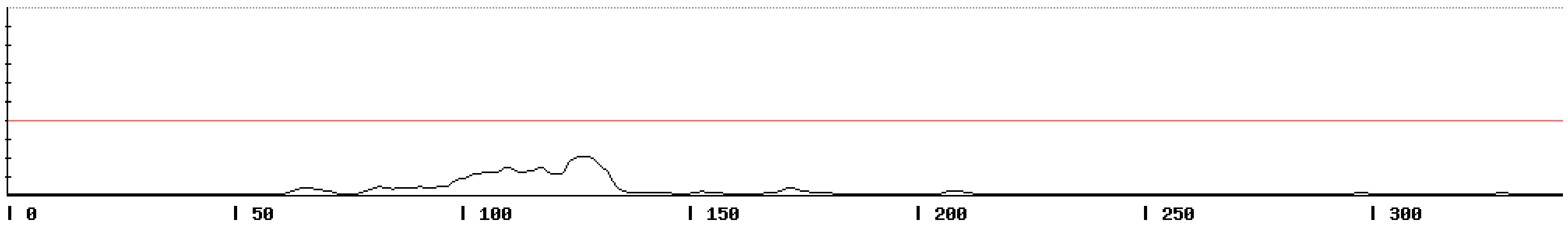

Predictions for Nicotianatabacum_XP_016439815.1

Viterbi Path

Viterbi Path

Posterior @ 0.4

Posterior @ 0.4

Posterior Graphic. Threshold Marked @ 0.4

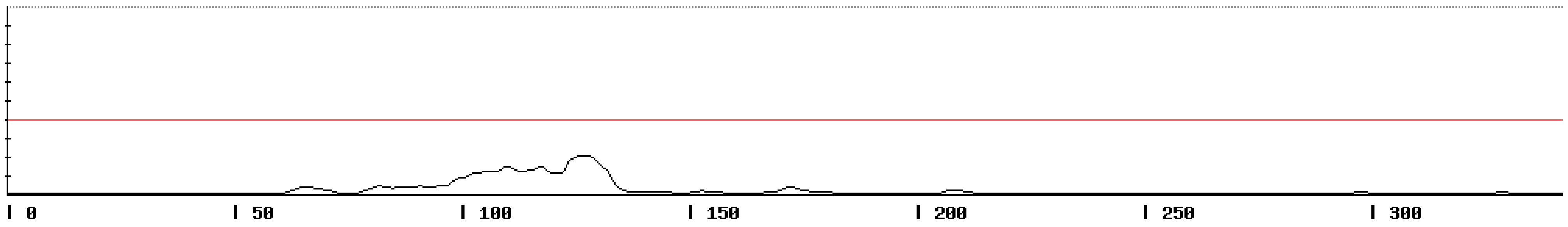

Predictions for Nicotianatabacum_XP_016439816.1

Viterbi Path

Viterbi Path

Posterior @ 0.4

Posterior @ 0.4

Posterior Graphic. Threshold Marked @ 0.4

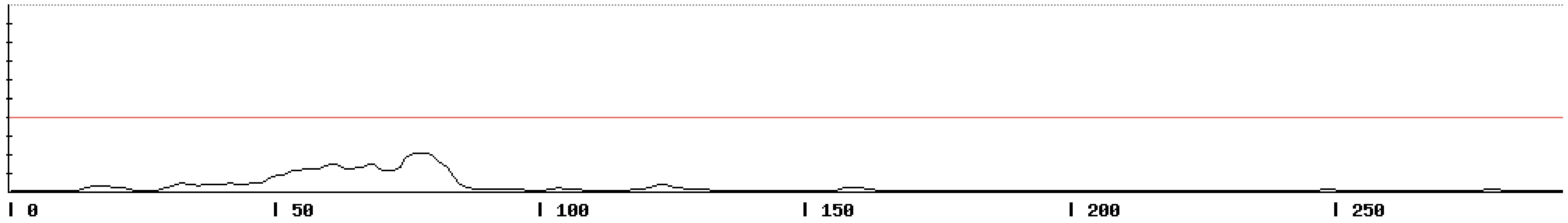

Predictions for Nicotianatabacum_XP_016440048.1

Viterbi Path

Viterbi Path

Posterior @ 0.4

Posterior @ 0.4

Posterior Graphic. Threshold Marked @ 0.4

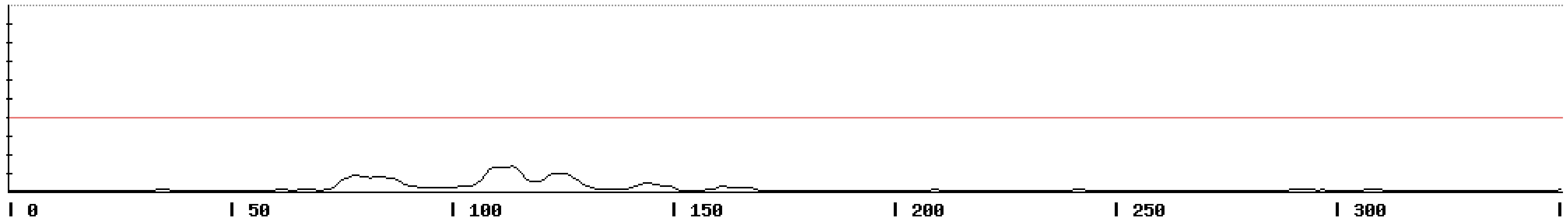

Predictions for Nicotianatabacum_XP_016440125.1

Viterbi Path

Viterbi Path

Posterior @ 0.4

Posterior @ 0.4

Posterior Graphic. Threshold Marked @ 0.4

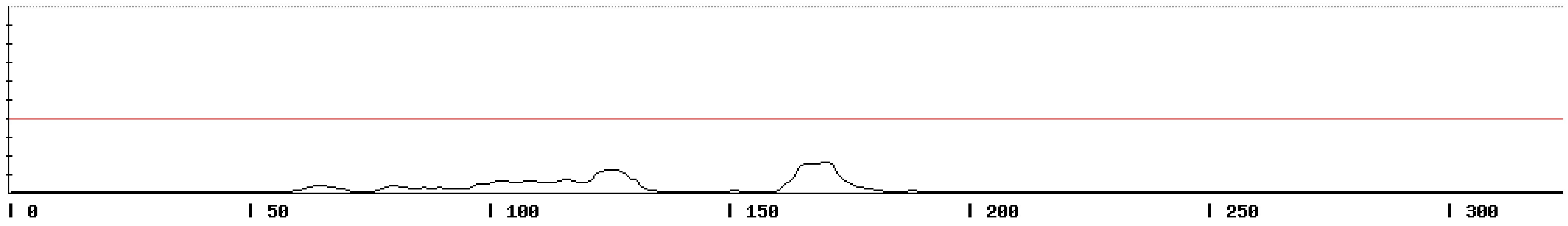

Predictions for Nicotianatabacum_XP_016440678.1

Viterbi Path

Viterbi Path

Posterior @ 0.4

Posterior @ 0.4

Posterior Graphic. Threshold Marked @ 0.4

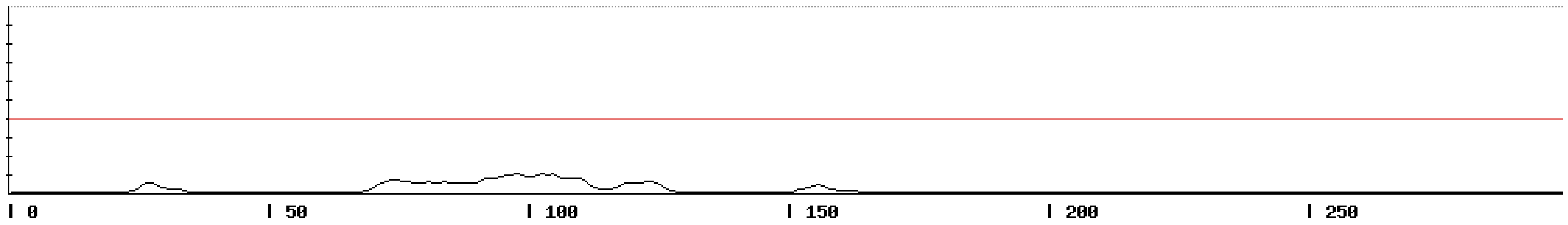

Predictions for Nicotianatabacum_XP_016441051.1

Viterbi Path	
Viterbi Path	
Posterior @ 0.4	
Posterior @ 0.4	
Posterior Graphic. Threshold Marked @ 0.4	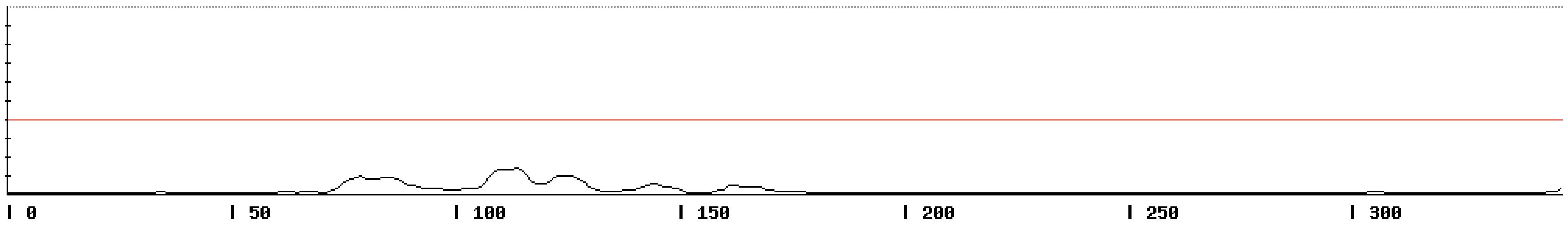

Predictions for Nicotianatabacum_XP_016441222.1

Viterbi Path	
Viterbi Path	
Posterior @ 0.4	
Posterior @ 0.4	
Posterior Graphic. Threshold Marked @ 0.4	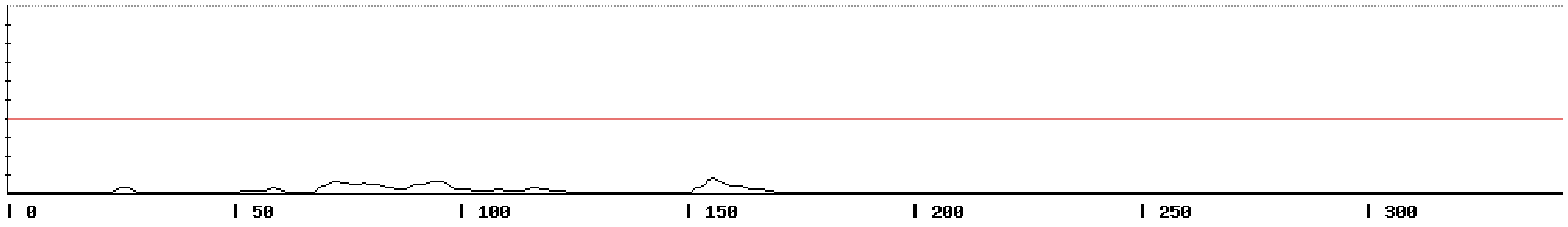

Predictions for Nicotianatabacum_XP_016441521.1	
Viterbi Path	
Viterbi Path	
Posterior @ 0.4	
Posterior @ 0.4	
Posterior Graphic. Threshold Marked @ 0.4	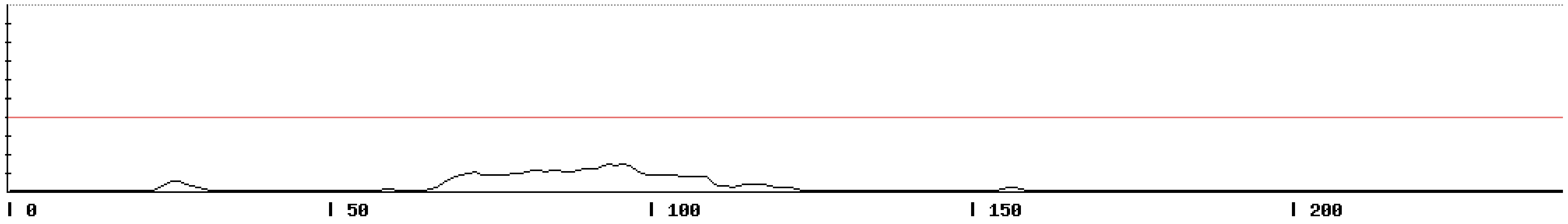

Predictions for Nicotianatabacum_XP_016441522.1	
Viterbi Path	
Viterbi Path	
Posterior @ 0.4	
Posterior @ 0.4	
Posterior Graphic. Threshold Marked @ 0.4	

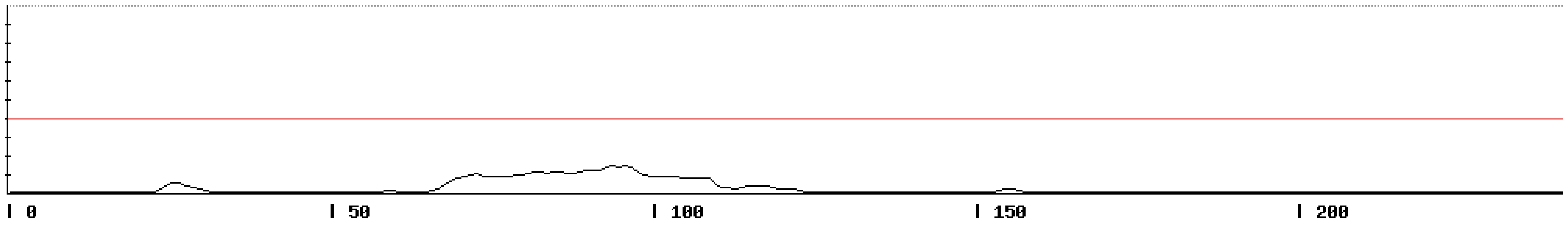

Predictions for Nicotianatabacum_XP_016441918.1

Viterbi Path

Viterbi Path

Posterior @ 0.4

Posterior @ 0.4

Posterior Graphic. Threshold Marked @ 0.4

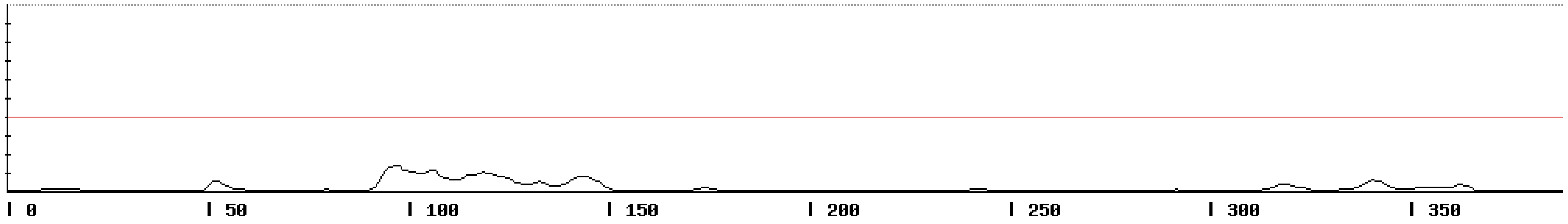

Predictions for Nicotianatabacum_XP_016441925.1

Viterbi Path

Viterbi Path

Posterior @ 0.4 — 91 - KKK - 93

Posterior @ 0.4

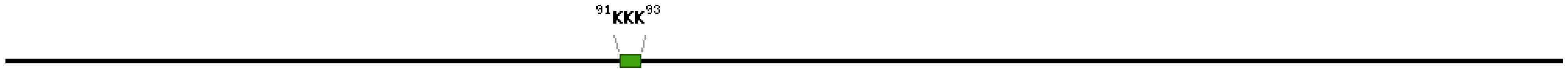

Posterior Graphic. Threshold Marked @ 0.4

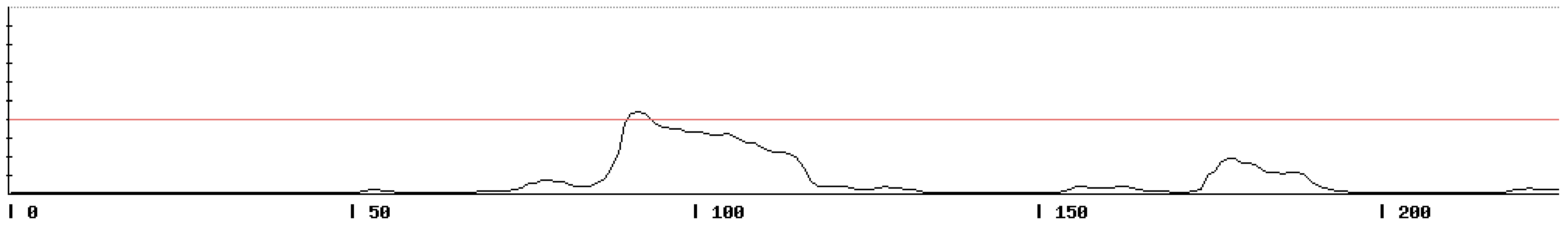

Predictions for Nicotianatabacum_XP_016441936.1

Viterbi Path

Viterbi Path

Posterior @ 0.4

Posterior @ 0.4

Posterior Graphic. Threshold Marked @ 0.4

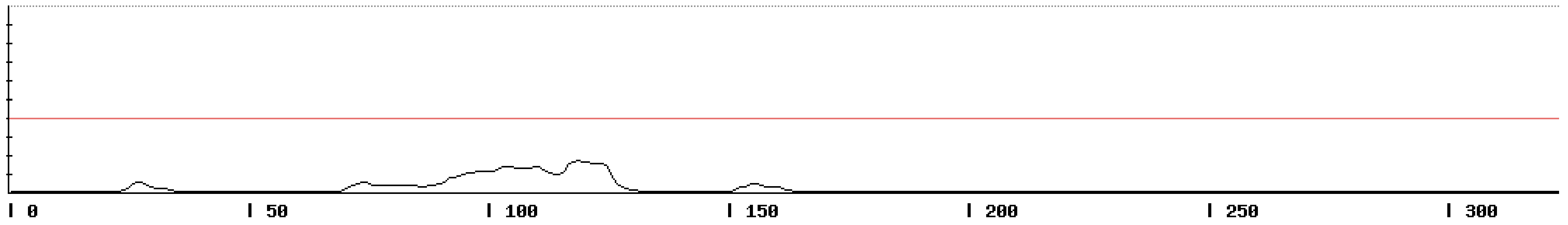

Predictions for Nicotianatabacum_XP_016441945.1

Viterbi Path

Viterbi Path

Posterior @ 0.4

Posterior @ 0.4

Posterior Graphic. Threshold Marked @ 0.4

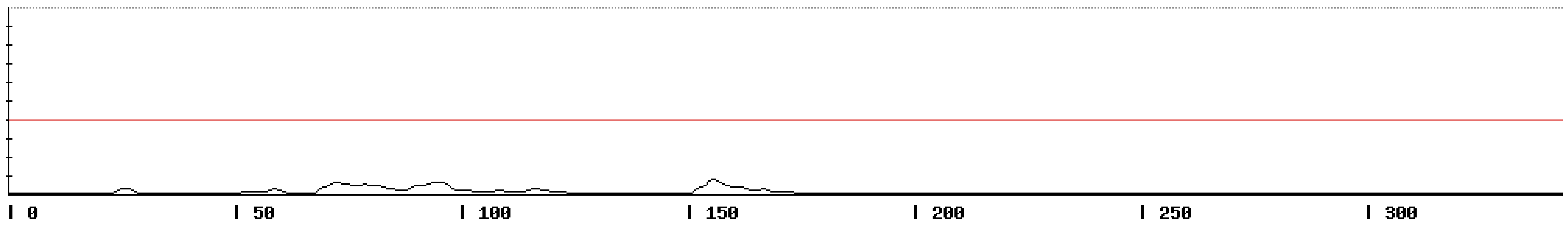

Predictions for Nicotianatabacum_XP_016442160.1

Viterbi Path

Viterbi Path

Posterior @ 0.4

Posterior @ 0.4

Posterior Graphic. Threshold Marked @ 0.4

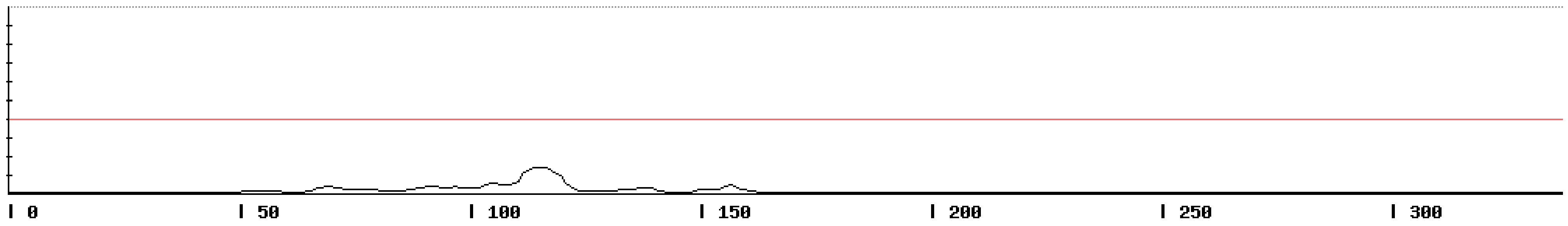

Predictions for Nicotianatabacum_XP_016442161.1

Viterbi Path

Viterbi Path

Posterior @ 0.4

Posterior @ 0.4

Posterior Graphic. Threshold Marked @ 0.4

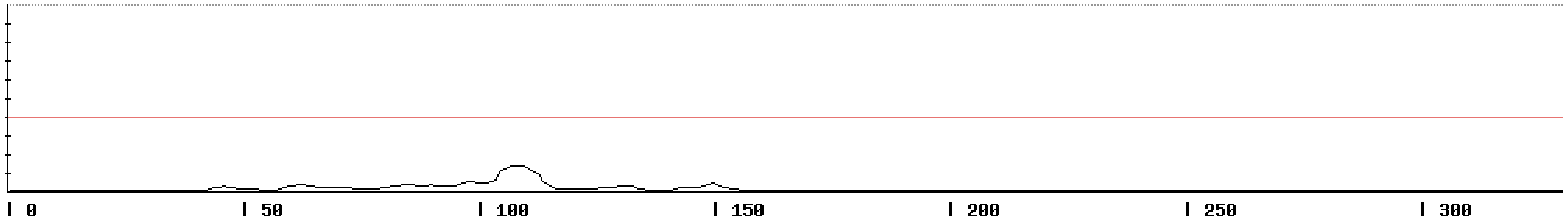

Predictions for Nicotianatabacum_XP_016442251.1

Viterbi Path

Viterbi Path

Posterior @ 0.4

Posterior @ 0.4

Posterior Graphic. Threshold Marked @ 0.4

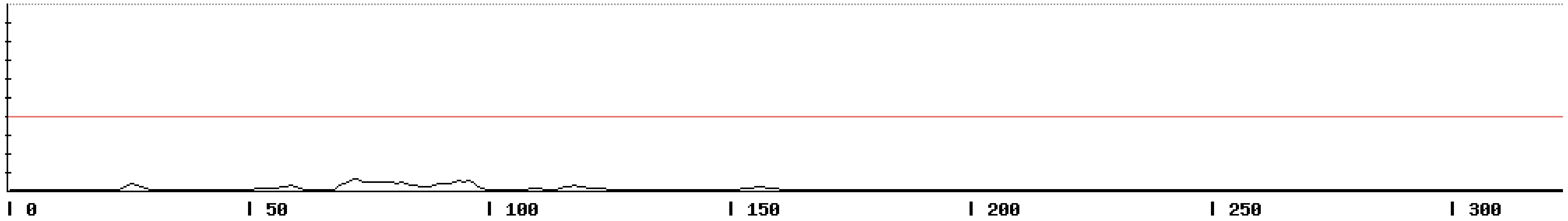

Predictions for Nicotianatabacum_XP_016442252.1

Viterbi Path

Viterbi Path

Posterior @ 0.4

Posterior @ 0.4

Posterior Graphic. Threshold Marked @ 0.4

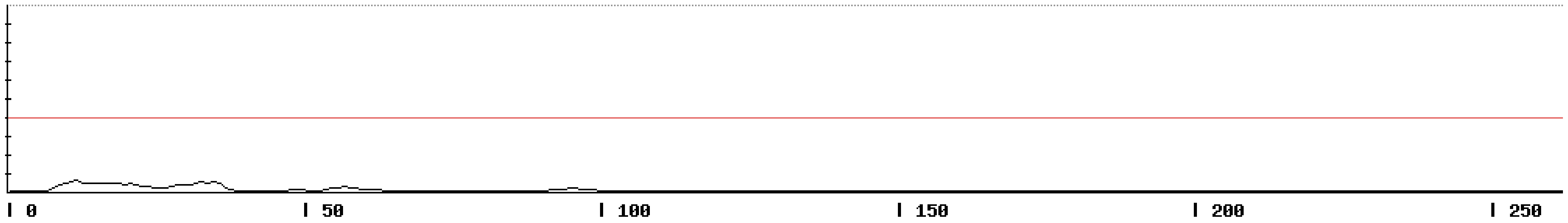

Predictions for Nicotianatabacum_XP_016442561.1

Viterbi Path

Viterbi Path

Posterior @ 0.4

Posterior @ 0.4

Posterior Graphic. Threshold Marked @ 0.4

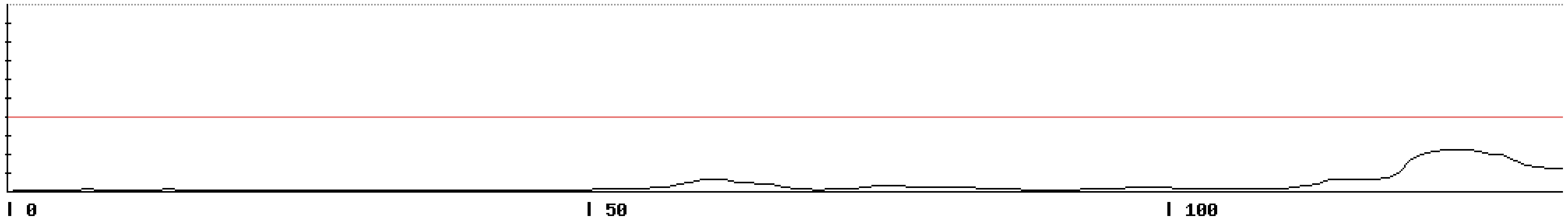

Predictions for Nicotianatabacum_XP_016443535.1

Viterbi Path	
Viterbi Path	
Posterior @ 0.4	82 - RVLKGGRRRAGGGYWKKRGRVR - 103
Posterior @ 0.4	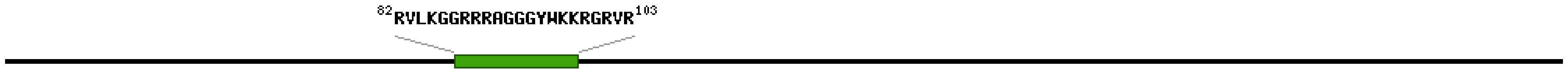
Posterior Graphic. Threshold Marked @ 0.4	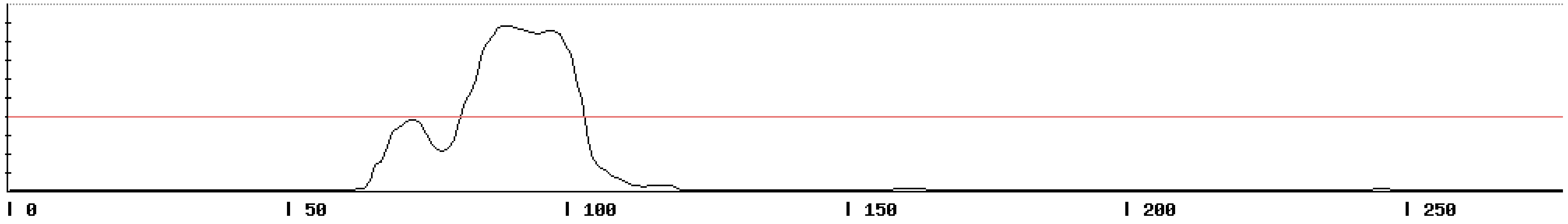

Predictions for Nicotianatabacum_XP_016443541.1

Viterbi Path	
Viterbi Path	
Posterior @ 0.4	82 - RVLKGGRRRAGGGYWKKRGRVR - 103
Posterior @ 0.4	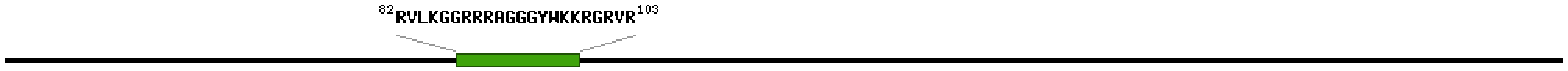
Posterior Graphic. Threshold Marked @ 0.4	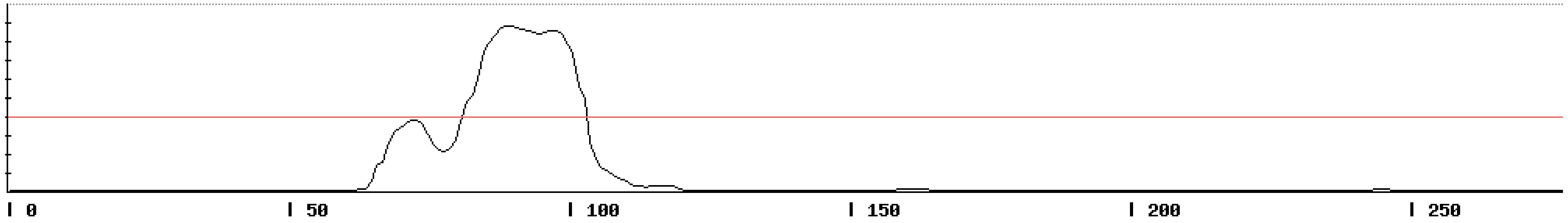

Predictions for Nicotianatabacum_XP_016443589.1

Viterbi Path

Viterbi Path

Posterior @ 0.4

Posterior @ 0.4

Posterior Graphic. Threshold Marked @ 0.4

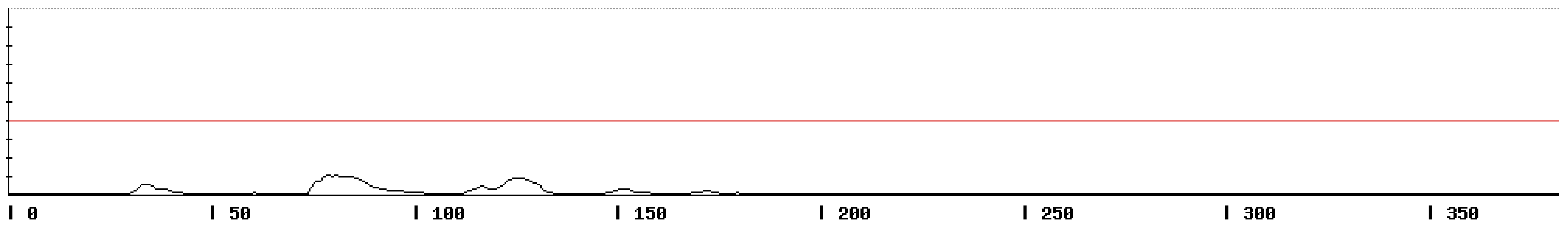

Predictions for Nicotianatabacum_XP_016443693.1

Viterbi Path

Viterbi Path

Posterior @ 0.4

Posterior @ 0.4

Posterior Graphic. Threshold Marked @ 0.4

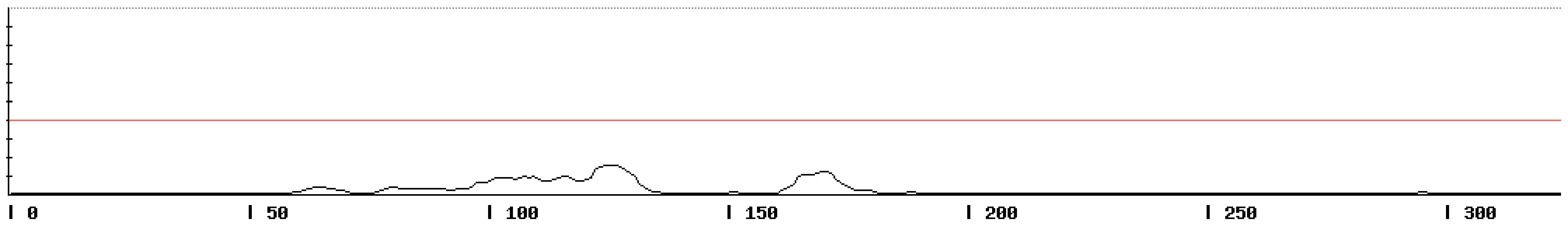

Predictions for Nicotianatabacum_XP_016443705.1

Viterbi Path

Viterbi Path

Posterior @ 0.4

Posterior @ 0.4

Posterior Graphic. Threshold Marked @ 0.4

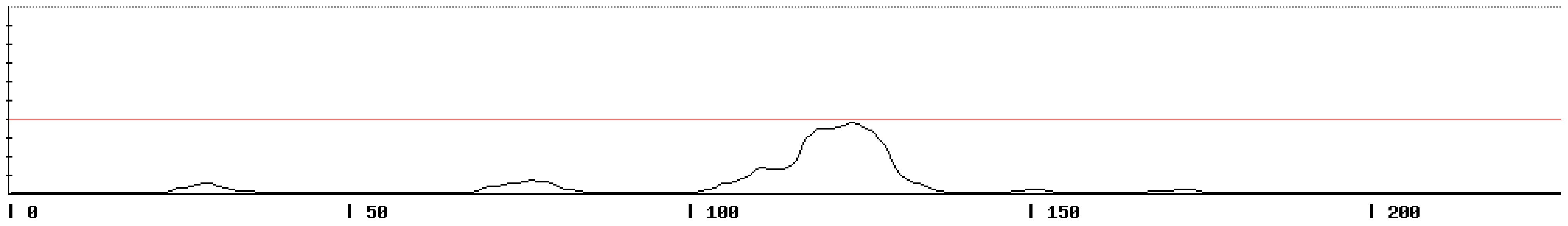

Predictions for Nicotianatabacum_XP_016443884.1

Viterbi Path

Viterbi Path

Posterior @ 0.4

Posterior @ 0.4

Posterior Graphic. Threshold Marked @ 0.4

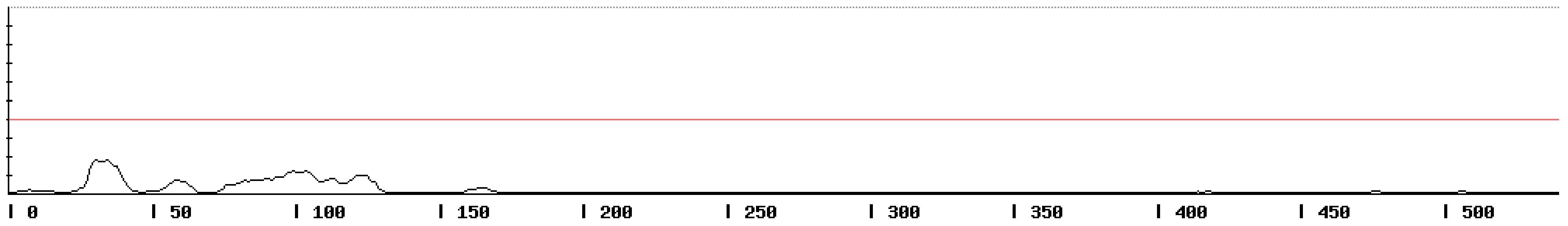

Predictions for Nicotianatabacum_XP_016444166.1

Viterbi Path

Viterbi Path

Posterior @ 0.4

131 - GKRKRRKI - 138

Posterior @ 0.4

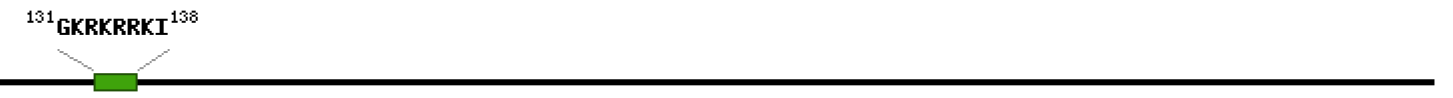

Posterior Graphic. Threshold Marked @ 0.4

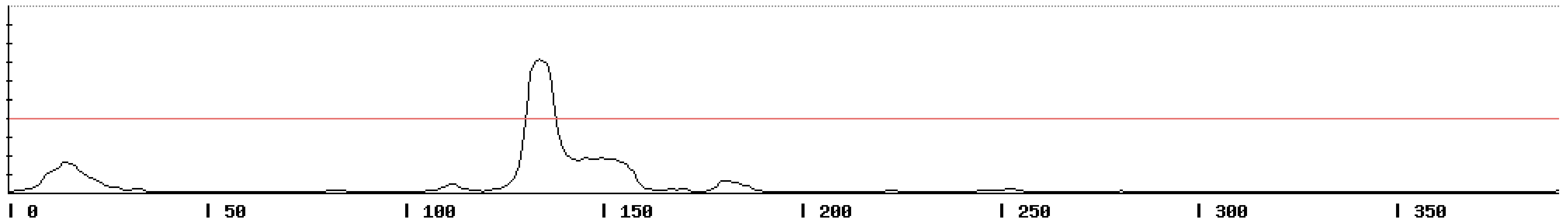

Predictions for Nicotianatabacum_XP_016444176.1

Viterbi Path

Viterbi Path

Posterior @ 0.4

Posterior @ 0.4

Posterior Graphic. Threshold Marked @ 0.4

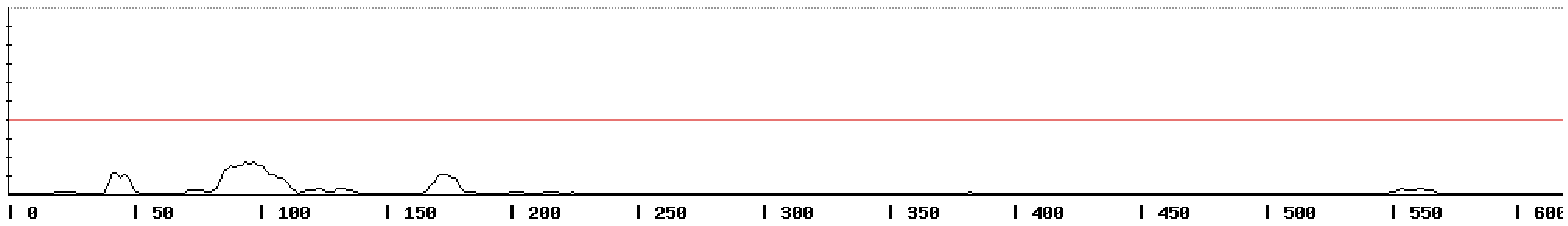

Predictions for Nicotianatabacum_XP_016444600.1

Viterbi Path

Viterbi Path

Posterior @ 0.4

Posterior @ 0.4

Posterior Graphic. Threshold Marked @ 0.4

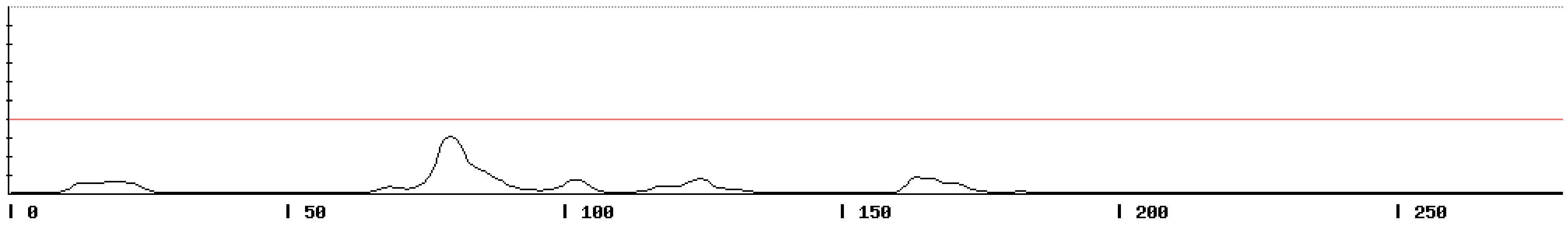

Predictions for Nicotianatabacum_XP_016444884.1

Viterbi Path

Viterbi Path

Posterior @ 0.4

Posterior @ 0.4

Posterior Graphic. Threshold Marked @ 0.4

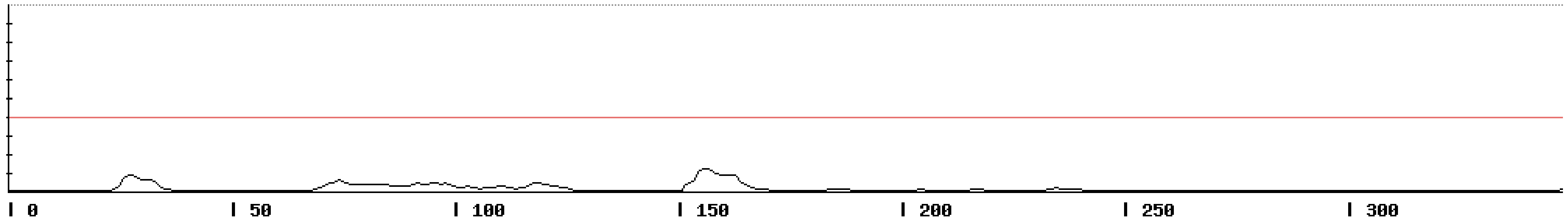

Predictions for Nicotianatabacum_XP_016445819.1

Viterbi Path

Viterbi Path

Posterior @ 0.4

Posterior @ 0.4

Posterior Graphic. Threshold Marked @ 0.4

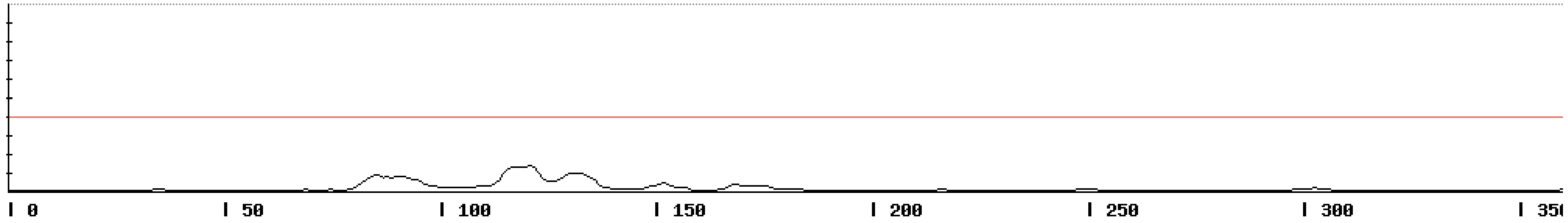

Predictions for Nicotianatabacum_XP_016445820.1

Viterbi Path

Viterbi Path

Posterior @ 0.4

Posterior @ 0.4

Posterior Graphic. Threshold Marked @ 0.4

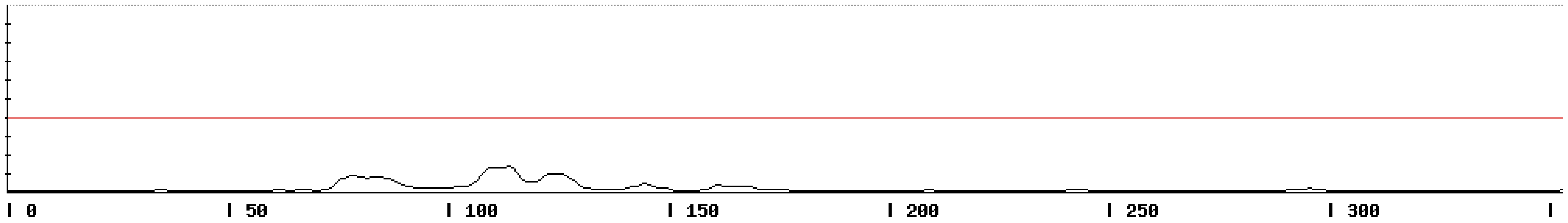

Predictions for Nicotianatabacum_XP_016446208.1

Viterbi Path

Viterbi Path

Posterior @ 0.4

Posterior @ 0.4

Posterior Graphic. Threshold Marked @ 0.4

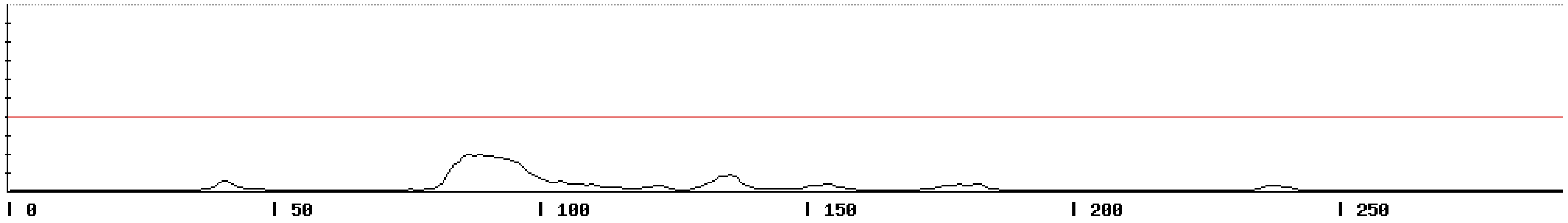

Predictions for Nicotianatabacum_XP_016446354.1

Viterbi Path	
Viterbi Path	
Posterior @ 0.4	81 - KKKSKA - 86
Posterior @ 0.4	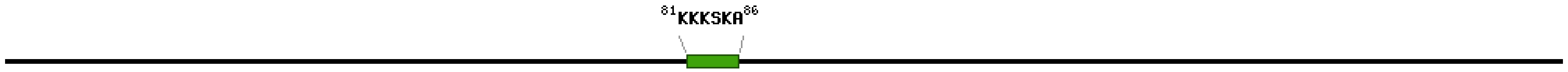
Posterior Graphic. Threshold Marked @ 0.4	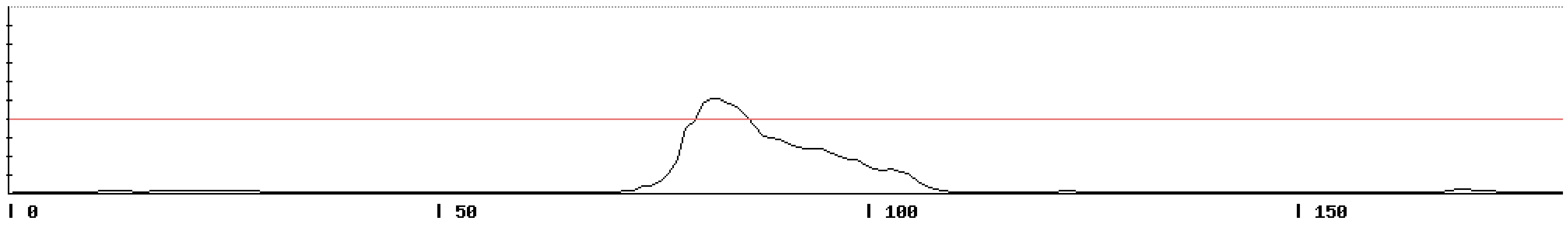

Predictions for Nicotianatabacum_XP_016446355.1

Viterbi Path	
Viterbi Path	
Posterior @ 0.4	50 - KKKSKA - 55
Posterior @ 0.4	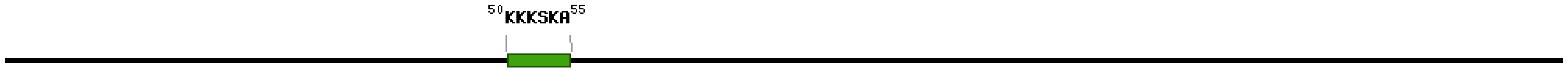
Posterior Graphic. Threshold Marked @ 0.4	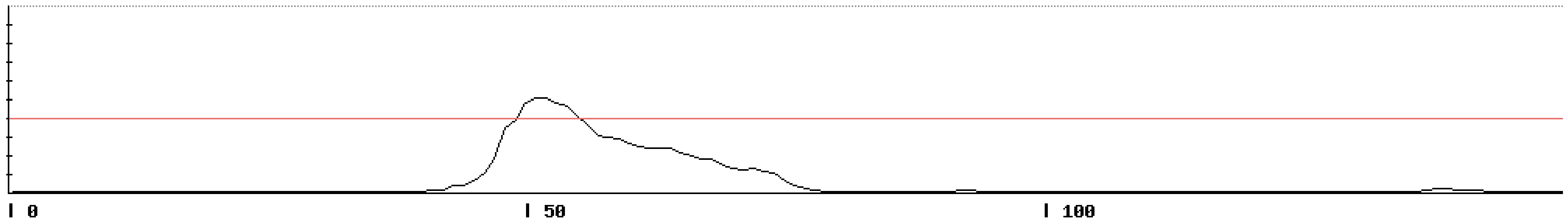

Predictions for Nicotianatabacum_XP_016446782.1	
Viterbi Path	
Viterbi Path	
Posterior @ 0.4	
Posterior @ 0.4	
Posterior Graphic. Threshold Marked @ 0.4	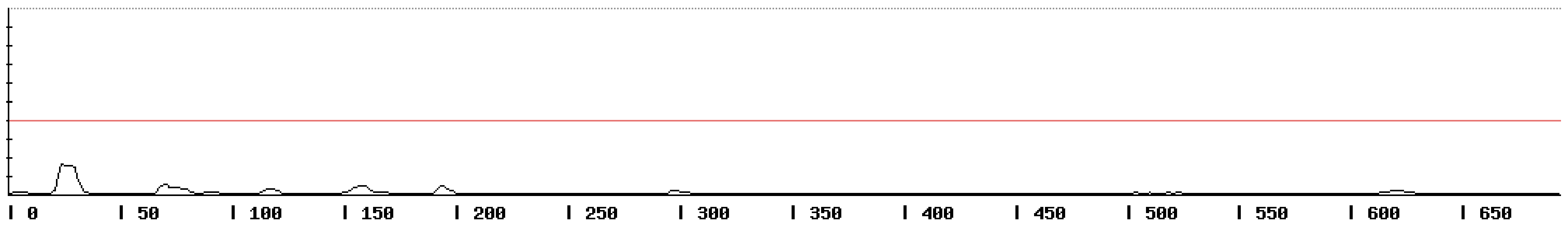

Predictions for Nicotianatabacum_XP_016446783.1	
Viterbi Path	
Viterbi Path	
Posterior @ 0.4	
Posterior @ 0.4	
Posterior Graphic. Threshold Marked @ 0.4	

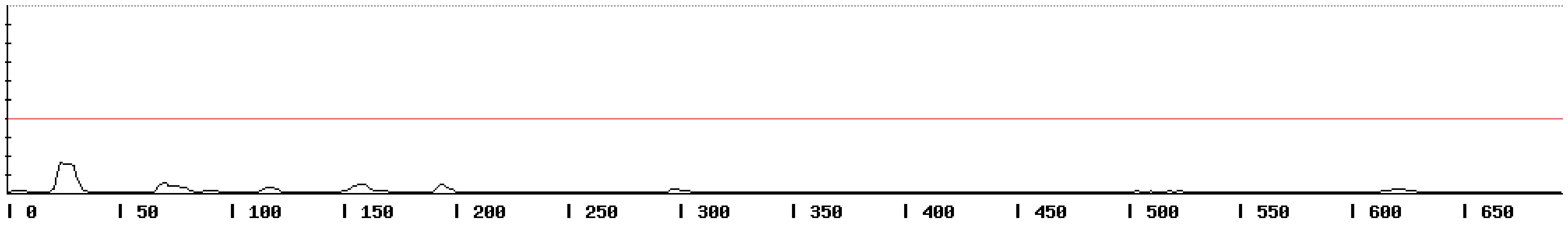

Predictions for Nicotianatabacum_XP_016447919.1

Viterbi Path	
Viterbi Path	
Posterior @ 0.4	67 - LKKRKESHKRFRR - 79
Posterior @ 0.4	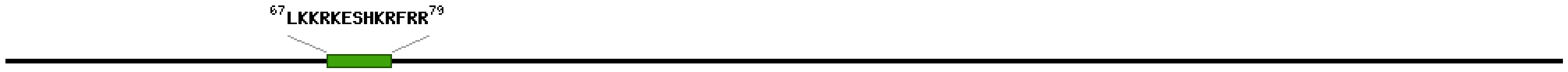
Posterior Graphic. Threshold Marked @ 0.4	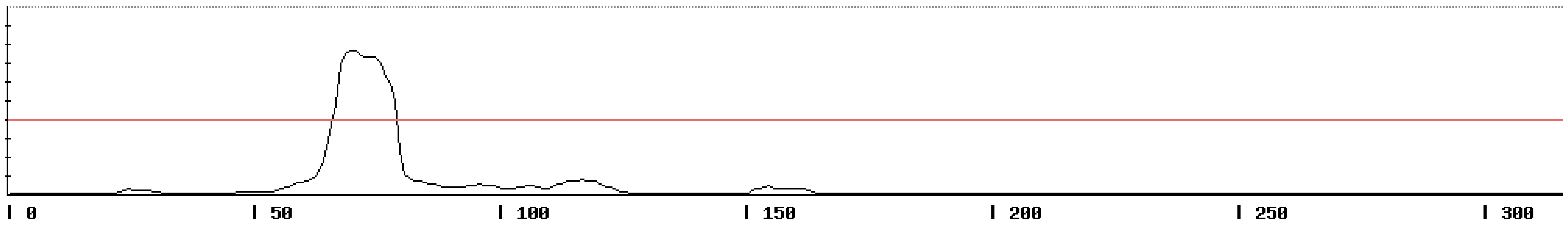

Predictions for Nicotianatabacum_XP_016448407.1

Viterbi Path	
Viterbi Path	
Posterior @ 0.4	
Posterior @ 0.4	

Posterior Graphic. Threshold Marked @ 0.4	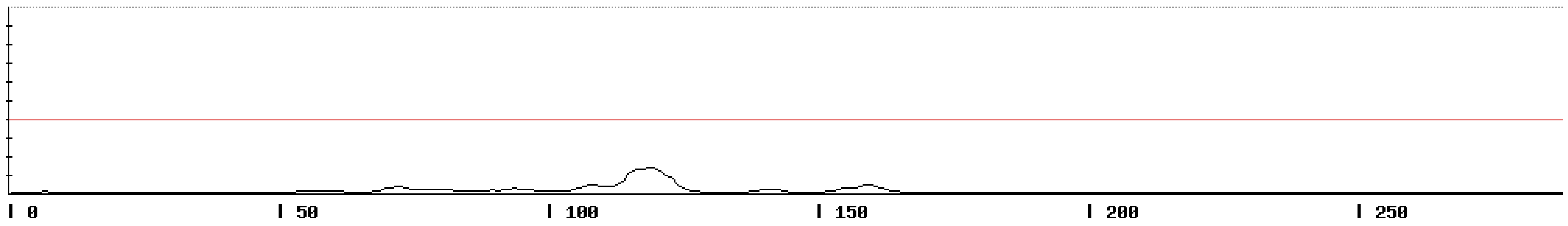

Predictions for Nicotianatabacum_XP_016448544.1	
Viterbi Path	
Viterbi Path	
Posterior @ 0.4	
Posterior @ 0.4	
Posterior Graphic. Threshold Marked @ 0.4	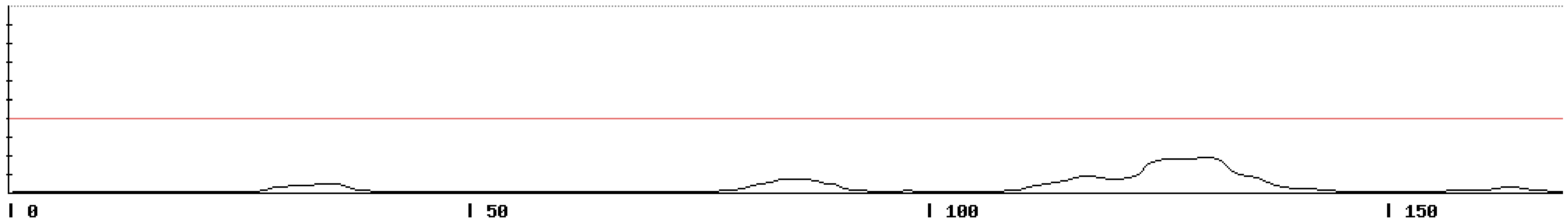

Predictions for Nicotianatabacum_XP_016448554.1	
Viterbi Path	
Viterbi Path	
Posterior @ 0.4	
Posterior @ 0.4	

Posterior Graphic. Threshold Marked @ 0.4

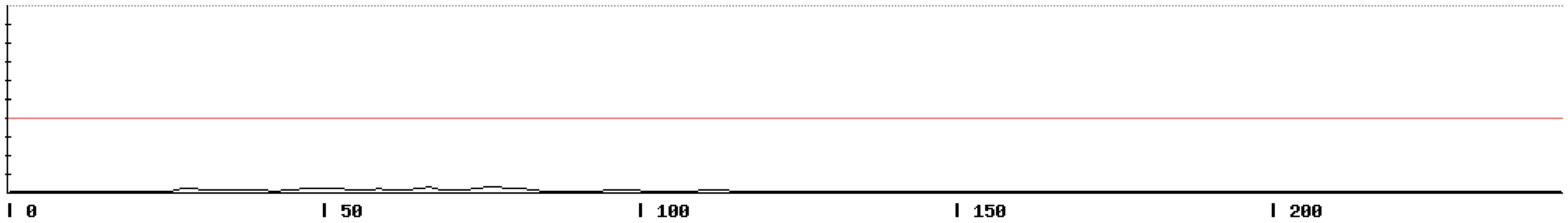

Predictions for Nicotianatabacum_XP_016449206.1

Viterbi Path

Viterbi Path

Posterior @ 0.4 — 372 - REEKRKKKLKK - 382

Posterior @ 0.4

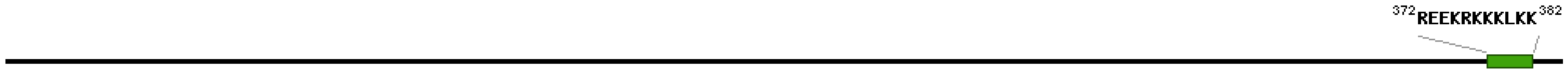

Posterior Graphic. Threshold Marked @ 0.4

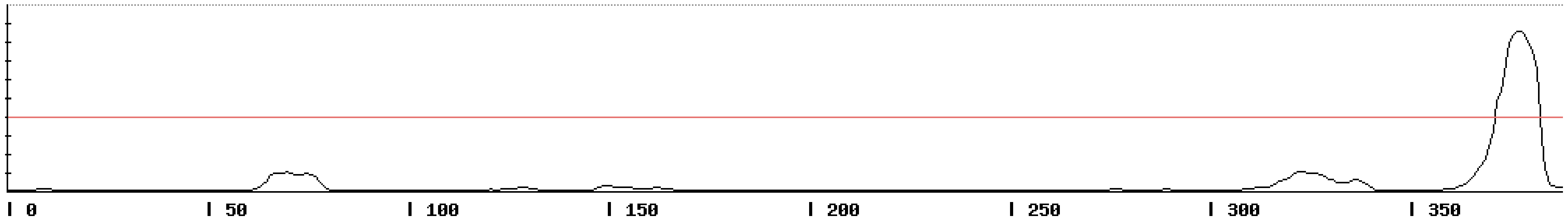

Predictions for Nicotianatabacum_XP_016449500.1

Viterbi Path — 162 - KIKKKGKPGDKKKG - 175

Viterbi Path

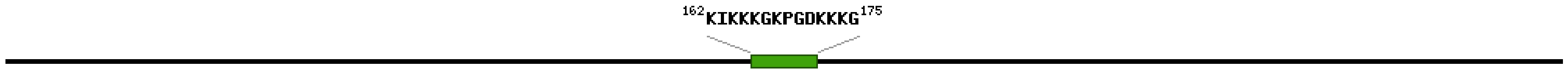

Posterior @ 0.4	79 - KKEKPTWKRVSRTVGKGTWKPQGKG - 103
	162 - KIKKKGKPGDKKKG - 175

Posterior @ 0.4

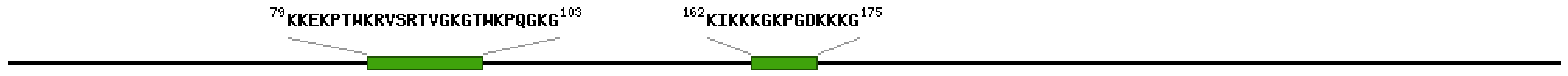

Posterior Graphic. Threshold Marked @ 0.4

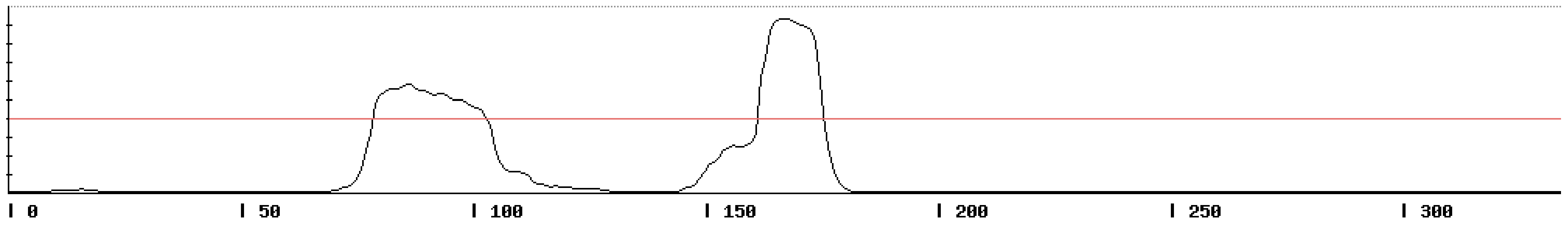

Predictions for Nicotianatabacum_XP_016449653.1

Viterbi Path

Viterbi Path

Posterior @ 0.4 — 81 - GRKRRYKNGRKPNRQVIGKKG - 101

Posterior @ 0.4

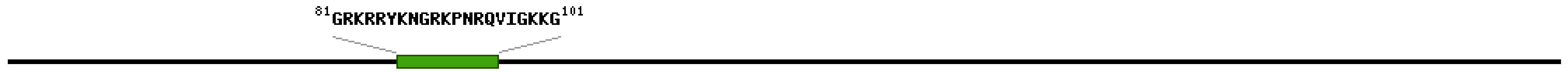

Posterior Graphic. Threshold Marked @ 0.4

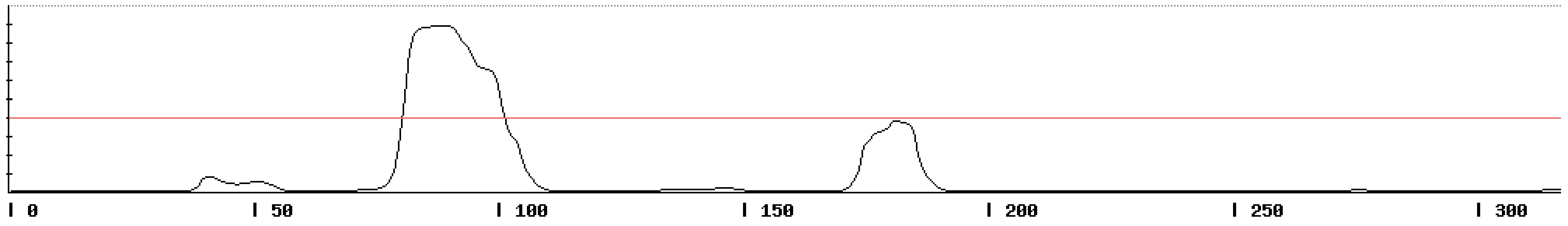

Predictions for Nicotianatabacum_XP_016449980.1

Viterbi Path

Viterbi Path

Posterior @ 0.4

Posterior @ 0.4

Posterior Graphic. Threshold Marked @ 0.4

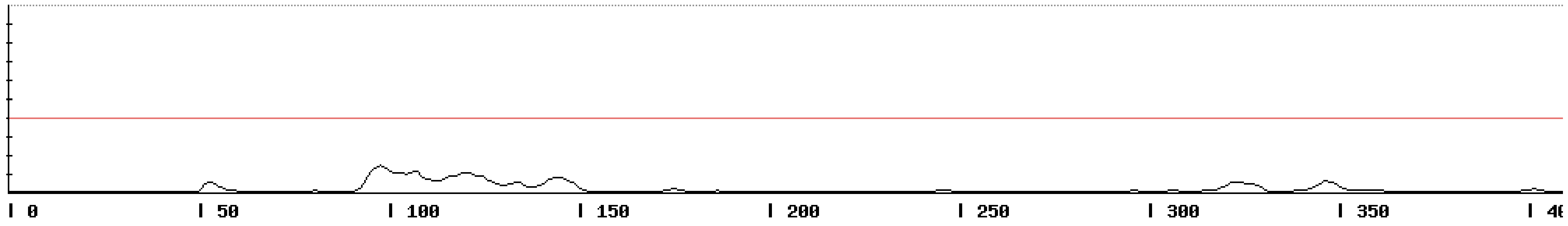

Predictions for Nicotianatabacum_XP_016450002.1

Viterbi Path

Viterbi Path

Posterior @ 0.4

Posterior @ 0.4

Posterior Graphic. Threshold Marked @ 0.4

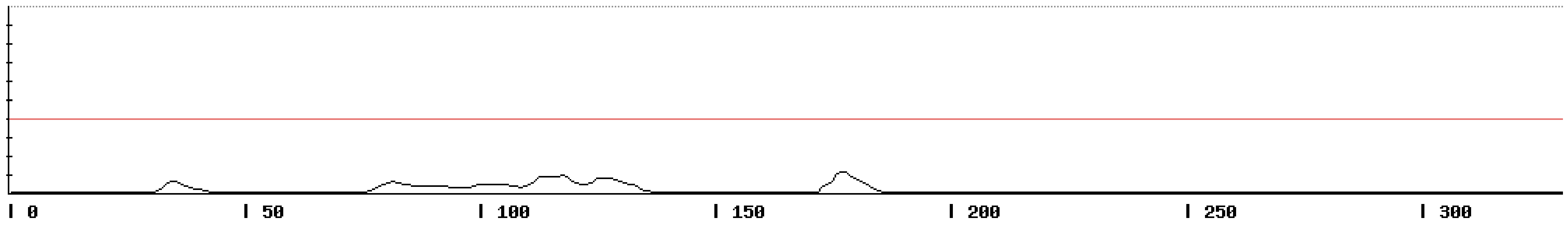

Predictions for Nicotianatabacum_XP_016450516.1

Viterbi Path

Viterbi Path

Posterior @ 0.4

Posterior @ 0.4

Posterior Graphic. Threshold Marked @ 0.4

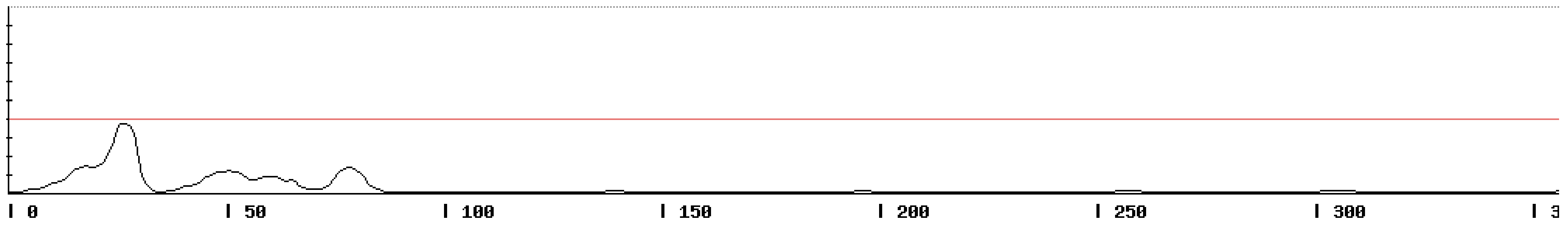

Predictions for Nicotianatabacum_XP_016451105.1

Viterbi Path

Viterbi Path

Posterior @ 0.4

Posterior @ 0.4

Posterior Graphic. Threshold Marked @ 0.4

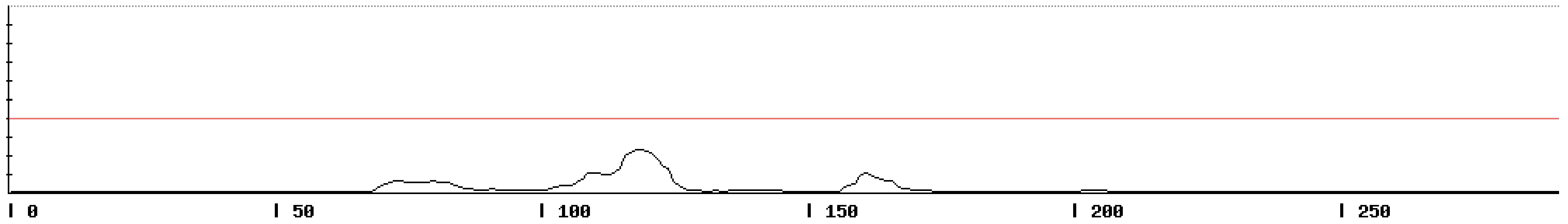

Predictions for Nicotianatabacum_XP_016451106.1	
Viterbi Path	
Viterbi Path	
Posterior @ 0.4	
Posterior @ 0.4	
Posterior Graphic. Threshold Marked @ 0.4	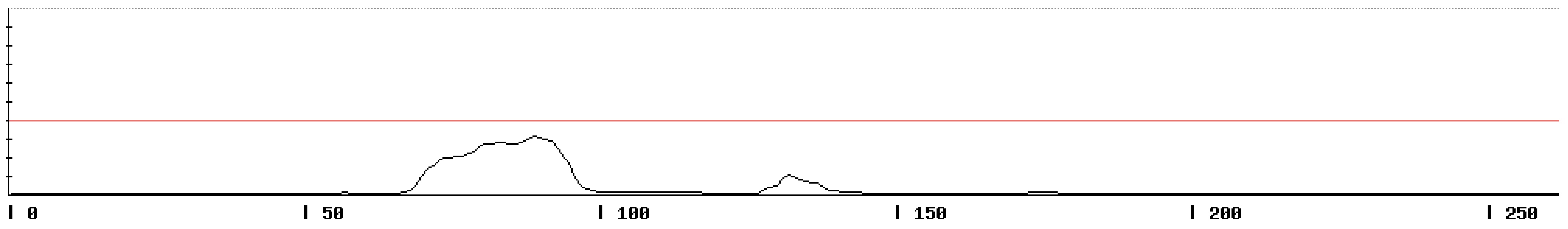

Predictions for Nicotianatabacum_XP_016451185.1	
Viterbi Path	
Viterbi Path	
Posterior @ 0.4	
Posterior @ 0.4	
Posterior Graphic. Threshold Marked @ 0.4	

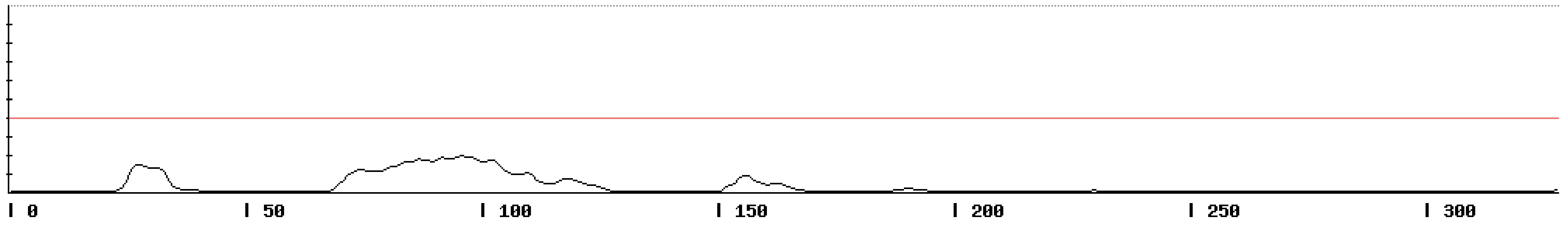

Predictions for Nicotianatabacum_XP_016451362.1

Viterbi Path

Viterbi Path

Posterior @ 0.4

Posterior @ 0.4

Posterior Graphic. Threshold Marked @ 0.4

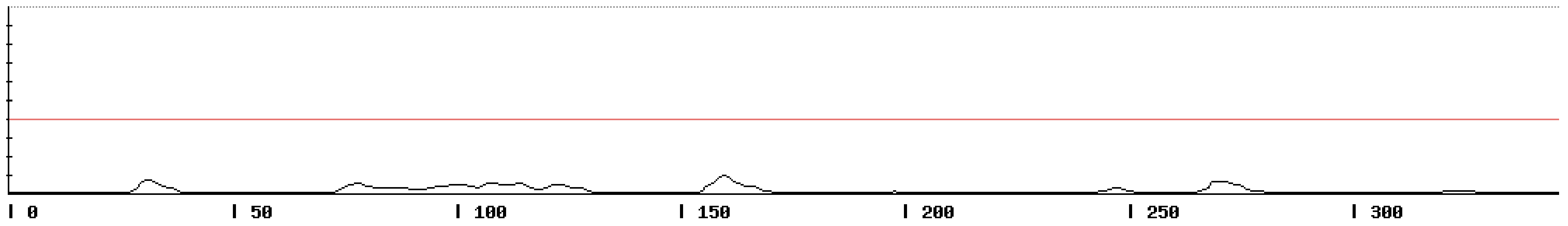

Predictions for Nicotianatabacum_XP_016451616.1

Viterbi Path

Viterbi Path

Posterior @ 0.4

Posterior @ 0.4

Posterior Graphic. Threshold Marked @ 0.4

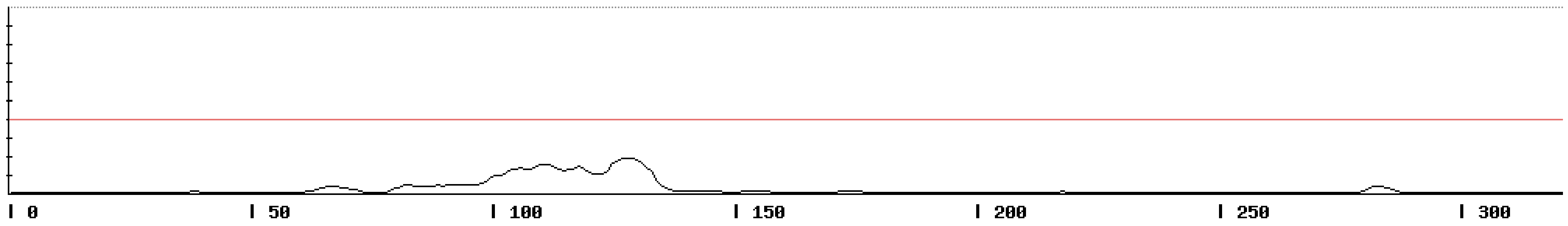

Predictions for Nicotianatabacum_XP_016451619.1

Viterbi Path

Viterbi Path

Posterior @ 0.4

Posterior @ 0.4

Posterior Graphic. Threshold Marked @ 0.4

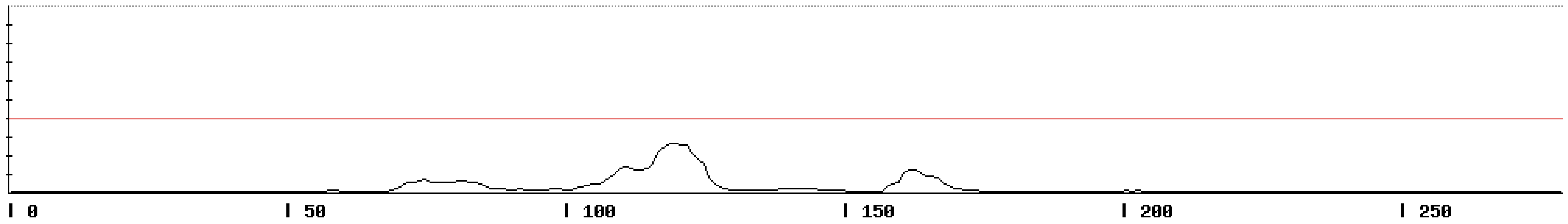

Predictions for Nicotianatabacum_XP_016451623.1

Viterbi Path

Viterbi Path

Posterior @ 0.4

Posterior @ 0.4

Posterior Graphic. Threshold Marked @ 0.4

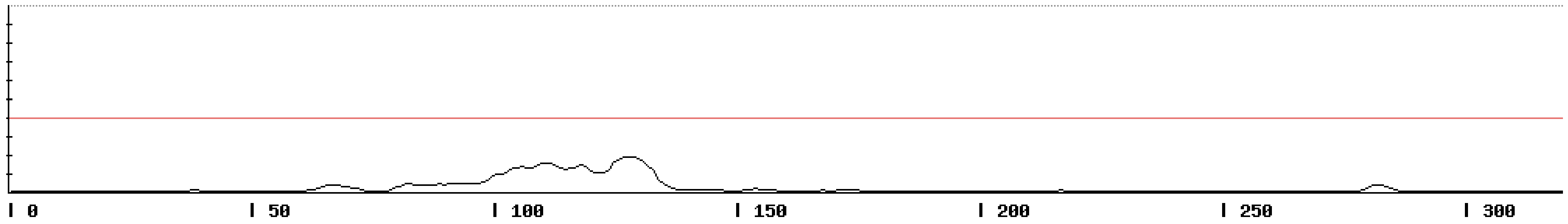

Predictions for Nicotianatabacum_XP_016452652.1

Viterbi Path

Viterbi Path

Posterior @ 0.4

Posterior @ 0.4

Posterior Graphic. Threshold Marked @ 0.4

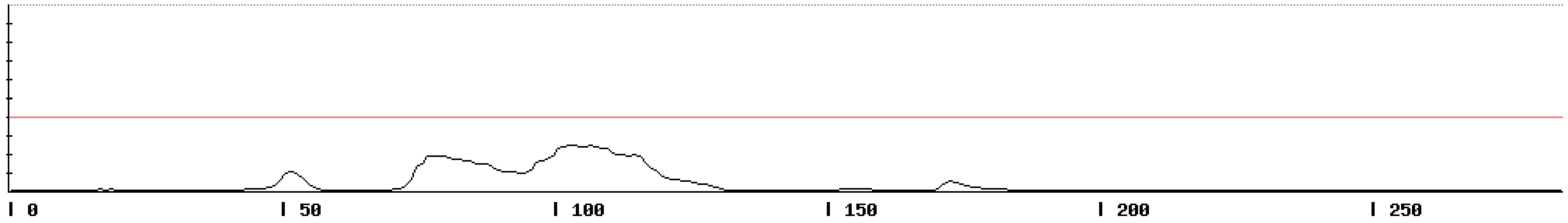

Predictions for Nicotianatabacum_XP_016452711.1

Viterbi Path

Viterbi Path

Posterior @ 0.4	370 - REEKRKKKLKK - 380
Posterior @ 0.4	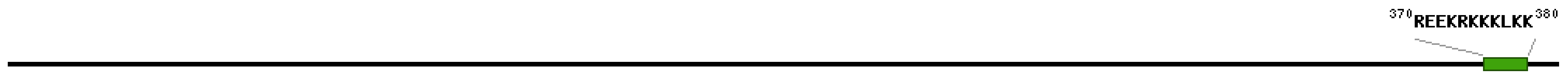
Posterior Graphic. Threshold Marked @ 0.4	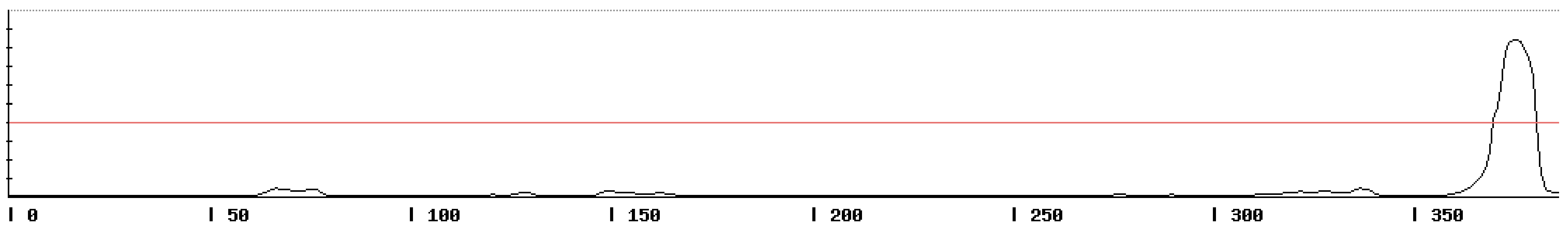

Predictions for Nicotianatabacum_XP_016453035.1

Viterbi Path	
Viterbi Path	
Posterior @ 0.4	99 - KK - 100
Posterior @ 0.4	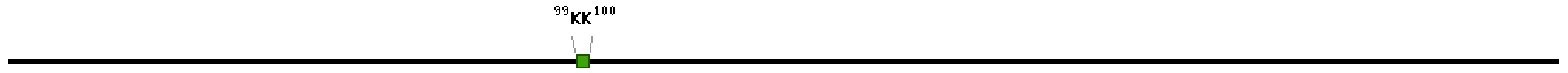
Posterior Graphic. Threshold Marked @ 0.4	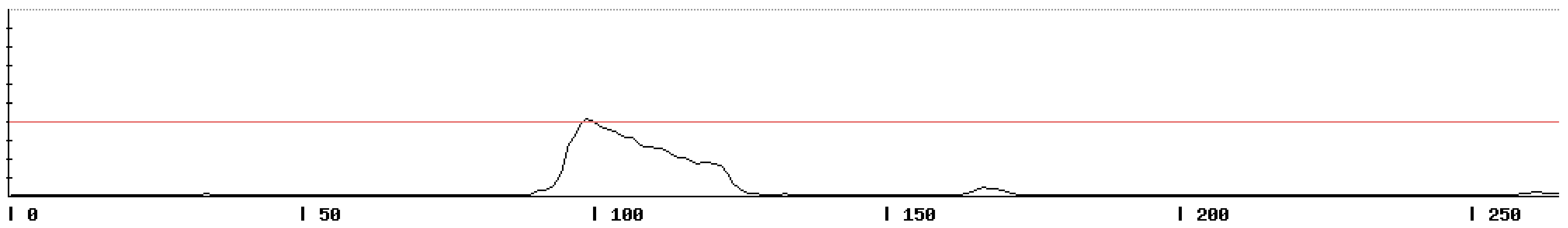

Predictions for Nicotianatabacum_XP_016453133.1

Viterbi Path	
Viterbi Path	

Posterior @ 0.4	131 - KRR - 133<br>147 - HKTGKTRPVVVKGKVKGHKK - 166
Posterior @ 0.4	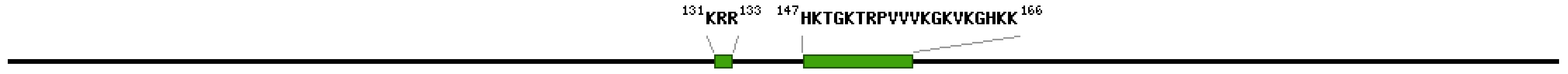
Posterior Graphic. Threshold Marked @ 0.4	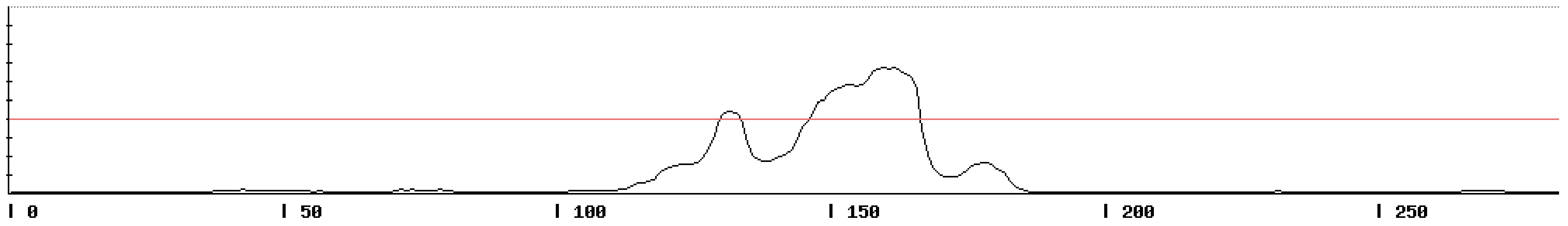

Predictions for Nicotianatabacum_XP_016454006.1

Viterbi Path	
Viterbi Path	
Posterior @ 0.4	
Posterior @ 0.4	
Posterior Graphic. Threshold Marked @ 0.4	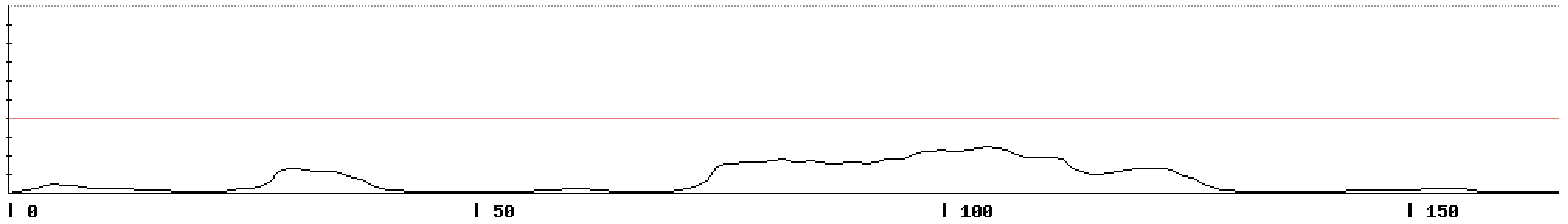

Predictions for Nicotianatabacum_XP_016454007.1	
Viterbi Path	
Viterbi Path	
Posterior @ 0.4	
Posterior @ 0.4	
Posterior Graphic. Threshold Marked @ 0.4	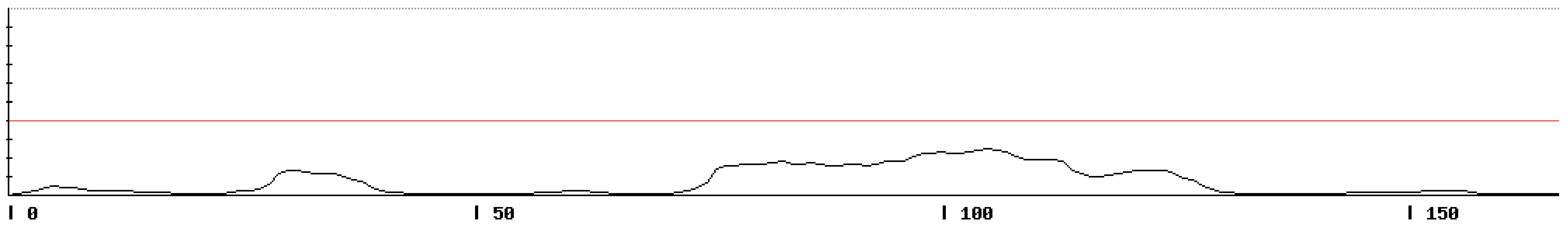

Predictions for Nicotianatabacum_XP_016454008.1	
Viterbi Path	
Viterbi Path	
Posterior @ 0.4	
Posterior @ 0.4	
Posterior Graphic. Threshold Marked @ 0.4	

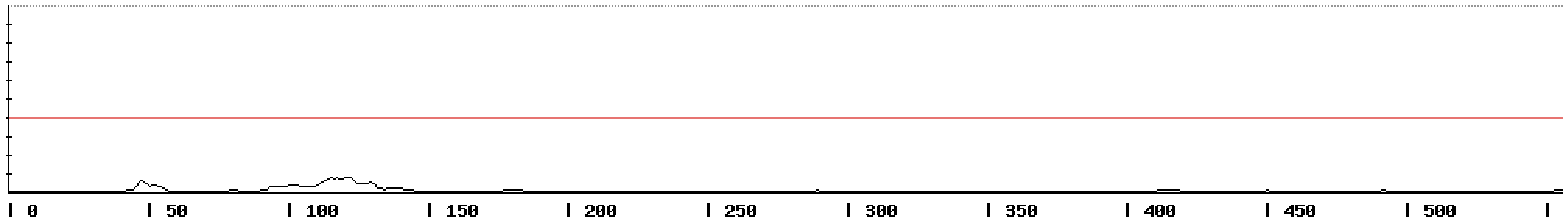

Predictions for Nicotianatabacum_XP_016454400.1

Viterbi Path	
Viterbi Path	
Posterior @ 0.4	
Posterior @ 0.4	
Posterior Graphic. Threshold Marked @ 0.4	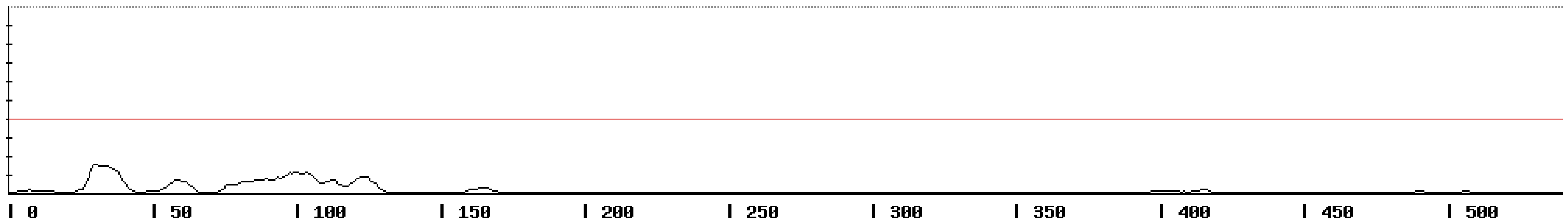

Predictions for Nicotianatabacum_XP_016454401.1

Viterbi Path	
Viterbi Path	
Posterior @ 0.4	
Posterior @ 0.4	

Posterior Graphic. Threshold Marked @ 0.4

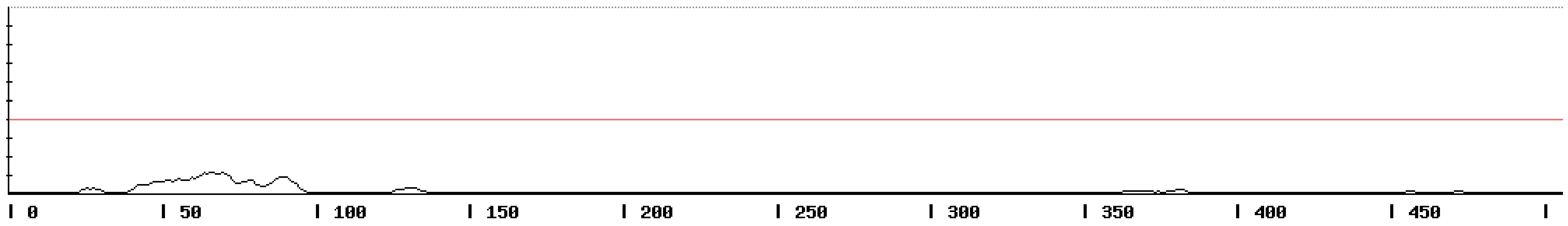

Predictions for Nicotianatabacum_XP_016454404.1

Viterbi Path

Viterbi Path

Posterior @ 0.4

Posterior @ 0.4

Posterior Graphic. Threshold Marked @ 0.4

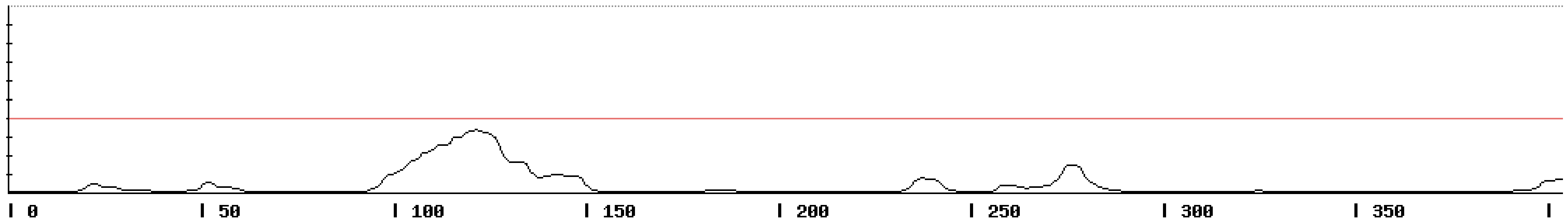

Predictions for Nicotianatabacum_XP_016454536.1

Viterbi Path

Viterbi Path

Posterior @ 0.4

Posterior @ 0.4

Posterior Graphic. Threshold Marked @ 0.4

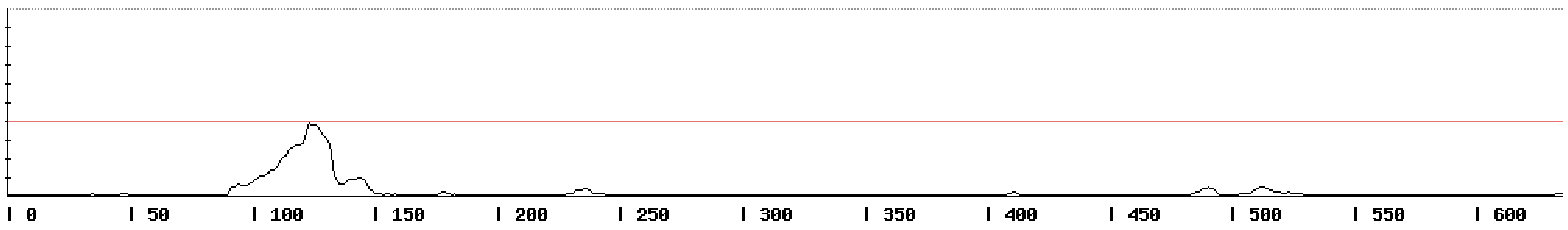

Predictions for Nicotianatabacum_XP_016454586.1

Viterbi Path

Viterbi Path

Posterior @ 0.4 — 68 - KKRKESDKRFR - 78

Posterior @ 0.4

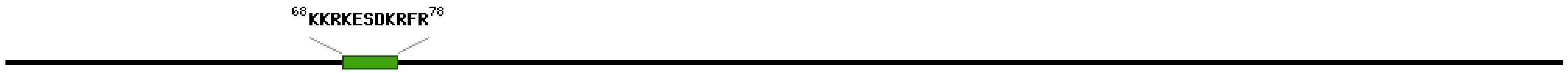

Posterior Graphic. Threshold Marked @ 0.4

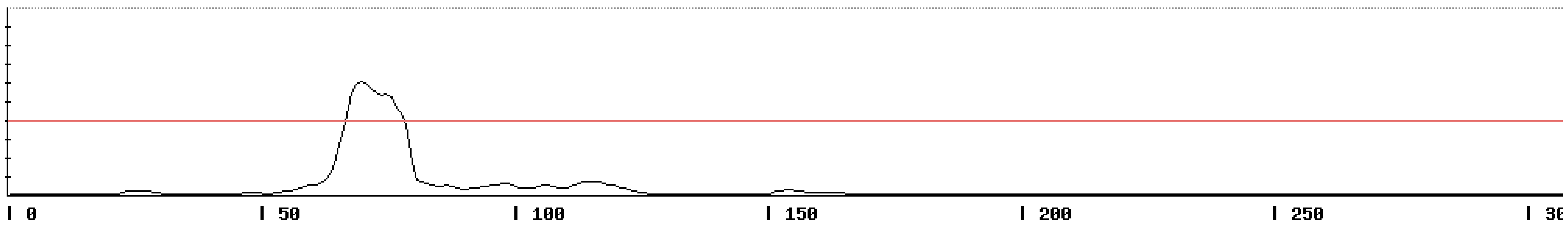

Predictions for Nicotianatabacum_XP_016455213.1

Viterbi Path

Viterbi Path

Posterior @ 0.4	
Posterior @ 0.4	
Posterior Graphic. Threshold Marked @ 0.4	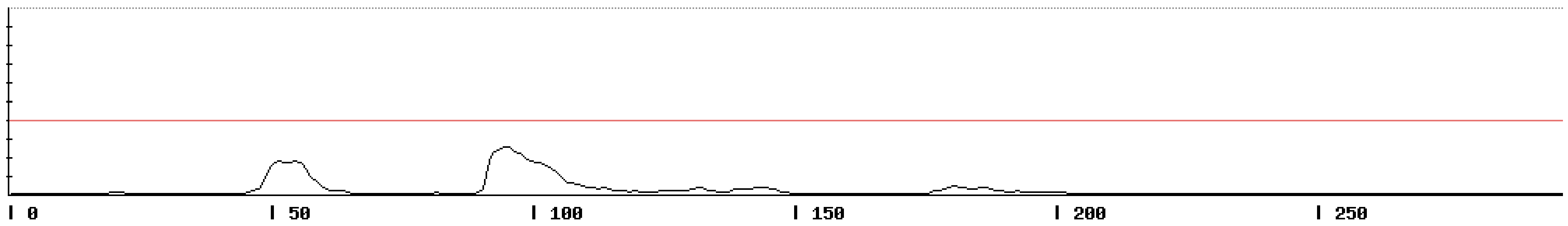

Predictions for Nicotianatabacum_XP_016455277.1

Viterbi Path	
Viterbi Path	
Posterior @ 0.4	
Posterior @ 0.4	
Posterior Graphic. Threshold Marked @ 0.4	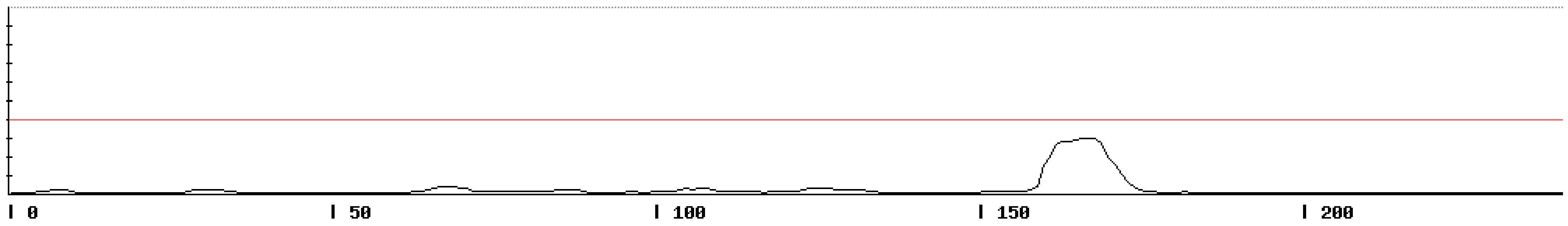

Predictions for Nicotianatabacum_XP_016455676.1

Viterbi Path	
Viterbi Path	

Posterior @ 0.4

Posterior @ 0.4

Posterior Graphic. Threshold Marked @ 0.4

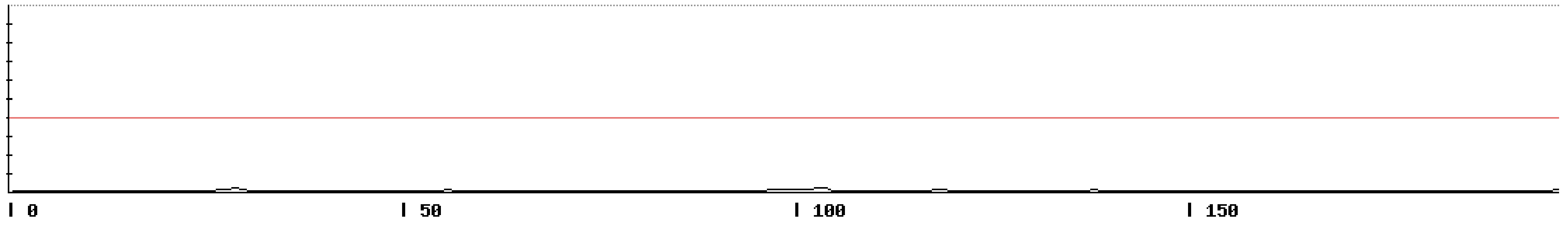

Predictions for Nicotianatabacum_XP_016455717.1

Viterbi Path

Viterbi Path

Posterior @ 0.4

Posterior @ 0.4

Posterior Graphic. Threshold Marked @ 0.4

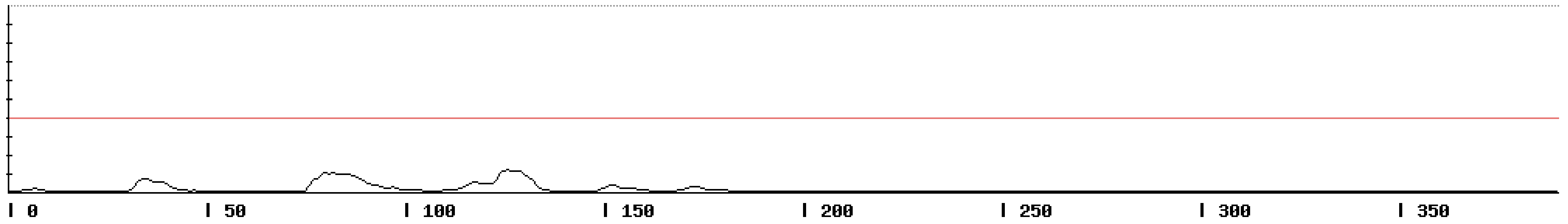

Predictions for Nicotianatabacum_XP_016455718.1

Viterbi Path

Viterbi Path

Posterior @ 0.4

Posterior @ 0.4

Posterior Graphic. Threshold Marked @ 0.4

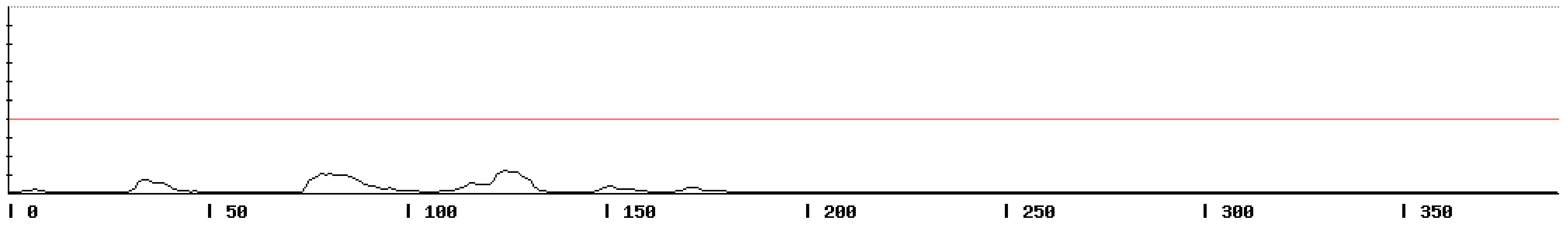

Predictions for Nicotianatabacum_XP_016455978.1

Viterbi Path

Viterbi Path

Posterior @ 0.4

Posterior @ 0.4

Posterior Graphic. Threshold Marked @ 0.4

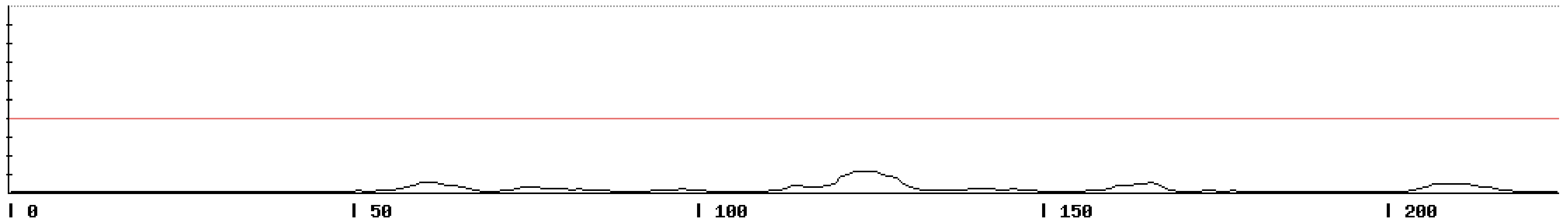

Predictions for Nicotianatabacum_XP_016455980.1

Viterbi Path	
Viterbi Path	
Posterior @ 0.4	
Posterior @ 0.4	
Posterior Graphic. Threshold Marked @ 0.4	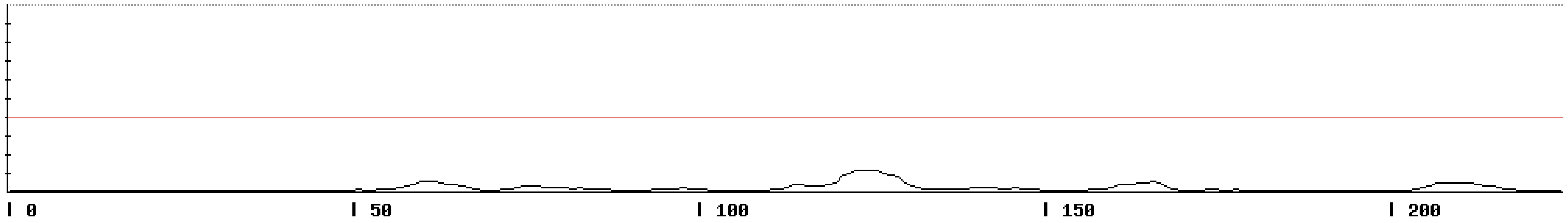

Predictions for Nicotianatabacum_XP_016456031.1

Viterbi Path	
Viterbi Path	
Posterior @ 0.4	
Posterior @ 0.4	
Posterior Graphic. Threshold Marked @ 0.4	

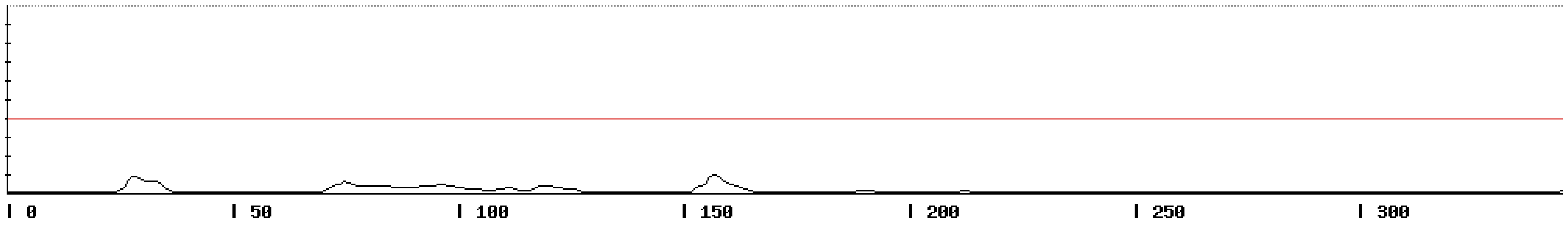

Predictions for Nicotianatabacum_XP_016456160.1

Viterbi Path

Viterbi Path

Posterior @ 0.4: 161 - RKRRK - 165

Posterior @ 0.4

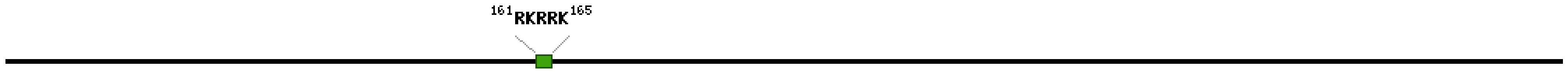

Posterior Graphic. Threshold Marked @ 0.4

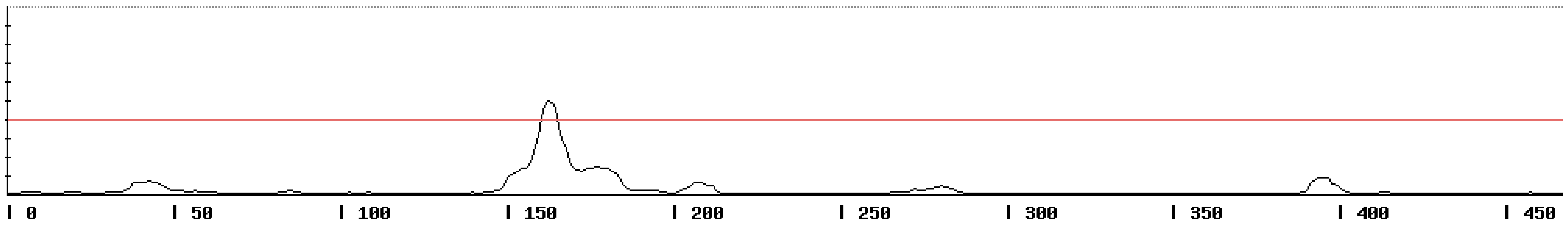

Predictions for Nicotianatabacum_XP_016456161.1

Viterbi Path

Viterbi Path

Posterior @ 0.4: 137 - RKRRK - 141

Posterior @ 0.4

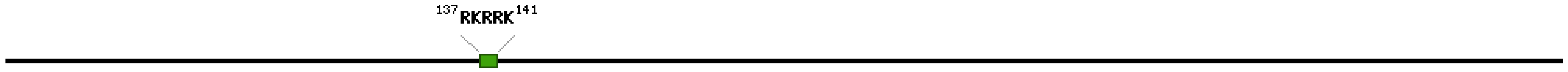

Posterior Graphic. Threshold Marked @ 0.4

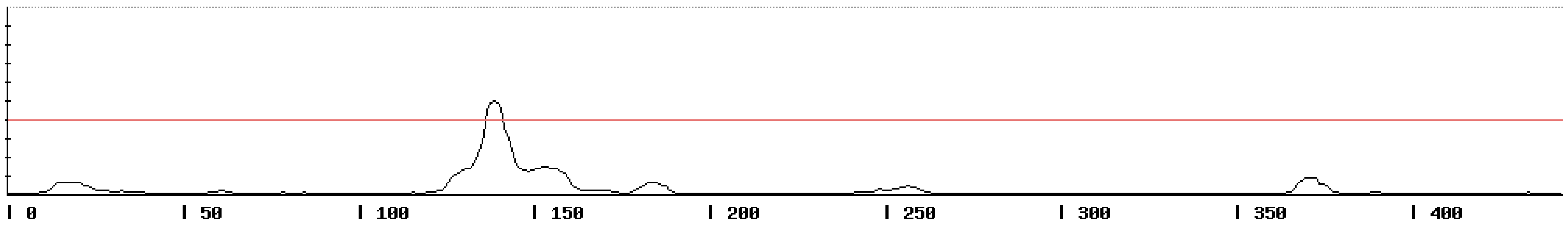

Predictions for Nicotianatabacum_XP_016456162.1

Viterbi Path	
Viterbi Path	
Posterior @ 0.4	134 - RKRRK - 138
Posterior @ 0.4	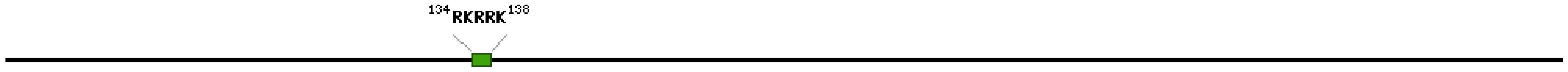
Posterior Graphic. Threshold Marked @ 0.4	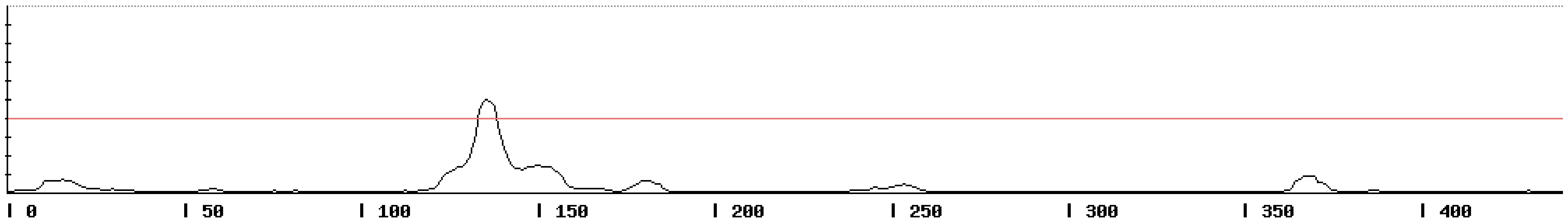

Predictions for Nicotianatabacum_XP_016456163.1

Viterbi Path	
Viterbi Path	
Posterior @ 0.4	133 - RKRRK - 137
Posterior @ 0.4	

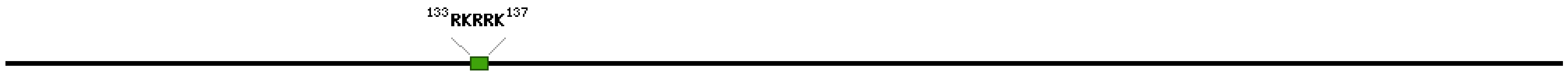

Posterior Graphic. Threshold Marked @ 0.4

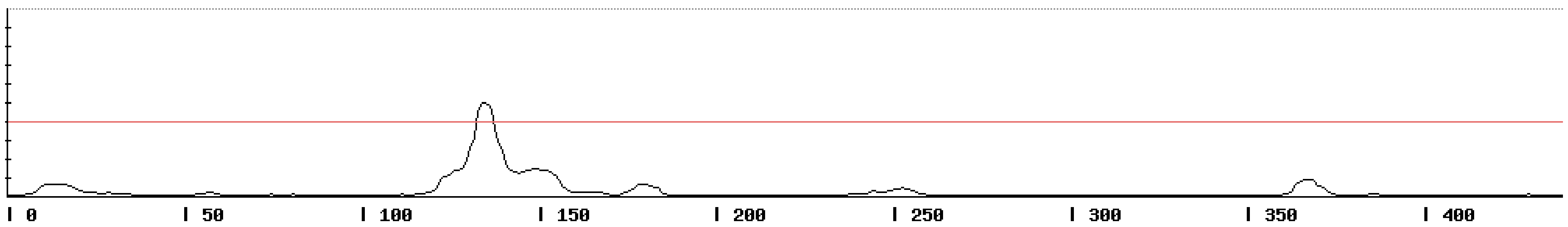

Predictions for Nicotianatabacum_XP_016456164.1

Viterbi Path

Viterbi Path

Posterior @ 0.4 — 133 - RKRRK - 137

Posterior @ 0.4

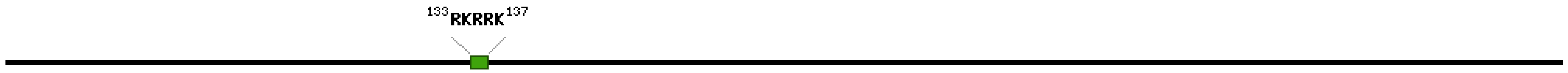

Posterior Graphic. Threshold Marked @ 0.4

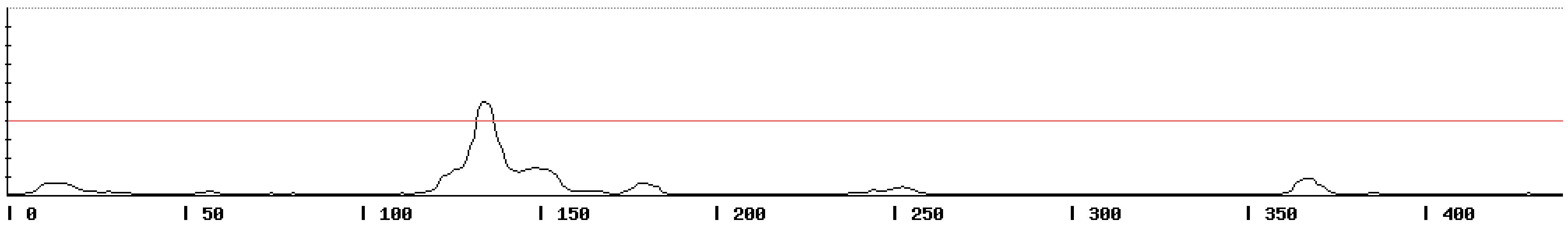

Predictions for Nicotianatabacum_XP_016456237.1

Viterbi Path

Viterbi Path

Posterior @ 0.4 32 - LKKRKESHKRFRR - 44

Posterior @ 0.4

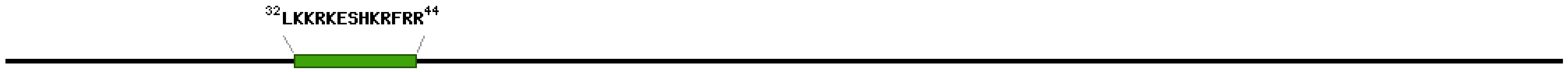

Posterior Graphic. Threshold Marked @ 0.4

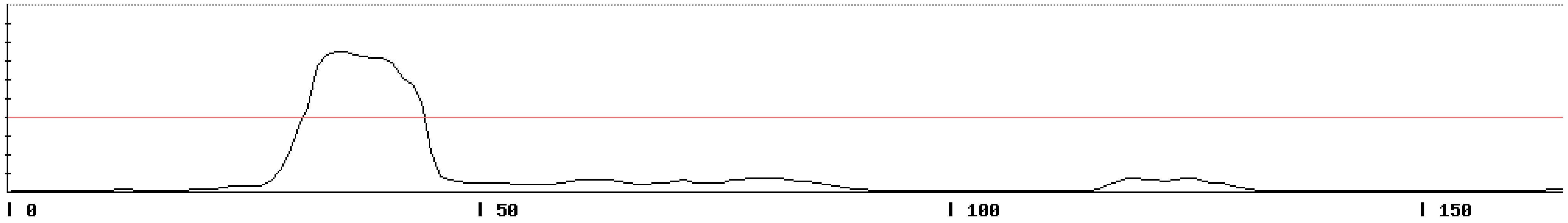

Predictions for Nicotianatabacum_XP_016456837.1

Viterbi Path

Viterbi Path

Posterior @ 0.4

Posterior @ 0.4

Posterior Graphic. Threshold Marked @ 0.4

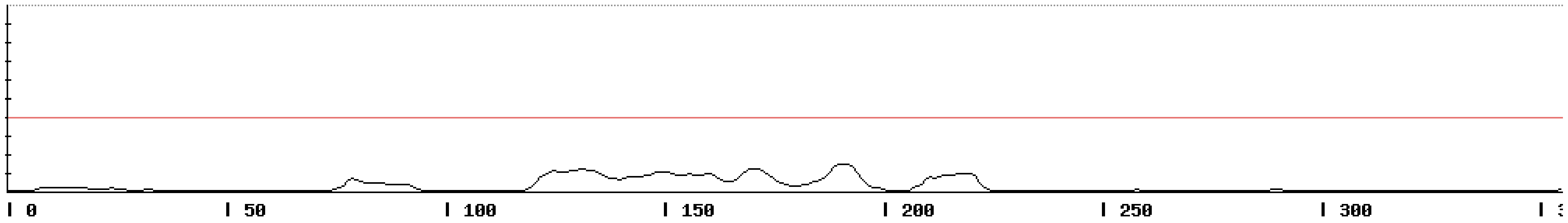

Predictions for Nicotianatabacum_XP_016457112.1

Viterbi Path

Viterbi Path

Posterior @ 0.4

Posterior @ 0.4

Posterior Graphic. Threshold Marked @ 0.4

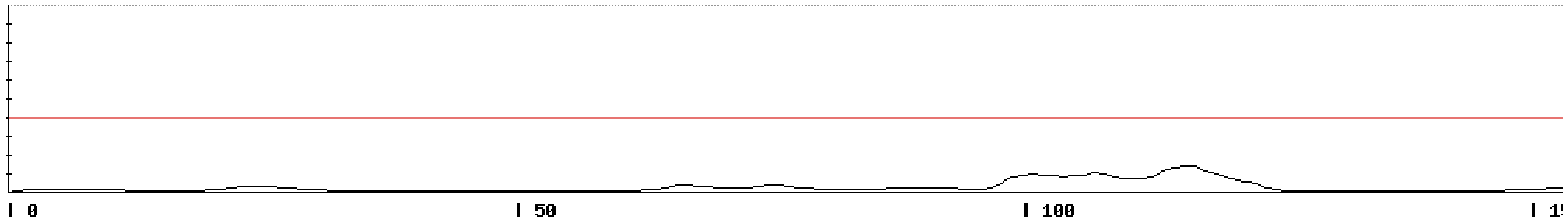

Predictions for Nicotianatabacum_XP_016457734.1

Viterbi Path

Viterbi Path

Posterior @ 0.4

Posterior @ 0.4

Posterior Graphic. Threshold Marked @ 0.4

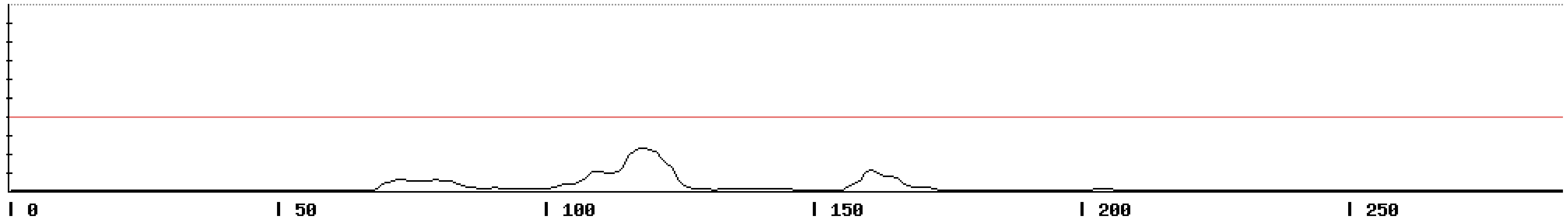

Predictions for Nicotianatabacum_XP_016457772.1

Viterbi Path	
Viterbi Path	
Posterior @ 0.4	
Posterior @ 0.4	
Posterior Graphic. Threshold Marked @ 0.4	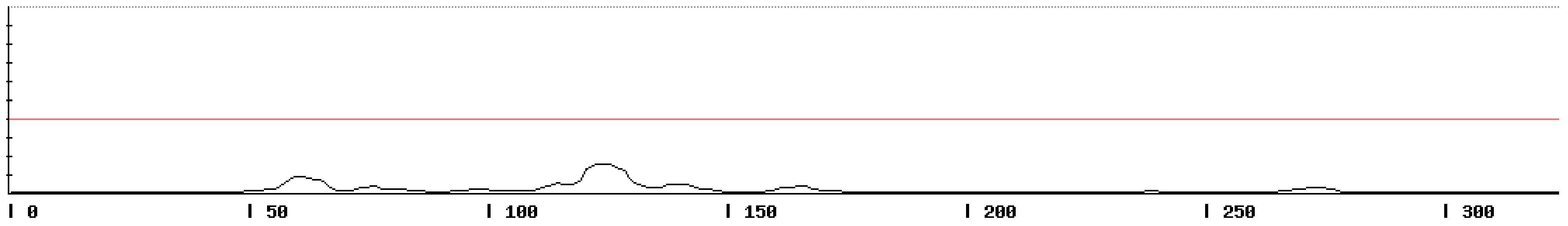

Predictions for Nicotianatabacum_XP_016457897.1

Viterbi Path	
Viterbi Path	
Posterior @ 0.4	
Posterior @ 0.4	
Posterior Graphic. Threshold Marked @ 0.4	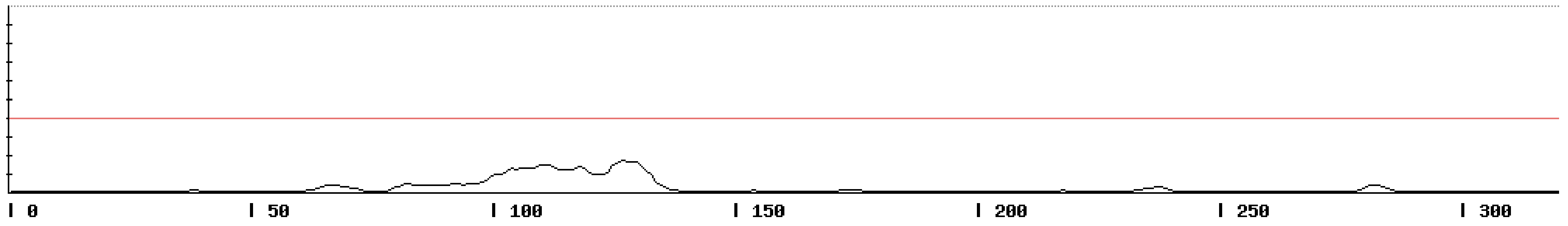

Predictions for Nicotianatabacum_XP_016457898.1	
Viterbi Path	
Viterbi Path	
Posterior @ 0.4	
Posterior @ 0.4	
Posterior Graphic. Threshold Marked @ 0.4	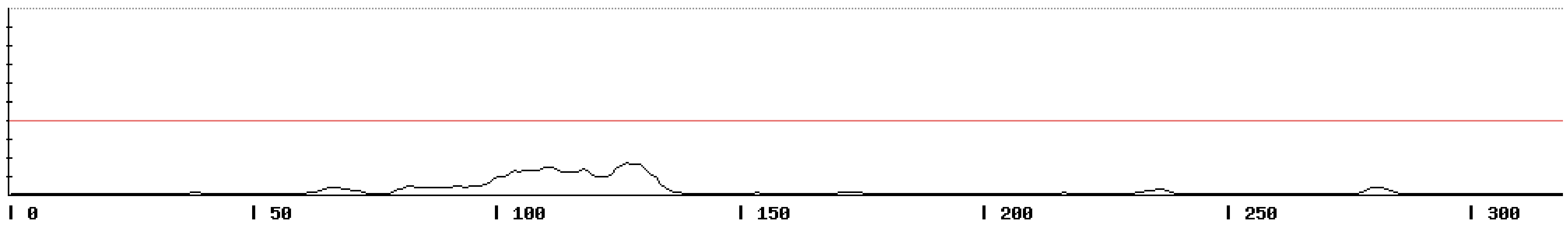

Predictions for Nicotianatabacum_XP_016457978.1	
Viterbi Path	
Viterbi Path	
Posterior @ 0.4	
Posterior @ 0.4	
Posterior Graphic. Threshold Marked @ 0.4	

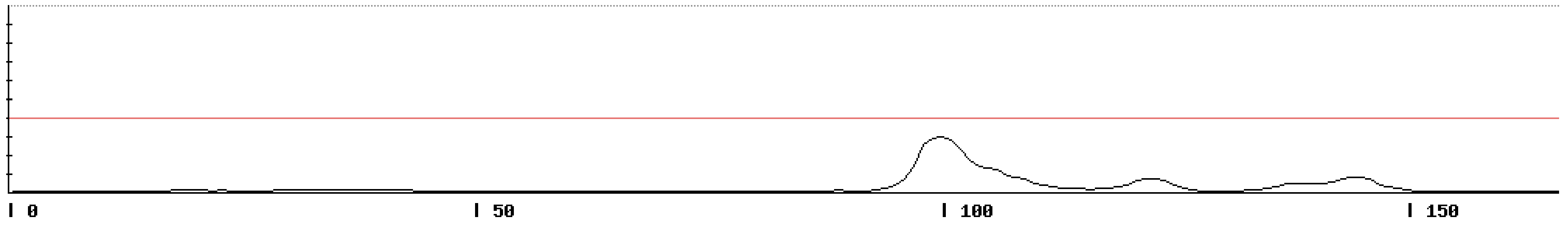

Predictions for Nicotianatabacum_XP_016458224.1

Viterbi Path

Viterbi Path

Posterior @ 0.4

Posterior @ 0.4

Posterior Graphic. Threshold Marked @ 0.4

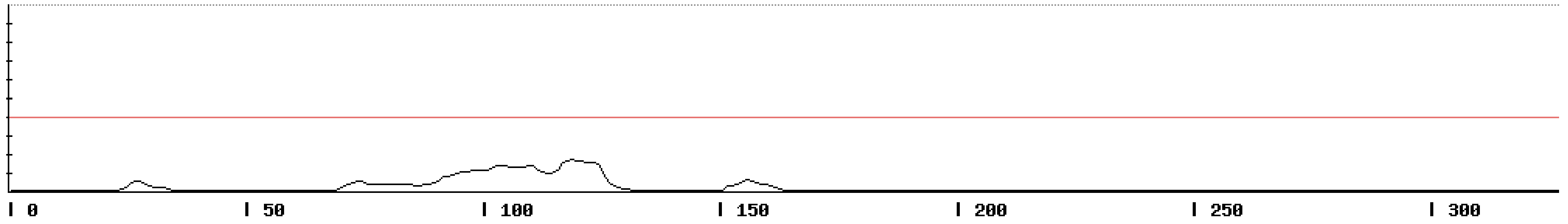

Predictions for Nicotianatabacum_XP_016458225.1

Viterbi Path

Viterbi Path

Posterior @ 0.4

Posterior @ 0.4

Posterior Graphic. Threshold Marked @ 0.4

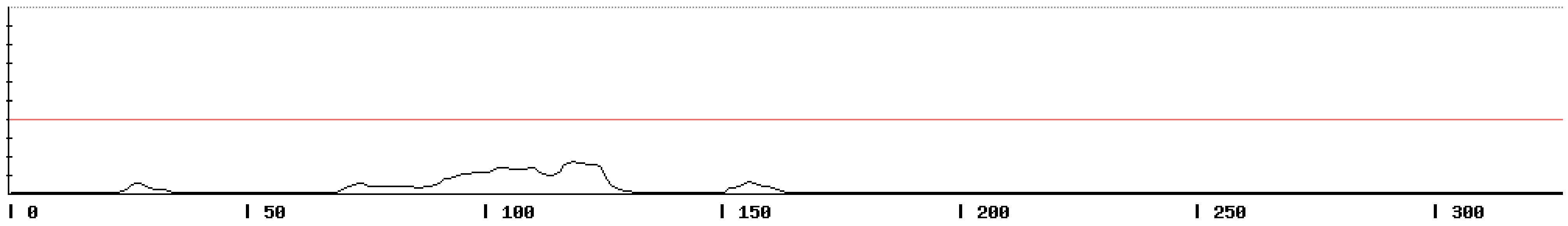

Predictions for Nicotianatabacum_XP_016458474.1

Viterbi Path

Viterbi Path

Posterior @ 0.4

Posterior @ 0.4

Posterior Graphic. Threshold Marked @ 0.4

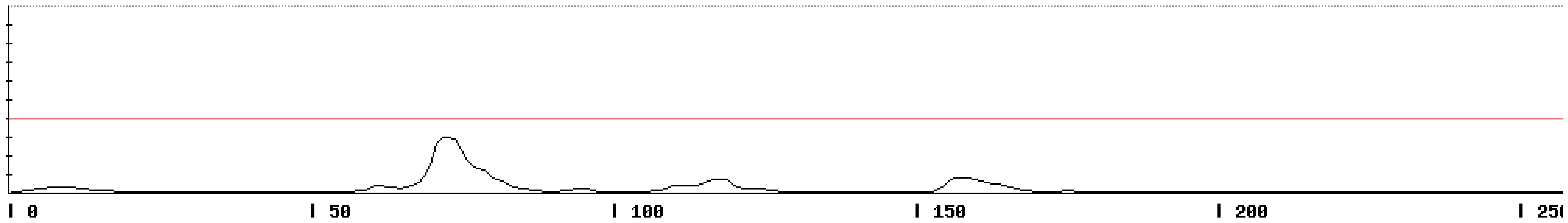

Predictions for Nicotianatabacum_XP_016458602.1

Viterbi Path

Viterbi Path

Posterior @ 0.4

Posterior @ 0.4

Posterior Graphic. Threshold Marked @ 0.4

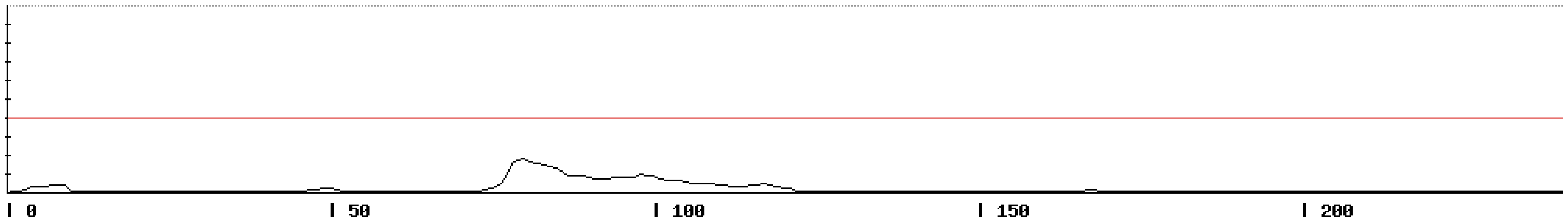

Predictions for Nicotianatabacum_XP_016458603.1

Viterbi Path

Viterbi Path

Posterior @ 0.4

272 - VRNKRPRRKRDSHPPP - 287

499 - KK - 500

Posterior @ 0.4

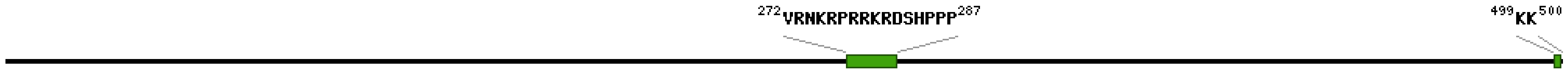

Posterior Graphic. Threshold Marked @ 0.4

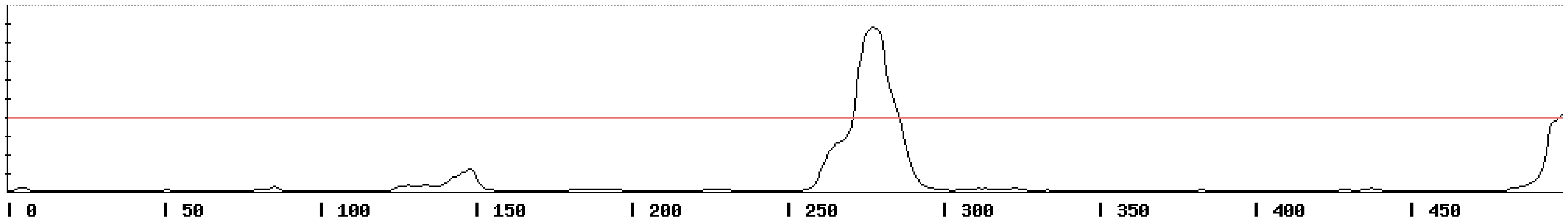

Predictions for Nicotianatabacum_XP_016458604.1

Viterbi Path

Viterbi Path

Posterior @ 0.4	272 - VRNKRPRRKRDSHPPP - 287
	499 - KK - 500

Posterior @ 0.4

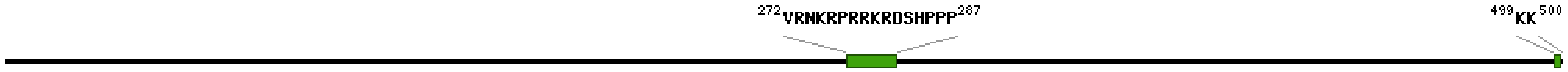

Posterior Graphic. Threshold Marked @ 0.4

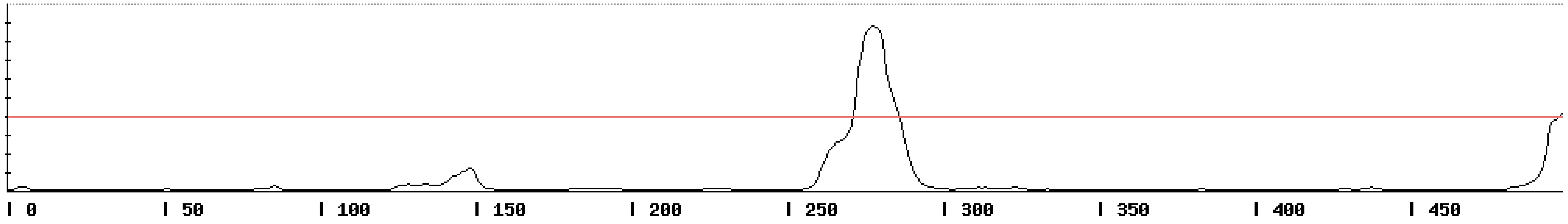

Predictions for Nicotianatabacum_XP_016458605.1

Viterbi Path

Viterbi Path

Posterior @ 0.4	272 - VRNKRPRRKRDSHPP - 286

Posterior @ 0.4

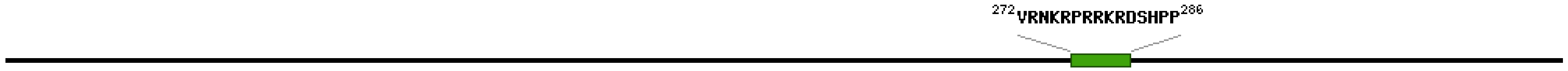

Posterior Graphic. Threshold Marked @ 0.4

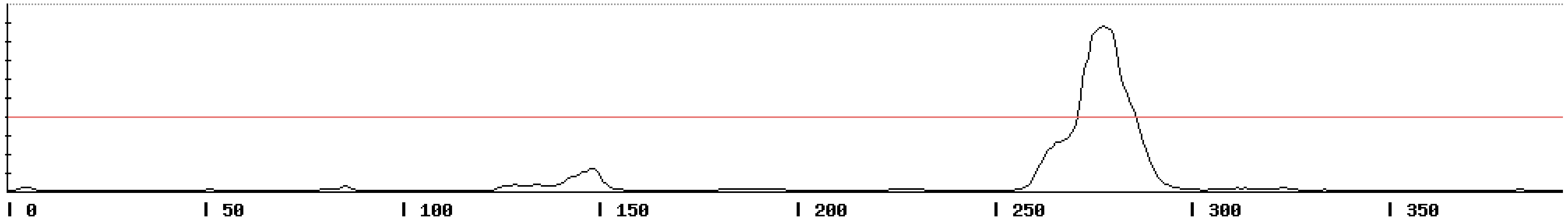

Predictions for Nicotianatabacum_XP_016458645.1	
Viterbi Path	
Viterbi Path	
Posterior @ 0.4	
Posterior @ 0.4	
Posterior Graphic. Threshold Marked @ 0.4	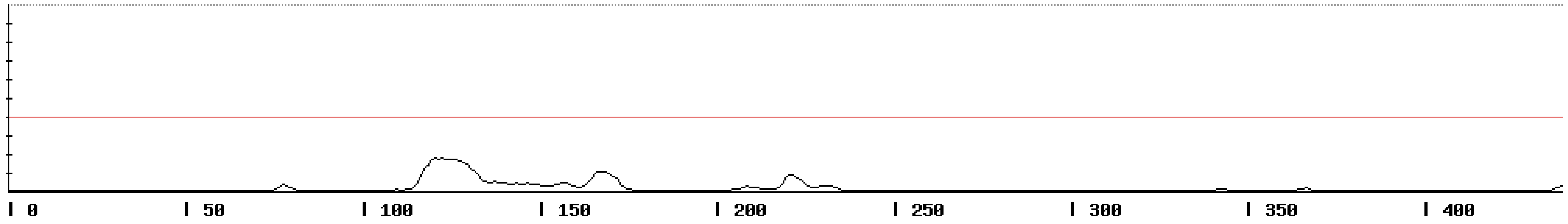

Predictions for Nicotianatabacum_XP_016458989.1	
Viterbi Path	
Viterbi Path	
Posterior @ 0.4	
Posterior @ 0.4	
Posterior Graphic. Threshold Marked @ 0.4	

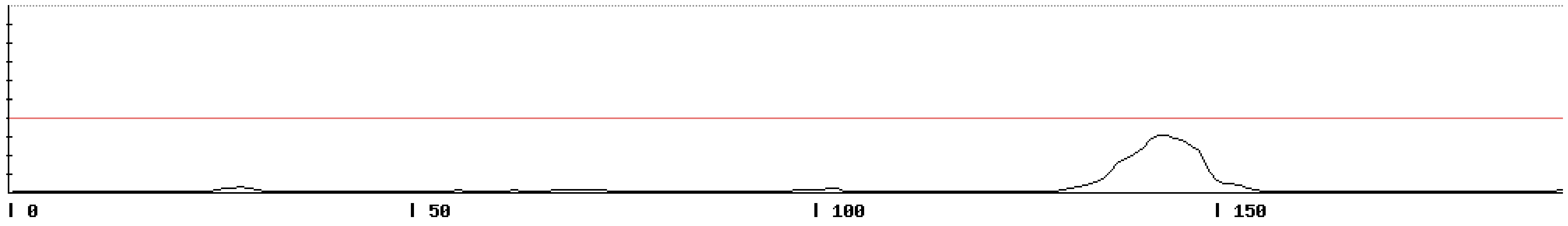

Predictions for Nicotianatabacum_XP_016459095.1	
Viterbi Path	
Viterbi Path	
Posterior @ 0.4	
Posterior @ 0.4	
Posterior Graphic. Threshold Marked @ 0.4	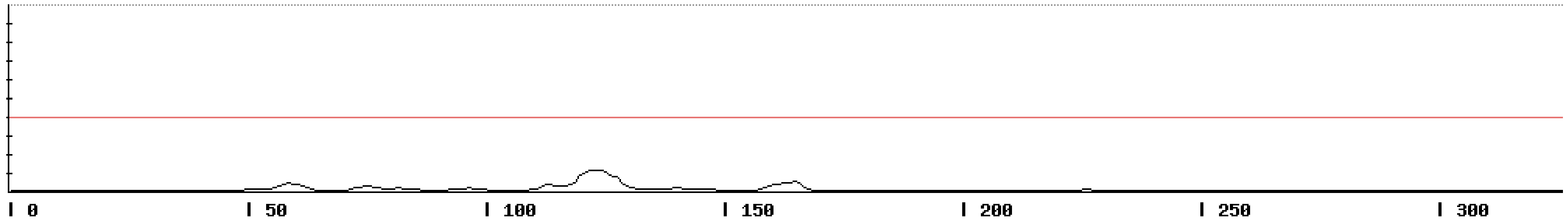

Predictions for Nicotianatabacum_XP_016459506.1	
Viterbi Path	
Viterbi Path	
Posterior @ 0.4	
Posterior @ 0.4	

Posterior Graphic. Threshold Marked @ 0.4

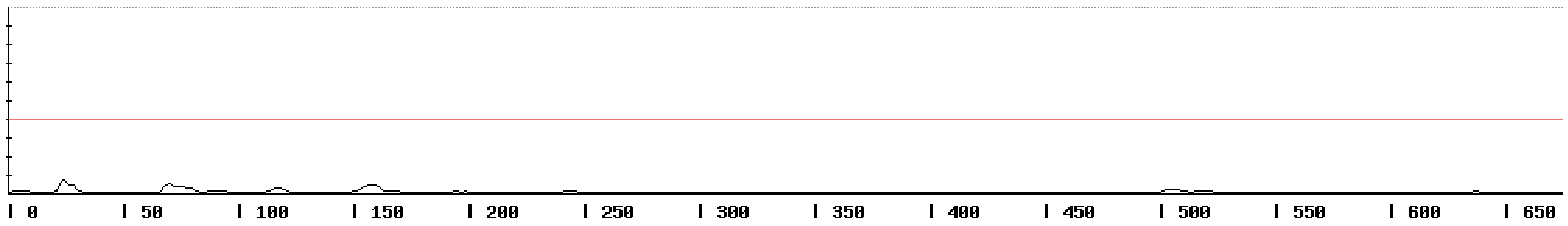

Predictions for Nicotianatabacum_XP_016459507.1

Viterbi Path

Viterbi Path

Posterior @ 0.4

Posterior @ 0.4

Posterior Graphic. Threshold Marked @ 0.4

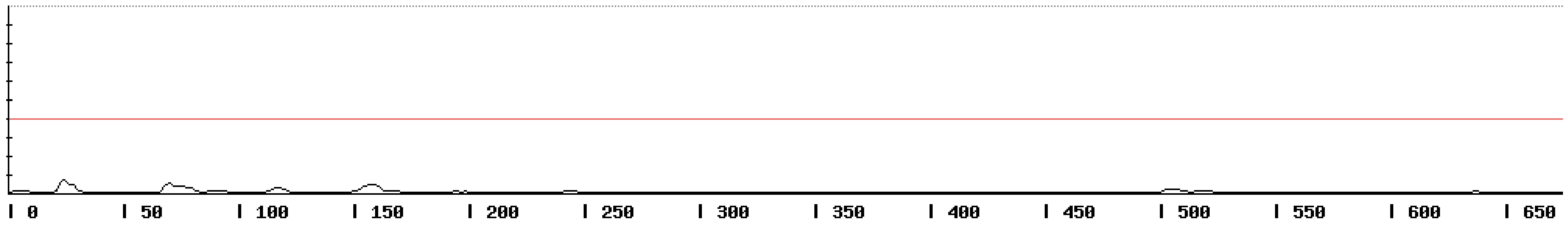

Predictions for Nicotianatabacum_XP_016459881.1

Viterbi Path

Viterbi Path

Posterior @ 0.4

Posterior @ 0.4

Posterior Graphic. Threshold Marked @ 0.4

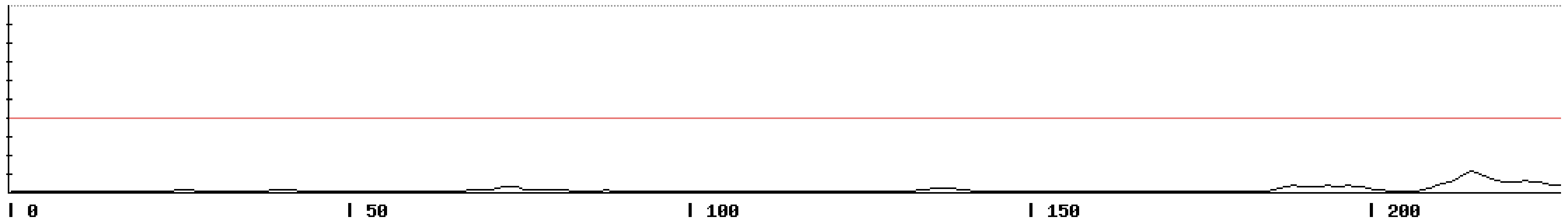

Predictions for Nicotianatabacum_XP_016459882.1

Viterbi Path

Viterbi Path

Posterior @ 0.4

Posterior @ 0.4

Posterior Graphic. Threshold Marked @ 0.4

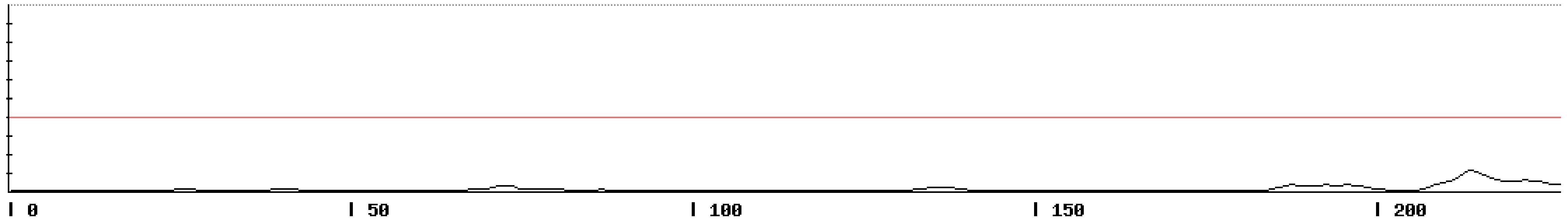

Predictions for Nicotianatabacum_XP_016460408.1

Viterbi Path

Viterbi Path

Posterior @ 0.4

Posterior @ 0.4

Posterior Graphic. Threshold Marked @ 0.4

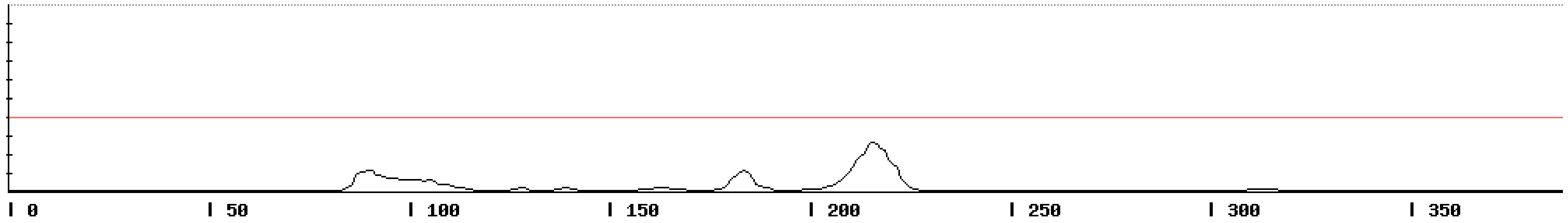

Predictions for Nicotianatabacum_XP_016460548.1

Viterbi Path

Viterbi Path

Posterior @ 0.4

Posterior @ 0.4

Posterior Graphic. Threshold Marked @ 0.4

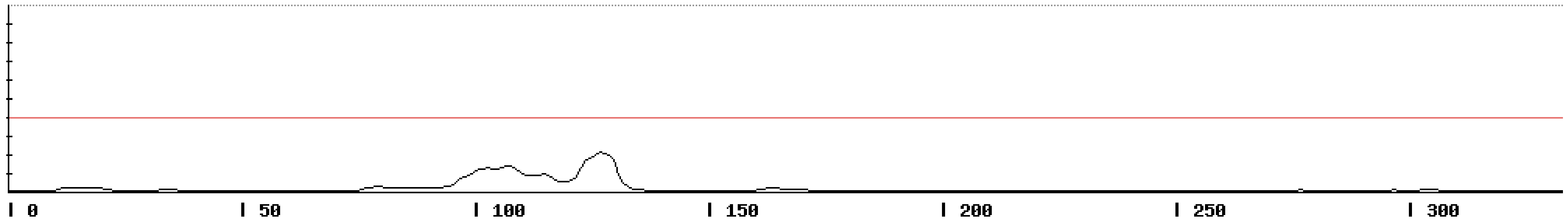

Predictions for Nicotianatabacum_XP_016460549.1

Viterbi Path

Viterbi Path

Posterior @ 0.4

Posterior @ 0.4

Posterior Graphic. Threshold Marked @ 0.4

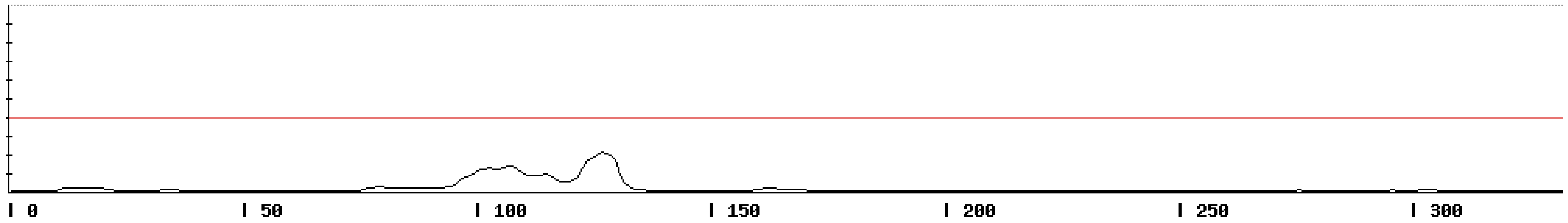

Predictions for Nicotianatabacum_XP_016461013.1

Viterbi Path

Viterbi Path

Posterior @ 0.4

Posterior @ 0.4

Posterior Graphic. Threshold Marked @ 0.4

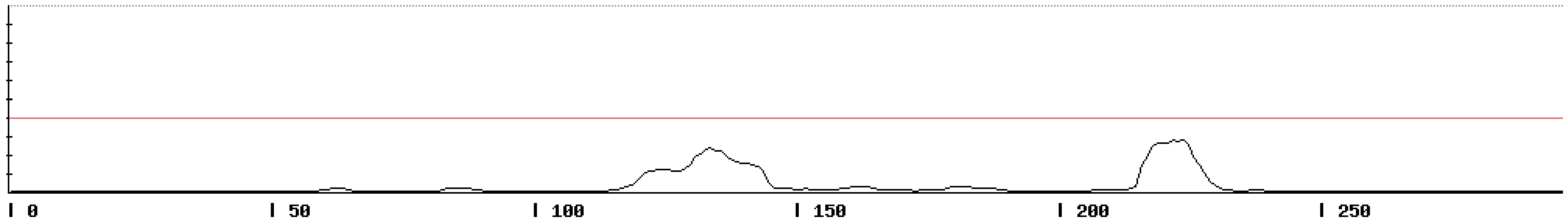

Predictions for Nicotianatabacum_XP_016461097.1

Viterbi Path	
Viterbi Path	
Posterior @ 0.4	92 - KKKKPT - 97
Posterior @ 0.4	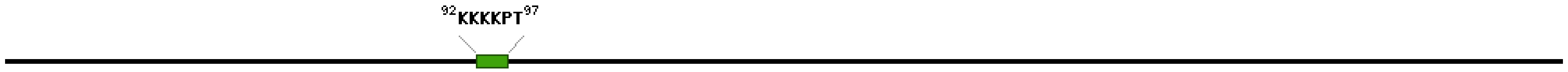
Posterior Graphic. Threshold Marked @ 0.4	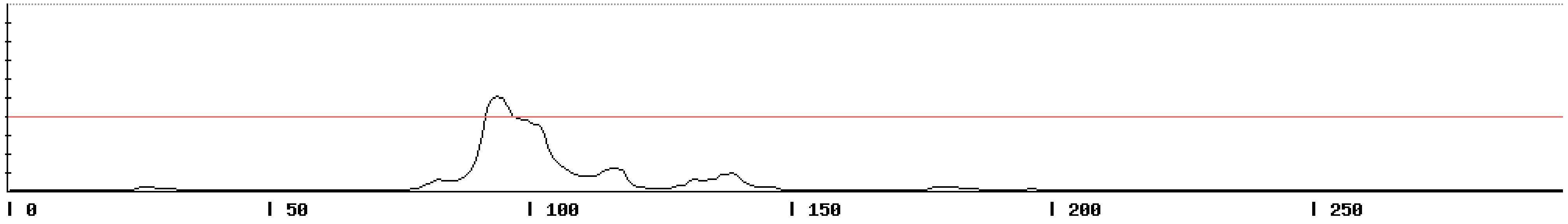

Predictions for Nicotianatabacum_XP_016461118.1

Viterbi Path	
Viterbi Path	
Posterior @ 0.4	
Posterior @ 0.4	
Posterior Graphic. Threshold Marked @ 0.4	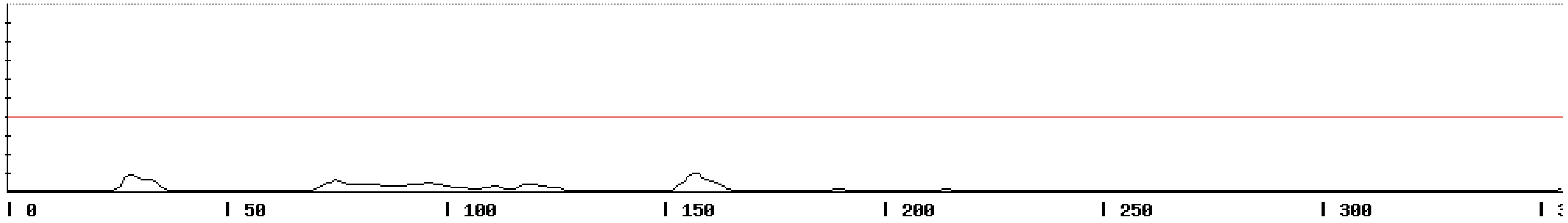

Predictions for Nicotianatabacum_XP_016461144.1	
Viterbi Path	
Viterbi Path	
Posterior @ 0.4	104 - VGKPK - 108
Posterior @ 0.4	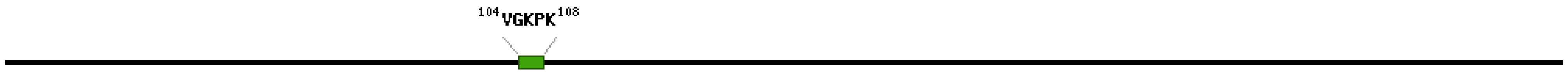
Posterior Graphic. Threshold Marked @ 0.4	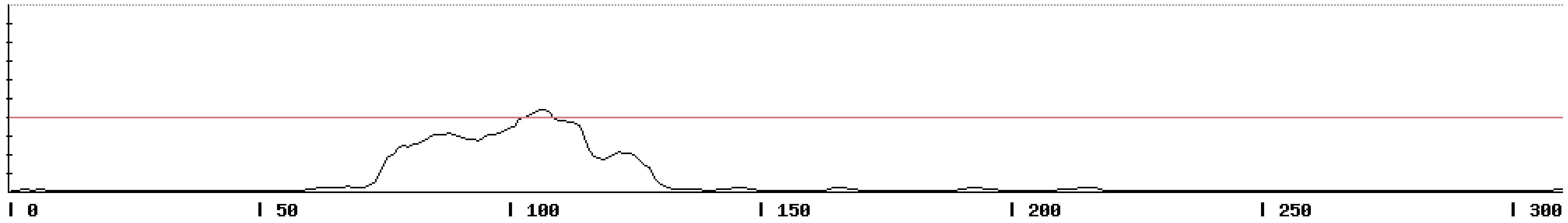

Predictions for Nicotianatabacum_XP_016462374.1	
Viterbi Path	
Viterbi Path	
Posterior @ 0.4	
Posterior @ 0.4	
Posterior Graphic. Threshold Marked @ 0.4	

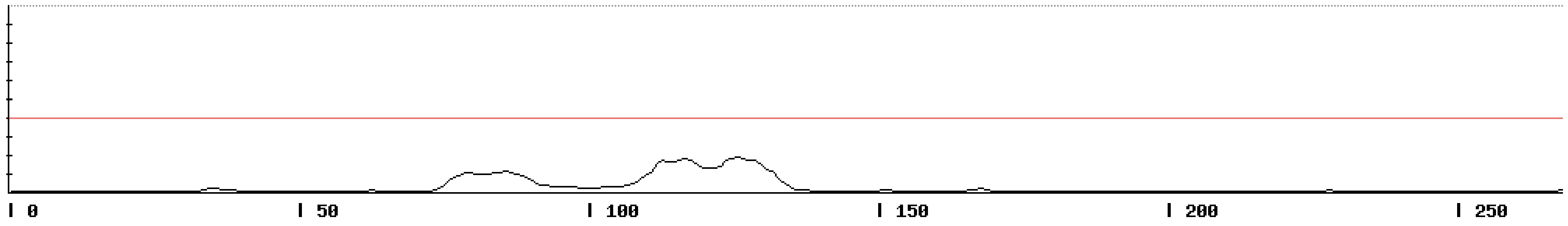

Predictions for Nicotianatabacum_XP_016462967.1	
Viterbi Path	
Viterbi Path	
Posterior @ 0.4	
Posterior @ 0.4	
Posterior Graphic. Threshold Marked @ 0.4	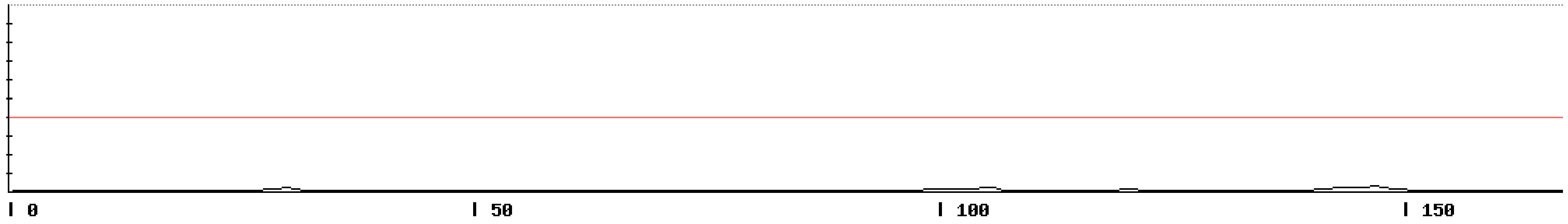

Predictions for Nicotianatabacum_XP_016463159.1	
Viterbi Path	
Viterbi Path	
Posterior @ 0.4	
Posterior @ 0.4	

Posterior Graphic. Threshold Marked @ 0.4

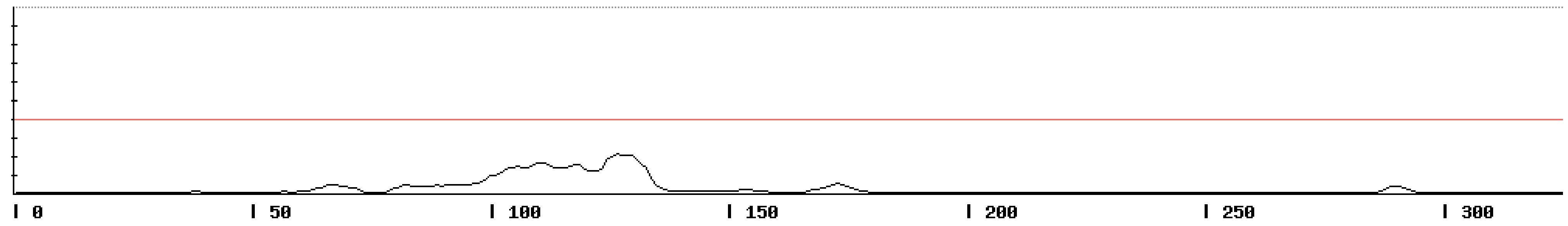

Predictions for Nicotianatabacum_XP_016463299.1

Viterbi Path

Viterbi Path

Posterior @ 0.4

Posterior @ 0.4

Posterior Graphic. Threshold Marked @ 0.4

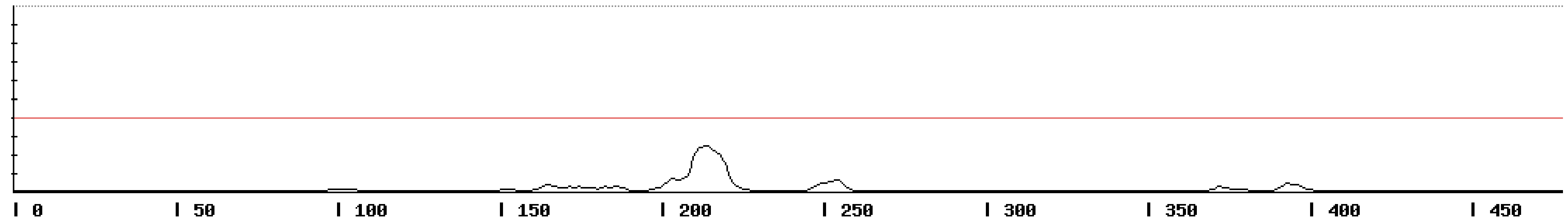

Predictions for Nicotianatabacum_XP_016463542.1

Viterbi Path

Viterbi Path

Posterior @ 0.4

Posterior @ 0.4

Posterior Graphic. Threshold Marked @ 0.4

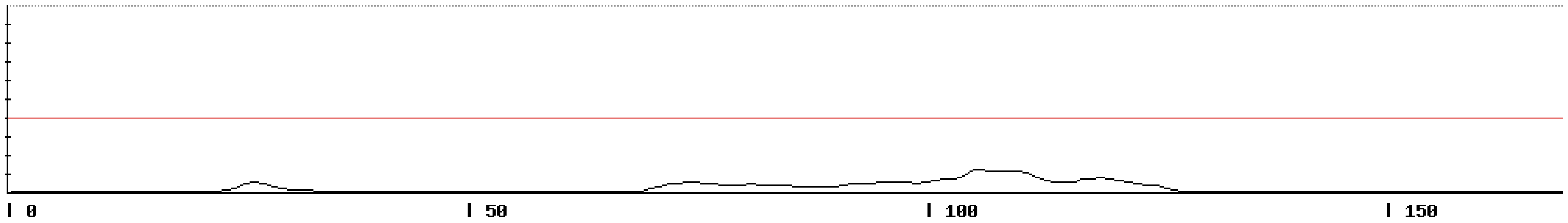

Predictions for Nicotianatabacum_XP_016464143.1

Viterbi Path

Viterbi Path

Posterior @ 0.4

Posterior @ 0.4

Posterior Graphic. Threshold Marked @ 0.4

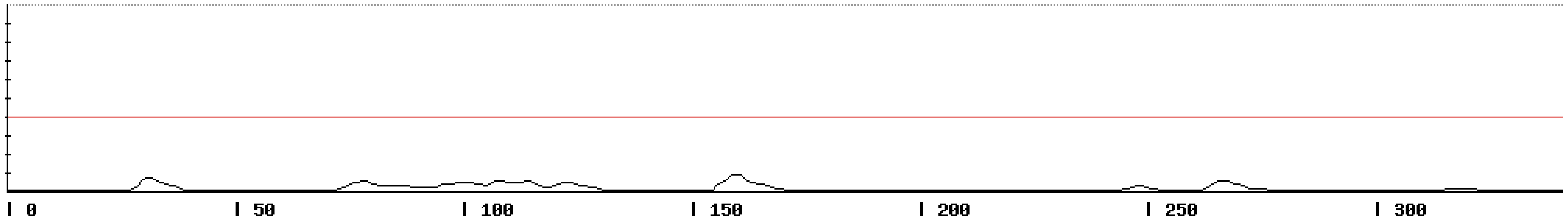

Predictions for Nicotianatabacum_XP_016464144.1

Viterbi Path

Viterbi Path

Posterior @ 0.4

Posterior @ 0.4

Posterior Graphic. Threshold Marked @ 0.4

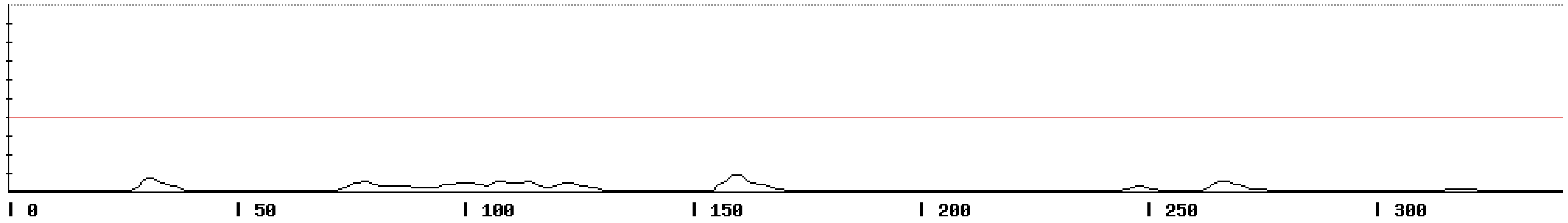

Predictions for Nicotianatabacum_XP_016464323.1

Viterbi Path

Viterbi Path

Posterior @ 0.4

Posterior @ 0.4

Posterior Graphic. Threshold Marked @ 0.4

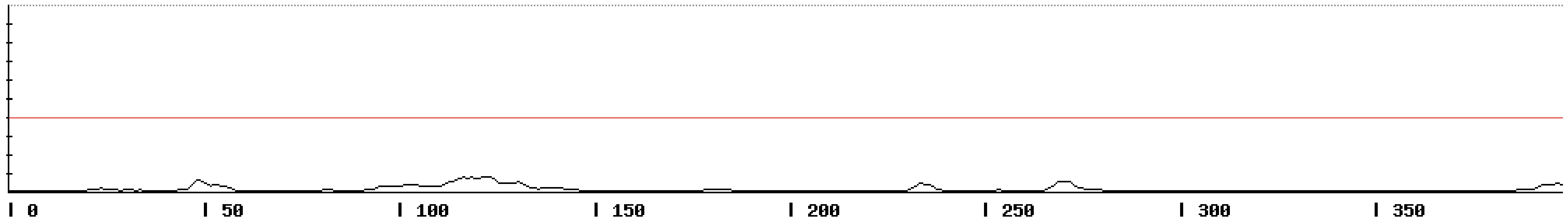

Predictions for Nicotianatabacum_XP_016466600.1

Viterbi Path

Viterbi Path

Posterior @ 0.4

Posterior @ 0.4

Posterior Graphic. Threshold Marked @ 0.4

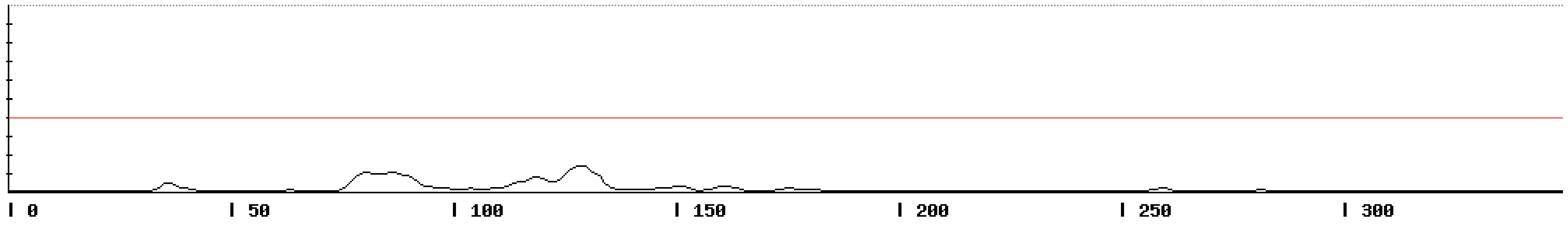

Predictions for Nicotianatabacum_XP_016466638.1

Viterbi Path

Viterbi Path

Posterior @ 0.4

Posterior @ 0.4

Posterior Graphic. Threshold Marked @ 0.4

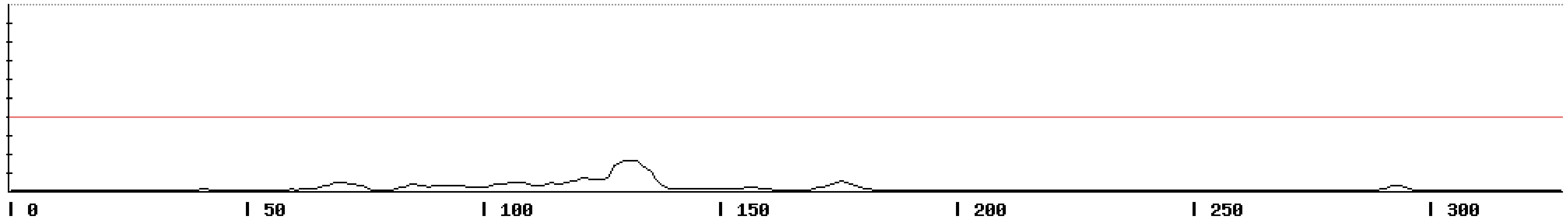

Predictions for Nicotianatabacum_XP_016466987.1

Viterbi Path

Viterbi Path

Posterior @ 0.4

Posterior @ 0.4

Posterior Graphic. Threshold Marked @ 0.4

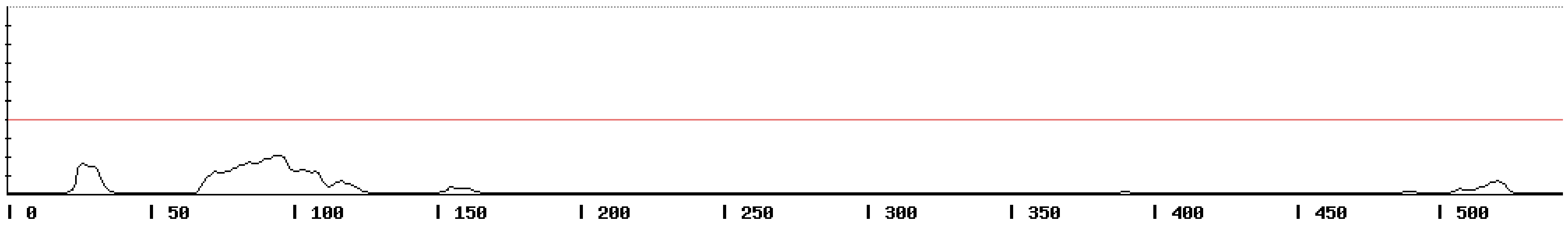

Predictions for Nicotianatabacum_XP_016466988.1

Viterbi Path

Viterbi Path

Posterior @ 0.4

Posterior @ 0.4

Posterior Graphic. Threshold Marked @ 0.4

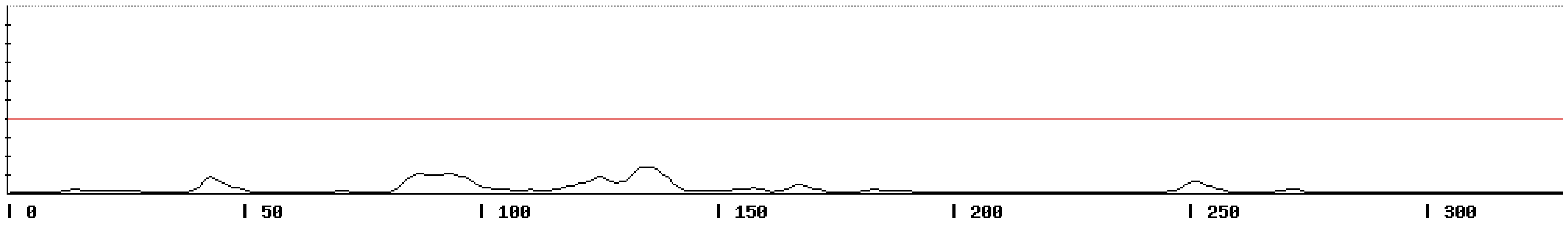

Predictions for Nicotianatabacum_XP_016467346.1	
Viterbi Path	
Viterbi Path	
Posterior @ 0.4	
Posterior @ 0.4	
Posterior Graphic. Threshold Marked @ 0.4	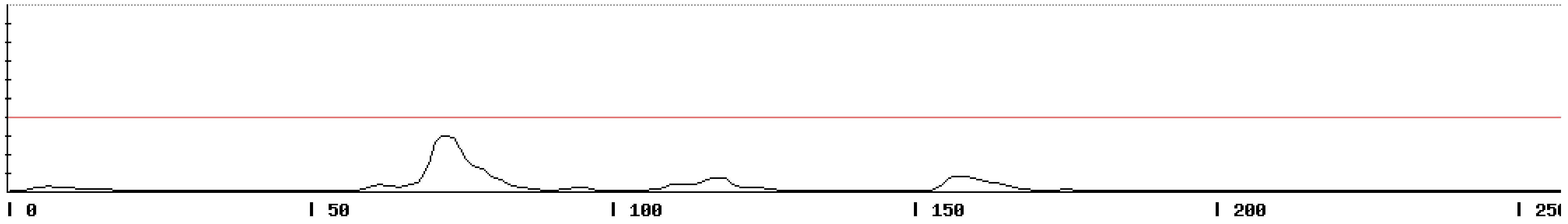

Predictions for Nicotianatabacum_XP_016467787.1	
Viterbi Path	
Viterbi Path	
Posterior @ 0.4	80 - NGAKTNRATKRGYWKTTGKDRAVHHKSK - 107
Posterior @ 0.4	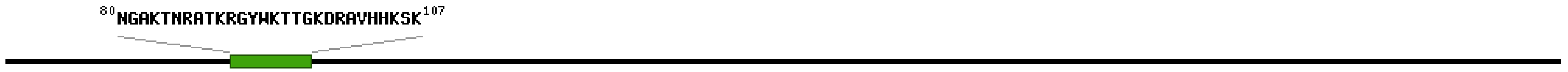
Posterior Graphic. Threshold Marked @ 0.4	

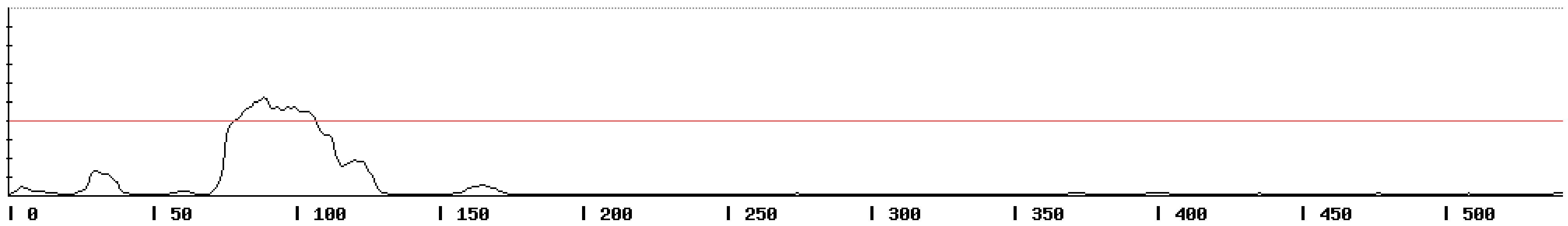

Predictions for Nicotianatabacum_XP_016468466.1

Viterbi Path

Viterbi Path

Posterior @ 0.4

Posterior @ 0.4

Posterior Graphic. Threshold Marked @ 0.4

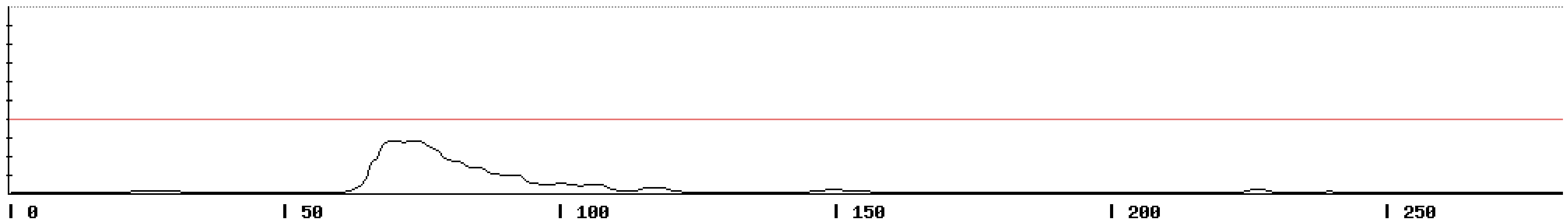

Predictions for Nicotianatabacum_XP_016468971.1

Viterbi Path

Viterbi Path

Posterior @ 0.4

Posterior @ 0.4

Posterior Graphic. Threshold Marked @ 0.4

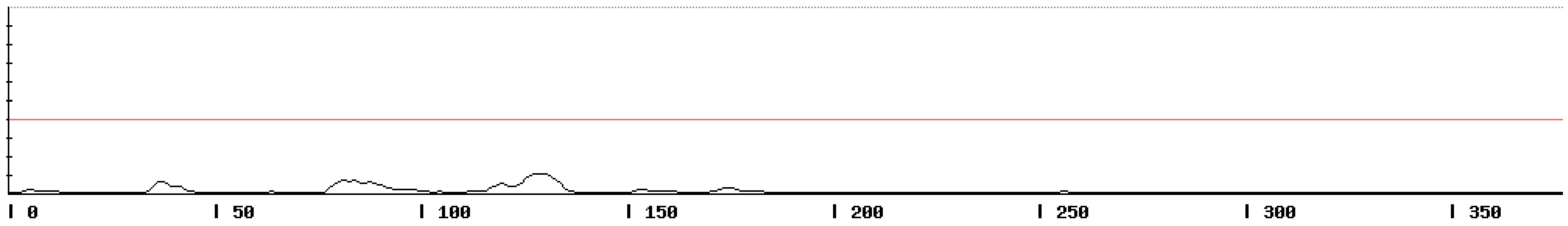

Predictions for Nicotianatabacum_XP_016469037.1

Viterbi Path

Viterbi Path

Posterior @ 0.4

Posterior @ 0.4

Posterior Graphic. Threshold Marked @ 0.4

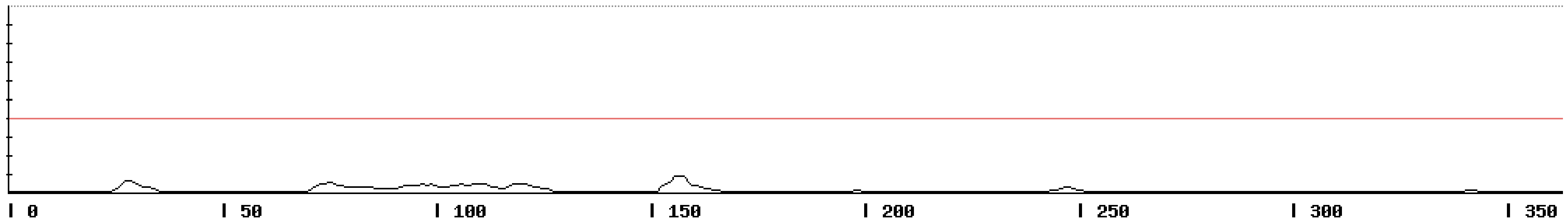

Predictions for Nicotianatabacum_XP_016469053.1

Viterbi Path

Viterbi Path

Posterior @ 0.4

Posterior @ 0.4

Posterior Graphic. Threshold Marked @ 0.4

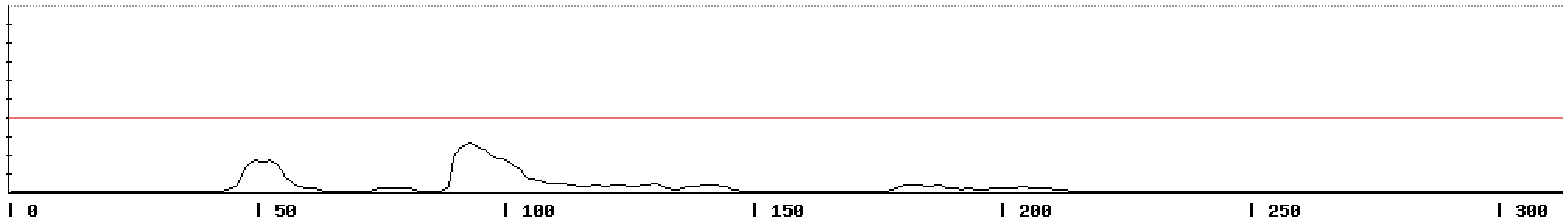

Predictions for Nicotianatabacum_XP_016469110.1

Viterbi Path

Viterbi Path

Posterior @ 0.4

Posterior @ 0.4

Posterior Graphic. Threshold Marked @ 0.4

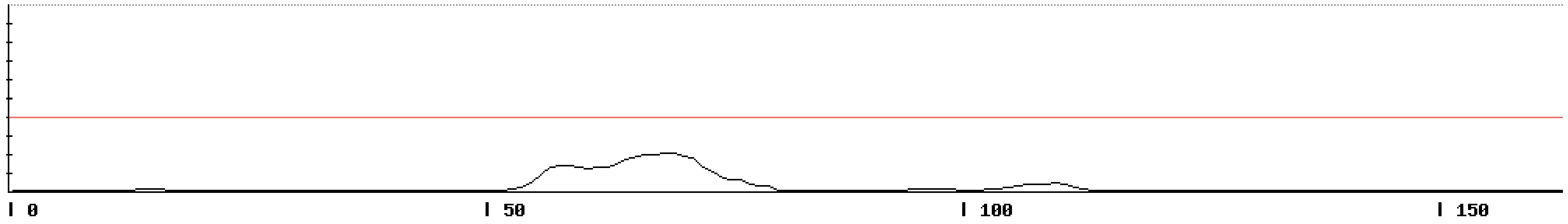

Predictions for Nicotianatabacum_XP_016469611.1

Viterbi Path

Viterbi Path

Posterior @ 0.4

Posterior @ 0.4

Posterior Graphic. Threshold Marked @ 0.4

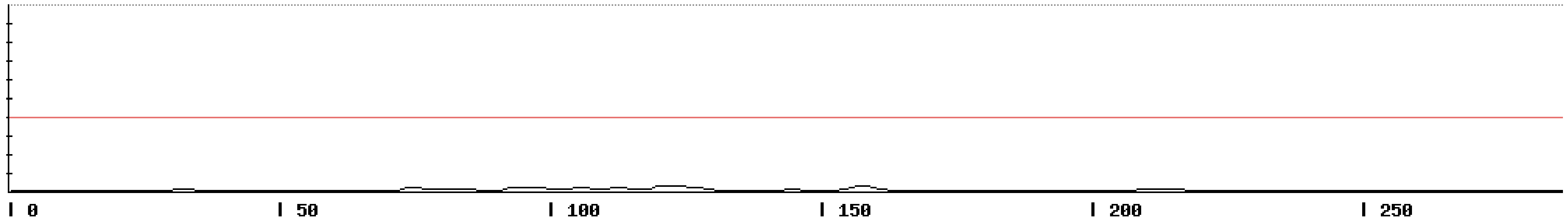

Predictions for Nicotianatabacum_XP_016470618.1

Viterbi Path

Viterbi Path

Posterior @ 0.4

Posterior @ 0.4

Posterior Graphic. Threshold Marked @ 0.4

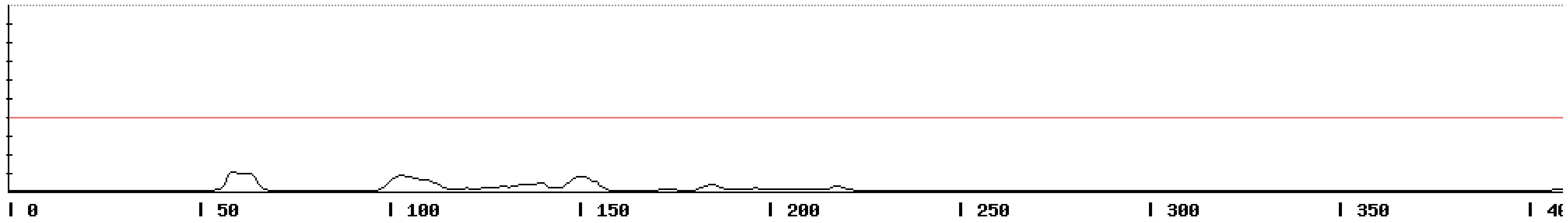

Predictions for Nicotianatabacum_XP_016471013.1

Viterbi Path

Viterbi Path

Posterior @ 0.4

Posterior @ 0.4

Posterior Graphic. Threshold Marked @ 0.4

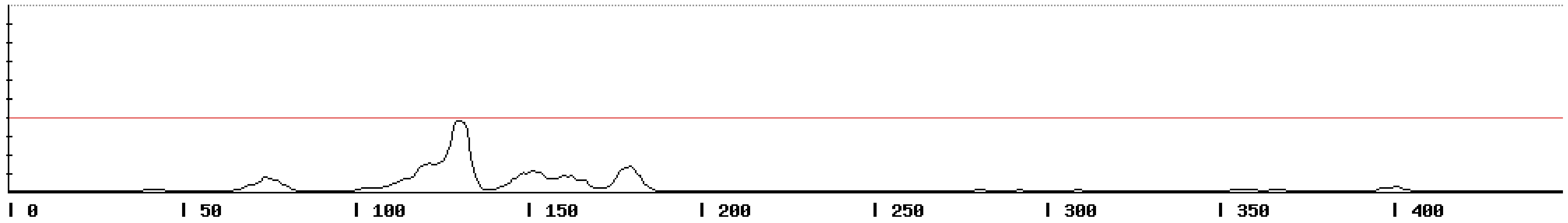

Predictions for Nicotianatabacum_XP_016471014.1

Viterbi Path

Viterbi Path

Posterior @ 0.4

Posterior @ 0.4

Posterior Graphic. Threshold Marked @ 0.4

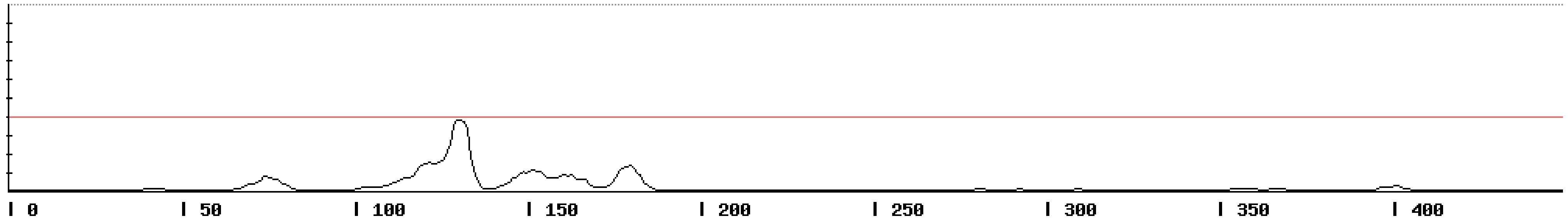

Predictions for Nicotianatabacum_XP_016471015.1

Viterbi Path

Viterbi Path

Posterior @ 0.4

Posterior @ 0.4

Posterior Graphic. Threshold Marked @ 0.4

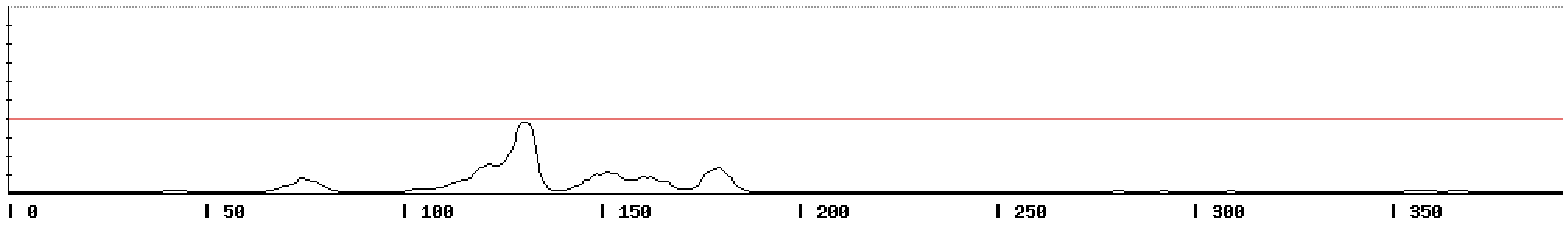

Predictions for Nicotianatabacum_XP_016471465.1

Viterbi Path

Viterbi Path

Posterior @ 0.4

Posterior @ 0.4

Posterior Graphic. Threshold Marked @ 0.4

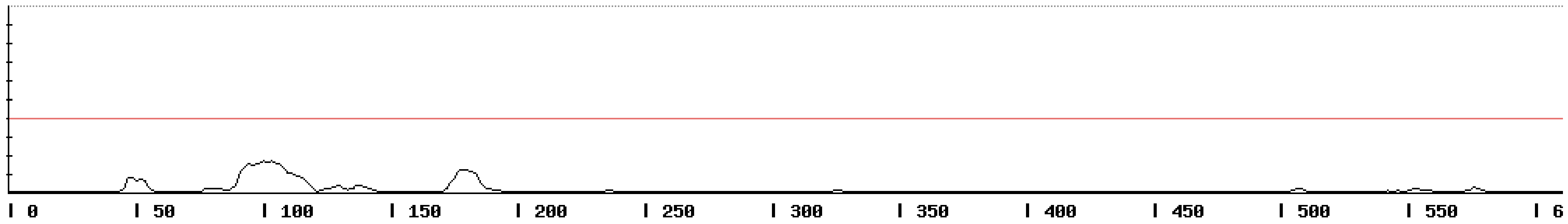

Predictions for Nicotianatabacum_XP_016471466.1

Viterbi Path	
Viterbi Path	
Posterior @ 0.4	
Posterior @ 0.4	
Posterior Graphic. Threshold Marked @ 0.4	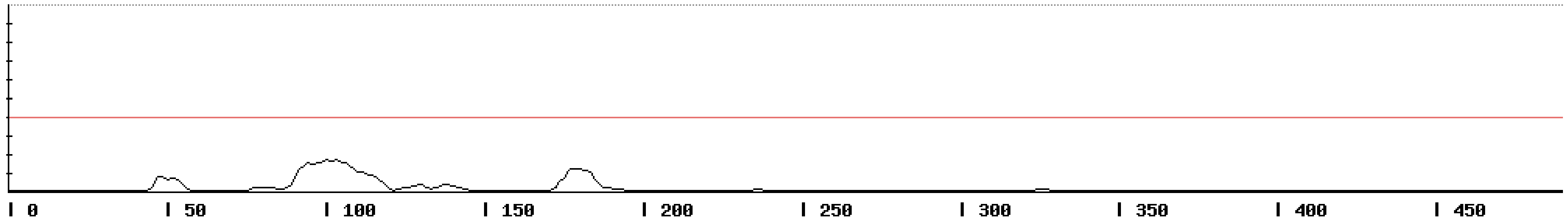

Predictions for Nicotianatabacum_XP_016471988.1

Viterbi Path	
Viterbi Path	
Posterior @ 0.4	136 - RKRRKV - 141<br>151 - RWHKTGKTRPVVVKGKVKGHKK - 172
Posterior @ 0.4	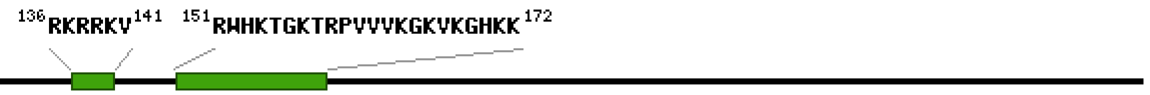
Posterior Graphic. Threshold Marked @ 0.4	

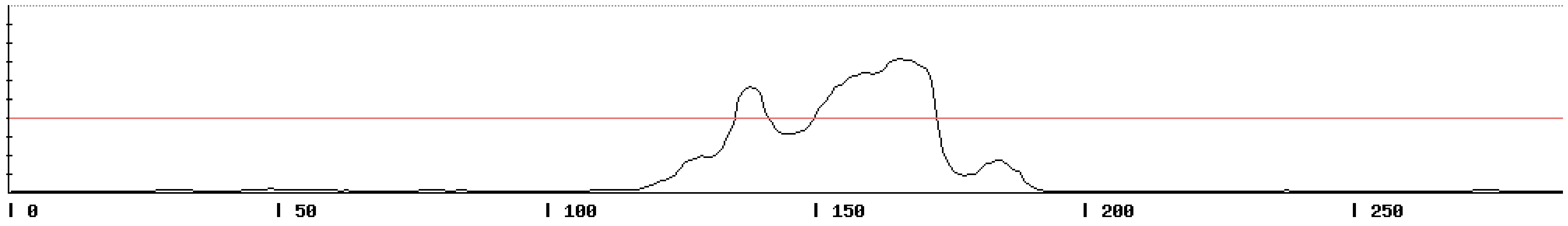

Predictions for Nicotianatabacum_XP_016471991.1	
Viterbi Path	
Viterbi Path	
Posterior @ 0.4	
Posterior @ 0.4	
Posterior Graphic. Threshold Marked @ 0.4	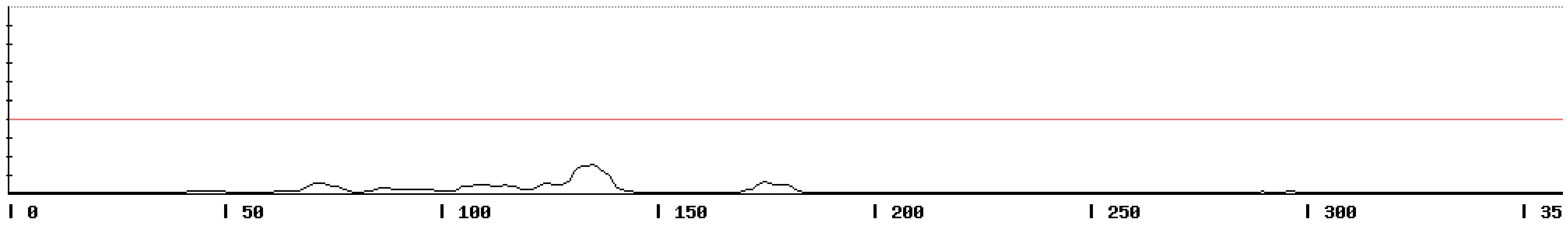

Predictions for Nicotianatabacum_XP_016472368.1	
Viterbi Path	
Viterbi Path	
Posterior @ 0.4	
Posterior @ 0.4	

Posterior Graphic. Threshold Marked @ 0.4

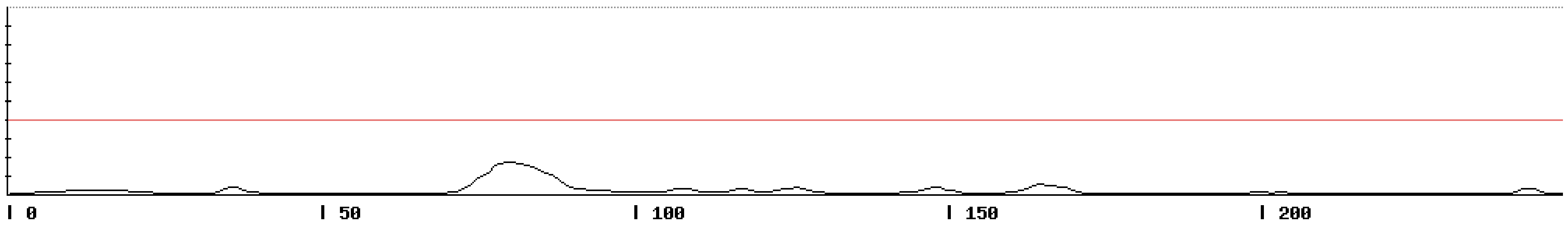

Predictions for Nicotianatabacum_XP_016473313.1

Viterbi Path

Viterbi Path

Posterior @ 0.4

Posterior @ 0.4

Posterior Graphic. Threshold Marked @ 0.4

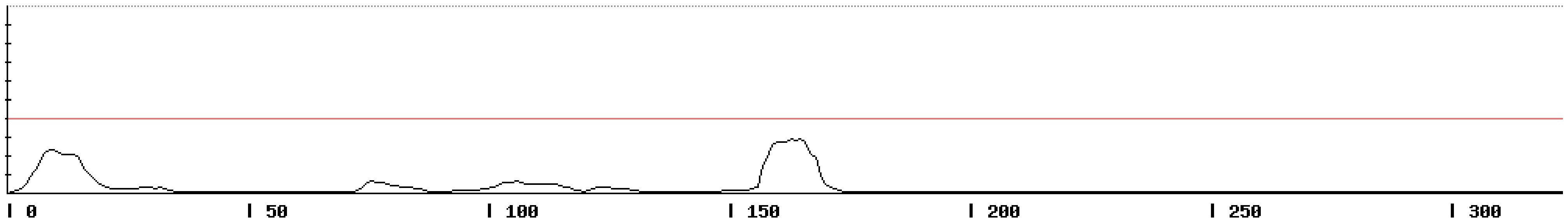

Predictions for Nicotianatabacum_XP_016473781.1

Viterbi Path

Viterbi Path

Posterior @ 0.4

Posterior @ 0.4

Posterior Graphic. Threshold Marked @ 0.4

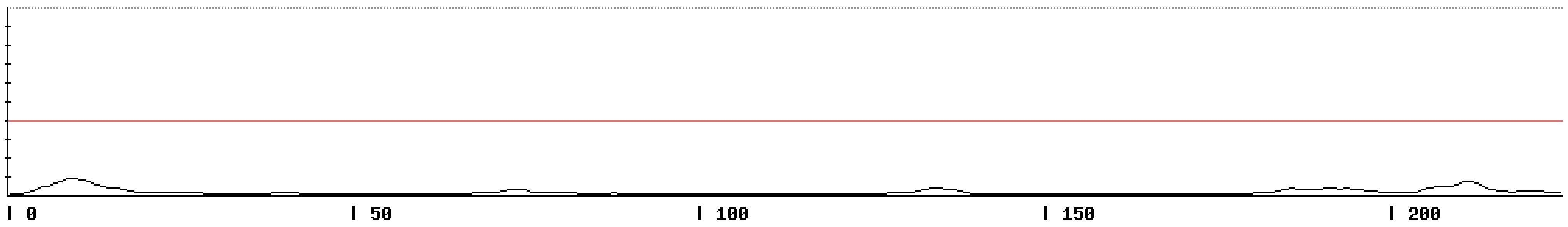

Predictions for Nicotianatabacum_XP_016473782.1

Viterbi Path

Viterbi Path

Posterior @ 0.4

Posterior @ 0.4

Posterior Graphic. Threshold Marked @ 0.4

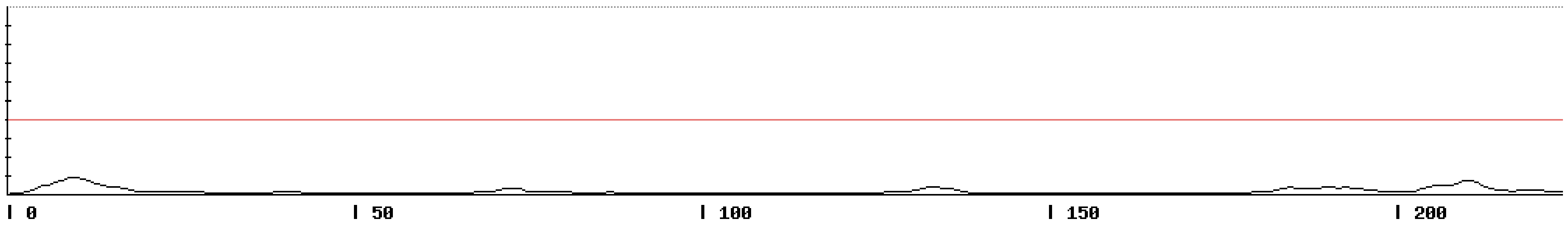

Predictions for Nicotianatabacum_XP_016473927.1

Viterbi Path

Viterbi Path

Posterior @ 0.4	79 - KRTKAGGLRFSRGLIGGGSWKGLDKRKPVFKGSKLVG - 115
Posterior @ 0.4	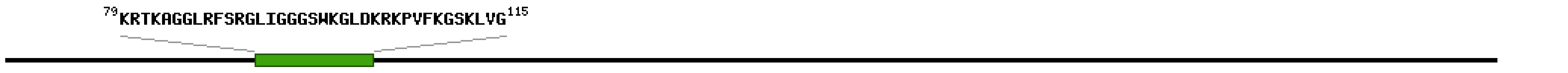
Posterior Graphic. Threshold Marked @ 0.4	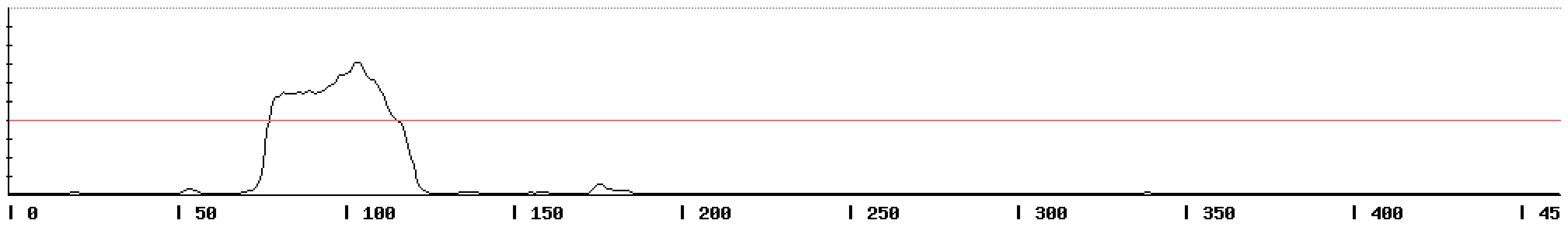

Predictions for Nicotianatabacum_XP_016474024.1

Viterbi Path	
Viterbi Path	
Posterior @ 0.4	91 - KKK - 93
Posterior @ 0.4	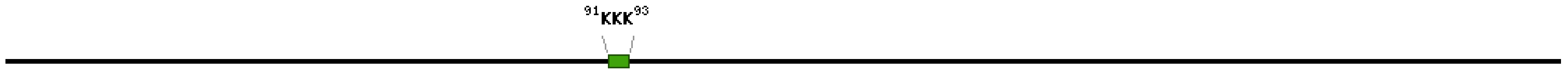
Posterior Graphic. Threshold Marked @ 0.4	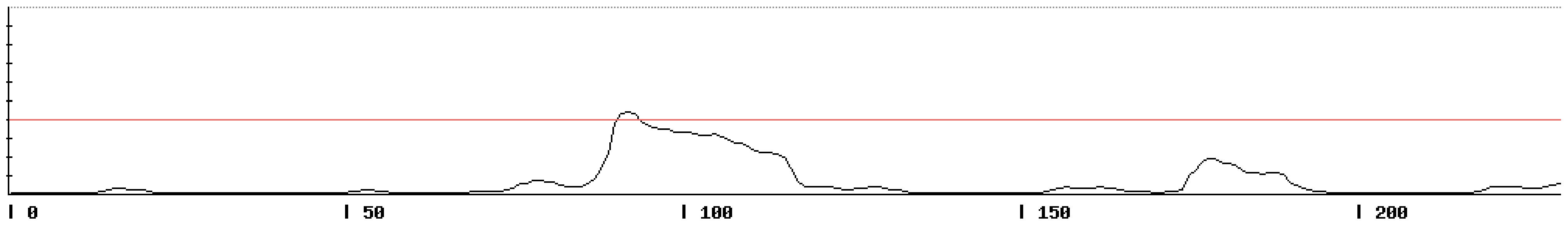

Predictions for Nicotianatabacum_XP_016474545.1

Viterbi Path	161 - KIKKKGKPGDKKKG - 174
Viterbi Path	

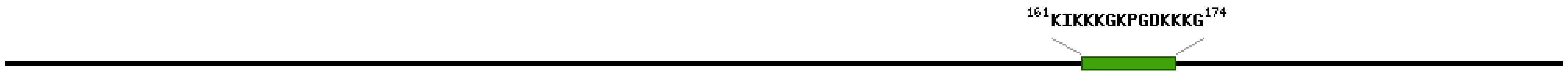

Posterior @ 0.4 — 161 - KIKKKGKPGDKKKG - 174

Posterior @ 0.4

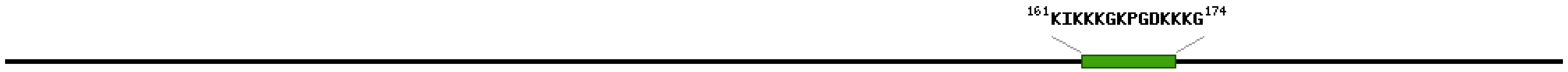

Posterior Graphic. Threshold Marked @ 0.4

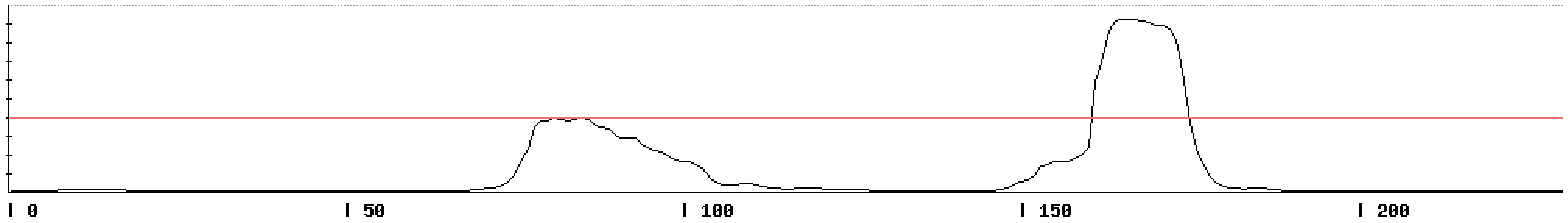

Predictions for Nicotianatabacum_XP_016475806.1

Viterbi Path

Viterbi Path

Posterior @ 0.4 — 76 - KLKKKNKARH - 85

Posterior @ 0.4

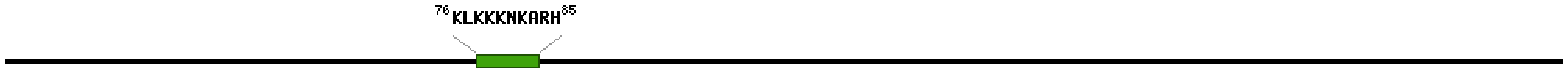

Posterior Graphic. Threshold Marked @ 0.4

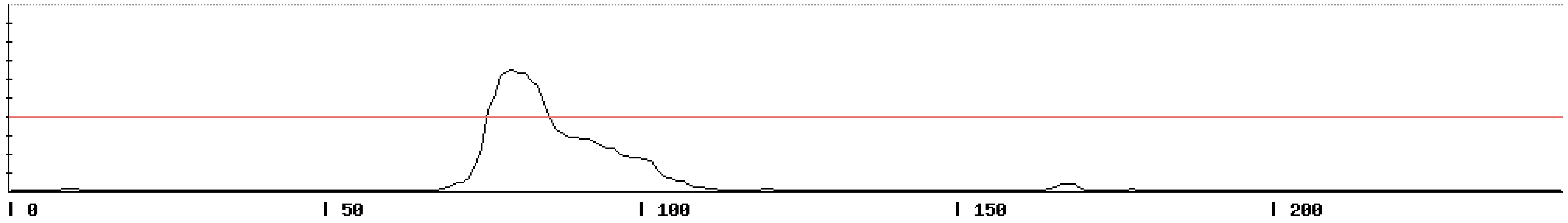

Predictions for Nicotianatabacum_XP_016476258.1

Viterbi Path	
Viterbi Path	
Posterior @ 0.4	
Posterior @ 0.4	
Posterior Graphic. Threshold Marked @ 0.4	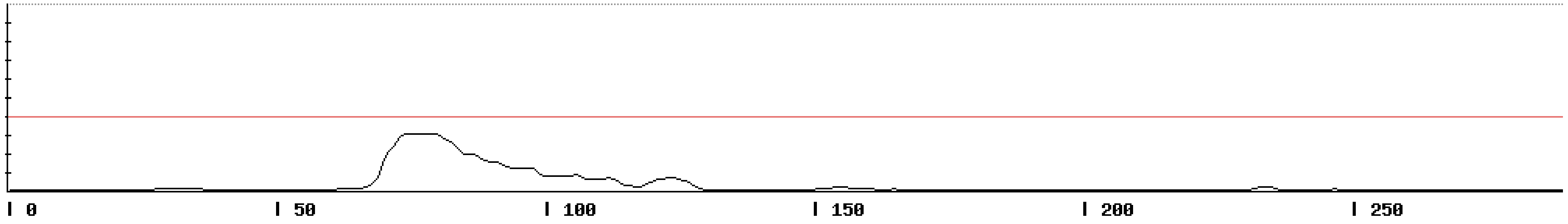

Predictions for Nicotianatabacum_XP_016476451.1

Viterbi Path	
Viterbi Path	
Posterior @ 0.4	68 - KLKRFKNSTKKFSRTVGQGTWKGQSGGKPIKHRSKNIIVGFRRSLKYKSGIKK - 120
Posterior @ 0.4	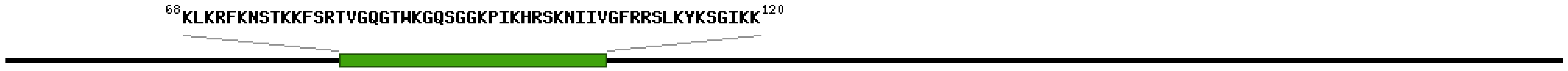
Posterior Graphic. Threshold Marked @ 0.4	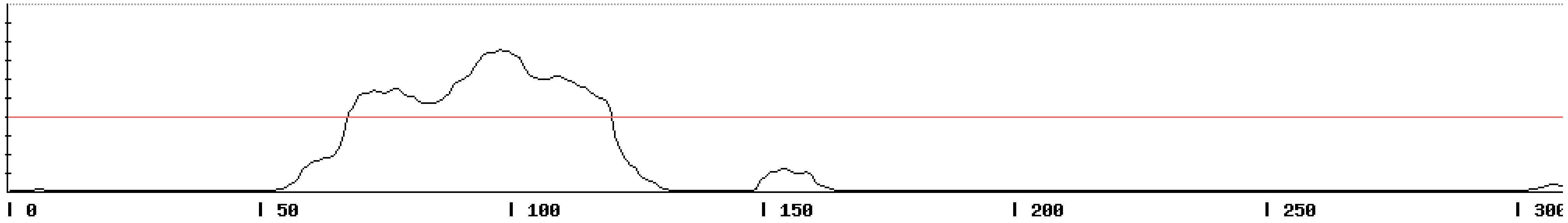

Predictions for Nicotianatabacum_XP_016476680.1	
Viterbi Path	
Viterbi Path	
Posterior @ 0.4	131 - GKRKRRKISDENNLRKEHVRWHK - 153
Posterior @ 0.4	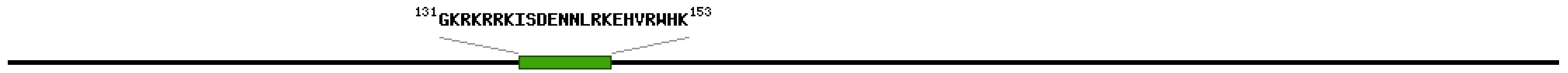
Posterior Graphic. Threshold Marked @ 0.4	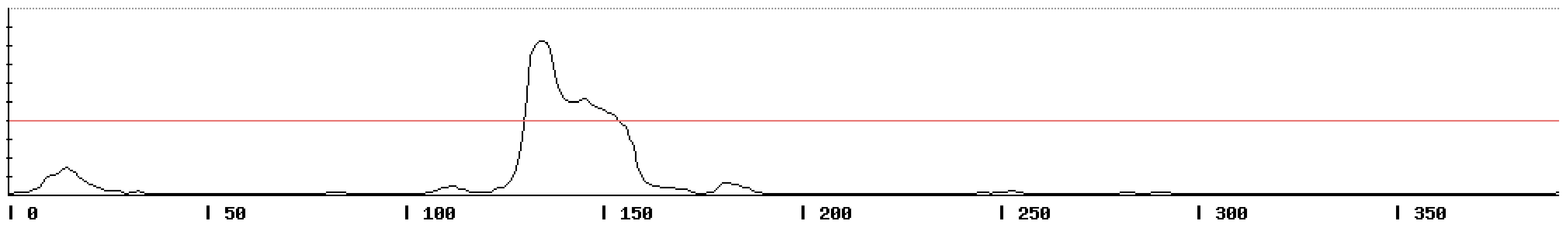

Predictions for Nicotianatabacum_XP_016477162.1	
Viterbi Path	162 - KIKKKGKPGDKKKG - 175
Viterbi Path	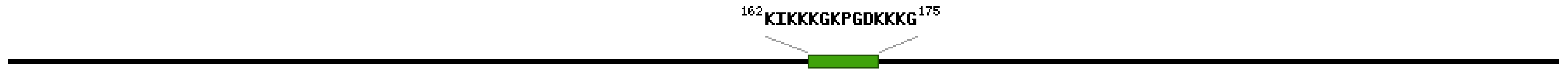
Posterior @ 0.4	79 - KKEKPTWKRVSRTVGKGTWKPQGKG - 103
	162 - KIKKKGKPGDKKKG - 175
Posterior @ 0.4	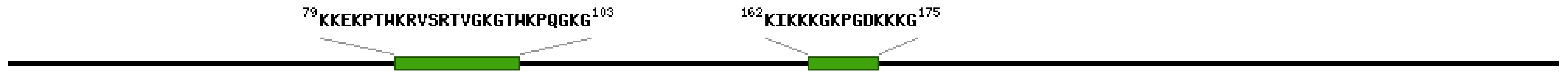
Posterior Graphic. Threshold Marked @ 0.4	

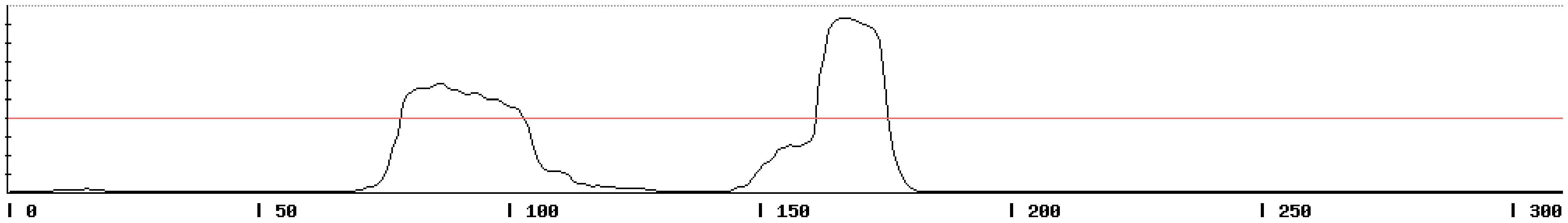

Predictions for Nicotianatabacum_XP_016478779.1	
Viterbi Path	
Viterbi Path	
Posterior @ 0.4	
Posterior @ 0.4	
Posterior Graphic. Threshold Marked @ 0.4	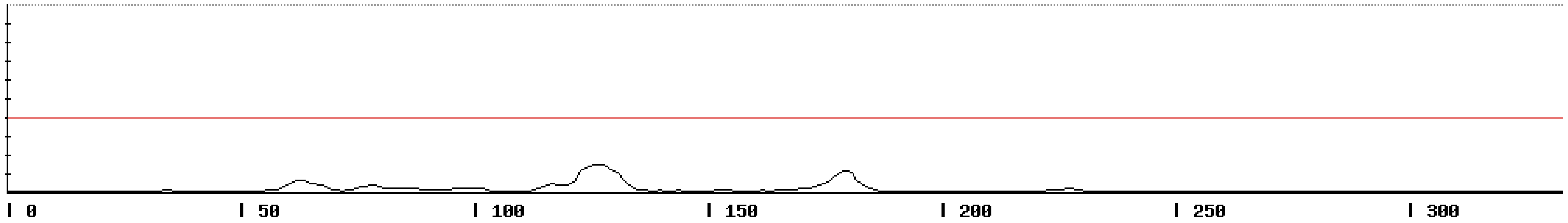

Predictions for Nicotianatabacum_XP_016478918.1	
Viterbi Path	
Viterbi Path	
Posterior @ 0.4	
Posterior @ 0.4	

Posterior Graphic. Threshold Marked @ 0.4

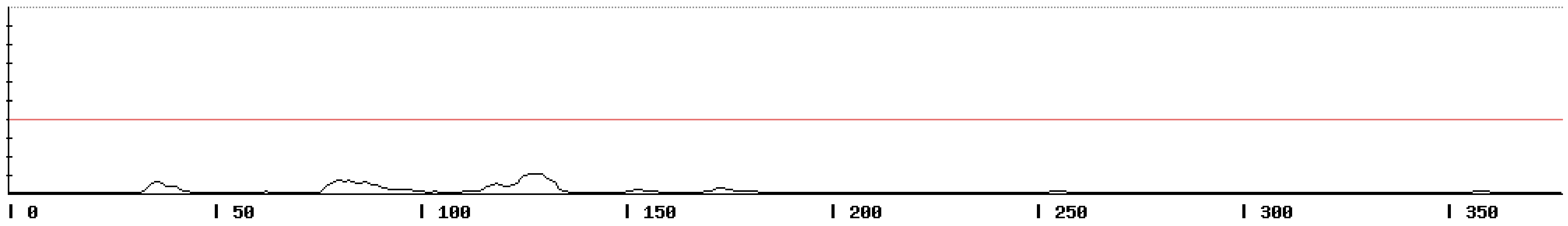

Predictions for Nicotianatabacum_XP_016480941.1

Viterbi Path

Viterbi Path

Posterior @ 0.4

Posterior @ 0.4

Posterior Graphic. Threshold Marked @ 0.4

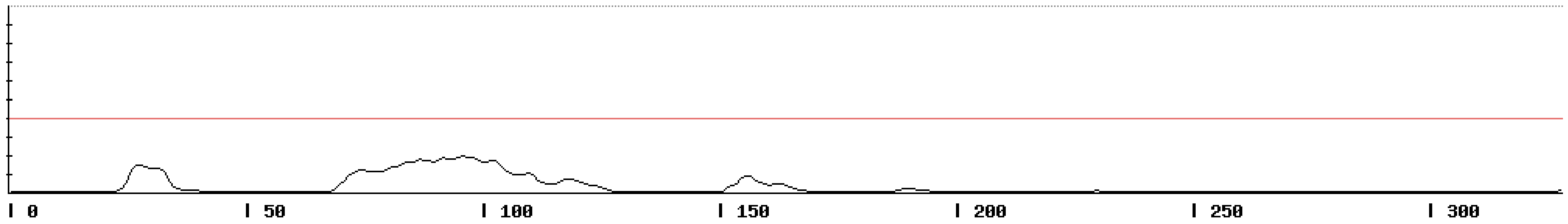

Predictions for Nicotianatabacum_XP_016481807.1

Viterbi Path

Viterbi Path

Posterior @ 0.4

Posterior @ 0.4

Posterior Graphic. Threshold Marked @ 0.4	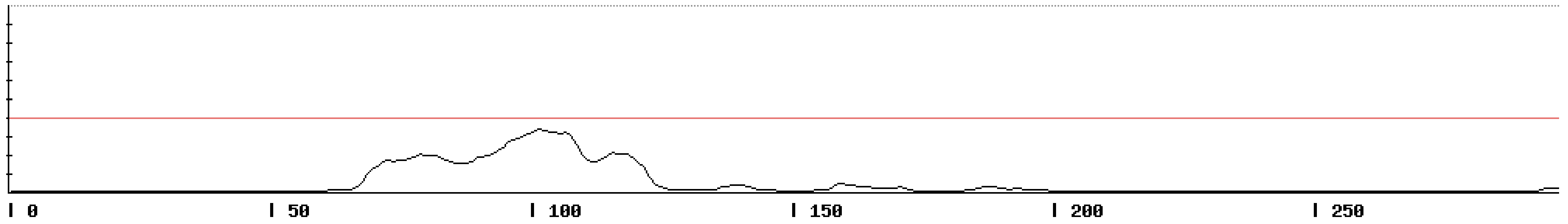

Predictions for Nicotianatabacum_XP_016482046.1

Viterbi Path	
Viterbi Path	
Posterior @ 0.4	
Posterior @ 0.4	
Posterior Graphic. Threshold Marked @ 0.4	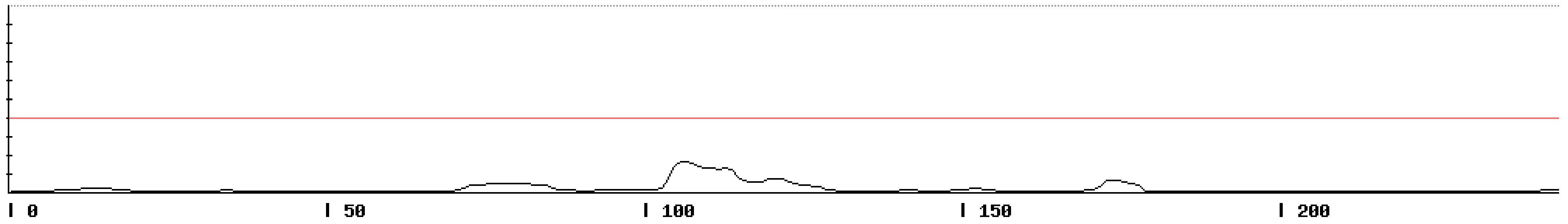

Predictions for Nicotianatabacum_XP_016482738.1

Viterbi Path	
Viterbi Path	

Posterior @ 0.4

Posterior @ 0.4

Posterior Graphic. Threshold Marked @ 0.4

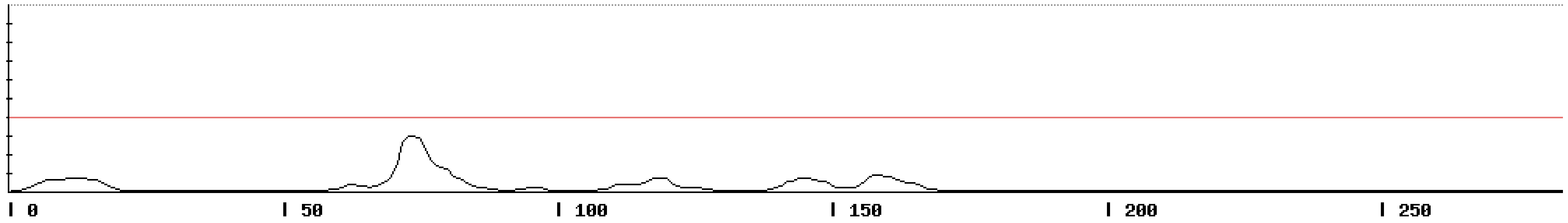

Predictions for Nicotianatabacum_XP_016482844.1

Viterbi Path

Viterbi Path

Posterior @ 0.4

Posterior @ 0.4

Posterior Graphic. Threshold Marked @ 0.4

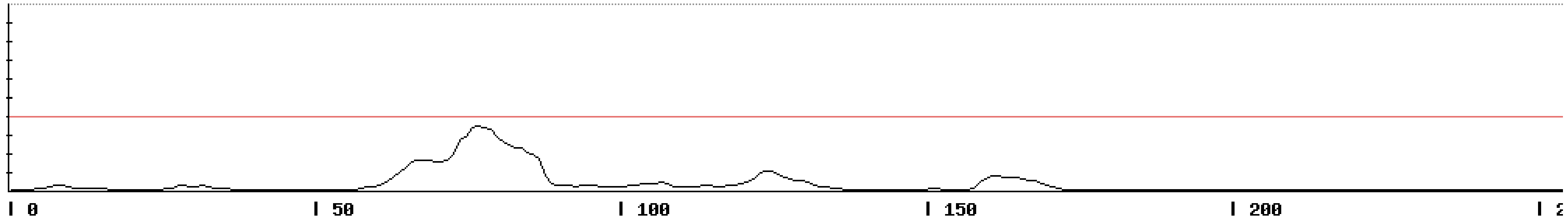

Predictions for Nicotianatabacum_XP_016482997.1

Viterbi Path

Viterbi Path

Posterior @ 0.4

Posterior @ 0.4

Posterior Graphic. Threshold Marked @ 0.4

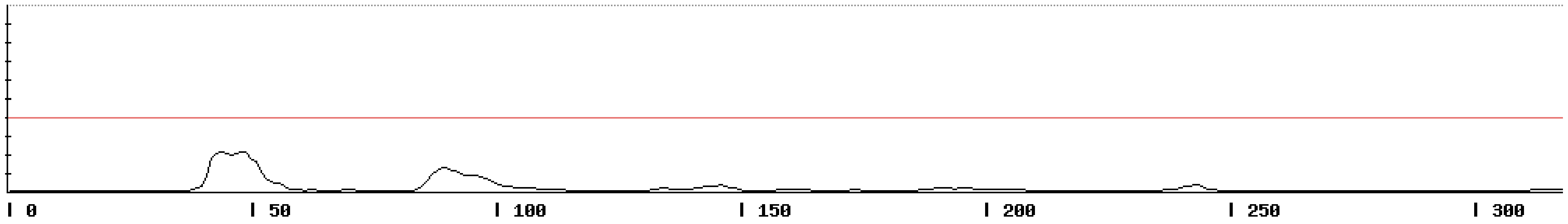

Predictions for Nicotianatabacum_XP_016483213.1

Viterbi Path

Viterbi Path

Posterior @ 0.4

Posterior @ 0.4

Posterior Graphic. Threshold Marked @ 0.4

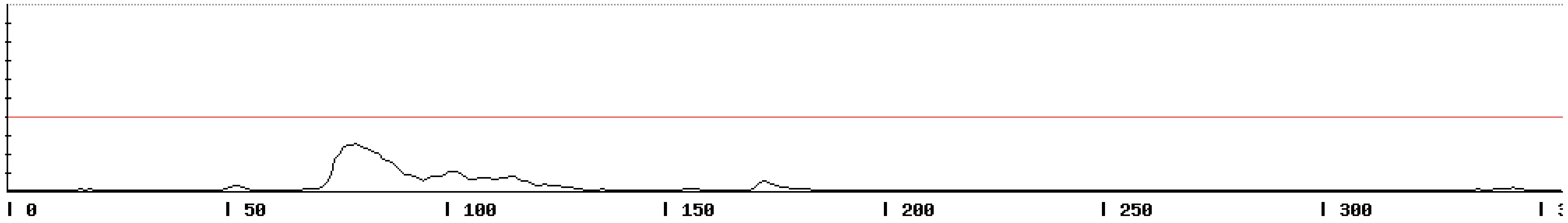

Predictions for Nicotianatabacum_XP_016483421.1

Viterbi Path	
Viterbi Path	
Posterior @ 0.4	
Posterior @ 0.4	
Posterior Graphic. Threshold Marked @ 0.4	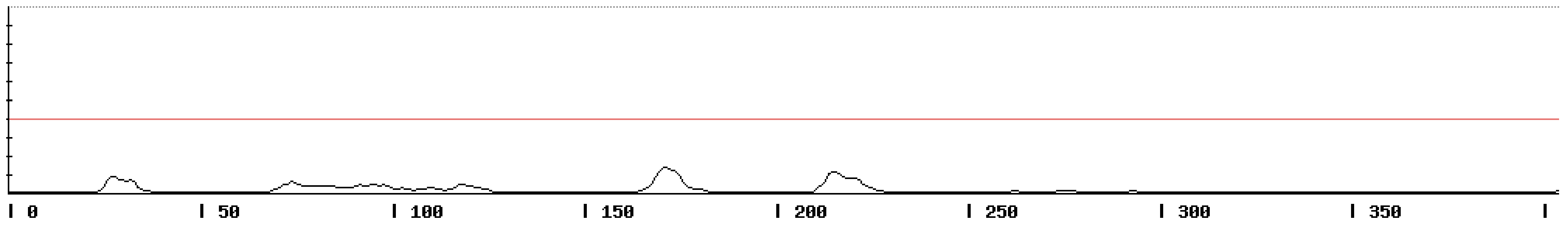

Predictions for Nicotianatabacum_XP_016483755.1

Viterbi Path	
Viterbi Path	
Posterior @ 0.4	
Posterior @ 0.4	
Posterior Graphic. Threshold Marked @ 0.4	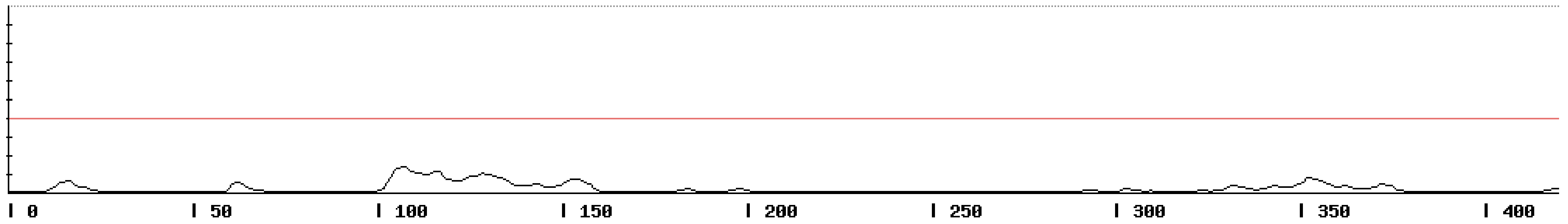

Predictions for Nicotianatabacum_XP_016484530.1

Viterbi Path

Viterbi Path

Posterior @ 0.4

Posterior @ 0.4

Posterior Graphic. Threshold Marked @ 0.4

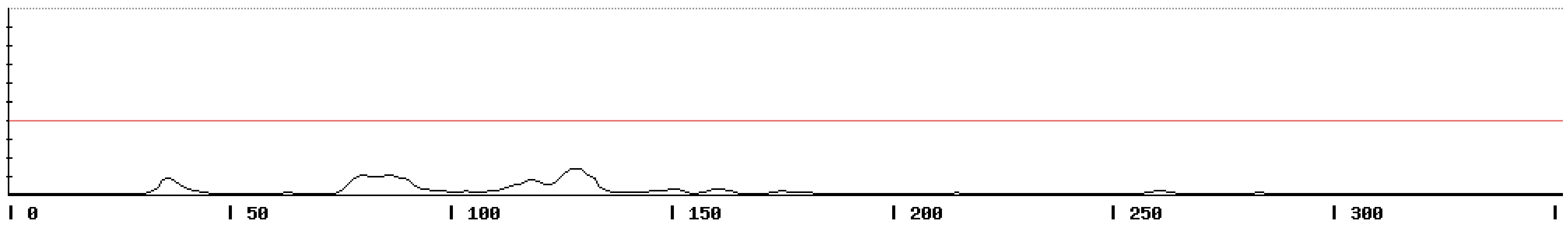

Predictions for Nicotianatabacum_XP_016484627.1

Viterbi Path

Viterbi Path

Posterior @ 0.4

Posterior @ 0.4

Posterior Graphic. Threshold Marked @ 0.4

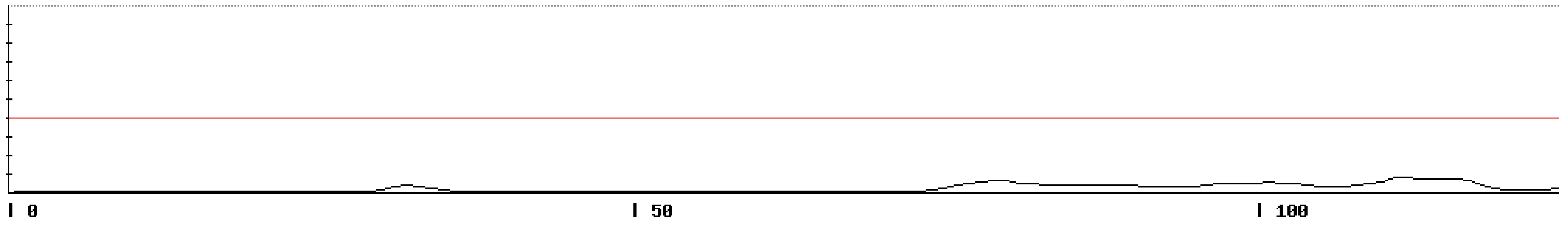

Predictions for Nicotianatabacum_XP_016484724.1

Viterbi Path

Viterbi Path

Posterior @ 0.4

Posterior @ 0.4

Posterior Graphic. Threshold Marked @ 0.4

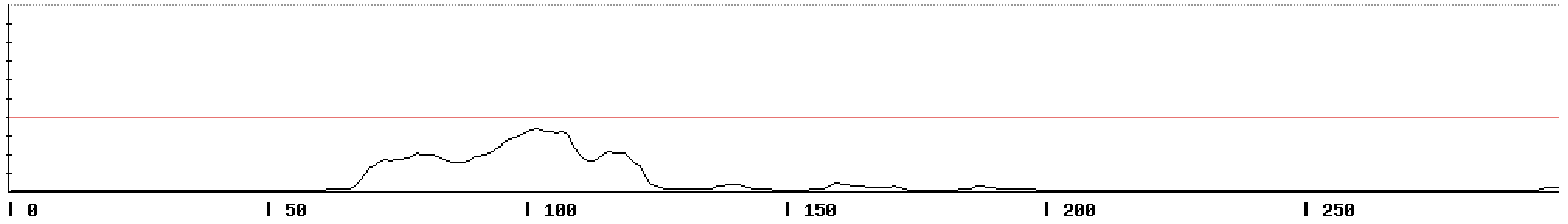

Predictions for Nicotianatabacum_XP_016484889.1

Viterbi Path

Viterbi Path

Posterior @ 0.4

Posterior @ 0.4

Posterior Graphic. Threshold Marked @ 0.4

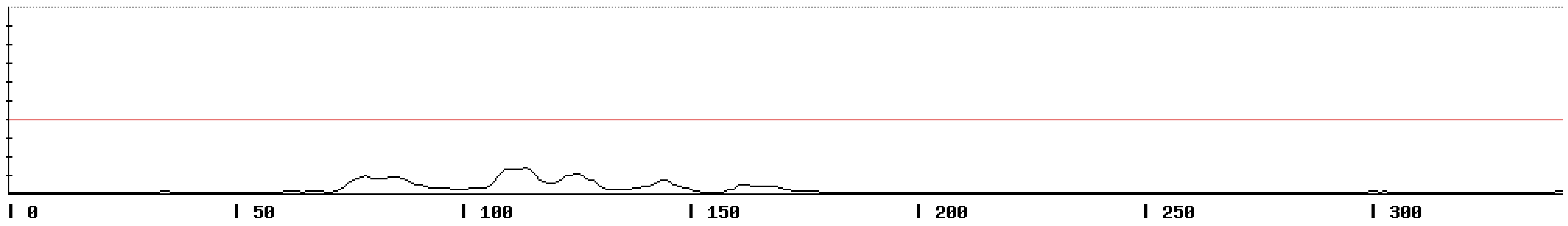

Predictions for Nicotianatabacum_XP_016485595.1

Viterbi Path

Viterbi Path

Posterior @ 0.4

Posterior @ 0.4

Posterior Graphic. Threshold Marked @ 0.4

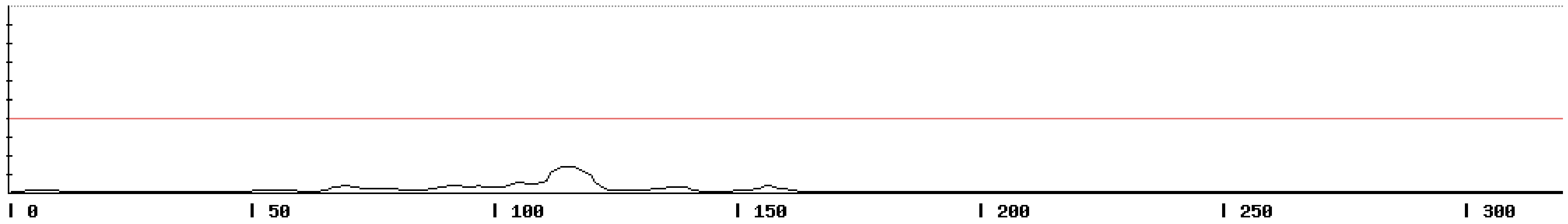

Predictions for Nicotianatabacum_XP_016485618.1

Viterbi Path

Viterbi Path

Posterior @ 0.4

Posterior @ 0.4

Posterior Graphic. Threshold Marked @ 0.4

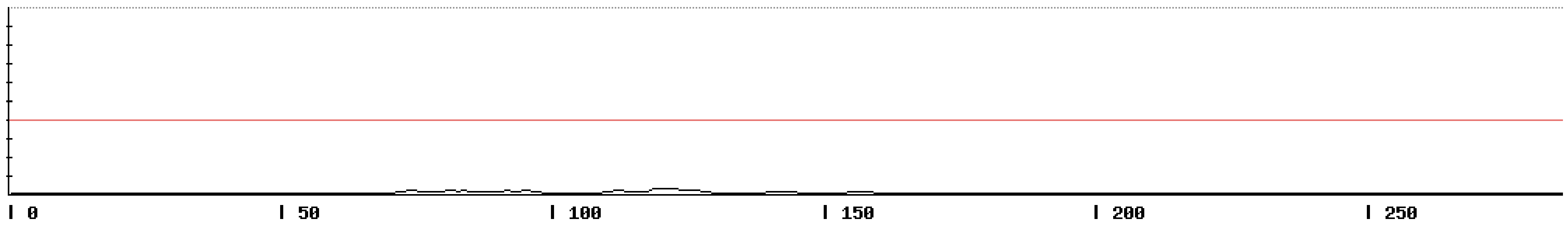

Predictions for Nicotianatabacum_XP_016486689.1

Viterbi Path

Viterbi Path

Posterior @ 0.4

Posterior @ 0.4

Posterior Graphic. Threshold Marked @ 0.4

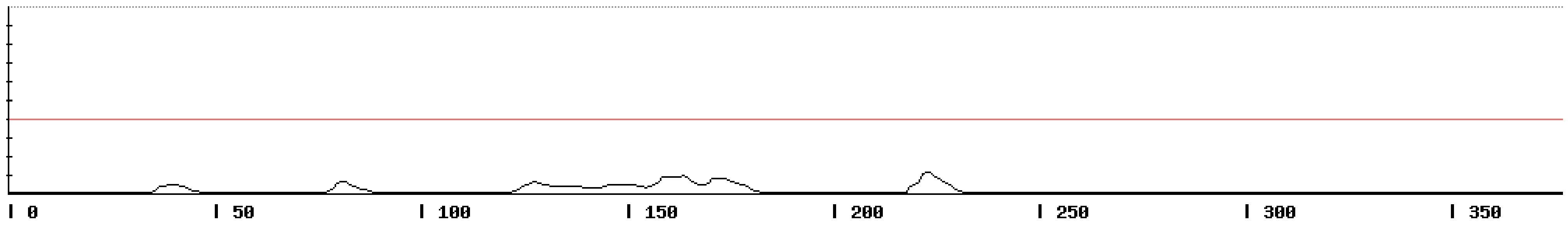

Predictions for Nicotianatabacum_XP_016487437.1

Viterbi Path

Viterbi Path

Posterior @ 0.4 | 83 - VLKGGRRRAGGGYWKKRGRIR - 103

Posterior @ 0.4

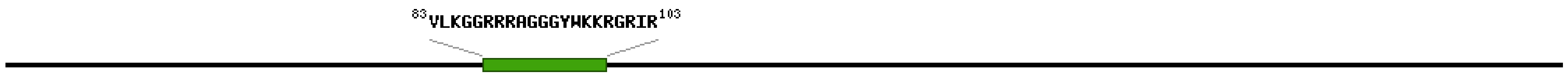

Posterior Graphic. Threshold Marked @ 0.4

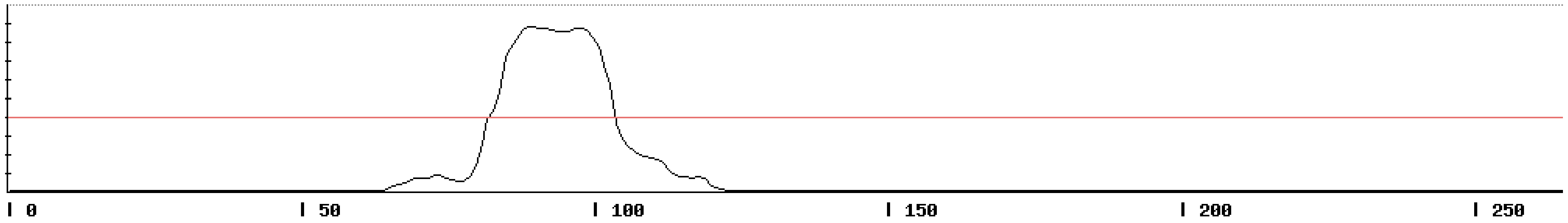

Predictions for Nicotianatabacum_XP_016487438.1

Viterbi Path

Viterbi Path

Posterior @ 0.4 | 83 - VLKGGRRRAGGGYWKKRGRIR - 103

Posterior @ 0.4

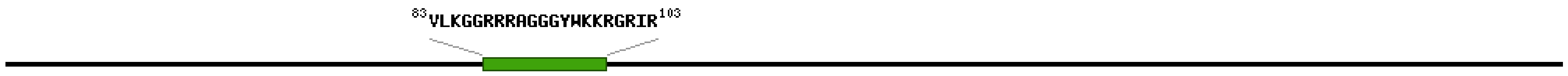

Posterior Graphic. Threshold Marked @ 0.4

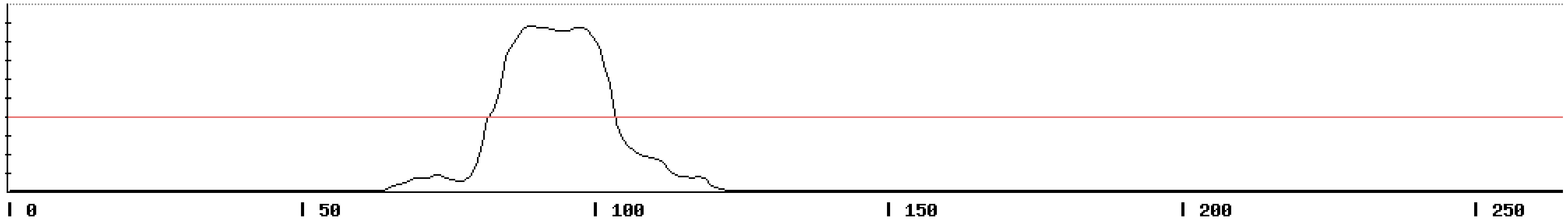

Predictions for Nicotianatabacum_XP_016487439.1

Viterbi Path

Viterbi Path

Posterior @ 0.4	83 - VLKGGRRRAGGGYWKKRGRIR - 103
Posterior @ 0.4	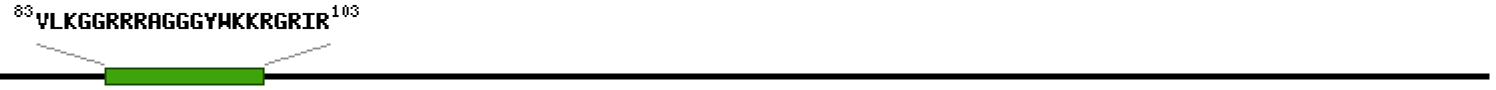
Posterior Graphic. Threshold Marked @ 0.4	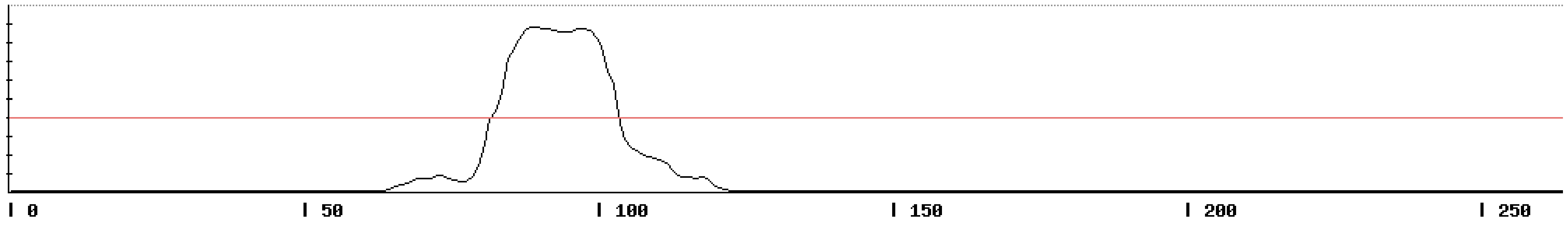

Predictions for Nicotianatabacum_XP_016487615.1

Viterbi Path	
Viterbi Path	
Posterior @ 0.4	
Posterior @ 0.4	
Posterior Graphic. Threshold Marked @ 0.4	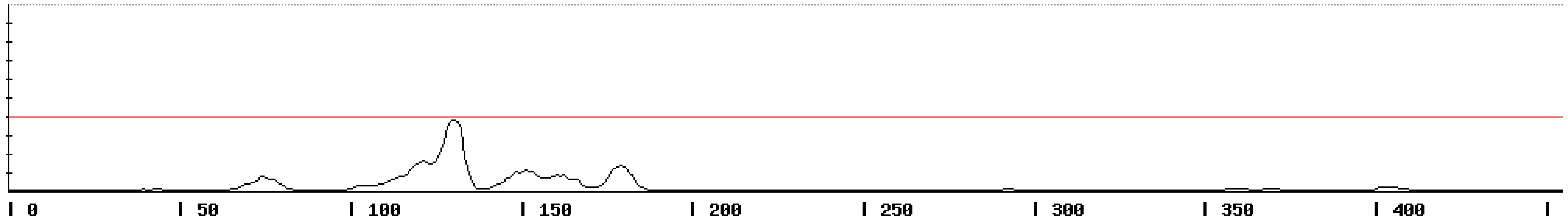

Predictions for Nicotianatabacum_XP_016487616.1

Viterbi Path

Viterbi Path

Posterior @ 0.4

Posterior @ 0.4

Posterior Graphic. Threshold Marked @ 0.4

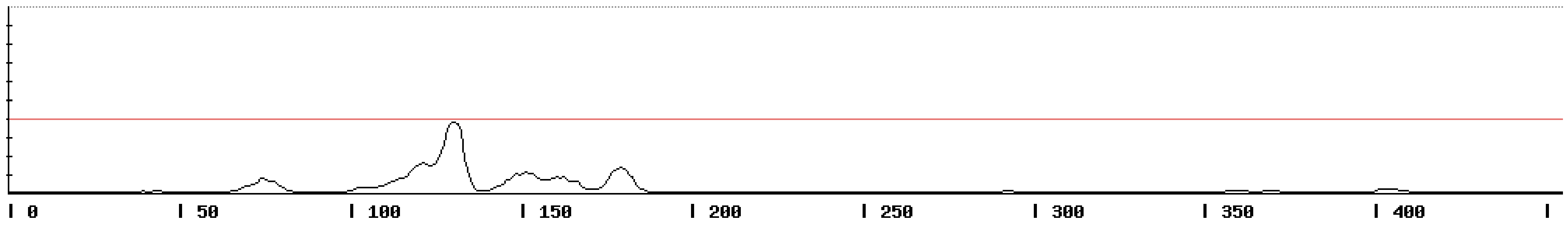

Predictions for Nicotianatabacum_XP_016487617.1

Viterbi Path

Viterbi Path

Posterior @ 0.4

Posterior @ 0.4

Posterior Graphic. Threshold Marked @ 0.4

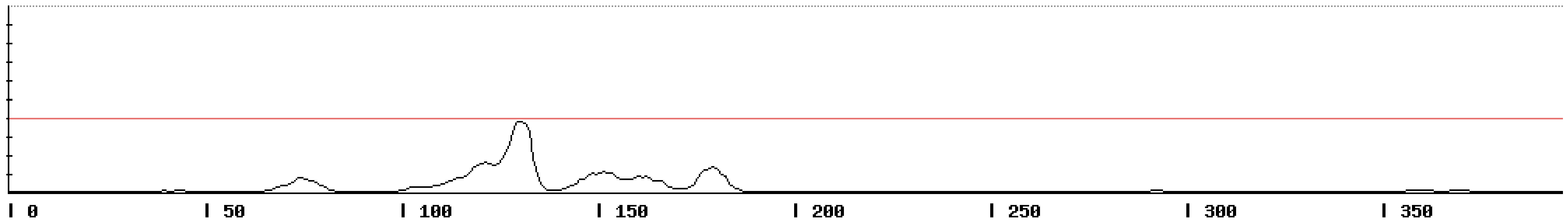

Predictions for Nicotianatabacum_XP_016487654.1	
Viterbi Path	
Viterbi Path	
Posterior @ 0.4	
Posterior @ 0.4	
Posterior Graphic. Threshold Marked @ 0.4	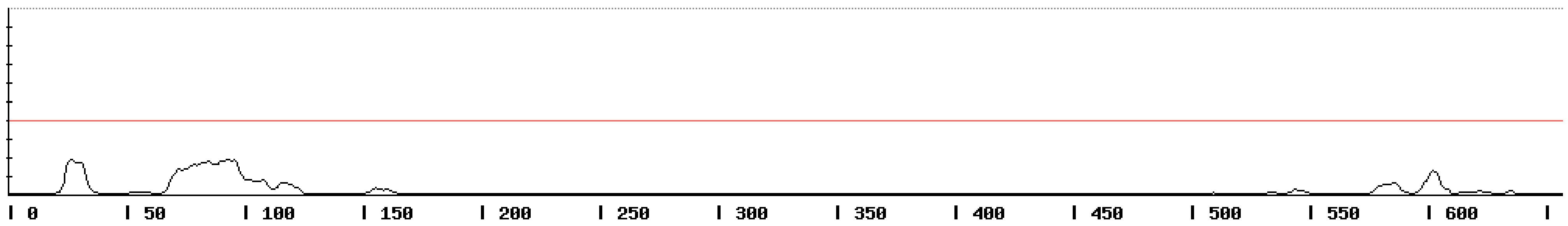

Predictions for Nicotianatabacum_XP_016487930.1	
Viterbi Path	
Viterbi Path	
Posterior @ 0.4	
Posterior @ 0.4	
Posterior Graphic. Threshold Marked @ 0.4	

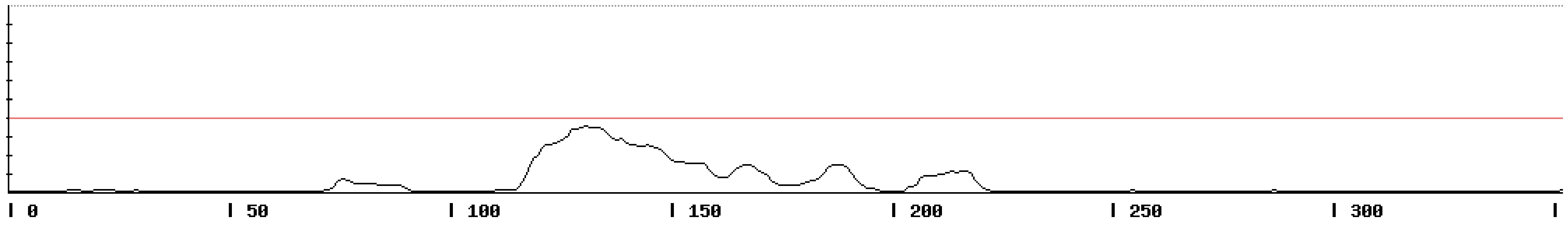

Predictions for Nicotianatabacum_XP_016488024.1

Viterbi Path	
Viterbi Path	
Posterior @ 0.4	79 - KRTKAGGLRFSRGLIGGGSWKGLDKRKPVFKGSKLVG - 115
Posterior @ 0.4	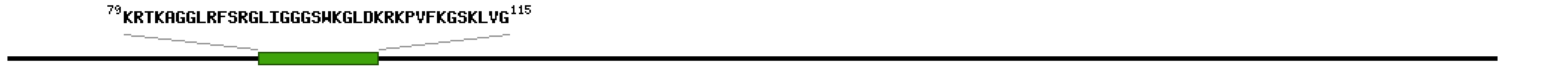
Posterior Graphic. Threshold Marked @ 0.4	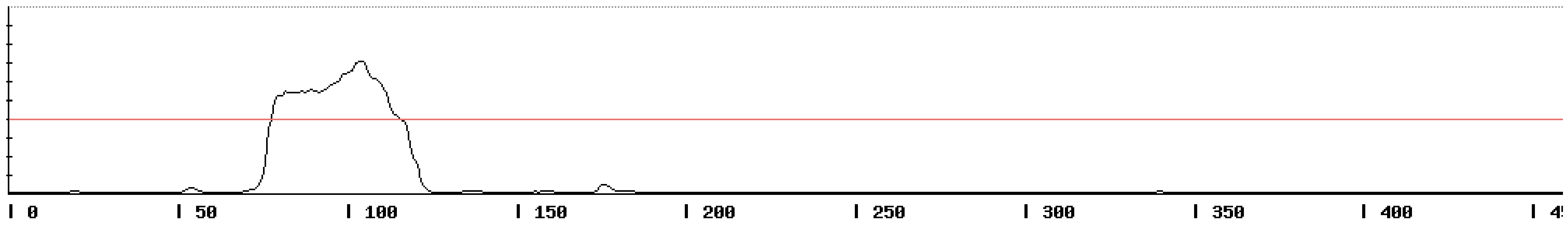

Predictions for Nicotianatabacum_XP_016488349.1

Viterbi Path	
Viterbi Path	
Posterior @ 0.4	
Posterior @ 0.4	

Posterior Graphic. Threshold Marked @ 0.4	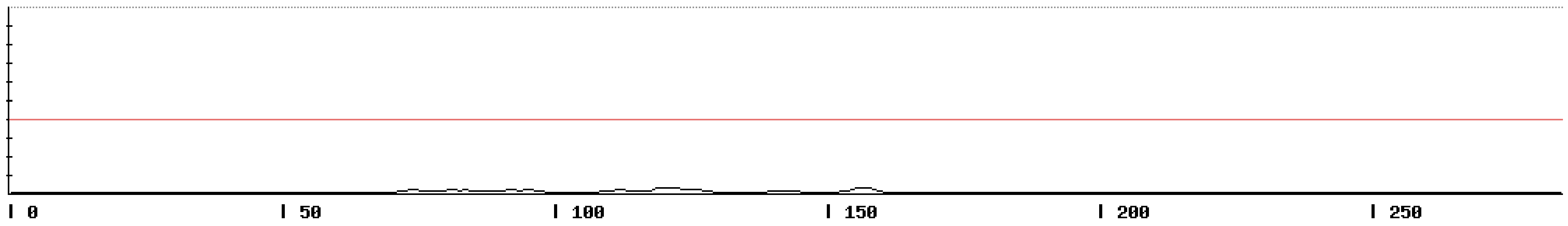

Predictions for Nicotianatabacum_XP_016488493.1	
Viterbi Path	
Viterbi Path	
Posterior @ 0.4	
Posterior @ 0.4	
Posterior Graphic. Threshold Marked @ 0.4	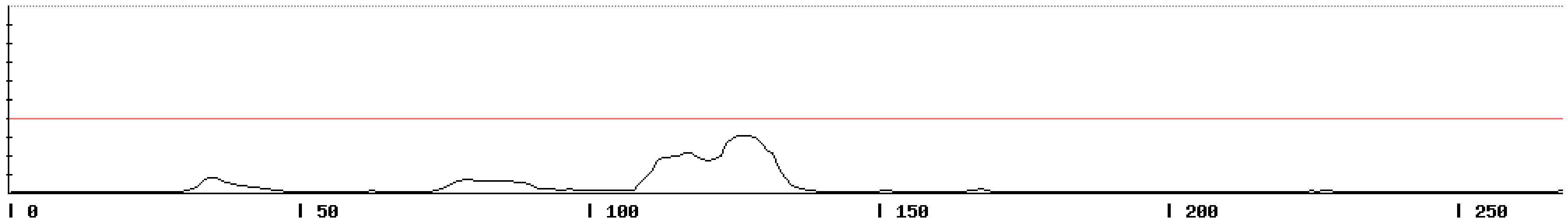

Predictions for Nicotianatabacum_XP_016489114.1	
Viterbi Path	
Viterbi Path	
Posterior @ 0.4	
Posterior @ 0.4	

Posterior Graphic. Threshold Marked @ 0.4

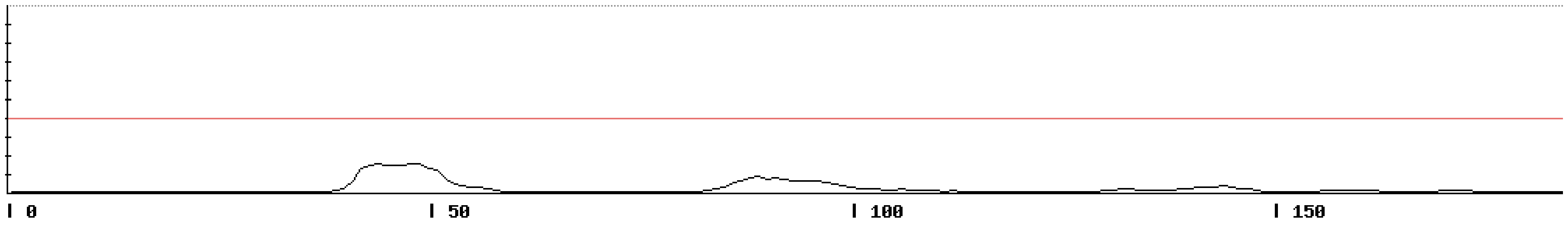

Predictions for Nicotianatabacum_XP_016489220.1

Viterbi Path

Viterbi Path

Posterior @ 0.4 | 140 - RKRRK - 144

Posterior @ 0.4

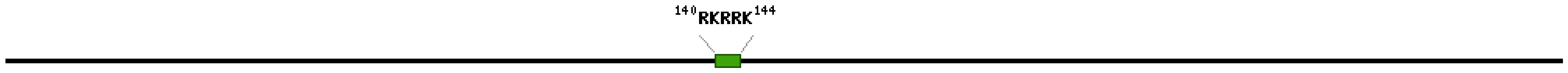

Posterior Graphic. Threshold Marked @ 0.4

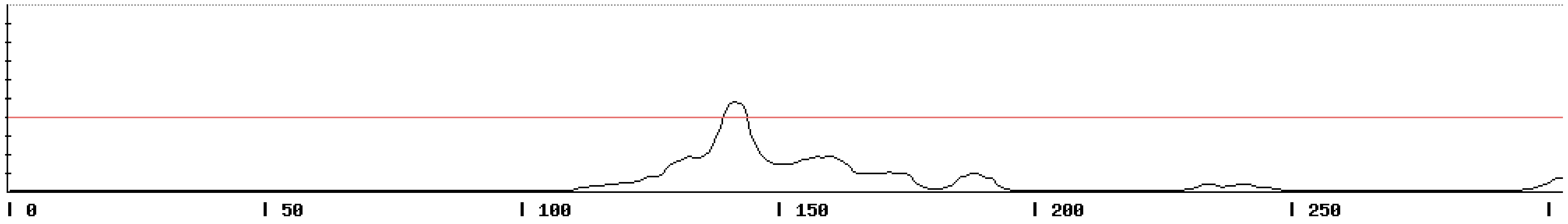

Predictions for Nicotianatabacum_XP_016489513.1

Viterbi Path

Viterbi Path

Posterior @ 0.4

Posterior @ 0.4

Posterior Graphic. Threshold Marked @ 0.4

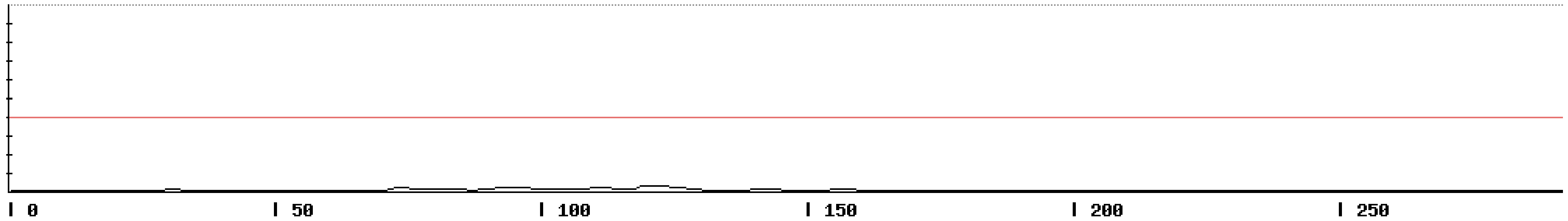

Predictions for Nicotianatabacum_XP_016489725.1

Viterbi Path

Viterbi Path

Posterior @ 0.4

Posterior @ 0.4

Posterior Graphic. Threshold Marked @ 0.4

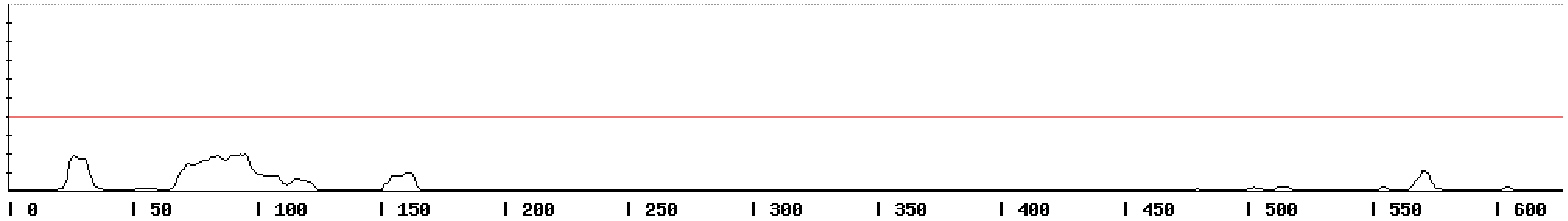

Predictions for Nicotianatabacum_XP_016490087.1

Viterbi Path

Viterbi Path

Posterior @ 0.4

Posterior @ 0.4

Posterior Graphic. Threshold Marked @ 0.4

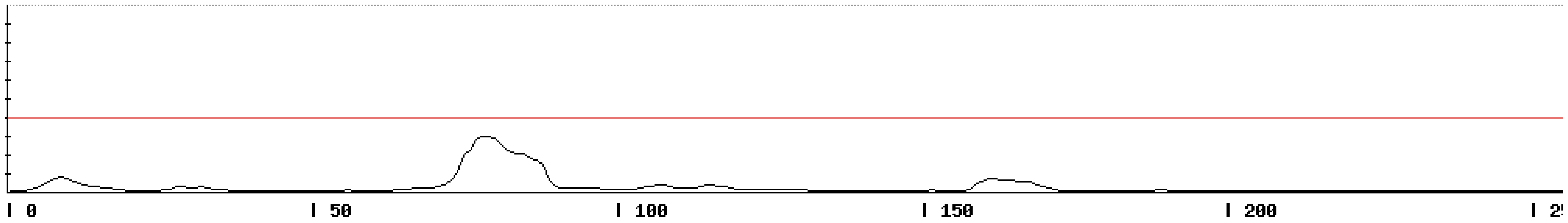

Predictions for Nicotianatabacum_XP_016490095.1

Viterbi Path

Viterbi Path

Posterior @ 0.4

Posterior @ 0.4

Posterior Graphic. Threshold Marked @ 0.4

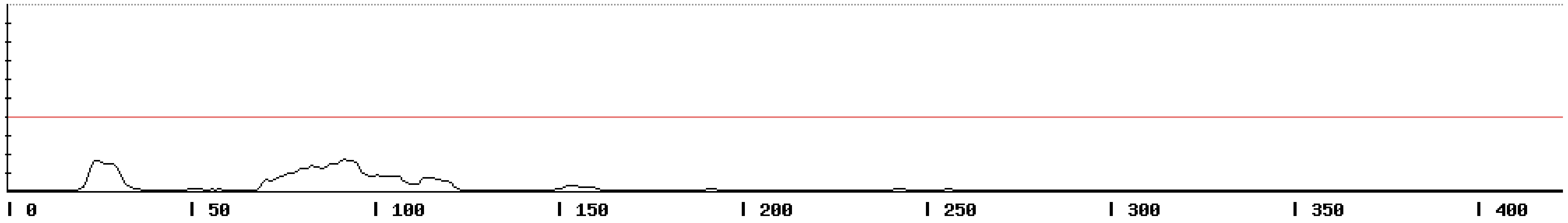

Predictions for Nicotianatabacum_XP_016490281.1

Viterbi Path

Viterbi Path

Posterior @ 0.4

Posterior @ 0.4

Posterior Graphic. Threshold Marked @ 0.4

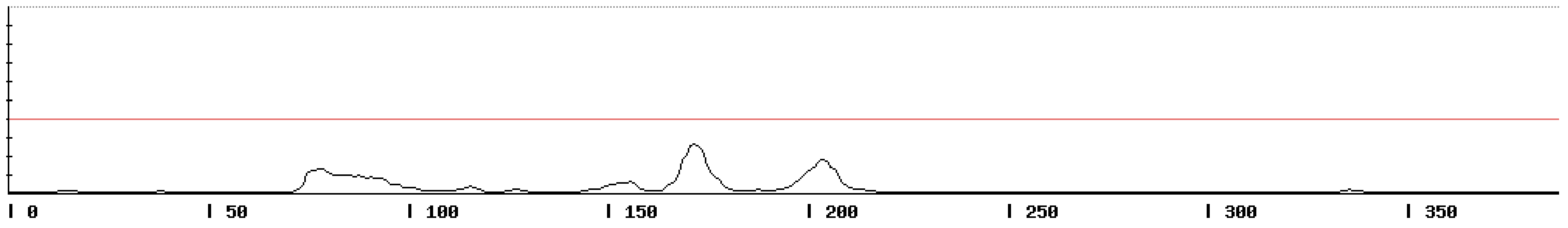

Predictions for Nicotianatabacum_XP_016490748.1

Viterbi Path

Viterbi Path

Posterior @ 0.4

Posterior @ 0.4

Posterior Graphic. Threshold Marked @ 0.4

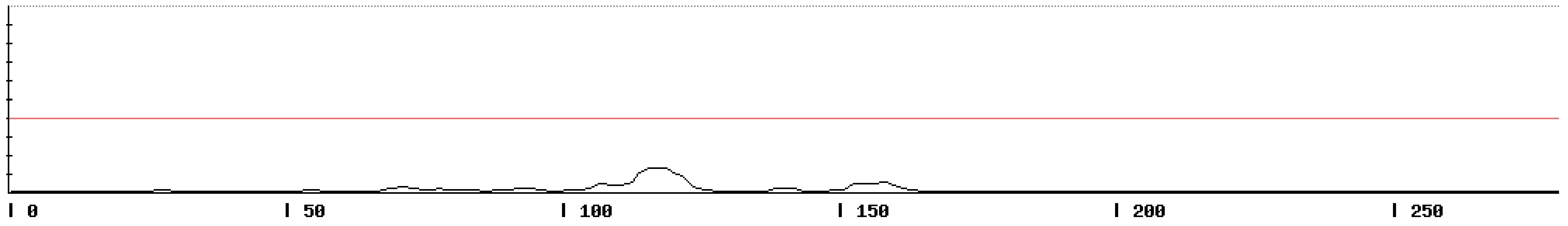

Predictions for Nicotianatabacum_XP_016490755.1	
Viterbi Path	162 - KIKKKGKPGDKKKG - 175<br>252 - KIKKKGKPGDKKKG - 265
Viterbi Path	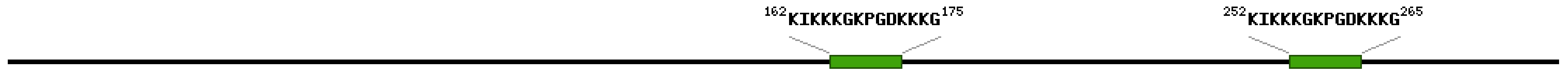
Posterior @ 0.4	79 - KKEKPTWKRVSRTVGKGTWKPQGKG - 103<br>162 - KIKKKGKPGDKKKG - 175<br>252 - KIKKKGKPGDKKKG - 265
Posterior @ 0.4	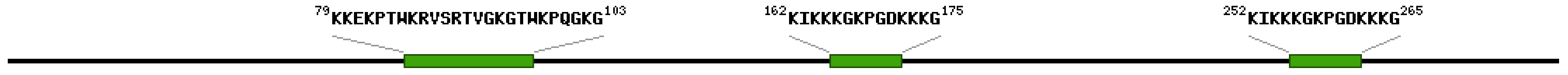
Posterior Graphic. Threshold Marked @ 0.4	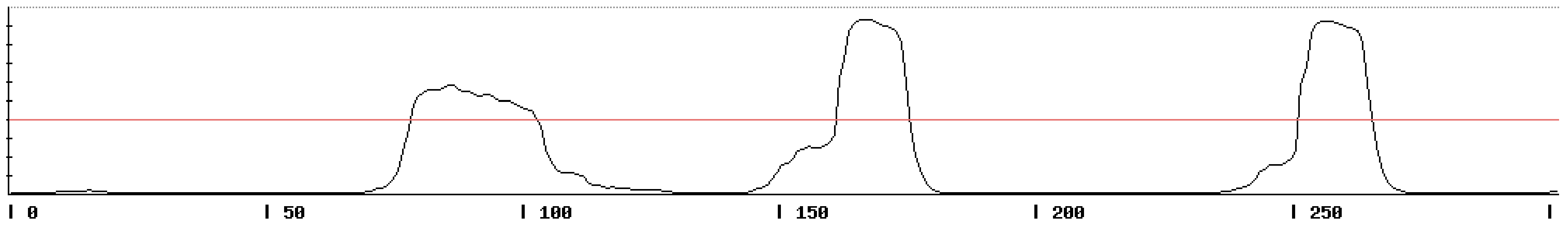

Predictions for Nicotianatabacum_XP_016490965.1	
Viterbi Path	
Viterbi Path	
Posterior @ 0.4	
Posterior @ 0.4	
Posterior Graphic. Threshold Marked @ 0.4	

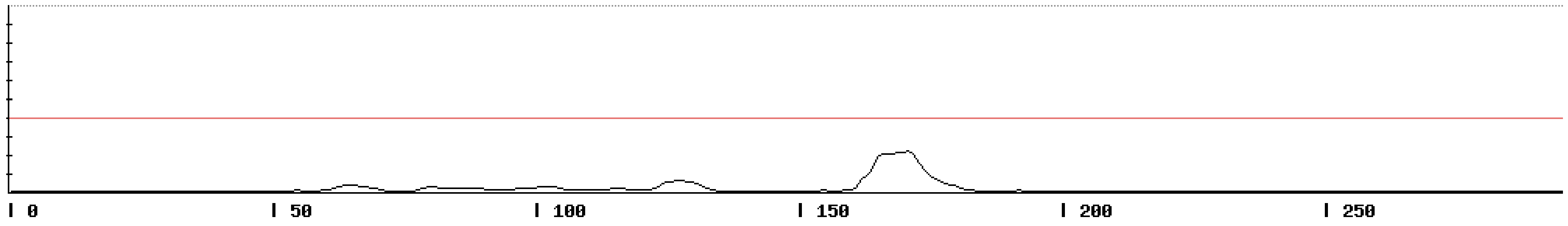

Predictions for Nicotianatabacum_XP_016491525.1

Viterbi Path	
Viterbi Path	
Posterior @ 0.4	141 - RKRRK - 145
Posterior @ 0.4	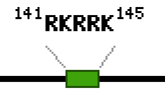
Posterior Graphic. Threshold Marked @ 0.4	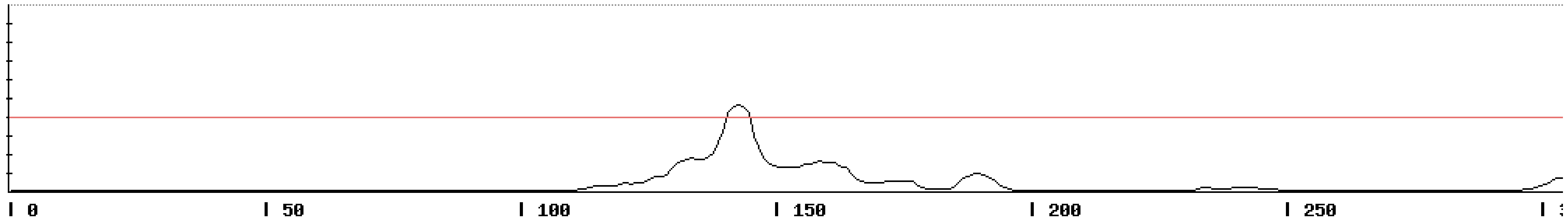

Predictions for Nicotianatabacum_XP_016491527.1

Viterbi Path	
Viterbi Path	
Posterior @ 0.4	
Posterior @ 0.4	

Posterior Graphic. Threshold Marked @ 0.4

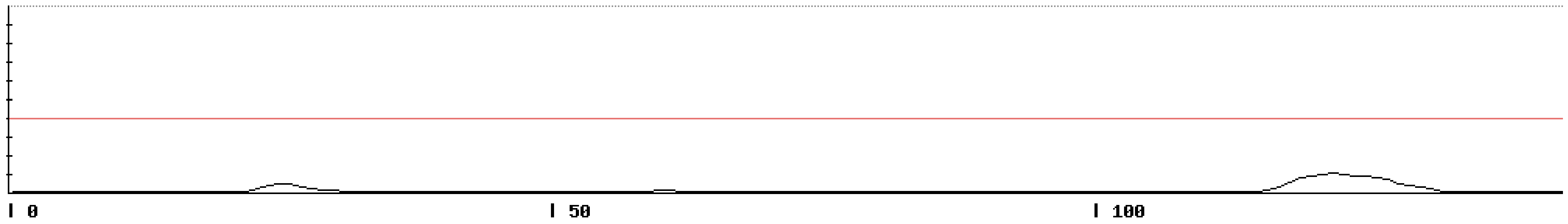

Predictions for Nicotianatabacum_XP_016491940.1

Viterbi Path

Viterbi Path

Posterior @ 0.4

Posterior @ 0.4

Posterior Graphic. Threshold Marked @ 0.4

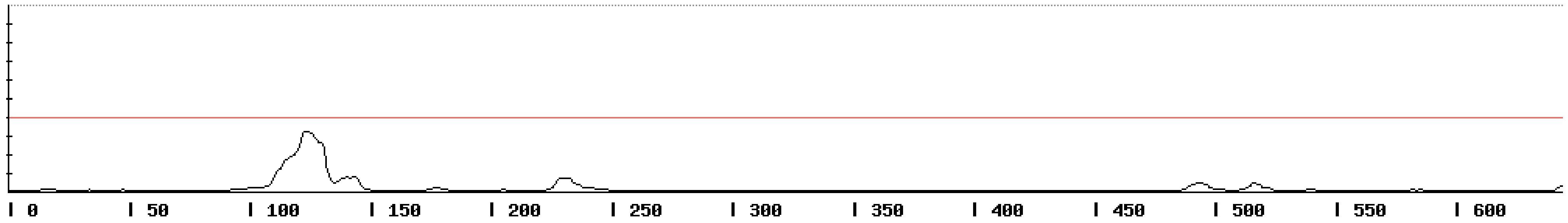

Predictions for Nicotianatabacum_XP_016491976.1

Viterbi Path

Viterbi Path

Posterior @ 0.4

Posterior @ 0.4

Posterior Graphic. Threshold Marked @ 0.4

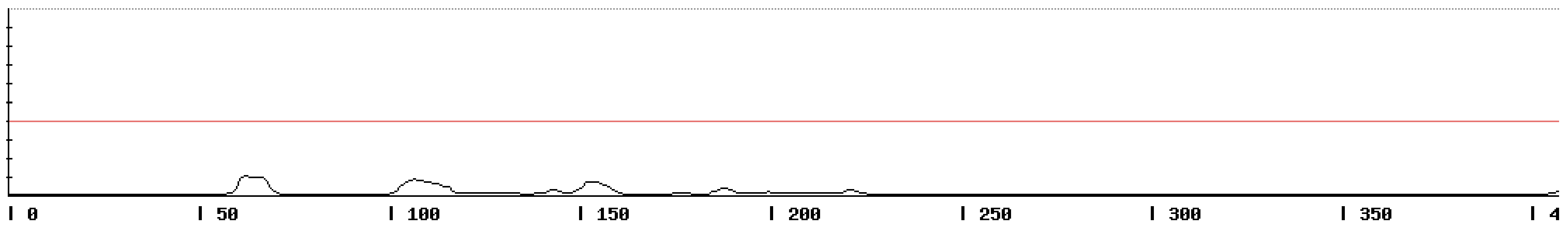

Predictions for Nicotianatabacum_XP_016491977.1

Viterbi Path	
Viterbi Path	
Posterior @ 0.4	
Posterior @ 0.4	
Posterior Graphic. Threshold Marked @ 0.4	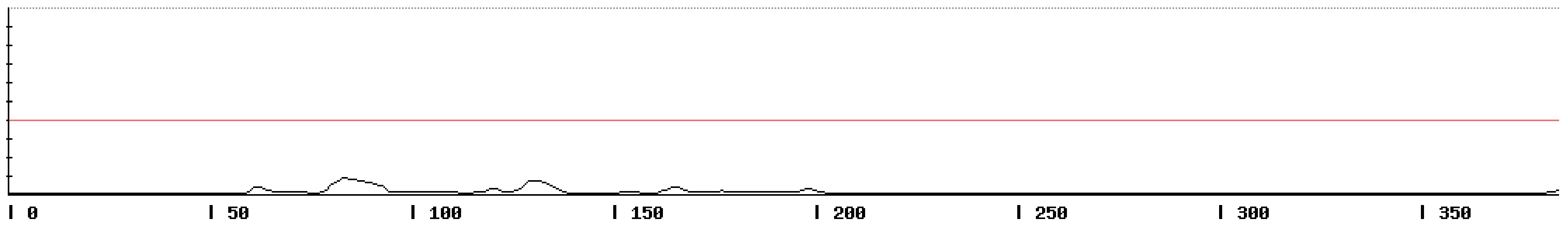

Predictions for Nicotianatabacum_XP_016492232.1

Viterbi Path	
Viterbi Path	

Posterior @ 0.4	
Posterior @ 0.4	
Posterior Graphic. Threshold Marked @ 0.4	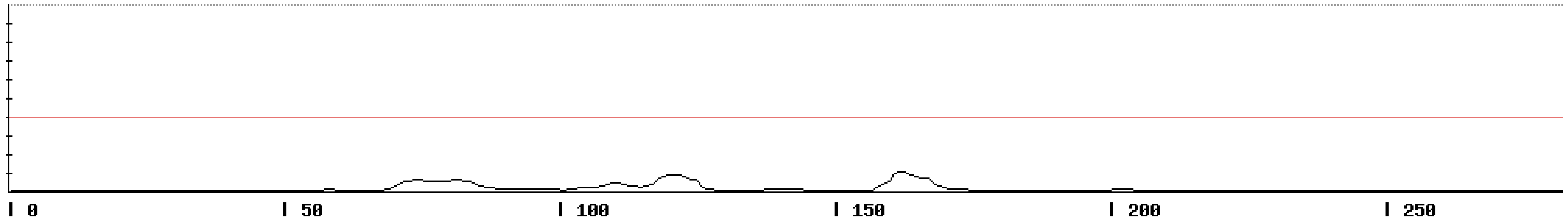

Predictions for Nicotianatabacum_XP_016493855.1	
Viterbi Path	
Viterbi Path	
Posterior @ 0.4	
Posterior @ 0.4	
Posterior Graphic. Threshold Marked @ 0.4	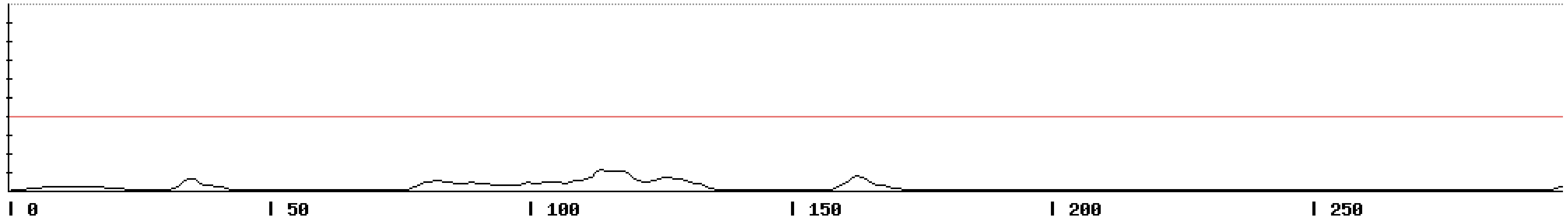

Predictions for Nicotianatabacum_XP_016494086.1	
Viterbi Path	
Viterbi Path	

Posterior @ 0.4

Posterior @ 0.4

Posterior Graphic. Threshold Marked @ 0.4

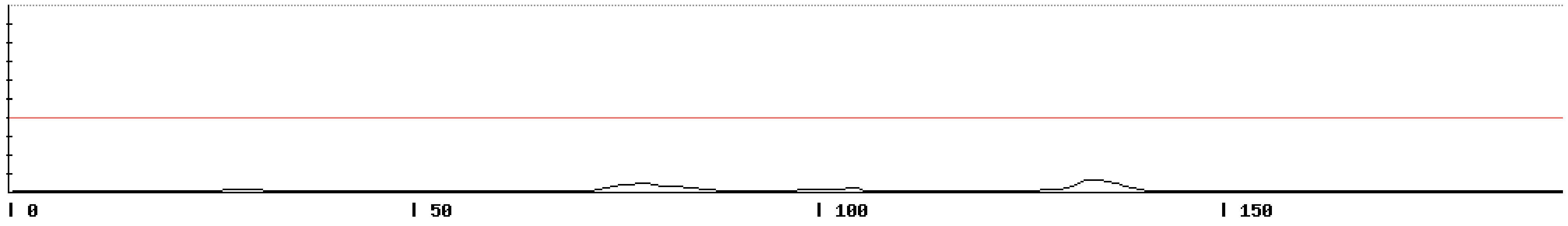

Predictions for Nicotianatabacum_XP_016494505.1

Viterbi Path

Viterbi Path

Posterior @ 0.4

Posterior @ 0.4

Posterior Graphic. Threshold Marked @ 0.4

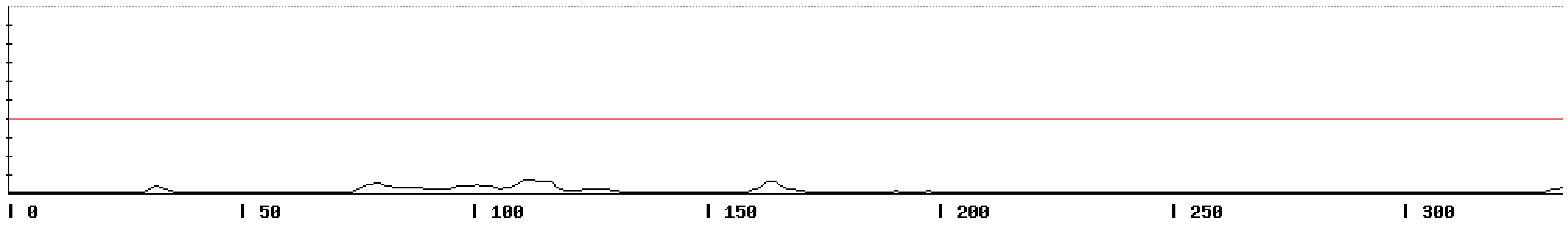

Predictions for Nicotianatabacum_XP_016494506.1

Viterbi Path

Viterbi Path

Posterior @ 0.4

Posterior @ 0.4

Posterior Graphic. Threshold Marked @ 0.4

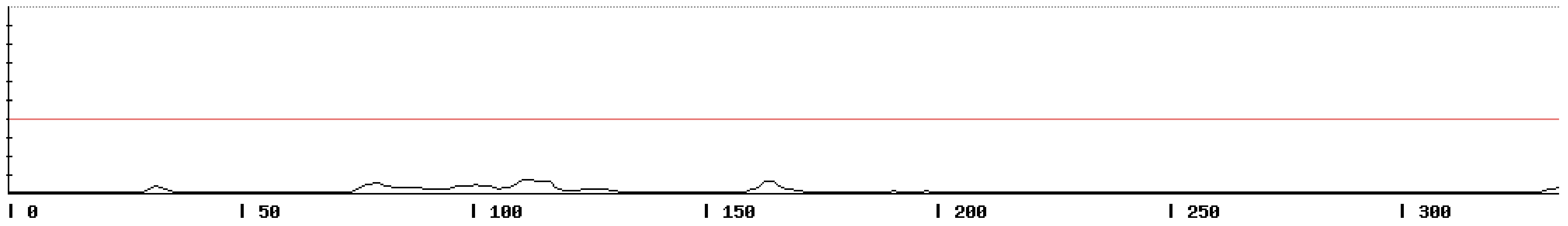

Predictions for Nicotianatabacum_XP_016494507.1

Viterbi Path

Viterbi Path

Posterior @ 0.4

Posterior @ 0.4

Posterior Graphic. Threshold Marked @ 0.4

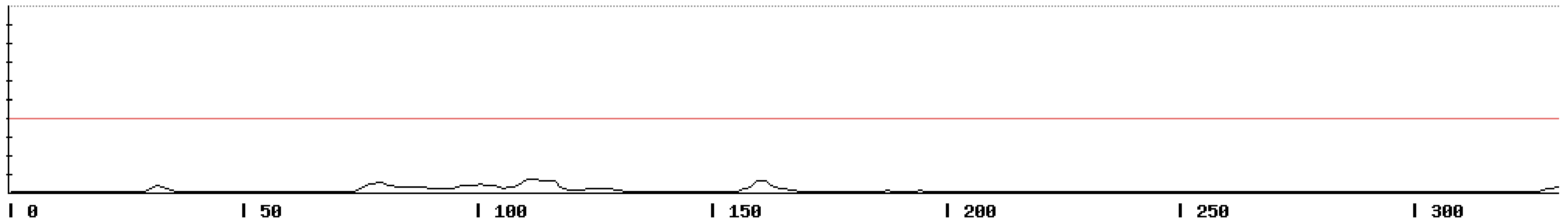

Predictions for Nicotianatabacum_XP_016494557.1	
Viterbi Path	
Viterbi Path	
Posterior @ 0.4	71 - RKHGPKGKPNR - 81
Posterior @ 0.4	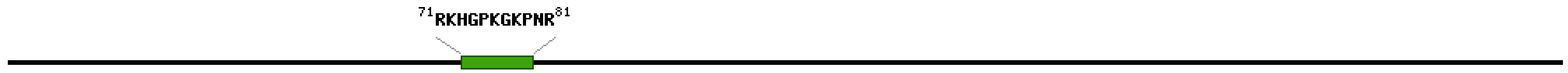
Posterior Graphic. Threshold Marked @ 0.4	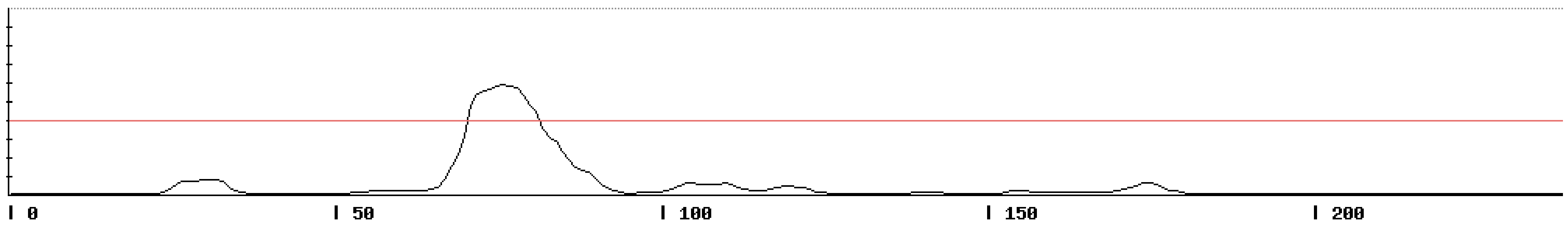

Predictions for Nicotianatabacum_XP_016496356.1	
Viterbi Path	
Viterbi Path	
Posterior @ 0.4	
Posterior @ 0.4	
Posterior Graphic. Threshold Marked @ 0.4	

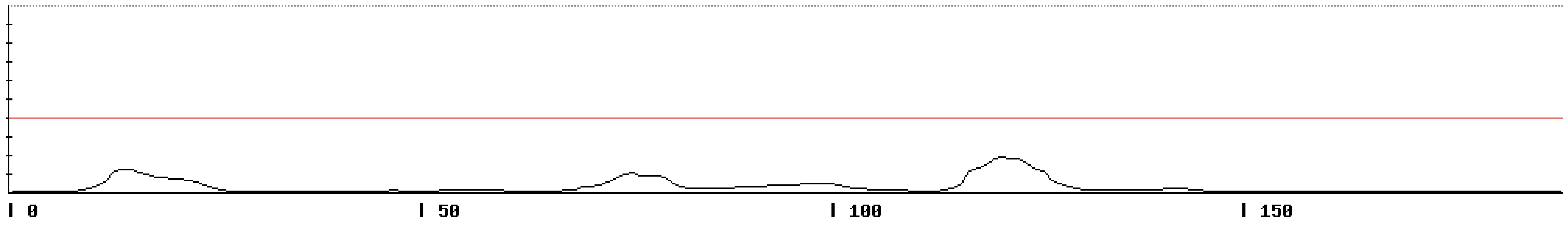

Predictions for Nicotianatabacum_XP_016497364.1	
Viterbi Path	
Viterbi Path	
Posterior @ 0.4	
Posterior @ 0.4	
Posterior Graphic. Threshold Marked @ 0.4	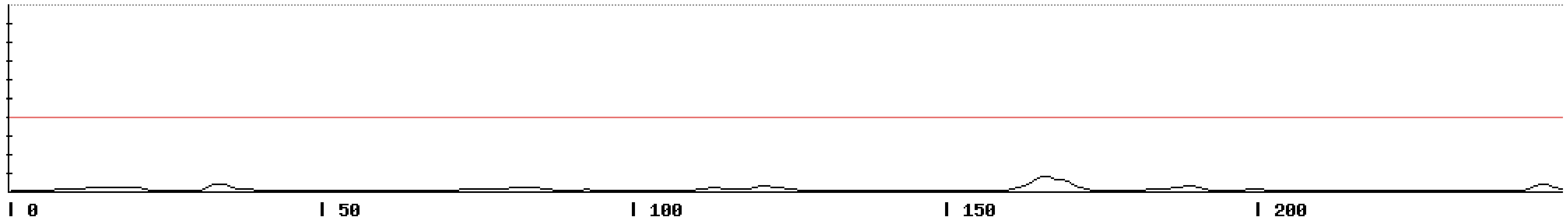

Predictions for Nicotianatabacum_XP_016497740.1	
Viterbi Path	
Viterbi Path	
Posterior @ 0.4	
Posterior @ 0.4	

Posterior Graphic. Threshold Marked @ 0.4

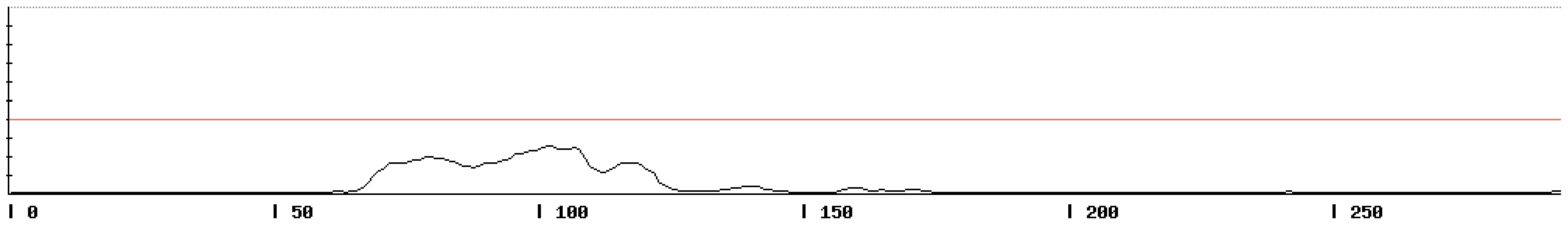

Predictions for Nicotianatabacum_XP_016498397.1

Viterbi Path

Viterbi Path

Posterior @ 0.4

Posterior @ 0.4

Posterior Graphic. Threshold Marked @ 0.4

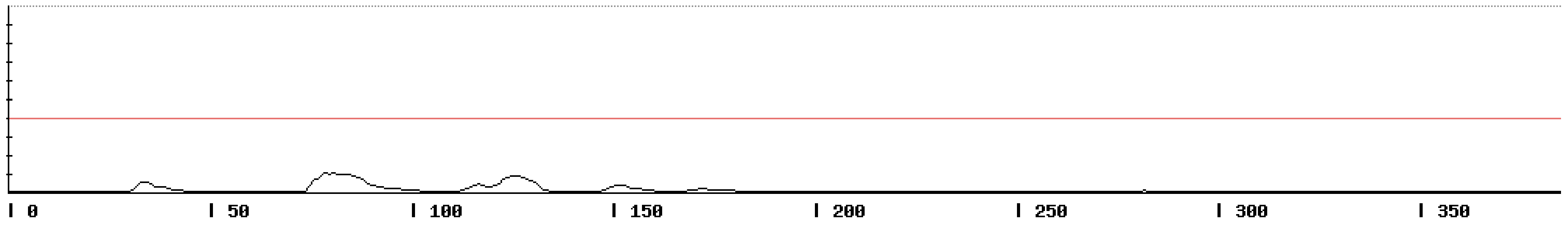

Predictions for Nicotianatabacum_XP_016500123.1

Viterbi Path

Viterbi Path

Posterior @ 0.4

Posterior @ 0.4

Posterior Graphic. Threshold Marked @ 0.4

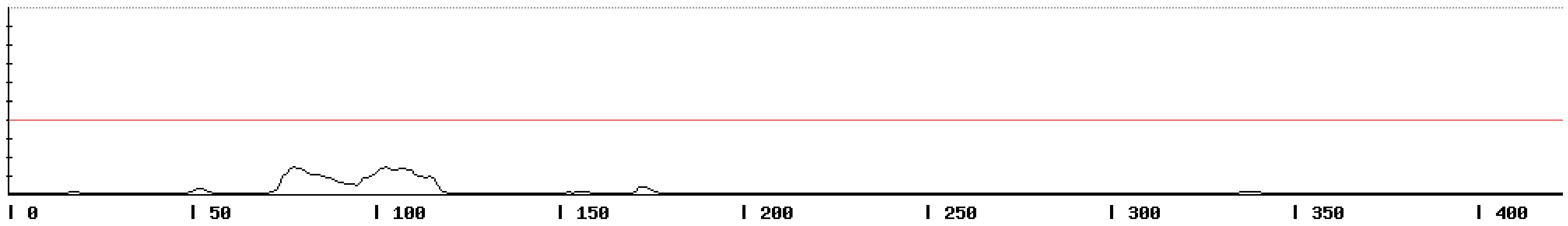

Predictions for Nicotianatabacum_XP_016500359.1

Viterbi Path

Viterbi Path

Posterior @ 0.4

Posterior @ 0.4

Posterior Graphic. Threshold Marked @ 0.4

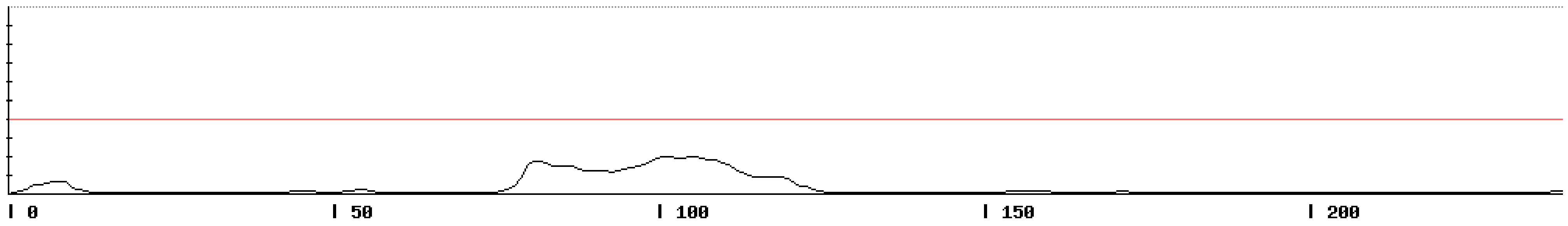

Predictions for Nicotianatabacum_XP_016500362.1

Viterbi Path

Viterbi Path

Posterior @ 0.4	266 - VRNKRPRRKRDSHPPP - 281
	492 - KK - 493
Posterior @ 0.4	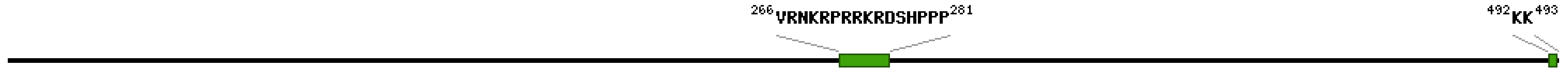
Posterior Graphic. Threshold Marked @ 0.4	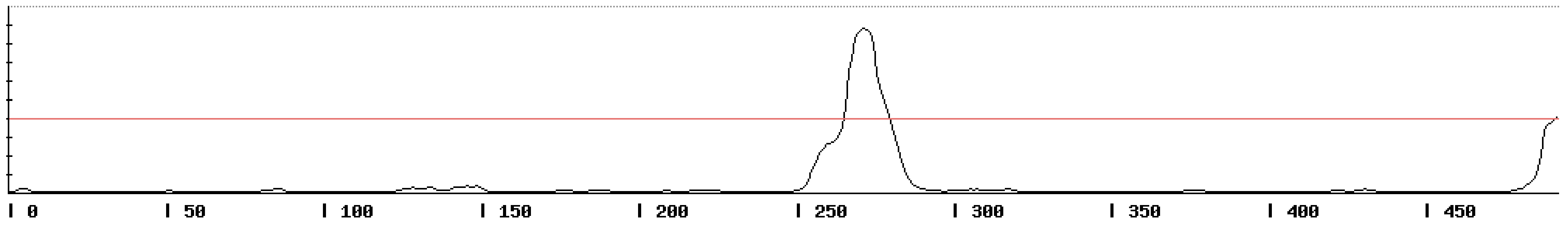

Predictions for Nicotianatabacum_XP_016500364.1

Viterbi Path	
Viterbi Path	
Posterior @ 0.4	266 - VRNKRPRRKRDSHPPP - 281
	492 - KK - 493
Posterior @ 0.4	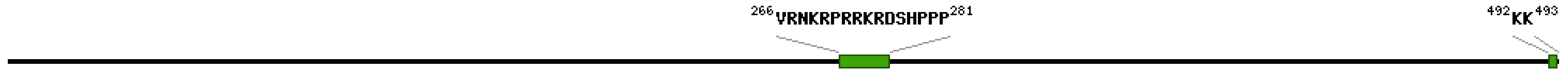
Posterior Graphic. Threshold Marked @ 0.4	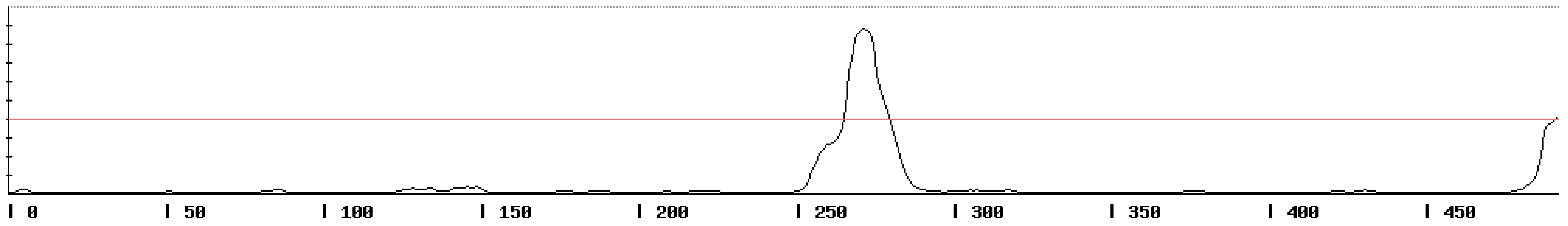

Predictions for Nicotianatabacum_XP_016500365.1

Viterbi Path	

Viterbi Path

Posterior @ 0.4 266 - VRNKRPRRKRDSHPP - 280

Posterior @ 0.4

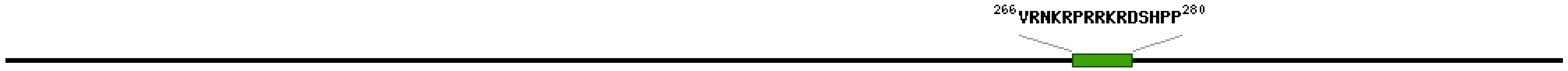

Posterior Graphic. Threshold Marked @ 0.4

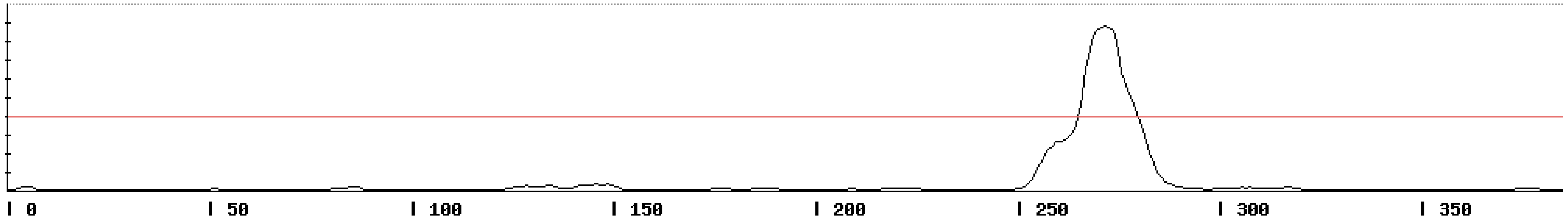

Predictions for Nicotianatabacum_XP_016500749.1

Viterbi Path

Viterbi Path

Posterior @ 0.4

Posterior @ 0.4

Posterior Graphic. Threshold Marked @ 0.4

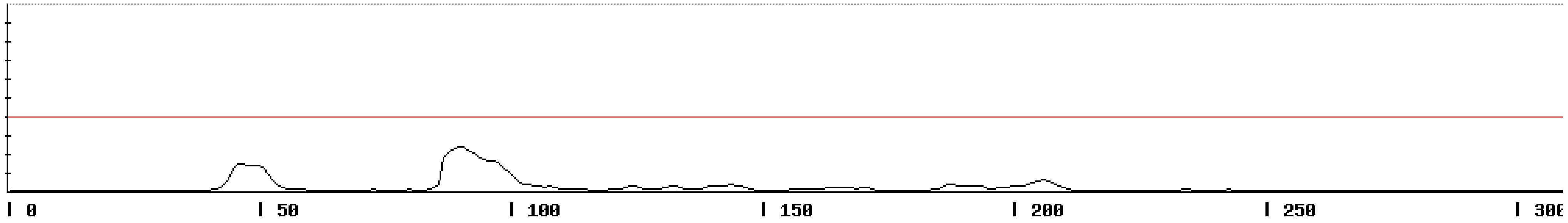

Predictions for Nicotianatabacum_XP_016501117.1

Viterbi Path

Viterbi Path

Posterior @ 0.4

Posterior @ 0.4

Posterior Graphic. Threshold Marked @ 0.4

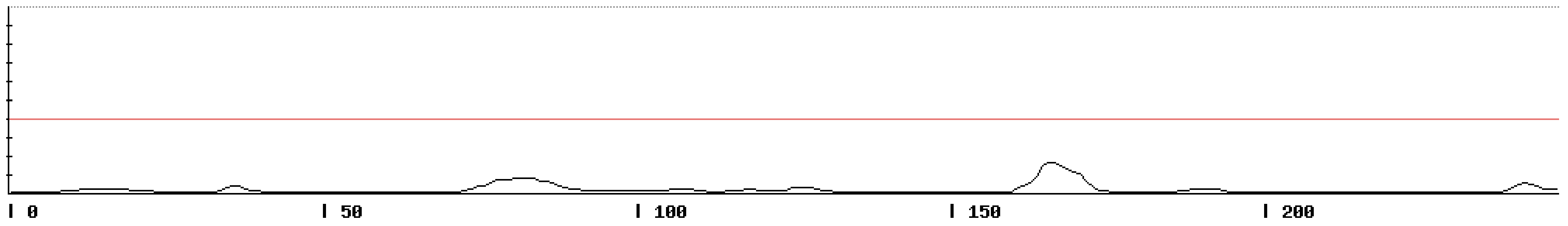

Predictions for Nicotianatabacum_XP_016501212.1

Viterbi Path

Viterbi Path

Posterior @ 0.4

Posterior @ 0.4

Posterior Graphic. Threshold Marked @ 0.4

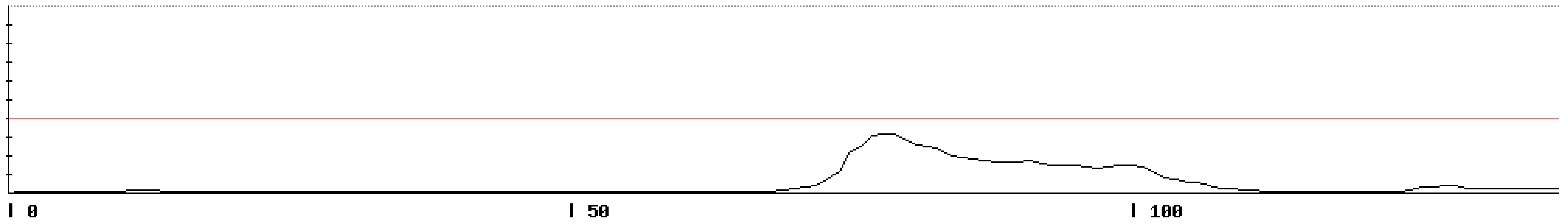

Predictions for Nicotianatabacum_XP_016501877.1	
Viterbi Path	
Viterbi Path	
Posterior @ 0.4	
Posterior @ 0.4	
Posterior Graphic. Threshold Marked @ 0.4	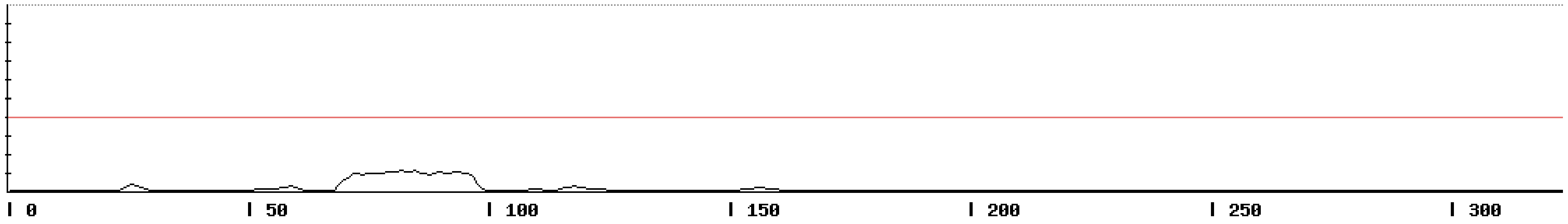

Predictions for Nicotianatabacum_XP_016502907.1	
Viterbi Path	
Viterbi Path	
Posterior @ 0.4	
Posterior @ 0.4	
Posterior Graphic. Threshold Marked @ 0.4	

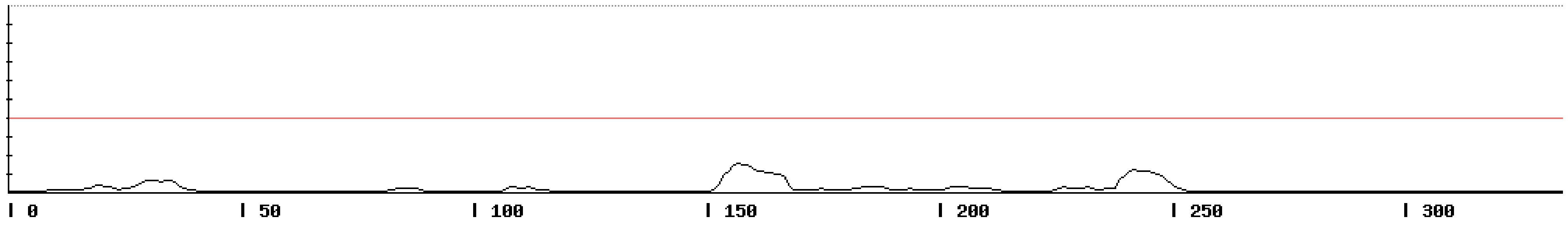

Predictions for Nicotianatabacum_XP_016504286.1

Viterbi Path

Viterbi Path

Posterior @ 0.4

Posterior @ 0.4

Posterior Graphic. Threshold Marked @ 0.4

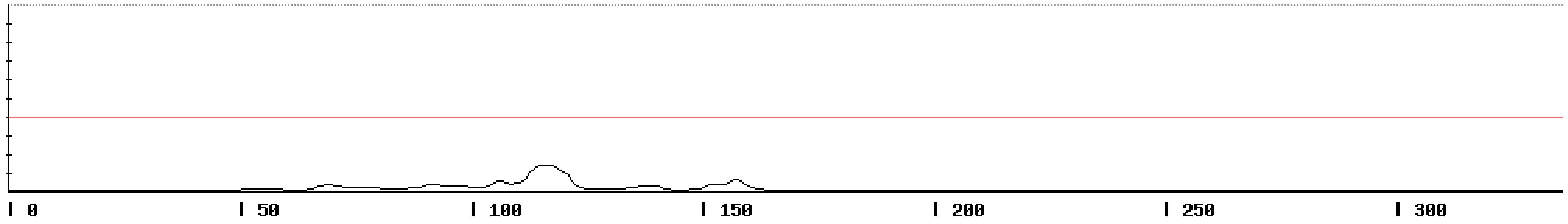

Predictions for Nicotianatabacum_XP_016504577.1

Viterbi Path

Viterbi Path

Posterior @ 0.4

71 - K - 71

75 - K - 75

151 - KIKKKGKPGDKKN - 163

Posterior @ 0.4

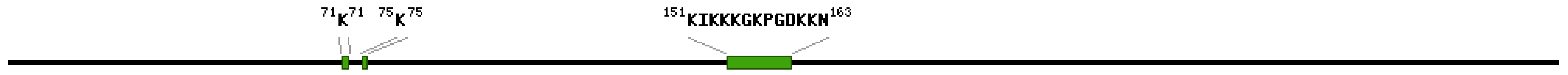

Posterior Graphic. Threshold Marked @ 0.4

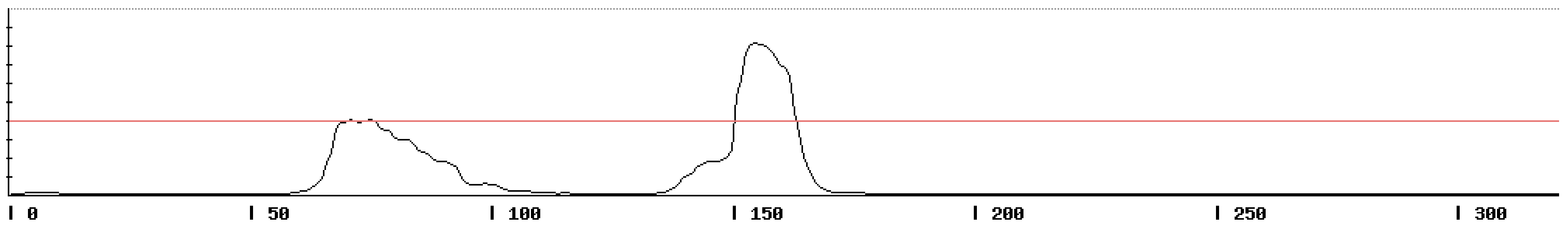

Predictions for Nicotianatabacum_XP_016505053.1

Viterbi Path

Viterbi Path

Posterior @ 0.4

Posterior @ 0.4

Posterior Graphic. Threshold Marked @ 0.4

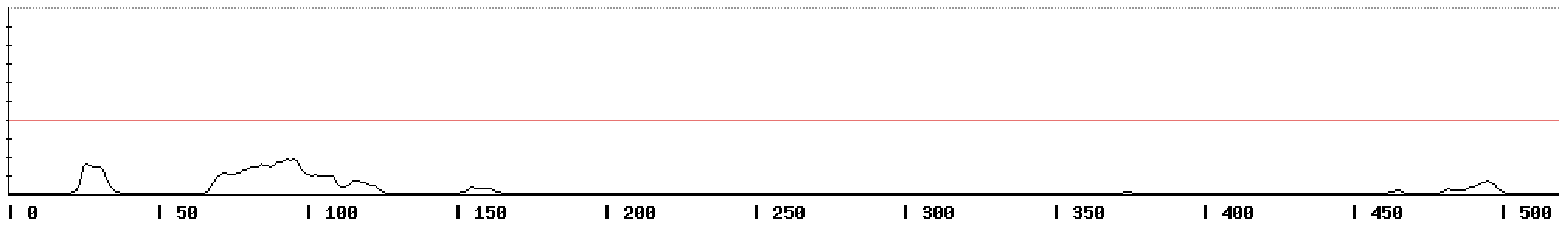

Predictions for Nicotianatabacum_XP_016505247.1

Viterbi Path

Viterbi Path

Posterior @ 0.4	88 - KKKSNARYHRSVRNK - 102
Posterior @ 0.4	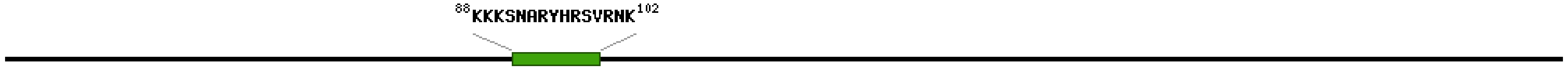
Posterior Graphic. Threshold Marked @ 0.4	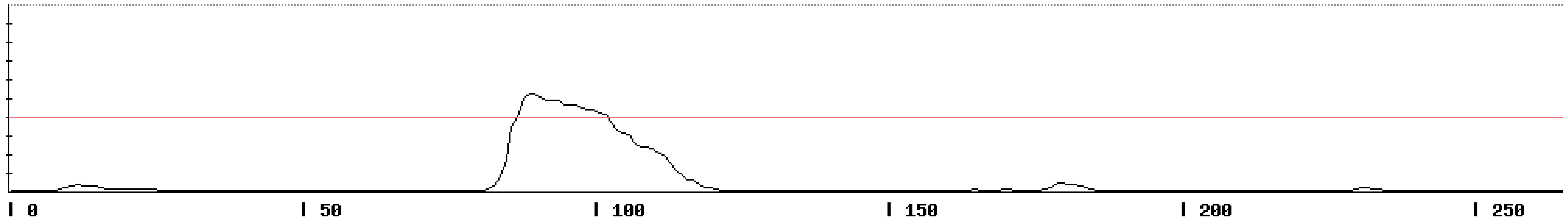

Predictions for Nicotianatabacum_XP_016505492.1

Viterbi Path	
Viterbi Path	
Posterior @ 0.4	
Posterior @ 0.4	
Posterior Graphic. Threshold Marked @ 0.4	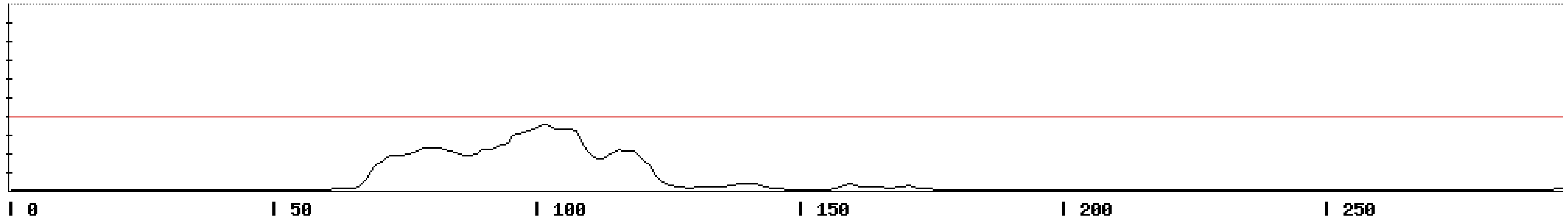

Predictions for Nicotianatabacum_XP_016505493.1

Viterbi Path	
Viterbi Path	

Posterior @ 0.4

Posterior @ 0.4

Posterior Graphic. Threshold Marked @ 0.4

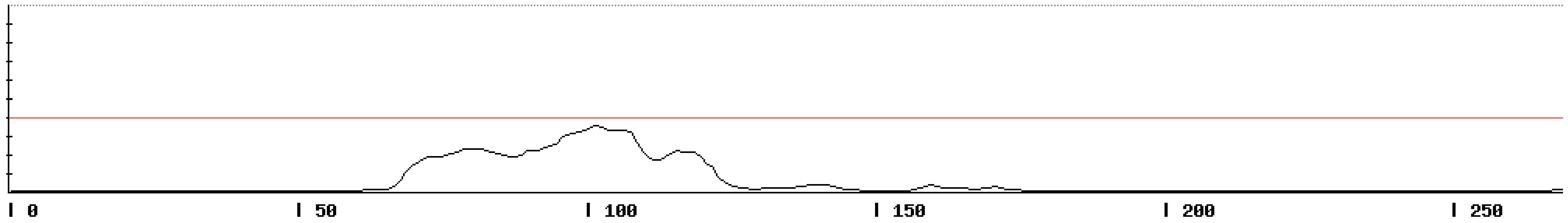

Predictions for Nicotianatabacum_XP_016506045.1

Viterbi Path

Viterbi Path

Posterior @ 0.4

Posterior @ 0.4

Posterior Graphic. Threshold Marked @ 0.4

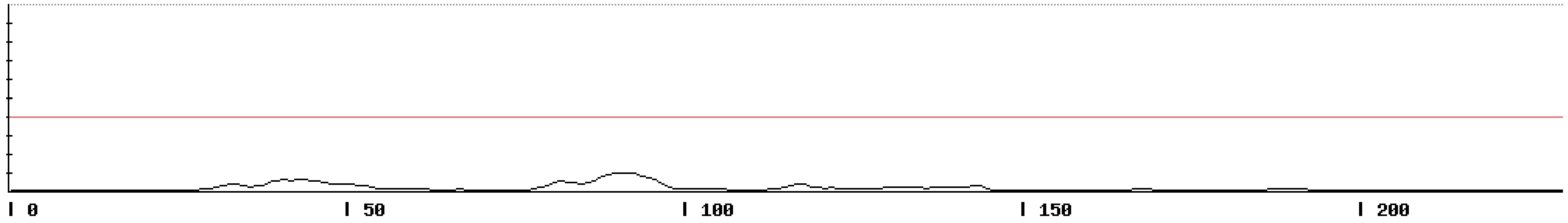

Predictions for Nicotianatabacum_XP_016506197.1

Viterbi Path	
Viterbi Path	
Posterior @ 0.4	
Posterior @ 0.4	
Posterior Graphic. Threshold Marked @ 0.4	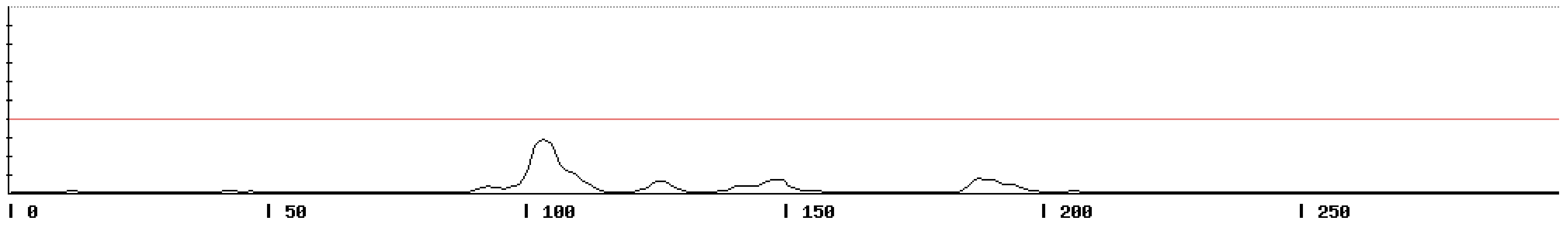

Predictions for Nicotianatabacum_XP_016506198.1

Viterbi Path	
Viterbi Path	
Posterior @ 0.4	
Posterior @ 0.4	
Posterior Graphic. Threshold Marked @ 0.4	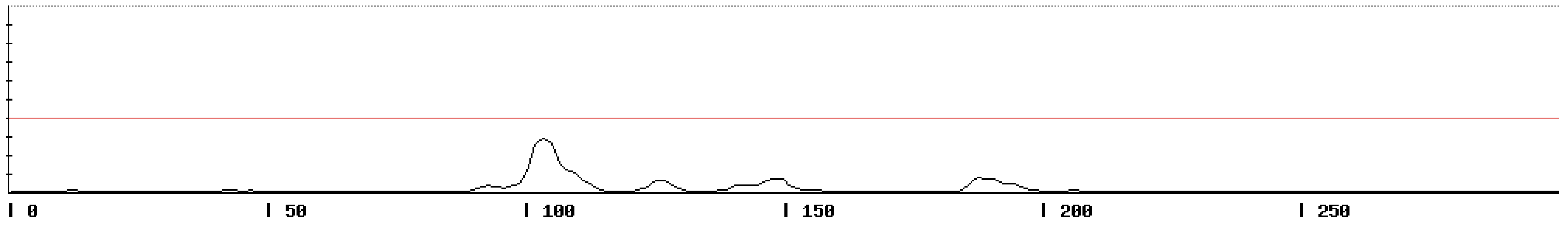

Predictions for Nicotianatabacum_XP_016506200.1	
Viterbi Path	
Viterbi Path	
Posterior @ 0.4	
Posterior @ 0.4	
Posterior Graphic. Threshold Marked @ 0.4	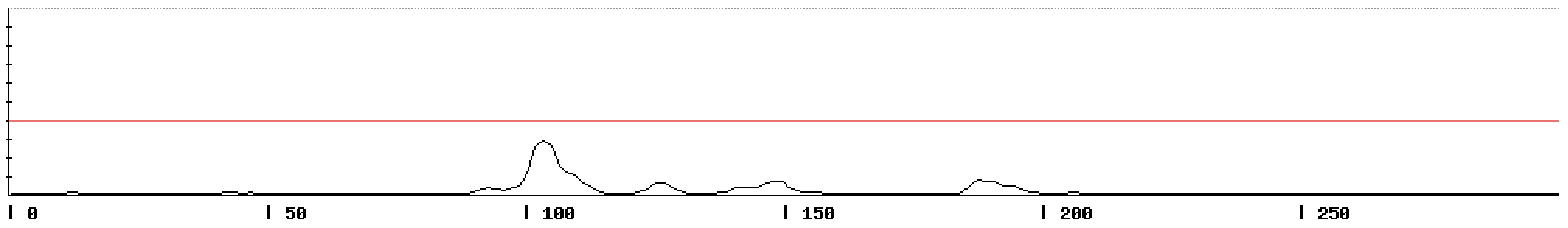

Predictions for Nicotianatabacum_XP_016506201.1	
Viterbi Path	
Viterbi Path	
Posterior @ 0.4	
Posterior @ 0.4	
Posterior Graphic. Threshold Marked @ 0.4	

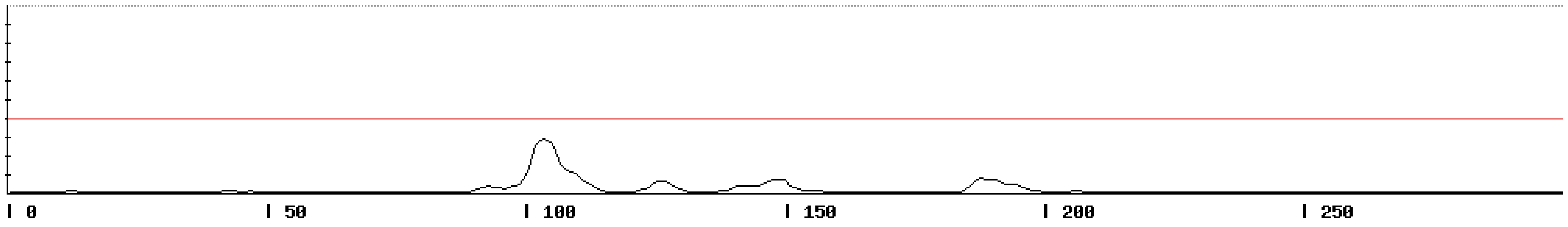

Predictions for Nicotianatabacum_XP_016506202.1

Viterbi Path

Viterbi Path

Posterior @ 0.4

Posterior @ 0.4

Posterior Graphic. Threshold Marked @ 0.4

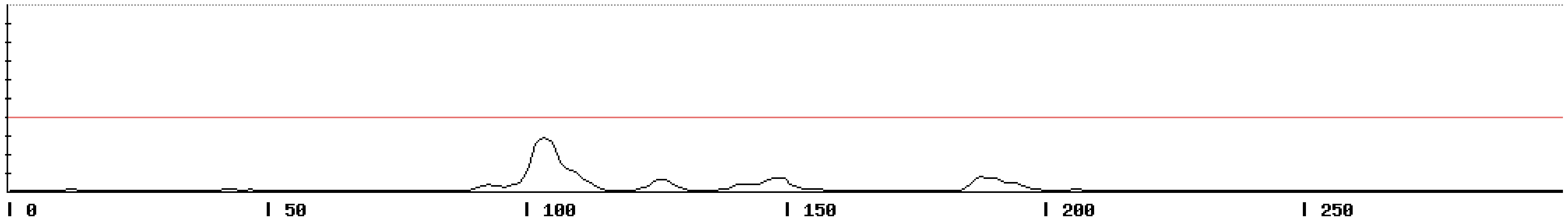

Predictions for Nicotianatabacum_XP_016506326.1

Viterbi Path

Viterbi Path

Posterior @ 0.4

Posterior @ 0.4

Posterior Graphic. Threshold Marked @ 0.4

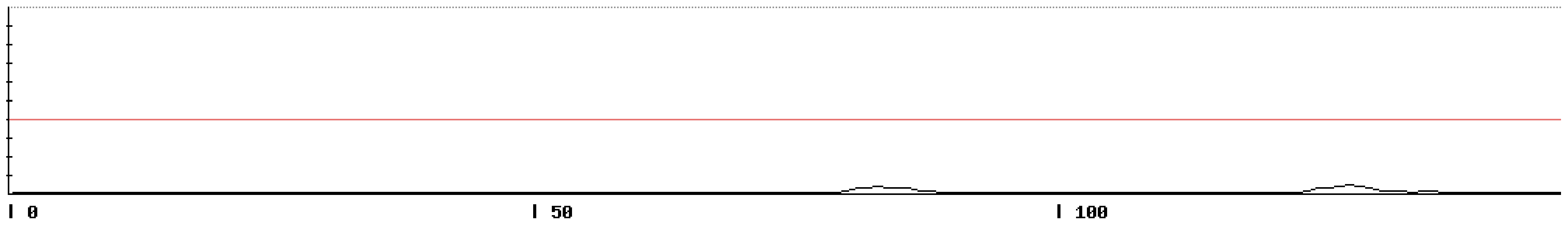

Predictions for Nicotianatabacum_XP_016506449.1

Viterbi Path

Viterbi Path

Posterior @ 0.4

Posterior @ 0.4

Posterior Graphic. Threshold Marked @ 0.4

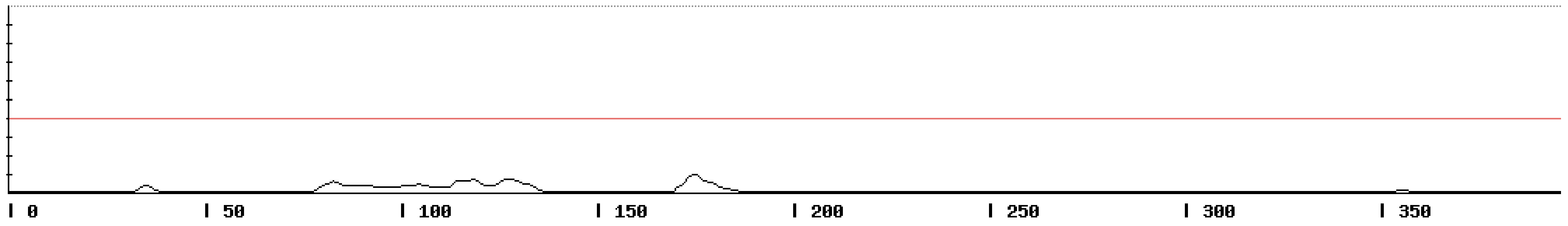

Predictions for Nicotianatabacum_XP_016506527.1

Viterbi Path

Viterbi Path

Posterior @ 0.4

Posterior @ 0.4

Posterior Graphic. Threshold Marked @ 0.4

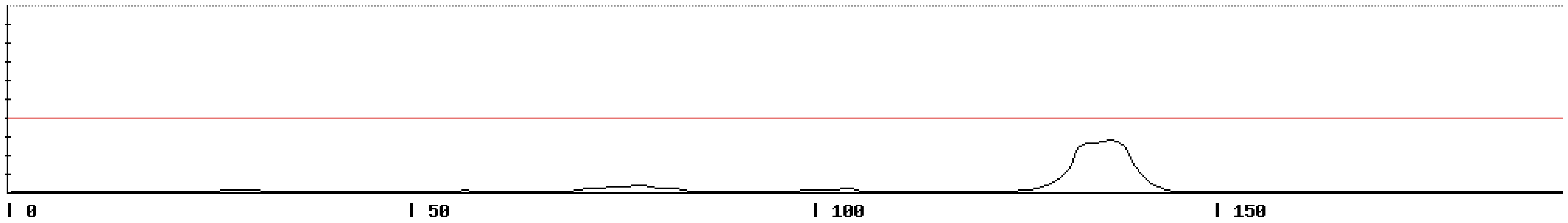

Predictions for Nicotianatabacum_XP_016507083.1

Viterbi Path

Viterbi Path

Posterior @ 0.4 — 80 - KKEKPTWKRVSRTVGKGTWKPQGKG - 104

Posterior @ 0.4

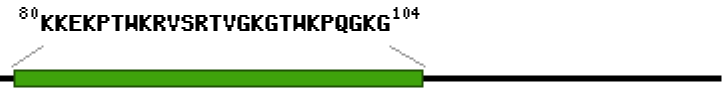

Posterior Graphic. Threshold Marked @ 0.4

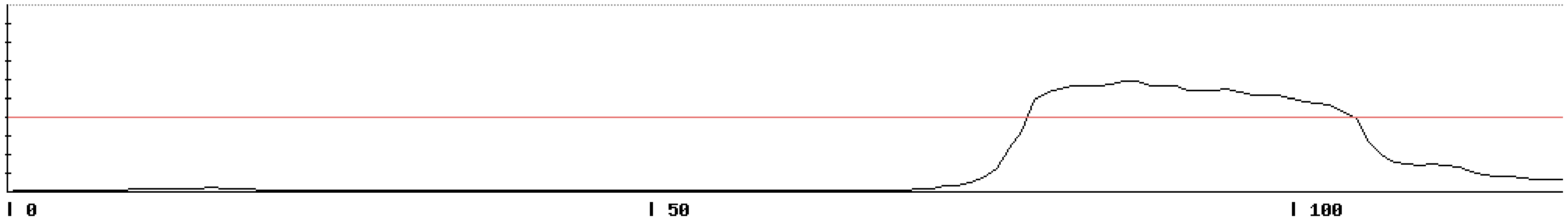

Predictions for Nicotianatabacum_XP_016507092.1

Viterbi Path

Viterbi Path

Posterior @ 0.4 — 81 - GRKRRYKNGRKPNRQ - 95

Posterior @ 0.4

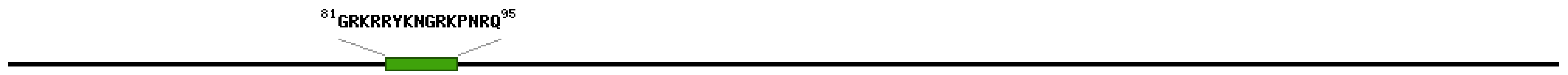

Posterior Graphic. Threshold Marked @ 0.4

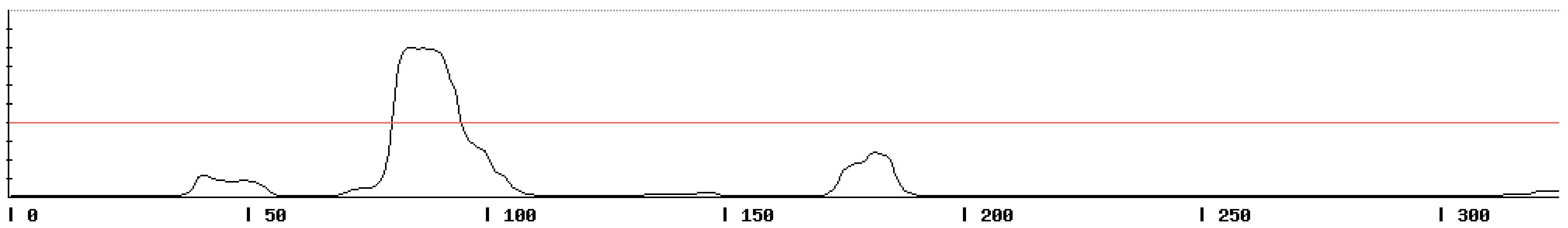

Predictions for Nicotianatabacum_XP_016507538.1

Viterbi Path

Viterbi Path

Posterior @ 0.4

Posterior @ 0.4

Posterior Graphic. Threshold Marked @ 0.4

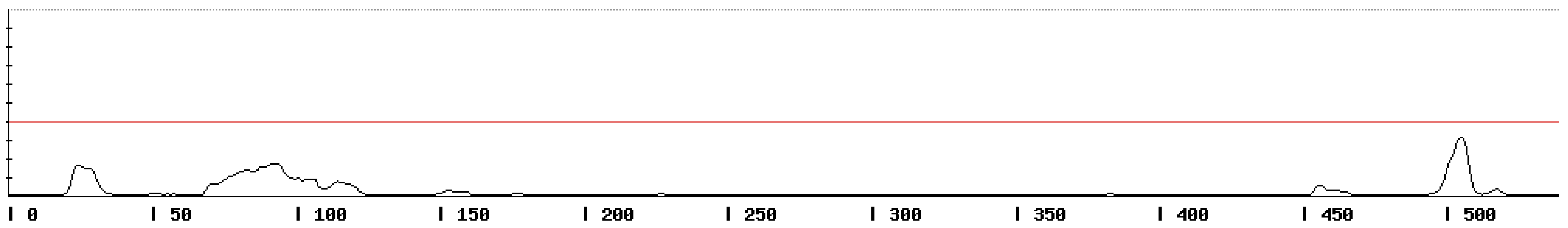

Predictions for Nicotianatabacum_XP_016507618.1

Viterbi Path

Viterbi Path

Posterior @ 0.4

Posterior @ 0.4

Posterior Graphic. Threshold Marked @ 0.4

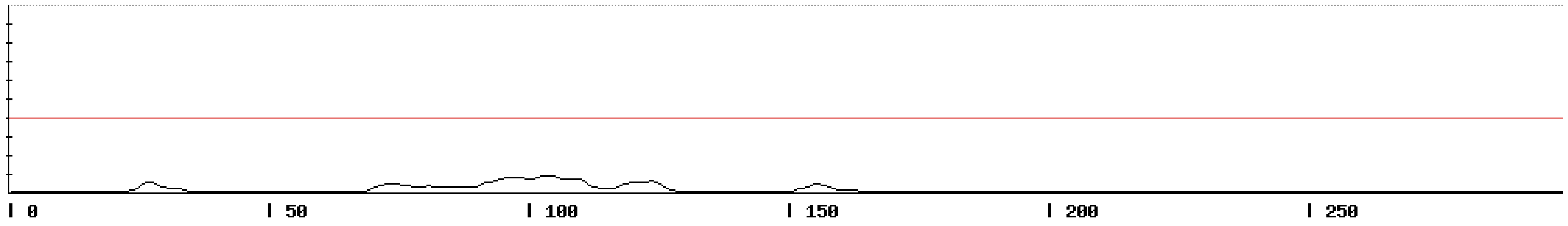

Predictions for Nicotianatabacum_XP_016509171.1

Viterbi Path

Viterbi Path

Posterior @ 0.4

Posterior @ 0.4

Posterior Graphic. Threshold Marked @ 0.4

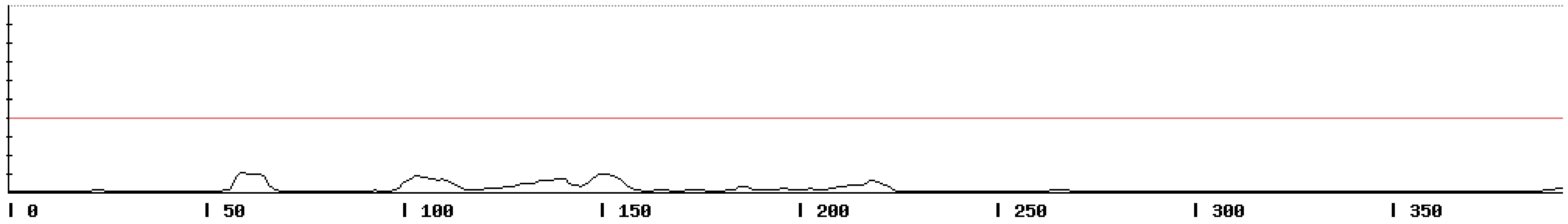

Predictions for Nicotianatabacum_XP_016509208.1

Viterbi Path

Viterbi Path

Posterior @ 0.4

Posterior @ 0.4

Posterior Graphic. Threshold Marked @ 0.4

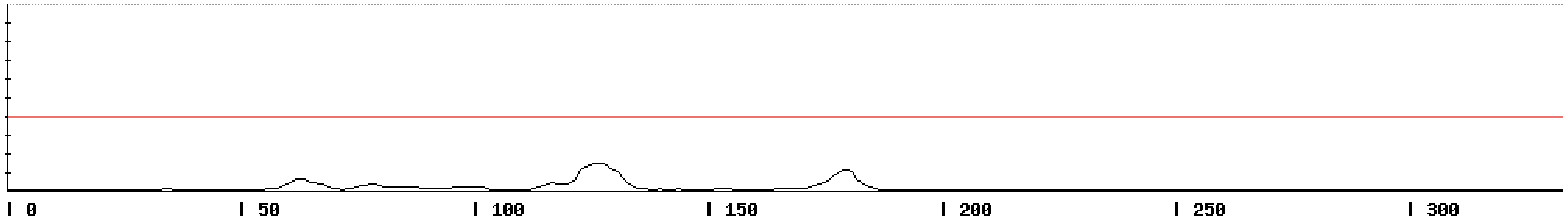

Predictions for Nicotianatabacum_XP_016509570.1

Viterbi Path

Viterbi Path

Posterior @ 0.4

72 - RKHGPKGKPNR - 82

Posterior @ 0.4

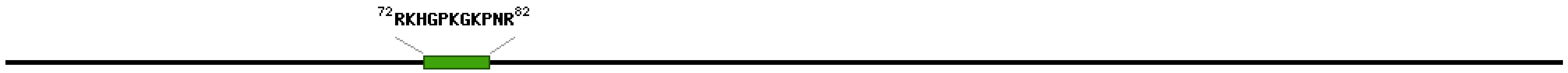

Posterior Graphic. Threshold Marked @ 0.4

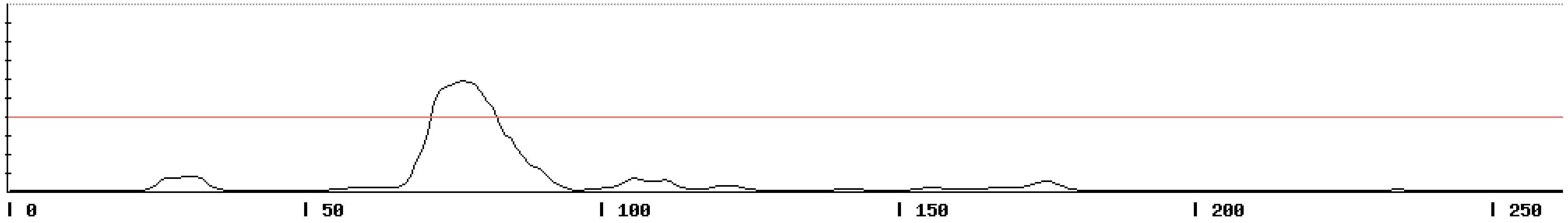

Predictions for Nicotianatabacum_XP_016509571.1	
Viterbi Path	
Viterbi Path	
Posterior @ 0.4	71 - RKHGPKGKPNR - 81
Posterior @ 0.4	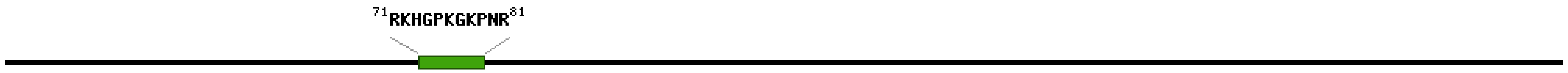
Posterior Graphic. Threshold Marked @ 0.4	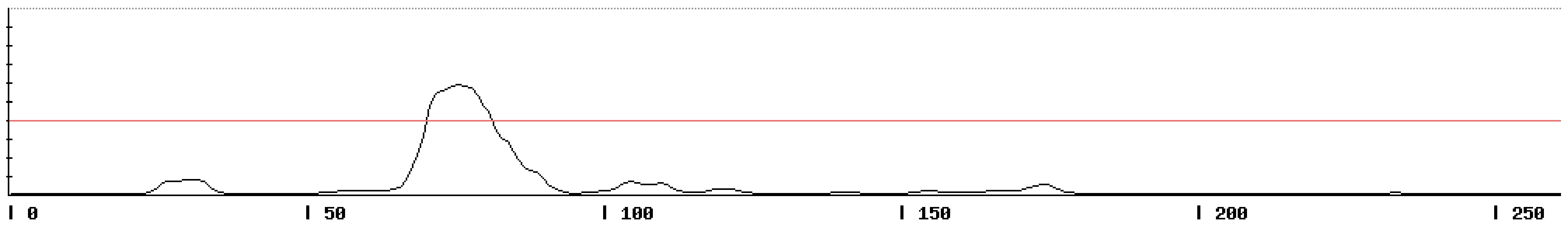

Predictions for Nicotianatabacum_XP_016510067.1	
Viterbi Path	
Viterbi Path	
Posterior @ 0.4	
Posterior @ 0.4	
Posterior Graphic. Threshold Marked @ 0.4	

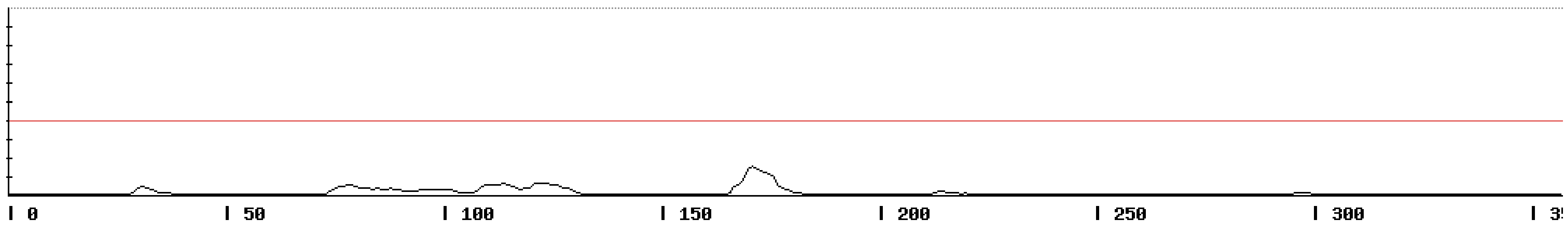

Predictions for Nicotianatabacum_XP_016510514.1

Viterbi Path	
Viterbi Path	
Posterior @ 0.4	
Posterior @ 0.4	
Posterior Graphic. Threshold Marked @ 0.4	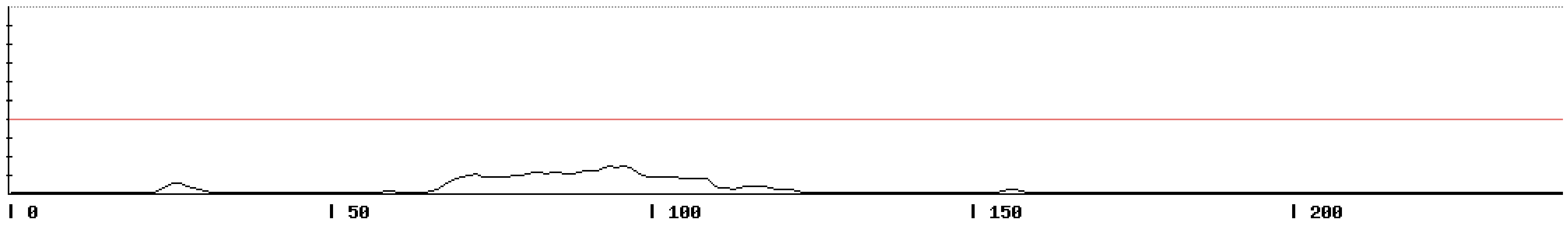

Predictions for Nicotianatabacum_XP_016510515.1

Viterbi Path	
Viterbi Path	
Posterior @ 0.4	
Posterior @ 0.4	

Posterior Graphic. Threshold Marked @ 0.4	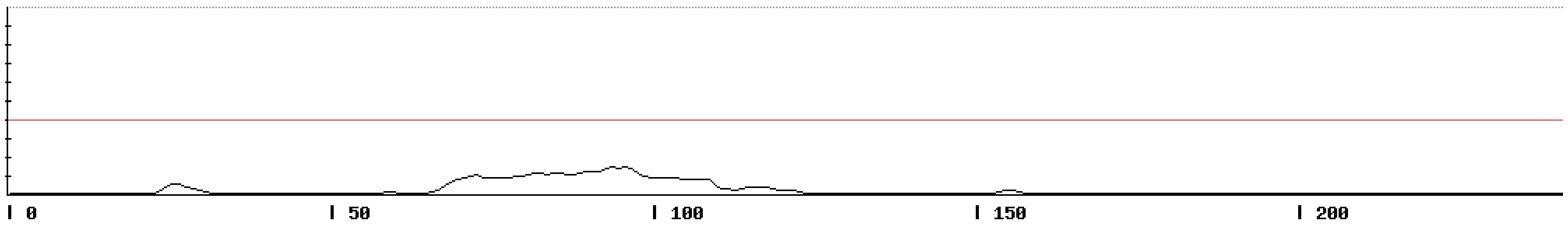

Predictions for Nicotianatabacum_XP_016510726.1

Viterbi Path	162 - KIKKKGKPGDKKKG - 175
Viterbi Path	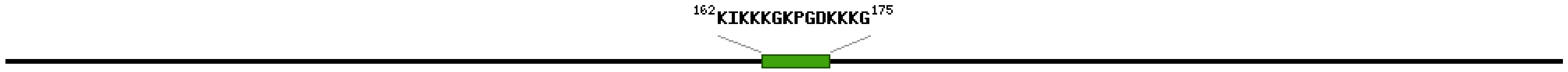

Posterior @ 0.4	79 - KKEKPTWKRVSRTVGKGTWKPQGKG - 103
	162 - KIKKKGKPGDKKKG - 175
Posterior @ 0.4	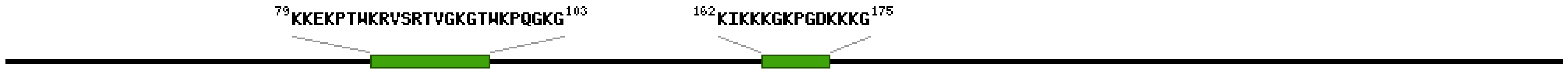

Posterior Graphic. Threshold Marked @ 0.4	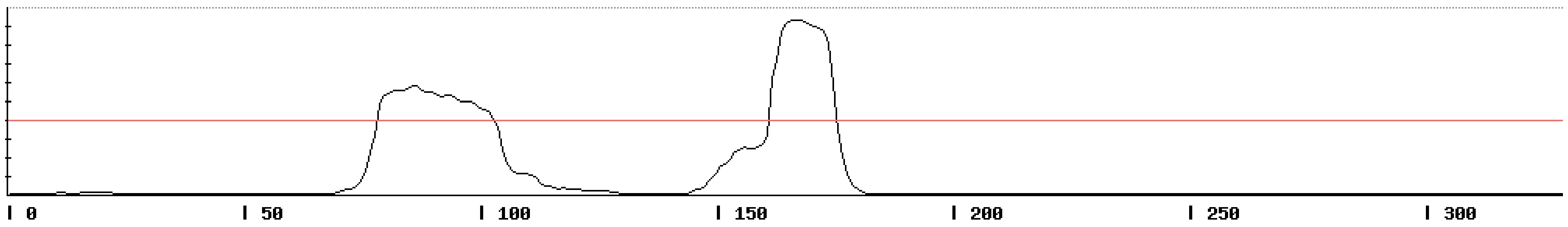

Predictions for Nicotianatabacum_XP_016510918.1

Viterbi Path	
Viterbi Path	
Posterior @ 0.4	
Posterior @ 0.4	

Posterior Graphic. Threshold Marked @ 0.4

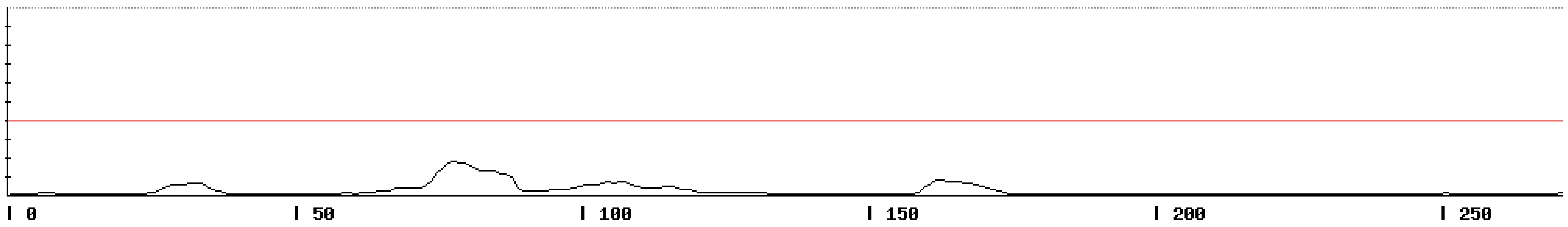

Predictions for Nicotianatabacum_XP_016511406.1

Viterbi Path

Viterbi Path

Posterior @ 0.4

Posterior @ 0.4

Posterior Graphic. Threshold Marked @ 0.4

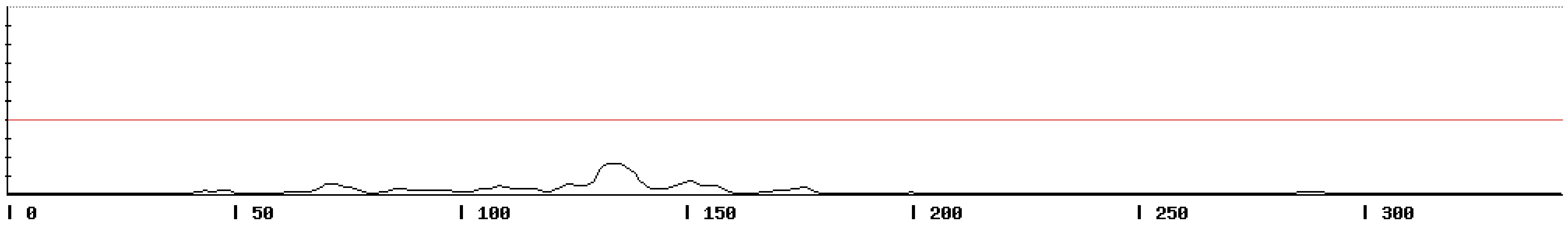

Predictions for Nicotianatabacum_XP_016511523.1

Viterbi Path

Viterbi Path

Posterior @ 0.4

Posterior @ 0.4

Posterior Graphic. Threshold Marked @ 0.4

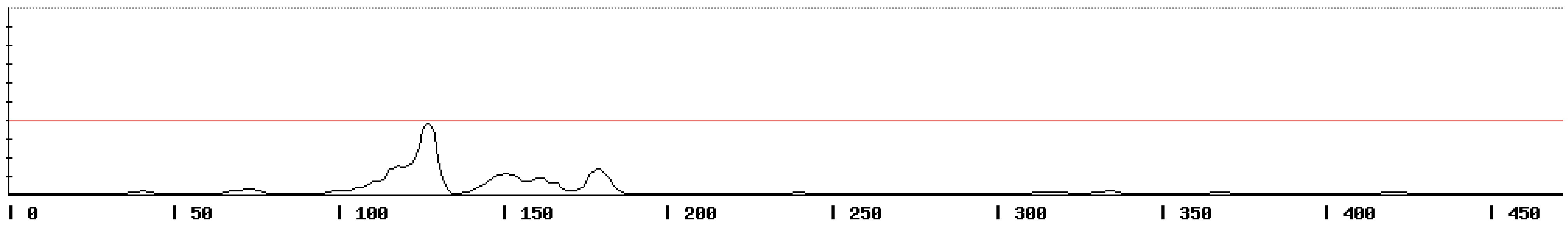

Predictions for Nicotianatabacum_XP_016511581.1

Viterbi Path

Viterbi Path

Posterior @ 0.4

Posterior @ 0.4

Posterior Graphic. Threshold Marked @ 0.4

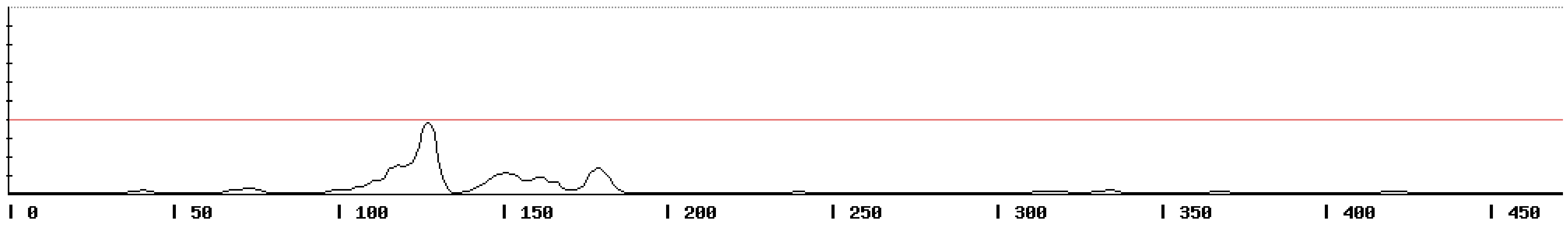

Predictions for Nicotianatabacum_XP_016511644.1

Viterbi Path

Viterbi Path

Posterior @ 0.4	
Posterior @ 0.4	
Posterior Graphic. Threshold Marked @ 0.4	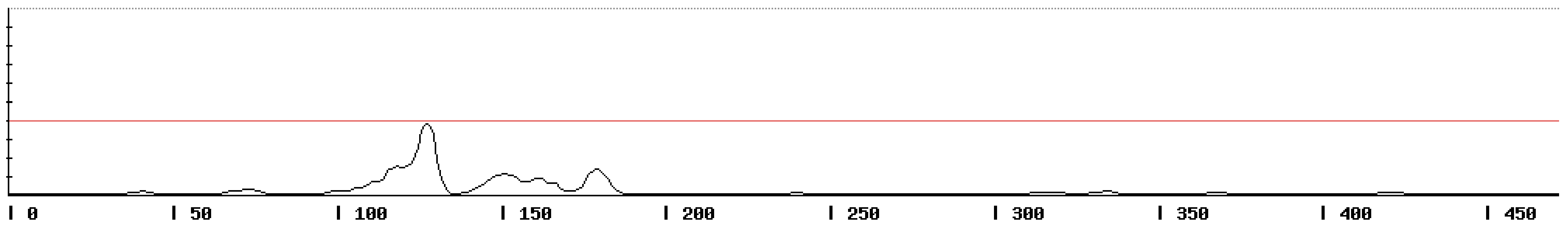

Predictions for Nicotianatabacum_XP_016512203.1

Viterbi Path	
Viterbi Path	
Posterior @ 0.4	
Posterior @ 0.4	
Posterior Graphic. Threshold Marked @ 0.4	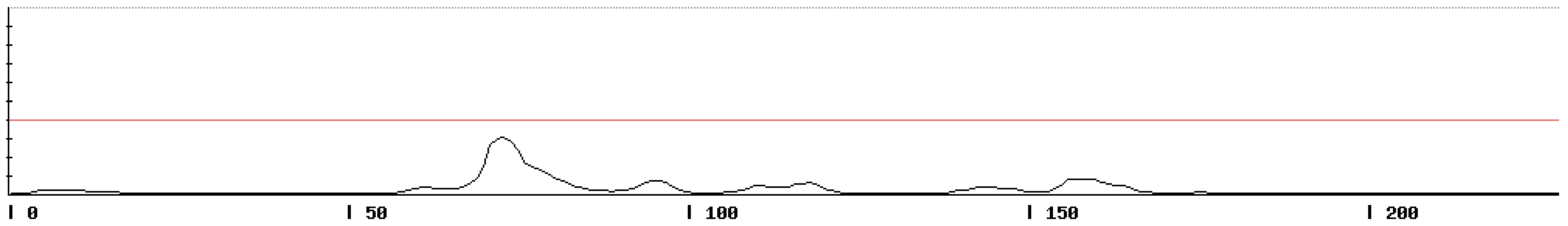

Predictions for Nicotianatabacum_XP_016512401.1

Viterbi Path

Viterbi Path

Posterior @ 0.4

Posterior @ 0.4

Posterior Graphic. Threshold Marked @ 0.4

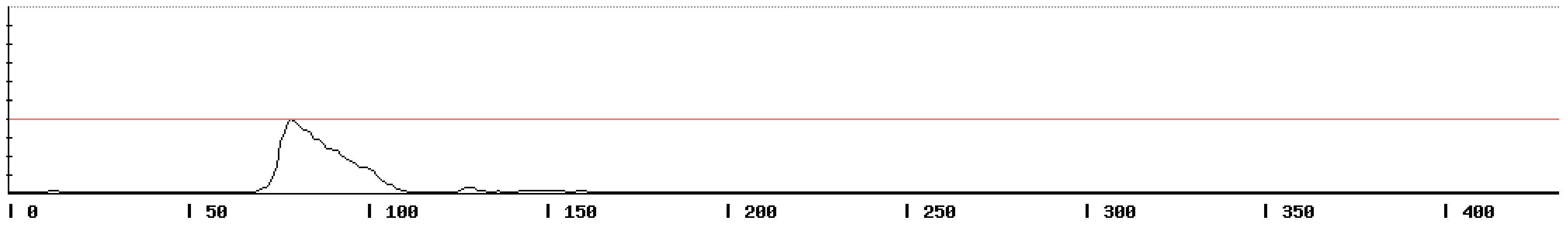

Predictions for Nicotianatabacum_XP_016512625.1

Viterbi Path

Viterbi Path

Posterior @ 0.4

Posterior @ 0.4

Posterior Graphic. Threshold Marked @ 0.4

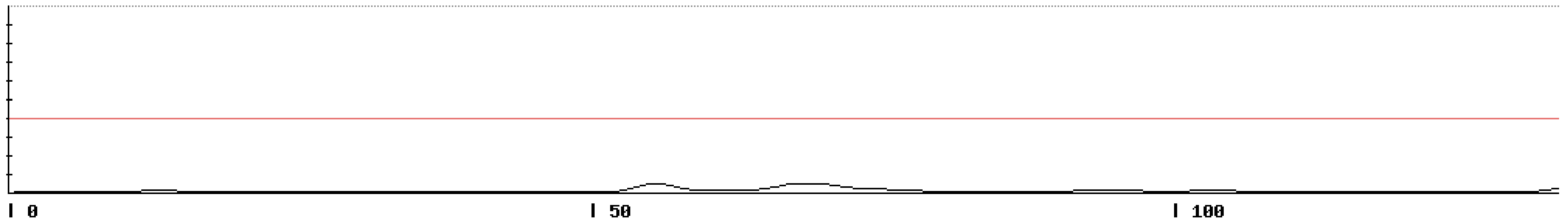

Predictions for Nicotianatabacum_XP_016512818.1

Viterbi Path	
Viterbi Path	
Posterior @ 0.4	
Posterior @ 0.4	
Posterior Graphic. Threshold Marked @ 0.4	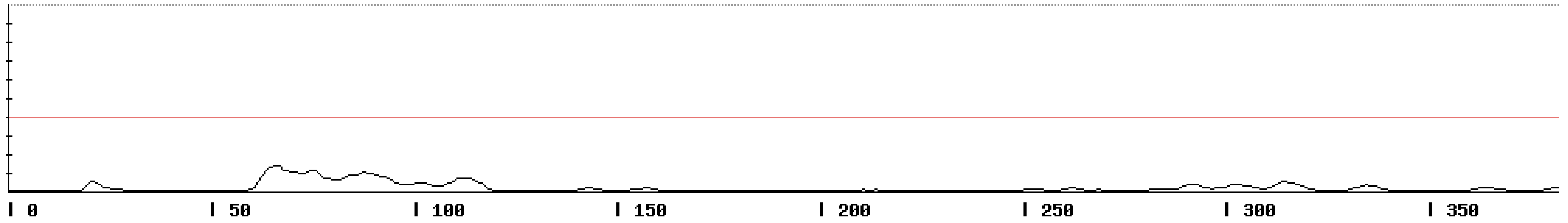

Predictions for Nicotianatabacum_XP_016512873.1

Viterbi Path	
Viterbi Path	
Posterior @ 0.4	
Posterior @ 0.4	
Posterior Graphic. Threshold Marked @ 0.4	

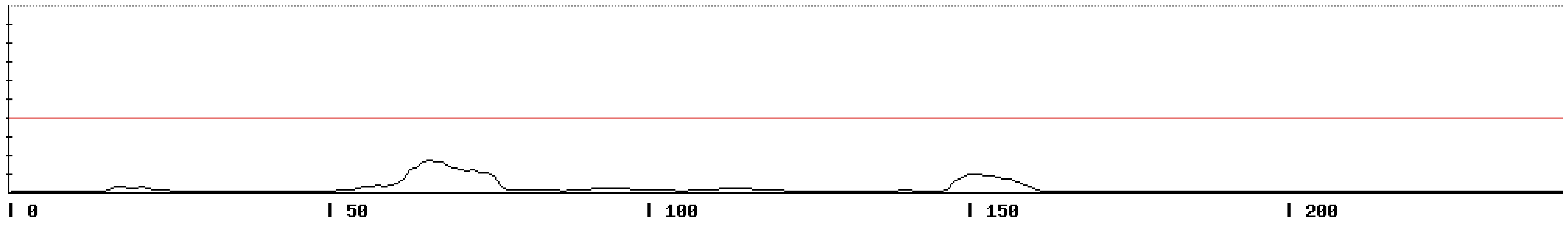

Predictions for Nicotianatabacum_XP_016512874.1

Viterbi Path	
Viterbi Path	
Posterior @ 0.4	189 - KKQKREHIR - 197
Posterior @ 0.4	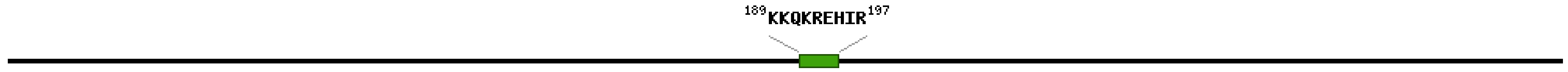
Posterior Graphic. Threshold Marked @ 0.4	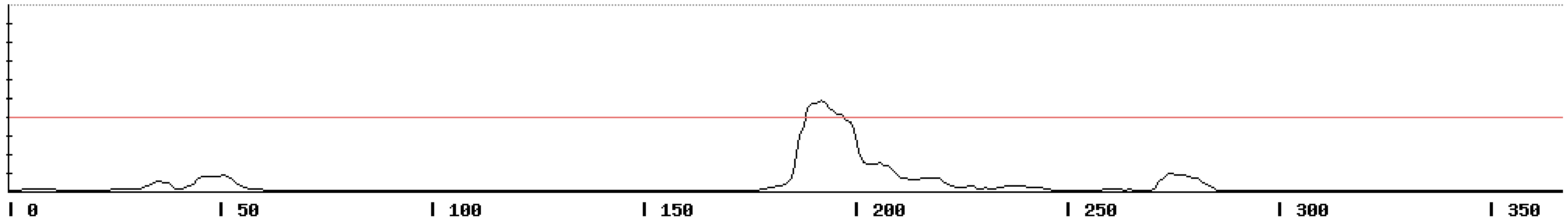

Predictions for Nicotianatabacum_XP_016513125.1

Viterbi Path	
Viterbi Path	
Posterior @ 0.4	
Posterior @ 0.4	

Posterior Graphic. Threshold Marked @ 0.4

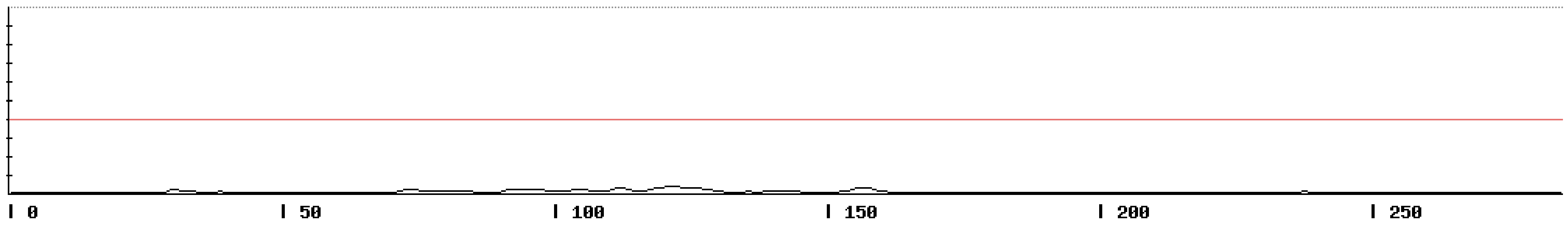

Predictions for Nicotianatabacum_XP_016514883.1

Viterbi Path

Viterbi Path

Posterior @ 0.4

Posterior @ 0.4

Posterior Graphic. Threshold Marked @ 0.4

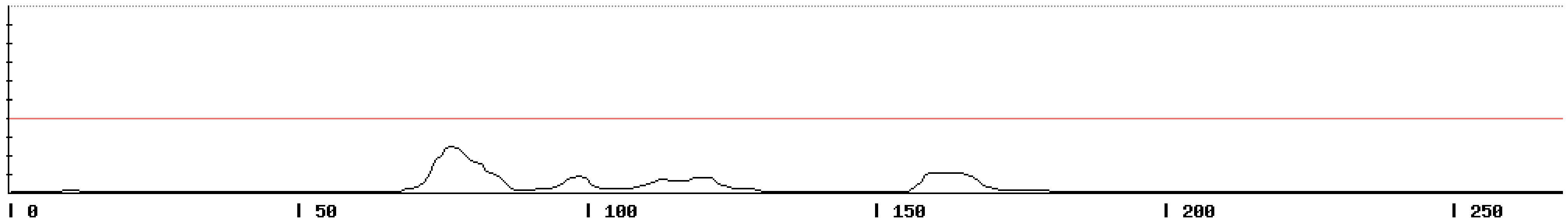

Predictions for Nicotianatabacum_XP_016515059.1

Viterbi Path

Viterbi Path

Posterior @ 0.4

Posterior @ 0.4

Posterior Graphic. Threshold Marked @ 0.4

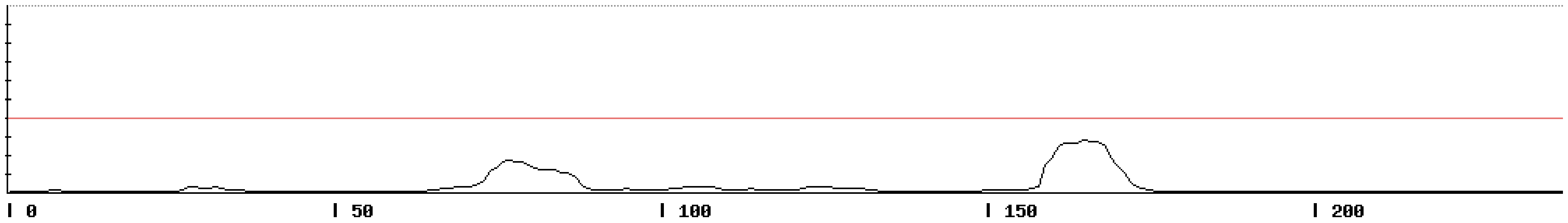

Predictions for Nicotianatabacum_XP_016515157.1

Viterbi Path

Viterbi Path

Posterior @ 0.4

Posterior @ 0.4

Posterior Graphic. Threshold Marked @ 0.4

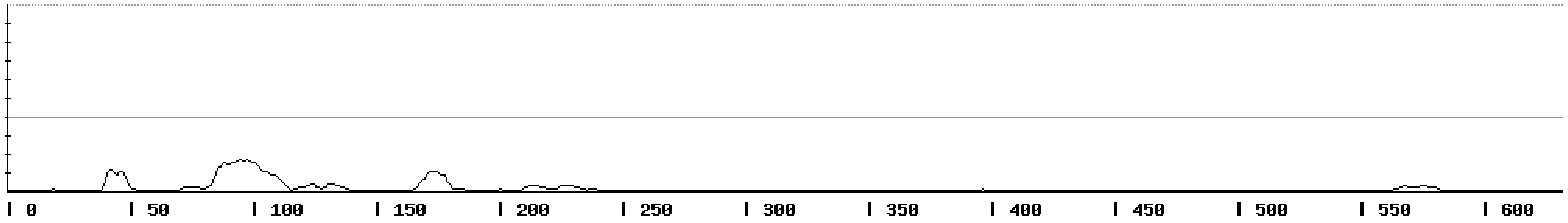

Predictions for Nicotianatabacum_XP_016515300.1

Viterbi Path

Viterbi Path

Posterior @ 0.4

Posterior @ 0.4

Posterior Graphic. Threshold Marked @ 0.4

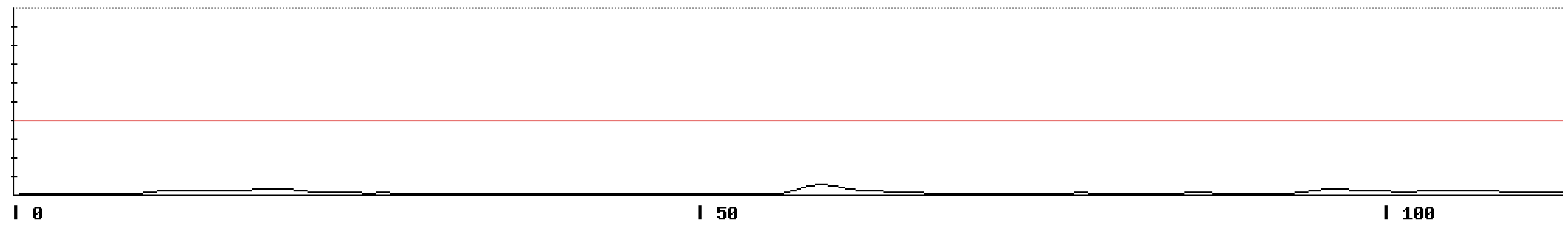

Predictions for Nicotianatabacum_XP_016515338.1

Viterbi Path

Viterbi Path

Posterior @ 0.4

Posterior @ 0.4

Posterior Graphic. Threshold Marked @ 0.4

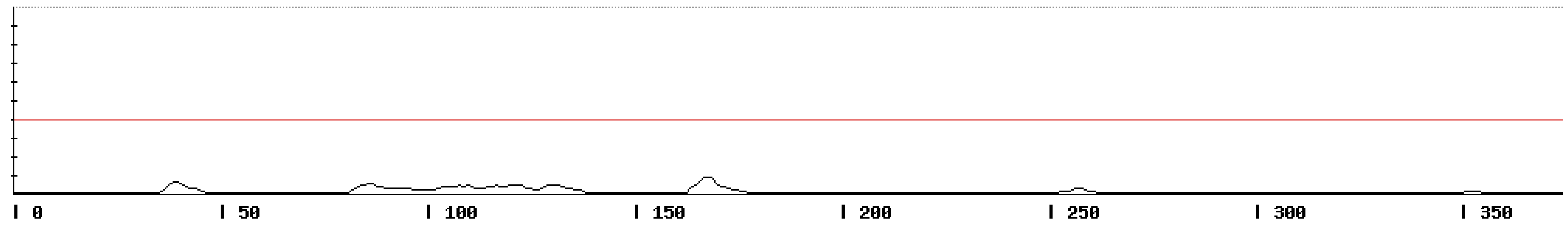

Predictions for Nicotianatabacum_XP_016515497.1

Viterbi Path

Viterbi Path

Posterior @ 0.4

Posterior @ 0.4

Posterior Graphic. Threshold Marked @ 0.4

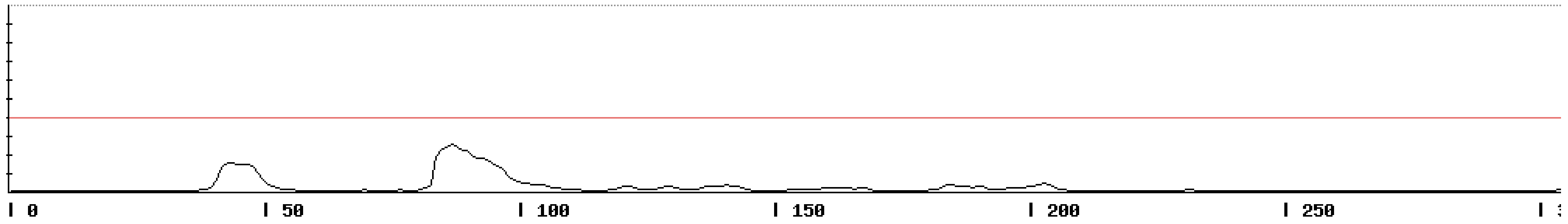

Predictions for Nicotianatabacum_XP_016516166.1

Viterbi Path

Viterbi Path

Posterior @ 0.4

Posterior @ 0.4

Posterior Graphic. Threshold Marked @ 0.4

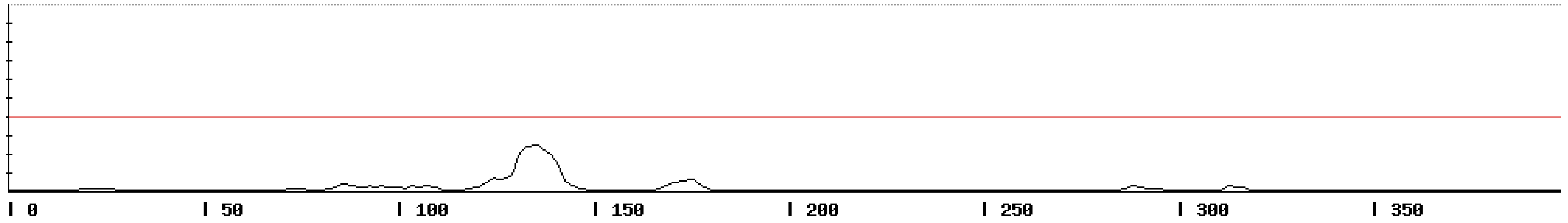

Intended to be used with Firefox

Viterbi Path

Posterior @ 0.4

Posterior @ 0.4

Posterior Graphic. Threshold Marked @ 0.4

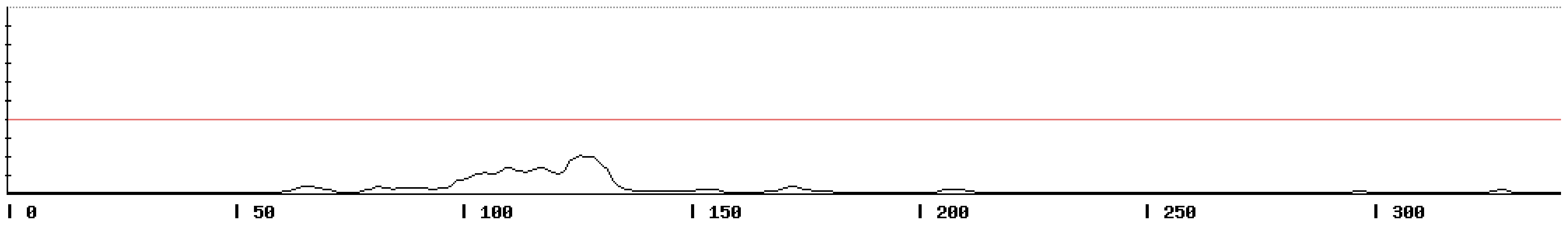

Predictions for Nicotianatomentosiformis_XP_009587307.1

Viterbi Path

Viterbi Path

Posterior @ 0.4

Posterior @ 0.4

Posterior Graphic. Threshold Marked @ 0.4

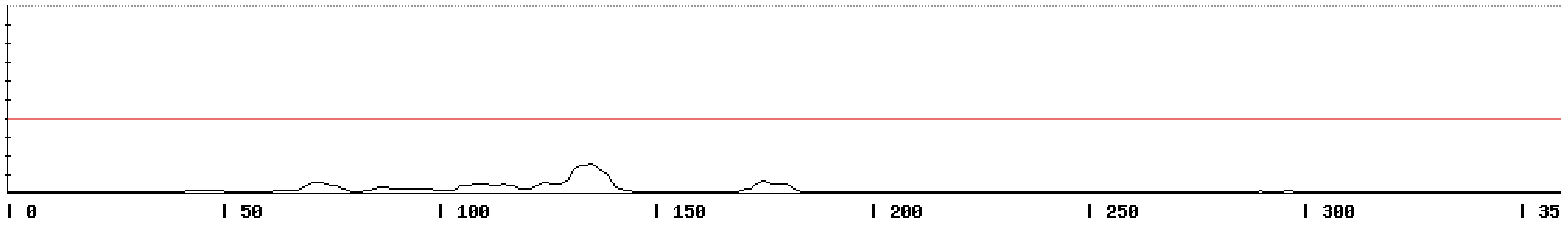

Predictions for Nicotianatomentosiformis_XP_009587632.1

Viterbi Path	
Viterbi Path	
Posterior @ 0.4	136 - RKRRKV - 141
	151 - RWHKTGKTRPVVVKGKVKGHKK - 172
Posterior @ 0.4	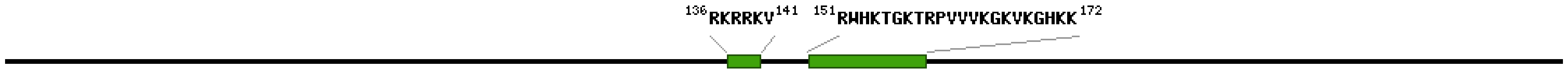
Posterior Graphic. Threshold Marked @ 0.4	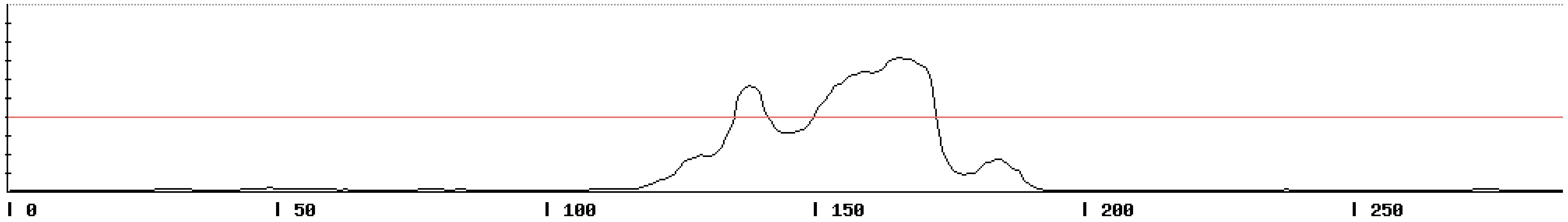

Predictions for Nicotianatomentosiformis_XP_009587883.1

Viterbi Path	
Viterbi Path	
Posterior @ 0.4	
Posterior @ 0.4	
Posterior Graphic. Threshold Marked @ 0.4	

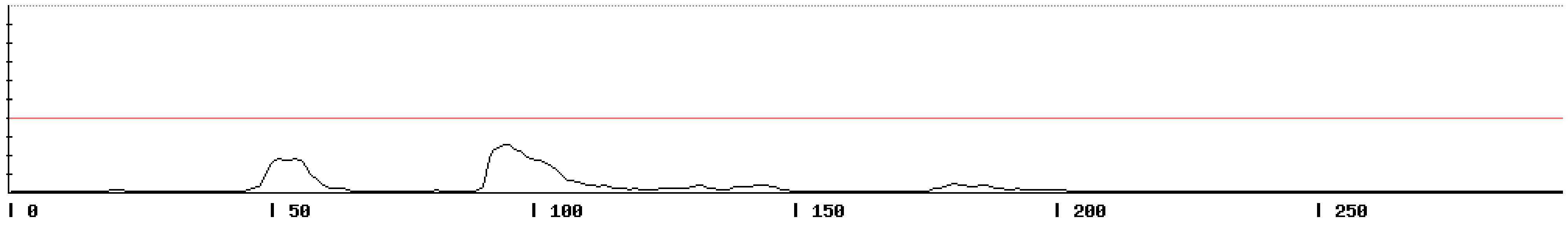

Predictions for Nicotianatomentosiformis_XP_009588904.1

Viterbi Path

Viterbi Path

Posterior @ 0.4

Posterior @ 0.4

Posterior Graphic. Threshold Marked @ 0.4

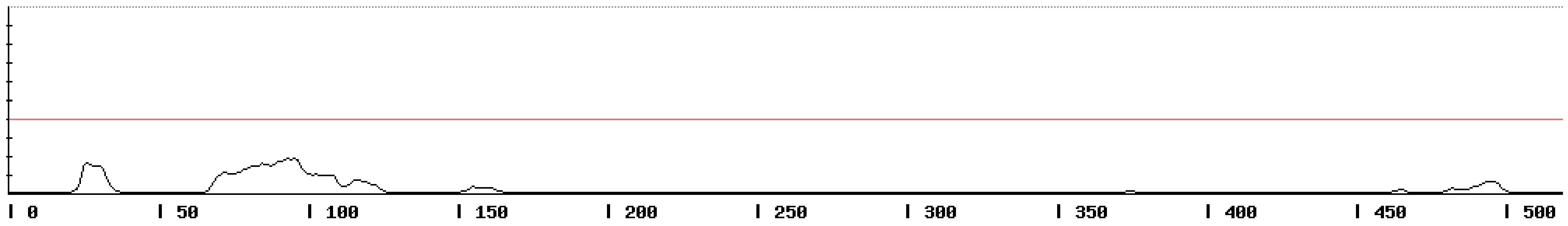

Predictions for Nicotianatomentosiformis_XP_009589119.1

Viterbi Path

Viterbi Path

Posterior @ 0.4

Posterior @ 0.4

Posterior Graphic. Threshold Marked @ 0.4	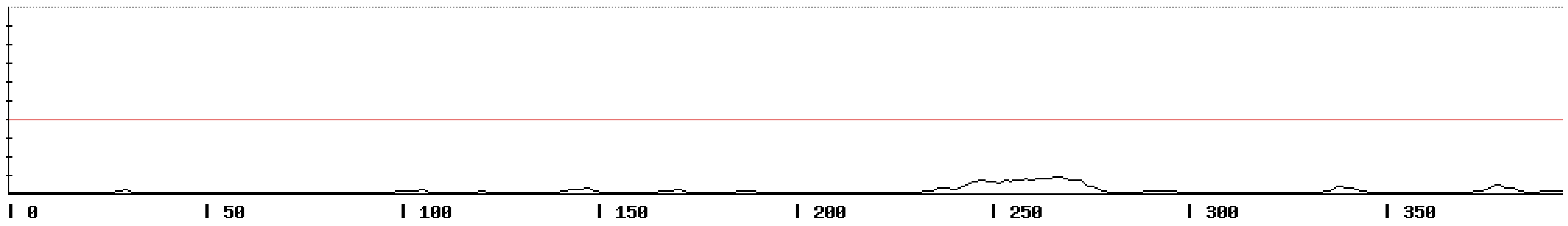

Predictions for Nicotianatomentosiformis_XP_009589539.1	
Viterbi Path	
Viterbi Path	
Posterior @ 0.4	80 - KKK - 82
Posterior @ 0.4	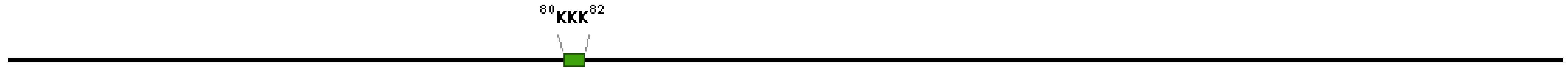
Posterior Graphic. Threshold Marked @ 0.4	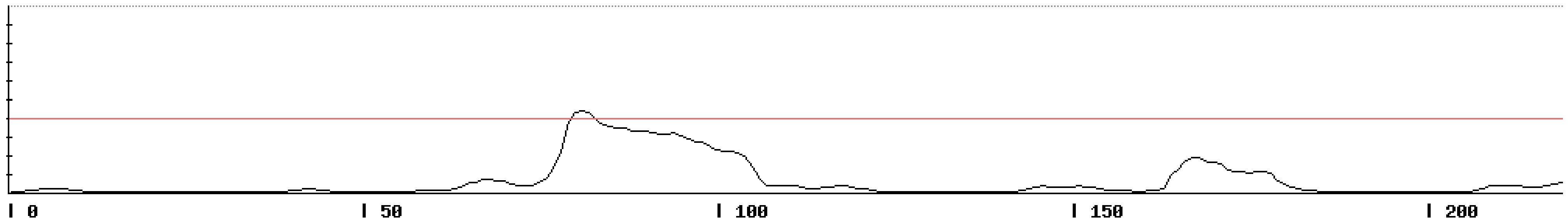

Predictions for Nicotianatomentosiformis_XP_009589729.1	
Viterbi Path	
Viterbi Path	
Posterior @ 0.4	
Posterior @ 0.4	

Posterior Graphic. Threshold Marked @ 0.4

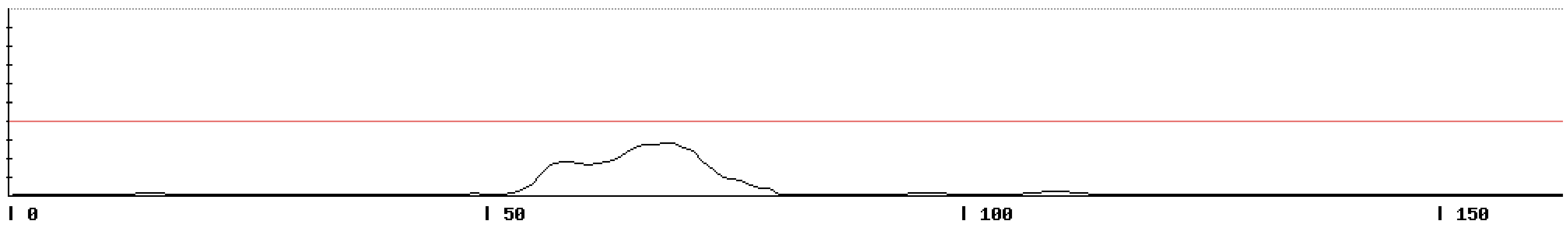

Predictions for Nicotianatomentosiformis_XP_009590031.1

Viterbi Path

Viterbi Path

Posterior @ 0.4

Posterior @ 0.4

Posterior Graphic. Threshold Marked @ 0.4

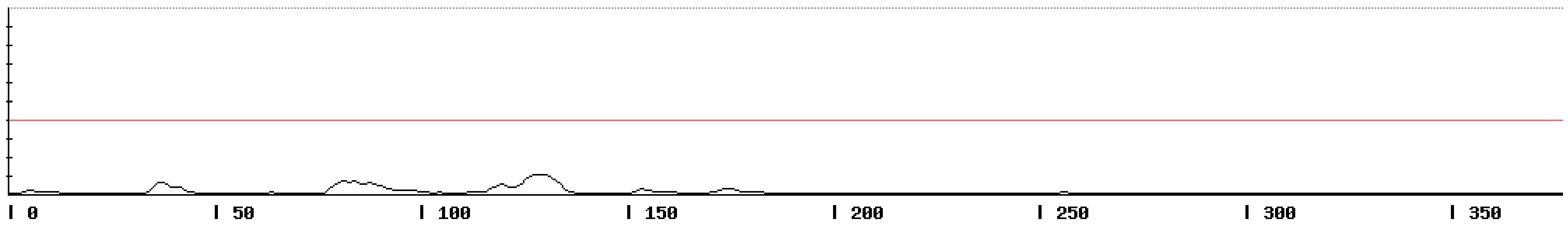

Predictions for Nicotianatomentosiformis_XP_009590289.1

Viterbi Path

Viterbi Path

Posterior @ 0.4

Posterior @ 0.4

Posterior Graphic. Threshold Marked @ 0.4

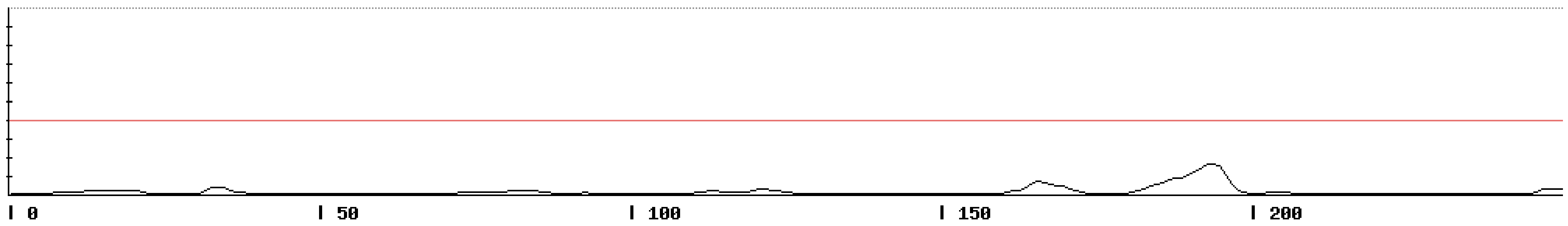

Predictions for Nicotianatomentosiformis_XP_009590290.1

Viterbi Path

Viterbi Path

Posterior @ 0.4

Posterior @ 0.4

Posterior Graphic. Threshold Marked @ 0.4

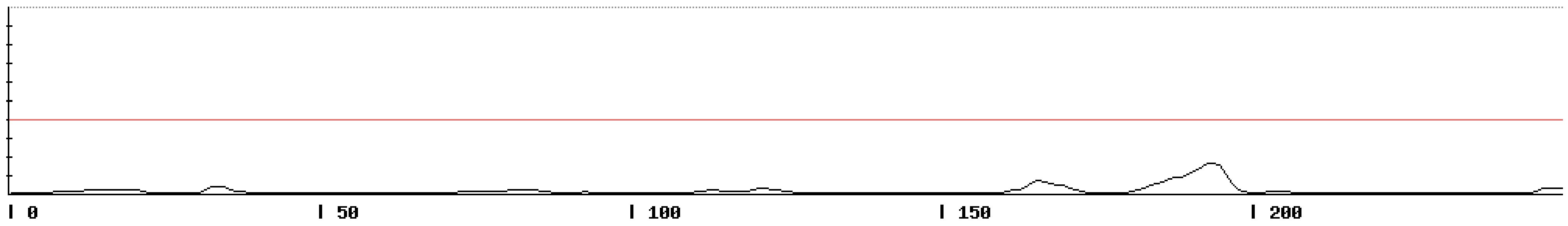

Predictions for Nicotianatomentosiformis_XP_009590494.1

Viterbi Path

Viterbi Path

Posterior @ 0.4	
Posterior @ 0.4	
Posterior Graphic. Threshold Marked @ 0.4	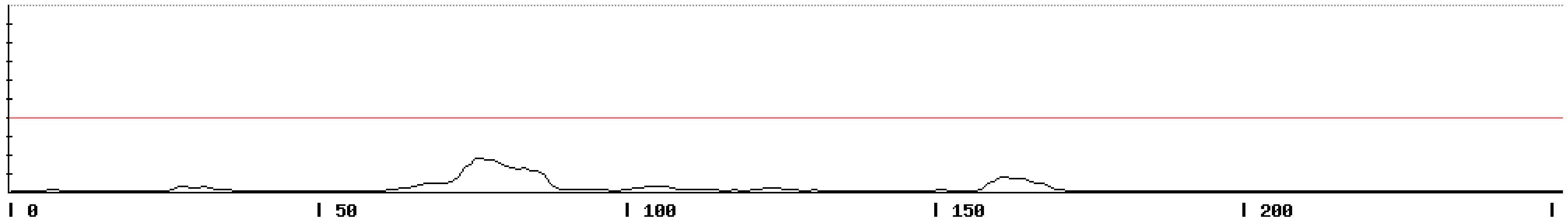

Predictions for Nicotianatomentosiformis_XP_009590635.1	
Viterbi Path	
Viterbi Path	
Posterior @ 0.4	
Posterior @ 0.4	
Posterior Graphic. Threshold Marked @ 0.4	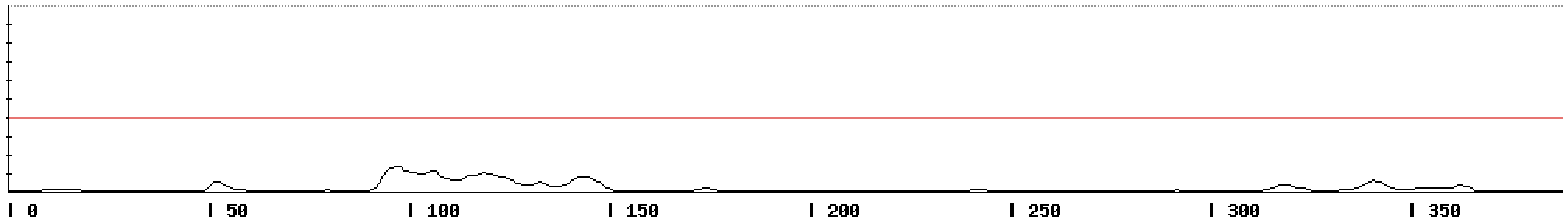

Predictions for Nicotianatomentosiformis_XP_009591047.1

Viterbi Path

Viterbi Path

Posterior @ 0.4

Posterior @ 0.4

Posterior Graphic. Threshold Marked @ 0.4

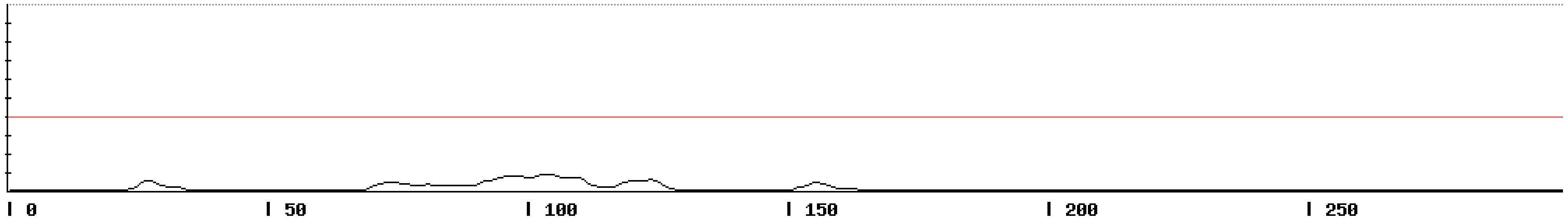

Predictions for Nicotianatomentosiformis_XP_009591137.1

Viterbi Path

Viterbi Path

Posterior @ 0.4

Posterior @ 0.4

Posterior Graphic. Threshold Marked @ 0.4

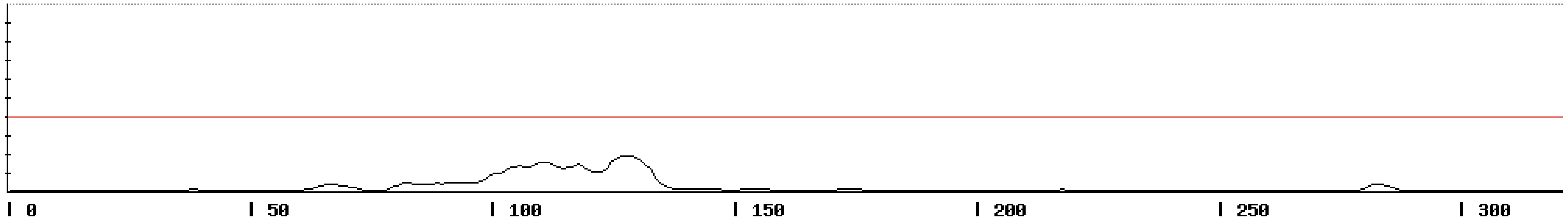

Predictions for Nicotianatomentosiformis_XP_009591138.1	
Viterbi Path	
Viterbi Path	
Posterior @ 0.4	
Posterior @ 0.4	
Posterior Graphic. Threshold Marked @ 0.4	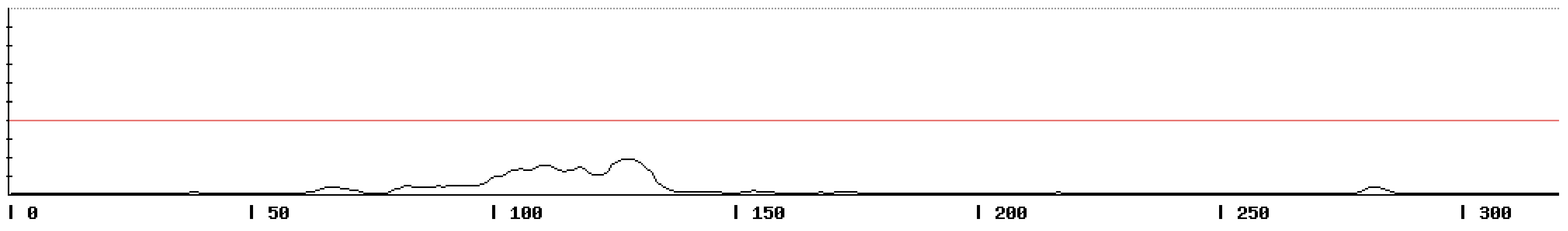

Predictions for Nicotianatomentosiformis_XP_009591286.1	
Viterbi Path	
Viterbi Path	
Posterior @ 0.4	
Posterior @ 0.4	
Posterior Graphic. Threshold Marked @ 0.4	

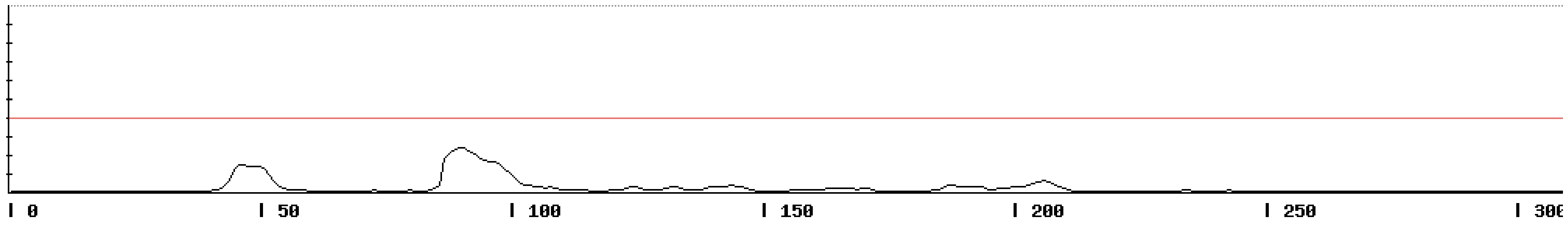

Predictions for Nicotianatomentosiformis_XP_009591317.1

Viterbi Path

Viterbi Path

Posterior @ 0.4

Posterior @ 0.4

Posterior Graphic. Threshold Marked @ 0.4

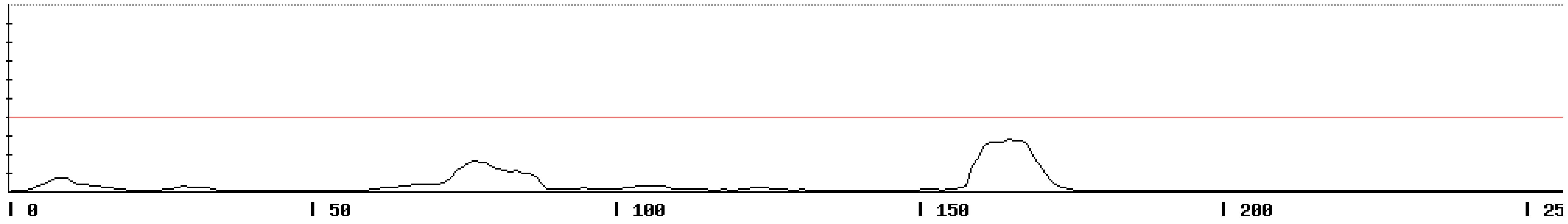

Predictions for Nicotianatomentosiformis_XP_009592902.1

Viterbi Path

Viterbi Path

Posterior @ 0.4

Posterior @ 0.4

Posterior Graphic. Threshold Marked @ 0.4

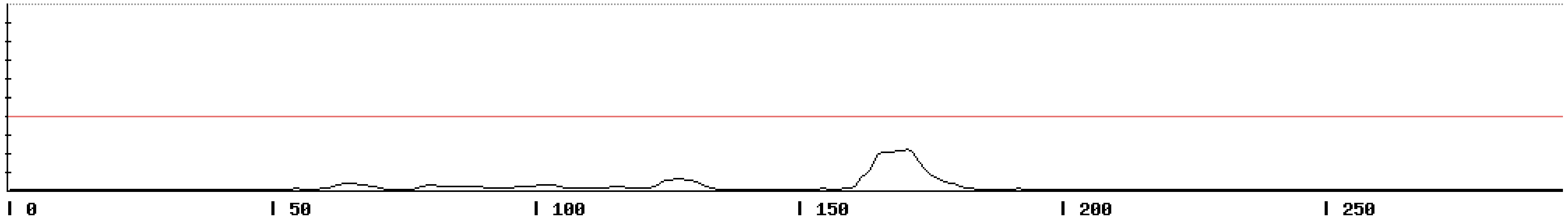

Predictions for Nicotianatomentosiformis_XP_009593583.1

Viterbi Path

Viterbi Path

Posterior @ 0.4

Posterior @ 0.4

Posterior Graphic. Threshold Marked @ 0.4

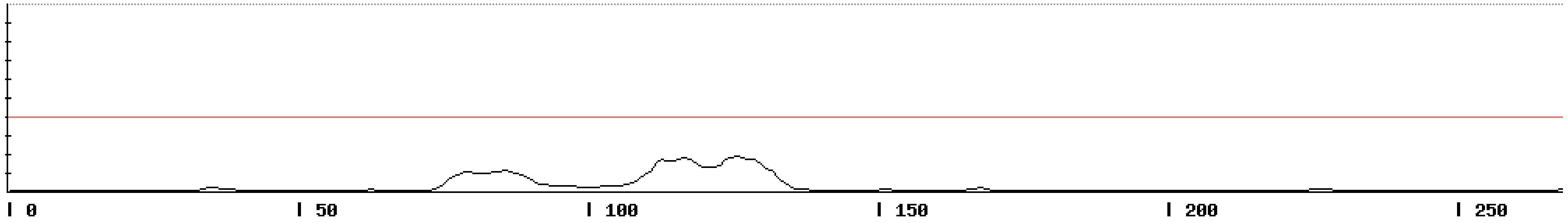

Predictions for Nicotianatomentosiformis_XP_009593584.1

Viterbi Path

Viterbi Path

Posterior @ 0.4

Posterior @ 0.4

Posterior Graphic. Threshold Marked @ 0.4

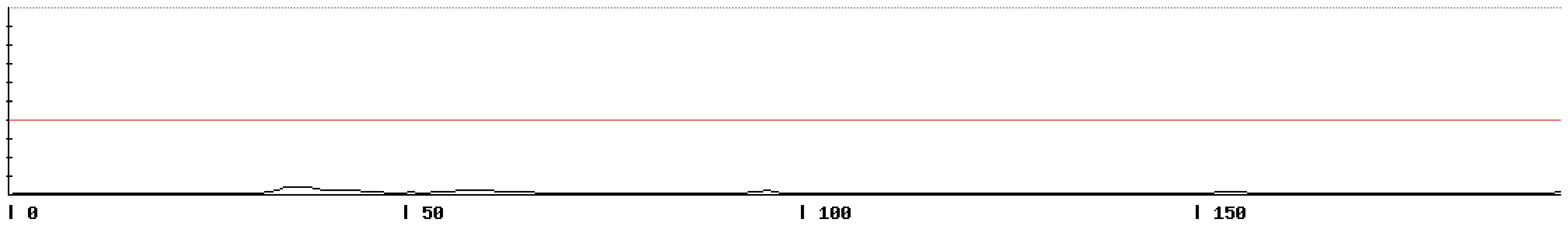

Predictions for Nicotianatomentosiformis_XP_009593586.1

Viterbi Path

Viterbi Path

Posterior @ 0.4

Posterior @ 0.4

Posterior Graphic. Threshold Marked @ 0.4

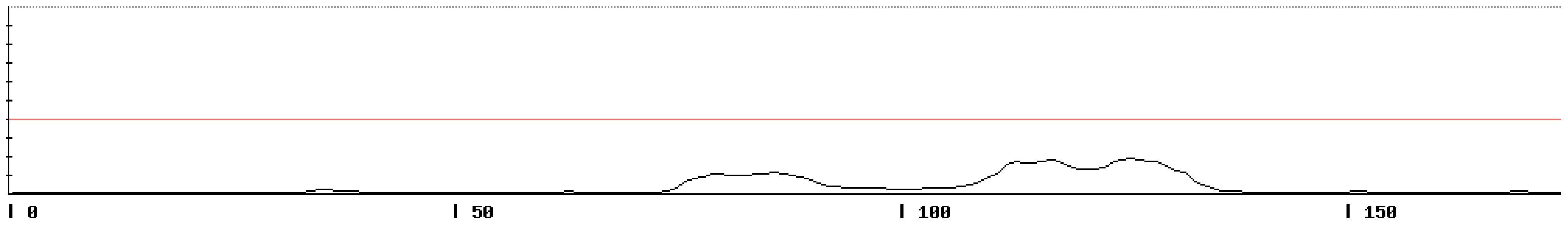

Predictions for Nicotianatomentosiformis_XP_009594217.1

Viterbi Path

Viterbi Path

Posterior @ 0.4	
Posterior @ 0.4	
Posterior Graphic. Threshold Marked @ 0.4	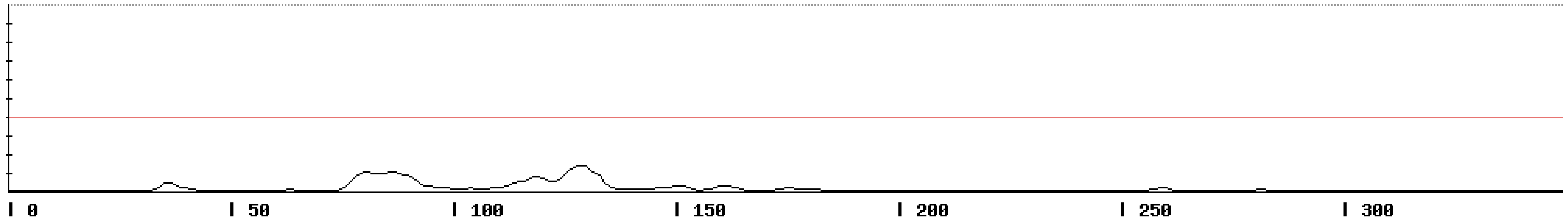

Predictions for Nicotianatomentosiformis_XP_009594219.1

Viterbi Path	
Viterbi Path	
Posterior @ 0.4	
Posterior @ 0.4	
Posterior Graphic. Threshold Marked @ 0.4	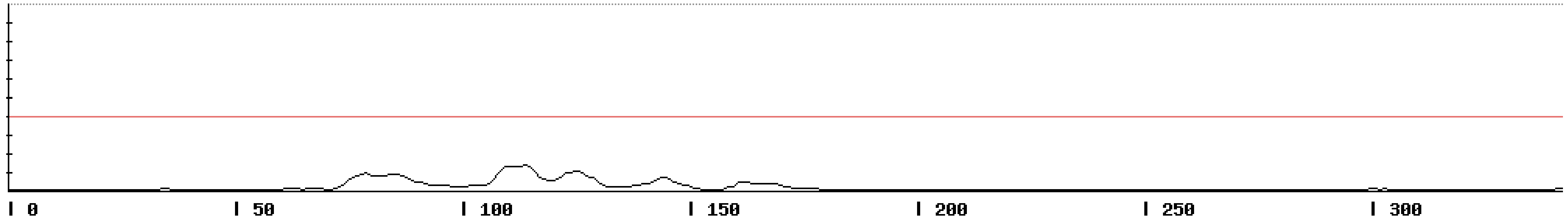

Predictions for Nicotianatomentosiformis_XP_009594623.1

Viterbi Path	
Viterbi Path	

Posterior @ 0.4	372 - REEKRKKKLKK - 382
Posterior @ 0.4	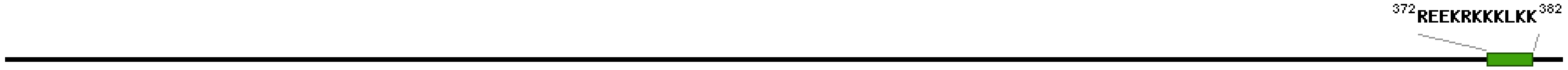
Posterior Graphic. Threshold Marked @ 0.4	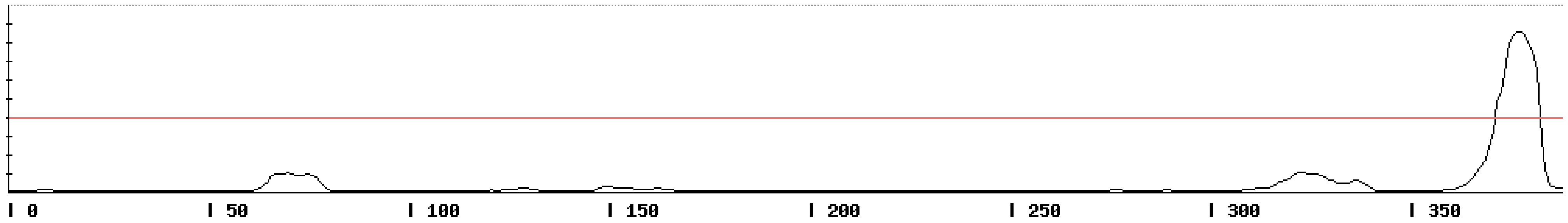

Predictions for Nicotianatomentosiformis_XP_009595150.1

Viterbi Path	
Viterbi Path	
Posterior @ 0.4	
Posterior @ 0.4	
Posterior Graphic. Threshold Marked @ 0.4	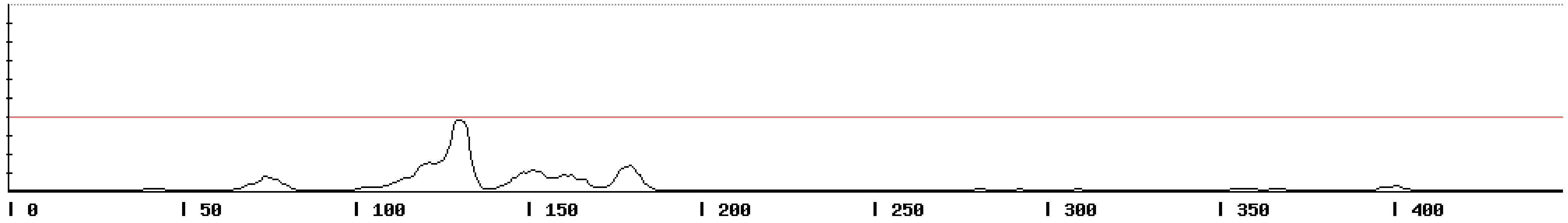

Predictions for Nicotianatomentosiformis_XP_009595151.1

Viterbi Path

Viterbi Path

Posterior @ 0.4

Posterior @ 0.4

Posterior Graphic. Threshold Marked @ 0.4

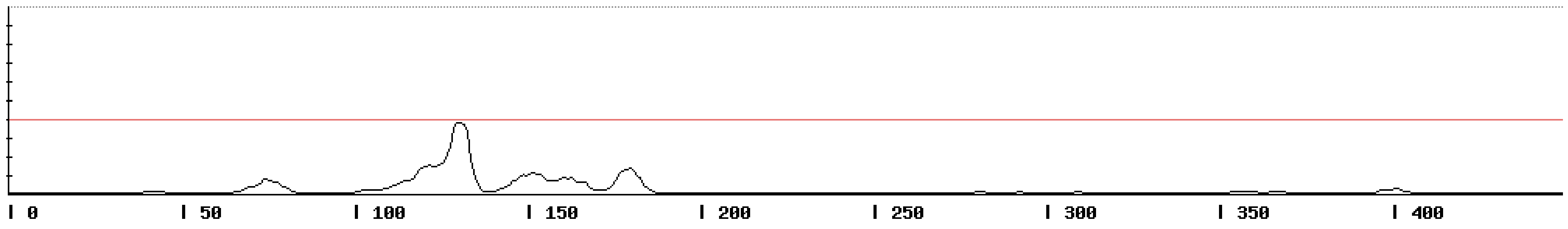

Predictions for Nicotianatomentosiformis_XP_009595153.1

Viterbi Path

Viterbi Path

Posterior @ 0.4

Posterior @ 0.4

Posterior Graphic. Threshold Marked @ 0.4

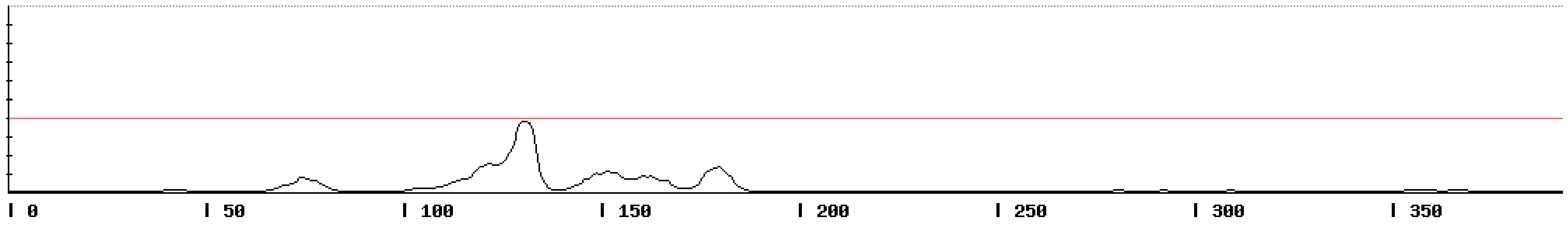

Predictions for Nicotianatomentosiformis_XP_009596681.1	
Viterbi Path	
Viterbi Path	
Posterior @ 0.4	
Posterior @ 0.4	
Posterior Graphic. Threshold Marked @ 0.4	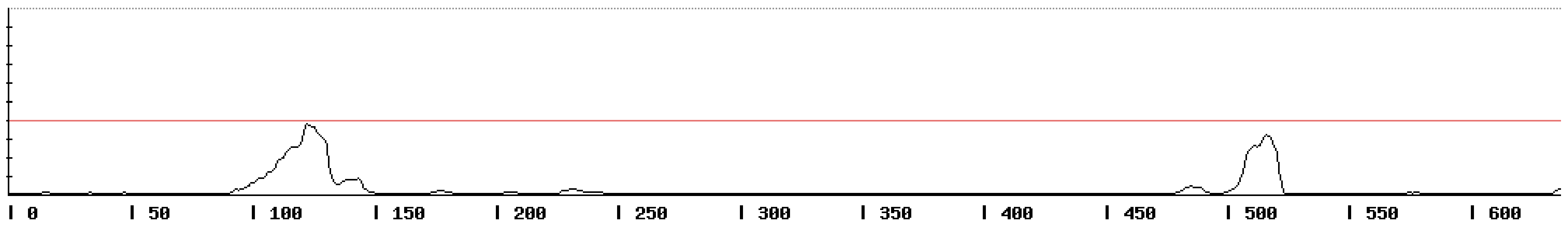

Predictions for Nicotianatomentosiformis_XP_009597016.1	
Viterbi Path	
Viterbi Path	
Posterior @ 0.4	
Posterior @ 0.4	
Posterior Graphic. Threshold Marked @ 0.4	

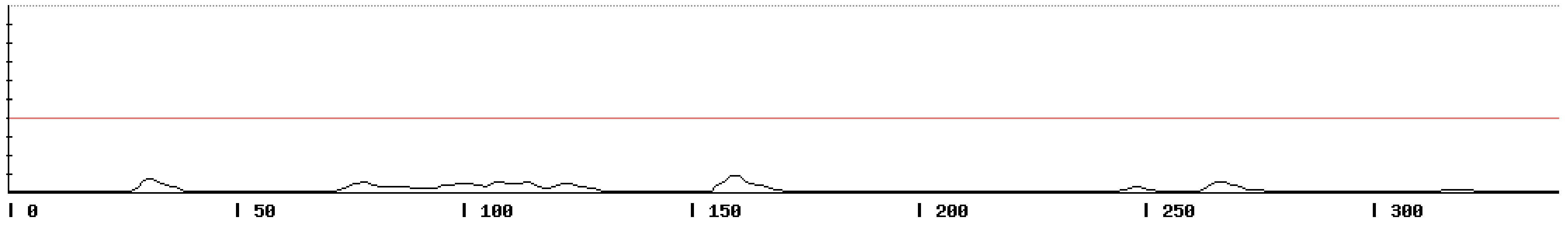

Predictions for Nicotianatomentosiformis_XP_009597017.1

Viterbi Path	
Viterbi Path	
Posterior @ 0.4	
Posterior @ 0.4	
Posterior Graphic. Threshold Marked @ 0.4	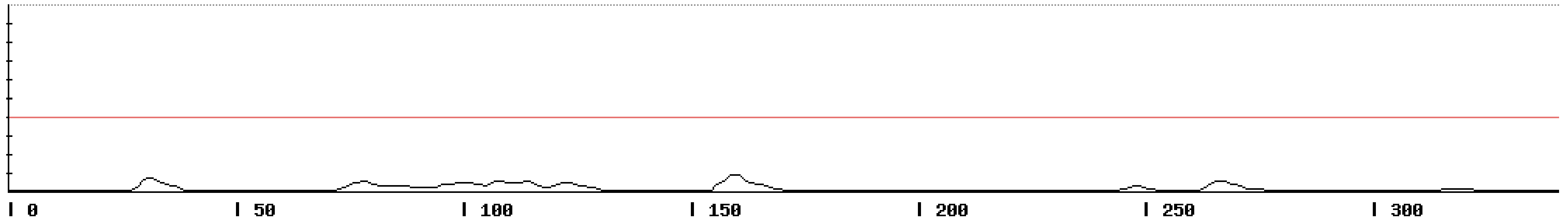

Predictions for Nicotianatomentosiformis_XP_009597084.1

Viterbi Path	
Viterbi Path	
Posterior @ 0.4	79 - KRTKAGGLRFSRGLIGGGSWKGLDKRKPVFKGSKLVG - 115
Posterior @ 0.4	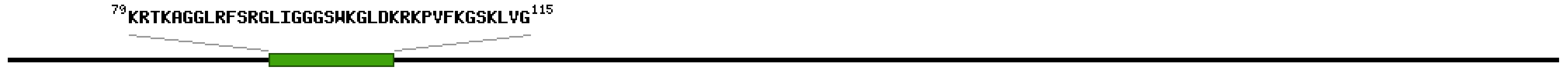

Posterior Graphic. Threshold Marked @ 0.4	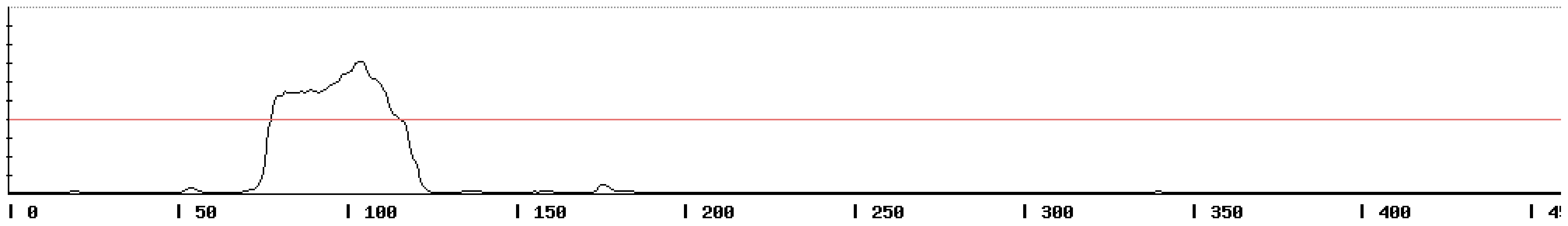

Predictions for Nicotianatomentosiformis_XP_009597132.1	
Viterbi Path	
Viterbi Path	
Posterior @ 0.4	
Posterior @ 0.4	
Posterior Graphic. Threshold Marked @ 0.4	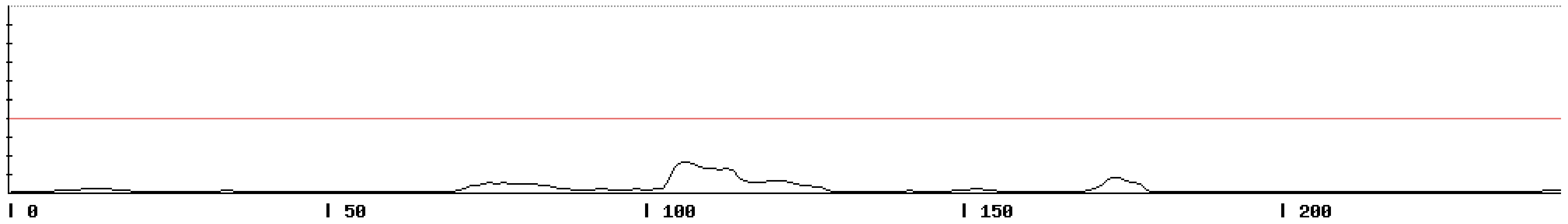

Predictions for Nicotianatomentosiformis_XP_009597635.1	
Viterbi Path	
Viterbi Path	
Posterior @ 0.4	
Posterior @ 0.4	

Posterior Graphic. Threshold Marked @ 0.4

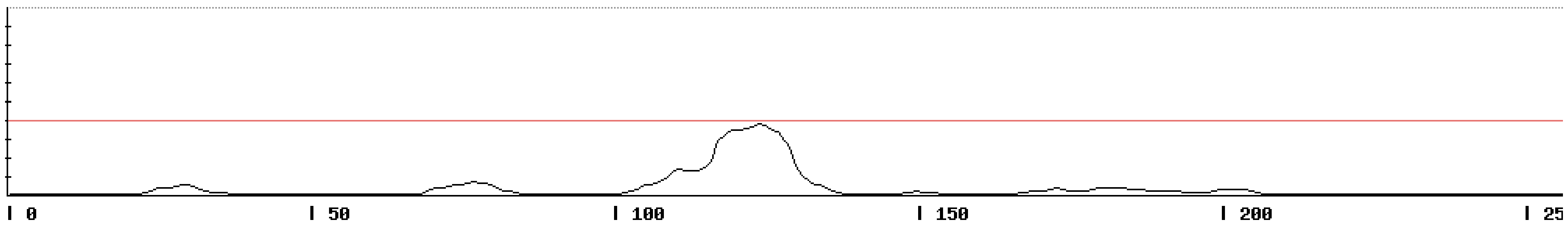

Predictions for Nicotianatomentosiformis_XP_009598091.1

Viterbi Path

Viterbi Path

Posterior @ 0.4

Posterior @ 0.4

Posterior Graphic. Threshold Marked @ 0.4

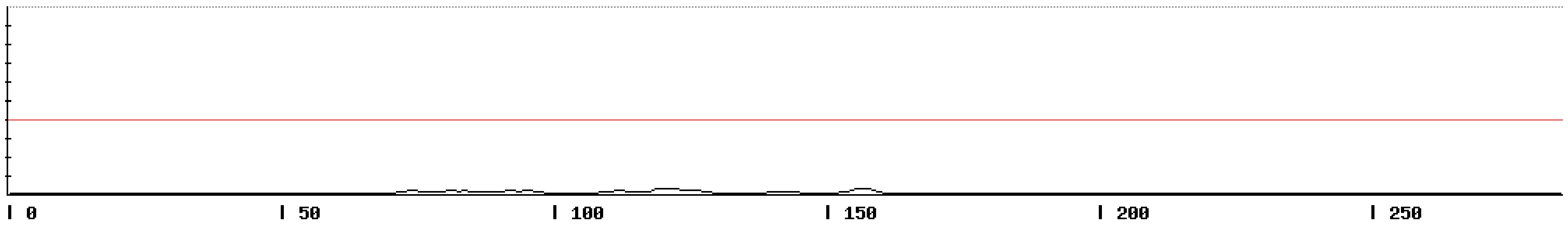

Predictions for Nicotianatomentosiformis_XP_009598687.1

Viterbi Path

Viterbi Path

Posterior @ 0.4

Posterior @ 0.4

Posterior Graphic. Threshold Marked @ 0.4

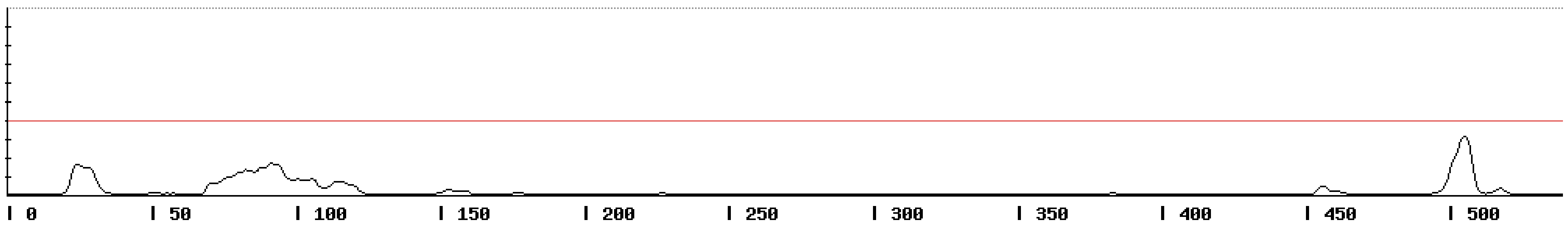

Predictions for Nicotianatomentosiformis_XP_009598688.1

Viterbi Path

Viterbi Path

Posterior @ 0.4

Posterior @ 0.4

Posterior Graphic. Threshold Marked @ 0.4

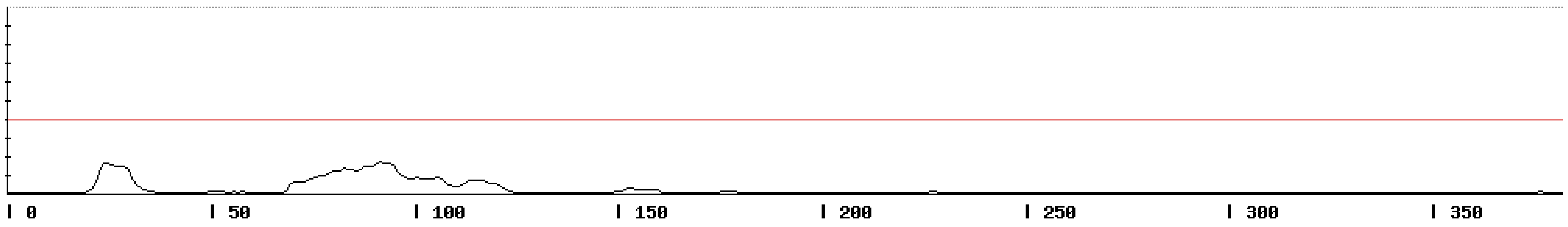

Predictions for Nicotianatomentosiformis_XP_009598748.1

Viterbi Path

Viterbi Path

Posterior @ 0.4	158 - IKKKENPDDKKKGK - 171

Posterior @ 0.4

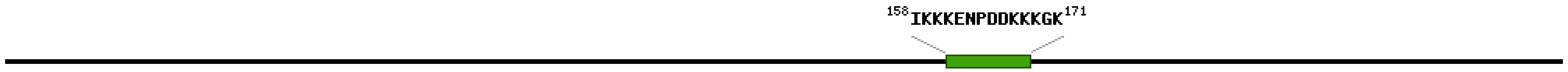

Posterior Graphic. Threshold Marked @ 0.4

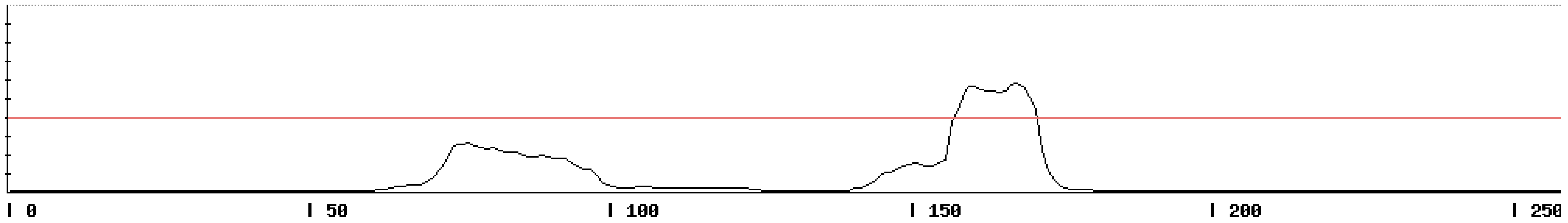

Predictions for Nicotianatomentosiformis_XP_009598866.1

Viterbi Path	
Viterbi Path	
Posterior @ 0.4	
Posterior @ 0.4	

Posterior Graphic. Threshold Marked @ 0.4

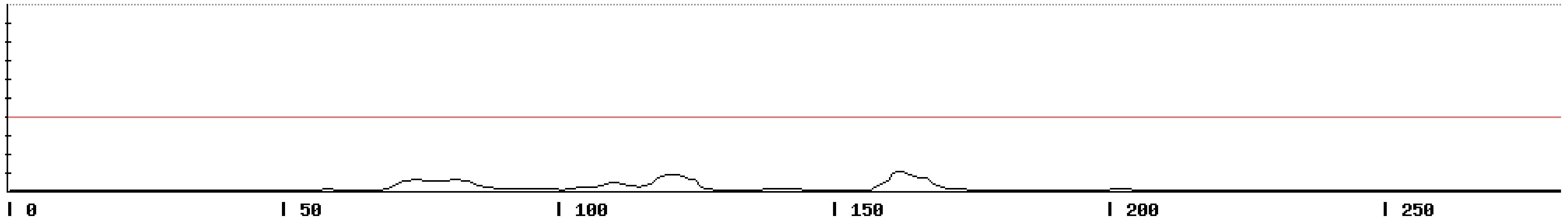

Predictions for Nicotianatomentosiformis_XP_009599075.1

Viterbi Path

Viterbi Path

Posterior @ 0.4

Posterior @ 0.4

Posterior Graphic. Threshold Marked @ 0.4

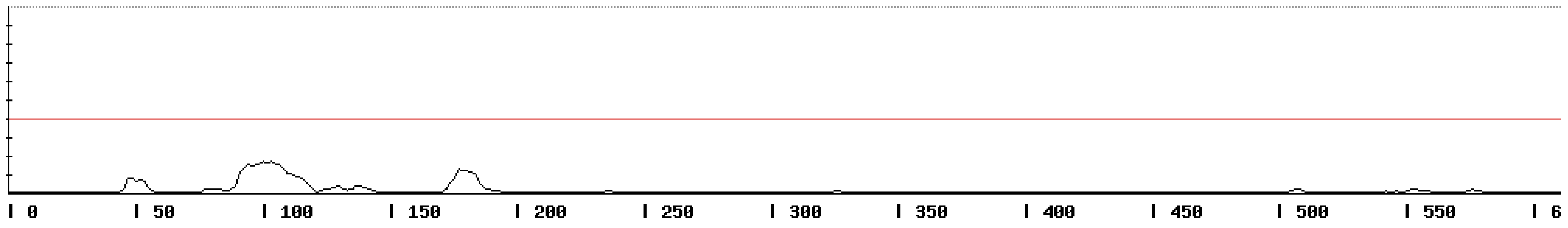

Predictions for Nicotianatomentosiformis_XP_009599412.1

Viterbi Path

Viterbi Path

Posterior @ 0.4

Posterior @ 0.4

Posterior Graphic. Threshold Marked @ 0.4

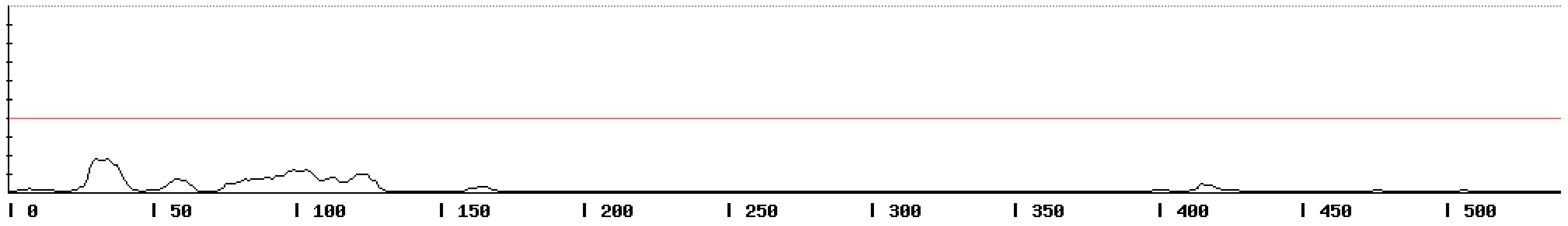

Predictions for Nicotianatomentosiformis_XP_009599621.1

Viterbi Path	
Viterbi Path	
Posterior @ 0.4	
Posterior @ 0.4	
Posterior Graphic. Threshold Marked @ 0.4	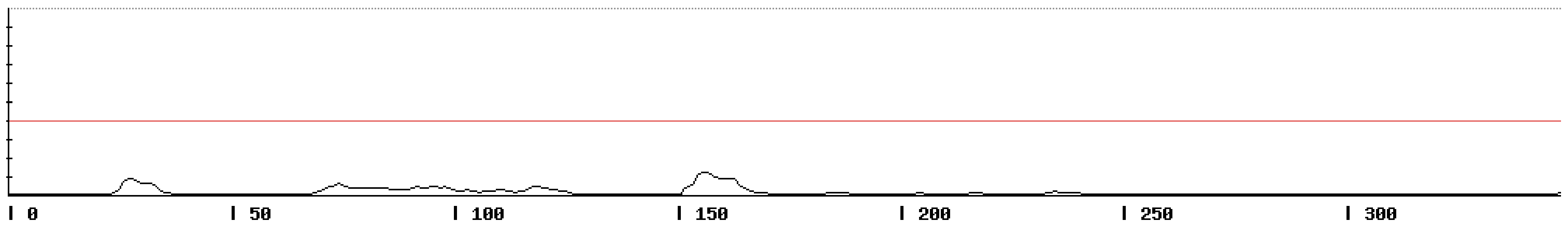

Predictions for Nicotianatomentosiformis_XP_009600037.1

Viterbi Path	
Viterbi Path	
Posterior @ 0.4	
Posterior @ 0.4	
Posterior Graphic. Threshold Marked @ 0.4	

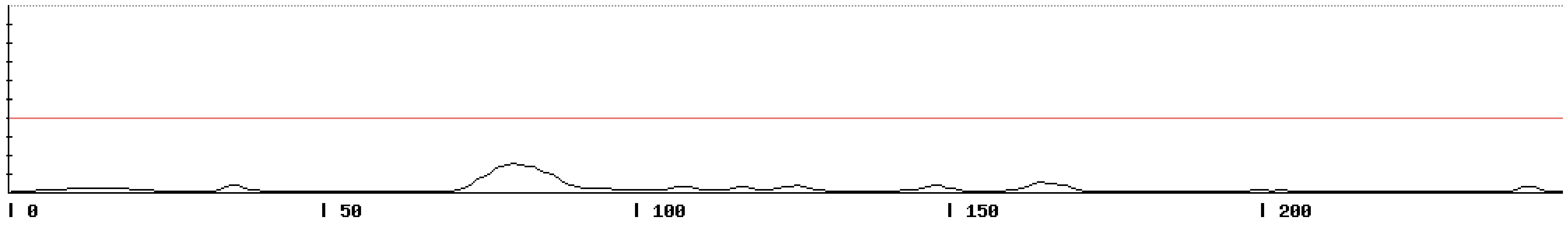

Predictions for Nicotianatomentosiformis_XP_009600052.1

Viterbi Path	
Viterbi Path	
Posterior @ 0.4	76 - LKKKSNARYHRAVGNKGS - 93
Posterior @ 0.4	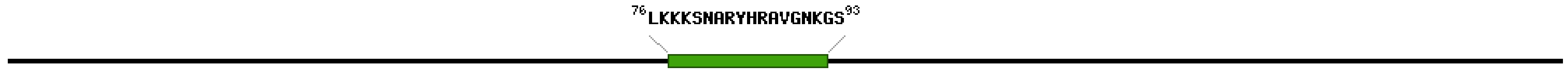
Posterior Graphic. Threshold Marked @ 0.4	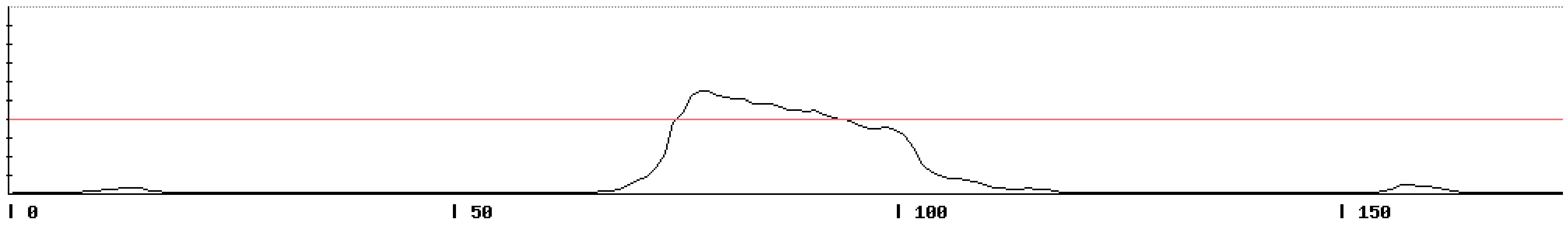

Predictions for Nicotianatomentosiformis_XP_009600309.1

Viterbi Path	
Viterbi Path	
Posterior @ 0.4	
Posterior @ 0.4	

Posterior Graphic. Threshold Marked @ 0.4

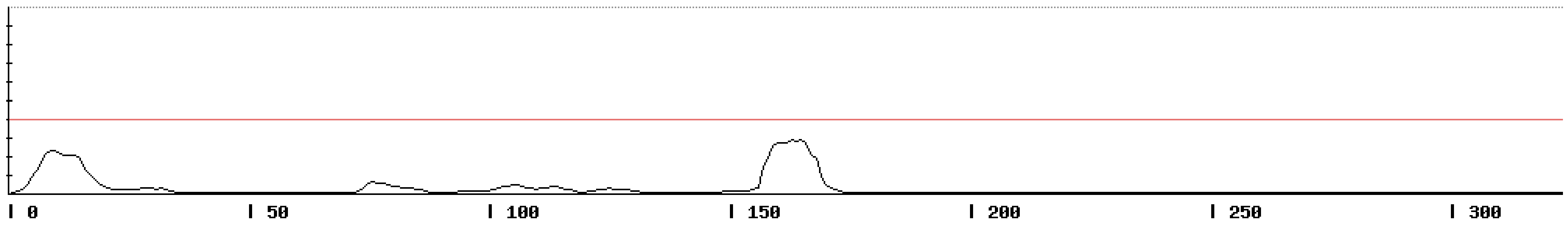

Predictions for Nicotianatomentosiformis_XP_009600694.1

Viterbi Path

Viterbi Path

Posterior @ 0.4

Posterior @ 0.4

Posterior Graphic. Threshold Marked @ 0.4

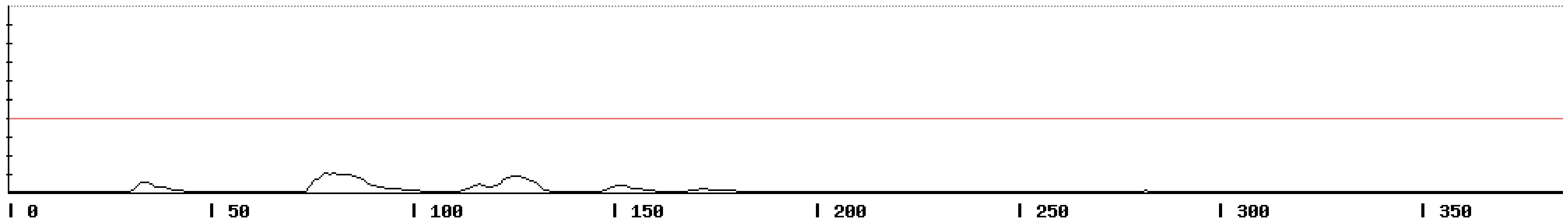

Predictions for Nicotianatomentosiformis_XP_009601216.1

Viterbi Path

Viterbi Path

Posterior @ 0.4

Posterior @ 0.4

Posterior Graphic. Threshold Marked @ 0.4

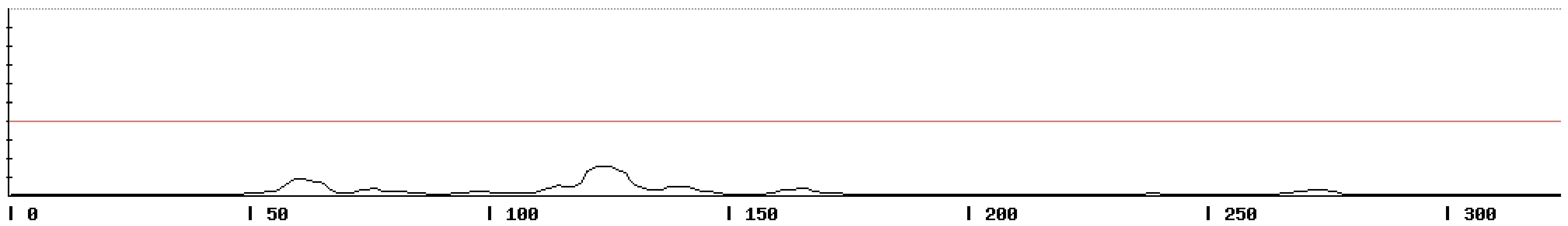

Predictions for Nicotianatomentosiformis_XP_009601217.1

Viterbi Path: 161 - KIKKKGKPGDKKKG - 174

Viterbi Path

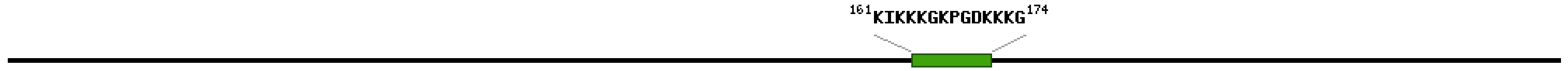

Posterior @ 0.4: 161 - KIKKKGKPGDKKKG - 174

Posterior @ 0.4

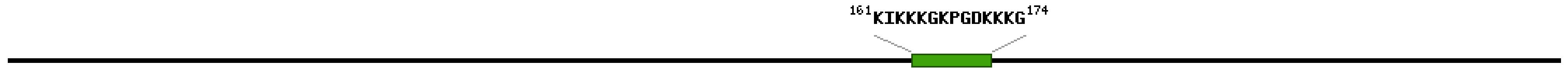

Posterior Graphic. Threshold Marked @ 0.4

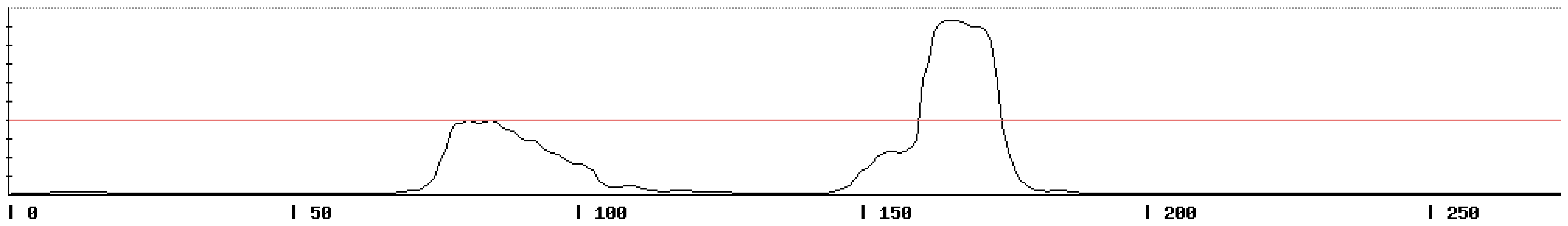

Predictions for Nicotianatomentosiformis_XP_009601554.1

Viterbi Path

Viterbi Path

Posterior @ 0.4

Posterior @ 0.4

Posterior Graphic. Threshold Marked @ 0.4

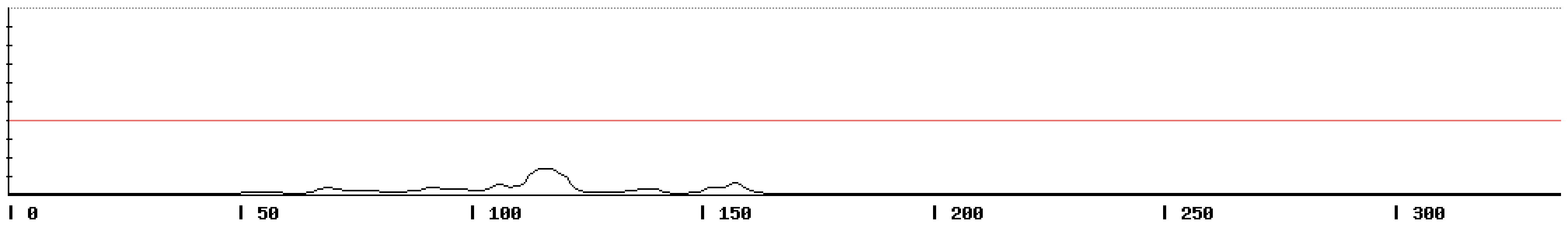

Predictions for Nicotianatomentosiformis_XP_009602081.1

Viterbi Path

Viterbi Path

Posterior @ 0.4

Posterior @ 0.4

Posterior Graphic. Threshold Marked @ 0.4

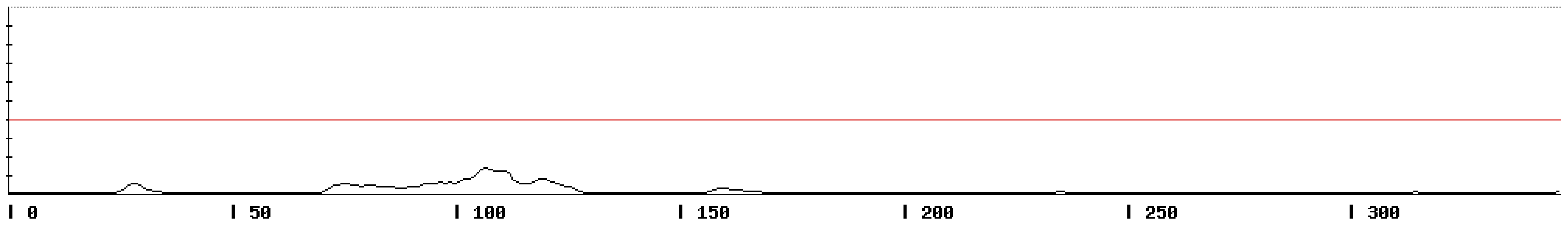

Predictions for Nicotianatomentosiformis_XP_009602357.1

Viterbi Path

Viterbi Path

Posterior @ 0.4

Posterior @ 0.4

Posterior Graphic. Threshold Marked @ 0.4

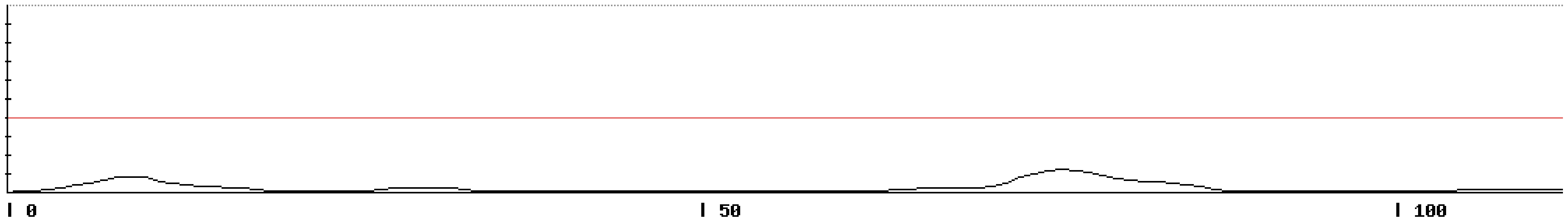

Predictions for Nicotianatomentosiformis_XP_009602844.1

Viterbi Path

Viterbi Path

Posterior @ 0.4

Posterior @ 0.4

Posterior Graphic. Threshold Marked @ 0.4

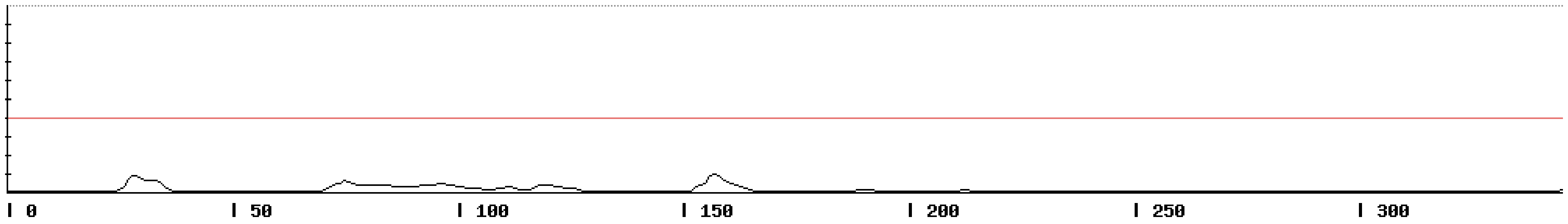

Predictions for Nicotianatomentosiformis_XP_009602846.1

Viterbi Path	
Viterbi Path	
Posterior @ 0.4	
Posterior @ 0.4	
Posterior Graphic. Threshold Marked @ 0.4	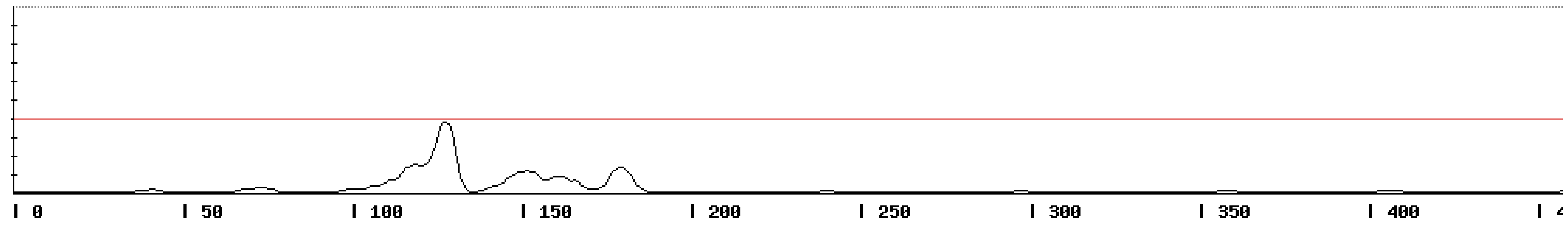

Predictions for Nicotianatomentosiformis_XP_009602847.1	
Viterbi Path	
Viterbi Path	
Posterior @ 0.4	
Posterior @ 0.4	
Posterior Graphic. Threshold Marked @ 0.4	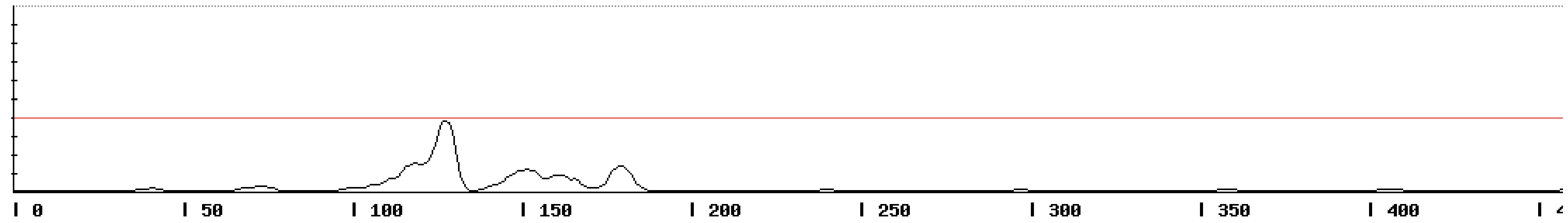

Predictions for Nicotianatomentosiformis_XP_009602848.1	
Viterbi Path	
Viterbi Path	
Posterior @ 0.4	
Posterior @ 0.4	
Posterior Graphic. Threshold Marked @ 0.4	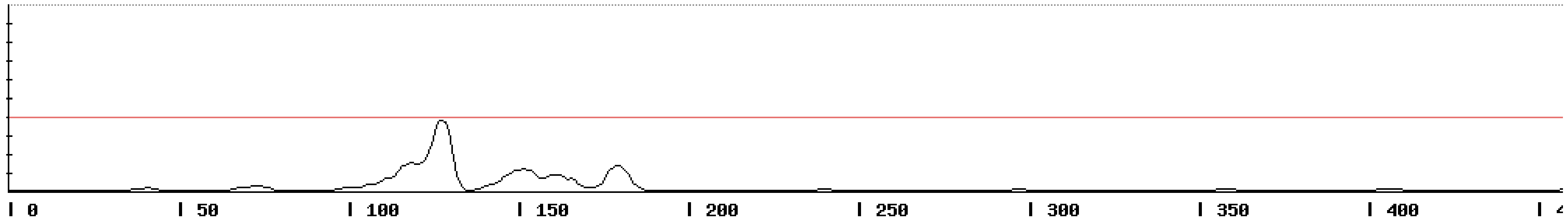

Predictions for Nicotianatomentosiformis_XP_009602849.1	
Viterbi Path	
Viterbi Path	
Posterior @ 0.4	
Posterior @ 0.4	
Posterior Graphic. Threshold Marked @ 0.4	

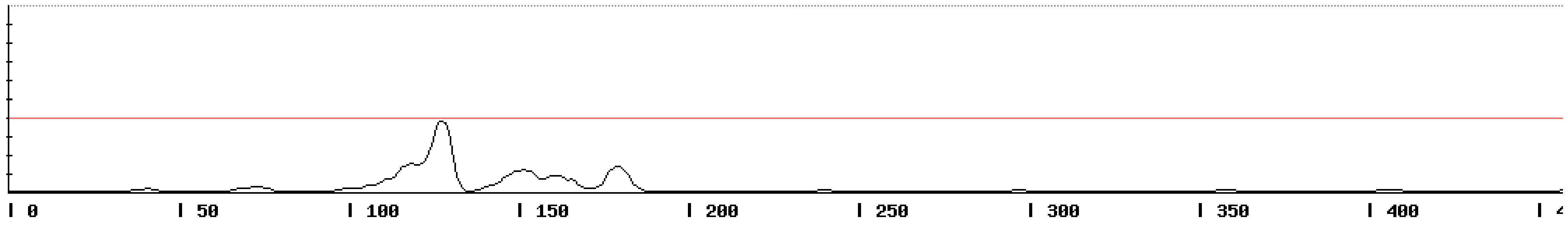

Predictions for Nicotianatomentosiformis_XP_009602850.1

Viterbi Path

Viterbi Path

Posterior @ 0.4

Posterior @ 0.4

Posterior Graphic. Threshold Marked @ 0.4

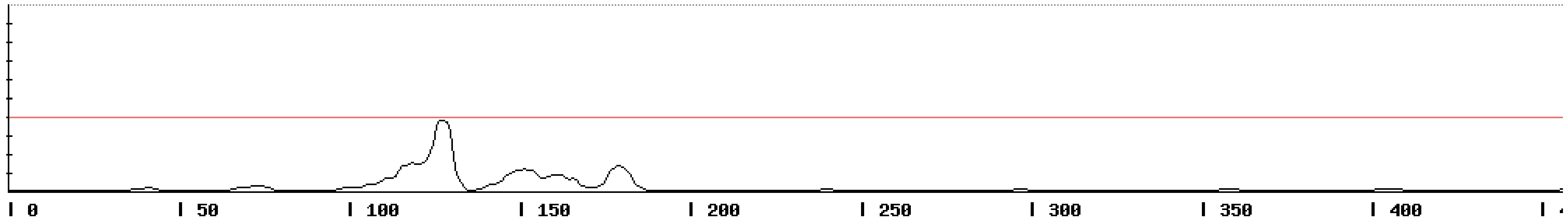

Predictions for Nicotianatomentosiformis_XP_009602851.1

Viterbi Path

Viterbi Path

Posterior @ 0.4

Posterior @ 0.4

Posterior Graphic. Threshold Marked @ 0.4

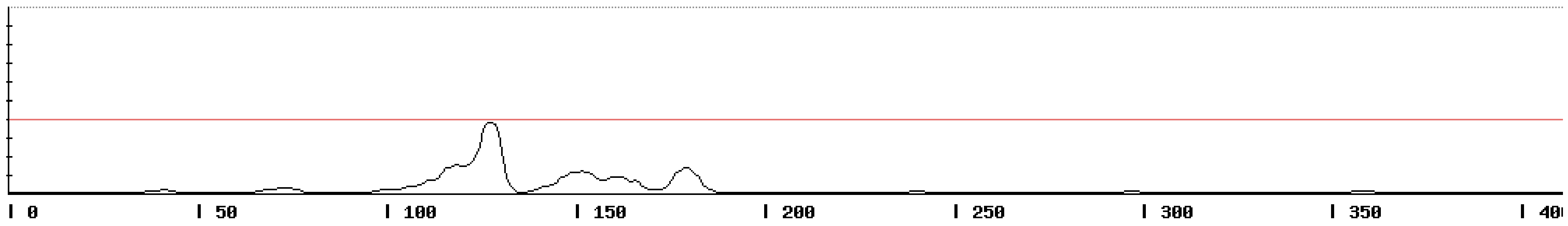

Predictions for Nicotianatomentosiformis_XP_009602879.1	
Viterbi Path	
Viterbi Path	
Posterior @ 0.4	163 - KIKKKGKPGDKKN - 175
Posterior @ 0.4	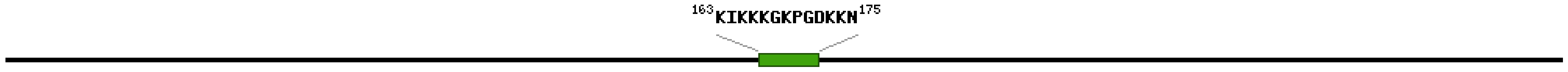
Posterior Graphic. Threshold Marked @ 0.4	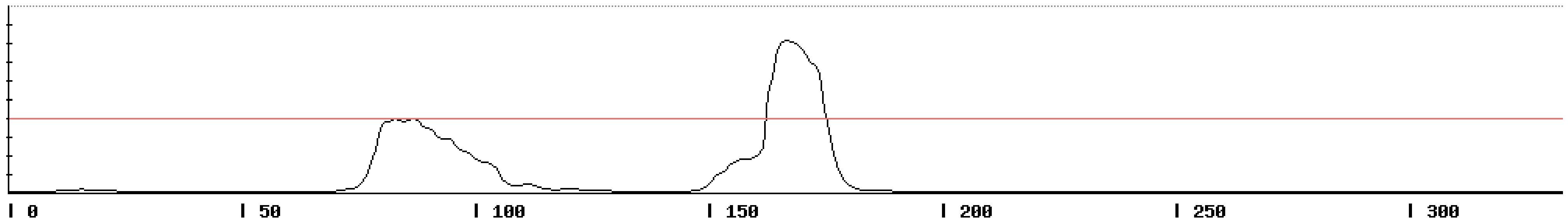

Predictions for Nicotianatomentosiformis_XP_009603097.1	
Viterbi Path	
Viterbi Path	
Posterior @ 0.4	
Posterior @ 0.4	

Posterior Graphic. Threshold Marked @ 0.4

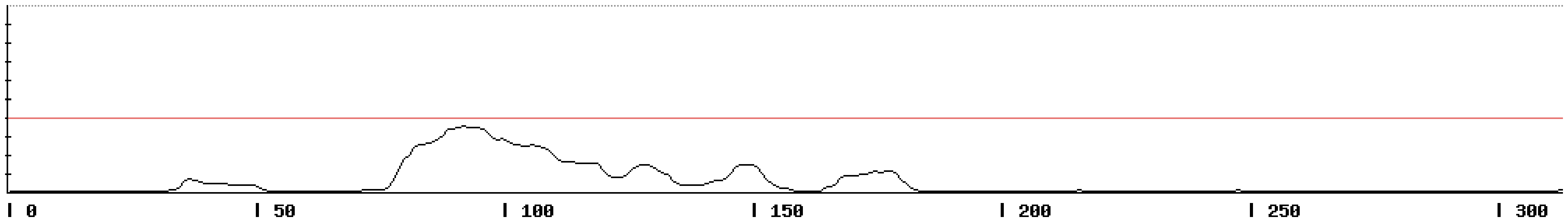

Predictions for Nicotianatomentosiformis_XP_009603640.1

Viterbi Path

Viterbi Path

Posterior @ 0.4

Posterior @ 0.4

Posterior Graphic. Threshold Marked @ 0.4

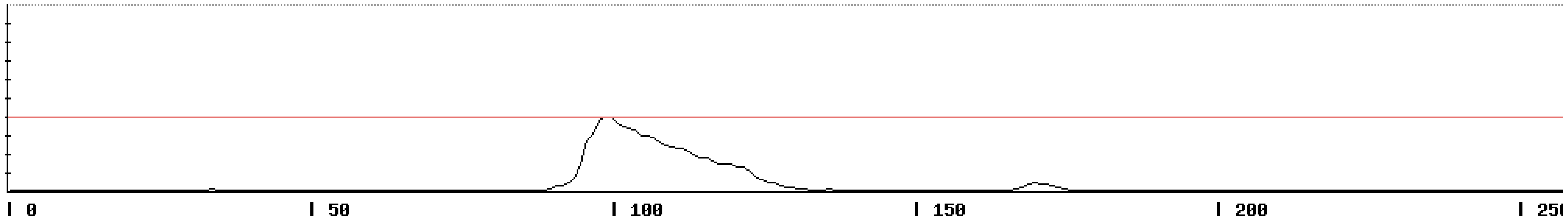

Predictions for Nicotianatomentosiformis_XP_009604550.1

Viterbi Path

Viterbi Path

Posterior @ 0.4	
Posterior @ 0.4	
Posterior Graphic. Threshold Marked @ 0.4	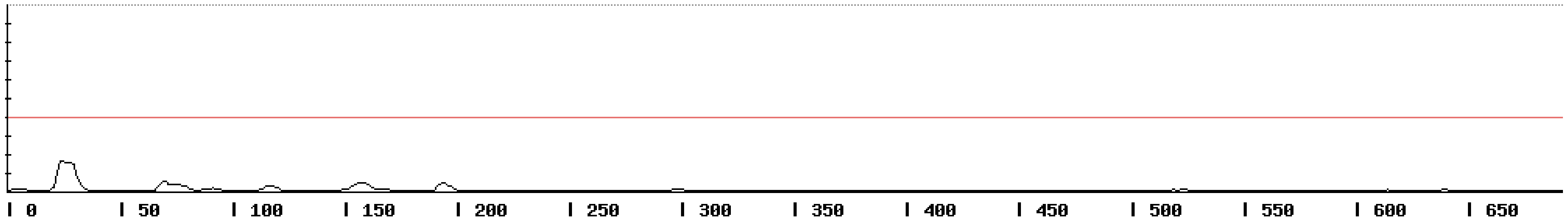

Predictions for Nicotianatomentosiformis_XP_009604551.1

Viterbi Path	
Viterbi Path	
Posterior @ 0.4	
Posterior @ 0.4	
Posterior Graphic. Threshold Marked @ 0.4	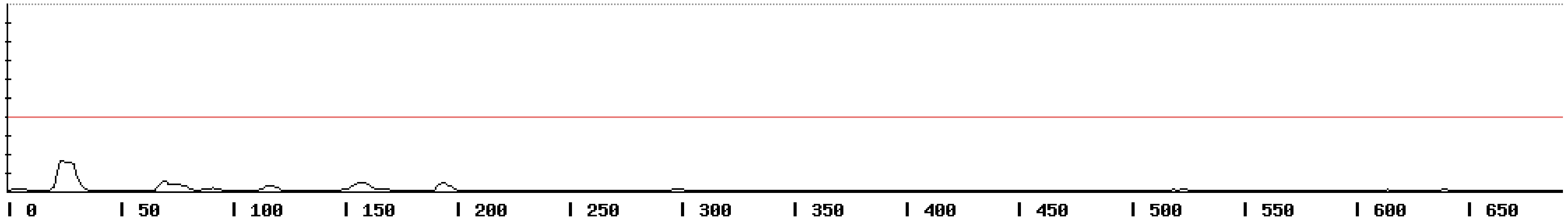

Predictions for Nicotianatomentosiformis_XP_009604784.1

Viterbi Path	
Viterbi Path	

Posterior @ 0.4

Posterior @ 0.4

Posterior Graphic. Threshold Marked @ 0.4

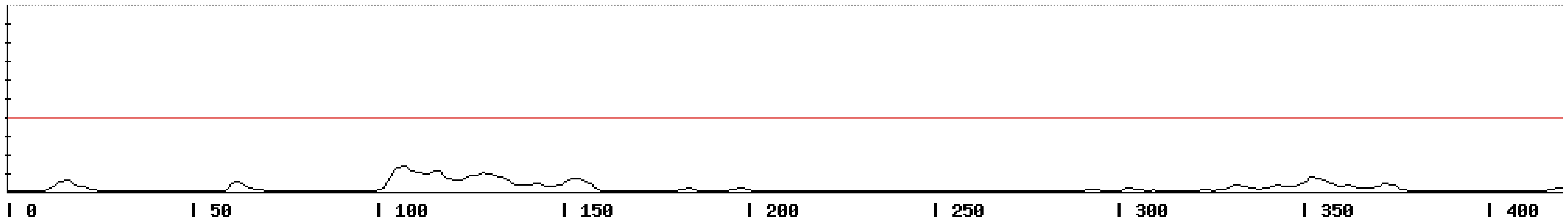

Predictions for Nicotianatomentosiformis_XP_009604942.1

Viterbi Path

Viterbi Path

Posterior @ 0.4 — 68 - KLKRFKNSTKKFSRTVGQGTWKGQSGGKPIKHRSKNIIVGFRRSLKYKSGIKK - 120

Posterior @ 0.4

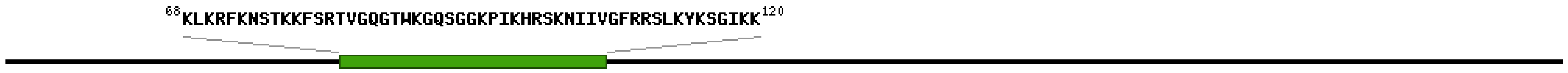

Posterior Graphic. Threshold Marked @ 0.4

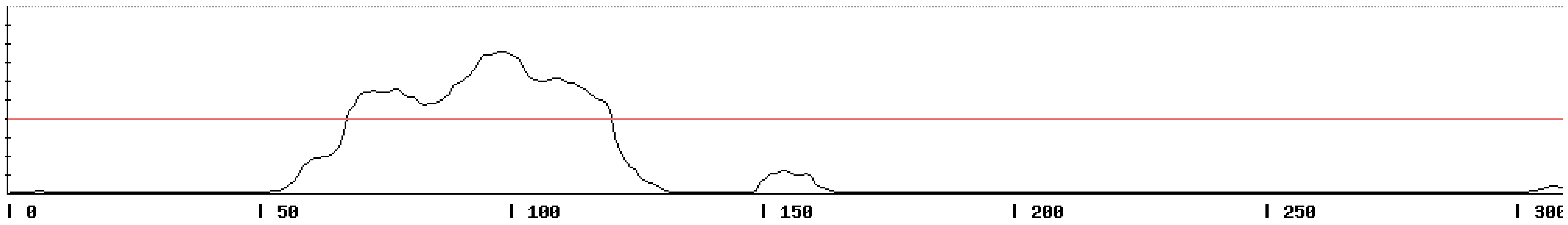

Predictions for Nicotianatomentosiformis_XP_009605650.1

Viterbi Path

Viterbi Path

Posterior @ 0.4

Posterior @ 0.4

Posterior Graphic. Threshold Marked @ 0.4

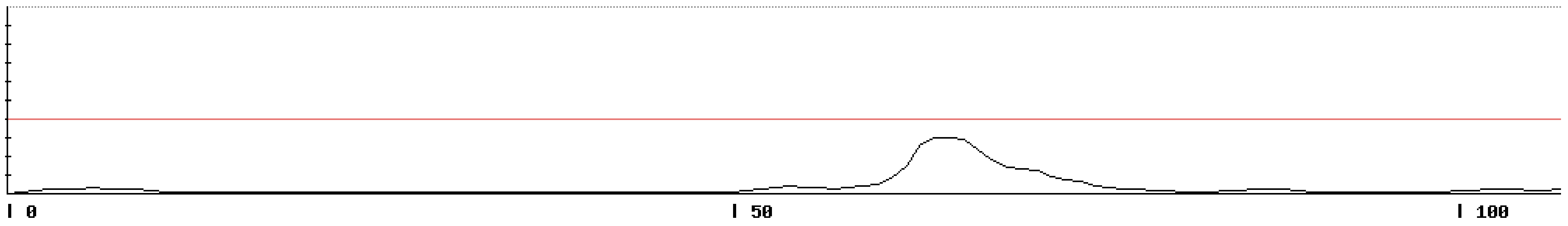

Predictions for Nicotianatomentosiformis_XP_009605813.1

Viterbi Path

Viterbi Path

Posterior @ 0.4

Posterior @ 0.4

Posterior Graphic. Threshold Marked @ 0.4

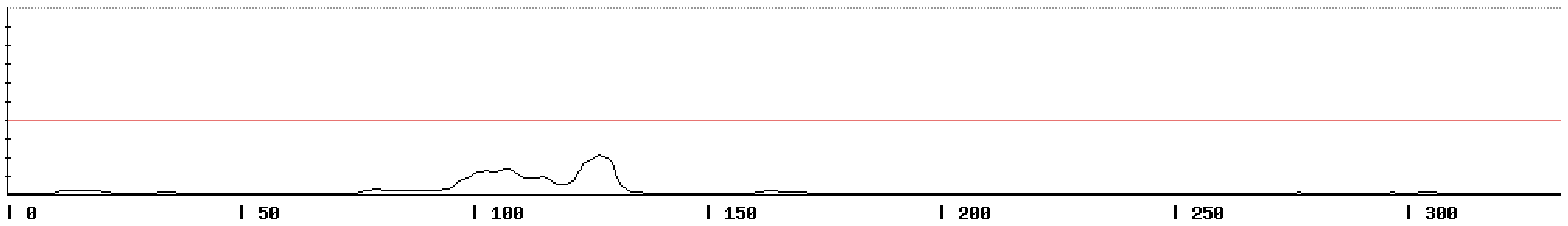

Predictions for Nicotianatomentosiformis_XP_009605814.1	
Viterbi Path	
Viterbi Path	
Posterior @ 0.4	
Posterior @ 0.4	
Posterior Graphic. Threshold Marked @ 0.4	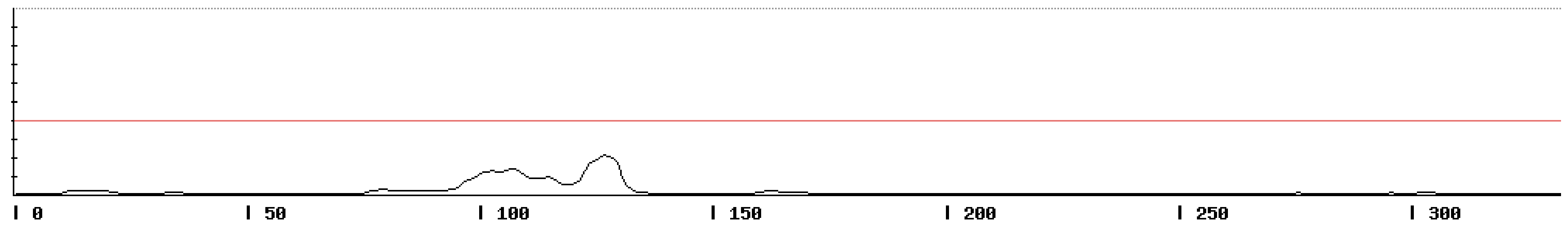

Predictions for Nicotianatomentosiformis_XP_009605868.1	
Viterbi Path	
Viterbi Path	
Posterior @ 0.4	
Posterior @ 0.4	
Posterior Graphic. Threshold Marked @ 0.4	

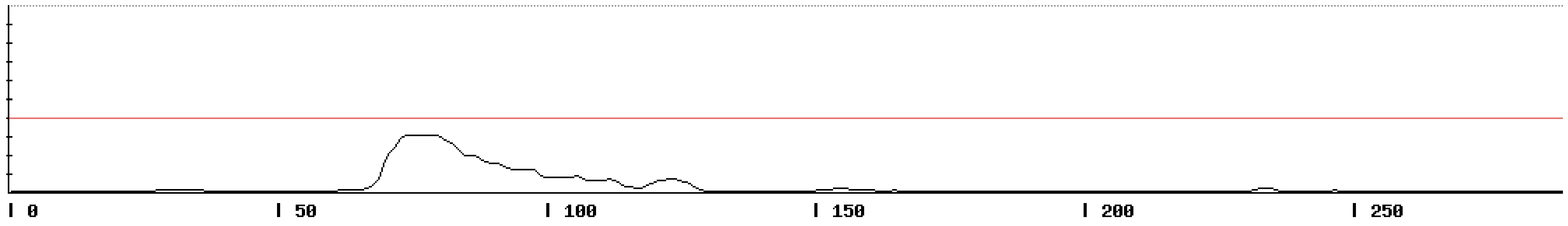

Predictions for Nicotianatomentosiformis_XP_009606198.1

Viterbi Path

Viterbi Path

Posterior @ 0.4

Posterior @ 0.4

Posterior Graphic. Threshold Marked @ 0.4

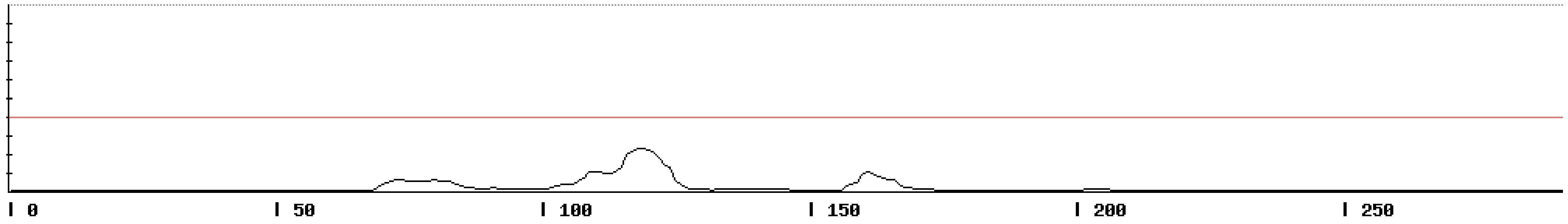

Predictions for Nicotianatomentosiformis_XP_009606805.1

Viterbi Path

Viterbi Path

Posterior @ 0.4: 71 - RKHGPKGKPNR - 81

Posterior @ 0.4

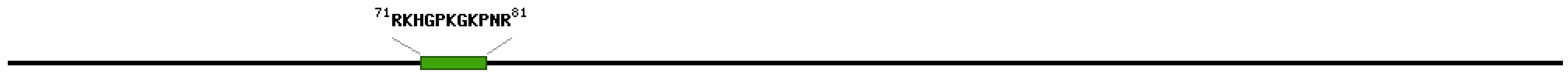

Posterior Graphic. Threshold Marked @ 0.4

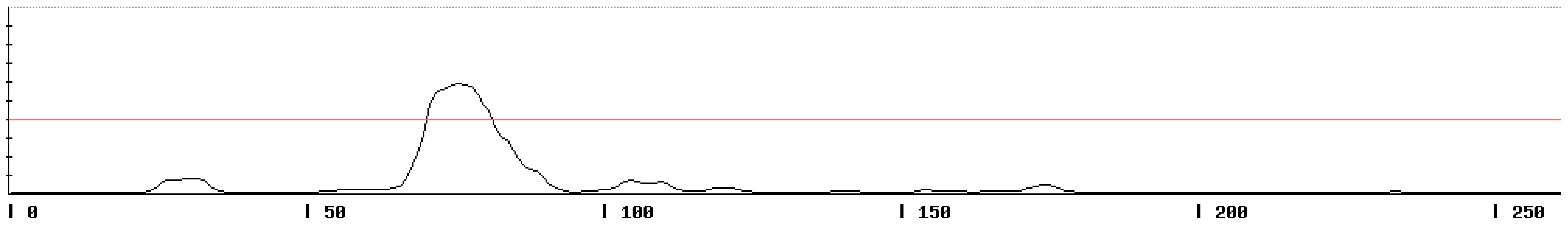

Predictions for Nicotianatomentosiformis_XP_009606924.1

Viterbi Path

Viterbi Path

Posterior @ 0.4

Posterior @ 0.4

Posterior Graphic. Threshold Marked @ 0.4

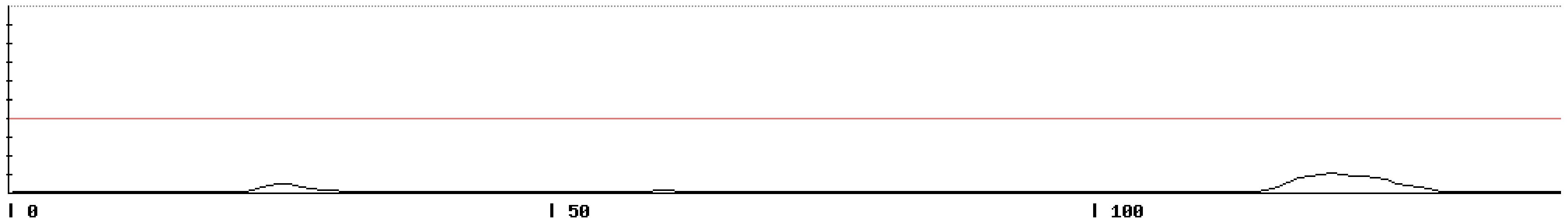

Predictions for Nicotianatomentosiformis_XP_009607149.1

Viterbi Path

Viterbi Path

Posterior @ 0.4

272 - VRNKRPRRKRDSHPPP - 287

499 - KK - 500

Posterior @ 0.4

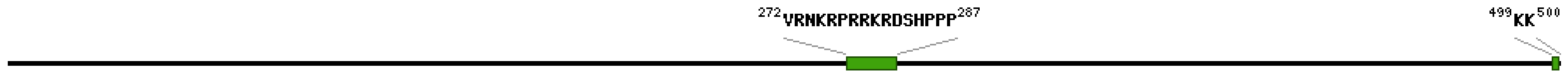

Posterior Graphic. Threshold Marked @ 0.4

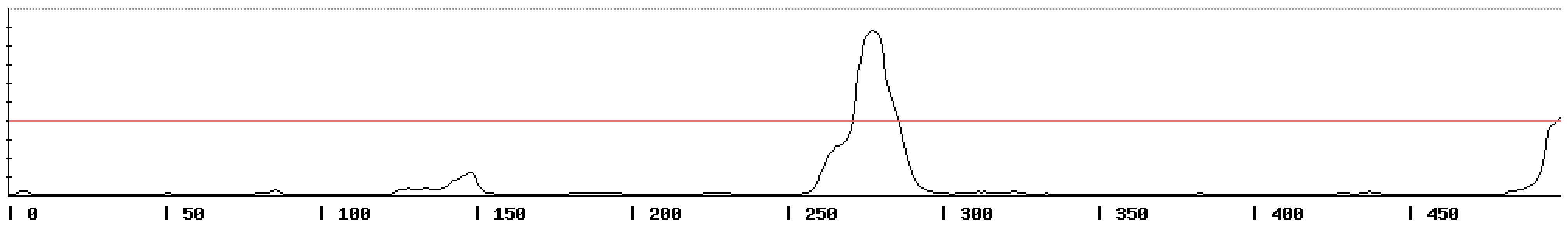

Predictions for Nicotianatomentosiformis_XP_009607150.1

Viterbi Path

Viterbi Path

Posterior @ 0.4

272 - VRNKRPRRKRDSHPPP - 287

499 - KK - 500

Posterior @ 0.4

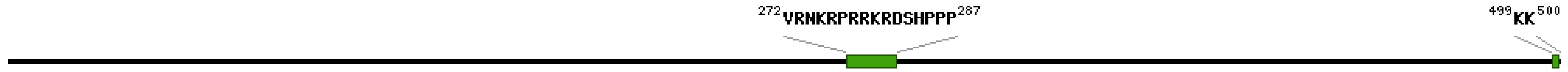

Posterior Graphic. Threshold Marked @ 0.4

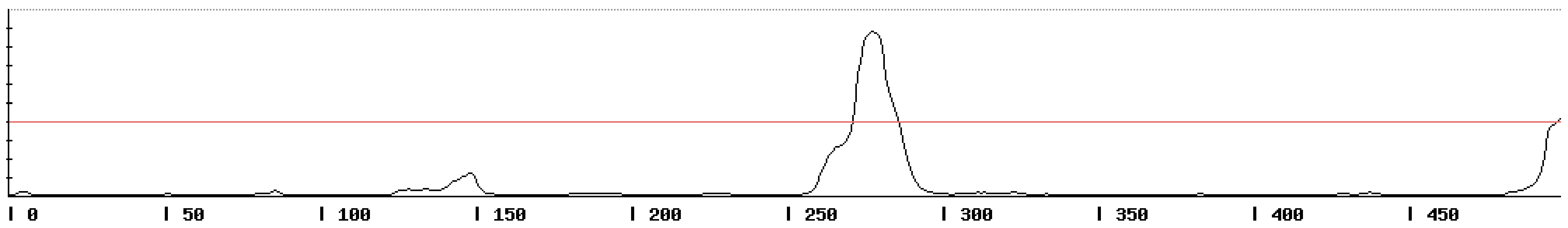

Predictions for Nicotianatomentosiformis_XP_009607151.1

Viterbi Path

Viterbi Path

Posterior @ 0.4	194 - VRNKRPRRKRDSHPPP - 209
	421 - KK - 422

Posterior @ 0.4

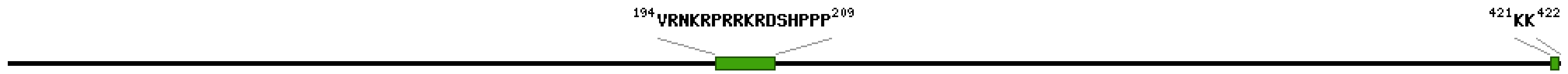

Posterior Graphic. Threshold Marked @ 0.4

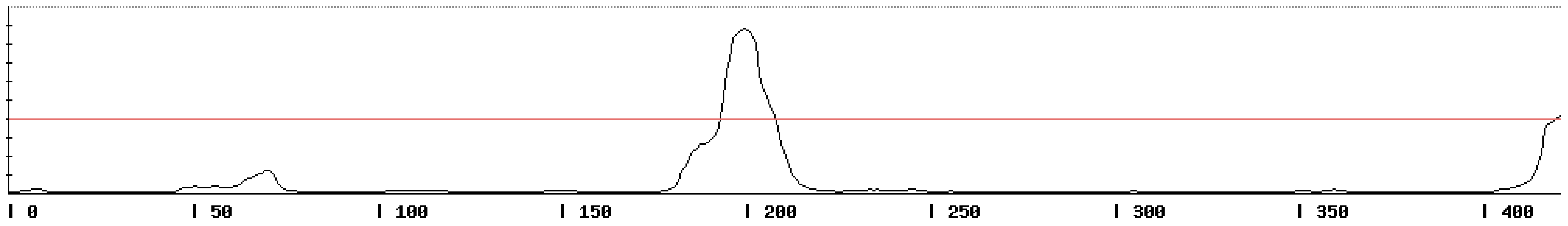

Predictions for Nicotianatomentosiformis_XP_009607152.1

Viterbi Path

Viterbi Path

Posterior @ 0.4	272 - VRNKRPRRKRDSHPP - 286

Posterior @ 0.4

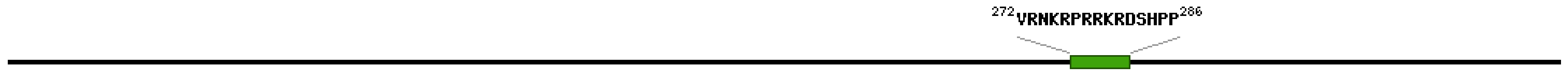

Posterior Graphic. Threshold Marked @ 0.4

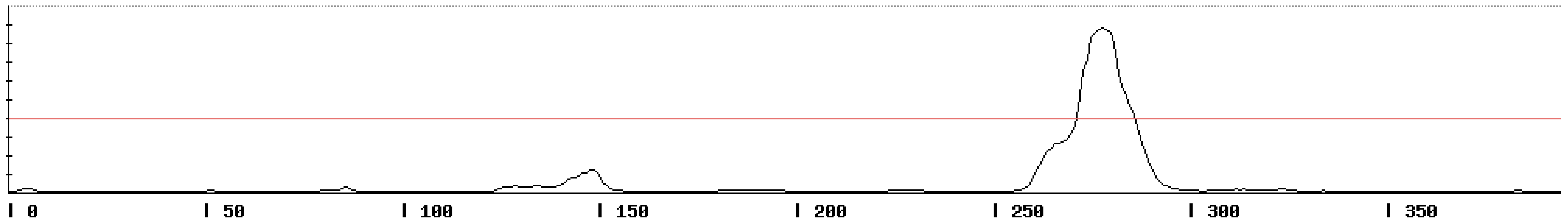

Predictions for Nicotianatomentosiformis_XP_009607153.1	
Viterbi Path	
Viterbi Path	
Posterior @ 0.4	
Posterior @ 0.4	
Posterior Graphic. Threshold Marked @ 0.4	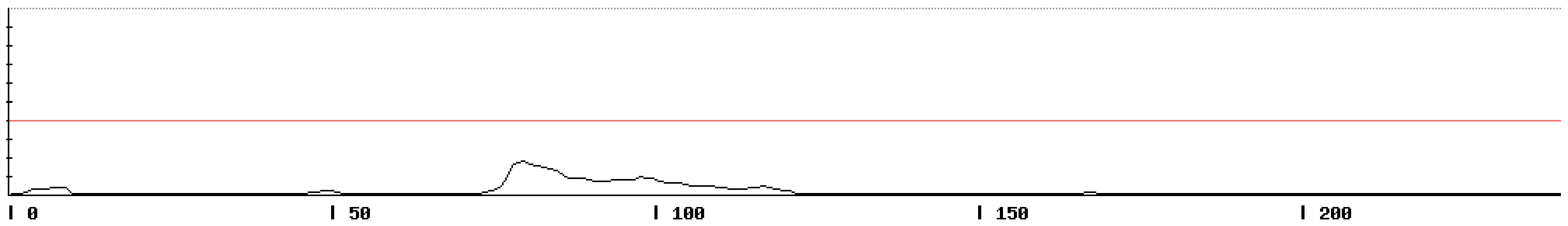

Predictions for Nicotianatomentosiformis_XP_009607557.1	
Viterbi Path	
Viterbi Path	
Posterior @ 0.4	
Posterior @ 0.4	
Posterior Graphic. Threshold Marked @ 0.4	

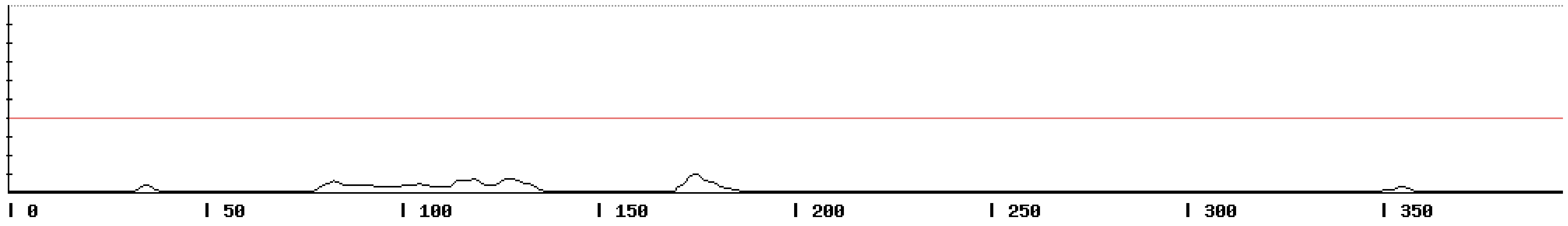

Predictions for Nicotianatomentosiformis_XP_009607926.1

Viterbi Path

Viterbi Path

Posterior @ 0.4

Posterior @ 0.4

Posterior Graphic. Threshold Marked @ 0.4

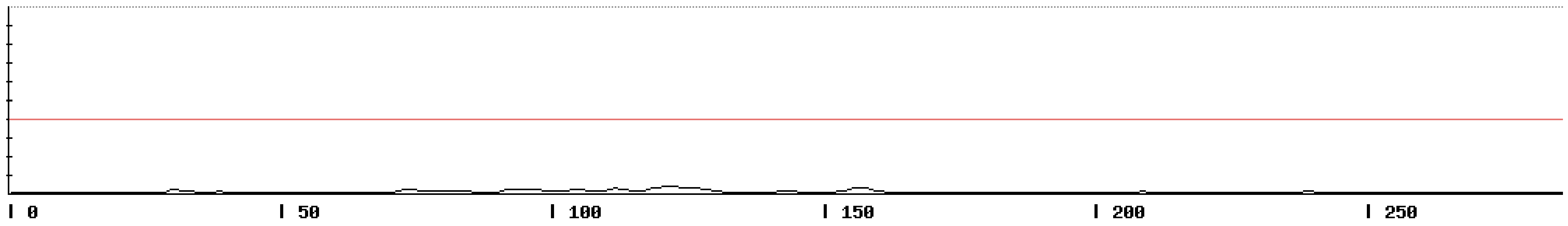

Predictions for Nicotianatomentosiformis_XP_009608176.1

Viterbi Path

Viterbi Path

Posterior @ 0.4

Posterior @ 0.4

Posterior Graphic. Threshold Marked @ 0.4

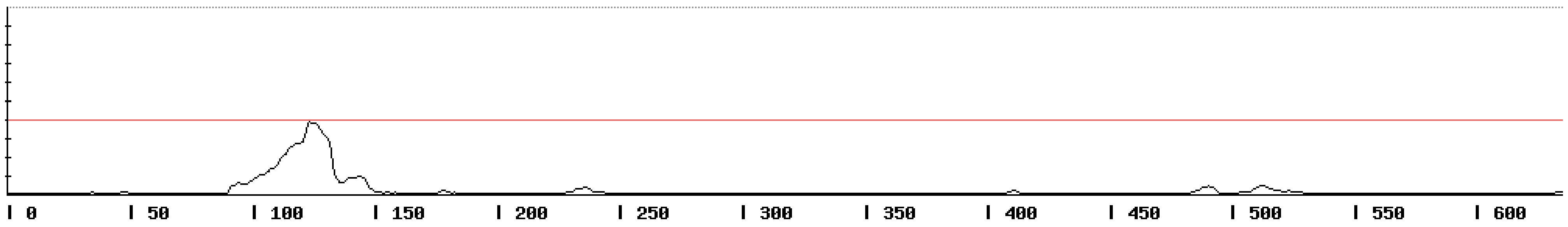

Predictions for Nicotianatomentosiformis_XP_009608935.1

Viterbi Path	
Viterbi Path	
Posterior @ 0.4	138 - RKRR - 141
Posterior @ 0.4	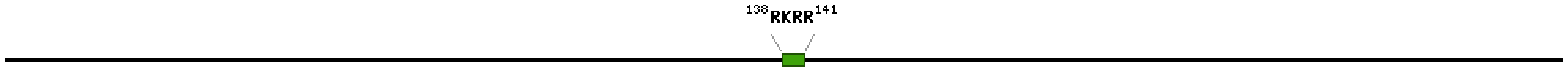
Posterior Graphic. Threshold Marked @ 0.4	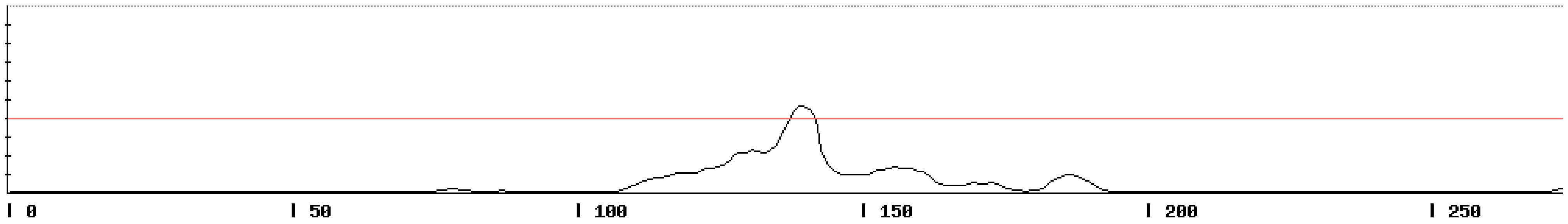

Predictions for Nicotianatomentosiformis_XP_009608936.1

Viterbi Path	
Viterbi Path	
Posterior @ 0.4	138 - RKRR - 141
Posterior @ 0.4	

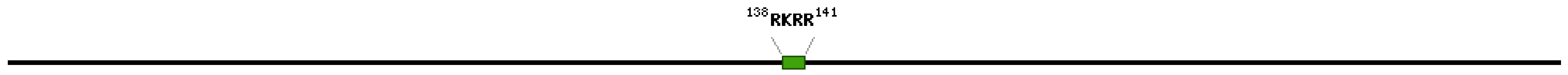

Posterior Graphic. Threshold Marked @ 0.4

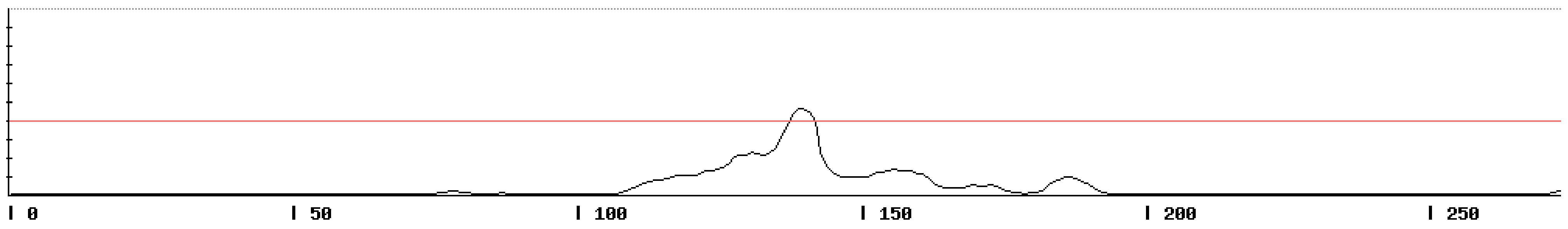

Predictions for Nicotianatomentosiformis_XP_009608937.1

Viterbi Path

Viterbi Path

Posterior @ 0.4

Posterior @ 0.4

Posterior Graphic. Threshold Marked @ 0.4

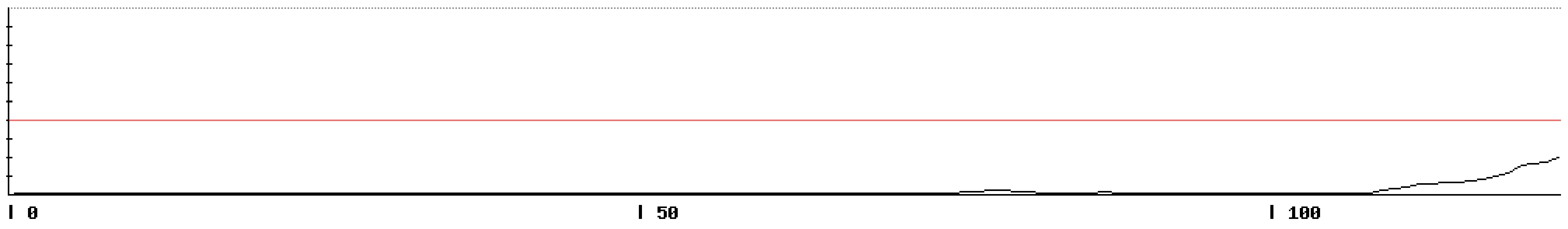

Predictions for Nicotianatomentosiformis_XP_009609037.1

Viterbi Path

Viterbi Path

Posterior @ 0.4

Posterior @ 0.4

Posterior Graphic. Threshold Marked @ 0.4

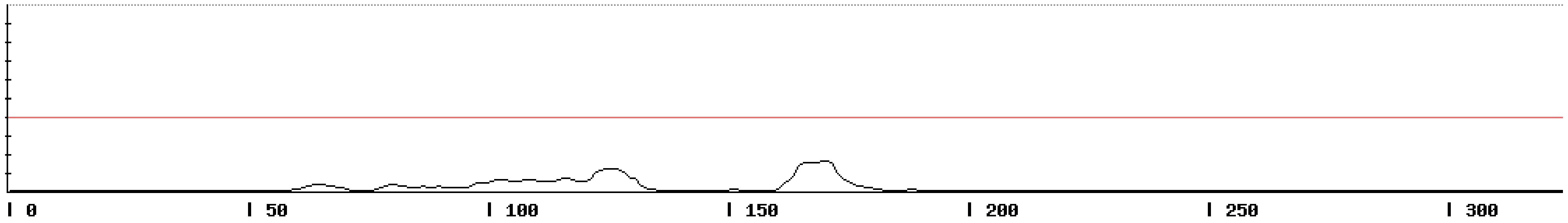

Predictions for Nicotianatomentosiformis_XP_009609295.1

Viterbi Path

Viterbi Path

Posterior @ 0.4

Posterior @ 0.4

Posterior Graphic. Threshold Marked @ 0.4

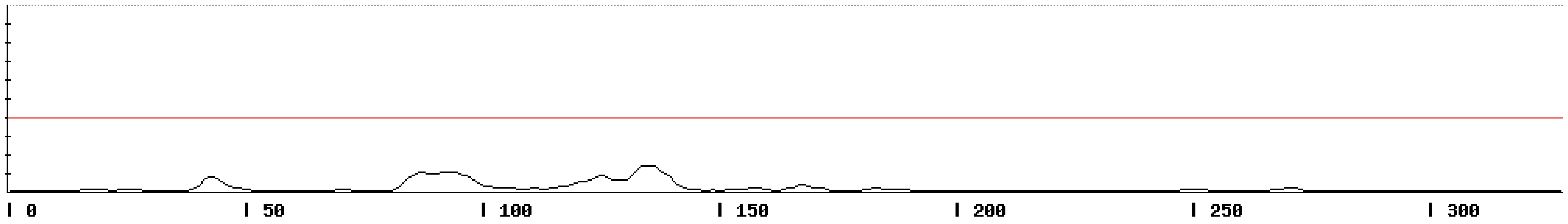

Predictions for Nicotianatomentosiformis_XP_009610416.1

Viterbi Path

Viterbi Path

Posterior @ 0.4

Posterior @ 0.4

Posterior Graphic. Threshold Marked @ 0.4

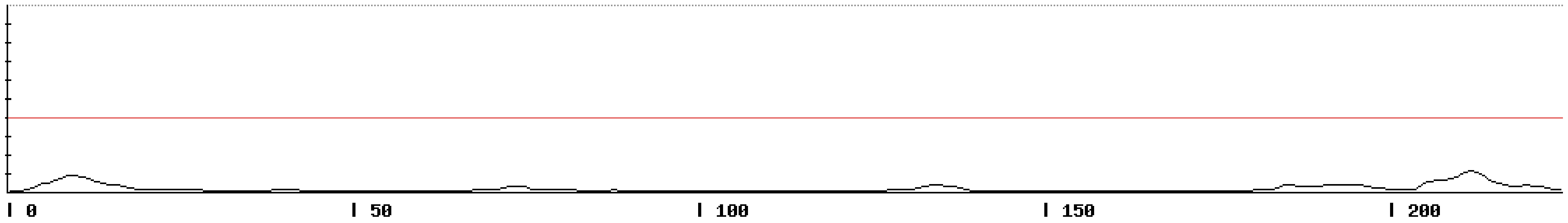

Predictions for Nicotianatomentosiformis_XP_009610417.1

Viterbi Path

Viterbi Path

Posterior @ 0.4

Posterior @ 0.4

Posterior Graphic. Threshold Marked @ 0.4

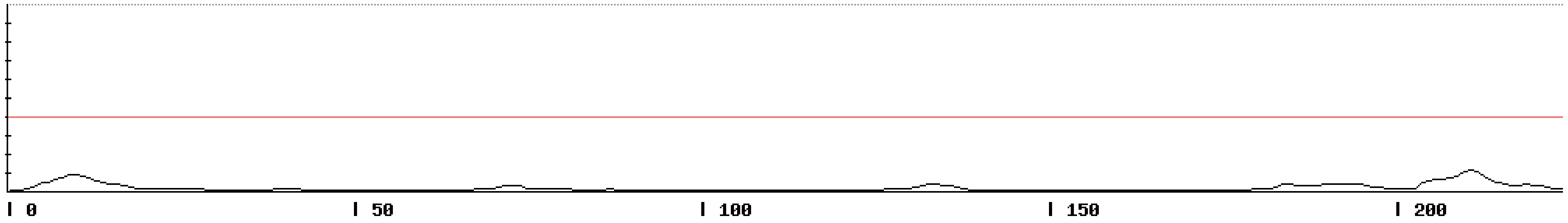

Predictions for Nicotianatomentosiformis_XP_009611016.1

Viterbi Path	
Viterbi Path	
Posterior @ 0.4	81 - GRKRRYKNGRKPNRQVIGKKG - 101
Posterior @ 0.4	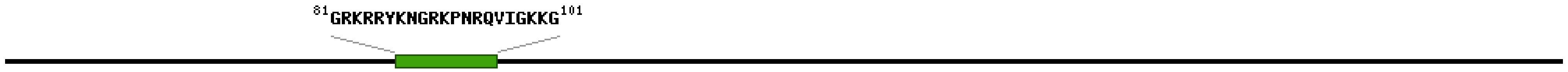
Posterior Graphic. Threshold Marked @ 0.4	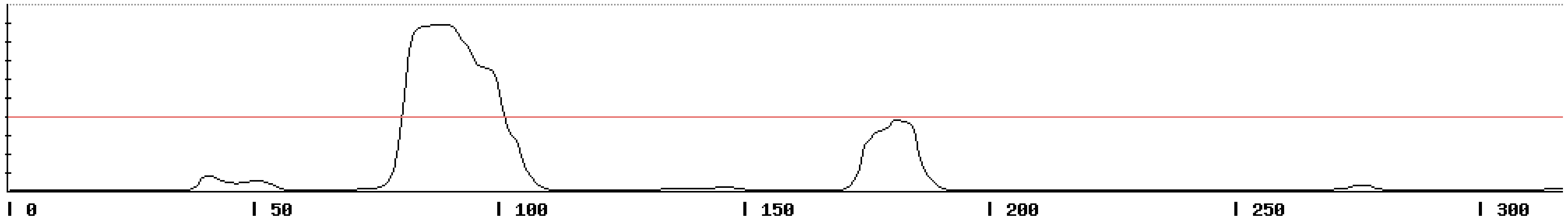

Predictions for Nicotianatomentosiformis_XP_009611780.1

Viterbi Path	
Viterbi Path	
Posterior @ 0.4	
Posterior @ 0.4	
Posterior Graphic. Threshold Marked @ 0.4	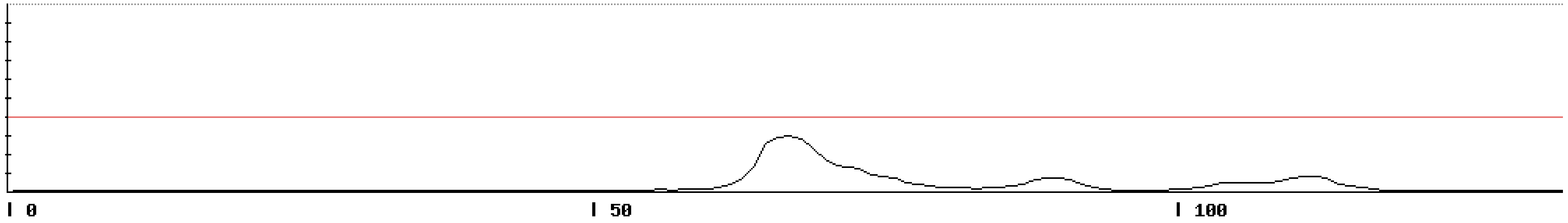

Predictions for Nicotianatomentosiformis_XP_009611974.1

Viterbi Path

Viterbi Path

Posterior @ 0.4

Posterior @ 0.4

Posterior Graphic. Threshold Marked @ 0.4

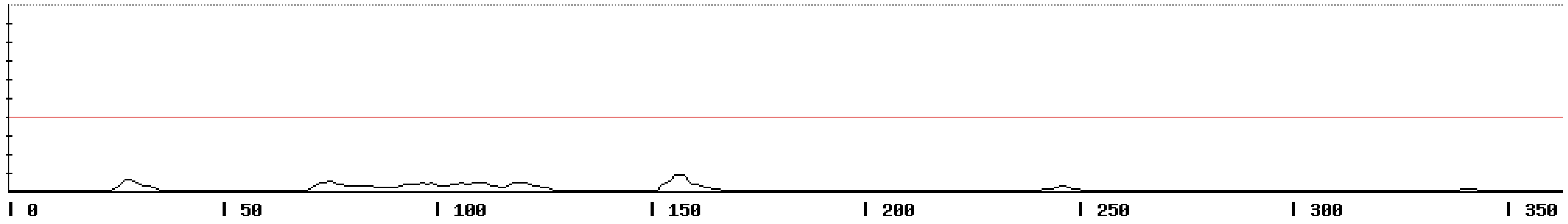

Predictions for Nicotianatomentosiformis_XP_009612311.1

Viterbi Path

Viterbi Path

Posterior @ 0.4

Posterior @ 0.4

Posterior Graphic. Threshold Marked @ 0.4

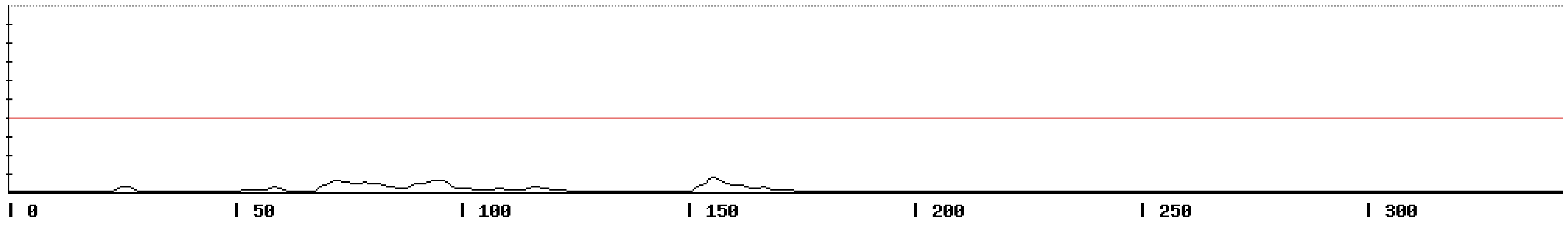

Predictions for Nicotianatomentosiformis_XP_009612742.1

Viterbi Path	
Viterbi Path	
Posterior @ 0.4	111 - LKKKSNARYHRAVGNKGS - 128
Posterior @ 0.4	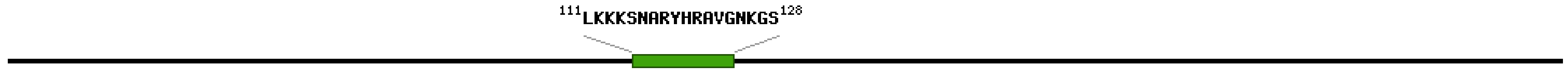
Posterior Graphic. Threshold Marked @ 0.4	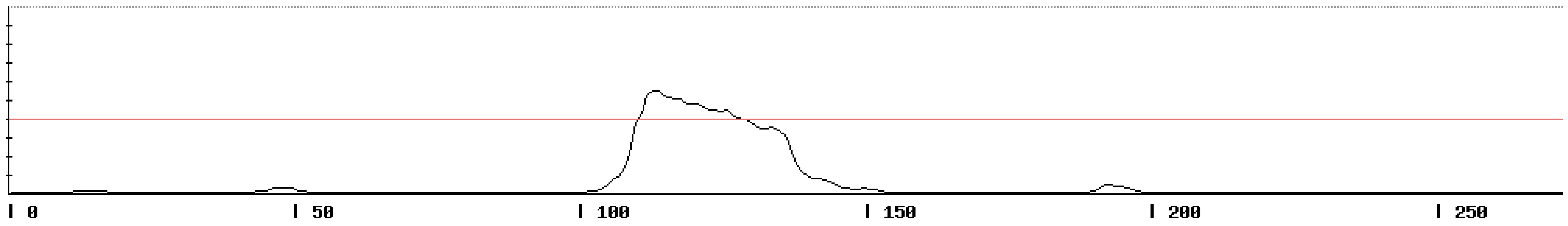

Predictions for Nicotianatomentosiformis_XP_009613092.1

Viterbi Path	
Viterbi Path	
Posterior @ 0.4	
Posterior @ 0.4	

Posterior Graphic. Threshold Marked @ 0.4	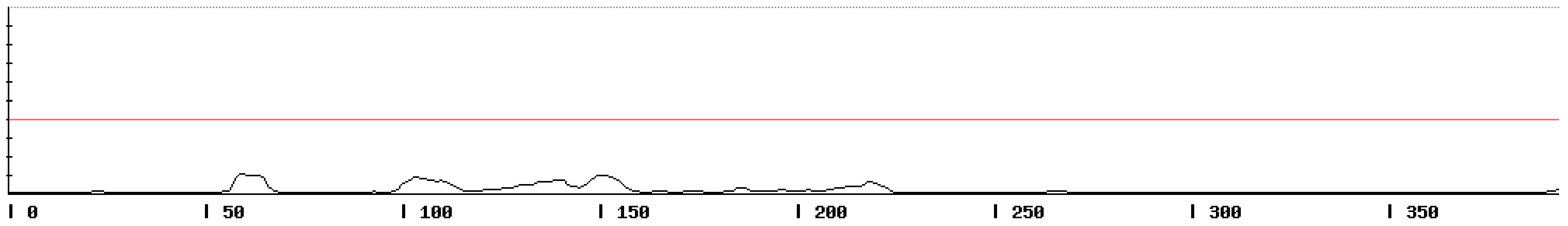

Predictions for Nicotianatomentosiformis_XP_009613291.1

Viterbi Path	
Viterbi Path	
Posterior @ 0.4	
Posterior @ 0.4	
Posterior Graphic. Threshold Marked @ 0.4	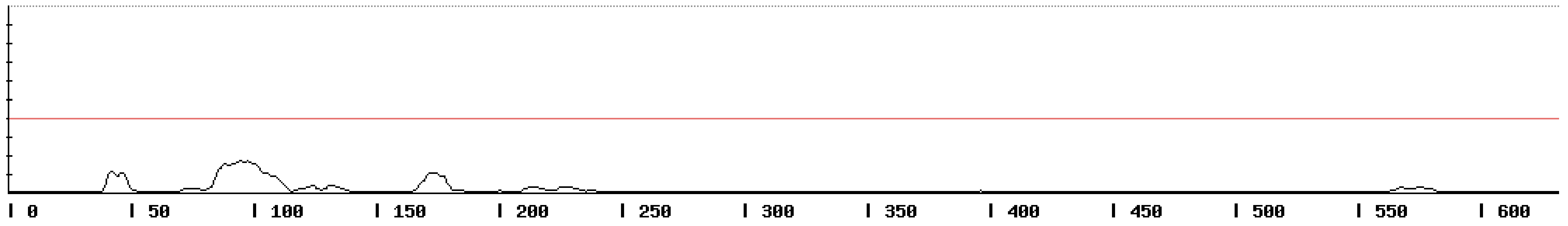

Predictions for Nicotianatomentosiformis_XP_009613292.1

Viterbi Path	
Viterbi Path	
Posterior @ 0.4	
Posterior @ 0.4	

Posterior Graphic. Threshold Marked @ 0.4

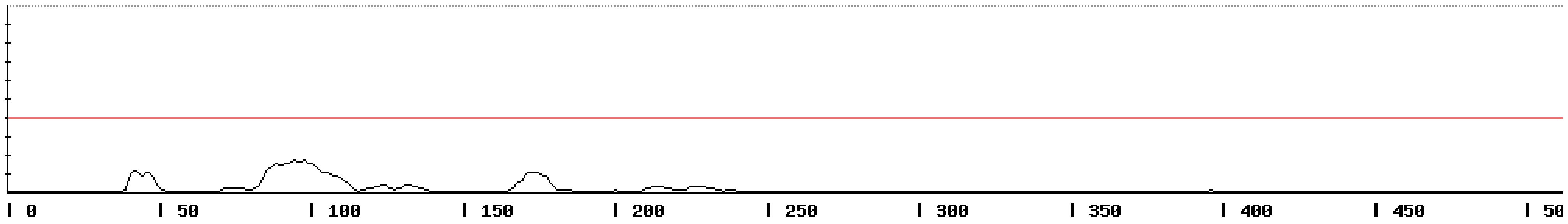

Predictions for Nicotianatomentosiformis_XP_009614314.1

Viterbi Path

Viterbi Path

Posterior @ 0.4 — 138 - RKRRK - 142

Posterior @ 0.4

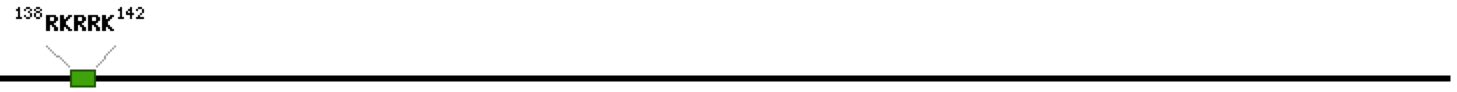

Posterior Graphic. Threshold Marked @ 0.4

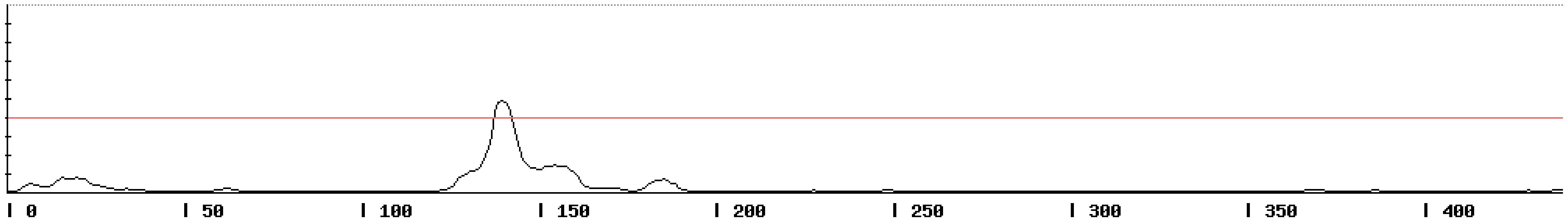

Predictions for Nicotianatomentosiformis_XP_009614315.1

Viterbi Path

Viterbi Path

Posterior @ 0.4	134 - RKRRK - 138
Posterior @ 0.4	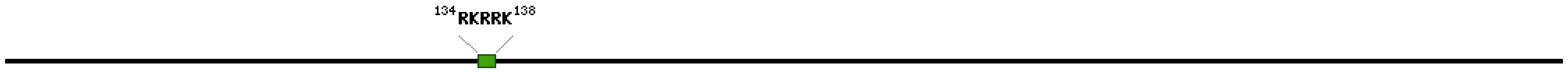
Posterior Graphic. Threshold Marked @ 0.4	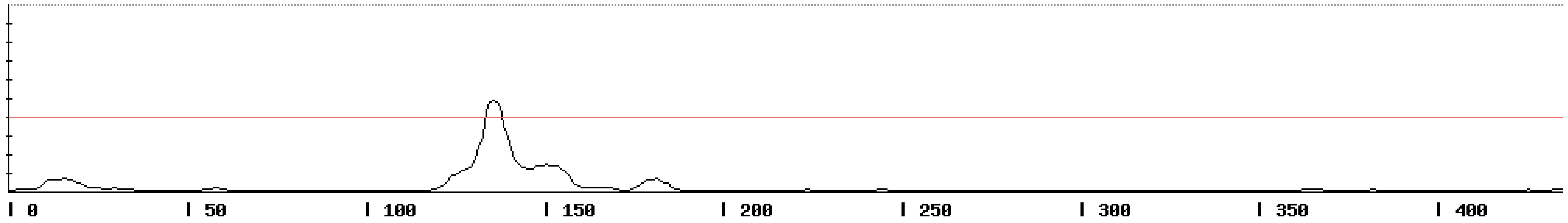

Predictions for Nicotianatomentosiformis_XP_009614316.1

Viterbi Path	
Viterbi Path	
Posterior @ 0.4	134 - RKRRK - 138
Posterior @ 0.4	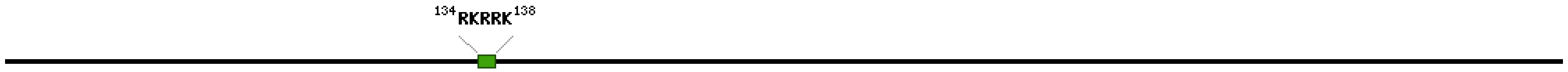
Posterior Graphic. Threshold Marked @ 0.4	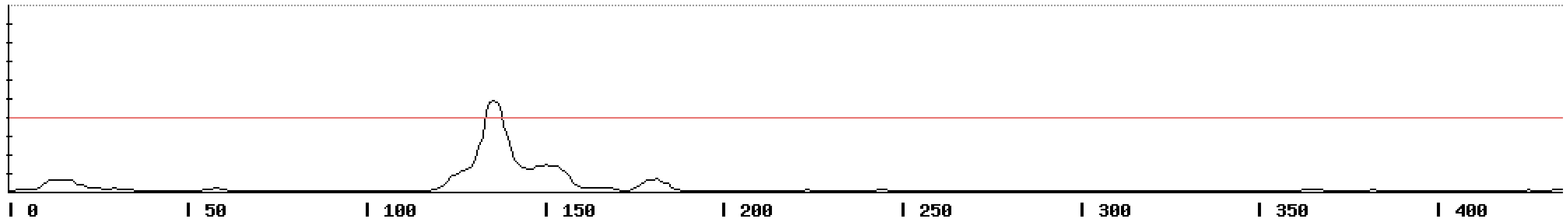

Predictions for Nicotianatomentosiformis_XP_009614700.1

Viterbi Path	
Viterbi Path	

Posterior @ 0.4

Posterior @ 0.4

Posterior Graphic. Threshold Marked @ 0.4

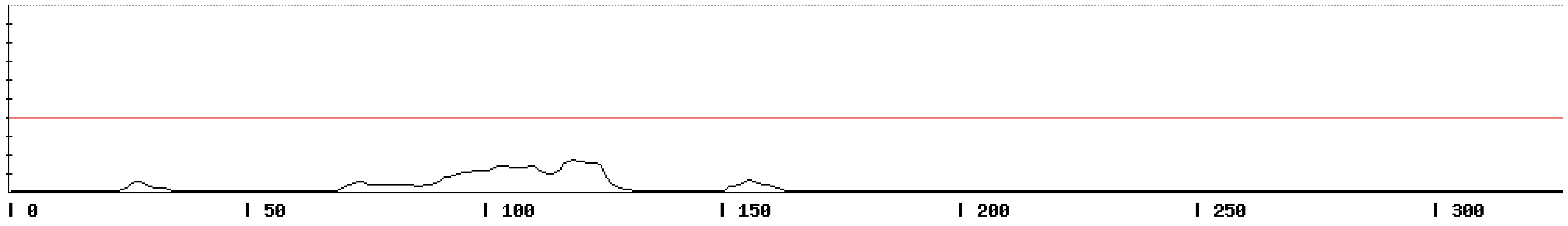

Predictions for Nicotianatomentosiformis_XP_009614701.1

Viterbi Path

Viterbi Path

Posterior @ 0.4

Posterior @ 0.4

Posterior Graphic. Threshold Marked @ 0.4

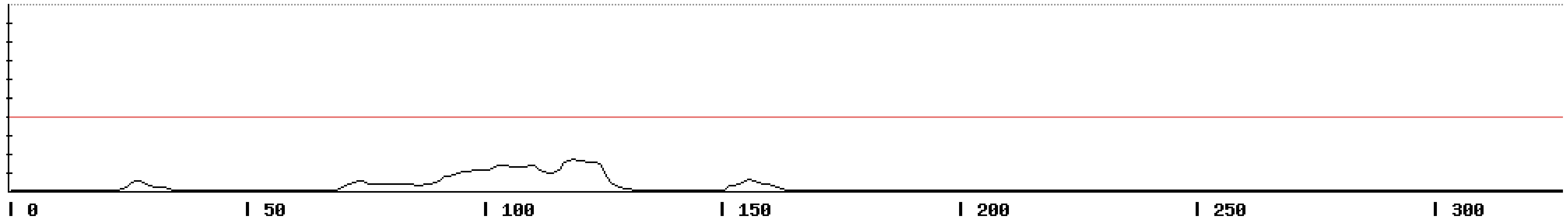

Predictions for Nicotianatomentosiformis_XP_009615029.1

Viterbi Path	
Viterbi Path	
Posterior @ 0.4	
Posterior @ 0.4	
Posterior Graphic. Threshold Marked @ 0.4	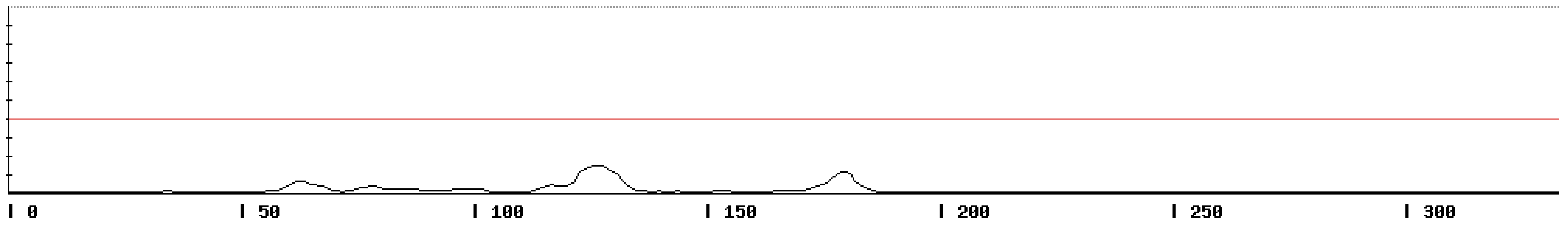

Predictions for Nicotianatomentosiformis_XP_009615269.1

Viterbi Path	
Viterbi Path	
Posterior @ 0.4	
Posterior @ 0.4	
Posterior Graphic. Threshold Marked @ 0.4	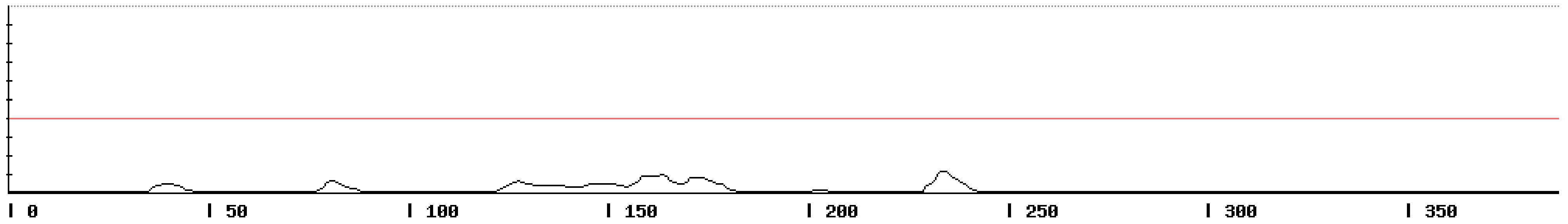

Predictions for Nicotianatomentosiformis_XP_009615270.1	
Viterbi Path	
Viterbi Path	
Posterior @ 0.4	
Posterior @ 0.4	
Posterior Graphic. Threshold Marked @ 0.4	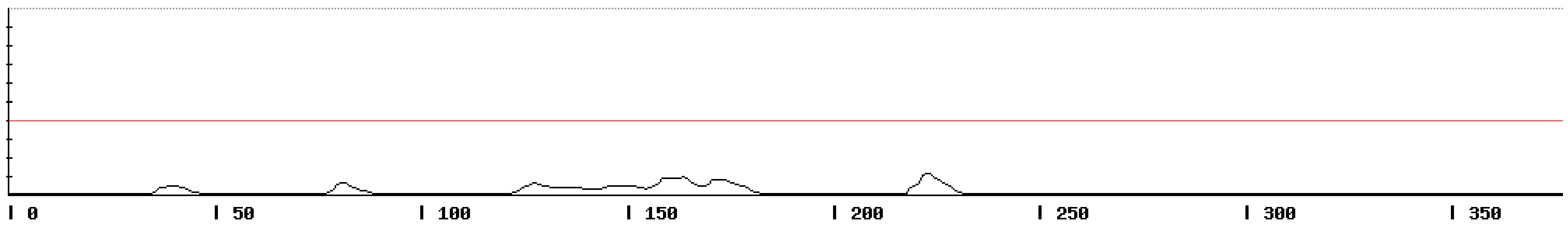

Predictions for Nicotianatomentosiformis_XP_009615559.1	
Viterbi Path	
Viterbi Path	
Posterior @ 0.4	
Posterior @ 0.4	
Posterior Graphic. Threshold Marked @ 0.4	

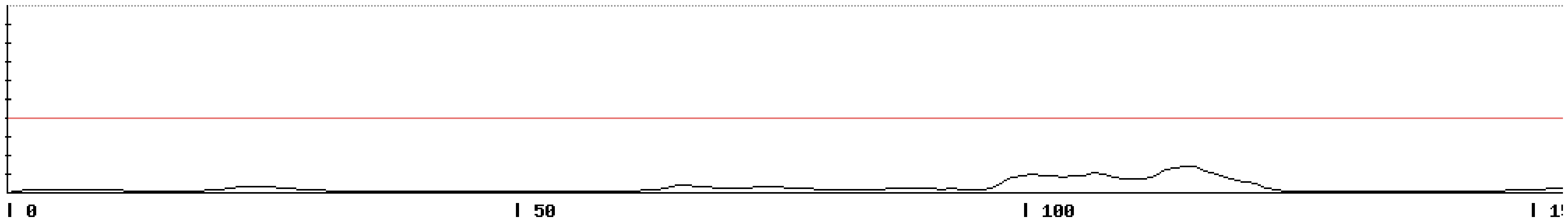

Predictions for Nicotianatomentosiformis_XP_009615661.1

Viterbi Path

Viterbi Path

Posterior @ 0.4

Posterior @ 0.4

Posterior Graphic. Threshold Marked @ 0.4

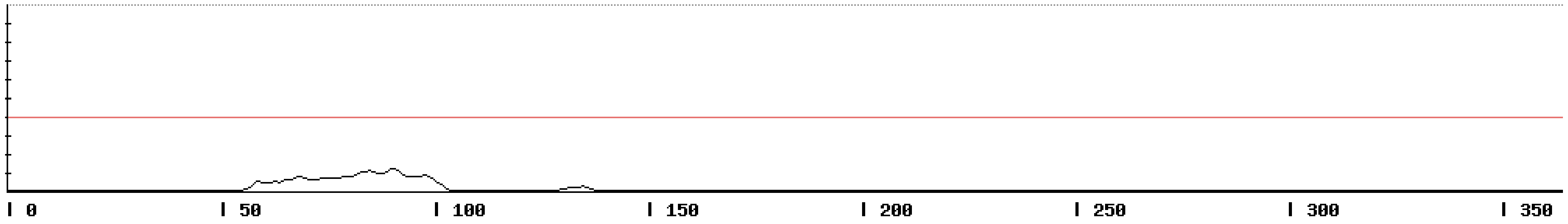

Predictions for Nicotianatomentosiformis_XP_009616121.1

Viterbi Path

Viterbi Path

Posterior @ 0.4

Posterior @ 0.4

Posterior Graphic. Threshold Marked @ 0.4

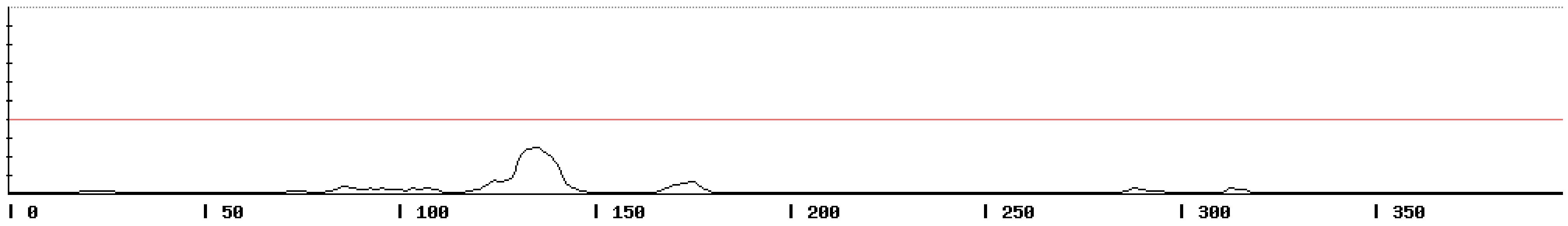

Predictions for Nicotianatomentosiformis_XP_009616658.1

Viterbi Path

Viterbi Path

Posterior @ 0.4

Posterior @ 0.4

Posterior Graphic. Threshold Marked @ 0.4

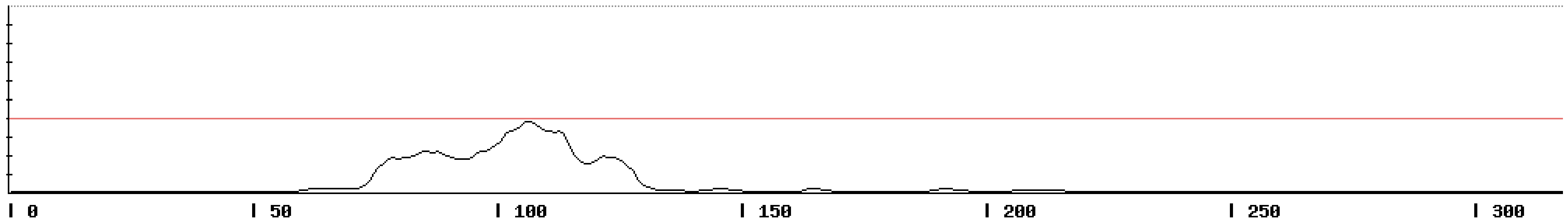

Predictions for Nicotianatomentosiformis_XP_009617292.1

Viterbi Path

Viterbi Path

Posterior @ 0.4

Posterior @ 0.4

Posterior Graphic. Threshold Marked @ 0.4

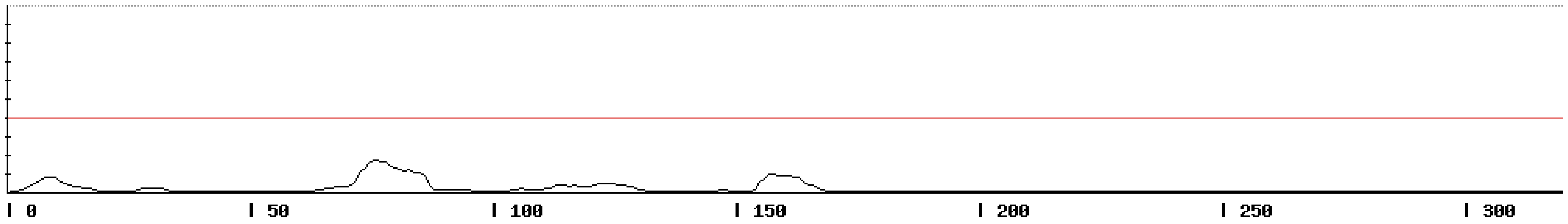

Predictions for Nicotianatomentosiformis_XP_009618124.1

Viterbi Path

Viterbi Path

Posterior @ 0.4

Posterior @ 0.4

Posterior Graphic. Threshold Marked @ 0.4

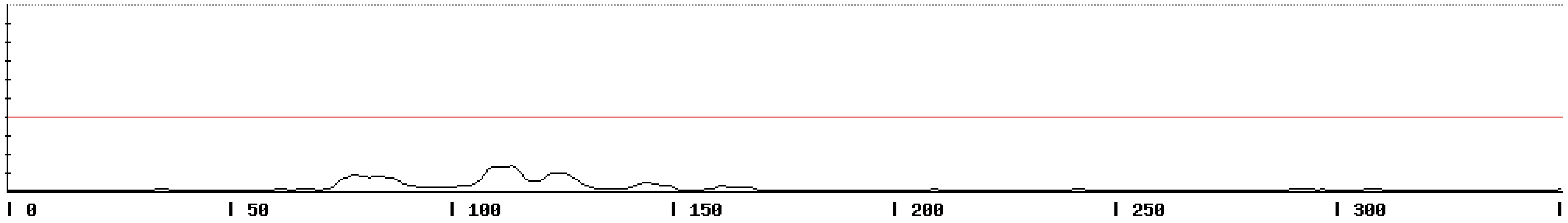

Predictions for Nicotianatomentosiformis_XP_009618174.1

Viterbi Path

Viterbi Path

Posterior @ 0.4 | 141 - RKRRK - 145

Posterior @ 0.4

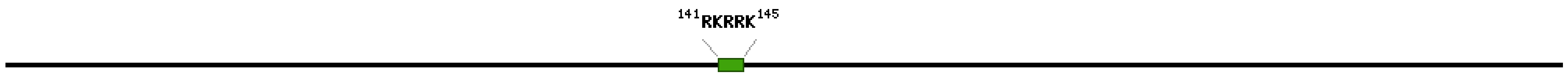

Posterior Graphic. Threshold Marked @ 0.4

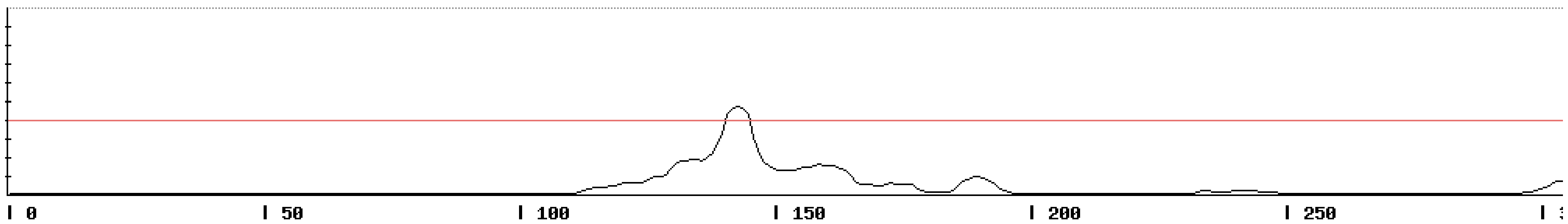

Predictions for Nicotianatomentosiformis_XP_009618980.1

Viterbi Path

Viterbi Path

Posterior @ 0.4 | 63 - KKKKPT - 68

Posterior @ 0.4

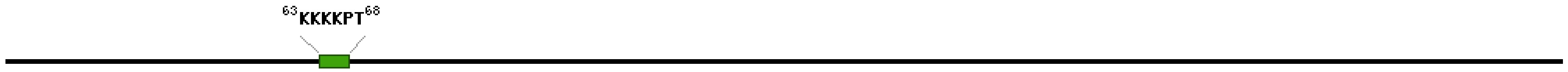

Posterior Graphic. Threshold Marked @ 0.4

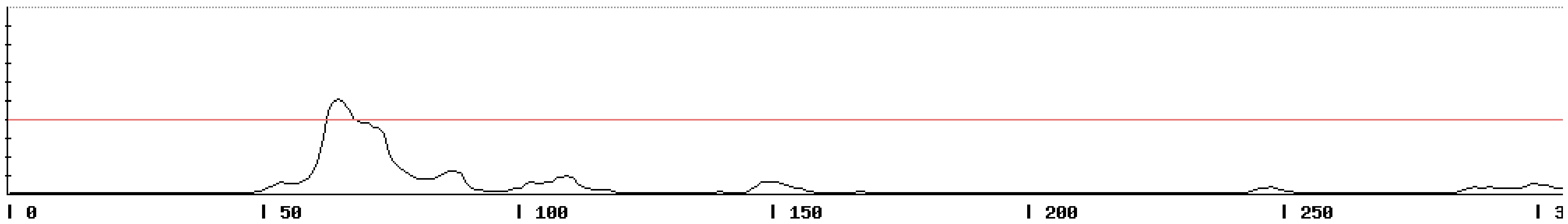

Predictions for Nicotianatomentosiformis_XP_009618981.1

Viterbi Path

Viterbi Path

Posterior @ 0.4	63 - KKKKPT - 68
Posterior @ 0.4	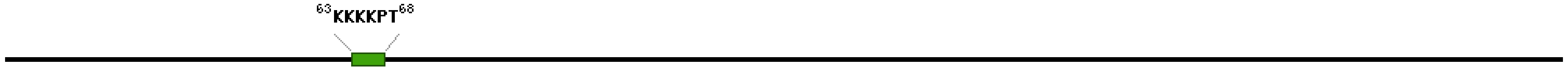
Posterior Graphic. Threshold Marked @ 0.4	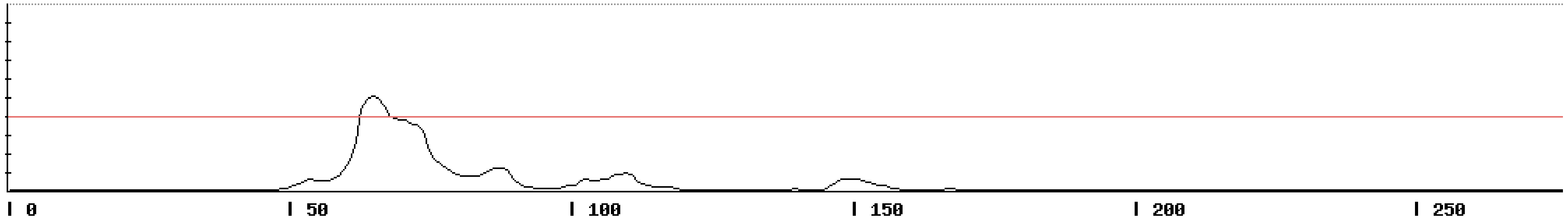

Predictions for Nicotianatomentosiformis_XP_009619245.1

Viterbi Path	
Viterbi Path	
Posterior @ 0.4	
Posterior @ 0.4	
Posterior Graphic. Threshold Marked @ 0.4	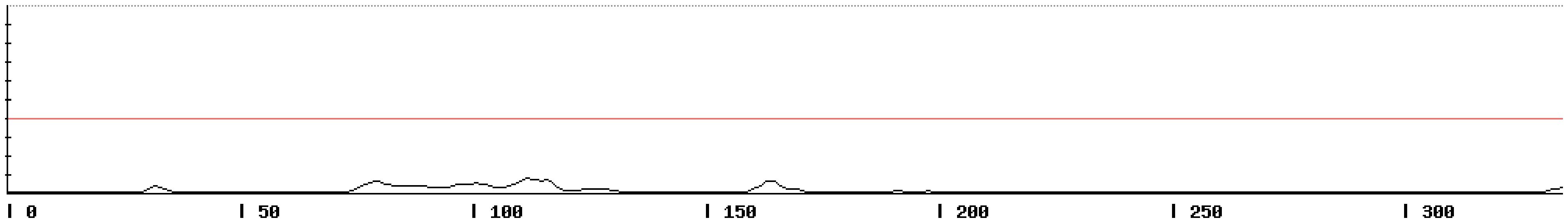

Predictions for Nicotianatomentosiformis_XP_009619246.1

Viterbi Path

Viterbi Path

Posterior @ 0.4

Posterior @ 0.4

Posterior Graphic. Threshold Marked @ 0.4

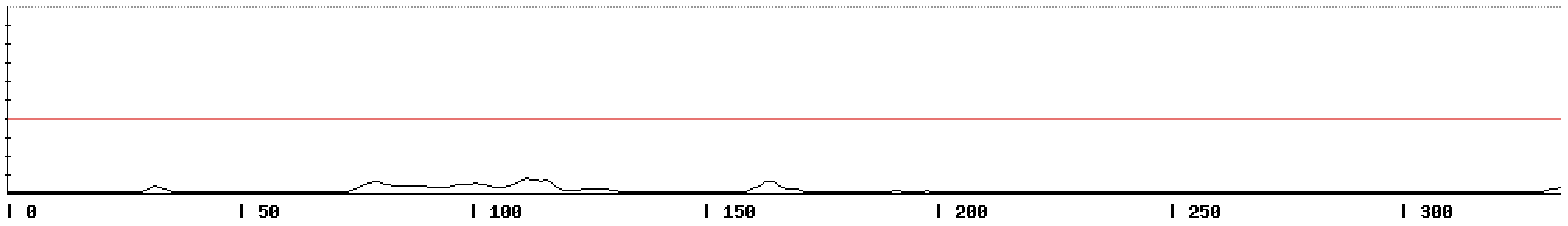

Predictions for Nicotianatomentosiformis_XP_009619248.1

Viterbi Path

Viterbi Path

Posterior @ 0.4

Posterior @ 0.4

Posterior Graphic. Threshold Marked @ 0.4

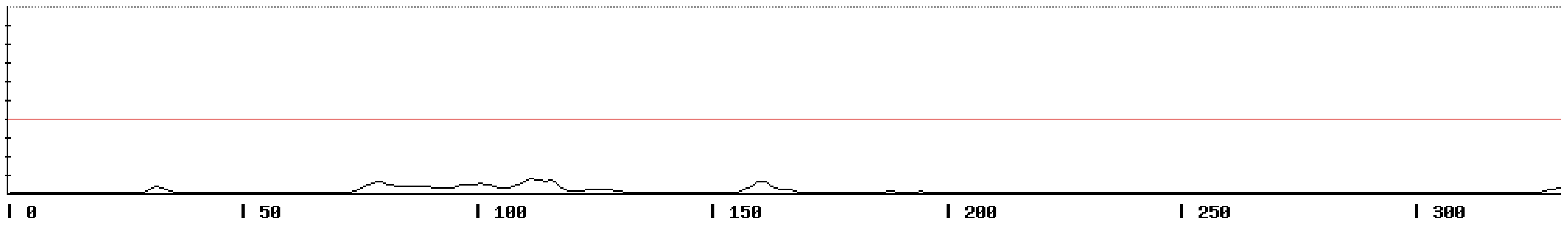

Predictions for Nicotianatomentosiformis_XP_009619467.1	
Viterbi Path	
Viterbi Path	
Posterior @ 0.4	
Posterior @ 0.4	
Posterior Graphic. Threshold Marked @ 0.4	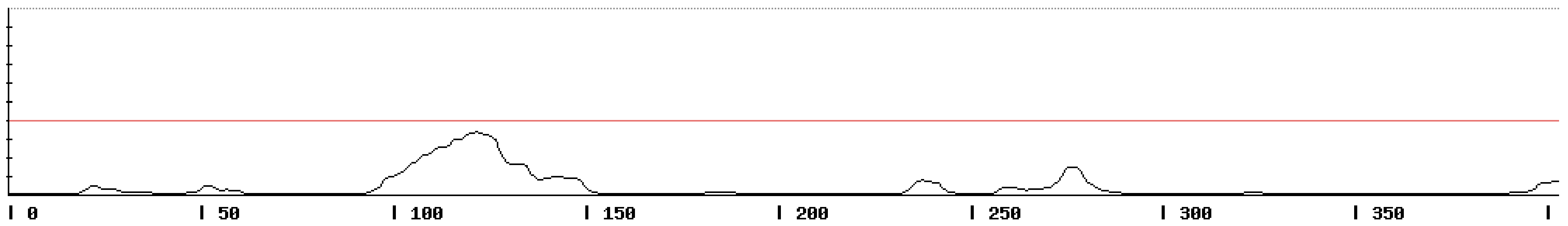

Predictions for Nicotianatomentosiformis_XP_009620409.1	
Viterbi Path	
Viterbi Path	
Posterior @ 0.4	
Posterior @ 0.4	
Posterior Graphic. Threshold Marked @ 0.4	

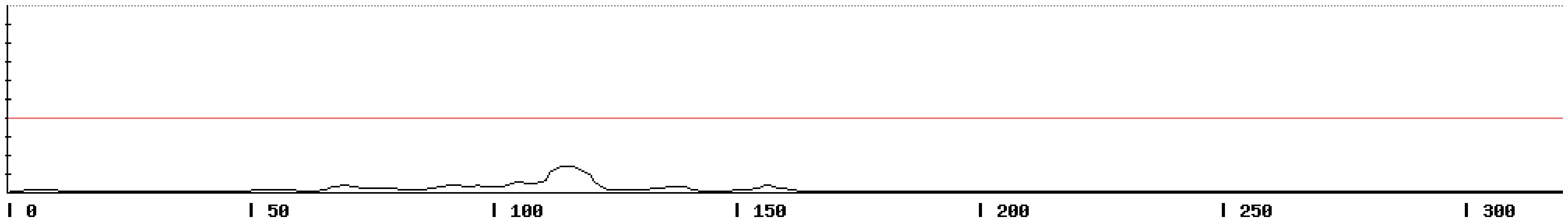

Predictions for Nicotianatomentosiformis_XP_009620854.1

Viterbi Path

Viterbi Path

Posterior @ 0.4

Posterior @ 0.4

Posterior Graphic. Threshold Marked @ 0.4

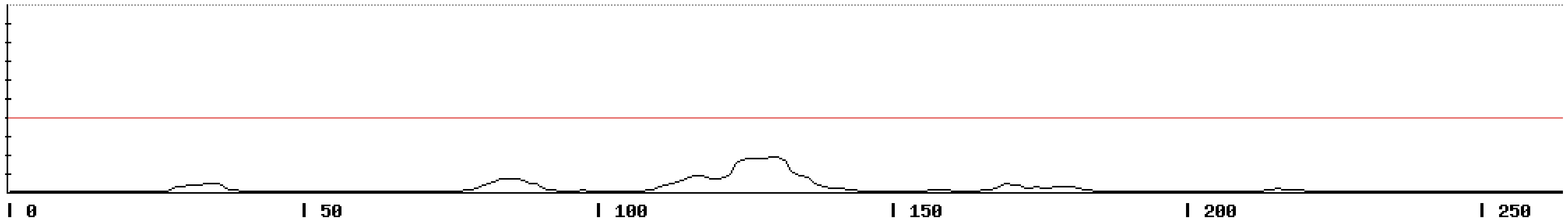

Predictions for Nicotianatomentosiformis_XP_009621551.1

Viterbi Path

Viterbi Path

Posterior @ 0.4

Posterior @ 0.4

Posterior Graphic. Threshold Marked @ 0.4

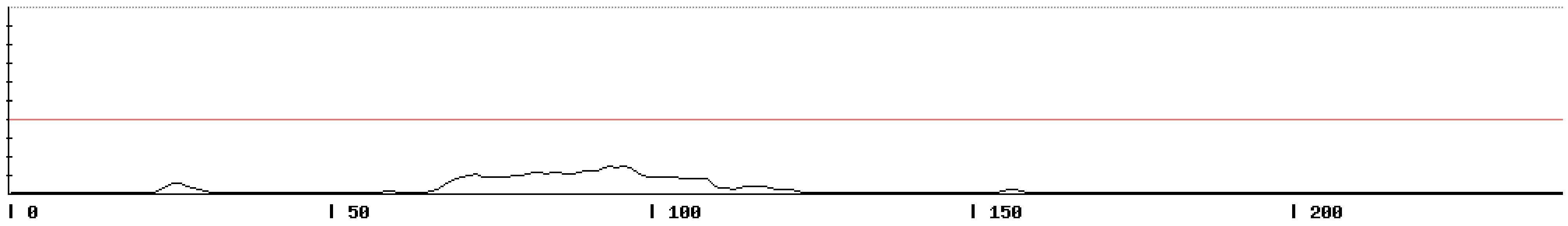

Predictions for Nicotianatomentosiformis_XP_009621552.1

Viterbi Path

Viterbi Path

Posterior @ 0.4

Posterior @ 0.4

Posterior Graphic. Threshold Marked @ 0.4

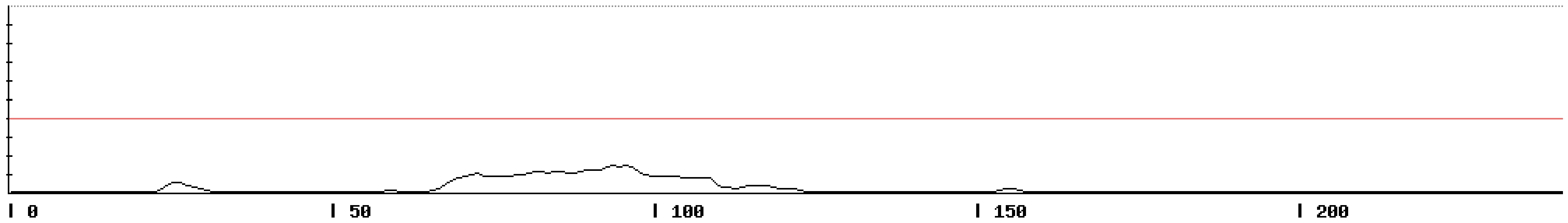

Predictions for Nicotianatomentosiformis_XP_009621672.1

Viterbi Path

Viterbi Path

Posterior @ 0.4

Posterior @ 0.4

Posterior Graphic. Threshold Marked @ 0.4

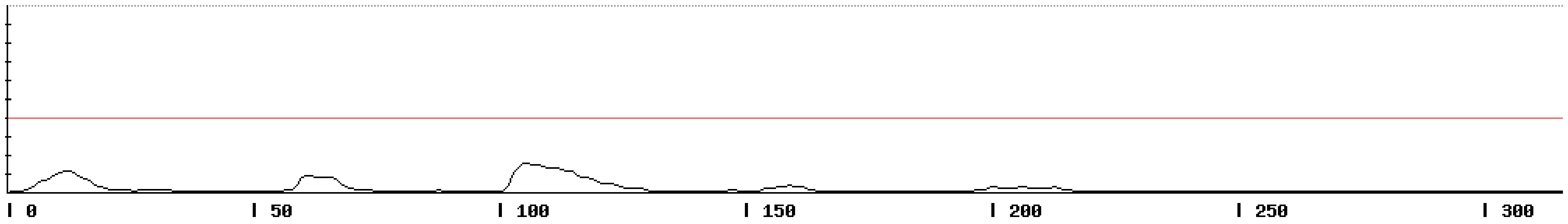

Predictions for Nicotianatomentosiformis_XP_009621726.1

Viterbi Path

Viterbi Path

Posterior @ 0.4

Posterior @ 0.4

Posterior Graphic. Threshold Marked @ 0.4

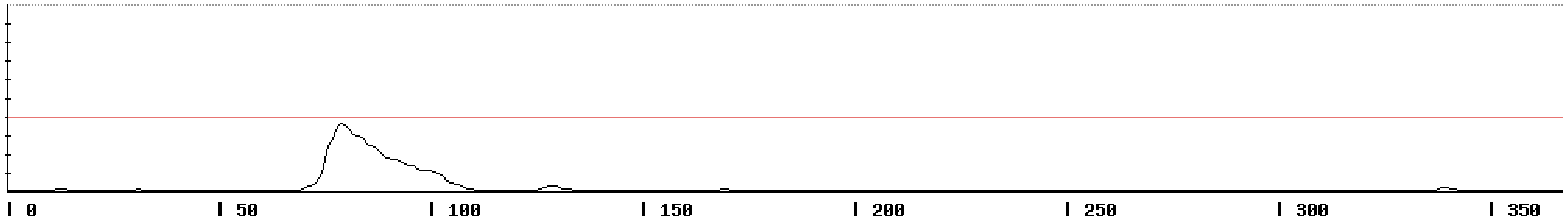

Predictions for Nicotianatomentosiformis_XP_009622295.1

Viterbi Path

Viterbi Path

Posterior @ 0.4

Posterior @ 0.4

Posterior Graphic. Threshold Marked @ 0.4

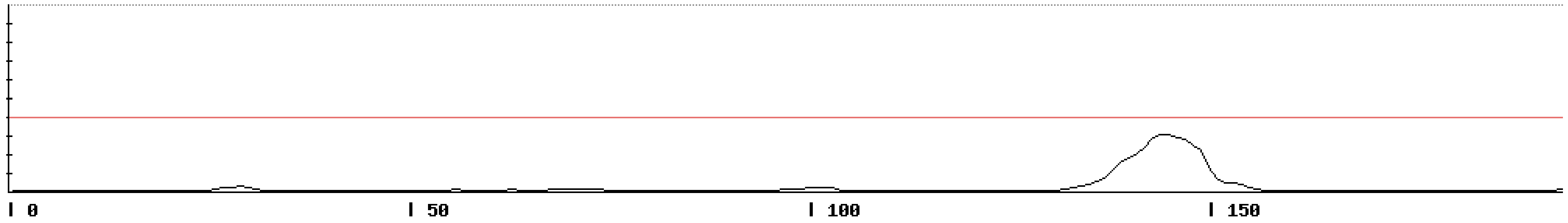

Predictions for Nicotianatomentosiformis_XP_009622296.1

Viterbi Path

Viterbi Path

Posterior @ 0.4

Posterior @ 0.4

Posterior Graphic. Threshold Marked @ 0.4

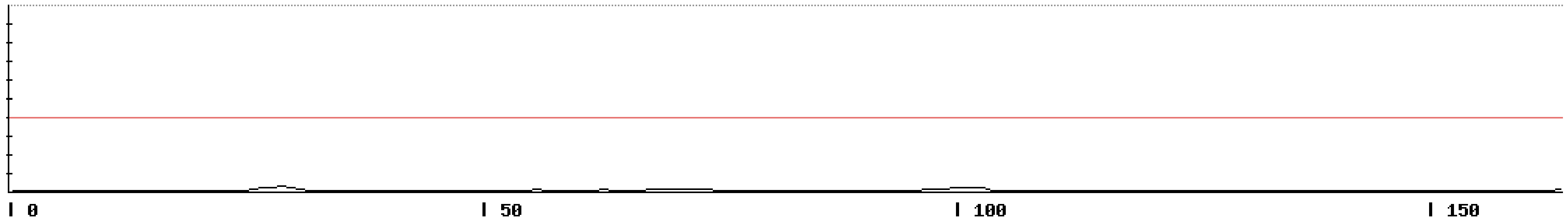

Predictions for Nicotianatomentosiformis_XP_009622368.1

Viterbi Path

Viterbi Path

Posterior @ 0.4	131 - GKRKRRKISDENNLRKEHVRWHK - 153
Posterior @ 0.4	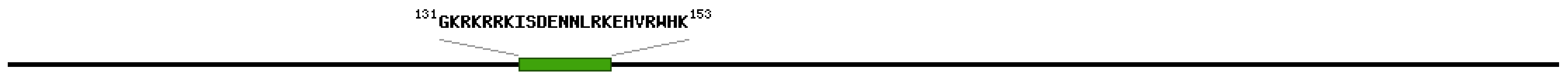
Posterior Graphic. Threshold Marked @ 0.4	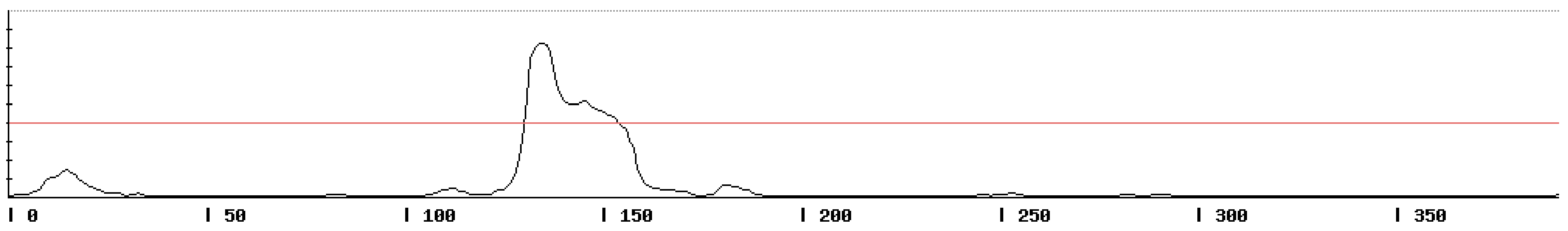

Predictions for Nicotianatomentosiformis_XP_009622624.1

Viterbi Path	
Viterbi Path	
Posterior @ 0.4	79 - PRKKNKGRFSRIVGKRGGTWKQ - 100
Posterior @ 0.4	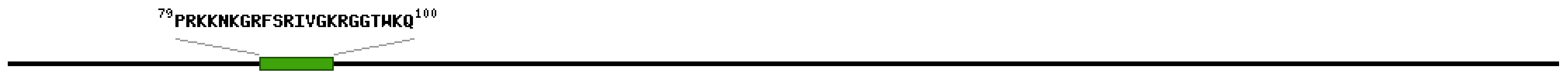
Posterior Graphic. Threshold Marked @ 0.4	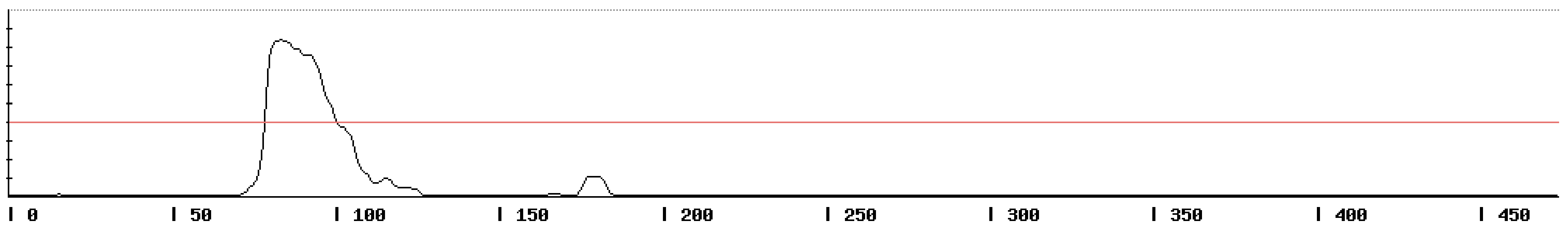

Predictions for Nicotianatomentosiformis_XP_009622905.1

Viterbi Path

Viterbi Path

Posterior @ 0.4

Posterior @ 0.4

Posterior Graphic. Threshold Marked @ 0.4

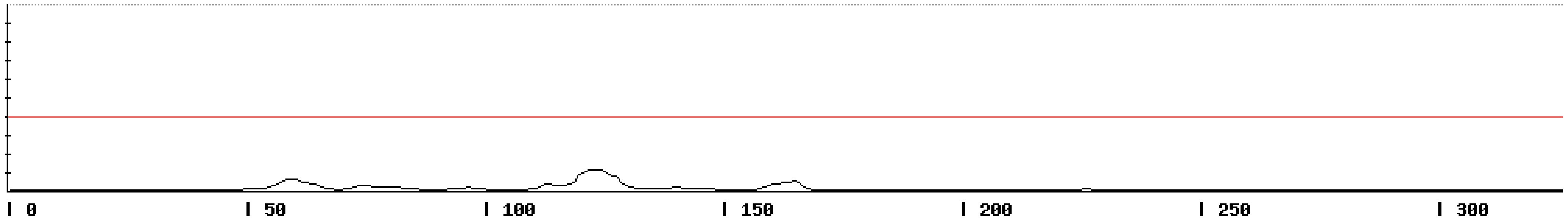

Predictions for Nicotianatomentosiformis_XP_009623547.1

Viterbi Path

Viterbi Path

Posterior @ 0.4

Posterior @ 0.4

Posterior Graphic. Threshold Marked @ 0.4

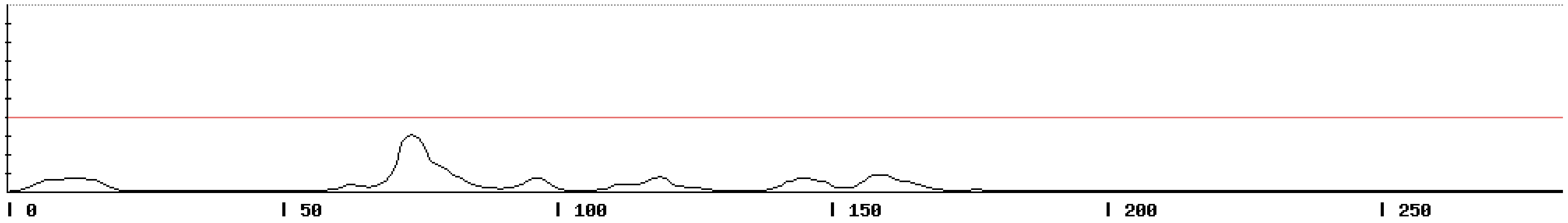

Predictions for Nicotianatomentosiformis_XP_009624128.1

Viterbi Path

Viterbi Path

Posterior @ 0.4

Posterior @ 0.4

Posterior Graphic. Threshold Marked @ 0.4

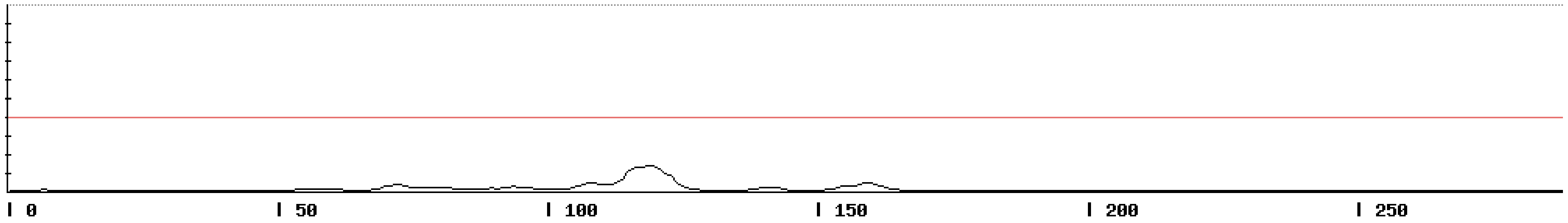

Predictions for Nicotianatomentosiformis_XP_009624151.1

Viterbi Path

Viterbi Path

Posterior @ 0.4

Posterior @ 0.4

Posterior Graphic. Threshold Marked @ 0.4

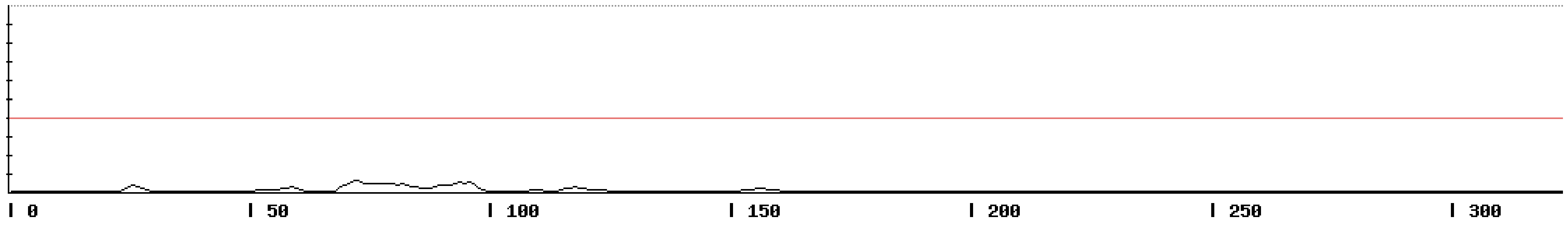

Predictions for Nicotianatomentosiformis_XP_009624737.1

Viterbi Path

Viterbi Path

Posterior @ 0.4

Posterior @ 0.4

Posterior Graphic. Threshold Marked @ 0.4

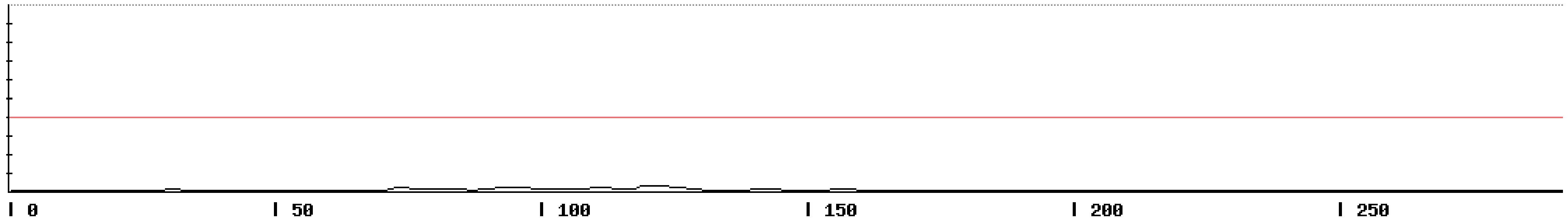

Predictions for Nicotianatomentosiformis_XP_009625026.1

Viterbi Path

Viterbi Path

Posterior @ 0.4

Posterior @ 0.4

Posterior Graphic. Threshold Marked @ 0.4

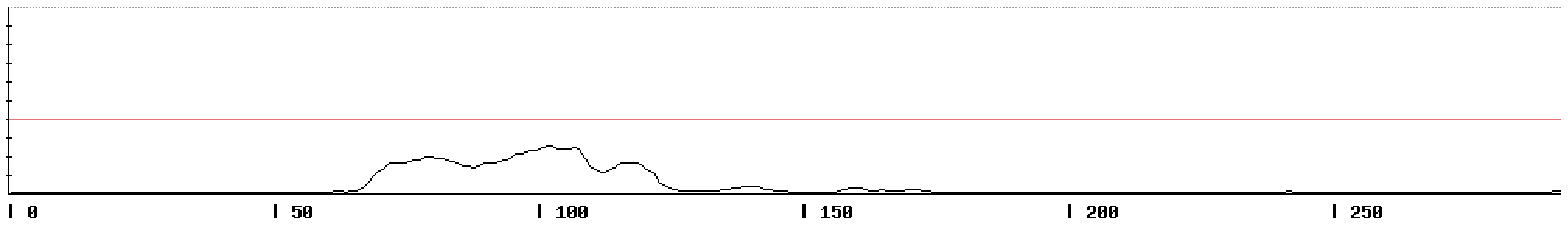

Predictions for Nicotianatomentosiformis_XP_009625360.1

Viterbi Path

Viterbi Path

Posterior @ 0.4

Posterior @ 0.4

Posterior Graphic. Threshold Marked @ 0.4

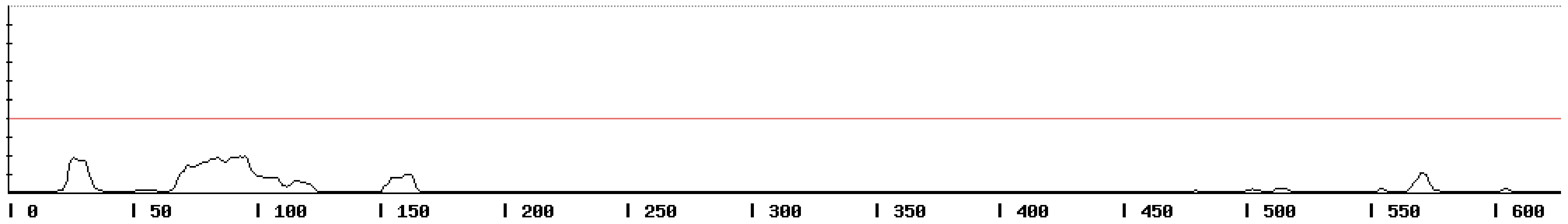

Predictions for Nicotianatomentosiformis_XP_009625395.1

Viterbi Path

Viterbi Path

Posterior @ 0.4

Posterior @ 0.4

Posterior Graphic. Threshold Marked @ 0.4

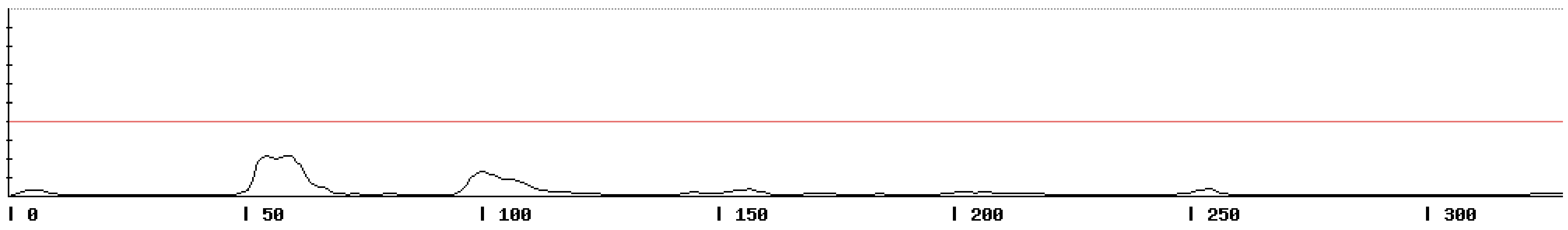

Predictions for Nicotianatomentosiformis_XP_009625396.1

Viterbi Path

Viterbi Path

Posterior @ 0.4

Posterior @ 0.4

Posterior Graphic. Threshold Marked @ 0.4

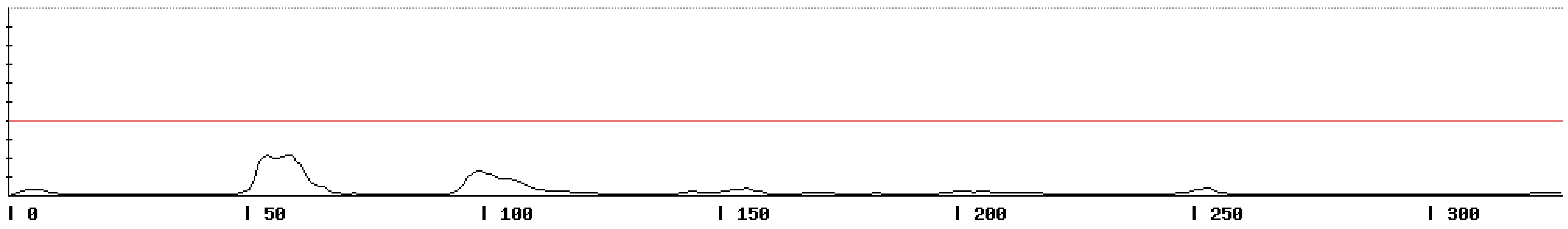

Predictions for Nicotianatomentosiformis_XP_009625486.1

Viterbi Path

Viterbi Path

Posterior @ 0.4

Posterior @ 0.4

Posterior Graphic. Threshold Marked @ 0.4

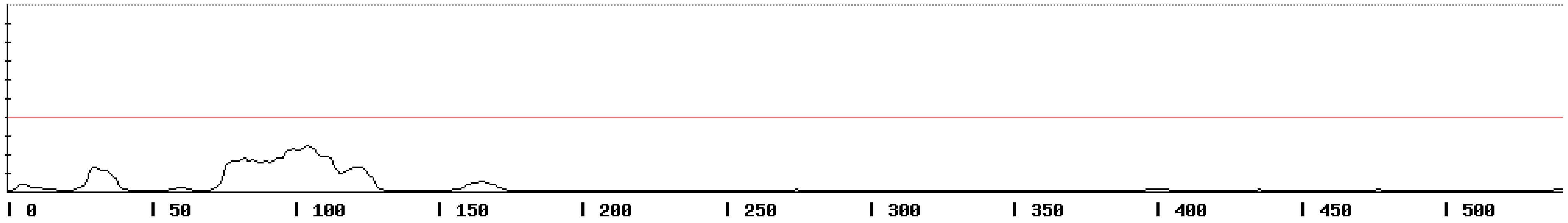

Predictions for Nicotianatomentosiformis_XP_009625487.1

Viterbi Path

Viterbi Path

Posterior @ 0.4

Posterior @ 0.4

Posterior Graphic. Threshold Marked @ 0.4

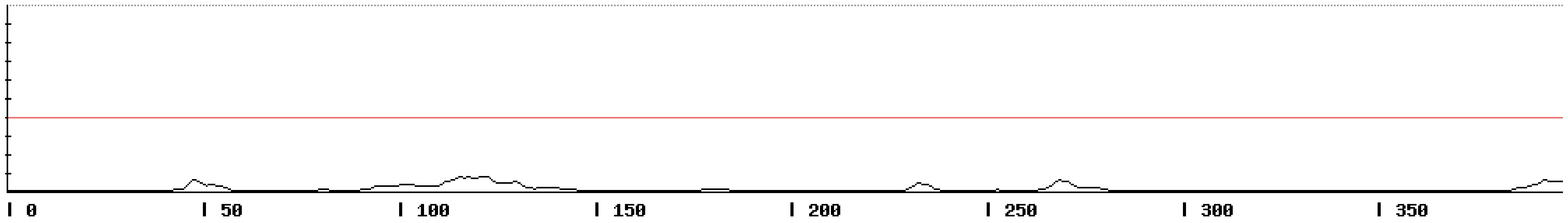

Predictions for Nicotianatomentosiformis_XP_009625550.1

Viterbi Path

Viterbi Path

Posterior @ 0.4

Posterior @ 0.4

Posterior Graphic. Threshold Marked @ 0.4

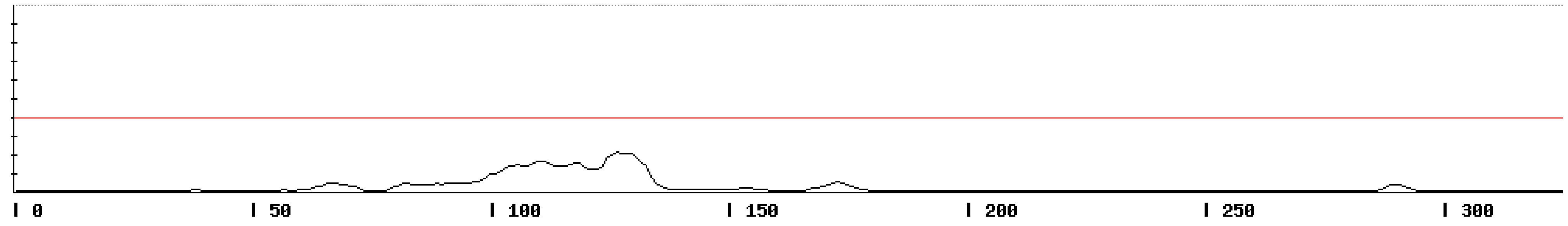

Predictions for Nicotianatomentosiformis_XP_009625571.1

Viterbi Path

Viterbi Path

Posterior @ 0.4

Posterior @ 0.4

Posterior Graphic. Threshold Marked @ 0.4

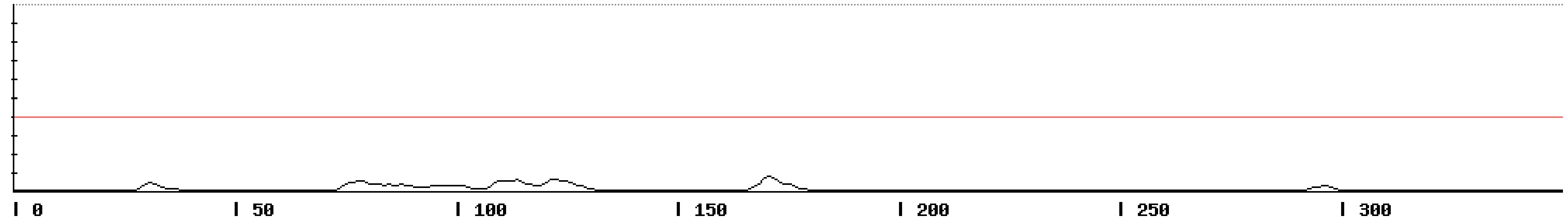

Predictions for Nicotianatomentosiformis_XP_009625611.1

Viterbi Path

Viterbi Path

Posterior @ 0.4

Posterior @ 0.4

Posterior Graphic. Threshold Marked @ 0.4

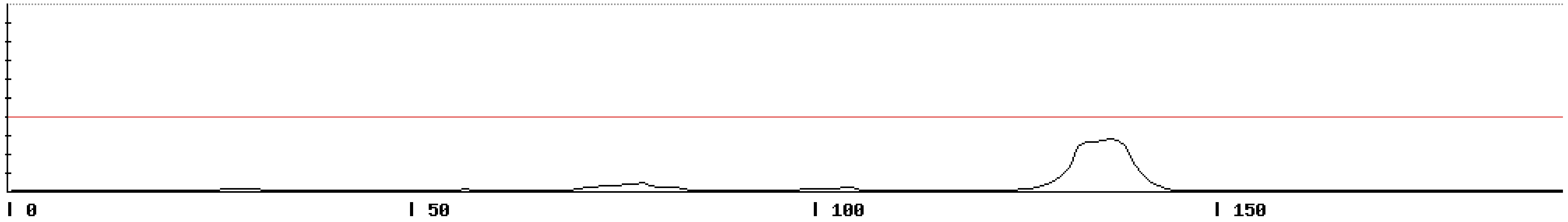

Predictions for Nicotianatomentosiformis_XP_009626107.1

Viterbi Path

Viterbi Path

Posterior @ 0.4

Posterior @ 0.4

Posterior Graphic. Threshold Marked @ 0.4

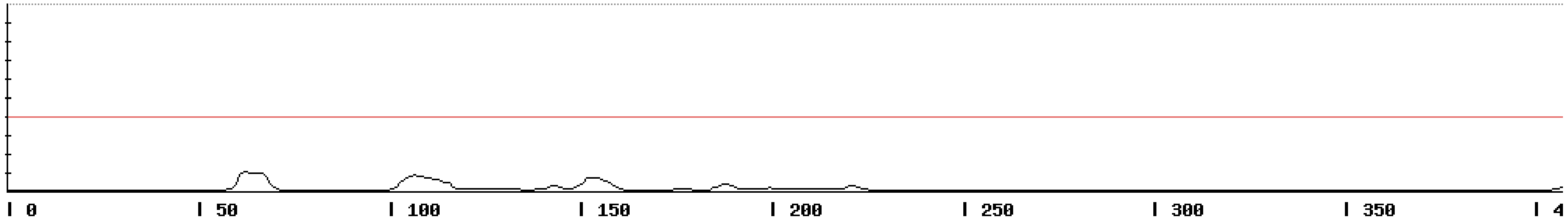

Predictions for Nicotianatomentosiformis_XP_009626108.1

Viterbi Path

Viterbi Path

Posterior @ 0.4

Posterior @ 0.4

Posterior Graphic. Threshold Marked @ 0.4

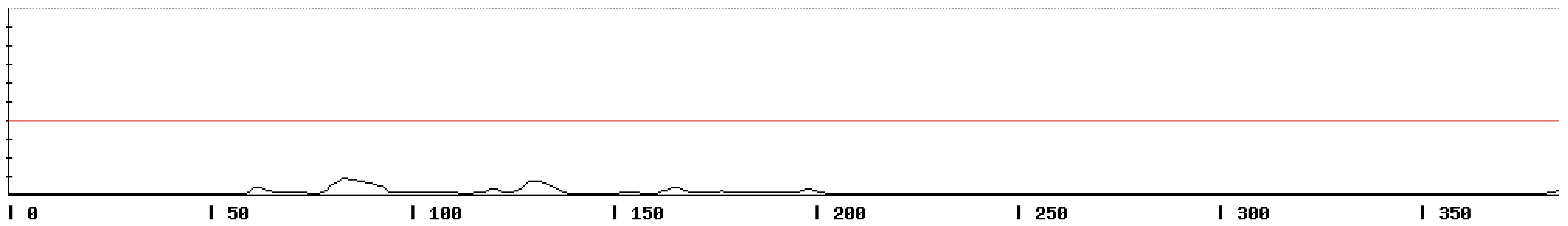

Predictions for Nicotianatomentosiformis_XP_009626689.1

Viterbi Path

Viterbi Path

Posterior @ 0.4

Posterior @ 0.4

Posterior Graphic. Threshold Marked @ 0.4

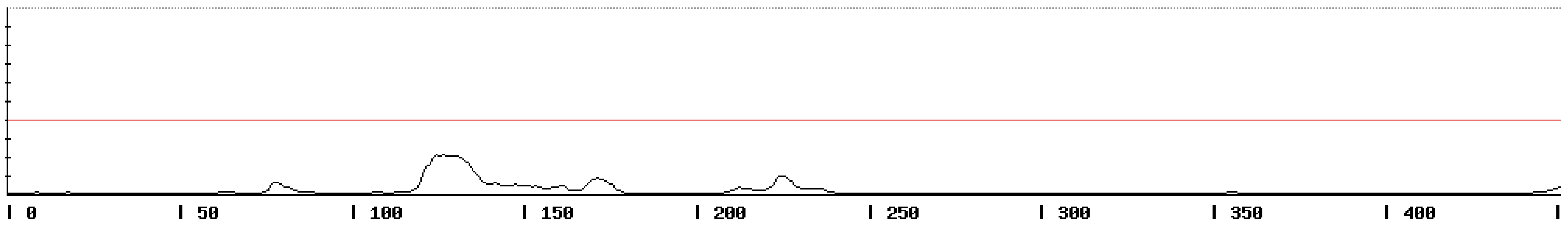

Predictions for Nicotianatomentosiformis_XP_009626970.1

Viterbi Path

Viterbi Path

Posterior @ 0.4: 372 - KK - 373

Posterior @ 0.4

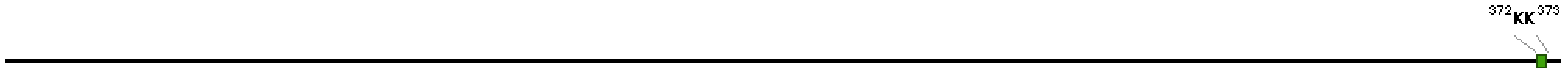

Posterior Graphic. Threshold Marked @ 0.4

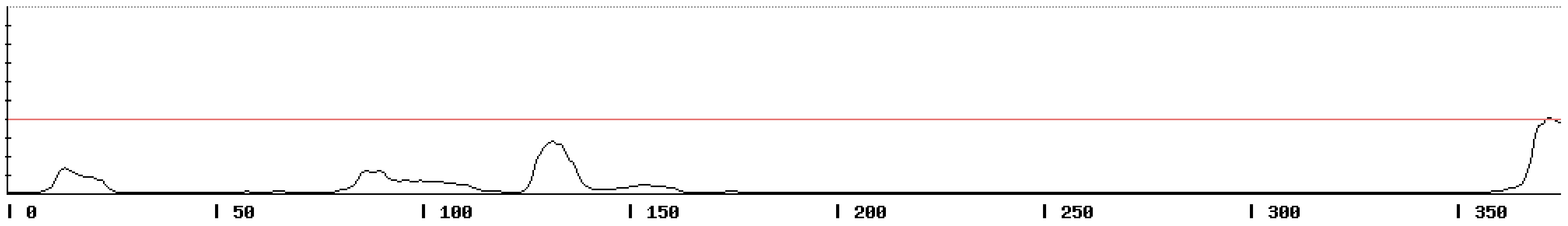

Predictions for Nicotianatomentosiformis_XP_009627220.1

Viterbi Path

Viterbi Path

Posterior @ 0.4: 83 - VLKGGRRRAGGGYWKKRGRIR - 103

Posterior @ 0.4

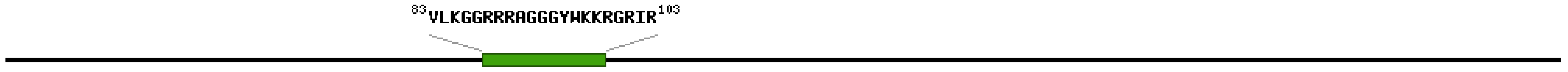

Posterior Graphic. Threshold Marked @ 0.4

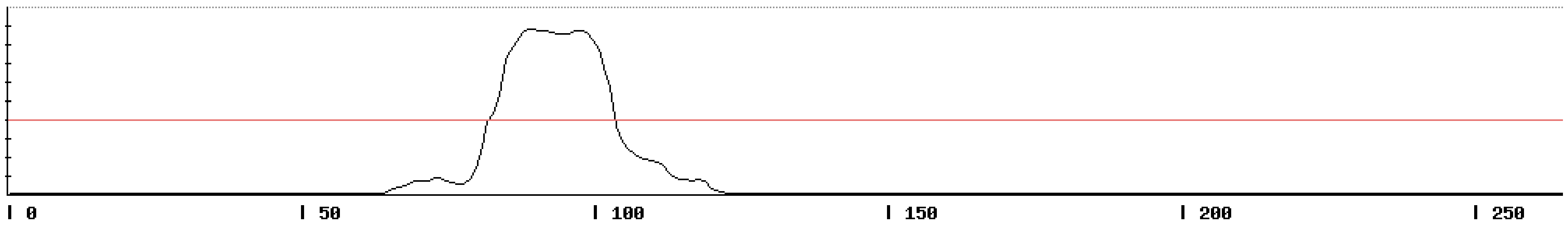

Predictions for Nicotianatomentosiformis_XP_009627221.1

Viterbi Path

Viterbi Path

Posterior @ 0.4

83 - VLKGGRRRAGGGYWKKRGRIR - 103

Posterior @ 0.4

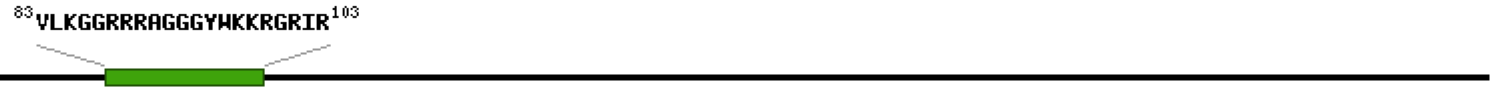

Posterior Graphic. Threshold Marked @ 0.4

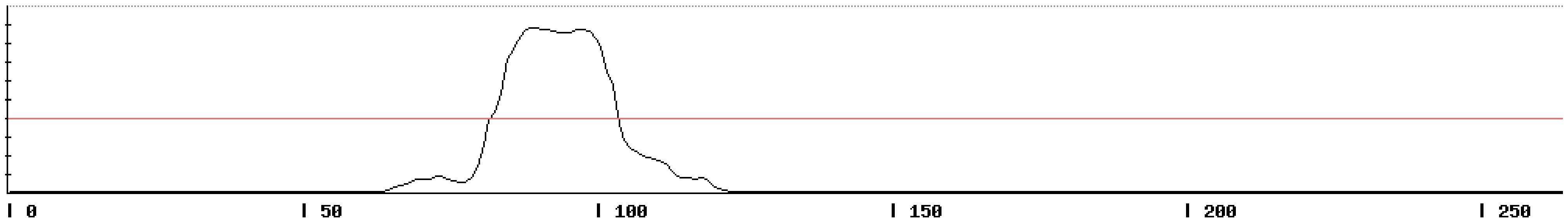

Predictions for Nicotianatomentosiformis_XP_009627656.1

Viterbi Path

Viterbi Path

Posterior @ 0.4

Posterior @ 0.4

Posterior Graphic. Threshold Marked @ 0.4

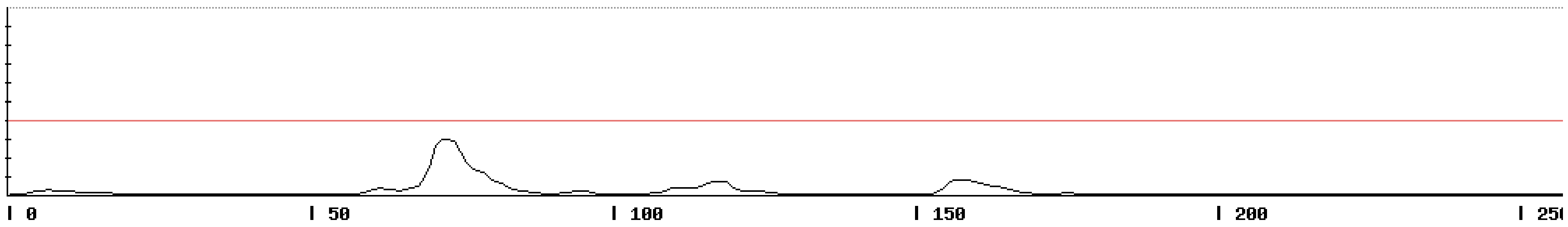

Predictions for Nicotianatomentosiformis_XP_009627678.1

Viterbi Path

Viterbi Path

Posterior @ 0.4

Posterior @ 0.4

Posterior Graphic. Threshold Marked @ 0.4

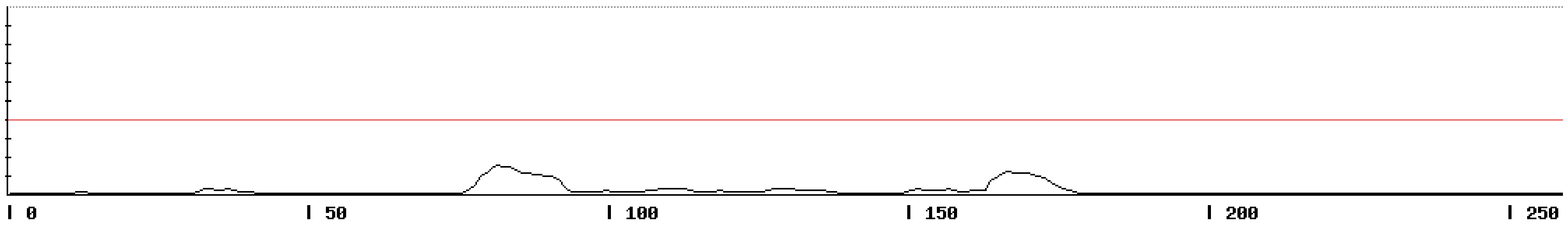

Predictions for Nicotianatomentosiformis_XP_009628603.1

Viterbi Path

Viterbi Path

Posterior @ 0.4

Posterior @ 0.4

Posterior Graphic. Threshold Marked @ 0.4

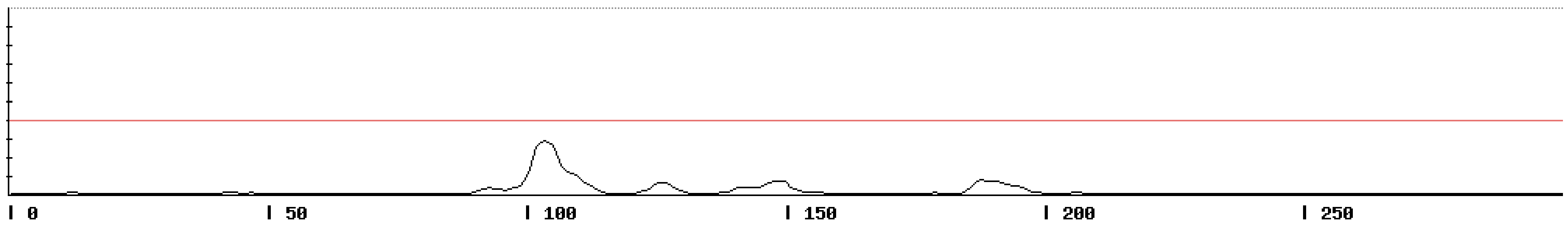

Predictions for Nicotianatomentosiformis_XP_009628604.1

Viterbi Path

Viterbi Path

Posterior @ 0.4

Posterior @ 0.4

Posterior Graphic. Threshold Marked @ 0.4

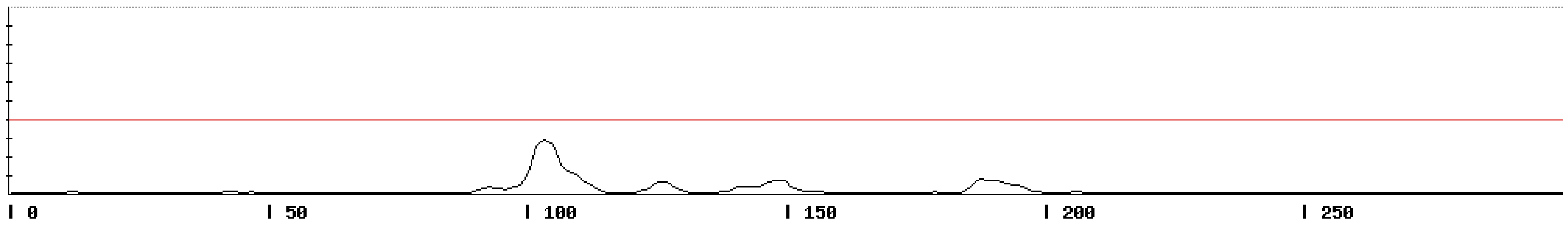

Predictions for Nicotianatomentosiformis_XP_009628605.1

Viterbi Path

Viterbi Path

Posterior @ 0.4	
Posterior @ 0.4	
Posterior Graphic. Threshold Marked @ 0.4	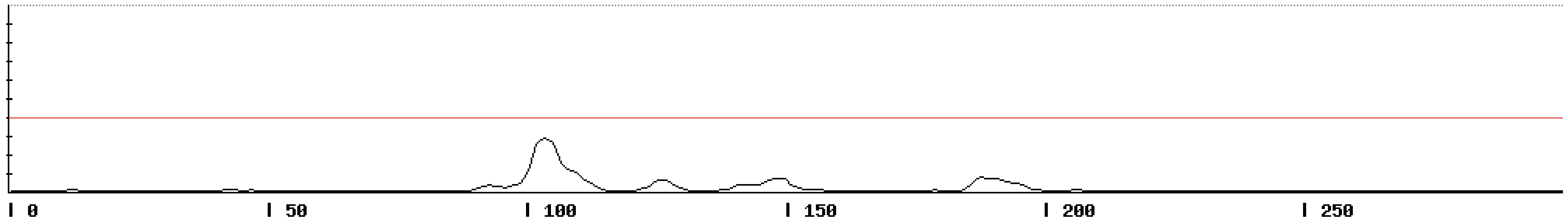

Predictions for Nicotianatomentosiformis_XP_009628606.1

Viterbi Path	
Viterbi Path	
Posterior @ 0.4	
Posterior @ 0.4	
Posterior Graphic. Threshold Marked @ 0.4	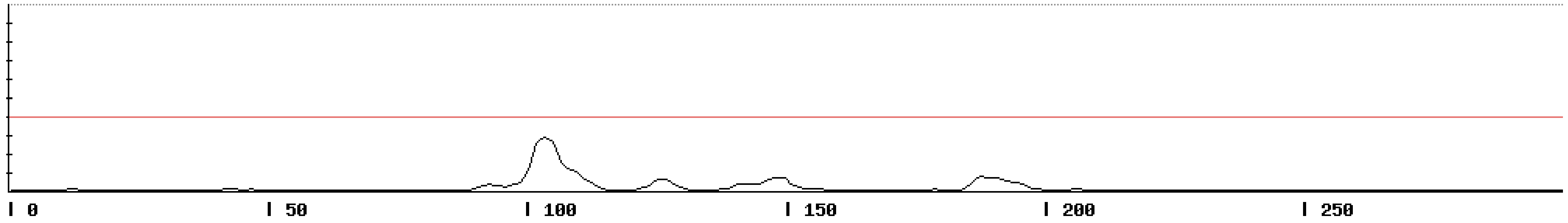

Predictions for Nicotianatomentosiformis_XP_009628607.1

Viterbi Path	
Viterbi Path	
Posterior @ 0.4	
Posterior @ 0.4	
Posterior Graphic. Threshold Marked @ 0.4	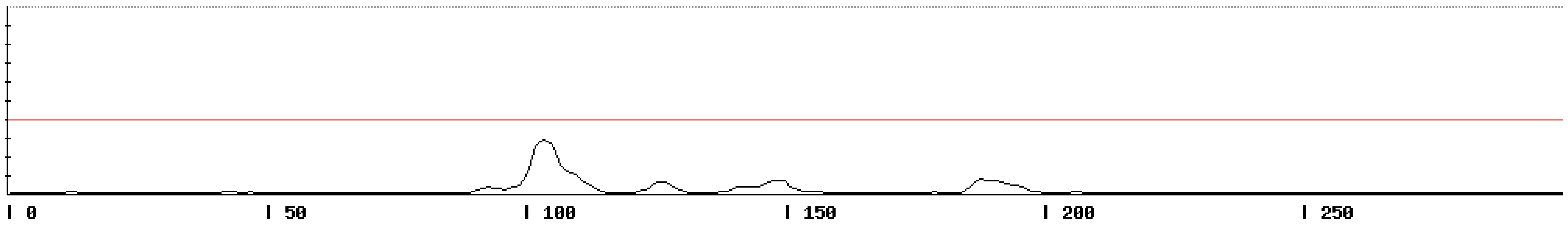

Predictions for Nicotianatomentosiformis_XP_009628610.1

Viterbi Path	
Viterbi Path	
Posterior @ 0.4	79 - KRKNKGT - 85
Posterior @ 0.4	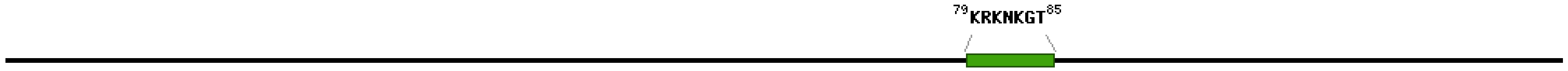
Posterior Graphic. Threshold Marked @ 0.4	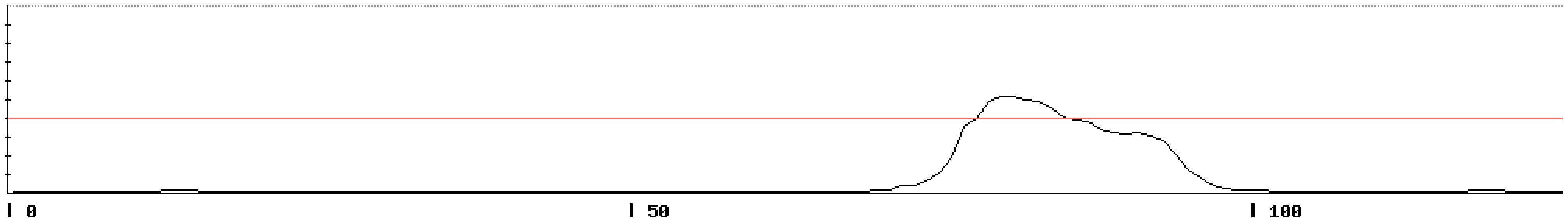

Predictions for Nicotianatomentosiformis_XP_009628837.1	
Viterbi Path	
Viterbi Path	
Posterior @ 0.4	94 - IKKKENPDDKKKGK - 107
Posterior @ 0.4	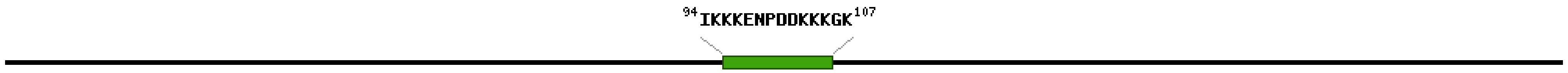
Posterior Graphic. Threshold Marked @ 0.4	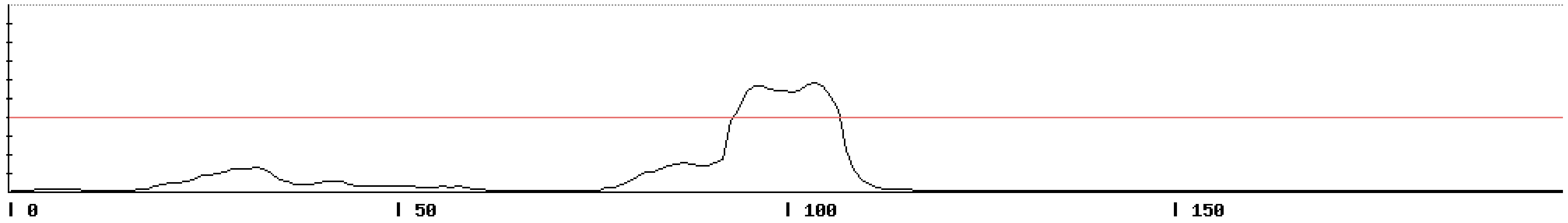

Predictions for Nicotianatomentosiformis_XP_009629334.1	
Viterbi Path	
Viterbi Path	
Posterior @ 0.4	
Posterior @ 0.4	
Posterior Graphic. Threshold Marked @ 0.4	

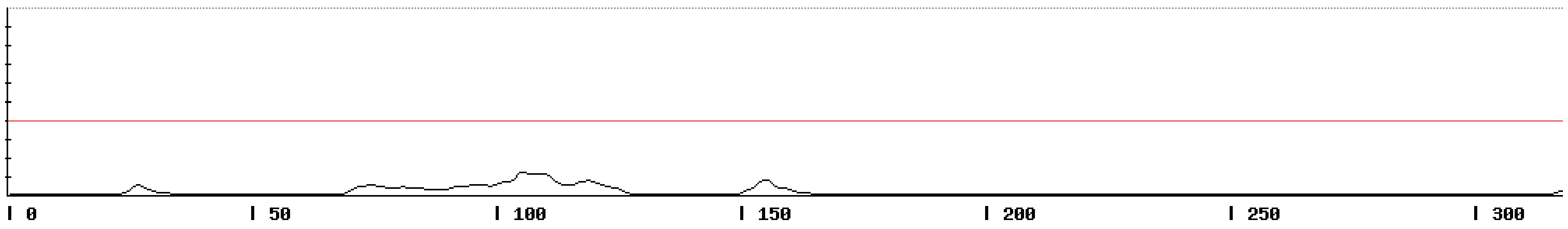

Predictions for Nicotianatomentosiformis_XP_009629524.1

Viterbi Path

Viterbi Path

Posterior @ 0.4

Posterior @ 0.4

Posterior Graphic. Threshold Marked @ 0.4

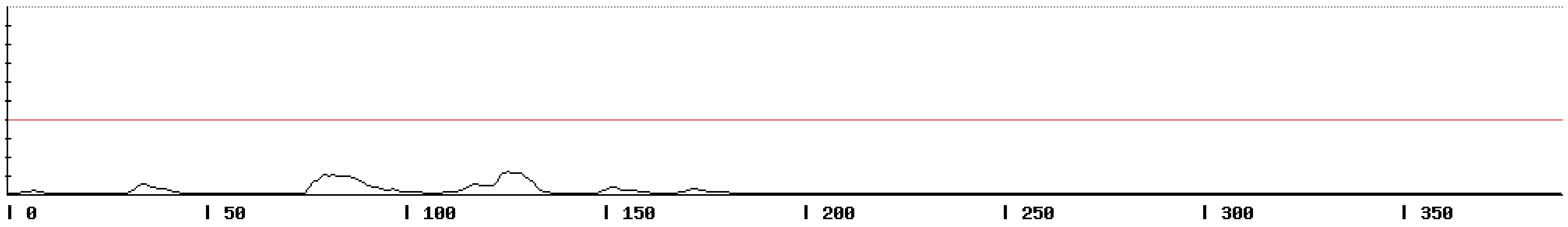

Predictions for Nicotianatomentosiformis_XP_009629525.1

Viterbi Path

Viterbi Path

Posterior @ 0.4

Posterior @ 0.4

Posterior Graphic. Threshold Marked @ 0.4	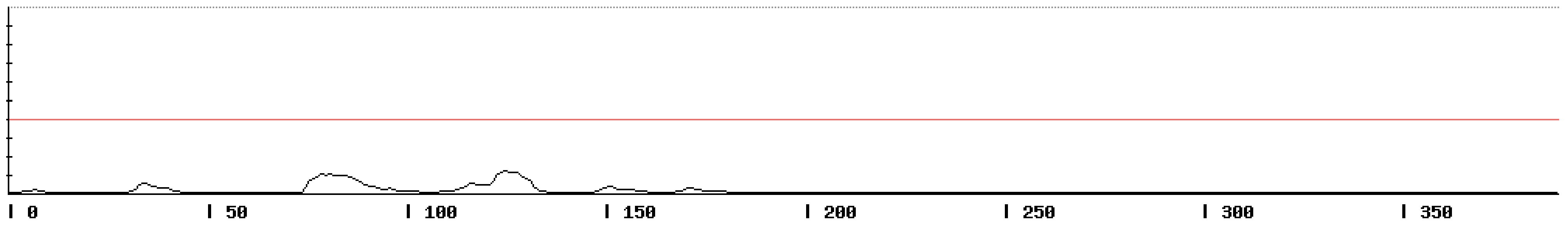

Predictions for Nicotianatomentosiformis_XP_009629774.1	
Viterbi Path	
Viterbi Path	
Posterior @ 0.4	
Posterior @ 0.4	
Posterior Graphic. Threshold Marked @ 0.4	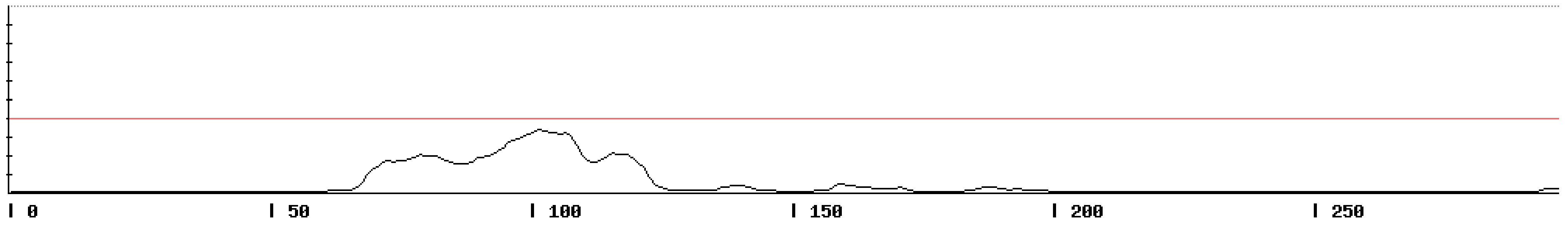

Predictions for Nicotianatomentosiformis_XP_009630787.1	
Viterbi Path	
Viterbi Path	
Posterior @ 0.4	
Posterior @ 0.4	

Posterior Graphic. Threshold Marked @ 0.4

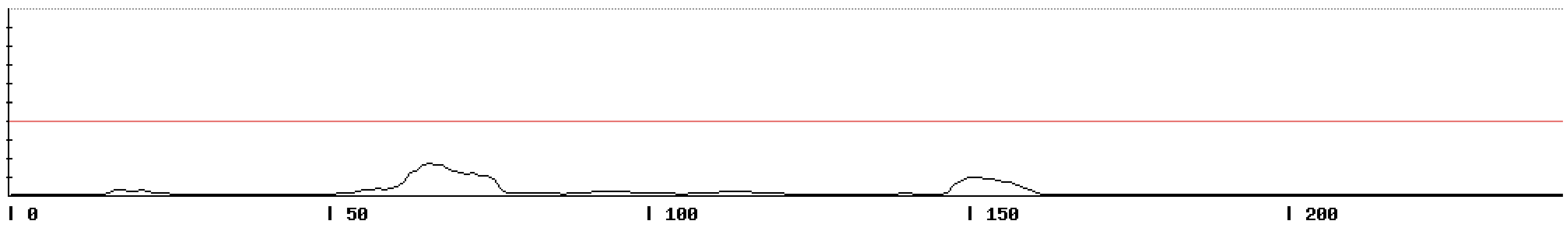

Predictions for Nicotianatomentosiformis_XP_009630818.1

Viterbi Path

Viterbi Path

Posterior @ 0.4

Posterior @ 0.4

Posterior Graphic. Threshold Marked @ 0.4

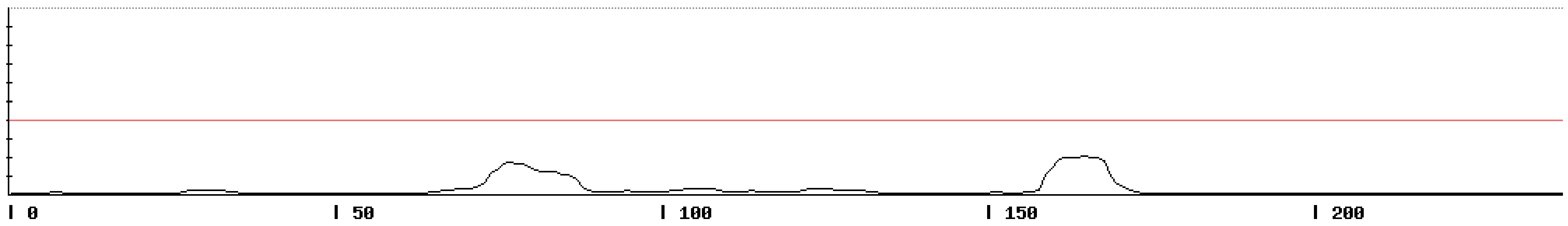

Predictions for Nicotianatomentosiformis_XP_009631312.1

Viterbi Path

Viterbi Path

Posterior @ 0.4	76 - KLKKKNKARH - 85
Posterior @ 0.4	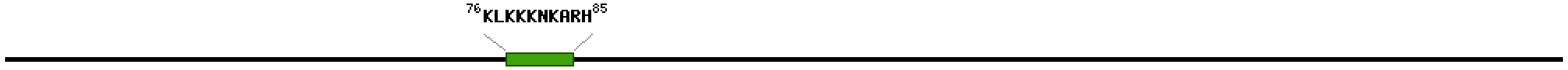
Posterior Graphic. Threshold Marked @ 0.4	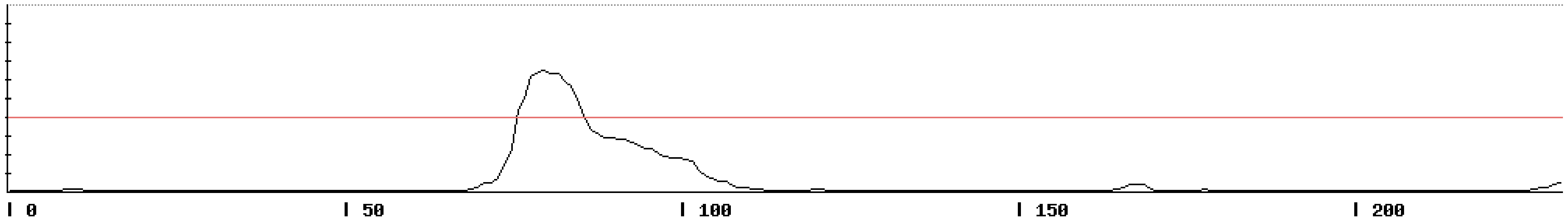

Predictions for Nicotianatomentosiformis_XP_009631313.1

Viterbi Path	
Viterbi Path	
Posterior @ 0.4	76 - KLKKKNKARH - 85
Posterior @ 0.4	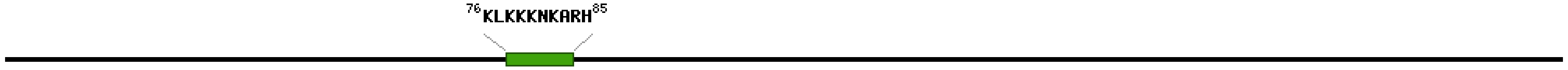
Posterior Graphic. Threshold Marked @ 0.4	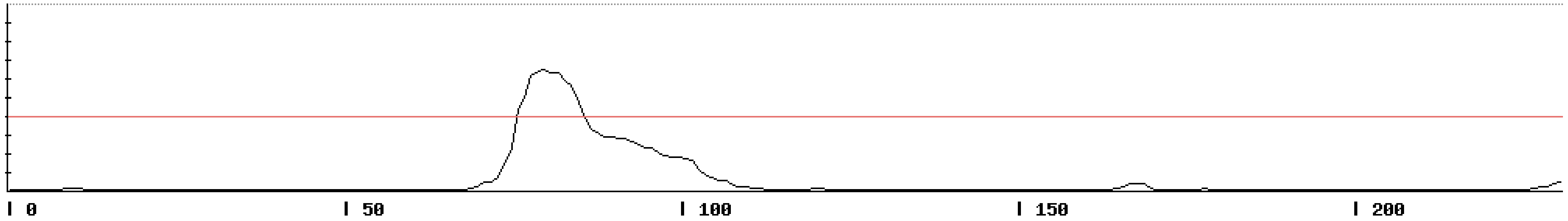

Predictions for Nicotianatomentosiformis_XP_009631314.1

Viterbi Path	
Viterbi Path	

Posterior @ 0.4	76 - KLKKKNKARH - 85
Posterior @ 0.4	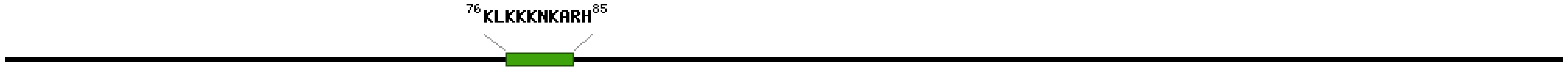
Posterior Graphic. Threshold Marked @ 0.4	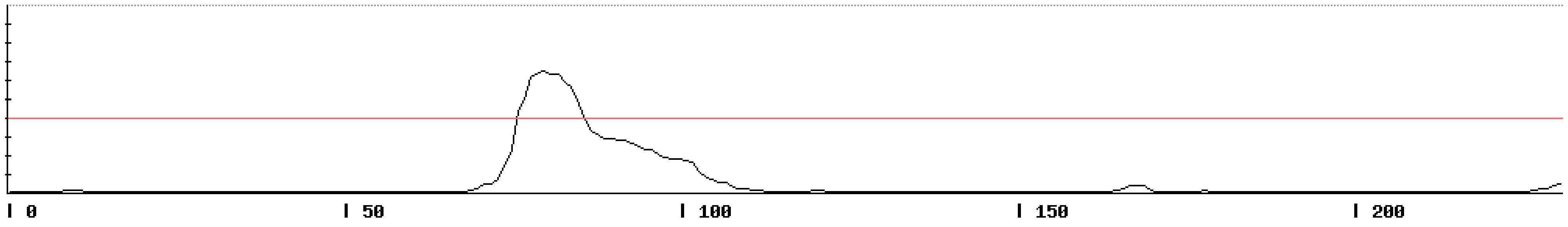

Predictions for Nicotianatomentosiformis_XP_009631315.1

Viterbi Path	
Viterbi Path	
Posterior @ 0.4	76 - KLKKKNKARH - 85
Posterior @ 0.4	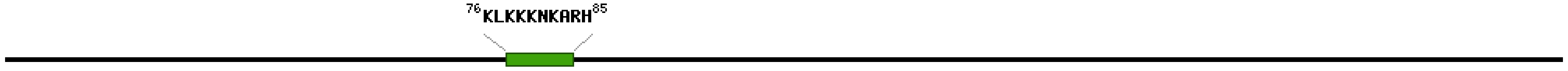
Posterior Graphic. Threshold Marked @ 0.4	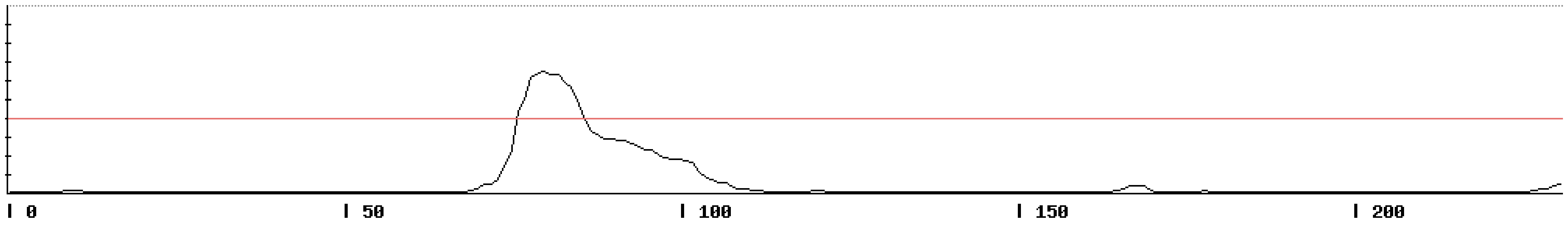

Predictions for Nicotianatomentosiformis_XP_009631912.1

Viterbi Path

Viterbi Path

Posterior @ 0.4

Posterior @ 0.4

Posterior Graphic. Threshold Marked @ 0.4

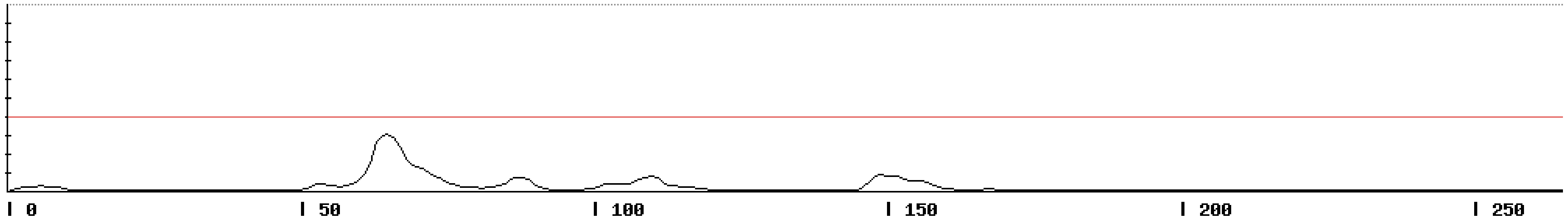

Predictions for Nicotianatomentosiformis_XP_009631913.1

Viterbi Path

Viterbi Path

Posterior @ 0.4

Posterior @ 0.4

Posterior Graphic. Threshold Marked @ 0.4

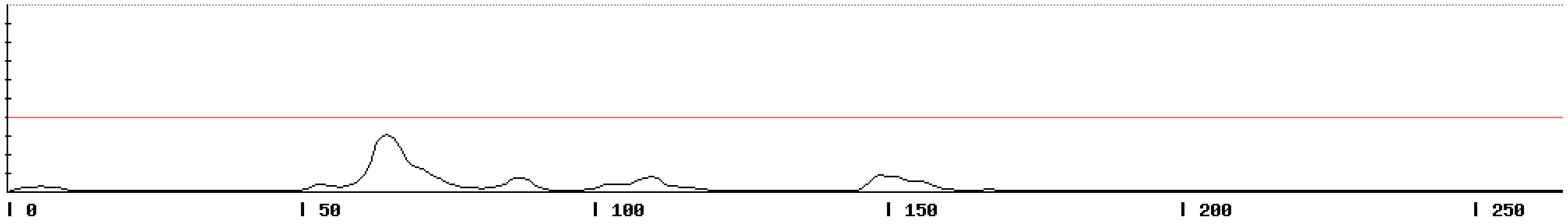

Predictions for Nicotianatomentosiformis_XP_009631914.1

Viterbi Path	
Viterbi Path	
Posterior @ 0.4	
Posterior @ 0.4	
Posterior Graphic. Threshold Marked @ 0.4	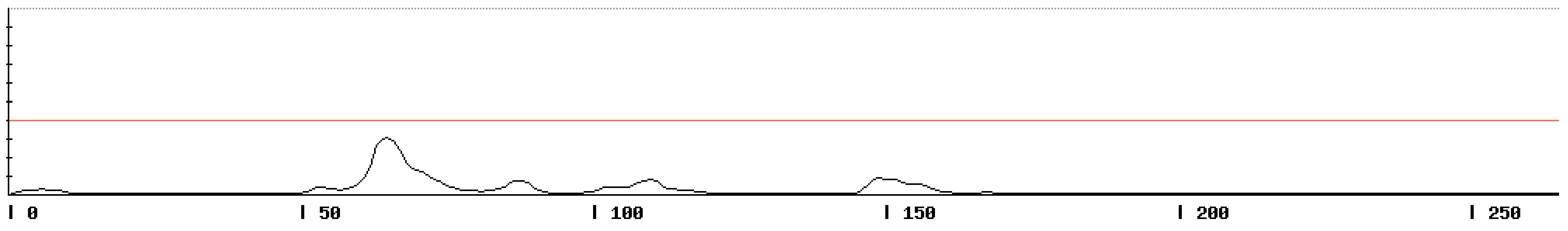

Predictions for Nicotianatomentosiformis_XP_009631915.1

Viterbi Path	
Viterbi Path	
Posterior @ 0.4	
Posterior @ 0.4	
Posterior Graphic. Threshold Marked @ 0.4	

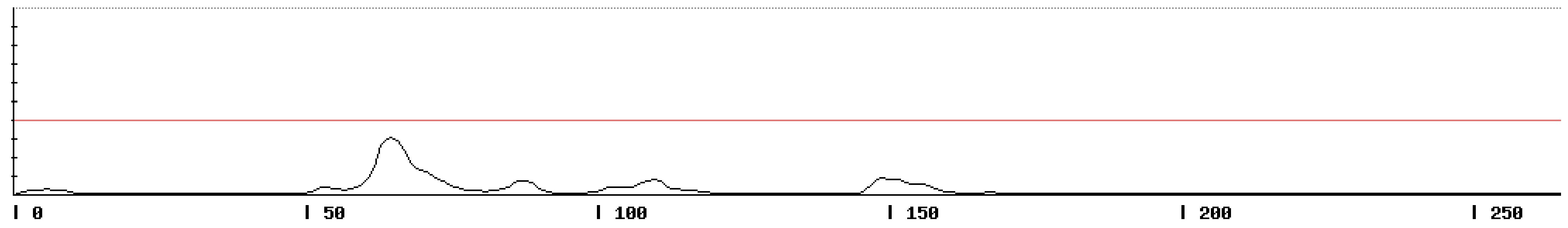

Predictions for Nicotianatomentosiformis_XP_009631955.1	
Viterbi Path	
Viterbi Path	
Posterior @ 0.4	
Posterior @ 0.4	
Posterior Graphic. Threshold Marked @ 0.4	

Revision r.9 - Last Updated on : February 24 2015 17:45:20. alex.nguyenba@utoronto.ca

Intended to be used with Firefox

Please cite this paper if NLStradamus was useful for your studies:
Nguyen Ba AN, Pogoutse A, Provart N, Moses AM. **NLStradamus: a simple Hidden Markov Model for nuclear localization signal prediction.** *BMC Bioinformatics.* 2009 Jun 29;**10(1)**:202.

The pubmed entry for this paper is found **here**.

- Program may resolve in an error if a large .fasta file is sent for prediction when images or graphics are desired. **Please only use the table display when using large predictions. (>100 sequences)**
- A standalone of the program is available **here**.
- Notice: NLStradamus is not a predictor of nuclear proteins; although better than random, it is a very poor classifier of nuclear proteins. Comparative approaches should be used for this problem. NLStradamus predicts NLSs in nuclear proteins that are transported by the import machinery of the cell.
- Click **here** for general help.

**Prediction Request**		
Sequence Text		Accepts :<br>- 1 protein sequence<br>- Multiple sequences in FASTA format.
File Upload	Choose File NLS.fasta	Accepts .fasta, .txt, and soon .gz, .bz2 and .zip.
**HMM state emission and transition frequencies.**		
Pre-loaded models	2 state HMM static / 2 state HMM dynamic / 4 state HMM static	Those are the models based on our characterized sequences.
**Prediction Type**		
Viterbi	✔	
Posterior	✔	0.4 Prediction Cutoff (]0,1])
**Prediction Display**		
Table	✔	A table that you can export with the start and stop positions of predicted NLS.
Image	✔	An image with the NLS boxed over a line representing the protein sequence.
Graphic	✔	Only applicable to posterior probability. A graphical representation of its distribution.
Predict		
Scroll down for predictions.		

**Predictions for Achn004841**	
Viterbi Path	

Viterbi Path

Posterior @ 0.4

Posterior @ 0.4

Posterior Graphic. Threshold Marked @ 0.4

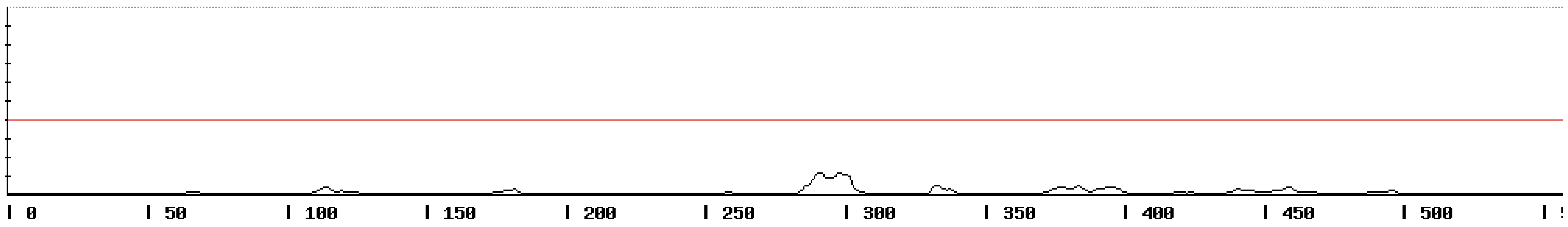

Predictions for Achn274891

Viterbi Path

Viterbi Path

Posterior @ 0.4

Posterior @ 0.4

Posterior Graphic. Threshold Marked @ 0.4

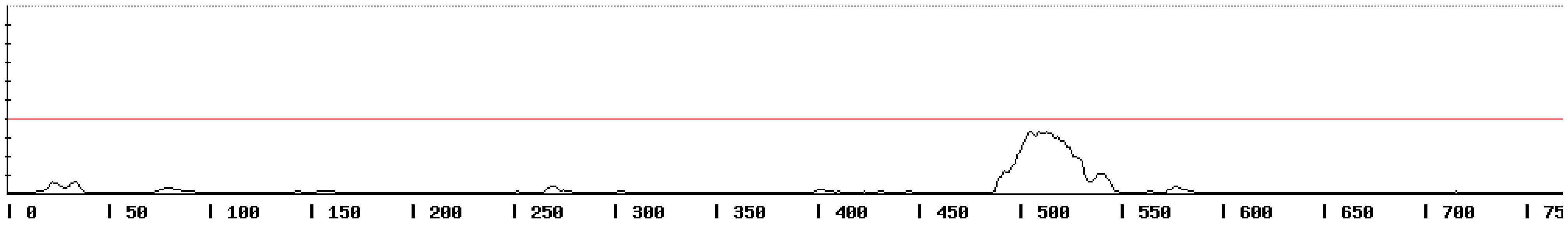

Predictions for Achn089081

Viterbi Path	646 - GRSMRSKVERRMRKESGKTLREIRRAKDLRKK - 677
Viterbi Path	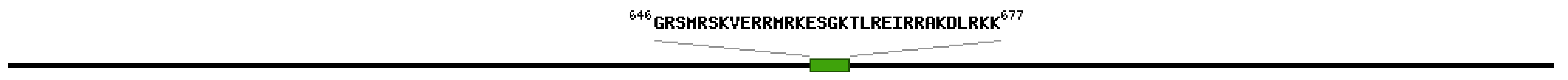
Posterior @ 0.4	347 - RKRRRIHNE - 355
	358 - MKKEG - 362
	647 - RSMRSKVERRMRKESGKTLREIRRAKDLRKKLM - 679
	689 - LRRAKKKV - 696
Posterior @ 0.4	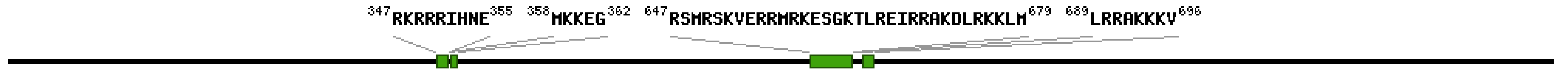
Posterior Graphic. Threshold Marked @ 0.4	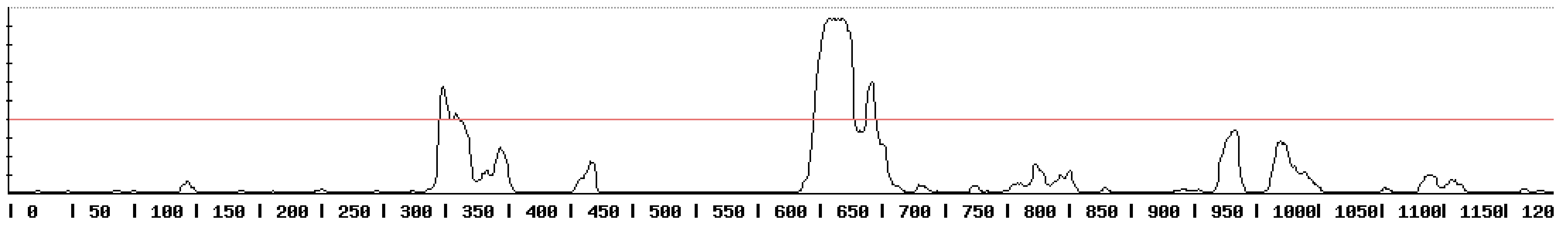

Predictions for Achn335281

Viterbi Path	
Viterbi Path	
Posterior @ 0.4	1181 - PSPKKSKGRTR - 1191
Posterior @ 0.4	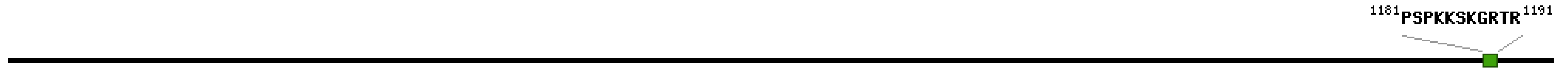
Posterior Graphic. Threshold Marked @ 0.4	

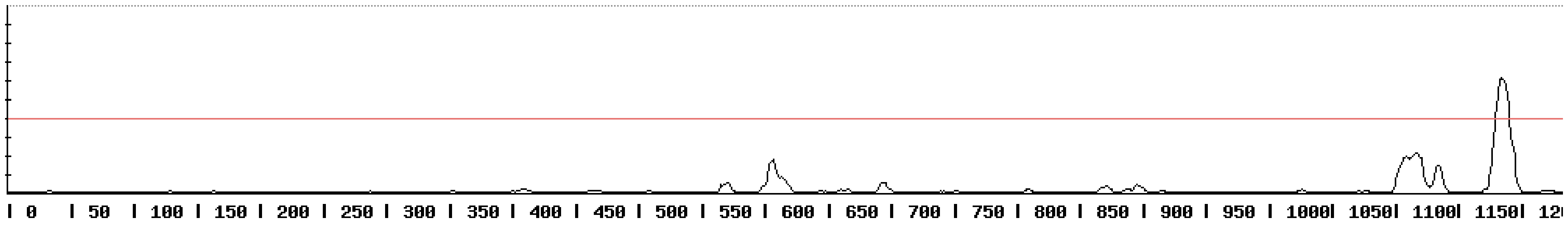

Predictions for Achn181121

Viterbi Path	
Viterbi Path	
Posterior @ 0.4	
Posterior @ 0.4	
Posterior Graphic. Threshold Marked @ 0.4	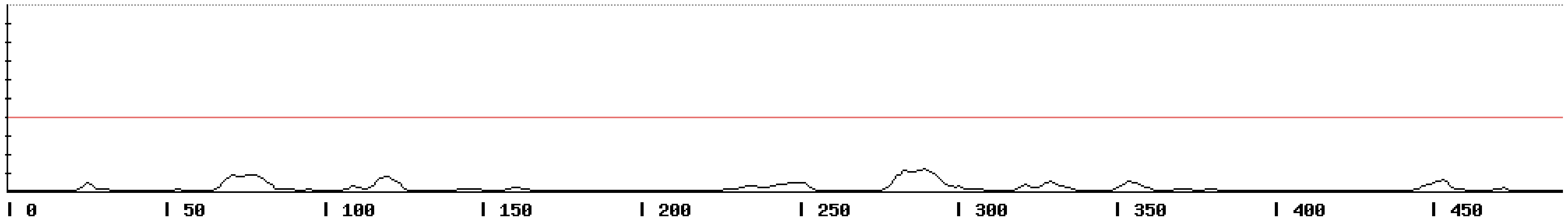

Predictions for Achn389881[Actinidia]

Viterbi Path	
Viterbi Path	
Posterior @ 0.4	345 - RKRRKIH - 351
Posterior @ 0.4	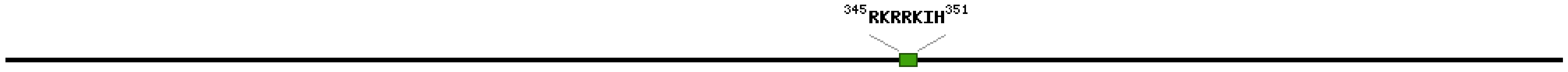

Posterior Graphic. Threshold Marked @ 0.4

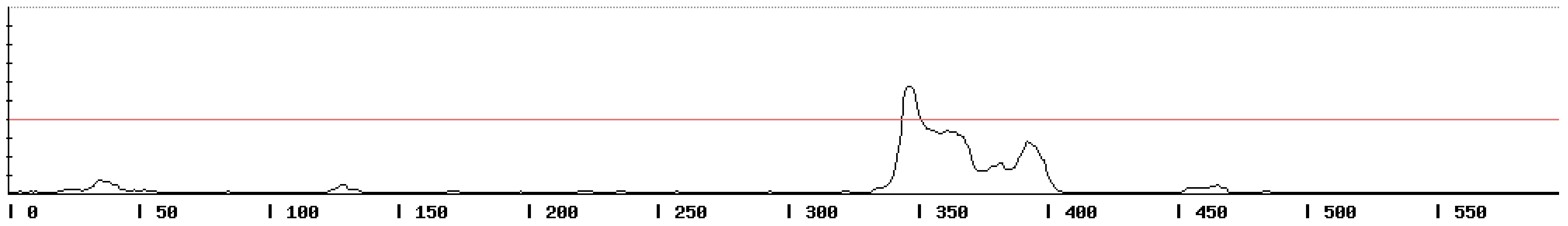

Predictions for AHYPO_009690

Viterbi Path

Viterbi Path

Posterior @ 0.4

Posterior @ 0.4

Posterior Graphic. Threshold Marked @ 0.4

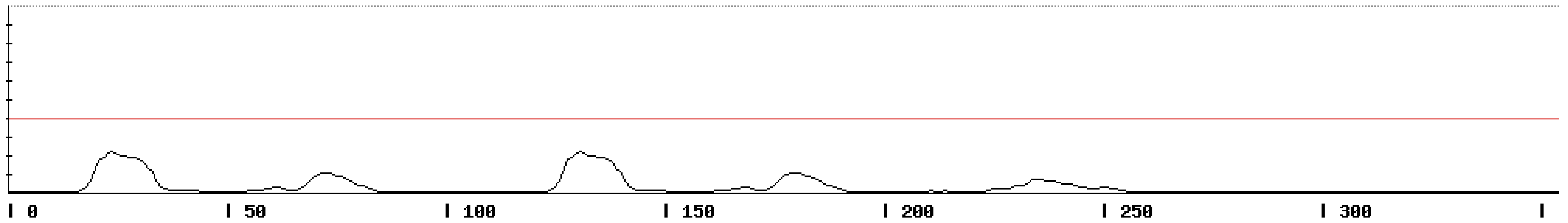

Predictions for EMT05508

Viterbi Path: 369 - PPRPHEPRQPRVNKKPAFPRPQEARKPQVIQKPPPPRALDKRRVPTPTQQAPKRRAPPIAEPPRPKRIR - 437

Viterbi Path

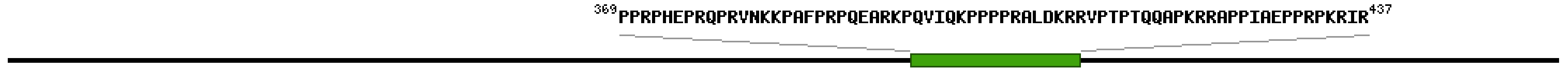

Posterior @ 0.4:

15 - KKARDKGTDDGLPAPAKEKRRWSFRRP - 41

369 - PPRPHEPRQPRVNKKPAFPRPQEARKPQVIQKPPPPRALDKRRVPTPTQQAPKRRAPPIAEPPRPKRIRAAA - 440

Posterior @ 0.4

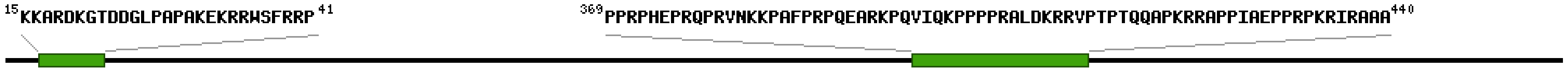

Posterior Graphic. Threshold Marked @ 0.4

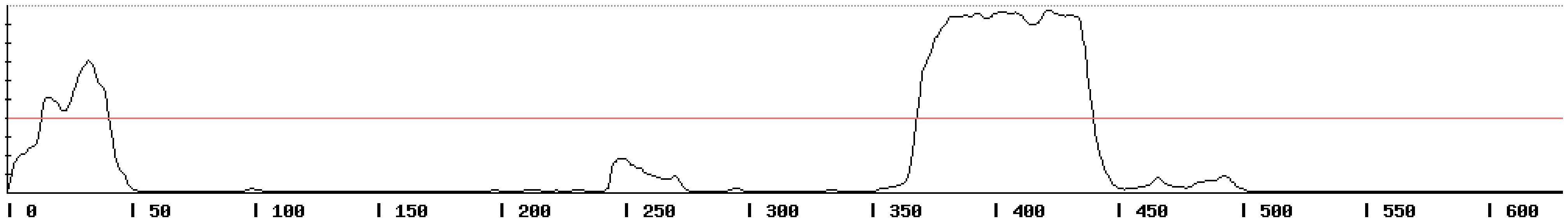

Predictions for EMT14186

Viterbi Path

Viterbi Path

Posterior @ 0.4

Posterior @ 0.4

Posterior Graphic. Threshold Marked @ 0.4

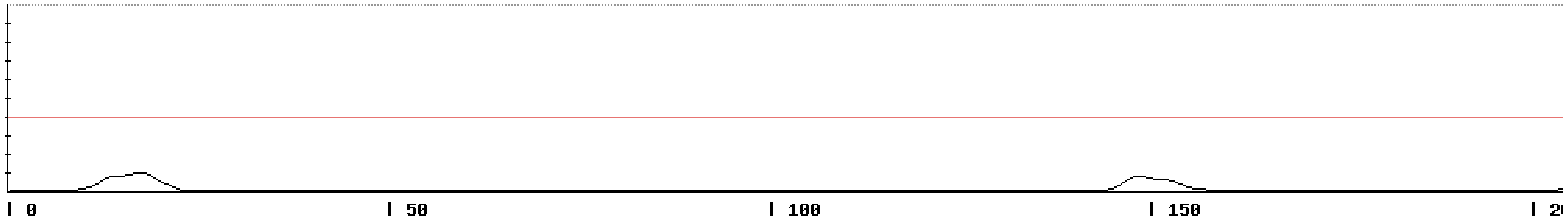

Predictions for Aegilopstauschii_EMT28787

Viterbi Path

Viterbi Path

Posterior @ 0.4 | 388 - KKRKK - 392

Posterior @ 0.4

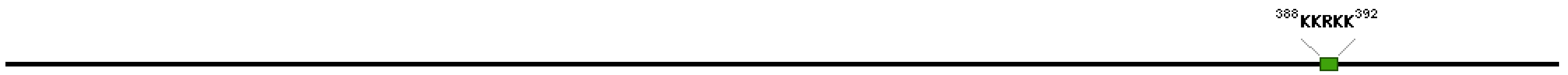

Posterior Graphic. Threshold Marked @ 0.4

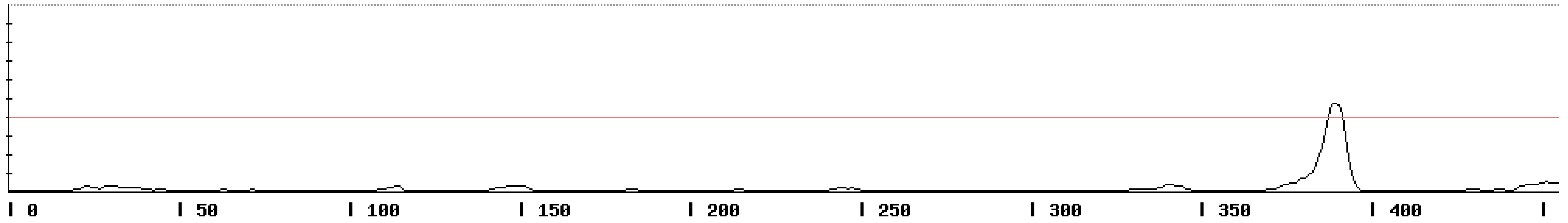

Predictions for Aegilopstauschii_EMT33859

Viterbi Path

Viterbi Path

Posterior @ 0.4

Posterior @ 0.4

Posterior Graphic. Threshold Marked @ 0.4

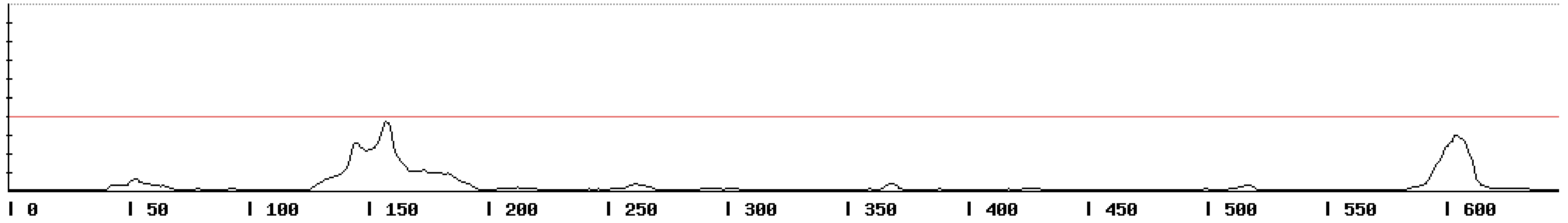

Predictions for Ananas

Viterbi Path

Viterbi Path

Posterior @ 0.4	
Posterior @ 0.4	
Posterior Graphic. Threshold Marked @ 0.4	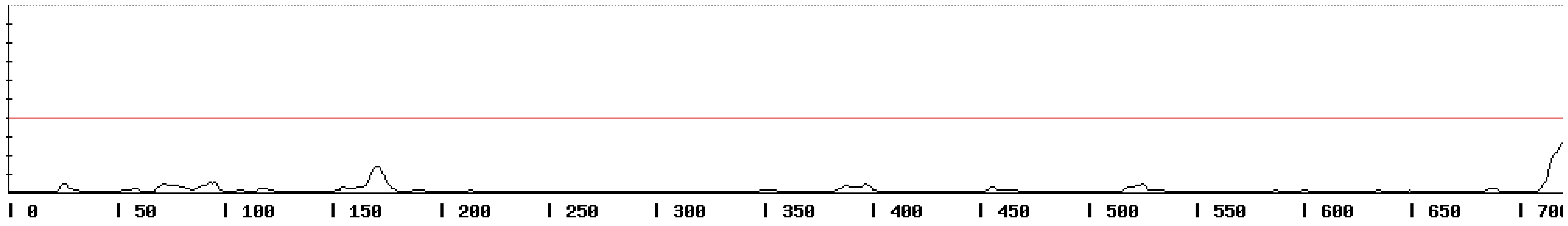

Predictions for 338342	
Viterbi Path	
Viterbi Path	
Posterior @ 0.4	
Posterior @ 0.4	
Posterior Graphic. Threshold Marked @ 0.4	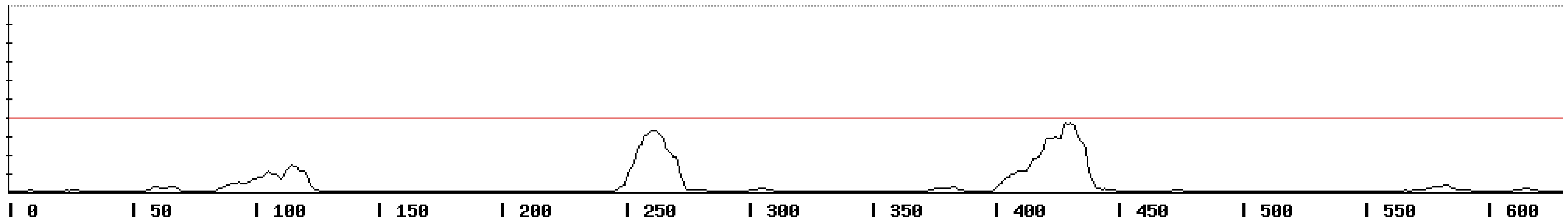

Predictions for 347836

Viterbi Path

Viterbi Path

Posterior @ 0.4

Posterior @ 0.4

Posterior Graphic. Threshold Marked @ 0.4

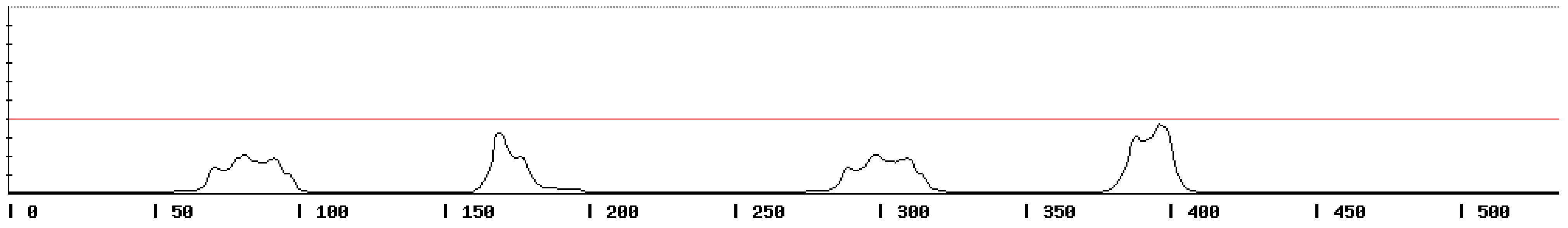

Predictions for 475292

Viterbi Path

Viterbi Path

Posterior @ 0.4

88 - GREKLIKSKKTGKILGFKKILKFCLKRKPREYKR - 121

Posterior @ 0.4

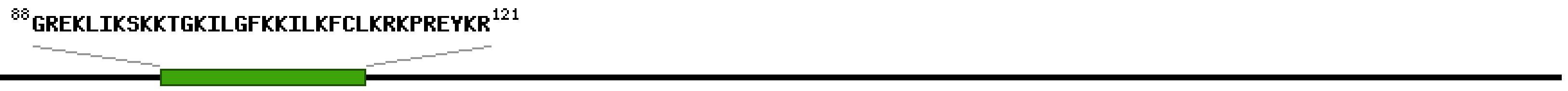

Posterior Graphic. Threshold Marked @ 0.4

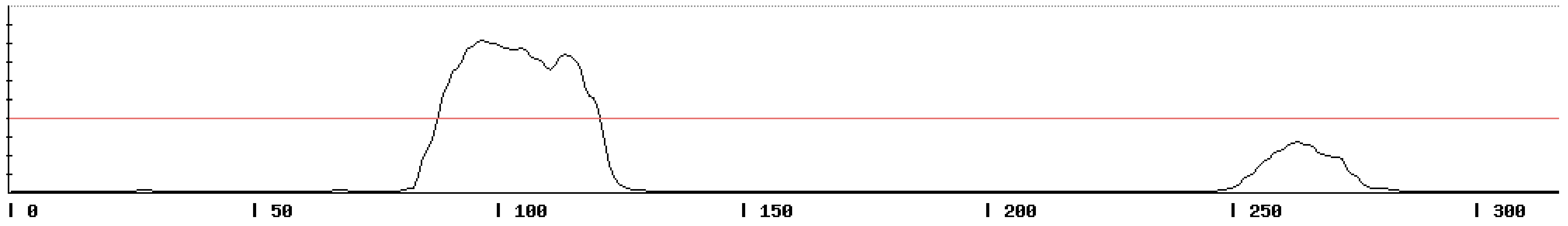

Predictions for Arabidopsislyrata_893515	
Viterbi Path	
Viterbi Path	
Posterior @ 0.4	
Posterior @ 0.4	
Posterior Graphic. Threshold Marked @ 0.4	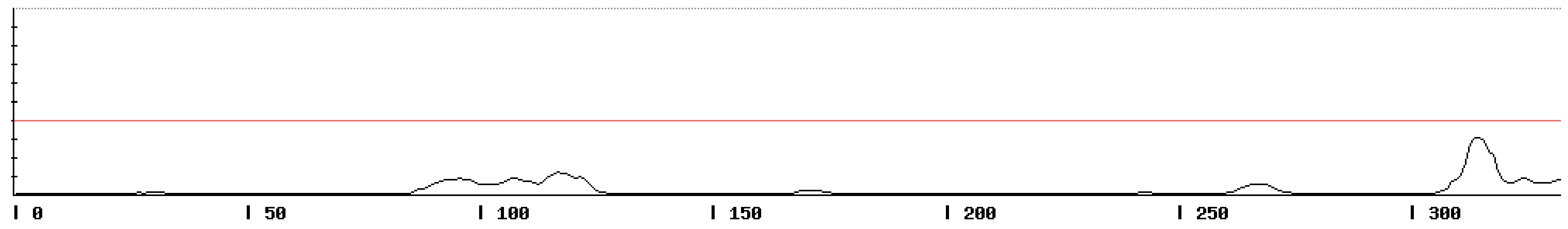

Predictions for Arabidopsislyrata_893532	
Viterbi Path	
Viterbi Path	
Posterior @ 0.4	91 - LIKSKATGKILGFKKILKFCKKTKPREY - 118
Posterior @ 0.4	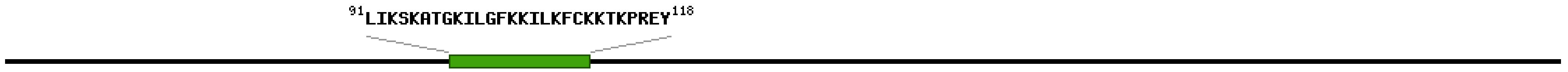
Posterior Graphic. Threshold Marked @ 0.4	

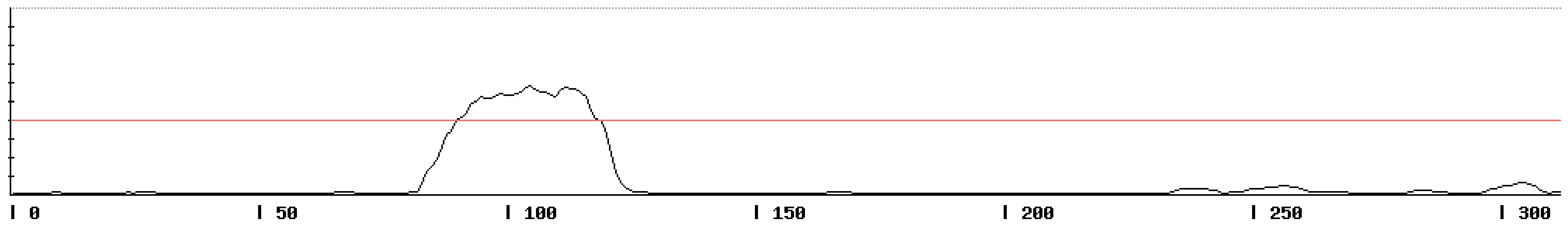

Predictions for Bradi4g03620.1

Viterbi Path	
Viterbi Path	
Posterior @ 0.4	
Posterior @ 0.4	
Posterior Graphic. Threshold Marked @ 0.4	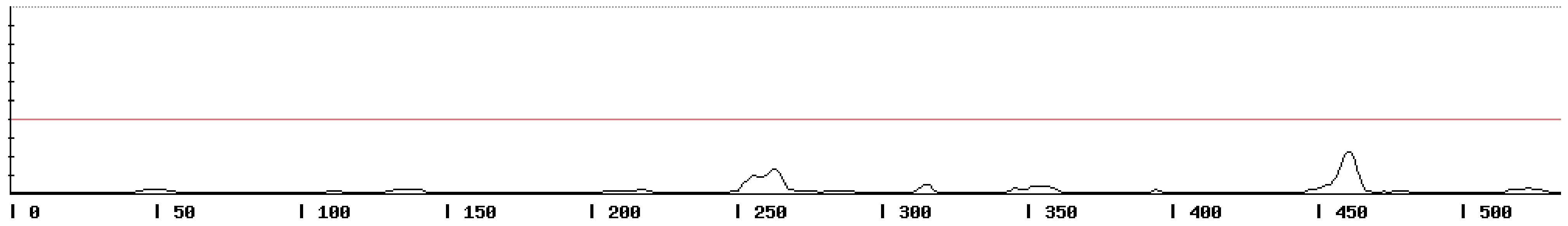

Predictions for Bradi2g46430.1

Viterbi Path	
Viterbi Path	
Posterior @ 0.4	77 - RAKSRRGQRKS - 87
	375 - RIRGQRR - 381
	478 - RKRKASEENSGAESPARGTKKSTAPAASGRKK - 509
Posterior @ 0.4	

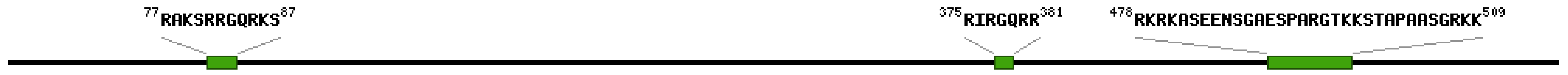

Posterior Graphic. Threshold Marked @ 0.4

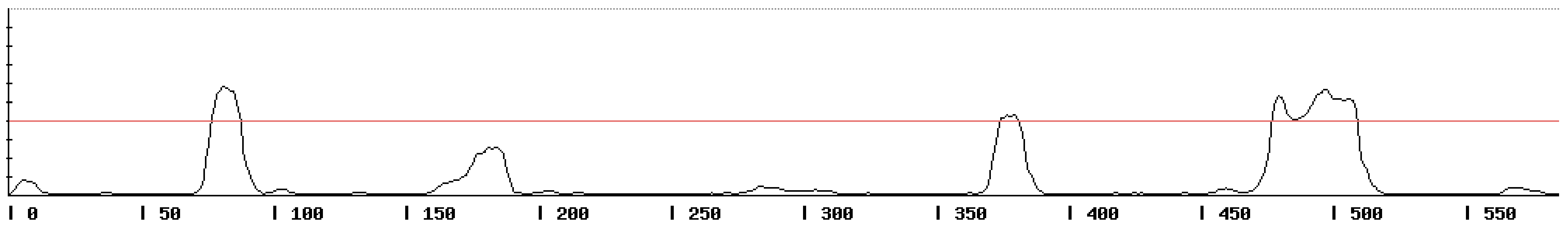

Predictions for Brachypodiumdistachyon_Bradi5g15230.1

Viterbi Path	
Viterbi Path	
Posterior @ 0.4	30 - KPRTRDKPPRTPPPR - 44
	291 - RRKRAANG - 298
	302 - ERPRR - 306

Posterior @ 0.4

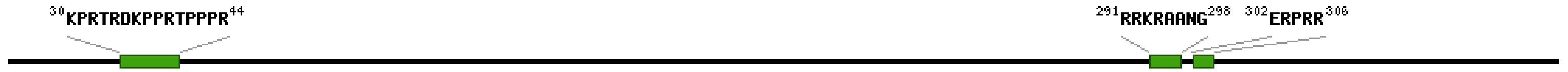

Posterior Graphic. Threshold Marked @ 0.4

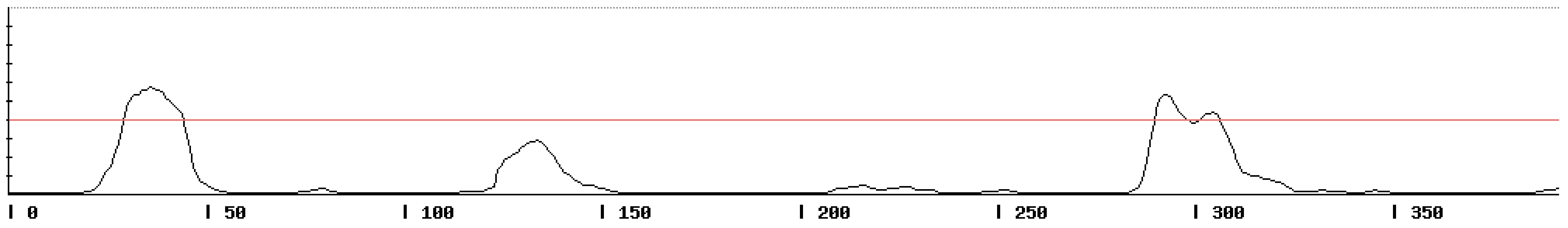

Predictions for Brassicanapus_Bna005178

Viterbi Path	
Viterbi Path	

Posterior @ 0.4

Posterior @ 0.4

Posterior Graphic. Threshold Marked @ 0.4

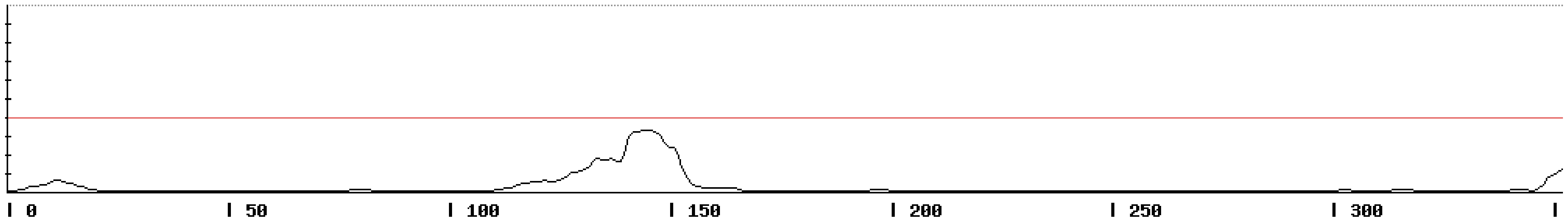

Predictions for Brassicarapa_Bra002857

Viterbi Path

Viterbi Path

Posterior @ 0.4

113 - KKTKRGR - 119

Posterior @ 0.4

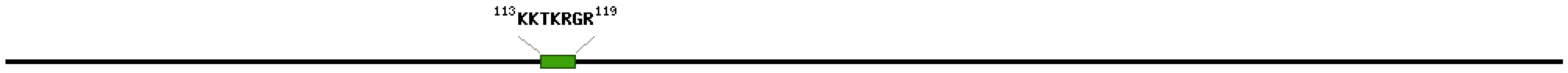

Posterior Graphic. Threshold Marked @ 0.4

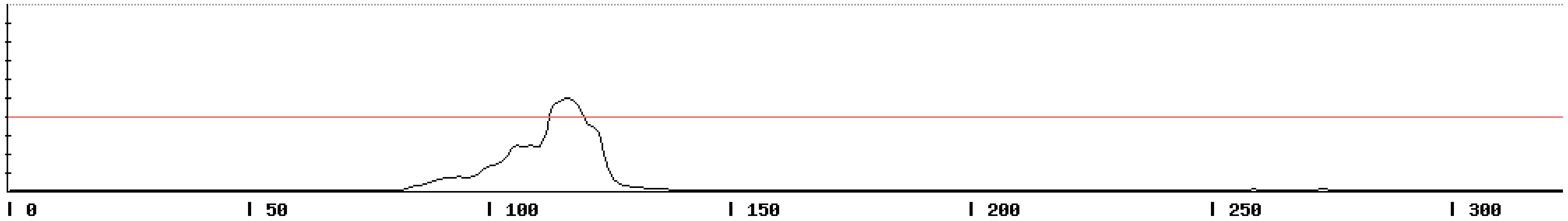

Predictions for Brassicarapa_Bra003244

Viterbi Path	
Viterbi Path	
Posterior @ 0.4	
Posterior @ 0.4	
Posterior Graphic. Threshold Marked @ 0.4	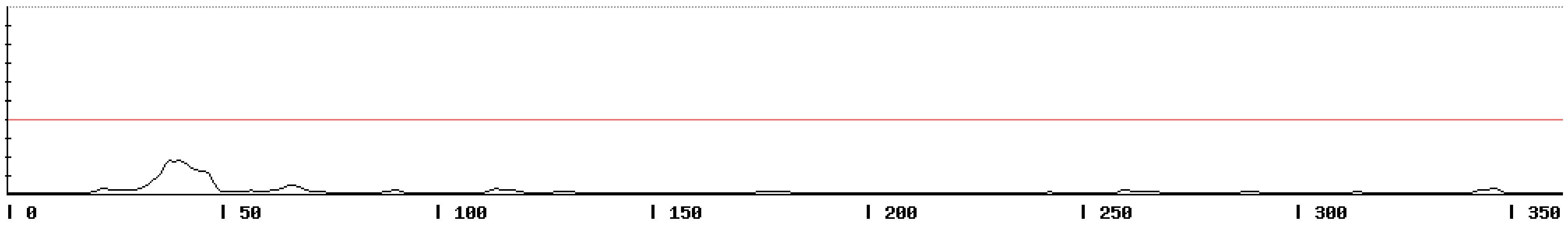

**Predictions for Brassicarapa_Bra012470**	
Viterbi Path	
Viterbi Path	
Posterior @ 0.4	416 - RGRGRGRGRGAGR - 428
Posterior @ 0.4	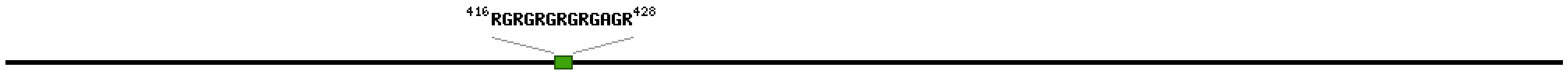
Posterior Graphic. Threshold Marked @ 0.4	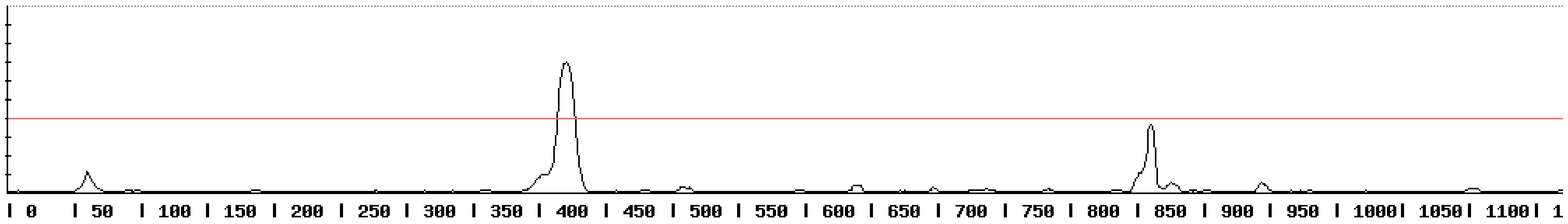

Predictions for Brassicarapa_Bra035412	
Viterbi Path	
Viterbi Path	
Posterior @ 0.4	
Posterior @ 0.4	
Posterior Graphic. Threshold Marked @ 0.4	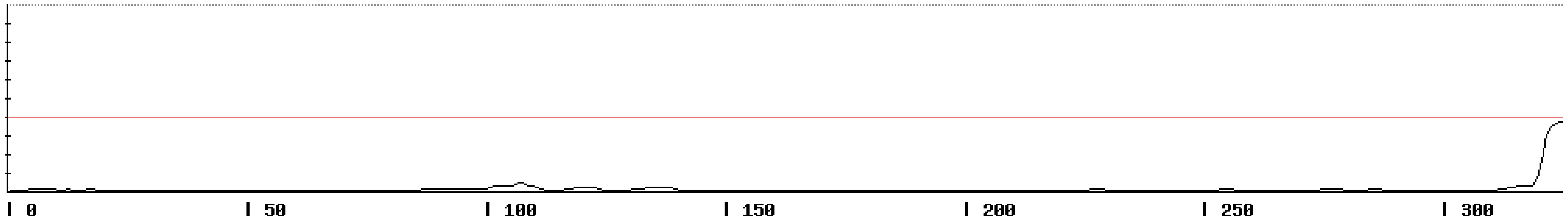

Predictions for Brassicarapa_Bra029571	
Viterbi Path	
Viterbi Path	
Posterior @ 0.4	
Posterior @ 0.4	
Posterior Graphic. Threshold Marked @ 0.4	

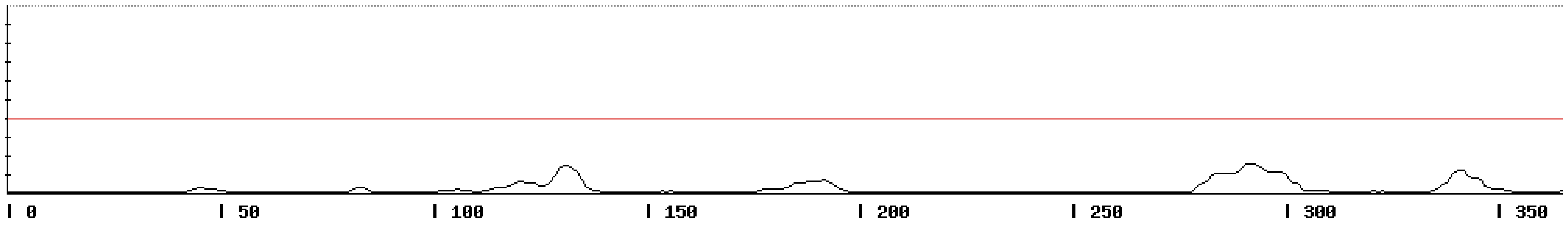

Predictions for Brassicarapa_Bra031518

Viterbi Path	
Viterbi Path	
Posterior @ 0.4	
Posterior @ 0.4	
Posterior Graphic. Threshold Marked @ 0.4	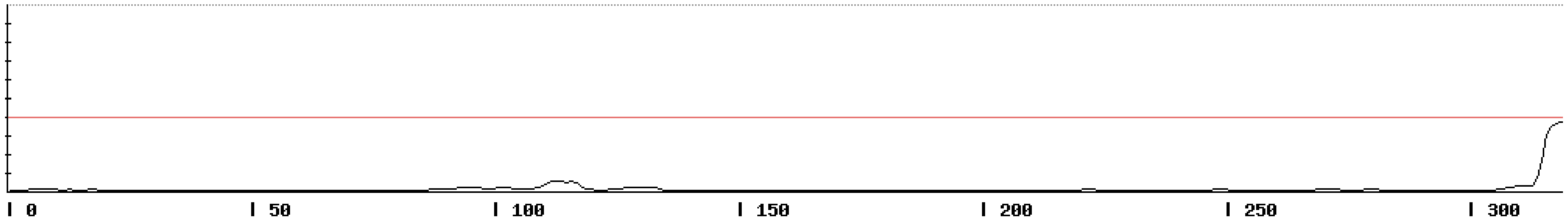

Predictions for Cagra.1301s0009

Viterbi Path	
Viterbi Path	
Posterior @ 0.4	76 - KK - 77
	83 - VKRKPRE - 89
Posterior @ 0.4	

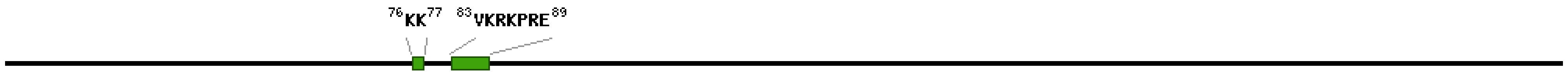

Posterior Graphic. Threshold Marked @ 0.4

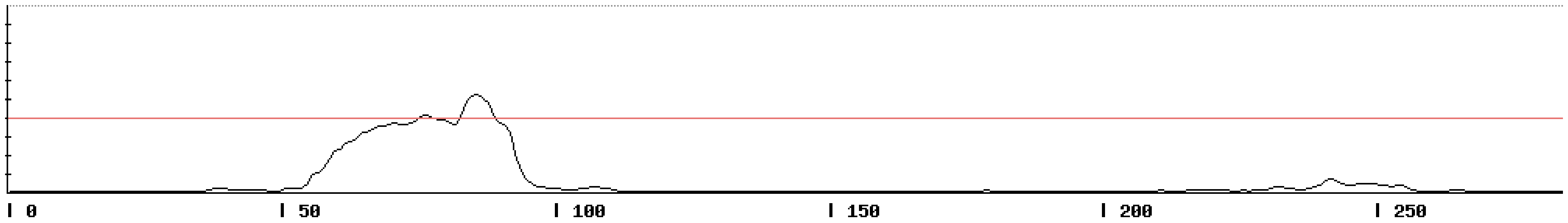

Predictions for Cagra.1301s0010

Viterbi Path

Viterbi Path

Posterior @ 0.4

Posterior @ 0.4

Posterior Graphic. Threshold Marked @ 0.4

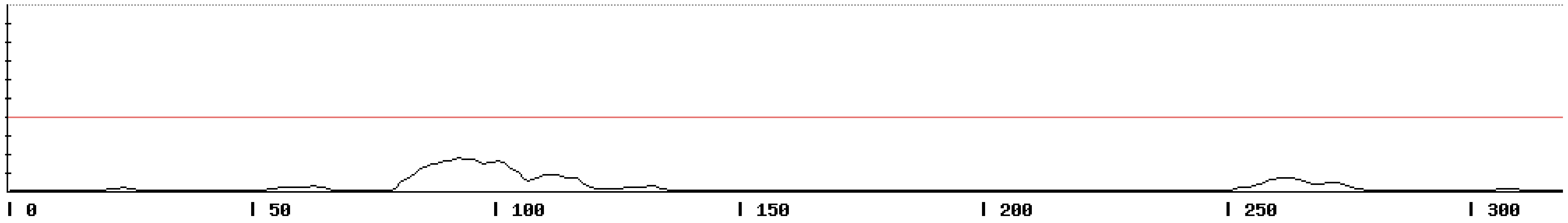

Predictions for Chenopodiumquinoa_AUR62022690

Viterbi Path

Viterbi Path

Posterior @ 0.4

Posterior @ 0.4

Posterior Graphic. Threshold Marked @ 0.4

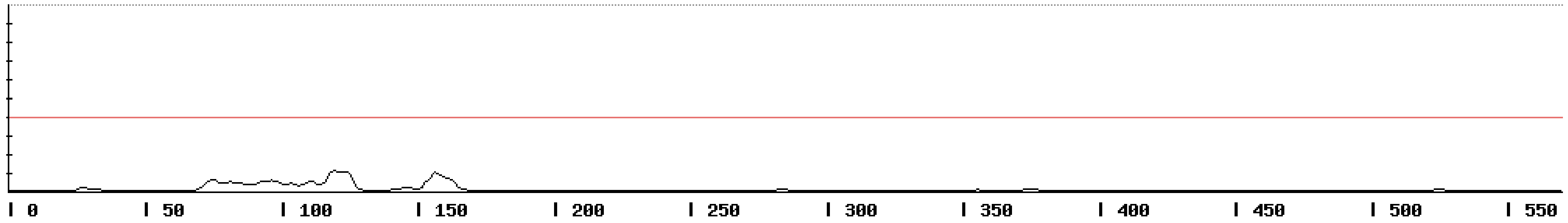

Predictions for Citrussinensis_orange1.1g044381m

Viterbi Path

Viterbi Path

Posterior @ 0.4

Posterior @ 0.4

Posterior Graphic. Threshold Marked @ 0.4

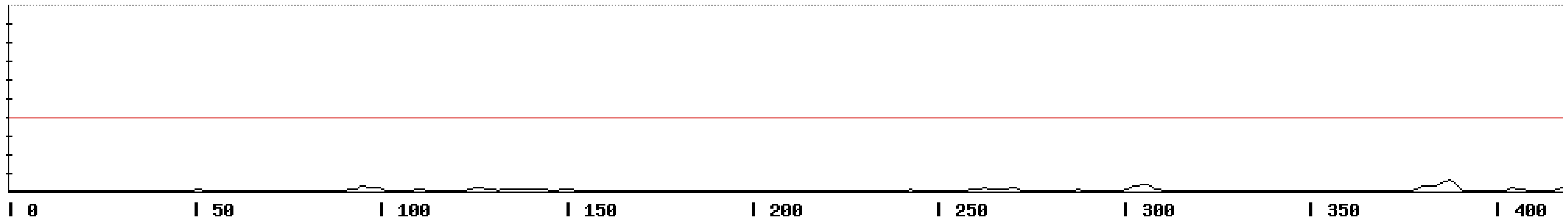

Predictions for Citrussinensis_orange1.1g045641m

Viterbi Path

Viterbi Path

Posterior @ 0.4	
Posterior @ 0.4	
Posterior Graphic. Threshold Marked @ 0.4	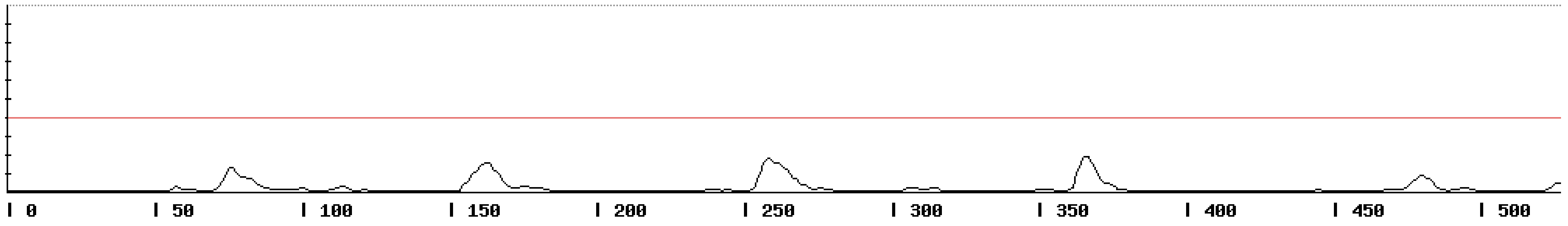

Predictions for Daucuscarota_DCAR_021729

Viterbi Path	505 - RGKSMRSKVETRMRKESGKTLREIRRSKKLRKK - 537
Viterbi Path	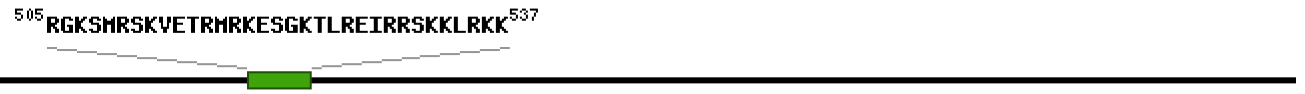
Posterior @ 0.4	133 - RKRRK - 137
	505 - RGKSMRSKVETRMRKESGKTLREIRRSKKLRKKMM - 539
	549 - LRRAKKKV - 556
Posterior @ 0.4	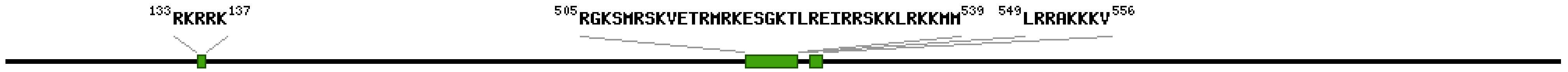
Posterior Graphic. Threshold Marked @ 0.4	

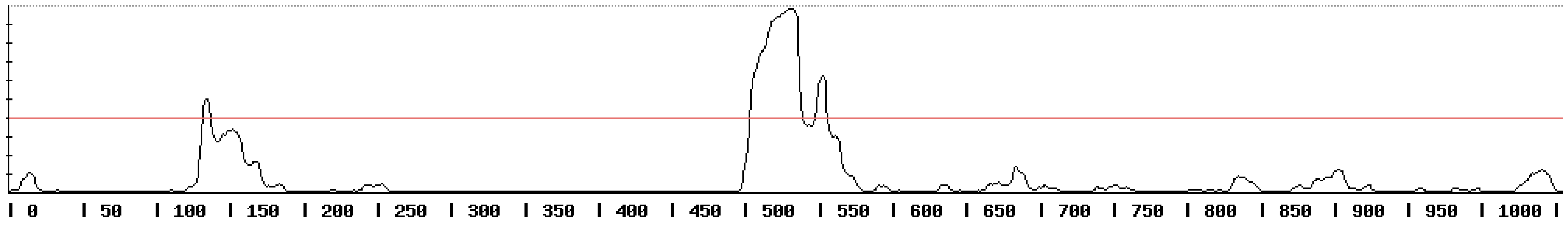

Predictions for Daucuscarota_DCAR_030911	
Viterbi Path	
Viterbi Path	
Posterior @ 0.4	
Posterior @ 0.4	
Posterior Graphic. Threshold Marked @ 0.4	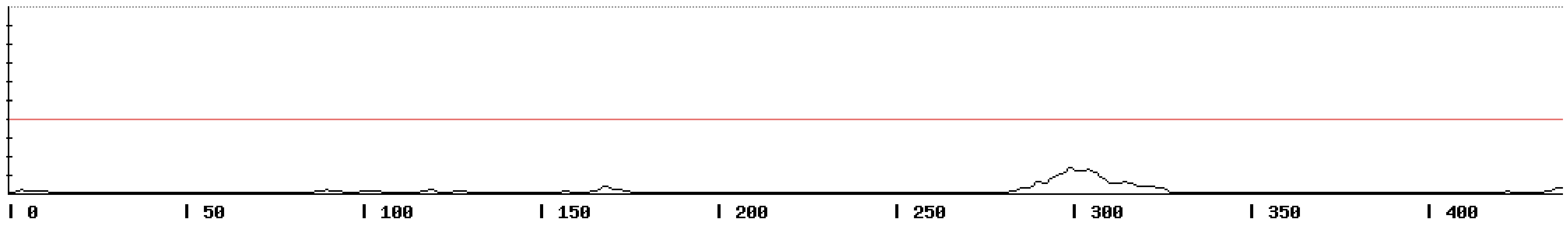

Predictions for Fragariavesca_mrna04303.1-v1.0-hybrid	
Viterbi Path	1941 - KRKRPAPRKGRKSGVR - 1956
Viterbi Path	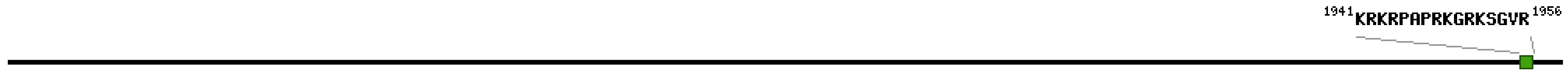
Posterior @ 0.4	414 - SRKPKAKRKA - 423
	1940 - VKRKRPAPRKGRKSG - 1954
Posterior @ 0.4	

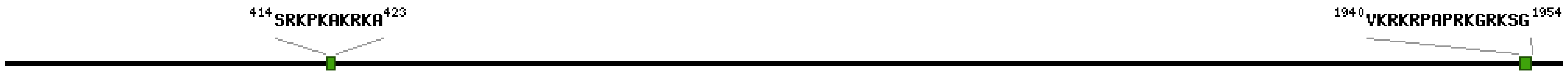

Posterior Graphic. Threshold Marked @ 0.4

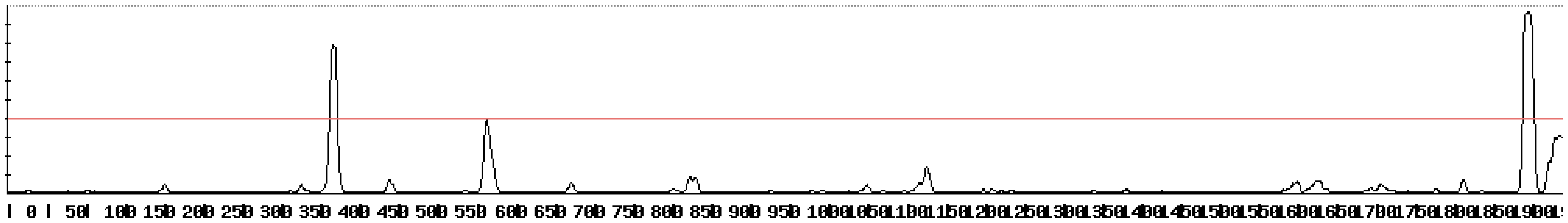

Predictions for Fragariavesca_mrna09127.1-v1.0-hybrid	
Viterbi Path	
Viterbi Path	
Posterior @ 0.4	
Posterior @ 0.4	
Posterior Graphic. Threshold Marked @ 0.4	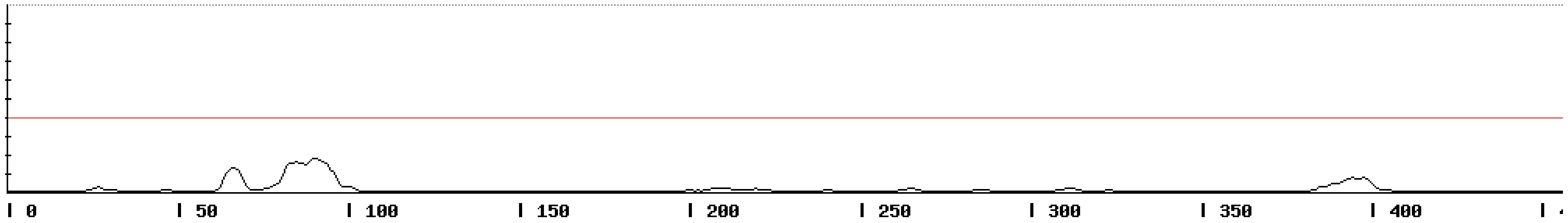

Predictions for Fragariavesca_mrna17720.1-v1.0-hybrid	
Viterbi Path	
Viterbi Path	

Posterior @ 0.4	106 - GEKKNKTPIGRKRKFR - 121
	285 - KKKDKGKEKK - 294
	407 - GRKRKL - 412
Posterior @ 0.4	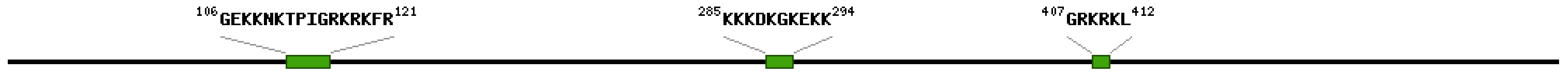
Posterior Graphic. Threshold Marked @ 0.4	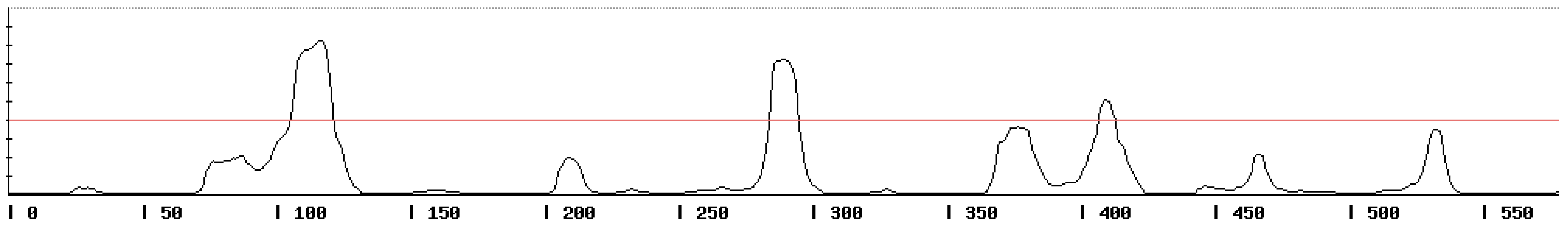

Predictions for Fragariavesca_mrna17721.1-v1.0-hybrid

Viterbi Path	
Viterbi Path	
Posterior @ 0.4	122 - KRKFR - 126
	161 - NIRKGRKRKE - 170
	283 - EAKQKHIGRRRKLR - 296
Posterior @ 0.4	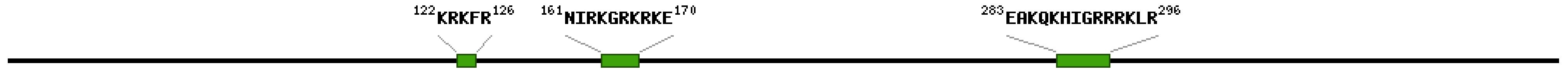
Posterior Graphic. Threshold Marked @ 0.4	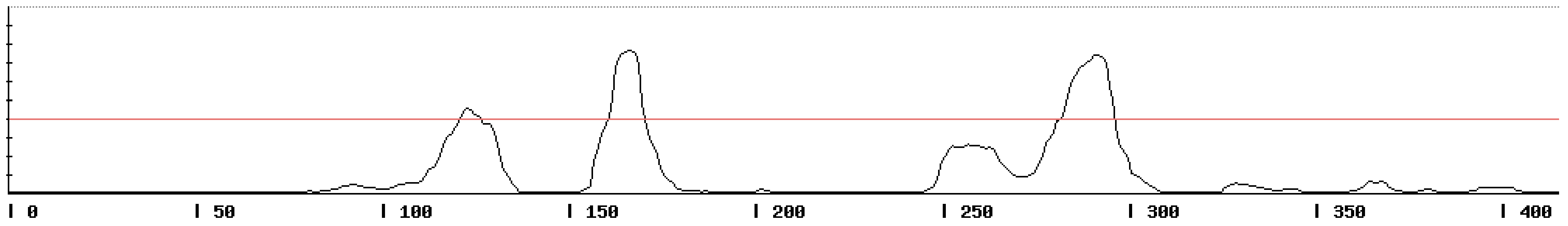

Predictions for Fragariavesca_mrna19382.1-v1.0-hybrid

Viterbi Path	298 - RKPKRIGKKRKLR - 310<br>357 - PKEKKRKRQQIDQLEDKKPKRKK - 379
Viterbi Path	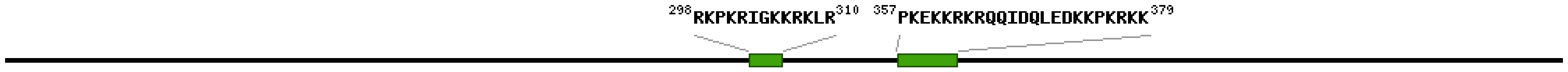
Posterior @ 0.4	297 - ERKPKRIGKKRKLR - 310<br>357 - PKEKKRKRQQIDQLEDKKPKRKKVV - 381
Posterior @ 0.4	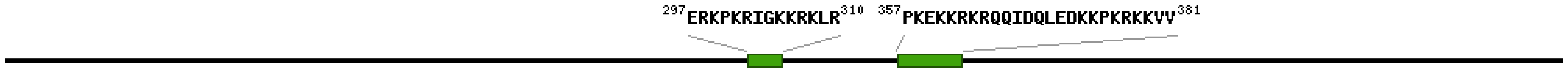
Posterior Graphic. Threshold Marked @ 0.4	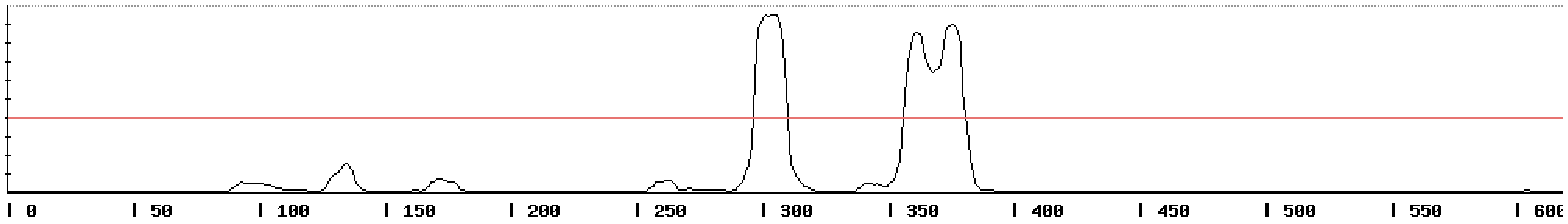

Predictions for Fragariavesca_mrna23394.1-v1.0-hybrid

Viterbi Path	
Viterbi Path	
Posterior @ 0.4	600 - VEREIKARRSKALIGKKR - 617
Posterior @ 0.4	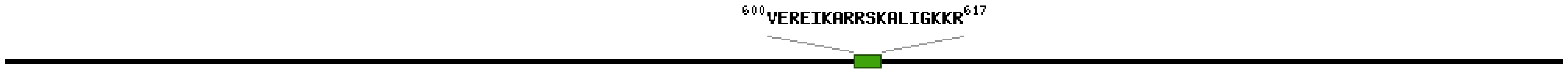
Posterior Graphic. Threshold Marked @ 0.4	

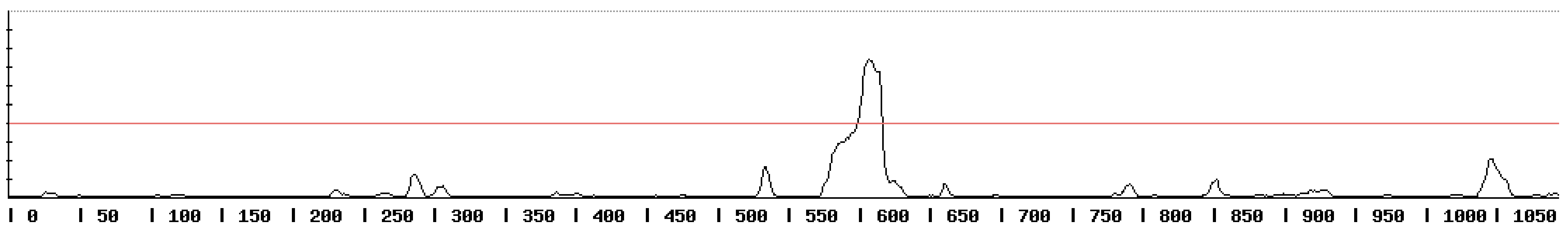

Predictions for Fragariavesca_mrna24179.1-v1.0-hybrid

Viterbi Path

Viterbi Path

Posterior @ 0.4
95 - KHIGKPKALGIKK - 107
656 - GRKPFKNIREVKK - 668
720 - SKKKKRKVR - 728

Posterior @ 0.4

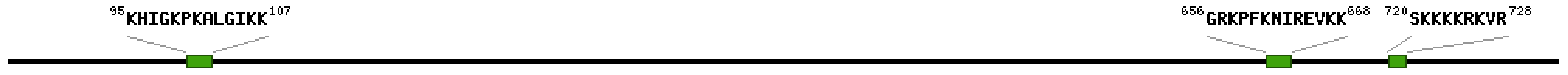

Posterior Graphic. Threshold Marked @ 0.4

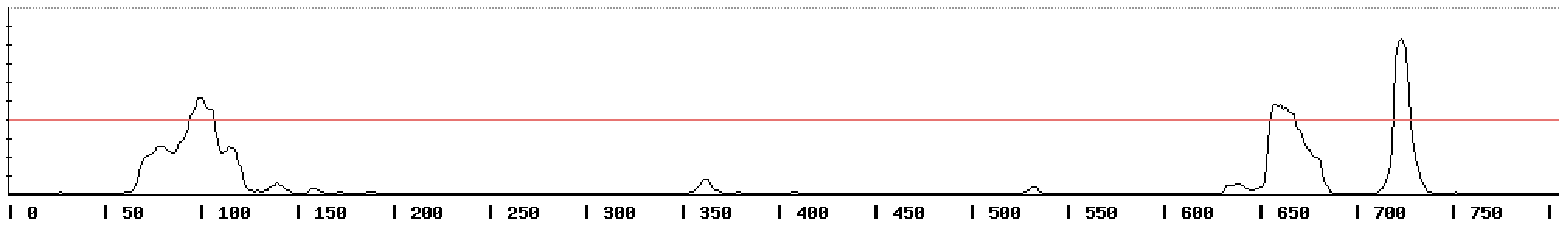

Predictions for Fragariavesca_mrna27940.1-v1.0-hybrid

Viterbi Path

Viterbi Path

Posterior @ 0.4
578 - RKRRKIDKE - 586
595 - RWHKTGKTKAIKGGVHPGWKK - 615

Posterior @ 0.4	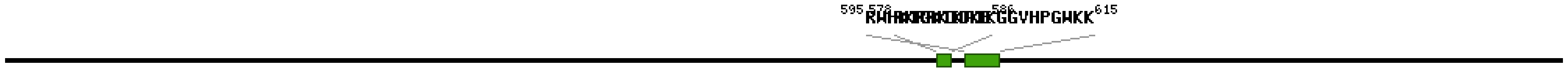
Posterior Graphic. Threshold Marked @ 0.4	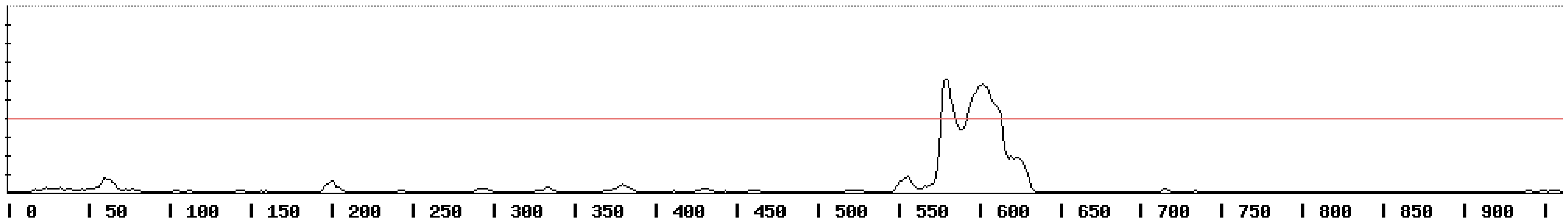

Predictions for Fragariavesca_mrna31175.1-v1.0-hybrid

Viterbi Path	
Viterbi Path	
Posterior @ 0.4	
Posterior @ 0.4	
Posterior Graphic. Threshold Marked @ 0.4	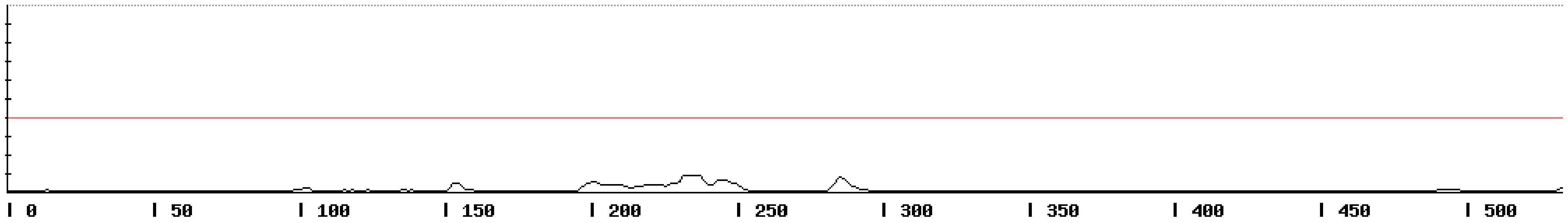

Predictions for Jatrophacurcas_Jcr4S16185.10

Viterbi Path	
Viterbi Path	

Posterior @ 0.4

Posterior @ 0.4

Posterior Graphic. Threshold Marked @ 0.4

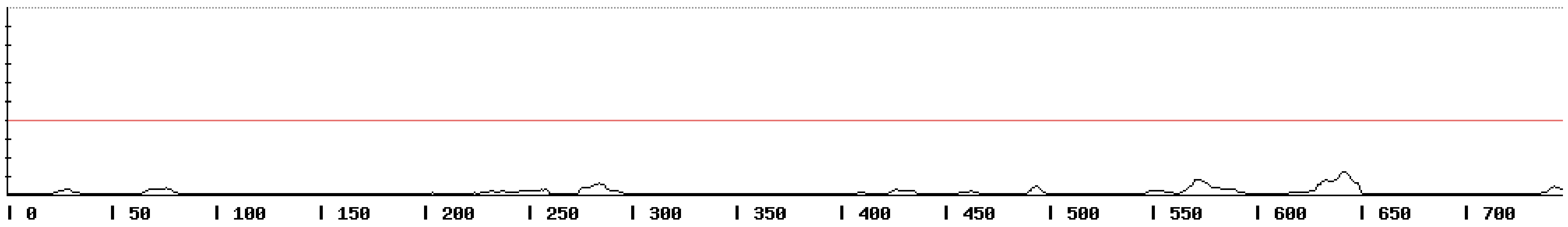

Predictions for Lotusjaponicus_chr1.CM0207.190.r2.m

Viterbi Path

Viterbi Path

Posterior @ 0.4

Posterior @ 0.4

Posterior Graphic. Threshold Marked @ 0.4

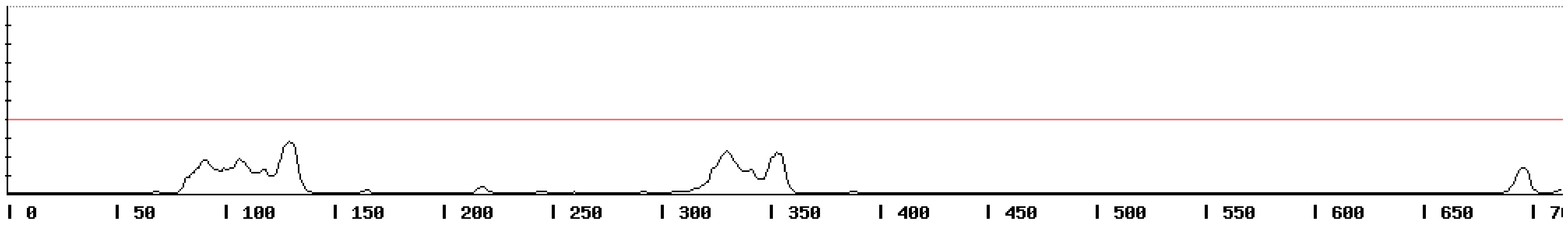

Predictions for Lotusjaponicus_chr3.CM0208.570.r2.m

Viterbi Path

Viterbi Path

Posterior @ 0.4

Posterior @ 0.4

Posterior Graphic. Threshold Marked @ 0.4

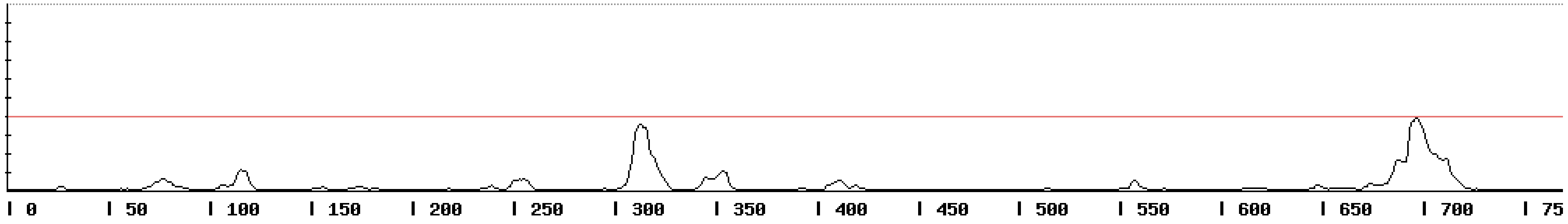

Predictions for Linumusitatissimum_Lus10002083

Viterbi Path

Viterbi Path

Posterior @ 0.4

Posterior @ 0.4

Posterior Graphic. Threshold Marked @ 0.4

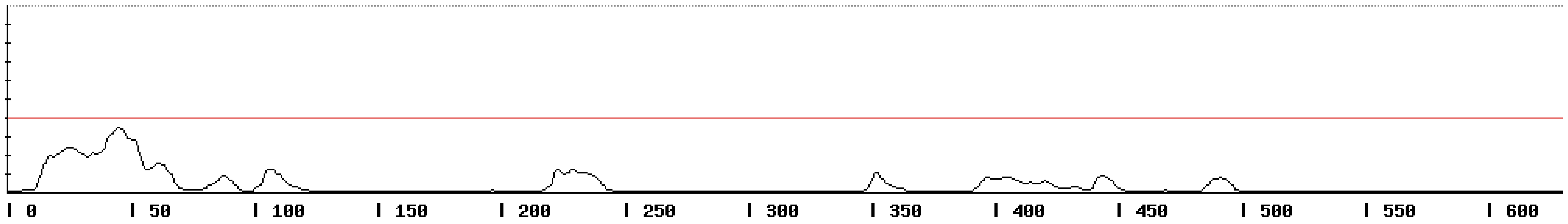

Predictions for Linumusitatissimum_Lus10003548

Viterbi Path	
Viterbi Path	
Posterior @ 0.4	101 - KERTGGENKKKKS - 113
Posterior @ 0.4	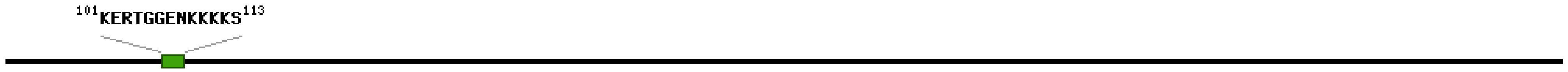
Posterior Graphic. Threshold Marked @ 0.4	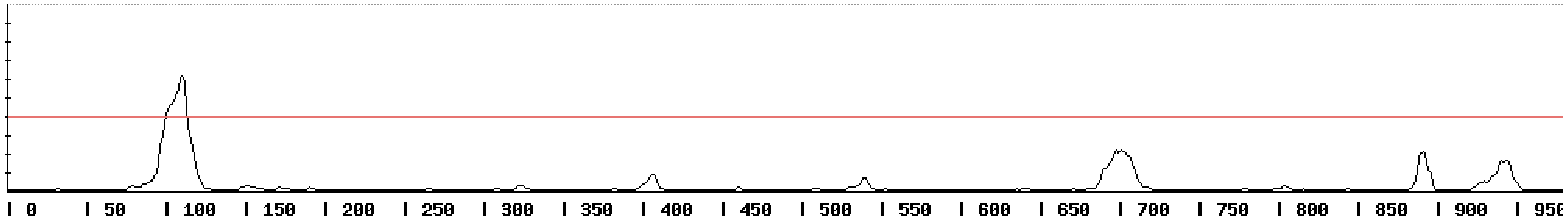

Predictions for Malusdomestica_MDP0000180046	
Viterbi Path	
Viterbi Path	
Posterior @ 0.4	
Posterior @ 0.4	
Posterior Graphic. Threshold Marked @ 0.4	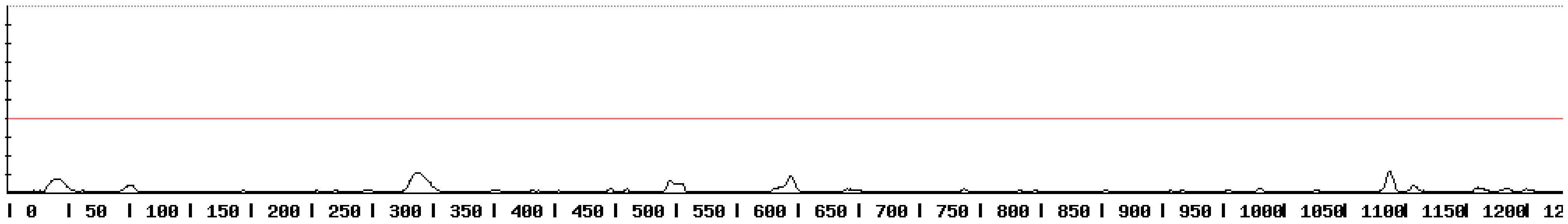

Predictions for Malusdomestica_MDP0000239596	
Viterbi Path	
Viterbi Path	
Posterior @ 0.4	
Posterior @ 0.4	
Posterior Graphic. Threshold Marked @ 0.4	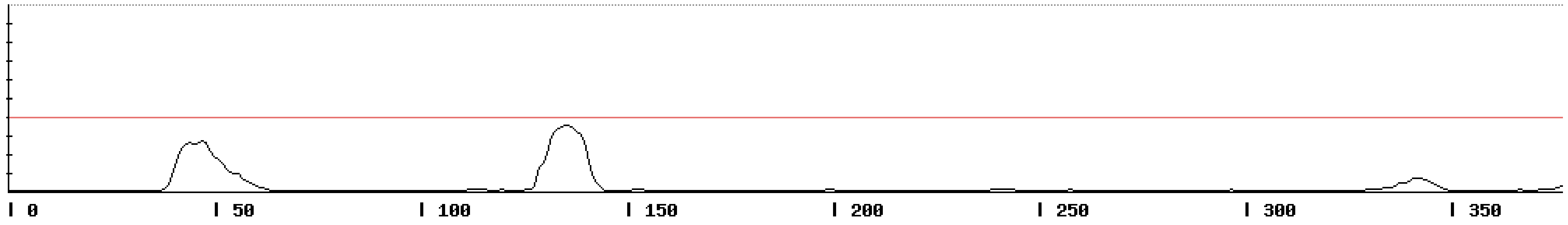

Predictions for Malusdomestica_MDP0000276278	
Viterbi Path	206 - PREERPIGMRGRRVLGRWGRRRRRRRRLQGERRVR - 240
Viterbi Path	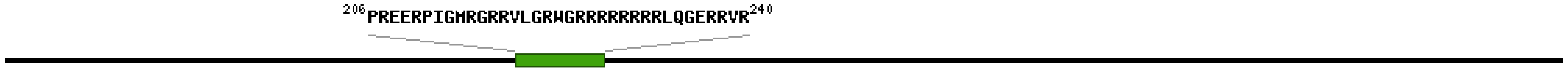
Posterior @ 0.4	207 - REERPIGMRGRRVLGRWGRRRRRRRRLQGERRVR - 240
	430 - RKRRK - 434
Posterior @ 0.4	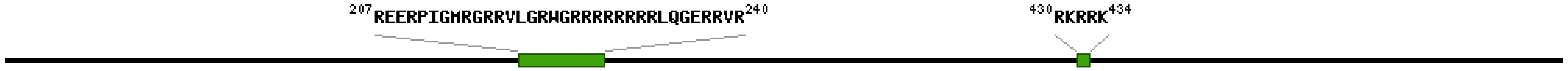
Posterior Graphic. Threshold Marked @ 0.4	

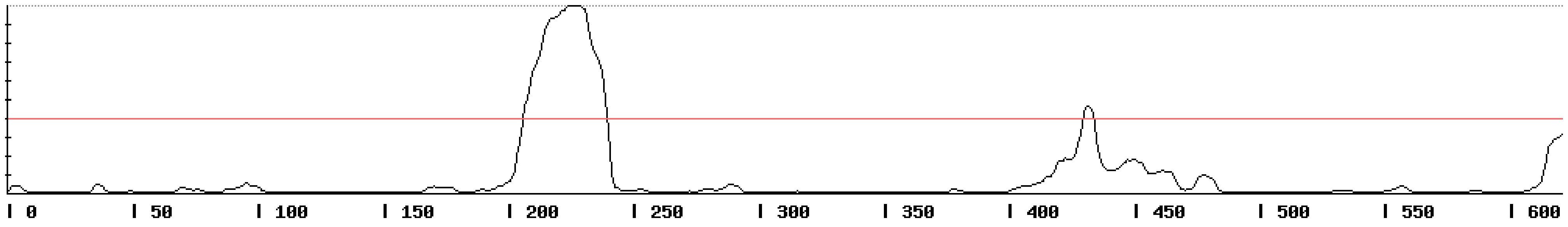

Predictions for Malusdomestica_MDP0000276982

Viterbi Path	
Viterbi Path	
Posterior @ 0.4	
Posterior @ 0.4	
Posterior Graphic. Threshold Marked @ 0.4	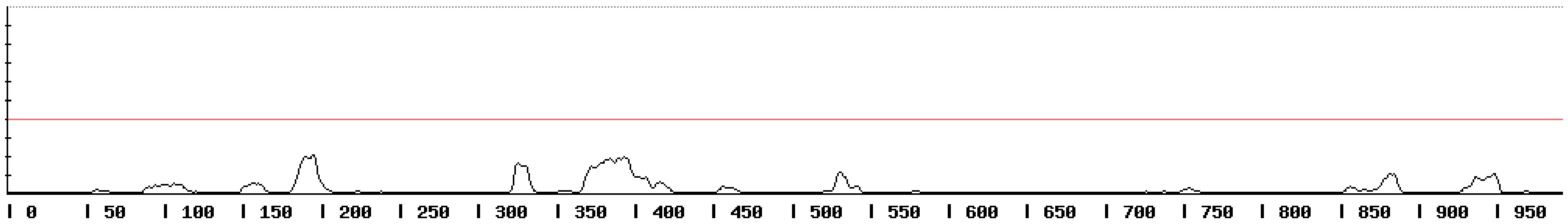

Predictions for Malusdomestica_MDP0000283975

Viterbi Path	
Viterbi Path	
Posterior @ 0.4	921 - KRR - 923
Posterior @ 0.4	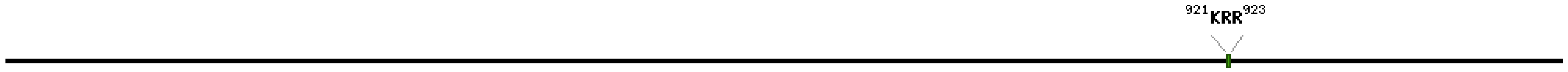

Posterior Graphic. Threshold Marked @ 0.4

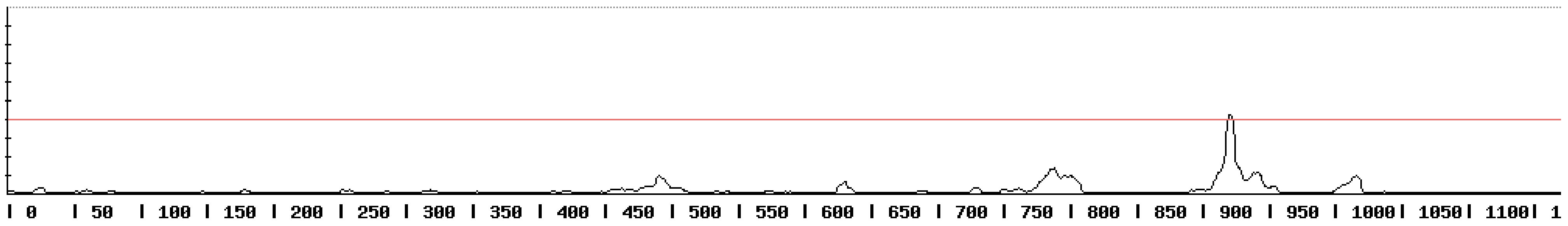

Predictions for Malusdomestica_MDP0000288624

Viterbi Path	
Viterbi Path	
Posterior @ 0.4	
Posterior @ 0.4	
Posterior Graphic. Threshold Marked @ 0.4	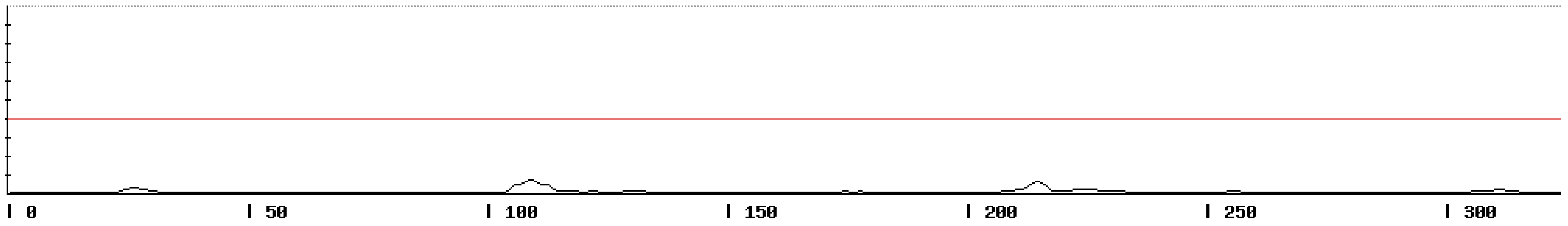

Predictions for Malusdomestica_MDP0000289955

Viterbi Path	194 - PAKKRARKEVKEKPAKKKARKEVKQKPAKKKAR - 226
Viterbi Path	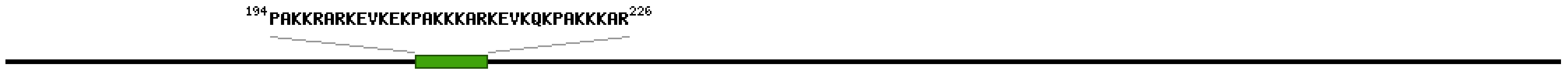
Posterior @ 0.4	194 - PAKKRARKEVKEKPAKKKARKEVKQKPAKKKARN - 227
Posterior @ 0.4	

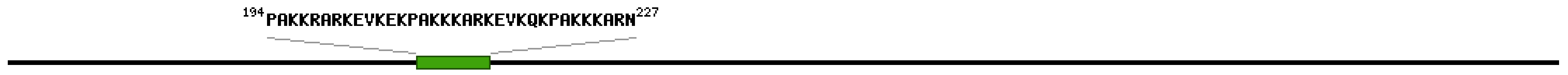

Posterior Graphic. Threshold Marked @ 0.4

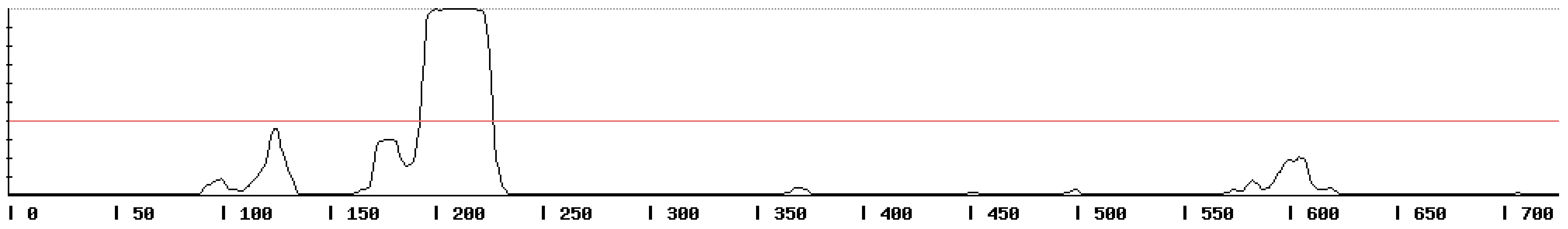

Predictions for Malusdomestica_MDP0000309351

Viterbi Path

Viterbi Path

Posterior @ 0.4 — 471 - RKRRK - 475

Posterior @ 0.4

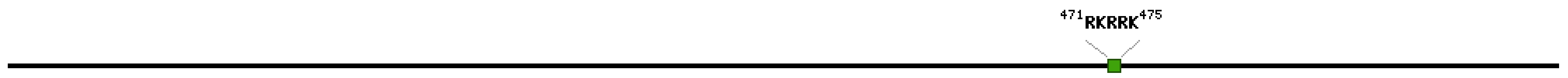

Posterior Graphic. Threshold Marked @ 0.4

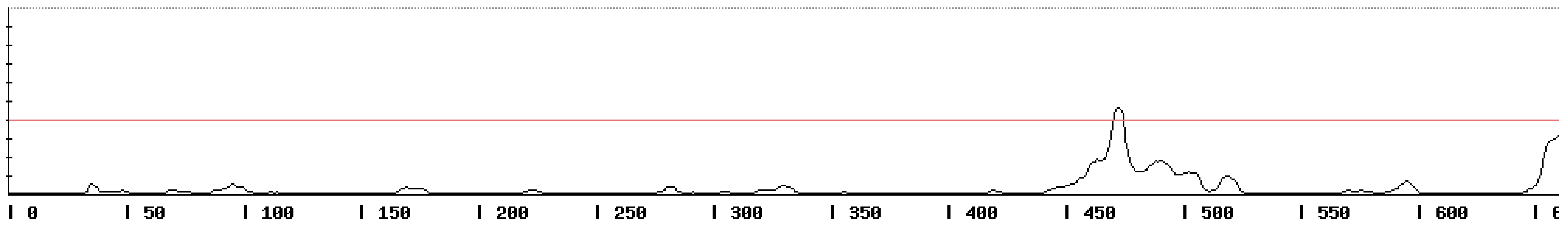

Predictions for Malusdomestica_MDP0000428265

Viterbi Path

Viterbi Path

Posterior @ 0.4

Posterior @ 0.4

Posterior Graphic. Threshold Marked @ 0.4

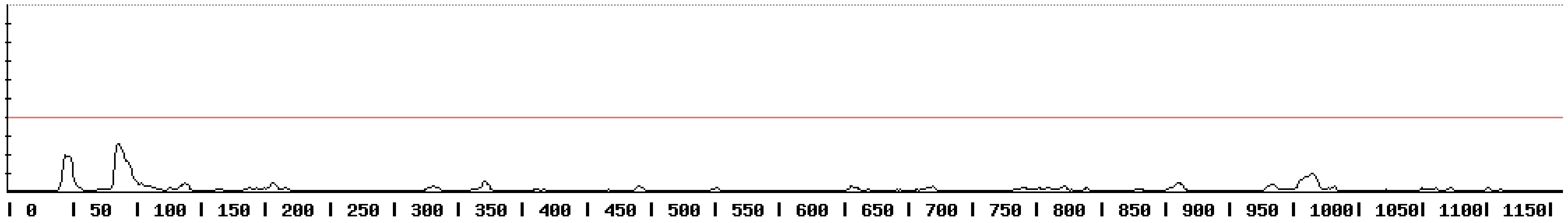

Predictions for Malusdomestica_MDP0000663564

Viterbi Path

Viterbi Path

Posterior @ 0.4

104 - RKRRK - 108

150 - KKG - 152

512 - MRSKVAKRMQKESGKTLREVRRAKKLQKKLMTENERLIYNLKRAKRKVALL - 562

565 - K - 565

Posterior @ 0.4

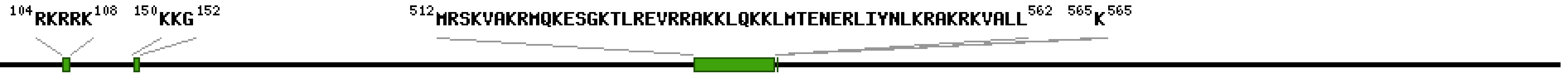

Posterior Graphic. Threshold Marked @ 0.4

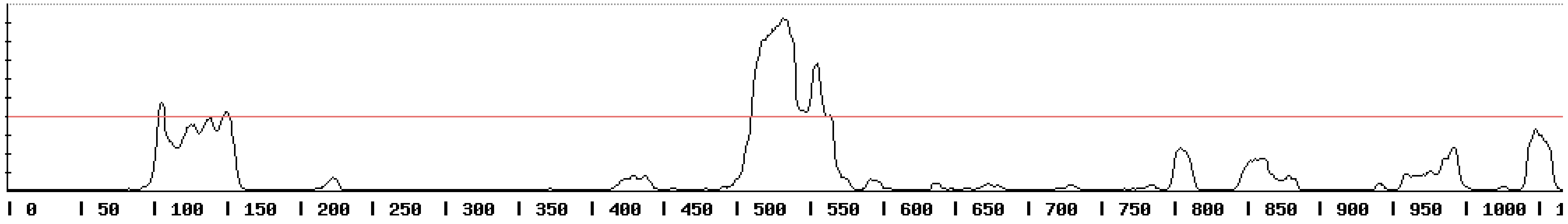

Predictions for Malusdomestica_MDP0000689991

Viterbi Path	
Viterbi Path	
Posterior @ 0.4	
Posterior @ 0.4	
Posterior Graphic. Threshold Marked @ 0.4	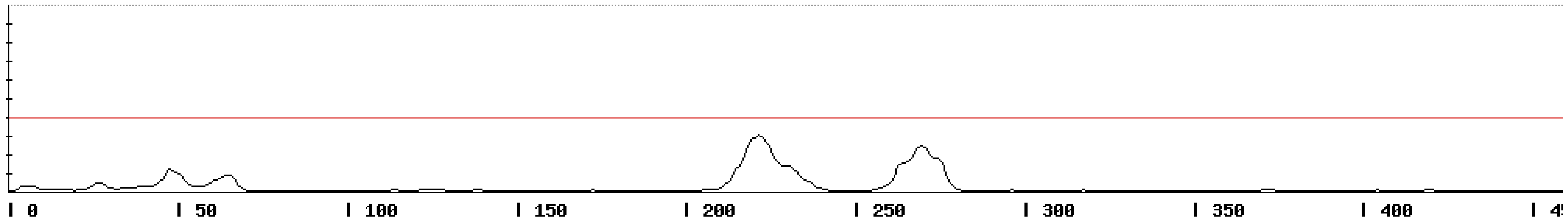

Predictions for Medicagotruncatula_AC233657_10.1

Viterbi Path	
Viterbi Path	
Posterior @ 0.4	
Posterior @ 0.4	
Posterior Graphic. Threshold Marked @ 0.4	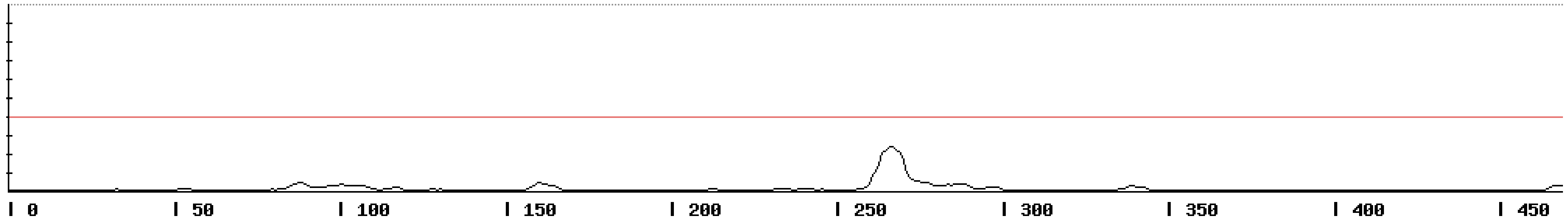

Predictions for Musaacuminata_GSMUA_Achr4P07150_001	
Viterbi Path	
Viterbi Path	
Posterior @ 0.4	1137 - QKNLRRYKAKK - 1147<br>1214 - RVARRE - 1219
Posterior @ 0.4	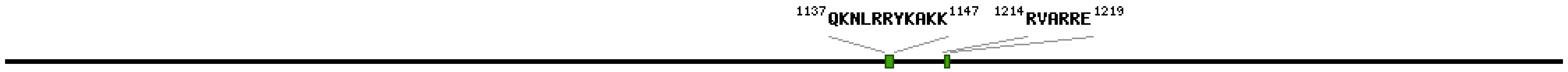
Posterior Graphic. Threshold Marked @ 0.4	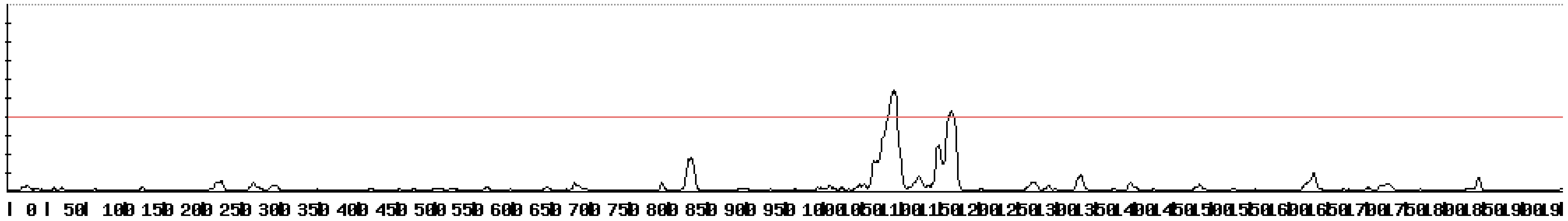

Predictions for Musaacuminata_GSMUA_Achr9P04960_001	
Viterbi Path	
Viterbi Path	
Posterior @ 0.4	
Posterior @ 0.4	
Posterior Graphic. Threshold Marked @ 0.4	

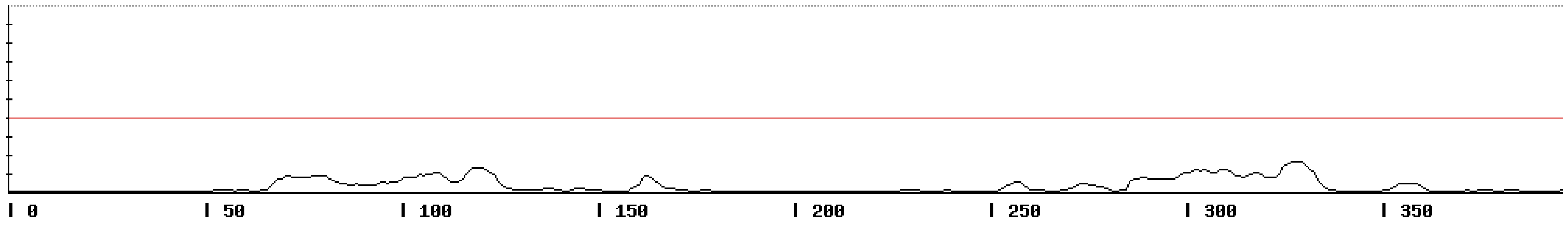

Predictions for Oropetiumthomaeum_Oropetium_20150105_15183

Viterbi Path

Viterbi Path

Posterior @ 0.4

Posterior @ 0.4

Posterior Graphic. Threshold Marked @ 0.4

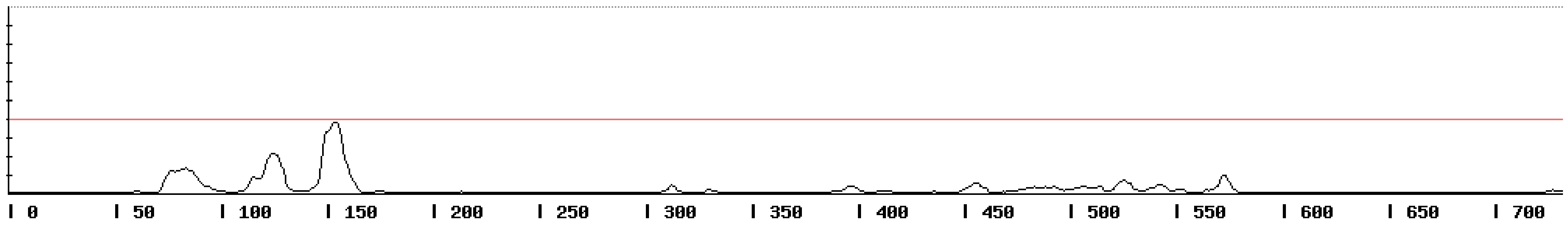

Predictions for Oryzabarthii_ObartAA03S_FGP0027

Viterbi Path

Viterbi Path

Posterior @ 0.4

Posterior @ 0.4

Posterior Graphic. Threshold Marked @ 0.4

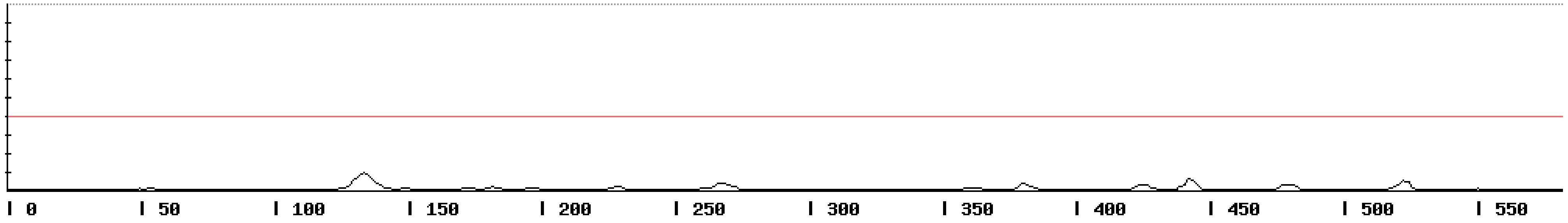

Predictions for Oryzabarthii_ObartAA03S_FGP21921

Viterbi Path

Viterbi Path

Posterior @ 0.4

Posterior @ 0.4

Posterior Graphic. Threshold Marked @ 0.4

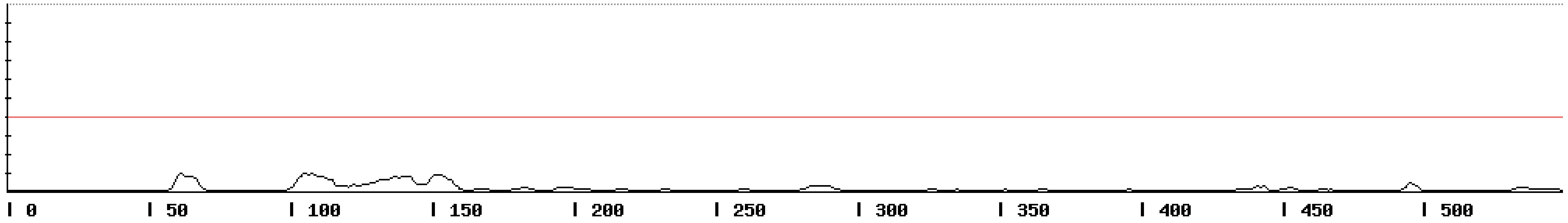

Predictions for Oryzabarthii_ObartAA03S_FGP31756

Viterbi Path

Viterbi Path

Posterior @ 0.4

Posterior @ 0.4

Posterior Graphic. Threshold Marked @ 0.4	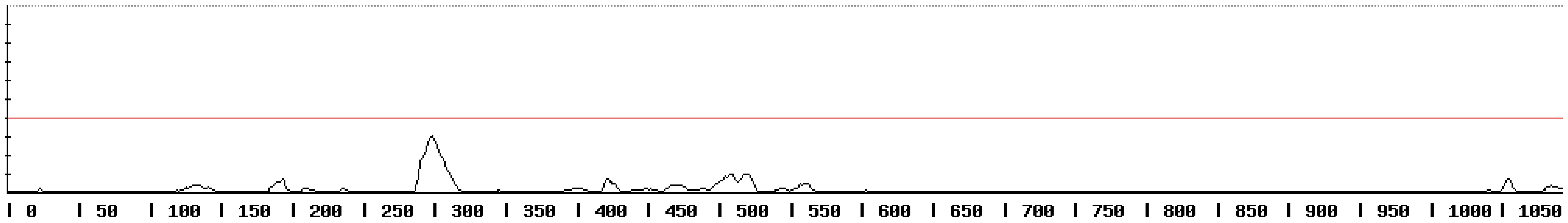

Predictions for Oryzabarthii_ObartAA03S_FGP8394

Viterbi Path	107 - RSIPHPRRKITRRRRRTTKMPPRLPDKLKQR - 137
Viterbi Path	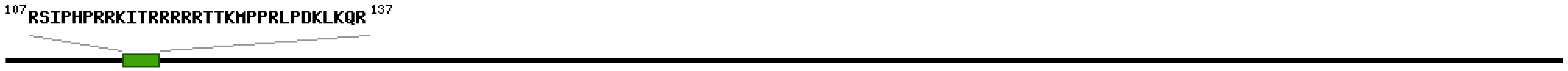
Posterior @ 0.4	108 - SIPHPRRKITRRRRRTTKMPPRLPDKLKQ - 136
Posterior @ 0.4	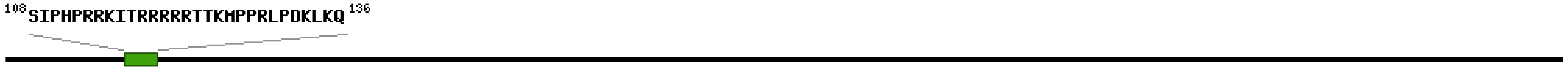
Posterior Graphic. Threshold Marked @ 0.4	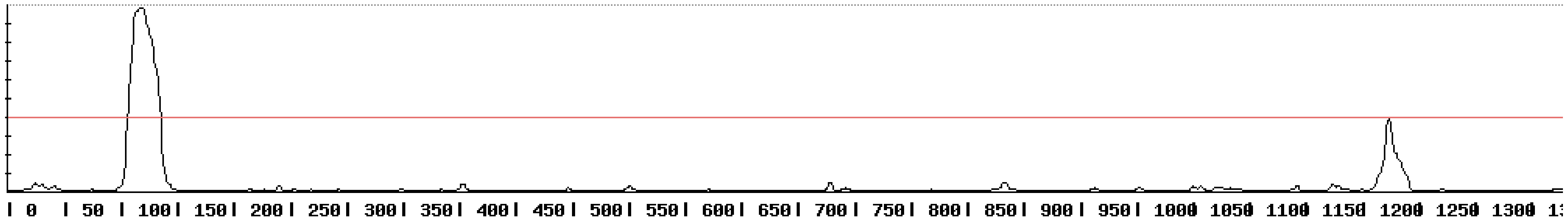

Predictions for Oryzabrachyantha_OB03G25660.1

Viterbi Path	
Viterbi Path	

Posterior @ 0.4	
Posterior @ 0.4	
Posterior Graphic. Threshold Marked @ 0.4	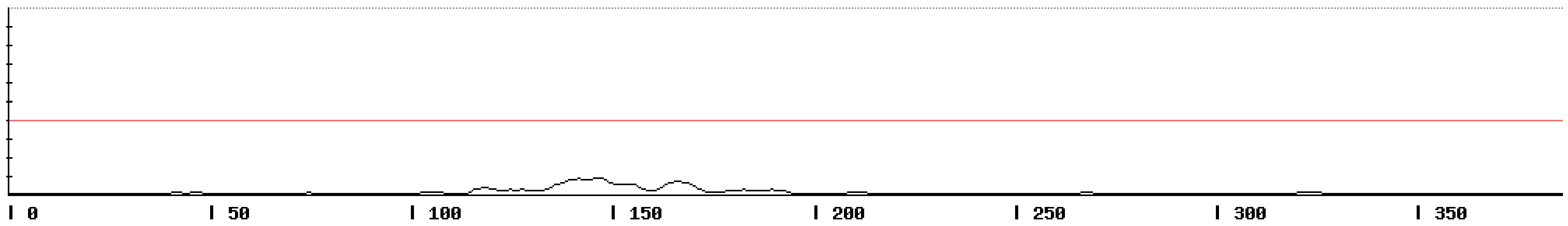

Predictions for Oryzaglaberrima_ORGLA04G0153400.1	
Viterbi Path	
Viterbi Path	
Posterior @ 0.4	302 - PPRPRKA - 308
Posterior @ 0.4	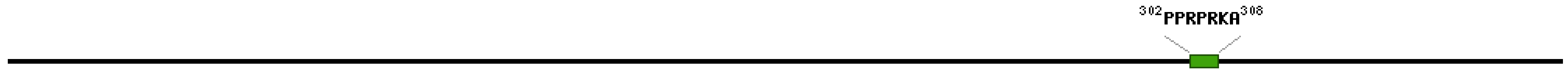
Posterior Graphic. Threshold Marked @ 0.4	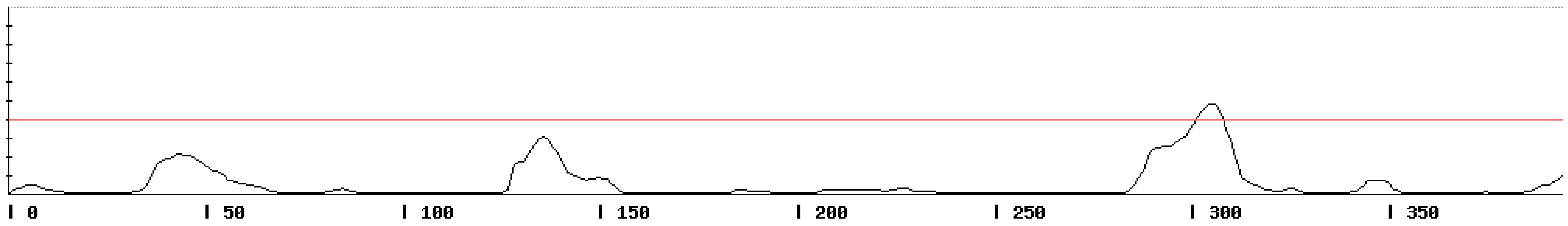

Predictions for Oryzasativa_LOC_Os04g42940.1	
Viterbi Path	
Viterbi Path	

Posterior @ 0.4	302 - PPRPRKA - 308
Posterior @ 0.4	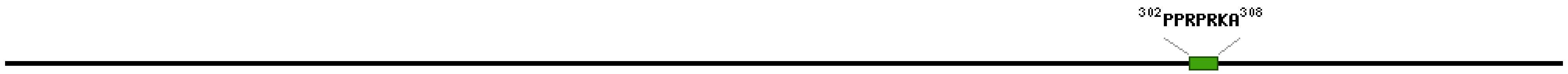
Posterior Graphic. Threshold Marked @ 0.4	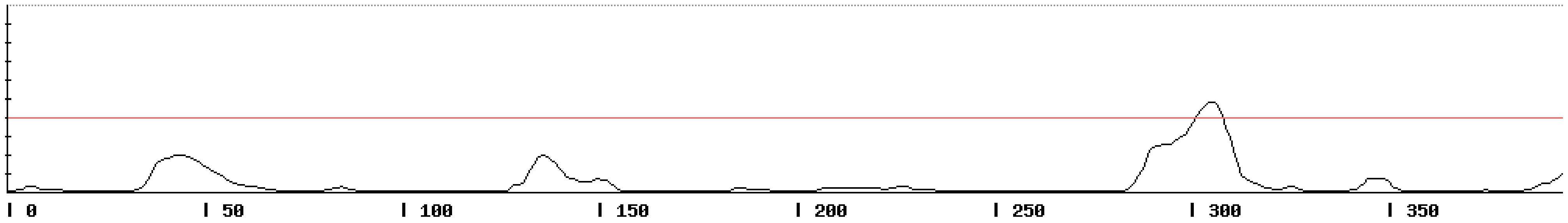

Predictions for Panicumhallii_Pahal.A02025	
Viterbi Path	
Viterbi Path	
Posterior @ 0.4	107 - GGGRGREAAWRKK - 119<br>329 - RFSGHGKKRKRA - 340
Posterior @ 0.4	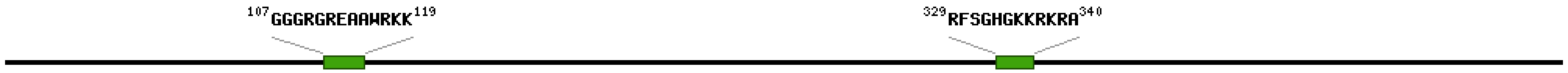
Posterior Graphic. Threshold Marked @ 0.4	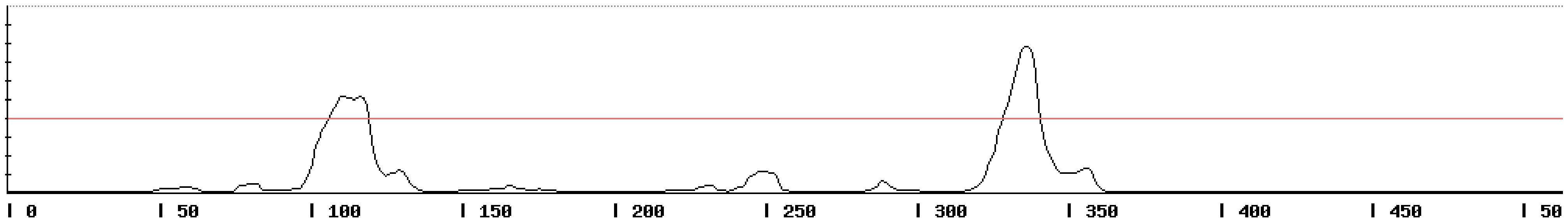

Predictions for Panicumhallii_Pahal.C04574

Viterbi Path

Viterbi Path

Posterior @ 0.4

Posterior @ 0.4

Posterior Graphic. Threshold Marked @ 0.4

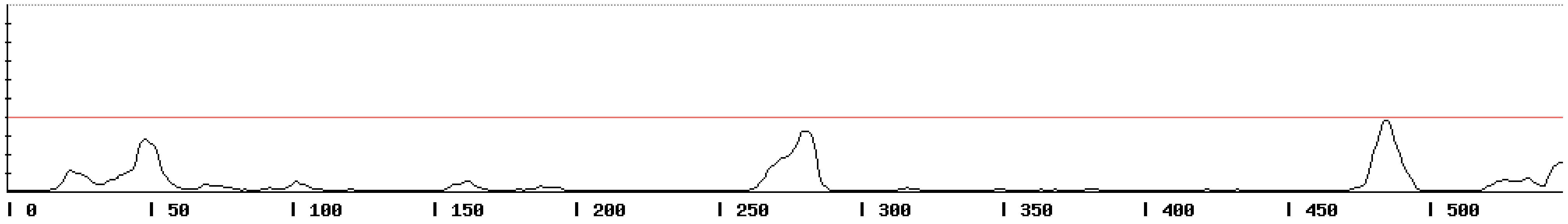

Predictions for Panicumhallii_Pahal.D02537

Viterbi Path

Viterbi Path

Posterior @ 0.4 318 - FSGHGKKRKRG - 328

Posterior @ 0.4

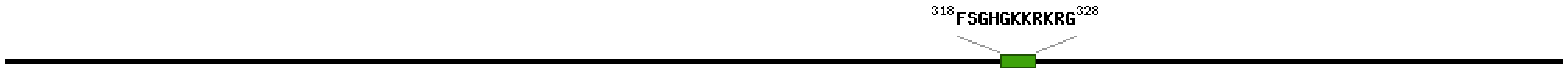

Posterior Graphic. Threshold Marked @ 0.4

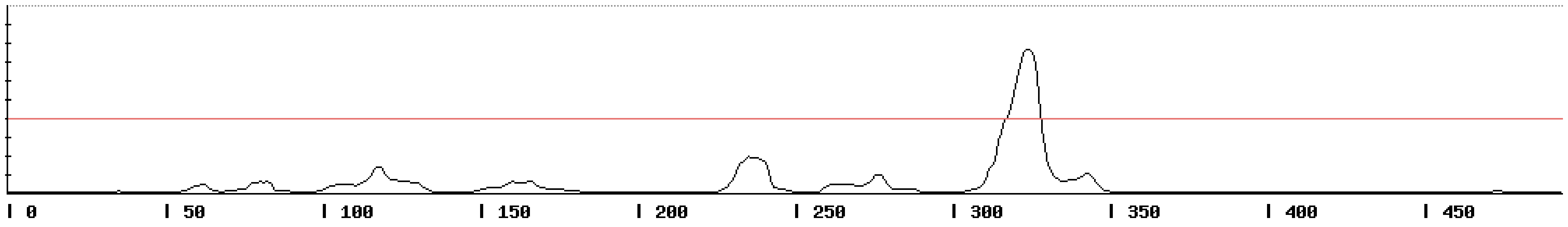

Predictions for Panicumvirgatum_Pavir.Bb00848

Viterbi Path

Viterbi Path

Posterior @ 0.4: 322 - HGKKRKRG - 329

Posterior @ 0.4

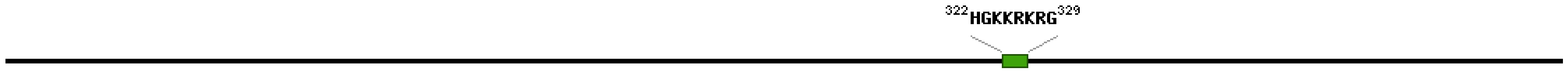

Posterior Graphic. Threshold Marked @ 0.4

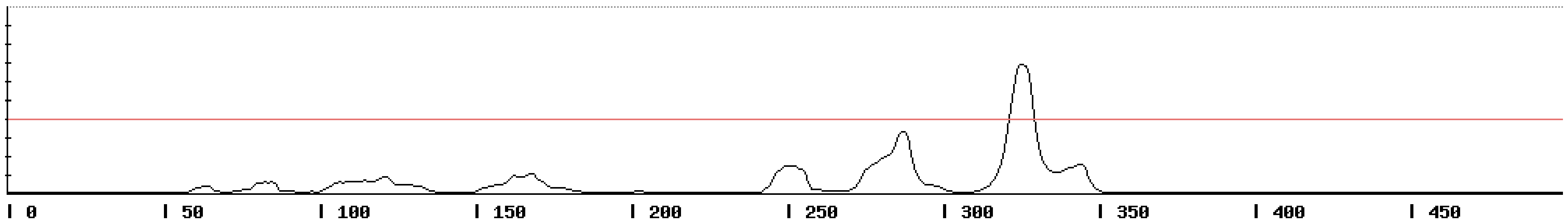

Predictions for Panicumvirgatum_Pavir.Db01755

Viterbi Path

Viterbi Path

Posterior @ 0.4: 251 - FSGHGKKRKRG - 261

Posterior @ 0.4

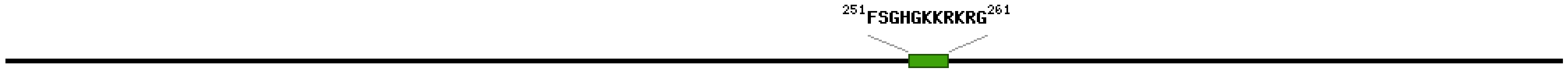

Posterior Graphic. Threshold Marked @ 0.4

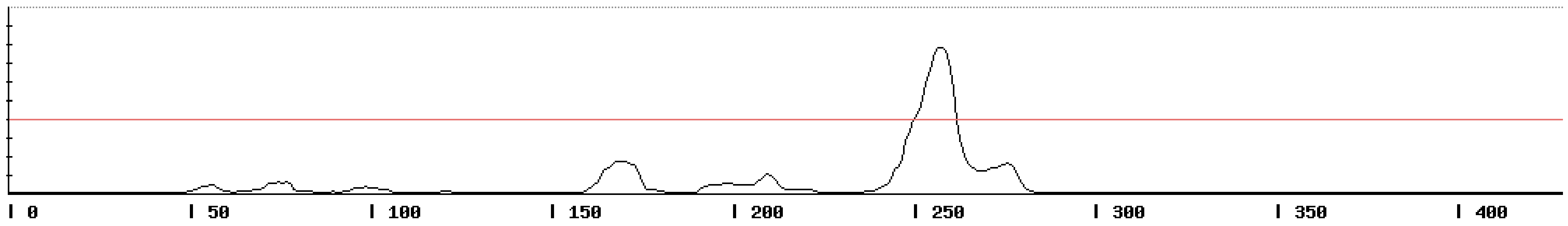

Predictions for Panicumvirgatum_Pavir.Db01806

Viterbi Path

Viterbi Path

Posterior @ 0.4

325 - RFSGHGKKRKRGEA - 338

349 - AARRPSPPPK - 358

Posterior @ 0.4

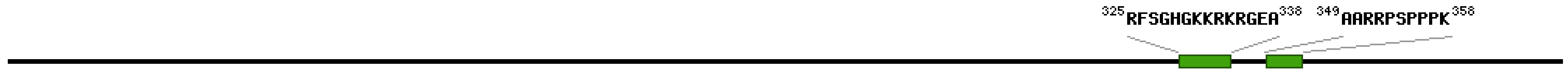

Posterior Graphic. Threshold Marked @ 0.4

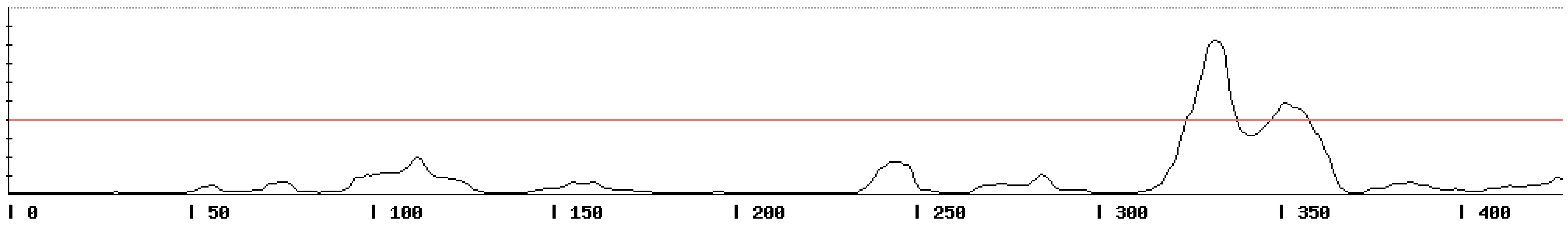

Predictions for Panicumvirgatum_Pavir.J34688

Viterbi Path

Viterbi Path

Posterior @ 0.4

193 - FSGHGKKRKRG - 203

372 - SRPRRRWRRRS - 382

Posterior @ 0.4

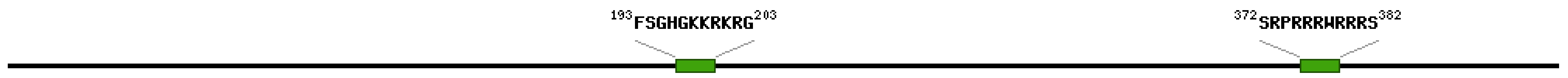

Posterior Graphic. Threshold Marked @ 0.4

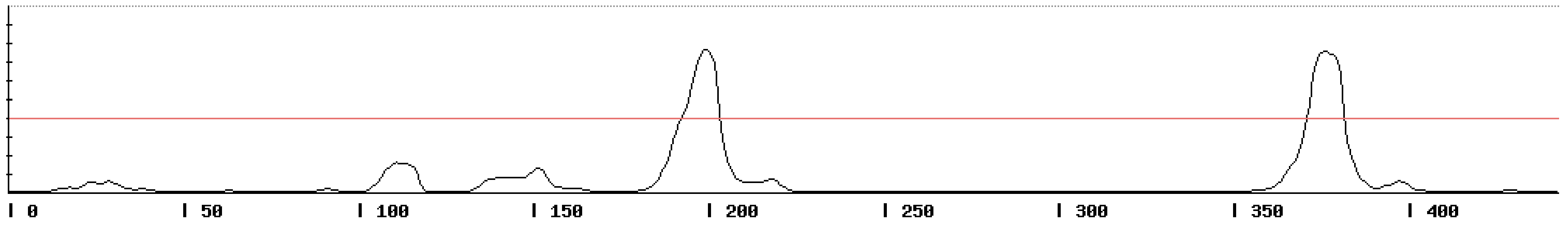

Predictions for Piceaabies_MA_10431997g0010

Viterbi Path

Viterbi Path

Posterior @ 0.4

85 - KKPKTTTKKKMKGNHK - 100

Posterior @ 0.4

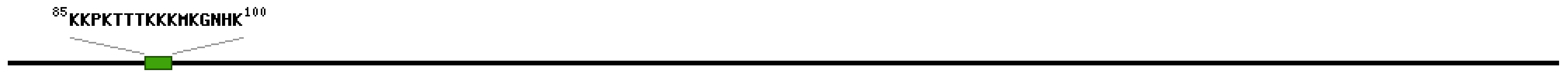

Posterior Graphic. Threshold Marked @ 0.4

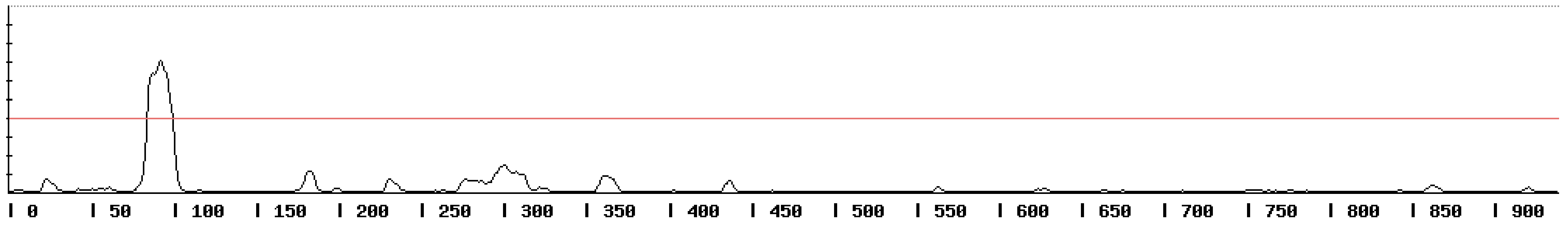

Predictions for Setariaitalica_Si008305m

Viterbi Path

Viterbi Path

Posterior @ 0.4	99 - RIPGGGGREAAWRKKA - 114
	307 - KKRKR - 311
Posterior @ 0.4	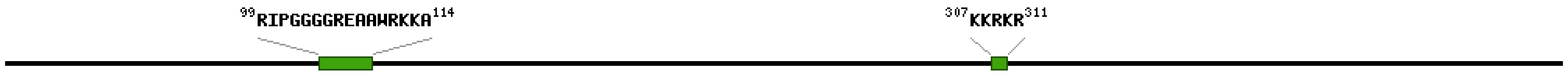
Posterior Graphic. Threshold Marked @ 0.4	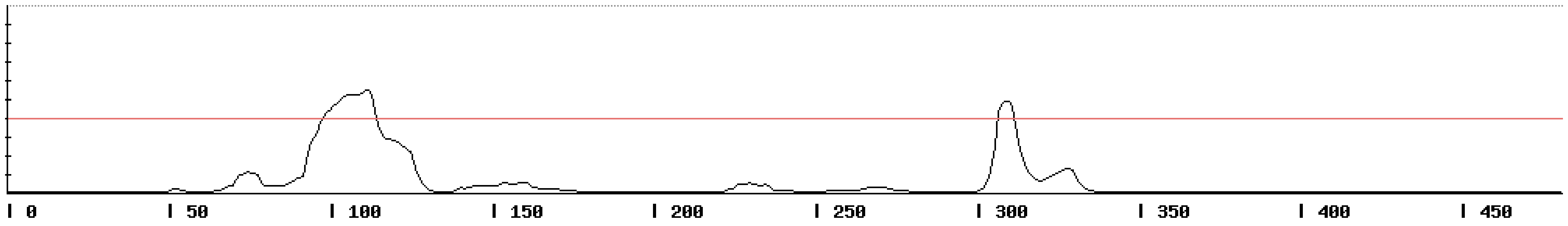

Predictions for Setariaitalica_Si011665m

Viterbi Path	
Viterbi Path	
Posterior @ 0.4	26 - RHGGRAGAGGRPPRPPRRGGNRPPGGQ - 52
Posterior @ 0.4	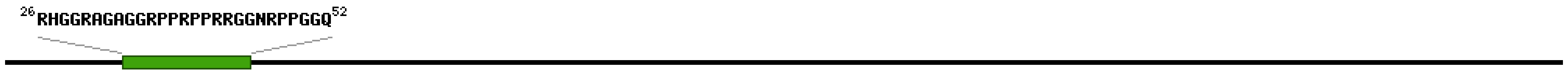
Posterior Graphic. Threshold Marked @ 0.4	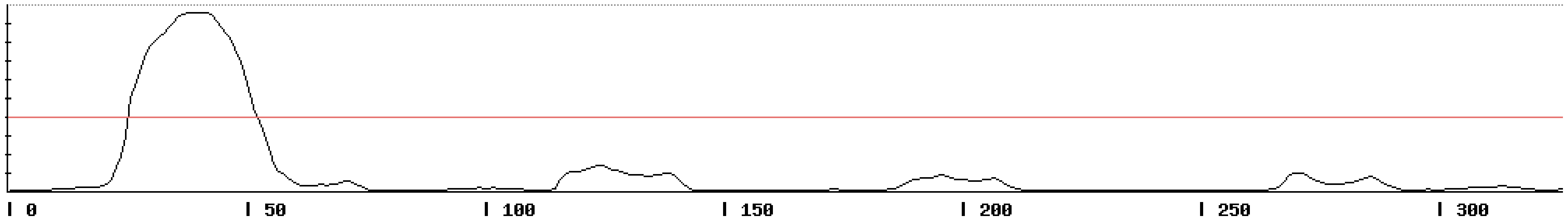

Predictions for Setariaitalica_Si019248m

Viterbi Path	
Viterbi Path	

Posterior @ 0.4	332 - GRKRKRG - 338
Posterior @ 0.4	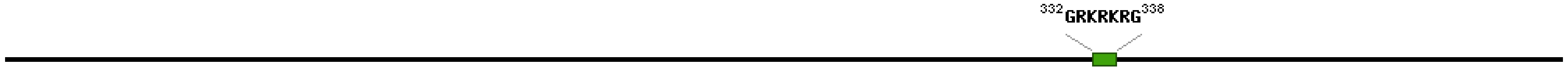
Posterior Graphic. Threshold Marked @ 0.4	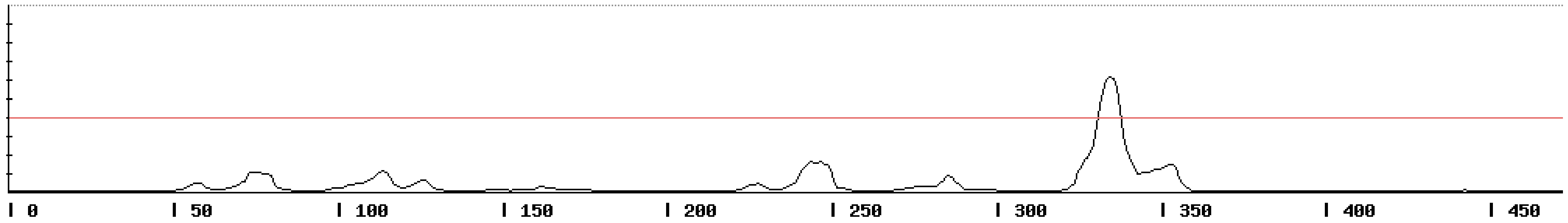

Predictions for Setariaitalica_Si019775m

Viterbi Path	
Viterbi Path	
Posterior @ 0.4	325 - GRKRKR - 330
Posterior @ 0.4	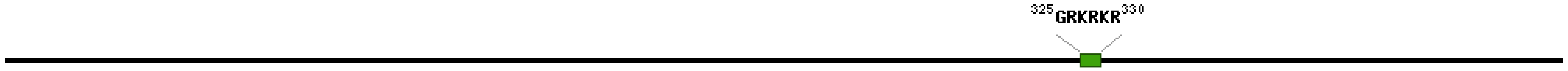
Posterior Graphic. Threshold Marked @ 0.4	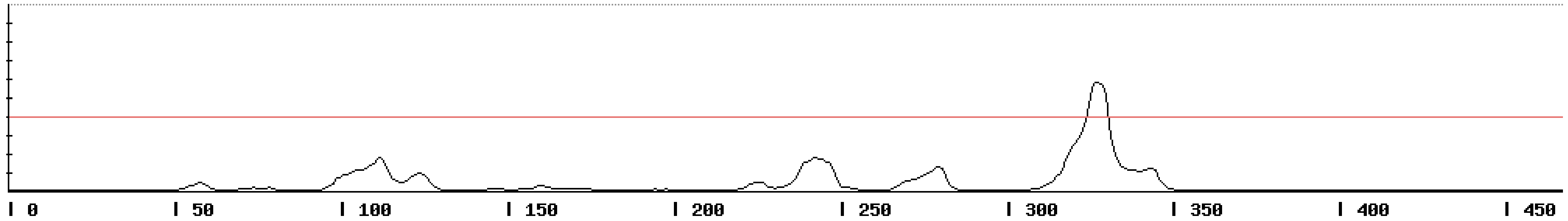

Predictions for Setariaviridis_Sevir.1G165700

Viterbi Path

Viterbi Path

Posterior @ 0.4

307 - GHGRKRKRE - 315

Posterior @ 0.4

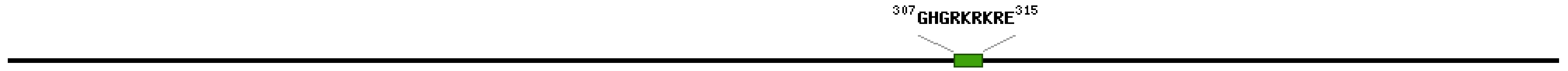

Posterior Graphic. Threshold Marked @ 0.4

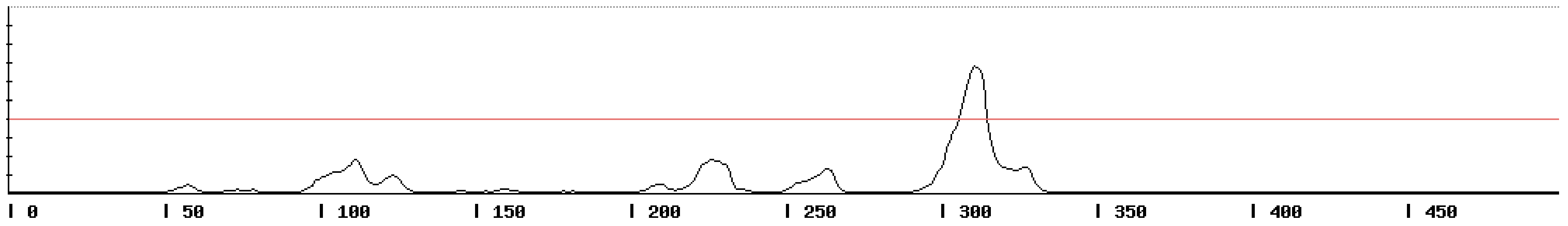

Predictions for Solanumtuberosum_PGSC0003DMP400054265

Viterbi Path

Viterbi Path

Posterior @ 0.4

Posterior @ 0.4

Posterior Graphic. Threshold Marked @ 0.4

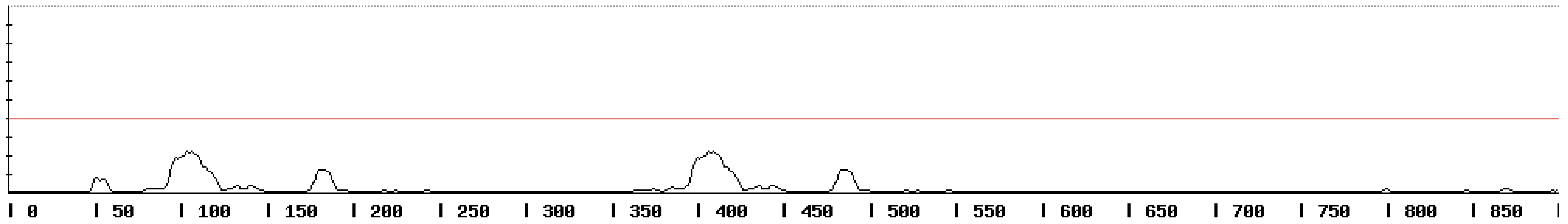

Predictions for Sorghumbicolor_Sobic.006G141900.1.p

Viterbi Path

Viterbi Path

Posterior @ 0.4

37 - GGGGGRPPRPPPPRRGTSRPSAGGER - 61

128 - RGLGGKRKAGRG - 139

Posterior @ 0.4

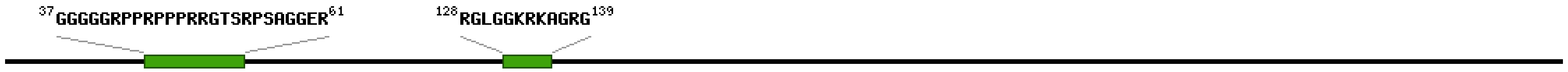

Posterior Graphic. Threshold Marked @ 0.4

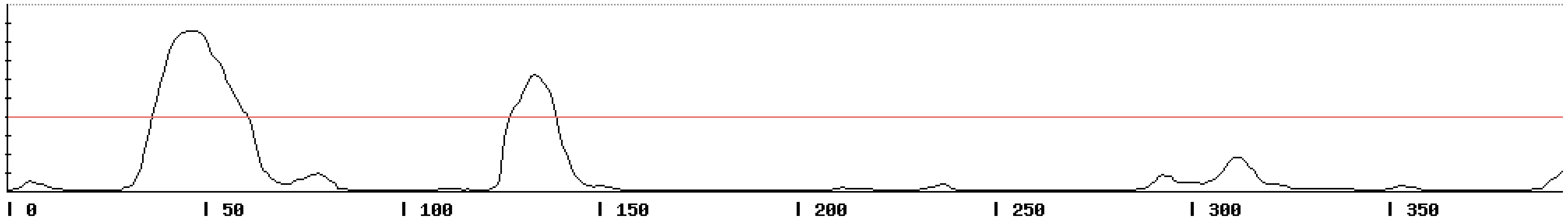

Predictions for Eutremasalsugineum_Thhalv10015599m

Viterbi Path

Viterbi Path

Posterior @ 0.4

22 - HKIPKINRSKKKDDRHRPRE - 41

62 - KRKLKGKSK - 70

156 - TKKTLTFTKSKNKKKKQK - 173

Posterior @ 0.4

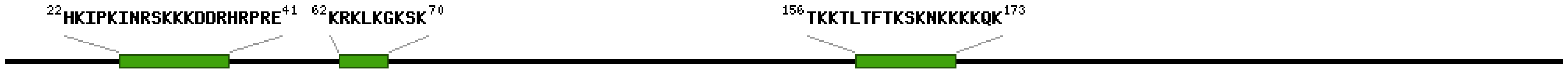

Posterior Graphic. Threshold Marked @ 0.4

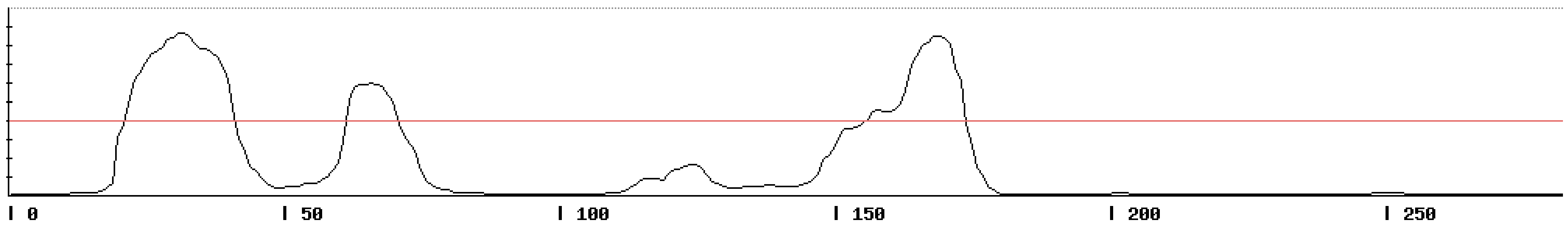

Predictions for Eutremasalsugineum_Thhalv10024006m

Viterbi Path

Viterbi Path

Posterior @ 0.4

Posterior @ 0.4

Posterior Graphic. Threshold Marked @ 0.4

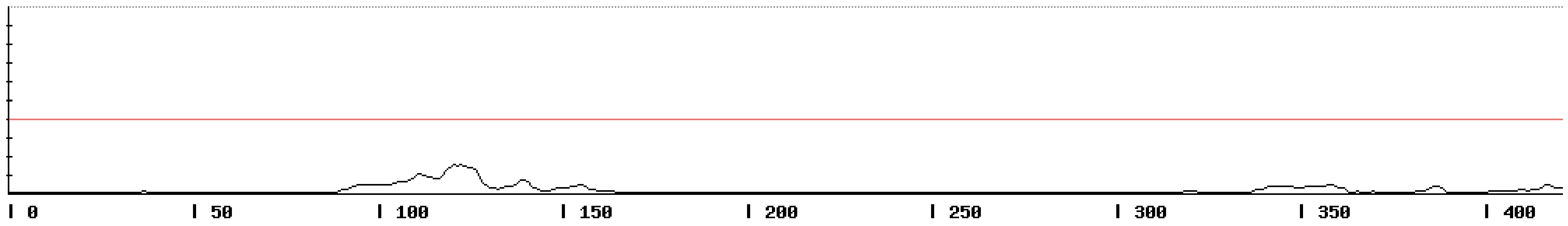

Predictions for Thellungiellaparvula_Tp2g04430

Viterbi Path

Viterbi Path

Posterior @ 0.4

Posterior @ 0.4

Posterior Graphic. Threshold Marked @ 0.4	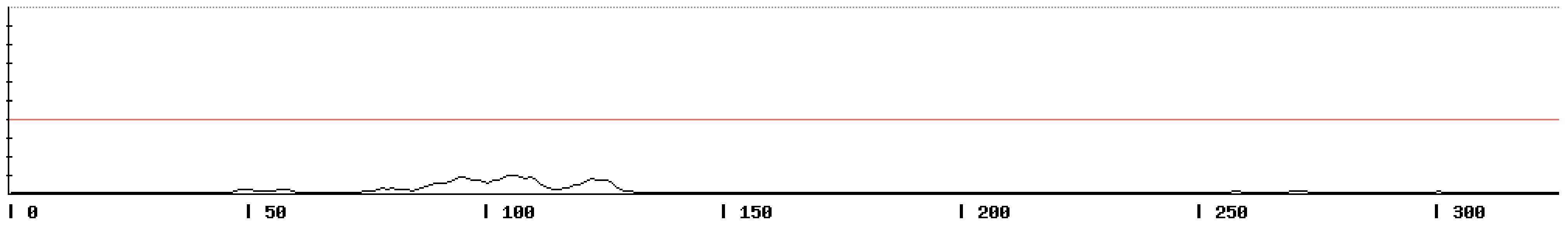

Predictions for Trifoliumpratense_Tp57577_TGAC_v2_gene15980

Viterbi Path	
Viterbi Path	
Posterior @ 0.4	82 - NRKAKGGYWKISGKGRIIKKKGTNNAIGRKK - 112
Posterior @ 0.4	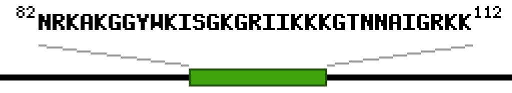
Posterior Graphic. Threshold Marked @ 0.4	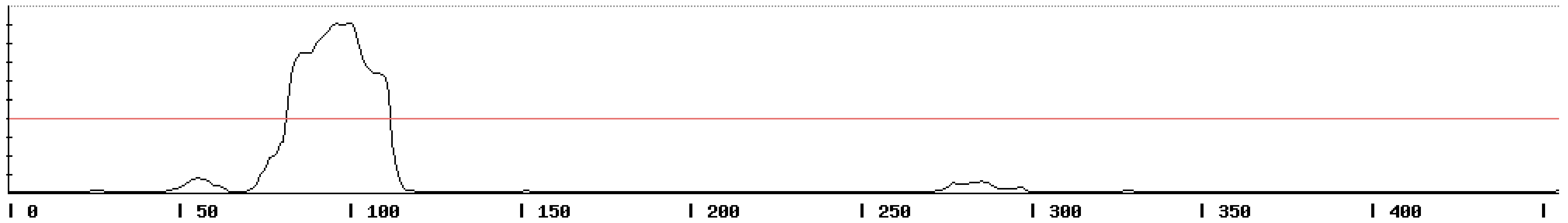

Predictions for Trifoliumpratense_Tp57577_TGAC_v2_gene30270

Viterbi Path	
Viterbi Path	
Posterior @ 0.4	83 - RKAKGGYWKISGKGRIIKKKGTNNVIGRKK - 112
Posterior @ 0.4	

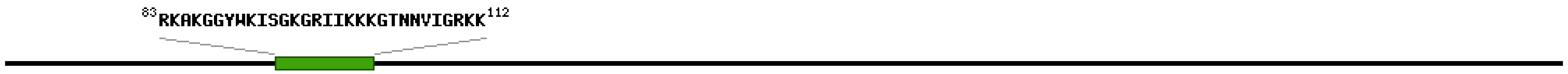

Posterior Graphic. Threshold Marked @ 0.4	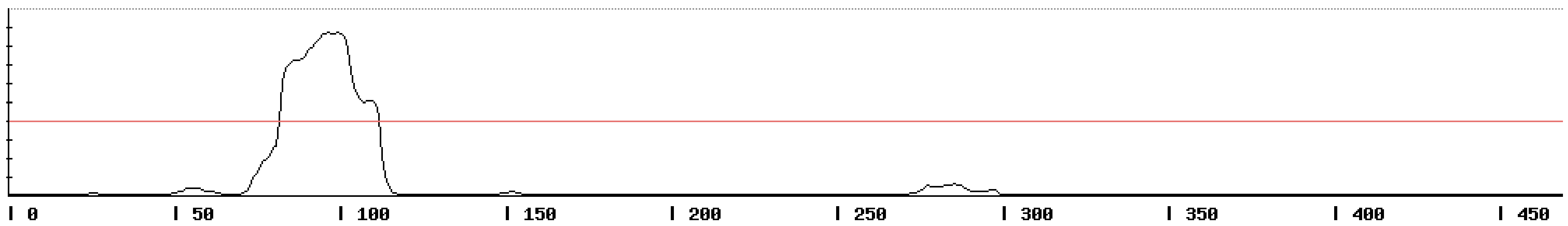

Predictions for Trifoliumpratense_Tp57577_TGAC_v2_gene37364

Viterbi Path	
Viterbi Path	
Posterior @ 0.4	735 - KRQKEKHPGT - 744
	982 - RKKEHR - 987
Posterior @ 0.4	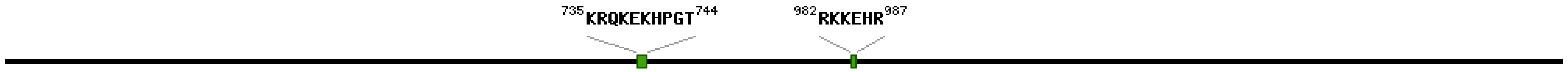
Posterior Graphic. Threshold Marked @ 0.4	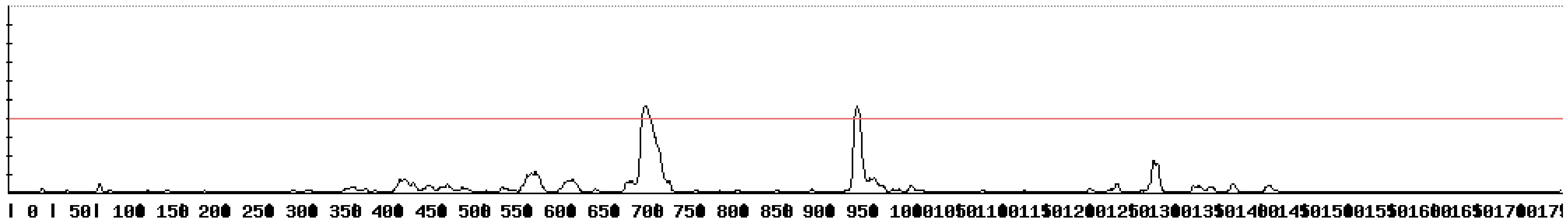

Predictions for Triticumurartu_EMS48536

Viterbi Path	
Viterbi Path	

Posterior @ 0.4	175 - KKRKK - 179
Posterior @ 0.4	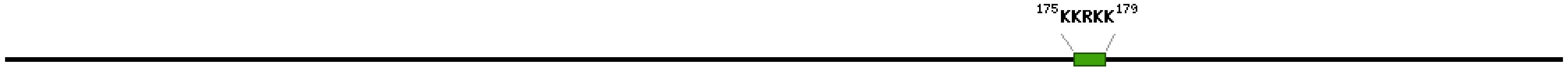
Posterior Graphic. Threshold Marked @ 0.4	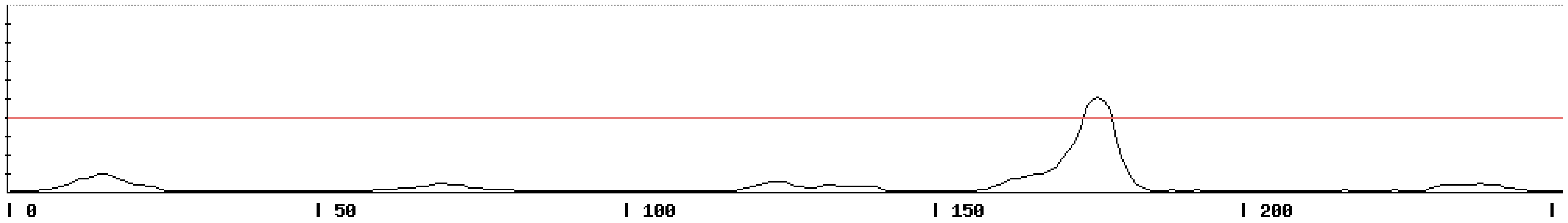

Predictions for Utriculariagibba_Scf00024.g3185.t1

Viterbi Path	
Viterbi Path	
Posterior @ 0.4	
Posterior @ 0.4	
Posterior Graphic. Threshold Marked @ 0.4	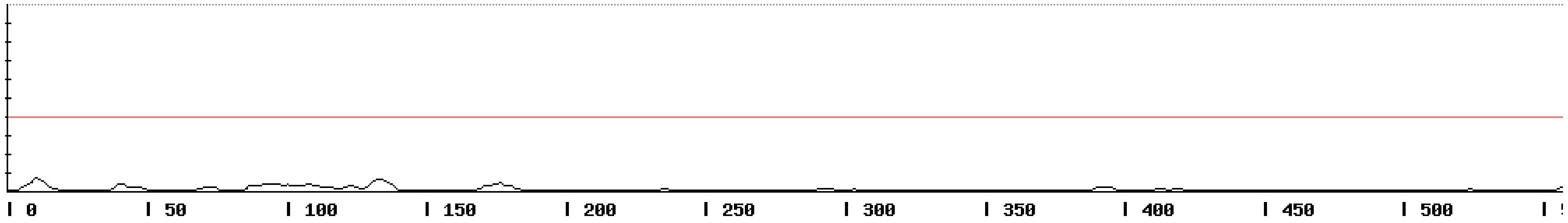

Predictions for Vitisvinifera_GSVIVT01027475001

Viterbi Path	
Viterbi Path	
Posterior @ 0.4	
Posterior @ 0.4	
Posterior Graphic. Threshold Marked @ 0.4	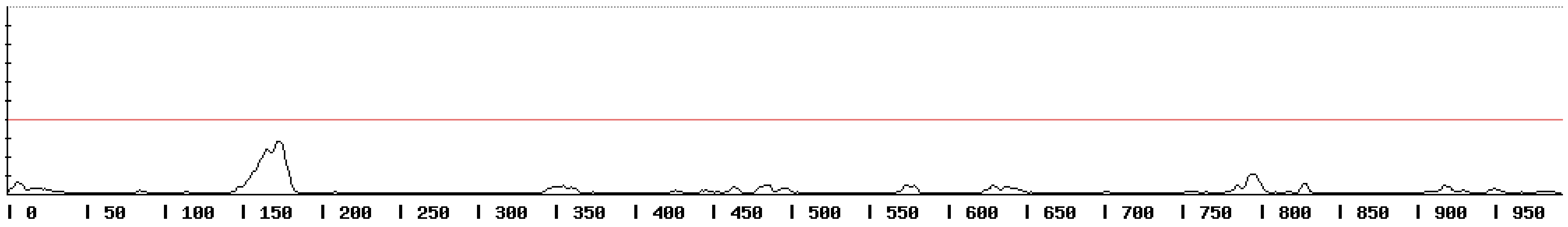

Predictions for Zeamays_AC208663.3_FGP002

Viterbi Path	
Viterbi Path	
Posterior @ 0.4	39 - PPRPPPRR - 46
	109 - RGLGGKRRAGRG - 120
Posterior @ 0.4	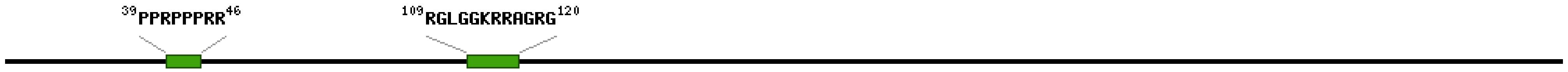
Posterior Graphic. Threshold Marked @ 0.4	

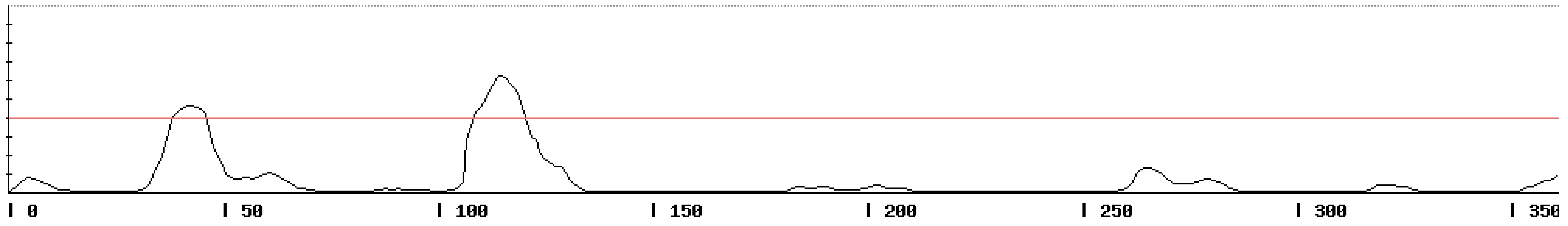

Predictions for Zeamays_GRMZM2G312201_P01

Viterbi Path	
Viterbi Path	
Posterior @ 0.4	
Posterior @ 0.4	
Posterior Graphic. Threshold Marked @ 0.4	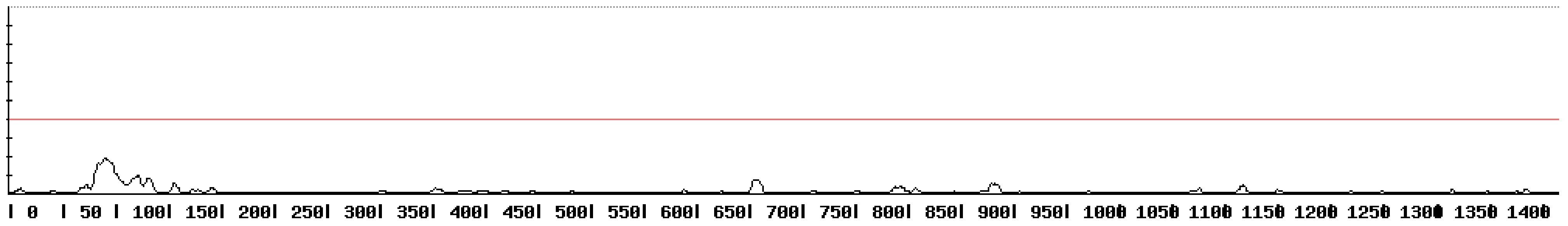

Predictions for Zosteramarina_Zosma250g00090

Viterbi Path	
Viterbi Path	
Posterior @ 0.4	
Posterior @ 0.4	

Posterior Graphic. Threshold Marked @ 0.4	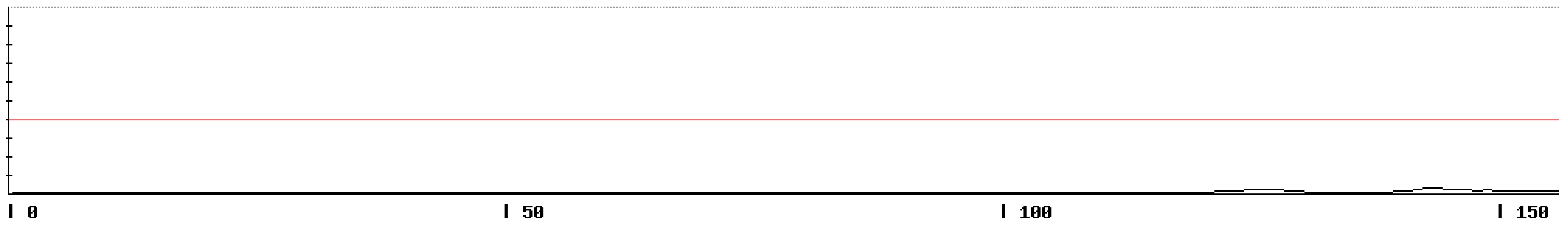

Predictions for Arabidopsisthaliana_AT1G60280.1

Viterbi Path	
Viterbi Path	
Posterior @ 0.4	114 - KETGKILGFKKILKFCLKKKPREYK - 138
Posterior @ 0.4	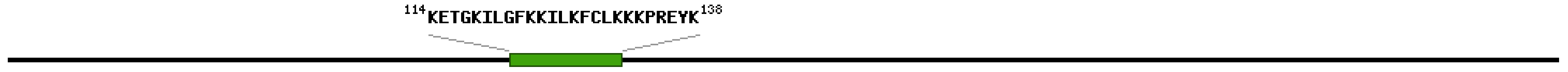
Posterior Graphic. Threshold Marked @ 0.4	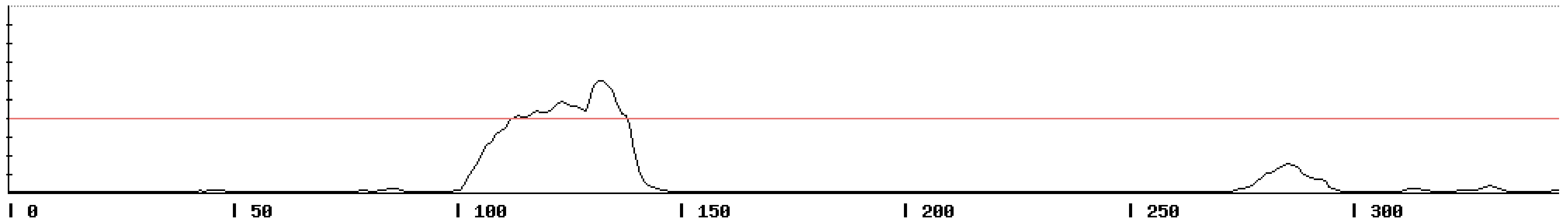

Predictions for Arabidopsisthaliana_AT1G60300.1

Viterbi Path	
Viterbi Path	
Posterior @ 0.4	
Posterior @ 0.4	

Posterior Graphic. Threshold Marked @ 0.4	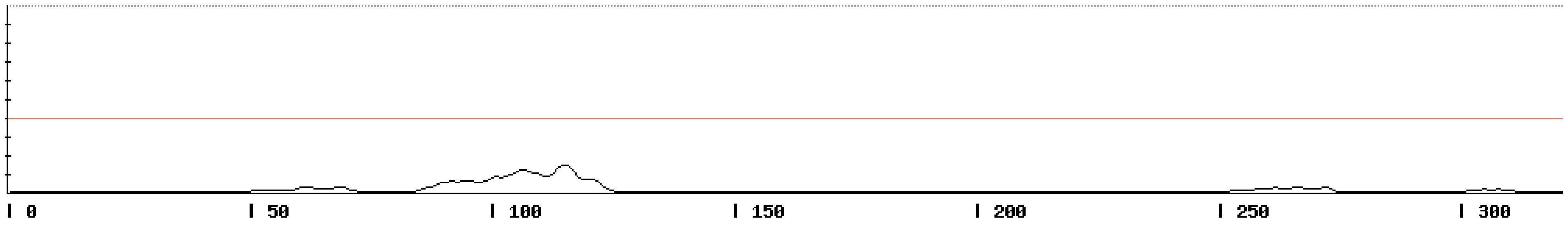

Predictions for Arabidopsisthaliana_AT1G60340.1

Viterbi Path	
Viterbi Path	
Posterior @ 0.4	
Posterior @ 0.4	
Posterior Graphic. Threshold Marked @ 0.4	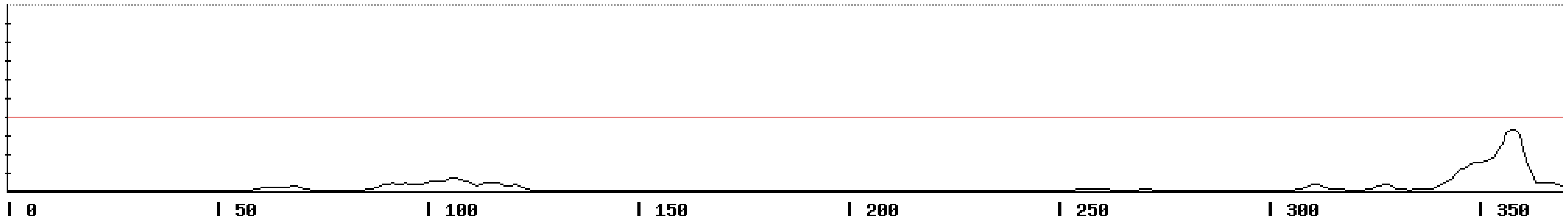

Predictions for Arabidopsisthaliana_AT1G60350.1

Viterbi Path	
Viterbi Path	

Posterior @ 0.4 113 - KRKPRGY - 119

Posterior @ 0.4

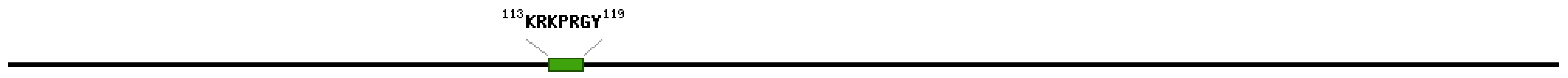

Posterior Graphic. Threshold Marked @ 0.4

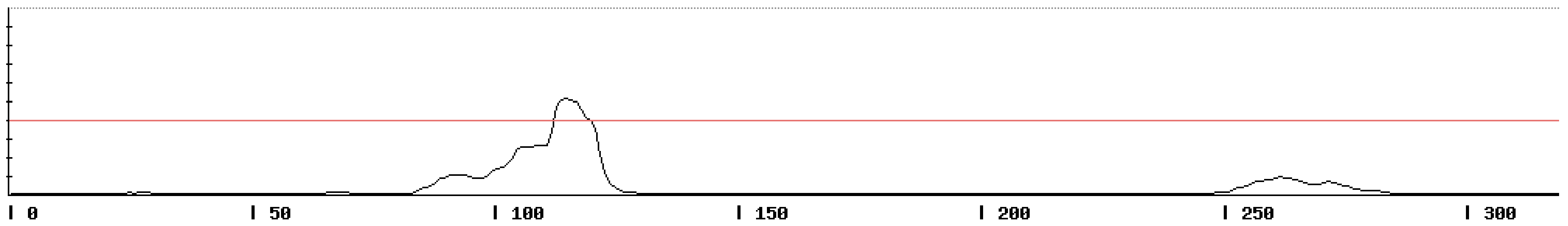

Predictions for Arabidopsisthaliana_AT1G60380.1

Viterbi Path

Viterbi Path

Posterior @ 0.4

Posterior @ 0.4

Posterior Graphic. Threshold Marked @ 0.4

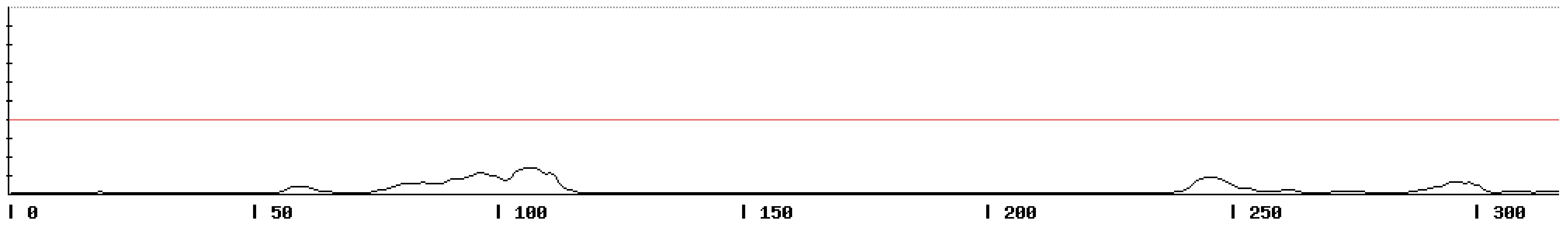

Predictions for Aethionemaarabicum_AA10G00196

Viterbi Path

Viterbi Path

Posterior @ 0.4

Posterior @ 0.4

Posterior Graphic. Threshold Marked @ 0.4

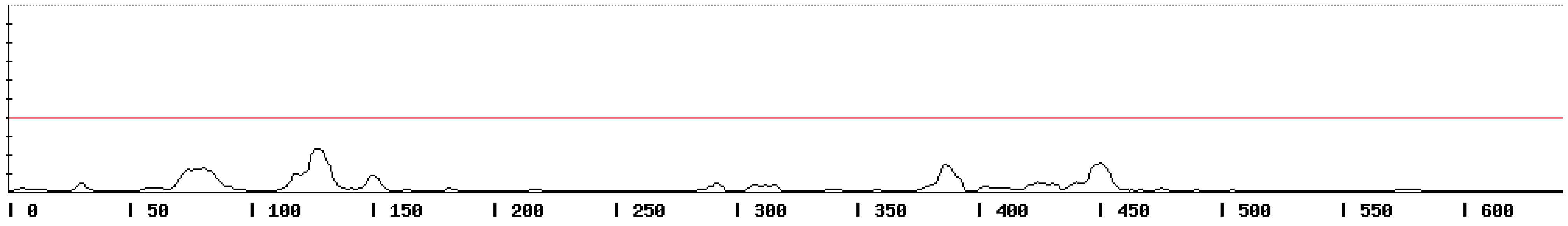

Predictions for Aethionemaarabicum_AA26G00262

Viterbi Path

Viterbi Path

Posterior @ 0.4

Posterior @ 0.4

Posterior Graphic. Threshold Marked @ 0.4

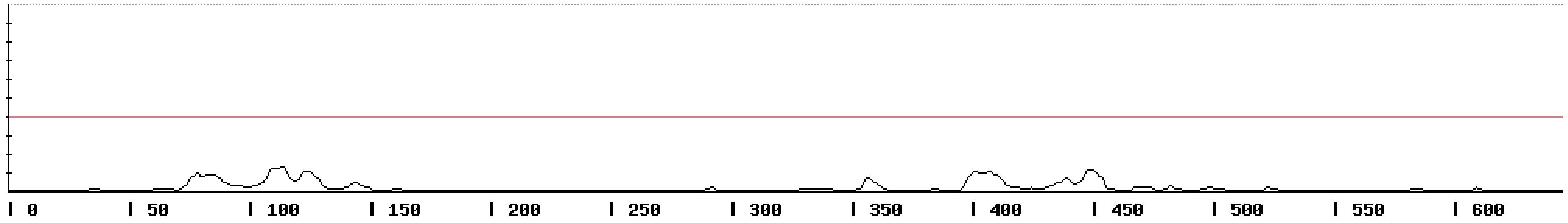

Predictions for Aethionemaarabicum_AA93G00231

Viterbi Path

Viterbi Path

Posterior @ 0.4

Posterior @ 0.4

Posterior Graphic. Threshold Marked @ 0.4

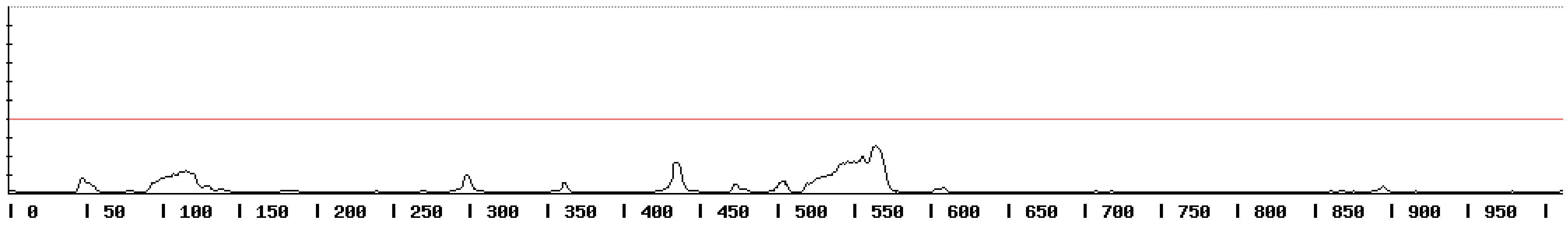

Predictions for Arabidopsishalleri_Araha.16028s0003.1.p

Viterbi Path

Viterbi Path

Posterior @ 0.4

82 - LIKSKATGKILGFKKILKFCKKTKPRE - 108

Posterior @ 0.4

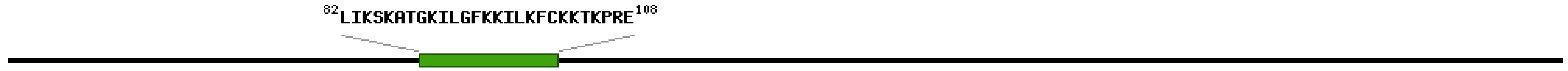

Posterior Graphic. Threshold Marked @ 0.4

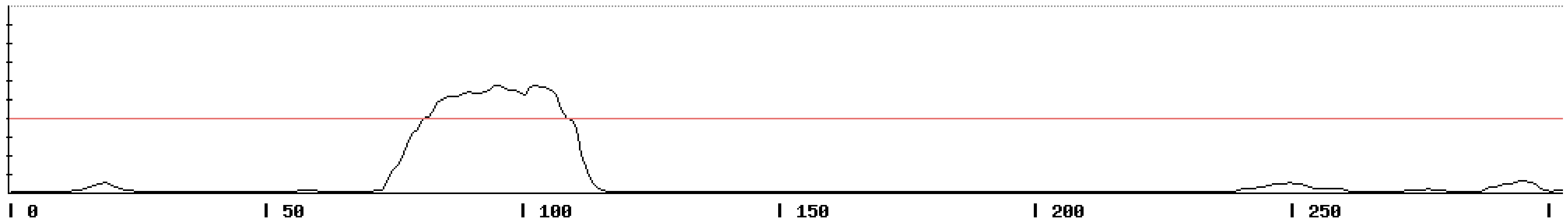

Predictions for Arabidopsishalleri_Araha.61839s0001.1.p

Viterbi Path	
Viterbi Path	
Posterior @ 0.4	96 - KSKETGKILGFKKILKFCKKTKPRE - 120
Posterior @ 0.4	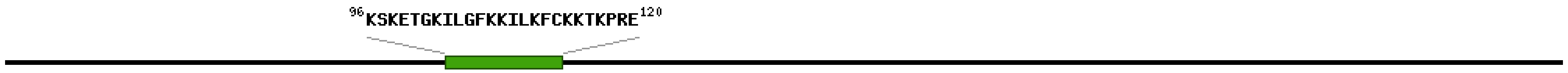
Posterior Graphic. Threshold Marked @ 0.4	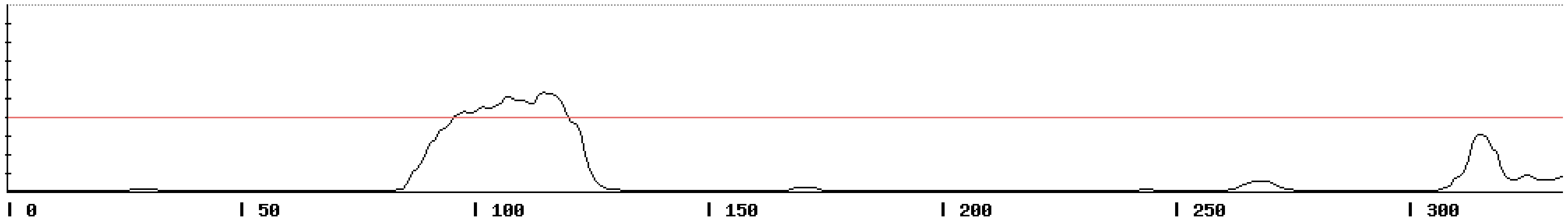

Predictions for Arabisalpina_KFK42489.1

Viterbi Path	
Viterbi Path	
Posterior @ 0.4	91 - KKTGKPTEIKRKSGNREK - 108
	286 - RKENKYNRGDRQIRKTKSGYWKKTGKPTEIKRKNGEKIGEK - 326
Posterior @ 0.4	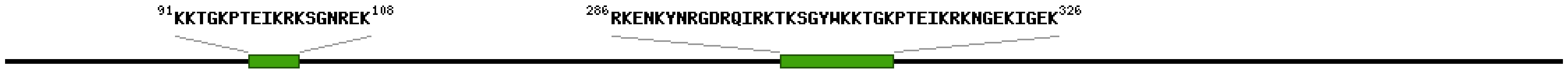
Posterior Graphic. Threshold Marked @ 0.4	

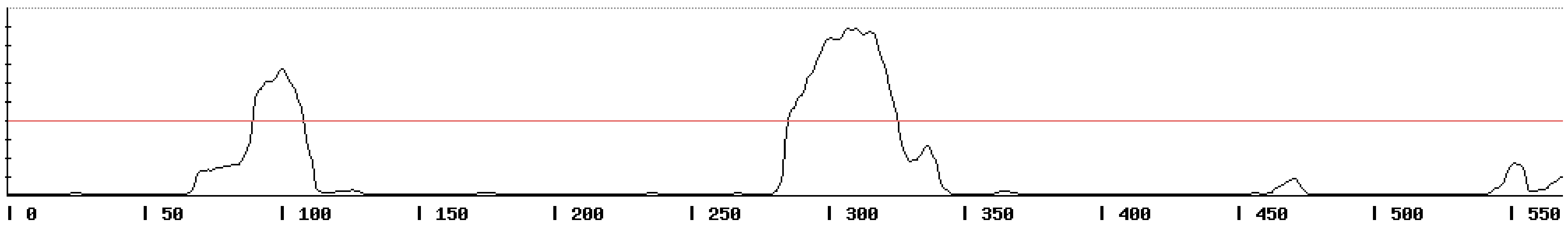

Predictions for Boecherastricta_Bostr.10040s0190.1.p

Viterbi Path

Viterbi Path

Posterior @ 0.4: 115 - KKKKPREYK - 123

Posterior @ 0.4

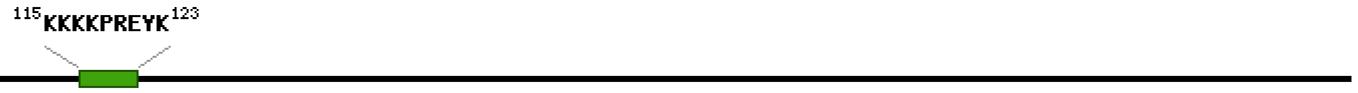

Posterior Graphic. Threshold Marked @ 0.4

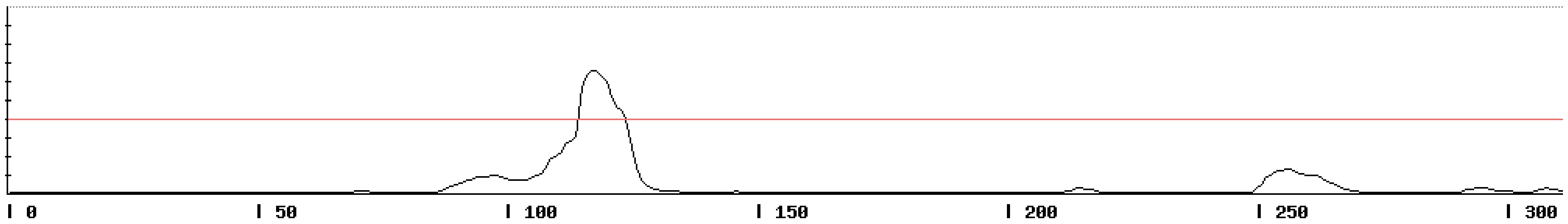

Predictions for Boecherastricta_Bostr.10040s0202.1.p

Viterbi Path

Viterbi Path

Posterior @ 0.4

75 - KKILK - 79

82 - EKRKPRE - 88

Posterior @ 0.4

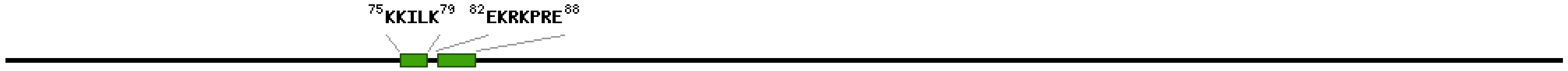

Posterior Graphic. Threshold Marked @ 0.4

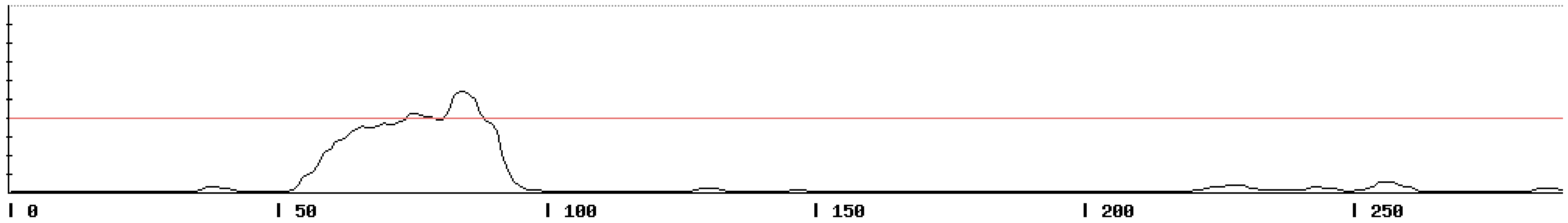

Predictions for Brachypodiumdistachyon_Bradi2g46427.1.p

Viterbi Path

Viterbi Path

Posterior @ 0.4

77 - RAKSRRGQRKS - 87

345 - RIRGQRR - 351

448 - RKRKASEENSGAESPARGTKKSTAPAASGRKK - 479

Posterior @ 0.4

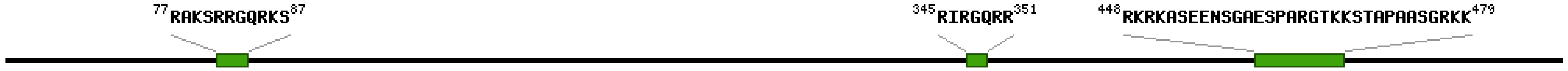

Posterior Graphic. Threshold Marked @ 0.4

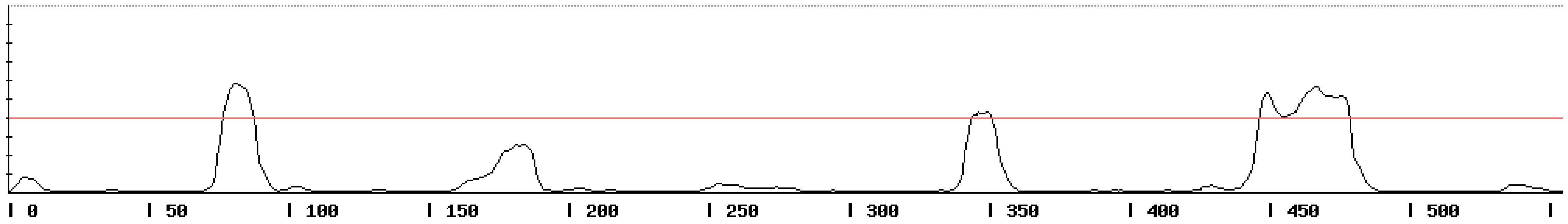

Predictions for Brachypodiumdistachyon_Bradi4g03620.1.p

Viterbi Path

Viterbi Path

Posterior @ 0.4

Posterior @ 0.4

Posterior Graphic. Threshold Marked @ 0.4

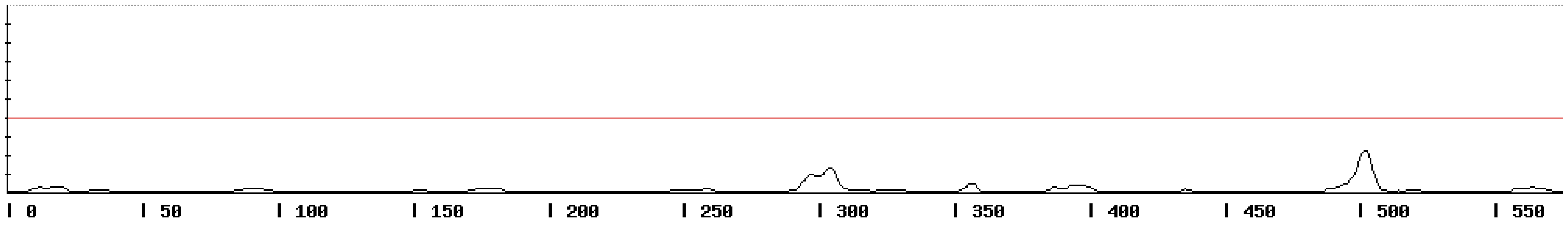

Predictions for Brachypodiumdistachyon_Bradi5g15230.1.p

Viterbi Path

Viterbi Path

Posterior @ 0.4

30 - KPRTRDKPPRTPPPR - 44

291 - RRKRAANG - 298

302 - ERPRR - 306

Posterior @ 0.4

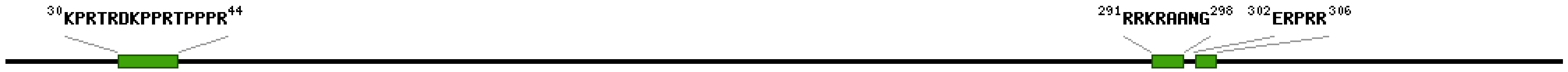

Posterior Graphic. Threshold Marked @ 0.4

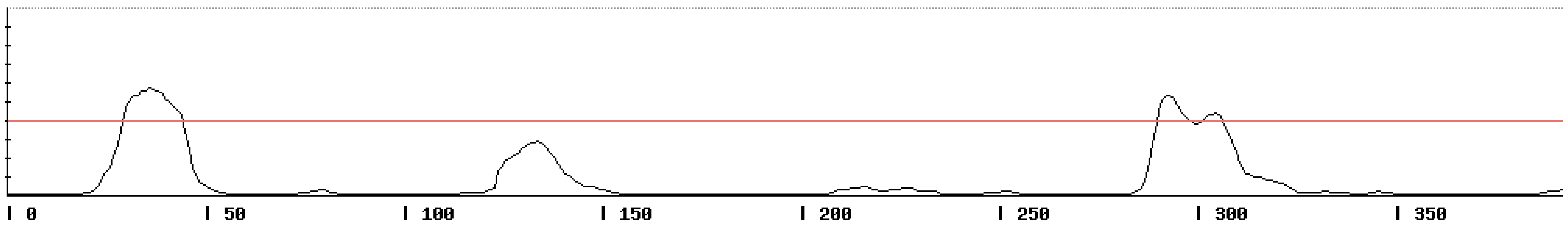

Predictions for Brachypodiumstacei_Brast09G117600.1.p

Viterbi Path: 221 - RRGGSRLPPRAARPPRPPKASKKPK - 245

Viterbi Path

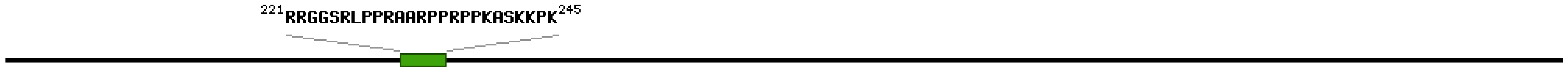

Posterior @ 0.4: 220 - LRRGGSRLPPRAARPPRPPKASKKPKVAPPPPAGPV - 255

Posterior @ 0.4

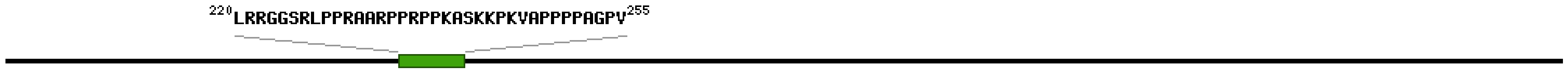

Posterior Graphic. Threshold Marked @ 0.4

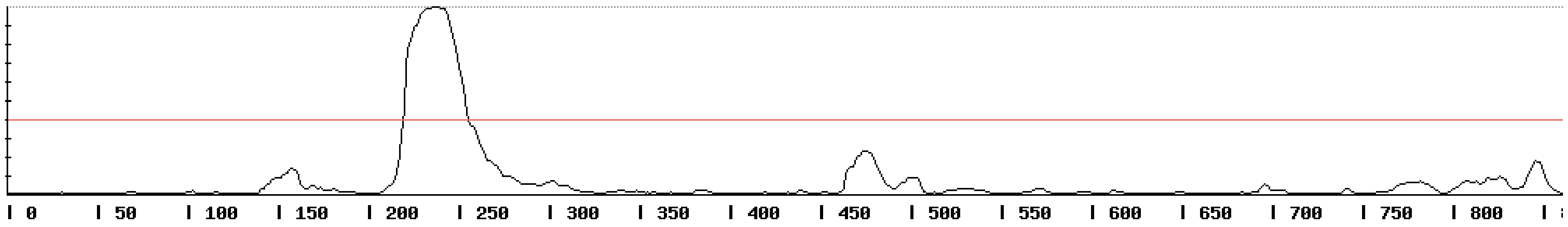

Predictions for Brachypodiumstacei_Brast09G138900.1.p

Viterbi Path

Viterbi Path

Posterior @ 0.4: 32 - PRGKPLRPRPR - 42

273 - ARRPKHKRAANGDEEERPRRA - 293

Posterior @ 0.4

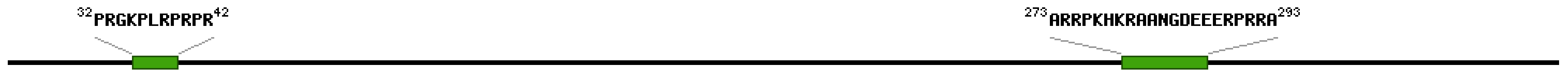

Posterior Graphic. Threshold Marked @ 0.4

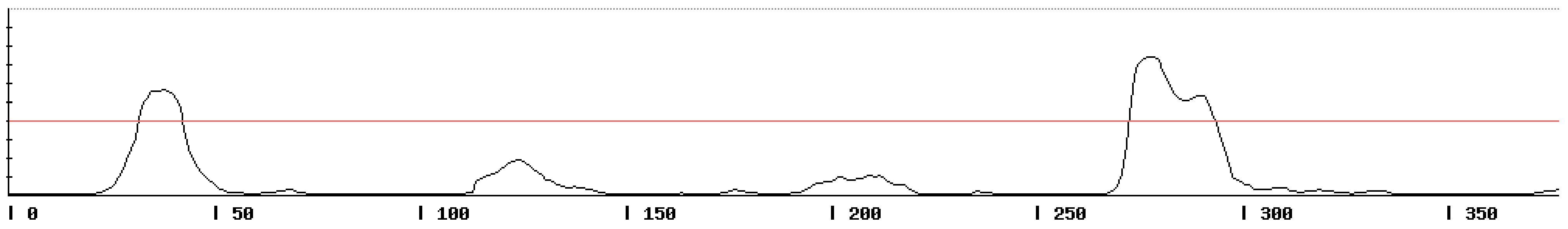

Predictions for Brassicanapus_GSBRNA2T00009829001

Viterbi Path

Viterbi Path

Posterior @ 0.4

Posterior @ 0.4

Posterior Graphic. Threshold Marked @ 0.4

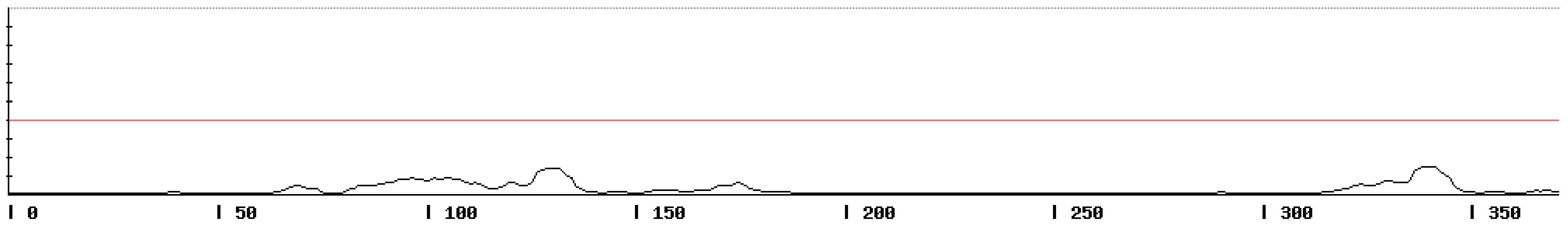

Predictions for Brassicanapus_GSBRNA2T00013424001

Viterbi Path

Viterbi Path

Posterior @ 0.4

Posterior @ 0.4

Posterior Graphic. Threshold Marked @ 0.4

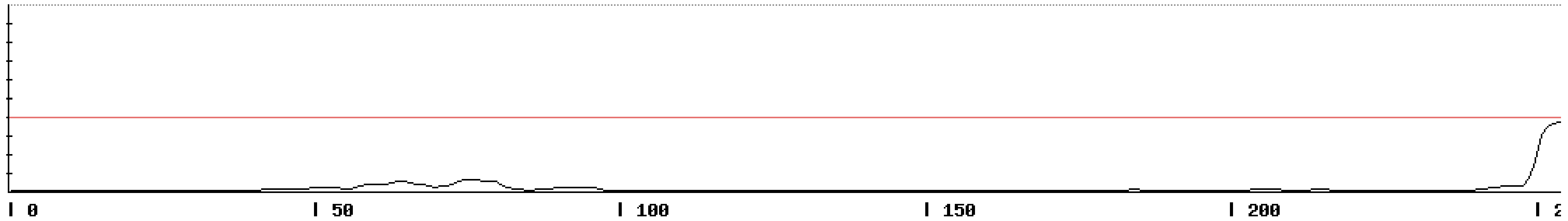

Predictions for Brassicanapus_GSBRNA2T00020965001

Viterbi Path

Viterbi Path

Posterior @ 0.4

Posterior @ 0.4

Posterior Graphic. Threshold Marked @ 0.4

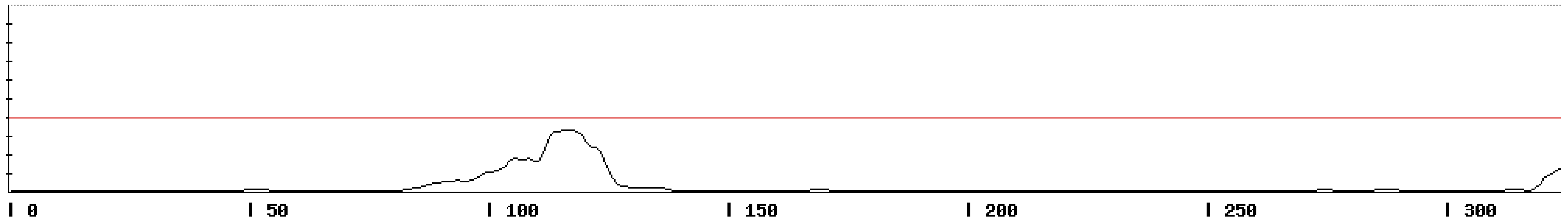

Predictions for Brassicanapus_GSBRNA2T00021663001

Viterbi Path

Viterbi Path

Posterior @ 0.4	233 - KKRLRRD - 239
Posterior @ 0.4	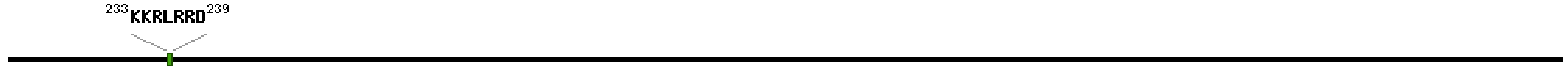
Posterior Graphic. Threshold Marked @ 0.4	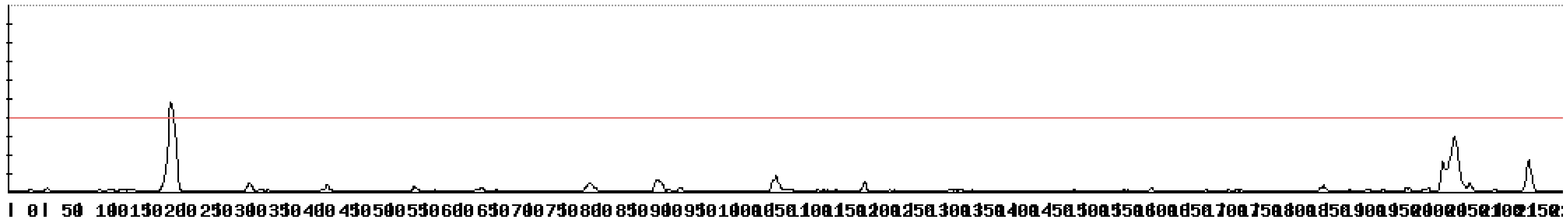

Predictions for Brassicanapus_GSBRNA2T00026805001

Viterbi Path	
Viterbi Path	
Posterior @ 0.4	
Posterior @ 0.4	
Posterior Graphic. Threshold Marked @ 0.4	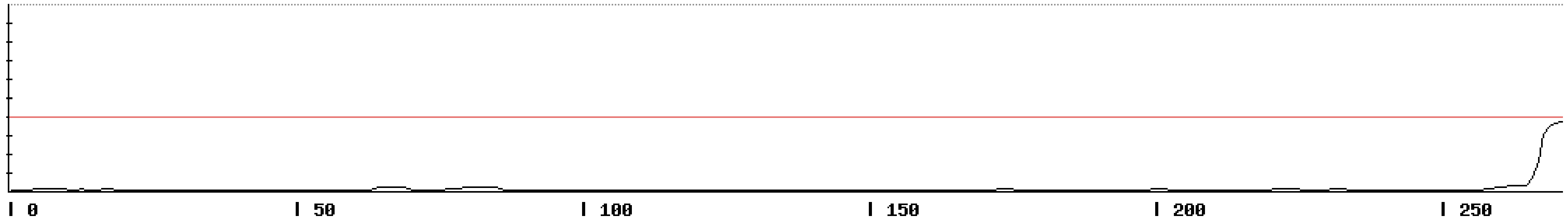

Predictions for Brassicanapus_GSBRNA2T00026833001

Viterbi Path	
Viterbi Path	
Posterior @ 0.4	
Posterior @ 0.4	
Posterior Graphic. Threshold Marked @ 0.4	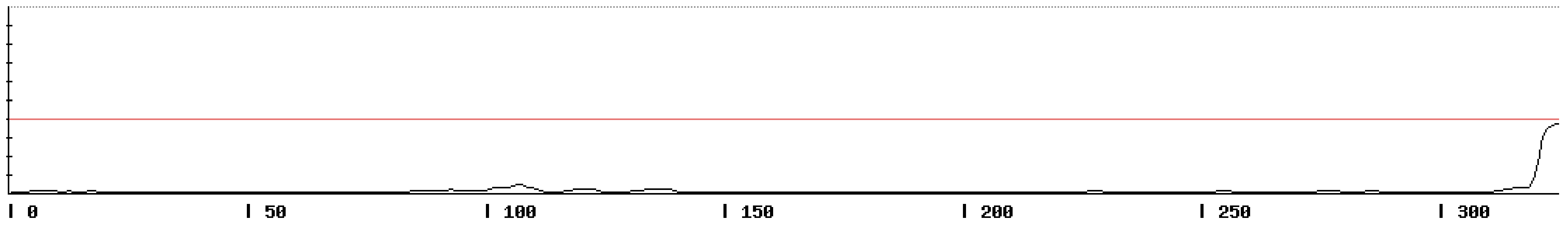

Predictions for Brassicanapus_GSBRNA2T00030633001

Viterbi Path	
Viterbi Path	
Posterior @ 0.4	
Posterior @ 0.4	
Posterior Graphic. Threshold Marked @ 0.4	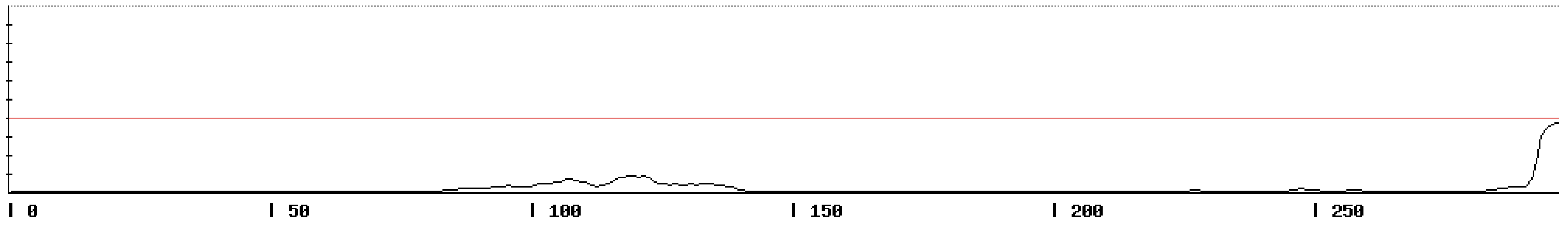

Predictions for Brassicanapus_GSBRNA2T00040675001

Viterbi Path	
Viterbi Path	
Posterior @ 0.4	
Posterior @ 0.4	
Posterior Graphic. Threshold Marked @ 0.4	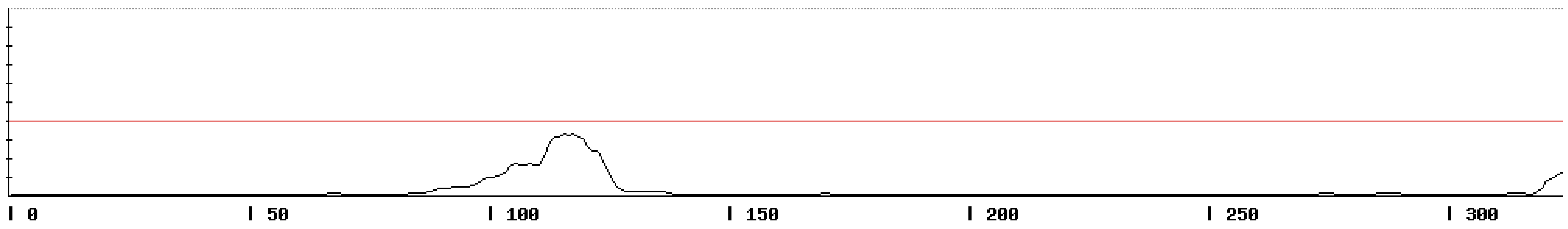

Predictions for Brassicanapus_GSBRNA2T00042092001

Viterbi Path	
Viterbi Path	
Posterior @ 0.4	
Posterior @ 0.4	
Posterior Graphic. Threshold Marked @ 0.4	

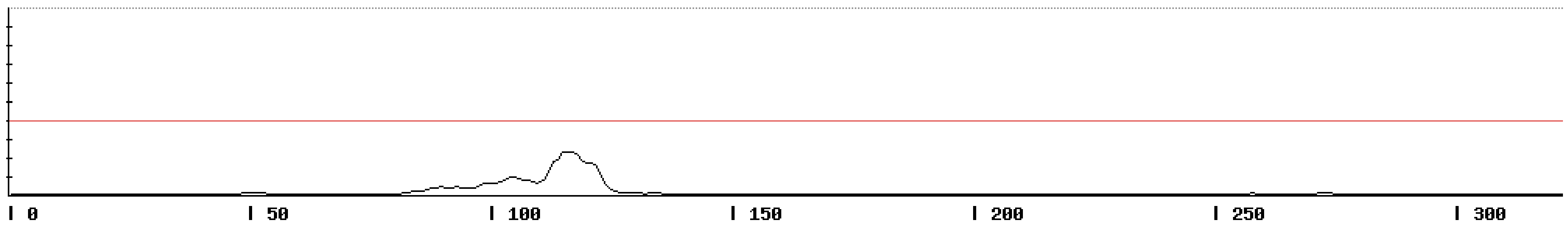

Predictions for Brassicanapus_GSBRNA2T00050859001

Viterbi Path

Viterbi Path

Posterior @ 0.4

Posterior @ 0.4

Posterior Graphic. Threshold Marked @ 0.4

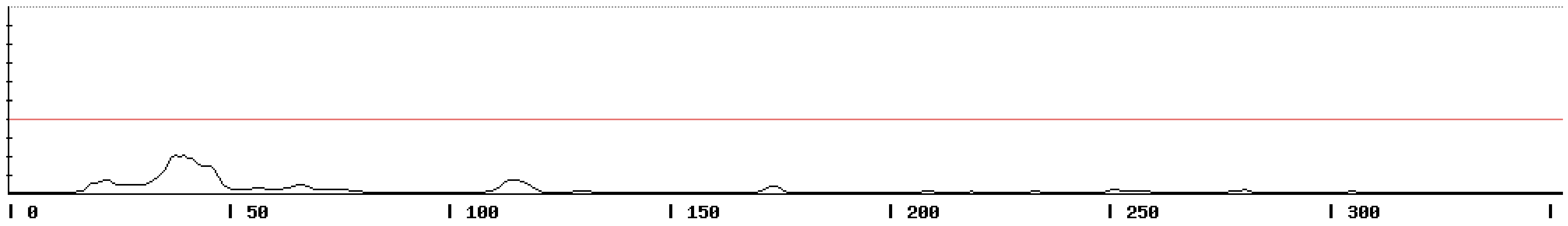

Predictions for Brassicanapus_GSBRNA2T00050907001

Viterbi Path

Viterbi Path

Posterior @ 0.4

Posterior @ 0.4

Posterior Graphic. Threshold Marked @ 0.4	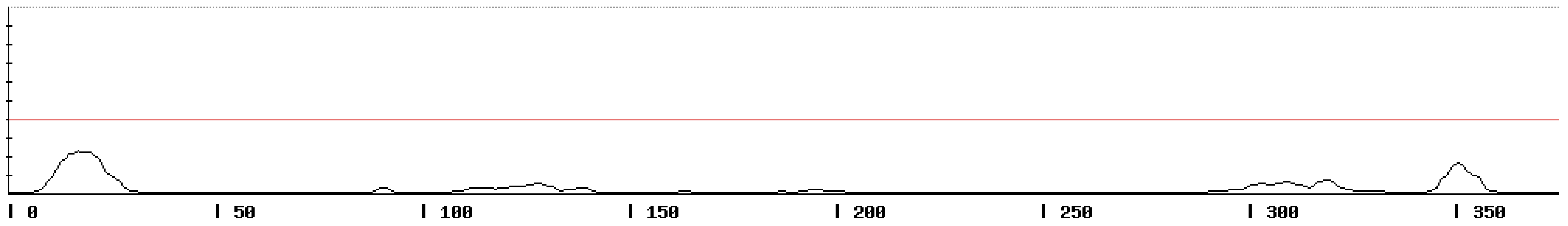

Predictions for Brassicanapus_GSBRNA2T00075489001

Viterbi Path	
Viterbi Path	
Posterior @ 0.4	305 - VKRPRGRPAGSKNKPKPP - 322
Posterior @ 0.4	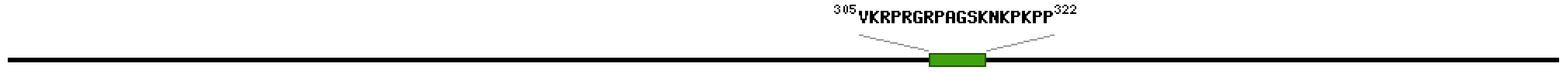
Posterior Graphic. Threshold Marked @ 0.4	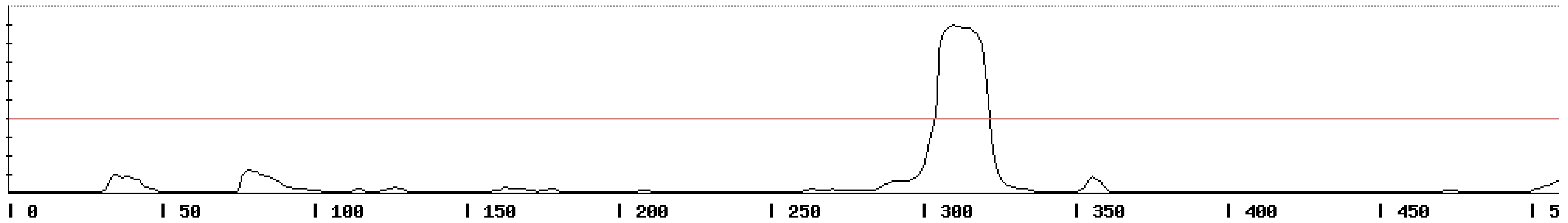

Predictions for Brassicanapus_GSBRNA2T00095486001

Viterbi Path	
Viterbi Path	
Posterior @ 0.4	113 - KKTKRGR - 119
Posterior @ 0.4	

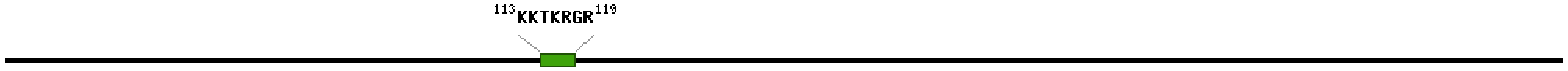

Posterior Graphic. Threshold Marked @ 0.4

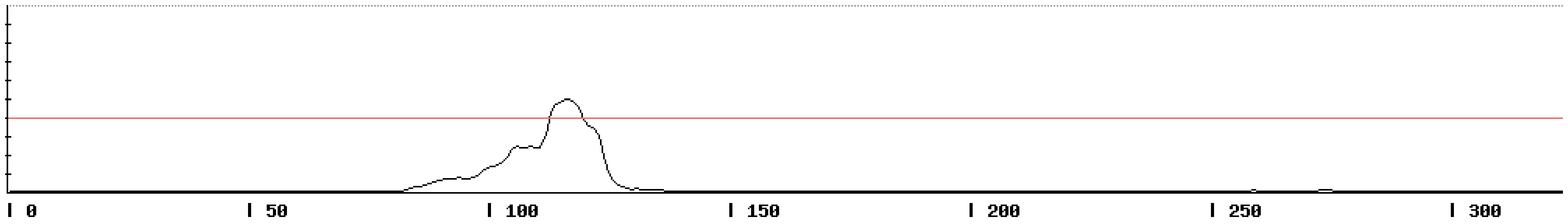

Predictions for Brassicanapus_GSBRNA2T00095503001

Viterbi Path

Viterbi Path

Posterior @ 0.4

Posterior @ 0.4

Posterior Graphic. Threshold Marked @ 0.4

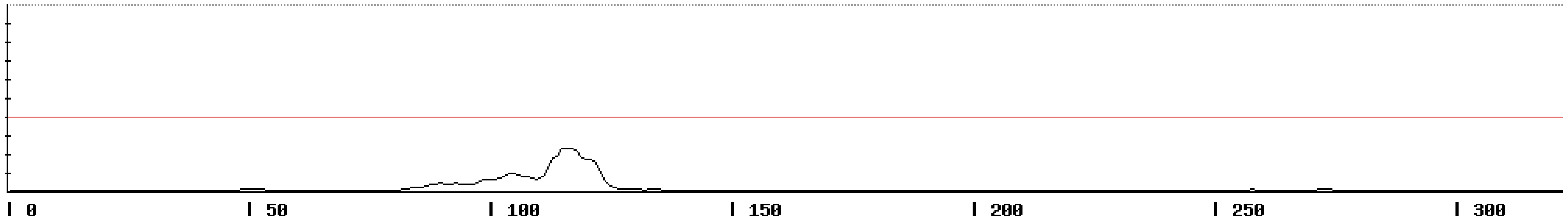

Predictions for Brassicanapus_GSBRNA2T00098300001

Viterbi Path

Viterbi Path

Posterior @ 0.4

Posterior @ 0.4

Posterior Graphic. Threshold Marked @ 0.4

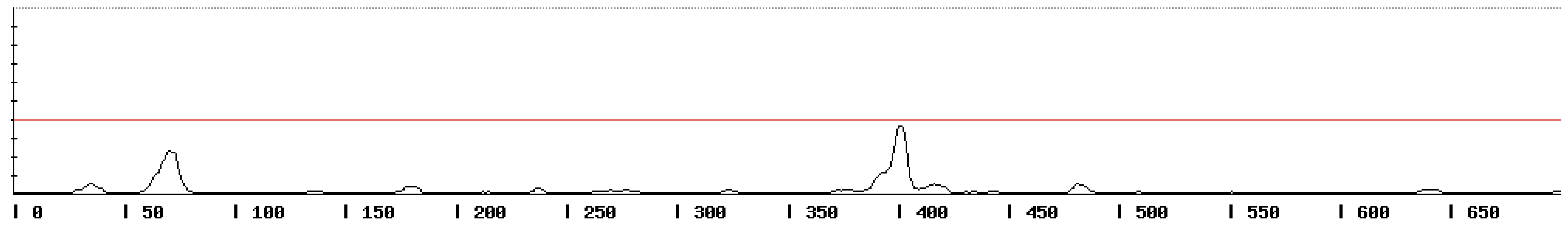

Predictions for Brassicanapus_GSBRNA2T00098817001

Viterbi Path

Viterbi Path

Posterior @ 0.4

Posterior @ 0.4

Posterior Graphic. Threshold Marked @ 0.4

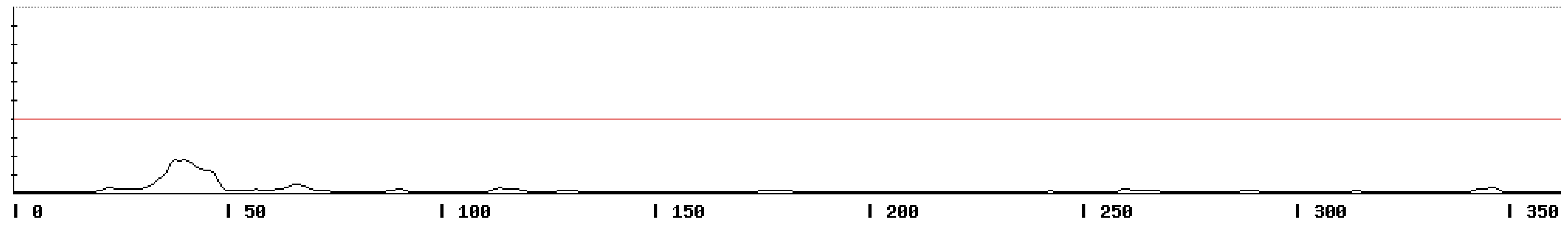

Predictions for Brassicanapus_GSBRNA2T00139105001

Viterbi Path

Viterbi Path

Posterior @ 0.4	305 - VKRPRGRPAGSKNKPKPP - 322
Posterior @ 0.4	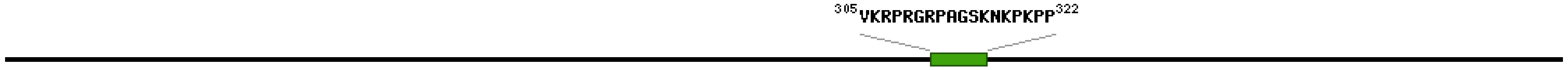
Posterior Graphic. Threshold Marked @ 0.4	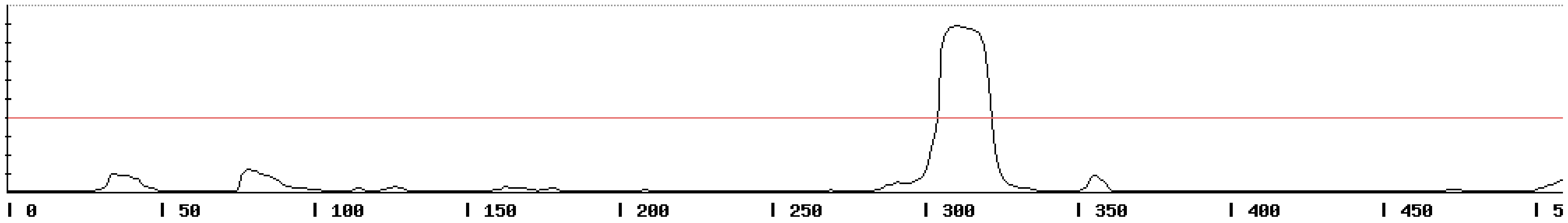

Predictions for Brassicanapus_GSBRNA2T00155943001

Viterbi Path	
Viterbi Path	
Posterior @ 0.4	335 - KKREKKK - 341
Posterior @ 0.4	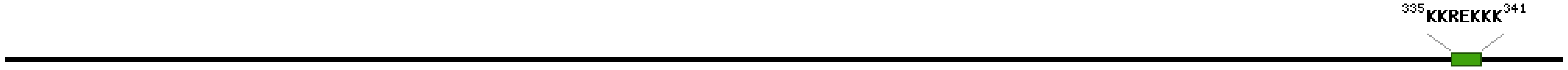
Posterior Graphic. Threshold Marked @ 0.4	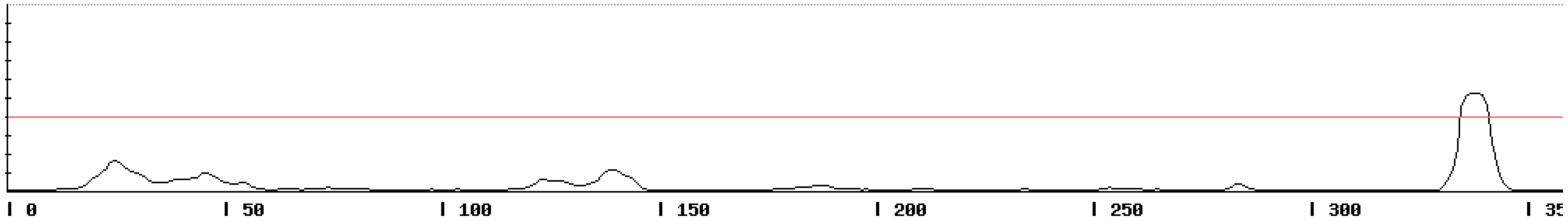

Predictions for Brassicaoleracea_XP_013589340.1

Viterbi Path

Viterbi Path

Posterior @ 0.4

Posterior @ 0.4

Posterior Graphic. Threshold Marked @ 0.4

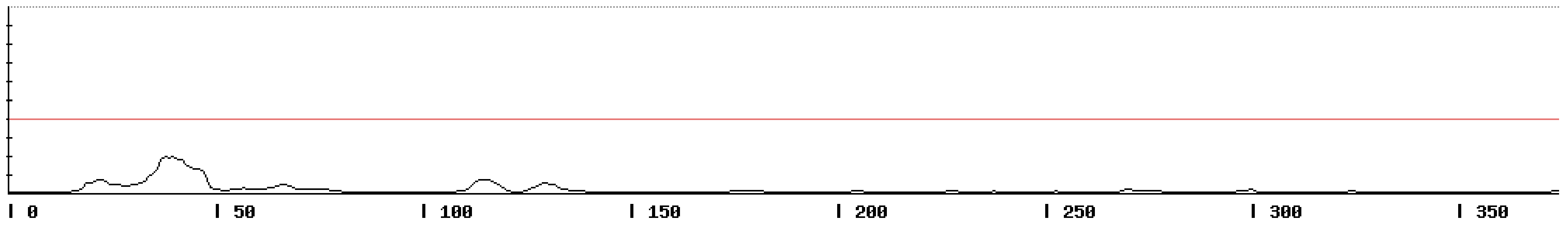

Predictions for Brassicaoleracea_XP_013594418.1

Viterbi Path

Viterbi Path

Posterior @ 0.4

Posterior @ 0.4

Posterior Graphic. Threshold Marked @ 0.4

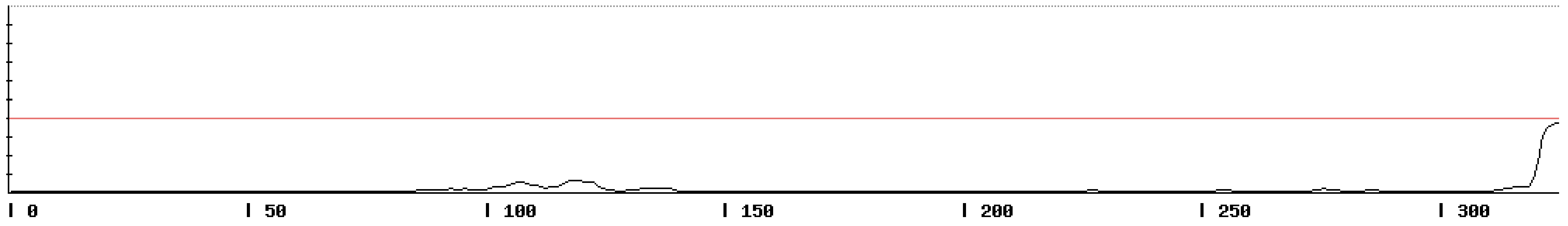

Predictions for Brassicaoleracea_XP_013601975.1	
Viterbi Path	
Viterbi Path	
Posterior @ 0.4	337 - KKREKKK - 343
Posterior @ 0.4	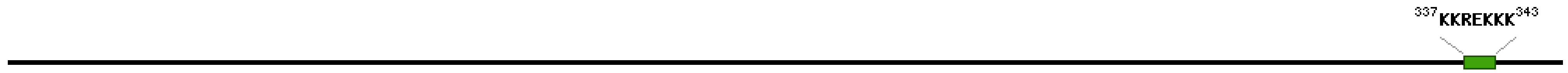
Posterior Graphic. Threshold Marked @ 0.4	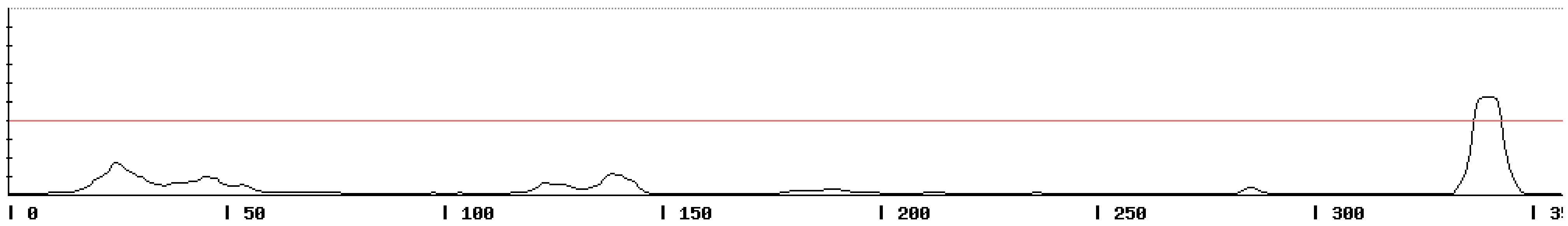

Predictions for Brassicaoleracea_XP_013608221.1	
Viterbi Path	
Viterbi Path	
Posterior @ 0.4	
Posterior @ 0.4	
Posterior Graphic. Threshold Marked @ 0.4	

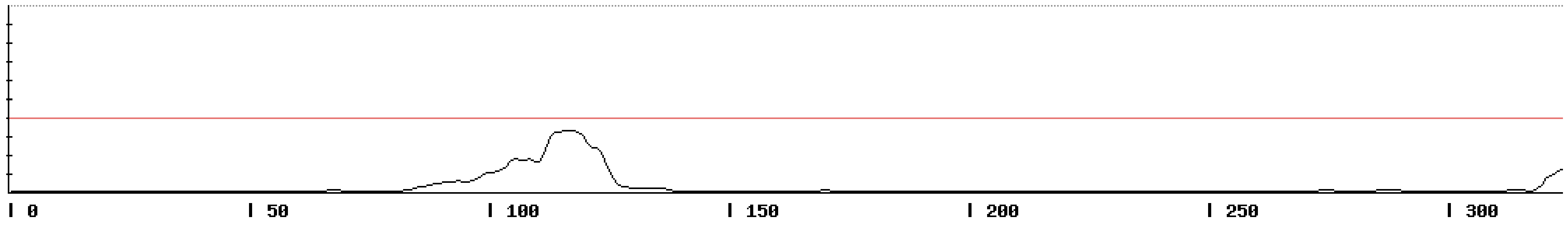

Predictions for Brassicaoleracea_XP_013610671.1	
Viterbi Path	
Viterbi Path	
Posterior @ 0.4	114 - KKTKRAR - 120
Posterior @ 0.4	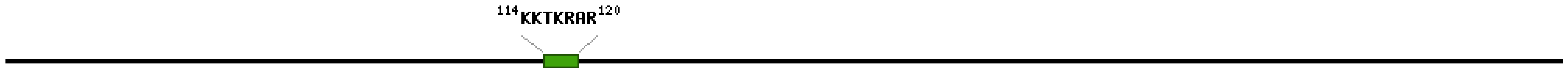
Posterior Graphic. Threshold Marked @ 0.4	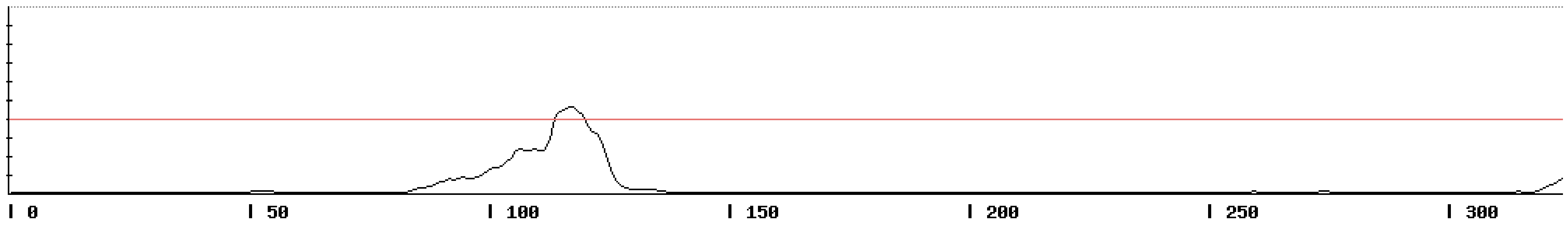

Predictions for Brassicaoleracea_XP_013632799.1	
Viterbi Path	
Viterbi Path	
Posterior @ 0.4	
Posterior @ 0.4	

Posterior Graphic. Threshold Marked @ 0.4

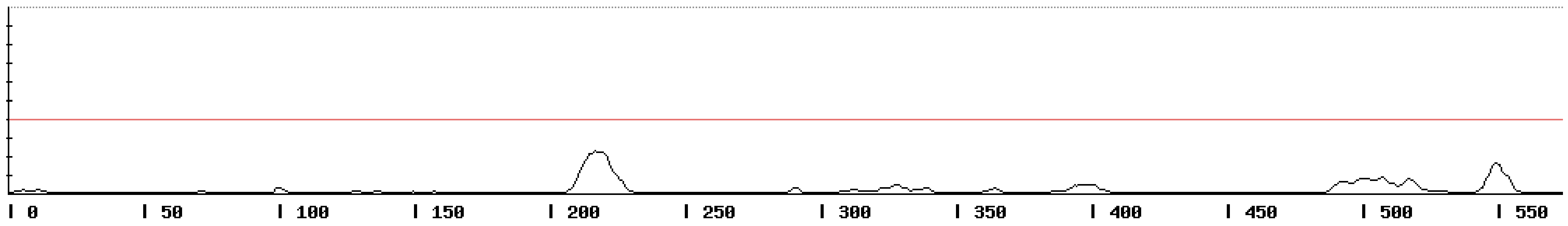

Predictions for Brassicarapa_XP_009104009.1

Viterbi Path

Viterbi Path

Posterior @ 0.4

Posterior @ 0.4

Posterior Graphic. Threshold Marked @ 0.4

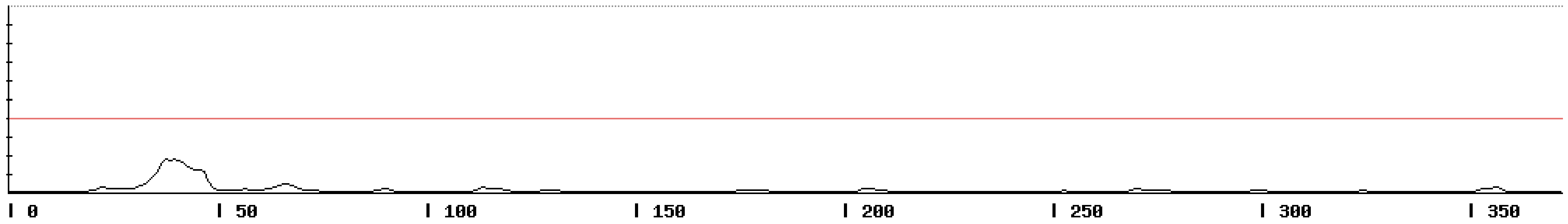

Predictions for Camelinasativa_Csa01g011380.1

Viterbi Path

Viterbi Path

Posterior @ 0.4

Posterior @ 0.4

Posterior Graphic. Threshold Marked @ 0.4

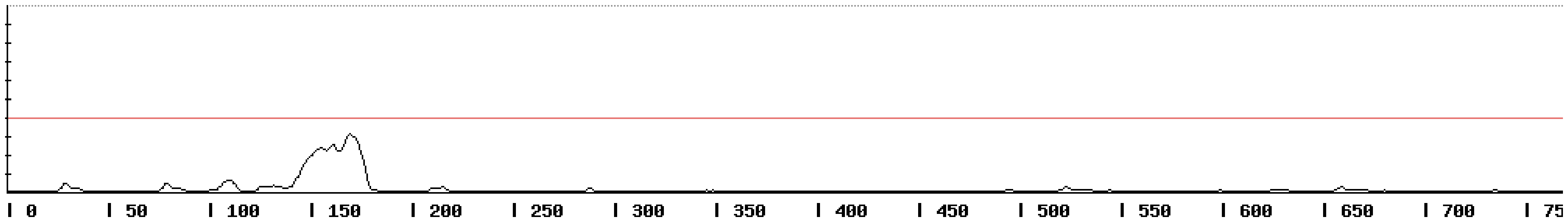

Predictions for Camelinasativa_Csa03g002220.1

Viterbi Path

Viterbi Path

Posterior @ 0.4

Posterior @ 0.4

Posterior Graphic. Threshold Marked @ 0.4

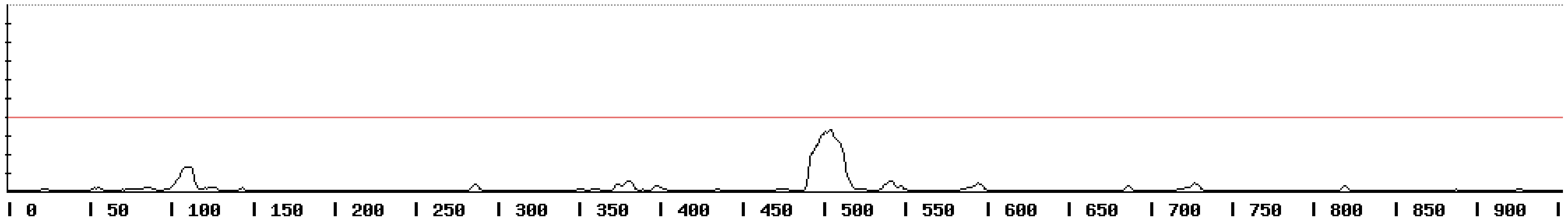

Predictions for Camelinasativa_Csa03g037070.1

Viterbi Path

Viterbi Path

Posterior @ 0.4

Posterior @ 0.4

Posterior Graphic. Threshold Marked @ 0.4

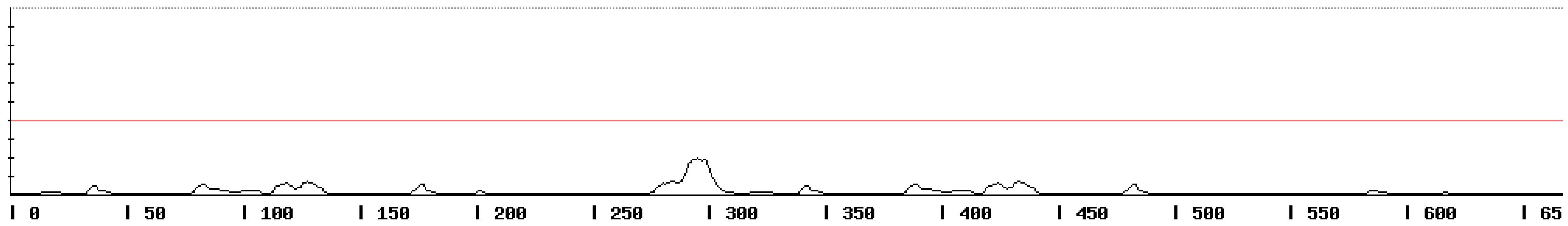

Predictions for Camelinasativa_Csa04g065900.1

Viterbi Path

Viterbi Path

Posterior @ 0.4

Posterior @ 0.4

Posterior Graphic. Threshold Marked @ 0.4

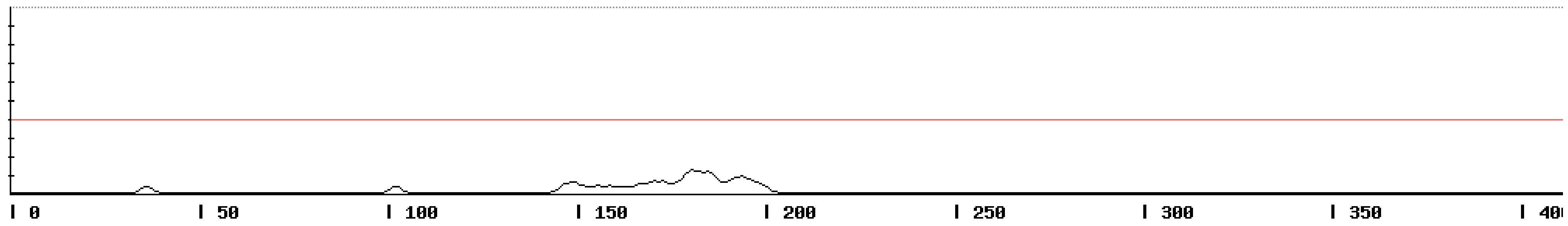

Predictions for Camelinasativa_Csa05g047220.1

Viterbi Path

Viterbi Path

Posterior @ 0.4

Posterior @ 0.4

Posterior Graphic. Threshold Marked @ 0.4

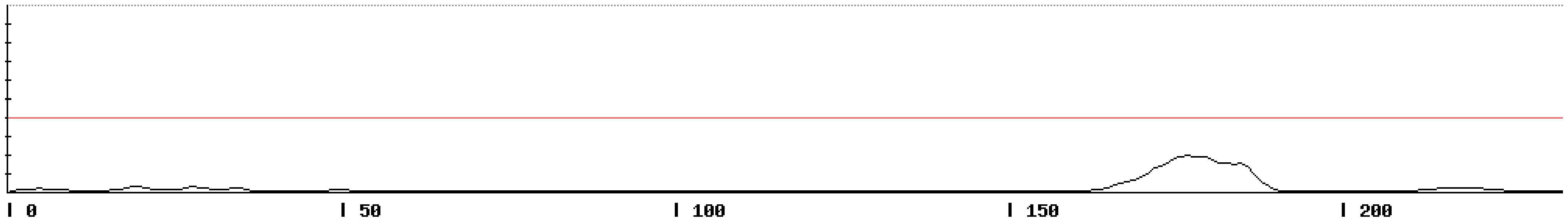

Predictions for Camelinasativa_Csa05g047230.1

Viterbi Path

Viterbi Path

Posterior @ 0.4 — 128 - KRKPR - 132

Posterior @ 0.4

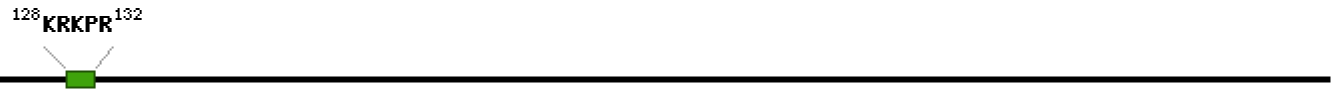

Posterior Graphic. Threshold Marked @ 0.4

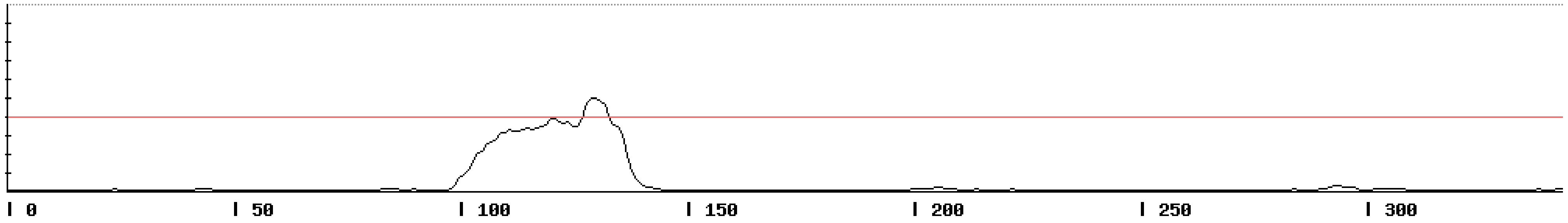

Predictions for Camelinasativa_Csa06g007190.1

Viterbi Path

Viterbi Path

Posterior @ 0.4

Posterior @ 0.4

Posterior Graphic. Threshold Marked @ 0.4

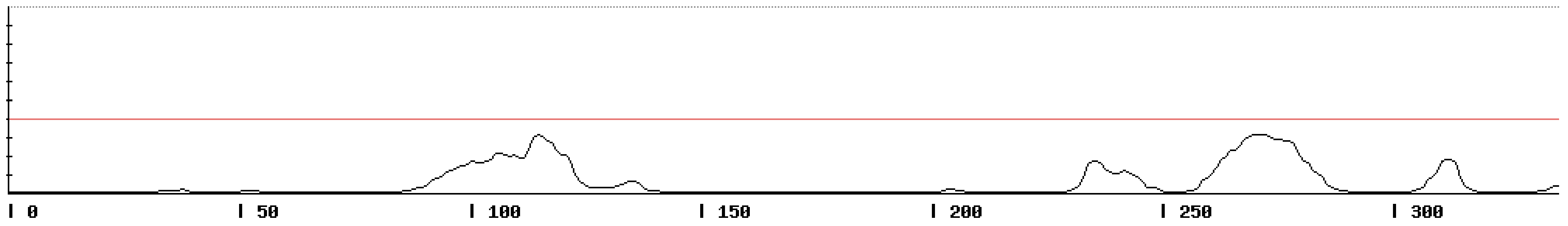

Predictions for Camelinasativa_Csa08g006010.1

Viterbi Path

Viterbi Path

Posterior @ 0.4

98 - RKGSKARKQRKSR - 110

284 - RKGSKARKQRKSR - 296

Posterior @ 0.4

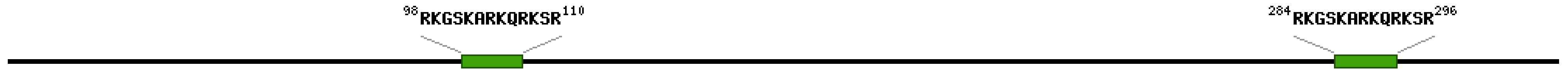

Posterior Graphic. Threshold Marked @ 0.4

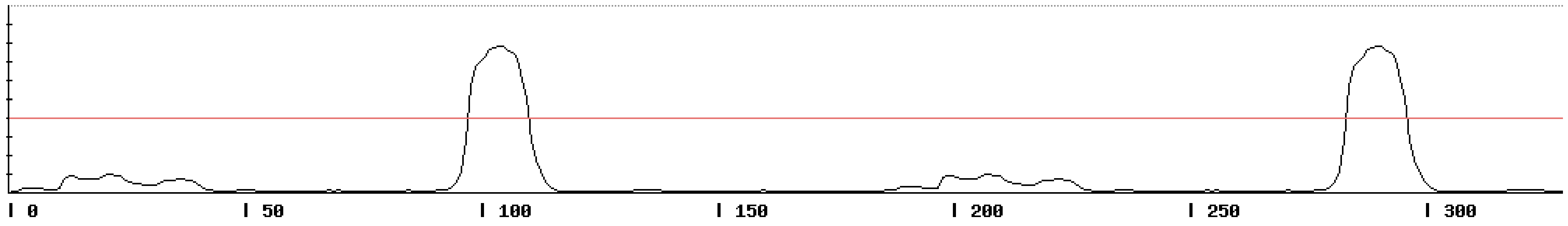

Predictions for Camelinasativa_Csa08g060070.1

Viterbi Path

Viterbi Path

Posterior @ 0.4

Posterior @ 0.4

Posterior Graphic. Threshold Marked @ 0.4

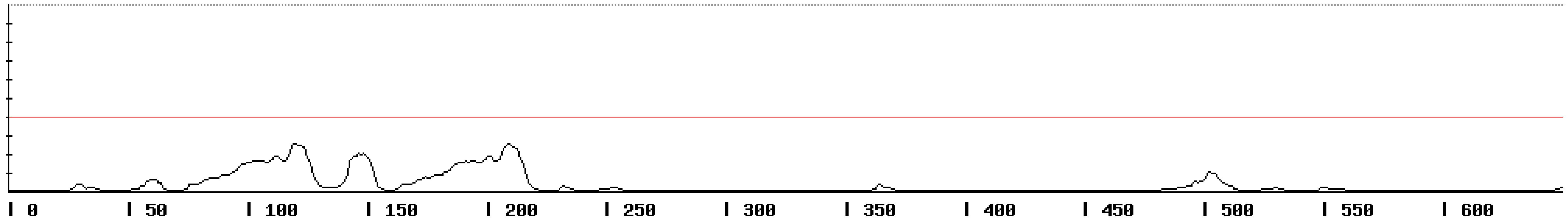

Predictions for Camelinasativa_Csa09g013080.1

Viterbi Path

Viterbi Path

Posterior @ 0.4

Posterior @ 0.4

Posterior Graphic. Threshold Marked @ 0.4

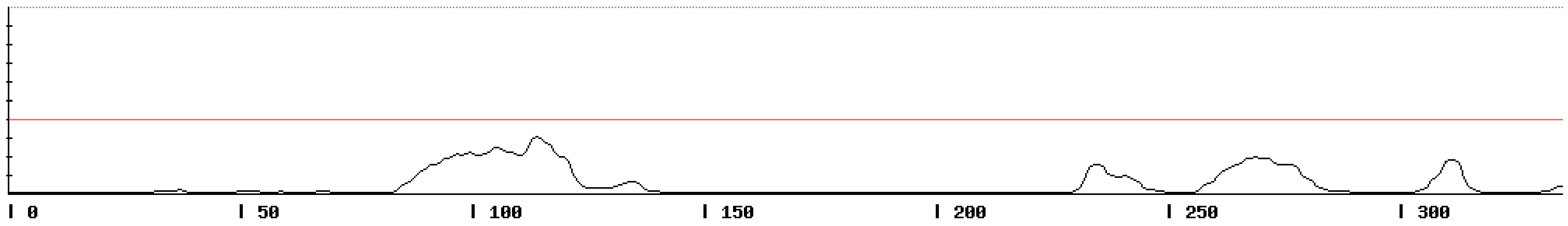

Predictions for Camelinasativa_Csa09g081160.1

Viterbi Path

Viterbi Path

Posterior @ 0.4

Posterior @ 0.4

Posterior Graphic. Threshold Marked @ 0.4

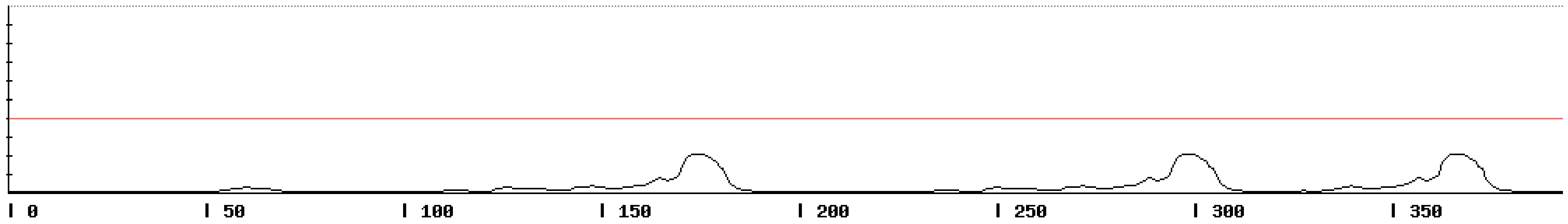

Predictions for Camelinasativa_Csa14g002180.1

Viterbi Path

Viterbi Path

Posterior @ 0.4

Posterior @ 0.4

Posterior Graphic. Threshold Marked @ 0.4

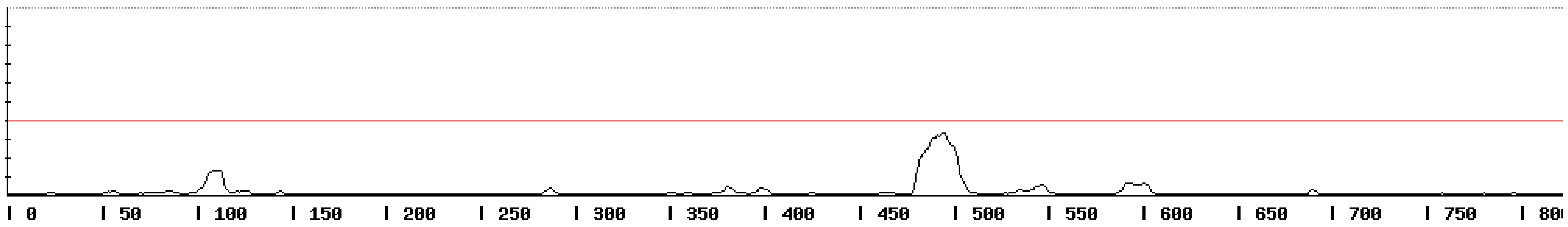

Predictions for Camelinasativa_Csa14g002590.1

Viterbi Path

Viterbi Path

Posterior @ 0.4

66 - TLKARKTPNRKV - 77

579 - RRKRRK - 584

Posterior @ 0.4

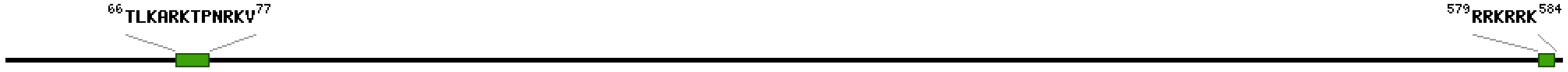

Posterior Graphic. Threshold Marked @ 0.4

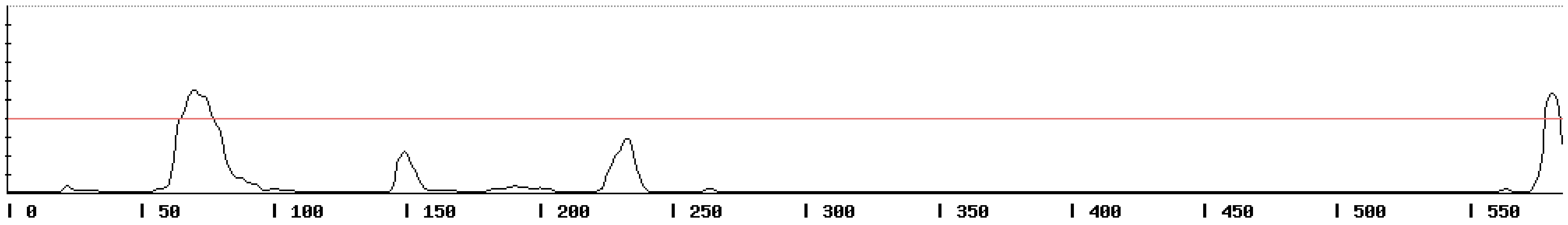

Predictions for Camelinasativa_Csa14g044030.1

Viterbi Path

Viterbi Path

Posterior @ 0.4

Posterior @ 0.4

Posterior Graphic. Threshold Marked @ 0.4

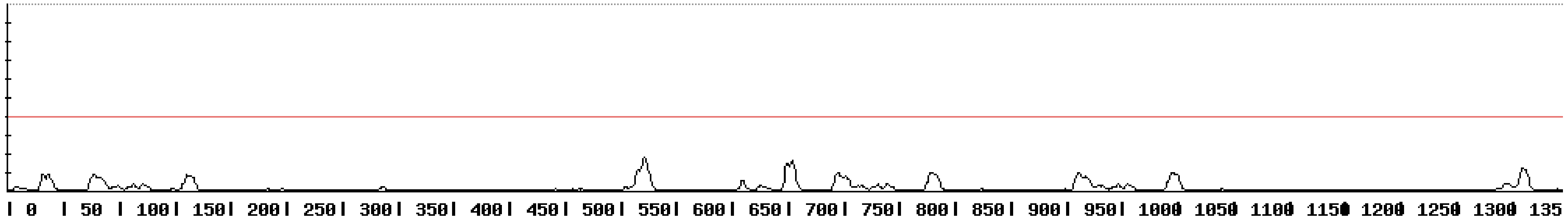

Predictions for Camelinasativa_Csa15g003940.1

Viterbi Path

Viterbi Path

Posterior @ 0.4

Posterior @ 0.4

Posterior Graphic. Threshold Marked @ 0.4

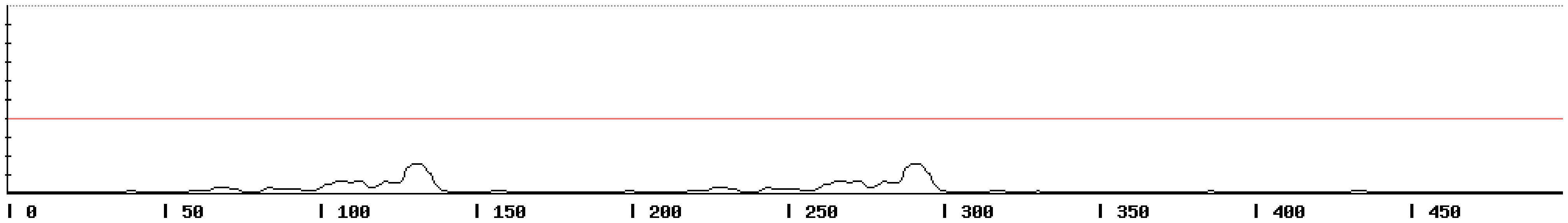

Predictions for Camelinasativa_Csa16g052260.1

Viterbi Path	
Viterbi Path	
Posterior @ 0.4	568 - KRKPR - 572
Posterior @ 0.4	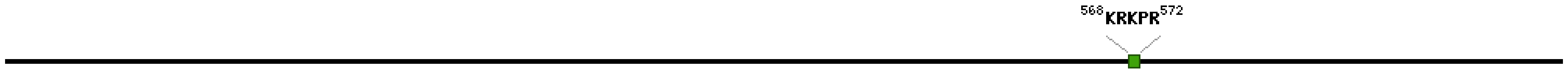
Posterior Graphic. Threshold Marked @ 0.4	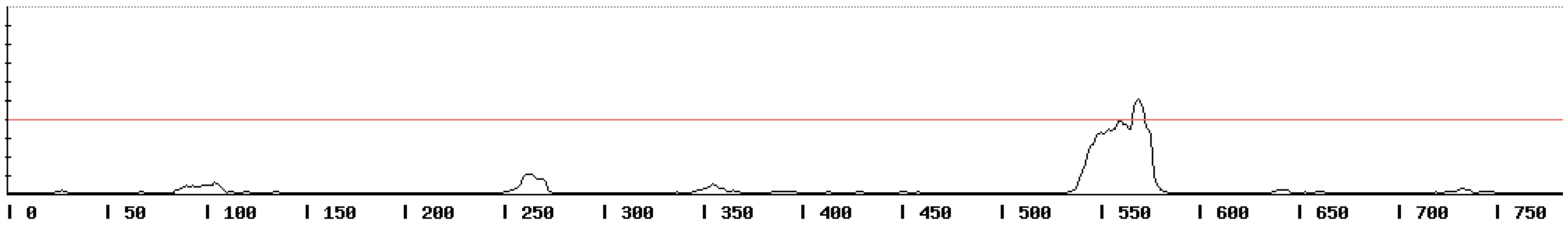

Predictions for Camelinasativa_Csa17g001330.1

Viterbi Path	
Viterbi Path	
Posterior @ 0.4	66 - TLKARKTPNRKV - 77
	684 - RRKRRK - 689
Posterior @ 0.4	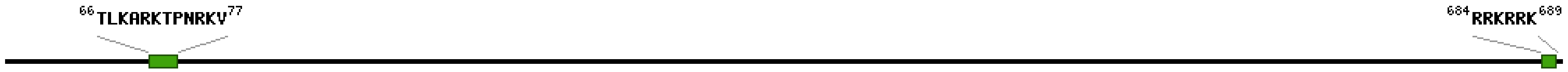
Posterior Graphic. Threshold Marked @ 0.4	

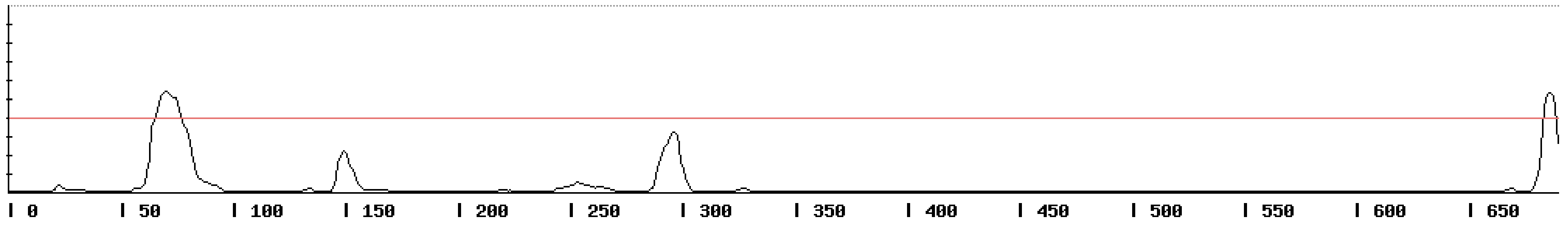

Predictions for Camelinasativa_Csa17g001480.1

Viterbi Path	
Viterbi Path	
Posterior @ 0.4	1021 - RLKKSKERSKKK - 1032
Posterior @ 0.4	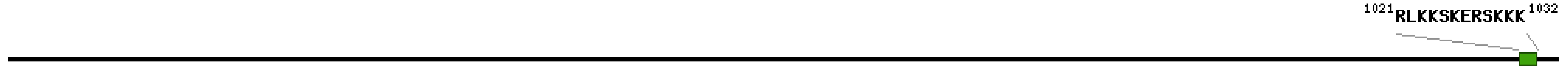
Posterior Graphic. Threshold Marked @ 0.4	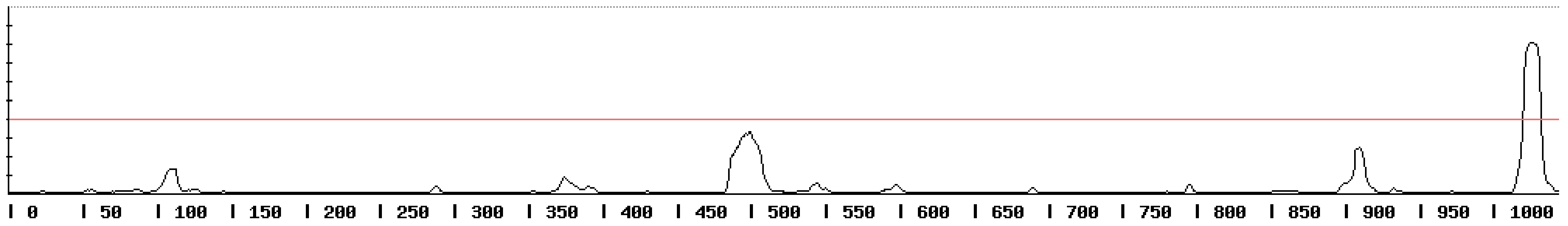

Predictions for Camelinasativa_Csa17g097090.1

Viterbi Path	
Viterbi Path	
Posterior @ 0.4	
Posterior @ 0.4	

Posterior Graphic. Threshold Marked @ 0.4

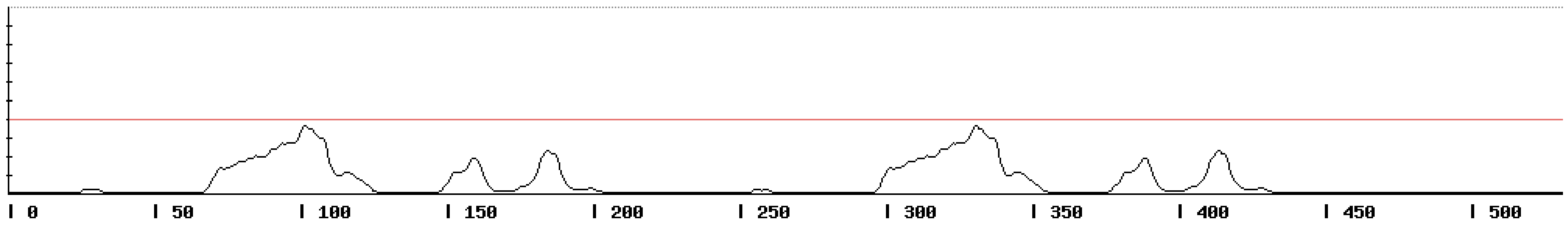

Predictions for Camelinasativa_Csa20g023680.1

Viterbi Path

Viterbi Path

Posterior @ 0.4

Posterior @ 0.4

Posterior Graphic. Threshold Marked @ 0.4

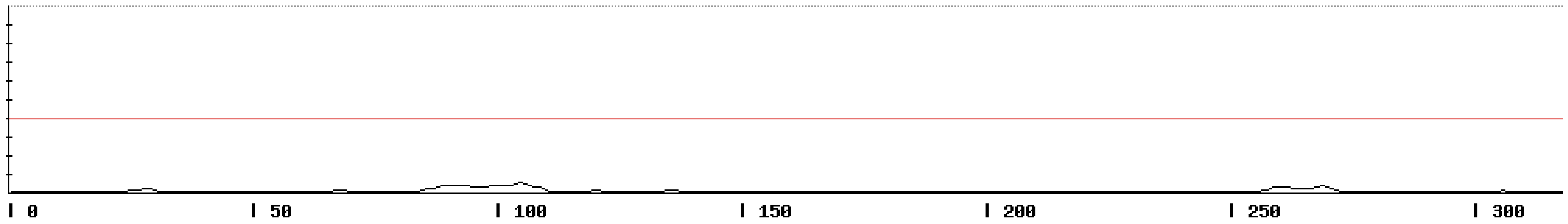

Predictions for Capsellarubella_Carubv10008035m

Viterbi Path

Viterbi Path

Posterior @ 0.4

Posterior @ 0.4

Posterior Graphic. Threshold Marked @ 0.4

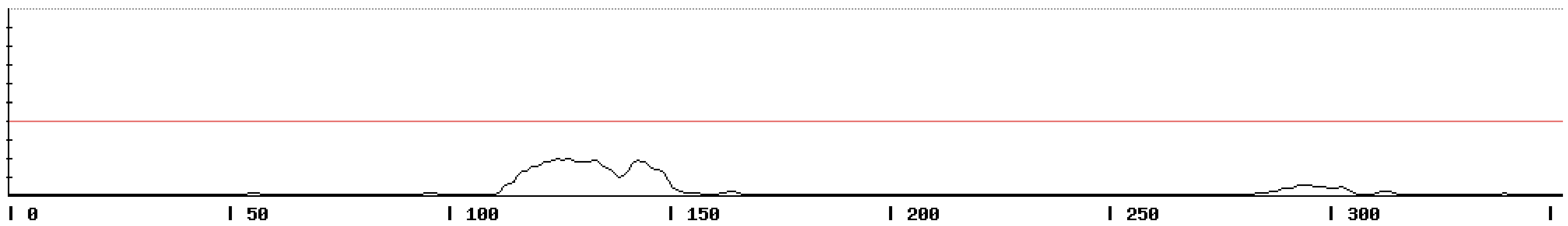

Predictions for Capsellarubella_Carubv10008036m

Viterbi Path

Viterbi Path

Posterior @ 0.4

Posterior @ 0.4

Posterior Graphic. Threshold Marked @ 0.4

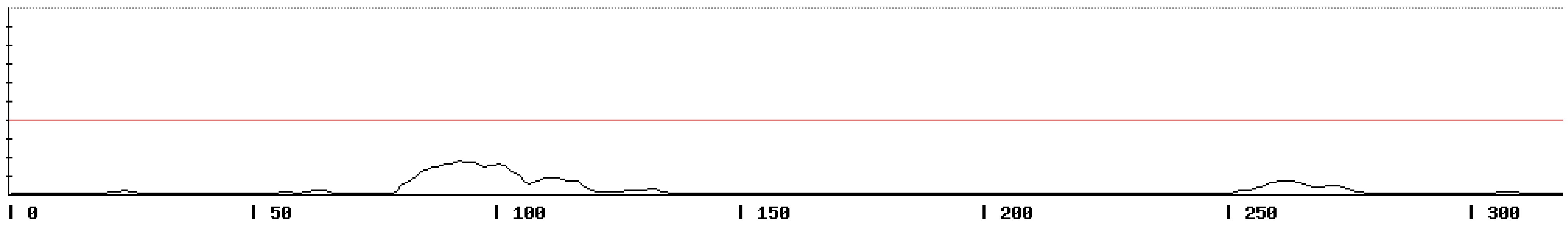

Predictions for Capsellarubella_Carubv10021319m

Viterbi Path

Viterbi Path

Posterior @ 0.4	115 - RKP - 117
Posterior @ 0.4	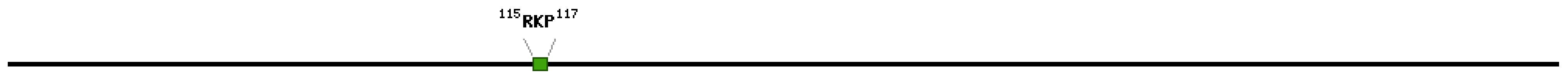
Posterior Graphic. Threshold Marked @ 0.4	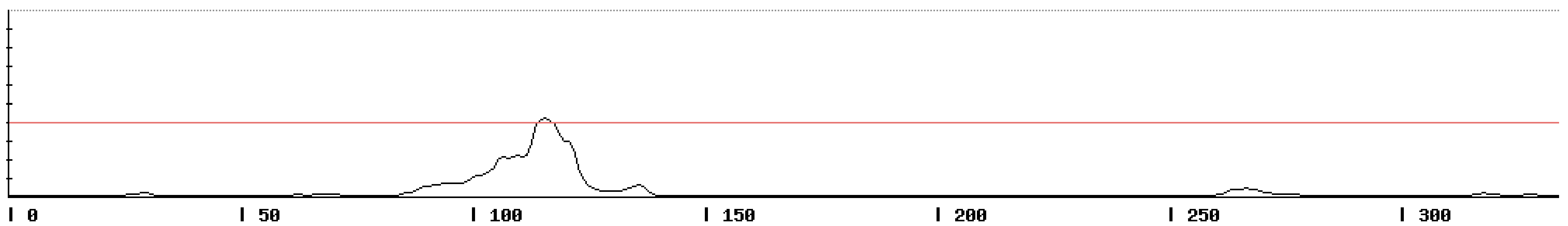

Predictions for Capsellarubella_Carubv10021818m

Viterbi Path	
Viterbi Path	
Posterior @ 0.4	
Posterior @ 0.4	
Posterior Graphic. Threshold Marked @ 0.4	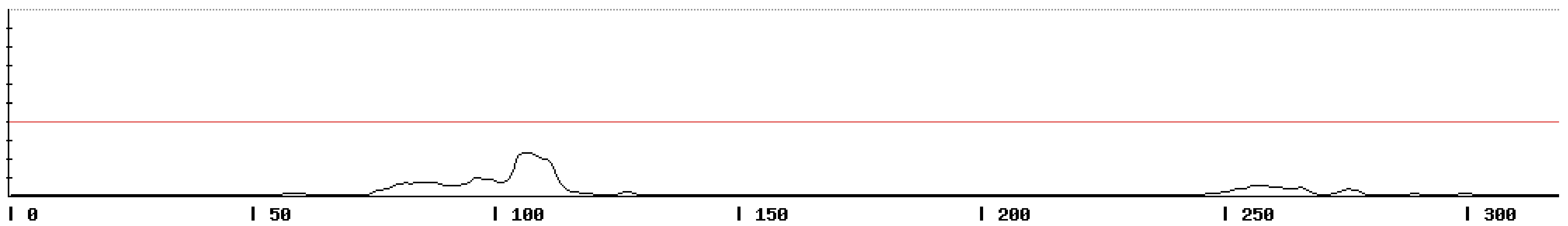

Predictions for Capsellarubella_Carubv10022099m

Viterbi Path	
Viterbi Path	

Posterior @ 0.4

Posterior @ 0.4

Posterior Graphic. Threshold Marked @ 0.4

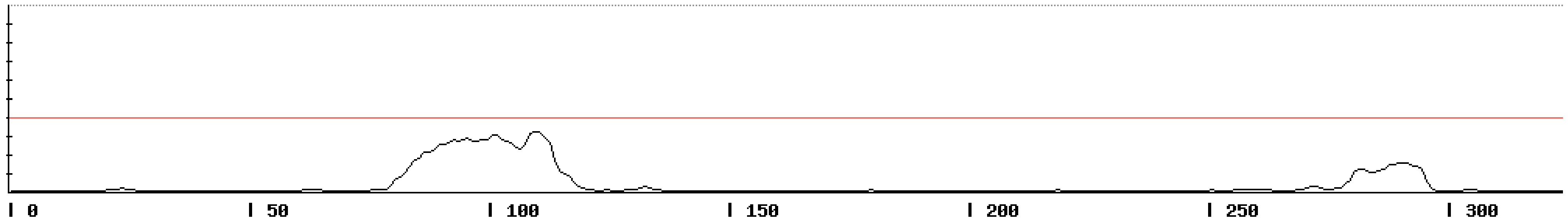

Predictions for Castaneamollissima_scaffold04550-snap-gene-0.24

Viterbi Path

Viterbi Path

Posterior @ 0.4 431 - KKIK - 434

Posterior @ 0.4

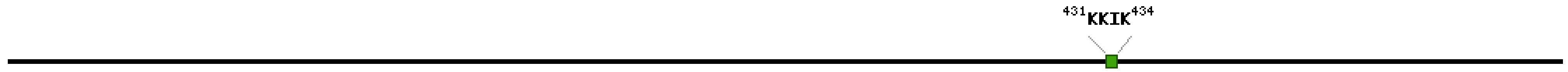

Posterior Graphic. Threshold Marked @ 0.4

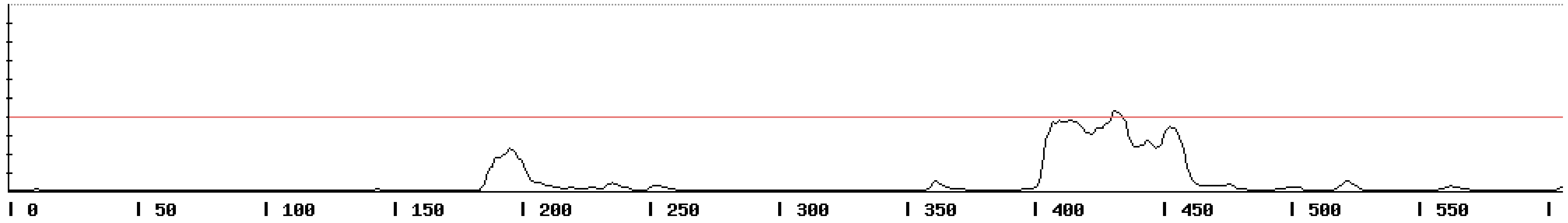

Predictions for Castaneamollissima_scaffold04583-augustus-gene-0.13

Viterbi Path

Viterbi Path

Posterior @ 0.4

Posterior @ 0.4

Posterior Graphic. Threshold Marked @ 0.4

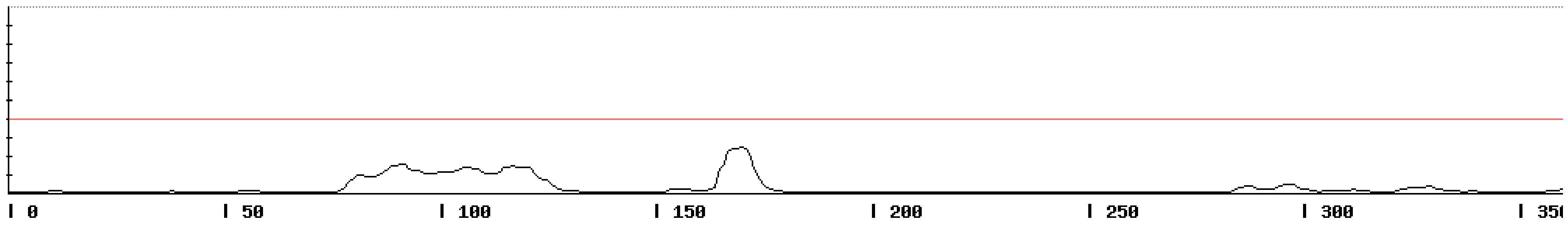

Predictions for Castaneamollissima_scaffold07067-augustus-gene-0.6

Viterbi Path

Viterbi Path

Posterior @ 0.4

104 - PKKRKRDQKQERVSRKTK - 121

618 - PKKRKRGQK - 626

Posterior @ 0.4

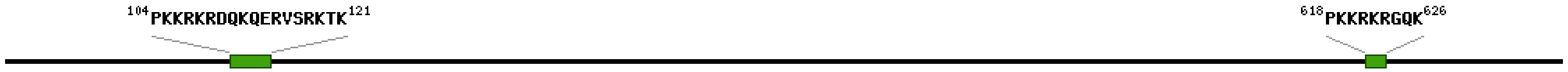

Posterior Graphic. Threshold Marked @ 0.4

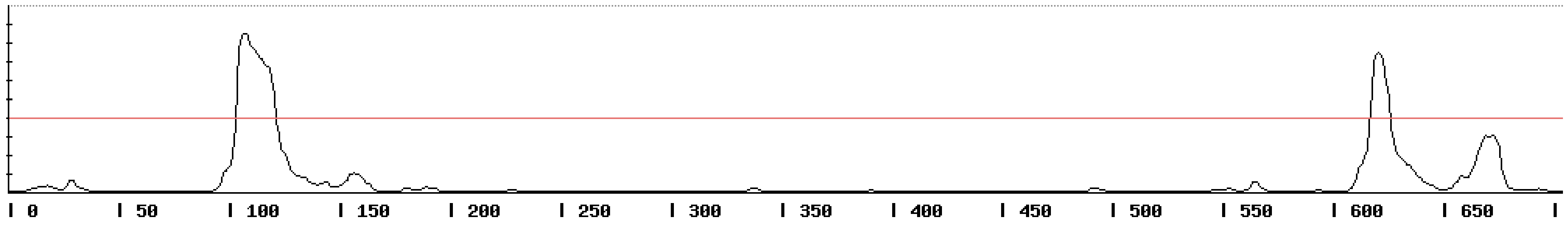

Predictions for Castaneamollissima_scaffold08137-snap-gene-0.8

Viterbi Path

Viterbi Path

Posterior @ 0.4 — 88 - RSRANRATKRGYWK - 101

Posterior @ 0.4

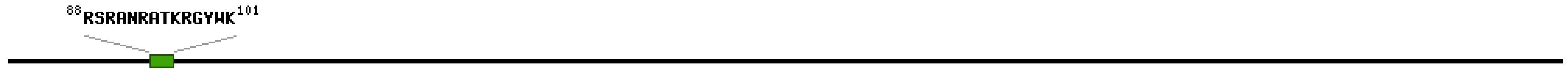

Posterior Graphic. Threshold Marked @ 0.4

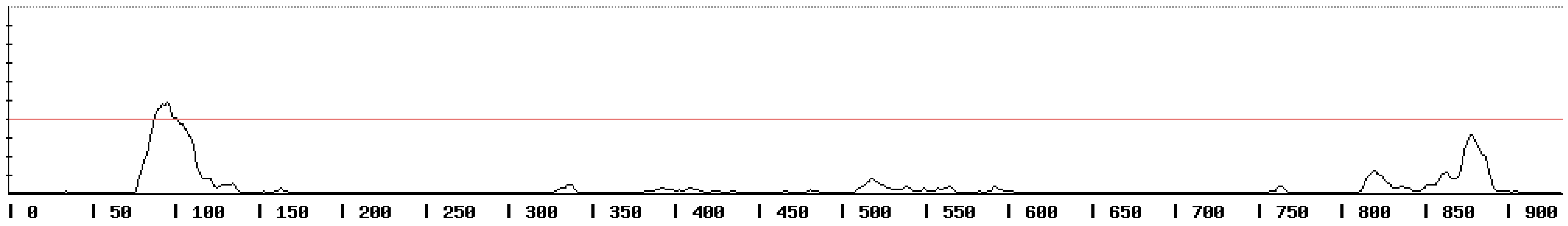

Predictions for Catharanthusroseus_cra_locus_1368_iso_3

Viterbi Path

Viterbi Path

Posterior @ 0.4 — 2 - RDRRRRQRNGYNRDRRPRGRQREPR - 26

Posterior @ 0.4

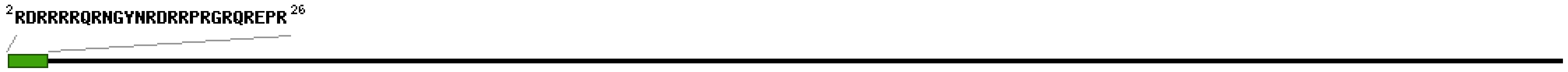

Posterior Graphic. Threshold Marked @ 0.4

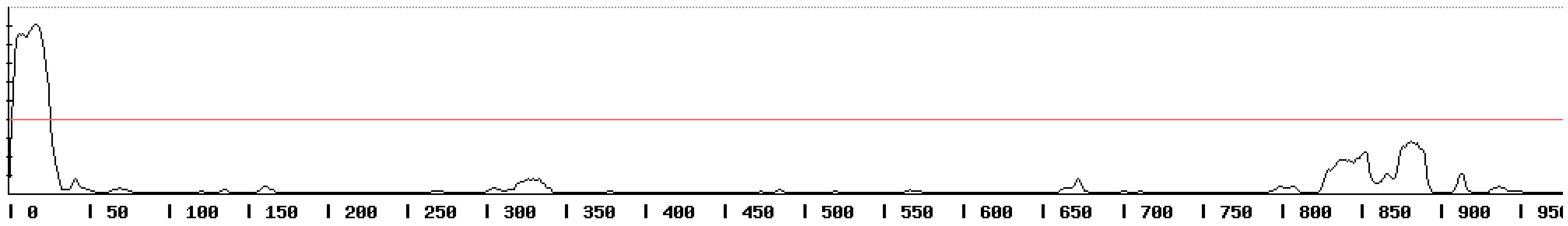

Predictions for Catharanthusroseus_cra_locus_1368_iso_4

Viterbi Path	
Viterbi Path	
Posterior @ 0.4	2 - RDRRRRQRNGYNRDRRPRGRQREPR - 26
Posterior @ 0.4	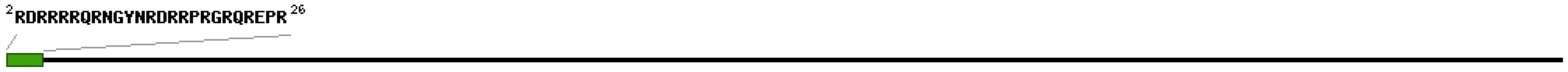
Posterior Graphic. Threshold Marked @ 0.4	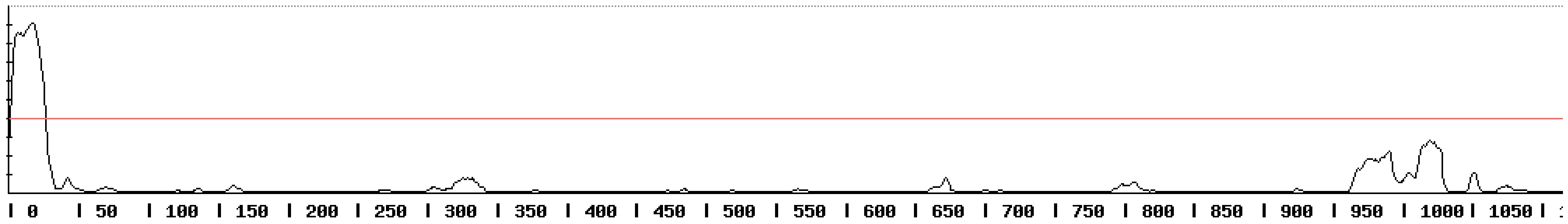

Predictions for Daucuscarota_DCAR_021729

Viterbi Path	505 - RGKSMRSKVETRMRKESGKTLREIRRSKKLRKK - 537
Viterbi Path	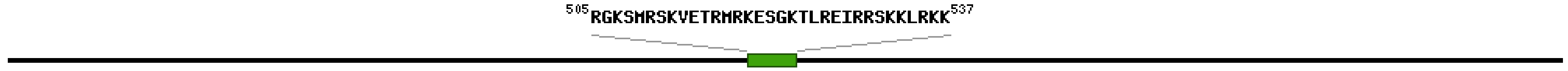
Posterior @ 0.4	133 - RKRRK - 137
	505 - RGKSMRSKVETRMRKESGKTLREIRRSKKLRKKMM - 539
	549 - LRRAKKKV - 556

Posterior @ 0.4

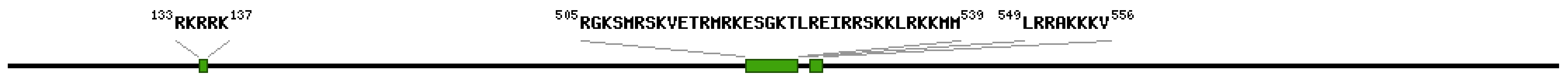

Posterior Graphic. Threshold Marked @ 0.4

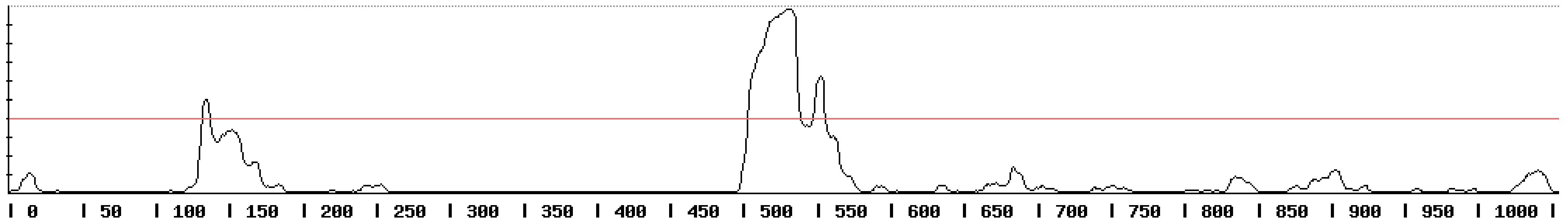

Predictions for Daucuscarota_DCAR_030911

Viterbi Path

Viterbi Path

Posterior @ 0.4

Posterior @ 0.4

Posterior Graphic. Threshold Marked @ 0.4

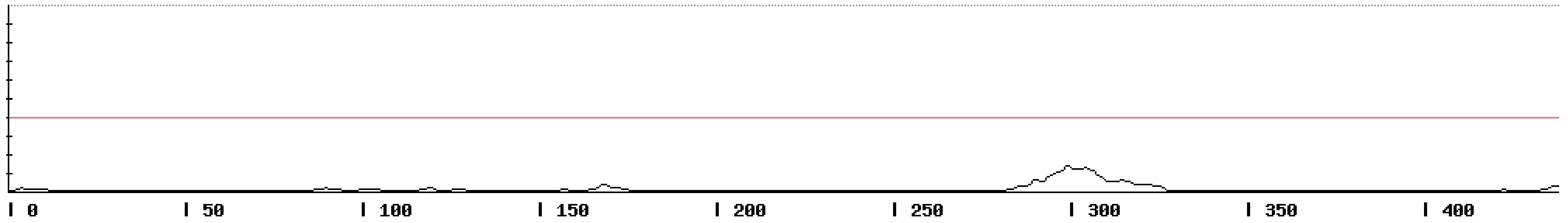

Predictions for Dichantheliumoligosanthes_Do002294.1

Viterbi Path

Viterbi Path

Posterior @ 0.4 | 104 - KLRIPGGGGKEAAWRKK - 120

333 - SGHGKKRKRG - 342

Posterior @ 0.4

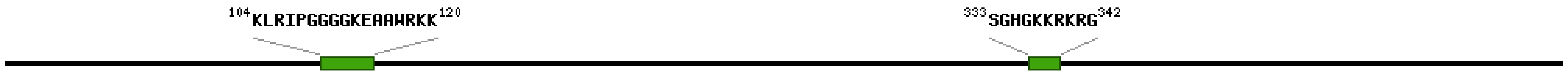

Posterior Graphic. Threshold Marked @ 0.4

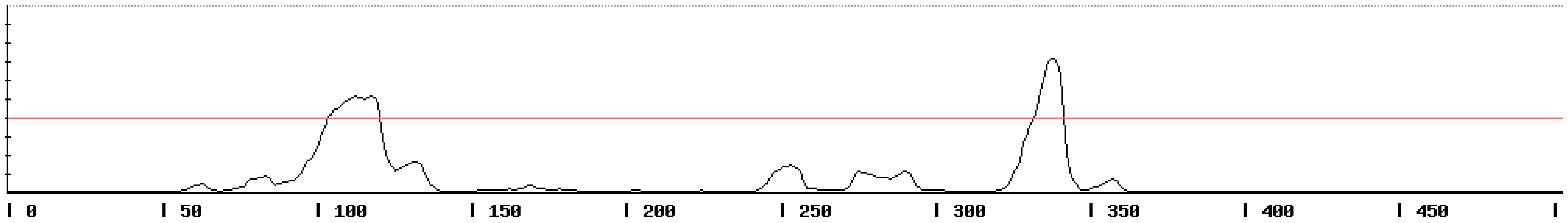

Predictions for Dichantheliumoligosanthes_Do002300.1

Viterbi Path

Viterbi Path

Posterior @ 0.4 | 330 - SGHGKKRKRG - 339

Posterior @ 0.4

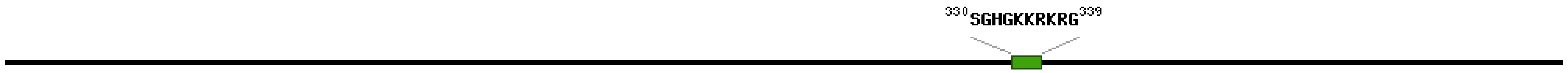

Posterior Graphic. Threshold Marked @ 0.4

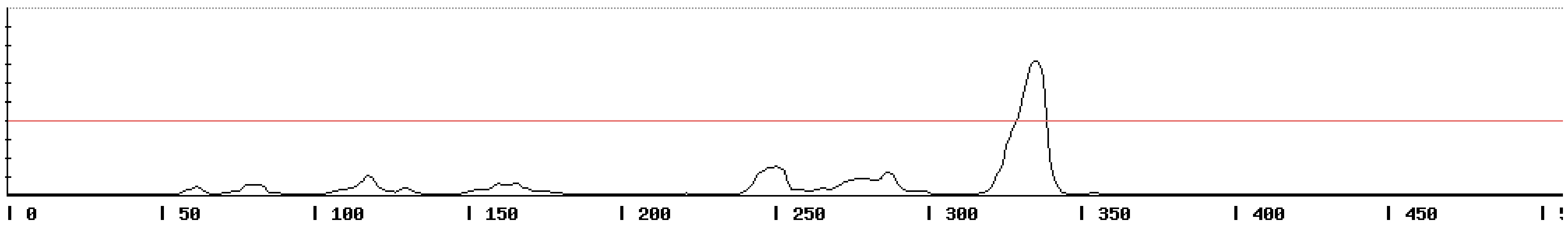

Predictions for Dichantheliumoligosanthes_Do009067.1

Viterbi Path | 524 - PRGPRLRRRRREDPARRAR - 542

Viterbi Path

	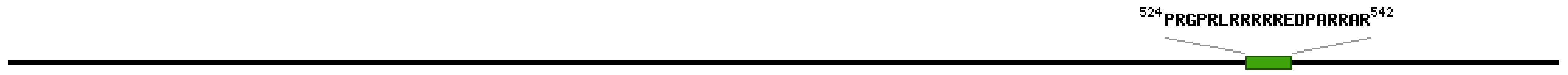
Posterior @ 0.4	517 - RSGATSSPRGPRLRRRRREDPARRARTTS - 545
Posterior @ 0.4	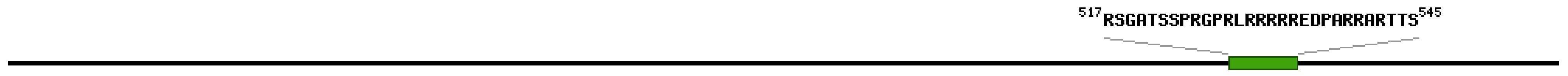
Posterior Graphic. Threshold Marked @ 0.4	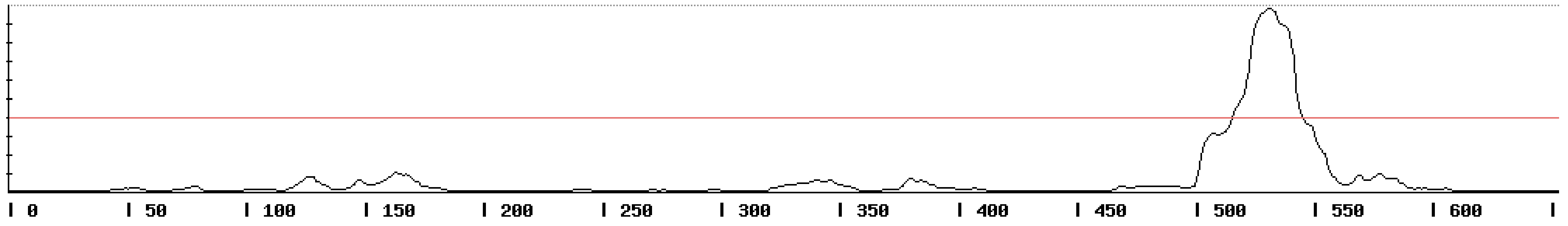

**Predictions for Dichantheliumoligosanthes_Do010830.1**	
Viterbi Path	
Viterbi Path	
Posterior @ 0.4	330 - SGHGKKRKRG - 339<br>730 - GHAKKRRL - 737
Posterior @ 0.4	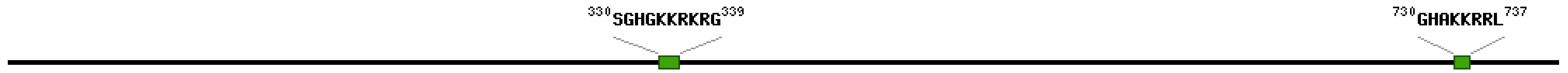
Posterior Graphic. Threshold Marked @ 0.4	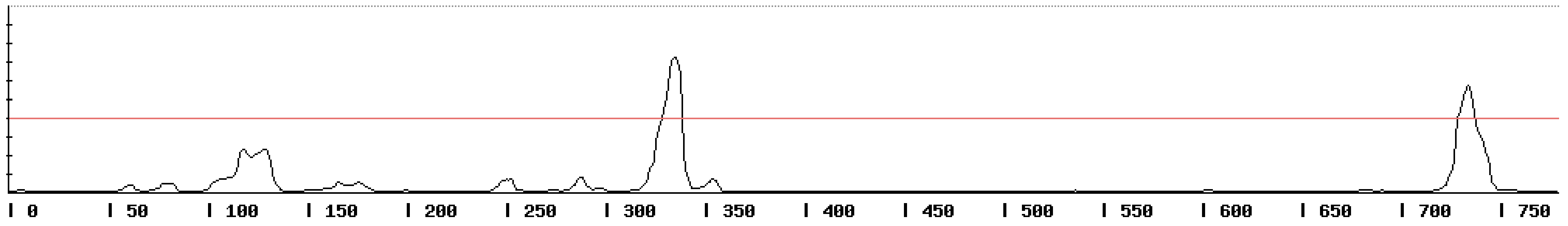

Predictions for Dichantheliumoligosanthes_Do011477.1

Viterbi Path

Viterbi Path

Posterior @ 0.4

80 - RHKSVRGKRKAR - 91

283 - PARKRKAP - 290

304 - PAPKRK - 309

602 - PRLRGRRTTSTLVRRQRHRLDPRRRPSVRALVRPRLGNSAMTGGPLLMLRRKPRPQQQRRSPKISPN - 668

685 - RKSRSPANPG - 694

Posterior @ 0.4

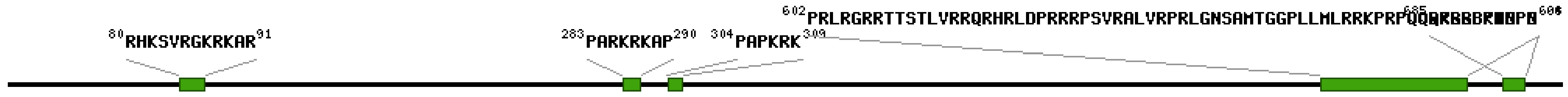

Posterior Graphic. Threshold Marked @ 0.4

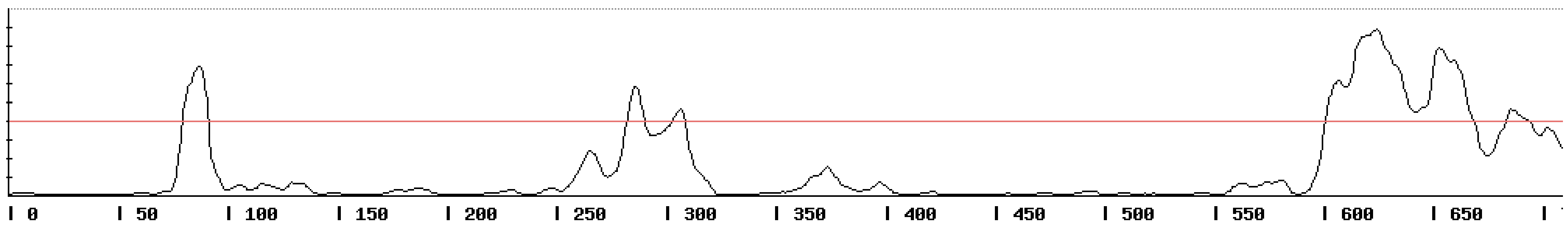

Predictions for Dichantheliumoligosanthes_Do015402.1

Viterbi Path

Viterbi Path

Posterior @ 0.4

177 - R - 177

558 - RG - 559

Posterior @ 0.4

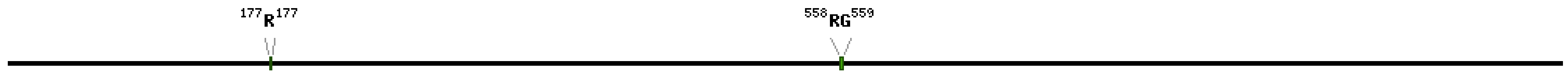

Posterior Graphic. Threshold Marked @ 0.4

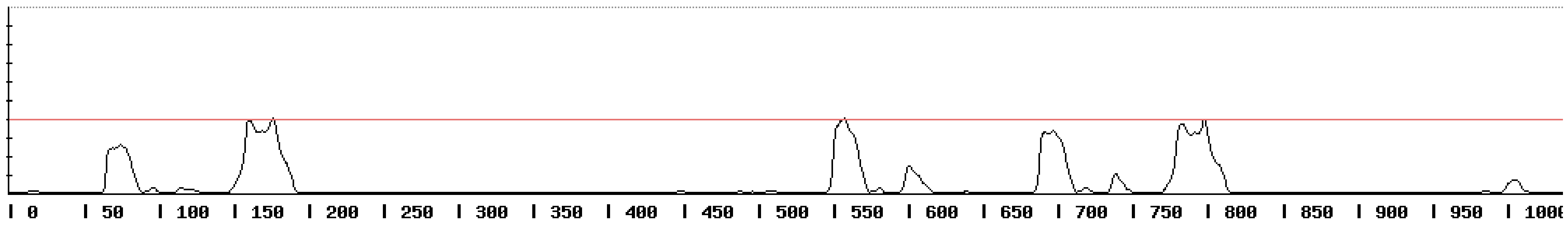

Predictions for Dichantheliumoligosanthes_Do015782.1

Viterbi Path	357 - RKKQGKGGLHPAKEPKKEQSNPTKKPSSSRRRR - 389
Viterbi Path	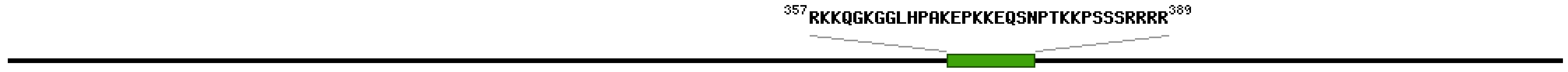
Posterior @ 0.4	163 - MLSRIRITGRAKKERKK - 179<br>356 - VRKKQGKGGLHPAKEPKKEQSNPTKKPSSSRRRRV - 390<br>563 - GRAKKER - 569
Posterior @ 0.4	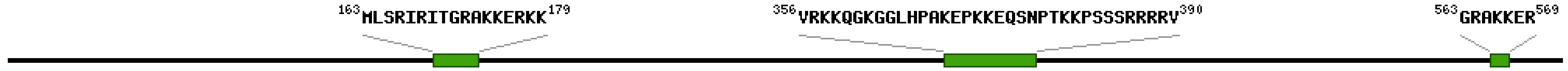
Posterior Graphic. Threshold Marked @ 0.4	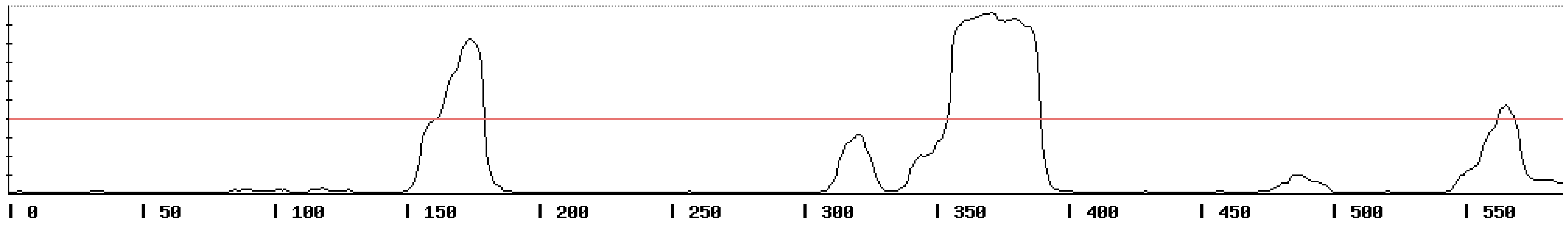

Predictions for Dichantheliumoligosanthes_Do020978.1

Viterbi Path	
Viterbi Path	
Posterior @ 0.4	

Posterior @ 0.4

Posterior Graphic. Threshold Marked @ 0.4

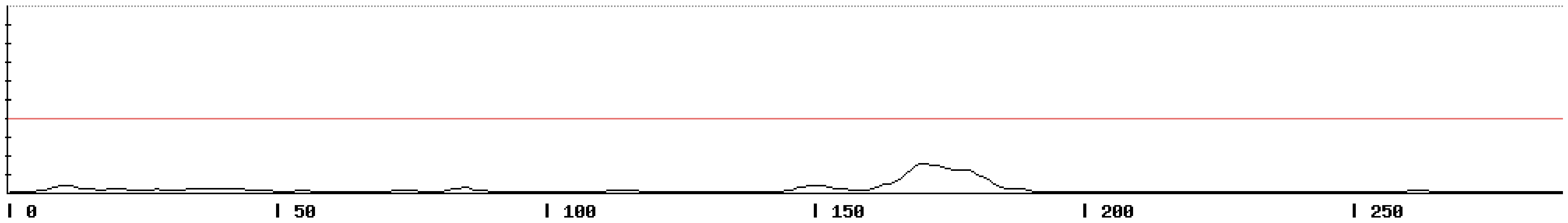

Predictions for Dichantheliumoligosanthes_Do022798.1

Viterbi Path

Viterbi Path

Posterior @ 0.4 606 - RSTRKRKP - 613

Posterior @ 0.4

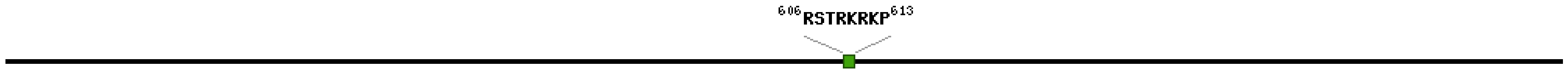

Posterior Graphic. Threshold Marked @ 0.4

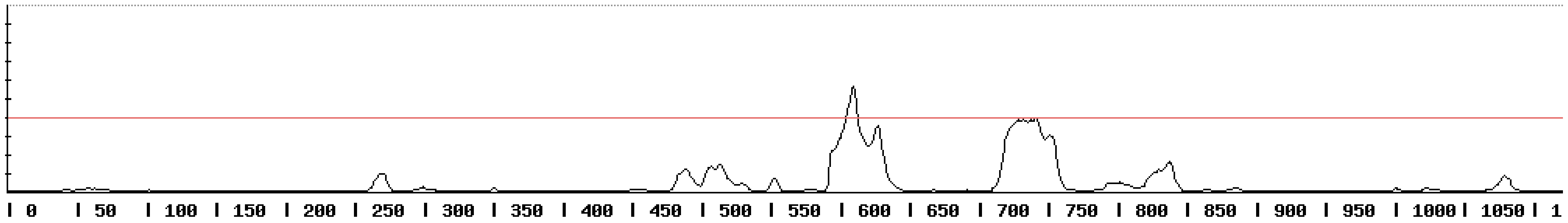

Predictions for Dichantheliumoligosanthes_Do004998.1

Viterbi Path

Viterbi Path

Posterior @ 0.4

Posterior @ 0.4

Posterior Graphic. Threshold Marked @ 0.4

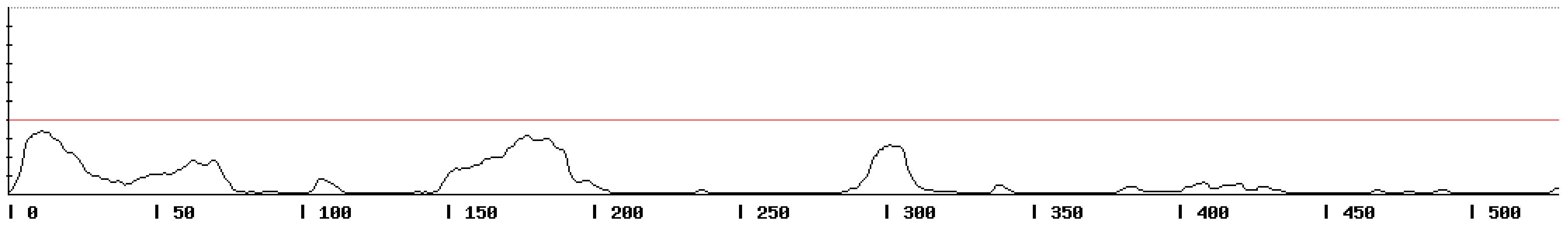

Predictions for Dorcocerashygrometricum_KZV39672.1

Viterbi Path

Viterbi Path

Posterior @ 0.4

Posterior @ 0.4

Posterior Graphic. Threshold Marked @ 0.4

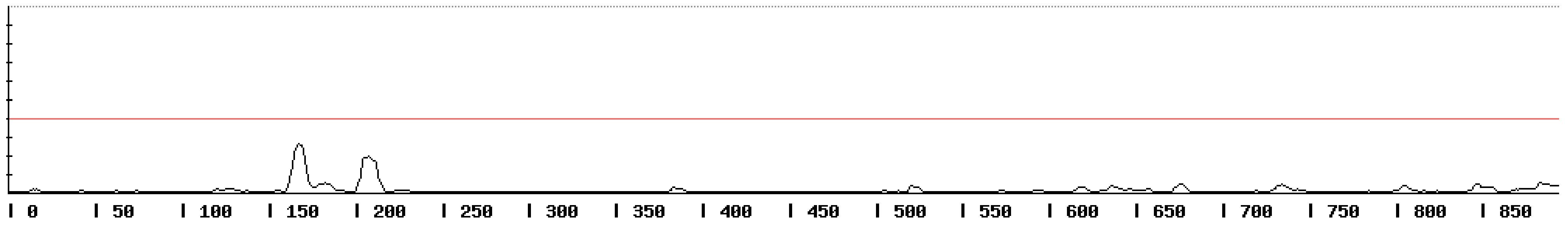

Predictions for Dorcocerashygrometricum_KZV46422.1

Viterbi Path

Viterbi Path

Posterior @ 0.4	372 - QRKRRRISGRESTIKAHVRWHKTGKTK - 398
Posterior @ 0.4	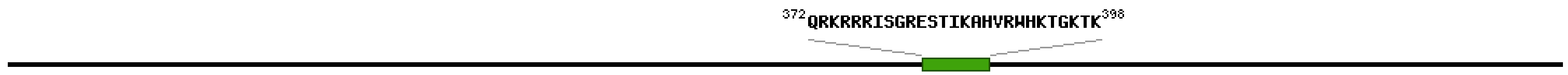
Posterior Graphic. Threshold Marked @ 0.4	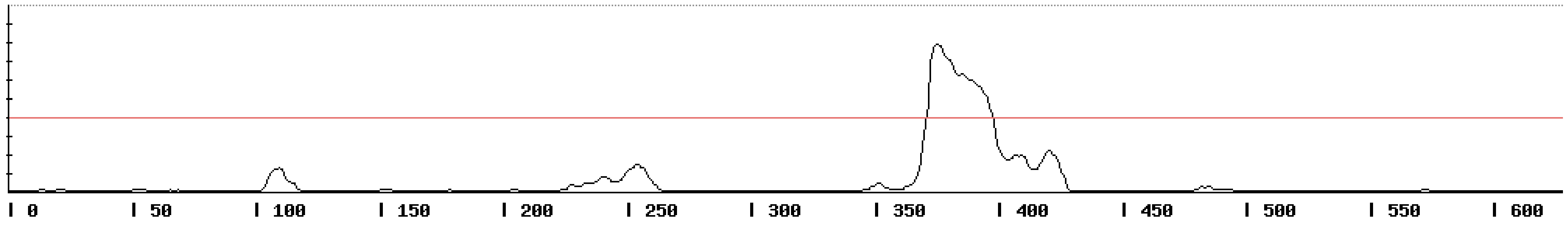

Predictions for Elaeisguineensis_XP_010916182.1

Viterbi Path	
Viterbi Path	
Posterior @ 0.4	197 - KKGKKP - 202<br>1005 - RRRRKK - 1010
Posterior @ 0.4	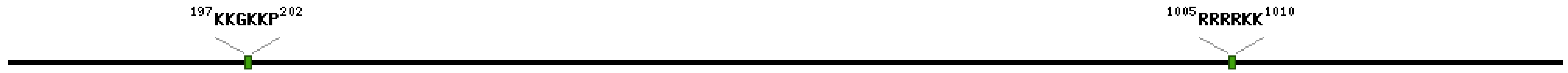
Posterior Graphic. Threshold Marked @ 0.4	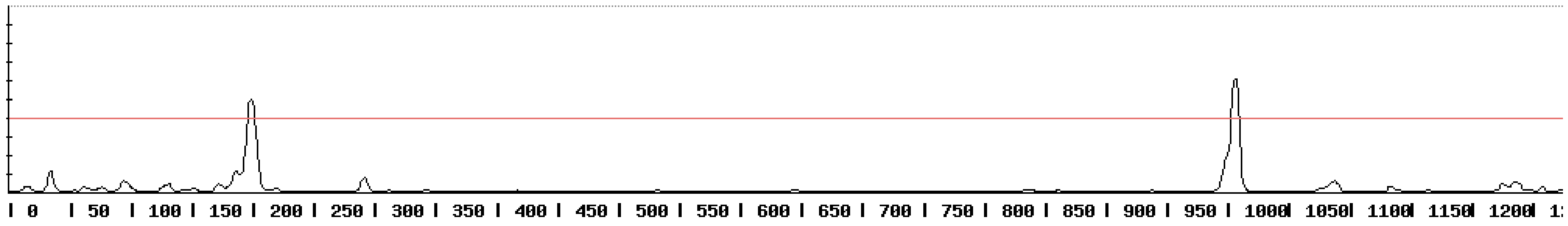

Predictions for Elaeisguineensis_XP_010927165.1	
Viterbi Path	
Viterbi Path	
Posterior @ 0.4	246 - PRKKKYPKGTRPARETNGGYWKASGRDMIIKDKNKKEIGHKK - 287
Posterior @ 0.4	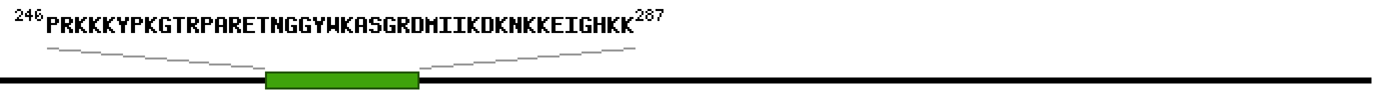
Posterior Graphic. Threshold Marked @ 0.4	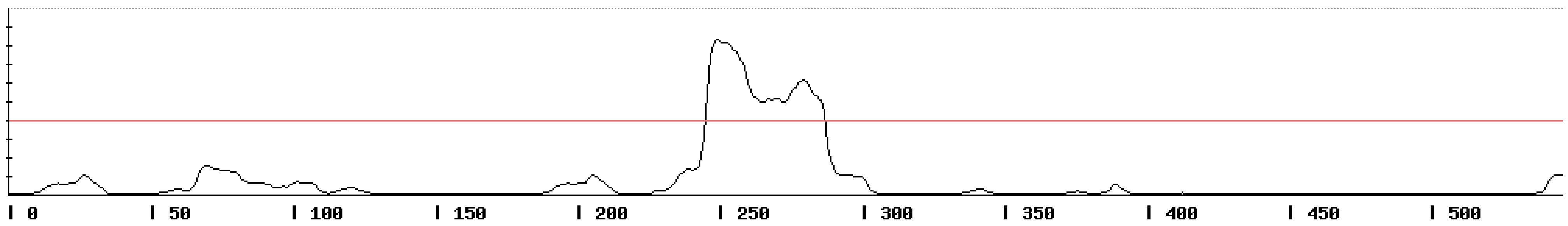

Predictions for Elaeisguineensis_XP_010904551.1	
Viterbi Path	
Viterbi Path	
Posterior @ 0.4	81 - RRAGLGR - 87
Posterior @ 0.4	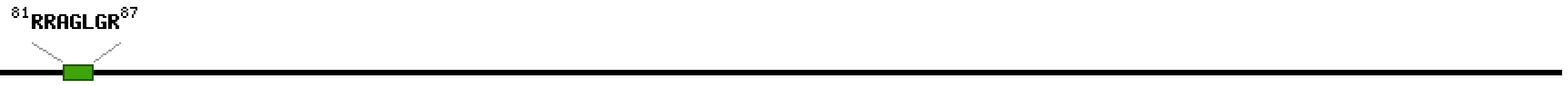
Posterior Graphic. Threshold Marked @ 0.4	

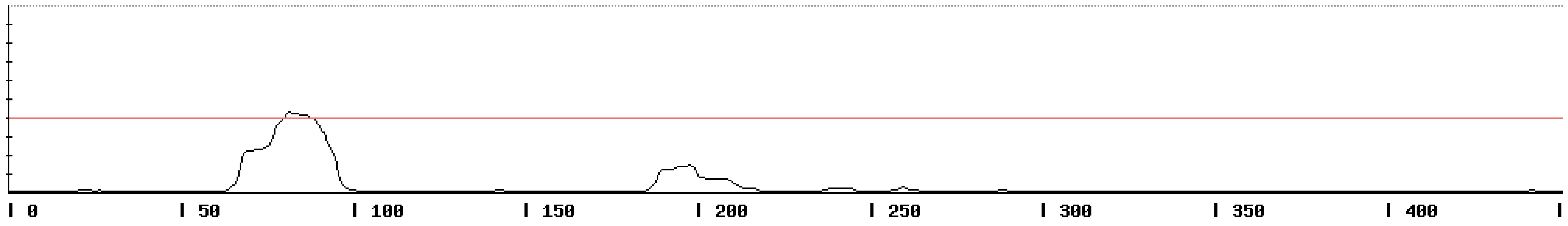

Predictions for Eragrostistef_462877018

Viterbi Path	
Viterbi Path	
Posterior @ 0.4	
Posterior @ 0.4	
Posterior Graphic. Threshold Marked @ 0.4	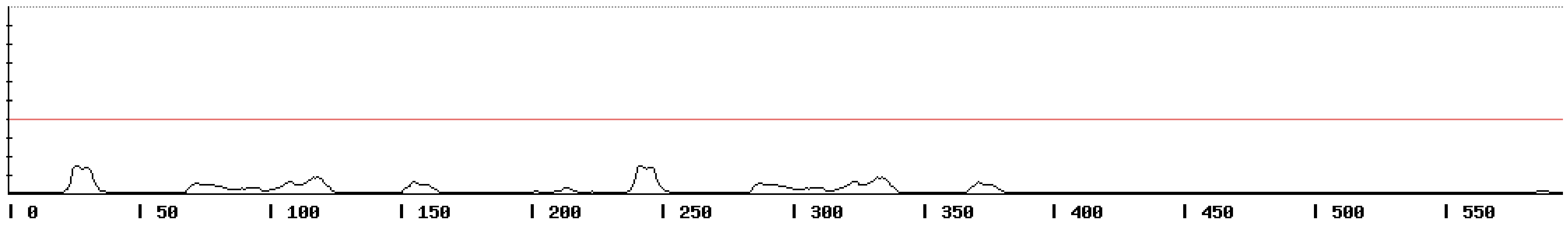

Predictions for Eragrostistef_462886127

Viterbi Path	
Viterbi Path	
Posterior @ 0.4	6 - RPRRQGRQTQRAAGGAGFVRHKLKRWRPPAAAPAVASRRR - 45
Posterior @ 0.4	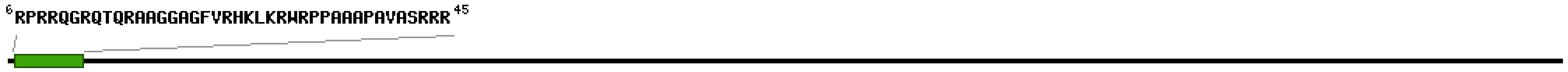

Posterior Graphic. Threshold Marked @ 0.4

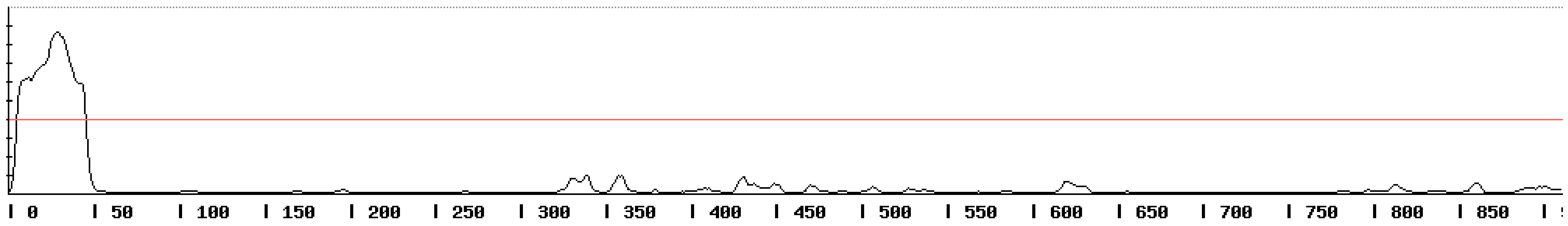

Predictions for Eragrostistef_462890696

Viterbi Path	
Viterbi Path	
Posterior @ 0.4	
Posterior @ 0.4	
Posterior Graphic. Threshold Marked @ 0.4	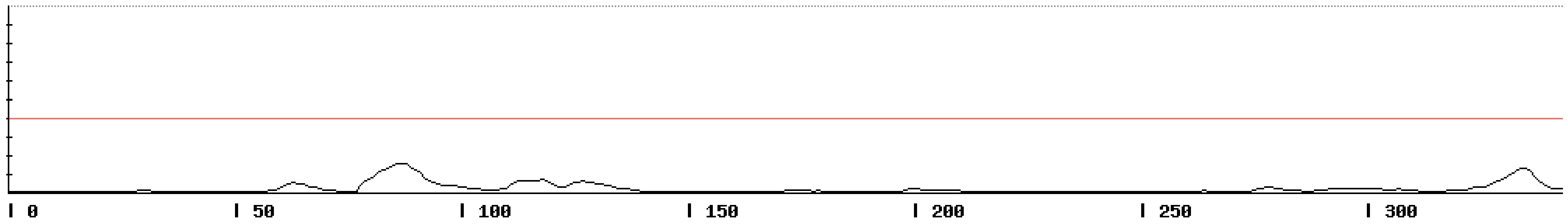

Predictions for Eragrostistef_462898851

Viterbi Path	
Viterbi Path	
Posterior @ 0.4	
Posterior @ 0.4	

Posterior Graphic. Threshold Marked @ 0.4

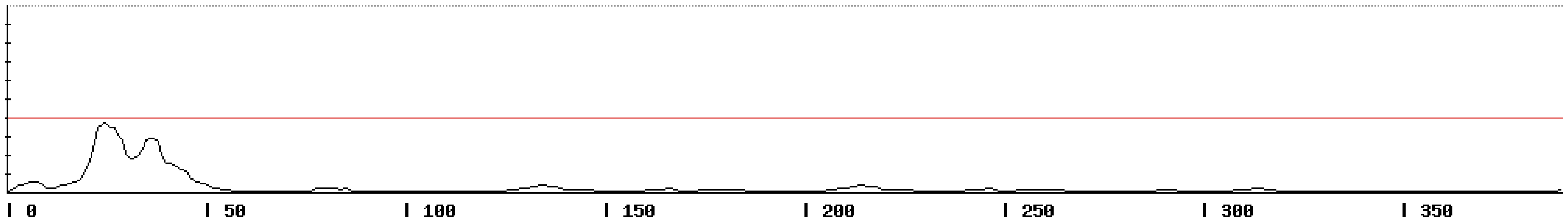

Predictions for Eragrostistef_462914386

Viterbi Path

Viterbi Path

Posterior @ 0.4

Posterior @ 0.4

Posterior Graphic. Threshold Marked @ 0.4

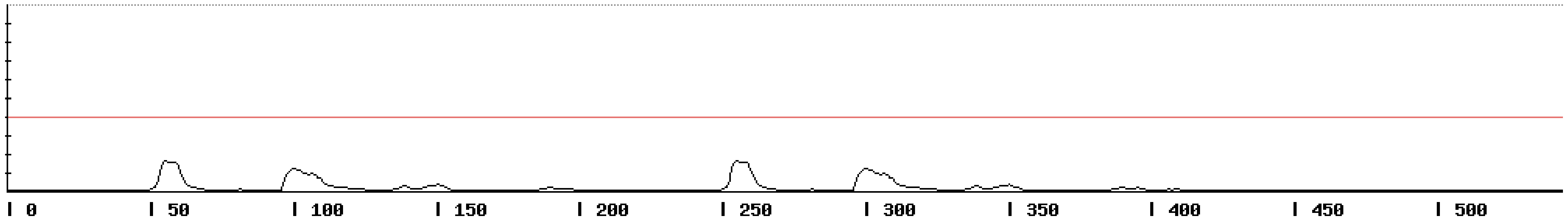

Predictions for Eragrostistef_462914484

Viterbi Path

Viterbi Path

Posterior @ 0.4	
Posterior @ 0.4	
Posterior Graphic. Threshold Marked @ 0.4	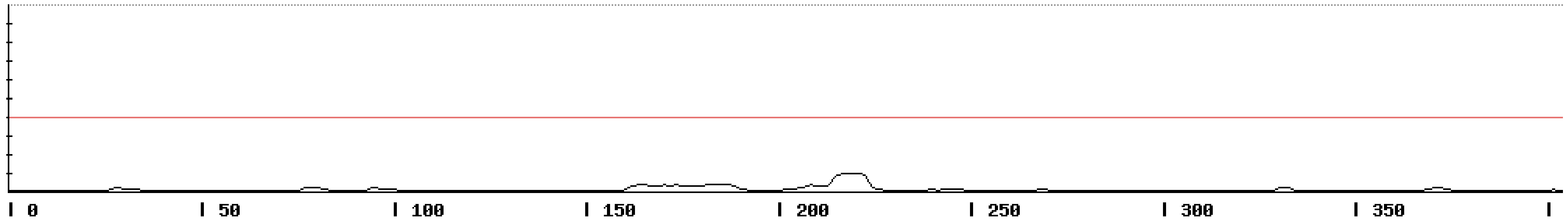

Predictions for Eragrostistef_462923579	
Viterbi Path	
Viterbi Path	
Posterior @ 0.4	
Posterior @ 0.4	
Posterior Graphic. Threshold Marked @ 0.4	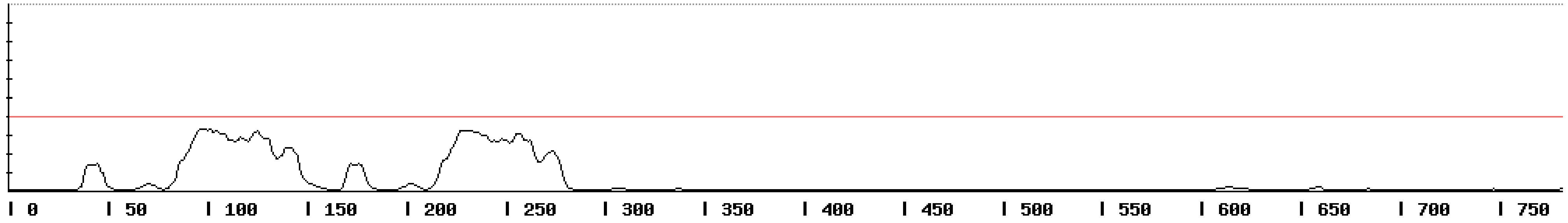

Predictions for Eragrostistef_462925604	
Viterbi Path	
Viterbi Path	

Posterior @ 0.4	
Posterior @ 0.4	
Posterior Graphic. Threshold Marked @ 0.4	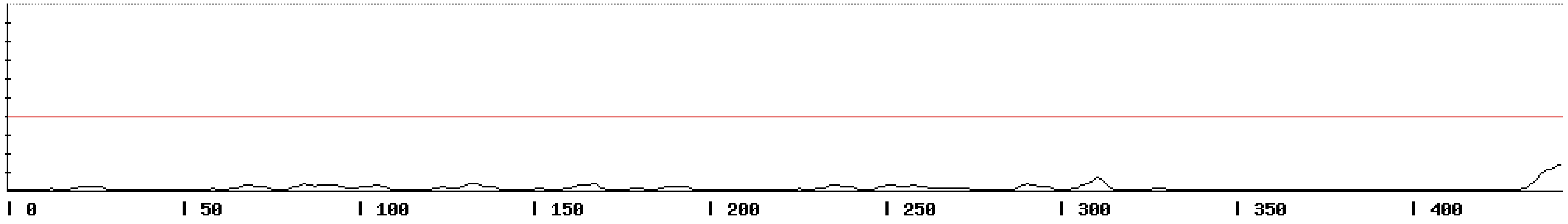

Predictions for Eragrostistef_462939746	
Viterbi Path	
Viterbi Path	
Posterior @ 0.4	
Posterior @ 0.4	
Posterior Graphic. Threshold Marked @ 0.4	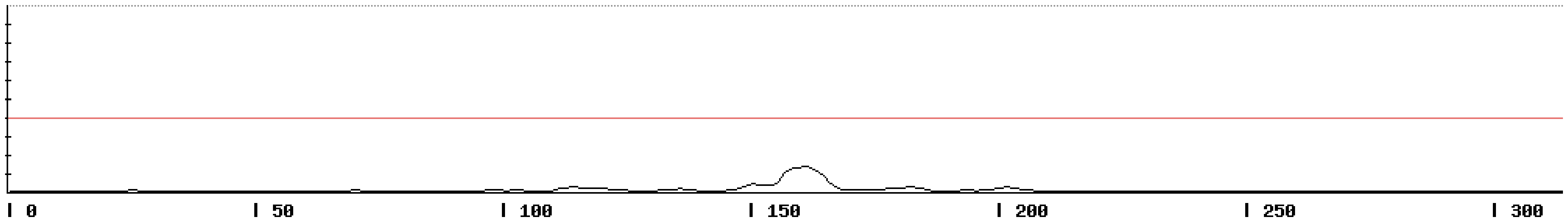

Predictions for Eragrostistef_462951506

Viterbi Path

Viterbi Path

Posterior @ 0.4

Posterior @ 0.4

Posterior Graphic. Threshold Marked @ 0.4

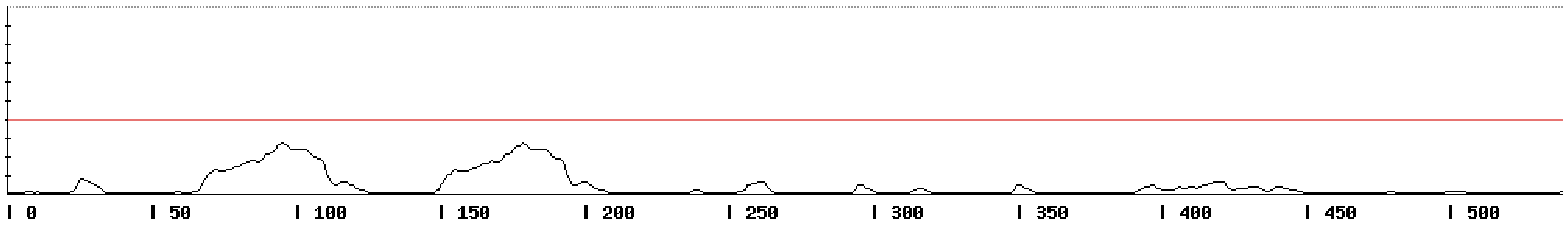

Predictions for Eragrostistef_462963614

Viterbi Path

Viterbi Path

Posterior @ 0.4

Posterior @ 0.4

Posterior Graphic. Threshold Marked @ 0.4

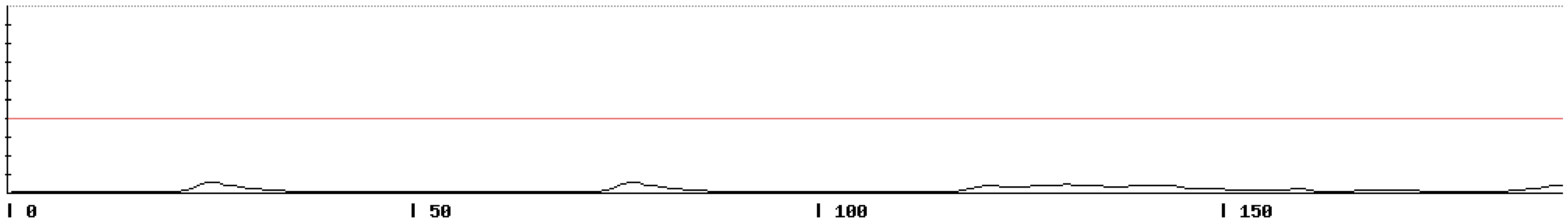

Predictions for Eutremasalsugineum_Thhalv10015599m

Viterbi Path

Viterbi Path

Posterior @ 0.4

22 - HKIPKINRSKKKDDRHRPRE - 41

62 - KRKLKGKSK - 70

156 - TKKTLTFTKSKNKKKKQK - 173

Posterior @ 0.4

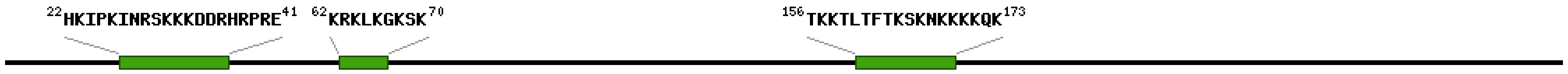

Posterior Graphic. Threshold Marked @ 0.4

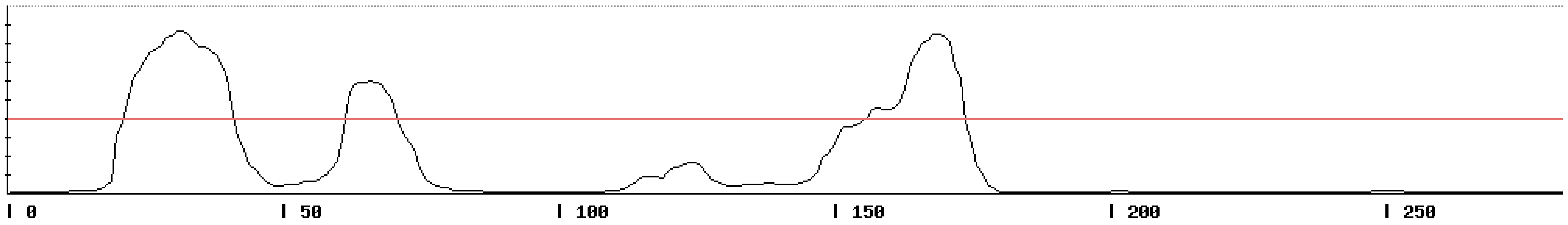

Predictions for Eutremasalsugineum_Thhalv10024006m

Viterbi Path

Viterbi Path

Posterior @ 0.4

Posterior @ 0.4

Posterior Graphic. Threshold Marked @ 0.4

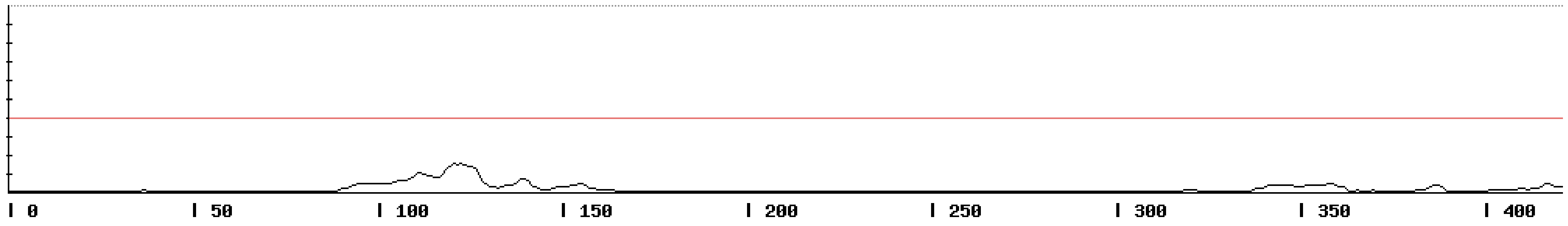

Predictions for FANhyb_icon00002021_a.1.g00001.1

Viterbi Path

298 - RKPKRIGKKRKLR - 310

357 - PKEKKRKRQQIDQLEDKKPKRKK - 379

Viterbi Path

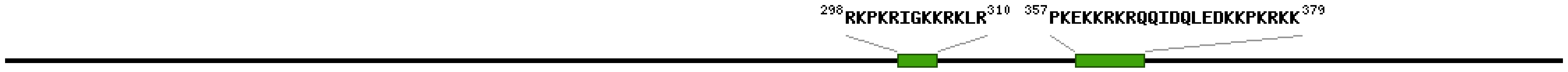

Posterior @ 0.4

297 - ERKPKRIGKKRKLR - 310

357 - PKEKKRKRQQIDQLEDKKPKRKKVV - 381

Posterior @ 0.4

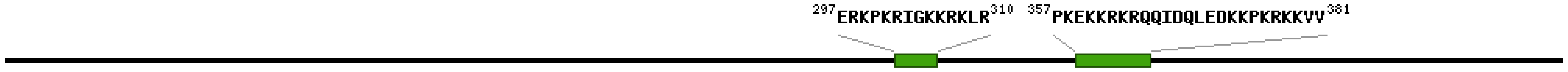

Posterior Graphic. Threshold Marked @ 0.4

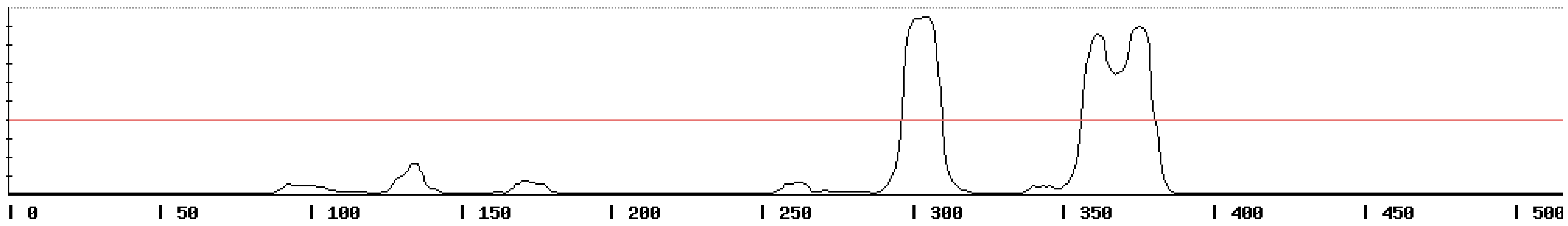

Predictions for FANhyb_rscf00000878.1.g00001.1

Viterbi Path

283 - KKKKEKGKEKK - 293

Viterbi Path

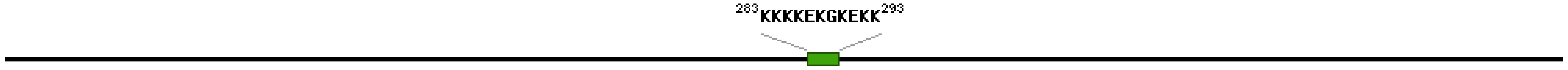

Posterior @ 0.4

106 - GEKKNKTPIGRKRKFR - 121

282 - EKKKKEKGKEKKT - 294

	406 - GRKRKL - 411
	495 - ASSKAKRRR - 503
Posterior @ 0.4	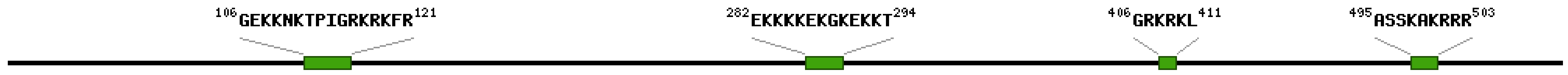
Posterior Graphic. Threshold Marked @ 0.4	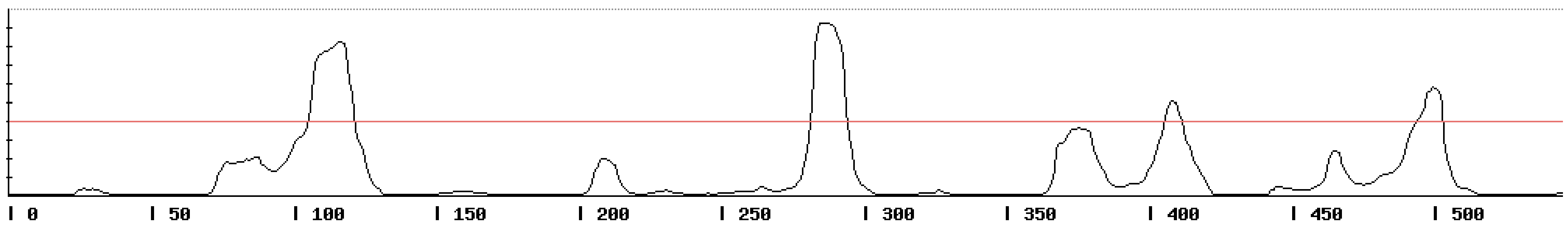

Predictions for FANhyb_rscf00001150.1.g00006.1

Viterbi Path	486 - KHGLRKMKRKKKIR - 499
Viterbi Path	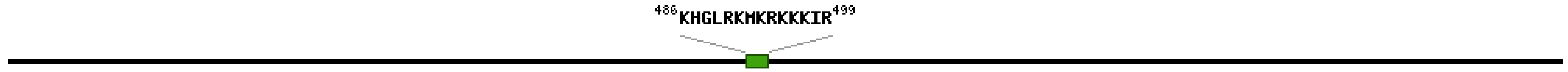
Posterior @ 0.4	486 - KHGLRKMKRKKKIR - 499
	529 - WKITGVEREIKARRSKALIGKKRP - 552
Posterior @ 0.4	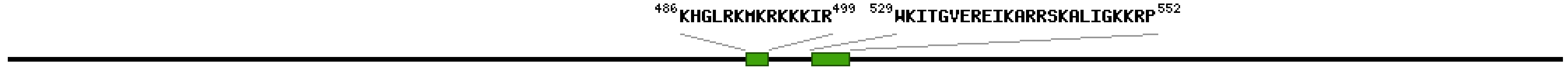
Posterior Graphic. Threshold Marked @ 0.4	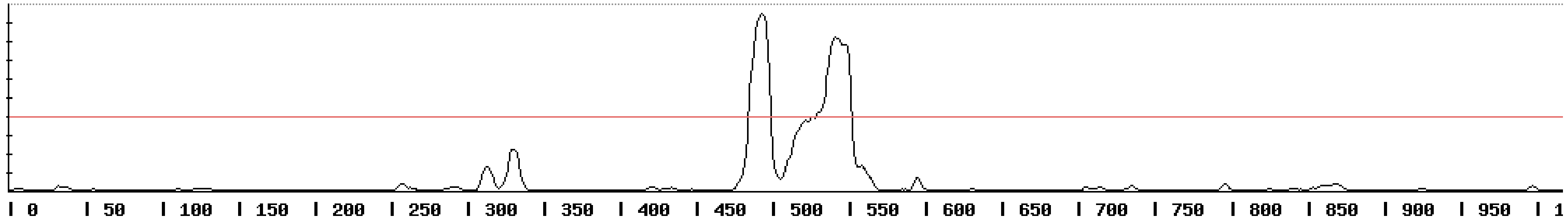

Predictions for Genliseaaurea_EPS67213.1

Viterbi Path

Viterbi Path

Posterior @ 0.4

Posterior @ 0.4

Posterior Graphic. Threshold Marked @ 0.4

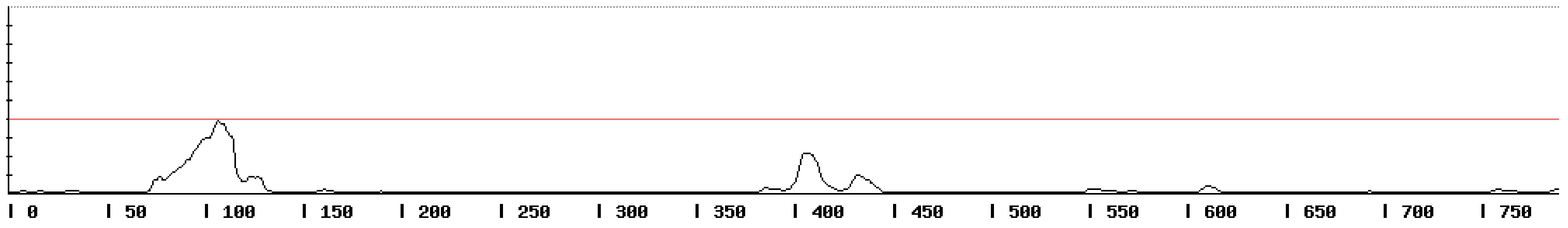

Predictions for Glycinesoja_KHN21163.1

Viterbi Path

Viterbi Path

Posterior @ 0.4

Posterior @ 0.4

Posterior Graphic. Threshold Marked @ 0.4

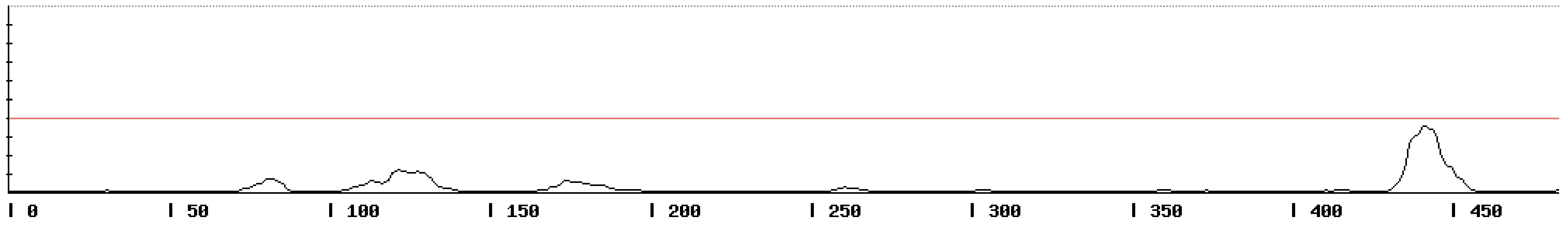

Predictions for Gossypiumhirsutum_Gh_A10G1944	
Viterbi Path	
Viterbi Path	
Posterior @ 0.4	282 - LKKKSKSR - 289
Posterior @ 0.4	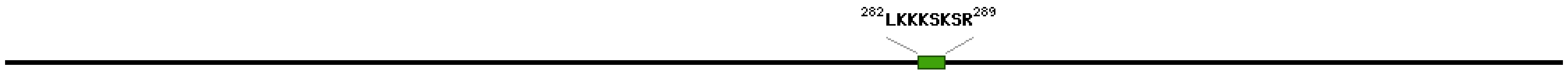
Posterior Graphic. Threshold Marked @ 0.4	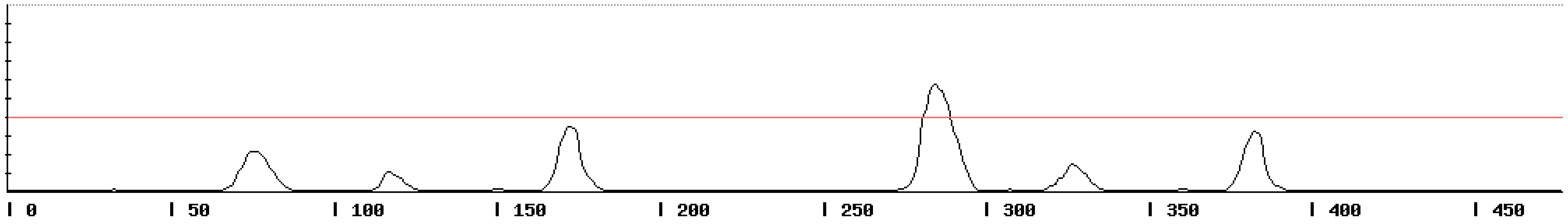

Predictions for Gossypiumhirsutum_Gh_A01G0250	
Viterbi Path	
Viterbi Path	
Posterior @ 0.4	
Posterior @ 0.4	
Posterior Graphic. Threshold Marked @ 0.4	

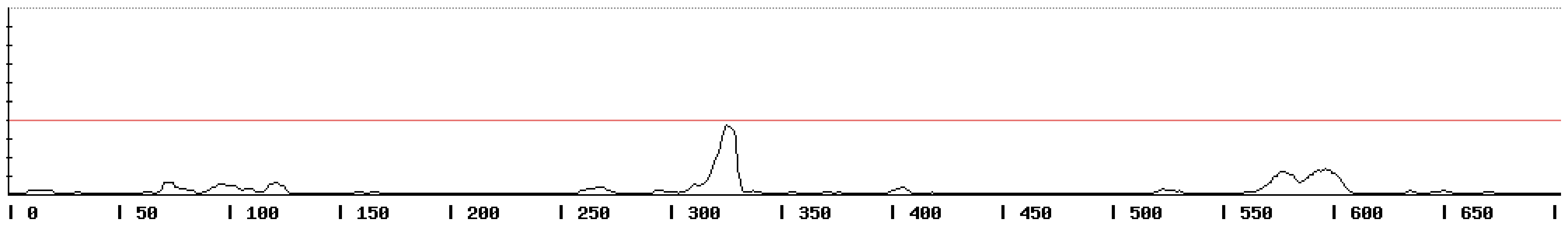

Predictions for Gossypiumhirsutum_Gh_D11G0701

Viterbi Path

Viterbi Path

Posterior @ 0.4

579 - PSKSKKEKKSKIK - 591

965 - RKRRRIQNRHS - 975

980 - HVRWHKTGKTKPVIENGVRKGWKKIMVLYKRSNRAHKP - 1017

Posterior @ 0.4

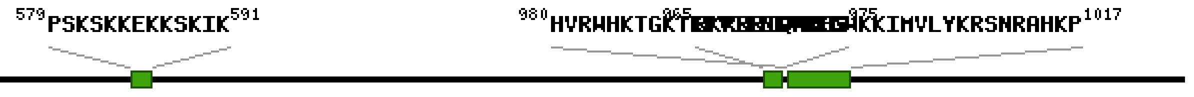

Posterior Graphic. Threshold Marked @ 0.4

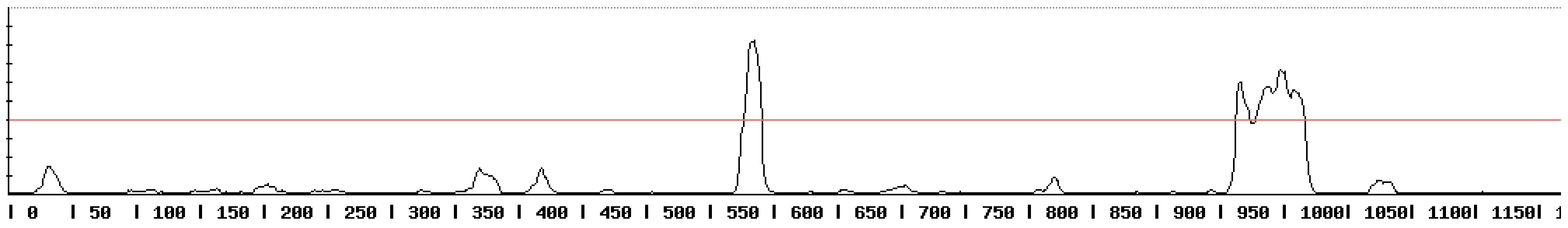

Predictions for Ipomoeatrifida_Itr_sc000185.1_g00020.1

Viterbi Path

Viterbi Path

Posterior @ 0.4

66 - KLKKKNPRNSRYRRSIGDGKGTWKAQDKGKAISRSGKIIGYKR - 108

Posterior @ 0.4

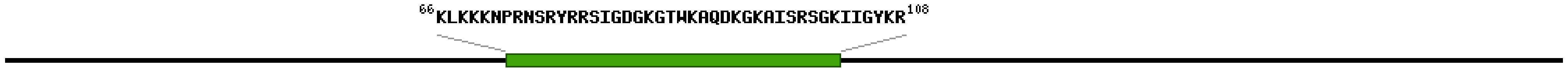

Posterior Graphic. Threshold Marked @ 0.4

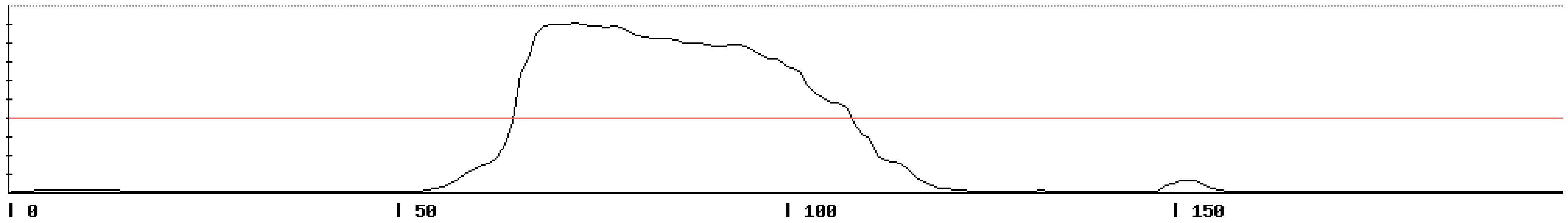

Predictions for Ipomoeatrifida_Itr_sc004419.1_g00003.1

Viterbi Path	985 - KKKNRRRGWKKYFKRSDKEKKDKGP - 1009
Viterbi Path	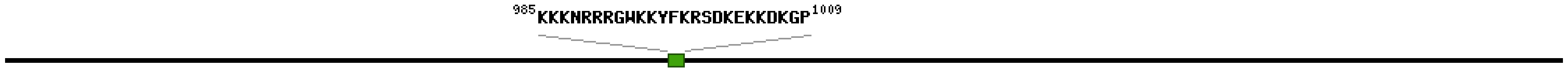
Posterior @ 0.4	729 - RAWAVRGSIREKKRSKPERR - 748
	983 - AEKKKNRRRGWKKYFKRSDKEKKDKGPE - 1010
Posterior @ 0.4	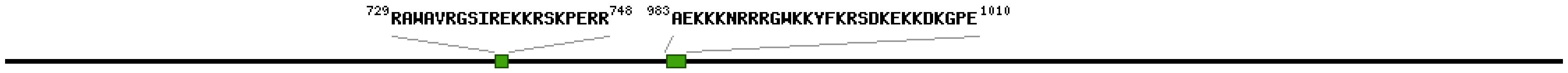
Posterior Graphic. Threshold Marked @ 0.4	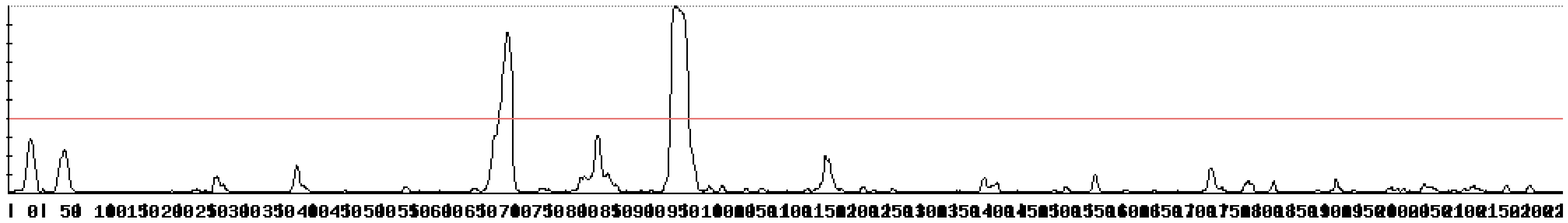

Predictions for Ipomoeatrifida_Itr_sc004968.1_g00002.1

Viterbi Path

Viterbi Path

Posterior @ 0.4	
Posterior @ 0.4	
Posterior Graphic. Threshold Marked @ 0.4	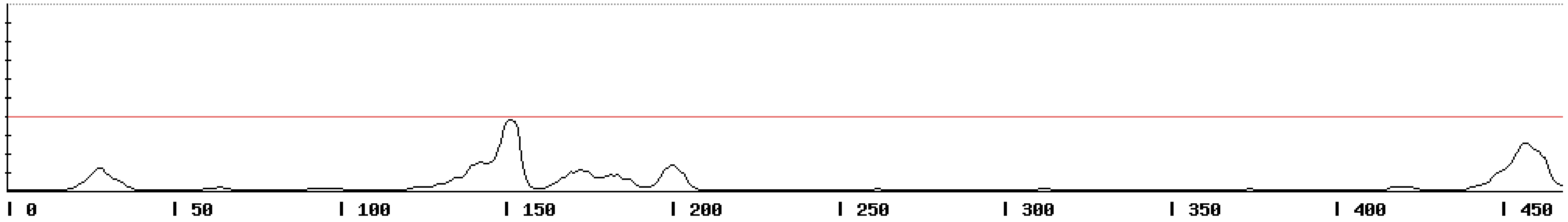

Predictions for Juglansregia_WALNUT_00018866-RA

Viterbi Path	
Viterbi Path	
Posterior @ 0.4	
Posterior @ 0.4	
Posterior Graphic. Threshold Marked @ 0.4	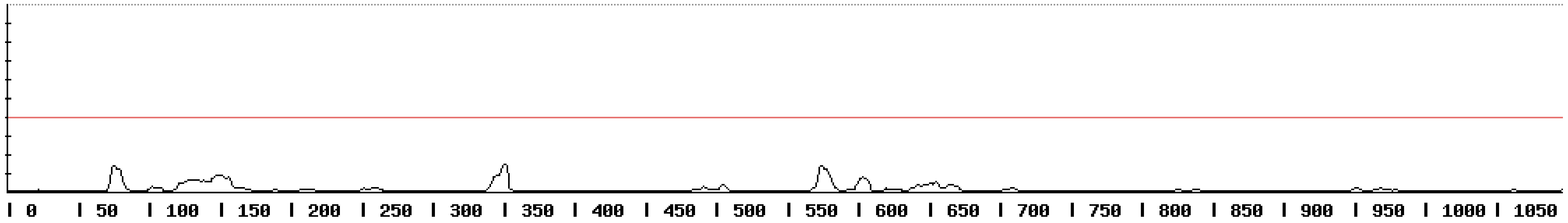

Predictions for Juglansregia_WALNUT_00025170-RA

Viterbi Path

Viterbi Path

Posterior @ 0.4

Posterior @ 0.4

Posterior Graphic. Threshold Marked @ 0.4

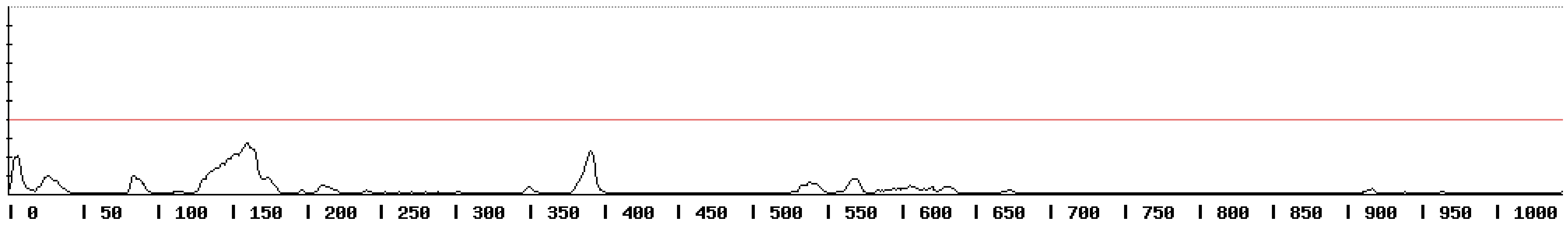

Predictions for Juglansregia_WALNUT_00031849-RA

Viterbi Path

Viterbi Path

Posterior @ 0.4

Posterior @ 0.4

Posterior Graphic. Threshold Marked @ 0.4

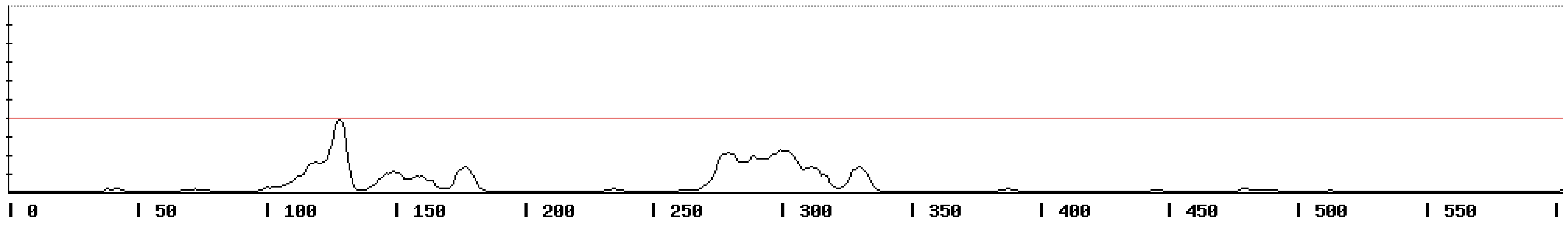

Predictions for Leersiaperrieri_LPERR04G10720.1

Viterbi Path	
Viterbi Path	
Posterior @ 0.4	
Posterior @ 0.4	
Posterior Graphic. Threshold Marked @ 0.4	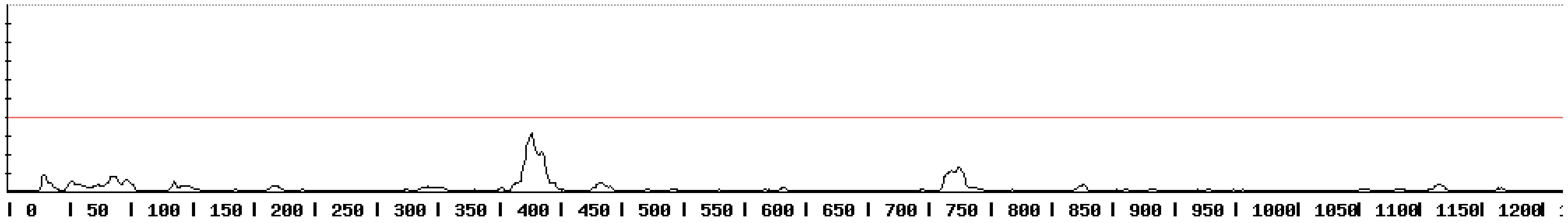

Predictions for Leersiaperrieri_LPERR04G12800.1

Viterbi Path	
Viterbi Path	
Posterior @ 0.4	184 - KRKRRKI - 190
Posterior @ 0.4	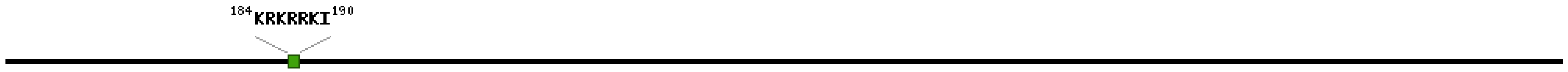
Posterior Graphic. Threshold Marked @ 0.4	

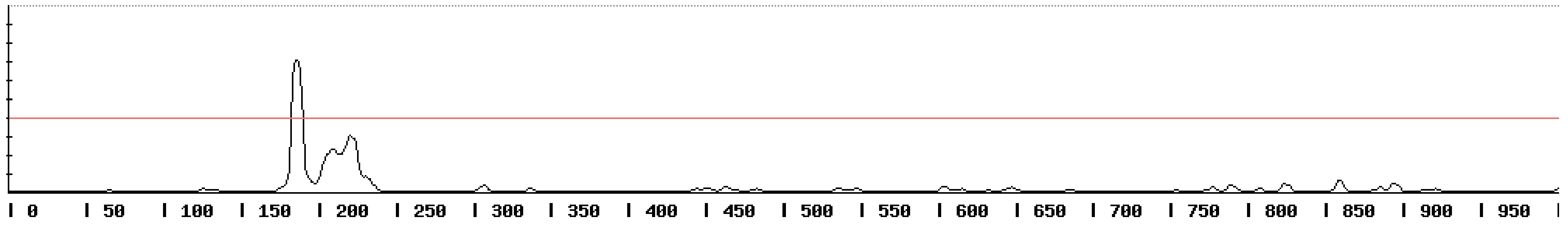

Predictions for Leersiaperrieri_LPERR04G14690.1	
Viterbi Path	
Viterbi Path	
Posterior @ 0.4	121 - RRRRCPGGRRRAGP - 134
	282 - RQRRRGEHGAARPRKAG - 298
Posterior @ 0.4	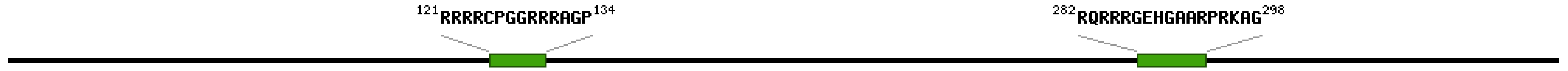
Posterior Graphic. Threshold Marked @ 0.4	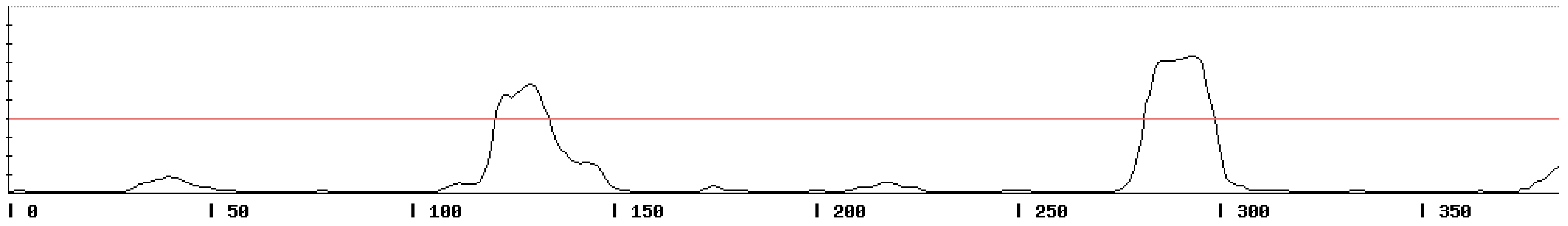

Predictions for Leersiaperrieri_LPERR07G05500.1	
Viterbi Path	
Viterbi Path	
Posterior @ 0.4	73 - RAKNGKGRRQKR - 84
	152 - RIRFSGHGRKRKREP - 166
	387 - RFSGHGKKRKREP - 399

Posterior @ 0.4

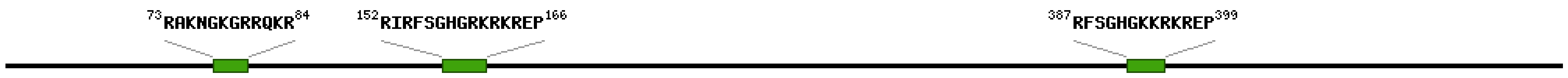

Posterior Graphic. Threshold Marked @ 0.4

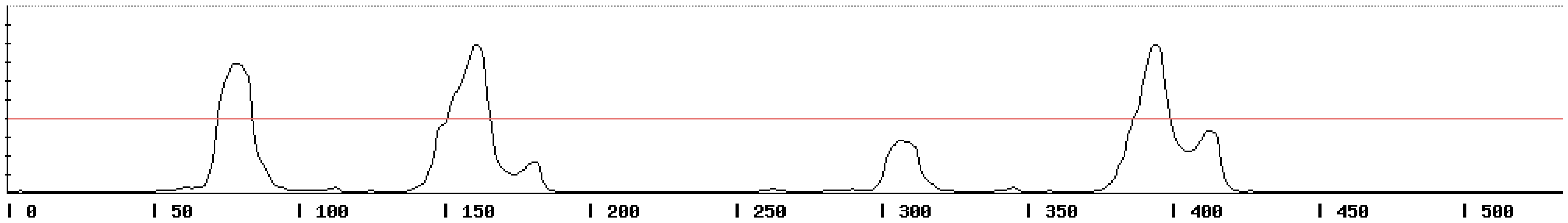

Predictions for Leersiaperrieri_LPERR07G05530.1

Viterbi Path

Viterbi Path

Posterior @ 0.4

71 - GKARAKNGKGSRQKR - 85

159 - RFSGHGKKRKREP - 171

393 - RAKNGKGRRQRR - 404

471 - YRIRFSGHGKKRKREP - 486

Posterior @ 0.4

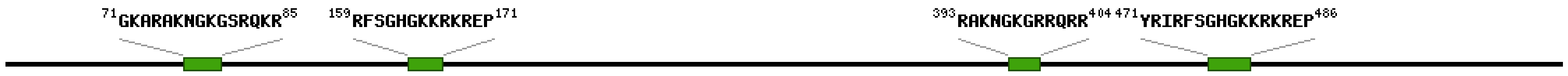

Posterior Graphic. Threshold Marked @ 0.4

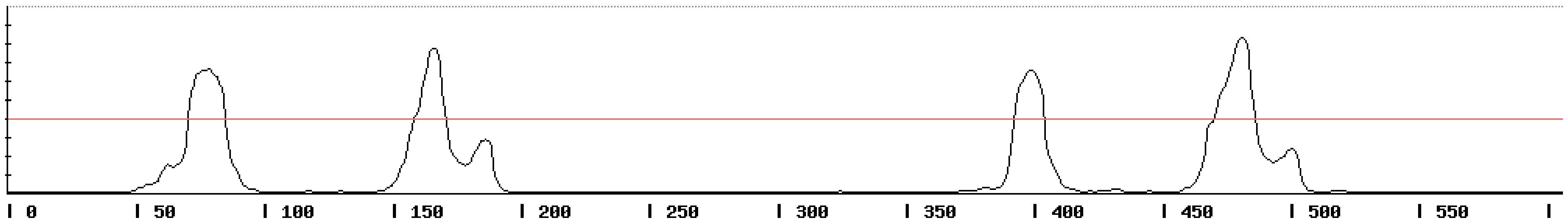

Predictions for Leersiaperrieri_LPERR07G08560.1

Viterbi Path

Viterbi Path

Posterior @ 0.4

75 - RAKNNKGKREKR - 86

154 - SGHGKKRRRE - 163

341 - TKGKRQKR - 348

Posterior @ 0.4

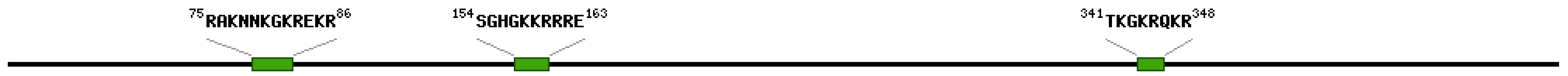

Posterior Graphic. Threshold Marked @ 0.4

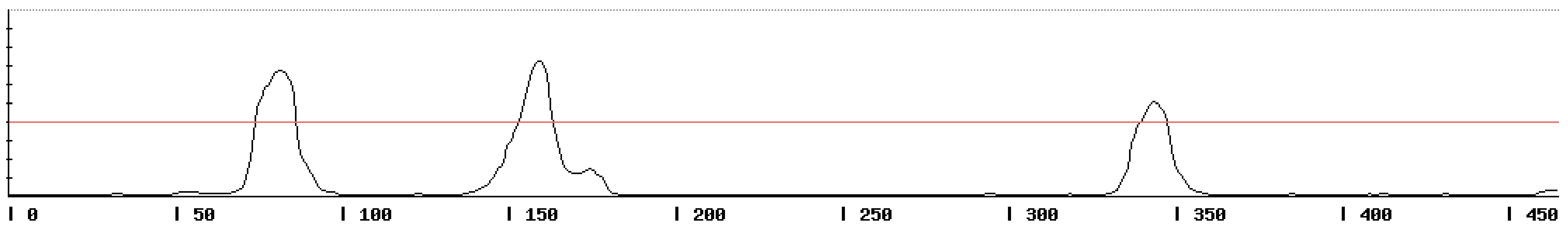

Predictions for Leersiaperrieri_LPERR07G12620.1

Viterbi Path

Viterbi Path

Posterior @ 0.4

Posterior @ 0.4

Posterior Graphic. Threshold Marked @ 0.4

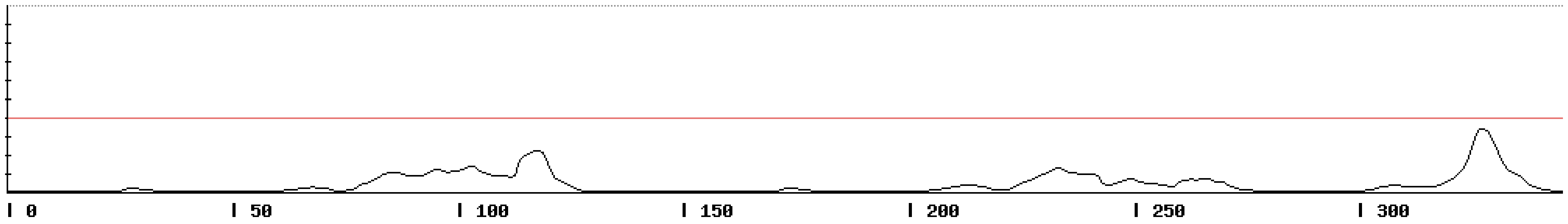

Predictions for Medicagotruncatula_Medtr8g023900.1	
Viterbi Path	
Viterbi Path	
Posterior @ 0.4	
Posterior @ 0.4	
Posterior Graphic. Threshold Marked @ 0.4	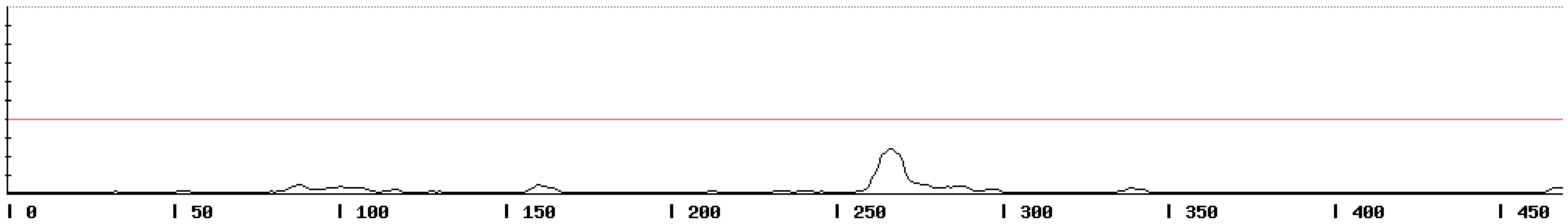

Predictions for Morusnotabilis_XP_010091280.1	
Viterbi Path	
Viterbi Path	
Posterior @ 0.4	
Posterior @ 0.4	

Posterior Graphic. Threshold Marked @ 0.4	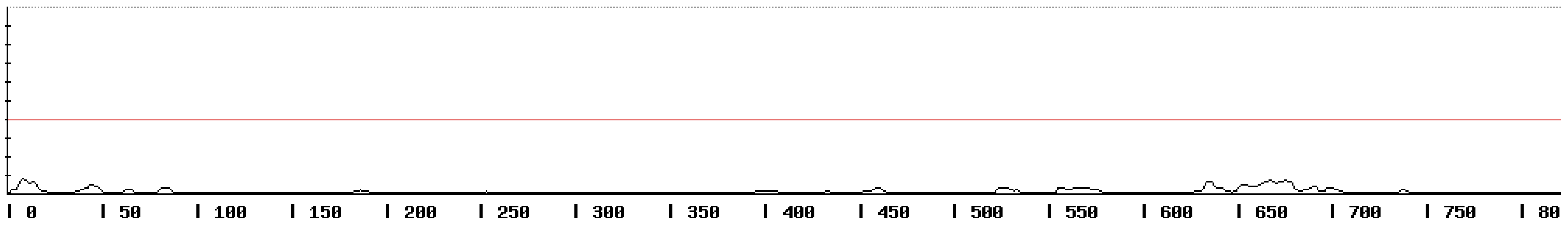

Predictions for Morusnotabilis_XP_010108843.1

Viterbi Path	538 - RGGSMRSKVEKRMKKESGKTLREIRRAKKIQKKLMTESERLIYNLKRAKKK - 588
Viterbi Path	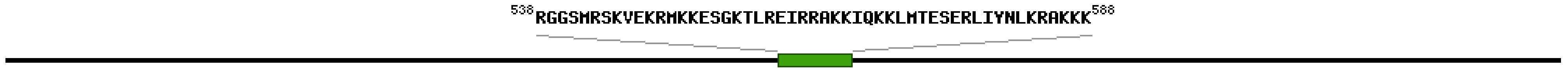
Posterior @ 0.4	161 - KRR - 163
	537 - ARGGSMRSKVEKRMKKESGKTLREIRRAKKIQKKLMTESERLIYNLKRAKKKVA - 590
Posterior @ 0.4	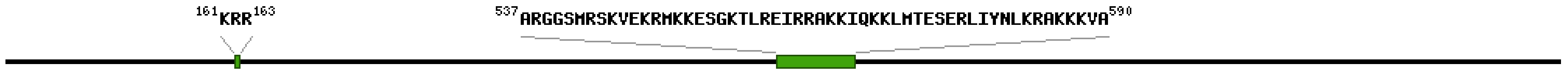
Posterior Graphic. Threshold Marked @ 0.4	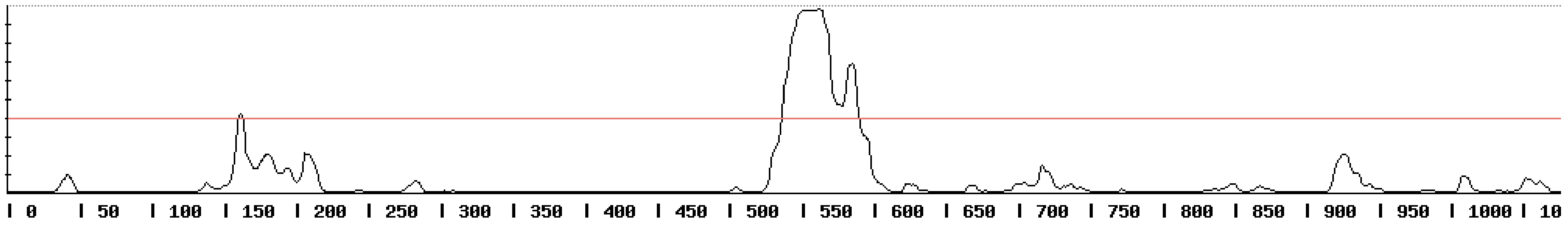

Predictions for Nicotianabenthamiana_Niben101Scf04745g02009.1

Viterbi Path	
Viterbi Path	
Posterior @ 0.4	
Posterior @ 0.4	

Posterior Graphic. Threshold Marked @ 0.4

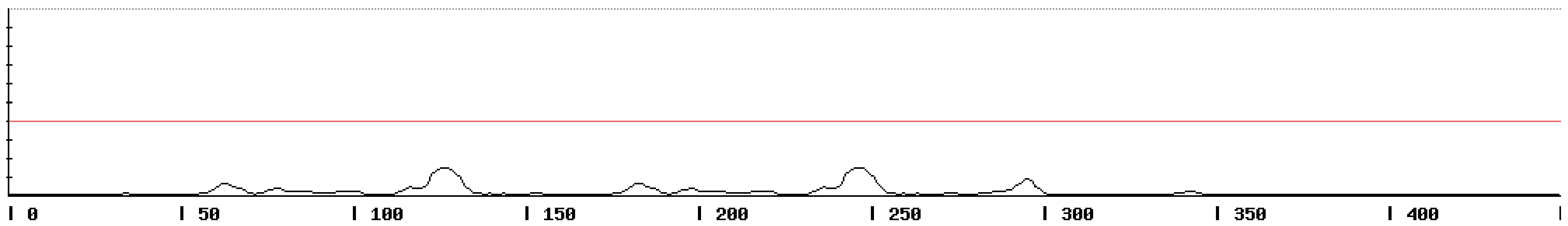

Predictions for Nicotianabenthamiana_Niben101Scf00193g01009.1

Viterbi Path

Viterbi Path

Posterior @ 0.4

Posterior @ 0.4

Posterior Graphic. Threshold Marked @ 0.4

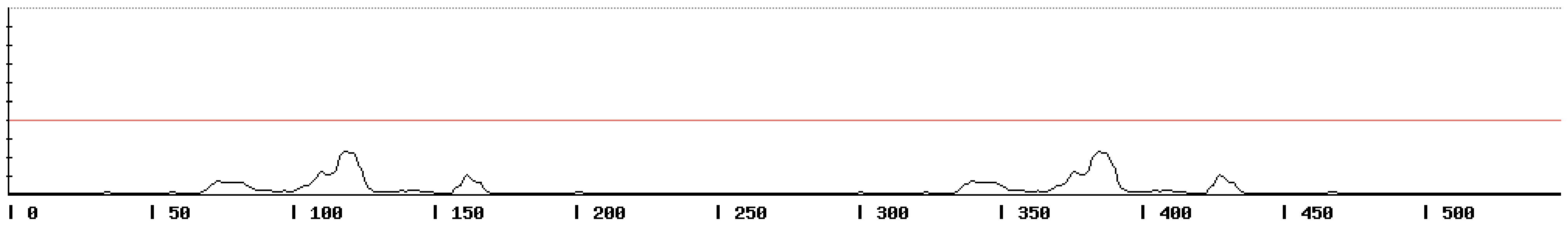

Predictions for Nicotianabenthamiana_Niben101Scf07168g02012.1

Viterbi Path

Viterbi Path

Posterior @ 0.4	78 - KKEKPTWKRVSRTVGKGTWKPQGKG - 102
	161 - KIKKKGKPGDKIK - 173
	307 - KKEKPTWKRVSRTVGKGTWKPQGKG - 331
	390 - KIKKKGKPGDKIK - 402
	568 - KKEKPTWKRVSRTVGKGTWKPQGKG - 592
	651 - KIKKKGKPGDKIK - 663
Posterior @ 0.4	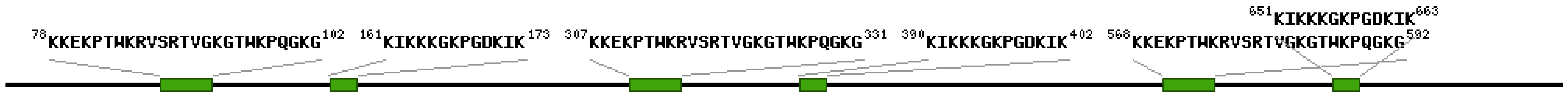
Posterior Graphic. Threshold Marked @ 0.4	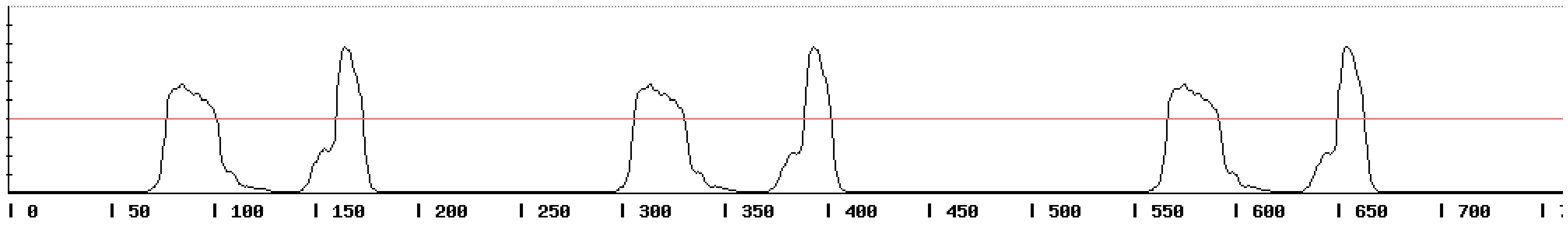

Predictions for Nicotianabenthamiana_Niben101Scf09065g00002.1

Viterbi Path	
Viterbi Path	
Posterior @ 0.4	298 - RLKKKKKEHSRVRR - 311
Posterior @ 0.4	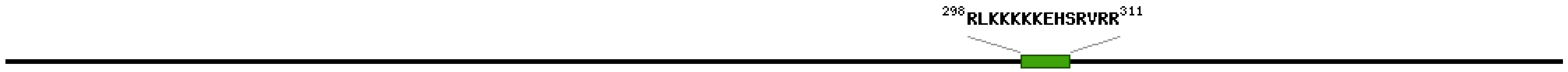
Posterior Graphic. Threshold Marked @ 0.4	

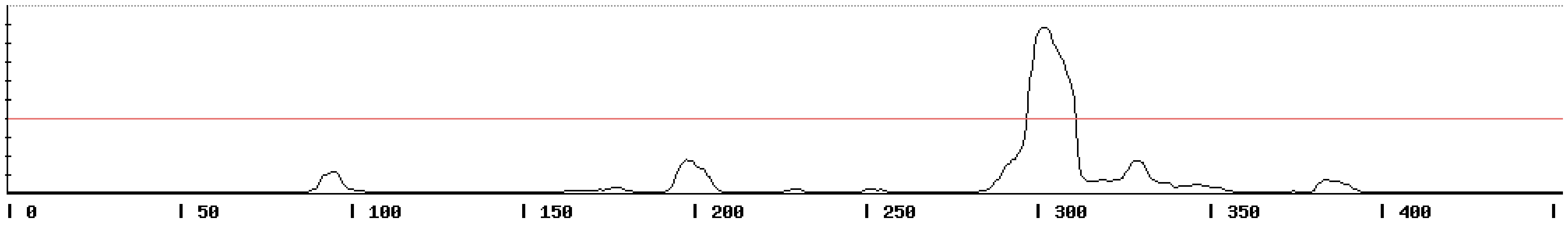

Predictions for Ocimumtenuiflorum_Ote100004440122

Viterbi Path

Viterbi Path

Posterior @ 0.4

389 - SKTGRKKPSR - 398

866 - RKWKMGKRGGGR - 877

Posterior @ 0.4

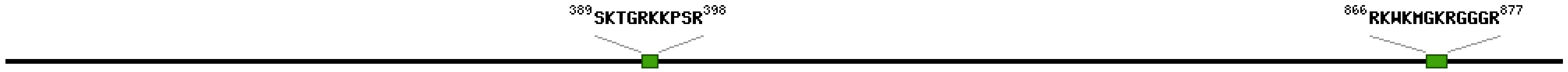

Posterior Graphic. Threshold Marked @ 0.4

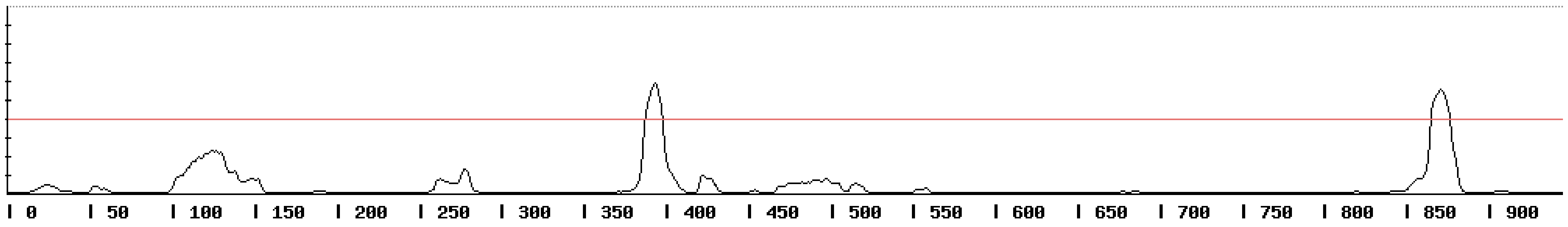

Predictions for Ocimumtenuiflorum_Ote100092950061

Viterbi Path

Viterbi Path

Posterior @ 0.4

868 - GRRKRRRIQDG - 878

Posterior @ 0.4

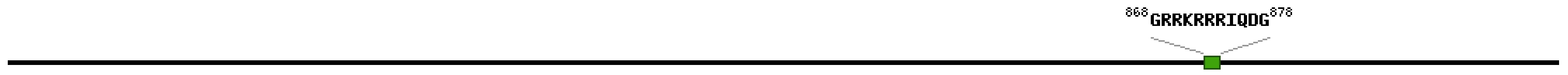

Posterior Graphic. Threshold Marked @ 0.4

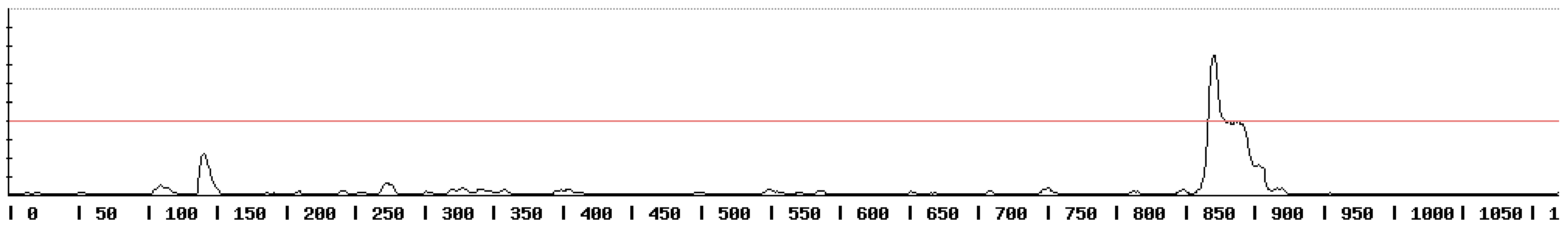

Predictions for Ocimumtenuiflorum_Ote100239950061

Viterbi Path

Viterbi Path

Posterior @ 0.4

Posterior @ 0.4

Posterior Graphic. Threshold Marked @ 0.4

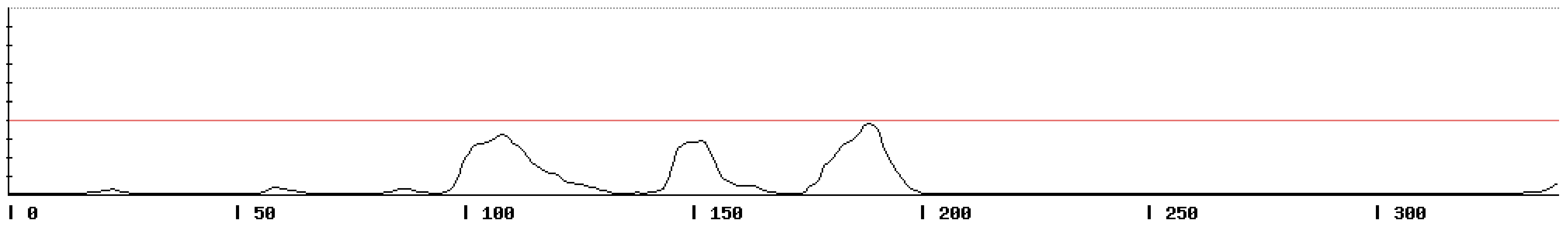

Predictions for Oropetiumthomaeum_20150105_15183A

Viterbi Path

Viterbi Path

Posterior @ 0.4

Posterior @ 0.4

Posterior Graphic. Threshold Marked @ 0.4

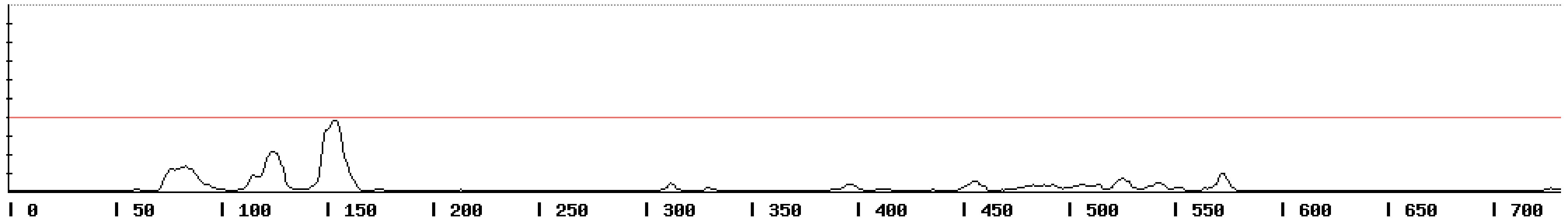

Predictions for Oryzabrachyantha_OB04G21050.1

Viterbi Path

Viterbi Path

Posterior @ 0.4

Posterior @ 0.4

Posterior Graphic. Threshold Marked @ 0.4

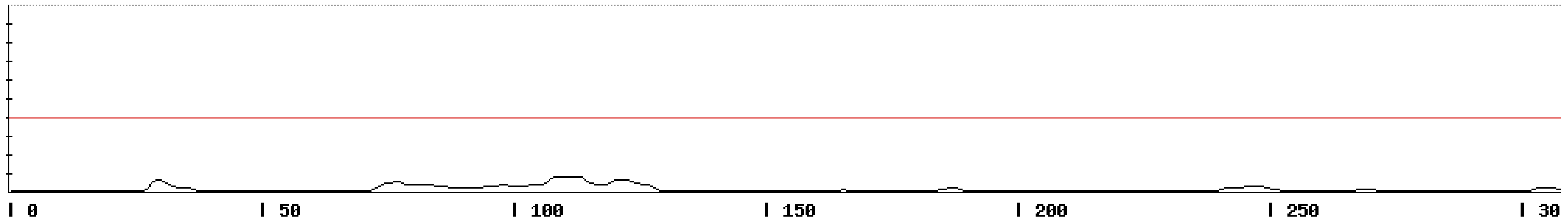

Predictions for Oryzabrachyantha_OB11G21460.1

Viterbi Path

Viterbi Path

Posterior @ 0.4 | 77 - RKERKHP - 83

Posterior @ 0.4

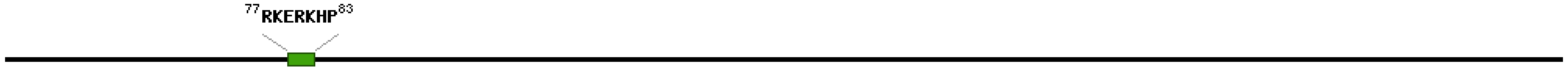

Posterior Graphic. Threshold Marked @ 0.4

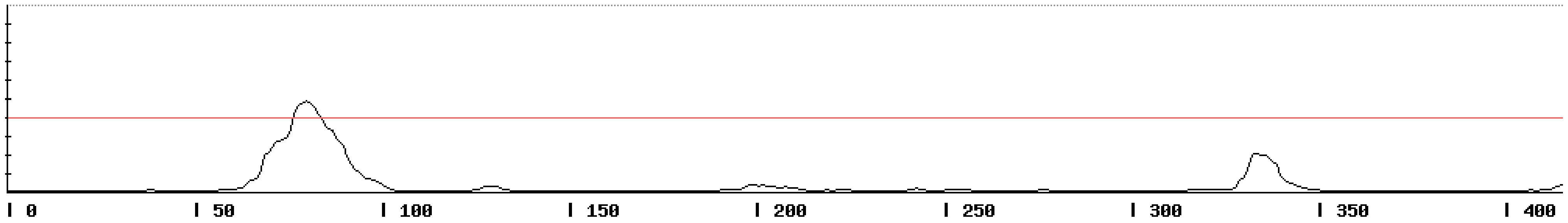

Predictions for Oryzaglumaepatula_OGLUM04G18300.1

Viterbi Path

Viterbi Path

Posterior @ 0.4 | 299 - PPRPRKA - 305

Posterior @ 0.4

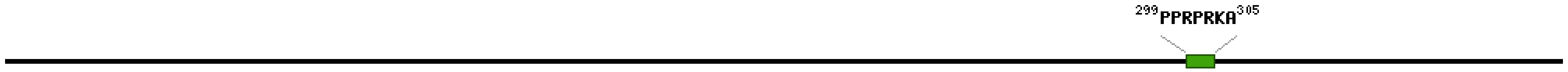

Posterior Graphic. Threshold Marked @ 0.4

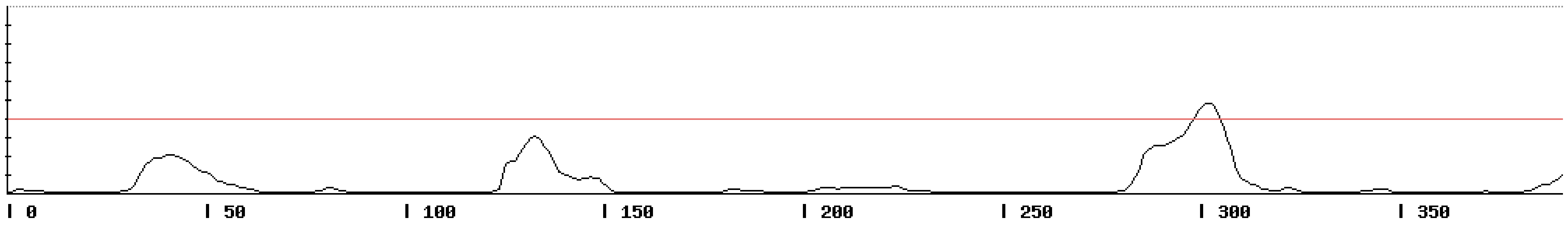

Predictions for Oryzaglumaepatula_OGLUM11G14240.1

Viterbi Path

Viterbi Path

Posterior @ 0.4

Posterior @ 0.4

Posterior Graphic. Threshold Marked @ 0.4

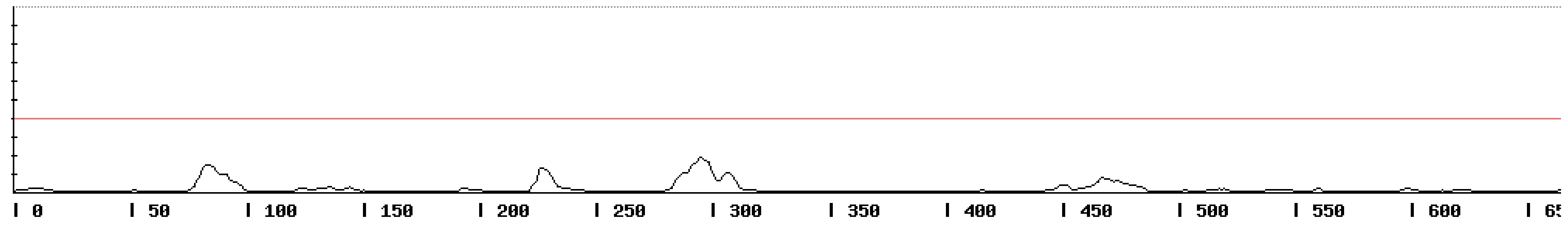

Predictions for Oryzalongistaminata_KN538956.1_FGP004

Viterbi Path

Viterbi Path

Posterior @ 0.4

Posterior @ 0.4

Posterior Graphic. Threshold Marked @ 0.4

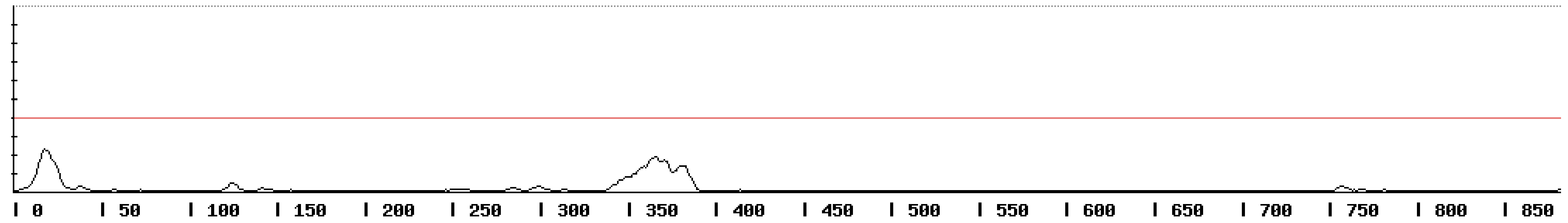

Predictions for Oryzalongistaminata_KN539106.1_FGP005

Viterbi Path

Viterbi Path

Posterior @ 0.4: 164 - RKRRK - 168

Posterior @ 0.4

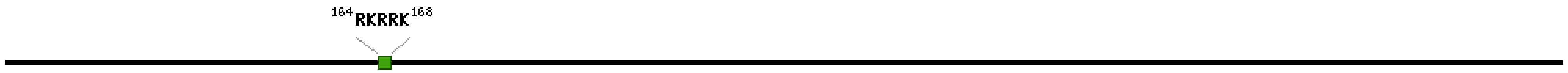

Posterior Graphic. Threshold Marked @ 0.4

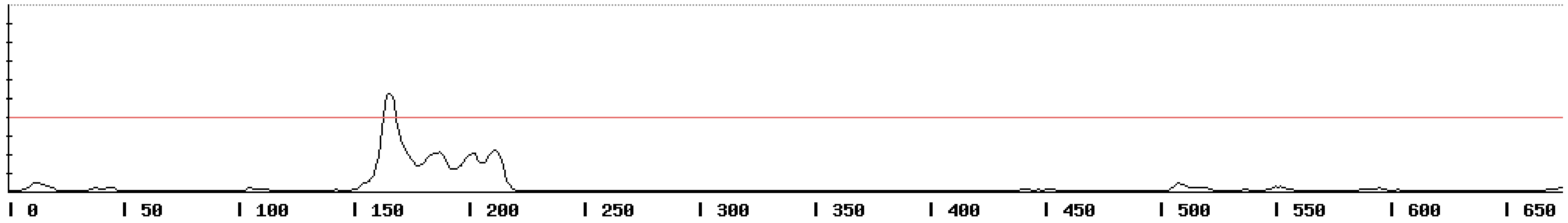

Predictions for Oryzalongistaminata_KN539151.1_FGP017

Viterbi Path

Viterbi Path

Posterior @ 0.4: 632 - KRKLSRTGSRLRGKSKA - 648

Posterior @ 0.4

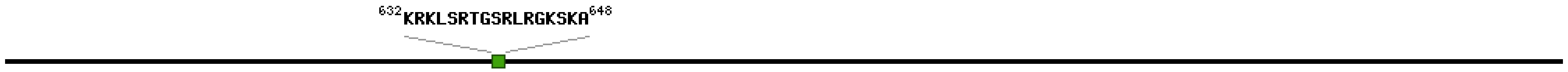

Posterior Graphic. Threshold Marked @ 0.4

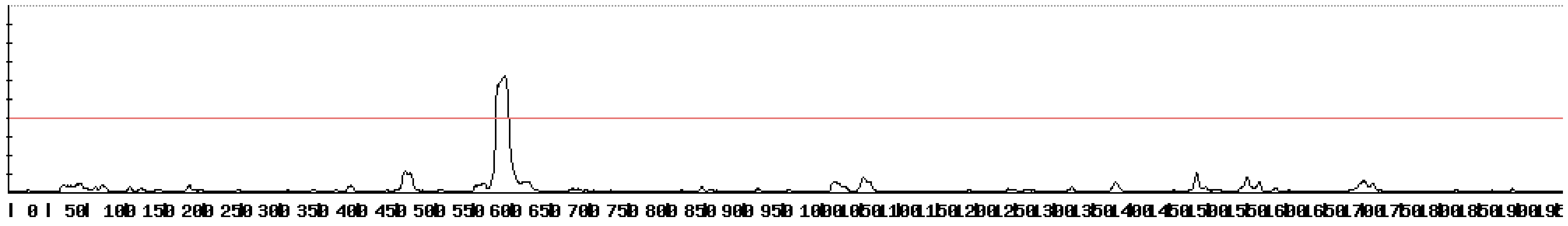

Predictions for Oryzalongistaminata_KN539501.1_FGP004

Viterbi Path

Viterbi Path

Posterior @ 0.4

Posterior @ 0.4

Posterior Graphic. Threshold Marked @ 0.4

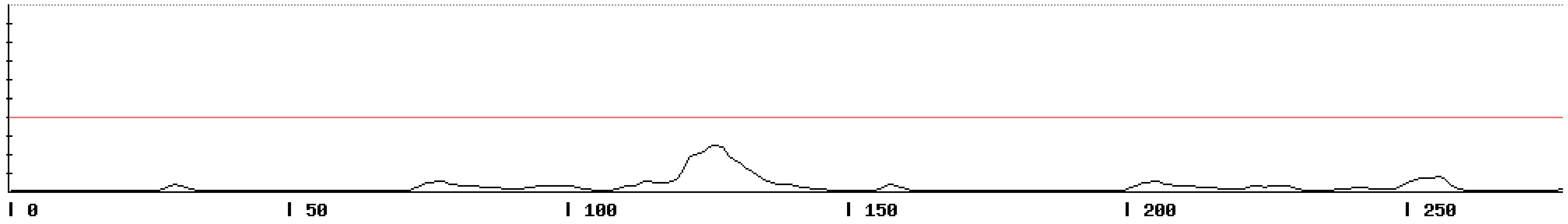

Predictions for Oryzalongistaminata_KN539582.1_FGP005

Viterbi Path

Viterbi Path

Posterior @ 0.4

Posterior @ 0.4

Posterior Graphic. Threshold Marked @ 0.4	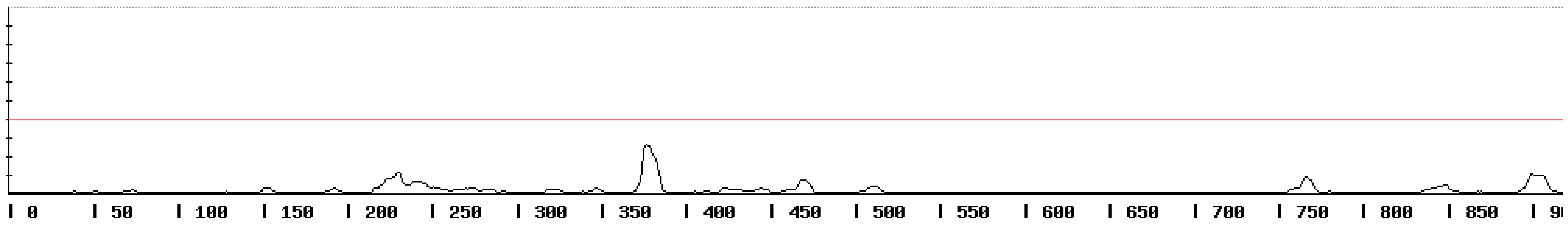

Predictions for Oryzalongistaminata_KN540176.1_FGP005

Viterbi Path	
Viterbi Path	
Posterior @ 0.4	
Posterior @ 0.4	
Posterior Graphic. Threshold Marked @ 0.4	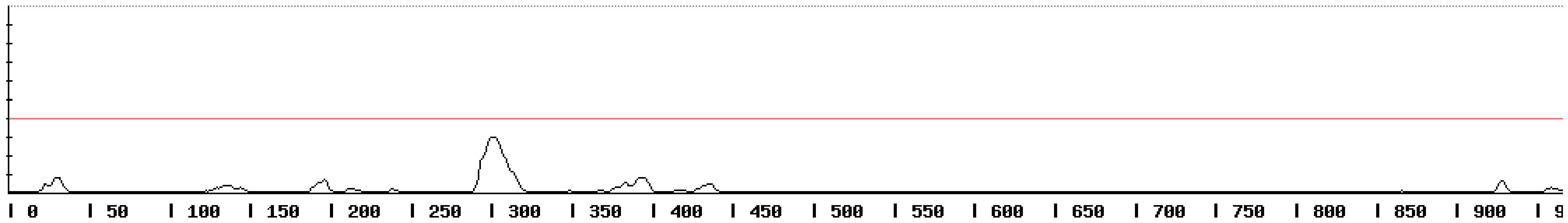

Predictions for Oryzalongistaminata_KN541034.1_FGP003

Viterbi Path	94 - RSIPHPRRKITRRRRRTTKMPPRLPDKLKQR - 124
Viterbi Path	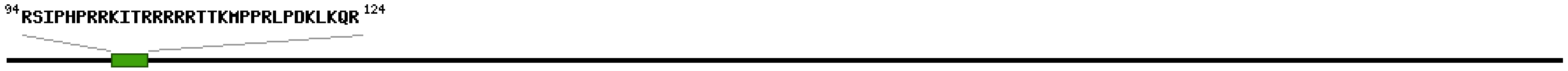
Posterior @ 0.4	95 - SIPHPRRKITRRRRRTTKMPPRLPDKLKQ - 123<br>1202 - KNAHKSRKRRATESR - 1216
Posterior @ 0.4	

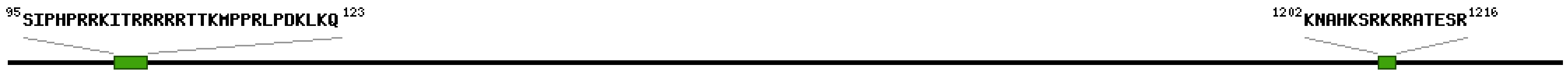

Posterior Graphic. Threshold Marked @ 0.4

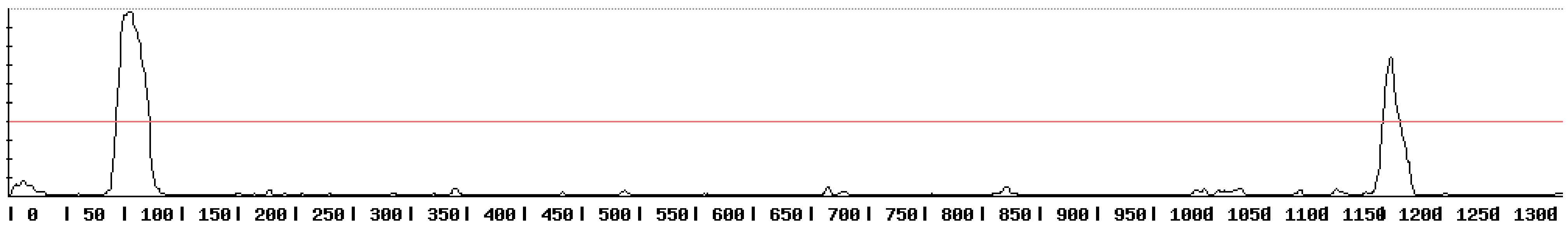

Predictions for Oryzameridionalis_OMERI04G12430.3

Viterbi Path

Viterbi Path

Posterior @ 0.4

Posterior @ 0.4

Posterior Graphic. Threshold Marked @ 0.4

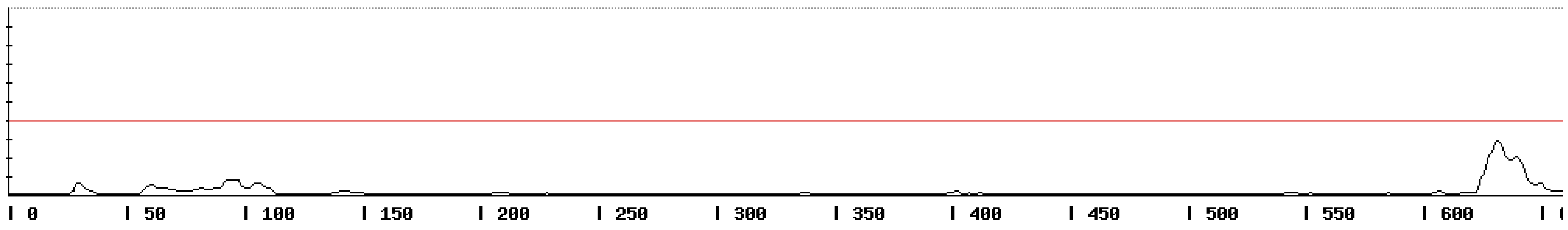

Predictions for Oryzameridionalis_OMERI04G15260.1

Viterbi Path

Viterbi Path

Posterior @ 0.4 248 - PRPRK - 252

Posterior @ 0.4

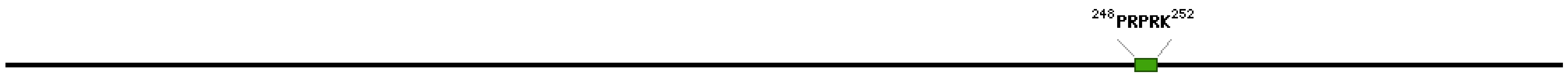

Posterior Graphic. Threshold Marked @ 0.4

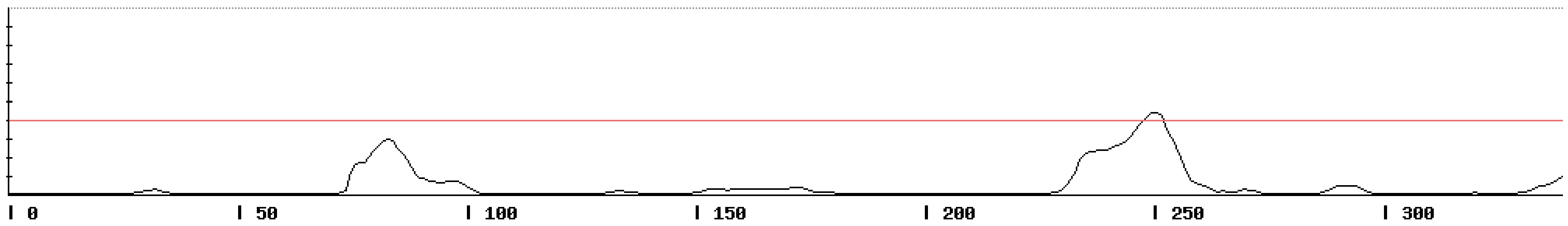

Predictions for Oryzameridionalis_OMERI04G25580.2

Viterbi Path

Viterbi Path

Posterior @ 0.4

Posterior @ 0.4

Posterior Graphic. Threshold Marked @ 0.4

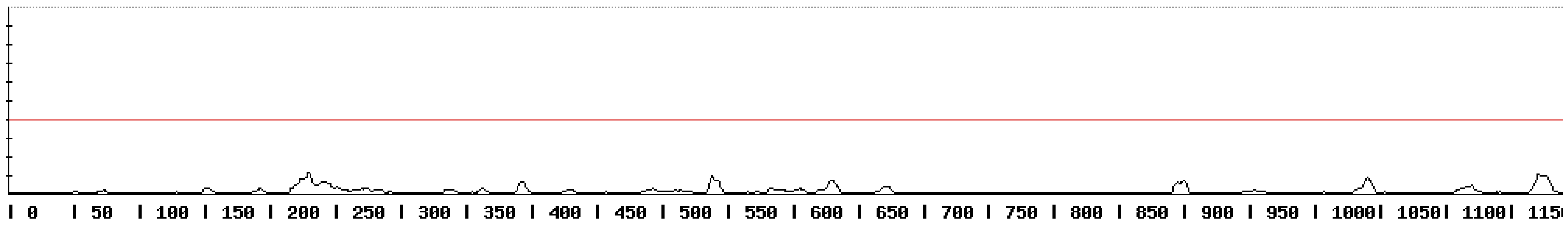

Predictions for Oryzameridionalis_OMERI12G00510.1

Viterbi Path

Viterbi Path

Posterior @ 0.4	
Posterior @ 0.4	
Posterior Graphic. Threshold Marked @ 0.4	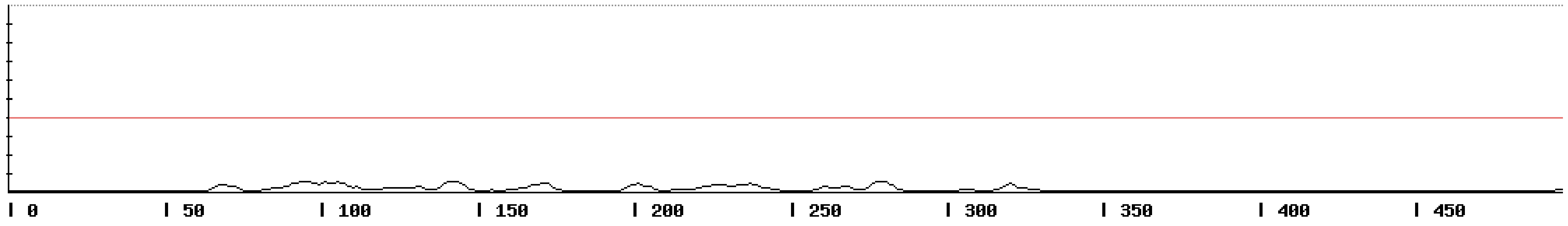

Predictions for Oryzanivara_ONIVA02G35680.1

Viterbi Path	
Viterbi Path	
Posterior @ 0.4	
Posterior @ 0.4	
Posterior Graphic. Threshold Marked @ 0.4	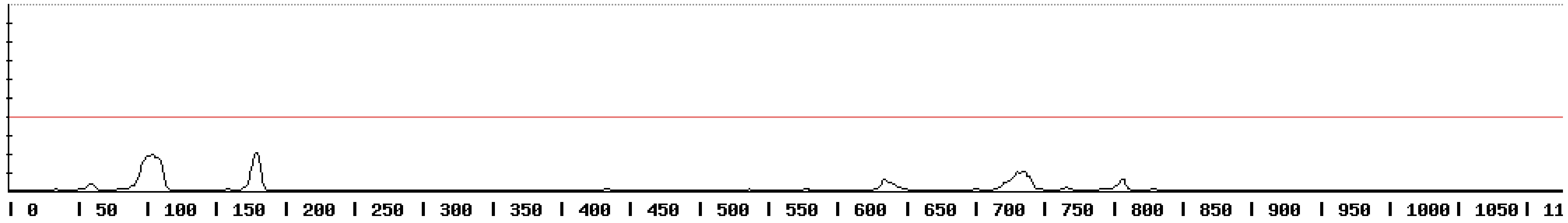

Predictions for Oryzanivara_ONIVA04G14230.1

Viterbi Path	
Viterbi Path	
Posterior @ 0.4	164 - RKRRK - 168
Posterior @ 0.4	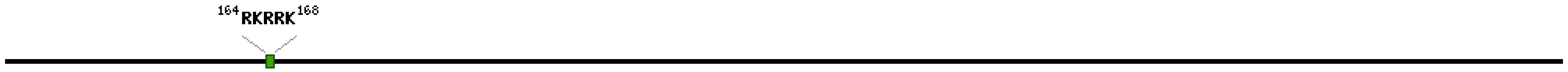
Posterior Graphic. Threshold Marked @ 0.4	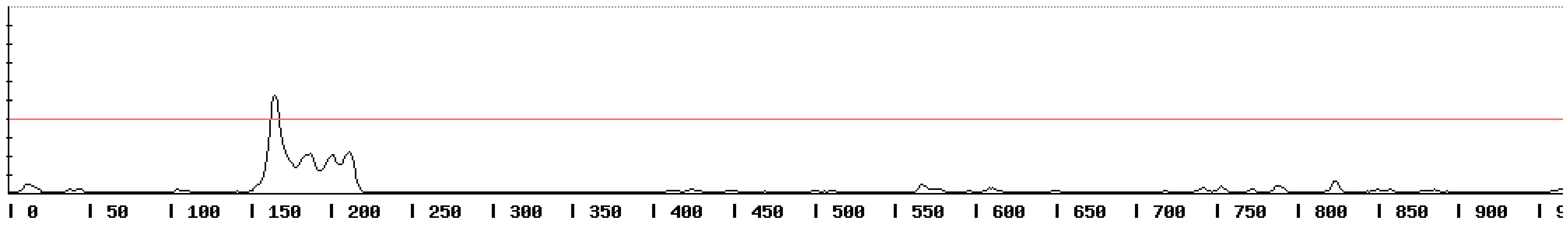

Predictions for Oryzanivara_ONIVA04G16580.1

Viterbi Path	
Viterbi Path	
Posterior @ 0.4	299 - PPRPRKA - 305
Posterior @ 0.4	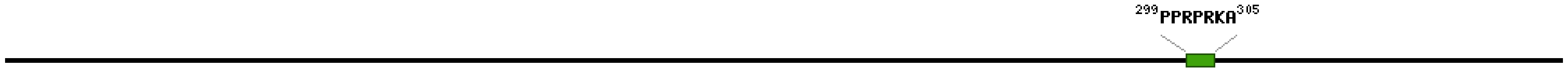
Posterior Graphic. Threshold Marked @ 0.4	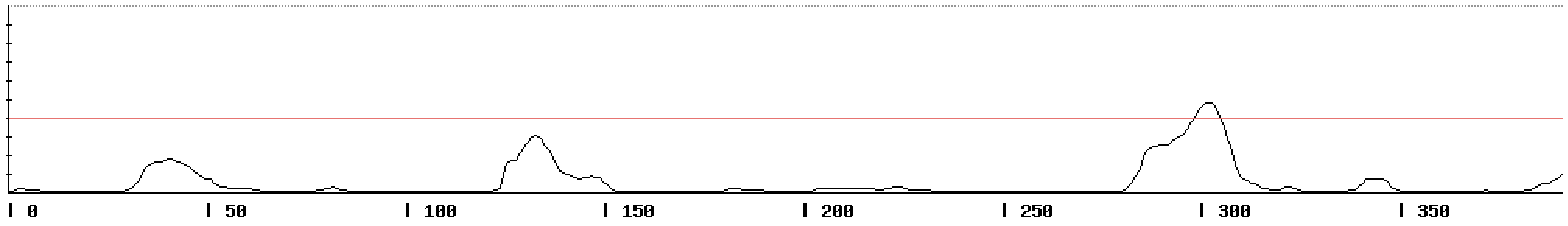

Predictions for Oryzanivara_ONIVA10G08840.1

Viterbi Path	
Viterbi Path	
Posterior @ 0.4	28 - GKWKGKEKVVPKHGKNR - 44
	140 - GNKRRPTQVAKGGT - 153
	155 - K - 155
	213 - KPGRRTKR - 220
Posterior @ 0.4	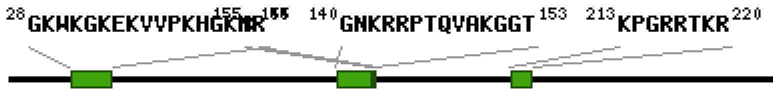
Posterior Graphic. Threshold Marked @ 0.4	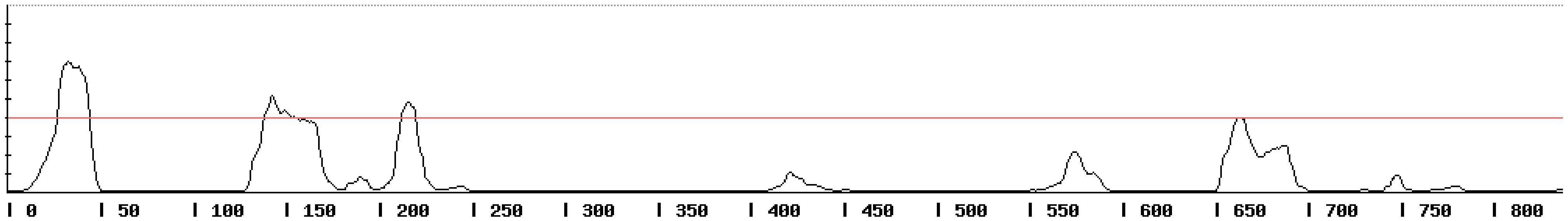

Predictions for Oryzanivara_ONIVA10G09230.1

Viterbi Path	
Viterbi Path	
Posterior @ 0.4	20 - KAAKPKQKKWPRRAAQGGRWK - 40
	839 - KAAKPKQKKWPRRAAQGGRWK - 859
Posterior @ 0.4	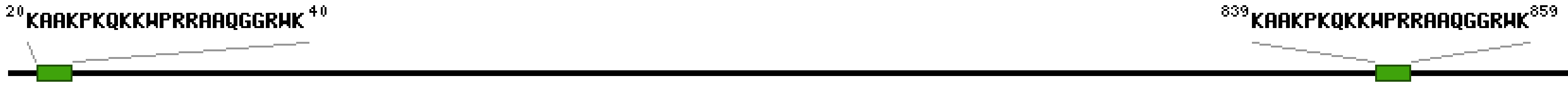
Posterior Graphic. Threshold Marked @ 0.4	

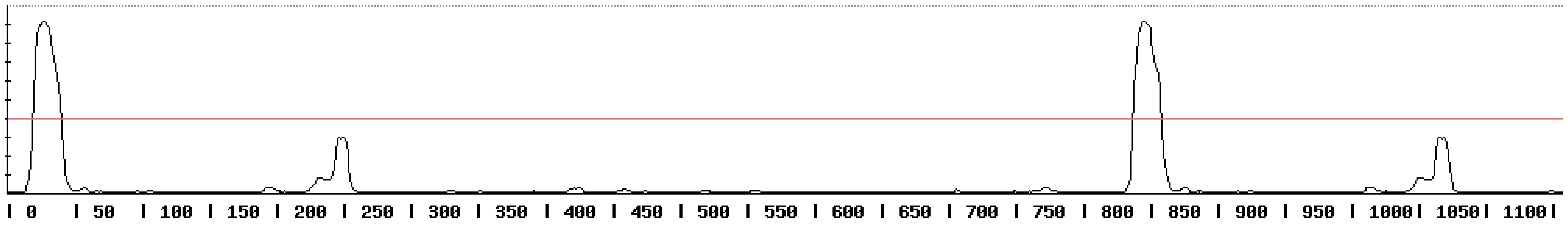

Predictions for Oryzapunctata_OPUNC04G11700.1

Viterbi Path	
Viterbi Path	
Posterior @ 0.4	
Posterior @ 0.4	
Posterior Graphic. Threshold Marked @ 0.4	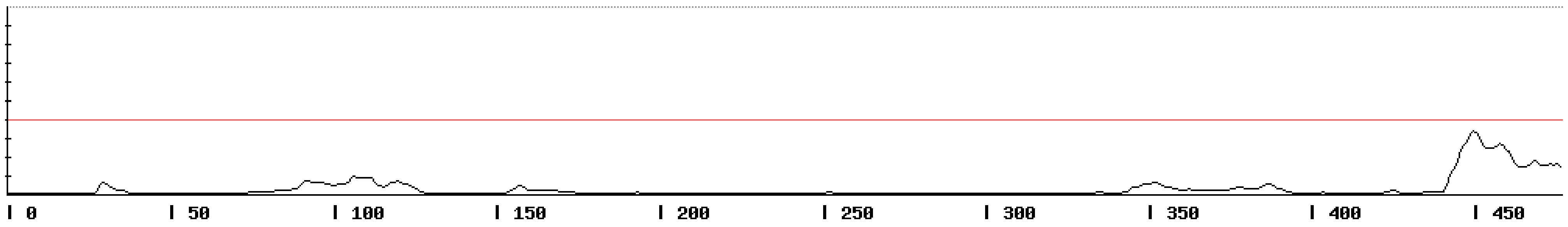

Predictions for Oryzapunctata_OPUNC04G15950.1

Viterbi Path	
Viterbi Path	
Posterior @ 0.4	277 - RRRGEHRDGASPRPRKAGP - 295
Posterior @ 0.4	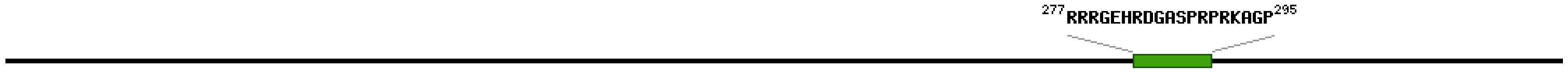

Posterior Graphic. Threshold Marked @ 0.4

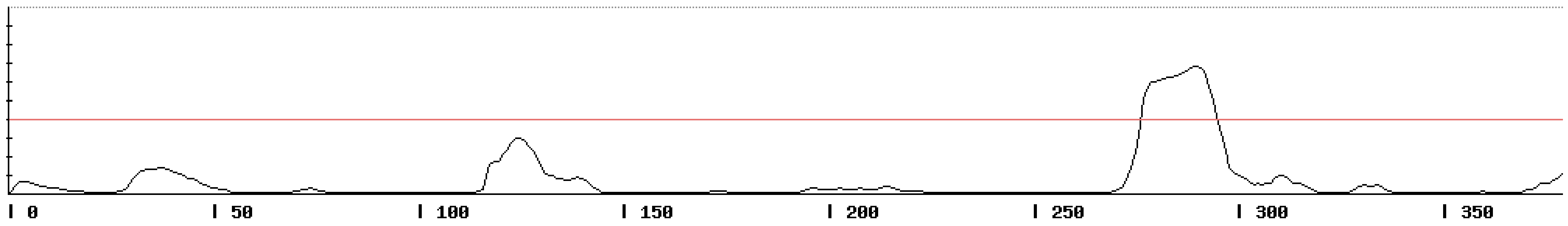

Predictions for Oryzapunctata_OPUNC06G17430.1

Viterbi Path

Viterbi Path

Posterior @ 0.4

156 - RFSGHGKKRKRE - 167

820 - RFSGHGKKRKREP - 832

Posterior @ 0.4

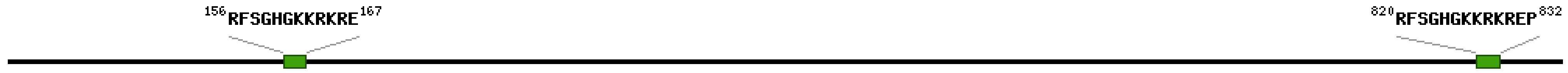

Posterior Graphic. Threshold Marked @ 0.4

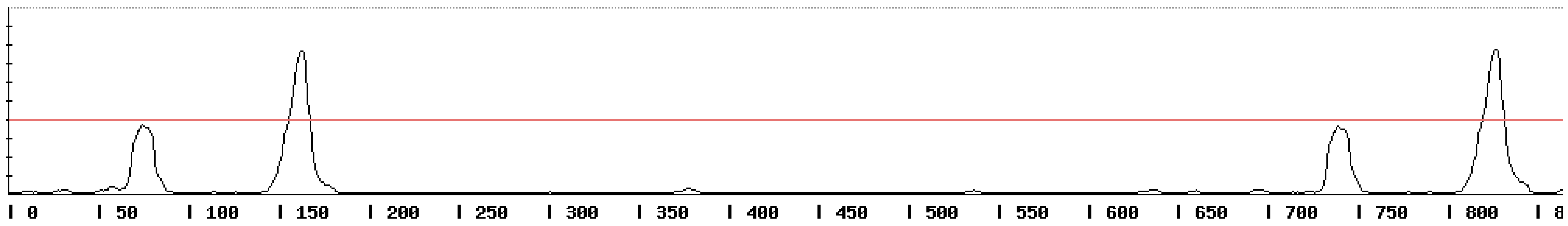

Predictions for Oryzapunctata_OPUNC09G17350.1

Viterbi Path

Viterbi Path

Posterior @ 0.4

Posterior @ 0.4

Posterior Graphic. Threshold Marked @ 0.4

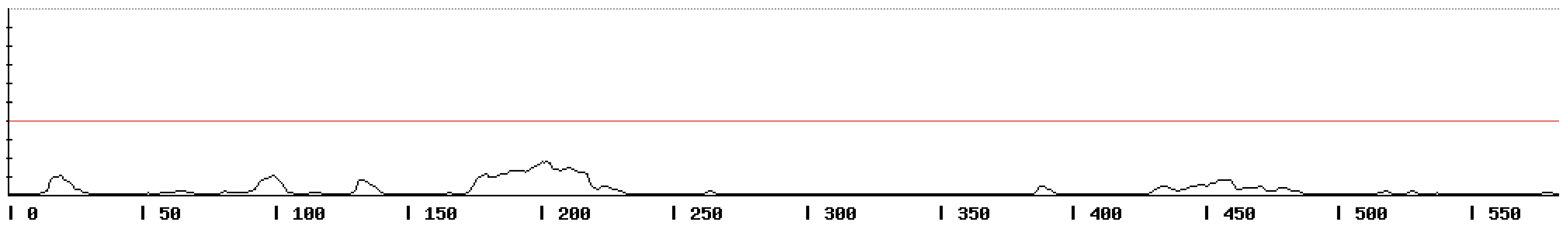

Predictions for Oryzapunctata_OPUNC10G08540.1

Viterbi Path

Viterbi Path

Posterior @ 0.4

18 - GERARRRRRVAQRLLPSG - 35

244 - LIKRKKKEEKKNRF - 257

Posterior @ 0.4

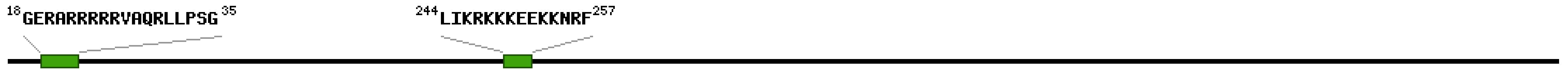

Posterior Graphic. Threshold Marked @ 0.4

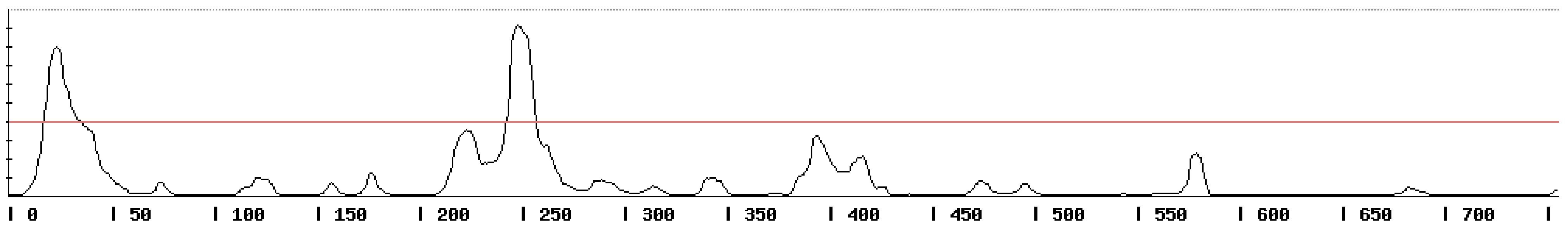

Predictions for Oryzapunctata_OPUNC11G12330.1

Viterbi Path

Viterbi Path

Posterior @ 0.4

Posterior @ 0.4

Posterior Graphic. Threshold Marked @ 0.4

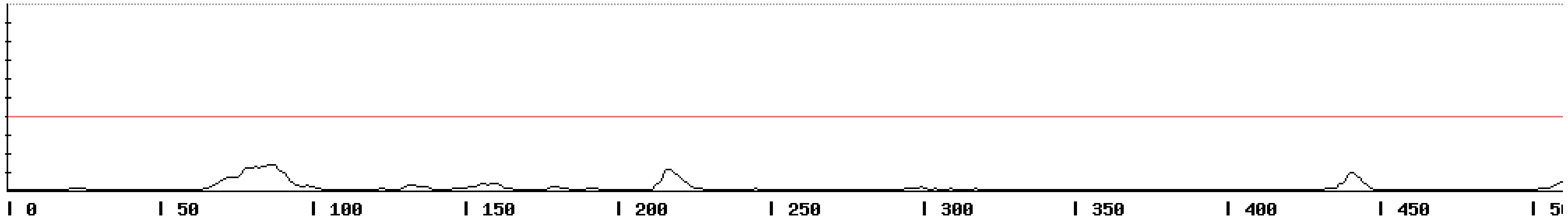

Predictions for Oryzapunctata_OPUNC11G12330.1

Viterbi Path

Viterbi Path

Posterior @ 0.4

Posterior @ 0.4

Posterior Graphic. Threshold Marked @ 0.4

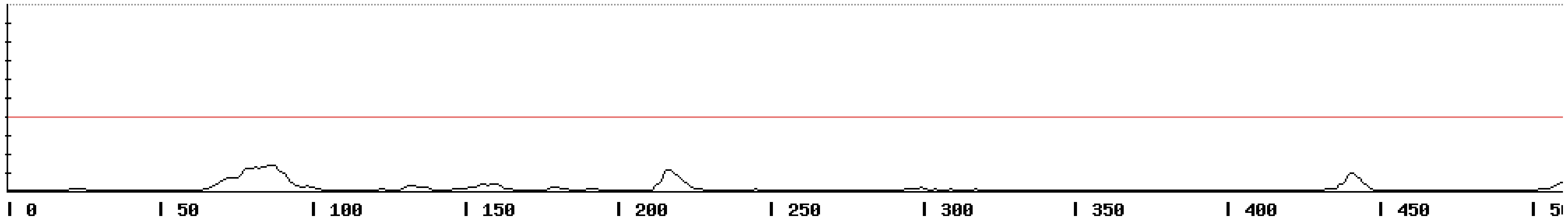

Predictions for Oryzarufipogon_ORUFI02G12670.1

Viterbi Path	
Viterbi Path	
Posterior @ 0.4	
Posterior @ 0.4	
Posterior Graphic. Threshold Marked @ 0.4	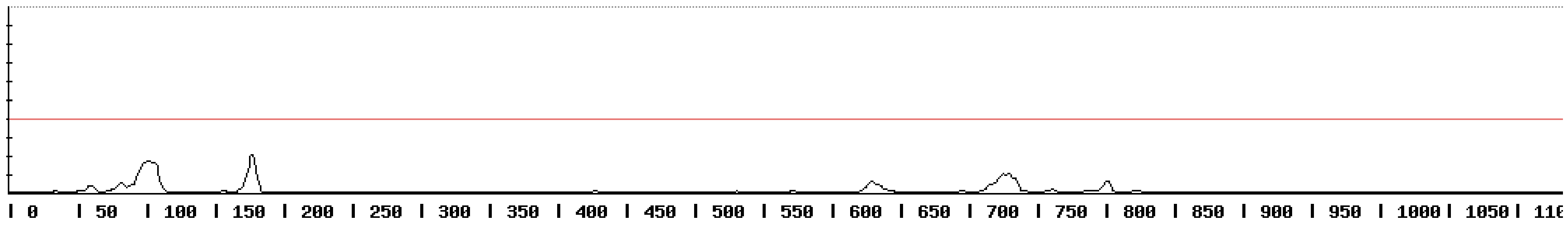

Predictions for Oryzarufipogon_ORUFI04G17470.1

Viterbi Path	
Viterbi Path	
Posterior @ 0.4	136 - RKRRK - 140
Posterior @ 0.4	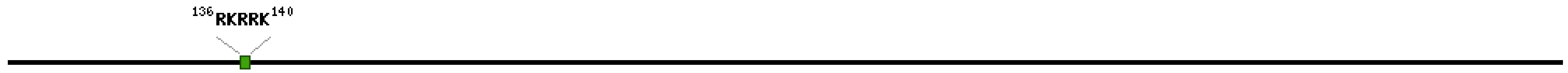
Posterior Graphic. Threshold Marked @ 0.4	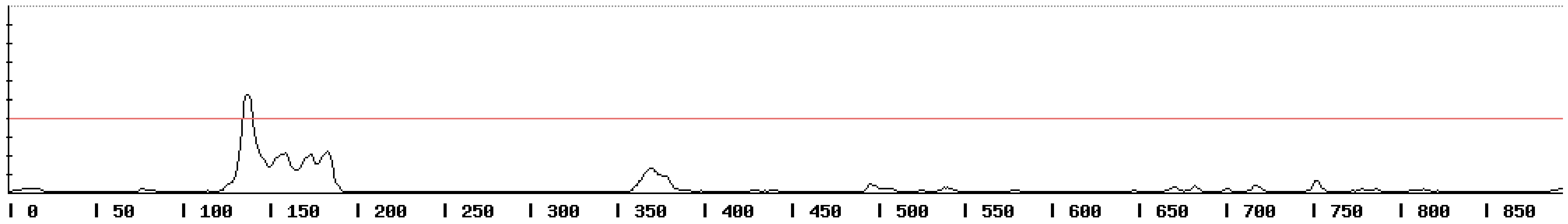

Predictions for Oryzarufipogon_ORUFI04G19680.1

Viterbi Path	
Viterbi Path	
Posterior @ 0.4	299 - PPRPRKA - 305
Posterior @ 0.4	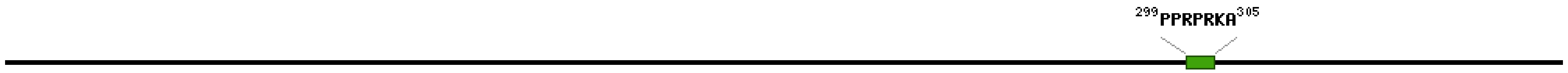
Posterior Graphic. Threshold Marked @ 0.4	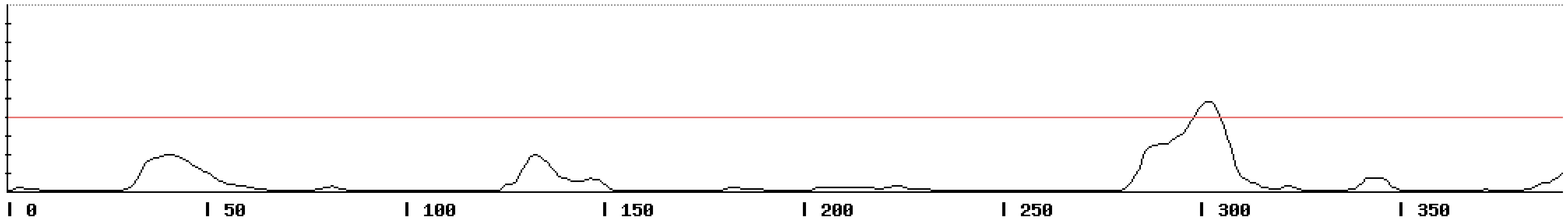

Predictions for Oryzarufipogon_ORUFI09G20530.1

Viterbi Path	
Viterbi Path	
Posterior @ 0.4	
Posterior @ 0.4	
Posterior Graphic. Threshold Marked @ 0.4	

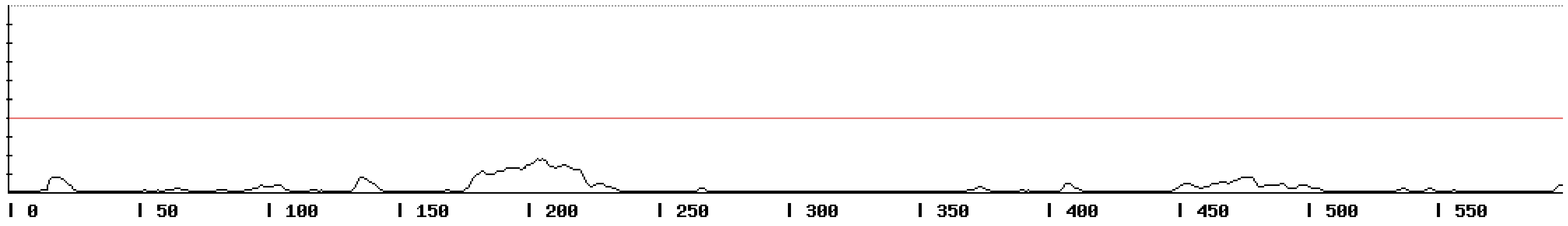

Predictions for Oryzarufipogon_ORUFI10G09270.1

Viterbi Path

Viterbi Path

Posterior @ 0.4

55 - KARNKRRPMRV - 65

384 - KG - 385

472 - AGNKRRPTRVTKDGTWKASGGSKTVRSK - 499

Posterior @ 0.4

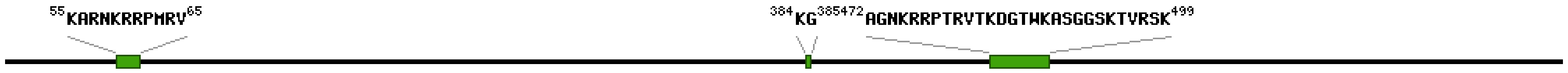

Posterior Graphic. Threshold Marked @ 0.4

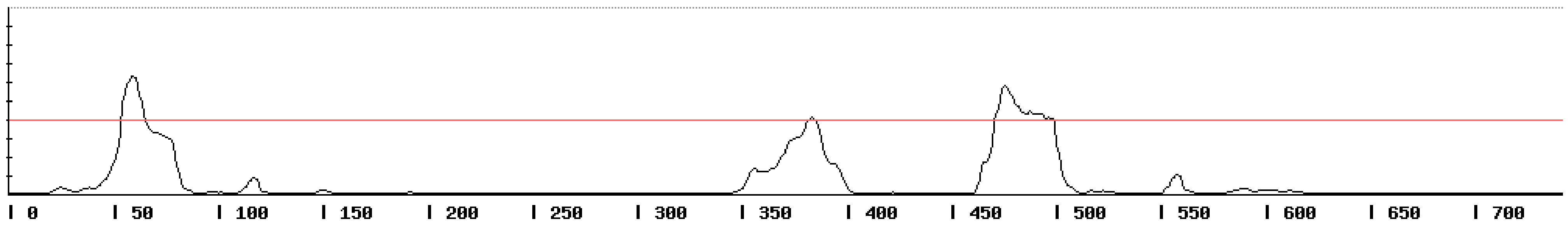

Predictions for Oryzarufipogon_ORUFI11G01500.1

Viterbi Path

Viterbi Path

Posterior @ 0.4

Posterior @ 0.4

Posterior Graphic. Threshold Marked @ 0.4	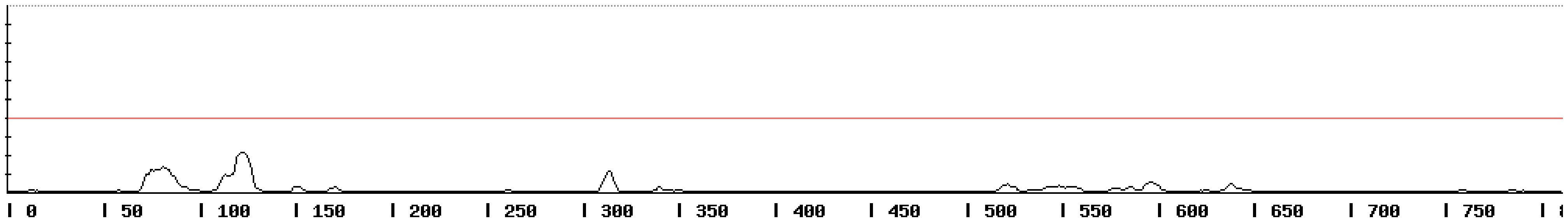

Predictions for Oryzarufipogon_ORUFI11G15730.1

Viterbi Path	
Viterbi Path	
Posterior @ 0.4	338 - KKNGKHFFTRKEKK - 351
Posterior @ 0.4	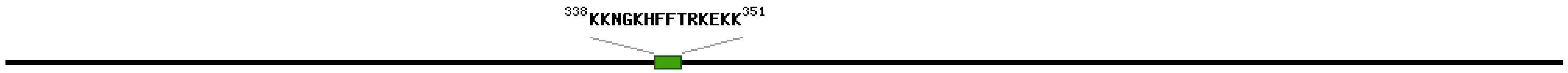
Posterior Graphic. Threshold Marked @ 0.4	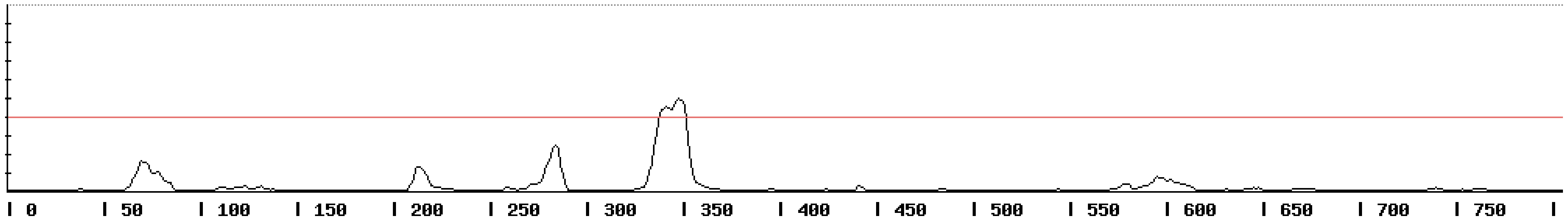

Predictions for Oryzasativa_indica_BGIOSGA014751-PA

Viterbi Path	
Viterbi Path	

Posterior @ 0.4	299 - PPRPRKA - 305
Posterior @ 0.4	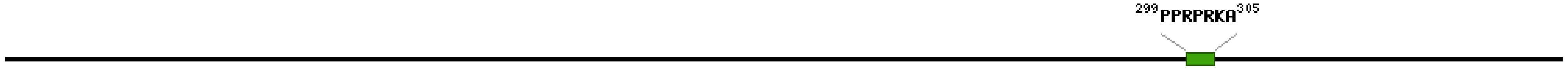
Posterior Graphic. Threshold Marked @ 0.4	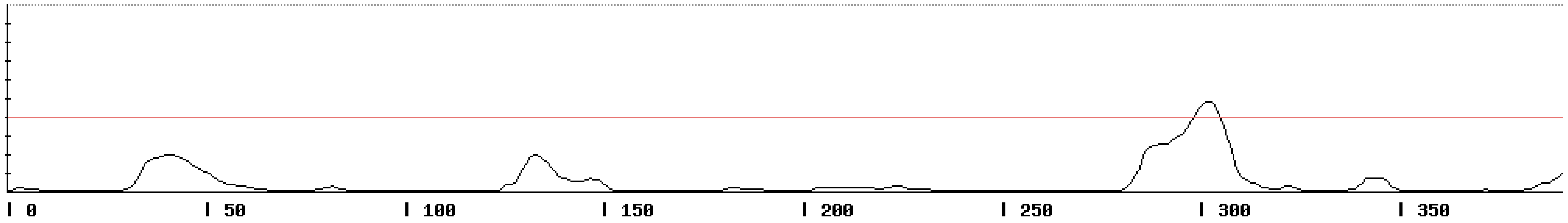

Predictions for Oryzasativa_indica_BGIOSGA015018-PA

Viterbi Path	
Viterbi Path	
Posterior @ 0.4	
Posterior @ 0.4	
Posterior Graphic. Threshold Marked @ 0.4	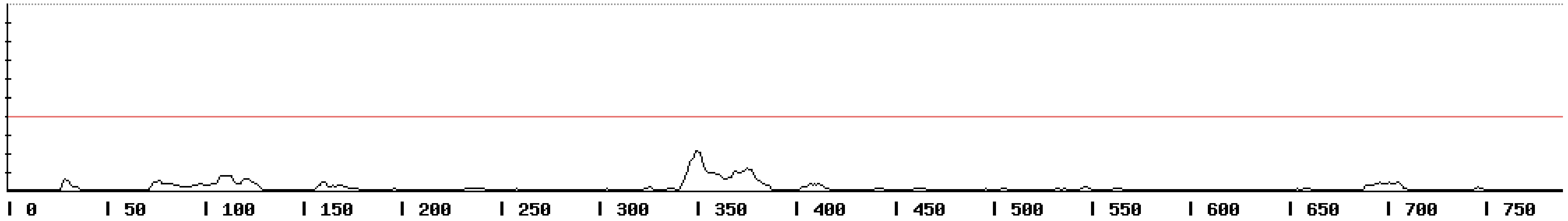

Predictions for Oryzasativa_indica_BGIOSGA034998-PA

Viterbi Path	
Viterbi Path	

Posterior @ 0.4

Posterior @ 0.4

Posterior Graphic. Threshold Marked @ 0.4

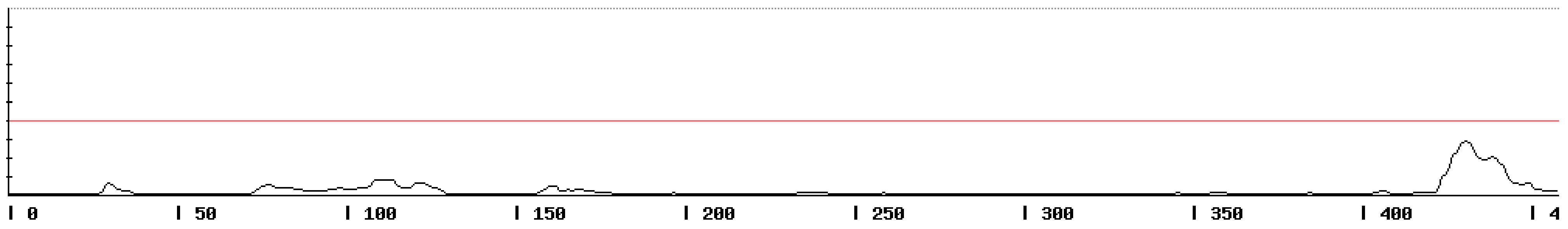

Predictions for Oryzasativa_indica_BGIOSGA036980-PA

Viterbi Path

Viterbi Path

Posterior @ 0.4

Posterior @ 0.4

Posterior Graphic. Threshold Marked @ 0.4

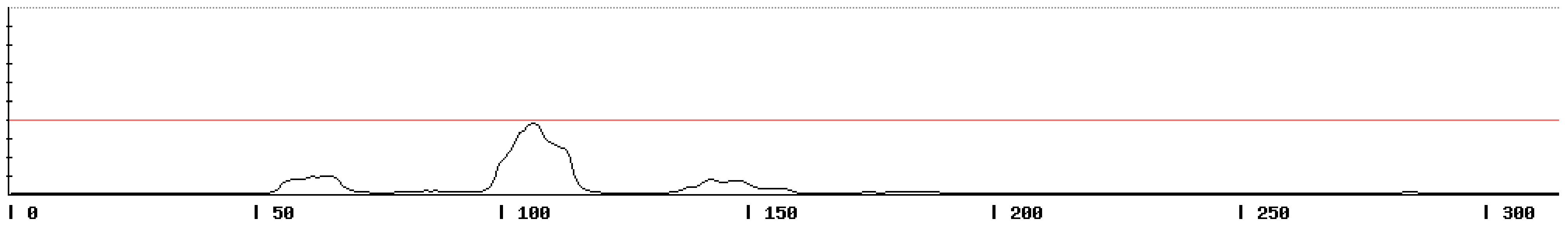

Predictions for Oryzasativa_japonica_LOC_Os04g42940.1

Viterbi Path

Viterbi Path

Posterior @ 0.4 302 - PPRPRKA - 308

Posterior @ 0.4

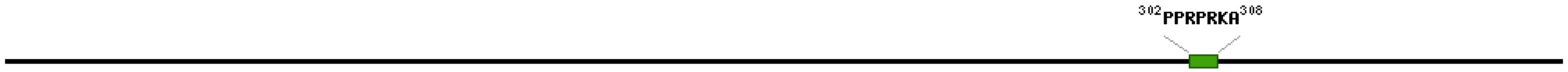

Posterior Graphic. Threshold Marked @ 0.4

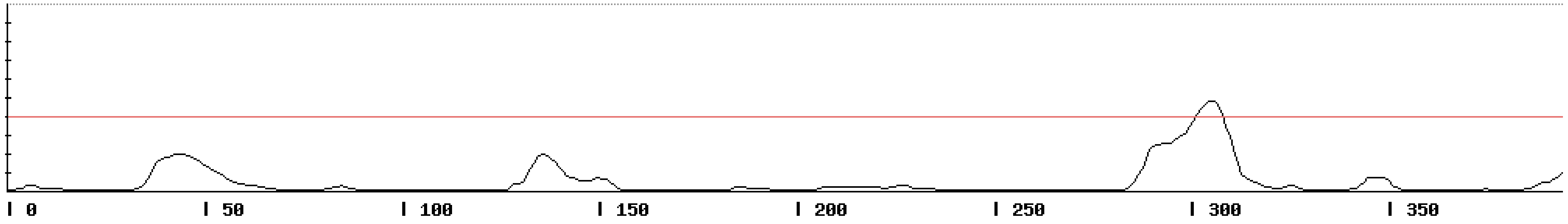

Predictions for Panicumhallii_Pahal.A02025.1

Viterbi Path

Viterbi Path

Posterior @ 0.4

107 - GGGRGREAAWRKK - 119

329 - RFSGHGKKRKRA - 340

Posterior @ 0.4

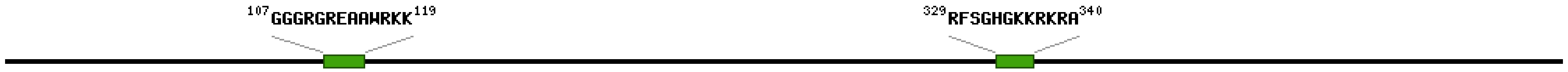

Posterior Graphic. Threshold Marked @ 0.4

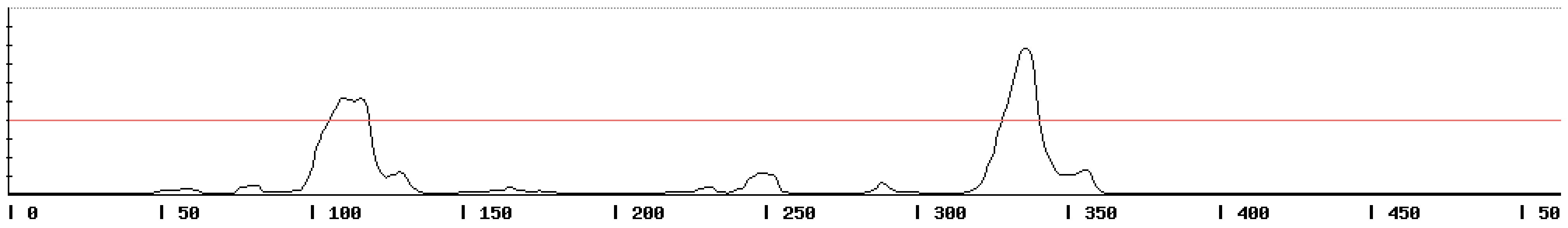

Predictions for Panicumhallii_Pahal.C04447.1

Viterbi Path

Viterbi Path

Posterior @ 0.4

Posterior @ 0.4

Posterior Graphic. Threshold Marked @ 0.4

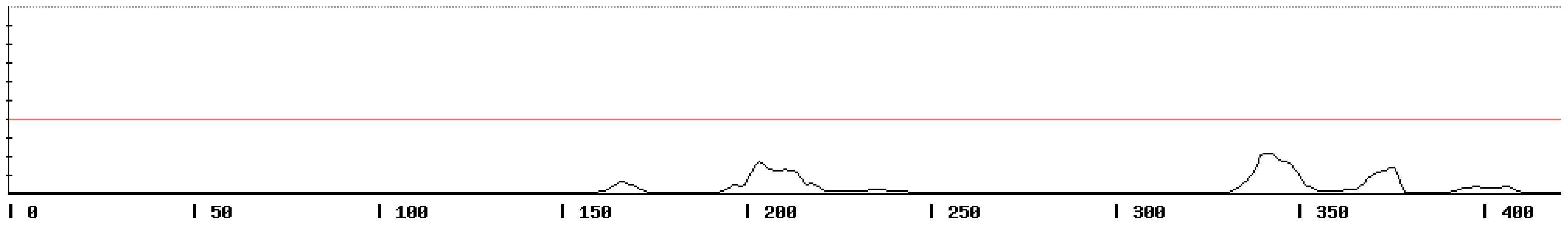

Predictions for Panicumhallii_Pahal.C04564.1

Viterbi Path

Viterbi Path

Posterior @ 0.4

134 - RKVFRKKRKDRVHSGLKR - 151

483 - GKRKRTG - 489

Posterior @ 0.4

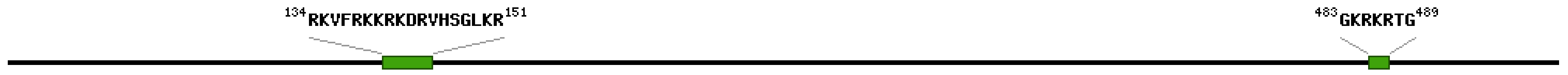

Posterior Graphic. Threshold Marked @ 0.4

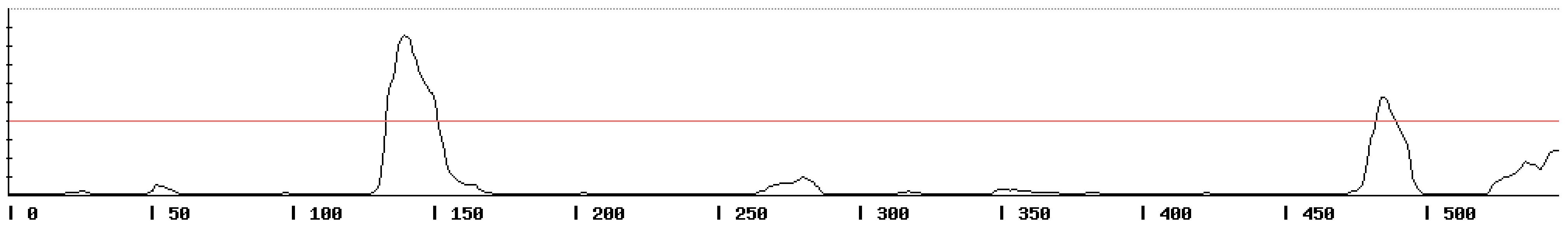

Predictions for Panicumhallii_Pahal.C04569.1

Viterbi Path

Viterbi Path

Posterior @ 0.4

Posterior @ 0.4

Posterior Graphic. Threshold Marked @ 0.4

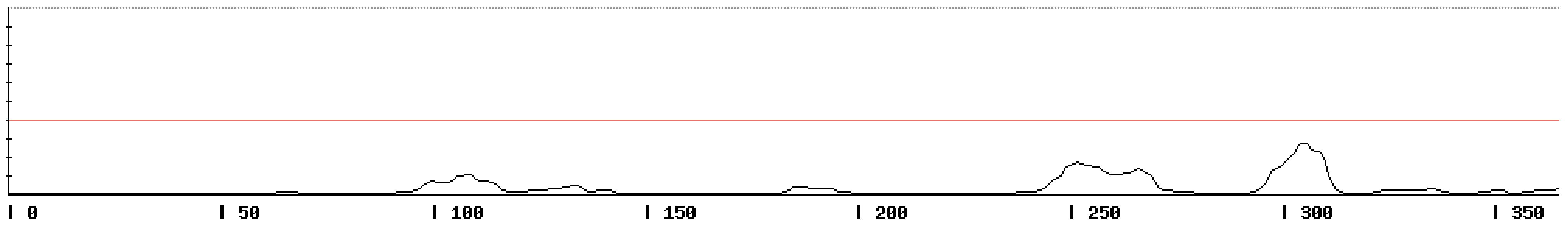

Predictions for Panicumhallii_Pahal.C04570.1

Viterbi Path

Viterbi Path

Posterior @ 0.4

Posterior @ 0.4

Posterior Graphic. Threshold Marked @ 0.4

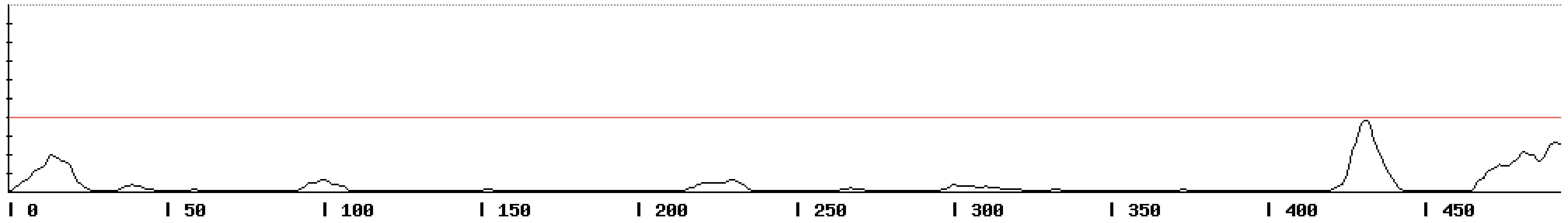

Predictions for Panicumhallii_Pahal.C04572.1

Viterbi Path

Viterbi Path

Posterior @ 0.4

Posterior @ 0.4

Posterior Graphic. Threshold Marked @ 0.4

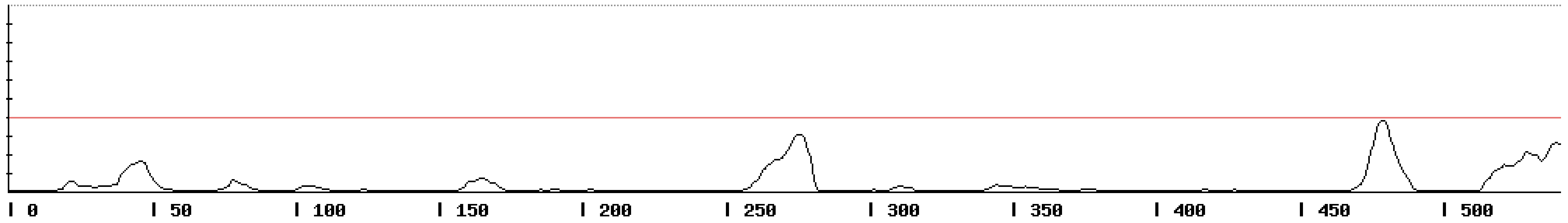

Predictions for Panicumhallii_Pahal.C04574.1

Viterbi Path

Viterbi Path

Posterior @ 0.4

Posterior @ 0.4

Posterior Graphic. Threshold Marked @ 0.4

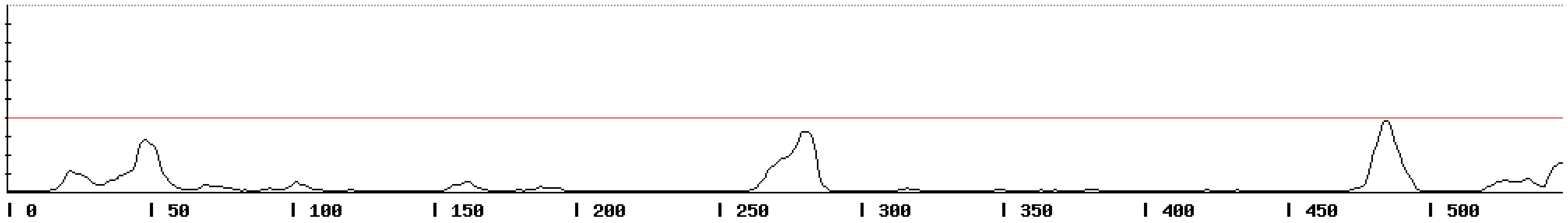

Predictions for Panicumhallii_Pahal.G00927.1

Viterbi Path

Viterbi Path

Posterior @ 0.4 6 - PGRRLRPSP - 14

Posterior @ 0.4

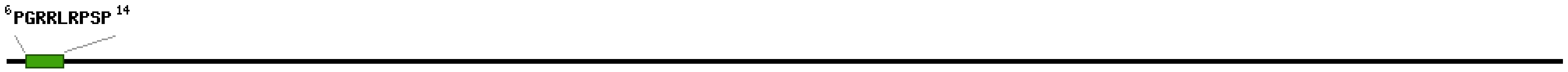

Posterior Graphic. Threshold Marked @ 0.4

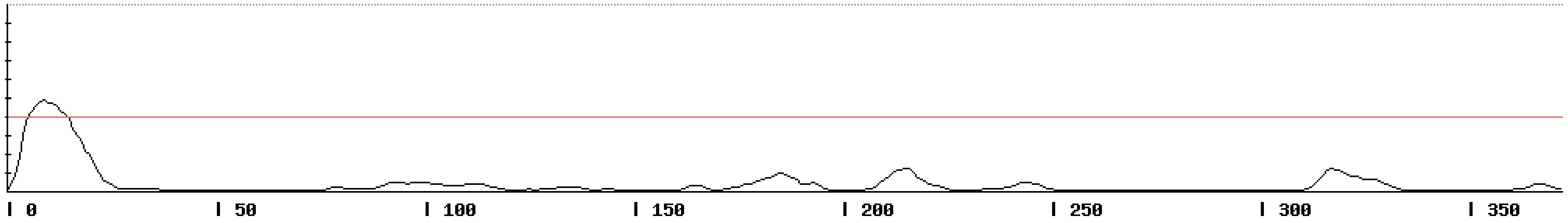

Predictions for Panicumhallii_Pahal.G01013.1

Viterbi Path

Viterbi Path

Posterior @ 0.4

87 - GRPLRPPRRGGNRP - 100

175 - GGKRRA - 180

Posterior @ 0.4

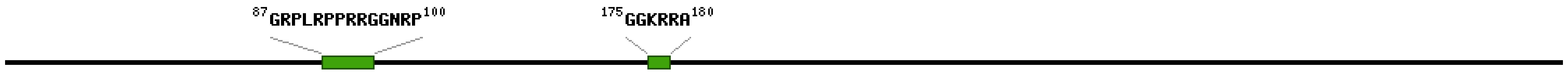

Posterior Graphic. Threshold Marked @ 0.4

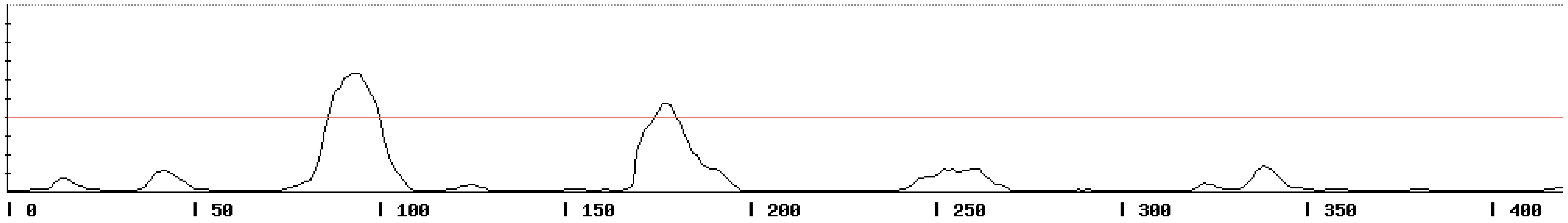

Predictions for Panicumhallii_Pahal.J01717.1

Viterbi Path

Viterbi Path

Posterior @ 0.4

Posterior @ 0.4

Posterior Graphic. Threshold Marked @ 0.4

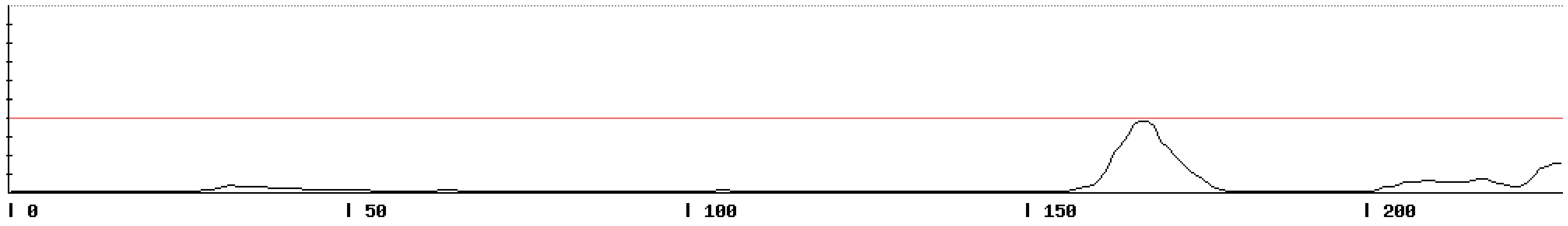

Predictions for Panicumvirgatum_Pavir.1KG322500.1.p

Viterbi Path

Viterbi Path

Posterior @ 0.4 320 - RFSGHGKKRKRGA - 332

Posterior @ 0.4

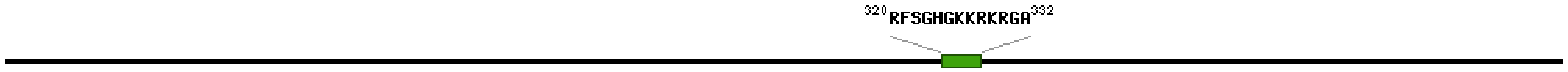

Posterior Graphic. Threshold Marked @ 0.4

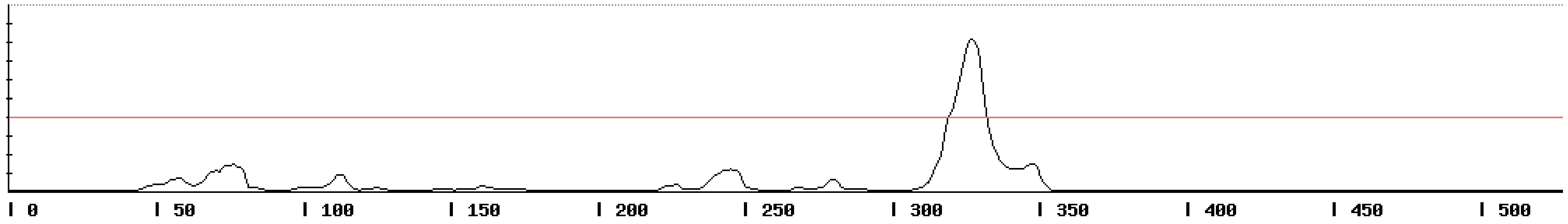

Predictions for Panicumvirgatum_Pavir.1KG445000.1.p

Viterbi Path

Viterbi Path

Posterior @ 0.4 193 - FSGHGKKRKRG - 203

Posterior @ 0.4

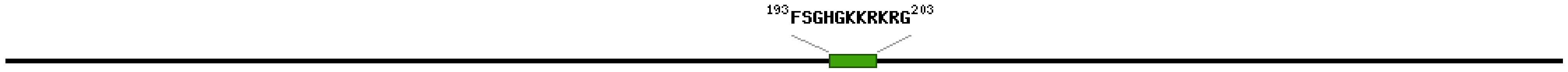

Posterior Graphic. Threshold Marked @ 0.4	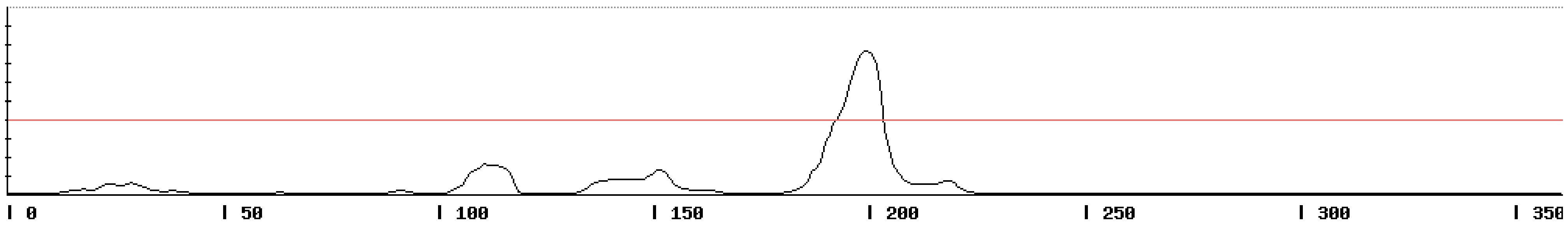

Predictions for Panicumvirgatum_Pavir.2KG138100.1.p

Viterbi Path	
Viterbi Path	
Posterior @ 0.4	320 - HGKKRKRG - 327
Posterior @ 0.4	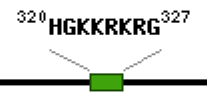
Posterior Graphic. Threshold Marked @ 0.4	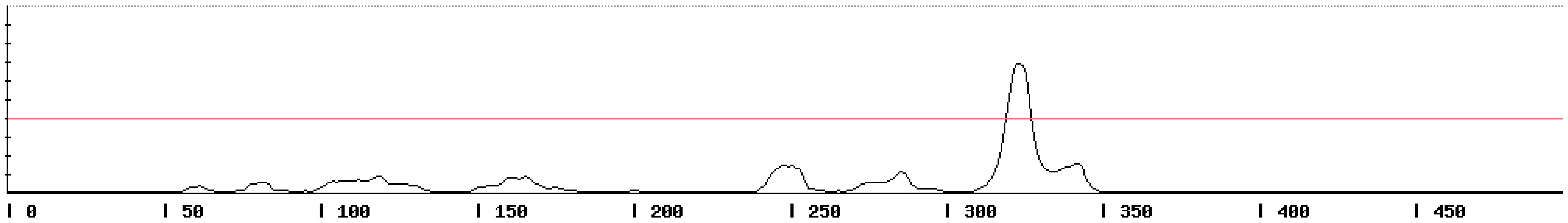

Predictions for Panicumvirgatum_Pavir.2KG140000.1.p

Viterbi Path	
Viterbi Path	
Posterior @ 0.4	
Posterior @ 0.4	

Posterior Graphic. Threshold Marked @ 0.4

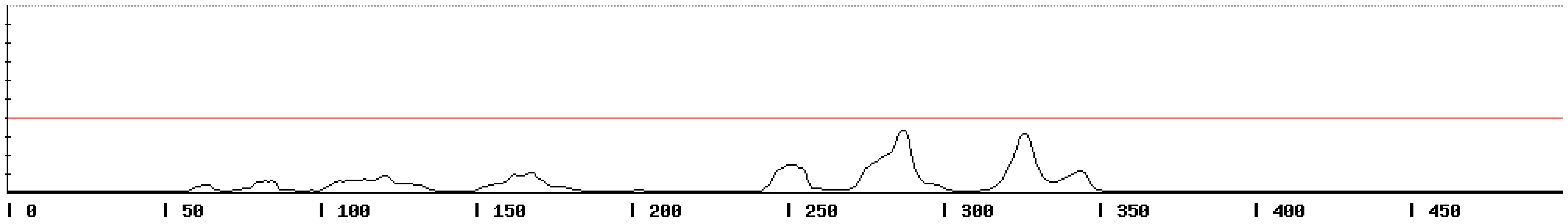

Predictions for Panicumvirgatum_Pavir.3KG480900.1.p

Viterbi Path

Viterbi Path

Posterior @ 0.4 475 - KRKR - 478

Posterior @ 0.4

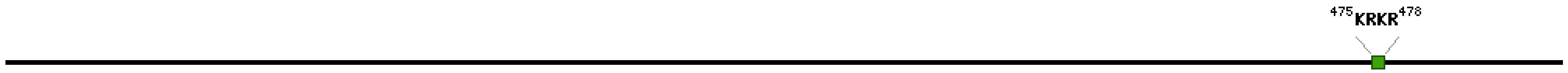

Posterior Graphic. Threshold Marked @ 0.4

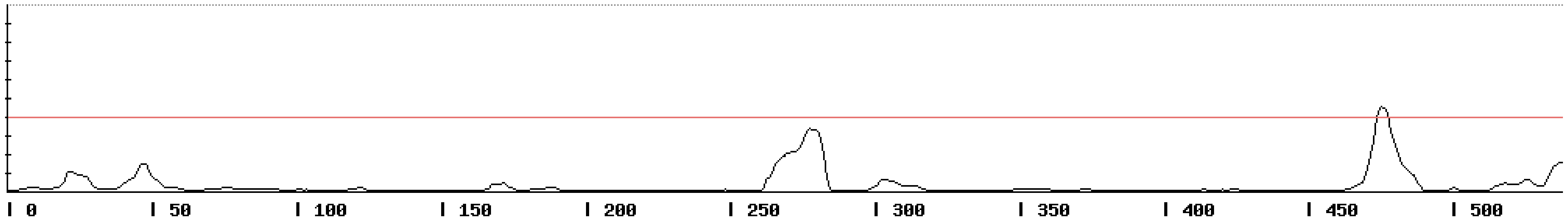

Predictions for Panicumvirgatum_Pavir.3KG499100.1.p

Viterbi Path

Viterbi Path

Posterior @ 0.4	
Posterior @ 0.4	
Posterior Graphic. Threshold Marked @ 0.4	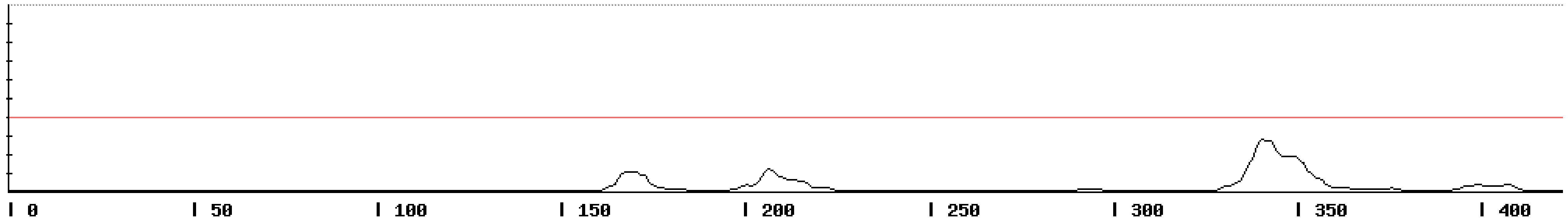

Predictions for Panicumvirgatum_Pavir.3NG254300.1.p

Viterbi Path	
Viterbi Path	
Posterior @ 0.4	464 - KRKR - 467
Posterior @ 0.4	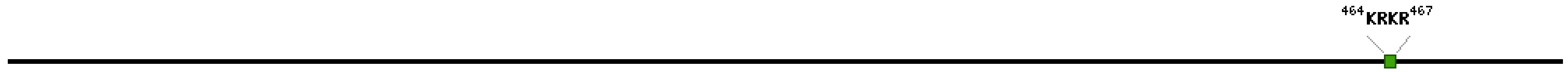
Posterior Graphic. Threshold Marked @ 0.4	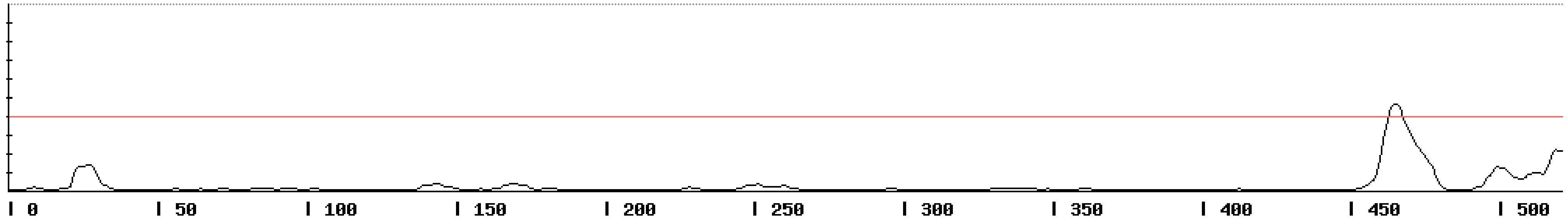

Predictions for Panicumvirgatum_Pavir.3NG302500.1.p

Viterbi Path	
Viterbi Path	

Posterior @ 0.4	
Posterior @ 0.4	
Posterior Graphic. Threshold Marked @ 0.4	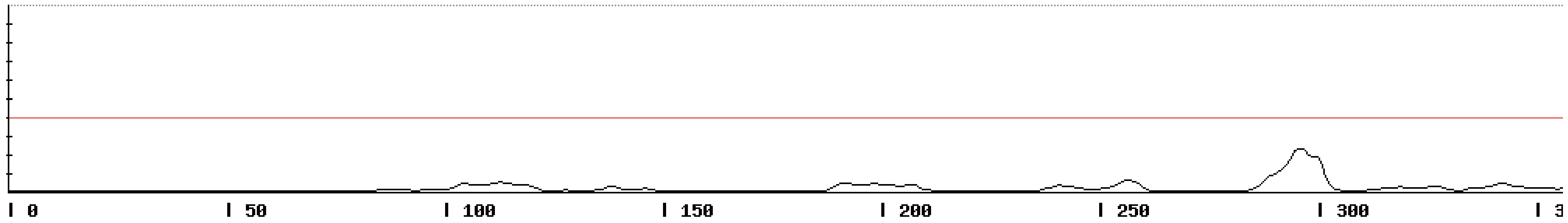

Predictions for Panicumvirgatum_Pavir.4NG078000.1.p

Viterbi Path	
Viterbi Path	
Posterior @ 0.4	194 - FSGHGKKRKRG - 204
Posterior @ 0.4	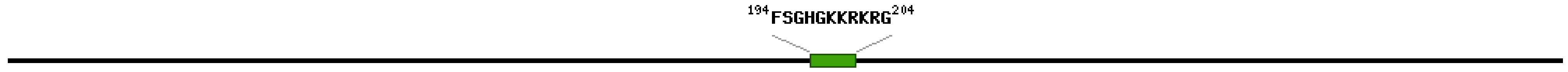
Posterior Graphic. Threshold Marked @ 0.4	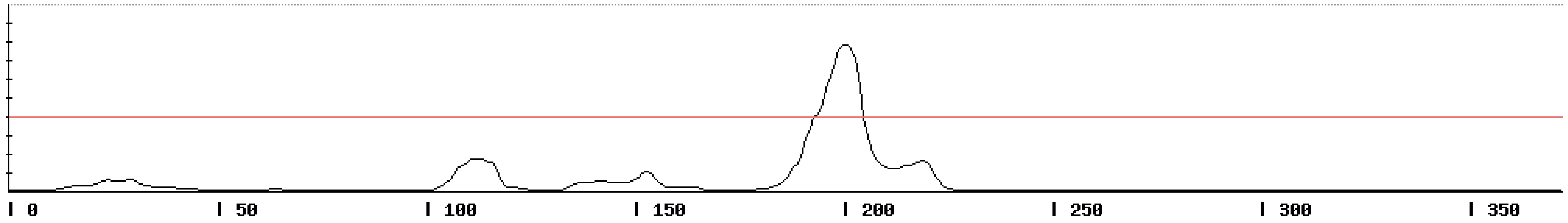

Predictions for Panicumvirgatum_Pavir.4NG078200.1.p

Viterbi Path

Viterbi Path

Posterior @ 0.4 | 332 - FSGHGKKRKRG - 342

Posterior @ 0.4

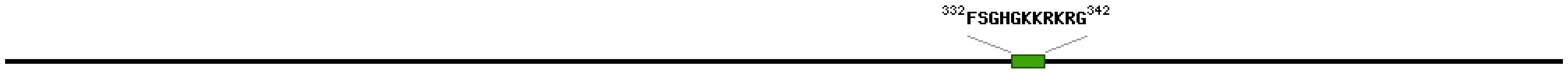

Posterior Graphic. Threshold Marked @ 0.4

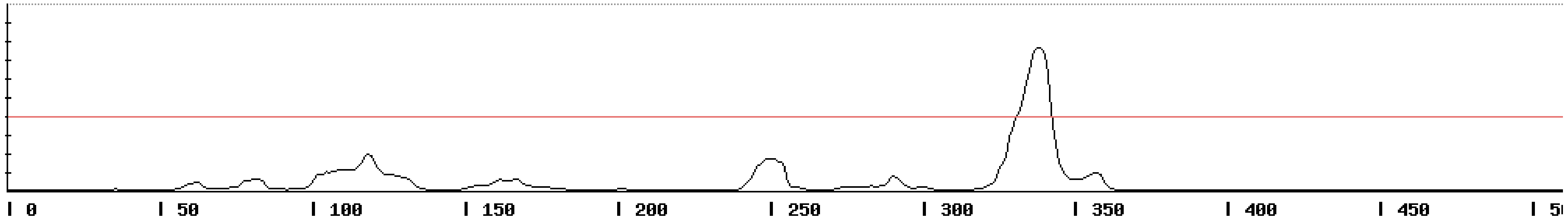

Predictions for Panicumvirgatum_Pavir.5NG430200.1.p

Viterbi Path

Viterbi Path

Posterior @ 0.4

Posterior @ 0.4

Posterior Graphic. Threshold Marked @ 0.4

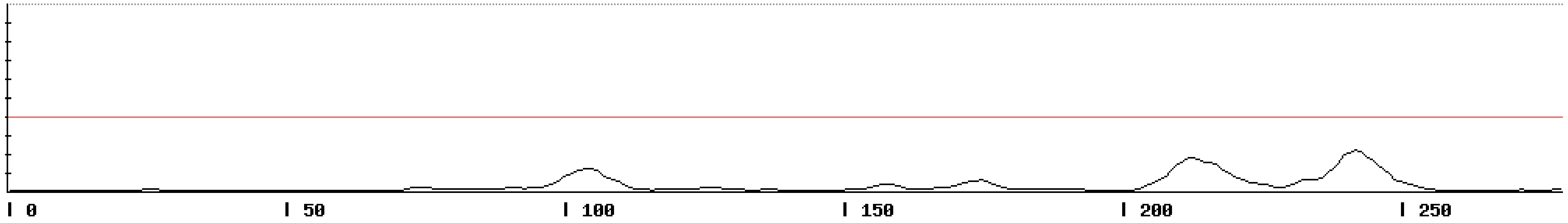

Predictions for Panicumvirgatum_Pavir.5NG465300.1.p	
Viterbi Path	
Viterbi Path	
Posterior @ 0.4	
Posterior @ 0.4	
Posterior Graphic. Threshold Marked @ 0.4	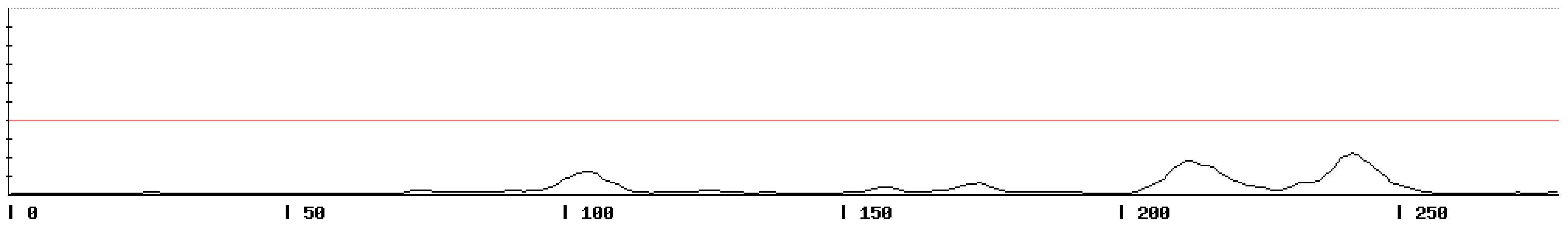

Predictions for Panicumvirgatum_Pavir.6NG320700.1.p	
Viterbi Path	
Viterbi Path	
Posterior @ 0.4	
Posterior @ 0.4	
Posterior Graphic. Threshold Marked @ 0.4	

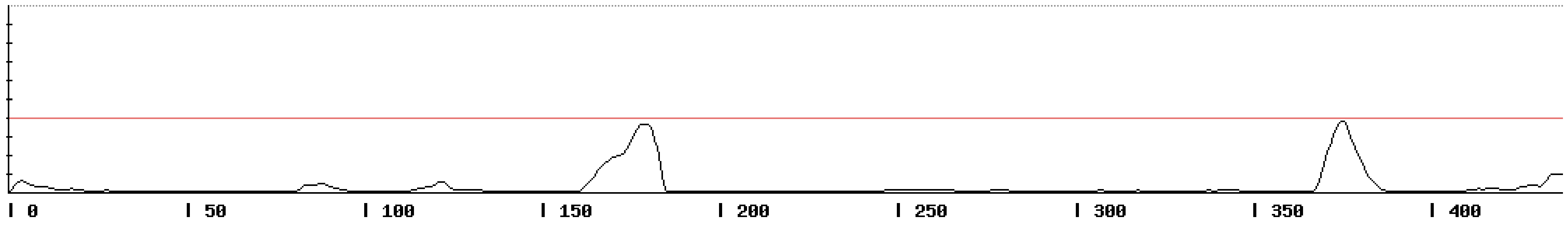

Predictions for Panicumvirgatum_Pavir.6NG334600.1.p

Viterbi Path	282 - KKHNAVNKKRKRTRPKSK - 299
Viterbi Path	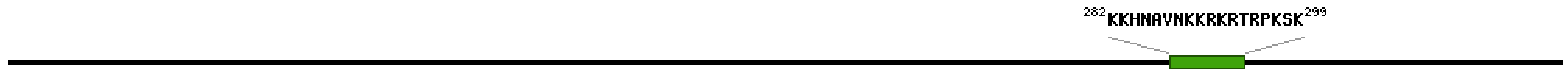
Posterior @ 0.4	282 - KKHNAVNKKRKRTRPKSK - 299
Posterior @ 0.4	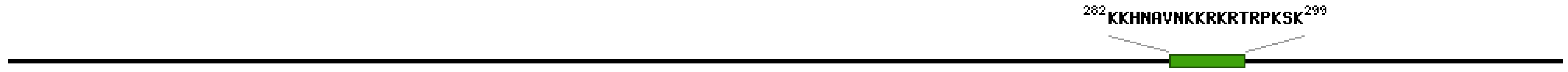
Posterior Graphic. Threshold Marked @ 0.4	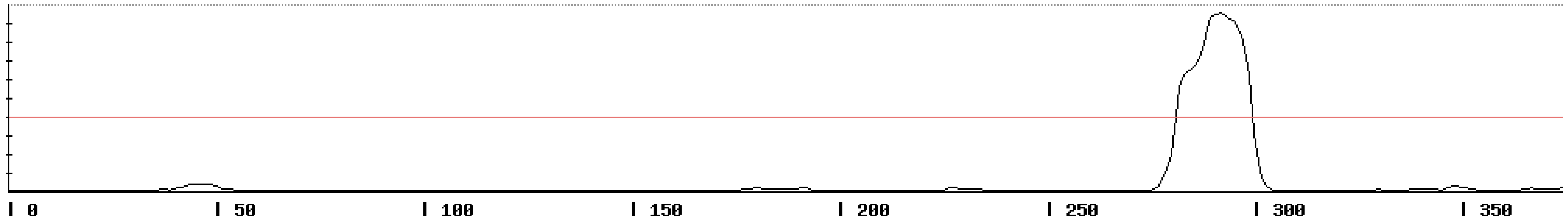

Predictions for Panicumvirgatum_Pavir.7KG245100.1.p

Viterbi Path	
Viterbi Path	
Posterior @ 0.4	39 - RPPRPPRRGGNRPPA - 53
	126 - GKRR - 129
Posterior @ 0.4	

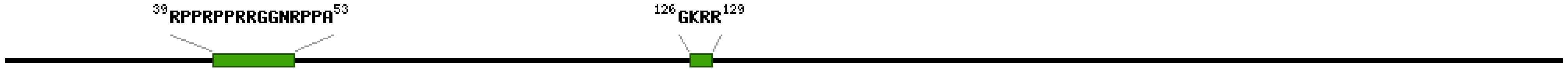

Posterior Graphic. Threshold Marked @ 0.4

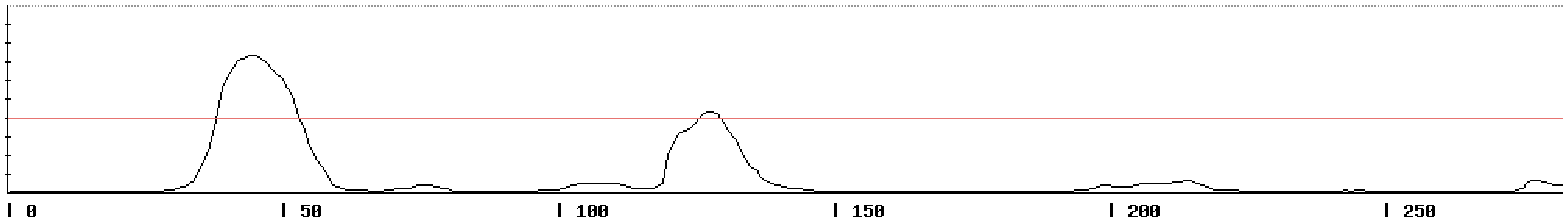

Predictions for Petuniaaxillaris_Peaxi162Scf00176g01638.1

Viterbi Path

Viterbi Path

Posterior @ 0.4

Posterior @ 0.4

Posterior Graphic. Threshold Marked @ 0.4

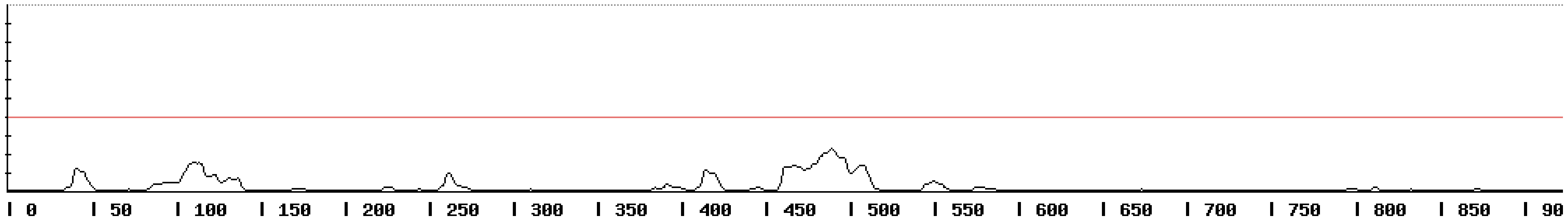

Predictions for Petuniaaxillaris_Peaxi162Scf01007g00210.1

Viterbi Path

Viterbi Path

Posterior @ 0.4	167 - KKKEKK - 172
	446 - KKKEKK - 451
Posterior @ 0.4	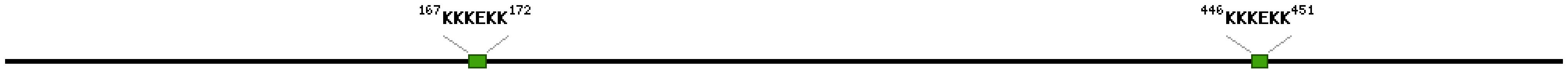
Posterior Graphic. Threshold Marked @ 0.4	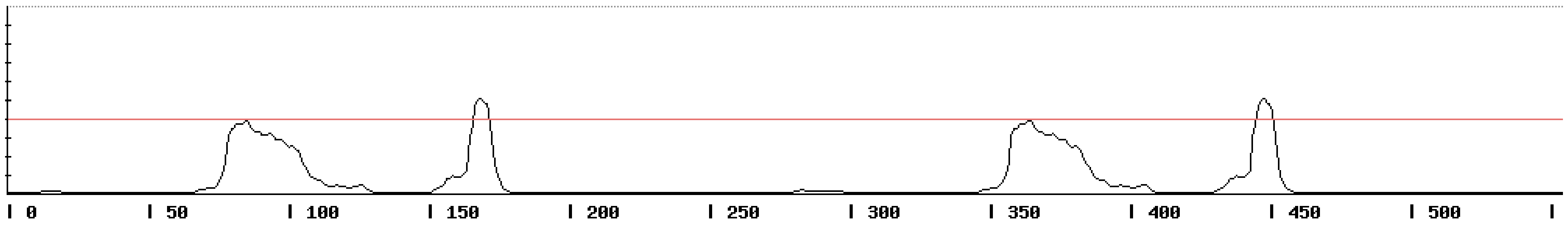

Predictions for Petuniaaxillaris_Peaxi162Scf00493g00439.1

Viterbi Path	
Viterbi Path	
Posterior @ 0.4	149 - KKKVKFRSKKFR - 160
Posterior @ 0.4	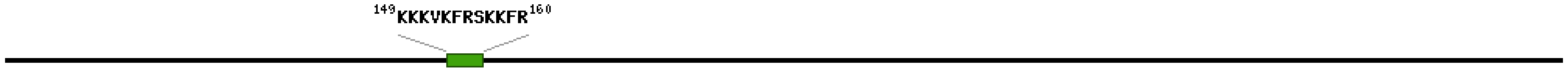
Posterior Graphic. Threshold Marked @ 0.4	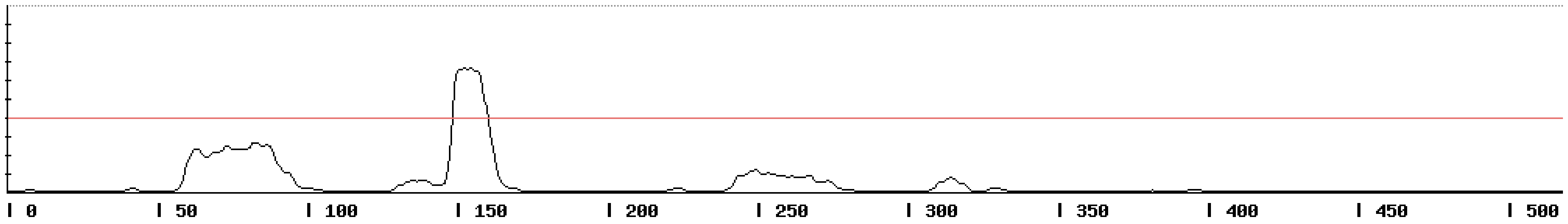

Predictions for Phoenixdactylifera_PDK_30s1165641g010

Viterbi Path	
Viterbi Path	

Posterior @ 0.4 | 109 - RKRKVG - 114

Posterior @ 0.4

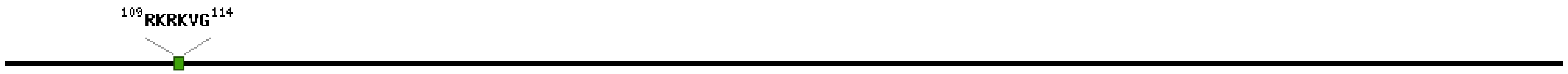

Posterior Graphic. Threshold Marked @ 0.4

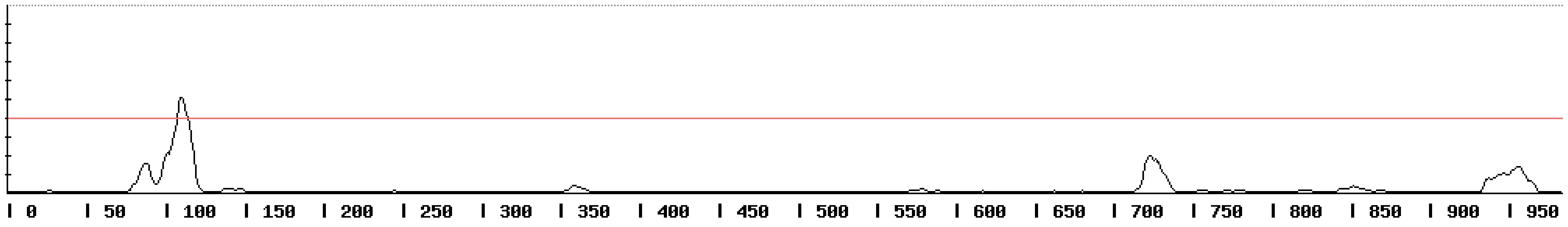

Predictions for Phoenixdactylifera_PDK_30s698051g001

Viterbi Path

Viterbi Path

Posterior @ 0.4 | 78 - RASRRAGLGRWKATK - 92

Posterior @ 0.4

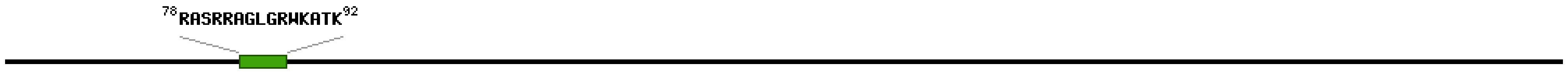

Posterior Graphic. Threshold Marked @ 0.4

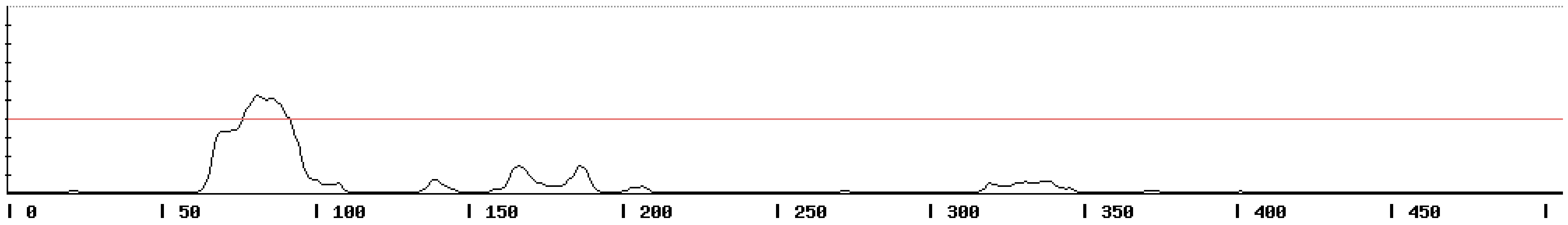

Predictions for Phoenixdactylifera_PDK_30s758201g003

Viterbi Path	
Viterbi Path	
Posterior @ 0.4	
Posterior @ 0.4	
Posterior Graphic. Threshold Marked @ 0.4	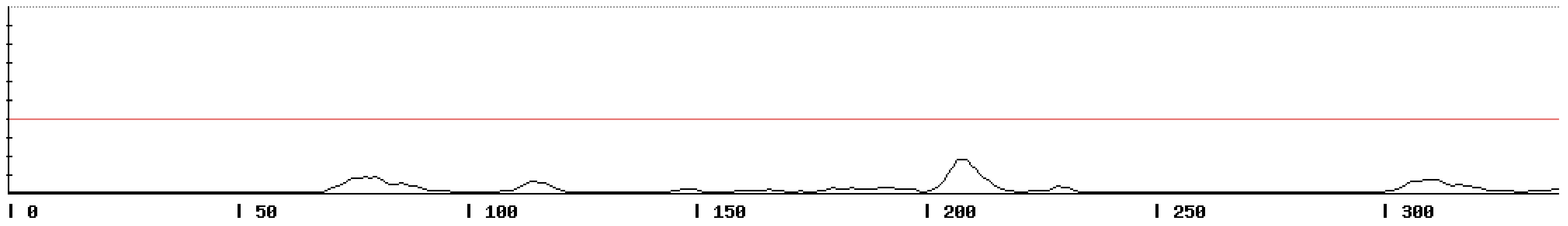

Predictions for Phoenixdactylifera_PDK_30s830451g001

Viterbi Path	
Viterbi Path	
Posterior @ 0.4	94 - RPARGTNRGYWKASGKDKIIRDKDSKE - 120
Posterior @ 0.4	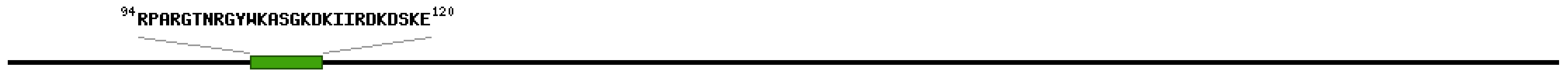
Posterior Graphic. Threshold Marked @ 0.4	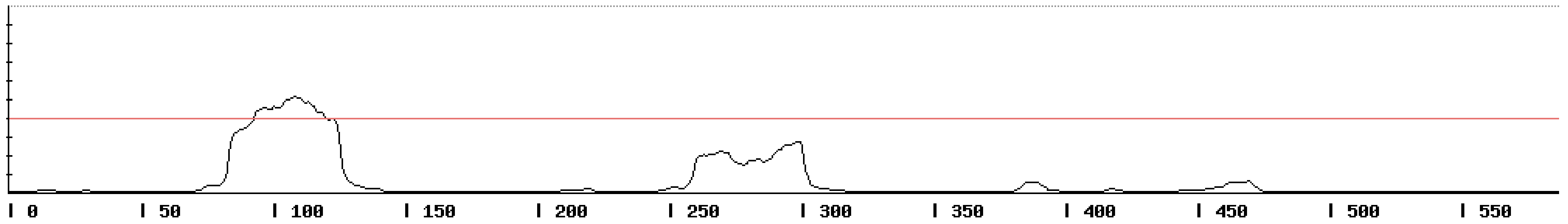

Predictions for Phyllostachysheterocycla_PH01000020G0110

Viterbi Path	
Viterbi Path	
Posterior @ 0.4	136 - KRRR - 139<br>167 - GVKKGWKKILVLYKGSKREG - 186
Posterior @ 0.4	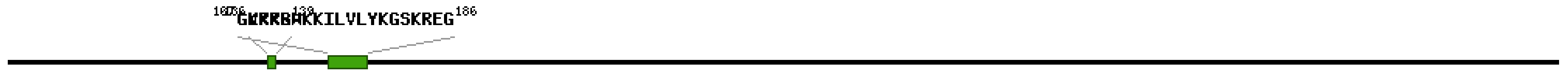
Posterior Graphic. Threshold Marked @ 0.4	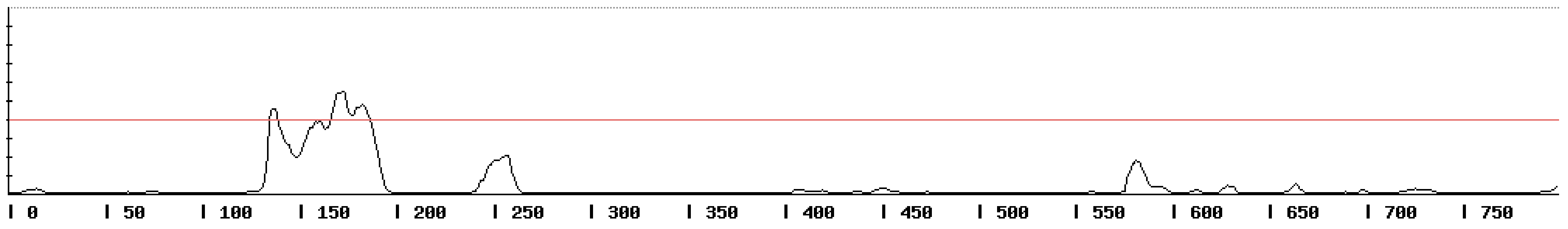

Predictions for Phyllostachysheterocycla_PH01000143G0710

Viterbi Path	
Viterbi Path	
Posterior @ 0.4	117 - PRRPNRGKDRVYVKSGQKGSGSKGG - 141<br>543 - ATGKDRKVNSQRR - 555
Posterior @ 0.4	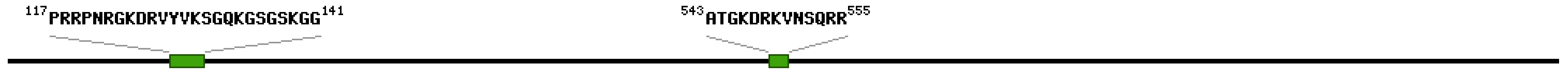
Posterior Graphic. Threshold Marked @ 0.4	

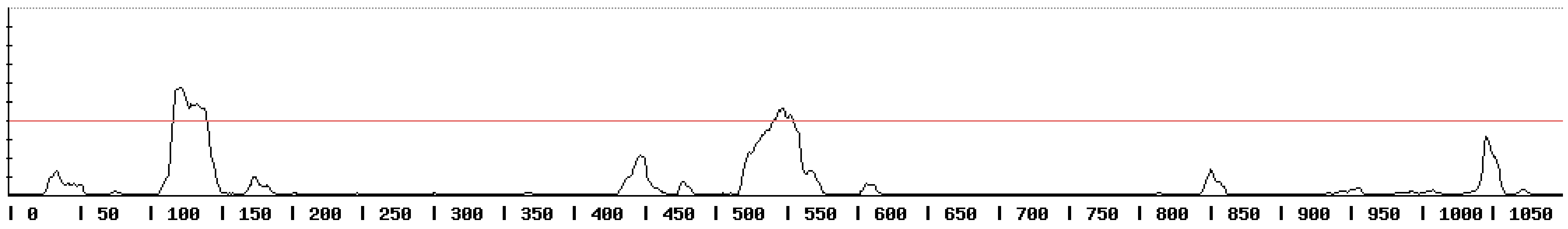

Predictions for Phyllostachysheterocycla_PH01000804G0640

Viterbi Path

Viterbi Path

Posterior @ 0.4

77 - KNARRGRKSR - 86

240 - KNARRGRKSR - 249

Posterior @ 0.4

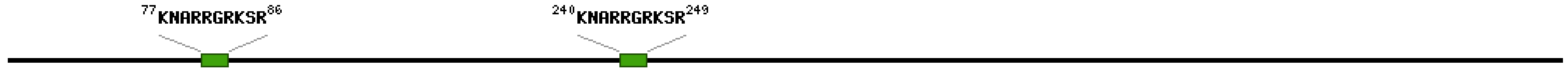

Posterior Graphic. Threshold Marked @ 0.4

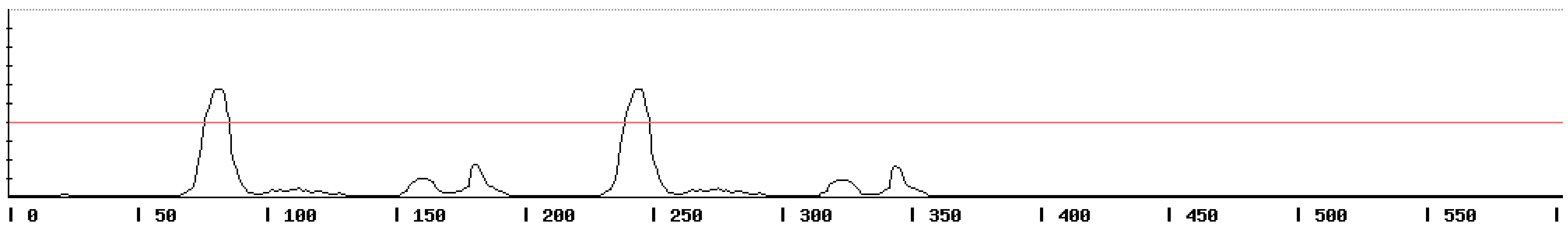

Predictions for Phyllostachysheterocycla_PH01001619G0180

Viterbi Path

Viterbi Path

Posterior @ 0.4

Posterior @ 0.4

Posterior Graphic. Threshold Marked @ 0.4	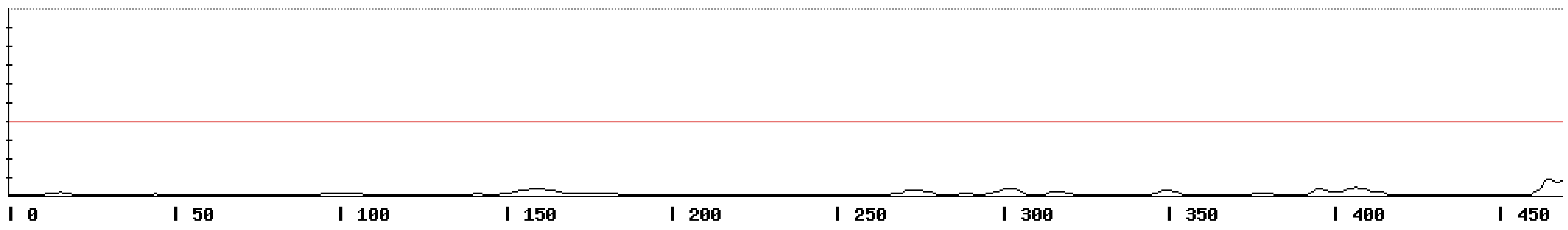

Predictions for Populuseuphratica_CCG001083.1

Viterbi Path	
Viterbi Path	
Posterior @ 0.4	865 - KRKLQRKK - 872
Posterior @ 0.4	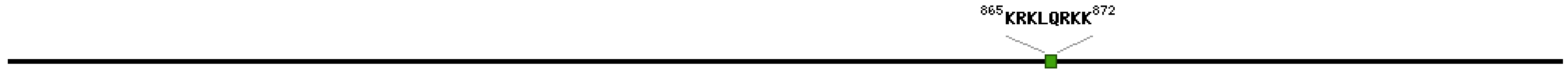
Posterior Graphic. Threshold Marked @ 0.4	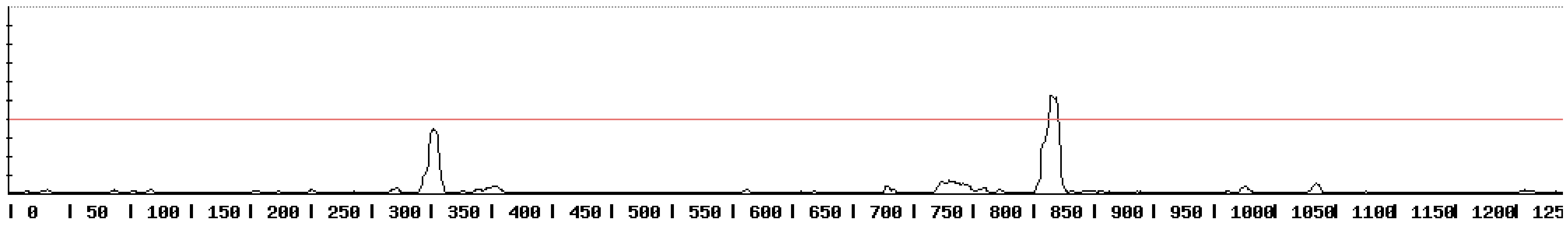

Predictions for Populuseuphratica_CCG003661.1

Viterbi Path	
Viterbi Path	

Posterior @ 0.4

Posterior @ 0.4

Posterior Graphic. Threshold Marked @ 0.4

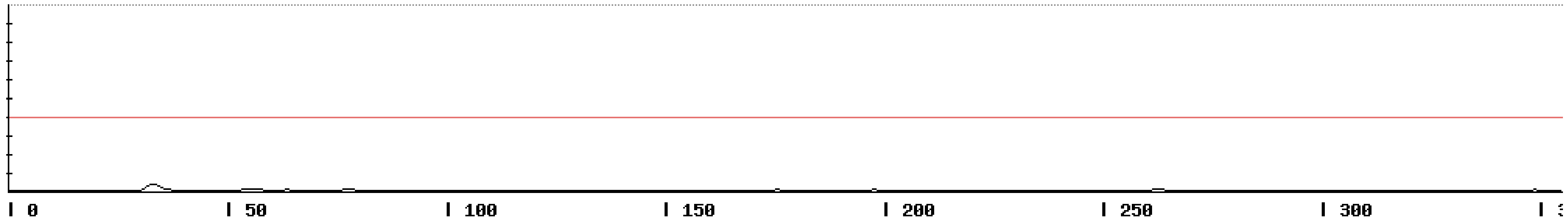

Predictions for Populuseuphratica_CCG010882.1

Viterbi Path

Viterbi Path

Posterior @ 0.4

Posterior @ 0.4

Posterior Graphic. Threshold Marked @ 0.4

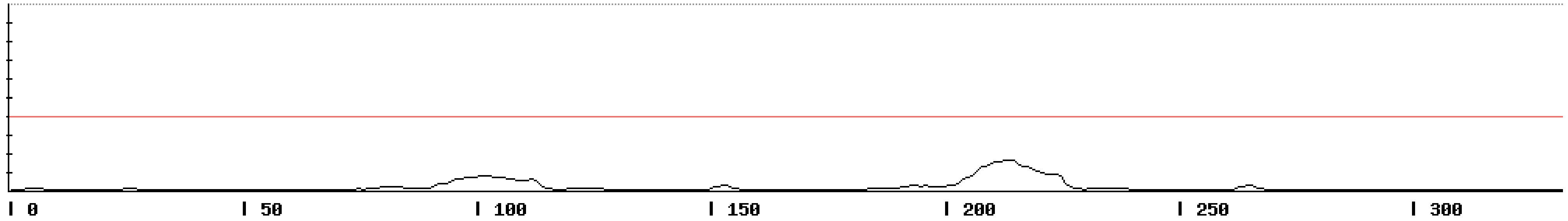

Predictions for Populuseuphratica_CCG015305.2

Viterbi Path

Viterbi Path

Posterior @ 0.4

Posterior @ 0.4

Posterior Graphic. Threshold Marked @ 0.4

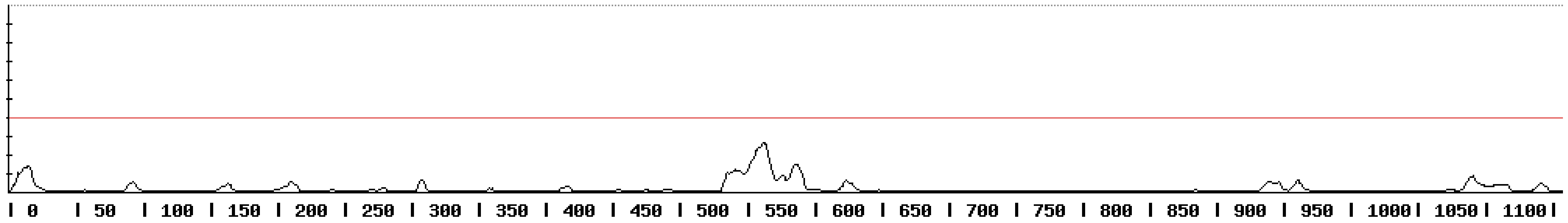

Predictions for Populuseuphratica_CCG017923.1

Viterbi Path

Viterbi Path

Posterior @ 0.4

Posterior @ 0.4

Posterior Graphic. Threshold Marked @ 0.4

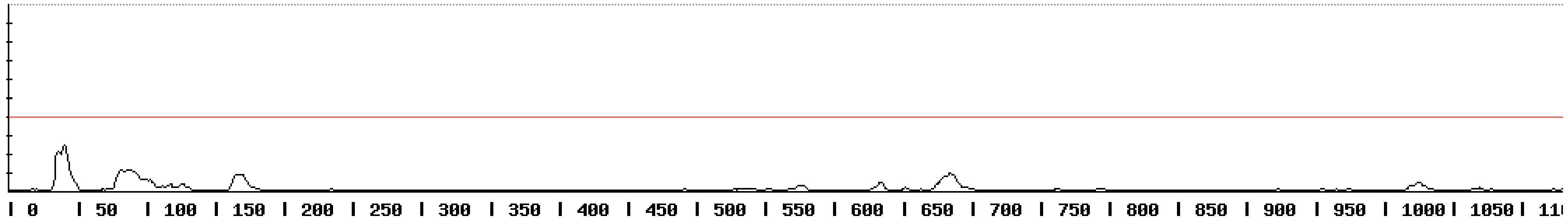

Predictions for Populustrichocarpa_Potri.006G051400.1

Viterbi Path

Viterbi Path

Posterior @ 0.4

Posterior @ 0.4

Posterior Graphic. Threshold Marked @ 0.4

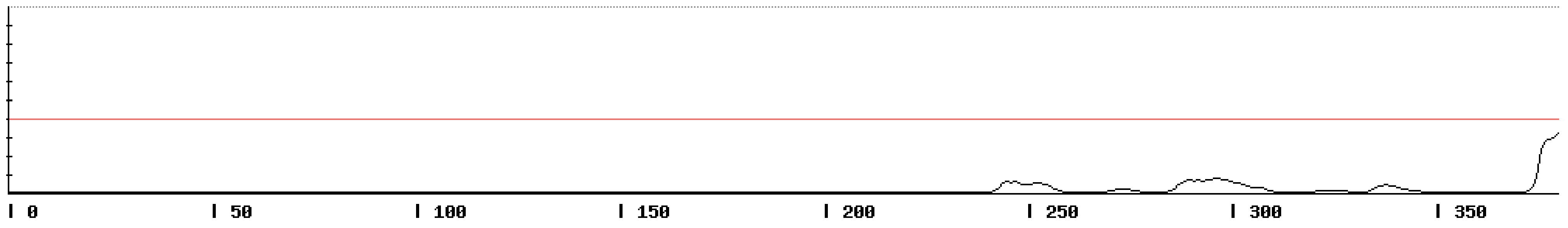

Predictions for Prunusmume_XP_016648008.1

Viterbi Path

Viterbi Path

Posterior @ 0.4

Posterior @ 0.4

Posterior Graphic. Threshold Marked @ 0.4

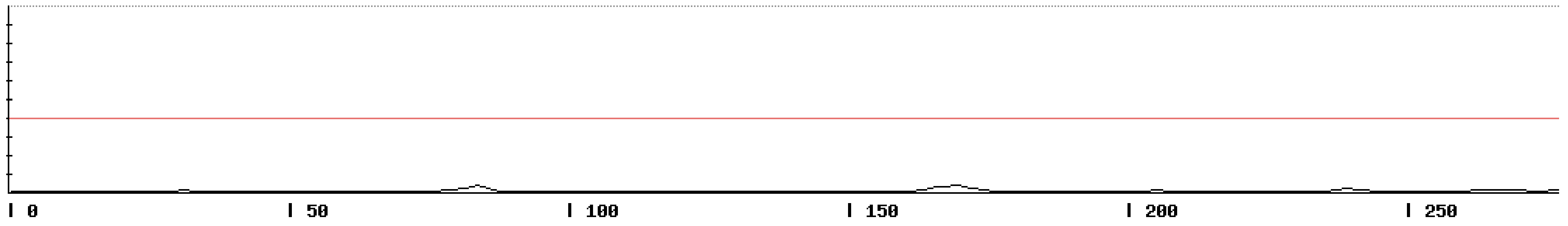

Predictions for Prunuspersica_Prupe.1G284800.1.p	
Viterbi Path	
Viterbi Path	
Posterior @ 0.4	
Posterior @ 0.4	
Posterior Graphic. Threshold Marked @ 0.4	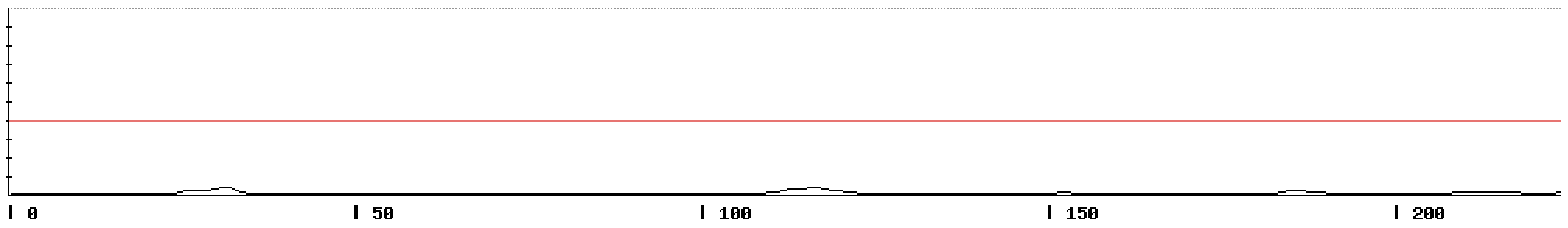

Predictions for Prunuspersica_Prupe.4G112200.1.p	
Viterbi Path	
Viterbi Path	
Posterior @ 0.4	
Posterior @ 0.4	
Posterior Graphic. Threshold Marked @ 0.4	

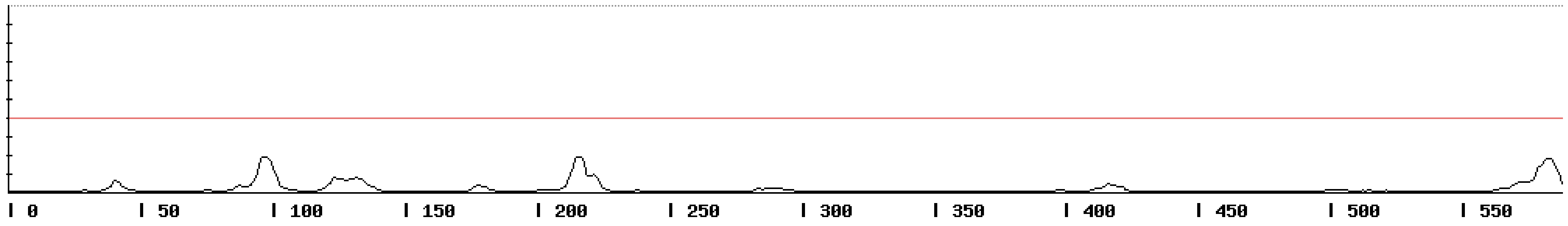

Predictions for Pseudotsugamenziesii_PSME_00001409-RA

Viterbi Path

Viterbi Path

Posterior @ 0.4

138 - GKAPKGKR - 145

589 - KAPKGK - 594

Posterior @ 0.4

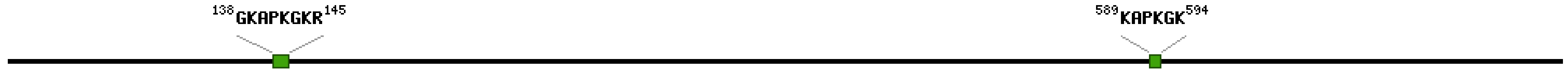

Posterior Graphic. Threshold Marked @ 0.4

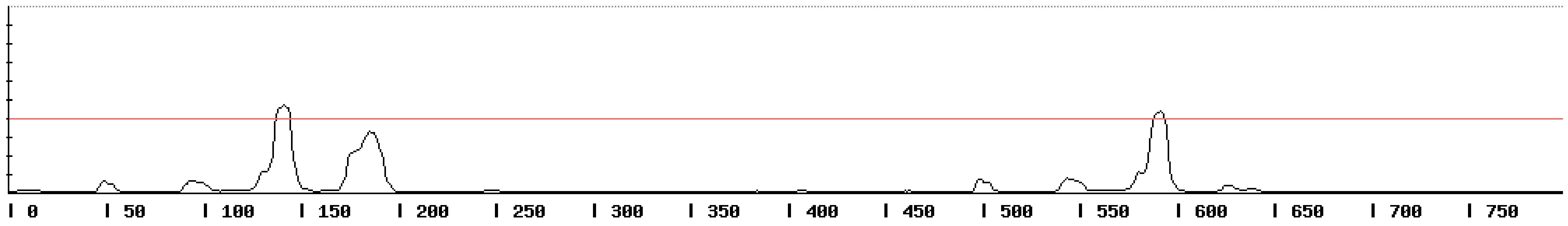

Predictions for Pseudotsugamenziesii_PSME_00011888-RA

Viterbi Path

Viterbi Path

Posterior @ 0.4

Posterior @ 0.4

Posterior Graphic. Threshold Marked @ 0.4

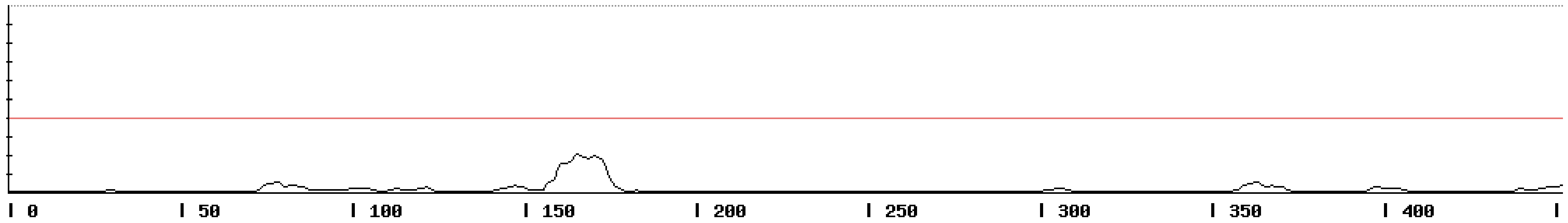

Predictions for Pseudotsugamenziesii_PSME_00012319-RA

Viterbi Path

Viterbi Path

Posterior @ 0.4

77 - GKKR - 80

409 - DKGGKKRVGIKK - 420

Posterior @ 0.4

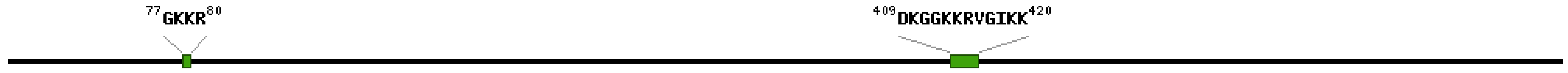

Posterior Graphic. Threshold Marked @ 0.4

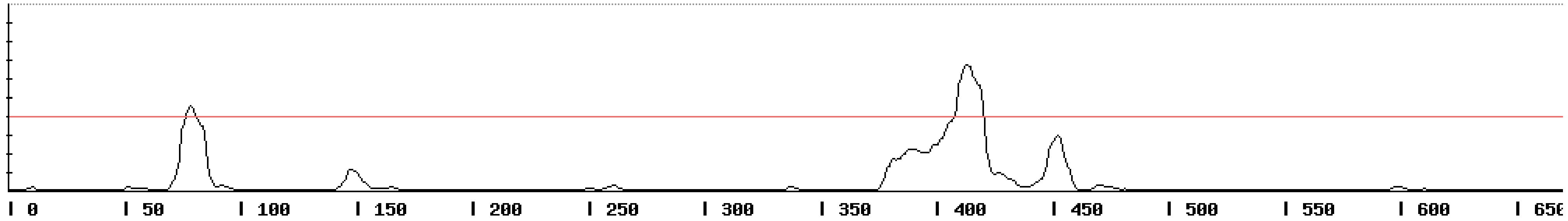

Predictions for Pseudotsugamenziesii_PSME_00016918-RA

Viterbi Path

Viterbi Path

Posterior @ 0.4

Posterior @ 0.4

Posterior Graphic. Threshold Marked @ 0.4

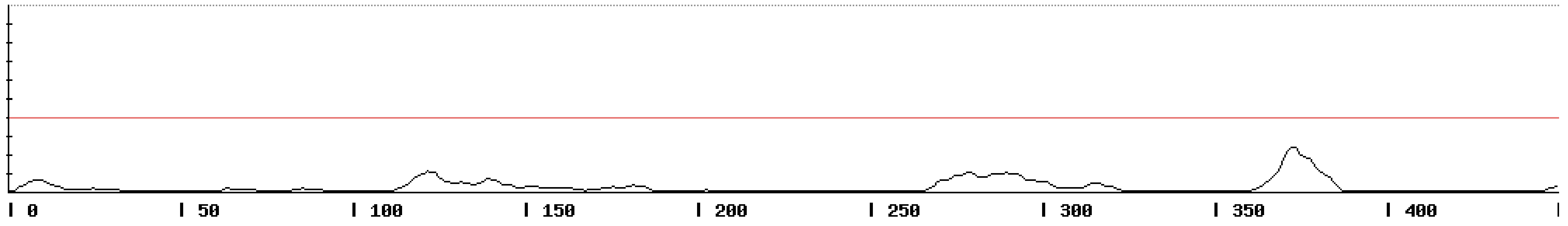

Predictions for Pseudotsugamenziesii_PSME_00017053-RA

Viterbi Path

Viterbi Path

Posterior @ 0.4

91 - RKYPNGSRPKRAAGSGYWKATGADKPINAKGGKKRVGIK - 129

281 - RRKR - 284

Posterior @ 0.4

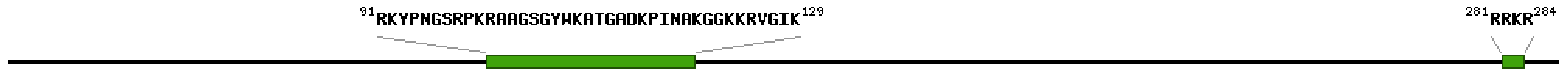

Posterior Graphic. Threshold Marked @ 0.4

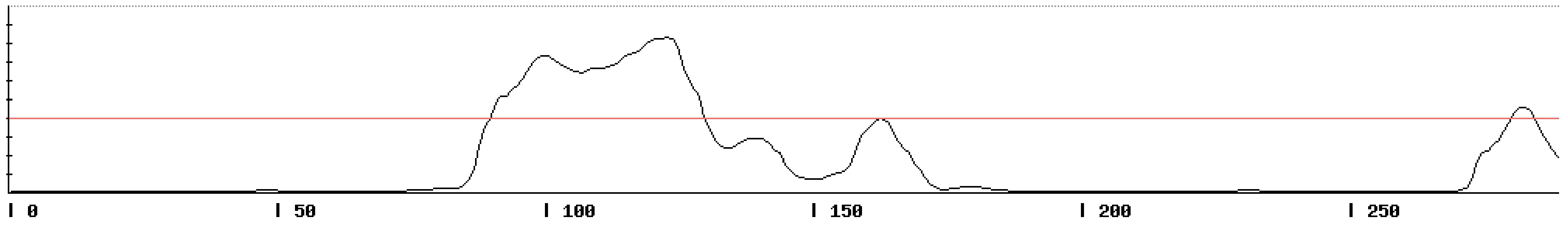

Predictions for Pseudotsugamenziesii_PSME_00020635-RA	
Viterbi Path	
Viterbi Path	
Posterior @ 0.4	67 - GSRPNKAAGSRYWKAIGADKPINAKGGKKRVGIKKALVFYAGKAPK - 112
Posterior @ 0.4	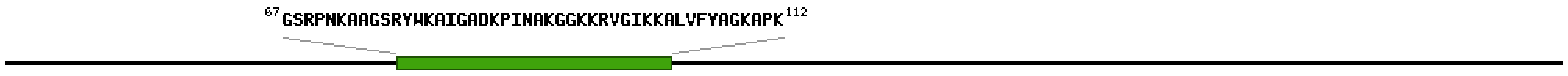
Posterior Graphic. Threshold Marked @ 0.4	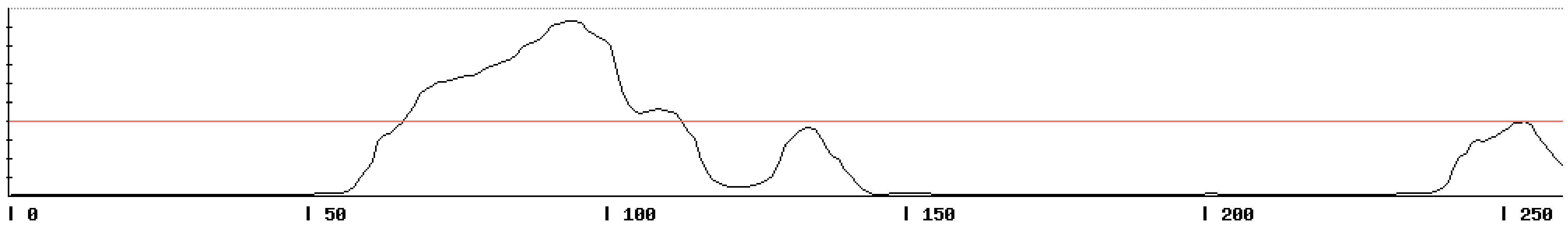

Predictions for Pseudotsugamenziesii_PSME_00024440-RA	
Viterbi Path	
Viterbi Path	
Posterior @ 0.4	222 - SRPKRAVGSRYWKATGADNPINAKGGKKRVGIKKALHPKAQRQ - 264
	277 - SRPARKKGSLRGK - 289
Posterior @ 0.4	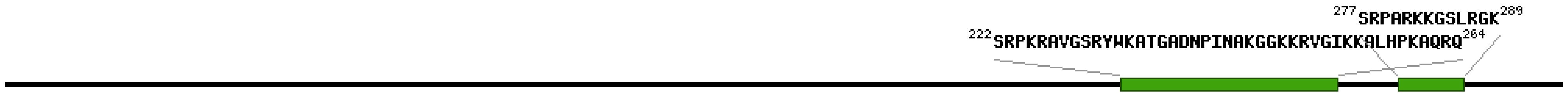
Posterior Graphic. Threshold Marked @ 0.4	

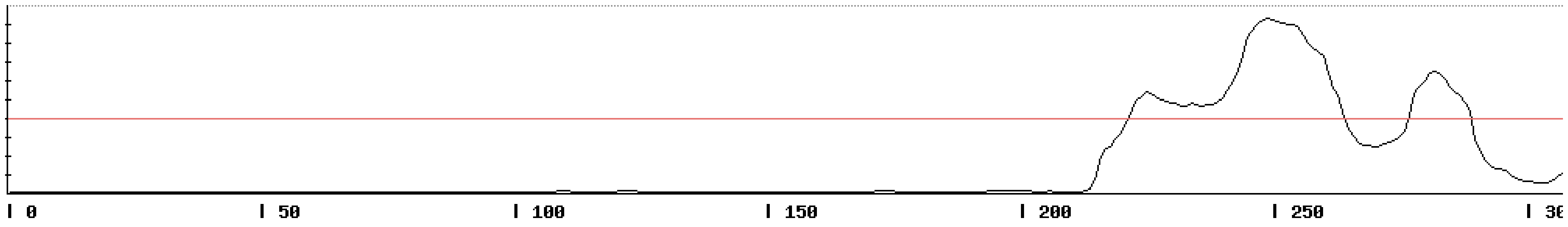

Predictions for Pseudotsugamenziesii_PSME_00026525-RA

Viterbi Path	
Viterbi Path	
Posterior @ 0.4	46 - K - 46
	100 - RKSRSKRKPRLPAAQRTKAR - 119
Posterior @ 0.4	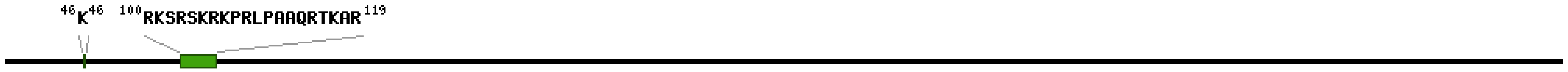
Posterior Graphic. Threshold Marked @ 0.4	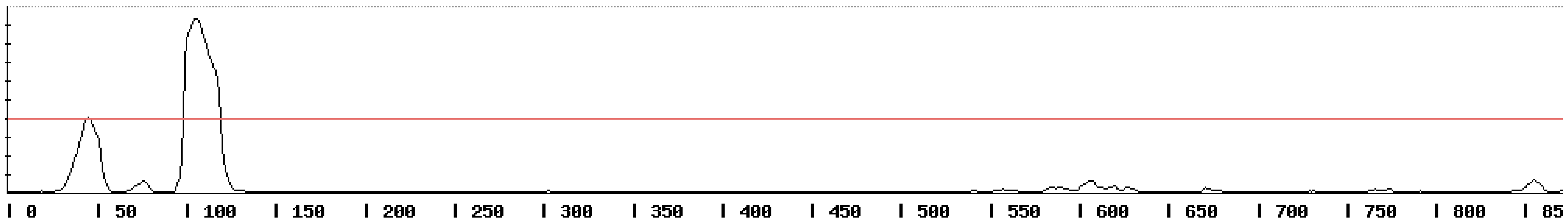

Predictions for Pyrusbretschneideri_Pbr004711.1

Viterbi Path	
Viterbi Path	
Posterior @ 0.4	
Posterior @ 0.4	

Posterior Graphic. Threshold Marked @ 0.4

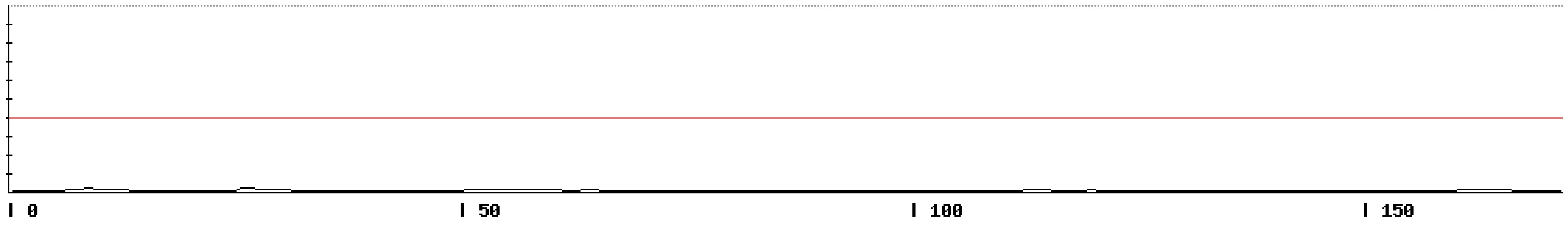

Predictions for Pyrusbretschneideri_Pbr038615.1

Viterbi Path

Viterbi Path

Posterior @ 0.4

Posterior @ 0.4

Posterior Graphic. Threshold Marked @ 0.4

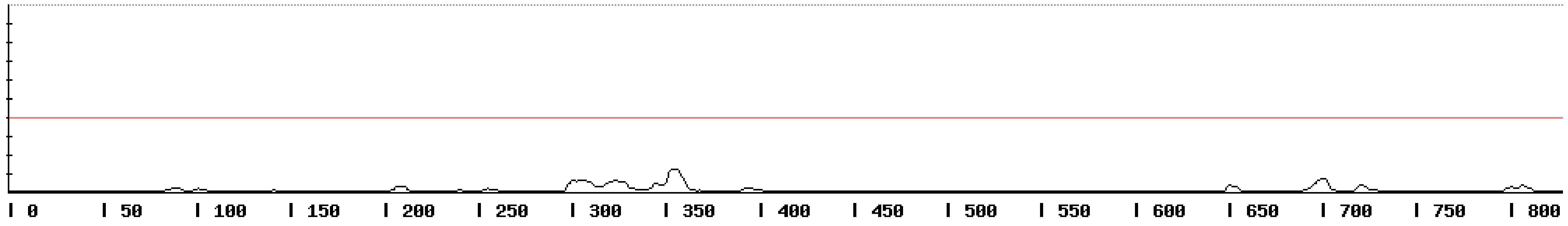

Predictions for Pyrusbretschneideri_Pbr009270.1

Viterbi Path

Viterbi Path

Posterior @ 0.4

Posterior @ 0.4

Posterior Graphic. Threshold Marked @ 0.4

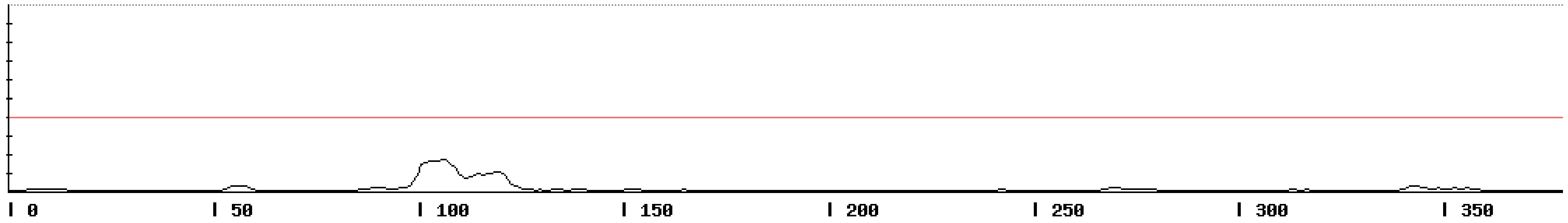

Predictions for Pyrusbretschneideri_Pbr012547.2

Viterbi Path

Viterbi Path

Posterior @ 0.4

135 - R - 135

502 - MRSKVAKRMQKESGKTLREVRRAKKLQKKLMTE - 534

537 - R - 537

541 - NLKRAKRKV - 549

1039 - EEKKGNRNKSTR - 1050

Posterior @ 0.4

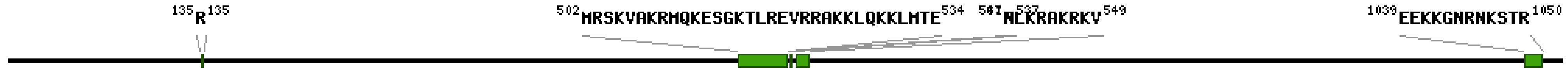

Posterior Graphic. Threshold Marked @ 0.4

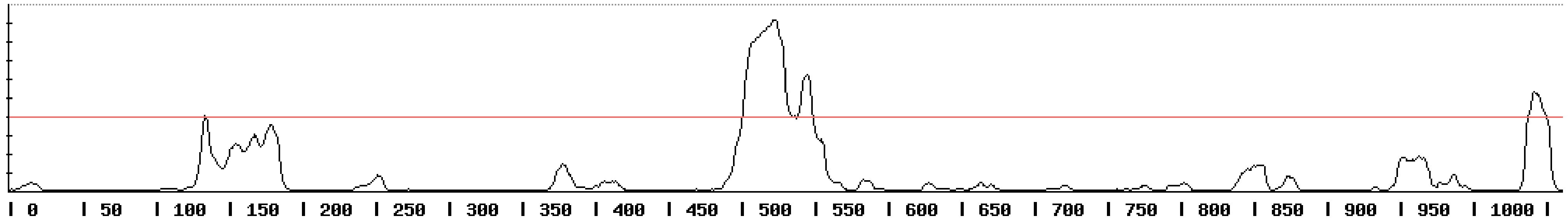

Predictions for Pyrusbretschneideri_Pbr014522.2	
Viterbi Path	
Viterbi Path	
Posterior @ 0.4	253 - PPPRRPKILSPNLRPGG - 269
	463 - R - 463
	830 - MRSKVAKRMQKESGKTLREVRRAKKLQKKLMTEN - 863
	865 - R - 865
	869 - NLKRAKRKV - 877
Posterior @ 0.4	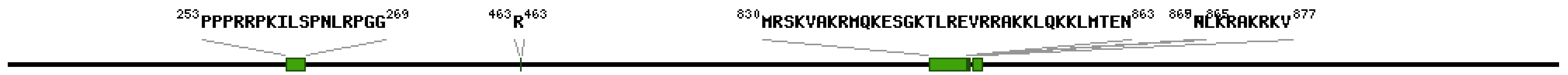
Posterior Graphic. Threshold Marked @ 0.4	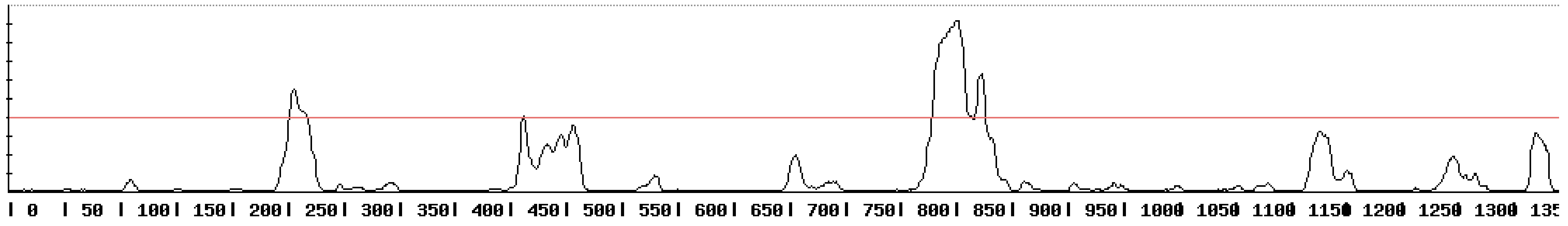

Predictions for Pyrusbretschneideri_Pbr019214.6	
Viterbi Path	
Viterbi Path	
Posterior @ 0.4	
Posterior @ 0.4	

Posterior Graphic. Threshold Marked @ 0.4

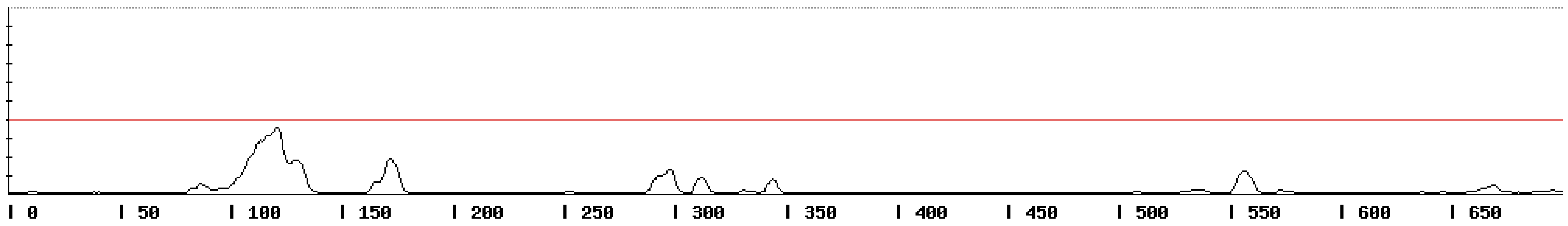

Predictions for Raphanusraphanistrum_RrC935_p1

Viterbi Path

Viterbi Path

Posterior @ 0.4

Posterior @ 0.4

Posterior Graphic. Threshold Marked @ 0.4

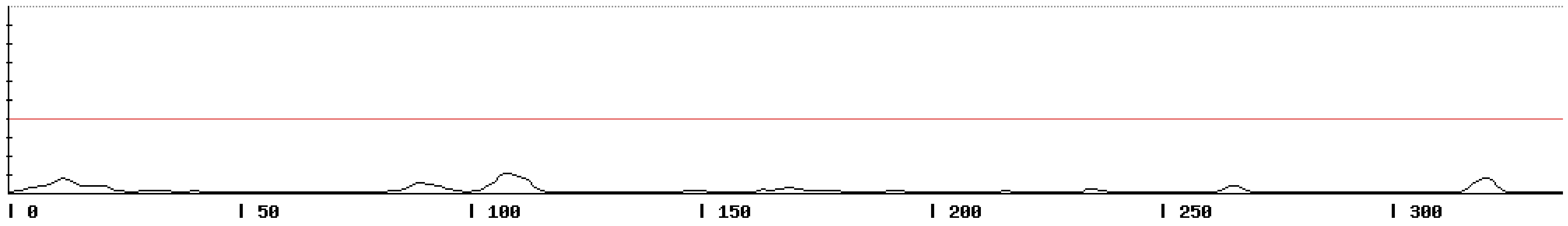

Predictions for Raphanusraphanistrum_RrC1847_p1

Viterbi Path

Viterbi Path

Posterior @ 0.4

Posterior @ 0.4

Posterior Graphic. Threshold Marked @ 0.4

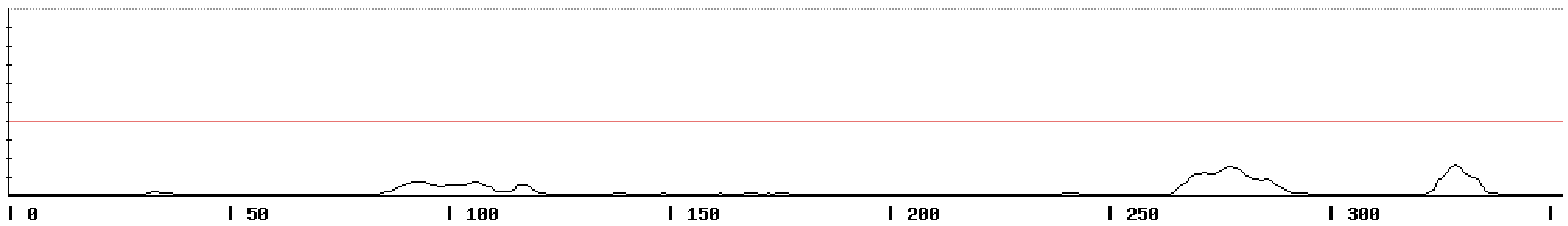

Predictions for Raphanusraphanistrum_RrC9803_p2

Viterbi Path	
Viterbi Path	
Posterior @ 0.4	
Posterior @ 0.4	
Posterior Graphic. Threshold Marked @ 0.4	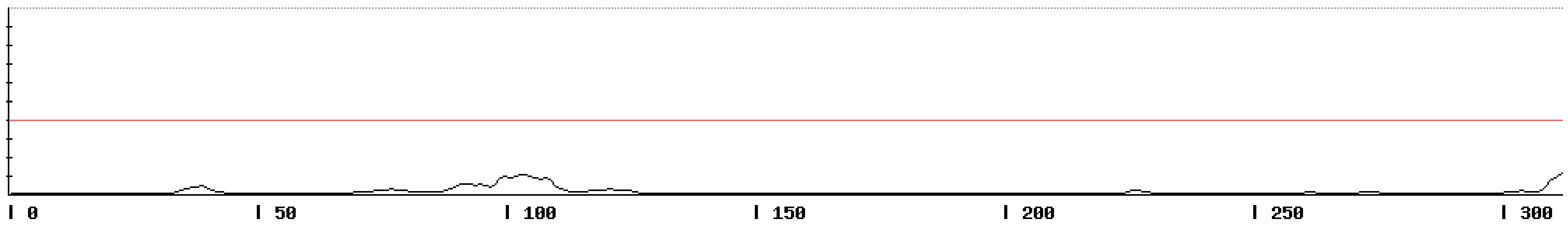

Predictions for Raphanusraphanistrum_RrC20665_p1

Viterbi Path	
Viterbi Path	
Posterior @ 0.4	433 - KR - 434
Posterior @ 0.4	

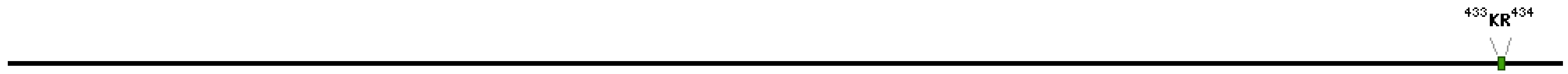

Posterior Graphic. Threshold Marked @ 0.4

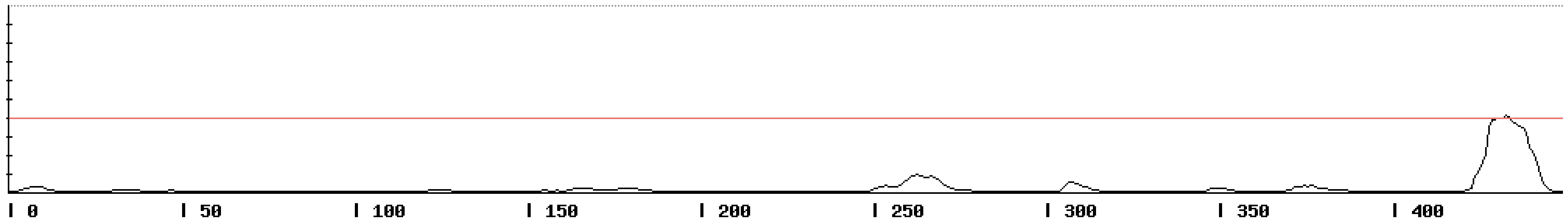

Predictions for Raphanusraphanistrum_RrC21868_p1

Viterbi Path

Viterbi Path

Posterior @ 0.4

Posterior @ 0.4

Posterior Graphic. Threshold Marked @ 0.4

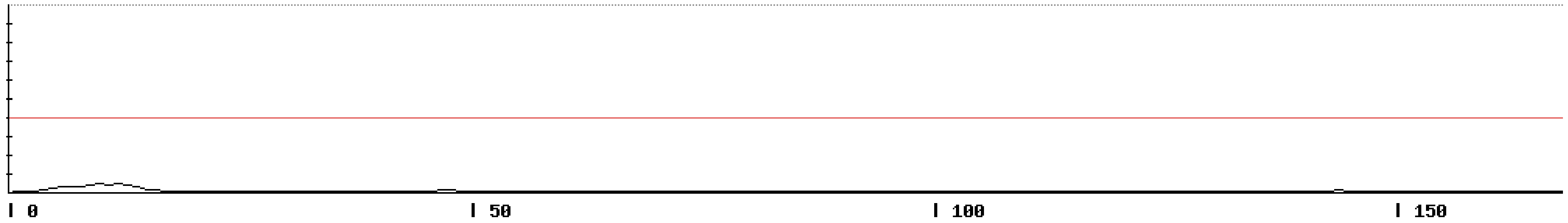

Predictions for Raphanusraphanistrum_RrC3821_p1

Viterbi Path

Viterbi Path

Posterior @ 0.4

Posterior @ 0.4

Posterior Graphic. Threshold Marked @ 0.4

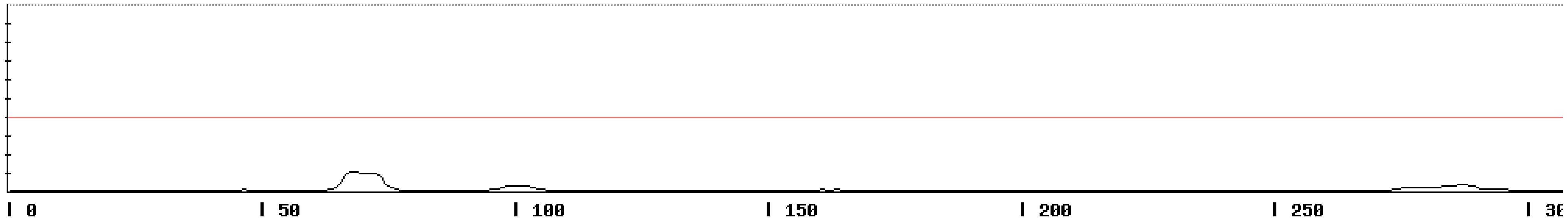

Predictions for Raphanusraphanistrum_RrC5114_p2

Viterbi Path

Viterbi Path

Posterior @ 0.4

Posterior @ 0.4

Posterior Graphic. Threshold Marked @ 0.4

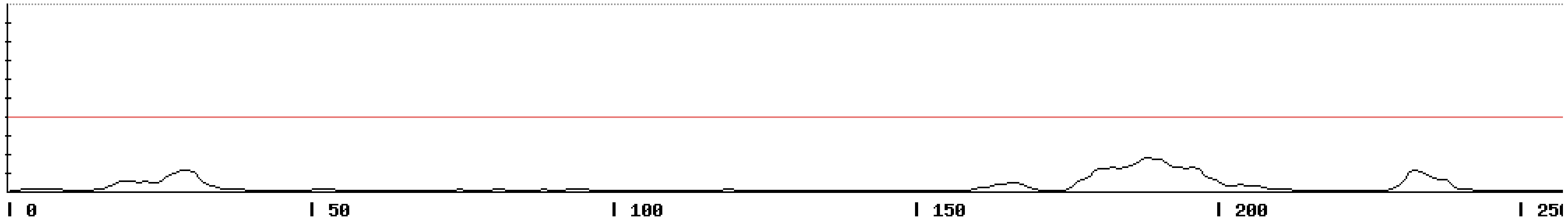

Predictions for Raphanussativus_Rsa1.0_00100.1_g00020.1

Viterbi Path

Viterbi Path

Posterior @ 0.4	
Posterior @ 0.4	
Posterior Graphic. Threshold Marked @ 0.4	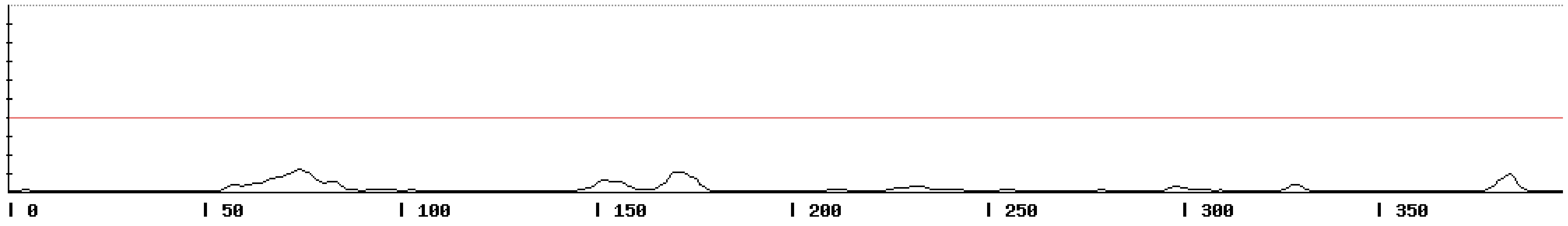

Predictions for Raphanussativus_Rsa1.0_06656.1_g00001.1

Viterbi Path	
Viterbi Path	
Posterior @ 0.4	
Posterior @ 0.4	
Posterior Graphic. Threshold Marked @ 0.4	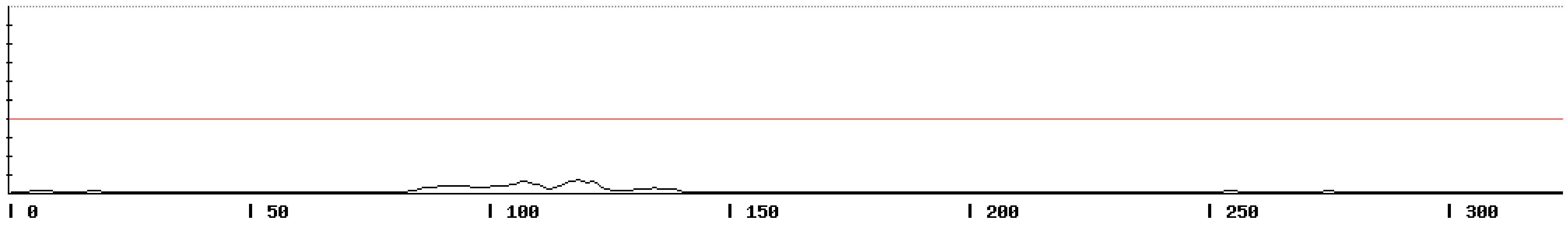

Predictions for Raphanussativus_Rsa1.0_00835.1_g00007.1

Viterbi Path

Viterbi Path

Posterior @ 0.4

Posterior @ 0.4

Posterior Graphic. Threshold Marked @ 0.4

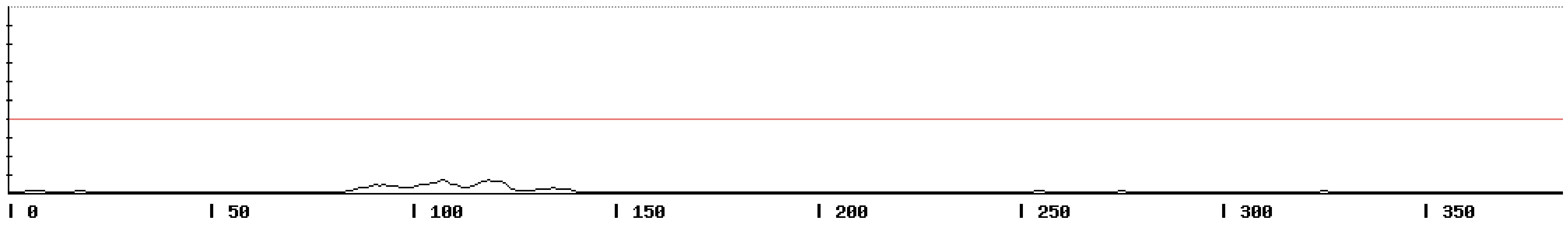

Predictions for Raphanussativus_Rsa1.0_01306.1_g00007.1

Viterbi Path

Viterbi Path

Posterior @ 0.4

Posterior @ 0.4

Posterior Graphic. Threshold Marked @ 0.4

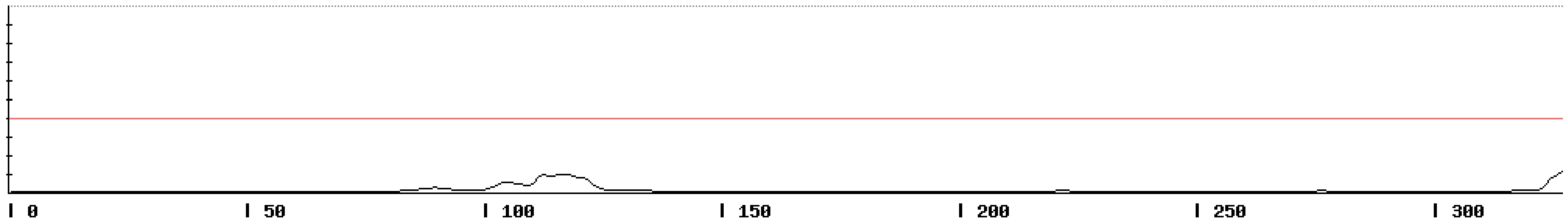

Predictions for Raphanussativus_Rsa1.0_01306.1_g00009.1	
Viterbi Path	
Viterbi Path	
Posterior @ 0.4	
Posterior @ 0.4	
Posterior Graphic. Threshold Marked @ 0.4	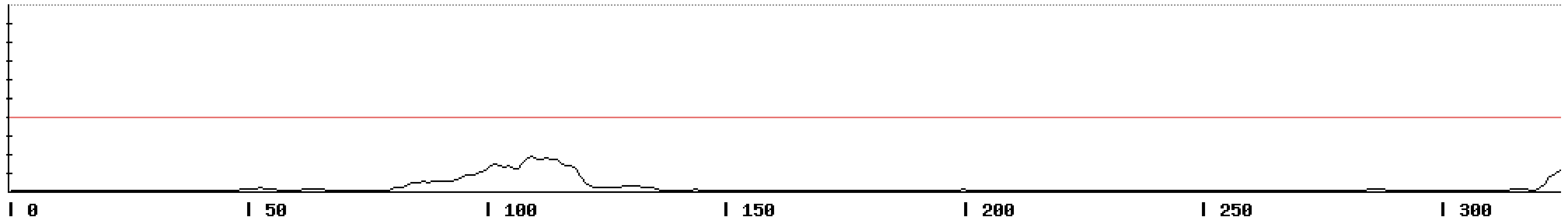

Predictions for Raphanussativus_Rsa1.0_01979.1_g00001.1	
Viterbi Path	
Viterbi Path	
Posterior @ 0.4	
Posterior @ 0.4	
Posterior Graphic. Threshold Marked @ 0.4	

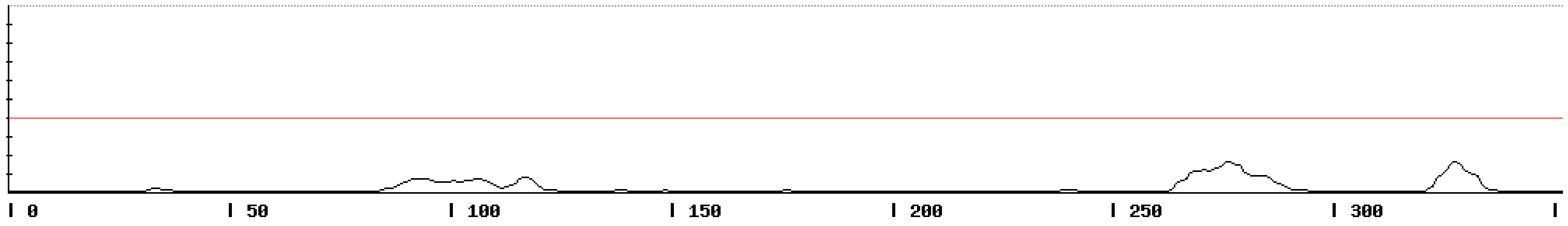

Predictions for Salviamiltiorrhiza_SMil_00004615-RA_Salv

Viterbi Path

Viterbi Path

Posterior @ 0.4

Posterior @ 0.4

Posterior Graphic. Threshold Marked @ 0.4

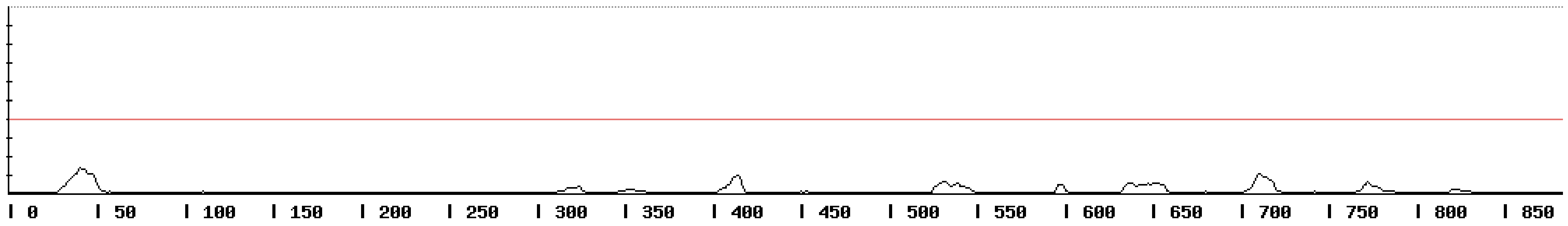

Predictions for Salviamiltiorrhiza_SMil_00008150-RA_Salv

Viterbi Path

Viterbi Path

Posterior @ 0.4

Posterior @ 0.4

Posterior Graphic. Threshold Marked @ 0.4

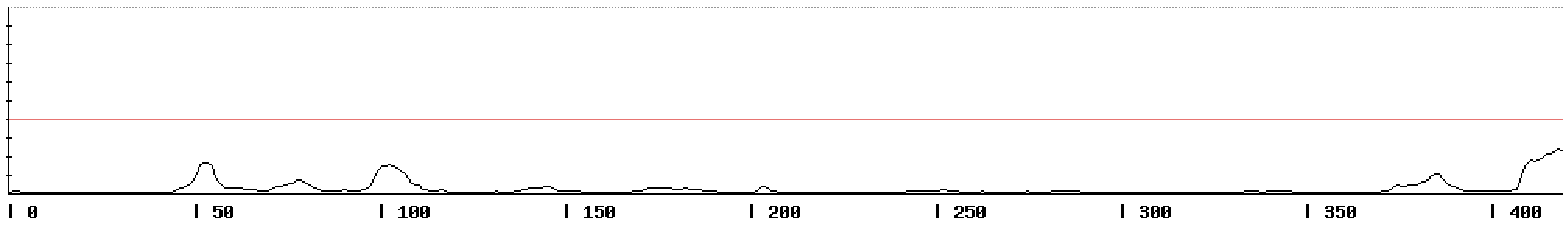

Predictions for Salviamiltiorrhiza_SMil_00028195-RA_Salv

Viterbi Path	
Viterbi Path	
Posterior @ 0.4	
Posterior @ 0.4	
Posterior Graphic. Threshold Marked @ 0.4	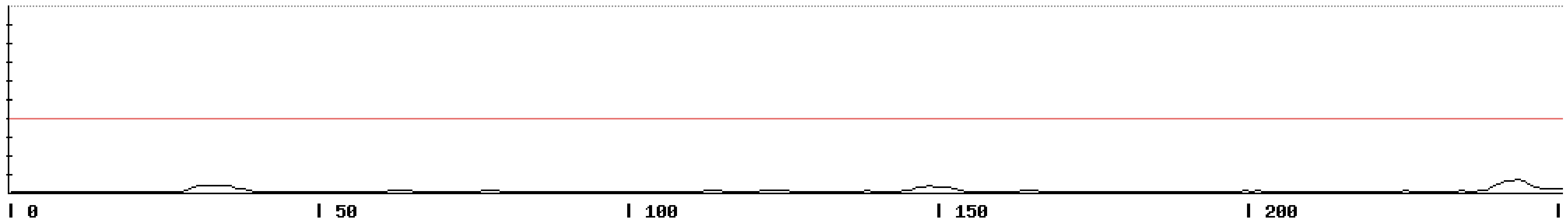

Predictions for Setariaitalica_Seita.1G163800.1.p

Viterbi Path	
Viterbi Path	
Posterior @ 0.4	309 - GRKRKR - 314
Posterior @ 0.4	

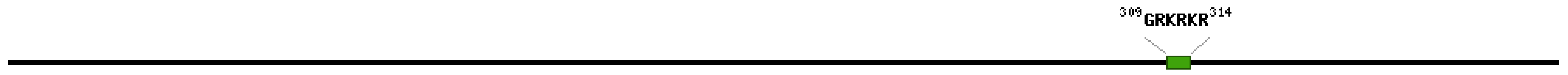

Posterior Graphic. Threshold Marked @ 0.4

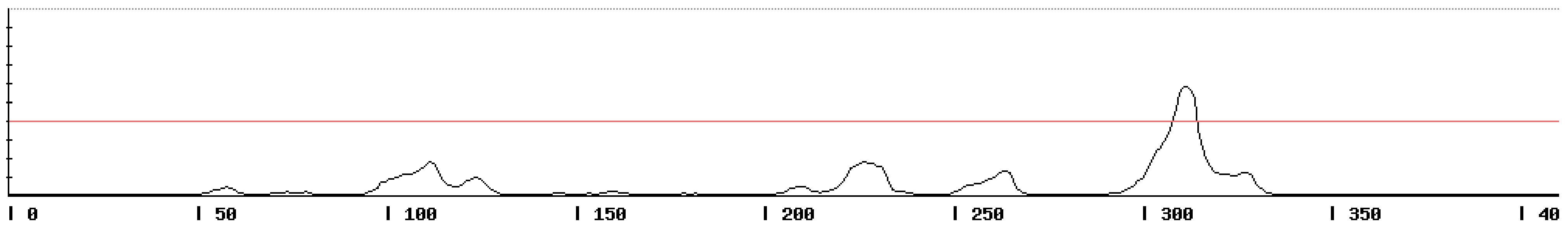

Predictions for Setariaitalica_Seita.1G183600.1.p

Viterbi Path

Viterbi Path

Posterior @ 0.4 316 - GRKRKRG - 322

Posterior @ 0.4

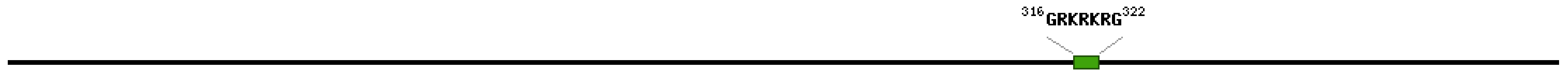

Posterior Graphic. Threshold Marked @ 0.4

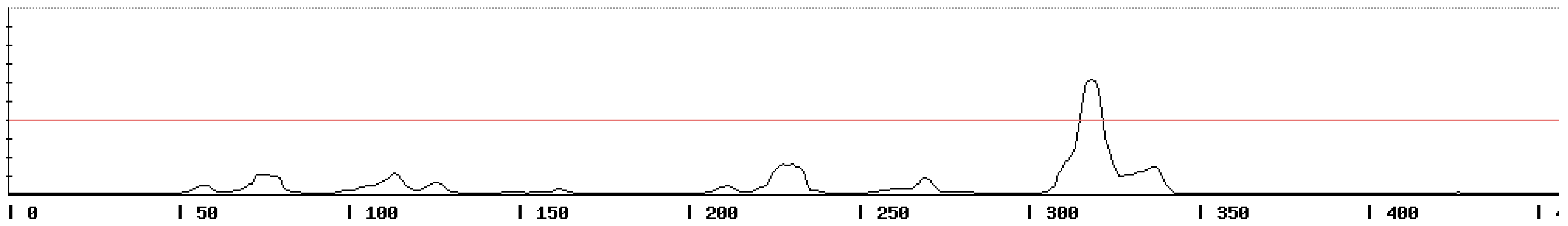

Predictions for Setariaitalica_Seita.4G094000.1.p

Viterbi Path

Viterbi Path

Posterior @ 0.4	99 - RIPGGGGREAAWRKKA - 114
	295 - KKRKR - 299
Posterior @ 0.4	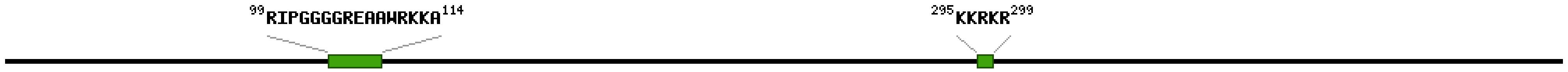
Posterior Graphic. Threshold Marked @ 0.4	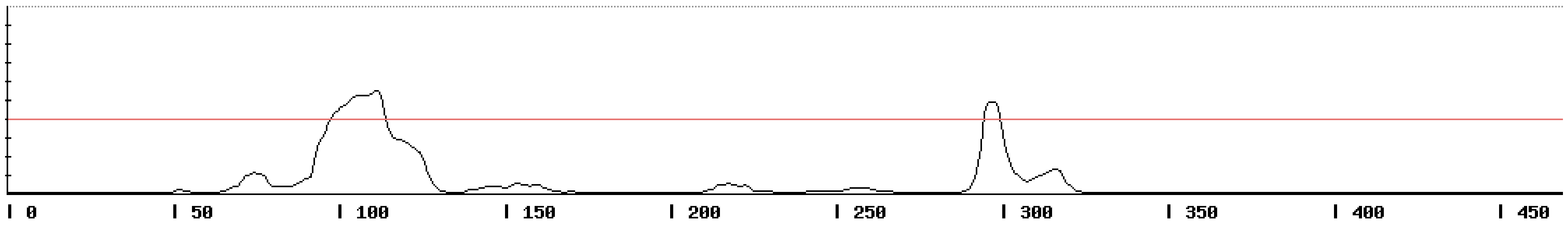

Predictions for Setariaitalica_Seita.7G161300.1.p

Viterbi Path	
Viterbi Path	
Posterior @ 0.4	26 - RHGGRAGAGGRPPRPPRRGGNRPPGGQ - 52
Posterior @ 0.4	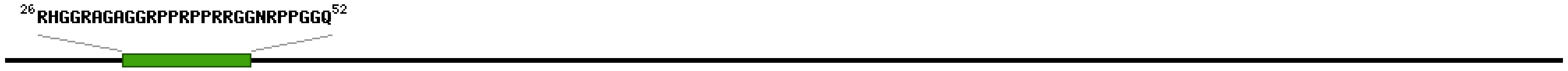
Posterior Graphic. Threshold Marked @ 0.4	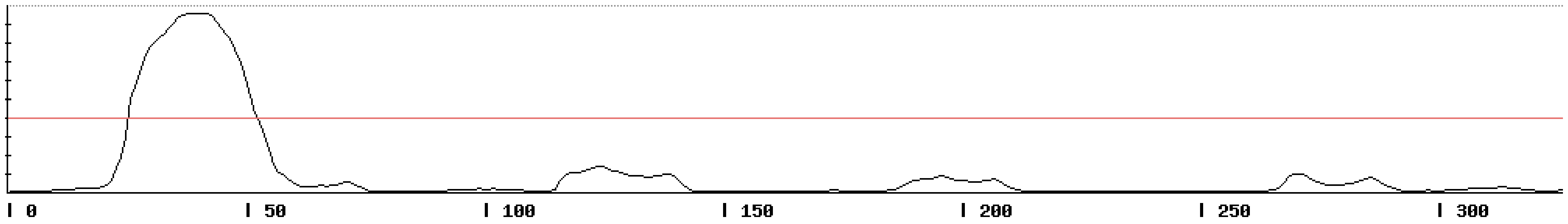

Predictions for Solanummelongena_Sme2.5_00096.1_g00018.1

Viterbi Path	
Viterbi Path	

Posterior @ 0.4

Posterior @ 0.4

Posterior Graphic. Threshold Marked @ 0.4

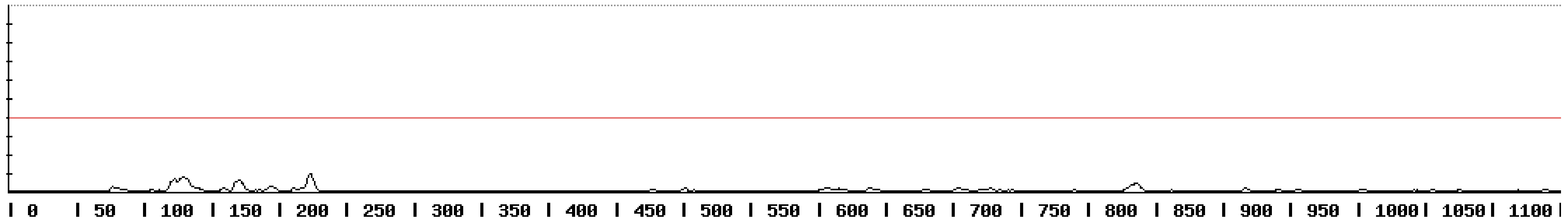

Predictions for Solanummelongena_Sme2.5_00142.1_g00013.1

Viterbi Path

Viterbi Path

Posterior @ 0.4

Posterior @ 0.4

Posterior Graphic. Threshold Marked @ 0.4

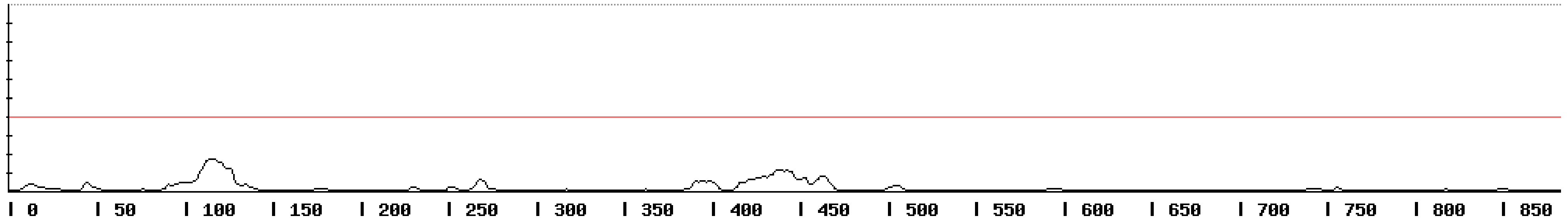

Predictions for Solanummelongena_Sme2.5_00756.1_g00007.1

Viterbi Path	
Viterbi Path	
Posterior @ 0.4	
Posterior @ 0.4	
Posterior Graphic. Threshold Marked @ 0.4	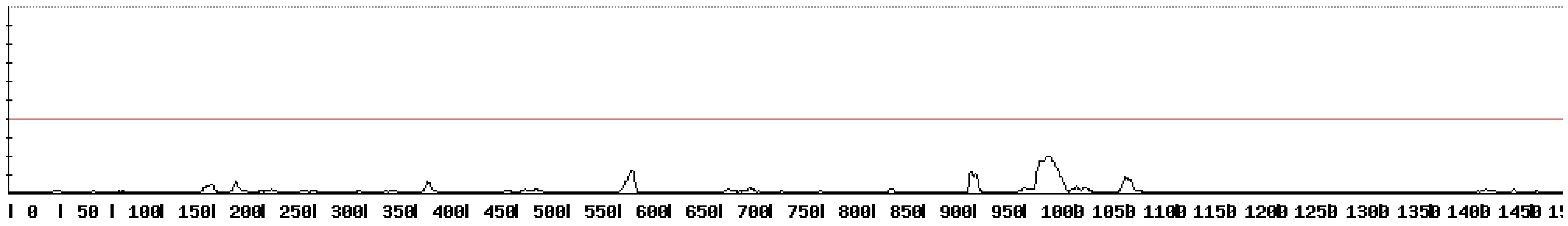

Predictions for Solanummelongena_Sme2.5_01725.1_g00009.1

Viterbi Path	
Viterbi Path	
Posterior @ 0.4	69 - RKRVKK - 74
	149 - HKKKGRR - 155
Posterior @ 0.4	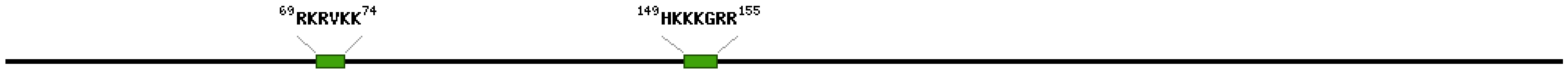
Posterior Graphic. Threshold Marked @ 0.4	

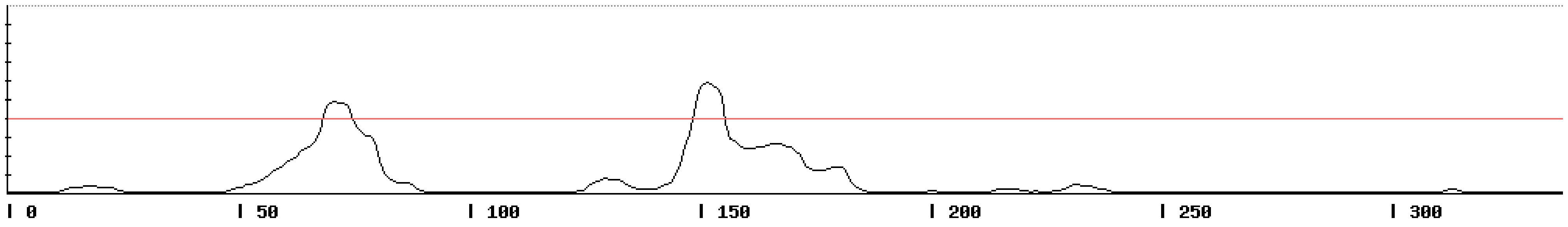

Predictions for Solanumpennellii_Sopen02g012300.1

Viterbi Path	
Viterbi Path	
Posterior @ 0.4	
Posterior @ 0.4	
Posterior Graphic. Threshold Marked @ 0.4	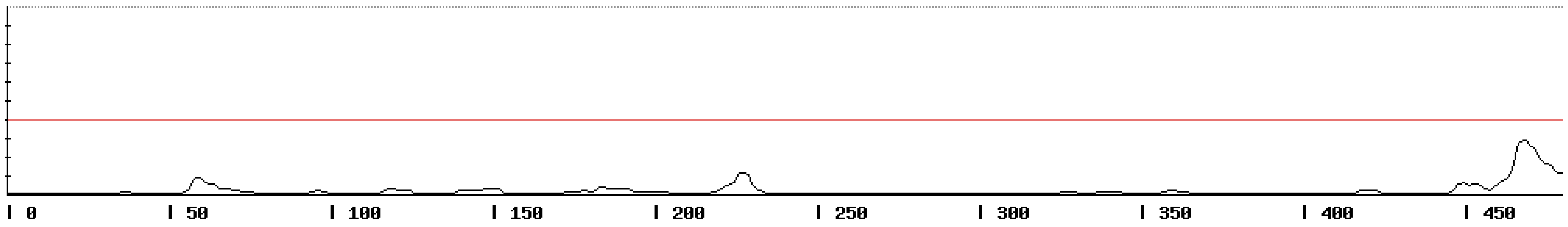

Predictions for Solanumpennellii_Sopen10g022760.1

Viterbi Path	
Viterbi Path	
Posterior @ 0.4	68 - KKRDTRFSRTLVGGGSWKGKSKGKPVGANKAGTK - 101
	532 - RGNNRGSGSRNRRRSQSKA - 550
	722 - KKNKVRFTESRGQKKVVFARKKPRATGQIQDERARKGSGRPVRG - 765
	774 - RASAKAPRR - 782

Posterior @ 0.4	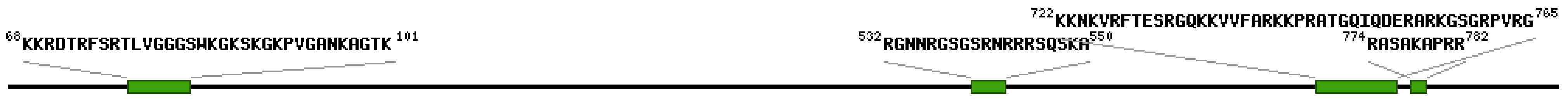
Posterior Graphic. Threshold Marked @ 0.4	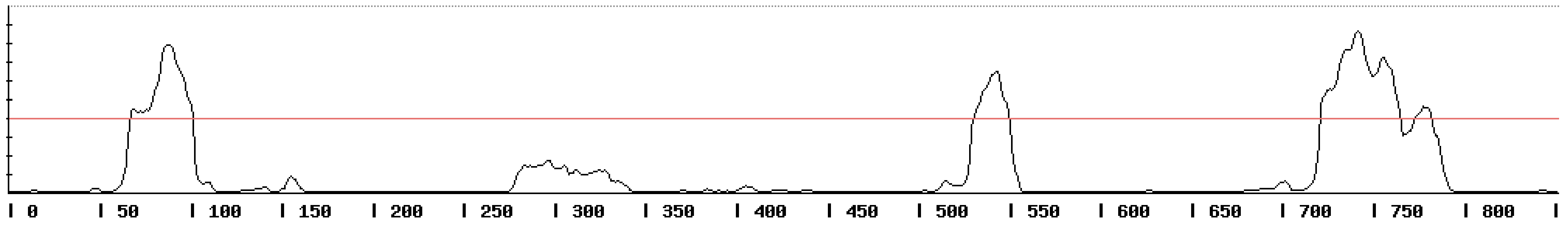

Predictions for Tarenayahassleriana_XP_010536267.1

Viterbi Path	
Viterbi Path	
Posterior @ 0.4	343 - HNSGRRKRKAFGGHWRATGKR - 363
	536 - RGKRKRNDGRP - 546
Posterior @ 0.4	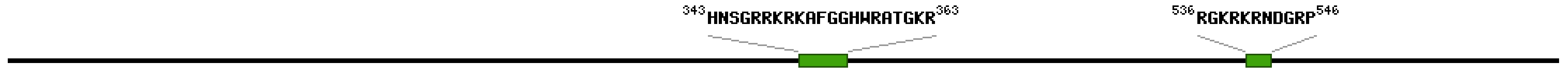
Posterior Graphic. Threshold Marked @ 0.4	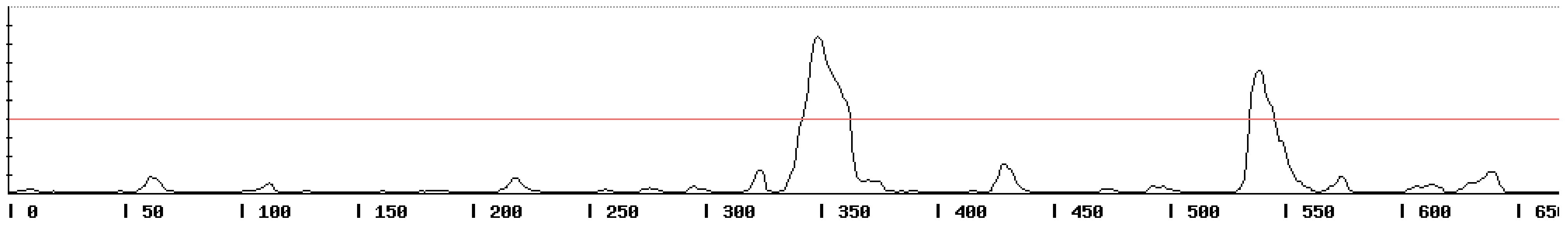

Predictions for Trifoliumpratense_Tp57577_TGAC_v2_mRNA14116

Viterbi Path	
Viterbi Path	

Posterior @ 0.4 | 1884 - RKKKGEK - 1890

Posterior @ 0.4

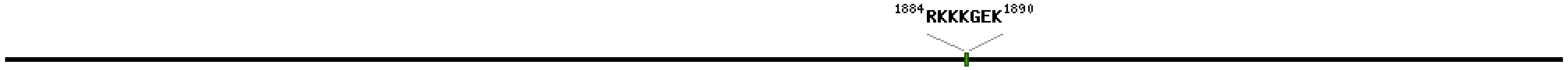

Posterior Graphic. Threshold Marked @ 0.4

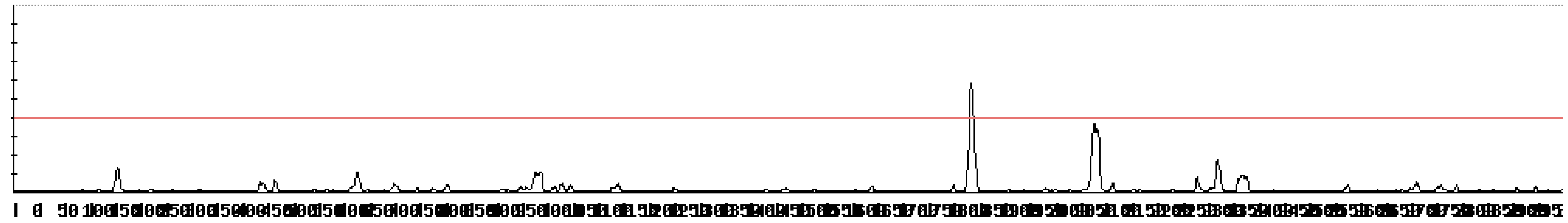

Predictions for Trifoliumpratense_Tp57577_TGAC_v2_mRNA16525

Viterbi Path

Viterbi Path

Posterior @ 0.4 | 82 - NRKAKGGYWKISGKGRIIKKKGTNNAIGRKK - 112

Posterior @ 0.4

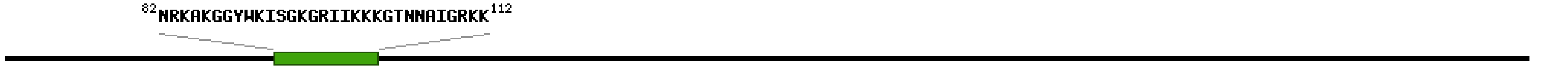

Posterior Graphic. Threshold Marked @ 0.4

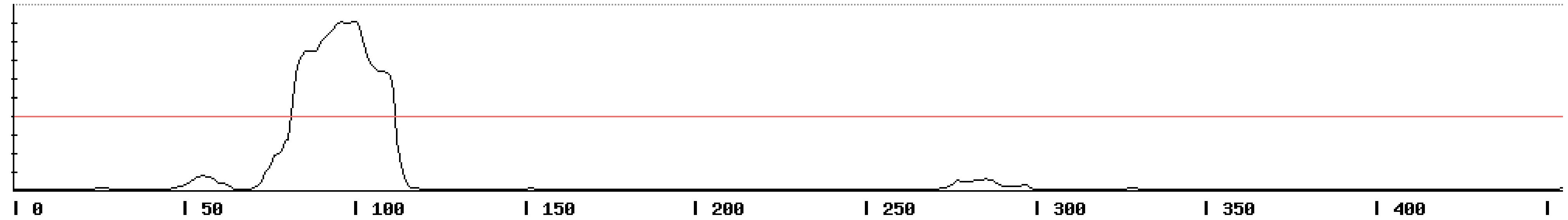

Predictions for Trifoliumpratense_Tp57577_TGAC_v2_mRNA31296

Viterbi Path

Viterbi Path

Posterior @ 0.4 | 83 - RKAKGGYWKISGKGRIIKKKGTNNVIGRKK - 112

Posterior @ 0.4

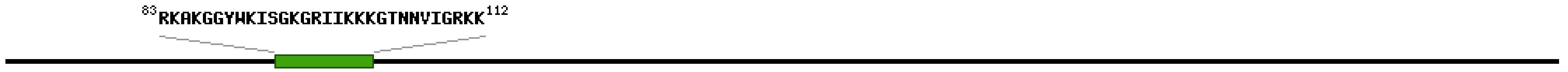

Posterior Graphic. Threshold Marked @ 0.4

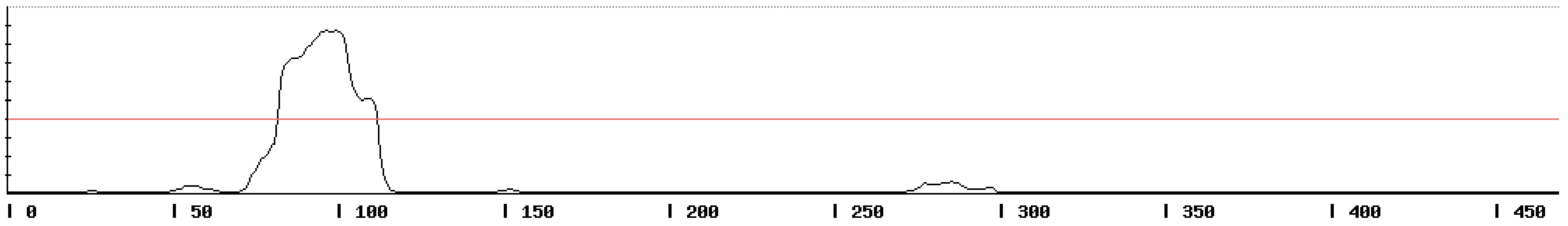

Predictions for Trifoliumpratense_Tp57577_TGAC_v2_mRNA38618

Viterbi Path

Viterbi Path

Posterior @ 0.4

735 - KRQKEKHPGT - 744

982 - RKKEHR - 987

Posterior @ 0.4

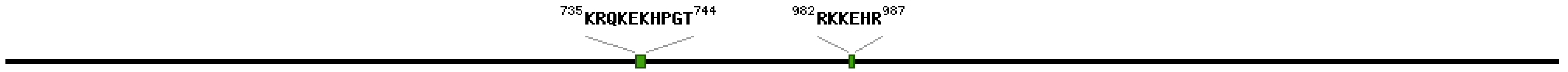

Posterior Graphic. Threshold Marked @ 0.4

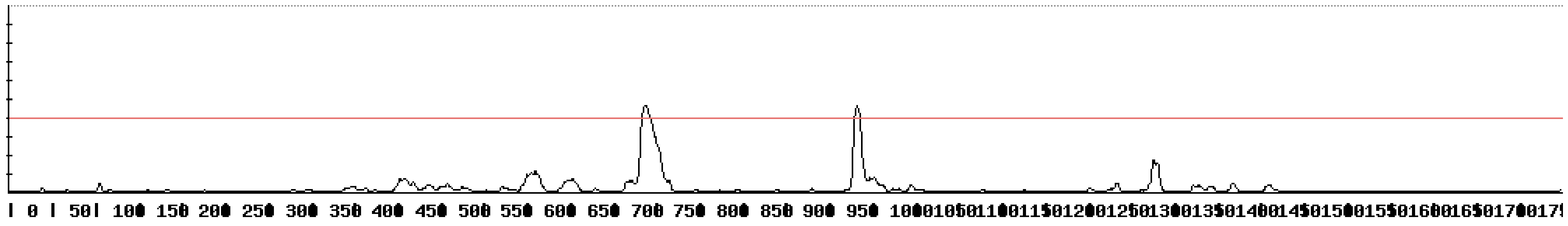

Predictions for Triticumaestivum_Traes_1DS_4A2001BAC.1

Viterbi Path

Viterbi Path

Posterior @ 0.4: 388 - KKRKK - 392

Posterior @ 0.4

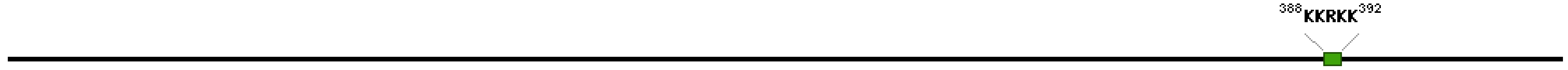

Posterior Graphic. Threshold Marked @ 0.4

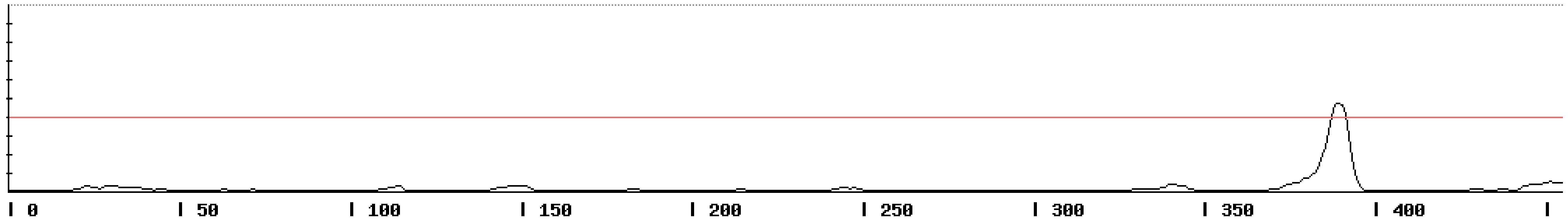

Predictions for Triticumaestivum_Traes_2BL_75FBE2E45.1

Viterbi Path

Viterbi Path

Posterior @ 0.4: 5 - KSGKPPRPPP - 14

Posterior @ 0.4

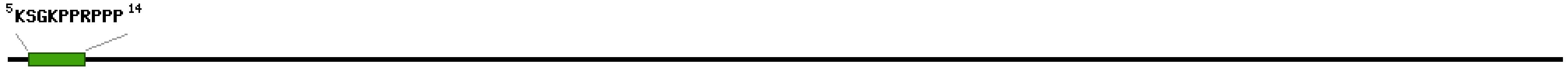

Posterior Graphic. Threshold Marked @ 0.4

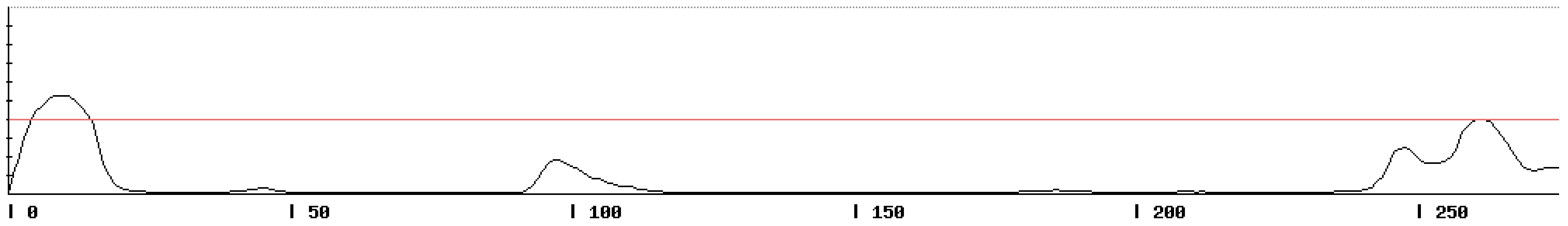

Predictions for Triticumaestivum_Traes3BF073600060CFD_t1

Viterbi Path

Viterbi Path

Posterior @ 0.4

78 - AKSRRGQRKS - 87

434 - AAGRKRK - 440

473 - GHKKRKTHSRNSGHVMRPARGVLKLTRPATSGRKK - 507

Posterior @ 0.4

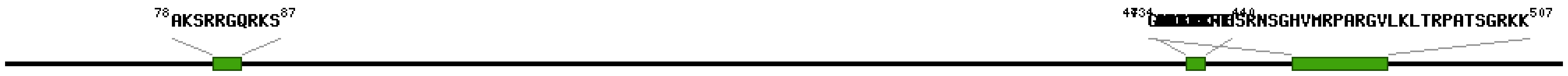

Posterior Graphic. Threshold Marked @ 0.4

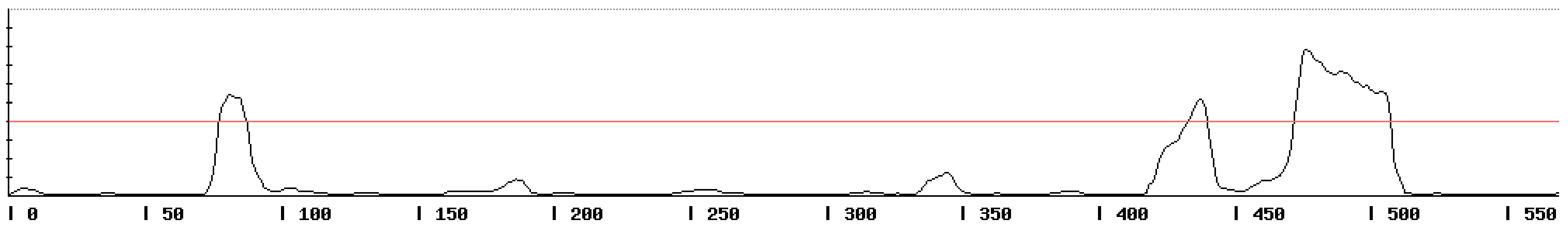

Predictions for Triticumaestivum_Traes3BF073600100CFD_t1

Viterbi Path

Viterbi Path

Posterior @ 0.4

Posterior @ 0.4	
Posterior Graphic. Threshold Marked @ 0.4	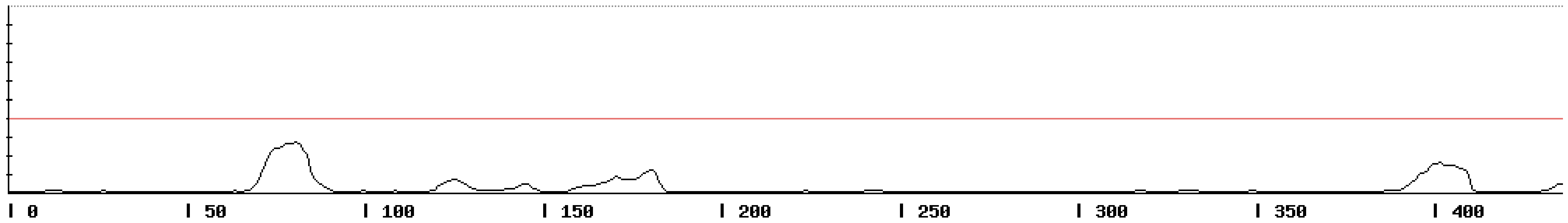

Predictions for Vignaradiata_Vradi0051s00540.1	
Viterbi Path	
Viterbi Path	
Posterior @ 0.4	69 - KKR - 71
	162 - TGADKPIGKPKALGIKKA - 179
	363 - RPNRAAGSGYWKATGADKPIGKPKALGIKKALKGKIEK - 400
Posterior @ 0.4	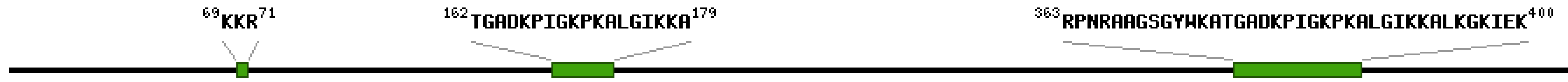
Posterior Graphic. Threshold Marked @ 0.4	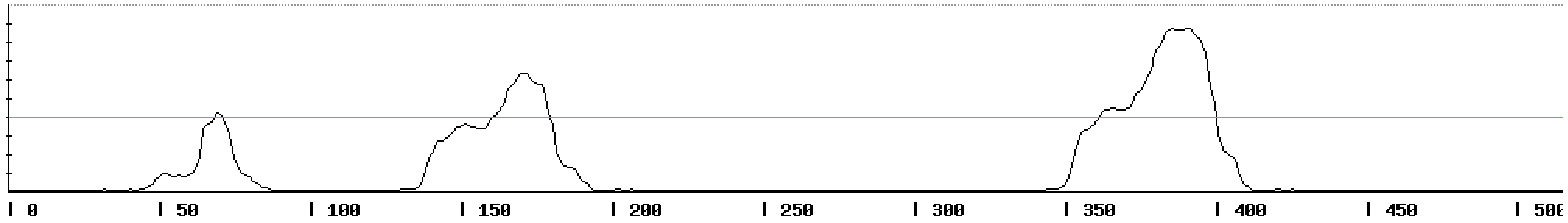

Predictions for Vignaradiata_Vradi08g15940.1	
Viterbi Path	
Viterbi Path	

Posterior @ 0.4	
Posterior @ 0.4	
Posterior Graphic. Threshold Marked @ 0.4	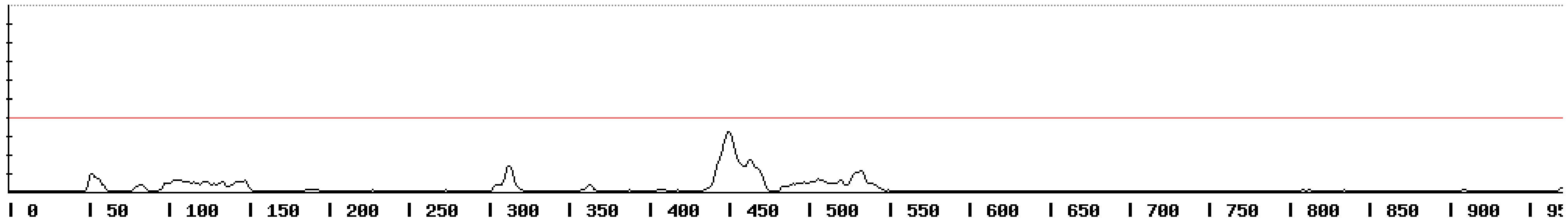

Predictions for Zoysiajaponica_Zjn_sc00007.1.g11670.1.am.mk	
Viterbi Path	
Viterbi Path	
Posterior @ 0.4	618 - PGRGGTRRRR - 627
Posterior @ 0.4	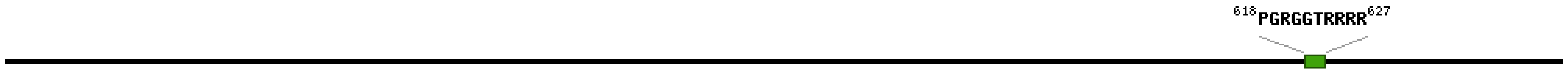
Posterior Graphic. Threshold Marked @ 0.4	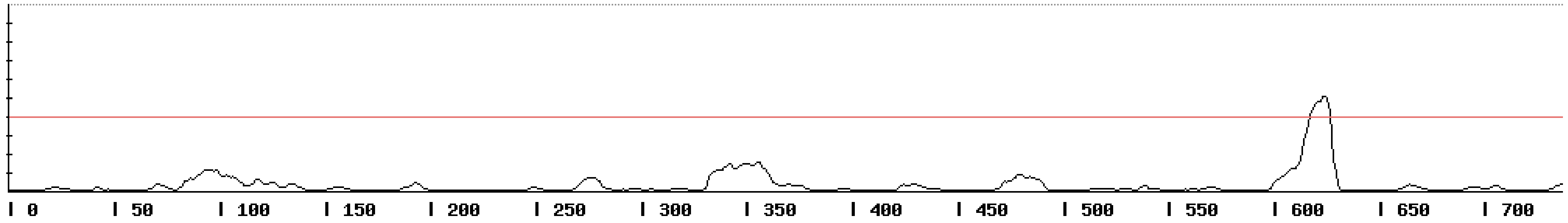

Predictions for Zoysiajaponica_Zjn_sc00028.1.g03130.1.am.mk

Viterbi Path

Viterbi Path

Posterior @ 0.4

Posterior @ 0.4

Posterior Graphic. Threshold Marked @ 0.4

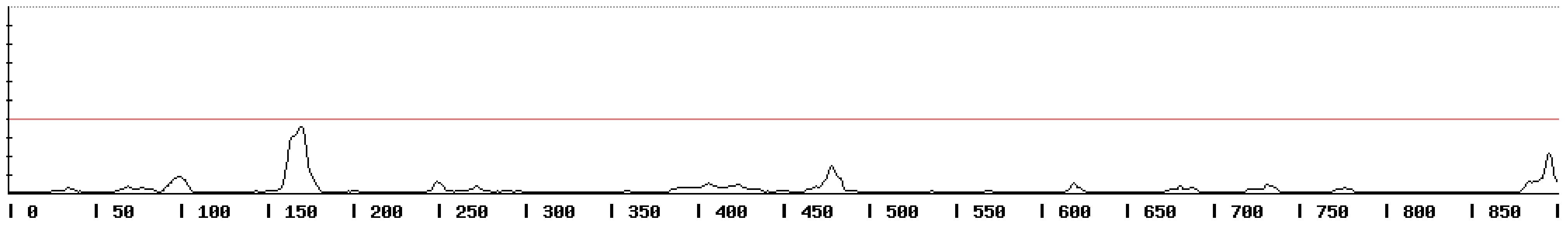

Predictions for Zoysiajaponica_Zjn_sc00067.1.g01060.1.am.mk

Viterbi Path

Viterbi Path

Posterior @ 0.4

304 - SAARRRKGSTAACPHRRARARH - 325

Posterior @ 0.4

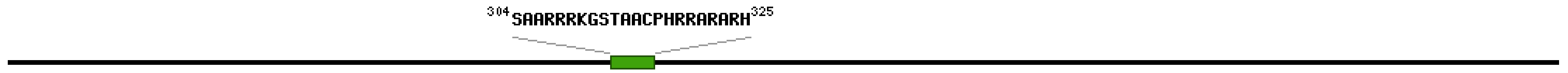

Posterior Graphic. Threshold Marked @ 0.4

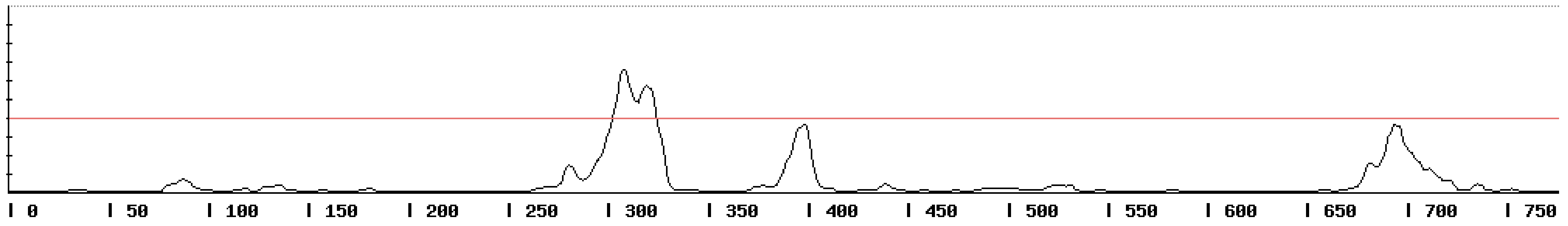

Predictions for Zoysiajaponica_Zjn_sc00138.1.g00550.1.am.mk

Viterbi Path

Viterbi Path

Posterior @ 0.4 — 603 - YRPRRNRARPPPRHPGAALP - 622

Posterior @ 0.4

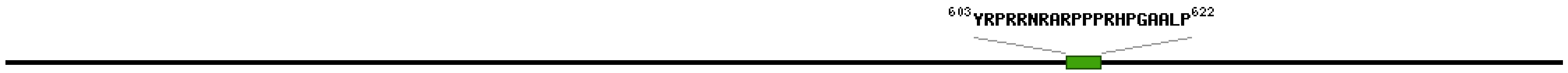

Posterior Graphic. Threshold Marked @ 0.4

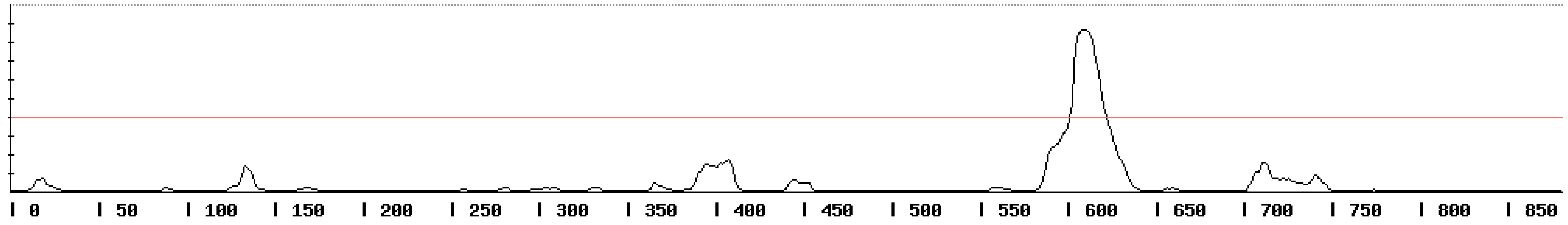

Predictions for Zoysiamatrella_Zmw_sc04707.1.g00090.1

Viterbi Path

Viterbi Path

Posterior @ 0.4 — 588 - YRPRRNRARPPPRHPGAALP - 607

Posterior @ 0.4

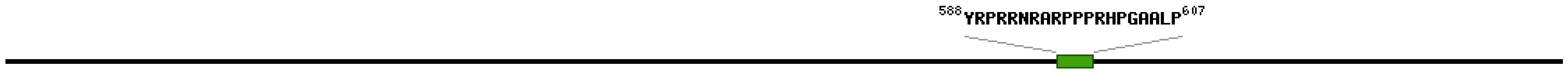

Posterior Graphic. Threshold Marked @ 0.4

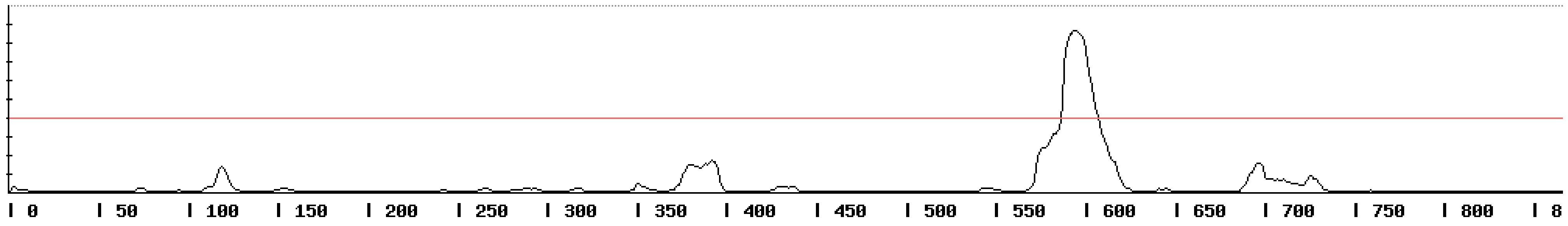

Predictions for Zoysiamatrella_Zmw_sc00723.1.g00180.1

Viterbi Path

Viterbi Path

Posterior @ 0.4: 747 - RRSRTQRRRAS - 757

Posterior @ 0.4

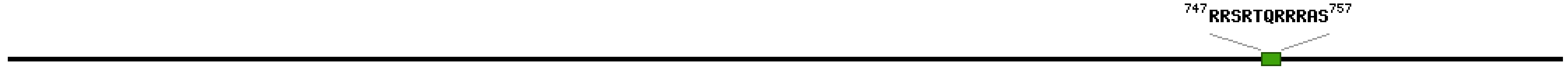

Posterior Graphic. Threshold Marked @ 0.4

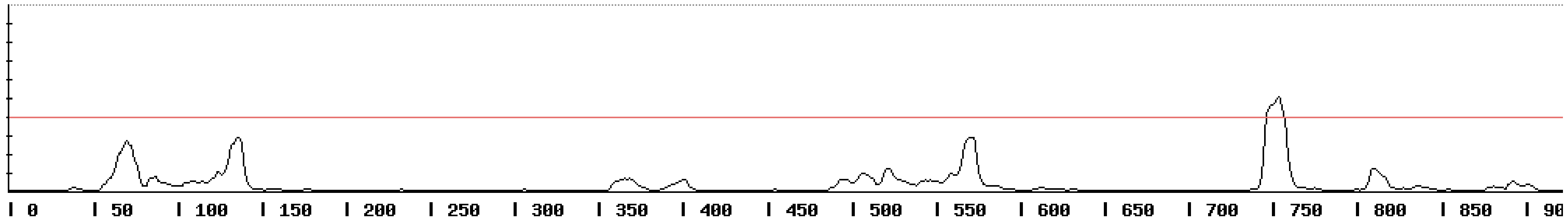

Predictions for Zoysiamatrella_Zmw_sc00881.1.g00060.1

Viterbi Path

Viterbi Path

Posterior @ 0.4: 295 - ARARGRGGRSGGARRLRR - 312

Posterior @ 0.4

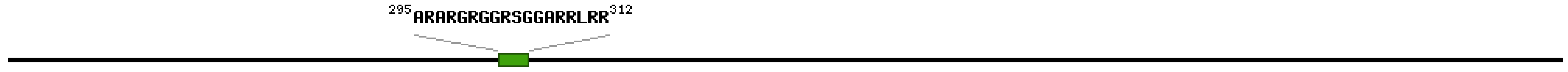

Posterior Graphic. Threshold Marked @ 0.4

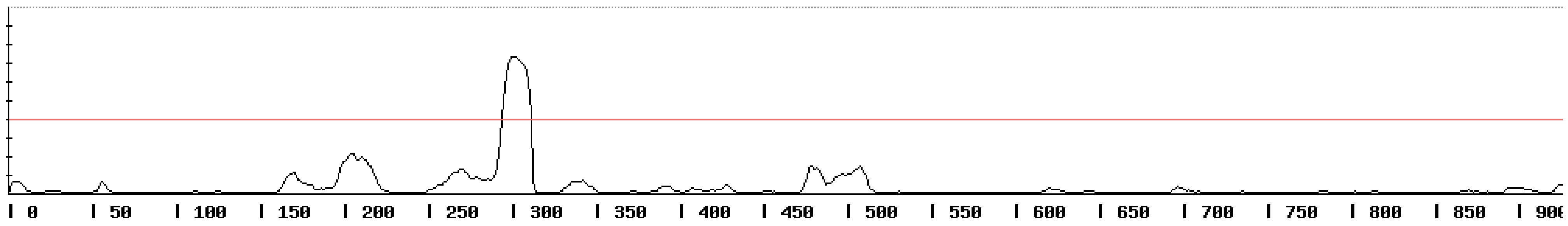

Predictions for Zoysiamatrella_Zmw_sc01551.1.g00060.1

Viterbi Path

Viterbi Path

Posterior @ 0.4

308 - SAARRRKGSTAACPHRRARARH - 329

Posterior @ 0.4

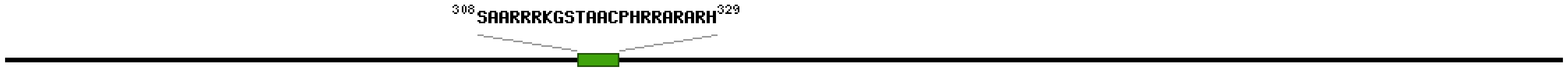

Posterior Graphic. Threshold Marked @ 0.4

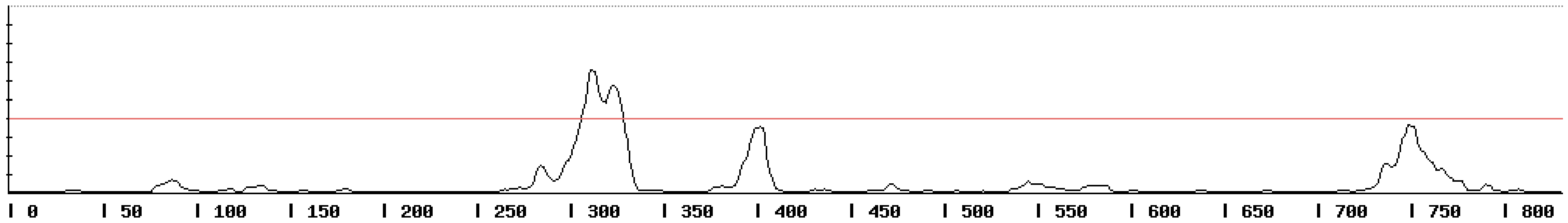

Predictions for Zoysiapacifica_Zpz_sc01162.1.g00050.1.am.mkhc

Viterbi Path

Viterbi Path

Posterior @ 0.4

213 - KATGKDRVIRSKGDKRKQHVIGMKKTLVFHKGRAPKGE - 250

Posterior @ 0.4

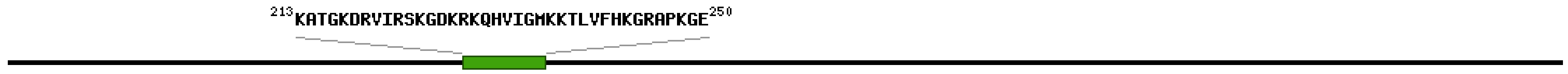

Posterior Graphic. Threshold Marked @ 0.4

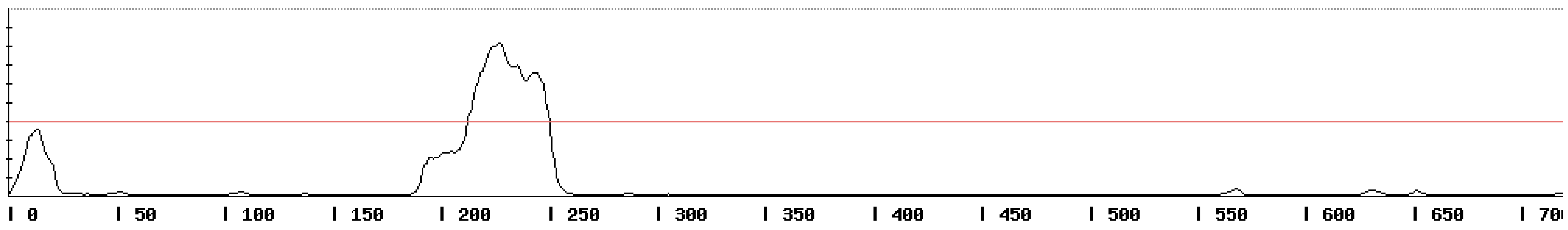

Predictions for Zoysiapacifica_Zpz_sc00130.1.g00590.1.am.mk

Viterbi Path

Viterbi Path

Posterior @ 0.4

323 - PKSKAVAPESAKPVMFKKRRLVKADLKKMK - 352

390 - KKEKMKGKEEERKQG - 404

422 - RNRAEKRSR - 430

Posterior @ 0.4

Revision r.9 - Last Updated on : February 24 2015 17:45:20. alex.nguyenba@utoronto.ca

Intended to be used with Firefox

Viterbi Path

Posterior @ 0.4

Posterior @ 0.4

Posterior Graphic. Threshold Marked @ 0.4

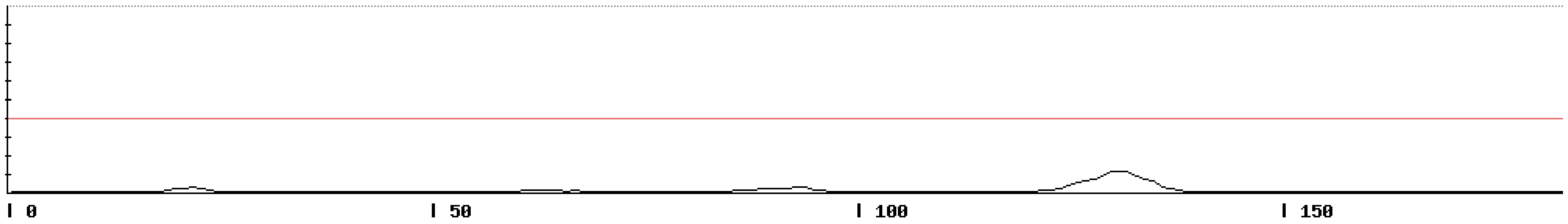

Predictions for Ocimumtenuiflorum_Ote100001860042

Viterbi Path

Viterbi Path

Posterior @ 0.4

Posterior @ 0.4

Posterior Graphic. Threshold Marked @ 0.4

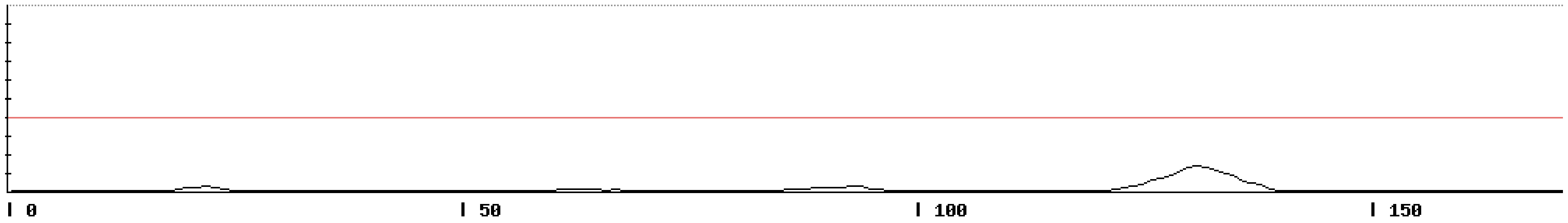

Predictions for Ocimumtenuiflorum_Ote100003460061

Viterbi Path

Viterbi Path

Posterior @ 0.4

Posterior @ 0.4

Posterior Graphic. Threshold Marked @ 0.4

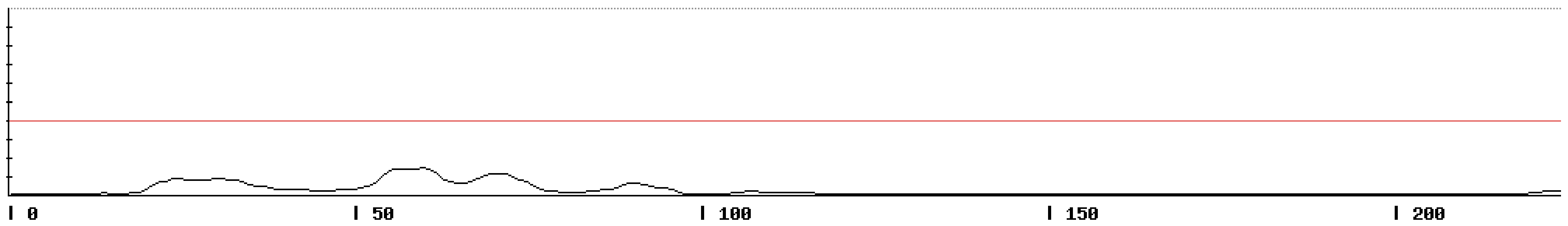

Predictions for Ocimumtenuiflorum_Ote100003460071

Viterbi Path

Viterbi Path

Posterior @ 0.4

Posterior @ 0.4

Posterior Graphic. Threshold Marked @ 0.4

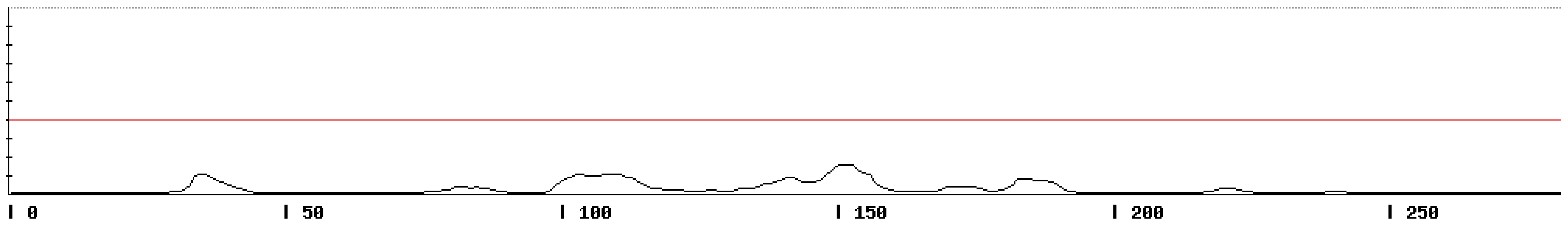

Predictions for Ocimumtenuiflorum_Ote100004440121

Viterbi Path	
Viterbi Path	
Posterior @ 0.4	8 - KK - 9<br>480 - RKWKMGKRGGGR - 491
Posterior @ 0.4	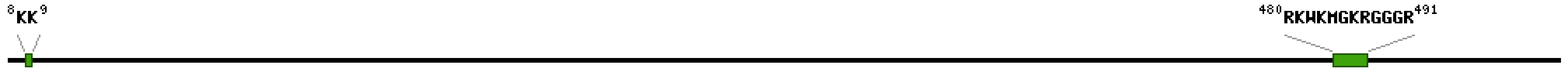
Posterior Graphic. Threshold Marked @ 0.4	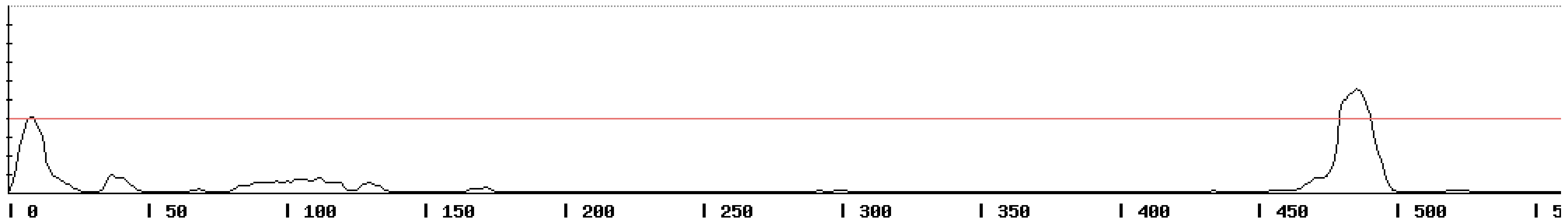

Predictions for Ocimumtenuiflorum_Ote100004440122

Viterbi Path	
Viterbi Path	
Posterior @ 0.4	389 - SKTGRKKPSR - 398<br>866 - RKWKMGKRGGGR - 877
Posterior @ 0.4	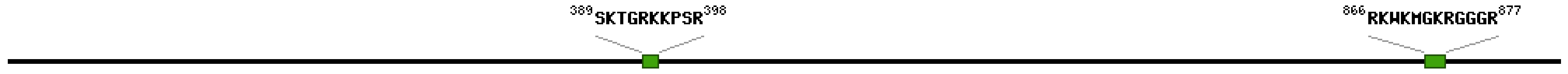
Posterior Graphic. Threshold Marked @ 0.4	

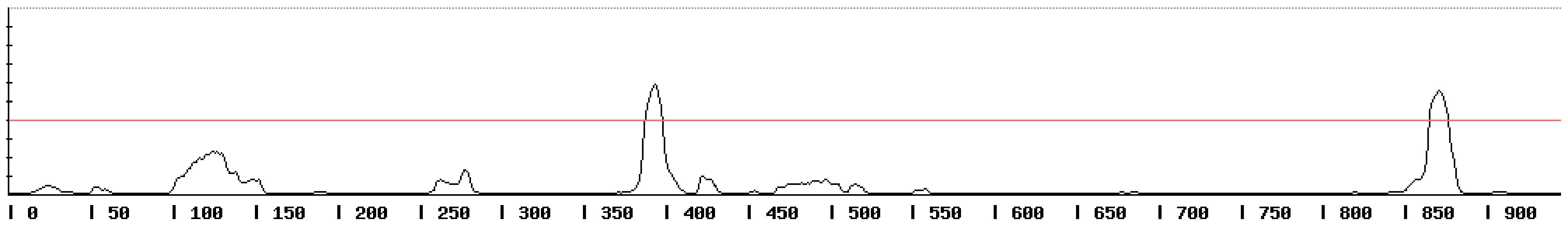

Predictions for Ocimumtenuiflorum_Ote100005370051

Viterbi Path

Viterbi Path

Posterior @ 0.4

Posterior @ 0.4

Posterior Graphic. Threshold Marked @ 0.4

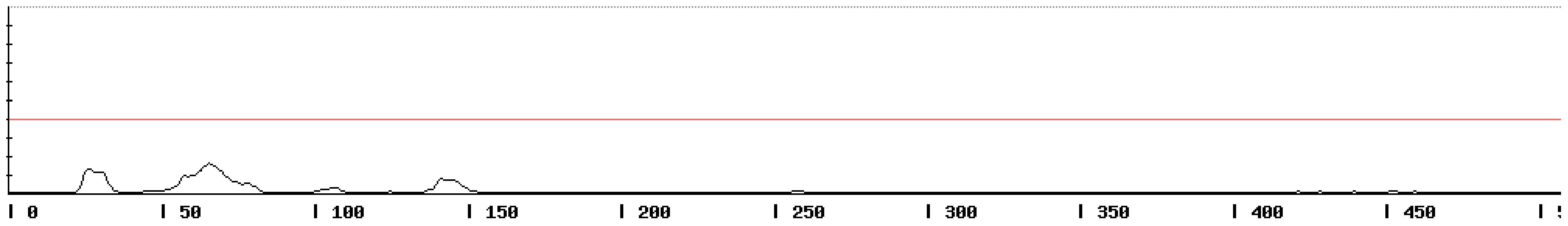

Predictions for Ocimumtenuiflorum_Ote100006770211

Viterbi Path

Viterbi Path

Posterior @ 0.4

Posterior @ 0.4

Posterior Graphic. Threshold Marked @ 0.4	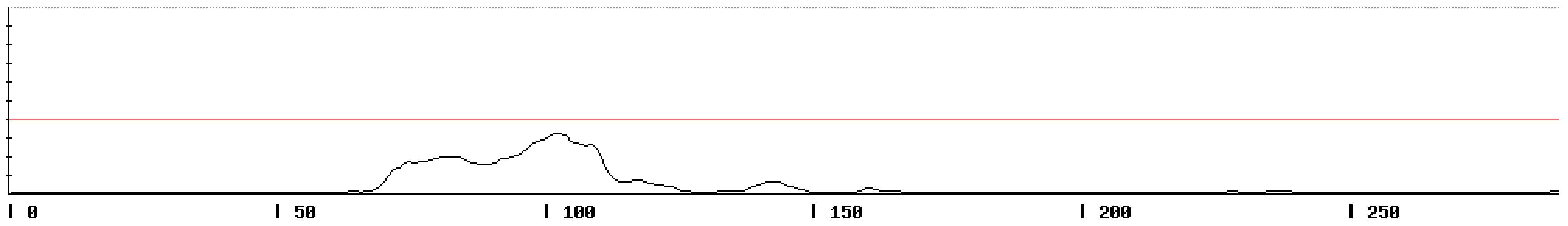

Predictions for Ocimumtenuiflorum_Ote100009660061	
Viterbi Path	
Viterbi Path	
Posterior @ 0.4	
Posterior @ 0.4	
Posterior Graphic. Threshold Marked @ 0.4	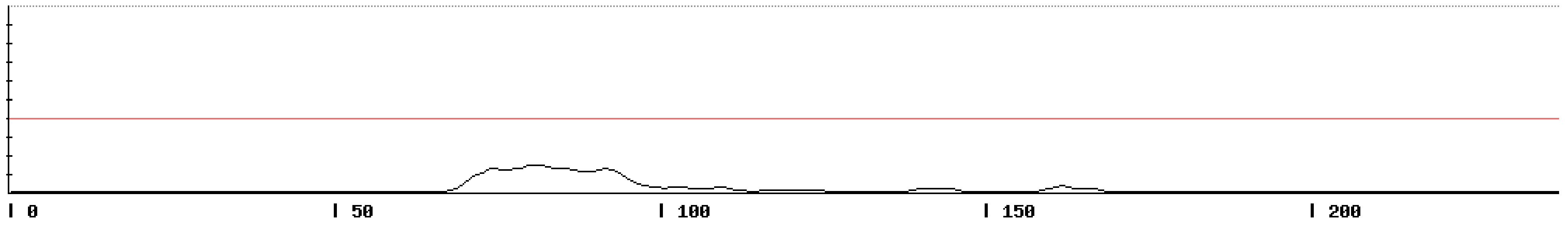

Predictions for Ocimumtenuiflorum_Ote100009660062	
Viterbi Path	
Viterbi Path	
Posterior @ 0.4	
Posterior @ 0.4	

Posterior Graphic. Threshold Marked @ 0.4

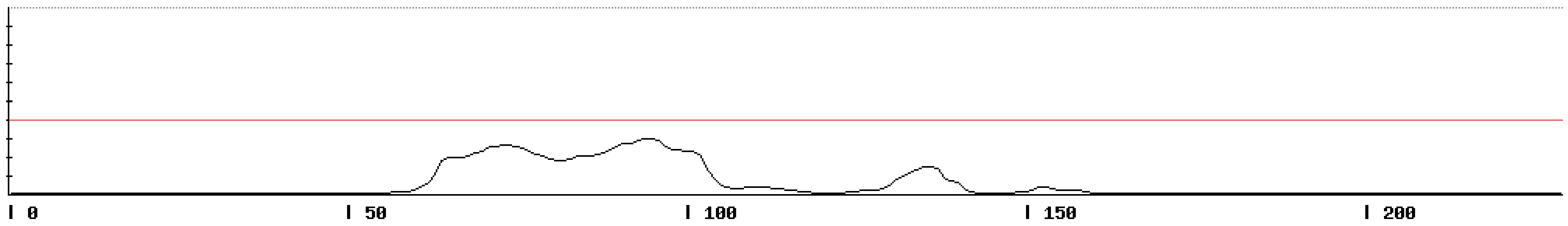

Predictions for Ocimumtenuiflorum_Ote100009830031

Viterbi Path

Viterbi Path

Posterior @ 0.4

Posterior @ 0.4

Posterior Graphic. Threshold Marked @ 0.4

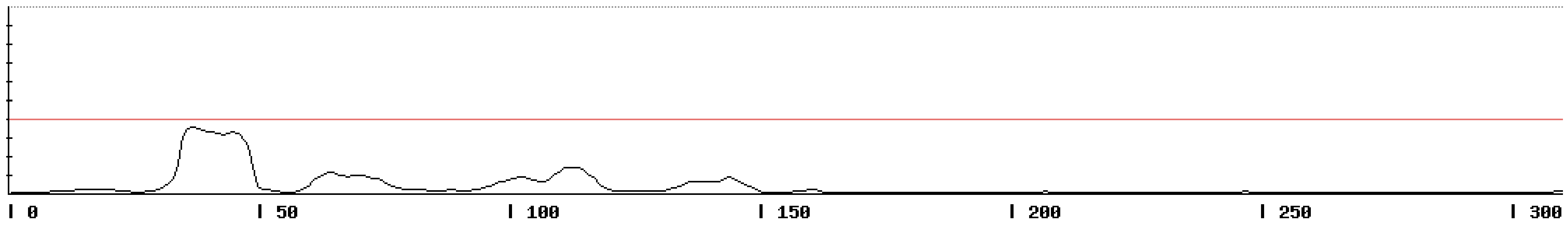

Predictions for Ocimumtenuiflorum_Ote100017900131

Viterbi Path

Viterbi Path

Posterior @ 0.4

Posterior @ 0.4

Posterior Graphic. Threshold Marked @ 0.4

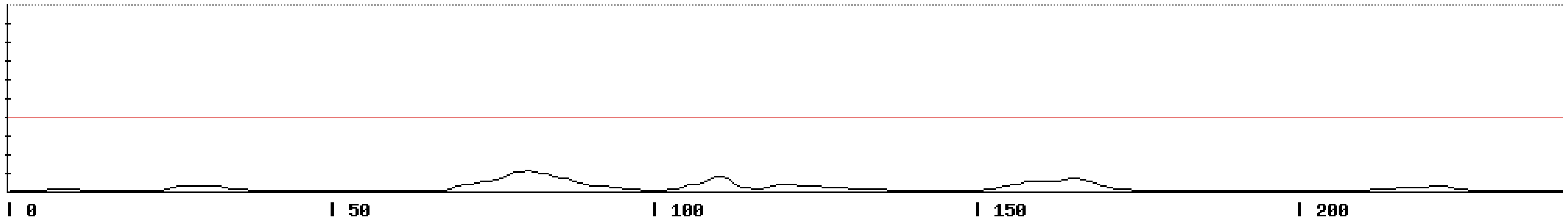

Predictions for Ocimumtenuiflorum_Ote100018490011

Viterbi Path

Viterbi Path

Posterior @ 0.4

Posterior @ 0.4

Posterior Graphic. Threshold Marked @ 0.4

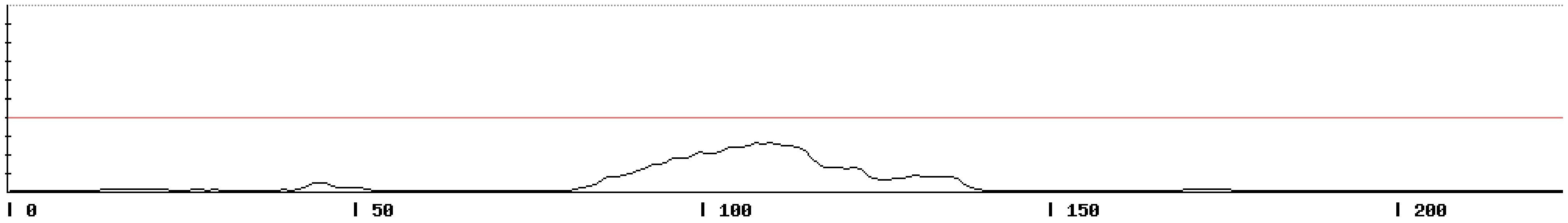

Predictions for Ocimumtenuiflorum_Ote100018490031

Viterbi Path

Viterbi Path

Posterior @ 0.4

Posterior @ 0.4

Posterior Graphic. Threshold Marked @ 0.4

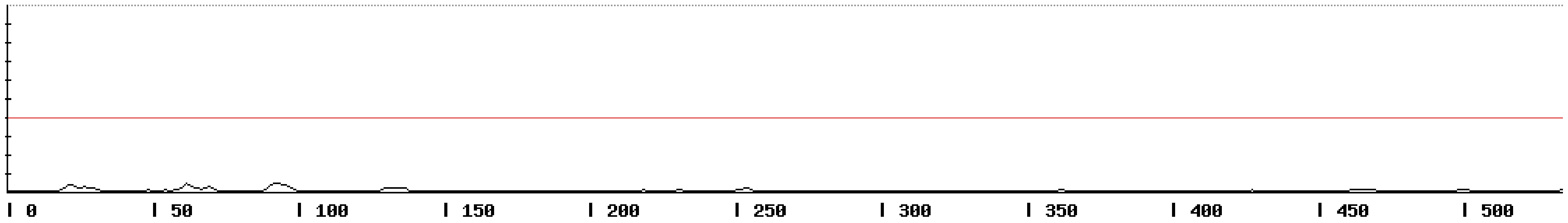

Predictions for Ocimumtenuiflorum_Ote100018500041

Viterbi Path

Viterbi Path

Posterior @ 0.4

Posterior @ 0.4

Posterior Graphic. Threshold Marked @ 0.4

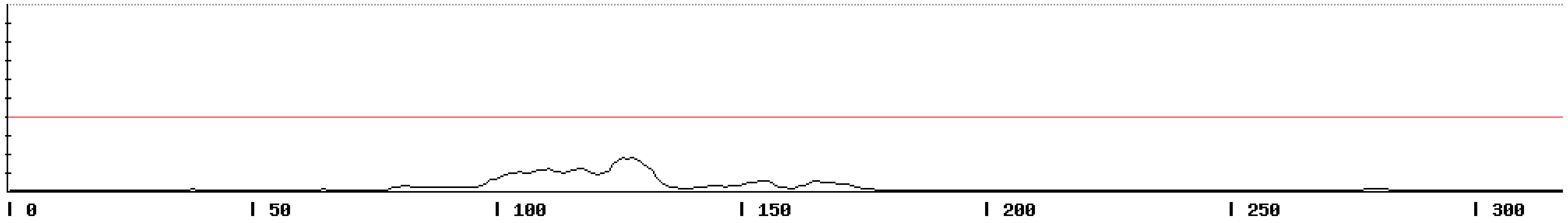

Predictions for Ocimumtenuiflorum_Ote100019360041

Viterbi Path

Viterbi Path

Posterior @ 0.4

Posterior @ 0.4

Posterior Graphic. Threshold Marked @ 0.4

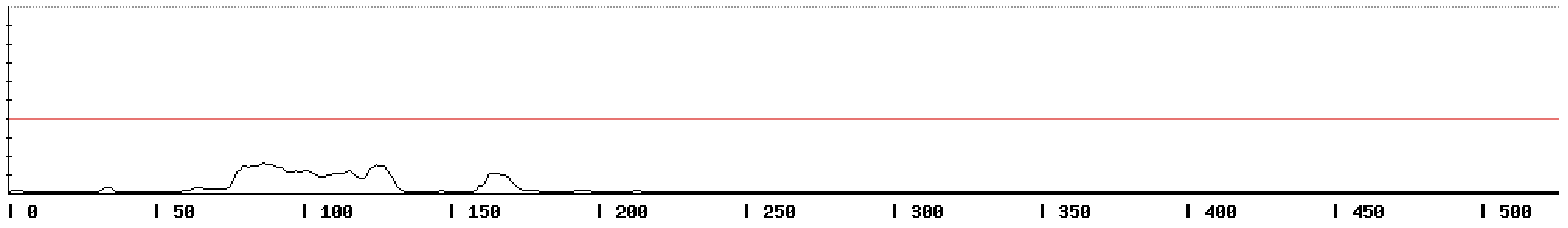

Predictions for Ocimumtenuiflorum_Ote100019360051

Viterbi Path

Viterbi Path

Posterior @ 0.4

Posterior @ 0.4

Posterior Graphic. Threshold Marked @ 0.4

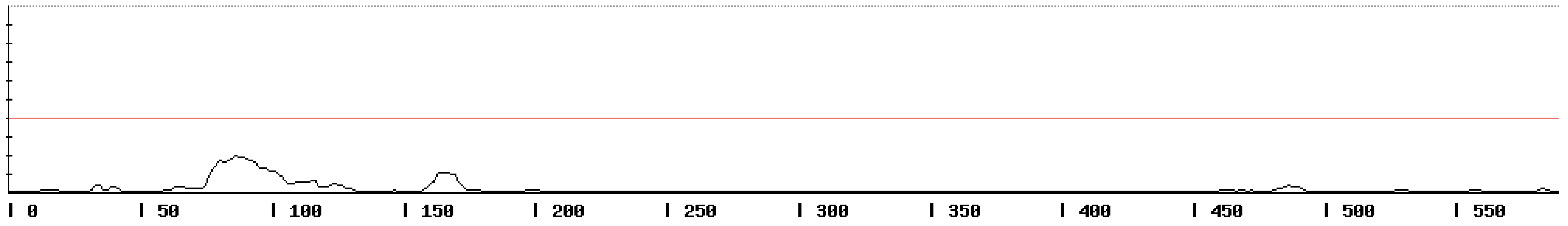

Predictions for Ocimumtenuiflorum_Ote100021820061

Viterbi Path	
Viterbi Path	
Posterior @ 0.4	
Posterior @ 0.4	
Posterior Graphic. Threshold Marked @ 0.4	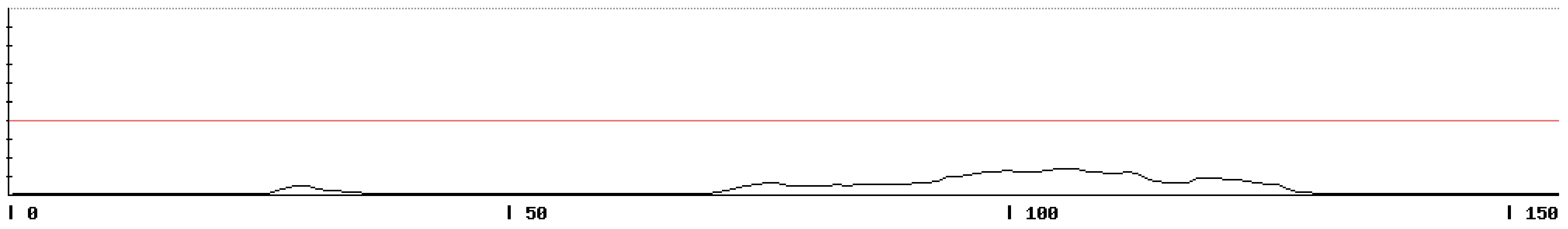

Predictions for Ocimumtenuiflorum_Ote100022610011

Viterbi Path	
Viterbi Path	
Posterior @ 0.4	
Posterior @ 0.4	
Posterior Graphic. Threshold Marked @ 0.4	

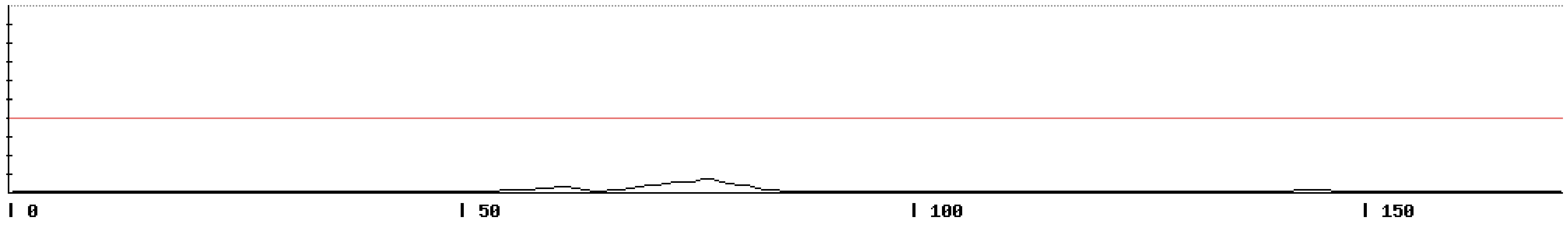

Predictions for Ocimumtenuiflorum_Ote100025110121

Viterbi Path

Viterbi Path

Posterior @ 0.4

128 - RRKRRR - 133

Posterior @ 0.4

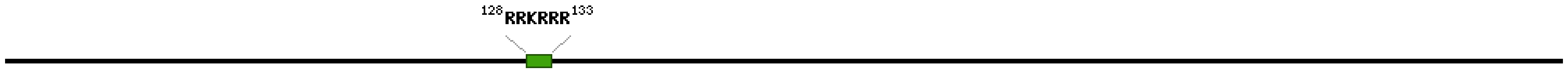

Posterior Graphic. Threshold Marked @ 0.4

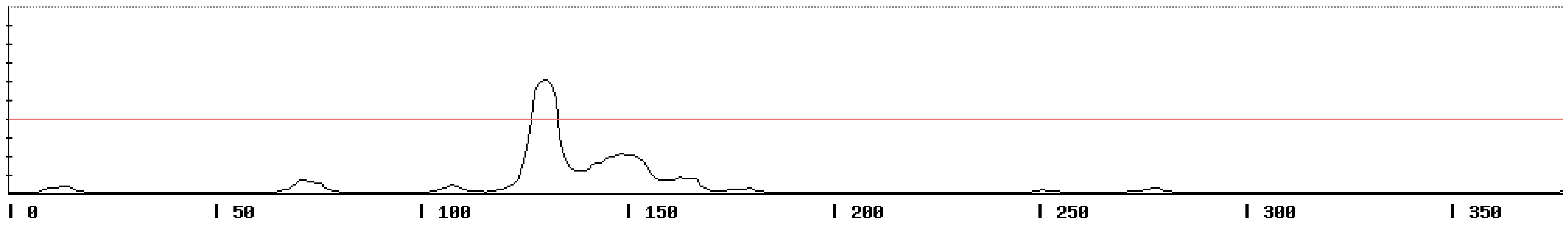

Predictions for Ocimumtenuiflorum_Ote100025110161

Viterbi Path

Viterbi Path

Posterior @ 0.4

79 - RRKRRR - 84

Posterior @ 0.4

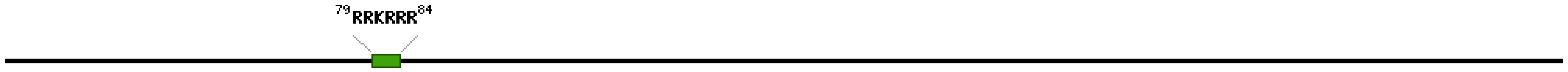

Posterior Graphic. Threshold Marked @ 0.4	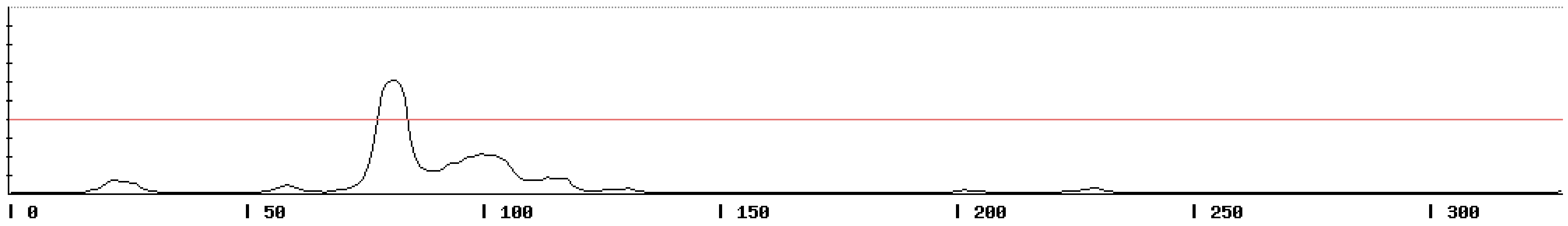

Predictions for Ocimumtenuiflorum_Ote100027510021

Viterbi Path	
Viterbi Path	
Posterior @ 0.4	
Posterior @ 0.4	
Posterior Graphic. Threshold Marked @ 0.4	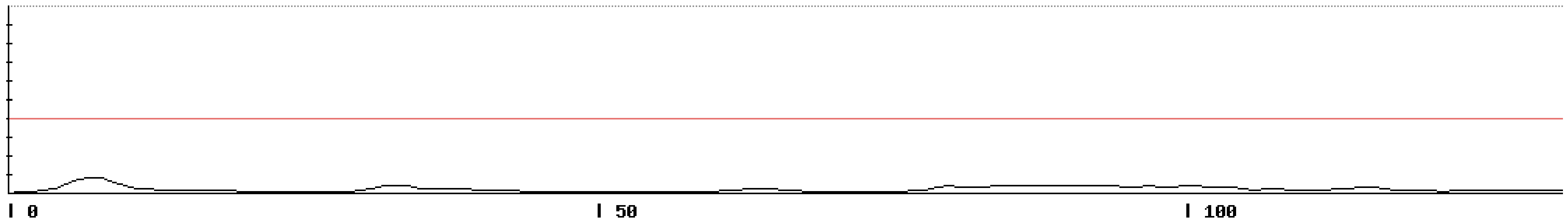

Predictions for Ocimumtenuiflorum_Ote100038860061

Viterbi Path	
Viterbi Path	
Posterior @ 0.4	
Posterior @ 0.4	

Posterior Graphic. Threshold Marked @ 0.4

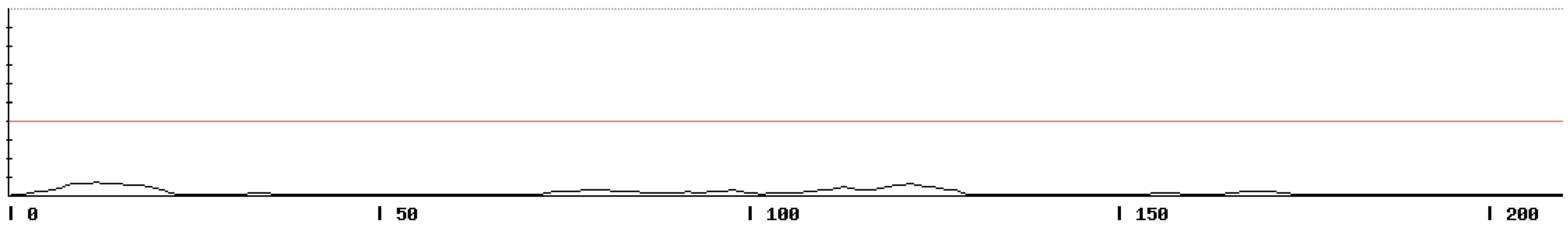

Predictions for Ocimumtenuiflorum_Ote100041470021

Viterbi Path

Viterbi Path

Posterior @ 0.4

161 - KRRAR - 165

Posterior @ 0.4

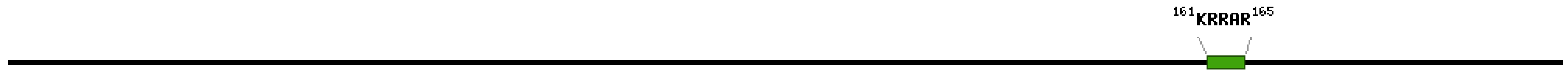

Posterior Graphic. Threshold Marked @ 0.4

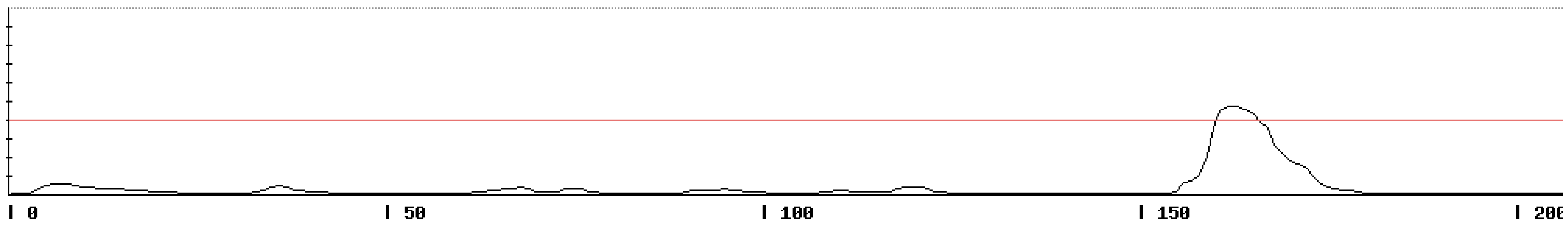

Predictions for Ocimumtenuiflorum_Ote100044030011

Viterbi Path

Viterbi Path

Posterior @ 0.4

Posterior @ 0.4

Posterior Graphic. Threshold Marked @ 0.4

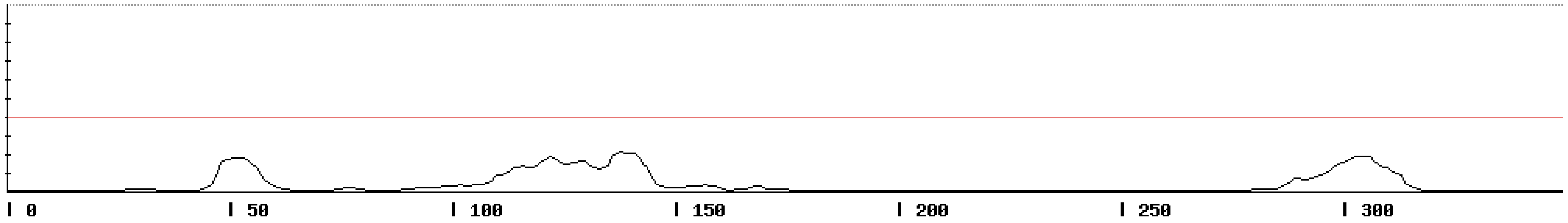

Predictions for Ocimumtenuiflorum_Ote100046160011

Viterbi Path

Viterbi Path

Posterior @ 0.4

Posterior @ 0.4

Posterior Graphic. Threshold Marked @ 0.4

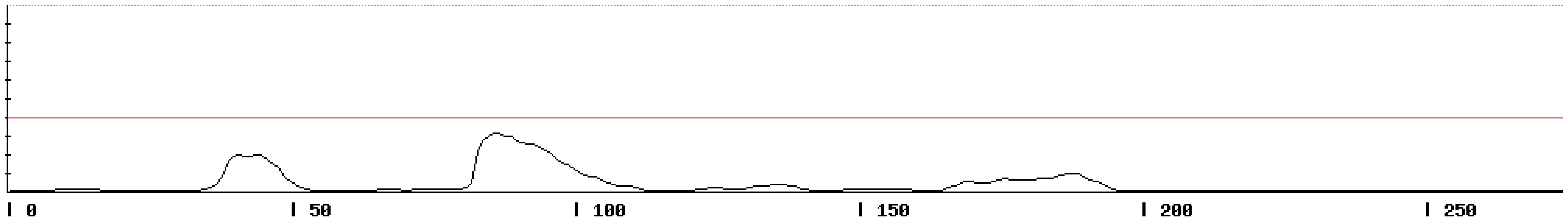

Predictions for Ocimumtenuiflorum_Ote100049580021

Viterbi Path

Viterbi Path

Posterior @ 0.4

Posterior @ 0.4

Posterior Graphic. Threshold Marked @ 0.4

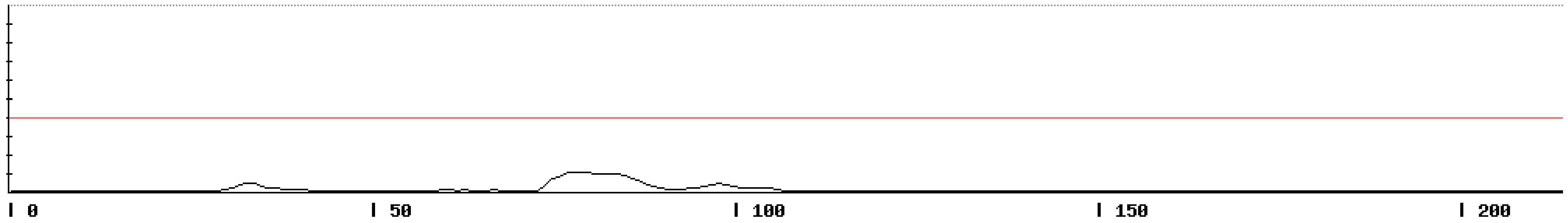

Predictions for Ocimumtenuiflorum_Ote100067080011

Viterbi Path

Viterbi Path

Posterior @ 0.4

Posterior @ 0.4

Posterior Graphic. Threshold Marked @ 0.4

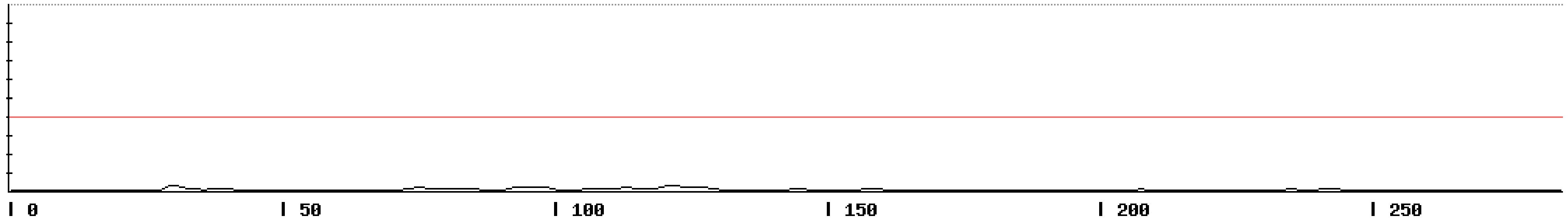

Predictions for Ocimumtenuiflorum_Ote100067460051

Viterbi Path

Viterbi Path

Posterior @ 0.4

Posterior @ 0.4

Posterior Graphic. Threshold Marked @ 0.4

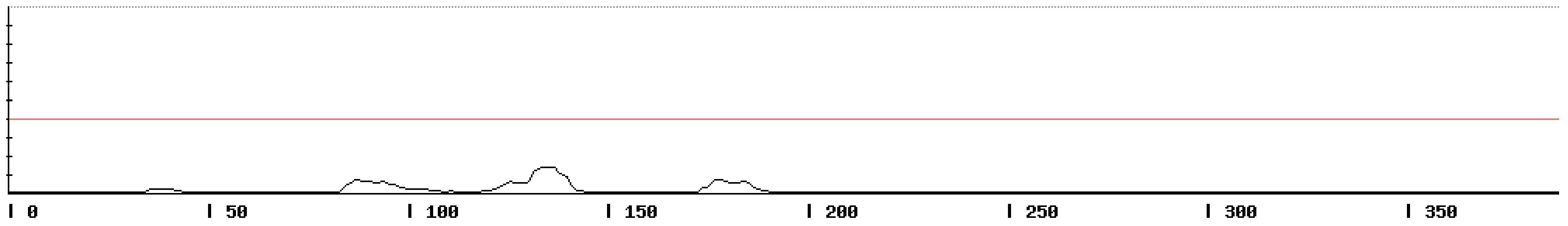

Predictions for Ocimumtenuiflorum_Ote100070460001

Viterbi Path

Viterbi Path

Posterior @ 0.4

Posterior @ 0.4

Posterior Graphic. Threshold Marked @ 0.4

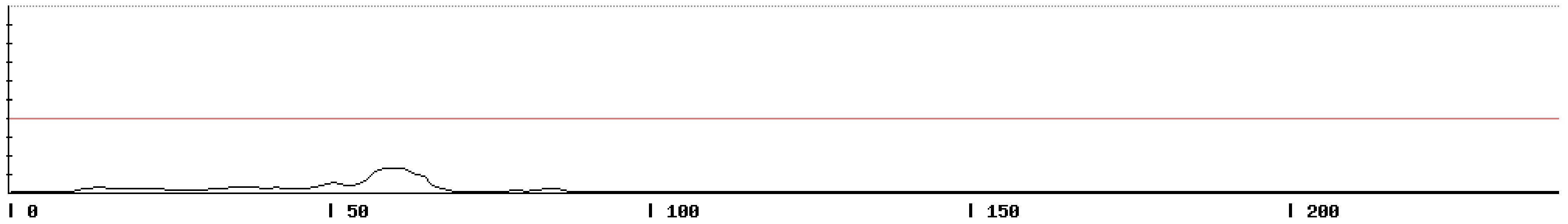

Predictions for Ocimumtenuiflorum_Ote100072310011	
Viterbi Path	
Viterbi Path	
Posterior @ 0.4	125 - RK - 126
Posterior @ 0.4	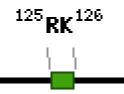
Posterior Graphic. Threshold Marked @ 0.4	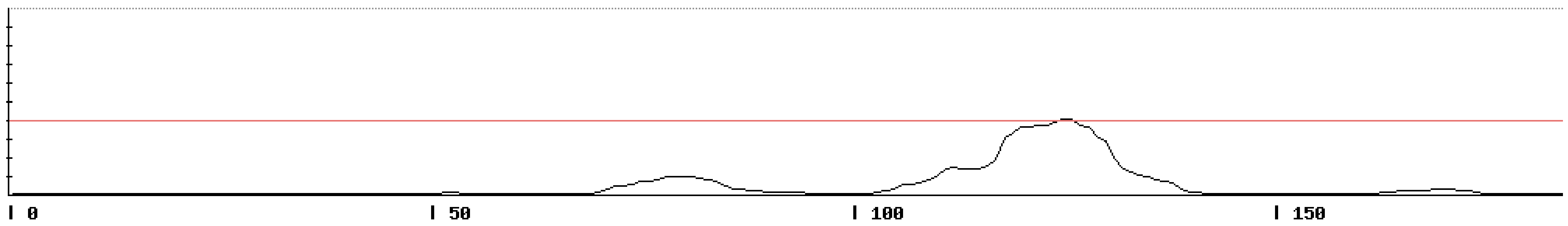

Predictions for Ocimumtenuiflorum_Ote100076500021	
Viterbi Path	
Viterbi Path	
Posterior @ 0.4	
Posterior @ 0.4	
Posterior Graphic. Threshold Marked @ 0.4	

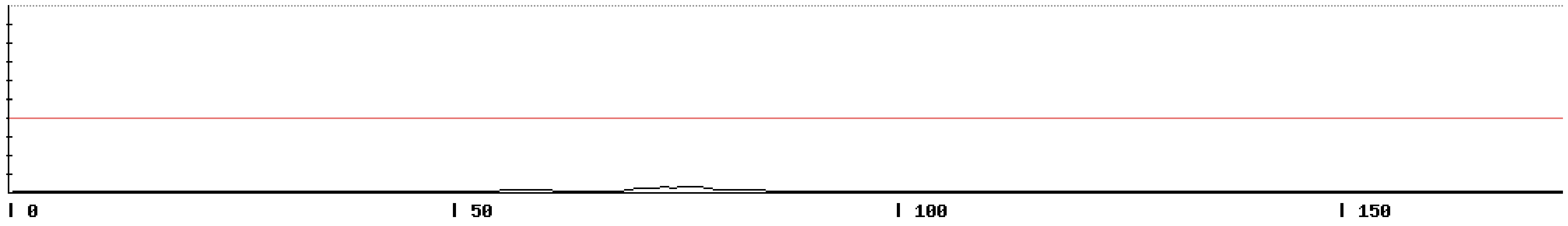

Predictions for Ocimumtenuiflorum_Ote100077000021

Viterbi Path	
Viterbi Path	
Posterior @ 0.4	
Posterior @ 0.4	
Posterior Graphic. Threshold Marked @ 0.4	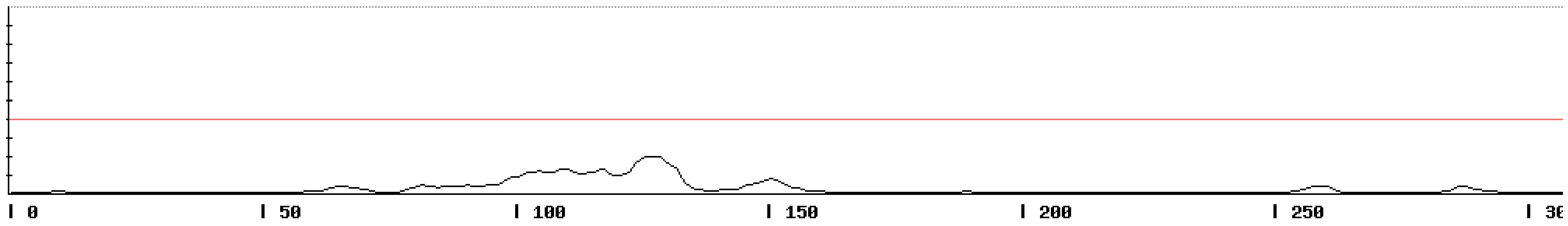

Predictions for Ocimumtenuiflorum_Ote100077950011

Viterbi Path	
Viterbi Path	
Posterior @ 0.4	
Posterior @ 0.4	

Posterior Graphic. Threshold Marked @ 0.4

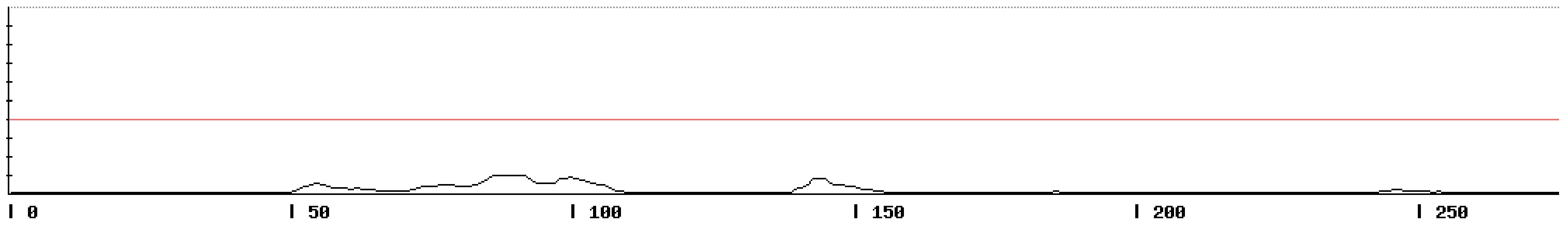

Predictions for Ocimumtenuiflorum_Ote100080930061

Viterbi Path

Viterbi Path

Posterior @ 0.4

Posterior @ 0.4

Posterior Graphic. Threshold Marked @ 0.4

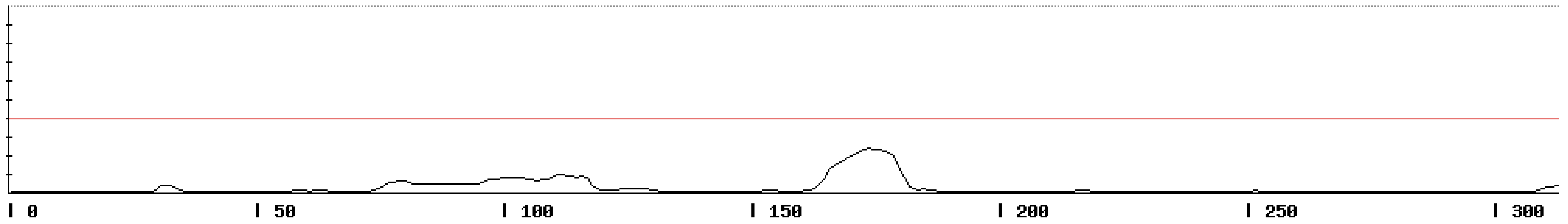

Predictions for Ocimumtenuiflorum_Ote100081090011

Viterbi Path

Viterbi Path

Posterior @ 0.4

Posterior @ 0.4

Posterior Graphic. Threshold Marked @ 0.4

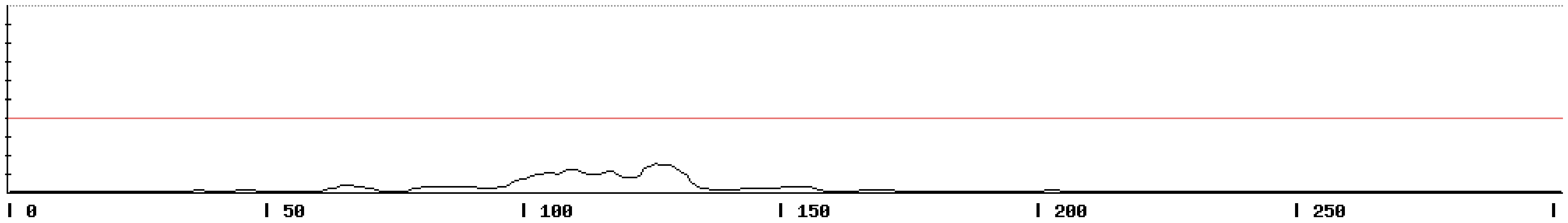

Predictions for Ocimumtenuiflorum_Ote100083700031

Viterbi Path

Viterbi Path

Posterior @ 0.4

Posterior @ 0.4

Posterior Graphic. Threshold Marked @ 0.4

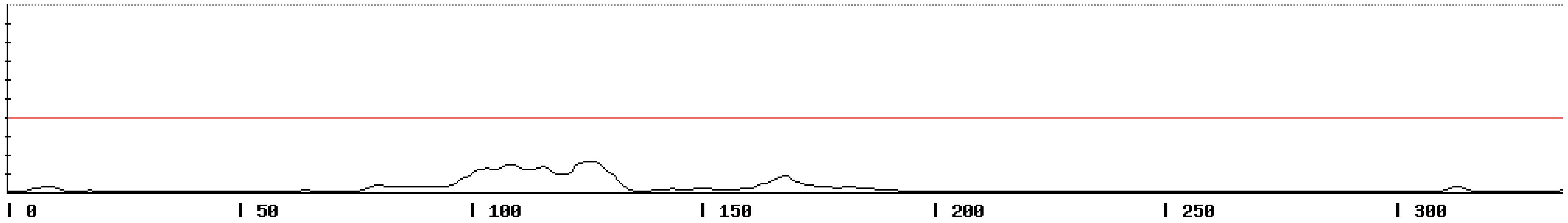

Predictions for Ocimumtenuiflorum_Ote100091280041

Viterbi Path

Viterbi Path

Posterior @ 0.4

Posterior @ 0.4

Posterior Graphic. Threshold Marked @ 0.4

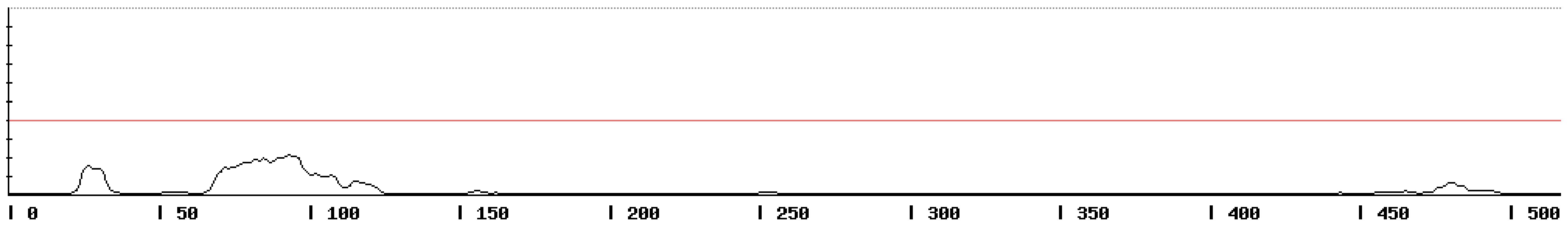

Predictions for Ocimumtenuiflorum_Ote100091280061

Viterbi Path

Viterbi Path

Posterior @ 0.4

Posterior @ 0.4

Posterior Graphic. Threshold Marked @ 0.4

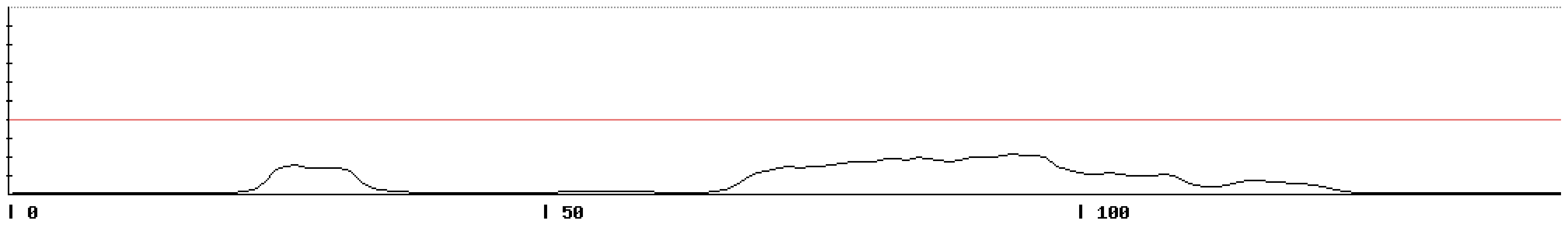

Predictions for Ocimumtenuiflorum_Ote100092010001

Viterbi Path

Viterbi Path

Posterior @ 0.4

Posterior @ 0.4

Posterior Graphic. Threshold Marked @ 0.4

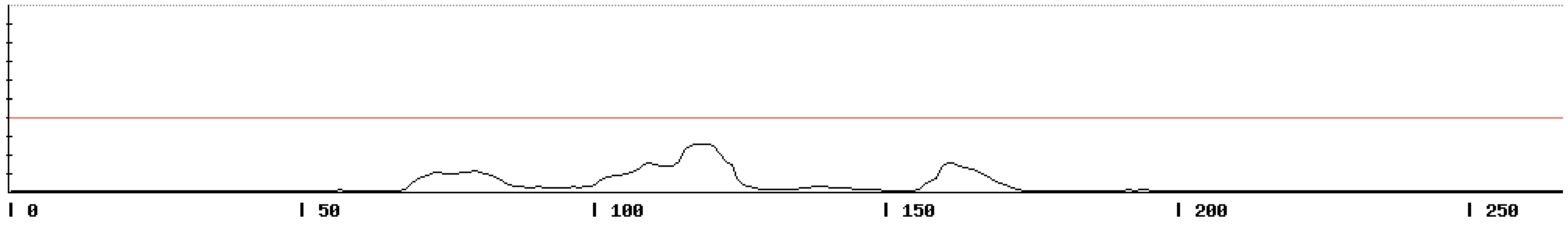

Predictions for Ocimumtenuiflorum_Ote100092950061

Viterbi Path

Viterbi Path

Posterior @ 0.4

868 - GRRKRRRIQDG - 878

Posterior @ 0.4

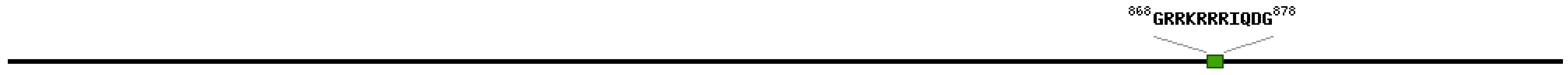

Posterior Graphic. Threshold Marked @ 0.4

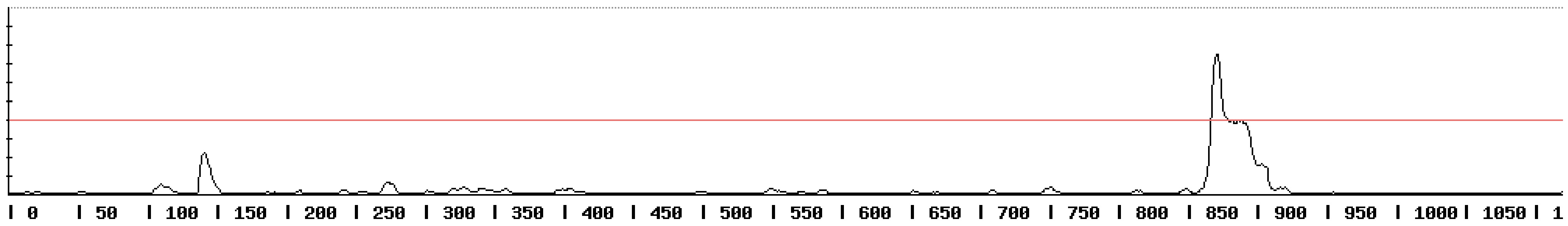

Predictions for Ocimumtenuiflorum_Ote100092950111

Viterbi Path

Viterbi Path

Posterior @ 0.4 | 83 - GRRKRRRIQDG - 93

Posterior @ 0.4

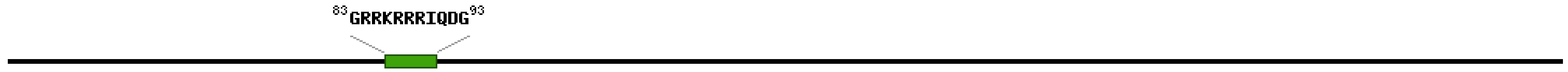

Posterior Graphic. Threshold Marked @ 0.4

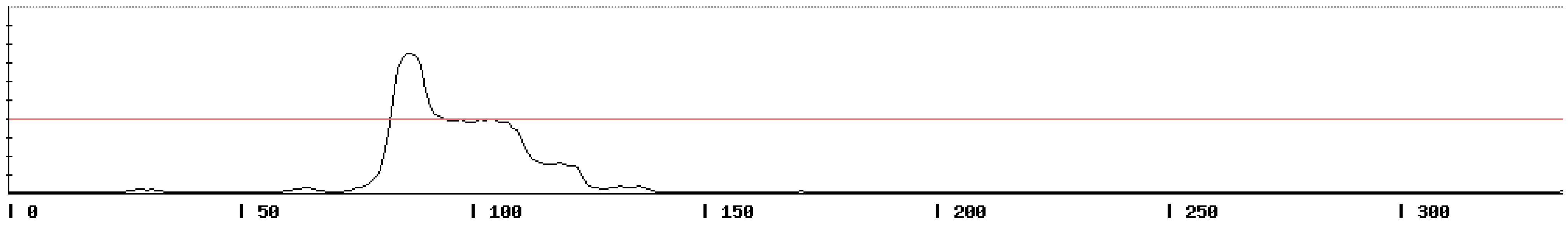

Predictions for Ocimumtenuiflorum_Ote100094600201

Viterbi Path

Viterbi Path

Posterior @ 0.4

Posterior @ 0.4

Posterior Graphic. Threshold Marked @ 0.4

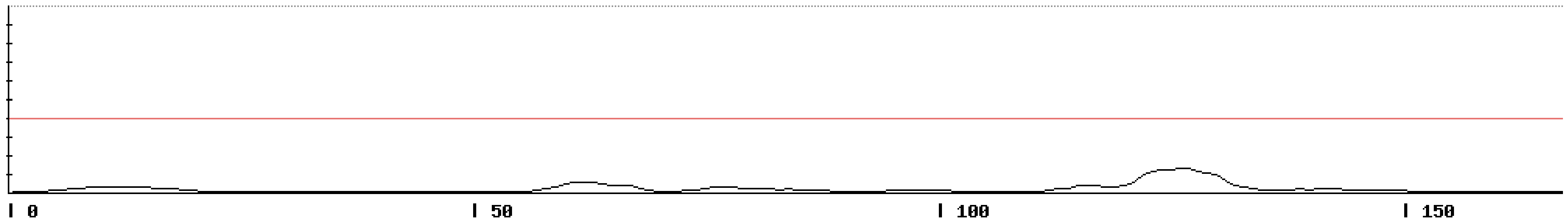

Predictions for Ocimumtenuiflorum_Ote100096990031	
Viterbi Path	
Viterbi Path	
Posterior @ 0.4	
Posterior @ 0.4	
Posterior Graphic. Threshold Marked @ 0.4	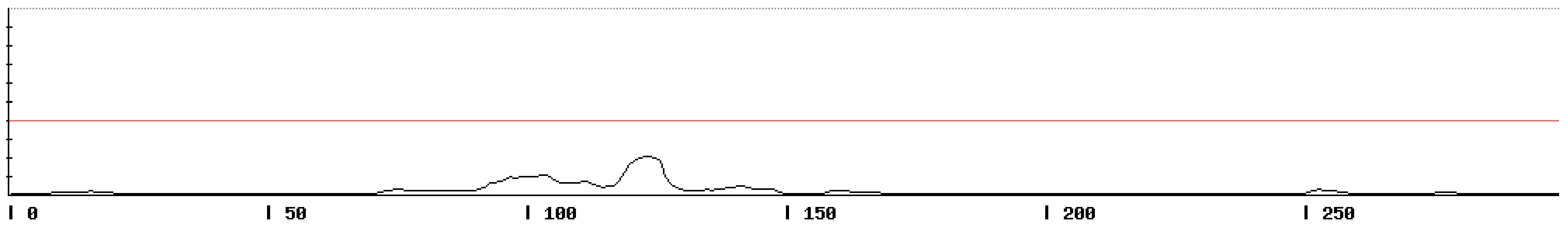

Predictions for Ocimumtenuiflorum_Ote100101960061	
Viterbi Path	
Viterbi Path	
Posterior @ 0.4	153 - RKRRK - 157
Posterior @ 0.4	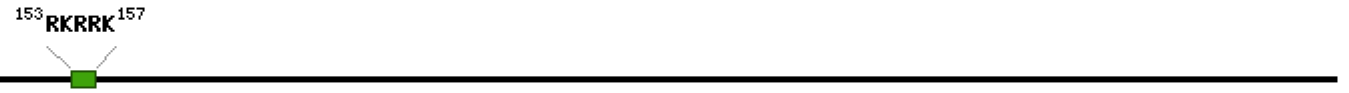
Posterior Graphic. Threshold Marked @ 0.4	

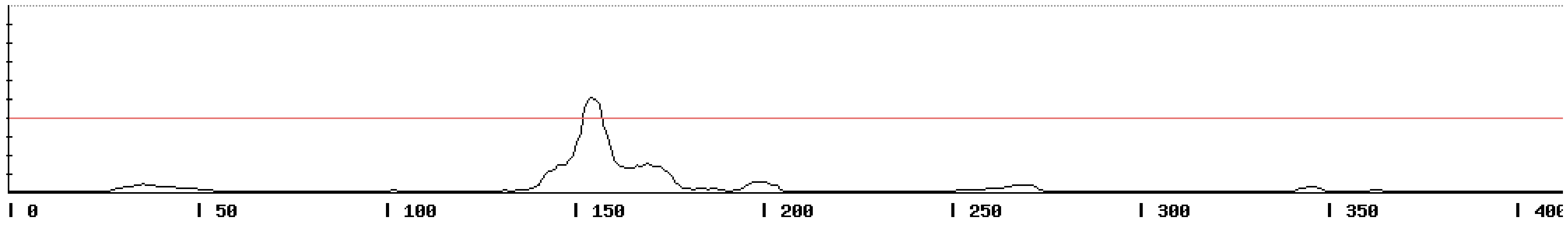

Predictions for Ocimumtenuiflorum_Ote100106510021

Viterbi Path

Viterbi Path

Posterior @ 0.4

Posterior @ 0.4

Posterior Graphic. Threshold Marked @ 0.4

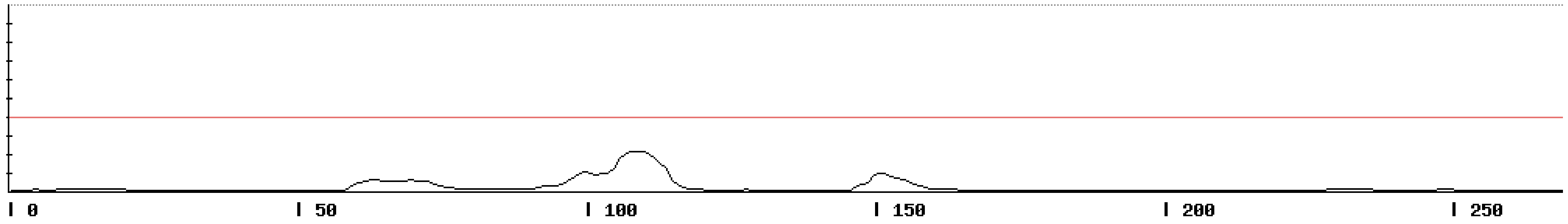

Predictions for Ocimumtenuiflorum_Ote100106760061

Viterbi Path

Viterbi Path

Posterior @ 0.4

Posterior @ 0.4

Posterior Graphic. Threshold Marked @ 0.4	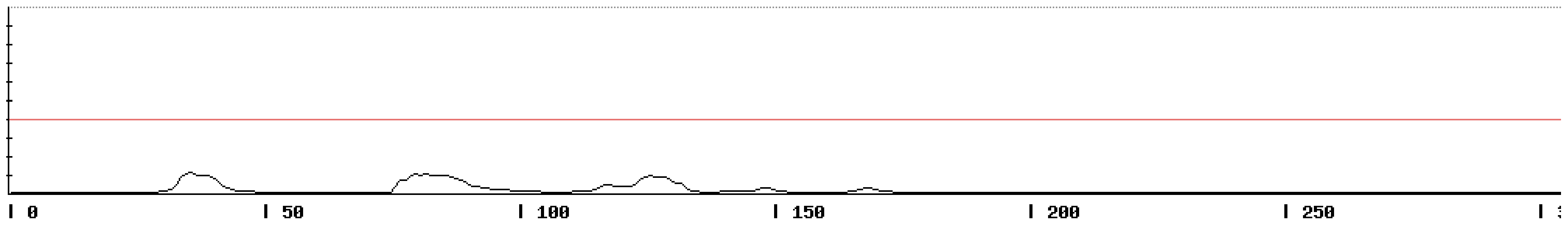

Predictions for Ocimumtenuiflorum_Ote100106760231

Viterbi Path	
Viterbi Path	
Posterior @ 0.4	
Posterior @ 0.4	
Posterior Graphic. Threshold Marked @ 0.4	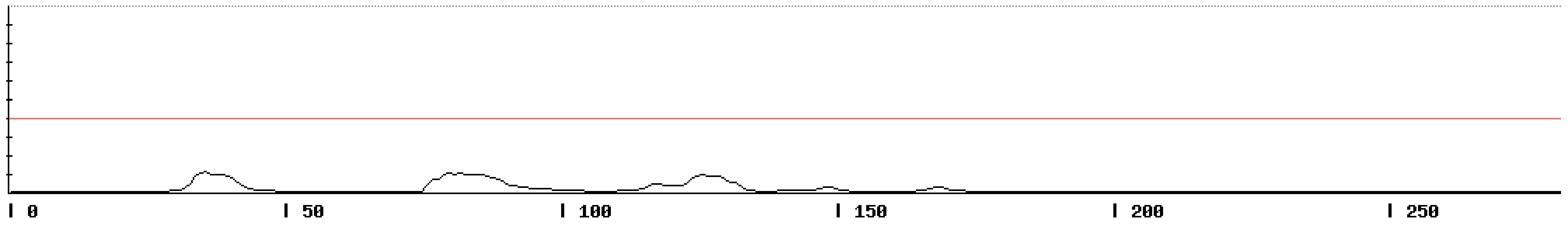

Predictions for Ocimumtenuiflorum_Ote100111460001

Viterbi Path	
Viterbi Path	
Posterior @ 0.4	
Posterior @ 0.4	

Posterior Graphic. Threshold Marked @ 0.4

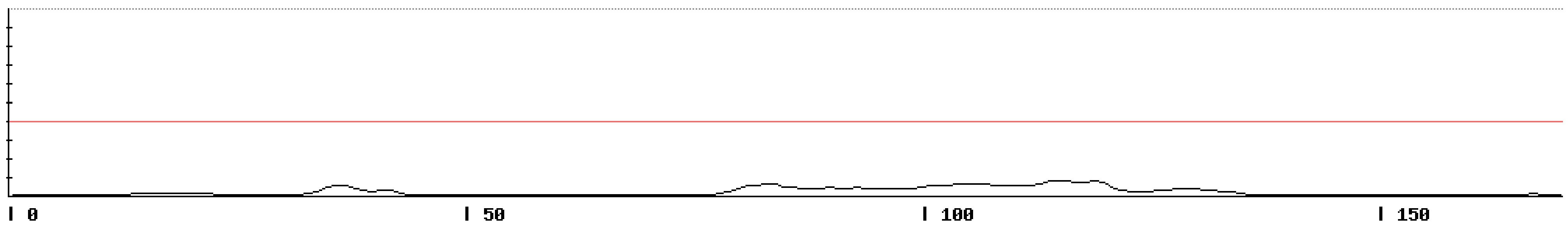

Predictions for Ocimumtenuiflorum_Ote100112500221

Viterbi Path

Viterbi Path

Posterior @ 0.4

Posterior @ 0.4

Posterior Graphic. Threshold Marked @ 0.4

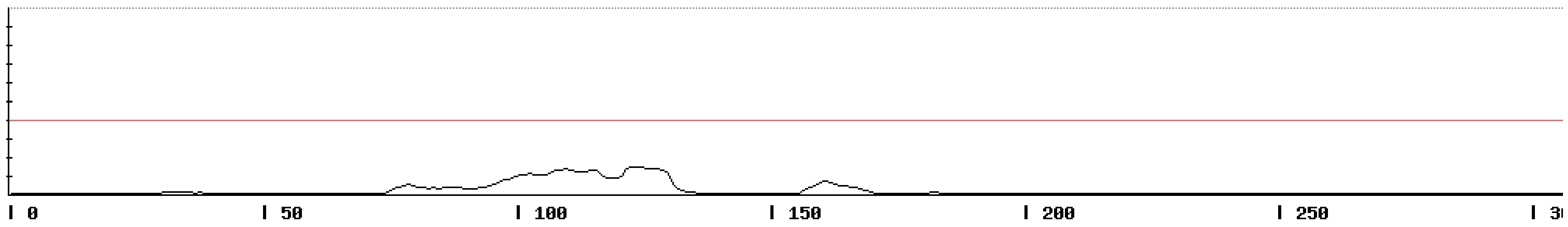

Predictions for Ocimumtenuiflorum_Ote100115880081

Viterbi Path

Viterbi Path

Posterior @ 0.4

Posterior @ 0.4

Posterior Graphic. Threshold Marked @ 0.4

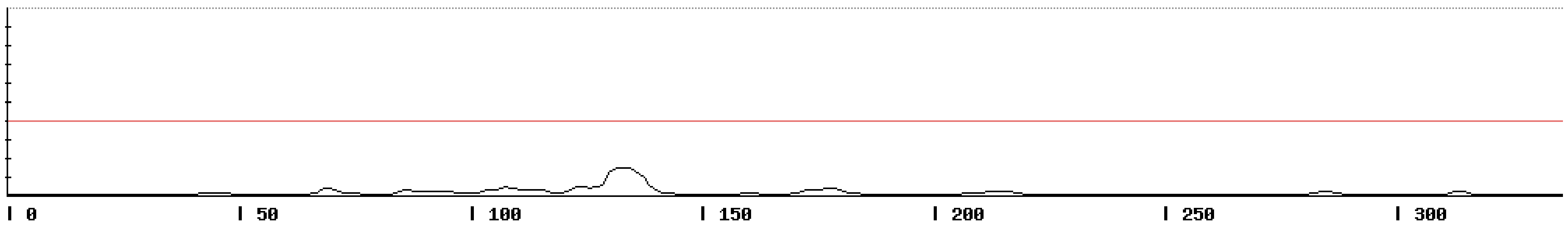

Predictions for Ocimumtenuiflorum_Ote100117380011

Viterbi Path

Viterbi Path

Posterior @ 0.4 | 74 - GRAPKGRK - 81

Posterior @ 0.4

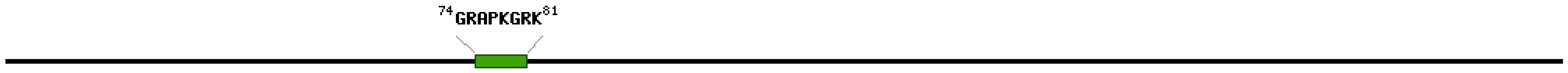

Posterior Graphic. Threshold Marked @ 0.4

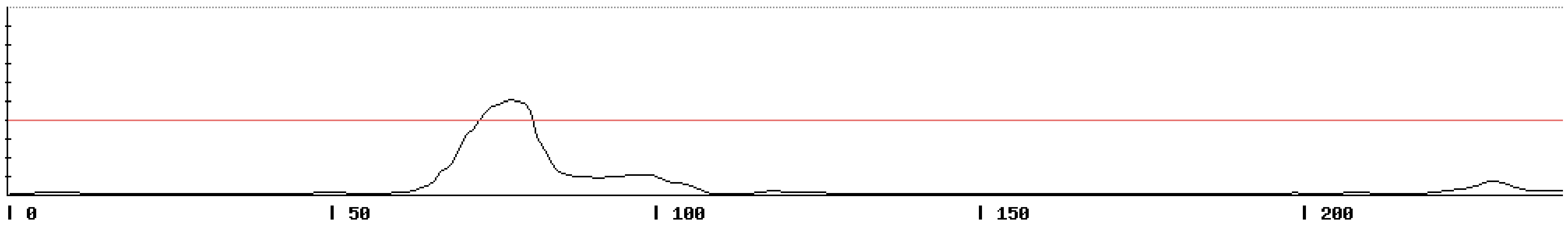

Predictions for Ocimumtenuiflorum_Ote100124420011

Viterbi Path

Viterbi Path

Posterior @ 0.4	
Posterior @ 0.4	
Posterior Graphic. Threshold Marked @ 0.4	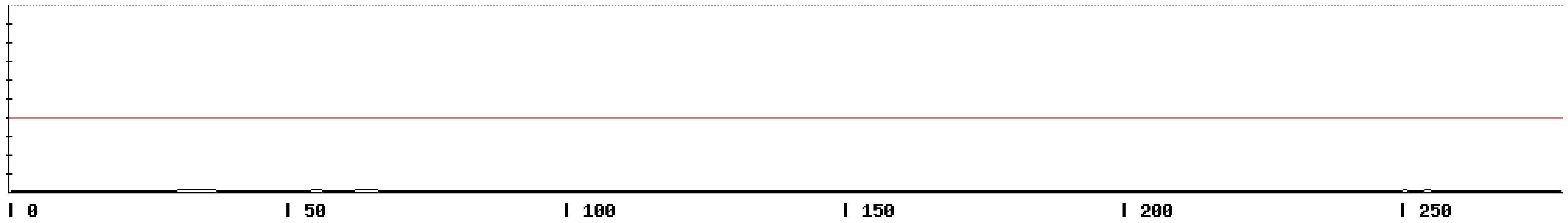

Predictions for Ocimumtenuiflorum_Ote100147760051	
Viterbi Path	
Viterbi Path	
Posterior @ 0.4	
Posterior @ 0.4	
Posterior Graphic. Threshold Marked @ 0.4	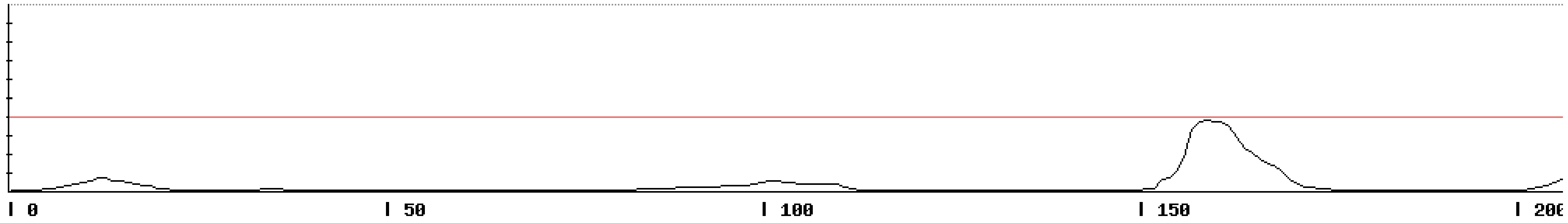

Predictions for Ocimumtenuiflorum_Ote100158560071

Viterbi Path	
Viterbi Path	
Posterior @ 0.4	
Posterior @ 0.4	
Posterior Graphic. Threshold Marked @ 0.4	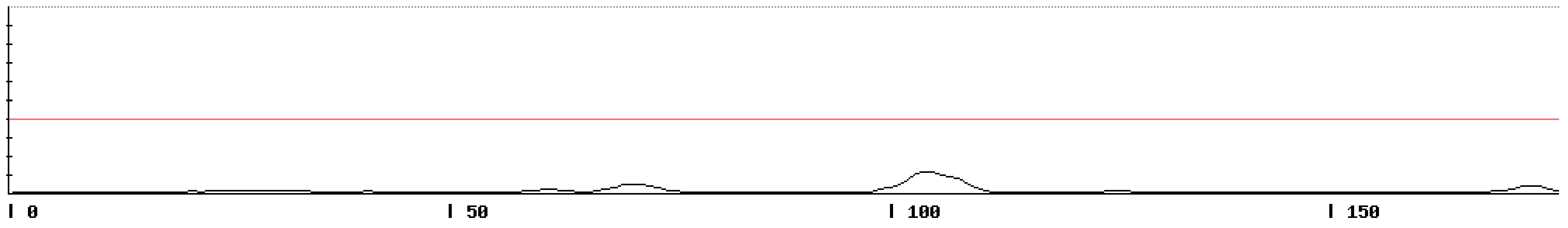

Predictions for Ocimumtenuiflorum_Ote100162300001

Viterbi Path	
Viterbi Path	
Posterior @ 0.4	118 - RTKRRRSGPA - 127
Posterior @ 0.4	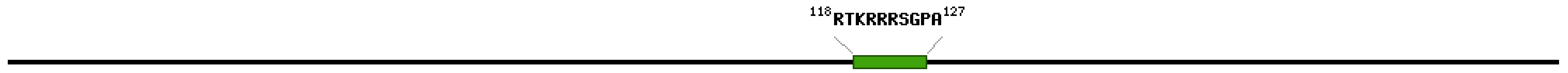
Posterior Graphic. Threshold Marked @ 0.4	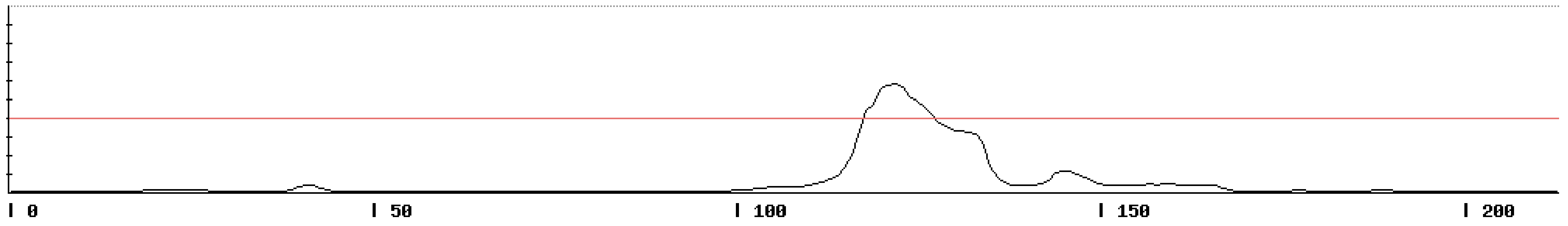

Predictions for Ocimumtenuiflorum_Ote100162300011	
Viterbi Path	
Viterbi Path	
Posterior @ 0.4	
Posterior @ 0.4	
Posterior Graphic. Threshold Marked @ 0.4	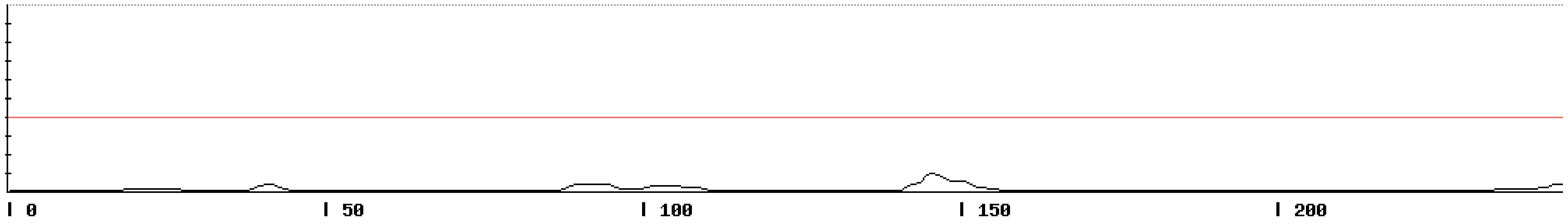

Predictions for Ocimumtenuiflorum_Ote100162870041	
Viterbi Path	
Viterbi Path	
Posterior @ 0.4	74 - VTGRRPGKPKTLGIKKA - 90
Posterior @ 0.4	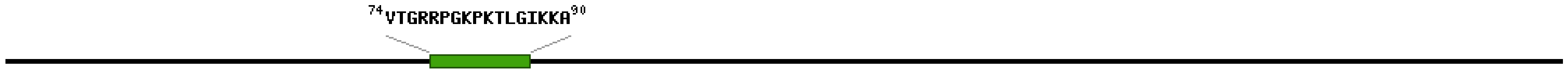
Posterior Graphic. Threshold Marked @ 0.4	

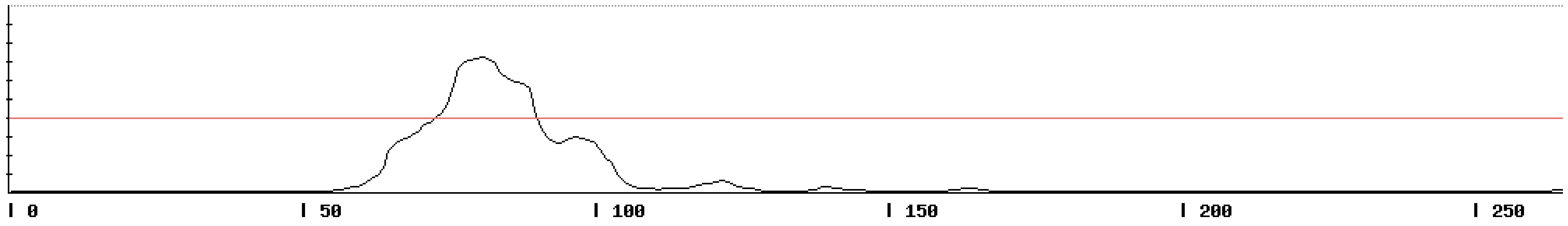

Predictions for Ocimumtenuiflorum_Ote100163870051

Viterbi Path

Viterbi Path

Posterior @ 0.4

Posterior @ 0.4

Posterior Graphic. Threshold Marked @ 0.4

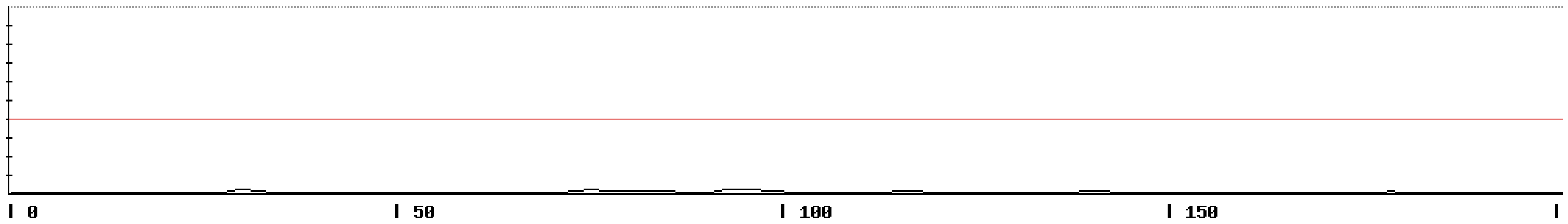

Predictions for Ocimumtenuiflorum_Ote100164310011

Viterbi Path

Viterbi Path

Posterior @ 0.4

Posterior @ 0.4

Posterior Graphic. Threshold Marked @ 0.4

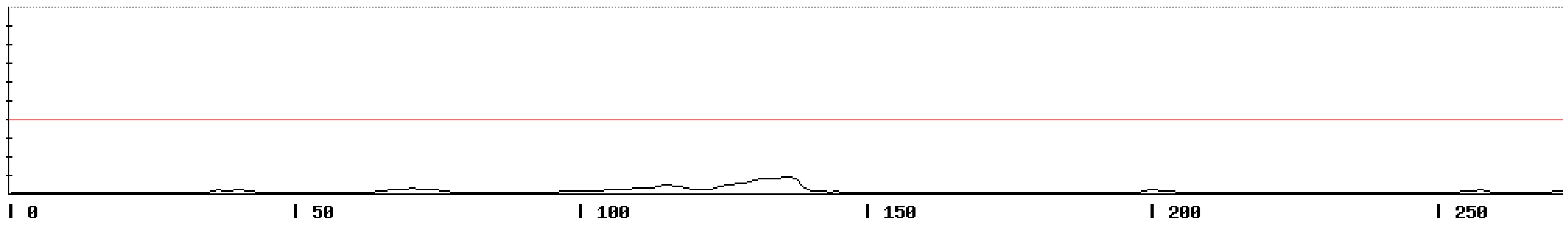

Predictions for Ocimumtenuiflorum_Ote100164310021

Viterbi Path

Viterbi Path

Posterior @ 0.4

Posterior @ 0.4

Posterior Graphic. Threshold Marked @ 0.4

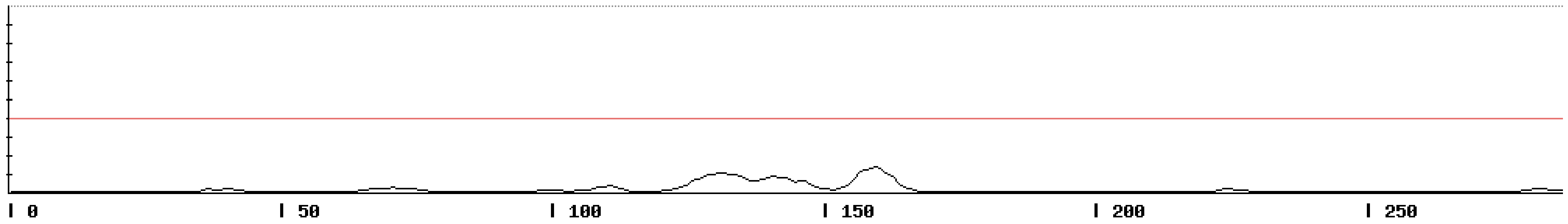

Predictions for Ocimumtenuiflorum_Ote100166350161

Viterbi Path

Viterbi Path

Posterior @ 0.4

Posterior @ 0.4

Posterior Graphic. Threshold Marked @ 0.4

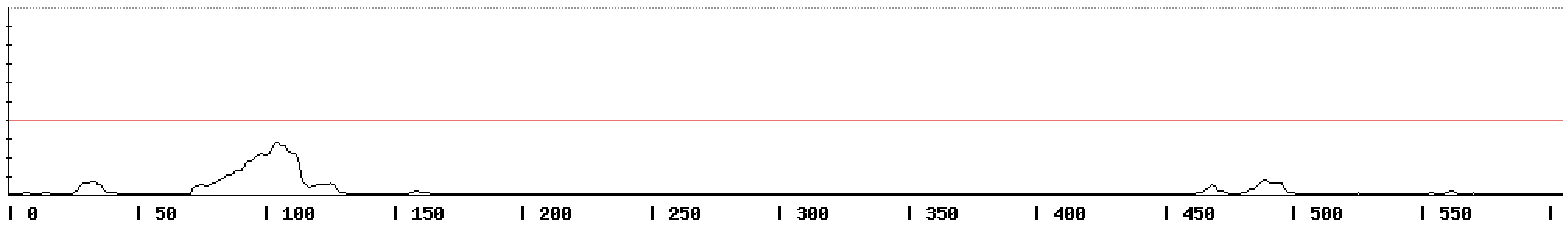

Predictions for Ocimumtenuiflorum_Ote100168000211

Viterbi Path

Viterbi Path

Posterior @ 0.4

Posterior @ 0.4

Posterior Graphic. Threshold Marked @ 0.4

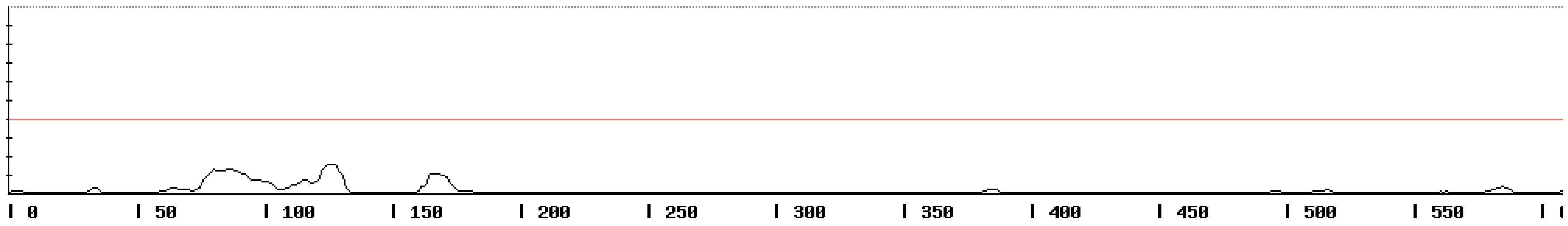

Predictions for Ocimumtenuiflorum_Ote100169980041

Viterbi Path

Viterbi Path

Posterior @ 0.4	94 - RK - 95
Posterior @ 0.4	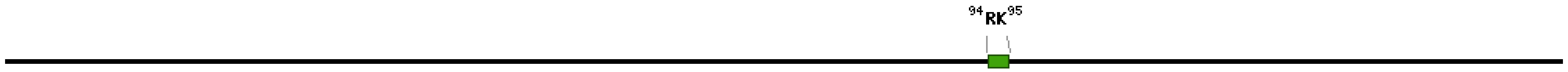
Posterior Graphic. Threshold Marked @ 0.4	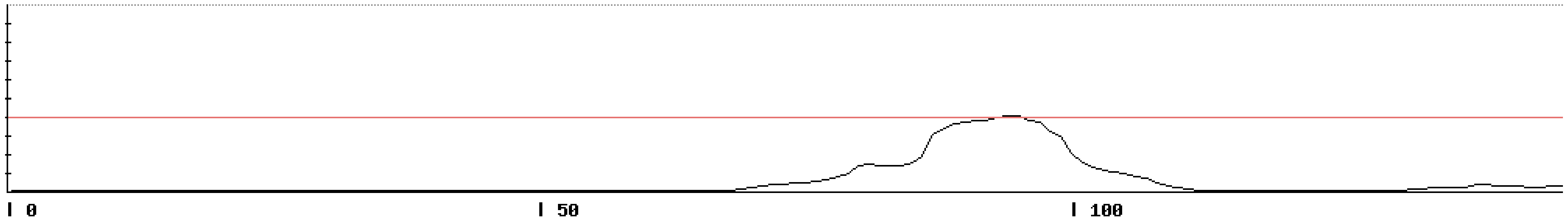

Predictions for Ocimumtenuiflorum_Ote100182980051

Viterbi Path	
Viterbi Path	
Posterior @ 0.4	
Posterior @ 0.4	
Posterior Graphic. Threshold Marked @ 0.4	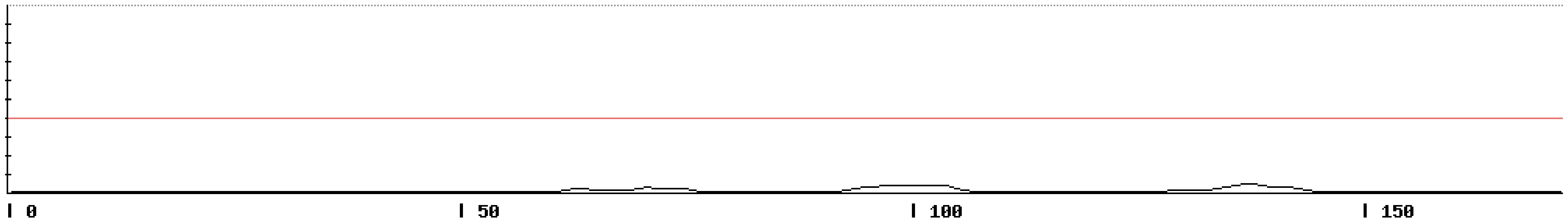

Predictions for Ocimumtenuiflorum_Ote100183750261

Viterbi Path	
Viterbi Path	

Posterior @ 0.4

Posterior @ 0.4

Posterior Graphic. Threshold Marked @ 0.4

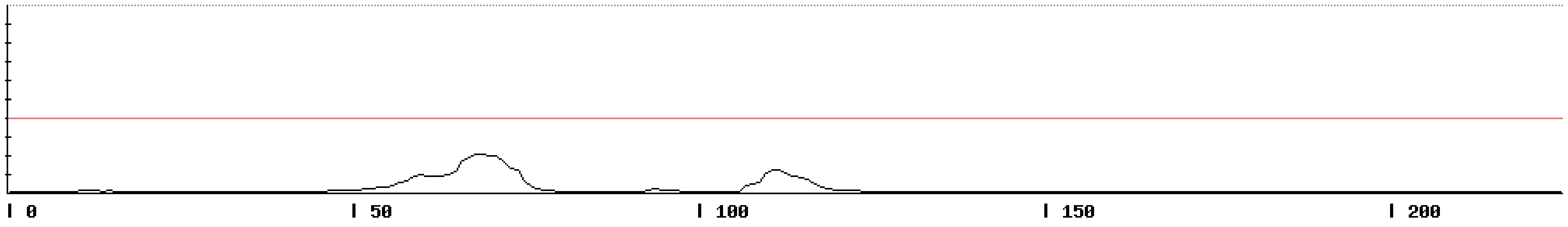

Predictions for Ocimumtenuiflorum_Ote100183810011

Viterbi Path

Viterbi Path

Posterior @ 0.4

Posterior @ 0.4

Posterior Graphic. Threshold Marked @ 0.4

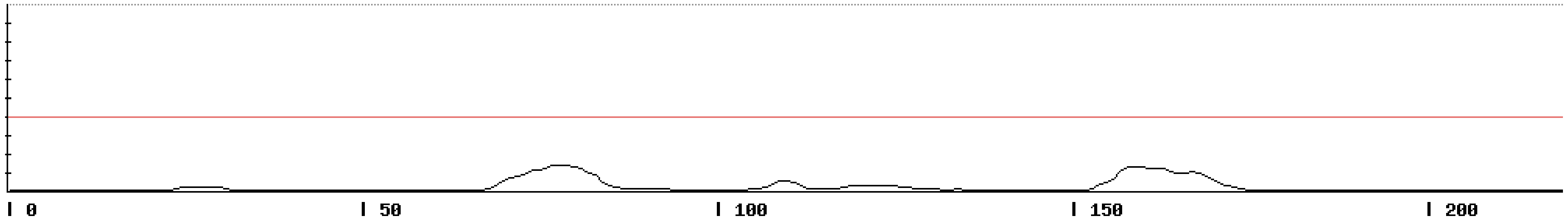

Predictions for Ocimumtenuiflorum_Ote100186430021

Viterbi Path

Viterbi Path

Posterior @ 0.4

Posterior @ 0.4

Posterior Graphic. Threshold Marked @ 0.4

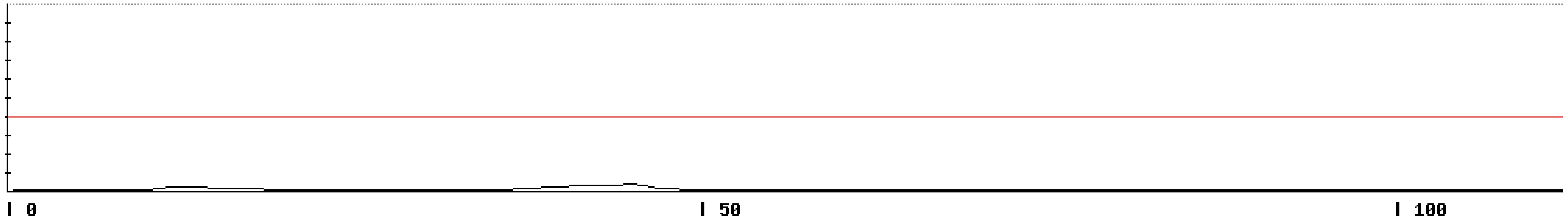

Predictions for Ocimumtenuiflorum_Ote100188410031

Viterbi Path

Viterbi Path

Posterior @ 0.4

Posterior @ 0.4

Posterior Graphic. Threshold Marked @ 0.4

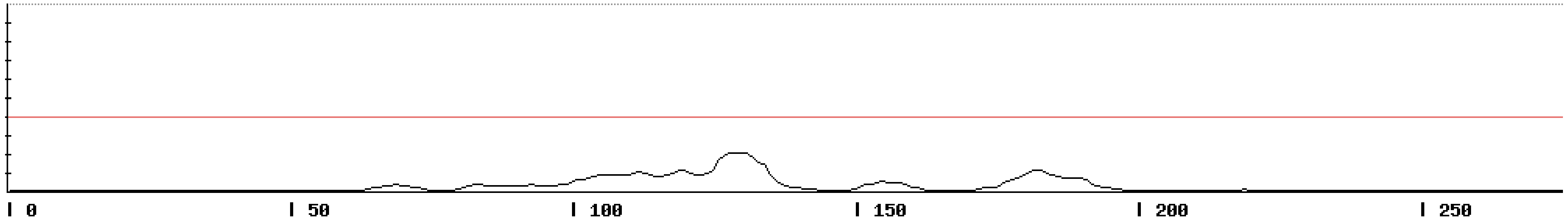

Predictions for Ocimumtenuiflorum_Ote100188410032	
Viterbi Path	
Viterbi Path	
Posterior @ 0.4	
Posterior @ 0.4	
Posterior Graphic. Threshold Marked @ 0.4	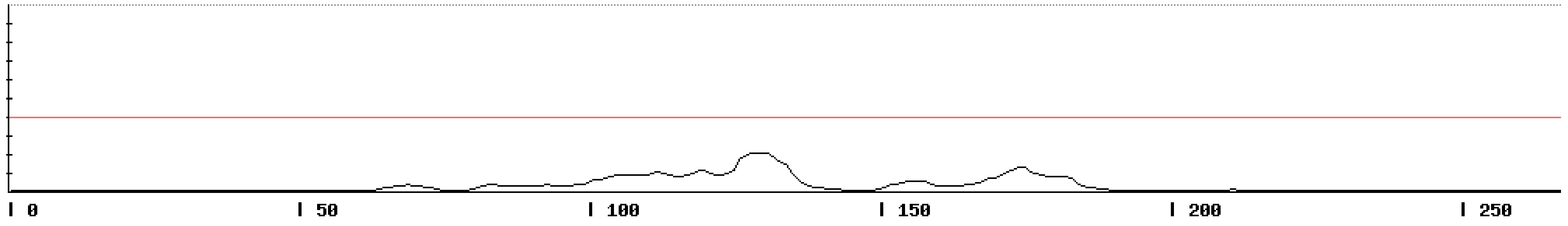

Predictions for Ocimumtenuiflorum_Ote100192480011	
Viterbi Path	
Viterbi Path	
Posterior @ 0.4	
Posterior @ 0.4	
Posterior Graphic. Threshold Marked @ 0.4	

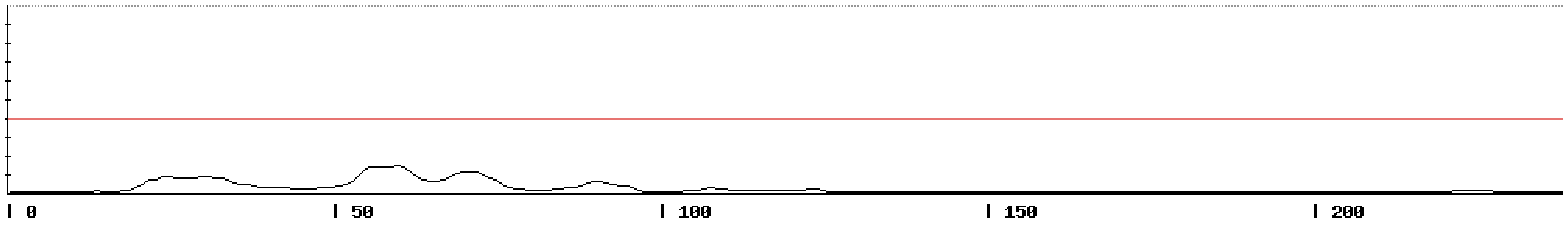

Predictions for Ocimumtenuiflorum_Ote100193880041

Viterbi Path

Viterbi Path

Posterior @ 0.4

Posterior @ 0.4

Posterior Graphic. Threshold Marked @ 0.4

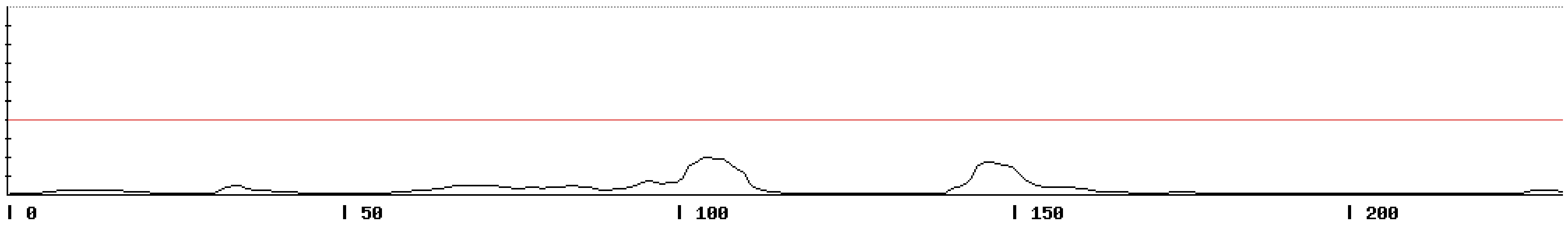

Predictions for Ocimumtenuiflorum_Ote100194770151

Viterbi Path

Viterbi Path

Posterior @ 0.4

Posterior @ 0.4

Posterior Graphic. Threshold Marked @ 0.4

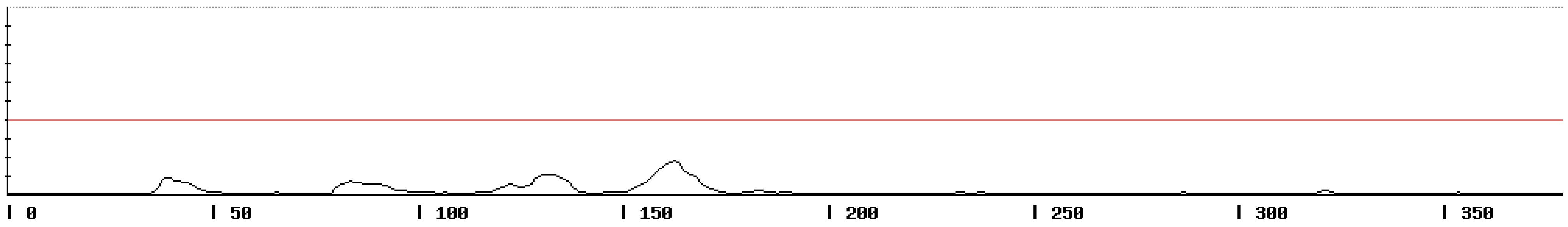

Predictions for Ocimumtenuiflorum_Ote100203500011

Viterbi Path

Viterbi Path

Posterior @ 0.4

Posterior @ 0.4

Posterior Graphic. Threshold Marked @ 0.4

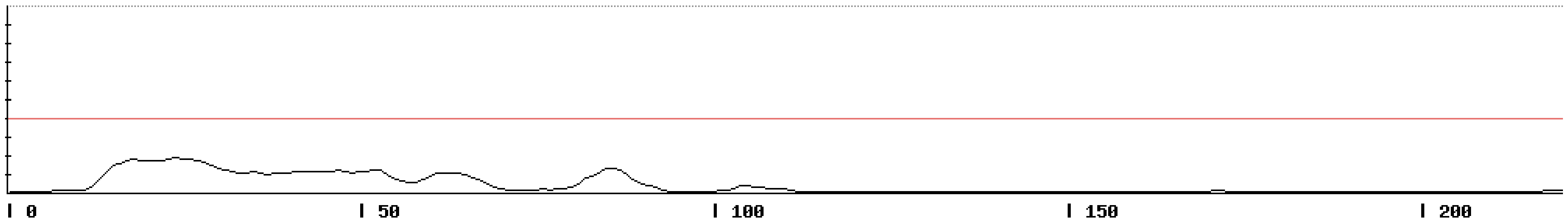

Predictions for Ocimumtenuiflorum_Ote100204950011

Viterbi Path

Viterbi Path

Posterior @ 0.4

Posterior @ 0.4

Posterior Graphic. Threshold Marked @ 0.4

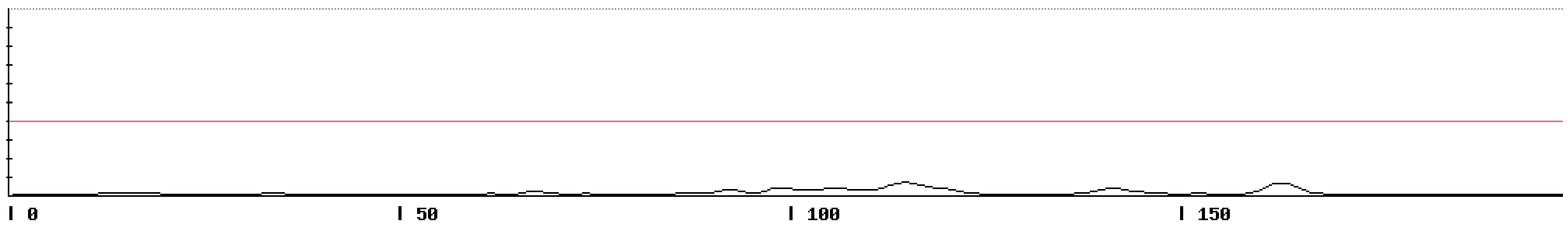

Predictions for Ocimumtenuiflorum_Ote100205630101

Viterbi Path

Viterbi Path

Posterior @ 0.4

Posterior @ 0.4

Posterior Graphic. Threshold Marked @ 0.4

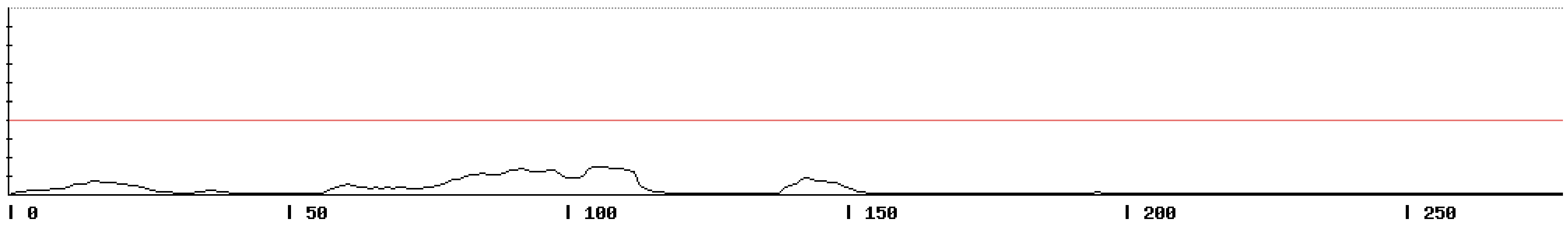

Predictions for Ocimumtenuiflorum_Ote100205730021

Viterbi Path

Viterbi Path

Posterior @ 0.4

Posterior @ 0.4

Posterior Graphic. Threshold Marked @ 0.4

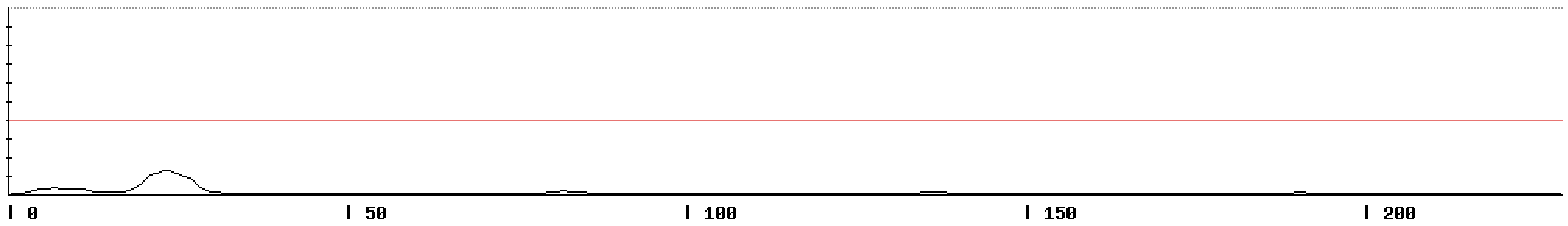

Predictions for Ocimumtenuiflorum_Ote100207980031

Viterbi Path

Viterbi Path

Posterior @ 0.4

Posterior @ 0.4

Posterior Graphic. Threshold Marked @ 0.4

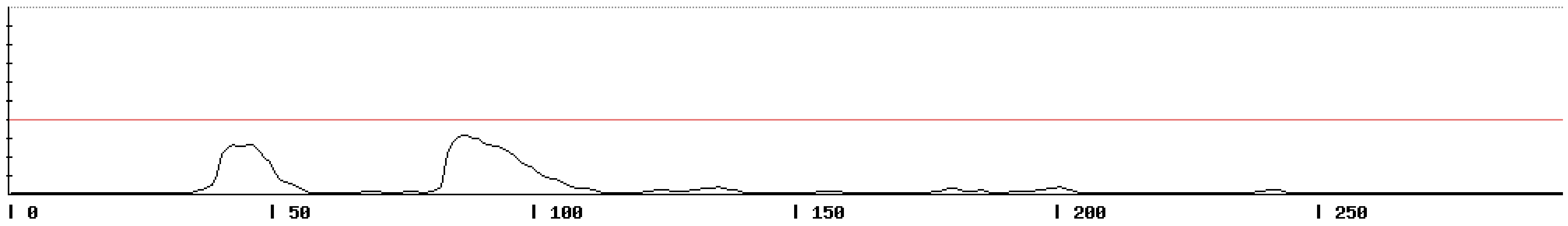

Predictions for Ocimumtenuiflorum_Ote100208250021

Viterbi Path

Viterbi Path

Posterior @ 0.4	
Posterior @ 0.4	
Posterior Graphic. Threshold Marked @ 0.4	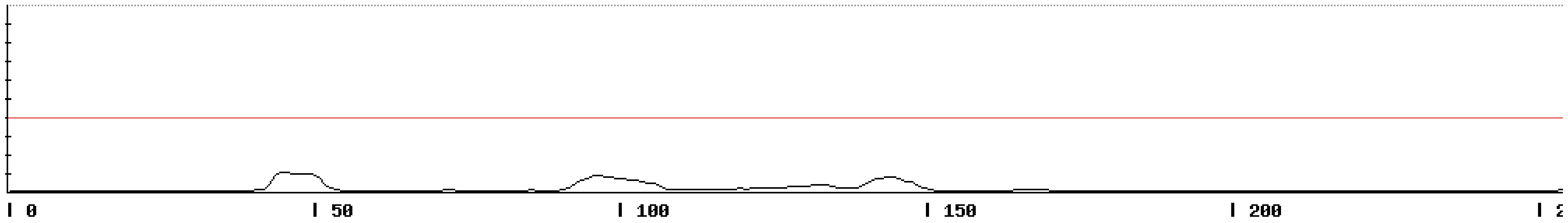

Predictions for Ocimumtenuiflorum_Ote100215050061

Viterbi Path	
Viterbi Path	
Posterior @ 0.4	
Posterior @ 0.4	
Posterior Graphic. Threshold Marked @ 0.4	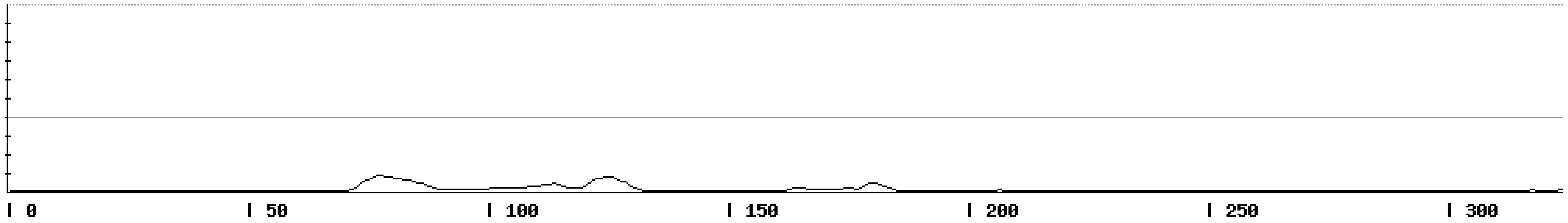

Predictions for Ocimumtenuiflorum_Ote100221540161

Viterbi Path

Viterbi Path

Posterior @ 0.4

Posterior @ 0.4

Posterior Graphic. Threshold Marked @ 0.4

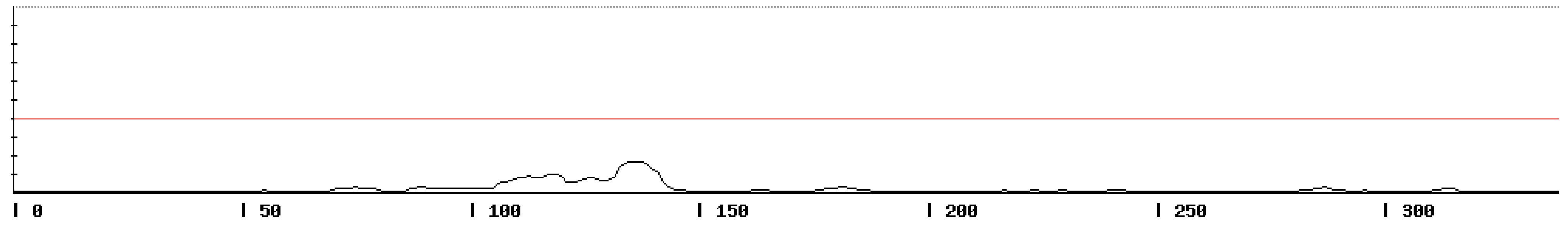

Predictions for Ocimumtenuiflorum_Ote100223590061

Viterbi Path

Viterbi Path

Posterior @ 0.4

Posterior @ 0.4

Posterior Graphic. Threshold Marked @ 0.4

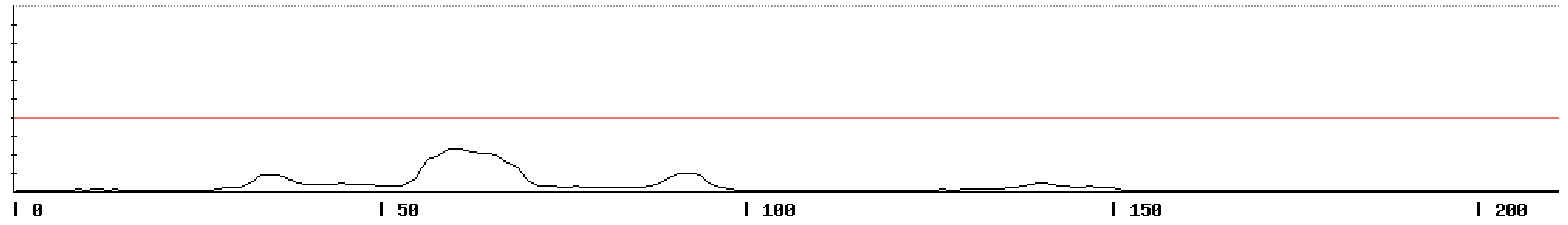

Predictions for Ocimumtenuiflorum_Ote100227540001

Viterbi Path

Viterbi Path

Posterior @ 0.4

Posterior @ 0.4

Posterior Graphic. Threshold Marked @ 0.4

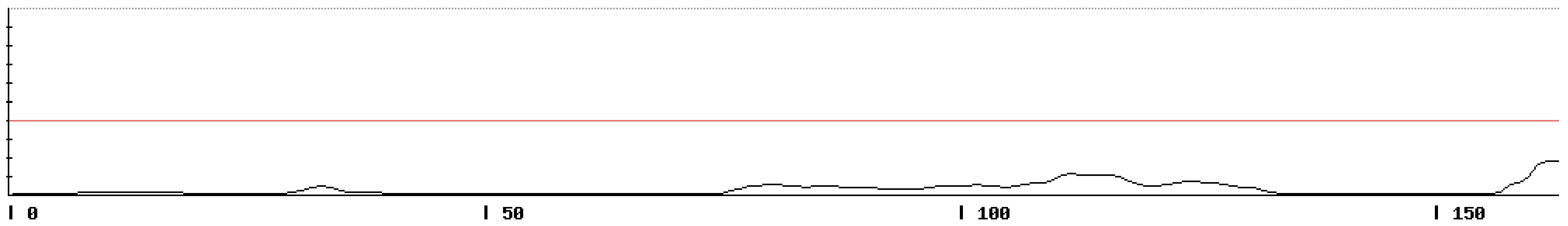

Predictions for Ocimumtenuiflorum_Ote100227540031

Viterbi Path

Viterbi Path

Posterior @ 0.4

Posterior @ 0.4

Posterior Graphic. Threshold Marked @ 0.4

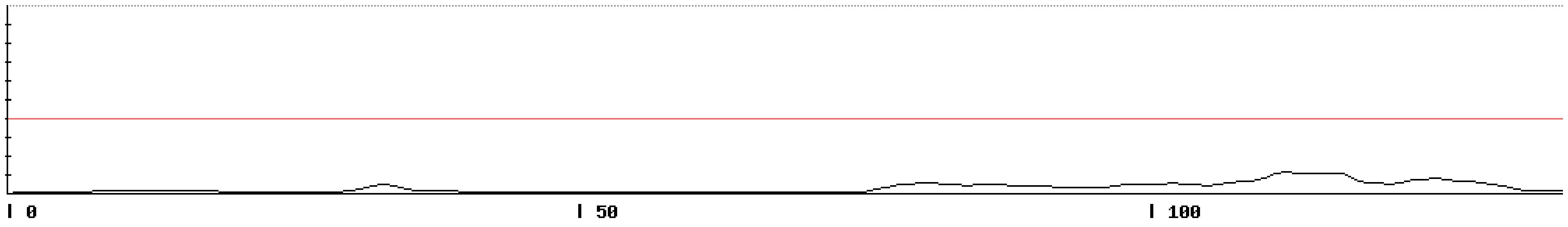

Predictions for Ocimumtenuiflorum_Ote100228250221

Viterbi Path	
Viterbi Path	
Posterior @ 0.4	
Posterior @ 0.4	
Posterior Graphic. Threshold Marked @ 0.4	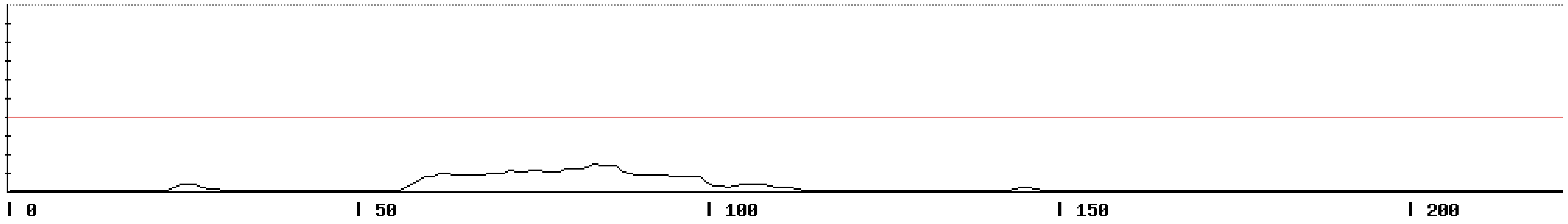

Predictions for Ocimumtenuiflorum_Ote100228750051

Viterbi Path	
Viterbi Path	
Posterior @ 0.4	
Posterior @ 0.4	

Posterior Graphic. Threshold Marked @ 0.4

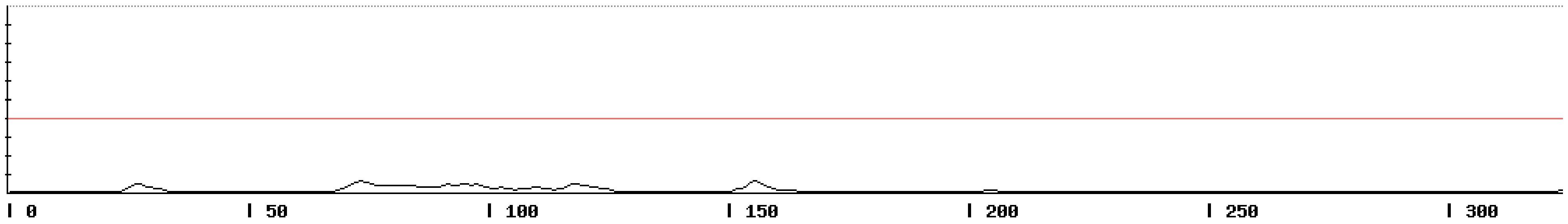

Predictions for Ocimumtenuiflorum_Ote100231490031

Viterbi Path

Viterbi Path

Posterior @ 0.4

Posterior @ 0.4

Posterior Graphic. Threshold Marked @ 0.4

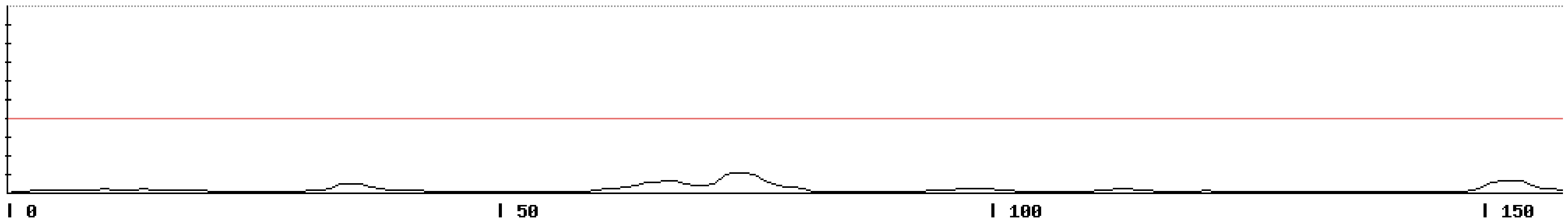

Predictions for Ocimumtenuiflorum_Ote100231490081

Viterbi Path

Viterbi Path

Posterior @ 0.4

Posterior @ 0.4

Posterior Graphic. Threshold Marked @ 0.4

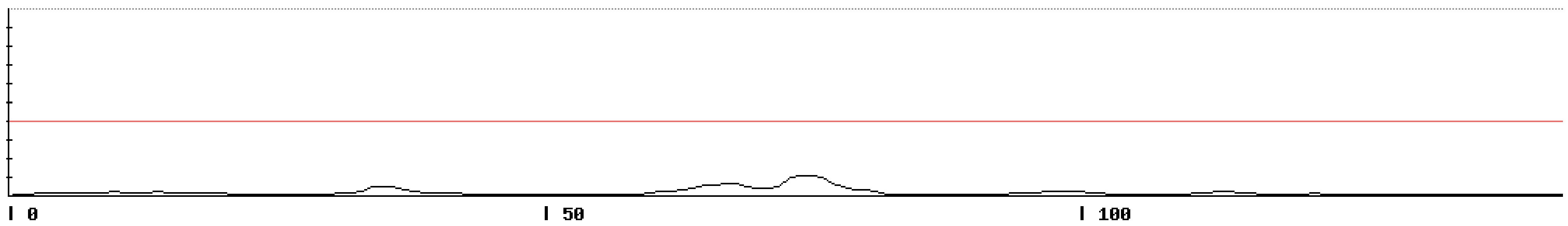

Predictions for Ocimumtenuiflorum_Ote100235590101

Viterbi Path

Viterbi Path

Posterior @ 0.4

Posterior @ 0.4

Posterior Graphic. Threshold Marked @ 0.4

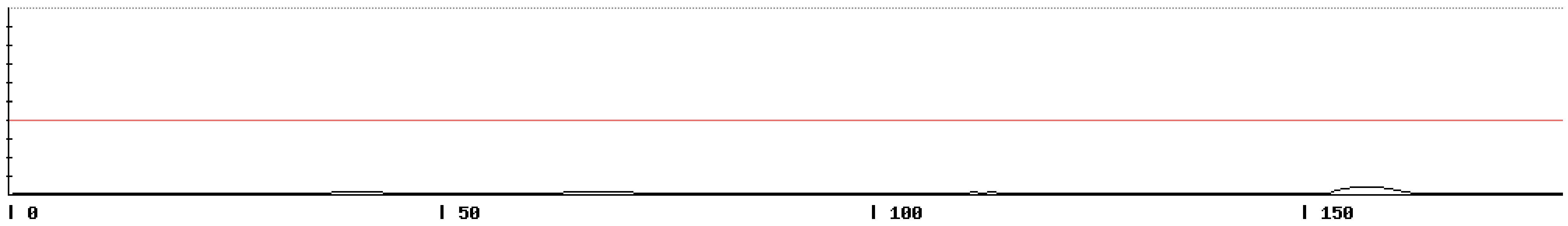

Predictions for Ocimumtenuiflorum_Ote100236530051

Viterbi Path

Viterbi Path

Posterior @ 0.4

Posterior @ 0.4

Posterior Graphic. Threshold Marked @ 0.4

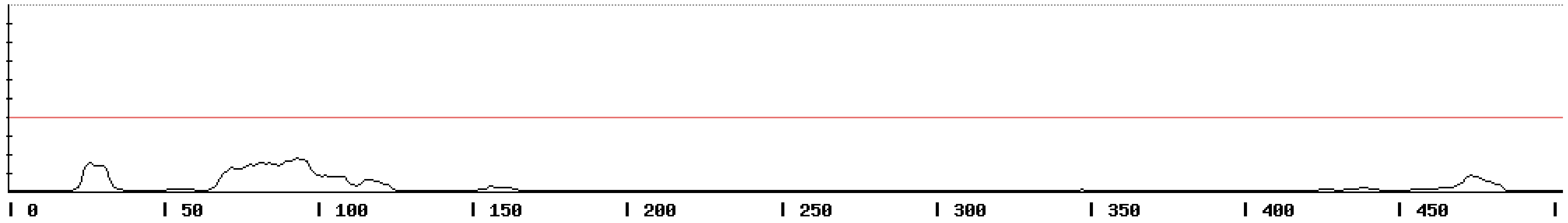

Predictions for Ocimumtenuiflorum_Ote100237400011

Viterbi Path

Viterbi Path

Posterior @ 0.4

Posterior @ 0.4

Posterior Graphic. Threshold Marked @ 0.4

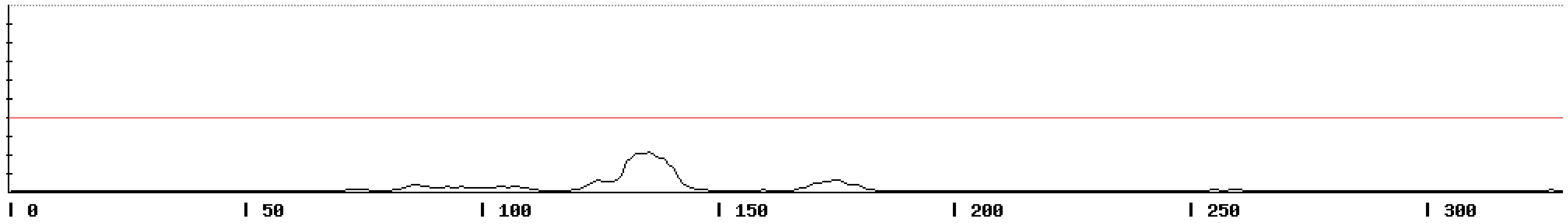

Predictions for Ocimumtenuiflorum_Ote100237740051

Viterbi Path

Viterbi Path

Posterior @ 0.4

Posterior @ 0.4

Posterior Graphic. Threshold Marked @ 0.4

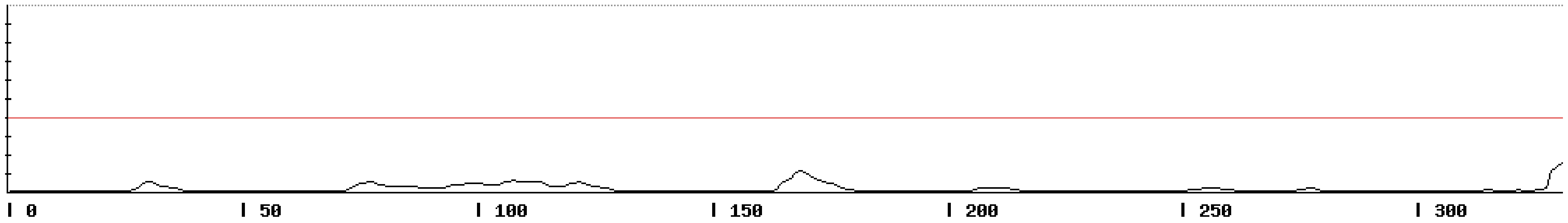

Predictions for Ocimumtenuiflorum_Ote100237740091

Viterbi Path

Viterbi Path

Posterior @ 0.4

Posterior @ 0.4

Posterior Graphic. Threshold Marked @ 0.4

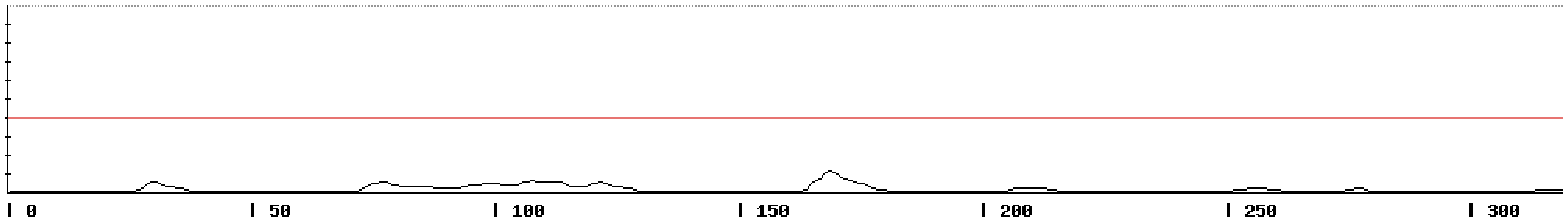

Predictions for Ocimumtenuiflorum_Ote100239950021

Viterbi Path

Viterbi Path

Posterior @ 0.4

Posterior @ 0.4

Posterior Graphic. Threshold Marked @ 0.4

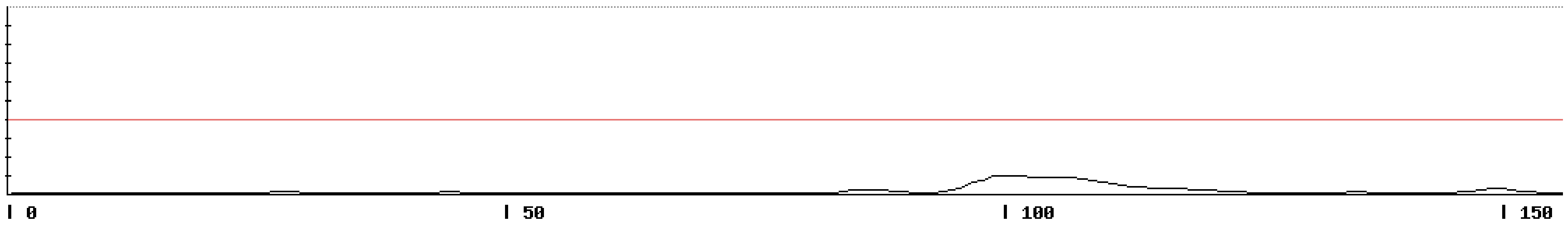

Predictions for Ocimumtenuiflorum_Ote100239950061

Viterbi Path

Viterbi Path

Posterior @ 0.4

Posterior @ 0.4

Posterior Graphic. Threshold Marked @ 0.4

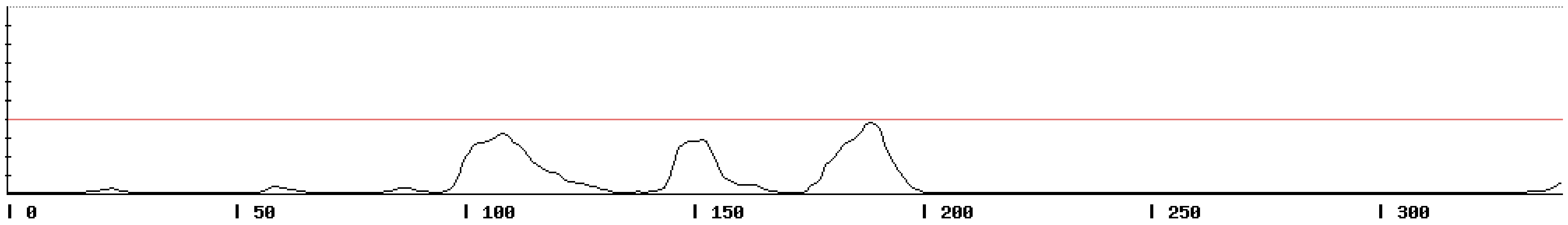

Predictions for Ocimumtenuiflorum_Ote100245930021	
Viterbi Path	
Viterbi Path	
Posterior @ 0.4	
Posterior @ 0.4	
Posterior Graphic. Threshold Marked @ 0.4	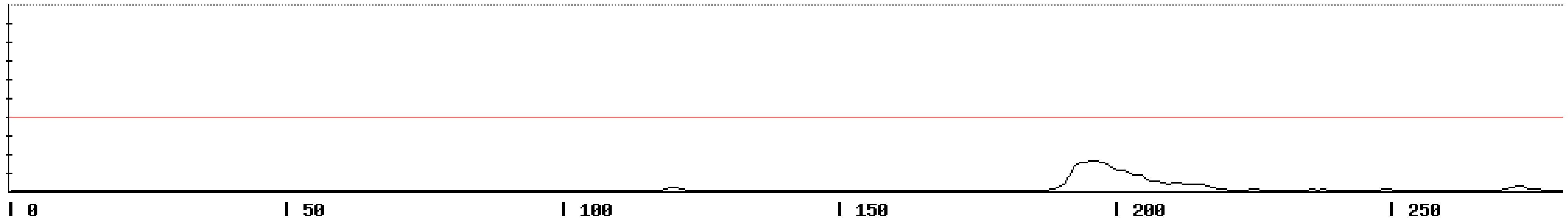

Predictions for Ocimumtenuiflorum_Ote100247120041	
Viterbi Path	
Viterbi Path	
Posterior @ 0.4	
Posterior @ 0.4	
Posterior Graphic. Threshold Marked @ 0.4	

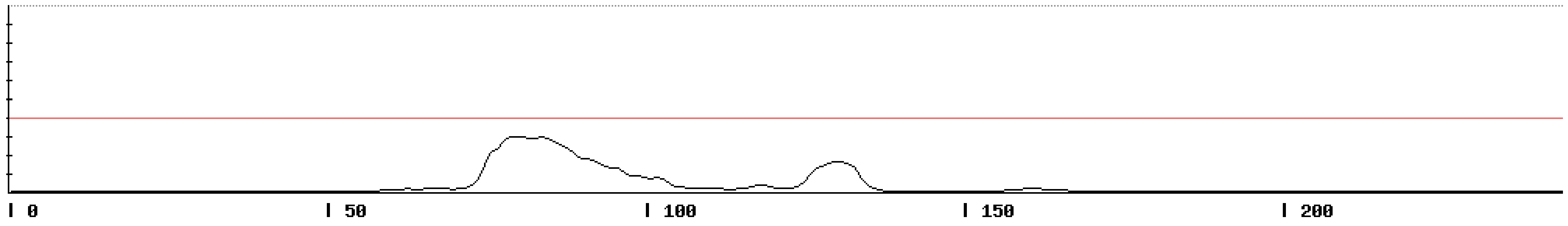

Predictions for Ocimumtenuiflorum_Ote100251470061

Viterbi Path	
Viterbi Path	
Posterior @ 0.4	
Posterior @ 0.4	
Posterior Graphic. Threshold Marked @ 0.4	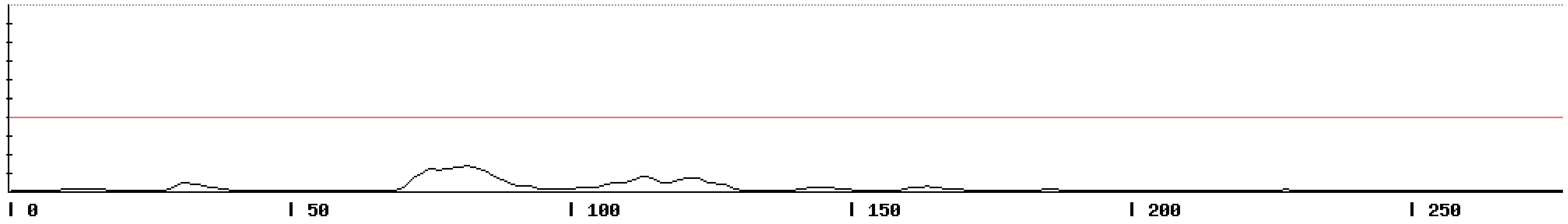

Predictions for Ocimumtenuiflorum_Ote100255260011

Viterbi Path	
Viterbi Path	
Posterior @ 0.4	
Posterior @ 0.4	

Posterior Graphic. Threshold Marked @ 0.4

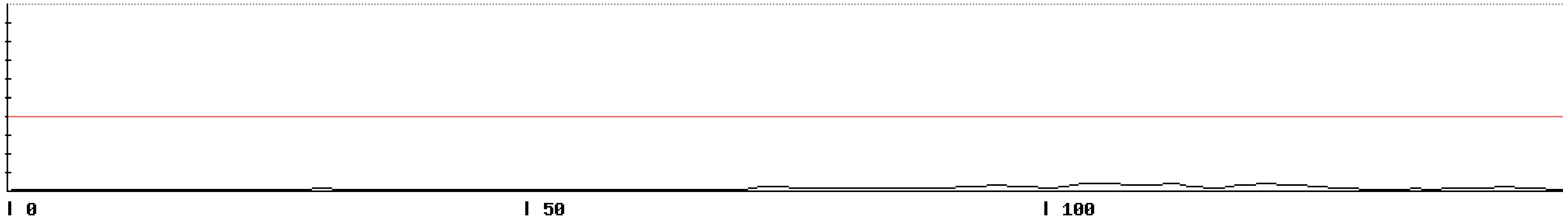

Predictions for Ocimumtenuiflorum_Ote100257070021

Viterbi Path	
Viterbi Path	
Posterior @ 0.4	
Posterior @ 0.4	
Posterior Graphic. Threshold Marked @ 0.4	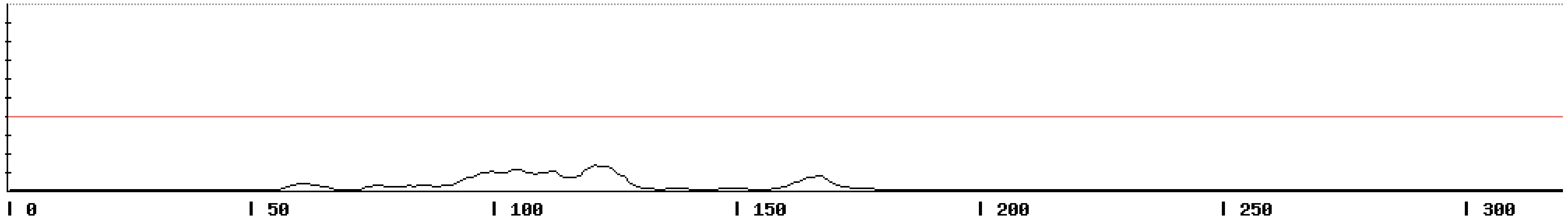

Predictions for Ocimumtenuiflorum_Ote100264090021

Viterbi Path	
Viterbi Path	
Posterior @ 0.4	
Posterior @ 0.4	

Posterior Graphic. Threshold Marked @ 0.4

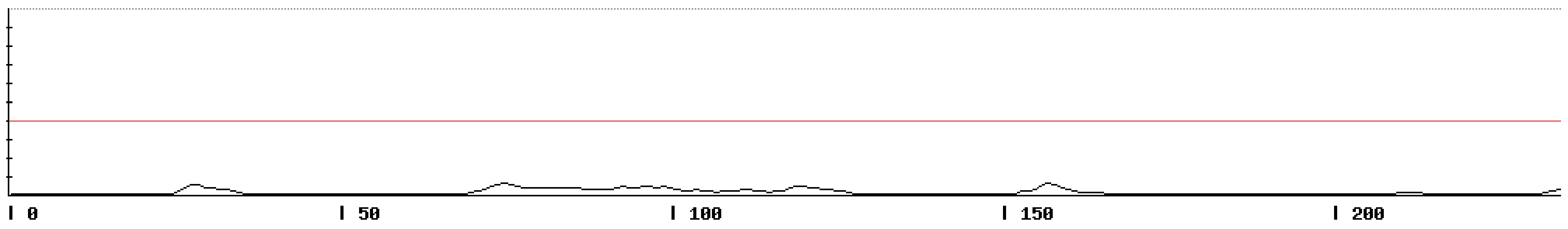

Predictions for Ocimumtenuiflorum_Ote100267660001

Viterbi Path

Viterbi Path

Posterior @ 0.4

Posterior @ 0.4

Posterior Graphic. Threshold Marked @ 0.4

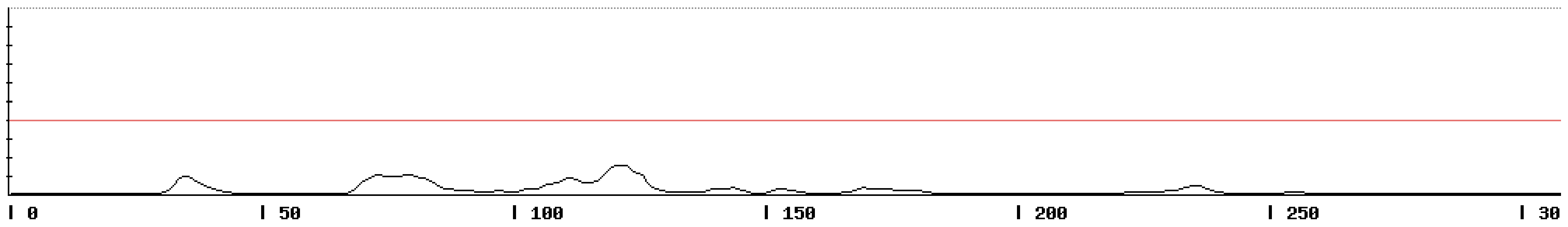

Predictions for Ocimumtenuiflorum_Ote100270600021

Viterbi Path

Viterbi Path

Posterior @ 0.4

Posterior @ 0.4

Posterior Graphic. Threshold Marked @ 0.4

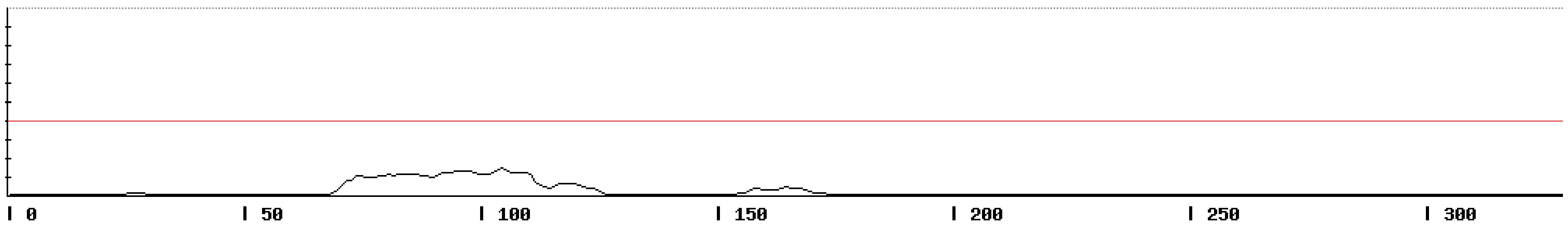

Predictions for Ocimumtenuiflorum_Ote100272800001

Viterbi Path

Viterbi Path

Posterior @ 0.4 — 132 - KRR - 134

Posterior @ 0.4

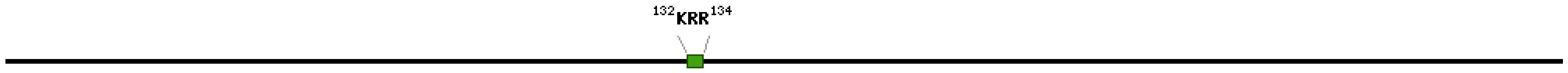

Posterior Graphic. Threshold Marked @ 0.4

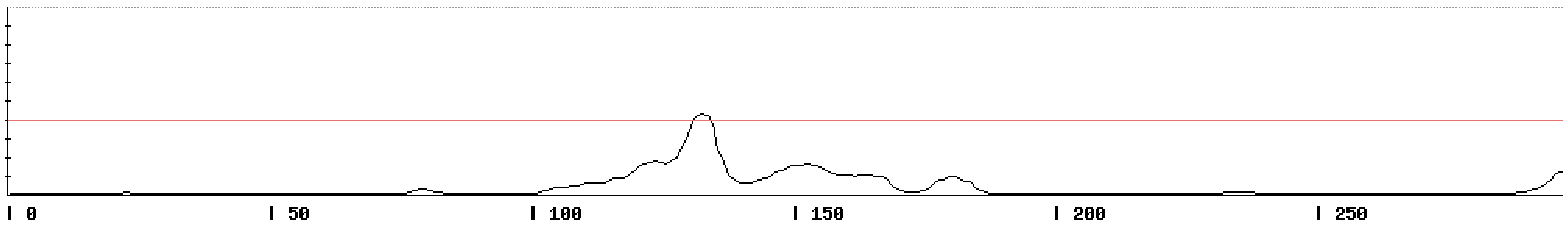

Predictions for Ocimumtenuiflorum_Ote100274130011

Viterbi Path

Viterbi Path

Posterior @ 0.4

Posterior @ 0.4

Posterior Graphic. Threshold Marked @ 0.4

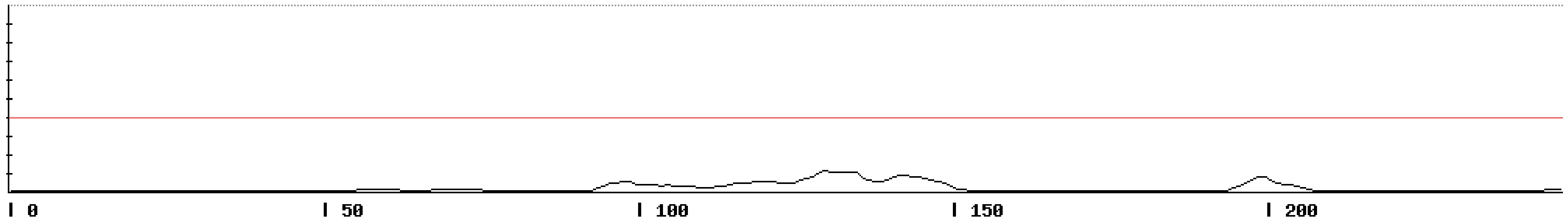

Predictions for Ocimumtenuiflorum_Ote100274130012

Viterbi Path

Viterbi Path

Posterior @ 0.4

Posterior @ 0.4

Posterior Graphic. Threshold Marked @ 0.4

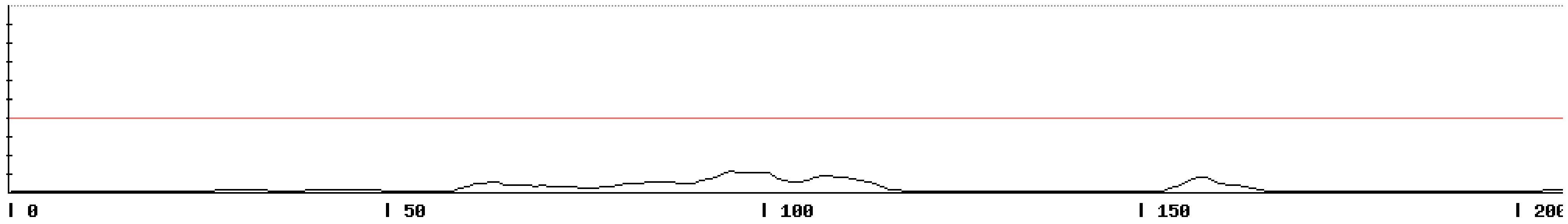

Predictions for Ocimumtenuiflorum_Ote100275050011

Viterbi Path	
Viterbi Path	
Posterior @ 0.4	
Posterior @ 0.4	
Posterior Graphic. Threshold Marked @ 0.4	

Revision r.9 - Last Updated on : February 24 2015 17:45:20. alex.nguyenba@utoronto.ca

Intended to be used with Firefox

Viterbi Path

Posterior @ 0.4

Posterior @ 0.4

Posterior Graphic. Threshold Marked @ 0.4

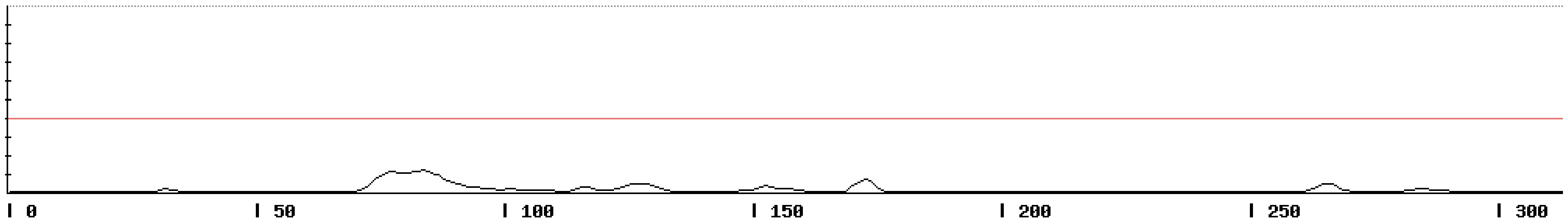

Predictions for Oropetiumthomaeum_Oropetium_20150105_01204A

Viterbi Path

Viterbi Path

Posterior @ 0.4

Posterior @ 0.4

Posterior Graphic. Threshold Marked @ 0.4

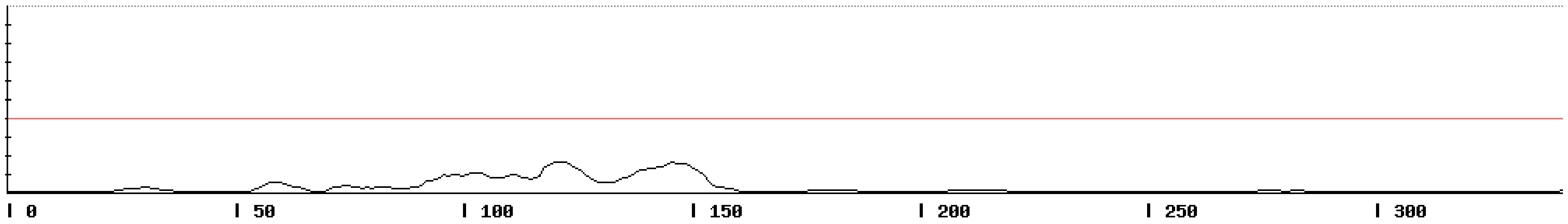

Predictions for Oropetiumthomaeum_Oropetium_20150105_01206A

Viterbi Path

Viterbi Path

Posterior @ 0.4

Posterior @ 0.4

Posterior Graphic. Threshold Marked @ 0.4

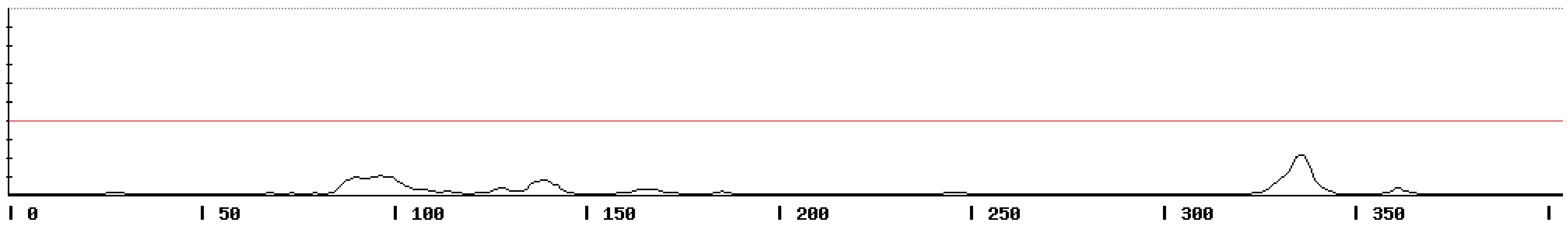

Predictions for Oropetiumthomaeum_Oropetium_20150105_01714A

Viterbi Path

Viterbi Path

Posterior @ 0.4

Posterior @ 0.4

Posterior Graphic. Threshold Marked @ 0.4

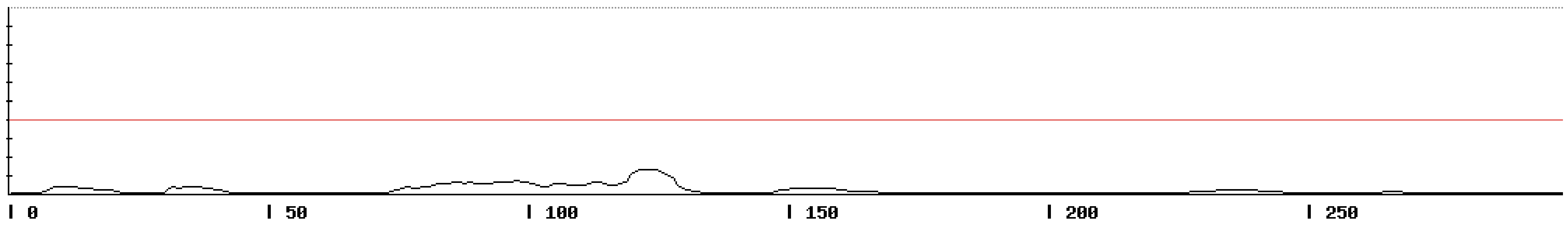

Predictions for Oropetiumthomaeum_Oropetium_20150105_02233A	
Viterbi Path	
Viterbi Path	
Posterior @ 0.4	
Posterior @ 0.4	
Posterior Graphic. Threshold Marked @ 0.4	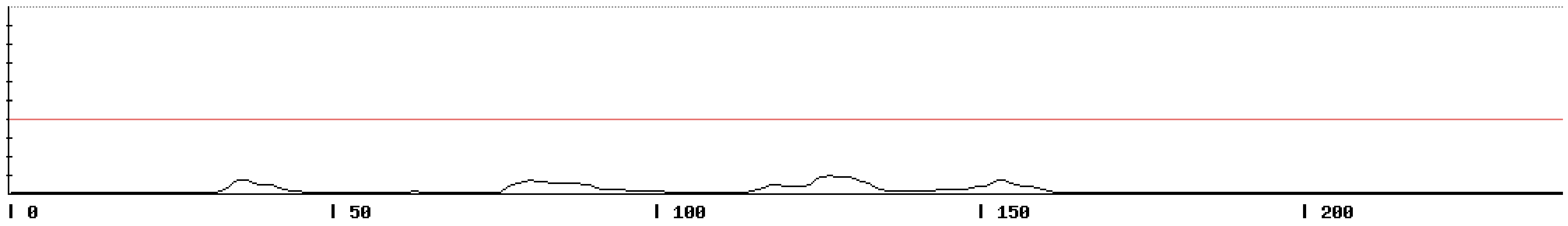

Predictions for Oropetiumthomaeum_Oropetium_20150105_02558A	
Viterbi Path	
Viterbi Path	
Posterior @ 0.4	
Posterior @ 0.4	
Posterior Graphic. Threshold Marked @ 0.4	

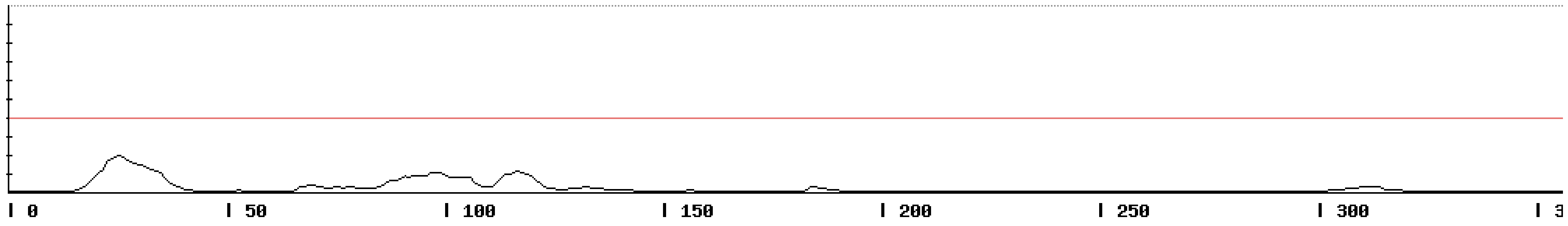

Predictions for Oropetiumthomaeum_Oropetium_20150105_02638A

Viterbi Path	
Viterbi Path	
Posterior @ 0.4	
Posterior @ 0.4	
Posterior Graphic. Threshold Marked @ 0.4	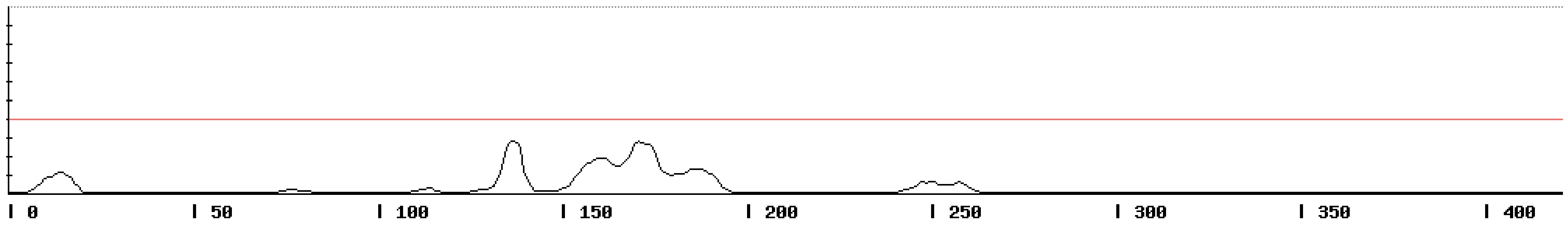

Predictions for Oropetiumthomaeum_Oropetium_20150105_02800A

Viterbi Path	
Viterbi Path	
Posterior @ 0.4	
Posterior @ 0.4	

Posterior Graphic. Threshold Marked @ 0.4	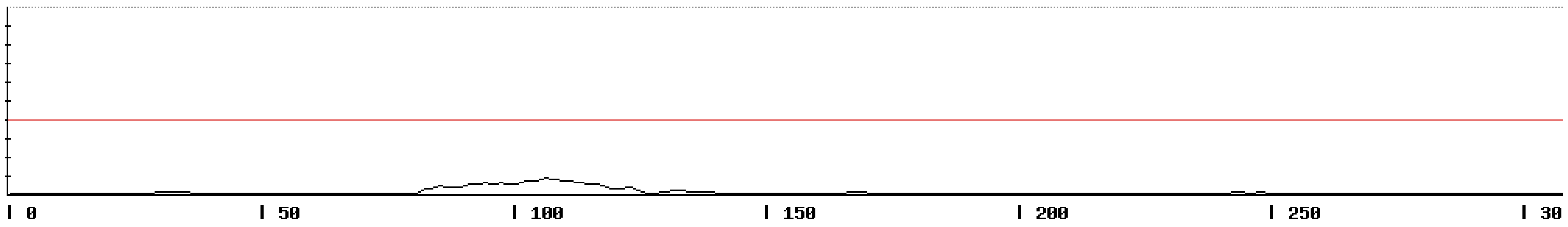

Predictions for Oropetiumthomaeum_Oropetium_20150105_02881A	
Viterbi Path	
Viterbi Path	
Posterior @ 0.4	
Posterior @ 0.4	
Posterior Graphic. Threshold Marked @ 0.4	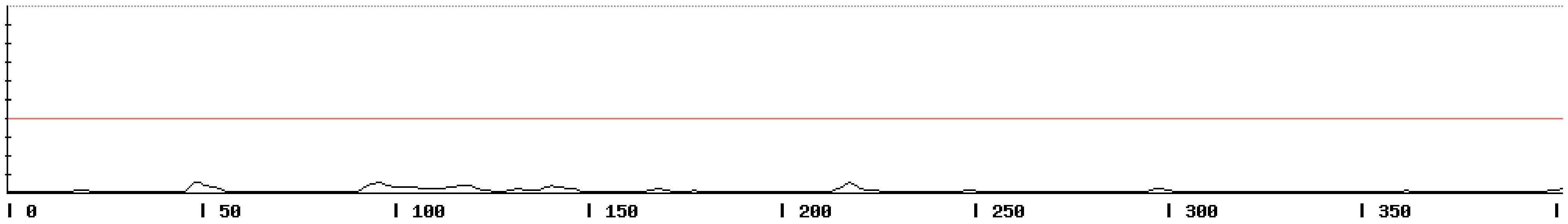

Predictions for Oropetiumthomaeum_Oropetium_20150105_03172A	
Viterbi Path	
Viterbi Path	
Posterior @ 0.4	124 - RKRRR - 128
Posterior @ 0.4	

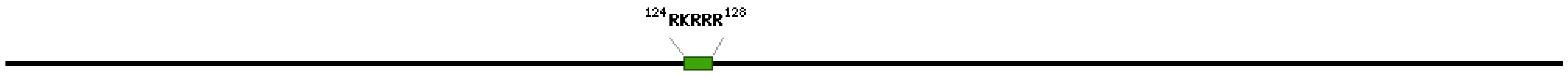

Posterior Graphic. Threshold Marked @ 0.4

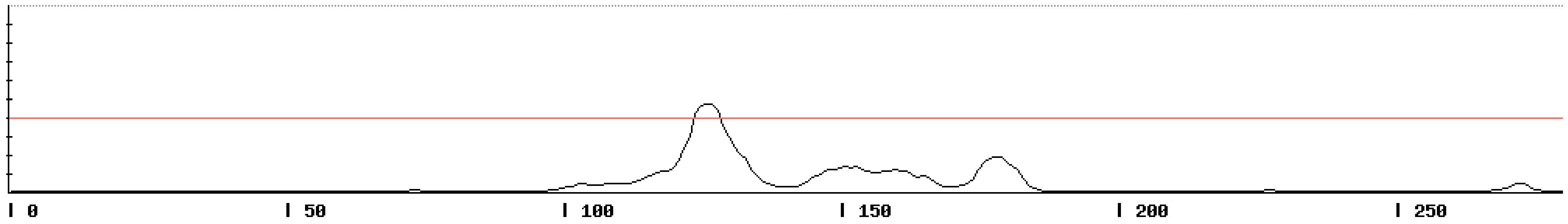

Predictions for Oropetiumthomaeum_Oropetium_20150105_03756A

Viterbi Path	192 - KPKQKRKRKEKTVNAEPDKKTTKRKKVVPARQKKK - 226
Viterbi Path	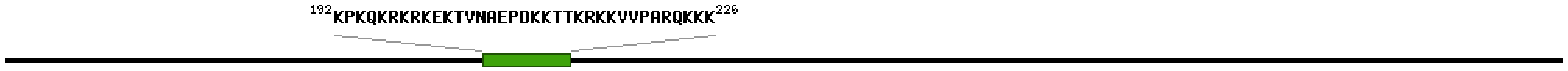
Posterior @ 0.4	191 - NKPKQKRKRKEKTVNAEPDKKTTKRKKVVPARQKKKQGAPP - 231
Posterior @ 0.4	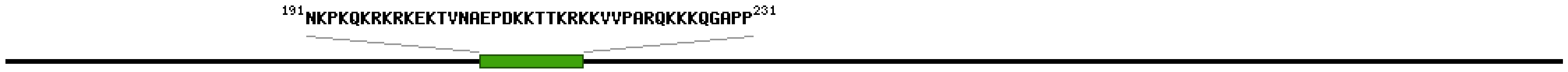
Posterior Graphic. Threshold Marked @ 0.4	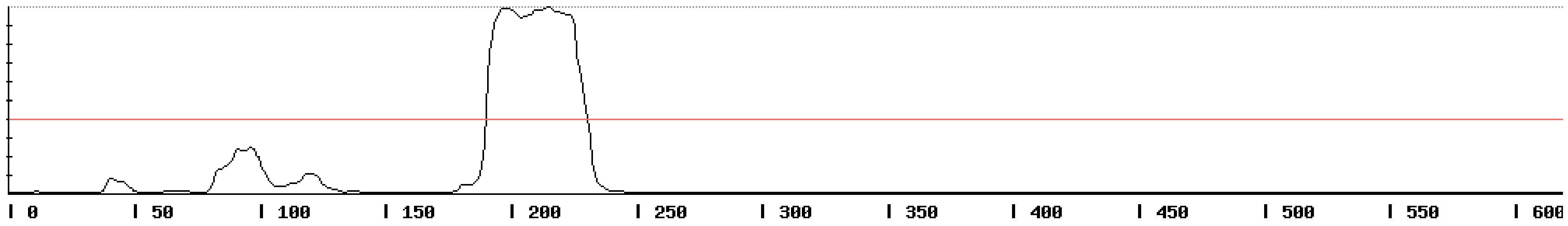

Predictions for Oropetiumthomaeum_Oropetium_20150105_03892A

Viterbi Path	
Viterbi Path	

Posterior @ 0.4 143 - RKRRK - 147

Posterior @ 0.4

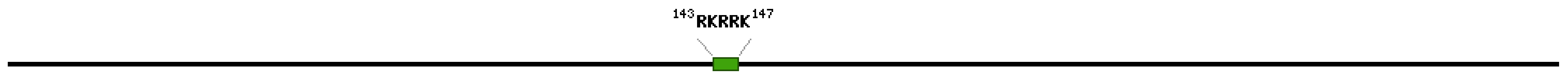

Posterior Graphic. Threshold Marked @ 0.4

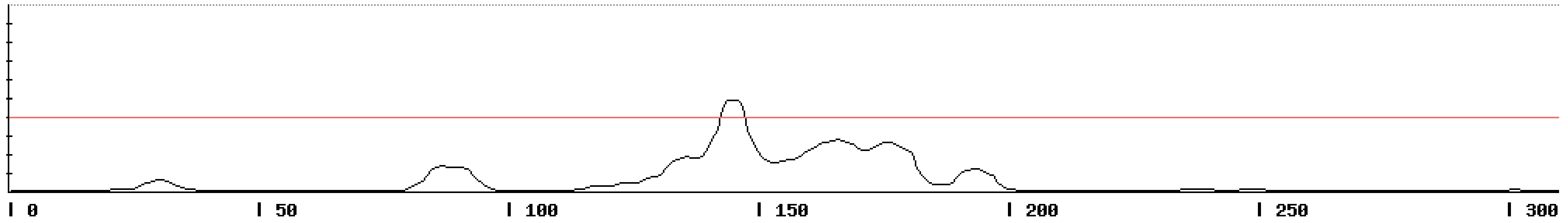

Predictions for Oropetiumthomaeum_Oropetium_20150105_04194A

Viterbi Path

Viterbi Path

Posterior @ 0.4

Posterior @ 0.4

Posterior Graphic. Threshold Marked @ 0.4

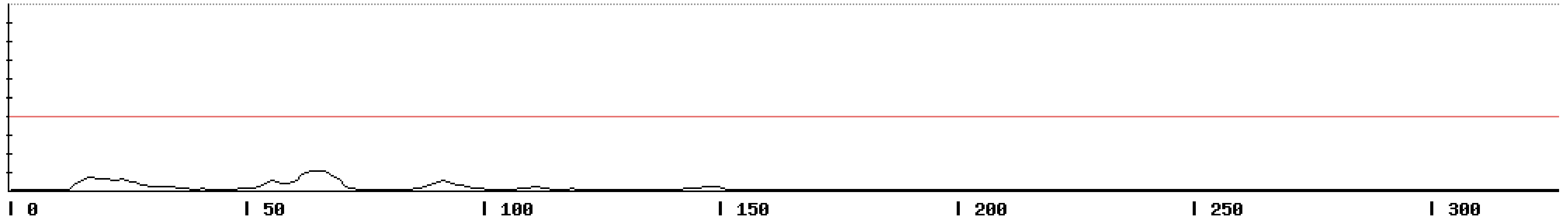

Predictions for Oropetiumthomaeum_Oropetium_20150105_04583A

Viterbi Path

Viterbi Path

Posterior @ 0.4

Posterior @ 0.4

Posterior Graphic. Threshold Marked @ 0.4

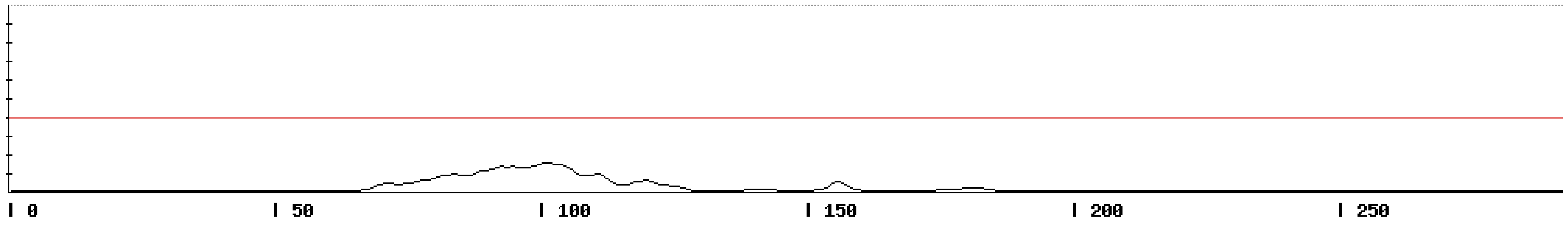

Predictions for Oropetiumthomaeum_Oropetium_20150105_04693A

Viterbi Path

Viterbi Path

Posterior @ 0.4

Posterior @ 0.4

Posterior Graphic. Threshold Marked @ 0.4

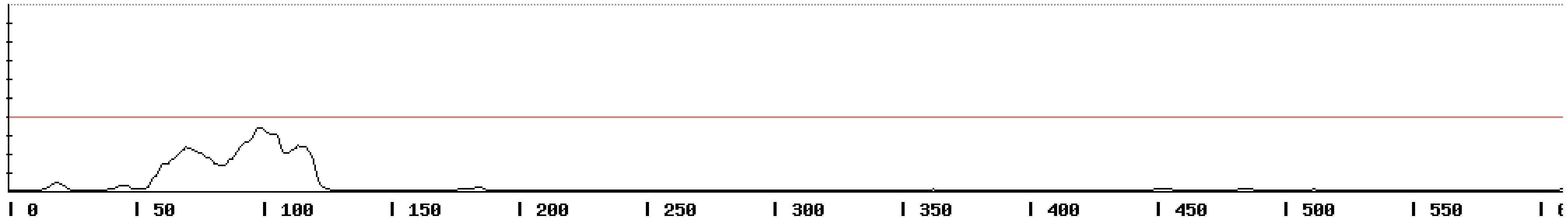

Predictions for Oropetiumthomaeum_Oropetium_20150105_04962A

Viterbi Path	
Viterbi Path	
Posterior @ 0.4	
Posterior @ 0.4	
Posterior Graphic. Threshold Marked @ 0.4	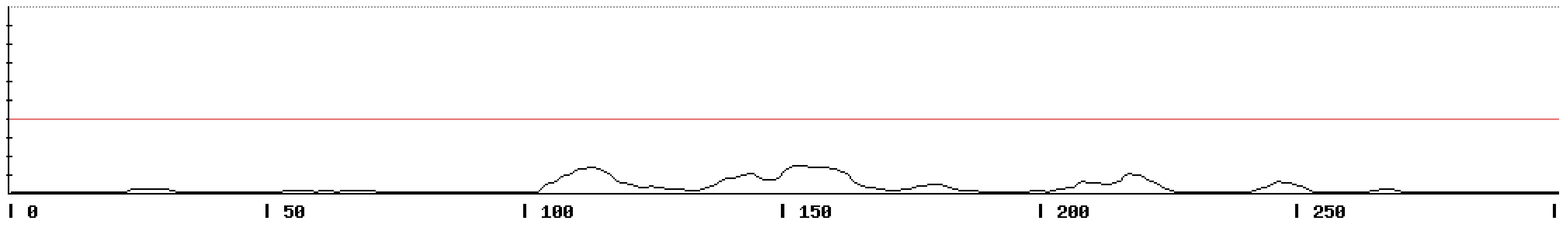

Predictions for Oropetiumthomaeum_Oropetium_20150105_05045A

Viterbi Path	
Viterbi Path	
Posterior @ 0.4	
Posterior @ 0.4	
Posterior Graphic. Threshold Marked @ 0.4	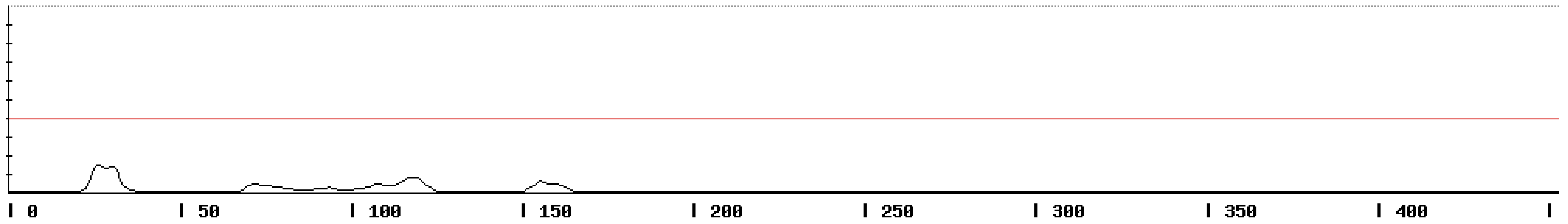

Predictions for Oropetiumthomaeum_Oropetium_20150105_05058A	
Viterbi Path	
Viterbi Path	
Posterior @ 0.4	
Posterior @ 0.4	
Posterior Graphic. Threshold Marked @ 0.4	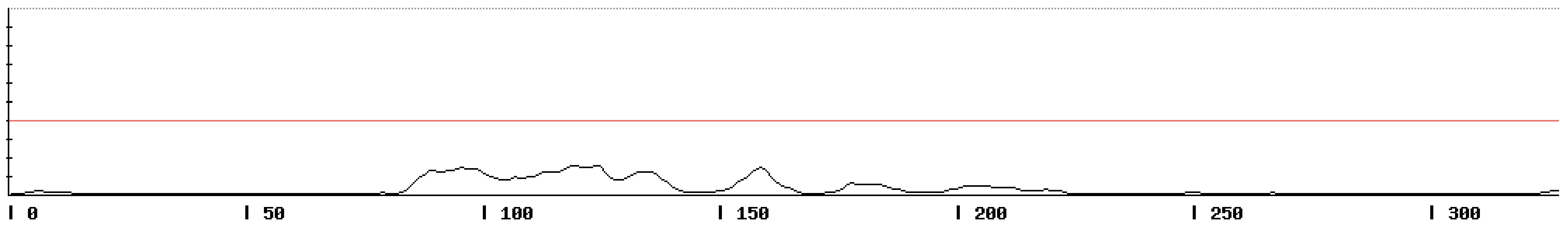

Predictions for Oropetiumthomaeum_Oropetium_20150105_05071A	
Viterbi Path	
Viterbi Path	
Posterior @ 0.4	
Posterior @ 0.4	
Posterior Graphic. Threshold Marked @ 0.4	

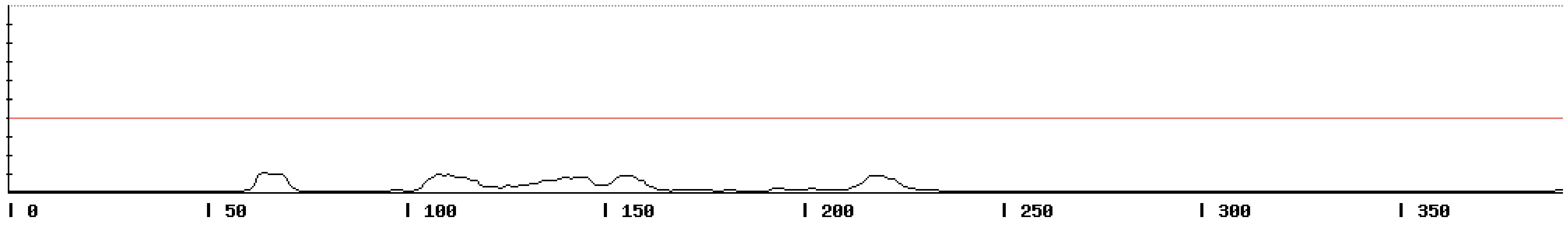

Predictions for Oropetiumthomaeum_Oropetium_20150105_05464A

Viterbi Path

Viterbi Path

Posterior @ 0.4

Posterior @ 0.4

Posterior Graphic. Threshold Marked @ 0.4

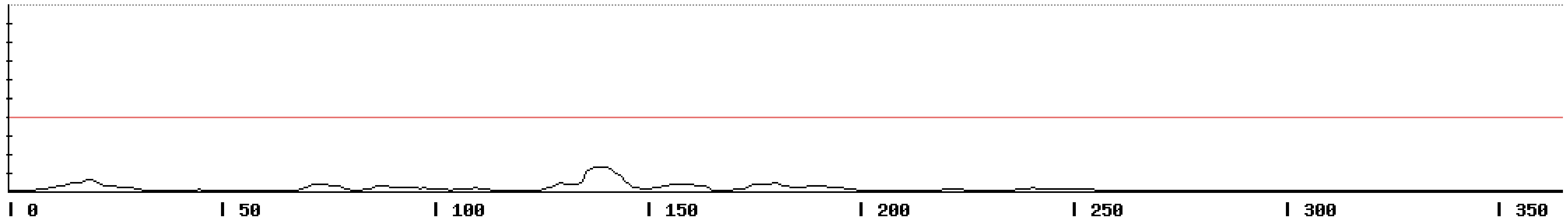

Predictions for Oropetiumthomaeum_Oropetium_20150105_05479A

Viterbi Path

Viterbi Path

Posterior @ 0.4

Posterior @ 0.4

Posterior Graphic. Threshold Marked @ 0.4

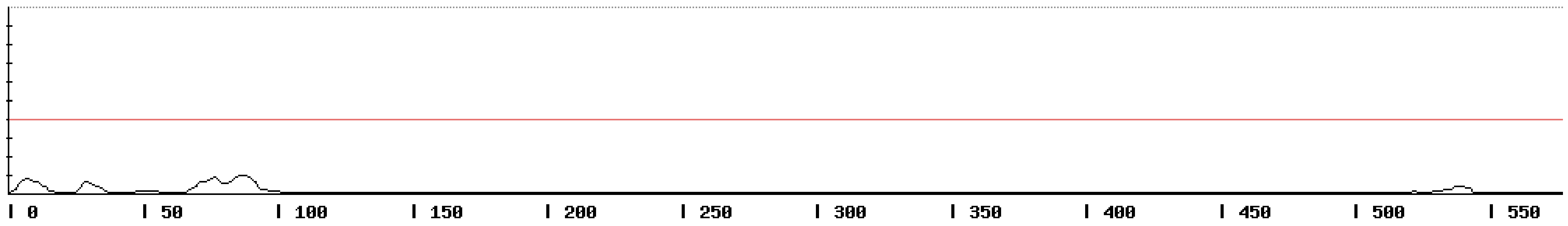

Predictions for Oropetiumthomaeum_Oropetium_20150105_06049A

Viterbi Path

Viterbi Path

Posterior @ 0.4

Posterior @ 0.4

Posterior Graphic. Threshold Marked @ 0.4

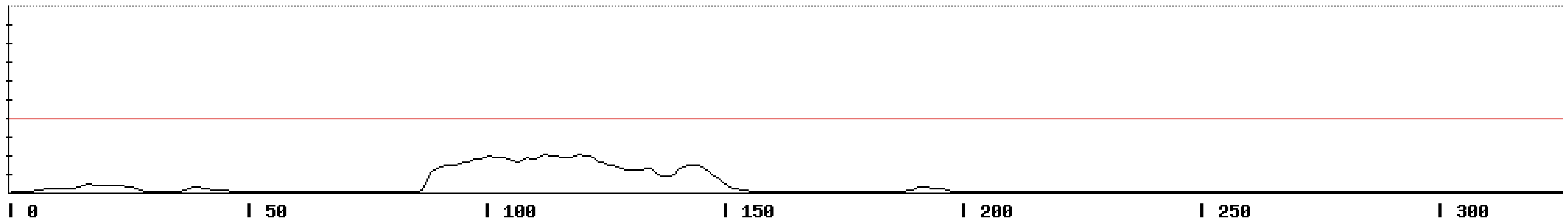

Predictions for Oropetiumthomaeum_Oropetium_20150105_06294A

Viterbi Path

Viterbi Path

Posterior @ 0.4 | 43 - RR - 44

Posterior @ 0.4

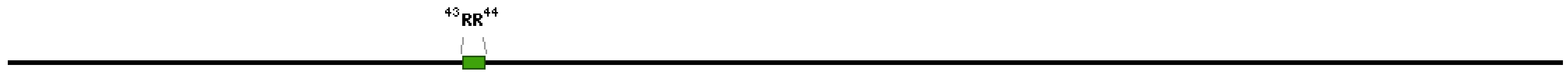

Posterior Graphic. Threshold Marked @ 0.4

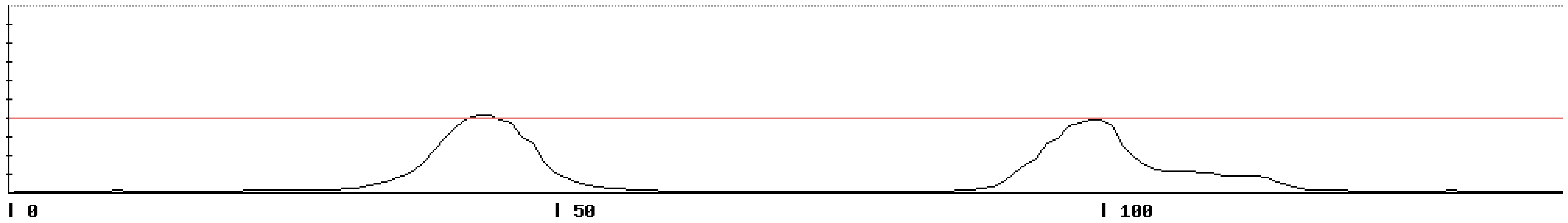

Predictions for Oropetiumthomaeum_Oropetium_20150105_06295A

Viterbi Path

Viterbi Path

Posterior @ 0.4

Posterior @ 0.4

Posterior Graphic. Threshold Marked @ 0.4

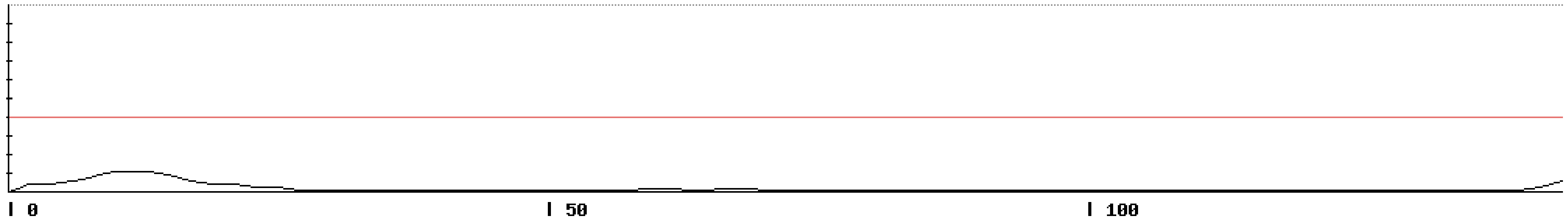

Predictions for Oropetiumthomaeum_Oropetium_20150105_06296A

Viterbi Path

Viterbi Path

Posterior @ 0.4	18 - GRR - 20
Posterior @ 0.4	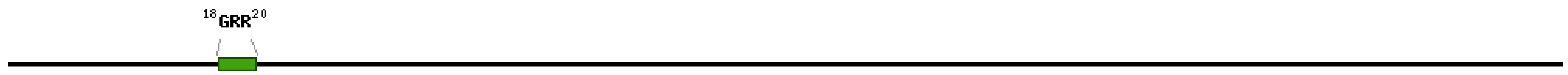
Posterior Graphic. Threshold Marked @ 0.4	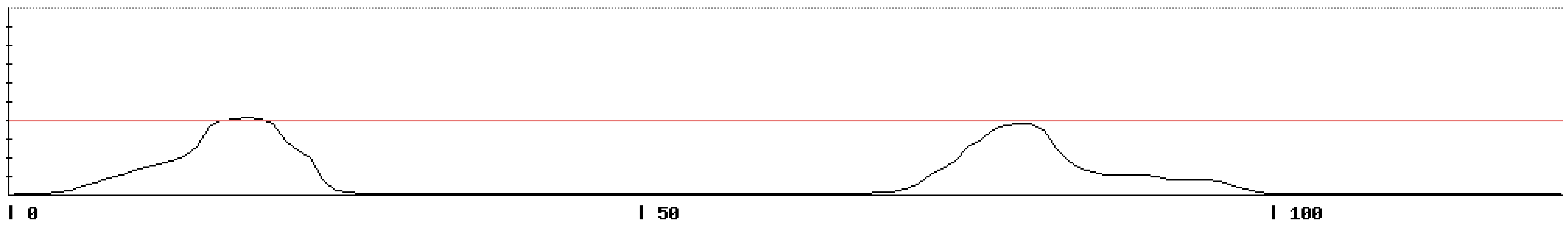

Predictions for Oropetiumthomaeum_Oropetium_20150105_06297A

Viterbi Path	
Viterbi Path	
Posterior @ 0.4	
Posterior @ 0.4	
Posterior Graphic. Threshold Marked @ 0.4	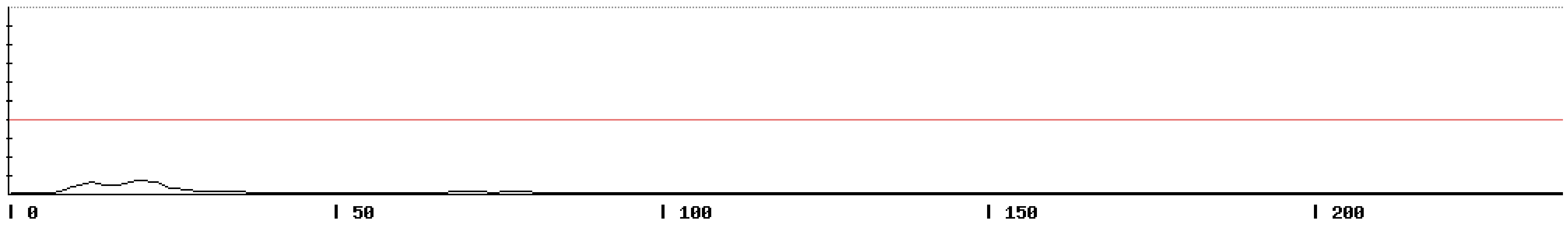

Predictions for Oropetiumthomaeum_Oropetium_20150105_06298A

Viterbi Path	
Viterbi Path	

Posterior @ 0.4

Posterior @ 0.4

Posterior Graphic. Threshold Marked @ 0.4

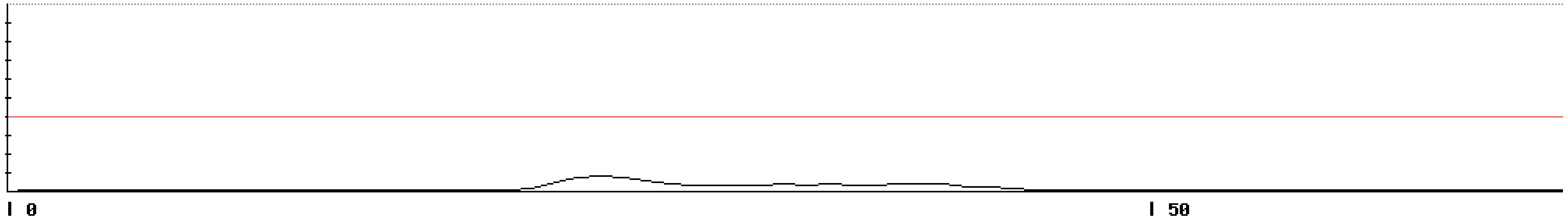

Predictions for Oropetiumthomaeum_Oropetium_20150105_06317A

Viterbi Path

Viterbi Path

Posterior @ 0.4

70 - HGHRRQR - 76

194 - APPRRRGRPRKNIAAATSSGNKRKKA - 219

290 - RPKKK - 294

Posterior @ 0.4

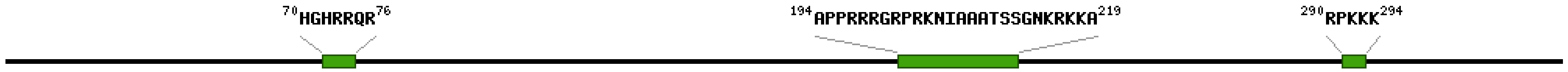

Posterior Graphic. Threshold Marked @ 0.4

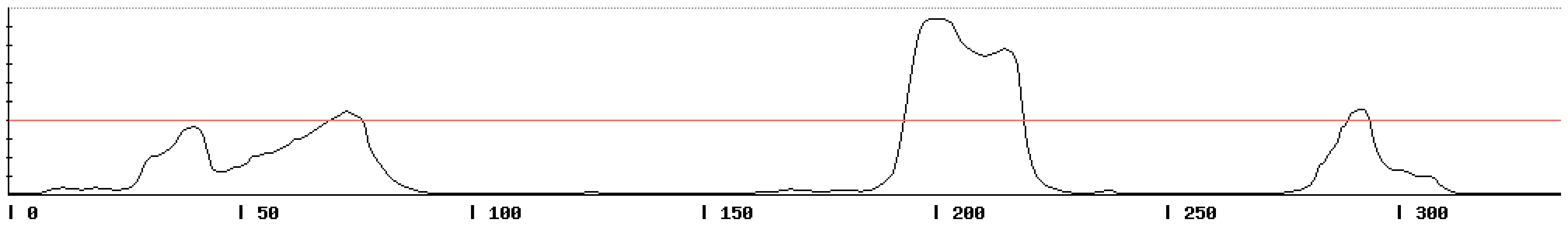

Predictions for Oropetiumthomaeum_Oropetium_20150105_06453A

Viterbi Path

Viterbi Path

Posterior @ 0.4

Posterior @ 0.4

Posterior Graphic. Threshold Marked @ 0.4

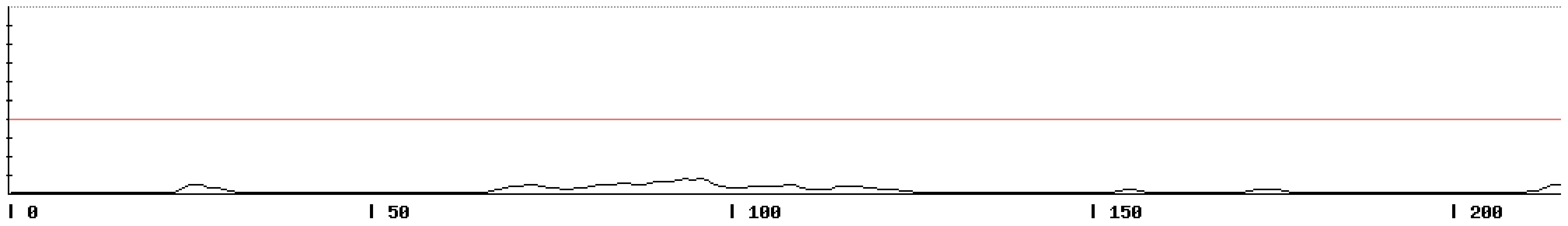

Predictions for Oropetiumthomaeum_Oropetium_20150105_06454A

Viterbi Path

Viterbi Path

Posterior @ 0.4

Posterior @ 0.4

Posterior Graphic. Threshold Marked @ 0.4

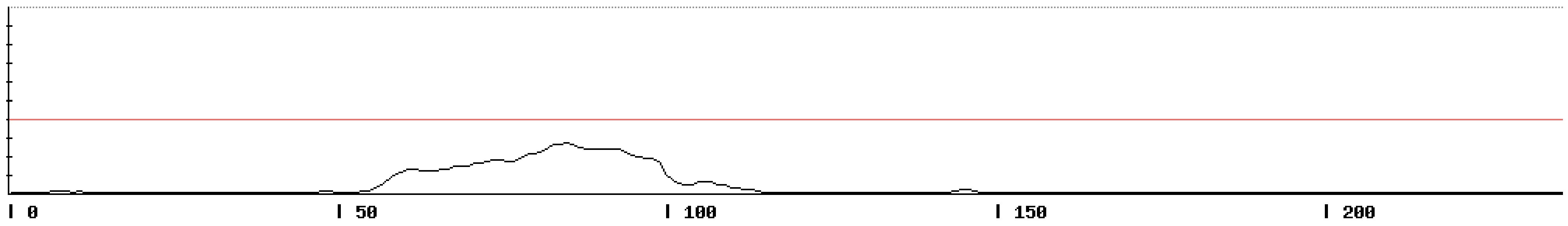

Predictions for Oropetiumthomaeum_Oropetium_20150105_06798A

Viterbi Path

Viterbi Path

Posterior @ 0.4

Posterior @ 0.4

Posterior Graphic. Threshold Marked @ 0.4

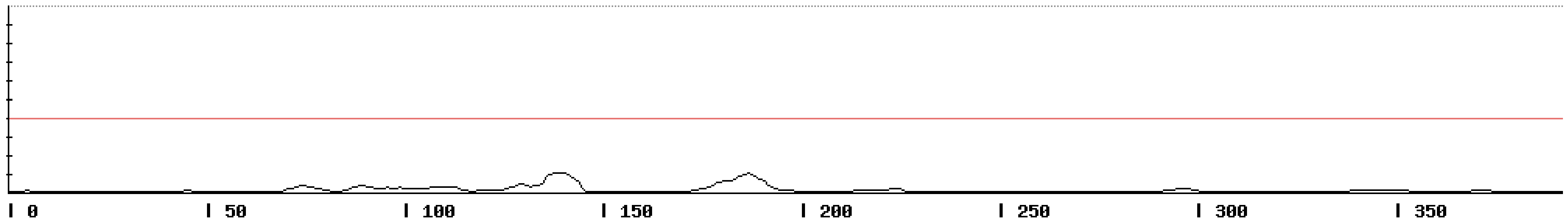

Predictions for Oropetiumthomaeum_Oropetium_20150105_06801A

Viterbi Path

Viterbi Path

Posterior @ 0.4

Posterior @ 0.4

Posterior Graphic. Threshold Marked @ 0.4

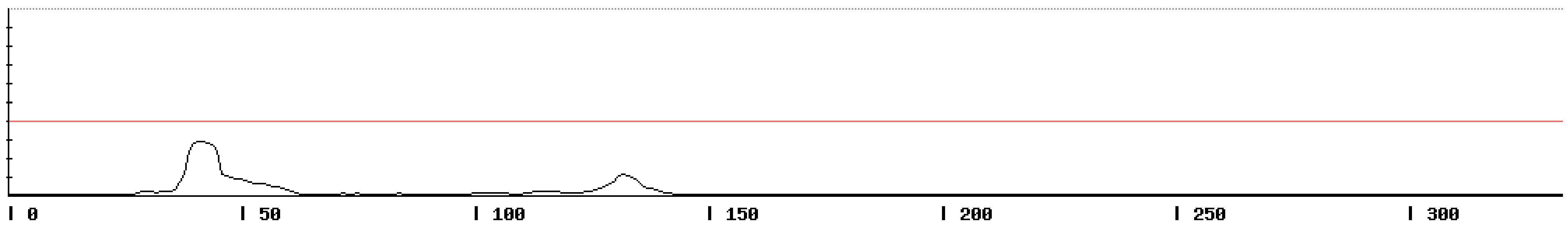

Predictions for Oropetiumthomaeum_Oropetium_20150105_06856A

Viterbi Path

Viterbi Path

Posterior @ 0.4

Posterior @ 0.4

Posterior Graphic. Threshold Marked @ 0.4

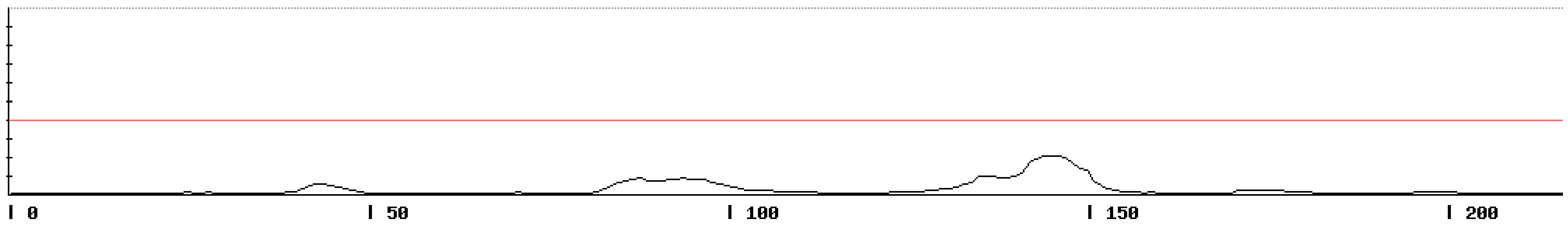

Predictions for Oropetiumthomaeum_Oropetium_20150105_06862A

Viterbi Path

Viterbi Path

Posterior @ 0.4

Posterior @ 0.4

Posterior Graphic. Threshold Marked @ 0.4

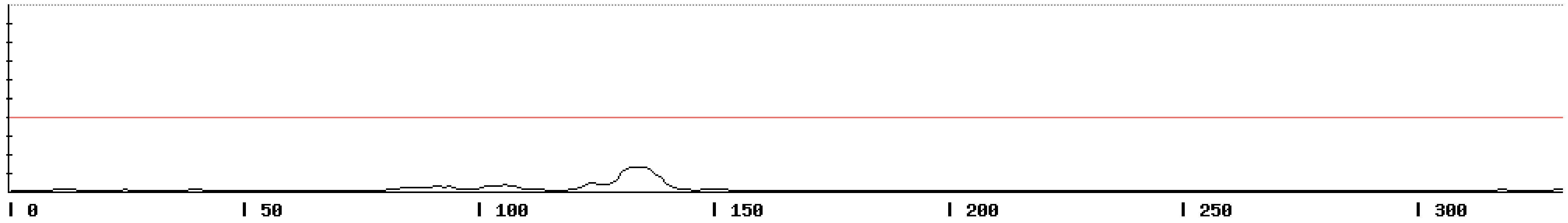

Predictions for Oropetiumthomaeum_Oropetium_20150105_07363A

Viterbi Path

Viterbi Path

Posterior @ 0.4 — 123 - R - 123

Posterior @ 0.4

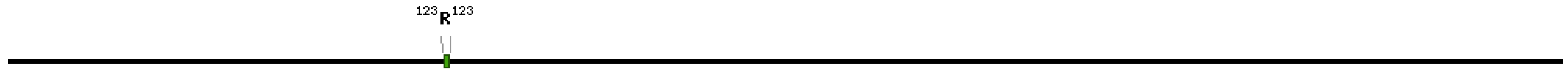

Posterior Graphic. Threshold Marked @ 0.4

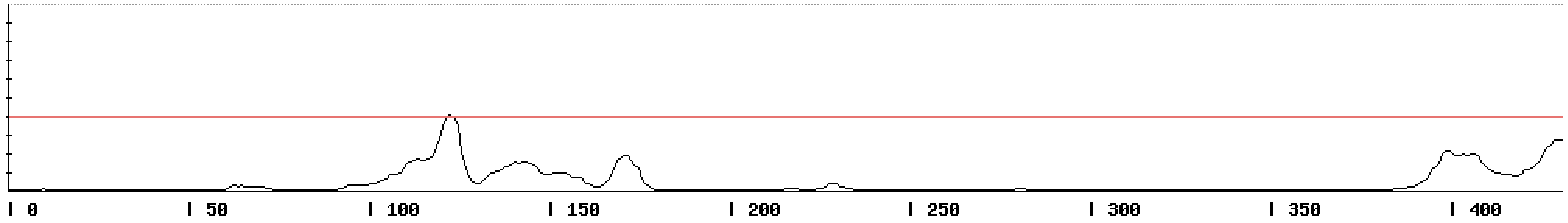

Predictions for Oropetiumthomaeum_Oropetium_20150105_07750A

Viterbi Path

Viterbi Path

Posterior @ 0.4

Posterior @ 0.4

Posterior Graphic. Threshold Marked @ 0.4

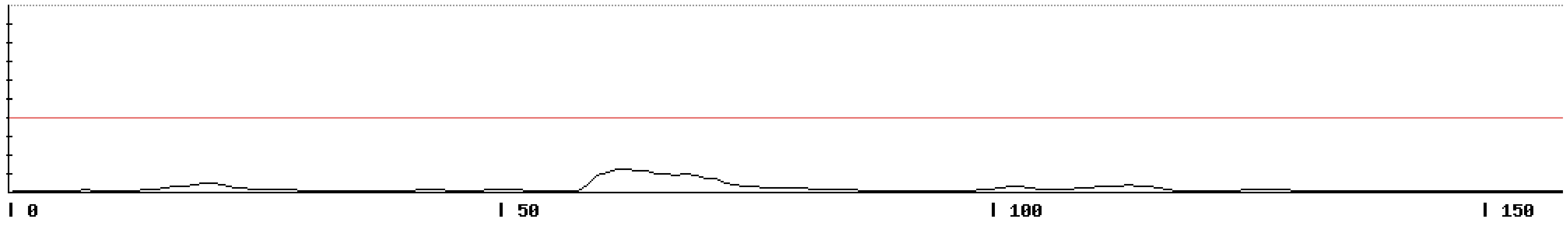

Predictions for Oropetiumthomaeum_Oropetium_20150105_08447A

Viterbi Path

Viterbi Path

Posterior @ 0.4

Posterior @ 0.4

Posterior Graphic. Threshold Marked @ 0.4

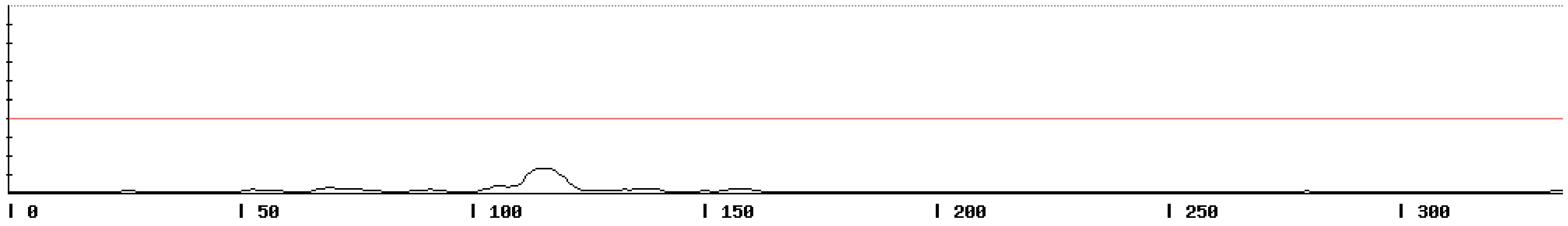

Predictions for Oropetiumthomaeum_Oropetium_20150105_09207A

Viterbi Path

Viterbi Path

Posterior @ 0.4

147 - GRPPRERPQIKTRTRRRRS - 165

Posterior @ 0.4

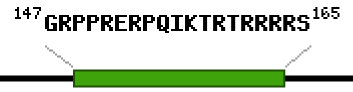

Posterior Graphic. Threshold Marked @ 0.4

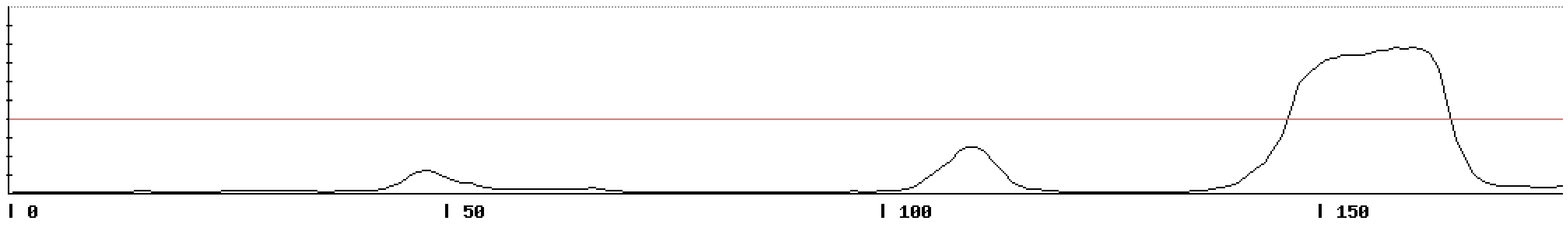

Predictions for Oropetiumthomaeum_Oropetium_20150105_09267A

Viterbi Path

Viterbi Path

Posterior @ 0.4

Posterior @ 0.4

Posterior Graphic. Threshold Marked @ 0.4

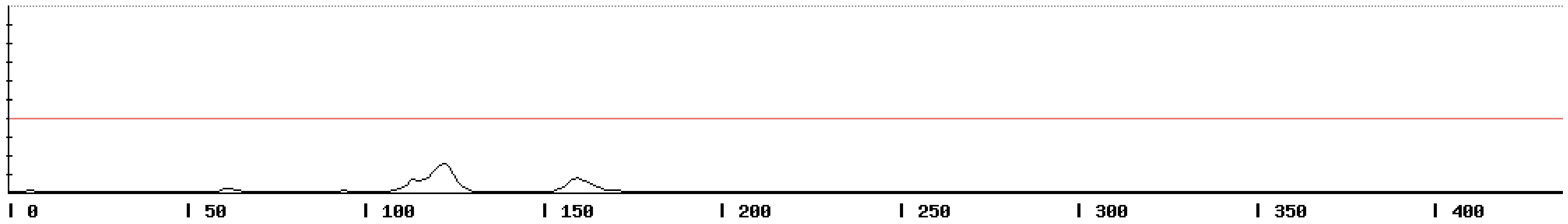

Predictions for Oropetiumthomaeum_Oropetium_20150105_09268A	
Viterbi Path	
Viterbi Path	
Posterior @ 0.4	
Posterior @ 0.4	
Posterior Graphic. Threshold Marked @ 0.4	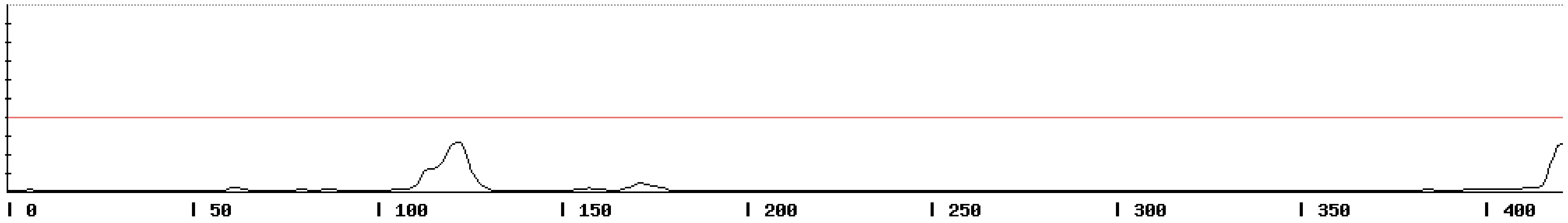

Predictions for Oropetiumthomaeum_Oropetium_20150105_09708A	
Viterbi Path	
Viterbi Path	
Posterior @ 0.4	99 - KSTGKDRAIKLKNR - 112
Posterior @ 0.4	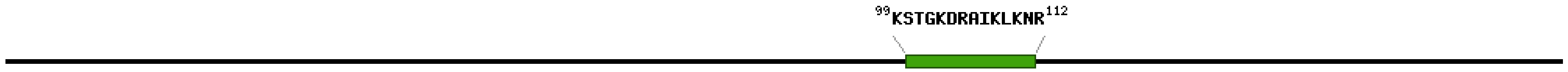
Posterior Graphic. Threshold Marked @ 0.4	

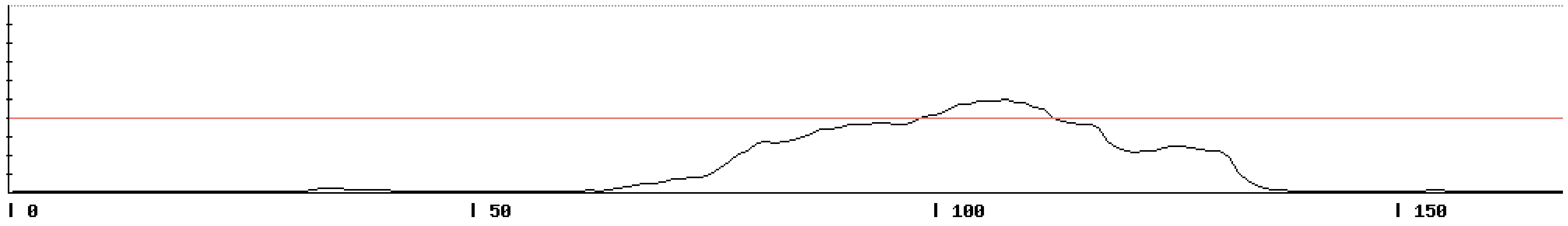

Predictions for Oropetiumthomaeum_Oropetium_20150105_09827A	
Viterbi Path	
Viterbi Path	
Posterior @ 0.4	
Posterior @ 0.4	
Posterior Graphic. Threshold Marked @ 0.4	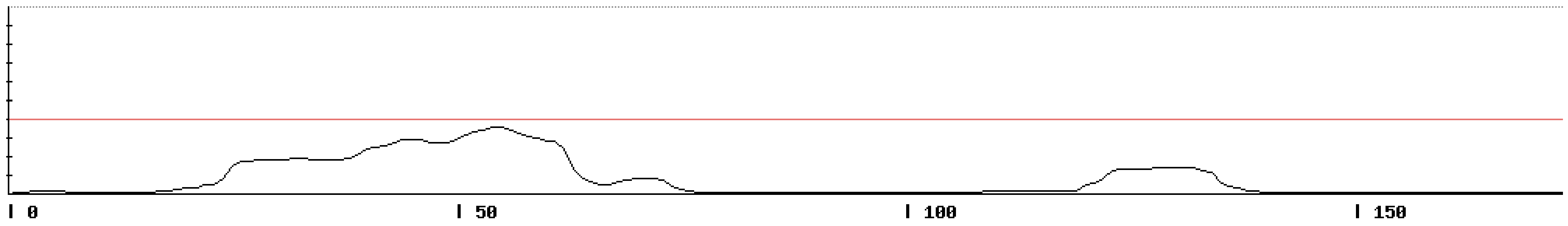

Predictions for Oropetiumthomaeum_Oropetium_20150105_09929A	
Viterbi Path	
Viterbi Path	
Posterior @ 0.4	
Posterior @ 0.4	

Posterior Graphic. Threshold Marked @ 0.4

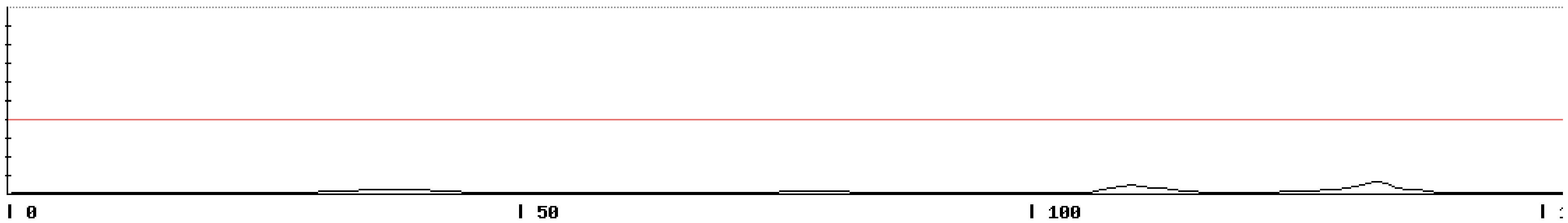

Predictions for Oropetiumthomaeum_Oropetium_20150105_10171A

Viterbi Path

Viterbi Path

Posterior @ 0.4

Posterior @ 0.4

Posterior Graphic. Threshold Marked @ 0.4

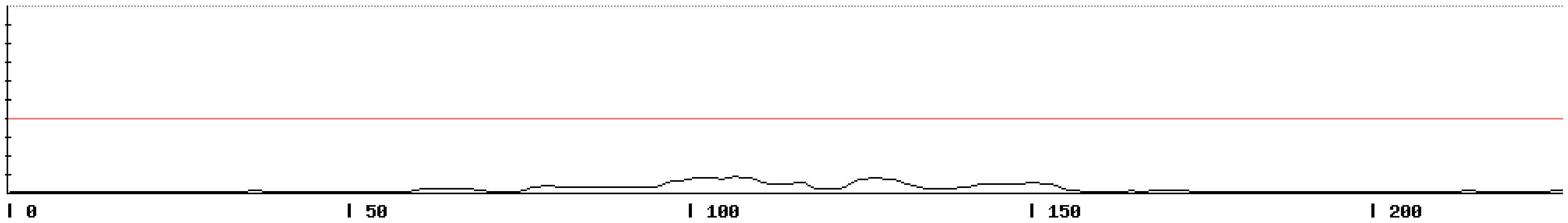

Predictions for Oropetiumthomaeum_Oropetium_20150105_10176A

Viterbi Path

Viterbi Path

Posterior @ 0.4

Posterior @ 0.4

Posterior Graphic. Threshold Marked @ 0.4

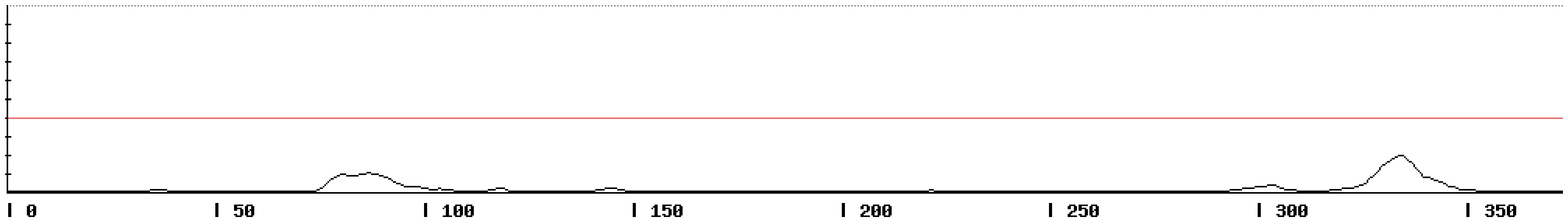

Predictions for Oropetiumthomaeum_Oropetium_20150105_10805A

Viterbi Path	
Viterbi Path	
Posterior @ 0.4	352 - REKKKKPRLTTRP - 364
Posterior @ 0.4	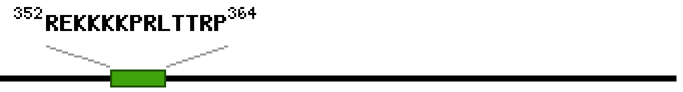
Posterior Graphic. Threshold Marked @ 0.4	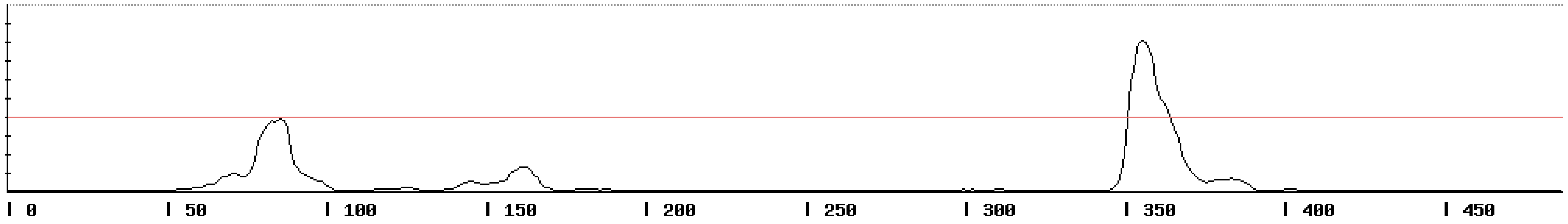

Predictions for Oropetiumthomaeum_Oropetium_20150105_11005A

Viterbi Path	
Viterbi Path	

Posterior @ 0.4	195 - PPPPEKAVTAHKKRPAAAPP - 214
Posterior @ 0.4	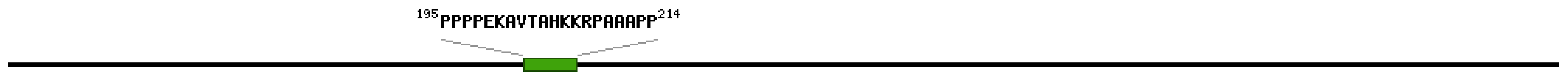
Posterior Graphic. Threshold Marked @ 0.4	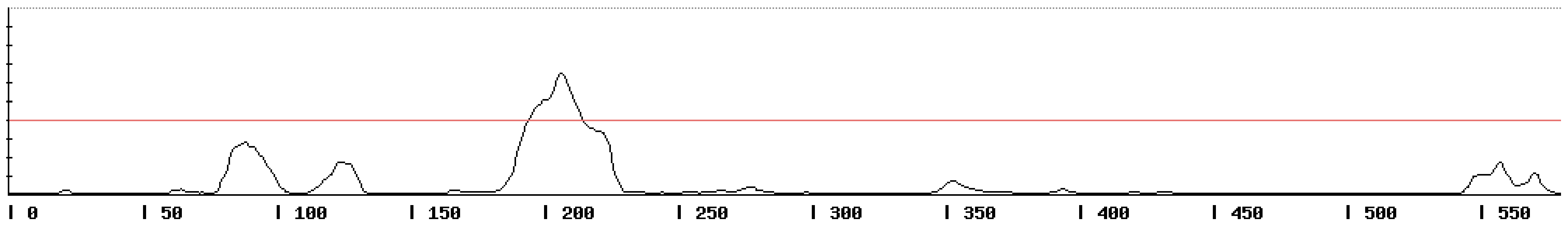

Predictions for Oropetiumthomaeum_Oropetium_20150105_11009A

Viterbi Path	
Viterbi Path	
Posterior @ 0.4	181 - PRKRI - 185
Posterior @ 0.4	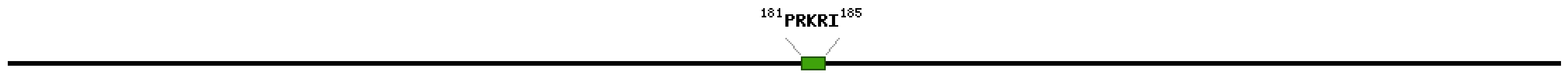
Posterior Graphic. Threshold Marked @ 0.4	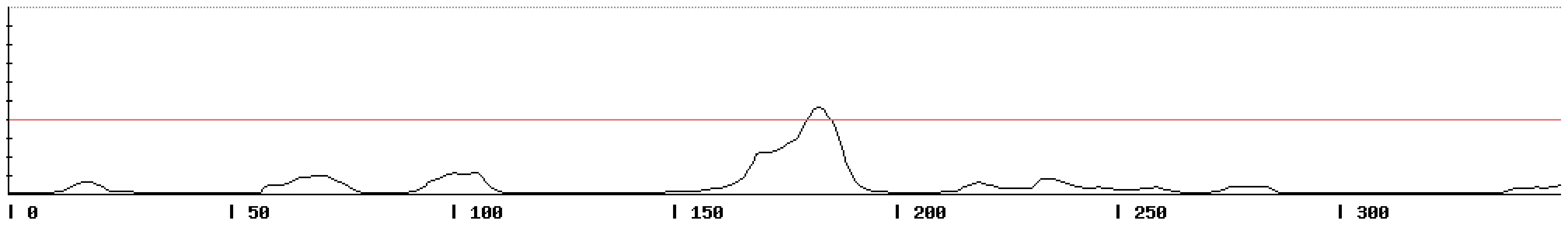

Predictions for Oropetiumthomaeum_Oropetium_20150105_11021A

Viterbi Path	
Viterbi Path	

Posterior @ 0.4

Posterior @ 0.4

Posterior Graphic. Threshold Marked @ 0.4

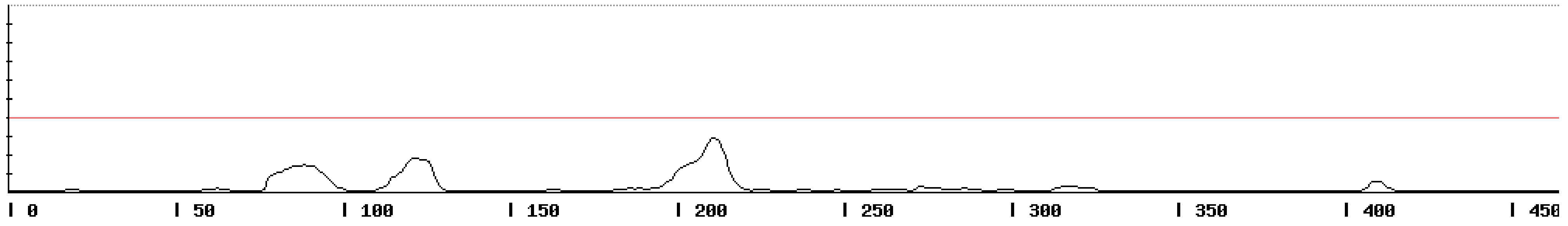

Predictions for Oropetiumthomaeum_Oropetium_20150105_11443A

Viterbi Path

Viterbi Path

Posterior @ 0.4

Posterior @ 0.4

Posterior Graphic. Threshold Marked @ 0.4

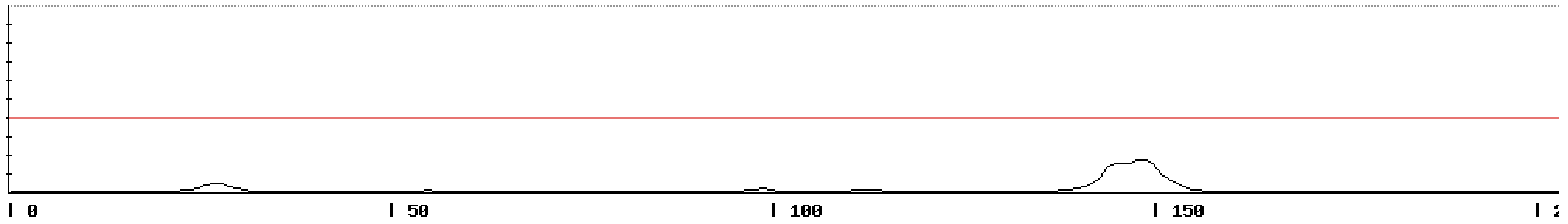

Predictions for Oropetiumthomaeum_Oropetium_20150105_11618A

Viterbi Path	
Viterbi Path	
Posterior @ 0.4	81 - LRRKSERGKRKVR - 93
	283 - K - 283
Posterior @ 0.4	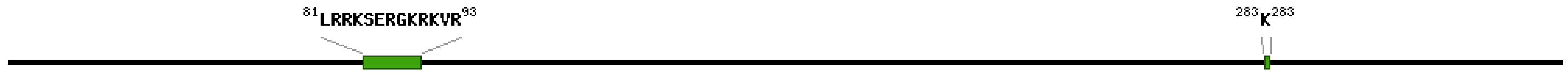
Posterior Graphic. Threshold Marked @ 0.4	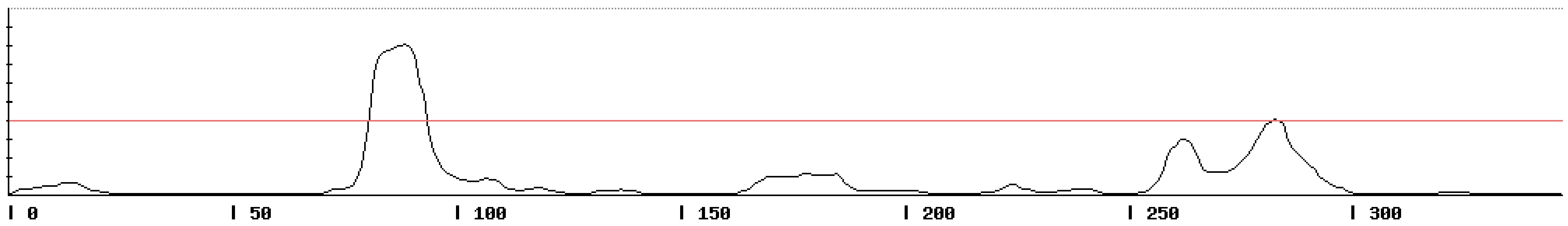

Predictions for Oropetiumthomaeum_Oropetium_20150105_11649A

Viterbi Path	
Viterbi Path	
Posterior @ 0.4	134 - RKRRK - 138
Posterior @ 0.4	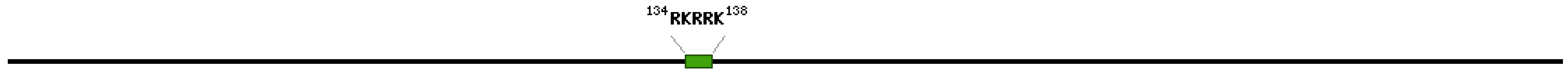
Posterior Graphic. Threshold Marked @ 0.4	

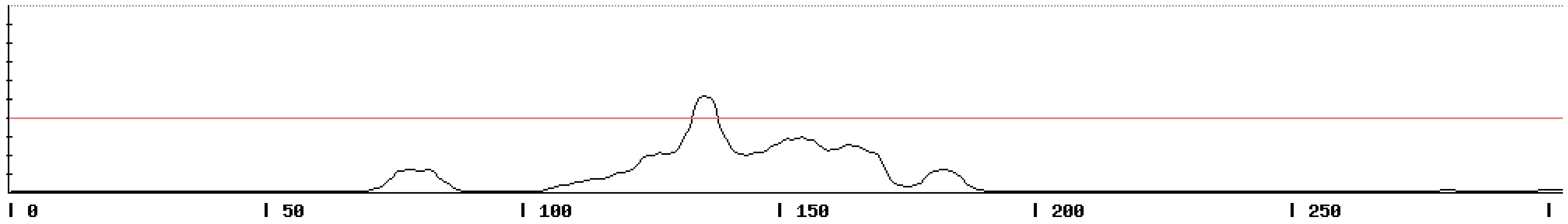

Predictions for Oropetiumthomaeum_Oropetium_20150105_12042A

Viterbi Path

Viterbi Path

Posterior @ 0.4

Posterior @ 0.4

Posterior Graphic. Threshold Marked @ 0.4

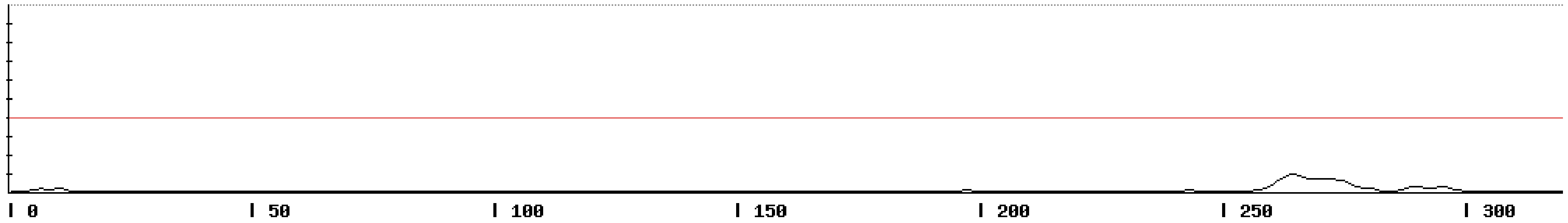

Predictions for Oropetiumthomaeum_Oropetium_20150105_12105A

Viterbi Path

Viterbi Path

Posterior @ 0.4

Posterior @ 0.4

Posterior Graphic. Threshold Marked @ 0.4

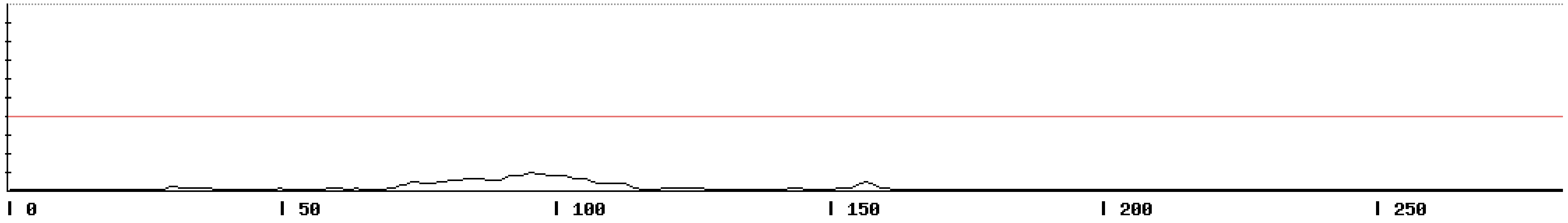

Predictions for Oropetiumthomaeum_Oropetium_20150105_12219A

Viterbi Path

Viterbi Path

Posterior @ 0.4

Posterior @ 0.4

Posterior Graphic. Threshold Marked @ 0.4

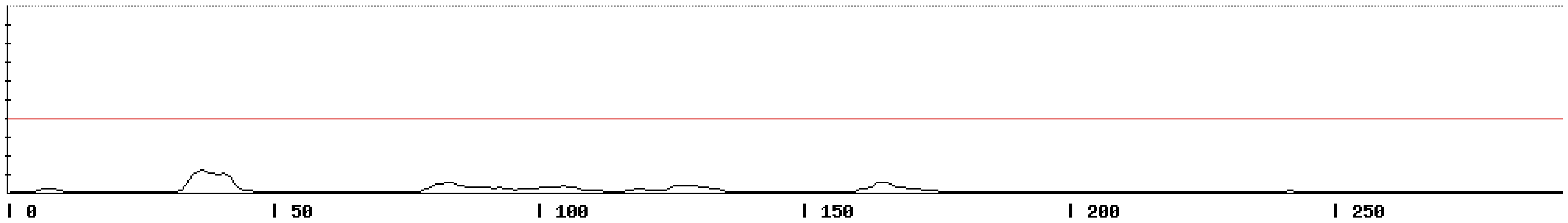

Predictions for Oropetiumthomaeum_Oropetium_20150105_12880A

Viterbi Path

Viterbi Path

Posterior @ 0.4

Posterior @ 0.4

Posterior Graphic. Threshold Marked @ 0.4

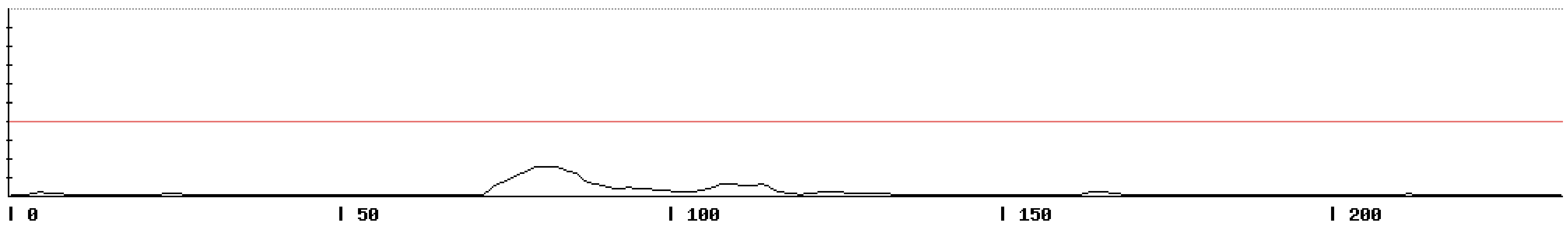

Predictions for Oropetiumthomaeum_Oropetium_20150105_13009A

Viterbi Path

Viterbi Path

Posterior @ 0.4

Posterior @ 0.4

Posterior Graphic. Threshold Marked @ 0.4

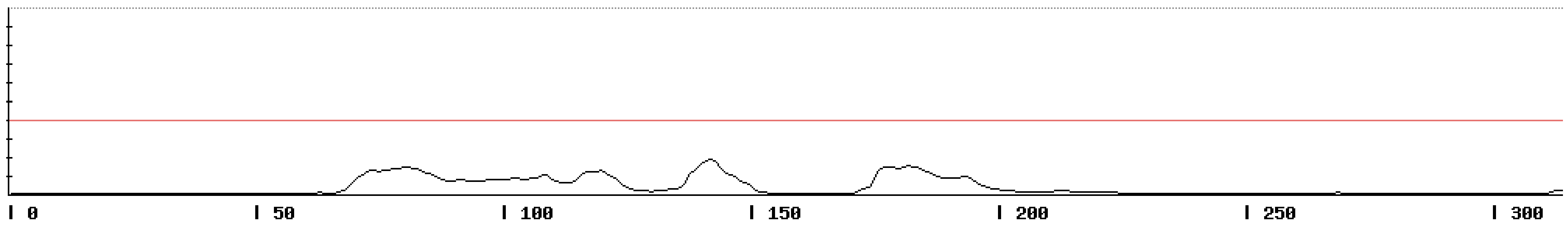

Predictions for Oropetiumthomaeum_Oropetium_20150105_13032A

Viterbi Path

Viterbi Path

Posterior @ 0.4

Posterior @ 0.4

Posterior Graphic. Threshold Marked @ 0.4

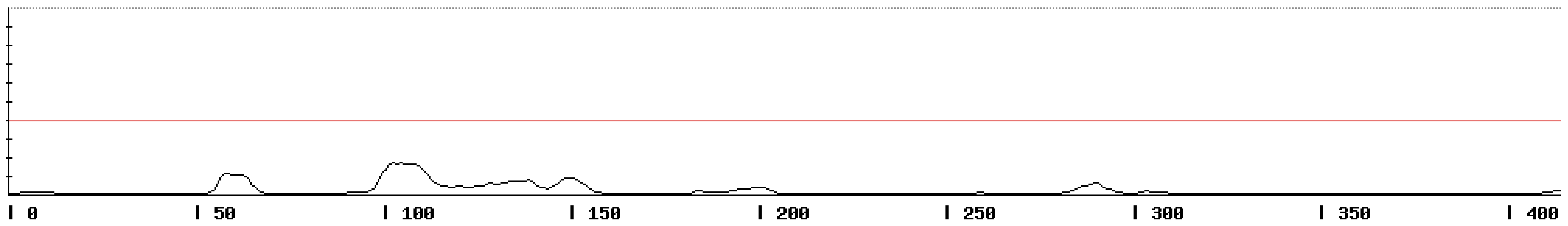

Predictions for Oropetiumthomaeum_Oropetium_20150105_13648A

Viterbi Path

Viterbi Path

Posterior @ 0.4

Posterior @ 0.4

Posterior Graphic. Threshold Marked @ 0.4

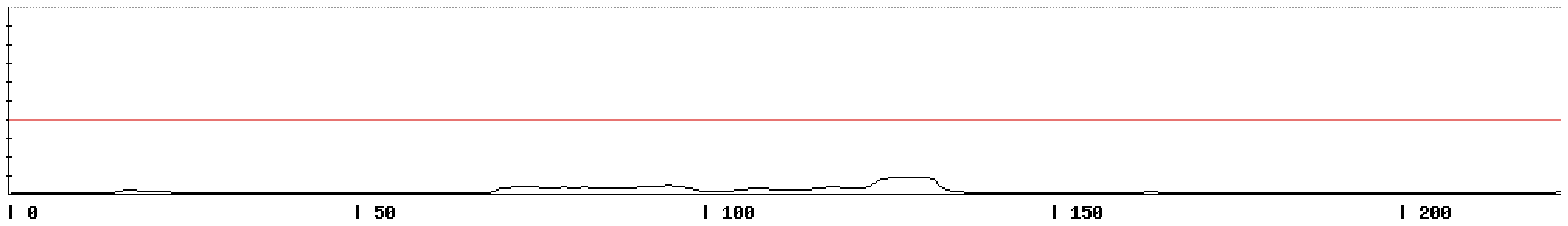

Predictions for Oropetiumthomaeum_Oropetium_20150105_13728A

Viterbi Path

Viterbi Path

Posterior @ 0.4	
Posterior @ 0.4	
Posterior Graphic. Threshold Marked @ 0.4	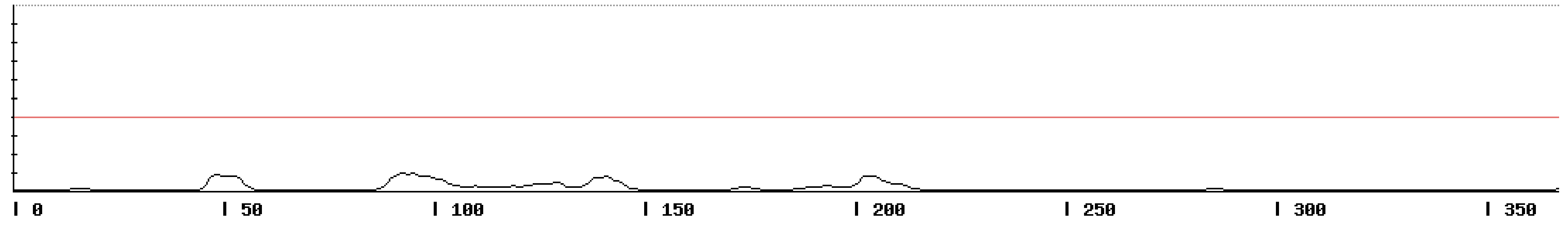

Predictions for Oropetiumthomaeum_Oropetium_20150105_13739A

Viterbi Path	
Viterbi Path	
Posterior @ 0.4	208 - KKHHHK - 213
Posterior @ 0.4	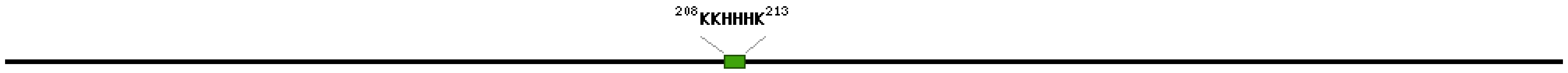
Posterior Graphic. Threshold Marked @ 0.4	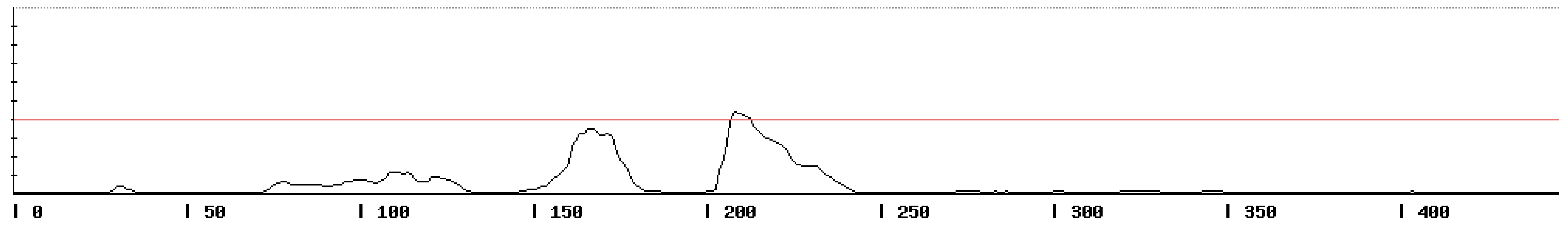

Predictions for Oropetiumthomaeum_Oropetium_20150105_14460A

Viterbi Path

Viterbi Path

Posterior @ 0.4

Posterior @ 0.4

Posterior Graphic. Threshold Marked @ 0.4

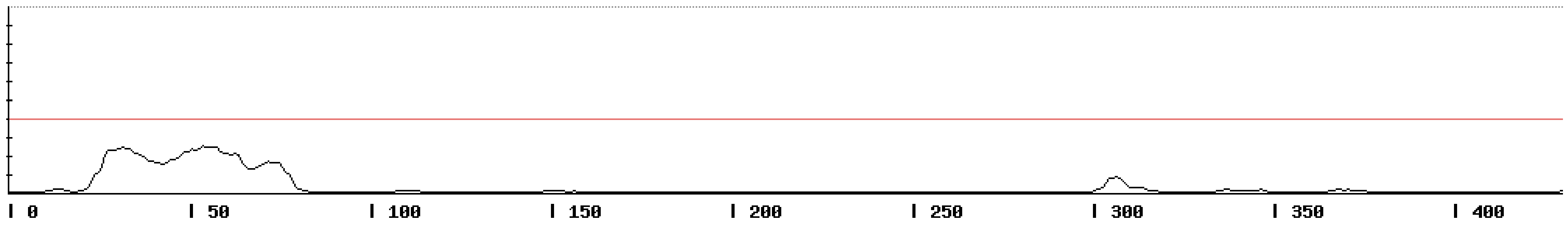

Predictions for Oropetiumthomaeum_Oropetium_20150105_14658A

Viterbi Path

Viterbi Path

Posterior @ 0.4

119 - KRALAFAPRRRPGKKKQ - 135

Posterior @ 0.4

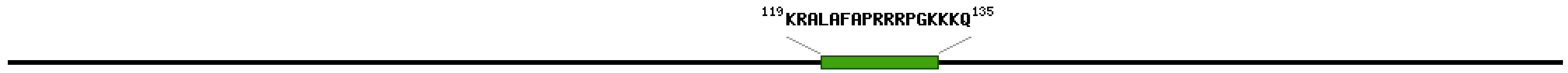

Posterior Graphic. Threshold Marked @ 0.4

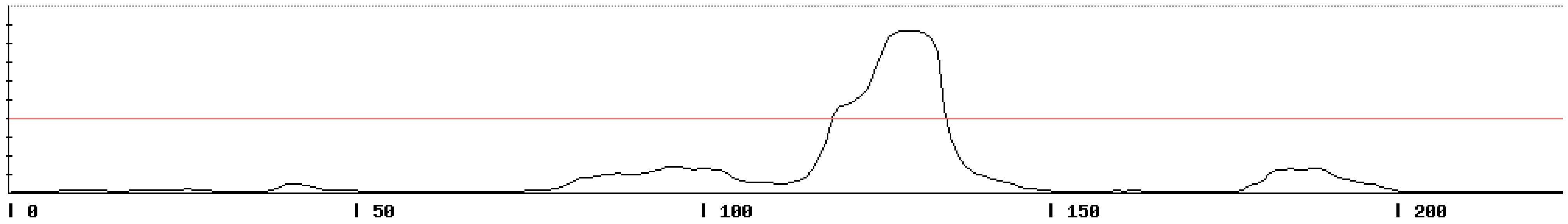

Predictions for Oropetiumthomaeum_Oropetium_20150105_14820A

Viterbi Path

Viterbi Path

Posterior @ 0.4

Posterior @ 0.4

Posterior Graphic. Threshold Marked @ 0.4

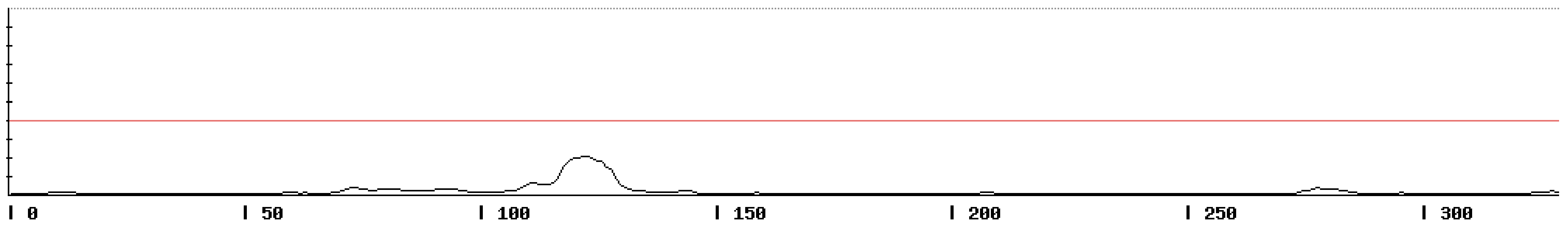

Predictions for Oropetiumthomaeum_Oropetium_20150105_14849A

Viterbi Path

Viterbi Path

Posterior @ 0.4

Posterior @ 0.4

Posterior Graphic. Threshold Marked @ 0.4

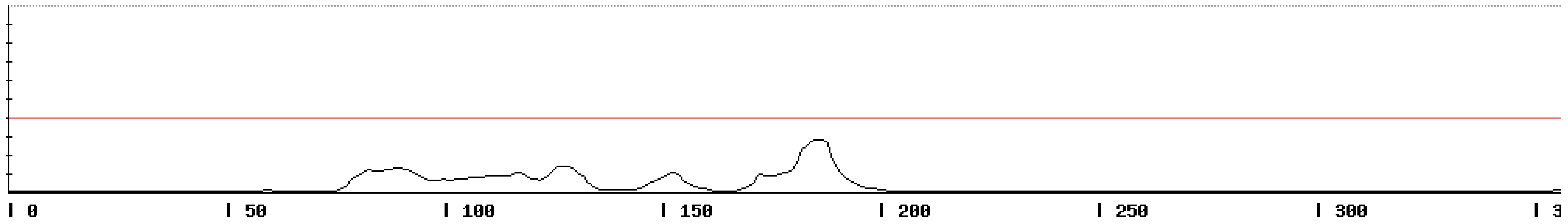

Predictions for Oropetiumthomaeum_Oropetium_20150105_14882A

Viterbi Path

Viterbi Path

Posterior @ 0.4

Posterior @ 0.4

Posterior Graphic. Threshold Marked @ 0.4

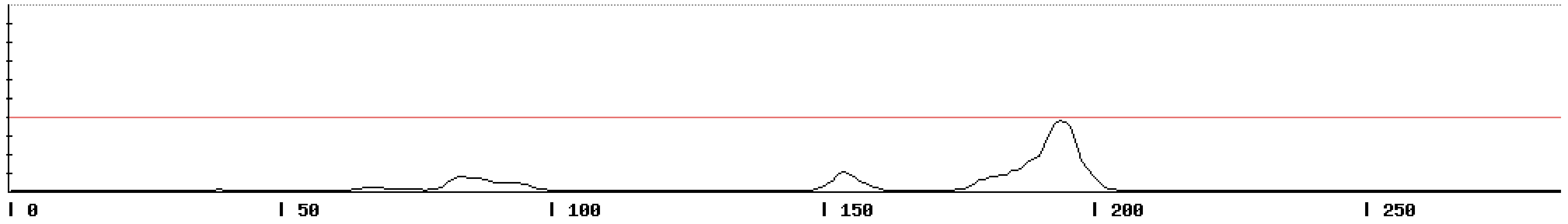

Predictions for Oropetiumthomaeum_Oropetium_20150105_14895A

Viterbi Path

Viterbi Path

Posterior @ 0.4

Posterior @ 0.4

Posterior Graphic. Threshold Marked @ 0.4

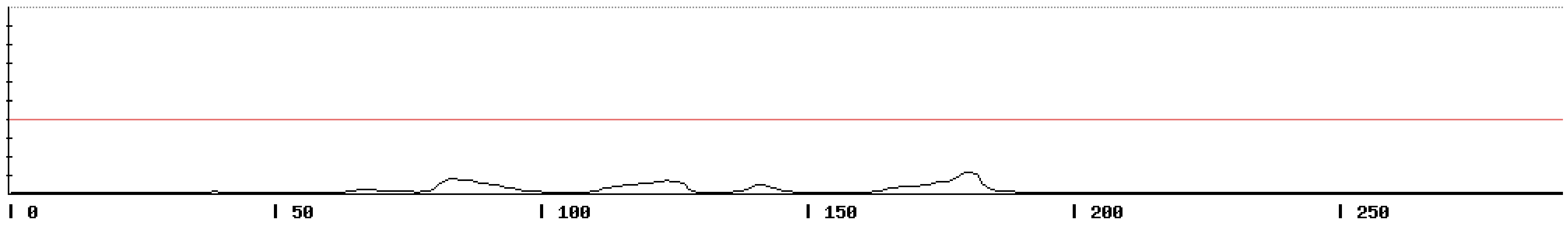

Predictions for Oropetiumthomaeum_Oropetium_20150105_15017A

Viterbi Path	
Viterbi Path	
Posterior @ 0.4	
Posterior @ 0.4	
Posterior Graphic. Threshold Marked @ 0.4	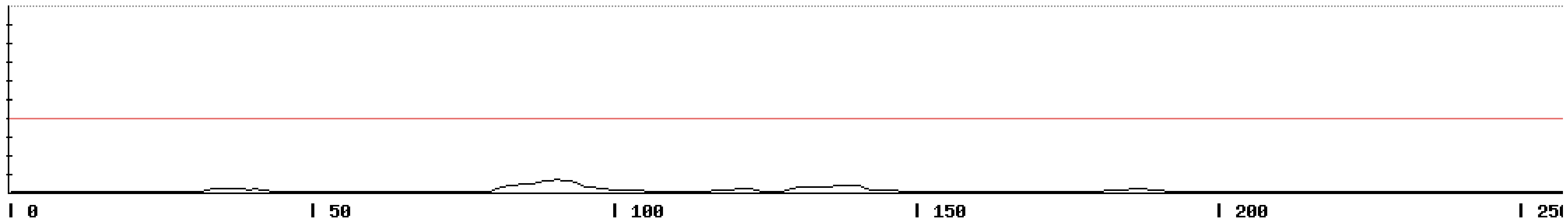

Predictions for Oropetiumthomaeum_Oropetium_20150105_15111A

Viterbi Path	
Viterbi Path	
Posterior @ 0.4	79 - RWKAGNKRKKFSRRVEGGGAWRGIGTSKP - 107
Posterior @ 0.4	

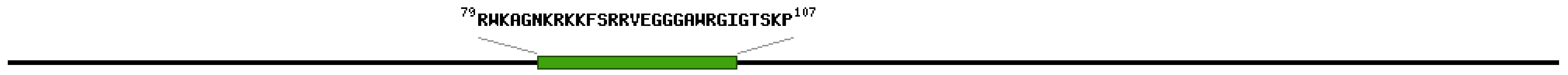

Posterior Graphic. Threshold Marked @ 0.4

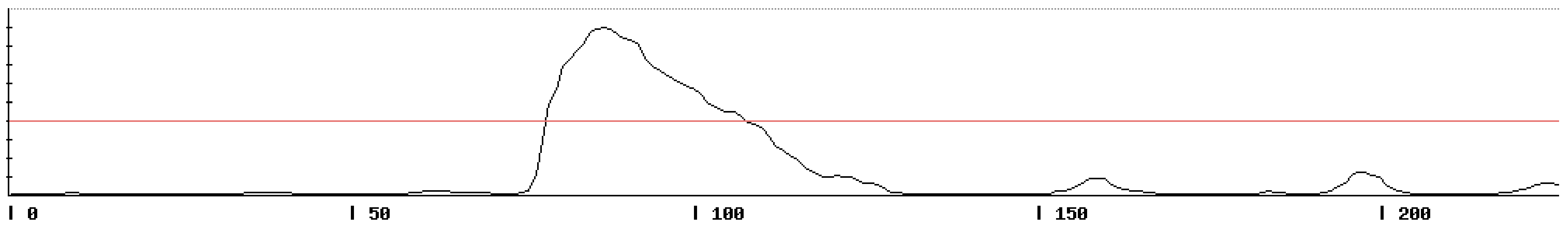

Predictions for Oropetiumthomaeum_Oropetium_20150105_15183A

Viterbi Path

Viterbi Path

Posterior @ 0.4

Posterior @ 0.4

Posterior Graphic. Threshold Marked @ 0.4

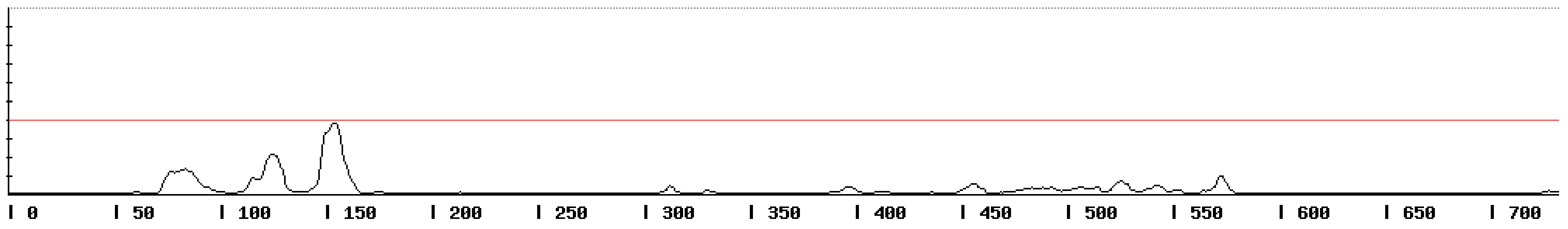

Predictions for Oropetiumthomaeum_Oropetium_20150105_15601A

Viterbi Path

Viterbi Path

Posterior @ 0.4	
Posterior @ 0.4	
Posterior Graphic. Threshold Marked @ 0.4	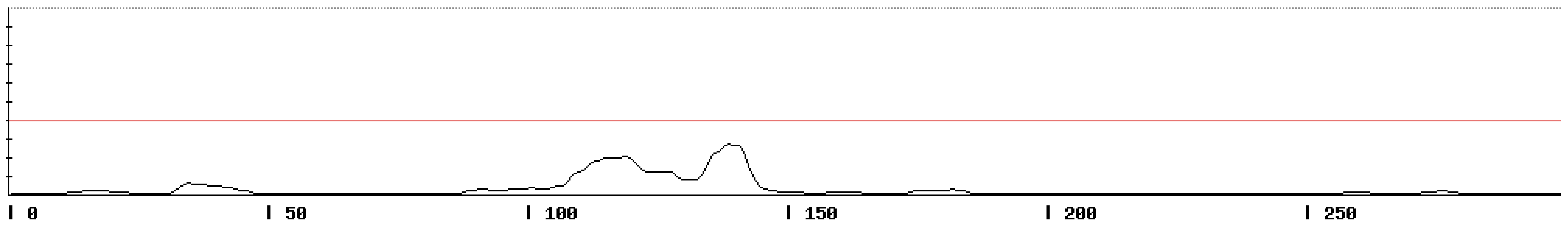

Predictions for Oropetiumthomaeum_Oropetium_20150105_16055A	
Viterbi Path	
Viterbi Path	
Posterior @ 0.4	
Posterior @ 0.4	
Posterior Graphic. Threshold Marked @ 0.4	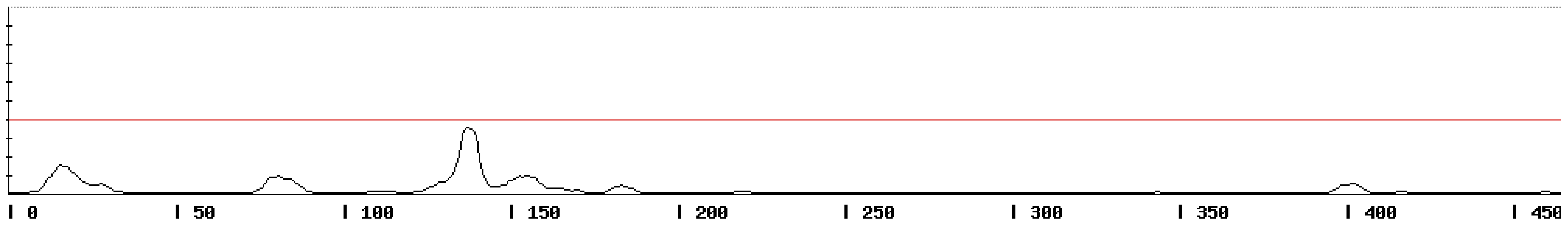

Predictions for Oropetiumthomaeum_Oropetium_20150105_16247A	
Viterbi Path	
Viterbi Path	

Posterior @ 0.4 | 81 - RRRGRP - 86

Posterior @ 0.4

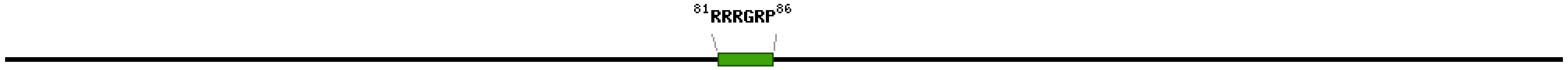

Posterior Graphic. Threshold Marked @ 0.4

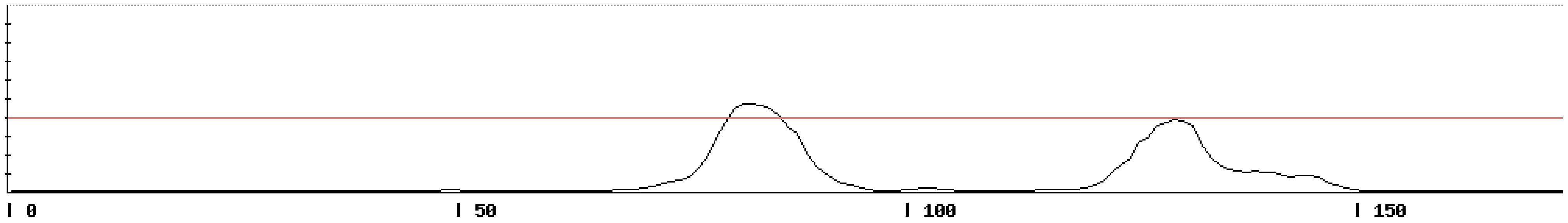

Predictions for Oropetiumthomaeum_Oropetium_20150105_16248A

Viterbi Path

Viterbi Path

Posterior @ 0.4

662 - KGRRVKTAMEKRSD - 675

678 - QPKKK - 682

699 - LEKRKSKV - 706

Posterior @ 0.4

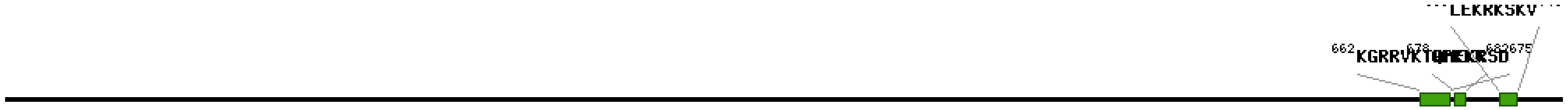

Posterior Graphic. Threshold Marked @ 0.4

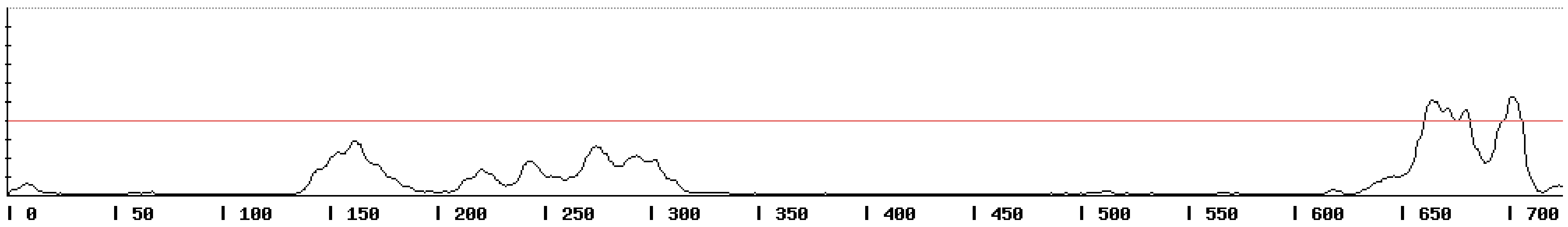

Predictions for Oropetiumthomaeum_Oropetium_20150105_16427A

Viterbi Path

Viterbi Path

Posterior @ 0.4

Posterior @ 0.4

Posterior Graphic. Threshold Marked @ 0.4

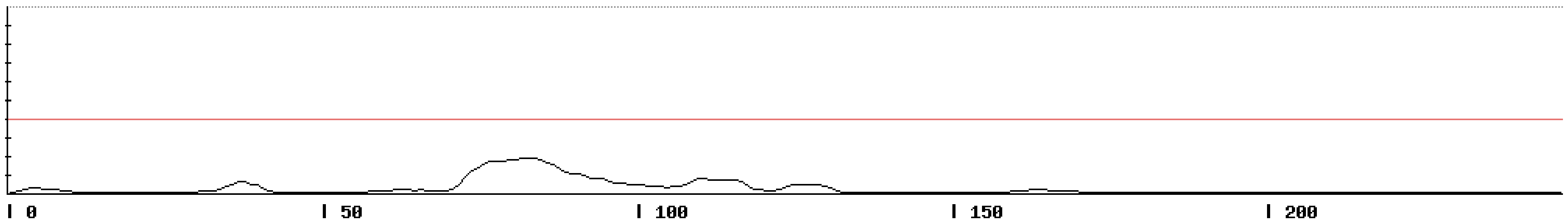

Predictions for Oropetiumthomaeum_Oropetium_20150105_16555A

Viterbi Path

Viterbi Path

Posterior @ 0.4

Posterior @ 0.4

Posterior Graphic. Threshold Marked @ 0.4	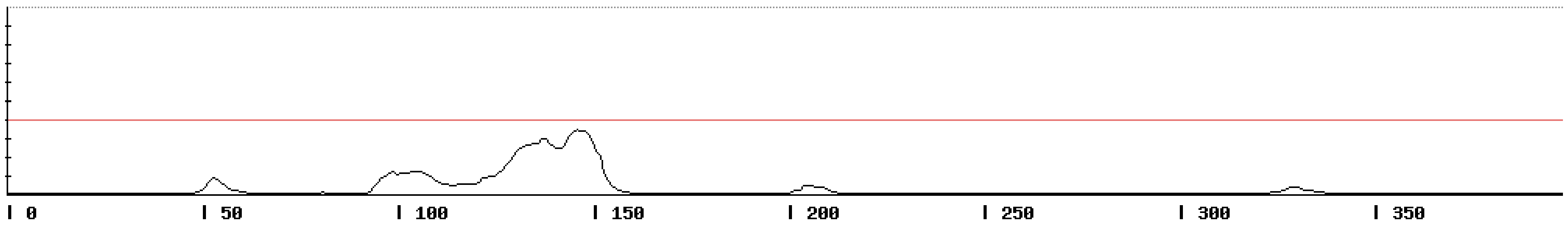

Predictions for Oropetiumthomaeum_Oropetium_20150105_16831A	
Viterbi Path	
Viterbi Path	
Posterior @ 0.4	65 - FHRGRAPRGRR - 75
Posterior @ 0.4	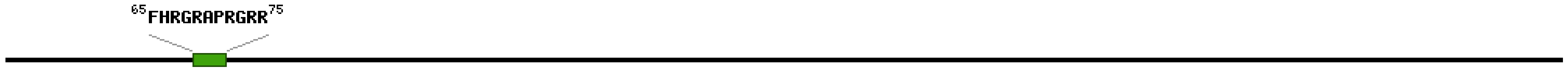
Posterior Graphic. Threshold Marked @ 0.4	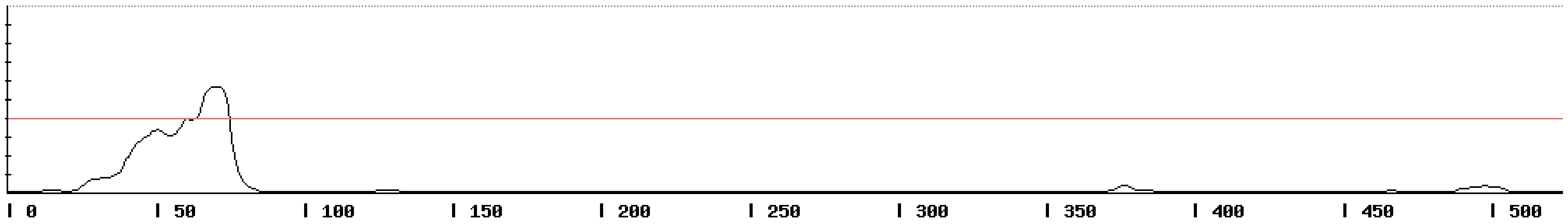

Predictions for Oropetiumthomaeum_Oropetium_20150105_16860A	
Viterbi Path	
Viterbi Path	
Posterior @ 0.4	
Posterior @ 0.4	

Posterior Graphic. Threshold Marked @ 0.4

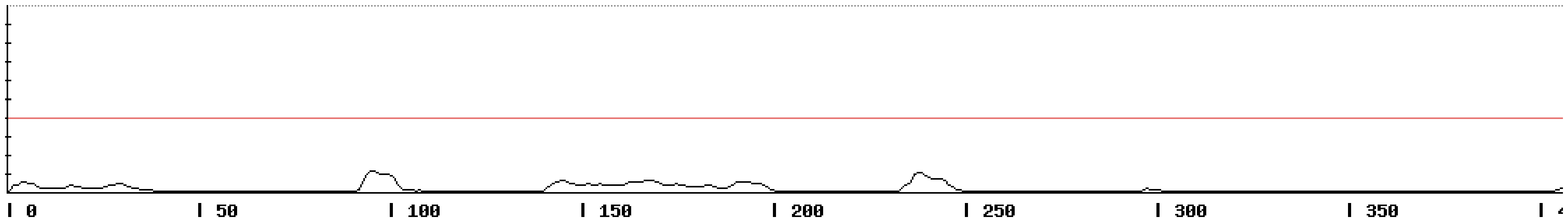

Predictions for Oropetiumthomaeum_Oropetium_20150105_17005A

Viterbi Path

Viterbi Path

Posterior @ 0.4

Posterior @ 0.4

Posterior Graphic. Threshold Marked @ 0.4

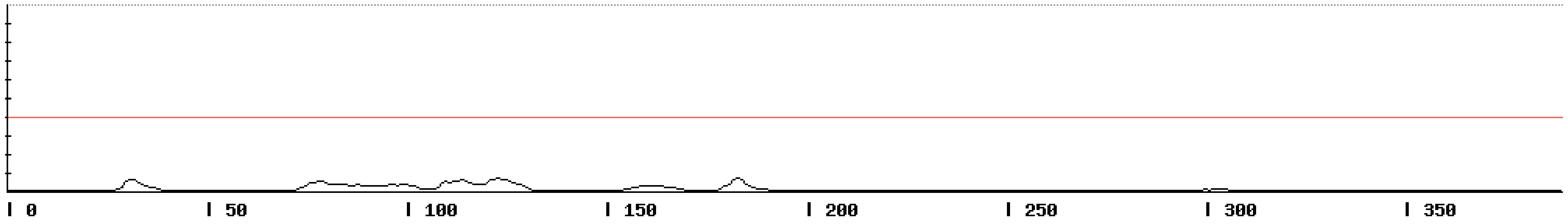

Predictions for Oropetiumthomaeum_Oropetium_20150105_17047A

Viterbi Path

Viterbi Path

Posterior @ 0.4

Posterior @ 0.4

Posterior Graphic. Threshold Marked @ 0.4

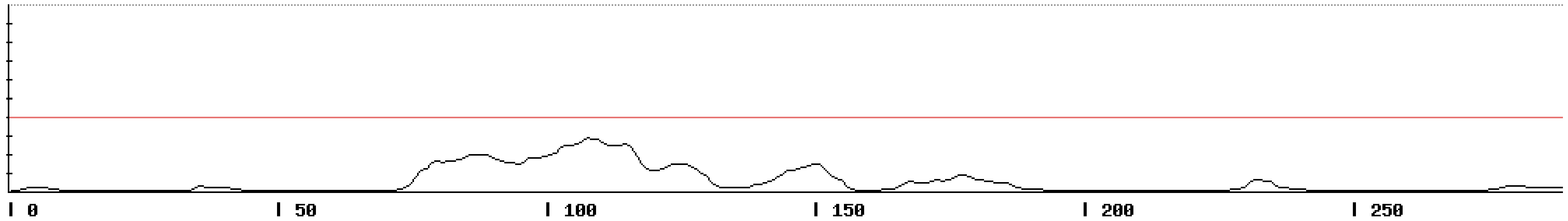

Predictions for Oropetiumthomaeum_Oropetium_20150105_17636A

Viterbi Path

Viterbi Path

Posterior @ 0.4 — 42 - RKRRK - 46

Posterior @ 0.4

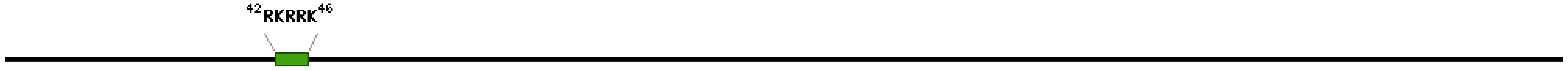

Posterior Graphic. Threshold Marked @ 0.4

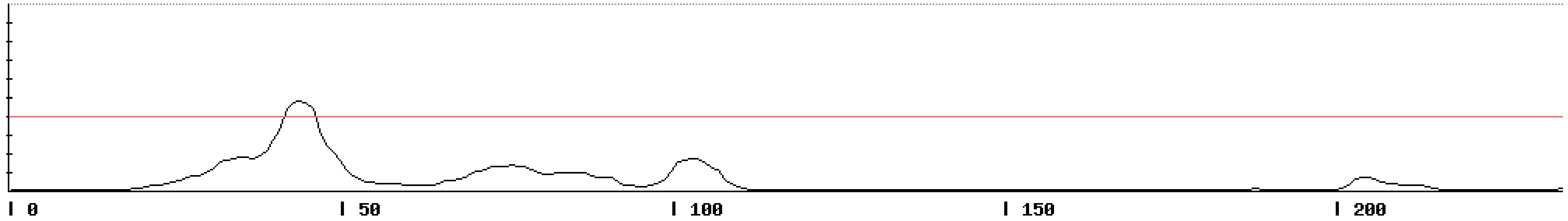

Predictions for Oropetiumthomaeum_Oropetium_20150105_17725A

Viterbi Path

Viterbi Path

Posterior @ 0.4

Posterior @ 0.4

Posterior Graphic. Threshold Marked @ 0.4

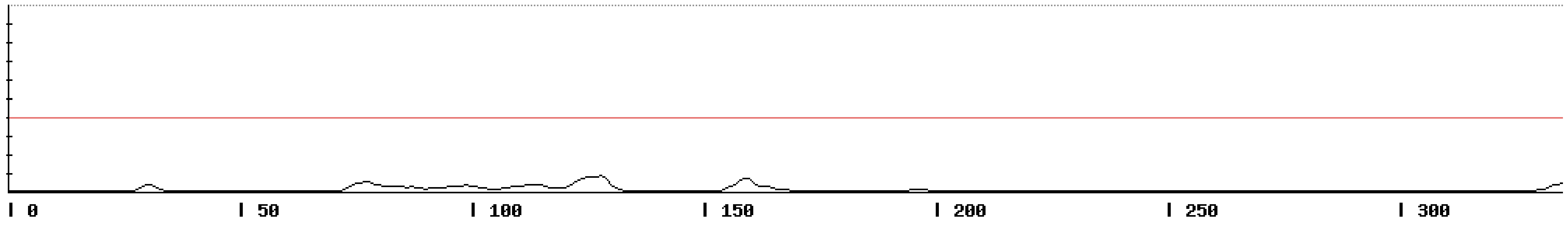

Predictions for Oropetiumthomaeum_Oropetium_20150105_18218A

Viterbi Path

Viterbi Path

Posterior @ 0.4

Posterior @ 0.4

Posterior Graphic. Threshold Marked @ 0.4

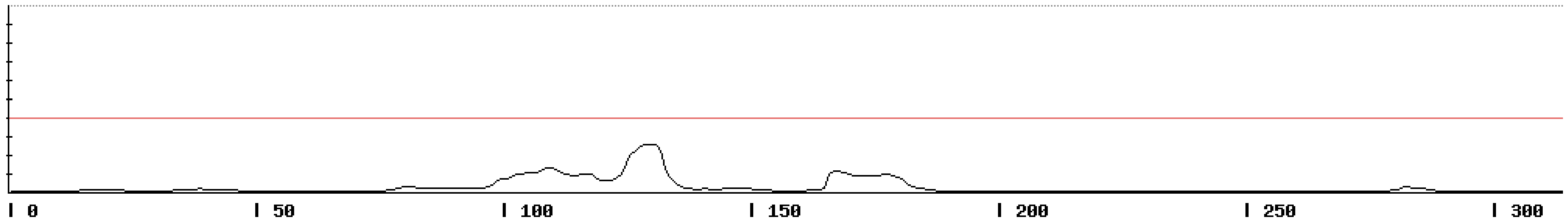

Predictions for Oropetiumthomaeum_Oropetium_20150105_18674A

Viterbi Path

Viterbi Path

Posterior @ 0.4

Posterior @ 0.4

Posterior Graphic. Threshold Marked @ 0.4

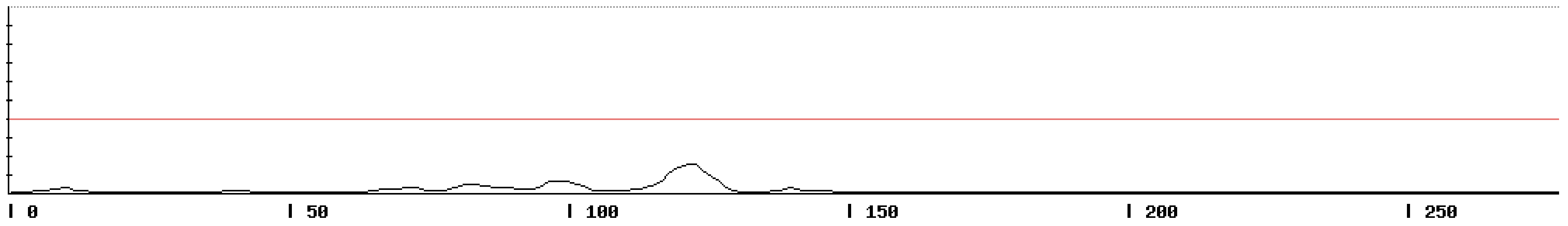

Predictions for Oropetiumthomaeum_Oropetium_20150105_19054A

Viterbi Path

Viterbi Path

Posterior @ 0.4

Posterior @ 0.4

Posterior Graphic. Threshold Marked @ 0.4

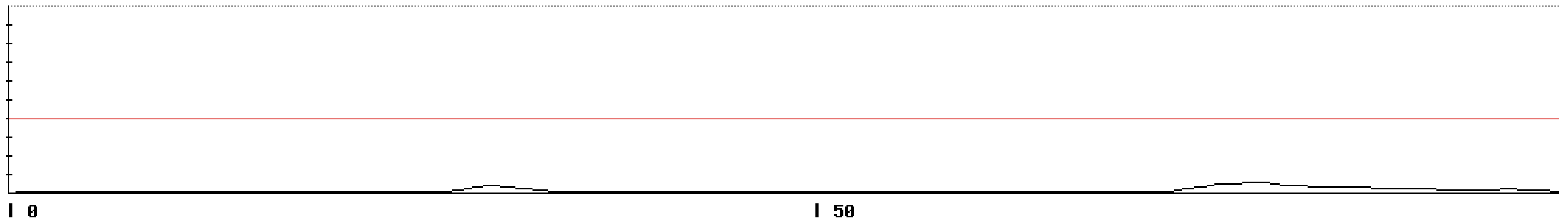

Predictions for Oropetiumthomaeum_Oropetium_20150105_19870A

Viterbi Path	
Viterbi Path	
Posterior @ 0.4	
Posterior @ 0.4	
Posterior Graphic. Threshold Marked @ 0.4	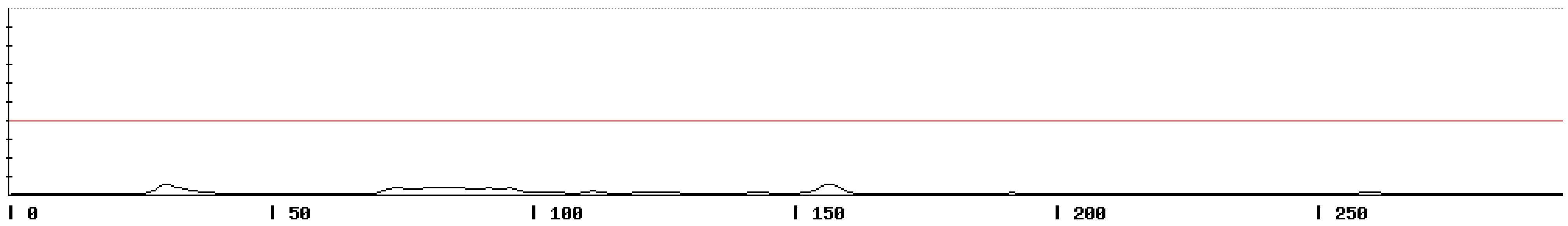

Predictions for Oropetiumthomaeum_Oropetium_20150105_20110A

Viterbi Path	
Viterbi Path	
Posterior @ 0.4	
Posterior @ 0.4	
Posterior Graphic. Threshold Marked @ 0.4	

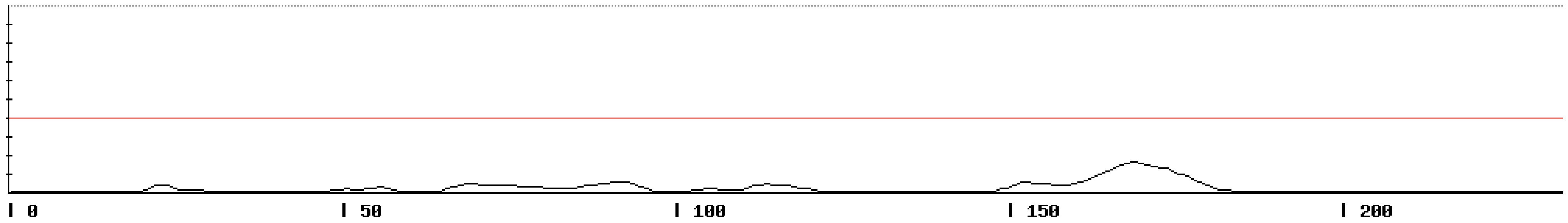

Predictions for Oropetiumthomaeum_Oropetium_20150105_20691A

Viterbi Path	
Viterbi Path	
Posterior @ 0.4	
Posterior @ 0.4	
Posterior Graphic. Threshold Marked @ 0.4	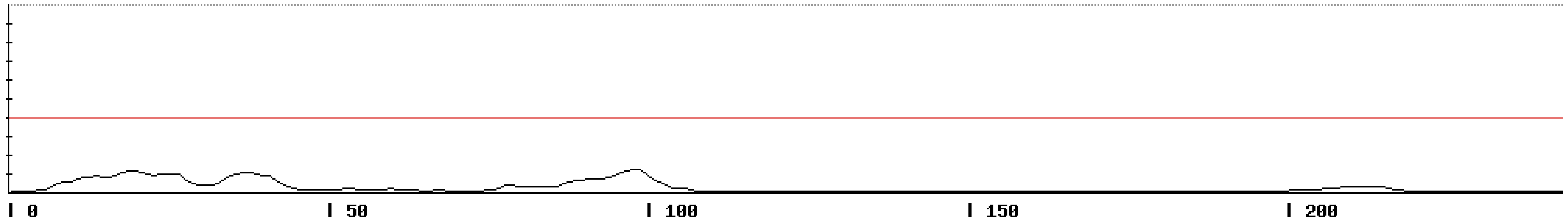

Predictions for Oropetiumthomaeum_Oropetium_20150105_20800A

Viterbi Path	
Viterbi Path	
Posterior @ 0.4	
Posterior @ 0.4	

Posterior Graphic. Threshold Marked @ 0.4	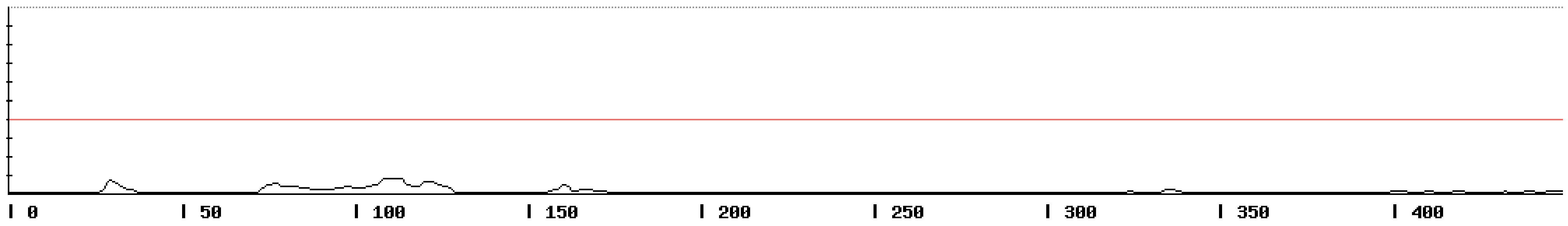

Predictions for Oropetiumthomaeum_Oropetium_20150105_20851A	
Viterbi Path	
Viterbi Path	
Posterior @ 0.4	
Posterior @ 0.4	
Posterior Graphic. Threshold Marked @ 0.4	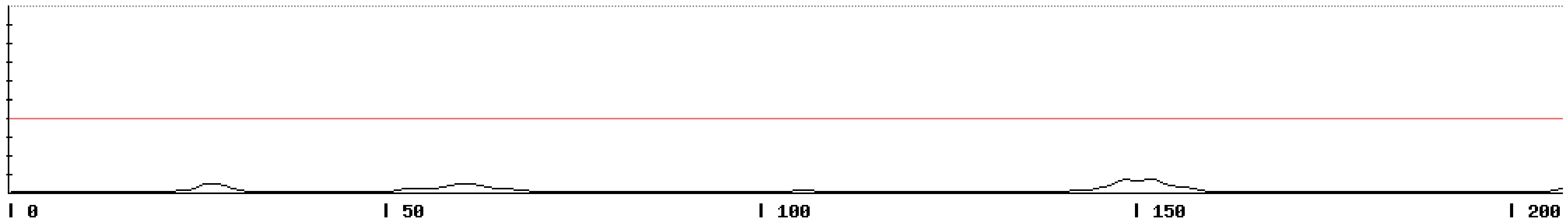

Predictions for Oropetiumthomaeum_Oropetium_20150105_21393A	
Viterbi Path	
Viterbi Path	
Posterior @ 0.4	90 - KATGKDRKVNSHRRA - 104
Posterior @ 0.4	

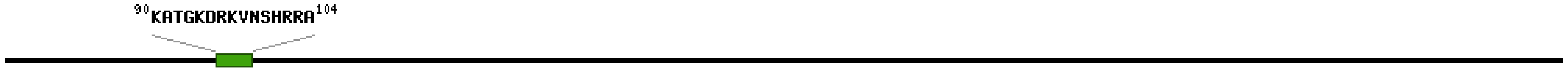

Posterior Graphic. Threshold Marked @ 0.4

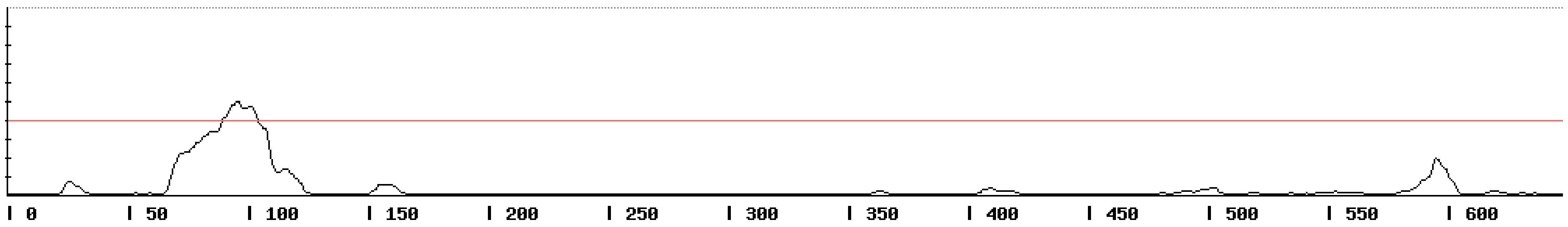

Predictions for Oropetiumthomaeum_Oropetium_20150105_21506A

Viterbi Path

Viterbi Path

Posterior @ 0.4

Posterior @ 0.4

Posterior Graphic. Threshold Marked @ 0.4

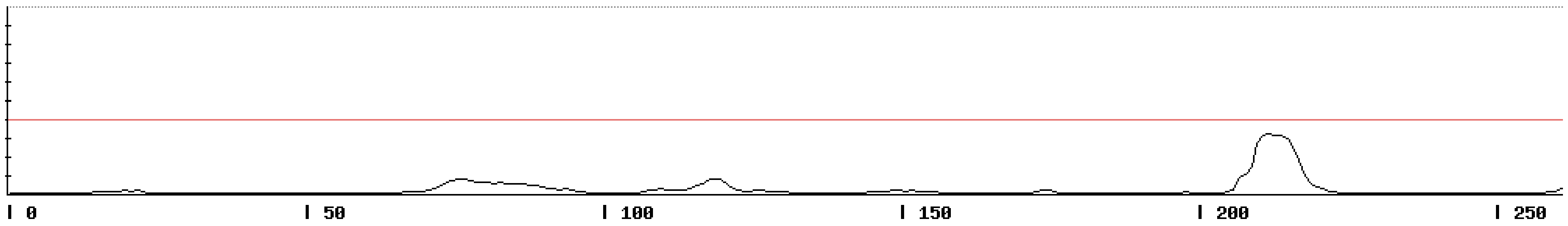

Predictions for Oropetiumthomaeum_Oropetium_20150105_21681A

Viterbi Path

Viterbi Path

Posterior @ 0.4

Posterior @ 0.4

Posterior Graphic. Threshold Marked @ 0.4

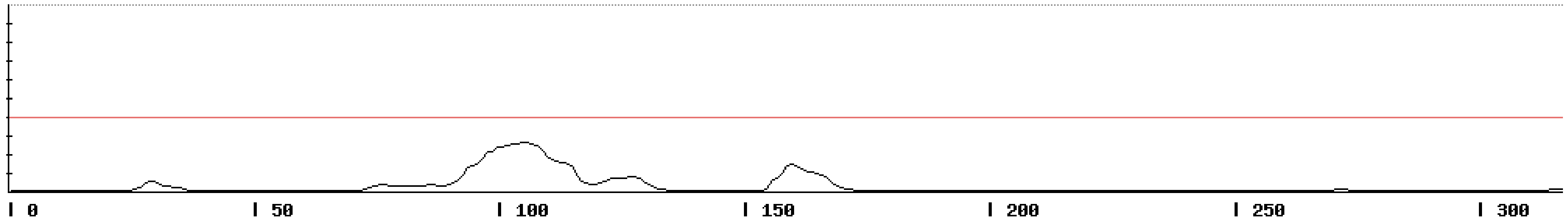

Predictions for Oropetiumthomaeum_Oropetium_20150105_22096A

Viterbi Path

Viterbi Path

Posterior @ 0.4

Posterior @ 0.4

Posterior Graphic. Threshold Marked @ 0.4

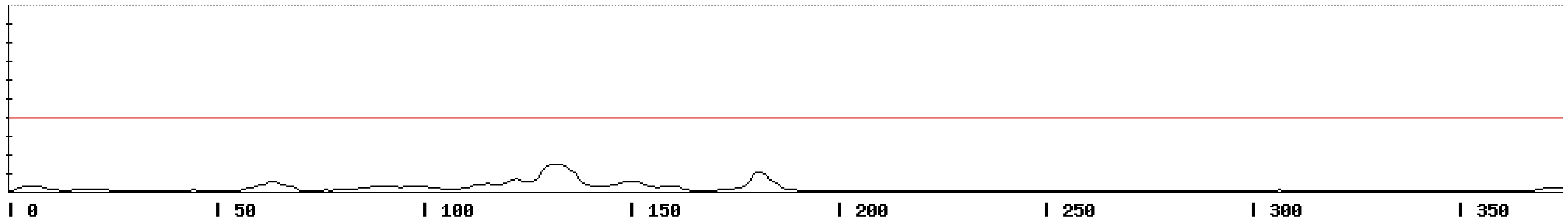

Predictions for Oropetiumthomaeum_Oropetium_20150105_22098A

Viterbi Path

Viterbi Path

Posterior @ 0.4

Posterior @ 0.4

Posterior Graphic. Threshold Marked @ 0.4

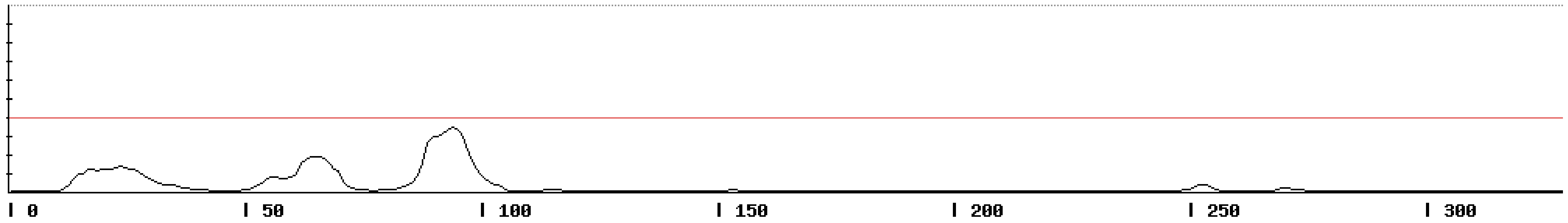

Predictions for Oropetiumthomaeum_Oropetium_20150105_22454A

Viterbi Path

Viterbi Path

Posterior @ 0.4

47 - ESNPSKRKPIRRR - 59

Posterior @ 0.4

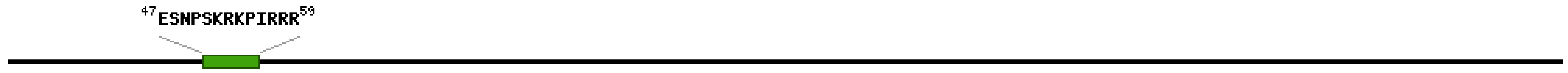

Posterior Graphic. Threshold Marked @ 0.4

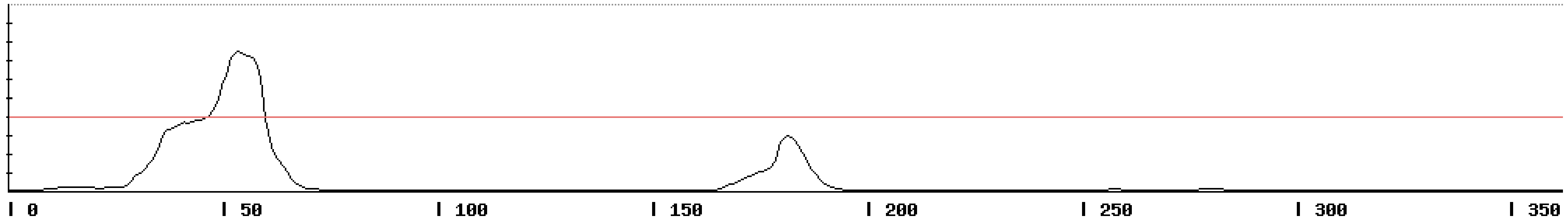

Predictions for Oropetiumthomaeum_Oropetium_20150105_22455A

Viterbi Path	
Viterbi Path	
Posterior @ 0.4	359 - PRKRKRA - 365
Posterior @ 0.4	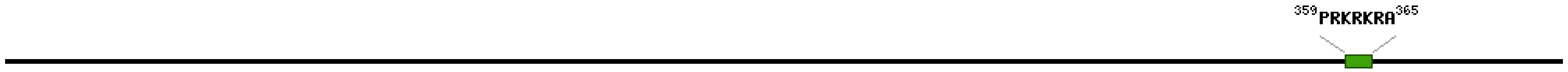
Posterior Graphic. Threshold Marked @ 0.4	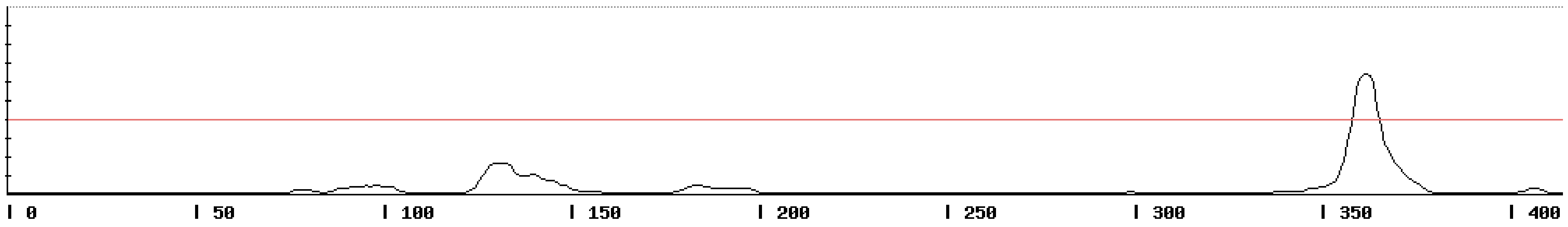

Predictions for Oropetiumthomaeum_Oropetium_20150105_22456A

Viterbi Path	
Viterbi Path	
Posterior @ 0.4	
Posterior @ 0.4	
Posterior Graphic. Threshold Marked @ 0.4	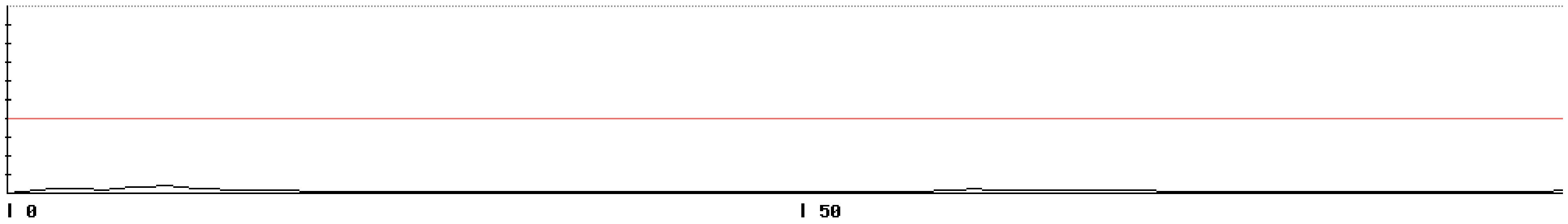

Predictions for Oropetiumthomaeum_Oropetium_20150105_22457A	
Viterbi Path	
Viterbi Path	
Posterior @ 0.4	237 - RLRARGGRRGQRR - 249
Posterior @ 0.4	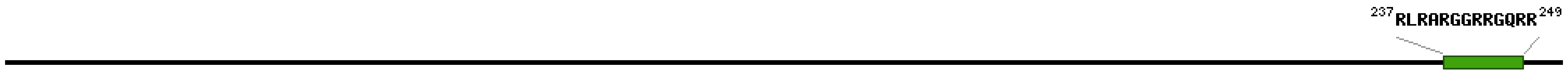
Posterior Graphic. Threshold Marked @ 0.4	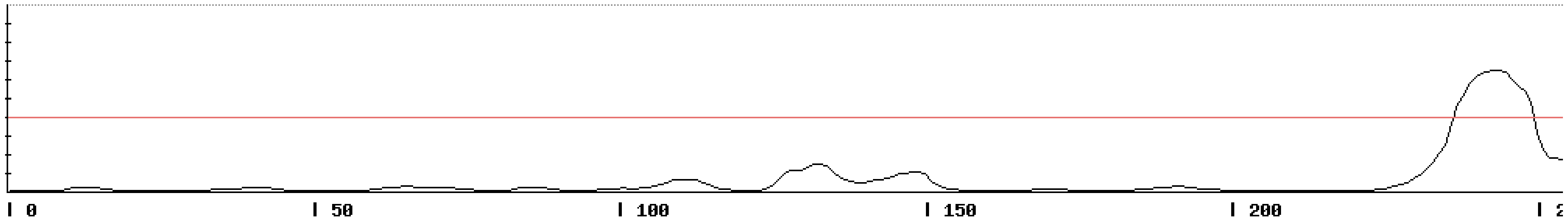

Predictions for Oropetiumthomaeum_Oropetium_20150105_22509A	
Viterbi Path	
Viterbi Path	
Posterior @ 0.4	
Posterior @ 0.4	
Posterior Graphic. Threshold Marked @ 0.4	

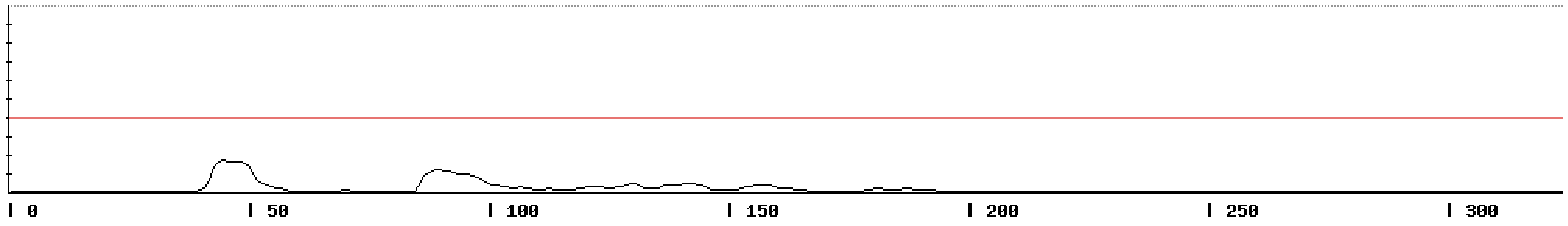

Predictions for Oropetiumthomaeum_Oropetium_20150105_22595A

Viterbi Path	
Viterbi Path	
Posterior @ 0.4	272 - GRRRSKKARA - 281
Posterior @ 0.4	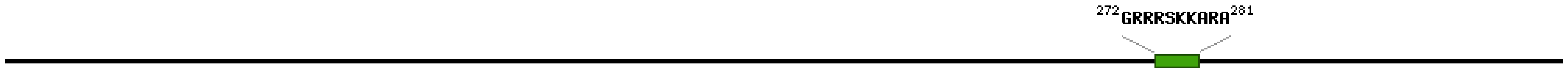
Posterior Graphic. Threshold Marked @ 0.4	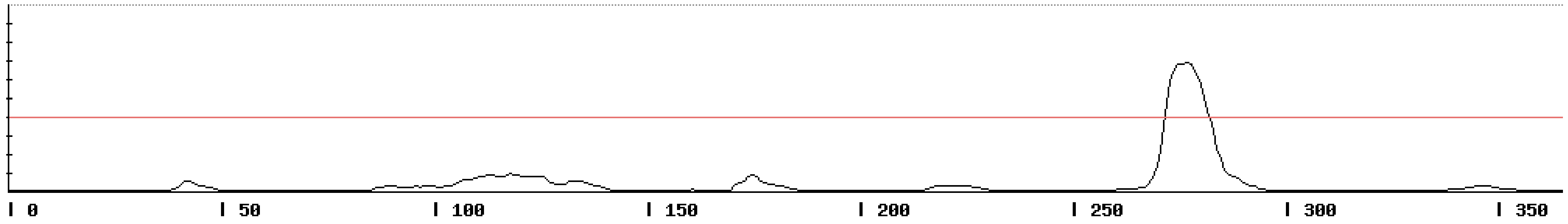

Predictions for Oropetiumthomaeum_Oropetium_20150105_22775A

Viterbi Path	
Viterbi Path	
Posterior @ 0.4	
Posterior @ 0.4	

Posterior Graphic. Threshold Marked @ 0.4

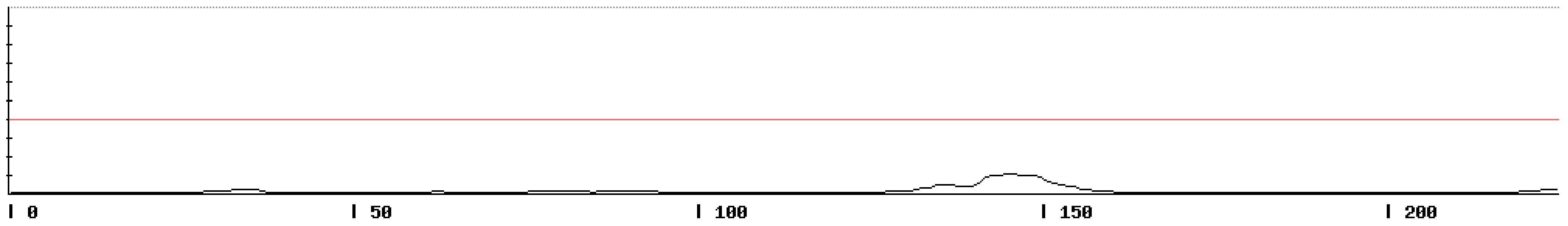

Predictions for Oropetiumthomaeum_Oropetium_20150105_22789A

Viterbi Path

Viterbi Path

Posterior @ 0.4

142 - RKRRKID - 148

156 - DKKVRWHKTGRSK - 168

176 - AKRGWKK - 182

Posterior @ 0.4

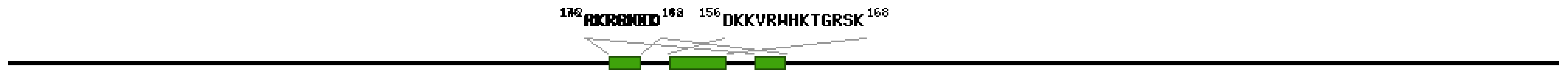

Posterior Graphic. Threshold Marked @ 0.4

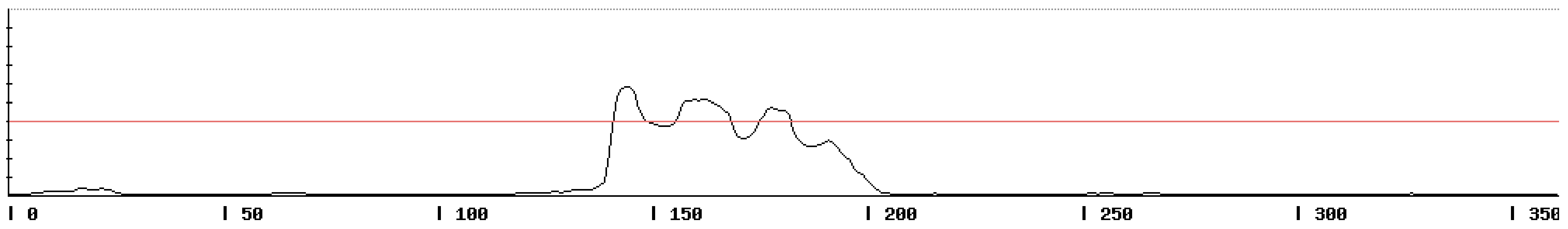

Predictions for Oropetiumthomaeum_Oropetium_20150105_23154A

Viterbi Path

Viterbi Path

Posterior @ 0.4

94 - KATGKDRVIKSKGDKRKQHIIGMKKTLVFHKGRAPKGER - 132

Posterior @ 0.4

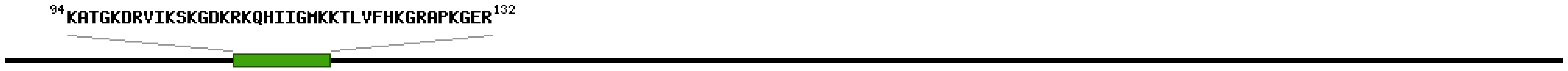

Posterior Graphic. Threshold Marked @ 0.4

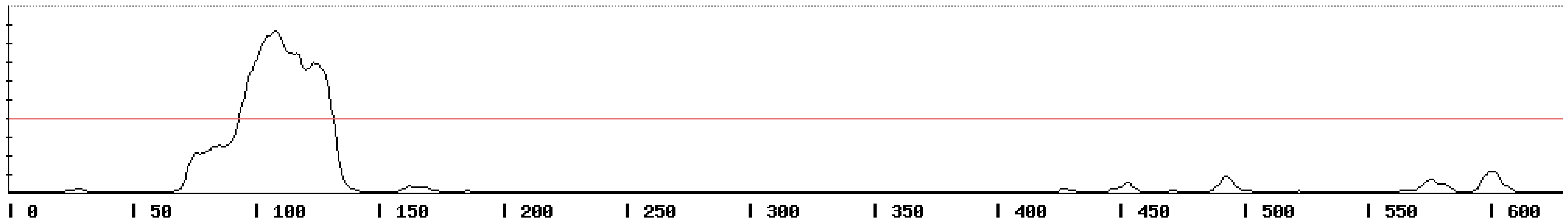

Predictions for Oropetiumthomaeum_Oropetium_20150105_23267A

Viterbi Path

Viterbi Path

Posterior @ 0.4

Posterior @ 0.4

Posterior Graphic. Threshold Marked @ 0.4

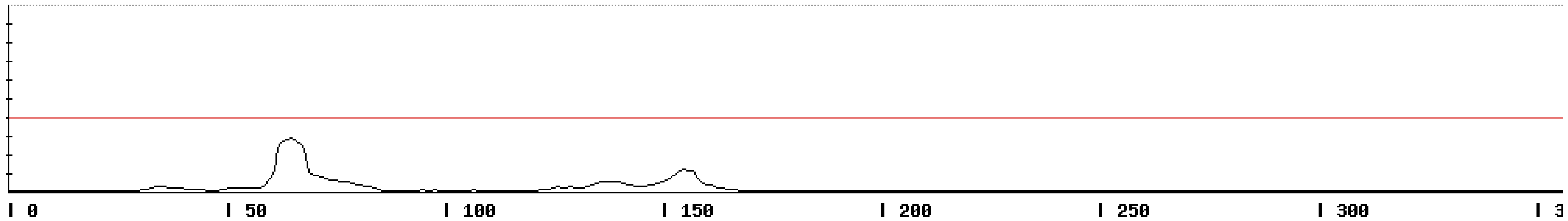

Predictions for Oropetiumthomaeum_Oropetium_20150105_24230A

Viterbi Path

Viterbi Path

Posterior @ 0.4	
Posterior @ 0.4	
Posterior Graphic. Threshold Marked @ 0.4	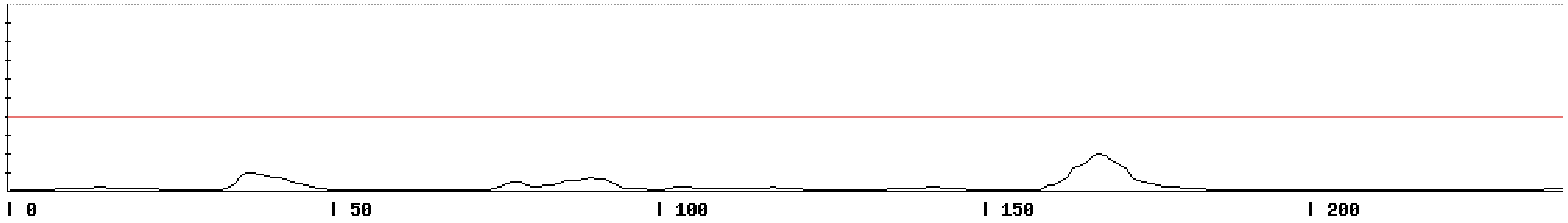

Predictions for Oropetiumthomaeum_Oropetium_20150105_24912A

Viterbi Path	
Viterbi Path	
Posterior @ 0.4	
Posterior @ 0.4	
Posterior Graphic. Threshold Marked @ 0.4	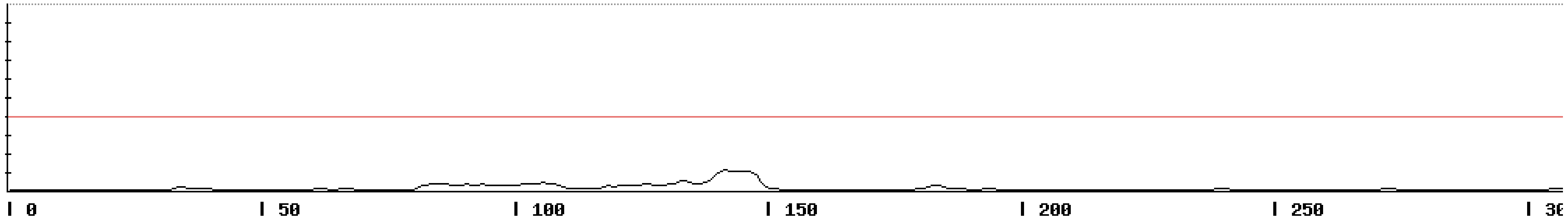

Predictions for Oropetiumthomaeum_Oropetium_20150105_24933A

Viterbi Path	
Viterbi Path	

Posterior @ 0.4

Posterior @ 0.4

Posterior Graphic. Threshold Marked @ 0.4

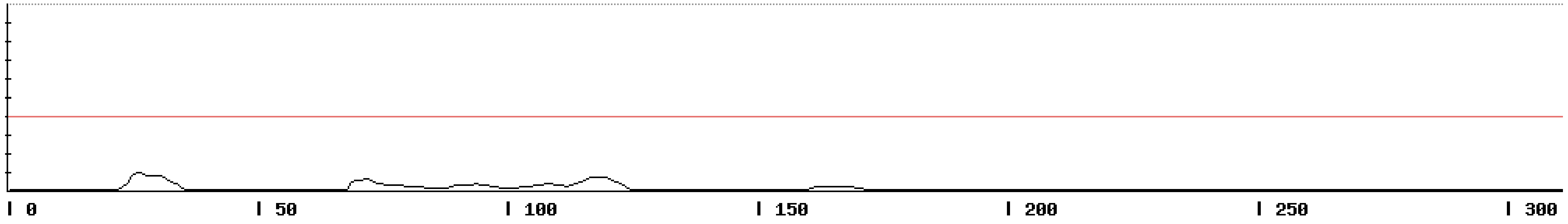

Predictions for Oropetiumthomaeum_Oropetium_20150105_26056A

Viterbi Path

Viterbi Path

Posterior @ 0.4

Posterior @ 0.4

Posterior Graphic. Threshold Marked @ 0.4

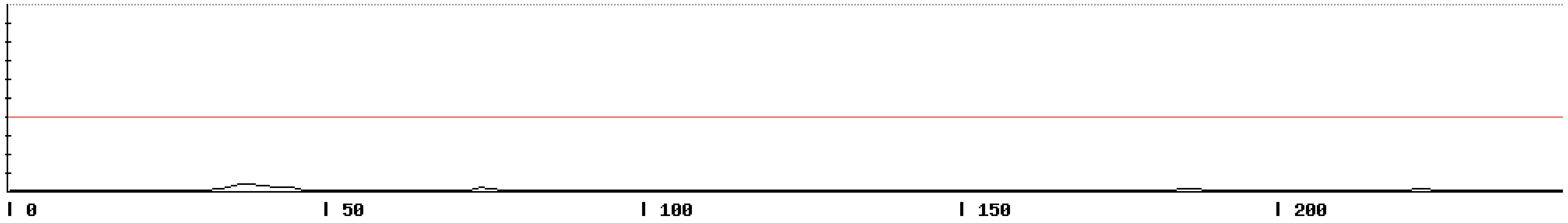

Predictions for Oropetiumthomaeum_Oropetium_20150105_26117A

Viterbi Path

Viterbi Path

Posterior @ 0.4

Posterior @ 0.4

Posterior Graphic. Threshold Marked @ 0.4

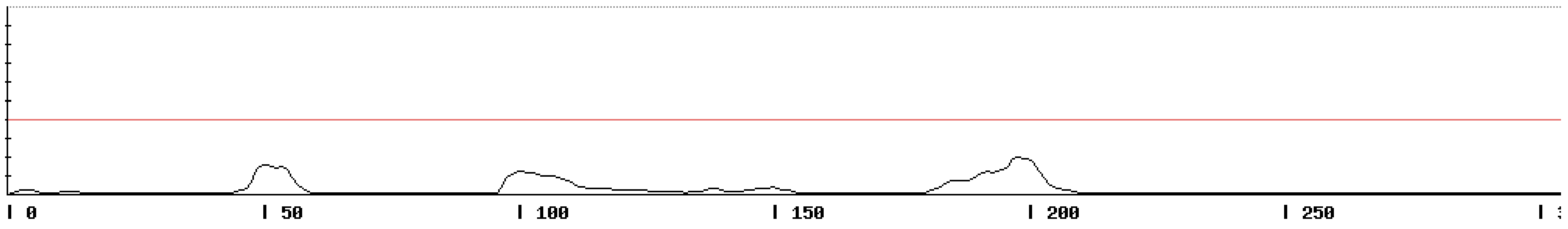

Predictions for Oropetiumthomaeum_Oropetium_20150105_26419A

Viterbi Path

Viterbi Path

Posterior @ 0.4

Posterior @ 0.4

Posterior Graphic. Threshold Marked @ 0.4

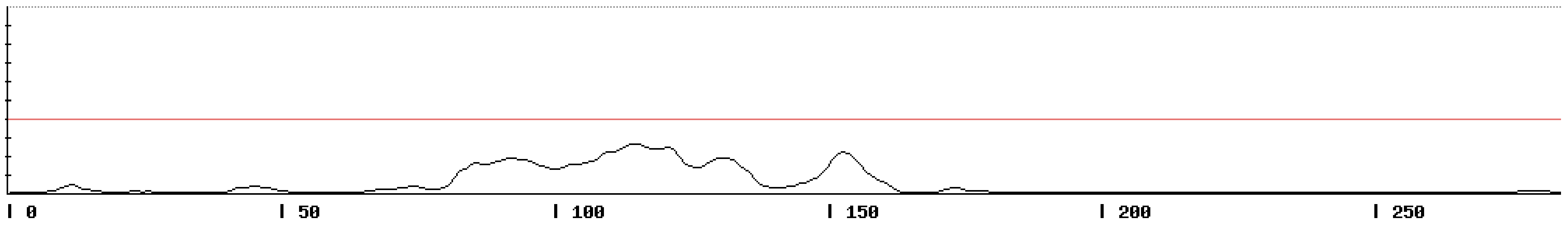

Predictions for Oropetiumthomaeum_Oropetium_20150105_26569A	
Viterbi Path	
Viterbi Path	
Posterior @ 0.4	
Posterior @ 0.4	
Posterior Graphic. Threshold Marked @ 0.4	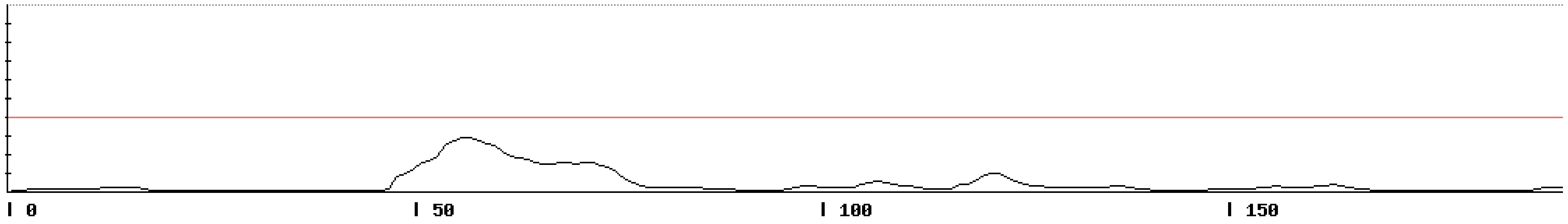

Predictions for Oropetiumthomaeum_Oropetium_20150105_26981A	
Viterbi Path	
Viterbi Path	
Posterior @ 0.4	204 - AHKKSTKR - 211
Posterior @ 0.4	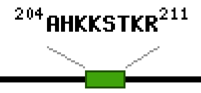
Posterior Graphic. Threshold Marked @ 0.4	

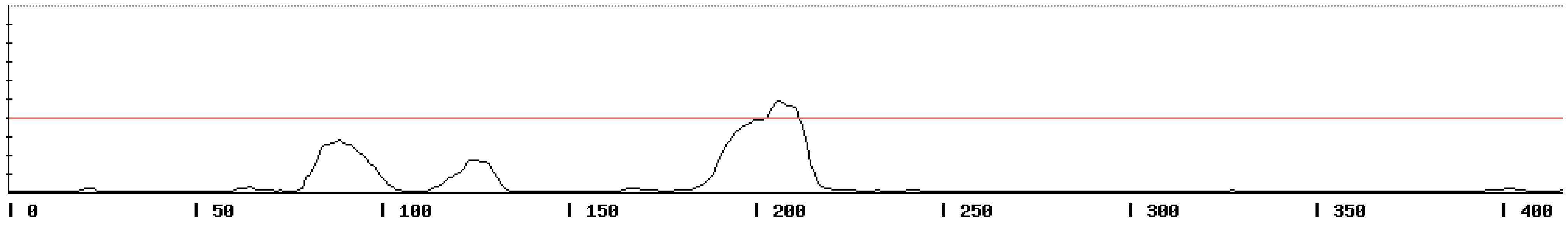

Predictions for Oropetiumthomaeum_Oropetium_20150105_26987A

Viterbi Path	
Viterbi Path	
Posterior @ 0.4	181 - PRKRI - 185
Posterior @ 0.4	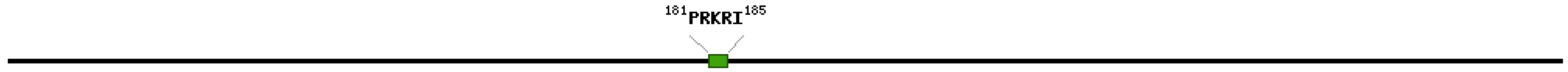
Posterior Graphic. Threshold Marked @ 0.4	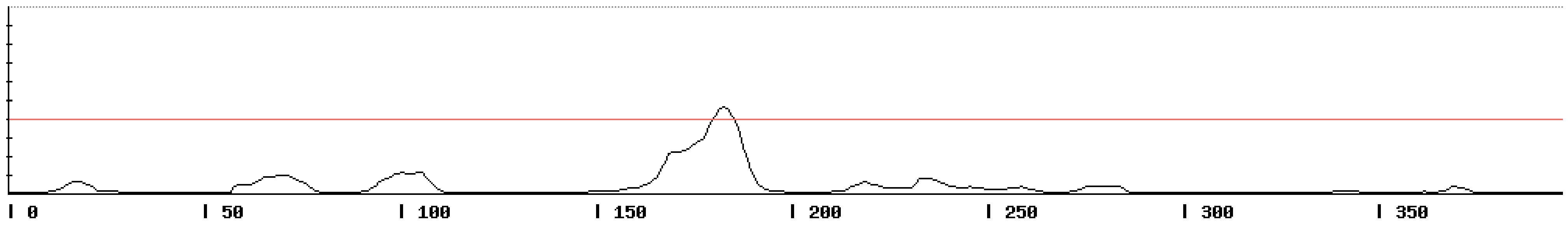

Predictions for Oropetiumthomaeum_Oropetium_20150105_26988A

Viterbi Path	
Viterbi Path	

Revision r.9 - Last Updated on : February 24 2015 17:45:20.

alex.nguyenba@utoronto.ca

Intended to be used with Firefox

Viterbi Path

Posterior @ 0.4

Posterior @ 0.4

Posterior Graphic. Threshold Marked @ 0.4

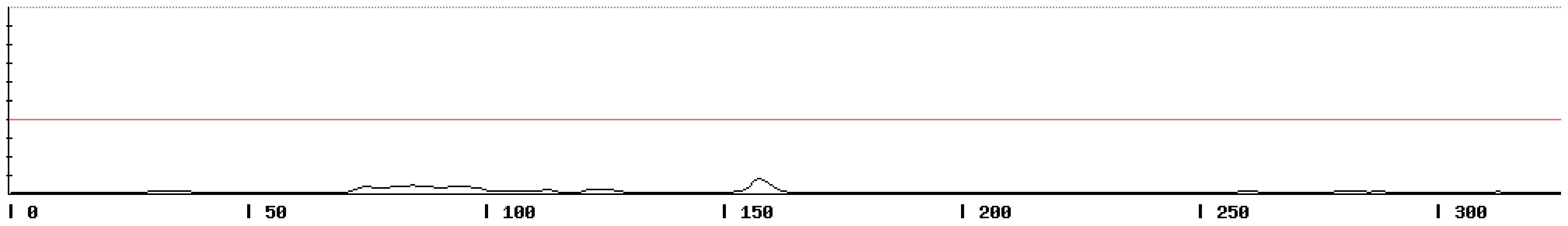

Predictions for Oryzabrachyantha_OB0134G10010.1

Viterbi Path

Viterbi Path

Posterior @ 0.4 96 - GRKRIIK - 102

Posterior @ 0.4

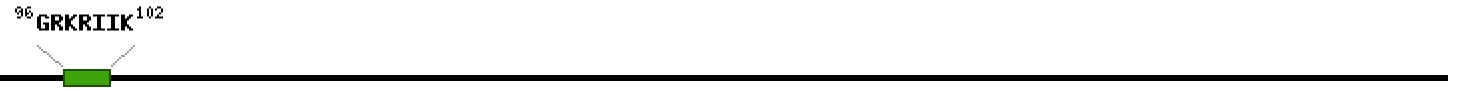

Posterior Graphic. Threshold Marked @ 0.4

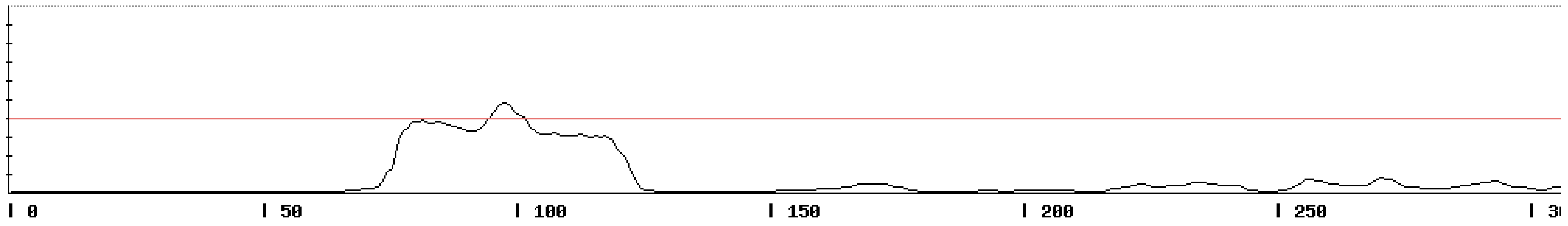

Predictions for Oryzabrachyantha_OB01G10300.1

Viterbi Path

Viterbi Path

Posterior @ 0.4

Posterior @ 0.4

Posterior Graphic. Threshold Marked @ 0.4

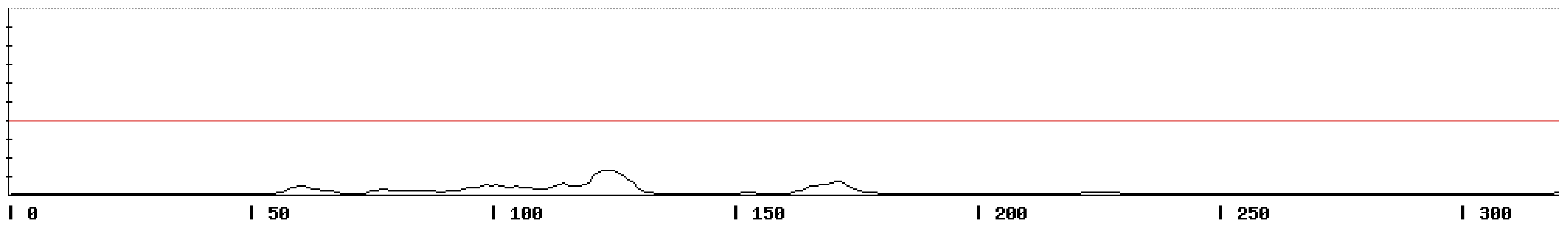

Predictions for Oryzabrachyantha_OB01G15780.1

Viterbi Path

Viterbi Path

Posterior @ 0.4

Posterior @ 0.4

Posterior Graphic. Threshold Marked @ 0.4

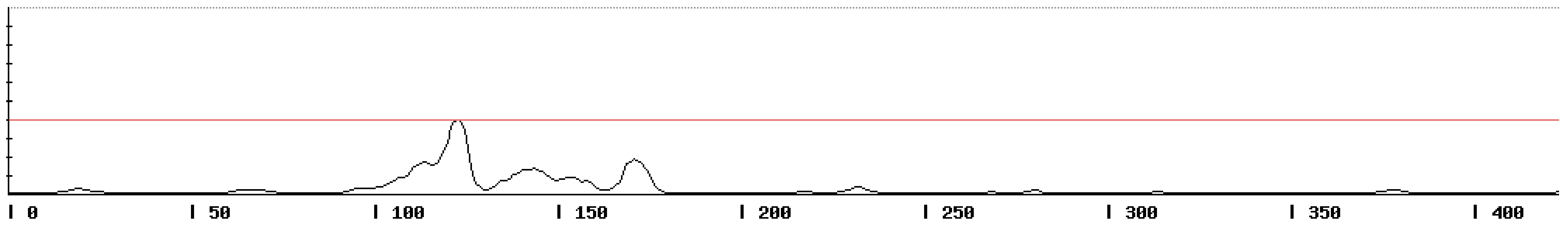

Predictions for Oryzabrachyantha_OB01G20290.1

Viterbi Path	
Viterbi Path	
Posterior @ 0.4	
Posterior @ 0.4	
Posterior Graphic. Threshold Marked @ 0.4	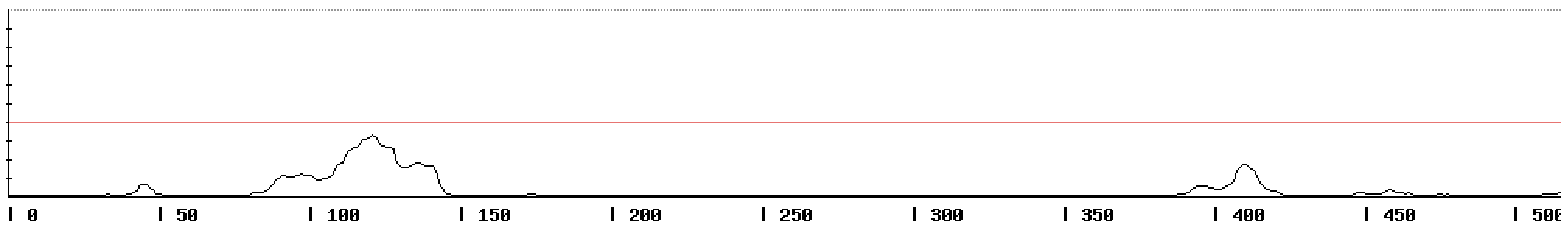

Predictions for Oryzabrachyantha_OB01G35210.1

Viterbi Path	
Viterbi Path	
Posterior @ 0.4	31 - RKRRK - 35
Posterior @ 0.4	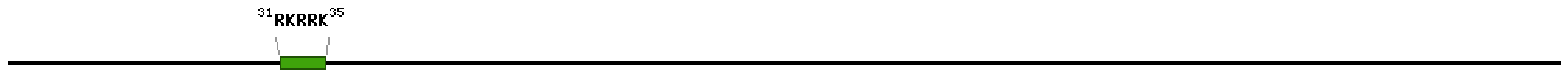
Posterior Graphic. Threshold Marked @ 0.4	

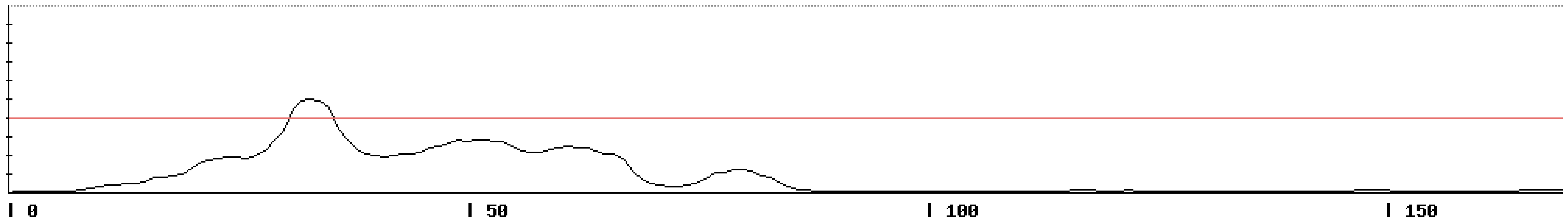

Predictions for Oryzabrachyantha_OB01G35220.1

Viterbi Path

Viterbi Path

Posterior @ 0.4

Posterior @ 0.4

Posterior Graphic. Threshold Marked @ 0.4

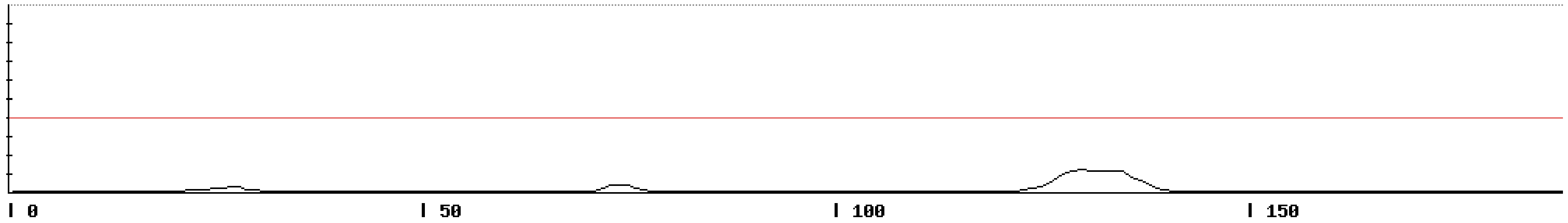

Predictions for Oryzabrachyantha_OB01G35490.1

Viterbi Path

Viterbi Path

Posterior @ 0.4

Posterior @ 0.4

Posterior Graphic. Threshold Marked @ 0.4

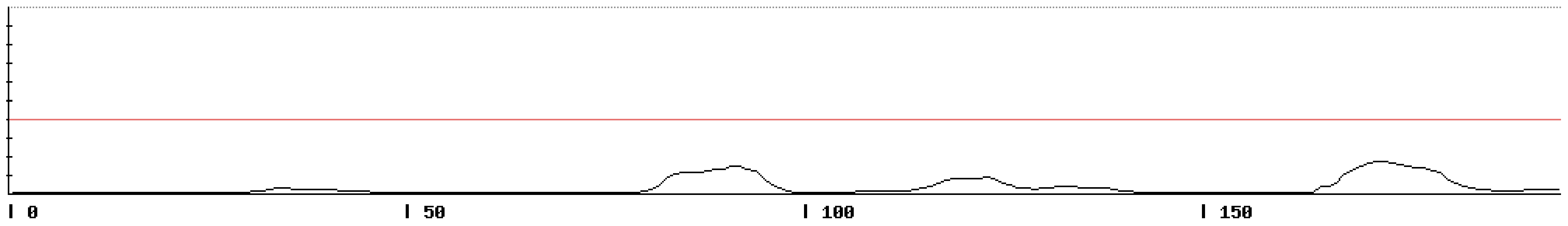

Predictions for Oryzabrachyantha_OB01G43540.1

Viterbi Path

Viterbi Path

Posterior @ 0.4

Posterior @ 0.4

Posterior Graphic. Threshold Marked @ 0.4

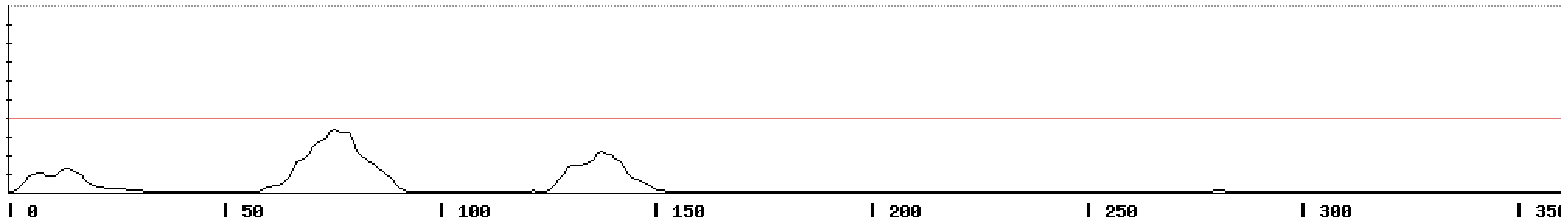

Predictions for Oryzabrachyantha_OB01G43840.1

Viterbi Path

Viterbi Path

Posterior @ 0.4 92 - RPNRAAGR - 99

Posterior @ 0.4

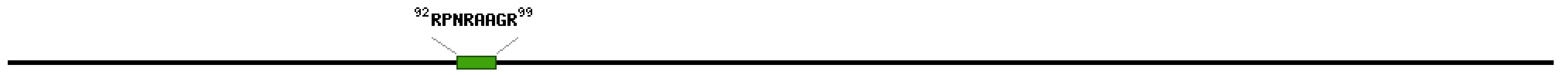

Posterior Graphic. Threshold Marked @ 0.4

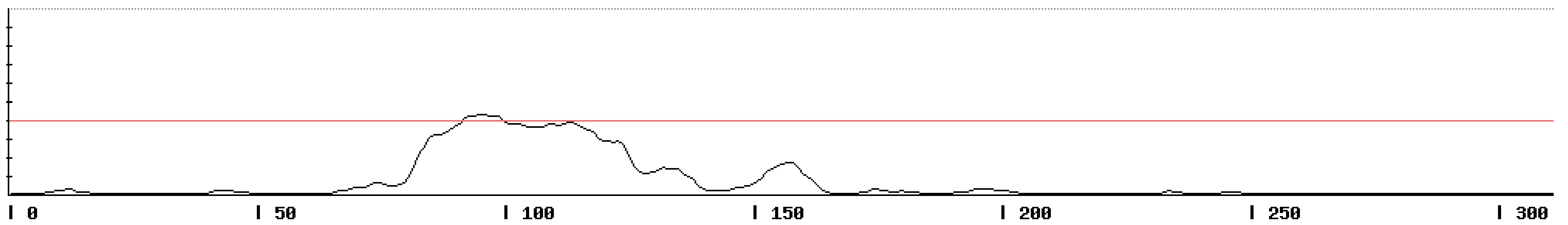

Predictions for Oryzabrachyantha_OB01G46930.1

Viterbi Path

Viterbi Path

Posterior @ 0.4 57 - ARRQRRRRRG - 66

Posterior @ 0.4

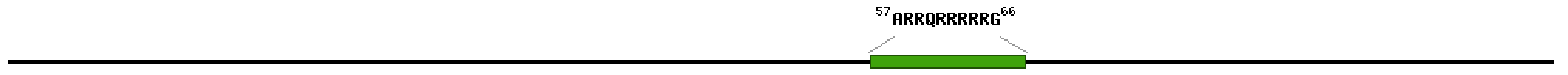

Posterior Graphic. Threshold Marked @ 0.4

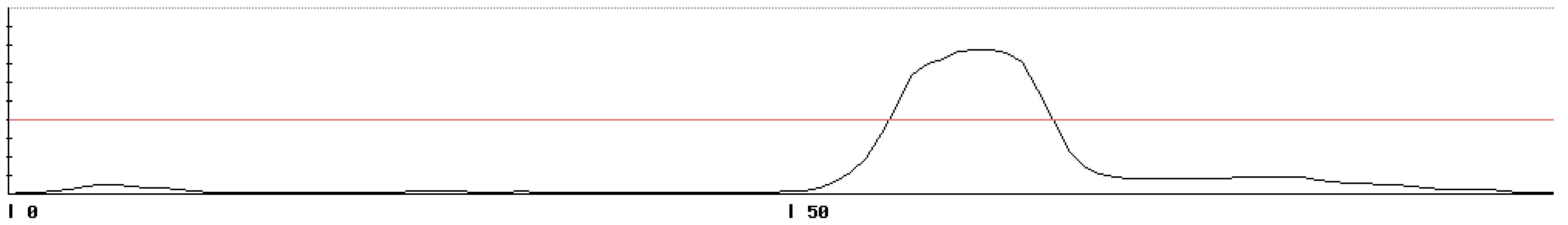

Predictions for Oryzabrachyantha_OB01G48340.1

Viterbi Path 248 - RRVGAHVRWRRRPGRLDAGPARPRRRRPAPPGHP - 281

Viterbi Path

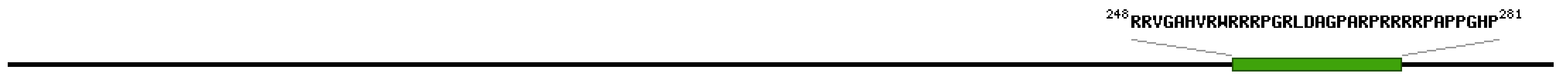

Posterior @ 0.4	248 - RRVGAHVRWRRRPGRLDAGPARPRRRRPAPPGH - 280
Posterior @ 0.4	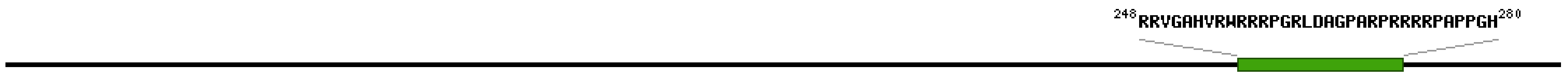
Posterior Graphic. Threshold Marked @ 0.4	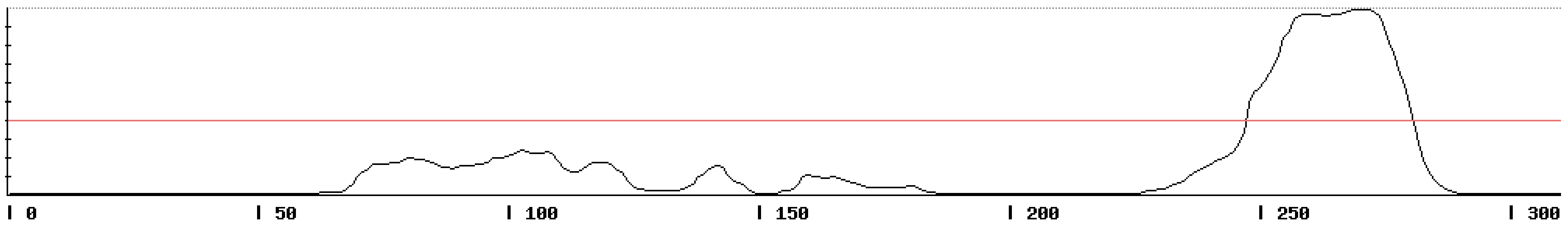

Predictions for Oryzabrachyantha_OB01G48600.1

Viterbi Path	
Viterbi Path	
Posterior @ 0.4	
Posterior @ 0.4	
Posterior Graphic. Threshold Marked @ 0.4	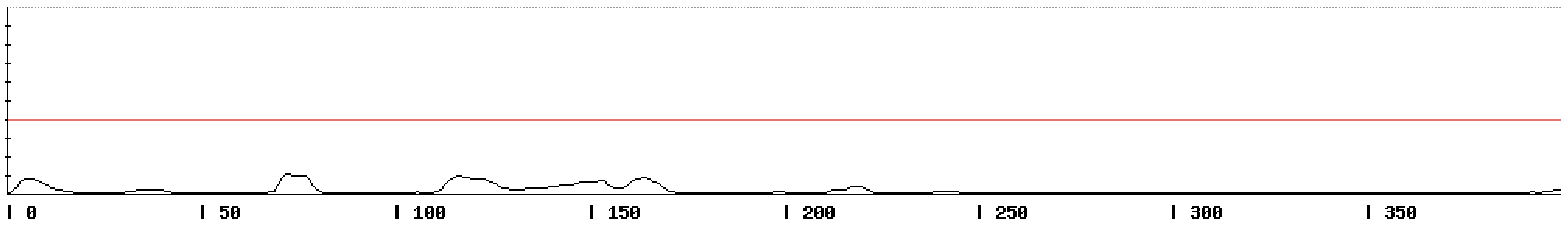

Predictions for Oryzabrachyantha_OB01G51370.1

Viterbi Path	
Viterbi Path	

Posterior @ 0.4	150 - FKRPTPAHRRSPPRIRAATARRR - 172
Posterior @ 0.4	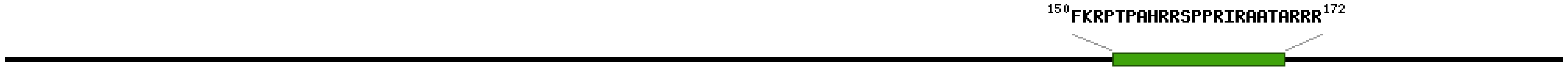
Posterior Graphic. Threshold Marked @ 0.4	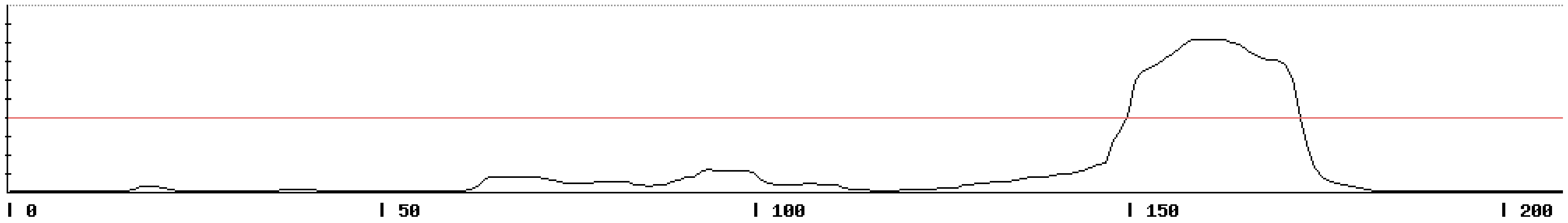

Predictions for Oryzabrachyantha_OB02G19660.1

Viterbi Path	
Viterbi Path	
Posterior @ 0.4	
Posterior @ 0.4	
Posterior Graphic. Threshold Marked @ 0.4	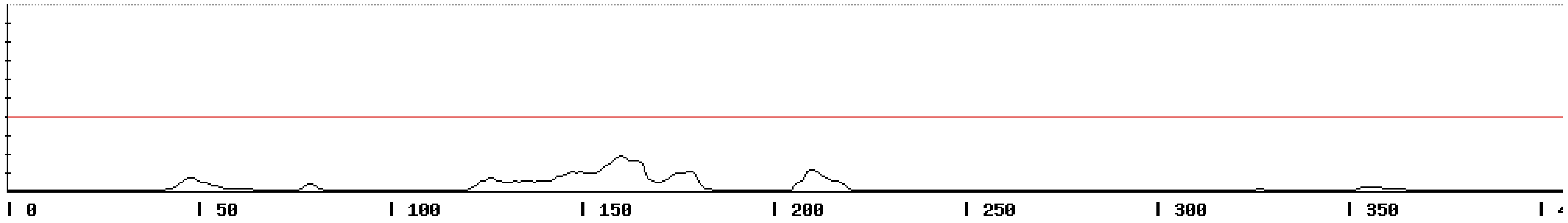

Predictions for Oryzabrachyantha_OB02G21080.1

Viterbi Path

Viterbi Path

Posterior @ 0.4

Posterior @ 0.4

Posterior Graphic. Threshold Marked @ 0.4

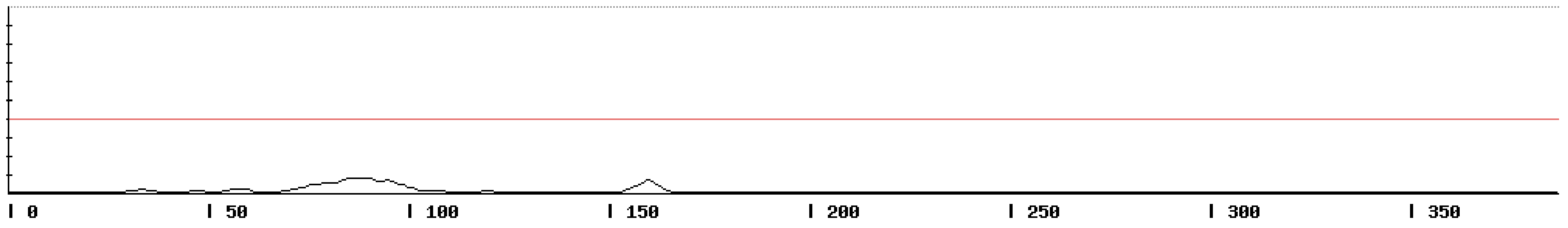

Predictions for Oryzabrachyantha_OB02G21090.1

Viterbi Path

Viterbi Path

Posterior @ 0.4

Posterior @ 0.4

Posterior Graphic. Threshold Marked @ 0.4

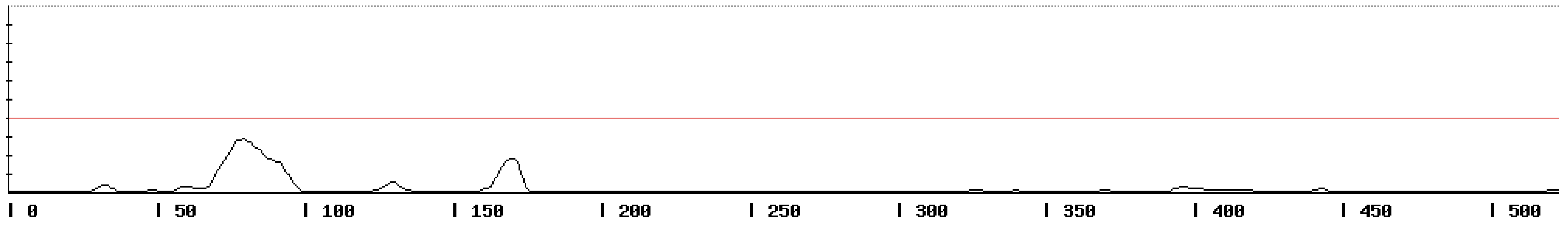

Predictions for Oryzabrachyantha_OB02G27960.1

Viterbi Path	
Viterbi Path	
Posterior @ 0.4	147 - SHRKR - 151
Posterior @ 0.4	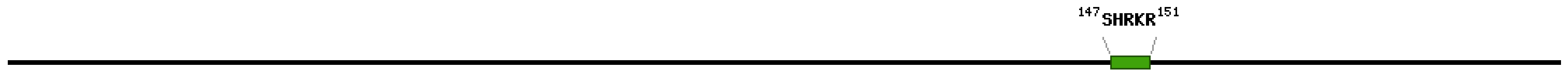
Posterior Graphic. Threshold Marked @ 0.4	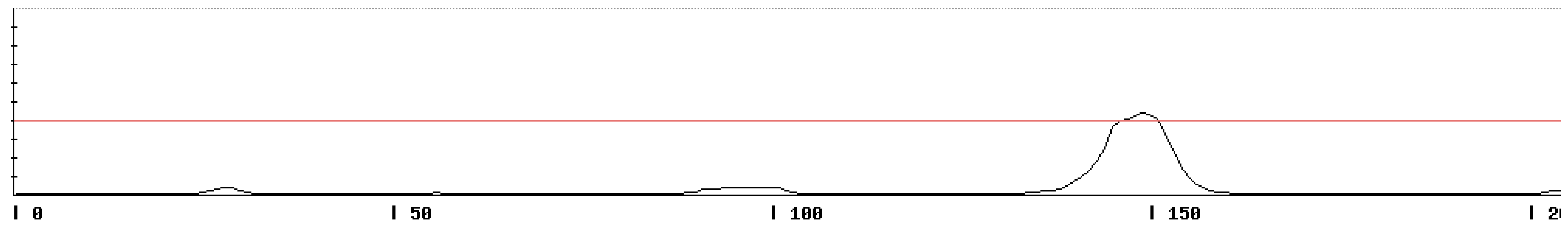

Predictions for Oryzabrachyantha_OB02G29280.1

Viterbi Path	
Viterbi Path	
Posterior @ 0.4	
Posterior @ 0.4	
Posterior Graphic. Threshold Marked @ 0.4	

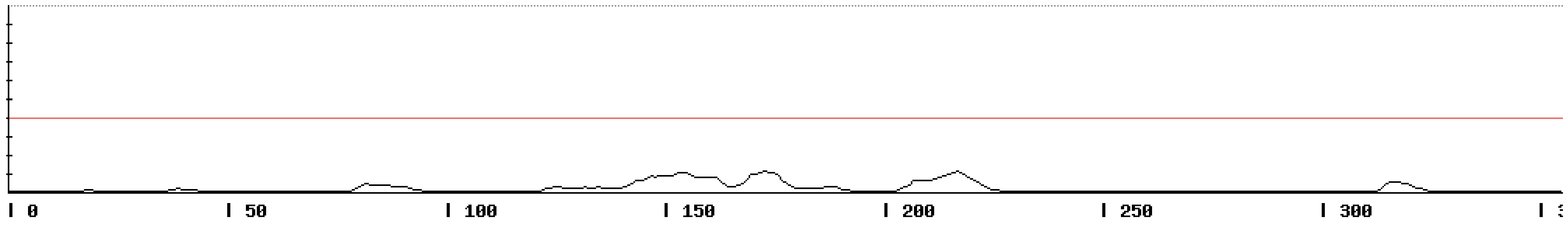

Predictions for Oryzabrachyantha_OB02G30060.1	
Viterbi Path	
Viterbi Path	
Posterior @ 0.4	
Posterior @ 0.4	
Posterior Graphic. Threshold Marked @ 0.4	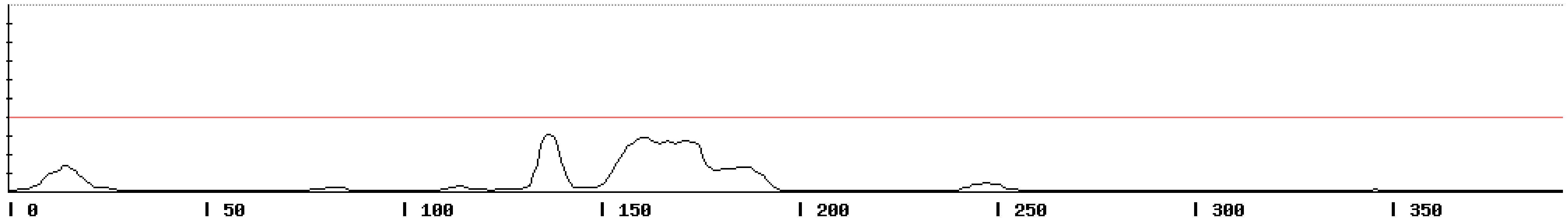

Predictions for Oryzabrachyantha_OB02G31950.1	
Viterbi Path	
Viterbi Path	
Posterior @ 0.4	
Posterior @ 0.4	

Posterior Graphic. Threshold Marked @ 0.4

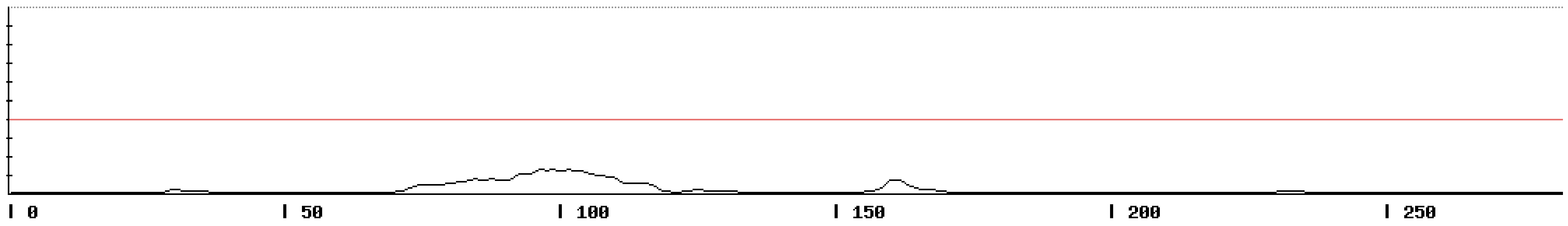

Predictions for Oryzabrachyantha_OB02G33070.1

Viterbi Path

Viterbi Path

Posterior @ 0.4

Posterior @ 0.4

Posterior Graphic. Threshold Marked @ 0.4

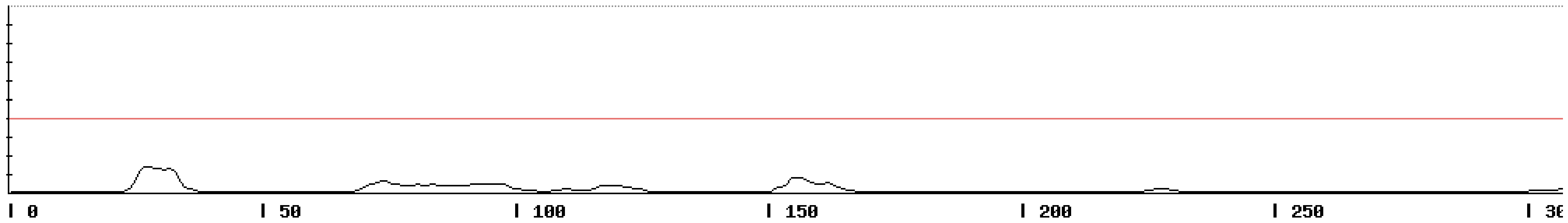

Predictions for Oryzabrachyantha_OB02G39340.1

Viterbi Path

Viterbi Path

Posterior @ 0.4

Posterior @ 0.4

Posterior Graphic. Threshold Marked @ 0.4

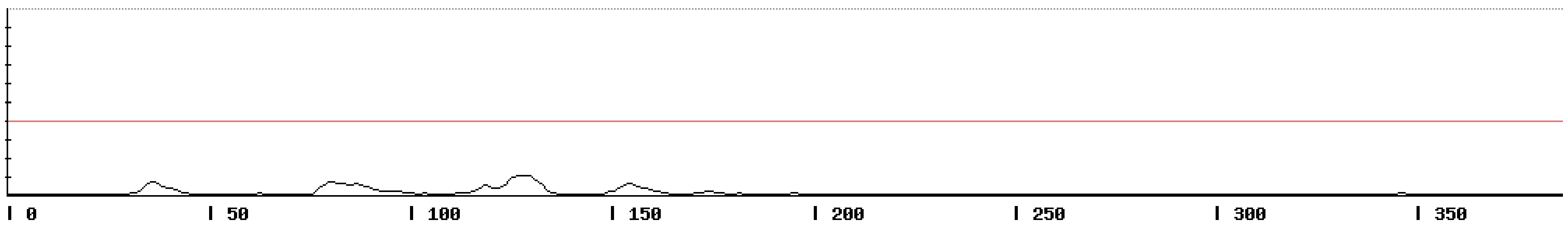

Predictions for Oryzabrachyantha_OB02G43380.1

Viterbi Path

Viterbi Path

Posterior @ 0.4

Posterior @ 0.4

Posterior Graphic. Threshold Marked @ 0.4

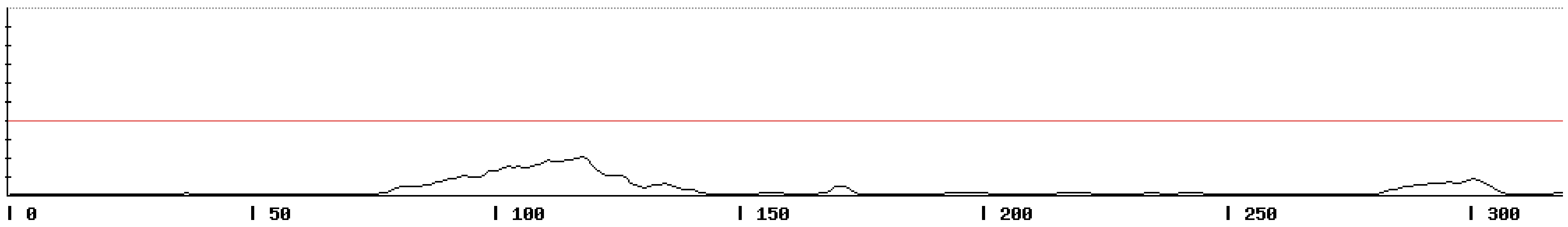

Predictions for Oryzabrachyantha_OB02G44540.1

Viterbi Path

Viterbi Path

Posterior @ 0.4

Posterior @ 0.4

Posterior Graphic. Threshold Marked @ 0.4

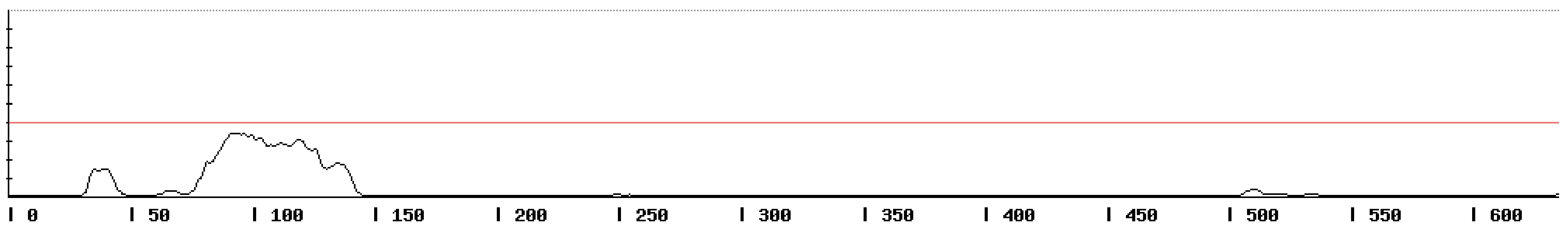

Predictions for Oryzabrachyantha_OB0325G10010.1

Viterbi Path

Viterbi Path

Posterior @ 0.4

85 - R - 85

101 - KNPQGAKIGESKNFRFKKKDGK - 122

Posterior @ 0.4

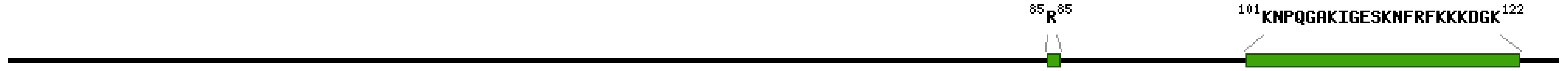

Posterior Graphic. Threshold Marked @ 0.4

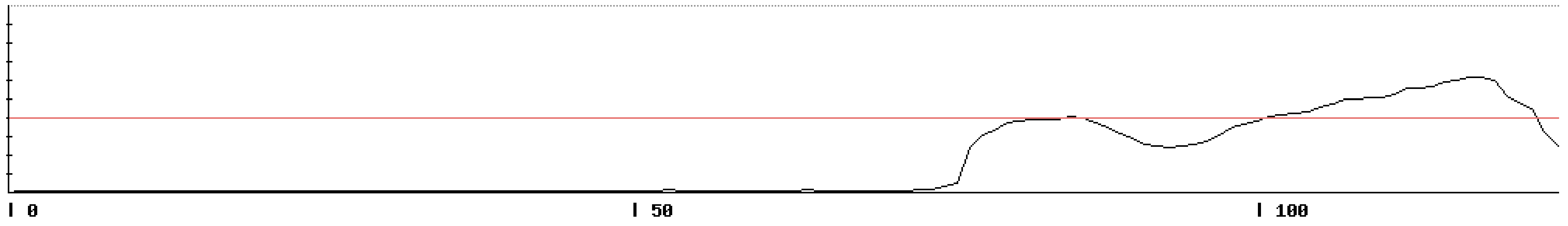

Predictions for Oryzabrachyantha_OB03G10720.1

Viterbi Path

Viterbi Path

Posterior @ 0.4

Posterior @ 0.4

Posterior Graphic. Threshold Marked @ 0.4

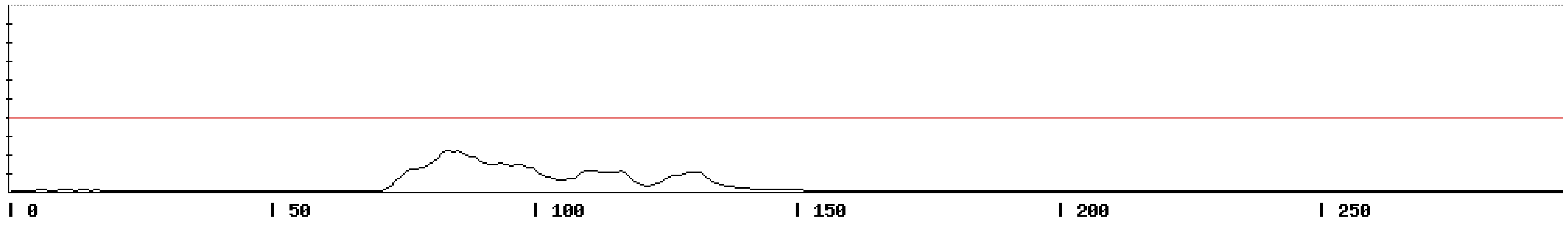

Predictions for Oryzabrachyantha_OB03G11530.1

Viterbi Path

Viterbi Path

Posterior @ 0.4	
	94 - K - 94
	96 - RK - 97
	596 - KKAK - 599

Posterior @ 0.4

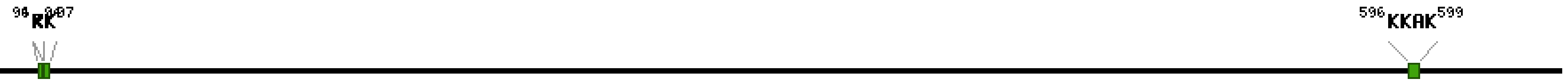

Posterior Graphic. Threshold Marked @ 0.4

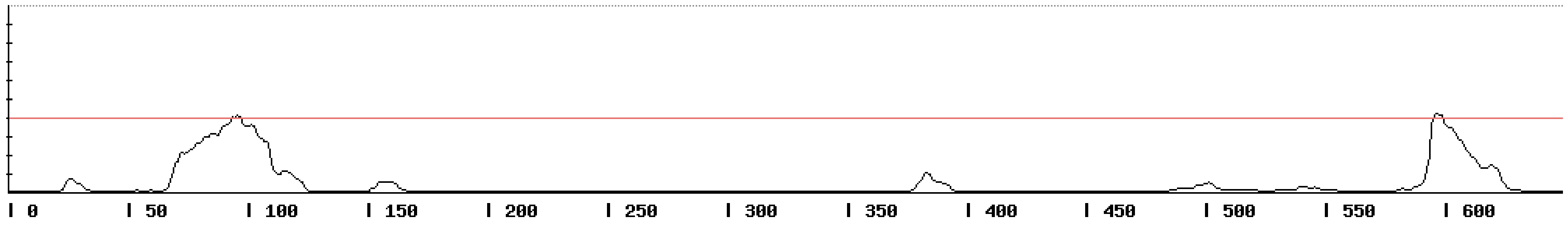

Predictions for Oryzabrachyantha_OB03G12100.1

Viterbi Path

Viterbi Path

Posterior @ 0.4: 246 - RRASKKPRG - 254

Posterior @ 0.4

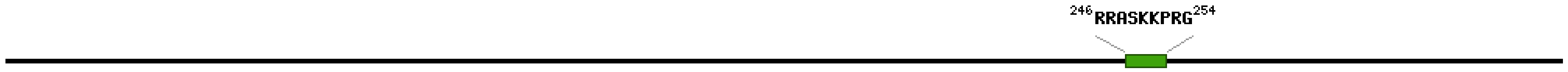

Posterior Graphic. Threshold Marked @ 0.4

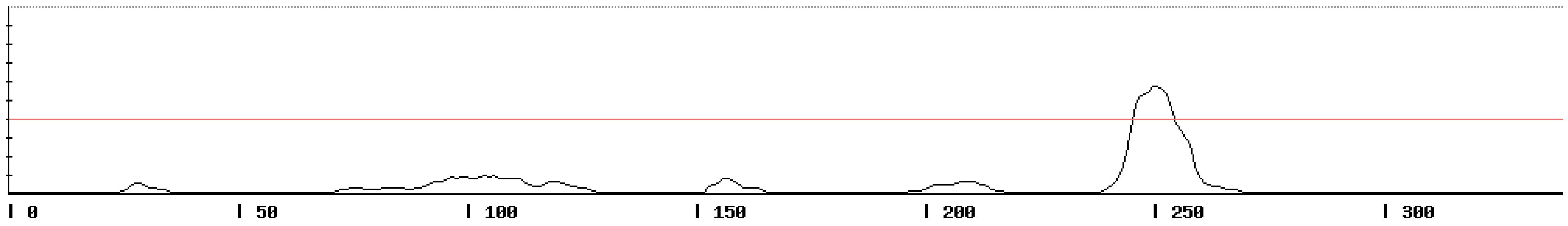

Predictions for Oryzabrachyantha_OB03G12640.1

Viterbi Path

Viterbi Path

Posterior @ 0.4

Posterior @ 0.4

Posterior Graphic. Threshold Marked @ 0.4

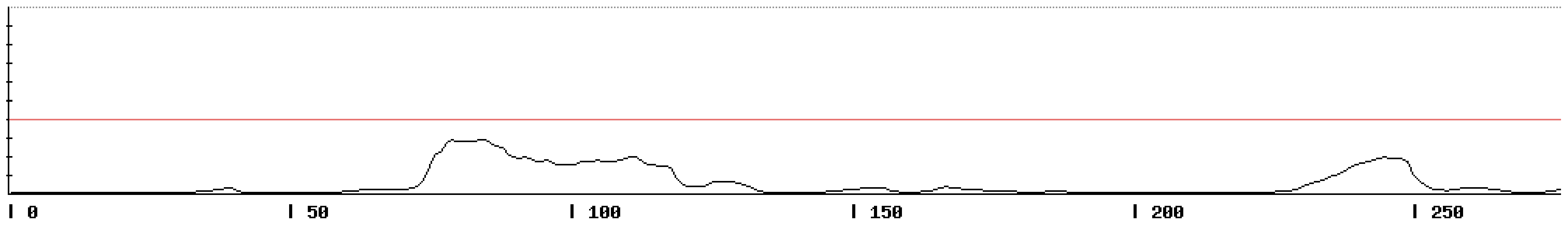

Predictions for Oryzabrachyantha_OB03G19030.1

Viterbi Path

Viterbi Path

Posterior @ 0.4

24 - ERRRRLLRPPVDSKPPALERREARPKA - 50

586 - SKRKRRKTGRDPS - 598

617 - SRRRRRRK - 624

Posterior @ 0.4

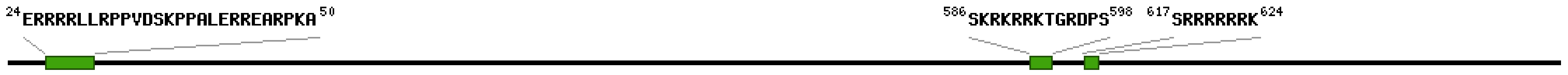

Posterior Graphic. Threshold Marked @ 0.4

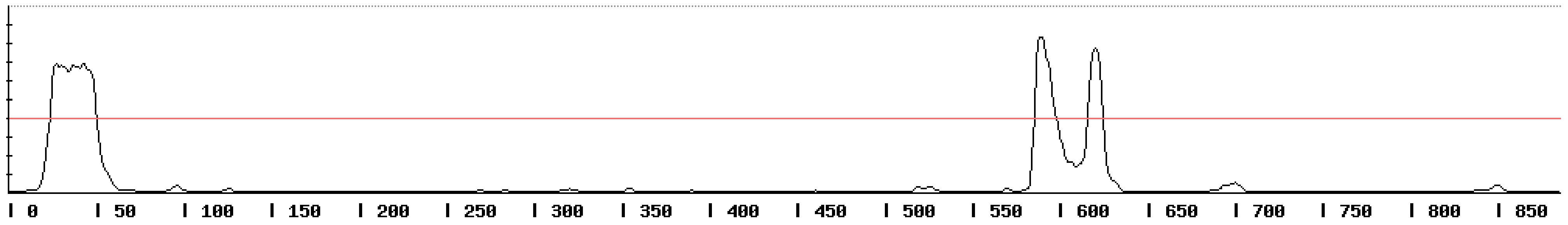

Predictions for Oryzabrachyantha_OB03G25660.1

Viterbi Path

Viterbi Path

Posterior @ 0.4

Posterior @ 0.4

Posterior Graphic. Threshold Marked @ 0.4

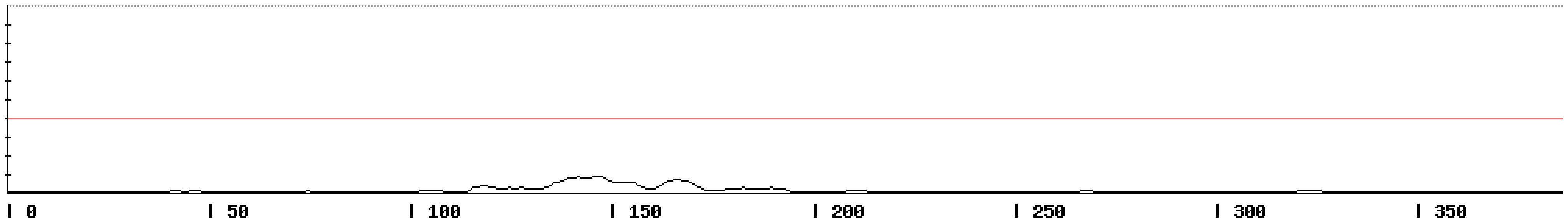

Predictions for Oryzabrachyantha_OB03G25680.1

Viterbi Path

Viterbi Path

Posterior @ 0.4

Posterior @ 0.4

Posterior Graphic. Threshold Marked @ 0.4

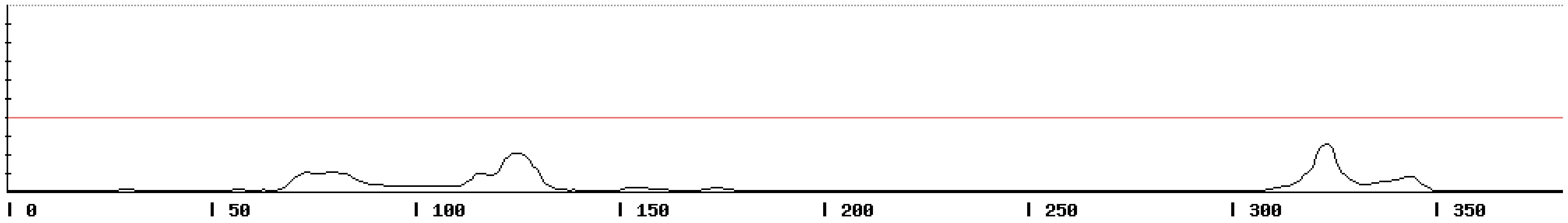

Predictions for Oryzabrachyantha_OB03G34470.1

Viterbi Path

Viterbi Path

Posterior @ 0.4

Posterior @ 0.4

Posterior Graphic. Threshold Marked @ 0.4

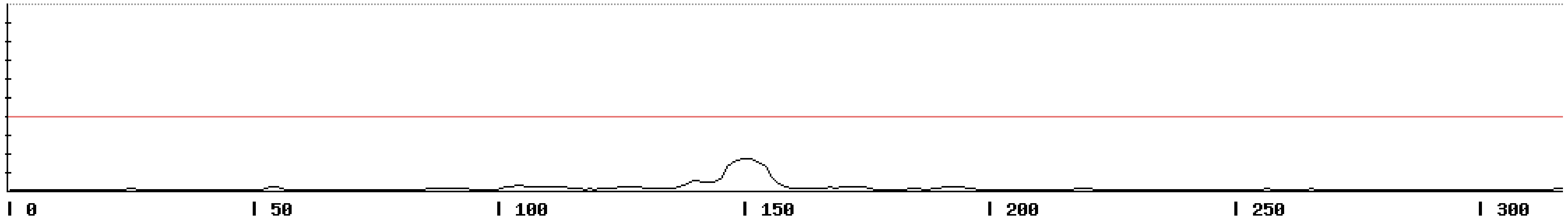

Predictions for Oryzabrachyantha_OB03G42970.1

Viterbi Path

Viterbi Path

Posterior @ 0.4

Posterior @ 0.4

Posterior Graphic. Threshold Marked @ 0.4

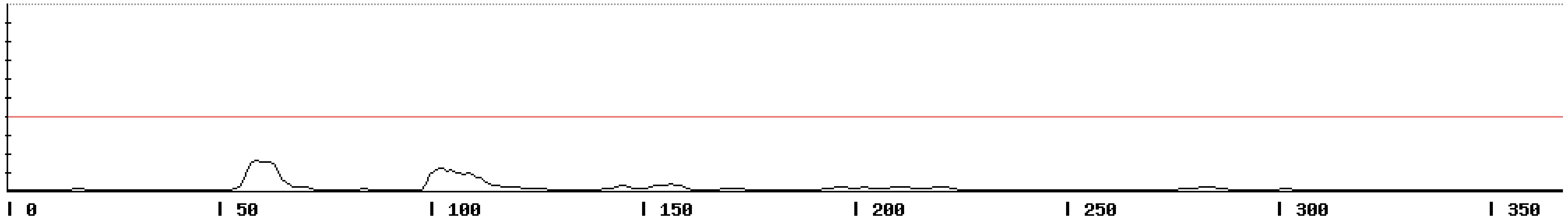

Predictions for Oryzabrachyantha_OB03G45600.1

Viterbi Path

Viterbi Path

Posterior @ 0.4

Posterior @ 0.4

Posterior Graphic. Threshold Marked @ 0.4

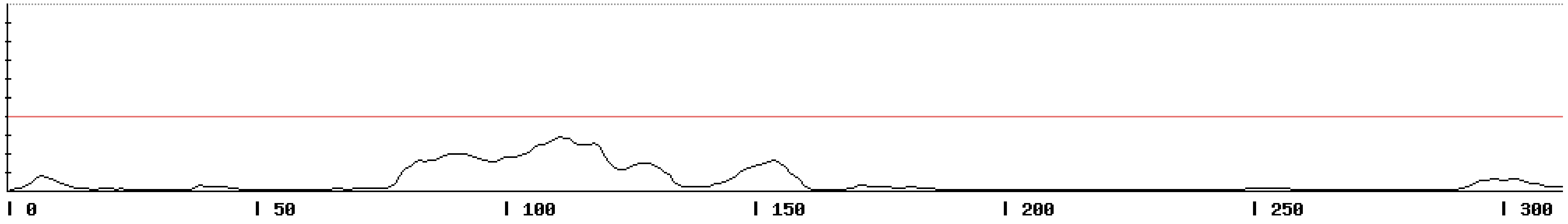

Predictions for Oryzabrachyantha_OB03G46690.1

Viterbi Path

Viterbi Path

Posterior @ 0.4

Posterior @ 0.4

Posterior Graphic. Threshold Marked @ 0.4

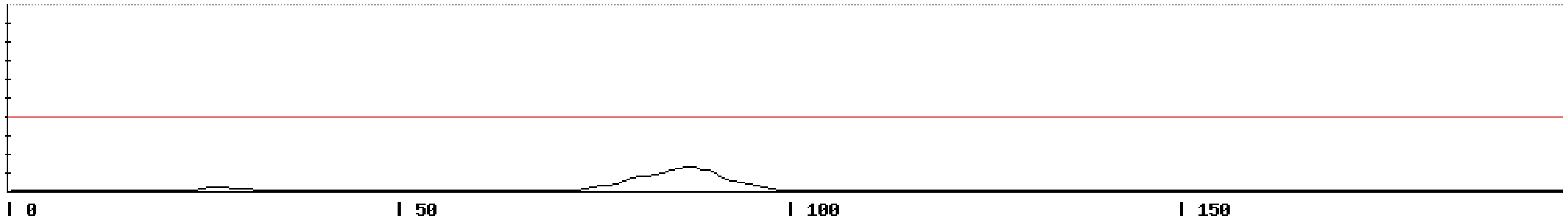

Predictions for Oryzabrachyantha_OB03G46930.1

Viterbi Path

Viterbi Path

Posterior @ 0.4

Posterior @ 0.4

Posterior Graphic. Threshold Marked @ 0.4

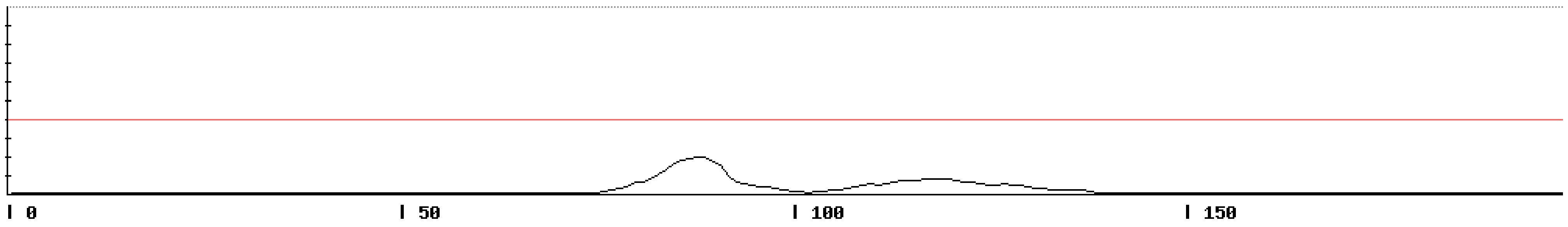

Predictions for Oryzabrachyantha_OB03G47140.1

Viterbi Path

Viterbi Path

Posterior @ 0.4 — 96 - GGKKPVKA - 103

Posterior @ 0.4

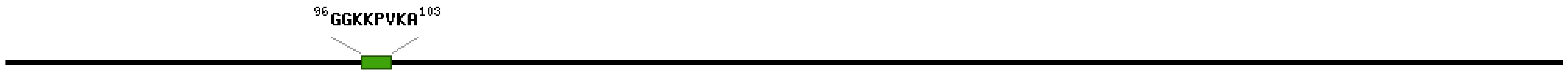

Posterior Graphic. Threshold Marked @ 0.4

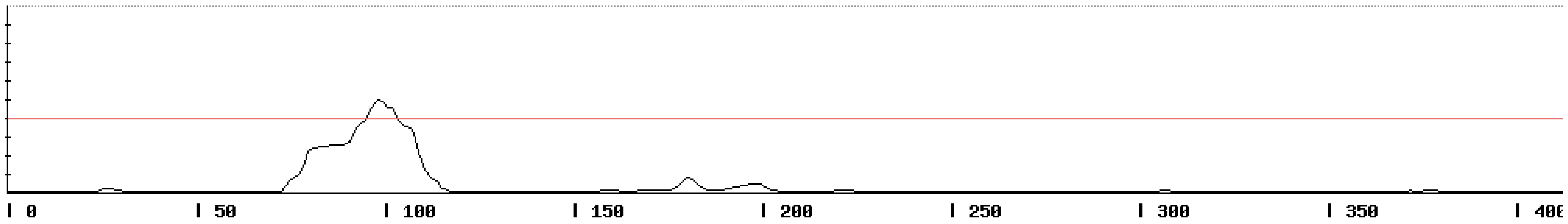

Predictions for Oryzabrachyantha_OB03G47560.1	
Viterbi Path	
Viterbi Path	
Posterior @ 0.4	80 - KRASSGGRRQRAVGGGGGGGESVWKSEGGKKPVKDADGRRVRPPRRLPPEV - 130<br>156 - SRRRRRRSREA - 166
Posterior @ 0.4	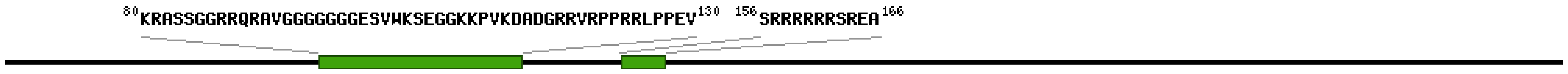
Posterior Graphic. Threshold Marked @ 0.4	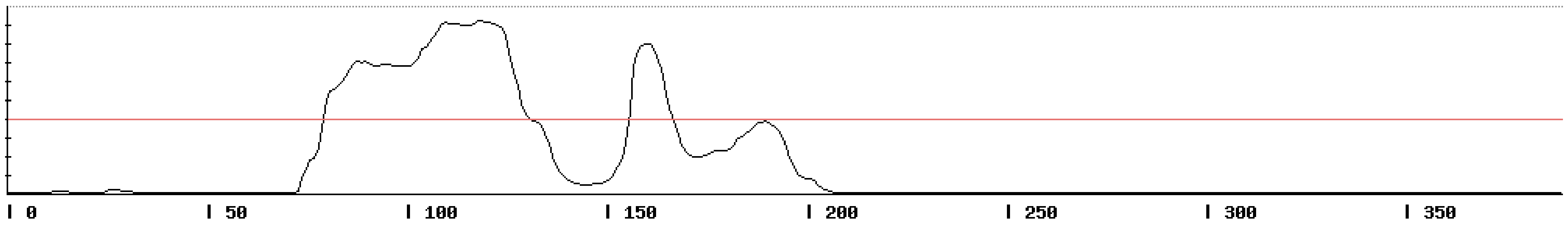

Predictions for Oryzabrachyantha_OB04G20560.1	
Viterbi Path	
Viterbi Path	
Posterior @ 0.4	
Posterior @ 0.4	
Posterior Graphic. Threshold Marked @ 0.4	

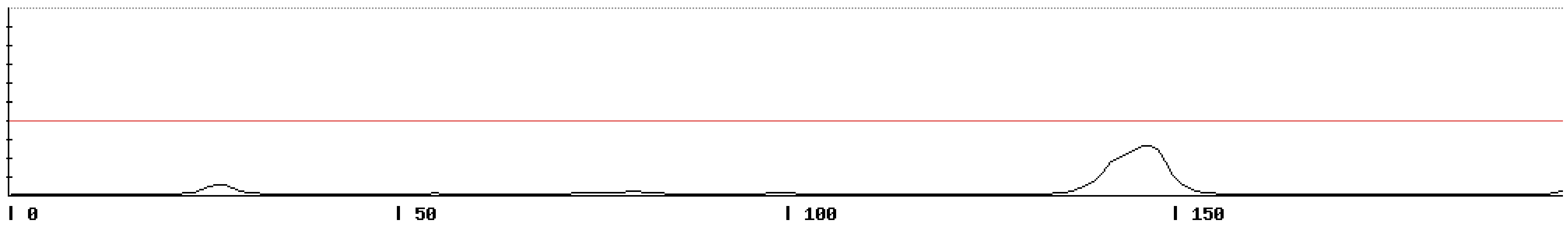

Predictions for Oryzabrachyantha_OB04G21050.1

Viterbi Path

Viterbi Path

Posterior @ 0.4

Posterior @ 0.4

Posterior Graphic. Threshold Marked @ 0.4

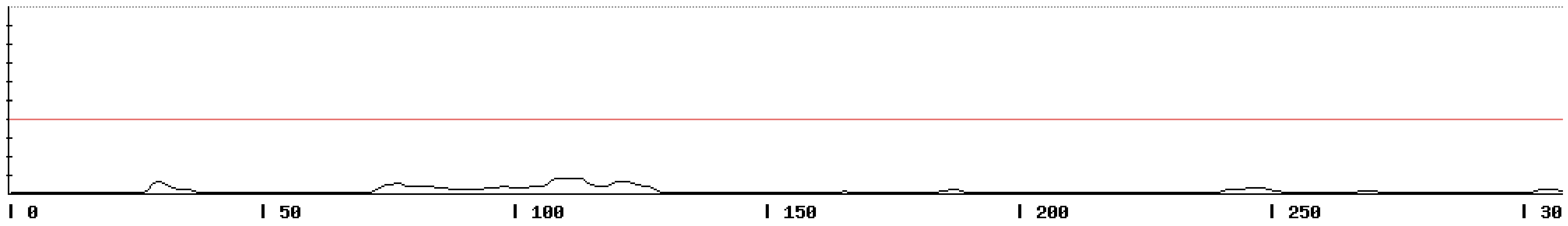

Predictions for Oryzabrachyantha_OB04G22290.1

Viterbi Path

Viterbi Path

Posterior @ 0.4

Posterior @ 0.4

Posterior Graphic. Threshold Marked @ 0.4

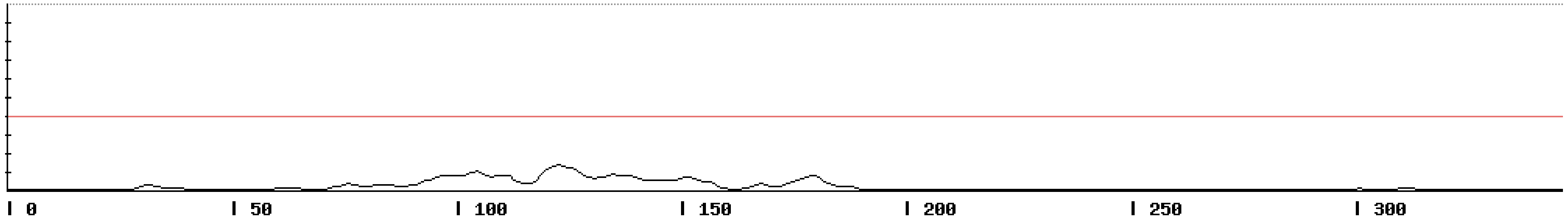

Predictions for Oryzabrachyantha_OB04G23300.1

Viterbi Path	
Viterbi Path	
Posterior @ 0.4	
Posterior @ 0.4	
Posterior Graphic. Threshold Marked @ 0.4	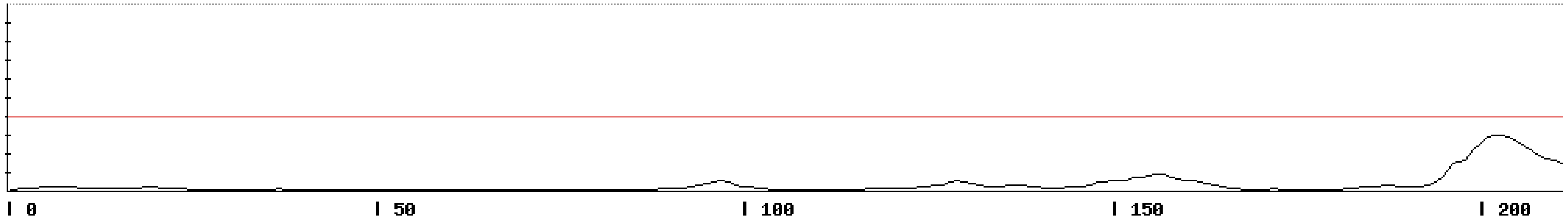

Predictions for Oryzabrachyantha_OB04G23430.1

Viterbi Path	
Viterbi Path	
Posterior @ 0.4	19 - GQRKRRKISHHKHSASDENIRWHKTGKSKEIYHNGVKKGWKKI - 61
Posterior @ 0.4	

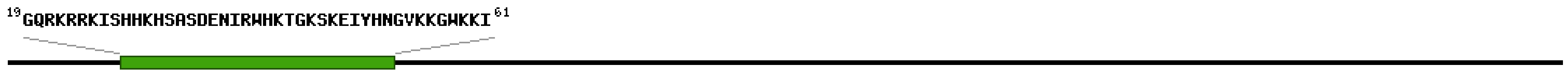

Posterior Graphic. Threshold Marked @ 0.4

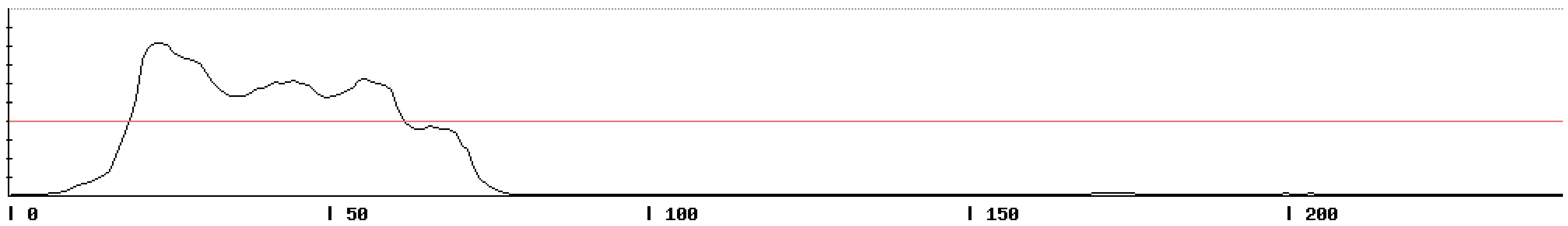

Predictions for Oryzabrachyantha_OB04G25790.1

Viterbi Path

Viterbi Path

Posterior @ 0.4

Posterior @ 0.4

Posterior Graphic. Threshold Marked @ 0.4

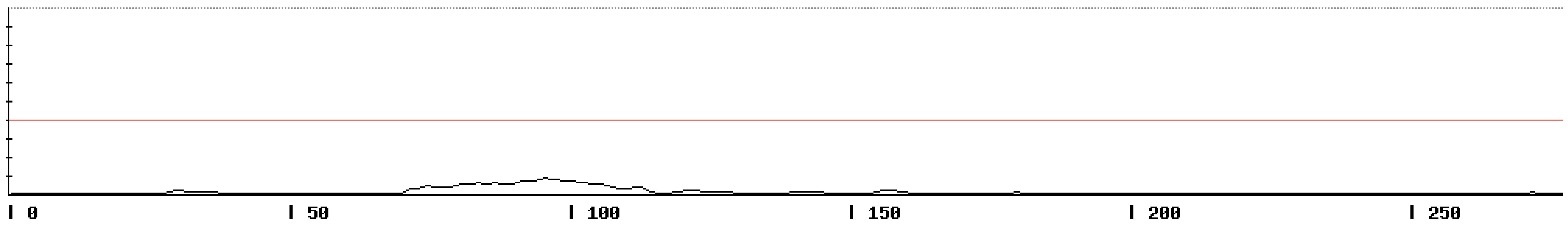

Predictions for Oryzabrachyantha_OB04G27030.1

Viterbi Path

Viterbi Path

Posterior @ 0.4	
Posterior @ 0.4	
Posterior Graphic. Threshold Marked @ 0.4	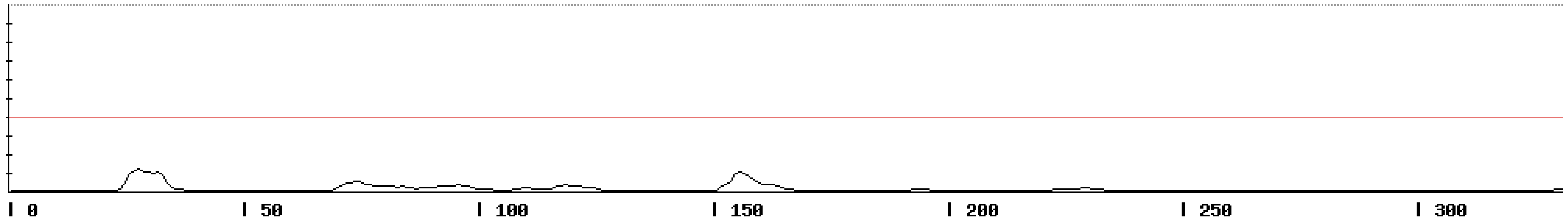

Predictions for Oryzabrachyantha_OB04G28320.1

Viterbi Path	
Viterbi Path	
Posterior @ 0.4	92 - KRGGGKHQHRRRA - 104
Posterior @ 0.4	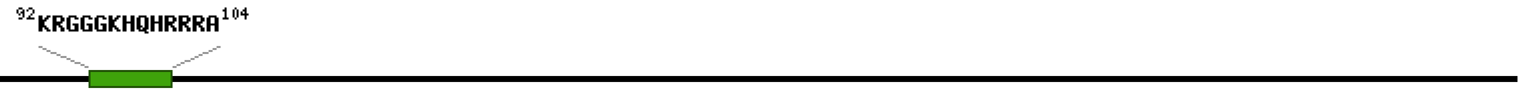

Posterior Graphic. Threshold Marked @ 0.4	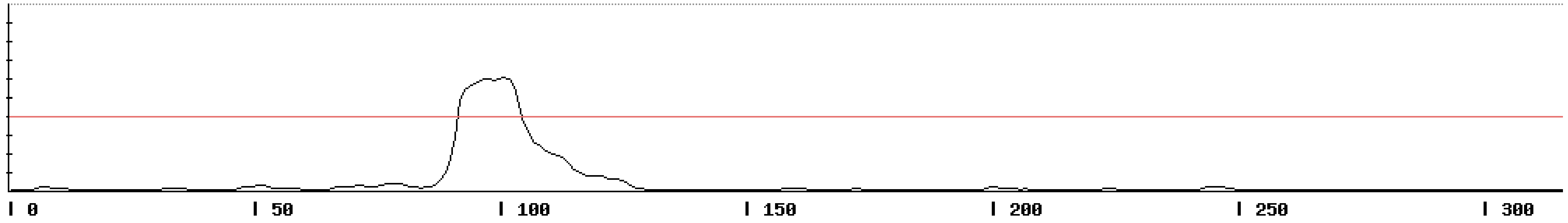

Predictions for Oryzabrachyantha_OB04G32440.1

[truncated: 573,589 more chars]
